# SIRT2 functions as a histone delactylase and inhibits the proliferation and migration of neuroblastoma cells

**DOI:** 10.1038/s41421-022-00398-y

**Published:** 2022-06-07

**Authors:** Hanxiao Zu, Chang Li, Chenrui Dai, Yi Pan, Chen Ding, Huiying Sun, Xuan Zhang, Xuebiao Yao, Jianye Zang, Xi Mo

**Affiliations:** 1 Department of Clinical Laboratory, the First Affiliated Hospital of USTC, MOE Key Laboratory for Membraneless Organelles and Cellular Dynamics, CAS Center for Excellence in Biomacromolecules, and School of Life Sciences, University of Science and Technology of China, 96 Jinzhai Road, Hefei, 230026 Anhui China; 2 Pediatric Translational Medicine Institute, Shanghai Children’s Medical Center, School of Medicine, Shanghai Jiao Tong University, Shanghai, 200127 China; 3 Academy of Medical Science, Zhengzhou University, Zhengzhou, 450052 Henan China; 4 State Key Laboratory of Cell Differentiation and Regulation, College of Life Science, Henan Normal University, Xinxiang, 453007 Henan China

**Keywords:** Acetylation, Acetylation

Dear Editor,

Histone lysine acylations are responsible for the regulation of gene transcription which are involved in the crosstalk between metabolism and epigenetics^1,2^. Lysine lactylation (Kla) was identified as a new type of histone acylation when breast cancer cells were under hypoxia or macrophages were exposed to bacterial challenge^3^. Similar to other fatty acids, lactate reacts with coenzyme A to form lactyl-CoA, and Kla deposition is then catalyzed by histone acetyltransferase (HAT) p300 from a lactyl-CoA substrate^3,4^, but no proteins are known to function as an “eraser” of histone lactylation modification. Sirtuins are a family of NAD^+^-dependent enzymes capable of removing acyl lysine modifications to dynamically regulate histone acylations in the context of different physiological and pathological cues^1^. A recent study showed that SIRT2 can remove the lactyl group from synthetic peptides related to pyruvate kinase M2 (PKM2)^5^. It is therefore possible that some sirtuins could function as delactylases for histone lactylation modifications.

To investigate whether sirtuins have the ability to catalyze the delactylation of histones, we designed an activity-based fluorescence probe bearing a lactylated lysine residue (Kla) and a nitrobenzoxadiazole (NBD) group (Fig. 1a). When the lactyl moiety is removed from the lysine residue, the free amine of lysine residue can react with the NBD group to turn on the fluorescence signal that can be recorded at 545 nm. Using this probe, we found that SIRT1, SIRT2, SIRT3, and SIRT5, but not SIRT6 or SIRT7, can catalyze the removal of the lactyl moiety (Fig. 1b). Comparison of the fluorescence intensities suggested that the catalytic activities of SIRT1 and SIRT2 are much higher than that of SIRT3 and SIRT5, with SIRT2 yielding the strongest signal under the same reaction conditions, which can be inhibited by inhibitor Tenovin-6 (Fig. 1b, c), confirming that the enzymatic activity of SIRT2 is required for delactylation of Kla.

**Fig. 1:**
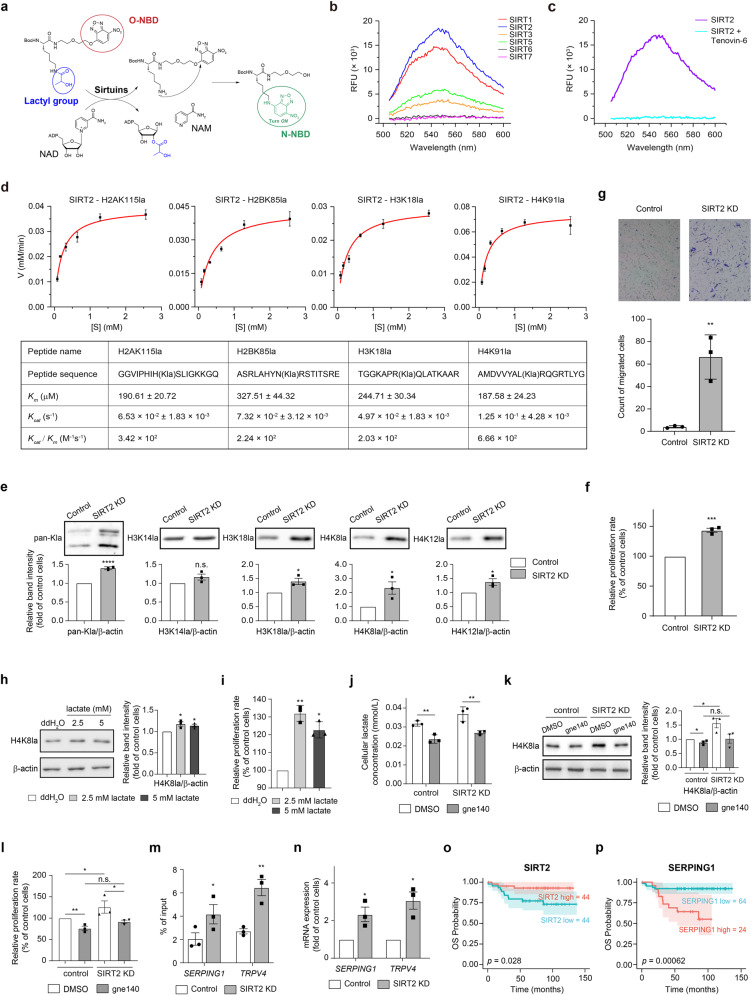
SIRT2 functions as an eraser of histone lactylation in neuroblastoma cells and increased histone lactylation promote cell proliferation and migration. **a** Schematic illustration of screening for the delactylase activity of sirtuins using a fluorescence probe. In the probe, NBD is covalently linked to an oxygen atom; when the lactyl group is removed (for example by some tested sirtuin proteins), the free amine group of lysine residue attacks the O-linked NBD group, and covalent linkage of the NBD group with a nitrogen atom turns on the fluorescence signal. The lactyl group is colored blue. The O-NBD group is indicated by a red circle. The N-NBD is colored green to indicate the fluorescence signal and is marked by a green circle. **b** Fluorescence assay of the probe with the indicated sirtuin proteins (λex = 480 nm, λem = 545 nm). **c** Fluorescence assay of the probe with SIRT2 (λex = 480 nm, λem = 545 nm) in the presence of the inhibitor Tenovin-6 (500 μM). **d** Michaelis-Menten plots for SIRT2 against the four peptides bearing H2AK115la, H2BK85la, H3K18la, or H4K91la modifications, respectively. The sequence of the peptides and kinetic parameters *K*_*m*_, *K*_cat_, and *K*_cat_*/K*_*m*_ are shown in the tables. **e** Pan and histone lactylation levels at the H3K14, H3K18, H4K8, and H4K12 sites in SIRT2 knockdown (KD) SH-SY5Y cells, as detected by immunoblotting. **f** Cell proliferation rate of SIRT2 KD cells was significantly increased, as measured using a BrdU assay (chemiluminescent). **g** Cell migration capacity of SIRT2 KD cells was significantly increased, as evaluated using Transwell assays. The number of stained cells which migrated to the lower chamber was counted using ImageJ. **h** Histone lactylation levels at the H4K8 site in SH-SY5Y cells upon lactate treatment, as detected by immunoblotting. **i** Cell proliferation rate of SH-SY5Y cells was significantly increased upon lactate treatment, as measured using a BrdU assay (chemiluminescent). **j** Intracellular lactate concentration of control or SIRT2 KD SH-SY5Y cells upon LDHA inhibitor gne140 treatment (10 μM), as detected by ELISA. **k** Histone lactylation levels at H4K8 site in control or SIRT2 KD cells upon LDHA inhibitor gen140 treatment (10 μM), as detected by immunoblotting. **l** Cell proliferation rate of control or SIRT2 KD cells upon LDHA inhibitor gen140 treatment (10 μM), as measured using a BrdU assay (chemiluminescent). **m** ChIP was performed with an anti-H4K8la antibody in control and SIRT2 KD cells. qPCR analysis of the ChIP precipitates was performed to assess the occupancy of H4K8la marks at the promoters of *SERPING1* and *TRPV4* genes shown to regulate tumor cell proliferation and migration. **n** qPCR analysis of *SERPING1* and *TRPV4* expression in control and SIRT2 KD cells. **o**, **p** The protein levels of SIRT2 (**o**) and SERPING1 (**p**) were quantified by mass spectrometry, and Kaplan–Meier (K-M) analysis of their levels with overall survival (OS) was conducted using the R package “survival”. The optimal cut-off value of each protein for the K-M plots was calculated using the “survminer” package. The SIRT2 KD cell line in the present figure refers to the SIRT2 KD-1 cell line in Supplementary Fig. S3. The complete data sets of SIRT2 KD cell lines with two shRNA are presented in Supplementary Fig. S3. The relative intensity of each band was quantified via densitometry, using ImageJ after normalization to β-actin, with the values expressed as the fold change versus the value detected for control SH-SY5Y cells. All data are presented as the means ± SEM, calculated from three independent experiments. *P < 0.05; **P < 0.01; ***P < 0.001; ****P < 0.0001; n.s., not significant as calculated by two-tailed student’s *t-*test.

Since SIRT2 exhibited the strongest delactylation activity, our follow-up studies only focused on SIRT2. We evaluated the enzymatic activity of SIRT2 against 26 synthetic histone peptides bearing lactyl group at the lysine site previously identified in HeLa cells (Supplementary Table S1)^3^, and found that SIRT2 was able to remove the lactyl moieties from many of the tested lysine sites (Supplementary Table S1). However, for substrates whose Kla sites were positioned immediately adjacent to glycine (G) or proline (P)—as with H2AK11la, H2BK11la, H4K5la, H4K8la, and H4K31la sites—the delactylation activities of these enzymes tended to be much weaker or even lost (Supplementary Table S1). This is consistent with a previous study showing that the identity of amino acids positioned next to a modified lysine residue impacts the desuccinylation activity of SIRT5^6^. In addition to the lactylated peptides, SIRT2 also showed delactylation activity against purified histone proteins and nucleosomes from HeLa cells as detected by immunoblotting using specific antibodies against four lactylated lysine residues in Histone H3 or H4 (Supplementary Fig. S1).

We next measured the kinetic parameters for SIRT2-mediated delactylation and deacetylation of these four peptides (Fig. 1d and Supplementary Fig. S2). The *Kcat*/*Km* values of SIRT2-mediated delactylation of histone peptides are more than 100-folds higher than that for D- and L-lactylated PKM2 (of 0.8 and 1.1 M^−1^S^−1^, respectively)^5^. Although SIRT2 showed a higher catalytic efficiency towards Kac-containing histone peptides compared to Kla-containing substrates in the in vitro assays, these data still confirmed SIRT2 as an efficient delactylase.

Given that histone lactylation can activate gene transcription and drive tumorigenesis (e.g., with ocular melanoma)^7^, we speculated that lowering the SIRT2 level, which should result in increased histone lactylation in cells, may influence tumor progression. SIRT2 has been reported as a tumor suppressor in studies of various human cancers, and among these cancers neuroblastoma (NB) is of particular interest to pediatric clinicians because it is the most common pediatric extra-cranial solid tumor and is the primary cause of death from pediatric cancers in children between 1 and 5 years old^8^.

Immunofluorescence staining of SH-SY5Y NB cells showed that a significant amount of SIRT2 was localized in the nucleus where H3K18la and H4K8la localized (Supplementary Fig. S3a), suggesting the potential interaction possibility of SIRT2 with these modifications. SIRT2 knockdown (KD) via shRNA (Supplementary Fig. S3b, c) induced a significant increase in the pan-lactylation level as well as the histone lactylation levels at H3K18, H4K8, and H4K12 sites in NB cells (Fig. 1e and Supplementary Fig. S3d), which can be rescued upon SIRT2 complementation (Supplementary Fig. S4a–c). Specific inhibitors of SIRT2 (e.g., AK7 and AK1) caused the increased H4K8la level in a dose-dependent manner (Supplementary Fig. S3e). Consistently, the SIRT2 catalytically dead mutation H187Y significantly hampered the delactylation activity of SIRT2 (Supplementary Fig. S3f), further confirming SIRT2 as a histone delactylase. Given SIRT2’s known function as a histone deacetylase, we also detected the acetylation levels in SIRT2 KD SH-SY5Y cells. Immunoblotting analysis showed a similar pattern of increased acetylation as the lactylation, but the extent of the increased acetylation at H4K8 sites was much reduced compared to the extent of increased lactylation (Supplementary Fig. S3g). Consistent with endogenous SIRT2, when 3× FLAG-tagged SIRT2 was stably overexpressed in SH-SY5Y cells (Supplementary Fig. S5a, b), we also detected localization of exogenously expressed SIRT2 in the nucleus (Supplementary Fig. S5c). SIRT2 overexpression (OE) led to significantly decreased lactylation levels at all of the lysine sites tested (Supplementary Fig. S5d), but no significant change in acetylation was observed except the H3K18ac site (Supplementary Fig. S5e). These results demonstrate that SIRT2 can remove lactylation modifications from histone lysine residues in NB cells.

Functional assays revealed that the SIRT2 KD cells displayed a significantly increased cell proliferation rate (Fig. 1f and Supplementary Fig. S3h) and cell migration capacity (Fig. 1g and Supplementary Fig. S3i), which can be rescued upon SIRT2 complementation (Supplementary Fig. S4d). Conversely, the SIRT2 OE SH-SY5Y cells had a significantly decreased proliferation rate (by 48.1%) (Supplementary Fig. S5f). To further confirm that the histone delactylation activity of SIRT2 contributes to NB cell proliferation, we treated the SH-SY5Y cells with sodium lactate, which was reported to increase cellular lactate concentration^3^. Both the H4K8la level and cell proliferation rate significantly increased upon lactate treatment (Fig. 1h, i), suggesting that increased H4K8la modification contributes to NB cell proliferation. In contrast, when the NB cells were treated with LDHA inhibitor gne140, the lactate production in both control and SIRT2 KD cells was significantly decreased (Fig. 1j). As a result, the increase of H4K8la modification level and cell proliferation rate caused by SIRT2 knockdown was no longer observed (Fig. 1k, l), suggesting that the increased cell proliferation rate caused by SIRT2 knockdown depends on the intracellular lactate level and H4K8la level.

To explore the mechanism underlying the apparent tumor-promoting effects resulted from SIRT2 knockdown, we performed RNA-seq (Supplementary Fig. S6 and Table S2) and ChIP-seq with anti-H4K8la and anti-H4K8ac antibodies (Supplementary Fig. S7a–c and Tables S3–S6) for control and SIRT2 KD cells. To distinguish different functional consequences of H4K8la from H4K8ac, we assessed our RNA-seq and ChIP-seq datasets looking for any genes which were (i) significantly upregulated upon SIRT2 knockdown and (ii) specifically called by anti-H4K8la antibody in SIRT2 KD cells (Supplementary Fig. S7a); there were eight such genes (*C1RL*, *DRAM1*, *DUSP2*, *NOXA1*, *PAQR6*, *SERPINF1*, *SERPING1,* and *TRPV4*). ChIP-seq and ChIP-qPCR of these loci showed that a significantly higher amount of DNA for *SERPING1* and *TRPV4* genes were detected with anti-H4K8la antibody in SIRT2 KD cells compared to control cells (Supplementary Fig. S7d and Fig. 1m), but such significant increase was not observed for anti-H4K8ac antibody (Supplementary Fig. S7d, e) or for anti-H3K18la antibody (Supplementary Fig. S7f). No such increase was observed for the other six genes. qPCR confirmed that the transcription of *SERPING1* and *TRPV4* was significantly increased in the SIRT2 KD cells compared to the control cells (Fig. 1n).

SERPING1 and TRPV4 have been shown to induce cell proliferation, migration, and metastasis in several types of tumors. Analysis of the public data in the TCGA database showed that relatively high *SERPING1* expression is related to poor prognosis in renal and colorectal cancers (Supplementary Fig. S8a), and relatively high *TRPV4* expression is related to poor prognosis in ovarian cancer (Supplementary Fig. S8b). There were no reported impacts from the *SERPING1* or *TRPV4* genes in NB. We therefore tried to quantify the protein levels of SIRT2, SERPING1, and TRPV4 in resected tumors from 88 newly diagnosed NB patients by mass spectrometry. Kaplan–Meier analysis showed that patients with relatively low SIRT2 protein levels had significantly poorer overall survival (Fig. 1o). This is consistent with a previous study that SUMOlytion of SIRT2 can suppress NB cell proliferation and migration by inhibiting the P38-mTORC2-AKT signaling axis^9^. Conversely, patients with relatively high SERPING1 levels had poorer overall survival outcomes (Fig. 1p). Overall, our results provide strong evidence that increased histone lactylation can promote NB progression and support that the elevated H4K8la modification levels in SIRT2 KD cells contribute to the transcriptional activation of *SERPING1* and *TRPV4*. It would be of great interest to characterize whether levels of H4K8la, TRPV4 and SERPING1 can be used as biomarkers to predict clinical outcomes and therapeutic targets for precision management of NB.

Although Moreno-Yruela et al. recently showed that HDAC1-3 and SIRT1-3 catalyzed the delactylation of histone peptides and/or the purified histone proteins, only HDAC1-3 exhibits low activity on specific site like histone H4K5 in HeLa cells^10^. In this study, we identified SIRT2 as an efficient “eraser” for multiple histone lactylation sites of synthetic histone peptides, purified histones and nucleosomes, and histones in NB cells. Moreover, we unraveled a potential regulation mechanism of aberrant histone lactylation on NB progression. Thus, agonizing SIRT2’s delactylation function may provide a unique niche for innovative antitumor therapies against NB.

## Supplementary information

Supplementary Figures and Tables

Supplementary Table S2

Supplementary Table S3

Supplementary Table S4

Supplementary Table S5

Supplementary Table S6
