# Supplementary material for: SIRT2 functions as a histone delactylase and inhibits the proliferation and migration of neuroblastoma cells
Source: Cell Discov. 2022 Jun 7;8:54. doi: 10.1038/s41421-022-00398-y (PMC9174446; doi:10.1038/s41421-022-00398-y)
Supplement: Supplementary file 1 — Supplementary Figures and Tables [file 41421_2022_398_MOESM1_ESM.docx]

**Supplementary materials**

**Supplementary methods**

**Reagents and Cell culture**

Fluorescence probe was synthesized at WuXi AppTec. All the histone peptides used in this study were synthesized at > 95% purity by Beijing SciLight Biotechnology Ltd. Co. Pan anti-Lactyl lysine (PTM-1404), anti-Lactyl Histone H3K14 (PTM-1414), anti-Lactyl Histone H3K18 (PTM-1406), anti-Lactyl Histone H4K8 (PTM-1415), anti-Lactyl Histone H4K12 (PTM-1411), and anti-Acetyl Histone H3K18 (PTM-158) antibodies were purchased from PTM Bio Inc. Pan anti-acetylated lysine (9441), anti-SIRT2 (12650), and anti-FLAG (8146) antibodies were purchased from Cell Signaling Technology. Anti-acetyl Histone H3K14 (ab203952), anti-acetyl Histone H4K8 (ab45166), anti-acetyl Histone H4K12 (ab177793), and Alexa Fluor 647 conjugated anti-SIRT2 (ab205831) antibodies were purchased from Abcam. β-nicotinamide adenine dinucleotide hydrate (NAD^+^) (NAD100-RO), spermidine (85578), spermine (85605), and Sodium L-lactate (L7022) were purchased from Sigma Aldrich. The sirtuin inhibitor Tenovin-6 (S4900) was purchased from Selleck. LDHA inhibitor (R)-GNE-140 (HY-100742A), SIRT2 specific inhibitor AK-7 (HY-166691) and AK-1 (HY-101465) were purchased from MedChem Express. Micrococcal Nuclease (2910A) was purchased from TAKARA.

HeLa cell line, HEK 293T cell line, and neuroblastoma cell line SH-SY5Y were purchased from the Cell Bank of Chinese Academy of Sciences. The HeLa and HEK 293T cells were cultured in DMEM and RPMI-1640 medium, respectively, supplemented with 10% fetal bovine serum (Ausbian), 1% penicillin-streptomycin (Gibco), and 1% GlutaMAX (Gibco); while the SH-SY5Y cells were cultured in Eagle’s minimum essential medium/F12 medium (1:1) supplemented with 15% fetal bovine serum (Ausbian), 1% penicillin-streptomycin (Gibco), and 1% GlutaMAX (Gibco). The cells were incubated at 37 ℃ in a humidiﬁed incubator with 5% CO_2_, and mycoplasma contamination was routinely tested.

**Protein expression and purification**

# Human sirtuin genes (except *sirt4*) were cloned into pET vectors and expressed in *Escherichia coli* strains in LB medium (Supplementary Table S7). cellswere induced with 0.4 mM IPTG and grown at 16 ℃ for an additional 20 h for protein expression. The Cells were harvested by centrifugation and resuspended in lysis buffer (50 mM Tris-HCl, pH 7.5, 500 mM NaCl, 5% glycerol, 5 mM imidazole), and lysed with a high pressure French Press (UH-06, Union-Biotech). The cell debris in lysate was removed by centrifugation, and the supernatant was loaded onto Ni-NTA affinity resin (GE Healthcare) pre-equilibrated with lysis buffer. The target protein was eluted by lysis buffer supplemented with 250 mM imidazole. The eluted protein from Ni-NTA column was concentrated and loaded onto a Superdex 200 increase 10/300 GL column (GE Healthcare) pre-equilibrated with SEC buffer [20 mM Tris-HCl, pH 7.5, 200 mM NaCl, 2 mM dithiothreitol (DTT)]. The eluted fractions containing sirtuins were concentrated for further use.

**Screening delactylation activity using fluorescent probe substrate**

To screen the delactylation activity of sirtuins, 500 μM fluorescent probe was incubated with 5 μM sirtuins at 37 °C for 2 h in the presence of 2.5 mM NAD^+^ in 100 μl reaction buffer (20 mM HEPES, pH 8.0, 50 mM NaCl, 2.7 mM KCl, and 1 mM MgCl_2_). The fluorescent signal was then measured by a Microplate Reader (CLARIOstar, BMG LABTECH) with excitation at 480 nm and emission at 505 to 600 nm. For enzymatic inhibition assays, the sirtuin inhibitor Tenovin-6 was added into the reaction system mentioned above.

**Detecting delactylation activity using lactylated histone peptide substrates**

For delactylation activity profiling studies, 100 μM of each synthesized histone peptide bearing a lactylated lysine residue was incubated with 10 μM SIRT2 in 50 μl reaction buffer (20 mM Tris-HCl, pH 7.5, and 1 mM DTT) supplemented with 5 mM NAD^+^ at 37 °C for 5h. The reaction was stopped by addition of 0.1% TFA. The delactylation of histone peptide was detected by MALDI-TOF mass spectrometry (MS) assay as previously described ^1^. Briefly, samples were desalted by ZipTip with C18 resin (Merck) which was activated by solution A (70% acetonitrile, 0.1% TFA) and pre-equilibrated with solution B (0.1% TFA), respectively. Peptide on Tips was washed three times using solution B and finally eluted by 10 μl solution A. The elution fractions were then analyzed by MALDI-TOF MS (Atouflex Speed).

**Detecting delactylation activity using purified histone as substrates**

To detect the delactylation activity of SIRT2 to histones, histones were extracted according to the methods reported previously ^2^. Briefly, HeLa cells were treated with or without 20 mM sodium lactate for 24 h and harvested by centrifugation. The cells were resuspended in cold extraction buffer (PBS Buffer with 0.5% Triton X-100 and 2 mM PMSF) at 4 ℃ for 10 min. The lysate was centrifuged at 6500 *g* at 4 ℃ for 5 min to remove cytosolic components in the supernatant. The pellets were collected and resuspended in PBS buffer, and the soluble nuclear components in supernatant were removed by centrifugation at 6500 *g* at 4 ℃ for 5 min. The proteins in the pellets were extracted with 0.4 M HCl at 4 ℃ for 12 h. The supernatant was centrifuged at 16000 *g* at 4 ℃ for 10 min. The histones in solution were precipitated by addition of 20% (V/V) trichloroacetic acid, washed with acetone. 4~6 μg histone extract was incubated with 10 μM SIRT2 in 100 μl PBS supplemented by 5 mM NAD^+^ at 37 °C for 5h. The lactylation levels at various histone lysine sites were detected by immunoblotting.

**Detecting delactylation activity using purified nucleosome as substrates**

To detect the delactylation activity of SIRT2 to nucleosomes, nucleosomes were purified according to the reported methods^3,4^. Briefly, HeLa cells were treated with or without 20 mM sodium lactate for 24 h and harvested by centrifugation. The cells were resuspended in cold extraction buffer (15 mM Tris-HCl, 15 mM NaCl, 60 mM KCl, 2 mM EDTA, 0.5 mM EGTA, 0.5 mM spermine, 0.15 mM spermidine, 0.34 M sucrose, 15 mM β-mercatoethanol, 0.5% TritonX-100, 0.2 mM PMSF, pH 7.5) at 4 ℃ for 10 min. The lysate was centrifuged at 2500 *g* at 4 ℃ for 10 min to remove the cytosolic components in the supernatant. The chromatin pellets were washed three times by washing buffer (15 mM Tris-HCl, 15 mM NaCl, 60 mM KCl, 2 mM EDTA, 0.5 mM EGTA, 0.5 mM spermine, 0.15 mM spermidine, 0.34 M sucrose, 15 mM β-mercatoethanol, 0.2 mM PMSF, pH 7.5) and resuspended in chromatin digestion buffer (4 mM MgCl_2_, 25 mM KCl, 1 mM CaCl_2_, 50 mM Tris, pH 7.5). The chromatin was digested by addition of Micrococcal Nuclease to a final concentration of 0.2 units/μl at 37 ℃ for 30 min. The mixture was centrifuged at 4000 *g*at 4 ℃ for 10 min. The pellets were collected and resolved in a dissolving buffer (1 mM Tris-HCl, 0.2 mM EDTA, 0.1 mM PMSF, pH 7.8) on ice for 1 h. The solution was centrifuged at 4000 *g* at 4 ℃ for 10 min and the supernatant containing nucleosomes was collected for delactylation reaction. ~ 7 μM nucleosomes were incubated with 10 μM SIRT2 in 20 μl reaction buffer (20 mM Tris-HCl, pH 7.8) supplemented by 5 mM NAD^+^ at 37 °C for 5h. The lactylation levels at various histone lysine sites in nucleosome were detected by immunoblotting.

**Extraction of DNA from nucleosome**

10 μl extraction buffer (phenol : chloroform : isoamyl alcohol = 25 : 24 : 1, pH 7.8) was added to 50 μl nucleosome solution. The upper layer solution was collected. The extraction process was repeated once. Precipitation buffer (0.1 volume of 3 M NaAc, pH 5.2 and 2.5 volume cold alcohol) was added to the collected buffer to precipitate DNA at -20 ℃ for 20 h. The mixture was centrifuged at 12, 000 *g* at 4 ℃ for 30 min. The pellet was collected and air dried for 5 min. The DNA pellet was solved in ddH_2_O and centrifuged at 12, 000 *g* at 4 ℃ for 30 min. The DNA solution was analyzed by 2% agarose gel.

**Enzyme-coupled assay**

The kinetic parameters were measured by enzyme-coupled assay as previously reported with slight modification ^5^. 20 to 640 μM lactylated histone peptide, 2.56 mM NAD^+^, 0.4 mM NADH, 1 mM DTT, 6.6 mM α-ketoglutarate, 10 μM nicotinamidase, 4 units of glutamate dehydrogenase from bovine liver, and 10 μM SIRT2 were added into PBS buffer (20 mM, pH 7.5) to a final volume of 100 μl. The reaction was carried out in a silica plate at 25 ℃ which was initiated by addition of SIRT2. The consumption of NADH reflecting the generation of nicotinamide was monitored at 304 nm using UV-Vis spectrophotometer (UVmini-1240, SHIMADZU) for 5 min with interval of 20 s. The kinetic parameters were calculated by Michaelies-Menten equation via plotting of steady-state nicotinamide generation rates versus substrate concentrations using non-linear regression fitting by Origin 2000. The experiments were repeated three times.

**Lentivirus-mediated SIRT2 knockdown and overexpression**

To generate SIRT2 knockdown (KD) SH-SY5Y cell line, shRNA targeting SIRT2 was synthesized by Cyagen (China), with the sequences of shRNA: TGCCCAAGATCG AGGTTATATCTCGAGATATAACCTCGATCTTGGGCA and GCCTGATCTATCTGGTTCAATCTCGAGATTGAACCAGATAGATCAGGC. shRNA was cloned into the pLVX-IRES-Puro vector (Cyagen, China), and co-transfected with two packing plasmids psPAX2 and pMD2.G into HEK 293T cells to generate lentivirus. SH-SY5Y cells were then transduced with the lentivirus for 48 h, and subjected to puromycin selection at 6 μg/ml for 7 days to obtain stable SIRT2 KD cell line.

To obtain SIRT2 complementation (SIRT2 KD + SIRT2) and SIRT2 overexpression (OE) SH-SY5Y cell lines, SIRT2 gene were amplified by PCR and cloned into the modified pLentiLox3.7 expression vector (kindly provided by Dr. Fan Yang, Shanghai Children’s Medical Center) with a C-terminal 3 × FLAG-tag. Lentivirus package and transduction were carried out as described above into SIRT2 KD and control SH-SY5Y cells. The cells were selected by blastcidin at 6 μg/ml for 14 days to obtain stable cell line. All cell lines were verified by immunoblotting and qPCR.

**Transient transfection of SIRT2**

Plasmids containing wild type or 3 × FLAG-tagged SIRT2 H187Y mutant were transiently transfected into SIRT2 KD SH-SY5Y cells using Lipofectamine 3000 transfection reagent (Invitrogen) according to the manufacturer’s instructions. Briefly, 7.5 μl Lipo3000 was incubated with 2500 ng plasmids and 5 μl p3000 in a total of 250 μl OPTI-MEM medium at room temperature for 15 min, and was then added to 5 × 10^5^ cells. The cells were collected 72 h after transfection and processed for immunoblotting analysis.

**Immunoblotting analysis**

Cells were lysed with lysis buffer (500 mM Tris-HCl, pH 6.8, 2% SDS, 10% Glycerol, 10% β-mercaptoethanol, and 0.02% bromophenol blue) supplemented with 1 × protease and phosphatase inhibitor cocktail (Thermo) on ice for 5 min. Total protein concentration was determined by NanoDrop 2000 (Thermo Fisher Scientific). 5 μg total proteins were separated on 15% SDS-polyacrylamide gel electrophoresis, transferred to a PVDF membrane, and immunoblotted with various primary antibodies as indicated at 4 ℃ overnight. The membrane was incubated with HRP-conjugated secondary antibody at room temperature for 1 h, and finally visualized using an enhanced chemiluminescence (ECL) substrate kit (Millipore) with ImageQuant LAS 4000 mini densitometer (GE Healthcare Life Science). The relative intensity of each band is quantitated by densitometry using ImageJ (1.53c version) after normalization to β-actin, and then expressed as the fold of that in the control cells.

**Immunofluorescence staining and confocal microscopy imaging**

Immunofluorescence staining and confocal microscopy imaging was performed. Briefly, cells were fixed with 4% paraformaldehyde, permeabilized with 0.5% Triton X-100, and blocked with 5% BSA and 5% goat serum. After staining with primary, secondary antibodies and DAPI, cells were dehydrated with ethanol and mounted with ProLong Diamond Antifade Mountant (Invitrogen). Images were acquired using a Leica TCS SP8 confocal microscope (Leica Microsystems) with a 63 × oil-immersed objective, and were analyzed with ImageJ.

**BrdU assay**

Cell proliferation was measured using a BrdU (chemiluminescent) kit (Roche Diagnostics) following the manufacturer's instructions. Briefly, cells were plated into an opaque 96-well microplate at the density of 5 × 10^3^ cells/well and cultured for 48 h. BrdU was added to each well at a final concentration of 10 μM at 37 ℃ for another 2 h-incubation. The cells were then fixed for 60 min and incubated with anti-BrdU-POD for 90 min at room temperature. After washing, the substrate was added, and light emission was recorded using a BioTek Synergy/2 microplate reader (BioTek).

**Cell migration**

Cell migration was detected using Transwell cell culture chambers (pore size, 8 μm, Corning). Briefly, 1 × 10^5^ cells were seeded into the upper chamber in serum free medium, while the lower chamber was filled with EMEM/F12 medium containing 15% FBS. After 48 h, migrated cells were fixed in 4% paraformaldehyde and stained with 0.1% crystal violet at room temperature. Images were captured using an inverted microscope (Olympus) and the number of migrated cells was counted in five randomly selected images using ImageJ.

**Lactate colorimetric assay**

Intracellular lactate level was determined by an L-lactate Colorimetric Assay kit (E-BC-K044-M, Elabscience, China) following the manufacturer’s manual. Briefly, the cells were sonicated in PBS, and enzyme solution (LDH, NAD^+^, PMS) and chromogenic agent (NBT) were then added to the supernatant. After incubation at 37 ℃ for 10 min, the stop buffer was added to stop the reaction. Lactate level was measured by a microplate reader with absorbance at 530 nm.

**RNA-seq and Quantitative real-time PCR (qPCR)**

Total RNA from various cell samples were extracted with TRIzol Reagent (Invitrogen) according to the manufacturer’s instructions. RNA purification, reverse transcription, library construction and sequencing were performed at Mingma Technologies Co., Ltd. according to the manufacturer’s instructions (Illumina). The rRNA-depleted sequencing libraries from total RNA were prepared using Illumina TruSeq^®^ Stranded Total RNA Gold preparation Kit. About 1 μg total RNA was used as input material, and Ribo-Zero Gold kit was used to remove both cytoplasmic and mitochondrial rRNA. Purified RNA was then fragmented into small pieces using divalent cations under elevated temperature. The cleaved RNA fragments were copied into first strand cDNA using reverse transcriptase and random primers, followed by second strand cDNA synthesis. These cDNA fragments were then subjected to end-repair, phosphorylation and 'A' base addition according to Illumina's library construction protocol. The products were purified and enriched with PCR, and the AMPure XP Beads (Beckmen) were used to clean up the amplified target fragments to create the final cDNA library. After library construction, Qubit 2.0 fluorimeter dsDNA HS Assay (Thermo Fisher Scientific) was used to quantify concentration of the resulting sequencing libraries, while the size distribution was analyzed using Agilent BioAnalyzer 2100 (Agilent). Sequencing was performed using an Illumina systems following Illumina-provided protocols for 2 × 150 paired-end sequencing in Mingma Technologies Co., Ltd.

For RNA-seq data processing, RNA-seq data were quantified using salmon, an alignment-free tool ^6^. DESeq2 package was used to test the differentially expressed genes ^7^. A fold change > 2 and adjusted *p* value < 0.05 was defined as the significantly differentially expressed genes between groups. The gene set enrichment analysis (GSEA) was performed using gene sets specific to the cell proliferation and cell migration with GSEA software ^8,9^.

To determine the transcription level of target genes, cDNA was synthesized using PrimeScript RT reagent kit (TAKARA) from total RNA extracted. The mRNA levels of target genes were quantified with Hieff SYBR Green Master Mix (Yeasen) using CFX96 Touch Real-Time PCR (Bio-Rad), under the following cycling conditions: 95 °C for 2 min, 40 amplification cycles of 95 °C for 10 s and 60 °C for 30 s, followed by a final cycle of 95 °C for 5 s and 65 °C for 5 s. The relative mRNA level of each target gene was analyzed as 2^-ΔΔCt^ with β-actin as an internal control, and the levels of these genes in control SH-SY5Y cells were set at 1 for normalization. qPCR for each gene was performed in technical triplicates in three independent experiments. Primer sequences for each target gene are listed in Supplementary Table S8.

**Chromatin immunoprecipitation sequencing (ChIP-seq) and quantitative PCR (ChIP-qPCR)**

ChIP assays were performed as previously described. Briefly ^10^, 2 × 10^7^ cells were fixed with 1% formaldehyde at room temperature for 10 min, and the fixation was stopped by 0.125 M glycine at room temperature for 5 min. The chromatin pellets were extracted from cells by lysis buffer (10 mM Tris-HCl, pH 7.4, 10 mM NaCl, 3 mM MgCl_2_, 0.5% NP-40), and re-suspended with 0.8 ml of RIPA buffer. The genomic DNA was fragmented by sonication at 25% amplitude for a total of 8 min (with intervals). Sonicated chromatin was incubated with 2 μg of anti-Histone H3K18la/ac and H4K8la/ac antibodies, or control IgG pre-coated on Dynabeads protein G (Life Technologies) at 4 ℃ overnight.

After washing the immunoprecipitants with RIPA buffer and LiCl washing buffer, the chromatin was crosslink reversed by protease K digestion at 65℃ overnight and purified using a QIAquick PCR purification kit (Qiagen). The immunoprecipitated DNA fragments were then used for library preparation of high-throughput sequencing (Novogene) or assessed by qPCR as described above with the primer information being listed in Supplementary Table S9.

For ChIP-seq data processing, the ChIP-seq reads were aligned to the hg19 reference genome using Burrows-Wheeler Alignment tool v0.7.12 ^11^. After filtering duplicates, multi-mappers, ENCODE blacklist regions, MACS2 2.7.1 ^12^ was used to call the peaks with the following parameters, -f BAMPE -g hs -B. Results from the ChIP-seq data were visualized in Integrative Genomics Viewer v2.8.1 ^13^, and the peaks were annotated by PAVIS ^14^. The promoter region was defined as ± 2kb around the known transcriptional start site..

**Patients and clinical samples**

A total of 88 neuroblastoma patients were recruited in Shanghai Children’s Medical Center (SCMC) from January 2001 to July 2016. According to the International Neuroblastoma Pathology Classification, all enrolled patients were newly diagnosed as neuroblastoma with histological provement, and had not received chemotherapy or radiotherapy.

Tumor tissues from surgical resection or biopsy were fixed with 10% neutral formalin, paraffin embedded, and then cut into 4-μm slices. All samples were histopathologically verified after hematoxylin-eosin (HE) and immunohistochemical staining under light microscope. Formalin-fixed paraffin-embedded (FFPE) samples were enrolled in the present study if: (1) samples are sufficient for further protein extraction; (2) with complete clinic information and ≥ 36 months follow-up.

The study was approved by the Institutional Review Board and the Ethics Committee of Shanghai Children’s Medical Center (SCMCIRB-K2017042), and formal written informed consents were obtained by the patients and/or their patients.

**Protein quantification via label-free-based mass spectrometry**

Proteins extracted from FFPE samples were submitted for MS detection using Q Exactive HF-X Hybrid Quadrupole-Orbitrap Mass Spectrometer (Thermo Fisher Scientific, Rockford, IL, USA) as previously described ^15^. MS raw files were searched against the human National Center for Biotechnology Information (NCBI) Refseq protein database (updated on 04-07-2013, 32,015 entries) by Mascot 2.3 (Matrix Science Inc) implemented on Proteome Discoverer 1.4 (Thermo Scientific). A mass tolerance of 20 ppm for precursor and 0.5 Da for production was allowed, and up to two missed cleavages were allowed. The search engine set cysteine carbamidomethylation as a fixed modification and N-acetylation, oxidation of methionine as variable modifications. For the quality control of proteins identification, the target-decoy based strategy was applied to confirm the FDR (False Discovery Rate) of both peptide and protein was lower than 1%. Precursor ion score charges were limited to +2, +3, and +4. Label-free protein quantifications were calculated using a label-free, intensity-based absolute quantification (iBAQ) approach. Identification results and the raw data from mzXML file was loaded. Then for each identified peptide of SIRT2, SERPING1, and LDHA proteins, the extracted-ion chromatogram (XIC) was extracted by searching against the MS1 based on its identification information, and the abundance was estimated by calculating the area under the extracted XIC curve (AUC). For protein abundance calculation of SIRT2, SERPING1, and LDHA, the nonredundant peptide list was used to assemble proteins following the parsimony principle. Then, the protein abundance was estimated with a traditional label-free, intensity-based absolute quantification (iBAQ) algorithm ^16^, which divided the protein abundance (derived from identified peptides’ intensities) by the number of theoretically observable peptides.

**Statistical analysis**

All data represented are calculated from at least three independent experiments and expressed as mean ± SD (stand deviation) or SEM (standard error of mean). All data were analyzed with the Shapiro–Wilk test for normal distribution prior to testing for differences between groups. Paired *t*-tests were used for normally distributed data, whereas Wilcoxon rank-sum tests were used for non-normally distributed data. All graphs were generated using GraphPad PRISM 6.0 (GraphPad Software, Inc., USA). The gene expression matrix and relevant clinic data were obtained from the Cancer Genome Atlas (TCGA, https://portal.godchild.cancer.gov). The Kaplan-Meier survival plots were generated in R. The surv_cutpoint function of the “survminer" R package was used to determin the optimal cut-off feature, and separation of the high and low expression group is based on maximally selected rank statistics (https://cran.r-project.org/web/packages/survminer/index.html). A *p* value < 0.05 is considered to be statistically significant.

**Reference**

1 Hang, T. *et al.* Structural insights into the molecular mechanism underlying Sirt5-catalyzed desuccinylation of histone peptides. *Biochem J* **476**, 211-223, doi:10.1042/BCJ20180745 (2019).

2 Shechter, D., Dormann, H. L., Allis, C. D. & Hake, S. B. Extraction, purification and analysis of histones. *Nat Protoc* **2**, 1445-1457, doi:10.1038/nprot.2007.202 (2007).

3 Arimura, Y., Shih, R. M., Froom, R. & Funabiki, H. Structural features of nucleosomes in interphase and metaphase chromosomes. *Mol Cell* **81**, 4377-4397 e4312, doi:10.1016/j.molcel.2021.08.010 (2021).

4 Whitlock, J. P., Jr. & Simpson, R. T. Removal of histone H1 exposes a fifty base pair DNA segment between nucleosomes. *Biochemistry* **15**, 3307-3314, doi:10.1021/bi00660a022 (1976).

5 Smith, B. C., Hallows, W. C. & Denu, J. M. A continuous microplate assay for sirtuins and nicotinamide-producing enzymes. *Anal Biochem* **394**, 101-109, doi:10.1016/j.ab.2009.07.019 (2009).

6 Patro, R., Duggal, G., Love, M. I., Irizarry, R. A. & Kingsford, C. Salmon provides fast and bias-aware quantification of transcript expression. *Nat Methods* **14**, 417-419, doi:10.1038/nmeth.4197 (2017).

7 Love, M. I., Huber, W. & Anders, S. Moderated estimation of fold change and dispersion for RNA-seq data with DESeq2. *Genome Biol* **15**, 550, doi:10.1186/s13059-014-0550-8 (2014).

8 Wu, Y., Siadaty, M. S., Berens, M. E., Hampton, G. M. & Theodorescu, D. Overlapping gene expression profiles of cell migration and tumor invasion in human bladder cancer identify metallothionein 1E and nicotinamide N-methyltransferase as novel regulators of cell migration. *Oncogene* **27**, 6679-6689, doi:10.1038/onc.2008.264 (2008).

9 Subramanian, A. *et al.* Gene set enrichment analysis: a knowledge-based approach for interpreting genome-wide expression profiles. *Proc Natl Acad Sci USA* **102**, 15545-15550, doi:10.1073/pnas.0506580102 (2005).

10 Zhao, J. *et al.* Structural insights into the recognition of histone H3Q5 serotonylation by WDR5. *Sci Adv* **7**, doi:10.1126/sciadv.abf4291 (2021).

11 Li, H. & Durbin, R. Fast and accurate short read alignment with Burrows-Wheeler transform. *Bioinformatics* **25**, 1754-1760, doi:10.1093/bioinformatics/btp324 (2009).

12 Zhang, Y. *et al.* Model-based analysis of ChIP-Seq (MACS). *Genome Biol* **9**, R137, doi:10.1186/gb-2008-9-9-r137 (2008).

13 Robinson, J. T. *et al.* Integrative genomics viewer. *Nat Biotechnol* **29**, 24-26, doi:10.1038/nbt.1754 (2011).

14 Huang, W., Loganantharaj, R., Schroeder, B., Fargo, D. & Li, L. PAVIS: a tool for Peak Annotation and Visualization. *Bioinformatics* **29**, 3097-3099, doi:10.1093/bioinformatics/btt520 (2013).

15 Wisniewski, J. R., Ostasiewicz, P. & Mann, M. High recovery FASP applied to the proteomic analysis of microdissected formalin fixed paraffin embedded cancer tissues retrieves known colon cancer markers. *J Proteome Res* **10**, 3040-3049, doi:10.1021/pr200019m (2011).

16 Schwanhausser, B. *et al.* Global quantification of mammalian gene expression control. *Nature* **473**, 337-342, doi:10.1038/nature10098 (2011).

**Supplementary Figures and Tables**

**
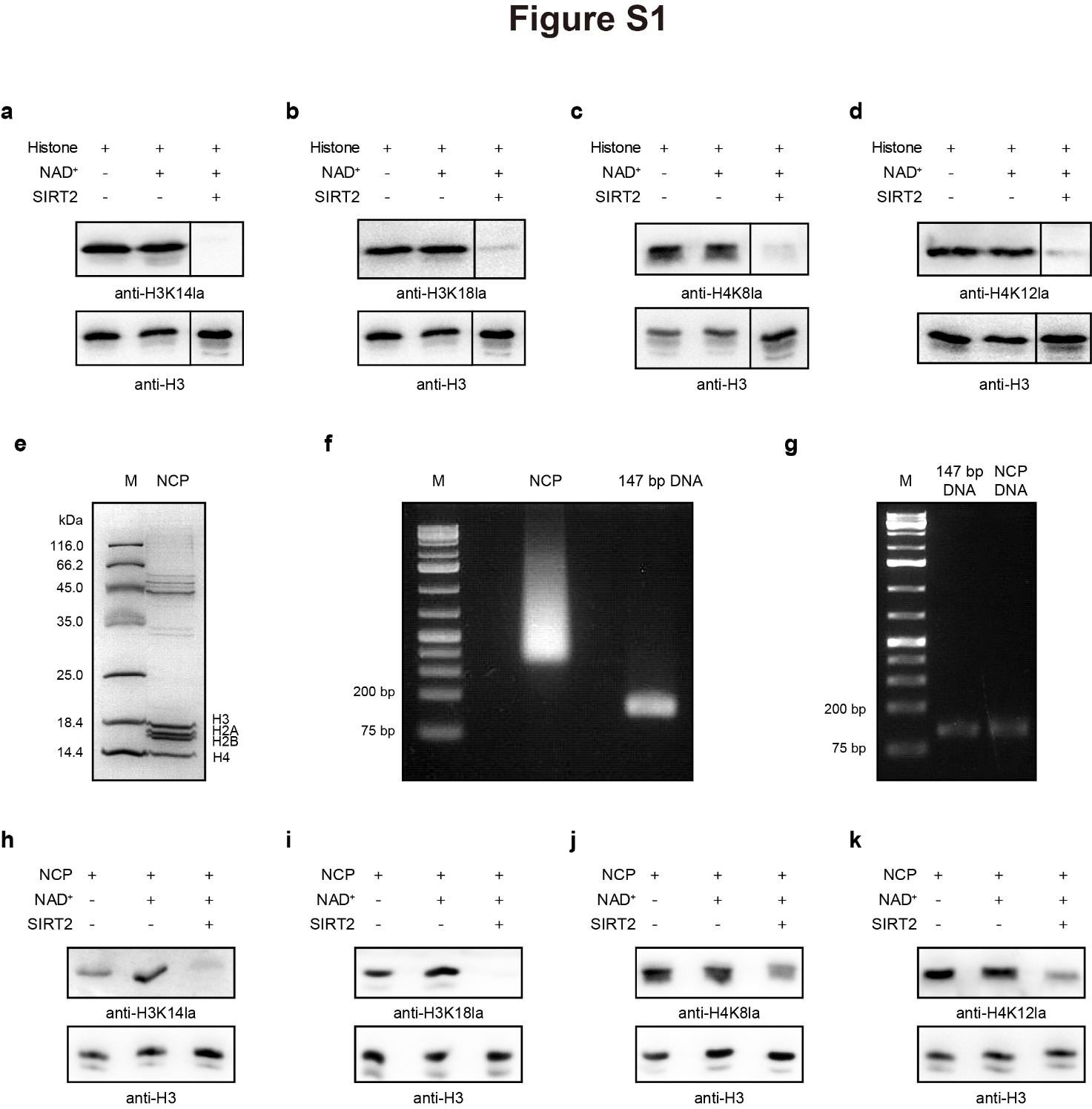
**

**Figure S1. Delactylation assays for SIRT2 using histone proteins and nucleosomes purified from HeLa cells as substrates.**

(**a-d**) Purified histone proteins (4~6 μg) were incubated with SIRT2 (10 μM) at 37 ℃ for 5 h in PBS buffer supplemented by 5 mM NAD^+^. The histone lactylation marks in the purified histone proteins were detected by immunoblotting using antibodies as follows: (**a**) anti-H3K14la, (**b**) anti-H3K18la, (**c)** anti-H4K8la, and (**d**) anti-H4K12la. The lanes for SIRT2 treatment were cut and spliced from the same immunoblotting PVDF membrane with the control lanes.

(**e-g**) Verification of purified nucleosomes. Purified nucleosomes were analyzed by SDS-PAGE (**e**) and 2% agarose gel (**f**) to detect histones and DNA, respectively. (**g**) DNA was extracted from the purified nucleosomes and analyzed by 2% agarose gel. NCP, nucleosome core particle.

(**h-k**) Purified nucleosomes (~ 7 μM) were incubated with SIRT2 (10 μM) at 37 ℃ for 5 h in 20 mM Tris-HCl buffer, pH 7.8, supplemented by 5 mM NAD^+^. The histone lactylation marks in the purified nucleosomes were detected by immunoblotting using antibodies as follows: (**h**) anti-H3K14la, (**i**) anti-H3K18la, (**j)** anti-H4K8la, and (**k**) anti-H4K12la.

**
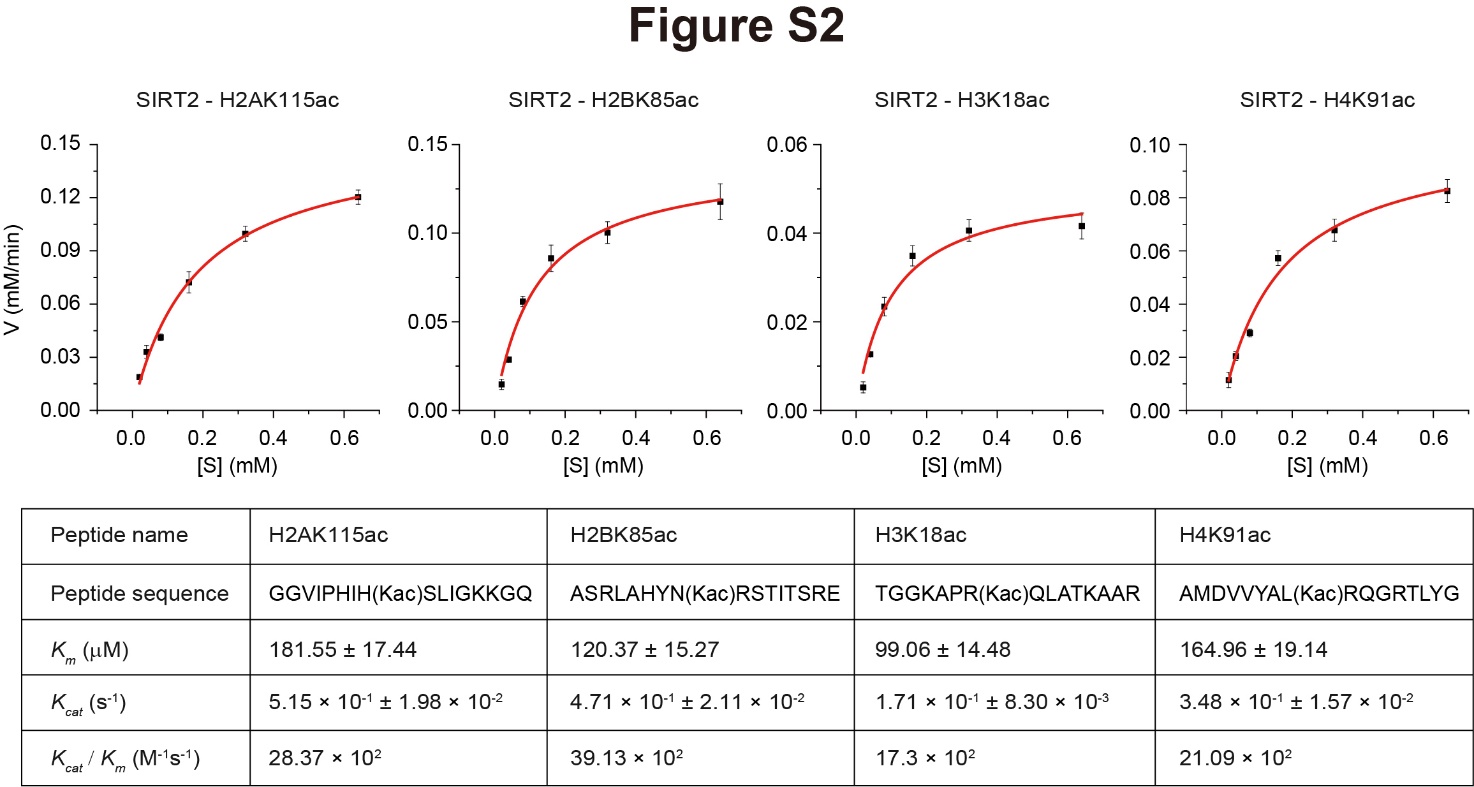
**

**Figure S2. Michaelis-Menten plots for SIRT2 against the four peptides containing acetylated lysine residues.** The sequence of the peptides and kinetic parameters *K_m_*, *K_cat_*, and *K_cat_/K_m_* are shown in the table. All data are presented as the mean ± SD, calculated from three independent experiments.


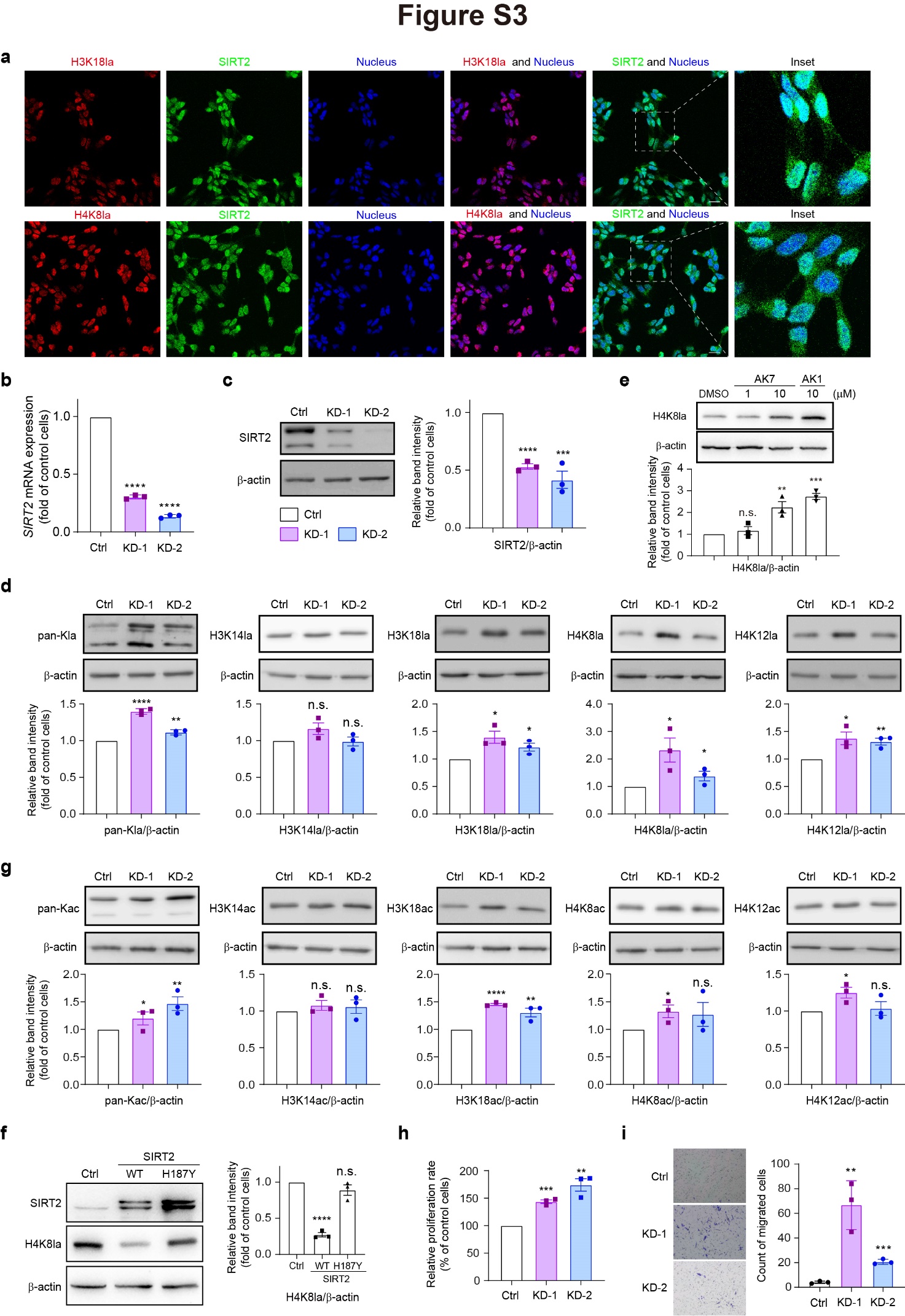


**Figure S3. SIRT2 functions as an eraser of histone lactylation in neuroblastoma cells as revealed by SIRT2 knockdown.**

**(a)** Confocal immunofluorescence analysis of endogenous SIRT2 (anti-SIRT2 antibody, green) with H3K18la or H4K8la modification (using anti-H3K18la or H4K8la antibody, red) in SH-SY5Y neuroblastoma cells. Scale bar, 5 μm.

**(b, c)** Establishment of SIRT2 knockdown SH-SY5Y cell lines. Two different shRNAs were synthesized, and the knockdown cell lines (SIRT2 KD-1 and KD-2) were established by lentivirus infection. *SIRT2* mRNA expression **(b)** and the SIRT2 protein level **(c)** were assessed by qPCR and immunoblotting analysis, respectively.

**(d)** Pan and histone lactylation levels at the H3K14, H3K18, H4K8, and H4K12 sites in SIRT2 KD cells, as detected by immunoblotting.

**(e)** Histone lactylation level at the H4K8 site in SH-SY5Y cells upon SIRT2 inhibitor AK1 or AK7 treatment for 24 h, as detected by immunoblotting.

**(f)** SIRT2 KD-1 cells were transiently transfected with wild type or SIRT2 H187Y mutant, and 72 h after transfection, the histone lactylation level at the H4K8 site was detected by immunoblotting.

**(g)** Pan and histone acetylation levels at the H3K14, H3K18, H4K8, and H4K12 sites in SIRT2 KD cells, as detected by immunoblotting.

**(h)** Cell proliferation rate of SIRT2 KD cells was significantly increased, as measured using a BrdU assay (chemiluminescent).

**(i)** Cell migration capacity of SIRT2 KD cells was significantly increased, as evaluated using Transwell assays. The number of stained cells which migrated to the lower chamber was counted using ImageJ.

The relative intensity of each band was quantified via densitometry, using ImageJ after normalization to β-actin, with the values expressed as the fold change versus the value detected for control SH-SY5Y cells. All data are presented as the mean ± SEM, calculated from three independent experiments. *, *p* < 0.05; **, *p* < 0.01; ***, *p* < 0.001; ****, *p* < 0.0001; n.s., not significant as calculated by two tailed student's *t* test. ctrl, control.

**
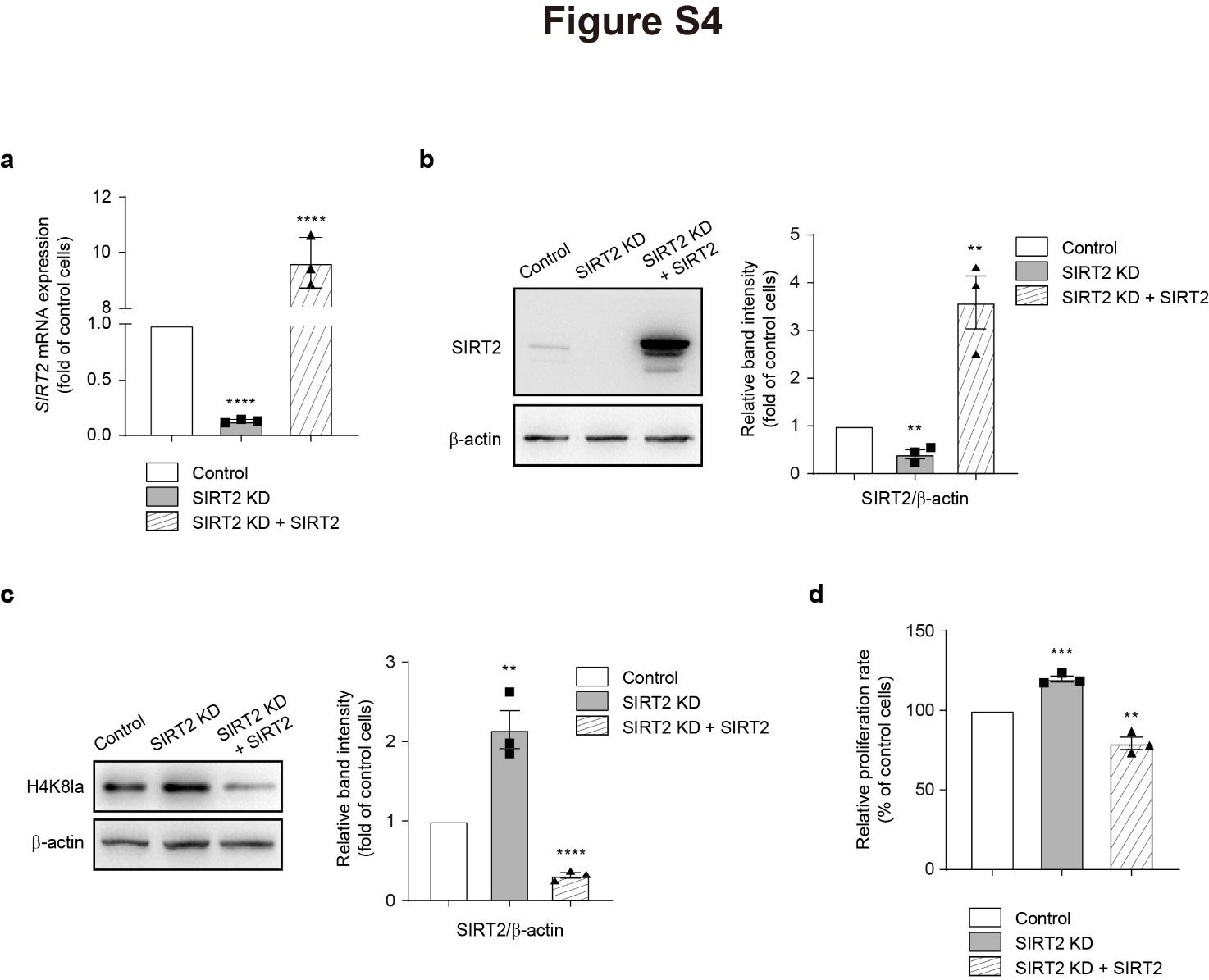
 Fig. S4 SIRT2 functions as an eraser of histone lactylation in neuroblastoma cells as revealed by SIRT2 complementation in SIRT2 knockdown cells.**

**(a, b)** Establishment of SIRT2 complementation cell line in SIRT2 knockdown (KD) SH-SY5Y cell lines (SIRT2 KD + SIRT2). A 3 × FLAG-tagged SIRT2 expressing plasmid was constructed, and the SIRT2 complementation cell line was established by lentivirus infection of SIRT2 KD cells. *SIRT2* mRNA expression **(a)** and the SIRT2 protein level **(b)** were assessed by qPCR and immunoblotting analysis, respectively.

**(c)** Histone lactylation levels at H4K8 site in control, SIRT2 KD, and SIRT2 KD + SIRT2 cells, as detected by immunoblotting.

**(d)** Cell proliferation rate of control, SIRT2 KD, and SIRT2 KD + SIRT2 cells was measured using a BrdU assay (chemiluminescent).

The relative intensity of each band was quantified via densitometry, using ImageJ after normalization to β-actin, with the values expressed as the fold change versus the value detected for control SH-SY5Y cells. All data are presented as the mean ± SEM, calculated from three independent experiments. **, *p* < 0.01; ***, *p* < 0.001; ****, *p* < 0.0001 as calculated by two tailed student's *t* test.

**
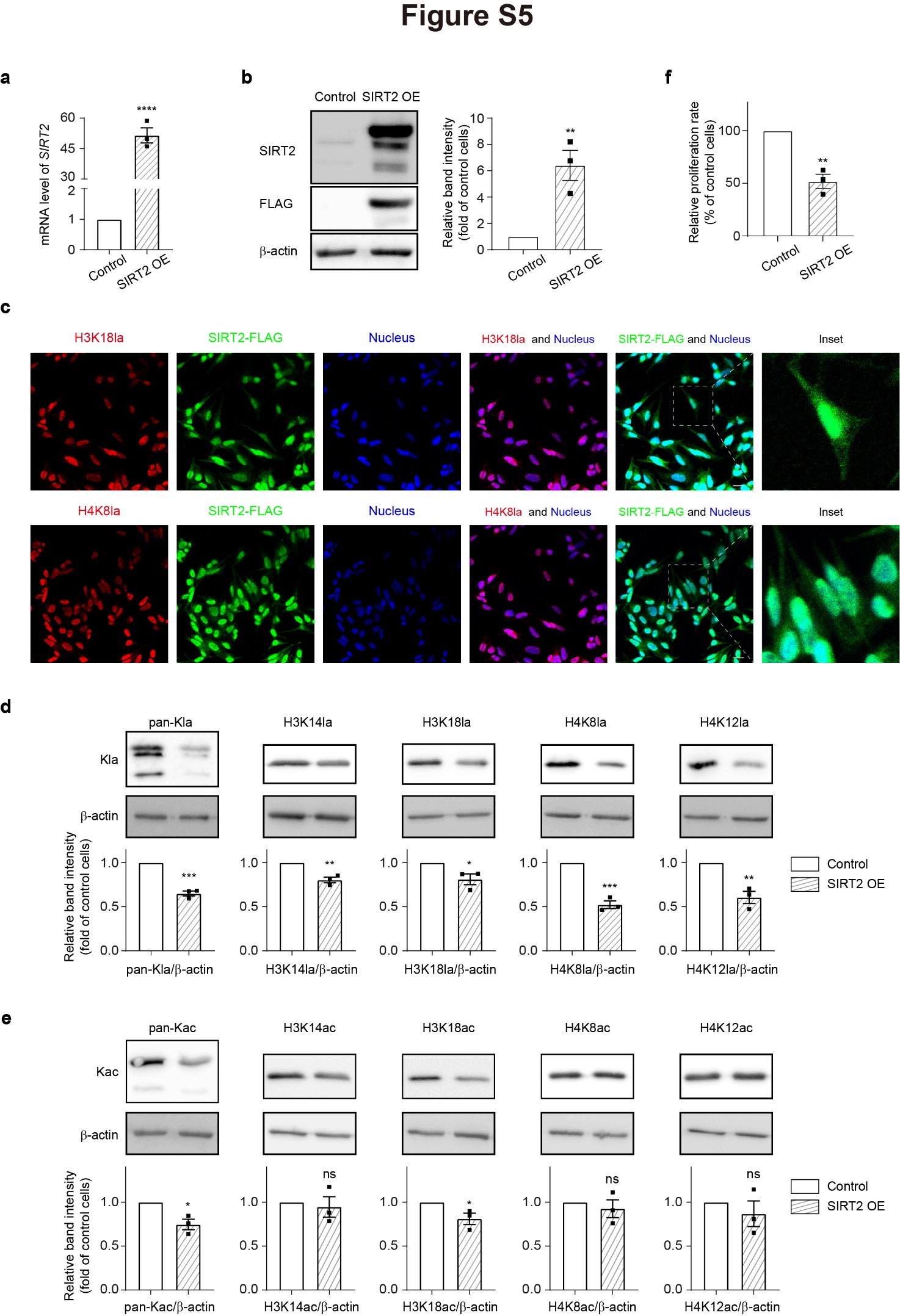
Figure S5. SIRT2 functions as an eraser of histone lactylation in neuroblastoma cells as revealed by SIRT2 overexpression.**

**(a, b)** Establishment of a SIRT2 overexpression SH-SY5Y cell line (SIRT2 OE). A 3 × FLAG-tagged SIRT2 expressing plasmid was constructed, and the overexpression cell line was established by lentivirus infection. *SIRT2* mRNA expression **(a)** and the SIRT2 protein level **(b)** were assessed by qPCR and immunoblotting analysis, respectively.

**(c)** Confocal immunofluorescence analysis of overexpressed FLAG-tagged SIRT2 (anti-FLAG tag antibody, green) with H3K18la or H4K8la modifications (using anti-H3K18la or H4K8la antibody, red) in SIRT2 OE cells. Scale bar, 5 μm.

**(d, e)** Pan and histone lactylation **(d)** or acetylation **(e)** levels at the H3K14, H3K18, H4K8, and H4K12 sites in SIRT2 OE cells, as detected by immunoblotting.

**(f)** Cell proliferation rate of SIRT2 OE cells was significantly decreased, as measured using a BrdU assay (chemiluminescent).

The relative intensity of each band was quantified via densitometry, using ImageJ after normalization to β-actin, with the values expressed as the fold change versus the value detected for control SH-SY5Y cells. All data are presented as the mean ± SEM, calculated from three independent experiments. *, *p* < 0.05; **, *p* < 0.01; ***, *p* < 0.001; ****, *p* < 0.0001; n.s., not significant as calculated by two tailed student's *t* test.

**
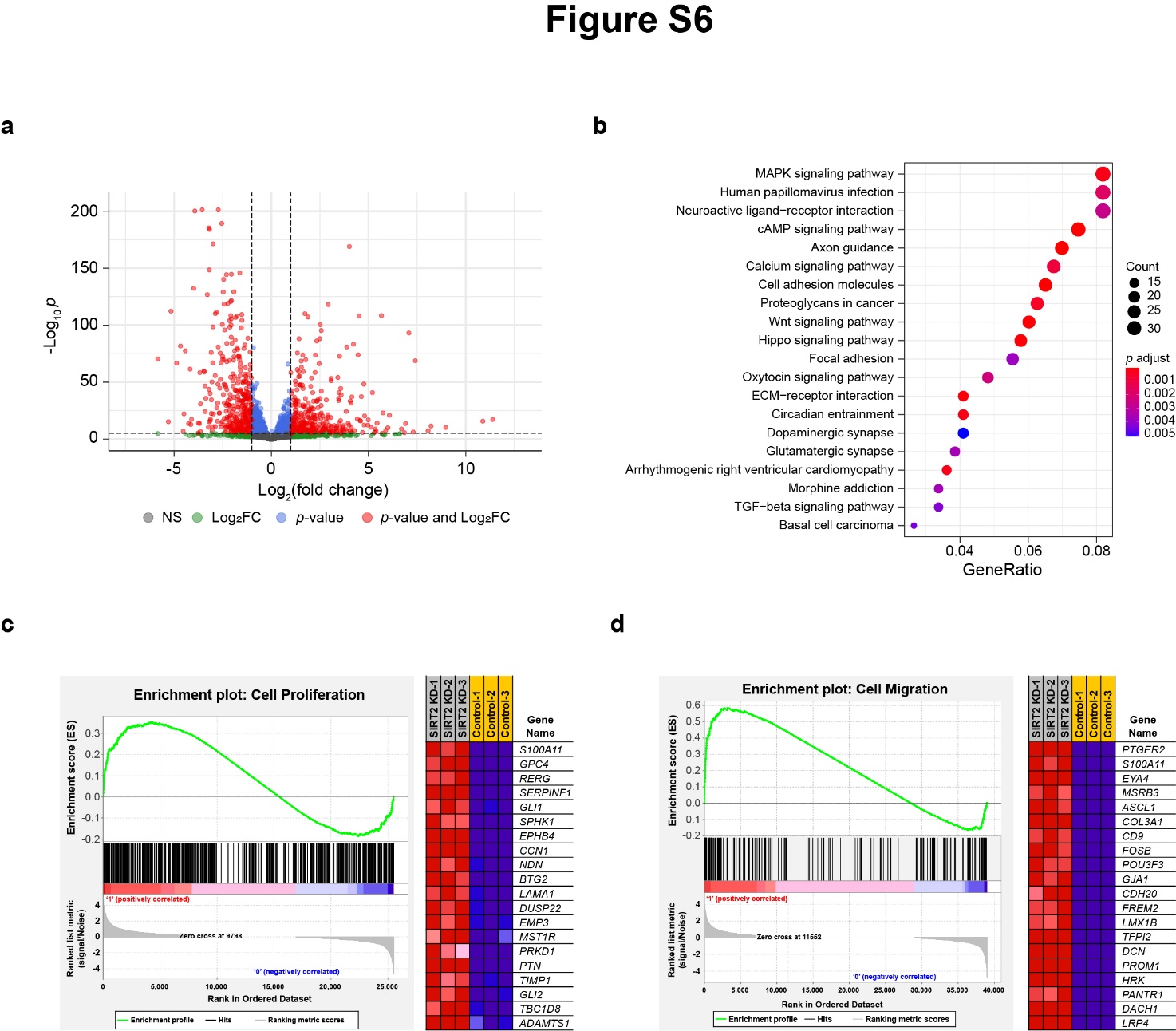
**

**Figure S6. RNA-seq analysis of control and SIRT2 knockdown SH-SY5Y cells.**

**(a)** Volcano plot comparing the gene expression between control and SIRT2 KD cells. Threshold of Log_10_ *p* value has been set as 10^-6^ and the log_2_FC is > |2|. FC, fold change.

**(b)** Functional enrichment analysis comparing the gene expression between control and SIRT2 KD cells. The differentially expressed genes were enriched in KEGG pathway database and visualized through dot plot using the R package clusterProfiler (https://doi.org/10.1016/j.xinn.2021.100141).

**(c, d)** Gene Set Enrichment Analysis (GSEA) for cell proliferation related-genes **(c)** and for cell migration related-genes **(d)**, analyzed in three-independent RNA-seq datasets from control *vs.* SIRT2 KD cells. Heatmaps for each analysis are presented in the right-hand panel.

**
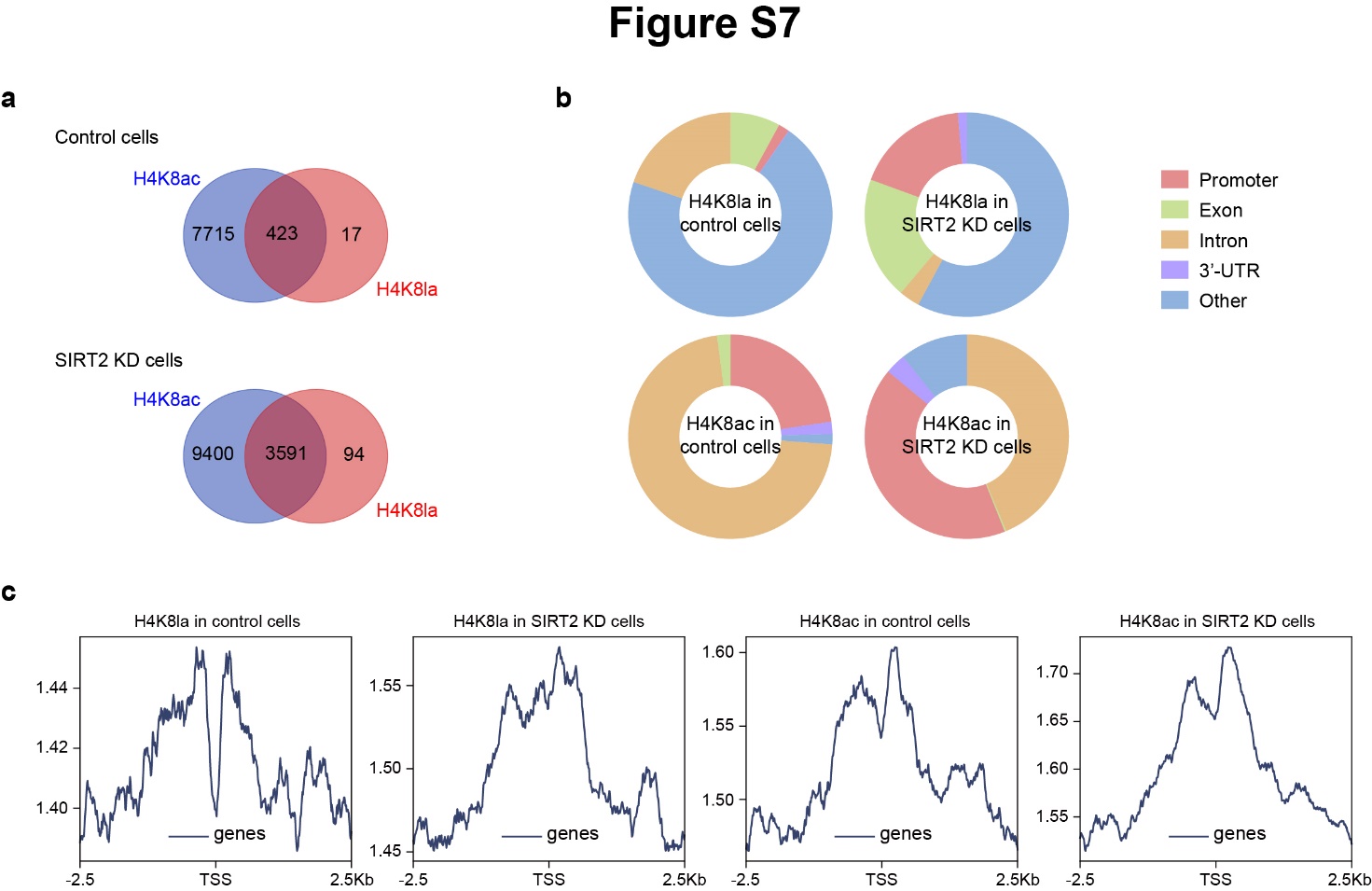
**

**
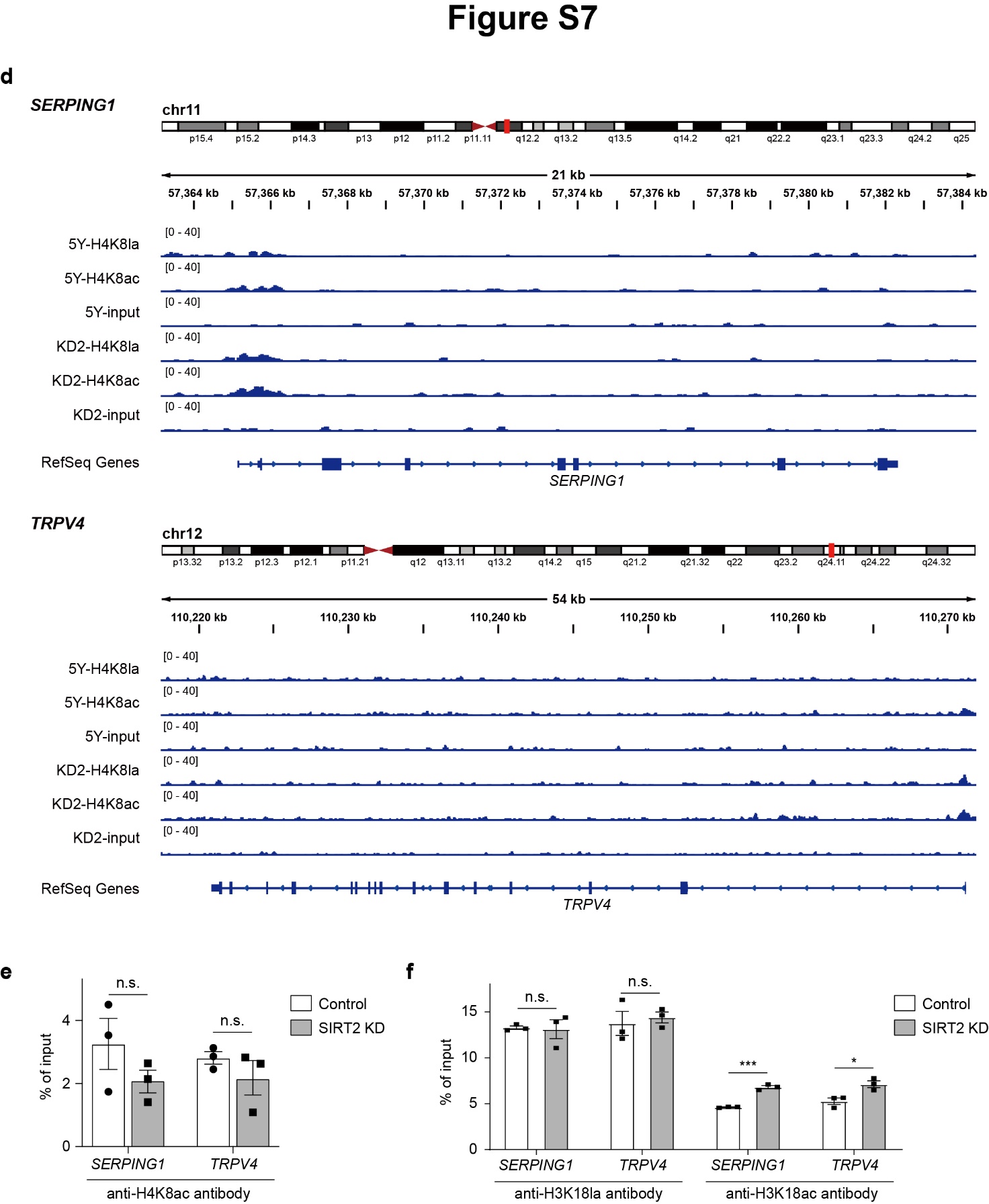
Figure S7. ChIP-seq analysis of H4K8la and H4K8ac in control and SIRT2 knockdown SH-SY5Y cells.**

**(a)** Venn diagram showing the number of nearest genes whose peaks were called by anti-H4K8la and anti-H4K8ac antibodies by ChIP-seq in control (upper) and SIRT2 KD (lower) cells.

**(b)** Donut chart showing the genomic distribution of all peaks called by anti-H4K8la or H4K8ac antibodies with annotated genomic regions in control and SIRT2 KD SH-SY5Y cells. A promoter is here defined as a region ± 2 kb of a known transcription start site (in the RefSeq database).

**(c)** Anchor plot of the H4K8la or H4K8ac ChIP-seq signal around TSSs, respectively, in control and SIRT2 KD cells. ChIP-seq SPMR (sequence tags per million reads) values for nucleotide positions between ± 2.5 kb were averaged and plotted. TSS, transcription start site.

**(d)** Genome browser representations of H4K8la and H4K8ac signals enriched at the *SERPING1* and *TRPV4* gene promoter loci in control and SIRT2 KD cells.

**(e, f)** ChIP was performed with an anti-H4K8ac antibody **(e)** or anti-H3K18la and anti-H3K18ac antibodies **(f)** in control and SIRT2 KD cells. qPCR analysis of the ChIP precipitates was performed to assess the occupancy of H4K8ac, H3K18la, and H3K18ac marks at the promoters of *SERPING1* and *TRPV4* genes shown to regulate tumor cell proliferation and migration. The data are presented as the mean ± SEM calculated from three independent experiments. *, *p* < 0.05; ***, *p* < 0.001; n.s., not significant as calculated by two tailed student's *t* test.

**
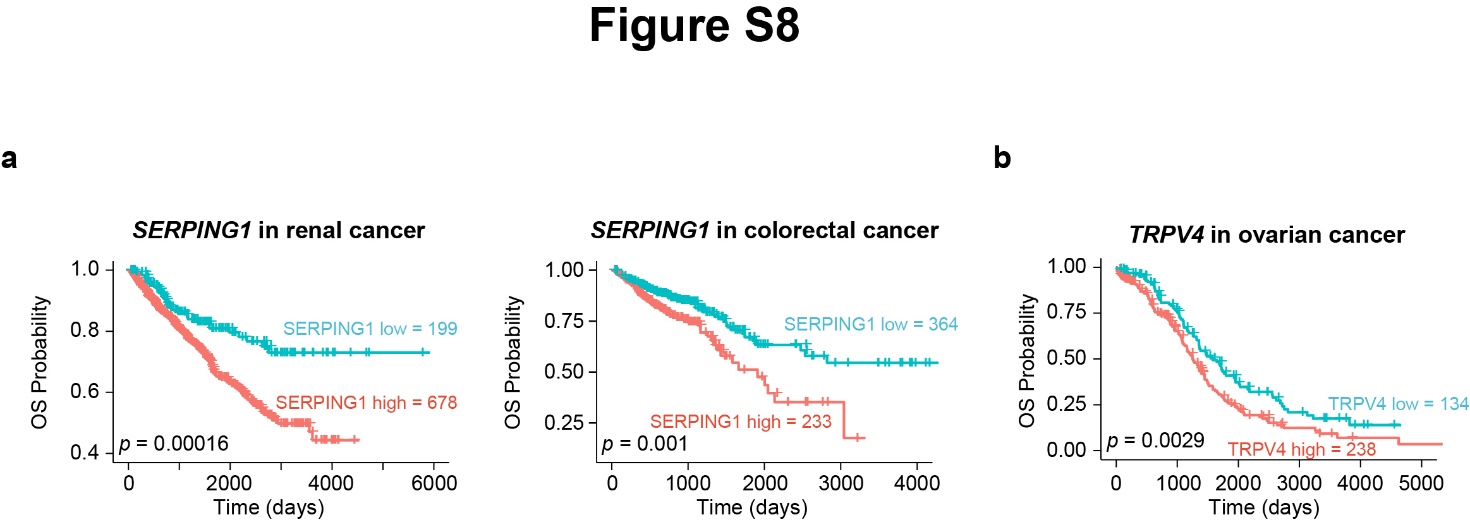
**

**Figure S8.** **SERPING1 and TRPV4 levels are related to tumor patient prognosis.**

The mRNA levels of *SERPING1* **(a)** and *TRPV4* **(b)** genes were obtained from TCGA, and Kaplan-Meier (K-M) analysis of their levels with the OS of different types of cancers were conducted using the R package “survival”. The optimal cut-off value for the K-M plots were calculated using the “survminer” package.

**Table S1. MALDI-TOF MS for delactylation activity of sirtuins on histone-lactyl peptides.**

| **Lactyl-peptide** | **Sequence** | **SIRT2** |
| --- | --- | --- |
| H3K9la (1-15) | ARTKQTAR(Kla)STGGKA | ＋ |
| H3K18la (11-25) | TGGKAPR(Kla)QLATKAA | ＋ |
| H3K23la (16-31) | PRKQLAT(Kla)AARKSAPA | ＋ |
| H3K27la (21-35) | ATKAAR(Kla)SAPATGGV | ＋ |
| H3K79la (70-87) | VREIAQDF(Kla)TDLRFQSS | ＋ |
| H4K5la (1-14) | SGRG(Kla)GGKGLGKGG | － |
| H4K8la (1-14) | SGRGKGG(Kla)GLGKGG | － |
| H4K12la (4-20) | GKGGKGLG(Kla)GGAKRHRK | ＋ |
| H4K16la (11-25) | GKGGA(Kla)RHRKVLRDN | ＋ |
| H4K31la (23-39) | RDNIQGIT(Kla)PAIRRLAR | － |
| H4K77la (69-85) | AVTYTEHA(Kla)RKTVTAMD | － |
| H4K91la (83-99) | AMDVVYAL(Kla)RQGRTLYG | ＋ |
| H2AK11la (4-19) | KAGKDSG(Kla)AKTKAVSR | － |
| H2AK13la (4-19) | KAGKDSGKA(Kla)TKAVSR | ＋ |
| H2AK115la (107-123) | GGVIPHIH(Kla)SLIGKKGQ | ＋ |
| H2BK5la (1-14) | PEPA(Kla)SAPAPKKGS | － |
| H2BK11la (3-19) | PAKSAPAP(Kla)KGSKKAVT | － |
| H2BK15la (7-23) | APAPKKGS(Kla)KAVTKAQK | － |
| H2BK16la (8-24) | PAPKKGSK(Kla)AVTKAQKK | ＋ |
| H2BK20la (12-28) | KGSKKAVT(Kla)AQKKDGKK | ＋ |
| H2BK23la (15-31) | KKAVTKAQ(Kla)KDGKKRKR | ＋ |
| H2BK43la (35-51) | ESYSVYVY(Kla)VLKQVHPD | － |
| H2BK85la (77-93) | ASRLAHYN(Kla)RSTITSRE | ＋ |
| H2BK108la (100-116) | LLLPGELA(Kla)HAVSEGTK | ＋ |
| H2BK116la (108-124) | KHAVSEGT(Kla)AVTKYTSS | ＋ |
| H2BK120la (108-125) | KHAVSEGTKAVT(Kla)YTSSK | ＋ |

100 μM of each synthesized histone peptide bearing a lactylated lysine residue was incubated with 10 μM SIRT2 at 37 °C for 5h, respectively, in 50 μl reaction buffer (20 mM Tris-HCl, pH 7.5 and 1 mM dithiothreitol) supplemented with 5 mM NAD^+^.

**Table S2. The differentially expressed genes in SIRT2 KD cells *vs.* control SH-SY5Y cells determined by RNA-seq.**

**Table S3. ChIP-seq peaks and annotated genes by anti-H4K8la antibody in control SH-SY5Y cells.**

**Table S4. ChIP-seq peaks and annotated genes by anti-H4K8ac antibody in control SH-SY5Y cells.**

**Table S5. ChIP-seq peaks and annotated genes by anti-H4K8la antibody in SIRT2 KD SH-SY5Y cells.**

**Table S6. ChIP-seq peaks and annotated genes by anti-H4K8ac antibody in SIRT2 KD SH-SY5Y cells.**

**Table S7. Constructs of sirtuin proteins cloned into expression vectors and the *E. coli* strains used to express these proteins.­­­**

| **Construct** | ***E. coli* strain** |
| --- | --- |
| pET-15b(+)-SIRT1 (183-664) | BL21-CodonPlus (DE3)-RIL (Agilent) |
| pET-28a(+)-SIRT2 (55-356) | BL21 (DE3) (Novagen) |
| pET-15b(+)-SIRT3 (102-399) | Rosetta™ 2 (DE3) (Novagen) |
| pET-28a(+)-SIRT5 (34-302) | Rosetta™ 2 (DE3) (Novagen) |
| pET-28a(+)-SIRT6 (1-314) | Rosetta™ 2 (DE3) (Novagen) |
| pET-28a(+)-SIRT7 (68-358) | BL21-CodonPlus (DE3)-RIL (Agilent) |

**Table S8. Sequences of the primers used for qPCR.**

| **Gene** | **Primer Sequence** |
| --- | --- |
| *SIRT2*-F | TGCGGAACTTATTCTCCCAGA |
| *SIRT2*-R | GAGAGCGAAAGTCGGGGAT |
| *SERPING1*-F | CTGGCTGGGGATAGAGCCT |
| *SERPING1*-R | GAGATAACTGTTGTTGCGACCT |
| *TRPV4*-F | GATGGGCGACCAAATCTGC |
| *TRPV4*-R | GAGGACTCATATAGGGTGGACTC |
| *β-actin*-F | CACCAACTGGGACGACAT |
| *β-actin*-R | ATACAGGGACAGCACAGC |

**Table S9. Sequences of the primers used for ChIP-qPCR.**

| **Gene** | **Primer Sequence** |
| --- | --- |
| *SERPING1*-F | GTTTCCCAAAGTCAATCTCTCAGG |
| *SERPING1*-R | ATCAAAGCGGGACCCACC |
| *TRPV4*-F | GGCATGATCACGGCTCACTA |
| *TRPV4*-R | CTGTACTCCCAGCTGCTCAG |
