# Supplementary material for: SIRT2 functions as a histone delactylase and inhibits the proliferation and migration of neuroblastoma cells
Source: Cell Discov. 2022 Jun 7;8:54. doi: 10.1038/s41421-022-00398-y (PMC9174446; doi:10.1038/s41421-022-00398-y)
Supplement: Supplementary file 3 — Supplementary Table S3 [file 41421_2022_398_MOESM3_ESM.pdf]

Table S3. ChIP-seq peaks and annotated genes by anti-H4K8la antibody in control SH-SY5Y cells.

chr	start	end	peakid	-LOG(pvalue)	gene
1	1310091	1310445	5Y-H4K8la_peak_1	7.16957	AURKAIP1_ENSG00000175756
1	1963748	1964083	5Y-H4K8la_peak_2	10.0574	
1	2246087	2246283	5Y-H4K8la_peak_3	7.1479	
1	2413810	2414189	5Y-H4K8la_peak_4	8.15295	
1	3087463	3087674	5Y-H4K8la_peak_5	6.53263	
1	3737152	3737388	5Y-H4K8la_peak_6	5.27516	
1	6420335	6420539	5Y-H4K8la_peak_7	5.78576	
1	7690798	7691257	5Y-H4K8la_peak_8	6.53263	
1	7740602	7740798	5Y-H4K8la_peak_9	11.11308	
1	7741024	7741625	5Y-H4K8la_peak_10	7.20156	
1	10961510	10961966	5Y-H4K8la_peak_11	7.42863	
1	10964767	10965025	5Y-H4K8la_peak_12	5.02936	
1	11001824	11002045	5Y-H4K8la_peak_13	7.31696	
1	11751314	11751562	5Y-H4K8la_peak_14	7.32908	MAD2L2_ENSG00000116670;DRAXIN_ENSG00000162490
1	14026852	14027106	5Y-H4K8la_peak_15	6.97847	PRDM2_ENSG00000116731
1	16010120	16010609	5Y-H4K8la_peak_16	8.34321	RP4-680D5.9_ENSG00000271742;PLEKHM2_ENSG00000116786;AL121992.1_ENSG00000264048
1	16692693	16693102	5Y-H4K8la_peak_17	10.12966	
1	16971762	16972198	5Y-H4K8la_peak_18	17.84833	CROCCP2_ENSG00000215908;MST1P2_ENSG00000186301
1	17306910	17307345	5Y-H4K8la_peak_19	11.50519	MFAP2_ENSG00000117122
1	20812884	20813235	5Y-H4K8la_peak_20	7.20129	CAMK2N1_ENSG00000162545
1	21503703	21503982	5Y-H4K8la_peak_21	6.9507	EIF4G3_ENSG00000075151;AL031005.1_ENSG00000266867
1	22378601	22378915	5Y-H4K8la_peak_22	10.17199	CDC42_ENSG00000070831
1	23961148	23961359	5Y-H4K8la_peak_23	9.00262	
1	25429666	25429869	5Y-H4K8la_peak_24	4.96761	
1	26125876	26126165	5Y-H4K8la_peak_25	10.44872	SEPN1_ENSG00000162430
1	26868246	26868463	5Y-H4K8la_peak_26	5.41836	
1	27023553	27023941	5Y-H4K8la_peak_27	9.61758	
1	27854486	27854742	5Y-H4K8la_peak_28	6.72615	
1	29138818	29139205	5Y-H4K8la_peak_29	13.42677	OPRD1_ENSG00000116329
1	29508056	29508323	5Y-H4K8la_peak_30	5.60833	SRSF4_ENSG00000116350
1	29797079	29797482	5Y-H4K8la_peak_31	12.63314	
1	29797994	29798209	5Y-H4K8la_peak_32	8.84908	
1	30180601	30180926	5Y-H4K8la_peak_33	5.82532	
1	32279617	32280307	5Y-H4K8la_peak_34	9.9061	
1	33438576	33438815	5Y-H4K8la_peak_35	8.03849	FKSG48_ENSG00000267885
1	35658036	35658533	5Y-H4K8la_peak_36	11.16506	SFPQ_ENSG00000116560
1	36173221	36173586	5Y-H4K8la_peak_37	10.01769	
1	36553954	36554200	5Y-H4K8la_peak_38	10.00392	ADPRHL2_ENSG00000116863
1	39491230	39491429	5Y-H4K8la_peak_39	8.89577	NDUFS5_ENSG00000168653
1	42921423	42921627	5Y-H4K8la_peak_40	5.02936	ZMYND12_ENSG00000066185;PPCS_ENSG00000127125
1	44445077	44445273	5Y-H4K8la_peak_41	11.69305	B4GALT2_ENSG00000117411
1	45204065	45204261	5Y-H4K8la_peak_42	9.24465	
1	49364629	49364832	5Y-H4K8la_peak_43	9.82869	
1	53098668	53099065	5Y-H4K8la_peak_44	7.69436	FAM159A_ENSG00000182183

1	53527328	53527591	5Y-H4K8la_peak_45	5.02936	PODN_ENSG00000174348
1	62827053	62827276	5Y-H4K8la_peak_46	6.03474	
1	64970907	64971169	5Y-H4K8la_peak_47	5.93696	
1	85313611	85313909	5Y-H4K8la_peak_48	7.39065	
1	96407471	96407679	5Y-H4K8la_peak_49	6.97847	
1	110603207	110603567	5Y-H4K8la_peak_50	6.09172	
1	116915375	116915642	5Y-H4K8la_peak_51	10.44872	ATP1A1_ENSG00000163399
1	117453028	117453271	5Y-H4K8la_peak_52	7.35213	RP4-753F5.1_ENSG00000272715;PTGFRN_ENSG00000134247
1	117893643	117893846	5Y-H4K8la_peak_53	6.46211	
1	150185421	150185656	5Y-H4K8la_peak_54	7.29177	
1	153509013	153509290	5Y-H4K8la_peak_55	6.4327	S100A6_ENSG00000197956
1	153962588	153962806	5Y-H4K8la_peak_56	7.08077	RPS27_ENSG00000177954
1	154976955	154977377	5Y-H4K8la_peak_57	8.11274	
1	155162974	155163315	5Y-H4K8la_peak_58	13.64172	MUC1_ENSG00000185499
1	155197628	155197874	5Y-H4K8la_peak_59	8.80583	GBAP1_ENSG00000160766
1	156883490	156883946	5Y-H4K8la_peak_60	8.14442	
1	160231846	160232146	5Y-H4K8la_peak_61	6.71102	RP11-574F21.2_ENSG00000228606
1	161068363	161068606	5Y-H4K8la_peak_62	7.47094	KLHDC9_ENSG00000162755
1	161171327	161171533	5Y-H4K8la_peak_63	5.60833	
1	162851375	162851793	5Y-H4K8la_peak_64	7.72393	
1	162887711	162887983	5Y-H4K8la_peak_65	6.89955	
1	166845113	166845332	5Y-H4K8la_peak_66	9.24465	TADA1_ENSG00000152382
1	170501434	170501760	5Y-H4K8la_peak_67	5.81948	RP11-576I22.2_ENSG00000231407;GORAB_ENSG00000120370
1	172419356	172419577	5Y-H4K8la_peak_68	8.2602	
1	180896297	180896495	5Y-H4K8la_peak_69	8.78149	
1	185285755	185286273	5Y-H4K8la_peak_70	9.00262	IVNS1ABP_ENSG00000116679;GS1-279B7.2_ENSG00000273004
1	193027866	193028320	5Y-H4K8la_peak_71	8.3797	UCHL5_ENSG00000116750;TROVE2_ENSG00000116747
1	203456393	203456764	5Y-H4K8la_peak_72	6.14037	
1	205473857	205474064	5Y-H4K8la_peak_73	9.99013	CDK18_ENSG00000117266
1	208137266	208137529	5Y-H4K8la_peak_74	10.48208	
1	210425072	210425480	5Y-H4K8la_peak_75	10.86348	
1	210743644	210743933	5Y-H4K8la_peak_76	8.53959	
1	211663563	211663806	5Y-H4K8la_peak_77	6.35224	
1	211664984	211665180	5Y-H4K8la_peak_78	5.17488	RP11-359E8.3_ENSG00000223649
1	211790121	211790424	5Y-H4K8la_peak_79	5.17488	
1	212731656	212731903	5Y-H4K8la_peak_80	7.5782	RP11-61J19.4_ENSG00000260805
1	224363777	224364030	5Y-H4K8la_peak_81	7.79008	RP11-365O16.1_ENSG00000236773;DEGS1_ENSG00000143753
1	226310130	226310345	5Y-H4K8la_peak_82	5.89028	
1	227127366	227127577	5Y-H4K8la_peak_83	6.66609	
1	227750667	227750947	5Y-H4K8la_peak_84	12.59446	ZNF678_ENSG00000181450
1	228074866	228075367	5Y-H4K8la_peak_85	10.77389	
1	230416827	230417024	5Y-H4K8la_peak_86	5.41836	RP5-956O18.3_ENSG00000224407
1	231004756	231005032	5Y-H4K8la_peak_87	7.09368	C1orf198_ENSG00000119280
1	233007170	233007426	5Y-H4K8la_peak_88	10.84781	
1	233430994	233431194	5Y-H4K8la_peak_89	6.11939	PCNXL2_ENSG00000135749;RP5-862P8.3_ENSG00000251508
1	234614148	234614387	5Y-H4K8la_peak_90	8.27701	TARBP1_ENSG00000059588

1	235183447	235183719	5Y-H4K8la_peak_91	9.93134	
1	243660184	243660388	5Y-H4K8la_peak_92	10.84781	
1	245916082	245916303	5Y-H4K8la_peak_93	9.56697	
10	1316974	1317298	5Y-H4K8la_peak_94	10.01769	
10	13389302	13389572	5Y-H4K8la_peak_95	10.84781	SEPHS1_ENSG00000086475
10	23003712	23004025	5Y-H4K8la_peak_96	8.13463	PIP4K2A_ENSG00000150867
10	43367703	43367949	5Y-H4K8la_peak_97	8.15295	RP11-124O11.1_ENSG00000234944
10	44143974	44144183	5Y-H4K8la_peak_98	7.79008	ZNF32_ENSG00000169740
10	46168569	46168861	5Y-H4K8la_peak_99	10.87965	ZFAND4_ENSG00000172671
10	46222243	46222513	5Y-H4K8la_peak_100	6.33552	FAM21FP_ENSG00000237840;RP11-671E7.1_ENSG00000228702;FAM21C_ENSG00000172661
10	62492811	62493069	5Y-H4K8la_peak_101	7.71476	ANK3_ENSG00000151150
10	65281298	65281521	5Y-H4K8la_peak_102	6.32424	REEP3_ENSG00000165476
10	72995166	72995492	5Y-H4K8la_peak_103	6.89955	
10	73019843	73020098	5Y-H4K8la_peak_104	6.11939	
10	73022976	73023172	5Y-H4K8la_peak_105	6.0804	
10	73804292	73804513	5Y-H4K8la_peak_106	8.24368	
10	74032986	74033261	5Y-H4K8la_peak_107	8.69005	DDIT4_ENSG00000168209
10	74082560	74082876	5Y-H4K8la_peak_108	5.1759	
10	74095232	74095498	5Y-H4K8la_peak_109	8.23998	
10	79471029	79471238	5Y-H4K8la_peak_110	7.57826	
10	79972520	79972792	5Y-H4K8la_peak_111	8.3797	
10	80416320	80416573	5Y-H4K8la_peak_112	8.15295	
10	80981738	80982148	5Y-H4K8la_peak_113	9.8767	
10	80982390	80983073	5Y-H4K8la_peak_114	8.41069	
10	81035066	81035344	5Y-H4K8la_peak_115	9.24465	
10	88026343	88026645	5Y-H4K8la_peak_116	5.78576	
10	94450014	94450241	5Y-H4K8la_peak_117	8.97426	
10	95653289	95653522	5Y-H4K8la_peak_118	7.1479	SLC35G1_ENSG00000176273
10	100027744	100027957	5Y-H4K8la_peak_119	6.88861	LOXL4_ENSG00000138131
10	102472751	102472960	5Y-H4K8la_peak_120	6.68187	
10	102473723	102473961	5Y-H4K8la_peak_121	9.8767	
10	103816406	103816730	5Y-H4K8la_peak_122	8.9585	C10orf76_ENSG00000120029
10	104387685	104387910	5Y-H4K8la_peak_123	11.39542	
10	111767021	111767233	5Y-H4K8la_peak_124	11.09733	
10	112403449	112403721	5Y-H4K8la_peak_125	7.95926	Y_RNA_ENSG00000223302;RBM20_ENSG00000203867
10	119805997	119806279	5Y-H4K8la_peak_126	14.48633	RAB11FIP2_ENSG00000107560;CASC2_ENSG00000177640
10	123900286	123900523	5Y-H4K8la_peak_127	10.78118	
10	131264859	131265125	5Y-H4K8la_peak_128	9.24465	MGMT_ENSG00000170430
11	355533	355973	5Y-H4K8la_peak_129	9.34762	
11	728475	728697	5Y-H4K8la_peak_130	7.16957	
11	855284	855539	5Y-H4K8la_peak_131	9.11759	
11	890225	890428	5Y-H4K8la_peak_132	6.09172	
11	1331312	1331523	5Y-H4K8la_peak_133	6.53263	TOLLIP_ENSG00000078902;TOLLIP-AS1_ENSG00000255153
11	1714824	1715316	5Y-H4K8la_peak_134	13.55423	
11	1897588	1897832	5Y-H4K8la_peak_135	11.27875	
11	1899087	1899458	5Y-H4K8la_peak_136	8.53671	

11	1968607	1969192	5Y-H4K8la_peak_137	14.92651	MRPL23_ENSG00000214026
11	1977938	1978246	5Y-H4K8la_peak_138	5.41836	
11	2000697	2001421	5Y-H4K8la_peak_139	9.30692	
11	2002335	2002899	5Y-H4K8la_peak_140	10.21971	
11	2003816	2004051	5Y-H4K8la_peak_141	9.62047	
11	2422086	2422309	5Y-H4K8la_peak_142	8.34321	TSSC4_ENSG00000184281
11	2799397	2799773	5Y-H4K8la_peak_143	10.00392	
11	8249799	8250071	5Y-H4K8la_peak_144	7.32908	
11	11991030	11991328	5Y-H4K8la_peak_145	6.65331	
11	16838513	16838749	5Y-H4K8la_peak_146	7.95926	
11	43946926	43947194	5Y-H4K8la_peak_147	6.11939	C11orf96_ENSG00000187479
11	46739689	46739888	5Y-H4K8la_peak_148	8.75054	F2_ENSG00000180210
11	55640444	55640755	5Y-H4K8la_peak_149	6.03474	
11	63685848	63686407	5Y-H4K8la_peak_150	7.69149	
11	63824133	63824436	5Y-H4K8la_peak_151	7.42863	
11	63917054	63917262	5Y-H4K8la_peak_152	5.89026	
11	64258225	64258437	5Y-H4K8la_peak_153	8.03849	
11	64334621	64334971	5Y-H4K8la_peak_154	6.53263	
11	64612219	64612523	5Y-H4K8la_peak_155	5.70363	CDC42BPG_ENSG00000171219
11	65679508	65679724	5Y-H4K8la_peak_156	9.8767	
11	66496022	66496395	5Y-H4K8la_peak_157	10.37149	SPTBN2_ENSG00000173898
11	66885852	66886087	5Y-H4K8la_peak_158	6.71638	KDM2A_ENSG00000173120
11	67771902	67772141	5Y-H4K8la_peak_159	5.82532	UNC93B1_ENSG00000110057
11	68427161	68427378	5Y-H4K8la_peak_160	6.53263	
11	68452271	68452475	5Y-H4K8la_peak_161	6.54023	
11	68459620	68459846	5Y-H4K8la_peak_162	7.79699	
11	68924385	68924675	5Y-H4K8la_peak_163	13.94218	
11	72859273	72859502	5Y-H4K8la_peak_164	7.46877	
11	75266286	75266606	5Y-H4K8la_peak_165	8.96376	
11	75294522	75294815	5Y-H4K8la_peak_166	9.06236	CTD-2530H12.4_ENSG00000255326
11	76327104	76327321	5Y-H4K8la_peak_167	6.28883	
11	78730362	78730584	5Y-H4K8la_peak_168	10.72928	
11	115529818	115530142	5Y-H4K8la_peak_169	12.27803	AP000797.3_ENSG00000256717
11	117186184	117186386	5Y-H4K8la_peak_170	5.27516	BACE1_ENSG00000186318;CEP164_ENSG00000110274
11	117314621	117314949	5Y-H4K8la_peak_171	9.10112	
11	118560052	118560318	5Y-H4K8la_peak_172	10.40816	AP002954.6_ENSG00000255239
11	124588058	124588287	5Y-H4K8la_peak_173	7.1479	
11	125820715	125820961	5Y-H4K8la_peak_174	9.03201	RP11-680F20.6_ENSG00000254967
11	125984559	125984983	5Y-H4K8la_peak_175	7.45648	
11	126413729	126413955	5Y-H4K8la_peak_176	8.20666	KIRREL3-AS1_ENSG00000257271
11	128106833	128107367	5Y-H4K8la_peak_177	11.71161	
11	129871884	129872116	5Y-H4K8la_peak_178	7.57826	PRDM10_ENSG00000170325;LINC00167_ENSG00000233220
12	6387406	6387893	5Y-H4K8la_peak_179	8.9585	
12	6721729	6722066	5Y-H4K8la_peak_180	8.03849	
12	6798265	6798506	5Y-H4K8la_peak_181	11.67604	ZNF384_ENSG00000126746
12	7046377	7046962	5Y-H4K8la_peak_182	5.41836	

12	12502790	12503027	5Y-H4K8la_peak_183	6.37554	MANSC1_ENSG00000111261
12	12869714	12869944	5Y-H4K8la_peak_184	8.03849	
12	22199261	22199486	5Y-H4K8la_peak_185	10.021	CMAS_ENSG00000111726
12	26379604	26379821	5Y-H4K8la_peak_186	6.9195	
12	29302424	29302680	5Y-H4K8la_peak_187	7.79008	RP11-946L16.1_ENSG00000257258;FAR2_ENSG00000064763
12	32440755	32440969	5Y-H4K8la_peak_188	8.97426	
12	46662890	46663099	5Y-H4K8la_peak_189	6.90369	SLC38A1_ENSG00000111371
12	48222964	48223168	5Y-H4K8la_peak_190	5.46366	
12	52242245	52242469	5Y-H4K8la_peak_191	10.67724	RP11-923I11.5_ENSG00000259887
12	52597479	52597749	5Y-H4K8la_peak_192	8.0821	
12	53441414	53441667	5Y-H4K8la_peak_193	8.15295	TENC1_ENSG00000111077
12	56321147	56321492	5Y-H4K8la_peak_194	6.84763	DGKA_ENSG00000065357
12	69202093	69202303	5Y-H4K8la_peak_195	6.97847	MDM2_ENSG00000135679
12	89739652	89739889	5Y-H4K8la_peak_196	8.03849	
12	104531237	104531654	5Y-H4K8la_peak_197	6.97847	NFYB_ENSG00000120837
12	109770175	109770492	5Y-H4K8la_peak_198	6.53263	
12	109970847	109971245	5Y-H4K8la_peak_199	6.53263	
12	112280216	112280436	5Y-H4K8la_peak_200	8.02705	MAPKAPK5-AS1_ENSG00000234608;MAPKAPK5_ENSG00000089022
12	120801150	120801379	5Y-H4K8la_peak_201	11.41209	
12	122238621	122238909	5Y-H4K8la_peak_202	6.14037	
12	127630810	127631108	5Y-H4K8la_peak_203	9.82869	RP11-575F12.2_ENSG00000256001
12	133758170	133758455	5Y-H4K8la_peak_204	9.8767	
13	25874953	25875235	5Y-H4K8la_peak_205	10.2251	RP11-271M24.2_ENSG00000260509;NUPL1_ENSG00000139496
13	46039174	46039415	5Y-H4K8la_peak_206	10.17199	COG3_ENSG00000136152
13	74709274	74709474	5Y-H4K8la_peak_207	10.44337	KLF12_ENSG00000118922
13	76209715	76210008	5Y-H4K8la_peak_208	8.90889	RP11-173B14.5_ENSG00000261105
13	96185985	96186184	5Y-H4K8la_peak_209	8.97426	CLDN10-AS1_ENSG00000223392
13	99852523	99852810	5Y-H4K8la_peak_210	9.0527	UBAC2-AS1_ENSG00000228889;UBAC2_ENSG00000134882
13	114829837	114830035	5Y-H4K8la_peak_211	6.53263	
13	115000502	115000713	5Y-H4K8la_peak_212	7.32908	CDC16_ENSG00000130177
14	21728876	21729104	5Y-H4K8la_peak_213	8.97426	
14	23789800	23789998	5Y-H4K8la_peak_214	6.11939	PABPN1_ENSG00000100836
14	24438741	24439037	5Y-H4K8la_peak_215	5.13147	DHRS4L2_ENSG00000187630
14	24520771	24520997	5Y-H4K8la_peak_216	7.46931	LRRC16B_ENSG00000186648
14	24838725	24838980	5Y-H4K8la_peak_217	7.47094	
14	54976721	54976951	5Y-H4K8la_peak_218	7.71476	CGRRF1_ENSG00000100532
14	61104125	61104377	5Y-H4K8la_peak_219	6.0804	
14	69262142	69262442	5Y-H4K8la_peak_220	8.03849	ZFP36L1_ENSG00000185650
14	69283034	69283460	5Y-H4K8la_peak_221	10.17199	
14	74253554	74253754	5Y-H4K8la_peak_222	5.5367	RP5-1021I20.1_ENSG00000259065
14	75078550	75078758	5Y-H4K8la_peak_223	10.18615	LTBP2_ENSG00000119681
14	77494652	77494852	5Y-H4K8la_peak_224	5.56085	IRF2BPL_ENSG00000119669
14	77499149	77499438	5Y-H4K8la_peak_225	8.15295	
14	77504379	77504635	5Y-H4K8la_peak_226	7.47283	
14	90917660	90917931	5Y-H4K8la_peak_227	9.34955	
14	91749646	91750149	5Y-H4K8la_peak_228	8.15295	

14	91884566	91884832	5Y-H4K8la_peak_229	6.9195	CCDC88C_ENSG00000015133;RP11-895M11.3_ENSG00000258798;RP11-895M11.2_ENSG00000258446
14	93384387	93384613	5Y-H4K8la_peak_230	12.39425	
14	93474192	93474618	5Y-H4K8la_peak_231	8.31681	
14	100039189	100039400	5Y-H4K8la_peak_232	8.15295	
14	100240163	100240652	5Y-H4K8la_peak_233	8.39578	
14	100751969	100752232	5Y-H4K8la_peak_234	6.62475	
14	100907695	100907894	5Y-H4K8la_peak_235	8.01893	
14	100910265	100910508	5Y-H4K8la_peak_236	13.09586	
14	101241986	101242264	5Y-H4K8la_peak_237	8.3797	
14	101242505	101242710	5Y-H4K8la_peak_238	7.73692	
14	101245823	101246445	5Y-H4K8la_peak_239	9.99013	MEG3_ENSG00000214548
14	101249539	101249912	5Y-H4K8la_peak_240	9.44926	
14	101251444	101251648	5Y-H4K8la_peak_241	7.53185	
14	101593150	101593365	5Y-H4K8la_peak_242	5.96979	
14	103213037	103213272	5Y-H4K8la_peak_243	6.22411	
14	103589219	103589585	5Y-H4K8la_peak_244	10.76437	LINC00677_ENSG00000259717;TNFAIP2_ENSG00000185215
14	104602176	104602398	5Y-H4K8la_peak_245	6.62448	
14	104678644	104678920	5Y-H4K8la_peak_246	8.70467	
14	104903214	104903808	5Y-H4K8la_peak_247	9.85416	
14	105218504	105218745	5Y-H4K8la_peak_248	7.20129	SIVA1_ENSG00000184990
15	27212901	27213121	5Y-H4K8la_peak_249	6.03474	
15	31507951	31508308	5Y-H4K8la_peak_250	5.02936	RP11-16E12.1_ENSG00000259448
15	31690712	31690990	5Y-H4K8la_peak_251	10.77389	
15	34331163	34331453	5Y-H4K8la_peak_252	11.09733	AVEN_ENSG00000169857
15	37388241	37388522	5Y-H4K8la_peak_253	6.39254	
15	38544783	38544979	5Y-H4K8la_peak_254	7.42863	SPRED1_ENSG00000166068
15	40728779	40728977	5Y-H4K8la_peak_255	10.10129	
15	41244671	41245085	5Y-H4K8la_peak_256	5.76537	CHAC1_ENSG00000128965
15	42186597	42186857	5Y-H4K8la_peak_257	6.53263	SPTBN5_ENSG00000137877
15	45458816	45459097	5Y-H4K8la_peak_258	10.21547	CTD-2651B20.1_ENSG00000259539
15	47857410	47857609	5Y-H4K8la_peak_259	6.03474	
15	57998219	57998434	5Y-H4K8la_peak_260	9.53456	
15	63797065	63797263	5Y-H4K8la_peak_261	8.69005	USP3_ENSG00000140455
15	73430980	73431202	5Y-H4K8la_peak_262	6.97847	
15	74429791	74430228	5Y-H4K8la_peak_263	5.76108	
15	74516324	74516642	5Y-H4K8la_peak_264	8.05492	
15	74584417	74584705	5Y-H4K8la_peak_265	10.77389	
15	74585002	74585236	5Y-H4K8la_peak_266	10.24966	
15	75182504	75182733	5Y-H4K8la_peak_267	5.76537	MPI_ENSG00000178802
15	78912969	78913197	5Y-H4K8la_peak_268	7.95919	CHRNA3_ENSG00000080644
15	79044016	79044303	5Y-H4K8la_peak_269	12.77906	RP11-160C18.4_ENSG00000238166
15	79051069	79051275	5Y-H4K8la_peak_270	7.71476	
15	79053515	79053985	5Y-H4K8la_peak_271	7.19867	
15	90293536	90294002	5Y-H4K8la_peak_272	6.04448	MESP1_ENSG00000166823
15	90319146	90319721	5Y-H4K8la_peak_273	9.9061	
15	93364290	93364573	5Y-H4K8la_peak_274	11.42306	

15	93821308	93821516	5Y-H4K8la_peak_275	6.75415	
15	101306981	101307177	5Y-H4K8la_peak_276	7.92423	
15	101404722	101404934	5Y-H4K8la_peak_277	8.3533	
15	101689030	101689415	5Y-H4K8la_peak_278	7.79008	
15	102192843	102193178	5Y-H4K8la_peak_279	5.56085	TM2D3_ENSG00000184277
15	102506188	102506443	5Y-H4K8la_peak_280	6.04448	
16	691449	691660	5Y-H4K8la_peak_281	7.91054	AL022341.1_ENSG00000197727;FAM195A_ENSG00000172366
16	1582038	1582318	5Y-H4K8la_peak_282	6.53263	
16	1979989	1980267	5Y-H4K8la_peak_283	12.77906	
16	2087947	2088160	5Y-H4K8la_peak_284	7.32908	
16	2301942	2302177	5Y-H4K8la_peak_285	7.54283	ECI1_ENSG00000167969
16	2827970	2828186	5Y-H4K8la_peak_286	6.62448	TCEB2_ENSG00000103363
16	4442950	4443175	5Y-H4K8la_peak_287	8.34321	
16	5083424	5083642	5Y-H4K8la_peak_288	7.71476	NAGPA_ENSG00000103174;ALG1_ENSG00000033011
16	15952262	15952471	5Y-H4K8la_peak_289	5.57055	
16	27280281	27280730	5Y-H4K8la_peak_290	5.82532	NSMCE1_ENSG00000169189;CTD-3203P2.2_ENSG00000245888
16	28525591	28525951	5Y-H4K8la_peak_291	6.75415	
16	28834610	28834987	5Y-H4K8la_peak_292	13.13483	ATXN2L_ENSG00000168488
16	29187984	29188209	5Y-H4K8la_peak_293	10.77389	
16	29875201	29875412	5Y-H4K8la_peak_294	12.008	CDIPT_ENSG00000103502;CDIPT-AS1_ENSG00000214725
16	30537534	30537757	5Y-H4K8la_peak_295	6.48484	ZNF768_ENSG00000169957
16	30938956	30939234	5Y-H4K8la_peak_296	10.0197	
16	47270133	47270360	5Y-H4K8la_peak_297	6.97847	
16	48573805	48574053	5Y-H4K8la_peak_298	5.81948	
16	57541962	57542632	5Y-H4K8la_peak_299	6.76954	
16	67596995	67597196	5Y-H4K8la_peak_300	8.3797	CTD-2012K14.6_ENSG00000261386;CTD-2012K14.7_ENSG00000259804;CTCF_ENSG00000102974
16	67816998	67817212	5Y-H4K8la_peak_301	7.1479	
16	70333141	70333441	5Y-H4K8la_peak_302	10.31591	RP11-529K1.3_ENSG00000260537
16	81764237	81764446	5Y-H4K8la_peak_303	7.54839	
16	82204085	82204361	5Y-H4K8la_peak_304	6.97847	MPHOSPH6_ENSG00000135698;CTD-2588J6.2_ENSG00000261029
16	85191831	85192476	5Y-H4K8la_peak_305	7.9701	
16	85253444	85253846	5Y-H4K8la_peak_306	9.17304	
16	85685175	85685985	5Y-H4K8la_peak_307	9.77417	
16	87903373	87903677	5Y-H4K8la_peak_308	6.22227	SLC7A5_ENSG00000103257
16	88126274	88126587	5Y-H4K8la_peak_309	9.00262	
16	89883323	89883638	5Y-H4K8la_peak_310	6.36955	SPIRE2_ENSG00000204991;FANCA_ENSG00000187741
16	90038353	90038736	5Y-H4K8la_peak_311	11.91101	CENPBD1_ENSG00000177946;AFG3L1P_ENSG00000223959
17	150758	150979	5Y-H4K8la_peak_312	6.62475	
17	1666244	1666458	5Y-H4K8la_peak_313	5.74411	SERPINF1_ENSG00000132386
17	2089021	2089303	5Y-H4K8la_peak_314	8.03849	
17	2283965	2284285	5Y-H4K8la_peak_315	7.1479	
17	2863731	2863947	5Y-H4K8la_peak_316	5.46366	
17	5014713	5014934	5Y-H4K8la_peak_317	5.46366	AC012146.7_ENSG00000234327
17	7452592	7452994	5Y-H4K8la_peak_318	6.53263	TNFSF12_ENSG00000239697;TNFSF12-TNFSF13_ENSG00000248871
17	7476258	7476495	5Y-H4K8la_peak_319	9.18087	EIF4A1_ENSG00000161960;SNORA67_ENSG00000264772
17	7486366	7486678	5Y-H4K8la_peak_320	9.18087	AC113189.5_ENSG00000233223;MPDU1_ENSG00000129255

17	16934502	16934800	5Y-H4K8la_peak_321	5.76537	
17	17584909	17585242	5Y-H4K8la_peak_322	12.91038	RAI1_ENSG00000108557
17	17739873	17740193	5Y-H4K8la_peak_323	11.33283	SREBF1_ENSG00000072310
17	18085897	18086201	5Y-H4K8la_peak_324	8.03849	ALKBH5_ENSG00000091542
17	19647737	19647959	5Y-H4K8la_peak_325	8.15295	
17	19912016	19912223	5Y-H4K8la_peak_326	7.39065	RP11-209D14.2_ENSG00000261033;SPECC1_ENSG00000128487
17	20945970	20946263	5Y-H4K8la_peak_327	9.00262	USP22_ENSG00000124422
17	27388182	27388406	5Y-H4K8la_peak_328	5.26936	
17	29152019	29152251	5Y-H4K8la_peak_329	8.3533	CRLF3_ENSG00000176390
17	29829000	29829203	5Y-H4K8la_peak_330	5.13147	
17	30608371	30608572	5Y-H4K8la_peak_331	6.30996	RP11-443G13.2_ENSG00000263674
17	34111993	34112205	5Y-H4K8la_peak_332	5.8673	
17	36571332	36571555	5Y-H4K8la_peak_333	9.61758	
17	36860456	36860652	5Y-H4K8la_peak_334	8.15295	
17	38507633	38507854	5Y-H4K8la_peak_335	9.17304	
17	40718276	40718803	5Y-H4K8la_peak_336	6.84763	MLX_ENSG00000108788
17	40832206	40832446	5Y-H4K8la_peak_337	5.43901	CTD-3193K9.4_ENSG00000267042
17	40976561	40976814	5Y-H4K8la_peak_338	7.46931	PSME3_ENSG00000131467
17	42061310	42061589	5Y-H4K8la_peak_339	9.06236	
17	43239644	43239850	5Y-H4K8la_peak_340	5.02936	AC002117.1_ENSG00000224505
17	43926790	43926995	5Y-H4K8la_peak_341	8.99659	
17	44270356	44270683	5Y-H4K8la_peak_342	10.21547	KANSL1-AS1_ENSG00000214401
17	46089119	46089322	5Y-H4K8la_peak_343	8.27752	
17	46090014	46090372	5Y-H4K8la_peak_344	8.79833	
17	46091609	46091935	5Y-H4K8la_peak_345	10.21971	
17	48239353	48239592	5Y-H4K8la_peak_346	7.9701	RP11-893F2.13_ENSG00000253730
17	49021533	49021862	5Y-H4K8la_peak_347	16.20851	RP11-700H6.1_ENSG00000247011;RP11-700H6.2_ENSG00000251665
17	55215589	55215789	5Y-H4K8la_peak_348	5.76537	
17	55624243	55624559	5Y-H4K8la_peak_349	10.13769	
17	55636660	55636972	5Y-H4K8la_peak_350	5.88707	
17	56159983	56160445	5Y-H4K8la_peak_351	13.719	RP11-159D12.10_ENSG00000266290;DYNLL2_ENSG00000264364
17	56401453	56401803	5Y-H4K8la_peak_352	9.32141	
17	59461497	59461693	5Y-H4K8la_peak_353	4.99806	
17	59494688	59494951	5Y-H4K8la_peak_354	9.31339	
17	59565638	59565837	5Y-H4K8la_peak_355	6.85433	
17	60730316	60730541	5Y-H4K8la_peak_356	9.43441	
17	61523861	61524561	5Y-H4K8la_peak_357	16.78473	CYB561_ENSG00000008283
17	61698923	61699171	5Y-H4K8la_peak_358	8.27701	MAP3K3_ENSG00000198909
17	63300162	63300395	5Y-H4K8la_peak_359	8.03849	
17	64960173	64960421	5Y-H4K8la_peak_360	5.44443	CACNG4_ENSG00000075461
17	64965372	64965595	5Y-H4K8la_peak_361	8.31614	
17	65014336	65014728	5Y-H4K8la_peak_362	4.99806	
17	65015907	65016331	5Y-H4K8la_peak_363	8.04592	
17	66031991	66032220	5Y-H4K8la_peak_364	6.11939	KPNA2_ENSG00000182481
17	67323605	67323898	5Y-H4K8la_peak_365	7.1479	ABCA5_ENSG00000154265
17	72239288	72239484	5Y-H4K8la_peak_366	6.50125	

17	73268312	73268583	5Y-H4K8la_peak_367	7.82398	RP11-649A18.12_ENSG00000263843
17	73750084	73750514	5Y-H4K8la_peak_368	9.19627	
17	73823926	73824122	5Y-H4K8la_peak_369	8.3797	
17	74033115	74033327	5Y-H4K8la_peak_370	8.85235	
17	74100167	74100659	5Y-H4K8la_peak_371	8.99659	
17	74117922	74118253	5Y-H4K8la_peak_372	6.11939	EXOC7_ENSG00000182473
17	74526283	74526533	5Y-H4K8la_peak_373	7.5782	
17	74534256	74534468	5Y-H4K8la_peak_374	8.29908	
17	74536439	74536677	5Y-H4K8la_peak_375	10.89523	
17	74540414	74540634	5Y-H4K8la_peak_376	12.20171	
17	74541169	74541549	5Y-H4K8la_peak_377	11.71161	
17	74542131	74542351	5Y-H4K8la_peak_378	9.29994	
17	74667175	74667444	5Y-H4K8la_peak_379	11.27875	RP11-318A15.2_ENSG00000261335
17	75084199	75084497	5Y-H4K8la_peak_380	19.2484	AC015815.3_ENSG00000203316
17	75429299	75429529	5Y-H4K8la_peak_381	8.96386	
17	75432618	75432836	5Y-H4K8la_peak_382	8.27752	
17	75860019	75860246	5Y-H4K8la_peak_383	12.60518	
17	76921665	76921922	5Y-H4K8la_peak_384	8.93736	TIMP2_ENSG00000035862
17	76976487	76976822	5Y-H4K8la_peak_385	5.44443	LGALS3BP_ENSG00000108679
17	77701822	77702160	5Y-H4K8la_peak_386	9.57151	
17	77702651	77702987	5Y-H4K8la_peak_387	16.35057	
17	77771770	77772133	5Y-H4K8la_peak_388	16.04433	
17	77787563	77787899	5Y-H4K8la_peak_389	17.18548	
17	77813579	77813850	5Y-H4K8la_peak_390	9.0527	CBX4_ENSG00000141582
17	77817958	77818347	5Y-H4K8la_peak_391	11.46595	
17	78233889	78234154	5Y-H4K8la_peak_392	18.72022	RNF213_ENSG00000173821
17	78419525	78419723	5Y-H4K8la_peak_393	5.82613	
17	78427671	78428442	5Y-H4K8la_peak_394	10.49072	CTD-2526A2.2_ENSG00000260369
17	78428858	78429164	5Y-H4K8la_peak_395	7.60153	CTD-2526A2.2_ENSG00000260369
17	78429751	78429947	5Y-H4K8la_peak_396	5.17488	
17	78833706	78834330	5Y-H4K8la_peak_397	14.41622	
17	78965828	78966175	5Y-H4K8la_peak_398	11.65167	CHMP6_ENSG00000176108
17	79139499	79139709	5Y-H4K8la_peak_399	6.48219	AATK_ENSG00000181409;AATK-AS1_ENSG00000225180
17	79283834	79284089	5Y-H4K8la_peak_400	11.13219	LINC00482_ENSG00000185168
17	79284305	79284505	5Y-H4K8la_peak_401	6.50125	
17	79288745	79289116	5Y-H4K8la_peak_402	8.66894	
17	79302391	79302717	5Y-H4K8la_peak_403	8.57784	
17	79304782	79305056	5Y-H4K8la_peak_404	6.95057	TMEM105_ENSG00000185332
17	79313277	79313759	5Y-H4K8la_peak_405	8.66743	
17	79315125	79315477	5Y-H4K8la_peak_406	9.04397	
17	79319457	79319708	5Y-H4K8la_peak_407	7.82398	
17	79480102	79480410	5Y-H4K8la_peak_408	11.54753	RP13-766D20.1_ENSG00000229947;RP13-766D20.2_ENSG00000229848
17	79633874	79634121	5Y-H4K8la_peak_409	7.53185	OXLD1_ENSG00000204237;CCDC137_ENSG00000185298
17	79829449	79830231	5Y-H4K8la_peak_410	9.43458	ARHGDIA_ENSG00000141522
17	79924479	79925717	5Y-H4K8la_peak_411	8.99659	RP11-498C9.17_ENSG00000264735
17	79950492	79950826	5Y-H4K8la_peak_412	5.60833	

17	79970587	79970811	5Y-H4K8la_peak_413	10.59072	
17	80023805	80024167	5Y-H4K8la_peak_414	19.91533	DUS1L_ENSG00000169718
17	80057029	80057344	5Y-H4K8la_peak_415	11.84648	FASN_ENSG00000169710
17	80057595	80057813	5Y-H4K8la_peak_416	9.49545	
17	80454802	80455011	5Y-H4K8la_peak_417	6.84763	
17	81010079	81010324	5Y-H4K8la_peak_418	9.24737	B3GNTL1_ENSG00000175711
18	3590534	3590733	5Y-H4K8la_peak_419	6.46211	
18	19180134	19180381	5Y-H4K8la_peak_420	6.12664	ESCO1_ENSG00000141446
18	46308980	46309182	5Y-H4K8la_peak_421	7.34702	
18	61090188	61090389	5Y-H4K8la_peak_422	9.07974	VPS4B_ENSG00000119541
18	61899211	61899442	5Y-H4K8la_peak_423	7.95926	
18	74534964	74535209	5Y-H4K8la_peak_424	5.46366	RP11-162A12.2_ENSG00000264278;ZNF236_ENSG00000130856
18	76828731	76829090	5Y-H4K8la_peak_425	19.32723	ATP9B_ENSG00000166377
19	524327	524664	5Y-H4K8la_peak_426	11.90661	
19	796293	796507	5Y-H4K8la_peak_427	8.34321	PTBP1_ENSG00000011304
19	940436	940736	5Y-H4K8la_peak_428	7.32908	
19	2221726	2221995	5Y-H4K8la_peak_429	8.13463	
19	2307647	2307876	5Y-H4K8la_peak_430	8.87282	LINGO3_ENSG00000220008
19	3441347	3441593	5Y-H4K8la_peak_431	11.22499	
19	3442085	3442329	5Y-H4K8la_peak_432	9.9061	
19	3990649	3990856	5Y-H4K8la_peak_433	9.82869	
19	4374719	4374929	5Y-H4K8la_peak_434	6.72615	
19	4791935	4792149	5Y-H4K8la_peak_435	9.9061	AC005523.3_ENSG00000268536;FEM1A_ENSG00000141965
19	4816223	4816614	5Y-H4K8la_peak_436	6.53263	
19	6739094	6739301	5Y-H4K8la_peak_437	5.67525	
19	8455344	8455543	5Y-H4K8la_peak_438	19.83344	RAB11B-AS1_ENSG00000269386;RAB11B_ENSG00000185236
19	12793037	12793401	5Y-H4K8la_peak_439	6.7474	DHPS_ENSG00000095059
19	13085782	13086259	5Y-H4K8la_peak_440	7.71476	
19	13201661	13201970	5Y-H4K8la_peak_441	6.90369	
19	13203452	13203672	5Y-H4K8la_peak_442	7.82398	
19	16221551	16221843	5Y-H4K8la_peak_443	6.84763	RAB8A_ENSG00000167461
19	17325194	17325542	5Y-H4K8la_peak_444	10.84781	USE1_ENSG00000053501
19	18335719	18335958	5Y-H4K8la_peak_445	6.28883	
19	22018657	22018862	5Y-H4K8la_peak_446	6.25718	
19	22816027	22816271	5Y-H4K8la_peak_447	7.95926	ZNF492_ENSG00000229676
19	32836696	32837014	5Y-H4K8la_peak_448	5.46366	ZNF507_ENSG00000168813
19	32896049	32896300	5Y-H4K8la_peak_449	12.59811	AC007773.2_ENSG00000267213;AC007773.3_ENSG00000269093;DPY19L3_ENSG00000178904
19	33361282	33361491	5Y-H4K8la_peak_450	5.46366	SLC7A9_ENSG00000021488
19	34359852	34360188	5Y-H4K8la_peak_451	5.17488	
19	35599758	35599965	5Y-H4K8la_peak_452	5.41648	
19	35615894	35616098	5Y-H4K8la_peak_453	7.82398	
19	39678362	39678559	5Y-H4K8la_peak_454	6.03474	
19	41081915	41082290	5Y-H4K8la_peak_455	11.09733	SHKBP1_ENSG00000160410
19	42574767	42574982	5Y-H4K8la_peak_456	7.32908	
19	46119675	46119969	5Y-H4K8la_peak_457	7.02081	
19	47134483	47134927	5Y-H4K8la_peak_458	9.46533	

19	47363818	47364030	5Y-H4K8la_peak_459	7.32908	
19	47615048	47615412	5Y-H4K8la_peak_460	9.18087	
19	51871825	51872026	5Y-H4K8la_peak_461	6.62475	CTD-2616J11.11_ENSG00000269403;CLDND2_ENSG00000160318
19	55668354	55668584	5Y-H4K8la_peak_462	8.3533	TNNI3_ENSG00000129991
19	55771236	55771555	5Y-H4K8la_peak_463	10.87965	PPP6R1_ENSG00000105063
19	56145731	56145928	5Y-H4K8la_peak_464	7.02081	ZNF580_ENSG00000213015;ZNF581_ENSG00000171425
19	56146480	56146939	5Y-H4K8la_peak_465	8.13463	ZNF580_ENSG00000213015;ZNF581_ENSG00000171425
19	57702429	57702633	5Y-H4K8la_peak_466	6.53263	ZNF264_ENSG00000083844
19	57922784	57923100	5Y-H4K8la_peak_467	5.41836	ZNF17_ENSG00000186272
19	58258437	58258714	5Y-H4K8la_peak_468	6.97847	ZNF776_ENSG00000152443
19	58897659	58897975	5Y-H4K8la_peak_469	7.32908	RPS5_ENSG00000083845;MIR4754_ENSG00000266640
19	59065868	59066102	5Y-H4K8la_peak_470	6.28883	CHMP2A_ENSG00000130724
2	3105881	3106530	5Y-H4K8la_peak_471	9.96103	
2	3129510	3130584	5Y-H4K8la_peak_472	10.38546	AC019118.2_ENSG00000234423
2	3307012	3307247	5Y-H4K8la_peak_473	10.79855	
2	3317441	3317837	5Y-H4K8la_peak_474	8.47909	
2	3318531	3318731	5Y-H4K8la_peak_475	5.17488	
2	3650931	3651234	5Y-H4K8la_peak_476	7.20129	AC010907.2_ENSG00000237370
2	3697986	3698286	5Y-H4K8la_peak_477	8.6002	
2	5260486	5260689	5Y-H4K8la_peak_478	11.97923	
2	10588815	10589158	5Y-H4K8la_peak_479	8.27752	ODC1_ENSG00000115758;RP11-320M2.1_ENSG00000257135
2	20368666	20368988	5Y-H4K8la_peak_480	6.37554	
2	20732659	20732860	5Y-H4K8la_peak_481	8.04355	
2	25050214	25050436	5Y-H4K8la_peak_482	7.92423	
2	26875707	26875999	5Y-H4K8la_peak_483	8.56836	
2	27294849	27295053	5Y-H4K8la_peak_484	8.04189	OST4_ENSG00000228474
2	29117706	29118131	5Y-H4K8la_peak_485	9.34955	WDR43_ENSG00000163811
2	29338401	29338912	5Y-H4K8la_peak_486	5.24601	
2	29495905	29496176	5Y-H4K8la_peak_487	10.84781	
2	29739187	29739424	5Y-H4K8la_peak_488	7.54283	
2	29804274	29804610	5Y-H4K8la_peak_489	9.68419	
2	36664245	36664476	5Y-H4K8la_peak_490	7.1479	
2	37899042	37899284	5Y-H4K8la_peak_491	9.59502	
2	42720622	42720857	5Y-H4K8la_peak_492	6.4327	KCNG3_ENSG00000171126;MTA3_ENSG00000057935
2	43038262	43038502	5Y-H4K8la_peak_493	5.41836	
2	43195291	43195572	5Y-H4K8la_peak_494	7.82398	
2	43273511	43273732	5Y-H4K8la_peak_495	7.21152	
2	44224104	44224353	5Y-H4K8la_peak_496	7.30826	LRPPRC_ENSG00000138095
2	47290927	47291263	5Y-H4K8la_peak_497	5.60833	
2	47306990	47307442	5Y-H4K8la_peak_498	7.71217	
2	47314292	47314603	5Y-H4K8la_peak_499	10.84781	
2	48339296	48339826	5Y-H4K8la_peak_500	8.76859	
2	48647381	48647622	5Y-H4K8la_peak_501	7.32908	
2	64246357	64246592	5Y-H4K8la_peak_502	6.86977	VPS54_ENSG00000143952
2	69170983	69171527	5Y-H4K8la_peak_503	14.48633	
2	70369171	70369391	5Y-H4K8la_peak_504	10.00392	

2	85645230	85645470	5Y-H4K8la_peak_505	14.21197	CAPG_ENSG00000042493;SH2D6_ENSG00000152292
2	104994899	104995169	5Y-H4K8la_peak_506	6.97847	
2	114341187	114341549	5Y-H4K8la_peak_507	24.94722	MIR1302-3_ENSG00000221055;WASH2P_ENSG00000146556
2	121584454	121584663	5Y-H4K8la_peak_508	6.36955	
2	129207774	129207999	5Y-H4K8la_peak_509	8.23102	
2	174313606	174313956	5Y-H4K8la_peak_510	7.95926	
2	176986567	176986767	5Y-H4K8la_peak_511	7.95919	HOXD9_ENSG00000128709
2	178077652	178077900	5Y-H4K8la_peak_512	6.11939	AC079305.8_ENSG00000229337;HNRNPA3_ENSG00000170144;MIR4444-2_ENSG00000263721
2	182548869	182549137	5Y-H4K8la_peak_513	8.8671	
2	202423405	202423601	5Y-H4K8la_peak_514	7.46931	
2	216176844	216177066	5Y-H4K8la_peak_515	7.32908	ATIC_ENSG00000138363
2	218868697	218868912	5Y-H4K8la_peak_516	6.51244	TNS1_ENSG00000079308
2	219745295	219745522	5Y-H4K8la_peak_517	9.06738	WNT10A_ENSG00000135925
2	219773456	219773668	5Y-H4K8la_peak_518	11.54902	
2	219885816	219886046	5Y-H4K8la_peak_519	6.53263	
2	220375170	220375474	5Y-H4K8la_peak_520	7.83001	
2	220492442	220492748	5Y-H4K8la_peak_521	10.01769	SLC4A3_ENSG00000114923
2	226146945	226147149	5Y-H4K8la_peak_522	6.97847	
2	227655889	227656141	5Y-H4K8la_peak_523	10.01769	
2	232253238	232253538	5Y-H4K8la_peak_524	10.44872	AC017104.6_ENSG00000224376
2	232260352	232260714	5Y-H4K8la_peak_525	8.01893	B3GNT7_ENSG00000156966
2	232478377	232479099	5Y-H4K8la_peak_526	10.62464	
2	232545252	232545712	5Y-H4K8la_peak_527	6.50125	
2	232546189	232546491	5Y-H4K8la_peak_528	4.99806	
2	233415494	233415696	5Y-H4K8la_peak_529	8.5108	TIGD1_ENSG00000221944;EIF4E2_ENSG00000135930;MIR5001_ENSG00000266620
2	238383714	238383949	5Y-H4K8la_peak_530	12.89902	
2	238794082	238795033	5Y-H4K8la_peak_531	10.86735	
2	241508103	241508360	5Y-H4K8la_peak_532	5.15153	ANKMY1_ENSG00000144504
2	242744324	242744564	5Y-H4K8la_peak_533	8.9585	
2	242752128	242752329	5Y-H4K8la_peak_534	8.84365	AC114730.3_ENSG00000224272
2	242823353	242823563	5Y-H4K8la_peak_535	5.29825	AC131097.3_ENSG00000233806
20	825767	826190	5Y-H4K8la_peak_536	5.44443	
20	2820607	2821144	5Y-H4K8la_peak_537	12.6484	PCED1A_ENSG00000132635;VPS16_ENSG00000215305
20	30225546	30226114	5Y-H4K8la_peak_538	13.02594	COX4I2_ENSG00000131055
20	30499081	30499430	5Y-H4K8la_peak_539	6.60529	
20	32599591	32599829	5Y-H4K8la_peak_540	6.03474	
20	34560797	34561022	5Y-H4K8la_peak_541	6.83084	
20	34823794	34824064	5Y-H4K8la_peak_542	7.15878	AAR2_ENSG00000131043
20	36024037	36024564	5Y-H4K8la_peak_543	9.56697	
20	36226413	36226611	5Y-H4K8la_peak_544	7.43825	
20	37075489	37075716	5Y-H4K8la_peak_545	9.46366	SNHG11_ENSG00000174365
20	37502689	37502915	5Y-H4K8la_peak_546	5.02936	
20	37504403	37504820	5Y-H4K8la_peak_547	7.17106	
20	37509759	37509969	5Y-H4K8la_peak_548	6.37554	
20	43159887	43160189	5Y-H4K8la_peak_549	6.6495	PKIG_ENSG00000168734
20	44400110	44400584	5Y-H4K8la_peak_550	15.39737	

20	45985777	45986069	5Y-H4K8la_peak_551	8.66894	ZMYND8_ENSG00000101040
20	48531528	48531783	5Y-H4K8la_peak_552	7.27616	SPATA2_ENSG00000158480
20	49462126	49462383	5Y-H4K8la_peak_553	7.40394	
20	49547088	49547349	5Y-H4K8la_peak_554	9.93066	ADNP_ENSG00000101126;RP5-914P20.5_ENSG00000259456
20	55965495	55965934	5Y-H4K8la_peak_555	9.11759	RBM38_ENSG00000132819
20	56595422	56595660	5Y-H4K8la_peak_556	10.40411	
20	56623048	56623249	5Y-H4K8la_peak_557	9.69532	
20	57226380	57226596	5Y-H4K8la_peak_558	8.23102	STX16_ENSG00000124222;STX16-NPEPL1_ENSG00000254995
20	62085704	62086403	5Y-H4K8la_peak_559	8.3797	
20	62086951	62087243	5Y-H4K8la_peak_560	8.20071	
20	62088806	62089126	5Y-H4K8la_peak_561	6.78126	
20	62089564	62090084	5Y-H4K8la_peak_562	7.24062	
20	62257768	62258003	5Y-H4K8la_peak_563	5.76537	GMEB2_ENSG00000101216;CTD-3184A7.4_ENSG00000232442
20	62673699	62674021	5Y-H4K8la_peak_564	6.11939	
21	10602380	10602611	5Y-H4K8la_peak_565	6.3274	
21	11157246	11157470	5Y-H4K8la_peak_566	7.08077	
21	30391328	30391544	5Y-H4K8la_peak_567	8.97426	RWDD2B_ENSG00000156253
21	30391896	30392243	5Y-H4K8la_peak_568	8.97426	RWDD2B_ENSG00000156253
21	43373441	43373644	5Y-H4K8la_peak_569	8.31121	C2CD2_ENSG00000157617
21	43933372	43933676	5Y-H4K8la_peak_570	8.2602	
21	44393769	44394231	5Y-H4K8la_peak_571	9.33999	PKNOX1_ENSG00000160199
21	44847436	44847934	5Y-H4K8la_peak_572	10.37149	SIK1_ENSG00000142178
21	44870776	44871032	5Y-H4K8la_peak_573	8.6002	
21	45285235	45285656	5Y-H4K8la_peak_574	8.03849	AGPAT3_ENSG00000160216
21	46676599	46676795	5Y-H4K8la_peak_575	8.54502	
21	47062166	47062457	5Y-H4K8la_peak_576	7.79008	
22	17680013	17680252	5Y-H4K8la_peak_577	9.46366	
22	19158748	19158967	5Y-H4K8la_peak_578	8.8671	AC004463.6_ENSG00000260924
22	19159528	19159733	5Y-H4K8la_peak_579	8.9585	AC004463.6_ENSG00000260924
22	20066729	20066962	5Y-H4K8la_peak_580	9.54093	DGCR8_ENSG00000128191
22	20747803	20748112	5Y-H4K8la_peak_581	13.57341	ZNF74_ENSG00000185252
22	21922093	21922305	5Y-H4K8la_peak_582	9.9061	
22	21983910	21984124	5Y-H4K8la_peak_583	10.00392	YDJC_ENSG00000161179
22	23745209	23745919	5Y-H4K8la_peak_584	14.21197	ZDHHC8P1_ENSG00000133519
22	24191540	24191788	5Y-H4K8la_peak_585	8.99659	
22	24667232	24667480	5Y-H4K8la_peak_586	8.14665	SPECC1L_ENSG00000100014;SPECC1L-ADORA2A_ENSG00000258555
22	24802252	24802505	5Y-H4K8la_peak_587	5.76537	
22	27654863	27655126	5Y-H4K8la_peak_588	6.11939	
22	29784179	29784400	5Y-H4K8la_peak_589	5.73215	
22	35863731	35864128	5Y-H4K8la_peak_590	12.61503	
22	36433277	36433649	5Y-H4K8la_peak_591	6.88861	
22	36878499	36878814	5Y-H4K8la_peak_592	6.11939	TXN2_ENSG00000100348
22	37562845	37563052	5Y-H4K8la_peak_593	10.35984	RP1-151B14.6_ENSG00000235237
22	44319950	44320163	5Y-H4K8la_peak_594	6.84763	PNPLA3_ENSG00000100344
22	45124974	45125208	5Y-H4K8la_peak_595	5.92398	
22	45125829	45126252	5Y-H4K8la_peak_596	6.9434	

22	46466113	46466319	5Y-H4K8la_peak_597	9.73458	RP6-109B7.4_ENSG00000235159
22	46646429	46646639	5Y-H4K8la_peak_598	8.65079	CDPF1_ENSG00000205643
22	46972441	46972677	5Y-H4K8la_peak_599	8.69005	GRAMD4_ENSG00000075240
22	47016604	47016827	5Y-H4K8la_peak_600	8.66894	
22	49547368	49547583	5Y-H4K8la_peak_601	7.6269	
22	50707734	50708294	5Y-H4K8la_peak_602	7.02081	MAPK11_ENSG00000185386
22	50709372	50709650	5Y-H4K8la_peak_603	7.98787	MAPK11_ENSG00000185386
22	50746470	50746758	5Y-H4K8la_peak_604	7.32908	PLXNB2_ENSG00000196576
22	50875034	50875295	5Y-H4K8la_peak_605	6.97847	
3	4344508	4344720	5Y-H4K8la_peak_606	5.13147	SETMAR_ENSG00000170364
3	4546916	4547218	5Y-H4K8la_peak_607	6.9507	
3	8665004	8665210	5Y-H4K8la_peak_608	7.32908	
3	9932508	9932774	5Y-H4K8la_peak_609	8.9585	JAGN1_ENSG00000171135
3	13462061	13462297	5Y-H4K8la_peak_610	5.80897	NUP210_ENSG00000132182
3	16646780	16646986	5Y-H4K8la_peak_611	6.03474	
3	24563141	24563487	5Y-H4K8la_peak_612	7.95926	MIR4792_ENSG00000265028
3	32858224	32858438	5Y-H4K8la_peak_613	5.81161	
3	42922103	42922499	5Y-H4K8la_peak_614	10.77389	
3	49203997	49204489	5Y-H4K8la_peak_615	7.20129	CCDC71_ENSG00000177352
3	50424665	50425180	5Y-H4K8la_peak_616	11.69305	
3	50465072	50465395	5Y-H4K8la_peak_617	5.41836	
3	52064557	52064806	5Y-H4K8la_peak_618	8.36909	
3	52102810	52103084	5Y-H4K8la_peak_619	6.11939	
3	52345906	52346206	5Y-H4K8la_peak_620	11.87772	
3	56716790	56717054	5Y-H4K8la_peak_621	5.32615	FAM208A_ENSG00000163946
3	73318214	73318433	5Y-H4K8la_peak_622	7.57826	
3	75057460	75057714	5Y-H4K8la_peak_623	5.81948	
3	115377519	115377806	5Y-H4K8la_peak_624	7.35213	RP11-326J18.1_ENSG00000241596
3	119013417	119013662	5Y-H4K8la_peak_625	7.46931	ARHGAP31_ENSG00000031081
3	119053062	119053267	5Y-H4K8la_peak_626	5.02936	
3	119378940	119379197	5Y-H4K8la_peak_627	10.47134	
3	126046177	126046395	5Y-H4K8la_peak_628	6.97847	
3	127416867	127417073	5Y-H4K8la_peak_629	8.2602	
3	128126623	128126907	5Y-H4K8la_peak_630	9.53858	
3	128128543	128129018	5Y-H4K8la_peak_631	10.51622	
3	128129789	128130099	5Y-H4K8la_peak_632	10.41836	
3	128131225	128131610	5Y-H4K8la_peak_633	8.42533	
3	128173276	128173480	5Y-H4K8la_peak_634	6.49152	
3	128175652	128175875	5Y-H4K8la_peak_635	9.18087	
3	128176370	128176605	5Y-H4K8la_peak_636	9.35387	
3	128176849	128177075	5Y-H4K8la_peak_637	6.84763	
3	128216043	128216398	5Y-H4K8la_peak_638	5.02936	
3	139062157	139062621	5Y-H4K8la_peak_639	8.03849	
3	193988457	193988885	5Y-H4K8la_peak_640	7.46931	
3	194901368	194901622	5Y-H4K8la_peak_641	6.62475	
3	195636962	195637181	5Y-H4K8la_peak_642	8.34321	

4	52100	52451	5Y-H4K8la_peak_643	10.596	ZNF595_ENSG00000197701
4	468418	468651	5Y-H4K8la_peak_644	10.021	ABCA11P_ENSG00000251595
4	967589	968016	5Y-H4K8la_peak_645	8.03849	
4	1685610	1685838	5Y-H4K8la_peak_646	5.56085	FAM53A_ENSG00000174137;Y_RNA_ENSG00000207009
4	1722228	1722438	5Y-H4K8la_peak_647	6.36955	TMEM129_ENSG00000168936;TACC3_ENSG00000013810
4	8442740	8442964	5Y-H4K8la_peak_648	7.46931	ACOX3_ENSG00000087008
4	9382719	9382979	5Y-H4K8la_peak_649	9.46366	
4	39182176	39182373	5Y-H4K8la_peak_650	7.83962	
4	40058604	40058812	5Y-H4K8la_peak_651	10.87965	RP11-333E13.4_ENSG00000205794;N4BP2_ENSG00000078177
4	41883637	41884230	5Y-H4K8la_peak_652	14.36156	LINC00682_ENSG00000245870
4	83294412	83294771	5Y-H4K8la_peak_653	5.5367	HNRNPD_ENSG00000138668;RP11-127B20.3_ENSG00000272677
4	148652823	148653041	5Y-H4K8la_peak_654	6.84763	ARHGAP10_ENSG00000071205
4	174452733	174452982	5Y-H4K8la_peak_655	7.61397	
5	34191636	34191942	5Y-H4K8la_peak_656	10.85054	
5	34192404	34193045	5Y-H4K8la_peak_657	17.31746	
5	50678656	50678940	5Y-H4K8la_peak_658	6.03474	CTD-2314G24.2_ENSG00000259663;ISL1_ENSG00000016082
5	67830349	67830623	5Y-H4K8la_peak_659	6.28883	CTC-537E7.2_ENSG00000248359
5	90104573	90104818	5Y-H4K8la_peak_660	5.5367	
5	90648118	90648434	5Y-H4K8la_peak_661	7.79008	
5	94955849	94956133	5Y-H4K8la_peak_662	5.46366	GPR150_ENSG00000178015
5	126113545	126113822	5Y-H4K8la_peak_663	5.76537	
5	130500523	130500753	5Y-H4K8la_peak_664	7.23632	
5	134735121	134735373	5Y-H4K8la_peak_665	7.16957	H2AFY_ENSG00000113648;CTC-203F4.2_ENSG00000270021
5	139089372	139089862	5Y-H4K8la_peak_666	8.86211	
5	141062133	141062332	5Y-H4K8la_peak_667	7.96941	ARAP3_ENSG00000120318
5	151150495	151150721	5Y-H4K8la_peak_668	6.30734	G3BP1_ENSG00000145907
5	174220326	174220528	5Y-H4K8la_peak_669	6.97847	
5	176560147	176560462	5Y-H4K8la_peak_670	8.79833	NSD1_ENSG00000165671
5	176900223	176900442	5Y-H4K8la_peak_671	6.11939	DBN1_ENSG00000113758
5	177775519	177775811	5Y-H4K8la_peak_672	8.15295	
5	178737073	178737309	5Y-H4K8la_peak_673	8.01893	
5	178752793	178753038	5Y-H4K8la_peak_674	9.66663	
5	179051313	179051511	5Y-H4K8la_peak_675	6.11154	
6	291691	292001	5Y-H4K8la_peak_676	5.82532	DUSP22_ENSG00000112679
6	22557824	22558043	5Y-H4K8la_peak_677	7.1479	
6	30685868	30686090	5Y-H4K8la_peak_678	8.54502	MDC1_ENSG00000137337
6	34394350	34394570	5Y-H4K8la_peak_679	7.95926	RPS10-NUDT3_ENSG00000270800;RPS10_ENSG00000124614
6	35465428	35465669	5Y-H4K8la_peak_680	7.92423	TEAD3_ENSG00000007866
6	36098895	36099259	5Y-H4K8la_peak_681	13.03385	
6	40688779	40688989	5Y-H4K8la_peak_682	6.68187	
6	40996037	40996293	5Y-H4K8la_peak_683	12.63314	
6	41040956	41041221	5Y-H4K8la_peak_684	9.8767	NFYA_ENSG00000001167
6	74231371	74231572	5Y-H4K8la_peak_685	7.95926	
6	99275497	99275729	5Y-H4K8la_peak_686	10.8901	
6	110679509	110679841	5Y-H4K8la_peak_687	7.1479	METTL24_ENSG00000053328
6	110864117	110864373	5Y-H4K8la_peak_688	6.97847	

6	137365687	137365891	5Y-H4K8la_peak_689	8.80583	IL20RA_ENSG00000016402
6	143832511	143832748	5Y-H4K8la_peak_690	7.32908	FUCA2_ENSG00000001036
6	146864405	146864619	5Y-H4K8la_peak_691	8.15295	RAB32_ENSG00000118508
6	149638878	149639174	5Y-H4K8la_peak_692	7.95926	
6	158243698	158244118	5Y-H4K8la_peak_693	13.09444	SNX9_ENSG00000130340
6	167032986	167033189	5Y-H4K8la_peak_694	9.38089	
6	167441588	167441797	5Y-H4K8la_peak_695	5.46366	
6	168216200	168216402	5Y-H4K8la_peak_696	6.03474	
6	170102508	170102726	5Y-H4K8la_peak_697	9.25671	WDR27_ENSG00000184465;C6orf120_ENSG00000185127
6	170124908	170125256	5Y-H4K8la_peak_698	10.87965	PHF10_ENSG00000130024;RP1-266L20.2_ENSG00000232640;RP1-266L20.4_ENSG00000227704
7	119224	119465	5Y-H4K8la_peak_699	7.32908	
7	149847	150646	5Y-H4K8la_peak_700	9.43441	AC093627.9_ENSG00000242474;AC093627.10_ENSG00000240859
7	751918	752150	5Y-H4K8la_peak_701	10.77389	
7	1513522	1513733	5Y-H4K8la_peak_702	8.76859	
7	1625862	1626089	5Y-H4K8la_peak_703	10.89943	
7	1682012	1682211	5Y-H4K8la_peak_704	5.31882	
7	1687291	1687841	5Y-H4K8la_peak_705	5.82532	
7	2491800	2491999	5Y-H4K8la_peak_706	5.55047	
7	3422737	3422992	5Y-H4K8la_peak_707	8.58413	
7	4183358	4183590	5Y-H4K8la_peak_708	6.50125	
7	5229200	5229550	5Y-H4K8la_peak_709	6.84763	WIPI2_ENSG00000157954
7	5569802	5570012	5Y-H4K8la_peak_710	10.89943	
7	5595793	5596056	5Y-H4K8la_peak_711	7.92423	CTB-161C1.1_ENSG00000272719
7	5614678	5615137	5Y-H4K8la_peak_712	7.85758	
7	6413538	6413790	5Y-H4K8la_peak_713	8.56836	RAC1_ENSG00000136238
7	6704044	6704247	5Y-H4K8la_peak_714	10.0197	AC073343.13_ENSG00000228010
7	8301509	8302096	5Y-H4K8la_peak_715	11.76723	ICA1_ENSG00000003147;AC007009.1_ENSG00000244239;AC007128.1_ENSG00000229970
7	11065444	11065645	5Y-H4K8la_peak_716	9.00262	
7	26438468	26438733	5Y-H4K8la_peak_717	9.24465	AC004540.5_ENSG00000214870
7	29287100	29287337	5Y-H4K8la_peak_718	7.26933	
7	30795022	30795223	5Y-H4K8la_peak_719	8.36751	
7	44143417	44143634	5Y-H4K8la_peak_720	8.27752	AEBP1_ENSG00000106624
7	44316044	44316349	5Y-H4K8la_peak_721	8.27752	
7	47576492	47576876	5Y-H4K8la_peak_722	10.79611	
7	47674739	47674938	5Y-H4K8la_peak_723	11.3563	
7	47802339	47802604	5Y-H4K8la_peak_724	8.3797	
7	50518348	50518622	5Y-H4K8la_peak_725	12.68645	FIGNL1_ENSG00000132436
7	50635766	50636073	5Y-H4K8la_peak_726	8.54843	
7	56032460	56032683	5Y-H4K8la_peak_727	7.32908	
7	64699701	64699949	5Y-H4K8la_peak_728	6.95057	
7	66162050	66162250	5Y-H4K8la_peak_729	5.76537	
7	69043149	69043406	5Y-H4K8la_peak_730	6.68187	
7	72843201	72843615	5Y-H4K8la_peak_731	9.43441	
7	73284282	73284494	5Y-H4K8la_peak_732	6.007	
7	73441929	73442148	5Y-H4K8la_peak_733	6.50125	ELN_ENSG00000049540
7	73507524	73507735	5Y-H4K8la_peak_734	7.82398	

7	73588751	73588964	5Y-H4K8la_peak_735	14.21033	EIF4H_ENSG00000106682
7	74072100	74072495	5Y-H4K8la_peak_736	8.27752	GTF2I_ENSG00000077809
7	74306324	74306540	5Y-H4K8la_peak_737	7.32908	STAG3L2_ENSG00000160828;PMS2P5_ENSG00000123965
7	75814573	75814795	5Y-H4K8la_peak_738	9.88272	
7	75923766	75924015	5Y-H4K8la_peak_739	12.62719	
7	75946754	75946991	5Y-H4K8la_peak_740	8.76859	
7	75987711	75987965	5Y-H4K8la_peak_741	5.50596	YWHAG_ENSG00000170027
7	77045805	77046061	5Y-H4K8la_peak_742	10.84781	GSAP_ENSG00000186088;RP5-899E9.1_ENSG00000273341
7	86849628	86849834	5Y-H4K8la_peak_743	8.15295	TMEM243_ENSG00000135185
7	87806101	87806338	5Y-H4K8la_peak_744	8.15295	
7	91195170	91195366	5Y-H4K8la_peak_745	5.02936	
7	94285191	94285392	5Y-H4K8la_peak_746	9.9061	SGCE_ENSG00000127990;PEG10_ENSG00000242265
7	98246076	98246313	5Y-H4K8la_peak_747	12.20171	NPTX2_ENSG00000106236
7	99177564	99177854	5Y-H4K8la_peak_748	5.41836	
7	99213715	99213926	5Y-H4K8la_peak_749	12.008	ZSCAN25_ENSG00000197037
7	99698650	99698896	5Y-H4K8la_peak_750	10.12311	MCM7_ENSG00000166508;AP4M1_ENSG00000221838
7	99725510	99725725	5Y-H4K8la_peak_751	5.26481	RP11-506M12.1_ENSG00000242798
7	100200135	100200399	5Y-H4K8la_peak_752	8.3797	PCOLCE_ENSG00000106333
7	100208892	100209376	5Y-H4K8la_peak_753	5.76537	MOSPD3_ENSG00000106330
7	100471697	100472144	5Y-H4K8la_peak_754	11.5543	SRRT_ENSG00000087087
7	100488504	100488811	5Y-H4K8la_peak_755	6.24256	
7	100796982	100797179	5Y-H4K8la_peak_756	7.5782	AP1S1_ENSG00000106367
7	100824100	100824298	5Y-H4K8la_peak_757	6.4137	NAT16_ENSG00000167011
7	100836145	100836543	5Y-H4K8la_peak_758	6.34953	
7	112430585	112430840	5Y-H4K8la_peak_759	7.99376	TMEM168_ENSG00000146802
7	123673375	123673603	5Y-H4K8la_peak_760	7.92423	TMEM229A_ENSG00000234224
7	127291024	127291223	5Y-H4K8la_peak_761	9.31722	AC073934.6_ENSG00000240790
7	127905329	127905555	5Y-H4K8la_peak_762	9.20868	RP11-62J1.4_ENSG00000272915
7	128001327	128001982	5Y-H4K8la_peak_763	17.12679	PRRT4_ENSG00000224940
7	128019104	128019349	5Y-H4K8la_peak_764	9.59502	
7	128020741	128021232	5Y-H4K8la_peak_765	10.61974	
7	131242594	131242876	5Y-H4K8la_peak_766	7.02042	PODXL_ENSG00000128567
7	133640548	133640861	5Y-H4K8la_peak_767	7.32908	
7	139691776	139691992	5Y-H4K8la_peak_768	7.32908	
7	149118910	149119452	5Y-H4K8la_peak_769	11.83122	RP11-143I21.1_ENSG00000261842
7	150948584	150948788	5Y-H4K8la_peak_770	8.23102	
7	155547005	155547239	5Y-H4K8la_peak_771	7.79699	
7	156432970	156433167	5Y-H4K8la_peak_772	11.26428	LINC01006_ENSG00000182648;C7orf13_ENSG00000244291;RNF32_ENSG00000105982
7	157903517	157903792	5Y-H4K8la_peak_773	11.83122	
8	11141448	11141691	5Y-H4K8la_peak_774	6.97847	MTMR9_ENSG00000104643
8	11471019	11471537	5Y-H4K8la_peak_775	10.77389	
8	21915168	21915404	5Y-H4K8la_peak_776	7.15878	
8	22069356	22069569	5Y-H4K8la_peak_777	7.42863	
8	22089232	22089436	5Y-H4K8la_peak_778	9.24465	PHYHIP_ENSG00000168490
8	22297987	22298224	5Y-H4K8la_peak_779	9.35159	PPP3CC_ENSG00000120910
8	22853240	22853494	5Y-H4K8la_peak_780	6.58555	

8	29230419	29231033	5Y-H4K8la_peak_781	7.31397	
8	48920368	48920741	5Y-H4K8la_peak_782	12.49539	UBE2V2_ENSG00000169139
8	72519367	72519584	5Y-H4K8la_peak_783	6.28883	
8	74005524	74005720	5Y-H4K8la_peak_784	10.01769	
8	80215818	80216056	5Y-H4K8la_peak_785	10.021	
8	80739845	80740070	5Y-H4K8la_peak_786	7.39065	
8	99306237	99306435	5Y-H4K8la_peak_787	6.97847	NIPAL2_ENSG00000104361
8	101942426	101942634	5Y-H4K8la_peak_788	6.03474	
8	142412416	142412617	5Y-H4K8la_peak_789	6.53263	
8	143864253	143864625	5Y-H4K8la_peak_790	7.32908	
8	144333238	144333456	5Y-H4K8la_peak_791	6.03474	
8	144427437	144427643	5Y-H4K8la_peak_792	9.12339	
8	144718347	144718669	5Y-H4K8la_peak_793	7.56321	ZNF623_ENSG00000183309
8	145330343	145330553	5Y-H4K8la_peak_794	7.95926	KM-PA-2_ENSG00000204775
8	145597048	145597284	5Y-H4K8la_peak_795	7.31397	ADCK5_ENSG00000173137
8	146053096	146053333	5Y-H4K8la_peak_796	9.9061	ZNF7_ENSG00000147789
9	6412446	6412819	5Y-H4K8la_peak_797	7.95926	UHRF2_ENSG00000147854
9	14314916	14315262	5Y-H4K8la_peak_798	10.57306	
9	14693499	14693718	5Y-H4K8la_peak_799	7.32908	ZDHHC21_ENSG00000175893
9	33817515	33817775	5Y-H4K8la_peak_800	10.77389	UBE2R2_ENSG00000107341
9	35675994	35676336	5Y-H4K8la_peak_801	6.84763	ARHGEF39_ENSG00000137135
9	38233041	38233261	5Y-H4K8la_peak_802	11.12382	
9	66458093	66458373	5Y-H4K8la_peak_803	8.10903	RP11-262H14.1_ENSG00000238113;RNA5SP283_ENSG00000202474
9	92716289	92716633	5Y-H4K8la_peak_804	7.1479	
9	94698083	94698280	5Y-H4K8la_peak_805	6.53263	
9	99179983	99180489	5Y-H4K8la_peak_806	10.2251	ZNF367_ENSG00000165244
9	100744944	100745249	5Y-H4K8la_peak_807	13.13483	ANP32B_ENSG00000136938
9	100746200	100746433	5Y-H4K8la_peak_808	9.8767	ANP32B_ENSG00000136938
9	127082351	127082576	5Y-H4K8la_peak_809	8.3797	
9	127239431	127239754	5Y-H4K8la_peak_810	12.61503	
9	127275211	127275456	5Y-H4K8la_peak_811	10.01769	
9	129270482	129270690	5Y-H4K8la_peak_812	7.60153	
9	129284077	129284849	5Y-H4K8la_peak_813	9.00262	
9	129293909	129294166	5Y-H4K8la_peak_814	6.53263	AL356309.1_ENSG00000221768
9	129295199	129295428	5Y-H4K8la_peak_815	10.77389	
9	129485057	129485446	5Y-H4K8la_peak_816	13.5242	
9	130185836	130186043	5Y-H4K8la_peak_817	7.50839	ZNF79_ENSG00000196152
9	132082582	132082800	5Y-H4K8la_peak_818	11.4564	C9orf106_ENSG00000179082
9	132222060	132222390	5Y-H4K8la_peak_819	7.79008	
9	132411864	132412233	5Y-H4K8la_peak_820	6.51199	
9	132460137	132460381	5Y-H4K8la_peak_821	8.15295	
9	133455184	133455383	5Y-H4K8la_peak_822	9.82869	FUBP3_ENSG00000107164
9	134153777	134154088	5Y-H4K8la_peak_823	10.17199	
9	134406903	134407187	5Y-H4K8la_peak_824	10.44872	UCK1_ENSG00000130717
9	136379847	136380098	5Y-H4K8la_peak_825	15.1968	
9	136380456	136380958	5Y-H4K8la_peak_826	6.4137	

9	136499475	136499737	5Y-H4K8la_peak_827	9.51625	
9	136500641	136500887	5Y-H4K8la_peak_828	11.0515	DBH_ENSG00000123454
9	136548090	136548527	5Y-H4K8la_peak_829	10.49857	
9	137028568	137028871	5Y-H4K8la_peak_830	10.44337	RNU6ATAC_ENSG00000221676
9	137347588	137348072	5Y-H4K8la_peak_831	11.54902	
9	137599742	137599988	5Y-H4K8la_peak_832	7.1479	
9	138884356	138884558	5Y-H4K8la_peak_833	9.93134	
9	139221274	139221494	5Y-H4K8la_peak_834	4.99806	GPSM1_ENSG00000160360
9	139560703	139560945	5Y-H4K8la_peak_835	6.9161	
9	139844754	139845384	5Y-H4K8la_peak_836	14.80859	LCN12_ENSG00000184925
9	139948803	139949060	5Y-H4K8la_peak_837	8.32039	ENTPD2_ENSG00000054179
9	139965866	139966186	5Y-H4K8la_peak_838	7.32908	SAPCD2_ENSG00000186193
9	139971350	139971578	5Y-H4K8la_peak_839	5.82532	UAP1L1_ENSG00000197355
9	140317255	140317525	5Y-H4K8la_peak_840	10.00392	EXD3_ENSG00000187609;NOXA1_ENSG00000188747
9	140771763	140771997	5Y-H4K8la_peak_841	8.3797	CACNA1B_ENSG00000148408
GL(	30187	30483	5Y-H4K8la_peak_842	4.96385	
GL(	111035	111255	5Y-H4K8la_peak_843	7.5579	
GL(	111534	111833	5Y-H4K8la_peak_844	6.33529	
GL(	135086	135303	5Y-H4K8la_peak_845	8.27951	
GL(	47559	47961	5Y-H4K8la_peak_846	6.97847	
GL(	99985	100352	5Y-H4K8la_peak_847	16.22176	
GL(	137550	137748	5Y-H4K8la_peak_848	17.50283	
GL(	145261	145508	5Y-H4K8la_peak_849	17.0743	
X	2418336	2418562	5Y-H4K8la_peak_850	8.9585	ZBED1_ENSG00000214717
X	9982662	9982863	5Y-H4K8la_peak_851	6.97847	WWC3_ENSG00000047644
X	115004625	115005182	5Y-H4K8la_peak_852	27.69216	
