# Supplementary material for: SIRT2 functions as a histone delactylase and inhibits the proliferation and migration of neuroblastoma cells
Source: Cell Discov. 2022 Jun 7;8:54. doi: 10.1038/s41421-022-00398-y (PMC9174446; doi:10.1038/s41421-022-00398-y)
Supplement: Supplementary file 4 — Supplementary Table S4 [file 41421_2022_398_MOESM4_ESM.pdf]

Table S4. ChIP-seq peaks and annotated genes by anti-H4K8ac antibody in control SH-SY5Y cells.

chr	start	end	peakid	-LOG(pvalue)	gene
1	713436	713628	5Y-H4K8ac_peak_1	8.16382	RP11-206L10.2_ENSG00000228327;RP11-206L10.9_ENSG00000237491
1	762556	763195	5Y-H4K8ac_peak_2	7.34185	LINC00115_ENSG00000225880;LINC01128_ENSG00000228794
1	839340	839886	5Y-H4K8ac_peak_3	6.77436	RP11-54O7.16_ENSG00000272438
1	853731	853935	5Y-H4K8ac_peak_4	5.92809	
1	875280	875501	5Y-H4K8ac_peak_5	6.10343	
1	877612	878346	5Y-H4K8ac_peak_6	9.64915	
1	896077	896542	5Y-H4K8ac_peak_7	8.14913	KLHL17_ENSG00000187961
1	911336	911557	5Y-H4K8ac_peak_8	4.36976	
1	935903	936103	5Y-H4K8ac_peak_9	8.46442	HES4_ENSG00000188290
1	936995	937243	5Y-H4K8ac_peak_10	8.78946	
1	949144	949536	5Y-H4K8ac_peak_11	5.39991	RP11-54O7.11_ENSG00000224969;ISG15_ENSG00000187608
1	994829	995171	5Y-H4K8ac_peak_12	5.56894	RP11-465B22.3_ENSG00000217801
1	1079541	1080571	5Y-H4K8ac_peak_13	9.78792	
1	1092995	1093365	5Y-H4K8ac_peak_14	9.79526	
1	1143726	1144076	5Y-H4K8ac_peak_15	8.47164	
1	1144555	1144805	5Y-H4K8ac_peak_16	5.12213	
1	1166987	1167299	5Y-H4K8ac_peak_17	9.30505	SDF4_ENSG00000078808;B3GALT6_ENSG00000176022
1	1167763	1168116	5Y-H4K8ac_peak_18	12.26606	SDF4_ENSG00000078808;B3GALT6_ENSG00000176022
1	1176444	1176663	5Y-H4K8ac_peak_19	6.50117	
1	1208894	1209167	5Y-H4K8ac_peak_20	4.29586	UBE2J2_ENSG00000160087
1	1209459	1210209	5Y-H4K8ac_peak_21	20.81622	UBE2J2_ENSG00000160087;RP5-902P8.10_ENSG00000230415
1	1239838	1240110	5Y-H4K8ac_peak_22	8.98784	
1	1240323	1241357	5Y-H4K8ac_peak_23	17.1861	
1	1243538	1243803	5Y-H4K8ac_peak_24	15.82359	PUSL1_ENSG00000169972
1	1295087	1295780	5Y-H4K8ac_peak_25	13.71233	
1	1310185	1310519	5Y-H4K8ac_peak_26	13.72401	AURKAIP1_ENSG00000175756
1	1311255	1311880	5Y-H4K8ac_peak_27	9.7353	AURKAIP1_ENSG00000175756
1	1334967	1335192	5Y-H4K8ac_peak_28	7.03573	CCNL2_ENSG00000221978;RP4-758J18.2_ENSG00000224870
1	1341865	1342578	5Y-H4K8ac_peak_29	7.89273	RN7SL657P_ENSG00000264293;MRPL20_ENSG00000242485
1	1342947	1343251	5Y-H4K8ac_peak_30	7.63144	MRPL20_ENSG00000242485
1	1354452	1354851	5Y-H4K8ac_peak_31	6.73047	RP4-758J18.7_ENSG00000225905
1	1355767	1355958	5Y-H4K8ac_peak_32	7.49746	RP4-758J18.7_ENSG00000225905
1	1362962	1363247	5Y-H4K8ac_peak_33	12.21176	
1	1367370	1367635	5Y-H4K8ac_peak_34	5.79005	
1	1397319	1397981	5Y-H4K8ac_peak_35	9.31608	
1	1446873	1447308	5Y-H4K8ac_peak_36	7.61515	ATAD3A_ENSG00000197785
1	1475626	1475825	5Y-H4K8ac_peak_37	7.24844	TMEM240_ENSG00000205090
1	1476180	1476384	5Y-H4K8ac_peak_38	7.59101	TMEM240_ENSG00000205090
1	1509652	1510180	5Y-H4K8ac_peak_39	9.58986	SSU72_ENSG00000160075;AL645728.1_ENSG00000215014
1	1534751	1534970	5Y-H4K8ac_peak_40	8.21582	C1orf233_ENSG00000228594
1	1590496	1590704	5Y-H4K8ac_peak_41	5.64909	CDK11B_ENSG00000248333;RP11-345P4.10_ENSG00000272004
1	1623766	1624326	5Y-H4K8ac_peak_42	12.71156	SLC35E2B_ENSG00000189339
1	1709492	1709841	5Y-H4K8ac_peak_43	5.9856	
1	1710450	1710711	5Y-H4K8ac_peak_44	11.71078	

1	1821861	1822413	5Y-H4K8ac_peak_45	9.58986	GNB1_ENSG00000078369;RP1-140A9.1_ENSG00000231050
1	1840865	1841064	5Y-H4K8ac_peak_46	4.1776	
1	1851203	1851488	5Y-H4K8ac_peak_47	9.48719	TMEM52_ENSG00000178821
1	1875023	1875509	5Y-H4K8ac_peak_48	9.02782	
1	1963786	1964848	5Y-H4K8ac_peak_49	10.4678	
1	1976035	1976390	5Y-H4K8ac_peak_50	9.89244	
1	1981130	1981511	5Y-H4K8ac_peak_51	6.64195	RP11-547D24.3_ENSG00000226969;PRKCZ_ENSG00000067606
1	2058331	2058562	5Y-H4K8ac_peak_52	7.31815	
1	2126347	2126543	5Y-H4K8ac_peak_53	7.60057	
1	2130403	2130622	5Y-H4K8ac_peak_54	11.09973	
1	2136841	2137321	5Y-H4K8ac_peak_55	5.87725	
1	2146000	2146297	5Y-H4K8ac_peak_56	6.50117	AL590822.1_ENSG00000203301
1	2158219	2158620	5Y-H4K8ac_peak_57	8.47129	
1	2201855	2202154	5Y-H4K8ac_peak_58	5.37237	
1	2245657	2246603	5Y-H4K8ac_peak_59	22.45371	
1	2246818	2247703	5Y-H4K8ac_peak_60	9.51254	
1	2257906	2258480	5Y-H4K8ac_peak_61	6.78128	RP4-713A8.1_ENSG00000272161;AL589739.1_ENSG00000269753
1	2343476	2343908	5Y-H4K8ac_peak_62	4.642	
1	2344316	2344658	5Y-H4K8ac_peak_63	5.89922	PEX10_ENSG00000157911
1	2399565	2399760	5Y-H4K8ac_peak_64	4.77126	
1	2401677	2402378	5Y-H4K8ac_peak_65	5.87725	
1	2404005	2404364	5Y-H4K8ac_peak_66	4.95697	
1	2405392	2405908	5Y-H4K8ac_peak_67	7.8267	
1	2406150	2407212	5Y-H4K8ac_peak_68	9.19793	
1	2411360	2411595	5Y-H4K8ac_peak_69	9.93116	
1	2411979	2412242	5Y-H4K8ac_peak_70	7.09658	
1	2412778	2413251	5Y-H4K8ac_peak_71	9.79526	
1	2413678	2414401	5Y-H4K8ac_peak_72	17.04999	
1	2473877	2474107	5Y-H4K8ac_peak_73	7.11863	
1	2474366	2476673	5Y-H4K8ac_peak_74	8.09118	
1	2477235	2477470	5Y-H4K8ac_peak_75	6.71016	RP3-395M20.12_ENSG00000272449
1	2517194	2517626	5Y-H4K8ac_peak_76	7.28311	FAM213B_ENSG00000157870
1	2543143	2543341	5Y-H4K8ac_peak_77	5.91107	
1	2574211	2574424	5Y-H4K8ac_peak_78	5.41472	
1	3073262	3073510	5Y-H4K8ac_peak_79	6.08523	
1	3086228	3086537	5Y-H4K8ac_peak_80	12.34314	
1	3086799	3087780	5Y-H4K8ac_peak_81	12.06963	
1	3375020	3375235	5Y-H4K8ac_peak_82	9.44864	
1	3375983	3376173	5Y-H4K8ac_peak_83	5.23083	
1	3419409	3419684	5Y-H4K8ac_peak_84	8.79957	
1	3433023	3434294	5Y-H4K8ac_peak_85	17.66193	
1	3462169	3462390	5Y-H4K8ac_peak_86	7.38046	
1	3489715	3490091	5Y-H4K8ac_peak_87	5.98695	
1	3490633	3490940	5Y-H4K8ac_peak_88	6.08523	
1	3497688	3497891	5Y-H4K8ac_peak_89	7.30348	
1	3515672	3515967	5Y-H4K8ac_peak_90	8.69112	

1	3566253	3566494	5Y-H4K8ac_peak_91	7.11863	
1	3568213	3568885	5Y-H4K8ac_peak_92	8.73985	WRAP73_ENSG00000116213;TP73_ENSG00000078900
1	3628890	3629089	5Y-H4K8ac_peak_93	6.98394	
1	3629443	3630021	5Y-H4K8ac_peak_94	7.89273	RP5-1092A11.2_ENSG00000235131
1	3630268	3630509	5Y-H4K8ac_peak_95	6.54441	RP5-1092A11.2_ENSG00000235131
1	3632478	3633451	5Y-H4K8ac_peak_96	5.64909	
1	3634163	3634580	5Y-H4K8ac_peak_97	10.58233	
1	3635199	3635432	5Y-H4K8ac_peak_98	8.43511	
1	3636213	3636436	5Y-H4K8ac_peak_99	9.18169	
1	3636762	3637078	5Y-H4K8ac_peak_100	13.00733	
1	3663346	3663864	5Y-H4K8ac_peak_101	7.7384	TP73-AS1_ENSG00000227372
1	3688717	3688957	5Y-H4K8ac_peak_102	8.69112	SMIM1_ENSG00000235169
1	3691568	3692518	5Y-H4K8ac_peak_103	5.24695	
1	3692840	3693120	5Y-H4K8ac_peak_104	6.60109	
1	3736187	3736513	5Y-H4K8ac_peak_105	3.95366	
1	3738137	3738381	5Y-H4K8ac_peak_106	12.26606	
1	3816205	3816774	5Y-H4K8ac_peak_107	10.74739	C1orf174_ENSG00000198912;LINC01134_ENSG00000236423
1	3817560	3817822	5Y-H4K8ac_peak_108	11.1169	C1orf174_ENSG00000198912;LINC01134_ENSG00000236423
1	3819782	3820095	5Y-H4K8ac_peak_109	5.87382	
1	4672108	4672793	5Y-H4K8ac_peak_110	5.98695	
1	4674037	4674443	5Y-H4K8ac_peak_111	5.48291	
1	5217703	5218172	5Y-H4K8ac_peak_112	9.26157	
1	5483599	5484080	5Y-H4K8ac_peak_113	8.4454	
1	5539625	5540036	5Y-H4K8ac_peak_114	8.43511	
1	5540380	5540606	5Y-H4K8ac_peak_115	5.65584	
1	5826246	5826443	5Y-H4K8ac_peak_116	4.79585	
1	5831032	5831286	5Y-H4K8ac_peak_117	8.24461	
1	5834572	5835025	5Y-H4K8ac_peak_118	8.31764	
1	6053444	6053665	5Y-H4K8ac_peak_119	6.5814	NPHP4_ENSG00000131697
1	6093770	6095291	5Y-H4K8ac_peak_120	9.30505	
1	6113375	6113724	5Y-H4K8ac_peak_121	6.82266	
1	6120272	6120512	5Y-H4K8ac_peak_122	4.728	
1	6231010	6231318	5Y-H4K8ac_peak_123	7.59101	
1	6268453	6268684	5Y-H4K8ac_peak_124	9.38276	RPL22_ENSG00000116251
1	6295615	6295938	5Y-H4K8ac_peak_125	10.1994	ICMT_ENSG00000116237;LINC00337_ENSG00000225077
1	6302214	6302694	5Y-H4K8ac_peak_126	6.73385	
1	6329745	6330219	5Y-H4K8ac_peak_127	6.84317	
1	6419528	6419740	5Y-H4K8ac_peak_128	4.51497	
1	6420216	6420597	5Y-H4K8ac_peak_129	7.07069	
1	6445332	6446364	5Y-H4K8ac_peak_130	15.71541	
1	6453650	6453848	5Y-H4K8ac_peak_131	8.2913	RP1-202O8.3_ENSG00000271746;ACOT7_ENSG00000097021
1	6544738	6545607	5Y-H4K8ac_peak_132	11.22005	
1	6545832	6546053	5Y-H4K8ac_peak_133	12.05638	
1	6550224	6551210	5Y-H4K8ac_peak_134	8.28468	
1	6567266	6567567	5Y-H4K8ac_peak_135	8.43511	
1	6567962	6568651	5Y-H4K8ac_peak_136	6.08523	

1	6571608	6571987	5Y-H4K8ac_peak_137	8.69112	
1	6572589	6572784	5Y-H4K8ac_peak_138	4.77126	
1	6573154	6573352	5Y-H4K8ac_peak_139	5.77617	
1	6614312	6614539	5Y-H4K8ac_peak_140	10.35586	NOL9_ENSG00000162408;TAS1R1_ENSG00000173662
1	6615215	6615714	5Y-H4K8ac_peak_141	8.62703	NOL9_ENSG00000162408;TAS1R1_ENSG00000173662
1	6639487	6639815	5Y-H4K8ac_peak_142	8.43511	ZBTB48_ENSG00000204859
1	6662599	6662852	5Y-H4K8ac_peak_143	10.90365	
1	6731716	6731907	5Y-H4K8ac_peak_144	6.50117	
1	6761351	6761715	5Y-H4K8ac_peak_145	8.73392	DNAJC11_ENSG00000007923
1	7104227	7104447	5Y-H4K8ac_peak_146	6.91702	
1	7352924	7353475	5Y-H4K8ac_peak_147	9.01738	
1	7401087	7401281	5Y-H4K8ac_peak_148	7.31102	
1	7448656	7448859	5Y-H4K8ac_peak_149	9.63153	RP3-453P22.2_ENSG00000237728
1	7449122	7449647	5Y-H4K8ac_peak_150	6.08523	RP3-453P22.2_ENSG00000237728
1	7523042	7523364	5Y-H4K8ac_peak_151	5.72848	
1	7536621	7537149	5Y-H4K8ac_peak_152	8.1667	
1	7537630	7538227	5Y-H4K8ac_peak_153	10.83079	
1	7554483	7554679	5Y-H4K8ac_peak_154	4.40052	
1	7569604	7569806	5Y-H4K8ac_peak_155	4.84727	
1	7584142	7584343	5Y-H4K8ac_peak_156	8.2913	
1	7584656	7584862	5Y-H4K8ac_peak_157	4.07874	
1	7618383	7618798	5Y-H4K8ac_peak_158	10.54669	
1	7619044	7619739	5Y-H4K8ac_peak_159	7.50148	
1	7645726	7645919	5Y-H4K8ac_peak_160	5.23083	
1	7648190	7648692	5Y-H4K8ac_peak_161	5.23083	
1	7688793	7689492	5Y-H4K8ac_peak_162	8.62703	
1	7690836	7691958	5Y-H4K8ac_peak_163	13.68097	
1	7706942	7707153	5Y-H4K8ac_peak_164	4.29586	
1	7727898	7728318	5Y-H4K8ac_peak_165	10.12457	
1	7728851	7730918	5Y-H4K8ac_peak_166	21.92939	
1	7731381	7731595	5Y-H4K8ac_peak_167	4.55128	
1	7732189	7732460	5Y-H4K8ac_peak_168	5.41472	
1	7738874	7739170	5Y-H4K8ac_peak_169	6.14981	
1	7739751	7739982	5Y-H4K8ac_peak_170	5.449	
1	7740189	7741350	5Y-H4K8ac_peak_171	24.88223	
1	7764319	7764553	5Y-H4K8ac_peak_172	7.60057	
1	8086577	8086953	5Y-H4K8ac_peak_173	11.22005	ERRFI1_ENSG00000116285;RP11-431K24.1_ENSG00000238290
1	8230263	8230812	5Y-H4K8ac_peak_174	4.50834	
1	8377747	8378042	5Y-H4K8ac_peak_175	7.29055	SLC45A1_ENSG00000162426
1	8440856	8441057	5Y-H4K8ac_peak_176	5.19372	
1	8938430	8938646	5Y-H4K8ac_peak_177	7.59101	ENO1_ENSG00000074800;ENO1-IT1_ENSG00000236269;ENO1-AS1_ENSG00000230679
1	8938926	8939225	5Y-H4K8ac_peak_178	10.1994	ENO1_ENSG00000074800;ENO1-IT1_ENSG00000236269;ENO1-AS1_ENSG00000230679
1	9131613	9131832	5Y-H4K8ac_peak_179	5.40331	
1	9189656	9189849	5Y-H4K8ac_peak_180	4.24869	GPR157_ENSG00000180758
1	9206922	9207117	5Y-H4K8ac_peak_181	5.40331	
1	9242544	9242792	5Y-H4K8ac_peak_182	8.69112	RP3-510D11.2_ENSG00000234546

1	9294864	9295357	5Y-H4K8ac_peak_183	6.47245	H6PD_ENSG00000049239
1	9353117	9353496	5Y-H4K8ac_peak_184	9.56187	SPSB1_ENSG00000171621
1	9445089	9445349	5Y-H4K8ac_peak_185	7.89273	
1	9597788	9598107	5Y-H4K8ac_peak_186	7.31815	
1	9599595	9600030	5Y-H4K8ac_peak_187	5.19372	SLC25A33_ENSG00000171612
1	9652307	9652559	5Y-H4K8ac_peak_188	6.37023	
1	9654083	9654343	5Y-H4K8ac_peak_189	5.91107	
1	9663190	9663421	5Y-H4K8ac_peak_190	8.43511	
1	9663690	9663989	5Y-H4K8ac_peak_191	7.89273	
1	9664669	9665033	5Y-H4K8ac_peak_192	5.41472	
1	9665905	9666769	5Y-H4K8ac_peak_193	8.08337	
1	9687300	9687579	5Y-H4K8ac_peak_194	10.1994	
1	9747297	9747504	5Y-H4K8ac_peak_195	7.30348	RP11-558F24.4_ENSG00000231789
1	9883617	9883850	5Y-H4K8ac_peak_196	4.15658	CLSTN1_ENSG00000171603
1	9884242	9884619	5Y-H4K8ac_peak_197	7.64648	CLSTN1_ENSG00000171603
1	9912908	9913234	5Y-H4K8ac_peak_198	7.50148	
1	10685348	10685538	5Y-H4K8ac_peak_199	6.86362	
1	10685750	10686499	5Y-H4K8ac_peak_200	4.90696	
1	10698297	10698629	5Y-H4K8ac_peak_201	5.23083	RP4-734G22.3_ENSG00000272078
1	10699898	10700352	5Y-H4K8ac_peak_202	4.95697	RP4-734G22.3_ENSG00000272078
1	10700631	10702077	5Y-H4K8ac_peak_203	12.05638	
1	10718176	10718373	5Y-H4K8ac_peak_204	5.12213	
1	10723367	10723754	5Y-H4K8ac_peak_205	5.35202	
1	10731829	10732045	5Y-H4K8ac_peak_206	7.89142	
1	10737817	10738028	5Y-H4K8ac_peak_207	4.50834	
1	10739043	10739247	5Y-H4K8ac_peak_208	8.24952	
1	10740618	10740812	5Y-H4K8ac_peak_209	6.49458	
1	10755136	10755464	5Y-H4K8ac_peak_210	6.43775	
1	10841573	10841776	5Y-H4K8ac_peak_211	5.65765	
1	10856343	10856648	5Y-H4K8ac_peak_212	4.50834	CASZ1_ENSG00000130940
1	10961090	10962090	5Y-H4K8ac_peak_213	10.54764	
1	10963656	10963858	5Y-H4K8ac_peak_214	5.83608	
1	10964092	10964301	5Y-H4K8ac_peak_215	7.73231	
1	10964530	10964887	5Y-H4K8ac_peak_216	10.19948	
1	10969559	10969758	5Y-H4K8ac_peak_217	5.99504	
1	10971679	10971879	5Y-H4K8ac_peak_218	4.97006	
1	10993989	10994231	5Y-H4K8ac_peak_219	3.91125	
1	10999979	11000219	5Y-H4K8ac_peak_220	10.6196	
1	11001859	11002131	5Y-H4K8ac_peak_221	10.43045	
1	11004848	11006175	5Y-H4K8ac_peak_222	9.63153	
1	11006651	11007201	5Y-H4K8ac_peak_223	7.89273	
1	11119599	11119919	5Y-H4K8ac_peak_224	8.73985	SRM_ENSG00000116649
1	11159559	11159805	5Y-H4K8ac_peak_225	10.4495	EXOSC10_ENSG00000171824;RP4-635E18.6_ENSG00000230337
1	11160198	11160522	5Y-H4K8ac_peak_226	7.72887	EXOSC10_ENSG00000171824;RP4-635E18.6_ENSG00000230337
1	11322241	11322522	5Y-H4K8ac_peak_227	8.69112	MTOR_ENSG00000198793
1	11333340	11333906	5Y-H4K8ac_peak_228	7.38046	UBIAD1_ENSG00000120942

1	11361993	11362269	5Y-H4K8ac_peak_229	11.80885	
1	11398403	11398636	5Y-H4K8ac_peak_230	6.81557	
1	11518744	11518938	5Y-H4K8ac_peak_231	9.01738	
1	11519241	11519631	5Y-H4K8ac_peak_232	12.65187	
1	11538596	11539154	5Y-H4K8ac_peak_233	9.32595	PTCHD2_ENSG00000204624
1	11623540	11623743	5Y-H4K8ac_peak_234	8.90774	
1	11689587	11689866	5Y-H4K8ac_peak_235	5.98695	
1	11750643	11751509	5Y-H4K8ac_peak_236	7.31102	MAD2L2_ENSG00000116670;DRAXIN_ENSG00000162490
1	11761734	11761958	5Y-H4K8ac_peak_237	6.73385	
1	11907122	11907411	5Y-H4K8ac_peak_238	6.37023	NPPA_ENSG00000175206
1	11907609	11907939	5Y-H4K8ac_peak_239	10.11191	NPPA_ENSG00000175206
1	11908569	11908913	5Y-H4K8ac_peak_240	5.98695	NPPA_ENSG00000175206
1	11919176	11919514	5Y-H4K8ac_peak_241	6.35402	NPPB_ENSG00000120937
1	11940317	11940517	5Y-H4K8ac_peak_242	4.0639	
1	11941556	11941784	5Y-H4K8ac_peak_243	10.6196	
1	11969250	11969447	5Y-H4K8ac_peak_244	8.4454	RNU5E-4P_ENSG00000201801
1	11991070	11991465	5Y-H4K8ac_peak_245	6.76096	
1	11994203	11994952	5Y-H4K8ac_peak_246	11.80885	PLOD1_ENSG00000083444
1	12004558	12005095	5Y-H4K8ac_peak_247	13.12848	
1	12039260	12039479	5Y-H4K8ac_peak_248	6.1654	MFN2_ENSG00000116688
1	12114138	12114353	5Y-H4K8ac_peak_249	11.28133	
1	12227100	12227486	5Y-H4K8ac_peak_250	7.55879	TNFRSF1B_ENSG00000028137
1	12244097	12245349	5Y-H4K8ac_peak_251	17.50049	
1	12289651	12289881	5Y-H4K8ac_peak_252	5.98695	VPS13D_ENSG00000048707
1	12404927	12405300	5Y-H4K8ac_peak_253	5.87382	
1	12654758	12655238	5Y-H4K8ac_peak_254	4.0639	
1	12656894	12657161	5Y-H4K8ac_peak_255	4.30961	
1	12677019	12677272	5Y-H4K8ac_peak_256	11.46984	DHRS3_ENSG00000162496
1	14026296	14027256	5Y-H4K8ac_peak_257	13.04415	PRDM2_ENSG00000116731
1	14075404	14075678	5Y-H4K8ac_peak_258	7.31815	
1	15270964	15271245	5Y-H4K8ac_peak_259	6.37023	
1	15272283	15272643	5Y-H4K8ac_peak_260	6.78128	
1	15294012	15294341	5Y-H4K8ac_peak_261	6.50117	
1	15467402	15467721	5Y-H4K8ac_peak_262	4.50834	
1	15479402	15479668	5Y-H4K8ac_peak_263	5.64909	TMEM51-AS1_ENSG00000175147;TMEM51_ENSG00000171729
1	15480393	15480596	5Y-H4K8ac_peak_264	4.29586	
1	15513167	15513965	5Y-H4K8ac_peak_265	8.16382	
1	15720367	15720773	5Y-H4K8ac_peak_266	4.15658	
1	15736511	15736745	5Y-H4K8ac_peak_267	7.49746	RP3-467K16.4_ENSG00000228140;EFHD2_ENSG00000142634
1	15911160	15911520	5Y-H4K8ac_peak_268	12.69256	AGMAT_ENSG00000116771
1	15930382	15930740	5Y-H4K8ac_peak_269	4.70501	RP4-680D5.2_ENSG00000237301;CHCHD2P6_ENSG00000235084
1	16009986	16010694	5Y-H4K8ac_peak_270	21.10958	RP4-680D5.9_ENSG00000271742;PLEKHM2_ENSG00000116786;AL121992.1_ENSG00000264048
1	16011094	16011423	5Y-H4K8ac_peak_271	4.84727	PLEKHM2_ENSG00000116786;AL121992.1_ENSG00000264048
1	16065660	16065869	5Y-H4K8ac_peak_272	5.99504	
1	16076108	16076369	5Y-H4K8ac_peak_273	5.64909	RP11-169K16.4_ENSG00000224459
1	16083546	16084024	5Y-H4K8ac_peak_274	6.50117	FBLIM1_ENSG00000162458

1	16085390	16086289	5Y-H4K8ac_peak_275	6.37023	
1	16161024	16161289	5Y-H4K8ac_peak_276	6.98416	
1	16161638	16162183	5Y-H4K8ac_peak_277	13.68515	
1	16339358	16339872	5Y-H4K8ac_peak_278	10.35586	
1	16341458	16341690	5Y-H4K8ac_peak_279	8.63295	
1	16344048	16344987	5Y-H4K8ac_peak_280	15.90073	CLCNKA_ENSG00000186510
1	16374722	16375193	5Y-H4K8ac_peak_281	7.9215	
1	16466640	16466877	5Y-H4K8ac_peak_282	5.30478	
1	16468040	16468743	5Y-H4K8ac_peak_283	11.04542	
1	16481448	16481756	5Y-H4K8ac_peak_284	6.38173	EPHA2_ENSG00000142627;RP11-276H7.2_ENSG00000227959
1	16482050	16482438	5Y-H4K8ac_peak_285	6.49458	EPHA2_ENSG00000142627;RP11-276H7.2_ENSG00000227959
1	16491934	16492153	5Y-H4K8ac_peak_286	9.45148	
1	16501498	16501768	5Y-H4K8ac_peak_287	4.95697	
1	16502157	16502604	5Y-H4K8ac_peak_288	8.62703	
1	16502887	16503416	5Y-H4K8ac_peak_289	12.05638	
1	16516317	16516512	5Y-H4K8ac_peak_290	6.78128	
1	16533348	16533718	5Y-H4K8ac_peak_291	5.98695	
1	16534503	16534832	5Y-H4K8ac_peak_292	7.59101	
1	16679147	16679344	5Y-H4K8ac_peak_293	10.69698	FBXO42_ENSG00000037637;SZRD1_ENSG00000055070
1	16692748	16693214	5Y-H4K8ac_peak_294	8.55081	
1	16766615	16767057	5Y-H4K8ac_peak_295	13.99721	NECAP2_ENSG00000157191
1	16825031	16825523	5Y-H4K8ac_peak_296	7.50148	CROCCP3_ENSG00000080947
1	16825923	16826213	5Y-H4K8ac_peak_297	7.58806	CROCCP3_ENSG00000080947
1	16940269	16940557	5Y-H4K8ac_peak_298	6.69371	NBPF1_ENSG00000219481
1	16970644	16972291	5Y-H4K8ac_peak_299	37.18359	CROCCP2_ENSG00000215908;MST1P2_ENSG00000186301
1	17026209	17026528	5Y-H4K8ac_peak_300	4.15658	
1	17030522	17031126	5Y-H4K8ac_peak_301	6.34245	
1	17201639	17201842	5Y-H4K8ac_peak_302	4.15658	
1	17230570	17230809	5Y-H4K8ac_peak_303	5.45148	RP11-108M9.6_ENSG00000272426
1	17231433	17231689	5Y-H4K8ac_peak_304	26.68337	RP11-108M9.6_ENSG00000272426
1	17240089	17240320	5Y-H4K8ac_peak_305	14.89891	
1	17240724	17241232	5Y-H4K8ac_peak_306	13.09548	
1	17247702	17247943	5Y-H4K8ac_peak_307	4.89347	
1	17248144	17249251	5Y-H4K8ac_peak_308	11.69482	
1	17287123	17287919	5Y-H4K8ac_peak_309	10.5157	
1	17304622	17305080	5Y-H4K8ac_peak_310	6.00747	RP1-37C10.3_ENSG00000226526
1	17306167	17307695	5Y-H4K8ac_peak_311	27.04863	MFAP2_ENSG00000117122;RP1-37C10.3_ENSG00000226526
1	17380998	17381287	5Y-H4K8ac_peak_312	6.73047	SDHB_ENSG00000117118
1	17488038	17488237	5Y-H4K8ac_peak_313	5.87382	
1	17865573	17866116	5Y-H4K8ac_peak_314	14.99673	ARHGEF10L_ENSG00000074964
1	17866422	17866707	5Y-H4K8ac_peak_315	7.50148	ARHGEF10L_ENSG00000074964
1	17877921	17878111	5Y-H4K8ac_peak_316	5.23083	
1	17919648	17920571	5Y-H4K8ac_peak_317	8.69112	
1	17920886	17921145	5Y-H4K8ac_peak_318	7.03573	
1	17921930	17922155	5Y-H4K8ac_peak_319	6.08523	
1	17928546	17929670	5Y-H4K8ac_peak_320	10.35586	

1	17968631	17969236	5Y-H4K8ac_peak_321	10.6196	
1	17997651	17998217	5Y-H4K8ac_peak_322	4.0639	
1	18009174	18009607	5Y-H4K8ac_peak_323	5.65584	
1	18009803	18010047	5Y-H4K8ac_peak_324	4.84727	
1	18100252	18100482	5Y-H4K8ac_peak_325	4.77126	
1	18459844	18460045	5Y-H4K8ac_peak_326	5.03917	
1	18516378	18516632	5Y-H4K8ac_peak_327	5.97532	
1	18525900	18526099	5Y-H4K8ac_peak_328	6.37023	
1	18609944	18610160	5Y-H4K8ac_peak_329	8.47164	
1	19122040	19122306	5Y-H4K8ac_peak_330	11.31171	
1	19248427	19248827	5Y-H4K8ac_peak_331	4.29586	RP13-279N23.2_ENSG00000255275
1	19392621	19392859	5Y-H4K8ac_peak_332	8.55081	
1	19638253	19638535	5Y-H4K8ac_peak_333	5.64909	AKR7A2_ENSG00000053371;PQLC2_ENSG00000040487
1	19639141	19639402	5Y-H4K8ac_peak_334	7.58806	AKR7A2_ENSG00000053371;PQLC2_ENSG00000040487
1	19657443	19657812	5Y-H4K8ac_peak_335	6.20989	
1	19664772	19665263	5Y-H4K8ac_peak_336	5.80888	
1	19679552	19679794	5Y-H4K8ac_peak_337	6.78815	
1	19698204	19698462	5Y-H4K8ac_peak_338	5.41472	
1	19722366	19722721	5Y-H4K8ac_peak_339	10.19948	
1	19774532	19774814	5Y-H4K8ac_peak_340	9.31608	
1	19778583	19779138	5Y-H4K8ac_peak_341	6.50117	
1	19811542	19811969	5Y-H4K8ac_peak_342	11.76668	CAPZB_ENSG00000077549
1	19923670	19923902	5Y-H4K8ac_peak_343	6.77436	RP5-1056L3.1_ENSG00000235185;MINOS1-NBL1_ENSG00000270136;MINOS1_ENSG00000173436
1	19970239	19970565	5Y-H4K8ac_peak_344	7.59101	
1	19971048	19971518	5Y-H4K8ac_peak_345	5.37237	
1	20030802	20031174	5Y-H4K8ac_peak_346	6.77436	
1	20126337	20126754	5Y-H4K8ac_peak_347	9.36633	TMCO4_ENSG00000162542
1	20478649	20478911	5Y-H4K8ac_peak_348	9.38276	
1	20609164	20609380	5Y-H4K8ac_peak_349	4.84727	
1	20635129	20635382	5Y-H4K8ac_peak_350	7.11863	
1	20668919	20669544	5Y-H4K8ac_peak_351	7.59101	
1	20692649	20693071	5Y-H4K8ac_peak_352	6.34245	
1	20703565	20703793	5Y-H4K8ac_peak_353	9.88125	
1	20713690	20713958	5Y-H4K8ac_peak_354	8.2913	
1	20770856	20771078	5Y-H4K8ac_peak_355	7.97699	
1	20802499	20804345	5Y-H4K8ac_peak_356	7.50148	
1	20811431	20811828	5Y-H4K8ac_peak_357	6.77436	CAMK2N1_ENSG00000162545
1	20812793	20813133	5Y-H4K8ac_peak_358	12.26606	CAMK2N1_ENSG00000162545
1	20820619	20820964	5Y-H4K8ac_peak_359	4.95697	
1	20940297	20940774	5Y-H4K8ac_peak_360	6.77436	
1	20988239	20988861	5Y-H4K8ac_peak_361	8.24461	DDOST_ENSG00000244038
1	21023211	21023740	5Y-H4K8ac_peak_362	7.09658	
1	21043803	21044024	5Y-H4K8ac_peak_363	9.84749	KIF17_ENSG00000117245
1	21503600	21503996	5Y-H4K8ac_peak_364	13.43531	EIF4G3_ENSG00000075151;AL031005.1_ENSG00000266867
1	21543974	21544228	5Y-H4K8ac_peak_365	6.73385	
1	21546620	21547449	5Y-H4K8ac_peak_366	10.54764	

1	21616027	21616256	5Y-H4K8ac_peak_367	6.77436	
1	21634758	21635398	5Y-H4K8ac_peak_368	5.50568	
1	21635614	21635956	5Y-H4K8ac_peak_369	4.29586	
1	21643802	21644279	5Y-H4K8ac_peak_370	6.22669	
1	21650678	21650924	5Y-H4K8ac_peak_371	10.508	
1	21670972	21671219	5Y-H4K8ac_peak_372	5.8635	ECE1_ENSG00000117298
1	21762972	21763864	5Y-H4K8ac_peak_373	11.35501	
1	21766262	21766497	5Y-H4K8ac_peak_374	4.77126	NBPF3_ENSG00000142794
1	21835173	21835635	5Y-H4K8ac_peak_375	8.28346	ALPL_ENSG00000162551
1	21835867	21836326	5Y-H4K8ac_peak_376	10.35586	ALPL_ENSG00000162551
1	21946613	21946988	5Y-H4K8ac_peak_377	5.59843	
1	21949193	21950161	5Y-H4K8ac_peak_378	18.10232	
1	21952625	21953099	5Y-H4K8ac_peak_379	4.95697	
1	21953352	21953767	5Y-H4K8ac_peak_380	5.98695	
1	21968545	21968834	5Y-H4K8ac_peak_381	4.84727	
1	21972030	21972220	5Y-H4K8ac_peak_382	6.50117	
1	21994987	21995385	5Y-H4K8ac_peak_383	5.77617	RAP1GAP_ENSG00000076864
1	21995628	21995824	5Y-H4K8ac_peak_384	7.20869	RAP1GAP_ENSG00000076864
1	22109190	22109514	5Y-H4K8ac_peak_385	4.83834	USP48_ENSG00000090686
1	22350779	22351014	5Y-H4K8ac_peak_386	6.77436	RP1-224A6.3_ENSG00000228397;LINC00339_ENSG00000218510
1	22351477	22351671	5Y-H4K8ac_peak_387	6.46053	RP1-224A6.3_ENSG00000228397;LINC00339_ENSG00000218510
1	22352058	22352294	5Y-H4K8ac_peak_388	7.60057	RP1-224A6.3_ENSG00000228397;LINC00339_ENSG00000218510
1	22378720	22378939	5Y-H4K8ac_peak_389	8.47164	CDC42_ENSG00000070831
1	22379268	22379622	5Y-H4K8ac_peak_390	7.89273	CDC42_ENSG00000070831
1	22487344	22487581	5Y-H4K8ac_peak_391	6.62622	
1	22641895	22642572	5Y-H4K8ac_peak_392	8.04693	
1	22655005	22655233	5Y-H4K8ac_peak_393	12.0443	
1	22655528	22655948	5Y-H4K8ac_peak_394	10.12457	
1	22667195	22667495	5Y-H4K8ac_peak_395	6.33308	
1	22668511	22669082	5Y-H4K8ac_peak_396	8.2913	
1	22676305	22677031	5Y-H4K8ac_peak_397	12.91364	
1	22677667	22678141	5Y-H4K8ac_peak_398	14.61459	
1	22777658	22778038	5Y-H4K8ac_peak_399	6.55841	ZBTB40_ENSG00000184677
1	22778557	22778904	5Y-H4K8ac_peak_400	14.39608	ZBTB40_ENSG00000184677
1	22883284	22883498	5Y-H4K8ac_peak_401	9.6443	
1	22999965	23000176	5Y-H4K8ac_peak_402	4.50302	
1	23036242	23036679	5Y-H4K8ac_peak_403	6.77436	EPHB2_ENSG00000133216
1	23153105	23153489	5Y-H4K8ac_peak_404	6.92707	
1	23504006	23504342	5Y-H4K8ac_peak_405	7.01266	LUZP1_ENSG00000169641
1	23670327	23670645	5Y-H4K8ac_peak_406	15.3483	HNRNPR_ENSG00000125944
1	23695053	23696066	5Y-H4K8ac_peak_407	10.90365	ZNF436_ENSG00000125945;C1orf213_ENSG00000249087;Y_RNA_ENSG00000201405
1	23823847	23824280	5Y-H4K8ac_peak_408	7.59101	
1	23857350	23857652	5Y-H4K8ac_peak_409	7.20869	E2F2_ENSG00000007968
1	23871529	23871837	5Y-H4K8ac_peak_410	5.23083	
1	23880581	23881363	5Y-H4K8ac_peak_411	19.8038	
1	23894062	23895124	5Y-H4K8ac_peak_412	16.99491	

1	23895450	23895764	5Y-H4K8ac_peak_413	6.19716	
1	23925107	23925575	5Y-H4K8ac_peak_414	7.52971	
1	23945987	23946293	5Y-H4K8ac_peak_415	7.59101	
1	23961108	23961575	5Y-H4K8ac_peak_416	8.21582	
1	23964169	23964429	5Y-H4K8ac_peak_417	4.70501	
1	24018419	24018682	5Y-H4K8ac_peak_418	6.78128	RPL11_ENSG00000142676
1	24069411	24070461	5Y-H4K8ac_peak_419	16.71988	TCEB3_ENSG00000011007
1	24104965	24105227	5Y-H4K8ac_peak_420	6.38173	RP5-886K2.3_ENSG00000236810;PITHD1_ENSG00000057757
1	24127468	24127671	5Y-H4K8ac_peak_421	9.44864	GALE_ENSG00000117308
1	24151661	24151861	5Y-H4K8ac_peak_422	7.11863	
1	24437635	24438114	5Y-H4K8ac_peak_423	7.41197	MYOM3_ENSG00000142661
1	24438553	24438951	5Y-H4K8ac_peak_424	4.71803	MYOM3_ENSG00000142661
1	24513492	24513703	5Y-H4K8ac_peak_425	10.1994	IFNLR1_ENSG00000185436
1	24739769	24740158	5Y-H4K8ac_peak_426	7.59101	
1	24740593	24740909	5Y-H4K8ac_peak_427	9.179	
1	24828827	24829037	5Y-H4K8ac_peak_428	8.97752	RCAN3_ENSG00000117602
1	24969045	24969274	5Y-H4K8ac_peak_429	9.60627	
1	24969826	24970016	5Y-H4K8ac_peak_430	7.01266	
1	25240781	25241079	5Y-H4K8ac_peak_431	9.30206	
1	25566279	25566473	5Y-H4K8ac_peak_432	8.69112	
1	25757578	25758100	5Y-H4K8ac_peak_433	7.59101	RHCE_ENSG00000188672;TMEM57_ENSG00000204178
1	25993117	25993548	5Y-H4K8ac_peak_434	6.34046	
1	26053894	26054295	5Y-H4K8ac_peak_435	11.22005	
1	26091785	26092211	5Y-H4K8ac_peak_436	4.84727	
1	26096729	26097120	5Y-H4K8ac_peak_437	5.59843	
1	26098176	26098392	5Y-H4K8ac_peak_438	9.37812	
1	26110196	26110390	5Y-H4K8ac_peak_439	4.07874	
1	26125959	26126519	5Y-H4K8ac_peak_440	12.21176	SEPN1_ENSG00000162430
1	26146465	26146892	5Y-H4K8ac_peak_441	4.44548	RP1-317E23.3_ENSG00000228172;AL020996.1_ENSG00000223474
1	26201062	26201309	5Y-H4K8ac_peak_442	7.31102	
1	26201540	26201942	5Y-H4K8ac_peak_443	5.12488	
1	26202145	26202676	5Y-H4K8ac_peak_444	8.90774	
1	26232259	26232724	5Y-H4K8ac_peak_445	6.55225	STMN1_ENSG00000117632;MIR3917_ENSG00000264021
1	26233072	26233545	5Y-H4K8ac_peak_446	13.0168	STMN1_ENSG00000117632;MIR3917_ENSG00000264021
1	26253459	26253931	5Y-H4K8ac_peak_447	7.87406	
1	26421510	26421742	5Y-H4K8ac_peak_448	5.35202	
1	26437594	26438062	5Y-H4K8ac_peak_449	13.99721	PDIK1L_ENSG00000175087
1	26482859	26483309	5Y-H4K8ac_peak_450	9.51254	
1	26487891	26488404	5Y-H4K8ac_peak_451	8.43511	
1	26490610	26490927	5Y-H4K8ac_peak_452	5.64909	
1	26491214	26491420	5Y-H4K8ac_peak_453	7.03573	
1	26495514	26495775	5Y-H4K8ac_peak_454	5.03917	ZNF593_ENSG00000142684
1	26496080	26496279	5Y-H4K8ac_peak_455	6.78128	ZNF593_ENSG00000142684
1	26560018	26560383	5Y-H4K8ac_peak_456	9.38276	CEP85_ENSG00000130695
1	26647781	26648010	5Y-H4K8ac_peak_457	6.46053	
1	26663164	26663360	5Y-H4K8ac_peak_458	6.34245	

1	26798207	26798736	5Y-H4K8ac_peak_459	16.50659	HMGN2_ENSG00000198830
1	26798985	26799318	5Y-H4K8ac_peak_460	23.51162	HMGN2_ENSG00000198830
1	26826929	26827149	5Y-H4K8ac_peak_461	8.7173	
1	26855792	26856075	5Y-H4K8ac_peak_462	12.05638	RPS6KA1_ENSG00000117676
1	26856311	26856512	5Y-H4K8ac_peak_463	5.64909	RPS6KA1_ENSG00000117676
1	26947582	26948119	5Y-H4K8ac_peak_464	8.5915	
1	27022584	27024086	5Y-H4K8ac_peak_465	13.12848	ARID1A_ENSG00000117713
1	27113935	27114248	5Y-H4K8ac_peak_466	6.86362	PIGV_ENSG00000060642
1	27114502	27115077	5Y-H4K8ac_peak_467	5.85188	PIGV_ENSG00000060642
1	27152691	27153051	5Y-H4K8ac_peak_468	11.04146	ZDHHC18_ENSG00000204160
1	27217131	27217352	5Y-H4K8ac_peak_469	6.03632	GPN2_ENSG00000142751
1	27226427	27226825	5Y-H4K8ac_peak_470	7.50148	GPATCH3_ENSG00000198746;NUDC_ENSG00000090273
1	27248323	27248733	5Y-H4K8ac_peak_471	7.89273	
1	27338860	27339328	5Y-H4K8ac_peak_472	8.21582	FAM46B_ENSG00000158246
1	27426507	27426834	5Y-H4K8ac_peak_473	11.68317	
1	27481124	27481564	5Y-H4K8ac_peak_474	5.23083	
1	27560464	27560773	5Y-H4K8ac_peak_475	8.2913	RP11-40H20.4_ENSG00000224311;WDTC1_ENSG00000142784
1	27624435	27624636	5Y-H4K8ac_peak_476	3.94555	
1	27625465	27625791	5Y-H4K8ac_peak_477	5.98695	
1	27626589	27627393	5Y-H4K8ac_peak_478	7.44077	
1	27644838	27645058	5Y-H4K8ac_peak_479	5.87382	
1	27648816	27649140	5Y-H4K8ac_peak_480	9.23159	TMEM222_ENSG00000186501
1	27675567	27675981	5Y-H4K8ac_peak_481	10.60083	
1	27676176	27676394	5Y-H4K8ac_peak_482	9.63153	
1	27676640	27677339	5Y-H4K8ac_peak_483	12.82593	
1	27693197	27693439	5Y-H4K8ac_peak_484	5.41472	MAP3K6_ENSG00000142733
1	27817033	27817573	5Y-H4K8ac_peak_485	10.17195	WASF2_ENSG00000158195
1	27831187	27831527	5Y-H4K8ac_peak_486	9.22011	
1	27845844	27846418	5Y-H4K8ac_peak_487	7.50148	
1	27854186	27854905	5Y-H4K8ac_peak_488	6.03632	
1	27955245	27955584	5Y-H4K8ac_peak_489	7.38046	
1	27963445	27963784	5Y-H4K8ac_peak_490	5.59843	
1	27986064	27986533	5Y-H4K8ac_peak_491	4.0639	
1	27989304	27989586	5Y-H4K8ac_peak_492	4.95697	RP11-288L9.1_ENSG00000233975
1	28086256	28086534	5Y-H4K8ac_peak_493	7.01856	
1	28156592	28156888	5Y-H4K8ac_peak_494	8.17203	PPP1R8_ENSG00000117751
1	28157451	28157840	5Y-H4K8ac_peak_495	7.11863	PPP1R8_ENSG00000117751
1	28205776	28206012	5Y-H4K8ac_peak_496	6.50117	
1	28500368	28500752	5Y-H4K8ac_peak_497	5.65584	
1	28561890	28562364	5Y-H4K8ac_peak_498	4.84727	ATPIF1_ENSG00000130770
1	28574664	28574942	5Y-H4K8ac_peak_499	8.43511	
1	28696312	28696522	5Y-H4K8ac_peak_500	7.08486	PHACTR4_ENSG00000204138
1	28831829	28832219	5Y-H4K8ac_peak_501	6.3265	RCC1_ENSG00000180198;SNHG3_ENSG00000242125
1	28974146	28974340	5Y-H4K8ac_peak_502	9.44864	RNU11_ENSG00000270103
1	28994186	28994981	5Y-H4K8ac_peak_503	4.29586	GMEB1_ENSG00000162419
1	28995594	28996191	5Y-H4K8ac_peak_504	13.92877	GMEB1_ENSG00000162419

1	29062834	29063138	5Y-H4K8ac_peak_505	7.4047	YTHDF2_ENSG00000198492
1	29063555	29064095	5Y-H4K8ac_peak_506	10.48476	YTHDF2_ENSG00000198492
1	29138477	29139317	5Y-H4K8ac_peak_507	20.32264	OPRD1_ENSG00000116329
1	29161391	29161784	5Y-H4K8ac_peak_508	8.43511	
1	29162239	29162445	5Y-H4K8ac_peak_509	6.76091	
1	29211129	29211345	5Y-H4K8ac_peak_510	5.89113	
1	29241214	29241411	5Y-H4K8ac_peak_511	5.34199	
1	29447892	29448506	5Y-H4K8ac_peak_512	6.54441	
1	29451461	29452152	5Y-H4K8ac_peak_513	14.19462	
1	29507922	29508391	5Y-H4K8ac_peak_514	9.89244	SRSF4_ENSG00000116350
1	29508769	29509351	5Y-H4K8ac_peak_515	8.16031	SRSF4_ENSG00000116350
1	29721197	29721509	5Y-H4K8ac_peak_516	7.63144	
1	29753826	29754230	5Y-H4K8ac_peak_517	7.38046	
1	29776756	29777026	5Y-H4K8ac_peak_518	5.23083	
1	29795053	29795244	5Y-H4K8ac_peak_519	4.79585	
1	29795611	29796421	5Y-H4K8ac_peak_520	8.69112	
1	29796973	29798405	5Y-H4K8ac_peak_521	19.61244	
1	29800111	29800761	5Y-H4K8ac_peak_522	11.22005	
1	29801670	29802124	5Y-H4K8ac_peak_523	9.51254	
1	29848288	29848691	5Y-H4K8ac_peak_524	5.449	
1	30102344	30102902	5Y-H4K8ac_peak_525	8.71064	
1	30105832	30106055	5Y-H4K8ac_peak_526	12.6675	
1	30106278	30106604	5Y-H4K8ac_peak_527	6.50117	
1	30107266	30108400	5Y-H4K8ac_peak_528	12.2918	
1	30115961	30116318	5Y-H4K8ac_peak_529	6.14981	
1	30124850	30125205	5Y-H4K8ac_peak_530	11.19336	
1	30155346	30155552	5Y-H4K8ac_peak_531	5.449	
1	30161868	30162257	5Y-H4K8ac_peak_532	7.30348	
1	30179477	30179874	5Y-H4K8ac_peak_533	5.16687	
1	30180521	30181467	5Y-H4K8ac_peak_534	21.41928	RP4-656G21.1_ENSG00000228176
1	30182236	30183149	5Y-H4K8ac_peak_535	15.28394	RP4-656G21.1_ENSG00000228176
1	30226643	30226875	5Y-H4K8ac_peak_536	6.47245	
1	30227130	30227688	5Y-H4K8ac_peak_537	7.69843	
1	30228185	30228662	5Y-H4K8ac_peak_538	5.24695	
1	30274392	30274647	5Y-H4K8ac_peak_539	6.34046	
1	30299326	30299638	5Y-H4K8ac_peak_540	6.43775	
1	30308895	30309139	5Y-H4K8ac_peak_541	5.13361	
1	30574014	30574571	5Y-H4K8ac_peak_542	5.59843	
1	30605382	30605703	5Y-H4K8ac_peak_543	11.39862	
1	30606120	30606713	5Y-H4K8ac_peak_544	5.41472	
1	30805596	30805964	5Y-H4K8ac_peak_545	5.72848	
1	30852985	30853325	5Y-H4K8ac_peak_546	8.43511	
1	31123233	31123434	5Y-H4K8ac_peak_547	9.33466	
1	31165069	31165543	5Y-H4K8ac_peak_548	6.50117	
1	31168639	31168934	5Y-H4K8ac_peak_549	4.84727	
1	31191698	31192093	5Y-H4K8ac_peak_550	11.19336	MATN1-AS1_ENSG00000186056

1	31361247	31361742	5Y-H4K8ac_peak_551	7.38046	
1	31381816	31382059	5Y-H4K8ac_peak_552	9.23159	SDC3_ENSG00000162512
1	31386026	31386349	5Y-H4K8ac_peak_553	8.43511	
1	31538926	31539253	5Y-H4K8ac_peak_554	4.15658	PUM1_ENSG00000134644
1	31574200	31574390	5Y-H4K8ac_peak_555	4.61652	
1	31627450	31627728	5Y-H4K8ac_peak_556	15.06486	
1	31628128	31628573	5Y-H4K8ac_peak_557	6.37023	
1	31712268	31712521	5Y-H4K8ac_peak_558	8.564	NKAIN1_ENSG00000084628
1	32045978	32046276	5Y-H4K8ac_peak_559	7.53429	
1	32079763	32080143	5Y-H4K8ac_peak_560	7.76645	
1	32083361	32083800	5Y-H4K8ac_peak_561	7.64648	HCRTR1_ENSG00000121764
1	32092508	32093524	5Y-H4K8ac_peak_562	4.642	
1	32095548	32095777	5Y-H4K8ac_peak_563	6.78318	
1	32109966	32110461	5Y-H4K8ac_peak_564	14.86775	PEF1_ENSG00000162517;RP11-73M7.6_ENSG00000235790;RP11-73M7.9_ENSG00000264078
1	32167199	32167418	5Y-H4K8ac_peak_565	7.41519	
1	32169222	32169873	5Y-H4K8ac_peak_566	10.4678	COL16A1_ENSG00000084636
1	32172198	32172476	5Y-H4K8ac_peak_567	6.00382	
1	32198265	32198693	5Y-H4K8ac_peak_568	8.63306	
1	32199145	32199499	5Y-H4K8ac_peak_569	9.26157	
1	32246742	32246940	5Y-H4K8ac_peak_570	6.19178	
1	32247281	32248048	5Y-H4K8ac_peak_571	11.64182	
1	32248480	32248841	5Y-H4K8ac_peak_572	5.449	
1	32264674	32264918	5Y-H4K8ac_peak_573	5.35202	
1	32279502	32280332	5Y-H4K8ac_peak_574	8.16031	
1	32404200	32404485	5Y-H4K8ac_peak_575	9.30505	
1	32420291	32420524	5Y-H4K8ac_peak_576	5.23083	
1	32479638	32480013	5Y-H4K8ac_peak_577	7.87406	KHDRBS1_ENSG00000121774
1	32538121	32538326	5Y-H4K8ac_peak_578	7.59101	RP11-277A4.4_ENSG00000203325;TMEM39B_ENSG00000121775
1	32573001	32573235	5Y-H4K8ac_peak_579	6.54441	KPNA6_ENSG00000025800
1	32596834	32597123	5Y-H4K8ac_peak_580	5.59837	
1	32705460	32705801	5Y-H4K8ac_peak_581	7.30348	
1	32801174	32801389	5Y-H4K8ac_peak_582	5.44849	MARCKSL1_ENSG00000175130
1	32817291	32817899	5Y-H4K8ac_peak_583	8.43511	TSSK3_ENSG00000162526
1	32866806	32867026	5Y-H4K8ac_peak_584	5.98695	
1	32892520	32892759	5Y-H4K8ac_peak_585	9.38203	LRRC37A12P_ENSG00000229259
1	32966010	32966467	5Y-H4K8ac_peak_586	14.86775	
1	33116834	33117363	5Y-H4K8ac_peak_587	12.26819	ZBTB8OS_ENSG00000176261;RBBP4_ENSG00000162521
1	33168752	33169171	5Y-H4K8ac_peak_588	5.23819	SYNC_ENSG00000162520
1	33206648	33207412	5Y-H4K8ac_peak_589	6.96612	KIAA1522_ENSG00000162522
1	33207687	33207985	5Y-H4K8ac_peak_590	9.89244	KIAA1522_ENSG00000162522
1	33282385	33282795	5Y-H4K8ac_peak_591	15.49523	YARS_ENSG00000134684;S100PBP_ENSG00000116497
1	33283392	33283753	5Y-H4K8ac_peak_592	8.90774	YARS_ENSG00000134684
1	33335776	33336144	5Y-H4K8ac_peak_593	5.52265	
1	33336534	33336970	5Y-H4K8ac_peak_594	5.23083	
1	33358927	33359132	5Y-H4K8ac_peak_595	7.11863	
1	33367211	33367452	5Y-H4K8ac_peak_596	7.34185	TMEM54_ENSG00000121900

1	33438352	33438894	5Y-H4K8ac_peak_597	13.48981	FKSG48_ENSG00000267885
1	33618678	33618933	5Y-H4K8ac_peak_598	8.69112	
1	33626306	33626963	5Y-H4K8ac_peak_599	6.37023	
1	33721301	33721728	5Y-H4K8ac_peak_600	8.69112	ZNF362_ENSG00000160094
1	33723893	33724121	5Y-H4K8ac_peak_601	5.90587	
1	33815035	33815266	5Y-H4K8ac_peak_602	5.16353	RP11-415J8.5_ENSG00000233246
1	33815733	33816214	5Y-H4K8ac_peak_603	8.2913	RP11-415J8.5_ENSG00000233246
1	33830171	33830516	5Y-H4K8ac_peak_604	8.42168	
1	34098891	34099176	5Y-H4K8ac_peak_605	15.27452	
1	34151940	34152442	5Y-H4K8ac_peak_606	6.6946	
1	34286287	34286547	5Y-H4K8ac_peak_607	6.78128	
1	35105183	35105487	5Y-H4K8ac_peak_608	5.34938	
1	35162369	35162692	5Y-H4K8ac_peak_609	6.73385	
1	35383723	35383967	5Y-H4K8ac_peak_610	5.12213	
1	35450581	35450827	5Y-H4K8ac_peak_611	7.11863	RP11-244H3.1_ENSG00000241014;ZMYM6NB_ENSG00000243749
1	35497301	35497539	5Y-H4K8ac_peak_612	13.2534	RP11-244H3.4_ENSG00000271741;ZMYM6_ENSG00000163867
1	35545021	35545273	5Y-H4K8ac_peak_613	7.31815	
1	35657847	35658684	5Y-H4K8ac_peak_614	11.72479	SFPQ_ENSG00000116560
1	35734383	35734853	5Y-H4K8ac_peak_615	22.04676	ZMYM4_ENSG00000146463
1	36022658	36023290	5Y-H4K8ac_peak_616	13.00733	KIAA0319L_ENSG00000142687;NCDN_ENSG00000020129
1	36023488	36023971	5Y-H4K8ac_peak_617	7.90236	KIAA0319L_ENSG00000142687;NCDN_ENSG00000020129
1	36172926	36174303	5Y-H4K8ac_peak_618	18.21442	
1	36184381	36184898	5Y-H4K8ac_peak_619	6.78128	C1orf216_ENSG00000142686
1	36273424	36273754	5Y-H4K8ac_peak_620	6.73385	AGO4_ENSG00000134698
1	36273949	36274342	5Y-H4K8ac_peak_621	6.37023	AGO4_ENSG00000134698
1	36348138	36348525	5Y-H4K8ac_peak_622	5.98695	
1	36396362	36396553	5Y-H4K8ac_peak_623	6.22904	RP4-789D17.5_ENSG00000271914;AGO3_ENSG00000126070
1	36396932	36397172	5Y-H4K8ac_peak_624	8.04693	AGO3_ENSG00000126070
1	36549419	36549816	5Y-H4K8ac_peak_625	8.47164	TEKT2_ENSG00000092850
1	36553969	36554296	5Y-H4K8ac_peak_626	9.36633	ADPRHL2_ENSG00000116863
1	36554591	36554840	5Y-H4K8ac_peak_627	6.88736	ADPRHL2_ENSG00000116863
1	36559813	36560277	5Y-H4K8ac_peak_628	7.72887	
1	36580250	36580883	5Y-H4K8ac_peak_629	8.43511	
1	36591106	36591416	5Y-H4K8ac_peak_630	6.37023	COL8A2_ENSG00000171812
1	36599190	36599402	5Y-H4K8ac_peak_631	4.50834	
1	36615948	36616488	5Y-H4K8ac_peak_632	9.63153	TRAPPC3_ENSG00000054116
1	36620431	36620634	5Y-H4K8ac_peak_633	7.0618	MAP7D1_ENSG00000116871
1	36621540	36621875	5Y-H4K8ac_peak_634	7.50148	MAP7D1_ENSG00000116871
1	36626439	36626872	5Y-H4K8ac_peak_635	11.86504	
1	36690100	36690445	5Y-H4K8ac_peak_636	6.2403	THRAP3_ENSG00000054118
1	36852338	36852549	5Y-H4K8ac_peak_637	7.58806	STK40_ENSG00000196182
1	36948259	36948559	5Y-H4K8ac_peak_638	6.86362	CSF3R_ENSG00000119535
1	37331454	37331831	5Y-H4K8ac_peak_639	7.50081	
1	37336758	37337032	5Y-H4K8ac_peak_640	5.25819	
1	37731776	37732158	5Y-H4K8ac_peak_641	4.8188	
1	38020218	38020438	5Y-H4K8ac_peak_642	9.15007	SNIP1_ENSG00000163877

1	38259021	38259348	5Y-H4K8ac_peak_643	11.22005	MANEAL_ENSG00000185090
1	38259637	38259957	5Y-H4K8ac_peak_644	9.7353	MANEAL_ENSG00000185090
1	38273502	38273773	5Y-H4K8ac_peak_645	7.50148	YRDC_ENSG00000196449;C1orf122_ENSG00000197982
1	38461966	38462167	5Y-H4K8ac_peak_646	5.13364	
1	38465452	38465813	5Y-H4K8ac_peak_647	10.23399	
1	38471583	38471962	5Y-H4K8ac_peak_648	6.40964	FHL3_ENSG00000183386
1	38478477	38478808	5Y-H4K8ac_peak_649	9.84749	
1	39338614	39338909	5Y-H4K8ac_peak_650	7.76232	
1	39491155	39491802	5Y-H4K8ac_peak_651	5.72233	NDUFS5_ENSG00000168653
1	39570639	39570880	5Y-H4K8ac_peak_652	9.30206	
1	40102929	40103151	5Y-H4K8ac_peak_653	5.67588	
1	40122525	40122762	5Y-H4K8ac_peak_654	6.00598	
1	40157687	40157918	5Y-H4K8ac_peak_655	7.11863	HPCAL4_ENSG00000116983;PPIE_ENSG00000084072
1	40158295	40158528	5Y-H4K8ac_peak_656	4.29586	HPCAL4_ENSG00000116983;PPIE_ENSG00000084072
1	40203962	40204272	5Y-H4K8ac_peak_657	6.77436	
1	40253792	40254289	5Y-H4K8ac_peak_658	7.9215	BMP8B_ENSG00000116985;RP1-118J21.25_ENSG00000261798
1	40348924	40349122	5Y-H4K8ac_peak_659	9.63153	TRIT1_ENSG00000043514
1	40367357	40367605	5Y-H4K8ac_peak_660	10.54764	MYCL_ENSG00000116990
1	40506483	40506690	5Y-H4K8ac_peak_661	9.26157	CAP1_ENSG00000131236
1	40626549	40626848	5Y-H4K8ac_peak_662	5.98695	RLF_ENSG00000117000
1	40780548	40781441	5Y-H4K8ac_peak_663	7.50148	
1	40782471	40782719	5Y-H4K8ac_peak_664	11.68317	COL9A2_ENSG00000049089
1	40916373	40916744	5Y-H4K8ac_peak_665	7.17184	ZFP69B_ENSG00000187801
1	40943210	40943448	5Y-H4K8ac_peak_666	6.34046	ZFP69_ENSG00000187815
1	41157395	41157664	5Y-H4K8ac_peak_667	16.13719	NFYC-AS1_ENSG00000272145;NFYC_ENSG00000066136
1	41314132	41314325	5Y-H4K8ac_peak_668	4.24332	
1	41445780	41446090	5Y-H4K8ac_peak_669	10.60083	CTPS1_ENSG00000171793
1	41708073	41708591	5Y-H4K8ac_peak_670	12.05638	SCMH1_ENSG00000010803;RP11-399E6.1_ENSG00000235358
1	41831694	41832094	5Y-H4K8ac_peak_671	14.77593	
1	41847338	41847698	5Y-H4K8ac_peak_672	6.08523	
1	41848054	41848420	5Y-H4K8ac_peak_673	4.77126	
1	41898899	41899269	5Y-H4K8ac_peak_674	6.31818	
1	41920331	41920569	5Y-H4K8ac_peak_675	8.69112	
1	41940546	41941118	5Y-H4K8ac_peak_676	4.48815	
1	41962419	41962645	5Y-H4K8ac_peak_677	10.19948	
1	41968271	41968573	5Y-H4K8ac_peak_678	10.69698	
1	41968976	41969317	5Y-H4K8ac_peak_679	5.89113	
1	41970515	41970715	5Y-H4K8ac_peak_680	7.9412	
1	41982070	41982465	5Y-H4K8ac_peak_681	9.44542	
1	42078391	42078788	5Y-H4K8ac_peak_682	6.78815	
1	42128113	42128565	5Y-H4K8ac_peak_683	6.50117	
1	42161203	42161423	5Y-H4K8ac_peak_684	9.63153	
1	42179409	42179710	5Y-H4K8ac_peak_685	10.35586	
1	42180092	42180376	5Y-H4K8ac_peak_686	6.10343	
1	42181509	42181921	5Y-H4K8ac_peak_687	11.36989	
1	42182278	42182826	5Y-H4K8ac_peak_688	7.50148	

1	42192979	42193273	5Y-H4K8ac_peak_689	4.96019	
1	42357185	42357375	5Y-H4K8ac_peak_690	6.73385	
1	42501873	42502180	5Y-H4K8ac_peak_691	7.59101	HIVEP3_ENSG00000127124
1	42630227	42630614	5Y-H4K8ac_peak_692	5.65584	GUCA2A_ENSG00000197273
1	42846618	42846877	5Y-H4K8ac_peak_693	6.08523	RIMKLA_ENSG00000177181
1	42921046	42921611	5Y-H4K8ac_peak_694	5.98695	ZMYND12_ENSG00000066185;PPCS_ENSG00000127125
1	42922359	42922811	5Y-H4K8ac_peak_695	11.28133	ZMYND12_ENSG00000066185;PPCS_ENSG00000127125
1	43124255	43124488	5Y-H4K8ac_peak_696	6.37023	PPIH_ENSG00000171960
1	43282823	43283034	5Y-H4K8ac_peak_697	11.42066	CCDC23_ENSG00000177868;ERMAP_ENSG00000164010
1	43450905	43451173	5Y-H4K8ac_peak_698	8.2913	
1	43470883	43471503	5Y-H4K8ac_peak_699	9.30505	
1	43524392	43524668	5Y-H4K8ac_peak_700	5.98695	
1	43533961	43534268	5Y-H4K8ac_peak_701	11.71078	
1	43814332	43814557	5Y-H4K8ac_peak_702	6.34245	
1	43824085	43824290	5Y-H4K8ac_peak_703	8.79957	RP1-92O14.3_ENSG00000234694;CDC20_ENSG00000117399
1	43833893	43834118	5Y-H4K8ac_peak_704	12.10416	ELOVL1_ENSG00000066322
1	43919073	43919818	5Y-H4K8ac_peak_705	7.31815	HYI_ENSG00000178922;HYI-AS1_ENSG00000229348
1	43996640	43996905	5Y-H4K8ac_peak_706	8.2913	
1	44173248	44173657	5Y-H4K8ac_peak_707	6.77436	KDM4A-AS1_ENSG00000236200
1	44378362	44378766	5Y-H4K8ac_peak_708	5.65584	
1	44379443	44379634	5Y-H4K8ac_peak_709	5.41472	
1	44401753	44401977	5Y-H4K8ac_peak_710	10.95042	
1	44402345	44402556	5Y-H4K8ac_peak_711	7.11863	
1	44412406	44412600	5Y-H4K8ac_peak_712	9.00954	RP11-7O11.3_ENSG00000237950;IPO13_ENSG00000117408
1	44440153	44440371	5Y-H4K8ac_peak_713	4.642	ATP6V0B_ENSG00000117410
1	44445135	44445391	5Y-H4K8ac_peak_714	13.0168	B4GALT2_ENSG00000117411
1	44457543	44457995	5Y-H4K8ac_peak_715	4.50302	CCDC24_ENSG00000159214
1	44461147	44461901	5Y-H4K8ac_peak_716	8.69112	
1	44462343	44462869	5Y-H4K8ac_peak_717	8.1667	
1	44463254	44463558	5Y-H4K8ac_peak_718	6.77436	
1	44495562	44495903	5Y-H4K8ac_peak_719	8.1667	
1	44496216	44496444	5Y-H4K8ac_peak_720	7.60057	SLC6A9_ENSG00000196517
1	44501290	44501596	5Y-H4K8ac_peak_721	5.3989	RP5-1198O20.4_ENSG00000230615
1	44678152	44678818	5Y-H4K8ac_peak_722	9.23159	DMAP1_ENSG00000178028
1	44679141	44679413	5Y-H4K8ac_peak_723	8.43511	DMAP1_ENSG00000178028
1	44820692	44820913	5Y-H4K8ac_peak_724	10.1994	ERI3_ENSG00000117419
1	45097022	45098360	5Y-H4K8ac_peak_725	21.35466	
1	45197384	45197598	5Y-H4K8ac_peak_726	6.43775	RNU5D-1_ENSG00000200169
1	45241414	45241854	5Y-H4K8ac_peak_727	5.03917	RP11-269F19.2_ENSG00000225721;RPS8_ENSG00000142937;SNORD55_ENSG00000264294;SNORD46_ENSG
1	45278749	45279755	5Y-H4K8ac_peak_728	11.1169	
1	45280332	45280791	5Y-H4K8ac_peak_729	4.77016	
1	45296482	45296766	5Y-H4K8ac_peak_730	5.23083	
1	45412710	45412948	5Y-H4K8ac_peak_731	5.65584	
1	45477372	45477589	5Y-H4K8ac_peak_732	5.70209	HECTD3_ENSG00000126107;UROD_ENSG00000126088
1	45769584	45770238	5Y-H4K8ac_peak_733	10.31981	
1	45792568	45792870	5Y-H4K8ac_peak_734	5.64909	HPDL_ENSG00000186603

1	46598882	46599281	5Y-H4K8ac_peak_735	14.43262	RP4-533D7.5_ENSG00000227857
1	46663787	46664497	5Y-H4K8ac_peak_736	9.88908	
1	46767504	46768142	5Y-H4K8ac_peak_737	5.89922	
1	46768482	46768852	5Y-H4K8ac_peak_738	10.23828	LRRC41_ENSG00000132128;UQCRH_ENSG00000173660
1	46805916	46806788	5Y-H4K8ac_peak_739	15.40182	NSUN4_ENSG00000117481
1	46871696	46871955	5Y-H4K8ac_peak_740	9.96543	
1	46931755	46932913	5Y-H4K8ac_peak_741	8.5915	
1	47081879	47082071	5Y-H4K8ac_peak_742	4.96019	MKNK1_ENSG00000079277;MOB3C_ENSG00000142961
1	47133608	47133889	5Y-H4K8ac_peak_743	6.78128	TEX38_ENSG00000186118
1	47184438	47184695	5Y-H4K8ac_peak_744	11.36844	EFCAB14_ENSG00000159658
1	47184920	47185153	5Y-H4K8ac_peak_745	8.2913	EFCAB14_ENSG00000159658
1	47780483	47780884	5Y-H4K8ac_peak_746	11.36844	STIL_ENSG00000123473
1	47973386	47974379	5Y-H4K8ac_peak_747	15.87289	
1	47999681	47999928	5Y-H4K8ac_peak_748	7.38046	
1	48044023	48044405	5Y-H4K8ac_peak_749	11.68317	
1	48242658	48243033	5Y-H4K8ac_peak_750	7.52971	
1	48360871	48361086	5Y-H4K8ac_peak_751	4.07874	
1	48853281	48853494	5Y-H4K8ac_peak_752	6.34046	
1	49242071	49242447	5Y-H4K8ac_peak_753	6.20875	BEND5_ENSG00000162373
1	49513566	49513818	5Y-H4K8ac_peak_754	7.76232	RP11-141A19.1_ENSG00000229846
1	50489814	50490075	5Y-H4K8ac_peak_755	4.3419	AGBL4_ENSG00000186094
1	50513726	50514479	5Y-H4K8ac_peak_756	10.5157	ELAVL4_ENSG00000162374
1	50730912	50731414	5Y-H4K8ac_peak_757	7.31815	
1	50738942	50739216	5Y-H4K8ac_peak_758	6.43775	
1	50741713	50742097	5Y-H4K8ac_peak_759	6.19178	
1	50743496	50743807	5Y-H4K8ac_peak_760	12.0443	
1	51443235	51443494	5Y-H4K8ac_peak_761	9.23159	
1	51443798	51444055	5Y-H4K8ac_peak_762	19.17092	
1	51702400	51702684	5Y-H4K8ac_peak_763	4.50834	RNF11_ENSG00000123091
1	51762483	51763421	5Y-H4K8ac_peak_764	11.19336	
1	51796313	51796787	5Y-H4K8ac_peak_765	8.69112	RP11-275F13.1_ENSG00000261664
1	51797053	51797495	5Y-H4K8ac_peak_766	8.8236	
1	52082816	52083008	5Y-H4K8ac_peak_767	9.23159	
1	52113839	52114029	5Y-H4K8ac_peak_768	6.77436	
1	52195000	52195217	5Y-H4K8ac_peak_769	11.19336	
1	52195565	52195827	5Y-H4K8ac_peak_770	10.36926	
1	52522055	52522375	5Y-H4K8ac_peak_771	13.92877	TXNDC12_ENSG00000117862;BTF3L4_ENSG00000134717
1	52607890	52608345	5Y-H4K8ac_peak_772	5.64909	ZFYVE9_ENSG00000157077
1	52814628	52814870	5Y-H4K8ac_peak_773	4.09853	
1	52831493	52831757	5Y-H4K8ac_peak_774	6.37023	CC2D1B_ENSG00000154222
1	53018358	53018638	5Y-H4K8ac_peak_775	11.09973	ZCCHC11_ENSG00000134744
1	53019230	53019793	5Y-H4K8ac_peak_776	12.6675	RP11-25O10.2_ENSG00000272371;ZCCHC11_ENSG00000134744
1	53067778	53067968	5Y-H4K8ac_peak_777	5.34294	GPX7_ENSG00000116157
1	53098468	53099208	5Y-H4K8ac_peak_778	8.73985	FAM159A_ENSG00000182183
1	53169262	53169496	5Y-H4K8ac_peak_779	5.91107	
1	53308712	53308986	5Y-H4K8ac_peak_780	4.95697	ZYG11A_ENSG00000203995

1	53387187	53387381	5Y-H4K8ac_peak_781	4.07874	
1	53527562	53528373	5Y-H4K8ac_peak_782	11.1169	PODN_ENSG00000174348
1	53611968	53612257	5Y-H4K8ac_peak_783	4.47071	
1	53686085	53687009	5Y-H4K8ac_peak_784	12.2918	C1orf123_ENSG00000162384;RP5-1024G6.7_ENSG00000259818
1	53704446	53704652	5Y-H4K8ac_peak_785	9.30505	MAGOH_ENSG00000162385;RP5-1024G6.5_ENSG00000226754
1	53753243	53753445	5Y-H4K8ac_peak_786	4.70571	RP4-784A16.2_ENSG00000228838
1	53760794	53760992	5Y-H4K8ac_peak_787	7.04158	
1	53780849	53781696	5Y-H4K8ac_peak_788	9.16473	
1	53782074	53782469	5Y-H4K8ac_peak_789	11.9002	
1	53783377	53783719	5Y-H4K8ac_peak_790	8.82628	
1	53793326	53793517	5Y-H4K8ac_peak_791	6.35402	LRP8_ENSG00000157193;RP4-784A16.5_ENSG00000225675
1	53793946	53794324	5Y-H4K8ac_peak_792	8.47164	LRP8_ENSG00000157193;RP4-784A16.5_ENSG00000225675
1	53833308	53833646	5Y-H4K8ac_peak_793	9.79526	RP11-117D22.2_ENSG00000230138
1	53834017	53834736	5Y-H4K8ac_peak_794	10.54764	RP11-117D22.2_ENSG00000230138
1	53837205	53837453	5Y-H4K8ac_peak_795	8.24952	
1	53858309	53858597	5Y-H4K8ac_peak_796	5.03335	
1	53875217	53875459	5Y-H4K8ac_peak_797	7.01266	
1	53978238	53978465	5Y-H4K8ac_peak_798	8.43511	
1	53979641	53979838	5Y-H4K8ac_peak_799	6.77436	
1	53992706	53993318	5Y-H4K8ac_peak_800	6.52331	
1	54056070	54056426	5Y-H4K8ac_peak_801	6.11316	
1	54058340	54058583	5Y-H4K8ac_peak_802	4.50834	
1	54121676	54121977	5Y-H4K8ac_peak_803	7.50148	
1	54151885	54152115	5Y-H4K8ac_peak_804	5.57299	
1	54303817	54304249	5Y-H4K8ac_peak_805	15.82713	NDC1_ENSG00000058804
1	54695614	54696007	5Y-H4K8ac_peak_806	7.30348	
1	54704087	54704452	5Y-H4K8ac_peak_807	4.84727	SSBP3-AS1_ENSG00000198711
1	54712656	54712909	5Y-H4K8ac_peak_808	7.24844	
1	54802079	54802294	5Y-H4K8ac_peak_809	6.49308	
1	54940664	54941739	5Y-H4K8ac_peak_810	6.77436	
1	54953358	54953995	5Y-H4K8ac_peak_811	6.08523	
1	54954485	54955416	5Y-H4K8ac_peak_812	9.30505	
1	54964827	54965625	5Y-H4K8ac_peak_813	13.61292	
1	55008436	55008807	5Y-H4K8ac_peak_814	5.41472	ACOT11_ENSG00000162390
1	55037338	55037670	5Y-H4K8ac_peak_815	5.12213	
1	55180587	55180971	5Y-H4K8ac_peak_816	5.65584	TTC4_ENSG00000243725
1	55181600	55181952	5Y-H4K8ac_peak_817	9.38203	TTC4_ENSG00000243725
1	55415899	55416964	5Y-H4K8ac_peak_818	8.24461	
1	55446269	55446600	5Y-H4K8ac_peak_819	5.91107	TMEM61_ENSG00000143001
1	55453923	55454171	5Y-H4K8ac_peak_820	8.47164	
1	55488039	55488301	5Y-H4K8ac_peak_821	6.34046	
1	55516023	55516470	5Y-H4K8ac_peak_822	6.73047	
1	55680612	55681471	5Y-H4K8ac_peak_823	13.85283	USP24_ENSG00000162402
1	55841157	55841491	5Y-H4K8ac_peak_824	7.34202	
1	55843244	55843770	5Y-H4K8ac_peak_825	6.52593	RN7SKP291_ENSG00000199831
1	55933781	55934018	5Y-H4K8ac_peak_826	5.93823	

1	56022937	56023444	5Y-H4K8ac_peak_827	7.97699	
1	56107054	56107579	5Y-H4K8ac_peak_828	5.98695	
1	56150696	56151048	5Y-H4K8ac_peak_829	9.38203	
1	56158859	56159367	5Y-H4K8ac_peak_830	5.98695	
1	56159560	56159842	5Y-H4K8ac_peak_831	8.564	
1	56160404	56160677	5Y-H4K8ac_peak_832	7.31807	
1	56232501	56232699	5Y-H4K8ac_peak_833	7.3889	
1	56666955	56667260	5Y-H4K8ac_peak_834	5.81062	
1	56828888	56829106	5Y-H4K8ac_peak_835	7.31815	
1	56842449	56842693	5Y-H4K8ac_peak_836	7.59101	AC119674.1_ENSG00000223307
1	56844928	56845335	5Y-H4K8ac_peak_837	6.55906	
1	56972079	56972303	5Y-H4K8ac_peak_838	5.65584	
1	58898597	58898820	5Y-H4K8ac_peak_839	9.30505	
1	59165365	59165663	5Y-H4K8ac_peak_840	13.99721	MYSM1_ENSG00000162601
1	59238803	59239301	5Y-H4K8ac_peak_841	9.83437	
1	59247795	59248615	5Y-H4K8ac_peak_842	7.38046	
1	59250094	59250824	5Y-H4K8ac_peak_843	8.1667	JUN_ENSG00000177606;LINC01135_ENSG00000234807
1	59282214	59282406	5Y-H4K8ac_peak_844	7.20869	
1	59369482	59369700	5Y-H4K8ac_peak_845	8.69112	
1	59762052	59762259	5Y-H4K8ac_peak_846	4.00285	FGGY_ENSG00000172456
1	59912607	59913077	5Y-H4K8ac_peak_847	7.04637	
1	59963009	59963207	5Y-H4K8ac_peak_848	5.23083	
1	60279920	60280201	5Y-H4K8ac_peak_849	11.19336	HOOK1_ENSG00000134709
1	60280645	60281024	5Y-H4K8ac_peak_850	9.23159	HOOK1_ENSG00000134709
1	61331479	61331756	5Y-H4K8ac_peak_851	8.33296	NFIA_ENSG00000162599
1	61351189	61351381	5Y-H4K8ac_peak_852	6.34046	
1	61354025	61354373	5Y-H4K8ac_peak_853	5.64909	
1	61362899	61363094	5Y-H4K8ac_peak_854	4.51076	
1	61369158	61369857	5Y-H4K8ac_peak_855	9.23159	
1	61370060	61370523	5Y-H4K8ac_peak_856	16.27715	
1	61400800	61401095	5Y-H4K8ac_peak_857	10.69698	
1	61516227	61516544	5Y-H4K8ac_peak_858	10.35586	
1	61523374	61523642	5Y-H4K8ac_peak_859	5.18494	
1	61549264	61549496	5Y-H4K8ac_peak_860	4.9885	AC096534.1_ENSG00000263380
1	62190522	62191132	5Y-H4K8ac_peak_861	7.89273	TM2D1_ENSG00000162604
1	62208043	62208785	5Y-H4K8ac_peak_862	10.21117	RP11-430G17.3_ENSG00000271200;INADL_ENSG00000132849
1	62272680	62272908	5Y-H4K8ac_peak_863	5.65584	
1	62737273	62737650	5Y-H4K8ac_peak_864	13.27281	
1	62738187	62738609	5Y-H4K8ac_peak_865	6.61077	
1	62738838	62739319	5Y-H4K8ac_peak_866	9.02782	
1	62740131	62740634	5Y-H4K8ac_peak_867	13.63503	
1	62774088	62774590	5Y-H4K8ac_peak_868	10.19948	
1	62793104	62793389	5Y-H4K8ac_peak_869	14.15915	
1	62827636	62828125	5Y-H4K8ac_peak_870	7.31815	
1	62849386	62849579	5Y-H4K8ac_peak_871	8.2913	
1	62861782	62862086	5Y-H4K8ac_peak_872	5.65584	

1	62901497	62901837	5Y-H4K8ac_peak_873	7.50501	USP1_ENSG00000162607
1	62902610	62902879	5Y-H4K8ac_peak_874	5.73251	USP1_ENSG00000162607
1	63153594	63154096	5Y-H4K8ac_peak_875	11.99586	DOCK7_ENSG00000116641;RP11-230B22.1_ENSG00000235545
1	63187200	63187401	5Y-H4K8ac_peak_876	5.65584	
1	63553808	63554012	5Y-H4K8ac_peak_877	4.07874	
1	63832439	63833090	5Y-H4K8ac_peak_878	11.48691	ALG6_ENSG00000088035
1	63833392	63833598	5Y-H4K8ac_peak_879	10.60083	ALG6_ENSG00000088035
1	64194035	64194231	5Y-H4K8ac_peak_880	4.07874	
1	64239783	64239986	5Y-H4K8ac_peak_881	4.79585	ROR1_ENSG00000185483
1	64575341	64575559	5Y-H4K8ac_peak_882	7.30348	
1	64661935	64662427	5Y-H4K8ac_peak_883	9.05168	
1	64749180	64749482	5Y-H4K8ac_peak_884	5.37137	
1	64754572	64754795	5Y-H4K8ac_peak_885	5.68769	
1	64812662	64812865	5Y-H4K8ac_peak_886	8.35139	
1	64935444	64935653	5Y-H4K8ac_peak_887	11.28133	CACHD1_ENSG00000158966
1	65061159	65061365	5Y-H4K8ac_peak_888	7.53429	
1	65090982	65091326	5Y-H4K8ac_peak_889	16.27715	
1	65094249	65094443	5Y-H4K8ac_peak_890	5.74859	
1	65210349	65210799	5Y-H4K8ac_peak_891	8.79957	RAVER2_ENSG00000162437
1	65532939	65533182	5Y-H4K8ac_peak_892	10.1994	RP4-535B20.1_ENSG00000231485
1	65533610	65533952	5Y-H4K8ac_peak_893	8.08688	RP4-535B20.1_ENSG00000231485
1	65731513	65731894	5Y-H4K8ac_peak_894	10.79655	
1	65775540	65775764	5Y-H4K8ac_peak_895	8.35139	
1	65886419	65886789	5Y-H4K8ac_peak_896	13.57931	LEPR_ENSG00000116678;LEPROT_ENSG00000213625
1	65991028	65991639	5Y-H4K8ac_peak_897	15.84891	
1	66998938	66999475	5Y-H4K8ac_peak_898	4.84727	SGIP1_ENSG00000118473
1	67519709	67520070	5Y-H4K8ac_peak_899	9.23159	SLC35D1_ENSG00000116704
1	67895591	67896038	5Y-H4K8ac_peak_900	15.28394	SERBP1_ENSG00000142864
1	68075975	68076279	5Y-H4K8ac_peak_901	8.43011	
1	68113174	68113378	5Y-H4K8ac_peak_902	6.3019	
1	68151546	68152013	5Y-H4K8ac_peak_903	4.29586	GADD45A_ENSG00000116717
1	68178611	68178858	5Y-H4K8ac_peak_904	6.50117	
1	68404810	68405418	5Y-H4K8ac_peak_905	5.98695	
1	68411446	68411718	5Y-H4K8ac_peak_906	6.20875	
1	68412859	68413060	5Y-H4K8ac_peak_907	6.79955	
1	68697974	68698195	5Y-H4K8ac_peak_908	5.65584	WLS_ENSG00000116729
1	70598871	70599079	5Y-H4K8ac_peak_909	9.38203	
1	74663321	74663648	5Y-H4K8ac_peak_910	6.1654	LRRIQ3_ENSG00000162620;FPGT_ENSG00000254685;FPGT-TNNI3K_ENSG00000259030;TNNI3K_ENSG000
1	76081400	76081782	5Y-H4K8ac_peak_911	8.24461	
1	76082035	76082520	5Y-H4K8ac_peak_912	16.38263	
1	76190236	76190603	5Y-H4K8ac_peak_913	5.65584	RP4-682C21.5_ENSG00000178193;ACADM_ENSG00000117054
1	76251430	76251652	5Y-H4K8ac_peak_914	5.23083	RABGGTB_ENSG00000137955
1	76513636	76513842	5Y-H4K8ac_peak_915	4.51076	
1	77230617	77230893	5Y-H4K8ac_peak_916	16.94054	
1	78148904	78149621	5Y-H4K8ac_peak_917	11.19336	ZZZ3_ENSG00000036549
1	78444326	78444644	5Y-H4K8ac_peak_918	9.34935	FUBP1_ENSG00000162613;DNAJB4_ENSG00000162616;GIPC2_ENSG00000137960

1	82267272	82267497	5Y-H4K8ac_peak_919	6.14981	
1	82268426	82268898	5Y-H4K8ac_peak_920	5.83797	
1	84157896	84158160	5Y-H4K8ac_peak_921	4.29586	
1	84160166	84160781	5Y-H4K8ac_peak_922	6.59249	
1	84167175	84167395	5Y-H4K8ac_peak_923	5.64909	
1	84543194	84543551	5Y-H4K8ac_peak_924	6.08523	RP11-486G15.2_ENSG00000271576;PRKACB_ENSG00000142875
1	84764293	84764529	5Y-H4K8ac_peak_925	8.46442	SAMD13_ENSG00000203943
1	84945006	84945299	5Y-H4K8ac_peak_926	7.38046	RPF1_ENSG00000117133
1	85155677	85156200	5Y-H4K8ac_peak_927	9.23159	SSX2IP_ENSG00000117155
1	85156552	85156793	5Y-H4K8ac_peak_928	6.50117	SSX2IP_ENSG00000117155
1	85313630	85314142	5Y-H4K8ac_peak_929	6.79955	
1	85410112	85410311	5Y-H4K8ac_peak_930	6.34046	
1	85930428	85930763	5Y-H4K8ac_peak_931	8.2913	
1	86041993	86042327	5Y-H4K8ac_peak_932	9.7353	
1	86046333	86046672	5Y-H4K8ac_peak_933	8.2913	CYR61_ENSG00000142871
1	86174286	86174767	5Y-H4K8ac_peak_934	7.3889	ZNHIT6_ENSG00000117174
1	86861373	86861602	5Y-H4K8ac_peak_935	6.50117	ODF2L_ENSG00000122417
1	87621420	87621680	5Y-H4K8ac_peak_936	4.50834	
1	87793760	87793971	5Y-H4K8ac_peak_937	12.21176	LMO4_ENSG00000143013
1	89989766	89990233	5Y-H4K8ac_peak_938	10.48476	LRRC8B_ENSG00000197147
1	90227875	90228188	5Y-H4K8ac_peak_939	4.8773	
1	90460895	90461141	5Y-H4K8ac_peak_940	5.64909	ZNF326_ENSG00000162664
1	91316592	91317144	5Y-H4K8ac_peak_941	5.98695	RP4-665J23.1_ENSG00000233593;RP4-665J23.2_ENSG00000225446
1	91487339	91488207	5Y-H4K8ac_peak_942	9.30505	ZNF644_ENSG00000122482
1	91870173	91870386	5Y-H4K8ac_peak_943	6.22669	HFM1_ENSG00000162669
1	92495018	92495665	5Y-H4K8ac_peak_944	9.30505	EPHX4_ENSG00000172031
1	92764742	92764985	5Y-H4K8ac_peak_945	7.03573	RPAP2_ENSG00000122484
1	93251209	93251416	5Y-H4K8ac_peak_946	8.08688	
1	93811609	93811895	5Y-H4K8ac_peak_947	4.0639	RP4-717I23.3_ENSG00000223745;DR1_ENSG00000117505
1	93913065	93913525	5Y-H4K8ac_peak_948	8.93304	FNBP1L_ENSG00000137942
1	93913801	93914161	5Y-H4K8ac_peak_949	8.47164	FNBP1L_ENSG00000137942
1	94041035	94041493	5Y-H4K8ac_peak_950	19.71555	
1	94073771	94074036	5Y-H4K8ac_peak_951	5.34407	
1	94074975	94075363	5Y-H4K8ac_peak_952	5.64909	
1	94082354	94082567	5Y-H4K8ac_peak_953	5.98695	
1	94082873	94083890	5Y-H4K8ac_peak_954	9.51254	
1	94283257	94283623	5Y-H4K8ac_peak_955	8.21582	
1	94293486	94293697	5Y-H4K8ac_peak_956	10.31981	
1	94312017	94312411	5Y-H4K8ac_peak_957	8.43511	BCAR3_ENSG00000137936;MIR760_ENSG00000211575;RP4-561L24.3_ENSG00000260464
1	94509863	94510128	5Y-H4K8ac_peak_958	4.77126	
1	94910932	94911231	5Y-H4K8ac_peak_959	9.56187	
1	95007739	95008071	5Y-H4K8ac_peak_960	9.05168	F3_ENSG00000117525
1	95285571	95286253	5Y-H4K8ac_peak_961	7.59101	LINC01057_ENSG00000224081;SLC44A3_ENSG00000143036
1	95392147	95392383	5Y-H4K8ac_peak_962	6.64195	CNN3_ENSG00000117519;RP4-639F20.1_ENSG00000235501
1	95392937	95393152	5Y-H4K8ac_peak_963	6.37023	CNN3_ENSG00000117519;RP4-639F20.1_ENSG00000235501
1	95414832	95415103	5Y-H4K8ac_peak_964	11.20652	

1	95468722	95468924	5Y-H4K8ac_peak_965	4.24332	
1	95773823	95774102	5Y-H4K8ac_peak_966	9.23159	
1	95774317	95774538	5Y-H4K8ac_peak_967	5.65584	
1	95790689	95791023	5Y-H4K8ac_peak_968	6.34046	
1	95846020	95846391	5Y-H4K8ac_peak_969	7.38046	RP11-14O19.2_ENSG00000237954
1	95851349	95851574	5Y-H4K8ac_peak_970	6.31594	
1	95862746	95862944	5Y-H4K8ac_peak_971	7.29091	
1	97187398	97187975	5Y-H4K8ac_peak_972	10.35586	PTBP2_ENSG00000117569
1	97454745	97455035	5Y-H4K8ac_peak_973	7.72345	
1	98518884	98519732	5Y-H4K8ac_peak_974	15.28394	
1	98918232	98918466	5Y-H4K8ac_peak_975	5.40331	
1	98927263	98927469	5Y-H4K8ac_peak_976	6.34046	
1	99469729	99470229	5Y-H4K8ac_peak_977	10.69698	LPPR5_ENSG00000117598;RP5-896L10.1_ENSG00000232825
1	99729583	99729909	5Y-H4K8ac_peak_978	5.40331	LPPR4_ENSG00000117600
1	100315009	100315979	5Y-H4K8ac_peak_979	13.00733	
1	100409134	100409349	5Y-H4K8ac_peak_980	5.40331	
1	100503817	100504057	5Y-H4K8ac_peak_981	7.64648	HIAT1_ENSG00000156875
1	101491493	101491942	5Y-H4K8ac_peak_982	10.46287	DPH5_ENSG00000117543;RP11-421L21.3_ENSG00000233184
1	105680555	105681154	5Y-H4K8ac_peak_983	13.72401	
1	107598749	107599077	5Y-H4K8ac_peak_984	7.01266	PRMT6_ENSG00000198890
1	107599381	107600097	5Y-H4K8ac_peak_985	15.77156	PRMT6_ENSG00000198890
1	108507160	108507564	5Y-H4K8ac_peak_986	7.30348	VAV3_ENSG00000134215;VAV3-AS1_ENSG00000230489
1	109101896	109102167	5Y-H4K8ac_peak_987	4.50834	FAM102B_ENSG00000162636
1	109234964	109235413	5Y-H4K8ac_peak_988	9.38276	PRPF38B_ENSG00000134186
1	109359566	109359885	5Y-H4K8ac_peak_989	4.15658	
1	109372781	109373427	5Y-H4K8ac_peak_990	10.19948	
1	109374374	109374606	5Y-H4K8ac_peak_991	7.3889	
1	109505732	109505940	5Y-H4K8ac_peak_992	7.38046	AKNAD1_ENSG00000162641;CLCC1_ENSG00000121940
1	109618844	109619498	5Y-H4K8ac_peak_993	14.86775	TAF13_ENSG00000197780
1	109642947	109643277	5Y-H4K8ac_peak_994	6.34245	SCARNA2_ENSG00000270066
1	109783368	109783648	5Y-H4K8ac_peak_995	4.79585	
1	109791523	109791722	5Y-H4K8ac_peak_996	7.11863	CELSR2_ENSG00000143126
1	109796335	109797244	5Y-H4K8ac_peak_997	9.47304	
1	109797578	109798092	5Y-H4K8ac_peak_998	6.19716	
1	109940880	109941100	5Y-H4K8ac_peak_999	6.78128	SORT1_ENSG00000134243
1	110026590	110027058	5Y-H4K8ac_peak_1000	16.15586	ATXN7L2_ENSG00000162650
1	110037173	110037366	5Y-H4K8ac_peak_1001	5.70209	CYB561D1_ENSG00000174151
1	110041686	110041898	5Y-H4K8ac_peak_1002	7.38046	
1	110073876	110074224	5Y-H4K8ac_peak_1003	10.55771	
1	110075218	110075438	5Y-H4K8ac_peak_1004	6.34245	
1	110183094	110183339	5Y-H4K8ac_peak_1005	6.78128	
1	110313216	110313446	5Y-H4K8ac_peak_1006	4.84727	
1	110526675	110527149	5Y-H4K8ac_peak_1007	11.01067	AHCYL1_ENSG00000168710
1	110593181	110593398	5Y-H4K8ac_peak_1008	5.23083	
1	110599919	110600501	5Y-H4K8ac_peak_1009	8.27643	RP4-773N10.4_ENSG00000258634
1	110601263	110601694	5Y-H4K8ac_peak_1010	7.65404	RP4-773N10.4_ENSG00000258634

1	110603026	110603355	5Y-H4K8ac_peak_1011	5.23083	
1	110604002	110604408	5Y-H4K8ac_peak_1012	6.49786	
1	110640619	110641116	5Y-H4K8ac_peak_1013	12.44818	
1	110649014	110649350	5Y-H4K8ac_peak_1014	10.35586	
1	110777757	110778320	5Y-H4K8ac_peak_1015	12.26606	
1	110824511	110824911	5Y-H4K8ac_peak_1016	6.34046	
1	110878457	110878674	5Y-H4K8ac_peak_1017	5.65584	
1	110950713	110950913	5Y-H4K8ac_peak_1018	6.77436	LAMTOR5_ENSG00000134248
1	111217753	111218008	5Y-H4K8ac_peak_1019	6.78128	KCNA3_ENSG00000177272
1	111506202	111506466	5Y-H4K8ac_peak_1020	7.58806	LRIF1_ENSG00000121931;RP11-96K19.5_ENSG00000273010
1	111682366	111682605	5Y-H4K8ac_peak_1021	7.3889	DRAM2_ENSG00000156171;CEPT1_ENSG00000134255
1	111746934	111747221	5Y-H4K8ac_peak_1022	7.13294	DENND2D_ENSG00000162777
1	112050575	112050933	5Y-H4K8ac_peak_1023	8.20773	
1	112298365	112298803	5Y-H4K8ac_peak_1024	20.19152	FAM212B_ENSG00000197852;DDX20_ENSG00000064703
1	113161815	113162215	5Y-H4K8ac_peak_1025	9.30206	CAPZA1_ENSG00000116489
1	113217692	113218230	5Y-H4K8ac_peak_1026	10.19948	
1	113392060	113392386	5Y-H4K8ac_peak_1027	10.35586	RP3-522D1.1_ENSG00000224167;RP11-426L16.8_ENSG00000215866
1	113498503	113498754	5Y-H4K8ac_peak_1028	9.96543	SLC16A1_ENSG00000155380;SLC16A1-AS1_ENSG00000226419
1	113683530	113683967	5Y-H4K8ac_peak_1029	10.19948	
1	113690435	113690856	5Y-H4K8ac_peak_1030	14.00129	
1	113691163	113691523	5Y-H4K8ac_peak_1031	7.88114	
1	114472410	114472795	5Y-H4K8ac_peak_1032	11.09973	RP5-1073O3.7_ENSG00000235527;HIPK1_ENSG00000163349
1	114888637	114889003	5Y-H4K8ac_peak_1033	11.1169	
1	115124618	115124857	5Y-H4K8ac_peak_1034	8.43511	BCAS2_ENSG00000116752
1	115300235	115300575	5Y-H4K8ac_peak_1035	23.16826	CSDE1_ENSG00000009307
1	115323514	115323811	5Y-H4K8ac_peak_1036	6.43775	SIKE1_ENSG00000052723
1	115793528	115793840	5Y-H4K8ac_peak_1037	9.38203	
1	115880036	115880775	5Y-H4K8ac_peak_1038	8.43511	NGF_ENSG00000134259
1	115906599	115906923	5Y-H4K8ac_peak_1039	9.30505	
1	116247646	116247864	5Y-H4K8ac_peak_1040	5.65584	
1	116371906	116372099	5Y-H4K8ac_peak_1041	4.95697	
1	116518520	116519545	5Y-H4K8ac_peak_1042	11.19336	SLC22A15_ENSG00000163393
1	116681411	116681611	5Y-H4K8ac_peak_1043	11.93212	
1	116915240	116915660	5Y-H4K8ac_peak_1044	8.35139	ATP1A1_ENSG00000163399
1	116916408	116916720	5Y-H4K8ac_peak_1045	8.33296	
1	116960313	116960507	5Y-H4K8ac_peak_1046	5.91107	ATP1A1OS_ENSG00000203865
1	116960857	116961155	5Y-H4K8ac_peak_1047	6.54441	ATP1A1OS_ENSG00000203865
1	117027521	117027973	5Y-H4K8ac_peak_1048	6.78128	
1	117058101	117058328	5Y-H4K8ac_peak_1049	6.20875	
1	117113223	117113570	5Y-H4K8ac_peak_1050	5.64909	CD58_ENSG00000116815
1	117113973	117114311	5Y-H4K8ac_peak_1051	10.35586	CD58_ENSG00000116815
1	117229471	117229790	5Y-H4K8ac_peak_1052	5.59843	
1	117230670	117231035	5Y-H4K8ac_peak_1053	9.30505	
1	117235683	117236269	5Y-H4K8ac_peak_1054	6.12014	C1orf137_ENSG00000203864
1	117250801	117251010	5Y-H4K8ac_peak_1055	7.50148	
1	117254243	117254509	5Y-H4K8ac_peak_1056	6.50117	

1	117367416	117367614	5Y-H4K8ac_peak_1057	9.96543	
1	117397832	117398382	5Y-H4K8ac_peak_1058	8.8021	
1	117422062	117422269	5Y-H4K8ac_peak_1059	6.43775	
1	117452635	117453209	5Y-H4K8ac_peak_1060	17.63051	RP4-753F5.1_ENSG00000272715;PTGFRN_ENSG00000134247
1	117664594	117665236	5Y-H4K8ac_peak_1061	12.44818	TRIM45_ENSG00000134253
1	118370889	118371263	5Y-H4K8ac_peak_1062	6.34046	
1	120254976	120255246	5Y-H4K8ac_peak_1063	6.79955	
1	120904719	120904981	5Y-H4K8ac_peak_1064	4.07874	RP11-439A17.4_ENSG00000227193;HIST2H3DP1_ENSG00000213244
1	142618507	142619318	5Y-H4K8ac_peak_1065	12.50545	RP11-417J8.3_ENSG00000230880
1	144018062	144018345	5Y-H4K8ac_peak_1066	6.84536	
1	144534209	144534578	5Y-H4K8ac_peak_1067	6.77436	RNU1-59P_ENSG00000201699
1	144931667	144931866	5Y-H4K8ac_peak_1068	6.35183	
1	145396049	145396262	5Y-H4K8ac_peak_1069	3.96434	
1	145437780	145438051	5Y-H4K8ac_peak_1070	9.62747	TXNIP_ENSG00000117289
1	145472533	145472768	5Y-H4K8ac_peak_1071	7.26691	
1	145474681	145474944	5Y-H4K8ac_peak_1072	13.45283	
1	145477299	145477501	5Y-H4K8ac_peak_1073	5.40265	LIX1L_ENSG00000152022
1	145506930	145507286	5Y-H4K8ac_peak_1074	6.73385	RBM8A_ENSG00000131795
1	145516623	145516851	5Y-H4K8ac_peak_1075	4.95697	GNRHR2_ENSG00000211451;PEX11B_ENSG00000131779
1	145713492	145714172	5Y-H4K8ac_peak_1076	10.60083	
1	145719049	145719264	5Y-H4K8ac_peak_1077	4.88619	
1	145721030	145721228	5Y-H4K8ac_peak_1078	5.99504	
1	145726670	145727468	5Y-H4K8ac_peak_1079	9.13842	PDZK1_ENSG00000174827
1	145826733	145826973	5Y-H4K8ac_peak_1080	6.19716	GPR89A_ENSG00000117262;WI2-925H4.1_ENSG00000235702
1	146643508	146643984	5Y-H4K8ac_peak_1081	4.48815	PRKAB2_ENSG00000131791;RP11-337C18.8_ENSG00000237188;RP11-337C18.10_ENSG00000273071
1	146644403	146644714	5Y-H4K8ac_peak_1082	5.60566	PRKAB2_ENSG00000131791;RP11-337C18.8_ENSG00000237188;RP11-337C18.10_ENSG00000273071
1	146714363	146714715	5Y-H4K8ac_peak_1083	20.56946	FMO5_ENSG00000131781;CHD1L_ENSG00000131778
1	147013816	147014047	5Y-H4K8ac_peak_1084	9.30505	BCL9_ENSG00000116128
1	147400609	147400952	5Y-H4K8ac_peak_1085	9.23159	GPR89B_ENSG00000188092
1	148000060	148000484	5Y-H4K8ac_peak_1086	6.34046	RP3-328E19.4_ENSG00000223491
1	148247321	148248353	5Y-H4K8ac_peak_1087	9.02938	RP11-89F3.2_ENSG00000236140
1	148598402	148598884	5Y-H4K8ac_peak_1088	5.87725	RP11-666A1.5_ENSG00000237253;RP11-666A1.3_ENSG00000226675
1	149223436	149223691	5Y-H4K8ac_peak_1089	9.14959	RNVU1-18_ENSG00000206737
1	149819119	149819386	5Y-H4K8ac_peak_1090	6.47245	
1	149859139	149859453	5Y-H4K8ac_peak_1091	12.15235	HIST2H2BE_ENSG00000184678;HIST2H2AC_ENSG00000184260;HIST2H2AB_ENSG00000184270;BOLA1_ENSG00000178096
1	149871306	149871691	5Y-H4K8ac_peak_1092	11.18476	
1	149889230	149889863	5Y-H4K8ac_peak_1093	7.21932	SV2A_ENSG00000159164
1	149900040	149900458	5Y-H4K8ac_peak_1094	7.70811	SF3B4_ENSG00000143368
1	149982190	149982496	5Y-H4K8ac_peak_1095	4.77126	OTUD7B_ENSG00000163113
1	149982877	149983185	5Y-H4K8ac_peak_1096	4.95697	OTUD7B_ENSG00000163113
1	150039461	150039717	5Y-H4K8ac_peak_1097	10.79081	VPS45_ENSG00000136631
1	150121639	150122689	5Y-H4K8ac_peak_1098	14.99673	PLEKHO1_ENSG00000023902
1	150185062	150185666	5Y-H4K8ac_peak_1099	6.77436	
1	150209527	150209743	5Y-H4K8ac_peak_1100	13.92877	RNU2-17P_ENSG00000222222
1	150228184	150228374	5Y-H4K8ac_peak_1101	6.89892	
1	150240993	150241266	5Y-H4K8ac_peak_1102	7.20869	APH1A_ENSG00000117362;C1orf54_ENSG00000118292

1	150293320	150293701	5Y-H4K8ac_peak_1103	4.48815	PRPF3_ENSG00000117360
1	150335456	150335672	5Y-H4K8ac_peak_1104	10.69239	RPRD2_ENSG00000163125
1	150458830	150459034	5Y-H4K8ac_peak_1105	9.23159	TARS2_ENSG00000143374
1	150460082	150460276	5Y-H4K8ac_peak_1106	6.31818	TARS2_ENSG00000143374
1	150488304	150488628	5Y-H4K8ac_peak_1107	6.03632	LINC00568_ENSG00000228126
1	150601812	150602004	5Y-H4K8ac_peak_1108	11.94897	ENSA_ENSG00000143420
1	150849455	150849737	5Y-H4K8ac_peak_1109	7.72887	ARNT_ENSG00000143437
1	150946559	150947204	5Y-H4K8ac_peak_1110	20.94053	CERS2_ENSG00000143418;RP11-316M1.3_ENSG00000231073
1	150947442	150947899	5Y-H4K8ac_peak_1111	7.90236	CERS2_ENSG00000143418
1	151042836	151043115	5Y-H4K8ac_peak_1112	5.93575	CDC42SE1_ENSG00000197622;GABPB2_ENSG00000143458
1	151137527	151137748	5Y-H4K8ac_peak_1113	9.51254	LYSMD1_ENSG00000163155
1	151162272	151162587	5Y-H4K8ac_peak_1114	7.90005	
1	151168512	151168721	5Y-H4K8ac_peak_1115	8.564	VPS72_ENSG00000163159
1	151226620	151227024	5Y-H4K8ac_peak_1116	6.80915	PSMD4_ENSG00000159352
1	151299789	151300015	5Y-H4K8ac_peak_1117	4.29586	PI4KB_ENSG00000143393;RP11-126K1.9_ENSG00000273481
1	151371429	151371890	5Y-H4K8ac_peak_1118	12.84845	PSMB4_ENSG00000159377
1	151372099	151372289	5Y-H4K8ac_peak_1119	10.31981	PSMB4_ENSG00000159377
1	151584670	151584940	5Y-H4K8ac_peak_1120	15.80786	RP11-404E16.1_ENSG00000250734;SNX27_ENSG00000143376
1	151702005	151702543	5Y-H4K8ac_peak_1121	8.42168	
1	151735463	151735798	5Y-H4K8ac_peak_1122	9.79526	MRPL9_ENSG00000143436;OAZ3_ENSG00000143450
1	151777681	151777924	5Y-H4K8ac_peak_1123	5.16741	LINGO4_ENSG00000213171
1	151795107	151795404	5Y-H4K8ac_peak_1124	11.30958	
1	151804139	151804390	5Y-H4K8ac_peak_1125	6.93228	RORC_ENSG00000143365
1	151882208	151882590	5Y-H4K8ac_peak_1126	8.36116	THEM4_ENSG00000159445
1	152020786	152021132	5Y-H4K8ac_peak_1127	6.78128	S100A11_ENSG00000163191
1	153497977	153498640	5Y-H4K8ac_peak_1128	9.05168	
1	153508676	153509099	5Y-H4K8ac_peak_1129	5.40584	S100A6_ENSG00000197956
1	153540843	153541209	5Y-H4K8ac_peak_1130	12.2918	S100A2_ENSG00000196754
1	153541427	153541652	5Y-H4K8ac_peak_1131	8.69112	
1	153555320	153555678	5Y-H4K8ac_peak_1132	4.13628	
1	153606003	153606255	5Y-H4K8ac_peak_1133	5.17251	S100A13_ENSG00000189171;RP1-178F15.5_ENSG00000271853;RP1-178F15.4_ENSG00000272030;CHTOP_ENSG00000160679
1	153606607	153606807	5Y-H4K8ac_peak_1134	9.02938	S100A13_ENSG00000189171;RP1-178F15.5_ENSG00000271853;RP1-178F15.4_ENSG00000272030;CHTOP_ENSG00000160679
1	153631182	153631402	5Y-H4K8ac_peak_1135	11.60058	SNAPIN_ENSG00000143553
1	153642969	153643429	5Y-H4K8ac_peak_1136	10.72427	ILF2_ENSG00000143621
1	153699816	153700119	5Y-H4K8ac_peak_1137	6.08523	INTS3_ENSG00000143624;Y_RNA_ENSG00000199565
1	153700589	153700959	5Y-H4K8ac_peak_1138	15.80786	INTS3_ENSG00000143624
1	153755288	153755705	5Y-H4K8ac_peak_1139	6.27467	
1	153755905	153756169	5Y-H4K8ac_peak_1140	9.58986	
1	153756460	153756927	5Y-H4K8ac_peak_1141	4.95697	
1	153853981	153854273	5Y-H4K8ac_peak_1142	7.09658	
1	153895956	153896277	5Y-H4K8ac_peak_1143	10.12457	GATAD2B_ENSG00000143614
1	153918739	153919068	5Y-H4K8ac_peak_1144	12.45678	DENND4B_ENSG00000198837
1	153935387	153935866	5Y-H4K8ac_peak_1145	5.24695	
1	153939715	153940116	5Y-H4K8ac_peak_1146	9.99838	SLC39A1_ENSG00000143570;RP11-422P24.10_ENSG00000273026;CREB3L4_ENSG00000143578
1	153957915	153958178	5Y-H4K8ac_peak_1147	9.51254	RAB13_ENSG00000143545
1	153959634	153959833	5Y-H4K8ac_peak_1148	9.31608	RAB13_ENSG00000143545

1	153962515	153962850	5Y-H4K8ac_peak_1149	12.65187	RPS27_ENSG00000177954
1	154156508	154156773	5Y-H4K8ac_peak_1150	6.98405	
1	154192659	154193038	5Y-H4K8ac_peak_1151	15.01223	C1orf43_ENSG00000143612;UBAP2L_ENSG00000143569
1	154193741	154194160	5Y-H4K8ac_peak_1152	7.90751	C1orf43_ENSG00000143612
1	154244764	154244956	5Y-H4K8ac_peak_1153	4.77126	HAX1_ENSG00000143575
1	154245202	154245518	5Y-H4K8ac_peak_1154	8.73392	HAX1_ENSG00000143575
1	154296708	154296969	5Y-H4K8ac_peak_1155	9.02782	
1	154297377	154297725	5Y-H4K8ac_peak_1156	6.78128	ATP8B2_ENSG00000143515
1	154391953	154392144	5Y-H4K8ac_peak_1157	6.4472	
1	154530709	154531065	5Y-H4K8ac_peak_1158	9.37812	UBE2Q1_ENSG00000160714
1	154531557	154532125	5Y-H4K8ac_peak_1159	15.25284	UBE2Q1_ENSG00000160714
1	154580367	154580917	5Y-H4K8ac_peak_1160	14.89891	
1	154600051	154600364	5Y-H4K8ac_peak_1161	19.58975	ADAR_ENSG00000160710
1	154719522	154719796	5Y-H4K8ac_peak_1162	5.17251	
1	154720286	154720608	5Y-H4K8ac_peak_1163	7.21761	
1	154733038	154733473	5Y-H4K8ac_peak_1164	9.02938	
1	154813332	154813580	5Y-H4K8ac_peak_1165	5.41472	
1	154908935	154909431	5Y-H4K8ac_peak_1166	12.50545	PMVK_ENSG00000163344;RP11-307C12.13_ENSG00000270361
1	154934327	154934936	5Y-H4K8ac_peak_1167	5.32952	RP11-307C12.12_ENSG00000271380
1	154942476	154942858	5Y-H4K8ac_peak_1168	4.55128	
1	154946358	154946863	5Y-H4K8ac_peak_1169	14.86775	SHC1_ENSG00000160691;CKS1B_ENSG00000173207
1	154955974	154956350	5Y-H4K8ac_peak_1170	4.90696	FLAD1_ENSG00000160688
1	154971788	154972790	5Y-H4K8ac_peak_1171	8.24461	
1	154975262	154975457	5Y-H4K8ac_peak_1172	8.95951	ZBTB7B_ENSG00000160685
1	154975873	154977601	5Y-H4K8ac_peak_1173	12.2918	ZBTB7B_ENSG00000160685
1	154989784	154989998	5Y-H4K8ac_peak_1174	6.08471	
1	155022303	155022691	5Y-H4K8ac_peak_1175	13.33225	ADAM15_ENSG00000143537
1	155023114	155023490	5Y-H4K8ac_peak_1176	5.40265	ADAM15_ENSG00000143537
1	155042966	155044054	5Y-H4K8ac_peak_1177	4.92482	
1	155050843	155051038	5Y-H4K8ac_peak_1178	4.75174	
1	155052751	155053341	5Y-H4K8ac_peak_1179	8.3285	
1	155058839	155059084	5Y-H4K8ac_peak_1180	8.90774	
1	155066203	155066458	5Y-H4K8ac_peak_1181	4.70545	
1	155099046	155099493	5Y-H4K8ac_peak_1182	6.98416	EFNA1_ENSG00000169242
1	155099929	155100255	5Y-H4K8ac_peak_1183	14.6127	EFNA1_ENSG00000169242
1	155101986	155102419	5Y-H4K8ac_peak_1184	8.27643	
1	155107807	155108130	5Y-H4K8ac_peak_1185	7.20869	SLC50A1_ENSG00000169241
1	155139312	155139898	5Y-H4K8ac_peak_1186	11.5813	
1	155140159	155140466	5Y-H4K8ac_peak_1187	7.90236	
1	155145300	155145630	5Y-H4K8ac_peak_1188	8.36116	KRTCAP2_ENSG00000163463;TRIM46_ENSG00000163462
1	155146403	155146838	5Y-H4K8ac_peak_1189	7.20869	KRTCAP2_ENSG00000163463;TRIM46_ENSG00000163462
1	155158596	155158944	5Y-H4K8ac_peak_1190	4.50834	RP11-201K10.3_ENSG00000273088
1	155162893	155163374	5Y-H4K8ac_peak_1191	17.55395	MUC1_ENSG00000185499
1	155163600	155164018	5Y-H4K8ac_peak_1192	6.78128	MUC1_ENSG00000185499;MIR92B_ENSG00000271748
1	155175821	155176056	5Y-H4K8ac_peak_1193	4.44204	
1	155177134	155178345	5Y-H4K8ac_peak_1194	6.08523	THBS3_ENSG00000169231;MTX1_ENSG00000173171

1	155178625	155178889	5Y-H4K8ac_peak_1195	13.92877	THBS3_ENSG00000169231;MTX1_ENSG00000173171
1	155179102	155179298	5Y-H4K8ac_peak_1196	5.51759	THBS3_ENSG00000169231;MTX1_ENSG00000173171
1	155197573	155197966	5Y-H4K8ac_peak_1197	12.06963	GBAP1_ENSG00000160766
1	155220239	155220484	5Y-H4K8ac_peak_1198	5.50568	
1	155231777	155232005	5Y-H4K8ac_peak_1199	8.14883	SCAMP3_ENSG00000116521
1	155232398	155232681	5Y-H4K8ac_peak_1200	5.70472	SCAMP3_ENSG00000116521
1	155242906	155243134	5Y-H4K8ac_peak_1201	16.39907	
1	155277529	155278297	5Y-H4K8ac_peak_1202	7.31674	FDPS_ENSG00000160752
1	155443676	155443866	5Y-H4K8ac_peak_1203	5.09997	
1	155532348	155532546	5Y-H4K8ac_peak_1204	6.78128	ASH1L-AS1_ENSG00000235919;ASH1L_ENSG00000116539
1	155532746	155532971	5Y-H4K8ac_peak_1205	11.94897	ASH1L-AS1_ENSG00000235919;ASH1L_ENSG00000116539
1	155657846	155658416	5Y-H4K8ac_peak_1206	5.84816	YY1AP1_ENSG00000163374;DAP3_ENSG00000132676
1	155714433	155714687	5Y-H4K8ac_peak_1207	6.43775	
1	155826656	155826910	5Y-H4K8ac_peak_1208	9.79526	
1	155830125	155830331	5Y-H4K8ac_peak_1209	6.4759	SYT11_ENSG00000132718;GON4L_ENSG00000116580
1	155904575	155904938	5Y-H4K8ac_peak_1210	8.18236	KIAA0907_ENSG00000132680
1	155909924	155910441	5Y-H4K8ac_peak_1211	8.69112	
1	155911009	155911310	5Y-H4K8ac_peak_1212	5.24695	RXFP4_ENSG00000173080
1	155948441	155948799	5Y-H4K8ac_peak_1213	4.97086	RP11-336K24.12_ENSG00000273002
1	156023730	156024173	5Y-H4K8ac_peak_1214	11.26379	UBQLN4_ENSG00000160803;LAMTOR2_ENSG00000116586
1	156024652	156024898	5Y-H4K8ac_peak_1215	6.78128	LAMTOR2_ENSG00000116586
1	156039763	156040677	5Y-H4K8ac_peak_1216	15.80786	
1	156051961	156052239	5Y-H4K8ac_peak_1217	14.0329	MEX3A_ENSG00000254726;LMNA_ENSG00000160789
1	156075691	156076194	5Y-H4K8ac_peak_1218	11.22005	
1	156083934	156084323	5Y-H4K8ac_peak_1219	6.40724	
1	156095915	156096171	5Y-H4K8ac_peak_1220	4.48815	
1	156116225	156116525	5Y-H4K8ac_peak_1221	5.01024	SEMA4A_ENSG00000196189
1	156162541	156162784	5Y-H4K8ac_peak_1222	5.98695	SNORA26_ENSG00000252236
1	156163334	156163564	5Y-H4K8ac_peak_1223	6.95285	SLC25A44_ENSG00000160785
1	156182828	156183022	5Y-H4K8ac_peak_1224	13.01447	PMF1_ENSG00000160783
1	156221776	156221966	5Y-H4K8ac_peak_1225	6.14551	
1	156252344	156252573	5Y-H4K8ac_peak_1226	15.03212	SMG5_ENSG00000198952;TMEM79_ENSG00000163472
1	156266006	156266221	5Y-H4K8ac_peak_1227	9.51254	C1orf85_ENSG00000198715
1	156307713	156307983	5Y-H4K8ac_peak_1228	7.89273	TSACC_ENSG00000163467
1	156390288	156390588	5Y-H4K8ac_peak_1229	4.70739	MIR9-1_ENSG00000207933
1	156390976	156391511	5Y-H4K8ac_peak_1230	10.4495	MIR9-1_ENSG00000207933
1	156406053	156406263	5Y-H4K8ac_peak_1231	4.00285	
1	156416112	156416448	5Y-H4K8ac_peak_1232	4.68359	
1	156425849	156426347	5Y-H4K8ac_peak_1233	7.90751	RP11-98G7.1_ENSG00000236947
1	156457735	156457992	5Y-H4K8ac_peak_1234	4.50302	
1	156470862	156471120	5Y-H4K8ac_peak_1235	10.60083	MEF2D_ENSG00000116604
1	156473875	156474184	5Y-H4K8ac_peak_1236	5.5089	
1	156474580	156474849	5Y-H4K8ac_peak_1237	14.40623	
1	156492858	156493055	5Y-H4K8ac_peak_1238	6.85744	
1	156561137	156561407	5Y-H4K8ac_peak_1239	7.11863	APOA1BP_ENSG00000163382
1	156571834	156572071	5Y-H4K8ac_peak_1240	5.69598	GPATCH4_ENSG00000160818

1	156594365	156594565	5Y-H4K8ac_peak_1241	6.55841	
1	156630160	156630893	5Y-H4K8ac_peak_1242	10.46949	RP11-284F21.7_ENSG00000229953
1	156631652	156631978	5Y-H4K8ac_peak_1243	7.89273	RP11-284F21.7_ENSG00000229953
1	156646807	156647038	5Y-H4K8ac_peak_1244	9.07085	NES_ENSG00000132688
1	156662020	156662267	5Y-H4K8ac_peak_1245	5.32676	RP11-66D17.3_ENSG00000237588
1	156674719	156674914	5Y-H4K8ac_peak_1246	6.80915	CRABP2_ENSG00000143320
1	156675549	156675845	5Y-H4K8ac_peak_1247	16.37893	CRABP2_ENSG00000143320
1	156676493	156677028	5Y-H4K8ac_peak_1248	7.87192	CRABP2_ENSG00000143320
1	156677343	156678160	5Y-H4K8ac_peak_1249	11.44642	
1	156679898	156680828	5Y-H4K8ac_peak_1250	10.50008	
1	156698771	156699018	5Y-H4K8ac_peak_1251	10.16277	ISG20L2_ENSG00000143319;RRNAD1_ENSG00000143303
1	156704041	156704273	5Y-H4K8ac_peak_1252	4.15658	
1	156710599	156710814	5Y-H4K8ac_peak_1253	7.16017	MRPL24_ENSG00000143314
1	156716642	156716904	5Y-H4K8ac_peak_1254	9.19793	
1	156717207	156717699	5Y-H4K8ac_peak_1255	6.86362	
1	156720939	156721480	5Y-H4K8ac_peak_1256	18.51582	PRCC_ENSG00000143294
1	156721740	156722162	5Y-H4K8ac_peak_1257	12.00715	
1	156736502	156736837	5Y-H4K8ac_peak_1258	11.30958	HDGF_ENSG00000143321
1	156737376	156737746	5Y-H4K8ac_peak_1259	12.26819	HDGF_ENSG00000143321
1	156771014	156771225	5Y-H4K8ac_peak_1260	5.50111	
1	156798268	156798475	5Y-H4K8ac_peak_1261	4.95697	
1	156821727	156822034	5Y-H4K8ac_peak_1262	9.74838	
1	156829297	156829611	5Y-H4K8ac_peak_1263	5.51759	INSRR_ENSG00000027644
1	156830778	156831106	5Y-H4K8ac_peak_1264	13.57316	
1	156852690	156852908	5Y-H4K8ac_peak_1265	8.27643	
1	156883068	156883978	5Y-H4K8ac_peak_1266	14.21916	
1	156884430	156884911	5Y-H4K8ac_peak_1267	11.5901	
1	157015174	157015437	5Y-H4K8ac_peak_1268	6.36723	ARHGEF11_ENSG00000132694
1	157015779	157016240	5Y-H4K8ac_peak_1269	8.82628	ARHGEF11_ENSG00000132694
1	157108414	157109471	5Y-H4K8ac_peak_1270	16.30401	ETV3_ENSG00000117036
1	157110922	157111224	5Y-H4K8ac_peak_1271	5.39991	
1	159892834	159893093	5Y-H4K8ac_peak_1272	5.24695	
1	159894055	159895155	5Y-H4K8ac_peak_1273	8.24461	
1	159915628	159916139	5Y-H4K8ac_peak_1274	6.53157	IGSF9_ENSG00000085552
1	160039618	160039852	5Y-H4K8ac_peak_1275	6.37023	KCNJ10_ENSG00000177807
1	160051326	160051614	5Y-H4K8ac_peak_1276	4.642	KCNJ9_ENSG00000162728
1	160163216	160163481	5Y-H4K8ac_peak_1277	13.99721	
1	160174228	160174952	5Y-H4K8ac_peak_1278	10.4689	PEA15_ENSG00000162734;AL121987.1_ENSG00000265381
1	160175292	160175542	5Y-H4K8ac_peak_1279	8.5159	PEA15_ENSG00000162734;AL121987.1_ENSG00000265381
1	160231638	160232183	5Y-H4K8ac_peak_1280	11.28133	RP11-574F21.2_ENSG00000228606
1	160313196	160313598	5Y-H4K8ac_peak_1281	11.94897	COPA_ENSG00000122218;NCSTN_ENSG00000162736
1	160369554	160370194	5Y-H4K8ac_peak_1282	14.17466	
1	160370548	160371091	5Y-H4K8ac_peak_1283	6.98416	
1	161015247	161015645	5Y-H4K8ac_peak_1284	6.53157	USF1_ENSG00000158773
1	161068381	161068587	5Y-H4K8ac_peak_1285	4.95697	KLHDC9_ENSG00000162755
1	161102324	161102544	5Y-H4K8ac_peak_1286	9.05168	DEDD_ENSG00000158796

1	161123943	161124447	5Y-H4K8ac_peak_1287	8.82628	
1	161136455	161136701	5Y-H4K8ac_peak_1288	7.0618	PPOX_ENSG00000143224
1	161166209	161167091	5Y-H4K8ac_peak_1289	6.67135	NDUFS2_ENSG00000158864
1	161167585	161168245	5Y-H4K8ac_peak_1290	6.67135	NDUFS2_ENSG00000158864;ADAMTS4_ENSG00000158859
1	161168941	161169199	5Y-H4K8ac_peak_1291	10.04821	ADAMTS4_ENSG00000158859
1	161171071	161171332	5Y-H4K8ac_peak_1292	6.69752	
1	161171576	161171910	5Y-H4K8ac_peak_1293	9.32595	
1	161172239	161172482	5Y-H4K8ac_peak_1294	10.90097	
1	161283535	161283786	5Y-H4K8ac_peak_1295	6.37023	SDHC_ENSG00000143252
1	161368161	161368562	5Y-H4K8ac_peak_1296	9.89244	
1	161368914	161369473	5Y-H4K8ac_peak_1297	13.89096	
1	161369680	161370027	5Y-H4K8ac_peak_1298	4.00285	
1	161410302	161410574	5Y-H4K8ac_peak_1299	6.98416	
1	161696261	161696629	5Y-H4K8ac_peak_1300	8.43511	
1	161719590	161720023	5Y-H4K8ac_peak_1301	12.52787	DUSP12_ENSG00000081721
1	161993226	161993624	5Y-H4K8ac_peak_1302	13.00733	OLFML2B_ENSG00000162745
1	162087352	162087614	5Y-H4K8ac_peak_1303	5.87725	
1	162466983	162467233	5Y-H4K8ac_peak_1304	5.98695	UHMK1_ENSG00000152332
1	162467675	162468007	5Y-H4K8ac_peak_1305	15.80786	UHMK1_ENSG00000152332
1	162530717	162530984	5Y-H4K8ac_peak_1306	5.449	RP11-359K18.3_ENSG00000259788;UAP1_ENSG00000117143
1	162760031	162760284	5Y-H4K8ac_peak_1307	8.2913	HSD17B7_ENSG00000132196
1	162851226	162851994	5Y-H4K8ac_peak_1308	11.23789	
1	162857722	162857972	5Y-H4K8ac_peak_1309	7.59101	
1	162860600	162860879	5Y-H4K8ac_peak_1310	7.50148	
1	162887161	162888127	5Y-H4K8ac_peak_1311	16.73191	
1	162888635	162889681	5Y-H4K8ac_peak_1312	8.56189	
1	163090456	163090711	5Y-H4K8ac_peak_1313	7.83553	
1	163125479	163125679	5Y-H4K8ac_peak_1314	5.65584	
1	163157355	163157741	5Y-H4K8ac_peak_1315	6.08523	
1	163166417	163167058	5Y-H4K8ac_peak_1316	4.29586	
1	163406490	163406700	5Y-H4K8ac_peak_1317	9.58986	
1	163408444	163408662	5Y-H4K8ac_peak_1318	5.49268	
1	163461853	163462067	5Y-H4K8ac_peak_1319	6.46053	
1	164183061	164183263	5Y-H4K8ac_peak_1320	6.6946	
1	164290568	164290888	5Y-H4K8ac_peak_1321	7.89273	
1	164679638	164679988	5Y-H4K8ac_peak_1322	7.65404	
1	164680482	164681073	5Y-H4K8ac_peak_1323	8.20773	
1	164682031	164682463	5Y-H4K8ac_peak_1324	8.5915	
1	164723151	164723385	5Y-H4K8ac_peak_1325	4.18845	
1	164724442	164724662	5Y-H4K8ac_peak_1326	7.10001	
1	164895887	164896122	5Y-H4K8ac_peak_1327	4.50834	
1	165737899	165738092	5Y-H4K8ac_peak_1328	10.60083	RP11-466F5.8_ENSG00000224358
1	165796858	165797722	5Y-H4K8ac_peak_1329	10.48476	TMCO1_ENSG00000143183;UCK2_ENSG00000143179
1	166845016	166845484	5Y-H4K8ac_peak_1330	14.99673	TADA1_ENSG00000152382
1	167090356	167090996	5Y-H4K8ac_peak_1331	6.08523	
1	167105524	167105741	5Y-H4K8ac_peak_1332	4.50834	

1	167190333	167190979	5Y-H4K8ac_peak_1333	7.18391	RP11-277B15.3_ENSG00000272205;POU2F1_ENSG00000143190
1	167683500	167683846	5Y-H4K8ac_peak_1334	4.95697	
1	168147500	168147842	5Y-H4K8ac_peak_1335	7.65114	TIPRL_ENSG00000143155
1	168193931	168194125	5Y-H4K8ac_peak_1336	5.60423	
1	168194434	168195370	5Y-H4K8ac_peak_1337	7.65114	SFT2D2_ENSG00000213064
1	168311737	168312072	5Y-H4K8ac_peak_1338	13.99721	
1	168435702	168436093	5Y-H4K8ac_peak_1339	7.79831	
1	168482183	168482441	5Y-H4K8ac_peak_1340	4.95697	
1	168482946	168483301	5Y-H4K8ac_peak_1341	10.4689	
1	168658830	168659020	5Y-H4K8ac_peak_1342	10.01583	
1	168660371	168660875	5Y-H4K8ac_peak_1343	8.01306	
1	168907996	168908196	5Y-H4K8ac_peak_1344	9.30206	RPL29P7_ENSG00000213063
1	169055109	169055319	5Y-H4K8ac_peak_1345	4.70545	LINC00970_ENSG00000203601
1	169074407	169075337	5Y-H4K8ac_peak_1346	16.13719	ATP1B1_ENSG00000143153
1	169076033	169076224	5Y-H4K8ac_peak_1347	5.12488	
1	169454867	169455077	5Y-H4K8ac_peak_1348	4.6766	SLC19A2_ENSG00000117479;AL021068.1_ENSG00000213062
1	169862446	169862998	5Y-H4K8ac_peak_1349	7.64648	SCYL3_ENSG00000000457
1	169863691	169863952	5Y-H4K8ac_peak_1350	9.58986	SCYL3_ENSG00000000457
1	170500780	170501045	5Y-H4K8ac_peak_1351	10.84616	RP11-576I22.2_ENSG00000231407;GORAB_ENSG00000120370
1	171454858	171455175	5Y-H4K8ac_peak_1352	7.96285	PRRC2C_ENSG00000117523
1	171710706	171711054	5Y-H4K8ac_peak_1353	7.02726	VAMP4_ENSG00000117533
1	171750152	171750358	5Y-H4K8ac_peak_1354	6.47245	METTL13_ENSG00000010165
1	172412771	172413041	5Y-H4K8ac_peak_1355	15.41988	PIGC_ENSG00000135845
1	172419472	172419831	5Y-H4K8ac_peak_1356	6.78318	
1	173445044	173445333	5Y-H4K8ac_peak_1357	5.67283	
1	173683726	173683929	5Y-H4K8ac_peak_1358	5.24695	KLHL20_ENSG00000076321
1	173684164	173684366	5Y-H4K8ac_peak_1359	10.35586	KLHL20_ENSG00000076321
1	173793809	173794148	5Y-H4K8ac_peak_1360	9.23159	CENPL_ENSG00000120334;DARS2_ENSG00000117593
1	173837498	173837819	5Y-H4K8ac_peak_1361	18.78631	GAS5_ENSG00000234741;ZBTB37_ENSG00000185278
1	173991215	173991901	5Y-H4K8ac_peak_1362	20.85916	RC3H1_ENSG00000135870;RP11-160H22.3_ENSG00000224977
1	174232147	174232393	5Y-H4K8ac_peak_1363	7.50148	
1	174967952	174968720	5Y-H4K8ac_peak_1364	8.46442	CACYBP_ENSG00000116161
1	174992789	174993669	5Y-H4K8ac_peak_1365	11.1169	MRPS14_ENSG00000120333
1	175343223	175343507	5Y-H4K8ac_peak_1366	5.78188	
1	175457959	175458156	5Y-H4K8ac_peak_1367	5.65584	
1	176176007	176176598	5Y-H4K8ac_peak_1368	11.04542	RFWD2_ENSG00000143207;RP11-195C7.1_ENSG00000236021
1	178510986	178511689	5Y-H4K8ac_peak_1369	13.89096	C1orf220_ENSG00000213057;C1ORF220_ENSG00000184909
1	178511959	178512244	5Y-H4K8ac_peak_1370	10.29016	C1orf220_ENSG00000213057;C1ORF220_ENSG00000184909
1	178693855	178694284	5Y-H4K8ac_peak_1371	8.43511	RALGPS2_ENSG00000116191
1	178694644	178694969	5Y-H4K8ac_peak_1372	4.95697	RP11-428K3.1_ENSG00000273062;RALGPS2_ENSG00000116191
1	179179192	179179460	5Y-H4K8ac_peak_1373	4.93618	
1	179198987	179199326	5Y-H4K8ac_peak_1374	9.179	ABL2_ENSG00000143322
1	179334333	179334633	5Y-H4K8ac_peak_1375	4.32488	RN7SL374P_ENSG00000263633;AXDND1_ENSG00000162779
1	179676236	179676535	5Y-H4K8ac_peak_1376	7.30348	
1	179711937	179712183	5Y-H4K8ac_peak_1377	7.17527	FAM163A_ENSG00000143340
1	179712405	179713844	5Y-H4K8ac_peak_1378	10.48476	FAM163A_ENSG00000143340

1	179735874	179736126	5Y-H4K8ac_peak_1379	6.37023	
1	179750669	179750964	5Y-H4K8ac_peak_1380	7.50148	
1	179751573	179751785	5Y-H4K8ac_peak_1381	11.04542	
1	179756705	179757164	5Y-H4K8ac_peak_1382	9.02938	
1	179786281	179786516	5Y-H4K8ac_peak_1383	4.95697	RP11-12M5.3_ENSG00000229407
1	179851962	179852428	5Y-H4K8ac_peak_1384	13.35169	RP11-533E19.7_ENSG00000272906;TOR1AIP1_ENSG00000143337
1	179923958	179924238	5Y-H4K8ac_peak_1385	12.34969	RP11-533E19.5_ENSG00000260360;CEP350_ENSG00000135837
1	180123620	180123852	5Y-H4K8ac_peak_1386	17.32847	QSOX1_ENSG00000116260
1	180151325	180151563	5Y-H4K8ac_peak_1387	5.34199	
1	180151972	180152381	5Y-H4K8ac_peak_1388	4.642	
1	180471198	180471909	5Y-H4K8ac_peak_1389	12.26819	ACBD6_ENSG00000135847
1	180869049	180869264	5Y-H4K8ac_peak_1390	4.90696	
1	180870269	180870629	5Y-H4K8ac_peak_1391	6.1654	
1	180890639	180890835	5Y-H4K8ac_peak_1392	6.64195	
1	180893443	180893774	5Y-H4K8ac_peak_1393	8.66094	
1	180894221	180895464	5Y-H4K8ac_peak_1394	19.24319	
1	180896169	180897820	5Y-H4K8ac_peak_1395	35.18826	
1	180898049	180899463	5Y-H4K8ac_peak_1396	10.86648	
1	180900144	180900660	5Y-H4K8ac_peak_1397	9.16197	
1	180900875	180901279	5Y-H4K8ac_peak_1398	10.66841	
1	180921798	180922004	5Y-H4K8ac_peak_1399	6.37023	
1	180925111	180925596	5Y-H4K8ac_peak_1400	6.36723	
1	180991365	180991843	5Y-H4K8ac_peak_1401	11.24252	STX6_ENSG00000135823
1	181073621	181074409	5Y-H4K8ac_peak_1402	15.03212	
1	181074746	181075080	5Y-H4K8ac_peak_1403	18.62331	
1	181082282	181082983	5Y-H4K8ac_peak_1404	9.74838	
1	181102892	181103275	5Y-H4K8ac_peak_1405	5.64909	
1	181103921	181104323	5Y-H4K8ac_peak_1406	6.37023	
1	181111370	181111708	5Y-H4K8ac_peak_1407	4.0639	
1	181998964	181999566	5Y-H4K8ac_peak_1408	16.17072	
1	182083341	182083554	5Y-H4K8ac_peak_1409	5.718	
1	182360240	182360583	5Y-H4K8ac_peak_1410	8.24461	
1	182361156	182361705	5Y-H4K8ac_peak_1411	22.3217	
1	182584109	182584946	5Y-H4K8ac_peak_1412	10.48476	RP11-317P15.4_ENSG00000261504
1	182600164	182600532	5Y-H4K8ac_peak_1413	8.43511	
1	182602639	182603035	5Y-H4K8ac_peak_1414	5.44849	
1	182758446	182759153	5Y-H4K8ac_peak_1415	17.76126	NPL_ENSG00000135838
1	182808494	182808705	5Y-H4K8ac_peak_1416	11.9002	DHX9_ENSG00000135829
1	182991861	182992180	5Y-H4K8ac_peak_1417	9.36633	LAMC1_ENSG00000135862
1	182992709	182992954	5Y-H4K8ac_peak_1418	11.04542	LAMC1_ENSG00000135862
1	183300514	183300848	5Y-H4K8ac_peak_1419	5.0078	
1	183386295	183387272	5Y-H4K8ac_peak_1420	10.77254	NMNAT2_ENSG00000157064
1	183411637	183411947	5Y-H4K8ac_peak_1421	5.98695	
1	183440489	183440778	5Y-H4K8ac_peak_1422	9.79526	SMG7-AS1_ENSG00000232860;SMG7_ENSG00000116698
1	183441173	183441958	5Y-H4K8ac_peak_1423	23.58923	SMG7-AS1_ENSG00000232860;SMG7_ENSG00000116698
1	184006417	184006660	5Y-H4K8ac_peak_1424	6.08523	COLGALT2_ENSG00000198756

1	184020920	184021191	5Y-H4K8ac_peak_1425	13.60583	TSEN15_ENSG00000198860
1	184134552	184134977	5Y-H4K8ac_peak_1426	12.47704	
1	184137142	184137336	5Y-H4K8ac_peak_1427	7.50044	
1	184267702	184268139	5Y-H4K8ac_peak_1428	7.20869	
1	184355300	184355823	5Y-H4K8ac_peak_1429	12.38385	RP11-382D12.2_ENSG00000271387;C1orf21_ENSG00000116667
1	185014100	185014360	5Y-H4K8ac_peak_1430	6.08523	RNF2_ENSG00000121481
1	185014670	185015208	5Y-H4K8ac_peak_1431	9.51254	RNF2_ENSG00000121481
1	185125648	185125971	5Y-H4K8ac_peak_1432	4.41844	TRMT1L_ENSG00000121486;SWT1_ENSG00000116668
1	185225993	185226271	5Y-H4K8ac_peak_1433	5.59843	Y_RNA_ENSG00000206640
1	185226594	185226914	5Y-H4K8ac_peak_1434	21.90754	Y_RNA_ENSG00000206640
1	185285520	185286161	5Y-H4K8ac_peak_1435	8.43511	IVNS1ABP_ENSG00000116679;GS1-279B7.2_ENSG00000273004
1	188405686	188405919	5Y-H4K8ac_peak_1436	6.50117	
1	192815405	192815810	5Y-H4K8ac_peak_1437	4.07874	
1	193027882	193028385	5Y-H4K8ac_peak_1438	7.11863	UCHL5_ENSG00000116750;TROVE2_ENSG00000116747
1	193090605	193090807	5Y-H4K8ac_peak_1439	7.50148	CDC73_ENSG00000134371
1	193854599	193854821	5Y-H4K8ac_peak_1440	4.07874	
1	197115524	197115774	5Y-H4K8ac_peak_1441	7.11863	ASPM_ENSG00000066279
1	197116507	197116771	5Y-H4K8ac_peak_1442	6.54441	ASPM_ENSG00000066279
1	197169985	197170322	5Y-H4K8ac_peak_1443	5.67283	ZBTB41_ENSG00000177888;CRB1_ENSG00000134376
1	197440249	197440784	5Y-H4K8ac_peak_1444	6.40964	
1	197654101	197654300	5Y-H4K8ac_peak_1445	7.31815	Y_RNA_ENSG00000207139
1	197744556	197744781	5Y-H4K8ac_peak_1446	10.72427	DENND1B_ENSG00000213047
1	197770952	197771143	5Y-H4K8ac_peak_1447	6.1654	
1	197871268	197871998	5Y-H4K8ac_peak_1448	12.00715	C1orf53_ENSG00000203724
1	198871783	198872091	5Y-H4K8ac_peak_1449	6.46053	
1	199829523	199829726	5Y-H4K8ac_peak_1450	5.23083	
1	199927316	199927578	5Y-H4K8ac_peak_1451	7.97699	
1	200270530	200270733	5Y-H4K8ac_peak_1452	4.0639	
1	200271803	200272097	5Y-H4K8ac_peak_1453	7.38046	
1	200638605	200638983	5Y-H4K8ac_peak_1454	11.69482	DDX59_ENSG00000118197;RP11-92G12.3_ENSG00000260088
1	200708179	200708895	5Y-H4K8ac_peak_1455	12.10416	CAMSAP2_ENSG00000118200
1	200860183	200860942	5Y-H4K8ac_peak_1456	9.86146	C1orf106_ENSG00000163362
1	200986154	200986744	5Y-H4K8ac_peak_1457	5.87725	
1	201083523	201084236	5Y-H4K8ac_peak_1458	14.89891	
1	201123286	201123479	5Y-H4K8ac_peak_1459	7.90236	
1	201123865	201124306	5Y-H4K8ac_peak_1460	7.89273	
1	201237596	201237910	5Y-H4K8ac_peak_1461	7.20869	
1	201395477	201395957	5Y-H4K8ac_peak_1462	7.92818	
1	201400179	201400422	5Y-H4K8ac_peak_1463	5.6957	
1	201405566	201405761	5Y-H4K8ac_peak_1464	7.63144	
1	201419902	201420321	5Y-H4K8ac_peak_1465	6.86362	
1	201437237	201437481	5Y-H4K8ac_peak_1466	8.43511	PHLDA3_ENSG00000174307
1	201437706	201438313	5Y-H4K8ac_peak_1467	14.01193	PHLDA3_ENSG00000174307
1	201451458	201451670	5Y-H4K8ac_peak_1468	7.02726	
1	201475954	201476638	5Y-H4K8ac_peak_1469	12.87684	RP11-134G8.7_ENSG00000224536
1	201508212	201508468	5Y-H4K8ac_peak_1470	8.24461	

1	201508823	201509304	5Y-H4K8ac_peak_1471	7.59101	
1	201528692	201528893	5Y-H4K8ac_peak_1472	8.24461	
1	201561862	201562166	5Y-H4K8ac_peak_1473	5.9192	
1	201563299	201563503	5Y-H4K8ac_peak_1474	4.93905	
1	201617855	201618050	5Y-H4K8ac_peak_1475	7.50148	
1	201664246	201664579	5Y-H4K8ac_peak_1476	6.43068	
1	201664778	201665305	5Y-H4K8ac_peak_1477	13.00733	
1	201798393	201798635	5Y-H4K8ac_peak_1478	10.72427	IPO9-AS1_ENSG00000231871;IPO9_ENSG00000198700
1	201857863	201858088	5Y-H4K8ac_peak_1479	5.45148	SHISA4_ENSG00000198892
1	201951933	201952548	5Y-H4K8ac_peak_1480	6.50031	RNPEP_ENSG00000176393
1	202091721	202092289	5Y-H4K8ac_peak_1481	6.10343	GPR37L1_ENSG00000170075
1	202099807	202100010	5Y-H4K8ac_peak_1482	7.21761	
1	202113536	202114087	5Y-H4K8ac_peak_1483	7.27671	ARL8A_ENSG00000143862
1	202133494	202133980	5Y-H4K8ac_peak_1484	5.41472	
1	202136972	202137362	5Y-H4K8ac_peak_1485	19.52902	PTPRVP_ENSG00000243323
1	202137701	202137906	5Y-H4K8ac_peak_1486	4.48815	PTPRVP_ENSG00000243323
1	202206060	202206346	5Y-H4K8ac_peak_1487	7.20869	
1	202210336	202210630	5Y-H4K8ac_peak_1488	10.1994	
1	202224324	202224566	5Y-H4K8ac_peak_1489	9.25361	
1	202273209	202273607	5Y-H4K8ac_peak_1490	5.99756	
1	202317988	202318223	5Y-H4K8ac_peak_1491	7.44077	PPP1R12B_ENSG00000077157
1	202584478	202584772	5Y-H4K8ac_peak_1492	5.64909	
1	202636267	202636471	5Y-H4K8ac_peak_1493	5.74271	
1	202776523	202776730	5Y-H4K8ac_peak_1494	10.4689	
1	202829631	202829982	5Y-H4K8ac_peak_1495	6.37023	RP11-480I12.5_ENSG00000214796;RP11-480I12.7_ENSG00000234996
1	202857716	202858212	5Y-H4K8ac_peak_1496	7.74473	RABIF_ENSG00000183155
1	202895999	202896262	5Y-H4K8ac_peak_1497	4.48815	
1	202896826	202897034	5Y-H4K8ac_peak_1498	4.90696	KLHL12_ENSG00000117153
1	202927059	202927371	5Y-H4K8ac_peak_1499	11.22005	ADIPOR1_ENSG00000159346
1	202936002	202936290	5Y-H4K8ac_peak_1500	9.00954	CYB5R1_ENSG00000159348
1	202975646	202976005	5Y-H4K8ac_peak_1501	7.59101	TMEM183A_ENSG00000163444
1	202976578	202976798	5Y-H4K8ac_peak_1502	9.32595	TMEM183A_ENSG00000163444
1	202995746	202996200	5Y-H4K8ac_peak_1503	7.50148	PPFIA4_ENSG00000143847
1	202998140	202998461	5Y-H4K8ac_peak_1504	4.55128	
1	203037692	203038137	5Y-H4K8ac_peak_1505	7.89142	
1	203097007	203097315	5Y-H4K8ac_peak_1506	4.41844	RP11-335O13.7_ENSG00000234775
1	203097757	203098102	5Y-H4K8ac_peak_1507	6.77365	RP11-335O13.7_ENSG00000234775
1	203242468	203242754	5Y-H4K8ac_peak_1508	8.69112	CHIT1_ENSG00000133063
1	203247585	203248298	5Y-H4K8ac_peak_1509	9.37812	
1	203256376	203256901	5Y-H4K8ac_peak_1510	9.51254	RP11-134P9.3_ENSG00000231507
1	203258875	203259145	5Y-H4K8ac_peak_1511	7.72887	
1	203259445	203260275	5Y-H4K8ac_peak_1512	10.14791	
1	203274140	203274396	5Y-H4K8ac_peak_1513	11.86704	LINC01136_ENSG00000233791;BTG2_ENSG00000159388
1	203296194	203296462	5Y-H4K8ac_peak_1514	6.34245	
1	203296776	203297407	5Y-H4K8ac_peak_1515	25.35292	
1	203333272	203333824	5Y-H4K8ac_peak_1516	6.37023	

1	203456334	203457412	5Y-H4K8ac_peak_1517	13.00733	
1	203524517	203525870	5Y-H4K8ac_peak_1518	7.90751	
1	203763988	203764247	5Y-H4K8ac_peak_1519	7.31102	ZC3H11A_ENSG00000058673
1	203830808	203831149	5Y-H4K8ac_peak_1520	7.00938	SNRPE_ENSG00000182004
1	204076770	204076971	5Y-H4K8ac_peak_1521	5.32832	
1	204095422	204095632	5Y-H4K8ac_peak_1522	7.50148	
1	204096169	204096400	5Y-H4K8ac_peak_1523	8.24461	
1	204098535	204098947	5Y-H4K8ac_peak_1524	8.24461	
1	204121647	204121911	5Y-H4K8ac_peak_1525	7.93983	ETNK2_ENSG00000143845
1	204182703	204183111	5Y-H4K8ac_peak_1526	7.02726	GOLT1A_ENSG00000174567
1	204303216	204303486	5Y-H4K8ac_peak_1527	7.65114	
1	204318120	204318397	5Y-H4K8ac_peak_1528	5.81898	
1	204332191	204332962	5Y-H4K8ac_peak_1529	11.4254	
1	204380538	204380850	5Y-H4K8ac_peak_1530	9.79526	PPP1R15B_ENSG00000158615
1	204381375	204381938	5Y-H4K8ac_peak_1531	5.87725	PPP1R15B_ENSG00000158615
1	204463458	204463769	5Y-H4K8ac_peak_1532	12.45678	PIK3C2B_ENSG00000133056
1	204485015	204485242	5Y-H4K8ac_peak_1533	8.6813	MDM4_ENSG00000198625
1	204485576	204485828	5Y-H4K8ac_peak_1534	18.60312	MDM4_ENSG00000198625
1	204599557	204599929	5Y-H4K8ac_peak_1535	8.54067	
1	204607910	204608138	5Y-H4K8ac_peak_1536	9.62747	
1	204643052	204643252	5Y-H4K8ac_peak_1537	8.10449	
1	204654166	204654365	5Y-H4K8ac_peak_1538	4.75174	LRRN2_ENSG00000170382
1	205012355	205012788	5Y-H4K8ac_peak_1539	10.69698	CNTN2_ENSG00000184144
1	205030034	205031185	5Y-H4K8ac_peak_1540	8.24461	AL583832.1_ENSG00000251696
1	205090751	205091061	5Y-H4K8ac_peak_1541	6.08523	RBBP5_ENSG00000117222
1	205092215	205092443	5Y-H4K8ac_peak_1542	5.03917	
1	205179998	205180445	5Y-H4K8ac_peak_1543	5.0789	DSTYK_ENSG00000133059
1	205313468	205313677	5Y-H4K8ac_peak_1544	7.41172	
1	205321273	205321668	5Y-H4K8ac_peak_1545	7.8267	SNRPGP10_ENSG00000235363
1	205426116	205426367	5Y-H4K8ac_peak_1546	3.96434	
1	205441999	205442213	5Y-H4K8ac_peak_1547	8.41685	
1	205470190	205470486	5Y-H4K8ac_peak_1548	8.20773	
1	205473841	205474062	5Y-H4K8ac_peak_1549	16.17363	CDK18_ENSG00000117266
1	205485293	205485848	5Y-H4K8ac_peak_1550	8.33454	
1	205497865	205498630	5Y-H4K8ac_peak_1551	8.52662	
1	205551629	205551980	5Y-H4K8ac_peak_1552	6.03632	
1	205561215	205561897	5Y-H4K8ac_peak_1553	5.87725	
1	205600714	205600996	5Y-H4K8ac_peak_1554	9.32595	ELK4_ENSG00000158711
1	205718767	205719098	5Y-H4K8ac_peak_1555	11.12941	NUCKS1_ENSG00000069275
1	205719684	205719990	5Y-H4K8ac_peak_1556	5.41472	NUCKS1_ENSG00000069275
1	205844601	205844828	5Y-H4K8ac_peak_1557	4.90471	
1	206136101	206136487	5Y-H4K8ac_peak_1558	10.46287	FAM72A_ENSG00000196550
1	206235451	206235681	5Y-H4K8ac_peak_1559	6.77436	
1	206235980	206236679	5Y-H4K8ac_peak_1560	12.78119	
1	206621042	206621238	5Y-H4K8ac_peak_1561	5.98695	
1	206663741	206664018	5Y-H4K8ac_peak_1562	4.642	

1	206730362	206730898	5Y-H4K8ac_peak_1563	11.69482	
1	206785379	206785722	5Y-H4K8ac_peak_1564	9.44542	EIF2D_ENSG00000143486
1	206837167	206837370	5Y-H4K8ac_peak_1565	5.98695	
1	207082723	207083330	5Y-H4K8ac_peak_1566	7.18391	
1	207224149	207224816	5Y-H4K8ac_peak_1567	9.00954	
1	207226597	207226827	5Y-H4K8ac_peak_1568	8.24461	YOD1_ENSG00000180667
1	207924770	207925153	5Y-H4K8ac_peak_1569	6.00747	CD46_ENSG00000117335
1	208042275	208042477	5Y-H4K8ac_peak_1570	9.87124	C1orf132_ENSG00000203709
1	208084018	208084509	5Y-H4K8ac_peak_1571	10.12457	CD34_ENSG00000174059
1	208136030	208136900	5Y-H4K8ac_peak_1572	14.99099	
1	208137238	208137698	5Y-H4K8ac_peak_1573	10.79063	
1	208230773	208230996	5Y-H4K8ac_peak_1574	10.50356	
1	208232068	208232315	5Y-H4K8ac_peak_1575	3.93392	
1	208233718	208234158	5Y-H4K8ac_peak_1576	7.82057	
1	208250723	208251096	5Y-H4K8ac_peak_1577	5.77617	
1	208256389	208256760	5Y-H4K8ac_peak_1578	11.94897	
1	208257519	208257844	5Y-H4K8ac_peak_1579	10.79081	
1	208333432	208333835	5Y-H4K8ac_peak_1580	4.52203	
1	208336641	208337322	5Y-H4K8ac_peak_1581	4.95697	
1	208348414	208348605	5Y-H4K8ac_peak_1582	4.62763	
1	208352862	208353085	5Y-H4K8ac_peak_1583	6.53157	
1	208561785	208562017	5Y-H4K8ac_peak_1584	4.79585	
1	208707195	208707409	5Y-H4K8ac_peak_1585	6.37023	
1	209008602	209008839	5Y-H4K8ac_peak_1586	6.50117	
1	209279045	209279383	5Y-H4K8ac_peak_1587	4.50834	
1	209380397	209381091	5Y-H4K8ac_peak_1588	16.13719	
1	209393162	209393432	5Y-H4K8ac_peak_1589	8.21582	
1	209475957	209476188	5Y-H4K8ac_peak_1590	4.55128	
1	209849187	209849474	5Y-H4K8ac_peak_1591	8.24461	G0S2_ENSG00000123689
1	209958163	209958730	5Y-H4K8ac_peak_1592	9.30206	C1orf74_ENSG00000162757
1	209989225	209989676	5Y-H4K8ac_peak_1593	6.37023	
1	210000678	210001170	5Y-H4K8ac_peak_1594	8.43511	DIEXF_ENSG00000117597
1	210111174	210112036	5Y-H4K8ac_peak_1595	14.17466	SYT14_ENSG00000143469
1	210406076	210406510	5Y-H4K8ac_peak_1596	12.34969	SERTAD4-AS1_ENSG00000203706;SERTAD4_ENSG00000082497
1	210406974	210407264	5Y-H4K8ac_peak_1597	11.01067	SERTAD4-AS1_ENSG00000203706;SERTAD4_ENSG00000082497
1	210424403	210425830	5Y-H4K8ac_peak_1598	27.35138	
1	210433804	210434087	5Y-H4K8ac_peak_1599	4.77126	
1	210465686	210466222	5Y-H4K8ac_peak_1600	8.09013	
1	210466514	210466828	5Y-H4K8ac_peak_1601	7.44077	
1	210485110	210485346	5Y-H4K8ac_peak_1602	5.76631	
1	210501976	210503351	5Y-H4K8ac_peak_1603	12.79198	HHAT_ENSG00000054392
1	210706786	210706976	5Y-H4K8ac_peak_1604	6.73047	
1	210707219	210707583	5Y-H4K8ac_peak_1605	9.26003	
1	210708908	210709135	5Y-H4K8ac_peak_1606	5.78188	
1	210710604	210711004	5Y-H4K8ac_peak_1607	8.11614	
1	210743529	210745085	5Y-H4K8ac_peak_1608	18.64118	

1	210745843	210746297	5Y-H4K8ac_peak_1609	12.50545	
1	210746596	210746830	5Y-H4K8ac_peak_1610	6.22904	
1	210757964	210758160	5Y-H4K8ac_peak_1611	7.24864	
1	210759102	210759366	5Y-H4K8ac_peak_1612	6.37023	
1	210821371	210821705	5Y-H4K8ac_peak_1613	4.29586	
1	210836291	210836698	5Y-H4K8ac_peak_1614	6.08523	
1	210837007	210837244	5Y-H4K8ac_peak_1615	11.1169	
1	211306694	211306923	5Y-H4K8ac_peak_1616	8.24461	KCNH1_ENSG00000143473;KCNH1-IT1_ENSG00000234233
1	211307187	211307468	5Y-H4K8ac_peak_1617	12.10416	KCNH1_ENSG00000143473;KCNH1-IT1_ENSG00000234233
1	211307750	211308265	5Y-H4K8ac_peak_1618	9.41701	KCNH1_ENSG00000143473
1	211431949	211432300	5Y-H4K8ac_peak_1619	7.50464	RCOR3_ENSG00000117625
1	211432821	211433073	5Y-H4K8ac_peak_1620	25.89424	
1	211499698	211500550	5Y-H4K8ac_peak_1621	8.75963	TRAF5_ENSG00000082512
1	211556247	211556515	5Y-H4K8ac_peak_1622	11.88337	LINC00467_ENSG00000153363
1	211589701	211590204	5Y-H4K8ac_peak_1623	5.64909	SNX25P1_ENSG00000236809
1	211662130	211662386	5Y-H4K8ac_peak_1624	7.11863	
1	211663382	211663901	5Y-H4K8ac_peak_1625	16.11404	
1	211664134	211665360	5Y-H4K8ac_peak_1626	16.79029	RD3_ENSG00000198570;RP11-359E8.3_ENSG00000223649
1	211665569	211665835	5Y-H4K8ac_peak_1627	10.15788	RD3_ENSG00000198570;RP11-359E8.3_ENSG00000223649
1	211666509	211667556	5Y-H4K8ac_peak_1628	10.08244	RD3_ENSG00000198570;RP11-359E8.3_ENSG00000223649
1	211689161	211689513	5Y-H4K8ac_peak_1629	7.48932	
1	211817050	211817445	5Y-H4K8ac_peak_1630	9.07085	RP11-354K1.2_ENSG00000228792
1	212003542	212004073	5Y-H4K8ac_peak_1631	6.88736	LPGAT1_ENSG00000123684;RP11-552D8.1_ENSG00000229258
1	212119402	212119652	5Y-H4K8ac_peak_1632	5.59837	
1	212376060	212376261	5Y-H4K8ac_peak_1633	6.50117	
1	212588748	212589099	5Y-H4K8ac_peak_1634	9.32639	TMEM206_ENSG00000065600
1	212659704	212659954	5Y-H4K8ac_peak_1635	5.81898	
1	212691861	212692507	5Y-H4K8ac_peak_1636	11.71078	
1	212731692	212732285	5Y-H4K8ac_peak_1637	5.93036	RP11-61J19.4_ENSG00000260805
1	212781268	212781573	5Y-H4K8ac_peak_1638	7.54409	
1	212782146	212782461	5Y-H4K8ac_peak_1639	11.64833	
1	212809159	212809368	5Y-H4K8ac_peak_1640	8.80077	
1	212809746	212809950	5Y-H4K8ac_peak_1641	7.14092	
1	212810394	212810712	5Y-H4K8ac_peak_1642	4.77126	
1	212964735	212965089	5Y-H4K8ac_peak_1643	10.1994	NSL1_ENSG00000117697;TATDN3_ENSG00000203705
1	212965292	212965678	5Y-H4K8ac_peak_1644	7.58806	NSL1_ENSG00000117697;TATDN3_ENSG00000203705
1	213031769	213032165	5Y-H4K8ac_peak_1645	6.37023	FLVCR1-AS1_ENSG00000198468;FLVCR1_ENSG00000162769
1	213101951	213102267	5Y-H4K8ac_peak_1646	4.642	
1	213188532	213188805	5Y-H4K8ac_peak_1647	10.4495	ANGEL2_ENSG00000174606
1	213189373	213189720	5Y-H4K8ac_peak_1648	14.5825	ANGEL2_ENSG00000174606
1	213224293	213224531	5Y-H4K8ac_peak_1649	6.43068	RPS6KC1_ENSG00000136643
1	214156097	214157063	5Y-H4K8ac_peak_1650	12.8095	PROX1_ENSG00000117707
1	214162528	214162751	5Y-H4K8ac_peak_1651	6.36723	
1	214193600	214193838	5Y-H4K8ac_peak_1652	4.77126	
1	214327641	214328143	5Y-H4K8ac_peak_1653	8.69112	
1	214328886	214329136	5Y-H4K8ac_peak_1654	5.23083	

1	214339726	214339946	5Y-H4K8ac_peak_1655	4.50834	
1	214453725	214454765	5Y-H4K8ac_peak_1656	19.72309	SMYD2_ENSG00000143499
1	214776606	214776941	5Y-H4K8ac_peak_1657	5.64909	CENPF_ENSG00000117724
1	215740598	215741067	5Y-H4K8ac_peak_1658	6.19716	KCTD3_ENSG00000136636
1	216227086	216227317	5Y-H4K8ac_peak_1659	6.50117	
1	216773543	216773787	5Y-H4K8ac_peak_1660	7.30348	
1	216774272	216774696	5Y-H4K8ac_peak_1661	12.65187	
1	217262959	217263191	5Y-H4K8ac_peak_1662	13.1133	
1	217263603	217263894	5Y-H4K8ac_peak_1663	11.23789	
1	217580728	217581128	5Y-H4K8ac_peak_1664	6.1654	
1	218337985	218339010	5Y-H4K8ac_peak_1665	13.72401	
1	218456853	218457455	5Y-H4K8ac_peak_1666	10.5157	
1	218457936	218458246	5Y-H4K8ac_peak_1667	15.80786	RRP15_ENSG00000067533
1	218458818	218459064	5Y-H4K8ac_peak_1668	6.47245	RRP15_ENSG00000067533
1	218497629	218497825	5Y-H4K8ac_peak_1669	5.12213	
1	219028831	219029055	5Y-H4K8ac_peak_1670	4.47071	
1	219347263	219347670	5Y-H4K8ac_peak_1671	11.68317	RP11-135J2.4_ENSG00000228063;LYPLAL1_ENSG00000143353
1	219967006	219967276	5Y-H4K8ac_peak_1672	4.96019	
1	220101166	220101779	5Y-H4K8ac_peak_1673	8.62703	
1	220220316	220221070	5Y-H4K8ac_peak_1674	8.47129	EPRS_ENSG00000136628
1	220267521	220267769	5Y-H4K8ac_peak_1675	11.86504	IARS2_ENSG00000067704
1	220445451	220445727	5Y-H4K8ac_peak_1676	12.2918	RAB3GAP2_ENSG00000118873
1	220585707	220586043	5Y-H4K8ac_peak_1677	4.79585	
1	220701868	220702530	5Y-H4K8ac_peak_1678	9.74838	MARK1_ENSG00000116141
1	220863771	220863988	5Y-H4K8ac_peak_1679	6.77436	C1orf115_ENSG00000162817
1	220906058	220906305	5Y-H4K8ac_peak_1680	8.86184	
1	220959547	220959876	5Y-H4K8ac_peak_1681	9.44542	MARC1_ENSG00000186205
1	220960261	220960664	5Y-H4K8ac_peak_1682	14.18025	MARC1_ENSG00000186205
1	222790920	222791111	5Y-H4K8ac_peak_1683	5.67588	MIA3_ENSG00000154305
1	222816719	222817462	5Y-H4K8ac_peak_1684	7.89273	
1	222885957	222886150	5Y-H4K8ac_peak_1685	9.00954	AIDA_ENSG00000186063;BROX_ENSG00000162819
1	222988410	222988627	5Y-H4K8ac_peak_1686	13.14766	RP11-452F19.3_ENSG00000228106;DISP1_ENSG00000154309
1	223196232	223197001	5Y-H4K8ac_peak_1687	5.89922	
1	223316234	223317042	5Y-H4K8ac_peak_1688	12.11208	TLR5_ENSG00000187554;RP11-239E10.2_ENSG00000236846
1	223886331	223886657	5Y-H4K8ac_peak_1689	11.12941	
1	223899913	223900629	5Y-H4K8ac_peak_1690	14.00559	
1	223914227	223914750	5Y-H4K8ac_peak_1691	9.38276	
1	224033099	224033600	5Y-H4K8ac_peak_1692	9.44542	TP53BP2_ENSG00000143514
1	224034024	224034837	5Y-H4K8ac_peak_1693	11.9002	TP53BP2_ENSG00000143514
1	224179153	224179576	5Y-H4K8ac_peak_1694	4.29586	
1	224301867	224302380	5Y-H4K8ac_peak_1695	6.67135	FBXO28_ENSG00000143756
1	224363519	224363866	5Y-H4K8ac_peak_1696	9.7353	RP11-365O16.1_ENSG00000236773;DEGS1_ENSG00000143753
1	224371136	224371698	5Y-H4K8ac_peak_1697	6.03632	
1	224517500	224517719	5Y-H4K8ac_peak_1698	7.59101	NVL_ENSG00000143748
1	224528586	224528825	5Y-H4K8ac_peak_1699	8.24461	
1	224540379	224540617	5Y-H4K8ac_peak_1700	4.50834	

1	224544691	224545156	5Y-H4K8ac_peak_1701	9.30505	CNIH4_ENSG00000143771
1	224622577	224622870	5Y-H4K8ac_peak_1702	15.82359	CNIH3_ENSG00000143786
1	224690468	224690671	5Y-H4K8ac_peak_1703	16.01457	
1	225117059	225117837	5Y-H4K8ac_peak_1704	7.64648	
1	225615974	225616477	5Y-H4K8ac_peak_1705	10.21117	LBR_ENSG00000143815
1	225662971	225663237	5Y-H4K8ac_peak_1706	4.75174	
1	225840259	225840769	5Y-H4K8ac_peak_1707	16.01457	ENAH_ENSG00000154380
1	225841191	225841534	5Y-H4K8ac_peak_1708	14.00559	ENAH_ENSG00000154380
1	226070204	226070685	5Y-H4K8ac_peak_1709	7.64648	TMEM63A_ENSG00000196187
1	226070977	226071186	5Y-H4K8ac_peak_1710	6.37023	TMEM63A_ENSG00000196187
1	226104420	226104664	5Y-H4K8ac_peak_1711	10.07378	
1	226111571	226111909	5Y-H4K8ac_peak_1712	8.57063	RP4-559A3.7_ENSG00000255835;PYCR2_ENSG00000143811
1	226112338	226112764	5Y-H4K8ac_peak_1713	9.38692	RP4-559A3.7_ENSG00000255835;PYCR2_ENSG00000143811
1	226135347	226135619	5Y-H4K8ac_peak_1714	4.94375	
1	226187395	226187702	5Y-H4K8ac_peak_1715	13.81505	SDE2_ENSG00000143751
1	226249496	226249859	5Y-H4K8ac_peak_1716	10.14791	RP11-396C23.4_ENSG00000272562;H3F3A_ENSG00000163041
1	226271339	226271604	5Y-H4K8ac_peak_1717	6.83052	RP11-396C23.2_ENSG00000225518
1	226297865	226298702	5Y-H4K8ac_peak_1718	15.90416	
1	226308343	226308601	5Y-H4K8ac_peak_1719	7.11753	
1	226308968	226310816	5Y-H4K8ac_peak_1720	13.82621	
1	226323051	226323255	5Y-H4K8ac_peak_1721	8.5915	
1	226373971	226374377	5Y-H4K8ac_peak_1722	10.15788	ACBD3_ENSG00000182827
1	226374633	226374937	5Y-H4K8ac_peak_1723	7.34185	ACBD3_ENSG00000182827
1	226411133	226411534	5Y-H4K8ac_peak_1724	8.36588	MIXL1_ENSG00000185155
1	226496396	226496809	5Y-H4K8ac_peak_1725	16.09729	LIN9_ENSG00000183814
1	226497059	226497371	5Y-H4K8ac_peak_1726	10.48476	LIN9_ENSG00000183814
1	226595356	226595725	5Y-H4K8ac_peak_1727	7.41197	PARP1_ENSG00000143799
1	226596178	226596433	5Y-H4K8ac_peak_1728	5.67517	PARP1_ENSG00000143799
1	226661638	226661897	5Y-H4K8ac_peak_1729	7.60057	
1	226821676	226821879	5Y-H4K8ac_peak_1730	4.95697	
1	226828287	226828834	5Y-H4K8ac_peak_1731	5.64909	
1	226890871	226891695	5Y-H4K8ac_peak_1732	21.88077	
1	227126597	227126792	5Y-H4K8ac_peak_1733	9.07585	
1	227127145	227127642	5Y-H4K8ac_peak_1734	13.00733	
1	227241066	227241268	5Y-H4K8ac_peak_1735	4.84727	
1	227469553	227469799	5Y-H4K8ac_peak_1736	7.59101	
1	227505483	227506096	5Y-H4K8ac_peak_1737	9.36633	CDC42BPA_ENSG00000143776
1	227506292	227506868	5Y-H4K8ac_peak_1738	4.642	CDC42BPA_ENSG00000143776
1	227729459	227730228	5Y-H4K8ac_peak_1739	14.21916	
1	227730557	227730992	5Y-H4K8ac_peak_1740	7.90751	
1	227922663	227922928	5Y-H4K8ac_peak_1741	9.45199	JMJD4_ENSG00000081692
1	227923194	227923462	5Y-H4K8ac_peak_1742	9.13842	JMJD4_ENSG00000081692
1	228074559	228075558	5Y-H4K8ac_peak_1743	10.35586	
1	228269807	228270148	5Y-H4K8ac_peak_1744	13.84318	ARF1_ENSG00000143761
1	228270540	228270904	5Y-H4K8ac_peak_1745	5.73383	ARF1_ENSG00000143761
1	228291469	228291662	5Y-H4K8ac_peak_1746	6.98416	

1	228296739	228296945	5Y-H4K8ac_peak_1747	12.42757	MRPL55_ENSG00000162910
1	228327184	228327578	5Y-H4K8ac_peak_1748	9.87097	GUK1_ENSG00000143774
1	228350602	228350922	5Y-H4K8ac_peak_1749	4.97006	
1	228353453	228353720	5Y-H4K8ac_peak_1750	28.84212	IBA57-AS1_ENSG00000203684;IBA57_ENSG00000181873
1	228565672	228565959	5Y-H4K8ac_peak_1751	7.80036	
1	228566281	228567194	5Y-H4K8ac_peak_1752	6.80915	
1	228604038	228604361	5Y-H4K8ac_peak_1753	12.05638	TRIM17_ENSG00000162931
1	228674097	228675016	5Y-H4K8ac_peak_1754	18.62331	RNF187_ENSG00000168159
1	228782770	228783103	5Y-H4K8ac_peak_1755	4.15658	RNA5S17_ENSG00000200370;RNA5SP18_ENSG00000212237
1	228871307	228871893	5Y-H4K8ac_peak_1756	7.50148	RHOU_ENSG00000116574
1	229002208	229002675	5Y-H4K8ac_peak_1757	11.4254	
1	229003774	229005069	5Y-H4K8ac_peak_1758	11.69482	
1	229007347	229007676	5Y-H4K8ac_peak_1759	5.83797	
1	229034324	229034714	5Y-H4K8ac_peak_1760	7.75012	
1	229035334	229035702	5Y-H4K8ac_peak_1761	8.24461	
1	229047126	229047430	5Y-H4K8ac_peak_1762	4.3419	
1	229114738	229115028	5Y-H4K8ac_peak_1763	5.77617	
1	229123518	229123762	5Y-H4K8ac_peak_1764	8.79172	
1	229126169	229126482	5Y-H4K8ac_peak_1765	7.46945	
1	229139345	229139599	5Y-H4K8ac_peak_1766	7.16882	
1	229193955	229194585	5Y-H4K8ac_peak_1767	10.12457	
1	229195371	229195819	5Y-H4K8ac_peak_1768	8.46442	
1	229197827	229198018	5Y-H4K8ac_peak_1769	8.43511	
1	229249096	229249488	5Y-H4K8ac_peak_1770	8.2581	
1	229253595	229253938	5Y-H4K8ac_peak_1771	6.97863	
1	229388696	229389028	5Y-H4K8ac_peak_1772	8.16382	
1	229406709	229407252	5Y-H4K8ac_peak_1773	11.19935	RP5-1061H20.4_ENSG00000177788;RP5-1061H20.3_ENSG00000240524;RAB4A_ENSG00000168118
1	229544186	229544564	5Y-H4K8ac_peak_1774	7.98754	
1	229545454	229546362	5Y-H4K8ac_peak_1775	10.60083	RN7SKP276_ENSG00000252051
1	229643734	229644041	5Y-H4K8ac_peak_1776	12.50545	NUP133_ENSG00000069248;RP4-613A2.1_ENSG00000223635
1	229714929	229715411	5Y-H4K8ac_peak_1777	8.69112	
1	229761658	229762417	5Y-H4K8ac_peak_1778	13.0168	TAF5L_ENSG00000135801;URB2_ENSG00000135763
1	229828590	229829061	5Y-H4K8ac_peak_1779	8.69112	
1	229829433	229830114	5Y-H4K8ac_peak_1780	7.74473	
1	229830721	229830969	5Y-H4K8ac_peak_1781	6.55841	
1	230078330	230078537	5Y-H4K8ac_peak_1782	5.21777	
1	230079414	230080066	5Y-H4K8ac_peak_1783	16.91444	
1	230403190	230403391	5Y-H4K8ac_peak_1784	6.47527	RP5-956O18.2_ENSG00000227006
1	230404282	230404773	5Y-H4K8ac_peak_1785	9.74838	RP5-956O18.2_ENSG00000227006
1	230405526	230405901	5Y-H4K8ac_peak_1786	7.89273	
1	230406854	230407656	5Y-H4K8ac_peak_1787	12.10416	
1	230416824	230417176	5Y-H4K8ac_peak_1788	8.80077	RP5-956O18.3_ENSG00000224407
1	230435011	230435547	5Y-H4K8ac_peak_1789	9.30505	
1	230435793	230436044	5Y-H4K8ac_peak_1790	6.73385	
1	230879171	230879515	5Y-H4K8ac_peak_1791	7.1449	
1	231004696	231005094	5Y-H4K8ac_peak_1792	12.50545	C1orf198_ENSG00000119280

1	231113992	231114319	5Y-H4K8ac_peak_1793	9.37812	TTC13_ENSG00000143643;ARV1_ENSG00000173409
1	231297923	231298471	5Y-H4K8ac_peak_1794	8.5159	TRIM67_ENSG00000119283
1	231298790	231299191	5Y-H4K8ac_peak_1795	5.40265	TRIM67_ENSG00000119283
1	231328421	231328613	5Y-H4K8ac_peak_1796	4.77126	
1	231376508	231376782	5Y-H4K8ac_peak_1797	4.0639	C1orf131_ENSG00000143633;GNPAT_ENSG00000116906
1	231557706	231557962	5Y-H4K8ac_peak_1798	7.89273	
1	231558169	231558552	5Y-H4K8ac_peak_1799	19.75467	
1	231663689	231663903	5Y-H4K8ac_peak_1800	7.87192	RP11-295G20.2_ENSG00000233461;TSNAX-DISC1_ENSG00000270106;TSNAX_ENSG00000116918
1	231762533	231763125	5Y-H4K8ac_peak_1801	9.36633	DISC1_ENSG00000162946
1	231829777	231831341	5Y-H4K8ac_peak_1802	19.99409	
1	231832714	231832962	5Y-H4K8ac_peak_1803	14.02873	
1	232071689	232071954	5Y-H4K8ac_peak_1804	9.66296	
1	232766148	232766389	5Y-H4K8ac_peak_1805	4.81081	
1	232941043	232941700	5Y-H4K8ac_peak_1806	14.89523	MAP10_ENSG00000212916
1	233006982	233007665	5Y-H4K8ac_peak_1807	13.12848	
1	233113368	233113741	5Y-H4K8ac_peak_1808	6.37023	
1	233464056	233464284	5Y-H4K8ac_peak_1809	5.0789	MLK4_ENSG00000143674
1	233582999	233583321	5Y-H4K8ac_peak_1810	7.50148	
1	233590359	233590987	5Y-H4K8ac_peak_1811	11.22005	
1	233750583	233750782	5Y-H4K8ac_peak_1812	4.50302	KCNK1_ENSG00000135750
1	234041232	234041463	5Y-H4K8ac_peak_1813	6.03632	SLC35F3_ENSG00000183780
1	234247706	234247909	5Y-H4K8ac_peak_1814	5.67283	
1	234381735	234382089	5Y-H4K8ac_peak_1815	4.79585	
1	234418045	234418307	5Y-H4K8ac_peak_1816	9.51254	
1	234445317	234445523	5Y-H4K8ac_peak_1817	5.98695	
1	234509261	234509652	5Y-H4K8ac_peak_1818	6.4759	RP5-827C21.4_ENSG00000231663;COA6_ENSG00000168275
1	234613941	234614855	5Y-H4K8ac_peak_1819	8.82628	TARBP1_ENSG00000059588
1	234634707	234635647	5Y-H4K8ac_peak_1820	14.72057	
1	234645085	234645736	5Y-H4K8ac_peak_1821	9.30505	
1	234670250	234670483	5Y-H4K8ac_peak_1822	10.35586	
1	234678025	234678246	5Y-H4K8ac_peak_1823	8.5915	
1	234679433	234679954	5Y-H4K8ac_peak_1824	7.11863	
1	234680692	234681284	5Y-H4K8ac_peak_1825	7.26691	
1	234688925	234689262	5Y-H4K8ac_peak_1826	12.00715	
1	234690459	234690653	5Y-H4K8ac_peak_1827	5.83797	
1	234694595	234694796	5Y-H4K8ac_peak_1828	7.59339	
1	234706680	234706905	5Y-H4K8ac_peak_1829	7.59101	
1	234708350	234708615	5Y-H4K8ac_peak_1830	7.59101	
1	234744731	234745270	5Y-H4K8ac_peak_1831	6.53157	IRF2BP2_ENSG00000168264
1	234746142	234746752	5Y-H4K8ac_peak_1832	16.92114	IRF2BP2_ENSG00000168264
1	234811536	234812417	5Y-H4K8ac_peak_1833	13.58472	
1	234860516	234861754	5Y-H4K8ac_peak_1834	12.72376	LINC01132_ENSG00000227630
1	234905786	234906154	5Y-H4K8ac_peak_1835	9.58986	
1	235044225	235044458	5Y-H4K8ac_peak_1836	8.31536	
1	235093224	235093827	5Y-H4K8ac_peak_1837	9.51254	RP11-443B7.2_ENSG00000237520
1	235105657	235105954	5Y-H4K8ac_peak_1838	15.49523	RP11-443B7.1_ENSG00000238005

1	235183543	235184002	5Y-H4K8ac_peak_1839	9.56702	
1	235184966	235185250	5Y-H4K8ac_peak_1840	10.79063	
1	235291843	235292053	5Y-H4K8ac_peak_1841	7.33686	TOMM20_ENSG00000173726;SNORA14B_ENSG00000207181
1	235490826	235491414	5Y-H4K8ac_peak_1842	9.51254	ARID4B_ENSG00000054267;GGPS1_ENSG00000152904
1	235668489	235668824	5Y-H4K8ac_peak_1843	8.24461	B3GALNT2_ENSG00000162885
1	235710250	235710550	5Y-H4K8ac_peak_1844	9.02782	
1	235711594	235712128	5Y-H4K8ac_peak_1845	7.20869	
1	235712400	235712838	5Y-H4K8ac_peak_1846	10.79081	
1	235713498	235713923	5Y-H4K8ac_peak_1847	6.08523	
1	235714320	235714570	5Y-H4K8ac_peak_1848	6.84536	
1	235715512	235716270	5Y-H4K8ac_peak_1849	11.37095	
1	235717353	235717909	5Y-H4K8ac_peak_1850	10.12457	
1	235812546	235813061	5Y-H4K8ac_peak_1851	5.87725	GNG4_ENSG00000168243
1	236030420	236030954	5Y-H4K8ac_peak_1852	8.62703	
1	236228250	236228842	5Y-H4K8ac_peak_1853	6.80915	NID1_ENSG00000116962
1	236304676	236305004	5Y-H4K8ac_peak_1854	7.1449	GPR137B_ENSG00000077585
1	236444315	236444539	5Y-H4K8ac_peak_1855	6.77436	ERO1LB_ENSG00000086619
1	236686671	236687743	5Y-H4K8ac_peak_1856	12.10416	LGALS8-AS1_ENSG00000223776
1	236767980	236768721	5Y-H4K8ac_peak_1857	14.86775	HEATR1_ENSG00000119285
1	236849845	236850323	5Y-H4K8ac_peak_1858	9.30206	ACTN2_ENSG00000077522
1	237008137	237008427	5Y-H4K8ac_peak_1859	8.2913	
1	237045786	237045986	5Y-H4K8ac_peak_1860	5.03438	
1	237056347	237056695	5Y-H4K8ac_peak_1861	5.72233	
1	237057609	237057837	5Y-H4K8ac_peak_1862	5.60566	
1	237058207	237058412	5Y-H4K8ac_peak_1863	10.23828	
1	237058611	237058931	5Y-H4K8ac_peak_1864	24.20876	
1	237059709	237060065	5Y-H4K8ac_peak_1865	5.67517	
1	237070125	237070574	5Y-H4K8ac_peak_1866	7.28023	
1	237076151	237076731	5Y-H4K8ac_peak_1867	7.11863	
1	237269541	237269747	5Y-H4K8ac_peak_1868	5.76631	
1	237997955	237998437	5Y-H4K8ac_peak_1869	5.23083	
1	238126682	238126897	5Y-H4K8ac_peak_1870	4.07874	
1	238133008	238133450	5Y-H4K8ac_peak_1871	6.82266	
1	239340972	239341208	5Y-H4K8ac_peak_1872	5.65584	
1	239550093	239550627	5Y-H4K8ac_peak_1873	7.72887	CHRM3_ENSG00000133019
1	239993841	239994740	5Y-H4K8ac_peak_1874	9.51254	
1	239995528	239995955	5Y-H4K8ac_peak_1875	12.44818	
1	239996160	239996843	5Y-H4K8ac_peak_1876	10.34353	
1	240007612	240007967	5Y-H4K8ac_peak_1877	5.39991	
1	240031270	240031551	5Y-H4K8ac_peak_1878	4.48815	
1	240031934	240032610	5Y-H4K8ac_peak_1879	15.54885	
1	240041215	240041640	5Y-H4K8ac_peak_1880	5.23083	
1	240042015	240042653	5Y-H4K8ac_peak_1881	10.14791	
1	240056630	240056848	5Y-H4K8ac_peak_1882	4.48815	
1	240059301	240059988	5Y-H4K8ac_peak_1883	9.59156	
1	240061015	240061218	5Y-H4K8ac_peak_1884	5.76631	

1	240128581	240128869	5Y-H4K8ac_peak_1885	7.11863	
1	240161240	240161533	5Y-H4K8ac_peak_1886	4.0639	
1	240308423	240308634	5Y-H4K8ac_peak_1887	5.65584	RP11-567G24.1_ENSG00000228818
1	241128873	241129078	5Y-H4K8ac_peak_1888	8.2913	
1	241520090	241520757	5Y-H4K8ac_peak_1889	7.50148	RGS7_ENSG00000182901
1	241587217	241588181	5Y-H4K8ac_peak_1890	13.92877	RP11-527D7.1_ENSG00000225554
1	241803877	241804488	5Y-H4K8ac_peak_1891	10.90365	
1	241826574	241826861	5Y-H4K8ac_peak_1892	6.5814	
1	242011253	242011754	5Y-H4K8ac_peak_1893	11.69482	EXO1_ENSG00000174371
1	242162314	242162521	5Y-H4K8ac_peak_1894	5.98695	MAP1LC3C_ENSG00000197769
1	242375396	242376048	5Y-H4K8ac_peak_1895	5.98695	
1	242609817	242610035	5Y-H4K8ac_peak_1896	8.47164	
1	242612496	242612736	5Y-H4K8ac_peak_1897	6.87095	
1	242687214	242687659	5Y-H4K8ac_peak_1898	4.0639	PLD5_ENSG00000180287
1	243140752	243140953	5Y-H4K8ac_peak_1899	5.07473	
1	243181219	243181457	5Y-H4K8ac_peak_1900	8.2913	
1	243257221	243257448	5Y-H4K8ac_peak_1901	8.84534	
1	243418209	243418401	5Y-H4K8ac_peak_1902	4.00285	CEP170_ENSG00000143702;SDCCAG8_ENSG00000054282
1	243645773	243646004	5Y-H4K8ac_peak_1903	6.78318	
1	243646274	243646794	5Y-H4K8ac_peak_1904	9.32595	
1	243658640	243659015	5Y-H4K8ac_peak_1905	10.60083	
1	243659980	243660422	5Y-H4K8ac_peak_1906	9.87097	
1	244013702	244014520	5Y-H4K8ac_peak_1907	12.65187	AKT3_ENSG00000117020
1	244069214	244069469	5Y-H4K8ac_peak_1908	9.02242	
1	244080441	244081083	5Y-H4K8ac_peak_1909	7.64648	RP11-278H7.1_ENSG00000226828
1	244218222	244218423	5Y-H4K8ac_peak_1910	5.59843	
1	244419529	244419725	5Y-H4K8ac_peak_1911	7.57406	
1	244614738	244615126	5Y-H4K8ac_peak_1912	8.36588	ADSS_ENSG00000035687
1	244624511	244625532	5Y-H4K8ac_peak_1913	14.26354	
1	244997291	244997517	5Y-H4K8ac_peak_1914	8.3285	
1	244998147	244998467	5Y-H4K8ac_peak_1915	9.02782	COX20_ENSG00000203667
1	244998660	244998854	5Y-H4K8ac_peak_1916	16.23405	COX20_ENSG00000203667
1	245026671	245027722	5Y-H4K8ac_peak_1917	13.00733	HNRNPU_ENSG00000153187;RP11-11N7.4_ENSG00000273175
1	245133150	245133882	5Y-H4K8ac_peak_1918	12.26606	RP11-156E8.1_ENSG00000272195;EFCAB2_ENSG00000203666
1	245134110	245134741	5Y-H4K8ac_peak_1919	22.9394	RP11-156E8.1_ENSG00000272195
1	245317203	245318035	5Y-H4K8ac_peak_1920	19.64191	KIF26B_ENSG00000162849
1	245320039	245320517	5Y-H4K8ac_peak_1921	15.41988	
1	245343228	245343442	5Y-H4K8ac_peak_1922	5.60566	
1	245364293	245364510	5Y-H4K8ac_peak_1923	8.31764	
1	245378610	245378846	5Y-H4K8ac_peak_1924	4.95697	
1	245404066	245404260	5Y-H4K8ac_peak_1925	5.12213	
1	245730719	245730988	5Y-H4K8ac_peak_1926	8.69112	
1	245762105	245762348	5Y-H4K8ac_peak_1927	5.51472	
1	245769258	245769456	5Y-H4K8ac_peak_1928	3.94247	
1	245777489	245778023	5Y-H4K8ac_peak_1929	8.08337	RP11-522M21.2_ENSG00000238224
1	245785806	245786071	5Y-H4K8ac_peak_1930	4.95697	

1	245846801	245847225	5Y-H4K8ac_peak_1931	8.69112	
1	245915057	245915399	5Y-H4K8ac_peak_1932	5.91107	
1	245916126	245916420	5Y-H4K8ac_peak_1933	8.27643	
1	245971769	245971994	5Y-H4K8ac_peak_1934	6.53157	
1	246036657	246037399	5Y-H4K8ac_peak_1935	8.43511	
1	246039131	246039410	5Y-H4K8ac_peak_1936	4.95697	
1	246887054	246888026	5Y-H4K8ac_peak_1937	16.51777	SCCPDH_ENSG00000143653
1	247171056	247171265	5Y-H4K8ac_peak_1938	8.1667	ZNF695_ENSG00000197472
1	247171849	247172079	5Y-H4K8ac_peak_1939	9.11392	ZNF695_ENSG00000197472
1	247241662	247242008	5Y-H4K8ac_peak_1940	7.02726	ZNF670_ENSG00000135747
1	247267370	247267612	5Y-H4K8ac_peak_1941	7.87192	ZNF669_ENSG00000188295
1	247268330	247268580	5Y-H4K8ac_peak_1942	7.89142	ZNF669_ENSG00000188295
1	247274309	247275180	5Y-H4K8ac_peak_1943	9.44542	C1orf229_ENSG00000221953
1	247275527	247275836	5Y-H4K8ac_peak_1944	8.26455	C1orf229_ENSG00000221953
1	247276203	247276411	5Y-H4K8ac_peak_1945	4.13628	C1orf229_ENSG00000221953
1	247373806	247374071	5Y-H4K8ac_peak_1946	3.96434	MIR3916_ENSG00000227671
1	247494642	247494940	5Y-H4K8ac_peak_1947	7.29055	ZNF496_ENSG00000162714
1	247495307	247495973	5Y-H4K8ac_peak_1948	6.19716	ZNF496_ENSG00000162714
1	247611339	247611529	5Y-H4K8ac_peak_1949	11.86504	
1	249132727	249132935	5Y-H4K8ac_peak_1950	5.26789	ZNF672_ENSG00000171161
1	249152100	249153040	5Y-H4K8ac_peak_1951	9.63153	ZNF692_ENSG00000171163;AL672294.1_ENSG00000227237
1	249153513	249154057	5Y-H4K8ac_peak_1952	9.63153	ZNF692_ENSG00000171163;AL672294.1_ENSG00000227237
1	249157664	249158023	5Y-H4K8ac_peak_1953	15.82359	
10	119801	120156	5Y-H4K8ac_peak_1954	17.50049	TUBB8_ENSG00000173876
10	122094	122954	5Y-H4K8ac_peak_1955	5.98695	
10	180603	181217	5Y-H4K8ac_peak_1956	7.20869	ZMYND11_ENSG00000015171
10	181559	181751	5Y-H4K8ac_peak_1957	7.34185	
10	414336	414930	5Y-H4K8ac_peak_1958	7.11863	
10	416237	416440	5Y-H4K8ac_peak_1959	12.69256	
10	418697	418896	5Y-H4K8ac_peak_1960	4.5753	
10	977946	978183	5Y-H4K8ac_peak_1961	10.46287	LARP4B_ENSG00000107929;RP11-363N22.2_ENSG00000229869
10	1034465	1034812	5Y-H4K8ac_peak_1962	7.90751	AL359878.1_ENSG00000205740;GTPBP4_ENSG00000107937
10	1102323	1102600	5Y-H4K8ac_peak_1963	16.01457	
10	1178580	1178783	5Y-H4K8ac_peak_1964	5.40331	
10	1313882	1314146	5Y-H4K8ac_peak_1965	6.75784	
10	1314922	1315245	5Y-H4K8ac_peak_1966	10.73603	
10	1315581	1315778	5Y-H4K8ac_peak_1967	6.53157	
10	1317002	1317636	5Y-H4K8ac_peak_1968	14.72057	
10	1321465	1321763	5Y-H4K8ac_peak_1969	8.564	
10	1330833	1331151	5Y-H4K8ac_peak_1970	7.76768	
10	1334034	1334351	5Y-H4K8ac_peak_1971	12.06254	
10	1336476	1336699	5Y-H4K8ac_peak_1972	5.60566	
10	1337035	1337310	5Y-H4K8ac_peak_1973	4.29586	
10	2267530	2267730	5Y-H4K8ac_peak_1974	4.77126	
10	2634160	2634358	5Y-H4K8ac_peak_1975	4.07874	
10	2654064	2654344	5Y-H4K8ac_peak_1976	4.14761	

10	2666009	2666230	5Y-H4K8ac_peak_1977	7.89142	
10	3115326	3115718	5Y-H4K8ac_peak_1978	4.84727	
10	3880450	3880791	5Y-H4K8ac_peak_1979	6.34046	
10	4867868	4868129	5Y-H4K8ac_peak_1980	14.19462	
10	5596125	5596338	5Y-H4K8ac_peak_1981	4.90471	
10	5625863	5626374	5Y-H4K8ac_peak_1982	6.77436	
10	5632921	5633138	5Y-H4K8ac_peak_1983	9.63153	
10	5637691	5637961	5Y-H4K8ac_peak_1984	5.65584	RP13-463N16.6_ENSG00000242147
10	5638893	5639519	5Y-H4K8ac_peak_1985	7.63144	RP13-463N16.6_ENSG00000242147
10	5726881	5727328	5Y-H4K8ac_peak_1986	5.65584	FAM208B_ENSG00000108021
10	5734525	5734814	5Y-H4K8ac_peak_1987	5.23083	
10	5854804	5855322	5Y-H4K8ac_peak_1988	5.98695	RP11-318E3.9_ENSG00000272764
10	5855668	5855963	5Y-H4K8ac_peak_1989	10.1994	RP11-318E3.9_ENSG00000272764
10	5917961	5918259	5Y-H4K8ac_peak_1990	4.84727	
10	6131012	6131254	5Y-H4K8ac_peak_1991	8.62703	RBM17_ENSG00000134453
10	7293702	7293954	5Y-H4K8ac_peak_1992	11.57334	
10	7294758	7294992	5Y-H4K8ac_peak_1993	8.04493	
10	7308187	7308802	5Y-H4K8ac_peak_1994	7.83553	
10	7309030	7309545	5Y-H4K8ac_peak_1995	8.71064	
10	7449973	7450259	5Y-H4K8ac_peak_1996	4.1776	
10	7453175	7453836	5Y-H4K8ac_peak_1997	12.24192	SFMBT2_ENSG00000198879
10	8090103	8090358	5Y-H4K8ac_peak_1998	8.69112	
10	8091466	8091699	5Y-H4K8ac_peak_1999	7.59101	
10	8091928	8093067	5Y-H4K8ac_peak_2000	9.31608	RP11-379F12.4_ENSG00000232638
10	8093461	8093673	5Y-H4K8ac_peak_2001	7.90751	RP11-379F12.4_ENSG00000232638
10	8096773	8097272	5Y-H4K8ac_peak_2002	5.98695	
10	8114082	8114435	5Y-H4K8ac_peak_2003	4.50834	
10	8137371	8138017	5Y-H4K8ac_peak_2004	5.64909	
10	8154218	8154615	5Y-H4K8ac_peak_2005	6.78128	
10	8158078	8158335	5Y-H4K8ac_peak_2006	6.73385	
10	8159526	8160024	5Y-H4K8ac_peak_2007	14.15915	
10	8340750	8341289	5Y-H4K8ac_peak_2008	8.17203	RP5-1119O21.2_ENSG00000226861
10	8342430	8342627	5Y-H4K8ac_peak_2009	7.76232	
10	8681547	8681813	5Y-H4K8ac_peak_2010	6.50117	
10	9284780	9285007	5Y-H4K8ac_peak_2011	4.50834	
10	11653193	11653390	5Y-H4K8ac_peak_2012	7.03573	USP6NL_ENSG00000148429;RP11-138I18.1_ENSG00000271360
10	11784606	11785018	5Y-H4K8ac_peak_2013	4.77126	ECHDC3_ENSG00000134463
10	12084956	12085155	5Y-H4K8ac_peak_2014	11.1169	UPF2_ENSG00000151461
10	12095924	12096120	5Y-H4K8ac_peak_2015	7.14616	
10	12110314	12110612	5Y-H4K8ac_peak_2016	6.22669	DHTKD1_ENSG00000181192
10	12111024	12111235	5Y-H4K8ac_peak_2017	13.73676	DHTKD1_ENSG00000181192
10	12305940	12306144	5Y-H4K8ac_peak_2018	5.87382	
10	13141561	13141829	5Y-H4K8ac_peak_2019	4.84727	CCDC3_ENSG00000151468;OPTN_ENSG00000123240
10	13341729	13342045	5Y-H4K8ac_peak_2020	9.31608	
10	13344084	13344353	5Y-H4K8ac_peak_2021	7.89142	PHYH_ENSG00000107537
10	13389295	13389648	5Y-H4K8ac_peak_2022	14.77644	SEPHS1_ENSG00000086475

10	13390500	13390774	5Y-H4K8ac_peak_2023	10.35586	SEPHS1_ENSG00000086475
10	13482388	13482618	5Y-H4K8ac_peak_2024	7.3889	
10	13571250	13571493	5Y-H4K8ac_peak_2025	8.30301	BEND7_ENSG00000165626;RP11-214D15.2_ENSG00000227175
10	13629015	13629225	5Y-H4K8ac_peak_2026	5.35202	PRPF18_ENSG00000165630
10	14131206	14131439	5Y-H4K8ac_peak_2027	5.98695	
10	14921014	14921318	5Y-H4K8ac_peak_2028	4.67245	RP11-398C13.6_ENSG00000272853;SUV39H2_ENSG00000152455
10	15002748	15002965	5Y-H4K8ac_peak_2029	6.77436	
10	15139174	15139650	5Y-H4K8ac_peak_2030	6.53157	C10orf111_ENSG00000176236;RPP38_ENSG00000152464
10	15412370	15412740	5Y-H4K8ac_peak_2031	12.98738	FAM171A1_ENSG00000148468
10	16562799	16563001	5Y-H4K8ac_peak_2032	6.37023	
10	17007163	17007431	5Y-H4K8ac_peak_2033	7.31815	
10	17243486	17243690	5Y-H4K8ac_peak_2034	13.03079	TRDMT1_ENSG00000107614
10	17270791	17271011	5Y-H4K8ac_peak_2035	6.77436	VIM-AS1_ENSG00000229124;VIM_ENSG00000026025
10	18448101	18448316	5Y-H4K8ac_peak_2036	8.2913	
10	18916346	18916687	5Y-H4K8ac_peak_2037	9.30505	
10	19391992	19392183	5Y-H4K8ac_peak_2038	6.34046	
10	20257199	20257624	5Y-H4K8ac_peak_2039	10.19948	
10	21243470	21243695	5Y-H4K8ac_peak_2040	7.38046	
10	21250745	21250943	5Y-H4K8ac_peak_2041	8.00339	
10	21357926	21358201	5Y-H4K8ac_peak_2042	7.9215	
10	21387597	21388009	5Y-H4K8ac_peak_2043	7.38046	
10	21389073	21389537	5Y-H4K8ac_peak_2044	5.67283	
10	21462408	21462746	5Y-H4K8ac_peak_2045	5.65584	NEBL_ENSG00000078114;NEBL-AS1_ENSG00000231920
10	21463327	21463689	5Y-H4K8ac_peak_2046	6.37023	NEBL_ENSG00000078114;NEBL-AS1_ENSG00000231920
10	21653206	21653439	5Y-H4K8ac_peak_2047	6.46053	
10	21785282	21785660	5Y-H4K8ac_peak_2048	9.23159	CASC10_ENSG00000204682;MIR1915_ENSG00000222071
10	21808964	21809255	5Y-H4K8ac_peak_2049	6.66026	
10	21813849	21814044	5Y-H4K8ac_peak_2050	7.76232	SKIDA1_ENSG00000180592
10	21814835	21815307	5Y-H4K8ac_peak_2051	4.95697	SKIDA1_ENSG00000180592
10	22541314	22541855	5Y-H4K8ac_peak_2052	5.41472	
10	22604869	22605064	5Y-H4K8ac_peak_2053	7.38046	COMMD3_ENSG00000148444;COMMD3-BMI1_ENSG00000269897
10	22605394	22605604	5Y-H4K8ac_peak_2054	13.8759	COMMD3_ENSG00000148444;COMMD3-BMI1_ENSG00000269897
10	22610190	22610717	5Y-H4K8ac_peak_2055	10.1994	BMI1_ENSG00000168283
10	22625079	22625383	5Y-H4K8ac_peak_2056	13.00733	
10	22629389	22629943	5Y-H4K8ac_peak_2057	13.85283	RP11-573G6.10_ENSG00000272366
10	22764955	22765238	5Y-H4K8ac_peak_2058	7.09658	
10	23003647	23004186	5Y-H4K8ac_peak_2059	12.11208	PIP4K2A_ENSG00000150867
10	23384537	23385099	5Y-H4K8ac_peak_2060	10.1994	MSRB2_ENSG00000148450
10	23408604	23409088	5Y-H4K8ac_peak_2061	4.51076	
10	24404513	24404726	5Y-H4K8ac_peak_2062	7.21112	
10	24544035	24544581	5Y-H4K8ac_peak_2063	6.77436	
10	25011658	25012511	5Y-H4K8ac_peak_2064	11.22005	ARHGAP21_ENSG00000107863
10	25241094	25242002	5Y-H4K8ac_peak_2065	13.85283	PRTFDC1_ENSG00000099256;RP11-165A20.3_ENSG00000273107
10	25305754	25305975	5Y-H4K8ac_peak_2066	9.93099	ENKUR_ENSG00000151023;THNSL1_ENSG00000185875
10	26985810	26986731	5Y-H4K8ac_peak_2067	8.69112	PDSS1_ENSG00000148459
10	27149302	27149746	5Y-H4K8ac_peak_2068	6.50117	ABI1_ENSG00000136754

10	27444383	27444618	5Y-H4K8ac_peak_2069	5.64909	YME1L1_ENSG00000136758;MASTL_ENSG00000120539
10	27793407	27793752	5Y-H4K8ac_peak_2070	5.41472	RAB18_ENSG00000099246
10	28821295	28821881	5Y-H4K8ac_peak_2071	9.51254	WAC-AS1_ENSG00000254635;WAC_ENSG00000095787
10	28822128	28822493	5Y-H4K8ac_peak_2072	8.35139	WAC-AS1_ENSG00000254635;WAC_ENSG00000095787
10	28965830	28966162	5Y-H4K8ac_peak_2073	9.05168	BAMBI_ENSG00000095739
10	29099407	29099886	5Y-H4K8ac_peak_2074	8.00339	
10	29121646	29121874	5Y-H4K8ac_peak_2075	9.11354	
10	29371272	29371593	5Y-H4K8ac_peak_2076	8.35139	
10	29541772	29542312	5Y-H4K8ac_peak_2077	8.564	
10	29543862	29544076	5Y-H4K8ac_peak_2078	9.05766	
10	29698004	29698923	5Y-H4K8ac_peak_2079	13.8759	PTCHD3P1_ENSG00000224597
10	29992416	29992631	5Y-H4K8ac_peak_2080	5.65584	
10	30638446	30639194	5Y-H4K8ac_peak_2081	8.2913	
10	30722439	30723391	5Y-H4K8ac_peak_2082	13.99721	MAP3K8_ENSG00000107968
10	30894017	30894399	5Y-H4K8ac_peak_2083	4.84727	
10	30994963	30995233	5Y-H4K8ac_peak_2084	7.31807	
10	31064685	31064894	5Y-H4K8ac_peak_2085	4.14761	
10	31073829	31074257	5Y-H4K8ac_peak_2086	8.73985	
10	31423232	31423606	5Y-H4K8ac_peak_2087	15.3483	
10	31608191	31608659	5Y-H4K8ac_peak_2088	8.04493	ZEB1-AS1_ENSG00000237036;ZEB1_ENSG00000148516
10	31871809	31872032	5Y-H4K8ac_peak_2089	7.66481	
10	31873569	31873851	5Y-H4K8ac_peak_2090	9.00954	
10	31892826	31893026	5Y-H4K8ac_peak_2091	5.8635	RP11-472N13.2_ENSG00000223834
10	31937793	31938046	5Y-H4K8ac_peak_2092	8.97752	
10	32039219	32039546	5Y-H4K8ac_peak_2093	6.31818	
10	32063436	32063775	5Y-H4K8ac_peak_2094	4.50834	
10	32344949	32345294	5Y-H4K8ac_peak_2095	10.69698	KIF5B_ENSG00000170759;Y_RNA_ENSG00000206660
10	32635391	32636043	5Y-H4K8ac_peak_2096	20.88791	RP11-135A24.4_ENSG00000233825;AL391839.1_ENSG00000222309;RP11-135A24.2_ENSG00000229327
10	32636336	32636648	5Y-H4K8ac_peak_2097	5.449	RP11-135A24.4_ENSG00000233825;AL391839.1_ENSG00000222309;RP11-135A24.2_ENSG00000229327
10	33246759	33247111	5Y-H4K8ac_peak_2098	4.51497	RP11-462L8.1_ENSG00000229656
10	33247455	33247833	5Y-H4K8ac_peak_2099	15.59156	RP11-462L8.1_ENSG00000229656
10	33269505	33269733	5Y-H4K8ac_peak_2100	4.29586	
10	33270664	33270882	5Y-H4K8ac_peak_2101	10.54764	
10	34502971	34503161	5Y-H4K8ac_peak_2102	5.40331	
10	35082049	35082281	5Y-H4K8ac_peak_2103	4.728	
10	35103861	35104117	5Y-H4K8ac_peak_2104	9.60627	PARD3_ENSG00000148498;PARD3-AS1_ENSG00000226386
10	35379348	35379560	5Y-H4K8ac_peak_2105	7.59101	CUL2_ENSG00000108094
10	35896660	35897094	5Y-H4K8ac_peak_2106	10.12234	RP11-425A6.5_ENSG00000273312
10	38146716	38146987	5Y-H4K8ac_peak_2107	23.70116	ZNF248_ENSG00000198105;RP11-162G10.5_ENSG00000236514
10	38299667	38299950	5Y-H4K8ac_peak_2108	9.02938	ZNF33A_ENSG00000189180
10	38382335	38382628	5Y-H4K8ac_peak_2109	9.15977	ZNF37A_ENSG00000075407
10	38691454	38691728	5Y-H4K8ac_peak_2110	6.77436	SEPT7P9_ENSG00000120555
10	42971139	42971504	5Y-H4K8ac_peak_2111	5.89922	LINC00839_ENSG00000185904
10	43133663	43133976	5Y-H4K8ac_peak_2112	16.38263	ZNF33B_ENSG00000272373;ZNF33B_ENSG00000196693
10	43277111	43277406	5Y-H4K8ac_peak_2113	5.97089	BMS1_ENSG00000165733
10	43277703	43278048	5Y-H4K8ac_peak_2114	7.61515	BMS1_ENSG00000165733

10	43331628	43331846	5Y-H4K8ac_peak_2115	8.23578	
10	43332504	43332784	5Y-H4K8ac_peak_2116	5.62788	
10	43361828	43362228	5Y-H4K8ac_peak_2117	10.60083	
10	43366293	43366816	5Y-H4K8ac_peak_2118	7.41197	RP11-124O11.1_ENSG00000234944
10	43367339	43368059	5Y-H4K8ac_peak_2119	10.94041	RP11-124O11.1_ENSG00000234944
10	43429756	43429982	5Y-H4K8ac_peak_2120	5.23083	
10	43456751	43458053	5Y-H4K8ac_peak_2121	9.23159	
10	43464824	43465663	5Y-H4K8ac_peak_2122	10.60083	
10	43466421	43467106	5Y-H4K8ac_peak_2123	8.76608	
10	43476461	43476975	5Y-H4K8ac_peak_2124	6.78318	RP11-124O11.2_ENSG00000229630
10	43477351	43477617	5Y-H4K8ac_peak_2125	6.53157	RP11-124O11.2_ENSG00000229630
10	43535179	43535470	5Y-H4K8ac_peak_2126	13.00733	
10	43552057	43552510	5Y-H4K8ac_peak_2127	10.1994	
10	43578895	43579217	5Y-H4K8ac_peak_2128	14.13577	
10	43588731	43588924	5Y-H4K8ac_peak_2129	4.8188	
10	43600735	43600962	5Y-H4K8ac_peak_2130	9.37812	
10	43633326	43633698	5Y-H4K8ac_peak_2131	5.24695	RP11-351D16.3_ENSG00000273008;CSGALNACT2_ENSG00000169826
10	43633964	43634207	5Y-H4K8ac_peak_2132	9.87097	RP11-351D16.3_ENSG00000273008;CSGALNACT2_ENSG00000169826
10	43714781	43715116	5Y-H4K8ac_peak_2133	7.07936	
10	43857376	43857577	5Y-H4K8ac_peak_2134	9.13842	
10	43858016	43858564	5Y-H4K8ac_peak_2135	12.00715	
10	43892442	43892689	5Y-H4K8ac_peak_2136	9.31608	
10	43902783	43903009	5Y-H4K8ac_peak_2137	8.69112	
10	43904918	43905445	5Y-H4K8ac_peak_2138	5.67517	HNRNPF_ENSG00000169813
10	43916099	43916782	5Y-H4K8ac_peak_2139	8.43511	RP11-517P14.2_ENSG00000230555
10	43951400	43951791	5Y-H4K8ac_peak_2140	7.89273	
10	44069507	44070027	5Y-H4K8ac_peak_2141	11.28133	ZNF239_ENSG00000196793
10	44101942	44102477	5Y-H4K8ac_peak_2142	10.12457	CAP1P2_ENSG00000232004;ZNF485_ENSG00000198298
10	44143543	44144221	5Y-H4K8ac_peak_2143	17.04999	ZNF32_ENSG00000169740
10	44184802	44185222	5Y-H4K8ac_peak_2144	4.95697	
10	44277155	44277419	5Y-H4K8ac_peak_2145	5.718	
10	44756318	44756595	5Y-H4K8ac_peak_2146	4.5753	RP11-20J15.2_ENSG00000237590
10	45469709	45470307	5Y-H4K8ac_peak_2147	12.71088	
10	45495784	45496951	5Y-H4K8ac_peak_2148	8.43511	C10orf25_ENSG00000165511;ZNF22_ENSG00000165512
10	46168403	46168998	5Y-H4K8ac_peak_2149	11.19336	ZFAND4_ENSG00000172671
10	46222124	46222995	5Y-H4K8ac_peak_2150	12.00715	FAM21FP_ENSG00000237840;RP11-671E7.1_ENSG00000228702;FAM21C_ENSG00000172661
10	46973982	46974735	5Y-H4K8ac_peak_2151	5.41472	
10	46975019	46975414	5Y-H4K8ac_peak_2152	10.14791	
10	46992923	46993681	5Y-H4K8ac_peak_2153	15.48671	GPRIN2_ENSG00000204175
10	47056505	47056984	5Y-H4K8ac_peak_2154	8.09118	
10	47057909	47058210	5Y-H4K8ac_peak_2155	4.44548	
10	47083446	47083741	5Y-H4K8ac_peak_2156	7.38868	NPY4R_ENSG00000204174
10	47618113	47618312	5Y-H4K8ac_peak_2157	5.64909	
10	47627016	47627235	5Y-H4K8ac_peak_2158	8.60027	
10	47653881	47654258	5Y-H4K8ac_peak_2159	7.11863	
10	47656047	47656257	5Y-H4K8ac_peak_2160	4.55128	RP11-292F22.7_ENSG00000259942

10	48354551	48354775	5Y-H4K8ac_peak_2161	7.11863	ZNF488_ENSG00000165388
10	48452081	48452451	5Y-H4K8ac_peak_2162	5.64909	
10	49514755	49515019	5Y-H4K8ac_peak_2163	10.48476	MAPK8_ENSG00000107643
10	49664381	49664683	5Y-H4K8ac_peak_2164	7.43638	
10	49863466	49863661	5Y-H4K8ac_peak_2165	5.40331	ARHGAP22_ENSG00000128805
10	49863988	49864220	5Y-H4K8ac_peak_2166	9.38203	ARHGAP22_ENSG00000128805
10	49864841	49865232	5Y-H4K8ac_peak_2167	5.23083	ARHGAP22_ENSG00000128805
10	49866627	49867072	5Y-H4K8ac_peak_2168	5.59843	
10	50209651	50209959	5Y-H4K8ac_peak_2169	6.34046	
10	50323022	50323329	5Y-H4K8ac_peak_2170	6.77436	VSTM4_ENSG00000165633
10	50506485	50506924	5Y-H4K8ac_peak_2171	4.50834	C10orf71-AS1_ENSG00000236208;C10orf71_ENSG00000177354
10	50507692	50507983	5Y-H4K8ac_peak_2172	8.24461	C10orf71-AS1_ENSG00000236208;C10orf71_ENSG00000177354
10	50508771	50509115	5Y-H4K8ac_peak_2173	6.35402	
10	50526151	50526476	5Y-H4K8ac_peak_2174	4.84727	
10	50567352	50567674	5Y-H4K8ac_peak_2175	14.99238	
10	50568859	50569582	5Y-H4K8ac_peak_2176	9.44542	
10	50569876	50570217	5Y-H4K8ac_peak_2177	7.11863	
10	50603588	50604069	5Y-H4K8ac_peak_2178	5.41472	DRGX_ENSG00000165606
10	50605216	50605582	5Y-H4K8ac_peak_2179	7.03573	
10	50606194	50606525	5Y-H4K8ac_peak_2180	4.642	
10	50625285	50625538	5Y-H4K8ac_peak_2181	6.43775	
10	50803185	50803433	5Y-H4K8ac_peak_2182	5.56912	
10	50841430	50841660	5Y-H4K8ac_peak_2183	7.63144	
10	50861668	50861929	5Y-H4K8ac_peak_2184	4.07874	
10	50872667	50873218	5Y-H4K8ac_peak_2185	7.03573	
10	50887747	50888018	5Y-H4K8ac_peak_2186	20.04267	C10orf53_ENSG00000178645
10	50899175	50899369	5Y-H4K8ac_peak_2187	6.77436	
10	50969878	50970214	5Y-H4K8ac_peak_2188	10.54764	OGDHL_ENSG00000197444
10	51489203	51489461	5Y-H4K8ac_peak_2189	6.80915	
10	51497842	51498054	5Y-H4K8ac_peak_2190	6.1654	
10	52384506	52385004	5Y-H4K8ac_peak_2191	7.90236	SGMS1_ENSG00000198964;RP11-50E11.3_ENSG00000226200
10	52498755	52499585	5Y-H4K8ac_peak_2192	5.64909	ASAH2B_ENSG00000204147
10	55163175	55163469	5Y-H4K8ac_peak_2193	7.31815	
10	55163860	55164133	5Y-H4K8ac_peak_2194	9.23159	
10	60094412	60094974	5Y-H4K8ac_peak_2195	10.19948	UBE2D1_ENSG00000072401
10	60145234	60145551	5Y-H4K8ac_peak_2196	9.31608	TFAM_ENSG00000108064
10	60409007	60409323	5Y-H4K8ac_peak_2197	7.31815	
10	60935960	60936185	5Y-H4K8ac_peak_2198	11.5813	PHYHIPL_ENSG00000165443
10	60936474	60937123	5Y-H4K8ac_peak_2199	8.69112	PHYHIPL_ENSG00000165443
10	61469101	61469553	5Y-H4K8ac_peak_2200	7.72887	
10	61469745	61469964	5Y-H4K8ac_peak_2201	3.96434	
10	61493260	61493456	5Y-H4K8ac_peak_2202	6.34046	
10	62492714	62493302	5Y-H4K8ac_peak_2203	8.69112	ANK3_ENSG00000151150;RP11-131N11.4_ENSG00000254271
10	62703708	62704654	5Y-H4K8ac_peak_2204	6.78128	
10	62761343	62761571	5Y-H4K8ac_peak_2205	12.57157	RHOBTB1_ENSG00000072422
10	63212038	63212261	5Y-H4K8ac_peak_2206	15.27452	TMEM26_ENSG00000196932;RP11-809M12.1_ENSG00000237233

10	64133888	64134851	5Y-H4K8ac_peak_2207	8.47164	ZNF365_ENSG00000138311
10	64563977	64564178	5Y-H4K8ac_peak_2208	11.4254	ADO_ENSG00000181915
10	64564647	64564959	5Y-H4K8ac_peak_2209	6.77436	RP11-436D10.3_ENSG00000238280;ADO_ENSG00000181915
10	65280689	65280935	5Y-H4K8ac_peak_2210	12.49811	REEP3_ENSG00000165476
10	65281271	65281600	5Y-H4K8ac_peak_2211	8.93923	REEP3_ENSG00000165476
10	65389387	65389880	5Y-H4K8ac_peak_2212	15.94046	
10	65470670	65470968	5Y-H4K8ac_peak_2213	4.0639	
10	65471348	65471599	5Y-H4K8ac_peak_2214	6.14981	
10	69598261	69598474	5Y-H4K8ac_peak_2215	7.31815	DNAJC12_ENSG00000108176
10	69609557	69609807	5Y-H4K8ac_peak_2216	7.17184	RP11-57G10.8_ENSG00000272892
10	69991533	69991790	5Y-H4K8ac_peak_2217	6.73047	ATOH7_ENSG00000179774
10	70090604	70090885	5Y-H4K8ac_peak_2218	13.2534	HNRNPH3_ENSG00000096746
10	70091101	70091321	5Y-H4K8ac_peak_2219	7.50148	HNRNPH3_ENSG00000096746
10	70092018	70092637	5Y-H4K8ac_peak_2220	11.86504	PBLD_ENSG00000108187
10	70166582	70167398	5Y-H4K8ac_peak_2221	13.99721	RUFY2_ENSG00000204130
10	70320194	70320390	5Y-H4K8ac_peak_2222	5.41472	TET1_ENSG00000138336
10	70359224	70359489	5Y-H4K8ac_peak_2223	8.16382	
10	70481099	70481482	5Y-H4K8ac_peak_2224	6.08523	CCAR1_ENSG00000060339
10	70586872	70587436	5Y-H4K8ac_peak_2225	6.77436	STOX1_ENSG00000165730
10	70660486	70660819	5Y-H4K8ac_peak_2226	15.84891	DDX50_ENSG00000107625
10	70661106	70661401	5Y-H4K8ac_peak_2227	10.69698	DDX50_ENSG00000107625
10	70714740	70715038	5Y-H4K8ac_peak_2228	6.37023	DDX21_ENSG00000165732
10	70716121	70716340	5Y-H4K8ac_peak_2229	14.43262	DDX21_ENSG00000165732
10	70940048	70940394	5Y-H4K8ac_peak_2230	10.12457	SUPV3L1_ENSG00000156502
10	70985322	70985578	5Y-H4K8ac_peak_2231	5.65584	
10	71077699	71078368	5Y-H4K8ac_peak_2232	10.44054	
10	71078909	71079146	5Y-H4K8ac_peak_2233	8.69895	
10	71094832	71095030	5Y-H4K8ac_peak_2234	8.35138	
10	71158633	71159168	5Y-H4K8ac_peak_2235	7.03573	
10	71211397	71211643	5Y-H4K8ac_peak_2236	7.89273	TSPAN15_ENSG00000099282
10	71267101	71267298	5Y-H4K8ac_peak_2237	10.46287	
10	71267507	71267769	5Y-H4K8ac_peak_2238	8.33296	
10	71389402	71389717	5Y-H4K8ac_peak_2239	8.73985	C10orf35_ENSG00000171224
10	71390063	71390495	5Y-H4K8ac_peak_2240	11.04542	C10orf35_ENSG00000171224
10	71446194	71446547	5Y-H4K8ac_peak_2241	7.46096	
10	71517022	71517717	5Y-H4K8ac_peak_2242	7.11863	
10	71518167	71518400	5Y-H4K8ac_peak_2243	6.73047	
10	71626485	71627303	5Y-H4K8ac_peak_2244	7.38046	
10	71812620	71813469	5Y-H4K8ac_peak_2245	11.5813	H2AFY2_ENSG00000099284
10	71892341	71892638	5Y-H4K8ac_peak_2246	5.72233	AIFM2_ENSG00000042286
10	71905968	71906271	5Y-H4K8ac_peak_2247	5.51767	TYSND1_ENSG00000156521
10	72123338	72124130	5Y-H4K8ac_peak_2248	9.34555	
10	72141932	72142246	5Y-H4K8ac_peak_2249	16.01457	LRRC20_ENSG00000172731
10	72218277	72218747	5Y-H4K8ac_peak_2250	12.21176	
10	72238360	72238577	5Y-H4K8ac_peak_2251	7.18391	PALD1_ENSG00000107719
10	72344242	72344710	5Y-H4K8ac_peak_2252	5.23083	

10	72450208	72450521	5Y-H4K8ac_peak_2253	5.40331	
10	72453391	72453638	5Y-H4K8ac_peak_2254	7.31102	
10	72455181	72455742	5Y-H4K8ac_peak_2255	4.98439	
10	72647931	72648199	5Y-H4K8ac_peak_2256	6.50117	PCBD1_ENSG00000166228
10	72951177	72951805	5Y-H4K8ac_peak_2257	4.00285	
10	72972516	72972778	5Y-H4K8ac_peak_2258	7.37325	UNC5B_ENSG00000107731
10	72993464	72993713	5Y-H4K8ac_peak_2259	6.53157	
10	72993926	72994194	5Y-H4K8ac_peak_2260	15.05532	
10	72994938	72995704	5Y-H4K8ac_peak_2261	19.58975	
10	72996031	72996852	5Y-H4K8ac_peak_2262	8.19376	
10	72999292	72999763	5Y-H4K8ac_peak_2263	8.9562	
10	72999980	73000754	5Y-H4K8ac_peak_2264	15.49948	
10	73000987	73001197	5Y-H4K8ac_peak_2265	8.22388	
10	73001695	73002574	5Y-H4K8ac_peak_2266	9.01738	
10	73003849	73004385	5Y-H4K8ac_peak_2267	8.76608	
10	73005093	73006027	5Y-H4K8ac_peak_2268	4.77126	
10	73007175	73007516	5Y-H4K8ac_peak_2269	6.31818	
10	73008997	73009334	5Y-H4K8ac_peak_2270	7.59101	
10	73009662	73010206	5Y-H4K8ac_peak_2271	13.0168	
10	73010693	73010916	5Y-H4K8ac_peak_2272	4.40975	
10	73012421	73014475	5Y-H4K8ac_peak_2273	9.30505	
10	73015606	73016066	5Y-H4K8ac_peak_2274	13.19488	
10	73016354	73017406	5Y-H4K8ac_peak_2275	12.65187	
10	73017911	73018162	5Y-H4K8ac_peak_2276	6.77436	
10	73018407	73019015	5Y-H4K8ac_peak_2277	8.564	
10	73019457	73020162	5Y-H4K8ac_peak_2278	7.89273	
10	73020567	73020873	5Y-H4K8ac_peak_2279	5.24695	
10	73022684	73023275	5Y-H4K8ac_peak_2280	8.21582	
10	73025085	73025718	5Y-H4K8ac_peak_2281	12.10416	
10	73025978	73026621	5Y-H4K8ac_peak_2282	12.10416	
10	73027534	73027913	5Y-H4K8ac_peak_2283	7.03573	
10	73030627	73031721	5Y-H4K8ac_peak_2284	10.0346	
10	73033272	73033542	5Y-H4K8ac_peak_2285	9.30505	
10	73037085	73037509	5Y-H4K8ac_peak_2286	5.12488	
10	73038247	73038638	5Y-H4K8ac_peak_2287	14.0329	
10	73039206	73039898	5Y-H4K8ac_peak_2288	17.8819	
10	73078256	73078746	5Y-H4K8ac_peak_2289	12.05638	SLC29A3_ENSG00000198246
10	73136781	73137149	5Y-H4K8ac_peak_2290	5.24695	
10	73156173	73156505	5Y-H4K8ac_peak_2291	10.19948	CDH23_ENSG00000107736
10	73485029	73485487	5Y-H4K8ac_peak_2292	13.99721	
10	73486382	73486607	5Y-H4K8ac_peak_2293	8.20773	
10	73487017	73487445	5Y-H4K8ac_peak_2294	14.20885	
10	73596039	73596254	5Y-H4K8ac_peak_2295	6.50117	
10	73610580	73610873	5Y-H4K8ac_peak_2296	10.44054	PSAP_ENSG00000197746
10	73611362	73611737	5Y-H4K8ac_peak_2297	9.60458	PSAP_ENSG00000197746
10	73640805	73641006	5Y-H4K8ac_peak_2298	8.43511	

10	73649235	73649491	5Y-H4K8ac_peak_2299	5.12213	
10	73723541	73724474	5Y-H4K8ac_peak_2300	12.06963	CHST3_ENSG00000122863
10	73741088	73741298	5Y-H4K8ac_peak_2301	5.65584	
10	73757667	73757898	5Y-H4K8ac_peak_2302	6.95844	
10	73758210	73758407	5Y-H4K8ac_peak_2303	9.79526	
10	73769084	73769968	5Y-H4K8ac_peak_2304	7.50148	
10	73804169	73804508	5Y-H4K8ac_peak_2305	8.43511	
10	73817293	73817493	5Y-H4K8ac_peak_2306	5.87382	
10	73828736	73828947	5Y-H4K8ac_peak_2307	4.55128	
10	73847398	73848100	5Y-H4K8ac_peak_2308	13.14766	SPOCK2_ENSG00000107742
10	74008423	74008628	5Y-H4K8ac_peak_2309	4.84727	
10	74014818	74015012	5Y-H4K8ac_peak_2310	6.08523	
10	74020784	74020987	5Y-H4K8ac_peak_2311	8.1667	
10	74030466	74030671	5Y-H4K8ac_peak_2312	4.29586	
10	74032868	74033342	5Y-H4K8ac_peak_2313	14.31711	DDIT4_ENSG00000168209
10	74057101	74057548	5Y-H4K8ac_peak_2314	6.37023	
10	74058098	74058688	5Y-H4K8ac_peak_2315	7.64716	
10	74068073	74068895	5Y-H4K8ac_peak_2316	11.19336	
10	74079007	74079328	5Y-H4K8ac_peak_2317	9.41701	
10	74079591	74079959	5Y-H4K8ac_peak_2318	11.22005	
10	74081156	74081410	5Y-H4K8ac_peak_2319	12.44818	
10	74081791	74082749	5Y-H4K8ac_peak_2320	12.44818	
10	74084687	74085411	5Y-H4K8ac_peak_2321	6.47527	
10	74090894	74091251	5Y-H4K8ac_peak_2322	8.73392	
10	74092242	74092454	5Y-H4K8ac_peak_2323	9.0011	
10	74093258	74093605	5Y-H4K8ac_peak_2324	6.90928	
10	74094411	74095505	5Y-H4K8ac_peak_2325	5.72233	
10	74114502	74114759	5Y-H4K8ac_peak_2326	13.94477	DNAJB12_ENSG00000148719
10	74713384	74713811	5Y-H4K8ac_peak_2327	8.8021	PLA2G12B_ENSG00000138308
10	74870842	74871055	5Y-H4K8ac_peak_2328	8.2913	NUDT13_ENSG00000166321
10	75006710	75006946	5Y-H4K8ac_peak_2329	12.10416	DNAJC9-AS1_ENSG00000236756
10	75118216	75118437	5Y-H4K8ac_peak_2330	7.76232	TTC18_ENSG00000156042
10	75256320	75256515	5Y-H4K8ac_peak_2331	5.10404	PPP3CB_ENSG00000107758
10	75385918	75386405	5Y-H4K8ac_peak_2332	10.46287	USP54_ENSG00000166348;RP11-464F9.20_ENSG00000268584
10	75406542	75407591	5Y-H4K8ac_peak_2333	12.05638	
10	75407876	75408296	5Y-H4K8ac_peak_2334	7.60057	
10	75634671	75634863	5Y-H4K8ac_peak_2335	7.59101	CAMK2G_ENSG00000148660
10	76585348	76585555	5Y-H4K8ac_peak_2336	8.43511	KAT6B_ENSG00000156650
10	76857984	76858208	5Y-H4K8ac_peak_2337	7.4047	
10	76870661	76870921	5Y-H4K8ac_peak_2338	5.64909	
10	76969893	76970114	5Y-H4K8ac_peak_2339	6.78128	VDAC2_ENSG00000165637
10	76970736	76970999	5Y-H4K8ac_peak_2340	7.89142	VDAC2_ENSG00000165637
10	76994739	76994965	5Y-H4K8ac_peak_2341	5.24695	COMTD1_ENSG00000165644
10	76998414	76998612	5Y-H4K8ac_peak_2342	7.44355	
10	77022508	77022807	5Y-H4K8ac_peak_2343	7.63144	
10	77053019	77053213	5Y-H4K8ac_peak_2344	7.05631	

10	77053557	77054398	5Y-H4K8ac_peak_2345	10.02453	
10	77054936	77055267	5Y-H4K8ac_peak_2346	5.98695	
10	77165212	77165406	5Y-H4K8ac_peak_2347	7.41519	
10	77169092	77169315	5Y-H4K8ac_peak_2348	4.50834	RP11-399K21.13_ENSG00000273248;RP11-399K21.14_ENSG00000272692
10	77190980	77191759	5Y-H4K8ac_peak_2349	7.9215	RP11-399K21.10_ENSG00000236842
10	77238579	77238807	5Y-H4K8ac_peak_2350	9.02242	
10	77523862	77524059	5Y-H4K8ac_peak_2351	4.36976	
10	78192592	78193294	5Y-H4K8ac_peak_2352	9.23159	
10	78729357	78729709	5Y-H4K8ac_peak_2353	11.72217	
10	78730374	78730592	5Y-H4K8ac_peak_2354	8.43511	
10	78731784	78731995	5Y-H4K8ac_peak_2355	8.2913	
10	78801231	78801523	5Y-H4K8ac_peak_2356	5.98695	
10	78850914	78851544	5Y-H4K8ac_peak_2357	11.19336	
10	78864424	78865002	5Y-H4K8ac_peak_2358	10.90097	
10	78866508	78866756	5Y-H4K8ac_peak_2359	7.56545	
10	79471178	79471693	5Y-H4K8ac_peak_2360	5.8635	
10	79563694	79563893	5Y-H4K8ac_peak_2361	4.27459	
10	79597559	79597945	5Y-H4K8ac_peak_2362	9.22312	
10	79606057	79606406	5Y-H4K8ac_peak_2363	8.564	
10	79632338	79632758	5Y-H4K8ac_peak_2364	5.98695	
10	79633113	79633329	5Y-H4K8ac_peak_2365	6.73385	
10	79637289	79637552	5Y-H4K8ac_peak_2366	7.89273	
10	79638075	79639142	5Y-H4K8ac_peak_2367	14.39608	
10	79639443	79640375	5Y-H4K8ac_peak_2368	4.95697	
10	79686464	79686817	5Y-H4K8ac_peak_2369	8.16382	DLG5_ENSG00000151208;DLG5-AS1_ENSG00000233871
10	79807508	79807727	5Y-H4K8ac_peak_2370	8.748	
10	79899132	79899931	5Y-H4K8ac_peak_2371	7.69843	
10	79954879	79955092	5Y-H4K8ac_peak_2372	5.87725	
10	79972532	79973009	5Y-H4K8ac_peak_2373	14.43262	
10	80053884	80054201	5Y-H4K8ac_peak_2374	5.78188	RP11-90J7.2_ENSG00000229543
10	80054818	80055040	5Y-H4K8ac_peak_2375	9.23159	
10	80055540	80055752	5Y-H4K8ac_peak_2376	6.50117	
10	80062410	80062991	5Y-H4K8ac_peak_2377	11.1169	
10	80076252	80076536	5Y-H4K8ac_peak_2378	4.24332	
10	80167328	80167640	5Y-H4K8ac_peak_2379	7.31102	
10	80192560	80193277	5Y-H4K8ac_peak_2380	8.69112	
10	80194748	80194979	5Y-H4K8ac_peak_2381	6.54441	
10	80195884	80196138	5Y-H4K8ac_peak_2382	7.09658	
10	80201755	80201979	5Y-H4K8ac_peak_2383	5.12213	
10	80202934	80203496	5Y-H4K8ac_peak_2384	9.72464	
10	80224436	80225019	5Y-H4K8ac_peak_2385	11.19336	
10	80230112	80230635	5Y-H4K8ac_peak_2386	7.46829	
10	80231014	80231601	5Y-H4K8ac_peak_2387	24.2291	
10	80232284	80232524	5Y-H4K8ac_peak_2388	17.64055	
10	80270424	80270647	5Y-H4K8ac_peak_2389	8.564	
10	80274982	80275176	5Y-H4K8ac_peak_2390	5.317	

10	80357690	80357964	5Y-H4K8ac_peak_2391	5.65584	
10	80386088	80386331	5Y-H4K8ac_peak_2392	8.43511	
10	80416100	80416936	5Y-H4K8ac_peak_2393	14.31711	
10	80448885	80449625	5Y-H4K8ac_peak_2394	12.21176	
10	80451145	80451536	5Y-H4K8ac_peak_2395	5.98695	
10	80500984	80501368	5Y-H4K8ac_peak_2396	6.11316	
10	80502247	80502813	5Y-H4K8ac_peak_2397	10.1046	
10	80503765	80505141	5Y-H4K8ac_peak_2398	9.74838	
10	80505687	80506340	5Y-H4K8ac_peak_2399	7.90751	
10	80506555	80507007	5Y-H4K8ac_peak_2400	7.90236	
10	80513662	80514378	5Y-H4K8ac_peak_2401	10.19948	
10	80517506	80517813	5Y-H4K8ac_peak_2402	4.50834	
10	80531512	80531926	5Y-H4K8ac_peak_2403	4.84727	
10	80562030	80562226	5Y-H4K8ac_peak_2404	7.11439	
10	80562510	80563121	5Y-H4K8ac_peak_2405	7.50148	
10	80564233	80564770	5Y-H4K8ac_peak_2406	7.11863	
10	80567348	80567901	5Y-H4K8ac_peak_2407	6.4221	
10	80568501	80568963	5Y-H4K8ac_peak_2408	7.34185	
10	80620401	80620594	5Y-H4K8ac_peak_2409	4.36976	
10	80621539	80621858	5Y-H4K8ac_peak_2410	4.00285	
10	80721934	80722573	5Y-H4K8ac_peak_2411	7.82914	
10	80723092	80723372	5Y-H4K8ac_peak_2412	3.94247	
10	80723757	80724002	5Y-H4K8ac_peak_2413	11.24252	
10	80724691	80725141	5Y-H4K8ac_peak_2414	6.53157	
10	80732397	80732682	5Y-H4K8ac_peak_2415	8.62703	
10	80733069	80733414	5Y-H4K8ac_peak_2416	6.98416	
10	80777979	80778313	5Y-H4K8ac_peak_2417	5.80888	
10	80787295	80787623	5Y-H4K8ac_peak_2418	8.43511	
10	80808264	80809064	5Y-H4K8ac_peak_2419	9.79526	
10	80809727	80809944	5Y-H4K8ac_peak_2420	6.3265	
10	80812147	80812531	5Y-H4K8ac_peak_2421	8.11614	
10	80826619	80826929	5Y-H4K8ac_peak_2422	9.51254	ZMIZ1-AS1_ENSG00000224596
10	80829203	80829427	5Y-H4K8ac_peak_2423	7.11863	ZMIZ1_ENSG00000108175
10	80842483	80842747	5Y-H4K8ac_peak_2424	8.16031	
10	80872745	80873426	5Y-H4K8ac_peak_2425	8.2913	
10	80889677	80890163	5Y-H4K8ac_peak_2426	4.29586	
10	80936969	80937592	5Y-H4K8ac_peak_2427	11.4254	
10	80937837	80938301	5Y-H4K8ac_peak_2428	6.08523	
10	80939075	80939506	5Y-H4K8ac_peak_2429	16.13719	
10	80954711	80954936	5Y-H4K8ac_peak_2430	6.89892	
10	80956891	80957257	5Y-H4K8ac_peak_2431	7.86017	
10	80962712	80962998	5Y-H4K8ac_peak_2432	9.79526	
10	80963191	80963493	5Y-H4K8ac_peak_2433	10.73603	
10	80963998	80964314	5Y-H4K8ac_peak_2434	7.90236	
10	80967010	80967582	5Y-H4K8ac_peak_2435	5.9726	
10	80980046	80980442	5Y-H4K8ac_peak_2436	5.23083	

10	80981006	80981970	5Y-H4K8ac_peak_2437	11.71078	
10	80982321	80983268	5Y-H4K8ac_peak_2438	14.00894	
10	80983490	80983854	5Y-H4K8ac_peak_2439	5.67283	
10	80984085	80984431	5Y-H4K8ac_peak_2440	8.43511	
10	80990581	80990777	5Y-H4K8ac_peak_2441	4.36976	
10	80995155	80995412	5Y-H4K8ac_peak_2442	7.98641	
10	80999245	80999489	5Y-H4K8ac_peak_2443	5.67283	
10	81020820	81021028	5Y-H4K8ac_peak_2444	10.90365	
10	81029509	81030327	5Y-H4K8ac_peak_2445	10.35586	
10	81031386	81031783	5Y-H4K8ac_peak_2446	9.41701	
10	81032177	81032371	5Y-H4K8ac_peak_2447	6.01077	
10	81032920	81033143	5Y-H4K8ac_peak_2448	9.00954	
10	81033432	81034567	5Y-H4K8ac_peak_2449	12.15659	
10	81034792	81036146	5Y-H4K8ac_peak_2450	6.37023	
10	81106296	81106716	5Y-H4K8ac_peak_2451	9.51254	PPIF_ENSG00000108179
10	81107427	81107943	5Y-H4K8ac_peak_2452	9.11629	PPIF_ENSG00000108179
10	81159283	81159516	5Y-H4K8ac_peak_2453	6.18374	
10	81160780	81161129	5Y-H4K8ac_peak_2454	10.11191	
10	81161650	81161965	5Y-H4K8ac_peak_2455	11.09973	
10	81664182	81664477	5Y-H4K8ac_peak_2456	6.18374	MBL1P_ENSG00000242600
10	81741659	81742175	5Y-H4K8ac_peak_2457	8.43511	SFTPD_ENSG00000133661;ZNRF2P3_ENSG00000235924;RP11-479O17.7_ENSG00000225100
10	81837856	81838804	5Y-H4K8ac_peak_2458	8.47164	TMEM254-AS1_ENSG00000230091;TMEM254_ENSG00000133678
10	81964656	81964892	5Y-H4K8ac_peak_2459	9.7353	ANXA11_ENSG00000122359
10	81965096	81965317	5Y-H4K8ac_peak_2460	6.77436	ANXA11_ENSG00000122359
10	82116131	82116579	5Y-H4K8ac_peak_2461	10.1994	DYDC1_ENSG00000170788
10	82117076	82117517	5Y-H4K8ac_peak_2462	8.17203	DYDC1_ENSG00000170788
10	82167115	82167376	5Y-H4K8ac_peak_2463	7.01856	FAM213A_ENSG00000122378
10	82202419	82202688	5Y-H4K8ac_peak_2464	4.07874	
10	82213510	82213828	5Y-H4K8ac_peak_2465	10.94674	TSPAN14_ENSG00000108219
10	82214172	82214743	5Y-H4K8ac_peak_2466	8.04493	TSPAN14_ENSG00000108219
10	82219375	82219771	5Y-H4K8ac_peak_2467	7.88114	
10	82220375	82220599	5Y-H4K8ac_peak_2468	6.50117	
10	83634614	83634869	5Y-H4K8ac_peak_2469	5.98695	NRG3_ENSG00000185737
10	85303925	85304195	5Y-H4K8ac_peak_2470	8.04493	
10	86302539	86302854	5Y-H4K8ac_peak_2471	6.08523	
10	87923970	87924196	5Y-H4K8ac_peak_2472	5.62788	
10	88011550	88011823	5Y-H4K8ac_peak_2473	7.50044	
10	88013514	88013883	5Y-H4K8ac_peak_2474	4.95697	
10	88026460	88026700	5Y-H4K8ac_peak_2475	8.05083	
10	88101108	88101511	5Y-H4K8ac_peak_2476	4.29586	
10	88126694	88127175	5Y-H4K8ac_peak_2477	10.1994	GRID1_ENSG00000182771
10	88136903	88137118	5Y-H4K8ac_peak_2478	9.23159	
10	88281122	88281459	5Y-H4K8ac_peak_2479	7.89273	WAPAL_ENSG00000062650;RP11-77P6.2_ENSG00000227896
10	88429227	88429486	5Y-H4K8ac_peak_2480	5.81394	
10	88432770	88432976	5Y-H4K8ac_peak_2481	7.9412	
10	88433442	88433678	5Y-H4K8ac_peak_2482	6.50117	

10	88433904	88434148	5Y-H4K8ac_peak_2483	5.23819	
10	88470258	88471590	5Y-H4K8ac_peak_2484	11.94897	
10	88471853	88472227	5Y-H4K8ac_peak_2485	8.50349	
10	88515845	88516069	5Y-H4K8ac_peak_2486	6.08523	RP11-359E3.4_ENSG00000272631;BMPR1A_ENSG00000107779
10	88655663	88655859	5Y-H4K8ac_peak_2487	5.40331	
10	88726546	88726823	5Y-H4K8ac_peak_2488	11.48679	
10	88777500	88777696	5Y-H4K8ac_peak_2489	6.53157	
10	88779235	88779911	5Y-H4K8ac_peak_2490	6.61077	FAM25A_ENSG00000188100
10	88854356	88854601	5Y-H4K8ac_peak_2491	7.38046	FAM35A_ENSG00000122376
10	88855068	88855262	5Y-H4K8ac_peak_2492	9.05168	
10	89623512	89623818	5Y-H4K8ac_peak_2493	6.18374	KLLN_ENSG00000227268;PTEN_ENSG00000171862
10	90640030	90640373	5Y-H4K8ac_peak_2494	5.12488	STAMBPL1_ENSG00000138134
10	90640594	90640839	5Y-H4K8ac_peak_2495	6.54441	
10	91044606	91044885	5Y-H4K8ac_peak_2496	4.8773	
10	91045228	91045557	5Y-H4K8ac_peak_2497	10.6025	
10	91174397	91174864	5Y-H4K8ac_peak_2498	7.38046	LIPA_ENSG00000107798;IFIT5_ENSG00000152778
10	91404192	91405009	5Y-H4K8ac_peak_2499	7.11863	PANK1_ENSG00000152782;RP11-80H5.5_ENSG00000249962;RP11-80H5.2_ENSG00000232936
10	92922869	92923323	5Y-H4K8ac_peak_2500	7.88114	
10	93169471	93169969	5Y-H4K8ac_peak_2501	13.35169	HECTD2_ENSG00000165338
10	93350547	93351044	5Y-H4K8ac_peak_2502	7.29055	
10	93430503	93430772	5Y-H4K8ac_peak_2503	5.23083	
10	93431641	93431985	5Y-H4K8ac_peak_2504	13.8759	
10	93558265	93558823	5Y-H4K8ac_peak_2505	10.5157	TNKS2-AS1_ENSG00000228701;TNKS2_ENSG00000107854
10	93646979	93647606	5Y-H4K8ac_peak_2506	12.71156	
10	94051037	94051463	5Y-H4K8ac_peak_2507	13.06587	CPEB3_ENSG00000107864;MARCH5_ENSG00000198060
10	94333297	94333737	5Y-H4K8ac_peak_2508	7.11863	IDE_ENSG00000119912
10	94333972	94334212	5Y-H4K8ac_peak_2509	6.6946	IDE_ENSG00000119912
10	94352995	94353305	5Y-H4K8ac_peak_2510	6.47245	KIF11_ENSG00000138160
10	94449248	94449482	5Y-H4K8ac_peak_2511	16.01457	
10	94449684	94450183	5Y-H4K8ac_peak_2512	15.06486	
10	94607984	94608853	5Y-H4K8ac_peak_2513	19.15843	
10	95653766	95653979	5Y-H4K8ac_peak_2514	10.19948	SLC35G1_ENSG00000176273
10	95753428	95753657	5Y-H4K8ac_peak_2515	9.7353	PLCE1_ENSG00000138193
10	96123160	96123489	5Y-H4K8ac_peak_2516	6.13909	NOC3L_ENSG00000173145
10	96161508	96161762	5Y-H4K8ac_peak_2517	6.14981	TBC1D12_ENSG00000108239
10	96304348	96304644	5Y-H4K8ac_peak_2518	8.63306	HELLS_ENSG00000119969
10	96305306	96305949	5Y-H4K8ac_peak_2519	8.2913	HELLS_ENSG00000119969
10	96943132	96943444	5Y-H4K8ac_peak_2520	5.449	PAWRP1_ENSG00000225533
10	97415866	97416278	5Y-H4K8ac_peak_2521	7.46096	ALDH18A1_ENSG00000059573
10	97802903	97803129	5Y-H4K8ac_peak_2522	9.02782	CCNJ_ENSG00000107443
10	97803378	97803774	5Y-H4K8ac_peak_2523	8.62703	CCNJ_ENSG00000107443
10	97849701	97850603	5Y-H4K8ac_peak_2524	9.52603	ENTPD1-AS1_ENSG00000226688
10	97889530	97889733	5Y-H4K8ac_peak_2525	7.34185	ZNF518A_ENSG00000177853
10	98134859	98135187	5Y-H4K8ac_peak_2526	7.79831	
10	98346454	98346710	5Y-H4K8ac_peak_2527	7.21591	TM9SF3_ENSG00000077147
10	98479857	98480610	5Y-H4K8ac_peak_2528	18.68977	PIK3AP1_ENSG00000155629

10	98591629	98592249	5Y-H4K8ac_peak_2529	17.63051	LCOR_ENSG00000196233
10	98734684	98734915	5Y-H4K8ac_peak_2530	4.50834	
10	98899536	98899726	5Y-H4K8ac_peak_2531	4.84727	
10	98900005	98900656	5Y-H4K8ac_peak_2532	10.19948	
10	98940463	98940776	5Y-H4K8ac_peak_2533	5.62406	
10	98941033	98941368	5Y-H4K8ac_peak_2534	6.46606	
10	98944705	98945762	5Y-H4K8ac_peak_2535	9.79526	SLIT1_ENSG00000187122
10	99078764	99078971	5Y-H4K8ac_peak_2536	12.21176	FRAT1_ENSG00000165879
10	99094474	99094737	5Y-H4K8ac_peak_2537	13.00733	FRAT2_ENSG00000181274;RP11-452K12.4_ENSG00000225850
10	99186084	99186340	5Y-H4K8ac_peak_2538	16.38263	AL355490.1_ENSG00000224474;PGAM1_ENSG00000171314
10	99257696	99258112	5Y-H4K8ac_peak_2539	9.51254	MMS19_ENSG00000155229;UBTD1_ENSG00000165886
10	99277349	99277553	5Y-H4K8ac_peak_2540	5.8635	
10	99277848	99278078	5Y-H4K8ac_peak_2541	5.74859	
10	99392830	99393114	5Y-H4K8ac_peak_2542	4.9885	MORN4_ENSG00000171160
10	99419892	99420148	5Y-H4K8ac_peak_2543	5.64909	
10	99473099	99473323	5Y-H4K8ac_peak_2544	16.38263	MARVELD1_ENSG00000155254
10	99474020	99474613	5Y-H4K8ac_peak_2545	15.01219	
10	99496017	99496787	5Y-H4K8ac_peak_2546	13.8759	ZFYVE27_ENSG00000155256
10	99540879	99541452	5Y-H4K8ac_peak_2547	15.92074	
10	99608848	99609069	5Y-H4K8ac_peak_2548	5.317	LINC00866_ENSG00000227356;GOLGA7B_ENSG00000155265
10	99628890	99629271	5Y-H4K8ac_peak_2549	7.89273	
10	99649057	99649674	5Y-H4K8ac_peak_2550	9.30505	
10	99674522	99674983	5Y-H4K8ac_peak_2551	8.47164	
10	100027630	100028083	5Y-H4K8ac_peak_2552	10.90365	LOXL4_ENSG00000138131
10	100068819	100069387	5Y-H4K8ac_peak_2553	11.68317	
10	100081184	100081528	5Y-H4K8ac_peak_2554	6.77436	
10	100174493	100174687	5Y-H4K8ac_peak_2555	8.46442	PYROXD2_ENSG00000119943
10	100206592	100206967	5Y-H4K8ac_peak_2556	6.78128	HPS1_ENSG00000107521
10	100227609	100227864	5Y-H4K8ac_peak_2557	7.20869	
10	100766829	100767048	5Y-H4K8ac_peak_2558	5.40331	
10	101056717	101057039	5Y-H4K8ac_peak_2559	9.78792	
10	101089345	101089875	5Y-H4K8ac_peak_2560	6.77436	CNNM1_ENSG00000119946
10	101190780	101190991	5Y-H4K8ac_peak_2561	5.64909	GOT1_ENSG00000120053;RP11-441O15.3_ENSG00000224934
10	101380658	101381032	5Y-H4K8ac_peak_2562	7.87406	SLC25A28_ENSG00000155287;RP11-85A1.3_ENSG00000260475
10	101381740	101382081	5Y-H4K8ac_peak_2563	5.98695	RP11-85A1.3_ENSG00000260475
10	101989490	101989905	5Y-H4K8ac_peak_2564	11.57334	CHUK_ENSG00000213341;RP11-316M21.6_ENSG00000227492
10	102027056	102027246	5Y-H4K8ac_peak_2565	8.33296	CWF19L1_ENSG00000095485
10	102046106	102046629	5Y-H4K8ac_peak_2566	8.2913	BLOC1S2_ENSG00000196072
10	102105384	102105578	5Y-H4K8ac_peak_2567	5.91107	RP11-34D15.2_ENSG00000231188
10	102106198	102106448	5Y-H4K8ac_peak_2568	5.70472	RP11-34D15.2_ENSG00000231188;SCD_ENSG00000099194
10	102289795	102290019	5Y-H4K8ac_peak_2569	7.04637	SEC31B_ENSG00000075826;NDUFB8_ENSG00000255339;NDUFB8_ENSG00000166136;HIF1AN_ENSG00000166135
10	102397496	102397850	5Y-H4K8ac_peak_2570	10.19948	
10	102441244	102441457	5Y-H4K8ac_peak_2571	8.43511	
10	102443102	102443667	5Y-H4K8ac_peak_2572	6.37023	
10	102444869	102445506	5Y-H4K8ac_peak_2573	14.10646	
10	102448005	102448239	5Y-H4K8ac_peak_2574	6.77436	

10	102470054	102470251	5Y-H4K8ac_peak_2575	5.87725	
10	102472836	102473279	5Y-H4K8ac_peak_2576	8.2913	
10	102568391	102568644	5Y-H4K8ac_peak_2577	6.82266	
10	102728827	102729021	5Y-H4K8ac_peak_2578	4.77126	SEMA4G_ENSG00000095539
10	102746744	102747188	5Y-H4K8ac_peak_2579	6.86362	MRPL43_ENSG00000055950;C10orf2_ENSG00000107815
10	102757561	102757866	5Y-H4K8ac_peak_2580	8.43511	
10	102758059	102758368	5Y-H4K8ac_peak_2581	6.43068	
10	102758680	102759322	5Y-H4K8ac_peak_2582	13.92877	
10	102778433	102778687	5Y-H4K8ac_peak_2583	5.91107	
10	102791821	102792030	5Y-H4K8ac_peak_2584	4.97086	PDZD7_ENSG00000186862;SFXN3_ENSG00000107819
10	102792665	102792875	5Y-H4K8ac_peak_2585	5.96059	
10	102820515	102821795	5Y-H4K8ac_peak_2586	22.54299	RP11-108L7.15_ENSG00000273162;KAZALD1_ENSG00000107821
10	102901661	102902125	5Y-H4K8ac_peak_2587	16.9826	
10	103051120	103051517	5Y-H4K8ac_peak_2588	13.00733	
10	103052581	103053062	5Y-H4K8ac_peak_2589	4.50834	
10	103070115	103070451	5Y-H4K8ac_peak_2590	9.79526	RP11-107I14.2_ENSG00000225208
10	103319492	103319825	5Y-H4K8ac_peak_2591	6.78128	
10	103347451	103347854	5Y-H4K8ac_peak_2592	6.37023	POLL_ENSG00000166169
10	103453919	103455214	5Y-H4K8ac_peak_2593	10.60083	FBXW4_ENSG00000107829
10	103590852	103591056	5Y-H4K8ac_peak_2594	8.43511	
10	103815466	103815860	5Y-H4K8ac_peak_2595	7.31102	C10orf76_ENSG00000120029
10	103816334	103816611	5Y-H4K8ac_peak_2596	8.2913	C10orf76_ENSG00000120029
10	103825132	103825579	5Y-H4K8ac_peak_2597	5.87725	HPS6_ENSG00000166189
10	103873904	103874456	5Y-H4K8ac_peak_2598	6.67017	
10	103876850	103877105	5Y-H4K8ac_peak_2599	12.05638	
10	103877797	103878048	5Y-H4K8ac_peak_2600	8.69112	
10	103881150	103881656	5Y-H4K8ac_peak_2601	9.96543	LDB1_ENSG00000198728
10	103892884	103893074	5Y-H4K8ac_peak_2602	6.22904	PPRC1_ENSG00000148840
10	103911469	103911695	5Y-H4K8ac_peak_2603	6.50117	NOLC1_ENSG00000166197
10	103929843	103930103	5Y-H4K8ac_peak_2604	8.93923	
10	104169106	104169372	5Y-H4K8ac_peak_2605	4.84727	
10	104179547	104179789	5Y-H4K8ac_peak_2606	8.43511	FBXL15_ENSG00000107872
10	104192861	104193064	5Y-H4K8ac_peak_2607	6.24511	CUEDC2_ENSG00000107874
10	104210440	104210860	5Y-H4K8ac_peak_2608	13.68515	RP11-18I14.10_ENSG00000269609;C10orf95_ENSG00000120055
10	104220130	104220381	5Y-H4K8ac_peak_2609	5.67283	TMEM180_ENSG00000138111
10	104387053	104388104	5Y-H4K8ac_peak_2610	18.22112	
10	104388411	104389407	5Y-H4K8ac_peak_2611	8.73985	
10	104390084	104390280	5Y-H4K8ac_peak_2612	6.52343	
10	104402146	104402535	5Y-H4K8ac_peak_2613	13.86507	
10	104420286	104421406	5Y-H4K8ac_peak_2614	9.30338	
10	104473472	104474011	5Y-H4K8ac_peak_2615	9.52603	ARL3_ENSG00000138175;SFXN2_ENSG00000156398
10	104629602	104629858	5Y-H4K8ac_peak_2616	7.63144	AS3MT_ENSG00000214435
10	104678289	104678499	5Y-H4K8ac_peak_2617	5.98695	CNNM2_ENSG00000148842
10	104953181	104953406	5Y-H4K8ac_peak_2618	11.99586	NT5C2_ENSG00000076685
10	105037019	105037218	5Y-H4K8ac_peak_2619	5.24695	INA_ENSG00000148798
10	105110348	105110563	5Y-H4K8ac_peak_2620	9.7353	PCGF6_ENSG00000156374

10	105126624	105126908	5Y-H4K8ac_peak_2621	6.49308	TAF5_ENSG00000148835
10	105261958	105262161	5Y-H4K8ac_peak_2622	4.29586	
10	105303832	105304038	5Y-H4K8ac_peak_2623	5.35202	
10	105315151	105315416	5Y-H4K8ac_peak_2624	4.07874	
10	105377846	105378398	5Y-H4K8ac_peak_2625	8.27643	
10	105480500	105480770	5Y-H4K8ac_peak_2626	5.99504	
10	105486639	105487588	5Y-H4K8ac_peak_2627	21.35466	
10	105492277	105492556	5Y-H4K8ac_peak_2628	9.05168	
10	105498192	105498403	5Y-H4K8ac_peak_2629	8.47164	
10	105539088	105539291	5Y-H4K8ac_peak_2630	5.449	
10	105564673	105565108	5Y-H4K8ac_peak_2631	7.07936	
10	105633530	105633749	5Y-H4K8ac_peak_2632	4.79658	
10	105881073	105881294	5Y-H4K8ac_peak_2633	7.59101	SFR1_ENSG00000156384
10	105881929	105882129	5Y-H4K8ac_peak_2634	10.1994	SFR1_ENSG00000156384
10	106014410	106015031	5Y-H4K8ac_peak_2635	8.62703	
10	106059352	106059710	5Y-H4K8ac_peak_2636	5.98695	
10	106060153	106060507	5Y-H4K8ac_peak_2637	7.97699	
10	106398425	106398626	5Y-H4K8ac_peak_2638	6.10478	
10	106492507	106492717	5Y-H4K8ac_peak_2639	6.79955	
10	108327247	108327444	5Y-H4K8ac_peak_2640	4.07874	
10	108328163	108328436	5Y-H4K8ac_peak_2641	6.34046	
10	108475378	108475584	5Y-H4K8ac_peak_2642	6.43775	
10	108602432	108602730	5Y-H4K8ac_peak_2643	14.81591	
10	108762687	108762929	5Y-H4K8ac_peak_2644	6.13909	
10	108924062	108924375	5Y-H4K8ac_peak_2645	6.53157	SORCS1_ENSG00000108018
10	109304553	109304759	5Y-H4K8ac_peak_2646	5.87382	
10	109305327	109305761	5Y-H4K8ac_peak_2647	11.42066	
10	109674044	109674509	5Y-H4K8ac_peak_2648	7.38046	
10	111766932	111767549	5Y-H4K8ac_peak_2649	13.2534	
10	111970059	111970508	5Y-H4K8ac_peak_2650	6.00382	
10	112219520	112220475	5Y-H4K8ac_peak_2651	8.75926	
10	112395927	112396272	5Y-H4K8ac_peak_2652	5.28616	
10	112403509	112404219	5Y-H4K8ac_peak_2653	11.53521	Y_RNA_ENSG00000223302;RBM20_ENSG00000203867
10	112541365	112541749	5Y-H4K8ac_peak_2654	5.03335	
10	112545848	112546441	5Y-H4K8ac_peak_2655	8.31252	
10	112549275	112549572	5Y-H4K8ac_peak_2656	5.64909	
10	112554566	112554899	5Y-H4K8ac_peak_2657	6.52593	
10	112580042	112580286	5Y-H4K8ac_peak_2658	6.20875	
10	112631836	112632049	5Y-H4K8ac_peak_2659	7.89273	PDCD4-AS1_ENSG00000203497;PDCD4_ENSG00000150593
10	113943077	113943433	5Y-H4K8ac_peak_2660	7.31815	
10	114207037	114207228	5Y-H4K8ac_peak_2661	7.97699	ZDHHC6_ENSG00000023041;VTI1A_ENSG00000151532
10	115613730	115614340	5Y-H4K8ac_peak_2662	8.30301	DCLRE1A_ENSG00000198924;NHLRC2_ENSG00000196865
10	115614540	115614992	5Y-H4K8ac_peak_2663	13.2534	DCLRE1A_ENSG00000198924;NHLRC2_ENSG00000196865
10	115707143	115707794	5Y-H4K8ac_peak_2664	9.79526	
10	115860615	115860849	5Y-H4K8ac_peak_2665	6.50117	
10	115933524	115933874	5Y-H4K8ac_peak_2666	5.91107	C10orf118_ENSG00000165813;MIR2110_ENSG00000238742

10	116070329	116070556	5Y-H4K8ac_peak_2667	5.8635	
10	116127237	116127503	5Y-H4K8ac_peak_2668	5.23083	
10	116527610	116527896	5Y-H4K8ac_peak_2669	4.07874	
10	116581591	116582108	5Y-H4K8ac_peak_2670	9.30505	FAM160B1_ENSG00000151553
10	117863391	117863703	5Y-H4K8ac_peak_2671	7.17184	
10	117945591	117945802	5Y-H4K8ac_peak_2672	6.34046	
10	118011165	118011389	5Y-H4K8ac_peak_2673	5.40331	
10	118481309	118482121	5Y-H4K8ac_peak_2674	8.43511	
10	118482962	118483394	5Y-H4K8ac_peak_2675	9.31608	
10	118501395	118501614	5Y-H4K8ac_peak_2676	5.12213	HSPA12A_ENSG00000165868
10	118502696	118502947	5Y-H4K8ac_peak_2677	10.11191	HSPA12A_ENSG00000165868
10	118595685	118595878	5Y-H4K8ac_peak_2678	6.34046	
10	118609087	118609490	5Y-H4K8ac_peak_2679	8.08688	RP11-539I5.1_ENSG00000225302;ENO4_ENSG00000188316
10	118764445	118764678	5Y-H4K8ac_peak_2680	7.60057	
10	118976479	118976996	5Y-H4K8ac_peak_2681	8.8021	
10	119135207	119135493	5Y-H4K8ac_peak_2682	6.75784	PDZD8_ENSG00000165650
10	119261195	119261405	5Y-H4K8ac_peak_2683	6.55906	
10	119262287	119262570	5Y-H4K8ac_peak_2684	9.89244	
10	119263090	119263479	5Y-H4K8ac_peak_2685	8.04943	
10	119806208	119806813	5Y-H4K8ac_peak_2686	12.71156	RAB11FIP2_ENSG00000107560;CASC2_ENSG00000177640
10	120101940	120102157	5Y-H4K8ac_peak_2687	5.91107	FAM204A_ENSG00000165669
10	120207039	120207333	5Y-H4K8ac_peak_2688	4.77126	
10	120369344	120369813	5Y-H4K8ac_peak_2689	5.59843	
10	120374783	120375081	5Y-H4K8ac_peak_2690	5.72848	
10	120429531	120430786	5Y-H4K8ac_peak_2691	13.12848	
10	120667885	120668496	5Y-H4K8ac_peak_2692	8.43511	
10	120782387	120782657	5Y-H4K8ac_peak_2693	5.54013	
10	120783128	120783357	5Y-H4K8ac_peak_2694	9.23159	
10	120784708	120785015	5Y-H4K8ac_peak_2695	8.69112	
10	120788408	120788774	5Y-H4K8ac_peak_2696	5.98695	NANOS1_ENSG00000188613
10	120839782	120840155	5Y-H4K8ac_peak_2697	10.1994	EIF3A_ENSG00000107581
10	120863687	120863995	5Y-H4K8ac_peak_2698	6.77436	FAM45A_ENSG00000119979
10	120966541	120966849	5Y-H4K8ac_peak_2699	11.4254	GRK5_ENSG00000198873
10	120982274	120982491	5Y-H4K8ac_peak_2700	5.23083	
10	121171596	121171802	5Y-H4K8ac_peak_2701	7.89273	
10	121173093	121173502	5Y-H4K8ac_peak_2702	6.50117	
10	121302562	121302900	5Y-H4K8ac_peak_2703	4.89128	RGS10_ENSG00000148908
10	121355747	121355971	5Y-H4K8ac_peak_2704	8.43511	TIAL1_ENSG00000151923
10	121356165	121356424	5Y-H4K8ac_peak_2705	10.54764	TIAL1_ENSG00000151923
10	121410474	121410690	5Y-H4K8ac_peak_2706	7.20869	BAG3_ENSG00000151929
10	121415918	121416139	5Y-H4K8ac_peak_2707	7.21406	
10	121484992	121485268	5Y-H4K8ac_peak_2708	13.27281	INPP5F_ENSG00000198825
10	121485651	121485950	5Y-H4K8ac_peak_2709	6.54441	INPP5F_ENSG00000198825
10	121631790	121632363	5Y-H4K8ac_peak_2710	14.31711	
10	121632665	121633083	5Y-H4K8ac_peak_2711	10.1994	
10	122610030	122610386	5Y-H4K8ac_peak_2712	11.36844	WDR11-AS1_ENSG00000227165;WDR11_ENSG00000120008

10	122610941	122611260	5Y-H4K8ac_peak_2713	4.29586	WDR11-AS1_ENSG00000227165;WDR11_ENSG00000120008
10	122802711	122802951	5Y-H4K8ac_peak_2714	5.65584	
10	123357434	123357815	5Y-H4K8ac_peak_2715	12.65187	FGFR2_ENSG00000066468
10	123368114	123368620	5Y-H4K8ac_peak_2716	9.44542	
10	123403881	123404153	5Y-H4K8ac_peak_2717	4.15658	
10	123427306	123427665	5Y-H4K8ac_peak_2718	9.19468	
10	123429272	123429469	5Y-H4K8ac_peak_2719	8.20773	
10	123469748	123470318	5Y-H4K8ac_peak_2720	5.41472	
10	123687033	123687488	5Y-H4K8ac_peak_2721	11.53136	ATE1_ENSG00000107669;ATE1-AS1_ENSG00000226864
10	123687741	123688036	5Y-H4K8ac_peak_2722	9.51254	ATE1_ENSG00000107669;ATE1-AS1_ENSG00000226864
10	123734459	123734760	5Y-H4K8ac_peak_2723	12.52787	NSMCE4A_ENSG00000107672
10	123872653	123873500	5Y-H4K8ac_peak_2724	7.65114	
10	123900196	123901073	5Y-H4K8ac_peak_2725	14.99673	
10	123901703	123902406	5Y-H4K8ac_peak_2726	12.05638	
10	123922523	123922737	5Y-H4K8ac_peak_2727	8.73392	
10	123923225	123923503	5Y-H4K8ac_peak_2728	13.56578	
10	123992810	123993475	5Y-H4K8ac_peak_2729	10.69698	
10	124058588	124058867	5Y-H4K8ac_peak_2730	10.4689	
10	124638859	124639424	5Y-H4K8ac_peak_2731	5.12213	CUZD1_ENSG00000138161;FAM24B_ENSG00000213185;RP11-564D11.3_ENSG00000255624
10	124739729	124740143	5Y-H4K8ac_peak_2732	15.06486	
10	124767767	124768244	5Y-H4K8ac_peak_2733	10.27264	IKZF5_ENSG00000095574;ACADSB_ENSG00000196177
10	124913932	124914192	5Y-H4K8ac_peak_2734	11.19336	BUB3_ENSG00000154473
10	125981513	125981757	5Y-H4K8ac_peak_2735	8.564	
10	126043114	126043475	5Y-H4K8ac_peak_2736	9.99623	
10	126185252	126185562	5Y-H4K8ac_peak_2737	4.29586	
10	126210961	126211226	5Y-H4K8ac_peak_2738	8.2913	
10	126213588	126214804	5Y-H4K8ac_peak_2739	13.99721	
10	126224509	126224792	5Y-H4K8ac_peak_2740	6.72926	
10	126228566	126228782	5Y-H4K8ac_peak_2741	6.12014	
10	126336449	126337280	5Y-H4K8ac_peak_2742	9.38276	
10	126337888	126338280	5Y-H4K8ac_peak_2743	7.37325	
10	126358696	126359020	5Y-H4K8ac_peak_2744	7.0618	
10	126359826	126360185	5Y-H4K8ac_peak_2745	5.90602	
10	126367150	126367375	5Y-H4K8ac_peak_2746	5.56894	
10	126431785	126432150	5Y-H4K8ac_peak_2747	6.36454	FAM53B_ENSG00000189319
10	126432406	126432669	5Y-H4K8ac_peak_2748	5.67283	FAM53B_ENSG00000189319
10	126479375	126479656	5Y-H4K8ac_peak_2749	6.50117	RP11-12J10.3_ENSG00000258539;METTL10_ENSG00000203791
10	126479910	126480629	5Y-H4K8ac_peak_2750	7.31102	RP11-12J10.3_ENSG00000258539;METTL10_ENSG00000203791
10	126481410	126481607	5Y-H4K8ac_peak_2751	4.29586	METTL10_ENSG00000203791
10	126490478	126490721	5Y-H4K8ac_peak_2752	8.76608	FAM175B_ENSG00000165660
10	126605910	126606208	5Y-H4K8ac_peak_2753	4.84727	RP11-298J20.4_ENSG00000249456
10	126703416	126703850	5Y-H4K8ac_peak_2754	5.57299	
10	126711079	126711870	5Y-H4K8ac_peak_2755	11.22005	
10	126745459	126745675	5Y-H4K8ac_peak_2756	8.83356	
10	126848286	126848488	5Y-H4K8ac_peak_2757	10.11191	
10	126848745	126849042	5Y-H4K8ac_peak_2758	4.84727	CTBP2_ENSG00000175029

10	126850317	126850955	5Y-H4K8ac_peak_2759	11.09973	CTBP2_ENSG00000175029
10	127202107	127202720	5Y-H4K8ac_peak_2760	6.50117	
10	127584324	127584685	5Y-H4K8ac_peak_2761	5.03564	DHX32_ENSG00000089876;FANK1_ENSG00000203780
10	127746262	127746863	5Y-H4K8ac_peak_2762	10.19948	
10	127923651	127924087	5Y-H4K8ac_peak_2763	9.23159	
10	127924349	127924652	5Y-H4K8ac_peak_2764	9.15007	
10	127947150	127947474	5Y-H4K8ac_peak_2765	6.34046	
10	128062541	128062825	5Y-H4K8ac_peak_2766	4.50834	
10	128077050	128077509	5Y-H4K8ac_peak_2767	6.50117	ADAM12_ENSG00000148848
10	128594421	128594612	5Y-H4K8ac_peak_2768	5.41472	DOCK1_ENSG00000150760
10	129185177	129185370	5Y-H4K8ac_peak_2769	6.34046	
10	129686199	129686522	5Y-H4K8ac_peak_2770	5.28616	
10	129948484	129948817	5Y-H4K8ac_peak_2771	10.86079	
10	130008906	130009626	5Y-H4K8ac_peak_2772	7.38046	
10	130010796	130011095	5Y-H4K8ac_peak_2773	5.51139	
10	130324329	130324553	5Y-H4K8ac_peak_2774	7.69843	
10	131264910	131265242	5Y-H4K8ac_peak_2775	17.04136	MGMT_ENSG00000170430
10	131265525	131265974	5Y-H4K8ac_peak_2776	14.49096	MGMT_ENSG00000170430
10	131366430	131366685	5Y-H4K8ac_peak_2777	6.77436	
10	131645324	131645517	5Y-H4K8ac_peak_2778	5.90587	
10	131661853	131662063	5Y-H4K8ac_peak_2779	4.79585	
10	131663791	131663990	5Y-H4K8ac_peak_2780	5.68769	
10	131668619	131669022	5Y-H4K8ac_peak_2781	7.89273	
10	131687385	131687854	5Y-H4K8ac_peak_2782	6.14981	
10	131748312	131748558	5Y-H4K8ac_peak_2783	9.78792	
10	131846550	131846792	5Y-H4K8ac_peak_2784	5.65584	
10	131909662	131910057	5Y-H4K8ac_peak_2785	7.30348	LINC00959_ENSG00000237489
10	131934130	131934443	5Y-H4K8ac_peak_2786	9.38276	
10	131989012	131989285	5Y-H4K8ac_peak_2787	10.79063	
10	132893080	132893323	5Y-H4K8ac_peak_2788	5.07473	TCERG1L-AS1_ENSG00000230098
10	132897238	132897455	5Y-H4K8ac_peak_2789	4.84727	
10	133794993	133795272	5Y-H4K8ac_peak_2790	9.15007	BNIP3_ENSG00000176171
10	133795758	133796357	5Y-H4K8ac_peak_2791	9.01738	BNIP3_ENSG00000176171
10	133999864	134000163	5Y-H4K8ac_peak_2792	9.38276	RP11-140A10.3_ENSG00000235010;DPYSL4_ENSG00000151640
10	134036909	134037362	5Y-H4K8ac_peak_2793	9.30505	
10	134050947	134051309	5Y-H4K8ac_peak_2794	12.3708	
10	134052484	134052750	5Y-H4K8ac_peak_2795	6.08523	
10	134052997	134053203	5Y-H4K8ac_peak_2796	4.55128	
10	134053619	134054002	5Y-H4K8ac_peak_2797	5.60566	
10	134078708	134079046	5Y-H4K8ac_peak_2798	4.00285	
10	134083285	134083606	5Y-H4K8ac_peak_2799	5.59843	
10	134095248	134095543	5Y-H4K8ac_peak_2800	5.68769	
10	134106606	134107818	5Y-H4K8ac_peak_2801	13.12848	
10	134110418	134110672	5Y-H4K8ac_peak_2802	10.1994	
10	134122147	134122434	5Y-H4K8ac_peak_2803	9.52603	
10	134210313	134210531	5Y-H4K8ac_peak_2804	7.47209	PWWP2B_ENSG00000171813

10	134330439	134331014	5Y-H4K8ac_peak_2805	6.08523	RP11-432J24.5_ENSG00000226900
10	134331214	134331404	5Y-H4K8ac_peak_2806	8.58926	RP11-432J24.5_ENSG00000226900
10	134350388	134350787	5Y-H4K8ac_peak_2807	9.23159	INPP5A_ENSG00000068383
10	134549896	134550168	5Y-H4K8ac_peak_2808	7.56545	
10	134755800	134756892	5Y-H4K8ac_peak_2809	10.19948	TTC40_ENSG00000171811
10	134800968	134801214	5Y-H4K8ac_peak_2810	7.87406	
10	134801786	134802186	5Y-H4K8ac_peak_2811	12.76485	
10	135132079	135132652	5Y-H4K8ac_peak_2812	20.04267	
10	135139412	135139725	5Y-H4K8ac_peak_2813	5.98695	
10	135171264	135171478	5Y-H4K8ac_peak_2814	8.79957	FUOM_ENSG00000148803
10	135171925	135172162	5Y-H4K8ac_peak_2815	9.62714	FUOM_ENSG00000148803
10	135186975	135187170	5Y-H4K8ac_peak_2816	6.53157	ECHS1_ENSG00000127884
10	135207844	135208182	5Y-H4K8ac_peak_2817	5.23083	MTG1_ENSG00000148824
11	208250	208695	5Y-H4K8ac_peak_2818	4.68359	BET1L_ENSG00000177951;RIC8A_ENSG00000177963
11	237051	237324	5Y-H4K8ac_peak_2819	9.78792	SIRT3_ENSG00000142082;PSMD13_ENSG00000185627
11	355560	356047	5Y-H4K8ac_peak_2820	14.31711	
11	396446	396932	5Y-H4K8ac_peak_2821	8.564	
11	506384	506739	5Y-H4K8ac_peak_2822	10.35586	RNH1_ENSG00000023191
11	534839	535164	5Y-H4K8ac_peak_2823	6.55225	
11	535870	536174	5Y-H4K8ac_peak_2824	6.69752	
11	568861	569220	5Y-H4K8ac_peak_2825	8.24461	MIR210HG_ENSG00000247095;MIR210_ENSG00000199038
11	576225	576680	5Y-H4K8ac_peak_2826	8.69112	PHRF1_ENSG00000070047
11	695868	696254	5Y-H4K8ac_peak_2827	6.64195	TMEM80_ENSG00000177042
11	721040	721445	5Y-H4K8ac_peak_2828	8.51022	
11	728318	728572	5Y-H4K8ac_peak_2829	8.79957	
11	746924	747223	5Y-H4K8ac_peak_2830	8.4454	TALDO1_ENSG00000177156
11	793213	793518	5Y-H4K8ac_peak_2831	6.37023	
11	804856	805192	5Y-H4K8ac_peak_2832	4.15658	
11	805454	805942	5Y-H4K8ac_peak_2833	6.78128	
11	808886	809779	5Y-H4K8ac_peak_2834	9.51254	PIDD_ENSG00000177595;RPLP2_ENSG00000177600
11	810195	810475	5Y-H4K8ac_peak_2835	6.77436	PIDD_ENSG00000177595;RPLP2_ENSG00000177600
11	818833	819383	5Y-H4K8ac_peak_2836	9.23159	PNPLA2_ENSG00000177666
11	855263	856116	5Y-H4K8ac_peak_2837	10.6523	
11	856309	856598	5Y-H4K8ac_peak_2838	5.89922	
11	856960	857188	5Y-H4K8ac_peak_2839	9.76277	
11	890145	890597	5Y-H4K8ac_peak_2840	6.52343	
11	911166	911713	5Y-H4K8ac_peak_2841	5.24695	
11	924836	925634	5Y-H4K8ac_peak_2842	9.51254	AP2A2_ENSG00000183020
11	1054083	1054358	5Y-H4K8ac_peak_2843	6.00382	
11	1331037	1331573	5Y-H4K8ac_peak_2844	17.04999	TOLLIP_ENSG00000078902;TOLLIP-AS1_ENSG00000255153
11	1403855	1404456	5Y-H4K8ac_peak_2845	7.20869	
11	1420050	1420424	5Y-H4K8ac_peak_2846	5.34199	
11	1522808	1523014	5Y-H4K8ac_peak_2847	6.57504	MOB2_ENSG00000182208
11	1567752	1569051	5Y-H4K8ac_peak_2848	21.33748	
11	1569461	1569832	5Y-H4K8ac_peak_2849	8.2913	
11	1592633	1592921	5Y-H4K8ac_peak_2850	6.50117	DUSP8_ENSG00000184545;KRTAP5-AS1_ENSG00000233930

11	1593718	1594158	5Y-H4K8ac_peak_2851	14.2053	DUSP8_ENSG00000184545
11	1649720	1650091	5Y-H4K8ac_peak_2852	6.78128	KRTAP5-5_ENSG00000185940
11	1661093	1661283	5Y-H4K8ac_peak_2853	6.43068	
11	1661630	1661971	5Y-H4K8ac_peak_2854	6.1654	
11	1674167	1674722	5Y-H4K8ac_peak_2855	7.89273	
11	1714736	1715891	5Y-H4K8ac_peak_2856	17.63051	
11	1765499	1766334	5Y-H4K8ac_peak_2857	13.99721	
11	1767730	1767954	5Y-H4K8ac_peak_2858	10.3915	
11	1769202	1769395	5Y-H4K8ac_peak_2859	7.50148	
11	1769712	1770433	5Y-H4K8ac_peak_2860	15.49668	
11	1771008	1771533	5Y-H4K8ac_peak_2861	5.98695	IFITM10_ENSG00000244242
11	1772148	1772569	5Y-H4K8ac_peak_2862	5.23083	IFITM10_ENSG00000244242
11	1773394	1773960	5Y-H4K8ac_peak_2863	7.09658	
11	1784825	1785070	5Y-H4K8ac_peak_2864	5.50568	CTSD_ENSG00000117984;AC068580.5_ENSG00000229512;AC068580.1_ENSG00000265587
11	1847823	1848537	5Y-H4K8ac_peak_2865	10.90365	SYT8_ENSG00000149043
11	1848804	1849376	5Y-H4K8ac_peak_2866	5.24695	SYT8_ENSG00000149043
11	1851666	1851890	5Y-H4K8ac_peak_2867	4.67245	
11	1856276	1856592	5Y-H4K8ac_peak_2868	4.642	
11	1885384	1885672	5Y-H4K8ac_peak_2869	16.50659	AC051649.12_ENSG00000249086
11	1895758	1896099	5Y-H4K8ac_peak_2870	4.90696	
11	1896678	1897885	5Y-H4K8ac_peak_2871	25.55525	
11	1898554	1899602	5Y-H4K8ac_peak_2872	15.20402	
11	1901940	1902342	5Y-H4K8ac_peak_2873	6.54915	
11	1928022	1928227	5Y-H4K8ac_peak_2874	4.84727	
11	1960381	1960681	5Y-H4K8ac_peak_2875	5.98695	
11	1967790	1968193	5Y-H4K8ac_peak_2876	11.28133	MRPL23_ENSG00000214026
11	1968594	1970293	5Y-H4K8ac_peak_2877	21.44514	MRPL23_ENSG00000214026
11	1975178	1976102	5Y-H4K8ac_peak_2878	8.24461	
11	1977189	1978199	5Y-H4K8ac_peak_2879	15.45397	
11	1988025	1988690	5Y-H4K8ac_peak_2880	11.67591	
11	1989485	1991458	5Y-H4K8ac_peak_2881	13.34872	
11	1997909	1998199	5Y-H4K8ac_peak_2882	9.99838	
11	1999034	1999489	5Y-H4K8ac_peak_2883	11.4254	
11	1999782	2000057	5Y-H4K8ac_peak_2884	8.27639	
11	2000696	2004937	5Y-H4K8ac_peak_2885	14.89891	
11	2006021	2006612	5Y-H4K8ac_peak_2886	5.57299	
11	2009107	2009793	5Y-H4K8ac_peak_2887	15.15651	
11	2010122	2010683	5Y-H4K8ac_peak_2888	9.31608	MRPL23-AS1_ENSG00000226416
11	2010978	2011187	5Y-H4K8ac_peak_2889	5.16687	MRPL23-AS1_ENSG00000226416
11	2011822	2012605	5Y-H4K8ac_peak_2890	9.93175	MRPL23-AS1_ENSG00000226416;AC051649.6_ENSG00000232987
11	2014704	2016045	5Y-H4K8ac_peak_2891	10.97738	
11	2017959	2018270	5Y-H4K8ac_peak_2892	7.04158	
11	2018523	2018942	5Y-H4K8ac_peak_2893	5.64909	
11	2190290	2190678	5Y-H4K8ac_peak_2894	5.54773	
11	2206456	2207143	5Y-H4K8ac_peak_2895	11.93212	
11	2211238	2211810	5Y-H4K8ac_peak_2896	11.39862	

11	2212012	2212872	5Y-H4K8ac_peak_2897	12.84845	
11	2213871	2214552	5Y-H4K8ac_peak_2898	12.00715	
11	2214818	2215225	5Y-H4K8ac_peak_2899	10.1994	
11	2215648	2216145	5Y-H4K8ac_peak_2900	13.94026	
11	2216466	2216711	5Y-H4K8ac_peak_2901	7.41197	
11	2233989	2234188	5Y-H4K8ac_peak_2902	5.23083	
11	2325280	2325471	5Y-H4K8ac_peak_2903	7.65114	
11	2326280	2326539	5Y-H4K8ac_peak_2904	5.56912	
11	2327554	2327838	5Y-H4K8ac_peak_2905	7.31807	
11	2336519	2336920	5Y-H4K8ac_peak_2906	12.50545	
11	2420610	2421412	5Y-H4K8ac_peak_2907	11.22005	TSSC4_ENSG00000184281
11	2422031	2422442	5Y-H4K8ac_peak_2908	16.00537	TSSC4_ENSG00000184281
11	2441354	2441866	5Y-H4K8ac_peak_2909	5.60566	
11	2603290	2603619	5Y-H4K8ac_peak_2910	5.99504	
11	2604200	2604451	5Y-H4K8ac_peak_2911	9.01738	
11	2729615	2729828	5Y-H4K8ac_peak_2912	6.49458	
11	2734643	2735512	5Y-H4K8ac_peak_2913	14.01193	
11	2735712	2735923	5Y-H4K8ac_peak_2914	6.54441	
11	2738186	2738380	5Y-H4K8ac_peak_2915	4.95697	
11	2769664	2769998	5Y-H4K8ac_peak_2916	5.59843	
11	2798011	2798255	5Y-H4K8ac_peak_2917	7.11863	
11	2798864	2799928	5Y-H4K8ac_peak_2918	23.07898	
11	2800546	2801580	5Y-H4K8ac_peak_2919	9.02938	
11	2801932	2802326	5Y-H4K8ac_peak_2920	10.4689	
11	2905591	2905992	5Y-H4K8ac_peak_2921	6.3265	
11	2907433	2908023	5Y-H4K8ac_peak_2922	10.69698	CDKN1C_ENSG00000129757
11	2913030	2913301	5Y-H4K8ac_peak_2923	4.8509	
11	2913496	2913793	5Y-H4K8ac_peak_2924	11.86504	
11	2923526	2923868	5Y-H4K8ac_peak_2925	12.09312	
11	3013840	3014262	5Y-H4K8ac_peak_2926	6.77436	NAP1L4_ENSG00000205531
11	3078252	3078564	5Y-H4K8ac_peak_2927	6.36723	CARS_ENSG00000110619
11	3078863	3079417	5Y-H4K8ac_peak_2928	7.50148	CARS_ENSG00000110619
11	3253574	3253820	5Y-H4K8ac_peak_2929	7.8267	MRGPRE_ENSG00000184350
11	3399910	3400333	5Y-H4K8ac_peak_2930	13.00733	ZNF195_ENSG00000005801
11	3401033	3401262	5Y-H4K8ac_peak_2931	4.69231	ZNF195_ENSG00000005801;TSSC2_ENSG00000223756
11	3443448	3443643	5Y-H4K8ac_peak_2932	7.46096	FAM86GP_ENSG00000166492
11	3819219	3819429	5Y-H4K8ac_peak_2933	8.21582	NUP98_ENSG00000110713;PGAP2_ENSG00000148985
11	3862533	3862739	5Y-H4K8ac_peak_2934	5.65765	RHOG_ENSG00000177105
11	3863373	3863626	5Y-H4K8ac_peak_2935	7.57144	
11	3875749	3875962	5Y-H4K8ac_peak_2936	6.78128	AC090587.5_ENSG00000228661;STIM1_ENSG00000167323;AC090587.4_ENSG00000229368
11	4115512	4115839	5Y-H4K8ac_peak_2937	6.08523	RRM1_ENSG00000167325
11	4414516	4414832	5Y-H4K8ac_peak_2938	6.79955	TRIM21_ENSG00000132109
11	4629021	4629368	5Y-H4K8ac_peak_2939	13.04415	TRIM68_ENSG00000167333
11	6598028	6598385	5Y-H4K8ac_peak_2940	5.40331	
11	6607940	6608426	5Y-H4K8ac_peak_2941	13.68515	
11	6633593	6633918	5Y-H4K8ac_peak_2942	9.02782	TAF10_ENSG00000166337

11	6668198	6668397	5Y-H4K8ac_peak_2943	9.05168	
11	6676764	6676998	5Y-H4K8ac_peak_2944	6.52593	DCHS1_ENSG00000166341
11	6704162	6704443	5Y-H4K8ac_peak_2945	8.24461	MRPL17_ENSG00000158042
11	7041012	7041390	5Y-H4K8ac_peak_2946	6.46053	ZNF214_ENSG00000149050;NLRP14_ENSG00000158077
11	7593318	7593594	5Y-H4K8ac_peak_2947	6.00382	CTD-2516F10.4_ENSG00000254864
11	7597765	7598189	5Y-H4K8ac_peak_2948	14.6127	
11	8008256	8008478	5Y-H4K8ac_peak_2949	7.11863	
11	8040776	8041178	5Y-H4K8ac_peak_2950	11.53136	TUB_ENSG00000166402
11	8087802	8088472	5Y-H4K8ac_peak_2951	6.1654	
11	8088713	8088918	5Y-H4K8ac_peak_2952	7.04637	
11	8090105	8090501	5Y-H4K8ac_peak_2953	14.77414	
11	8100944	8101141	5Y-H4K8ac_peak_2954	4.12295	
11	8102281	8102487	5Y-H4K8ac_peak_2955	10.95042	
11	8102979	8103392	5Y-H4K8ac_peak_2956	11.22005	
11	8134980	8135220	5Y-H4K8ac_peak_2957	7.49746	
11	8190363	8191311	5Y-H4K8ac_peak_2958	8.24461	RIC3_ENSG00000166405;RP11-379P15.1_ENSG00000246820
11	8228466	8228896	5Y-H4K8ac_peak_2959	11.1169	
11	8249777	8250588	5Y-H4K8ac_peak_2960	8.43511	
11	8251440	8251653	5Y-H4K8ac_peak_2961	6.54441	
11	8251946	8253032	5Y-H4K8ac_peak_2962	11.05199	
11	8253685	8255225	5Y-H4K8ac_peak_2963	9.30505	
11	8255933	8256648	5Y-H4K8ac_peak_2964	20.25522	
11	8257593	8257913	5Y-H4K8ac_peak_2965	9.96543	
11	8259085	8259320	5Y-H4K8ac_peak_2966	7.11863	
11	8264235	8265061	5Y-H4K8ac_peak_2967	7.64648	
11	8265289	8265828	5Y-H4K8ac_peak_2968	9.22478	
11	8283928	8285035	5Y-H4K8ac_peak_2969	18.22112	
11	8289538	8290216	5Y-H4K8ac_peak_2970	13.61292	LMO1_ENSG00000166407
11	8305417	8305787	5Y-H4K8ac_peak_2971	13.0168	
11	8350697	8351162	5Y-H4K8ac_peak_2972	12.10416	
11	8365108	8365376	5Y-H4K8ac_peak_2973	5.29015	
11	8376354	8376834	5Y-H4K8ac_peak_2974	9.58986	
11	8615482	8615759	5Y-H4K8ac_peak_2975	5.41472	STK33_ENSG00000130413
11	8703891	8704145	5Y-H4K8ac_peak_2976	14.87721	RPL27A_ENSG00000166441
11	8704402	8704769	5Y-H4K8ac_peak_2977	14.19462	RPL27A_ENSG00000166441
11	8892274	8892610	5Y-H4K8ac_peak_2978	6.22669	
11	8932855	8933161	5Y-H4K8ac_peak_2979	4.95697	ST5_ENSG00000166444;AKIP1_ENSG00000166452
11	9025134	9026097	5Y-H4K8ac_peak_2980	6.08523	NRIP3_ENSG00000175352;RP11-467K18.2_ENSG00000253973
11	9336772	9337053	5Y-H4K8ac_peak_2981	12.09312	TMEM41B_ENSG00000166471
11	9385588	9385937	5Y-H4K8ac_peak_2982	7.59101	
11	9406240	9406536	5Y-H4K8ac_peak_2983	8.09118	IPO7_ENSG00000205339
11	9595279	9595568	5Y-H4K8ac_peak_2984	6.75784	WEE1_ENSG00000166483
11	9779525	9779755	5Y-H4K8ac_peak_2985	14.72057	SBF2-AS1_ENSG00000246273
11	9779960	9781058	5Y-H4K8ac_peak_2986	11.12941	SBF2-AS1_ENSG00000246273;RP11-540A21.2_ENSG00000245522
11	10315889	10316302	5Y-H4K8ac_peak_2987	9.01738	SBF2_ENSG00000133812
11	10324082	10324509	5Y-H4K8ac_peak_2988	4.50834	RP11-351I24.1_ENSG00000254554

11	10326195	10326432	5Y-H4K8ac_peak_2989	11.1169	RP11-351I24.1_ENSG00000254554;ADM_ENSG00000148926
11	10429277	10429784	5Y-H4K8ac_peak_2990	6.50117	
11	10476660	10476853	5Y-H4K8ac_peak_2991	7.01266	
11	10562192	10562625	5Y-H4K8ac_peak_2992	8.21582	RNF141_ENSG00000110315;MRVI1-AS1_ENSG00000177112
11	10772911	10773123	5Y-H4K8ac_peak_2993	6.78128	CTR9_ENSG00000198730
11	10829510	10830110	5Y-H4K8ac_peak_2994	11.1169	RP11-685M7.3_ENSG00000246308;EIF4G2_ENSG00000110321
11	10830841	10831149	5Y-H4K8ac_peak_2995	6.37023	RP11-685M7.3_ENSG00000246308;EIF4G2_ENSG00000110321
11	10878955	10879264	5Y-H4K8ac_peak_2996	6.73385	ZBED5-AS1_ENSG00000247271
11	10880116	10880379	5Y-H4K8ac_peak_2997	5.5192	ZBED5_ENSG00000236287;ZBED5-AS1_ENSG00000247271
11	10952365	10952647	5Y-H4K8ac_peak_2998	6.77436	
11	11602216	11602487	5Y-H4K8ac_peak_2999	5.91107	
11	11603671	11603940	5Y-H4K8ac_peak_3000	8.8236	
11	11643916	11644108	5Y-H4K8ac_peak_3001	6.73047	GALNT18_ENSG00000110328
11	11862979	11863319	5Y-H4K8ac_peak_3002	6.54441	USP47_ENSG00000170242
11	11986963	11987420	5Y-H4K8ac_peak_3003	6.50117	
11	11989905	11990133	5Y-H4K8ac_peak_3004	3.96434	
11	11990607	11991643	5Y-H4K8ac_peak_3005	12.05638	
11	12000869	12001204	5Y-H4K8ac_peak_3006	4.29586	
11	12001453	12001939	5Y-H4K8ac_peak_3007	6.38173	
11	12398831	12399368	5Y-H4K8ac_peak_3008	7.50148	PARVA_ENSG00000197702
11	12528695	12529208	5Y-H4K8ac_peak_3009	6.20875	
11	12695430	12695644	5Y-H4K8ac_peak_3010	13.0168	TEAD1_ENSG00000187079
11	12696622	12696832	5Y-H4K8ac_peak_3011	10.46287	TEAD1_ENSG00000187079
11	12714171	12714601	5Y-H4K8ac_peak_3012	5.23083	
11	12822104	12822627	5Y-H4K8ac_peak_3013	5.13364	
11	12834839	12835316	5Y-H4K8ac_peak_3014	4.07874	
11	12835724	12836170	5Y-H4K8ac_peak_3015	5.98695	
11	12868451	12868710	5Y-H4K8ac_peak_3016	7.09444	
11	13126563	13126785	5Y-H4K8ac_peak_3017	6.79955	
11	13316907	13317129	5Y-H4K8ac_peak_3018	6.00388	
11	13317553	13317774	5Y-H4K8ac_peak_3019	4.93237	
11	13320920	13321206	5Y-H4K8ac_peak_3020	9.23159	
11	13350793	13351220	5Y-H4K8ac_peak_3021	6.54441	
11	13352405	13352764	5Y-H4K8ac_peak_3022	8.51022	
11	13689565	13689765	5Y-H4K8ac_peak_3023	9.38276	FAR1_ENSG00000197601
11	13690334	13690575	5Y-H4K8ac_peak_3024	5.29015	FAR1_ENSG00000197601;FAR1-IT1_ENSG00000254791
11	14380380	14380642	5Y-H4K8ac_peak_3025	5.65584	
11	14402520	14403064	5Y-H4K8ac_peak_3026	13.0168	
11	14541530	14541843	5Y-H4K8ac_peak_3027	4.50834	PSMA1_ENSG00000256206
11	14542129	14542471	5Y-H4K8ac_peak_3028	9.38203	PSMA1_ENSG00000256206
11	14542914	14543209	5Y-H4K8ac_peak_3029	5.40331	
11	14665353	14665762	5Y-H4K8ac_peak_3030	9.78792	PSMA1_ENSG00000129084;PDE3B_ENSG00000152270
11	14913386	14913656	5Y-H4K8ac_peak_3031	9.30505	CYP2R1_ENSG00000186104
11	15136030	15136284	5Y-H4K8ac_peak_3032	10.99773	
11	15643811	15644100	5Y-H4K8ac_peak_3033	8.88899	RP11-531H8.2_ENSG00000254789
11	15864217	15864439	5Y-H4K8ac_peak_3034	7.3889	

11	15907399	15907735	5Y-H4K8ac_peak_3035	6.42825	
11	17036083	17036644	5Y-H4K8ac_peak_3036	13.2534	PLEKHA7_ENSG00000166689;OR7E14P_ENSG00000184669
11	17099503	17100008	5Y-H4K8ac_peak_3037	15.06486	RPS13_ENSG00000110700
11	17297872	17298375	5Y-H4K8ac_peak_3038	8.21582	
11	17373092	17373485	5Y-H4K8ac_peak_3039	9.30505	NCR3LG1_ENSG00000188211
11	17373816	17374190	5Y-H4K8ac_peak_3040	12.71156	NCR3LG1_ENSG00000188211
11	17375024	17375241	5Y-H4K8ac_peak_3041	7.3889	
11	17426754	17427080	5Y-H4K8ac_peak_3042	9.33466	
11	17500722	17501113	5Y-H4K8ac_peak_3043	5.15917	
11	17757369	17757710	5Y-H4K8ac_peak_3044	6.73047	
11	17790308	17790505	5Y-H4K8ac_peak_3045	5.65765	
11	18034049	18034266	5Y-H4K8ac_peak_3046	10.11191	SERGEF_ENSG00000129158
11	18034919	18035214	5Y-H4K8ac_peak_3047	7.38046	SERGEF_ENSG00000129158
11	18127290	18127629	5Y-H4K8ac_peak_3048	9.00954	SAAL1_ENSG00000166788;HIGD1AP5_ENSG00000255254
11	18405461	18405914	5Y-H4K8ac_peak_3049	6.31818	
11	18406193	18406427	5Y-H4K8ac_peak_3050	10.07308	
11	18548203	18548398	5Y-H4K8ac_peak_3051	9.89244	TSG101_ENSG00000074319
11	18727466	18727759	5Y-H4K8ac_peak_3052	4.79585	RP11-1081L13.4_ENSG00000254966
11	19262861	19263257	5Y-H4K8ac_peak_3053	10.14566	E2F8_ENSG00000129173
11	19586392	19586594	5Y-H4K8ac_peak_3054	5.64909	
11	19617784	19618336	5Y-H4K8ac_peak_3055	11.1169	
11	19654944	19655217	5Y-H4K8ac_peak_3056	8.2913	
11	19701834	19702182	5Y-H4K8ac_peak_3057	10.11191	
11	19733541	19733804	5Y-H4K8ac_peak_3058	7.59101	RP11-359E10.1_ENSG00000270607
11	19758493	19759217	5Y-H4K8ac_peak_3059	7.97699	
11	19760110	19760398	5Y-H4K8ac_peak_3060	5.64909	
11	19828975	19829239	5Y-H4K8ac_peak_3061	11.61993	
11	20408484	20408947	5Y-H4K8ac_peak_3062	7.38046	PRMT3_ENSG00000185238
11	20409259	20409772	5Y-H4K8ac_peak_3063	9.15007	PRMT3_ENSG00000185238
11	20520906	20521102	5Y-H4K8ac_peak_3064	4.07874	
11	20630212	20630405	5Y-H4K8ac_peak_3065	5.23083	
11	20639328	20639532	5Y-H4K8ac_peak_3066	5.29015	
11	22151811	22152268	5Y-H4K8ac_peak_3067	4.50834	
11	22215014	22215328	5Y-H4K8ac_peak_3068	10.12234	ANO5_ENSG00000171714
11	22646857	22647344	5Y-H4K8ac_peak_3069	9.38203	FANCF_ENSG00000183161;AC103801.2_ENSG00000229387;GAS2_ENSG00000148935
11	22688317	22688846	5Y-H4K8ac_peak_3070	7.3889	
11	23134684	23134923	5Y-H4K8ac_peak_3071	6.50117	
11	26974618	26975177	5Y-H4K8ac_peak_3072	5.65584	
11	27493899	27494155	5Y-H4K8ac_peak_3073	5.23083	LGR4_ENSG00000205213;RP11-159H22.2_ENSG00000254862
11	27740500	27740892	5Y-H4K8ac_peak_3074	14.86775	
11	30606867	30607097	5Y-H4K8ac_peak_3075	6.77436	
11	32604585	32604928	5Y-H4K8ac_peak_3076	7.38046	EIF3M_ENSG00000149100
11	32605450	32605709	5Y-H4K8ac_peak_3077	5.35202	EIF3M_ENSG00000149100
11	32914799	32915446	5Y-H4K8ac_peak_3078	4.77126	QSER1_ENSG00000060749
11	33279009	33279630	5Y-H4K8ac_peak_3079	13.72401	HIPK3_ENSG00000110422
11	33722699	33722889	5Y-H4K8ac_peak_3080	6.5814	C11orf91_ENSG00000205177

11	34011530	34011726	5Y-H4K8ac_peak_3081	6.34046	
11	34073996	34074211	5Y-H4K8ac_peak_3082	7.28023	CAPRIN1_ENSG00000135387
11	34181347	34181868	5Y-H4K8ac_peak_3083	11.09973	
11	34937692	34937891	5Y-H4K8ac_peak_3084	8.43511	APIP_ENSG00000149089;PDHX_ENSG00000110435
11	34938270	34938506	5Y-H4K8ac_peak_3085	7.89142	APIP_ENSG00000149089;PDHX_ENSG00000110435
11	35160698	35161096	5Y-H4K8ac_peak_3086	7.31815	CD44_ENSG00000026508
11	35441824	35442224	5Y-H4K8ac_peak_3087	8.12305	SLC1A2_ENSG00000110436
11	35684000	35684862	5Y-H4K8ac_peak_3088	15.19225	TRIM44_ENSG00000166326
11	35949308	35949583	5Y-H4K8ac_peak_3089	13.8759	
11	35966213	35966546	5Y-H4K8ac_peak_3090	5.67283	LDLRAD3_ENSG00000179241
11	36181291	36181596	5Y-H4K8ac_peak_3091	11.1169	
11	36273363	36273794	5Y-H4K8ac_peak_3092	7.3889	
11	36588187	36588384	5Y-H4K8ac_peak_3093	8.16031	
11	36640401	36640687	5Y-H4K8ac_peak_3094	11.19336	
11	36649656	36650028	5Y-H4K8ac_peak_3095	13.0168	
11	36774067	36774536	5Y-H4K8ac_peak_3096	8.2913	
11	36970361	36970587	5Y-H4K8ac_peak_3097	7.38046	
11	36977969	36978389	5Y-H4K8ac_peak_3098	6.20875	
11	43380532	43381052	5Y-H4K8ac_peak_3099	9.00954	RP11-484D2.2_ENSG00000254907;TTC17_ENSG00000052841
11	43665825	43666161	5Y-H4K8ac_peak_3100	5.65584	
11	43902459	43902753	5Y-H4K8ac_peak_3101	5.87725	RP11-613D13.5_ENSG00000246250;ALKBH3_ENSG00000166199
11	43945909	43946347	5Y-H4K8ac_peak_3102	3.96434	C11orf96_ENSG00000187479
11	43946829	43947430	5Y-H4K8ac_peak_3103	14.44021	C11orf96_ENSG00000187479
11	43948319	43948575	5Y-H4K8ac_peak_3104	10.0695	
11	43956110	43956350	5Y-H4K8ac_peak_3105	5.98695	
11	43962848	43963078	5Y-H4K8ac_peak_3106	7.11863	
11	43963754	43964164	5Y-H4K8ac_peak_3107	11.22005	
11	43964472	43965192	5Y-H4K8ac_peak_3108	7.89273	
11	44008829	44009072	5Y-H4K8ac_peak_3109	12.65187	
11	44009823	44010116	5Y-H4K8ac_peak_3110	4.50834	
11	44010575	44010911	5Y-H4K8ac_peak_3111	6.1654	
11	44026799	44026997	5Y-H4K8ac_peak_3112	8.47164	
11	44299727	44299931	5Y-H4K8ac_peak_3113	6.59249	
11	44337801	44338152	5Y-H4K8ac_peak_3114	5.67283	
11	44617634	44617977	5Y-H4K8ac_peak_3115	9.48719	
11	44621485	44621887	5Y-H4K8ac_peak_3116	6.35402	
11	44656042	44656255	5Y-H4K8ac_peak_3117	5.98695	
11	44656552	44656771	5Y-H4K8ac_peak_3118	6.43775	
11	44675478	44675724	5Y-H4K8ac_peak_3119	4.66683	
11	44715585	44716076	5Y-H4K8ac_peak_3120	10.31981	
11	44716853	44717294	5Y-H4K8ac_peak_3121	6.77436	RP11-45A12.1_ENSG00000255079
11	44724715	44724928	5Y-H4K8ac_peak_3122	6.50117	
11	44732705	44733164	5Y-H4K8ac_peak_3123	6.77436	
11	44748073	44748344	5Y-H4K8ac_peak_3124	8.2913	TSPAN18_ENSG00000157570
11	44750002	44750204	5Y-H4K8ac_peak_3125	4.29586	
11	44750847	44751041	5Y-H4K8ac_peak_3126	7.87406	

11	44765153	44765375	5Y-H4K8ac_peak_3127	4.51497	
11	44774728	44775178	5Y-H4K8ac_peak_3128	8.45687	
11	44776088	44776365	5Y-H4K8ac_peak_3129	7.11863	
11	44777292	44777630	5Y-H4K8ac_peak_3130	10.1994	
11	44802691	44802908	5Y-H4K8ac_peak_3131	6.60109	
11	44804964	44805854	5Y-H4K8ac_peak_3132	8.43511	
11	44807297	44807541	5Y-H4K8ac_peak_3133	4.95697	
11	44807875	44808087	5Y-H4K8ac_peak_3134	7.65114	
11	44889965	44890477	5Y-H4K8ac_peak_3135	7.46945	
11	44971291	44972325	5Y-H4K8ac_peak_3136	9.7353	TP53I11_ENSG00000175274
11	45011850	45012443	5Y-H4K8ac_peak_3137	11.28133	
11	45012805	45013013	5Y-H4K8ac_peak_3138	6.0873	
11	45047345	45048141	5Y-H4K8ac_peak_3139	14.99673	
11	45113483	45113826	5Y-H4K8ac_peak_3140	5.87382	
11	45126979	45127175	5Y-H4K8ac_peak_3141	5.84208	
11	45167656	45168180	5Y-H4K8ac_peak_3142	8.16382	
11	45168906	45169272	5Y-H4K8ac_peak_3143	15.3483	
11	45201798	45202065	5Y-H4K8ac_peak_3144	6.86362	
11	45202311	45202702	5Y-H4K8ac_peak_3145	10.12457	
11	45307601	45307803	5Y-H4K8ac_peak_3146	7.38046	SYT13_ENSG00000019505
11	45433056	45433316	5Y-H4K8ac_peak_3147	11.82621	
11	45582316	45582579	5Y-H4K8ac_peak_3148	6.77436	
11	45680840	45681042	5Y-H4K8ac_peak_3149	5.89922	
11	45903762	45904092	5Y-H4K8ac_peak_3150	4.50834	
11	45904365	45904613	5Y-H4K8ac_peak_3151	5.77617	
11	45906760	45907034	5Y-H4K8ac_peak_3152	7.89142	MAPK8IP1_ENSG00000121653
11	45919237	45919583	5Y-H4K8ac_peak_3153	7.17997	
11	45943733	45944819	5Y-H4K8ac_peak_3154	13.00733	GYLTL1B_ENSG00000165905
11	46143230	46143732	5Y-H4K8ac_peak_3155	8.51022	PHF21A_ENSG00000135365
11	46259596	46259846	5Y-H4K8ac_peak_3156	10.07308	CTD-2589M5.5_ENSG00000254639
11	46264462	46264869	5Y-H4K8ac_peak_3157	8.43511	
11	46383098	46383481	5Y-H4K8ac_peak_3158	11.12941	
11	46383775	46383970	5Y-H4K8ac_peak_3159	4.15658	
11	46401398	46401742	5Y-H4K8ac_peak_3160	9.36633	MDK_ENSG00000110492
11	46402412	46402921	5Y-H4K8ac_peak_3161	15.82359	MDK_ENSG00000110492
11	46403409	46404020	5Y-H4K8ac_peak_3162	6.98416	
11	46639223	46639456	5Y-H4K8ac_peak_3163	4.71803	HARBI1_ENSG00000180423;ATG13_ENSG00000175224
11	46721947	46722144	5Y-H4K8ac_peak_3164	12.44818	ARHGAP1_ENSG00000175220;ZNF408_ENSG00000175213
11	46722806	46723189	5Y-H4K8ac_peak_3165	5.64909	ARHGAP1_ENSG00000175220;ZNF408_ENSG00000175213
11	46739655	46740181	5Y-H4K8ac_peak_3166	6.17374	F2_ENSG00000180210
11	46867448	46867772	5Y-H4K8ac_peak_3167	8.16382	CKAP5_ENSG00000175216;LRP4-AS1_ENSG00000247675
11	47198055	47198380	5Y-H4K8ac_peak_3168	10.16277	ARFGAP2_ENSG00000149182
11	47207486	47207928	5Y-H4K8ac_peak_3169	6.37023	PACSIN3_ENSG00000165912
11	47236698	47237043	5Y-H4K8ac_peak_3170	12.05638	DDB2_ENSG00000134574
11	47270172	47271258	5Y-H4K8ac_peak_3171	12.10416	ACP2_ENSG00000134575;NR1H3_ENSG00000025434
11	47290152	47291116	5Y-H4K8ac_peak_3172	5.67283	MADD_ENSG00000110514

11	47376693	47377180	5Y-H4K8ac_peak_3173	5.98695	
11	47421450	47421765	5Y-H4K8ac_peak_3174	8.78565	MIR4487_ENSG00000264583
11	47429364	47429837	5Y-H4K8ac_peak_3175	4.29586	SLC39A13_ENSG00000165915;RP11-750H9.5_ENSG00000255197
11	47430263	47430602	5Y-H4K8ac_peak_3176	5.23083	RP11-750H9.5_ENSG00000255197
11	47447622	47447978	5Y-H4K8ac_peak_3177	8.1667	PSMC3_ENSG00000165916
11	47586585	47586947	5Y-H4K8ac_peak_3178	8.43511	CELF1_ENSG00000149187;NDUFS3_ENSG00000213619;PTPMT1_ENSG00000110536
11	47587206	47587527	5Y-H4K8ac_peak_3179	5.56894	CELF1_ENSG00000149187;NDUFS3_ENSG00000213619;PTPMT1_ENSG00000110536
11	47789291	47789568	5Y-H4K8ac_peak_3180	5.64909	FNBP4_ENSG00000109920
11	47790374	47790694	5Y-H4K8ac_peak_3181	10.19948	
11	47869631	47870015	5Y-H4K8ac_peak_3182	14.72546	NUP160_ENSG00000030066
11	48001572	48001835	5Y-H4K8ac_peak_3183	6.50117	PTPRJ_ENSG00000149177
11	55640523	55640823	5Y-H4K8ac_peak_3184	7.31815	
11	56612564	56613199	5Y-H4K8ac_peak_3185	9.23159	
11	56613578	56613818	5Y-H4K8ac_peak_3186	5.23083	
11	56867179	56867370	5Y-H4K8ac_peak_3187	5.40331	
11	56921874	56922067	5Y-H4K8ac_peak_3188	6.98118	
11	57008907	57009309	5Y-H4K8ac_peak_3189	12.11208	
11	57009804	57010018	5Y-H4K8ac_peak_3190	5.68769	
11	57102895	57103231	5Y-H4K8ac_peak_3191	10.1994	SSRP1_ENSG00000149136
11	57243630	57244531	5Y-H4K8ac_peak_3192	9.38276	RP11-624G17.3_ENSG00000255301
11	57249770	57250157	5Y-H4K8ac_peak_3193	9.36633	
11	57261215	57261687	5Y-H4K8ac_peak_3194	5.65584	
11	57281998	57283163	5Y-H4K8ac_peak_3195	10.54764	SLC43A1_ENSG00000149150
11	57405127	57405668	5Y-H4K8ac_peak_3196	18.77281	AP000662.4_ENSG00000254602
11	57405993	57406221	5Y-H4K8ac_peak_3197	4.50834	AP000662.4_ENSG00000254602
11	57417779	57417997	5Y-H4K8ac_peak_3198	7.09444	YPEL4_ENSG00000166793
11	57434429	57434700	5Y-H4K8ac_peak_3199	9.26157	ZDHHC5_ENSG00000156599
11	57480228	57480438	5Y-H4K8ac_peak_3200	6.34046	MED19_ENSG00000156603;TMX2_ENSG00000213593;TMX2-CTNND1_ENSG00000254462
11	57509741	57509971	5Y-H4K8ac_peak_3201	5.56912	RP11-691N7.6_ENSG00000254732;C11orf31_ENSG00000211450
11	58346016	58346317	5Y-H4K8ac_peak_3202	13.0168	LPXN_ENSG00000110031;ZFP91_ENSG00000186660;ZFP91-CNTF_ENSG00000255073
11	58346666	58347074	5Y-H4K8ac_peak_3203	21.45231	LPXN_ENSG00000110031;ZFP91_ENSG00000186660;ZFP91-CNTF_ENSG00000255073
11	59332797	59333048	5Y-H4K8ac_peak_3204	9.02242	AP000442.4_ENSG00000255008
11	59522611	59523144	5Y-H4K8ac_peak_3205	8.2913	AP000640.10_ENSG00000254477
11	59577544	59577770	5Y-H4K8ac_peak_3206	6.46053	MRPL16_ENSG00000166902
11	60608813	60609334	5Y-H4K8ac_peak_3207	8.60024	CCDC86_ENSG00000110104
11	60673741	60674012	5Y-H4K8ac_peak_3208	6.37023	PRPF19_ENSG00000110107;RP11-881M11.2_ENSG00000257052
11	60681461	60681942	5Y-H4K8ac_peak_3209	6.77436	RP11-881M11.1_ENSG00000256944;TMEM109_ENSG00000110108
11	60691975	60692246	5Y-H4K8ac_peak_3210	10.72427	RP11-881M11.4_ENSG00000256196;TMEM132A_ENSG00000006118
11	60745747	60745944	5Y-H4K8ac_peak_3211	6.43775	
11	60775043	60775434	5Y-H4K8ac_peak_3212	12.05638	
11	60835643	60835887	5Y-H4K8ac_peak_3213	6.37023	
11	60849759	60850014	5Y-H4K8ac_peak_3214	11.57334	
11	60893965	60894309	5Y-H4K8ac_peak_3215	7.3889	
11	60928705	60929126	5Y-H4K8ac_peak_3216	9.00954	VPS37C_ENSG00000167987
11	61061799	61062861	5Y-H4K8ac_peak_3217	6.53157	VWCE_ENSG00000167992
11	61104244	61104525	5Y-H4K8ac_peak_3218	6.77436	

11	61112449	61112653	5Y-H4K8ac_peak_3219	8.60024	
11	61112952	61113325	5Y-H4K8ac_peak_3220	9.7353	
11	61129458	61129695	5Y-H4K8ac_peak_3221	13.00733	CYB561A3_ENSG00000162144;TMEM138_ENSG00000149483
11	61159350	61159903	5Y-H4K8ac_peak_3222	5.41472	TMEM216_ENSG00000187049
11	61197021	61197300	5Y-H4K8ac_peak_3223	7.90751	CPSF7_ENSG00000149532;RP11-286N22.8_ENSG00000256591;SDHAF2_ENSG00000167985
11	61198489	61198680	5Y-H4K8ac_peak_3224	5.97532	CPSF7_ENSG00000149532;SDHAF2_ENSG00000167985
11	61284734	61284975	5Y-H4K8ac_peak_3225	9.34555	
11	61322670	61322900	5Y-H4K8ac_peak_3226	8.21582	
11	61366793	61367179	5Y-H4K8ac_peak_3227	4.50834	
11	61447995	61448199	5Y-H4K8ac_peak_3228	4.83834	
11	61485595	61485904	5Y-H4K8ac_peak_3229	11.51936	
11	61486334	61486566	5Y-H4K8ac_peak_3230	7.03573	
11	61515083	61515297	5Y-H4K8ac_peak_3231	8.1667	
11	61520169	61520417	5Y-H4K8ac_peak_3232	4.8188	MYRF_ENSG00000124920
11	61559782	61560551	5Y-H4K8ac_peak_3233	16.33454	TMEM258_ENSG00000134825;MIR611_ENSG00000207601;FEN1_ENSG00000168496;FADS2_ENSG00000134824
11	61582980	61584304	5Y-H4K8ac_peak_3234	11.22005	MIR1908_ENSG00000222326
11	61594331	61595034	5Y-H4K8ac_peak_3235	12.69256	
11	61595628	61596168	5Y-H4K8ac_peak_3236	11.36989	FADS1_ENSG00000149485
11	61658628	61658925	5Y-H4K8ac_peak_3237	7.56545	FADS3_ENSG00000221968
11	61684719	61685354	5Y-H4K8ac_peak_3238	9.52603	
11	61690904	61691586	5Y-H4K8ac_peak_3239	7.50148	
11	61735701	61736022	5Y-H4K8ac_peak_3240	8.43511	FTH1_ENSG00000167996;AP003733.1_ENSG00000269089
11	61879812	61880360	5Y-H4K8ac_peak_3241	18.10232	
11	62168579	62168778	5Y-H4K8ac_peak_3242	5.21093	
11	62310497	62310700	5Y-H4K8ac_peak_3243	8.43511	
11	62359424	62359704	5Y-H4K8ac_peak_3244	4.15658	MIR3654_ENSG00000255508;TUT1_ENSG00000149016
11	62368797	62369240	5Y-H4K8ac_peak_3245	10.60083	MTA2_ENSG00000149480
11	62370057	62370319	5Y-H4K8ac_peak_3246	7.82914	MTA2_ENSG00000149480
11	62379284	62379500	5Y-H4K8ac_peak_3247	6.77436	EML3_ENSG00000149499;ROM1_ENSG00000149489
11	62379702	62380131	5Y-H4K8ac_peak_3248	8.43511	EML3_ENSG00000149499;ROM1_ENSG00000149489
11	62413751	62413979	5Y-H4K8ac_peak_3249	11.19336	GANAB_ENSG00000089597
11	62420356	62420689	5Y-H4K8ac_peak_3250	7.59101	INTS5_ENSG00000185085
11	62439376	62439566	5Y-H4K8ac_peak_3251	8.43511	C11orf48_ENSG00000162194
11	62446180	62446848	5Y-H4K8ac_peak_3252	6.08523	UBXN1_ENSG00000162191
11	62494724	62494929	5Y-H4K8ac_peak_3253	10.35586	HNRNPUL2-BSCL2_ENSG00000234857;HNRNPUL2_ENSG00000214753;TTC9C_ENSG00000162222
11	62521949	62522333	5Y-H4K8ac_peak_3254	9.23159	ZBTB3_ENSG00000185670
11	62606694	62606980	5Y-H4K8ac_peak_3255	7.89273	
11	62607545	62607774	5Y-H4K8ac_peak_3256	12.05638	
11	62609613	62610100	5Y-H4K8ac_peak_3257	10.11191	WDR74_ENSG00000133316;RNU2-2P_ENSG00000222328
11	62648474	62648936	5Y-H4K8ac_peak_3258	5.98695	
11	62695848	62696203	5Y-H4K8ac_peak_3259	10.36926	
11	63438330	63438937	5Y-H4K8ac_peak_3260	8.62703	ATL3_ENSG00000184743
11	63536323	63537060	5Y-H4K8ac_peak_3261	12.76485	C11orf95_ENSG00000188070;RP11-466C23.4_ENSG00000255651
11	63551520	63551778	5Y-H4K8ac_peak_3262	11.85279	
11	63683295	63683605	5Y-H4K8ac_peak_3263	4.95697	RCOR2_ENSG00000167771
11	63683965	63684373	5Y-H4K8ac_peak_3264	7.38046	RCOR2_ENSG00000167771

11	63684815	63685331	5Y-H4K8ac_peak_3265	17.04999	RCOR2_ENSG00000167771
11	63685688	63686949	5Y-H4K8ac_peak_3266	14.00894	
11	63729798	63729996	5Y-H4K8ac_peak_3267	6.43775	
11	63742168	63742368	5Y-H4K8ac_peak_3268	5.65584	COX8A_ENSG00000176340;AP000721.4_ENSG00000256100
11	63754088	63754402	5Y-H4K8ac_peak_3269	5.24695	OTUB1_ENSG00000167770
11	63810595	63810801	5Y-H4K8ac_peak_3270	5.37237	
11	63812971	63813176	5Y-H4K8ac_peak_3271	7.50501	
11	63820028	63820827	5Y-H4K8ac_peak_3272	10.75877	
11	63823480	63823693	5Y-H4K8ac_peak_3273	7.20869	
11	63824028	63825952	5Y-H4K8ac_peak_3274	9.44542	
11	63826874	63827099	5Y-H4K8ac_peak_3275	6.34245	
11	63827809	63828609	5Y-H4K8ac_peak_3276	9.13495	
11	63834987	63835275	5Y-H4K8ac_peak_3277	5.79005	
11	63841124	63841365	5Y-H4K8ac_peak_3278	5.64909	
11	63845474	63845749	5Y-H4K8ac_peak_3279	6.16949	
11	63846027	63846443	5Y-H4K8ac_peak_3280	7.07275	
11	63846915	63847150	5Y-H4K8ac_peak_3281	5.64909	
11	63851771	63852776	5Y-H4K8ac_peak_3282	9.9863	
11	63853524	63853901	5Y-H4K8ac_peak_3283	13.61292	
11	63864094	63864399	5Y-H4K8ac_peak_3284	8.20773	
11	63864689	63864892	5Y-H4K8ac_peak_3285	4.44548	
11	63868431	63868837	5Y-H4K8ac_peak_3286	9.30505	
11	63900410	63901274	5Y-H4K8ac_peak_3287	10.26997	
11	63911600	63911808	5Y-H4K8ac_peak_3288	5.14442	
11	63912215	63913231	5Y-H4K8ac_peak_3289	4.77126	
11	63915391	63915701	5Y-H4K8ac_peak_3290	5.98695	
11	63916386	63917283	5Y-H4K8ac_peak_3291	8.43511	
11	63918258	63918708	5Y-H4K8ac_peak_3292	9.02782	
11	63933154	63933349	5Y-H4K8ac_peak_3293	4.50834	MACROD1_ENSG00000133315
11	63933752	63934103	5Y-H4K8ac_peak_3294	7.11863	MACROD1_ENSG00000133315
11	64007982	64008321	5Y-H4K8ac_peak_3295	8.24461	FKBP2_ENSG00000173486
11	64013923	64014246	5Y-H4K8ac_peak_3296	9.67903	PPP1R14B_ENSG00000173457;RP11-783K16.5_ENSG00000256940
11	64014497	64014751	5Y-H4K8ac_peak_3297	7.2422	PPP1R14B_ENSG00000173457
11	64015086	64015539	5Y-H4K8ac_peak_3298	11.57236	PPP1R14B_ENSG00000173457
11	64036583	64037354	5Y-H4K8ac_peak_3299	22.01094	GPR137_ENSG00000173264
11	64051704	64051933	5Y-H4K8ac_peak_3300	6.03632	BAD_ENSG00000002330
11	64052661	64053084	5Y-H4K8ac_peak_3301	8.73392	BAD_ENSG00000002330
11	64059255	64059460	5Y-H4K8ac_peak_3302	7.11863	KCNK4_ENSG00000182450;RP11-783K16.10_ENSG00000257069
11	64084326	64084978	5Y-H4K8ac_peak_3303	10.54764	TRMT112_ENSG00000173113;PRDX5_ENSG00000126432
11	64086451	64086711	5Y-H4K8ac_peak_3304	5.70209	TRMT112_ENSG00000173113;PRDX5_ENSG00000126432
11	64126169	64126478	5Y-H4K8ac_peak_3305	5.98695	RPS6KA4_ENSG00000162302
11	64126729	64126945	5Y-H4K8ac_peak_3306	8.16382	RPS6KA4_ENSG00000162302
11	64333777	64333982	5Y-H4K8ac_peak_3307	4.44712	
11	64334328	64335349	5Y-H4K8ac_peak_3308	12.05638	
11	64387744	64388142	5Y-H4K8ac_peak_3309	7.01266	
11	64404500	64404774	5Y-H4K8ac_peak_3310	9.89244	

11	64405331	64405805	5Y-H4K8ac_peak_3311	17.50049	
11	64410453	64410679	5Y-H4K8ac_peak_3312	6.39626	
11	64455630	64455834	5Y-H4K8ac_peak_3313	9.38276	
11	64461536	64462370	5Y-H4K8ac_peak_3314	12.10416	
11	64479498	64479806	5Y-H4K8ac_peak_3315	5.65584	
11	64490780	64491308	5Y-H4K8ac_peak_3316	21.88077	NRXN2_ENSG00000110076
11	64512480	64512926	5Y-H4K8ac_peak_3317	9.179	RASGRP2_ENSG00000068831
11	64545656	64546094	5Y-H4K8ac_peak_3318	14.78466	SF1_ENSG00000168066;AP001462.6_ENSG00000269038
11	64611884	64612538	5Y-H4K8ac_peak_3319	7.89273	CDC42BPG_ENSG00000171219
11	64614803	64615035	5Y-H4K8ac_peak_3320	7.38046	
11	64655261	64655467	5Y-H4K8ac_peak_3321	12.14232	EHD1_ENSG00000110047
11	64655672	64655946	5Y-H4K8ac_peak_3322	6.50117	EHD1_ENSG00000110047
11	64690993	64691507	5Y-H4K8ac_peak_3323	7.89273	
11	64781735	64782554	5Y-H4K8ac_peak_3324	10.90365	ARL2_ENSG00000213465;RP11-399J13.3_ENSG00000273003
11	64863175	64863490	5Y-H4K8ac_peak_3325	6.08523	
11	64863744	64864180	5Y-H4K8ac_peak_3326	9.51254	
11	64883998	64885102	5Y-H4K8ac_peak_3327	14.19462	ZNHIT2_ENSG00000174276;AP003068.12_ENSG00000255173
11	64901778	64902436	5Y-H4K8ac_peak_3328	9.51254	SYVN1_ENSG00000162298
11	64918386	64918775	5Y-H4K8ac_peak_3329	13.27648	
11	64937354	64937831	5Y-H4K8ac_peak_3330	5.40331	SPDYC_ENSG00000204710
11	64949427	64949643	5Y-H4K8ac_peak_3331	16.01457	AP003068.23_ENSG00000254614
11	64992488	64992684	5Y-H4K8ac_peak_3332	5.64909	
11	64993351	64993865	5Y-H4K8ac_peak_3333	5.72233	
11	65029572	65029831	5Y-H4K8ac_peak_3334	7.72887	POLA2_ENSG00000014138
11	65066529	65067087	5Y-H4K8ac_peak_3335	11.36844	
11	65100788	65101179	5Y-H4K8ac_peak_3336	9.15007	DPF2_ENSG00000133884
11	65149279	65149763	5Y-H4K8ac_peak_3337	10.86711	
11	65149956	65150404	5Y-H4K8ac_peak_3338	7.11863	SLC25A45_ENSG00000162241
11	65153630	65154310	5Y-H4K8ac_peak_3339	14.67885	FRMD8_ENSG00000126391
11	65183569	65183843	5Y-H4K8ac_peak_3340	5.98695	
11	65189421	65189647	5Y-H4K8ac_peak_3341	7.61515	NEAT1_ENSG00000245532
11	65220123	65220736	5Y-H4K8ac_peak_3342	5.74859	
11	65244485	65244942	5Y-H4K8ac_peak_3343	13.18589	
11	65245305	65245505	5Y-H4K8ac_peak_3344	9.30505	
11	65255475	65256207	5Y-H4K8ac_peak_3345	8.24461	
11	65265319	65266085	5Y-H4K8ac_peak_3346	7.44077	MALAT1_ENSG00000251562;AP000769.7_ENSG00000270117
11	65292624	65292851	5Y-H4K8ac_peak_3347	8.90223	SCYL1_ENSG00000142186
11	65325620	65325891	5Y-H4K8ac_peak_3348	8.62703	LTBP3_ENSG00000168056
11	65326134	65326846	5Y-H4K8ac_peak_3349	11.08964	LTBP3_ENSG00000168056
11	65419671	65419970	5Y-H4K8ac_peak_3350	6.35402	
11	65420607	65420921	5Y-H4K8ac_peak_3351	7.18918	
11	65430003	65430229	5Y-H4K8ac_peak_3352	4.26524	RELA_ENSG00000173039
11	65430610	65430881	5Y-H4K8ac_peak_3353	4.77126	RELA_ENSG00000173039
11	65478267	65478794	5Y-H4K8ac_peak_3354	7.28023	KAT5_ENSG00000172977
11	65479529	65479892	5Y-H4K8ac_peak_3355	10.44054	KAT5_ENSG00000172977
11	65547863	65548247	5Y-H4K8ac_peak_3356	14.01193	AP5B1_ENSG00000254470;AP001266.1_ENSG00000175827

11	65585345	65585657	5Y-H4K8ac_peak_3357	9.23159	
11	65600713	65601291	5Y-H4K8ac_peak_3358	10.60083	SNX32_ENSG00000172803
11	65627095	65627470	5Y-H4K8ac_peak_3359	6.77436	
11	65639531	65640318	5Y-H4K8ac_peak_3360	6.69752	EFEMP2_ENSG00000172638
11	65658345	65658554	5Y-H4K8ac_peak_3361	4.77126	CCDC85B_ENSG00000175602
11	65666715	65666944	5Y-H4K8ac_peak_3362	5.98695	
11	65679084	65679360	5Y-H4K8ac_peak_3363	7.38046	
11	65686052	65686360	5Y-H4K8ac_peak_3364	13.12848	C11orf68_ENSG00000175573;DRAP1_ENSG00000175550
11	65728524	65728832	5Y-H4K8ac_peak_3365	7.89142	SART1_ENSG00000175467
11	65769755	65770102	5Y-H4K8ac_peak_3366	12.05638	EIF1AD_ENSG00000175376;BANF1_ENSG00000175334
11	65789801	65790018	5Y-H4K8ac_peak_3367	9.74189	
11	65816810	65817009	5Y-H4K8ac_peak_3368	7.59101	GAL3ST3_ENSG00000175229
11	65819297	65819530	5Y-H4K8ac_peak_3369	6.08471	
11	65838082	65838330	5Y-H4K8ac_peak_3370	9.52603	RP11-1167A19.2_ENSG00000255038;PACS1_ENSG00000175115
11	66024746	66025060	5Y-H4K8ac_peak_3371	7.09658	KLC2_ENSG00000174996
11	66050326	66050586	5Y-H4K8ac_peak_3372	8.51022	
11	66059436	66059760	5Y-H4K8ac_peak_3373	5.91107	TMEM151A_ENSG00000179292
11	66080637	66081562	5Y-H4K8ac_peak_3374	11.4254	RP11-867G23.13_ENSG00000254458
11	66084234	66084514	5Y-H4K8ac_peak_3375	9.32595	CD248_ENSG00000174807
11	66084740	66085339	5Y-H4K8ac_peak_3376	13.89642	CD248_ENSG00000174807
11	66094107	66094418	5Y-H4K8ac_peak_3377	13.0168	
11	66095740	66095936	5Y-H4K8ac_peak_3378	8.62703	
11	66103540	66104178	5Y-H4K8ac_peak_3379	12.21176	RIN1_ENSG00000174791
11	66104532	66104989	5Y-H4K8ac_peak_3380	14.10494	RIN1_ENSG00000174791
11	66105404	66105603	5Y-H4K8ac_peak_3381	4.29586	
11	66138489	66139137	5Y-H4K8ac_peak_3382	9.69901	SLC29A2_ENSG00000174669
11	66208429	66208660	5Y-H4K8ac_peak_3383	4.03329	
11	66234898	66235096	5Y-H4K8ac_peak_3384	8.67356	MRPL11_ENSG00000174547;PELI3_ENSG00000174516
11	66247112	66247553	5Y-H4K8ac_peak_3385	13.23366	CTD-3074O7.5_ENSG00000255517;DPP3_ENSG00000254986
11	66335771	66336007	5Y-H4K8ac_peak_3386	7.89273	CTSF_ENSG00000174080
11	66383529	66383817	5Y-H4K8ac_peak_3387	14.59308	RNU4-39P_ENSG00000199325;RBM14_ENSG00000239306;RBM4_ENSG00000173933;RBM14-RBM4_ENSG00000248643
11	66405421	66405611	5Y-H4K8ac_peak_3388	4.50834	
11	66495162	66495369	5Y-H4K8ac_peak_3389	10.1994	
11	66495946	66496475	5Y-H4K8ac_peak_3390	16.45525	SPTBN2_ENSG00000173898
11	66511780	66512073	5Y-H4K8ac_peak_3391	8.24461	C11orf80_ENSG00000173715
11	66611005	66611434	5Y-H4K8ac_peak_3392	7.30348	RCE1_ENSG00000173653
11	66623471	66624167	5Y-H4K8ac_peak_3393	10.07308	LRFN4_ENSG00000173621
11	66649729	66650714	5Y-H4K8ac_peak_3394	12.20141	
11	66719173	66719445	5Y-H4K8ac_peak_3395	5.03917	
11	66816872	66817115	5Y-H4K8ac_peak_3396	6.20989	
11	66883101	66883423	5Y-H4K8ac_peak_3397	5.84208	
11	66885788	66886182	5Y-H4K8ac_peak_3398	13.0168	KDM2A_ENSG00000173120
11	67033447	67033658	5Y-H4K8ac_peak_3399	5.98695	ADRBK1_ENSG00000173020
11	67055635	67055929	5Y-H4K8ac_peak_3400	11.30548	ANKRD13D_ENSG00000172932
11	67056147	67056378	5Y-H4K8ac_peak_3401	6.53157	ANKRD13D_ENSG00000172932
11	67070568	67071696	5Y-H4K8ac_peak_3402	10.35586	SSH3_ENSG00000172830

11	67121385	67121639	5Y-H4K8ac_peak_3403	6.89892	
11	67139812	67140117	5Y-H4K8ac_peak_3404	7.89273	AP003419.11_ENSG00000256514
11	67158976	67159256	5Y-H4K8ac_peak_3405	12.50545	RAD9A_ENSG00000172613
11	67168644	67169205	5Y-H4K8ac_peak_3406	8.24461	
11	67169657	67169848	5Y-H4K8ac_peak_3407	7.59101	
11	67194704	67195046	5Y-H4K8ac_peak_3408	8.35207	RPS6KB2_ENSG00000175634
11	67232348	67232773	5Y-H4K8ac_peak_3409	14.87721	
11	67236960	67237169	5Y-H4K8ac_peak_3410	10.19948	TMEM134_ENSG00000172663
11	67276337	67276566	5Y-H4K8ac_peak_3411	7.59101	CDK2AP2_ENSG00000167797
11	67499404	67499742	5Y-H4K8ac_peak_3412	9.30505	
11	67573732	67573963	5Y-H4K8ac_peak_3413	6.82266	FAM86C2P_ENSG00000160172
11	67722536	67722913	5Y-H4K8ac_peak_3414	4.70501	
11	67771144	67771587	5Y-H4K8ac_peak_3415	6.73385	UNC93B1_ENSG00000110057
11	67787928	67788454	5Y-H4K8ac_peak_3416	6.1654	
11	67807673	67808492	5Y-H4K8ac_peak_3417	10.11191	
11	67889627	67889873	5Y-H4K8ac_peak_3418	6.46606	CHKA_ENSG00000110721;CTD-2655K5.1_ENSG00000255236
11	67895411	67895620	5Y-H4K8ac_peak_3419	5.41472	
11	67980874	67982178	5Y-H4K8ac_peak_3420	16.41521	SUV420H1_ENSG00000110066
11	68039658	68039909	5Y-H4K8ac_peak_3421	7.20869	C11orf24_ENSG00000171067
11	68064936	68065162	5Y-H4K8ac_peak_3422	9.23159	
11	68080152	68080416	5Y-H4K8ac_peak_3423	7.59832	LRP5_ENSG00000162337
11	68140207	68141085	5Y-H4K8ac_peak_3424	11.1169	
11	68142309	68142994	5Y-H4K8ac_peak_3425	21.48346	
11	68144511	68144730	5Y-H4K8ac_peak_3426	7.11863	
11	68210152	68210432	5Y-H4K8ac_peak_3427	4.84727	
11	68427016	68427986	5Y-H4K8ac_peak_3428	7.90236	
11	68428452	68429500	5Y-H4K8ac_peak_3429	3.94247	
11	68451460	68452640	5Y-H4K8ac_peak_3430	14.00894	GAL_ENSG00000069482
11	68518723	68518964	5Y-H4K8ac_peak_3431	18.33883	MTL5_ENSG00000132749
11	68532203	68532410	5Y-H4K8ac_peak_3432	6.79955	
11	68671085	68671683	5Y-H4K8ac_peak_3433	23.3512	MRPL21_ENSG00000197345;IGHMBP2_ENSG00000132740
11	68766240	68766441	5Y-H4K8ac_peak_3434	9.15007	
11	68769399	68769630	5Y-H4K8ac_peak_3435	4.36976	RP11-554A11.4_ENSG00000261625
11	68780534	68781238	5Y-H4K8ac_peak_3436	6.37023	MRGPRF_ENSG00000172935;RP11-554A11.6_ENSG00000256508
11	68874193	68874564	5Y-H4K8ac_peak_3437	8.24461	
11	68899327	68899642	5Y-H4K8ac_peak_3438	8.564	
11	68923799	68924691	5Y-H4K8ac_peak_3439	15.08122	RP11-554A11.9_ENSG00000259799
11	68924902	68925750	5Y-H4K8ac_peak_3440	14.92663	
11	68933926	68934408	5Y-H4K8ac_peak_3441	6.11316	
11	68950950	68951419	5Y-H4K8ac_peak_3442	7.82914	
11	68953391	68953706	5Y-H4K8ac_peak_3443	12.10416	
11	68959800	68960236	5Y-H4K8ac_peak_3444	8.08337	
11	68960436	68960641	5Y-H4K8ac_peak_3445	7.31102	
11	68964382	68964580	5Y-H4K8ac_peak_3446	5.98695	
11	68967905	68968312	5Y-H4K8ac_peak_3447	10.4689	
11	68979767	68980021	5Y-H4K8ac_peak_3448	10.95042	

11	69195233	69195513	5Y-H4K8ac_peak_3449	7.59101	
11	69257456	69257976	5Y-H4K8ac_peak_3450	9.48616	
11	69258386	69259243	5Y-H4K8ac_peak_3451	10.54764	AP000439.2_ENSG00000255606
11	69263831	69264262	5Y-H4K8ac_peak_3452	6.36723	
11	69264866	69265226	5Y-H4K8ac_peak_3453	9.23159	
11	69452794	69453358	5Y-H4K8ac_peak_3454	8.43511	
11	69455061	69455607	5Y-H4K8ac_peak_3455	5.65584	CCND1_ENSG00000110092
11	69456044	69456357	5Y-H4K8ac_peak_3456	6.79169	CCND1_ENSG00000110092
11	69456889	69457161	5Y-H4K8ac_peak_3457	14.31711	
11	69457705	69458113	5Y-H4K8ac_peak_3458	5.78188	
11	69458811	69459140	5Y-H4K8ac_peak_3459	10.35586	
11	69518856	69519359	5Y-H4K8ac_peak_3460	12.21176	FGF19_ENSG00000162344
11	69555896	69556101	5Y-H4K8ac_peak_3461	5.97089	
11	69707292	69708110	5Y-H4K8ac_peak_3462	10.79063	
11	69708454	69708779	5Y-H4K8ac_peak_3463	8.24461	
11	70244675	70244955	5Y-H4K8ac_peak_3464	11.68317	AP000487.5_ENSG00000246889;CTTN_ENSG00000085733
11	70428030	70428243	5Y-H4K8ac_peak_3465	7.31102	
11	70430644	70430877	5Y-H4K8ac_peak_3466	4.98439	
11	70431149	70431391	5Y-H4K8ac_peak_3467	6.82266	
11	70456401	70456644	5Y-H4K8ac_peak_3468	9.41037	
11	70487918	70488224	5Y-H4K8ac_peak_3469	13.33225	
11	70508310	70508526	5Y-H4K8ac_peak_3470	11.09973	
11	70508871	70509474	5Y-H4K8ac_peak_3471	13.92877	
11	70516634	70516848	5Y-H4K8ac_peak_3472	5.35202	
11	71199653	71199936	5Y-H4K8ac_peak_3473	12.09463	
11	71200428	71200665	5Y-H4K8ac_peak_3474	4.9885	
11	71497490	71497741	5Y-H4K8ac_peak_3475	6.77436	FAM86C1_ENSG00000158483
11	71524403	71524886	5Y-H4K8ac_peak_3476	12.21176	
11	71639069	71639940	5Y-H4K8ac_peak_3477	10.36926	RP11-849H4.2_ENSG00000254469;RNF121_ENSG00000137522
11	71752437	71753323	5Y-H4K8ac_peak_3478	10.19948	
11	71792024	71792351	5Y-H4K8ac_peak_3479	11.1169	NUMA1_ENSG00000137497;LRTOMT_ENSG00000184154
11	71813955	71814293	5Y-H4K8ac_peak_3480	12.21176	LAMTOR1_ENSG00000149357;snoU13_ENSG00000238768
11	71814646	71814968	5Y-H4K8ac_peak_3481	9.26157	LAMTOR1_ENSG00000149357;snoU13_ENSG00000238768
11	71824387	71824823	5Y-H4K8ac_peak_3482	10.89497	ANAPC15_ENSG00000110200
11	71934737	71935193	5Y-H4K8ac_peak_3483	7.27597	INPPL1_ENSG00000165458
11	71948375	71948819	5Y-H4K8ac_peak_3484	5.98695	
11	71951734	71952115	5Y-H4K8ac_peak_3485	7.89273	
11	71955936	71956349	5Y-H4K8ac_peak_3486	8.69112	PHOX2A_ENSG00000165462
11	72017347	72018201	5Y-H4K8ac_peak_3487	7.89273	
11	72018617	72019587	5Y-H4K8ac_peak_3488	7.20869	
11	72022736	72022951	5Y-H4K8ac_peak_3489	8.43511	
11	72030939	72031196	5Y-H4K8ac_peak_3490	10.90543	
11	72032799	72033422	5Y-H4K8ac_peak_3491	8.28346	
11	72046313	72047274	5Y-H4K8ac_peak_3492	7.97699	
11	72053358	72053593	5Y-H4K8ac_peak_3493	4.50834	
11	72055153	72055397	5Y-H4K8ac_peak_3494	5.65584	

11	72060426	72060837	5Y-H4K8ac_peak_3495	8.79957	
11	72067042	72067257	5Y-H4K8ac_peak_3496	6.20989	
11	72068060	72068587	5Y-H4K8ac_peak_3497	7.80086	
11	72068872	72069660	5Y-H4K8ac_peak_3498	10.35586	
11	72071022	72071330	5Y-H4K8ac_peak_3499	11.19336	
11	72081664	72081940	5Y-H4K8ac_peak_3500	4.8509	
11	72122320	72122710	5Y-H4K8ac_peak_3501	5.03335	RP11-7N14.1_ENSG00000256403
11	72124915	72125221	5Y-H4K8ac_peak_3502	6.50117	
11	72336592	72336842	5Y-H4K8ac_peak_3503	4.67245	
11	72339884	72340526	5Y-H4K8ac_peak_3504	9.79526	
11	72341112	72341391	5Y-H4K8ac_peak_3505	7.387	
11	72348337	72348567	5Y-H4K8ac_peak_3506	4.50302	
11	72385939	72386359	5Y-H4K8ac_peak_3507	10.19948	PDE2A_ENSG00000186642
11	72424101	72424345	5Y-H4K8ac_peak_3508	7.01364	
11	72433830	72434063	5Y-H4K8ac_peak_3509	8.11614	
11	72462771	72463446	5Y-H4K8ac_peak_3510	6.50117	
11	72492357	72492569	5Y-H4K8ac_peak_3511	7.04158	
11	72504055	72504245	5Y-H4K8ac_peak_3512	5.77617	ARAP1_ENSG00000186635;STARD10_ENSG00000214530
11	72525150	72525352	5Y-H4K8ac_peak_3513	8.90774	ATG16L2_ENSG00000168010
11	72532619	72533162	5Y-H4K8ac_peak_3514	16.79029	
11	72944377	72944568	5Y-H4K8ac_peak_3515	6.17973	
11	72953349	72953774	5Y-H4K8ac_peak_3516	7.63144	
11	72981791	72982594	5Y-H4K8ac_peak_3517	7.59101	
11	73019331	73019902	5Y-H4K8ac_peak_3518	9.7353	RP11-800A3.7_ENSG00000257038;ARHGEF17_ENSG00000110237
11	73024146	73024676	5Y-H4K8ac_peak_3519	6.92707	
11	73032998	73033628	5Y-H4K8ac_peak_3520	8.73392	
11	73045457	73046404	5Y-H4K8ac_peak_3521	6.53157	
11	73053978	73054365	5Y-H4K8ac_peak_3522	6.4472	
11	73086634	73087202	5Y-H4K8ac_peak_3523	11.12941	RELT_ENSG00000054967
11	73309804	73310005	5Y-H4K8ac_peak_3524	10.55504	FAM168A_ENSG00000054965
11	73371662	73371903	5Y-H4K8ac_peak_3525	6.43775	
11	73440862	73441071	5Y-H4K8ac_peak_3526	6.7002	
11	73472744	73473089	5Y-H4K8ac_peak_3527	10.27264	RAB6A_ENSG00000175582
11	74108944	74109245	5Y-H4K8ac_peak_3528	5.22422	PGM2L1_ENSG00000165434;RP11-702H23.4_ENSG00000254631
11	74204462	74204752	5Y-H4K8ac_peak_3529	10.44054	LIPT2_ENSG00000175536;AP001372.2_ENSG00000254837;POLD3_ENSG00000077514
11	74303779	74304008	5Y-H4K8ac_peak_3530	5.77617	
11	74383809	74384036	5Y-H4K8ac_peak_3531	11.19336	
11	74394337	74394595	5Y-H4K8ac_peak_3532	9.07085	
11	74402203	74402407	5Y-H4K8ac_peak_3533	4.9885	
11	74906591	74906937	5Y-H4K8ac_peak_3534	4.84727	
11	74952501	74952719	5Y-H4K8ac_peak_3535	8.1667	CTD-2562J17.4_ENSG00000255136
11	75012288	75012786	5Y-H4K8ac_peak_3536	6.43068	
11	75015400	75015707	5Y-H4K8ac_peak_3537	6.3265	
11	75022928	75023130	5Y-H4K8ac_peak_3538	6.34245	
11	75050763	75051439	5Y-H4K8ac_peak_3539	5.64909	
11	75051639	75051871	5Y-H4K8ac_peak_3540	6.06162	

11	75060776	75061051	5Y-H4K8ac_peak_3541	8.18236	
11	75062392	75062635	5Y-H4K8ac_peak_3542	9.31608	ARRB1_ENSG00000137486
11	75062827	75063302	5Y-H4K8ac_peak_3543	7.20869	ARRB1_ENSG00000137486
11	75110612	75111135	5Y-H4K8ac_peak_3544	11.22005	RPS3_ENSG00000149273;SNORD15A_ENSG00000206941
11	75141325	75141857	5Y-H4K8ac_peak_3545	13.03079	KLHL35_ENSG00000149243
11	75180269	75180465	5Y-H4K8ac_peak_3546	4.07874	
11	75218385	75218648	5Y-H4K8ac_peak_3547	4.71803	RP11-939C17.4_ENSG00000254460
11	75265857	75266780	5Y-H4K8ac_peak_3548	16.13719	
11	75273154	75273577	5Y-H4K8ac_peak_3549	12.42757	SERPINH1_ENSG00000149257
11	75274084	75274536	5Y-H4K8ac_peak_3550	6.10343	SERPINH1_ENSG00000149257
11	75293979	75294962	5Y-H4K8ac_peak_3551	23.51162	CTD-2530H12.4_ENSG00000255326
11	75295179	75295643	5Y-H4K8ac_peak_3552	6.77436	CTD-2530H12.4_ENSG00000255326
11	75336108	75336314	5Y-H4K8ac_peak_3553	5.65584	
11	75338863	75339682	5Y-H4K8ac_peak_3554	9.15007	
11	75378386	75378649	5Y-H4K8ac_peak_3555	5.70472	
11	75417599	75417840	5Y-H4K8ac_peak_3556	8.564	
11	75425213	75425441	5Y-H4K8ac_peak_3557	5.34938	
11	75444288	75444763	5Y-H4K8ac_peak_3558	8.2913	
11	75479146	75480042	5Y-H4K8ac_peak_3559	16.94054	CTD-2530H12.1_ENSG00000247867
11	75871722	75872964	5Y-H4K8ac_peak_3560	9.51254	
11	75873158	75873641	5Y-H4K8ac_peak_3561	5.23083	
11	75891705	75891931	5Y-H4K8ac_peak_3562	5.74859	
11	75945122	75945319	5Y-H4K8ac_peak_3563	7.59101	
11	75945641	75946018	5Y-H4K8ac_peak_3564	7.11863	
11	76310382	76310646	5Y-H4K8ac_peak_3565	7.30348	
11	76314063	76314454	5Y-H4K8ac_peak_3566	6.17973	
11	76314786	76315388	5Y-H4K8ac_peak_3567	11.99586	
11	76321875	76322342	5Y-H4K8ac_peak_3568	6.77436	
11	76326798	76327169	5Y-H4K8ac_peak_3569	7.01364	
11	76382127	76382331	5Y-H4K8ac_peak_3570	3.99744	LRRC32_ENSG00000137507
11	76432642	76432894	5Y-H4K8ac_peak_3571	8.97839	
11	76494413	76494835	5Y-H4K8ac_peak_3572	11.36989	RP11-21L23.3_ENSG00000255100
11	76777095	76777307	5Y-H4K8ac_peak_3573	7.25081	CAPN5_ENSG00000149260
11	76784542	76785686	5Y-H4K8ac_peak_3574	11.1169	
11	76793886	76794122	5Y-H4K8ac_peak_3575	6.73385	
11	76837847	76838477	5Y-H4K8ac_peak_3576	8.2913	MYO7A_ENSG00000137474
11	76839357	76839623	5Y-H4K8ac_peak_3577	13.2534	MYO7A_ENSG00000137474
11	77184328	77184729	5Y-H4K8ac_peak_3578	11.53136	PAK1_ENSG00000149269;DKFZP434E1119_ENSG00000268635
11	77348490	77348773	5Y-H4K8ac_peak_3579	7.59101	CLNS1A_ENSG00000074201
11	77531324	77531799	5Y-H4K8ac_peak_3580	7.72887	RSF1_ENSG00000048649;AAMDC_ENSG00000087884
11	77741133	77741589	5Y-H4K8ac_peak_3581	8.27399	
11	77850316	77850597	5Y-H4K8ac_peak_3582	8.24461	ALG8_ENSG00000159063;KCTD21-AS1_ENSG00000246174
11	78063161	78063352	5Y-H4K8ac_peak_3583	6.31818	
11	78064284	78064506	5Y-H4K8ac_peak_3584	8.17203	
11	78128185	78128789	5Y-H4K8ac_peak_3585	9.88125	GAB2_ENSG00000033327
11	78129129	78129412	5Y-H4K8ac_peak_3586	4.55128	GAB2_ENSG00000033327

11	78131000	78131425	5Y-H4K8ac_peak_3587	11.32351	
11	78285540	78285733	5Y-H4K8ac_peak_3588	4.95697	NARS2_ENSG00000137513
11	78448229	78448468	5Y-H4K8ac_peak_3589	5.65584	
11	78450917	78451230	5Y-H4K8ac_peak_3590	13.63503	
11	78453656	78453931	5Y-H4K8ac_peak_3591	5.35202	
11	78563438	78563927	5Y-H4K8ac_peak_3592	8.43511	
11	78652499	78652885	5Y-H4K8ac_peak_3593	7.09444	
11	78655558	78655765	5Y-H4K8ac_peak_3594	5.38149	
11	78661104	78661391	5Y-H4K8ac_peak_3595	5.98695	
11	78661996	78662188	5Y-H4K8ac_peak_3596	4.03329	
11	78692726	78692936	5Y-H4K8ac_peak_3597	6.94416	
11	78694100	78694522	5Y-H4K8ac_peak_3598	5.87382	
11	78701380	78701641	5Y-H4K8ac_peak_3599	6.34046	
11	78729984	78730532	5Y-H4K8ac_peak_3600	5.65584	
11	78757160	78757557	5Y-H4K8ac_peak_3601	9.58986	
11	78773795	78774003	5Y-H4K8ac_peak_3602	5.56894	
11	78788892	78789217	5Y-H4K8ac_peak_3603	5.35202	
11	78790291	78790516	5Y-H4K8ac_peak_3604	8.71064	
11	78798831	78799089	5Y-H4K8ac_peak_3605	8.43511	
11	78799319	78799515	5Y-H4K8ac_peak_3606	5.65765	
11	79148581	79148962	5Y-H4K8ac_peak_3607	7.89142	
11	82444636	82444868	5Y-H4K8ac_peak_3608	7.11863	FAM181B_ENSG00000182103
11	82461598	82461822	5Y-H4K8ac_peak_3609	9.38203	
11	82502836	82503051	5Y-H4K8ac_peak_3610	7.76232	RP11-718B12.2_ENSG00000255382
11	85566285	85566487	5Y-H4K8ac_peak_3611	8.47164	AP000974.1_ENSG00000215504;CCDC83_ENSG00000150676
11	86013399	86013716	5Y-H4K8ac_peak_3612	7.97699	C11orf73_ENSG00000149196
11	86383575	86383776	5Y-H4K8ac_peak_3613	5.65584	ME3_ENSG00000151376
11	86748415	86748710	5Y-H4K8ac_peak_3614	8.21582	TMEM135_ENSG00000166575
11	88070605	88070836	5Y-H4K8ac_peak_3615	6.22669	CTSC_ENSG00000109861
11	89224389	89224813	5Y-H4K8ac_peak_3616	5.65584	
11	89955787	89956123	5Y-H4K8ac_peak_3617	10.79063	CHORDC1_ENSG00000110172
11	92930779	92931033	5Y-H4K8ac_peak_3618	6.08523	SLC36A4_ENSG00000180773
11	93063750	93064116	5Y-H4K8ac_peak_3619	14.59308	CCDC67_ENSG00000165325
11	93517517	93518081	5Y-H4K8ac_peak_3620	17.04999	TAF1D_ENSG00000166012;MED17_ENSG00000042429
11	93583078	93583720	5Y-H4K8ac_peak_3621	7.89273	VSTM5_ENSG00000214376
11	93861466	93861788	5Y-H4K8ac_peak_3622	5.81062	PANX1_ENSG00000110218
11	93862068	93862549	5Y-H4K8ac_peak_3623	5.64909	PANX1_ENSG00000110218
11	94501785	94502195	5Y-H4K8ac_peak_3624	5.87725	
11	94800215	94800906	5Y-H4K8ac_peak_3625	13.04415	SRSF8_ENSG00000180771;SRSF8_ENSG00000271885
11	94822637	94822829	5Y-H4K8ac_peak_3626	8.35139	ENDOD1_ENSG00000149218
11	95656954	95657230	5Y-H4K8ac_peak_3627	16.01457	
11	95976430	95976676	5Y-H4K8ac_peak_3628	7.3889	
11	103197061	103197307	5Y-H4K8ac_peak_3629	7.38046	
11	103398069	103398283	5Y-H4K8ac_peak_3630	7.31815	
11	103406988	103407644	5Y-H4K8ac_peak_3631	9.83437	
11	105948582	105948783	5Y-H4K8ac_peak_3632	6.43775	KBTBD3_ENSG00000182359

11	107328290	107328501	5Y-H4K8ac_peak_3633	10.19948	CWF19L2_ENSG00000152404
11	107879698	107880349	5Y-H4K8ac_peak_3634	25.80181	CUL5_ENSG00000166266
11	108336963	108337245	5Y-H4K8ac_peak_3635	5.40331	
11	108369331	108369643	5Y-H4K8ac_peak_3636	7.34185	KDELC2_ENSG00000178202
11	109963884	109964589	5Y-H4K8ac_peak_3637	7.64648	ZC3H12C_ENSG00000149289
11	110166671	110167280	5Y-H4K8ac_peak_3638	8.14883	RDX_ENSG00000137710
11	111101560	111101855	5Y-H4K8ac_peak_3639	7.30348	
11	111383325	111383593	5Y-H4K8ac_peak_3640	6.49308	BTG4_ENSG00000137707;MIR34B_ENSG00000207811;MIR34C_ENSG00000207562
11	111750051	111750491	5Y-H4K8ac_peak_3641	5.35202	ALG9_ENSG00000258529;C11orf1_ENSG00000137720
11	111895846	111896356	5Y-H4K8ac_peak_3642	9.30206	DLAT_ENSG00000150768
11	111945293	111945486	5Y-H4K8ac_peak_3643	8.2913	PIH1D2_ENSG00000150773;C11orf57_ENSG00000150776
11	111957231	111957459	5Y-H4K8ac_peak_3644	9.38276	TIMM8B_ENSG00000150779;SDHD_ENSG00000204370;SDHD_ENSG00000255292
11	112160497	112160776	5Y-H4K8ac_peak_3645	6.1654	
11	112647775	112648349	5Y-H4K8ac_peak_3646	11.1169	
11	112704895	112705139	5Y-H4K8ac_peak_3647	4.84193	
11	112705894	112706133	5Y-H4K8ac_peak_3648	6.38173	
11	112707063	112707334	5Y-H4K8ac_peak_3649	5.70472	
11	112886224	112886591	5Y-H4K8ac_peak_3650	6.34046	
11	113063723	113064194	5Y-H4K8ac_peak_3651	4.84727	
11	113185413	113185818	5Y-H4K8ac_peak_3652	6.37023	RP11-839D17.3_ENSG00000255129;TTC12_ENSG00000149292
11	113241318	113241522	5Y-H4K8ac_peak_3653	7.21112	
11	113308271	113308467	5Y-H4K8ac_peak_3654	4.07874	
11	113318313	113319076	5Y-H4K8ac_peak_3655	6.24411	
11	113319286	113319692	5Y-H4K8ac_peak_3656	4.84727	
11	113420442	113420841	5Y-H4K8ac_peak_3657	9.15007	
11	113448115	113448331	5Y-H4K8ac_peak_3658	7.46829	
11	113461129	113461334	5Y-H4K8ac_peak_3659	4.95697	
11	113644031	113644355	5Y-H4K8ac_peak_3660	5.40331	ZW10_ENSG00000086827
11	114270841	114271105	5Y-H4K8ac_peak_3661	7.93983	C11orf71_ENSG00000180425;RBM7_ENSG00000076053;RP11-212D19.4_ENSG00000255663
11	115451404	115451607	5Y-H4K8ac_peak_3662	4.642	
11	115452585	115452896	5Y-H4K8ac_peak_3663	6.39626	RP11-136I14.5_ENSG00000255689
11	115477389	115477654	5Y-H4K8ac_peak_3664	4.07874	
11	115529855	115530769	5Y-H4K8ac_peak_3665	11.19336	AP000797.3_ENSG00000256717
11	115540502	115540839	5Y-H4K8ac_peak_3666	6.94416	
11	115541041	115541279	5Y-H4K8ac_peak_3667	7.01266	
11	115608627	115609000	5Y-H4K8ac_peak_3668	5.84208	
11	115615491	115615865	5Y-H4K8ac_peak_3669	5.34199	
11	115617785	115618079	5Y-H4K8ac_peak_3670	5.64982	
11	115619492	115619822	5Y-H4K8ac_peak_3671	7.87406	
11	115932872	115933196	5Y-H4K8ac_peak_3672	9.32595	
11	115935675	115935953	5Y-H4K8ac_peak_3673	8.97183	
11	115949120	115949403	5Y-H4K8ac_peak_3674	6.77436	
11	116051134	116051392	5Y-H4K8ac_peak_3675	11.1169	
11	116064255	116064534	5Y-H4K8ac_peak_3676	9.22011	
11	116078464	116079249	5Y-H4K8ac_peak_3677	11.1169	
11	116147535	116147991	5Y-H4K8ac_peak_3678	4.15658	

11	116182070	116182379	5Y-H4K8ac_peak_3679	5.18558	
11	116182713	116183675	5Y-H4K8ac_peak_3680	5.98695	
11	116184133	116184388	5Y-H4K8ac_peak_3681	7.37325	
11	116200323	116200565	5Y-H4K8ac_peak_3682	7.25081	
11	116254377	116254616	5Y-H4K8ac_peak_3683	9.48616	
11	116255496	116255731	5Y-H4K8ac_peak_3684	6.34245	
11	116256544	116256760	5Y-H4K8ac_peak_3685	5.67517	
11	116293793	116294021	5Y-H4K8ac_peak_3686	8.79957	
11	116294831	116295208	5Y-H4K8ac_peak_3687	8.73227	
11	116299362	116300346	5Y-H4K8ac_peak_3688	7.09658	
11	116369026	116369370	5Y-H4K8ac_peak_3689	5.96059	
11	116370819	116371250	5Y-H4K8ac_peak_3690	6.34245	AP001891.1_ENSG00000236437
11	116373217	116373678	5Y-H4K8ac_peak_3691	10.4689	
11	116375417	116375835	5Y-H4K8ac_peak_3692	6.67135	
11	116427875	116428212	5Y-H4K8ac_peak_3693	5.98695	
11	116430845	116431079	5Y-H4K8ac_peak_3694	9.23159	
11	116511866	116512079	5Y-H4K8ac_peak_3695	4.79585	
11	116512271	116512786	5Y-H4K8ac_peak_3696	12.24192	
11	116574446	116574990	5Y-H4K8ac_peak_3697	7.01266	
11	116643364	116643656	5Y-H4K8ac_peak_3698	8.24461	BUD13_ENSG00000137656;AP006216.10_ENSG00000226645
11	116662248	116662505	5Y-H4K8ac_peak_3699	9.01738	APOA5_ENSG00000110243
11	116699534	116700179	5Y-H4K8ac_peak_3700	10.8165	APOC3_ENSG00000110245
11	117103045	117103242	5Y-H4K8ac_peak_3701	8.43511	PCSK7_ENSG00000160613;RNF214_ENSG00000167257
11	117103726	117103969	5Y-H4K8ac_peak_3702	9.23159	PCSK7_ENSG00000160613;RNF214_ENSG00000167257
11	117281658	117281894	5Y-H4K8ac_peak_3703	7.38046	
11	117313651	117315108	5Y-H4K8ac_peak_3704	19.93272	
11	117315713	117316325	5Y-H4K8ac_peak_3705	11.1169	
11	117360503	117360757	5Y-H4K8ac_peak_3706	6.77436	
11	117361607	117361875	5Y-H4K8ac_peak_3707	6.27097	
11	117406945	117407163	5Y-H4K8ac_peak_3708	6.62622	
11	117489827	117490106	5Y-H4K8ac_peak_3709	4.07874	
11	117534056	117534255	5Y-H4K8ac_peak_3710	4.29586	
11	117595797	117596046	5Y-H4K8ac_peak_3711	7.89273	
11	117679766	117680098	5Y-H4K8ac_peak_3712	5.03335	
11	117687007	117688008	5Y-H4K8ac_peak_3713	7.44355	DSCAML1_ENSG00000177103
11	118016392	118016910	5Y-H4K8ac_peak_3714	7.35333	
11	118122614	118123081	5Y-H4K8ac_peak_3715	7.89273	MPZL3_ENSG00000160588
11	118271536	118271925	5Y-H4K8ac_peak_3716	7.59101	RP11-770J1.5_ENSG00000254873;ATP5L_ENSG00000167283
11	118272369	118272564	5Y-H4K8ac_peak_3717	6.77436	RP11-770J1.5_ENSG00000254873;ATP5L_ENSG00000167283
11	118305577	118305993	5Y-H4K8ac_peak_3718	17.50049	RP11-770J1.4_ENSG00000255384
11	118401274	118401794	5Y-H4K8ac_peak_3719	8.2913	RP11-770J1.3_ENSG00000255435;TMEM25_ENSG00000149582
11	118436438	118436637	5Y-H4K8ac_peak_3720	7.31102	
11	118437012	118437223	5Y-H4K8ac_peak_3721	6.1654	
11	118443178	118443445	5Y-H4K8ac_peak_3722	7.90751	IFT46_ENSG00000118096;ARCN1_ENSG00000095139
11	118493791	118494380	5Y-H4K8ac_peak_3723	5.64909	
11	118560151	118560405	5Y-H4K8ac_peak_3724	8.43511	AP002954.6_ENSG00000255239

11	118561355	118561769	5Y-H4K8ac_peak_3725	7.92287	AP002954.6_ENSG00000255239
11	118576802	118577290	5Y-H4K8ac_peak_3726	7.76232	
11	118661378	118662577	5Y-H4K8ac_peak_3727	9.00954	DDX6_ENSG00000110367
11	118691177	118691386	5Y-H4K8ac_peak_3728	7.38046	
11	118778028	118778488	5Y-H4K8ac_peak_3729	9.63153	
11	118780330	118780605	5Y-H4K8ac_peak_3730	4.642	MIR4492_ENSG00000264211
11	118789464	118789661	5Y-H4K8ac_peak_3731	6.73385	
11	118868865	118869334	5Y-H4K8ac_peak_3732	9.38276	RP11-110I1.12_ENSG00000255121;CCDC84_ENSG00000186166
11	118927587	118927786	5Y-H4K8ac_peak_3733	5.64909	HYOU1_ENSG00000149428
11	118937700	118938067	5Y-H4K8ac_peak_3734	6.77436	RP11-110I1.13_ENSG00000272186;VPS11_ENSG00000160695
11	118966781	118967011	5Y-H4K8ac_peak_3735	11.28133	H2AFX_ENSG00000188486
11	118977604	118977801	5Y-H4K8ac_peak_3736	8.1667	
11	119019659	119019861	5Y-H4K8ac_peak_3737	4.90696	ABCG4_ENSG00000172350
11	119039539	119040224	5Y-H4K8ac_peak_3738	9.02938	
11	119205277	119205626	5Y-H4K8ac_peak_3739	8.2913	RNF26_ENSG00000173456
11	119210419	119210669	5Y-H4K8ac_peak_3740	4.52203	
11	119210910	119211241	5Y-H4K8ac_peak_3741	6.98416	
11	119238174	119238404	5Y-H4K8ac_peak_3742	4.70501	
11	119239659	119239924	5Y-H4K8ac_peak_3743	9.24789	
11	119246015	119247029	5Y-H4K8ac_peak_3744	8.04693	
11	119294105	119294679	5Y-H4K8ac_peak_3745	5.87725	
11	119438338	119438528	5Y-H4K8ac_peak_3746	5.40331	
11	119454802	119455008	5Y-H4K8ac_peak_3747	5.91107	
11	119514682	119515092	5Y-H4K8ac_peak_3748	7.04637	
11	119542152	119542532	5Y-H4K8ac_peak_3749	5.64909	
11	119543171	119543605	5Y-H4K8ac_peak_3750	7.24967	
11	119554550	119555000	5Y-H4K8ac_peak_3751	6.37023	
11	119555223	119555584	5Y-H4K8ac_peak_3752	7.41197	
11	119555944	119556417	5Y-H4K8ac_peak_3753	9.30505	
11	119571520	119571788	5Y-H4K8ac_peak_3754	7.01266	
11	119572108	119572300	5Y-H4K8ac_peak_3755	6.43775	
11	119573586	119574112	5Y-H4K8ac_peak_3756	9.56516	
11	119575162	119575601	5Y-H4K8ac_peak_3757	9.56516	
11	119591772	119592097	5Y-H4K8ac_peak_3758	5.47303	
11	119594099	119594306	5Y-H4K8ac_peak_3759	6.61077	
11	119600523	119600723	5Y-H4K8ac_peak_3760	7.58806	PVRL1_ENSG00000110400;CTD-2523D13.2_ENSG00000254854
11	119613521	119613868	5Y-H4K8ac_peak_3761	6.69752	
11	119620759	119620952	5Y-H4K8ac_peak_3762	5.65584	
11	119635088	119635888	5Y-H4K8ac_peak_3763	4.95697	
11	119649313	119649953	5Y-H4K8ac_peak_3764	7.50148	
11	119650553	119651418	5Y-H4K8ac_peak_3765	11.64182	
11	119653310	119653517	5Y-H4K8ac_peak_3766	7.11863	
11	119658015	119658297	5Y-H4K8ac_peak_3767	5.94703	
11	119659832	119660265	5Y-H4K8ac_peak_3768	6.12014	
11	119667270	119667649	5Y-H4K8ac_peak_3769	6.77436	
11	119702733	119703098	5Y-H4K8ac_peak_3770	6.95541	

11	119736197	119736407	5Y-H4K8ac_peak_3771	8.2913	
11	119746543	119746770	5Y-H4K8ac_peak_3772	9.05168	
11	119760262	119760672	5Y-H4K8ac_peak_3773	9.87097	
11	119761344	119761599	5Y-H4K8ac_peak_3774	7.31674	
11	119779168	119779443	5Y-H4K8ac_peak_3775	13.72401	
11	119884249	119884532	5Y-H4K8ac_peak_3776	6.78318	
11	120039603	120039810	5Y-H4K8ac_peak_3777	7.89273	AP000679.2_ENSG00000176984
11	120040111	120040302	5Y-H4K8ac_peak_3778	9.00954	AP000679.2_ENSG00000176984
11	120055109	120055483	5Y-H4K8ac_peak_3779	4.15658	TRIM29_ENSG00000137699
11	120056996	120057900	5Y-H4K8ac_peak_3780	6.53157	TRIM29_ENSG00000137699
11	120064069	120064282	5Y-H4K8ac_peak_3781	5.23083	
11	120081013	120081248	5Y-H4K8ac_peak_3782	8.2913	OAF_ENSG00000184232
11	120081799	120082122	5Y-H4K8ac_peak_3783	8.2913	OAF_ENSG00000184232
11	120195639	120196339	5Y-H4K8ac_peak_3784	21.88077	TMEM136_ENSG00000181264
11	120206527	120206821	5Y-H4K8ac_peak_3785	4.0639	ARHGEF12_ENSG00000196914
11	120207167	120207429	5Y-H4K8ac_peak_3786	11.22005	ARHGEF12_ENSG00000196914
11	120495023	120495241	5Y-H4K8ac_peak_3787	3.95677	
11	120611000	120611212	5Y-H4K8ac_peak_3788	5.41472	
11	120618817	120619223	5Y-H4K8ac_peak_3789	5.57299	
11	120656591	120656913	5Y-H4K8ac_peak_3790	5.37237	
11	120769099	120769357	5Y-H4K8ac_peak_3791	6.37023	
11	120795631	120795847	5Y-H4K8ac_peak_3792	7.97699	
11	120803641	120803888	5Y-H4K8ac_peak_3793	4.93618	
11	120824130	120824622	5Y-H4K8ac_peak_3794	6.54441	
11	120856588	120856856	5Y-H4K8ac_peak_3795	5.98695	
11	120894847	120895058	5Y-H4K8ac_peak_3796	9.30505	TBCEL_ENSG00000154114
11	121323071	121323320	5Y-H4K8ac_peak_3797	13.04415	RP11-730K11.1_ENSG00000246790;SORL1_ENSG00000137642
11	121336432	121336783	5Y-H4K8ac_peak_3798	8.21582	
11	121526592	121526834	5Y-H4K8ac_peak_3799	12.10416	
11	121527256	121527855	5Y-H4K8ac_peak_3800	15.82713	
11	121543723	121543968	5Y-H4K8ac_peak_3801	9.62747	
11	121593589	121594250	5Y-H4K8ac_peak_3802	10.19948	
11	122029875	122030100	5Y-H4K8ac_peak_3803	7.31815	
11	122376041	122376446	5Y-H4K8ac_peak_3804	7.38046	
11	122386797	122387072	5Y-H4K8ac_peak_3805	7.21112	
11	122395883	122396138	5Y-H4K8ac_peak_3806	6.34046	
11	122753731	122754101	5Y-H4K8ac_peak_3807	6.43775	C11orf63_ENSG00000109944
11	122855193	122855408	5Y-H4K8ac_peak_3808	5.41472	
11	122933454	122933782	5Y-H4K8ac_peak_3809	9.74838	HSPA8_ENSG00000109971
11	123008026	123008445	5Y-H4K8ac_peak_3810	8.4454	RP11-762B21.5_ENSG00000255342
11	123065386	123066269	5Y-H4K8ac_peak_3811	11.22005	CLMP_ENSG00000166250
11	123066595	123067052	5Y-H4K8ac_peak_3812	4.642	CLMP_ENSG00000166250
11	123072849	123073335	5Y-H4K8ac_peak_3813	5.87382	
11	123073686	123073894	5Y-H4K8ac_peak_3814	4.95697	
11	123131952	123132426	5Y-H4K8ac_peak_3815	7.49746	
11	123229172	123229582	5Y-H4K8ac_peak_3816	6.1654	

11	123348728	123348936	5Y-H4K8ac_peak_3817	6.77436	
11	123380766	123381107	5Y-H4K8ac_peak_3818	5.87725	
11	123389674	123389940	5Y-H4K8ac_peak_3819	9.43096	
11	123553584	123553785	5Y-H4K8ac_peak_3820	4.84727	
11	123612545	123612841	5Y-H4K8ac_peak_3821	5.98695	ZNF202_ENSG00000166261
11	124543244	124543551	5Y-H4K8ac_peak_3822	6.37023	SPA17_ENSG00000064199
11	124588297	124588487	5Y-H4K8ac_peak_3823	9.60627	
11	124616090	124616513	5Y-H4K8ac_peak_3824	6.50117	RP11-677M14.2_ENSG00000255045
11	124669561	124670829	5Y-H4K8ac_peak_3825	9.30505	MSANTD2_ENSG00000120458;RP11-677M14.7_ENSG00000245498
11	124712844	124713333	5Y-H4K8ac_peak_3826	8.30301	AP000866.1_ENSG00000215942
11	124732738	124732940	5Y-H4K8ac_peak_3827	7.49746	
11	124745836	124746514	5Y-H4K8ac_peak_3828	12.05638	
11	124747058	124747373	5Y-H4K8ac_peak_3829	6.50117	
11	124790250	124790491	5Y-H4K8ac_peak_3830	14.12043	
11	124933500	124933741	5Y-H4K8ac_peak_3831	6.50117	SLC37A2_ENSG00000134955
11	125274552	125274838	5Y-H4K8ac_peak_3832	4.71803	
11	125365244	125365460	5Y-H4K8ac_peak_3833	5.35202	FEZ1_ENSG00000149557;AP000708.1_ENSG00000255537
11	125461809	125462096	5Y-H4K8ac_peak_3834	5.98695	STT3A-AS1_ENSG00000254671;STT3A_ENSG00000134910
11	125757648	125757991	5Y-H4K8ac_peak_3835	11.01287	
11	125758288	125758510	5Y-H4K8ac_peak_3836	4.07874	
11	125774402	125774813	5Y-H4K8ac_peak_3837	9.78792	
11	125820688	125820956	5Y-H4K8ac_peak_3838	10.16277	RP11-680F20.6_ENSG00000254967
11	125821771	125822062	5Y-H4K8ac_peak_3839	4.76429	
11	125932458	125932660	5Y-H4K8ac_peak_3840	5.35202	CDON_ENSG00000064309
11	125932972	125933659	5Y-H4K8ac_peak_3841	12.49811	CDON_ENSG00000064309
11	125980724	125980923	5Y-H4K8ac_peak_3842	6.22904	
11	125981744	125982442	5Y-H4K8ac_peak_3843	10.60083	
11	125982843	125983204	5Y-H4K8ac_peak_3844	6.78128	
11	125983397	125983638	5Y-H4K8ac_peak_3845	10.86711	
11	125983956	125984505	5Y-H4K8ac_peak_3846	6.78318	
11	125984824	125985093	5Y-H4K8ac_peak_3847	14.65178	
11	125985632	125985831	5Y-H4K8ac_peak_3848	4.642	
11	126011203	126011519	5Y-H4K8ac_peak_3849	7.04158	
11	126152696	126153262	5Y-H4K8ac_peak_3850	12.05638	TIRAP_ENSG00000150455
11	126173634	126173826	5Y-H4K8ac_peak_3851	5.24695	RP11-712L6.5_ENSG00000255062;DCPS_ENSG00000110063
11	126224879	126225410	5Y-H4K8ac_peak_3852	19.00192	ST3GAL4_ENSG00000110080
11	126225615	126225852	5Y-H4K8ac_peak_3853	9.63153	ST3GAL4_ENSG00000110080
11	126264381	126264576	5Y-H4K8ac_peak_3854	5.99504	
11	126312558	126312847	5Y-H4K8ac_peak_3855	9.51254	
11	126313571	126313796	5Y-H4K8ac_peak_3856	5.12213	
11	126349754	126350017	5Y-H4K8ac_peak_3857	5.51139	
11	126350351	126350760	5Y-H4K8ac_peak_3858	16.97602	
11	126388827	126389108	5Y-H4K8ac_peak_3859	12.52787	
11	126391185	126391408	5Y-H4K8ac_peak_3860	5.77617	
11	126413488	126414127	5Y-H4K8ac_peak_3861	19.39444	KIRREL3-AS1_ENSG00000257271
11	126728730	126729204	5Y-H4K8ac_peak_3862	5.29015	

11	126859946	126860203	5Y-H4K8ac_peak_3863	7.34185	
11	126872899	126873376	5Y-H4K8ac_peak_3864	5.64909	KIRREL3_ENSG00000149571;KIRREL3-AS3_ENSG00000218109;AP002833.1_ENSG00000256527
11	126873664	126874031	5Y-H4K8ac_peak_3865	10.54764	KIRREL3_ENSG00000149571;KIRREL3-AS3_ENSG00000218109;AP002833.1_ENSG00000256527
11	127052273	127052671	5Y-H4K8ac_peak_3866	5.40331	
11	127063719	127063984	5Y-H4K8ac_peak_3867	6.43775	
11	127923336	127923530	5Y-H4K8ac_peak_3868	6.00388	
11	128106824	128107481	5Y-H4K8ac_peak_3869	16.74969	
11	128291019	128292021	5Y-H4K8ac_peak_3870	6.40964	
11	128325451	128325915	5Y-H4K8ac_peak_3871	6.50117	
11	128326201	128326713	5Y-H4K8ac_peak_3872	9.15977	
11	128418424	128418770	5Y-H4K8ac_peak_3873	23.16993	
11	128646683	128646993	5Y-H4K8ac_peak_3874	7.50148	
11	129620408	129620780	5Y-H4K8ac_peak_3875	5.56912	
11	129685585	129686115	5Y-H4K8ac_peak_3876	9.51254	TMEM45B_ENSG00000151715
11	129838556	129838933	5Y-H4K8ac_peak_3877	6.94416	
11	129871633	129872320	5Y-H4K8ac_peak_3878	14.72547	PRDM10_ENSG00000170325;LINC00167_ENSG00000233220
11	129872593	129872818	5Y-H4K8ac_peak_3879	5.91107	PRDM10_ENSG00000170325;LINC00167_ENSG00000233220
11	130185669	130185867	5Y-H4K8ac_peak_3880	9.78792	RP11-121M22.1_ENSG00000175773
11	130785706	130786281	5Y-H4K8ac_peak_3881	6.94416	SNX19_ENSG00000120451
11	133098495	133098752	5Y-H4K8ac_peak_3882	7.89142	
11	133797906	133798112	5Y-H4K8ac_peak_3883	7.387	
11	133805393	133805831	5Y-H4K8ac_peak_3884	8.5915	
11	133806443	133806739	5Y-H4K8ac_peak_3885	8.62703	
11	133807663	133807883	5Y-H4K8ac_peak_3886	5.32103	
11	133815448	133815743	5Y-H4K8ac_peak_3887	5.23083	
11	133818716	133818943	5Y-H4K8ac_peak_3888	4.15658	
11	133827254	133827574	5Y-H4K8ac_peak_3889	6.64036	IGSF9B_ENSG00000080854;AP000911.1_ENSG00000264674
11	133835787	133836598	5Y-H4K8ac_peak_3890	8.2913	
11	133902567	133903304	5Y-H4K8ac_peak_3891	6.08523	RP11-713P17.3_ENSG00000204241
11	133904588	133905051	5Y-H4K8ac_peak_3892	7.50081	
11	133990931	133991279	5Y-H4K8ac_peak_3893	6.14981	
11	134146807	134147154	5Y-H4K8ac_peak_3894	4.29586	
11	134281671	134281892	5Y-H4K8ac_peak_3895	10.4326	B3GAT1_ENSG00000109956
11	134443184	134443512	5Y-H4K8ac_peak_3896	7.35333	
11	134874864	134875145	5Y-H4K8ac_peak_3897	5.59843	
12	73701	74098	5Y-H4K8ac_peak_3898	21.56204	AC215219.1_ENSG00000238823;ABC7-42389800N19.1_ENSG00000226210
12	74600	74793	5Y-H4K8ac_peak_3899	10.46287	ABC7-42389800N19.1_ENSG00000226210
12	187056	187258	5Y-H4K8ac_peak_3900	6.79955	
12	191950	192285	5Y-H4K8ac_peak_3901	5.83608	
12	281691	282572	5Y-H4K8ac_peak_3902	7.89273	
12	284187	284830	5Y-H4K8ac_peak_3903	6.27467	
12	285175	285751	5Y-H4K8ac_peak_3904	9.02782	
12	286614	287269	5Y-H4K8ac_peak_3905	14.26354	
12	312601	312884	5Y-H4K8ac_peak_3906	7.11863	RP11-283I3.2_ENSG00000256577
12	329449	329676	5Y-H4K8ac_peak_3907	5.51139	
12	498926	499197	5Y-H4K8ac_peak_3908	7.3889	KDM5A_ENSG00000073614;CCDC77_ENSG00000120647

12	569653	570131	5Y-H4K8ac_peak_3909	5.23819	B4GALNT3_ENSG00000139044
12	685044	685420	5Y-H4K8ac_peak_3910	9.00954	
12	701497	701826	5Y-H4K8ac_peak_3911	13.76914	
12	768493	768794	5Y-H4K8ac_peak_3912	7.05631	
12	861876	862075	5Y-H4K8ac_peak_3913	4.51497	WNK1_ENSG00000060237
12	1058556	1058797	5Y-H4K8ac_peak_3914	9.23159	
12	1059309	1059579	5Y-H4K8ac_peak_3915	7.63144	
12	1099685	1099882	5Y-H4K8ac_peak_3916	4.0639	RAD52_ENSG00000002016;RP11-359B12.2_ENSG00000250132;ERC1_ENSG00000082805
12	1640450	1640665	5Y-H4K8ac_peak_3917	7.04637	
12	1703305	1703521	5Y-H4K8ac_peak_3918	8.2913	FBXL14_ENSG00000171823
12	1739093	1739515	5Y-H4K8ac_peak_3919	7.18391	
12	1770631	1770926	5Y-H4K8ac_peak_3920	14.24171	
12	1771163	1771490	5Y-H4K8ac_peak_3921	12.31844	
12	1905632	1906015	5Y-H4K8ac_peak_3922	5.87725	
12	1909886	1910202	5Y-H4K8ac_peak_3923	6.73385	
12	1913935	1914533	5Y-H4K8ac_peak_3924	6.34245	
12	1935742	1935984	5Y-H4K8ac_peak_3925	5.23083	
12	1939365	1939613	5Y-H4K8ac_peak_3926	7.31102	
12	1945249	1946889	5Y-H4K8ac_peak_3927	9.00954	
12	1947973	1948561	5Y-H4K8ac_peak_3928	9.95485	
12	2033196	2033460	5Y-H4K8ac_peak_3929	4.86522	
12	2113238	2113602	5Y-H4K8ac_peak_3930	15.19225	DCP1B_ENSG00000151065;RP5-1096D14.6_ENSG00000203593
12	2144238	2144762	5Y-H4K8ac_peak_3931	11.1169	
12	2322192	2322418	5Y-H4K8ac_peak_3932	6.13909	
12	2348534	2348739	5Y-H4K8ac_peak_3933	8.4454	
12	2392861	2393220	5Y-H4K8ac_peak_3934	7.56545	
12	2893021	2893213	5Y-H4K8ac_peak_3935	6.37023	
12	2985747	2986102	5Y-H4K8ac_peak_3936	5.317	FOXM1_ENSG00000111206;RHNO1_ENSG00000171792;TULP3_ENSG00000078246
12	2999102	2999375	5Y-H4K8ac_peak_3937	6.86362	
12	3085748	3085990	5Y-H4K8ac_peak_3938	6.17374	
12	3185924	3186380	5Y-H4K8ac_peak_3939	15.15055	TSPAN9_ENSG00000011105
12	3206844	3207187	5Y-H4K8ac_peak_3940	4.8509	
12	3213613	3213896	5Y-H4K8ac_peak_3941	6.47245	
12	3214615	3214810	5Y-H4K8ac_peak_3942	9.96543	
12	3314723	3315110	5Y-H4K8ac_peak_3943	14.31711	
12	3333338	3333822	5Y-H4K8ac_peak_3944	6.1654	
12	3373244	3373435	5Y-H4K8ac_peak_3945	5.74859	
12	3412990	3413632	5Y-H4K8ac_peak_3946	7.12953	
12	3451467	3451739	5Y-H4K8ac_peak_3947	8.564	
12	3474855	3475472	5Y-H4K8ac_peak_3948	6.37023	
12	3529822	3530070	5Y-H4K8ac_peak_3949	4.51076	
12	3713371	3714254	5Y-H4K8ac_peak_3950	8.2913	
12	3795968	3796202	5Y-H4K8ac_peak_3951	6.34046	
12	3862000	3862416	5Y-H4K8ac_peak_3952	9.23159	
12	3981821	3982112	5Y-H4K8ac_peak_3953	5.59843	PARP11_ENSG00000111224;RP11-664D1.1_ENSG00000256862
12	4714134	4714459	5Y-H4K8ac_peak_3954	6.50117	RP11-500M8.7_ENSG00000272921

12	6310158	6310355	5Y-H4K8ac_peak_3955	5.13364	
12	6387365	6387913	5Y-H4K8ac_peak_3956	7.97699	
12	6560325	6560517	5Y-H4K8ac_peak_3957	9.05168	CD27-AS1_ENSG00000215039;TAPBPL_ENSG00000139192
12	6601921	6602296	5Y-H4K8ac_peak_3958	8.33296	MRPL51_ENSG00000111639;NCAPD2_ENSG00000010292
12	6642517	6643409	5Y-H4K8ac_peak_3959	15.09867	RP5-940J5.3_ENSG00000255966;GAPDH_ENSG00000111640
12	6722514	6722738	5Y-H4K8ac_peak_3960	18.10232	
12	6723396	6723665	5Y-H4K8ac_peak_3961	9.01738	
12	6797849	6798560	5Y-H4K8ac_peak_3962	22.66	ZNF384_ENSG00000126746
12	6833626	6833867	5Y-H4K8ac_peak_3963	10.11191	COPS7A_ENSG00000111652
12	6863166	6863487	5Y-H4K8ac_peak_3964	6.1654	
12	6874350	6874631	5Y-H4K8ac_peak_3965	4.47077	PTMS_ENSG00000159335
12	6936098	6936523	5Y-H4K8ac_peak_3966	10.60003	
12	6938053	6938365	5Y-H4K8ac_peak_3967	6.78128	LEPREL2_ENSG00000110811
12	6976068	6976259	5Y-H4K8ac_peak_3968	4.50834	TPI1_ENSG00000111669
12	7000681	7000995	5Y-H4K8ac_peak_3969	5.98695	
12	7013340	7013776	5Y-H4K8ac_peak_3970	5.03335	
12	7023179	7023445	5Y-H4K8ac_peak_3971	10.35586	ENO2_ENSG00000111674
12	7046337	7047106	5Y-H4K8ac_peak_3972	18.78631	
12	7047740	7047996	5Y-H4K8ac_peak_3973	8.43511	
12	7052075	7052677	5Y-H4K8ac_peak_3974	7.86017	C12orf57_ENSG00000111678;RNU7-1_ENSG00000238923
12	7053274	7053482	5Y-H4K8ac_peak_3975	10.21117	U47924.31_ENSG00000272173;RNU7-1_ENSG00000238923
12	7071062	7071264	5Y-H4K8ac_peak_3976	4.8509	
12	7079180	7079769	5Y-H4K8ac_peak_3977	10.60083	PHB2_ENSG00000215021
12	7080122	7080758	5Y-H4K8ac_peak_3978	11.19336	PHB2_ENSG00000215021
12	7082391	7082619	5Y-H4K8ac_peak_3979	4.84727	
12	7125751	7125985	5Y-H4K8ac_peak_3980	4.50834	LPCAT3_ENSG00000111684
12	8332957	8333286	5Y-H4K8ac_peak_3981	7.20869	FAM66C_ENSG00000226711
12	9217103	9217385	5Y-H4K8ac_peak_3982	7.31102	LINC00612_ENSG00000214851;A2M-AS1_ENSG00000245105
12	9800476	9800837	5Y-H4K8ac_peak_3983	9.96543	RP11-705C15.4_ENSG00000256442
12	11323809	11324015	5Y-H4K8ac_peak_3984	11.1169	PRR4_ENSG00000111215;TAS2R14_ENSG00000212127;RP11-785H5.2_ENSG00000256712;RP11-785H5.1_ENSG00000256537
12	11698559	11698959	5Y-H4K8ac_peak_3985	5.40331	RNU7-60P_ENSG00000251747
12	11801620	11802056	5Y-H4K8ac_peak_3986	11.12941	ETV6_ENSG00000139083
12	11802856	11803112	5Y-H4K8ac_peak_3987	7.72345	ETV6_ENSG00000139083
12	12419991	12420187	5Y-H4K8ac_peak_3988	8.20773	LRP6_ENSG00000070018
12	12459291	12459481	5Y-H4K8ac_peak_3989	6.34046	
12	12502791	12503032	5Y-H4K8ac_peak_3990	9.63153	MANSC1_ENSG00000111261
12	12664094	12664309	5Y-H4K8ac_peak_3991	4.84727	
12	12714891	12715139	5Y-H4K8ac_peak_3992	5.65584	DUSP16_ENSG00000111266
12	12869645	12869996	5Y-H4K8ac_peak_3993	22.60818	
12	12876761	12877567	5Y-H4K8ac_peak_3994	7.50148	RP11-180M15.4_ENSG00000256658
12	12940019	12940364	5Y-H4K8ac_peak_3995	4.00285	
12	13154488	13154810	5Y-H4K8ac_peak_3996	6.59249	
12	13254512	13254878	5Y-H4K8ac_peak_3997	8.2913	
12	14720388	14720711	5Y-H4K8ac_peak_3998	7.30348	PLBD1_ENSG00000121316;RP11-695J4.2_ENSG00000256751
12	14924327	14924538	5Y-H4K8ac_peak_3999	5.87382	HIST4H4_ENSG00000197837
12	14926645	14926839	5Y-H4K8ac_peak_4000	4.12295	H2AFJ_ENSG00000246705

12	15475618	15476024	5Y-H4K8ac_peak_4001	8.47164	PTPRO_ENSG00000151490
12	15942535	15942819	5Y-H4K8ac_peak_4002	7.34185	
12	16064289	16064496	5Y-H4K8ac_peak_4003	8.43511	DERA_ENSG00000023697
12	19282835	19283687	5Y-H4K8ac_peak_4004	11.93212	PLEKHA5_ENSG00000052126
12	19592598	19592856	5Y-H4K8ac_peak_4005	11.57334	
12	20098058	20098464	5Y-H4K8ac_peak_4006	6.50117	
12	20522215	20522606	5Y-H4K8ac_peak_4007	13.99721	RP11-284H19.1_ENSG00000256879;PDE3A_ENSG00000172572
12	20741632	20742062	5Y-H4K8ac_peak_4008	7.01856	
12	22199213	22199536	5Y-H4K8ac_peak_4009	12.71156	CMAS_ENSG00000111726
12	22696952	22697341	5Y-H4K8ac_peak_4010	5.41472	C2CD5_ENSG00000111731
12	22777423	22778000	5Y-H4K8ac_peak_4011	8.43511	RP11-268P4.4_ENSG00000257023;ETNK1_ENSG00000139163
12	22778270	22778846	5Y-H4K8ac_peak_4012	4.50834	RP11-268P4.4_ENSG00000257023;ETNK1_ENSG00000139163
12	25101475	25102007	5Y-H4K8ac_peak_4013	5.23083	BCAT1_ENSG00000060982;RP11-662I13.2_ENSG00000255921
12	25404189	25404396	5Y-H4K8ac_peak_4014	9.78792	KRAS_ENSG00000133703
12	25803352	25803616	5Y-H4K8ac_peak_4015	7.01856	
12	26266952	26267153	5Y-H4K8ac_peak_4016	5.23083	
12	26348418	26348852	5Y-H4K8ac_peak_4017	4.29586	
12	26379499	26379935	5Y-H4K8ac_peak_4018	17.63051	
12	27006498	27006757	5Y-H4K8ac_peak_4019	4.50834	
12	27396803	27397020	5Y-H4K8ac_peak_4020	7.38046	STK38L_ENSG00000211455
12	27485437	27486277	5Y-H4K8ac_peak_4021	7.89273	ARNTL2_ENSG00000029153
12	27932398	27933561	5Y-H4K8ac_peak_4022	9.02938	KLHL42_ENSG00000087448;RP11-860B13.1_ENSG00000256747
12	28123467	28123662	5Y-H4K8ac_peak_4023	6.1654	
12	29301480	29302693	5Y-H4K8ac_peak_4024	7.89273	RP11-946L16.1_ENSG00000257258;FAR2_ENSG00000064763
12	29533737	29533987	5Y-H4K8ac_peak_4025	5.98695	ERGIC2_ENSG00000087502
12	29936377	29936797	5Y-H4K8ac_peak_4026	7.76232	TMTC1_ENSG00000133687
12	30848940	30849245	5Y-H4K8ac_peak_4027	8.43511	IPO8_ENSG00000133704
12	30949260	30949643	5Y-H4K8ac_peak_4028	9.38203	
12	31078600	31079091	5Y-H4K8ac_peak_4029	7.59101	TSPAN11_ENSG00000110900
12	31079542	31080062	5Y-H4K8ac_peak_4030	4.77126	TSPAN11_ENSG00000110900
12	31226419	31226649	5Y-H4K8ac_peak_4031	7.11863	DDX11-AS1_ENSG00000245614;DDX11_ENSG00000013573
12	31270250	31270547	5Y-H4K8ac_peak_4032	5.65584	
12	31388126	31388317	5Y-H4K8ac_peak_4033	5.59837	
12	31478172	31478526	5Y-H4K8ac_peak_4034	4.3419	AC024940.1_ENSG00000177340
12	32112374	32112803	5Y-H4K8ac_peak_4035	10.69698	KIAA1551_ENSG00000174718
12	32259154	32259568	5Y-H4K8ac_peak_4036	26.24446	RP11-843B15.2_ENSG00000257530;BICD1_ENSG00000151746
12	32831305	32831717	5Y-H4K8ac_peak_4037	9.60627	DNM1L_ENSG00000087470
12	32908327	32908758	5Y-H4K8ac_peak_4038	9.79526	YARS2_ENSG00000139131
12	34175551	34175798	5Y-H4K8ac_peak_4039	11.57334	ALG10_ENSG00000139133
12	34496532	34496838	5Y-H4K8ac_peak_4040	7.38046	
12	37937570	37937838	5Y-H4K8ac_peak_4041	5.25503	RP11-125N22.3_ENSG00000257899;RP11-125N22.1_ENSG00000257933
12	38710599	38711088	5Y-H4K8ac_peak_4042	16.27715	ALG10B_ENSG00000175548
12	39299892	39300546	5Y-H4K8ac_peak_4043	11.57334	CPNE8_ENSG00000139117;RP11-396F22.1_ENSG00000257718
12	39785633	39785855	5Y-H4K8ac_peak_4044	7.53429	
12	40618385	40618871	5Y-H4K8ac_peak_4045	6.53157	AC079630.4_ENSG00000225342
12	41039500	41039704	5Y-H4K8ac_peak_4046	6.20875	

12	42325892	42326325	5Y-H4K8ac_peak_4047	10.1994	RP11-630C16.2_ENSG00000257239
12	42538306	42538501	5Y-H4K8ac_peak_4048	4.29586	GXYLT1_ENSG00000151233
12	44229466	44230187	5Y-H4K8ac_peak_4049	4.77126	TMEM117_ENSG00000139173
12	46004998	46005208	5Y-H4K8ac_peak_4050	4.51076	RP11-352M15.1_ENSG00000257657
12	46122871	46123085	5Y-H4K8ac_peak_4051	4.24332	ARID2_ENSG00000189079
12	46123642	46124488	5Y-H4K8ac_peak_4052	10.22205	ARID2_ENSG00000189079
12	46662926	46663757	5Y-H4K8ac_peak_4053	13.27281	SLC38A1_ENSG00000111371
12	46765970	46766195	5Y-H4K8ac_peak_4054	8.75926	SLC38A2_ENSG00000134294;RP11-474P2.2_ENSG00000258096
12	46776693	46777074	5Y-H4K8ac_peak_4055	22.6507	RP11-96H19.1_ENSG00000257261
12	48111598	48111807	5Y-H4K8ac_peak_4056	5.29015	
12	48131103	48131668	5Y-H4K8ac_peak_4057	8.43511	
12	48134658	48135257	5Y-H4K8ac_peak_4058	13.72401	
12	48212843	48213485	5Y-H4K8ac_peak_4059	15.40182	
12	48213827	48214312	5Y-H4K8ac_peak_4060	8.24461	
12	48222851	48223420	5Y-H4K8ac_peak_4061	11.56948	
12	48357497	48357732	5Y-H4K8ac_peak_4062	4.48815	TMEM106C_ENSG00000134291
12	48384359	48385265	5Y-H4K8ac_peak_4063	7.90236	
12	48499425	48500144	5Y-H4K8ac_peak_4064	14.99673	SENP1_ENSG00000079387;PFKM_ENSG00000152556
12	48500449	48500811	5Y-H4K8ac_peak_4065	6.73047	SENP1_ENSG00000079387
12	48592665	48593010	5Y-H4K8ac_peak_4066	4.70501	DKFZP779L1853_ENSG00000269514
12	49075553	49075895	5Y-H4K8ac_peak_4067	10.1994	KANSL2_ENSG00000139620
12	49110915	49111579	5Y-H4K8ac_peak_4068	12.61054	CCNT1_ENSG00000129315
12	49182917	49183282	5Y-H4K8ac_peak_4069	14.86775	ADCY6_ENSG00000174233;RP11-579D7.4_ENSG00000257660
12	49183707	49183965	5Y-H4K8ac_peak_4070	7.11863	ADCY6_ENSG00000174233;RP11-579D7.4_ENSG00000257660
12	49208086	49208307	5Y-H4K8ac_peak_4071	4.67245	CACNB3_ENSG00000167535
12	49208773	49209013	5Y-H4K8ac_peak_4072	6.96612	
12	49211634	49211916	5Y-H4K8ac_peak_4073	8.46442	
12	49259921	49260116	5Y-H4K8ac_peak_4074	8.2589	RND1_ENSG00000172602
12	49350922	49351184	5Y-H4K8ac_peak_4075	11.42066	RP11-302B13.5_ENSG00000272822;ARF3_ENSG00000134287
12	49391468	49391672	5Y-H4K8ac_peak_4076	5.98695	RP11-386G11.5_ENSG00000257913
12	49391945	49392373	5Y-H4K8ac_peak_4077	5.23083	DDN_ENSG00000181418;RP11-386G11.5_ENSG00000257913
12	49411827	49412446	5Y-H4K8ac_peak_4078	4.0639	PRKAG1_ENSG00000181929
12	49454001	49454566	5Y-H4K8ac_peak_4079	14.2053	KMT2D_ENSG00000167548
12	49524714	49525127	5Y-H4K8ac_peak_4080	12.00715	TUBA1B_ENSG00000123416
12	49582322	49582617	5Y-H4K8ac_peak_4081	5.98695	TUBA1A_ENSG00000167552;TUBA1C_ENSG00000167553
12	49688163	49688373	5Y-H4K8ac_peak_4082	5.64909	
12	49717350	49717550	5Y-H4K8ac_peak_4083	9.93099	RP11-161H23.9_ENSG00000258334;TROAP_ENSG00000135451
12	49730300	49730959	5Y-H4K8ac_peak_4084	7.88114	C1QL4_ENSG00000186897
12	49740954	49741184	5Y-H4K8ac_peak_4085	6.54441	DNAJC22_ENSG00000178401
12	49760708	49760957	5Y-H4K8ac_peak_4086	18.10232	SPATS2_ENSG00000123352
12	49961553	49961807	5Y-H4K8ac_peak_4087	4.77126	MCRS1_ENSG00000187778;PRPF40B_ENSG00000110844
12	50017654	50017854	5Y-H4K8ac_peak_4088	10.46287	
12	50067271	50067645	5Y-H4K8ac_peak_4089	9.30206	
12	50222454	50222658	5Y-H4K8ac_peak_4090	5.56912	NCKAP5L_ENSG00000167566;BCDIN3D-AS1_ENSG00000258057
12	50262094	50262543	5Y-H4K8ac_peak_4091	9.59918	
12	50263628	50264117	5Y-H4K8ac_peak_4092	5.77617	

12	50264900	50265160	5Y-H4K8ac_peak_4093	4.83834	
12	50297931	50298336	5Y-H4K8ac_peak_4094	7.60057	FAIM2_ENSG00000135472
12	50368220	50368439	5Y-H4K8ac_peak_4095	8.73392	
12	50369142	50369479	5Y-H4K8ac_peak_4096	8.43511	
12	50371942	50372211	5Y-H4K8ac_peak_4097	10.21117	
12	50374010	50374327	5Y-H4K8ac_peak_4098	5.41472	
12	50375454	50375773	5Y-H4K8ac_peak_4099	8.49231	
12	50377290	50377575	5Y-H4K8ac_peak_4100	11.19336	
12	50402342	50402825	5Y-H4K8ac_peak_4101	4.50834	
12	50442313	50442509	5Y-H4K8ac_peak_4102	4.9885	
12	50444257	50444517	5Y-H4K8ac_peak_4103	8.2913	
12	50444997	50445272	5Y-H4K8ac_peak_4104	8.1667	
12	50504663	50505442	5Y-H4K8ac_peak_4105	11.75922	COX14_ENSG00000178449;RP4-605O3.4_ENSG00000272368
12	50560595	50560792	5Y-H4K8ac_peak_4106	7.58806	CERS5_ENSG00000139624
12	50561537	50561834	5Y-H4K8ac_peak_4107	5.65584	CERS5_ENSG00000139624
12	50677503	50677874	5Y-H4K8ac_peak_4108	9.38203	LIMA1_ENSG00000050405;AC008147.2_ENSG00000203433
12	50793448	50793778	5Y-H4K8ac_peak_4109	4.47071	
12	50899235	50899455	5Y-H4K8ac_peak_4110	7.87406	DIP2B_ENSG00000066084
12	51157884	51158124	5Y-H4K8ac_peak_4111	14.2053	ATF1_ENSG00000123268
12	51477042	51477244	5Y-H4K8ac_peak_4112	7.31815	CSRNP2_ENSG00000110925
12	51611481	51611755	5Y-H4K8ac_peak_4113	7.50148	POU6F1_ENSG00000184271
12	51611949	51612178	5Y-H4K8ac_peak_4114	11.9478	POU6F1_ENSG00000184271
12	51632647	51632877	5Y-H4K8ac_peak_4115	14.31711	DAZAP2_ENSG00000183283
12	51784946	51785840	5Y-H4K8ac_peak_4116	7.89273	GALNT6_ENSG00000139629;SLC4A8_ENSG00000050438
12	51818704	51818957	5Y-H4K8ac_peak_4117	9.36633	RP11-607P23.1_ENSG00000271065
12	51984160	51984497	5Y-H4K8ac_peak_4118	5.23083	SCN8A_ENSG00000196876
12	51985085	51985363	5Y-H4K8ac_peak_4119	7.89273	
12	52208094	52208543	5Y-H4K8ac_peak_4120	8.90774	
12	52214888	52215176	5Y-H4K8ac_peak_4121	10.48476	
12	52240981	52241220	5Y-H4K8ac_peak_4122	4.56708	RP11-923I11.5_ENSG00000259887
12	52242194	52242451	5Y-H4K8ac_peak_4123	12.29232	RP11-923I11.5_ENSG00000259887
12	52262472	52263162	5Y-H4K8ac_peak_4124	9.62747	
12	52263377	52263621	5Y-H4K8ac_peak_4125	12.87247	
12	52305691	52306341	5Y-H4K8ac_peak_4126	12.21176	
12	52311571	52312024	5Y-H4K8ac_peak_4127	7.59101	
12	52334952	52335308	5Y-H4K8ac_peak_4128	5.51139	
12	52345563	52345924	5Y-H4K8ac_peak_4129	8.69112	ACVR1B_ENSG00000135503
12	52431105	52431354	5Y-H4K8ac_peak_4130	5.5592	
12	52444414	52444876	5Y-H4K8ac_peak_4131	8.43511	
12	52456644	52456919	5Y-H4K8ac_peak_4132	6.47245	
12	52463565	52464000	5Y-H4K8ac_peak_4133	11.86504	C12orf44_ENSG00000123395
12	52577526	52578243	5Y-H4K8ac_peak_4134	10.31981	
12	52579174	52579367	5Y-H4K8ac_peak_4135	8.47164	
12	52593373	52594050	5Y-H4K8ac_peak_4136	10.54764	
12	52594906	52595535	5Y-H4K8ac_peak_4137	8.69112	
12	52596179	52596970	5Y-H4K8ac_peak_4138	9.24789	

12	52597390	52597827	5Y-H4K8ac_peak_4139	8.63295	
12	52637993	52638417	5Y-H4K8ac_peak_4140	6.08523	
12	53400424	53400625	5Y-H4K8ac_peak_4141	5.29015	EIF4B_ENSG00000063046
12	53441440	53441843	5Y-H4K8ac_peak_4142	7.58806	TENC1_ENSG00000111077
12	53443192	53443647	5Y-H4K8ac_peak_4143	6.22904	
12	53444010	53444209	5Y-H4K8ac_peak_4144	5.69598	
12	53448026	53448547	5Y-H4K8ac_peak_4145	9.96543	RP11-983P16.4_ENSG00000257337
12	53490948	53492238	5Y-H4K8ac_peak_4146	9.23159	IGFBP6_ENSG00000167779
12	53553554	53553813	5Y-H4K8ac_peak_4147	7.34185	RP11-1136G11.8_ENSG00000257808
12	53565535	53565735	5Y-H4K8ac_peak_4148	5.3176	
12	53616334	53616795	5Y-H4K8ac_peak_4149	5.12213	
12	53625445	53625974	5Y-H4K8ac_peak_4150	9.51254	RARG_ENSG00000172819
12	53628431	53628629	5Y-H4K8ac_peak_4151	6.09071	
12	53645961	53646242	5Y-H4K8ac_peak_4152	8.43511	MFSD5_ENSG00000182544
12	53689416	53689637	5Y-H4K8ac_peak_4153	6.76425	PFDN5_ENSG00000123349
12	53693615	53693992	5Y-H4K8ac_peak_4154	6.43775	RP11-680A11.5_ENSG00000257605;C12orf10_ENSG00000139637
12	53718854	53719617	5Y-H4K8ac_peak_4155	8.24461	AAAS_ENSG00000094914
12	53738271	53738569	5Y-H4K8ac_peak_4156	7.66481	SP7_ENSG00000170374
12	53774215	53774406	5Y-H4K8ac_peak_4157	4.68359	SP1_ENSG00000185591
12	53808585	53808790	5Y-H4K8ac_peak_4158	8.17203	
12	53895523	53896027	5Y-H4K8ac_peak_4159	12.05638	RP11-793H13.11_ENSG00000270175;TARBP2_ENSG00000139546
12	54070296	54070584	5Y-H4K8ac_peak_4160	8.5159	ATP5G2_ENSG00000135390
12	54393421	54393650	5Y-H4K8ac_peak_4161	11.75922	HOXC-AS1_ENSG00000250451
12	54393965	54394447	5Y-H4K8ac_peak_4162	13.78819	HOXC-AS1_ENSG00000250451
12	54411955	54412609	5Y-H4K8ac_peak_4163	18.10232	
12	54413735	54413931	5Y-H4K8ac_peak_4164	6.33308	AC012531.25_ENSG00000260597
12	54415842	54416072	5Y-H4K8ac_peak_4165	4.07874	
12	54428309	54428656	5Y-H4K8ac_peak_4166	9.52107	MIR615_ENSG00000207571
12	54445389	54445729	5Y-H4K8ac_peak_4167	7.34202	
12	54446364	54446712	5Y-H4K8ac_peak_4168	9.07085	HOXC4_ENSG00000273266
12	54447243	54447443	5Y-H4K8ac_peak_4169	7.11863	HOXC4_ENSG00000273266
12	54447802	54447995	5Y-H4K8ac_peak_4170	6.40964	HOXC4_ENSG00000273266
12	54473191	54473392	5Y-H4K8ac_peak_4171	5.35202	
12	54484078	54484345	5Y-H4K8ac_peak_4172	6.08523	
12	54506895	54507087	5Y-H4K8ac_peak_4173	8.748	
12	54519882	54520252	5Y-H4K8ac_peak_4174	7.11863	RP11-834C11.5_ENSG00000250432;RP11-834C11.4_ENSG00000250742
12	54529157	54529499	5Y-H4K8ac_peak_4175	7.37325	
12	54558178	54558448	5Y-H4K8ac_peak_4176	7.60057	RP11-834C11.10_ENSG00000257534
12	54567081	54567347	5Y-H4K8ac_peak_4177	8.24461	
12	54591129	54591480	5Y-H4K8ac_peak_4178	5.64909	
12	54654040	54654296	5Y-H4K8ac_peak_4179	8.35138	
12	54656824	54657031	5Y-H4K8ac_peak_4180	6.49308	RP11-968A15.2_ENSG00000257596
12	54745800	54746109	5Y-H4K8ac_peak_4181	6.78128	
12	54747381	54747695	5Y-H4K8ac_peak_4182	8.43511	RP11-753H16.3_ENSG00000258137;RP11-753H16.5_ENSG00000258086
12	54753372	54753689	5Y-H4K8ac_peak_4183	9.30505	
12	54772705	54772974	5Y-H4K8ac_peak_4184	11.9478	

12	54779158	54779518	5Y-H4K8ac_peak_4185	11.22005	
12	54784890	54785086	5Y-H4K8ac_peak_4186	7.38046	ZNF385A_ENSG00000161642
12	56241420	56241620	5Y-H4K8ac_peak_4187	8.4454	
12	56320639	56320856	5Y-H4K8ac_peak_4188	8.93923	DGKA_ENSG00000065357
12	56321122	56321683	5Y-H4K8ac_peak_4189	11.22005	DGKA_ENSG00000065357
12	56367952	56368232	5Y-H4K8ac_peak_4190	8.73392	PMEL_ENSG00000185664;RAB5B_ENSG00000111540
12	56401275	56401530	5Y-H4K8ac_peak_4191	12.84845	IKZF4_ENSG00000123411
12	56415127	56415400	5Y-H4K8ac_peak_4192	11.09973	
12	56435767	56436261	5Y-H4K8ac_peak_4193	10.69698	RP11-603J24.4_ENSG00000257449;RPS26_ENSG00000197728
12	56473788	56474137	5Y-H4K8ac_peak_4194	7.89142	ERBB3_ENSG00000065361
12	56512094	56512400	5Y-H4K8ac_peak_4195	4.55128	ZC3H10_ENSG00000135482;ESYT1_ENSG00000139641
12	56618291	56618513	5Y-H4K8ac_peak_4196	9.51254	
12	56652296	56652504	5Y-H4K8ac_peak_4197	7.89273	ANKRD52_ENSG00000139645
12	56709478	56709703	5Y-H4K8ac_peak_4198	6.78318	RP11-977G19.10_ENSG00000144785;CNPY2_ENSG00000257727
12	56726984	56727422	5Y-H4K8ac_peak_4199	10.21117	PAN2_ENSG00000135473
12	56728116	56728435	5Y-H4K8ac_peak_4200	7.89142	PAN2_ENSG00000135473
12	56881585	56882157	5Y-H4K8ac_peak_4201	9.15007	
12	56882362	56882620	5Y-H4K8ac_peak_4202	6.77436	
12	57028543	57028776	5Y-H4K8ac_peak_4203	8.43511	
12	57039522	57039712	5Y-H4K8ac_peak_4204	7.89142	ATP5B_ENSG00000110955;SNORD59A_ENSG00000207031
12	57081643	57082027	5Y-H4K8ac_peak_4205	7.00004	PTGES3_ENSG00000110958
12	57082397	57082744	5Y-H4K8ac_peak_4206	9.51254	PTGES3_ENSG00000110958
12	57118412	57119036	5Y-H4K8ac_peak_4207	7.50148	
12	57420496	57420737	5Y-H4K8ac_peak_4208	6.31818	
12	57423664	57423913	5Y-H4K8ac_peak_4209	5.12213	TAC3_ENSG00000166863
12	57481254	57481477	5Y-H4K8ac_peak_4210	10.19948	TMEM194A_ENSG00000166881
12	57498065	57498274	5Y-H4K8ac_peak_4211	7.53429	
12	57545127	57545561	5Y-H4K8ac_peak_4212	10.69698	
12	57546126	57546712	5Y-H4K8ac_peak_4213	9.52107	
12	57548235	57548537	5Y-H4K8ac_peak_4214	7.9592	
12	57569357	57569718	5Y-H4K8ac_peak_4215	10.35586	
12	57633651	57633922	5Y-H4K8ac_peak_4216	8.69112	NDUFA4L2_ENSG00000185633
12	57847087	57847420	5Y-H4K8ac_peak_4217	5.87382	INHBE_ENSG00000139269
12	57848376	57848596	5Y-H4K8ac_peak_4218	4.77016	
12	57881937	57882182	5Y-H4K8ac_peak_4219	13.63503	ARHGAP9_ENSG00000123329
12	57914843	57915081	5Y-H4K8ac_peak_4220	5.75508	DDIT3_ENSG00000175197;MBD6_ENSG00000166987
12	57940653	57940938	5Y-H4K8ac_peak_4221	11.68317	DCTN2_ENSG00000175203
12	57998171	57999160	5Y-H4K8ac_peak_4222	14.77644	DTX3_ENSG00000178498
12	58004671	58005434	5Y-H4K8ac_peak_4223	16.01457	ARHGEF25_ENSG00000240771
12	58026112	58026387	5Y-H4K8ac_peak_4224	7.9057	B4GALNT1_ENSG00000135454
12	58026704	58027183	5Y-H4K8ac_peak_4225	9.23159	B4GALNT1_ENSG00000135454
12	58119647	58120499	5Y-H4K8ac_peak_4226	14.72057	RP11-571M6.8_ENSG00000257499;AGAP2-AS1_ENSG00000255737
12	58120888	58121174	5Y-H4K8ac_peak_4227	12.57157	AGAP2-AS1_ENSG00000255737
12	58148916	58149106	5Y-H4K8ac_peak_4228	7.24844	CDK4_ENSG00000135446;MARCH9_ENSG00000139266
12	58166423	58166623	5Y-H4K8ac_peak_4229	5.24695	METTL1_ENSG00000037897;RP11-571M6.15_ENSG00000257921
12	58262510	58262825	5Y-H4K8ac_peak_4230	8.79957	

12	58280618	58280907	5Y-H4K8ac_peak_4231	7.04637	
12	58282441	58282674	5Y-H4K8ac_peak_4232	9.30505	
12	58287024	58287613	5Y-H4K8ac_peak_4233	9.93099	
12	58289929	58290793	5Y-H4K8ac_peak_4234	20.85916	RP11-620J15.2_ENSG00000245651
12	58291474	58291673	5Y-H4K8ac_peak_4235	6.79955	
12	59448643	59448846	5Y-H4K8ac_peak_4236	4.07874	RP11-557F20.2_ENSG00000257288
12	62860636	62861086	5Y-H4K8ac_peak_4237	13.2534	MON2_ENSG00000061987
12	62996713	62997001	5Y-H4K8ac_peak_4238	10.18556	C12orf61_ENSG00000221949;RP11-631N16.2_ENSG00000257354;MIRLET7I_ENSG00000199179
12	63025750	63026591	5Y-H4K8ac_peak_4239	7.8267	
12	64333676	64333997	5Y-H4K8ac_peak_4240	7.42477	
12	64616177	64616736	5Y-H4K8ac_peak_4241	9.78792	C12orf66_ENSG00000174206;RPS11P6_ENSG00000243024
12	64798350	64798897	5Y-H4K8ac_peak_4242	6.78128	XPOT_ENSG00000184575
12	64936462	64936666	5Y-H4K8ac_peak_4243	4.84727	
12	65003725	65003931	5Y-H4K8ac_peak_4244	8.1667	RP11-338E21.1_ENSG00000256670;RASSF3_ENSG00000153179
12	65152943	65153135	5Y-H4K8ac_peak_4245	5.87725	GNS_ENSG00000135677;RP11-629N8.3_ENSG00000215159
12	65563072	65563640	5Y-H4K8ac_peak_4246	6.37023	LEMD3_ENSG00000174106
12	66563197	66563750	5Y-H4K8ac_peak_4247	14.1808	TMBIM4_ENSG00000228144;TMBIM4_ENSG00000155957
12	66564017	66564386	5Y-H4K8ac_peak_4248	7.84116	TMBIM4_ENSG00000228144;TMBIM4_ENSG00000155957
12	68043026	68043483	5Y-H4K8ac_peak_4249	6.78318	DYRK2_ENSG00000127334
12	68043700	68043968	5Y-H4K8ac_peak_4250	7.58806	
12	68639343	68639634	5Y-H4K8ac_peak_4251	5.87382	
12	69080824	69081060	5Y-H4K8ac_peak_4252	8.73392	RP11-637A17.2_ENSG00000247363;NUP107_ENSG00000111581
12	69202000	69202395	5Y-H4K8ac_peak_4253	22.6507	MDM2_ENSG00000135679
12	69633525	69634008	5Y-H4K8ac_peak_4254	9.62747	CPSF6_ENSG00000111605
12	69724787	69724980	5Y-H4K8ac_peak_4255	7.31815	
12	69753554	69753808	5Y-H4K8ac_peak_4256	8.5159	YEATS4_ENSG00000127337
12	69978190	69978395	5Y-H4K8ac_peak_4257	7.17184	MIR3913-1_ENSG00000264405;CCT2_ENSG00000166226
12	69979312	69979539	5Y-H4K8ac_peak_4258	13.2534	MIR3913-1_ENSG00000264405;CCT2_ENSG00000166226
12	70330074	70330280	5Y-H4K8ac_peak_4259	6.34046	
12	70636804	70636999	5Y-H4K8ac_peak_4260	7.50148	RP11-611E13.2_ENSG00000257815;CNOT2_ENSG00000111596
12	70637248	70637730	5Y-H4K8ac_peak_4261	9.15007	RP11-611E13.2_ENSG00000257815;CNOT2_ENSG00000111596
12	70760654	70761071	5Y-H4K8ac_peak_4262	7.17184	KCNMB4_ENSG00000135643
12	71003494	71004046	5Y-H4K8ac_peak_4263	4.77126	
12	72056848	72057560	5Y-H4K8ac_peak_4264	10.46287	THAP2_ENSG00000173451
12	75785155	75785422	5Y-H4K8ac_peak_4265	6.50117	CAPS2_ENSG00000180881
12	75973819	75974024	5Y-H4K8ac_peak_4266	5.40331	
12	76178882	76179133	5Y-H4K8ac_peak_4267	8.21582	
12	76195083	76195296	5Y-H4K8ac_peak_4268	5.56912	
12	76397661	76397858	5Y-H4K8ac_peak_4269	4.07874	
12	76742394	76742822	5Y-H4K8ac_peak_4270	6.54441	BBS10_ENSG00000179941
12	77458830	77459265	5Y-H4K8ac_peak_4271	8.63306	E2F7_ENSG00000165891
12	77459621	77459875	5Y-H4K8ac_peak_4272	7.31102	E2F7_ENSG00000165891
12	78490275	78490643	5Y-H4K8ac_peak_4273	10.36926	
12	83102276	83102605	5Y-H4K8ac_peak_4274	5.03335	
12	83234507	83234708	5Y-H4K8ac_peak_4275	5.56912	
12	88536197	88536442	5Y-H4K8ac_peak_4276	7.89142	CEP290_ENSG00000198707;TMTC3_ENSG00000139324

12	89739628	89740096	5Y-H4K8ac_peak_4277	8.2913	
12	89747128	89748493	5Y-H4K8ac_peak_4278	12.84845	DUSP6_ENSG00000139318
12	89919256	89919919	5Y-H4K8ac_peak_4279	9.38276	POC1B_ENSG00000139323;GALNT4_ENSG00000257594;POC1B-GALNT4_ENSG00000259075;RP11-734K2.4_ENSG00000270344
12	91950867	91951105	5Y-H4K8ac_peak_4280	4.79585	
12	92757356	92757649	5Y-H4K8ac_peak_4281	7.31815	
12	92758172	92758396	5Y-H4K8ac_peak_4282	5.65584	
12	93323377	93323635	5Y-H4K8ac_peak_4283	6.79955	EEA1_ENSG00000102189
12	93692701	93692921	5Y-H4K8ac_peak_4284	6.34046	
12	93693666	93694077	5Y-H4K8ac_peak_4285	6.77436	
12	93861330	93861691	5Y-H4K8ac_peak_4286	13.06587	MRPL42_ENSG00000198015
12	93964810	93965512	5Y-H4K8ac_peak_4287	11.09973	SOCS2-AS1_ENSG00000246985
12	93965720	93966022	5Y-H4K8ac_peak_4288	12.11208	SOCS2-AS1_ENSG00000246985
12	94070171	94070649	5Y-H4K8ac_peak_4289	15.34073	CRADD_ENSG00000169372
12	94071258	94071767	5Y-H4K8ac_peak_4290	9.30505	CRADD_ENSG00000169372
12	94179134	94179364	5Y-H4K8ac_peak_4291	7.72345	
12	94288248	94288509	5Y-H4K8ac_peak_4292	4.07874	
12	94853376	94853585	5Y-H4K8ac_peak_4293	12.49199	CCDC41_ENSG00000173588
12	94853892	94854403	5Y-H4K8ac_peak_4294	12.05638	CCDC41_ENSG00000173588
12	94926850	94927282	5Y-H4K8ac_peak_4295	7.58806	
12	95266952	95267515	5Y-H4K8ac_peak_4296	8.24461	
12	95467947	95468152	5Y-H4K8ac_peak_4297	7.38046	NR2C1_ENSG00000120798
12	95611635	95611846	5Y-H4K8ac_peak_4298	8.69112	FGD6_ENSG00000180263;VEZT_ENSG00000028203
12	95941649	95941914	5Y-H4K8ac_peak_4299	4.15658	
12	96336287	96336694	5Y-H4K8ac_peak_4300	20.27109	CCDC38_ENSG00000165972;AMDHD1_ENSG00000139344
12	96545911	96546262	5Y-H4K8ac_peak_4301	9.15977	
12	96587116	96587534	5Y-H4K8ac_peak_4302	7.17184	ELK3_ENSG00000111145
12	96587725	96588018	5Y-H4K8ac_peak_4303	16.52279	ELK3_ENSG00000111145
12	97300752	97301212	5Y-H4K8ac_peak_4304	8.69112	NEDD1_ENSG00000139350
12	98909488	98910238	5Y-H4K8ac_peak_4305	12.69256	TMPO-AS1_ENSG00000257167;TMPO_ENSG00000120802
12	101801888	101802180	5Y-H4K8ac_peak_4306	6.77436	ARL1_ENSG00000120805;RP11-321F8.4_ENSG00000257543
12	102091687	102092269	5Y-H4K8ac_peak_4307	10.42746	CHPT1_ENSG00000111666
12	102270929	102271421	5Y-H4K8ac_peak_4308	4.70501	DRAM1_ENSG00000136048
12	102455439	102455743	5Y-H4K8ac_peak_4309	11.19336	CCDC53_ENSG00000120860
12	104323106	104323452	5Y-H4K8ac_peak_4310	9.58986	RP11-642P15.1_ENSG00000214198;HSP90B1_ENSG00000166598;MIR3652_ENSG00000265072
12	104350412	104350860	5Y-H4K8ac_peak_4311	7.04637	
12	104359060	104359367	5Y-H4K8ac_peak_4312	6.78318	C12orf73_ENSG00000204954;TDG_ENSG00000139372
12	104359720	104359922	5Y-H4K8ac_peak_4313	9.74838	C12orf73_ENSG00000204954;TDG_ENSG00000139372
12	104457761	104458106	5Y-H4K8ac_peak_4314	8.47164	HCFC2_ENSG00000111727
12	104531084	104531752	5Y-H4K8ac_peak_4315	29.46868	NFYB_ENSG00000120837
12	104532165	104532557	5Y-H4K8ac_peak_4316	11.19336	NFYB_ENSG00000120837
12	104697691	104697979	5Y-H4K8ac_peak_4317	7.29091	EID3_ENSG00000255150
12	105062569	105062805	5Y-H4K8ac_peak_4318	6.54441	
12	105351675	105351898	5Y-H4K8ac_peak_4319	7.59101	SLC41A2_ENSG00000136052
12	105352218	105352589	5Y-H4K8ac_peak_4320	7.89273	SLC41A2_ENSG00000136052
12	105352864	105353058	5Y-H4K8ac_peak_4321	7.53429	SLC41A2_ENSG00000136052
12	105629523	105629852	5Y-H4K8ac_peak_4322	10.19948	APPL2_ENSG00000136044;C12orf75_ENSG00000235162

12	105724539	105725012	5Y-H4K8ac_peak_4323	7.3889	
12	106532588	106533727	5Y-H4K8ac_peak_4324	15.03212	NUAK1_ENSG00000074590
12	106641515	106641775	5Y-H4K8ac_peak_4325	15.87289	RP11-651L5.2_ENSG00000258355
12	106642088	106642499	5Y-H4K8ac_peak_4326	5.74859	
12	106696317	106696528	5Y-H4K8ac_peak_4327	8.24461	TCP11L2_ENSG00000166046
12	107524032	107524393	5Y-H4K8ac_peak_4328	8.33296	
12	107525844	107526389	5Y-H4K8ac_peak_4329	6.34046	
12	107767483	107768188	5Y-H4K8ac_peak_4330	10.79063	SNORD74_ENSG00000200897
12	107775100	107775311	5Y-H4K8ac_peak_4331	6.06152	
12	107776727	107777012	5Y-H4K8ac_peak_4332	8.2913	
12	107799510	107799726	5Y-H4K8ac_peak_4333	4.9603	
12	108001148	108001339	5Y-H4K8ac_peak_4334	9.23159	
12	108001594	108001804	5Y-H4K8ac_peak_4335	5.59843	
12	108039560	108039751	5Y-H4K8ac_peak_4336	4.95697	
12	108236709	108236910	5Y-H4K8ac_peak_4337	4.24332	
12	108239081	108239367	5Y-H4K8ac_peak_4338	4.15658	
12	108646844	108647187	5Y-H4K8ac_peak_4339	7.97699	
12	108698495	108698727	5Y-H4K8ac_peak_4340	9.66296	
12	108787089	108787424	5Y-H4K8ac_peak_4341	4.93237	
12	108839979	108840337	5Y-H4K8ac_peak_4342	4.84727	
12	108954747	108955123	5Y-H4K8ac_peak_4343	4.15658	SART3_ENSG00000075856
12	109003320	109003533	5Y-H4K8ac_peak_4344	7.63144	
12	109085829	109086094	5Y-H4K8ac_peak_4345	8.564	
12	109244638	109245005	5Y-H4K8ac_peak_4346	5.64909	
12	109251563	109252150	5Y-H4K8ac_peak_4347	4.95697	SSH1_ENSG00000084112;DAO_ENSG00000110887
12	109489514	109489716	5Y-H4K8ac_peak_4348	7.38046	
12	109535482	109535704	5Y-H4K8ac_peak_4349	10.73603	UNG_ENSG00000076248
12	109553603	109553842	5Y-H4K8ac_peak_4350	6.50117	ACACB_ENSG00000076555
12	109725826	109726062	5Y-H4K8ac_peak_4351	8.45687	
12	109728057	109728953	5Y-H4K8ac_peak_4352	7.56545	
12	109729698	109730069	5Y-H4K8ac_peak_4353	8.24461	
12	109747911	109748257	5Y-H4K8ac_peak_4354	6.31818	FOXN4_ENSG00000139445
12	109770046	109770687	5Y-H4K8ac_peak_4355	5.64909	
12	109915208	109915807	5Y-H4K8ac_peak_4356	9.32595	KCTD10_ENSG00000110906;UBE3B_ENSG00000151148
12	109959911	109960477	5Y-H4K8ac_peak_4357	8.43511	
12	109961380	109961680	5Y-H4K8ac_peak_4358	4.642	
12	109969827	109970079	5Y-H4K8ac_peak_4359	8.2913	
12	109970560	109970954	5Y-H4K8ac_peak_4360	9.56516	
12	109971241	109971638	5Y-H4K8ac_peak_4361	5.64909	
12	109976466	109976857	5Y-H4K8ac_peak_4362	5.23083	
12	109978643	109979079	5Y-H4K8ac_peak_4363	5.12488	
12	110044969	110045277	5Y-H4K8ac_peak_4364	11.94897	
12	110049563	110049793	5Y-H4K8ac_peak_4365	5.98695	
12	110061614	110062108	5Y-H4K8ac_peak_4366	7.11863	
12	110064249	110064492	5Y-H4K8ac_peak_4367	4.00285	
12	110092035	110092450	5Y-H4K8ac_peak_4368	10.93537	

12	110093215	110093455	5Y-H4K8ac_peak_4369	7.01266	
12	110093851	110094239	5Y-H4K8ac_peak_4370	8.35207	
12	110157478	110157712	5Y-H4K8ac_peak_4371	7.28023	
12	110158466	110158699	5Y-H4K8ac_peak_4372	5.59843	
12	110164520	110165234	5Y-H4K8ac_peak_4373	7.89273	
12	110169329	110169631	5Y-H4K8ac_peak_4374	9.79526	
12	110171856	110172167	5Y-H4K8ac_peak_4375	5.54882	
12	110176005	110176365	5Y-H4K8ac_peak_4376	4.642	
12	110196831	110197102	5Y-H4K8ac_peak_4377	4.15658	
12	110198191	110198383	5Y-H4K8ac_peak_4378	8.63295	
12	110283030	110283572	5Y-H4K8ac_peak_4379	7.31815	
12	110318599	110318792	5Y-H4K8ac_peak_4380	5.72848	RP1-7G5.6_ENSG00000249094
12	110338070	110338426	5Y-H4K8ac_peak_4381	10.90543	TCHP_ENSG00000139437
12	110562268	110562476	5Y-H4K8ac_peak_4382	5.64909	IFT81_ENSG00000122970
12	110719470	110719724	5Y-H4K8ac_peak_4383	10.19948	ATP2A2_ENSG00000174437
12	110887766	110888070	5Y-H4K8ac_peak_4384	11.86504	ARPC3_ENSG00000111229
12	110906711	110906918	5Y-H4K8ac_peak_4385	7.64648	GPN3_ENSG00000111231;FAM216A_ENSG00000204856
12	110940126	110940350	5Y-H4K8ac_peak_4386	4.15658	VPS29_ENSG00000111237;RAD9B_ENSG00000151164
12	111137559	111137901	5Y-H4K8ac_peak_4387	9.15007	
12	111181431	111181666	5Y-H4K8ac_peak_4388	6.73385	PPP1CC_ENSG00000186298
12	111472054	111472854	5Y-H4K8ac_peak_4389	8.43511	CUX2_ENSG00000111249
12	111474093	111474312	5Y-H4K8ac_peak_4390	5.65584	
12	111497334	111497615	5Y-H4K8ac_peak_4391	7.31807	
12	111498490	111498685	5Y-H4K8ac_peak_4392	6.50117	
12	111616758	111617834	5Y-H4K8ac_peak_4393	12.64868	
12	111620083	111620378	5Y-H4K8ac_peak_4394	6.08523	
12	111621088	111621361	5Y-H4K8ac_peak_4395	4.31078	
12	111623976	111624502	5Y-H4K8ac_peak_4396	7.43638	
12	111639230	111639444	5Y-H4K8ac_peak_4397	9.80622	
12	111641666	111642218	5Y-H4K8ac_peak_4398	16.78315	
12	111645763	111645958	5Y-H4K8ac_peak_4399	6.20989	
12	111646934	111647530	5Y-H4K8ac_peak_4400	6.52593	
12	111663203	111663475	5Y-H4K8ac_peak_4401	5.65584	
12	111867576	111868028	5Y-H4K8ac_peak_4402	7.4047	
12	111882833	111883058	5Y-H4K8ac_peak_4403	5.449	
12	112036736	112037404	5Y-H4K8ac_peak_4404	17.76126	ATXN2_ENSG00000204842;RP11-686G8.2_ENSG00000258099
12	112279335	112279587	5Y-H4K8ac_peak_4405	7.64675	AC003029.1_ENSG00000248594;MAPKAPK5_ENSG00000089022
12	112279868	112280668	5Y-H4K8ac_peak_4406	12.21176	MAPKAPK5-AS1_ENSG00000234608;AC003029.1_ENSG00000248594;MAPKAPK5_ENSG00000089022
12	112450418	112450827	5Y-H4K8ac_peak_4407	10.54764	TMEM116_ENSG00000198270;ERP29_ENSG00000089248
12	112451280	112451575	5Y-H4K8ac_peak_4408	13.61292	TMEM116_ENSG00000198270;ERP29_ENSG00000089248
12	112553919	112554357	5Y-H4K8ac_peak_4409	5.91107	
12	112846627	112847359	5Y-H4K8ac_peak_4410	5.87725	
12	112856759	112857021	5Y-H4K8ac_peak_4411	12.06963	RPL6_ENSG00000089009;PTPN11_ENSG00000179295
12	113246288	113246721	5Y-H4K8ac_peak_4412	7.59101	
12	113247617	113248106	5Y-H4K8ac_peak_4413	5.23083	
12	113252583	113253263	5Y-H4K8ac_peak_4414	8.2913	

12	113262582	113262805	5Y-H4K8ac_peak_4415	5.449	
12	113494482	113495133	5Y-H4K8ac_peak_4416	11.09973	DTX1_ENSG00000135144
12	113554855	113555113	5Y-H4K8ac_peak_4417	7.93065	
12	113568321	113568588	5Y-H4K8ac_peak_4418	6.43775	
12	113622847	113623518	5Y-H4K8ac_peak_4419	12.10416	DDX54_ENSG00000123064;RITA1_ENSG00000139405
12	113683136	113683525	5Y-H4K8ac_peak_4420	9.63153	
12	113684242	113684912	5Y-H4K8ac_peak_4421	9.51254	
12	114203767	114204100	5Y-H4K8ac_peak_4422	10.15101	
12	114403724	114403966	5Y-H4K8ac_peak_4423	9.7353	RBM19_ENSG00000122965
12	115112136	115112463	5Y-H4K8ac_peak_4424	5.04571	
12	115122488	115122802	5Y-H4K8ac_peak_4425	6.75784	TBX3_ENSG00000135111
12	115450585	115450954	5Y-H4K8ac_peak_4426	8.59175	
12	115451151	115451423	5Y-H4K8ac_peak_4427	5.29015	
12	115648918	115649125	5Y-H4K8ac_peak_4428	6.07082	
12	116715757	116716119	5Y-H4K8ac_peak_4429	8.43511	MED13L_ENSG00000123066
12	117131293	117131677	5Y-H4K8ac_peak_4430	5.89113	
12	117256808	117257229	5Y-H4K8ac_peak_4431	8.43511	
12	117348855	117349149	5Y-H4K8ac_peak_4432	16.27715	FBXW8_ENSG00000174989
12	117408047	117408268	5Y-H4K8ac_peak_4433	8.43511	
12	117483371	117483689	5Y-H4K8ac_peak_4434	5.24695	
12	117537093	117537806	5Y-H4K8ac_peak_4435	5.87725	TESC_ENSG00000088992;RP11-103B5.2_ENSG00000258285
12	117846094	117846354	5Y-H4K8ac_peak_4436	10.27264	
12	117876679	117877743	5Y-H4K8ac_peak_4437	11.1169	
12	117930126	117930352	5Y-H4K8ac_peak_4438	8.63306	
12	118541361	118542201	5Y-H4K8ac_peak_4439	10.89622	
12	118763555	118763812	5Y-H4K8ac_peak_4440	4.51076	
12	119446828	119447239	5Y-H4K8ac_peak_4441	9.68432	
12	119511326	119511545	5Y-H4K8ac_peak_4442	8.21582	
12	119616396	119616863	5Y-H4K8ac_peak_4443	5.65584	HSPB8_ENSG00000152137
12	120105799	120106027	5Y-H4K8ac_peak_4444	9.30505	RP11-768F21.1_ENSG00000248636;PRKAB1_ENSG00000111725
12	120314819	120315016	5Y-H4K8ac_peak_4445	9.38276	CIT_ENSG00000122966
12	120531002	120531372	5Y-H4K8ac_peak_4446	12.24654	
12	120554772	120555147	5Y-H4K8ac_peak_4447	8.8021	RAB35_ENSG00000111737
12	120632158	120633133	5Y-H4K8ac_peak_4448	12.50545	GCN1L1_ENSG00000089154
12	120654856	120655199	5Y-H4K8ac_peak_4449	7.24844	
12	120668155	120668392	5Y-H4K8ac_peak_4450	8.43511	
12	120799161	120799626	5Y-H4K8ac_peak_4451	6.98416	
12	120799847	120800401	5Y-H4K8ac_peak_4452	9.87097	
12	120807123	120807483	5Y-H4K8ac_peak_4453	16.19089	MSI1_ENSG00000135097;RPS27P25_ENSG00000239881
12	120884280	120884585	5Y-H4K8ac_peak_4454	11.22005	TRIAP1_ENSG00000170855;GATC_ENSG00000257218
12	120907247	120907482	5Y-H4K8ac_peak_4455	15.45397	SRSF9_ENSG00000111786;DYNLL1_ENSG00000088986
12	121022405	121022603	5Y-H4K8ac_peak_4456	5.93823	
12	121079161	121079417	5Y-H4K8ac_peak_4457	5.65584	RP11-728G15.1_ENSG00000256008;CABP1_ENSG00000157782
12	121124422	121124625	5Y-H4K8ac_peak_4458	6.54441	MLEC_ENSG00000110917
12	121163671	121163872	5Y-H4K8ac_peak_4459	7.89142	ACADS_ENSG00000122971
12	121342349	121342540	5Y-H4K8ac_peak_4460	7.41172	SPPL3_ENSG00000157837

12	121453611	121453823	5Y-H4K8ac_peak_4461	9.05168	C12orf43_ENSG00000157895
12	121571101	121571320	5Y-H4K8ac_peak_4462	11.86504	P2RX7_ENSG00000089041
12	121648043	121648435	5Y-H4K8ac_peak_4463	13.4304	P2RX4_ENSG00000135124
12	121734897	121735521	5Y-H4K8ac_peak_4464	7.65114	CAMKK2_ENSG00000110931
12	121838058	121838309	5Y-H4K8ac_peak_4465	6.73385	ANAPC5_ENSG00000089053;RNF34_ENSG00000170633
12	121904296	121904517	5Y-H4K8ac_peak_4466	8.63306	
12	121905472	121905809	5Y-H4K8ac_peak_4467	7.59101	
12	121976054	121976416	5Y-H4K8ac_peak_4468	5.99504	
12	122016994	122017486	5Y-H4K8ac_peak_4469	13.22169	
12	122110749	122111256	5Y-H4K8ac_peak_4470	6.77436	MORN3_ENSG00000139714
12	122119720	122119939	5Y-H4K8ac_peak_4471	8.21582	
12	122125229	122125724	5Y-H4K8ac_peak_4472	16.50659	
12	122204659	122205273	5Y-H4K8ac_peak_4473	8.66584	
12	122231309	122231520	5Y-H4K8ac_peak_4474	6.77436	
12	122237720	122239385	5Y-H4K8ac_peak_4475	20.25522	
12	122239722	122239957	5Y-H4K8ac_peak_4476	4.63981	
12	122241850	122242155	5Y-H4K8ac_peak_4477	11.8513	SETD1B_ENSG00000139718;RHOF_ENSG00000139725;AC084018.1_ENSG00000212694
12	122249543	122249756	5Y-H4K8ac_peak_4478	4.50834	
12	122279009	122279244	5Y-H4K8ac_peak_4479	6.20989	
12	122310807	122311296	5Y-H4K8ac_peak_4480	8.55081	
12	122326127	122326332	5Y-H4K8ac_peak_4481	9.15977	PSMD9_ENSG00000110801;RP11-87C12.2_ENSG00000256950
12	122459304	122459674	5Y-H4K8ac_peak_4482	5.03564	
12	122667804	122668148	5Y-H4K8ac_peak_4483	13.85283	
12	122712088	122712416	5Y-H4K8ac_peak_4484	4.91235	DIABLO_ENSG00000184047
12	122713452	122713809	5Y-H4K8ac_peak_4485	9.23159	
12	122750669	122750917	5Y-H4K8ac_peak_4486	9.01738	RP11-512M8.5_ENSG00000256861;VPS33A_ENSG00000139719
12	122906568	122906983	5Y-H4K8ac_peak_4487	9.89244	CLIP1_ENSG00000130779
12	122985123	122985339	5Y-H4K8ac_peak_4488	5.64909	ZCCHC8_ENSG00000033030
12	122986513	122986706	5Y-H4K8ac_peak_4489	4.93237	ZCCHC8_ENSG00000033030
12	123237417	123237679	5Y-H4K8ac_peak_4490	8.93923	DENR_ENSG00000139726
12	123258977	123259352	5Y-H4K8ac_peak_4491	6.50117	CCDC62_ENSG00000130783
12	123356448	123356656	5Y-H4K8ac_peak_4492	6.73047	
12	123380816	123381043	5Y-H4K8ac_peak_4493	5.8635	VPS37B_ENSG00000139722
12	123459414	123459680	5Y-H4K8ac_peak_4494	8.75963	OGFOD2_ENSG00000111325;RP11-197N18.2_ENSG00000256028
12	123465290	123465833	5Y-H4K8ac_peak_4495	9.66296	ABCB9_ENSG00000150967;ARL6IP4_ENSG00000182196
12	123634853	123635197	5Y-H4K8ac_peak_4496	4.15658	PITPNM2_ENSG00000090975
12	123718051	123718309	5Y-H4K8ac_peak_4497	11.68317	C12orf65_ENSG00000130921
12	123752335	123754098	5Y-H4K8ac_peak_4498	14.43262	
12	123754459	123754862	5Y-H4K8ac_peak_4499	11.98202	
12	123755179	123755505	5Y-H4K8ac_peak_4500	5.24695	
12	123755819	123756634	5Y-H4K8ac_peak_4501	12.11208	CDK2AP1_ENSG00000111328
12	123920764	123921119	5Y-H4K8ac_peak_4502	4.95697	RILPL2_ENSG00000150977
12	123942814	123943094	5Y-H4K8ac_peak_4503	6.22669	SNRNP35_ENSG00000184209
12	123946353	123946614	5Y-H4K8ac_peak_4504	4.50834	
12	124017727	124018078	5Y-H4K8ac_peak_4505	7.42477	RILPL1_ENSG00000188026
12	124018403	124018863	5Y-H4K8ac_peak_4506	6.37023	RILPL1_ENSG00000188026

12	124055519	124055729	5Y-H4K8ac_peak_4507	5.23083	
12	124086353	124086556	5Y-H4K8ac_peak_4508	7.29055	DDX55_ENSG00000111364
12	124086848	124087053	5Y-H4K8ac_peak_4509	8.5915	DDX55_ENSG00000111364
12	124456874	124457117	5Y-H4K8ac_peak_4510	6.64195	CCDC92_ENSG00000119242;RP11-214K3.18_ENSG00000270095;ZNF664_ENSG00000179195;FAM101A_ENSG00000178882
12	124773801	124774247	5Y-H4K8ac_peak_4511	7.58806	
12	124854279	124854808	5Y-H4K8ac_peak_4512	8.24461	
12	124876923	124877359	5Y-H4K8ac_peak_4513	5.60566	
12	124878404	124878652	5Y-H4K8ac_peak_4514	9.24789	
12	124881125	124881322	5Y-H4K8ac_peak_4515	9.32595	
12	124895181	124895432	5Y-H4K8ac_peak_4516	7.01266	
12	124907453	124907839	5Y-H4K8ac_peak_4517	19.11901	
12	124911772	124912430	5Y-H4K8ac_peak_4518	9.08389	
12	124913919	124914309	5Y-H4K8ac_peak_4519	8.04693	
12	124989776	124990122	5Y-H4K8ac_peak_4520	6.50117	
12	124990319	124990877	5Y-H4K8ac_peak_4521	10.93537	
12	124992020	124992344	5Y-H4K8ac_peak_4522	7.64648	
12	124994559	124995399	5Y-H4K8ac_peak_4523	8.69112	
12	125021989	125022332	5Y-H4K8ac_peak_4524	5.23819	
12	125023867	125024182	5Y-H4K8ac_peak_4525	4.95697	
12	125028009	125028467	5Y-H4K8ac_peak_4526	5.77617	
12	125041060	125041279	5Y-H4K8ac_peak_4527	5.03335	
12	125051773	125051971	5Y-H4K8ac_peak_4528	8.43511	NCOR2_ENSG00000196498
12	125053429	125053970	5Y-H4K8ac_peak_4529	9.7353	
12	125066637	125066885	5Y-H4K8ac_peak_4530	6.6946	
12	125087539	125088464	5Y-H4K8ac_peak_4531	11.22005	
12	125088736	125089300	5Y-H4K8ac_peak_4532	5.98695	
12	125089688	125089973	5Y-H4K8ac_peak_4533	4.95697	
12	125139236	125139696	5Y-H4K8ac_peak_4534	11.05822	
12	125140277	125140937	5Y-H4K8ac_peak_4535	9.96543	
12	125158682	125159232	5Y-H4K8ac_peak_4536	8.43511	
12	125166060	125166429	5Y-H4K8ac_peak_4537	5.75508	
12	125198111	125198333	5Y-H4K8ac_peak_4538	9.15007	
12	125199837	125200047	5Y-H4K8ac_peak_4539	9.15007	
12	125204584	125204994	5Y-H4K8ac_peak_4540	10.07308	
12	125226912	125227438	5Y-H4K8ac_peak_4541	11.12941	
12	125230300	125230578	5Y-H4K8ac_peak_4542	12.20141	
12	125270639	125271144	5Y-H4K8ac_peak_4543	7.63144	
12	125318570	125319022	5Y-H4K8ac_peak_4544	4.50834	
12	125326371	125327540	5Y-H4K8ac_peak_4545	13.29029	
12	125348634	125349209	5Y-H4K8ac_peak_4546	9.96543	
12	125383123	125383338	5Y-H4K8ac_peak_4547	5.65584	
12	125398994	125399669	5Y-H4K8ac_peak_4548	12.84845	MIR5188_ENSG00000265345
12	125474062	125474301	5Y-H4K8ac_peak_4549	6.27984	DHX37_ENSG00000150990
12	125477639	125477914	5Y-H4K8ac_peak_4550	4.69231	BRI3BP_ENSG00000184992
12	125478294	125478793	5Y-H4K8ac_peak_4551	9.34555	BRI3BP_ENSG00000184992
12	125479083	125479306	5Y-H4K8ac_peak_4552	8.2913	BRI3BP_ENSG00000184992

12	127630780	127631184	5Y-H4K8ac_peak_4553	13.64163	RP11-575F12.2_ENSG00000256001
12	129308756	129308979	5Y-H4K8ac_peak_4554	6.64195	SLC15A4_ENSG00000139370
12	129309222	129309423	5Y-H4K8ac_peak_4555	3.96434	SLC15A4_ENSG00000139370
12	130875357	130875829	5Y-H4K8ac_peak_4556	4.95697	
12	130969008	130969243	5Y-H4K8ac_peak_4557	5.65584	
12	131041698	131042111	5Y-H4K8ac_peak_4558	7.89142	
12	131056838	131057055	5Y-H4K8ac_peak_4559	6.22669	
12	131061155	131061555	5Y-H4K8ac_peak_4560	9.52603	
12	131104516	131104918	5Y-H4K8ac_peak_4561	12.69256	
12	131105400	131105603	5Y-H4K8ac_peak_4562	8.24461	
12	131118204	131118691	5Y-H4K8ac_peak_4563	8.2913	
12	131170600	131170988	5Y-H4K8ac_peak_4564	6.08523	
12	131356087	131356314	5Y-H4K8ac_peak_4565	7.89273	RAN_ENSG00000132341
12	131356782	131357228	5Y-H4K8ac_peak_4566	7.89273	RAN_ENSG00000132341
12	131436254	131436670	5Y-H4K8ac_peak_4567	7.04637	
12	131714016	131714218	5Y-H4K8ac_peak_4568	7.76232	
12	131714802	131715162	5Y-H4K8ac_peak_4569	4.84727	
12	131851931	131852442	5Y-H4K8ac_peak_4570	11.43902	RP13-507P19.1_ENSG00000256484
12	131852957	131853157	5Y-H4K8ac_peak_4571	8.43511	
12	131871895	131872143	5Y-H4K8ac_peak_4572	6.50117	
12	131872786	131873032	5Y-H4K8ac_peak_4573	7.24844	
12	131930806	131930997	5Y-H4K8ac_peak_4574	4.84727	
12	131939825	131940131	5Y-H4K8ac_peak_4575	6.77436	
12	132040477	132040722	5Y-H4K8ac_peak_4576	8.2913	
12	132058737	132059150	5Y-H4K8ac_peak_4577	5.77617	
12	132124128	132124362	5Y-H4K8ac_peak_4578	9.30505	
12	132124846	132125151	5Y-H4K8ac_peak_4579	10.39333	
12	132125918	132126261	5Y-H4K8ac_peak_4580	8.3285	
12	132194961	132196186	5Y-H4K8ac_peak_4581	12.19856	SFSWAP_ENSG00000061936
12	132314824	132315642	5Y-H4K8ac_peak_4582	7.89273	
12	132318295	132318700	5Y-H4K8ac_peak_4583	7.92287	
12	132336910	132337357	5Y-H4K8ac_peak_4584	6.14981	
12	132345327	132345668	5Y-H4K8ac_peak_4585	16.67379	
12	132433930	132434167	5Y-H4K8ac_peak_4586	12.11208	EP400_ENSG00000183495
12	132434600	132434881	5Y-H4K8ac_peak_4587	11.04542	EP400_ENSG00000183495
12	133136705	133137791	5Y-H4K8ac_peak_4588	5.64909	
12	133180623	133180833	5Y-H4K8ac_peak_4589	4.83834	
12	133264219	133264520	5Y-H4K8ac_peak_4590	7.34185	POLE_ENSG00000177084;PXMP2_ENSG00000176894;RP13-672B3.2_ENSG00000256632
12	133337885	133338378	5Y-H4K8ac_peak_4591	13.76914	ANKLE2_ENSG00000176915
12	133338569	133339124	5Y-H4K8ac_peak_4592	5.98695	ANKLE2_ENSG00000176915
12	133405904	133406191	5Y-H4K8ac_peak_4593	4.95697	GOLGA3_ENSG00000090615
12	133464524	133465009	5Y-H4K8ac_peak_4594	7.65114	RP11-46H11.12_ENSG00000236617
12	133706503	133706861	5Y-H4K8ac_peak_4595	12.26819	ZNF891_ENSG00000214029;ZNF10_ENSG00000256223;ZNF268_ENSG00000090612;CTD-2140B24.4_ENSG00000256825
12	133758223	133758455	5Y-H4K8ac_peak_4596	5.23819	
13	20176107	20176812	5Y-H4K8ac_peak_4597	8.79957	
13	20356618	20356908	5Y-H4K8ac_peak_4598	7.59101	PSPC1_ENSG00000121390

13	20357284	20357732	5Y-H4K8ac_peak_4599	11.1169	PSPC1_ENSG00000121390
13	20438087	20439293	5Y-H4K8ac_peak_4600	15.82359	ZMYM5_ENSG00000132950
13	20531739	20532336	5Y-H4K8ac_peak_4601	18.78631	ZMYM2_ENSG00000121741
13	20533429	20533844	5Y-H4K8ac_peak_4602	9.38203	ZMYM2_ENSG00000121741
13	20692475	20692813	5Y-H4K8ac_peak_4603	6.43775	
13	21099643	21099903	5Y-H4K8ac_peak_4604	6.50117	CRYL1_ENSG00000165475
13	21277174	21277433	5Y-H4K8ac_peak_4605	6.43775	IL17D_ENSG00000172458;AL161772.1_ENSG00000265710
13	21347457	21347874	5Y-H4K8ac_peak_4606	7.90751	N6AMT2_ENSG00000150456
13	21348311	21348547	5Y-H4K8ac_peak_4607	4.50834	N6AMT2_ENSG00000150456
13	21476594	21477336	5Y-H4K8ac_peak_4608	17.63051	XPO4_ENSG00000132953
13	21750844	21751256	5Y-H4K8ac_peak_4609	7.59101	SKA3_ENSG00000165480;MRP63_ENSG00000173141
13	22177996	22178247	5Y-H4K8ac_peak_4610	10.19948	MICU2_ENSG00000165487
13	24040339	24040982	5Y-H4K8ac_peak_4611	12.0443	LINC00327_ENSG00000232977
13	25080913	25081127	5Y-H4K8ac_peak_4612	5.93823	
13	25086530	25087294	5Y-H4K8ac_peak_4613	8.2913	PARP4_ENSG00000102699
13	25875020	25875471	5Y-H4K8ac_peak_4614	17.56739	RP11-271M24.2_ENSG00000260509;NUPL1_ENSG00000139496
13	26547982	26548317	5Y-H4K8ac_peak_4615	6.34046	
13	26548666	26548966	5Y-H4K8ac_peak_4616	4.84727	
13	26797017	26797247	5Y-H4K8ac_peak_4617	4.5753	RNF6_ENSG00000127870
13	26828035	26828504	5Y-H4K8ac_peak_4618	11.4254	CDK8_ENSG00000132964
13	27075019	27075275	5Y-H4K8ac_peak_4619	8.39058	
13	27131890	27132585	5Y-H4K8ac_peak_4620	9.30505	WASF3_ENSG00000132970
13	27137253	27137465	5Y-H4K8ac_peak_4621	4.74398	
13	27825839	27826194	5Y-H4K8ac_peak_4622	8.43511	RPL21_ENSG00000122026
13	28024405	28025280	5Y-H4K8ac_peak_4623	12.21176	MTIF3_ENSG00000122033
13	28713119	28713346	5Y-H4K8ac_peak_4624	5.98695	PAN3-AS1_ENSG00000261485;PAN3_ENSG00000152520
13	29233383	29233617	5Y-H4K8ac_peak_4625	5.59843	POMP_ENSG00000132963
13	29329098	29329336	5Y-H4K8ac_peak_4626	7.59101	
13	30168900	30169743	5Y-H4K8ac_peak_4627	12.2918	SLC7A1_ENSG00000139514
13	30717404	30717607	5Y-H4K8ac_peak_4628	9.02782	
13	31038660	31038890	5Y-H4K8ac_peak_4629	7.76232	
13	31039702	31039969	5Y-H4K8ac_peak_4630	11.53136	
13	31248219	31248413	5Y-H4K8ac_peak_4631	9.23159	
13	31270481	31270692	5Y-H4K8ac_peak_4632	5.40331	
13	31384553	31384745	5Y-H4K8ac_peak_4633	4.79585	
13	31407878	31408107	5Y-H4K8ac_peak_4634	6.14981	
13	31457106	31457333	5Y-H4K8ac_peak_4635	6.77436	LINC00545_ENSG00000236094
13	31481577	31481767	5Y-H4K8ac_peak_4636	5.65584	
13	31572934	31573389	5Y-H4K8ac_peak_4637	4.642	
13	31618322	31618663	5Y-H4K8ac_peak_4638	4.40812	
13	31782909	31783279	5Y-H4K8ac_peak_4639	10.69698	
13	33160734	33161012	5Y-H4K8ac_peak_4640	10.46287	PDS5B_ENSG00000083642
13	33515522	33515749	5Y-H4K8ac_peak_4641	6.50117	
13	34459051	34459261	5Y-H4K8ac_peak_4642	8.33296	
13	34931950	34932143	5Y-H4K8ac_peak_4643	4.07874	
13	34993749	34994095	5Y-H4K8ac_peak_4644	6.14981	

13	36052486	36053059	5Y-H4K8ac_peak_4645	12.10416	
13	36196211	36196421	5Y-H4K8ac_peak_4646	7.31815	
13	36287873	36288095	5Y-H4K8ac_peak_4647	4.03329	
13	36339623	36339819	5Y-H4K8ac_peak_4648	4.84727	
13	36359101	36359791	5Y-H4K8ac_peak_4649	5.64909	
13	36384818	36385147	5Y-H4K8ac_peak_4650	6.79955	
13	36705794	36706066	5Y-H4K8ac_peak_4651	7.38046	DCLK1_ENSG00000133083
13	36871650	36872194	5Y-H4K8ac_peak_4652	9.15007	SOHLH2_ENSG00000120669;CCDC169-SOHLH2_ENSG00000250709;CCDC169_ENSG00000242715
13	36920393	36920754	5Y-H4K8ac_peak_4653	15.82359	SPG20OS_ENSG00000120664
13	37493792	37494039	5Y-H4K8ac_peak_4654	5.40331	SMAD9_ENSG00000120693
13	37494395	37495001	5Y-H4K8ac_peak_4655	17.51157	SMAD9_ENSG00000120693
13	37574059	37574419	5Y-H4K8ac_peak_4656	12.1498	ALG5_ENSG00000120697
13	37574741	37575569	5Y-H4K8ac_peak_4657	6.43775	ALG5_ENSG00000120697
13	39611679	39612098	5Y-H4K8ac_peak_4658	9.23159	PROSER1_ENSG00000120685;NHLRC3_ENSG00000188811
13	41346209	41346462	5Y-H4K8ac_peak_4659	6.13909	MRPS31_ENSG00000102738
13	41363707	41364211	5Y-H4K8ac_peak_4660	6.77436	SLC25A15_ENSG00000102743
13	41496018	41496406	5Y-H4K8ac_peak_4661	8.33296	TPTE2P5_ENSG00000168852;SUGT1P3_ENSG00000239827
13	41768003	41768363	5Y-H4K8ac_peak_4662	12.21176	KBTBD7_ENSG00000120696
13	42534807	42535754	5Y-H4K8ac_peak_4663	9.30505	VWA8_ENSG00000102763
13	42614236	42614499	5Y-H4K8ac_peak_4664	6.37023	DGKH_ENSG00000102780
13	42622658	42623020	5Y-H4K8ac_peak_4665	5.56912	
13	42845290	42846052	5Y-H4K8ac_peak_4666	10.79655	AKAP11_ENSG00000023516
13	44361302	44361603	5Y-H4K8ac_peak_4667	6.08471	ENOX1_ENSG00000120658;RP11-168P13.1_ENSG00000270522
13	44453100	44453623	5Y-H4K8ac_peak_4668	11.86504	CCDC122_ENSG00000151773;LACC1_ENSG00000179630
13	44715857	44716054	5Y-H4K8ac_peak_4669	6.50117	SMIM2-AS1_ENSG00000227258
13	44846233	44846568	5Y-H4K8ac_peak_4670	6.50117	
13	44905691	44905915	5Y-H4K8ac_peak_4671	7.58806	
13	44946943	44947195	5Y-H4K8ac_peak_4672	5.03335	SERP2_ENSG00000151778
13	44947505	44948150	5Y-H4K8ac_peak_4673	13.61292	SERP2_ENSG00000151778
13	44977126	44977343	5Y-H4K8ac_peak_4674	6.50117	
13	44978339	44979009	5Y-H4K8ac_peak_4675	9.30505	
13	45151861	45152304	5Y-H4K8ac_peak_4676	15.80786	TSC22D1_ENSG00000102804
13	45308511	45308987	5Y-H4K8ac_peak_4677	6.78128	
13	45504407	45504750	5Y-H4K8ac_peak_4678	4.90471	
13	45563165	45563484	5Y-H4K8ac_peak_4679	8.28346	NUFIP1_ENSG00000083635;GPALPP1_ENSG00000133114
13	45694653	45694871	5Y-H4K8ac_peak_4680	9.15007	GTF2F2_ENSG00000188342
13	45914933	45915192	5Y-H4K8ac_peak_4681	10.69698	TPT1_ENSG00000133112;RP11-290D2.6_ENSG00000273149;TPT1-AS1_ENSG00000170919
13	46039101	46039507	5Y-H4K8ac_peak_4682	11.68317	COG3_ENSG00000136152
13	46626486	46626804	5Y-H4K8ac_peak_4683	14.74967	ZC3H13_ENSG00000123200;CPB2-AS1_ENSG00000235903
13	47127929	47128128	5Y-H4K8ac_peak_4684	5.24695	LRCH1_ENSG00000136141
13	47187304	47187496	5Y-H4K8ac_peak_4685	6.79955	
13	47203676	47204021	5Y-H4K8ac_peak_4686	6.77436	
13	47323034	47323302	5Y-H4K8ac_peak_4687	6.39626	
13	48574905	48575391	5Y-H4K8ac_peak_4688	6.08523	SUCLA2-AS1_ENSG00000227848
13	48668857	48669091	5Y-H4K8ac_peak_4689	13.60015	MED4_ENSG00000136146
13	48877250	48877581	5Y-H4K8ac_peak_4690	10.36926	LINC00441_ENSG00000231473;RB1_ENSG00000139687

13	49106975	49107200	5Y-H4K8ac_peak_4691	11.22005	RCBTB2_ENSG00000136161
13	49549798	49549989	5Y-H4K8ac_peak_4692	7.65114	FNDC3A_ENSG00000102531
13	50159860	50160153	5Y-H4K8ac_peak_4693	11.68317	RCBTB1_ENSG00000136144
13	50422356	50422572	5Y-H4K8ac_peak_4694	9.20645	
13	50570337	50570928	5Y-H4K8ac_peak_4695	10.42746	TRIM13_ENSG00000204977;MIR3613_ENSG00000264864
13	50655512	50656009	5Y-H4K8ac_peak_4696	7.11863	DLEU1_ENSG00000176124
13	50699009	50700358	5Y-H4K8ac_peak_4697	21.10958	DLEU2_ENSG00000231607
13	51483977	51484615	5Y-H4K8ac_peak_4698	12.72376	RNASEH2B-AS1_ENSG00000233672;RNASEH2B_ENSG00000136104
13	52026092	52026832	5Y-H4K8ac_peak_4699	12.11208	INTS6-AS1_ENSG00000236778
13	52027631	52028014	5Y-H4K8ac_peak_4700	8.2913	INTS6_ENSG00000102786;RPS4XP16_ENSG00000224892
13	52157603	52157895	5Y-H4K8ac_peak_4701	5.64909	WDFY2_ENSG00000139668
13	52194652	52194855	5Y-H4K8ac_peak_4702	4.84727	
13	52195363	52195608	5Y-H4K8ac_peak_4703	7.09444	
13	52207217	52207507	5Y-H4K8ac_peak_4704	8.39058	
13	52209107	52209349	5Y-H4K8ac_peak_4705	5.59843	
13	52311909	52312121	5Y-H4K8ac_peak_4706	5.87382	
13	52356252	52356451	5Y-H4K8ac_peak_4707	4.50834	
13	52361420	52361724	5Y-H4K8ac_peak_4708	18.54495	
13	52378037	52378247	5Y-H4K8ac_peak_4709	7.11863	DHRS12_ENSG00000102796;RP11-327P2.5_ENSG00000231856
13	52378469	52378758	5Y-H4K8ac_peak_4710	5.23819	DHRS12_ENSG00000102796;RP11-327P2.5_ENSG00000231856
13	52733292	52733682	5Y-H4K8ac_peak_4711	12.21176	NEK3_ENSG00000136098
13	52769571	52769775	5Y-H4K8ac_peak_4712	4.07874	MRPS31P5_ENSG00000243406
13	53024368	53024708	5Y-H4K8ac_peak_4713	13.1133	VPS36_ENSG00000136100
13	53029742	53029992	5Y-H4K8ac_peak_4714	7.63144	CKAP2_ENSG00000136108
13	60218908	60219132	5Y-H4K8ac_peak_4715	10.46135	
13	60737678	60737955	5Y-H4K8ac_peak_4716	7.97699	DIAPH3_ENSG00000139734
13	60971094	60971292	5Y-H4K8ac_peak_4717	7.89273	TDRD3_ENSG00000083544
13	71199021	71199226	5Y-H4K8ac_peak_4718	9.38203	
13	73301558	73301749	5Y-H4K8ac_peak_4719	7.89273	MZT1_ENSG00000204899;BORA_ENSG00000136122
13	73355688	73355937	5Y-H4K8ac_peak_4720	8.93923	DIS3_ENSG00000083520;PIBF1_ENSG00000083535
13	74339304	74339577	5Y-H4K8ac_peak_4721	6.90749	
13	74621440	74621668	5Y-H4K8ac_peak_4722	6.34046	
13	74708738	74709594	5Y-H4K8ac_peak_4723	15.17389	KLF12_ENSG00000118922
13	74709827	74710073	5Y-H4K8ac_peak_4724	7.11863	
13	74862749	74863078	5Y-H4K8ac_peak_4725	5.56912	RNY1P5_ENSG00000206617
13	75129221	75129565	5Y-H4K8ac_peak_4726	6.43775	
13	75149600	75149885	5Y-H4K8ac_peak_4727	6.22669	
13	75150223	75150463	5Y-H4K8ac_peak_4728	3.96434	
13	75150720	75150959	5Y-H4K8ac_peak_4729	4.36976	
13	76209751	76210336	5Y-H4K8ac_peak_4730	12.21176	RP11-173B14.5_ENSG00000261105
13	76210940	76211158	5Y-H4K8ac_peak_4731	4.95697	RP11-173B14.5_ENSG00000261105
13	77459567	77459966	5Y-H4K8ac_peak_4732	5.98695	KCTD12_ENSG00000178695;AC000403.1_ENSG00000264908
13	77600960	77601285	5Y-H4K8ac_peak_4733	11.1169	FBXL3_ENSG00000005812
13	79057881	79058123	5Y-H4K8ac_peak_4734	7.17184	
13	79979943	79980149	5Y-H4K8ac_peak_4735	9.15007	RBM26_ENSG00000139746;RBM26-AS1_ENSG00000227354
13	79980566	79980801	5Y-H4K8ac_peak_4736	8.69112	RBM26_ENSG00000139746;RBM26-AS1_ENSG00000227354

13	80054975	80055197	5Y-H4K8ac_peak_4737	8.93304	NDFIP2-AS1_ENSG00000232132;NDFIP2_ENSG00000102471
13	80055557	80055852	5Y-H4K8ac_peak_4738	6.77436	NDFIP2-AS1_ENSG00000232132;NDFIP2_ENSG00000102471
13	80232616	80233011	5Y-H4K8ac_peak_4739	9.38203	
13	80247492	80247761	5Y-H4K8ac_peak_4740	6.34046	
13	80250205	80250469	5Y-H4K8ac_peak_4741	8.33296	
13	80399922	80400116	5Y-H4K8ac_peak_4742	7.31815	
13	80408403	80408624	5Y-H4K8ac_peak_4743	8.33296	
13	86395188	86395500	5Y-H4K8ac_peak_4744	4.84727	
13	91079714	91080022	5Y-H4K8ac_peak_4745	4.84727	
13	91999982	92001309	5Y-H4K8ac_peak_4746	28.49394	MIR17HG_ENSG00000215417
13	92051115	92051585	5Y-H4K8ac_peak_4747	8.33296	GPC5_ENSG00000179399
13	92274181	92274377	5Y-H4K8ac_peak_4748	6.00388	
13	93879706	93880106	5Y-H4K8ac_peak_4749	7.87406	GPC6_ENSG00000183098
13	95202248	95202446	5Y-H4K8ac_peak_4750	6.53157	
13	95248806	95249324	5Y-H4K8ac_peak_4751	8.79957	TGDS_ENSG00000088451
13	95512573	95512793	5Y-H4K8ac_peak_4752	5.26353	
13	95953281	95953997	5Y-H4K8ac_peak_4753	5.98695	ABCC4_ENSG00000125257
13	96296389	96296658	5Y-H4K8ac_peak_4754	8.21582	DZIP1_ENSG00000134874
13	96328887	96329129	5Y-H4K8ac_peak_4755	7.31815	DNAJC3-AS1_ENSG00000247400;DNAJC3_ENSG00000102580
13	96329561	96329798	5Y-H4K8ac_peak_4756	8.2913	DNAJC3-AS1_ENSG00000247400;DNAJC3_ENSG00000102580
13	96705304	96705537	5Y-H4K8ac_peak_4757	7.58806	UGGT2_ENSG00000102595
13	97176842	97177050	5Y-H4K8ac_peak_4758	7.31815	
13	97450425	97450618	5Y-H4K8ac_peak_4759	7.31815	
13	98085888	98086126	5Y-H4K8ac_peak_4760	4.29586	RAP2A_ENSG00000125249
13	98627776	98628569	5Y-H4K8ac_peak_4761	14.81591	
13	98628938	98629318	5Y-H4K8ac_peak_4762	8.79957	
13	98794504	98795176	5Y-H4K8ac_peak_4763	5.91107	FARP1_ENSG00000152767
13	99229528	99229871	5Y-H4K8ac_peak_4764	4.642	STK24_ENSG00000102572;STK24-AS1_ENSG00000224418
13	99275301	99275494	5Y-H4K8ac_peak_4765	5.65584	
13	99610457	99610694	5Y-H4K8ac_peak_4766	6.79955	
13	99738296	99738497	5Y-H4K8ac_peak_4767	7.73231	DOCK9_ENSG00000088387
13	99852398	99852889	5Y-H4K8ac_peak_4768	9.89244	UBAC2-AS1_ENSG00000228889;UBAC2_ENSG00000134882
13	99853162	99853521	5Y-H4K8ac_peak_4769	5.24695	UBAC2-AS1_ENSG00000228889;UBAC2_ENSG00000134882
13	99965214	99965454	5Y-H4K8ac_peak_4770	6.59249	
13	100259016	100259246	5Y-H4K8ac_peak_4771	11.57334	CLYBL_ENSG00000125246
13	100741489	100741716	5Y-H4K8ac_peak_4772	8.21582	PCCA_ENSG00000175198
13	101240646	101241060	5Y-H4K8ac_peak_4773	13.27281	GGACT_ENSG00000134864
13	101326620	101327050	5Y-H4K8ac_peak_4774	9.23159	TMTC4_ENSG00000125247
13	101327495	101327771	5Y-H4K8ac_peak_4775	8.43511	TMTC4_ENSG00000125247
13	103047018	103047356	5Y-H4K8ac_peak_4776	9.63153	FGF14-IT1_ENSG00000243319;FGF14-AS2_ENSG00000272143
13	103208786	103209102	5Y-H4K8ac_peak_4777	5.03335	
13	103426508	103426857	5Y-H4K8ac_peak_4778	7.11863	TEX30_ENSG00000151287
13	103497881	103498323	5Y-H4K8ac_peak_4779	8.35139	ERCC5_ENSG00000134899
13	104659730	104660023	5Y-H4K8ac_peak_4780	6.34046	
13	107220167	107220357	5Y-H4K8ac_peak_4781	4.50834	
13	107570051	107570518	5Y-H4K8ac_peak_4782	18.31877	

13	108870915	108871218	5Y-H4K8ac_peak_4783	6.77436	LIG4_ENSG00000174405;ABHD13_ENSG00000139826
13	109985032	109985270	5Y-H4K8ac_peak_4784	4.16635	
13	109986719	109987038	5Y-H4K8ac_peak_4785	10.1994	
13	110021970	110022440	5Y-H4K8ac_peak_4786	12.06963	
13	110308047	110308314	5Y-H4K8ac_peak_4787	9.30505	
13	110319111	110319311	5Y-H4K8ac_peak_4788	4.8773	
13	110321579	110321842	5Y-H4K8ac_peak_4789	6.50117	
13	110322343	110322657	5Y-H4K8ac_peak_4790	12.82003	
13	110434677	110434995	5Y-H4K8ac_peak_4791	7.58806	
13	110439514	110439725	5Y-H4K8ac_peak_4792	4.50834	IRS2_ENSG00000185950
13	110790834	110791343	5Y-H4K8ac_peak_4793	12.71156	
13	110958775	110959834	5Y-H4K8ac_peak_4794	13.85283	COL4A1_ENSG00000187498;COL4A2_ENSG00000134871
13	111267362	111267659	5Y-H4K8ac_peak_4795	10.79063	CARKD_ENSG00000213995
13	111364964	111365227	5Y-H4K8ac_peak_4796	13.12848	CARS2_ENSG00000134905;ING1_ENSG00000153487
13	111566498	111566722	5Y-H4K8ac_peak_4797	7.03573	ANKRD10_ENSG00000088448
13	111567083	111567332	5Y-H4K8ac_peak_4798	8.62703	ANKRD10_ENSG00000088448
13	111568038	111568252	5Y-H4K8ac_peak_4799	7.89273	ANKRD10_ENSG00000088448
13	111767947	111768154	5Y-H4K8ac_peak_4800	7.38046	ARHGEF7-AS2_ENSG00000235875
13	111805509	111805838	5Y-H4K8ac_peak_4801	9.38203	
13	111935206	111935648	5Y-H4K8ac_peak_4802	7.01856	
13	113187438	113187736	5Y-H4K8ac_peak_4803	7.89273	
13	113242031	113242935	5Y-H4K8ac_peak_4804	16.83629	TUBGCP3_ENSG00000126216
13	113343893	113344132	5Y-H4K8ac_peak_4805	7.89273	ATP11A_ENSG00000068650
13	113344491	113345042	5Y-H4K8ac_peak_4806	8.55081	ATP11A_ENSG00000068650
13	113436408	113436621	5Y-H4K8ac_peak_4807	6.77436	
13	113548848	113549088	5Y-H4K8ac_peak_4808	6.73385	MCF2L_ENSG00000126217
13	113598001	113598340	5Y-H4K8ac_peak_4809	7.87406	
13	113623138	113623377	5Y-H4K8ac_peak_4810	5.64909	MCF2L-AS1_ENSG00000235280
13	113654821	113655221	5Y-H4K8ac_peak_4811	6.77436	
13	113656119	113656942	5Y-H4K8ac_peak_4812	9.79526	
13	113863087	113863762	5Y-H4K8ac_peak_4813	9.58986	PCID2_ENSG00000126226;CUL4A_ENSG00000139842
13	113864022	113864304	5Y-H4K8ac_peak_4814	6.50117	PCID2_ENSG00000126226
13	113950605	113951158	5Y-H4K8ac_peak_4815	9.01738	LAMP1_ENSG00000185896
13	114061911	114062458	5Y-H4K8ac_peak_4816	9.74838	
13	114067805	114068064	5Y-H4K8ac_peak_4817	4.33206	
13	114076330	114076544	5Y-H4K8ac_peak_4818	6.77436	
13	114077237	114077440	5Y-H4K8ac_peak_4819	7.59101	
13	114198379	114198594	5Y-H4K8ac_peak_4820	7.90751	
13	114199903	114200258	5Y-H4K8ac_peak_4821	10.69698	
13	114203621	114203975	5Y-H4K8ac_peak_4822	11.28133	
13	114238478	114238734	5Y-H4K8ac_peak_4823	7.76232	TFDP1_ENSG00000198176
13	114239148	114239800	5Y-H4K8ac_peak_4824	8.67475	TFDP1_ENSG00000198176
13	114273548	114274199	5Y-H4K8ac_peak_4825	7.59101	
13	114307735	114307982	5Y-H4K8ac_peak_4826	6.03718	
13	114450174	114450402	5Y-H4K8ac_peak_4827	10.86079	
13	114461848	114462080	5Y-H4K8ac_peak_4828	7.59101	TMEM255B_ENSG00000184497

13	114504644	114506021	5Y-H4K8ac_peak_4829	19.93272	
13	114515370	114515653	5Y-H4K8ac_peak_4830	4.90696	
13	114540284	114541105	5Y-H4K8ac_peak_4831	10.69698	
13	114631951	114632357	5Y-H4K8ac_peak_4832	5.23083	LINC00565_ENSG00000260910
13	114829737	114830514	5Y-H4K8ac_peak_4833	9.05168	
13	114830808	114831184	5Y-H4K8ac_peak_4834	5.60566	
13	114863370	114863665	5Y-H4K8ac_peak_4835	9.93099	
13	114864705	114864964	5Y-H4K8ac_peak_4836	7.21406	
13	114875426	114875786	5Y-H4K8ac_peak_4837	4.90696	
13	114999979	115000260	5Y-H4K8ac_peak_4838	10.19948	CDC16_ENSG00000130177
13	115000493	115000756	5Y-H4K8ac_peak_4839	6.50117	CDC16_ENSG00000130177
13	115046630	115047273	5Y-H4K8ac_peak_4840	8.24461	UPF3A_ENSG00000169062
13	115075338	115075778	5Y-H4K8ac_peak_4841	6.50117	
13	115078952	115079387	5Y-H4K8ac_peak_4842	6.50117	CHAMP1_ENSG00000198824
14	20881758	20882099	5Y-H4K8ac_peak_4843	6.17374	TEP1_ENSG00000129566
14	21537471	21538117	5Y-H4K8ac_peak_4844	5.64909	NDRG2_ENSG00000165795;ARHGEF40_ENSG00000165801
14	21538424	21538684	5Y-H4K8ac_peak_4845	12.06963	NDRG2_ENSG00000165795;ARHGEF40_ENSG00000165801
14	21560260	21560489	5Y-H4K8ac_peak_4846	7.35333	
14	21565845	21566066	5Y-H4K8ac_peak_4847	6.07082	
14	21575328	21575557	5Y-H4K8ac_peak_4848	4.29586	RP11-998D10.4_ENSG00000178107
14	21769063	21769533	5Y-H4K8ac_peak_4849	7.31102	
14	21978923	21979350	5Y-H4K8ac_peak_4850	8.17203	METTL3_ENSG00000165819
14	23025129	23025658	5Y-H4K8ac_peak_4851	6.43775	AE000662.93_ENSG00000259054;AE000662.92_ENSG00000259003
14	23026110	23026447	5Y-H4K8ac_peak_4852	5.24695	AE000662.93_ENSG00000259054;AE000662.92_ENSG00000259003
14	23236046	23236315	5Y-H4K8ac_peak_4853	10.1994	CTD-2555K7.2_ENSG00000258458;OXA1L_ENSG00000155463
14	23291585	23291813	5Y-H4K8ac_peak_4854	8.2913	AL135998.1_ENSG00000215306
14	23298360	23298638	5Y-H4K8ac_peak_4855	6.72926	MRPL52_ENSG00000172590;SLC7A7_ENSG00000155465
14	23317759	23319015	5Y-H4K8ac_peak_4856	7.9057	
14	23341276	23341514	5Y-H4K8ac_peak_4857	5.35202	LRP10_ENSG00000197324
14	23355817	23356402	5Y-H4K8ac_peak_4858	9.36633	
14	23450950	23451187	5Y-H4K8ac_peak_4859	4.55128	RP11-298I3.5_ENSG00000259132;AJUBA_ENSG00000129474;RP11-298I3.4_ENSG00000258457
14	23476003	23476297	5Y-H4K8ac_peak_4860	12.44818	
14	23771347	23771773	5Y-H4K8ac_peak_4861	7.59101	PPP1R3E_ENSG00000235194
14	23775609	23775897	5Y-H4K8ac_peak_4862	7.18918	BCL2L2-PABPN1_ENSG00000258643
14	23776088	23776374	5Y-H4K8ac_peak_4863	12.05638	BCL2L2-PABPN1_ENSG00000258643
14	23790799	23791055	5Y-H4K8ac_peak_4864	7.89273	AL049829.1_ENSG00000268702;PABPN1_ENSG00000100836
14	23834116	23835472	5Y-H4K8ac_peak_4865	7.90005	EFS_ENSG00000100842
14	23835830	23836252	5Y-H4K8ac_peak_4866	7.92287	EFS_ENSG00000100842
14	23938129	23938352	5Y-H4K8ac_peak_4867	8.35139	NGDN_ENSG00000129460
14	23981119	23981609	5Y-H4K8ac_peak_4868	6.34046	RP11-66N24.4_ENSG00000157306
14	23982167	23982359	5Y-H4K8ac_peak_4869	5.23083	
14	23982646	23983149	5Y-H4K8ac_peak_4870	6.50117	
14	24021120	24021644	5Y-H4K8ac_peak_4871	9.30338	
14	24025182	24025405	5Y-H4K8ac_peak_4872	10.35586	ZFHX2_ENSG00000136367;THTPA_ENSG00000259431
14	24046550	24046802	5Y-H4K8ac_peak_4873	4.79658	
14	24048416	24048657	5Y-H4K8ac_peak_4874	4.29586	JPH4_ENSG00000092051

14	24049672	24049981	5Y-H4K8ac_peak_4875	6.50117	
14	24422981	24423225	5Y-H4K8ac_peak_4876	12.21176	DHRS4_ENSG00000157326
14	24438736	24439424	5Y-H4K8ac_peak_4877	11.57334	DHRS4L2_ENSG00000187630
14	24476350	24476637	5Y-H4K8ac_peak_4878	11.01287	
14	24505582	24505815	5Y-H4K8ac_peak_4879	8.24461	RP11-468E2.9_ENSG00000225766
14	24520584	24521111	5Y-H4K8ac_peak_4880	19.79054	LRRC16B_ENSG00000186648
14	24521360	24521685	5Y-H4K8ac_peak_4881	11.19336	LRRC16B_ENSG00000186648
14	24521911	24522182	5Y-H4K8ac_peak_4882	8.71064	LRRC16B_ENSG00000186648
14	24549491	24549834	5Y-H4K8ac_peak_4883	5.15917	
14	24550460	24551137	5Y-H4K8ac_peak_4884	6.78128	
14	24551859	24552071	5Y-H4K8ac_peak_4885	5.94703	
14	24562752	24563078	5Y-H4K8ac_peak_4886	8.36588	PCK2_ENSG00000100889
14	24616227	24616453	5Y-H4K8ac_peak_4887	9.63153	PSME2_ENSG00000100911;RNF31_ENSG00000092098
14	24657695	24657886	5Y-H4K8ac_peak_4888	5.65584	IPO4_ENSG00000196497
14	24664264	24664873	5Y-H4K8ac_peak_4889	17.67055	
14	24740006	24740200	5Y-H4K8ac_peak_4890	8.47164	RABGGTA_ENSG00000100949
14	24740456	24740724	5Y-H4K8ac_peak_4891	17.18444	RABGGTA_ENSG00000100949
14	24836716	24837075	5Y-H4K8ac_peak_4892	12.10275	
14	24838560	24838854	5Y-H4K8ac_peak_4893	6.54441	
14	24860813	24861022	5Y-H4K8ac_peak_4894	8.12924	
14	24867572	24867772	5Y-H4K8ac_peak_4895	10.27264	NYNRIN_ENSG00000205978
14	24868245	24868563	5Y-H4K8ac_peak_4896	6.79955	NYNRIN_ENSG00000205978
14	24898471	24899455	5Y-H4K8ac_peak_4897	12.69256	CBLN3_ENSG00000139899;KHNYN_ENSG00000100441
14	24911672	24911903	5Y-H4K8ac_peak_4898	10.35586	SDR39U1_ENSG00000100445
14	31342973	31344252	5Y-H4K8ac_peak_4899	7.90005	COCH_ENSG00000100473
14	31495103	31495416	5Y-H4K8ac_peak_4900	4.642	STRN3_ENSG00000196792;AP4S1_ENSG00000100478
14	31676359	31676824	5Y-H4K8ac_peak_4901	5.18558	HECTD1_ENSG00000092148
14	32420508	32420914	5Y-H4K8ac_peak_4902	12.43358	
14	32546761	32547071	5Y-H4K8ac_peak_4903	4.84727	ARHGAP5-AS1_ENSG00000258655
14	34407502	34407859	5Y-H4K8ac_peak_4904	6.08523	
14	34529166	34529813	5Y-H4K8ac_peak_4905	10.15788	
14	35098851	35099253	5Y-H4K8ac_peak_4906	4.50834	SNX6_ENSG00000129515
14	35099445	35099723	5Y-H4K8ac_peak_4907	9.30505	SNX6_ENSG00000129515
14	35183506	35184090	5Y-H4K8ac_peak_4908	9.20645	CFL2_ENSG00000165410
14	35591457	35591724	5Y-H4K8ac_peak_4909	7.65114	PPP2R3C_ENSG00000092020;KIAA0391_ENSG00000100890;KIAA0391_ENSG00000258790
14	35760711	35760914	5Y-H4K8ac_peak_4910	5.34938	AL121594.1_ENSG00000265530
14	35761657	35762044	5Y-H4K8ac_peak_4911	10.11191	AL121594.1_ENSG00000265530
14	36002748	36002991	5Y-H4K8ac_peak_4912	6.55841	INSM2_ENSG00000168348
14	36277924	36278179	5Y-H4K8ac_peak_4913	8.75926	RALGAPA1_ENSG00000174373;AL162311.1_ENSG00000266264
14	36295619	36295866	5Y-H4K8ac_peak_4914	8.69112	RP11-317N8.5_ENSG00000258938;BRMS1L_ENSG00000100916
14	50065280	50066035	5Y-H4K8ac_peak_4915	12.69256	RPS29_ENSG00000213741;AL139099.1_ENSG00000253459;LRR1_ENSG00000165501;RHOQP1_ENSG00000258568
14	50100350	50100801	5Y-H4K8ac_peak_4916	9.51254	
14	50159645	50160250	5Y-H4K8ac_peak_4917	12.0443	KLHDC1_ENSG00000197776
14	50357841	50358037	5Y-H4K8ac_peak_4918	7.14616	
14	50469945	50470443	5Y-H4K8ac_peak_4919	9.38276	
14	50998842	50999130	5Y-H4K8ac_peak_4920	9.7353	ATL1_ENSG00000198513

14	51027303	51027870	5Y-H4K8ac_peak_4921	12.05638	MAP4K5_ENSG00000012983
14	51297546	51297806	5Y-H4K8ac_peak_4922	10.1994	NIN_ENSG00000100503
14	51326124	51326455	5Y-H4K8ac_peak_4923	6.34046	
14	51562074	51562760	5Y-H4K8ac_peak_4924	7.89273	TRIM9_ENSG00000100505
14	51974722	51975031	5Y-H4K8ac_peak_4925	11.09973	
14	52117970	52118360	5Y-H4K8ac_peak_4926	12.21176	
14	52242716	52242926	5Y-H4K8ac_peak_4927	5.40331	
14	52456296	52456508	5Y-H4K8ac_peak_4928	10.1994	C14orf166_ENSG00000087302
14	53172461	53172656	5Y-H4K8ac_peak_4929	7.38046	
14	53258715	53258984	5Y-H4K8ac_peak_4930	5.23083	GNPNAT1_ENSG00000100522
14	53418150	53418357	5Y-H4K8ac_peak_4931	8.24461	FERMT2_ENSG00000073712
14	53620137	53620519	5Y-H4K8ac_peak_4932	4.29586	DDHD1_ENSG00000100523;AL356020.1_ENSG00000266552;RP11-547D23.1_ENSG00000258731
14	54811226	54811575	5Y-H4K8ac_peak_4933	8.69112	
14	54955306	54955652	5Y-H4K8ac_peak_4934	7.20869	GMFB_ENSG00000197045
14	55251302	55251591	5Y-H4K8ac_peak_4935	5.10825	
14	55276138	55276338	5Y-H4K8ac_peak_4936	8.14913	
14	55282173	55282505	5Y-H4K8ac_peak_4937	7.10001	
14	55284186	55285192	5Y-H4K8ac_peak_4938	13.03079	
14	55286307	55286832	5Y-H4K8ac_peak_4939	5.23083	
14	55303583	55303804	5Y-H4K8ac_peak_4940	7.31815	
14	55337797	55337997	5Y-H4K8ac_peak_4941	4.47071	
14	55344422	55344992	5Y-H4K8ac_peak_4942	6.77436	MIR4308_ENSG00000265432
14	55350379	55350642	5Y-H4K8ac_peak_4943	5.65584	
14	55352224	55352645	5Y-H4K8ac_peak_4944	9.38807	FDPSP3_ENSG00000258872
14	55369225	55369431	5Y-H4K8ac_peak_4945	7.38046	GCH1_ENSG00000131979
14	55517598	55518027	5Y-H4K8ac_peak_4946	8.75926	MAPK1IP1L_ENSG00000168175
14	55518425	55518721	5Y-H4K8ac_peak_4947	15.84891	MAPK1IP1L_ENSG00000168175
14	55878145	55878808	5Y-H4K8ac_peak_4948	12.05638	ATG14_ENSG00000126775
14	57046309	57046515	5Y-H4K8ac_peak_4949	10.19948	
14	57735236	57735530	5Y-H4K8ac_peak_4950	8.69112	EXOC5_ENSG00000070367;AP5M1_ENSG00000053770
14	57856911	57857170	5Y-H4K8ac_peak_4951	7.31815	NAA30_ENSG00000139977
14	58862232	58863090	5Y-H4K8ac_peak_4952	12.14222	RP11-517O13.3_ENSG00000258658;TOMM20L_ENSG00000196860
14	59930954	59931256	5Y-H4K8ac_peak_4953	4.94258	GPR135_ENSG00000181619
14	59950841	59951037	5Y-H4K8ac_peak_4954	7.87406	L3HYPDH_ENSG00000126790;JKAMP_ENSG00000050130
14	60558716	60558972	5Y-H4K8ac_peak_4955	6.53157	RP11-16B13.1_ENSG00000258553;PCNXL4_ENSG00000126773
14	60715394	60715672	5Y-H4K8ac_peak_4956	11.1169	CTD-2184C24.2_ENSG00000254718
14	60716133	60716440	5Y-H4K8ac_peak_4957	11.53521	CTD-2184C24.2_ENSG00000254718
14	60780857	60781116	5Y-H4K8ac_peak_4958	7.53429	
14	60816445	60816680	5Y-H4K8ac_peak_4959	6.50117	
14	60981479	60982189	5Y-H4K8ac_peak_4960	11.48691	C14orf39_ENSG00000179008;RP11-1042B17.3_ENSG00000258670
14	61104028	61104631	5Y-H4K8ac_peak_4961	17.50045	
14	61747448	61748337	5Y-H4K8ac_peak_4962	12.71156	TMEM30B_ENSG00000182107
14	61936747	61937070	5Y-H4K8ac_peak_4963	4.84727	
14	61937661	61937990	5Y-H4K8ac_peak_4964	6.88532	
14	61939899	61940200	5Y-H4K8ac_peak_4965	7.18391	
14	61950666	61950880	5Y-H4K8ac_peak_4966	4.33574	

14	62028455	62028838	5Y-H4K8ac_peak_4967	5.12213	
14	62043081	62043397	5Y-H4K8ac_peak_4968	7.46096	
14	62074660	62074945	5Y-H4K8ac_peak_4969	5.24695	
14	62162463	62163073	5Y-H4K8ac_peak_4970	7.11863	HIF1A-AS1_ENSG00000258777;HIF1A_ENSG00000100644
14	62228583	62228826	5Y-H4K8ac_peak_4971	6.50117	SNAPC1_ENSG00000023608
14	62278867	62279198	5Y-H4K8ac_peak_4972	6.50117	CTD-2277K2.1_ENSG00000258882
14	62279716	62280028	5Y-H4K8ac_peak_4973	7.11863	
14	64009651	64009895	5Y-H4K8ac_peak_4974	8.43511	PPP2R5E_ENSG00000154001;CTD-2302E22.4_ENSG00000261242
14	64320140	64320363	5Y-H4K8ac_peak_4975	7.89142	SYNE2_ENSG00000054654
14	64804633	64804905	5Y-H4K8ac_peak_4976	5.64909	ESR2_ENSG00000140009;RP11-544I20.2_ENSG00000214770
14	64805736	64806028	5Y-H4K8ac_peak_4977	9.69628	ESR2_ENSG00000140009;RP11-544I20.2_ENSG00000214770
14	64932263	64932455	5Y-H4K8ac_peak_4978	8.08609	AKAP5_ENSG00000179841
14	64970758	64971916	5Y-H4K8ac_peak_4979	15.45397	ZBTB25_ENSG00000089775;ZBTB1_ENSG00000126804
14	65006247	65006809	5Y-H4K8ac_peak_4980	12.69256	RP11-973N13.4_ENSG00000259116
14	65103144	65103381	5Y-H4K8ac_peak_4981	9.97632	
14	65170731	65171027	5Y-H4K8ac_peak_4982	13.4304	PLEKHG3_ENSG00000126822
14	65228107	65228483	5Y-H4K8ac_peak_4983	6.1654	
14	65229110	65229351	5Y-H4K8ac_peak_4984	5.65584	
14	65229739	65230060	5Y-H4K8ac_peak_4985	7.63144	
14	65230757	65231444	5Y-H4K8ac_peak_4986	7.20869	
14	65231887	65232299	5Y-H4K8ac_peak_4987	14.61459	
14	65290156	65290503	5Y-H4K8ac_peak_4988	14.87721	
14	65438715	65438915	5Y-H4K8ac_peak_4989	5.449	RAB15_ENSG00000139998
14	65439120	65439421	5Y-H4K8ac_peak_4990	7.38046	RAB15_ENSG00000139998
14	65696414	65696649	5Y-H4K8ac_peak_4991	11.19336	
14	65878713	65879132	5Y-H4K8ac_peak_4992	6.78318	
14	65936780	65937011	5Y-H4K8ac_peak_4993	7.76232	MIR625_ENSG00000207781
14	66699528	66700003	5Y-H4K8ac_peak_4994	5.23083	
14	66700781	66701065	5Y-H4K8ac_peak_4995	8.17203	
14	67981651	67981877	5Y-H4K8ac_peak_4996	10.60083	
14	67999510	67999755	5Y-H4K8ac_peak_4997	10.35586	TMEM229B_ENSG00000198133;PLEKHH1_ENSG00000054690
14	68067158	68067512	5Y-H4K8ac_peak_4998	8.73392	PIGH_ENSG00000100564
14	68141033	68141621	5Y-H4K8ac_peak_4999	16.01457	VTI1B_ENSG00000100568
14	68141812	68142097	5Y-H4K8ac_peak_5000	5.65584	VTI1B_ENSG00000100568
14	68285839	68286128	5Y-H4K8ac_peak_5001	10.19948	RAD51B_ENSG00000182185
14	69051915	69052216	5Y-H4K8ac_peak_5002	7.69843	
14	69121039	69121774	5Y-H4K8ac_peak_5003	11.22005	
14	69122190	69122981	5Y-H4K8ac_peak_5004	9.45148	
14	69155927	69156220	5Y-H4K8ac_peak_5005	5.35202	
14	69156524	69157090	5Y-H4K8ac_peak_5006	5.65584	
14	69157306	69158068	5Y-H4K8ac_peak_5007	8.43511	
14	69262135	69263165	5Y-H4K8ac_peak_5008	13.00733	ZFP36L1_ENSG00000185650
14	69273958	69274305	5Y-H4K8ac_peak_5009	7.38046	
14	69282798	69283541	5Y-H4K8ac_peak_5010	14.99673	
14	69290865	69291519	5Y-H4K8ac_peak_5011	7.59101	
14	69404424	69404622	5Y-H4K8ac_peak_5012	7.03573	

14	69405737	69406261	5Y-H4K8ac_peak_5013	10.72893	
14	69620210	69620432	5Y-H4K8ac_peak_5014	5.87382	DCAF5_ENSG00000139990
14	69658401	69658718	5Y-H4K8ac_peak_5015	6.77436	EXD2_ENSG00000081177
14	69754859	69755100	5Y-H4K8ac_peak_5016	4.50834	
14	70038977	70039205	5Y-H4K8ac_peak_5017	6.96612	CCDC177_ENSG00000255994
14	70193444	70193673	5Y-H4K8ac_peak_5018	10.90365	SRSF5_ENSG00000100650
14	70512623	70512821	5Y-H4K8ac_peak_5019	5.64909	
14	70513016	70513259	5Y-H4K8ac_peak_5020	8.43511	
14	70560678	70561298	5Y-H4K8ac_peak_5021	5.78188	
14	70654621	70654869	5Y-H4K8ac_peak_5022	6.50117	SLC8A3_ENSG00000100678;RP11-486O13.2_ENSG00000258422
14	71108691	71108885	5Y-H4K8ac_peak_5023	4.29586	CTD-2540L5.5_ENSG00000259115;CTD-2540L5.6_ENSG00000245466;TTC9_ENSG00000133985
14	71288524	71288926	5Y-H4K8ac_peak_5024	7.38046	
14	71703728	71703932	5Y-H4K8ac_peak_5025	6.50117	
14	71787431	71787756	5Y-H4K8ac_peak_5026	7.89273	RP1-261D10.2_ENSG00000259146;SIPA1L1_ENSG00000197555
14	72062903	72063108	5Y-H4K8ac_peak_5027	6.34046	
14	73026994	73027228	5Y-H4K8ac_peak_5028	8.39058	
14	73204841	73205287	5Y-H4K8ac_peak_5029	8.21582	
14	73207194	73207463	5Y-H4K8ac_peak_5030	9.26003	
14	73208543	73209308	5Y-H4K8ac_peak_5031	12.26606	
14	74036124	74036651	5Y-H4K8ac_peak_5032	7.27671	
14	74058425	74058913	5Y-H4K8ac_peak_5033	7.65114	ACOT4_ENSG00000177465
14	74110649	74110854	5Y-H4K8ac_peak_5034	9.23159	DNAL1_ENSG00000119661
14	74253417	74254234	5Y-H4K8ac_peak_5035	5.98695	RP5-1021I20.1_ENSG00000259065
14	74352972	74353168	5Y-H4K8ac_peak_5036	6.00388	ZNF410_ENSG00000119725
14	74353604	74354037	5Y-H4K8ac_peak_5037	7.59101	ZNF410_ENSG00000119725
14	74417035	74417356	5Y-H4K8ac_peak_5038	9.30505	FAM161B_ENSG00000156050;COQ6_ENSG00000119723
14	74723874	74724189	5Y-H4K8ac_peak_5039	5.87725	
14	74769311	74769591	5Y-H4K8ac_peak_5040	7.34185	ABCD4_ENSG00000119688;VRTN_ENSG00000133980
14	74869242	74869590	5Y-H4K8ac_peak_5041	6.00382	
14	75078467	75079024	5Y-H4K8ac_peak_5042	11.19336	LTBP2_ENSG00000119681
14	75103403	75104180	5Y-H4K8ac_peak_5043	9.56516	
14	75348731	75349022	5Y-H4K8ac_peak_5044	8.79957	DLST_ENSG00000119689
14	75389404	75389982	5Y-H4K8ac_peak_5045	12.21176	RPS6KL1_ENSG00000198208
14	75421947	75422169	5Y-H4K8ac_peak_5046	9.38276	PGF_ENSG00000119630
14	75439906	75440336	5Y-H4K8ac_peak_5047	5.91107	
14	75444049	75445014	5Y-H4K8ac_peak_5048	9.01966	
14	75446487	75447018	5Y-H4K8ac_peak_5049	10.69698	
14	75469715	75469955	5Y-H4K8ac_peak_5050	6.31818	EIF2B2_ENSG00000119718
14	75536401	75536663	5Y-H4K8ac_peak_5051	11.12941	ACYP1_ENSG00000119640
14	75593180	75594026	5Y-H4K8ac_peak_5052	7.50148	NEK9_ENSG00000119638;RP11-950C14.7_ENSG00000259138
14	75594238	75594434	5Y-H4K8ac_peak_5053	5.64909	NEK9_ENSG00000119638;RP11-950C14.7_ENSG00000259138
14	75642872	75643201	5Y-H4K8ac_peak_5054	11.57334	TMED10_ENSG00000170348
14	75703860	75704104	5Y-H4K8ac_peak_5055	3.93411	
14	75770610	75770807	5Y-H4K8ac_peak_5056	7.38046	
14	75777504	75777795	5Y-H4K8ac_peak_5057	5.65584	
14	76445387	76445762	5Y-H4K8ac_peak_5058	4.29586	

14	76448559	76448849	5Y-H4K8ac_peak_5059	9.52603	TGFB3_ENSG00000119699
14	76605038	76605268	5Y-H4K8ac_peak_5060	5.91897	
14	77227371	77227632	5Y-H4K8ac_peak_5061	10.54321	RP11-99E15.2_ENSG00000258569;VASH1_ENSG00000071246
14	77228385	77228614	5Y-H4K8ac_peak_5062	7.44077	RP11-99E15.2_ENSG00000258569;VASH1_ENSG00000071246
14	77278838	77279087	5Y-H4K8ac_peak_5063	7.09658	
14	77370799	77371501	5Y-H4K8ac_peak_5064	7.38046	
14	77385144	77386324	5Y-H4K8ac_peak_5065	13.0168	
14	77386685	77387456	5Y-H4K8ac_peak_5066	12.10416	
14	77391027	77391546	5Y-H4K8ac_peak_5067	6.77436	RP11-488C13.4_ENSG00000259058
14	77393327	77393527	5Y-H4K8ac_peak_5068	6.14981	
14	77412861	77413585	5Y-H4K8ac_peak_5069	10.12457	
14	77414706	77415087	5Y-H4K8ac_peak_5070	5.41472	
14	77419659	77420209	5Y-H4K8ac_peak_5071	12.10416	
14	77421183	77421843	5Y-H4K8ac_peak_5072	9.41549	
14	77422559	77422953	5Y-H4K8ac_peak_5073	11.22005	
14	77459930	77460215	5Y-H4K8ac_peak_5074	5.77946	
14	77494347	77494735	5Y-H4K8ac_peak_5075	4.77126	IRF2BPL_ENSG00000119669
14	77498012	77498327	5Y-H4K8ac_peak_5076	4.728	
14	77499043	77499511	5Y-H4K8ac_peak_5077	17.04999	
14	77500090	77500450	5Y-H4K8ac_peak_5078	8.73985	
14	77503747	77503940	5Y-H4K8ac_peak_5079	5.23083	
14	77504203	77504716	5Y-H4K8ac_peak_5080	15.10396	
14	77506097	77506484	5Y-H4K8ac_peak_5081	6.20989	RP11-7F17.5_ENSG00000246548
14	77507031	77507687	5Y-H4K8ac_peak_5082	17.04999	RP11-7F17.5_ENSG00000246548
14	77517663	77517867	5Y-H4K8ac_peak_5083	7.20869	
14	77519201	77519444	5Y-H4K8ac_peak_5084	8.04693	
14	77607747	77608062	5Y-H4K8ac_peak_5085	7.31102	AC007375.1_ENSG00000269883
14	77608289	77608523	5Y-H4K8ac_peak_5086	7.84116	ZDHHC22_ENSG00000177108
14	77612521	77612731	5Y-H4K8ac_peak_5087	4.9603	
14	77624370	77624689	5Y-H4K8ac_peak_5088	7.38046	
14	77624946	77625478	5Y-H4K8ac_peak_5089	11.19336	
14	77648356	77648965	5Y-H4K8ac_peak_5090	11.39862	
14	77924164	77924857	5Y-H4K8ac_peak_5091	10.10203	VIPAS39_ENSG00000151445;AHSA1_ENSG00000100591
14	78266499	78267003	5Y-H4K8ac_peak_5092	9.00954	ADCK1_ENSG00000063761
14	78348793	78349107	5Y-H4K8ac_peak_5093	9.30505	
14	78349922	78350434	5Y-H4K8ac_peak_5094	17.50049	
14	78350631	78350863	5Y-H4K8ac_peak_5095	6.50117	
14	78447341	78447546	5Y-H4K8ac_peak_5096	4.84727	
14	78630118	78630429	5Y-H4K8ac_peak_5097	5.23083	
14	78715542	78715786	5Y-H4K8ac_peak_5098	5.97089	
14	78787369	78787618	5Y-H4K8ac_peak_5099	4.74398	
14	81636443	81636691	5Y-H4K8ac_peak_5100	12.26606	RP11-114N19.3_ENSG00000258999
14	88458692	88459263	5Y-H4K8ac_peak_5101	7.01266	GALC_ENSG00000054983
14	88459733	88460202	5Y-H4K8ac_peak_5102	4.77126	GALC_ENSG00000054983
14	89258432	89258707	5Y-H4K8ac_peak_5103	8.47164	EML5_ENSG00000165521
14	89291122	89291319	5Y-H4K8ac_peak_5104	7.03573	TTC8_ENSG00000165533

14	89292365	89292660	5Y-H4K8ac_peak_5105	9.78792	
14	89371482	89372049	5Y-H4K8ac_peak_5106	13.10145	
14	89451103	89451473	5Y-H4K8ac_peak_5107	9.11392	
14	89452710	89453296	5Y-H4K8ac_peak_5108	9.95819	
14	89629002	89629202	5Y-H4K8ac_peak_5109	4.07874	
14	89882836	89883243	5Y-H4K8ac_peak_5110	6.73385	FOXN3-AS1_ENSG00000258920
14	89895217	89895463	5Y-H4K8ac_peak_5111	4.77016	
14	90091239	90091522	5Y-H4K8ac_peak_5112	6.20875	
14	90091918	90092132	5Y-H4K8ac_peak_5113	11.19336	
14	90125582	90125974	5Y-H4K8ac_peak_5114	12.71156	
14	90127009	90127259	5Y-H4K8ac_peak_5115	7.57144	
14	90797335	90797650	5Y-H4K8ac_peak_5116	8.2913	NRDE2_ENSG00000119720
14	90798031	90798327	5Y-H4K8ac_peak_5117	7.11863	NRDE2_ENSG00000119720
14	90848635	90848910	5Y-H4K8ac_peak_5118	6.73047	
14	90849186	90849483	5Y-H4K8ac_peak_5119	7.83553	
14	90849829	90850441	5Y-H4K8ac_peak_5120	8.62703	
14	90909254	90909899	5Y-H4K8ac_peak_5121	12.34314	
14	90916565	90916788	5Y-H4K8ac_peak_5122	11.22005	
14	90917387	90918218	5Y-H4K8ac_peak_5123	12.52787	
14	90918760	90918995	5Y-H4K8ac_peak_5124	9.69677	
14	90919263	90919612	5Y-H4K8ac_peak_5125	9.00954	
14	90919909	90920718	5Y-H4K8ac_peak_5126	14.99673	RP11-1078H9.1_ENSG00000258678;LINC00642_ENSG00000233208
14	90920949	90921985	5Y-H4K8ac_peak_5127	17.34187	RP11-1078H9.1_ENSG00000258678;LINC00642_ENSG00000233208
14	90958049	90958419	5Y-H4K8ac_peak_5128	8.20773	RP11-1078H9.6_ENSG00000259789
14	90978246	90978686	5Y-H4K8ac_peak_5129	5.91107	
14	90988687	90989365	5Y-H4K8ac_peak_5130	13.12848	
14	91011846	91012217	5Y-H4K8ac_peak_5131	9.01738	
14	91012718	91013032	5Y-H4K8ac_peak_5132	5.99504	
14	91018363	91018679	5Y-H4K8ac_peak_5133	6.68024	
14	91082261	91082660	5Y-H4K8ac_peak_5134	6.94897	
14	91096041	91096244	5Y-H4K8ac_peak_5135	6.46606	
14	91283125	91283326	5Y-H4K8ac_peak_5136	9.6443	TTC7B_ENSG00000165914
14	91589140	91589479	5Y-H4K8ac_peak_5137	8.43511	
14	91716109	91716410	5Y-H4K8ac_peak_5138	7.31102	
14	91719459	91720702	5Y-H4K8ac_peak_5139	14.86775	GPR68_ENSG00000119714
14	91730439	91731296	5Y-H4K8ac_peak_5140	4.95697	
14	91749511	91750155	5Y-H4K8ac_peak_5141	8.73985	
14	91787346	91787690	5Y-H4K8ac_peak_5142	8.43511	
14	91838527	91838753	5Y-H4K8ac_peak_5143	7.03573	
14	91843443	91843746	5Y-H4K8ac_peak_5144	11.8513	
14	91844077	91844317	5Y-H4K8ac_peak_5145	7.04158	
14	91845601	91846290	5Y-H4K8ac_peak_5146	5.24695	
14	91859528	91859795	5Y-H4K8ac_peak_5147	7.01856	
14	91860688	91860886	5Y-H4K8ac_peak_5148	7.57144	
14	91865238	91865764	5Y-H4K8ac_peak_5149	5.98695	
14	91883780	91884976	5Y-H4K8ac_peak_5150	9.96543	CCDC88C_ENSG00000015133;RP11-895M11.3_ENSG00000258798;RP11-895M11.2_ENSG00000258446

14	91976105	91976327	5Y-H4K8ac_peak_5151	7.89142	SMEK1_ENSG00000100796
14	91976604	91977325	5Y-H4K8ac_peak_5152	10.60083	SMEK1_ENSG00000100796
14	92572679	92572875	5Y-H4K8ac_peak_5153	10.1994	ATXN3_ENSG00000066427
14	92573387	92573611	5Y-H4K8ac_peak_5154	8.33296	ATXN3_ENSG00000066427
14	93382519	93382740	5Y-H4K8ac_peak_5155	9.30505	
14	93383000	93383602	5Y-H4K8ac_peak_5156	8.79957	
14	93384205	93384739	5Y-H4K8ac_peak_5157	9.52603	
14	93389128	93389336	5Y-H4K8ac_peak_5158	5.64909	CHGA_ENSG00000100604
14	93389631	93389979	5Y-H4K8ac_peak_5159	5.449	CHGA_ENSG00000100604
14	93426293	93426798	5Y-H4K8ac_peak_5160	6.01761	
14	93453649	93454153	5Y-H4K8ac_peak_5161	6.78128	
14	93473945	93474481	5Y-H4K8ac_peak_5162	10.19948	
14	93475666	93475871	5Y-H4K8ac_peak_5163	9.44864	
14	93476350	93477169	5Y-H4K8ac_peak_5164	6.49458	
14	93484309	93484536	5Y-H4K8ac_peak_5165	6.46053	
14	93485499	93486048	5Y-H4K8ac_peak_5166	12.09312	
14	93490479	93490721	5Y-H4K8ac_peak_5167	8.53555	
14	93581903	93582194	5Y-H4K8ac_peak_5168	12.87098	ITPK1_ENSG00000100605
14	93644794	93644997	5Y-H4K8ac_peak_5169	6.50117	
14	93650925	93651120	5Y-H4K8ac_peak_5170	11.62198	MOAP1_ENSG00000165943;TMEM251_ENSG00000153485;RP11-371E8.4_ENSG00000259066
14	93697123	93697693	5Y-H4K8ac_peak_5171	9.58986	
14	93799090	93799305	5Y-H4K8ac_peak_5172	8.43511	BTBD7_ENSG00000011114;UNC79_ENSG00000133958
14	93896776	93896975	5Y-H4K8ac_peak_5173	7.50148	
14	94211782	94212055	5Y-H4K8ac_peak_5174	7.72887	
14	94214644	94214948	5Y-H4K8ac_peak_5175	10.1994	
14	94215296	94215790	5Y-H4K8ac_peak_5176	9.01966	
14	94216026	94216716	5Y-H4K8ac_peak_5177	7.387	
14	94226060	94226479	5Y-H4K8ac_peak_5178	4.95697	
14	94254520	94255321	5Y-H4K8ac_peak_5179	9.36633	PRIMA1_ENSG00000175785
14	94255575	94256031	5Y-H4K8ac_peak_5180	9.00954	PRIMA1_ENSG00000175785
14	94358649	94358855	5Y-H4K8ac_peak_5181	4.17859	
14	94359070	94359316	5Y-H4K8ac_peak_5182	8.21582	
14	94360629	94360843	5Y-H4K8ac_peak_5183	7.11863	
14	94372953	94373171	5Y-H4K8ac_peak_5184	8.12305	
14	94492925	94493556	5Y-H4K8ac_peak_5185	16.50659	OTUB2_ENSG00000089723
14	95156135	95156408	5Y-H4K8ac_peak_5186	12.11208	
14	95236064	95236346	5Y-H4K8ac_peak_5187	7.59101	GSC_ENSG00000133937
14	95403331	95403562	5Y-H4K8ac_peak_5188	10.1994	
14	95536910	95537151	5Y-H4K8ac_peak_5189	8.97752	
14	95623211	95623646	5Y-H4K8ac_peak_5190	7.50148	DICER1_ENSG00000100697;DICER1-AS1_ENSG00000235706
14	95624876	95625087	5Y-H4K8ac_peak_5191	5.56912	DICER1_ENSG00000100697;DICER1-AS1_ENSG00000235706
14	95880412	95880697	5Y-H4K8ac_peak_5192	5.97089	
14	95909482	95909708	5Y-H4K8ac_peak_5193	7.38046	
14	95948328	95948555	5Y-H4K8ac_peak_5194	8.16031	
14	95982601	95983260	5Y-H4K8ac_peak_5195	8.73985	RP11-1070N10.3_ENSG00000258572
14	95986769	95987050	5Y-H4K8ac_peak_5196	4.07435	

14	96000665	96000879	5Y-H4K8ac_peak_5197	9.7353	SNHG10_ENSG00000247092;SCARNA13_ENSG00000252481
14	96126376	96126577	5Y-H4K8ac_peak_5198	6.34046	
14	96343099	96343562	5Y-H4K8ac_peak_5199	7.38046	LINC00617_ENSG00000250366
14	96353087	96353301	5Y-H4K8ac_peak_5200	6.1654	
14	96505279	96505497	5Y-H4K8ac_peak_5201	7.89142	C14orf132_ENSG00000227051
14	96505893	96506310	5Y-H4K8ac_peak_5202	8.24461	C14orf132_ENSG00000227051
14	96734290	96734509	5Y-H4K8ac_peak_5203	6.43775	RP11-404P21.3_ENSG00000258793
14	96839108	96839442	5Y-H4K8ac_peak_5204	8.33296	
14	96968845	96969097	5Y-H4K8ac_peak_5205	9.51254	RP11-872J21.3_ENSG00000260806
14	97059095	97059548	5Y-H4K8ac_peak_5206	7.59101	RP11-433J8.1_ENSG00000258702
14	99947807	99947997	5Y-H4K8ac_peak_5207	8.62703	SETD3_ENSG00000183576;CCNK_ENSG00000090061
14	99981702	99981941	5Y-H4K8ac_peak_5208	5.65584	
14	100029844	100030123	5Y-H4K8ac_peak_5209	7.29176	
14	100030557	100030757	5Y-H4K8ac_peak_5210	8.30418	
14	100035473	100035818	5Y-H4K8ac_peak_5211	5.47078	
14	100038210	100038670	5Y-H4K8ac_peak_5212	7.27597	
14	100039208	100039568	5Y-H4K8ac_peak_5213	7.03573	
14	100041224	100041724	5Y-H4K8ac_peak_5214	8.73985	
14	100042417	100042791	5Y-H4K8ac_peak_5215	5.64909	
14	100057541	100057949	5Y-H4K8ac_peak_5216	6.36723	
14	100071036	100071574	5Y-H4K8ac_peak_5217	20.96132	CCDC85C_ENSG00000205476;RP11-543C4.1_ENSG00000247970
14	100204075	100204395	5Y-H4K8ac_peak_5218	6.43775	EML1_ENSG00000066629
14	100239337	100240729	5Y-H4K8ac_peak_5219	14.77644	
14	100259065	100259518	5Y-H4K8ac_peak_5220	18.77281	
14	100432985	100433181	5Y-H4K8ac_peak_5221	6.50117	
14	100437412	100437637	5Y-H4K8ac_peak_5222	10.1994	EVL_ENSG00000196405
14	100438473	100438844	5Y-H4K8ac_peak_5223	15.16038	EVL_ENSG00000196405
14	100609542	100609874	5Y-H4K8ac_peak_5224	9.52107	
14	100658669	100659226	5Y-H4K8ac_peak_5225	13.92877	
14	100659573	100660256	5Y-H4K8ac_peak_5226	9.99623	
14	100680152	100680750	5Y-H4K8ac_peak_5227	6.78128	
14	100751515	100752582	5Y-H4K8ac_peak_5228	17.50045	
14	100772500	100772716	5Y-H4K8ac_peak_5229	5.41472	SLC25A29_ENSG00000197119
14	100773095	100773626	5Y-H4K8ac_peak_5230	11.1169	SLC25A29_ENSG00000197119;MIR345_ENSG00000198984
14	100856119	100856318	5Y-H4K8ac_peak_5231	7.89142	
14	100867553	100867898	5Y-H4K8ac_peak_5232	12.52787	
14	100886339	100886563	5Y-H4K8ac_peak_5233	4.15658	
14	100889575	100889768	5Y-H4K8ac_peak_5234	6.85744	
14	100890247	100891318	5Y-H4K8ac_peak_5235	12.10416	
14	100894153	100895000	5Y-H4K8ac_peak_5236	9.99623	
14	100895493	100895740	5Y-H4K8ac_peak_5237	12.11208	
14	100902905	100903192	5Y-H4K8ac_peak_5238	4.77126	
14	100905156	100905691	5Y-H4K8ac_peak_5239	14.77644	
14	100906681	100907047	5Y-H4K8ac_peak_5240	5.80888	
14	100907630	100908779	5Y-H4K8ac_peak_5241	17.55091	
14	100910209	100910697	5Y-H4K8ac_peak_5242	11.8513	

14	100952000	100952236	5Y-H4K8ac_peak_5243	4.79585	
14	100952879	100953136	5Y-H4K8ac_peak_5244	9.30505	RN7SKP92_ENSG00000253075
14	101016469	101016677	5Y-H4K8ac_peak_5245	6.68024	
14	101033941	101034171	5Y-H4K8ac_peak_5246	4.77126	
14	101034862	101035082	5Y-H4K8ac_peak_5247	10.60083	
14	101052414	101053701	5Y-H4K8ac_peak_5248	12.05638	BEGAIN_ENSG00000183092
14	101054133	101054562	5Y-H4K8ac_peak_5249	10.1994	BEGAIN_ENSG00000183092
14	101055133	101055549	5Y-H4K8ac_peak_5250	9.45148	CTD-2644I21.1_ENSG00000258576
14	101075295	101075579	5Y-H4K8ac_peak_5251	4.50834	
14	101080126	101080337	5Y-H4K8ac_peak_5252	4.50834	
14	101106957	101107166	5Y-H4K8ac_peak_5253	5.59843	
14	101120128	101120498	5Y-H4K8ac_peak_5254	7.89273	
14	101120949	101121756	5Y-H4K8ac_peak_5255	8.04693	
14	101123092	101123296	5Y-H4K8ac_peak_5256	4.15907	LINC00523_ENSG00000196273
14	101124603	101125039	5Y-H4K8ac_peak_5257	5.18558	
14	101125968	101126405	5Y-H4K8ac_peak_5258	4.15658	
14	101131177	101131447	5Y-H4K8ac_peak_5259	8.73227	
14	101134042	101134258	5Y-H4K8ac_peak_5260	6.37023	
14	101138723	101139947	5Y-H4K8ac_peak_5261	15.59156	
14	101140602	101141588	5Y-H4K8ac_peak_5262	13.00733	
14	101142066	101143359	5Y-H4K8ac_peak_5263	9.79526	
14	101143587	101144971	5Y-H4K8ac_peak_5264	13.59183	
14	101145210	101145413	5Y-H4K8ac_peak_5265	6.34245	RP11-566J3.2_ENSG00000258717
14	101146106	101146345	5Y-H4K8ac_peak_5266	15.39698	RP11-566J3.2_ENSG00000258717
14	101147003	101147581	5Y-H4K8ac_peak_5267	11.60073	RP11-566J3.2_ENSG00000258717
14	101157324	101157536	5Y-H4K8ac_peak_5268	4.15658	
14	101159220	101159521	5Y-H4K8ac_peak_5269	9.05168	
14	101162562	101162772	5Y-H4K8ac_peak_5270	12.69256	
14	101163052	101163433	5Y-H4K8ac_peak_5271	7.21932	
14	101165305	101165895	5Y-H4K8ac_peak_5272	8.52802	
14	101172323	101172594	5Y-H4K8ac_peak_5273	7.12953	
14	101175286	101175872	5Y-H4K8ac_peak_5274	4.55128	
14	101192630	101192862	5Y-H4K8ac_peak_5275	4.29586	DLK1_ENSG00000185559
14	101204606	101204860	5Y-H4K8ac_peak_5276	7.31102	RP11-566J3.4_ENSG00000273087
14	101236746	101237455	5Y-H4K8ac_peak_5277	11.04542	
14	101238381	101239505	5Y-H4K8ac_peak_5278	16.51777	
14	101239824	101240748	5Y-H4K8ac_peak_5279	15.03212	
14	101241901	101244058	5Y-H4K8ac_peak_5280	17.78344	
14	101244853	101246789	5Y-H4K8ac_peak_5281	15.27603	MEG3_ENSG00000214548
14	101247174	101247653	5Y-H4K8ac_peak_5282	6.19716	
14	101248182	101248966	5Y-H4K8ac_peak_5283	10.35586	
14	101249389	101250246	5Y-H4K8ac_peak_5284	21.95908	
14	101250854	101251788	5Y-H4K8ac_peak_5285	11.4254	
14	101251991	101252790	5Y-H4K8ac_peak_5286	12.44818	
14	101254759	101255155	5Y-H4K8ac_peak_5287	7.0618	
14	101256474	101257005	5Y-H4K8ac_peak_5288	4.50834	

14	101307750	101308049	5Y-H4K8ac_peak_5289	4.98439	
14	101363061	101363278	5Y-H4K8ac_peak_5290	6.77436	
14	101365317	101365904	5Y-H4K8ac_peak_5291	10.58128	
14	101366876	101367117	5Y-H4K8ac_peak_5292	10.97738	
14	101367519	101369136	5Y-H4K8ac_peak_5293	7.44077	AL117190.1_ENSG00000221077
14	101543273	101543500	5Y-H4K8ac_peak_5294	6.78318	AL132709.1_ENSG00000230805
14	101593103	101593534	5Y-H4K8ac_peak_5295	7.17997	
14	101597164	101597417	5Y-H4K8ac_peak_5296	6.49458	
14	101642546	101642872	5Y-H4K8ac_peak_5297	5.98695	
14	101697121	101697327	5Y-H4K8ac_peak_5298	5.23083	
14	101700942	101701211	5Y-H4K8ac_peak_5299	15.62779	
14	101701472	101702035	5Y-H4K8ac_peak_5300	6.73047	
14	101702924	101703142	5Y-H4K8ac_peak_5301	5.25503	
14	101925580	101926033	5Y-H4K8ac_peak_5302	14.30055	
14	102054306	102054870	5Y-H4K8ac_peak_5303	6.77436	
14	102056216	102056496	5Y-H4K8ac_peak_5304	8.63295	
14	102062977	102063243	5Y-H4K8ac_peak_5305	4.84727	
14	102094125	102094451	5Y-H4K8ac_peak_5306	11.20395	
14	102095078	102095411	5Y-H4K8ac_peak_5307	7.20869	
14	102096539	102096995	5Y-H4K8ac_peak_5308	7.20869	
14	102247418	102248287	5Y-H4K8ac_peak_5309	7.38046	
14	102276342	102276574	5Y-H4K8ac_peak_5310	8.63306	CTD-2017C7.2_ENSG00000259088
14	102304965	102305262	5Y-H4K8ac_peak_5311	8.86184	CTD-2017C7.1_ENSG00000256705
14	102307888	102308366	5Y-H4K8ac_peak_5312	10.21117	
14	102343516	102343832	5Y-H4K8ac_peak_5313	4.84727	
14	102413433	102414305	5Y-H4K8ac_peak_5314	6.77436	RP11-1017G21.5_ENSG00000271780
14	102414820	102415417	5Y-H4K8ac_peak_5315	7.89273	RP11-1017G21.5_ENSG00000271780
14	102552959	102553291	5Y-H4K8ac_peak_5316	13.2534	
14	102553703	102554011	5Y-H4K8ac_peak_5317	6.98416	
14	102656759	102657239	5Y-H4K8ac_peak_5318	6.59249	
14	102783694	102783889	5Y-H4K8ac_peak_5319	7.59101	ZNF839_ENSG00000022976
14	102786342	102786709	5Y-H4K8ac_peak_5320	16.41521	
14	102983519	102984229	5Y-H4K8ac_peak_5321	7.44077	
14	102990382	102990869	5Y-H4K8ac_peak_5322	12.26606	
14	103006632	103007071	5Y-H4K8ac_peak_5323	12.87098	MIR4309_ENSG00000266015
14	103011716	103012178	5Y-H4K8ac_peak_5324	6.27097	CTD-2555C10.3_ENSG00000259230
14	103059056	103059266	5Y-H4K8ac_peak_5325	10.90365	RCOR1_ENSG00000089902
14	103244258	103244463	5Y-H4K8ac_peak_5326	5.98695	TRAF3_ENSG00000131323
14	103291683	103291890	5Y-H4K8ac_peak_5327	4.8773	
14	103315812	103316191	5Y-H4K8ac_peak_5328	6.03718	
14	103317876	103318143	5Y-H4K8ac_peak_5329	5.23083	
14	103557566	103558308	5Y-H4K8ac_peak_5330	13.33225	
14	103578597	103579002	5Y-H4K8ac_peak_5331	5.98695	
14	103579255	103579534	5Y-H4K8ac_peak_5332	11.19336	
14	103588693	103589403	5Y-H4K8ac_peak_5333	18.71546	LINC00677_ENSG00000259717;TNFAIP2_ENSG00000185215
14	103593047	103593958	5Y-H4K8ac_peak_5334	10.4689	

14	103622061	103622252	5Y-H4K8ac_peak_5335	5.51139	
14	103673775	103674333	5Y-H4K8ac_peak_5336	9.58986	RP11-736N17.9_ENSG00000270705
14	103702286	103702476	5Y-H4K8ac_peak_5337	8.43511	
14	103726324	103726729	5Y-H4K8ac_peak_5338	4.50834	
14	103800610	103800932	5Y-H4K8ac_peak_5339	7.90751	EIF5_ENSG00000100664
14	103851194	103851407	5Y-H4K8ac_peak_5340	6.00747	MARK3_ENSG00000075413
14	103989369	103989729	5Y-H4K8ac_peak_5341	6.37023	CKB_ENSG00000166165
14	104008927	104009152	5Y-H4K8ac_peak_5342	7.52971	
14	104028772	104029372	5Y-H4K8ac_peak_5343	15.17389	BAG5_ENSG00000166170;KLC1_ENSG00000126214;RP11-73M18.2_ENSG00000256500;APOPT1_ENSG00000256053
14	104094455	104095867	5Y-H4K8ac_peak_5344	20.44546	
14	104314097	104314915	5Y-H4K8ac_peak_5345	22.1484	PPP1R13B_ENSG00000088808;LINC00637_ENSG00000258735
14	104321796	104322137	5Y-H4K8ac_peak_5346	9.7353	
14	104346425	104348608	5Y-H4K8ac_peak_5347	8.62703	CTD-2134A5.4_ENSG00000258534
14	104349191	104350116	5Y-H4K8ac_peak_5348	5.41472	
14	104392106	104392366	5Y-H4K8ac_peak_5349	5.35202	
14	104516512	104516713	5Y-H4K8ac_peak_5350	5.87382	
14	104549125	104549460	5Y-H4K8ac_peak_5351	5.07473	
14	104570519	104570723	5Y-H4K8ac_peak_5352	4.8509	
14	104615042	104616431	5Y-H4K8ac_peak_5353	14.97676	
14	104617099	104617582	5Y-H4K8ac_peak_5354	7.59101	
14	104625508	104625843	5Y-H4K8ac_peak_5355	7.08486	
14	104667943	104668160	5Y-H4K8ac_peak_5356	4.642	
14	104677685	104677877	5Y-H4K8ac_peak_5357	4.50834	
14	104686668	104686914	5Y-H4K8ac_peak_5358	5.37237	
14	104687481	104688165	5Y-H4K8ac_peak_5359	6.78128	
14	104688986	104689627	5Y-H4K8ac_peak_5360	12.87247	RP11-260M19.2_ENSG00000258913
14	104689972	104690372	5Y-H4K8ac_peak_5361	13.81505	RP11-260M19.2_ENSG00000258913
14	104690719	104691596	5Y-H4K8ac_peak_5362	14.59308	RP11-260M19.2_ENSG00000258913
14	104695125	104695761	5Y-H4K8ac_peak_5363	4.66162	
14	104696911	104697143	5Y-H4K8ac_peak_5364	7.28023	
14	104719911	104720239	5Y-H4K8ac_peak_5365	4.9885	
14	104746998	104747227	5Y-H4K8ac_peak_5366	7.9592	
14	104750780	104750989	5Y-H4K8ac_peak_5367	7.95453	
14	104757161	104757354	5Y-H4K8ac_peak_5368	4.04213	
14	104758846	104759127	5Y-H4K8ac_peak_5369	9.08389	
14	104759712	104759998	5Y-H4K8ac_peak_5370	6.08523	
14	104761179	104761870	5Y-H4K8ac_peak_5371	6.98416	
14	104762231	104763005	5Y-H4K8ac_peak_5372	16.07596	
14	104763275	104763694	5Y-H4K8ac_peak_5373	8.1667	
14	104835223	104835430	5Y-H4K8ac_peak_5374	8.86778	
14	104889606	104890267	5Y-H4K8ac_peak_5375	8.2913	
14	104890618	104890893	5Y-H4K8ac_peak_5376	4.95697	
14	104893589	104893807	5Y-H4K8ac_peak_5377	6.88532	
14	104901800	104902196	5Y-H4K8ac_peak_5378	5.64909	
14	104902780	104903896	5Y-H4K8ac_peak_5379	18.60331	
14	104927462	104927682	5Y-H4K8ac_peak_5380	6.37023	

14	104973374	104973593	5Y-H4K8ac_peak_5381	5.87725	
14	104976175	104976437	5Y-H4K8ac_peak_5382	8.05083	
14	104977652	104977878	5Y-H4K8ac_peak_5383	7.18391	
14	105029644	105030756	5Y-H4K8ac_peak_5384	11.36989	
14	105046719	105046918	5Y-H4K8ac_peak_5385	8.43511	C14orf180_ENSG00000184601
14	105048020	105048238	5Y-H4K8ac_peak_5386	7.11863	
14	105048612	105048948	5Y-H4K8ac_peak_5387	7.73538	
14	105050487	105050684	5Y-H4K8ac_peak_5388	5.90602	
14	105053532	105053843	5Y-H4K8ac_peak_5389	9.02938	
14	105054745	105055152	5Y-H4K8ac_peak_5390	4.95697	
14	105055377	105055672	5Y-H4K8ac_peak_5391	9.07085	RP11-614O9.1_ENSG00000259037
14	105064883	105065441	5Y-H4K8ac_peak_5392	5.83799	
14	105070066	105070439	5Y-H4K8ac_peak_5393	10.07308	
14	105070697	105070892	5Y-H4K8ac_peak_5394	4.95697	
14	105071535	105071737	5Y-H4K8ac_peak_5395	5.94703	TMEM179_ENSG00000258986
14	105147178	105147607	5Y-H4K8ac_peak_5396	4.84727	RP11-982M15.6_ENSG00000256050
14	105154647	105156357	5Y-H4K8ac_peak_5397	12.1498	INF2_ENSG00000203485
14	105218334	105218849	5Y-H4K8ac_peak_5398	7.11863	SIVA1_ENSG00000184990
14	105266174	105266825	5Y-H4K8ac_peak_5399	9.95819	ZBTB42_ENSG00000179627
14	105282553	105283133	5Y-H4K8ac_peak_5400	7.90751	
14	105292581	105292858	5Y-H4K8ac_peak_5401	9.179	
14	105297162	105297388	5Y-H4K8ac_peak_5402	7.13294	RPS26P49_ENSG00000239365
14	105331041	105331315	5Y-H4K8ac_peak_5403	10.94674	CEP170B_ENSG00000099814
14	105399533	105399874	5Y-H4K8ac_peak_5404	9.79526	
14	105434389	105434961	5Y-H4K8ac_peak_5405	6.77436	
14	105559214	105559505	5Y-H4K8ac_peak_5406	9.87124	RP11-44N21.1_ENSG00000257556
14	105634039	105634351	5Y-H4K8ac_peak_5407	4.99136	JAG2_ENSG00000184916
14	105646935	105647214	5Y-H4K8ac_peak_5408	7.59101	RP11-44N21.4_ENSG00000257622;NUDT14_ENSG00000183828
14	105659801	105660111	5Y-H4K8ac_peak_5409	6.73047	
14	105660525	105660847	5Y-H4K8ac_peak_5410	6.12014	
14	105714443	105714744	5Y-H4K8ac_peak_5411	5.13361	BTBD6_ENSG00000184887
14	105759101	105759609	5Y-H4K8ac_peak_5412	11.69482	
14	105759821	105760180	5Y-H4K8ac_peak_5413	14.43262	
14	105760375	105760653	5Y-H4K8ac_peak_5414	11.68317	
14	105761802	105762022	5Y-H4K8ac_peak_5415	5.49775	
14	105766840	105767228	5Y-H4K8ac_peak_5416	5.77617	PACS2_ENSG00000179364
14	105863781	105865578	5Y-H4K8ac_peak_5417	12.06963	TEX22_ENSG00000226174
14	105877441	105877661	5Y-H4K8ac_peak_5418	6.55841	
14	105880769	105881546	5Y-H4K8ac_peak_5419	8.47164	
14	105885697	105886140	5Y-H4K8ac_peak_5420	15.59156	RP11-521B24.3_ENSG00000251602;MTA1_ENSG00000182979
14	105886700	105887112	5Y-H4K8ac_peak_5421	6.53157	RP11-521B24.3_ENSG00000251602;MTA1_ENSG00000182979
14	105887433	105888826	5Y-H4K8ac_peak_5422	6.61077	
14	105937319	105937600	5Y-H4K8ac_peak_5423	4.0639	RP11-521B24.5_ENSG00000257270
14	105940688	105940895	5Y-H4K8ac_peak_5424	6.62731	
14	105943079	105943505	5Y-H4K8ac_peak_5425	4.15658	
14	105946869	105947191	5Y-H4K8ac_peak_5426	9.96543	

14	105947412	105947992	5Y-H4K8ac_peak_5427	9.00954	
14	105948768	105949100	5Y-H4K8ac_peak_5428	5.87725	
14	105952177	105952808	5Y-H4K8ac_peak_5429	9.92042	CRIP1_ENSG00000213145;CRIP1_ENSG00000257341
14	105956248	105956914	5Y-H4K8ac_peak_5430	9.66296	C14orf80_ENSG00000185347
14	105993329	105993595	5Y-H4K8ac_peak_5431	5.62406	TMEM121_ENSG00000184986
14	105994211	105994720	5Y-H4K8ac_peak_5432	7.90751	
14	106025741	106026083	5Y-H4K8ac_peak_5433	8.2913	
14	106040145	106040387	5Y-H4K8ac_peak_5434	4.07874	
14	106318573	106318903	5Y-H4K8ac_peak_5435	8.31252	
14	106345664	106345859	5Y-H4K8ac_peak_5436	4.29586	
14	106938565	106939000	5Y-H4K8ac_peak_5437	10.11191	LINC00221_ENSG00000187156
14	107253018	107253212	5Y-H4K8ac_peak_5438	6.43775	
15	22832842	22833052	5Y-H4K8ac_peak_5439	9.47421	TUBGCP5_ENSG00000153575
15	22833568	22833785	5Y-H4K8ac_peak_5440	8.43511	TUBGCP5_ENSG00000153575
15	22892126	22892530	5Y-H4K8ac_peak_5441	10.90365	CYFIP1_ENSG00000068793
15	23035067	23035273	5Y-H4K8ac_peak_5442	4.50834	NIPA2_ENSG00000140157
15	23810915	23811333	5Y-H4K8ac_peak_5443	9.30505	MKRN3_ENSG00000179455
15	27018518	27018819	5Y-H4K8ac_peak_5444	7.11863	
15	27136982	27137190	5Y-H4K8ac_peak_5445	9.36633	
15	27342189	27342418	5Y-H4K8ac_peak_5446	4.40812	
15	28235093	28235365	5Y-H4K8ac_peak_5447	7.38046	
15	28265861	28266156	5Y-H4K8ac_peak_5448	6.20875	
15	28266522	28266873	5Y-H4K8ac_peak_5449	9.25361	
15	28351623	28351846	5Y-H4K8ac_peak_5450	6.13909	
15	28699830	28700155	5Y-H4K8ac_peak_5451	5.84208	
15	28834216	28834417	5Y-H4K8ac_peak_5452	19.93272	HERC2P9_ENSG00000206149
15	28982806	28983304	5Y-H4K8ac_peak_5453	9.30505	GOLGA8M_ENSG00000188626;RP11-578F21.6_ENSG00000261480;WHAMMP2_ENSG00000248334
15	29130292	29132084	5Y-H4K8ac_peak_5454	18.63671	APBA2_ENSG00000034053
15	29155538	29155809	5Y-H4K8ac_peak_5455	5.13364	
15	29157566	29157778	5Y-H4K8ac_peak_5456	5.65584	
15	29405069	29405274	5Y-H4K8ac_peak_5457	4.79585	
15	29519275	29519792	5Y-H4K8ac_peak_5458	7.38046	
15	30260395	30261075	5Y-H4K8ac_peak_5459	12.71156	TJP1_ENSG00000104067
15	30488320	30488680	5Y-H4K8ac_peak_5460	12.05638	AC026150.5_ENSG00000225930
15	30504473	30504678	5Y-H4K8ac_peak_5461	4.84727	
15	30659346	30659741	5Y-H4K8ac_peak_5462	11.57334	
15	31284086	31284388	5Y-H4K8ac_peak_5463	9.63153	MTMR10_ENSG00000166912
15	31508024	31508288	5Y-H4K8ac_peak_5464	7.87048	RP11-16E12.1_ENSG00000259448
15	31556008	31557023	5Y-H4K8ac_peak_5465	8.69112	
15	31572299	31572686	5Y-H4K8ac_peak_5466	7.89273	
15	31593144	31593756	5Y-H4K8ac_peak_5467	10.11191	
15	31596659	31597108	5Y-H4K8ac_peak_5468	7.79831	
15	31598311	31598707	5Y-H4K8ac_peak_5469	9.89244	
15	31617688	31617897	5Y-H4K8ac_peak_5470	9.52603	
15	31664341	31664560	5Y-H4K8ac_peak_5471	7.50148	
15	31665134	31665392	5Y-H4K8ac_peak_5472	8.73985	

15	31665808	31666010	5Y-H4K8ac_peak_5473	7.89142	
15	31690530	31691140	5Y-H4K8ac_peak_5474	7.60057	
15	31732716	31733725	5Y-H4K8ac_peak_5475	12.20548	
15	32163185	32163559	5Y-H4K8ac_peak_5476	7.76232	OTUD7A_ENSG00000169918
15	32269704	32269989	5Y-H4K8ac_peak_5477	4.00285	
15	32385624	32385831	5Y-H4K8ac_peak_5478	7.17184	
15	32389171	32389487	5Y-H4K8ac_peak_5479	4.5753	
15	32412746	32413105	5Y-H4K8ac_peak_5480	5.12213	
15	32426345	32426540	5Y-H4K8ac_peak_5481	7.04637	
15	32455513	32455730	5Y-H4K8ac_peak_5482	6.34046	
15	33453843	33454037	5Y-H4K8ac_peak_5483	7.60893	
15	34330564	34331493	5Y-H4K8ac_peak_5484	16.27715	AVEN_ENSG00000169857
15	34501885	34502139	5Y-H4K8ac_peak_5485	6.98118	KATNBL1_ENSG00000134152
15	34517308	34517525	5Y-H4K8ac_peak_5486	5.99504	EMC4_ENSG00000128463
15	34658514	34658706	5Y-H4K8ac_peak_5487	6.77436	LPCAT4_ENSG00000176454
15	34659579	34659777	5Y-H4K8ac_peak_5488	6.98416	LPCAT4_ENSG00000176454
15	34875848	34876350	5Y-H4K8ac_peak_5489	5.64909	
15	34880639	34880853	5Y-H4K8ac_peak_5490	6.90749	GOLGA8A_ENSG00000175265;GOLGA8B_ENSG00000215252
15	35280630	35281001	5Y-H4K8ac_peak_5491	11.57334	ZNF770_ENSG00000198146
15	35837871	35838675	5Y-H4K8ac_peak_5492	12.50545	DPH6_ENSG00000134146;DPH6-AS1_ENSG00000248079
15	37389796	37389992	5Y-H4K8ac_peak_5493	7.58806	
15	37391151	37391553	5Y-H4K8ac_peak_5494	6.85387	RP11-128A17.1_ENSG00000259460
15	37402495	37402743	5Y-H4K8ac_peak_5495	9.83437	
15	37970402	37970617	5Y-H4K8ac_peak_5496	7.31815	
15	38113523	38113730	5Y-H4K8ac_peak_5497	8.93923	
15	38365034	38365503	5Y-H4K8ac_peak_5498	13.14766	RP11-1008C21.2_ENSG00000236914
15	38544742	38545035	5Y-H4K8ac_peak_5499	5.23083	SPRED1_ENSG00000166068
15	38856329	38856584	5Y-H4K8ac_peak_5500	10.19948	
15	38988420	38988802	5Y-H4K8ac_peak_5501	10.27264	C15orf53_ENSG00000175779
15	38994992	38995253	5Y-H4K8ac_peak_5502	6.24411	
15	39172636	39172888	5Y-H4K8ac_peak_5503	5.65584	
15	39177113	39177318	5Y-H4K8ac_peak_5504	10.69866	
15	40330812	40331302	5Y-H4K8ac_peak_5505	14.00129	SRP14_ENSG00000140319;SRP14-AS1_ENSG00000248508
15	40361786	40362221	5Y-H4K8ac_peak_5506	10.60083	
15	40374169	40374632	5Y-H4K8ac_peak_5507	7.59101	
15	40395145	40395350	5Y-H4K8ac_peak_5508	4.51497	
15	40531112	40531786	5Y-H4K8ac_peak_5509	15.84891	
15	40540387	40540611	5Y-H4K8ac_peak_5510	6.15578	
15	40545059	40545495	5Y-H4K8ac_peak_5511	4.15658	C15orf56_ENSG00000176753
15	40574920	40575304	5Y-H4K8ac_peak_5512	6.08523	ANKRD63_ENSG00000230778
15	40583240	40583843	5Y-H4K8ac_peak_5513	12.05638	
15	40594294	40594951	5Y-H4K8ac_peak_5514	16.41092	
15	40630814	40631453	5Y-H4K8ac_peak_5515	6.4221	
15	40632383	40633082	5Y-H4K8ac_peak_5516	15.80786	C15orf52_ENSG00000188549
15	40636988	40637336	5Y-H4K8ac_peak_5517	10.85484	
15	40646842	40647183	5Y-H4K8ac_peak_5518	13.00733	

15	40674679	40674999	5Y-H4K8ac_peak_5519	7.59101	KNSTRN_ENSG00000128944
15	40675387	40675659	5Y-H4K8ac_peak_5520	11.19336	KNSTRN_ENSG00000128944
15	40727902	40729038	5Y-H4K8ac_peak_5521	12.21176	
15	40762694	40762904	5Y-H4K8ac_peak_5522	11.68317	CHST14_ENSG00000169105
15	40763483	40763870	5Y-H4K8ac_peak_5523	9.09778	CHST14_ENSG00000169105
15	40799286	40799622	5Y-H4K8ac_peak_5524	6.37023	
15	40856948	40857145	5Y-H4K8ac_peak_5525	7.30348	C15orf57_ENSG00000128891
15	41049718	41050207	5Y-H4K8ac_peak_5526	9.31608	
15	41059472	41059915	5Y-H4K8ac_peak_5527	4.76429	
15	41061000	41062611	5Y-H4K8ac_peak_5528	10.1046	C15orf62_ENSG00000188277
15	41136539	41136813	5Y-H4K8ac_peak_5529	5.99504	RP11-532F12.5_ENSG00000261183;SPINT1_ENSG00000166145
15	41165806	41166419	5Y-H4K8ac_peak_5530	8.24461	RHOV_ENSG00000104140
15	41198836	41199178	5Y-H4K8ac_peak_5531	11.5813	RP11-540O11.1_ENSG00000251161
15	41199525	41200083	5Y-H4K8ac_peak_5532	7.59101	RP11-540O11.1_ENSG00000251161
15	41207001	41207297	5Y-H4K8ac_peak_5533	7.31102	
15	41232621	41233072	5Y-H4K8ac_peak_5534	10.65816	
15	41233747	41234583	5Y-H4K8ac_peak_5535	9.36633	
15	41244550	41245367	5Y-H4K8ac_peak_5536	20.32264	CHAC1_ENSG00000128965
15	41245794	41246001	5Y-H4K8ac_peak_5537	10.97604	CHAC1_ENSG00000128965
15	41407854	41408660	5Y-H4K8ac_peak_5538	9.51254	INO80_ENSG00000128908
15	41575427	41575782	5Y-H4K8ac_peak_5539	10.21117	OIP5-AS1_ENSG00000247556
15	41694988	41695552	5Y-H4K8ac_peak_5540	8.21525	NDUFAF1_ENSG00000137806
15	41835976	41836340	5Y-H4K8ac_peak_5541	5.23083	RPAP1_ENSG00000103932
15	41851800	41851991	5Y-H4K8ac_peak_5542	5.91107	
15	41913217	41913483	5Y-H4K8ac_peak_5543	6.47245	MGA_ENSG00000174197
15	42096763	42096967	5Y-H4K8ac_peak_5544	8.21582	
15	42186157	42187366	5Y-H4K8ac_peak_5545	14.99673	SPTBN5_ENSG00000137877
15	42281637	42281834	5Y-H4K8ac_peak_5546	7.60893	
15	42349005	42349209	5Y-H4K8ac_peak_5547	4.66683	
15	42356405	42356672	5Y-H4K8ac_peak_5548	4.07874	
15	42399830	42400035	5Y-H4K8ac_peak_5549	8.33296	
15	42500109	42500448	5Y-H4K8ac_peak_5550	8.2913	VPS39_ENSG00000166887;RP11-546B15.1_ENSG00000261002
15	42565842	42566274	5Y-H4K8ac_peak_5551	7.89142	TMEM87A_ENSG00000103978;GANC_ENSG00000214013
15	42783421	42783665	5Y-H4K8ac_peak_5552	9.51254	ZNF106_ENSG00000103994;SNAP23_ENSG00000092531
15	43212678	43212879	5Y-H4K8ac_peak_5553	6.73047	TTBK2_ENSG00000128881
15	43425859	43426296	5Y-H4K8ac_peak_5554	13.81505	
15	43622006	43622418	5Y-H4K8ac_peak_5555	6.53157	LCMT2_ENSG00000168806;ADAL_ENSG00000168803
15	43663401	43663625	5Y-H4K8ac_peak_5556	9.23159	ZSCAN29_ENSG00000140265
15	44037915	44038418	5Y-H4K8ac_peak_5557	7.11863	PDIA3_ENSG00000167004;CATSPER2P1_ENSG00000205771
15	44083343	44083571	5Y-H4K8ac_peak_5558	7.57144	
15	44084650	44084902	5Y-H4K8ac_peak_5559	4.77126	
15	44486481	44487089	5Y-H4K8ac_peak_5560	6.20875	FRMD5_ENSG00000171877
15	44487744	44488062	5Y-H4K8ac_peak_5561	11.19336	FRMD5_ENSG00000171877
15	44955460	44955744	5Y-H4K8ac_peak_5562	11.69482	SPG11_ENSG00000104133
15	45076331	45076538	5Y-H4K8ac_peak_5563	4.29586	
15	45111563	45111846	5Y-H4K8ac_peak_5564	4.84727	

15	45114654	45114975	5Y-H4K8ac_peak_5565	6.20875	
15	45314706	45315185	5Y-H4K8ac_peak_5566	9.78792	SORD_ENSG00000140263
15	45458816	45459442	5Y-H4K8ac_peak_5567	18.33806	CTD-2651B20.1_ENSG00000259539
15	45460793	45461857	5Y-H4K8ac_peak_5568	9.79526	RP11-519G16.2_ENSG00000259519
15	45479145	45479951	5Y-H4K8ac_peak_5569	7.59101	
15	45572007	45572288	5Y-H4K8ac_peak_5570	6.20875	CTD-2651B20.3_ENSG00000259520
15	45670513	45670971	5Y-H4K8ac_peak_5571	8.4454	
15	45878406	45878825	5Y-H4K8ac_peak_5572	7.24844	BLOC1S6_ENSG00000104164;RP11-96O20.4_ENSG00000260170
15	45926960	45927209	5Y-H4K8ac_peak_5573	7.11863	
15	46007216	46007459	5Y-H4K8ac_peak_5574	5.38149	
15	46373051	46373457	5Y-H4K8ac_peak_5575	4.51076	
15	47477090	47477474	5Y-H4K8ac_peak_5576	5.98695	SEMA6D_ENSG00000137872
15	47657583	47657984	5Y-H4K8ac_peak_5577	8.08688	
15	48470001	48470854	5Y-H4K8ac_peak_5578	19.17092	MYEF2_ENSG00000104177
15	48838846	48839079	5Y-H4K8ac_peak_5579	8.33296	
15	48938223	48938655	5Y-H4K8ac_peak_5580	8.69112	FBN1_ENSG00000166147;RP11-227D13.1_ENSG00000259705
15	49170386	49170728	5Y-H4K8ac_peak_5581	8.93923	EID1_ENSG00000255302;AC012379.1_ENSG00000235883
15	49268188	49268519	5Y-H4K8ac_peak_5582	6.78128	
15	49447324	49447661	5Y-H4K8ac_peak_5583	5.23819	NDUFAF4P1_ENSG00000259467;COPS2_ENSG00000166200;GALK2_ENSG00000156958
15	50474362	50474624	5Y-H4K8ac_peak_5584	11.19336	ATP8B4_ENSG00000104043;SLC27A2_ENSG00000140284
15	50646385	50647282	5Y-H4K8ac_peak_5585	13.99721	GABPB1_ENSG00000104064;GABPB1-AS1_ENSG00000244879
15	50715778	50715978	5Y-H4K8ac_peak_5586	4.1776	USP8_ENSG00000138592
15	51057720	51058173	5Y-H4K8ac_peak_5587	9.15007	SPPL2A_ENSG00000138600
15	51633962	51634192	5Y-H4K8ac_peak_5588	6.49308	
15	51973479	51973689	5Y-H4K8ac_peak_5589	4.50834	SCG3_ENSG00000104112
15	52587401	52587925	5Y-H4K8ac_peak_5590	7.89273	MYO5C_ENSG00000128833
15	52821352	52821584	5Y-H4K8ac_peak_5591	7.65114	MYO5A_ENSG00000197535
15	53097956	53098161	5Y-H4K8ac_peak_5592	5.98695	RP11-209K10.2_ENSG00000259203
15	55489446	55489849	5Y-H4K8ac_peak_5593	6.46053	RSL24D1_ENSG00000137876
15	55582957	55583221	5Y-H4K8ac_peak_5594	10.17195	
15	55611444	55611738	5Y-H4K8ac_peak_5595	7.89142	RAB27A_ENSG00000069974;RP11-139H15.1_ENSG00000225973;PIGB_ENSG00000069943
15	55673041	55673245	5Y-H4K8ac_peak_5596	7.17184	
15	55790848	55791132	5Y-H4K8ac_peak_5597	9.05168	DYX1C1-CCPG1_ENSG00000261771
15	55880757	55881088	5Y-H4K8ac_peak_5598	7.3889	PYGO1_ENSG00000171016
15	56035403	56035740	5Y-H4K8ac_peak_5599	14.00559	PRTG_ENSG00000166450
15	56535570	56535872	5Y-H4K8ac_peak_5600	12.71156	RFX7_ENSG00000181827;TEX9_ENSG00000151575
15	56536070	56536311	5Y-H4K8ac_peak_5601	10.22205	RFX7_ENSG00000181827;TEX9_ENSG00000151575
15	57025273	57026126	5Y-H4K8ac_peak_5602	13.0168	
15	57179661	57179953	5Y-H4K8ac_peak_5603	8.2913	
15	57209972	57210619	5Y-H4K8ac_peak_5604	7.59101	ZNF280D_ENSG00000137871;TCF12_ENSG00000140262
15	57210937	57212038	5Y-H4K8ac_peak_5605	17.72591	ZNF280D_ENSG00000137871;TCF12_ENSG00000140262
15	57598551	57598803	5Y-H4K8ac_peak_5606	10.32379	
15	57668178	57668867	5Y-H4K8ac_peak_5607	10.32379	CGNL1_ENSG00000128849
15	57789145	57789342	5Y-H4K8ac_peak_5608	7.59101	
15	57918995	57919365	5Y-H4K8ac_peak_5609	8.33296	
15	57998285	57998569	5Y-H4K8ac_peak_5610	6.50117	

15	57998949	57999324	5Y-H4K8ac_peak_5611	7.90751	
15	58358002	58358252	5Y-H4K8ac_peak_5612	6.22669	CTD-2330J20.2_ENSG00000259285
15	59225228	59225580	5Y-H4K8ac_peak_5613	11.4254	SLTM_ENSG00000137776
15	59462071	59462717	5Y-H4K8ac_peak_5614	11.42066	MIR2116_ENSG00000253030
15	59463253	59463535	5Y-H4K8ac_peak_5615	6.98118	MIR2116_ENSG00000253030
15	59730484	59730791	5Y-H4K8ac_peak_5616	4.84727	
15	59981274	59981494	5Y-H4K8ac_peak_5617	7.31102	RP11-361D15.2_ENSG00000259238;BNIP2_ENSG00000140299
15	60666897	60667152	5Y-H4K8ac_peak_5618	11.93212	
15	61377422	61377621	5Y-H4K8ac_peak_5619	7.97699	
15	61480258	61480488	5Y-H4K8ac_peak_5620	6.34046	
15	61497471	61497761	5Y-H4K8ac_peak_5621	6.00382	
15	62207283	62207572	5Y-H4K8ac_peak_5622	8.75926	RN7SL613P_ENSG00000264376
15	62360433	62360712	5Y-H4K8ac_peak_5623	17.50049	
15	62403522	62403756	5Y-H4K8ac_peak_5624	4.07874	
15	62456210	62456416	5Y-H4K8ac_peak_5625	4.07874	
15	62456615	62456848	5Y-H4K8ac_peak_5626	7.97699	C2CD4B_ENSG00000205502
15	62682287	62682730	5Y-H4K8ac_peak_5627	19.43764	TLN2_ENSG00000171914
15	63227630	63227889	5Y-H4K8ac_peak_5628	12.71599	
15	63340850	63341087	5Y-H4K8ac_peak_5629	10.18556	RP11-244F12.3_ENSG00000259498
15	63413575	63413777	5Y-H4K8ac_peak_5630	4.84727	LACTB_ENSG00000103642
15	63449830	63450022	5Y-H4K8ac_peak_5631	7.96285	RPS27L_ENSG00000185088
15	63481093	63481361	5Y-H4K8ac_peak_5632	5.29015	RAB8B_ENSG00000166128
15	63796879	63797711	5Y-H4K8ac_peak_5633	13.27281	USP3_ENSG00000140455
15	63893546	63893822	5Y-H4K8ac_peak_5634	5.64988	USP3-AS1_ENSG00000259248
15	64205685	64205905	5Y-H4K8ac_peak_5635	7.49746	
15	64237409	64237912	5Y-H4K8ac_peak_5636	6.37023	
15	64338821	64339037	5Y-H4K8ac_peak_5637	5.89113	
15	64388263	64388469	5Y-H4K8ac_peak_5638	6.39626	
15	64680122	64680358	5Y-H4K8ac_peak_5639	7.61515	KIAA0101_ENSG00000166803;TRIP4_ENSG00000103671
15	65067086	65067304	5Y-H4K8ac_peak_5640	4.70501	RBPMS2_ENSG00000166831
15	65137615	65137844	5Y-H4K8ac_peak_5641	3.91471	
15	65203605	65203918	5Y-H4K8ac_peak_5642	7.46829	ANKDD1A_ENSG00000166839
15	65425713	65425986	5Y-H4K8ac_peak_5643	8.16382	PDCD7_ENSG00000090470
15	65503958	65504418	5Y-H4K8ac_peak_5644	5.64909	CILP_ENSG00000138615
15	65578574	65579008	5Y-H4K8ac_peak_5645	14.31711	SNORA24_ENSG00000206903
15	65596039	65596319	5Y-H4K8ac_peak_5646	6.14981	RNU5B-1_ENSG00000200156
15	65713538	65713858	5Y-H4K8ac_peak_5647	4.07874	
15	65822959	65823481	5Y-H4K8ac_peak_5648	9.30505	PTPLAD1_ENSG00000074696
15	65904222	65904473	5Y-H4K8ac_peak_5649	5.41472	VWA9_ENSG00000138614;SLC24A1_ENSG00000074621
15	66161921	66162176	5Y-H4K8ac_peak_5650	9.15977	
15	66504494	66504878	5Y-H4K8ac_peak_5651	6.77436	
15	66585670	66586461	5Y-H4K8ac_peak_5652	11.18077	RP11-653J6.1_ENSG00000261318;DIS3L_ENSG00000166938
15	66678290	66678521	5Y-H4K8ac_peak_5653	6.34046	TIPIN_ENSG00000075131;MAP2K1_ENSG00000169032
15	66796901	66797115	5Y-H4K8ac_peak_5654	7.38046	ZWILCH_ENSG00000174442
15	66923318	66923670	5Y-H4K8ac_peak_5655	16.73191	
15	66924591	66924793	5Y-H4K8ac_peak_5656	11.1169	

15	66971887	66972458	5Y-H4K8ac_peak_5657	12.11208	
15	67037455	67037691	5Y-H4K8ac_peak_5658	4.84727	
15	67066436	67066751	5Y-H4K8ac_peak_5659	8.21582	
15	67142479	67142906	5Y-H4K8ac_peak_5660	8.19789	
15	67318094	67318379	5Y-H4K8ac_peak_5661	6.77436	
15	67357222	67357720	5Y-H4K8ac_peak_5662	8.43511	
15	67384293	67384483	5Y-H4K8ac_peak_5663	6.50117	
15	67413529	67413777	5Y-H4K8ac_peak_5664	9.26157	
15	67480868	67481381	5Y-H4K8ac_peak_5665	6.50117	
15	67481574	67481961	5Y-H4K8ac_peak_5666	10.46287	
15	67813623	67813883	5Y-H4K8ac_peak_5667	9.51254	IQCH-AS1_ENSG00000259673;C15orf61_ENSG00000189227
15	68097171	68097640	5Y-H4K8ac_peak_5668	8.2913	
15	68132828	68133046	5Y-H4K8ac_peak_5669	5.26353	RNU6-1_ENSG00000206625
15	68260128	68260414	5Y-H4K8ac_peak_5670	6.37023	
15	68502897	68503146	5Y-H4K8ac_peak_5671	5.449	
15	68521560	68521967	5Y-H4K8ac_peak_5672	6.50117	RP11-315D16.2_ENSG00000260007;AC107871.1_ENSG00000264779
15	68568677	68568929	5Y-H4K8ac_peak_5673	7.73231	
15	68569257	68570585	5Y-H4K8ac_peak_5674	12.69256	RP11-315D16.4_ENSG00000260657;FEM1B_ENSG00000169018
15	68992440	68992965	5Y-H4K8ac_peak_5675	5.23083	
15	69110574	69111320	5Y-H4K8ac_peak_5676	6.37023	SPESP1_ENSG00000258484
15	69327776	69328111	5Y-H4K8ac_peak_5677	5.48291	
15	69366610	69366933	5Y-H4K8ac_peak_5678	8.90038	
15	69618834	69619033	5Y-H4K8ac_peak_5679	8.33296	
15	69754435	69754822	5Y-H4K8ac_peak_5680	8.71681	RP11-253M7.4_ENSG00000259215;RP11-279F6.1_ENSG00000245750
15	70168205	70168579	5Y-H4K8ac_peak_5681	4.84727	
15	70169125	70169351	5Y-H4K8ac_peak_5682	7.38046	
15	70289438	70289802	5Y-H4K8ac_peak_5683	6.34046	
15	70389506	70389881	5Y-H4K8ac_peak_5684	4.95697	TLE3_ENSG00000140332
15	70391028	70391638	5Y-H4K8ac_peak_5685	8.76581	TLE3_ENSG00000140332
15	70477151	70477412	5Y-H4K8ac_peak_5686	4.84727	
15	70478054	70478285	5Y-H4K8ac_peak_5687	6.14981	
15	70487690	70488075	5Y-H4K8ac_peak_5688	6.14981	
15	70488747	70489029	5Y-H4K8ac_peak_5689	9.38276	
15	70492091	70492361	5Y-H4K8ac_peak_5690	8.43511	
15	70632729	70632919	5Y-H4K8ac_peak_5691	5.81394	
15	70641390	70641829	5Y-H4K8ac_peak_5692	8.93923	
15	70677638	70678294	5Y-H4K8ac_peak_5693	13.43594	
15	70679346	70679863	5Y-H4K8ac_peak_5694	5.98695	
15	70731002	70731381	5Y-H4K8ac_peak_5695	7.38046	
15	70740366	70740601	5Y-H4K8ac_peak_5696	4.0639	
15	70755620	70755995	5Y-H4K8ac_peak_5697	5.32832	
15	70819701	70820086	5Y-H4K8ac_peak_5698	9.60627	
15	70851612	70852014	5Y-H4K8ac_peak_5699	8.17203	
15	70920107	70920340	5Y-H4K8ac_peak_5700	9.30338	
15	70921000	70921231	5Y-H4K8ac_peak_5701	5.23083	
15	70962581	70962787	5Y-H4K8ac_peak_5702	4.07874	

15	71184375	71184606	5Y-H4K8ac_peak_5703	8.43511	THAP10_ENSG00000129028
15	72410821	72411021	5Y-H4K8ac_peak_5704	7.89142	MYO9A_ENSG00000066933
15	72489856	72490101	5Y-H4K8ac_peak_5705	7.9057	GRAMD2_ENSG00000175318
15	72522940	72523494	5Y-H4K8ac_peak_5706	8.43511	PKM_ENSG00000067225
15	72766135	72766455	5Y-H4K8ac_peak_5707	9.30505	ARIH1_ENSG00000166233
15	72929441	72929660	5Y-H4K8ac_peak_5708	15.06486	RP11-1006G14.2_ENSG00000259783
15	73075704	73075974	5Y-H4K8ac_peak_5709	5.23083	ADPGK-AS1_ENSG00000260898
15	73303345	73303538	5Y-H4K8ac_peak_5710	4.24332	
15	73343859	73344310	5Y-H4K8ac_peak_5711	10.11191	NEO1_ENSG00000067141
15	73429826	73430030	5Y-H4K8ac_peak_5712	6.34046	
15	73615407	73615737	5Y-H4K8ac_peak_5713	4.93237	
15	73925261	73925468	5Y-H4K8ac_peak_5714	6.77436	
15	73976626	73977274	5Y-H4K8ac_peak_5715	12.50545	CD276_ENSG00000103855
15	74045067	74045455	5Y-H4K8ac_peak_5716	6.73385	C15orf59_ENSG00000205363
15	74110557	74110936	5Y-H4K8ac_peak_5717	8.28346	
15	74189832	74190172	5Y-H4K8ac_peak_5718	5.56912	
15	74191498	74191817	5Y-H4K8ac_peak_5719	9.43096	
15	74236405	74236983	5Y-H4K8ac_peak_5720	7.20869	
15	74243684	74244469	5Y-H4K8ac_peak_5721	8.24461	
15	74244665	74244906	5Y-H4K8ac_peak_5722	6.70695	
15	74265777	74266162	5Y-H4K8ac_peak_5723	7.11863	
15	74284819	74285153	5Y-H4K8ac_peak_5724	8.16382	
15	74420535	74422390	5Y-H4K8ac_peak_5725	16.41521	RP11-247C2.2_ENSG00000248540
15	74422734	74423034	5Y-H4K8ac_peak_5726	10.31981	
15	74427456	74428122	5Y-H4K8ac_peak_5727	7.89273	
15	74428376	74428765	5Y-H4K8ac_peak_5728	15.82359	
15	74429026	74429422	5Y-H4K8ac_peak_5729	7.89273	
15	74429684	74430175	5Y-H4K8ac_peak_5730	7.9412	
15	74449961	74450158	5Y-H4K8ac_peak_5731	4.84727	
15	74468145	74468362	5Y-H4K8ac_peak_5732	4.16635	
15	74468776	74469223	5Y-H4K8ac_peak_5733	6.69752	
15	74493691	74493943	5Y-H4K8ac_peak_5734	5.18558	
15	74494974	74495198	5Y-H4K8ac_peak_5735	8.1667	RP11-60L3.1_ENSG00000259264
15	74513863	74514490	5Y-H4K8ac_peak_5736	5.60566	
15	74516303	74516750	5Y-H4K8ac_peak_5737	13.0168	
15	74519782	74520027	5Y-H4K8ac_peak_5738	9.24789	
15	74520275	74521031	5Y-H4K8ac_peak_5739	13.12848	
15	74521291	74522254	5Y-H4K8ac_peak_5740	8.0168	
15	74523972	74524194	5Y-H4K8ac_peak_5741	6.47245	
15	74526450	74527090	5Y-H4K8ac_peak_5742	5.12488	
15	74531784	74532166	5Y-H4K8ac_peak_5743	9.87097	
15	74534545	74534888	5Y-H4K8ac_peak_5744	6.24511	
15	74535640	74536029	5Y-H4K8ac_peak_5745	5.37588	
15	74537281	74537489	5Y-H4K8ac_peak_5746	7.10001	
15	74543579	74543890	5Y-H4K8ac_peak_5747	6.96612	
15	74551253	74551745	5Y-H4K8ac_peak_5748	13.23366	

15	74552429	74552737	5Y-H4K8ac_peak_5749	6.32323	
15	74579496	74579744	5Y-H4K8ac_peak_5750	9.34873	
15	74581614	74582372	5Y-H4K8ac_peak_5751	7.92287	
15	74582725	74583260	5Y-H4K8ac_peak_5752	10.1994	
15	74584089	74585345	5Y-H4K8ac_peak_5753	20.38467	
15	74585931	74586492	5Y-H4K8ac_peak_5754	5.23083	
15	74587727	74588216	5Y-H4K8ac_peak_5755	7.89142	
15	74589260	74590275	5Y-H4K8ac_peak_5756	7.89273	
15	74593707	74594045	5Y-H4K8ac_peak_5757	7.30348	
15	74594582	74596090	5Y-H4K8ac_peak_5758	15.17389	RP11-60L3.2_ENSG00000261384
15	74597075	74597715	5Y-H4K8ac_peak_5759	5.41472	
15	74600384	74600578	5Y-H4K8ac_peak_5760	9.66405	
15	74601231	74601476	5Y-H4K8ac_peak_5761	7.11863	
15	74609927	74610117	5Y-H4K8ac_peak_5762	6.27205	
15	74610564	74610814	5Y-H4K8ac_peak_5763	13.81774	
15	74611900	74612598	5Y-H4K8ac_peak_5764	9.84563	
15	74667172	74667506	5Y-H4K8ac_peak_5765	5.40331	
15	74833614	74833850	5Y-H4K8ac_peak_5766	7.11863	ARID3B_ENSG00000179361
15	74877160	74877461	5Y-H4K8ac_peak_5767	4.84727	
15	74907216	74908003	5Y-H4K8ac_peak_5768	11.29141	CTD-3154N5.1_ENSG00000260919
15	74908252	74908487	5Y-H4K8ac_peak_5769	9.51254	CTD-3154N5.1_ENSG00000260919
15	74988399	74988604	5Y-H4K8ac_peak_5770	6.08523	EDC3_ENSG00000179151
15	75074504	75074700	5Y-H4K8ac_peak_5771	5.81394	CSK_ENSG00000103653
15	75115553	75115748	5Y-H4K8ac_peak_5772	6.09071	
15	75135130	75135479	5Y-H4K8ac_peak_5773	13.0168	ULK3_ENSG00000140474
15	75136220	75136499	5Y-H4K8ac_peak_5774	9.30505	ULK3_ENSG00000140474
15	75181765	75181987	5Y-H4K8ac_peak_5775	8.97839	MPI_ENSG00000178802
15	75182437	75182876	5Y-H4K8ac_peak_5776	9.23159	MPI_ENSG00000178802
15	75230735	75231293	5Y-H4K8ac_peak_5777	11.09973	COX5A_ENSG00000178741
15	75248167	75248370	5Y-H4K8ac_peak_5778	8.47164	
15	75248770	75249033	5Y-H4K8ac_peak_5779	10.11191	RPP25_ENSG00000178718;SCAMP5_ENSG00000198794
15	75249245	75250269	5Y-H4K8ac_peak_5780	6.78128	RPP25_ENSG00000178718;SCAMP5_ENSG00000198794
15	75287944	75288290	5Y-H4K8ac_peak_5781	11.30958	
15	75316028	75316254	5Y-H4K8ac_peak_5782	15.28394	RP11-151H2.1_ENSG00000260483;PPCDC_ENSG00000138621
15	75339733	75340088	5Y-H4K8ac_peak_5783	7.65885	
15	75472737	75473071	5Y-H4K8ac_peak_5784	10.21121	
15	75488056	75488761	5Y-H4K8ac_peak_5785	10.21117	C15orf39_ENSG00000167173
15	75628435	75628880	5Y-H4K8ac_peak_5786	11.19336	COMMD4_ENSG00000140365
15	75640374	75640693	5Y-H4K8ac_peak_5787	14.19462	
15	75660492	75660804	5Y-H4K8ac_peak_5788	7.27671	MAN2C1_ENSG00000140400;RP11-817O13.8_ENSG00000260274
15	75918206	75918650	5Y-H4K8ac_peak_5789	8.43511	SNUPN_ENSG00000169371
15	75939870	75940093	5Y-H4K8ac_peak_5790	6.1654	IMP3_ENSG00000177971;SNX33_ENSG00000173548
15	75986340	75986611	5Y-H4K8ac_peak_5791	10.21117	
15	75992786	75993114	5Y-H4K8ac_peak_5792	5.41472	
15	75995705	75995960	5Y-H4K8ac_peak_5793	6.40964	
15	76001648	76002165	5Y-H4K8ac_peak_5794	5.65584	

15	76031217	76031643	5Y-H4K8ac_peak_5795	7.76645	DNM1P35_ENSG00000246877;AC019294.1_ENSG00000256530
15	76050988	76051432	5Y-H4K8ac_peak_5796	9.30505	
15	76136213	76136568	5Y-H4K8ac_peak_5797	5.41472	UBE2Q2_ENSG00000140367
15	76195807	76196022	5Y-H4K8ac_peak_5798	4.77126	FBXO22_ENSG00000167196
15	76488507	76488698	5Y-H4K8ac_peak_5799	5.0078	
15	76627598	76627823	5Y-H4K8ac_peak_5800	6.50117	
15	76628256	76628483	5Y-H4K8ac_peak_5801	8.52802	ISL2_ENSG00000159556
15	77287381	77288189	5Y-H4K8ac_peak_5802	14.2053	
15	77362933	77363379	5Y-H4K8ac_peak_5803	5.70472	
15	77820332	77820540	5Y-H4K8ac_peak_5804	6.73385	
15	77835389	77836207	5Y-H4K8ac_peak_5805	6.53157	
15	77896493	77896804	5Y-H4K8ac_peak_5806	5.87725	
15	77898673	77898904	5Y-H4K8ac_peak_5807	4.77126	
15	77899533	77899911	5Y-H4K8ac_peak_5808	6.57504	
15	77925004	77925371	5Y-H4K8ac_peak_5809	8.21582	
15	77987975	77988456	5Y-H4K8ac_peak_5810	6.52593	
15	78010058	78010356	5Y-H4K8ac_peak_5811	6.54441	
15	78031252	78031505	5Y-H4K8ac_peak_5812	4.8188	
15	78241425	78241814	5Y-H4K8ac_peak_5813	4.95697	
15	78266222	78266517	5Y-H4K8ac_peak_5814	5.41472	
15	78267354	78267629	5Y-H4K8ac_peak_5815	7.60057	ADAMTS7P3_ENSG00000261143
15	78270767	78271014	5Y-H4K8ac_peak_5816	4.68359	
15	78280632	78281091	5Y-H4K8ac_peak_5817	6.77436	
15	78283210	78283776	5Y-H4K8ac_peak_5818	9.13842	
15	78284563	78284867	5Y-H4K8ac_peak_5819	4.90696	RP11-114H24.6_ENSG00000259792
15	78285725	78286826	5Y-H4K8ac_peak_5820	13.00733	RP11-114H24.6_ENSG00000259792
15	78290660	78291498	5Y-H4K8ac_peak_5821	9.02782	
15	78423013	78423291	5Y-H4K8ac_peak_5822	4.95697	CIB2_ENSG00000136425;IDH3A_ENSG00000166411
15	78424116	78424362	5Y-H4K8ac_peak_5823	5.56894	CIB2_ENSG00000136425;IDH3A_ENSG00000166411
15	78608077	78608617	5Y-H4K8ac_peak_5824	8.47164	
15	78715027	78715281	5Y-H4K8ac_peak_5825	5.23083	
15	78729372	78729714	5Y-H4K8ac_peak_5826	6.39626	IREB2_ENSG00000136381
15	78800039	78800230	5Y-H4K8ac_peak_5827	7.01266	HYKK_ENSG00000188266
15	78912183	78913806	5Y-H4K8ac_peak_5828	11.18363	CHRNA3_ENSG00000080644
15	78929942	78930315	5Y-H4K8ac_peak_5829	7.64675	
15	78930553	78931187	5Y-H4K8ac_peak_5830	8.75926	
15	78932538	78932930	5Y-H4K8ac_peak_5831	7.31102	
15	78933504	78934329	5Y-H4K8ac_peak_5832	8.69112	
15	78944496	78944761	5Y-H4K8ac_peak_5833	5.74859	
15	78960923	78961201	5Y-H4K8ac_peak_5834	7.11262	
15	78963191	78963467	5Y-H4K8ac_peak_5835	8.2913	
15	78964776	78965162	5Y-H4K8ac_peak_5836	6.80915	
15	78965720	78966293	5Y-H4K8ac_peak_5837	8.73392	
15	78971191	78972297	5Y-H4K8ac_peak_5838	15.45397	
15	78973025	78973337	5Y-H4K8ac_peak_5839	5.36617	
15	78974604	78975083	5Y-H4K8ac_peak_5840	9.66296	

15	78975443	78975861	5Y-H4K8ac_peak_5841	19.85404	
15	79002012	79002378	5Y-H4K8ac_peak_5842	7.14616	
15	79043974	79044758	5Y-H4K8ac_peak_5843	11.22005	RP11-160C18.4_ENSG00000238166
15	79045135	79045425	5Y-H4K8ac_peak_5844	14.86775	RP11-160C18.4_ENSG00000238166
15	79046037	79046786	5Y-H4K8ac_peak_5845	9.51254	
15	79049210	79049931	5Y-H4K8ac_peak_5846	7.89273	
15	79050427	79050717	5Y-H4K8ac_peak_5847	7.52971	
15	79051047	79051381	5Y-H4K8ac_peak_5848	11.12941	
15	79051704	79052474	5Y-H4K8ac_peak_5849	15.35687	
15	79052988	79053921	5Y-H4K8ac_peak_5850	31.89748	
15	79054260	79054743	5Y-H4K8ac_peak_5851	12.05638	
15	79056827	79057035	5Y-H4K8ac_peak_5852	6.86362	
15	79057377	79057724	5Y-H4K8ac_peak_5853	6.37023	
15	79104070	79104639	5Y-H4K8ac_peak_5854	11.22005	ADAMTS7_ENSG00000136378
15	79165386	79165598	5Y-H4K8ac_peak_5855	9.00954	
15	79237156	79237349	5Y-H4K8ac_peak_5856	10.1994	
15	79271180	79271502	5Y-H4K8ac_peak_5857	6.47245	RP11-16K12.1_ENSG00000177699
15	79271837	79272177	5Y-H4K8ac_peak_5858	12.26606	RP11-16K12.1_ENSG00000177699
15	79290280	79290512	5Y-H4K8ac_peak_5859	6.94897	
15	79603540	79603778	5Y-H4K8ac_peak_5860	8.79957	TMED3_ENSG00000166557
15	80189192	80189669	5Y-H4K8ac_peak_5861	10.46287	MTHFS_ENSG00000136371
15	80696225	80697053	5Y-H4K8ac_peak_5862	12.2918	RP11-210M15.2_ENSG00000259495;ARNT2_ENSG00000172379
15	80987098	80987339	5Y-H4K8ac_peak_5863	7.59101	
15	81127899	81128194	5Y-H4K8ac_peak_5864	4.29586	
15	81292585	81293114	5Y-H4K8ac_peak_5865	18.09952	MESDC1_ENSG00000140406
15	81293326	81293709	5Y-H4K8ac_peak_5866	8.1667	MESDC1_ENSG00000140406
15	81452605	81452969	5Y-H4K8ac_peak_5867	6.50117	IL16_ENSG00000172349
15	81453435	81453658	5Y-H4K8ac_peak_5868	5.65584	
15	81616139	81616410	5Y-H4K8ac_peak_5869	6.79955	STARD5_ENSG00000172345;RP11-761I4.3_ENSG00000259343
15	81616829	81617028	5Y-H4K8ac_peak_5870	7.04637	STARD5_ENSG00000172345;RP11-761I4.3_ENSG00000259343
15	82042325	82042605	5Y-H4K8ac_peak_5871	5.23819	
15	82043724	82043956	5Y-H4K8ac_peak_5872	5.23083	
15	82201815	82202011	5Y-H4K8ac_peak_5873	5.45466	
15	82206537	82206804	5Y-H4K8ac_peak_5874	6.43068	
15	82338845	82339297	5Y-H4K8ac_peak_5875	18.77281	MEX3B_ENSG00000183496
15	82399214	82399502	5Y-H4K8ac_peak_5876	7.38046	
15	83315870	83316705	5Y-H4K8ac_peak_5877	10.79063	CPEB1_ENSG00000214575;RP11-752G15.3_ENSG00000259462
15	83378801	83379314	5Y-H4K8ac_peak_5878	7.34185	AP3B2_ENSG00000103723
15	83519471	83519676	5Y-H4K8ac_peak_5879	8.43511	
15	83654288	83654806	5Y-H4K8ac_peak_5880	6.53157	HOMER2_ENSG00000103942;FAM103A1_ENSG00000169612
15	83679975	83680299	5Y-H4K8ac_peak_5881	11.22005	C15orf40_ENSG00000169609
15	83680515	83681227	5Y-H4K8ac_peak_5882	10.69698	C15orf40_ENSG00000169609;RP11-382A20.7_ENSG00000260608
15	83735617	83735950	5Y-H4K8ac_peak_5883	7.20869	BTBD1_ENSG00000064726;MIR4515_ENSG00000263643
15	83736280	83736850	5Y-H4K8ac_peak_5884	6.73047	BTBD1_ENSG00000064726;MIR4515_ENSG00000263643
15	83876728	83877153	5Y-H4K8ac_peak_5885	15.40182	HDGFRP3_ENSG00000166503
15	84864121	84864412	5Y-H4K8ac_peak_5886	5.87725	

15	84865368	84865921	5Y-H4K8ac_peak_5887	14.31711	
15	85113514	85113886	5Y-H4K8ac_peak_5888	5.89922	LINC00933_ENSG00000259728;UBE2Q2P1_ENSG00000189136
15	85174783	85174975	5Y-H4K8ac_peak_5889	8.17203	SCAND2P_ENSG00000176700
15	85291201	85291437	5Y-H4K8ac_peak_5890	7.59101	ZNF592_ENSG00000166716
15	85291824	85292064	5Y-H4K8ac_peak_5891	11.1169	ZNF592_ENSG00000166716
15	85458784	85459397	5Y-H4K8ac_peak_5892	10.55504	
15	85498812	85499090	5Y-H4K8ac_peak_5893	8.43511	
15	85522999	85523202	5Y-H4K8ac_peak_5894	7.38046	PDE8A_ENSG00000073417
15	85799634	85799900	5Y-H4K8ac_peak_5895	4.50834	
15	88418436	88418634	5Y-H4K8ac_peak_5896	5.65584	
15	89114832	89115088	5Y-H4K8ac_peak_5897	8.93923	
15	89191057	89191286	5Y-H4K8ac_peak_5898	10.68564	
15	89561574	89561887	5Y-H4K8ac_peak_5899	8.79957	
15	89630923	89631184	5Y-H4K8ac_peak_5900	7.90236	RP11-326A19.3_ENSG00000261407;ABHD2_ENSG00000140526
15	89688338	89688537	5Y-H4K8ac_peak_5901	5.84208	
15	89786541	89787011	5Y-H4K8ac_peak_5902	15.40182	FANCI_ENSG00000140525
15	89902017	89902713	5Y-H4K8ac_peak_5903	14.10646	
15	89904899	89905101	5Y-H4K8ac_peak_5904	7.30348	LINC00925_ENSG00000255571
15	89910976	89911307	5Y-H4K8ac_peak_5905	10.73606	MIR9-3_ENSG00000207819
15	89927112	89927308	5Y-H4K8ac_peak_5906	6.1654	
15	89942576	89943173	5Y-H4K8ac_peak_5907	11.5813	
15	89943473	89943957	5Y-H4K8ac_peak_5908	7.11863	
15	89992587	89992954	5Y-H4K8ac_peak_5909	9.79526	
15	90118962	90119192	5Y-H4K8ac_peak_5910	8.4454	TICRR_ENSG00000140534
15	90208910	90209647	5Y-H4K8ac_peak_5911	9.62747	
15	90293570	90294944	5Y-H4K8ac_peak_5912	18.68977	MESP1_ENSG00000166823;MRPL15P1_ENSG00000259441
15	90319245	90320575	5Y-H4K8ac_peak_5913	13.99776	
15	90358211	90358458	5Y-H4K8ac_peak_5914	5.41472	ANPEP_ENSG00000166825
15	90358736	90358959	5Y-H4K8ac_peak_5915	4.29586	ANPEP_ENSG00000166825
15	90456046	90456532	5Y-H4K8ac_peak_5916	9.44542	C15orf38-AP3S2_ENSG00000250021;C15orf38_ENSG00000242498
15	90544471	90544703	5Y-H4K8ac_peak_5917	6.31818	ZNF710_ENSG00000140548
15	90545232	90545490	5Y-H4K8ac_peak_5918	10.55771	ZNF710_ENSG00000140548
15	90718506	90718795	5Y-H4K8ac_peak_5919	6.50117	
15	90736204	90736586	5Y-H4K8ac_peak_5920	7.64716	
15	90808429	90809178	5Y-H4K8ac_peak_5921	9.36633	NGRN_ENSG00000182768
15	90844234	90844498	5Y-H4K8ac_peak_5922	11.22005	
15	90894986	90895241	5Y-H4K8ac_peak_5923	5.67283	ZNF774_ENSG00000196391
15	91072689	91073721	5Y-H4K8ac_peak_5924	6.78128	CRTC3_ENSG00000140577
15	91208662	91209098	5Y-H4K8ac_peak_5925	10.79081	
15	91366970	91367232	5Y-H4K8ac_peak_5926	4.84727	
15	91415253	91415480	5Y-H4K8ac_peak_5927	10.11191	
15	91438453	91438820	5Y-H4K8ac_peak_5928	8.46442	
15	91446160	91446778	5Y-H4K8ac_peak_5929	8.55081	MAN2A2_ENSG00000196547
15	91475273	91475730	5Y-H4K8ac_peak_5930	7.03573	HDDC3_ENSG00000184508
15	91498301	91498672	5Y-H4K8ac_peak_5931	5.24695	AC068831.6_ENSG00000258384;RCCD1_ENSG00000166965
15	91500047	91500612	5Y-H4K8ac_peak_5932	7.03573	

15	91538993	91539271	5Y-H4K8ac_peak_5933	5.68769	PRC1_ENSG00000198901
15	92040234	92040649	5Y-H4K8ac_peak_5934	4.84727	
15	92937134	92937664	5Y-H4K8ac_peak_5935	7.18918	ST8SIA2_ENSG00000140557
15	92937939	92938453	5Y-H4K8ac_peak_5936	9.92042	ST8SIA2_ENSG00000140557
15	93042463	93042814	5Y-H4K8ac_peak_5937	5.65584	
15	93161069	93161260	5Y-H4K8ac_peak_5938	10.19948	
15	93257756	93258113	5Y-H4K8ac_peak_5939	12.21176	
15	93277017	93277281	5Y-H4K8ac_peak_5940	8.33296	RP11-386M24.4_ENSG00000258741
15	93364171	93364510	5Y-H4K8ac_peak_5941	8.17203	
15	93426392	93426741	5Y-H4K8ac_peak_5942	10.46287	AC013394.2_ENSG00000272888;CHD2_ENSG00000173575
15	99190591	99191107	5Y-H4K8ac_peak_5943	8.2913	RP11-35O15.1_ENSG00000259424
15	99192235	99192572	5Y-H4K8ac_peak_5944	7.76232	IGF1R_ENSG00000140443
15	99270722	99271075	5Y-H4K8ac_peak_5945	6.87117	
15	99271395	99271976	5Y-H4K8ac_peak_5946	5.41472	
15	99279955	99280205	5Y-H4K8ac_peak_5947	6.14981	
15	99280957	99281168	5Y-H4K8ac_peak_5948	8.33296	
15	99434542	99434793	5Y-H4K8ac_peak_5949	6.34046	
15	99558044	99558415	5Y-H4K8ac_peak_5950	5.64909	
15	99602523	99602818	5Y-H4K8ac_peak_5951	9.79526	
15	99640629	99640858	5Y-H4K8ac_peak_5952	8.2913	
15	99645446	99645663	5Y-H4K8ac_peak_5953	7.38046	RP11-654A16.3_ENSG00000259475
15	99663610	99663945	5Y-H4K8ac_peak_5954	15.82359	
15	100027214	100027747	5Y-H4K8ac_peak_5955	9.38203	
15	100105259	100105873	5Y-H4K8ac_peak_5956	8.69112	
15	100273931	100274404	5Y-H4K8ac_peak_5957	12.82003	LYSMD4_ENSG00000183060
15	100890076	100890336	5Y-H4K8ac_peak_5958	9.51254	SPATA41_ENSG00000189419
15	100913337	100913787	5Y-H4K8ac_peak_5959	5.65584	RP11-168G16.2_ENSG00000259430
15	101207952	101208161	5Y-H4K8ac_peak_5960	7.01364	
15	101209497	101209791	5Y-H4K8ac_peak_5961	11.09973	
15	101254533	101254774	5Y-H4K8ac_peak_5962	4.24869	
15	101305194	101305547	5Y-H4K8ac_peak_5963	6.22904	
15	101305861	101306412	5Y-H4K8ac_peak_5964	16.6076	
15	101310041	101310400	5Y-H4K8ac_peak_5965	4.95697	
15	101311211	101311999	5Y-H4K8ac_peak_5966	5.41472	
15	101314252	101314677	5Y-H4K8ac_peak_5967	7.59101	
15	101315467	101315722	5Y-H4K8ac_peak_5968	5.18494	
15	101321321	101321675	5Y-H4K8ac_peak_5969	14.89891	
15	101322119	101322473	5Y-H4K8ac_peak_5970	6.37023	
15	101328443	101328649	5Y-H4K8ac_peak_5971	7.42477	
15	101370849	101371040	5Y-H4K8ac_peak_5972	5.5192	
15	101419224	101420171	5Y-H4K8ac_peak_5973	22.01193	
15	101440769	101441296	5Y-H4K8ac_peak_5974	8.43511	
15	101459613	101460206	5Y-H4K8ac_peak_5975	9.51254	RP11-66B24.4_ENSG00000259583;LRRK1_ENSG00000154237
15	101666842	101667377	5Y-H4K8ac_peak_5976	10.11191	
15	101674056	101674348	5Y-H4K8ac_peak_5977	6.66026	
15	101688907	101689547	5Y-H4K8ac_peak_5978	17.63051	

15	101690709	101691012	5Y-H4K8ac_peak_5979	4.40052	
15	101835174	101835407	5Y-H4K8ac_peak_5980	12.69256	SNRPA1_ENSG00000131876;RP11-299G20.2_ENSG00000259172
15	101990107	101991359	5Y-H4K8ac_peak_5981	4.15658	
15	102041816	102042190	5Y-H4K8ac_peak_5982	7.46096	
15	102192840	102193310	5Y-H4K8ac_peak_5983	5.91107	TM2D3_ENSG00000184277
15	102264145	102264442	5Y-H4K8ac_peak_5984	4.50834	TARSL2_ENSG00000185418
15	102506189	102506477	5Y-H4K8ac_peak_5985	4.48815	
16	128305	128526	5Y-H4K8ac_peak_5986	7.18391	
16	188839	189071	5Y-H4K8ac_peak_5987	10.1994	NPRL3_ENSG00000103148
16	231236	231457	5Y-H4K8ac_peak_5988	7.09658	HBQ1_ENSG00000086506
16	279196	279406	5Y-H4K8ac_peak_5989	11.04542	LUC7L_ENSG00000007392
16	324958	325265	5Y-H4K8ac_peak_5990	4.95697	RGS11_ENSG00000076344
16	330759	331253	5Y-H4K8ac_peak_5991	9.23159	
16	431538	431807	5Y-H4K8ac_peak_5992	10.90543	Z97634.3_ENSG00000236829
16	432263	432461	5Y-H4K8ac_peak_5993	6.50117	Z97634.3_ENSG00000236829
16	447234	447518	5Y-H4K8ac_peak_5994	14.10646	NME4_ENSG00000103202
16	476001	476397	5Y-H4K8ac_peak_5995	9.93099	RAB11FIP3_ENSG00000090565
16	508110	508318	5Y-H4K8ac_peak_5996	8.19789	
16	576931	577351	5Y-H4K8ac_peak_5997	4.95697	CAPN15_ENSG00000103326
16	614769	615062	5Y-H4K8ac_peak_5998	4.29586	
16	617014	617424	5Y-H4K8ac_peak_5999	8.43511	PIGQ_ENSG00000007541;NHLRC4_ENSG00000257108
16	619348	619836	5Y-H4K8ac_peak_6000	8.24461	
16	620055	620275	5Y-H4K8ac_peak_6001	6.37023	
16	638586	639820	5Y-H4K8ac_peak_6002	13.9425	RAB40C_ENSG00000197562
16	685979	686250	5Y-H4K8ac_peak_6003	9.51254	C16orf13_ENSG00000130731
16	691506	691805	5Y-H4K8ac_peak_6004	6.50117	AL022341.1_ENSG00000197727;FAM195A_ENSG00000172366
16	692035	692372	5Y-H4K8ac_peak_6005	9.51785	AL022341.1_ENSG00000197727;FAM195A_ENSG00000172366
16	698712	699096	5Y-H4K8ac_peak_6006	13.10659	AL022341.3_ENSG00000228201;WDR90_ENSG00000161996
16	699466	699793	5Y-H4K8ac_peak_6007	7.15925	AL022341.3_ENSG00000228201;WDR90_ENSG00000161996
16	710061	710543	5Y-H4K8ac_peak_6008	8.82628	
16	733743	734265	5Y-H4K8ac_peak_6009	8.73227	JMJD8_ENSG00000161999
16	739746	740348	5Y-H4K8ac_peak_6010	11.22005	WDR24_ENSG00000127580;LA16c-313D11.12_ENSG00000261659
16	740639	741192	5Y-H4K8ac_peak_6011	10.90365	WDR24_ENSG00000127580
16	759875	760315	5Y-H4K8ac_peak_6012	8.19789	LA16c-380A1.1_ENSG00000259840
16	760958	761150	5Y-H4K8ac_peak_6013	5.41472	LA16c-380A1.1_ENSG00000259840
16	770042	770245	5Y-H4K8ac_peak_6014	7.19446	FAM173A_ENSG00000103254
16	770646	771352	5Y-H4K8ac_peak_6015	7.50501	FAM173A_ENSG00000103254
16	791216	791573	5Y-H4K8ac_peak_6016	7.60057	NARFL_ENSG00000103245
16	857339	857768	5Y-H4K8ac_peak_6017	9.38276	
16	858022	858269	5Y-H4K8ac_peak_6018	10.1994	
16	1020518	1020812	5Y-H4K8ac_peak_6019	5.87725	
16	1358688	1358899	5Y-H4K8ac_peak_6020	11.48679	
16	1359122	1359913	5Y-H4K8ac_peak_6021	20.93228	
16	1401450	1401799	5Y-H4K8ac_peak_6022	9.23159	TSR3_ENSG00000007520;GNPTG_ENSG00000090581
16	1402081	1402324	5Y-H4K8ac_peak_6023	4.29586	TSR3_ENSG00000007520;GNPTG_ENSG00000090581
16	1471395	1472122	5Y-H4K8ac_peak_6024	7.04637	

16	1480305	1480676	5Y-H4K8ac_peak_6025	5.50568	C16orf91_ENSG00000174109
16	1487511	1487870	5Y-H4K8ac_peak_6026	6.50117	
16	1524496	1524710	5Y-H4K8ac_peak_6027	5.23819	CLCN7_ENSG00000103249
16	1525145	1525543	5Y-H4K8ac_peak_6028	18.78631	CLCN7_ENSG00000103249
16	1543399	1543797	5Y-H4K8ac_peak_6029	4.95697	TELO2_ENSG00000100726
16	1580664	1581003	5Y-H4K8ac_peak_6030	4.70545	
16	1581490	1581774	5Y-H4K8ac_peak_6031	6.43068	
16	1755672	1755988	5Y-H4K8ac_peak_6032	14.81591	MAPK8IP3_ENSG00000138834
16	1820813	1821056	5Y-H4K8ac_peak_6033	5.77617	NME3_ENSG00000103024
16	1823239	1823491	5Y-H4K8ac_peak_6034	8.09118	MRPS34_ENSG00000074071;EME2_ENSG00000197774
16	1922588	1922791	5Y-H4K8ac_peak_6035	5.28616	
16	1946305	1946580	5Y-H4K8ac_peak_6036	4.84727	
16	1952760	1952970	5Y-H4K8ac_peak_6037	6.14981	
16	1953837	1954248	5Y-H4K8ac_peak_6038	10.54764	
16	1954466	1954725	5Y-H4K8ac_peak_6039	8.71064	
16	1956444	1956772	5Y-H4K8ac_peak_6040	5.05812	
16	1957587	1957901	5Y-H4K8ac_peak_6041	6.77436	
16	1958442	1958797	5Y-H4K8ac_peak_6042	4.95697	
16	1979942	1980397	5Y-H4K8ac_peak_6043	10.35586	
16	2004039	2004493	5Y-H4K8ac_peak_6044	10.69698	
16	2008567	2009176	5Y-H4K8ac_peak_6045	10.99576	RPL3L_ENSG00000140986;NDUFB10_ENSG00000140990
16	2009613	2009916	5Y-H4K8ac_peak_6046	12.21176	NDUFB10_ENSG00000140990
16	2014382	2014792	5Y-H4K8ac_peak_6047	15.39044	AC005363.9_ENSG00000255513;RPS2_ENSG00000140988;SNHG9_ENSG00000255198
16	2015070	2015527	5Y-H4K8ac_peak_6048	11.19336	AC005363.9_ENSG00000255513;RPS2_ENSG00000140988;SNHG9_ENSG00000255198
16	2059399	2059593	5Y-H4K8ac_peak_6049	6.24511	ZNF598_ENSG00000167962;NPW_ENSG00000183971
16	2083259	2083870	5Y-H4K8ac_peak_6050	9.7353	
16	2085430	2085823	5Y-H4K8ac_peak_6051	5.23083	
16	2086286	2086525	5Y-H4K8ac_peak_6052	5.67283	
16	2088070	2088278	5Y-H4K8ac_peak_6053	6.77436	
16	2097476	2097739	5Y-H4K8ac_peak_6054	8.43511	NTHL1_ENSG00000065057;TSC2_ENSG00000103197
16	2171976	2172597	5Y-H4K8ac_peak_6055	9.62747	
16	2172814	2173359	5Y-H4K8ac_peak_6056	9.51254	
16	2173720	2173915	5Y-H4K8ac_peak_6057	8.24461	
16	2174142	2175077	5Y-H4K8ac_peak_6058	13.12848	
16	2175418	2176376	5Y-H4K8ac_peak_6059	9.79526	
16	2176914	2177129	5Y-H4K8ac_peak_6060	7.50148	
16	2197556	2197890	5Y-H4K8ac_peak_6061	6.86362	
16	2198291	2198512	5Y-H4K8ac_peak_6062	7.90236	
16	2198978	2199210	5Y-H4K8ac_peak_6063	5.41472	
16	2246838	2247033	5Y-H4K8ac_peak_6064	8.05083	CASKIN1_ENSG00000167971
16	2255166	2255953	5Y-H4K8ac_peak_6065	18.12001	MLST8_ENSG00000167965;AC009065.3_ENSG00000252958
16	2265749	2266109	5Y-H4K8ac_peak_6066	4.642	PGP_ENSG00000184207
16	2273664	2273892	5Y-H4K8ac_peak_6067	11.1169	E4F1_ENSG00000167967
16	2274116	2274388	5Y-H4K8ac_peak_6068	4.50834	E4F1_ENSG00000167967
16	2301251	2302173	5Y-H4K8ac_peak_6069	10.1994	ECI1_ENSG00000167969
16	2317751	2318110	5Y-H4K8ac_peak_6070	13.14766	RNPS1_ENSG00000205937;AC009065.2_ENSG00000207715;MIR940_ENSG00000260778

16	2391055	2391362	5Y-H4K8ac_peak_6071	11.1169	ABCA3_ENSG00000167972
16	2478570	2479007	5Y-H4K8ac_peak_6072	4.97086	CCNF_ENSG00000162063
16	2517352	2517544	5Y-H4K8ac_peak_6073	7.65114	RP11-715J22.2_ENSG00000259895
16	2517805	2518571	5Y-H4K8ac_peak_6074	20.56946	RP11-715J22.2_ENSG00000259895
16	2518961	2520589	5Y-H4K8ac_peak_6075	11.12941	RP11-715J22.2_ENSG00000259895;NTN3_ENSG00000162068
16	2521474	2522072	5Y-H4K8ac_peak_6076	8.5915	NTN3_ENSG00000162068
16	2523679	2524006	5Y-H4K8ac_peak_6077	7.50148	
16	2524555	2524928	5Y-H4K8ac_peak_6078	6.40724	TBC1D24_ENSG00000162065
16	2532239	2532697	5Y-H4K8ac_peak_6079	10.03452	
16	2563742	2563956	5Y-H4K8ac_peak_6080	9.51254	ATP6V0C_ENSG00000185883;ATP6C_ENSG00000259784
16	2570732	2571043	5Y-H4K8ac_peak_6081	5.3989	AMDHD2_ENSG00000162066
16	2581594	2581849	5Y-H4K8ac_peak_6082	9.30505	CEMP1_ENSG00000205923;MIR3178_ENSG00000266232
16	2653471	2653749	5Y-H4K8ac_peak_6083	8.69112	AC141586.5_ENSG00000215154
16	2732581	2732906	5Y-H4K8ac_peak_6084	7.11863	KCTD5_ENSG00000167977
16	2770114	2771067	5Y-H4K8ac_peak_6085	8.20773	PRSS27_ENSG00000172382
16	2801799	2802893	5Y-H4K8ac_peak_6086	20.83471	SRRM2-AS1_ENSG00000205913;SRRM2_ENSG00000167978
16	2826827	2827066	5Y-H4K8ac_peak_6087	6.77436	TCEB2_ENSG00000103363
16	2827416	2828116	5Y-H4K8ac_peak_6088	12.65187	TCEB2_ENSG00000103363
16	2870305	2870549	5Y-H4K8ac_peak_6089	6.62622	
16	2902362	2902690	5Y-H4K8ac_peak_6090	9.15007	
16	2917944	2918478	5Y-H4K8ac_peak_6091	6.08471	LA16c-325D7.2_ENSG00000263280
16	2918814	2919005	5Y-H4K8ac_peak_6092	4.67245	
16	2954540	2954754	5Y-H4K8ac_peak_6093	8.52802	
16	3018631	3018957	5Y-H4K8ac_peak_6094	7.59101	PAQR4_ENSG00000162073
16	3019722	3019914	5Y-H4K8ac_peak_6095	4.90696	PAQR4_ENSG00000162073
16	3030164	3030419	5Y-H4K8ac_peak_6096	16.01457	PKMYT1_ENSG00000127564
16	3030650	3030873	5Y-H4K8ac_peak_6097	13.0168	PKMYT1_ENSG00000127564
16	3033951	3034244	5Y-H4K8ac_peak_6098	10.54764	
16	3109593	3109992	5Y-H4K8ac_peak_6099	7.11863	RP11-473M20.7_ENSG00000261971
16	3162411	3162918	5Y-H4K8ac_peak_6100	7.59101	ZNF205_ENSG00000122386
16	3184373	3184772	5Y-H4K8ac_peak_6101	8.01306	RP11-473M20.14_ENSG00000263072
16	3199565	3199771	5Y-H4K8ac_peak_6102	8.86184	
16	3238293	3238581	5Y-H4K8ac_peak_6103	5.17251	AJ003147.9_ENSG00000262668
16	3332788	3333276	5Y-H4K8ac_peak_6104	12.44818	
16	3333540	3333766	5Y-H4K8ac_peak_6105	9.05168	
16	3355584	3356081	5Y-H4K8ac_peak_6106	10.69698	TIGD7_ENSG00000140993;ZNF75A_ENSG00000162086
16	3507114	3507384	5Y-H4K8ac_peak_6107	9.38203	LA16c-306E5.3_ENSG00000263212
16	3507700	3507943	5Y-H4K8ac_peak_6108	11.57334	LA16c-306E5.3_ENSG00000263212
16	3508302	3508517	5Y-H4K8ac_peak_6109	9.23159	LA16c-306E5.3_ENSG00000263212
16	3767703	3768001	5Y-H4K8ac_peak_6110	10.35586	TRAP1_ENSG00000126602
16	3930187	3930404	5Y-H4K8ac_peak_6111	10.69698	CREBBP_ENSG00000005339
16	3930909	3931648	5Y-H4K8ac_peak_6112	19.17092	CREBBP_ENSG00000005339
16	3988998	3989376	5Y-H4K8ac_peak_6113	7.63144	
16	4233196	4233843	5Y-H4K8ac_peak_6114	12.11208	RP11-95P2.3_ENSG00000263105
16	4234252	4234455	5Y-H4K8ac_peak_6115	8.47164	RP11-95P2.3_ENSG00000263105
16	4321156	4322596	5Y-H4K8ac_peak_6116	10.60083	TFAP4_ENSG00000090447

16	4357834	4358250	5Y-H4K8ac_peak_6117	9.23159	
16	4365077	4365709	5Y-H4K8ac_peak_6118	13.77072	
16	4378387	4378874	5Y-H4K8ac_peak_6119	10.90097	AC005356.1_ENSG00000262686
16	4400976	4401196	5Y-H4K8ac_peak_6120	6.37023	
16	4433744	4434252	5Y-H4K8ac_peak_6121	5.23083	
16	4436294	4436585	5Y-H4K8ac_peak_6122	5.98695	
16	4441611	4442540	5Y-H4K8ac_peak_6123	8.62703	
16	4442859	4443218	5Y-H4K8ac_peak_6124	11.68317	
16	4443664	4444308	5Y-H4K8ac_peak_6125	7.98641	
16	4444531	4444942	5Y-H4K8ac_peak_6126	13.00792	
16	4466194	4466482	5Y-H4K8ac_peak_6127	8.16382	
16	4475579	4476144	5Y-H4K8ac_peak_6128	10.18964	CORO7_ENSG00000262246;DNAJA3_ENSG00000103423
16	4664357	4664762	5Y-H4K8ac_peak_6129	6.54441	UBALD1_ENSG00000153443
16	4665737	4666175	5Y-H4K8ac_peak_6130	8.97839	UBALD1_ENSG00000153443;MGRN1_ENSG00000102858
16	4666618	4667108	5Y-H4K8ac_peak_6131	6.47245	MGRN1_ENSG00000102858
16	4795429	4795658	5Y-H4K8ac_peak_6132	5.40331	
16	4797368	4797571	5Y-H4K8ac_peak_6133	4.79585	
16	4819332	4819545	5Y-H4K8ac_peak_6134	5.98695	
16	4822092	4822449	5Y-H4K8ac_peak_6135	5.29015	
16	4845663	4846149	5Y-H4K8ac_peak_6136	5.59837	RP11-127I20.5_ENSG00000267077
16	4896864	4897262	5Y-H4K8ac_peak_6137	7.01266	UBN1_ENSG00000118900
16	5083432	5083863	5Y-H4K8ac_peak_6138	9.30505	NAGPA_ENSG00000103174;ALG1_ENSG00000033011
16	5121821	5122202	5Y-H4K8ac_peak_6139	7.38046	
16	5555854	5556355	5Y-H4K8ac_peak_6140	8.4454	
16	5665834	5666216	5Y-H4K8ac_peak_6141	12.10416	RP11-124K4.1_ENSG00000260338
16	8715585	8715902	5Y-H4K8ac_peak_6142	7.59101	METTL22_ENSG00000067365
16	8806867	8807077	5Y-H4K8ac_peak_6143	8.24461	
16	8860101	8860936	5Y-H4K8ac_peak_6144	8.24461	
16	8891739	8892128	5Y-H4K8ac_peak_6145	13.12848	TMEM186_ENSG00000184857
16	8892354	8892609	5Y-H4K8ac_peak_6146	9.97324	TMEM186_ENSG00000184857
16	8915660	8915869	5Y-H4K8ac_peak_6147	5.29015	
16	8918994	8919192	5Y-H4K8ac_peak_6148	5.81062	
16	9184218	9184652	5Y-H4K8ac_peak_6149	6.43068	C16orf72_ENSG00000182831
16	9186012	9186604	5Y-H4K8ac_peak_6150	10.54764	C16orf72_ENSG00000182831
16	10674745	10674992	5Y-H4K8ac_peak_6151	4.15658	EMP2_ENSG00000213853
16	10710610	10711176	5Y-H4K8ac_peak_6152	8.2913	
16	10747054	10747272	5Y-H4K8ac_peak_6153	4.15658	
16	10837689	10837900	5Y-H4K8ac_peak_6154	9.23159	NUBP1_ENSG00000103274
16	10838808	10839038	5Y-H4K8ac_peak_6155	5.97532	
16	11225023	11225445	5Y-H4K8ac_peak_6156	7.38046	
16	11349079	11349399	5Y-H4K8ac_peak_6157	4.95697	SOCS1_ENSG00000185338
16	11396927	11397157	5Y-H4K8ac_peak_6158	8.04943	
16	11397505	11397776	5Y-H4K8ac_peak_6159	11.08752	
16	11439413	11439645	5Y-H4K8ac_peak_6160	13.00733	RP11-485G7.5_ENSG00000263080
16	11490419	11490671	5Y-H4K8ac_peak_6161	7.11863	
16	11504647	11504844	5Y-H4K8ac_peak_6162	4.00596	

16	11505216	11505413	5Y-H4K8ac_peak_6163	5.87725	
16	11597298	11597917	5Y-H4K8ac_peak_6164	6.46053	
16	11680082	11680648	5Y-H4K8ac_peak_6165	8.2913	
16	11680991	11681315	5Y-H4K8ac_peak_6166	5.56894	
16	11712190	11712393	5Y-H4K8ac_peak_6167	8.35138	
16	11891454	11891803	5Y-H4K8ac_peak_6168	9.30505	ZC3H7A_ENSG00000122299
16	12070670	12070872	5Y-H4K8ac_peak_6169	9.65108	RP11-166B2.1_ENSG00000234719;SNX29_ENSG00000048471;RP11-166B2.7_ENSG00000260488
16	12897017	12897730	5Y-H4K8ac_peak_6170	14.81591	CPPED1_ENSG00000103381
16	14014106	14014527	5Y-H4K8ac_peak_6171	9.01738	ERCC4_ENSG00000175595
16	14164534	14165016	5Y-H4K8ac_peak_6172	10.21117	MKL2_ENSG00000186260
16	14404615	14404858	5Y-H4K8ac_peak_6173	7.68492	
16	14726884	14727197	5Y-H4K8ac_peak_6174	8.21582	PARN_ENSG00000140694;BFAR_ENSG00000103429
16	15122796	15123146	5Y-H4K8ac_peak_6175	5.41472	
16	15234634	15234833	5Y-H4K8ac_peak_6176	7.30348	
16	15235417	15235677	5Y-H4K8ac_peak_6177	6.73385	
16	15236180	15236503	5Y-H4K8ac_peak_6178	9.26003	
16	15236751	15238118	5Y-H4K8ac_peak_6179	9.02938	
16	15238548	15239454	5Y-H4K8ac_peak_6180	5.87725	
16	15240327	15240568	5Y-H4K8ac_peak_6181	9.92042	
16	15489509	15489820	5Y-H4K8ac_peak_6182	6.77436	RP11-1021N1.1_ENSG00000261130;MPV17L_ENSG00000156968
16	15490015	15490392	5Y-H4K8ac_peak_6183	10.19948	RP11-1021N1.1_ENSG00000261130;MPV17L_ENSG00000156968
16	15528749	15528991	5Y-H4K8ac_peak_6184	7.31815	C16orf45_ENSG00000166780
16	15616613	15616870	5Y-H4K8ac_peak_6185	4.95697	
16	15736687	15736887	5Y-H4K8ac_peak_6186	10.54764	KIAA0430_ENSG00000166783;NDE1_ENSG00000072864;MIR484_ENSG00000272213
16	15737172	15737417	5Y-H4K8ac_peak_6187	7.60893	KIAA0430_ENSG00000166783;NDE1_ENSG00000072864;MIR484_ENSG00000272213
16	15952084	15952385	5Y-H4K8ac_peak_6188	6.34046	
16	16043708	16044059	5Y-H4K8ac_peak_6189	4.77126	ABCC1_ENSG00000103222
16	16244547	16244750	5Y-H4K8ac_peak_6190	8.2913	
16	16317334	16317739	5Y-H4K8ac_peak_6191	10.46287	ABCC6_ENSG00000091262;RP11-517A5.7_ENSG00000262332
16	17363730	17364325	5Y-H4K8ac_peak_6192	6.83519	
16	17430347	17430544	5Y-H4K8ac_peak_6193	4.15658	
16	17685091	17685340	5Y-H4K8ac_peak_6194	8.46442	
16	17686382	17686776	5Y-H4K8ac_peak_6195	4.84727	
16	17842320	17842556	5Y-H4K8ac_peak_6196	7.97699	
16	18064943	18065144	5Y-H4K8ac_peak_6197	6.39626	
16	18066118	18066606	5Y-H4K8ac_peak_6198	10.31981	
16	18801929	18802263	5Y-H4K8ac_peak_6199	4.71803	RPS15A_ENSG00000134419
16	18813234	18813431	5Y-H4K8ac_peak_6200	9.89244	RP11-1035H13.3_ENSG00000260342;ARL6IP1_ENSG00000170540;RP11-1035H13.2_ENSG00000260017
16	19097697	19097943	5Y-H4K8ac_peak_6201	13.0168	RP11-626G11.4_ENSG00000260430
16	19125642	19126101	5Y-H4K8ac_peak_6202	13.4304	ITPRIPL2_ENSG00000205730;CTD-2349B8.1_ENSG00000261427
16	19566333	19566654	5Y-H4K8ac_peak_6203	12.26606	C16orf62_ENSG00000103544
16	19567240	19567441	5Y-H4K8ac_peak_6204	4.0639	C16orf62_ENSG00000103544
16	19729065	19729399	5Y-H4K8ac_peak_6205	5.23819	KNOP1_ENSG00000103550
16	20817162	20817723	5Y-H4K8ac_peak_6206	10.95042	AC004381.6_ENSG00000005189
16	20818385	20818590	5Y-H4K8ac_peak_6207	4.9885	AC004381.6_ENSG00000005189
16	20912224	20912532	5Y-H4K8ac_peak_6208	11.94897	ERI2_ENSG00000196678;DCUN1D3_ENSG00000188215

16	21170071	21170593	5Y-H4K8ac_peak_6209	4.50834	DNAH3_ENSG00000158486;TMEM159_ENSG00000011638
16	21532020	21532548	5Y-H4K8ac_peak_6210	12.74275	CTD-2547E10.2_ENSG00000180747;SLC7A5P2_ENSG00000258186
16	21565803	21566090	5Y-H4K8ac_peak_6211	7.00004	
16	21566420	21566721	5Y-H4K8ac_peak_6212	7.3297	
16	21568353	21568616	5Y-H4K8ac_peak_6213	10.48476	
16	21610356	21610588	5Y-H4K8ac_peak_6214	7.50148	
16	21831379	21831612	5Y-H4K8ac_peak_6215	4.03329	RRN3P1_ENSG00000248124
16	21964766	21965137	5Y-H4K8ac_peak_6216	8.73392	UQCRC2_ENSG00000140740
16	22018887	22019352	5Y-H4K8ac_peak_6217	13.14766	C16orf52_ENSG00000185716
16	22308843	22309287	5Y-H4K8ac_peak_6218	7.59101	POLR3E_ENSG00000058600
16	22774563	22775174	5Y-H4K8ac_peak_6219	5.41472	
16	22967750	22968301	5Y-H4K8ac_peak_6220	4.95697	
16	22981791	22982099	5Y-H4K8ac_peak_6221	4.50834	
16	23568450	23568666	5Y-H4K8ac_peak_6222	11.09973	EARS2_ENSG00000103356;UBFD1_ENSG00000103353
16	23569107	23569495	5Y-H4K8ac_peak_6223	6.78128	EARS2_ENSG00000103356;UBFD1_ENSG00000103353
16	23607383	23607573	5Y-H4K8ac_peak_6224	4.642	NDUFAB1_ENSG00000004779
16	23690282	23690479	5Y-H4K8ac_peak_6225	7.03573	
16	24550940	24551178	5Y-H4K8ac_peak_6226	11.1169	
16	24740213	24740585	5Y-H4K8ac_peak_6227	9.23159	TNRC6A_ENSG00000090905
16	24741050	24741337	5Y-H4K8ac_peak_6228	4.95697	TNRC6A_ENSG00000090905
16	24864870	24865256	5Y-H4K8ac_peak_6229	6.08523	
16	25026830	25027030	5Y-H4K8ac_peak_6230	6.08523	ARHGAP17_ENSG00000140750;RP11-266L9.1_ENSG00000260714
16	25078282	25078481	5Y-H4K8ac_peak_6231	10.1994	RP11-266L9.5_ENSG00000262155
16	25118140	25118419	5Y-H4K8ac_peak_6232	14.87721	
16	25269412	25269887	5Y-H4K8ac_peak_6233	4.77126	ZKSCAN2_ENSG00000155592
16	27215435	27215660	5Y-H4K8ac_peak_6234	8.24461	KDM8_ENSG00000155666
16	27234597	27235101	5Y-H4K8ac_peak_6235	6.36454	
16	27280233	27280818	5Y-H4K8ac_peak_6236	9.51254	NSMCE1_ENSG00000169189;CTD-3203P2.2_ENSG00000245888
16	27711857	27712061	5Y-H4K8ac_peak_6237	4.84727	
16	27795442	27795941	5Y-H4K8ac_peak_6238	9.58986	
16	27797872	27798077	5Y-H4K8ac_peak_6239	6.46606	
16	27991371	27991588	5Y-H4K8ac_peak_6240	6.24411	
16	28289280	28289516	5Y-H4K8ac_peak_6241	6.78318	
16	28302333	28302569	5Y-H4K8ac_peak_6242	10.69698	RP11-57A19.2_ENSG00000246465
16	28303103	28303424	5Y-H4K8ac_peak_6243	5.39991	RP11-57A19.2_ENSG00000246465;SBK1_ENSG00000188322
16	28304856	28305069	5Y-H4K8ac_peak_6244	4.642	
16	28502837	28503268	5Y-H4K8ac_peak_6245	7.90236	CLN3_ENSG00000261832
16	28508240	28508448	5Y-H4K8ac_peak_6246	5.28616	
16	28564741	28564970	5Y-H4K8ac_peak_6247	10.32379	CCDC101_ENSG00000176476
16	28565946	28566162	5Y-H4K8ac_peak_6248	5.48291	CCDC101_ENSG00000176476
16	28761416	28761622	5Y-H4K8ac_peak_6249	8.33296	RP11-1348G14.6_ENSG00000270424
16	28834585	28835135	5Y-H4K8ac_peak_6250	21.85855	ATXN2L_ENSG00000168488
16	28857414	28857612	5Y-H4K8ac_peak_6251	5.41472	TUFM_ENSG00000178952;SH2B1_ENSG00000178188
16	28873573	28873923	5Y-H4K8ac_peak_6252	6.12014	RP11-22P6.2_ENSG00000261766
16	28874140	28874505	5Y-H4K8ac_peak_6253	5.65584	RP11-22P6.2_ENSG00000261766
16	28875370	28875777	5Y-H4K8ac_peak_6254	6.77436	RP11-22P6.2_ENSG00000261766

16	28890794	28891017	5Y-H4K8ac_peak_6255	7.03573	RP11-22P6.3_ENSG00000260442
16	28891451	28891839	5Y-H4K8ac_peak_6256	14.6127	RP11-22P6.3_ENSG00000260442
16	28936367	28937118	5Y-H4K8ac_peak_6257	11.42066	
16	28956632	28956867	5Y-H4K8ac_peak_6258	5.72233	
16	28961306	28961897	5Y-H4K8ac_peak_6259	8.4454	NFATC2IP_ENSG00000176953
16	29142552	29143252	5Y-H4K8ac_peak_6260	8.43511	
16	29144038	29144402	5Y-H4K8ac_peak_6261	7.04637	
16	29169310	29170395	5Y-H4K8ac_peak_6262	11.19336	
16	29180639	29180833	5Y-H4K8ac_peak_6263	9.7353	
16	29181094	29181878	5Y-H4K8ac_peak_6264	10.60083	
16	29183084	29183436	5Y-H4K8ac_peak_6265	7.80086	
16	29186782	29186984	5Y-H4K8ac_peak_6266	6.7002	
16	29188539	29189552	5Y-H4K8ac_peak_6267	16.17072	
16	29190789	29191329	5Y-H4K8ac_peak_6268	8.40854	
16	29191726	29192030	5Y-H4K8ac_peak_6269	10.4678	
16	29194012	29194272	5Y-H4K8ac_peak_6270	9.30505	
16	29202391	29202772	5Y-H4K8ac_peak_6271	7.59101	
16	29212515	29212979	5Y-H4K8ac_peak_6272	7.60057	
16	29214328	29214559	5Y-H4K8ac_peak_6273	5.98695	
16	29231520	29231908	5Y-H4K8ac_peak_6274	5.67283	RP11-426C22.4_ENSG00000259807
16	29239831	29240244	5Y-H4K8ac_peak_6275	4.55128	
16	29270272	29270637	5Y-H4K8ac_peak_6276	10.61721	
16	29271334	29271791	5Y-H4K8ac_peak_6277	14.89891	
16	29272013	29272235	5Y-H4K8ac_peak_6278	7.11863	
16	29272754	29273010	5Y-H4K8ac_peak_6279	8.0168	
16	29273246	29273774	5Y-H4K8ac_peak_6280	8.62703	
16	29275525	29275746	5Y-H4K8ac_peak_6281	5.64982	RP11-426C22.6_ENSG00000260953
16	29275988	29276182	5Y-H4K8ac_peak_6282	7.59101	RP11-426C22.6_ENSG00000260953
16	29278454	29278822	5Y-H4K8ac_peak_6283	5.57299	
16	29303270	29303651	5Y-H4K8ac_peak_6284	5.29015	
16	29625338	29625812	5Y-H4K8ac_peak_6285	10.36926	SLC7A5P1_ENSG00000260727
16	29689835	29690858	5Y-H4K8ac_peak_6286	13.29029	
16	29732733	29733002	5Y-H4K8ac_peak_6287	10.11191	
16	29801330	29801575	5Y-H4K8ac_peak_6288	4.68335	KIF22_ENSG00000079616
16	29818516	29818808	5Y-H4K8ac_peak_6289	7.11863	AC009133.15_ENSG00000259952
16	29827790	29828110	5Y-H4K8ac_peak_6290	5.67483	PAGR1_ENSG00000185928;PAGR1_ENSG00000263136
16	29875049	29875547	5Y-H4K8ac_peak_6291	4.77126	CDIPT_ENSG00000103502;CDIPT-AS1_ENSG00000214725
16	29985769	29985967	5Y-H4K8ac_peak_6292	4.50834	TAOK2_ENSG00000149930
16	30006500	30006907	5Y-H4K8ac_peak_6293	16.23118	HIRIP3_ENSG00000149929;INO80E_ENSG00000169592
16	30021360	30021638	5Y-H4K8ac_peak_6294	4.95697	
16	30022103	30022307	5Y-H4K8ac_peak_6295	6.08523	
16	30022928	30023382	5Y-H4K8ac_peak_6296	10.72893	
16	30042492	30043243	5Y-H4K8ac_peak_6297	5.24695	
16	30064058	30064254	5Y-H4K8ac_peak_6298	5.8635	FAM57B_ENSG00000149926;ALDOA_ENSG00000149925
16	30074980	30075285	5Y-H4K8ac_peak_6299	5.87725	
16	30076497	30076874	5Y-H4K8ac_peak_6300	8.27643	

16	30102828	30103253	5Y-H4K8ac_peak_6301	11.22005	TBX6_ENSG00000149922
16	30107819	30108348	5Y-H4K8ac_peak_6302	17.55091	YPEL3_ENSG00000090238;RP11-455F5.3_ENSG00000250616
16	30133933	30134300	5Y-H4K8ac_peak_6303	7.89273	MAPK3_ENSG00000102882
16	30135077	30135312	5Y-H4K8ac_peak_6304	7.87406	MAPK3_ENSG00000102882
16	30194139	30194579	5Y-H4K8ac_peak_6305	12.87098	CORO1A_ENSG00000102879
16	30217318	30217535	5Y-H4K8ac_peak_6306	8.33296	
16	30366293	30366552	5Y-H4K8ac_peak_6307	16.6076	CD2BP2_ENSG00000169217;RP11-347C12.10_ENSG00000260219
16	30366885	30367467	5Y-H4K8ac_peak_6308	11.68317	CD2BP2_ENSG00000169217;RP11-347C12.10_ENSG00000260219
16	30381428	30381665	5Y-H4K8ac_peak_6309	8.16382	TBC1D10B_ENSG00000169221;MYLPF_ENSG00000180209
16	30382868	30383095	5Y-H4K8ac_peak_6310	4.66162	MYLPF_ENSG00000180209
16	30388290	30388765	5Y-H4K8ac_peak_6311	7.59101	ZNF48_ENSG00000180035
16	30389304	30389515	5Y-H4K8ac_peak_6312	15.19225	ZNF48_ENSG00000180035
16	30404945	30405160	5Y-H4K8ac_peak_6313	5.70209	
16	30405659	30406141	5Y-H4K8ac_peak_6314	11.31171	
16	30406336	30406581	5Y-H4K8ac_peak_6315	13.14766	SEPT1_ENSG00000180096
16	30411575	30412314	5Y-H4K8ac_peak_6316	10.03452	
16	30428867	30429117	5Y-H4K8ac_peak_6317	15.84891	
16	30441773	30442110	5Y-H4K8ac_peak_6318	5.87725	DCTPP1_ENSG00000179958
16	30456847	30457145	5Y-H4K8ac_peak_6319	11.22005	SEPHS2_ENSG00000179918
16	30537506	30537859	5Y-H4K8ac_peak_6320	8.66584	ZNF768_ENSG00000169957
16	30538234	30538635	5Y-H4K8ac_peak_6321	13.12848	ZNF768_ENSG00000169957
16	30545179	30545407	5Y-H4K8ac_peak_6322	7.59101	ZNF747_ENSG00000261459;AC002310.12_ENSG00000235560
16	30545693	30546142	5Y-H4K8ac_peak_6323	12.26606	ZNF747_ENSG00000169955;ZNF747_ENSG00000261459;AC002310.12_ENSG00000235560
16	30547020	30547518	5Y-H4K8ac_peak_6324	7.01266	ZNF747_ENSG00000169955;ZNF747_ENSG00000261459;AC002310.12_ENSG00000235560
16	30569042	30569667	5Y-H4K8ac_peak_6325	8.90774	AC002310.13_ENSG00000260869;ZNF764_ENSG00000169951
16	30570251	30570444	5Y-H4K8ac_peak_6326	6.78128	AC002310.13_ENSG00000260869;ZNF764_ENSG00000169951
16	30582445	30583174	5Y-H4K8ac_peak_6327	12.84845	ZNF688_ENSG00000229809;AC002310.7_ENSG00000239791
16	30596597	30596928	5Y-H4K8ac_peak_6328	7.90751	ZNF785_ENSG00000197162;RP11-146F11.5_ENSG00000260167
16	30597524	30597800	5Y-H4K8ac_peak_6329	9.8829	ZNF785_ENSG00000197162;RP11-146F11.5_ENSG00000260167
16	30621155	30621641	5Y-H4K8ac_peak_6330	21.35466	
16	30661243	30661906	5Y-H4K8ac_peak_6331	14.86775	PRR14_ENSG00000156858
16	30662297	30662512	5Y-H4K8ac_peak_6332	6.61077	PRR14_ENSG00000156858
16	30709571	30710333	5Y-H4K8ac_peak_6333	16.51777	RP11-146F11.1_ENSG00000261840;SRCAP_ENSG00000080603
16	30786852	30787275	5Y-H4K8ac_peak_6334	7.44077	
16	30798271	30799164	5Y-H4K8ac_peak_6335	10.4689	ZNF629_ENSG00000102870
16	30816971	30817392	5Y-H4K8ac_peak_6336	12.57157	
16	30825473	30825675	5Y-H4K8ac_peak_6337	9.79526	
16	30886349	30886605	5Y-H4K8ac_peak_6338	15.27603	MIR4519_ENSG00000260083;MIR4519_ENSG00000265991
16	30887297	30887667	5Y-H4K8ac_peak_6339	9.30505	MIR4519_ENSG00000260083;MIR4519_ENSG00000265991
16	30888113	30888367	5Y-H4K8ac_peak_6340	6.08523	
16	30905661	30905862	5Y-H4K8ac_peak_6341	7.34185	BCL7C_ENSG00000099385;AC106782.20_ENSG00000262721;MIR762_ENSG00000211591
16	30907978	30908238	5Y-H4K8ac_peak_6342	7.89273	CTF1_ENSG00000150281
16	30913224	30913516	5Y-H4K8ac_peak_6343	4.29586	
16	30915285	30915487	5Y-H4K8ac_peak_6344	9.01738	
16	30935518	30935761	5Y-H4K8ac_peak_6345	7.61752	FBXL19-AS1_ENSG00000260852
16	30938942	30939208	5Y-H4K8ac_peak_6346	5.25819	

16	30967915	30968333	5Y-H4K8ac_peak_6347	4.15658	SETD1A_ENSG00000099381
16	30968609	30968869	5Y-H4K8ac_peak_6348	10.73603	SETD1A_ENSG00000099381
16	30969074	30969512	5Y-H4K8ac_peak_6349	10.90097	SETD1A_ENSG00000099381
16	31044223	31044533	5Y-H4K8ac_peak_6350	10.23399	STX4_ENSG00000103496
16	31084144	31084468	5Y-H4K8ac_peak_6351	5.64909	
16	31084673	31085327	5Y-H4K8ac_peak_6352	5.64909	ZNF668_ENSG00000167394;ZNF646_ENSG00000167395
16	31105675	31106114	5Y-H4K8ac_peak_6353	4.95697	RP11-196G11.1_ENSG00000255439
16	31119380	31119624	5Y-H4K8ac_peak_6354	8.564	AC135050.1_ENSG00000252809
16	31119858	31120230	5Y-H4K8ac_peak_6355	10.1994	AC135050.1_ENSG00000252809
16	31145031	31145436	5Y-H4K8ac_peak_6356	4.85014	
16	31152824	31153343	5Y-H4K8ac_peak_6357	4.77126	
16	31153975	31154353	5Y-H4K8ac_peak_6358	8.43511	
16	31191264	31191708	5Y-H4K8ac_peak_6359	9.18646	FUS_ENSG00000089280
16	31439364	31439575	5Y-H4K8ac_peak_6360	5.98695	COX6A2_ENSG00000156885
16	31454611	31454811	5Y-H4K8ac_peak_6361	7.31815	ZNF843_ENSG00000176723
16	31470414	31471028	5Y-H4K8ac_peak_6362	11.88337	RP11-452L6.5_ENSG00000260267
16	31471802	31472005	5Y-H4K8ac_peak_6363	5.59837	RP11-452L6.5_ENSG00000260267
16	31483070	31483336	5Y-H4K8ac_peak_6364	6.6946	TGFB1I1_ENSG00000140682
16	31483606	31484175	5Y-H4K8ac_peak_6365	8.67475	TGFB1I1_ENSG00000140682
16	31519330	31519606	5Y-H4K8ac_peak_6366	4.77126	RP11-452L6.7_ENSG00000260625
16	31885162	31885388	5Y-H4K8ac_peak_6367	10.19948	ZNF267_ENSG00000185947
16	32265588	32265925	5Y-H4K8ac_peak_6368	7.59101	RP11-56L13.7_ENSG00000260575
16	33319020	33319210	5Y-H4K8ac_peak_6369	6.37023	
16	33509198	33509646	5Y-H4K8ac_peak_6370	11.28133	
16	34441331	34441756	5Y-H4K8ac_peak_6371	11.09973	RP11-244B22.5_ENSG00000261274;RP11-488I20.8_ENSG00000260958
16	46824005	46824331	5Y-H4K8ac_peak_6372	9.23159	MYLK3_ENSG00000140795;RP11-480G7.2_ENSG00000260782
16	47007714	47007923	5Y-H4K8ac_peak_6373	6.78128	DNAJA2_ENSG00000069345;RP11-169E6.1_ENSG00000261173
16	47176811	47177812	5Y-H4K8ac_peak_6374	16.27715	NETO2_ENSG00000171208;RP11-329J18.2_ENSG00000260281
16	47494741	47495152	5Y-H4K8ac_peak_6375	9.62747	PHKB_ENSG00000102893
16	47746553	47746770	5Y-H4K8ac_peak_6376	8.35139	
16	48151084	48151459	5Y-H4K8ac_peak_6377	7.11262	
16	48199325	48199545	5Y-H4K8ac_peak_6378	9.38203	
16	48418962	48419231	5Y-H4K8ac_peak_6379	15.82359	
16	48419499	48420330	5Y-H4K8ac_peak_6380	10.1994	
16	48427889	48428139	5Y-H4K8ac_peak_6381	9.30505	
16	48565362	48565649	5Y-H4K8ac_peak_6382	6.47245	
16	48644352	48644589	5Y-H4K8ac_peak_6383	5.23083	
16	49474783	49475195	5Y-H4K8ac_peak_6384	7.04637	
16	49526777	49527289	5Y-H4K8ac_peak_6385	6.33308	
16	49527866	49528169	5Y-H4K8ac_peak_6386	5.62788	
16	49557549	49558518	5Y-H4K8ac_peak_6387	9.31608	
16	49558889	49559202	5Y-H4K8ac_peak_6388	11.4254	
16	49560225	49560425	5Y-H4K8ac_peak_6389	8.86778	
16	49561044	49561437	5Y-H4K8ac_peak_6390	4.50834	
16	49563497	49563764	5Y-H4K8ac_peak_6391	6.67017	
16	49564694	49565431	5Y-H4K8ac_peak_6392	9.30505	

16	49566280	49566501	5Y-H4K8ac_peak_6393	8.18236	
16	49581341	49581644	5Y-H4K8ac_peak_6394	8.04493	
16	49628728	49629075	5Y-H4K8ac_peak_6395	10.79063	
16	49634800	49635118	5Y-H4K8ac_peak_6396	8.38675	
16	49636203	49636509	5Y-H4K8ac_peak_6397	7.56545	
16	49638140	49638336	5Y-H4K8ac_peak_6398	4.84727	
16	49671907	49672389	5Y-H4K8ac_peak_6399	6.46606	
16	49695340	49695773	5Y-H4K8ac_peak_6400	6.43775	
16	49697496	49699616	5Y-H4K8ac_peak_6401	18.62331	
16	49730759	49731172	5Y-H4K8ac_peak_6402	5.98695	
16	49731498	49731842	5Y-H4K8ac_peak_6403	10.11191	
16	49733002	49733378	5Y-H4K8ac_peak_6404	7.01266	
16	49733794	49734041	5Y-H4K8ac_peak_6405	10.1994	
16	49734279	49734777	5Y-H4K8ac_peak_6406	13.2534	
16	49739650	49740534	5Y-H4K8ac_peak_6407	12.05638	
16	49740954	49741199	5Y-H4K8ac_peak_6408	8.21582	MRPS21P7_ENSG00000260024
16	49788988	49789179	5Y-H4K8ac_peak_6409	10.35586	
16	49834595	49834830	5Y-H4K8ac_peak_6410	4.24869	
16	49843583	49843796	5Y-H4K8ac_peak_6411	4.00285	
16	49845185	49845425	5Y-H4K8ac_peak_6412	5.64909	
16	49855473	49855876	5Y-H4K8ac_peak_6413	11.86504	
16	49868527	49868887	5Y-H4K8ac_peak_6414	9.7353	
16	49869411	49869728	5Y-H4K8ac_peak_6415	5.87725	
16	49888365	49889385	5Y-H4K8ac_peak_6416	11.78766	
16	49891829	49892079	5Y-H4K8ac_peak_6417	7.90751	ZNF423_ENSG00000102935
16	49900321	49900701	5Y-H4K8ac_peak_6418	12.79198	
16	49955587	49955895	5Y-H4K8ac_peak_6419	14.31711	
16	50059234	50059506	5Y-H4K8ac_peak_6420	7.30348	CNEP1R1_ENSG00000205423
16	50099996	50100221	5Y-H4K8ac_peak_6421	7.11863	RP11-429P3.3_ENSG00000259843;HEATR3_ENSG00000155393
16	50279558	50279790	5Y-H4K8ac_peak_6422	7.87406	ADCY7_ENSG00000121281
16	50281111	50281314	5Y-H4K8ac_peak_6423	4.24332	
16	50318062	50318301	5Y-H4K8ac_peak_6424	5.10825	
16	50349189	50349434	5Y-H4K8ac_peak_6425	5.23083	
16	50402487	50402855	5Y-H4K8ac_peak_6426	7.38046	BRD7_ENSG00000166164;RP11-21B23.1_ENSG00000261393
16	50423786	50424908	5Y-H4K8ac_peak_6427	11.1169	
16	50502310	50502524	5Y-H4K8ac_peak_6428	7.50148	
16	50503338	50503746	5Y-H4K8ac_peak_6429	6.43775	
16	50625910	50626230	5Y-H4K8ac_peak_6430	6.36454	
16	50727224	50727677	5Y-H4K8ac_peak_6431	9.30505	NOD2_ENSG00000167207
16	53088359	53088597	5Y-H4K8ac_peak_6432	8.73392	CHD9_ENSG00000177200
16	53105663	53105980	5Y-H4K8ac_peak_6433	5.84208	
16	53117896	53118098	5Y-H4K8ac_peak_6434	7.04158	
16	53125023	53125586	5Y-H4K8ac_peak_6435	7.87048	
16	53132885	53133105	5Y-H4K8ac_peak_6436	6.1654	
16	53133510	53134016	5Y-H4K8ac_peak_6437	6.34245	
16	53163913	53164200	5Y-H4K8ac_peak_6438	4.95697	

16	54096379	54096666	5Y-H4K8ac_peak_6439	4.70501	
16	54209275	54209475	5Y-H4K8ac_peak_6440	5.26353	
16	54211169	54211461	5Y-H4K8ac_peak_6441	4.95697	
16	54227865	54228571	5Y-H4K8ac_peak_6442	11.12941	
16	54319568	54320147	5Y-H4K8ac_peak_6443	9.23159	IRX3_ENSG00000177508
16	54321026	54321505	5Y-H4K8ac_peak_6444	11.22005	IRX3_ENSG00000177508
16	54516553	54516785	5Y-H4K8ac_peak_6445	6.00388	
16	54666655	54666907	5Y-H4K8ac_peak_6446	6.90749	
16	54962408	54962768	5Y-H4K8ac_peak_6447	13.2534	CRNDE_ENSG00000245694
16	54964921	54965407	5Y-H4K8ac_peak_6448	5.59843	IRX5_ENSG00000176842
16	55090469	55090750	5Y-H4K8ac_peak_6449	7.31807	
16	55358731	55358937	5Y-H4K8ac_peak_6450	8.75926	
16	55407643	55407848	5Y-H4K8ac_peak_6451	6.1654	
16	55685093	55685325	5Y-H4K8ac_peak_6452	5.07797	
16	55690614	55690891	5Y-H4K8ac_peak_6453	6.37023	
16	56080682	56081016	5Y-H4K8ac_peak_6454	8.51022	
16	56135585	56135849	5Y-H4K8ac_peak_6455	9.52603	
16	56291701	56291902	5Y-H4K8ac_peak_6456	5.98695	
16	56297732	56298097	5Y-H4K8ac_peak_6457	5.98695	
16	56352029	56352319	5Y-H4K8ac_peak_6458	8.79957	
16	56554680	56555015	5Y-H4K8ac_peak_6459	6.77436	BBS2_ENSG00000125124
16	56742905	56743100	5Y-H4K8ac_peak_6460	4.12295	
16	56894250	56894600	5Y-H4K8ac_peak_6461	4.50834	
16	56908033	56908859	5Y-H4K8ac_peak_6462	8.38675	
16	56965267	56965826	5Y-H4K8ac_peak_6463	18.63671	HERPUD1_ENSG00000051108
16	56966127	56966317	5Y-H4K8ac_peak_6464	8.1667	HERPUD1_ENSG00000051108
16	57094765	57094975	5Y-H4K8ac_peak_6465	7.50148	
16	57095225	57095805	5Y-H4K8ac_peak_6466	11.01067	
16	57125780	57126333	5Y-H4K8ac_peak_6467	17.56739	RP11-407G23.1_ENSG00000260148;CPNE2_ENSG00000140848
16	57150497	57150710	5Y-H4K8ac_peak_6468	4.84193	
16	57219503	57219808	5Y-H4K8ac_peak_6469	4.642	FAM192A_ENSG00000172775;RSPRY1_ENSG00000159579
16	57279233	57279462	5Y-H4K8ac_peak_6470	10.21117	ARL2BP_ENSG00000102931
16	57296782	57297028	5Y-H4K8ac_peak_6471	6.20989	
16	57297766	57298116	5Y-H4K8ac_peak_6472	4.48815	
16	57318278	57318926	5Y-H4K8ac_peak_6473	9.59236	PLLP_ENSG00000102934
16	57405365	57405710	5Y-H4K8ac_peak_6474	8.47164	CX3CL1_ENSG00000006210
16	57406049	57406240	5Y-H4K8ac_peak_6475	6.51488	CX3CL1_ENSG00000006210
16	57406445	57406777	5Y-H4K8ac_peak_6476	5.87725	CX3CL1_ENSG00000006210
16	57450166	57451403	5Y-H4K8ac_peak_6477	9.30505	
16	57507134	57507957	5Y-H4K8ac_peak_6478	11.22005	
16	57513287	57513717	5Y-H4K8ac_peak_6479	5.87725	
16	57514053	57514714	5Y-H4K8ac_peak_6480	9.32595	
16	57515267	57515480	5Y-H4K8ac_peak_6481	5.95028	
16	57518317	57518694	5Y-H4K8ac_peak_6482	5.41472	
16	57519069	57519298	5Y-H4K8ac_peak_6483	4.6766	
16	57521495	57521695	5Y-H4K8ac_peak_6484	7.03573	DOK4_ENSG00000125170

16	57541065	57542646	5Y-H4K8ac_peak_6485	17.32862	
16	57543550	57544143	5Y-H4K8ac_peak_6486	9.38276	
16	57544576	57545160	5Y-H4K8ac_peak_6487	11.22005	
16	57552121	57552378	5Y-H4K8ac_peak_6488	8.16031	
16	57570264	57571003	5Y-H4K8ac_peak_6489	14.86775	CCDC102A_ENSG00000135736
16	57662279	57662607	5Y-H4K8ac_peak_6490	6.37023	
16	57768646	57769102	5Y-H4K8ac_peak_6491	8.69112	KATNB1_ENSG00000140854
16	58018474	58018688	5Y-H4K8ac_peak_6492	6.37023	
16	58033764	58034411	5Y-H4K8ac_peak_6493	6.64195	ZNF319_ENSG00000166188;USB1_ENSG00000103005
16	58497102	58497333	5Y-H4K8ac_peak_6494	7.69843	NDRG4_ENSG00000103034
16	58521558	58521972	5Y-H4K8ac_peak_6495	6.73385	
16	58529754	58530278	5Y-H4K8ac_peak_6496	10.69698	RNU6-103P_ENSG00000200556
16	58533304	58533634	5Y-H4K8ac_peak_6497	7.89273	
16	58663488	58663703	5Y-H4K8ac_peak_6498	9.30505	CNOT1_ENSG00000125107
16	58672834	58673039	5Y-H4K8ac_peak_6499	6.49308	
16	58686605	58686997	5Y-H4K8ac_peak_6500	4.07874	
16	58718208	58718538	5Y-H4K8ac_peak_6501	10.19948	SLC38A7_ENSG00000103042
16	58767454	58768117	5Y-H4K8ac_peak_6502	9.23159	GOT2_ENSG00000125166;RP11-410D17.2_ENSG00000245768
16	62069283	62069642	5Y-H4K8ac_peak_6503	6.34046	
16	64187221	64187419	5Y-H4K8ac_peak_6504	6.34046	
16	64470874	64471127	5Y-H4K8ac_peak_6505	7.31815	
16	65156587	65156915	5Y-H4K8ac_peak_6506	12.11208	
16	66304124	66304432	5Y-H4K8ac_peak_6507	9.74838	
16	66512859	66513195	5Y-H4K8ac_peak_6508	4.29586	
16	66583265	66584019	5Y-H4K8ac_peak_6509	11.71078	Y_RNA_ENSG00000261519
16	66729992	66730409	5Y-H4K8ac_peak_6510	11.4254	CMTM4_ENSG00000183723
16	66785264	66785699	5Y-H4K8ac_peak_6511	8.69112	DYNC1LI2_ENSG00000135720;RP11-61A14.4_ENSG00000246777
16	66864401	66864792	5Y-H4K8ac_peak_6512	10.79063	
16	66906911	66907123	5Y-H4K8ac_peak_6513	10.19038	NAE1_ENSG00000159593
16	66913966	66914256	5Y-H4K8ac_peak_6514	7.34185	
16	66933446	66933845	5Y-H4K8ac_peak_6515	10.69698	
16	66934143	66934628	5Y-H4K8ac_peak_6516	9.88125	
16	67011229	67011530	5Y-H4K8ac_peak_6517	6.22669	
16	67034162	67034424	5Y-H4K8ac_peak_6518	12.65187	
16	67047395	67048053	5Y-H4K8ac_peak_6519	13.68515	
16	67048288	67048514	5Y-H4K8ac_peak_6520	8.76608	
16	67063186	67063485	5Y-H4K8ac_peak_6521	6.43775	CBFB_ENSG00000067955
16	67143274	67143605	5Y-H4K8ac_peak_6522	5.59843	C16orf70_ENSG00000125149
16	67196771	67197000	5Y-H4K8ac_peak_6523	4.15658	HSF4_ENSG00000102878;RP11-5A19.5_ENSG00000265690
16	67197266	67197480	5Y-H4K8ac_peak_6524	5.64909	HSF4_ENSG00000102878;RP11-5A19.5_ENSG00000265690
16	67198607	67199019	5Y-H4K8ac_peak_6525	13.86507	
16	67203475	67203699	5Y-H4K8ac_peak_6526	6.77436	NOL3_ENSG00000140939
16	67204735	67204999	5Y-H4K8ac_peak_6527	7.20869	NOL3_ENSG00000140939
16	67207550	67208047	5Y-H4K8ac_peak_6528	5.98695	
16	67208435	67208642	5Y-H4K8ac_peak_6529	6.34245	
16	67217675	67217875	5Y-H4K8ac_peak_6530	10.11191	KIAA0895L_ENSG00000196123

16	67241164	67241825	5Y-H4K8ac_peak_6531	12.11208	
16	67261191	67261434	5Y-H4K8ac_peak_6532	7.87406	LRRC29_ENSG00000125122;AC040160.1_ENSG00000237102;TMEM208_ENSG00000168701
16	67282404	67282741	5Y-H4K8ac_peak_6533	5.64909	FHOD1_ENSG00000135723
16	67282950	67283449	5Y-H4K8ac_peak_6534	5.91107	
16	67313560	67313996	5Y-H4K8ac_peak_6535	9.37812	
16	67360259	67360749	5Y-H4K8ac_peak_6536	6.34245	KCTD19_ENSG00000168676;LRRC36_ENSG00000159708
16	67450059	67450324	5Y-H4K8ac_peak_6537	9.79526	ZDHHC1_ENSG00000159714
16	67515494	67515694	5Y-H4K8ac_peak_6538	6.1654	ATP6V0D1_ENSG00000159720;RP11-297D21.4_ENSG00000270049
16	67516005	67516774	5Y-H4K8ac_peak_6539	5.23083	ATP6V0D1_ENSG00000159720;RP11-297D21.4_ENSG00000270049;AGRP_ENSG00000159723
16	67555320	67555539	5Y-H4K8ac_peak_6540	5.91107	
16	67563041	67563304	5Y-H4K8ac_peak_6541	5.23083	
16	67596984	67597414	5Y-H4K8ac_peak_6542	11.68317	CTD-2012K14.6_ENSG00000261386;CTD-2012K14.7_ENSG00000259804;CTCF_ENSG00000102974
16	67678804	67679314	5Y-H4K8ac_peak_6543	8.69112	RLTPR_ENSG00000159753
16	67686448	67686745	5Y-H4K8ac_peak_6544	14.99238	
16	67687070	67687318	5Y-H4K8ac_peak_6545	4.95697	
16	67875680	67876675	5Y-H4K8ac_peak_6546	7.11863	THAP11_ENSG00000168286
16	67907080	67907294	5Y-H4K8ac_peak_6547	6.43775	EDC4_ENSG00000038358
16	67927253	67927445	5Y-H4K8ac_peak_6548	7.11863	PSKH1_ENSG00000159792
16	68001905	68002421	5Y-H4K8ac_peak_6549	6.78318	
16	68002804	68003078	5Y-H4K8ac_peak_6550	6.53157	SLC12A4_ENSG00000124067
16	68026741	68027600	5Y-H4K8ac_peak_6551	5.72233	
16	68117994	68118471	5Y-H4K8ac_peak_6552	14.0329	NFATC3_ENSG00000072736
16	68118715	68118924	5Y-H4K8ac_peak_6553	5.87725	NFATC3_ENSG00000072736
16	68122508	68122703	5Y-H4K8ac_peak_6554	5.10404	Y_RNA_ENSG00000201850
16	68271788	68272357	5Y-H4K8ac_peak_6555	15.17389	ESRP2_ENSG00000103067;RP11-96D1.6_ENSG00000261469
16	68298089	68298314	5Y-H4K8ac_peak_6556	7.58806	SLC7A6_ENSG00000103064
16	68344296	68344740	5Y-H4K8ac_peak_6557	6.77436	SLC7A6OS_ENSG00000103061;PRMT7_ENSG00000132600
16	68344966	68345180	5Y-H4K8ac_peak_6558	7.38046	SLC7A6OS_ENSG00000103061;PRMT7_ENSG00000132600
16	68480651	68480911	5Y-H4K8ac_peak_6559	11.86504	
16	68679272	68679811	5Y-H4K8ac_peak_6560	4.36976	RP11-615I2.2_ENSG00000260577
16	68877457	68877918	5Y-H4K8ac_peak_6561	13.00733	TANGO6_ENSG00000103047
16	69165859	69166127	5Y-H4K8ac_peak_6562	10.84616	CHTF8_ENSG00000168802;CIRH1A_ENSG00000141076
16	69166551	69166809	5Y-H4K8ac_peak_6563	15.34073	CHTF8_ENSG00000168802
16	69363929	69364449	5Y-H4K8ac_peak_6564	9.38276	PDF_ENSG00000258429
16	69373802	69374067	5Y-H4K8ac_peak_6565	5.64909	COG8_ENSG00000213380;NIP7_ENSG00000132603
16	69419580	69419838	5Y-H4K8ac_peak_6566	5.41472	
16	69529157	69529946	5Y-H4K8ac_peak_6567	9.23159	
16	69794758	69794955	5Y-H4K8ac_peak_6568	4.79585	
16	69864009	69864590	5Y-H4K8ac_peak_6569	7.38046	
16	69923918	69924152	5Y-H4K8ac_peak_6570	5.8635	
16	70285250	70285783	5Y-H4K8ac_peak_6571	6.77436	EXOSC6_ENSG00000223496
16	70323087	70323351	5Y-H4K8ac_peak_6572	14.87721	AARS_ENSG00000090861;DDX19B_ENSG00000157349
16	70323784	70324418	5Y-H4K8ac_peak_6573	9.23159	AARS_ENSG00000090861;DDX19B_ENSG00000157349
16	70333172	70333438	5Y-H4K8ac_peak_6574	6.98118	RP11-529K1.3_ENSG00000260537
16	70379807	70380585	5Y-H4K8ac_peak_6575	7.58806	RP11-529K1.2_ENSG00000261777;DDX19A_ENSG00000168872
16	70414937	70415239	5Y-H4K8ac_peak_6576	9.51254	

16	70464531	70464733	5Y-H4K8ac_peak_6577	6.08523	
16	70473372	70473699	5Y-H4K8ac_peak_6578	8.09118	ST3GAL2_ENSG00000157350
16	70488286	70488923	5Y-H4K8ac_peak_6579	13.06587	FUK_ENSG00000157353
16	70557234	70557426	5Y-H4K8ac_peak_6580	12.69256	COG4_ENSG00000103051;SF3B3_ENSG00000189091
16	70557797	70558109	5Y-H4K8ac_peak_6581	11.12941	COG4_ENSG00000103051;SF3B3_ENSG00000189091
16	70680590	70680875	5Y-H4K8ac_peak_6582	6.08523	
16	70687932	70688296	5Y-H4K8ac_peak_6583	7.63144	
16	70721841	70722173	5Y-H4K8ac_peak_6584	5.98695	
16	70722917	70723126	5Y-H4K8ac_peak_6585	6.4221	
16	70725351	70725774	5Y-H4K8ac_peak_6586	7.20869	
16	70729621	70730010	5Y-H4K8ac_peak_6587	6.81557	
16	70737308	70738403	5Y-H4K8ac_peak_6588	6.29918	
16	70748834	70749070	5Y-H4K8ac_peak_6589	6.19716	
16	70770864	70771366	5Y-H4K8ac_peak_6590	7.50148	
16	70771851	70772498	5Y-H4K8ac_peak_6591	7.50148	
16	70834635	70835009	5Y-H4K8ac_peak_6592	11.22005	VAC14_ENSG00000103043
16	71264175	71264403	5Y-H4K8ac_peak_6593	5.87382	HYDIN_ENSG00000157423
16	71495752	71496089	5Y-H4K8ac_peak_6594	10.60083	ZNF23_ENSG00000167377;RP11-510M2.2_ENSG00000247324
16	71518087	71518434	5Y-H4K8ac_peak_6595	10.69698	
16	71518964	71519343	5Y-H4K8ac_peak_6596	5.40331	
16	71879447	71879693	5Y-H4K8ac_peak_6597	14.86775	ATXN1L_ENSG00000224470;IST1_ENSG00000182149
16	71917549	71918038	5Y-H4K8ac_peak_6598	14.00559	
16	72231094	72231701	5Y-H4K8ac_peak_6599	7.59101	
16	72232389	72232628	5Y-H4K8ac_peak_6600	8.21582	
16	72233162	72233466	5Y-H4K8ac_peak_6601	8.21582	
16	72233686	72234123	5Y-H4K8ac_peak_6602	7.89273	
16	72981936	72982150	5Y-H4K8ac_peak_6603	6.88532	
16	72996350	72996563	5Y-H4K8ac_peak_6604	4.36976	
16	73082158	73082528	5Y-H4K8ac_peak_6605	11.80583	
16	73091653	73091883	5Y-H4K8ac_peak_6606	10.1994	
16	73092124	73092599	5Y-H4K8ac_peak_6607	9.23159	ZFHX3_ENSG00000140836
16	73204434	73204697	5Y-H4K8ac_peak_6608	8.33296	
16	73216936	73217170	5Y-H4K8ac_peak_6609	5.64909	
16	73338105	73338311	5Y-H4K8ac_peak_6610	4.642	
16	74330790	74331058	5Y-H4K8ac_peak_6611	6.22669	AC009120.4_ENSG00000261404;PSMD7_ENSG00000103035
16	74700271	74700583	5Y-H4K8ac_peak_6612	4.84727	RFWD3_ENSG00000168411;RP11-144N1.1_ENSG00000262904
16	74734158	74734440	5Y-H4K8ac_peak_6613	4.71803	MLKL_ENSG00000168404
16	75032220	75032540	5Y-H4K8ac_peak_6614	5.16353	ZNRF1_ENSG00000186187
16	75032963	75033384	5Y-H4K8ac_peak_6615	10.89622	WDR59_ENSG00000103091;ZNRF1_ENSG00000186187
16	75103274	75103538	5Y-H4K8ac_peak_6616	13.0168	
16	75139200	75139415	5Y-H4K8ac_peak_6617	6.73385	
16	75139826	75140070	5Y-H4K8ac_peak_6618	8.35138	
16	75182418	75182818	5Y-H4K8ac_peak_6619	23.33415	ZFP1_ENSG00000184517
16	75290544	75290759	5Y-H4K8ac_peak_6620	5.94703	
16	75298915	75299648	5Y-H4K8ac_peak_6621	14.2053	
16	75299995	75300263	5Y-H4K8ac_peak_6622	13.03079	

16	75300828	75301047	5Y-H4K8ac_peak_6623	7.50148	BCAR1_ENSG00000050820
16	75467001	75467233	5Y-H4K8ac_peak_6624	7.90236	CFDP1_ENSG00000153774
16	75550940	75551136	5Y-H4K8ac_peak_6625	6.78128	RP11-77K12.5_ENSG00000262583
16	75551619	75551839	5Y-H4K8ac_peak_6626	8.2913	
16	75658118	75658323	5Y-H4K8ac_peak_6627	7.31815	ADAT1_ENSG00000065457
16	75681157	75681450	5Y-H4K8ac_peak_6628	8.90038	TERF2IP_ENSG00000166848
16	76691661	76691865	5Y-H4K8ac_peak_6629	4.07874	
16	77246365	77246886	5Y-H4K8ac_peak_6630	5.64909	
16	77756490	77756772	5Y-H4K8ac_peak_6631	4.95697	NUDT7_ENSG00000140876
16	77821873	77822145	5Y-H4K8ac_peak_6632	7.64675	VAT1L_ENSG00000171724
16	78036486	78036686	5Y-H4K8ac_peak_6633	5.22422	
16	79463728	79463936	5Y-H4K8ac_peak_6634	4.30075	
16	81040326	81040753	5Y-H4K8ac_peak_6635	14.86775	CENPN_ENSG00000166451
16	81110984	81111384	5Y-H4K8ac_peak_6636	7.49746	C16orf46_ENSG00000166455;RP11-303E16.7_ENSG00000245059
16	81349070	81349339	5Y-H4K8ac_peak_6637	5.81062	GAN_ENSG00000261609
16	81477650	81478330	5Y-H4K8ac_peak_6638	10.1994	CMIP_ENSG00000153815
16	81549332	81549945	5Y-H4K8ac_peak_6639	6.08523	
16	81763976	81764526	5Y-H4K8ac_peak_6640	20.51189	
16	82203418	82204314	5Y-H4K8ac_peak_6641	8.2913	MPHOSPH6_ENSG00000135698;CTD-2588J6.2_ENSG00000261029
16	83841040	83841336	5Y-H4K8ac_peak_6642	6.31818	RP11-483P21.2_ENSG00000260228;HSBP1_ENSG00000230989
16	83841717	83842003	5Y-H4K8ac_peak_6643	6.86362	RP11-483P21.2_ENSG00000260228;HSBP1_ENSG00000230989
16	84268153	84268612	5Y-H4K8ac_peak_6644	6.1654	
16	84538622	84539222	5Y-H4K8ac_peak_6645	7.87406	
16	84860056	84860423	5Y-H4K8ac_peak_6646	11.36989	
16	84861065	84861559	5Y-H4K8ac_peak_6647	6.78128	RP11-254F19.2_ENSG00000260859
16	85045328	85045539	5Y-H4K8ac_peak_6648	7.90236	ZDHHC7_ENSG00000153786
16	85146051	85146394	5Y-H4K8ac_peak_6649	11.20395	FAM92B_ENSG00000153789
16	85170713	85170999	5Y-H4K8ac_peak_6650	10.16277	
16	85178135	85178486	5Y-H4K8ac_peak_6651	4.93618	
16	85180484	85180758	5Y-H4K8ac_peak_6652	6.47245	
16	85185060	85185257	5Y-H4K8ac_peak_6653	7.11863	
16	85185677	85186344	5Y-H4K8ac_peak_6654	8.69112	
16	85191396	85192744	5Y-H4K8ac_peak_6655	12.10416	
16	85203295	85203736	5Y-H4K8ac_peak_6656	6.83255	
16	85235619	85236471	5Y-H4K8ac_peak_6657	9.44542	
16	85236735	85237006	5Y-H4K8ac_peak_6658	4.29586	
16	85239220	85240273	5Y-H4K8ac_peak_6659	14.30055	
16	85240703	85241492	5Y-H4K8ac_peak_6660	7.20869	
16	85241848	85242101	5Y-H4K8ac_peak_6661	6.73047	
16	85253343	85253873	5Y-H4K8ac_peak_6662	16.05821	
16	85254655	85255413	5Y-H4K8ac_peak_6663	11.18363	
16	85268570	85268955	5Y-H4K8ac_peak_6664	7.87406	
16	85269194	85269479	5Y-H4K8ac_peak_6665	6.81557	
16	85269698	85269917	5Y-H4K8ac_peak_6666	6.73385	
16	85292623	85293119	5Y-H4K8ac_peak_6667	7.31102	
16	85299729	85300022	5Y-H4K8ac_peak_6668	7.08802	

16	85300718	85301083	5Y-H4K8ac_peak_6669	9.59236	
16	85334536	85334781	5Y-H4K8ac_peak_6670	8.89349	
16	85335405	85335846	5Y-H4K8ac_peak_6671	7.90227	
16	85382083	85382280	5Y-H4K8ac_peak_6672	7.31102	
16	85383577	85384145	5Y-H4K8ac_peak_6673	12.96197	
16	85389894	85390098	5Y-H4K8ac_peak_6674	7.41519	RP11-680G10.1_ENSG00000261567
16	85390693	85390894	5Y-H4K8ac_peak_6675	5.0561	RP11-680G10.1_ENSG00000261567
16	85395070	85395802	5Y-H4K8ac_peak_6676	9.04239	
16	85398386	85398762	5Y-H4K8ac_peak_6677	6.92707	
16	85399493	85399798	5Y-H4K8ac_peak_6678	9.30505	
16	85400649	85401248	5Y-H4K8ac_peak_6679	12.44818	
16	85404622	85404967	5Y-H4K8ac_peak_6680	8.46442	
16	85409856	85410272	5Y-H4K8ac_peak_6681	4.15658	
16	85411190	85412131	5Y-H4K8ac_peak_6682	13.4304	
16	85412325	85412928	5Y-H4K8ac_peak_6683	5.23083	
16	85415092	85415388	5Y-H4K8ac_peak_6684	4.77307	
16	85415688	85416229	5Y-H4K8ac_peak_6685	10.79063	
16	85417285	85417986	5Y-H4K8ac_peak_6686	7.76645	
16	85419243	85419606	5Y-H4K8ac_peak_6687	7.21406	
16	85429546	85429981	5Y-H4K8ac_peak_6688	14.59308	
16	85449109	85449305	5Y-H4K8ac_peak_6689	5.77946	
16	85470485	85470710	5Y-H4K8ac_peak_6690	5.91107	
16	85472599	85473004	5Y-H4K8ac_peak_6691	6.08523	
16	85477845	85478150	5Y-H4K8ac_peak_6692	4.53347	
16	85478356	85478894	5Y-H4K8ac_peak_6693	8.24461	
16	85479356	85479637	5Y-H4K8ac_peak_6694	11.4254	
16	85480038	85480811	5Y-H4K8ac_peak_6695	12.15235	
16	85481278	85482237	5Y-H4K8ac_peak_6696	5.24695	
16	85482588	85482816	5Y-H4K8ac_peak_6697	6.29621	
16	85483025	85483544	5Y-H4K8ac_peak_6698	5.12488	
16	85495489	85495719	5Y-H4K8ac_peak_6699	6.37023	
16	85496446	85496844	5Y-H4K8ac_peak_6700	9.89244	
16	85497367	85497669	5Y-H4K8ac_peak_6701	7.50148	
16	85499955	85500146	5Y-H4K8ac_peak_6702	6.00747	
16	85508686	85508887	5Y-H4K8ac_peak_6703	5.52265	
16	85548819	85549110	5Y-H4K8ac_peak_6704	9.23159	
16	85551613	85551965	5Y-H4K8ac_peak_6705	9.93099	
16	85557313	85557891	5Y-H4K8ac_peak_6706	5.99504	
16	85558357	85558865	5Y-H4K8ac_peak_6707	7.89142	
16	85560808	85561247	5Y-H4K8ac_peak_6708	7.98641	
16	85568818	85569186	5Y-H4K8ac_peak_6709	4.09461	
16	85607982	85608459	5Y-H4K8ac_peak_6710	6.77436	
16	85616147	85616363	5Y-H4K8ac_peak_6711	5.5592	RP11-118F19.1_ENSG00000270124
16	85627594	85628585	5Y-H4K8ac_peak_6712	8.93923	
16	85640081	85640403	5Y-H4K8ac_peak_6713	7.89273	
16	85641305	85641518	5Y-H4K8ac_peak_6714	6.53157	

16	85644461	85644794	5Y-H4K8ac_peak_6715	10.6523	GSE1_ENSG00000131149
16	85645022	85645302	5Y-H4K8ac_peak_6716	13.14841	GSE1_ENSG00000131149
16	85645559	85645794	5Y-H4K8ac_peak_6717	14.99673	GSE1_ENSG00000131149
16	85646980	85647237	5Y-H4K8ac_peak_6718	8.8624	
16	85659895	85660300	5Y-H4K8ac_peak_6719	6.52593	
16	85680662	85680944	5Y-H4K8ac_peak_6720	5.23083	
16	85683202	85684325	5Y-H4K8ac_peak_6721	7.89273	
16	85684767	85686726	5Y-H4K8ac_peak_6722	16.60632	
16	85722962	85723202	5Y-H4K8ac_peak_6723	6.3265	GINS2_ENSG00000131153
16	85784320	85784588	5Y-H4K8ac_peak_6724	5.18558	C16orf74_ENSG00000154102
16	85832855	85833049	5Y-H4K8ac_peak_6725	7.03573	EMC8_ENSG00000131148;COX4I1_ENSG00000131143
16	85833423	85833617	5Y-H4K8ac_peak_6726	8.1667	EMC8_ENSG00000131148
16	86588416	86588685	5Y-H4K8ac_peak_6727	7.89273	MTHFSD_ENSG00000103248
16	87358674	87358864	5Y-H4K8ac_peak_6728	7.89142	
16	87385508	87385705	5Y-H4K8ac_peak_6729	7.31102	
16	87403689	87403881	5Y-H4K8ac_peak_6730	5.64909	
16	87417867	87418398	5Y-H4K8ac_peak_6731	13.99721	MAP1LC3B_ENSG00000140941
16	87491829	87492041	5Y-H4K8ac_peak_6732	11.48691	
16	87498439	87498760	5Y-H4K8ac_peak_6733	6.73385	
16	87526891	87527233	5Y-H4K8ac_peak_6734	15.3483	RP11-482M8.1_ENSG00000260750
16	87590861	87591213	5Y-H4K8ac_peak_6735	7.87406	
16	87604048	87604717	5Y-H4K8ac_peak_6736	10.5157	
16	87755918	87756769	5Y-H4K8ac_peak_6737	8.24461	
16	87799675	87800450	5Y-H4K8ac_peak_6738	10.73603	KLHDC4_ENSG00000104731
16	87812252	87813662	5Y-H4K8ac_peak_6739	14.00559	RP4-536B24.4_ENSG00000260498
16	87839951	87841106	5Y-H4K8ac_peak_6740	13.00733	RP4-536B24.3_ENSG00000260177
16	87845801	87845998	5Y-H4K8ac_peak_6741	5.41472	
16	87869341	87869795	5Y-H4K8ac_peak_6742	5.65584	RP4-536B24.2_ENSG00000260466
16	87903303	87903591	5Y-H4K8ac_peak_6743	8.43511	SLC7A5_ENSG00000103257
16	87904909	87905126	5Y-H4K8ac_peak_6744	5.64909	
16	87905574	87905830	5Y-H4K8ac_peak_6745	6.61077	
16	87963883	87964230	5Y-H4K8ac_peak_6746	13.19488	
16	87965697	87966273	5Y-H4K8ac_peak_6747	6.50117	
16	87979107	87979574	5Y-H4K8ac_peak_6748	7.38046	CTD-3057O21.1_ENSG00000260443
16	87984202	87984460	5Y-H4K8ac_peak_6749	9.07803	
16	87985241	87985617	5Y-H4K8ac_peak_6750	15.90416	
16	88076770	88076967	5Y-H4K8ac_peak_6751	7.63144	
16	88096246	88096948	5Y-H4K8ac_peak_6752	7.50148	
16	88110102	88110468	5Y-H4K8ac_peak_6753	8.43511	
16	88111052	88111619	5Y-H4K8ac_peak_6754	13.23366	
16	88125116	88125416	5Y-H4K8ac_peak_6755	6.37023	
16	88126236	88126578	5Y-H4K8ac_peak_6756	12.21176	
16	88152989	88153341	5Y-H4K8ac_peak_6757	5.12213	
16	88153676	88153870	5Y-H4K8ac_peak_6758	7.89273	
16	88165142	88165439	5Y-H4K8ac_peak_6759	6.79169	
16	88165970	88166707	5Y-H4K8ac_peak_6760	7.11863	

16	88175160	88175376	5Y-H4K8ac_peak_6761	9.3149	
16	88176298	88176601	5Y-H4K8ac_peak_6762	6.37023	
16	88177438	88178130	5Y-H4K8ac_peak_6763	14.43262	
16	88179073	88179349	5Y-H4K8ac_peak_6764	5.449	
16	88179566	88179812	5Y-H4K8ac_peak_6765	9.31608	
16	88180018	88180451	5Y-H4K8ac_peak_6766	9.30505	
16	88182221	88182654	5Y-H4K8ac_peak_6767	7.79831	
16	88192101	88192313	5Y-H4K8ac_peak_6768	5.35202	
16	88263843	88264133	5Y-H4K8ac_peak_6769	6.33308	
16	88266184	88266544	5Y-H4K8ac_peak_6770	7.89142	
16	88267181	88267403	5Y-H4K8ac_peak_6771	5.17251	LA16c-444G7.1_ENSG00000261273
16	88268079	88268406	5Y-H4K8ac_peak_6772	13.72401	LA16c-444G7.1_ENSG00000261273
16	88268603	88269115	5Y-H4K8ac_peak_6773	9.23159	LA16c-444G7.1_ENSG00000261273
16	88271766	88272219	5Y-H4K8ac_peak_6774	6.54441	
16	88278866	88279222	5Y-H4K8ac_peak_6775	7.59101	
16	88302297	88302526	5Y-H4K8ac_peak_6776	4.8773	
16	88303844	88304079	5Y-H4K8ac_peak_6777	7.14616	
16	88308092	88308470	5Y-H4K8ac_peak_6778	8.1667	
16	88309052	88309768	5Y-H4K8ac_peak_6779	14.99673	
16	88315878	88316159	5Y-H4K8ac_peak_6780	6.77436	
16	88323567	88323763	5Y-H4K8ac_peak_6781	4.84727	
16	88328445	88328700	5Y-H4K8ac_peak_6782	5.51139	
16	88330244	88330709	5Y-H4K8ac_peak_6783	4.29586	
16	88443193	88443696	5Y-H4K8ac_peak_6784	7.3889	
16	88449308	88450865	5Y-H4K8ac_peak_6785	15.68832	
16	88471787	88472334	5Y-H4K8ac_peak_6786	9.58986	
16	88472554	88473047	5Y-H4K8ac_peak_6787	9.7353	
16	88473394	88473599	5Y-H4K8ac_peak_6788	8.31764	
16	88545750	88545967	5Y-H4K8ac_peak_6789	10.90365	
16	88554248	88554470	5Y-H4K8ac_peak_6790	6.77436	
16	88572226	88572453	5Y-H4K8ac_peak_6791	7.66481	
16	88577060	88577402	5Y-H4K8ac_peak_6792	5.64988	
16	88577841	88578059	5Y-H4K8ac_peak_6793	6.55225	
16	88578664	88579499	5Y-H4K8ac_peak_6794	13.0168	
16	88579983	88581009	5Y-H4K8ac_peak_6795	11.86504	
16	88636905	88637131	5Y-H4K8ac_peak_6796	5.37237	ZC3H18_ENSG00000158545
16	88772299	88772756	5Y-H4K8ac_peak_6797	7.89273	RNF166_ENSG00000158717;CTU2_ENSG00000174177
16	88773130	88773431	5Y-H4K8ac_peak_6798	9.20645	RNF166_ENSG00000158717;CTU2_ENSG00000174177
16	88797341	88797744	5Y-H4K8ac_peak_6799	7.30348	RP5-1142A6.2_ENSG00000224888
16	88803970	88804368	5Y-H4K8ac_peak_6800	8.43511	
16	88837355	88837601	5Y-H4K8ac_peak_6801	5.35202	
16	88851693	88851919	5Y-H4K8ac_peak_6802	5.91107	PIEZO1_ENSG00000103335
16	88869572	88869887	5Y-H4K8ac_peak_6803	7.34185	CDT1_ENSG00000167513
16	88870178	88870455	5Y-H4K8ac_peak_6804	16.01457	CDT1_ENSG00000167513
16	89120148	89120360	5Y-H4K8ac_peak_6805	4.95697	CTD-2555A7.2_ENSG00000256982
16	89137756	89137978	5Y-H4K8ac_peak_6806	5.98695	

16	89138327	89138606	5Y-H4K8ac_peak_6807	9.51254	
16	89159864	89160089	5Y-H4K8ac_peak_6808	6.37023	
16	89160518	89160716	5Y-H4K8ac_peak_6809	7.34185	
16	89216714	89216990	5Y-H4K8ac_peak_6810	5.72233	
16	89239605	89239810	5Y-H4K8ac_peak_6811	4.95697	
16	89277618	89277971	5Y-H4K8ac_peak_6812	4.29586	
16	89307019	89307417	5Y-H4K8ac_peak_6813	11.99586	
16	89313346	89313659	5Y-H4K8ac_peak_6814	7.59101	
16	89574187	89574624	5Y-H4K8ac_peak_6815	9.00699	
16	89627128	89627609	5Y-H4K8ac_peak_6816	14.59308	RPL13_ENSG00000167526;SNORD68_ENSG00000200084
16	89787559	89787806	5Y-H4K8ac_peak_6817	6.98394	VPS9D1_ENSG00000075399;ZNF276_ENSG00000158805
16	89846067	89846270	5Y-H4K8ac_peak_6818	5.65584	
16	89868644	89868899	5Y-H4K8ac_peak_6819	8.60024	
16	89882762	89883001	5Y-H4K8ac_peak_6820	13.2534	FANCA_ENSG00000187741
16	89883204	89883808	5Y-H4K8ac_peak_6821	14.15915	SPIRE2_ENSG00000204991;FANCA_ENSG00000187741
16	89939490	89939863	5Y-H4K8ac_peak_6822	18.10232	TCF25_ENSG00000141002
16	89977075	89977395	5Y-H4K8ac_peak_6823	4.5753	
16	89984488	89985240	5Y-H4K8ac_peak_6824	7.65114	AC092143.1_ENSG00000256390;TUBB3_ENSG00000198211
16	89989246	89989446	5Y-H4K8ac_peak_6825	7.90227	RP11-566K11.4_ENSG00000259006
16	90014588	90014984	5Y-H4K8ac_peak_6826	5.65584	DEF8_ENSG00000140995
16	90038426	90039493	5Y-H4K8ac_peak_6827	19.93272	CENPBD1_ENSG00000177946;AFG3L1P_ENSG00000223959
16	90085516	90085740	5Y-H4K8ac_peak_6828	8.20773	DBNDD1_ENSG00000003249;GAS8_ENSG00000141013
16	90113511	90114053	5Y-H4K8ac_peak_6829	13.0168	URAHP_ENSG00000222019
16	90144049	90144893	5Y-H4K8ac_peak_6830	9.38276	
17	29868	30172	5Y-H4K8ac_peak_6831	6.54441	
17	30485	30973	5Y-H4K8ac_peak_6832	5.98695	DOC2B_ENSG00000272636
17	148697	149011	5Y-H4K8ac_peak_6833	7.57144	
17	150581	150791	5Y-H4K8ac_peak_6834	8.43511	
17	183923	184116	5Y-H4K8ac_peak_6835	8.2913	
17	258919	259151	5Y-H4K8ac_peak_6836	5.37237	C17orf97_ENSG00000187624
17	502252	502546	5Y-H4K8ac_peak_6837	13.22169	
17	635259	635487	5Y-H4K8ac_peak_6838	9.30505	FAM57A_ENSG00000167695
17	655096	655309	5Y-H4K8ac_peak_6839	6.339	DBIL5P_ENSG00000231784
17	655650	656370	5Y-H4K8ac_peak_6840	7.44077	GEMIN4_ENSG00000179409;DBIL5P_ENSG00000231784
17	883630	883986	5Y-H4K8ac_peak_6841	5.68769	NXN_ENSG00000167693
17	900297	900666	5Y-H4K8ac_peak_6842	5.98695	TIMM22_ENSG00000177370
17	973783	973998	5Y-H4K8ac_peak_6843	4.642	
17	974675	975115	5Y-H4K8ac_peak_6844	8.04943	
17	981729	981975	5Y-H4K8ac_peak_6845	4.29586	
17	982364	982896	5Y-H4K8ac_peak_6846	9.05766	
17	983374	983617	5Y-H4K8ac_peak_6847	4.85014	
17	1012559	1012910	5Y-H4K8ac_peak_6848	10.35586	
17	1057369	1057942	5Y-H4K8ac_peak_6849	15.3483	
17	1083522	1083761	5Y-H4K8ac_peak_6850	7.89142	
17	1171676	1172140	5Y-H4K8ac_peak_6851	12.44818	
17	1302892	1303266	5Y-H4K8ac_peak_6852	6.03632	YWHAE_ENSG00000108953

17	1388755	1389143	5Y-H4K8ac_peak_6853	7.28023	
17	1389541	1389786	5Y-H4K8ac_peak_6854	9.30505	
17	1395087	1395408	5Y-H4K8ac_peak_6855	11.67591	MYO1C_ENSG00000197879
17	1466321	1466536	5Y-H4K8ac_peak_6856	5.67283	PITPNA_ENSG00000174238
17	1478252	1478476	5Y-H4K8ac_peak_6857	5.98695	
17	1478904	1479117	5Y-H4K8ac_peak_6858	5.56912	
17	1533002	1533197	5Y-H4K8ac_peak_6859	5.41472	SLC43A2_ENSG00000167703
17	1551811	1552195	5Y-H4K8ac_peak_6860	5.98695	
17	1587831	1588089	5Y-H4K8ac_peak_6861	6.73385	PRPF8_ENSG00000174231
17	1613300	1613565	5Y-H4K8ac_peak_6862	8.43011	TLCD2_ENSG00000185561
17	1620058	1620312	5Y-H4K8ac_peak_6863	4.0639	MIR22HG_ENSG00000186594;WDR81_ENSG00000167716
17	1628107	1628537	5Y-H4K8ac_peak_6864	7.89273	
17	1658622	1659231	5Y-H4K8ac_peak_6865	9.51254	
17	1665658	1666479	5Y-H4K8ac_peak_6866	6.98416	SERPINF1_ENSG00000132386
17	1666755	1668011	5Y-H4K8ac_peak_6867	7.90751	
17	1802225	1802473	5Y-H4K8ac_peak_6868	12.11208	
17	1846226	1846747	5Y-H4K8ac_peak_6869	10.31981	
17	1899163	1899607	5Y-H4K8ac_peak_6870	8.43511	CTD-2545H1.2_ENSG00000262445
17	1945873	1946078	5Y-H4K8ac_peak_6871	7.59101	OVCA2_ENSG00000262664;RP11-667K14.4_ENSG00000262533
17	1953905	1954262	5Y-H4K8ac_peak_6872	10.1994	MIR132_ENSG00000267200;MIR212_ENSG00000267195
17	1958429	1958947	5Y-H4K8ac_peak_6873	9.23159	HIC1_ENSG00000177374
17	1977339	1977700	5Y-H4K8ac_peak_6874	6.40964	
17	1978723	1979191	5Y-H4K8ac_peak_6875	9.01738	
17	1980130	1980374	5Y-H4K8ac_peak_6876	13.99721	
17	1985827	1986038	5Y-H4K8ac_peak_6877	9.63153	
17	1987395	1987596	5Y-H4K8ac_peak_6878	10.41081	
17	2058404	2058620	5Y-H4K8ac_peak_6879	7.31815	
17	2082292	2082519	5Y-H4K8ac_peak_6880	6.77436	
17	2087544	2088139	5Y-H4K8ac_peak_6881	7.59101	
17	2094469	2094812	5Y-H4K8ac_peak_6882	5.84208	
17	2116583	2116782	5Y-H4K8ac_peak_6883	7.53429	AC090617.1_ENSG00000236838
17	2239098	2239663	5Y-H4K8ac_peak_6884	14.30055	
17	2283743	2284238	5Y-H4K8ac_peak_6885	12.05638	
17	2296682	2297093	5Y-H4K8ac_peak_6886	13.00733	
17	2302478	2302685	5Y-H4K8ac_peak_6887	7.6267	
17	2496490	2496736	5Y-H4K8ac_peak_6888	7.17184	PAFAH1B1_ENSG00000007168
17	2497245	2497736	5Y-H4K8ac_peak_6889	11.19336	PAFAH1B1_ENSG00000007168
17	2615222	2615610	5Y-H4K8ac_peak_6890	8.75963	CLUH_ENSG00000132361;RP11-74E22.3_ENSG00000262050
17	2653062	2653295	5Y-H4K8ac_peak_6891	9.00954	RP11-74E22.4_ENSG00000261963
17	2659054	2659366	5Y-H4K8ac_peak_6892	6.77436	
17	2679507	2679937	5Y-H4K8ac_peak_6893	10.86648	RAP1GAP2_ENSG00000132359
17	2756321	2756765	5Y-H4K8ac_peak_6894	7.59101	
17	2787040	2787244	5Y-H4K8ac_peak_6895	7.31815	
17	2801681	2802130	5Y-H4K8ac_peak_6896	6.20989	
17	2805727	2805952	5Y-H4K8ac_peak_6897	6.38173	
17	2831573	2831911	5Y-H4K8ac_peak_6898	4.84727	

17	2832474	2832871	5Y-H4K8ac_peak_6899	8.39058	
17	2862342	2862544	5Y-H4K8ac_peak_6900	6.39626	
17	2863599	2864077	5Y-H4K8ac_peak_6901	9.93099	
17	2905840	2907209	5Y-H4K8ac_peak_6902	8.79957	
17	2907778	2908091	5Y-H4K8ac_peak_6903	21.48346	
17	3438917	3439266	5Y-H4K8ac_peak_6904	5.40331	
17	3572164	3572378	5Y-H4K8ac_peak_6905	11.57714	TAX1BP3_ENSG00000213977;EMC6_ENSG00000127774
17	3598286	3599074	5Y-H4K8ac_peak_6906	10.5157	P2RX5-TAX1BP3_ENSG00000257950;P2RX5_ENSG00000083454
17	3599288	3599720	5Y-H4K8ac_peak_6907	9.51254	P2RX5-TAX1BP3_ENSG00000257950;P2RX5_ENSG00000083454
17	3749127	3749399	5Y-H4K8ac_peak_6908	5.24695	C17orf85_ENSG00000074356
17	3782814	3783673	5Y-H4K8ac_peak_6909	7.50148	
17	3793180	3793407	5Y-H4K8ac_peak_6910	5.98695	
17	3795985	3796189	5Y-H4K8ac_peak_6911	6.37023	
17	3796546	3797028	5Y-H4K8ac_peak_6912	9.7353	
17	3848319	3848997	5Y-H4K8ac_peak_6913	9.36633	
17	4378125	4379164	5Y-H4K8ac_peak_6914	10.35586	
17	4437291	4438024	5Y-H4K8ac_peak_6915	9.79526	
17	4459259	4459479	5Y-H4K8ac_peak_6916	11.1169	MYBBP1A_ENSG00000132382
17	4607054	4607323	5Y-H4K8ac_peak_6917	5.23083	PELP1_ENSG00000141456;RP11-314A20.2_ENSG00000244184
17	4607632	4607910	5Y-H4K8ac_peak_6918	5.40331	PELP1_ENSG00000141456;RP11-314A20.2_ENSG00000244184;RP11-314A20.1_ENSG00000213939
17	4612278	4612758	5Y-H4K8ac_peak_6919	13.27446	
17	4736777	4737092	5Y-H4K8ac_peak_6920	11.99586	MINK1_ENSG00000141503
17	4812672	4813405	5Y-H4K8ac_peak_6921	14.24171	
17	4842721	4843214	5Y-H4K8ac_peak_6922	12.43028	SLC25A11_ENSG00000108528;RNF167_ENSG00000108523
17	4843543	4843799	5Y-H4K8ac_peak_6923	5.98695	SLC25A11_ENSG00000108528;RNF167_ENSG00000108523
17	4851380	4851773	5Y-H4K8ac_peak_6924	7.03573	PFN1_ENSG00000108518;ENO3_ENSG00000108515
17	4852495	4853273	5Y-H4K8ac_peak_6925	16.79568	PFN1_ENSG00000108518
17	4853471	4853713	5Y-H4K8ac_peak_6926	7.60057	
17	4854658	4854916	5Y-H4K8ac_peak_6927	9.51254	
17	4870032	4870269	5Y-H4K8ac_peak_6928	8.69112	SPAG7_ENSG00000091640
17	4890441	4891330	5Y-H4K8ac_peak_6929	10.35586	CAMTA2_ENSG00000108509;RP5-1050D4.4_ENSG00000262678;AC004771.1_ENSG00000203562;RP5-1050D4.5_ENSG00000262227
17	4935286	4935599	5Y-H4K8ac_peak_6930	4.07874	
17	4980965	4981668	5Y-H4K8ac_peak_6931	7.11863	RP11-46I8.3_ENSG00000262693;ZFP3_ENSG00000180787
17	5014685	5015124	5Y-H4K8ac_peak_6932	16.01457	AC012146.7_ENSG00000234327
17	5016030	5016227	5Y-H4K8ac_peak_6933	9.11392	
17	5026009	5026296	5Y-H4K8ac_peak_6934	8.2913	ZNF232_ENSG00000167840
17	5094648	5095100	5Y-H4K8ac_peak_6935	11.19336	ZNF594_ENSG00000180626;RP11-333E1.1_ENSG00000261879
17	5323485	5323704	5Y-H4K8ac_peak_6936	6.37023	NUP88_ENSG00000108559;RPAIN_ENSG00000129197
17	5342959	5343229	5Y-H4K8ac_peak_6937	11.04146	
17	5372618	5372841	5Y-H4K8ac_peak_6938	6.86362	DHX33_ENSG00000005100;CTC-524C5.5_ENSG00000262099
17	6357095	6357303	5Y-H4K8ac_peak_6939	10.35586	
17	6358042	6358881	5Y-H4K8ac_peak_6940	13.81774	
17	6542945	6543623	5Y-H4K8ac_peak_6941	5.56894	KIAA0753_ENSG00000198920;TXNDC17_ENSG00000129235
17	6550037	6550291	5Y-H4K8ac_peak_6942	5.40331	
17	6555095	6555594	5Y-H4K8ac_peak_6943	18.60331	MED31_ENSG00000108590;C17orf100_ENSG00000212734;AC004706.1_ENSG00000256806
17	6915706	6916168	5Y-H4K8ac_peak_6944	16.01457	AC027763.2_ENSG00000215067;RNASEK_ENSG00000219200;RNASEK-C17orf49_ENSG00000161939

17	6921703	6921923	5Y-H4K8ac_peak_6945	5.25819	RP11-589P10.7_ENSG00000267047
17	6922723	6923387	5Y-H4K8ac_peak_6946	19.71555	RP11-589P10.7_ENSG00000267047;MIR497HG_ENSG00000267532
17	6923588	6923804	5Y-H4K8ac_peak_6947	8.56781	RP11-589P10.7_ENSG00000267047;MIR497HG_ENSG00000267532
17	6925209	6925460	5Y-H4K8ac_peak_6948	10.90365	BCL6B_ENSG00000161940
17	6939386	6939639	5Y-H4K8ac_peak_6949	6.90749	SLC16A13_ENSG00000174327
17	7081581	7081784	5Y-H4K8ac_peak_6950	6.77436	
17	7118720	7118931	5Y-H4K8ac_peak_6951	6.47245	
17	7121125	7121327	5Y-H4K8ac_peak_6952	5.98695	ACADVL_ENSG00000072778
17	7121686	7121907	5Y-H4K8ac_peak_6953	6.79169	
17	7154350	7155269	5Y-H4K8ac_peak_6954	18.77281	CTDNEP1_ENSG00000175826;ELP5_ENSG00000170291
17	7164160	7164888	5Y-H4K8ac_peak_6955	11.19336	RP1-4G17.5_ENSG00000262302
17	7197494	7197979	5Y-H4K8ac_peak_6956	7.92287	YBX2_ENSG00000006047
17	7211216	7211869	5Y-H4K8ac_peak_6957	9.56187	EIF5A_ENSG00000132507
17	7218814	7219177	5Y-H4K8ac_peak_6958	8.73985	GPS2_ENSG00000132522
17	7233926	7234272	5Y-H4K8ac_peak_6959	6.29918	AC026954.6_ENSG00000224647
17	7254500	7254930	5Y-H4K8ac_peak_6960	4.29586	KCTD11_ENSG00000213859
17	7255213	7255660	5Y-H4K8ac_peak_6961	7.41197	KCTD11_ENSG00000213859
17	7287144	7287918	5Y-H4K8ac_peak_6962	7.20869	
17	7308641	7308935	5Y-H4K8ac_peak_6963	7.11863	NLGN2_ENSG00000169992
17	7342917	7343219	5Y-H4K8ac_peak_6964	8.25419	RP11-104H15.10_ENSG00000272884
17	7343598	7344269	5Y-H4K8ac_peak_6965	7.11863	RP11-104H15.10_ENSG00000272884
17	7382543	7382789	5Y-H4K8ac_peak_6966	11.75922	
17	7385937	7386202	5Y-H4K8ac_peak_6967	5.24695	
17	7386878	7387148	5Y-H4K8ac_peak_6968	6.78318	ZBTB4_ENSG00000174282;POLR2A_ENSG00000181222
17	7452428	7453087	5Y-H4K8ac_peak_6969	16.01457	TNFSF12_ENSG00000239697;TNFSF12-TNFSF13_ENSG00000248871
17	7460648	7461062	5Y-H4K8ac_peak_6970	5.98695	TNFSF13_ENSG00000161955
17	7465361	7465680	5Y-H4K8ac_peak_6971	7.34185	SENP3_ENSG00000161956;SENP3-EIF4A1_ENSG00000265500
17	7476230	7476685	5Y-H4K8ac_peak_6972	13.0168	EIF4A1_ENSG00000161960;SNORA67_ENSG00000264772
17	7486099	7486831	5Y-H4K8ac_peak_6973	9.58986	AC113189.5_ENSG00000233223;MPDU1_ENSG00000129255
17	7492416	7492685	5Y-H4K8ac_peak_6974	6.38173	SOX15_ENSG00000129194
17	7590059	7590250	5Y-H4K8ac_peak_6975	6.81557	TP53_ENSG00000141510;RP11-199F11.2_ENSG00000262251;WRAP53_ENSG00000141499
17	7620186	7620424	5Y-H4K8ac_peak_6976	11.48691	DNAH2_ENSG00000183914
17	7620788	7620992	5Y-H4K8ac_peak_6977	9.23159	DNAH2_ENSG00000183914
17	7739203	7739531	5Y-H4K8ac_peak_6978	5.98695	
17	7745167	7745396	5Y-H4K8ac_peak_6979	6.03632	
17	7788327	7789223	5Y-H4K8ac_peak_6980	13.4304	LSMD1_ENSG00000183011;CHD3_ENSG00000170004
17	7791283	7791620	5Y-H4K8ac_peak_6981	5.12488	
17	7791830	7792126	5Y-H4K8ac_peak_6982	8.69112	
17	7819465	7819857	5Y-H4K8ac_peak_6983	8.69112	AC025335.1_ENSG00000179859
17	8065812	8066196	5Y-H4K8ac_peak_6984	11.69482	RP11-599B13.6_ENSG00000263620;VAMP2_ENSG00000220205
17	8066630	8067845	5Y-H4K8ac_peak_6985	14.31711	RP11-599B13.6_ENSG00000263620;VAMP2_ENSG00000220205
17	8079944	8080422	5Y-H4K8ac_peak_6986	9.51254	TMEM107_ENSG00000179029;RP11-599B13.7_ENSG00000266824
17	8122732	8123002	5Y-H4K8ac_peak_6987	7.72345	
17	8126910	8127122	5Y-H4K8ac_peak_6988	10.31981	LINC00324_ENSG00000178977
17	8191361	8191552	5Y-H4K8ac_peak_6989	4.95697	RANGRF_ENSG00000108961
17	8192110	8192370	5Y-H4K8ac_peak_6990	9.7353	RANGRF_ENSG00000108961

17	8230304	8230513	5Y-H4K8ac_peak_6991	4.95697	
17	8287014	8287261	5Y-H4K8ac_peak_6992	9.23159	RP11-849F2.7_ENSG00000263809;RPL26_ENSG00000161970
17	8339291	8339599	5Y-H4K8ac_peak_6993	7.38046	
17	8534438	8534959	5Y-H4K8ac_peak_6994	9.38203	MYH10_ENSG00000133026
17	8584302	8584551	5Y-H4K8ac_peak_6995	9.38203	
17	8585167	8585383	5Y-H4K8ac_peak_6996	9.30505	
17	8600268	8600512	5Y-H4K8ac_peak_6997	5.16353	
17	9143593	9143851	5Y-H4K8ac_peak_6998	4.77126	
17	10600399	10600794	5Y-H4K8ac_peak_6999	8.43511	SCO1_ENSG00000133028;ADPRM_ENSG00000170222
17	10632706	10633357	5Y-H4K8ac_peak_7000	6.43775	TMEM220_ENSG00000187824;CTC-297N7.5_ENSG00000263400
17	11900235	11900682	5Y-H4K8ac_peak_7001	7.20869	ZNF18_ENSG00000154957
17	11901216	11901669	5Y-H4K8ac_peak_7002	4.50834	ZNF18_ENSG00000154957
17	11924333	11924709	5Y-H4K8ac_peak_7003	11.57334	RPL21P122_ENSG00000241185;MAP2K4_ENSG00000065559
17	12692861	12693061	5Y-H4K8ac_peak_7004	5.23083	RP11-1090M7.1_ENSG00000265489;ARHGAP44_ENSG00000006740
17	13973016	13973233	5Y-H4K8ac_peak_7005	8.20773	COX10-AS1_ENSG00000236088;COX10_ENSG00000006695
17	15302546	15303220	5Y-H4K8ac_peak_7006	11.57334	
17	15587108	15587550	5Y-H4K8ac_peak_7007	9.23159	TRIM16_ENSG00000221926
17	15603128	15603409	5Y-H4K8ac_peak_7008	12.71156	ZNF286A_ENSG00000187607;ZNF286A_ENSG00000255104
17	15689221	15689945	5Y-H4K8ac_peak_7009	8.69112	MEIS3P1_ENSG00000179277
17	15847680	15848004	5Y-H4K8ac_peak_7010	9.30206	ADORA2B_ENSG00000170425
17	15848840	15849268	5Y-H4K8ac_peak_7011	6.1654	ADORA2B_ENSG00000170425
17	15902714	15902962	5Y-H4K8ac_peak_7012	7.31815	ZSWIM7_ENSG00000214941;TTC19_ENSG00000011295
17	16118565	16118774	5Y-H4K8ac_peak_7013	11.62198	
17	16119759	16120006	5Y-H4K8ac_peak_7014	5.99504	
17	16256164	16256623	5Y-H4K8ac_peak_7015	11.09973	CENPV_ENSG00000166582
17	16256987	16257223	5Y-H4K8ac_peak_7016	6.39626	CENPV_ENSG00000166582
17	16283408	16283906	5Y-H4K8ac_peak_7017	4.95697	UBB_ENSG00000170315
17	16311398	16311591	5Y-H4K8ac_peak_7018	6.79955	
17	16365355	16365569	5Y-H4K8ac_peak_7019	5.94703	
17	16395604	16395819	5Y-H4K8ac_peak_7020	8.08337	FAM211A_ENSG00000181350
17	16556763	16557017	5Y-H4K8ac_peak_7021	7.50148	ZNF624_ENSG00000197566;RP11-92B11.4_ENSG00000264765
17	16928003	16928313	5Y-H4K8ac_peak_7022	15.75437	
17	16932763	16933052	5Y-H4K8ac_peak_7023	4.642	
17	16933385	16933750	5Y-H4K8ac_peak_7024	9.96543	
17	16934489	16934871	5Y-H4K8ac_peak_7025	9.04239	
17	16935643	16936041	5Y-H4K8ac_peak_7026	6.34245	
17	17109102	17109411	5Y-H4K8ac_peak_7027	9.30505	PLD6_ENSG00000179598
17	17140215	17140760	5Y-H4K8ac_peak_7028	18.77281	RP11-45M22.4_ENSG00000264187;FLCN_ENSG00000154803;RP11-45M22.5_ENSG00000266498
17	17206303	17206891	5Y-H4K8ac_peak_7029	11.72217	NT5M_ENSG00000205309
17	17207128	17207335	5Y-H4K8ac_peak_7030	10.508	NT5M_ENSG00000205309
17	17254527	17255140	5Y-H4K8ac_peak_7031	8.18236	
17	17255384	17256155	5Y-H4K8ac_peak_7032	10.03452	
17	17256540	17256910	5Y-H4K8ac_peak_7033	9.30505	
17	17288291	17288556	5Y-H4K8ac_peak_7034	7.87406	RPL13P12_ENSG00000215030
17	17306241	17306450	5Y-H4K8ac_peak_7035	4.69231	
17	17306761	17306970	5Y-H4K8ac_peak_7036	6.77436	

17	17307179	17307913	5Y-H4K8ac_peak_7037	4.00285	
17	17317588	17317805	5Y-H4K8ac_peak_7038	4.50834	
17	17379786	17380106	5Y-H4K8ac_peak_7039	10.99773	MED9_ENSG00000141026
17	17380337	17380741	5Y-H4K8ac_peak_7040	5.98695	MED9_ENSG00000141026
17	17399836	17400165	5Y-H4K8ac_peak_7041	7.11863	RASD1_ENSG00000108551
17	17400391	17400673	5Y-H4K8ac_peak_7042	4.642	RASD1_ENSG00000108551
17	17418604	17419046	5Y-H4K8ac_peak_7043	5.96059	
17	17444542	17444742	5Y-H4K8ac_peak_7044	9.01738	
17	17450202	17450647	5Y-H4K8ac_peak_7045	10.94041	
17	17451384	17451637	5Y-H4K8ac_peak_7046	9.89244	
17	17566152	17566576	5Y-H4K8ac_peak_7047	5.23083	
17	17572533	17572885	5Y-H4K8ac_peak_7048	7.53429	
17	17584727	17585215	5Y-H4K8ac_peak_7049	13.45646	RAI1_ENSG00000108557
17	17585428	17586154	5Y-H4K8ac_peak_7050	7.64648	RAI1_ENSG00000108557
17	17586641	17586846	5Y-H4K8ac_peak_7051	7.38046	
17	17588942	17589322	5Y-H4K8ac_peak_7052	7.63144	
17	17596461	17597183	5Y-H4K8ac_peak_7053	10.68758	
17	17643047	17643801	5Y-H4K8ac_peak_7054	13.96066	
17	17649454	17649871	5Y-H4K8ac_peak_7055	5.83799	
17	17652078	17652357	5Y-H4K8ac_peak_7056	7.30348	
17	17655994	17656251	5Y-H4K8ac_peak_7057	4.50834	
17	17688131	17688405	5Y-H4K8ac_peak_7058	7.39248	
17	17688870	17689068	5Y-H4K8ac_peak_7059	4.19948	
17	17693054	17693421	5Y-H4K8ac_peak_7060	8.65166	
17	17694179	17695310	5Y-H4K8ac_peak_7061	7.64648	
17	17722729	17722978	5Y-H4K8ac_peak_7062	7.15925	
17	17727666	17728105	5Y-H4K8ac_peak_7063	5.44849	
17	17735344	17736158	5Y-H4K8ac_peak_7064	7.30348	
17	17738913	17739392	5Y-H4K8ac_peak_7065	4.29586	SREBF1_ENSG00000072310
17	17739754	17741143	5Y-H4K8ac_peak_7066	9.77183	SREBF1_ENSG00000072310
17	17742893	17744304	5Y-H4K8ac_peak_7067	9.00954	
17	17750486	17750817	5Y-H4K8ac_peak_7068	6.08523	
17	17764116	17764331	5Y-H4K8ac_peak_7069	3.95366	
17	17780662	17780852	5Y-H4K8ac_peak_7070	6.43775	
17	17815510	17815792	5Y-H4K8ac_peak_7071	8.75926	
17	17817404	17817735	5Y-H4K8ac_peak_7072	4.24332	
17	17929296	17929524	5Y-H4K8ac_peak_7073	6.46053	
17	17991332	17991584	5Y-H4K8ac_peak_7074	11.1169	DRG2_ENSG00000108591
17	18007991	18008204	5Y-H4K8ac_peak_7075	5.37237	
17	18056612	18057005	5Y-H4K8ac_peak_7076	5.65765	
17	18085946	18086554	5Y-H4K8ac_peak_7077	10.10203	ALKBH5_ENSG00000091542
17	18127974	18128173	5Y-H4K8ac_peak_7078	8.16382	
17	18217915	18218229	5Y-H4K8ac_peak_7079	5.03564	TOP3A_ENSG00000177302;SMCR8_ENSG00000176994
17	18218646	18219211	5Y-H4K8ac_peak_7080	8.43511	TOP3A_ENSG00000177302;SMCR8_ENSG00000176994
17	18266518	18266751	5Y-H4K8ac_peak_7081	7.11863	SHMT1_ENSG00000176974
17	18281219	18281414	5Y-H4K8ac_peak_7082	4.5753	EVPLL_ENSG00000214860

17	18601384	18601719	5Y-H4K8ac_peak_7083	7.58806	TRIM16L_ENSG00000108448
17	18761501	18761746	5Y-H4K8ac_peak_7084	12.82003	
17	18864433	18864975	5Y-H4K8ac_peak_7085	11.66706	
17	18907809	18908238	5Y-H4K8ac_peak_7086	5.41472	FAM83G_ENSG00000188522
17	19140761	19141010	5Y-H4K8ac_peak_7087	8.08688	
17	19268001	19268712	5Y-H4K8ac_peak_7088	5.64909	
17	19281085	19281434	5Y-H4K8ac_peak_7089	4.77126	B9D1_ENSG00000108641;MAPK7_ENSG00000166484
17	19289099	19289331	5Y-H4K8ac_peak_7090	6.10343	
17	19290862	19291132	5Y-H4K8ac_peak_7091	14.55234	MFAP4_ENSG00000166482
17	19291590	19292139	5Y-H4K8ac_peak_7092	15.67761	
17	19305903	19306143	5Y-H4K8ac_peak_7093	7.59832	
17	19314226	19314710	5Y-H4K8ac_peak_7094	5.64909	RNF112_ENSG00000128482
17	19410369	19410713	5Y-H4K8ac_peak_7095	4.69231	
17	19483148	19484048	5Y-H4K8ac_peak_7096	10.31981	AC025627.7_ENSG00000228983
17	19551193	19551875	5Y-H4K8ac_peak_7097	12.05638	ALDH3A2_ENSG00000072210
17	19647766	19648174	5Y-H4K8ac_peak_7098	16.01457	
17	19648456	19648676	5Y-H4K8ac_peak_7099	5.12488	
17	19770534	19771771	5Y-H4K8ac_peak_7100	18.21442	ULK2_ENSG00000083290
17	19881284	19881885	5Y-H4K8ac_peak_7101	13.12848	AKAP10_ENSG00000108599
17	19911989	19912459	5Y-H4K8ac_peak_7102	14.31711	RP11-209D14.2_ENSG00000261033;SPECC1_ENSG00000128487
17	20224009	20224404	5Y-H4K8ac_peak_7103	7.89142	CCDC144CP_ENSG00000154898
17	20771051	20771429	5Y-H4K8ac_peak_7104	11.19336	RP11-344E13.3_ENSG00000233098
17	20810992	20811380	5Y-H4K8ac_peak_7105	9.51254	
17	20945857	20946292	5Y-H4K8ac_peak_7106	20.25522	USP22_ENSG00000124422
17	21030336	21030556	5Y-H4K8ac_peak_7107	7.90751	
17	21178929	21179133	5Y-H4K8ac_peak_7108	7.59101	
17	21190743	21190936	5Y-H4K8ac_peak_7109	4.82467	
17	21357383	21357575	5Y-H4K8ac_peak_7110	7.38046	
17	21825338	21825534	5Y-H4K8ac_peak_7111	8.2913	FAM27L_ENSG00000178130;RP11-1109M24.14_ENSG00000264811
17	25680390	25680798	5Y-H4K8ac_peak_7112	6.78318	RP11-173M1.5_ENSG00000265683
17	25681098	25681410	5Y-H4K8ac_peak_7113	10.36926	
17	25708306	25708602	5Y-H4K8ac_peak_7114	4.47071	
17	25782798	25783349	5Y-H4K8ac_peak_7115	10.95042	KSR1_ENSG00000141068
17	25981321	25981734	5Y-H4K8ac_peak_7116	6.43775	
17	26120412	26120650	5Y-H4K8ac_peak_7117	9.30505	
17	26132375	26133316	5Y-H4K8ac_peak_7118	8.97752	
17	26662871	26663140	5Y-H4K8ac_peak_7119	4.77126	IFT20_ENSG00000109083;TNFAIP1_ENSG00000109079
17	26683938	26684463	5Y-H4K8ac_peak_7120	6.34245	POLDIP2_ENSG00000004142;TMEM199_ENSG00000244045;CTB-96E2.3_ENSG00000258924
17	26698874	26699292	5Y-H4K8ac_peak_7121	6.77436	VTN_ENSG00000109072
17	26732025	26733504	5Y-H4K8ac_peak_7122	13.2534	SLC46A1_ENSG00000076351;CTD-2350C19.2_ENSG00000265254
17	26853511	26853839	5Y-H4K8ac_peak_7123	4.84727	
17	26973658	26973931	5Y-H4K8ac_peak_7124	4.8509	
17	26988700	26989153	5Y-H4K8ac_peak_7125	7.89273	SDF2_ENSG00000132581;SUPT6H_ENSG00000109111
17	27038561	27038983	5Y-H4K8ac_peak_7126	7.87406	PROCA1_ENSG00000167525
17	27047073	27047425	5Y-H4K8ac_peak_7127	6.08523	RPL23A_ENSG00000198242;SNORD42B_ENSG00000238423
17	27052645	27053008	5Y-H4K8ac_peak_7128	7.96285	NEK8_ENSG00000160602

17	27055592	27055909	5Y-H4K8ac_peak_7129	4.95697	TLCD1_ENSG00000160606
17	27140948	27141157	5Y-H4K8ac_peak_7130	7.31807	
17	27170086	27170342	5Y-H4K8ac_peak_7131	5.99504	
17	27181112	27181630	5Y-H4K8ac_peak_7132	9.23159	FAM222B_ENSG00000173065;ERAL1_ENSG00000132591
17	27224198	27224966	5Y-H4K8ac_peak_7133	10.12457	FLOT2_ENSG00000132589;RP11-20B24.4_ENSG00000266642
17	27229348	27229596	5Y-H4K8ac_peak_7134	5.64909	DHRS13_ENSG00000167536
17	27370020	27370251	5Y-H4K8ac_peak_7135	7.20869	
17	27375889	27376250	5Y-H4K8ac_peak_7136	11.94897	
17	27378318	27378703	5Y-H4K8ac_peak_7137	4.74441	
17	27381801	27382177	5Y-H4K8ac_peak_7138	9.7353	
17	27387742	27388120	5Y-H4K8ac_peak_7139	11.09973	
17	27412294	27412548	5Y-H4K8ac_peak_7140	6.52331	
17	27429594	27430348	5Y-H4K8ac_peak_7141	6.37023	
17	27476083	27476678	5Y-H4K8ac_peak_7142	5.29015	
17	27506744	27507016	5Y-H4K8ac_peak_7143	4.50834	MYO18A_ENSG00000196535
17	27507621	27507836	5Y-H4K8ac_peak_7144	6.75784	MYO18A_ENSG00000196535
17	27568264	27568482	5Y-H4K8ac_peak_7145	8.88899	
17	27717400	27717718	5Y-H4K8ac_peak_7146	9.23159	MIR4523_ENSG00000264808;TAOK1_ENSG00000160551;MIR4523_ENSG00000263719
17	27918181	27918393	5Y-H4K8ac_peak_7147	4.642	RP11-68I3.7_ENSG00000264647
17	27919636	27919957	5Y-H4K8ac_peak_7148	4.29586	RP11-68I3.7_ENSG00000264647
17	28053155	28053387	5Y-H4K8ac_peak_7149	9.38203	
17	28256587	28256945	5Y-H4K8ac_peak_7150	7.04637	SSH2_ENSG00000141298;EFCAB5_ENSG00000176927
17	28432121	28432520	5Y-H4K8ac_peak_7151	6.50117	
17	28619140	28619406	5Y-H4K8ac_peak_7152	9.05168	BLMH_ENSG00000108578
17	28927908	28928193	5Y-H4K8ac_peak_7153	5.28616	SMURF2P1_ENSG00000248121
17	28973701	28973910	5Y-H4K8ac_peak_7154	4.51076	
17	28994178	28994476	5Y-H4K8ac_peak_7155	6.78128	
17	28994810	28995134	5Y-H4K8ac_peak_7156	16.50659	
17	29036819	29037204	5Y-H4K8ac_peak_7157	7.65114	SUZ12P_ENSG00000264538
17	29057839	29058059	5Y-H4K8ac_peak_7158	5.98695	
17	29151222	29151653	5Y-H4K8ac_peak_7159	9.23159	CRLF3_ENSG00000176390
17	29151948	29152245	5Y-H4K8ac_peak_7160	8.75926	CRLF3_ENSG00000176390
17	29157846	29158130	5Y-H4K8ac_peak_7161	8.08688	CTD-2349P21.1_ENSG00000242439;ATAD5_ENSG00000176208
17	29422250	29422440	5Y-H4K8ac_peak_7162	9.78792	RP11-848P1.5_ENSG00000264107;MIR4733_ENSG00000265444;NF1_ENSG00000196712
17	29718016	29718507	5Y-H4K8ac_peak_7163	8.69112	RAB11FIP4_ENSG00000131242
17	29733287	29733478	5Y-H4K8ac_peak_7164	9.84749	
17	29752613	29753230	5Y-H4K8ac_peak_7165	10.19948	
17	29754890	29755189	5Y-H4K8ac_peak_7166	4.50834	
17	29876336	29876937	5Y-H4K8ac_peak_7167	10.19948	
17	29877270	29877720	5Y-H4K8ac_peak_7168	7.59101	
17	29886310	29886587	5Y-H4K8ac_peak_7169	15.34073	MIR193A_ENSG00000207614
17	29908323	29908651	5Y-H4K8ac_peak_7170	8.5159	
17	30263384	30263588	5Y-H4K8ac_peak_7171	4.95697	SUZ12_ENSG00000178691
17	30264106	30264437	5Y-H4K8ac_peak_7172	9.63153	SUZ12_ENSG00000178691
17	30406783	30407379	5Y-H4K8ac_peak_7173	5.23083	
17	30410689	30411135	5Y-H4K8ac_peak_7174	9.30505	RP11-640N20.6_ENSG00000264164

17	30454477	30454676	5Y-H4K8ac_peak_7175	4.5753	
17	30469558	30469904	5Y-H4K8ac_peak_7176	8.2913	AC090616.2_ENSG00000214708;RHOT1_ENSG00000126858
17	30592278	30592534	5Y-H4K8ac_peak_7177	4.95697	RHBDL3_ENSG00000141314
17	30607553	30608668	5Y-H4K8ac_peak_7178	14.66739	RP11-443G13.2_ENSG00000263674
17	30650843	30651053	5Y-H4K8ac_peak_7179	7.58806	RP11-227G15.3_ENSG00000265794
17	30668365	30669128	5Y-H4K8ac_peak_7180	7.89273	
17	30770975	30771189	5Y-H4K8ac_peak_7181	6.50117	PSMD11_ENSG00000108671
17	30810729	30810993	5Y-H4K8ac_peak_7182	8.1667	
17	30813046	30814297	5Y-H4K8ac_peak_7183	14.99673	CDK5R1_ENSG00000176749
17	30821977	30822480	5Y-H4K8ac_peak_7184	9.89244	RP11-466A19.1_ENSG00000266718
17	30846066	30846476	5Y-H4K8ac_peak_7185	7.20869	RP11-466A19.6_ENSG00000263717;RP11-466A19.3_ENSG00000266599
17	31254861	31255135	5Y-H4K8ac_peak_7186	12.26606	TMEM98_ENSG00000006042
17	32940621	32941058	5Y-H4K8ac_peak_7187	10.65037	
17	32943119	32944048	5Y-H4K8ac_peak_7188	14.31711	
17	32992968	32993332	5Y-H4K8ac_peak_7189	5.64909	
17	33288092	33288449	5Y-H4K8ac_peak_7190	7.89273	ZNF830_ENSG00000198783
17	33307633	33308201	5Y-H4K8ac_peak_7191	7.90236	LIG3_ENSG00000005156;CCT6B_ENSG00000132141
17	33415957	33416173	5Y-H4K8ac_peak_7192	7.18391	RFFL_ENSG00000092871
17	33446326	33447401	5Y-H4K8ac_peak_7193	15.06486	
17	33914391	33914847	5Y-H4K8ac_peak_7194	7.38046	
17	34077064	34077267	5Y-H4K8ac_peak_7195	7.89273	
17	34092652	34092940	5Y-H4K8ac_peak_7196	8.79957	
17	34111956	34112151	5Y-H4K8ac_peak_7197	5.25503	
17	34114524	34114757	5Y-H4K8ac_peak_7198	5.87382	
17	34122165	34122545	5Y-H4K8ac_peak_7199	9.34555	MMP28_ENSG00000129270
17	34838593	34838884	5Y-H4K8ac_peak_7200	9.30505	
17	34839097	34839377	5Y-H4K8ac_peak_7201	8.9562	
17	34890172	34890646	5Y-H4K8ac_peak_7202	7.59101	PIGW_ENSG00000184886
17	34891078	34891275	5Y-H4K8ac_peak_7203	8.43011	PIGW_ENSG00000184886
17	34900246	34900659	5Y-H4K8ac_peak_7204	12.26819	MYO19_ENSG00000141140;GGNBP2_ENSG00000005955
17	34900911	34901162	5Y-H4K8ac_peak_7205	8.43511	GGNBP2_ENSG00000005955
17	34997874	34998784	5Y-H4K8ac_peak_7206	10.48476	
17	35036274	35036639	5Y-H4K8ac_peak_7207	9.32595	
17	35036846	35037158	5Y-H4K8ac_peak_7208	7.50148	
17	35083255	35084377	5Y-H4K8ac_peak_7209	11.32039	
17	35085155	35085479	5Y-H4K8ac_peak_7210	12.19856	
17	35086288	35087004	5Y-H4K8ac_peak_7211	6.47245	
17	35087392	35087893	5Y-H4K8ac_peak_7212	11.24252	
17	35098705	35098991	5Y-H4K8ac_peak_7213	6.50117	
17	35154731	35154921	5Y-H4K8ac_peak_7214	4.84727	
17	35165270	35166186	5Y-H4K8ac_peak_7215	14.89891	
17	35242007	35242293	5Y-H4K8ac_peak_7216	5.00877	
17	35716535	35716764	5Y-H4K8ac_peak_7217	4.50834	
17	35766491	35766894	5Y-H4K8ac_peak_7218	13.2534	ACACA_ENSG00000132142;TADA2A_ENSG00000108264
17	35767323	35768135	5Y-H4K8ac_peak_7219	9.43096	RP11-378E13.4_ENSG00000267613;ACACA_ENSG00000132142;TADA2A_ENSG00000108264
17	35849238	35849686	5Y-H4K8ac_peak_7220	7.89142	DUSP14_ENSG00000161326

17	35969140	35969361	5Y-H4K8ac_peak_7221	9.79526	SYNRG_ENSG00000006114;RP11-697E22.1_ENSG00000267542
17	36003087	36003441	5Y-H4K8ac_peak_7222	7.11863	DDX52_ENSG00000141141;RP11-697E22.2_ENSG00000267668
17	36413512	36413727	5Y-H4K8ac_peak_7223	10.46287	
17	36433090	36433327	5Y-H4K8ac_peak_7224	5.97089	
17	36452320	36452686	5Y-H4K8ac_peak_7225	8.35139	MRPL45_ENSG00000174100
17	36570900	36572331	5Y-H4K8ac_peak_7226	25.87888	
17	36572590	36572833	5Y-H4K8ac_peak_7227	6.87095	
17	36666039	36666667	5Y-H4K8ac_peak_7228	19.79054	
17	36735044	36735254	5Y-H4K8ac_peak_7229	8.47164	
17	36850275	36850669	5Y-H4K8ac_peak_7230	8.79957	
17	36858875	36859133	5Y-H4K8ac_peak_7231	6.39716	MIR4734_ENSG00000265930;CTB-58E17.1_ENSG00000261005
17	36860320	36860598	5Y-H4K8ac_peak_7232	12.05638	
17	36861001	36861306	5Y-H4K8ac_peak_7233	6.78128	MLLT6_ENSG00000108292
17	36881772	36882178	5Y-H4K8ac_peak_7234	14.2053	
17	36886674	36886912	5Y-H4K8ac_peak_7235	8.69112	CISD3_ENSG00000230055
17	36903964	36904320	5Y-H4K8ac_peak_7236	8.08595	
17	36955984	36956191	5Y-H4K8ac_peak_7237	10.07308	PIP4K2B_ENSG00000141720
17	37009699	37009930	5Y-H4K8ac_peak_7238	13.14766	RPL23_ENSG00000125691;SNORA21_ENSG00000199293
17	37025660	37026091	5Y-H4K8ac_peak_7239	7.59101	LASP1_ENSG00000002834
17	37311971	37312216	5Y-H4K8ac_peak_7240	5.98695	
17	37321359	37321611	5Y-H4K8ac_peak_7241	13.81505	
17	37354159	37354387	5Y-H4K8ac_peak_7242	9.32595	CACNB1_ENSG00000067191
17	37356437	37356735	5Y-H4K8ac_peak_7243	4.93618	RPL19_ENSG00000108298
17	37365922	37366435	5Y-H4K8ac_peak_7244	8.27643	
17	37387335	37387729	5Y-H4K8ac_peak_7245	5.98695	
17	37387944	37388267	5Y-H4K8ac_peak_7246	4.07874	
17	37607939	37608477	5Y-H4K8ac_peak_7247	7.87406	MED1_ENSG00000125686
17	37617064	37617429	5Y-H4K8ac_peak_7248	7.90751	CDK12_ENSG00000167258
17	37773513	37773802	5Y-H4K8ac_peak_7249	4.51497	
17	37774856	37775625	5Y-H4K8ac_peak_7250	9.96543	AC087491.2_ENSG00000214546
17	37792785	37793235	5Y-H4K8ac_peak_7251	8.76581	STARD3_ENSG00000131748
17	37823397	37823847	5Y-H4K8ac_peak_7252	7.31102	PNMT_ENSG00000141744
17	37824322	37824677	5Y-H4K8ac_peak_7253	7.11863	PNMT_ENSG00000141744
17	37844113	37844921	5Y-H4K8ac_peak_7254	10.19948	ERBB2_ENSG00000141736
17	37886710	37887021	5Y-H4K8ac_peak_7255	5.41472	MIEN1_ENSG00000141741
17	37910031	37910488	5Y-H4K8ac_peak_7256	10.15788	
17	37910811	37911069	5Y-H4K8ac_peak_7257	5.24695	
17	38084200	38084531	5Y-H4K8ac_peak_7258	7.89142	ORMDL3_ENSG00000172057;RP11-387H17.4_ENSG00000264968
17	38109632	38110241	5Y-H4K8ac_peak_7259	10.35586	
17	38115485	38115712	5Y-H4K8ac_peak_7260	6.20989	
17	38210792	38211025	5Y-H4K8ac_peak_7261	9.7353	
17	38219308	38219598	5Y-H4K8ac_peak_7262	6.50117	
17	38228849	38229330	5Y-H4K8ac_peak_7263	8.25419	
17	38231420	38231822	5Y-H4K8ac_peak_7264	5.23083	
17	38264484	38264724	5Y-H4K8ac_peak_7265	6.17973	
17	38268990	38269242	5Y-H4K8ac_peak_7266	6.50117	

17	38278516	38278726	5Y-H4K8ac_peak_7267	12.82003	MSL1_ENSG00000188895
17	38348180	38348449	5Y-H4K8ac_peak_7268	5.64909	
17	38444326	38444601	5Y-H4K8ac_peak_7269	4.50834	CDC6_ENSG00000094804
17	38461004	38461299	5Y-H4K8ac_peak_7270	4.77126	
17	38462825	38463264	5Y-H4K8ac_peak_7271	7.59101	
17	38466322	38466520	5Y-H4K8ac_peak_7272	6.73047	RARA_ENSG00000131759
17	38467550	38468114	5Y-H4K8ac_peak_7273	8.2913	
17	38473175	38473629	5Y-H4K8ac_peak_7274	10.11191	
17	38501446	38501759	5Y-H4K8ac_peak_7275	7.30348	
17	38505897	38506123	5Y-H4K8ac_peak_7276	5.41472	
17	38507660	38507884	5Y-H4K8ac_peak_7277	10.6196	
17	38508103	38508457	5Y-H4K8ac_peak_7278	6.87095	
17	38574565	38575014	5Y-H4K8ac_peak_7279	7.31102	TOP2A_ENSG00000131747
17	38588323	38588520	5Y-H4K8ac_peak_7280	7.35333	
17	38589807	38590018	5Y-H4K8ac_peak_7281	11.01287	
17	38599128	38600369	5Y-H4K8ac_peak_7282	14.49348	IGFBP4_ENSG00000141753
17	38614388	38614639	5Y-H4K8ac_peak_7283	4.55128	
17	38618016	38618721	5Y-H4K8ac_peak_7284	7.89273	
17	38646475	38646853	5Y-H4K8ac_peak_7285	10.03452	
17	38697752	38698911	5Y-H4K8ac_peak_7286	14.99673	
17	38700225	38700556	5Y-H4K8ac_peak_7287	8.56781	
17	38703967	38704224	5Y-H4K8ac_peak_7288	4.95697	
17	38705279	38706074	5Y-H4K8ac_peak_7289	6.08523	
17	38712978	38713306	5Y-H4K8ac_peak_7290	10.65816	
17	38713831	38714352	5Y-H4K8ac_peak_7291	6.70695	
17	38803685	38804034	5Y-H4K8ac_peak_7292	12.05638	SMARCE1_ENSG00000073584
17	38804317	38804835	5Y-H4K8ac_peak_7293	11.5813	SMARCE1_ENSG00000073584
17	39677476	39677666	5Y-H4K8ac_peak_7294	6.81557	
17	39686232	39686465	5Y-H4K8ac_peak_7295	6.31818	
17	39687151	39687527	5Y-H4K8ac_peak_7296	8.21582	
17	39705023	39705260	5Y-H4K8ac_peak_7297	6.69752	
17	39894529	39895069	5Y-H4K8ac_peak_7298	8.20773	
17	39968047	39968719	5Y-H4K8ac_peak_7299	5.34199	LEPREL4_ENSG00000141696;FKBP10_ENSG00000141756
17	39992197	39992469	5Y-H4K8ac_peak_7300	10.90097	KLHL10_ENSG00000161594;NT5C3B_ENSG00000141698
17	40021267	40021590	5Y-H4K8ac_peak_7301	7.90236	KLHL11_ENSG00000178502
17	40052419	40052706	5Y-H4K8ac_peak_7302	5.35202	
17	40118892	40119163	5Y-H4K8ac_peak_7303	4.15658	CNP_ENSG00000173786
17	40168997	40169392	5Y-H4K8ac_peak_7304	13.45283	
17	40202241	40203081	5Y-H4K8ac_peak_7305	9.93099	
17	40250213	40250481	5Y-H4K8ac_peak_7306	9.33466	
17	40306606	40306908	5Y-H4K8ac_peak_7307	9.05168	CTD-2132N18.3_ENSG00000267261;RAB5C_ENSG00000108774;RP11-358B23.1_ENSG00000267658
17	40307368	40307657	5Y-H4K8ac_peak_7308	6.31818	CTD-2132N18.3_ENSG00000267261;RAB5C_ENSG00000108774;RP11-358B23.1_ENSG00000267658
17	40428119	40428395	5Y-H4K8ac_peak_7309	7.30348	STAT5B_ENSG00000173757
17	40428593	40429105	5Y-H4K8ac_peak_7310	13.61292	STAT5B_ENSG00000173757
17	40463320	40463680	5Y-H4K8ac_peak_7311	8.564	
17	40688330	40688658	5Y-H4K8ac_peak_7312	12.69256	RP11-400F19.8_ENSG00000266929

17	40713343	40713852	5Y-H4K8ac_peak_7313	8.2913	COASY_ENSG00000068120
17	40718568	40718800	5Y-H4K8ac_peak_7314	5.98695	MLX_ENSG00000108788
17	40830679	40832518	5Y-H4K8ac_peak_7315	11.01287	CTD-3193K9.4_ENSG00000267042
17	40834271	40834497	5Y-H4K8ac_peak_7316	5.47303	CNTNAP1_ENSG00000108797
17	40896501	40896937	5Y-H4K8ac_peak_7317	7.80156	EZH1_ENSG00000108799
17	40897672	40898065	5Y-H4K8ac_peak_7318	4.71803	EZH1_ENSG00000108799
17	40912771	40913717	5Y-H4K8ac_peak_7319	13.00733	RAMP2-AS1_ENSG00000197291
17	40950935	40951185	5Y-H4K8ac_peak_7320	8.4454	COA3_ENSG00000183978;CNTD1_ENSG00000176563
17	40976462	40976967	5Y-H4K8ac_peak_7321	13.85283	PSME3_ENSG00000131467
17	41116753	41116965	5Y-H4K8ac_peak_7322	4.79585	AARSD1_ENSG00000266967
17	41173107	41173517	5Y-H4K8ac_peak_7323	3.96434	
17	41392478	41392720	5Y-H4K8ac_peak_7324	8.47164	
17	41411402	41411672	5Y-H4K8ac_peak_7325	4.07874	
17	41437214	41437482	5Y-H4K8ac_peak_7326	5.8635	
17	41439417	41439666	5Y-H4K8ac_peak_7327	7.38046	
17	41445881	41446078	5Y-H4K8ac_peak_7328	6.27097	
17	41446426	41447116	5Y-H4K8ac_peak_7329	5.72233	
17	41560664	41560865	5Y-H4K8ac_peak_7330	7.87406	DHX8_ENSG00000067596
17	41669626	41669896	5Y-H4K8ac_peak_7331	5.91107	
17	41755041	41755313	5Y-H4K8ac_peak_7332	9.7353	
17	41790993	41791608	5Y-H4K8ac_peak_7333	6.08523	
17	41797759	41798210	5Y-H4K8ac_peak_7334	13.2534	RP11-209M4.1_ENSG00000267253
17	41855939	41856203	5Y-H4K8ac_peak_7335	10.31981	DUSP3_ENSG00000108861
17	42051016	42051224	5Y-H4K8ac_peak_7336	5.42666	
17	42060970	42061641	5Y-H4K8ac_peak_7337	11.53136	
17	42072042	42072341	5Y-H4K8ac_peak_7338	10.16277	
17	42091871	42092300	5Y-H4K8ac_peak_7339	17.50049	
17	42143695	42143942	5Y-H4K8ac_peak_7340	8.35139	
17	42144310	42144647	5Y-H4K8ac_peak_7341	11.56948	LSM12_ENSG00000161654
17	42147483	42147732	5Y-H4K8ac_peak_7342	8.2913	G6PC3_ENSG00000141349
17	42148356	42148576	5Y-H4K8ac_peak_7343	5.64909	G6PC3_ENSG00000141349
17	42173178	42173577	5Y-H4K8ac_peak_7344	10.46135	
17	42218847	42219047	5Y-H4K8ac_peak_7345	6.78318	C17orf53_ENSG00000125319
17	42277851	42278279	5Y-H4K8ac_peak_7346	18.63671	ATXN7L3_ENSG00000087152
17	42295399	42295791	5Y-H4K8ac_peak_7347	5.13361	
17	42296709	42296940	5Y-H4K8ac_peak_7348	9.00954	
17	42462965	42463515	5Y-H4K8ac_peak_7349	8.69112	
17	42685190	42687194	5Y-H4K8ac_peak_7350	9.23159	
17	42766848	42767057	5Y-H4K8ac_peak_7351	7.87192	CCDC43_ENSG00000180329
17	42835933	42836317	5Y-H4K8ac_peak_7352	8.69112	ADAM11_ENSG00000073670
17	42857026	42857309	5Y-H4K8ac_peak_7353	4.55128	
17	42906947	42907556	5Y-H4K8ac_peak_7354	8.1667	GJC1_ENSG00000182963
17	42976843	42977222	5Y-H4K8ac_peak_7355	8.2913	EFTUD2_ENSG00000108883;CCDC103_ENSG00000167131;FAM187A_ENSG00000214447
17	43045075	43045551	5Y-H4K8ac_peak_7356	7.61752	C1QL1_ENSG00000131094
17	43047482	43047893	5Y-H4K8ac_peak_7357	4.77126	
17	43108055	43108350	5Y-H4K8ac_peak_7358	4.15658	

17	43109683	43109967	5Y-H4K8ac_peak_7359	9.30206	
17	43127330	43127877	5Y-H4K8ac_peak_7360	7.9215	
17	43213093	43213393	5Y-H4K8ac_peak_7361	4.15658	
17	43224515	43225336	5Y-H4K8ac_peak_7362	7.89273	HEXIM1_ENSG00000186834
17	43238456	43238843	5Y-H4K8ac_peak_7363	10.98149	AC002117.1_ENSG00000224505;HEXIM2_ENSG00000168517
17	43239422	43240138	5Y-H4K8ac_peak_7364	7.24967	AC002117.1_ENSG00000224505
17	43249899	43250437	5Y-H4K8ac_peak_7365	9.30505	RP13-890H12.2_ENSG00000267288
17	43274444	43274853	5Y-H4K8ac_peak_7366	5.91107	
17	43318454	43319060	5Y-H4K8ac_peak_7367	10.16277	CTD-2020K17.3_ENSG00000233175
17	43325411	43325751	5Y-H4K8ac_peak_7368	8.73392	CTD-2020K17.4_ENSG00000233483;MAP3K14-AS1_ENSG00000267278
17	43365864	43366285	5Y-H4K8ac_peak_7369	9.89244	
17	43367349	43367592	5Y-H4K8ac_peak_7370	8.43999	
17	43371266	43371512	5Y-H4K8ac_peak_7371	10.11191	
17	43394097	43394359	5Y-H4K8ac_peak_7372	7.56545	MAP3K14_ENSG00000006062
17	43449056	43449276	5Y-H4K8ac_peak_7373	5.91107	CTB-39G8.2_ENSG00000267446
17	43482382	43483399	5Y-H4K8ac_peak_7374	10.35586	
17	43508624	43509004	5Y-H4K8ac_peak_7375	4.79658	
17	43514246	43514732	5Y-H4K8ac_peak_7376	20.27109	
17	43661815	43662877	5Y-H4K8ac_peak_7377	5.78084	DND1P1_ENSG00000264070
17	43663942	43664153	5Y-H4K8ac_peak_7378	8.27643	DND1P1_ENSG00000264070
17	43697915	43698123	5Y-H4K8ac_peak_7379	6.46053	CRHR1-IT1_ENSG00000204650
17	43861722	43861921	5Y-H4K8ac_peak_7380	6.43068	
17	43862660	43862920	5Y-H4K8ac_peak_7381	4.15658	
17	43886498	43886711	5Y-H4K8ac_peak_7382	4.0639	
17	43892452	43892797	5Y-H4K8ac_peak_7383	8.3285	
17	43926156	43926974	5Y-H4K8ac_peak_7384	15.93748	
17	43937835	43938098	5Y-H4K8ac_peak_7385	5.67483	
17	43938336	43938577	5Y-H4K8ac_peak_7386	4.642	
17	43971330	43971602	5Y-H4K8ac_peak_7387	10.90097	MAPT_ENSG00000186868
17	44270208	44270759	5Y-H4K8ac_peak_7388	33.58964	KANSL1-AS1_ENSG00000214401
17	44271040	44271273	5Y-H4K8ac_peak_7389	6.78128	KANSL1-AS1_ENSG00000214401
17	44302303	44302629	5Y-H4K8ac_peak_7390	7.90751	KANSL1_ENSG00000120071
17	44343754	44344114	5Y-H4K8ac_peak_7391	10.15788	RP11-259G18.1_ENSG00000261575
17	44848059	44848994	5Y-H4K8ac_peak_7392	14.89891	
17	44849343	44849893	5Y-H4K8ac_peak_7393	11.60058	
17	45000320	45000840	5Y-H4K8ac_peak_7394	14.43262	RP11-63A1.1_ENSG00000261886;GOSR2_ENSG00000108433;RP11-156P1.2_ENSG00000262633
17	45079207	45079425	5Y-H4K8ac_peak_7395	10.93537	
17	45144655	45144862	5Y-H4K8ac_peak_7396	12.73673	
17	45177207	45177599	5Y-H4K8ac_peak_7397	10.35586	RP11-156P1.3_ENSG00000262879
17	45352804	45353110	5Y-H4K8ac_peak_7398	6.77436	
17	45400777	45401202	5Y-H4K8ac_peak_7399	8.69112	RP11-290H9.4_ENSG00000263293;EFCAB13_ENSG00000178852
17	45570294	45570631	5Y-H4K8ac_peak_7400	8.69895	MRPL45P2_ENSG00000228782
17	45607801	45608063	5Y-H4K8ac_peak_7401	7.59101	
17	45726608	45726836	5Y-H4K8ac_peak_7402	6.37023	RP11-580I16.2_ENSG00000263766;KPNB1_ENSG00000108424
17	45727651	45727862	5Y-H4K8ac_peak_7403	11.09973	RP11-580I16.2_ENSG00000263766;KPNB1_ENSG00000108424
17	45728204	45728412	5Y-H4K8ac_peak_7404	10.1994	

17	45804277	45804536	5Y-H4K8ac_peak_7405	9.74838	
17	45866911	45867778	5Y-H4K8ac_peak_7406	7.64648	
17	45898741	45898948	5Y-H4K8ac_peak_7407	6.59112	OSBPL7_ENSG00000006025
17	45918134	45918356	5Y-H4K8ac_peak_7408	3.93392	SCRN2_ENSG00000141295
17	45918548	45919263	5Y-H4K8ac_peak_7409	13.60583	SCRN2_ENSG00000141295
17	45972704	45972912	5Y-H4K8ac_peak_7410	3.96434	RP11-6N17.4_ENSG00000264920;SP2_ENSG00000167182
17	45973609	45974163	5Y-H4K8ac_peak_7411	20.47833	RP11-6N17.4_ENSG00000264920;SP2_ENSG00000167182
17	46018022	46018726	5Y-H4K8ac_peak_7412	15.17389	AC003665.1_ENSG00000234494;PNPO_ENSG00000108439
17	46048525	46048900	5Y-H4K8ac_peak_7413	10.72427	
17	46059373	46059695	5Y-H4K8ac_peak_7414	10.68758	
17	46059920	46060887	5Y-H4K8ac_peak_7415	6.37023	
17	46064407	46065761	5Y-H4K8ac_peak_7416	12.93132	
17	46068441	46069127	5Y-H4K8ac_peak_7417	14.99673	
17	46072240	46072437	5Y-H4K8ac_peak_7418	8.73392	
17	46073451	46073748	5Y-H4K8ac_peak_7419	13.29549	RP11-6N17.10_ENSG00000264701
17	46074008	46074324	5Y-H4K8ac_peak_7420	10.60083	RP11-6N17.10_ENSG00000264701
17	46079282	46080084	5Y-H4K8ac_peak_7421	9.00954	
17	46080471	46081152	5Y-H4K8ac_peak_7422	8.3285	
17	46081862	46082165	5Y-H4K8ac_peak_7423	12.06963	
17	46082583	46082808	5Y-H4K8ac_peak_7424	7.49539	
17	46088812	46089464	5Y-H4K8ac_peak_7425	21.56204	
17	46089944	46090791	5Y-H4K8ac_peak_7426	16.71698	
17	46091299	46091986	5Y-H4K8ac_peak_7427	3.96434	
17	46092610	46092804	5Y-H4K8ac_peak_7428	4.48815	
17	46125205	46125400	5Y-H4K8ac_peak_7429	8.3285	RP5-890E16.2_ENSG00000263412;NFE2L1_ENSG00000082641
17	46125746	46125936	5Y-H4K8ac_peak_7430	11.94897	RP5-890E16.2_ENSG00000263412;NFE2L1_ENSG00000082641
17	46178529	46178765	5Y-H4K8ac_peak_7431	10.90097	CBX1_ENSG00000108468
17	46185376	46185629	5Y-H4K8ac_peak_7432	10.73603	
17	46908627	46909019	5Y-H4K8ac_peak_7433	7.21591	CALCOCO2_ENSG00000136436
17	46969256	46969591	5Y-H4K8ac_peak_7434	8.56121	ATP5G1_ENSG00000159199
17	46986005	46986198	5Y-H4K8ac_peak_7435	10.1994	SUMO2P17_ENSG00000248278;UBE2Z_ENSG00000159202
17	47022284	47022556	5Y-H4K8ac_peak_7436	9.28683	SNF8_ENSG00000159210
17	47269628	47269832	5Y-H4K8ac_peak_7437	9.02938	
17	47438964	47439412	5Y-H4K8ac_peak_7438	15.80786	ZNF652_ENSG00000198740;RP11-1079K10.3_ENSG00000248714
17	47439884	47440212	5Y-H4K8ac_peak_7439	5.23083	ZNF652_ENSG00000198740
17	47532460	47532753	5Y-H4K8ac_peak_7440	10.31981	
17	47548690	47548996	5Y-H4K8ac_peak_7441	7.17527	
17	47549696	47550078	5Y-H4K8ac_peak_7442	8.47129	
17	47592617	47593199	5Y-H4K8ac_peak_7443	15.19225	
17	47596948	47597278	5Y-H4K8ac_peak_7444	8.41209	
17	47633260	47634073	5Y-H4K8ac_peak_7445	7.90751	
17	47785076	47785537	5Y-H4K8ac_peak_7446	16.93245	SLC35B1_ENSG00000121073;RP11-613C6.2_ENSG00000250751
17	47864968	47865608	5Y-H4K8ac_peak_7447	10.8904	FAM117A_ENSG00000121104;KAT7_ENSG00000136504
17	47866076	47866349	5Y-H4K8ac_peak_7448	15.48671	FAM117A_ENSG00000121104;KAT7_ENSG00000136504
17	48046117	48046402	5Y-H4K8ac_peak_7449	6.31818	DLX4_ENSG00000108813
17	48104575	48105013	5Y-H4K8ac_peak_7450	6.34245	

17	48134097	48134448	5Y-H4K8ac_peak_7451	7.27386	RP11-1094H24.4_ENSG00000246640;ITGA3_ENSG00000005884
17	48189240	48189668	5Y-H4K8ac_peak_7452	6.339	
17	48189976	48190570	5Y-H4K8ac_peak_7453	5.24695	
17	48206782	48207299	5Y-H4K8ac_peak_7454	10.07378	SAMD14_ENSG00000167100
17	48207595	48207839	5Y-H4K8ac_peak_7455	9.3585	SAMD14_ENSG00000167100
17	48228709	48229084	5Y-H4K8ac_peak_7456	8.33454	PPP1R9B_ENSG00000108819
17	48229573	48230380	5Y-H4K8ac_peak_7457	17.67055	
17	48239286	48239562	5Y-H4K8ac_peak_7458	12.79198	RP11-893F2.13_ENSG00000253730
17	48250375	48250866	5Y-H4K8ac_peak_7459	8.51726	
17	48251106	48251388	5Y-H4K8ac_peak_7460	6.62037	
17	48254421	48254649	5Y-H4K8ac_peak_7461	5.22001	
17	48255211	48256316	5Y-H4K8ac_peak_7462	17.13377	
17	48260706	48261113	5Y-H4K8ac_peak_7463	4.25295	
17	48278356	48278588	5Y-H4K8ac_peak_7464	4.642	COL1A1_ENSG00000108821
17	48285275	48285567	5Y-H4K8ac_peak_7465	6.96612	RP11-893F2.5_ENSG00000249406
17	48287253	48287470	5Y-H4K8ac_peak_7466	6.35459	RP11-893F2.5_ENSG00000249406
17	48287818	48288509	5Y-H4K8ac_peak_7467	9.13842	
17	48343847	48344070	5Y-H4K8ac_peak_7468	5.83797	
17	48350412	48350614	5Y-H4K8ac_peak_7469	10.75771	
17	48423018	48423209	5Y-H4K8ac_peak_7470	5.60566	XYLT2_ENSG00000015532
17	48423555	48423806	5Y-H4K8ac_peak_7471	5.22001	XYLT2_ENSG00000015532
17	48450292	48450857	5Y-H4K8ac_peak_7472	14.84178	MRPL27_ENSG00000108826;EME1_ENSG00000154920
17	48474573	48474812	5Y-H4K8ac_peak_7473	8.39119	LRRC59_ENSG00000108829;RP1-117B12.4_ENSG00000253102
17	48475097	48475540	5Y-H4K8ac_peak_7474	12.06963	LRRC59_ENSG00000108829
17	48502789	48503284	5Y-H4K8ac_peak_7475	15.82359	ACSF2_ENSG00000167107
17	48503596	48504056	5Y-H4K8ac_peak_7476	6.08523	ACSF2_ENSG00000167107
17	48555417	48556069	5Y-H4K8ac_peak_7477	7.89273	RSAD1_ENSG00000136444
17	48585282	48585878	5Y-H4K8ac_peak_7478	16.57564	RP11-94C24.6_ENSG00000249451;MYCBPAP_ENSG00000136449
17	48610174	48610425	5Y-H4K8ac_peak_7479	7.18391	EPN3_ENSG00000049283
17	48624565	48624947	5Y-H4K8ac_peak_7480	12.93794	
17	48634036	48634270	5Y-H4K8ac_peak_7481	7.8267	
17	48638558	48638796	5Y-H4K8ac_peak_7482	6.53157	CACNA1G-AS1_ENSG00000250107;CACNA1G_ENSG00000006283
17	48641964	48643247	5Y-H4K8ac_peak_7483	13.92877	RP11-94C24.11_ENSG00000250976
17	48647112	48647393	5Y-H4K8ac_peak_7484	5.69194	
17	48670470	48670705	5Y-H4K8ac_peak_7485	7.96285	
17	48679294	48679946	5Y-H4K8ac_peak_7486	10.86648	
17	48681271	48681853	5Y-H4K8ac_peak_7487	9.68742	
17	48683019	48683547	5Y-H4K8ac_peak_7488	6.16949	
17	48686424	48686828	5Y-H4K8ac_peak_7489	7.44077	
17	48703582	48703913	5Y-H4K8ac_peak_7490	6.10343	
17	48705573	48705829	5Y-H4K8ac_peak_7491	4.0639	
17	48737668	48737869	5Y-H4K8ac_peak_7492	5.60566	
17	48767730	48767991	5Y-H4K8ac_peak_7493	13.10899	
17	48784747	48785241	5Y-H4K8ac_peak_7494	14.19462	ANKRD40_ENSG00000154945
17	48786416	48786823	5Y-H4K8ac_peak_7495	4.77126	
17	48796264	48796740	5Y-H4K8ac_peak_7496	7.89273	LUC7L3_ENSG00000108848

17	48797058	48797435	5Y-H4K8ac_peak_7497	11.67326	LUC7L3_ENSG00000108848
17	48910760	48911010	5Y-H4K8ac_peak_7498	6.98416	
17	48911406	48911604	5Y-H4K8ac_peak_7499	7.07378	WFIKKN2_ENSG00000173714
17	48944135	48944513	5Y-H4K8ac_peak_7500	10.72427	TOB1_ENSG00000141232;TOB1-AS1_ENSG00000229980
17	48982077	48983095	5Y-H4K8ac_peak_7501	10.98149	
17	48995921	48997029	5Y-H4K8ac_peak_7502	12.708	
17	48997404	48997696	5Y-H4K8ac_peak_7503	9.11686	
17	49007216	49007536	5Y-H4K8ac_peak_7504	6.03632	
17	49008317	49009242	5Y-H4K8ac_peak_7505	21.45803	
17	49021416	49021955	5Y-H4K8ac_peak_7506	12.79198	RP11-700H6.1_ENSG00000247011;RP11-700H6.2_ENSG00000251665
17	49025449	49025687	5Y-H4K8ac_peak_7507	7.91521	
17	49026968	49027557	5Y-H4K8ac_peak_7508	6.17806	
17	49198635	49198837	5Y-H4K8ac_peak_7509	7.11863	SPAG9_ENSG00000008294
17	49199233	49199493	5Y-H4K8ac_peak_7510	7.59101	
17	49229718	49229950	5Y-H4K8ac_peak_7511	8.24461	NME1_ENSG00000239672;NME1-NME2_ENSG00000243678
17	49230977	49231392	5Y-H4K8ac_peak_7512	8.62008	NME1_ENSG00000239672;NME1-NME2_ENSG00000243678;NME2_ENSG00000011052
17	49244266	49244503	5Y-H4K8ac_peak_7513	8.06489	
17	49432510	49433021	5Y-H4K8ac_peak_7514	6.03632	
17	53046322	53046596	5Y-H4K8ac_peak_7515	6.73385	COX11_ENSG00000166260;STXBP4_ENSG00000166263
17	53828376	53828843	5Y-H4K8ac_peak_7516	13.59183	PCTP_ENSG00000141179
17	54911516	54911740	5Y-H4K8ac_peak_7517	8.62703	DGKE_ENSG00000153933
17	54991544	54991769	5Y-H4K8ac_peak_7518	15.41988	TRIM25_ENSG00000121060
17	55037032	55037584	5Y-H4K8ac_peak_7519	7.47209	COIL_ENSG00000121058
17	55038111	55038367	5Y-H4K8ac_peak_7520	14.86775	COIL_ENSG00000121058
17	55162685	55162912	5Y-H4K8ac_peak_7521	9.02938	RP11-166P13.3_ENSG00000263004;AKAP1_ENSG00000121057
17	55213480	55213707	5Y-H4K8ac_peak_7522	4.95697	
17	55223839	55224141	5Y-H4K8ac_peak_7523	7.89273	
17	55333109	55333599	5Y-H4K8ac_peak_7524	10.21117	MSI2_ENSG00000153944
17	55498742	55498965	5Y-H4K8ac_peak_7525	8.24461	
17	55515415	55515612	5Y-H4K8ac_peak_7526	5.87725	
17	55520630	55520964	5Y-H4K8ac_peak_7527	7.6267	
17	55573849	55574075	5Y-H4K8ac_peak_7528	6.37023	
17	55583875	55584158	5Y-H4K8ac_peak_7529	8.24461	
17	55586014	55586329	5Y-H4K8ac_peak_7530	8.69112	
17	55588449	55588660	5Y-H4K8ac_peak_7531	5.99504	
17	55603383	55603613	5Y-H4K8ac_peak_7532	5.57299	
17	55614365	55614866	5Y-H4K8ac_peak_7533	8.3285	
17	55615130	55615709	5Y-H4K8ac_peak_7534	10.86648	
17	55619978	55620283	5Y-H4K8ac_peak_7535	9.58986	
17	55620692	55620889	5Y-H4K8ac_peak_7536	10.8904	
17	55622668	55622870	5Y-H4K8ac_peak_7537	6.78128	
17	55623929	55624591	5Y-H4K8ac_peak_7538	16.73191	
17	55628632	55628908	5Y-H4K8ac_peak_7539	8.1667	
17	55629274	55629543	5Y-H4K8ac_peak_7540	4.95697	
17	55636134	55636331	5Y-H4K8ac_peak_7541	4.92207	
17	55636596	55637224	5Y-H4K8ac_peak_7542	4.97086	

17	55639833	55640272	5Y-H4K8ac_peak_7543	6.97863	
17	55641190	55641381	5Y-H4K8ac_peak_7544	5.41472	
17	55642647	55642859	5Y-H4K8ac_peak_7545	5.24695	
17	55643194	55643399	5Y-H4K8ac_peak_7546	8.03282	
17	55643937	55644364	5Y-H4K8ac_peak_7547	6.4298	
17	55644958	55645588	5Y-H4K8ac_peak_7548	12.26819	
17	55661026	55661427	5Y-H4K8ac_peak_7549	9.61943	
17	55662028	55662257	5Y-H4K8ac_peak_7550	4.642	
17	55663006	55663295	5Y-H4K8ac_peak_7551	4.75174	
17	55664008	55664710	5Y-H4K8ac_peak_7552	10.4495	
17	55665346	55665594	5Y-H4K8ac_peak_7553	8.90224	
17	55671300	55671797	5Y-H4K8ac_peak_7554	5.98695	
17	55673288	55673957	5Y-H4K8ac_peak_7555	8.24461	
17	55674230	55674522	5Y-H4K8ac_peak_7556	9.31608	
17	55676032	55677245	5Y-H4K8ac_peak_7557	16.47427	
17	55680443	55680876	5Y-H4K8ac_peak_7558	7.50148	
17	55682630	55682988	5Y-H4K8ac_peak_7559	7.83354	
17	55685529	55685926	5Y-H4K8ac_peak_7560	5.24064	RP11-118E18.4_ENSG00000263499
17	55686957	55687179	5Y-H4K8ac_peak_7561	4.31838	
17	55699422	55699630	5Y-H4K8ac_peak_7562	6.08523	
17	55701630	55702923	5Y-H4K8ac_peak_7563	8.62703	
17	55703215	55703728	5Y-H4K8ac_peak_7564	6.78128	
17	55713735	55714208	5Y-H4K8ac_peak_7565	9.07585	
17	55716655	55716941	5Y-H4K8ac_peak_7566	5.5369	
17	55717243	55717462	5Y-H4K8ac_peak_7567	6.13242	
17	55738888	55739357	5Y-H4K8ac_peak_7568	6.71016	
17	55742392	55742585	5Y-H4K8ac_peak_7569	7.72887	
17	55746483	55746782	5Y-H4K8ac_peak_7570	5.87725	
17	55747642	55747858	5Y-H4K8ac_peak_7571	7.21591	
17	55777000	55777268	5Y-H4K8ac_peak_7572	9.69677	
17	55785323	55785544	5Y-H4K8ac_peak_7573	4.50834	
17	55817651	55817879	5Y-H4K8ac_peak_7574	6.08523	
17	55828009	55828600	5Y-H4K8ac_peak_7575	5.12488	
17	55829123	55829479	5Y-H4K8ac_peak_7576	11.22005	
17	55859651	55860645	5Y-H4K8ac_peak_7577	10.31072	
17	55887371	55887662	5Y-H4K8ac_peak_7578	7.18391	
17	55888300	55888639	5Y-H4K8ac_peak_7579	9.93175	
17	55981952	55982208	5Y-H4K8ac_peak_7580	6.78128	
17	55982750	55982999	5Y-H4K8ac_peak_7581	11.1169	
17	55993502	55994015	5Y-H4K8ac_peak_7582	6.86362	
17	56031997	56032383	5Y-H4K8ac_peak_7583	9.00954	CUEDC1_ENSG00000180891
17	56065189	56066106	5Y-H4K8ac_peak_7584	23.60332	VEZF1_ENSG00000136451
17	56070562	56070769	5Y-H4K8ac_peak_7585	4.92498	
17	56084037	56084561	5Y-H4K8ac_peak_7586	13.48948	SRSF1_ENSG00000136450
17	56160038	56160575	5Y-H4K8ac_peak_7587	32.18204	RP11-159D12.10_ENSG00000266290;DYNLL2_ENSG00000264364
17	56296937	56297269	5Y-H4K8ac_peak_7588	5.12488	MKS1_ENSG00000011143

17	56401370	56402561	5Y-H4K8ac_peak_7589	17.87582	BZRAP1-AS1_ENSG00000265148
17	56429701	56430012	5Y-H4K8ac_peak_7590	7.51382	SUPT4H1_ENSG00000213246
17	56591676	56591915	5Y-H4K8ac_peak_7591	13.92877	
17	56594929	56595201	5Y-H4K8ac_peak_7592	9.68742	MTMR4_ENSG00000108389
17	56596314	56597020	5Y-H4K8ac_peak_7593	7.20869	RP11-112H10.4_ENSG00000264672
17	56709760	56710061	5Y-H4K8ac_peak_7594	6.96612	U3_ENSG00000212195
17	56769124	56769740	5Y-H4K8ac_peak_7595	9.89244	TEX14_ENSG00000121101;RAD51C_ENSG00000108384
17	56770013	56770247	5Y-H4K8ac_peak_7596	5.24695	TEX14_ENSG00000121101;RAD51C_ENSG00000108384
17	56833262	56833742	5Y-H4K8ac_peak_7597	11.71078	PPM1E_ENSG00000175175
17	56863451	56863678	5Y-H4K8ac_peak_7598	5.39991	
17	57092578	57092792	5Y-H4K8ac_peak_7599	6.27097	
17	57184478	57184681	5Y-H4K8ac_peak_7600	4.00285	TRIM37_ENSG00000108395;AC099850.1_ENSG00000224738
17	57231868	57232508	5Y-H4K8ac_peak_7601	8.69112	SKA2_ENSG00000182628;PRR11_ENSG00000068489
17	57287420	57287959	5Y-H4K8ac_peak_7602	9.02938	SMG8_ENSG00000167447
17	57296828	57297096	5Y-H4K8ac_peak_7603	5.449	GDPD1_ENSG00000153982
17	57405862	57406108	5Y-H4K8ac_peak_7604	7.2422	
17	57464033	57464251	5Y-H4K8ac_peak_7605	5.77617	
17	57465346	57465639	5Y-H4K8ac_peak_7606	5.03917	
17	57484753	57485102	5Y-H4K8ac_peak_7607	11.8592	
17	57562000	57562213	5Y-H4K8ac_peak_7608	5.21093	
17	57591121	57591481	5Y-H4K8ac_peak_7609	6.15578	
17	57592286	57592480	5Y-H4K8ac_peak_7610	8.07508	
17	57623783	57624075	5Y-H4K8ac_peak_7611	10.37708	
17	57625429	57625639	5Y-H4K8ac_peak_7612	5.77617	
17	57696706	57697564	5Y-H4K8ac_peak_7613	9.79526	CLTC_ENSG00000141367
17	57784191	57784556	5Y-H4K8ac_peak_7614	7.50148	PTRH2_ENSG00000141378;VMP1_ENSG00000062716
17	57970532	57970884	5Y-H4K8ac_peak_7615	8.99358	TUBD1_ENSG00000108423;RPS6KB1_ENSG00000108443
17	58042610	58042904	5Y-H4K8ac_peak_7616	9.51254	RNFT1_ENSG00000189050;RP11-178C3.2_ENSG00000267302
17	58469839	58470293	5Y-H4K8ac_peak_7617	9.51254	C17orf64_ENSG00000141371
17	58498852	58499274	5Y-H4K8ac_peak_7618	19.26009	USP32_ENSG00000170832
17	58603316	58603525	5Y-H4K8ac_peak_7619	10.12457	APPBP2_ENSG00000062725;RP11-15E18.1_ENSG00000259349
17	58677059	58677363	5Y-H4K8ac_peak_7620	12.00715	PPM1D_ENSG00000170836
17	59223335	59223618	5Y-H4K8ac_peak_7621	9.38276	
17	59252345	59252625	5Y-H4K8ac_peak_7622	9.96543	
17	59272850	59273118	5Y-H4K8ac_peak_7623	6.77436	
17	59391404	59391853	5Y-H4K8ac_peak_7624	5.88408	
17	59458616	59458825	5Y-H4K8ac_peak_7625	7.64648	
17	59459065	59459529	5Y-H4K8ac_peak_7626	7.25122	RP11-332H18.3_ENSG00000266934
17	59460156	59461029	5Y-H4K8ac_peak_7627	12.15235	RP11-332H18.3_ENSG00000266934
17	59461242	59462252	5Y-H4K8ac_peak_7628	25.65133	
17	59462843	59463165	5Y-H4K8ac_peak_7629	14.82927	
17	59463545	59464057	5Y-H4K8ac_peak_7630	11.69482	
17	59464457	59464877	5Y-H4K8ac_peak_7631	7.20869	
17	59468992	59469252	5Y-H4K8ac_peak_7632	7.89273	
17	59473835	59475107	5Y-H4K8ac_peak_7633	10.30425	
17	59475454	59475682	5Y-H4K8ac_peak_7634	6.08523	

17	59476343	59476884	5Y-H4K8ac_peak_7635	9.76673	RP11-332H18.5_ENSG00000267131;TBX2_ENSG00000121068
17	59487713	59488089	5Y-H4K8ac_peak_7636	4.15658	RP11-332H18.4_ENSG00000267280
17	59488600	59489683	5Y-H4K8ac_peak_7637	11.79142	RP11-332H18.4_ENSG00000267280;C17orf82_ENSG00000187013
17	59490016	59490635	5Y-H4K8ac_peak_7638	10.48476	C17orf82_ENSG00000187013
17	59491615	59492122	5Y-H4K8ac_peak_7639	6.27435	
17	59493528	59494228	5Y-H4K8ac_peak_7640	13.33225	
17	59540013	59540521	5Y-H4K8ac_peak_7641	4.00285	RP11-15K2.2_ENSG00000267137
17	59553983	59554188	5Y-H4K8ac_peak_7642	4.55128	
17	59554923	59555119	5Y-H4K8ac_peak_7643	10.35586	
17	59562663	59563094	5Y-H4K8ac_peak_7644	8.62703	
17	59563582	59564230	5Y-H4K8ac_peak_7645	9.64738	
17	59565326	59565766	5Y-H4K8ac_peak_7646	22.81411	
17	59573926	59574252	5Y-H4K8ac_peak_7647	5.64909	
17	59628911	59629338	5Y-H4K8ac_peak_7648	7.31102	
17	60004874	60005271	5Y-H4K8ac_peak_7649	16.23405	INTS2_ENSG00000108506
17	60215502	60216480	5Y-H4K8ac_peak_7650	9.22492	
17	60501419	60501821	5Y-H4K8ac_peak_7651	6.53157	METTL2A_ENSG00000087995
17	60549502	60549751	5Y-H4K8ac_peak_7652	5.5592	
17	60555337	60555914	5Y-H4K8ac_peak_7653	7.89273	
17	60556168	60556928	5Y-H4K8ac_peak_7654	16.15771	
17	60706395	60706668	5Y-H4K8ac_peak_7655	6.88736	
17	60729588	60730615	5Y-H4K8ac_peak_7656	15.80786	
17	60781375	60781583	5Y-H4K8ac_peak_7657	7.33686	
17	60781865	60782087	5Y-H4K8ac_peak_7658	11.79376	
17	60827310	60827809	5Y-H4K8ac_peak_7659	7.11863	
17	60948204	60948399	5Y-H4K8ac_peak_7660	7.387	
17	60975870	60976088	5Y-H4K8ac_peak_7661	8.31536	
17	61000366	61000723	5Y-H4K8ac_peak_7662	10.35586	
17	61143359	61143576	5Y-H4K8ac_peak_7663	5.23083	
17	61296878	61297075	5Y-H4K8ac_peak_7664	5.37137	
17	61503429	61503804	5Y-H4K8ac_peak_7665	6.06178	
17	61505840	61506095	5Y-H4K8ac_peak_7666	4.0639	
17	61508352	61508561	5Y-H4K8ac_peak_7667	5.87725	RP11-269G24.3_ENSG00000263644
17	61509978	61510619	5Y-H4K8ac_peak_7668	11.21854	RP11-269G24.4_ENSG00000265282
17	61510895	61511436	5Y-H4K8ac_peak_7669	10.54669	
17	61511717	61512083	5Y-H4K8ac_peak_7670	5.51759	
17	61512345	61512854	5Y-H4K8ac_peak_7671	10.48476	
17	61513361	61513752	5Y-H4K8ac_peak_7672	7.38868	
17	61515995	61516664	5Y-H4K8ac_peak_7673	11.94533	
17	61517588	61519191	5Y-H4K8ac_peak_7674	15.90416	
17	61519483	61520088	5Y-H4K8ac_peak_7675	9.87097	
17	61520535	61521167	5Y-H4K8ac_peak_7676	7.50148	
17	61522114	61522729	5Y-H4K8ac_peak_7677	11.40596	
17	61522962	61525267	5Y-H4K8ac_peak_7678	25.44315	CYB561_ENSG00000008283
17	61527788	61527983	5Y-H4K8ac_peak_7679	6.55841	
17	61532095	61532540	5Y-H4K8ac_peak_7680	5.51759	RP11-269G24.6_ENSG00000265971

17	61534052	61534259	5Y-H4K8ac_peak_7681	6.78318	
17	61553237	61553622	5Y-H4K8ac_peak_7682	6.98416	ACE_ENSG00000159640
17	61554019	61555591	5Y-H4K8ac_peak_7683	9.32595	ACE_ENSG00000159640
17	61558750	61559031	5Y-H4K8ac_peak_7684	6.20944	
17	61615229	61615457	5Y-H4K8ac_peak_7685	10.18556	
17	61619275	61619481	5Y-H4K8ac_peak_7686	5.72376	
17	61627135	61627345	5Y-H4K8ac_peak_7687	8.09118	DCAF7_ENSG00000136485
17	61678271	61678788	5Y-H4K8ac_peak_7688	10.7427	TACO1_ENSG00000136463
17	61698984	61699262	5Y-H4K8ac_peak_7689	13.60583	MAP3K3_ENSG00000198909
17	61776618	61777096	5Y-H4K8ac_peak_7690	12.29582	
17	61777296	61778536	5Y-H4K8ac_peak_7691	26.34191	LIMD2_ENSG00000136490
17	61851686	61851970	5Y-H4K8ac_peak_7692	12.87247	DDX42_ENSG00000198231
17	61904845	61905236	5Y-H4K8ac_peak_7693	8.24461	PSMC5_ENSG00000087191
17	61919621	61919995	5Y-H4K8ac_peak_7694	8.54067	SMARCD2_ENSG00000108604
17	61920562	61921140	5Y-H4K8ac_peak_7695	8.84335	SMARCD2_ENSG00000108604
17	61959356	61959549	5Y-H4K8ac_peak_7696	4.9885	GH2_ENSG00000136487
17	61966601	61966808	5Y-H4K8ac_peak_7697	5.40331	
17	62207789	62208002	5Y-H4K8ac_peak_7698	6.37023	ERN1_ENSG00000178607
17	62255499	62255760	5Y-H4K8ac_peak_7699	8.79399	
17	62256146	62256356	5Y-H4K8ac_peak_7700	4.642	
17	62293891	62294330	5Y-H4K8ac_peak_7701	7.15925	
17	62294614	62294927	5Y-H4K8ac_peak_7702	5.98695	
17	62340836	62341388	5Y-H4K8ac_peak_7703	8.69112	TEX2_ENSG00000136478
17	62493399	62493775	5Y-H4K8ac_peak_7704	5.23083	POLG2_ENSG00000256525
17	62501731	62501993	5Y-H4K8ac_peak_7705	5.24695	CEP95_ENSG00000258890
17	62503159	62503493	5Y-H4K8ac_peak_7706	6.66983	CEP95_ENSG00000258890;DDX5_ENSG00000108654
17	62658048	62658401	5Y-H4K8ac_peak_7707	17.15332	SMURF2_ENSG00000108854
17	62772479	62772939	5Y-H4K8ac_peak_7708	14.70562	
17	62778002	62778314	5Y-H4K8ac_peak_7709	10.21121	hsa-mir-6080_ENSG00000215769
17	62915758	62916436	5Y-H4K8ac_peak_7710	11.35213	LRRC37A3_ENSG00000176809
17	62962009	62962227	5Y-H4K8ac_peak_7711	9.36633	
17	62971236	62971592	5Y-H4K8ac_peak_7712	5.60566	AMZ2P1_ENSG00000214174
17	63052513	63052858	5Y-H4K8ac_peak_7713	13.00733	GNA13_ENSG00000120063
17	63053083	63053832	5Y-H4K8ac_peak_7714	10.14791	GNA13_ENSG00000120063
17	63075836	63076086	5Y-H4K8ac_peak_7715	7.87406	
17	63118828	63119509	5Y-H4K8ac_peak_7716	7.50148	
17	63119838	63120090	5Y-H4K8ac_peak_7717	8.1667	
17	63133523	63133733	5Y-H4K8ac_peak_7718	8.36588	RGS9_ENSG00000108370
17	63170113	63170432	5Y-H4K8ac_peak_7719	5.37237	
17	63175823	63176015	5Y-H4K8ac_peak_7720	7.16761	
17	63179776	63180034	5Y-H4K8ac_peak_7721	6.95844	
17	63198930	63199121	5Y-H4K8ac_peak_7722	5.49775	
17	63290062	63290395	5Y-H4K8ac_peak_7723	6.81557	
17	63435186	63435385	5Y-H4K8ac_peak_7724	5.99504	
17	63556709	63556984	5Y-H4K8ac_peak_7725	5.59311	AXIN2_ENSG00000168646
17	64244238	64244438	5Y-H4K8ac_peak_7726	6.5814	

17	64298082	64298362	5Y-H4K8ac_peak_7727	11.48691	PRKCA_ENSG00000154229
17	64401084	64401477	5Y-H4K8ac_peak_7728	6.37023	
17	64659948	64660615	5Y-H4K8ac_peak_7729	10.03452	
17	64769814	64770028	5Y-H4K8ac_peak_7730	5.14442	
17	64772058	64772488	5Y-H4K8ac_peak_7731	8.69112	
17	64830988	64831622	5Y-H4K8ac_peak_7732	11.69482	CACNG5_ENSG00000075429
17	64899942	64900196	5Y-H4K8ac_peak_7733	6.37023	
17	64900941	64901516	5Y-H4K8ac_peak_7734	7.95414	
17	64949301	64949655	5Y-H4K8ac_peak_7735	7.64648	
17	64950125	64950389	5Y-H4K8ac_peak_7736	4.642	
17	64960152	64960467	5Y-H4K8ac_peak_7737	9.02938	CACNG4_ENSG00000075461
17	64960785	64961153	5Y-H4K8ac_peak_7738	5.449	CACNG4_ENSG00000075461
17	64961353	64961653	5Y-H4K8ac_peak_7739	16.30551	CACNG4_ENSG00000075461
17	64961970	64962407	5Y-H4K8ac_peak_7740	6.67135	CACNG4_ENSG00000075461
17	64963424	64964389	5Y-H4K8ac_peak_7741	8.20773	
17	64965241	64966583	5Y-H4K8ac_peak_7742	17.0779	
17	64967170	64967379	5Y-H4K8ac_peak_7743	9.02938	
17	64968238	64969005	5Y-H4K8ac_peak_7744	19.41527	
17	64969486	64969980	5Y-H4K8ac_peak_7745	10.60083	
17	64970460	64970972	5Y-H4K8ac_peak_7746	8.56853	
17	64971792	64972387	5Y-H4K8ac_peak_7747	6.78128	
17	64973679	64974593	5Y-H4K8ac_peak_7748	12.99645	
17	64975024	64975244	5Y-H4K8ac_peak_7749	9.74838	
17	64979356	64979644	5Y-H4K8ac_peak_7750	5.03564	
17	64980956	64981277	5Y-H4K8ac_peak_7751	14.6127	
17	64982098	64982679	5Y-H4K8ac_peak_7752	14.68223	
17	64983778	64984841	5Y-H4K8ac_peak_7753	12.79198	
17	64988619	64988832	5Y-H4K8ac_peak_7754	4.97086	
17	64989062	64989287	5Y-H4K8ac_peak_7755	7.7384	
17	64991350	64991628	5Y-H4K8ac_peak_7756	5.37237	
17	65012548	65016520	5Y-H4K8ac_peak_7757	25.65408	
17	65017101	65018958	5Y-H4K8ac_peak_7758	12.77513	RP11-349A8.3_ENSG00000264491
17	65019773	65020027	5Y-H4K8ac_peak_7759	6.37023	
17	65021673	65021910	5Y-H4K8ac_peak_7760	4.642	
17	65118256	65118531	5Y-H4K8ac_peak_7761	8.04693	
17	65122639	65122914	5Y-H4K8ac_peak_7762	14.30055	
17	65147039	65147257	5Y-H4K8ac_peak_7763	5.56912	
17	65240782	65241232	5Y-H4K8ac_peak_7764	6.08523	HELZ_ENSG00000198265;RP11-401F2.3_ENSG00000266473
17	65241540	65242089	5Y-H4K8ac_peak_7765	6.08523	HELZ_ENSG00000198265;RP11-401F2.3_ENSG00000266473
17	65362339	65362655	5Y-H4K8ac_peak_7766	15.35687	PSMD12_ENSG00000197170
17	65373422	65373667	5Y-H4K8ac_peak_7767	12.14222	PITPNC1_ENSG00000154217
17	65434560	65434750	5Y-H4K8ac_peak_7768	5.30478	
17	65439227	65439556	5Y-H4K8ac_peak_7769	6.08523	
17	65524940	65525244	5Y-H4K8ac_peak_7770	15.92074	
17	65989396	65989600	5Y-H4K8ac_peak_7771	7.87406	C17orf58_ENSG00000186665
17	65990054	65990405	5Y-H4K8ac_peak_7772	9.74838	C17orf58_ENSG00000186665

17	66016301	66016520	5Y-H4K8ac_peak_7773	12.2918	
17	66031888	66032378	5Y-H4K8ac_peak_7774	12.15659	KPNA2_ENSG00000182481
17	66097969	66098179	5Y-H4K8ac_peak_7775	10.60083	AC145343.2_ENSG00000265055;LINC00674_ENSG00000237854
17	66197253	66197510	5Y-H4K8ac_peak_7776	7.72887	
17	66243513	66243954	5Y-H4K8ac_peak_7777	14.53477	RP11-147L13.2_ENSG00000265100;AMZ2_ENSG00000196704
17	66244250	66244552	5Y-H4K8ac_peak_7778	13.03153	RP11-147L13.2_ENSG00000265100;AMZ2_ENSG00000196704
17	66508656	66508919	5Y-H4K8ac_peak_7779	6.31818	PRKAR1A_ENSG00000108946
17	67323512	67323785	5Y-H4K8ac_peak_7780	9.7353	ABCA5_ENSG00000154265
17	67410080	67410359	5Y-H4K8ac_peak_7781	6.43775	MAP2K6_ENSG00000108984
17	68648123	68648334	5Y-H4K8ac_peak_7782	4.64156	
17	69283550	69283820	5Y-H4K8ac_peak_7783	5.59843	
17	70216503	70216697	5Y-H4K8ac_peak_7784	4.79585	
17	70374693	70374932	5Y-H4K8ac_peak_7785	6.78128	
17	70378217	70378423	5Y-H4K8ac_peak_7786	4.0639	
17	71007881	71008100	5Y-H4K8ac_peak_7787	7.65114	
17	71089145	71089374	5Y-H4K8ac_peak_7788	11.04356	SLC39A11_ENSG00000133195
17	71114786	71114991	5Y-H4K8ac_peak_7789	5.87725	
17	71130634	71130861	5Y-H4K8ac_peak_7790	7.89142	
17	71188129	71188321	5Y-H4K8ac_peak_7791	4.50834	COG1_ENSG00000166685
17	71189238	71189553	5Y-H4K8ac_peak_7792	5.87725	COG1_ENSG00000166685
17	71281186	71281701	5Y-H4K8ac_peak_7793	6.96612	
17	71306035	71306697	5Y-H4K8ac_peak_7794	13.92877	
17	71308372	71308604	5Y-H4K8ac_peak_7795	7.44077	CDC42EP4_ENSG00000179604
17	71337546	71337990	5Y-H4K8ac_peak_7796	11.67591	
17	71338396	71338883	5Y-H4K8ac_peak_7797	10.90097	
17	71339154	71340300	5Y-H4K8ac_peak_7798	8.6437	
17	71345119	71345845	5Y-H4K8ac_peak_7799	6.37023	
17	71352914	71353200	5Y-H4K8ac_peak_7800	11.88337	
17	71353904	71354181	5Y-H4K8ac_peak_7801	9.86146	
17	71365635	71366068	5Y-H4K8ac_peak_7802	4.642	
17	71471241	71471450	5Y-H4K8ac_peak_7803	6.03632	
17	71488242	71488871	5Y-H4K8ac_peak_7804	10.0444	
17	71490316	71490628	5Y-H4K8ac_peak_7805	8.27643	
17	71529134	71529533	5Y-H4K8ac_peak_7806	7.8267	
17	71715246	71715943	5Y-H4K8ac_peak_7807	17.55091	
17	71716248	71716888	5Y-H4K8ac_peak_7808	12.10416	
17	71766412	71766731	5Y-H4K8ac_peak_7809	5.24695	
17	71948693	71949008	5Y-H4K8ac_peak_7810	5.12488	
17	72121520	72121936	5Y-H4K8ac_peak_7811	4.97006	
17	72143594	72143891	5Y-H4K8ac_peak_7812	5.64909	
17	72144099	72144352	5Y-H4K8ac_peak_7813	7.89273	
17	72178045	72178396	5Y-H4K8ac_peak_7814	5.71283	
17	72199926	72200205	5Y-H4K8ac_peak_7815	3.94247	RPL38_ENSG00000172809
17	72208990	72209468	5Y-H4K8ac_peak_7816	8.9562	CTD-2514K5.2_ENSG00000246731;TTYH2_ENSG00000141540
17	72210086	72210302	5Y-H4K8ac_peak_7817	7.65404	CTD-2514K5.2_ENSG00000246731;TTYH2_ENSG00000141540
17	72239195	72239639	5Y-H4K8ac_peak_7818	8.69112	

17	72240403	72240618	5Y-H4K8ac_peak_7819	5.27937	
17	72253514	72253748	5Y-H4K8ac_peak_7820	5.89113	
17	72321948	72322212	5Y-H4K8ac_peak_7821	6.10343	KIF19_ENSG00000196169
17	72375819	72376180	5Y-H4K8ac_peak_7822	6.96612	
17	72449823	72450128	5Y-H4K8ac_peak_7823	14.26354	
17	72450424	72451022	5Y-H4K8ac_peak_7824	11.09673	
17	72453483	72453686	5Y-H4K8ac_peak_7825	5.67483	
17	72595368	72595597	5Y-H4K8ac_peak_7826	12.19856	CTD-2006K23.1_ENSG00000261222
17	72628766	72628980	5Y-H4K8ac_peak_7827	7.50148	
17	72744225	72744510	5Y-H4K8ac_peak_7828	11.40439	MIR3615_ENSG00000266036;MIR3615_ENSG00000264624;SLC9A3R1_ENSG00000109062
17	72754230	72754506	5Y-H4K8ac_peak_7829	7.11863	
17	72754885	72755094	5Y-H4K8ac_peak_7830	5.03564	
17	72755327	72755904	5Y-H4K8ac_peak_7831	5.12488	
17	72756190	72756785	5Y-H4K8ac_peak_7832	15.80786	
17	72757317	72757712	5Y-H4K8ac_peak_7833	8.34719	
17	72838548	72838993	5Y-H4K8ac_peak_7834	9.07085	
17	72839237	72839570	5Y-H4K8ac_peak_7835	5.17251	
17	72951467	72951949	5Y-H4K8ac_peak_7836	6.25061	
17	72954286	72954557	5Y-H4K8ac_peak_7837	10.44473	
17	72967730	72968093	5Y-H4K8ac_peak_7838	11.22005	RP11-309N17.4_ENSG00000263586
17	72977531	72978345	5Y-H4K8ac_peak_7839	12.00715	
17	72978600	72978927	5Y-H4K8ac_peak_7840	12.00715	
17	72985179	72985377	5Y-H4K8ac_peak_7841	3.94247	
17	72986713	72986937	5Y-H4K8ac_peak_7842	6.08523	
17	73008315	73009377	5Y-H4K8ac_peak_7843	8.19942	ICT1_ENSG00000167862
17	73030651	73031163	5Y-H4K8ac_peak_7844	16.79029	
17	73031763	73032136	5Y-H4K8ac_peak_7845	8.06489	
17	73043521	73043797	5Y-H4K8ac_peak_7846	5.12488	ATP5H_ENSG00000167863
17	73087079	73087348	5Y-H4K8ac_peak_7847	12.05638	
17	73105234	73105799	5Y-H4K8ac_peak_7848	7.90751	ARMC7_ENSG00000125449
17	73127367	73128331	5Y-H4K8ac_peak_7849	7.27671	NT5C_ENSG00000125458
17	73149758	73150196	5Y-H4K8ac_peak_7850	6.10343	
17	73178346	73179030	5Y-H4K8ac_peak_7851	16.17072	SUMO2_ENSG00000188612
17	73179371	73179856	5Y-H4K8ac_peak_7852	13.23366	SUMO2_ENSG00000188612
17	73200826	73201044	5Y-H4K8ac_peak_7853	5.87725	NUP85_ENSG00000125450
17	73201558	73202021	5Y-H4K8ac_peak_7854	13.89096	NUP85_ENSG00000125450
17	73240326	73240553	5Y-H4K8ac_peak_7855	6.78128	
17	73241786	73242169	5Y-H4K8ac_peak_7856	8.36588	
17	73257335	73258487	5Y-H4K8ac_peak_7857	9.8327	GGA3_ENSG00000125447;MRPS7_ENSG00000125445
17	73266883	73267195	5Y-H4K8ac_peak_7858	7.90751	MIF4GD_ENSG00000125457;RP11-649A18.12_ENSG00000263843
17	73267542	73268341	5Y-H4K8ac_peak_7859	13.33225	MIF4GD_ENSG00000125457;RP11-649A18.12_ENSG00000263843
17	73268555	73268848	5Y-H4K8ac_peak_7860	6.19716	
17	73401372	73401703	5Y-H4K8ac_peak_7861	9.93175	GRB2_ENSG00000177885;MIR3678_ENSG00000264511
17	73452817	73453447	5Y-H4K8ac_peak_7862	8.09118	
17	73511336	73511567	5Y-H4K8ac_peak_7863	8.73392	CASKIN2_ENSG00000177303;TSEN54_ENSG00000182173
17	73512142	73512410	5Y-H4K8ac_peak_7864	11.62198	CASKIN2_ENSG00000177303;TSEN54_ENSG00000182173

17	73512672	73512972	5Y-H4K8ac_peak_7865	7.8267	TSEN54_ENSG00000182173
17	73548885	73549106	5Y-H4K8ac_peak_7866	6.73385	
17	73553384	73553759	5Y-H4K8ac_peak_7867	8.04693	
17	73554197	73554521	5Y-H4K8ac_peak_7868	5.37237	
17	73584271	73585242	5Y-H4K8ac_peak_7869	12.79198	MYO15B_ENSG00000266714
17	73585573	73586219	5Y-H4K8ac_peak_7870	11.26379	
17	73616447	73616689	5Y-H4K8ac_peak_7871	8.76608	
17	73629265	73629493	5Y-H4K8ac_peak_7872	7.72887	SMIM5_ENSG00000204323
17	73662905	73663181	5Y-H4K8ac_peak_7873	14.99673	RECQL5_ENSG00000108469;SAP30BP_ENSG00000161526
17	73745726	73746076	5Y-H4K8ac_peak_7874	12.00715	
17	73749283	73750508	5Y-H4K8ac_peak_7875	21.41794	
17	73760582	73761166	5Y-H4K8ac_peak_7876	15.41988	GALK1_ENSG00000108479
17	73775233	73775730	5Y-H4K8ac_peak_7877	7.72887	
17	73780217	73780616	5Y-H4K8ac_peak_7878	8.26455	MIR4738_ENSG00000263565;UNK_ENSG00000132478
17	73781207	73781614	5Y-H4K8ac_peak_7879	17.69008	H3F3B_ENSG00000132475;MIR4738_ENSG00000263565;UNK_ENSG00000132478
17	73822771	73823202	5Y-H4K8ac_peak_7880	10.55714	
17	73823883	73824376	5Y-H4K8ac_peak_7881	17.89499	
17	73824884	73825108	5Y-H4K8ac_peak_7882	5.87725	
17	73839698	73840115	5Y-H4K8ac_peak_7883	5.51759	UNC13D_ENSG00000092929
17	73857207	73857481	5Y-H4K8ac_peak_7884	8.16382	
17	73872087	73872402	5Y-H4K8ac_peak_7885	8.3285	RP11-552F3.9_ENSG00000267801
17	73874350	73874616	5Y-H4K8ac_peak_7886	6.80915	TRIM47_ENSG00000132481
17	73892329	73892931	5Y-H4K8ac_peak_7887	10.14791	TRIM65_ENSG00000141569;RP11-552F3.10_ENSG00000267342
17	73900532	73900790	5Y-H4K8ac_peak_7888	7.50501	
17	73901330	73901644	5Y-H4K8ac_peak_7889	7.20869	
17	73906014	73906246	5Y-H4K8ac_peak_7890	3.93327	MRPL38_ENSG00000204316
17	73975307	73976186	5Y-H4K8ac_peak_7891	11.34483	ACOX1_ENSG00000161533;TEN1_ENSG00000257949;TEN1-CDK3_ENSG00000261408
17	74010098	74010343	5Y-H4K8ac_peak_7892	7.7384	
17	74033037	74033333	5Y-H4K8ac_peak_7893	8.49078	
17	74068198	74068469	5Y-H4K8ac_peak_7894	9.02782	SRP68_ENSG00000167881;ZACN_ENSG00000186919
17	74068748	74069047	5Y-H4K8ac_peak_7895	11.26379	SRP68_ENSG00000167881;ZACN_ENSG00000186919
17	74099672	74100849	5Y-H4K8ac_peak_7896	13.33225	
17	74235797	74236231	5Y-H4K8ac_peak_7897	8.09118	RNF157_ENSG00000141576
17	74236898	74237181	5Y-H4K8ac_peak_7898	10.0444	RNF157_ENSG00000141576
17	74261654	74261865	5Y-H4K8ac_peak_7899	8.36027	UBALD2_ENSG00000185262
17	74350041	74350257	5Y-H4K8ac_peak_7900	12.51536	
17	74364145	74364553	5Y-H4K8ac_peak_7901	8.22388	
17	74382201	74382458	5Y-H4K8ac_peak_7902	12.34969	
17	74493890	74494236	5Y-H4K8ac_peak_7903	5.24695	
17	74496998	74498041	5Y-H4K8ac_peak_7904	7.20869	RHBDF2_ENSG00000129667
17	74502228	74502938	5Y-H4K8ac_peak_7905	11.86704	
17	74525694	74526014	5Y-H4K8ac_peak_7906	6.08523	
17	74526209	74527032	5Y-H4K8ac_peak_7907	11.23789	
17	74534155	74535186	5Y-H4K8ac_peak_7908	15.81923	
17	74536129	74536966	5Y-H4K8ac_peak_7909	14.86775	
17	74537265	74537727	5Y-H4K8ac_peak_7910	5.72233	

17	74538245	74539961	5Y-H4K8ac_peak_7911	23.29116	
17	74540337	74541684	5Y-H4K8ac_peak_7912	19.8038	
17	74548585	74548781	5Y-H4K8ac_peak_7913	5.24695	
17	74549795	74550033	5Y-H4K8ac_peak_7914	9.79526	
17	74553328	74553642	5Y-H4K8ac_peak_7915	4.642	RP11-666A8.8_ENSG00000267546;SNHG16_ENSG00000163597
17	74632436	74632770	5Y-H4K8ac_peak_7916	5.65584	
17	74667035	74667707	5Y-H4K8ac_peak_7917	16.01457	RP11-318A15.2_ENSG00000261335
17	74668158	74668357	5Y-H4K8ac_peak_7918	8.1667	RP11-318A15.2_ENSG00000261335
17	74705654	74706025	5Y-H4K8ac_peak_7919	7.59101	
17	74706362	74706608	5Y-H4K8ac_peak_7920	7.89273	MXRA7_ENSG00000182534
17	74722361	74722552	5Y-H4K8ac_peak_7921	7.00004	JMJD6_ENSG00000070495;METTL23_ENSG00000181038
17	74722991	74723319	5Y-H4K8ac_peak_7922	13.82621	JMJD6_ENSG00000070495;METTL23_ENSG00000181038
17	74732672	74733367	5Y-H4K8ac_peak_7923	11.4254	SRSF2_ENSG00000161547;MFSD11_ENSG00000092931;MIR636_ENSG00000207556
17	74733774	74734024	5Y-H4K8ac_peak_7924	12.26819	SRSF2_ENSG00000161547
17	74816148	74816402	5Y-H4K8ac_peak_7925	4.642	
17	74816839	74817053	5Y-H4K8ac_peak_7926	7.82914	
17	74837709	74838141	5Y-H4K8ac_peak_7927	11.22005	
17	74864778	74865006	5Y-H4K8ac_peak_7928	7.08486	MGAT5B_ENSG00000167889
17	74865950	74866383	5Y-H4K8ac_peak_7929	6.78128	
17	74965195	74965622	5Y-H4K8ac_peak_7930	11.40439	RP11-87G24.3_ENSG00000267568
17	74966149	74966350	5Y-H4K8ac_peak_7931	9.36633	RP11-87G24.3_ENSG00000267568
17	75081576	75082156	5Y-H4K8ac_peak_7932	9.44016	LINC00338_ENSG00000234912;SEC14L1_ENSG00000129657
17	75083744	75084535	5Y-H4K8ac_peak_7933	13.00733	LINC00338_ENSG00000234912;SEC14L1_ENSG00000129657;AC015815.3_ENSG00000203316
17	75084812	75085209	5Y-H4K8ac_peak_7934	23.16211	
17	75242849	75243598	5Y-H4K8ac_peak_7935	10.56003	
17	75260639	75260987	5Y-H4K8ac_peak_7936	8.69112	RP11-285E9.5_ENSG00000265121
17	75274104	75274358	5Y-H4K8ac_peak_7937	5.27937	
17	75277540	75277828	5Y-H4K8ac_peak_7938	6.80915	RP11-285E9.6_ENSG00000263718;SEPT9_ENSG00000184640
17	75278885	75279130	5Y-H4K8ac_peak_7939	5.87725	RP11-285E9.6_ENSG00000263718
17	75283494	75284684	5Y-H4K8ac_peak_7940	9.88714	
17	75302301	75302807	5Y-H4K8ac_peak_7941	5.60566	
17	75303046	75303621	5Y-H4K8ac_peak_7942	9.19793	
17	75304782	75305537	5Y-H4K8ac_peak_7943	6.35459	
17	75306198	75306829	5Y-H4K8ac_peak_7944	6.53157	
17	75307042	75307239	5Y-H4K8ac_peak_7945	7.20726	
17	75309231	75309489	5Y-H4K8ac_peak_7946	7.72887	
17	75310725	75311155	5Y-H4K8ac_peak_7947	9.79526	
17	75311753	75312234	5Y-H4K8ac_peak_7948	7.68528	
17	75316734	75317113	5Y-H4K8ac_peak_7949	7.00889	
17	75318758	75319071	5Y-H4K8ac_peak_7950	7.3565	
17	75347565	75347823	5Y-H4K8ac_peak_7951	5.60566	
17	75350530	75350789	5Y-H4K8ac_peak_7952	7.8511	
17	75359078	75359357	5Y-H4K8ac_peak_7953	12.26819	
17	75359730	75359941	5Y-H4K8ac_peak_7954	3.93392	
17	75360288	75360931	5Y-H4K8ac_peak_7955	9.98604	
17	75370050	75370400	5Y-H4K8ac_peak_7956	3.94247	

17	75372818	75373150	5Y-H4K8ac_peak_7957	9.02938	RP11-936I5.1_ENSG00000266998
17	75373608	75373985	5Y-H4K8ac_peak_7958	13.59183	RP11-936I5.1_ENSG00000266998
17	75384118	75384397	5Y-H4K8ac_peak_7959	8.13307	
17	75385029	75385800	5Y-H4K8ac_peak_7960	16.41608	
17	75386768	75387222	5Y-H4K8ac_peak_7961	7.11863	
17	75420413	75420637	5Y-H4K8ac_peak_7962	9.31478	
17	75421439	75422286	5Y-H4K8ac_peak_7963	8.09118	
17	75429015	75429846	5Y-H4K8ac_peak_7964	20.22935	
17	75430275	75431102	5Y-H4K8ac_peak_7965	9.45199	
17	75432673	75433303	5Y-H4K8ac_peak_7966	8.3438	
17	75446624	75446907	5Y-H4K8ac_peak_7967	5.46519	
17	75447199	75447406	5Y-H4K8ac_peak_7968	6.53157	
17	75447626	75448023	5Y-H4K8ac_peak_7969	20.79772	
17	75476073	75476954	5Y-H4K8ac_peak_7970	8.09118	
17	75524296	75525114	5Y-H4K8ac_peak_7971	9.44542	
17	75686942	75687786	5Y-H4K8ac_peak_7972	8.38845	
17	75696613	75696908	5Y-H4K8ac_peak_7973	7.59101	
17	75719859	75720049	5Y-H4K8ac_peak_7974	6.54441	
17	75730223	75730715	5Y-H4K8ac_peak_7975	7.89273	
17	75731185	75731833	5Y-H4K8ac_peak_7976	9.87124	
17	75732306	75732532	5Y-H4K8ac_peak_7977	6.37023	
17	75775462	75776277	5Y-H4K8ac_peak_7978	15.90416	
17	75777982	75778242	5Y-H4K8ac_peak_7979	6.78318	
17	75798309	75798560	5Y-H4K8ac_peak_7980	9.30505	
17	75798759	75799093	5Y-H4K8ac_peak_7981	8.73985	
17	75799324	75799565	5Y-H4K8ac_peak_7982	6.31818	
17	75860023	75860473	5Y-H4K8ac_peak_7983	15.82359	
17	75861460	75861730	5Y-H4K8ac_peak_7984	13.00733	
17	75869840	75870483	5Y-H4K8ac_peak_7985	15.82359	
17	75870968	75871279	5Y-H4K8ac_peak_7986	4.6766	
17	75876394	75876589	5Y-H4K8ac_peak_7987	8.62703	
17	75877092	75877688	5Y-H4K8ac_peak_7988	11.86704	FLJ45079_ENSG00000204283
17	75878719	75879368	5Y-H4K8ac_peak_7989	9.79526	FLJ45079_ENSG00000204283
17	75924127	75924380	5Y-H4K8ac_peak_7990	7.65404	
17	75927389	75927775	5Y-H4K8ac_peak_7991	6.20292	
17	75953969	75954427	5Y-H4K8ac_peak_7992	11.22005	
17	76037008	76037299	5Y-H4K8ac_peak_7993	5.12488	
17	76113131	76114047	5Y-H4K8ac_peak_7994	6.9958	
17	76121678	76122244	5Y-H4K8ac_peak_7995	4.0639	
17	76124549	76124788	5Y-H4K8ac_peak_7996	7.20869	
17	76128141	76128412	5Y-H4K8ac_peak_7997	9.30206	TMC6_ENSG00000141524
17	76164086	76164512	5Y-H4K8ac_peak_7998	12.2918	SYNGR2_ENSG00000108639
17	76210417	76210799	5Y-H4K8ac_peak_7999	8.20773	BIRC5_ENSG00000089685
17	76219523	76219750	5Y-H4K8ac_peak_8000	4.97086	AC087645.1_ENSG00000268310
17	76221225	76221848	5Y-H4K8ac_peak_8001	9.758	AC087645.1_ENSG00000268310
17	76227733	76228020	5Y-H4K8ac_peak_8002	12.67841	TMEM235_ENSG00000204278

17	76230610	76230984	5Y-H4K8ac_peak_8003	6.78128	
17	76233934	76234554	5Y-H4K8ac_peak_8004	6.55841	
17	76246677	76246950	5Y-H4K8ac_peak_8005	7.96285	
17	76249905	76250577	5Y-H4K8ac_peak_8006	10.74494	THA1P_ENSG00000267676
17	76252357	76252735	5Y-H4K8ac_peak_8007	5.73383	
17	76253061	76253438	5Y-H4K8ac_peak_8008	9.01738	
17	76253822	76254037	5Y-H4K8ac_peak_8009	12.42757	
17	76254485	76254797	5Y-H4K8ac_peak_8010	14.93667	
17	76309868	76310632	5Y-H4K8ac_peak_8011	10.35586	
17	76320815	76321015	5Y-H4K8ac_peak_8012	5.84816	
17	76337643	76338307	5Y-H4K8ac_peak_8013	11.23789	
17	76349149	76349381	5Y-H4K8ac_peak_8014	9.00954	
17	76355792	76356179	5Y-H4K8ac_peak_8015	8.62703	SOCS3_ENSG00000184557;RP11-806H10.4_ENSG00000266970
17	76365653	76366192	5Y-H4K8ac_peak_8016	10.37549	
17	76373900	76374135	5Y-H4K8ac_peak_8017	5.18558	PGS1_ENSG00000087157
17	76374435	76375067	5Y-H4K8ac_peak_8018	12.26819	PGS1_ENSG00000087157
17	76525740	76525981	5Y-H4K8ac_peak_8019	8.24461	
17	76712855	76713450	5Y-H4K8ac_peak_8020	10.1994	
17	76719833	76720309	5Y-H4K8ac_peak_8021	6.16671	
17	76836469	76836779	5Y-H4K8ac_peak_8022	5.87918	USP36_ENSG00000055483
17	76837290	76837538	5Y-H4K8ac_peak_8023	10.12457	USP36_ENSG00000055483
17	76878271	76879115	5Y-H4K8ac_peak_8024	12.50545	
17	76879355	76880615	5Y-H4K8ac_peak_8025	11.30548	
17	76887559	76888188	5Y-H4K8ac_peak_8026	4.55128	
17	76888494	76888782	5Y-H4K8ac_peak_8027	6.03632	
17	76912797	76912997	5Y-H4K8ac_peak_8028	4.00285	
17	76921610	76921993	5Y-H4K8ac_peak_8029	38.70009	TIMP2_ENSG00000035862
17	76922265	76922647	5Y-H4K8ac_peak_8030	10.35586	TIMP2_ENSG00000035862
17	76929263	76930308	5Y-H4K8ac_peak_8031	11.67272	
17	76930755	76931487	5Y-H4K8ac_peak_8032	13.1133	
17	76931958	76932190	5Y-H4K8ac_peak_8033	8.46718	
17	76932626	76932820	5Y-H4K8ac_peak_8034	8.32351	
17	76970735	76971007	5Y-H4K8ac_peak_8035	11.03999	
17	76975375	76975965	5Y-H4K8ac_peak_8036	10.18556	LGALS3BP_ENSG00000108679
17	76976217	76977058	5Y-H4K8ac_peak_8037	14.40623	LGALS3BP_ENSG00000108679
17	77005455	77005807	5Y-H4K8ac_peak_8038	7.54409	CANT1_ENSG00000171302
17	77006023	77006486	5Y-H4K8ac_peak_8039	9.13842	CANT1_ENSG00000171302
17	77019977	77020450	5Y-H4K8ac_peak_8040	12.34969	
17	77021034	77021251	5Y-H4K8ac_peak_8041	6.75784	
17	77023303	77023554	5Y-H4K8ac_peak_8042	5.60566	C1QTNF1-AS1_ENSG00000265096
17	77023884	77024251	5Y-H4K8ac_peak_8043	9.69453	C1QTNF1-AS1_ENSG00000265096
17	77042632	77043089	5Y-H4K8ac_peak_8044	4.44548	
17	77043533	77043847	5Y-H4K8ac_peak_8045	8.67963	
17	77071170	77071512	5Y-H4K8ac_peak_8046	6.78128	ENGASE_ENSG00000167280
17	77210557	77210764	5Y-H4K8ac_peak_8047	5.12488	
17	77211102	77211447	5Y-H4K8ac_peak_8048	7.44077	

17	77242723	77242916	5Y-H4K8ac_peak_8049	7.65114	
17	77255403	77255739	5Y-H4K8ac_peak_8050	7.02726	
17	77301734	77301956	5Y-H4K8ac_peak_8051	4.18845	
17	77302270	77302938	5Y-H4K8ac_peak_8052	10.12457	
17	77701756	77702442	5Y-H4K8ac_peak_8053	19.99409	
17	77702642	77703124	5Y-H4K8ac_peak_8054	15.2347	
17	77751372	77751630	5Y-H4K8ac_peak_8055	5.03564	CBX2_ENSG00000173894
17	77752045	77752501	5Y-H4K8ac_peak_8056	15.54885	CBX2_ENSG00000173894
17	77771717	77772270	5Y-H4K8ac_peak_8057	35.28442	
17	77783239	77783512	5Y-H4K8ac_peak_8058	8.24461	AC100791.2_ENSG00000238331
17	77783746	77784003	5Y-H4K8ac_peak_8059	13.27075	AC100791.2_ENSG00000238331
17	77784782	77785984	5Y-H4K8ac_peak_8060	11.26379	AC100791.2_ENSG00000238331
17	77786251	77787962	5Y-H4K8ac_peak_8061	43.68056	
17	77805113	77805593	5Y-H4K8ac_peak_8062	10.23828	
17	77805840	77806587	5Y-H4K8ac_peak_8063	15.17389	
17	77813468	77813825	5Y-H4K8ac_peak_8064	13.27075	CBX4_ENSG00000141582
17	77817314	77818377	5Y-H4K8ac_peak_8065	18.65264	
17	77818773	77819106	5Y-H4K8ac_peak_8066	9.44542	
17	77893877	77894094	5Y-H4K8ac_peak_8067	4.47015	RP11-353N14.4_ENSG00000262188
17	77904117	77904521	5Y-H4K8ac_peak_8068	6.78128	
17	78009820	78010261	5Y-H4K8ac_peak_8069	10.35586	TBC1D16_ENSG00000167291;CCDC40_ENSG00000141519
17	78070576	78071146	5Y-H4K8ac_peak_8070	11.60058	
17	78074978	78075193	5Y-H4K8ac_peak_8071	12.71428	GAA_ENSG00000171298
17	78120487	78120737	5Y-H4K8ac_peak_8072	6.37023	EIF4A3_ENSG00000141543
17	78193742	78194101	5Y-H4K8ac_peak_8073	8.62703	SGSH_ENSG00000181523;SLC26A11_ENSG00000181045
17	78233646	78234289	5Y-H4K8ac_peak_8074	17.34187	RNF213_ENSG00000173821
17	78235148	78235623	5Y-H4K8ac_peak_8075	4.56212	RNF213_ENSG00000173821
17	78236568	78237090	5Y-H4K8ac_peak_8076	4.72465	
17	78362902	78363474	5Y-H4K8ac_peak_8077	5.87725	
17	78413607	78413886	5Y-H4K8ac_peak_8078	11.36989	
17	78417719	78418310	5Y-H4K8ac_peak_8079	8.77023	
17	78418728	78419259	5Y-H4K8ac_peak_8080	6.78128	
17	78419502	78419830	5Y-H4K8ac_peak_8081	5.42736	
17	78426856	78427318	5Y-H4K8ac_peak_8082	7.6757	
17	78427620	78428491	5Y-H4K8ac_peak_8083	26.34191	CTD-2526A2.2_ENSG00000260369
17	78428773	78429339	5Y-H4K8ac_peak_8084	18.31883	CTD-2526A2.2_ENSG00000260369
17	78465833	78466027	5Y-H4K8ac_peak_8085	6.73047	
17	78517863	78518176	5Y-H4K8ac_peak_8086	8.69112	RPTOR_ENSG00000141564
17	78518699	78519064	5Y-H4K8ac_peak_8087	10.54669	RPTOR_ENSG00000141564
17	78670402	78670700	5Y-H4K8ac_peak_8088	4.29586	
17	78800495	78800687	5Y-H4K8ac_peak_8089	7.60057	
17	78806806	78807025	5Y-H4K8ac_peak_8090	11.4254	
17	78808437	78808664	5Y-H4K8ac_peak_8091	4.77126	
17	78810206	78810511	5Y-H4K8ac_peak_8092	4.89128	
17	78832782	78833171	5Y-H4K8ac_peak_8093	13.86769	
17	78833601	78834470	5Y-H4K8ac_peak_8094	22.3803	

17	78835407	78836193	5Y-H4K8ac_peak_8095	6.34245	
17	78965401	78966463	5Y-H4K8ac_peak_8096	11.69482	CHMP6_ENSG00000176108
17	79009377	79009567	5Y-H4K8ac_peak_8097	7.89273	BAIAP2-AS1_ENSG00000226137;BAIAP2_ENSG00000175866
17	79031002	79031194	5Y-H4K8ac_peak_8098	8.5159	
17	79038181	79038430	5Y-H4K8ac_peak_8099	10.48695	
17	79038736	79039271	5Y-H4K8ac_peak_8100	8.24461	
17	79040948	79041163	5Y-H4K8ac_peak_8101	4.44729	
17	79041486	79041862	5Y-H4K8ac_peak_8102	4.97086	
17	79049173	79049664	5Y-H4K8ac_peak_8103	7.93983	
17	79050548	79050754	5Y-H4K8ac_peak_8104	4.48815	
17	79056846	79057043	5Y-H4K8ac_peak_8105	7.18391	
17	79088522	79088746	5Y-H4K8ac_peak_8106	6.78128	
17	79088956	79089497	5Y-H4K8ac_peak_8107	9.79526	
17	79090077	79090421	5Y-H4K8ac_peak_8108	6.98416	
17	79138508	79138798	5Y-H4K8ac_peak_8109	7.11863	AATK-AS1_ENSG00000225180
17	79139104	79139834	5Y-H4K8ac_peak_8110	10.48476	AATK_ENSG00000181409;AATK-AS1_ENSG00000225180
17	79140393	79140716	5Y-H4K8ac_peak_8111	7.50148	AATK_ENSG00000181409
17	79213147	79213532	5Y-H4K8ac_peak_8112	17.69008	ENTHD2_ENSG00000167302;C17orf89_ENSG00000224877
17	79226067	79227035	5Y-H4K8ac_peak_8113	11.16796	
17	79268576	79268927	5Y-H4K8ac_peak_8114	6.56228	SLC38A10_ENSG00000157637
17	79283444	79284926	5Y-H4K8ac_peak_8115	15.2347	LINC00482_ENSG00000185168
17	79285162	79286013	5Y-H4K8ac_peak_8116	10.48476	
17	79287462	79288000	5Y-H4K8ac_peak_8117	7.67774	
17	79288232	79289155	5Y-H4K8ac_peak_8118	14.00559	
17	79290436	79290721	5Y-H4K8ac_peak_8119	4.92207	
17	79291857	79292083	5Y-H4K8ac_peak_8120	10.60083	
17	79296273	79296635	5Y-H4K8ac_peak_8121	6.339	
17	79297922	79298317	5Y-H4K8ac_peak_8122	8.01363	
17	79302268	79303017	5Y-H4K8ac_peak_8123	19.8038	
17	79303221	79306253	5Y-H4K8ac_peak_8124	16.73191	TMEM105_ENSG00000185332
17	79308401	79308770	5Y-H4K8ac_peak_8125	6.55841	
17	79312247	79312705	5Y-H4K8ac_peak_8126	5.41191	
17	79313290	79313853	5Y-H4K8ac_peak_8127	10.89466	
17	79314215	79315882	5Y-H4K8ac_peak_8128	15.41988	
17	79316568	79316994	5Y-H4K8ac_peak_8129	9.64738	
17	79317229	79317601	5Y-H4K8ac_peak_8130	13.89357	
17	79317923	79318223	5Y-H4K8ac_peak_8131	8.24461	
17	79319260	79319785	5Y-H4K8ac_peak_8132	16.86751	
17	79320290	79320550	5Y-H4K8ac_peak_8133	8.22976	
17	79366904	79367192	5Y-H4K8ac_peak_8134	5.60566	
17	79367434	79367769	5Y-H4K8ac_peak_8135	7.90005	
17	79373605	79373851	5Y-H4K8ac_peak_8136	4.90516	RP11-1055B8.7_ENSG00000171282;MIR4740_ENSG00000266392
17	79392957	79393941	5Y-H4K8ac_peak_8137	7.44077	
17	79438712	79439040	5Y-H4K8ac_peak_8138	9.02938	
17	79440142	79440372	5Y-H4K8ac_peak_8139	4.37349	
17	79446527	79446766	5Y-H4K8ac_peak_8140	9.11495	

17	79448744	79449022	5Y-H4K8ac_peak_8141	6.80915	
17	79449963	79450309	5Y-H4K8ac_peak_8142	5.83797	
17	79450527	79451226	5Y-H4K8ac_peak_8143	4.83889	
17	79451547	79452172	5Y-H4K8ac_peak_8144	11.41007	
17	79454645	79455646	5Y-H4K8ac_peak_8145	13.99124	
17	79480118	79480652	5Y-H4K8ac_peak_8146	12.00715	RP13-766D20.1_ENSG00000229947;RP13-766D20.2_ENSG00000229848
17	79481235	79482352	5Y-H4K8ac_peak_8147	12.26819	RP13-766D20.2_ENSG00000229848
17	79490977	79491311	5Y-H4K8ac_peak_8148	5.80081	ACTG1_ENSG00000184009
17	79519133	79519370	5Y-H4K8ac_peak_8149	10.29016	
17	79519752	79519957	5Y-H4K8ac_peak_8150	6.80915	
17	79520865	79521267	5Y-H4K8ac_peak_8151	11.5813	C17orf70_ENSG00000185504
17	79603683	79604060	5Y-H4K8ac_peak_8152	9.30505	TSPAN10_ENSG00000182612
17	79632201	79632417	5Y-H4K8ac_peak_8153	4.77126	CCDC137_ENSG00000185298
17	79633816	79634622	5Y-H4K8ac_peak_8154	13.45283	OXLD1_ENSG00000204237;CCDC137_ENSG00000185298
17	79650147	79650699	5Y-H4K8ac_peak_8155	8.3285	ARL16_ENSG00000214087;HGS_ENSG00000185359
17	79651140	79651416	5Y-H4K8ac_peak_8156	9.79526	ARL16_ENSG00000214087;HGS_ENSG00000185359
17	79669941	79670193	5Y-H4K8ac_peak_8157	10.35586	RP13-1032I1.7_ENSG00000262049;MRPL12_ENSG00000262814;SLC25A10_ENSG00000262660;SLC25A10_ENSG00000183048
17	79677827	79678339	5Y-H4K8ac_peak_8158	5.64815	
17	79679449	79679642	5Y-H4K8ac_peak_8159	6.34245	
17	79772731	79773140	5Y-H4K8ac_peak_8160	5.92809	
17	79791297	79791867	5Y-H4K8ac_peak_8161	13.33225	FAM195B_ENSG00000225663
17	79818167	79818358	5Y-H4K8ac_peak_8162	7.44077	P4HB_ENSG00000185624
17	79819539	79819740	5Y-H4K8ac_peak_8163	8.24461	P4HB_ENSG00000185624
17	79825962	79826172	5Y-H4K8ac_peak_8164	5.24064	RP11-498C9.3_ENSG00000262413
17	79828430	79828733	5Y-H4K8ac_peak_8165	3.94247	ARHGDIA_ENSG00000141522
17	79829316	79830313	5Y-H4K8ac_peak_8166	28.91936	ARHGDIA_ENSG00000141522
17	79848733	79849045	5Y-H4K8ac_peak_8167	11.18846	ALYREF_ENSG00000183684;ANAPC11_ENSG00000141552
17	79849696	79850163	5Y-H4K8ac_peak_8168	3.94247	ALYREF_ENSG00000183684
17	79860321	79860516	5Y-H4K8ac_peak_8169	5.31535	
17	79886083	79886947	5Y-H4K8ac_peak_8170	9.96717	MAFG_ENSG00000197063;MAFG-AS1_ENSG00000265688
17	79894557	79895046	5Y-H4K8ac_peak_8171	5.99756	
17	79895347	79896346	5Y-H4K8ac_peak_8172	6.17806	
17	79917059	79917997	5Y-H4K8ac_peak_8173	10.15788	
17	79924216	79925913	5Y-H4K8ac_peak_8174	28.41881	RP11-498C9.17_ENSG00000264735
17	79926225	79926869	5Y-H4K8ac_peak_8175	9.30505	
17	79934453	79934844	5Y-H4K8ac_peak_8176	5.87725	ASPSCR1_ENSG00000169696
17	79935042	79935300	5Y-H4K8ac_peak_8177	15.35687	ASPSCR1_ENSG00000169696
17	79935516	79936791	5Y-H4K8ac_peak_8178	17.37977	ASPSCR1_ENSG00000169696
17	79949981	79951215	5Y-H4K8ac_peak_8179	8.62703	
17	79964637	79964895	5Y-H4K8ac_peak_8180	7.61515	
17	79965316	79965940	5Y-H4K8ac_peak_8181	8.68553	
17	79970379	79971213	5Y-H4K8ac_peak_8182	5.03564	
17	79981360	79981621	5Y-H4K8ac_peak_8183	8.24461	STRA13_ENSG00000169689;LRRC45_ENSG00000169683
17	80009074	80009445	5Y-H4K8ac_peak_8184	12.10416	RFNG_ENSG00000169733;GPS1_ENSG00000169727
17	80023745	80024681	5Y-H4K8ac_peak_8185	23.58923	DUS1L_ENSG00000169718
17	80055591	80055945	5Y-H4K8ac_peak_8186	8.47129	FASN_ENSG00000169710

17	80056851	80057862	5Y-H4K8ac_peak_8187	26.70591	FASN_ENSG00000169710
17	80058116	80058309	5Y-H4K8ac_peak_8188	7.20869	
17	80059605	80060039	5Y-H4K8ac_peak_8189	5.85463	
17	80062690	80063144	5Y-H4K8ac_peak_8190	7.44077	
17	80063466	80064078	5Y-H4K8ac_peak_8191	14.68695	
17	80064514	80065064	5Y-H4K8ac_peak_8192	7.64648	
17	80170825	80171538	5Y-H4K8ac_peak_8193	16.0768	CCDC57_ENSG00000176155;RP13-516M14.2_ENSG00000264548
17	80199385	80199720	5Y-H4K8ac_peak_8194	5.03564	
17	80231040	80231487	5Y-H4K8ac_peak_8195	9.51254	CSNK1D_ENSG00000141551
17	80231805	80232051	5Y-H4K8ac_peak_8196	9.02938	CSNK1D_ENSG00000141551
17	80241542	80241919	5Y-H4K8ac_peak_8197	10.0861	
17	80250228	80250618	5Y-H4K8ac_peak_8198	17.29652	RP13-516M14.4_ENSG00000265692;RP13-516M14.1_ENSG00000260563
17	80255754	80256026	5Y-H4K8ac_peak_8199	10.01993	
17	80256320	80256646	5Y-H4K8ac_peak_8200	11.05566	
17	80316462	80317288	5Y-H4K8ac_peak_8201	8.62703	TEX19_ENSG00000182459
17	80341818	80342095	5Y-H4K8ac_peak_8202	7.89273	
17	80357784	80357979	5Y-H4K8ac_peak_8203	10.72427	
17	80408230	80408503	5Y-H4K8ac_peak_8204	8.43511	C17orf62_ENSG00000178927
17	80416049	80416392	5Y-H4K8ac_peak_8205	41.21512	RP13-20L14.6_ENSG00000265458;NARF_ENSG00000141562
17	80451992	80452311	5Y-H4K8ac_peak_8206	6.19716	
17	80454565	80455084	5Y-H4K8ac_peak_8207	13.0871	
17	80456023	80456238	5Y-H4K8ac_peak_8208	6.36248	
17	80477890	80478141	5Y-H4K8ac_peak_8209	7.50148	FOXK2_ENSG00000141568
17	80551380	80551585	5Y-H4K8ac_peak_8210	4.97086	
17	80605846	80606213	5Y-H4K8ac_peak_8211	6.08523	WDR45B_ENSG00000141580
17	80606888	80607081	5Y-H4K8ac_peak_8212	6.78318	WDR45B_ENSG00000141580
17	80673940	80674201	5Y-H4K8ac_peak_8213	5.64909	RP11-388C12.1_ENSG00000263063;FN3KRP_ENSG00000141560
17	80693114	80693398	5Y-H4K8ac_peak_8214	10.30425	FN3K_ENSG00000167363
17	80751345	80751561	5Y-H4K8ac_peak_8215	6.78128	
17	80751846	80752061	5Y-H4K8ac_peak_8216	7.65114	
17	80787966	80788225	5Y-H4K8ac_peak_8217	5.34199	
17	80788469	80788661	5Y-H4K8ac_peak_8218	8.3285	
17	80824339	80824661	5Y-H4K8ac_peak_8219	6.71125	
17	80825866	80826149	5Y-H4K8ac_peak_8220	10.35586	
17	81009911	81010405	5Y-H4K8ac_peak_8221	23.03824	B3GNTL1_ENSG00000175711
18	267700	267947	5Y-H4K8ac_peak_8222	5.91107	THOC1_ENSG00000079134;RP11-705O1.8_ENSG00000263884
18	268245	268515	5Y-H4K8ac_peak_8223	4.77126	THOC1_ENSG00000079134;RP11-705O1.8_ENSG00000263884
18	268755	269230	5Y-H4K8ac_peak_8224	6.34046	THOC1_ENSG00000079134;RP11-705O1.8_ENSG00000263884
18	2655004	2655393	5Y-H4K8ac_peak_8225	4.50834	CBX3P2_ENSG00000266405;SMCHD1_ENSG00000101596
18	2906232	2907128	5Y-H4K8ac_peak_8226	13.63503	
18	3012630	3013095	5Y-H4K8ac_peak_8227	7.59101	LPIN2_ENSG00000101577
18	3247910	3248282	5Y-H4K8ac_peak_8228	9.05168	RP13-270P17.3_ENSG00000272688;MYL12A_ENSG00000101608
18	3261893	3262256	5Y-H4K8ac_peak_8229	7.38046	RP13-270P17.1_ENSG00000264235;MYL12B_ENSG00000118680
18	3592456	3592765	5Y-H4K8ac_peak_8230	5.87382	DLGAP1-AS1_ENSG00000177337
18	3593375	3593580	5Y-H4K8ac_peak_8231	4.29586	DLGAP1-AS1_ENSG00000177337
18	3594531	3594828	5Y-H4K8ac_peak_8232	6.55906	DLGAP1-AS1_ENSG00000177337

18	3623389	3623722	5Y-H4K8ac_peak_8233	5.23819	
18	5237162	5237526	5Y-H4K8ac_peak_8234	7.38046	RP11-835E18.5_ENSG00000265091
18	5237827	5238314	5Y-H4K8ac_peak_8235	11.1169	RP11-835E18.5_ENSG00000265091;LINC00526_ENSG00000264575
18	5295635	5295902	5Y-H4K8ac_peak_8236	9.38203	
18	5890799	5891237	5Y-H4K8ac_peak_8237	6.77436	
18	6414385	6414879	5Y-H4K8ac_peak_8238	8.24461	L3MBTL4_ENSG00000154655
18	8257044	8257321	5Y-H4K8ac_peak_8239	5.98695	
18	8259808	8260184	5Y-H4K8ac_peak_8240	4.84727	
18	8260706	8260961	5Y-H4K8ac_peak_8241	11.1169	
18	8263328	8263895	5Y-H4K8ac_peak_8242	4.07874	
18	8264434	8264770	5Y-H4K8ac_peak_8243	19.33296	
18	8608799	8609123	5Y-H4K8ac_peak_8244	8.30301	RAB12_ENSG00000206418
18	8661853	8662061	5Y-H4K8ac_peak_8245	5.40331	
18	8765414	8765679	5Y-H4K8ac_peak_8246	9.63153	
18	8794413	8794609	5Y-H4K8ac_peak_8247	9.22011	
18	9016920	9017304	5Y-H4K8ac_peak_8248	8.67475	
18	9101285	9101503	5Y-H4K8ac_peak_8249	4.51076	
18	9102693	9102988	5Y-H4K8ac_peak_8250	20.96132	NDUFV2_ENSG00000178127;RP11-21J18.1_ENSG00000265257
18	9136198	9136485	5Y-H4K8ac_peak_8251	4.95697	RP11-143J12.2_ENSG00000266053;ANKRD12_ENSG00000101745
18	9474542	9475149	5Y-H4K8ac_peak_8252	8.43511	RP11-61L19.2_ENSG00000273335;RALBP1_ENSG00000017797
18	9614744	9615363	5Y-H4K8ac_peak_8253	11.53136	PPP4R1_ENSG00000154845;RP11-692N5.1_ENSG00000263627
18	9708043	9708789	5Y-H4K8ac_peak_8254	9.38276	RAB31_ENSG00000168461
18	11851457	11851652	5Y-H4K8ac_peak_8255	5.23083	CHMP1B_ENSG00000255112
18	12076422	12076767	5Y-H4K8ac_peak_8256	10.1994	RP11-815J4.6_ENSG00000256616
18	12271402	12271747	5Y-H4K8ac_peak_8257	5.04571	
18	12376755	12377043	5Y-H4K8ac_peak_8258	5.23083	AFG3L2_ENSG00000141385
18	12420038	12420565	5Y-H4K8ac_peak_8259	5.98695	
18	12420871	12421348	5Y-H4K8ac_peak_8260	10.508	
18	12702296	12703019	5Y-H4K8ac_peak_8261	11.69482	CEP76_ENSG00000101624
18	12884001	12884236	5Y-H4K8ac_peak_8262	10.11191	
18	12948021	12948247	5Y-H4K8ac_peak_8263	6.6946	SEH1L_ENSG00000085415
18	13136601	13137122	5Y-H4K8ac_peak_8264	9.36633	
18	13217915	13218501	5Y-H4K8ac_peak_8265	8.73392	LDLRAD4_ENSG00000168675
18	13222702	13222956	5Y-H4K8ac_peak_8266	5.56894	
18	13223289	13223700	5Y-H4K8ac_peak_8267	5.56894	
18	13223905	13224272	5Y-H4K8ac_peak_8268	6.50117	
18	13446611	13446906	5Y-H4K8ac_peak_8269	8.43511	
18	13448161	13448459	5Y-H4K8ac_peak_8270	5.94703	
18	13489411	13489620	5Y-H4K8ac_peak_8271	4.07874	
18	13500181	13500682	5Y-H4K8ac_peak_8272	9.179	RP11-53B2.5_ENSG00000267366
18	13502050	13502277	5Y-H4K8ac_peak_8273	4.66683	RP11-53B2.5_ENSG00000267366
18	13583865	13584148	5Y-H4K8ac_peak_8274	7.63144	
18	13627772	13628010	5Y-H4K8ac_peak_8275	4.03329	
18	14132960	14133223	5Y-H4K8ac_peak_8276	6.34046	ZNF519_ENSG00000175322
18	19179954	19180471	5Y-H4K8ac_peak_8277	9.88125	ESCO1_ENSG00000141446
18	19320813	19321234	5Y-H4K8ac_peak_8278	9.23159	

18	19321674	19321872	5Y-H4K8ac_peak_8279	6.77436	
18	20513303	20513960	5Y-H4K8ac_peak_8280	14.31711	RP11-739L10.1_ENSG00000265943;MIR4741_ENSG00000264817
18	20723707	20724111	5Y-H4K8ac_peak_8281	7.41197	
18	20772469	20772684	5Y-H4K8ac_peak_8282	5.64909	
18	20839082	20839382	5Y-H4K8ac_peak_8283	9.179	RP11-17J14.2_ENSG00000266495
18	20839622	20840137	5Y-H4K8ac_peak_8284	12.05638	RP11-17J14.2_ENSG00000266495
18	20840927	20841379	5Y-H4K8ac_peak_8285	5.65584	RP11-17J14.2_ENSG00000266495
18	20860088	20860479	5Y-H4K8ac_peak_8286	5.65584	
18	21018666	21018902	5Y-H4K8ac_peak_8287	5.65584	TMEM241_ENSG00000134490
18	21033464	21033664	5Y-H4K8ac_peak_8288	5.35202	RIOK3_ENSG00000101782
18	21082704	21083239	5Y-H4K8ac_peak_8289	7.50148	C18orf8_ENSG00000141452
18	21242754	21242976	5Y-H4K8ac_peak_8290	4.51076	ANKRD29_ENSG00000154065
18	21269583	21270140	5Y-H4K8ac_peak_8291	6.08523	LAMA3_ENSG00000053747
18	21594135	21594455	5Y-H4K8ac_peak_8292	9.32595	RP11-403A21.2_ENSG00000264745
18	21594851	21595379	5Y-H4K8ac_peak_8293	4.77126	RP11-403A21.2_ENSG00000264745
18	22006325	22006520	5Y-H4K8ac_peak_8294	12.10416	IMPACT_ENSG00000154059
18	22006756	22007129	5Y-H4K8ac_peak_8295	14.86775	IMPACT_ENSG00000154059
18	22250935	22251147	5Y-H4K8ac_peak_8296	4.07874	
18	22251412	22251720	5Y-H4K8ac_peak_8297	6.14981	
18	22929478	22929896	5Y-H4K8ac_peak_8298	11.36844	
18	23184814	23185175	5Y-H4K8ac_peak_8299	16.38263	
18	23406202	23406417	5Y-H4K8ac_peak_8300	7.31815	
18	23670583	23670842	5Y-H4K8ac_peak_8301	18.06205	SS18_ENSG00000141380
18	23671110	23671458	5Y-H4K8ac_peak_8302	6.79955	SS18_ENSG00000141380
18	23806544	23806819	5Y-H4K8ac_peak_8303	5.03917	TAF4B_ENSG00000141384
18	24084058	24084690	5Y-H4K8ac_peak_8304	6.50117	
18	24128251	24128523	5Y-H4K8ac_peak_8305	14.31711	
18	24235607	24235814	5Y-H4K8ac_peak_8306	7.31815	AQP4-AS1_ENSG00000260372
18	24236364	24237296	5Y-H4K8ac_peak_8307	30.87849	KCTD1_ENSG00000134504;AQP4-AS1_ENSG00000260372
18	24443152	24443428	5Y-H4K8ac_peak_8308	5.23083	
18	24443635	24443898	5Y-H4K8ac_peak_8309	5.56912	
18	25757410	25757890	5Y-H4K8ac_peak_8310	14.1808	CDH2_ENSG00000170558
18	29264788	29265303	5Y-H4K8ac_peak_8311	8.75926	B4GALT6_ENSG00000118276;RP11-549B18.1_ENSG00000259985
18	29265714	29265985	5Y-H4K8ac_peak_8312	7.01266	B4GALT6_ENSG00000118276;RP11-549B18.1_ENSG00000259985
18	29522505	29522926	5Y-H4K8ac_peak_8313	5.64909	RP11-326K13.4_ENSG00000263823
18	29671903	29672323	5Y-H4K8ac_peak_8314	15.40182	RP11-53I6.3_ENSG00000265008;RNF138_ENSG00000134758
18	30050219	30050790	5Y-H4K8ac_peak_8315	9.38203	GAREM_ENSG00000141441
18	30350155	30350404	5Y-H4K8ac_peak_8316	5.449	AC012123.1_ENSG00000228835
18	30350747	30351166	5Y-H4K8ac_peak_8317	9.30505	AC012123.1_ENSG00000228835
18	31020500	31020882	5Y-H4K8ac_peak_8318	13.8759	CCDC178_ENSG00000166960
18	31157812	31158142	5Y-H4K8ac_peak_8319	6.77436	RP11-258B16.1_ENSG00000267642;ASXL3_ENSG00000141431
18	31158672	31158938	5Y-H4K8ac_peak_8320	4.77126	RP11-258B16.1_ENSG00000267642;ASXL3_ENSG00000141431
18	31739065	31739992	5Y-H4K8ac_peak_8321	13.2534	
18	31802555	31802873	5Y-H4K8ac_peak_8322	12.50545	RP11-379L18.1_ENSG00000267746
18	32702852	32703136	5Y-H4K8ac_peak_8323	5.35202	
18	32820581	32821242	5Y-H4K8ac_peak_8324	12.71156	ZNF397_ENSG00000186812

18	32870398	32870643	5Y-H4K8ac_peak_8325	9.38203	ZSCAN30_ENSG00000186814;RP11-158H5.7_ENSG00000268573;ZNF271_ENSG00000257267
18	32924022	32924379	5Y-H4K8ac_peak_8326	22.85923	
18	33161079	33161395	5Y-H4K8ac_peak_8327	11.22005	GALNT1_ENSG00000141429
18	33530133	33530396	5Y-H4K8ac_peak_8328	10.1994	
18	33709498	33709740	5Y-H4K8ac_peak_8329	16.79568	SLC39A6_ENSG00000141424;ELP2_ENSG00000134759
18	33709964	33710164	5Y-H4K8ac_peak_8330	7.97699	SLC39A6_ENSG00000141424;ELP2_ENSG00000134759
18	33767447	33767652	5Y-H4K8ac_peak_8331	7.3889	RP11-49I11.1_ENSG00000260552;MOCOS_ENSG00000075643
18	33944730	33944984	5Y-H4K8ac_peak_8332	6.20875	
18	33961962	33962296	5Y-H4K8ac_peak_8333	6.50117	
18	34408597	34408920	5Y-H4K8ac_peak_8334	7.89273	TPGS2_ENSG00000134779;KIAA1328_ENSG00000150477
18	35020202	35020430	5Y-H4K8ac_peak_8335	4.47071	
18	35025135	35025409	5Y-H4K8ac_peak_8336	4.36976	
18	35028458	35028882	5Y-H4K8ac_peak_8337	4.84727	
18	35029860	35030052	5Y-H4K8ac_peak_8338	7.11863	
18	35031582	35031949	5Y-H4K8ac_peak_8339	4.50834	
18	35082587	35082796	5Y-H4K8ac_peak_8340	6.47245	
18	35095495	35095768	5Y-H4K8ac_peak_8341	5.59843	
18	35097019	35097266	5Y-H4K8ac_peak_8342	8.79957	
18	35114948	35115215	5Y-H4K8ac_peak_8343	7.59101	
18	35130463	35130668	5Y-H4K8ac_peak_8344	4.07874	
18	35132299	35132557	5Y-H4K8ac_peak_8345	8.8021	
18	35146682	35147016	5Y-H4K8ac_peak_8346	14.31711	CELF4_ENSG00000101489
18	35297600	35297823	5Y-H4K8ac_peak_8347	4.84727	
18	37421193	37421447	5Y-H4K8ac_peak_8348	10.19948	
18	38971914	38972115	5Y-H4K8ac_peak_8349	5.04571	
18	39178798	39179022	5Y-H4K8ac_peak_8350	4.84727	
18	39252973	39253183	5Y-H4K8ac_peak_8351	7.31815	
18	39693890	39694082	5Y-H4K8ac_peak_8352	10.46287	
18	40857242	40857614	5Y-H4K8ac_peak_8353	7.31815	SYT4_ENSG00000132872
18	41170385	41170583	5Y-H4K8ac_peak_8354	6.73047	
18	41175363	41175580	5Y-H4K8ac_peak_8355	8.21525	
18	42259178	42259683	5Y-H4K8ac_peak_8356	13.2534	RP11-456K23.1_ENSG00000267414;SETBP1_ENSG00000152217
18	42260269	42260659	5Y-H4K8ac_peak_8357	10.69698	RP11-456K23.1_ENSG00000267414;SETBP1_ENSG00000152217
18	42323427	42324154	5Y-H4K8ac_peak_8358	10.3915	
18	42324350	42324603	5Y-H4K8ac_peak_8359	6.73385	
18	42596253	42596469	5Y-H4K8ac_peak_8360	7.38046	
18	43417275	43418109	5Y-H4K8ac_peak_8361	6.50117	
18	43418871	43419137	5Y-H4K8ac_peak_8362	4.95697	
18	43546994	43547184	5Y-H4K8ac_peak_8363	5.8635	EPG5_ENSG00000152223
18	43607910	43608477	5Y-H4K8ac_peak_8364	8.43511	RP11-8H2.1_ENSG00000267293
18	43652034	43652489	5Y-H4K8ac_peak_8365	6.08523	PSTPIP2_ENSG00000152229
18	43684436	43684710	5Y-H4K8ac_peak_8366	5.65584	ATP5A1_ENSG00000152234;HAUS1_ENSG00000152240
18	43753549	43753740	5Y-H4K8ac_peak_8367	6.53157	C18orf25_ENSG00000152242
18	43913433	43913812	5Y-H4K8ac_peak_8368	10.36926	
18	43914324	43914518	5Y-H4K8ac_peak_8369	9.15007	
18	44203461	44203843	5Y-H4K8ac_peak_8370	8.12305	

18	44292450	44292739	5Y-H4K8ac_peak_8371	6.49308	
18	44497609	44497857	5Y-H4K8ac_peak_8372	13.61292	KATNAL2_ENSG00000167216
18	45456661	45457416	5Y-H4K8ac_peak_8373	8.21582	SMAD2_ENSG00000175387
18	45457714	45457937	5Y-H4K8ac_peak_8374	7.03573	SMAD2_ENSG00000175387
18	45533905	45534661	5Y-H4K8ac_peak_8375	6.53157	
18	45548961	45549204	5Y-H4K8ac_peak_8376	7.63144	
18	45550119	45550715	5Y-H4K8ac_peak_8377	8.2913	
18	45560964	45561170	5Y-H4K8ac_peak_8378	5.64909	
18	45561661	45562826	5Y-H4K8ac_peak_8379	7.11863	
18	45564137	45564423	5Y-H4K8ac_peak_8380	4.84727	
18	45587071	45587457	5Y-H4K8ac_peak_8381	7.09444	
18	45591963	45592197	5Y-H4K8ac_peak_8382	5.64909	
18	45599062	45599311	5Y-H4K8ac_peak_8383	10.54764	
18	45599871	45600856	5Y-H4K8ac_peak_8384	7.89273	
18	45663068	45663261	5Y-H4K8ac_peak_8385	9.38276	
18	45688175	45688510	5Y-H4K8ac_peak_8386	8.564	
18	45712423	45712630	5Y-H4K8ac_peak_8387	10.31981	
18	45847208	45847621	5Y-H4K8ac_peak_8388	7.87406	
18	45868640	45869031	5Y-H4K8ac_peak_8389	8.564	
18	45972814	45973032	5Y-H4K8ac_peak_8390	8.43511	
18	46203519	46203746	5Y-H4K8ac_peak_8391	8.18236	
18	46278652	46279018	5Y-H4K8ac_peak_8392	4.07874	
18	46307137	46307336	5Y-H4K8ac_peak_8393	6.78318	
18	46308392	46308590	5Y-H4K8ac_peak_8394	10.39333	
18	46359534	46359752	5Y-H4K8ac_peak_8395	5.8635	
18	46385390	46388174	5Y-H4K8ac_peak_8396	10.4689	
18	46478663	46479442	5Y-H4K8ac_peak_8397	14.45841	
18	46501811	46502171	5Y-H4K8ac_peak_8398	12.21176	
18	46502429	46502993	5Y-H4K8ac_peak_8399	14.26884	
18	46544743	46544941	5Y-H4K8ac_peak_8400	4.82324	
18	46552038	46552354	5Y-H4K8ac_peak_8401	7.04637	
18	46578991	46579347	5Y-H4K8ac_peak_8402	5.64909	
18	46587248	46588125	5Y-H4K8ac_peak_8403	11.4254	
18	46588784	46589387	5Y-H4K8ac_peak_8404	9.38807	
18	46986697	46986946	5Y-H4K8ac_peak_8405	5.65584	DYM_ENSG00000141627
18	47339495	47339809	5Y-H4K8ac_peak_8406	4.24332	ACAA2_ENSG00000167315;SCARNA17_ENSG00000267322;SCARNA17_ENSG00000251992;SCARNA18_ENSG00000252139
18	47435929	47436164	5Y-H4K8ac_peak_8407	4.90471	
18	47807579	47808080	5Y-H4K8ac_peak_8408	4.0639	MBD1_ENSG00000141644
18	47808369	47808651	5Y-H4K8ac_peak_8409	5.5192	MBD1_ENSG00000141644
18	48085839	48086191	5Y-H4K8ac_peak_8410	7.31815	MAPK4_ENSG00000141639
18	48086510	48087354	5Y-H4K8ac_peak_8411	7.59101	MAPK4_ENSG00000141639
18	48254468	48254816	5Y-H4K8ac_peak_8412	4.84727	
18	48493608	48494066	5Y-H4K8ac_peak_8413	9.34555	ELAC1_ENSG00000141642;RP11-729L2.2_ENSG00000267699;SMAD4_ENSG00000141646
18	48494473	48494772	5Y-H4K8ac_peak_8414	10.90097	ELAC1_ENSG00000141642;RP11-729L2.2_ENSG00000267699;SMAD4_ENSG00000141646
18	48556646	48556929	5Y-H4K8ac_peak_8415	11.71078	
18	48636364	48636795	5Y-H4K8ac_peak_8416	6.98118	

18	48723318	48723565	5Y-H4K8ac_peak_8417	10.46287	
18	48724856	48725114	5Y-H4K8ac_peak_8418	8.21582	
18	52442225	52442435	5Y-H4K8ac_peak_8419	6.62622	
18	52968554	52968775	5Y-H4K8ac_peak_8420	11.1169	
18	52989770	52990125	5Y-H4K8ac_peak_8421	25.32943	
18	53005180	53005600	5Y-H4K8ac_peak_8422	6.34046	
18	53255229	53255444	5Y-H4K8ac_peak_8423	9.38203	
18	53257300	53257715	5Y-H4K8ac_peak_8424	8.69112	
18	53447003	53447796	5Y-H4K8ac_peak_8425	15.06486	
18	54305431	54305726	5Y-H4K8ac_peak_8426	4.15658	
18	54318723	54318981	5Y-H4K8ac_peak_8427	5.23819	TXNL1_ENSG00000091164;WDR7_ENSG00000091157
18	54715695	54716358	5Y-H4K8ac_peak_8428	6.49308	
18	55018978	55019398	5Y-H4K8ac_peak_8429	5.65584	ST8SIA3_ENSG00000177511
18	55020146	55020557	5Y-H4K8ac_peak_8430	7.38046	
18	55021053	55021687	5Y-H4K8ac_peak_8431	7.50148	
18	55253594	55254181	5Y-H4K8ac_peak_8432	6.08523	FECH_ENSG00000066926
18	55289258	55289460	5Y-H4K8ac_peak_8433	8.59175	NARS_ENSG00000134440
18	55297167	55297384	5Y-H4K8ac_peak_8434	10.69698	RP11-35G9.3_ENSG00000267040
18	55583419	55583729	5Y-H4K8ac_peak_8435	9.30505	
18	56451946	56452238	5Y-H4K8ac_peak_8436	4.5753	
18	56530891	56531329	5Y-H4K8ac_peak_8437	7.64648	
18	57137979	57138260	5Y-H4K8ac_peak_8438	4.84727	
18	57367440	57367684	5Y-H4K8ac_peak_8439	8.93923	
18	57386739	57386929	5Y-H4K8ac_peak_8440	4.84727	
18	57566854	57567049	5Y-H4K8ac_peak_8441	6.53157	PMAIP1_ENSG00000141682
18	59001770	59001972	5Y-H4K8ac_peak_8442	5.98695	CDH20_ENSG00000101542
18	59249083	59249487	5Y-H4K8ac_peak_8443	6.34046	
18	59465672	59465919	5Y-H4K8ac_peak_8444	6.34046	
18	59466126	59466366	5Y-H4K8ac_peak_8445	4.84727	
18	59490920	59491129	5Y-H4K8ac_peak_8446	4.14761	
18	59561943	59562169	5Y-H4K8ac_peak_8447	4.50834	RNF152_ENSG00000176641
18	60755463	60756009	5Y-H4K8ac_peak_8448	5.65584	
18	60987692	60987956	5Y-H4K8ac_peak_8449	18.76707	BCL2_ENSG00000171791
18	60988163	60988526	5Y-H4K8ac_peak_8450	8.33296	BCL2_ENSG00000171791
18	61089322	61089628	5Y-H4K8ac_peak_8451	9.78792	VPS4B_ENSG00000119541
18	61089943	61090191	5Y-H4K8ac_peak_8452	8.67475	VPS4B_ENSG00000119541
18	61603767	61604126	5Y-H4K8ac_peak_8453	9.31608	
18	61637327	61637718	5Y-H4K8ac_peak_8454	11.71078	SERPINB8_ENSG00000166401
18	66381962	66382260	5Y-H4K8ac_peak_8455	11.36844	TMX3_ENSG00000166479;CCDC102B_ENSG00000150636
18	67067662	67068036	5Y-H4K8ac_peak_8456	16.38263	DOK6_ENSG00000206052
18	67955632	67956028	5Y-H4K8ac_peak_8457	7.38046	SOCS6_ENSG00000170677
18	67956245	67957032	5Y-H4K8ac_peak_8458	6.08523	SOCS6_ENSG00000170677
18	68138518	68138750	5Y-H4K8ac_peak_8459	5.03335	
18	68650658	68650982	5Y-H4K8ac_peak_8460	7.01266	
18	68695550	68695876	5Y-H4K8ac_peak_8461	9.30505	RP11-529J17.1_ENSG00000265639
18	68696208	68696828	5Y-H4K8ac_peak_8462	12.55063	RP11-529J17.1_ENSG00000265639

18	70210998	70211189	5Y-H4K8ac_peak_8463	8.47164	
18	72162900	72163172	5Y-H4K8ac_peak_8464	7.17184	CNDP2_ENSG00000133313
18	72264532	72264900	5Y-H4K8ac_peak_8465	8.73985	LINC00909_ENSG00000264247;ZNF407_ENSG00000215421
18	72837317	72838131	5Y-H4K8ac_peak_8466	7.20869	
18	72916010	72916259	5Y-H4K8ac_peak_8467	6.50117	
18	72960804	72961191	5Y-H4K8ac_peak_8468	11.1169	
18	73627955	73628350	5Y-H4K8ac_peak_8469	5.10404	
18	74397908	74398099	5Y-H4K8ac_peak_8470	6.34046	
18	74533922	74534175	5Y-H4K8ac_peak_8471	6.34046	RP11-162A12.2_ENSG00000264278;ZNF236_ENSG00000130856
18	74534395	74534685	5Y-H4K8ac_peak_8472	17.51157	RP11-162A12.2_ENSG00000264278;ZNF236_ENSG00000130856
18	74535017	74535350	5Y-H4K8ac_peak_8473	8.43511	RP11-162A12.2_ENSG00000264278;ZNF236_ENSG00000130856
18	76828644	76829039	5Y-H4K8ac_peak_8474	15.06486	ATP9B_ENSG00000166377
18	76829460	76829796	5Y-H4K8ac_peak_8475	6.34046	ATP9B_ENSG00000166377
18	77439021	77439217	5Y-H4K8ac_peak_8476	5.85188	RP11-567M16.3_ENSG00000178412;CTDP1_ENSG00000060069
18	77711851	77712497	5Y-H4K8ac_peak_8477	8.69112	PQLC1_ENSG00000122490
18	77724009	77724714	5Y-H4K8ac_peak_8478	8.43511	HSBP1L1_ENSG00000226742
18	77748706	77748946	5Y-H4K8ac_peak_8479	10.69698	
18	77866916	77867300	5Y-H4K8ac_peak_8480	15.82359	ADNP2_ENSG00000101544
19	267244	267772	5Y-H4K8ac_peak_8481	7.89273	
19	290713	291151	5Y-H4K8ac_peak_8482	9.51254	PPAP2C_ENSG00000141934
19	291740	292232	5Y-H4K8ac_peak_8483	8.53555	PPAP2C_ENSG00000141934
19	344603	345401	5Y-H4K8ac_peak_8484	7.11863	MIER2_ENSG00000105556
19	408776	409079	5Y-H4K8ac_peak_8485	15.44907	C2CD4C_ENSG00000183186
19	460825	461271	5Y-H4K8ac_peak_8486	7.38046	SHC2_ENSG00000129946
19	488538	488873	5Y-H4K8ac_peak_8487	11.22005	MADCAM1_ENSG00000099866
19	489552	489909	5Y-H4K8ac_peak_8488	7.59101	MADCAM1_ENSG00000099866
19	524127	524797	5Y-H4K8ac_peak_8489	11.37188	
19	531244	531545	5Y-H4K8ac_peak_8490	6.37023	CDC34_ENSG00000099804
19	532124	532467	5Y-H4K8ac_peak_8491	6.13919	CDC34_ENSG00000099804
19	572716	573186	5Y-H4K8ac_peak_8492	11.1169	AC009005.2_ENSG00000267751
19	608684	608930	5Y-H4K8ac_peak_8493	7.89273	
19	609301	609521	5Y-H4K8ac_peak_8494	6.08523	
19	610935	611513	5Y-H4K8ac_peak_8495	8.43511	
19	633189	633463	5Y-H4K8ac_peak_8496	11.1169	POLRMT_ENSG00000099821
19	664709	664900	5Y-H4K8ac_peak_8497	5.59843	
19	665176	665377	5Y-H4K8ac_peak_8498	8.49231	
19	680116	680320	5Y-H4K8ac_peak_8499	11.48691	
19	681363	681560	5Y-H4K8ac_peak_8500	4.50834	
19	708230	708703	5Y-H4K8ac_peak_8501	10.19948	PALM_ENSG00000099864
19	719634	719890	5Y-H4K8ac_peak_8502	7.18391	
19	788818	790074	5Y-H4K8ac_peak_8503	8.62703	
19	796144	796891	5Y-H4K8ac_peak_8504	13.27443	PTBP1_ENSG00000011304
19	821344	821553	5Y-H4K8ac_peak_8505	9.52603	LPPR3_ENSG00000129951
19	821828	822154	5Y-H4K8ac_peak_8506	6.37023	LPPR3_ENSG00000129951
19	913180	913756	5Y-H4K8ac_peak_8507	6.77436	R3HDM4_ENSG00000198858
19	917248	917586	5Y-H4K8ac_peak_8508	4.29586	KISS1R_ENSG00000116014

19	924841	925090	5Y-H4K8ac_peak_8509	9.30505	ARID3A_ENSG00000116017
19	925513	925704	5Y-H4K8ac_peak_8510	9.30206	ARID3A_ENSG00000116017
19	926183	926810	5Y-H4K8ac_peak_8511	8.43511	ARID3A_ENSG00000116017
19	932588	933309	5Y-H4K8ac_peak_8512	8.62703	
19	933508	933760	5Y-H4K8ac_peak_8513	9.48719	
19	934131	934503	5Y-H4K8ac_peak_8514	4.29586	
19	935015	936122	5Y-H4K8ac_peak_8515	11.31171	
19	940314	941290	5Y-H4K8ac_peak_8516	9.30505	
19	944460	944860	5Y-H4K8ac_peak_8517	17.32862	
19	945443	945903	5Y-H4K8ac_peak_8518	6.37023	
19	956525	956874	5Y-H4K8ac_peak_8519	7.18391	
19	983597	984173	5Y-H4K8ac_peak_8520	6.65369	WDR18_ENSG00000065268
19	1026281	1026490	5Y-H4K8ac_peak_8521	9.02782	CNN2_ENSG00000064666
19	1026998	1027221	5Y-H4K8ac_peak_8522	5.449	CNN2_ENSG00000064666
19	1027895	1028090	5Y-H4K8ac_peak_8523	6.77436	
19	1028789	1029060	5Y-H4K8ac_peak_8524	8.63676	
19	1074430	1074796	5Y-H4K8ac_peak_8525	14.00559	
19	1074994	1075226	5Y-H4K8ac_peak_8526	7.10001	
19	1105060	1105253	5Y-H4K8ac_peak_8527	5.98695	
19	1203055	1203331	5Y-H4K8ac_peak_8528	6.76096	HMGB2P1_ENSG00000267736
19	1237518	1237892	5Y-H4K8ac_peak_8529	7.82914	C19orf26_ENSG00000099625;AC004221.2_ENSG00000267778
19	1239903	1240225	5Y-H4K8ac_peak_8530	12.22486	
19	1247995	1249571	5Y-H4K8ac_peak_8531	16.79568	MIDN_ENSG00000167470
19	1251125	1251354	5Y-H4K8ac_peak_8532	10.58128	
19	1252728	1252958	5Y-H4K8ac_peak_8533	6.34245	
19	1261346	1261645	5Y-H4K8ac_peak_8534	5.87725	
19	1269376	1269631	5Y-H4K8ac_peak_8535	12.52787	CIRBP-AS1_ENSG00000267493
19	1275099	1275339	5Y-H4K8ac_peak_8536	6.10343	C19orf24_ENSG00000228300
19	1285479	1285715	5Y-H4K8ac_peak_8537	10.74494	MUM1_ENSG00000160953;EFNA2_ENSG00000099617
19	1286443	1286699	5Y-H4K8ac_peak_8538	5.5369	MUM1_ENSG00000160953;EFNA2_ENSG00000099617
19	1324881	1325104	5Y-H4K8ac_peak_8539	5.23083	
19	1325472	1325676	5Y-H4K8ac_peak_8540	5.64909	
19	1354219	1354745	5Y-H4K8ac_peak_8541	22.1484	
19	1383948	1384452	5Y-H4K8ac_peak_8542	11.09973	NDUFS7_ENSG00000115286
19	1401698	1401910	5Y-H4K8ac_peak_8543	8.82628	GAMT_ENSG00000130005
19	1407787	1408317	5Y-H4K8ac_peak_8544	9.72967	DAZAP1_ENSG00000071626
19	1448954	1449441	5Y-H4K8ac_peak_8545	8.18236	
19	1450164	1450885	5Y-H4K8ac_peak_8546	12.11208	
19	1473386	1473853	5Y-H4K8ac_peak_8547	6.62622	
19	1479469	1479846	5Y-H4K8ac_peak_8548	7.11863	C19orf25_ENSG00000119559
19	1503273	1503664	5Y-H4K8ac_peak_8549	6.03632	
19	1566848	1567357	5Y-H4K8ac_peak_8550	8.37729	MEX3D_ENSG00000181588
19	1568563	1568805	5Y-H4K8ac_peak_8551	12.02397	MEX3D_ENSG00000181588
19	1591923	1592312	5Y-H4K8ac_peak_8552	15.92074	MBD3_ENSG00000071655
19	1605082	1605366	5Y-H4K8ac_peak_8553	6.31818	UQCR11_ENSG00000267059;UQCR11_ENSG00000127540
19	1651541	1652255	5Y-H4K8ac_peak_8554	12.64868	TCF3_ENSG00000071564

19	1652681	1652907	5Y-H4K8ac_peak_8555	26.19116	TCF3_ENSG00000071564
19	1653242	1653432	5Y-H4K8ac_peak_8556	6.87095	TCF3_ENSG00000071564
19	1667432	1667672	5Y-H4K8ac_peak_8557	6.50117	
19	1725253	1725665	5Y-H4K8ac_peak_8558	13.99721	
19	1725880	1726272	5Y-H4K8ac_peak_8559	6.77436	
19	1848033	1848717	5Y-H4K8ac_peak_8560	18.10232	REXO1_ENSG00000079313
19	1855603	1856036	5Y-H4K8ac_peak_8561	8.43511	
19	1864711	1865540	5Y-H4K8ac_peak_8562	6.49458	
19	1905429	1906348	5Y-H4K8ac_peak_8563	9.7353	SCAMP4_ENSG00000227500;ADAT3_ENSG00000213638
19	2014801	2015055	5Y-H4K8ac_peak_8564	6.38173	
19	2032350	2032959	5Y-H4K8ac_peak_8565	11.9478	
19	2041648	2042051	5Y-H4K8ac_peak_8566	7.11863	
19	2042372	2042836	5Y-H4K8ac_peak_8567	5.41472	
19	2051506	2051767	5Y-H4K8ac_peak_8568	4.0639	MKNK2_ENSG00000099875
19	2051978	2052212	5Y-H4K8ac_peak_8569	10.6196	MKNK2_ENSG00000099875
19	2055937	2056626	5Y-H4K8ac_peak_8570	13.99721	
19	2059480	2060247	5Y-H4K8ac_peak_8571	11.22005	
19	2060753	2061375	5Y-H4K8ac_peak_8572	12.10416	
19	2062170	2062444	5Y-H4K8ac_peak_8573	7.87406	
19	2096081	2096559	5Y-H4K8ac_peak_8574	12.57157	MOB3A_ENSG00000172081;IZUMO4_ENSG00000099840
19	2163200	2163622	5Y-H4K8ac_peak_8575	12.11208	AP3D1_ENSG00000065000;DOT1L_ENSG00000104885
19	2164284	2164543	5Y-H4K8ac_peak_8576	8.43511	AP3D1_ENSG00000065000;DOT1L_ENSG00000104885
19	2221642	2222134	5Y-H4K8ac_peak_8577	14.30055	
19	2235760	2235989	5Y-H4K8ac_peak_8578	4.94889	SF3A2_ENSG00000104897
19	2236907	2237466	5Y-H4K8ac_peak_8579	10.0695	PLEKHJ1_ENSG00000104886;SF3A2_ENSG00000104897
19	2243107	2243568	5Y-H4K8ac_peak_8580	11.02589	
19	2307742	2308003	5Y-H4K8ac_peak_8581	7.46829	LINGO3_ENSG00000220008
19	2308262	2308666	5Y-H4K8ac_peak_8582	13.10076	LINGO3_ENSG00000220008
19	2427142	2427895	5Y-H4K8ac_peak_8583	16.94054	TIMM13_ENSG00000099800
19	2462013	2462516	5Y-H4K8ac_peak_8584	9.02782	AC005624.2_ENSG00000267201
19	2543317	2544183	5Y-H4K8ac_peak_8585	7.89273	
19	2721107	2721323	5Y-H4K8ac_peak_8586	9.38276	DIRAS1_ENSG00000176490
19	2739393	2740032	5Y-H4K8ac_peak_8587	6.34046	AC006538.4_ENSG00000267001;SLC39A3_ENSG00000141873
19	2740273	2740824	5Y-H4K8ac_peak_8588	4.50834	AC006538.4_ENSG00000267001;SLC39A3_ENSG00000141873
19	2785580	2785816	5Y-H4K8ac_peak_8589	6.22904	THOP1_ENSG00000172009
19	2812736	2813647	5Y-H4K8ac_peak_8590	10.90365	
19	2841017	2841219	5Y-H4K8ac_peak_8591	9.36633	ZNF555_ENSG00000186300
19	2841531	2841955	5Y-H4K8ac_peak_8592	4.8509	ZNF555_ENSG00000186300
19	2982641	2982857	5Y-H4K8ac_peak_8593	8.12305	
19	2983562	2983907	5Y-H4K8ac_peak_8594	9.7353	
19	2987199	2987426	5Y-H4K8ac_peak_8595	5.89922	
19	3005708	3006035	5Y-H4K8ac_peak_8596	6.50117	
19	3034786	3035176	5Y-H4K8ac_peak_8597	5.77617	
19	3035962	3036163	5Y-H4K8ac_peak_8598	4.36976	
19	3046610	3046820	5Y-H4K8ac_peak_8599	8.60024	TLE2_ENSG00000065717
19	3061547	3061774	5Y-H4K8ac_peak_8600	4.642	

19	3062572	3062907	5Y-H4K8ac_peak_8601	8.45687	AES_ENSG00000104964
19	3067679	3067944	5Y-H4K8ac_peak_8602	8.79957	
19	3076212	3076492	5Y-H4K8ac_peak_8603	4.27459	
19	3079707	3079916	5Y-H4K8ac_peak_8604	4.84727	
19	3093585	3094109	5Y-H4K8ac_peak_8605	6.06152	GNA11_ENSG00000088256
19	3196929	3197137	5Y-H4K8ac_peak_8606	5.41472	
19	3336700	3337556	5Y-H4K8ac_peak_8607	11.09973	
19	3358190	3358485	5Y-H4K8ac_peak_8608	9.30505	
19	3359811	3360075	5Y-H4K8ac_peak_8609	13.99721	NFIC_ENSG00000141905
19	3365674	3365884	5Y-H4K8ac_peak_8610	9.30505	
19	3366753	3367592	5Y-H4K8ac_peak_8611	15.88359	
19	3386578	3386813	5Y-H4K8ac_peak_8612	11.28133	
19	3387148	3387765	5Y-H4K8ac_peak_8613	5.72233	
19	3388182	3388562	5Y-H4K8ac_peak_8614	9.87097	
19	3404933	3405315	5Y-H4K8ac_peak_8615	5.65584	
19	3428520	3428719	5Y-H4K8ac_peak_8616	5.98695	
19	3434610	3435266	5Y-H4K8ac_peak_8617	10.1994	
19	3435472	3435928	5Y-H4K8ac_peak_8618	6.1654	
19	3441392	3442354	5Y-H4K8ac_peak_8619	12.21176	
19	3500454	3500858	5Y-H4K8ac_peak_8620	8.24461	DOHH_ENSG00000129932;RN7SL866P_ENSG00000264159
19	3537099	3537295	5Y-H4K8ac_peak_8621	8.67475	
19	3573097	3573509	5Y-H4K8ac_peak_8622	6.78318	MFSD12_ENSG00000161091;HMG20B_ENSG00000064961
19	3585824	3586054	5Y-H4K8ac_peak_8623	4.29586	GIPC3_ENSG00000179855
19	3606252	3606523	5Y-H4K8ac_peak_8624	9.18646	TBXA2R_ENSG00000006638;CACTIN-AS1_ENSG00000226800
19	3626452	3626721	5Y-H4K8ac_peak_8625	8.79399	CACTIN_ENSG00000105298
19	3801323	3801748	5Y-H4K8ac_peak_8626	5.03335	MATK_ENSG00000007264
19	3825958	3826768	5Y-H4K8ac_peak_8627	8.93923	
19	3868781	3869253	5Y-H4K8ac_peak_8628	5.67588	ZFR2_ENSG00000105278
19	3988176	3988542	5Y-H4K8ac_peak_8629	6.94897	
19	3990524	3990919	5Y-H4K8ac_peak_8630	7.38046	
19	4007781	4008018	5Y-H4K8ac_peak_8631	9.87097	PIAS4_ENSG00000105229
19	4014759	4016384	5Y-H4K8ac_peak_8632	15.84891	
19	4066330	4066622	5Y-H4K8ac_peak_8633	8.90223	ZBTB7A_ENSG00000178951
19	4140646	4141116	5Y-H4K8ac_peak_8634	9.11354	
19	4181916	4182496	5Y-H4K8ac_peak_8635	14.19462	SIRT6_ENSG00000077463;ANKRD24_ENSG00000089847
19	4232895	4233191	5Y-H4K8ac_peak_8636	7.63144	
19	4246531	4246764	5Y-H4K8ac_peak_8637	4.95697	CCDC94_ENSG00000105248
19	4267585	4267786	5Y-H4K8ac_peak_8638	9.15977	
19	4277793	4278458	5Y-H4K8ac_peak_8639	12.57157	SHD_ENSG00000105251
19	4278792	4280028	5Y-H4K8ac_peak_8640	15.82359	SHD_ENSG00000105251
19	4302034	4302448	5Y-H4K8ac_peak_8641	11.19336	TMIGD2_ENSG00000167664
19	4342106	4342647	5Y-H4K8ac_peak_8642	7.89273	STAP2_ENSG00000178078;MPND_ENSG00000008382
19	4374139	4374788	5Y-H4K8ac_peak_8643	11.04542	
19	4375959	4376382	5Y-H4K8ac_peak_8644	4.70571	
19	4386105	4386315	5Y-H4K8ac_peak_8645	5.22422	
19	4470697	4471192	5Y-H4K8ac_peak_8646	6.73385	CTB-50L17.16_ENSG00000267011

19	4472594	4472795	5Y-H4K8ac_peak_8647	6.03255	HDGFRP2_ENSG00000167674
19	4474923	4475243	5Y-H4K8ac_peak_8648	10.23399	
19	4531700	4531924	5Y-H4K8ac_peak_8649	6.98118	
19	4584361	4584865	5Y-H4K8ac_peak_8650	11.5813	
19	4636082	4636341	5Y-H4K8ac_peak_8651	14.31711	
19	4685115	4685341	5Y-H4K8ac_peak_8652	3.91125	
19	4738466	4738941	5Y-H4K8ac_peak_8653	9.02242	
19	4742752	4743178	5Y-H4K8ac_peak_8654	7.89273	
19	4760104	4760323	5Y-H4K8ac_peak_8655	9.22011	
19	4791768	4792388	5Y-H4K8ac_peak_8656	18.28763	AC005523.3_ENSG00000268536;FEM1A_ENSG00000141965
19	4811585	4811820	5Y-H4K8ac_peak_8657	5.98695	
19	4816015	4816822	5Y-H4K8ac_peak_8658	9.30505	
19	4830892	4831200	5Y-H4K8ac_peak_8659	4.71803	TICAM1_ENSG00000127666
19	4831494	4831915	5Y-H4K8ac_peak_8660	6.37023	TICAM1_ENSG00000127666
19	4908893	4909273	5Y-H4K8ac_peak_8661	12.45737	
19	5036424	5036795	5Y-H4K8ac_peak_8662	5.60566	
19	5038707	5038990	5Y-H4K8ac_peak_8663	5.34199	
19	5144733	5144929	5Y-H4K8ac_peak_8664	4.35388	
19	5308409	5308695	5Y-H4K8ac_peak_8665	6.78128	
19	5436090	5436326	5Y-H4K8ac_peak_8666	10.74739	
19	5539284	5539542	5Y-H4K8ac_peak_8667	9.31608	
19	5687470	5687840	5Y-H4K8ac_peak_8668	7.09658	
19	5719735	5720113	5Y-H4K8ac_peak_8669	9.15007	LONP1_ENSG00000196365;CATSPERD_ENSG00000174898
19	5772866	5773107	5Y-H4K8ac_peak_8670	9.01738	
19	5773404	5773899	5Y-H4K8ac_peak_8671	6.08523	
19	5774128	5774340	5Y-H4K8ac_peak_8672	7.84116	
19	5790875	5791141	5Y-H4K8ac_peak_8673	6.08471	DUS3L_ENSG00000141994
19	5799515	5800311	5Y-H4K8ac_peak_8674	12.05638	
19	5826903	5827227	5Y-H4K8ac_peak_8675	5.35202	
19	5827425	5827670	5Y-H4K8ac_peak_8676	7.38046	
19	5828470	5829285	5Y-H4K8ac_peak_8677	9.07085	
19	5876624	5876891	5Y-H4K8ac_peak_8678	4.77126	
19	5892037	5892557	5Y-H4K8ac_peak_8679	5.449	
19	5894275	5894479	5Y-H4K8ac_peak_8680	5.98695	
19	5895089	5895493	5Y-H4K8ac_peak_8681	5.64909	
19	5927614	5927972	5Y-H4K8ac_peak_8682	5.98695	
19	5933056	5933816	5Y-H4K8ac_peak_8683	5.64909	
19	5935896	5936458	5Y-H4K8ac_peak_8684	9.77183	
19	5951943	5952234	5Y-H4K8ac_peak_8685	6.1654	
19	5992965	5993201	5Y-H4K8ac_peak_8686	6.34245	
19	5994935	5995405	5Y-H4K8ac_peak_8687	8.43511	
19	6241511	6242018	5Y-H4K8ac_peak_8688	5.64909	
19	6361582	6361817	5Y-H4K8ac_peak_8689	10.21117	CTB-180A7.3_ENSG00000269802;CLPP_ENSG00000125656
19	6393599	6394316	5Y-H4K8ac_peak_8690	12.52787	GTF2F1_ENSG00000125651;CTB-180A7.8_ENSG00000214347
19	6425661	6426116	5Y-H4K8ac_peak_8691	8.47164	KHSRP_ENSG00000088247
19	6459004	6459735	5Y-H4K8ac_peak_8692	10.47399	

19	6475730	6475988	5Y-H4K8ac_peak_8693	4.07874	
19	6476392	6476614	5Y-H4K8ac_peak_8694	11.36844	
19	6737289	6737562	5Y-H4K8ac_peak_8695	19.26368	GPR108_ENSG00000125734;TRIP10_ENSG00000125733
19	6738243	6738487	5Y-H4K8ac_peak_8696	7.89273	GPR108_ENSG00000125734;TRIP10_ENSG00000125733
19	6738935	6739377	5Y-H4K8ac_peak_8697	6.06152	TRIP10_ENSG00000125733
19	7069491	7069935	5Y-H4K8ac_peak_8698	8.43511	ZNF557_ENSG00000130544
19	7098822	7099199	5Y-H4K8ac_peak_8699	8.84534	
19	7197056	7197422	5Y-H4K8ac_peak_8700	6.50117	
19	7444831	7445118	5Y-H4K8ac_peak_8701	5.23083	CTD-2207O23.3_ENSG00000268861
19	7460070	7460424	5Y-H4K8ac_peak_8702	12.21176	CTB-133G6.2_ENSG00000267852;ARHGEF18_ENSG00000104880
19	7548238	7548456	5Y-H4K8ac_peak_8703	5.24695	
19	7559128	7559807	5Y-H4K8ac_peak_8704	13.06587	
19	7566206	7566527	5Y-H4K8ac_peak_8705	8.19789	
19	7570723	7570937	5Y-H4K8ac_peak_8706	4.69231	
19	7580223	7580885	5Y-H4K8ac_peak_8707	14.31711	ZNF358_ENSG00000198816
19	7581248	7581616	5Y-H4K8ac_peak_8708	8.24461	
19	7581813	7582039	5Y-H4K8ac_peak_8709	6.77436	
19	7598443	7598887	5Y-H4K8ac_peak_8710	6.96612	CTD-2207O23.10_ENSG00000268614;PNPLA6_ENSG00000032444
19	7600829	7601210	5Y-H4K8ac_peak_8711	4.90696	
19	7615752	7615967	5Y-H4K8ac_peak_8712	10.27264	
19	7616580	7616854	5Y-H4K8ac_peak_8713	7.38046	
19	7682625	7683117	5Y-H4K8ac_peak_8714	9.22312	
19	7745767	7746183	5Y-H4K8ac_peak_8715	11.86504	TRAPPC5_ENSG00000181029
19	7938981	7939936	5Y-H4K8ac_peak_8716	12.11208	CTD-3193O13.9_ENSG00000183248
19	7967870	7968319	5Y-H4K8ac_peak_8717	8.16382	AC010336.1_ENSG00000214248;RN7SL115P_ENSG00000264166;MAP2K7_ENSG00000076984
19	7981440	7981711	5Y-H4K8ac_peak_8718	4.95697	TGFBR3L_ENSG00000260001
19	7984619	7984911	5Y-H4K8ac_peak_8719	4.50834	CTD-3193O13.1_ENSG00000260500;SNAPC2_ENSG00000104976
19	8008032	8008467	5Y-H4K8ac_peak_8720	11.1169	TIMM44_ENSG00000104980
19	8115130	8115474	5Y-H4K8ac_peak_8721	9.23159	
19	8273363	8273870	5Y-H4K8ac_peak_8722	15.21338	
19	8432037	8432230	5Y-H4K8ac_peak_8723	8.08609	
19	8455315	8455524	5Y-H4K8ac_peak_8724	12.00715	RAB11B-AS1_ENSG00000269386;RAB11B_ENSG00000185236
19	8478215	8478511	5Y-H4K8ac_peak_8725	11.1169	MARCH2_ENSG00000099785
19	8509984	8510359	5Y-H4K8ac_peak_8726	10.69698	HNRNPM_ENSG00000099783
19	8577649	8578080	5Y-H4K8ac_peak_8727	7.34185	ZNF414_ENSG00000133250
19	8579304	8579530	5Y-H4K8ac_peak_8728	5.98695	ZNF414_ENSG00000133250
19	8674248	8674530	5Y-H4K8ac_peak_8729	7.11863	
19	9394073	9394594	5Y-H4K8ac_peak_8730	6.50117	
19	9419898	9420216	5Y-H4K8ac_peak_8731	4.03329	ZNF699_ENSG00000196110
19	9546313	9546525	5Y-H4K8ac_peak_8732	9.89244	ZNF266_ENSG00000174652
19	9649030	9649602	5Y-H4K8ac_peak_8733	8.69112	ZNF426_ENSG00000130818
19	9694943	9695144	5Y-H4K8ac_peak_8734	10.15788	ZNF121_ENSG00000197961
19	9731614	9732381	5Y-H4K8ac_peak_8735	6.37023	ZNF561_ENSG00000171469;C19orf82_ENSG00000267106
19	9785906	9786168	5Y-H4K8ac_peak_8736	9.01738	ZNF562_ENSG00000171466
19	9878860	9879073	5Y-H4K8ac_peak_8737	4.90696	
19	9903697	9903912	5Y-H4K8ac_peak_8738	5.23083	ZNF846_ENSG00000196605;CTD-2623N2.5_ENSG00000266950

19	9929498	9929706	5Y-H4K8ac_peak_8739	10.12457	SNORA70_ENSG00000200237
19	9946172	9946367	5Y-H4K8ac_peak_8740	5.77617	CTD-2623N2.11_ENSG00000267289;PIN1_ENSG00000127445
19	10056090	10056946	5Y-H4K8ac_peak_8741	7.50148	
19	10197590	10197991	5Y-H4K8ac_peak_8742	7.24844	C19orf66_ENSG00000130813
19	10207113	10207596	5Y-H4K8ac_peak_8743	11.88337	
19	10216435	10216691	5Y-H4K8ac_peak_8744	6.53157	PPAN-P2RY11_ENSG00000243207;PPAN_ENSG00000130810
19	10217064	10217280	5Y-H4K8ac_peak_8745	7.64648	PPAN-P2RY11_ENSG00000243207;PPAN_ENSG00000130810
19	10248860	10249519	5Y-H4K8ac_peak_8746	13.03079	
19	10304977	10305600	5Y-H4K8ac_peak_8747	10.1994	
19	10337297	10337516	5Y-H4K8ac_peak_8748	6.88532	
19	10337961	10338272	5Y-H4K8ac_peak_8749	6.77436	
19	10341223	10341927	5Y-H4K8ac_peak_8750	8.3285	DNMT1_ENSG00000130816;S1PR2_ENSG00000267534;MIR4322_ENSG00000264266
19	10515556	10516028	5Y-H4K8ac_peak_8751	7.01266	
19	10613070	10614436	5Y-H4K8ac_peak_8752	21.45562	KEAP1_ENSG00000079999
19	10654156	10654398	5Y-H4K8ac_peak_8753	7.59101	ATG4D_ENSG00000130734
19	10654625	10654857	5Y-H4K8ac_peak_8754	10.35586	ATG4D_ENSG00000130734
19	10676189	10676496	5Y-H4K8ac_peak_8755	6.37023	KRI1_ENSG00000129347
19	10679958	10680403	5Y-H4K8ac_peak_8756	10.69698	CDKN2D_ENSG00000129355
19	10713753	10713996	5Y-H4K8ac_peak_8757	5.98695	SLC44A2_ENSG00000129353
19	10735922	10736415	5Y-H4K8ac_peak_8758	6.77436	
19	10754934	10755400	5Y-H4K8ac_peak_8759	7.11863	
19	10765032	10765289	5Y-H4K8ac_peak_8760	14.59308	ILF3-AS1_ENSG00000267100;ILF3_ENSG00000129351
19	10827475	10827796	5Y-H4K8ac_peak_8761	9.96543	DNM2_ENSG00000079805
19	10928072	10928332	5Y-H4K8ac_peak_8762	10.1994	MIR199A1_ENSG00000207752
19	10945654	10945875	5Y-H4K8ac_peak_8763	7.31807	
19	10946682	10946902	5Y-H4K8ac_peak_8764	7.89273	TMED1_ENSG00000099203;C19orf38_ENSG00000214212
19	10947242	10947819	5Y-H4K8ac_peak_8765	13.2534	TMED1_ENSG00000099203;C19orf38_ENSG00000214212
19	10974839	10975141	5Y-H4K8ac_peak_8766	8.67475	
19	10975428	10975818	5Y-H4K8ac_peak_8767	7.38046	
19	10980685	10980893	5Y-H4K8ac_peak_8768	5.28616	
19	10989653	10989875	5Y-H4K8ac_peak_8769	9.23159	
19	10998632	10998835	5Y-H4K8ac_peak_8770	4.29586	
19	10999622	10999831	5Y-H4K8ac_peak_8771	5.98695	
19	11047624	11047868	5Y-H4K8ac_peak_8772	6.37023	
19	11070934	11071191	5Y-H4K8ac_peak_8773	4.15658	SMARCA4_ENSG00000127616
19	11253454	11253993	5Y-H4K8ac_peak_8774	11.09673	
19	11354275	11354565	5Y-H4K8ac_peak_8775	7.31102	
19	11465594	11465890	5Y-H4K8ac_peak_8776	8.79957	DKFZP761J1410_ENSG00000105520
19	11564000	11564205	5Y-H4K8ac_peak_8777	5.97532	
19	11590898	11591222	5Y-H4K8ac_peak_8778	5.24695	ELAVL3_ENSG00000196361
19	11593130	11593415	5Y-H4K8ac_peak_8779	9.51254	
19	11616243	11616584	5Y-H4K8ac_peak_8780	14.31711	CTC-398G3.6_ENSG00000267477;ZNF653_ENSG00000161914
19	11616951	11617188	5Y-H4K8ac_peak_8781	9.68432	CTC-398G3.6_ENSG00000267477;ZNF653_ENSG00000161914
19	11639544	11639940	5Y-H4K8ac_peak_8782	13.71233	ECSIT_ENSG00000130159
19	11640260	11640502	5Y-H4K8ac_peak_8783	5.98695	ECSIT_ENSG00000130159
19	11650703	11650922	5Y-H4K8ac_peak_8784	8.2913	

19	11698987	11699180	5Y-H4K8ac_peak_8785	4.50834	
19	11708311	11708722	5Y-H4K8ac_peak_8786	13.33225	
19	11850033	11850569	5Y-H4K8ac_peak_8787	11.68317	ZNF823_ENSG00000197933
19	11909534	11909774	5Y-H4K8ac_peak_8788	6.14981	CTC-499B15.7_ENSG00000267646
19	12035944	12036294	5Y-H4K8ac_peak_8789	9.30505	ZNF700_ENSG00000196757;ZNF763_ENSG00000197054;ZNF763_ENSG00000267179
19	12076032	12076292	5Y-H4K8ac_peak_8790	5.23083	
19	12444727	12445003	5Y-H4K8ac_peak_8791	8.35139	ZNF563_ENSG00000188868
19	12445230	12445542	5Y-H4K8ac_peak_8792	6.27984	ZNF563_ENSG00000188868
19	12675291	12675497	5Y-H4K8ac_peak_8793	4.12295	
19	12779967	12780177	5Y-H4K8ac_peak_8794	7.50148	CTD-2192J16.24_ENSG00000269590
19	12792344	12792692	5Y-H4K8ac_peak_8795	10.60083	DHPS_ENSG00000095059
19	12792987	12793409	5Y-H4K8ac_peak_8796	11.18077	DHPS_ENSG00000095059
19	12808392	12808727	5Y-H4K8ac_peak_8797	4.50834	FBXW9_ENSG00000132004
19	12845080	12845359	5Y-H4K8ac_peak_8798	12.60508	C19orf43_ENSG00000123144
19	12845823	12846261	5Y-H4K8ac_peak_8799	7.59101	C19orf43_ENSG00000123144
19	12868749	12869009	5Y-H4K8ac_peak_8800	10.73603	
19	12889449	12890485	5Y-H4K8ac_peak_8801	6.08523	
19	12893091	12894080	5Y-H4K8ac_peak_8802	15.95288	
19	12895006	12895801	5Y-H4K8ac_peak_8803	8.67356	CTD-2659N19.9_ENSG00000267212
19	12896595	12897762	5Y-H4K8ac_peak_8804	12.3912	CTD-2659N19.9_ENSG00000267212;MIR5684_ENSG00000263800
19	12899681	12899957	5Y-H4K8ac_peak_8805	12.05638	
19	12900309	12900591	5Y-H4K8ac_peak_8806	8.4454	
19	12912167	12912594	5Y-H4K8ac_peak_8807	9.37812	PRDX2_ENSG00000167815
19	12916863	12917223	5Y-H4K8ac_peak_8808	7.89142	RNASEH2A_ENSG00000104889
19	12944854	12945149	5Y-H4K8ac_peak_8809	9.69901	MAST1_ENSG00000105613
19	12996214	12996809	5Y-H4K8ac_peak_8810	7.31102	
19	13023460	13024426	5Y-H4K8ac_peak_8811	8.79957	
19	13044687	13044979	5Y-H4K8ac_peak_8812	9.7353	FARSA_ENSG00000179115
19	13048827	13049059	5Y-H4K8ac_peak_8813	9.00954	CALR_ENSG00000179218
19	13056105	13056325	5Y-H4K8ac_peak_8814	5.65584	CTC-425F1.4_ENSG00000267458;RAD23A_ENSG00000179262
19	13056807	13057144	5Y-H4K8ac_peak_8815	9.23159	RAD23A_ENSG00000179262
19	13063444	13063700	5Y-H4K8ac_peak_8816	5.91107	
19	13064355	13064643	5Y-H4K8ac_peak_8817	11.1169	
19	13079801	13080935	5Y-H4K8ac_peak_8818	14.66739	
19	13081628	13082039	5Y-H4K8ac_peak_8819	8.71064	
19	13082954	13083390	5Y-H4K8ac_peak_8820	5.03335	
19	13085136	13086766	5Y-H4K8ac_peak_8821	18.78631	
19	13086995	13087623	5Y-H4K8ac_peak_8822	7.72345	
19	13088935	13089197	5Y-H4K8ac_peak_8823	6.1654	
19	13133131	13133558	5Y-H4K8ac_peak_8824	7.11863	
19	13164477	13164871	5Y-H4K8ac_peak_8825	6.77436	
19	13165220	13165869	5Y-H4K8ac_peak_8826	9.89244	
19	13166179	13166854	5Y-H4K8ac_peak_8827	19.17092	
19	13170907	13171599	5Y-H4K8ac_peak_8828	10.69698	
19	13180036	13180879	5Y-H4K8ac_peak_8829	10.35586	
19	13200445	13200916	5Y-H4K8ac_peak_8830	6.53157	

19	13201119	13201983	5Y-H4K8ac_peak_8831	7.90236	
19	13202444	13203698	5Y-H4K8ac_peak_8832	12.14222	
19	13211523	13211831	5Y-H4K8ac_peak_8833	5.10825	
19	13213134	13214186	5Y-H4K8ac_peak_8834	7.50148	LYL1_ENSG00000104903
19	13273566	13273774	5Y-H4K8ac_peak_8835	10.44054	
19	13274131	13274740	5Y-H4K8ac_peak_8836	15.80786	
19	13275115	13276223	5Y-H4K8ac_peak_8837	10.90365	
19	13278568	13278759	5Y-H4K8ac_peak_8838	5.91107	
19	13279071	13279262	5Y-H4K8ac_peak_8839	8.21582	
19	13282969	13283263	5Y-H4K8ac_peak_8840	7.87406	
19	13291929	13292210	5Y-H4K8ac_peak_8841	5.94703	
19	13842632	13842849	5Y-H4K8ac_peak_8842	10.1994	CCDC130_ENSG00000104957
19	13947368	13947575	5Y-H4K8ac_peak_8843	6.77436	MIR24-2_ENSG00000267519;MIR27A_ENSG00000207808;MIR23A_ENSG00000207980
19	13949503	13949695	5Y-H4K8ac_peak_8844	4.50302	
19	13950035	13950519	5Y-H4K8ac_peak_8845	8.09118	
19	13951149	13951479	5Y-H4K8ac_peak_8846	5.64909	
19	13952061	13952413	5Y-H4K8ac_peak_8847	5.23083	
19	13953974	13954190	5Y-H4K8ac_peak_8848	9.30505	
19	13957537	13958402	5Y-H4K8ac_peak_8849	15.82359	
19	13960965	13961363	5Y-H4K8ac_peak_8850	10.8165	
19	13961570	13961948	5Y-H4K8ac_peak_8851	8.564	
19	13962270	13962553	5Y-H4K8ac_peak_8852	4.29586	
19	13972415	13972880	5Y-H4K8ac_peak_8853	7.31102	NANOS3_ENSG00000187556
19	13975656	13975963	5Y-H4K8ac_peak_8854	8.18236	
19	13976754	13976955	5Y-H4K8ac_peak_8855	5.67283	
19	14017134	14017387	5Y-H4K8ac_peak_8856	6.96612	C19orf57_ENSG00000132016;CC2D1A_ENSG00000132024
19	14048479	14048756	5Y-H4K8ac_peak_8857	6.77436	
19	14096634	14097342	5Y-H4K8ac_peak_8858	14.00559	
19	14097566	14098436	5Y-H4K8ac_peak_8859	7.20869	
19	14117572	14118066	5Y-H4K8ac_peak_8860	20.25522	RFX1_ENSG00000132005
19	14142033	14142882	5Y-H4K8ac_peak_8861	7.64648	CTB-55O6.4_ENSG00000267670;IL27RA_ENSG00000104998
19	14182629	14182979	5Y-H4K8ac_peak_8862	5.94703	hsa-mir-1199_ENSG00000141854
19	14183514	14183795	5Y-H4K8ac_peak_8863	7.89142	hsa-mir-1199_ENSG00000141854
19	14191222	14191429	5Y-H4K8ac_peak_8864	5.65584	
19	14201977	14202286	5Y-H4K8ac_peak_8865	9.15007	SAMD1_ENSG00000141858
19	14229224	14229434	5Y-H4K8ac_peak_8866	6.37023	PRKACA_ENSG00000072062;CTB-55O6.10_ENSG00000267783
19	14316722	14316927	5Y-H4K8ac_peak_8867	7.80086	LPHN1_ENSG00000072071
19	14317708	14318125	5Y-H4K8ac_peak_8868	18.09952	LPHN1_ENSG00000072071
19	14318447	14318813	5Y-H4K8ac_peak_8869	6.08523	
19	14320243	14320977	5Y-H4K8ac_peak_8870	11.68317	
19	14358867	14359279	5Y-H4K8ac_peak_8871	12.26819	
19	14359657	14360370	5Y-H4K8ac_peak_8872	14.17466	
19	14492428	14492627	5Y-H4K8ac_peak_8873	6.08523	
19	14530538	14530744	5Y-H4K8ac_peak_8874	4.16635	DDX39A_ENSG00000123136
19	14543789	14544080	5Y-H4K8ac_peak_8875	14.05114	PKN1_ENSG00000123143
19	14544318	14544829	5Y-H4K8ac_peak_8876	7.50148	PKN1_ENSG00000123143

19	14545481	14545671	5Y-H4K8ac_peak_8877	5.65584	
19	14583230	14583759	5Y-H4K8ac_peak_8878	9.97324	
19	14586223	14586764	5Y-H4K8ac_peak_8879	4.56212	PTGER1_ENSG00000160951
19	14628790	14629097	5Y-H4K8ac_peak_8880	11.27554	TECR_ENSG00000099797
19	15217847	15219012	5Y-H4K8ac_peak_8881	13.99721	SYDE1_ENSG00000105137
19	15305934	15306171	5Y-H4K8ac_peak_8882	7.46829	
19	15310482	15310807	5Y-H4K8ac_peak_8883	5.98695	NOTCH3_ENSG00000074181
19	15333062	15333945	5Y-H4K8ac_peak_8884	6.50117	AC004257.1_ENSG00000269635
19	15442512	15443143	5Y-H4K8ac_peak_8885	12.71156	BRD4_ENSG00000141867
19	15489992	15490397	5Y-H4K8ac_peak_8886	6.22904	AKAP8_ENSG00000105127;AC005785.2_ENSG00000268189
19	15490991	15491241	5Y-H4K8ac_peak_8887	5.77617	AKAP8_ENSG00000105127
19	15532072	15532293	5Y-H4K8ac_peak_8888	8.2913	
19	15560113	15560326	5Y-H4K8ac_peak_8889	5.75508	WIZ_ENSG00000011451;MIR1470_ENSG00000269782
19	15561102	15561322	5Y-H4K8ac_peak_8890	8.11614	WIZ_ENSG00000011451;MIR1470_ENSG00000269782
19	16187373	16187763	5Y-H4K8ac_peak_8891	11.28133	
19	16188246	16188988	5Y-H4K8ac_peak_8892	8.2913	
19	16221490	16221834	5Y-H4K8ac_peak_8893	16.49365	RAB8A_ENSG00000167461
19	16295651	16295916	5Y-H4K8ac_peak_8894	10.74494	FAM32A_ENSG00000105058
19	16308826	16309115	5Y-H4K8ac_peak_8895	6.37023	AP1M1_ENSG00000072958
19	16568681	16569114	5Y-H4K8ac_peak_8896	5.24695	
19	16582273	16582532	5Y-H4K8ac_peak_8897	6.78128	EPS15L1_ENSG00000127527
19	16682823	16683062	5Y-H4K8ac_peak_8898	6.64195	SLC35E1_ENSG00000127526
19	16739365	16740042	5Y-H4K8ac_peak_8899	10.1994	CTD-3222D19.2_ENSG00000141979;MED26_ENSG00000105085
19	16935828	16936128	5Y-H4K8ac_peak_8900	4.51076	
19	16999382	16999573	5Y-H4K8ac_peak_8901	7.38046	F2RL3_ENSG00000127533
19	17245589	17245885	5Y-H4K8ac_peak_8902	7.24844	
19	17246972	17247220	5Y-H4K8ac_peak_8903	10.19948	
19	17325075	17325730	5Y-H4K8ac_peak_8904	7.90236	USE1_ENSG00000053501
19	17326194	17326391	5Y-H4K8ac_peak_8905	10.35586	USE1_ENSG00000053501
19	17337435	17337900	5Y-H4K8ac_peak_8906	7.58806	OCEL1_ENSG00000099330
19	17357356	17357898	5Y-H4K8ac_peak_8907	6.77436	NR2F6_ENSG00000160113
19	17392213	17392650	5Y-H4K8ac_peak_8908	9.15007	USHBP1_ENSG00000130307;ANKLE1_ENSG00000160117
19	17413405	17414109	5Y-H4K8ac_peak_8909	6.43068	
19	17416988	17417262	5Y-H4K8ac_peak_8910	4.67245	
19	17444664	17444854	5Y-H4K8ac_peak_8911	5.98695	ANO8_ENSG00000074855;GTPBP3_ENSG00000130299
19	17447243	17447648	5Y-H4K8ac_peak_8912	4.75045	
19	17458927	17459250	5Y-H4K8ac_peak_8913	6.55906	
19	17459510	17459879	5Y-H4K8ac_peak_8914	9.43096	
19	17501443	17501799	5Y-H4K8ac_peak_8915	4.95697	
19	17516593	17516829	5Y-H4K8ac_peak_8916	4.8773	BST2_ENSG00000130303;CTD-2521M24.9_ENSG00000269640;MVB12A_ENSG00000141971
19	17529889	17530171	5Y-H4K8ac_peak_8917	5.59837	CTD-2521M24.4_ENSG00000254503;CTD-2521M24.8_ENSG00000269053
19	17530977	17531448	5Y-H4K8ac_peak_8918	5.72233	CTD-2521M24.8_ENSG00000269053
19	17622471	17622714	5Y-H4K8ac_peak_8919	15.82359	CTD-3131K8.2_ENSG00000269439
19	17666505	17666856	5Y-H4K8ac_peak_8920	5.98695	COLGALT1_ENSG00000130309
19	17716412	17716670	5Y-H4K8ac_peak_8921	11.53136	
19	17817364	17817640	5Y-H4K8ac_peak_8922	8.564	

19	17830340	17830663	5Y-H4K8ac_peak_8923	8.93923	MAP1S_ENSG00000130479
19	17873651	17873845	5Y-H4K8ac_peak_8924	8.93923	
19	17889257	17889535	5Y-H4K8ac_peak_8925	6.46053	
19	17901710	17901942	5Y-H4K8ac_peak_8926	4.95697	
19	17902976	17903246	5Y-H4K8ac_peak_8927	8.24952	
19	17940591	17940874	5Y-H4K8ac_peak_8928	4.84727	
19	17941234	17941476	5Y-H4K8ac_peak_8929	8.23578	
19	17953701	17953941	5Y-H4K8ac_peak_8930	5.67283	
19	17958622	17959107	5Y-H4K8ac_peak_8931	12.64868	JAK3_ENSG00000105639
19	17970699	17971305	5Y-H4K8ac_peak_8932	13.77072	RPL18A_ENSG00000105640
19	18042650	18042940	5Y-H4K8ac_peak_8933	5.62788	CCDC124_ENSG00000007080
19	18043186	18043411	5Y-H4K8ac_peak_8934	7.97699	CCDC124_ENSG00000007080
19	18043853	18044168	5Y-H4K8ac_peak_8935	10.90365	CCDC124_ENSG00000007080
19	18111843	18112540	5Y-H4K8ac_peak_8936	11.19336	ARRDC2_ENSG00000105643
19	18119100	18119307	5Y-H4K8ac_peak_8937	7.65114	
19	18133538	18133992	5Y-H4K8ac_peak_8938	4.77016	CTB-52I2.4_ENSG00000268032
19	18169019	18169255	5Y-H4K8ac_peak_8939	7.87406	
19	18169472	18169675	5Y-H4K8ac_peak_8940	4.69231	
19	18271708	18272207	5Y-H4K8ac_peak_8941	12.06963	
19	18334820	18335257	5Y-H4K8ac_peak_8942	9.96543	
19	18335635	18336105	5Y-H4K8ac_peak_8943	7.30348	
19	18336609	18337253	5Y-H4K8ac_peak_8944	13.0168	
19	18391870	18392093	5Y-H4K8ac_peak_8945	6.67017	JUND_ENSG00000130522;MIR3188_ENSG00000267959
19	18392876	18393077	5Y-H4K8ac_peak_8946	9.51254	JUND_ENSG00000130522;MIR3188_ENSG00000267959
19	18402211	18402537	5Y-H4K8ac_peak_8947	7.08486	
19	18402889	18403464	5Y-H4K8ac_peak_8948	10.79063	
19	18403869	18404326	5Y-H4K8ac_peak_8949	4.50834	
19	18415311	18416308	5Y-H4K8ac_peak_8950	11.8513	
19	18428887	18429298	5Y-H4K8ac_peak_8951	6.78128	
19	18433450	18433786	5Y-H4K8ac_peak_8952	9.96543	LSM4_ENSG00000130520
19	18475169	18475369	5Y-H4K8ac_peak_8953	9.66296	
19	18484474	18484845	5Y-H4K8ac_peak_8954	9.05168	GDF15_ENSG00000130513
19	18499053	18499260	5Y-H4K8ac_peak_8955	7.59101	
19	18527368	18527691	5Y-H4K8ac_peak_8956	5.03917	
19	18528029	18528442	5Y-H4K8ac_peak_8957	6.37023	
19	18528729	18528990	5Y-H4K8ac_peak_8958	13.00792	SSBP4_ENSG00000130511
19	18529261	18529638	5Y-H4K8ac_peak_8959	4.61816	SSBP4_ENSG00000130511
19	18530259	18530557	5Y-H4K8ac_peak_8960	6.37023	SSBP4_ENSG00000130511
19	18543746	18544404	5Y-H4K8ac_peak_8961	7.34185	
19	18548631	18548876	5Y-H4K8ac_peak_8962	11.19336	ISYNA1_ENSG00000105655
19	18667394	18667741	5Y-H4K8ac_peak_8963	4.50834	KXD1_ENSG00000105700
19	18668768	18668980	5Y-H4K8ac_peak_8964	9.23159	KXD1_ENSG00000105700
19	18699156	18699888	5Y-H4K8ac_peak_8965	5.72233	C19orf60_ENSG00000006015
19	18722600	18722842	5Y-H4K8ac_peak_8966	4.16635	
19	18723445	18723639	5Y-H4K8ac_peak_8967	7.8267	
19	18747902	18748107	5Y-H4K8ac_peak_8968	7.58806	KLHL26_ENSG00000167487

19	18771068	18771299	5Y-H4K8ac_peak_8969	4.79585	
19	18793831	18794272	5Y-H4K8ac_peak_8970	13.60015	CRTC1_ENSG00000105662
19	18830461	18830844	5Y-H4K8ac_peak_8971	8.79957	
19	18846717	18847441	5Y-H4K8ac_peak_8972	11.71078	
19	18853336	18854128	5Y-H4K8ac_peak_8973	16.05821	
19	18854446	18855130	5Y-H4K8ac_peak_8974	10.90365	
19	18872444	18873285	5Y-H4K8ac_peak_8975	7.50148	
19	18874642	18874924	5Y-H4K8ac_peak_8976	9.01966	
19	18942803	18943156	5Y-H4K8ac_peak_8977	9.15007	UPF1_ENSG00000005007
19	19006513	19006804	5Y-H4K8ac_peak_8978	9.01787	CERS1_ENSG00000223802;GDF1_ENSG00000130283
19	19144442	19144658	5Y-H4K8ac_peak_8979	16.01457	SUGP2_ENSG00000064607;ARMC6_ENSG00000105676
19	19221205	19221615	5Y-H4K8ac_peak_8980	5.74859	
19	19248976	19249215	5Y-H4K8ac_peak_8981	6.54441	TMEM161A_ENSG00000064545
19	19256596	19256964	5Y-H4K8ac_peak_8982	11.4254	
19	19302610	19302843	5Y-H4K8ac_peak_8983	7.90236	MEF2BNB-MEF2B_ENSG00000064489;MEF2B_ENSG00000213999;MEF2BNB_ENSG00000254901;RFXANK_ENSG00000064490
19	19303057	19303311	5Y-H4K8ac_peak_8984	8.75963	MEF2BNB-MEF2B_ENSG00000064489;MEF2B_ENSG00000213999;MEF2BNB_ENSG00000254901;RFXANK_ENSG00000064490
19	19372206	19372474	5Y-H4K8ac_peak_8985	7.87048	
19	19383942	19384163	5Y-H4K8ac_peak_8986	7.9057	AC138430.4_ENSG00000267629;TM6SF2_ENSG00000213996
19	19431042	19431243	5Y-H4K8ac_peak_8987	9.51254	SUGP1_ENSG00000105705;MAU2_ENSG00000129933
19	19431685	19432006	5Y-H4K8ac_peak_8988	13.10659	SUGP1_ENSG00000105705;MAU2_ENSG00000129933
19	19477433	19477708	5Y-H4K8ac_peak_8989	11.57334	
19	19495808	19496245	5Y-H4K8ac_peak_8990	12.21176	GATAD2A_ENSG00000167491
19	19496856	19497227	5Y-H4K8ac_peak_8991	8.24461	GATAD2A_ENSG00000167491
19	19516789	19517112	5Y-H4K8ac_peak_8992	10.55771	
19	19626254	19626878	5Y-H4K8ac_peak_8993	14.01193	TSSK6_ENSG00000178093;NDUFA13_ENSG00000186010;YJEFN3_ENSG00000250067;CTC-260F20.3_ENSG00000258674
19	19627150	19627650	5Y-H4K8ac_peak_8994	6.2403	TSSK6_ENSG00000178093;NDUFA13_ENSG00000186010;YJEFN3_ENSG00000250067;CTC-260F20.3_ENSG00000258674
19	19738684	19739008	5Y-H4K8ac_peak_8995	10.60083	LPAR2_ENSG00000064547
19	19739587	19740025	5Y-H4K8ac_peak_8996	5.98695	LPAR2_ENSG00000064547
19	19740313	19740550	5Y-H4K8ac_peak_8997	5.37137	LPAR2_ENSG00000064547
19	19754051	19754300	5Y-H4K8ac_peak_8998	6.37023	GMIP_ENSG00000089639
19	19774019	19774714	5Y-H4K8ac_peak_8999	15.17389	ATP13A1_ENSG00000105726
19	19779670	19780094	5Y-H4K8ac_peak_9000	4.50834	ZNF101_ENSG00000181896
19	19843606	19843849	5Y-H4K8ac_peak_9001	10.11191	ZNF14_ENSG00000105708
19	19932108	19932476	5Y-H4K8ac_peak_9002	4.0639	CTC-559E9.4_ENSG00000267581;ZNF506_ENSG00000081665
19	20149970	20150208	5Y-H4K8ac_peak_9003	8.21582	ZNF682_ENSG00000197124
19	20162511	20162768	5Y-H4K8ac_peak_9004	6.34046	
19	20349703	20349953	5Y-H4K8ac_peak_9005	5.65584	
19	20607339	20607721	5Y-H4K8ac_peak_9006	7.11863	ZNF826P_ENSG00000231205
19	20843996	20844319	5Y-H4K8ac_peak_9007	8.97752	CTC-513N18.7_ENSG00000269110;ZNF626_ENSG00000188171
19	21203527	21203717	5Y-H4K8ac_peak_9008	5.65584	ZNF430_ENSG00000118620
19	21324881	21325237	5Y-H4K8ac_peak_9009	6.31818	ZNF431_ENSG00000196705
19	21511887	21512099	5Y-H4K8ac_peak_9010	4.84727	ZNF708_ENSG00000182141
19	21512487	21512717	5Y-H4K8ac_peak_9011	6.73047	ZNF708_ENSG00000182141
19	21646387	21646897	5Y-H4K8ac_peak_9012	6.50117	CTD-2561J22.5_ENSG00000268119
19	21786230	21786479	5Y-H4K8ac_peak_9013	8.79957	
19	22018633	22018934	5Y-H4K8ac_peak_9014	7.57144	

19	22816065	22816603	5Y-H4K8ac_peak_9015	6.34046	ZNF492_ENSG00000229676
19	23258301	23258684	5Y-H4K8ac_peak_9016	5.65584	CTD-2291D10.4_ENSG00000267886;ZNF730_ENSG00000183850
19	23299910	23300217	5Y-H4K8ac_peak_9017	7.97699	
19	23945930	23946136	5Y-H4K8ac_peak_9018	5.35202	RP11-255H23.2_ENSG00000233836;RPSAP58_ENSG00000205246
19	24097125	24097383	5Y-H4K8ac_peak_9019	8.97752	ZNF726_ENSG00000213967
19	24224107	24224443	5Y-H4K8ac_peak_9020	4.51076	
19	29703604	29703893	5Y-H4K8ac_peak_9021	7.59101	UQCRFS1_ENSG00000169021;CTB-32O4.2_ENSG00000267498
19	30206218	30206857	5Y-H4K8ac_peak_9022	16.65971	C19orf12_ENSG00000131943
19	30301899	30302449	5Y-H4K8ac_peak_9023	11.42066	CCNE1_ENSG00000105173
19	30303331	30303562	5Y-H4K8ac_peak_9024	5.98695	CCNE1_ENSG00000105173
19	30364028	30364520	5Y-H4K8ac_peak_9025	12.26819	
19	30384406	30384720	5Y-H4K8ac_peak_9026	7.76232	
19	30433490	30433699	5Y-H4K8ac_peak_9027	5.28616	
19	30583820	30584264	5Y-H4K8ac_peak_9028	4.84727	
19	30602411	30602783	5Y-H4K8ac_peak_9029	9.01738	
19	30643689	30644173	5Y-H4K8ac_peak_9030	15.17973	
19	30865739	30866384	5Y-H4K8ac_peak_9031	5.77617	
19	31057703	31057929	5Y-H4K8ac_peak_9032	8.47164	
19	31160182	31160429	5Y-H4K8ac_peak_9033	7.09658	CTC-565M22.1_ENSG00000267760
19	31182715	31182935	5Y-H4K8ac_peak_9034	6.09071	
19	31210761	31211149	5Y-H4K8ac_peak_9035	7.09658	
19	31234276	31234978	5Y-H4K8ac_peak_9036	8.43511	
19	31807939	31808424	5Y-H4K8ac_peak_9037	5.23083	
19	31830059	31830405	5Y-H4K8ac_peak_9038	7.04637	
19	31831604	31831858	5Y-H4K8ac_peak_9039	10.46287	
19	31835458	31835711	5Y-H4K8ac_peak_9040	8.75926	
19	31842224	31842509	5Y-H4K8ac_peak_9041	10.31981	
19	32514329	32514646	5Y-H4K8ac_peak_9042	7.84116	
19	32692524	32692978	5Y-H4K8ac_peak_9043	7.31815	
19	32835991	32836305	5Y-H4K8ac_peak_9044	7.17184	ZNF507_ENSG00000168813
19	32836584	32836910	5Y-H4K8ac_peak_9045	13.64163	ZNF507_ENSG00000168813
19	32895956	32896378	5Y-H4K8ac_peak_9046	14.26884	AC007773.2_ENSG00000267213;AC007773.3_ENSG00000269093;DPY19L3_ENSG00000178904
19	33182717	33183004	5Y-H4K8ac_peak_9047	9.13842	CTD-2538C1.2_ENSG00000267475;NUDT19_ENSG00000213965
19	33667334	33667710	5Y-H4K8ac_peak_9048	7.04637	AC008738.2_ENSG00000264355;LRP3_ENSG00000130881
19	33685515	33685750	5Y-H4K8ac_peak_9049	4.91235	
19	33784504	33784913	5Y-H4K8ac_peak_9050	8.90038	
19	33793003	33793373	5Y-H4K8ac_peak_9051	9.93099	CEBPA_ENSG00000245848;AC008738.1_ENSG00000230259;CTD-2540B15.7_ENSG00000267727;CEBPA-AS1_ENSG00000267296;CEBPA-AS1_ENSG00000178863
19	33793733	33794032	5Y-H4K8ac_peak_9052	7.09658	CEBPA_ENSG00000245848;AC008738.1_ENSG00000230259;CEBPA-AS1_ENSG00000267296;CEBPA-AS1_ENSG00000178863
19	33864075	33864272	5Y-H4K8ac_peak_9053	9.51254	CEBPG_ENSG00000153879
19	33864690	33864929	5Y-H4K8ac_peak_9054	12.81408	CEBPG_ENSG00000153879
19	33897453	33897768	5Y-H4K8ac_peak_9055	7.11863	
19	33898039	33898410	5Y-H4K8ac_peak_9056	5.23083	
19	33925862	33926148	5Y-H4K8ac_peak_9057	6.34245	
19	33926404	33926964	5Y-H4K8ac_peak_9058	14.00559	
19	33927175	33928000	5Y-H4K8ac_peak_9059	8.73985	
19	33929304	33929545	5Y-H4K8ac_peak_9060	6.37023	

19	33995256	33995533	5Y-H4K8ac_peak_9061	8.69112	
19	33996227	33996510	5Y-H4K8ac_peak_9062	14.30055	
19	33998015	33998255	5Y-H4K8ac_peak_9063	6.37023	
19	33998917	33999609	5Y-H4K8ac_peak_9064	5.51759	
19	34013118	34013440	5Y-H4K8ac_peak_9065	9.15007	PEPD_ENSG00000124299
19	34168377	34168833	5Y-H4K8ac_peak_9066	6.50117	
19	34175809	34176002	5Y-H4K8ac_peak_9067	10.1994	
19	34177954	34178282	5Y-H4K8ac_peak_9068	8.1667	
19	34178667	34178869	5Y-H4K8ac_peak_9069	6.53157	
19	34254308	34254543	5Y-H4K8ac_peak_9070	12.44818	
19	34285716	34285918	5Y-H4K8ac_peak_9071	8.24461	KCTD15_ENSG00000153885
19	34286505	34287534	5Y-H4K8ac_peak_9072	19.41832	KCTD15_ENSG00000153885
19	34287816	34288090	5Y-H4K8ac_peak_9073	9.30505	KCTD15_ENSG00000153885
19	34303705	34303922	5Y-H4K8ac_peak_9074	7.28023	
19	34359742	34360193	5Y-H4K8ac_peak_9075	7.89273	
19	34396571	34396990	5Y-H4K8ac_peak_9076	8.24461	
19	34662885	34663222	5Y-H4K8ac_peak_9077	8.3285	LSM14A_ENSG00000257103
19	34745153	34745344	5Y-H4K8ac_peak_9078	7.38046	KIAA0355_ENSG00000166398
19	34895383	34895575	5Y-H4K8ac_peak_9079	7.89273	PDCD2L_ENSG00000126249
19	34919046	34919707	5Y-H4K8ac_peak_9080	9.78792	CTD-2588C8.8_ENSG00000267024;UBA2_ENSG00000126261
19	35136938	35137267	5Y-H4K8ac_peak_9081	8.2913	SCGB2B3P_ENSG00000269811
19	35168694	35168897	5Y-H4K8ac_peak_9082	8.24461	AC020910.2_ENSG00000256383;ZNF302_ENSG00000089335
19	35225205	35225465	5Y-H4K8ac_peak_9083	7.90751	ZNF181_ENSG00000197841
19	35328736	35329385	5Y-H4K8ac_peak_9084	6.76425	CTC-523E23.5_ENSG00000269086
19	35454656	35454924	5Y-H4K8ac_peak_9085	10.79063	ZNF792_ENSG00000180884
19	35584886	35585176	5Y-H4K8ac_peak_9086	4.66683	
19	35758370	35758572	5Y-H4K8ac_peak_9087	6.43775	
19	36103665	36103917	5Y-H4K8ac_peak_9088	11.5813	AC002115.9_ENSG00000267626;HAUS5_ENSG00000249115
19	36120041	36120439	5Y-H4K8ac_peak_9089	5.98695	RBM42_ENSG00000126254
19	36192708	36193256	5Y-H4K8ac_peak_9090	15.40182	
19	36206837	36207388	5Y-H4K8ac_peak_9091	6.37023	
19	36208071	36208316	5Y-H4K8ac_peak_9092	7.03573	KMT2B_ENSG00000105663;KMT2B_ENSG00000272333
19	36236056	36236259	5Y-H4K8ac_peak_9093	9.30505	AD000671.6_ENSG00000267120;U2AF1L4_ENSG00000161265;PSENEN_ENSG00000205155;AC002398.9_ENSG00000188223
19	36248142	36248343	5Y-H4K8ac_peak_9094	4.00285	HSPB6_ENSG00000004776;C19orf55_ENSG00000167595
19	36249052	36249403	5Y-H4K8ac_peak_9095	8.69112	HSPB6_ENSG00000004776;C19orf55_ENSG00000167595
19	36267180	36267388	5Y-H4K8ac_peak_9096	4.84727	
19	36358971	36359313	5Y-H4K8ac_peak_9097	4.50834	NPHS1_ENSG00000161270;APLP1_ENSG00000105290
19	36478582	36478893	5Y-H4K8ac_peak_9098	7.46096	
19	36484822	36485253	5Y-H4K8ac_peak_9099	7.01364	SDHAF1_ENSG00000205138
19	36486142	36486469	5Y-H4K8ac_peak_9100	5.64909	SDHAF1_ENSG00000205138
19	36500102	36500374	5Y-H4K8ac_peak_9101	8.16382	SYNE4_ENSG00000181392
19	36523922	36524161	5Y-H4K8ac_peak_9102	10.65037	CLIP3_ENSG00000105270
19	36602098	36602290	5Y-H4K8ac_peak_9103	6.17973	OVOL3_ENSG00000105261
19	36705125	36705347	5Y-H4K8ac_peak_9104	4.93237	ZNF146_ENSG00000167635
19	36748276	36748718	5Y-H4K8ac_peak_9105	6.75784	
19	36869527	36870050	5Y-H4K8ac_peak_9106	10.19948	ZFP14_ENSG00000142065;CTD-3162L10.3_ENSG00000266973

19	36870359	36870657	5Y-H4K8ac_peak_9107	7.04637	ZFP14_ENSG00000142065
19	36908931	36909473	5Y-H4K8ac_peak_9108	7.31102	ZFP82_ENSG00000181007
19	37018994	37019290	5Y-H4K8ac_peak_9109	7.3889	ZNF260_ENSG00000254004;AC092295.4_ENSG00000228629
19	37064378	37064833	5Y-H4K8ac_peak_9110	6.73385	AC092295.7_ENSG00000233527
19	37096305	37096569	5Y-H4K8ac_peak_9111	11.86504	ZNF529_ENSG00000186020;ZNF382_ENSG00000161298
19	37178613	37178814	5Y-H4K8ac_peak_9112	7.11863	AC074138.3_ENSG00000225975;ZNF567_ENSG00000189042
19	37263768	37264264	5Y-H4K8ac_peak_9113	6.50117	ZNF850_ENSG00000267041;CTD-2162K18.4_ENSG00000267260
19	37329402	37329608	5Y-H4K8ac_peak_9114	10.19948	
19	37406610	37407197	5Y-H4K8ac_peak_9115	12.10416	ZNF829_ENSG00000185869;ZNF568_ENSG00000198453
19	37463423	37464241	5Y-H4K8ac_peak_9116	16.50659	
19	37708841	37709124	5Y-H4K8ac_peak_9117	9.38203	ZNF585B_ENSG00000245680;ZNF383_ENSG00000188283
19	38145963	38146590	5Y-H4K8ac_peak_9118	10.4689	
19	38183659	38183851	5Y-H4K8ac_peak_9119	6.47245	ZFP30_ENSG00000120784;ZNF781_ENSG00000196381;CTD-2528L19.3_ENSG00000267319
19	38210324	38210540	5Y-H4K8ac_peak_9120	10.1994	CTD-2528L19.4_ENSG00000267552;ZNF607_ENSG00000198182
19	38397072	38397513	5Y-H4K8ac_peak_9121	6.78128	WDR87_ENSG00000171804;SIPA1L3_ENSG00000105738
19	38494819	38495082	5Y-H4K8ac_peak_9122	6.50117	
19	38584110	38584645	5Y-H4K8ac_peak_9123	9.41037	
19	38642786	38643663	5Y-H4K8ac_peak_9124	10.31981	
19	38664216	38664778	5Y-H4K8ac_peak_9125	8.43511	
19	38700584	38700878	5Y-H4K8ac_peak_9126	6.34046	
19	38724649	38724950	5Y-H4K8ac_peak_9127	8.43511	
19	38734684	38735215	5Y-H4K8ac_peak_9128	6.1654	SPINT2_ENSG00000167642
19	38741911	38742109	5Y-H4K8ac_peak_9129	4.84727	
19	38743776	38744175	5Y-H4K8ac_peak_9130	11.99586	
19	38754479	38755136	5Y-H4K8ac_peak_9131	9.05168	
19	38806932	38807248	5Y-H4K8ac_peak_9132	6.27205	YIF1B_ENSG00000167645
19	38810082	38810391	5Y-H4K8ac_peak_9133	7.28023	KCNK6_ENSG00000099337
19	38825591	38826089	5Y-H4K8ac_peak_9134	8.79957	CATSPERG_ENSG00000099338
19	38893945	38894238	5Y-H4K8ac_peak_9135	13.29029	FAM98C_ENSG00000130244
19	39005944	39006172	5Y-H4K8ac_peak_9136	9.23159	
19	39109189	39109414	5Y-H4K8ac_peak_9137	7.50501	EIF3K_ENSG00000178982;MAP4K1_ENSG00000104814
19	39210404	39210609	5Y-H4K8ac_peak_9138	5.18494	
19	39225963	39226183	5Y-H4K8ac_peak_9139	8.564	
19	39321984	39322295	5Y-H4K8ac_peak_9140	8.21582	ECH1_ENSG00000104823
19	39322774	39322997	5Y-H4K8ac_peak_9141	4.50834	ECH1_ENSG00000104823
19	39340262	39340570	5Y-H4K8ac_peak_9142	26.19116	
19	39341474	39341685	5Y-H4K8ac_peak_9143	6.43775	
19	39341972	39342339	5Y-H4K8ac_peak_9144	5.42666	HNRNPL_ENSG00000104824
19	39368758	39369391	5Y-H4K8ac_peak_9145	10.69698	RINL_ENSG00000187994
19	39407744	39408255	5Y-H4K8ac_peak_9146	8.55081	
19	39521723	39521942	5Y-H4K8ac_peak_9147	8.08688	CTB-189B5.3_ENSG00000267992
19	39522582	39522785	5Y-H4K8ac_peak_9148	5.24695	FBXO27_ENSG00000161243
19	39616452	39616699	5Y-H4K8ac_peak_9149	10.35586	PAK4_ENSG00000130669
19	39657454	39657804	5Y-H4K8ac_peak_9150	7.87406	
19	39658185	39658427	5Y-H4K8ac_peak_9151	4.642	
19	39811156	39811387	5Y-H4K8ac_peak_9152	6.37023	CTC-246B18.8_ENSG00000268262

19	39893595	39893805	5Y-H4K8ac_peak_9153	6.73385	
19	39900569	39900800	5Y-H4K8ac_peak_9154	10.77539	MIR4530_ENSG00000266559
19	39903736	39903958	5Y-H4K8ac_peak_9155	4.29586	PLEKHG2_ENSG00000090924
19	39904240	39904661	5Y-H4K8ac_peak_9156	6.54441	
19	39926216	39926525	5Y-H4K8ac_peak_9157	10.1994	RPS16_ENSG00000105193;SUPT5H_ENSG00000196235
19	39927151	39927351	5Y-H4K8ac_peak_9158	5.65584	RPS16_ENSG00000105193;SUPT5H_ENSG00000196235
19	39936357	39936655	5Y-H4K8ac_peak_9159	9.38276	
19	39971545	39971835	5Y-H4K8ac_peak_9160	8.1667	TIMM50_ENSG00000105197
19	40005867	40006428	5Y-H4K8ac_peak_9161	9.15007	SELV_ENSG00000186838
19	40030399	40030738	5Y-H4K8ac_peak_9162	8.69112	EID2_ENSG00000176396
19	40315605	40315902	5Y-H4K8ac_peak_9163	7.11863	
19	40316164	40316478	5Y-H4K8ac_peak_9164	6.73047	
19	40323846	40324078	5Y-H4K8ac_peak_9165	11.09973	DYRK1B_ENSG00000105204
19	40324481	40324675	5Y-H4K8ac_peak_9166	6.50117	DYRK1B_ENSG00000105204
19	40365973	40366348	5Y-H4K8ac_peak_9167	6.98416	
19	40397790	40398060	5Y-H4K8ac_peak_9168	10.19948	
19	40696988	40697259	5Y-H4K8ac_peak_9169	9.15007	MAP3K10_ENSG00000130758
19	40697484	40697991	5Y-H4K8ac_peak_9170	8.78565	MAP3K10_ENSG00000130758
19	40723247	40723680	5Y-H4K8ac_peak_9171	7.31815	TTC9B_ENSG00000174521
19	40724018	40724287	5Y-H4K8ac_peak_9172	11.57334	TTC9B_ENSG00000174521
19	40790668	40791261	5Y-H4K8ac_peak_9173	8.69112	AKT2_ENSG00000105221
19	40928687	40928935	5Y-H4K8ac_peak_9174	13.27281	
19	40931398	40931651	5Y-H4K8ac_peak_9175	6.77436	SERTAD1_ENSG00000197019
19	40939183	40940188	5Y-H4K8ac_peak_9176	13.27281	
19	41035705	41036245	5Y-H4K8ac_peak_9177	7.59101	
19	41054959	41055226	5Y-H4K8ac_peak_9178	10.11191	
19	41081933	41082353	5Y-H4K8ac_peak_9179	10.46287	SHKBP1_ENSG00000160410
19	41083080	41083299	5Y-H4K8ac_peak_9180	8.33296	SHKBP1_ENSG00000160410
19	41116436	41116747	5Y-H4K8ac_peak_9181	8.83356	
19	41196814	41197409	5Y-H4K8ac_peak_9182	9.23159	NUMBL_ENSG00000105245
19	41197651	41198089	5Y-H4K8ac_peak_9183	10.19948	NUMBL_ENSG00000105245
19	41220415	41220671	5Y-H4K8ac_peak_9184	7.3889	
19	41221201	41221523	5Y-H4K8ac_peak_9185	7.63144	
19	41224920	41225173	5Y-H4K8ac_peak_9186	7.38046	ADCK4_ENSG00000123815
19	41255899	41256215	5Y-H4K8ac_peak_9187	7.38046	SNRPA_ENSG00000077312
19	41282921	41283225	5Y-H4K8ac_peak_9188	5.80888	RAB4B_ENSG00000167578
19	41770380	41770841	5Y-H4K8ac_peak_9189	14.59308	
19	41815852	41816225	5Y-H4K8ac_peak_9190	7.60057	CCDC97_ENSG00000142039
19	41882408	41882857	5Y-H4K8ac_peak_9191	8.93923	
19	41933953	41934220	5Y-H4K8ac_peak_9192	11.36989	B3GNT8_ENSG00000177191
19	42386895	42387107	5Y-H4K8ac_peak_9193	10.16277	ARHGEF1_ENSG00000076928
19	42443992	42444345	5Y-H4K8ac_peak_9194	4.77126	
19	42444689	42445340	5Y-H4K8ac_peak_9195	7.50148	
19	42463150	42463349	5Y-H4K8ac_peak_9196	7.30348	RABAC1_ENSG00000105404
19	42500535	42501639	5Y-H4K8ac_peak_9197	7.08486	ATP1A3_ENSG00000105409
19	42502303	42503456	5Y-H4K8ac_peak_9198	10.12457	ATP1A3_ENSG00000105409

19	42573901	42574398	5Y-H4K8ac_peak_9199	13.0168	GRIK5_ENSG00000105737
19	42574597	42575129	5Y-H4K8ac_peak_9200	7.73726	GRIK5_ENSG00000105737
19	42579294	42579950	5Y-H4K8ac_peak_9201	9.79526	
19	42595776	42596276	5Y-H4K8ac_peak_9202	7.05631	
19	42633460	42633750	5Y-H4K8ac_peak_9203	5.5192	
19	42648455	42648775	5Y-H4K8ac_peak_9204	9.76527	SNORD112_ENSG00000252356
19	42657872	42658080	5Y-H4K8ac_peak_9205	4.67245	
19	42746322	42746681	5Y-H4K8ac_peak_9206	8.43511	AC006486.1_ENSG00000204957;GSK3A_ENSG00000105723
19	42759861	42760143	5Y-H4K8ac_peak_9207	11.01699	AC006486.9_ENSG00000268643;ERF_ENSG00000105722
19	42781929	42782310	5Y-H4K8ac_peak_9208	8.56121	
19	42783138	42783391	5Y-H4K8ac_peak_9209	6.54441	
19	42784334	42784529	5Y-H4K8ac_peak_9210	5.24695	
19	42785359	42785596	5Y-H4K8ac_peak_9211	5.98695	
19	42807407	42807726	5Y-H4K8ac_peak_9212	6.77436	PAFAH1B3_ENSG00000079462
19	42891547	42891801	5Y-H4K8ac_peak_9213	4.50834	
19	42927909	42928476	5Y-H4K8ac_peak_9214	11.57714	CTB-50E14.4_ENSG00000268605
19	44008713	44008950	5Y-H4K8ac_peak_9215	4.95697	PHLDB3_ENSG00000176531
19	44123208	44123678	5Y-H4K8ac_peak_9216	11.69482	ZNF428_ENSG00000131116
19	44258870	44259776	5Y-H4K8ac_peak_9217	15.66931	SMG9_ENSG00000105771
19	44289337	44289537	5Y-H4K8ac_peak_9218	9.06776	
19	44331146	44331339	5Y-H4K8ac_peak_9219	5.87725	LYPD5_ENSG00000159871;ZNF283_ENSG00000167637
19	44529579	44529820	5Y-H4K8ac_peak_9220	11.68317	AC067968.3_ENSG00000267144;ZNF222_ENSG00000159885;ZNF223_ENSG00000267022
19	44645773	44646104	5Y-H4K8ac_peak_9221	14.1808	ZNF234_ENSG00000263002
19	44668596	44668801	5Y-H4K8ac_peak_9222	10.46287	ZNF226_ENSG00000167380
19	44669301	44669507	5Y-H4K8ac_peak_9223	8.33296	ZNF226_ENSG00000167380
19	44764077	44764342	5Y-H4K8ac_peak_9224	7.17184	
19	44808904	44809098	5Y-H4K8ac_peak_9225	10.46287	ZNF235_ENSG00000159917
19	45004656	45004950	5Y-H4K8ac_peak_9226	9.63153	ZNF180_ENSG00000167384
19	45146740	45147052	5Y-H4K8ac_peak_9227	12.05638	PVR_ENSG00000073008
19	45394635	45394892	5Y-H4K8ac_peak_9228	9.23159	CTB-129P6.4_ENSG00000267282;TOMM40_ENSG00000130204
19	45417793	45418145	5Y-H4K8ac_peak_9229	7.18391	APOC1_ENSG00000130208
19	45430093	45430345	5Y-H4K8ac_peak_9230	10.73603	APOC1P1_ENSG00000214855
19	45542418	45542608	5Y-H4K8ac_peak_9231	11.48691	CLASRP_ENSG00000104859
19	45581919	45582337	5Y-H4K8ac_peak_9232	10.36926	GEMIN7_ENSG00000142252;MARK4_ENSG00000007047
19	45665009	45665226	5Y-H4K8ac_peak_9233	4.86522	
19	45682551	45682809	5Y-H4K8ac_peak_9234	5.23819	BLOC1S3_ENSG00000189114;AC005779.2_ENSG00000267545
19	45694270	45694496	5Y-H4K8ac_peak_9235	6.76425	
19	45827066	45827381	5Y-H4K8ac_peak_9236	7.9057	CKM_ENSG00000104879
19	45829241	45829501	5Y-H4K8ac_peak_9237	10.69866	
19	45874194	45874496	5Y-H4K8ac_peak_9238	9.7353	ERCC2_ENSG00000104884
19	45908151	45908637	5Y-H4K8ac_peak_9239	5.64909	PPP1R13L_ENSG00000104881;CD3EAP_ENSG00000117877
19	45909106	45909311	5Y-H4K8ac_peak_9240	5.64909	PPP1R13L_ENSG00000104881;CD3EAP_ENSG00000117877
19	45947747	45947971	5Y-H4K8ac_peak_9241	6.98394	
19	46110769	46111091	5Y-H4K8ac_peak_9242	12.69256	
19	46119604	46120111	5Y-H4K8ac_peak_9243	9.38807	
19	46148280	46148650	5Y-H4K8ac_peak_9244	7.59101	EML2_ENSG00000125746

19	46184428	46184749	5Y-H4K8ac_peak_9245	9.96543	
19	46234674	46234895	5Y-H4K8ac_peak_9246	4.29586	FBXO46_ENSG00000177051
19	46365544	46366173	5Y-H4K8ac_peak_9247	7.01266	SYMPK_ENSG00000125755
19	46367267	46367672	5Y-H4K8ac_peak_9248	12.06963	SYMPK_ENSG00000125755;FOXA3_ENSG00000170608
19	46388772	46389257	5Y-H4K8ac_peak_9249	7.38046	IRF2BP1_ENSG00000170604
19	46390210	46390527	5Y-H4K8ac_peak_9250	9.00954	IRF2BP1_ENSG00000170604
19	46457363	46457617	5Y-H4K8ac_peak_9251	5.65584	
19	46476415	46476651	5Y-H4K8ac_peak_9252	6.08523	NOVA2_ENSG00000104967
19	46850332	46850558	5Y-H4K8ac_peak_9253	12.2918	PPP5C_ENSG00000011485
19	46999320	46999538	5Y-H4K8ac_peak_9254	7.12953	PNMAL2_ENSG00000204851
19	47104761	47105043	5Y-H4K8ac_peak_9255	4.642	PPP5D1_ENSG00000230510;AC011551.3_ENSG00000268423;CALM3_ENSG00000160014
19	47123112	47123404	5Y-H4K8ac_peak_9256	5.64909	
19	47123880	47124178	5Y-H4K8ac_peak_9257	9.63153	
19	47124416	47125219	5Y-H4K8ac_peak_9258	5.41472	
19	47129206	47129883	5Y-H4K8ac_peak_9259	8.43999	PTGIR_ENSG00000160013
19	47130214	47130460	5Y-H4K8ac_peak_9260	4.00285	
19	47133266	47133523	5Y-H4K8ac_peak_9261	13.99721	
19	47133788	47134203	5Y-H4K8ac_peak_9262	12.78119	
19	47134420	47135304	5Y-H4K8ac_peak_9263	13.81774	
19	47137959	47138645	5Y-H4K8ac_peak_9264	8.69112	GNG8_ENSG00000167414
19	47142610	47142804	5Y-H4K8ac_peak_9265	8.69112	
19	47143076	47143401	5Y-H4K8ac_peak_9266	9.59236	
19	47217830	47218074	5Y-H4K8ac_peak_9267	9.22312	
19	47249411	47249650	5Y-H4K8ac_peak_9268	11.88337	STRN4_ENSG00000090372;FKRP_ENSG00000181027
19	47249884	47250190	5Y-H4K8ac_peak_9269	8.3285	STRN4_ENSG00000090372;FKRP_ENSG00000181027
19	47353624	47353957	5Y-H4K8ac_peak_9270	4.95697	AP2S1_ENSG00000042753
19	47363692	47364059	5Y-H4K8ac_peak_9271	14.31711	
19	47364347	47364562	5Y-H4K8ac_peak_9272	7.38046	
19	47481182	47481410	5Y-H4K8ac_peak_9273	5.91107	
19	47496127	47496317	5Y-H4K8ac_peak_9274	6.94897	
19	47552506	47552725	5Y-H4K8ac_peak_9275	8.86184	TMEM160_ENSG00000130748
19	47614247	47616389	5Y-H4K8ac_peak_9276	21.35466	ZC3H4_ENSG00000130749;SAE1_ENSG00000142230
19	47616643	47616992	5Y-H4K8ac_peak_9277	10.11191	ZC3H4_ENSG00000130749;SAE1_ENSG00000142230
19	47733994	47734223	5Y-H4K8ac_peak_9278	4.36976	
19	47747835	47748049	5Y-H4K8ac_peak_9279	5.18558	
19	47759120	47759855	5Y-H4K8ac_peak_9280	9.79526	CCDC9_ENSG00000105321
19	47922356	47922659	5Y-H4K8ac_peak_9281	7.31807	MEIS3_ENSG00000105419
19	47922969	47923395	5Y-H4K8ac_peak_9282	6.77436	MEIS3_ENSG00000105419
19	47924400	47924789	5Y-H4K8ac_peak_9283	4.44548	
19	47986659	47986997	5Y-H4K8ac_peak_9284	6.86362	KPTN_ENSG00000118162;NAPA-AS1_ENSG00000268061
19	47987298	47987713	5Y-H4K8ac_peak_9285	6.77436	KPTN_ENSG00000118162;NAPA-AS1_ENSG00000268061
19	47996524	47997889	5Y-H4K8ac_peak_9286	8.3285	
19	47998493	47998766	5Y-H4K8ac_peak_9287	11.68317	
19	47999036	47999246	5Y-H4K8ac_peak_9288	5.64909	
19	48000348	48000618	5Y-H4K8ac_peak_9289	8.46442	
19	48018931	48019178	5Y-H4K8ac_peak_9290	7.89142	NAPA_ENSG00000105402

19	48113212	48113455	5Y-H4K8ac_peak_9291	10.2787	
19	48222145	48222453	5Y-H4K8ac_peak_9292	8.69112	
19	48246266	48246642	5Y-H4K8ac_peak_9293	8.24461	
19	48281706	48281918	5Y-H4K8ac_peak_9294	13.92877	SEPW1_ENSG00000178980
19	48673262	48673842	5Y-H4K8ac_peak_9295	7.59101	LIG1_ENSG00000105486;C19orf68_ENSG00000185453
19	48759585	48759798	5Y-H4K8ac_peak_9296	4.24332	CARD8_ENSG00000105483;CTC-241F20.3_ENSG00000268001
19	48775292	48775514	5Y-H4K8ac_peak_9297	8.21582	CTC-241F20.4_ENSG00000268186
19	48866669	48867107	5Y-H4K8ac_peak_9298	11.48691	TMEM143_ENSG00000161558;SYNGR4_ENSG00000105467
19	48867680	48867970	5Y-H4K8ac_peak_9299	7.65114	TMEM143_ENSG00000161558;SYNGR4_ENSG00000105467
19	48894143	48894438	5Y-H4K8ac_peak_9300	9.96543	KDELR1_ENSG00000105438
19	48896614	48896923	5Y-H4K8ac_peak_9301	8.21582	
19	48899500	48899838	5Y-H4K8ac_peak_9302	5.47303	
19	48901371	48901878	5Y-H4K8ac_peak_9303	5.72848	
19	48972677	48972916	5Y-H4K8ac_peak_9304	12.50545	CTC-273B12.5_ENSG00000268530;CYTH2_ENSG00000105443
19	49000802	49001180	5Y-H4K8ac_peak_9305	6.78128	
19	49013608	49013841	5Y-H4K8ac_peak_9306	5.91107	
19	49016629	49017141	5Y-H4K8ac_peak_9307	6.77436	LMTK3_ENSG00000142235;CTC-273B12.10_ENSG00000269814
19	49017333	49017718	5Y-H4K8ac_peak_9308	8.31252	LMTK3_ENSG00000142235;CTC-273B12.10_ENSG00000269814
19	49077575	49077828	5Y-H4K8ac_peak_9309	8.69112	
19	49111958	49112371	5Y-H4K8ac_peak_9310	5.98695	
19	49127706	49127980	5Y-H4K8ac_peak_9311	6.3019	AC022154.7_ENSG00000268093
19	49138057	49138356	5Y-H4K8ac_peak_9312	8.20773	
19	49147646	49147938	5Y-H4K8ac_peak_9313	4.84727	
19	49149550	49149923	5Y-H4K8ac_peak_9314	9.51254	CA11_ENSG00000063180
19	49157545	49158340	5Y-H4K8ac_peak_9315	6.87117	
19	49223396	49223793	5Y-H4K8ac_peak_9316	8.38675	MAMSTR_ENSG00000176909
19	49224047	49224325	5Y-H4K8ac_peak_9317	7.56545	
19	49242362	49242815	5Y-H4K8ac_peak_9318	6.37023	
19	49255662	49256449	5Y-H4K8ac_peak_9319	8.1667	
19	49285472	49285745	5Y-H4K8ac_peak_9320	7.59101	
19	49338741	49338996	5Y-H4K8ac_peak_9321	5.41472	HSD17B14_ENSG00000087076
19	49371806	49372063	5Y-H4K8ac_peak_9322	10.11191	PLEKHA4_ENSG00000105559
19	49375763	49376016	5Y-H4K8ac_peak_9323	6.38173	PPP1R15A_ENSG00000087074
19	49399155	49399571	5Y-H4K8ac_peak_9324	14.31711	
19	49399817	49400120	5Y-H4K8ac_peak_9325	4.36976	
19	49436809	49437019	5Y-H4K8ac_peak_9326	5.77946	DHDH_ENSG00000104808
19	49458391	49458601	5Y-H4K8ac_peak_9327	4.15658	BAX_ENSG00000087088
19	49467882	49468230	5Y-H4K8ac_peak_9328	5.21093	CTD-2639E6.9_ENSG00000267898;FTL_ENSG00000087086
19	49522397	49522645	5Y-H4K8ac_peak_9329	5.23083	CTB-60B18.10_ENSG00000268655
19	49522989	49523180	5Y-H4K8ac_peak_9330	6.27097	CTB-60B18.10_ENSG00000268655
19	49524360	49524556	5Y-H4K8ac_peak_9331	4.82324	
19	49588742	49589017	5Y-H4K8ac_peak_9332	12.05638	SNRNP70_ENSG00000104852
19	49653934	49654162	5Y-H4K8ac_peak_9333	7.89273	
19	49957438	49957762	5Y-H4K8ac_peak_9334	7.11262	PIH1D1_ENSG00000104872
19	49977191	49978000	5Y-H4K8ac_peak_9335	13.48981	FLT3LG_ENSG00000090554;CTD-3148I10.15_ENSG00000273189
19	49989794	49990537	5Y-H4K8ac_peak_9336	16.72648	RPL13A_ENSG00000142541

19	49991042	49991246	5Y-H4K8ac_peak_9337	5.37237	RPL13A_ENSG00000142541
19	49998588	49999165	5Y-H4K8ac_peak_9338	8.22388	RPS11_ENSG00000142534
19	50015705	50016034	5Y-H4K8ac_peak_9339	6.50117	
19	50030587	50030833	5Y-H4K8ac_peak_9340	12.20548	RCN3_ENSG00000142552
19	50084283	50084661	5Y-H4K8ac_peak_9341	6.43775	PRRG2_ENSG00000126460
19	50094484	50094680	5Y-H4K8ac_peak_9342	11.22796	NOSIP_ENSG00000142546;PRR12_ENSG00000126464
19	50144031	50144708	5Y-H4K8ac_peak_9343	8.66584	RRAS_ENSG00000126458;SCAF1_ENSG00000126461
19	50145545	50145763	5Y-H4K8ac_peak_9344	8.1667	SCAF1_ENSG00000126461
19	50180527	50180717	5Y-H4K8ac_peak_9345	9.7353	
19	50181058	50181257	5Y-H4K8ac_peak_9346	4.1776	
19	50195418	50195898	5Y-H4K8ac_peak_9347	7.11863	
19	50204624	50204880	5Y-H4K8ac_peak_9348	4.79658	
19	50268873	50269154	5Y-H4K8ac_peak_9349	8.33296	RNU6-841P_ENSG00000206599
19	50305060	50305380	5Y-H4K8ac_peak_9350	7.50044	
19	50306111	50306378	5Y-H4K8ac_peak_9351	4.29586	
19	50315998	50316343	5Y-H4K8ac_peak_9352	7.8267	
19	50379592	50379869	5Y-H4K8ac_peak_9353	19.49156	TBC1D17_ENSG00000104946
19	50432263	50433084	5Y-H4K8ac_peak_9354	10.35586	IL4I1_ENSG00000104951;NUP62_ENSG00000213024;ATF5_ENSG00000169136
19	50501237	50501608	5Y-H4K8ac_peak_9355	7.97699	
19	50529434	50529633	5Y-H4K8ac_peak_9356	6.54441	VRK3_ENSG00000105053;ZNF473_ENSG00000142528
19	50818540	50818751	5Y-H4K8ac_peak_9357	8.2913	
19	50819058	50819913	5Y-H4K8ac_peak_9358	8.33296	
19	50831626	50831830	5Y-H4K8ac_peak_9359	4.5753	
19	50836420	50836730	5Y-H4K8ac_peak_9360	9.63153	KCNC3_ENSG00000131398
19	50872093	50872513	5Y-H4K8ac_peak_9361	5.84208	CTB-191K22.6_ENSG00000269392
19	50873009	50873367	5Y-H4K8ac_peak_9362	12.03996	CTB-191K22.6_ENSG00000269392
19	50934317	50934768	5Y-H4K8ac_peak_9363	6.1654	
19	50979159	50979505	5Y-H4K8ac_peak_9364	7.50148	FAM71E1_ENSG00000142530;EMC10_ENSG00000161671
19	51015328	51015605	5Y-H4K8ac_peak_9365	11.13396	JOSD2_ENSG00000161677
19	51015882	51016211	5Y-H4K8ac_peak_9366	7.97699	
19	51016662	51017900	5Y-H4K8ac_peak_9367	13.10076	ASPDH_ENSG00000204653
19	51018195	51018551	5Y-H4K8ac_peak_9368	3.96434	ASPDH_ENSG00000204653
19	51109039	51109348	5Y-H4K8ac_peak_9369	4.642	
19	51153060	51153419	5Y-H4K8ac_peak_9370	5.67283	C19orf81_ENSG00000235034
19	51161102	51161317	5Y-H4K8ac_peak_9371	9.15007	
19	51198901	51199215	5Y-H4K8ac_peak_9372	7.64648	
19	51227369	51227594	5Y-H4K8ac_peak_9373	6.77436	CLEC11A_ENSG00000105472
19	51339591	51339832	5Y-H4K8ac_peak_9374	7.34185	KLK15_ENSG00000174562
19	51612192	51612429	5Y-H4K8ac_peak_9375	7.59101	CTU1_ENSG00000142544
19	51626158	51626352	5Y-H4K8ac_peak_9376	4.84727	
19	51815265	51815482	5Y-H4K8ac_peak_9377	6.37023	
19	51843492	51844093	5Y-H4K8ac_peak_9378	5.41472	CTD-2616J11.16_ENSG00000267905
19	51871223	51872151	5Y-H4K8ac_peak_9379	5.12488	CTD-2616J11.11_ENSG00000269403;CLDND2_ENSG00000160318
19	51896938	51897213	5Y-H4K8ac_peak_9380	8.43511	CTD-2616J11.14_ENSG00000268889
19	51963894	51964213	5Y-H4K8ac_peak_9381	5.65584	
19	52096973	52097483	5Y-H4K8ac_peak_9382	7.88114	AC018755.1_ENSG00000167765;AC018755.16_ENSG00000269388

19	52097842	52098186	5Y-H4K8ac_peak_9383	6.78128	AC018755.1_ENSG00000167765;AC018755.16_ENSG00000269388
19	52193556	52193986	5Y-H4K8ac_peak_9384	7.04637	
19	52207585	52207804	5Y-H4K8ac_peak_9385	7.59101	
19	52511564	52511935	5Y-H4K8ac_peak_9386	7.76232	ZNF615_ENSG00000197619
19	52531350	52531958	5Y-H4K8ac_peak_9387	11.57334	
19	52552247	52552484	5Y-H4K8ac_peak_9388	8.33296	
19	52773000	52773296	5Y-H4K8ac_peak_9389	5.87725	ZNF766_ENSG00000196214
19	52800517	52800718	5Y-H4K8ac_peak_9390	10.1994	CTD-2525I3.5_ENSG00000269102;ZNF480_ENSG00000198464
19	52901199	52901734	5Y-H4K8ac_peak_9391	10.46287	CTD-3018O17.3_ENSG00000269834;ZNF528_ENSG00000167555
19	53140898	53141094	5Y-H4K8ac_peak_9392	6.66026	
19	53194011	53194281	5Y-H4K8ac_peak_9393	10.36926	ZNF83_ENSG00000167766
19	53400469	53400730	5Y-H4K8ac_peak_9394	9.23159	ZNF320_ENSG00000182986
19	53426105	53426592	5Y-H4K8ac_peak_9395	6.34046	ZNF888_ENSG00000213793;CTD-2331H12.7_ENSG00000269646
19	53445536	53445856	5Y-H4K8ac_peak_9396	7.31815	ZNF816-ZNF321P_ENSG00000213801
19	53606342	53606589	5Y-H4K8ac_peak_9397	12.11208	ZNF160_ENSG00000170949
19	53696887	53697142	5Y-H4K8ac_peak_9398	7.29091	ZNF665_ENSG00000197497
19	53868337	53868612	5Y-H4K8ac_peak_9399	10.37534	ZNF525_ENSG00000203326
19	53897756	53897976	5Y-H4K8ac_peak_9400	7.76232	
19	53971174	53971390	5Y-H4K8ac_peak_9401	7.04637	ZNF813_ENSG00000198346
19	54023317	54023780	5Y-H4K8ac_peak_9402	11.57334	ZNF331_ENSG00000130844
19	54040549	54040794	5Y-H4K8ac_peak_9403	4.77126	
19	54372312	54372517	5Y-H4K8ac_peak_9404	6.96612	AC008753.6_ENSG00000228323
19	54385538	54385765	5Y-H4K8ac_peak_9405	4.95697	
19	54392802	54393045	5Y-H4K8ac_peak_9406	7.3889	
19	54463262	54463601	5Y-H4K8ac_peak_9407	4.84727	
19	54641565	54642061	5Y-H4K8ac_peak_9408	9.96543	CNOT3_ENSG00000088038
19	54664019	54664349	5Y-H4K8ac_peak_9409	5.42666	LENG1_ENSG00000105617
19	54974332	54974827	5Y-H4K8ac_peak_9410	7.89273	LENG9_ENSG00000182909
19	54976560	54976782	5Y-H4K8ac_peak_9411	6.77436	
19	55629955	55630184	5Y-H4K8ac_peak_9412	7.50501	
19	55653253	55653559	5Y-H4K8ac_peak_9413	6.73047	
19	55658133	55658535	5Y-H4K8ac_peak_9414	4.77126	
19	55739676	55739933	5Y-H4K8ac_peak_9415	6.61077	
19	55770022	55770342	5Y-H4K8ac_peak_9416	11.86504	PPP6R1_ENSG00000105063
19	55771185	55771643	5Y-H4K8ac_peak_9417	11.19336	PPP6R1_ENSG00000105063
19	55791234	55791434	5Y-H4K8ac_peak_9418	6.78128	HSPBP1_ENSG00000133265
19	55791956	55792284	5Y-H4K8ac_peak_9419	13.2534	HSPBP1_ENSG00000133265
19	55795507	55795823	5Y-H4K8ac_peak_9420	19.19899	
19	55856550	55856851	5Y-H4K8ac_peak_9421	7.64716	
19	55894692	55894981	5Y-H4K8ac_peak_9422	7.61752	TMEM238_ENSG00000233493
19	55895384	55895846	5Y-H4K8ac_peak_9423	11.22005	TMEM238_ENSG00000233493;RPL28_ENSG00000108107
19	55919810	55920276	5Y-H4K8ac_peak_9424	14.15915	UBE2S_ENSG00000108106
19	55951957	55952150	5Y-H4K8ac_peak_9425	6.77436	
19	55972660	55972955	5Y-H4K8ac_peak_9426	10.19948	ISOC2_ENSG00000063241
19	55979466	55979873	5Y-H4K8ac_peak_9427	5.23712	
19	55987227	55987649	5Y-H4K8ac_peak_9428	7.44077	CTD-2537I9.16_ENSG00000269859;ZNF628_ENSG00000197483

19	55996181	55996458	5Y-H4K8ac_peak_9429	7.34185	NAT14_ENSG00000090971
19	55997213	55998340	5Y-H4K8ac_peak_9430	11.55992	NAT14_ENSG00000090971
19	56014890	56015127	5Y-H4K8ac_peak_9431	9.87097	
19	56028609	56028833	5Y-H4K8ac_peak_9432	7.97699	
19	56039437	56039658	5Y-H4K8ac_peak_9433	7.63144	
19	56057946	56058151	5Y-H4K8ac_peak_9434	4.84727	
19	56058544	56058869	5Y-H4K8ac_peak_9435	6.34046	
19	56061249	56061513	5Y-H4K8ac_peak_9436	5.98695	
19	56092465	56092779	5Y-H4K8ac_peak_9437	8.71064	ZNF579_ENSG00000218891
19	56110614	56110874	5Y-H4K8ac_peak_9438	10.35586	ZNF524_ENSG00000171443
19	56115631	56115821	5Y-H4K8ac_peak_9439	6.77436	ZNF865_ENSG00000261221
19	56117183	56117434	5Y-H4K8ac_peak_9440	6.37023	ZNF865_ENSG00000261221
19	56146442	56146906	5Y-H4K8ac_peak_9441	6.94897	ZNF580_ENSG00000213015;ZNF581_ENSG00000171425
19	56151682	56152831	5Y-H4K8ac_peak_9442	7.27671	CCDC106_ENSG00000173581
19	56155000	56155374	5Y-H4K8ac_peak_9443	4.728	
19	56159476	56160304	5Y-H4K8ac_peak_9444	7.29055	
19	56166411	56166842	5Y-H4K8ac_peak_9445	9.51254	U2AF2_ENSG00000063244
19	56631807	56632021	5Y-H4K8ac_peak_9446	9.23159	ZNF787_ENSG00000142409
19	56905110	56905340	5Y-H4K8ac_peak_9447	6.73385	ZNF582_ENSG00000018869;ZNF582-AS1_ENSG00000267454
19	57049510	57050060	5Y-H4K8ac_peak_9448	9.23159	ZFP28_ENSG00000196867;AC005498.3_ENSG00000267421
19	57106656	57107024	5Y-H4K8ac_peak_9449	15.34073	ZNF71_ENSG00000197951
19	57182624	57183087	5Y-H4K8ac_peak_9450	10.69698	ZNF835_ENSG00000127903;AC007228.5_ENSG00000268352
19	57183374	57183695	5Y-H4K8ac_peak_9451	8.16382	ZNF835_ENSG00000127903;AC007228.5_ENSG00000268352
19	57702223	57702616	5Y-H4K8ac_peak_9452	7.31102	ZNF264_ENSG00000083844
19	57791432	57791684	5Y-H4K8ac_peak_9453	4.15658	CTC-444N24.6_ENSG00000267871;ZNF460_ENSG00000197714
19	57831493	57831687	5Y-H4K8ac_peak_9454	10.46287	ZNF543_ENSG00000178229
19	57901515	57901748	5Y-H4K8ac_peak_9455	7.31102	ZNF548_ENSG00000188785;AC003002.6_ENSG00000269533
19	57922056	57922312	5Y-H4K8ac_peak_9456	4.71803	ZNF17_ENSG00000186272
19	57988596	57988807	5Y-H4K8ac_peak_9457	6.78128	AC004076.9_ENSG00000268163;ZNF772_ENSG00000197128;AC003005.2_ENSG00000268266
19	57999138	57999456	5Y-H4K8ac_peak_9458	10.11191	ZNF419_ENSG00000105136;AC003005.4_ENSG00000268107
19	58011377	58011591	5Y-H4K8ac_peak_9459	7.11863	ZNF773_ENSG00000152439
19	58125711	58126043	5Y-H4K8ac_peak_9460	6.96612	ZNF134_ENSG00000213762
19	58193519	58193813	5Y-H4K8ac_peak_9461	5.87725	ZNF551_ENSG00000204519;AC003006.7_ENSG00000269026
19	58258317	58258571	5Y-H4K8ac_peak_9462	7.31815	ZNF776_ENSG00000152443
19	58315188	58315432	5Y-H4K8ac_peak_9463	7.31815	
19	58331140	58331420	5Y-H4K8ac_peak_9464	10.60083	CTD-2583A14.10_ENSG00000268750;ZNF587B_ENSG00000269343
19	58427661	58427887	5Y-H4K8ac_peak_9465	6.34046	CTD-2583A14.9_ENSG00000269476;ZNF417_ENSG00000173480
19	58458728	58459010	5Y-H4K8ac_peak_9466	5.64909	ZNF256_ENSG00000152454
19	58513608	58514093	5Y-H4K8ac_peak_9467	10.12457	ZNF606_ENSG00000166704;CTD-2368P22.1_ENSG00000176593
19	58609281	58609516	5Y-H4K8ac_peak_9468	8.69112	
19	58666134	58666427	5Y-H4K8ac_peak_9469	19.59417	ZNF329_ENSG00000181894
19	58694457	58694690	5Y-H4K8ac_peak_9470	11.48691	ZNF274_ENSG00000171606
19	58790412	58790898	5Y-H4K8ac_peak_9471	8.16382	CTD-3138B18.5_ENSG00000268516;ZNF8_ENSG00000083842;ZNF8_ENSG00000273439;AC010642.1_ENSG00000267216
19	58872787	58872986	5Y-H4K8ac_peak_9472	6.47245	CTD-2619J13.3_ENSG00000269054
19	58873187	58873462	5Y-H4K8ac_peak_9473	14.2053	CTD-2619J13.8_ENSG00000268230;ZNF497_ENSG00000174586;CTD-2619J13.3_ENSG00000269054
19	58897162	58898083	5Y-H4K8ac_peak_9474	20.85916	RPS5_ENSG00000083845;MIR4754_ENSG00000266640

19	58898747	58899023	5Y-H4K8ac_peak_9475	5.65584	RPS5_ENSG00000083845;MIR4754_ENSG00000266640
19	58951008	58951509	5Y-H4K8ac_peak_9476	14.15915	ZNF132_ENSG00000131849;CTD-2619J13.19_ENSG00000269473
19	58951881	58952242	5Y-H4K8ac_peak_9477	6.1654	ZNF132_ENSG00000131849;CTD-2619J13.19_ENSG00000269473
19	59011387	59011580	5Y-H4K8ac_peak_9478	4.79585	
19	59025032	59025305	5Y-H4K8ac_peak_9479	5.87725	
19	59030504	59031108	5Y-H4K8ac_peak_9480	11.1169	
19	59054781	59055209	5Y-H4K8ac_peak_9481	8.564	RN7SL525P_ENSG00000264910;TRIM28_ENSG00000130726
19	59055827	59056036	5Y-H4K8ac_peak_9482	12.21176	RN7SL525P_ENSG00000264910;TRIM28_ENSG00000130726
19	59065870	59066294	5Y-H4K8ac_peak_9483	7.59101	CHMP2A_ENSG00000130724
19	59066706	59067054	5Y-H4K8ac_peak_9484	6.50117	CHMP2A_ENSG00000130724
19	59070059	59070266	5Y-H4K8ac_peak_9485	12.51872	UBE2M_ENSG00000130725;AC016629.8_ENSG00000267858
19	59071118	59071621	5Y-H4K8ac_peak_9486	8.69112	UBE2M_ENSG00000130725;AC016629.8_ENSG00000267858
19	59074386	59075003	5Y-H4K8ac_peak_9487	10.72427	
19	59083771	59084068	5Y-H4K8ac_peak_9488	6.78318	MZF1_ENSG00000099326;CENPBD1P1_ENSG00000213753
19	59084317	59084614	5Y-H4K8ac_peak_9489	3.94247	MZF1_ENSG00000099326;CENPBD1P1_ENSG00000213753
2	263929	264528	5Y-H4K8ac_peak_9490	15.82359	ACP1_ENSG00000143727
2	468358	468559	5Y-H4K8ac_peak_9491	7.73231	
2	579525	579723	5Y-H4K8ac_peak_9492	6.50117	
2	636602	637121	5Y-H4K8ac_peak_9493	10.60083	
2	677166	677389	5Y-H4K8ac_peak_9494	16.51777	TMEM18_ENSG00000151353;AC092159.2_ENSG00000233296
2	677629	677856	5Y-H4K8ac_peak_9495	8.43511	TMEM18_ENSG00000151353;AC092159.2_ENSG00000233296
2	854261	854527	5Y-H4K8ac_peak_9496	5.98695	
2	945730	945998	5Y-H4K8ac_peak_9497	4.95697	AC116614.1_ENSG00000235688;SNTG2_ENSG00000172554
2	1286618	1286952	5Y-H4K8ac_peak_9498	8.43511	
2	1545715	1546041	5Y-H4K8ac_peak_9499	7.89273	
2	1546687	1546910	5Y-H4K8ac_peak_9500	7.95414	
2	1598265	1598890	5Y-H4K8ac_peak_9501	6.08471	
2	1608866	1609057	5Y-H4K8ac_peak_9502	5.12488	
2	1628946	1629295	5Y-H4K8ac_peak_9503	5.12488	AC144450.2_ENSG00000203635
2	1669916	1670163	5Y-H4K8ac_peak_9504	5.18947	
2	1711948	1712475	5Y-H4K8ac_peak_9505	7.8267	
2	1717242	1717592	5Y-H4K8ac_peak_9506	11.29141	
2	1717976	1718404	5Y-H4K8ac_peak_9507	8.04693	
2	1719283	1720018	5Y-H4K8ac_peak_9508	10.31981	
2	1735164	1735487	5Y-H4K8ac_peak_9509	13.80949	
2	1747668	1748651	5Y-H4K8ac_peak_9510	12.01623	PXDN_ENSG00000130508
2	1812346	1812617	5Y-H4K8ac_peak_9511	4.00285	
2	1994850	1995140	5Y-H4K8ac_peak_9512	8.69112	
2	2117075	2117271	5Y-H4K8ac_peak_9513	7.00198	
2	2186633	2187093	5Y-H4K8ac_peak_9514	5.99504	
2	2196456	2196655	5Y-H4K8ac_peak_9515	8.47164	
2	2297356	2297548	5Y-H4K8ac_peak_9516	6.37023	
2	2325032	2325258	5Y-H4K8ac_peak_9517	4.50834	
2	2329823	2330063	5Y-H4K8ac_peak_9518	6.78128	
2	2337851	2338114	5Y-H4K8ac_peak_9519	7.89273	
2	2467646	2468466	5Y-H4K8ac_peak_9520	11.68317	

2	2528486	2528679	5Y-H4K8ac_peak_9521	7.59101	
2	2585504	2585772	5Y-H4K8ac_peak_9522	9.59236	
2	2616858	2617152	5Y-H4K8ac_peak_9523	10.32379	
2	2712708	2713315	5Y-H4K8ac_peak_9524	7.20869	
2	2721313	2721607	5Y-H4K8ac_peak_9525	8.31764	
2	2773901	2774319	5Y-H4K8ac_peak_9526	8.27643	
2	2791005	2791196	5Y-H4K8ac_peak_9527	3.93327	
2	2791740	2792184	5Y-H4K8ac_peak_9528	5.12488	
2	2849947	2850922	5Y-H4K8ac_peak_9529	12.71428	
2	2852188	2852491	5Y-H4K8ac_peak_9530	6.78128	
2	2852827	2855514	5Y-H4K8ac_peak_9531	19.58975	
2	2873340	2873677	5Y-H4K8ac_peak_9532	6.43068	
2	2879239	2879440	5Y-H4K8ac_peak_9533	4.70501	
2	2922849	2923223	5Y-H4K8ac_peak_9534	6.53157	
2	2936556	2936851	5Y-H4K8ac_peak_9535	7.52147	
2	2937736	2938200	5Y-H4K8ac_peak_9536	7.89273	
2	2941931	2942527	5Y-H4K8ac_peak_9537	6.08523	
2	3045930	3046429	5Y-H4K8ac_peak_9538	9.11686	
2	3046624	3047013	5Y-H4K8ac_peak_9539	7.90751	
2	3080614	3081030	5Y-H4K8ac_peak_9540	7.50148	
2	3103598	3103804	5Y-H4K8ac_peak_9541	4.0639	
2	3104238	3105302	5Y-H4K8ac_peak_9542	21.41794	
2	3105599	3107216	5Y-H4K8ac_peak_9543	20.07459	
2	3107967	3108886	5Y-H4K8ac_peak_9544	9.33021	
2	3109497	3109706	5Y-H4K8ac_peak_9545	6.88736	
2	3111403	3111602	5Y-H4K8ac_peak_9546	4.0639	
2	3129081	3131071	5Y-H4K8ac_peak_9547	22.59279	AC019118.2_ENSG00000234423
2	3131589	3132004	5Y-H4K8ac_peak_9548	21.6611	
2	3132423	3132916	5Y-H4K8ac_peak_9549	8.49078	
2	3133901	3134182	5Y-H4K8ac_peak_9550	5.95943	
2	3134824	3135248	5Y-H4K8ac_peak_9551	12.87247	
2	3135464	3135705	5Y-H4K8ac_peak_9552	7.89273	
2	3187624	3187819	5Y-H4K8ac_peak_9553	6.53157	
2	3193714	3193989	5Y-H4K8ac_peak_9554	6.75784	
2	3203986	3204279	5Y-H4K8ac_peak_9555	5.54882	
2	3207713	3207927	5Y-H4K8ac_peak_9556	4.95697	
2	3282062	3282288	5Y-H4K8ac_peak_9557	4.15658	
2	3286887	3287632	5Y-H4K8ac_peak_9558	12.60556	
2	3297076	3297330	5Y-H4K8ac_peak_9559	5.18558	
2	3306313	3307354	5Y-H4K8ac_peak_9560	29.54963	
2	3307820	3308079	5Y-H4K8ac_peak_9561	11.05199	
2	3316839	3317990	5Y-H4K8ac_peak_9562	8.43511	
2	3318352	3318711	5Y-H4K8ac_peak_9563	6.08523	
2	3381984	3382419	5Y-H4K8ac_peak_9564	9.37812	TSSC1_ENSG00000032389
2	3383297	3383730	5Y-H4K8ac_peak_9565	7.20869	TRAPPC12_ENSG00000171853
2	3403752	3403942	5Y-H4K8ac_peak_9566	9.04239	

2	3452211	3452644	5Y-H4K8ac_peak_9567	11.22005	
2	3463489	3463689	5Y-H4K8ac_peak_9568	5.64909	
2	3464739	3465117	5Y-H4K8ac_peak_9569	4.15658	
2	3522262	3522567	5Y-H4K8ac_peak_9570	8.04693	ADI1_ENSG00000182551;AC142528.1_ENSG00000235078
2	3523098	3523585	5Y-H4K8ac_peak_9571	19.06379	ADI1_ENSG00000182551;AC142528.1_ENSG00000235078
2	3584370	3584563	5Y-H4K8ac_peak_9572	5.449	AC108488.4_ENSG00000242282
2	3605453	3605840	5Y-H4K8ac_peak_9573	6.34245	RNASEH1_ENSG00000171865;RNASEH1-AS1_ENSG00000234171
2	3606182	3606615	5Y-H4K8ac_peak_9574	9.40888	RNASEH1_ENSG00000171865;RNASEH1-AS1_ENSG00000234171
2	3622386	3622631	5Y-H4K8ac_peak_9575	6.53157	RPS7_ENSG00000171863
2	3632415	3632646	5Y-H4K8ac_peak_9576	7.29176	
2	3633050	3633328	5Y-H4K8ac_peak_9577	6.78128	
2	3642412	3643265	5Y-H4K8ac_peak_9578	9.79526	COLEC11_ENSG00000118004
2	3650961	3651840	5Y-H4K8ac_peak_9579	12.00715	AC010907.2_ENSG00000237370
2	3653774	3654246	5Y-H4K8ac_peak_9580	15.25284	
2	3654520	3654711	5Y-H4K8ac_peak_9581	8.4382	
2	3698145	3698350	5Y-H4K8ac_peak_9582	5.69598	
2	3699523	3699936	5Y-H4K8ac_peak_9583	7.89273	
2	3976060	3976526	5Y-H4K8ac_peak_9584	7.3889	
2	5813266	5813722	5Y-H4K8ac_peak_9585	9.58986	
2	5831666	5832328	5Y-H4K8ac_peak_9586	12.72596	AC107057.2_ENSG00000224128;AC108025.2_ENSG00000230090;SOX11_ENSG00000176887
2	6052716	6053296	5Y-H4K8ac_peak_9587	8.22388	
2	6120862	6121191	5Y-H4K8ac_peak_9588	13.0728	
2	6121492	6122186	5Y-H4K8ac_peak_9589	9.02938	
2	6214460	6214709	5Y-H4K8ac_peak_9590	8.25419	
2	6220646	6221036	5Y-H4K8ac_peak_9591	10.72427	
2	6258660	6258853	5Y-H4K8ac_peak_9592	5.84208	
2	6429588	6429976	5Y-H4K8ac_peak_9593	5.24695	
2	6430523	6431714	5Y-H4K8ac_peak_9594	9.59156	
2	6450920	6451141	5Y-H4K8ac_peak_9595	10.1994	
2	6665582	6665818	5Y-H4K8ac_peak_9596	9.30505	
2	7005410	7006454	5Y-H4K8ac_peak_9597	3.93392	CMPK2_ENSG00000134326;RSAD2_ENSG00000134321
2	7133394	7133817	5Y-H4K8ac_peak_9598	14.39337	
2	7148096	7148526	5Y-H4K8ac_peak_9599	9.02938	
2	7148736	7149295	5Y-H4K8ac_peak_9600	5.51767	
2	7157071	7157420	5Y-H4K8ac_peak_9601	12.80227	
2	7237818	7238088	5Y-H4K8ac_peak_9602	5.41472	
2	8665854	8666177	5Y-H4K8ac_peak_9603	9.45199	
2	8667144	8667357	5Y-H4K8ac_peak_9604	5.47078	
2	8675247	8675469	5Y-H4K8ac_peak_9605	7.90751	
2	8678889	8679317	5Y-H4K8ac_peak_9606	6.78128	
2	8679820	8680068	5Y-H4K8ac_peak_9607	6.40778	
2	8684124	8684739	5Y-H4K8ac_peak_9608	6.80915	
2	8688518	8688708	5Y-H4K8ac_peak_9609	5.77617	
2	8714521	8715021	5Y-H4K8ac_peak_9610	6.86362	AC011747.3_ENSG00000231435
2	8715438	8715678	5Y-H4K8ac_peak_9611	5.98695	AC011747.3_ENSG00000231435
2	8817350	8817769	5Y-H4K8ac_peak_9612	6.78318	

2	8818022	8818685	5Y-H4K8ac_peak_9613	7.61752	ID2_ENSG00000115738
2	8819100	8819557	5Y-H4K8ac_peak_9614	22.60484	ID2_ENSG00000115738
2	9144239	9144603	5Y-H4K8ac_peak_9615	15.03212	MBOAT2_ENSG00000143797
2	9246092	9246292	5Y-H4K8ac_peak_9616	4.95697	RP11-734K21.5_ENSG00000261104
2	9347539	9347933	5Y-H4K8ac_peak_9617	9.02938	ASAP2_ENSG00000151693
2	9371962	9372232	5Y-H4K8ac_peak_9618	7.53011	
2	9375780	9376364	5Y-H4K8ac_peak_9619	10.72427	
2	9387008	9387316	5Y-H4K8ac_peak_9620	7.41172	
2	9408843	9409078	5Y-H4K8ac_peak_9621	6.59112	
2	9563293	9563575	5Y-H4K8ac_peak_9622	12.26819	ITGB1BP1_ENSG00000119185;CPSF3_ENSG00000119203
2	9563849	9564041	5Y-H4K8ac_peak_9623	6.53157	ITGB1BP1_ENSG00000119185;CPSF3_ENSG00000119203
2	9614362	9615180	5Y-H4K8ac_peak_9624	12.26819	IAH1_ENSG00000134330
2	9770408	9771072	5Y-H4K8ac_peak_9625	9.87097	YWHAQ_ENSG00000134308
2	9883779	9884003	5Y-H4K8ac_peak_9626	3.90241	
2	9892848	9893087	5Y-H4K8ac_peak_9627	4.50834	
2	9910838	9911074	5Y-H4K8ac_peak_9628	4.642	RP11-521D12.5_ENSG00000243491
2	9983164	9983396	5Y-H4K8ac_peak_9629	15.75437	TAF1B_ENSG00000115750
2	9983633	9983892	5Y-H4K8ac_peak_9630	8.3285	TAF1B_ENSG00000115750
2	10149820	10150434	5Y-H4K8ac_peak_9631	15.2347	
2	10168899	10169135	5Y-H4K8ac_peak_9632	3.94555	
2	10176536	10176898	5Y-H4K8ac_peak_9633	8.31536	
2	10183785	10184165	5Y-H4K8ac_peak_9634	9.36633	KLF11_ENSG00000172059
2	10261781	10262660	5Y-H4K8ac_peak_9635	13.33225	RP11-254F7.4_ENSG00000272524;RRM2_ENSG00000171848
2	10262932	10263264	5Y-H4K8ac_peak_9636	6.08523	RRM2_ENSG00000171848
2	10311128	10311491	5Y-H4K8ac_peak_9637	7.21591	
2	10410187	10410621	5Y-H4K8ac_peak_9638	4.76429	
2	10425781	10427465	5Y-H4K8ac_peak_9639	14.17466	
2	10466341	10466772	5Y-H4K8ac_peak_9640	5.51767	
2	10467030	10467310	5Y-H4K8ac_peak_9641	5.24695	
2	10470945	10471136	5Y-H4K8ac_peak_9642	4.00596	
2	10498372	10498586	5Y-H4K8ac_peak_9643	5.30014	
2	10500727	10501312	5Y-H4K8ac_peak_9644	6.34027	
2	10513150	10513359	5Y-H4K8ac_peak_9645	5.50568	
2	10513899	10514093	5Y-H4K8ac_peak_9646	6.88736	
2	10530861	10531422	5Y-H4K8ac_peak_9647	4.48815	
2	10531866	10532528	5Y-H4K8ac_peak_9648	8.62703	
2	10532869	10533158	5Y-H4K8ac_peak_9649	13.92701	
2	10533767	10534023	5Y-H4K8ac_peak_9650	9.87124	
2	10534324	10534617	5Y-H4K8ac_peak_9651	4.0639	
2	10536945	10537220	5Y-H4K8ac_peak_9652	7.72519	
2	10537589	10537885	5Y-H4K8ac_peak_9653	4.6766	
2	10544589	10544995	5Y-H4K8ac_peak_9654	9.79526	
2	10545439	10545721	5Y-H4K8ac_peak_9655	6.20938	
2	10546406	10546601	5Y-H4K8ac_peak_9656	4.15658	
2	10588133	10588652	5Y-H4K8ac_peak_9657	10.86648	ODC1_ENSG00000115758;RP11-320M2.1_ENSG00000257135
2	10588871	10589218	5Y-H4K8ac_peak_9658	14.00559	ODC1_ENSG00000115758;RP11-320M2.1_ENSG00000257135

2	10593991	10594403	5Y-H4K8ac_peak_9659	7.29055	
2	10688718	10689013	5Y-H4K8ac_peak_9660	11.69482	
2	10899661	10899860	5Y-H4K8ac_peak_9661	6.77436	
2	10951888	10952158	5Y-H4K8ac_peak_9662	4.61721	
2	10954448	10954671	5Y-H4K8ac_peak_9663	8.24461	
2	10954873	10955206	5Y-H4K8ac_peak_9664	11.22005	RNU7-176P_ENSG00000238962
2	11008940	11009269	5Y-H4K8ac_peak_9665	10.86648	
2	11294809	11295006	5Y-H4K8ac_peak_9666	6.08523	PQLC3_ENSG00000162976
2	11295668	11296007	5Y-H4K8ac_peak_9667	7.11439	PQLC3_ENSG00000162976
2	11485001	11485714	5Y-H4K8ac_peak_9668	13.12848	
2	11605728	11606207	5Y-H4K8ac_peak_9669	20.32089	E2F6_ENSG00000169016
2	11606534	11606833	5Y-H4K8ac_peak_9670	5.64909	E2F6_ENSG00000169016
2	11622131	11622500	5Y-H4K8ac_peak_9671	8.24461	
2	11623070	11623300	5Y-H4K8ac_peak_9672	11.29141	
2	11821980	11822209	5Y-H4K8ac_peak_9673	5.00069	AC106875.1_ENSG00000228496
2	11836898	11837801	5Y-H4K8ac_peak_9674	6.67135	
2	11883420	11884194	5Y-H4K8ac_peak_9675	8.95951	
2	11886219	11886489	5Y-H4K8ac_peak_9676	6.43068	AC012456.4_ENSG00000230790
2	11970172	11970451	5Y-H4K8ac_peak_9677	4.97086	
2	12223846	12224038	5Y-H4K8ac_peak_9678	5.37237	
2	12316316	12316745	5Y-H4K8ac_peak_9679	7.01364	
2	12856590	12856836	5Y-H4K8ac_peak_9680	5.93036	TRIB2_ENSG00000071575
2	12858913	12859314	5Y-H4K8ac_peak_9681	7.59101	
2	13087752	13087982	5Y-H4K8ac_peak_9682	6.37023	
2	13088864	13089506	5Y-H4K8ac_peak_9683	6.77436	
2	13091268	13091529	5Y-H4K8ac_peak_9684	8.35138	
2	13105142	13105389	5Y-H4K8ac_peak_9685	8.43511	
2	13120841	13121249	5Y-H4K8ac_peak_9686	8.31252	
2	13124107	13124298	5Y-H4K8ac_peak_9687	5.42666	
2	14772269	14772565	5Y-H4K8ac_peak_9688	8.62703	FAM84A_ENSG00000162981
2	15732114	15732445	5Y-H4K8ac_peak_9689	13.1133	DDX1_ENSG00000079785
2	15982727	15983576	5Y-H4K8ac_peak_9690	14.99099	
2	16079728	16079926	5Y-H4K8ac_peak_9691	11.34154	MYCN_ENSG00000134323
2	16080161	16080440	5Y-H4K8ac_peak_9692	10.1994	MYCN_ENSG00000134323
2	16124280	16124787	5Y-H4K8ac_peak_9693	4.86161	
2	16234168	16235200	5Y-H4K8ac_peak_9694	6.78128	
2	16378911	16379329	5Y-H4K8ac_peak_9695	8.24461	
2	16688896	16689441	5Y-H4K8ac_peak_9696	8.82628	
2	17721358	17721696	5Y-H4K8ac_peak_9697	9.00954	VSNL1_ENSG00000163032
2	18741262	18742275	5Y-H4K8ac_peak_9698	12.05638	RDH14_ENSG00000240857
2	19547405	19547696	5Y-H4K8ac_peak_9699	5.51759	MIR4757_ENSG00000266738;AC092594.1_ENSG00000236204
2	20101058	20101706	5Y-H4K8ac_peak_9700	13.99721	TTC32_ENSG00000183891;RP11-79O8.1_ENSG00000271991
2	20211898	20212318	5Y-H4K8ac_peak_9701	6.67135	MATN3_ENSG00000132031
2	20212538	20212862	5Y-H4K8ac_peak_9702	24.56462	MATN3_ENSG00000132031
2	20270601	20270940	5Y-H4K8ac_peak_9703	7.50148	
2	20271132	20271624	5Y-H4K8ac_peak_9704	8.73392	

2	20271848	20272098	5Y-H4K8ac_peak_9705	6.49786	
2	20272399	20272765	5Y-H4K8ac_peak_9706	7.19446	
2	20273190	20273666	5Y-H4K8ac_peak_9707	4.67245	
2	20274087	20274290	5Y-H4K8ac_peak_9708	7.20869	
2	20274551	20274901	5Y-H4K8ac_peak_9709	6.00747	
2	20367375	20368063	5Y-H4K8ac_peak_9710	6.34245	
2	20368574	20369333	5Y-H4K8ac_peak_9711	17.64055	
2	20423304	20424568	5Y-H4K8ac_peak_9712	11.60058	SDC1_ENSG00000115884
2	20425394	20425825	5Y-H4K8ac_peak_9713	6.34245	SDC1_ENSG00000115884
2	20550042	20550435	5Y-H4K8ac_peak_9714	20.25522	
2	20550816	20551530	5Y-H4K8ac_peak_9715	10.90097	PUM2_ENSG00000055917
2	20624643	20625039	5Y-H4K8ac_peak_9716	7.50148	
2	20646096	20646295	5Y-H4K8ac_peak_9717	8.24461	RHOB_ENSG00000143878
2	20646964	20647340	5Y-H4K8ac_peak_9718	10.4495	RHOB_ENSG00000143878
2	20798727	20799022	5Y-H4K8ac_peak_9719	5.23083	
2	20835875	20836136	5Y-H4K8ac_peak_9720	6.75871	
2	20850565	20850816	5Y-H4K8ac_peak_9721	12.42952	HS1BP3_ENSG00000118960
2	20851275	20851618	5Y-H4K8ac_peak_9722	6.75784	HS1BP3_ENSG00000118960
2	21212715	21213215	5Y-H4K8ac_peak_9723	9.22492	
2	21490412	21490644	5Y-H4K8ac_peak_9724	4.62763	
2	21498298	21498621	5Y-H4K8ac_peak_9725	7.98792	
2	21502165	21502658	5Y-H4K8ac_peak_9726	4.95697	
2	23551424	23551621	5Y-H4K8ac_peak_9727	8.43511	
2	23573783	23575015	5Y-H4K8ac_peak_9728	15.01223	
2	23607585	23607835	5Y-H4K8ac_peak_9729	5.41472	KLHL29_ENSG00000119771
2	23859896	23860286	5Y-H4K8ac_peak_9730	6.19716	
2	23892369	23892620	5Y-H4K8ac_peak_9731	8.69112	
2	24149557	24149808	5Y-H4K8ac_peak_9732	7.49287	ATAD2B_ENSG00000119778;UBXN2A_ENSG00000173960
2	24232619	24233066	5Y-H4K8ac_peak_9733	9.89244	MFSD2B_ENSG00000205639
2	24270659	24270899	5Y-H4K8ac_peak_9734	11.57236	
2	24306371	24306630	5Y-H4K8ac_peak_9735	6.00747	
2	24307405	24307656	5Y-H4K8ac_peak_9736	9.87124	
2	24346371	24347010	5Y-H4K8ac_peak_9737	8.75963	PFN4_ENSG00000176732;RP11-507M3.1_ENSG00000266118
2	24392808	24393026	5Y-H4K8ac_peak_9738	4.50834	
2	24582871	24583270	5Y-H4K8ac_peak_9739	5.12488	ITSN2_ENSG00000198399
2	24583471	24584112	5Y-H4K8ac_peak_9740	10.31072	ITSN2_ENSG00000198399
2	24625534	24625964	5Y-H4K8ac_peak_9741	11.64182	
2	24713516	24713969	5Y-H4K8ac_peak_9742	7.89273	NCOA1_ENSG00000084676
2	25142665	25143043	5Y-H4K8ac_peak_9743	4.55128	ADCY3_ENSG00000138031
2	25143396	25143685	5Y-H4K8ac_peak_9744	8.69112	ADCY3_ENSG00000138031
2	25194217	25194857	5Y-H4K8ac_peak_9745	16.35065	DNAJC27_ENSG00000115137;DNAJC27-AS1_ENSG00000224165
2	25195114	25195342	5Y-H4K8ac_peak_9746	6.00598	DNAJC27_ENSG00000115137;DNAJC27-AS1_ENSG00000224165
2	25436093	25436356	5Y-H4K8ac_peak_9747	8.79957	
2	25474178	25475022	5Y-H4K8ac_peak_9748	27.05751	
2	25481909	25482190	5Y-H4K8ac_peak_9749	4.0639	
2	25512462	25512675	5Y-H4K8ac_peak_9750	5.72233	

2	25513758	25513977	5Y-H4K8ac_peak_9751	9.74838	
2	25514277	25514905	5Y-H4K8ac_peak_9752	8.54067	
2	25517591	25518211	5Y-H4K8ac_peak_9753	10.62421	
2	25533330	25533880	5Y-H4K8ac_peak_9754	16.1726	
2	25534839	25535939	5Y-H4K8ac_peak_9755	11.69482	
2	25536792	25537245	5Y-H4K8ac_peak_9756	6.33649	
2	25537531	25537841	5Y-H4K8ac_peak_9757	8.47417	
2	25563226	25563666	5Y-H4K8ac_peak_9758	15.55712	
2	25564236	25564713	5Y-H4K8ac_peak_9759	11.57236	DNMT3A_ENSG00000119772
2	25565127	25565761	5Y-H4K8ac_peak_9760	8.62703	DNMT3A_ENSG00000119772
2	25590713	25590962	5Y-H4K8ac_peak_9761	9.51254	
2	25599153	25599642	5Y-H4K8ac_peak_9762	13.57316	AC012074.2_ENSG00000235072
2	25600028	25600539	5Y-H4K8ac_peak_9763	14.00559	
2	25896069	25896421	5Y-H4K8ac_peak_9764	9.51254	DTNB_ENSG00000138101
2	25896646	25896967	5Y-H4K8ac_peak_9765	17.78344	DTNB_ENSG00000138101
2	26100902	26101145	5Y-H4K8ac_peak_9766	5.41472	ASXL2_ENSG00000143970
2	26160046	26160376	5Y-H4K8ac_peak_9767	5.42736	
2	26174530	26174967	5Y-H4K8ac_peak_9768	7.89273	
2	26204645	26204943	5Y-H4K8ac_peak_9769	6.19716	KIF3C_ENSG00000084731
2	26205731	26206285	5Y-H4K8ac_peak_9770	20.46741	KIF3C_ENSG00000084731
2	26256100	26256585	5Y-H4K8ac_peak_9771	6.53157	RAB10_ENSG00000084733
2	26257027	26257242	5Y-H4K8ac_peak_9772	7.03859	RAB10_ENSG00000084733
2	26467225	26467702	5Y-H4K8ac_peak_9773	13.03153	HADHA_ENSG00000084754
2	26686827	26687133	5Y-H4K8ac_peak_9774	4.86927	
2	26785028	26785226	5Y-H4K8ac_peak_9775	6.54441	C2orf70_ENSG00000173557
2	26838940	26839203	5Y-H4K8ac_peak_9776	5.41472	
2	26875600	26876123	5Y-H4K8ac_peak_9777	16.9826	
2	26876889	26877130	5Y-H4K8ac_peak_9778	6.03611	
2	26914487	26914937	5Y-H4K8ac_peak_9779	12.10416	KCNK3_ENSG00000171303
2	26986416	26986868	5Y-H4K8ac_peak_9780	6.88736	SLC35F6_ENSG00000213699;CENPA_ENSG00000115163
2	27009031	27009342	5Y-H4K8ac_peak_9781	6.78128	
2	27070447	27070746	5Y-H4K8ac_peak_9782	8.26455	DPYSL5_ENSG00000157851
2	27071036	27071367	5Y-H4K8ac_peak_9783	5.03564	DPYSL5_ENSG00000157851
2	27072312	27072609	5Y-H4K8ac_peak_9784	9.10532	
2	27255973	27256232	5Y-H4K8ac_peak_9785	4.642	TMEM214_ENSG00000119777
2	27274167	27274595	5Y-H4K8ac_peak_9786	10.43761	
2	27294767	27295821	5Y-H4K8ac_peak_9787	14.54171	OST4_ENSG00000228474
2	27299975	27300944	5Y-H4K8ac_peak_9788	11.30548	EMILIN1_ENSG00000138080
2	27301539	27302347	5Y-H4K8ac_peak_9789	9.68742	EMILIN1_ENSG00000138080
2	27308923	27309178	5Y-H4K8ac_peak_9790	8.23286	KHK_ENSG00000138030
2	27318425	27318649	5Y-H4K8ac_peak_9791	5.53736	
2	27318921	27319245	5Y-H4K8ac_peak_9792	5.87725	
2	27341327	27341986	5Y-H4K8ac_peak_9793	14.97676	CGREF1_ENSG00000138028
2	27346479	27347062	5Y-H4K8ac_peak_9794	23.10777	ABHD1_ENSG00000143994
2	27357772	27358092	5Y-H4K8ac_peak_9795	12.45678	PREB_ENSG00000138073
2	27440376	27440777	5Y-H4K8ac_peak_9796	8.62703	CAD_ENSG00000084774

2	27487408	27487940	5Y-H4K8ac_peak_9797	9.56315	
2	27526034	27526321	5Y-H4K8ac_peak_9798	12.05638	
2	27529449	27529686	5Y-H4K8ac_peak_9799	6.25061	
2	27545318	27545578	5Y-H4K8ac_peak_9800	10.8904	
2	27546083	27546337	5Y-H4K8ac_peak_9801	10.90097	
2	27579281	27579565	5Y-H4K8ac_peak_9802	6.80915	GTF3C2_ENSG00000115207;AC074117.10_ENSG00000234072
2	27580048	27580238	5Y-H4K8ac_peak_9803	7.54409	GTF3C2_ENSG00000115207;AC074117.10_ENSG00000234072
2	27601287	27601581	5Y-H4K8ac_peak_9804	5.01024	
2	27603910	27604319	5Y-H4K8ac_peak_9805	9.37812	ZNF513_ENSG00000163795
2	27632018	27632391	5Y-H4K8ac_peak_9806	6.78128	PPM1G_ENSG00000115241
2	27804998	27805544	5Y-H4K8ac_peak_9807	8.7173	AC074091.1_ENSG00000221531;ZNF512_ENSG00000243943;RP11-158I13.2_ENSG00000259080
2	27852603	27852801	5Y-H4K8ac_peak_9808	3.97334	CCDC121_ENSG00000176714
2	27886179	27886468	5Y-H4K8ac_peak_9809	13.12848	SUPT7L_ENSG00000119760;SLC4A1AP_ENSG00000163798
2	27886827	27887167	5Y-H4K8ac_peak_9810	4.00147	SUPT7L_ENSG00000119760;SLC4A1AP_ENSG00000163798
2	27937826	27938620	5Y-H4K8ac_peak_9811	7.64648	AC074091.13_ENSG00000205334
2	27958304	27958584	5Y-H4K8ac_peak_9812	4.77126	
2	27961122	27961441	5Y-H4K8ac_peak_9813	12.50545	
2	28113694	28114090	5Y-H4K8ac_peak_9814	8.24461	BRE_ENSG00000158019;RBKS_ENSG00000171174
2	28557231	28557441	5Y-H4K8ac_peak_9815	4.55128	
2	28578049	28579099	5Y-H4K8ac_peak_9816	12.87883	
2	28579553	28580118	5Y-H4K8ac_peak_9817	6.34245	
2	28613204	28614136	5Y-H4K8ac_peak_9818	12.60556	
2	28659566	28659877	5Y-H4K8ac_peak_9819	6.78128	
2	28887466	28887731	5Y-H4K8ac_peak_9820	12.79198	AC074011.2_ENSG00000230730
2	28914754	28914993	5Y-H4K8ac_peak_9821	7.59101	
2	28974965	28975365	5Y-H4K8ac_peak_9822	6.08471	PPP1CB_ENSG00000213639
2	29033741	29034114	5Y-H4K8ac_peak_9823	11.60143	RP11-713D19.1_ENSG00000273233
2	29116902	29117184	5Y-H4K8ac_peak_9824	14.11825	WDR43_ENSG00000163811
2	29117650	29118293	5Y-H4K8ac_peak_9825	19.06379	WDR43_ENSG00000163811
2	29287780	29288294	5Y-H4K8ac_peak_9826	4.55128	
2	29294117	29294424	5Y-H4K8ac_peak_9827	9.25505	
2	29338263	29338953	5Y-H4K8ac_peak_9828	20.51174	
2	29388191	29388403	5Y-H4K8ac_peak_9829	7.63144	
2	29469313	29469786	5Y-H4K8ac_peak_9830	6.37023	
2	29472016	29472753	5Y-H4K8ac_peak_9831	9.00574	
2	29473350	29473621	5Y-H4K8ac_peak_9832	4.23251	
2	29473855	29474417	5Y-H4K8ac_peak_9833	9.59236	
2	29483425	29483677	5Y-H4K8ac_peak_9834	5.41472	
2	29484898	29485373	5Y-H4K8ac_peak_9835	8.24461	
2	29486161	29487240	5Y-H4K8ac_peak_9836	8.26455	
2	29489602	29490496	5Y-H4K8ac_peak_9837	12.26819	
2	29491106	29491523	5Y-H4K8ac_peak_9838	10.07545	
2	29491794	29492584	5Y-H4K8ac_peak_9839	7.50501	
2	29493329	29493522	5Y-H4K8ac_peak_9840	5.41472	
2	29493814	29494062	5Y-H4K8ac_peak_9841	7.90751	
2	29494459	29496575	5Y-H4K8ac_peak_9842	16.79568	

2	29497259	29498424	5Y-H4K8ac_peak_9843	12.77513	
2	29504066	29504387	5Y-H4K8ac_peak_9844	10.56003	
2	29613784	29614005	5Y-H4K8ac_peak_9845	5.98695	
2	29620983	29621753	5Y-H4K8ac_peak_9846	5.60566	
2	29666297	29666533	5Y-H4K8ac_peak_9847	8.60027	
2	29738704	29739684	5Y-H4K8ac_peak_9848	17.82613	
2	29749622	29749852	5Y-H4K8ac_peak_9849	7.65114	
2	29750488	29750786	5Y-H4K8ac_peak_9850	4.89128	
2	29751219	29751822	5Y-H4K8ac_peak_9851	7.90236	
2	29752425	29752856	5Y-H4K8ac_peak_9852	11.62198	
2	29753180	29753435	5Y-H4K8ac_peak_9853	7.98023	
2	29784636	29785064	5Y-H4K8ac_peak_9854	8.22125	
2	29796981	29797406	5Y-H4K8ac_peak_9855	6.64195	
2	29798631	29798980	5Y-H4K8ac_peak_9856	6.47214	
2	29799172	29799566	5Y-H4K8ac_peak_9857	18.03442	
2	29801492	29802117	5Y-H4K8ac_peak_9858	5.60566	
2	29802537	29802742	5Y-H4K8ac_peak_9859	7.41172	
2	29803871	29804814	5Y-H4K8ac_peak_9860	14.89335	
2	29805549	29806039	5Y-H4K8ac_peak_9861	5.86226	
2	29832602	29833148	5Y-H4K8ac_peak_9862	8.0168	
2	29833880	29834196	5Y-H4K8ac_peak_9863	12.68559	
2	29835236	29835814	5Y-H4K8ac_peak_9864	7.59101	
2	29837205	29838018	5Y-H4K8ac_peak_9865	7.00004	
2	29838887	29839460	5Y-H4K8ac_peak_9866	10.08165	
2	30247999	30248345	5Y-H4K8ac_peak_9867	5.40265	
2	30294849	30295148	5Y-H4K8ac_peak_9868	5.77617	
2	30369902	30370101	5Y-H4K8ac_peak_9869	8.73392	YPEL5_ENSG00000119801
2	30426125	30426319	5Y-H4K8ac_peak_9870	4.84727	
2	30522973	30523385	5Y-H4K8ac_peak_9871	4.95697	
2	30644409	30644670	5Y-H4K8ac_peak_9872	9.05168	
2	30670121	30670430	5Y-H4K8ac_peak_9873	8.3285	LCLAT1_ENSG00000172954
2	30978490	30978937	5Y-H4K8ac_peak_9874	4.48815	
2	30979271	30979773	5Y-H4K8ac_peak_9875	6.95844	
2	30980007	30980313	5Y-H4K8ac_peak_9876	11.94897	
2	30980954	30981586	5Y-H4K8ac_peak_9877	6.85744	
2	30993736	30993966	5Y-H4K8ac_peak_9878	4.2721	
2	31088439	31089053	5Y-H4K8ac_peak_9879	4.29586	
2	31095701	31095924	5Y-H4K8ac_peak_9880	5.64909	
2	31202907	31203171	5Y-H4K8ac_peak_9881	10.11377	
2	31213401	31213601	5Y-H4K8ac_peak_9882	7.33115	
2	31216350	31217163	5Y-H4K8ac_peak_9883	11.63557	
2	31229427	31229663	5Y-H4K8ac_peak_9884	6.20292	
2	31229998	31230272	5Y-H4K8ac_peak_9885	4.15658	
2	31230866	31231500	5Y-H4K8ac_peak_9886	8.43511	
2	31234552	31234771	5Y-H4K8ac_peak_9887	11.12941	
2	31353186	31353479	5Y-H4K8ac_peak_9888	5.65584	

2	31360350	31360879	5Y-H4K8ac_peak_9889	9.76673	
2	31361104	31361822	5Y-H4K8ac_peak_9890	9.44542	
2	31525569	31526081	5Y-H4K8ac_peak_9891	5.23083	
2	32501873	32502107	5Y-H4K8ac_peak_9892	6.37237	YIPF4_ENSG00000119820
2	32503192	32503487	5Y-H4K8ac_peak_9893	6.03632	YIPF4_ENSG00000119820
2	32582623	32582839	5Y-H4K8ac_peak_9894	6.78128	BIRC6_ENSG00000115760
2	36824989	36825215	5Y-H4K8ac_peak_9895	11.93705	
2	36846202	36846454	5Y-H4K8ac_peak_9896	4.75045	
2	37383772	37384370	5Y-H4K8ac_peak_9897	14.26354	EIF2AK2_ENSG00000055332
2	37423013	37423216	5Y-H4K8ac_peak_9898	7.89273	SULT6B1_ENSG00000138068;CEBPZ-AS1_ENSG00000218739
2	37551539	37551877	5Y-H4K8ac_peak_9899	4.42435	PRKD3_ENSG00000115825;AC007391.2_ENSG00000232028
2	37617647	37617858	5Y-H4K8ac_peak_9900	4.70571	
2	37734109	37734336	5Y-H4K8ac_peak_9901	5.65584	
2	37898447	37898756	5Y-H4K8ac_peak_9902	5.41472	
2	37899036	37899300	5Y-H4K8ac_peak_9903	9.01738	
2	37899522	37899794	5Y-H4K8ac_peak_9904	5.87725	
2	38082515	38083267	5Y-H4K8ac_peak_9905	16.9826	
2	38083649	38084174	5Y-H4K8ac_peak_9906	21.54805	
2	38084662	38085098	5Y-H4K8ac_peak_9907	7.59101	
2	38152184	38152577	5Y-H4K8ac_peak_9908	16.81039	
2	38152815	38153221	5Y-H4K8ac_peak_9909	7.90751	
2	38302950	38304639	5Y-H4K8ac_peak_9910	13.35169	CYP1B1-AS1_ENSG00000232973
2	38332774	38333259	5Y-H4K8ac_peak_9911	9.36633	
2	38445377	38445569	5Y-H4K8ac_peak_9912	9.8829	
2	38603996	38604354	5Y-H4K8ac_peak_9913	20.97372	ATL2_ENSG00000119787
2	38763091	38763650	5Y-H4K8ac_peak_9914	14.89891	RP11-541E12.1_ENSG00000271443
2	38829367	38829960	5Y-H4K8ac_peak_9915	10.86648	AC011247.3_ENSG00000235586;HNRNPLL_ENSG00000143889
2	38977706	38978457	5Y-H4K8ac_peak_9916	14.26354	SRSF7_ENSG00000115875;GEMIN6_ENSG00000152147
2	39347838	39348440	5Y-H4K8ac_peak_9917	12.2918	
2	39350901	39351391	5Y-H4K8ac_peak_9918	6.78128	SOS1_ENSG00000115904
2	39471555	39471839	5Y-H4K8ac_peak_9919	7.89273	
2	39893040	39893665	5Y-H4K8ac_peak_9920	8.3285	TMEM178A_ENSG00000152154
2	40006097	40006348	5Y-H4K8ac_peak_9921	14.40623	THUMPD2_ENSG00000138050
2	40679397	40679611	5Y-H4K8ac_peak_9922	7.64648	
2	42068349	42068606	5Y-H4K8ac_peak_9923	8.3285	
2	42112933	42113123	5Y-H4K8ac_peak_9924	4.29586	
2	42113986	42114205	5Y-H4K8ac_peak_9925	7.59101	
2	42182011	42182468	5Y-H4K8ac_peak_9926	7.90005	C2orf91_ENSG00000205086
2	42252478	42252765	5Y-H4K8ac_peak_9927	5.64909	AC013480.2_ENSG00000226398
2	42274437	42274848	5Y-H4K8ac_peak_9928	5.99756	PKDCC_ENSG00000162878
2	42275603	42275983	5Y-H4K8ac_peak_9929	6.08523	PKDCC_ENSG00000162878
2	42359922	42360187	5Y-H4K8ac_peak_9930	7.29055	
2	42361144	42361431	5Y-H4K8ac_peak_9931	14.50475	
2	42361675	42361930	5Y-H4K8ac_peak_9932	9.87097	
2	42364559	42364790	5Y-H4K8ac_peak_9933	10.35586	
2	42396066	42396288	5Y-H4K8ac_peak_9934	6.67135	EML4_ENSG00000143924

2	42459909	42460177	5Y-H4K8ac_peak_9935	4.00314	
2	42720530	42721032	5Y-H4K8ac_peak_9936	16.74969	KCNG3_ENSG00000171126;MTA3_ENSG00000057935
2	42721344	42721586	5Y-H4K8ac_peak_9937	9.23858	KCNG3_ENSG00000171126;MTA3_ENSG00000057935
2	42795130	42795506	5Y-H4K8ac_peak_9938	9.02938	
2	42795792	42796560	5Y-H4K8ac_peak_9939	22.81411	
2	42981486	42981799	5Y-H4K8ac_peak_9940	4.642	
2	42982576	42982843	5Y-H4K8ac_peak_9941	6.86362	
2	42998708	42999025	5Y-H4K8ac_peak_9942	9.28129	
2	43013900	43014155	5Y-H4K8ac_peak_9943	6.08523	
2	43036943	43037599	5Y-H4K8ac_peak_9944	17.67055	
2	43037858	43038657	5Y-H4K8ac_peak_9945	14.59308	
2	43038916	43039378	5Y-H4K8ac_peak_9946	6.77436	
2	43195129	43195580	5Y-H4K8ac_peak_9947	10.74494	
2	43202112	43202660	5Y-H4K8ac_peak_9948	13.84142	
2	43268571	43268899	5Y-H4K8ac_peak_9949	6.75784	
2	43273348	43273770	5Y-H4K8ac_peak_9950	11.06021	
2	43307673	43307956	5Y-H4K8ac_peak_9951	7.72887	
2	43309163	43309716	5Y-H4K8ac_peak_9952	5.99756	
2	43309994	43310243	5Y-H4K8ac_peak_9953	7.87192	
2	43311338	43312076	5Y-H4K8ac_peak_9954	10.79676	
2	43367476	43367752	5Y-H4K8ac_peak_9955	5.95028	
2	43395511	43396015	5Y-H4K8ac_peak_9956	4.642	
2	43444723	43445017	5Y-H4K8ac_peak_9957	7.89273	
2	43445579	43446109	5Y-H4K8ac_peak_9958	8.8315	
2	43446716	43447596	5Y-H4K8ac_peak_9959	6.98416	
2	43452864	43453692	5Y-H4K8ac_peak_9960	7.80036	ZFP36L2_ENSG00000152518
2	43454064	43454306	5Y-H4K8ac_peak_9961	10.18556	ZFP36L2_ENSG00000152518
2	43454721	43454918	5Y-H4K8ac_peak_9962	3.96434	ZFP36L2_ENSG00000152518
2	43540832	43541063	5Y-H4K8ac_peak_9963	5.24695	
2	43555676	43555955	5Y-H4K8ac_peak_9964	4.48815	
2	43822953	43823150	5Y-H4K8ac_peak_9965	8.62703	THADA_ENSG00000115970
2	43823377	43823619	5Y-H4K8ac_peak_9966	9.36633	THADA_ENSG00000115970
2	43903413	43903615	5Y-H4K8ac_peak_9967	4.84727	AC011242.6_ENSG00000223658
2	44222703	44223131	5Y-H4K8ac_peak_9968	9.30206	LRPPRC_ENSG00000138095
2	44271532	44272048	5Y-H4K8ac_peak_9969	16.79568	
2	44314884	44315144	5Y-H4K8ac_peak_9970	5.01024	
2	44906056	44906341	5Y-H4K8ac_peak_9971	5.49775	
2	44932412	44932837	5Y-H4K8ac_peak_9972	11.67083	
2	45161363	45161558	5Y-H4K8ac_peak_9973	4.48815	
2	45162087	45162397	5Y-H4K8ac_peak_9974	7.21591	
2	45166888	45167081	5Y-H4K8ac_peak_9975	7.2696	RP11-89K21.1_ENSG00000259439
2	45168111	45168438	5Y-H4K8ac_peak_9976	11.4254	SIX3-AS1_ENSG00000236502;SIX3_ENSG00000138083
2	45182430	45182633	5Y-H4K8ac_peak_9977	8.82628	AC012354.6_ENSG00000225156
2	45236278	45236494	5Y-H4K8ac_peak_9978	7.57115	SIX2_ENSG00000170577
2	45240813	45241287	5Y-H4K8ac_peak_9979	10.48476	AC093702.1_ENSG00000231156
2	45282230	45282467	5Y-H4K8ac_peak_9980	11.28133	

2	45396733	45397290	5Y-H4K8ac_peak_9981	11.04356	
2	45417572	45417983	5Y-H4K8ac_peak_9982	10.1994	
2	45447279	45447988	5Y-H4K8ac_peak_9983	11.62198	
2	45488546	45488870	5Y-H4K8ac_peak_9984	4.80442	
2	45490639	45490991	5Y-H4K8ac_peak_9985	6.80779	
2	45870566	45870821	5Y-H4K8ac_peak_9986	6.84536	
2	45878420	45878678	5Y-H4K8ac_peak_9987	11.62198	PRKCE_ENSG00000171132
2	45971903	45972124	5Y-H4K8ac_peak_9988	5.23083	
2	46040448	46040859	5Y-H4K8ac_peak_9989	8.1667	
2	46048672	46049077	5Y-H4K8ac_peak_9990	5.23083	
2	46050851	46051051	5Y-H4K8ac_peak_9991	7.03573	
2	46463358	46463559	5Y-H4K8ac_peak_9992	6.34245	
2	46463854	46464179	5Y-H4K8ac_peak_9993	5.64909	
2	46523760	46524275	5Y-H4K8ac_peak_9994	7.07275	
2	46557808	46558091	5Y-H4K8ac_peak_9995	9.47304	
2	46558912	46559256	5Y-H4K8ac_peak_9996	5.71283	
2	46560081	46560272	5Y-H4K8ac_peak_9997	6.78128	
2	46560605	46560911	5Y-H4K8ac_peak_9998	16.74969	
2	46562927	46563245	5Y-H4K8ac_peak_9999	6.08523	
2	46565479	46565975	5Y-H4K8ac_peak_10000	10.07378	
2	46566748	46567092	5Y-H4K8ac_peak_10001	16.29955	
2	46567687	46568384	5Y-H4K8ac_peak_10002	17.50136	
2	46569182	46569396	5Y-H4K8ac_peak_10003	3.96434	
2	46576505	46576714	5Y-H4K8ac_peak_10004	5.5192	
2	46768705	46769684	5Y-H4K8ac_peak_10005	13.01516	ATP6V1E2_ENSG00000250565;RHOQ_ENSG00000119729
2	47039655	47039875	5Y-H4K8ac_peak_10006	5.36319	
2	47073719	47074370	5Y-H4K8ac_peak_10007	7.90236	
2	47074738	47075445	5Y-H4K8ac_peak_10008	9.64915	
2	47075729	47076089	5Y-H4K8ac_peak_10009	11.23789	
2	47142524	47142862	5Y-H4K8ac_peak_10010	9.74838	TTC7A_ENSG00000068724
2	47210377	47210773	5Y-H4K8ac_peak_10011	6.5677	
2	47213820	47214235	5Y-H4K8ac_peak_10012	5.87725	
2	47215055	47215279	5Y-H4K8ac_peak_10013	7.18391	
2	47227783	47228098	5Y-H4K8ac_peak_10014	7.02726	
2	47229972	47230575	5Y-H4K8ac_peak_10015	10.85667	
2	47240575	47240770	5Y-H4K8ac_peak_10016	4.95697	
2	47241441	47242017	5Y-H4K8ac_peak_10017	4.15658	
2	47260319	47261086	5Y-H4K8ac_peak_10018	12.1872	
2	47261301	47261517	5Y-H4K8ac_peak_10019	11.01067	
2	47269966	47270210	5Y-H4K8ac_peak_10020	13.12848	
2	47270702	47271263	5Y-H4K8ac_peak_10021	8.69112	
2	47282758	47283676	5Y-H4K8ac_peak_10022	12.42289	
2	47283907	47284823	5Y-H4K8ac_peak_10023	12.64789	
2	47285135	47286060	5Y-H4K8ac_peak_10024	4.89347	
2	47288018	47288750	5Y-H4K8ac_peak_10025	11.4254	
2	47290716	47291956	5Y-H4K8ac_peak_10026	13.59183	

2	47299076	47299504	5Y-H4K8ac_peak_10027	13.24325	
2	47306876	47307440	5Y-H4K8ac_peak_10028	5.12488	
2	47307985	47308349	5Y-H4K8ac_peak_10029	6.62037	
2	47310215	47310783	5Y-H4K8ac_peak_10030	4.48815	
2	47311090	47311448	5Y-H4K8ac_peak_10031	7.20869	
2	47313007	47313821	5Y-H4K8ac_peak_10032	7.90005	
2	47314304	47314718	5Y-H4K8ac_peak_10033	21.80568	
2	47315975	47316732	5Y-H4K8ac_peak_10034	11.22005	
2	47403043	47403361	5Y-H4K8ac_peak_10035	8.62703	RP11-761B3.1_ENSG00000273269;CALM2_ENSG00000143933
2	47546603	47547047	5Y-H4K8ac_peak_10036	9.30206	
2	47548747	47549047	5Y-H4K8ac_peak_10037	7.60057	
2	47596183	47597072	5Y-H4K8ac_peak_10038	8.62703	
2	47629225	47629475	5Y-H4K8ac_peak_10039	5.89922	MSH2_ENSG00000095002
2	47630483	47630679	5Y-H4K8ac_peak_10040	4.87387	MSH2_ENSG00000095002
2	47747549	47748193	5Y-H4K8ac_peak_10041	13.86751	
2	47748595	47749148	5Y-H4K8ac_peak_10042	19.06379	
2	48009772	48010048	5Y-H4K8ac_peak_10043	6.34245	
2	48010349	48010885	5Y-H4K8ac_peak_10044	20.40213	
2	48132099	48132339	5Y-H4K8ac_peak_10045	7.96285	FBXO11_ENSG00000138081;AC079807.2_ENSG00000233230
2	48133410	48133618	5Y-H4K8ac_peak_10046	11.04542	FBXO11_ENSG00000138081;AC079807.2_ENSG00000233230
2	48339152	48339771	5Y-H4K8ac_peak_10047	10.12457	
2	48647453	48647711	5Y-H4K8ac_peak_10048	6.08523	
2	48667523	48667731	5Y-H4K8ac_peak_10049	11.43902	RP11-191L17.1_ENSG00000272663;PPP1R21_ENSG00000162869
2	48757411	48757641	5Y-H4K8ac_peak_10050	8.97752	STON1_ENSG00000243244;STON1-GTF2A1L_ENSG00000068781
2	51259462	51259869	5Y-H4K8ac_peak_10051	4.29586	NRXN1_ENSG00000179915;AC007682.1_ENSG00000231918
2	53995195	53995420	5Y-H4K8ac_peak_10052	6.50117	CHAC2_ENSG00000143942
2	54013674	54013953	5Y-H4K8ac_peak_10053	7.38046	ERLEC1_ENSG00000068912
2	54197606	54197819	5Y-H4K8ac_peak_10054	5.98695	PSME4_ENSG00000068878;ACYP2_ENSG00000170634
2	54198356	54198556	5Y-H4K8ac_peak_10055	8.2913	PSME4_ENSG00000068878;ACYP2_ENSG00000170634
2	54342588	54342932	5Y-H4K8ac_peak_10056	20.88791	
2	54682993	54683260	5Y-H4K8ac_peak_10057	13.63503	SPTBN1_ENSG00000115306
2	54742110	54742313	5Y-H4K8ac_peak_10058	5.07473	
2	54785944	54786261	5Y-H4K8ac_peak_10059	5.51139	
2	55276388	55276654	5Y-H4K8ac_peak_10060	6.37023	
2	55496095	55496304	5Y-H4K8ac_peak_10061	6.34046	MTIF2_ENSG00000085760
2	55844413	55844657	5Y-H4K8ac_peak_10062	5.91107	RP11-554J4.1_ENSG00000272606
2	56235992	56236198	5Y-H4K8ac_peak_10063	7.31815	
2	58654878	58655100	5Y-H4K8ac_peak_10064	6.20875	LINC01122_ENSG00000233723
2	58655933	58656437	5Y-H4K8ac_peak_10065	8.33296	LINC01122_ENSG00000233723
2	60808744	60809034	5Y-H4K8ac_peak_10066	9.00954	
2	61108121	61108396	5Y-H4K8ac_peak_10067	8.43511	AC010733.4_ENSG00000228414;REL_ENSG00000162924
2	61404079	61404305	5Y-H4K8ac_peak_10068	8.69112	AHSA2_ENSG00000173209
2	61404710	61405238	5Y-H4K8ac_peak_10069	15.84891	AHSA2_ENSG00000173209
2	61697375	61697816	5Y-H4K8ac_peak_10070	8.75926	USP34_ENSG00000115464;RP11-355B11.2_ENSG00000270820
2	61764953	61765245	5Y-H4K8ac_peak_10071	7.17184	XPO1_ENSG00000082898
2	61766045	61766476	5Y-H4K8ac_peak_10072	6.43775	XPO1_ENSG00000082898

2	61922070	61922300	5Y-H4K8ac_peak_10073	7.31815	
2	61991642	61991862	5Y-H4K8ac_peak_10074	10.95042	
2	62115368	62115964	5Y-H4K8ac_peak_10075	9.74189	CCT4_ENSG00000115484;COMMD1_ENSG00000173163
2	62132941	62133193	5Y-H4K8ac_peak_10076	4.77126	
2	62403923	62404159	5Y-H4K8ac_peak_10077	9.43096	
2	62422718	62423066	5Y-H4K8ac_peak_10078	9.51254	B3GNT2_ENSG00000170340
2	62536403	62537117	5Y-H4K8ac_peak_10079	10.86079	
2	62568366	62568634	5Y-H4K8ac_peak_10080	10.46135	
2	62638926	62639118	5Y-H4K8ac_peak_10081	9.52603	
2	62684397	62684606	5Y-H4K8ac_peak_10082	9.89244	
2	62702406	62702651	5Y-H4K8ac_peak_10083	4.0639	
2	64067445	64067778	5Y-H4K8ac_peak_10084	6.98118	UGP2_ENSG00000169764
2	64068237	64068686	5Y-H4K8ac_peak_10085	5.98695	UGP2_ENSG00000169764
2	64246014	64247061	5Y-H4K8ac_peak_10086	12.49811	VPS54_ENSG00000143952
2	64501087	64501289	5Y-H4K8ac_peak_10087	7.05631	
2	64681257	64681469	5Y-H4K8ac_peak_10088	9.34555	AC008074.3_ENSG00000223935;LGALSL_ENSG00000119862
2	64681669	64681972	5Y-H4K8ac_peak_10089	11.93212	AC008074.3_ENSG00000223935;LGALSL_ENSG00000119862
2	64881429	64881620	5Y-H4K8ac_peak_10090	7.50148	
2	64995072	64995563	5Y-H4K8ac_peak_10091	10.95042	
2	65215501	65216049	5Y-H4K8ac_peak_10092	11.36989	SLC1A4_ENSG00000115902
2	65282959	65283176	5Y-H4K8ac_peak_10093	6.03718	CEP68_ENSG00000011523
2	65357876	65358272	5Y-H4K8ac_peak_10094	5.48291	RAB1A_ENSG00000138069
2	65454978	65455214	5Y-H4K8ac_peak_10095	9.63153	ACTR2_ENSG00000138071
2	65664691	65664965	5Y-H4K8ac_peak_10096	8.16031	AC074391.1_ENSG00000204929
2	66297072	66297409	5Y-H4K8ac_peak_10097	7.59101	
2	66652925	66653255	5Y-H4K8ac_peak_10098	7.58806	
2	66659270	66659473	5Y-H4K8ac_peak_10099	6.77436	
2	66660028	66660264	5Y-H4K8ac_peak_10100	4.84727	MEIS1-AS3_ENSG00000226819;MEIS1_ENSG00000143995
2	66660948	66661959	5Y-H4K8ac_peak_10101	13.2534	MEIS1-AS3_ENSG00000226819;MEIS1_ENSG00000143995
2	66665296	66665572	5Y-H4K8ac_peak_10102	9.38276	
2	66667940	66668201	5Y-H4K8ac_peak_10103	8.12924	AC092669.1_ENSG00000244522;MEIS1-AS2_ENSG00000230749
2	67440540	67440851	5Y-H4K8ac_peak_10104	10.36926	
2	67543434	67543692	5Y-H4K8ac_peak_10105	7.57144	
2	67552071	67552315	5Y-H4K8ac_peak_10106	5.98695	AC023115.4_ENSG00000236605
2	67624621	67624898	5Y-H4K8ac_peak_10107	11.68317	ETAA1_ENSG00000143971
2	67876919	67877239	5Y-H4K8ac_peak_10108	8.2913	
2	68073336	68073573	5Y-H4K8ac_peak_10109	4.47071	
2	68107619	68107858	5Y-H4K8ac_peak_10110	8.2913	
2	68271666	68271862	5Y-H4K8ac_peak_10111	9.20645	
2	68384322	68384533	5Y-H4K8ac_peak_10112	4.8509	WDR92_ENSG00000243667;PNO1_ENSG00000115946
2	68479096	68479516	5Y-H4K8ac_peak_10113	6.77436	RP11-474G23.3_ENSG00000273064
2	68546189	68546787	5Y-H4K8ac_peak_10114	6.78128	CNRIP1_ENSG00000119865
2	68870452	68870722	5Y-H4K8ac_peak_10115	4.15658	PROKR1_ENSG00000169618
2	69027312	69027611	5Y-H4K8ac_peak_10116	5.40331	
2	69170884	69171547	5Y-H4K8ac_peak_10117	17.51157	
2	69240508	69240935	5Y-H4K8ac_peak_10118	4.50834	ANTXR1_ENSG00000169604

2	69440987	69441274	5Y-H4K8ac_peak_10119	11.22005	
2	69533692	69533922	5Y-H4K8ac_peak_10120	9.30505	
2	69664222	69664442	5Y-H4K8ac_peak_10121	5.87725	NFU1_ENSG00000169599
2	69870970	69871235	5Y-H4K8ac_peak_10122	16.03077	ANXA4_ENSG00000196975
2	69968854	69969547	5Y-H4K8ac_peak_10123	9.23159	
2	70120649	70120954	5Y-H4K8ac_peak_10124	7.3889	SNRNP27_ENSG00000124380
2	70141360	70141587	5Y-H4K8ac_peak_10125	7.89273	
2	70299005	70299239	5Y-H4K8ac_peak_10126	5.29015	
2	70313509	70314383	5Y-H4K8ac_peak_10127	20.21988	PCBP1_ENSG00000169564
2	70314880	70315448	5Y-H4K8ac_peak_10128	5.98695	PCBP1-AS1_ENSG00000179818;PCBP1_ENSG00000169564
2	70336274	70336477	5Y-H4K8ac_peak_10129	4.9885	
2	70351341	70351557	5Y-H4K8ac_peak_10130	6.53157	AC016700.5_ENSG00000231327
2	70351932	70352451	5Y-H4K8ac_peak_10131	13.2534	AC016700.5_ENSG00000231327
2	70359984	70360186	5Y-H4K8ac_peak_10132	7.01266	
2	70368760	70369577	5Y-H4K8ac_peak_10133	20.25522	
2	70475417	70475713	5Y-H4K8ac_peak_10134	15.40182	C2orf42_ENSG00000115998;TIA1_ENSG00000116001
2	70476683	70476916	5Y-H4K8ac_peak_10135	8.75926	C2orf42_ENSG00000115998;TIA1_ENSG00000116001
2	70520518	70520719	5Y-H4K8ac_peak_10136	6.77436	SNRPG_ENSG00000143977
2	70528693	70528975	5Y-H4K8ac_peak_10137	5.60566	FAM136A_ENSG00000035141;AC022201.5_ENSG00000233849
2	70780790	70781275	5Y-H4K8ac_peak_10138	4.15658	TGFA_ENSG00000163235
2	70994743	70995288	5Y-H4K8ac_peak_10139	6.50117	ADD2_ENSG00000075340
2	71228029	71228329	5Y-H4K8ac_peak_10140	5.24695	
2	71228609	71228912	5Y-H4K8ac_peak_10141	7.31102	
2	71270589	71270831	5Y-H4K8ac_peak_10142	11.36844	
2	71294818	71295166	5Y-H4K8ac_peak_10143	6.08523	RP11-467P9.1_ENSG00000272735
2	71558457	71558655	5Y-H4K8ac_peak_10144	7.76232	
2	71683290	71683727	5Y-H4K8ac_peak_10145	5.12213	
2	72079721	72080149	5Y-H4K8ac_peak_10146	8.1667	
2	73089893	73090185	5Y-H4K8ac_peak_10147	5.98695	
2	73340488	73340682	5Y-H4K8ac_peak_10148	5.23083	RP11-44N22.3_ENSG00000272702
2	73461539	73461915	5Y-H4K8ac_peak_10149	14.31711	CCT7_ENSG00000135624
2	73964826	73965491	5Y-H4K8ac_peak_10150	11.99586	TPRKB_ENSG00000144034
2	74056196	74056585	5Y-H4K8ac_peak_10151	6.77436	STAMBP_ENSG00000124356
2	74153304	74153539	5Y-H4K8ac_peak_10152	4.84727	DGUOK_ENSG00000114956
2	74211440	74212293	5Y-H4K8ac_peak_10153	19.93272	AC073046.25_ENSG00000235499
2	74212600	74213028	5Y-H4K8ac_peak_10154	8.35207	AC073046.25_ENSG00000235499;RP11-711M9.1_ENSG00000255989
2	74347502	74347791	5Y-H4K8ac_peak_10155	12.10416	FNBP1P1_ENSG00000257800
2	74374549	74374959	5Y-H4K8ac_peak_10156	4.15658	BOLA3_ENSG00000163170;BOLA3-AS1_ENSG00000225439
2	74425799	74426246	5Y-H4K8ac_peak_10157	14.99673	MTHFD2_ENSG00000065911
2	74618650	74618907	5Y-H4K8ac_peak_10158	6.31818	DCTN1_ENSG00000204843
2	74648930	74649827	5Y-H4K8ac_peak_10159	4.95697	C2orf81_ENSG00000159239;WDR54_ENSG00000005448
2	74667697	74668167	5Y-H4K8ac_peak_10160	12.26819	
2	74699226	74699438	5Y-H4K8ac_peak_10161	4.95697	
2	74734615	74734813	5Y-H4K8ac_peak_10162	11.1169	PCGF1_ENSG00000115289
2	74735042	74735347	5Y-H4K8ac_peak_10163	26.97738	PCGF1_ENSG00000115289
2	74739986	74740228	5Y-H4K8ac_peak_10164	5.77617	TLX2_ENSG00000115297

2	74740694	74741187	5Y-H4K8ac_peak_10165	7.58806	TLX2_ENSG00000115297
2	74741751	74742068	5Y-H4K8ac_peak_10166	4.95697	
2	74775902	74776548	5Y-H4K8ac_peak_10167	8.73392	DOK1_ENSG00000115325
2	74781279	74781765	5Y-H4K8ac_peak_10168	13.31667	
2	75006177	75006479	5Y-H4K8ac_peak_10169	9.30505	
2	75185349	75185577	5Y-H4K8ac_peak_10170	3.96434	POLE4_ENSG00000115350
2	75873974	75874405	5Y-H4K8ac_peak_10171	5.41472	MRPL19_ENSG00000115364
2	75937559	75937787	5Y-H4K8ac_peak_10172	7.50148	GCFC2_ENSG00000005436
2	75938067	75938383	5Y-H4K8ac_peak_10173	4.0639	GCFC2_ENSG00000005436
2	76897793	76897993	5Y-H4K8ac_peak_10174	6.34046	
2	76918184	76918380	5Y-H4K8ac_peak_10175	8.33296	
2	77035165	77035378	5Y-H4K8ac_peak_10176	7.31815	
2	78768641	78768914	5Y-H4K8ac_peak_10177	4.51076	
2	79740139	79740329	5Y-H4K8ac_peak_10178	8.33296	
2	85132828	85133029	5Y-H4K8ac_peak_10179	8.36588	TMSB10_ENSG00000034510
2	85443243	85443797	5Y-H4K8ac_peak_10180	6.43775	
2	85482375	85482582	5Y-H4K8ac_peak_10181	6.08523	
2	85484684	85484917	5Y-H4K8ac_peak_10182	9.96543	
2	85485380	85485712	5Y-H4K8ac_peak_10183	8.43511	
2	85500905	85501235	5Y-H4K8ac_peak_10184	7.56545	
2	85501445	85501703	5Y-H4K8ac_peak_10185	10.11191	
2	85645050	85646408	5Y-H4K8ac_peak_10186	19.75467	CAPG_ENSG00000042493;SH2D6_ENSG00000152292
2	85648105	85648358	5Y-H4K8ac_peak_10187	7.8267	
2	85658965	85659178	5Y-H4K8ac_peak_10188	7.50044	
2	85660331	85660883	5Y-H4K8ac_peak_10189	9.51254	Y_RNA_ENSG00000207207
2	85661560	85662002	5Y-H4K8ac_peak_10190	8.45687	Y_RNA_ENSG00000207207
2	85662690	85662970	5Y-H4K8ac_peak_10191	7.89142	
2	85663714	85664371	5Y-H4K8ac_peak_10192	7.11863	
2	85665458	85665758	5Y-H4K8ac_peak_10193	16.38263	
2	85672902	85673724	5Y-H4K8ac_peak_10194	5.99756	
2	85674062	85674277	5Y-H4K8ac_peak_10195	6.62731	
2	85750200	85750623	5Y-H4K8ac_peak_10196	6.66026	
2	85765342	85765750	5Y-H4K8ac_peak_10197	10.4689	MAT2A_ENSG00000168906
2	85766347	85766559	5Y-H4K8ac_peak_10198	7.89142	MAT2A_ENSG00000168906
2	85811505	85812073	5Y-H4K8ac_peak_10199	10.90365	VAMP5_ENSG00000168899
2	85838757	85839010	5Y-H4K8ac_peak_10200	10.95042	C2orf68_ENSG00000168887
2	85839394	85840084	5Y-H4K8ac_peak_10201	7.59101	C2orf68_ENSG00000168887
2	85842648	85843042	5Y-H4K8ac_peak_10202	8.01306	
2	85843336	85843574	5Y-H4K8ac_peak_10203	9.96543	
2	85968287	85968827	5Y-H4K8ac_peak_10204	9.07085	
2	85969077	85969326	5Y-H4K8ac_peak_10205	4.95697	
2	85969979	85970310	5Y-H4K8ac_peak_10206	9.66296	
2	86000162	86000763	5Y-H4K8ac_peak_10207	9.93175	
2	86021904	86022347	5Y-H4K8ac_peak_10208	8.73392	
2	86054829	86055101	5Y-H4K8ac_peak_10209	10.11191	
2	86263021	86263222	5Y-H4K8ac_peak_10210	8.28468	

2	86263818	86264648	5Y-H4K8ac_peak_10211	13.40522	
2	86422272	86422467	5Y-H4K8ac_peak_10212	11.04542	IMMT_ENSG00000132305;RP11-301O19.1_ENSG00000273080
2	86422875	86423211	5Y-H4K8ac_peak_10213	8.62703	IMMT_ENSG00000132305;RP11-301O19.1_ENSG00000273080
2	86450386	86450775	5Y-H4K8ac_peak_10214	8.00339	
2	86478575	86478785	5Y-H4K8ac_peak_10215	8.55081	
2	86789937	86790508	5Y-H4K8ac_peak_10216	5.64909	AC015971.2_ENSG00000228363
2	86850056	86850736	5Y-H4K8ac_peak_10217	7.3889	RNF103_ENSG00000239305
2	86860359	86860647	5Y-H4K8ac_peak_10218	6.20875	
2	86947490	86947741	5Y-H4K8ac_peak_10219	7.50148	CHMP3_ENSG00000115561;RNF103-CHMP3_ENSG00000249884;RMND5A_ENSG00000153561
2	87033678	87033902	5Y-H4K8ac_peak_10220	7.01266	
2	87035027	87035361	5Y-H4K8ac_peak_10221	6.79955	CD8A_ENSG00000153563
2	87036670	87036861	5Y-H4K8ac_peak_10222	7.59101	
2	87510745	87511194	5Y-H4K8ac_peak_10223	4.07874	
2	87568981	87569202	5Y-H4K8ac_peak_10224	8.24461	
2	87569427	87569665	5Y-H4K8ac_peak_10225	7.50148	
2	87893508	87893704	5Y-H4K8ac_peak_10226	8.2913	
2	87894185	87894927	5Y-H4K8ac_peak_10227	7.59101	
2	87934092	87934693	5Y-H4K8ac_peak_10228	11.53136	
2	88354860	88355245	5Y-H4K8ac_peak_10229	14.1808	KRCC1_ENSG00000172086
2	88500465	88500706	5Y-H4K8ac_peak_10230	7.38046	
2	88564590	88564803	5Y-H4K8ac_peak_10231	7.31815	
2	88650094	88650650	5Y-H4K8ac_peak_10232	10.84616	
2	88676136	88676336	5Y-H4K8ac_peak_10233	5.16353	
2	88802724	88803068	5Y-H4K8ac_peak_10234	10.11191	
2	88901389	88901582	5Y-H4K8ac_peak_10235	8.33296	
2	88991265	88991489	5Y-H4K8ac_peak_10236	5.64909	RPIA_ENSG00000153574
2	91757479	91757702	5Y-H4K8ac_peak_10237	5.51759	
2	91761826	91762329	5Y-H4K8ac_peak_10238	9.59331	
2	91845736	91845957	5Y-H4K8ac_peak_10239	6.96612	
2	91847128	91847901	5Y-H4K8ac_peak_10240	12.14222	AC027612.6_ENSG00000143429
2	91848176	91848388	5Y-H4K8ac_peak_10241	7.29055	AC027612.6_ENSG00000143429
2	95787295	95787739	5Y-H4K8ac_peak_10242	10.60083	
2	95872975	95873634	5Y-H4K8ac_peak_10243	6.78128	AC092835.2_ENSG00000233757
2	95968884	95969119	5Y-H4K8ac_peak_10244	8.564	
2	96068146	96068712	5Y-H4K8ac_peak_10245	11.1169	FAHD2A_ENSG00000115042
2	96675764	96676233	5Y-H4K8ac_peak_10246	10.16277	FAHD2CP_ENSG00000231584
2	96737253	96737816	5Y-H4K8ac_peak_10247	10.46287	
2	96755401	96755616	5Y-H4K8ac_peak_10248	5.23083	
2	96810443	96810762	5Y-H4K8ac_peak_10249	6.76096	DUSP2_ENSG00000158050
2	96812313	96812937	5Y-H4K8ac_peak_10250	8.69112	AC012307.2_ENSG00000228873
2	96813553	96813988	5Y-H4K8ac_peak_10251	13.14766	AC012307.2_ENSG00000228873
2	96874161	96874504	5Y-H4K8ac_peak_10252	7.57144	STARD7_ENSG00000084090;STARD7-AS1_ENSG00000204685
2	96875194	96875448	5Y-H4K8ac_peak_10253	5.65584	STARD7_ENSG00000084090
2	96971826	96972086	5Y-H4K8ac_peak_10254	9.30206	SNRNP200_ENSG00000144028
2	96986883	96987298	5Y-H4K8ac_peak_10255	8.2913	AC021188.4_ENSG00000230747
2	97073316	97073628	5Y-H4K8ac_peak_10256	4.36976	

2	97219370	97219627	5Y-H4K8ac_peak_10257	7.76768	
2	97426044	97426440	5Y-H4K8ac_peak_10258	9.23159	CNNM4_ENSG00000158158
2	97505708	97505929	5Y-H4K8ac_peak_10259	4.56708	
2	97523313	97523558	5Y-H4K8ac_peak_10260	8.4454	ANKRD23_ENSG00000163126;ANKRD39_ENSG00000213337
2	97534067	97534277	5Y-H4K8ac_peak_10261	7.87406	
2	97534534	97536321	5Y-H4K8ac_peak_10262	10.60083	SEMA4C_ENSG00000168758
2	97572857	97573267	5Y-H4K8ac_peak_10263	7.89273	
2	97573468	97574016	5Y-H4K8ac_peak_10264	5.64909	
2	97778407	97778675	5Y-H4K8ac_peak_10265	7.11863	ANKRD36_ENSG00000135976
2	97778915	97779135	5Y-H4K8ac_peak_10266	8.21582	ANKRD36_ENSG00000135976
2	98207037	98207260	5Y-H4K8ac_peak_10267	8.24461	ANKRD36B_ENSG00000196912
2	98280054	98280276	5Y-H4K8ac_peak_10268	6.86362	ACTR1B_ENSG00000115073;LINC01125_ENSG00000228486;RNU4-8P_ENSG00000201806
2	98611959	98612163	5Y-H4K8ac_peak_10269	5.98695	TMEM131_ENSG00000075568
2	99086855	99087057	5Y-H4K8ac_peak_10270	4.07874	
2	99096273	99096482	5Y-H4K8ac_peak_10271	4.79585	
2	99153922	99154126	5Y-H4K8ac_peak_10272	5.77617	
2	99225180	99225609	5Y-H4K8ac_peak_10273	7.38046	COA5_ENSG00000183513;UNC50_ENSG00000115446
2	99347744	99348065	5Y-H4K8ac_peak_10274	10.27264	MGAT4A_ENSG00000071073
2	99422226	99422476	5Y-H4K8ac_peak_10275	5.15917	
2	99439116	99439852	5Y-H4K8ac_peak_10276	13.45283	
2	99952508	99952707	5Y-H4K8ac_peak_10277	8.564	
2	100261507	100261996	5Y-H4K8ac_peak_10278	8.75926	
2	100454070	100454336	5Y-H4K8ac_peak_10279	6.27097	
2	100859214	100859441	5Y-H4K8ac_peak_10280	4.84727	
2	100937998	100938635	5Y-H4K8ac_peak_10281	9.38203	LONRF2_ENSG00000170500
2	101033770	101034101	5Y-H4K8ac_peak_10282	8.16382	CHST10_ENSG00000115526
2	101178366	101178682	5Y-H4K8ac_peak_10283	8.88899	PDCL3_ENSG00000115539
2	101436892	101437149	5Y-H4K8ac_peak_10284	9.38203	NPAS2_ENSG00000170485
2	101618190	101618555	5Y-H4K8ac_peak_10285	11.1169	AC016738.4_ENSG00000223947;RPL31_ENSG00000071082
2	101618865	101619158	5Y-H4K8ac_peak_10286	5.72233	AC016738.4_ENSG00000223947;RPL31_ENSG00000071082
2	101869464	101869728	5Y-H4K8ac_peak_10287	4.00285	TBC1D8_ENSG00000204634;CNOT11_ENSG00000158435
2	101924576	101925018	5Y-H4K8ac_peak_10288	8.2913	MIR5696_ENSG00000264857;RNF149_ENSG00000163162
2	101925351	101925580	5Y-H4K8ac_peak_10289	8.24461	MIR5696_ENSG00000264857;RNF149_ENSG00000163162
2	102090404	102090982	5Y-H4K8ac_peak_10290	11.42348	RFX8_ENSG00000196460
2	102096152	102096426	5Y-H4K8ac_peak_10291	4.35388	AC092570.2_ENSG00000223826
2	102313533	102313975	5Y-H4K8ac_peak_10292	12.71156	MAP4K4_ENSG00000071054
2	102314367	102314789	5Y-H4K8ac_peak_10293	8.33296	
2	104994914	104995255	5Y-H4K8ac_peak_10294	7.31815	
2	104995710	104996024	5Y-H4K8ac_peak_10295	8.33296	
2	105274994	105275187	5Y-H4K8ac_peak_10296	8.33296	AC013402.5_ENSG00000230690
2	105275403	105276212	5Y-H4K8ac_peak_10297	8.33296	AC013402.5_ENSG00000230690
2	105946279	105946830	5Y-H4K8ac_peak_10298	10.32379	TGFBRAP1_ENSG00000135966
2	105953716	105954007	5Y-H4K8ac_peak_10299	21.33748	RP11-332H14.2_ENSG00000272994;C2orf49_ENSG00000135974
2	106681821	106682316	5Y-H4K8ac_peak_10300	13.92877	
2	107503242	107503485	5Y-H4K8ac_peak_10301	7.31102	ST6GAL2_ENSG00000144057
2	107503724	107504236	5Y-H4K8ac_peak_10302	5.41472	ST6GAL2_ENSG00000144057

2	108208849	108209071	5Y-H4K8ac_peak_10303	6.50117	
2	109065280	109065480	5Y-H4K8ac_peak_10304	5.98695	GCC2_ENSG00000135968
2	109335023	109335554	5Y-H4K8ac_peak_10305	9.38203	RANBP2_ENSG00000153201
2	109345146	109345351	5Y-H4K8ac_peak_10306	6.34046	
2	109403375	109403626	5Y-H4K8ac_peak_10307	4.07874	CCDC138_ENSG00000163006
2	109746073	109746334	5Y-H4K8ac_peak_10308	4.90696	SH3RF3-AS1_ENSG00000259863;SH3RF3_ENSG00000172985
2	109787995	109788482	5Y-H4K8ac_peak_10309	5.41472	
2	110090831	110091252	5Y-H4K8ac_peak_10310	8.24461	
2	110858282	110858948	5Y-H4K8ac_peak_10311	9.51254	
2	111596320	111596515	5Y-H4K8ac_peak_10312	7.13294	
2	112020363	112020704	5Y-H4K8ac_peak_10313	4.07874	
2	112070559	112070797	5Y-H4K8ac_peak_10314	8.43511	
2	112123953	112124163	5Y-H4K8ac_peak_10315	11.57334	
2	112221207	112221413	5Y-H4K8ac_peak_10316	7.31815	
2	112230663	112230899	5Y-H4K8ac_peak_10317	8.17203	
2	112238975	112239501	5Y-H4K8ac_peak_10318	6.34046	
2	112268413	112268712	5Y-H4K8ac_peak_10319	5.65584	
2	112269459	112269657	5Y-H4K8ac_peak_10320	8.33296	
2	112271224	112271553	5Y-H4K8ac_peak_10321	6.34046	
2	112280657	112280880	5Y-H4K8ac_peak_10322	8.23578	
2	112323731	112324038	5Y-H4K8ac_peak_10323	4.07874	
2	112641627	112642097	5Y-H4K8ac_peak_10324	9.20645	ANAPC1_ENSG00000153107
2	113033192	113033402	5Y-H4K8ac_peak_10325	11.71078	ZC3H6_ENSG00000188177
2	113299107	113299506	5Y-H4K8ac_peak_10326	5.98695	POLR1B_ENSG00000125630
2	113299787	113300199	5Y-H4K8ac_peak_10327	10.79063	POLR1B_ENSG00000125630
2	113384342	113384735	5Y-H4K8ac_peak_10328	9.23159	
2	113403550	113403910	5Y-H4K8ac_peak_10329	8.73392	AC079922.3_ENSG00000237753;SLC20A1_ENSG00000144136
2	113484000	113484378	5Y-H4K8ac_peak_10330	7.11863	
2	114195573	114195782	5Y-H4K8ac_peak_10331	6.34046	CBWD2_ENSG00000136682
2	114341177	114341602	5Y-H4K8ac_peak_10332	42.57279	MIR1302-3_ENSG00000221055;WASH2P_ENSG00000146556
2	114341947	114342175	5Y-H4K8ac_peak_10333	10.84616	WASH2P_ENSG00000146556
2	114361491	114361701	5Y-H4K8ac_peak_10334	11.19336	
2	114646770	114647227	5Y-H4K8ac_peak_10335	7.31815	RP11-141B14.1_ENSG00000270019;ACTR3_ENSG00000115091
2	118394712	118394924	5Y-H4K8ac_peak_10336	5.74859	
2	118476696	118477280	5Y-H4K8ac_peak_10337	9.38203	
2	118477805	118478205	5Y-H4K8ac_peak_10338	10.95042	
2	118479301	118479670	5Y-H4K8ac_peak_10339	7.3889	
2	118572321	118572629	5Y-H4K8ac_peak_10340	5.98695	DDX18_ENSG00000088205
2	118771217	118771436	5Y-H4K8ac_peak_10341	6.53157	CCDC93_ENSG00000125633
2	118944134	118944461	5Y-H4K8ac_peak_10342	11.99586	AC093901.1_ENSG00000226856
2	119981065	119981264	5Y-H4K8ac_peak_10343	5.5192	STEAP3_ENSG00000115107
2	119981800	119982087	5Y-H4K8ac_peak_10344	5.59843	STEAP3_ENSG00000115107
2	119998013	119998314	5Y-H4K8ac_peak_10345	6.14981	
2	120123967	120124195	5Y-H4K8ac_peak_10346	5.59843	C2orf76_ENSG00000186132;DBI_ENSG00000155368
2	120124599	120124974	5Y-H4K8ac_peak_10347	8.75926	C2orf76_ENSG00000186132;DBI_ENSG00000155368
2	120189126	120189356	5Y-H4K8ac_peak_10348	9.30505	

2	120239584	120239775	5Y-H4K8ac_peak_10349	7.3889	
2	120240060	120240360	5Y-H4K8ac_peak_10350	9.23159	
2	120240882	120241087	5Y-H4K8ac_peak_10351	8.17203	
2	120441329	120441786	5Y-H4K8ac_peak_10352	7.17997	
2	120517540	120518005	5Y-H4K8ac_peak_10353	4.0639	PTPN4_ENSG00000088179
2	120980336	120980960	5Y-H4K8ac_peak_10354	9.87124	TMEM185B_ENSG00000226479
2	121010561	121010816	5Y-H4K8ac_peak_10355	5.70472	
2	121076239	121076450	5Y-H4K8ac_peak_10356	7.31815	AC012363.13_ENSG00000235840
2	121334240	121334451	5Y-H4K8ac_peak_10357	4.95697	
2	121334760	121334986	5Y-H4K8ac_peak_10358	4.03329	
2	121499322	121499668	5Y-H4K8ac_peak_10359	11.22005	
2	121577981	121578174	5Y-H4K8ac_peak_10360	5.54773	
2	121660936	121661305	5Y-H4K8ac_peak_10361	4.14065	
2	122363170	122363477	5Y-H4K8ac_peak_10362	7.04637	
2	122530150	122530435	5Y-H4K8ac_peak_10363	7.37325	
2	123289420	123289649	5Y-H4K8ac_peak_10364	9.38203	
2	124671162	124671390	5Y-H4K8ac_peak_10365	4.40812	
2	127733976	127734610	5Y-H4K8ac_peak_10366	8.93923	
2	127783092	127783516	5Y-H4K8ac_peak_10367	5.64909	RP11-521O16.1_ENSG00000260634;RP11-521O16.2_ENSG00000260163
2	127807320	127807606	5Y-H4K8ac_peak_10368	7.59101	
2	127829615	127830042	5Y-H4K8ac_peak_10369	8.43011	
2	127863017	127863476	5Y-H4K8ac_peak_10370	11.09973	
2	127977599	127977960	5Y-H4K8ac_peak_10371	7.38046	CYP27C1_ENSG00000186684
2	128145066	128145444	5Y-H4K8ac_peak_10372	13.2534	MAP3K2_ENSG00000169967
2	128165801	128166368	5Y-H4K8ac_peak_10373	6.77436	
2	128173466	128173657	5Y-H4K8ac_peak_10374	4.84727	
2	128406953	128407175	5Y-H4K8ac_peak_10375	9.51254	
2	128409092	128409604	5Y-H4K8ac_peak_10376	4.55128	
2	128410287	128410907	5Y-H4K8ac_peak_10377	9.10532	
2	128453027	128453431	5Y-H4K8ac_peak_10378	8.00339	
2	128459103	128459453	5Y-H4K8ac_peak_10379	18.62509	SFT2D3_ENSG00000173349
2	128568992	128569226	5Y-H4K8ac_peak_10380	5.12488	WDR33_ENSG00000136709
2	129039885	129040536	5Y-H4K8ac_peak_10381	9.78792	
2	129076650	129076951	5Y-H4K8ac_peak_10382	5.41472	HS6ST1_ENSG00000136720
2	129079663	129080175	5Y-H4K8ac_peak_10383	17.55395	
2	129104641	129105234	5Y-H4K8ac_peak_10384	5.23083	
2	129119972	129120243	5Y-H4K8ac_peak_10385	4.9885	
2	129124606	129125125	5Y-H4K8ac_peak_10386	6.34046	
2	129169514	129169908	5Y-H4K8ac_peak_10387	6.52331	
2	129170970	129171189	5Y-H4K8ac_peak_10388	8.43511	
2	129181545	129181747	5Y-H4K8ac_peak_10389	6.24511	
2	129206740	129206976	5Y-H4K8ac_peak_10390	8.47164	
2	129207220	129208120	5Y-H4K8ac_peak_10391	8.43511	
2	129361063	129361320	5Y-H4K8ac_peak_10392	6.14981	
2	129396403	129396644	5Y-H4K8ac_peak_10393	8.33296	
2	129493625	129494070	5Y-H4K8ac_peak_10394	7.38046	

2	130538043	130538249	5Y-H4K8ac_peak_10395	4.84727	
2	130901825	130902090	5Y-H4K8ac_peak_10396	5.40331	CCDC74B_ENSG00000152076
2	130938923	130939702	5Y-H4K8ac_peak_10397	14.05114	MZT2B_ENSG00000152082;SMPD4_ENSG00000136699
2	131100477	131100815	5Y-H4K8ac_peak_10398	7.59101	CCDC115_ENSG00000136710;IMP4_ENSG00000136718
2	131130390	131130833	5Y-H4K8ac_peak_10399	6.08523	
2	131148991	131149426	5Y-H4K8ac_peak_10400	11.22005	
2	131485589	131486066	5Y-H4K8ac_peak_10401	7.03573	GPR148_ENSG00000173302
2	131502548	131502863	5Y-H4K8ac_peak_10402	7.59101	
2	131513226	131514260	5Y-H4K8ac_peak_10403	6.77436	AMER3_ENSG00000178171;AC140481.8_ENSG00000229797
2	131555359	131555576	5Y-H4K8ac_peak_10404	4.642	
2	131792430	131793257	5Y-H4K8ac_peak_10405	4.95697	
2	131793456	131793779	5Y-H4K8ac_peak_10406	4.29586	
2	131850518	131850963	5Y-H4K8ac_peak_10407	8.69112	FAM168B_ENSG00000152102
2	132182346	132182654	5Y-H4K8ac_peak_10408	8.43511	GNAQP1_ENSG00000214077
2	132249815	132250248	5Y-H4K8ac_peak_10409	6.37023	MZT2A_ENSG00000173272;AC093838.4_ENSG00000152117
2	132439402	132439885	5Y-H4K8ac_peak_10410	9.38276	
2	133015229	133015622	5Y-H4K8ac_peak_10411	7.11863	ANKRD30BL_ENSG00000163046;MIR663B_ENSG00000221288
2	133062678	133062927	5Y-H4K8ac_peak_10412	8.43511	
2	133105170	133105437	5Y-H4K8ac_peak_10413	7.20869	RP11-725P16.2_ENSG00000272769
2	133110406	133111061	5Y-H4K8ac_peak_10414	20.72418	FAM201B_ENSG00000230992
2	134024234	134024451	5Y-H4K8ac_peak_10415	8.33296	
2	134917807	134918049	5Y-H4K8ac_peak_10416	5.54773	
2	135117539	135117898	5Y-H4K8ac_peak_10417	6.90749	
2	135118570	135118973	5Y-H4K8ac_peak_10418	5.64909	
2	136633592	136633855	5Y-H4K8ac_peak_10419	5.91107	MCM6_ENSG00000076003
2	136634221	136634477	5Y-H4K8ac_peak_10420	4.71803	MCM6_ENSG00000076003
2	136742733	136743047	5Y-H4K8ac_peak_10421	8.24461	DARS_ENSG00000115866;AC093391.2_ENSG00000231890
2	136743406	136743671	5Y-H4K8ac_peak_10422	7.41197	DARS_ENSG00000115866;AC093391.2_ENSG00000231890
2	136824919	136825109	5Y-H4K8ac_peak_10423	5.62788	
2	137084775	137085171	5Y-H4K8ac_peak_10424	6.79955	
2	137179348	137179553	5Y-H4K8ac_peak_10425	5.65584	
2	139148292	139148486	5Y-H4K8ac_peak_10426	4.51076	
2	139258763	139259201	5Y-H4K8ac_peak_10427	6.08523	AC097721.2_ENSG00000228043;SPOPL_ENSG00000144228
2	139259578	139259786	5Y-H4K8ac_peak_10428	6.73385	AC097721.2_ENSG00000228043;SPOPL_ENSG00000144228
2	144694215	144694765	5Y-H4K8ac_peak_10429	9.15007	AC016910.1_ENSG00000232377
2	144694979	144695174	5Y-H4K8ac_peak_10430	6.22669	AC016910.1_ENSG00000232377
2	145089399	145089698	5Y-H4K8ac_peak_10431	7.38046	GTDC1_ENSG00000121964
2	145188833	145189029	5Y-H4K8ac_peak_10432	4.51076	
2	145274274	145274892	5Y-H4K8ac_peak_10433	9.38203	ZEB2-AS1_ENSG00000238057
2	145280379	145280574	5Y-H4K8ac_peak_10434	5.64909	
2	148601380	148601664	5Y-H4K8ac_peak_10435	4.95697	ACVR2A_ENSG00000121989
2	149401857	149402120	5Y-H4K8ac_peak_10436	14.31711	EPC2_ENSG00000135999
2	149402352	149402745	5Y-H4K8ac_peak_10437	7.11863	EPC2_ENSG00000135999
2	149569626	149569949	5Y-H4K8ac_peak_10438	5.29015	
2	149645601	149646143	5Y-H4K8ac_peak_10439	11.36844	AC105402.4_ENSG00000231079
2	149895132	149895419	5Y-H4K8ac_peak_10440	8.08609	LYPD6B_ENSG00000150556

2	150186023	150186227	5Y-H4K8ac_peak_10441	7.17184	LYPD6_ENSG00000187123
2	150443806	150444149	5Y-H4K8ac_peak_10442	9.23159	MMADHC_ENSG00000168288;AC144449.1_ENSG00000231969
2	151184262	151184497	5Y-H4K8ac_peak_10443	5.65584	
2	152117919	152118607	5Y-H4K8ac_peak_10444	4.77126	RBM43_ENSG00000184898
2	152146027	152146309	5Y-H4K8ac_peak_10445	5.23083	NMI_ENSG00000123609
2	152216007	152216237	5Y-H4K8ac_peak_10446	6.77436	
2	152216562	152216807	5Y-H4K8ac_peak_10447	6.50117	
2	152266625	152266815	5Y-H4K8ac_peak_10448	5.77617	RIF1_ENSG00000080345
2	153574793	153575007	5Y-H4K8ac_peak_10449	13.35169	PRPF40A_ENSG00000196504;ARL6IP6_ENSG00000177917
2	154079063	154079412	5Y-H4K8ac_peak_10450	13.2534	
2	157292007	157292753	5Y-H4K8ac_peak_10451	12.87247	GPD2_ENSG00000115159
2	158858922	158859322	5Y-H4K8ac_peak_10452	6.73047	
2	159824488	159825024	5Y-H4K8ac_peak_10453	12.69256	TANC1_ENSG00000115183
2	160472352	160472686	5Y-H4K8ac_peak_10454	7.59101	BAZ2B_ENSG00000123636;AC009506.1_ENSG00000224152
2	160569087	160569375	5Y-H4K8ac_peak_10455	7.03573	AC009961.3_ENSG00000226266;MARCH7_ENSG00000136536
2	161099746	161099952	5Y-H4K8ac_peak_10456	5.07473	
2	161335664	161336102	5Y-H4K8ac_peak_10457	4.07874	
2	162016978	162017202	5Y-H4K8ac_peak_10458	5.65584	AC009313.1_ENSG00000224467
2	162095084	162095579	5Y-H4K8ac_peak_10459	9.51254	
2	162165033	162165244	5Y-H4K8ac_peak_10460	7.50148	PSMD14_ENSG00000115233
2	164592689	164593324	5Y-H4K8ac_peak_10461	16.38263	FIGN_ENSG00000182263
2	165478168	165478590	5Y-H4K8ac_peak_10462	4.90471	GRB14_ENSG00000115290
2	165698295	165698559	5Y-H4K8ac_peak_10463	12.72376	
2	166619376	166619701	5Y-H4K8ac_peak_10464	8.33296	
2	168149107	168149303	5Y-H4K8ac_peak_10465	11.12941	AC074363.1_ENSG00000228222
2	169104221	169104478	5Y-H4K8ac_peak_10466	5.87725	STK39_ENSG00000198648
2	169312435	169312628	5Y-H4K8ac_peak_10467	11.1169	CERS6_ENSG00000172292
2	169312896	169313513	5Y-H4K8ac_peak_10468	7.30348	CERS6_ENSG00000172292
2	170335574	170335866	5Y-H4K8ac_peak_10469	4.95697	BBS5_ENSG00000163093;RP11-724O16.1_ENSG00000251569
2	170430008	170430207	5Y-H4K8ac_peak_10470	6.34046	FASTKD1_ENSG00000138399
2	170440889	170441273	5Y-H4K8ac_peak_10471	10.1994	PPIG_ENSG00000138398
2	170551078	170551388	5Y-H4K8ac_peak_10472	7.38046	CCDC173_ENSG00000154479;PHOSPHO2_ENSG00000144362;KLHL23_ENSG00000213160
2	170573003	170573806	5Y-H4K8ac_peak_10473	19.18935	
2	170574145	170574444	5Y-H4K8ac_peak_10474	7.58806	
2	170590393	170590905	5Y-H4K8ac_peak_10475	7.64648	
2	171785810	171786247	5Y-H4K8ac_peak_10476	7.30348	GORASP2_ENSG00000115806
2	171829865	171830774	5Y-H4K8ac_peak_10477	18.10232	
2	172290826	172291104	5Y-H4K8ac_peak_10478	13.27281	METTL8_ENSG00000123600;DCAF17_ENSG00000115827
2	172379457	172380299	5Y-H4K8ac_peak_10479	6.77436	CYBRD1_ENSG00000071967
2	172778199	172778420	5Y-H4K8ac_peak_10480	4.84727	HAT1_ENSG00000128708
2	172779170	172779390	5Y-H4K8ac_peak_10481	4.95697	HAT1_ENSG00000128708
2	173099520	173099780	5Y-H4K8ac_peak_10482	4.36976	
2	173941235	173941487	5Y-H4K8ac_peak_10483	5.65584	
2	174219287	174219877	5Y-H4K8ac_peak_10484	6.08523	CDCA7_ENSG00000144354
2	174313738	174314058	5Y-H4K8ac_peak_10485	13.2534	
2	174829723	174829987	5Y-H4K8ac_peak_10486	7.11863	SP3_ENSG00000172845

2	174830311	174830562	5Y-H4K8ac_peak_10487	11.22005	SP3_ENSG00000172845
2	174904417	174904660	5Y-H4K8ac_peak_10488	9.32595	
2	175113574	175113806	5Y-H4K8ac_peak_10489	9.00954	OLA1_ENSG00000138430
2	175527822	175528056	5Y-H4K8ac_peak_10490	7.64675	
2	175546979	175548028	5Y-H4K8ac_peak_10491	14.99673	WIPF1_ENSG00000115935
2	175636951	175637221	5Y-H4K8ac_peak_10492	4.9885	
2	175652240	175652485	5Y-H4K8ac_peak_10493	9.38203	AC018890.4_ENSG00000237104
2	175869447	175870027	5Y-H4K8ac_peak_10494	14.39608	CHN1_ENSG00000128656
2	176032584	176032789	5Y-H4K8ac_peak_10495	5.85188	ATF2_ENSG00000115966;MIR933_ENSG00000215973;AC096649.2_ENSG00000229750
2	176756553	176756756	5Y-H4K8ac_peak_10496	6.50117	
2	176866691	176866914	5Y-H4K8ac_peak_10497	8.43511	KIAA1715_ENSG00000144320
2	176986514	176986770	5Y-H4K8ac_peak_10498	7.50044	HOXD9_ENSG00000128709
2	176987533	176987875	5Y-H4K8ac_peak_10499	8.73985	HOXD9_ENSG00000128709
2	176994165	176994411	5Y-H4K8ac_peak_10500	7.09658	HOXD8_ENSG00000175879
2	177001266	177001851	5Y-H4K8ac_peak_10501	9.36633	HOXD-AS2_ENSG00000237380;HOXD3_ENSG00000128652
2	177021119	177021399	5Y-H4K8ac_peak_10502	6.54441	
2	177021840	177022628	5Y-H4K8ac_peak_10503	12.57157	
2	177053030	177053355	5Y-H4K8ac_peak_10504	9.96543	HOXD-AS1_ENSG00000224189;HOXD1_ENSG00000128645
2	177053687	177054095	5Y-H4K8ac_peak_10505	6.37023	HOXD-AS1_ENSG00000224189;HOXD1_ENSG00000128645
2	177133968	177134575	5Y-H4K8ac_peak_10506	9.51254	MTX2_ENSG00000128654
2	177502319	177502843	5Y-H4K8ac_peak_10507	8.24461	LINC01116_ENSG00000163364;LINC01117_ENSG00000224577
2	178077483	178077903	5Y-H4K8ac_peak_10508	12.21176	AC079305.8_ENSG00000229337;HNRNPA3_ENSG00000170144;MIR4444-2_ENSG00000263721
2	178128618	178129369	5Y-H4K8ac_peak_10509	5.12488	AC079305.10_ENSG00000222043
2	178256891	178257841	5Y-H4K8ac_peak_10510	14.2053	NFE2L2_ENSG00000116044;AC074286.1_ENSG00000213963;AGPS_ENSG00000018510
2	178417176	178417875	5Y-H4K8ac_peak_10511	9.02242	TTC30B_ENSG00000196659
2	178483275	178483706	5Y-H4K8ac_peak_10512	11.99586	TTC30A_ENSG00000197557
2	179058715	179058950	5Y-H4K8ac_peak_10513	8.21582	OSBPL6_ENSG00000079156
2	179277976	179278232	5Y-H4K8ac_peak_10514	10.79063	AC009948.5_ENSG00000223960
2	179315678	179315878	5Y-H4K8ac_peak_10515	7.31102	PRKRA_ENSG00000180228;DFNB59_ENSG00000204311
2	179316143	179316859	5Y-H4K8ac_peak_10516	15.06486	PRKRA_ENSG00000180228;DFNB59_ENSG00000204311
2	180128947	180129206	5Y-H4K8ac_peak_10517	5.35202	SESTD1_ENSG00000187231
2	180725652	180726025	5Y-H4K8ac_peak_10518	10.5157	ZNF385B_ENSG00000144331;MIR1258_ENSG00000221240
2	180871548	180871776	5Y-H4K8ac_peak_10519	6.77436	CWC22_ENSG00000163510
2	181844791	181845447	5Y-H4K8ac_peak_10520	13.92877	AC104076.3_ENSG00000236153
2	181845840	181846067	5Y-H4K8ac_peak_10521	8.33296	
2	182321712	182322650	5Y-H4K8ac_peak_10522	13.63503	ITGA4_ENSG00000115232
2	182545739	182545931	5Y-H4K8ac_peak_10523	5.8635	CERKL_ENSG00000188452;NEUROD1_ENSG00000162992
2	182548312	182549151	5Y-H4K8ac_peak_10524	17.10724	AC013733.3_ENSG00000234595
2	183580865	183581201	5Y-H4K8ac_peak_10525	4.70501	DNAJC10_ENSG00000077232
2	183902375	183903055	5Y-H4K8ac_peak_10526	9.23159	NCKAP1_ENSG00000061676
2	183989210	183989527	5Y-H4K8ac_peak_10527	9.51254	
2	187350553	187350776	5Y-H4K8ac_peak_10528	9.38203	ZC3H15_ENSG00000065548
2	187351026	187351399	5Y-H4K8ac_peak_10529	5.40331	ZC3H15_ENSG00000065548
2	187454284	187455185	5Y-H4K8ac_peak_10530	15.06486	ITGAV_ENSG00000138448
2	187558068	187558383	5Y-H4K8ac_peak_10531	4.50834	FAM171B_ENSG00000144369
2	188859070	188859431	5Y-H4K8ac_peak_10532	6.1654	

2	190305371	190305686	5Y-H4K8ac_peak_10533	7.31102	WDR75_ENSG00000115368
2	190445620	190445933	5Y-H4K8ac_peak_10534	13.85283	
2	190539278	190539581	5Y-H4K8ac_peak_10535	5.23083	ANKAR_ENSG00000151687
2	190627094	190627403	5Y-H4K8ac_peak_10536	5.16353	OSGEPL1_ENSG00000128694;OSGEPL1-AS1_ENSG00000253559
2	191045030	191045317	5Y-H4K8ac_peak_10537	4.15658	
2	191208263	191208463	5Y-H4K8ac_peak_10538	7.59101	HIBCH_ENSG00000198130;INPP1_ENSG00000151689
2	191272586	191273009	5Y-H4K8ac_peak_10539	14.31711	MFSD6_ENSG00000151690
2	191399245	191399779	5Y-H4K8ac_peak_10540	9.15007	TMEM194B_ENSG00000189362;AC093388.3_ENSG00000233654
2	191745067	191745267	5Y-H4K8ac_peak_10541	5.65584	
2	191746240	191746665	5Y-H4K8ac_peak_10542	9.78792	AC005540.3_ENSG00000235852
2	191885865	191886132	5Y-H4K8ac_peak_10543	6.72926	STAT1_ENSG00000115415;AC067945.4_ENSG00000231858
2	192344884	192345248	5Y-H4K8ac_peak_10544	4.79658	
2	192365814	192366109	5Y-H4K8ac_peak_10545	6.43775	
2	195937671	195938019	5Y-H4K8ac_peak_10546	8.33296	
2	197457818	197458380	5Y-H4K8ac_peak_10547	14.31711	HECW2_ENSG00000138411
2	197663889	197664301	5Y-H4K8ac_peak_10548	5.65584	GTF3C3_ENSG00000119041
2	197664675	197665192	5Y-H4K8ac_peak_10549	11.57334	GTF3C3_ENSG00000119041
2	197915844	197916266	5Y-H4K8ac_peak_10550	9.74189	
2	197917604	197917887	5Y-H4K8ac_peak_10551	14.36519	
2	197955920	197956248	5Y-H4K8ac_peak_10552	8.33296	
2	197958833	197959023	5Y-H4K8ac_peak_10553	5.65584	
2	198070070	198070261	5Y-H4K8ac_peak_10554	7.3889	
2	198144475	198144782	5Y-H4K8ac_peak_10555	6.34046	
2	198224682	198225343	5Y-H4K8ac_peak_10556	11.19336	
2	198235497	198235743	5Y-H4K8ac_peak_10557	8.33296	
2	198243509	198243936	5Y-H4K8ac_peak_10558	7.38046	
2	198299368	198299681	5Y-H4K8ac_peak_10559	16.38263	SF3B1_ENSG00000115524
2	198380788	198381130	5Y-H4K8ac_peak_10560	8.35139	HSPD1_ENSG00000144381;MOB4_ENSG00000115540
2	200776135	200776393	5Y-H4K8ac_peak_10561	6.08523	AC073043.1_ENSG00000232732;C2orf69_ENSG00000178074
2	200820292	200820896	5Y-H4K8ac_peak_10562	8.69112	TYW5_ENSG00000162971;C2orf47_ENSG00000162972
2	201172039	201172378	5Y-H4K8ac_peak_10563	5.64909	
2	201374364	201374807	5Y-H4K8ac_peak_10564	7.3889	SGOL2_ENSG00000163535
2	201375004	201375480	5Y-H4K8ac_peak_10565	4.50834	SGOL2_ENSG00000163535
2	201728803	201729279	5Y-H4K8ac_peak_10566	11.19336	CLK1_ENSG00000013441;Y_RNA_ENSG00000252759
2	201729671	201729946	5Y-H4K8ac_peak_10567	5.65584	CLK1_ENSG00000013441
2	201754188	201754509	5Y-H4K8ac_peak_10568	4.29586	PPIL3_ENSG00000240344;NIF3L1_ENSG00000196290
2	201980578	201981728	5Y-H4K8ac_peak_10569	8.35139	CFLAR_ENSG00000003402
2	201982566	201982889	5Y-H4K8ac_peak_10570	4.84727	
2	202009622	202009912	5Y-H4K8ac_peak_10571	6.83519	
2	202298560	202298805	5Y-H4K8ac_peak_10572	6.46053	
2	202507582	202507981	5Y-H4K8ac_peak_10573	10.60083	TMEM237_ENSG00000155755
2	202508805	202509081	5Y-H4K8ac_peak_10574	7.11262	TMEM237_ENSG00000155755
2	202645355	202645582	5Y-H4K8ac_peak_10575	11.19336	ALS2_ENSG00000003393
2	202831419	202831760	5Y-H4K8ac_peak_10576	6.47245	
2	202832147	202832634	5Y-H4K8ac_peak_10577	10.90365	
2	202862440	202862639	5Y-H4K8ac_peak_10578	7.37325	

2	202881386	202881610	5Y-H4K8ac_peak_10579	6.55906	
2	202884218	202884415	5Y-H4K8ac_peak_10580	5.26353	
2	202897594	202897875	5Y-H4K8ac_peak_10581	7.46096	RP11-107N15.1_ENSG00000273209
2	203103811	203104209	5Y-H4K8ac_peak_10582	8.35139	SUMO1_ENSG00000116030;AC079354.2_ENSG00000267889
2	203130038	203130283	5Y-H4K8ac_peak_10583	7.11863	NOP58_ENSG00000055044
2	203240437	203240665	5Y-H4K8ac_peak_10584	5.87725	RP11-686O6.2_ENSG00000273456;BMPR2_ENSG00000204217
2	203453605	203454035	5Y-H4K8ac_peak_10585	9.38276	
2	203499980	203500420	5Y-H4K8ac_peak_10586	7.01266	FAM117B_ENSG00000138439
2	203736278	203736536	5Y-H4K8ac_peak_10587	8.33296	ICA1L_ENSG00000163596
2	204103711	204104172	5Y-H4K8ac_peak_10588	18.09952	CYP20A1_ENSG00000119004
2	204191641	204191831	5Y-H4K8ac_peak_10589	4.29586	
2	204192476	204192708	5Y-H4K8ac_peak_10590	7.59101	ABI2_ENSG00000138443
2	204193296	204193497	5Y-H4K8ac_peak_10591	7.04637	ABI2_ENSG00000138443;RP11-363J17.1_ENSG00000256458
2	204399421	204399897	5Y-H4K8ac_peak_10592	7.59101	RAPH1_ENSG00000173166
2	204650504	204650718	5Y-H4K8ac_peak_10593	5.28616	
2	205409777	205410159	5Y-H4K8ac_peak_10594	7.01266	PARD3B_ENSG00000116117
2	205410356	205410971	5Y-H4K8ac_peak_10595	9.63153	PARD3B_ENSG00000116117
2	206594750	206594998	5Y-H4K8ac_peak_10596	12.49811	
2	206949874	206950802	5Y-H4K8ac_peak_10597	14.31711	INO80D_ENSG00000114933;AC007383.3_ENSG00000227946
2	207139807	207140000	5Y-H4K8ac_peak_10598	6.50117	ZDBF2_ENSG00000204186
2	208031105	208031578	5Y-H4K8ac_peak_10599	29.7739	KLF7_ENSG00000118263
2	208277932	208278133	5Y-H4K8ac_peak_10600	4.9603	
2	208489649	208489965	5Y-H4K8ac_peak_10601	6.34245	METTL21A_ENSG00000144401
2	208490280	208491353	5Y-H4K8ac_peak_10602	4.15658	METTL21A_ENSG00000144401
2	208575221	208575442	5Y-H4K8ac_peak_10603	6.72926	CCNYL1_ENSG00000163249
2	208889981	208890221	5Y-H4K8ac_peak_10604	10.1994	PLEKHM3_ENSG00000178385
2	208994108	208994311	5Y-H4K8ac_peak_10605	8.17203	CRYGC_ENSG00000163254
2	209225011	209225215	5Y-H4K8ac_peak_10606	8.2913	AC012362.3_ENSG00000213081;PTH2R_ENSG00000144407
2	210288291	210288535	5Y-H4K8ac_peak_10607	8.35139	MAP2_ENSG00000078018
2	210636374	210636994	5Y-H4K8ac_peak_10608	7.59101	UNC80_ENSG00000144406
2	210866801	210867020	5Y-H4K8ac_peak_10609	5.59843	RPE_ENSG00000197713
2	210867468	210867675	5Y-H4K8ac_peak_10610	8.47164	RPE_ENSG00000197713
2	211035645	211035931	5Y-H4K8ac_peak_10611	14.99673	KANSL1L_ENSG00000144445;AC006994.3_ENSG00000263530;AC006994.2_ENSG00000231294
2	216176017	216176585	5Y-H4K8ac_peak_10612	10.27264	ATIC_ENSG00000138363
2	216176844	216177169	5Y-H4K8ac_peak_10613	10.19948	ATIC_ENSG00000138363
2	216946201	216946482	5Y-H4K8ac_peak_10614	10.55504	TMEM169_ENSG00000163449
2	216979775	216980194	5Y-H4K8ac_peak_10615	5.87382	
2	217068116	217068308	5Y-H4K8ac_peak_10616	8.33296	
2	217083637	217083925	5Y-H4K8ac_peak_10617	5.40331	
2	217144711	217145020	5Y-H4K8ac_peak_10618	8.35138	
2	217236073	217236446	5Y-H4K8ac_peak_10619	4.50834	MARCH4_ENSG00000144583
2	217236987	217237209	5Y-H4K8ac_peak_10620	10.11191	MARCH4_ENSG00000144583
2	217398384	217398574	5Y-H4K8ac_peak_10621	9.30505	
2	217459958	217460571	5Y-H4K8ac_peak_10622	6.46053	
2	217483871	217484099	5Y-H4K8ac_peak_10623	7.90236	
2	217484812	217485271	5Y-H4K8ac_peak_10624	12.52413	

2	217497672	217498003	5Y-H4K8ac_peak_10625	9.30505	IGFBP2_ENSG00000115457
2	217500572	217501013	5Y-H4K8ac_peak_10626	8.31252	
2	217558996	217559752	5Y-H4K8ac_peak_10627	5.89113	IGFBP5_ENSG00000115461;AC007563.5_ENSG00000236886
2	217656645	217656907	5Y-H4K8ac_peak_10628	5.64909	
2	218593992	218594262	5Y-H4K8ac_peak_10629	6.43775	
2	218672625	218672838	5Y-H4K8ac_peak_10630	6.14981	
2	218786062	218786312	5Y-H4K8ac_peak_10631	6.1654	
2	218798312	218798582	5Y-H4K8ac_peak_10632	12.21176	
2	218802626	218802835	5Y-H4K8ac_peak_10633	5.65584	
2	218869746	218870041	5Y-H4K8ac_peak_10634	6.06162	
2	218874456	218874659	5Y-H4K8ac_peak_10635	7.30348	
2	218875186	218875467	5Y-H4K8ac_peak_10636	5.91107	
2	218881655	218882346	5Y-H4K8ac_peak_10637	10.11191	
2	218900127	218900349	5Y-H4K8ac_peak_10638	5.74859	RUFY4_ENSG00000188282
2	219081387	219081678	5Y-H4K8ac_peak_10639	4.84727	ARPC2_ENSG00000163466
2	219124353	219124585	5Y-H4K8ac_peak_10640	5.64909	GPBAR1_ENSG00000179921
2	219135391	219135634	5Y-H4K8ac_peak_10641	9.7353	AAMP_ENSG00000127837;PNKD_ENSG00000127838
2	219146788	219147622	5Y-H4K8ac_peak_10642	11.93705	
2	219232373	219232941	5Y-H4K8ac_peak_10643	10.35586	AC021016.6_ENSG00000225062
2	219263513	219263982	5Y-H4K8ac_peak_10644	6.46606	CTDSP1_ENSG00000144579;RP11-378A13.2_ENSG00000273361
2	219271476	219272319	5Y-H4K8ac_peak_10645	7.01266	
2	219432743	219432995	5Y-H4K8ac_peak_10646	11.08752	USP37_ENSG00000135913;RQCD1_ENSG00000144580
2	219433356	219434038	5Y-H4K8ac_peak_10647	9.7353	USP37_ENSG00000135913;RQCD1_ENSG00000144580
2	219472906	219473098	5Y-H4K8ac_peak_10648	5.56894	PLCD4_ENSG00000115556
2	219537154	219537387	5Y-H4K8ac_peak_10649	6.37023	RNF25_ENSG00000163481;STK36_ENSG00000163482
2	219722879	219723205	5Y-H4K8ac_peak_10650	4.95697	
2	219724662	219725262	5Y-H4K8ac_peak_10651	7.38046	WNT6_ENSG00000115596
2	219744796	219745091	5Y-H4K8ac_peak_10652	7.92287	WNT10A_ENSG00000135925
2	219757316	219758059	5Y-H4K8ac_peak_10653	14.99673	
2	219773566	219774131	5Y-H4K8ac_peak_10654	10.19948	
2	219823774	219824137	5Y-H4K8ac_peak_10655	8.16382	CDK5R2_ENSG00000171450
2	219827391	219827765	5Y-H4K8ac_peak_10656	10.58128	AC097468.7_ENSG00000235024
2	219848833	219850034	5Y-H4K8ac_peak_10657	11.86504	FEV_ENSG00000163497
2	219850919	219851148	5Y-H4K8ac_peak_10658	7.58806	FEV_ENSG00000163497
2	219863138	219863595	5Y-H4K8ac_peak_10659	6.29888	
2	219863873	219864330	5Y-H4K8ac_peak_10660	8.69112	
2	219865144	219865579	5Y-H4K8ac_peak_10661	6.75871	MIR375_ENSG00000198973
2	219866473	219866667	5Y-H4K8ac_peak_10662	7.09658	MIR375_ENSG00000198973;AC097468.4_ENSG00000224090
2	219867350	219867901	5Y-H4K8ac_peak_10663	9.32595	MIR375_ENSG00000198973;AC097468.4_ENSG00000224090
2	219884115	219884550	5Y-H4K8ac_peak_10664	4.8509	
2	219885950	219886150	5Y-H4K8ac_peak_10665	8.56781	
2	219886423	219886659	5Y-H4K8ac_peak_10666	7.70703	
2	220071018	220071356	5Y-H4K8ac_peak_10667	6.22669	
2	220083211	220083623	5Y-H4K8ac_peak_10668	10.1994	ABCB6_ENSG00000115657
2	220093740	220093997	5Y-H4K8ac_peak_10669	6.98118	ATG9A_ENSG00000198925;ANKZF1_ENSG00000163516
2	220109901	220110091	5Y-H4K8ac_peak_10670	10.31072	STK16_ENSG00000115661

2	220110370	220110580	5Y-H4K8ac_peak_10671	3.93392	STK16_ENSG00000115661
2	220134334	220134624	5Y-H4K8ac_peak_10672	8.19789	
2	220142615	220142905	5Y-H4K8ac_peak_10673	6.37023	TUBA4A_ENSG00000127824
2	220159206	220159636	5Y-H4K8ac_peak_10674	11.22005	MIR153-1_ENSG00000207647
2	220306200	220307082	5Y-H4K8ac_peak_10675	10.11191	
2	220318122	220318334	5Y-H4K8ac_peak_10676	5.3989	
2	220362469	220363078	5Y-H4K8ac_peak_10677	6.37023	RP11-316O14.1_ENSG00000268603;GMPPA_ENSG00000144591
2	220369721	220369914	5Y-H4K8ac_peak_10678	4.75045	
2	220370660	220372115	5Y-H4K8ac_peak_10679	13.86751	
2	220373162	220373434	5Y-H4K8ac_peak_10680	11.86504	
2	220373746	220374062	5Y-H4K8ac_peak_10681	9.21468	
2	220374669	220374903	5Y-H4K8ac_peak_10682	7.79037	
2	220375116	220375601	5Y-H4K8ac_peak_10683	8.33212	
2	220377421	220377810	5Y-H4K8ac_peak_10684	8.73985	
2	220378723	220379416	5Y-H4K8ac_peak_10685	9.01738	ASIC4_ENSG00000072182
2	220386306	220386570	5Y-H4K8ac_peak_10686	7.38046	
2	220387389	220387587	5Y-H4K8ac_peak_10687	5.44849	
2	220394685	220394958	5Y-H4K8ac_peak_10688	10.6196	
2	220408092	220408412	5Y-H4K8ac_peak_10689	6.43775	CHPF_ENSG00000123989;TMEM198_ENSG00000188760
2	220435378	220435825	5Y-H4K8ac_peak_10690	4.50834	OBSL1_ENSG00000124006
2	220436376	220436651	5Y-H4K8ac_peak_10691	10.1994	OBSL1_ENSG00000124006
2	220437018	220437353	5Y-H4K8ac_peak_10692	9.80622	OBSL1_ENSG00000124006
2	220476389	220476580	5Y-H4K8ac_peak_10693	4.40812	
2	220491809	220493092	5Y-H4K8ac_peak_10694	13.2534	AC009955.8_ENSG00000228973;SLC4A3_ENSG00000114923
2	223183636	223183996	5Y-H4K8ac_peak_10695	7.76232	RP11-384O8.1_ENSG00000267034;AC010980.2_ENSG00000237732
2	223536115	223536610	5Y-H4K8ac_peak_10696	8.73985	MOGAT1_ENSG00000124003
2	223615648	223615991	5Y-H4K8ac_peak_10697	7.31815	
2	224478133	224478374	5Y-H4K8ac_peak_10698	5.40331	
2	224702487	224702693	5Y-H4K8ac_peak_10699	5.8635	AP1S3_ENSG00000152056
2	224809659	224809927	5Y-H4K8ac_peak_10700	5.5192	WDFY1_ENSG00000085449
2	224822245	224822547	5Y-H4K8ac_peak_10701	6.96612	MRPL44_ENSG00000135900
2	225307117	225307575	5Y-H4K8ac_peak_10702	6.34046	
2	225906692	225906949	5Y-H4K8ac_peak_10703	5.40331	DOCK10_ENSG00000135905
2	227655952	227656178	5Y-H4K8ac_peak_10704	7.97699	
2	227700259	227700564	5Y-H4K8ac_peak_10705	8.43511	RHBDD1_ENSG00000144468
2	227700809	227701127	5Y-H4K8ac_peak_10706	8.2913	RHBDD1_ENSG00000144468
2	228189998	228190420	5Y-H4K8ac_peak_10707	7.89273	AC097662.2_ENSG00000236432;MFF_ENSG00000168958
2	230786297	230786538	5Y-H4K8ac_peak_10708	11.09973	FBXO36_ENSG00000153832
2	230786869	230787171	5Y-H4K8ac_peak_10709	9.60627	TRIP12_ENSG00000153827;FBXO36_ENSG00000153832
2	230845627	230845853	5Y-H4K8ac_peak_10710	6.34046	
2	230932603	230932910	5Y-H4K8ac_peak_10711	7.50148	SLC16A14_ENSG00000163053
2	230933102	230933507	5Y-H4K8ac_peak_10712	6.77436	SLC16A14_ENSG00000163053
2	231729109	231729367	5Y-H4K8ac_peak_10713	5.16687	ITM2C_ENSG00000135916
2	231733425	231733678	5Y-H4K8ac_peak_10714	9.51254	
2	231734681	231735415	5Y-H4K8ac_peak_10715	9.00954	
2	231736196	231736906	5Y-H4K8ac_peak_10716	9.01738	

2	231917457	231917651	5Y-H4K8ac_peak_10717	10.15788	RP11-223I10.1_ENSG00000261829
2	231918046	231918354	5Y-H4K8ac_peak_10718	5.23083	
2	231921417	231921910	5Y-H4K8ac_peak_10719	7.50148	PSMD1_ENSG00000173692
2	232057161	232057351	5Y-H4K8ac_peak_10720	7.53429	
2	232224684	232224961	5Y-H4K8ac_peak_10721	5.64909	
2	232229417	232230226	5Y-H4K8ac_peak_10722	11.68317	
2	232230540	232230861	5Y-H4K8ac_peak_10723	7.66481	
2	232252789	232253776	5Y-H4K8ac_peak_10724	12.57157	AC017104.6_ENSG00000224376
2	232260301	232260606	5Y-H4K8ac_peak_10725	4.77307	B3GNT7_ENSG00000156966
2	232276898	232277096	5Y-H4K8ac_peak_10726	5.17251	
2	232277388	232277670	5Y-H4K8ac_peak_10727	11.19336	
2	232329565	232330466	5Y-H4K8ac_peak_10728	6.08523	
2	232347166	232347431	5Y-H4K8ac_peak_10729	7.88114	NCL_ENSG00000115053
2	232347697	232348063	5Y-H4K8ac_peak_10730	7.89273	NCL_ENSG00000115053
2	232348399	232348770	5Y-H4K8ac_peak_10731	5.35202	NCL_ENSG00000115053
2	232379292	232379681	5Y-H4K8ac_peak_10732	5.87725	LINC00471_ENSG00000181798
2	232394798	232395081	5Y-H4K8ac_peak_10733	9.79526	NMUR1_ENSG00000171596
2	232400755	232400974	5Y-H4K8ac_peak_10734	5.3176	
2	232458877	232459234	5Y-H4K8ac_peak_10735	6.17973	
2	232459787	232459993	5Y-H4K8ac_peak_10736	7.58806	
2	232468588	232469369	5Y-H4K8ac_peak_10737	19.26368	
2	232469654	232470046	5Y-H4K8ac_peak_10738	7.58806	
2	232476931	232477243	5Y-H4K8ac_peak_10739	4.95697	
2	232478234	232479108	5Y-H4K8ac_peak_10740	11.10114	
2	232479335	232479619	5Y-H4K8ac_peak_10741	5.98695	
2	232545090	232546543	5Y-H4K8ac_peak_10742	11.22005	
2	232552188	232552480	5Y-H4K8ac_peak_10743	12.84845	
2	232571186	232571616	5Y-H4K8ac_peak_10744	6.22904	MGC4771_ENSG00000269363;PTMA_ENSG00000187514
2	232571969	232572837	5Y-H4K8ac_peak_10745	15.80786	MGC4771_ENSG00000269363;PTMA_ENSG00000187514
2	232573570	232574056	5Y-H4K8ac_peak_10746	11.01067	
2	232825713	232826906	5Y-H4K8ac_peak_10747	9.00954	DIS3L2_ENSG00000144535
2	233367968	233368324	5Y-H4K8ac_peak_10748	6.34245	
2	233371614	233371996	5Y-H4K8ac_peak_10749	6.03255	
2	233415471	233415814	5Y-H4K8ac_peak_10750	12.65187	TIGD1_ENSG00000221944;EIF4E2_ENSG00000135930;MIR5001_ENSG00000266620
2	233498167	233498411	5Y-H4K8ac_peak_10751	4.8509	
2	233561458	233561731	5Y-H4K8ac_peak_10752	5.98695	GIGYF2_ENSG00000204120
2	233741023	233741811	5Y-H4K8ac_peak_10753	7.59101	
2	234378325	234378687	5Y-H4K8ac_peak_10754	6.50117	
2	235242273	235242539	5Y-H4K8ac_peak_10755	4.84727	
2	235244825	235245331	5Y-H4K8ac_peak_10756	15.34073	
2	235405989	235406310	5Y-H4K8ac_peak_10757	14.30055	ARL4C_ENSG00000188042
2	235465891	235466209	5Y-H4K8ac_peak_10758	4.84727	
2	235909287	235909564	5Y-H4K8ac_peak_10759	4.0639	
2	235958485	235959057	5Y-H4K8ac_peak_10760	13.11741	
2	236174284	236174605	5Y-H4K8ac_peak_10761	4.84727	
2	236401695	236402097	5Y-H4K8ac_peak_10762	5.89922	AGAP1_ENSG00000157985

2	236403464	236403689	5Y-H4K8ac_peak_10763	6.52593	AGAP1_ENSG00000157985
2	236570366	236570876	5Y-H4K8ac_peak_10764	11.1169	
2	236578386	236578686	5Y-H4K8ac_peak_10765	9.23159	
2	236579814	236580049	5Y-H4K8ac_peak_10766	5.16353	
2	238117677	238117985	5Y-H4K8ac_peak_10767	7.59101	
2	238316943	238317137	5Y-H4K8ac_peak_10768	7.38046	
2	238350416	238350759	5Y-H4K8ac_peak_10769	5.65584	
2	238360127	238360551	5Y-H4K8ac_peak_10770	9.7353	
2	238360839	238361381	5Y-H4K8ac_peak_10771	12.21176	
2	238382374	238382707	5Y-H4K8ac_peak_10772	6.55906	
2	238383091	238384110	5Y-H4K8ac_peak_10773	16.01457	
2	238481464	238481677	5Y-H4K8ac_peak_10774	9.38203	
2	238521698	238521931	5Y-H4K8ac_peak_10775	3.91125	AC096574.5_ENSG00000227107
2	238535696	238535955	5Y-H4K8ac_peak_10776	5.41472	LRRFIP1_ENSG00000124831
2	238536343	238536609	5Y-H4K8ac_peak_10777	6.77436	LRRFIP1_ENSG00000124831
2	238600120	238600360	5Y-H4K8ac_peak_10778	10.1994	
2	238601143	238601374	5Y-H4K8ac_peak_10779	4.36976	
2	238641697	238642023	5Y-H4K8ac_peak_10780	7.31815	
2	238767639	238768422	5Y-H4K8ac_peak_10781	13.0168	RAMP1_ENSG00000132329
2	238773217	238773472	5Y-H4K8ac_peak_10782	7.50148	
2	238775513	238775763	5Y-H4K8ac_peak_10783	4.28733	
2	238780790	238781244	5Y-H4K8ac_peak_10784	7.677	
2	238784883	238785201	5Y-H4K8ac_peak_10785	5.24695	
2	238791043	238792326	5Y-H4K8ac_peak_10786	13.86507	
2	238793198	238795284	5Y-H4K8ac_peak_10787	13.1133	
2	238795951	238796264	5Y-H4K8ac_peak_10788	4.15658	
2	238803227	238803525	5Y-H4K8ac_peak_10789	9.05168	
2	238804145	238805297	5Y-H4K8ac_peak_10790	10.1994	
2	238805637	238805862	5Y-H4K8ac_peak_10791	8.43511	
2	238807173	238807496	5Y-H4K8ac_peak_10792	6.37023	
2	238831754	238832075	5Y-H4K8ac_peak_10793	10.44054	
2	238864675	238864932	5Y-H4K8ac_peak_10794	4.79658	
2	239047059	239047274	5Y-H4K8ac_peak_10795	5.64988	KLHL30_ENSG00000168427
2	239067588	239067915	5Y-H4K8ac_peak_10796	12.24654	FAM132B_ENSG00000178752
2	239149114	239149371	5Y-H4K8ac_peak_10797	14.45841	HES6_ENSG00000144485
2	239335159	239335449	5Y-H4K8ac_peak_10798	9.88714	ASB1_ENSG00000065802;AC016999.2_ENSG00000229915
2	239335704	239336188	5Y-H4K8ac_peak_10799	13.00733	ASB1_ENSG00000065802;AC016999.2_ENSG00000229915
2	240000359	240000657	5Y-H4K8ac_peak_10800	9.30338	
2	240003867	240004291	5Y-H4K8ac_peak_10801	8.43511	
2	240014097	240014405	5Y-H4K8ac_peak_10802	8.76581	
2	240015033	240015237	5Y-H4K8ac_peak_10803	6.89892	
2	240017077	240017451	5Y-H4K8ac_peak_10804	6.22669	
2	240018685	240019031	5Y-H4K8ac_peak_10805	6.78128	
2	240043426	240043703	5Y-H4K8ac_peak_10806	5.04571	
2	240055156	240055358	5Y-H4K8ac_peak_10807	7.31815	
2	240148737	240148944	5Y-H4K8ac_peak_10808	5.98695	

2	240161544	240161789	5Y-H4K8ac_peak_10809	6.08523	
2	240193153	240193522	5Y-H4K8ac_peak_10810	10.69698	
2	240196118	240196357	5Y-H4K8ac_peak_10811	6.54915	
2	240197219	240197523	5Y-H4K8ac_peak_10812	16.23405	
2	240197889	240198493	5Y-H4K8ac_peak_10813	12.26819	
2	240198915	240199418	5Y-H4K8ac_peak_10814	8.73985	
2	240271473	240271792	5Y-H4K8ac_peak_10815	10.79655	
2	240323102	240323756	5Y-H4K8ac_peak_10816	13.99721	HDAC4_ENSG00000068024;AC062017.1_ENSG00000222020
2	240638159	240638503	5Y-H4K8ac_peak_10817	4.90471	
2	240964322	240964561	5Y-H4K8ac_peak_10818	9.93099	NDUFA10_ENSG00000130414
2	240964851	240965300	5Y-H4K8ac_peak_10819	21.14873	NDUFA10_ENSG00000130414
2	241075430	241075648	5Y-H4K8ac_peak_10820	5.44849	MYEOV2_ENSG00000172428
2	241076032	241076247	5Y-H4K8ac_peak_10821	7.11863	MYEOV2_ENSG00000172428
2	241172264	241172619	5Y-H4K8ac_peak_10822	7.17184	
2	241291248	241291450	5Y-H4K8ac_peak_10823	7.41519	
2	241292303	241292971	5Y-H4K8ac_peak_10824	6.78128	
2	241499789	241500465	5Y-H4K8ac_peak_10825	13.92877	DUSP28_ENSG00000188542
2	241505518	241505859	5Y-H4K8ac_peak_10826	4.77126	RNPEPL1_ENSG00000142327
2	241543911	241544379	5Y-H4K8ac_peak_10827	5.74859	GPR35_ENSG00000178623
2	241759107	241759315	5Y-H4K8ac_peak_10828	6.96612	KIF1A_ENSG00000130294
2	241760156	241761258	5Y-H4K8ac_peak_10829	6.19716	KIF1A_ENSG00000130294
2	241905419	241905703	5Y-H4K8ac_peak_10830	4.84727	
2	241926391	241926581	5Y-H4K8ac_peak_10831	7.59101	AC104809.2_ENSG00000223991
2	241949558	241949917	5Y-H4K8ac_peak_10832	8.69112	AC093585.6_ENSG00000229996
2	242041312	242042053	5Y-H4K8ac_peak_10833	7.11863	MTERFD2_ENSG00000122085
2	242156800	242157025	5Y-H4K8ac_peak_10834	8.79957	
2	242242383	242242972	5Y-H4K8ac_peak_10835	6.14981	
2	242244515	242244768	5Y-H4K8ac_peak_10836	5.87382	
2	242254192	242254518	5Y-H4K8ac_peak_10837	7.58806	SEPT2_ENSG00000168385
2	242430269	242430508	5Y-H4K8ac_peak_10838	7.97699	
2	242431507	242431730	5Y-H4K8ac_peak_10839	6.62622	
2	242448343	242448890	5Y-H4K8ac_peak_10840	13.92877	STK25_ENSG00000115694
2	242556753	242557526	5Y-H4K8ac_peak_10841	10.0732	
2	242576502	242576771	5Y-H4K8ac_peak_10842	8.69112	THAP4_ENSG00000176946;ATG4B_ENSG00000168397
2	242606319	242606577	5Y-H4K8ac_peak_10843	13.14766	
2	242626503	242626821	5Y-H4K8ac_peak_10844	8.43511	DTYMK_ENSG00000168393
2	242640940	242641205	5Y-H4K8ac_peak_10845	6.53157	ING5_ENSG00000168395
2	242673520	242673844	5Y-H4K8ac_peak_10846	10.54764	D2HGDH_ENSG00000180902
2	242674105	242674600	5Y-H4K8ac_peak_10847	5.12488	D2HGDH_ENSG00000180902;AC114730.8_ENSG00000215692
2	242703876	242704532	5Y-H4K8ac_peak_10848	6.50117	
2	242743574	242744690	5Y-H4K8ac_peak_10849	17.63051	
2	242749833	242750209	5Y-H4K8ac_peak_10850	5.91107	NEU4_ENSG00000204099
2	242786266	242786539	5Y-H4K8ac_peak_10851	14.31711	AC114730.2_ENSG00000235151
2	242796633	242796879	5Y-H4K8ac_peak_10852	4.5753	
2	242802629	242803143	5Y-H4K8ac_peak_10853	5.65584	
2	242809986	242810633	5Y-H4K8ac_peak_10854	8.33296	

2	242822619	242823140	5Y-H4K8ac_peak_10855	8.2913	AC131097.3_ENSG00000233806
2	242827210	242827737	5Y-H4K8ac_peak_10856	7.38046	
2	242893198	242893535	5Y-H4K8ac_peak_10857	11.04919	
20	270750	271099	5Y-H4K8ac_peak_10858	9.23159	C20orf96_ENSG00000196476
20	278271	278595	5Y-H4K8ac_peak_10859	6.86362	ZCCHC3_ENSG00000177764;AL034548.1_ENSG00000247315
20	306564	306843	5Y-H4K8ac_peak_10860	9.96543	SOX12_ENSG00000177732
20	328035	328278	5Y-H4K8ac_peak_10861	11.68317	RP5-1103G7.4_ENSG00000225377;NRSN2_ENSG00000125841
20	344298	344490	5Y-H4K8ac_peak_10862	7.28311	
20	352502	352838	5Y-H4K8ac_peak_10863	3.97334	
20	360785	361094	5Y-H4K8ac_peak_10864	6.46053	TRIB3_ENSG00000101255
20	361925	362500	5Y-H4K8ac_peak_10865	8.12924	TRIB3_ENSG00000101255
20	524060	524329	5Y-H4K8ac_peak_10866	13.92877	CSNK2A1_ENSG00000101266
20	524903	525269	5Y-H4K8ac_peak_10867	6.1654	CSNK2A1_ENSG00000101266
20	633455	634391	5Y-H4K8ac_peak_10868	10.1994	SRXN1_ENSG00000271303
20	655960	656399	5Y-H4K8ac_peak_10869	6.19716	RP5-850E9.3_ENSG00000270299;SCRT2_ENSG00000215397
20	657459	657680	5Y-H4K8ac_peak_10870	10.18556	SCRT2_ENSG00000215397
20	680047	680342	5Y-H4K8ac_peak_10871	6.10343	
20	680812	681257	5Y-H4K8ac_peak_10872	7.89273	
20	690581	690858	5Y-H4K8ac_peak_10873	7.89273	
20	814321	814562	5Y-H4K8ac_peak_10874	17.63051	FAM110A_ENSG00000125898
20	825421	826071	5Y-H4K8ac_peak_10875	6.37023	
20	983093	983539	5Y-H4K8ac_peak_10876	6.37023	RSPO4_ENSG00000101282
20	1233967	1234157	5Y-H4K8ac_peak_10877	6.34046	
20	1294017	1294219	5Y-H4K8ac_peak_10878	7.21112	
20	1306212	1306697	5Y-H4K8ac_peak_10879	5.5192	SDCBP2-AS1_ENSG00000234684
20	1757570	1757988	5Y-H4K8ac_peak_10880	10.69866	
20	2082814	2083615	5Y-H4K8ac_peak_10881	7.18391	STK35_ENSG00000125834
20	2187528	2188150	5Y-H4K8ac_peak_10882	9.05168	RP11-128M1.1_ENSG00000226644
20	2450999	2451304	5Y-H4K8ac_peak_10883	4.97086	SNRPB_ENSG00000125835
20	2489343	2490127	5Y-H4K8ac_peak_10884	8.4454	RP4-734P14.4_ENSG00000256566
20	2672863	2673140	5Y-H4K8ac_peak_10885	10.1994	EBF4_ENSG00000088881
20	2780772	2781522	5Y-H4K8ac_peak_10886	11.12941	CPXM1_ENSG00000088882
20	2781810	2782150	5Y-H4K8ac_peak_10887	7.03573	CPXM1_ENSG00000088882
20	2820422	2821363	5Y-H4K8ac_peak_10888	25.39295	PCED1A_ENSG00000132635;VPS16_ENSG00000215305
20	2853494	2853948	5Y-H4K8ac_peak_10889	7.11863	
20	2854244	2854640	5Y-H4K8ac_peak_10890	11.86504	
20	3025639	3025969	5Y-H4K8ac_peak_10891	5.32832	MRPS26_ENSG00000125901
20	3026380	3027230	5Y-H4K8ac_peak_10892	12.51872	MRPS26_ENSG00000125901
20	3084322	3084537	5Y-H4K8ac_peak_10893	5.51139	
20	3140169	3140473	5Y-H4K8ac_peak_10894	8.2913	UBOX5_ENSG00000185019;FASTKD5_ENSG00000215251
20	3140707	3140905	5Y-H4K8ac_peak_10895	7.11262	UBOX5_ENSG00000185019;FASTKD5_ENSG00000215251
20	3141212	3141455	5Y-H4K8ac_peak_10896	6.3019	UBOX5_ENSG00000185019;FASTKD5_ENSG00000215251
20	3144414	3144687	5Y-H4K8ac_peak_10897	4.24332	
20	3154863	3155261	5Y-H4K8ac_peak_10898	6.36454	LZTS3_ENSG00000088899
20	3189979	3190365	5Y-H4K8ac_peak_10899	8.47164	ITPA_ENSG00000125877
20	3263850	3264130	5Y-H4K8ac_peak_10900	8.8021	

20	3389748	3390316	5Y-H4K8ac_peak_10901	5.64909	
20	3722689	3723004	5Y-H4K8ac_peak_10902	5.41472	
20	3748762	3749236	5Y-H4K8ac_peak_10903	6.78128	C20orf27_ENSG00000101220
20	3758102	3758436	5Y-H4K8ac_peak_10904	8.2913	
20	3758740	3759098	5Y-H4K8ac_peak_10905	5.18558	
20	3776530	3776733	5Y-H4K8ac_peak_10906	7.03573	
20	3777207	3777475	5Y-H4K8ac_peak_10907	7.59101	
20	3792645	3793329	5Y-H4K8ac_peak_10908	5.41472	
20	3799479	3799694	5Y-H4K8ac_peak_10909	5.41472	
20	3801144	3801345	5Y-H4K8ac_peak_10910	6.78128	AP5S1_ENSG00000125843
20	3869448	3869709	5Y-H4K8ac_peak_10911	9.87097	RP11-119B16.2_ENSG00000229539;PANK2_ENSG00000125779
20	3995612	3995985	5Y-H4K8ac_peak_10912	9.63153	RNF24_ENSG00000101236
20	4044836	4045160	5Y-H4K8ac_peak_10913	5.40331	
20	4088997	4089290	5Y-H4K8ac_peak_10914	5.65584	
20	4093890	4094106	5Y-H4K8ac_peak_10915	7.97699	
20	4152811	4153136	5Y-H4K8ac_peak_10916	8.79957	
20	5383772	5383966	5Y-H4K8ac_peak_10917	4.84727	
20	5390715	5390915	5Y-H4K8ac_peak_10918	6.77436	
20	5392177	5392486	5Y-H4K8ac_peak_10919	10.4689	
20	5428624	5428854	5Y-H4K8ac_peak_10920	7.31815	
20	5484839	5485294	5Y-H4K8ac_peak_10921	7.29091	LINC00654_ENSG00000205181
20	5730328	5730858	5Y-H4K8ac_peak_10922	4.15658	C20orf196_ENSG00000171984
20	5892162	5892474	5Y-H4K8ac_peak_10923	7.89273	CHGB_ENSG00000089199
20	5930847	5931073	5Y-H4K8ac_peak_10924	13.64163	TRMT6_ENSG00000089195;MCM8_ENSG00000125885
20	7999926	8000319	5Y-H4K8ac_peak_10925	7.90236	TMX4_ENSG00000125827;RP5-971N18.3_ENSG00000229766
20	9048039	9048301	5Y-H4K8ac_peak_10926	7.01266	AL121898.1_ENSG00000252819
20	9496413	9496890	5Y-H4K8ac_peak_10927	7.59101	RP5-1119D9.4_ENSG00000225988
20	9513523	9513740	5Y-H4K8ac_peak_10928	7.3889	
20	9820032	9820275	5Y-H4K8ac_peak_10929	10.90365	PAK7_ENSG00000101349
20	10153023	10153314	5Y-H4K8ac_peak_10930	7.31815	RP11-416N4.4_ENSG00000230506
20	10198392	10198684	5Y-H4K8ac_peak_10931	11.20652	SNAP25_ENSG00000132639
20	10414585	10414797	5Y-H4K8ac_peak_10932	8.79957	MKKS_ENSG00000125863
20	10654855	10655381	5Y-H4K8ac_peak_10933	6.64195	JAG1_ENSG00000101384
20	11871551	11871946	5Y-H4K8ac_peak_10934	10.5157	BTBD3_ENSG00000132640
20	12906144	12906697	5Y-H4K8ac_peak_10935	4.50834	
20	13765864	13766174	5Y-H4K8ac_peak_10936	6.77436	ESF1_ENSG00000089048;NDUFAF5_ENSG00000101247
20	16088041	16088282	5Y-H4K8ac_peak_10937	7.63144	
20	16227545	16227817	5Y-H4K8ac_peak_10938	5.718	
20	16330932	16331144	5Y-H4K8ac_peak_10939	7.38046	
20	16423255	16423510	5Y-H4K8ac_peak_10940	6.07082	
20	16555048	16555391	5Y-H4K8ac_peak_10941	7.57144	KIF16B_ENSG00000089177
20	16631792	16632181	5Y-H4K8ac_peak_10942	6.20989	
20	16635017	16635319	5Y-H4K8ac_peak_10943	7.59101	
20	16636265	16636782	5Y-H4K8ac_peak_10944	5.98695	
20	16651441	16651787	5Y-H4K8ac_peak_10945	7.13294	Y_RNA_ENSG00000200494
20	16710764	16711192	5Y-H4K8ac_peak_10946	8.20773	SNRPB2_ENSG00000125870;RP4-705D16.3_ENSG00000228809

20	17485076	17485352	5Y-H4K8ac_peak_10947	5.23083	
20	17511619	17512063	5Y-H4K8ac_peak_10948	6.43068	
20	17854416	17854707	5Y-H4K8ac_peak_10949	7.58806	
20	17856638	17857034	5Y-H4K8ac_peak_10950	9.38276	
20	17948900	17949430	5Y-H4K8ac_peak_10951	13.00733	SNX5_ENSG00000089006;MGME1_ENSG00000125871
20	17949634	17950036	5Y-H4K8ac_peak_10952	8.21582	SNX5_ENSG00000089006;MGME1_ENSG00000125871
20	17988226	17988631	5Y-H4K8ac_peak_10953	6.79955	
20	18118052	18118355	5Y-H4K8ac_peak_10954	9.30505	PET117_ENSG00000232838;CSRP2BP_ENSG00000149474
20	18118547	18118929	5Y-H4K8ac_peak_10955	7.11863	PET117_ENSG00000232838;CSRP2BP_ENSG00000149474
20	18549406	18549602	5Y-H4K8ac_peak_10956	4.95697	
20	18568749	18569027	5Y-H4K8ac_peak_10957	10.4689	DTD1_ENSG00000125821
20	18774983	18775293	5Y-H4K8ac_peak_10958	5.40331	LINC00652_ENSG00000179935;RP5-1068E13.7_ENSG00000273148
20	19738091	19738530	5Y-H4K8ac_peak_10959	5.23083	AL121761.2_ENSG00000268628;RP1-122P22.2_ENSG00000233895
20	19738899	19739347	5Y-H4K8ac_peak_10960	8.43511	AL121761.2_ENSG00000268628;RP1-122P22.2_ENSG00000233895
20	19998388	19998588	5Y-H4K8ac_peak_10961	5.41472	NAA20_ENSG00000173418
20	20032769	20033014	5Y-H4K8ac_peak_10962	12.05638	C20orf26_ENSG00000089101
20	20184467	20184841	5Y-H4K8ac_peak_10963	8.21525	
20	21082639	21082890	5Y-H4K8ac_peak_10964	7.42477	
20	21272365	21272994	5Y-H4K8ac_peak_10965	9.38276	
20	23342206	23342441	5Y-H4K8ac_peak_10966	7.11863	GZF1_ENSG00000125812
20	23342879	23343281	5Y-H4K8ac_peak_10967	7.63144	GZF1_ENSG00000125812
20	23401696	23401985	5Y-H4K8ac_peak_10968	10.36926	NAPB_ENSG00000125814
20	23402304	23402528	5Y-H4K8ac_peak_10969	8.93923	NAPB_ENSG00000125814
20	23618152	23618469	5Y-H4K8ac_peak_10970	7.63144	CST3_ENSG00000101439
20	24973554	24973772	5Y-H4K8ac_peak_10971	14.99673	APMAP_ENSG00000101474
20	25227993	25228269	5Y-H4K8ac_peak_10972	6.79955	PYGB_ENSG00000100994
20	25565758	25566124	5Y-H4K8ac_peak_10973	10.4689	NINL_ENSG00000101004
20	25676928	25677439	5Y-H4K8ac_peak_10974	9.15007	ZNF337_ENSG00000130684
20	25834446	25834756	5Y-H4K8ac_peak_10975	8.78565	
20	25967619	25967918	5Y-H4K8ac_peak_10976	5.59843	
20	30157650	30158105	5Y-H4K8ac_peak_10977	8.4454	
20	30160790	30161023	5Y-H4K8ac_peak_10978	6.78318	HM13-AS1_ENSG00000230613
20	30191825	30192052	5Y-H4K8ac_peak_10979	4.8887	
20	30225348	30226113	5Y-H4K8ac_peak_10980	11.19336	COX4I2_ENSG00000131055
20	30226466	30227011	5Y-H4K8ac_peak_10981	11.36844	COX4I2_ENSG00000131055
20	30310435	30310633	5Y-H4K8ac_peak_10982	8.78565	
20	30310838	30311311	5Y-H4K8ac_peak_10983	13.72401	BCL2L1_ENSG00000171552
20	30449648	30449841	5Y-H4K8ac_peak_10984	7.20869	
20	30458098	30458674	5Y-H4K8ac_peak_10985	7.11863	DUSP15_ENSG00000149599;TTLL9_ENSG00000131044
20	30468290	30468547	5Y-H4K8ac_peak_10986	6.73047	
20	30498867	30499329	5Y-H4K8ac_peak_10987	8.93304	
20	30555401	30556248	5Y-H4K8ac_peak_10988	13.05418	XKR7_ENSG00000260903
20	30680058	30680264	5Y-H4K8ac_peak_10989	6.07082	
20	30697392	30697888	5Y-H4K8ac_peak_10990	19.72309	TM9SF4_ENSG00000101337
20	30738286	30738476	5Y-H4K8ac_peak_10991	5.19372	
20	30777888	30778079	5Y-H4K8ac_peak_10992	6.54441	TSPY26P_ENSG00000235217

20	30795160	30795375	5Y-H4K8ac_peak_10993	6.53157	POFUT1_ENSG00000101346
20	30795779	30795970	5Y-H4K8ac_peak_10994	6.96612	POFUT1_ENSG00000101346
20	30946157	30946357	5Y-H4K8ac_peak_10995	7.59832	ASXL1_ENSG00000171456
20	31044311	31044845	5Y-H4K8ac_peak_10996	9.89244	
20	31070452	31070647	5Y-H4K8ac_peak_10997	6.50117	
20	31071691	31072110	5Y-H4K8ac_peak_10998	27.06242	
20	31072376	31072729	5Y-H4K8ac_peak_10999	5.87725	
20	31082719	31083233	5Y-H4K8ac_peak_11000	8.2913	
20	31084004	31084707	5Y-H4K8ac_peak_11001	4.0639	
20	31085322	31085943	5Y-H4K8ac_peak_11002	9.7353	
20	31096907	31097259	5Y-H4K8ac_peak_11003	8.27399	
20	31104589	31105094	5Y-H4K8ac_peak_11004	14.39608	
20	31105872	31106128	5Y-H4K8ac_peak_11005	6.00598	
20	31110410	31111002	5Y-H4K8ac_peak_11006	7.41172	
20	31112992	31113223	5Y-H4K8ac_peak_11007	6.90765	
20	31114923	31115683	5Y-H4K8ac_peak_11008	8.66584	
20	31115948	31116547	5Y-H4K8ac_peak_11009	12.69256	
20	31123673	31124210	5Y-H4K8ac_peak_11010	10.55771	
20	31124963	31125275	5Y-H4K8ac_peak_11011	6.55225	
20	31170850	31171112	5Y-H4K8ac_peak_11012	9.29721	
20	31171316	31171730	5Y-H4K8ac_peak_11013	6.14981	
20	31198750	31198953	5Y-H4K8ac_peak_11014	8.46999	
20	31214578	31215669	5Y-H4K8ac_peak_11015	10.90097	
20	31226534	31226752	5Y-H4K8ac_peak_11016	8.73985	
20	31330661	31330975	5Y-H4K8ac_peak_11017	9.15977	COMMD7_ENSG00000149600
20	31331237	31331611	5Y-H4K8ac_peak_11018	13.33591	COMMD7_ENSG00000149600
20	31349490	31349900	5Y-H4K8ac_peak_11019	4.5753	DNMT3B_ENSG00000088305
20	31350492	31350767	5Y-H4K8ac_peak_11020	5.12488	DNMT3B_ENSG00000088305
20	31407058	31407453	5Y-H4K8ac_peak_11021	11.19336	MAPRE1_ENSG00000101367
20	31490560	31491167	5Y-H4K8ac_peak_11022	5.41472	
20	32031230	32031612	5Y-H4K8ac_peak_11023	4.5753	SNTA1_ENSG00000101400
20	32078099	32078434	5Y-H4K8ac_peak_11024	7.59101	CBFA2T2_ENSG00000078699
20	32086568	32086802	5Y-H4K8ac_peak_11025	5.67588	
20	32099773	32099966	5Y-H4K8ac_peak_11026	5.97089	
20	32121507	32121796	5Y-H4K8ac_peak_11027	5.40331	
20	32237904	32238168	5Y-H4K8ac_peak_11028	11.22005	
20	32254175	32254437	5Y-H4K8ac_peak_11029	9.05168	ACTL10_ENSG00000182584
20	32261811	32262006	5Y-H4K8ac_peak_11030	4.59665	NECAB3_ENSG00000125967;RP1-63M2.5_ENSG00000271803
20	32272742	32272949	5Y-H4K8ac_peak_11031	3.93411	
20	32273729	32274126	5Y-H4K8ac_peak_11032	6.47245	E2F1_ENSG00000101412
20	32320006	32320261	5Y-H4K8ac_peak_11033	6.31818	RP4-553F4.2_ENSG00000229188;ZNF341_ENSG00000131061
20	32377104	32377327	5Y-H4K8ac_peak_11034	7.90751	
20	32399393	32399592	5Y-H4K8ac_peak_11035	7.18391	RP4-553F4.6_ENSG00000230753;CHMP4B_ENSG00000101421
20	32582302	32582538	5Y-H4K8ac_peak_11036	6.46053	RP5-1125A11.1_ENSG00000228265;RALY_ENSG00000125970
20	32699607	32700042	5Y-H4K8ac_peak_11037	17.67751	EIF2S2_ENSG00000125977
20	32900398	32900799	5Y-H4K8ac_peak_11038	9.78792	AHCY_ENSG00000101444

20	32951252	32951733	5Y-H4K8ac_peak_11039	8.69112	ITCH_ENSG00000078747
20	33103683	33104022	5Y-H4K8ac_peak_11040	5.91107	DYNLRB1_ENSG00000125971
20	33104276	33104489	5Y-H4K8ac_peak_11041	6.77436	DYNLRB1_ENSG00000125971
20	33292209	33292752	5Y-H4K8ac_peak_11042	9.01738	TP53INP2_ENSG00000078804
20	33296720	33297523	5Y-H4K8ac_peak_11043	5.98695	
20	33413042	33413309	5Y-H4K8ac_peak_11044	4.77126	NCOA6_ENSG00000198646
20	33543182	33543462	5Y-H4K8ac_peak_11045	5.65584	GSS_ENSG00000100983
20	33543733	33544109	5Y-H4K8ac_peak_11046	7.59101	GSS_ENSG00000100983
20	33680833	33681114	5Y-H4K8ac_peak_11047	5.98695	TRPC4AP_ENSG00000100991
20	33734852	33735054	5Y-H4K8ac_peak_11048	11.61993	
20	33872188	33872441	5Y-H4K8ac_peak_11049	9.23159	EIF6_ENSG00000242372;FAM83C-AS1_ENSG00000235214
20	33872717	33873196	5Y-H4K8ac_peak_11050	7.59101	EIF6_ENSG00000242372;FAM83C-AS1_ENSG00000235214
20	33878138	33878509	5Y-H4K8ac_peak_11051	10.19948	
20	33884096	33884536	5Y-H4K8ac_peak_11052	7.03573	
20	33999409	33999676	5Y-H4K8ac_peak_11053	8.79957	UQCC1_ENSG00000101019
20	34000083	34000310	5Y-H4K8ac_peak_11054	5.77617	UQCC1_ENSG00000101019
20	34042105	34042491	5Y-H4K8ac_peak_11055	4.07874	GDF5_ENSG00000125965;MIR1289-1_ENSG00000221763;CEP250_ENSG00000126001
20	34129927	34130235	5Y-H4K8ac_peak_11056	9.62747	ERGIC3_ENSG00000125991
20	34203751	34204016	5Y-H4K8ac_peak_11057	6.77436	SPAG4_ENSG00000061656
20	34252264	34252810	5Y-H4K8ac_peak_11058	13.03079	CPNE1_ENSG00000214078;RBM12_ENSG00000244462
20	34287418	34287757	5Y-H4K8ac_peak_11059	11.01287	NFS1_ENSG00000244005;ROMO1_ENSG00000125995
20	34359969	34360437	5Y-H4K8ac_peak_11060	7.59101	PHF20_ENSG00000025293
20	34474081	34474299	5Y-H4K8ac_peak_11061	8.97839	
20	34541838	34542301	5Y-H4K8ac_peak_11062	12.10416	
20	34560562	34560812	5Y-H4K8ac_peak_11063	4.50834	
20	34638517	34638838	5Y-H4K8ac_peak_11064	10.69698	LINC00657_ENSG00000260032
20	34639045	34639639	5Y-H4K8ac_peak_11065	10.35586	LINC00657_ENSG00000260032
20	34652237	34652517	5Y-H4K8ac_peak_11066	8.79957	
20	34742709	34742901	5Y-H4K8ac_peak_11067	4.50834	RP11-234K24.3_ENSG00000232406
20	34823838	34824116	5Y-H4K8ac_peak_11068	6.78318	AAR2_ENSG00000131043
20	34863128	34863376	5Y-H4K8ac_peak_11069	8.51022	
20	34893797	34894008	5Y-H4K8ac_peak_11070	4.50834	DLGAP4_ENSG00000080845
20	35064374	35064731	5Y-H4K8ac_peak_11071	6.34245	
20	35089122	35089534	5Y-H4K8ac_peak_11072	13.0168	
20	35090236	35090520	5Y-H4K8ac_peak_11073	16.01457	
20	35112075	35112432	5Y-H4K8ac_peak_11074	6.77436	
20	35201335	35201736	5Y-H4K8ac_peak_11075	8.27643	RP5-977B1.7_ENSG00000232907;TGIF2_ENSG00000118707
20	35202317	35203340	5Y-H4K8ac_peak_11076	11.12751	RP5-977B1.7_ENSG00000232907;TGIF2_ENSG00000118707;TGIF2-C20orf24_ENSG00000259399
20	35233578	35233849	5Y-H4K8ac_peak_11077	8.24461	RP5-977B1.11_ENSG00000259716;C20orf24_ENSG00000101084
20	35401832	35402051	5Y-H4K8ac_peak_11078	8.16382	DSN1_ENSG00000149636
20	35402402	35402617	5Y-H4K8ac_peak_11079	9.89244	DSN1_ENSG00000149636
20	35492396	35492691	5Y-H4K8ac_peak_11080	4.95697	SOGA1_ENSG00000149639
20	35849692	35849912	5Y-H4K8ac_peak_11081	4.64156	
20	35860555	35860805	5Y-H4K8ac_peak_11082	9.05168	
20	35863119	35863334	5Y-H4K8ac_peak_11083	7.11863	
20	35864112	35864322	5Y-H4K8ac_peak_11084	6.78128	

20	35865928	35866383	5Y-H4K8ac_peak_11085	10.19948	
20	35871665	35872580	5Y-H4K8ac_peak_11086	12.05638	
20	35896343	35896851	5Y-H4K8ac_peak_11087	7.07936	
20	35904789	35905209	5Y-H4K8ac_peak_11088	5.98695	
20	35908293	35908858	5Y-H4K8ac_peak_11089	8.43011	
20	35918223	35918473	5Y-H4K8ac_peak_11090	10.11191	MANBAL_ENSG00000101363
20	35942591	35942904	5Y-H4K8ac_peak_11091	7.87406	
20	35944070	35944566	5Y-H4K8ac_peak_11092	6.34245	
20	35946608	35947067	5Y-H4K8ac_peak_11093	6.4472	
20	35954557	35954831	5Y-H4K8ac_peak_11094	11.46984	
20	35956981	35957202	5Y-H4K8ac_peak_11095	4.50834	
20	35959676	35960581	5Y-H4K8ac_peak_11096	8.69112	
20	35960877	35961270	5Y-H4K8ac_peak_11097	14.16341	
20	35962153	35962498	5Y-H4K8ac_peak_11098	5.77617	
20	35964638	35964895	5Y-H4K8ac_peak_11099	11.86504	
20	35974328	35974729	5Y-H4K8ac_peak_11100	16.49371	
20	35974938	35975369	5Y-H4K8ac_peak_11101	8.31764	
20	35984457	35984774	5Y-H4K8ac_peak_11102	4.15658	
20	36013358	36013748	5Y-H4K8ac_peak_11103	5.75508	
20	36021156	36021686	5Y-H4K8ac_peak_11104	10.0695	
20	36022080	36022995	5Y-H4K8ac_peak_11105	11.69482	
20	36023644	36024747	5Y-H4K8ac_peak_11106	20.99383	
20	36024950	36025648	5Y-H4K8ac_peak_11107	13.12848	
20	36034786	36035089	5Y-H4K8ac_peak_11108	8.09118	
20	36036254	36036517	5Y-H4K8ac_peak_11109	5.18558	
20	36037114	36037699	5Y-H4K8ac_peak_11110	13.81774	
20	36041916	36042179	5Y-H4K8ac_peak_11111	5.39991	
20	36226376	36226769	5Y-H4K8ac_peak_11112	12.81408	
20	36298225	36298426	5Y-H4K8ac_peak_11113	5.65584	
20	36340375	36340594	5Y-H4K8ac_peak_11114	8.33296	
20	36497903	36498105	5Y-H4K8ac_peak_11115	7.89273	
20	36510038	36510247	5Y-H4K8ac_peak_11116	4.36976	
20	36510594	36510983	5Y-H4K8ac_peak_11117	5.67588	
20	36531532	36531792	5Y-H4K8ac_peak_11118	11.01067	VSTM2L_ENSG00000132821
20	36540794	36541211	5Y-H4K8ac_peak_11119	4.642	
20	36543564	36543988	5Y-H4K8ac_peak_11120	11.60073	
20	36661928	36662325	5Y-H4K8ac_peak_11121	15.75437	TTI1_ENSG00000101407;RPRD1B_ENSG00000101413
20	36736330	36736589	5Y-H4K8ac_peak_11122	4.84727	
20	36772310	36772504	5Y-H4K8ac_peak_11123	6.38173	
20	36780965	36781301	5Y-H4K8ac_peak_11124	7.38046	
20	36888705	36889028	5Y-H4K8ac_peak_11125	4.50834	KIAA1755_ENSG00000149633;BPI_ENSG00000101425
20	36927056	36927377	5Y-H4K8ac_peak_11126	7.3889	
20	37063584	37063791	5Y-H4K8ac_peak_11127	6.53157	SNHG17_ENSG00000196756;SNORA71D_ENSG00000200354
20	37075342	37075710	5Y-H4K8ac_peak_11128	15.40182	SNHG11_ENSG00000174365
20	37258167	37258412	5Y-H4K8ac_peak_11129	4.95697	
20	37287380	37288141	5Y-H4K8ac_peak_11130	5.718	

20	37302753	37302998	5Y-H4K8ac_peak_11131	7.50148	
20	37495085	37495370	5Y-H4K8ac_peak_11132	9.30505	
20	37495831	37496090	5Y-H4K8ac_peak_11133	6.08523	
20	37501175	37501433	5Y-H4K8ac_peak_11134	5.87725	RN7SL116P_ENSG00000240474
20	37502758	37503475	5Y-H4K8ac_peak_11135	14.25523	
20	37504255	37504911	5Y-H4K8ac_peak_11136	7.59101	
20	37509662	37510081	5Y-H4K8ac_peak_11137	4.77126	
20	37510773	37511972	5Y-H4K8ac_peak_11138	9.79526	
20	37512698	37512945	5Y-H4K8ac_peak_11139	12.45737	
20	37513182	37513401	5Y-H4K8ac_peak_11140	8.43511	
20	37516966	37517309	5Y-H4K8ac_peak_11141	5.64909	
20	37522288	37522646	5Y-H4K8ac_peak_11142	11.19336	
20	37677910	37678814	5Y-H4K8ac_peak_11143	16.1136	
20	39525331	39525628	5Y-H4K8ac_peak_11144	6.73047	
20	39620326	39620619	5Y-H4K8ac_peak_11145	4.84727	
20	39658227	39658425	5Y-H4K8ac_peak_11146	10.79655	TOP1_ENSG00000198900
20	39765636	39766541	5Y-H4K8ac_peak_11147	22.46875	RP1-1J6.2_ENSG00000226648;PLCG1_ENSG00000124181
20	40016068	40016372	5Y-H4K8ac_peak_11148	8.2913	
20	40321271	40321500	5Y-H4K8ac_peak_11149	8.4454	
20	40680766	40681353	5Y-H4K8ac_peak_11150	7.64675	
20	40753412	40753675	5Y-H4K8ac_peak_11151	6.00388	
20	42018764	42019023	5Y-H4K8ac_peak_11152	5.40331	
20	42070911	42071203	5Y-H4K8ac_peak_11153	4.79585	
20	42073866	42075143	5Y-H4K8ac_peak_11154	4.77126	
20	42075577	42075970	5Y-H4K8ac_peak_11155	9.15007	
20	42086573	42086894	5Y-H4K8ac_peak_11156	25.09495	SRSF6_ENSG00000124193
20	42285916	42286141	5Y-H4K8ac_peak_11157	12.2918	
20	42296016	42296215	5Y-H4K8ac_peak_11158	5.98695	MYBL2_ENSG00000101057
20	42395355	42395831	5Y-H4K8ac_peak_11159	6.20875	
20	42542937	42543221	5Y-H4K8ac_peak_11160	9.30338	TOX2_ENSG00000124191
20	42587164	42587992	5Y-H4K8ac_peak_11161	8.00339	
20	42590013	42590427	5Y-H4K8ac_peak_11162	7.76232	
20	42590912	42591400	5Y-H4K8ac_peak_11163	7.59101	
20	42611068	42611310	5Y-H4K8ac_peak_11164	5.98695	
20	42623440	42623662	5Y-H4K8ac_peak_11165	8.88899	
20	42637570	42637856	5Y-H4K8ac_peak_11166	7.87406	
20	42638315	42638607	5Y-H4K8ac_peak_11167	8.43511	
20	42641485	42641774	5Y-H4K8ac_peak_11168	8.62703	
20	42642305	42642653	5Y-H4K8ac_peak_11169	7.15925	
20	42643287	42643815	5Y-H4K8ac_peak_11170	9.22312	
20	42644746	42645291	5Y-H4K8ac_peak_11171	14.87721	
20	42645679	42646351	5Y-H4K8ac_peak_11172	12.21176	
20	42647262	42647618	5Y-H4K8ac_peak_11173	11.9478	
20	42648567	42648996	5Y-H4K8ac_peak_11174	7.30348	
20	42649283	42649583	5Y-H4K8ac_peak_11175	5.18558	
20	42655310	42655525	5Y-H4K8ac_peak_11176	8.11614	

20	42657553	42657788	5Y-H4K8ac_peak_11177	8.79957	
20	42658524	42658797	5Y-H4K8ac_peak_11178	4.04213	
20	42660791	42661066	5Y-H4K8ac_peak_11179	9.21468	
20	42662543	42662979	5Y-H4K8ac_peak_11180	8.37729	
20	42663907	42664403	5Y-H4K8ac_peak_11181	5.23083	
20	42667792	42668260	5Y-H4K8ac_peak_11182	6.95541	
20	42703641	42704218	5Y-H4K8ac_peak_11183	13.33225	
20	42710159	42710490	5Y-H4K8ac_peak_11184	4.29586	
20	42810942	42811145	5Y-H4K8ac_peak_11185	6.34046	
20	42839029	42839342	5Y-H4K8ac_peak_11186	10.12234	OSER1_ENSG00000132823;OSER1-AS1_ENSG00000223891
20	42875405	42876199	5Y-H4K8ac_peak_11187	7.11863	GDAP1L1_ENSG00000124194
20	43104554	43104860	5Y-H4K8ac_peak_11188	6.53157	TTPAL_ENSG00000124120
20	43160191	43160767	5Y-H4K8ac_peak_11189	23.17788	PKIG_ENSG00000168734
20	43237834	43238318	5Y-H4K8ac_peak_11190	5.23083	
20	43280104	43280304	5Y-H4K8ac_peak_11191	9.05168	ADA_ENSG00000196839
20	43374450	43375018	5Y-H4K8ac_peak_11192	7.89273	RP11-445H22.4_ENSG00000244558;KCNK15_ENSG00000124249
20	43390040	43390251	5Y-H4K8ac_peak_11193	6.13919	
20	43390789	43390993	5Y-H4K8ac_peak_11194	5.41472	
20	43439300	43439705	5Y-H4K8ac_peak_11195	6.50117	RIMS4_ENSG00000101098
20	43514425	43514698	5Y-H4K8ac_peak_11196	9.51254	YWHAB_ENSG00000166913
20	44034724	44035134	5Y-H4K8ac_peak_11197	10.11191	DBNDD2_ENSG00000244274
20	44035366	44035794	5Y-H4K8ac_peak_11198	7.3565	TP53TG5_ENSG00000124251;DBNDD2_ENSG00000244274
20	44044396	44044589	5Y-H4K8ac_peak_11199	6.50117	PIGT_ENSG00000124155
20	44400120	44400747	5Y-H4K8ac_peak_11200	11.1169	
20	44420646	44420836	5Y-H4K8ac_peak_11201	6.22904	WFDC3_ENSG00000124116;DNTTIP1_ENSG00000101457
20	44462191	44463058	5Y-H4K8ac_peak_11202	9.89244	TNNC2_ENSG00000101470;SNX21_ENSG00000124104
20	44470250	44470456	5Y-H4K8ac_peak_11203	6.34046	
20	44519029	44519844	5Y-H4K8ac_peak_11204	5.72233	NEURL2_ENSG00000124257;CTSA_ENSG00000064601
20	44546754	44546949	5Y-H4K8ac_peak_11205	5.68769	
20	44563379	44563642	5Y-H4K8ac_peak_11206	16.33454	PCIF1_ENSG00000100982
20	44600633	44600941	5Y-H4K8ac_peak_11207	7.27671	ZNF335_ENSG00000198026
20	44650325	44650529	5Y-H4K8ac_peak_11208	11.53136	RP11-465L10.10_ENSG00000204044;SLC12A5_ENSG00000124140
20	44718323	44718578	5Y-H4K8ac_peak_11209	13.85283	NCOA5_ENSG00000124160
20	44757519	44757910	5Y-H4K8ac_peak_11210	5.59843	
20	44767723	44768388	5Y-H4K8ac_peak_11211	15.40182	
20	44768656	44768878	5Y-H4K8ac_peak_11212	8.2913	
20	44844361	44844581	5Y-H4K8ac_peak_11213	8.24461	
20	44844898	44845228	5Y-H4K8ac_peak_11214	11.48691	
20	44960722	44961174	5Y-H4K8ac_peak_11215	7.52971	
20	44961676	44961950	5Y-H4K8ac_peak_11216	5.81394	
20	44992709	44993000	5Y-H4K8ac_peak_11217	14.89891	SLC35C2_ENSG00000080189
20	45141863	45142142	5Y-H4K8ac_peak_11218	12.10416	ZNF334_ENSG00000198185
20	45337988	45338719	5Y-H4K8ac_peak_11219	16.01457	SLC2A10_ENSG00000197496
20	45440124	45440320	5Y-H4K8ac_peak_11220	7.31815	AL031055.1_ENSG00000266136
20	45523246	45523487	5Y-H4K8ac_peak_11221	10.27264	EYA2_ENSG00000064655
20	45592320	45592529	5Y-H4K8ac_peak_11222	5.64909	

20	45659051	45660154	5Y-H4K8ac_peak_11223	10.19948	
20	45770813	45771022	5Y-H4K8ac_peak_11224	11.53521	
20	45897232	45897446	5Y-H4K8ac_peak_11225	5.23083	
20	45985819	45986863	5Y-H4K8ac_peak_11226	14.89891	ZMYND8_ENSG00000101040
20	45987467	45987682	5Y-H4K8ac_peak_11227	7.03573	
20	46130725	46130980	5Y-H4K8ac_peak_11228	9.06776	NCOA3_ENSG00000124151
20	46335330	46335521	5Y-H4K8ac_peak_11229	8.49231	
20	46336979	46337180	5Y-H4K8ac_peak_11230	6.31818	
20	46338120	46339631	5Y-H4K8ac_peak_11231	11.99586	
20	46339980	46340315	5Y-H4K8ac_peak_11232	6.77436	
20	46340886	46341146	5Y-H4K8ac_peak_11233	10.19948	
20	46342659	46342954	5Y-H4K8ac_peak_11234	7.31815	
20	46586423	46586705	5Y-H4K8ac_peak_11235	7.59101	RP11-347D21.2_ENSG00000228503
20	46591376	46591732	5Y-H4K8ac_peak_11236	11.62149	
20	46653658	46654040	5Y-H4K8ac_peak_11237	7.31815	
20	46680226	46680578	5Y-H4K8ac_peak_11238	4.9885	
20	46725020	46725253	5Y-H4K8ac_peak_11239	5.81062	
20	46823605	46823802	5Y-H4K8ac_peak_11240	7.31807	
20	46928625	46928846	5Y-H4K8ac_peak_11241	7.53429	
20	46996420	46996635	5Y-H4K8ac_peak_11242	9.84749	
20	47026513	47026842	5Y-H4K8ac_peak_11243	12.52787	
20	47029404	47029754	5Y-H4K8ac_peak_11244	6.46606	
20	47236602	47236975	5Y-H4K8ac_peak_11245	10.79063	
20	47319036	47319239	5Y-H4K8ac_peak_11246	8.86184	
20	47538337	47538932	5Y-H4K8ac_peak_11247	8.62703	ARFGEF2_ENSG00000124198
20	47835985	47836432	5Y-H4K8ac_peak_11248	9.97632	DDX27_ENSG00000124228
20	47894393	47894973	5Y-H4K8ac_peak_11249	10.99773	ZNFX1_ENSG00000124201;ZFAS1_ENSG00000177410;SNORD12C_ENSG00000209042
20	47996830	47997034	5Y-H4K8ac_peak_11250	4.77126	
20	48100377	48100604	5Y-H4K8ac_peak_11251	5.23083	
20	48125368	48125671	5Y-H4K8ac_peak_11252	8.43511	
20	48184068	48184659	5Y-H4K8ac_peak_11253	18.77281	PTGIS_ENSG00000124212
20	48364509	48364739	5Y-H4K8ac_peak_11254	7.89142	
20	48531569	48532601	5Y-H4K8ac_peak_11255	13.2534	SPATA2_ENSG00000158480
20	48605582	48606070	5Y-H4K8ac_peak_11256	5.98695	
20	48606698	48607174	5Y-H4K8ac_peak_11257	12.61054	
20	48630930	48631160	5Y-H4K8ac_peak_11258	6.77436	
20	48631659	48631970	5Y-H4K8ac_peak_11259	5.98695	
20	48632242	48633013	5Y-H4K8ac_peak_11260	9.7353	
20	48646513	48646703	5Y-H4K8ac_peak_11261	5.44849	
20	48729217	48729628	5Y-H4K8ac_peak_11262	18.22239	
20	48769770	48770212	5Y-H4K8ac_peak_11263	14.1808	TMEM189-UBE2V1_ENSG00000124208;TMEM189_ENSG00000240849
20	48770427	48770840	5Y-H4K8ac_peak_11264	16.01457	TMEM189-UBE2V1_ENSG00000124208;TMEM189_ENSG00000240849
20	48775303	48775517	5Y-H4K8ac_peak_11265	7.21406	
20	48808013	48808415	5Y-H4K8ac_peak_11266	5.23083	CEBPB_ENSG00000172216
20	48859779	48859987	5Y-H4K8ac_peak_11267	4.84727	
20	48901785	48902055	5Y-H4K8ac_peak_11268	4.15658	

20	48920687	48921274	5Y-H4K8ac_peak_11269	8.28346	
20	48921853	48922192	5Y-H4K8ac_peak_11270	7.30348	
20	48923013	48923243	5Y-H4K8ac_peak_11271	4.95697	
20	48925521	48925844	5Y-H4K8ac_peak_11272	7.11863	
20	48962452	48962654	5Y-H4K8ac_peak_11273	7.89273	
20	49032138	49032332	5Y-H4K8ac_peak_11274	8.59175	
20	49233388	49233608	5Y-H4K8ac_peak_11275	5.16353	
20	49244543	49245008	5Y-H4K8ac_peak_11276	5.91107	
20	49245407	49245615	5Y-H4K8ac_peak_11277	7.50148	
20	49347161	49347500	5Y-H4K8ac_peak_11278	5.51759	PARD6B_ENSG00000124171
20	49406973	49407650	5Y-H4K8ac_peak_11279	13.0168	
20	49435017	49435241	5Y-H4K8ac_peak_11280	5.22422	
20	49462067	49462502	5Y-H4K8ac_peak_11281	8.748	
20	49546968	49547754	5Y-H4K8ac_peak_11282	19.90109	ADNP_ENSG00000101126;RP5-914P20.5_ENSG00000259456
20	49548394	49548779	5Y-H4K8ac_peak_11283	5.41472	ADNP_ENSG00000101126;RP5-914P20.5_ENSG00000259456
20	49575414	49575725	5Y-H4K8ac_peak_11284	8.21582	DPM1_ENSG00000000419;MOCS3_ENSG00000124217
20	49639150	49639444	5Y-H4K8ac_peak_11285	6.10343	KCNG1_ENSG00000026559
20	49742557	49743037	5Y-H4K8ac_peak_11286	7.68492	
20	49747442	49748000	5Y-H4K8ac_peak_11287	11.18077	RPSAP1_ENSG00000228820
20	50028682	50029344	5Y-H4K8ac_peak_11288	8.2913	
20	50073163	50073404	5Y-H4K8ac_peak_11289	7.90236	
20	50529262	50529681	5Y-H4K8ac_peak_11290	14.30317	
20	50807782	50809200	5Y-H4K8ac_peak_11291	24.88295	
20	51791841	51792067	5Y-H4K8ac_peak_11292	6.34046	
20	52209567	52209871	5Y-H4K8ac_peak_11293	9.38203	
20	52240220	52240577	5Y-H4K8ac_peak_11294	9.13822	
20	52532112	52532321	5Y-H4K8ac_peak_11295	5.03335	
20	52824633	52824850	5Y-H4K8ac_peak_11296	6.98416	PFDN4_ENSG00000101132
20	54096819	54097442	5Y-H4K8ac_peak_11297	13.60015	
20	54580219	54580607	5Y-H4K8ac_peak_11298	10.11191	CBLN4_ENSG00000054803
20	54736234	54736465	5Y-H4K8ac_peak_11299	5.25503	
20	54741331	54741540	5Y-H4K8ac_peak_11300	5.40331	
20	54934126	54934335	5Y-H4K8ac_peak_11301	5.35202	FAM210B_ENSG00000124098
20	55717085	55717640	5Y-H4K8ac_peak_11302	7.73231	
20	55717895	55718112	5Y-H4K8ac_peak_11303	10.69698	
20	55799561	55799753	5Y-H4K8ac_peak_11304	8.21582	
20	55817166	55817556	5Y-H4K8ac_peak_11305	9.96543	
20	55817871	55818250	5Y-H4K8ac_peak_11306	6.76091	
20	55818694	55819028	5Y-H4K8ac_peak_11307	7.30348	
20	55819225	55819715	5Y-H4K8ac_peak_11308	5.41472	
20	55826585	55826797	5Y-H4K8ac_peak_11309	5.91107	
20	55840399	55841068	5Y-H4K8ac_peak_11310	9.3149	BMP7_ENSG00000101144;RP4-813D12.3_ENSG00000226308
20	55841508	55841721	5Y-H4K8ac_peak_11311	12.06963	BMP7_ENSG00000101144;RP4-813D12.3_ENSG00000226308
20	55926370	55926840	5Y-H4K8ac_peak_11312	14.31711	RAE1_ENSG00000101146
20	55965640	55965857	5Y-H4K8ac_peak_11313	4.50834	RBM38_ENSG00000132819
20	55983891	55984174	5Y-H4K8ac_peak_11314	7.21932	

20	56015770	56016585	5Y-H4K8ac_peak_11315	10.35586	
20	56045623	56046296	5Y-H4K8ac_peak_11316	6.62622	
20	56171821	56172044	5Y-H4K8ac_peak_11317	5.35202	
20	56228327	56228518	5Y-H4K8ac_peak_11318	6.77436	
20	56241586	56241827	5Y-H4K8ac_peak_11319	5.79005	
20	56247321	56247823	5Y-H4K8ac_peak_11320	9.01966	
20	56257444	56257652	5Y-H4K8ac_peak_11321	6.31818	
20	56258770	56259211	5Y-H4K8ac_peak_11322	6.77436	
20	56260959	56261347	5Y-H4K8ac_peak_11323	7.12953	
20	56323127	56323378	5Y-H4K8ac_peak_11324	6.73047	
20	56323970	56324455	5Y-H4K8ac_peak_11325	15.28394	
20	56378279	56378479	5Y-H4K8ac_peak_11326	5.24695	
20	56412359	56412990	5Y-H4K8ac_peak_11327	8.41685	
20	56414427	56415144	5Y-H4K8ac_peak_11328	11.01067	
20	56457646	56458088	5Y-H4K8ac_peak_11329	7.09658	
20	56483333	56483725	5Y-H4K8ac_peak_11330	7.59101	
20	56568884	56569287	5Y-H4K8ac_peak_11331	8.43511	
20	56573818	56574306	5Y-H4K8ac_peak_11332	6.77436	
20	56574524	56574855	5Y-H4K8ac_peak_11333	7.59101	
20	56575316	56575594	5Y-H4K8ac_peak_11334	5.52265	
20	56575842	56576170	5Y-H4K8ac_peak_11335	9.01738	
20	56576857	56577423	5Y-H4K8ac_peak_11336	7.09658	
20	56578614	56578828	5Y-H4K8ac_peak_11337	6.73385	
20	56594865	56595064	5Y-H4K8ac_peak_11338	6.27097	
20	56595301	56595521	5Y-H4K8ac_peak_11339	5.65584	
20	56596577	56596834	5Y-H4K8ac_peak_11340	7.83553	
20	56612738	56613306	5Y-H4K8ac_peak_11341	7.59832	
20	56615163	56616358	5Y-H4K8ac_peak_11342	13.00733	
20	56618095	56618441	5Y-H4K8ac_peak_11343	7.14092	
20	56622646	56623206	5Y-H4K8ac_peak_11344	9.22312	
20	56650803	56651386	5Y-H4K8ac_peak_11345	10.1994	
20	56749599	56750197	5Y-H4K8ac_peak_11346	10.54321	
20	56751966	56752193	5Y-H4K8ac_peak_11347	6.21836	
20	56754548	56755097	5Y-H4K8ac_peak_11348	10.21117	
20	56964434	56964804	5Y-H4K8ac_peak_11349	16.52279	VAPB_ENSG00000124164
20	57046213	57046456	5Y-H4K8ac_peak_11350	5.98695	
20	57225935	57226669	5Y-H4K8ac_peak_11351	20.83544	STX16_ENSG00000124222;STX16-NPEPL1_ENSG00000254995
20	57273655	57273975	5Y-H4K8ac_peak_11352	4.84727	
20	57292063	57292268	5Y-H4K8ac_peak_11353	6.55841	
20	57607057	57607340	5Y-H4K8ac_peak_11354	15.92074	ATP5E_ENSG00000124172
20	57618172	57618684	5Y-H4K8ac_peak_11355	8.43511	SLMO2_ENSG00000101166
20	57797268	57797608	5Y-H4K8ac_peak_11356	7.59101	
20	57978428	57978816	5Y-H4K8ac_peak_11357	8.20773	
20	58152227	58152730	5Y-H4K8ac_peak_11358	5.23083	PHACTR3_ENSG00000087495
20	58178879	58179287	5Y-H4K8ac_peak_11359	4.95697	
20	58180326	58180705	5Y-H4K8ac_peak_11360	4.0639	

20	58508286	58508690	5Y-H4K8ac_peak_11361	12.10416	SYCP2_ENSG00000196074;FAM217B_ENSG00000196227
20	58508992	58509282	5Y-H4K8ac_peak_11362	15.3483	SYCP2_ENSG00000196074;FAM217B_ENSG00000196227
20	60717633	60717872	5Y-H4K8ac_peak_11363	5.41472	PSMA7_ENSG00000101182;SS18L1_ENSG00000184402
20	60718141	60718360	5Y-H4K8ac_peak_11364	7.89273	PSMA7_ENSG00000101182;SS18L1_ENSG00000184402
20	60718564	60718770	5Y-H4K8ac_peak_11365	16.01457	PSMA7_ENSG00000101182;SS18L1_ENSG00000184402
20	60751502	60751823	5Y-H4K8ac_peak_11366	9.23159	
20	60752131	60752390	5Y-H4K8ac_peak_11367	7.97699	
20	60758150	60758345	5Y-H4K8ac_peak_11368	6.78128	MTG2_ENSG00000101181
20	60785833	60786051	5Y-H4K8ac_peak_11369	7.31102	
20	60787508	60787767	5Y-H4K8ac_peak_11370	8.24952	
20	60878263	60878517	5Y-H4K8ac_peak_11371	10.69698	
20	60927356	60927555	5Y-H4K8ac_peak_11372	10.15788	RP11-157P1.5_ENSG00000228812
20	60928062	60928938	5Y-H4K8ac_peak_11373	10.54764	RP11-157P1.5_ENSG00000228812
20	60930175	60931122	5Y-H4K8ac_peak_11374	16.49365	
20	60931660	60932099	5Y-H4K8ac_peak_11375	9.51254	
20	60932863	60933645	5Y-H4K8ac_peak_11376	9.02782	
20	60938143	60938342	5Y-H4K8ac_peak_11377	5.75508	
20	60942089	60942319	5Y-H4K8ac_peak_11378	7.89273	LAMA5_ENSG00000130702
20	60942514	60942778	5Y-H4K8ac_peak_11379	11.60073	LAMA5_ENSG00000130702
20	60942981	60943189	5Y-H4K8ac_peak_11380	4.00285	LAMA5_ENSG00000130702
20	60943472	60943938	5Y-H4K8ac_peak_11381	6.78318	
20	60945091	60945851	5Y-H4K8ac_peak_11382	7.59101	
20	60946108	60946492	5Y-H4K8ac_peak_11383	7.60057	
20	60961656	60961936	5Y-H4K8ac_peak_11384	9.45199	RPS21_ENSG00000171858
20	60982528	60983450	5Y-H4K8ac_peak_11385	7.50148	CABLES2_ENSG00000149679
20	60985226	60985652	5Y-H4K8ac_peak_11386	11.68317	
20	61068392	61068649	5Y-H4K8ac_peak_11387	4.07874	
20	61264419	61264665	5Y-H4K8ac_peak_11388	4.50834	RP13-30A9.2_ENSG00000229882
20	61273234	61273669	5Y-H4K8ac_peak_11389	9.45199	RP11-93B14.6_ENSG00000167046;SLCO4A1_ENSG00000101187
20	61274175	61274393	5Y-H4K8ac_peak_11390	5.67283	RP11-93B14.6_ENSG00000167046;SLCO4A1_ENSG00000101187
20	61279832	61280221	5Y-H4K8ac_peak_11391	6.37023	
20	61281380	61281589	5Y-H4K8ac_peak_11392	5.07797	
20	61281811	61282035	5Y-H4K8ac_peak_11393	4.0639	
20	61283198	61283904	5Y-H4K8ac_peak_11394	11.82624	
20	61306565	61307122	5Y-H4K8ac_peak_11395	6.46053	
20	61323341	61323764	5Y-H4K8ac_peak_11396	7.11262	
20	61343056	61343287	5Y-H4K8ac_peak_11397	6.54441	
20	61392025	61392355	5Y-H4K8ac_peak_11398	11.08752	
20	61397358	61397560	5Y-H4K8ac_peak_11399	4.728	
20	61408033	61408268	5Y-H4K8ac_peak_11400	11.1169	
20	61425199	61425488	5Y-H4K8ac_peak_11401	4.0639	
20	61426846	61427078	5Y-H4K8ac_peak_11402	6.34245	MRGBP_ENSG00000101189
20	61435547	61435763	5Y-H4K8ac_peak_11403	7.83553	OGFR_ENSG00000060491
20	61449529	61449829	5Y-H4K8ac_peak_11404	6.19716	
20	61450094	61451763	5Y-H4K8ac_peak_11405	13.86751	
20	61455760	61456359	5Y-H4K8ac_peak_11406	9.05168	

20	61456588	61457017	5Y-H4K8ac_peak_11407	12.30751	
20	61461567	61461757	5Y-H4K8ac_peak_11408	3.93411	
20	61464479	61464700	5Y-H4K8ac_peak_11409	4.77126	
20	61505725	61505992	5Y-H4K8ac_peak_11410	6.66026	
20	61557242	61557840	5Y-H4K8ac_peak_11411	9.30505	
20	61568853	61569097	5Y-H4K8ac_peak_11412	12.05638	DIDO1_ENSG00000101191;GID8_ENSG00000101193
20	61847112	61847305	5Y-H4K8ac_peak_11413	6.77436	YTHDF1_ENSG00000149658
20	61847838	61848047	5Y-H4K8ac_peak_11414	11.08964	YTHDF1_ENSG00000149658
20	62041056	62041298	5Y-H4K8ac_peak_11415	5.45466	
20	62047636	62047847	5Y-H4K8ac_peak_11416	4.6766	
20	62048425	62048783	5Y-H4K8ac_peak_11417	8.5915	
20	62049283	62049550	5Y-H4K8ac_peak_11418	5.67517	
20	62083144	62083380	5Y-H4K8ac_peak_11419	6.54441	
20	62083902	62084165	5Y-H4K8ac_peak_11420	6.37023	
20	62084911	62085367	5Y-H4K8ac_peak_11421	7.72887	
20	62085579	62086501	5Y-H4K8ac_peak_11422	20.32089	
20	62086699	62087820	5Y-H4K8ac_peak_11423	16.01457	
20	62088621	62089364	5Y-H4K8ac_peak_11424	13.38311	
20	62089577	62089847	5Y-H4K8ac_peak_11425	15.17389	
20	62095693	62095954	5Y-H4K8ac_peak_11426	4.55128	
20	62099792	62100057	5Y-H4K8ac_peak_11427	9.50323	
20	62102648	62102987	5Y-H4K8ac_peak_11428	6.27467	
20	62103212	62103766	5Y-H4K8ac_peak_11429	11.22005	KCNQ2_ENSG00000075043
20	62104448	62105030	5Y-H4K8ac_peak_11430	6.59112	KCNQ2_ENSG00000075043
20	62113658	62114580	5Y-H4K8ac_peak_11431	14.15571	
20	62114868	62115305	5Y-H4K8ac_peak_11432	5.35202	
20	62130070	62130362	5Y-H4K8ac_peak_11433	7.89273	EEF1A2_ENSG00000101210
20	62133481	62134865	5Y-H4K8ac_peak_11434	12.11208	RP4-697K14.3_ENSG00000230226
20	62149808	62150088	5Y-H4K8ac_peak_11435	8.51022	
20	62151109	62151520	5Y-H4K8ac_peak_11436	8.78565	PPDPF_ENSG00000125534
20	62168118	62169367	5Y-H4K8ac_peak_11437	13.99721	PTK6_ENSG00000101213
20	62205701	62206215	5Y-H4K8ac_peak_11438	15.08825	HELZ2_ENSG00000130589
20	62257759	62258050	5Y-H4K8ac_peak_11439	14.99673	GMEB2_ENSG00000101216;CTD-3184A7.4_ENSG00000232442
20	62258700	62258950	5Y-H4K8ac_peak_11440	5.98695	GMEB2_ENSG00000101216;CTD-3184A7.4_ENSG00000232442
20	62284405	62284641	5Y-H4K8ac_peak_11441	5.64909	STMN3_ENSG00000197457
20	62285304	62285583	5Y-H4K8ac_peak_11442	10.16277	STMN3_ENSG00000197457
20	62288909	62289489	5Y-H4K8ac_peak_11443	13.00733	RTEL1_ENSG00000258366
20	62338877	62339263	5Y-H4K8ac_peak_11444	10.54764	ARFRP1_ENSG00000101246;ZGPAT_ENSG00000197114;RP4-583P15.15_ENSG00000273154
20	62486078	62486746	5Y-H4K8ac_peak_11445	7.50044	
20	62496707	62496921	5Y-H4K8ac_peak_11446	4.50834	TPD52L2_ENSG00000101150
20	62600639	62600948	5Y-H4K8ac_peak_11447	9.23159	
20	62609867	62610379	5Y-H4K8ac_peak_11448	5.52265	SAMD10_ENSG00000130590
20	62610762	62611654	5Y-H4K8ac_peak_11449	7.31102	SAMD10_ENSG00000130590;PRPF6_ENSG00000101161
20	62669857	62670145	5Y-H4K8ac_peak_11450	4.50834	
20	62670710	62670990	5Y-H4K8ac_peak_11451	9.31608	
20	62673431	62674007	5Y-H4K8ac_peak_11452	13.00733	

20	62684197	62684388	5Y-H4K8ac_peak_11453	6.52593	
20	62693668	62694282	5Y-H4K8ac_peak_11454	13.0168	
20	62710936	62711209	5Y-H4K8ac_peak_11455	7.64648	RGS19_ENSG00000171700;OPRL1_ENSG00000125510
20	62711588	62711818	5Y-H4K8ac_peak_11456	7.89273	RGS19_ENSG00000171700;OPRL1_ENSG00000125510
20	62715218	62715613	5Y-H4K8ac_peak_11457	9.22312	C20orf201_ENSG00000171695
20	62733649	62733910	5Y-H4K8ac_peak_11458	4.77126	
20	62786977	62787293	5Y-H4K8ac_peak_11459	5.91107	
20	62787670	62787993	5Y-H4K8ac_peak_11460	6.08523	
20	62886551	62886934	5Y-H4K8ac_peak_11461	9.00954	PCMTD2_ENSG00000203880
21	9882115	9882318	5Y-H4K8ac_peak_11462	11.22005	
21	10597416	10598403	5Y-H4K8ac_peak_11463	8.82355	
21	10602286	10602741	5Y-H4K8ac_peak_11464	9.14624	
21	18885383	18885718	5Y-H4K8ac_peak_11465	9.37812	CXADR_ENSG00000154639
21	18985405	18985674	5Y-H4K8ac_peak_11466	15.40182	BTG3_ENSG00000154640
21	22370087	22370545	5Y-H4K8ac_peak_11467	21.90754	NCAM2_ENSG00000154654
21	27011690	27012101	5Y-H4K8ac_peak_11468	7.09658	JAM2_ENSG00000154721
21	27107496	27107963	5Y-H4K8ac_peak_11469	27.2273	ATP5J_ENSG00000154723;GABPA_ENSG00000154727
21	27542393	27542720	5Y-H4K8ac_peak_11470	6.43068	APP_ENSG00000142192;AP000230.1_ENSG00000273492
21	27760857	27761052	5Y-H4K8ac_peak_11471	11.57334	
21	27961072	27961299	5Y-H4K8ac_peak_11472	5.54013	
21	28216734	28216984	5Y-H4K8ac_peak_11473	4.95697	ADAMTS1_ENSG00000154734
21	28218157	28218349	5Y-H4K8ac_peak_11474	6.31818	ADAMTS1_ENSG00000154734
21	28337899	28338403	5Y-H4K8ac_peak_11475	5.87382	ADAMTS5_ENSG00000154736
21	30391242	30391535	5Y-H4K8ac_peak_11476	9.38203	RWDD2B_ENSG00000156253
21	30391735	30392341	5Y-H4K8ac_peak_11477	8.33296	RWDD2B_ENSG00000156253
21	30396287	30396544	5Y-H4K8ac_peak_11478	7.38046	RP1-100J12.1_ENSG00000273254;USP16_ENSG00000156256
21	30670650	30671023	5Y-H4K8ac_peak_11479	10.36926	
21	32546326	32546602	5Y-H4K8ac_peak_11480	9.23159	
21	32547017	32547347	5Y-H4K8ac_peak_11481	8.47164	
21	32931098	32931330	5Y-H4K8ac_peak_11482	12.21176	TIAM1_ENSG00000156299;AP000251.3_ENSG00000237594
21	32932081	32932325	5Y-H4K8ac_peak_11483	7.31102	TIAM1_ENSG00000156299;AP000251.3_ENSG00000237594
21	33104624	33105035	5Y-H4K8ac_peak_11484	7.90236	SCAF4_ENSG00000156304
21	33246093	33246443	5Y-H4K8ac_peak_11485	5.67283	HUNK_ENSG00000142149
21	33247256	33247490	5Y-H4K8ac_peak_11486	6.55906	
21	33448752	33449409	5Y-H4K8ac_peak_11487	12.98738	
21	33472641	33472881	5Y-H4K8ac_peak_11488	5.84208	
21	33764930	33765138	5Y-H4K8ac_peak_11489	6.77436	URB1_ENSG00000142207;C21orf119_ENSG00000256073
21	33765448	33766014	5Y-H4K8ac_peak_11490	7.59101	URB1_ENSG00000142207;C21orf119_ENSG00000256073
21	33784772	33785320	5Y-H4K8ac_peak_11491	8.69112	EVA1C_ENSG00000166979
21	33848869	33849111	5Y-H4K8ac_peak_11492	5.28616	
21	33894327	33894683	5Y-H4K8ac_peak_11493	6.59249	
21	33986016	33986414	5Y-H4K8ac_peak_11494	10.54764	C21orf59_ENSG00000159079
21	34143741	34143991	5Y-H4K8ac_peak_11495	12.10416	PAXBP1_ENSG00000159086;C21orf49_ENSG00000205930
21	34696638	34697014	5Y-H4K8ac_peak_11496	10.46287	IFNAR1_ENSG00000142166
21	34697386	34697720	5Y-H4K8ac_peak_11497	10.46287	IFNAR1_ENSG00000142166
21	34775806	34776340	5Y-H4K8ac_peak_11498	4.15658	IFNGR2_ENSG00000159128

21	34914599	34915021	5Y-H4K8ac_peak_11499	9.51254	GART_ENSG00000159131;SON_ENSG00000159140
21	35267270	35267572	5Y-H4K8ac_peak_11500	6.90749	
21	35287705	35287957	5Y-H4K8ac_peak_11501	7.65114	ATP5O_ENSG00000241837;LINC00649_ENSG00000237945
21	35288526	35288787	5Y-H4K8ac_peak_11502	4.1776	ATP5O_ENSG00000241837;LINC00649_ENSG00000237945
21	35573609	35573897	5Y-H4K8ac_peak_11503	5.56912	
21	35747135	35747652	5Y-H4K8ac_peak_11504	12.49811	AP000320.6_ENSG00000225555;SMIM11_ENSG00000205670
21	37407376	37407688	5Y-H4K8ac_peak_11505	5.65584	
21	37485580	37485864	5Y-H4K8ac_peak_11506	15.87386	
21	37691548	37691982	5Y-H4K8ac_peak_11507	7.34185	AP000692.10_ENSG00000273199;MORC3_ENSG00000159256
21	37692775	37693097	5Y-H4K8ac_peak_11508	5.37237	AP000692.10_ENSG00000273199;MORC3_ENSG00000159256
21	38120350	38120764	5Y-H4K8ac_peak_11509	4.50834	
21	38338289	38338732	5Y-H4K8ac_peak_11510	11.57334	AP000704.5_ENSG00000224790
21	38361790	38362854	5Y-H4K8ac_peak_11511	14.05114	HLCS_ENSG00000159267
21	38444850	38445292	5Y-H4K8ac_peak_11512	7.11863	PIGP_ENSG00000185808;TTC3_ENSG00000182670
21	38593100	38593536	5Y-H4K8ac_peak_11513	6.37023	AP001432.14_ENSG00000242553
21	38640175	38640408	5Y-H4K8ac_peak_11514	9.00954	DSCR3_ENSG00000157538;AP001412.1_ENSG00000272948
21	38738053	38738550	5Y-H4K8ac_peak_11515	9.65108	AP001437.1_ENSG00000273210;DYRK1A_ENSG00000157540
21	38739179	38739600	5Y-H4K8ac_peak_11516	15.82359	AP001437.1_ENSG00000273210
21	38903845	38904042	5Y-H4K8ac_peak_11517	9.52603	
21	38936876	38937306	5Y-H4K8ac_peak_11518	6.50117	
21	39083410	39083710	5Y-H4K8ac_peak_11519	6.50117	
21	40124654	40124948	5Y-H4K8ac_peak_11520	5.28616	
21	40170402	40170592	5Y-H4K8ac_peak_11521	6.35402	
21	40177988	40178188	5Y-H4K8ac_peak_11522	5.65584	ETS2_ENSG00000157557
21	40376614	40376920	5Y-H4K8ac_peak_11523	8.67475	
21	40554986	40555260	5Y-H4K8ac_peak_11524	5.41472	PSMG1_ENSG00000183527
21	40720622	40720965	5Y-H4K8ac_peak_11525	13.0168	HMGN1_ENSG00000205581
21	40721541	40721960	5Y-H4K8ac_peak_11526	7.97699	HMGN1_ENSG00000205581
21	43242549	43242836	5Y-H4K8ac_peak_11527	4.69231	
21	43298569	43298870	5Y-H4K8ac_peak_11528	8.04493	PRDM15_ENSG00000141956;AP001619.2_ENSG00000227698
21	43299530	43299916	5Y-H4K8ac_peak_11529	9.30505	PRDM15_ENSG00000141956
21	43373555	43373912	5Y-H4K8ac_peak_11530	7.31102	C2CD2_ENSG00000157617
21	43374327	43374626	5Y-H4K8ac_peak_11531	6.22669	C2CD2_ENSG00000157617
21	43430707	43431283	5Y-H4K8ac_peak_11532	8.73985	ZBTB21_ENSG00000173276
21	43576931	43577223	5Y-H4K8ac_peak_11533	4.07874	
21	43655113	43655455	5Y-H4K8ac_peak_11534	4.95697	
21	44072941	44073545	5Y-H4K8ac_peak_11535	8.69112	PDE9A_ENSG00000160191
21	44201492	44202031	5Y-H4K8ac_peak_11536	7.59101	AP001628.7_ENSG00000233754
21	44299122	44299562	5Y-H4K8ac_peak_11537	13.60015	WDR4_ENSG00000160193;NDUFV3_ENSG00000160194
21	44311961	44312241	5Y-H4K8ac_peak_11538	7.88114	
21	44344258	44344724	5Y-H4K8ac_peak_11539	8.75926	
21	44393808	44394258	5Y-H4K8ac_peak_11540	11.90011	PKNOX1_ENSG00000160199
21	44394817	44395030	5Y-H4K8ac_peak_11541	11.19336	PKNOX1_ENSG00000160199
21	44478760	44478983	5Y-H4K8ac_peak_11542	6.00382	
21	44496441	44497084	5Y-H4K8ac_peak_11543	7.59101	CBS_ENSG00000160200
21	44527315	44527548	5Y-H4K8ac_peak_11544	7.01266	U2AF1_ENSG00000160201

21	44527884	44528441	5Y-H4K8ac_peak_11545	9.30505	U2AF1_ENSG00000160201
21	44598860	44599251	5Y-H4K8ac_peak_11546	9.05766	
21	44781453	44781769	5Y-H4K8ac_peak_11547	7.01266	AP001046.5_ENSG00000237989
21	44783496	44783805	5Y-H4K8ac_peak_11548	10.72893	AP001046.6_ENSG00000225637
21	44786590	44787293	5Y-H4K8ac_peak_11549	11.31171	
21	44787507	44787890	5Y-H4K8ac_peak_11550	5.64909	
21	44847442	44847960	5Y-H4K8ac_peak_11551	11.93212	SIK1_ENSG00000142178
21	44870654	44871726	5Y-H4K8ac_peak_11552	9.30505	
21	45023106	45023513	5Y-H4K8ac_peak_11553	4.95697	
21	45023769	45024004	5Y-H4K8ac_peak_11554	7.01266	
21	45149325	45149536	5Y-H4K8ac_peak_11555	5.67283	
21	45195881	45196212	5Y-H4K8ac_peak_11556	9.56187	CSTB_ENSG00000160213
21	45209577	45209795	5Y-H4K8ac_peak_11557	8.2913	RRP1_ENSG00000160214
21	45284297	45284621	5Y-H4K8ac_peak_11558	9.38203	AGPAT3_ENSG00000160216
21	45285137	45285679	5Y-H4K8ac_peak_11559	13.00733	AGPAT3_ENSG00000160216
21	45431672	45432057	5Y-H4K8ac_peak_11560	9.30505	TRAPPC10_ENSG00000160218
21	45553558	45553887	5Y-H4K8ac_peak_11561	10.15788	C21orf33_ENSG00000160221
21	45758998	45759571	5Y-H4K8ac_peak_11562	17.15915	C21orf2_ENSG00000160226;AP001062.9_ENSG00000232969
21	45856856	45857140	5Y-H4K8ac_peak_11563	5.15917	snoZ6_ENSG00000266692
21	45857891	45858321	5Y-H4K8ac_peak_11564	8.69112	snoZ6_ENSG00000266692;snoZ6_ENSG00000264452
21	45875587	45875802	5Y-H4K8ac_peak_11565	5.03917	LRRC3-AS1_ENSG00000229356;LRRC3_ENSG00000160233
21	46221347	46222442	5Y-H4K8ac_peak_11566	8.43511	UBE2G2_ENSG00000184787;AL773604.8_ENSG00000236519
21	46237861	46238547	5Y-H4K8ac_peak_11567	6.53157	SUMO3_ENSG00000184900
21	46331578	46331844	5Y-H4K8ac_peak_11568	15.3483	
21	46352542	46352997	5Y-H4K8ac_peak_11569	6.82266	ITGB2_ENSG00000160255
21	46359986	46360355	5Y-H4K8ac_peak_11570	11.22005	C21orf67_ENSG00000183250;FAM207A_ENSG00000160256
21	46404516	46404784	5Y-H4K8ac_peak_11571	8.69112	
21	46404986	46405566	5Y-H4K8ac_peak_11572	14.00559	
21	46406474	46406898	5Y-H4K8ac_peak_11573	5.18947	
21	46408712	46409219	5Y-H4K8ac_peak_11574	8.5159	
21	46410029	46411417	5Y-H4K8ac_peak_11575	11.09472	
21	46413207	46413406	5Y-H4K8ac_peak_11576	6.04031	LINC00163_ENSG00000234880
21	46438197	46439303	5Y-H4K8ac_peak_11577	9.52107	
21	46494090	46494334	5Y-H4K8ac_peak_11578	10.79655	SSR4P1_ENSG00000235374;ADARB1_ENSG00000197381
21	46494662	46495003	5Y-H4K8ac_peak_11579	8.8021	SSR4P1_ENSG00000235374;ADARB1_ENSG00000197381
21	46572378	46572574	5Y-H4K8ac_peak_11580	4.84193	
21	46575236	46575444	5Y-H4K8ac_peak_11581	7.11863	
21	46677095	46677289	5Y-H4K8ac_peak_11582	7.87048	
21	46712142	46712386	5Y-H4K8ac_peak_11583	9.15007	LINC00205_ENSG00000223768
21	46745827	46746298	5Y-H4K8ac_peak_11584	5.64988	
21	46773253	46773656	5Y-H4K8ac_peak_11585	10.11377	
21	46779757	46780011	5Y-H4K8ac_peak_11586	11.24252	
21	46787105	46787326	5Y-H4K8ac_peak_11587	7.80086	
21	46828702	46828922	5Y-H4K8ac_peak_11588	6.40964	
21	46885518	46885897	5Y-H4K8ac_peak_11589	5.74859	
21	46886350	46886615	5Y-H4K8ac_peak_11590	5.65584	

21	46886963	46887598	5Y-H4K8ac_peak_11591	12.86367	
21	46897783	46898105	5Y-H4K8ac_peak_11592	6.78128	
21	46898609	46898941	5Y-H4K8ac_peak_11593	5.65584	
21	46962799	46963258	5Y-H4K8ac_peak_11594	10.07308	
21	47059712	47060119	5Y-H4K8ac_peak_11595	8.24461	
21	47060343	47061519	5Y-H4K8ac_peak_11596	10.31981	
21	47062323	47062605	5Y-H4K8ac_peak_11597	5.64988	
21	47123998	47124363	5Y-H4K8ac_peak_11598	5.44849	
21	47125095	47125439	5Y-H4K8ac_peak_11599	9.68742	
21	47126107	47126349	5Y-H4K8ac_peak_11600	4.0639	
21	47145849	47146183	5Y-H4K8ac_peak_11601	6.73047	
21	47183429	47183650	5Y-H4K8ac_peak_11602	8.4454	
21	47239914	47240450	5Y-H4K8ac_peak_11603	5.12488	
21	47308294	47308509	5Y-H4K8ac_peak_11604	8.564	
21	47311972	47312549	5Y-H4K8ac_peak_11605	6.98118	
21	47312793	47313026	5Y-H4K8ac_peak_11606	5.35202	
21	47313790	47314064	5Y-H4K8ac_peak_11607	9.179	
21	47314408	47314598	5Y-H4K8ac_peak_11608	5.51139	
21	47390285	47390657	5Y-H4K8ac_peak_11609	7.89273	
21	47390948	47391586	5Y-H4K8ac_peak_11610	8.3285	
21	47392109	47392953	5Y-H4K8ac_peak_11611	14.19462	
21	47393237	47393491	5Y-H4K8ac_peak_11612	9.32595	
21	47393819	47394301	5Y-H4K8ac_peak_11613	8.5159	
21	47394534	47394739	5Y-H4K8ac_peak_11614	6.55841	
21	47401777	47402061	5Y-H4K8ac_peak_11615	7.11863	COL6A1_ENSG00000142156
21	47475720	47476100	5Y-H4K8ac_peak_11616	6.07082	AP001476.3_ENSG00000226115
21	47499603	47500417	5Y-H4K8ac_peak_11617	10.73603	
21	47518177	47518522	5Y-H4K8ac_peak_11618	5.91107	AP001471.1_ENSG00000227438;COL6A2_ENSG00000142173
21	47573525	47573746	5Y-H4K8ac_peak_11619	4.15658	
21	47614301	47614746	5Y-H4K8ac_peak_11620	5.64909	AP001468.1_ENSG00000235878
21	47715063	47715429	5Y-H4K8ac_peak_11621	6.00388	
21	47744070	47744343	5Y-H4K8ac_peak_11622	14.26354	PCNT_ENSG00000160299;C21orf58_ENSG00000160298
21	47744653	47745121	5Y-H4K8ac_peak_11623	7.59832	PCNT_ENSG00000160299;C21orf58_ENSG00000160298
21	47813508	47813749	5Y-H4K8ac_peak_11624	4.29586	
21	48055616	48055846	5Y-H4K8ac_peak_11625	10.4495	PRMT2_ENSG00000160310
22	16201894	16202092	5Y-H4K8ac_peak_11626	8.24461	
22	17601784	17602180	5Y-H4K8ac_peak_11627	7.11863	CECR6_ENSG00000183307;AC006946.15_ENSG00000235478
22	17602459	17602996	5Y-H4K8ac_peak_11628	13.60015	CECR6_ENSG00000183307;AC006946.15_ENSG00000235478
22	17639540	17640127	5Y-H4K8ac_peak_11629	8.2913	CECR5-AS1_ENSG00000185837
22	17640319	17640590	5Y-H4K8ac_peak_11630	7.76232	CECR5-AS1_ENSG00000185837
22	17652247	17652709	5Y-H4K8ac_peak_11631	11.01067	
22	17653022	17653353	5Y-H4K8ac_peak_11632	6.77436	
22	17679957	17680177	5Y-H4K8ac_peak_11633	11.42066	
22	17739433	17739675	5Y-H4K8ac_peak_11634	4.59169	
22	18120999	18121375	5Y-H4K8ac_peak_11635	5.23083	
22	18121597	18121900	5Y-H4K8ac_peak_11636	13.72401	

22	18256978	18257203	5Y-H4K8ac_peak_11637	9.15007	BID_ENSG00000015475
22	18560855	18561300	5Y-H4K8ac_peak_11638	7.31102	XXbac-B476C20.9_ENSG00000225335;PEX26_ENSG00000215193
22	18632232	18632558	5Y-H4K8ac_peak_11639	6.34046	USP18_ENSG00000184979
22	18893290	18893564	5Y-H4K8ac_peak_11640	6.34245	DGCR6_ENSG00000183628
22	18954373	18955020	5Y-H4K8ac_peak_11641	13.43531	
22	18955631	18955937	5Y-H4K8ac_peak_11642	11.1169	
22	18957190	18957513	5Y-H4K8ac_peak_11643	5.08308	DGCR5_ENSG00000237517
22	18958024	18958703	5Y-H4K8ac_peak_11644	13.16935	DGCR5_ENSG00000237517
22	19158710	19159075	5Y-H4K8ac_peak_11645	14.60852	AC004463.6_ENSG00000260924
22	19159269	19159631	5Y-H4K8ac_peak_11646	12.21176	AC004463.6_ENSG00000260924
22	19279546	19279848	5Y-H4K8ac_peak_11647	6.46053	CLTCL1_ENSG00000070371
22	19419456	19419824	5Y-H4K8ac_peak_11648	7.87406	MRPL40_ENSG00000185608
22	19650717	19650959	5Y-H4K8ac_peak_11649	6.46053	
22	19700846	19701286	5Y-H4K8ac_peak_11650	9.21468	SEPT5_ENSG00000184702
22	19701554	19701906	5Y-H4K8ac_peak_11651	6.77436	SEPT5_ENSG00000184702
22	19702154	19702658	5Y-H4K8ac_peak_11652	4.95697	SEPT5_ENSG00000184702
22	19733134	19733395	5Y-H4K8ac_peak_11653	6.22904	
22	19842607	19843142	5Y-H4K8ac_peak_11654	12.19856	GNB1L_ENSG00000185838;C22orf29_ENSG00000215012
22	19879849	19880236	5Y-H4K8ac_peak_11655	7.27671	
22	19892950	19893273	5Y-H4K8ac_peak_11656	9.23159	
22	19928989	19929861	5Y-H4K8ac_peak_11657	19.17092	TXNRD2_ENSG00000184470;COMT_ENSG00000093010
22	19974448	19974645	5Y-H4K8ac_peak_11658	7.11863	
22	19983432	19983769	5Y-H4K8ac_peak_11659	8.86184	
22	19985936	19986657	5Y-H4K8ac_peak_11660	11.04542	
22	19987694	19987894	5Y-H4K8ac_peak_11661	7.59101	
22	19995974	19996213	5Y-H4K8ac_peak_11662	6.89892	
22	19998399	19998602	5Y-H4K8ac_peak_11663	8.4454	
22	20008170	20008380	5Y-H4K8ac_peak_11664	8.69112	
22	20008776	20008991	5Y-H4K8ac_peak_11665	6.89892	
22	20066692	20067344	5Y-H4K8ac_peak_11666	15.12815	DGCR8_ENSG00000128191
22	20067841	20068250	5Y-H4K8ac_peak_11667	9.51254	DGCR8_ENSG00000128191
22	20105134	20105506	5Y-H4K8ac_peak_11668	13.00733	TRMT2A_ENSG00000099899
22	20118177	20118476	5Y-H4K8ac_peak_11669	7.59101	
22	20118828	20119142	5Y-H4K8ac_peak_11670	8.24461	
22	20142455	20142656	5Y-H4K8ac_peak_11671	8.76608	
22	20143664	20143878	5Y-H4K8ac_peak_11672	11.51936	
22	20218418	20218640	5Y-H4K8ac_peak_11673	4.07874	
22	20226518	20226802	5Y-H4K8ac_peak_11674	4.95697	
22	20236085	20236391	5Y-H4K8ac_peak_11675	5.62696	MIR1286_ENSG00000221039
22	20255277	20255522	5Y-H4K8ac_peak_11676	8.24952	
22	20256119	20256726	5Y-H4K8ac_peak_11677	7.34202	
22	20257861	20258460	5Y-H4K8ac_peak_11678	13.99721	
22	20307818	20308231	5Y-H4K8ac_peak_11679	10.19948	DGCR6L_ENSG00000128185;XXbac-B444P24.14_ENSG00000273139
22	20747692	20748143	5Y-H4K8ac_peak_11680	15.27452	ZNF74_ENSG00000185252
22	20772691	20772995	5Y-H4K8ac_peak_11681	7.24864	
22	20777237	20777667	5Y-H4K8ac_peak_11682	4.87387	

22	20778054	20778515	5Y-H4K8ac_peak_11683	5.69598	
22	20791724	20791927	5Y-H4K8ac_peak_11684	10.31981	SCARF2_ENSG00000244486
22	20849521	20849803	5Y-H4K8ac_peak_11685	5.84816	KLHL22_ENSG00000099910;MED15_ENSG00000099917
22	20861971	20862304	5Y-H4K8ac_peak_11686	8.69112	
22	20886934	20887462	5Y-H4K8ac_peak_11687	7.03573	
22	20888620	20889054	5Y-H4K8ac_peak_11688	6.35402	
22	20889257	20889475	5Y-H4K8ac_peak_11689	6.40964	
22	20890348	20890558	5Y-H4K8ac_peak_11690	10.77539	
22	21322565	21322788	5Y-H4K8ac_peak_11691	5.42666	
22	21335311	21335546	5Y-H4K8ac_peak_11692	5.64909	XXbac-B135H6.18_ENSG00000272829
22	21355612	21356367	5Y-H4K8ac_peak_11693	13.12848	THAP7_ENSG00000184436;THAP7-AS1_ENSG00000230513
22	21357042	21357377	5Y-H4K8ac_peak_11694	5.90587	THAP7_ENSG00000184436;THAP7-AS1_ENSG00000230513
22	21368200	21368495	5Y-H4K8ac_peak_11695	12.11208	TUBA3FP_ENSG00000161149
22	21369778	21370100	5Y-H4K8ac_peak_11696	10.1994	
22	21921695	21922483	5Y-H4K8ac_peak_11697	9.51254	
22	21984002	21984274	5Y-H4K8ac_peak_11698	11.22005	YDJC_ENSG00000161179
22	22005966	22006175	5Y-H4K8ac_peak_11699	9.34873	PPIL2_ENSG00000100023
22	22006853	22007350	5Y-H4K8ac_peak_11700	8.73392	PPIL2_ENSG00000100023;MIR301B_ENSG00000212102;MIR130B_ENSG00000207751
22	22019723	22020187	5Y-H4K8ac_peak_11701	6.54441	
22	22292737	22292954	5Y-H4K8ac_peak_11702	6.78128	LL22NC03-86G7.1_ENSG00000224086
22	23411777	23412237	5Y-H4K8ac_peak_11703	7.89273	GNAZ_ENSG00000128266
22	23483854	23484069	5Y-H4K8ac_peak_11704	7.53283	
22	23647829	23648071	5Y-H4K8ac_peak_11705	7.57144	
22	23725672	23726354	5Y-H4K8ac_peak_11706	8.51022	
22	23744501	23744804	5Y-H4K8ac_peak_11707	10.60083	ZDHHC8P1_ENSG00000133519
22	23745027	23745763	5Y-H4K8ac_peak_11708	22.46875	ZDHHC8P1_ENSG00000133519
22	23760382	23760670	5Y-H4K8ac_peak_11709	6.50117	
22	23778208	23778595	5Y-H4K8ac_peak_11710	10.50538	
22	23779066	23779304	5Y-H4K8ac_peak_11711	11.94897	
22	23799068	23799407	5Y-H4K8ac_peak_11712	10.4326	
22	23800202	23800677	5Y-H4K8ac_peak_11713	7.59101	
22	23861052	23861414	5Y-H4K8ac_peak_11714	6.47245	
22	23862142	23862332	5Y-H4K8ac_peak_11715	6.77436	
22	23862707	23863960	5Y-H4K8ac_peak_11716	14.6127	
22	23864784	23865562	5Y-H4K8ac_peak_11717	16.62144	
22	24059684	24060042	5Y-H4K8ac_peak_11718	10.44054	GUSBP11_ENSG00000228315;KB-1572G7.2_ENSG00000273000;AP000347.2_ENSG00000272578
22	24114602	24114902	5Y-H4K8ac_peak_11719	8.43511	
22	24115098	24115442	5Y-H4K8ac_peak_11720	6.34245	
22	24128693	24129451	5Y-H4K8ac_peak_11721	12.45737	SMARCB1_ENSG00000099956
22	24187813	24188068	5Y-H4K8ac_peak_11722	6.61077	
22	24191383	24192112	5Y-H4K8ac_peak_11723	7.64648	
22	24199388	24199770	5Y-H4K8ac_peak_11724	9.93099	KB-1125A3.11_ENSG00000272973;SLC2A11_ENSG00000133460
22	24236274	24236543	5Y-H4K8ac_peak_11725	9.63153	MIF_ENSG00000240972
22	24255672	24256123	5Y-H4K8ac_peak_11726	9.79526	
22	24359608	24359961	5Y-H4K8ac_peak_11727	8.33296	
22	24360432	24360753	5Y-H4K8ac_peak_11728	4.51076	

22	24407287	24407483	5Y-H4K8ac_peak_11729	7.35333	CABIN1_ENSG00000099991
22	24638627	24638883	5Y-H4K8ac_peak_11730	11.1169	
22	24639510	24640177	5Y-H4K8ac_peak_11731	11.1169	GGT5_ENSG00000099998
22	24640449	24640724	5Y-H4K8ac_peak_11732	9.92042	GGT5_ENSG00000099998
22	24641857	24642201	5Y-H4K8ac_peak_11733	8.43511	GGT5_ENSG00000099998
22	24643142	24643394	5Y-H4K8ac_peak_11734	6.08523	
22	24665792	24666074	5Y-H4K8ac_peak_11735	9.23159	AP000354.2_ENSG00000215464;SPECC1L_ENSG00000100014;SPECC1L-ADORA2A_ENSG00000258555
22	24666851	24667403	5Y-H4K8ac_peak_11736	9.00954	AP000354.2_ENSG00000215464;SPECC1L_ENSG00000100014;SPECC1L-ADORA2A_ENSG00000258555
22	24789231	24789442	5Y-H4K8ac_peak_11737	3.97985	
22	24802341	24802737	5Y-H4K8ac_peak_11738	6.3265	
22	24812609	24812824	5Y-H4K8ac_peak_11739	5.98695	
22	24820073	24820764	5Y-H4K8ac_peak_11740	9.30206	
22	24837563	24837864	5Y-H4K8ac_peak_11741	7.81811	
22	24840303	24840966	5Y-H4K8ac_peak_11742	6.54441	
22	24847040	24847329	5Y-H4K8ac_peak_11743	4.07874	
22	24918012	24918418	5Y-H4K8ac_peak_11744	6.64195	
22	24919029	24919382	5Y-H4K8ac_peak_11745	5.47303	
22	24920281	24920504	5Y-H4K8ac_peak_11746	5.64909	
22	24951466	24951812	5Y-H4K8ac_peak_11747	12.05638	SNRPD3_ENSG00000100028;GUCD1_ENSG00000138867
22	24988646	24989371	5Y-H4K8ac_peak_11748	7.89273	FAM211B_ENSG00000178026
22	25028400	25028661	5Y-H4K8ac_peak_11749	4.95697	BCRP3_ENSG00000215481
22	25081812	25082051	5Y-H4K8ac_peak_11750	9.00954	ARL5AP4_ENSG00000224806;AP000357.4_ENSG00000224334
22	25290959	25291373	5Y-H4K8ac_peak_11751	5.67588	
22	25348238	25348521	5Y-H4K8ac_peak_11752	11.22005	KIAA1671_ENSG00000197077
22	25365039	25365275	5Y-H4K8ac_peak_11753	4.71803	
22	25365697	25366324	5Y-H4K8ac_peak_11754	9.23159	
22	25371891	25372177	5Y-H4K8ac_peak_11755	5.59843	
22	25466023	25466214	5Y-H4K8ac_peak_11756	8.16382	
22	25888621	25889009	5Y-H4K8ac_peak_11757	5.40331	
22	25890022	25890659	5Y-H4K8ac_peak_11758	8.33296	
22	25961011	25961410	5Y-H4K8ac_peak_11759	8.2913	CTA-407F11.8_ENSG00000234884;ADRBK2_ENSG00000100077
22	26091997	26092228	5Y-H4K8ac_peak_11760	8.42168	
22	26093315	26093676	5Y-H4K8ac_peak_11761	6.85387	
22	26142774	26143114	5Y-H4K8ac_peak_11762	5.65584	
22	26157132	26157349	5Y-H4K8ac_peak_11763	6.73047	
22	26175972	26176436	5Y-H4K8ac_peak_11764	11.22005	
22	26177750	26177968	5Y-H4K8ac_peak_11765	6.77436	
22	26339187	26339462	5Y-H4K8ac_peak_11766	6.50117	
22	26565438	26565779	5Y-H4K8ac_peak_11767	13.2534	SEZ6L_ENSG00000100095
22	26801592	26801787	5Y-H4K8ac_peak_11768	5.28616	
22	26824390	26824633	5Y-H4K8ac_peak_11769	7.29055	ASPHD2_ENSG00000128203
22	26825350	26825680	5Y-H4K8ac_peak_11770	11.72217	ASPHD2_ENSG00000128203
22	26879412	26879710	5Y-H4K8ac_peak_11771	5.77617	HPS4_ENSG00000100099;SRRD_ENSG00000100104
22	27041052	27041284	5Y-H4K8ac_peak_11772	7.3889	ISCA2P1_ENSG00000226912
22	27042006	27042334	5Y-H4K8ac_peak_11773	6.07082	ISCA2P1_ENSG00000226912;MIAT_ENSG00000225783
22	27053544	27053825	5Y-H4K8ac_peak_11774	9.92856	

22	27068168	27068513	5Y-H4K8ac_peak_11775	10.99576	CTA-373H7.7_ENSG00000206028;CTA-211A9.5_ENSG00000244625
22	27069080	27069455	5Y-H4K8ac_peak_11776	26.15879	CTA-373H7.7_ENSG00000206028;CTA-211A9.5_ENSG00000244625
22	27428188	27428743	5Y-H4K8ac_peak_11777	11.68317	
22	27504520	27504750	5Y-H4K8ac_peak_11778	8.75926	
22	27507144	27507496	5Y-H4K8ac_peak_11779	13.2534	
22	27533774	27534015	5Y-H4K8ac_peak_11780	7.50044	
22	27582139	27582335	5Y-H4K8ac_peak_11781	5.98695	
22	27637996	27638300	5Y-H4K8ac_peak_11782	8.2913	
22	27655282	27655895	5Y-H4K8ac_peak_11783	8.43511	
22	27831663	27831856	5Y-H4K8ac_peak_11784	5.87725	
22	28010466	28010826	5Y-H4K8ac_peak_11785	9.88908	
22	28197870	28198419	5Y-H4K8ac_peak_11786	8.5915	MN1_ENSG00000169184
22	28412491	28412955	5Y-H4K8ac_peak_11787	4.642	
22	28416881	28417268	5Y-H4K8ac_peak_11788	7.58806	
22	28420677	28421033	5Y-H4K8ac_peak_11789	8.21582	
22	28423594	28423826	5Y-H4K8ac_peak_11790	4.84727	
22	28425383	28425902	5Y-H4K8ac_peak_11791	6.77436	
22	28838088	28838621	5Y-H4K8ac_peak_11792	8.33296	
22	29076153	29076377	5Y-H4K8ac_peak_11793	8.43511	TTC28_ENSG00000100154
22	29168738	29168958	5Y-H4K8ac_peak_11794	15.01219	CCDC117_ENSG00000159873
22	29196269	29196469	5Y-H4K8ac_peak_11795	8.4454	XBP1_ENSG00000100219;CTA-292E10.6_ENSG00000226471
22	29426855	29427168	5Y-H4K8ac_peak_11796	6.3019	ZNRF3-AS1_ENSG00000177993
22	29427467	29427659	5Y-H4K8ac_peak_11797	4.29586	ZNRF3-AS1_ENSG00000177993
22	29601558	29601755	5Y-H4K8ac_peak_11798	7.90236	EMID1_ENSG00000186998
22	29601999	29602320	5Y-H4K8ac_peak_11799	6.1654	EMID1_ENSG00000186998
22	29621681	29621947	5Y-H4K8ac_peak_11800	4.77016	
22	29664438	29664907	5Y-H4K8ac_peak_11801	6.19716	RHBDD3_ENSG00000100263;EWSR1_ENSG00000182944
22	29702450	29702950	5Y-H4K8ac_peak_11802	7.20869	GAS2L1_ENSG00000185340
22	29784054	29784439	5Y-H4K8ac_peak_11803	14.42778	
22	29787778	29787987	5Y-H4K8ac_peak_11804	6.87117	
22	29876610	29876931	5Y-H4K8ac_peak_11805	9.15007	NEFH_ENSG00000100285
22	29948223	29948740	5Y-H4K8ac_peak_11806	10.79655	
22	29949326	29949580	5Y-H4K8ac_peak_11807	8.2913	
22	29976577	29977057	5Y-H4K8ac_peak_11808	8.564	NIPSNAP1_ENSG00000184117
22	30106387	30106957	5Y-H4K8ac_peak_11809	10.60083	
22	30107590	30107816	5Y-H4K8ac_peak_11810	8.89349	
22	30116412	30116795	5Y-H4K8ac_peak_11811	6.73047	RP1-76B20.11_ENSG00000232396;RP1-76B20.12_ENSG00000239446;CABP7_ENSG00000100314
22	30130374	30130587	5Y-H4K8ac_peak_11812	5.17251	
22	30162643	30162872	5Y-H4K8ac_peak_11813	7.31102	ZMAT5_ENSG00000100319;UQCR10_ENSG00000184076
22	30600205	30600438	5Y-H4K8ac_peak_11814	10.11191	
22	30601228	30601559	5Y-H4K8ac_peak_11815	6.77436	
22	30604938	30605132	5Y-H4K8ac_peak_11816	7.89142	
22	30641975	30642624	5Y-H4K8ac_peak_11817	13.12848	LIF_ENSG00000128342;RP1-102K2.8_ENSG00000268812
22	30643270	30643467	5Y-H4K8ac_peak_11818	5.57299	LIF_ENSG00000128342
22	30648673	30649191	5Y-H4K8ac_peak_11819	7.89273	
22	30686962	30687190	5Y-H4K8ac_peak_11820	4.95697	

22	30819423	30819763	5Y-H4K8ac_peak_11821	8.24461	
22	31001202	31001486	5Y-H4K8ac_peak_11822	6.1654	
22	31002211	31002593	5Y-H4K8ac_peak_11823	9.30338	PES1_ENSG00000100029;TCN2_ENSG00000185339
22	31002925	31003204	5Y-H4K8ac_peak_11824	4.77126	PES1_ENSG00000100029;TCN2_ENSG00000185339
22	31030789	31031385	5Y-H4K8ac_peak_11825	18.09952	SLC35E4_ENSG00000100036
22	31064130	31064370	5Y-H4K8ac_peak_11826	9.7353	DUSP18_ENSG00000167065
22	31090225	31090561	5Y-H4K8ac_peak_11827	8.21582	OSBP2_ENSG00000184792
22	31091017	31091266	5Y-H4K8ac_peak_11828	7.11863	
22	31199303	31199502	5Y-H4K8ac_peak_11829	4.79585	
22	31273949	31274953	5Y-H4K8ac_peak_11830	5.72233	
22	31364514	31364998	5Y-H4K8ac_peak_11831	10.36926	MORC2_ENSG00000133422
22	31518325	31518862	5Y-H4K8ac_peak_11832	8.69112	INPP5J_ENSG00000185133
22	31555621	31556043	5Y-H4K8ac_peak_11833	7.97699	MIR3928_ENSG00000264141;RNF185_ENSG00000138942
22	31625496	31625779	5Y-H4K8ac_peak_11834	10.41511	Y_RNA_ENSG00000202019
22	31639551	31640152	5Y-H4K8ac_peak_11835	8.84534	
22	31641144	31641335	5Y-H4K8ac_peak_11836	5.8635	
22	31742910	31743506	5Y-H4K8ac_peak_11837	16.79568	PATZ1_ENSG00000100105;AC005003.1_ENSG00000213888
22	31892539	31892963	5Y-H4K8ac_peak_11838	10.5157	EIF4ENIF1_ENSG00000184708
22	32148358	32148560	5Y-H4K8ac_peak_11839	5.83608	
22	32149991	32150267	5Y-H4K8ac_peak_11840	9.8829	DEPDC5_ENSG00000100150
22	32325269	32326062	5Y-H4K8ac_peak_11841	7.46096	
22	32339398	32339672	5Y-H4K8ac_peak_11842	4.77126	YWHAH_ENSG00000128245
22	32340582	32341016	5Y-H4K8ac_peak_11843	6.53157	YWHAH_ENSG00000128245;C22orf24_ENSG00000128254
22	32870769	32871154	5Y-H4K8ac_peak_11844	7.58806	FBXO7_ENSG00000100225
22	32928199	32928803	5Y-H4K8ac_peak_11845	11.71297	
22	32973164	32973404	5Y-H4K8ac_peak_11846	4.50834	
22	33276177	33276949	5Y-H4K8ac_peak_11847	7.11863	
22	33277502	33277952	5Y-H4K8ac_peak_11848	6.78128	
22	33279489	33279980	5Y-H4K8ac_peak_11849	9.07803	
22	33307466	33307760	5Y-H4K8ac_peak_11850	7.34185	
22	33313841	33314050	5Y-H4K8ac_peak_11851	11.68317	
22	33346608	33347447	5Y-H4K8ac_peak_11852	22.31349	
22	33353115	33353376	5Y-H4K8ac_peak_11853	4.95697	
22	33353792	33353991	5Y-H4K8ac_peak_11854	6.79955	
22	33387756	33387979	5Y-H4K8ac_peak_11855	4.40052	
22	33606603	33606903	5Y-H4K8ac_peak_11856	4.07874	
22	35747243	35747645	5Y-H4K8ac_peak_11857	6.64195	
22	35777124	35777344	5Y-H4K8ac_peak_11858	4.642	HMOX1_ENSG00000100292
22	35794882	35795855	5Y-H4K8ac_peak_11859	9.15007	MCM5_ENSG00000100297
22	35863663	35864179	5Y-H4K8ac_peak_11860	14.49096	
22	35936163	35936392	5Y-H4K8ac_peak_11861	4.55128	RASD2_ENSG00000100302
22	35988521	35988876	5Y-H4K8ac_peak_11862	6.77436	
22	36001840	36002126	5Y-H4K8ac_peak_11863	12.65187	
22	36002428	36002880	5Y-H4K8ac_peak_11864	9.05168	
22	36003222	36003466	5Y-H4K8ac_peak_11865	7.21932	
22	36018919	36019151	5Y-H4K8ac_peak_11866	7.12953	

22	36433327	36433776	5Y-H4K8ac_peak_11867	8.4454	
22	36456328	36456535	5Y-H4K8ac_peak_11868	4.90471	
22	36506375	36506619	5Y-H4K8ac_peak_11869	5.64909	
22	36633831	36634081	5Y-H4K8ac_peak_11870	5.74859	
22	36635167	36635389	5Y-H4K8ac_peak_11871	5.8635	APOL2_ENSG00000128335
22	36850834	36851163	5Y-H4K8ac_peak_11872	16.94054	RP5-1119A7.14_ENSG00000228719
22	36851402	36851916	5Y-H4K8ac_peak_11873	5.87725	RP5-1119A7.14_ENSG00000228719
22	36877452	36877645	5Y-H4K8ac_peak_11874	14.2053	TXN2_ENSG00000100348
22	36902359	36903515	5Y-H4K8ac_peak_11875	11.12941	FOXRED2_ENSG00000100350
22	36925669	36926136	5Y-H4K8ac_peak_11876	8.21582	EIF3D_ENSG00000100353
22	36944747	36945040	5Y-H4K8ac_peak_11877	4.84727	
22	36965974	36966615	5Y-H4K8ac_peak_11878	6.50117	
22	37056194	37056511	5Y-H4K8ac_peak_11879	10.36926	
22	37099404	37099719	5Y-H4K8ac_peak_11880	10.11191	CACNG2_ENSG00000166862;RP1-293L6.1_ENSG00000234688
22	37153437	37153700	5Y-H4K8ac_peak_11881	5.28616	
22	37172944	37173218	5Y-H4K8ac_peak_11882	7.63144	IFT27_ENSG00000100360
22	37242606	37242827	5Y-H4K8ac_peak_11883	7.38046	
22	37373519	37373928	5Y-H4K8ac_peak_11884	7.89273	
22	37415512	37415763	5Y-H4K8ac_peak_11885	12.65187	TST_ENSG00000128311;MPST_ENSG00000128309
22	37562840	37563308	5Y-H4K8ac_peak_11886	9.84749	RP1-151B14.6_ENSG00000235237
22	37584516	37584892	5Y-H4K8ac_peak_11887	8.1667	
22	37594897	37595372	5Y-H4K8ac_peak_11888	13.0168	C1QTNF6_ENSG00000133466
22	37595705	37596126	5Y-H4K8ac_peak_11889	8.36588	C1QTNF6_ENSG00000133466
22	37703298	37703504	5Y-H4K8ac_peak_11890	10.26997	
22	37705444	37705697	5Y-H4K8ac_peak_11891	5.57299	
22	37705948	37706200	5Y-H4K8ac_peak_11892	5.98695	
22	37707099	37707339	5Y-H4K8ac_peak_11893	4.79585	
22	37740790	37741213	5Y-H4K8ac_peak_11894	9.96543	
22	37741519	37741846	5Y-H4K8ac_peak_11895	9.23159	
22	37816472	37816672	5Y-H4K8ac_peak_11896	5.67283	
22	37823687	37823990	5Y-H4K8ac_peak_11897	4.84727	RP1-63G5.5_ENSG00000243902;ELFN2_ENSG00000166897
22	37857831	37858134	5Y-H4K8ac_peak_11898	5.59843	
22	37858877	37859161	5Y-H4K8ac_peak_11899	8.47164	
22	37882549	37882896	5Y-H4K8ac_peak_11900	6.06152	MFNG_ENSG00000100060
22	37904479	37905667	5Y-H4K8ac_peak_11901	6.77436	
22	37915098	37915354	5Y-H4K8ac_peak_11902	10.15788	CARD10_ENSG00000100065
22	37934593	37934853	5Y-H4K8ac_peak_11903	9.78792	
22	37935047	37935267	5Y-H4K8ac_peak_11904	10.74739	
22	37956178	37956373	5Y-H4K8ac_peak_11905	5.98695	CDC42EP1_ENSG00000128283
22	37956683	37957121	5Y-H4K8ac_peak_11906	5.03611	CDC42EP1_ENSG00000128283
22	38029676	38030065	5Y-H4K8ac_peak_11907	10.16277	SH3BP1_ENSG00000100092
22	38030455	38030804	5Y-H4K8ac_peak_11908	8.13965	SH3BP1_ENSG00000100092
22	38053846	38054236	5Y-H4K8ac_peak_11909	4.79585	Z83844.1_ENSG00000233360;PDXP_ENSG00000241360
22	38070573	38071360	5Y-H4K8ac_peak_11910	11.1169	LGALS1_ENSG00000100097
22	38142366	38142820	5Y-H4K8ac_peak_11911	8.69112	
22	38147550	38147822	5Y-H4K8ac_peak_11912	6.75784	

22	38198164	38198683	5Y-H4K8ac_peak_11913	8.4454	
22	38200057	38200418	5Y-H4K8ac_peak_11914	11.1169	H1F0_ENSG00000189060
22	38200670	38201133	5Y-H4K8ac_peak_11915	8.73392	H1F0_ENSG00000189060
22	38301251	38301645	5Y-H4K8ac_peak_11916	4.95697	MICALL1_ENSG00000100139
22	38301848	38302817	5Y-H4K8ac_peak_11917	11.28133	MICALL1_ENSG00000100139
22	38577238	38577451	5Y-H4K8ac_peak_11918	5.40331	
22	38712817	38713234	5Y-H4K8ac_peak_11919	6.34046	
22	38902036	38902247	5Y-H4K8ac_peak_11920	6.50117	
22	39078008	39078218	5Y-H4K8ac_peak_11921	6.50117	RP3-508I15.9_ENSG00000228274;TOMM22_ENSG00000100216
22	39268875	39269085	5Y-H4K8ac_peak_11922	6.43775	CBX6_ENSG00000183741
22	39853505	39854067	5Y-H4K8ac_peak_11923	6.34046	MGAT3_ENSG00000128268
22	39867025	39867237	5Y-H4K8ac_peak_11924	6.50117	
22	40355454	40355661	5Y-H4K8ac_peak_11925	8.33296	RP3-370M22.8_ENSG00000225528
22	41417624	41417933	5Y-H4K8ac_peak_11926	9.38203	
22	41487996	41488406	5Y-H4K8ac_peak_11927	5.65584	EP300_ENSG00000100393;MIR1281_ENSG00000221160
22	41843716	41844115	5Y-H4K8ac_peak_11928	6.37023	TOB2_ENSG00000183864
22	41865182	41865385	5Y-H4K8ac_peak_11929	10.46287	PHF5A_ENSG00000100410;ACO2_ENSG00000100412
22	41951589	41951819	5Y-H4K8ac_peak_11930	6.34046	
22	42469872	42470126	5Y-H4K8ac_peak_11931	8.43511	FAM109B_ENSG00000177096
22	42470367	42470687	5Y-H4K8ac_peak_11932	8.21525	FAM109B_ENSG00000177096
22	42740591	42740905	5Y-H4K8ac_peak_11933	6.34046	TCF20_ENSG00000100207
22	42772075	42772269	5Y-H4K8ac_peak_11934	6.34046	
22	42934888	42935323	5Y-H4K8ac_peak_11935	8.33296	
22	42949290	42949724	5Y-H4K8ac_peak_11936	10.46287	SERHL2_ENSG00000183569
22	43010639	43010831	5Y-H4K8ac_peak_11937	6.79955	POLDIP3_ENSG00000100227;RNU12_ENSG00000270022
22	44350872	44351080	5Y-H4K8ac_peak_11938	6.53157	SAMM50_ENSG00000100347
22	44419491	44419990	5Y-H4K8ac_peak_11939	9.30505	
22	44420194	44420756	5Y-H4K8ac_peak_11940	15.87289	
22	44421016	44421337	5Y-H4K8ac_peak_11941	6.46053	
22	44893522	44894007	5Y-H4K8ac_peak_11942	9.01738	LDOC1L_ENSG00000188636
22	45016226	45016689	5Y-H4K8ac_peak_11943	6.77436	
22	45045010	45045331	5Y-H4K8ac_peak_11944	5.90587	
22	45072770	45073096	5Y-H4K8ac_peak_11945	5.12213	
22	45081609	45081825	5Y-H4K8ac_peak_11946	5.64909	
22	45125056	45126414	5Y-H4K8ac_peak_11947	10.12457	
22	45244988	45245180	5Y-H4K8ac_peak_11948	4.64156	
22	45404933	45405282	5Y-H4K8ac_peak_11949	6.54441	PHF21B_ENSG00000056487
22	45559945	45560212	5Y-H4K8ac_peak_11950	9.96543	CTA-217C2.1_ENSG00000226328;NUP50_ENSG00000093000
22	45607830	45608490	5Y-H4K8ac_peak_11951	8.50349	
22	45681223	45681499	5Y-H4K8ac_peak_11952	5.25503	UPK3A_ENSG00000100373
22	45705190	45705418	5Y-H4K8ac_peak_11953	8.24461	FAM118A_ENSG00000100376
22	45705969	45706210	5Y-H4K8ac_peak_11954	8.47164	
22	45709979	45710440	5Y-H4K8ac_peak_11955	6.79169	
22	45724356	45724656	5Y-H4K8ac_peak_11956	5.96059	
22	45733577	45733773	5Y-H4K8ac_peak_11957	6.14981	
22	45856423	45856744	5Y-H4K8ac_peak_11958	4.84727	

22	45898199	45898935	5Y-H4K8ac_peak_11959	9.79526	FBLN1_ENSG00000077942
22	45900824	45901379	5Y-H4K8ac_peak_11960	4.29586	
22	46067230	46068558	5Y-H4K8ac_peak_11961	18.36893	ATXN10_ENSG00000130638
22	46281181	46281503	5Y-H4K8ac_peak_11962	10.23004	
22	46281737	46281987	5Y-H4K8ac_peak_11963	6.7002	
22	46390995	46391455	5Y-H4K8ac_peak_11964	8.69112	
22	46393028	46393241	5Y-H4K8ac_peak_11965	8.73392	
22	46402704	46403074	5Y-H4K8ac_peak_11966	5.60566	
22	46403356	46403818	5Y-H4K8ac_peak_11967	13.12848	
22	46404015	46404405	5Y-H4K8ac_peak_11968	7.62723	
22	46409683	46410145	5Y-H4K8ac_peak_11969	6.78128	CITF22-92A6.1_ENSG00000273145
22	46410710	46411039	5Y-H4K8ac_peak_11970	4.8188	
22	46432872	46433126	5Y-H4K8ac_peak_11971	5.01024	
22	46439792	46440377	5Y-H4K8ac_peak_11972	8.93923	LINC00899_ENSG00000231711
22	46448767	46448983	5Y-H4K8ac_peak_11973	6.78128	FLJ27365_ENSG00000197182;RP6-109B7.5_ENSG00000273289;RP6-109B7.3_ENSG00000241990
22	46449404	46450886	5Y-H4K8ac_peak_11974	12.82593	C22orf26_ENSG00000182257;FLJ27365_ENSG00000197182;RP6-109B7.5_ENSG00000273289;RP6-109B7.3_ENSG00000241990
22	46454750	46455098	5Y-H4K8ac_peak_11975	6.77436	RP6-109B7.2_ENSG00000231010
22	46460319	46460514	5Y-H4K8ac_peak_11976	12.44818	
22	46463657	46463900	5Y-H4K8ac_peak_11977	8.49231	
22	46465988	46466736	5Y-H4K8ac_peak_11978	12.72376	RP6-109B7.4_ENSG00000235159
22	46467351	46468529	5Y-H4K8ac_peak_11979	15.01219	
22	46474257	46474666	5Y-H4K8ac_peak_11980	7.58806	
22	46475483	46475899	5Y-H4K8ac_peak_11981	11.1169	
22	46481062	46481522	5Y-H4K8ac_peak_11982	6.57504	
22	46481772	46482326	5Y-H4K8ac_peak_11983	7.87406	
22	46482934	46483655	5Y-H4K8ac_peak_11984	8.62703	
22	46485042	46485511	5Y-H4K8ac_peak_11985	5.41472	
22	46489729	46489974	5Y-H4K8ac_peak_11986	5.88655	
22	46518617	46518818	5Y-H4K8ac_peak_11987	7.03573	
22	46545956	46546176	5Y-H4K8ac_peak_11988	8.28346	PPARA_ENSG00000186951
22	46625229	46625456	5Y-H4K8ac_peak_11989	6.50117	
22	46638040	46638265	5Y-H4K8ac_peak_11990	11.26871	
22	46640615	46641025	5Y-H4K8ac_peak_11991	5.98695	
22	46641480	46641839	5Y-H4K8ac_peak_11992	9.89244	
22	46645709	46646030	5Y-H4K8ac_peak_11993	4.77126	CDPF1_ENSG00000205643
22	46646398	46647070	5Y-H4K8ac_peak_11994	9.74838	CDPF1_ENSG00000205643
22	46664018	46664242	5Y-H4K8ac_peak_11995	8.4454	TTC38_ENSG00000075234
22	46691845	46692500	5Y-H4K8ac_peak_11996	15.45397	GTSE1_ENSG00000075218
22	46731201	46732083	5Y-H4K8ac_peak_11997	7.97699	
22	46906408	46906650	5Y-H4K8ac_peak_11998	4.9885	
22	46959479	46959883	5Y-H4K8ac_peak_11999	10.32379	
22	46971014	46971324	5Y-H4K8ac_peak_12000	6.22669	GRAMD4_ENSG00000075240
22	46972345	46972802	5Y-H4K8ac_peak_12001	15.84891	GRAMD4_ENSG00000075240
22	46985593	46985884	5Y-H4K8ac_peak_12002	7.01364	
22	46991556	46991913	5Y-H4K8ac_peak_12003	5.23083	
22	47008680	47008925	5Y-H4K8ac_peak_12004	7.31807	

22	47014670	47015074	5Y-H4K8ac_peak_12005	6.22669	
22	47015293	47015485	5Y-H4K8ac_peak_12006	5.41472	
22	47016143	47016351	5Y-H4K8ac_peak_12007	10.1994	
22	47057318	47057541	5Y-H4K8ac_peak_12008	5.47303	
22	47124783	47124979	5Y-H4K8ac_peak_12009	10.95042	
22	47127039	47127632	5Y-H4K8ac_peak_12010	8.49231	
22	47133615	47133932	5Y-H4K8ac_peak_12011	4.99136	CERK_ENSG00000100422
22	47158577	47158793	5Y-H4K8ac_peak_12012	9.30505	CTA-29F11.1_ENSG00000260708;TBC1D22A_ENSG00000054611
22	48027567	48027759	5Y-H4K8ac_peak_12013	5.87382	LINC00898_ENSG00000205634;RP11-191L9.4_ENSG00000224271
22	49408135	49408437	5Y-H4K8ac_peak_12014	5.41472	
22	49411243	49411451	5Y-H4K8ac_peak_12015	11.56948	
22	49442663	49442936	5Y-H4K8ac_peak_12016	6.73385	
22	49472983	49473196	5Y-H4K8ac_peak_12017	6.22669	
22	49546872	49547137	5Y-H4K8ac_peak_12018	4.6766	
22	49547336	49547688	5Y-H4K8ac_peak_12019	11.01699	
22	50051387	50051665	5Y-H4K8ac_peak_12020	4.07874	C22orf34_ENSG00000188511
22	50221590	50221916	5Y-H4K8ac_peak_12021	7.89273	BRD1_ENSG00000100425
22	50328554	50328979	5Y-H4K8ac_peak_12022	7.90751	
22	50329738	50330189	5Y-H4K8ac_peak_12023	5.23083	
22	50353538	50353781	5Y-H4K8ac_peak_12024	6.88736	PIM3_ENSG00000198355
22	50354163	50354617	5Y-H4K8ac_peak_12025	14.97676	PIM3_ENSG00000198355
22	50438328	50438544	5Y-H4K8ac_peak_12026	5.65584	
22	50497065	50497261	5Y-H4K8ac_peak_12027	7.84116	
22	50553078	50553384	5Y-H4K8ac_peak_12028	5.29015	
22	50553884	50554243	5Y-H4K8ac_peak_12029	5.68769	
22	50607860	50608410	5Y-H4K8ac_peak_12030	11.13396	PANX2_ENSG00000073150
22	50707441	50708740	5Y-H4K8ac_peak_12031	11.61993	MAPK11_ENSG00000185386
22	50708992	50710107	5Y-H4K8ac_peak_12032	12.26819	MAPK11_ENSG00000185386
22	50745728	50745943	5Y-H4K8ac_peak_12033	9.30206	PLXNB2_ENSG00000196576
22	50746396	50746685	5Y-H4K8ac_peak_12034	10.1994	PLXNB2_ENSG00000196576
22	50765660	50766425	5Y-H4K8ac_peak_12035	13.92877	DENND6B_ENSG00000205593
22	50780410	50780769	5Y-H4K8ac_peak_12036	4.50834	PPP6R2_ENSG00000100239
22	50781828	50782022	5Y-H4K8ac_peak_12037	10.21117	PPP6R2_ENSG00000100239
22	50913679	50914001	5Y-H4K8ac_peak_12038	5.98695	SBF1_ENSG00000100241
22	50919561	50919939	5Y-H4K8ac_peak_12039	6.53157	ADM2_ENSG00000128165
22	50946874	50947071	5Y-H4K8ac_peak_12040	5.32832	LMF2_ENSG00000100258;NCAPH2_ENSG00000025770
22	50963494	50963941	5Y-H4K8ac_peak_12041	9.30505	SCO2_ENSG00000130489
22	50964314	50964888	5Y-H4K8ac_peak_12042	13.00733	SCO2_ENSG00000130489
22	50965163	50965433	5Y-H4K8ac_peak_12043	9.51254	SCO2_ENSG00000130489
22	51020442	51021233	5Y-H4K8ac_peak_12044	13.81505	CHKB-CPT1B_ENSG00000254413;CHKB-AS1_ENSG00000205559
3	2140570	2140954	5Y-H4K8ac_peak_12045	13.61292	CNTN4_ENSG00000144619
3	2339442	2339688	5Y-H4K8ac_peak_12046	5.28616	
3	3167616	3167810	5Y-H4K8ac_peak_12047	7.03573	IL5RA_ENSG00000091181;TRNT1_ENSG00000072756
3	3168719	3169239	5Y-H4K8ac_peak_12048	4.642	IL5RA_ENSG00000091181;TRNT1_ENSG00000072756
3	3221069	3221339	5Y-H4K8ac_peak_12049	12.05638	CRBN_ENSG00000113851
3	4345225	4345613	5Y-H4K8ac_peak_12050	9.78792	SETMAR_ENSG00000170364

3	4508557	4508777	5Y-H4K8ac_peak_12051	8.76608	SUMF1_ENSG00000144455
3	4546750	4547077	5Y-H4K8ac_peak_12052	8.564	
3	4553110	4553300	5Y-H4K8ac_peak_12053	6.27097	
3	4574992	4575210	5Y-H4K8ac_peak_12054	8.69112	
3	5019966	5020357	5Y-H4K8ac_peak_12055	9.30124	BHLHE40_ENSG00000134107
3	5068303	5068646	5Y-H4K8ac_peak_12056	7.89142	
3	5069301	5069703	5Y-H4K8ac_peak_12057	6.6946	
3	5164022	5164472	5Y-H4K8ac_peak_12058	12.71156	ARL8B_ENSG00000134108
3	5229544	5229745	5Y-H4K8ac_peak_12059	6.77436	AC026202.3_ENSG00000233912;AC026202.1_ENSG00000268509;EDEM1_ENSG00000134109
3	8664957	8665362	5Y-H4K8ac_peak_12060	7.53283	
3	8690477	8690720	5Y-H4K8ac_peak_12061	7.08486	
3	8691369	8691730	5Y-H4K8ac_peak_12062	7.89142	
3	8693046	8693715	5Y-H4K8ac_peak_12063	5.64909	
3	8700004	8700232	5Y-H4K8ac_peak_12064	8.2913	
3	8705513	8706023	5Y-H4K8ac_peak_12065	13.51759	
3	8834352	8834984	5Y-H4K8ac_peak_12066	11.1169	
3	9004880	9005075	5Y-H4K8ac_peak_12067	8.35138	RAD18_ENSG00000070950
3	9177651	9177924	5Y-H4K8ac_peak_12068	4.84727	
3	9356872	9357200	5Y-H4K8ac_peak_12069	9.52603	
3	9404499	9405005	5Y-H4K8ac_peak_12070	4.50834	SRGAP3_ENSG00000196220;RP11-380O24.1_ENSG00000254485;THUMPD3_ENSG00000134077
3	9595593	9595822	5Y-H4K8ac_peak_12071	9.43096	LHFPL4_ENSG00000156959
3	9791053	9791289	5Y-H4K8ac_peak_12072	10.21117	OGG1_ENSG00000114026
3	9811935	9812180	5Y-H4K8ac_peak_12073	5.41472	CAMK1_ENSG00000134072
3	9833664	9834598	5Y-H4K8ac_peak_12074	8.69112	TADA3_ENSG00000171148;ARPC4_ENSG00000241553;ARPC4-TTLL3_ENSG00000250151
3	9905933	9906235	5Y-H4K8ac_peak_12075	4.69231	
3	9929947	9930151	5Y-H4K8ac_peak_12076	6.73047	
3	9932350	9932732	5Y-H4K8ac_peak_12077	15.40182	JAGN1_ENSG00000171135
3	9957075	9957556	5Y-H4K8ac_peak_12078	4.97086	
3	9993645	9994050	5Y-H4K8ac_peak_12079	6.37023	PRRT3_ENSG00000163704
3	10028267	10028457	5Y-H4K8ac_peak_12080	4.95697	EMC3-AS1_ENSG00000180385
3	10183560	10183801	5Y-H4K8ac_peak_12081	8.79957	VHL_ENSG00000134086;snoU13_ENSG00000238345
3	10206711	10206926	5Y-H4K8ac_peak_12082	5.64909	IRAK2_ENSG00000134070
3	10474372	10474682	5Y-H4K8ac_peak_12083	7.66481	
3	10642269	10643094	5Y-H4K8ac_peak_12084	6.37023	
3	10857284	10858110	5Y-H4K8ac_peak_12085	11.99586	SLC6A11_ENSG00000132164
3	10968976	10969367	5Y-H4K8ac_peak_12086	6.1654	
3	10969960	10970349	5Y-H4K8ac_peak_12087	5.77617	
3	10970728	10970994	5Y-H4K8ac_peak_12088	6.88532	
3	11133914	11134166	5Y-H4K8ac_peak_12089	5.26353	
3	11138516	11138852	5Y-H4K8ac_peak_12090	11.86504	
3	11760559	11760937	5Y-H4K8ac_peak_12091	4.95697	
3	11762145	11762397	5Y-H4K8ac_peak_12092	8.75926	
3	11812673	11812872	5Y-H4K8ac_peak_12093	7.17184	
3	11887974	11888252	5Y-H4K8ac_peak_12094	5.99504	TAMM41_ENSG00000144559
3	12521644	12521882	5Y-H4K8ac_peak_12095	7.59832	
3	12526053	12526338	5Y-H4K8ac_peak_12096	8.69112	TSEN2_ENSG00000154743

3	12595458	12595906	5Y-H4K8ac_peak_12097	8.43511	
3	12705200	12705555	5Y-H4K8ac_peak_12098	12.10416	RAF1_ENSG00000132155
3	12835734	12835993	5Y-H4K8ac_peak_12099	6.79955	
3	12837864	12838550	5Y-H4K8ac_peak_12100	13.72401	CAND2_ENSG00000144712
3	12883551	12883948	5Y-H4K8ac_peak_12101	7.53429	RPL32_ENSG00000144713
3	12884321	12884627	5Y-H4K8ac_peak_12102	4.07874	
3	12917202	12917772	5Y-H4K8ac_peak_12103	12.05638	
3	12985970	12986254	5Y-H4K8ac_peak_12104	4.79585	
3	12987698	12987909	5Y-H4K8ac_peak_12105	5.13364	
3	12988945	12989280	5Y-H4K8ac_peak_12106	8.97752	
3	13063666	13063881	5Y-H4K8ac_peak_12107	6.64195	
3	13083744	13084082	5Y-H4K8ac_peak_12108	6.10343	
3	13084823	13085016	5Y-H4K8ac_peak_12109	5.37237	
3	13235600	13236046	5Y-H4K8ac_peak_12110	7.14616	
3	13461956	13462307	5Y-H4K8ac_peak_12111	11.94897	NUP210_ENSG00000132182
3	13468167	13468399	5Y-H4K8ac_peak_12112	4.9885	
3	13560883	13561196	5Y-H4K8ac_peak_12113	5.65584	
3	13566318	13566645	5Y-H4K8ac_peak_12114	4.64156	
3	13567432	13567736	5Y-H4K8ac_peak_12115	9.31608	
3	13577718	13577986	5Y-H4K8ac_peak_12116	4.24332	
3	13590287	13591008	5Y-H4K8ac_peak_12117	6.77436	
3	13607168	13607647	5Y-H4K8ac_peak_12118	7.38046	
3	13608021	13608227	5Y-H4K8ac_peak_12119	4.15658	
3	13614930	13615160	5Y-H4K8ac_peak_12120	9.24789	
3	13615626	13615902	5Y-H4K8ac_peak_12121	13.92877	
3	13616121	13616688	5Y-H4K8ac_peak_12122	13.61292	
3	13696696	13697225	5Y-H4K8ac_peak_12123	5.98695	
3	13824061	13824506	5Y-H4K8ac_peak_12124	5.23083	
3	13864625	13864829	5Y-H4K8ac_peak_12125	4.82324	
3	13936388	13936916	5Y-H4K8ac_peak_12126	5.41472	
3	14165913	14166120	5Y-H4K8ac_peak_12127	6.78128	CHCHD4_ENSG00000163528;TMEM43_ENSG00000170876
3	14361398	14361950	5Y-H4K8ac_peak_12128	6.46053	
3	14383030	14383346	5Y-H4K8ac_peak_12129	8.69112	
3	14443531	14443944	5Y-H4K8ac_peak_12130	14.89891	SLC6A6_ENSG00000131389
3	14493870	14494060	5Y-H4K8ac_peak_12131	4.03329	
3	14644060	14644436	5Y-H4K8ac_peak_12132	9.13822	
3	14832787	14833214	5Y-H4K8ac_peak_12133	7.49746	
3	14845794	14846266	5Y-H4K8ac_peak_12134	7.60893	
3	14879536	14879730	5Y-H4K8ac_peak_12135	6.43775	
3	14889549	14889842	5Y-H4K8ac_peak_12136	5.75508	
3	14920846	14921093	5Y-H4K8ac_peak_12137	5.51139	
3	14989251	14989523	5Y-H4K8ac_peak_12138	6.88736	FGD5-AS1_ENSG00000225733;NR2C2_ENSG00000177463
3	15106557	15106755	5Y-H4K8ac_peak_12139	8.73392	MRPS25_ENSG00000131368
3	15141268	15141466	5Y-H4K8ac_peak_12140	5.65584	ZFYVE20_ENSG00000131381
3	15468620	15468810	5Y-H4K8ac_peak_12141	6.64195	EAF1_ENSG00000144597
3	15469222	15469441	5Y-H4K8ac_peak_12142	4.95697	EAF1_ENSG00000144597

3	15688548	15688753	5Y-H4K8ac_peak_12143	7.59101	
3	16554382	16554621	5Y-H4K8ac_peak_12144	11.09973	RFTN1_ENSG00000131378
3	16554831	16555546	5Y-H4K8ac_peak_12145	8.16031	RFTN1_ENSG00000131378
3	16557860	16558051	5Y-H4K8ac_peak_12146	5.24695	
3	16591946	16592155	5Y-H4K8ac_peak_12147	8.43511	
3	16592555	16592891	5Y-H4K8ac_peak_12148	12.21176	
3	16925840	16926271	5Y-H4K8ac_peak_12149	14.30055	
3	17783485	17783992	5Y-H4K8ac_peak_12150	6.80915	
3	18167308	18167691	5Y-H4K8ac_peak_12151	14.55472	
3	18170013	18170226	5Y-H4K8ac_peak_12152	5.40331	
3	18180622	18180853	5Y-H4K8ac_peak_12153	9.20645	
3	18466615	18466826	5Y-H4K8ac_peak_12154	6.20875	
3	18467417	18467749	5Y-H4K8ac_peak_12155	11.93212	
3	19188761	19188986	5Y-H4K8ac_peak_12156	4.5753	KCNH8_ENSG00000183960
3	19189315	19189807	5Y-H4K8ac_peak_12157	6.08523	KCNH8_ENSG00000183960
3	20082001	20082340	5Y-H4K8ac_peak_12158	6.50117	KAT2B_ENSG00000114166
3	20227284	20227665	5Y-H4K8ac_peak_12159	11.19336	SGOL1_ENSG00000129810
3	23154247	23154475	5Y-H4K8ac_peak_12160	10.46287	
3	23753122	23753317	5Y-H4K8ac_peak_12161	5.23083	
3	23802435	23802734	5Y-H4K8ac_peak_12162	5.07473	
3	23816837	23817031	5Y-H4K8ac_peak_12163	6.50117	
3	23847472	23847812	5Y-H4K8ac_peak_12164	5.59837	UBE2E1-AS1_ENSG00000223791;UBE2E1_ENSG00000170142
3	23851892	23852309	5Y-H4K8ac_peak_12165	11.30958	
3	23958825	23959065	5Y-H4K8ac_peak_12166	6.64195	RPL15_ENSG00000174748
3	23986911	23987152	5Y-H4K8ac_peak_12167	6.50117	NKIRAS1_ENSG00000197885;NR1D2_ENSG00000174738
3	23987651	23987880	5Y-H4K8ac_peak_12168	11.19336	NKIRAS1_ENSG00000197885;NR1D2_ENSG00000174738
3	24563028	24563733	5Y-H4K8ac_peak_12169	10.46287	MIR4792_ENSG00000265028
3	24870323	24870751	5Y-H4K8ac_peak_12170	8.2589	
3	27524743	27525090	5Y-H4K8ac_peak_12171	4.77126	SLC4A7_ENSG00000033867
3	27609398	27609616	5Y-H4K8ac_peak_12172	5.01697	
3	27673974	27674305	5Y-H4K8ac_peak_12173	6.46053	AC098614.2_ENSG00000213846
3	28283234	28283456	5Y-H4K8ac_peak_12174	9.23159	CMC1_ENSG00000187118
3	28390035	28390446	5Y-H4K8ac_peak_12175	6.08523	AZI2_ENSG00000163512;ZCWPW2_ENSG00000206559
3	30648059	30648544	5Y-H4K8ac_peak_12176	8.35139	TGFBR2_ENSG00000163513
3	30732807	30733213	5Y-H4K8ac_peak_12177	5.65584	
3	31465922	31466121	5Y-H4K8ac_peak_12178	5.65584	
3	31574189	31574627	5Y-H4K8ac_peak_12179	4.77126	STT3B_ENSG00000163527
3	32147197	32147436	5Y-H4K8ac_peak_12180	5.04571	GPD1L_ENSG00000152642
3	32147657	32147874	5Y-H4K8ac_peak_12181	12.26606	GPD1L_ENSG00000152642
3	32148199	32148396	5Y-H4K8ac_peak_12182	9.30505	
3	32362745	32362935	5Y-H4K8ac_peak_12183	7.38046	
3	32443451	32443672	5Y-H4K8ac_peak_12184	5.99504	
3	32611834	32612230	5Y-H4K8ac_peak_12185	13.60015	DYNC1LI1_ENSG00000144635
3	32858132	32858521	5Y-H4K8ac_peak_12186	10.19948	TRIM71_ENSG00000206557
3	33138315	33138561	5Y-H4K8ac_peak_12187	7.50148	TMPPE_ENSG00000188167
3	33155519	33155869	5Y-H4K8ac_peak_12188	6.54441	CRTAP_ENSG00000170275

3	33482573	33483189	5Y-H4K8ac_peak_12189	10.2787	UBP1_ENSG00000153560
3	37034087	37034462	5Y-H4K8ac_peak_12190	6.20875	EPM2AIP1_ENSG00000178567;MLH1_ENSG00000076242
3	37217909	37218144	5Y-H4K8ac_peak_12191	10.1994	
3	37284202	37284669	5Y-H4K8ac_peak_12192	15.93543	RP11-259K5.2_ENSG00000270194;GOLGA4_ENSG00000144674
3	37562127	37562456	5Y-H4K8ac_peak_12193	11.71078	
3	37579133	37579671	5Y-H4K8ac_peak_12194	5.91107	
3	37743023	37743274	5Y-H4K8ac_peak_12195	5.98695	
3	37962338	37962607	5Y-H4K8ac_peak_12196	9.33466	
3	37963494	37963992	5Y-H4K8ac_peak_12197	5.23083	
3	38035679	38036048	5Y-H4K8ac_peak_12198	8.24461	
3	38180306	38180654	5Y-H4K8ac_peak_12199	5.24695	MYD88_ENSG00000172936
3	38355871	38356126	5Y-H4K8ac_peak_12200	9.23159	
3	38495630	38496161	5Y-H4K8ac_peak_12201	11.22005	ACVR2B-AS1_ENSG00000229589;ACVR2B_ENSG00000114739
3	38496613	38496810	5Y-H4K8ac_peak_12202	7.87406	ACVR2B-AS1_ENSG00000229589
3	38620970	38621667	5Y-H4K8ac_peak_12203	12.2918	
3	38622187	38622421	5Y-H4K8ac_peak_12204	11.1169	
3	39092881	39093740	5Y-H4K8ac_peak_12205	5.12488	WDR48_ENSG00000114742
3	39188942	39189360	5Y-H4K8ac_peak_12206	7.64675	
3	39543737	39544381	5Y-H4K8ac_peak_12207	10.37534	
3	39851267	39851626	5Y-H4K8ac_peak_12208	6.77436	MYRIP_ENSG00000170011
3	40351281	40351611	5Y-H4K8ac_peak_12209	7.72887	EIF1B_ENSG00000114784
3	40494197	40494556	5Y-H4K8ac_peak_12210	7.76232	ENTPD3-AS1_ENSG00000223797
3	40518383	40518891	5Y-H4K8ac_peak_12211	13.60015	ZNF619_ENSG00000177873
3	40566554	40566969	5Y-H4K8ac_peak_12212	5.41472	ZNF621_ENSG00000172888
3	40656686	40657073	5Y-H4K8ac_peak_12213	5.23083	
3	41240318	41240730	5Y-H4K8ac_peak_12214	7.30348	
3	41241067	41241301	5Y-H4K8ac_peak_12215	8.43511	
3	42054615	42054936	5Y-H4K8ac_peak_12216	9.30505	TRAK1_ENSG00000182606
3	42623200	42623413	5Y-H4K8ac_peak_12217	7.89273	SS18L2_ENSG00000008324
3	42632613	42632836	5Y-H4K8ac_peak_12218	4.77126	
3	42642242	42642669	5Y-H4K8ac_peak_12219	7.50148	SEC22C_ENSG00000093183;NKTR_ENSG00000114857
3	42696069	42696342	5Y-H4K8ac_peak_12220	13.00733	ZBTB47_ENSG00000114853;RP4-613B23.1_ENSG00000230084
3	42888393	42888645	5Y-H4K8ac_peak_12221	7.29091	
3	42922021	42923015	5Y-H4K8ac_peak_12222	18.10232	
3	42947434	42947719	5Y-H4K8ac_peak_12223	5.23083	ZNF662_ENSG00000182983
3	43327097	43327363	5Y-H4K8ac_peak_12224	4.24332	SNRK_ENSG00000163788
3	43731807	43732027	5Y-H4K8ac_peak_12225	7.90236	ABHD5_ENSG00000011198
3	43732587	43732968	5Y-H4K8ac_peak_12226	13.2534	ANO10_ENSG00000160746;ABHD5_ENSG00000011198
3	43811136	43811359	5Y-H4K8ac_peak_12227	12.05638	
3	43811560	43812099	5Y-H4K8ac_peak_12228	10.15788	
3	43812775	43813133	5Y-H4K8ac_peak_12229	6.53157	
3	43814971	43815181	5Y-H4K8ac_peak_12230	12.10416	
3	44001097	44001547	5Y-H4K8ac_peak_12231	6.36454	
3	44036739	44037257	5Y-H4K8ac_peak_12232	7.11863	
3	44040004	44040587	5Y-H4K8ac_peak_12233	12.10416	RP4-555D20.4_ENSG00000272121
3	44063022	44063546	5Y-H4K8ac_peak_12234	6.34046	

3	44379683	44380161	5Y-H4K8ac_peak_12235	6.53157	RP11-424N24.2_ENSG00000271937;TCAIM_ENSG00000179152
3	44519197	44519840	5Y-H4K8ac_peak_12236	19.2548	ZNF445_ENSG00000185219
3	44596340	44596603	5Y-H4K8ac_peak_12237	8.24461	ZKSCAN7_ENSG00000196345
3	44625888	44626148	5Y-H4K8ac_peak_12238	6.17374	ZNF197_ENSG00000186448
3	44626427	44626643	5Y-H4K8ac_peak_12239	11.1169	ZNF197_ENSG00000186448
3	44690360	44690584	5Y-H4K8ac_peak_12240	5.98695	ZNF35_ENSG00000169981
3	44726855	44727049	5Y-H4K8ac_peak_12241	5.64909	RP11-944L7.4_ENSG00000236869
3	44770561	44770955	5Y-H4K8ac_peak_12242	9.20645	ZNF501_ENSG00000186446
3	45208825	45209099	5Y-H4K8ac_peak_12243	8.21525	
3	45589213	45589565	5Y-H4K8ac_peak_12244	5.98695	
3	45624631	45624849	5Y-H4K8ac_peak_12245	4.29586	
3	45635300	45635493	5Y-H4K8ac_peak_12246	7.17527	
3	45883777	45884119	5Y-H4K8ac_peak_12247	7.76232	
3	46599553	46599748	5Y-H4K8ac_peak_12248	4.07874	LUZPP1_ENSG00000268324
3	46607454	46607789	5Y-H4K8ac_peak_12249	9.30206	
3	46701686	46701883	5Y-H4K8ac_peak_12250	5.94703	
3	46703981	46704399	5Y-H4K8ac_peak_12251	8.31252	
3	46704662	46705013	5Y-H4K8ac_peak_12252	4.74398	
3	46735347	46735567	5Y-H4K8ac_peak_12253	5.98695	ALS2CL_ENSG00000178038
3	46741690	46742145	5Y-H4K8ac_peak_12254	4.52203	TMIE_ENSG00000181585
3	46742931	46743190	5Y-H4K8ac_peak_12255	10.21117	TMIE_ENSG00000181585
3	46886582	46887416	5Y-H4K8ac_peak_12256	7.90751	
3	46887719	46888105	5Y-H4K8ac_peak_12257	12.84845	
3	46906406	46906617	5Y-H4K8ac_peak_12258	5.78188	
3	46951406	46951654	5Y-H4K8ac_peak_12259	7.31102	
3	46989837	46990265	5Y-H4K8ac_peak_12260	7.24844	
3	47021589	47021813	5Y-H4K8ac_peak_12261	8.2913	NBEAL2_ENSG00000160796
3	47412258	47412623	5Y-H4K8ac_peak_12262	5.77946	
3	47421155	47421491	5Y-H4K8ac_peak_12263	6.73385	RP11-708J19.1_ENSG00000260236
3	47554534	47554770	5Y-H4K8ac_peak_12264	4.70501	ELP6_ENSG00000163832
3	47555747	47556202	5Y-H4K8ac_peak_12265	4.95697	ELP6_ENSG00000163832
3	47563899	47564109	5Y-H4K8ac_peak_12266	5.64909	
3	47620504	47620792	5Y-H4K8ac_peak_12267	5.65584	
3	47621088	47621296	5Y-H4K8ac_peak_12268	5.23083	CSPG5_ENSG00000114646
3	47822906	47823341	5Y-H4K8ac_peak_12269	21.37039	SMARCC1_ENSG00000173473
3	47823544	47823983	5Y-H4K8ac_peak_12270	10.6025	SMARCC1_ENSG00000173473
3	47844209	47845172	5Y-H4K8ac_peak_12271	12.26606	DHX30_ENSG00000132153
3	48130658	48130874	5Y-H4K8ac_peak_12272	4.07874	MAP4_ENSG00000047849
3	48230221	48230573	5Y-H4K8ac_peak_12273	20.25522	CDC25A_ENSG00000164045
3	48282727	48282935	5Y-H4K8ac_peak_12274	6.73385	ZNF589_ENSG00000164048
3	48342637	48342863	5Y-H4K8ac_peak_12275	9.00954	NME6_ENSG00000172113
3	48470280	48471370	5Y-H4K8ac_peak_12276	12.10416	PLXNB1_ENSG00000164050
3	48507274	48507740	5Y-H4K8ac_peak_12277	7.58806	TREX1_ENSG00000213689
3	48540484	48540730	5Y-H4K8ac_peak_12278	4.29586	
3	48540922	48541514	5Y-H4K8ac_peak_12279	15.40182	SHISA5_ENSG00000164054
3	48632489	48632691	5Y-H4K8ac_peak_12280	7.38046	COL7A1_ENSG00000114270

3	48647230	48647939	5Y-H4K8ac_peak_12281	9.63153	UQCRC1_ENSG00000010256
3	48672386	48672591	5Y-H4K8ac_peak_12282	6.22669	SLC26A6_ENSG00000225697
3	48700590	48701071	5Y-H4K8ac_peak_12283	13.12848	CELSR3_ENSG00000008300;RP11-148G20.1_ENSG00000228350
3	48754120	48754487	5Y-H4K8ac_peak_12284	13.2534	
3	48777750	48777993	5Y-H4K8ac_peak_12285	7.64648	IP6K2_ENSG00000068745
3	49035994	49036313	5Y-H4K8ac_peak_12286	7.50501	
3	49044113	49044348	5Y-H4K8ac_peak_12287	5.87725	WDR6_ENSG00000178252
3	49044855	49045252	5Y-H4K8ac_peak_12288	14.43262	WDR6_ENSG00000178252
3	49059129	49059387	5Y-H4K8ac_peak_12289	9.68742	DALRD3_ENSG00000178149;MIR191_ENSG00000207605
3	49059667	49060117	5Y-H4K8ac_peak_12290	5.23083	DALRD3_ENSG00000178149
3	49131110	49131423	5Y-H4K8ac_peak_12291	5.35202	QRICH1_ENSG00000198218
3	49141944	49142144	5Y-H4K8ac_peak_12292	9.00954	QARS_ENSG00000172053
3	49157844	49158818	5Y-H4K8ac_peak_12293	18.62509	USP19_ENSG00000172046
3	49169633	49170086	5Y-H4K8ac_peak_12294	4.29586	LAMB2_ENSG00000172037
3	49203139	49204465	5Y-H4K8ac_peak_12295	13.92877	CCDC71_ENSG00000177352
3	49208520	49208812	5Y-H4K8ac_peak_12296	4.0639	KLHDC8B_ENSG00000185909
3	49210152	49210609	5Y-H4K8ac_peak_12297	10.24719	
3	49377804	49378782	5Y-H4K8ac_peak_12298	6.08523	USP4_ENSG00000114316
3	49395275	49396361	5Y-H4K8ac_peak_12299	8.69112	GPX1_ENSG00000233276
3	49449827	49450060	5Y-H4K8ac_peak_12300	10.19948	RHOA_ENSG00000067560;TCTA_ENSG00000145022
3	49459413	49459617	5Y-H4K8ac_peak_12301	6.50117	AMT_ENSG00000145020;NICN1-AS1_ENSG00000235261
3	49576976	49577197	5Y-H4K8ac_peak_12302	6.14981	
3	49577574	49577810	5Y-H4K8ac_peak_12303	6.78128	
3	49591446	49591739	5Y-H4K8ac_peak_12304	6.37023	BSN-AS2_ENSG00000226913;BSN_ENSG00000164061
3	49710743	49711111	5Y-H4K8ac_peak_12305	6.37023	APEH_ENSG00000164062
3	49711457	49711656	5Y-H4K8ac_peak_12306	9.02782	APEH_ENSG00000164062
3	49724379	49724586	5Y-H4K8ac_peak_12307	4.77126	
3	49726313	49726707	5Y-H4K8ac_peak_12308	7.61515	MST1_ENSG00000173531;RNF123_ENSG00000164068
3	49824252	49824857	5Y-H4K8ac_peak_12309	17.18444	IP6K1_ENSG00000176095
3	49843091	49843513	5Y-H4K8ac_peak_12310	15.82359	MIR5193_ENSG00000263506
3	49843944	49844473	5Y-H4K8ac_peak_12311	7.89273	MIR5193_ENSG00000263506
3	49844827	49845989	5Y-H4K8ac_peak_12312	8.00646	
3	49907016	49907305	5Y-H4K8ac_peak_12313	10.31019	CAMKV_ENSG00000164076
3	49910923	49911154	5Y-H4K8ac_peak_12314	9.23159	
3	49976988	49977278	5Y-H4K8ac_peak_12315	8.33296	RBM6_ENSG00000004534
3	50126421	50126726	5Y-H4K8ac_peak_12316	7.18391	RBM5_ENSG00000003756
3	50175748	50176339	5Y-H4K8ac_peak_12317	5.37137	
3	50188693	50188891	5Y-H4K8ac_peak_12318	4.29586	
3	50242169	50242476	5Y-H4K8ac_peak_12319	5.83797	SLC38A3_ENSG00000188338
3	50263862	50264265	5Y-H4K8ac_peak_12320	5.75508	GNAI2_ENSG00000114353
3	50265151	50265391	5Y-H4K8ac_peak_12321	10.16277	
3	50283218	50283731	5Y-H4K8ac_peak_12322	15.82359	
3	50283925	50284127	5Y-H4K8ac_peak_12323	4.77126	
3	50297739	50298542	5Y-H4K8ac_peak_12324	22.46875	U73166.2_ENSG00000230454
3	50330280	50330590	5Y-H4K8ac_peak_12325	11.48691	IFRD2_ENSG00000214706
3	50340870	50341075	5Y-H4K8ac_peak_12326	8.43511	

3	50358629	50359684	5Y-H4K8ac_peak_12327	14.99673	HYAL2_ENSG00000068001
3	50375153	50375429	5Y-H4K8ac_peak_12328	8.46442	
3	50375818	50376020	5Y-H4K8ac_peak_12329	8.38675	
3	50396651	50396927	5Y-H4K8ac_peak_12330	5.56894	TMEM115_ENSG00000126062
3	50408359	50408740	5Y-H4K8ac_peak_12331	10.69698	
3	50413716	50413970	5Y-H4K8ac_peak_12332	6.19178	
3	50415101	50415602	5Y-H4K8ac_peak_12333	5.12213	
3	50416495	50417441	5Y-H4K8ac_peak_12334	10.31981	
3	50418114	50418855	5Y-H4K8ac_peak_12335	13.35169	
3	50419529	50419917	5Y-H4K8ac_peak_12336	7.89273	
3	50422757	50423209	5Y-H4K8ac_peak_12337	7.50148	
3	50423522	50424110	5Y-H4K8ac_peak_12338	12.05638	
3	50424714	50425414	5Y-H4K8ac_peak_12339	19.26009	
3	50425783	50426626	5Y-H4K8ac_peak_12340	10.35586	
3	50426956	50427196	5Y-H4K8ac_peak_12341	9.51254	
3	50427797	50428255	5Y-H4K8ac_peak_12342	15.25579	
3	50428699	50429581	5Y-H4K8ac_peak_12343	11.30958	
3	50449195	50449492	5Y-H4K8ac_peak_12344	6.1654	
3	50463333	50463527	5Y-H4K8ac_peak_12345	5.87725	
3	50463752	50464845	5Y-H4K8ac_peak_12346	12.1432	
3	50465061	50465691	5Y-H4K8ac_peak_12347	16.56941	
3	50466113	50466640	5Y-H4K8ac_peak_12348	11.94897	
3	50467391	50468259	5Y-H4K8ac_peak_12349	12.79198	
3	50468931	50469291	5Y-H4K8ac_peak_12350	8.31536	
3	50469739	50470393	5Y-H4K8ac_peak_12351	16.41521	
3	50470833	50471039	5Y-H4K8ac_peak_12352	6.00747	
3	50472010	50472819	5Y-H4K8ac_peak_12353	7.54769	
3	50473281	50473488	5Y-H4K8ac_peak_12354	8.73392	
3	50475539	50475885	5Y-H4K8ac_peak_12355	6.03632	
3	50540487	50540681	5Y-H4K8ac_peak_12356	6.31818	CACNA2D2_ENSG00000007402
3	50541234	50541673	5Y-H4K8ac_peak_12357	6.43775	CACNA2D2_ENSG00000007402
3	50551864	50552987	5Y-H4K8ac_peak_12358	7.59101	
3	50553692	50553907	5Y-H4K8ac_peak_12359	5.41472	
3	50554180	50554541	5Y-H4K8ac_peak_12360	11.09973	
3	50554864	50555127	5Y-H4K8ac_peak_12361	10.35586	
3	50563483	50563806	5Y-H4K8ac_peak_12362	4.36976	
3	50571349	50571712	5Y-H4K8ac_peak_12363	6.00382	
3	50579621	50579839	5Y-H4K8ac_peak_12364	10.31981	
3	50605369	50605739	5Y-H4K8ac_peak_12365	4.5753	HEMK1_ENSG00000114735
3	50606633	50607068	5Y-H4K8ac_peak_12366	7.90236	HEMK1_ENSG00000114735
3	50638494	50639254	5Y-H4K8ac_peak_12367	10.54764	
3	51421734	51422372	5Y-H4K8ac_peak_12368	15.92074	MANF_ENSG00000145050
3	51428852	51429417	5Y-H4K8ac_peak_12369	7.90751	RBM15B_ENSG00000179837
3	51571662	51572214	5Y-H4K8ac_peak_12370	6.55906	
3	51572831	51573161	5Y-H4K8ac_peak_12371	7.59101	
3	51705233	51705913	5Y-H4K8ac_peak_12372	7.11863	

3	51846492	51846682	5Y-H4K8ac_peak_12373	4.84727	
3	51848239	51848529	5Y-H4K8ac_peak_12374	5.5192	
3	51976490	51977221	5Y-H4K8ac_peak_12375	9.36633	RRP9_ENSG00000114767;PARP3_ENSG00000041880
3	51990078	51990374	5Y-H4K8ac_peak_12376	5.98695	GPR62_ENSG00000180929
3	52000121	52000531	5Y-H4K8ac_peak_12377	8.12305	
3	52000954	52001357	5Y-H4K8ac_peak_12378	5.87725	
3	52001661	52002319	5Y-H4K8ac_peak_12379	17.97634	PCBP4_ENSG00000090097
3	52009090	52009345	5Y-H4K8ac_peak_12380	4.67245	ACY1_ENSG00000243989;ABHD14A-ACY1_ENSG00000114786
3	52017230	52017714	5Y-H4K8ac_peak_12381	5.0789	ABHD14B_ENSG00000114779
3	52029361	52029856	5Y-H4K8ac_peak_12382	9.179	RPL29_ENSG00000162244
3	52030148	52030349	5Y-H4K8ac_peak_12383	6.50117	RPL29_ENSG00000162244
3	52040575	52040968	5Y-H4K8ac_peak_12384	8.82628	
3	52049385	52049584	5Y-H4K8ac_peak_12385	5.87725	
3	52050074	52050469	5Y-H4K8ac_peak_12386	4.0639	
3	52053170	52053385	5Y-H4K8ac_peak_12387	6.59112	
3	52056435	52056645	5Y-H4K8ac_peak_12388	7.59101	
3	52058143	52058357	5Y-H4K8ac_peak_12389	4.88619	
3	52059086	52059587	5Y-H4K8ac_peak_12390	8.19376	
3	52062203	52063184	5Y-H4K8ac_peak_12391	18.78631	
3	52063526	52064951	5Y-H4K8ac_peak_12392	16.79568	
3	52065713	52066175	5Y-H4K8ac_peak_12393	8.62703	
3	52068524	52068789	5Y-H4K8ac_peak_12394	6.2526	
3	52070015	52070370	5Y-H4K8ac_peak_12395	11.17333	
3	52072154	52072393	5Y-H4K8ac_peak_12396	6.37023	
3	52075824	52076088	5Y-H4K8ac_peak_12397	11.1169	
3	52078298	52078524	5Y-H4K8ac_peak_12398	7.37325	
3	52079279	52079557	5Y-H4K8ac_peak_12399	7.73231	
3	52090691	52090932	5Y-H4K8ac_peak_12400	9.92042	DUSP7_ENSG00000164086
3	52091687	52091910	5Y-H4K8ac_peak_12401	9.36633	
3	52100920	52101537	5Y-H4K8ac_peak_12402	16.51777	
3	52102462	52103261	5Y-H4K8ac_peak_12403	15.82359	
3	52231667	52231896	5Y-H4K8ac_peak_12404	5.51759	ALAS1_ENSG00000023330
3	52273346	52274153	5Y-H4K8ac_peak_12405	13.4304	TLR9_ENSG00000239732;TWF2_ENSG00000247596;RP5-1157M23.2_ENSG00000243224
3	52274391	52274583	5Y-H4K8ac_peak_12406	8.43999	
3	52312298	52312620	5Y-H4K8ac_peak_12407	9.00954	
3	52312902	52313286	5Y-H4K8ac_peak_12408	6.73047	
3	52320698	52320928	5Y-H4K8ac_peak_12409	6.22669	
3	52321771	52322110	5Y-H4K8ac_peak_12410	24.73897	WDR82_ENSG00000164091
3	52333206	52333713	5Y-H4K8ac_peak_12411	10.1994	
3	52342062	52342512	5Y-H4K8ac_peak_12412	7.38046	
3	52345077	52346350	5Y-H4K8ac_peak_12413	16.50659	
3	52347820	52348021	5Y-H4K8ac_peak_12414	4.96019	
3	52443580	52443922	5Y-H4K8ac_peak_12415	7.38046	BAP1_ENSG00000163930;PHF7_ENSG00000010318
3	52444642	52444851	5Y-H4K8ac_peak_12416	7.9412	BAP1_ENSG00000163930;PHF7_ENSG00000010318
3	52489193	52489464	5Y-H4K8ac_peak_12417	8.62703	NISCH_ENSG00000010322
3	52568085	52568322	5Y-H4K8ac_peak_12418	9.58986	NT5DC2_ENSG00000168268;SMIM4_ENSG00000168273

3	52740222	52740440	5Y-H4K8ac_peak_12419	6.43775	
3	53079055	53079250	5Y-H4K8ac_peak_12420	8.43511	
3	53079694	53079980	5Y-H4K8ac_peak_12421	9.60627	SFMBT1_ENSG00000163935
3	53080187	53080579	5Y-H4K8ac_peak_12422	8.2913	SFMBT1_ENSG00000163935
3	53164136	53164383	5Y-H4K8ac_peak_12423	10.12457	RFT1_ENSG00000163933
3	53229829	53230031	5Y-H4K8ac_peak_12424	4.77126	
3	53304225	53304553	5Y-H4K8ac_peak_12425	10.11191	
3	53381802	53382003	5Y-H4K8ac_peak_12426	11.48691	DCP1A_ENSG00000162290
3	53528188	53528532	5Y-H4K8ac_peak_12427	5.23083	CACNA1D_ENSG00000157388
3	53568415	53568675	5Y-H4K8ac_peak_12428	5.8635	
3	53584115	53584393	5Y-H4K8ac_peak_12429	5.93823	
3	53807407	53807602	5Y-H4K8ac_peak_12430	10.10203	
3	53879950	53880835	5Y-H4K8ac_peak_12431	17.56739	CHDH_ENSG00000016391;IL17RB_ENSG00000056736
3	53915910	53916100	5Y-H4K8ac_peak_12432	11.88337	ACTR8_ENSG00000113812;AC012467.1_ENSG00000222888
3	55634852	55635055	5Y-H4K8ac_peak_12433	7.34185	
3	56716678	56717669	5Y-H4K8ac_peak_12434	11.69482	FAM208A_ENSG00000163946
3	56840154	56840460	5Y-H4K8ac_peak_12435	5.23083	
3	57010803	57011010	5Y-H4K8ac_peak_12436	8.2913	
3	57011950	57012160	5Y-H4K8ac_peak_12437	7.46096	
3	57019759	57020138	5Y-H4K8ac_peak_12438	7.64675	
3	57153824	57154071	5Y-H4K8ac_peak_12439	9.34555	
3	57155567	57155880	5Y-H4K8ac_peak_12440	5.00877	
3	57157132	57157333	5Y-H4K8ac_peak_12441	7.30348	
3	57158939	57159234	5Y-H4K8ac_peak_12442	6.7002	
3	57161615	57161842	5Y-H4K8ac_peak_12443	6.19178	
3	57163449	57164028	5Y-H4K8ac_peak_12444	19.11901	
3	57164223	57164721	5Y-H4K8ac_peak_12445	12.19856	
3	57165317	57165510	5Y-H4K8ac_peak_12446	8.4454	
3	57166086	57166416	5Y-H4K8ac_peak_12447	4.50834	
3	57169072	57169398	5Y-H4K8ac_peak_12448	4.77126	RP11-157F20.1_ENSG00000229863
3	57177190	57177566	5Y-H4K8ac_peak_12449	6.50117	
3	57180422	57180614	5Y-H4K8ac_peak_12450	6.98118	
3	57186179	57186407	5Y-H4K8ac_peak_12451	7.3889	
3	57191215	57191681	5Y-H4K8ac_peak_12452	4.15658	
3	57198464	57199302	5Y-H4K8ac_peak_12453	12.76485	
3	57203934	57204229	5Y-H4K8ac_peak_12454	10.46287	IL17RD_ENSG00000144730
3	57542072	57542370	5Y-H4K8ac_peak_12455	9.02782	PDE12_ENSG00000174840
3	58000252	58000887	5Y-H4K8ac_peak_12456	8.21582	
3	58163486	58163787	5Y-H4K8ac_peak_12457	7.11863	
3	58222835	58223079	5Y-H4K8ac_peak_12458	8.01306	ABHD6_ENSG00000163686
3	58318087	58318491	5Y-H4K8ac_peak_12459	11.53136	PXK_ENSG00000168297
3	58477010	58477466	5Y-H4K8ac_peak_12460	8.35139	RP11-359I18.5_ENSG00000272360;KCTD6_ENSG00000168301
3	58542410	58542623	5Y-H4K8ac_peak_12461	4.84727	
3	58566780	58567065	5Y-H4K8ac_peak_12462	9.93099	
3	59035359	59035686	5Y-H4K8ac_peak_12463	9.79526	C3orf67_ENSG00000163689
3	61235820	61236084	5Y-H4K8ac_peak_12464	4.8773	

3	62174815	62175097	5Y-H4K8ac_peak_12465	8.17203	
3	62573297	62573776	5Y-H4K8ac_peak_12466	5.65584	
3	63848902	63849458	5Y-H4K8ac_peak_12467	9.01738	THOC7_ENSG00000163634;ATXN7_ENSG00000163635
3	63849770	63849997	5Y-H4K8ac_peak_12468	7.58806	THOC7_ENSG00000163634;ATXN7_ENSG00000163635
3	64008694	64009016	5Y-H4K8ac_peak_12469	5.99504	PSMD6_ENSG00000163636
3	64661046	64661318	5Y-H4K8ac_peak_12470	12.21176	
3	64662496	64662910	5Y-H4K8ac_peak_12471	10.32373	
3	64673034	64673323	5Y-H4K8ac_peak_12472	8.43511	ADAMTS9_ENSG00000163638
3	64841422	64841622	5Y-H4K8ac_peak_12473	10.46287	
3	64842175	64842411	5Y-H4K8ac_peak_12474	6.20875	
3	64843302	64843512	5Y-H4K8ac_peak_12475	9.30505	
3	65221045	65221264	5Y-H4K8ac_peak_12476	5.98695	
3	65551231	65551457	5Y-H4K8ac_peak_12477	7.72345	
3	66025002	66025556	5Y-H4K8ac_peak_12478	12.21176	MAGI1_ENSG00000151276
3	66093397	66093671	5Y-H4K8ac_peak_12479	9.13822	
3	67704751	67705545	5Y-H4K8ac_peak_12480	15.80786	SUCLG2_ENSG00000172340;RP11-81N13.1_ENSG00000241316
3	68981404	68981645	5Y-H4K8ac_peak_12481	8.17203	FAM19A4_ENSG00000163377
3	69062385	69062804	5Y-H4K8ac_peak_12482	13.00733	EOGT_ENSG00000163378;CTD-2013N24.2_ENSG00000244513
3	69134550	69134977	5Y-H4K8ac_peak_12483	6.50117	ARL6IP5_ENSG00000144746
3	71114420	71114676	5Y-H4K8ac_peak_12484	7.59101	
3	71632877	71633095	5Y-H4K8ac_peak_12485	9.56187	FOXP1_ENSG00000114861;RP11-154H23.3_ENSG00000270562
3	71774665	71775043	5Y-H4K8ac_peak_12486	13.2534	
3	71802945	71803152	5Y-H4K8ac_peak_12487	4.29586	EIF4E3_ENSG00000163412;GPR27_ENSG00000170837
3	71834307	71834614	5Y-H4K8ac_peak_12488	8.00339	PROK2_ENSG00000163421
3	72005666	72005857	5Y-H4K8ac_peak_12489	4.84727	
3	72294119	72294314	5Y-H4K8ac_peak_12490	7.01266	
3	72304622	72305130	5Y-H4K8ac_peak_12491	7.04637	
3	72307881	72308296	5Y-H4K8ac_peak_12492	6.49308	
3	72338037	72338841	5Y-H4K8ac_peak_12493	8.90038	
3	72353180	72353378	5Y-H4K8ac_peak_12494	6.77436	
3	72378421	72378709	5Y-H4K8ac_peak_12495	5.67283	
3	72496566	72496927	5Y-H4K8ac_peak_12496	5.41472	RYBP_ENSG00000163602
3	72787623	72787892	5Y-H4K8ac_peak_12497	6.79955	
3	72788101	72788823	5Y-H4K8ac_peak_12498	10.46287	
3	72846253	72846670	5Y-H4K8ac_peak_12499	5.40331	
3	73045382	73045775	5Y-H4K8ac_peak_12500	5.91107	PPP4R2_ENSG00000163605
3	73321782	73321991	5Y-H4K8ac_peak_12501	6.98118	
3	73402770	73403061	5Y-H4K8ac_peak_12502	4.9885	
3	73673520	73674127	5Y-H4K8ac_peak_12503	14.31711	PDZRN3_ENSG00000121440
3	75834365	75834649	5Y-H4K8ac_peak_12504	11.57334	ZNF717_ENSG00000227124
3	85553763	85554118	5Y-H4K8ac_peak_12505	12.49811	
3	93560343	93560558	5Y-H4K8ac_peak_12506	8.33296	
3	93781267	93781480	5Y-H4K8ac_peak_12507	4.24869	DHFRL1_ENSG00000178700;NSUN3_ENSG00000178694
3	93781913	93782121	5Y-H4K8ac_peak_12508	10.1994	DHFRL1_ENSG00000178700;NSUN3_ENSG00000178694
3	94131927	94132119	5Y-H4K8ac_peak_12509	6.50117	
3	96532252	96532661	5Y-H4K8ac_peak_12510	5.12488	EPHA6_ENSG00000080224

3	97540865	97541187	5Y-H4K8ac_peak_12511	10.95042	CRYBG3_ENSG00000233280
3	98241388	98241705	5Y-H4K8ac_peak_12512	6.50117	CLDND1_ENSG00000080822;RP11-227H4.5_ENSG00000248839
3	98312119	98312724	5Y-H4K8ac_peak_12513	8.4454	CPOX_ENSG00000080819
3	98451376	98451579	5Y-H4K8ac_peak_12514	7.01266	ST3GAL6-AS1_ENSG00000239445;ST3GAL6_ENSG00000064225
3	99979897	99980238	5Y-H4K8ac_peak_12515	10.35586	TBC1D23_ENSG00000036054
3	100119487	100119819	5Y-H4K8ac_peak_12516	6.54441	TOMM70A_ENSG00000154174;LNP1_ENSG00000206535
3	100122407	100122599	5Y-H4K8ac_peak_12517	8.93304	
3	100210881	100211218	5Y-H4K8ac_peak_12518	5.23083	TMEM45A_ENSG00000181458
3	101231665	101231986	5Y-H4K8ac_peak_12519	9.38203	SENP7_ENSG00000138468
3	101280308	101280560	5Y-H4K8ac_peak_12520	12.21176	TRMT10C_ENSG00000174173
3	101293187	101293454	5Y-H4K8ac_peak_12521	11.36989	PCNP_ENSG00000081154
3	101405143	101405373	5Y-H4K8ac_peak_12522	4.9885	RPL24_ENSG00000114391
3	101405887	101406133	5Y-H4K8ac_peak_12523	8.79957	RPL24_ENSG00000114391
3	101567723	101568117	5Y-H4K8ac_peak_12524	11.19336	
3	105072616	105072853	5Y-H4K8ac_peak_12525	6.94416	
3	105223428	105223684	5Y-H4K8ac_peak_12526	7.31815	
3	107149819	107150823	5Y-H4K8ac_peak_12527	14.99673	RP11-115H18.1_ENSG00000273125
3	107700609	107700911	5Y-H4K8ac_peak_12528	6.34046	
3	107809975	107810591	5Y-H4K8ac_peak_12529	15.82713	CD47_ENSG00000196776
3	107940814	107941038	5Y-H4K8ac_peak_12530	10.46287	IFT57_ENSG00000114446
3	111697941	111698206	5Y-H4K8ac_peak_12531	4.642	ABHD10_ENSG00000144827
3	112280491	112280711	5Y-H4K8ac_peak_12532	7.76232	ATG3_ENSG00000144848;SLC35A5_ENSG00000138459
3	112738122	112738500	5Y-H4K8ac_peak_12533	14.43262	C3orf17_ENSG00000163608;RP11-572M11.4_ENSG00000240057
3	112866820	112867082	5Y-H4K8ac_peak_12534	7.11863	
3	112930461	112931210	5Y-H4K8ac_peak_12535	9.93099	BOC_ENSG00000144857
3	112940695	112940953	5Y-H4K8ac_peak_12536	9.30505	
3	113008450	113008721	5Y-H4K8ac_peak_12537	10.12234	
3	113234179	113234379	5Y-H4K8ac_peak_12538	5.8635	SPICE1_ENSG00000163611
3	113464504	113464919	5Y-H4K8ac_peak_12539	6.73385	NAA50_ENSG00000121579;ATP6V1A_ENSG00000114573
3	113666456	113666830	5Y-H4K8ac_peak_12540	7.3889	RP11-255E6.6_ENSG00000273394;ZDHHC23_ENSG00000184307
3	114958557	114958823	5Y-H4K8ac_peak_12541	7.31815	
3	115231459	115231782	5Y-H4K8ac_peak_12542	5.56912	
3	115237843	115238070	5Y-H4K8ac_peak_12543	4.84727	
3	115377126	115377930	5Y-H4K8ac_peak_12544	21.28918	RP11-326J18.1_ENSG00000241596
3	115503003	115503264	5Y-H4K8ac_peak_12545	14.30055	
3	116163793	116164008	5Y-H4K8ac_peak_12546	9.38203	
3	116193384	116193601	5Y-H4K8ac_peak_12547	4.9885	
3	117345057	117345291	5Y-H4K8ac_peak_12548	6.50117	
3	117632670	117632903	5Y-H4K8ac_peak_12549	9.20645	
3	117635035	117635239	5Y-H4K8ac_peak_12550	8.2913	
3	119011675	119011895	5Y-H4K8ac_peak_12551	7.21112	
3	119013220	119013867	5Y-H4K8ac_peak_12552	7.89273	ARHGAP31_ENSG00000031081
3	119016367	119016563	5Y-H4K8ac_peak_12553	5.62788	RPS26P21_ENSG00000242829
3	119041369	119042321	5Y-H4K8ac_peak_12554	15.84891	ARHGAP31-AS1_ENSG00000241155
3	119050995	119051321	5Y-H4K8ac_peak_12555	7.11863	
3	119052783	119053501	5Y-H4K8ac_peak_12556	14.99673	

3	119182152	119182490	5Y-H4K8ac_peak_12557	7.59101	
3	119187657	119188059	5Y-H4K8ac_peak_12558	7.89273	TMEM39A_ENSG00000176142
3	119217499	119217704	5Y-H4K8ac_peak_12559	5.70472	RP11-190C22.8_ENSG00000272662;TIMMDC1_ENSG00000113845
3	119218190	119218418	5Y-H4K8ac_peak_12560	5.77946	TIMMDC1_ENSG00000113845
3	119298284	119298781	5Y-H4K8ac_peak_12561	3.93327	RP11-190C22.9_ENSG00000272967;ADPRH_ENSG00000144843
3	119352058	119352250	5Y-H4K8ac_peak_12562	5.26353	
3	119356522	119356739	5Y-H4K8ac_peak_12563	10.69698	
3	119357286	119357670	5Y-H4K8ac_peak_12564	6.50117	
3	119378724	119379267	5Y-H4K8ac_peak_12565	16.59095	
3	119396006	119396241	5Y-H4K8ac_peak_12566	5.77946	COX17_ENSG00000138495
3	119814112	119814589	5Y-H4K8ac_peak_12567	18.06205	GSK3B_ENSG00000082701;RP11-18H7.1_ENSG00000242622
3	120003987	120004378	5Y-H4K8ac_peak_12568	16.52279	GPR156_ENSG00000175697
3	120067782	120068152	5Y-H4K8ac_peak_12569	4.95697	LRRC58_ENSG00000163428;RP11-174O3.3_ENSG00000240661
3	120170016	120170476	5Y-H4K8ac_peak_12570	7.89273	FSTL1_ENSG00000163430
3	120278001	120278330	5Y-H4K8ac_peak_12571	9.38203	
3	120315360	120315565	5Y-H4K8ac_peak_12572	5.87725	NDUFB4_ENSG00000065518
3	121264403	121264760	5Y-H4K8ac_peak_12573	5.65584	POLQ_ENSG00000051341
3	122282970	122283183	5Y-H4K8ac_peak_12574	7.20869	PARP9_ENSG00000138496;DTX3L_ENSG00000163840
3	122283378	122283604	5Y-H4K8ac_peak_12575	11.62198	PARP9_ENSG00000138496;DTX3L_ENSG00000163840
3	122656223	122656501	5Y-H4K8ac_peak_12576	8.73985	
3	122656776	122656977	5Y-H4K8ac_peak_12577	7.11863	
3	122660065	122660318	5Y-H4K8ac_peak_12578	10.31019	
3	122662146	122662538	5Y-H4K8ac_peak_12579	10.69698	
3	122693269	122693513	5Y-H4K8ac_peak_12580	10.1994	
3	122693863	122694193	5Y-H4K8ac_peak_12581	13.99721	
3	122695031	122695328	5Y-H4K8ac_peak_12582	7.59101	
3	122711712	122712056	5Y-H4K8ac_peak_12583	10.1994	
3	122714822	122715056	5Y-H4K8ac_peak_12584	4.728	
3	122744908	122745175	5Y-H4K8ac_peak_12585	5.35202	
3	122745981	122746276	5Y-H4K8ac_peak_12586	7.50148	
3	122746864	122747282	5Y-H4K8ac_peak_12587	7.01266	SEMA5B_ENSG00000082684
3	122765620	122765826	5Y-H4K8ac_peak_12588	4.50834	
3	122804433	122804648	5Y-H4K8ac_peak_12589	4.95697	
3	122859453	122859749	5Y-H4K8ac_peak_12590	4.00285	
3	123064020	123064246	5Y-H4K8ac_peak_12591	4.95697	
3	123602805	123603015	5Y-H4K8ac_peak_12592	7.17184	MYLK_ENSG00000065534
3	123751527	123751983	5Y-H4K8ac_peak_12593	9.63153	
3	123752223	123752645	5Y-H4K8ac_peak_12594	10.64267	
3	123892458	123892859	5Y-H4K8ac_peak_12595	8.2913	
3	123893938	123894496	5Y-H4K8ac_peak_12596	7.46829	
3	123921103	123921522	5Y-H4K8ac_peak_12597	11.9858	
3	123967047	123967256	5Y-H4K8ac_peak_12598	5.91107	
3	124001444	124001966	5Y-H4K8ac_peak_12599	10.21117	
3	124017789	124018156	5Y-H4K8ac_peak_12600	7.01266	
3	124133912	124134113	5Y-H4K8ac_peak_12601	4.84727	
3	124490228	124490541	5Y-H4K8ac_peak_12602	7.34185	

3	124553675	124554031	5Y-H4K8ac_peak_12603	4.29586	
3	124554258	124554462	5Y-H4K8ac_peak_12604	7.60057	
3	124554713	124555244	5Y-H4K8ac_peak_12605	7.31102	
3	124556011	124556303	5Y-H4K8ac_peak_12606	5.77946	
3	124604766	124605081	5Y-H4K8ac_peak_12607	7.57144	
3	124606080	124606508	5Y-H4K8ac_peak_12608	6.00388	
3	124620010	124620226	5Y-H4K8ac_peak_12609	5.84208	ITGB5_ENSG00000082781
3	124839569	124839903	5Y-H4K8ac_peak_12610	11.09973	
3	124849688	124850027	5Y-H4K8ac_peak_12611	5.40331	
3	124860264	124860677	5Y-H4K8ac_peak_12612	7.11863	
3	124931737	124932205	5Y-H4K8ac_peak_12613	6.79955	
3	124938269	124938495	5Y-H4K8ac_peak_12614	6.03718	
3	125094287	125094493	5Y-H4K8ac_peak_12615	11.62198	ZNF148_ENSG00000163848
3	125314046	125314262	5Y-H4K8ac_peak_12616	9.05168	OSBPL11_ENSG00000144909
3	125314600	125314853	5Y-H4K8ac_peak_12617	4.71803	OSBPL11_ENSG00000144909
3	125679472	125679668	5Y-H4K8ac_peak_12618	4.9885	
3	125931540	125932695	5Y-H4K8ac_peak_12619	10.17195	
3	126074265	126074721	5Y-H4K8ac_peak_12620	7.63144	
3	126194413	126194641	5Y-H4K8ac_peak_12621	7.89273	ZXDC_ENSG00000070476
3	126200200	126200790	5Y-H4K8ac_peak_12622	11.37188	
3	126248665	126248949	5Y-H4K8ac_peak_12623	5.98695	
3	126398402	126399103	5Y-H4K8ac_peak_12624	7.59101	
3	126400969	126401232	5Y-H4K8ac_peak_12625	6.78128	
3	126646031	126646231	5Y-H4K8ac_peak_12626	6.38173	
3	126701090	126701307	5Y-H4K8ac_peak_12627	7.19446	
3	126701556	126701759	5Y-H4K8ac_peak_12628	8.92475	
3	126720402	126720601	5Y-H4K8ac_peak_12629	7.90236	
3	126729863	126730178	5Y-H4K8ac_peak_12630	8.22388	
3	126733667	126733967	5Y-H4K8ac_peak_12631	6.77436	
3	126734881	126735093	5Y-H4K8ac_peak_12632	7.59101	
3	126852966	126853685	5Y-H4K8ac_peak_12633	9.51254	
3	126875852	126876358	5Y-H4K8ac_peak_12634	9.31608	
3	126878019	126878421	5Y-H4K8ac_peak_12635	5.8635	
3	126890008	126891250	5Y-H4K8ac_peak_12636	7.50148	
3	126891561	126891892	5Y-H4K8ac_peak_12637	7.00004	
3	126892429	126892979	5Y-H4K8ac_peak_12638	6.08523	
3	126893600	126893826	5Y-H4K8ac_peak_12639	8.43511	
3	126927527	126927753	5Y-H4K8ac_peak_12640	4.50834	
3	127090719	127091269	5Y-H4K8ac_peak_12641	4.07874	
3	127173885	127174484	5Y-H4K8ac_peak_12642	9.30505	
3	127174682	127175009	5Y-H4K8ac_peak_12643	13.92877	
3	127266107	127266526	5Y-H4K8ac_peak_12644	8.05083	
3	127309976	127310259	5Y-H4K8ac_peak_12645	9.05766	
3	127316752	127317005	5Y-H4K8ac_peak_12646	9.58986	TPRA1_ENSG00000163870;MCM2_ENSG00000073111
3	127317354	127317628	5Y-H4K8ac_peak_12647	10.4495	TPRA1_ENSG00000163870;MCM2_ENSG00000073111
3	127347528	127347775	5Y-H4K8ac_peak_12648	8.84534	PODXL2_ENSG00000114631

3	127348046	127348543	5Y-H4K8ac_peak_12649	25.5258	PODXL2_ENSG00000114631
3	127407492	127408410	5Y-H4K8ac_peak_12650	10.36926	
3	127409303	127409972	5Y-H4K8ac_peak_12651	7.87406	
3	127415467	127416358	5Y-H4K8ac_peak_12652	12.57157	
3	127416652	127417201	5Y-H4K8ac_peak_12653	7.89273	
3	127462636	127462835	5Y-H4K8ac_peak_12654	8.39058	
3	127473056	127473274	5Y-H4K8ac_peak_12655	4.71803	
3	127505618	127506146	5Y-H4K8ac_peak_12656	7.20869	
3	127508066	127508335	5Y-H4K8ac_peak_12657	5.86219	
3	127680060	127680280	5Y-H4K8ac_peak_12658	6.34046	
3	127892822	127893028	5Y-H4K8ac_peak_12659	7.31815	
3	127955554	127955834	5Y-H4K8ac_peak_12660	6.0873	
3	127957338	127957611	5Y-H4K8ac_peak_12661	5.449	
3	127957912	127958352	5Y-H4K8ac_peak_12662	8.43511	
3	127995771	127995992	5Y-H4K8ac_peak_12663	7.50148	
3	127997425	127997687	5Y-H4K8ac_peak_12664	7.64648	
3	127998303	127998643	5Y-H4K8ac_peak_12665	11.41382	
3	127999279	127999679	5Y-H4K8ac_peak_12666	13.0168	
3	128001000	128001204	5Y-H4K8ac_peak_12667	6.77436	
3	128001600	128002059	5Y-H4K8ac_peak_12668	8.69112	
3	128036639	128036987	5Y-H4K8ac_peak_12669	8.62703	
3	128037195	128037971	5Y-H4K8ac_peak_12670	9.38276	
3	128040175	128040383	5Y-H4K8ac_peak_12671	5.51472	
3	128048441	128048631	5Y-H4K8ac_peak_12672	4.77126	
3	128090493	128090732	5Y-H4K8ac_peak_12673	6.29918	
3	128113519	128113972	5Y-H4K8ac_peak_12674	7.50148	
3	128116684	128116964	5Y-H4K8ac_peak_12675	8.82628	
3	128126135	128126425	5Y-H4K8ac_peak_12676	7.98023	
3	128126736	128127889	5Y-H4K8ac_peak_12677	14.40573	
3	128128347	128130425	5Y-H4K8ac_peak_12678	14.97676	
3	128130960	128131931	5Y-H4K8ac_peak_12679	24.04427	
3	128132186	128132989	5Y-H4K8ac_peak_12680	8.20773	
3	128134452	128135469	5Y-H4K8ac_peak_12681	10.58233	
3	128144964	128145255	5Y-H4K8ac_peak_12682	13.03079	
3	128145459	128145732	5Y-H4K8ac_peak_12683	5.64909	
3	128154141	128154385	5Y-H4K8ac_peak_12684	6.54441	
3	128172465	128172976	5Y-H4K8ac_peak_12685	6.2403	
3	128173379	128173695	5Y-H4K8ac_peak_12686	6.88532	
3	128174072	128174264	5Y-H4K8ac_peak_12687	7.63144	
3	128174681	128175965	5Y-H4K8ac_peak_12688	16.79568	
3	128176428	128178979	5Y-H4K8ac_peak_12689	18.10232	
3	128186552	128187148	5Y-H4K8ac_peak_12690	12.79198	DNAJB8_ENSG00000179407
3	128187551	128188518	5Y-H4K8ac_peak_12691	13.4304	
3	128189639	128190090	5Y-H4K8ac_peak_12692	9.30206	
3	128207784	128208304	5Y-H4K8ac_peak_12693	7.50148	RP11-475N22.4_ENSG00000244300
3	128208689	128208891	5Y-H4K8ac_peak_12694	6.64195	RP11-475N22.4_ENSG00000244300

3	128210930	128211453	5Y-H4K8ac_peak_12695	9.36633	GATA2_ENSG00000179348
3	128211649	128211888	5Y-H4K8ac_peak_12696	8.27643	GATA2_ENSG00000179348
3	128212439	128212793	5Y-H4K8ac_peak_12697	5.36319	GATA2_ENSG00000179348
3	128215090	128215384	5Y-H4K8ac_peak_12698	19.17092	
3	128215667	128216492	5Y-H4K8ac_peak_12699	13.85283	
3	128256069	128256303	5Y-H4K8ac_peak_12700	4.14761	TMED10P2_ENSG00000239405
3	128302898	128303278	5Y-H4K8ac_peak_12701	5.87725	
3	128370420	128370636	5Y-H4K8ac_peak_12702	6.43775	
3	128398909	128399105	5Y-H4K8ac_peak_12703	7.30348	RPN1_ENSG00000163902
3	128419956	128420575	5Y-H4K8ac_peak_12704	7.59101	
3	128444304	128444766	5Y-H4K8ac_peak_12705	8.33296	RAB7A_ENSG00000075785
3	128445076	128445658	5Y-H4K8ac_peak_12706	6.46053	RAB7A_ENSG00000075785
3	128492171	128492366	5Y-H4K8ac_peak_12707	6.49308	
3	128568627	128569105	5Y-H4K8ac_peak_12708	5.23083	RP11-221E20.4_ENSG00000243022
3	128583858	128584127	5Y-H4K8ac_peak_12709	6.09071	
3	128746252	128746534	5Y-H4K8ac_peak_12710	7.90236	
3	128758254	128758773	5Y-H4K8ac_peak_12711	6.66026	
3	128771764	128771959	5Y-H4K8ac_peak_12712	6.34046	
3	128780290	128781015	5Y-H4K8ac_peak_12713	8.51022	GP9_ENSG00000169704
3	128880392	128880898	5Y-H4K8ac_peak_12714	4.8509	ISY1-RAB43_ENSG00000261796;ISY1_ENSG00000240682
3	128902232	128902693	5Y-H4K8ac_peak_12715	11.29141	CNBP_ENSG00000169714
3	128943927	128944119	5Y-H4K8ac_peak_12716	5.97089	
3	128998053	128998289	5Y-H4K8ac_peak_12717	6.46053	HMCES_ENSG00000183624
3	129036006	129036308	5Y-H4K8ac_peak_12718	5.64909	H1FX_ENSG00000184897
3	129066317	129066635	5Y-H4K8ac_peak_12719	11.19336	
3	129118014	129118411	5Y-H4K8ac_peak_12720	5.23083	RPL32P3_ENSG00000251474
3	129148158	129148616	5Y-H4K8ac_peak_12721	7.42477	EFCAB12_ENSG00000172771
3	129232547	129232783	5Y-H4K8ac_peak_12722	4.07874	
3	129277091	129277291	5Y-H4K8ac_peak_12723	4.5753	
3	129277734	129277987	5Y-H4K8ac_peak_12724	7.11863	
3	129279144	129279594	5Y-H4K8ac_peak_12725	6.29888	
3	129280055	129280512	5Y-H4K8ac_peak_12726	7.50148	
3	129295846	129296099	5Y-H4K8ac_peak_12727	7.38046	
3	129315355	129315626	5Y-H4K8ac_peak_12728	4.90696	
3	129325824	129326031	5Y-H4K8ac_peak_12729	7.38046	PLXND1_ENSG00000004399
3	129721616	129721927	5Y-H4K8ac_peak_12730	6.73385	
3	131100787	131101158	5Y-H4K8ac_peak_12731	5.41472	RP11-933H2.4_ENSG00000250608;NUDT16_ENSG00000198585
3	131222090	131222343	5Y-H4K8ac_peak_12732	5.40331	MRPL3_ENSG00000114686
3	132136401	132136634	5Y-H4K8ac_peak_12733	9.79526	DNAJC13_ENSG00000138246
3	132379058	132379423	5Y-H4K8ac_peak_12734	7.59101	ACAD11_ENSG00000240303
3	132757285	132757543	5Y-H4K8ac_peak_12735	9.30505	TMEM108_ENSG00000144868
3	132986617	132986891	5Y-H4K8ac_peak_12736	4.02665	
3	133292697	133293093	5Y-H4K8ac_peak_12737	8.43511	CDV3_ENSG00000091527
3	133380374	133380680	5Y-H4K8ac_peak_12738	16.95374	TOPBP1_ENSG00000163781;TFP1_ENSG00000242337
3	133465296	133465486	5Y-H4K8ac_peak_12739	10.84616	TF_ENSG00000091513
3	133614346	133614666	5Y-H4K8ac_peak_12740	12.11208	RAB6B_ENSG00000154917

3	133614987	133615250	5Y-H4K8ac_peak_12741	8.69112	RAB6B_ENSG00000154917
3	133646143	133646352	5Y-H4K8ac_peak_12742	4.50834	
3	133675079	133675335	5Y-H4K8ac_peak_12743	7.97699	
3	134010475	134010779	5Y-H4K8ac_peak_12744	6.79955	
3	134082578	134082898	5Y-H4K8ac_peak_12745	14.00559	
3	134093039	134093643	5Y-H4K8ac_peak_12746	6.08523	AMOTL2_ENSG00000114019
3	134125196	134125486	5Y-H4K8ac_peak_12747	12.21176	
3	134125791	134126194	5Y-H4K8ac_peak_12748	9.05168	
3	134205095	134205494	5Y-H4K8ac_peak_12749	7.90751	ANAPC13_ENSG00000129055;CEP63_ENSG00000182923
3	134321402	134321666	5Y-H4K8ac_peak_12750	5.98695	
3	134337851	134338350	5Y-H4K8ac_peak_12751	6.49308	
3	135914905	135915132	5Y-H4K8ac_peak_12752	9.07085	MSL2_ENSG00000174579
3	135915532	135916224	5Y-H4K8ac_peak_12753	12.11208	MSL2_ENSG00000174579
3	136470486	136471159	5Y-H4K8ac_peak_12754	13.60015	STAG1_ENSG00000118007;RP11-102M11.2_ENSG00000261758
3	136537025	136537417	5Y-H4K8ac_peak_12755	9.34555	SLC35G2_ENSG00000168917
3	136538152	136538347	5Y-H4K8ac_peak_12756	8.2913	SLC35G2_ENSG00000168917
3	136581102	136581368	5Y-H4K8ac_peak_12757	8.2913	RP11-85F14.5_ENSG00000239213;NCK1_ENSG00000158092
3	137906293	137906768	5Y-H4K8ac_peak_12758	13.45283	ARMC8_ENSG00000114098
3	138067015	138067868	5Y-H4K8ac_peak_12759	10.36926	MRAS_ENSG00000158186
3	138068151	138068489	5Y-H4K8ac_peak_12760	8.17203	
3	138327308	138328245	5Y-H4K8ac_peak_12761	9.79526	FAIM_ENSG00000158234
3	138553347	138553550	5Y-H4K8ac_peak_12762	3.99744	PIK3CB_ENSG00000051382
3	139047871	139048282	5Y-H4K8ac_peak_12763	6.79955	
3	139062228	139062655	5Y-H4K8ac_peak_12764	7.31807	
3	139108839	139109063	5Y-H4K8ac_peak_12765	10.31981	COPB2_ENSG00000184432;RP11-319G6.1_ENSG00000248932
3	139252919	139253170	5Y-H4K8ac_peak_12766	10.1994	
3	139253410	139253635	5Y-H4K8ac_peak_12767	6.77436	
3	139258688	139259168	5Y-H4K8ac_peak_12768	6.77436	RBP1_ENSG00000114115
3	139260085	139260428	5Y-H4K8ac_peak_12769	9.38276	
3	139392780	139393006	5Y-H4K8ac_peak_12770	5.98695	
3	139740872	139741475	5Y-H4K8ac_peak_12771	8.51022	
3	140769625	140769921	5Y-H4K8ac_peak_12772	6.37023	SPSB4_ENSG00000175093
3	140802729	140803033	5Y-H4K8ac_peak_12773	5.91897	
3	140950239	140950463	5Y-H4K8ac_peak_12774	5.98695	
3	140950716	140950998	5Y-H4K8ac_peak_12775	9.30505	
3	141030132	141030530	5Y-H4K8ac_peak_12776	10.21117	
3	141112304	141112822	5Y-H4K8ac_peak_12777	11.19336	
3	141120727	141121198	5Y-H4K8ac_peak_12778	8.43511	
3	141121449	141121743	5Y-H4K8ac_peak_12779	9.15007	
3	141205992	141206329	5Y-H4K8ac_peak_12780	7.18391	RASA2_ENSG00000155903
3	141378925	141379320	5Y-H4K8ac_peak_12781	4.77126	
3	141450911	141451116	5Y-H4K8ac_peak_12782	5.40331	
3	141595663	141595854	5Y-H4K8ac_peak_12783	5.18558	ATP1B3_ENSG00000069849
3	141944617	141944880	5Y-H4K8ac_peak_12784	12.05638	GK5_ENSG00000175066
3	142297245	142297471	5Y-H4K8ac_peak_12785	7.58806	ATR_ENSG00000175054
3	142442965	142443228	5Y-H4K8ac_peak_12786	9.23159	TRPC1_ENSG00000144935

3	142443507	142443784	5Y-H4K8ac_peak_12787	5.01697	TRPC1_ENSG00000144935
3	142682460	142682791	5Y-H4K8ac_peak_12788	9.38203	PAQR9_ENSG00000188582;RP11-372E1.6_ENSG00000241570;U2SURP_ENSG00000163714
3	142720660	142720867	5Y-H4K8ac_peak_12789	8.21582	RP11-91G21.1_ENSG00000268129
3	147111229	147111652	5Y-H4K8ac_peak_12790	13.00733	ZIC1_ENSG00000152977
3	147127699	147128082	5Y-H4K8ac_peak_12791	6.34046	
3	149470036	149470234	5Y-H4K8ac_peak_12792	10.46287	COMMD2_ENSG00000114744
3	149530406	149530886	5Y-H4K8ac_peak_12793	8.62703	RNF13_ENSG00000082996
3	149688375	149688566	5Y-H4K8ac_peak_12794	5.98695	AC117395.1_ENSG00000268175
3	149689139	149689333	5Y-H4K8ac_peak_12795	10.1994	AC117395.1_ENSG00000268175
3	149865174	149865364	5Y-H4K8ac_peak_12796	4.51076	
3	150126281	150126711	5Y-H4K8ac_peak_12797	4.0639	TSC22D2_ENSG00000196428
3	150321197	150321443	5Y-H4K8ac_peak_12798	5.98695	SERP1_ENSG00000120742;SELT_ENSG00000198843
3	150329647	150329845	5Y-H4K8ac_peak_12799	4.07874	RP11-392O18.2_ENSG00000271711
3	150480641	150480868	5Y-H4K8ac_peak_12800	7.05631	SIAH2_ENSG00000181788;SIAH2-AS1_ENSG00000244265
3	150481639	150481866	5Y-H4K8ac_peak_12801	8.17203	SIAH2_ENSG00000181788
3	150803069	150803385	5Y-H4K8ac_peak_12802	12.09463	MED12L_ENSG00000144893
3	150803650	150804025	5Y-H4K8ac_peak_12803	7.80156	MED12L_ENSG00000144893
3	151986159	151986508	5Y-H4K8ac_peak_12804	5.8635	MBNL1-AS1_ENSG00000229619
3	151987090	151987285	5Y-H4K8ac_peak_12805	4.84727	MBNL1-AS1_ENSG00000229619
3	154041812	154042225	5Y-H4K8ac_peak_12806	9.15007	DHX36_ENSG00000174953
3	155523615	155524026	5Y-H4K8ac_peak_12807	7.50148	C3orf33_ENSG00000174928
3	155588539	155589135	5Y-H4K8ac_peak_12808	17.63051	GMPS_ENSG00000163655
3	156534207	156534528	5Y-H4K8ac_peak_12809	6.98416	LINC00886_ENSG00000240875
3	156544312	156544502	5Y-H4K8ac_peak_12810	7.41197	
3	156799780	156799975	5Y-H4K8ac_peak_12811	9.02242	RP11-6F2.5_ENSG00000241544
3	156807615	156807807	5Y-H4K8ac_peak_12812	4.0639	LINC00881_ENSG00000241135
3	156878153	156878630	5Y-H4K8ac_peak_12813	9.34555	CCNL1_ENSG00000163660
3	157155370	157155734	5Y-H4K8ac_peak_12814	5.64909	PTX3_ENSG00000163661
3	157350996	157351192	5Y-H4K8ac_peak_12815	4.35388	
3	158362315	158362641	5Y-H4K8ac_peak_12816	13.2534	GFM1_ENSG00000168827
3	158450088	158450392	5Y-H4K8ac_peak_12817	5.12488	RARRES1_ENSG00000118849;MFSD1_ENSG00000118855;RP11-379F4.4_ENSG00000240207
3	158450612	158450817	5Y-H4K8ac_peak_12818	5.64909	RARRES1_ENSG00000118849;MFSD1_ENSG00000118855;RP11-379F4.4_ENSG00000240207
3	159482403	159482951	5Y-H4K8ac_peak_12819	7.97699	
3	159570722	159570979	5Y-H4K8ac_peak_12820	4.50834	
3	160472376	160472637	5Y-H4K8ac_peak_12821	5.98695	RP11-16N11.2_ENSG00000260572;PPM1L_ENSG00000163590
3	161090469	161091150	5Y-H4K8ac_peak_12822	8.73985	SPTSSB_ENSG00000196542
3	169530217	169530728	5Y-H4K8ac_peak_12823	7.59101	LRRC34_ENSG00000171757
3	169683978	169684408	5Y-H4K8ac_peak_12824	5.64909	RP11-379K17.4_ENSG00000239219;SEC62_ENSG00000008952
3	169939886	169940117	5Y-H4K8ac_peak_12825	9.11354	PRKCI_ENSG00000163558
3	170074275	170074817	5Y-H4K8ac_peak_12826	7.38046	SKIL_ENSG00000136603
3	170075672	170076063	5Y-H4K8ac_peak_12827	9.96543	SKIL_ENSG00000136603
3	174158785	174159008	5Y-H4K8ac_peak_12828	6.1654	
3	176914482	176914692	5Y-H4K8ac_peak_12829	4.84727	TBL1XR1_ENSG00000177565
3	176915523	176915840	5Y-H4K8ac_peak_12830	22.30274	TBL1XR1_ENSG00000177565
3	177076790	177077667	5Y-H4K8ac_peak_12831	11.71078	
3	177208427	177208636	5Y-H4K8ac_peak_12832	6.20875	

3	177471467	177471749	5Y-H4K8ac_peak_12833	5.87382	
3	178011509	178011837	5Y-H4K8ac_peak_12834	9.83437	
3	178363116	178363454	5Y-H4K8ac_peak_12835	4.84727	
3	178789711	178790301	5Y-H4K8ac_peak_12836	11.1169	ZMAT3_ENSG00000172667
3	178866287	178866689	5Y-H4K8ac_peak_12837	5.23083	RP11-360P21.2_ENSG00000229102;PIK3CA_ENSG00000121879
3	178978902	178979193	5Y-H4K8ac_peak_12838	5.64909	LRRFIP1P1_ENSG00000240429
3	179040277	179040619	5Y-H4K8ac_peak_12839	4.00285	ZNF639_ENSG00000121864
3	179041294	179041534	5Y-H4K8ac_peak_12840	6.50117	ZNF639_ENSG00000121864
3	179322051	179322337	5Y-H4K8ac_peak_12841	8.73985	MRPL47_ENSG00000136522;NDUFB5_ENSG00000136521
3	180319423	180319659	5Y-H4K8ac_peak_12842	9.20645	RP11-496B10.3_ENSG00000239774;TTC14_ENSG00000163728
3	180320034	180320258	5Y-H4K8ac_peak_12843	5.56894	RP11-496B10.3_ENSG00000239774;TTC14_ENSG00000163728
3	180630526	180630731	5Y-H4K8ac_peak_12844	8.2913	
3	180707111	180707474	5Y-H4K8ac_peak_12845	6.77436	DNAJC19_ENSG00000205981;SOX2-OT_ENSG00000242808
3	182400429	182400772	5Y-H4K8ac_peak_12846	9.7353	
3	182443784	182444077	5Y-H4K8ac_peak_12847	6.52593	
3	182510906	182511165	5Y-H4K8ac_peak_12848	13.88421	RP11-225N10.1_ENSG00000240063;ATP11B_ENSG00000058063
3	182698626	182699126	5Y-H4K8ac_peak_12849	8.564	
3	182880272	182880995	5Y-H4K8ac_peak_12850	8.43511	LAMP3_ENSG00000078081
3	182971991	182972574	5Y-H4K8ac_peak_12851	9.36633	B3GNT5_ENSG00000176597
3	183301576	183301968	5Y-H4K8ac_peak_12852	5.65584	
3	183622091	183622370	5Y-H4K8ac_peak_12853	5.29015	
3	183735415	183735626	5Y-H4K8ac_peak_12854	10.79063	ABCC5_ENSG00000114770
3	183853194	183853540	5Y-H4K8ac_peak_12855	11.09973	RP11-778D9.12_ENSG00000272721;EIF2B5_ENSG00000145191
3	183862695	183862933	5Y-H4K8ac_peak_12856	4.84727	
3	183903365	183903593	5Y-H4K8ac_peak_12857	7.20869	ABCF3_ENSG00000161204
3	183966849	183967157	5Y-H4K8ac_peak_12858	6.37023	ALG3_ENSG00000214160;ECE2_ENSG00000145194
3	184032610	184033149	5Y-H4K8ac_peak_12859	11.1169	EIF4G1_ENSG00000114867
3	184080592	184080961	5Y-H4K8ac_peak_12860	14.00559	
3	184081438	184081772	5Y-H4K8ac_peak_12861	5.65765	
3	184243124	184243948	5Y-H4K8ac_peak_12862	12.05638	
3	184286069	184286496	5Y-H4K8ac_peak_12863	4.55128	
3	184286985	184287738	5Y-H4K8ac_peak_12864	13.00733	
3	184292879	184293221	5Y-H4K8ac_peak_12865	5.72233	
3	184308538	184308995	5Y-H4K8ac_peak_12866	10.77254	
3	184309720	184310034	5Y-H4K8ac_peak_12867	9.60458	
3	184310304	184310656	5Y-H4K8ac_peak_12868	4.3419	
3	184311456	184311793	5Y-H4K8ac_peak_12869	7.30348	
3	184315516	184315814	5Y-H4K8ac_peak_12870	8.27643	
3	184352814	184353390	5Y-H4K8ac_peak_12871	6.08523	
3	184353583	184353827	5Y-H4K8ac_peak_12872	9.51254	
3	184376372	184376568	5Y-H4K8ac_peak_12873	7.72345	
3	184396560	184397335	5Y-H4K8ac_peak_12874	13.99721	
3	184409184	184409799	5Y-H4K8ac_peak_12875	6.06152	
3	184429118	184429717	5Y-H4K8ac_peak_12876	7.20869	MAGEF1_ENSG00000177383
3	184491187	184491598	5Y-H4K8ac_peak_12877	4.95697	RP11-329B9.1_ENSG00000229433
3	184513190	184513423	5Y-H4K8ac_peak_12878	10.1994	

3	184893477	184893686	5Y-H4K8ac_peak_12879	9.38203	
3	184971601	184971810	5Y-H4K8ac_peak_12880	5.29015	MIR5588_ENSG00000264614
3	185216173	185216490	5Y-H4K8ac_peak_12881	5.449	TMEM41A_ENSG00000163900
3	185277106	185277310	5Y-H4K8ac_peak_12882	5.59843	
3	185304169	185304432	5Y-H4K8ac_peak_12883	9.30505	
3	185655360	185655682	5Y-H4K8ac_peak_12884	10.27264	TRA2B_ENSG00000136527
3	185661187	185661397	5Y-H4K8ac_peak_12885	6.54441	
3	185826552	185826742	5Y-H4K8ac_peak_12886	5.91107	
3	185927890	185928370	5Y-H4K8ac_peak_12887	7.01266	
3	186250245	186250585	5Y-H4K8ac_peak_12888	8.33296	
3	186501122	186501863	5Y-H4K8ac_peak_12889	7.66481	RP11-573D15.9_ENSG00000263826;EIF4A2_ENSG00000156976;SNORD2_ENSG00000238942
3	186524405	186524661	5Y-H4K8ac_peak_12890	7.90751	RFC4_ENSG00000163918;RP11-573D15.3_ENSG00000231724
3	186648416	186648624	5Y-H4K8ac_peak_12891	6.14981	ST6GAL1_ENSG00000073849
3	186856985	186857595	5Y-H4K8ac_peak_12892	7.38046	
3	191046888	191047401	5Y-H4K8ac_peak_12893	11.99586	UTS2B_ENSG00000188958;CCDC50_ENSG00000152492
3	193462281	193462504	5Y-H4K8ac_peak_12894	4.5753	
3	193534581	193535253	5Y-H4K8ac_peak_12895	8.2913	
3	193720625	193721605	5Y-H4K8ac_peak_12896	13.0168	RP11-699L21.1_ENSG00000214146
3	193787969	193788962	5Y-H4K8ac_peak_12897	12.26606	RP11-407B7.1_ENSG00000230102
3	193851606	193851870	5Y-H4K8ac_peak_12898	9.23159	
3	193852183	193852635	5Y-H4K8ac_peak_12899	11.19336	
3	193855281	193855529	5Y-H4K8ac_peak_12900	4.642	
3	193858534	193859158	5Y-H4K8ac_peak_12901	6.78128	
3	193859517	193859986	5Y-H4K8ac_peak_12902	7.44077	
3	193923461	193923669	5Y-H4K8ac_peak_12903	5.97532	
3	193967269	193967566	5Y-H4K8ac_peak_12904	4.77126	RP11-513G11.4_ENSG00000225742
3	193982965	193983466	5Y-H4K8ac_peak_12905	11.19336	
3	193985733	193985983	5Y-H4K8ac_peak_12906	7.21932	
3	193987541	193987734	5Y-H4K8ac_peak_12907	5.12488	
3	193988348	193989102	5Y-H4K8ac_peak_12908	13.0168	
3	193989351	193989639	5Y-H4K8ac_peak_12909	4.29586	
3	193990056	193990388	5Y-H4K8ac_peak_12910	9.58986	
3	193991108	193991365	5Y-H4K8ac_peak_12911	13.12848	
3	193991750	193992102	5Y-H4K8ac_peak_12912	6.47245	
3	193995141	193995503	5Y-H4K8ac_peak_12913	5.87725	RP11-513G11.2_ENSG00000228271
3	194016524	194016939	5Y-H4K8ac_peak_12914	5.23083	
3	194018080	194018282	5Y-H4K8ac_peak_12915	5.65584	
3	194208136	194208447	5Y-H4K8ac_peak_12916	6.47245	LINC00884_ENSG00000233058
3	194353598	194354163	5Y-H4K8ac_peak_12917	6.50117	TMEM44_ENSG00000145014;AC046143.3_ENSG00000229334
3	194408754	194409016	5Y-H4K8ac_peak_12918	8.71064	
3	194574149	194574339	5Y-H4K8ac_peak_12919	6.17973	
3	194592406	194593238	5Y-H4K8ac_peak_12920	5.41472	
3	194629268	194629472	5Y-H4K8ac_peak_12921	4.84727	
3	194796252	194796688	5Y-H4K8ac_peak_12922	7.59101	
3	194821754	194821971	5Y-H4K8ac_peak_12923	7.80156	
3	194873676	194873880	5Y-H4K8ac_peak_12924	4.50834	

3	194882535	194882768	5Y-H4K8ac_peak_12925	8.47164	
3	194899087	194899897	5Y-H4K8ac_peak_12926	6.77436	
3	194900762	194901583	5Y-H4K8ac_peak_12927	9.63153	
3	194930208	194930456	5Y-H4K8ac_peak_12928	5.64909	
3	194980383	194980821	5Y-H4K8ac_peak_12929	10.31981	
3	195139280	195139473	5Y-H4K8ac_peak_12930	6.98118	Y_RNA_ENSG00000207368
3	195164390	195164680	5Y-H4K8ac_peak_12931	4.22304	ACAP2_ENSG00000114331
3	195270917	195271140	5Y-H4K8ac_peak_12932	4.5753	PPP1R2_ENSG00000184203;AC091633.3_ENSG00000223711
3	195384528	195384723	5Y-H4K8ac_peak_12933	4.84727	AC069513.4_ENSG00000229178;LINC00969_ENSG00000242086;SDHAP2_ENSG00000215837
3	195474452	195474759	5Y-H4K8ac_peak_12934	5.41472	
3	195474967	195475250	5Y-H4K8ac_peak_12935	11.1169	
3	195618949	195619179	5Y-H4K8ac_peak_12936	7.07069	
3	195622058	195622252	5Y-H4K8ac_peak_12937	6.49458	
3	195622958	195623169	5Y-H4K8ac_peak_12938	4.40362	
3	195636959	195637319	5Y-H4K8ac_peak_12939	5.23083	
3	195808550	195808905	5Y-H4K8ac_peak_12940	19.26368	TFRC_ENSG00000072274
3	195913966	195914426	5Y-H4K8ac_peak_12941	5.91107	
3	195920614	195921110	5Y-H4K8ac_peak_12942	8.21582	
3	195921320	195921549	5Y-H4K8ac_peak_12943	7.89273	
3	195934047	195934309	5Y-H4K8ac_peak_12944	7.13294	
3	195946865	195947058	5Y-H4K8ac_peak_12945	5.41472	
3	196014713	196015176	5Y-H4K8ac_peak_12946	16.01349	PCYT1A_ENSG00000161217
3	196044662	196044853	5Y-H4K8ac_peak_12947	5.98695	RP11-447L10.1_ENSG00000272741;TCTEX1D2_ENSG00000213123;TM4SF19-AS1_ENSG00000235897
3	196045274	196045536	5Y-H4K8ac_peak_12948	10.5157	RP11-447L10.1_ENSG00000272741;TCTEX1D2_ENSG00000213123;TM4SF19-AS1_ENSG00000235897
3	196046131	196046389	5Y-H4K8ac_peak_12949	6.43775	TCTEX1D2_ENSG00000213123;TM4SF19-AS1_ENSG00000235897
3	196159029	196159221	5Y-H4K8ac_peak_12950	5.65584	UBXN7_ENSG00000163960;UBXN7-AS1_ENSG00000225822
3	196359087	196359452	5Y-H4K8ac_peak_12951	9.78792	LINC01063_ENSG00000232065
3	196359678	196360260	5Y-H4K8ac_peak_12952	10.12234	LINC01063_ENSG00000232065
3	196366681	196367006	5Y-H4K8ac_peak_12953	7.11863	NRROS_ENSG00000174004;PIGX_ENSG00000163964
3	196594866	196595467	5Y-H4K8ac_peak_12954	7.38046	SENP5_ENSG00000119231
3	196668860	196669261	5Y-H4K8ac_peak_12955	9.30505	NCBP2_ENSG00000114503;NCBP2-AS2_ENSG00000270170
3	196729410	196730220	5Y-H4K8ac_peak_12956	11.88337	
3	196730439	196730924	5Y-H4K8ac_peak_12957	10.1994	
3	196756181	196756670	5Y-H4K8ac_peak_12958	10.35586	MFI2_ENSG00000163975
3	197024574	197024941	5Y-H4K8ac_peak_12959	14.43262	DLG1-AS1_ENSG00000227375
3	197066564	197066755	5Y-H4K8ac_peak_12960	6.77436	snoU13_ENSG00000238491
3	197067172	197067416	5Y-H4K8ac_peak_12961	4.95697	snoU13_ENSG00000238491
3	197081592	197082169	5Y-H4K8ac_peak_12962	9.20645	
3	197096958	197097170	5Y-H4K8ac_peak_12963	5.29015	
3	197151347	197151569	5Y-H4K8ac_peak_12964	4.64156	
3	197191512	197191905	5Y-H4K8ac_peak_12965	5.718	
3	197194480	197195219	5Y-H4K8ac_peak_12966	7.01266	
3	197211621	197212059	5Y-H4K8ac_peak_12967	8.62703	
3	197214285	197214544	5Y-H4K8ac_peak_12968	7.35899	
3	197217036	197217466	5Y-H4K8ac_peak_12969	8.60027	
3	197219298	197219570	5Y-H4K8ac_peak_12970	3.9664	

3	197220025	197220236	5Y-H4K8ac_peak_12971	8.69112	
3	197230664	197230945	5Y-H4K8ac_peak_12972	5.41472	
3	197282586	197283463	5Y-H4K8ac_peak_12973	10.4495	
3	197355049	197355258	5Y-H4K8ac_peak_12974	7.38046	AC024560.3_ENSG00000214135
3	197476154	197476511	5Y-H4K8ac_peak_12975	4.84727	KIAA0226_ENSG00000145016
4	52182	52468	5Y-H4K8ac_peak_12976	7.11613	ZNF595_ENSG00000197701
4	53395	53621	5Y-H4K8ac_peak_12977	6.35775	ZNF595_ENSG00000197701
4	206467	206843	5Y-H4K8ac_peak_12978	4.642	ZNF876P_ENSG00000198155
4	330197	330388	5Y-H4K8ac_peak_12979	5.37237	RP11-478C6.1_ENSG00000251535
4	330925	331145	5Y-H4K8ac_peak_12980	10.3507	RP11-478C6.1_ENSG00000251535;ZNF141_ENSG00000131127
4	467421	467899	5Y-H4K8ac_peak_12981	12.21176	ABCA11P_ENSG00000251595
4	493103	493401	5Y-H4K8ac_peak_12982	9.51254	ZNF721_ENSG00000182903;PIGG_ENSG00000174227
4	657207	658759	5Y-H4K8ac_peak_12983	11.22005	
4	668346	668649	5Y-H4K8ac_peak_12984	15.45397	ATP5I_ENSG00000169020;MYL5_ENSG00000215375
4	675588	675814	5Y-H4K8ac_peak_12985	5.64909	
4	678020	678514	5Y-H4K8ac_peak_12986	8.69112	
4	699672	700259	5Y-H4K8ac_peak_12987	9.30505	PCGF3_ENSG00000185619
4	775329	775552	5Y-H4K8ac_peak_12988	10.11191	RP11-440L14.1_ENSG00000249592
4	829401	829720	5Y-H4K8ac_peak_12989	13.00733	
4	967606	968013	5Y-H4K8ac_peak_12990	13.2534	
4	1161215	1161408	5Y-H4K8ac_peak_12991	6.73047	RP11-20I20.4_ENSG00000273179
4	1187738	1188726	5Y-H4K8ac_peak_12992	16.79568	
4	1188934	1189166	5Y-H4K8ac_peak_12993	6.62622	
4	1194988	1195230	5Y-H4K8ac_peak_12994	6.50117	
4	1199092	1200267	5Y-H4K8ac_peak_12995	7.69843	
4	1201551	1201841	5Y-H4K8ac_peak_12996	10.95042	SPON2_ENSG00000159674
4	1241717	1242470	5Y-H4K8ac_peak_12997	11.01699	CTBP1-AS2_ENSG00000196810
4	1243202	1244286	5Y-H4K8ac_peak_12998	11.1169	CTBP1_ENSG00000159692;CTBP1-AS2_ENSG00000196810
4	1283760	1284023	5Y-H4K8ac_peak_12999	7.50148	MAEA_ENSG00000090316
4	1341116	1341445	5Y-H4K8ac_peak_13000	13.0168	UVSSA_ENSG00000163945
4	1345536	1345774	5Y-H4K8ac_peak_13001	8.47164	
4	1594700	1595061	5Y-H4K8ac_peak_13002	6.37023	
4	1646646	1647002	5Y-H4K8ac_peak_13003	5.23083	
4	1685456	1685902	5Y-H4K8ac_peak_13004	10.90365	FAM53A_ENSG00000174137;Y_RNA_ENSG00000207009
4	1686310	1686922	5Y-H4K8ac_peak_13005	15.28394	FAM53A_ENSG00000174137
4	1713828	1714362	5Y-H4K8ac_peak_13006	23.27921	SLBP_ENSG00000163950;RP11-572O17.1_ENSG00000270195
4	1721819	1722529	5Y-H4K8ac_peak_13007	8.21582	TMEM129_ENSG00000168936;TACC3_ENSG00000013810
4	1755811	1756260	5Y-H4K8ac_peak_13008	8.49231	
4	1767844	1768100	5Y-H4K8ac_peak_13009	4.05358	
4	1858243	1858771	5Y-H4K8ac_peak_13010	11.1169	LETM1_ENSG00000168924
4	1873201	1873521	5Y-H4K8ac_peak_13011	12.11208	WHSC1_ENSG00000109685
4	2061525	2061785	5Y-H4K8ac_peak_13012	7.07936	NAT8L_ENSG00000185818
4	2264312	2264593	5Y-H4K8ac_peak_13013	6.19716	MXD4_ENSG00000123933
4	2415327	2415634	5Y-H4K8ac_peak_13014	8.73392	
4	2469902	2470508	5Y-H4K8ac_peak_13015	6.86362	
4	2536954	2537627	5Y-H4K8ac_peak_13016	5.24695	

4	2789625	2790045	5Y-H4K8ac_peak_13017	9.23159	
4	2790240	2790574	5Y-H4K8ac_peak_13018	7.38046	
4	2800487	2800894	5Y-H4K8ac_peak_13019	10.4689	
4	2802853	2803084	5Y-H4K8ac_peak_13020	6.4221	
4	2808471	2808737	5Y-H4K8ac_peak_13021	5.96059	
4	2813825	2814087	5Y-H4K8ac_peak_13022	4.55128	
4	2819358	2819713	5Y-H4K8ac_peak_13023	8.24461	
4	2820088	2820510	5Y-H4K8ac_peak_13024	12.10416	
4	2936732	2936943	5Y-H4K8ac_peak_13025	5.60566	MFSD10_ENSG00000109736;NOP14-AS1_ENSG00000249673
4	3278655	3278893	5Y-H4K8ac_peak_13026	4.51076	
4	3494843	3495499	5Y-H4K8ac_peak_13027	9.51254	
4	3587068	3587261	5Y-H4K8ac_peak_13028	6.36723	
4	3590533	3590725	5Y-H4K8ac_peak_13029	6.10343	
4	3616165	3616560	5Y-H4K8ac_peak_13030	5.03564	
4	3617112	3617370	5Y-H4K8ac_peak_13031	8.73227	
4	3659793	3660269	5Y-H4K8ac_peak_13032	8.2913	
4	3708772	3709395	5Y-H4K8ac_peak_13033	7.11863	
4	3709862	3710061	5Y-H4K8ac_peak_13034	9.7353	
4	3710348	3710576	5Y-H4K8ac_peak_13035	6.1654	
4	3781887	3782403	5Y-H4K8ac_peak_13036	4.50834	
4	3787542	3788059	5Y-H4K8ac_peak_13037	13.23366	
4	3829476	3829667	5Y-H4K8ac_peak_13038	6.53157	
4	3829877	3830172	5Y-H4K8ac_peak_13039	4.67245	
4	3832531	3833044	5Y-H4K8ac_peak_13040	6.7002	
4	3956838	3957111	5Y-H4K8ac_peak_13041	7.11863	AC226119.5_ENSG00000253917;FAM86EP_ENSG00000251669
4	4329447	4329638	5Y-H4K8ac_peak_13042	6.98118	
4	4388236	4388591	5Y-H4K8ac_peak_13043	13.2534	
4	4388889	4389097	5Y-H4K8ac_peak_13044	9.23159	
4	4407793	4408021	5Y-H4K8ac_peak_13045	4.77126	
4	4408898	4409218	5Y-H4K8ac_peak_13046	9.51254	
4	4543354	4543608	5Y-H4K8ac_peak_13047	7.87192	STX18_ENSG00000168818;STX18-AS1_ENSG00000247708
4	4765775	4765981	5Y-H4K8ac_peak_13048	6.09071	
4	4785823	4786308	5Y-H4K8ac_peak_13049	4.642	
4	5710506	5710891	5Y-H4K8ac_peak_13050	6.37023	EVC2_ENSG00000173040
4	5889599	5890168	5Y-H4K8ac_peak_13051	10.1994	
4	5957990	5958223	5Y-H4K8ac_peak_13052	9.23159	
4	5958486	5958794	5Y-H4K8ac_peak_13053	5.62406	
4	6173418	6173621	5Y-H4K8ac_peak_13054	8.2913	
4	6202400	6202679	5Y-H4K8ac_peak_13055	16.50659	JAKMIP1_ENSG00000152969;RP11-586D19.1_ENSG00000249896
4	6223920	6224181	5Y-H4K8ac_peak_13056	4.15658	
4	6472513	6472956	5Y-H4K8ac_peak_13057	7.11863	
4	6577057	6577405	5Y-H4K8ac_peak_13058	11.53136	MAN2B2_ENSG00000013288
4	6641918	6642188	5Y-H4K8ac_peak_13059	4.29586	MRFAP1_ENSG00000179010
4	6675857	6676360	5Y-H4K8ac_peak_13060	4.77126	RP11-539L10.3_ENSG00000251580;AC093323.3_ENSG00000170846
4	6717943	6718322	5Y-H4K8ac_peak_13061	7.20869	BLOC1S4_ENSG00000186222
4	6763103	6763872	5Y-H4K8ac_peak_13062	6.08523	

4	6783928	6784200	5Y-H4K8ac_peak_13063	4.55128	KIAA0232_ENSG00000170871
4	6784492	6785290	5Y-H4K8ac_peak_13064	8.93923	
4	6911199	6911395	5Y-H4K8ac_peak_13065	9.51254	TBC1D14_ENSG00000132405
4	7103479	7103799	5Y-H4K8ac_peak_13066	10.4689	
4	7105404	7105612	5Y-H4K8ac_peak_13067	6.17973	RP11-367J11.3_ENSG00000245468
4	7208959	7209237	5Y-H4K8ac_peak_13068	7.01266	
4	7227225	7228624	5Y-H4K8ac_peak_13069	12.69256	
4	7253737	7254134	5Y-H4K8ac_peak_13070	4.77126	
4	7287606	7287797	5Y-H4K8ac_peak_13071	5.37588	
4	7414353	7414545	5Y-H4K8ac_peak_13072	6.77436	
4	7517269	7517605	5Y-H4K8ac_peak_13073	10.69698	
4	7540977	7541212	5Y-H4K8ac_peak_13074	7.01266	
4	7542077	7542346	5Y-H4K8ac_peak_13075	6.77436	
4	7693646	7693972	5Y-H4K8ac_peak_13076	4.15658	
4	7888838	7889067	5Y-H4K8ac_peak_13077	5.65584	
4	7889592	7889831	5Y-H4K8ac_peak_13078	4.14761	
4	7893576	7894210	5Y-H4K8ac_peak_13079	6.94416	
4	7896175	7896448	5Y-H4K8ac_peak_13080	6.78128	
4	7902816	7903366	5Y-H4K8ac_peak_13081	6.50117	
4	7904298	7904570	5Y-H4K8ac_peak_13082	6.12014	
4	7904997	7905366	5Y-H4K8ac_peak_13083	9.23159	
4	7912254	7912741	5Y-H4K8ac_peak_13084	6.78128	
4	7912997	7913249	5Y-H4K8ac_peak_13085	6.1654	
4	7941042	7941447	5Y-H4K8ac_peak_13086	7.50148	AFAP1_ENSG00000196526;AC097381.1_ENSG00000228919
4	7950285	7950567	5Y-H4K8ac_peak_13087	8.79957	
4	8052135	8052381	5Y-H4K8ac_peak_13088	10.1994	
4	8052664	8052965	5Y-H4K8ac_peak_13089	10.35586	
4	8122821	8123248	5Y-H4K8ac_peak_13090	9.13822	
4	8160946	8161158	5Y-H4K8ac_peak_13091	8.1667	ABLIM2_ENSG00000163995
4	8200132	8200689	5Y-H4K8ac_peak_13092	12.06963	
4	8201080	8202301	5Y-H4K8ac_peak_13093	9.51254	
4	8202889	8203308	5Y-H4K8ac_peak_13094	7.15925	
4	8207619	8208269	5Y-H4K8ac_peak_13095	7.60893	
4	8230266	8230531	5Y-H4K8ac_peak_13096	4.79585	
4	8244245	8244648	5Y-H4K8ac_peak_13097	9.05168	
4	8264585	8264941	5Y-H4K8ac_peak_13098	5.00877	
4	8274706	8275108	5Y-H4K8ac_peak_13099	8.47164	
4	8276914	8277332	5Y-H4K8ac_peak_13100	6.34245	
4	8429811	8430038	5Y-H4K8ac_peak_13101	4.84727	
4	8442603	8442794	5Y-H4K8ac_peak_13102	6.77436	ACOX3_ENSG00000087008
4	8599746	8600187	5Y-H4K8ac_peak_13103	5.23083	
4	8781902	8782281	5Y-H4K8ac_peak_13104	5.87382	
4	8795535	8795869	5Y-H4K8ac_peak_13105	10.19948	
4	8913112	8913594	5Y-H4K8ac_peak_13106	10.1994	
4	8913785	8914163	5Y-H4K8ac_peak_13107	6.78128	
4	9155095	9155311	5Y-H4K8ac_peak_13108	8.24461	FAM86KP_ENSG00000163612

4	9382734	9383064	5Y-H4K8ac_peak_13109	17.63051	
4	10010744	10010934	5Y-H4K8ac_peak_13110	8.55081	
4	10118079	10118351	5Y-H4K8ac_peak_13111	6.50117	WDR1_ENSG00000071127;RNA5SP155_ENSG00000223086
4	13485531	13485764	5Y-H4K8ac_peak_13112	10.19948	RAB28_ENSG00000157869
4	13629204	13629959	5Y-H4K8ac_peak_13113	11.19336	BOD1L1_ENSG00000038219;MIR5091_ENSG00000266240
4	15657231	15657693	5Y-H4K8ac_peak_13114	11.36989	
4	16227733	16227982	5Y-H4K8ac_peak_13115	7.38046	TAPT1-AS1_ENSG00000263327
4	16228291	16228930	5Y-H4K8ac_peak_13116	13.2534	TAPT1_ENSG00000169762;TAPT1-AS1_ENSG00000263327
4	17491524	17492256	5Y-H4K8ac_peak_13117	9.56516	
4	17579205	17579490	5Y-H4K8ac_peak_13118	7.38046	LAP3_ENSG00000002549
4	17616318	17616515	5Y-H4K8ac_peak_13119	5.84208	AC006160.5_ENSG00000249502;MED28_ENSG00000118579
4	17672749	17673143	5Y-H4K8ac_peak_13120	7.38046	
4	17812078	17812288	5Y-H4K8ac_peak_13121	11.57334	DCAF16_ENSG00000163257;NCAPG_ENSG00000109805
4	18022704	18023144	5Y-H4K8ac_peak_13122	8.17203	LCORL_ENSG00000178177
4	22517201	22517648	5Y-H4K8ac_peak_13123	6.50117	GPR125_ENSG00000152990
4	24585684	24585974	5Y-H4K8ac_peak_13124	5.37237	DHX15_ENSG00000109606
4	24586648	24586877	5Y-H4K8ac_peak_13125	5.03917	DHX15_ENSG00000109606
4	25161763	25162011	5Y-H4K8ac_peak_13126	8.564	SEPSECS_ENSG00000109618;PI4K2B_ENSG00000038210
4	25378346	25378537	5Y-H4K8ac_peak_13127	9.02242	ANAPC4_ENSG00000053900
4	25378986	25379286	5Y-H4K8ac_peak_13128	4.50834	ANAPC4_ENSG00000053900
4	26320798	26320998	5Y-H4K8ac_peak_13129	6.73047	
4	26859428	26859735	5Y-H4K8ac_peak_13130	4.50834	STIM2_ENSG00000109689
4	37664545	37664839	5Y-H4K8ac_peak_13131	5.40331	
4	37892509	37893036	5Y-H4K8ac_peak_13132	8.33296	TBC1D1_ENSG00000065882
4	37978538	37978800	5Y-H4K8ac_peak_13133	13.06587	
4	37979066	37979497	5Y-H4K8ac_peak_13134	5.41472	
4	38664649	38664990	5Y-H4K8ac_peak_13135	5.98695	KLF3_ENSG00000109787
4	38665895	38666122	5Y-H4K8ac_peak_13136	7.31815	RP11-617D20.1_ENSG00000231160;AC021860.1_ENSG00000196355;KLF3_ENSG00000109787
4	38869556	38869906	5Y-H4K8ac_peak_13137	7.04637	FAM114A1_ENSG00000197712;MIR574_ENSG00000207944
4	39460192	39460398	5Y-H4K8ac_peak_13138	6.43068	RPL9_ENSG00000163682;LIAS_ENSG00000121897
4	39528637	39529028	5Y-H4K8ac_peak_13139	11.42066	UGDH_ENSG00000109814;UGDH-AS1_ENSG00000249348
4	39529359	39529853	5Y-H4K8ac_peak_13140	13.0168	UGDH_ENSG00000109814;UGDH-AS1_ENSG00000249348
4	39699832	39700421	5Y-H4K8ac_peak_13141	7.97699	UBE2K_ENSG00000078140
4	39817060	39817404	5Y-H4K8ac_peak_13142	8.1667	
4	39978361	39978811	5Y-H4K8ac_peak_13143	8.69112	PDS5A_ENSG00000121892
4	39979104	39979498	5Y-H4K8ac_peak_13144	15.93543	PDS5A_ENSG00000121892
4	39979758	39980028	5Y-H4K8ac_peak_13145	8.2913	PDS5A_ENSG00000121892
4	40057665	40058933	5Y-H4K8ac_peak_13146	14.99673	RP11-333E13.4_ENSG00000205794;N4BP2_ENSG00000078177
4	40267284	40267560	5Y-H4K8ac_peak_13147	9.23159	RP11-395I6.2_ENSG00000250338
4	40321959	40322347	5Y-H4K8ac_peak_13148	4.84727	
4	40752039	40752323	5Y-H4K8ac_peak_13149	7.87406	NSUN7_ENSG00000179299
4	41218541	41218733	5Y-H4K8ac_peak_13150	10.55771	APBB2_ENSG00000163697
4	41361162	41361403	5Y-H4K8ac_peak_13151	8.21582	LIMCH1_ENSG00000064042
4	41362393	41362684	5Y-H4K8ac_peak_13152	8.564	LIMCH1_ENSG00000064042
4	41748083	41748368	5Y-H4K8ac_peak_13153	7.55879	
4	41752287	41752520	5Y-H4K8ac_peak_13154	9.00699	RP11-227F19.2_ENSG00000249122

4	41752745	41753718	5Y-H4K8ac_peak_13155	9.8829	RP11-227F19.2_ENSG00000249122
4	41836771	41836970	5Y-H4K8ac_peak_13156	6.50117	
4	41875411	41875804	5Y-H4K8ac_peak_13157	9.30505	
4	41876499	41876776	5Y-H4K8ac_peak_13158	9.63153	
4	41879709	41880019	5Y-H4K8ac_peak_13159	6.55841	
4	41880263	41880841	5Y-H4K8ac_peak_13160	13.4304	
4	41882097	41883247	5Y-H4K8ac_peak_13161	13.33225	
4	41883454	41884601	5Y-H4K8ac_peak_13162	28.14579	LINC00682_ENSG00000245870
4	41885406	41885650	5Y-H4K8ac_peak_13163	6.7002	LINC00682_ENSG00000245870
4	41888280	41888566	5Y-H4K8ac_peak_13164	6.42825	
4	42307970	42308463	5Y-H4K8ac_peak_13165	6.42825	
4	42309749	42310036	5Y-H4K8ac_peak_13166	9.78792	
4	42310841	42311354	5Y-H4K8ac_peak_13167	5.77946	
4	42312541	42313134	5Y-H4K8ac_peak_13168	5.0078	
4	44680552	44680849	5Y-H4K8ac_peak_13169	4.95697	YIPF7_ENSG00000177752;GUF1_ENSG00000151806
4	44728236	44728497	5Y-H4K8ac_peak_13170	8.21582	GNPDA2_ENSG00000163281
4	48343794	48344177	5Y-H4K8ac_peak_13171	7.09658	SLAIN2_ENSG00000109171
4	48485354	48485979	5Y-H4K8ac_peak_13172	7.90751	SLC10A4_ENSG00000145248
4	48833158	48833531	5Y-H4K8ac_peak_13173	12.52787	
4	48908224	48909229	5Y-H4K8ac_peak_13174	15.87289	OCIAD2_ENSG00000145247
4	52904543	52904836	5Y-H4K8ac_peak_13175	10.21117	SGCB_ENSG00000163069
4	53126872	53127107	5Y-H4K8ac_peak_13176	7.31815	
4	53525027	53525338	5Y-H4K8ac_peak_13177	13.88421	USP46_ENSG00000109189;USP46-AS1_ENSG00000248866
4	53578055	53578304	5Y-H4K8ac_peak_13178	8.93923	DANCR_ENSG00000226950;MIR4449_ENSG00000264585
4	53617221	53617517	5Y-H4K8ac_peak_13179	8.21582	ERVMER34-1_ENSG00000226887;AC104066.1_ENSG00000266656
4	53706793	53707016	5Y-H4K8ac_peak_13180	10.94674	
4	53736422	53736713	5Y-H4K8ac_peak_13181	11.46984	
4	53878297	53878499	5Y-H4K8ac_peak_13182	7.80156	
4	53880662	53880947	5Y-H4K8ac_peak_13183	11.31394	
4	53912963	53913164	5Y-H4K8ac_peak_13184	8.21525	
4	53914680	53914991	5Y-H4K8ac_peak_13185	6.31818	
4	53915401	53915769	5Y-H4K8ac_peak_13186	8.2589	
4	53917253	53917723	5Y-H4K8ac_peak_13187	7.04637	
4	53920622	53920884	5Y-H4K8ac_peak_13188	4.07874	
4	53921484	53922110	5Y-H4K8ac_peak_13189	6.59249	
4	54568665	54569359	5Y-H4K8ac_peak_13190	13.99721	
4	54958174	54958555	5Y-H4K8ac_peak_13191	8.17203	
4	56212564	56213199	5Y-H4K8ac_peak_13192	13.4304	SRD5A3_ENSG00000128039
4	56719897	56720127	5Y-H4K8ac_peak_13193	8.43511	EXOC1_ENSG00000090989
4	56915410	56915946	5Y-H4K8ac_peak_13194	10.19948	
4	57108780	57109122	5Y-H4K8ac_peak_13195	5.40331	
4	57687577	57687870	5Y-H4K8ac_peak_13196	7.59101	SPINK2_ENSG00000128040
4	57737781	57738212	5Y-H4K8ac_peak_13197	4.50834	
4	57774605	57775287	5Y-H4K8ac_peak_13198	7.38046	REST_ENSG00000084093
4	57921865	57922183	5Y-H4K8ac_peak_13199	12.49199	
4	58030003	58030587	5Y-H4K8ac_peak_13200	8.43511	

4	62065745	62065976	5Y-H4K8ac_peak_13201	7.63144	
4	69216121	69216403	5Y-H4K8ac_peak_13202	5.40331	YTHDC1_ENSG00000083896
4	71569743	71569985	5Y-H4K8ac_peak_13203	5.718	RNU6-784P_ENSG00000207058;RP11-46J23.1_ENSG00000272986;RUFY3_ENSG00000018189
4	71859519	71859709	5Y-H4K8ac_peak_13204	4.8547	
4	71925205	71925479	5Y-H4K8ac_peak_13205	5.65584	
4	73295663	73296050	5Y-H4K8ac_peak_13206	5.40331	
4	73434764	73434969	5Y-H4K8ac_peak_13207	5.93823	ADAMTS3_ENSG00000156140
4	74124619	74125163	5Y-H4K8ac_peak_13208	12.71156	ANKRD17_ENSG00000132466;RP11-692D12.1_ENSG00000250220
4	75023967	75024181	5Y-H4K8ac_peak_13209	9.64738	AC093677.1_ENSG00000269559
4	76373322	76373520	5Y-H4K8ac_peak_13210	7.31815	
4	76598127	76598371	5Y-H4K8ac_peak_13211	4.29586	
4	76598991	76599252	5Y-H4K8ac_peak_13212	7.18391	
4	76650057	76650305	5Y-H4K8ac_peak_13213	6.77436	G3BP2_ENSG00000138757;USO1_ENSG00000138768
4	76912349	76912637	5Y-H4K8ac_peak_13214	16.52279	SDAD1_ENSG00000198301
4	77135159	77135792	5Y-H4K8ac_peak_13215	8.43511	SCARB2_ENSG00000138760;FAM47E_ENSG00000189157
4	77155161	77155406	5Y-H4K8ac_peak_13216	9.34555	
4	77172864	77173063	5Y-H4K8ac_peak_13217	6.22669	FAM47E-STBD1_ENSG00000272414;FAM47E-STBD1_ENSG00000118804
4	77342035	77342734	5Y-H4K8ac_peak_13218	8.43511	CCDC158_ENSG00000163749
4	77610442	77610994	5Y-H4K8ac_peak_13219	13.72401	
4	77996675	77996915	5Y-H4K8ac_peak_13220	11.87893	CCNI_ENSG00000118816
4	78740471	78740709	5Y-H4K8ac_peak_13221	5.98695	CNOT6L_ENSG00000138767
4	78952174	78952525	5Y-H4K8ac_peak_13222	5.65584	
4	78953414	78953629	5Y-H4K8ac_peak_13223	5.56912	
4	78980144	78980453	5Y-H4K8ac_peak_13224	8.21582	
4	79696550	79697045	5Y-H4K8ac_peak_13225	8.69112	RP11-109G23.3_ENSG00000260278;BMP2K_ENSG00000138756
4	79860123	79860512	5Y-H4K8ac_peak_13226	8.88899	PAQR3_ENSG00000163291
4	80644728	80645042	5Y-H4K8ac_peak_13227	7.31815	
4	80993584	80993799	5Y-H4K8ac_peak_13228	5.89922	
4	81187368	81187634	5Y-H4K8ac_peak_13229	4.36976	FGF5_ENSG00000138675
4	81187887	81188098	5Y-H4K8ac_peak_13230	4.77126	FGF5_ENSG00000138675
4	83294298	83295076	5Y-H4K8ac_peak_13231	7.89142	HNRNPD_ENSG00000138668;RP11-127B20.3_ENSG00000272677
4	83350850	83351352	5Y-H4K8ac_peak_13232	12.21176	HNRNPDL_ENSG00000152795;ENOPH1_ENSG00000145293
4	83483198	83483525	5Y-H4K8ac_peak_13233	11.71297	TMEM150C_ENSG00000249242
4	83719616	83720237	5Y-H4K8ac_peak_13234	7.20869	SCD5_ENSG00000145284
4	83955528	83955833	5Y-H4K8ac_peak_13235	5.23083	COPS4_ENSG00000138663
4	85887274	85887466	5Y-H4K8ac_peak_13236	8.82628	WDFY3_ENSG00000163625;WDFY3-AS2_ENSG00000180769
4	86049048	86049312	5Y-H4K8ac_peak_13237	7.31815	
4	87515568	87515825	5Y-H4K8ac_peak_13238	5.23083	MAPK10_ENSG00000109339;PTPN13_ENSG00000163629
4	87813175	87813914	5Y-H4K8ac_peak_13239	10.54764	
4	87856334	87856606	5Y-H4K8ac_peak_13240	7.76232	C4orf36_ENSG00000163633;AFF1_ENSG00000172493
4	87990066	87990287	5Y-H4K8ac_peak_13241	5.98695	
4	88142127	88142335	5Y-H4K8ac_peak_13242	5.40331	
4	88928435	88928684	5Y-H4K8ac_peak_13243	4.95697	PKD2_ENSG00000118762
4	89300114	89300351	5Y-H4K8ac_peak_13244	5.98695	HERC6_ENSG00000138642
4	89444579	89444841	5Y-H4K8ac_peak_13245	5.34294	PYURF_ENSG00000145337
4	90032736	90032936	5Y-H4K8ac_peak_13246	8.24461	FAM13A_ENSG00000138640;RP11-84C13.1_ENSG00000271359

4	91049500	91049874	5Y-H4K8ac_peak_13247	7.3889	CCSER1_ENSG00000184305
4	95128859	95129231	5Y-H4K8ac_peak_13248	6.46053	RP11-363G15.2_ENSG00000246541;SMARCAD1_ENSG00000163104
4	95570131	95570496	5Y-H4K8ac_peak_13249	4.50834	
4	95602707	95603191	5Y-H4K8ac_peak_13250	7.31815	
4	95678733	95678934	5Y-H4K8ac_peak_13251	9.15977	RP11-168E14.1_ENSG00000249599;BMPR1B_ENSG00000138696
4	95679126	95679843	5Y-H4K8ac_peak_13252	19.41185	RP11-168E14.1_ENSG00000249599;BMPR1B_ENSG00000138696
4	95680134	95680357	5Y-H4K8ac_peak_13253	4.95697	
4	95681652	95681915	5Y-H4K8ac_peak_13254	7.80156	
4	95728900	95729095	5Y-H4K8ac_peak_13255	8.33296	
4	96080747	96080967	5Y-H4K8ac_peak_13256	6.50117	
4	96435930	96436129	5Y-H4K8ac_peak_13257	7.31815	
4	96438683	96438899	5Y-H4K8ac_peak_13258	5.40331	
4	96468644	96468918	5Y-H4K8ac_peak_13259	5.03917	
4	96469176	96469510	5Y-H4K8ac_peak_13260	5.12213	UNC5C_ENSG00000182168;RP11-710C12.1_ENSG00000271474
4	96469719	96470113	5Y-H4K8ac_peak_13261	7.57144	UNC5C_ENSG00000182168;RP11-710C12.1_ENSG00000271474
4	96470527	96471019	5Y-H4K8ac_peak_13262	12.10416	UNC5C_ENSG00000182168;RP11-710C12.1_ENSG00000271474
4	99181455	99181747	5Y-H4K8ac_peak_13263	6.1654	RAP1GDS1_ENSG00000138698
4	99580079	99580401	5Y-H4K8ac_peak_13264	9.38203	TSPAN5_ENSG00000168785;RP11-1299A16.3_ENSG00000260641
4	99590488	99590682	5Y-H4K8ac_peak_13265	3.9339	
4	99850010	99850226	5Y-H4K8ac_peak_13266	12.84845	RP11-571L19.7_ENSG00000263923;AC019131.1_ENSG00000238449
4	99916917	99917576	5Y-H4K8ac_peak_13267	6.34245	METAP1_ENSG00000164024;MIR3684_ENSG00000265213
4	100457432	100457665	5Y-H4K8ac_peak_13268	5.87382	
4	100484052	100484346	5Y-H4K8ac_peak_13269	7.97699	TRMT10A_ENSG00000145331;MTTP_ENSG00000138823
4	100484567	100484764	5Y-H4K8ac_peak_13270	9.23159	TRMT10A_ENSG00000145331;MTTP_ENSG00000138823
4	100867398	100867800	5Y-H4K8ac_peak_13271	6.96612	DNAJB14_ENSG00000164031
4	102826379	102826575	5Y-H4K8ac_peak_13272	10.36926	
4	103265653	103266153	5Y-H4K8ac_peak_13273	7.03573	
4	103422988	103423283	5Y-H4K8ac_peak_13274	7.38046	AF213884.2_ENSG00000260651;NFKB1_ENSG00000109320
4	103748402	103748652	5Y-H4K8ac_peak_13275	9.51254	RP11-10L12.4_ENSG00000246560
4	103997593	103997935	5Y-H4K8ac_peak_13276	10.35586	
4	106067701	106068011	5Y-H4K8ac_peak_13277	4.84727	TET2_ENSG00000168769
4	106394726	106395208	5Y-H4K8ac_peak_13278	11.68317	PPA2_ENSG00000138777
4	107236996	107237218	5Y-H4K8ac_peak_13279	5.449	AIMP1_ENSG00000164022
4	107446404	107446820	5Y-H4K8ac_peak_13280	6.20875	
4	108393258	108393708	5Y-H4K8ac_peak_13281	9.34555	
4	109093836	109094111	5Y-H4K8ac_peak_13282	5.5192	
4	109541921	109542136	5Y-H4K8ac_peak_13283	7.17184	RPL34-AS1_ENSG00000234492;RPL34_ENSG00000109475
4	110480756	110481166	5Y-H4K8ac_peak_13284	23.98158	CCDC109B_ENSG00000005059
4	110624116	110624954	5Y-H4K8ac_peak_13285	7.3889	CASP6_ENSG00000138794
4	111117807	111118400	5Y-H4K8ac_peak_13286	8.69112	
4	113431033	113431286	5Y-H4K8ac_peak_13287	6.08523	
4	113431921	113432332	5Y-H4K8ac_peak_13288	7.90751	
4	113436042	113436528	5Y-H4K8ac_peak_13289	5.67283	NEUROG2_ENSG00000178403;RP11-402J6.1_ENSG00000249509
4	113436779	113437263	5Y-H4K8ac_peak_13290	7.59101	NEUROG2_ENSG00000178403;RP11-402J6.1_ENSG00000249509
4	113438359	113438751	5Y-H4K8ac_peak_13291	8.43511	
4	113451087	113451277	5Y-H4K8ac_peak_13292	5.23083	RP11-402J6.2_ENSG00000248439

4	113483164	113483359	5Y-H4K8ac_peak_13293	8.27399	
4	113814939	113815410	5Y-H4K8ac_peak_13294	4.07874	
4	113817035	113817538	5Y-H4K8ac_peak_13295	7.31815	
4	114683351	114683615	5Y-H4K8ac_peak_13296	10.54764	CAMK2D_ENSG00000145349
4	117847036	117847516	5Y-H4K8ac_peak_13297	11.19336	
4	118006324	118006593	5Y-H4K8ac_peak_13298	5.23083	TRAM1L1_ENSG00000174599
4	118006979	118007299	5Y-H4K8ac_peak_13299	8.33296	TRAM1L1_ENSG00000174599
4	118954484	118954997	5Y-H4K8ac_peak_13300	4.15658	NDST3_ENSG00000164100
4	119186617	119187041	5Y-H4K8ac_peak_13301	4.24332	
4	119248427	119248634	5Y-H4K8ac_peak_13302	7.31815	
4	119273237	119273791	5Y-H4K8ac_peak_13303	8.2913	PRSS12_ENSG00000164099
4	119824922	119825128	5Y-H4K8ac_peak_13304	7.38046	
4	119828056	119828262	5Y-H4K8ac_peak_13305	5.87382	
4	120548653	120548862	5Y-H4K8ac_peak_13306	5.23819	
4	121843454	121844267	5Y-H4K8ac_peak_13307	8.20773	PRDM5_ENSG00000138738
4	122617559	122618072	5Y-H4K8ac_peak_13308	4.84727	ANXA5_ENSG00000164111
4	122632847	122633344	5Y-H4K8ac_peak_13309	5.23083	
4	122744615	122744955	5Y-H4K8ac_peak_13310	5.98695	CCNA2_ENSG00000145386
4	123748016	123748416	5Y-H4K8ac_peak_13311	6.33308	FGF2_ENSG00000138685;AC021205.1_ENSG00000253069
4	123843324	123843748	5Y-H4K8ac_peak_13312	7.80156	NUDT6_ENSG00000170917;SPATA5_ENSG00000145375
4	124318746	124319255	5Y-H4K8ac_peak_13313	8.00339	SPRY1_ENSG00000164056
4	124319631	124319902	5Y-H4K8ac_peak_13314	6.54441	
4	124521154	124521407	5Y-H4K8ac_peak_13315	7.76232	
4	125633333	125633653	5Y-H4K8ac_peak_13316	5.97089	ANKRD50_ENSG00000151458
4	128543849	128544058	5Y-H4K8ac_peak_13317	5.23083	INTU_ENSG00000164066
4	128702763	128703177	5Y-H4K8ac_peak_13318	6.37023	HSPA4L_ENSG00000164070
4	128801077	128801290	5Y-H4K8ac_peak_13319	5.40331	PLK4_ENSG00000142731
4	128802105	128802443	5Y-H4K8ac_peak_13320	10.35586	PLK4_ENSG00000142731
4	128981787	128982171	5Y-H4K8ac_peak_13321	9.02938	LARP1B_ENSG00000138709
4	128982952	128983251	5Y-H4K8ac_peak_13322	5.12213	LARP1B_ENSG00000138709
4	129474418	129474620	5Y-H4K8ac_peak_13323	8.2913	RP11-130C6.1_ENSG00000273077
4	130014301	130014582	5Y-H4K8ac_peak_13324	8.33296	SCLT1_ENSG00000151466;C4orf33_ENSG00000151470
4	135122580	135122843	5Y-H4K8ac_peak_13325	6.34046	PABPC4L_ENSG00000254535
4	139832375	139833058	5Y-H4K8ac_peak_13326	11.1169	
4	139836412	139836668	5Y-H4K8ac_peak_13327	6.50117	
4	139837476	139837798	5Y-H4K8ac_peak_13328	8.78565	
4	139940179	139940448	5Y-H4K8ac_peak_13329	4.50834	
4	140216732	140216922	5Y-H4K8ac_peak_13330	8.62703	
4	140222930	140223263	5Y-H4K8ac_peak_13331	9.79526	NDUFC1_ENSG00000109390;NAA15_ENSG00000164134
4	140476567	140477458	5Y-H4K8ac_peak_13332	7.90236	RP11-342I1.2_ENSG00000272717
4	140478086	140478305	5Y-H4K8ac_peak_13333	7.30348	RP11-342I1.2_ENSG00000272717
4	140656968	140657190	5Y-H4K8ac_peak_13334	10.35586	
4	140661934	140662184	5Y-H4K8ac_peak_13335	11.28133	
4	140704730	140704985	5Y-H4K8ac_peak_13336	14.2053	
4	140747145	140747392	5Y-H4K8ac_peak_13337	5.65584	
4	140767540	140767826	5Y-H4K8ac_peak_13338	5.87382	

4	140786875	140787323	5Y-H4K8ac_peak_13339	11.19336	
4	140787537	140787770	5Y-H4K8ac_peak_13340	8.2913	
4	140810473	140810701	5Y-H4K8ac_peak_13341	8.43511	
4	140811341	140811615	5Y-H4K8ac_peak_13342	11.57334	
4	140823472	140823854	5Y-H4K8ac_peak_13343	4.12295	
4	140824266	140824503	5Y-H4K8ac_peak_13344	6.07082	
4	140842505	140843065	5Y-H4K8ac_peak_13345	6.1654	
4	140862862	140863169	5Y-H4K8ac_peak_13346	6.77436	
4	140925771	140925996	5Y-H4K8ac_peak_13347	5.98695	
4	140970172	140970416	5Y-H4K8ac_peak_13348	5.65584	
4	141016072	141016323	5Y-H4K8ac_peak_13349	7.42477	
4	141072245	141072765	5Y-H4K8ac_peak_13350	11.04542	
4	141073799	141074314	5Y-H4K8ac_peak_13351	9.52603	
4	141157780	141158056	5Y-H4K8ac_peak_13352	8.27399	
4	141158608	141158829	5Y-H4K8ac_peak_13353	6.50117	
4	141159798	141160106	5Y-H4K8ac_peak_13354	11.71078	
4	141173328	141173542	5Y-H4K8ac_peak_13355	6.12014	
4	141173785	141174362	5Y-H4K8ac_peak_13356	27.2273	
4	141348541	141348737	5Y-H4K8ac_peak_13357	5.91107	CLGN_ENSG00000153132
4	141445387	141445630	5Y-H4K8ac_peak_13358	7.90751	ELMOD2_ENSG00000179387
4	141609301	141609641	5Y-H4K8ac_peak_13359	4.50834	
4	141677242	141677780	5Y-H4K8ac_peak_13360	7.65114	TBC1D9_ENSG00000109436;RP11-102N12.3_ENSG00000273472
4	141823912	141824223	5Y-H4K8ac_peak_13361	6.77436	
4	141826576	141826777	5Y-H4K8ac_peak_13362	5.65584	
4	142526674	142526942	5Y-H4K8ac_peak_13363	7.31815	
4	144434688	144434937	5Y-H4K8ac_peak_13364	9.15007	SMARCA5_ENSG00000153147;SMARCA5-AS1_ENSG00000245112
4	144480357	144480715	5Y-H4K8ac_peak_13365	7.35333	GUSBP5_ENSG00000236296
4	146403493	146403851	5Y-H4K8ac_peak_13366	9.38203	
4	146539817	146540466	5Y-H4K8ac_peak_13367	8.33296	MMAA_ENSG00000151611
4	146654353	146654627	5Y-H4K8ac_peak_13368	16.50659	
4	146856933	146857448	5Y-H4K8ac_peak_13369	7.76232	
4	147096930	147097464	5Y-H4K8ac_peak_13370	11.19336	LSM6_ENSG00000164167
4	147163441	147163723	5Y-H4K8ac_peak_13371	7.89273	RP11-6L6.7_ENSG00000251010
4	148538642	148539003	5Y-H4K8ac_peak_13372	6.34046	RP11-425A23.1_ENSG00000251298;TMEM184C_ENSG00000164168
4	148652654	148653063	5Y-H4K8ac_peak_13373	9.51254	ARHGAP10_ENSG00000071205
4	150999856	151000561	5Y-H4K8ac_peak_13374	9.60627	DCLK2_ENSG00000170390
4	151789853	151790113	5Y-H4K8ac_peak_13375	4.07874	
4	151936231	151936798	5Y-H4K8ac_peak_13376	12.05638	LRBA_ENSG00000198589
4	152089967	152090172	5Y-H4K8ac_peak_13377	5.65584	
4	152246266	152246644	5Y-H4K8ac_peak_13378	9.30505	SH3D19_ENSG00000109686
4	152329880	152330161	5Y-H4K8ac_peak_13379	5.35202	RP11-610P16.1_ENSG00000251611;FAM160A1_ENSG00000164142
4	152681884	152682093	5Y-H4K8ac_peak_13380	7.59101	PET112_ENSG00000059691
4	153456882	153457093	5Y-H4K8ac_peak_13381	6.94416	FBXW7_ENSG00000109670;MIR4453_ENSG00000268471
4	154074182	154074969	5Y-H4K8ac_peak_13382	7.11863	TRIM2_ENSG00000109654
4	154076032	154076288	5Y-H4K8ac_peak_13383	8.43511	
4	154265984	154266256	5Y-H4K8ac_peak_13384	5.72233	MND1_ENSG00000121211

4	154386931	154387246	5Y-H4K8ac_peak_13385	5.65584	KIAA0922_ENSG00000121210
4	154387564	154388106	5Y-H4K8ac_peak_13386	12.87532	KIAA0922_ENSG00000121210
4	154680805	154681125	5Y-H4K8ac_peak_13387	6.78128	RNF175_ENSG00000145428
4	156588345	156588725	5Y-H4K8ac_peak_13388	4.00285	GUCY1A3_ENSG00000164116
4	156874558	156875584	5Y-H4K8ac_peak_13389	11.57334	CTSO_ENSG00000256043
4	159644137	159644414	5Y-H4K8ac_peak_13390	6.79955	PPID_ENSG00000171497
4	159645024	159645328	5Y-H4K8ac_peak_13391	7.38046	PPID_ENSG00000171497
4	159689671	159689908	5Y-H4K8ac_peak_13392	4.84727	FNIP2_ENSG00000052795
4	159791250	159791512	5Y-H4K8ac_peak_13393	6.46053	
4	160023703	160024035	5Y-H4K8ac_peak_13394	5.64909	
4	164088269	164088493	5Y-H4K8ac_peak_13395	4.50834	NAF1_ENSG00000145414
4	165878005	165878324	5Y-H4K8ac_peak_13396	8.33296	FAM218A_ENSG00000250486
4	166033537	166034010	5Y-H4K8ac_peak_13397	8.33296	
4	166128791	166129092	5Y-H4K8ac_peak_13398	12.71156	TMEM192_ENSG00000170088;KLHL2_ENSG00000109466
4	169239507	169239973	5Y-H4K8ac_peak_13399	12.10416	DDX60_ENSG00000137628
4	169753397	169753637	5Y-H4K8ac_peak_13400	7.04637	RP11-635L1.3_ENSG00000249609
4	169840413	169840671	5Y-H4K8ac_peak_13401	9.34555	
4	169930588	169931026	5Y-H4K8ac_peak_13402	8.2913	CBR4_ENSG00000145439;RP11-483A20.3_ENSG00000251445
4	170192312	170192852	5Y-H4K8ac_peak_13403	14.00559	SH3RF1_ENSG00000154447
4	170678733	170679011	5Y-H4K8ac_peak_13404	8.93923	C4orf27_ENSG00000056050
4	170947002	170947485	5Y-H4K8ac_peak_13405	10.36926	
4	171010904	171011396	5Y-H4K8ac_peak_13406	11.22005	
4	174090034	174090840	5Y-H4K8ac_peak_13407	5.98695	RP11-10K16.1_ENSG00000245213;GALNT7_ENSG00000109586
4	174255088	174255392	5Y-H4K8ac_peak_13408	9.34555	HMGB2_ENSG00000164104
4	174255981	174256250	5Y-H4K8ac_peak_13409	8.24461	HMGB2_ENSG00000164104
4	174427862	174428218	5Y-H4K8ac_peak_13410	6.09071	
4	174428506	174428732	5Y-H4K8ac_peak_13411	5.65584	
4	174430347	174431033	5Y-H4K8ac_peak_13412	13.85283	
4	174438073	174438400	5Y-H4K8ac_peak_13413	12.11208	
4	174438962	174439268	5Y-H4K8ac_peak_13414	9.52603	
4	174439600	174440237	5Y-H4K8ac_peak_13415	15.40182	
4	174442629	174442896	5Y-H4K8ac_peak_13416	8.20773	
4	174443768	174444259	5Y-H4K8ac_peak_13417	9.30505	
4	174448574	174448788	5Y-H4K8ac_peak_13418	5.23083	HAND2-AS1_ENSG00000237125
4	174450640	174450889	5Y-H4K8ac_peak_13419	5.65584	HAND2_ENSG00000164107
4	174452402	174453008	5Y-H4K8ac_peak_13420	11.99586	
4	174458862	174460110	5Y-H4K8ac_peak_13421	12.69256	
4	174757264	174757456	5Y-H4K8ac_peak_13422	8.17203	
4	174990020	174990275	5Y-H4K8ac_peak_13423	5.98695	
4	178363179	178363570	5Y-H4K8ac_peak_13424	13.8759	AGA_ENSG00000038002
4	178364174	178364405	5Y-H4K8ac_peak_13425	4.84727	AGA_ENSG00000038002
4	183065435	183066351	5Y-H4K8ac_peak_13426	7.50148	AC108142.1_ENSG00000177822;TENM3_ENSG00000218336;RP11-402C9.1_ENSG00000248266
4	183369354	183369860	5Y-H4K8ac_peak_13427	7.89273	
4	183497934	183498210	5Y-H4K8ac_peak_13428	4.95697	
4	183515927	183516153	5Y-H4K8ac_peak_13429	6.13909	
4	183708503	183708718	5Y-H4K8ac_peak_13430	4.07874	

4	183801238	183801471	5Y-H4K8ac_peak_13431	8.04943	
4	183801690	183802537	5Y-H4K8ac_peak_13432	6.77436	RP11-188P17.2_ENSG00000272646
4	183837735	183838471	5Y-H4K8ac_peak_13433	9.30505	DCTD_ENSG00000129187
4	183838679	183839176	5Y-H4K8ac_peak_13434	14.72547	DCTD_ENSG00000129187
4	184318977	184319906	5Y-H4K8ac_peak_13435	18.78631	
4	184320453	184320671	5Y-H4K8ac_peak_13436	10.6025	
4	184365077	184365604	5Y-H4K8ac_peak_13437	4.0639	CDKN2AIP_ENSG00000168564
4	184366011	184366363	5Y-H4K8ac_peak_13438	6.47245	CDKN2AIP_ENSG00000168564
4	184642281	184642480	5Y-H4K8ac_peak_13439	5.13361	
4	184643982	184644403	5Y-H4K8ac_peak_13440	6.53157	
4	184718356	184718848	5Y-H4K8ac_peak_13441	6.90749	
4	184780293	184780651	5Y-H4K8ac_peak_13442	6.34046	
4	184797770	184798061	5Y-H4K8ac_peak_13443	6.78128	
4	185025468	185025726	5Y-H4K8ac_peak_13444	5.70209	
4	185393929	185394131	5Y-H4K8ac_peak_13445	6.78318	RP11-326I11.5_ENSG00000270426
4	185468316	185468585	5Y-H4K8ac_peak_13446	11.19336	
4	185470118	185470318	5Y-H4K8ac_peak_13447	4.84727	
4	185571040	185571327	5Y-H4K8ac_peak_13448	7.38046	CASP3_ENSG00000164305;PRIMPOL_ENSG00000164306
4	185704183	185704578	5Y-H4K8ac_peak_13449	4.5753	
4	185747396	185747783	5Y-H4K8ac_peak_13450	7.03573	ACSL1_ENSG00000151726
4	186023105	186023309	5Y-H4K8ac_peak_13451	6.94416	
4	186039559	186039867	5Y-H4K8ac_peak_13452	5.3176	
4	186048607	186049159	5Y-H4K8ac_peak_13453	14.01193	
4	186049411	186049730	5Y-H4K8ac_peak_13454	5.12213	
4	186123378	186123603	5Y-H4K8ac_peak_13455	5.65584	
4	186124692	186125430	5Y-H4K8ac_peak_13456	8.69112	SNX25_ENSG00000109762
4	186317219	186317560	5Y-H4K8ac_peak_13457	11.23789	LRP2BP_ENSG00000109771;ANKRD37_ENSG00000186352
4	186392860	186393053	5Y-H4K8ac_peak_13458	8.35139	CCDC110_ENSG00000168491;RP11-279O9.4_ENSG00000249679
4	186456284	186456528	5Y-H4K8ac_peak_13459	6.31818	PDLIM3_ENSG00000154553
4	186491592	186491834	5Y-H4K8ac_peak_13460	5.98695	
4	186494792	186495223	5Y-H4K8ac_peak_13461	10.0732	
4	186759300	186759548	5Y-H4K8ac_peak_13462	5.40331	
4	187026025	187026516	5Y-H4K8ac_peak_13463	9.51254	FAM149A_ENSG00000109794
4	187112443	187113238	5Y-H4K8ac_peak_13464	13.99721	AC110771.1_ENSG00000269302;CYP4V2_ENSG00000145476
4	190642607	190642818	5Y-H4K8ac_peak_13465	6.0552	
4	190861400	190861757	5Y-H4K8ac_peak_13466	10.15788	AF146191.4_ENSG00000245685;FRG1_ENSG00000109536
5	90865	92367	5Y-H4K8ac_peak_13467	19.79054	CTD-2231H16.1_ENSG00000249430
5	190926	191705	5Y-H4K8ac_peak_13468	8.2913	LRRC14B_ENSG00000185028
5	373790	374476	5Y-H4K8ac_peak_13469	11.36989	
5	442708	443168	5Y-H4K8ac_peak_13470	7.59101	C5orf55_ENSG00000221990;EXOC3_ENSG00000180104
5	472239	473104	5Y-H4K8ac_peak_13471	24.88223	CTD-2228K2.5_ENSG00000188242;CTD-2228K2.7_ENSG00000225138
5	473413	473772	5Y-H4K8ac_peak_13472	10.19948	CTD-2228K2.5_ENSG00000188242;CTD-2228K2.7_ENSG00000225138
5	784389	784651	5Y-H4K8ac_peak_13473	6.86362	
5	784991	785375	5Y-H4K8ac_peak_13474	7.89273	
5	892122	892897	5Y-H4K8ac_peak_13475	11.1169	BRD9_ENSG00000028310;TRIP13_ENSG00000071539
5	1345573	1345801	5Y-H4K8ac_peak_13476	6.78128	CLPTM1L_ENSG00000049656

5	1524220	1524949	5Y-H4K8ac_peak_13477	11.4254	LPCAT1_ENSG00000153395
5	1594850	1595261	5Y-H4K8ac_peak_13478	9.52603	SDHAP3_ENSG00000185986;CTD-2012J19.3_ENSG00000271119
5	1633494	1634024	5Y-H4K8ac_peak_13479	10.74739	RP11-43F13.1_ENSG00000188002
5	1635138	1635340	5Y-H4K8ac_peak_13480	6.72926	
5	1799336	1799603	5Y-H4K8ac_peak_13481	5.68769	
5	1801446	1801866	5Y-H4K8ac_peak_13482	10.79063	MRPL36_ENSG00000171421;NDUFS6_ENSG00000145494
5	4912421	4912674	5Y-H4K8ac_peak_13483	8.08688	
5	5026274	5026529	5Y-H4K8ac_peak_13484	8.16382	
5	5318142	5318350	5Y-H4K8ac_peak_13485	4.84727	
5	5319524	5319852	5Y-H4K8ac_peak_13486	6.43775	
5	5712799	5713352	5Y-H4K8ac_peak_13487	7.50148	
5	6202463	6202653	5Y-H4K8ac_peak_13488	6.46053	
5	6359632	6359879	5Y-H4K8ac_peak_13489	4.07874	
5	6378996	6379225	5Y-H4K8ac_peak_13490	10.19948	MED10_ENSG00000133398
5	6632957	6633392	5Y-H4K8ac_peak_13491	7.65114	NSUN2_ENSG00000037474;SRD5A1_ENSG00000145545
5	6712648	6712918	5Y-H4K8ac_peak_13492	7.30348	
5	9715415	9715622	5Y-H4K8ac_peak_13493	5.8635	
5	10248997	10249330	5Y-H4K8ac_peak_13494	9.23159	FAM173B_ENSG00000150756;CTD-2256P15.1_ENSG00000248968;CCT5_ENSG00000150753
5	10249721	10249911	5Y-H4K8ac_peak_13495	8.2913	FAM173B_ENSG00000150756;CCT5_ENSG00000150753
5	10250478	10250919	5Y-H4K8ac_peak_13496	9.02938	FAM173B_ENSG00000150756;CCT5_ENSG00000150753
5	10353300	10353646	5Y-H4K8ac_peak_13497	14.99673	CTD-2256P15.2_ENSG00000259802;MARCH6_ENSG00000145495
5	10353854	10354421	5Y-H4K8ac_peak_13498	12.05638	CTD-2256P15.2_ENSG00000259802;MARCH6_ENSG00000145495
5	10501647	10501842	5Y-H4K8ac_peak_13499	6.20875	RP11-1C1.4_ENSG00000249396
5	10655435	10655661	5Y-H4K8ac_peak_13500	7.38046	RP11-54F2.1_ENSG00000251196
5	10761599	10762014	5Y-H4K8ac_peak_13501	9.30505	DAP_ENSG00000112977;CTD-2154B17.4_ENSG00000272324
5	14251569	14251896	5Y-H4K8ac_peak_13502	4.95697	
5	14418114	14418456	5Y-H4K8ac_peak_13503	13.14766	
5	14419860	14420052	5Y-H4K8ac_peak_13504	4.95697	
5	14441156	14441692	5Y-H4K8ac_peak_13505	5.98695	
5	14443679	14443886	5Y-H4K8ac_peak_13506	7.38046	
5	14445914	14446516	5Y-H4K8ac_peak_13507	6.77436	
5	14448460	14448799	5Y-H4K8ac_peak_13508	11.19336	
5	14581561	14581761	5Y-H4K8ac_peak_13509	13.0168	FAM105A_ENSG00000145569
5	14591476	14591699	5Y-H4K8ac_peak_13510	7.09658	
5	14871380	14871734	5Y-H4K8ac_peak_13511	6.10478	ANKH_ENSG00000154122;CTB-40H15.4_ENSG00000272057
5	14872277	14872683	5Y-H4K8ac_peak_13512	6.50117	ANKH_ENSG00000154122;CTB-40H15.4_ENSG00000272057
5	15500478	15500837	5Y-H4K8ac_peak_13513	9.38807	FBXL7_ENSG00000183580
5	15580302	15580650	5Y-H4K8ac_peak_13514	8.46999	
5	16179242	16179559	5Y-H4K8ac_peak_13515	7.01266	RP11-19O2.2_ENSG00000250448
5	16180085	16180523	5Y-H4K8ac_peak_13516	10.19948	MARCH11_ENSG00000183654;RP11-19O2.2_ENSG00000250448
5	16420394	16420597	5Y-H4K8ac_peak_13517	9.23159	
5	16420830	16421034	5Y-H4K8ac_peak_13518	7.01364	
5	16616605	16617677	5Y-H4K8ac_peak_13519	7.31102	FAM134B_ENSG00000154153;RP11-260E18.1_ENSG00000246214;CTC-461F20.1_ENSG00000250415
5	16866902	16867179	5Y-H4K8ac_peak_13520	8.93923	
5	21481731	21482207	5Y-H4K8ac_peak_13521	6.75261	
5	21482609	21482822	5Y-H4K8ac_peak_13522	10.66356	

5	31700981	31701698	5Y-H4K8ac_peak_13523	8.93923	
5	32174033	32174372	5Y-H4K8ac_peak_13524	13.85283	GOLPH3_ENSG00000113384;CTD-2186M15.3_ENSG00000272086
5	32174601	32174964	5Y-H4K8ac_peak_13525	11.19336	GOLPH3_ENSG00000113384;CTD-2186M15.3_ENSG00000272086
5	32444461	32444738	5Y-H4K8ac_peak_13526	5.91107	ZFR_ENSG00000056097
5	32444966	32445370	5Y-H4K8ac_peak_13527	5.64909	ZFR_ENSG00000056097
5	34008378	34008609	5Y-H4K8ac_peak_13528	8.75926	AMACR_ENSG00000242110
5	34047301	34047507	5Y-H4K8ac_peak_13529	6.46053	
5	34054186	34054482	5Y-H4K8ac_peak_13530	5.90587	
5	34191394	34192074	5Y-H4K8ac_peak_13531	20.39519	
5	34192285	34193721	5Y-H4K8ac_peak_13532	36.69449	
5	34838707	34839239	5Y-H4K8ac_peak_13533	4.90696	CTD-2517O10.6_ENSG00000272323;TTC23L_ENSG00000205838
5	36690190	36690505	5Y-H4K8ac_peak_13534	11.82621	
5	37249639	37249949	5Y-H4K8ac_peak_13535	5.85188	C5orf42_ENSG00000197603
5	37371643	37371841	5Y-H4K8ac_peak_13536	7.04637	NUP155_ENSG00000113569
5	39074145	39074430	5Y-H4K8ac_peak_13537	9.34555	RICTOR_ENSG00000164327
5	40798525	40798736	5Y-H4K8ac_peak_13538	11.48691	PRKAA1_ENSG00000132356
5	40834901	40835262	5Y-H4K8ac_peak_13539	9.51254	RPL37_ENSG00000145592
5	40835633	40835851	5Y-H4K8ac_peak_13540	10.11191	RPL37_ENSG00000145592
5	40975580	40975803	5Y-H4K8ac_peak_13541	7.68492	
5	41263481	41264041	5Y-H4K8ac_peak_13542	6.62622	
5	41287322	41287662	5Y-H4K8ac_peak_13543	4.29586	
5	41791835	41792134	5Y-H4K8ac_peak_13544	8.33296	
5	41925392	41925868	5Y-H4K8ac_peak_13545	8.73392	FBXO4_ENSG00000151876
5	43066221	43067023	5Y-H4K8ac_peak_13546	5.64909	CTD-2201E18.3_ENSG00000177738;ZNF131_ENSG00000172262
5	43067397	43067613	5Y-H4K8ac_peak_13547	7.64648	CTD-2201E18.3_ENSG00000177738
5	43556482	43556721	5Y-H4K8ac_peak_13548	6.53157	
5	43557059	43557621	5Y-H4K8ac_peak_13549	5.98695	PAIP1_ENSG00000172239
5	43602844	43603078	5Y-H4K8ac_peak_13550	14.12043	NNT-AS1_ENSG00000248092;NNT_ENSG00000112992
5	49736929	49737461	5Y-H4K8ac_peak_13551	8.43511	
5	49962225	49962614	5Y-H4K8ac_peak_13552	9.34555	PARP8_ENSG00000151883
5	49963317	49963599	5Y-H4K8ac_peak_13553	8.79957	
5	50259074	50259476	5Y-H4K8ac_peak_13554	8.79957	
5	50259813	50260190	5Y-H4K8ac_peak_13555	10.74739	
5	50261445	50261765	5Y-H4K8ac_peak_13556	6.50117	
5	50264333	50264604	5Y-H4K8ac_peak_13557	8.33296	CTD-2089N3.3_ENSG00000248918
5	50265114	50265894	5Y-H4K8ac_peak_13558	11.22005	CTD-2089N3.2_ENSG00000251573;CTD-2089N3.3_ENSG00000248918
5	50673494	50673828	5Y-H4K8ac_peak_13559	6.34046	
5	50678576	50679064	5Y-H4K8ac_peak_13560	20.04267	CTD-2314G24.2_ENSG00000259663;ISL1_ENSG00000016082
5	50686159	50686359	5Y-H4K8ac_peak_13561	4.1776	
5	50694782	50695026	5Y-H4K8ac_peak_13562	8.79957	
5	51118930	51119123	5Y-H4K8ac_peak_13563	5.65584	
5	51283556	51283919	5Y-H4K8ac_peak_13564	5.65584	
5	51288338	51288587	5Y-H4K8ac_peak_13565	8.17203	
5	51291059	51291323	5Y-H4K8ac_peak_13566	11.32351	
5	52405998	52406238	5Y-H4K8ac_peak_13567	5.65584	MOCS2_ENSG00000164172;CTD-2366F13.1_ENSG00000247796
5	55117582	55117975	5Y-H4K8ac_peak_13568	7.80156	

5	55291160	55291444	5Y-H4K8ac_peak_13569	10.11191	IL6ST_ENSG00000134352;CTD-2031P19.3_ENSG00000227908
5	55566992	55567399	5Y-H4K8ac_peak_13570	11.42066	
5	55604179	55604472	5Y-H4K8ac_peak_13571	5.29015	
5	55606213	55606486	5Y-H4K8ac_peak_13572	7.89142	
5	55622704	55622994	5Y-H4K8ac_peak_13573	7.31815	
5	55775803	55776352	5Y-H4K8ac_peak_13574	6.94897	
5	55777515	55777709	5Y-H4K8ac_peak_13575	8.1667	CTC-236F12.4_ENSG00000248727
5	55793335	55793604	5Y-H4K8ac_peak_13576	8.47164	
5	55794450	55794832	5Y-H4K8ac_peak_13577	10.1994	
5	56011151	56011368	5Y-H4K8ac_peak_13578	6.98118	
5	56058774	56059096	5Y-H4K8ac_peak_13579	7.31815	
5	56111424	56111692	5Y-H4K8ac_peak_13580	9.62747	MAP3K1_ENSG00000095015
5	56118760	56119146	5Y-H4K8ac_peak_13581	5.40331	
5	56247517	56247878	5Y-H4K8ac_peak_13582	9.93175	
5	56248173	56248383	5Y-H4K8ac_peak_13583	8.43511	
5	57877897	57878193	5Y-H4K8ac_peak_13584	9.38276	RAB3C_ENSG00000152932
5	60241360	60241550	5Y-H4K8ac_peak_13585	6.34046	ERCC8_ENSG00000049167;NDUFAF2_ENSG00000164182
5	60458455	60458816	5Y-H4K8ac_peak_13586	11.30958	SMIM15_ENSG00000188725;CTC-436P18.1_ENSG00000251279
5	60626452	60626725	5Y-H4K8ac_peak_13587	7.59101	
5	61601267	61601720	5Y-H4K8ac_peak_13588	7.04637	KIF2A_ENSG00000068796
5	61602375	61602598	5Y-H4K8ac_peak_13589	7.59101	KIF2A_ENSG00000068796
5	64331472	64331824	5Y-H4K8ac_peak_13590	4.91235	
5	64398607	64399230	5Y-H4K8ac_peak_13591	5.65584	
5	65220893	65221221	5Y-H4K8ac_peak_13592	4.642	CTD-2033C11.1_ENSG00000269961
5	65221917	65222187	5Y-H4K8ac_peak_13593	4.15658	CTD-2033C11.1_ENSG00000269961;ERBB2IP_ENSG00000112851
5	65440128	65440578	5Y-H4K8ac_peak_13594	10.35586	AC025442.3_ENSG00000253744
5	66412048	66412479	5Y-H4K8ac_peak_13595	5.65584	
5	66499586	66499797	5Y-H4K8ac_peak_13596	5.8635	
5	66504141	66504338	5Y-H4K8ac_peak_13597	5.23083	
5	66511634	66511948	5Y-H4K8ac_peak_13598	13.8759	
5	66563629	66564023	5Y-H4K8ac_peak_13599	8.51022	CTD-2187J20.1_ENSG00000251206
5	66565187	66565482	5Y-H4K8ac_peak_13600	7.59101	
5	67294667	67294935	5Y-H4K8ac_peak_13601	5.40331	
5	67511014	67511313	5Y-H4K8ac_peak_13602	7.14616	PIK3R1_ENSG00000145675
5	67552773	67553261	5Y-H4K8ac_peak_13603	14.15915	
5	67583835	67584039	5Y-H4K8ac_peak_13604	11.19336	
5	67584682	67584881	5Y-H4K8ac_peak_13605	4.50834	
5	67702698	67702947	5Y-H4K8ac_peak_13606	4.84727	
5	67731319	67731517	5Y-H4K8ac_peak_13607	4.85014	
5	67830263	67831172	5Y-H4K8ac_peak_13608	21.01499	CTC-537E7.2_ENSG00000248359
5	67953011	67953226	5Y-H4K8ac_peak_13609	6.50117	
5	68332000	68332212	5Y-H4K8ac_peak_13610	6.43775	
5	68389953	68390362	5Y-H4K8ac_peak_13611	7.41197	SLC30A5_ENSG00000145740
5	68628369	68628612	5Y-H4K8ac_peak_13612	11.12941	CCDC125_ENSG00000183323
5	68665311	68665596	5Y-H4K8ac_peak_13613	19.93272	TAF9_ENSG00000085231;RAD17_ENSG00000152942
5	68855285	68855853	5Y-H4K8ac_peak_13614	8.75926	GTF2H2C_ENSG00000183474

5	69785868	69786150	5Y-H4K8ac_peak_13615	16.50659	
5	70882018	70882432	5Y-H4K8ac_peak_13616	8.00339	MCCC2_ENSG00000131844
5	71078137	71078664	5Y-H4K8ac_peak_13617	6.34046	
5	71402617	71402935	5Y-H4K8ac_peak_13618	7.38046	MAP1B_ENSG00000131711
5	71403273	71403500	5Y-H4K8ac_peak_13619	5.99504	MAP1B_ENSG00000131711
5	71403819	71404021	5Y-H4K8ac_peak_13620	6.73385	MAP1B_ENSG00000131711
5	71404337	71404745	5Y-H4K8ac_peak_13621	10.31981	
5	71482312	71482511	5Y-H4K8ac_peak_13622	6.88532	
5	71487254	71487465	5Y-H4K8ac_peak_13623	7.38046	
5	71615596	71616020	5Y-H4K8ac_peak_13624	6.79955	MRPS27_ENSG00000113048;PTCD2_ENSG00000049883
5	72112081	72112287	5Y-H4K8ac_peak_13625	10.31981	CTD-2631K10.1_ENSG00000249085;TNPO1_ENSG00000083312
5	72251249	72251607	5Y-H4K8ac_peak_13626	10.1994	CTD-2376I4.2_ENSG00000272081;FCHO2_ENSG00000157107
5	72793849	72794104	5Y-H4K8ac_peak_13627	7.11863	RP11-79P5.9_ENSG00000272525;BTF3_ENSG00000145741
5	72794371	72794860	5Y-H4K8ac_peak_13628	9.79526	RP11-79P5.9_ENSG00000272525;BTF3_ENSG00000145741
5	72861713	72861970	5Y-H4K8ac_peak_13629	5.91107	ANKRA2_ENSG00000164331;UTP15_ENSG00000164338
5	73685724	73685946	5Y-H4K8ac_peak_13630	4.50834	
5	73936785	73937055	5Y-H4K8ac_peak_13631	8.24461	ENC1_ENSG00000171617;HEXB_ENSG00000049860
5	73980624	73981294	5Y-H4K8ac_peak_13632	8.2913	
5	74162338	74162744	5Y-H4K8ac_peak_13633	12.21176	FAM169A_ENSG00000198780;CTD-2377O17.1_ENSG00000271714
5	74532013	74532598	5Y-H4K8ac_peak_13634	7.3889	ANKRD31_ENSG00000145700
5	75379246	75379555	5Y-H4K8ac_peak_13635	7.38046	CTC-235G5.3_ENSG00000248127;SV2C_ENSG00000122012
5	75380686	75380959	5Y-H4K8ac_peak_13636	5.65584	CTC-235G5.3_ENSG00000248127
5	76012049	76012290	5Y-H4K8ac_peak_13637	6.37023	CTD-2384B11.2_ENSG00000225407;F2R_ENSG00000181104
5	76326357	76326570	5Y-H4K8ac_peak_13638	6.14981	
5	76373132	76373544	5Y-H4K8ac_peak_13639	9.93099	
5	76382792	76383177	5Y-H4K8ac_peak_13640	8.69112	ZBED3_ENSG00000132846;ZBED3-AS1_ENSG00000250802
5	76476143	76476386	5Y-H4K8ac_peak_13641	8.24461	
5	77147161	77147563	5Y-H4K8ac_peak_13642	9.00954	
5	77148411	77148672	5Y-H4K8ac_peak_13643	7.57144	
5	77655953	77656174	5Y-H4K8ac_peak_13644	6.13909	CTD-2037K23.2_ENSG00000245556;SCAMP1_ENSG00000085365
5	77944174	77944449	5Y-H4K8ac_peak_13645	11.12941	
5	77944717	77945130	5Y-H4K8ac_peak_13646	13.27281	
5	77974605	77974921	5Y-H4K8ac_peak_13647	9.25361	
5	78101531	78101724	5Y-H4K8ac_peak_13648	5.98695	
5	78104383	78104726	5Y-H4K8ac_peak_13649	10.43045	
5	78105677	78106078	5Y-H4K8ac_peak_13650	8.14913	
5	78205428	78205644	5Y-H4K8ac_peak_13651	6.34046	
5	78208488	78208706	5Y-H4K8ac_peak_13652	8.30301	
5	78280693	78281260	5Y-H4K8ac_peak_13653	8.69112	ARSB_ENSG00000113273
5	78443861	78444082	5Y-H4K8ac_peak_13654	7.31815	
5	78531551	78531781	5Y-H4K8ac_peak_13655	9.52603	DMGDH_ENSG00000132837;JMY_ENSG00000152409
5	78556233	78556468	5Y-H4K8ac_peak_13656	4.24332	
5	78810251	78810632	5Y-H4K8ac_peak_13657	5.35202	HOMER1_ENSG00000152413
5	78907820	78908058	5Y-H4K8ac_peak_13658	7.31102	PAPD4_ENSG00000164329
5	79286622	79286913	5Y-H4K8ac_peak_13659	7.59101	MTX3_ENSG00000177034;THBS4_ENSG00000113296
5	79330823	79331548	5Y-H4K8ac_peak_13660	11.1169	

5	79465169	79465372	5Y-H4K8ac_peak_13661	8.17203	
5	79703437	79703647	5Y-H4K8ac_peak_13662	11.19336	ZFYVE16_ENSG00000039319
5	80256489	80256780	5Y-H4K8ac_peak_13663	7.59101	CTC-459I6.1_ENSG00000251450;RASGRF2_ENSG00000113319
5	80318230	80318695	5Y-H4K8ac_peak_13664	5.03335	
5	80320680	80320981	5Y-H4K8ac_peak_13665	9.15007	
5	80436512	80436752	5Y-H4K8ac_peak_13666	5.40331	
5	80506621	80506822	5Y-H4K8ac_peak_13667	6.31594	
5	80597485	80597846	5Y-H4K8ac_peak_13668	8.69112	CKMT2-AS1_ENSG00000247572;ZCCHC9_ENSG00000131732
5	80689660	80689899	5Y-H4K8ac_peak_13669	7.97699	ACOT12_ENSG00000172497
5	81047409	81048251	5Y-H4K8ac_peak_13670	23.16826	SSBP2_ENSG00000145687
5	81146774	81146964	5Y-H4K8ac_peak_13671	5.64909	CTD-2249K22.1_ENSG00000249483
5	81148222	81148457	5Y-H4K8ac_peak_13672	5.718	CTD-2249K22.1_ENSG00000249483
5	81267911	81268439	5Y-H4K8ac_peak_13673	9.51254	ATG10_ENSG00000152348
5	81684223	81684417	5Y-H4K8ac_peak_13674	5.65584	
5	82768347	82768610	5Y-H4K8ac_peak_13675	4.29586	
5	86806949	86807190	5Y-H4K8ac_peak_13676	4.84727	
5	87564208	87564618	5Y-H4K8ac_peak_13677	13.8759	TMEM161B_ENSG00000164180;TMEM161B-AS1_ENSG00000247828
5	87970131	87970981	5Y-H4K8ac_peak_13678	7.89273	
5	90650367	90650642	5Y-H4K8ac_peak_13679	4.95697	CTD-2061E19.1_ENSG00000240388;CTD-2061E19.6_ENSG00000241059
5	90676321	90676623	5Y-H4K8ac_peak_13680	7.59101	
5	90987942	90988138	5Y-H4K8ac_peak_13681	5.65584	
5	91970997	91971195	5Y-H4K8ac_peak_13682	6.14981	
5	92585472	92585690	5Y-H4K8ac_peak_13683	7.90751	
5	92905892	92906593	5Y-H4K8ac_peak_13684	7.76232	
5	92914963	92915208	5Y-H4K8ac_peak_13685	8.97839	
5	92915708	92916108	5Y-H4K8ac_peak_13686	5.34199	
5	92956549	92956821	5Y-H4K8ac_peak_13687	17.23322	MIR2277_ENSG00000251725
5	93954415	93954748	5Y-H4K8ac_peak_13688	6.08523	KIAA0825_ENSG00000185261;CTC-303L1.2_ENSG00000270133;ANKRD32_ENSG00000133302
5	94955823	94957147	5Y-H4K8ac_peak_13689	10.90097	GPR150_ENSG00000178015
5	94981875	94982091	5Y-H4K8ac_peak_13690	5.68769	RFESD_ENSG00000175449
5	96142857	96143724	5Y-H4K8ac_peak_13691	22.31046	ERAP1_ENSG00000164307
5	96271849	96272204	5Y-H4K8ac_peak_13692	4.9885	LNPEP_ENSG00000113441;CTD-2260A17.2_ENSG00000247121
5	98264046	98264444	5Y-H4K8ac_peak_13693	4.0639	CTD-2007H13.3_ENSG00000248489
5	101631420	101631889	5Y-H4K8ac_peak_13694	7.38046	SLCO4C1_ENSG00000173930
5	102089972	102090789	5Y-H4K8ac_peak_13695	6.77436	PAM_ENSG00000145730
5	102594557	102595377	5Y-H4K8ac_peak_13696	4.95697	C5orf30_ENSG00000181751
5	106851051	106851254	5Y-H4K8ac_peak_13697	6.14981	
5	107006739	107006966	5Y-H4K8ac_peak_13698	7.01266	EFNA5_ENSG00000184349
5	107009130	107009353	5Y-H4K8ac_peak_13699	9.51254	
5	107717396	107718022	5Y-H4K8ac_peak_13700	10.54764	FBXL17_ENSG00000145743
5	108063221	108063932	5Y-H4K8ac_peak_13701	10.19948	LINC01023_ENSG00000272523
5	108083989	108084229	5Y-H4K8ac_peak_13702	5.64909	FER_ENSG00000151422
5	109025201	109025448	5Y-H4K8ac_peak_13703	9.51254	MAN2A1_ENSG00000112893
5	110074928	110075169	5Y-H4K8ac_peak_13704	8.2913	TMEM232_ENSG00000186952
5	110427702	110428214	5Y-H4K8ac_peak_13705	7.31102	CTC-551A13.2_ENSG00000253613;WDR36_ENSG00000134987
5	110559601	110559873	5Y-H4K8ac_peak_13706	4.77126	CAMK4_ENSG00000152495

5	110721311	110721669	5Y-H4K8ac_peak_13707	8.33296	
5	111093166	111093915	5Y-H4K8ac_peak_13708	14.1808	
5	111874824	111875225	5Y-H4K8ac_peak_13709	5.56912	
5	112043308	112043721	5Y-H4K8ac_peak_13710	5.23083	APC_ENSG00000134982
5	112196129	112196784	5Y-H4K8ac_peak_13711	13.51759	CTC-487M23.8_ENSG00000272869;SRP19_ENSG00000153037
5	112379064	112379574	5Y-H4K8ac_peak_13712	5.07473	
5	112394500	112394740	5Y-H4K8ac_peak_13713	4.50834	
5	112395367	112395685	5Y-H4K8ac_peak_13714	13.24242	
5	112824244	112824785	5Y-H4K8ac_peak_13715	10.36926	MCC_ENSG00000171444
5	114515099	114515543	5Y-H4K8ac_peak_13716	8.16382	TRIM36_ENSG00000152503
5	114961274	114961615	5Y-H4K8ac_peak_13717	7.97699	TICAM2_ENSG00000243414;TMED7-TICAM2_ENSG00000251201
5	115152507	115152749	5Y-H4K8ac_peak_13718	6.10343	CDO1_ENSG00000129596
5	115420209	115420553	5Y-H4K8ac_peak_13719	7.76232	CTD-2287O16.5_ENSG00000271918;COMMD10_ENSG00000145781
5	115697158	115697413	5Y-H4K8ac_peak_13720	5.29015	
5	115697639	115697832	5Y-H4K8ac_peak_13721	7.38046	
5	115908162	115908360	5Y-H4K8ac_peak_13722	7.9215	
5	115908572	115909459	5Y-H4K8ac_peak_13723	5.37237	CTB-118N6.2_ENSG00000249167
5	118405426	118405618	5Y-H4K8ac_peak_13724	8.2589	snoU13_ENSG00000239011;CTB-161M19.4_ENSG00000249494
5	118787853	118788079	5Y-H4K8ac_peak_13725	7.03573	HSD17B4_ENSG00000133835
5	121412876	121413226	5Y-H4K8ac_peak_13726	7.63144	LOX_ENSG00000113083
5	122180894	122181718	5Y-H4K8ac_peak_13727	14.99673	
5	122848127	122848326	5Y-H4K8ac_peak_13728	9.89244	CSNK1G3_ENSG00000151292
5	123121509	123121917	5Y-H4K8ac_peak_13729	6.34046	
5	123211349	123211618	5Y-H4K8ac_peak_13730	3.88879	
5	123392397	123392613	5Y-H4K8ac_peak_13731	6.77436	
5	123461159	123461392	5Y-H4K8ac_peak_13732	5.98695	
5	123471669	123471919	5Y-H4K8ac_peak_13733	7.38046	
5	123730451	123730657	5Y-H4K8ac_peak_13734	7.38046	CTC-369A16.2_ENSG00000251421;CTC-369A16.3_ENSG00000251662
5	123984513	123984885	5Y-H4K8ac_peak_13735	10.1994	
5	123987514	123987817	5Y-H4K8ac_peak_13736	13.27446	
5	123993771	123994063	5Y-H4K8ac_peak_13737	9.22011	
5	124042850	124043192	5Y-H4K8ac_peak_13738	5.98695	RP11-43D2.2_ENSG00000249112
5	124747063	124747397	5Y-H4K8ac_peak_13739	5.22422	
5	124886790	124886992	5Y-H4K8ac_peak_13740	6.98118	
5	125434948	125435145	5Y-H4K8ac_peak_13741	7.64675	
5	126014888	126015329	5Y-H4K8ac_peak_13742	7.38046	
5	126016182	126016484	5Y-H4K8ac_peak_13743	7.3889	
5	126113011	126113829	5Y-H4K8ac_peak_13744	10.1994	RP11-434D11.4_ENSG00000251072;LMNB1_ENSG00000113368
5	126255333	126255591	5Y-H4K8ac_peak_13745	7.53283	
5	127419045	127419289	5Y-H4K8ac_peak_13746	8.43511	SLC12A2_ENSG00000064651;CTC-228N24.3_ENSG00000245937
5	128430723	128430949	5Y-H4K8ac_peak_13747	5.23083	ISOC1_ENSG00000066583
5	130496950	130497149	5Y-H4K8ac_peak_13748	6.34046	
5	130500320	130500787	5Y-H4K8ac_peak_13749	8.43511	
5	130505977	130506197	5Y-H4K8ac_peak_13750	6.87117	LYRM7_ENSG00000186687
5	131132318	131132600	5Y-H4K8ac_peak_13751	6.64195	CTC-432M15.3_ENSG00000273217;FNIP1_ENSG00000217128
5	131335233	131335906	5Y-H4K8ac_peak_13752	7.60893	

5	131346917	131347146	5Y-H4K8ac_peak_13753	9.52603	ACSL6_ENSG00000164398;AC034228.2_ENSG00000231585
5	131347348	131347583	5Y-H4K8ac_peak_13754	6.43775	ACSL6_ENSG00000164398;AC034228.2_ENSG00000231585
5	131563219	131563477	5Y-H4K8ac_peak_13755	6.47245	
5	131563801	131564010	5Y-H4K8ac_peak_13756	5.24695	
5	131592693	131592919	5Y-H4K8ac_peak_13757	6.78128	PDLIM4_ENSG00000131435
5	131595764	131595967	5Y-H4K8ac_peak_13758	7.87406	
5	131629100	131629963	5Y-H4K8ac_peak_13759	5.12488	SLC22A4_ENSG00000197208
5	131705183	131705439	5Y-H4K8ac_peak_13760	4.00285	AC034220.3_ENSG00000233006;SLC22A5_ENSG00000197375
5	131705759	131706100	5Y-H4K8ac_peak_13761	9.15007	AC034220.3_ENSG00000233006;SLC22A5_ENSG00000197375
5	131746127	131747314	5Y-H4K8ac_peak_13762	7.96285	C5orf56_ENSG00000197536
5	131800180	131800598	5Y-H4K8ac_peak_13763	7.31102	
5	131832698	131832903	5Y-H4K8ac_peak_13764	7.50148	
5	131991759	131992668	5Y-H4K8ac_peak_13765	16.52279	IL13_ENSG00000169194
5	132072817	132073107	5Y-H4K8ac_peak_13766	9.30206	KIF3A_ENSG00000131437
5	132082937	132083708	5Y-H4K8ac_peak_13767	5.64909	CCNI2_ENSG00000205089
5	132112927	132113335	5Y-H4K8ac_peak_13768	6.78128	
5	132149036	132149226	5Y-H4K8ac_peak_13769	6.43775	SOWAHA_ENSG00000198944
5	132149479	132149712	5Y-H4K8ac_peak_13770	11.72217	SOWAHA_ENSG00000198944
5	132165482	132166266	5Y-H4K8ac_peak_13771	8.24461	SHROOM1_ENSG00000164403
5	132201795	132202016	5Y-H4K8ac_peak_13772	8.43511	GDF9_ENSG00000164404;UQCRQ_ENSG00000164405
5	132202426	132202829	5Y-H4K8ac_peak_13773	7.11863	GDF9_ENSG00000164404;UQCRQ_ENSG00000164405
5	132299461	132300029	5Y-H4K8ac_peak_13774	13.4304	AFF4_ENSG00000072364;CTC-350I8.1_ENSG00000272023
5	132361767	132362197	5Y-H4K8ac_peak_13775	12.20548	ZCCHC10_ENSG00000155329
5	132386849	132387494	5Y-H4K8ac_peak_13776	10.16277	HSPA4_ENSG00000170606
5	132846830	132847189	5Y-H4K8ac_peak_13777	6.20875	
5	132947716	132948029	5Y-H4K8ac_peak_13778	7.89142	FSTL4_ENSG00000053108
5	133016606	133016816	5Y-H4K8ac_peak_13779	8.69112	
5	133039341	133039579	5Y-H4K8ac_peak_13780	8.2913	
5	133040381	133040711	5Y-H4K8ac_peak_13781	4.29586	
5	133045639	133046101	5Y-H4K8ac_peak_13782	4.07874	
5	133236737	133237221	5Y-H4K8ac_peak_13783	4.50834	
5	133242770	133243023	5Y-H4K8ac_peak_13784	8.93691	
5	133260151	133260484	5Y-H4K8ac_peak_13785	9.36633	
5	133261813	133262599	5Y-H4K8ac_peak_13786	13.00733	
5	133339845	133340368	5Y-H4K8ac_peak_13787	9.96543	VDAC1_ENSG00000213585;CTB-113I20.2_ENSG00000271737
5	133423959	133424180	5Y-H4K8ac_peak_13788	7.89273	
5	133439834	133440033	5Y-H4K8ac_peak_13789	5.37588	
5	133442463	133442993	5Y-H4K8ac_peak_13790	16.01457	
5	133443409	133443890	5Y-H4K8ac_peak_13791	7.89142	
5	133448794	133449010	5Y-H4K8ac_peak_13792	5.94703	
5	133449880	133450228	5Y-H4K8ac_peak_13793	11.1169	TCF7_ENSG00000081059
5	133450987	133451267	5Y-H4K8ac_peak_13794	4.77126	TCF7_ENSG00000081059
5	133460264	133460864	5Y-H4K8ac_peak_13795	6.77436	
5	133706879	133707122	5Y-H4K8ac_peak_13796	14.88279	CDKL3_ENSG00000006837;CTD-2410N18.4_ENSG00000273345;UBE2B_ENSG00000119048
5	133707366	133707681	5Y-H4K8ac_peak_13797	8.17203	CDKL3_ENSG00000006837;CTD-2410N18.4_ENSG00000273345;UBE2B_ENSG00000119048
5	133801670	133802215	5Y-H4K8ac_peak_13798	7.60057	

5	133860278	133860561	5Y-H4K8ac_peak_13799	9.60627	JADE2_ENSG00000043143
5	133891010	133891331	5Y-H4K8ac_peak_13800	5.24695	
5	133891565	133891780	5Y-H4K8ac_peak_13801	11.12941	
5	133904703	133905044	5Y-H4K8ac_peak_13802	4.15658	
5	134181738	134182312	5Y-H4K8ac_peak_13803	12.21176	C5orf24_ENSG00000181904
5	134209367	134209895	5Y-H4K8ac_peak_13804	10.60083	TXNDC15_ENSG00000113621
5	134210107	134210433	5Y-H4K8ac_peak_13805	4.77126	TXNDC15_ENSG00000113621
5	134273607	134273980	5Y-H4K8ac_peak_13806	8.04493	
5	134734464	134735426	5Y-H4K8ac_peak_13807	15.84891	H2AFY_ENSG00000113648;CTC-203F4.2_ENSG00000270021
5	135266136	135266397	5Y-H4K8ac_peak_13808	7.59101	FBXL21_ENSG00000164616
5	136733160	136733486	5Y-H4K8ac_peak_13809	5.29015	
5	136834593	136834963	5Y-H4K8ac_peak_13810	6.08523	
5	136900609	136900879	5Y-H4K8ac_peak_13811	6.49458	
5	136901352	136901605	5Y-H4K8ac_peak_13812	5.23083	
5	136901827	136902025	5Y-H4K8ac_peak_13813	6.08523	
5	136933852	136934069	5Y-H4K8ac_peak_13814	5.67283	SPOCK1_ENSG00000152377
5	137090577	137090973	5Y-H4K8ac_peak_13815	9.7353	HNRNPA0_ENSG00000177733
5	137224503	137224752	5Y-H4K8ac_peak_13816	6.47245	RP11-381K20.2_ENSG00000250159;PKD2L2_ENSG00000078795
5	137225351	137225873	5Y-H4K8ac_peak_13817	6.03632	RP11-381K20.2_ENSG00000250159
5	137368403	137368681	5Y-H4K8ac_peak_13818	9.74838	RP11-325L7.1_ENSG00000246323
5	137577400	137577728	5Y-H4K8ac_peak_13819	13.72401	
5	137609864	137610486	5Y-H4K8ac_peak_13820	9.51254	GFRA3_ENSG00000146013
5	137610876	137611133	5Y-H4K8ac_peak_13821	5.23083	GFRA3_ENSG00000146013
5	137673455	137674211	5Y-H4K8ac_peak_13822	17.63051	CDC25C_ENSG00000158402
5	137775483	137775927	5Y-H4K8ac_peak_13823	4.95697	REEP2_ENSG00000132563
5	137785513	137785719	5Y-H4K8ac_peak_13824	9.59236	
5	137826994	137827187	5Y-H4K8ac_peak_13825	5.12488	
5	137827414	137827702	5Y-H4K8ac_peak_13826	8.27643	
5	137828026	137828315	5Y-H4K8ac_peak_13827	18.78851	
5	137828596	137828837	5Y-H4K8ac_peak_13828	8.24461	
5	137910890	137911355	5Y-H4K8ac_peak_13829	15.35687	HSPA9_ENSG00000113013
5	137911855	137912252	5Y-H4K8ac_peak_13830	13.27281	HSPA9_ENSG00000113013
5	137938828	137939040	5Y-H4K8ac_peak_13831	4.29586	
5	137945558	137945803	5Y-H4K8ac_peak_13832	5.56912	CTNNA1_ENSG00000044115
5	137946434	137946822	5Y-H4K8ac_peak_13833	8.97752	CTNNA1_ENSG00000044115
5	138088722	138088925	5Y-H4K8ac_peak_13834	7.60057	AC034243.1_ENSG00000253404
5	138279391	138279668	5Y-H4K8ac_peak_13835	6.64036	
5	138281488	138282319	5Y-H4K8ac_peak_13836	8.73227	
5	138284136	138284416	5Y-H4K8ac_peak_13837	5.60566	
5	138285023	138285254	5Y-H4K8ac_peak_13838	6.27467	
5	138286042	138286519	5Y-H4K8ac_peak_13839	11.56082	
5	138286856	138287167	5Y-H4K8ac_peak_13840	4.58963	
5	138288545	138289230	5Y-H4K8ac_peak_13841	10.90365	
5	138289459	138289839	5Y-H4K8ac_peak_13842	11.1169	
5	138290199	138290408	5Y-H4K8ac_peak_13843	7.03573	
5	138304441	138304655	5Y-H4K8ac_peak_13844	10.31981	

5	138533719	138533930	5Y-H4K8ac_peak_13845	7.50148	
5	138534300	138534536	5Y-H4K8ac_peak_13846	6.14981	
5	138629466	138630076	5Y-H4K8ac_peak_13847	12.11208	SIL1_ENSG00000120725
5	138861257	138861543	5Y-H4K8ac_peak_13848	4.95697	TMEM173_ENSG00000184584
5	138897266	138897926	5Y-H4K8ac_peak_13849	8.90774	
5	138940044	138940638	5Y-H4K8ac_peak_13850	11.68317	
5	139017204	139018185	5Y-H4K8ac_peak_13851	7.58806	
5	139018487	139019073	5Y-H4K8ac_peak_13852	13.0168	
5	139028065	139028351	5Y-H4K8ac_peak_13853	6.34772	CTD-3224K15.2_ENSG00000250635
5	139039061	139039523	5Y-H4K8ac_peak_13854	10.64597	
5	139047593	139048408	5Y-H4K8ac_peak_13855	9.76277	
5	139049862	139050123	5Y-H4K8ac_peak_13856	6.31818	
5	139055420	139055652	5Y-H4K8ac_peak_13857	6.13242	
5	139056267	139056738	5Y-H4K8ac_peak_13858	7.65114	
5	139069175	139069553	5Y-H4K8ac_peak_13859	10.1994	
5	139076621	139077014	5Y-H4K8ac_peak_13860	9.79526	
5	139077243	139077770	5Y-H4K8ac_peak_13861	6.37023	
5	139085315	139085709	5Y-H4K8ac_peak_13862	8.2913	
5	139088056	139088586	5Y-H4K8ac_peak_13863	6.08523	
5	139089176	139090869	5Y-H4K8ac_peak_13864	23.24344	
5	139091157	139091707	5Y-H4K8ac_peak_13865	9.30206	
5	139125440	139125744	5Y-H4K8ac_peak_13866	4.50834	CTB-35F21.2_ENSG00000249131
5	139126192	139126661	5Y-H4K8ac_peak_13867	13.0168	CTB-35F21.2_ENSG00000249131
5	139126873	139127971	5Y-H4K8ac_peak_13868	10.60083	
5	139138575	139138791	5Y-H4K8ac_peak_13869	6.54441	
5	139167924	139168292	5Y-H4K8ac_peak_13870	8.16031	
5	139273033	139273292	5Y-H4K8ac_peak_13871	7.35333	
5	139274365	139274871	5Y-H4K8ac_peak_13872	7.11863	
5	139362685	139362980	5Y-H4K8ac_peak_13873	10.36926	
5	139423032	139423619	5Y-H4K8ac_peak_13874	9.38807	NRG2_ENSG00000158458
5	139486783	139487121	5Y-H4K8ac_peak_13875	14.30055	PURA_ENSG00000185129
5	139494059	139494326	5Y-H4K8ac_peak_13876	7.59101	
5	139525258	139525838	5Y-H4K8ac_peak_13877	6.77436	
5	139536450	139536708	5Y-H4K8ac_peak_13878	4.47071	CTB-131B5.5_ENSG00000254363
5	139725615	139725837	5Y-H4K8ac_peak_13879	4.95697	HBEGF_ENSG00000113070
5	139937210	139937879	5Y-H4K8ac_peak_13880	8.35139	SRA1_ENSG00000213523
5	139943761	139944030	5Y-H4K8ac_peak_13881	6.77436	SLC35A4_ENSG00000176087
5	140043379	140043787	5Y-H4K8ac_peak_13882	6.79955	WDR55_ENSG00000120314
5	140700599	140701049	5Y-H4K8ac_peak_13883	9.65108	TAF7_ENSG00000178913;AC005618.1_ENSG00000255729
5	140887324	140888006	5Y-H4K8ac_peak_13884	12.21176	
5	140892827	140893222	5Y-H4K8ac_peak_13885	9.30505	
5	140904590	140904890	5Y-H4K8ac_peak_13886	6.08523	
5	140905444	140905809	5Y-H4K8ac_peak_13887	5.23083	
5	140997999	140998382	5Y-H4K8ac_peak_13888	5.87382	DIAPH1_ENSG00000131504;AC008781.7_ENSG00000228737
5	141017366	141017623	5Y-H4K8ac_peak_13889	11.48679	HDAC3_ENSG00000171720;RELL2_ENSG00000164620
5	141059799	141060383	5Y-H4K8ac_peak_13890	4.95697	

5	141060667	141061096	5Y-H4K8ac_peak_13891	8.75926	ARAP3_ENSG00000120318
5	141061962	141062628	5Y-H4K8ac_peak_13892	9.01738	ARAP3_ENSG00000120318
5	141108911	141109108	5Y-H4K8ac_peak_13893	9.23159	
5	141110072	141110444	5Y-H4K8ac_peak_13894	5.98695	
5	141111233	141111645	5Y-H4K8ac_peak_13895	6.64036	
5	141126413	141126747	5Y-H4K8ac_peak_13896	7.87406	
5	141191643	141191880	5Y-H4K8ac_peak_13897	9.96543	
5	141226023	141226249	5Y-H4K8ac_peak_13898	6.70695	
5	141229668	141229886	5Y-H4K8ac_peak_13899	6.22669	
5	141262648	141263201	5Y-H4K8ac_peak_13900	10.4689	
5	141404405	141404604	5Y-H4K8ac_peak_13901	10.46287	
5	141444538	141444736	5Y-H4K8ac_peak_13902	8.43511	
5	141445106	141445351	5Y-H4K8ac_peak_13903	10.95042	
5	141488468	141488790	5Y-H4K8ac_peak_13904	10.90365	NDFIP1_ENSG00000131507
5	141704839	141705145	5Y-H4K8ac_peak_13905	13.81505	SPRY4_ENSG00000187678;AC005592.2_ENSG00000231185
5	141705487	141706281	5Y-H4K8ac_peak_13906	13.35169	SPRY4_ENSG00000187678;AC005592.2_ENSG00000231185
5	141735649	141735911	5Y-H4K8ac_peak_13907	5.22422	
5	141736268	141737000	5Y-H4K8ac_peak_13908	16.50659	
5	141740442	141740735	5Y-H4K8ac_peak_13909	6.91702	
5	141742609	141742810	5Y-H4K8ac_peak_13910	4.29586	
5	141931319	141931570	5Y-H4K8ac_peak_13911	12.26606	
5	142065810	142066291	5Y-H4K8ac_peak_13912	5.24695	
5	142190547	142190845	5Y-H4K8ac_peak_13913	6.59249	
5	142251306	142251703	5Y-H4K8ac_peak_13914	8.27399	
5	142399681	142399923	5Y-H4K8ac_peak_13915	8.33296	
5	142784558	142785231	5Y-H4K8ac_peak_13916	10.3507	
5	143408845	143409050	5Y-H4K8ac_peak_13917	7.31815	
5	145826951	145827420	5Y-H4K8ac_peak_13918	16.38263	TCERG1_ENSG00000113649
5	146028261	146028454	5Y-H4K8ac_peak_13919	4.22304	
5	146258409	146258753	5Y-H4K8ac_peak_13920	5.29015	
5	146833752	146833947	5Y-H4K8ac_peak_13921	8.43511	
5	147699603	147699945	5Y-H4K8ac_peak_13922	9.38203	SPINK9_ENSG00000204909
5	148697219	148697536	5Y-H4K8ac_peak_13923	11.39862	
5	148700055	148700333	5Y-H4K8ac_peak_13924	4.77126	
5	148724022	148724227	5Y-H4K8ac_peak_13925	7.38046	GRPEL2_ENSG00000164284
5	148725138	148725550	5Y-H4K8ac_peak_13926	10.19948	GRPEL2_ENSG00000164284
5	148737646	148737851	5Y-H4K8ac_peak_13927	7.50148	GRPEL2-AS1_ENSG00000253618;PCYOX1L_ENSG00000145882
5	148761022	148761547	5Y-H4K8ac_peak_13928	11.22005	
5	148786379	148786680	5Y-H4K8ac_peak_13929	5.98695	MIR143HG_ENSG00000249669;AC131025.8_ENSG00000253864
5	148799427	148799748	5Y-H4K8ac_peak_13930	5.9726	
5	148802658	148802909	5Y-H4K8ac_peak_13931	4.89128	
5	148803313	148804101	5Y-H4K8ac_peak_13932	11.30958	
5	148808381	148808787	5Y-H4K8ac_peak_13933	9.38203	MIR143_ENSG00000208035
5	148821987	148822251	5Y-H4K8ac_peak_13934	6.1654	
5	148822516	148822729	5Y-H4K8ac_peak_13935	4.95697	
5	148928061	148928286	5Y-H4K8ac_peak_13936	6.55225	

5	148930654	148930897	5Y-H4K8ac_peak_13937	8.73985	CSNK1A1_ENSG00000113712;ARHGEF37_ENSG00000183111
5	148960745	148961312	5Y-H4K8ac_peak_13938	13.0168	
5	148961691	148961973	5Y-H4K8ac_peak_13939	9.34555	
5	149340626	149340830	5Y-H4K8ac_peak_13940	7.11863	SLC26A2_ENSG00000155850
5	149380004	149380712	5Y-H4K8ac_peak_13941	10.32379	TIGD6_ENSG00000164296;HMGXB3_ENSG00000113716
5	149438224	149438419	5Y-H4K8ac_peak_13942	4.02665	
5	149490825	149491031	5Y-H4K8ac_peak_13943	5.62788	
5	149524152	149524378	5Y-H4K8ac_peak_13944	9.14889	
5	149535146	149535336	5Y-H4K8ac_peak_13945	7.30348	PDGFRB_ENSG00000113721
5	149539452	149539839	5Y-H4K8ac_peak_13946	6.5677	
5	149737336	149737565	5Y-H4K8ac_peak_13947	10.16277	TCOF1_ENSG00000070814
5	149864569	149864762	5Y-H4K8ac_peak_13948	8.08337	CTC-367J11.1_ENSG00000254333;NDST1_ENSG00000070614
5	149949821	149950368	5Y-H4K8ac_peak_13949	7.07936	
5	149953713	149954100	5Y-H4K8ac_peak_13950	5.87725	
5	149954623	149955094	5Y-H4K8ac_peak_13951	4.642	
5	149955293	149956107	5Y-H4K8ac_peak_13952	7.89273	
5	149961615	149962390	5Y-H4K8ac_peak_13953	7.89273	
5	149970700	149971113	5Y-H4K8ac_peak_13954	4.642	
5	149973740	149974114	5Y-H4K8ac_peak_13955	4.77126	
5	149977699	149978045	5Y-H4K8ac_peak_13956	6.08523	
5	149978924	149979603	5Y-H4K8ac_peak_13957	5.69598	
5	149979898	149980335	5Y-H4K8ac_peak_13958	12.93132	SYNPO_ENSG00000171992
5	149980529	149981749	5Y-H4K8ac_peak_13959	21.70076	SYNPO_ENSG00000171992
5	149994975	149995189	5Y-H4K8ac_peak_13960	5.23083	CTB-140J7.2_ENSG00000253852
5	150035775	150036167	5Y-H4K8ac_peak_13961	9.51254	
5	150051242	150051794	5Y-H4K8ac_peak_13962	9.79526	CTC-345K18.2_ENSG00000250309
5	150603489	150603702	5Y-H4K8ac_peak_13963	5.59843	CCDC69_ENSG00000198624
5	150632834	150633093	5Y-H4K8ac_peak_13964	5.35202	
5	151066273	151066474	5Y-H4K8ac_peak_13965	6.50117	SPARC_ENSG00000113140
5	151138596	151138854	5Y-H4K8ac_peak_13966	7.01266	
5	151150038	151150864	5Y-H4K8ac_peak_13967	13.10659	G3BP1_ENSG00000145907
5	153570006	153570540	5Y-H4K8ac_peak_13968	8.2913	GALNT10_ENSG00000164574
5	153857004	153857205	5Y-H4K8ac_peak_13969	9.07085	HAND1_ENSG00000113196
5	153858861	153859262	5Y-H4K8ac_peak_13970	7.677	
5	153862663	153862916	5Y-H4K8ac_peak_13971	5.87725	
5	153863449	153864533	5Y-H4K8ac_peak_13972	14.2053	
5	153864986	153865311	5Y-H4K8ac_peak_13973	4.84727	CTB-158E9.1_ENSG00000254293
5	153885397	153885919	5Y-H4K8ac_peak_13974	11.09973	
5	153896314	153896586	5Y-H4K8ac_peak_13975	15.63754	
5	153898615	153898979	5Y-H4K8ac_peak_13976	4.55128	
5	153901070	153901297	5Y-H4K8ac_peak_13977	5.64909	
5	153901522	153901713	5Y-H4K8ac_peak_13978	5.03917	
5	153903545	153903945	5Y-H4K8ac_peak_13979	8.43511	
5	153907114	153907716	5Y-H4K8ac_peak_13980	5.98695	
5	153948351	153948634	5Y-H4K8ac_peak_13981	11.80583	
5	153988041	153988320	5Y-H4K8ac_peak_13982	8.21582	

5	153989275	153989695	5Y-H4K8ac_peak_13983	6.54441	
5	153990168	153990589	5Y-H4K8ac_peak_13984	10.1994	
5	154033498	154033798	5Y-H4K8ac_peak_13985	9.05168	
5	154134076	154134471	5Y-H4K8ac_peak_13986	17.78344	
5	154135092	154135320	5Y-H4K8ac_peak_13987	5.98695	
5	154135581	154135787	5Y-H4K8ac_peak_13988	6.78318	
5	154215508	154215883	5Y-H4K8ac_peak_13989	6.66026	
5	154236739	154237322	5Y-H4K8ac_peak_13990	14.30055	CNOT8_ENSG00000155508
5	154320432	154321208	5Y-H4K8ac_peak_13991	11.57334	MRPL22_ENSG00000082515
5	156787288	156787764	5Y-H4K8ac_peak_13992	6.43775	
5	156811604	156811853	5Y-H4K8ac_peak_13993	11.57334	CTB-47B11.3_ENSG00000248544
5	156813344	156813734	5Y-H4K8ac_peak_13994	5.29015	
5	156816233	156816520	5Y-H4K8ac_peak_13995	7.11863	
5	156874245	156874458	5Y-H4K8ac_peak_13996	7.31807	
5	156876532	156876738	5Y-H4K8ac_peak_13997	5.23083	
5	156886959	156887532	5Y-H4K8ac_peak_13998	10.12457	CTB-109A12.1_ENSG00000251405;NIPAL4_ENSG00000172548
5	157098607	157098830	5Y-H4K8ac_peak_13999	9.38203	SOX30_ENSG00000039600;C5orf52_ENSG00000187658
5	158522200	158522518	5Y-H4K8ac_peak_14000	5.65584	
5	158533688	158533945	5Y-H4K8ac_peak_14001	5.23083	
5	158689980	158690338	5Y-H4K8ac_peak_14002	7.59101	UBLCP1_ENSG00000164332
5	159435529	159435851	5Y-H4K8ac_peak_14003	16.01349	Y_RNA_ENSG00000199398;TTC1_ENSG00000113312
5	159545914	159546308	5Y-H4K8ac_peak_14004	7.01266	PWWP2A_ENSG00000170234
5	159591593	159592174	5Y-H4K8ac_peak_14005	4.77126	
5	159602590	159602811	5Y-H4K8ac_peak_14006	4.95697	
5	159713041	159713342	5Y-H4K8ac_peak_14007	4.84727	
5	159714011	159714367	5Y-H4K8ac_peak_14008	8.43511	
5	159739694	159739928	5Y-H4K8ac_peak_14009	6.50117	
5	159771884	159772128	5Y-H4K8ac_peak_14010	7.01266	CCNJL_ENSG00000135083
5	159797426	159797793	5Y-H4K8ac_peak_14011	7.97699	C1QTNF2_ENSG00000145861
5	159826589	159827028	5Y-H4K8ac_peak_14012	8.43511	C5orf54_ENSG00000221886
5	162886854	162887191	5Y-H4K8ac_peak_14013	4.29586	NUDCD2_ENSG00000170584;HMMR_ENSG00000072571
5	164006672	164006915	5Y-H4K8ac_peak_14014	8.2913	
5	166405226	166405851	5Y-H4K8ac_peak_14015	5.59843	
5	167343516	167343737	5Y-H4K8ac_peak_14016	4.24332	
5	167589369	167589617	5Y-H4K8ac_peak_14017	10.65037	
5	167718435	167718738	5Y-H4K8ac_peak_14018	8.33296	WWC1_ENSG00000113645
5	167718956	167719541	5Y-H4K8ac_peak_14019	6.79955	WWC1_ENSG00000113645
5	167955647	167955837	5Y-H4K8ac_peak_14020	9.89244	FBLL1_ENSG00000188573
5	167956120	167956433	5Y-H4K8ac_peak_14021	4.77126	FBLL1_ENSG00000188573
5	167956791	167957190	5Y-H4K8ac_peak_14022	4.95697	FBLL1_ENSG00000188573
5	168006140	168006349	5Y-H4K8ac_peak_14023	9.51254	PANK3_ENSG00000120137
5	168079669	168080247	5Y-H4K8ac_peak_14024	7.9215	
5	168085889	168086124	5Y-H4K8ac_peak_14025	7.3889	
5	168182503	168183472	5Y-H4K8ac_peak_14026	4.44548	
5	168194905	168195226	5Y-H4K8ac_peak_14027	8.79957	MIR218-2_ENSG00000207739
5	168196444	168196727	5Y-H4K8ac_peak_14028	5.64909	

5	168198058	168198915	5Y-H4K8ac_peak_14029	11.1169	
5	168199153	168199369	5Y-H4K8ac_peak_14030	5.58165	
5	168199965	168200304	5Y-H4K8ac_peak_14031	10.18556	
5	168256822	168257129	5Y-H4K8ac_peak_14032	6.10343	
5	168266699	168266990	5Y-H4K8ac_peak_14033	4.84727	
5	168271538	168271819	5Y-H4K8ac_peak_14034	8.73392	
5	168309929	168310213	5Y-H4K8ac_peak_14035	5.67283	
5	168311860	168312217	5Y-H4K8ac_peak_14036	8.69112	
5	168314352	168314570	5Y-H4K8ac_peak_14037	6.3265	
5	168321500	168321699	5Y-H4K8ac_peak_14038	8.63306	
5	168323105	168323378	5Y-H4K8ac_peak_14039	4.07874	
5	168324768	168325086	5Y-H4K8ac_peak_14040	13.8759	
5	168343304	168343586	5Y-H4K8ac_peak_14041	4.51076	
5	168373344	168373818	5Y-H4K8ac_peak_14042	5.98695	
5	168526767	168526996	5Y-H4K8ac_peak_14043	6.27984	
5	168578617	168578839	5Y-H4K8ac_peak_14044	5.97532	
5	168727703	168727995	5Y-H4K8ac_peak_14045	6.08523	SLIT3_ENSG00000184347
5	169581186	169581725	5Y-H4K8ac_peak_14046	5.40331	
5	169853850	169854080	5Y-H4K8ac_peak_14047	7.59101	
5	170011472	170011684	5Y-H4K8ac_peak_14048	6.34046	
5	170031698	170032020	5Y-H4K8ac_peak_14049	4.50834	
5	170173342	170173841	5Y-H4K8ac_peak_14050	9.58986	
5	170179607	170179802	5Y-H4K8ac_peak_14051	7.89273	
5	170735364	170735737	5Y-H4K8ac_peak_14052	5.65584	TLX3_ENSG00000164438
5	170736453	170737077	5Y-H4K8ac_peak_14053	6.54441	TLX3_ENSG00000164438
5	170815038	170815405	5Y-H4K8ac_peak_14054	8.33296	NPM1_ENSG00000181163
5	171432837	171433038	5Y-H4K8ac_peak_14055	9.68432	FBXW11_ENSG00000072803
5	171571739	171572068	5Y-H4K8ac_peak_14056	7.89142	
5	171671788	171672133	5Y-H4K8ac_peak_14057	5.29015	
5	171865064	171865419	5Y-H4K8ac_peak_14058	7.89273	
5	172139467	172139762	5Y-H4K8ac_peak_14059	5.65584	
5	172197940	172198138	5Y-H4K8ac_peak_14060	5.5192	DUSP1_ENSG00000120129
5	172198505	172198705	5Y-H4K8ac_peak_14061	7.09658	DUSP1_ENSG00000120129
5	172711062	172711388	5Y-H4K8ac_peak_14062	8.04493	
5	172716924	172717461	5Y-H4K8ac_peak_14063	6.50117	
5	172719254	172719575	5Y-H4K8ac_peak_14064	5.12213	RNA5SP200_ENSG00000252169
5	172750553	172750770	5Y-H4K8ac_peak_14065	7.87406	
5	172756392	172756726	5Y-H4K8ac_peak_14066	8.20773	STC2_ENSG00000113739
5	173070665	173070979	5Y-H4K8ac_peak_14067	5.26353	
5	173098095	173098806	5Y-H4K8ac_peak_14068	9.78792	
5	173116909	173117271	5Y-H4K8ac_peak_14069	7.13294	CTB-43E15.2_ENSG00000253428
5	173125240	173125605	5Y-H4K8ac_peak_14070	8.47164	
5	173134615	173134808	5Y-H4K8ac_peak_14071	7.63144	
5	173147924	173148280	5Y-H4K8ac_peak_14072	4.95697	
5	173155815	173156213	5Y-H4K8ac_peak_14073	8.93923	AC008674.1_ENSG00000263401
5	173180460	173180716	5Y-H4K8ac_peak_14074	3.96434	

5	173314549	173315072	5Y-H4K8ac_peak_14075	13.2534	CPEB4_ENSG00000113742
5	173510061	173511020	5Y-H4K8ac_peak_14076	10.19948	
5	173511387	173511700	5Y-H4K8ac_peak_14077	7.11863	
5	173572149	173572519	5Y-H4K8ac_peak_14078	8.04493	
5	173650090	173650412	5Y-H4K8ac_peak_14079	5.65584	
5	173660003	173660387	5Y-H4K8ac_peak_14080	7.31815	
5	173661563	173661786	5Y-H4K8ac_peak_14081	7.09444	
5	173663808	173664178	5Y-H4K8ac_peak_14082	7.3889	
5	173679912	173680104	5Y-H4K8ac_peak_14083	9.38203	
5	173736547	173736792	5Y-H4K8ac_peak_14084	7.50148	
5	173738452	173739065	5Y-H4K8ac_peak_14085	7.44077	
5	173739275	173739649	5Y-H4K8ac_peak_14086	5.24695	
5	173755920	173756155	5Y-H4K8ac_peak_14087	6.77436	CTC-430J12.2_ENSG00000249722
5	173763960	173764240	5Y-H4K8ac_peak_14088	6.77436	RP11-267A15.1_ENSG00000249306
5	174178199	174178497	5Y-H4K8ac_peak_14089	7.11863	CTD-2532K18.1_ENSG00000251670;MIR4634_ENSG00000266890
5	174220502	174220837	5Y-H4K8ac_peak_14090	9.38203	
5	174905630	174906234	5Y-H4K8ac_peak_14091	10.5157	
5	175223645	175224474	5Y-H4K8ac_peak_14092	6.78128	CPLX2_ENSG00000145920
5	175263822	175264266	5Y-H4K8ac_peak_14093	5.67283	
5	175287930	175288791	5Y-H4K8ac_peak_14094	8.93923	
5	175788271	175788714	5Y-H4K8ac_peak_14095	6.14981	KIAA1191_ENSG00000122203
5	175792607	175792988	5Y-H4K8ac_peak_14096	8.47164	ARL10_ENSG00000175414
5	175843074	175843406	5Y-H4K8ac_peak_14097	4.90696	CLTB_ENSG00000175416
5	175875429	175875621	5Y-H4K8ac_peak_14098	7.01266	FAF2_ENSG00000113194
5	175963138	175964179	5Y-H4K8ac_peak_14099	23.71361	RNF44_ENSG00000146083
5	176037282	176037475	5Y-H4K8ac_peak_14100	10.43045	GPRIN1_ENSG00000169258
5	176152820	176153130	5Y-H4K8ac_peak_14101	4.51497	RP11-375B1.1_ENSG00000248484
5	176220176	176220662	5Y-H4K8ac_peak_14102	10.11191	
5	176236997	176237411	5Y-H4K8ac_peak_14103	7.11863	UNC5A_ENSG00000113763
5	176244775	176245238	5Y-H4K8ac_peak_14104	7.59101	
5	176249554	176249873	5Y-H4K8ac_peak_14105	6.08523	
5	176258471	176258696	5Y-H4K8ac_peak_14106	6.37023	
5	176261247	176261483	5Y-H4K8ac_peak_14107	5.08308	
5	176261877	176262211	5Y-H4K8ac_peak_14108	4.642	
5	176265995	176266185	5Y-H4K8ac_peak_14109	4.50834	
5	176267134	176267364	5Y-H4K8ac_peak_14110	6.37023	
5	176271752	176272140	5Y-H4K8ac_peak_14111	7.50148	
5	176289627	176289987	5Y-H4K8ac_peak_14112	7.70703	
5	176310885	176311089	5Y-H4K8ac_peak_14113	6.1654	
5	176433283	176433604	5Y-H4K8ac_peak_14114	8.2913	
5	176449789	176450053	5Y-H4K8ac_peak_14115	9.00954	UIMC1_ENSG00000087206;ZNF346_ENSG00000113761
5	176514013	176514244	5Y-H4K8ac_peak_14116	4.77126	FGFR4_ENSG00000160867
5	176525410	176526015	5Y-H4K8ac_peak_14117	9.23159	
5	176560068	176560982	5Y-H4K8ac_peak_14118	8.14883	NSD1_ENSG00000165671
5	176779092	176779743	5Y-H4K8ac_peak_14119	8.69112	LMAN2_ENSG00000169223
5	176829180	176829456	5Y-H4K8ac_peak_14120	5.12488	GRK6_ENSG00000198055

5	176830342	176830755	5Y-H4K8ac_peak_14121	9.15007	GRK6_ENSG00000198055
5	176831121	176832001	5Y-H4K8ac_peak_14122	15.82713	GRK6_ENSG00000198055
5	176873874	176874402	5Y-H4K8ac_peak_14123	13.10076	PRR7-AS1_ENSG00000246334;PRR7_ENSG00000131188
5	176874943	176875289	5Y-H4K8ac_peak_14124	8.79957	PRR7-AS1_ENSG00000246334
5	176882772	176883046	5Y-H4K8ac_peak_14125	7.28023	
5	176899537	176899880	5Y-H4K8ac_peak_14126	8.37729	
5	176900117	176900577	5Y-H4K8ac_peak_14127	8.24461	DBN1_ENSG00000113758
5	176905097	176905535	5Y-H4K8ac_peak_14128	7.89142	
5	176905917	176906349	5Y-H4K8ac_peak_14129	6.10343	
5	176906978	176907172	5Y-H4K8ac_peak_14130	4.77307	
5	176922398	176922588	5Y-H4K8ac_peak_14131	4.07402	RP11-1334A24.6_ENSG00000248996
5	176981786	176981993	5Y-H4K8ac_peak_14132	7.38046	FAM193B_ENSG00000146067;RP11-1277A3.3_ENSG00000272459
5	177026629	177026970	5Y-H4K8ac_peak_14133	9.26157	B4GALT7_ENSG00000027847
5	177027185	177027413	5Y-H4K8ac_peak_14134	10.19948	B4GALT7_ENSG00000027847
5	177551116	177551527	5Y-H4K8ac_peak_14135	5.449	
5	177557536	177557821	5Y-H4K8ac_peak_14136	12.05638	RMND5B_ENSG00000145916
5	177631820	177632151	5Y-H4K8ac_peak_14137	7.09658	HNRNPAB_ENSG00000197451
5	177659213	177660178	5Y-H4K8ac_peak_14138	7.50148	PHYKPL_ENSG00000175309
5	177765525	177765755	5Y-H4K8ac_peak_14139	6.50117	
5	177775030	177776005	5Y-H4K8ac_peak_14140	10.19948	
5	177776673	177777024	5Y-H4K8ac_peak_14141	8.79957	RP11-1259L22.1_ENSG00000253698
5	177777973	177778169	5Y-H4K8ac_peak_14142	6.77436	RP11-1259L22.1_ENSG00000253698
5	177783598	177783816	5Y-H4K8ac_peak_14143	6.46053	
5	177845552	177845808	5Y-H4K8ac_peak_14144	4.9885	
5	177855497	177856626	5Y-H4K8ac_peak_14145	8.69112	
5	177861566	177861774	5Y-H4K8ac_peak_14146	8.24461	
5	177869519	177869907	5Y-H4K8ac_peak_14147	8.33296	CTB-26E19.1_ENSG00000245688
5	178054311	178054536	5Y-H4K8ac_peak_14148	7.11863	
5	178323060	178323352	5Y-H4K8ac_peak_14149	8.47164	ZFP2_ENSG00000198939
5	178450920	178451257	5Y-H4K8ac_peak_14150	5.23083	ZNF879_ENSG00000234284
5	178487472	178488165	5Y-H4K8ac_peak_14151	13.0168	ZNF354C_ENSG00000177932
5	178593676	178594199	5Y-H4K8ac_peak_14152	7.59101	
5	178618748	178619419	5Y-H4K8ac_peak_14153	13.92877	
5	178688788	178689129	5Y-H4K8ac_peak_14154	7.30348	
5	178692133	178693063	5Y-H4K8ac_peak_14155	10.54764	
5	178693840	178694071	5Y-H4K8ac_peak_14156	6.47245	
5	178698210	178698669	5Y-H4K8ac_peak_14157	6.95844	
5	178714711	178715209	5Y-H4K8ac_peak_14158	6.00598	
5	178715743	178716234	5Y-H4K8ac_peak_14159	9.28129	
5	178720977	178721253	5Y-H4K8ac_peak_14160	6.73047	
5	178721607	178722067	5Y-H4K8ac_peak_14161	10.1994	
5	178722663	178723043	5Y-H4K8ac_peak_14162	5.24695	
5	178736042	178736344	5Y-H4K8ac_peak_14163	6.43068	
5	178736902	178737418	5Y-H4K8ac_peak_14164	8.79957	
5	178737676	178737991	5Y-H4K8ac_peak_14165	11.36989	
5	178740933	178741195	5Y-H4K8ac_peak_14166	6.75784	

5	178741487	178741927	5Y-H4K8ac_peak_14167	12.26819	
5	178749720	178750001	5Y-H4K8ac_peak_14168	7.50148	
5	178750329	178750555	5Y-H4K8ac_peak_14169	9.04239	
5	178752395	178753175	5Y-H4K8ac_peak_14170	6.64195	
5	178757630	178757865	5Y-H4K8ac_peak_14171	7.04637	
5	178862981	178863260	5Y-H4K8ac_peak_14172	7.90236	
5	178864145	178864946	5Y-H4K8ac_peak_14173	12.79198	
5	178865326	178866144	5Y-H4K8ac_peak_14174	17.67751	
5	178977269	178978204	5Y-H4K8ac_peak_14175	16.50659	RUFY1_ENSG00000176783
5	179050119	179050599	5Y-H4K8ac_peak_14176	13.0168	
5	179051009	179051752	5Y-H4K8ac_peak_14177	19.52902	
5	179105187	179105490	5Y-H4K8ac_peak_14178	13.06587	CANX_ENSG00000127022;HMGB3P22_ENSG00000225051
5	179105753	179106457	5Y-H4K8ac_peak_14179	7.90751	CANX_ENSG00000127022;HMGB3P22_ENSG00000225051
5	179125344	179125559	5Y-H4K8ac_peak_14180	8.47164	
5	179159893	179160122	5Y-H4K8ac_peak_14181	8.75926	MAML1_ENSG00000161021
5	179234165	179234357	5Y-H4K8ac_peak_14182	8.24461	MGAT4B_ENSG00000161013;SQSTM1_ENSG00000161011
5	179247093	179247730	5Y-H4K8ac_peak_14183	13.85283	
5	179286131	179286423	5Y-H4K8ac_peak_14184	8.5915	CTC-241N9.1_ENSG00000245317
5	179498101	179499116	5Y-H4K8ac_peak_14185	9.31608	RNF130_ENSG00000113269
5	179517186	179517376	5Y-H4K8ac_peak_14186	4.71803	
5	179588699	179588972	5Y-H4K8ac_peak_14187	7.50148	
5	179603856	179604560	5Y-H4K8ac_peak_14188	9.01738	
5	179719328	179719945	5Y-H4K8ac_peak_14189	8.43011	MAPK9_ENSG00000050748;CTB-129O4.1_ENSG00000248367
5	179808369	179808580	5Y-H4K8ac_peak_14190	10.19948	
5	179808847	179809058	5Y-H4K8ac_peak_14191	7.58806	
5	179921436	179921916	5Y-H4K8ac_peak_14192	11.19336	CNOT6_ENSG00000113300
5	180257758	180258408	5Y-H4K8ac_peak_14193	11.09973	LINC00847_ENSG00000245060
5	180287887	180288750	5Y-H4K8ac_peak_14194	9.23159	ZFP62_ENSG00000196670
5	180588323	180588629	5Y-H4K8ac_peak_14195	8.27399	
5	180618319	180618523	5Y-H4K8ac_peak_14196	6.06162	CTC-338M12.2_ENSG00000248473;CTC-338M12.5_ENSG00000250222
5	180632056	180632311	5Y-H4K8ac_peak_14197	6.14981	TRIM7_ENSG00000146054;CTC-338M12.1_ENSG00000248514
5	180670645	180670876	5Y-H4K8ac_peak_14198	5.24695	SNORD95_ENSG00000264549
5	180688288	180688830	5Y-H4K8ac_peak_14199	12.26606	TRIM52_ENSG00000183718;TRIM52-AS1_ENSG00000248275
6	291659	292341	5Y-H4K8ac_peak_14200	14.86775	DUSP22_ENSG00000112679
6	1614211	1614740	5Y-H4K8ac_peak_14201	6.73047	
6	1904783	1905390	5Y-H4K8ac_peak_14202	11.09973	
6	2634330	2634689	5Y-H4K8ac_peak_14203	10.95042	C6orf195_ENSG00000164385
6	2765801	2766093	5Y-H4K8ac_peak_14204	9.23159	WRNIP1_ENSG00000124535
6	2903440	2903679	5Y-H4K8ac_peak_14205	5.23083	SERPINB9_ENSG00000170542
6	2971194	2971578	5Y-H4K8ac_peak_14206	12.9311	SERPINB6_ENSG00000124570
6	2971838	2972348	5Y-H4K8ac_peak_14207	6.37023	SERPINB6_ENSG00000124570
6	2989038	2989260	5Y-H4K8ac_peak_14208	9.30505	LINC01011_ENSG00000244041;NQO2_ENSG00000124588
6	3054151	3054361	5Y-H4K8ac_peak_14209	10.36926	
6	3063974	3064287	5Y-H4K8ac_peak_14210	4.32718	RIPK1_ENSG00000137275
6	3068894	3069120	5Y-H4K8ac_peak_14211	6.64195	RP1-40E16.12_ENSG00000272277
6	3069355	3069566	5Y-H4K8ac_peak_14212	4.09853	RP1-40E16.12_ENSG00000272277

6	3157988	3158199	5Y-H4K8ac_peak_14213	9.51254	TUBB2A_ENSG00000137267
6	3163070	3163267	5Y-H4K8ac_peak_14214	6.77436	
6	3180498	3180793	5Y-H4K8ac_peak_14215	7.31815	TUBB2BP1_ENSG00000216819
6	3228308	3228733	5Y-H4K8ac_peak_14216	7.59101	
6	3229382	3230155	5Y-H4K8ac_peak_14217	9.30505	
6	3231452	3231692	5Y-H4K8ac_peak_14218	8.24461	TUBB2B_ENSG00000137285;PSMG4_ENSG00000180822
6	3232158	3232371	5Y-H4K8ac_peak_14219	9.96543	TUBB2B_ENSG00000137285;PSMG4_ENSG00000180822
6	3826633	3826836	5Y-H4K8ac_peak_14220	6.77436	
6	3849190	3849400	5Y-H4K8ac_peak_14221	6.50117	FAM50B_ENSG00000145945
6	4018506	4018809	5Y-H4K8ac_peak_14222	7.11863	RP3-406P24.4_ENSG00000272248
6	4021679	4021899	5Y-H4K8ac_peak_14223	5.12213	RP3-406P24.3_ENSG00000230648;PRPF4B_ENSG00000112739
6	4775290	4775487	5Y-H4K8ac_peak_14224	4.84727	RP3-430A16.1_ENSG00000236336
6	5261025	5261403	5Y-H4K8ac_peak_14225	10.90365	LYRM4_ENSG00000214113;FARS2_ENSG00000145982
6	5261613	5261948	5Y-H4K8ac_peak_14226	8.75926	LYRM4_ENSG00000214113;FARS2_ENSG00000145982
6	5664084	5664331	5Y-H4K8ac_peak_14227	10.46287	
6	5749258	5749463	5Y-H4K8ac_peak_14228	6.50117	
6	7313863	7314252	5Y-H4K8ac_peak_14229	8.84534	
6	7910288	7910806	5Y-H4K8ac_peak_14230	8.43011	
6	7911203	7911469	5Y-H4K8ac_peak_14231	12.05638	
6	8065020	8065226	5Y-H4K8ac_peak_14232	6.1654	BLOC1S5-TXNDC5_ENSG00000259040;BLOC1S5_ENSG00000188428
6	8102521	8102724	5Y-H4K8ac_peak_14233	6.50117	EEF1E1-BLOC1S5_ENSG00000265818;EEF1E1_ENSG00000124802
6	8278057	8278247	5Y-H4K8ac_peak_14234	7.72345	
6	8278501	8278715	5Y-H4K8ac_peak_14235	8.2913	
6	10404931	10405237	5Y-H4K8ac_peak_14236	7.50501	
6	10660050	10660339	5Y-H4K8ac_peak_14237	5.23083	
6	10720269	10720602	5Y-H4K8ac_peak_14238	9.30505	
6	10720864	10721198	5Y-H4K8ac_peak_14239	8.97752	
6	11094077	11094324	5Y-H4K8ac_peak_14240	11.1169	SMIM13_ENSG00000224531
6	13558823	13559132	5Y-H4K8ac_peak_14241	4.84727	
6	13574903	13575461	5Y-H4K8ac_peak_14242	4.50834	SIRT5_ENSG00000124523
6	13712211	13712528	5Y-H4K8ac_peak_14243	9.93099	RANBP9_ENSG00000010017
6	13860043	13860288	5Y-H4K8ac_peak_14244	5.90587	
6	13873783	13874213	5Y-H4K8ac_peak_14245	11.1169	
6	15244975	15245337	5Y-H4K8ac_peak_14246	8.35139	RP11-560J1.2_ENSG00000271888
6	15245905	15246109	5Y-H4K8ac_peak_14247	7.59101	RP11-560J1.2_ENSG00000271888;JARID2_ENSG00000008083
6	15444524	15444814	5Y-H4K8ac_peak_14248	11.19336	
6	15662909	15663120	5Y-H4K8ac_peak_14249	5.8635	DTNBP1_ENSG00000047579
6	17580480	17580726	5Y-H4K8ac_peak_14250	7.38046	
6	17581077	17581332	5Y-H4K8ac_peak_14251	11.1169	SUMO2P13_ENSG00000218359
6	17706816	17707072	5Y-H4K8ac_peak_14252	8.79957	NUP153_ENSG00000124789;RP11-500C11.3_ENSG00000272269
6	18264223	18264696	5Y-H4K8ac_peak_14253	4.29586	DEK_ENSG00000124795
6	20127354	20127623	5Y-H4K8ac_peak_14254	8.17203	
6	20212327	20212655	5Y-H4K8ac_peak_14255	10.11191	MBOAT1_ENSG00000172197;RP11-239H6.2_ENSG00000227803
6	20402748	20403027	5Y-H4K8ac_peak_14256	9.02782	E2F3_ENSG00000112242
6	20403289	20403597	5Y-H4K8ac_peak_14257	6.73385	E2F3_ENSG00000112242
6	21199645	21199914	5Y-H4K8ac_peak_14258	7.01266	

6	21207208	21207403	5Y-H4K8ac_peak_14259	6.52593	
6	21223951	21224146	5Y-H4K8ac_peak_14260	6.90749	
6	21244472	21244671	5Y-H4K8ac_peak_14261	5.98695	
6	21383214	21383430	5Y-H4K8ac_peak_14262	9.15007	
6	21523180	21524144	5Y-H4K8ac_peak_14263	9.78792	RP11-204E9.1_ENSG00000231754
6	21587787	21588219	5Y-H4K8ac_peak_14264	8.24461	
6	21588500	21588948	5Y-H4K8ac_peak_14265	7.11863	
6	21594543	21595288	5Y-H4K8ac_peak_14266	11.04919	SOX4_ENSG00000124766
6	21665891	21666440	5Y-H4K8ac_peak_14267	6.43775	CASC15_ENSG00000272168
6	22146846	22147311	5Y-H4K8ac_peak_14268	18.2299	CASC14_ENSG00000260455
6	22828588	22828997	5Y-H4K8ac_peak_14269	8.33296	
6	23283261	23283459	5Y-H4K8ac_peak_14270	6.83519	
6	23446957	23447166	5Y-H4K8ac_peak_14271	4.07874	
6	23656715	23656928	5Y-H4K8ac_peak_14272	7.31807	
6	24403335	24403561	5Y-H4K8ac_peak_14273	4.642	MRS2_ENSG00000124532
6	24495318	24495756	5Y-H4K8ac_peak_14274	8.47164	GPLD1_ENSG00000112293;ALDH5A1_ENSG00000112294
6	24720800	24721020	5Y-H4K8ac_peak_14275	7.11863	C6orf62_ENSG00000112308
6	24775213	24775516	5Y-H4K8ac_peak_14276	13.0168	GMNN_ENSG00000112312
6	24855905	24856122	5Y-H4K8ac_peak_14277	5.98695	
6	25218429	25218694	5Y-H4K8ac_peak_14278	6.20875	RP11-191A15.4_ENSG00000219681
6	26034214	26034566	5Y-H4K8ac_peak_14279	5.54013	HIST1H2AB_ENSG00000137259
6	26043231	26043651	5Y-H4K8ac_peak_14280	6.59249	HIST1H2BB_ENSG00000196226;U91328.2_ENSG00000234816
6	26184188	26184425	5Y-H4K8ac_peak_14281	11.19336	HIST1H2BE_ENSG00000197697
6	26271254	26271478	5Y-H4K8ac_peak_14282	8.33296	HIST1H3G_ENSG00000256018
6	26272523	26272754	5Y-H4K8ac_peak_14283	8.93923	HIST1H3G_ENSG00000256018;HIST1H2APS4_ENSG00000218690;HIST1H2BI_ENSG00000168242
6	26383936	26384160	5Y-H4K8ac_peak_14284	8.564	BTN2A2_ENSG00000124508
6	26458395	26458614	5Y-H4K8ac_peak_14285	6.73047	BTN2A1_ENSG00000112763
6	26538388	26538696	5Y-H4K8ac_peak_14286	5.23083	HMGN4_ENSG00000182952
6	26550851	26551151	5Y-H4K8ac_peak_14287	4.29586	
6	27064571	27064791	5Y-H4K8ac_peak_14288	10.46287	
6	27342673	27343023	5Y-H4K8ac_peak_14289	7.01266	ZNF391_ENSG00000124613
6	27440481	27440735	5Y-H4K8ac_peak_14290	11.57334	ZNF184_ENSG00000096654
6	27661968	27662348	5Y-H4K8ac_peak_14291	7.38046	
6	27794414	27794789	5Y-H4K8ac_peak_14292	8.33296	
6	27805584	27806022	5Y-H4K8ac_peak_14293	12.43358	HIST1H2AK_ENSG00000184348;HIST1H2BN_ENSG00000233822
6	27831778	27832151	5Y-H4K8ac_peak_14294	5.29015	HIST1H2BPS2_ENSG00000217646;HIST1H2AL_ENSG00000198374
6	27861484	27861706	5Y-H4K8ac_peak_14295	8.43011	HIST1H3J_ENSG00000197153;HIST1H2AM_ENSG00000233224;HIST1H2BO_ENSG00000196331
6	28048773	28049335	5Y-H4K8ac_peak_14296	7.60893	RP1-313I6.12_ENSG00000272009;ZNF165_ENSG00000197279
6	28059015	28059216	5Y-H4K8ac_peak_14297	7.17184	ZSCAN12P1_ENSG00000219891
6	28104715	28105160	5Y-H4K8ac_peak_14298	11.19336	ZSCAN16-AS1_ENSG00000269293;RP1-265C24.8_ENSG00000261839
6	28129381	28129846	5Y-H4K8ac_peak_14299	9.34555	ZNF192P1_ENSG00000226314
6	28185994	28186320	5Y-H4K8ac_peak_14300	6.73385	TOB2P1_ENSG00000176933
6	28187139	28187395	5Y-H4K8ac_peak_14301	6.24411	TOB2P1_ENSG00000176933
6	28220248	28220708	5Y-H4K8ac_peak_14302	5.64909	
6	28367721	28367936	5Y-H4K8ac_peak_14303	9.23159	ZSCAN12_ENSG00000158691
6	28414375	28415151	5Y-H4K8ac_peak_14304	4.84727	COX11P1_ENSG00000231162

6	28589605	28589857	5Y-H4K8ac_peak_14305	5.77617	
6	28805532	28805751	5Y-H4K8ac_peak_14306	4.9885	
6	28834401	28834724	5Y-H4K8ac_peak_14307	7.03573	
6	28862218	28862411	5Y-H4K8ac_peak_14308	5.40331	
6	28863635	28863904	5Y-H4K8ac_peak_14309	10.19948	HCG14_ENSG00000224157
6	28891370	28891617	5Y-H4K8ac_peak_14310	7.89273	TRIM27_ENSG00000204713
6	28953737	28954044	5Y-H4K8ac_peak_14311	6.34046	HCG15_ENSG00000227214;HCG16_ENSG00000244349
6	29595543	29595751	5Y-H4K8ac_peak_14312	8.04943	
6	29618142	29618365	5Y-H4K8ac_peak_14313	4.07874	
6	30294256	30294624	5Y-H4K8ac_peak_14314	10.1994	HCG17_ENSG00000270604;HCG18_ENSG00000231074;TRIM39_ENSG00000204599
6	30524164	30524622	5Y-H4K8ac_peak_14315	7.01266	GNL1_ENSG00000204590;PRR3_ENSG00000204576
6	30525136	30525400	5Y-H4K8ac_peak_14316	6.78128	GNL1_ENSG00000204590;PRR3_ENSG00000204576
6	30539236	30539446	5Y-H4K8ac_peak_14317	13.06587	ABCF1_ENSG00000204574
6	30583732	30584079	5Y-H4K8ac_peak_14318	6.76425	AL662800.1_ENSG00000222894
6	30594866	30595175	5Y-H4K8ac_peak_14319	5.65584	ATAT1_ENSG00000137343
6	30614925	30615536	5Y-H4K8ac_peak_14320	11.53136	C6orf136_ENSG00000204564;AL662800.2_ENSG00000266183
6	30655319	30655695	5Y-H4K8ac_peak_14321	5.98695	PPP1R18_ENSG00000146112
6	30684138	30684384	5Y-H4K8ac_peak_14322	4.50834	
6	30684597	30684889	5Y-H4K8ac_peak_14323	6.73385	MDC1_ENSG00000137337
6	30747517	30747844	5Y-H4K8ac_peak_14324	8.75926	
6	30845290	30845484	5Y-H4K8ac_peak_14325	4.50834	
6	30851612	30852029	5Y-H4K8ac_peak_14326	8.4454	
6	30852377	30852578	5Y-H4K8ac_peak_14327	6.03255	
6	30875410	30875669	5Y-H4K8ac_peak_14328	5.23083	RN7SL175P_ENSG00000264731;GTF2H4_ENSG00000213780;VARS2_ENSG00000137411
6	30882649	30882898	5Y-H4K8ac_peak_14329	10.19948	
6	31105684	31106003	5Y-H4K8ac_peak_14330	8.2913	
6	31165083	31165308	5Y-H4K8ac_peak_14331	5.18558	XXbac-BPG299F13.17_ENSG00000272501;HCG27_ENSG00000206344
6	31509365	31509632	5Y-H4K8ac_peak_14332	6.73385	DDX39B_ENSG00000198563;SNORD84_ENSG00000265236;DDX39B-AS1_ENSG00000234006
6	31515952	31516142	5Y-H4K8ac_peak_14333	7.89273	ATP6V1G2_ENSG00000213760
6	31548479	31549144	5Y-H4K8ac_peak_14334	11.1169	
6	31587245	31587489	5Y-H4K8ac_peak_14335	10.37534	
6	31588537	31588929	5Y-H4K8ac_peak_14336	12.06963	PRRC2A_ENSG00000204469
6	31620177	31620676	5Y-H4K8ac_peak_14337	11.46984	BAG6_ENSG00000204463;APOM_ENSG00000204444
6	31632872	31633177	5Y-H4K8ac_peak_14338	6.73385	GPANK1_ENSG00000204438;CSNK2B_ENSG00000204435;CSNK2B-LY6G5B-1181_ENSG00000263020
6	31648975	31649532	5Y-H4K8ac_peak_14339	8.2913	
6	31671408	31671606	5Y-H4K8ac_peak_14340	8.47164	ABHD16A_ENSG00000204427
6	31679062	31679261	5Y-H4K8ac_peak_14341	5.84208	
6	31704144	31704442	5Y-H4K8ac_peak_14342	6.14981	
6	31707834	31708542	5Y-H4K8ac_peak_14343	9.23159	CLIC1_ENSG00000213719;MSH5_ENSG00000204410;MSH5-SAPCD1_ENSG00000255152
6	31762671	31762941	5Y-H4K8ac_peak_14344	9.48719	VARS_ENSG00000204394
6	31763167	31763676	5Y-H4K8ac_peak_14345	4.44548	VARS_ENSG00000204394
6	31763946	31764289	5Y-H4K8ac_peak_14346	10.4689	VARS_ENSG00000204394
6	31940234	31940465	5Y-H4K8ac_peak_14347	4.5753	DXO_ENSG00000204348
6	32016205	32016479	5Y-H4K8ac_peak_14348	7.38046	
6	32121051	32121331	5Y-H4K8ac_peak_14349	8.12305	PRRT1_ENSG00000204314;PPT2_ENSG00000221988;PPT2-EGFL8_ENSG00000258388
6	32157031	32157687	5Y-H4K8ac_peak_14350	10.99773	PBX2_ENSG00000204304

6	32158105	32158506	5Y-H4K8ac_peak_14351	10.1994	PBX2_ENSG00000204304
6	32163821	32164246	5Y-H4K8ac_peak_14352	8.69112	GPSM3_ENSG00000213654
6	32164666	32165114	5Y-H4K8ac_peak_14353	5.449	
6	32805766	32805990	5Y-H4K8ac_peak_14354	13.06587	TAP2_ENSG00000250264;TAP2_ENSG00000204267
6	32935870	32936132	5Y-H4K8ac_peak_14355	6.77436	HLA-DMA_ENSG00000204257;BRD2_ENSG00000204256
6	32937060	32938521	5Y-H4K8ac_peak_14356	8.43511	HLA-DMA_ENSG00000204257;BRD2_ENSG00000204256;BRD2-IT1_ENSG00000223837
6	33129548	33129887	5Y-H4K8ac_peak_14357	7.11863	
6	33159384	33159695	5Y-H4K8ac_peak_14358	4.71803	COL11A2_ENSG00000204248
6	33168018	33168319	5Y-H4K8ac_peak_14359	6.53157	RXRB_ENSG00000204231;RNY4P10_ENSG00000202441;SLC39A7_ENSG00000112473
6	33175784	33176200	5Y-H4K8ac_peak_14360	4.50834	MIR219-1_ENSG00000199036;RING1_ENSG00000204227
6	33239143	33239618	5Y-H4K8ac_peak_14361	9.179	VPS52_ENSG00000223501;RPS18_ENSG00000231500
6	33291187	33291815	5Y-H4K8ac_peak_14362	11.61993	
6	33332933	33333270	5Y-H4K8ac_peak_14363	6.76425	LYPLA2P1_ENSG00000228285
6	33396060	33396311	5Y-H4K8ac_peak_14364	13.8759	
6	33548293	33548513	5Y-H4K8ac_peak_14365	10.21117	BAK1_ENSG00000030110
6	33579029	33579266	5Y-H4K8ac_peak_14366	6.37023	
6	33588655	33588883	5Y-H4K8ac_peak_14367	4.03329	ITPR3_ENSG00000096433
6	33636713	33636954	5Y-H4K8ac_peak_14368	5.19372	
6	33678886	33679420	5Y-H4K8ac_peak_14369	7.89273	UQCC2_ENSG00000137288
6	33713986	33714194	5Y-H4K8ac_peak_14370	5.23083	IP6K3_ENSG00000161896
6	33756148	33756673	5Y-H4K8ac_peak_14371	12.05638	LEMD2_ENSG00000161904
6	33757264	33757679	5Y-H4K8ac_peak_14372	9.30505	LEMD2_ENSG00000161904
6	33772969	33773159	5Y-H4K8ac_peak_14373	5.29015	
6	33783554	33783759	5Y-H4K8ac_peak_14374	11.71297	
6	34163968	34164724	5Y-H4K8ac_peak_14375	9.34555	
6	34205430	34205985	5Y-H4K8ac_peak_14376	8.24461	HMGA1_ENSG00000137309
6	34393437	34393671	5Y-H4K8ac_peak_14377	6.08523	RPS10-NUDT3_ENSG00000270800;RPS10_ENSG00000124614
6	34493190	34493816	5Y-H4K8ac_peak_14378	12.05638	
6	34664796	34665113	5Y-H4K8ac_peak_14379	8.8021	C6orf106_ENSG00000196821;RP11-140K17.3_ENSG00000272288
6	34724574	34724769	5Y-H4K8ac_peak_14380	5.41472	SNRPC_ENSG00000124562
6	34759343	34759568	5Y-H4K8ac_peak_14381	7.76232	UHRF1BP1_ENSG00000065060
6	34760112	34760433	5Y-H4K8ac_peak_14382	4.29586	UHRF1BP1_ENSG00000065060
6	34857105	34857331	5Y-H4K8ac_peak_14383	9.7353	ANKS1A_ENSG00000064999
6	35149940	35150278	5Y-H4K8ac_peak_14384	7.42477	
6	35331151	35331381	5Y-H4K8ac_peak_14385	7.59101	
6	35436224	35436673	5Y-H4K8ac_peak_14386	12.99645	RPL10A_ENSG00000198755
6	35656314	35656539	5Y-H4K8ac_peak_14387	8.35139	
6	35656992	35657235	5Y-H4K8ac_peak_14388	8.35139	
6	35695338	35696428	5Y-H4K8ac_peak_14389	14.86775	FKBP5_ENSG00000096060
6	35699354	35700081	5Y-H4K8ac_peak_14390	17.04963	
6	35704620	35705109	5Y-H4K8ac_peak_14391	8.76608	RP3-510O8.4_ENSG00000232909;ARMC12_ENSG00000157343
6	35705738	35705942	5Y-H4K8ac_peak_14392	7.59101	ARMC12_ENSG00000157343
6	35772988	35773447	5Y-H4K8ac_peak_14393	4.77126	LHFPL5_ENSG00000197753
6	35994918	35995155	5Y-H4K8ac_peak_14394	6.37023	MAPK14_ENSG00000112062
6	35995670	35995904	5Y-H4K8ac_peak_14395	5.5192	MAPK14_ENSG00000112062
6	36067676	36068233	5Y-H4K8ac_peak_14396	6.39626	

6	36098927	36099365	5Y-H4K8ac_peak_14397	16.38263	
6	36099851	36100152	5Y-H4K8ac_peak_14398	13.45283	
6	36100345	36100558	5Y-H4K8ac_peak_14399	5.24695	
6	36164559	36164792	5Y-H4K8ac_peak_14400	11.1169	RP1-179N16.6_ENSG00000246982;BRPF3_ENSG00000096070
6	36561119	36561447	5Y-H4K8ac_peak_14401	9.92042	SRSF3_ENSG00000112081
6	36562194	36562437	5Y-H4K8ac_peak_14402	7.89142	SRSF3_ENSG00000112081
6	36617554	36617773	5Y-H4K8ac_peak_14403	10.31981	
6	36643983	36644342	5Y-H4K8ac_peak_14404	10.12457	CDKN1A_ENSG00000124762
6	36761645	36761836	5Y-H4K8ac_peak_14405	6.31818	
6	36762116	36762778	5Y-H4K8ac_peak_14406	6.98416	
6	36853231	36853444	5Y-H4K8ac_peak_14407	7.58806	
6	36954092	36954455	5Y-H4K8ac_peak_14408	6.31818	MTCH1_ENSG00000137409
6	37060744	37061115	5Y-H4K8ac_peak_14409	7.05631	
6	37071861	37072132	5Y-H4K8ac_peak_14410	5.28616	
6	37189768	37190045	5Y-H4K8ac_peak_14411	8.4454	
6	37225610	37225850	5Y-H4K8ac_peak_14412	8.69112	TMEM217_ENSG00000172738;TBC1D22B_ENSG00000065491
6	37401123	37401321	5Y-H4K8ac_peak_14413	6.34245	CMTR1_ENSG00000137200
6	37457043	37457409	5Y-H4K8ac_peak_14414	5.37237	
6	37484119	37484385	5Y-H4K8ac_peak_14415	9.62747	
6	37484581	37485127	5Y-H4K8ac_peak_14416	13.99721	
6	37504141	37504653	5Y-H4K8ac_peak_14417	7.93983	
6	37533685	37534135	5Y-H4K8ac_peak_14418	8.21582	
6	37543533	37543756	5Y-H4K8ac_peak_14419	5.64909	
6	37592344	37593340	5Y-H4K8ac_peak_14420	12.69256	
6	37786649	37787480	5Y-H4K8ac_peak_14421	16.50659	RP3-441A12.1_ENSG00000225945;ZFAND3_ENSG00000156639
6	38137797	38138045	5Y-H4K8ac_peak_14422	4.07874	
6	38176765	38176964	5Y-H4K8ac_peak_14423	7.42477	
6	38235231	38235595	5Y-H4K8ac_peak_14424	9.15007	
6	38326835	38327226	5Y-H4K8ac_peak_14425	6.34046	AL031905.1_ENSG00000238716
6	38607297	38608245	5Y-H4K8ac_peak_14426	8.43511	BTBD9_ENSG00000183826
6	39016112	39016794	5Y-H4K8ac_peak_14427	9.89244	
6	39083105	39083330	5Y-H4K8ac_peak_14428	5.24695	SAYSD1_ENSG00000112167
6	39196990	39197185	5Y-H4K8ac_peak_14429	5.56912	KCNK5_ENSG00000164626
6	39399397	39399814	5Y-H4K8ac_peak_14430	8.23578	
6	39693431	39693786	5Y-H4K8ac_peak_14431	10.46287	KIF6_ENSG00000164627
6	39759764	39760232	5Y-H4K8ac_peak_14432	4.95697	DAAM2_ENSG00000146122
6	39901759	39902118	5Y-H4K8ac_peak_14433	9.15007	MOCS1_ENSG00000124615
6	40377414	40378016	5Y-H4K8ac_peak_14434	10.60003	
6	40379042	40379606	5Y-H4K8ac_peak_14435	9.74838	
6	40379854	40380259	5Y-H4K8ac_peak_14436	4.00285	
6	40380741	40381206	5Y-H4K8ac_peak_14437	9.23159	
6	40388387	40388612	5Y-H4K8ac_peak_14438	5.54773	
6	40392481	40393201	5Y-H4K8ac_peak_14439	7.89273	
6	40399582	40400049	5Y-H4K8ac_peak_14440	7.76645	
6	40405919	40406142	5Y-H4K8ac_peak_14441	4.36976	
6	40407295	40407564	5Y-H4K8ac_peak_14442	6.21836	

6	40409016	40409251	5Y-H4K8ac_peak_14443	6.0873	
6	40410230	40410440	5Y-H4K8ac_peak_14444	9.30505	
6	40452155	40452530	5Y-H4K8ac_peak_14445	10.35586	
6	40453473	40453817	5Y-H4K8ac_peak_14446	6.40964	
6	40457186	40458238	5Y-H4K8ac_peak_14447	13.03079	
6	40458745	40459296	5Y-H4K8ac_peak_14448	9.34555	
6	40460357	40461087	5Y-H4K8ac_peak_14449	12.14232	
6	40467586	40467955	5Y-H4K8ac_peak_14450	8.28346	
6	40504391	40504616	5Y-H4K8ac_peak_14451	7.31815	
6	40597556	40597840	5Y-H4K8ac_peak_14452	7.42477	
6	40677983	40678439	5Y-H4K8ac_peak_14453	10.69698	
6	40681379	40681703	5Y-H4K8ac_peak_14454	4.15658	
6	40686941	40687393	5Y-H4K8ac_peak_14455	7.38046	
6	40687748	40688049	5Y-H4K8ac_peak_14456	18.77281	
6	40688555	40689056	5Y-H4K8ac_peak_14457	11.75922	
6	40689470	40690294	5Y-H4K8ac_peak_14458	6.37023	
6	40691122	40691642	5Y-H4K8ac_peak_14459	11.1169	
6	40708741	40709406	5Y-H4K8ac_peak_14460	6.77436	
6	40764422	40764964	5Y-H4K8ac_peak_14461	5.98695	
6	40837055	40837309	5Y-H4K8ac_peak_14462	6.36454	
6	40879872	40880081	5Y-H4K8ac_peak_14463	9.43096	
6	40995908	40996329	5Y-H4K8ac_peak_14464	13.85283	
6	41040949	41041363	5Y-H4K8ac_peak_14465	11.09973	NFYA_ENSG00000001167
6	41455772	41456006	5Y-H4K8ac_peak_14466	5.24695	
6	41471595	41472107	5Y-H4K8ac_peak_14467	9.96543	
6	41513428	41513706	5Y-H4K8ac_peak_14468	5.72233	FOXP4_ENSG00000137166
6	41570136	41570496	5Y-H4K8ac_peak_14469	4.79585	
6	41602189	41602396	5Y-H4K8ac_peak_14470	7.80086	
6	41603029	41603227	5Y-H4K8ac_peak_14471	6.50117	
6	41604630	41604858	5Y-H4K8ac_peak_14472	4.77307	MDFI_ENSG00000112559
6	41605193	41606028	5Y-H4K8ac_peak_14473	6.06162	MDFI_ENSG00000112559
6	41641497	41641688	5Y-H4K8ac_peak_14474	7.35333	
6	41642548	41642977	5Y-H4K8ac_peak_14475	6.77436	
6	41701261	41701494	5Y-H4K8ac_peak_14476	6.7002	
6	41734477	41734856	5Y-H4K8ac_peak_14477	7.90236	
6	41754052	41754280	5Y-H4K8ac_peak_14478	6.08523	FRS3_ENSG00000137218
6	42016393	42016694	5Y-H4K8ac_peak_14479	5.23083	
6	42104523	42104734	5Y-H4K8ac_peak_14480	7.89273	
6	42220052	42220365	5Y-H4K8ac_peak_14481	5.23083	
6	42420148	42420789	5Y-H4K8ac_peak_14482	12.05638	TRERF1_ENSG00000124496
6	42515528	42516192	5Y-H4K8ac_peak_14483	8.2913	
6	42714206	42714441	5Y-H4K8ac_peak_14484	7.3889	TBCC_ENSG00000124659
6	42750352	42751700	5Y-H4K8ac_peak_14485	13.46013	
6	42847781	42847978	5Y-H4K8ac_peak_14486	8.45687	RPL7L1_ENSG00000146223
6	42848349	42848555	5Y-H4K8ac_peak_14487	5.89113	RPL7L1_ENSG00000146223
6	42858241	42858523	5Y-H4K8ac_peak_14488	13.0168	C6orf226_ENSG00000221821

6	42928322	42928690	5Y-H4K8ac_peak_14489	4.79585	GNMT_ENSG00000124713
6	42946480	42946844	5Y-H4K8ac_peak_14490	5.91897	PEX6_ENSG00000124587
6	42988930	42989250	5Y-H4K8ac_peak_14491	6.08523	RRP36_ENSG00000124541
6	43021281	43021571	5Y-H4K8ac_peak_14492	8.43511	CUL7_ENSG00000044090
6	43081864	43082297	5Y-H4K8ac_peak_14493	5.19372	
6	43082537	43082778	5Y-H4K8ac_peak_14494	8.88899	
6	43089962	43090276	5Y-H4K8ac_peak_14495	6.77436	
6	43091575	43091912	5Y-H4K8ac_peak_14496	6.08523	
6	43110923	43111182	5Y-H4K8ac_peak_14497	6.50117	
6	43139190	43139460	5Y-H4K8ac_peak_14498	6.54441	SRF_ENSG00000112658
6	43196808	43197124	5Y-H4K8ac_peak_14499	8.62703	DNPH1_ENSG00000112667
6	43336983	43337222	5Y-H4K8ac_peak_14500	6.03632	ZNF318_ENSG00000171467
6	43337719	43338061	5Y-H4K8ac_peak_14501	6.54441	ZNF318_ENSG00000171467
6	43338411	43338628	5Y-H4K8ac_peak_14502	7.9412	
6	43359093	43359836	5Y-H4K8ac_peak_14503	11.77396	
6	43394869	43395188	5Y-H4K8ac_peak_14504	11.68317	ABCC10_ENSG00000124574
6	43456546	43457343	5Y-H4K8ac_peak_14505	9.7353	
6	43457754	43457958	5Y-H4K8ac_peak_14506	5.18654	
6	43602839	43603406	5Y-H4K8ac_peak_14507	15.93543	
6	43603636	43603827	5Y-H4K8ac_peak_14508	9.15007	
6	43655139	43655946	5Y-H4K8ac_peak_14509	11.19336	MRPS18A_ENSG00000096080
6	43739738	43740102	5Y-H4K8ac_peak_14510	13.99721	
6	43742885	43743174	5Y-H4K8ac_peak_14511	5.64909	
6	43868609	43868821	5Y-H4K8ac_peak_14512	9.95819	
6	43893885	43894245	5Y-H4K8ac_peak_14513	7.31102	
6	43895040	43895375	5Y-H4K8ac_peak_14514	4.29586	
6	43905494	43905830	5Y-H4K8ac_peak_14515	12.64868	
6	43907165	43907764	5Y-H4K8ac_peak_14516	6.37023	
6	43907981	43908219	5Y-H4K8ac_peak_14517	6.34772	
6	43908940	43909330	5Y-H4K8ac_peak_14518	7.59101	
6	43910070	43910551	5Y-H4K8ac_peak_14519	5.23083	
6	43921530	43921781	5Y-H4K8ac_peak_14520	7.01266	
6	43957660	43959067	5Y-H4K8ac_peak_14521	6.78128	
6	43970759	43971014	5Y-H4K8ac_peak_14522	6.5814	
6	44011516	44011744	5Y-H4K8ac_peak_14523	9.7353	
6	44019334	44019595	5Y-H4K8ac_peak_14524	6.96612	
6	44022420	44022863	5Y-H4K8ac_peak_14525	6.73437	
6	44024245	44025455	5Y-H4K8ac_peak_14526	11.36989	
6	44026753	44027039	5Y-H4K8ac_peak_14527	7.11863	
6	44043023	44043436	5Y-H4K8ac_peak_14528	6.13919	RP5-1120P11.1_ENSG00000237686
6	44044733	44044962	5Y-H4K8ac_peak_14529	9.00954	
6	44134066	44134265	5Y-H4K8ac_peak_14530	6.00388	
6	44187023	44187243	5Y-H4K8ac_peak_14531	5.24695	SLC29A1_ENSG00000112759
6	44189626	44189876	5Y-H4K8ac_peak_14532	8.69112	
6	44213806	44214008	5Y-H4K8ac_peak_14533	7.8267	HSP90AB1_ENSG00000096384
6	44310254	44310749	5Y-H4K8ac_peak_14534	11.1169	SPATS1_ENSG00000249481

6	44355426	44355630	5Y-H4K8ac_peak_14535	8.43511	CDC5L_ENSG00000096401
6	44443730	44443960	5Y-H4K8ac_peak_14536	10.79063	
6	45345230	45345549	5Y-H4K8ac_peak_14537	8.17203	SUPT3H_ENSG00000196284
6	45346017	45346367	5Y-H4K8ac_peak_14538	5.65584	SUPT3H_ENSG00000196284
6	47445019	47445392	5Y-H4K8ac_peak_14539	8.17203	RP11-385F7.1_ENSG00000270761;CD2AP_ENSG00000198087
6	49429038	49429316	5Y-H4K8ac_peak_14540	5.40331	
6	49431217	49431465	5Y-H4K8ac_peak_14541	5.29015	MUT_ENSG00000146085;CENPQ_ENSG00000031691
6	50692179	50692545	5Y-H4K8ac_peak_14542	7.38046	
6	50787337	50788144	5Y-H4K8ac_peak_14543	8.3285	TFAP2B_ENSG00000008196
6	51130294	51130521	5Y-H4K8ac_peak_14544	10.1994	
6	51418551	51418886	5Y-H4K8ac_peak_14545	9.38276	
6	51857847	51858080	5Y-H4K8ac_peak_14546	4.84727	
6	51873808	51874162	5Y-H4K8ac_peak_14547	7.13294	
6	51883979	51884269	5Y-H4K8ac_peak_14548	5.12213	
6	52227253	52227500	5Y-H4K8ac_peak_14549	7.50148	
6	52284630	52284885	5Y-H4K8ac_peak_14550	13.2534	EFHC1_ENSG00000096093
6	52382816	52383064	5Y-H4K8ac_peak_14551	6.22669	
6	52441166	52441434	5Y-H4K8ac_peak_14552	4.95697	TRAM2_ENSG00000065308;TRAM2-AS1_ENSG00000225791
6	52442013	52442455	5Y-H4K8ac_peak_14553	8.69112	TRAM2_ENSG00000065308;TRAM2-AS1_ENSG00000225791
6	52529390	52529618	5Y-H4K8ac_peak_14554	6.73385	RP1-152L7.5_ENSG00000216775
6	52859565	52859920	5Y-H4K8ac_peak_14555	9.34935	GSTA4_ENSG00000170899;RN7SK_ENSG00000202198
6	52930220	52930418	5Y-H4K8ac_peak_14556	5.24695	
6	53212866	53213613	5Y-H4K8ac_peak_14557	4.55128	ELOVL5_ENSG00000012660
6	53223621	53224177	5Y-H4K8ac_peak_14558	9.79526	
6	53516500	53516707	5Y-H4K8ac_peak_14559	5.56894	
6	53658935	53659316	5Y-H4K8ac_peak_14560	7.31102	RP13-476E20.1_ENSG00000228614;LRRC1_ENSG00000137269
6	56716128	56716346	5Y-H4K8ac_peak_14561	6.34046	
6	56819390	56819947	5Y-H4K8ac_peak_14562	10.95042	DST_ENSG00000151914;BEND6_ENSG00000151917
6	57037243	57037771	5Y-H4K8ac_peak_14563	15.3483	BAG2_ENSG00000112208
6	57086579	57087037	5Y-H4K8ac_peak_14564	5.99504	RAB23_ENSG00000112210
6	58288110	58288383	5Y-H4K8ac_peak_14565	5.19372	LINC00680_ENSG00000215190
6	64346148	64346566	5Y-H4K8ac_peak_14566	13.60015	PHF3_ENSG00000118482
6	70311203	70311405	5Y-H4K8ac_peak_14567	6.34046	
6	70577059	70577503	5Y-H4K8ac_peak_14568	9.30505	COL19A1_ENSG00000082293
6	70615435	70615658	5Y-H4K8ac_peak_14569	7.31815	
6	71121846	71122041	5Y-H4K8ac_peak_14570	4.84727	FAM135A_ENSG00000082269
6	71122615	71122846	5Y-H4K8ac_peak_14571	13.8759	RP11-462G2.2_ENSG00000224349;FAM135A_ENSG00000082269
6	71276696	71277071	5Y-H4K8ac_peak_14572	9.78792	C6orf57_ENSG00000154079
6	73972967	73973372	5Y-H4K8ac_peak_14573	15.16038	AC019205.1_ENSG00000263378;RP11-257K9.8_ENSG00000243501;RP11-398K22.12_ENSG00000229852
6	74230097	74230693	5Y-H4K8ac_peak_14574	9.96543	
6	74231131	74231575	5Y-H4K8ac_peak_14575	11.57334	
6	74289093	74289442	5Y-H4K8ac_peak_14576	5.65584	
6	75993988	75994446	5Y-H4K8ac_peak_14577	10.27264	TMEM30A_ENSG00000112697;RP1-234P15.4_ENSG00000225793
6	76311674	76312215	5Y-H4K8ac_peak_14578	11.1169	SENP6_ENSG00000112701
6	76459012	76459245	5Y-H4K8ac_peak_14579	14.15915	MYO6_ENSG00000196586
6	80341051	80341252	5Y-H4K8ac_peak_14580	6.08523	SH3BGRL2_ENSG00000198478

6	80521873	80522312	5Y-H4K8ac_peak_14581	7.3889	
6	80531669	80531982	5Y-H4K8ac_peak_14582	8.33296	
6	80657440	80657684	5Y-H4K8ac_peak_14583	9.51254	ELOVL4_ENSG00000118402
6	83445680	83445885	5Y-H4K8ac_peak_14584	7.31815	
6	84140858	84141121	5Y-H4K8ac_peak_14585	9.07085	ME1_ENSG00000065833
6	84221787	84222122	5Y-H4K8ac_peak_14586	8.17203	PRSS35_ENSG00000146250
6	84562744	84562953	5Y-H4K8ac_peak_14587	6.78318	RIPPLY2_ENSG00000203877
6	84563427	84563719	5Y-H4K8ac_peak_14588	7.89273	RIPPLY2_ENSG00000203877
6	84742989	84743440	5Y-H4K8ac_peak_14589	10.01583	MRAP2_ENSG00000135324
6	86351978	86352585	5Y-H4K8ac_peak_14590	11.67083	SYNCRIP_ENSG00000135316
6	86352809	86352999	5Y-H4K8ac_peak_14591	9.65108	SYNCRIP_ENSG00000135316
6	87647047	87647237	5Y-H4K8ac_peak_14592	5.23083	HTR1E_ENSG00000168830
6	87861483	87862040	5Y-H4K8ac_peak_14593	13.92877	ZNF292_ENSG00000188994
6	87865328	87865740	5Y-H4K8ac_peak_14594	5.60566	RP11-393I2.4_ENSG00000272008
6	88181797	88182016	5Y-H4K8ac_peak_14595	7.31815	
6	88182428	88182623	5Y-H4K8ac_peak_14596	10.11191	
6	88876615	88876835	5Y-H4K8ac_peak_14597	5.65584	CNR1_ENSG00000118432
6	89790769	89791077	5Y-H4K8ac_peak_14598	8.43511	RP11-63L7.5_ENSG00000271931;PNRC1_ENSG00000146278
6	89827246	89827595	5Y-H4K8ac_peak_14599	6.1654	SRSF12_ENSG00000154548
6	89855809	89856438	5Y-H4K8ac_peak_14600	7.34185	PM20D2_ENSG00000146281
6	90021560	90021915	5Y-H4K8ac_peak_14601	6.14981	
6	90240311	90240560	5Y-H4K8ac_peak_14602	6.50117	
6	90348261	90348457	5Y-H4K8ac_peak_14603	12.05638	LYRM2_ENSG00000083099
6	90679081	90679440	5Y-H4K8ac_peak_14604	4.71803	
6	90681552	90681954	5Y-H4K8ac_peak_14605	5.65584	
6	90688536	90688809	5Y-H4K8ac_peak_14606	4.79585	
6	90689751	90690043	5Y-H4K8ac_peak_14607	8.14913	
6	90695430	90695830	5Y-H4K8ac_peak_14608	15.94046	
6	90734400	90734967	5Y-H4K8ac_peak_14609	10.22205	
6	91006851	91007360	5Y-H4K8ac_peak_14610	10.35586	BACH2_ENSG00000112182
6	91149646	91149930	5Y-H4K8ac_peak_14611	4.50834	
6	91179255	91179475	5Y-H4K8ac_peak_14612	5.54013	
6	91296324	91297287	5Y-H4K8ac_peak_14613	7.59101	MAP3K7_ENSG00000135341
6	96025608	96026034	5Y-H4K8ac_peak_14614	6.1654	MANEA-AS1_ENSG00000261366;MANEA_ENSG00000172469
6	96463333	96463686	5Y-H4K8ac_peak_14615	6.50117	FUT9_ENSG00000172461
6	96463946	96464214	5Y-H4K8ac_peak_14616	6.77436	FUT9_ENSG00000172461
6	96969770	96970022	5Y-H4K8ac_peak_14617	6.53157	UFL1-AS1_ENSG00000233797;UFL1_ENSG00000014123
6	97285571	97285926	5Y-H4K8ac_peak_14618	13.00733	GPR63_ENSG00000112218
6	99272355	99272693	5Y-H4K8ac_peak_14619	7.31815	
6	99395508	99395761	5Y-H4K8ac_peak_14620	5.23083	FBXL4_ENSG00000112234
6	99797186	99797759	5Y-H4K8ac_peak_14621	5.64909	FAXC_ENSG00000146267
6	99872777	99873113	5Y-H4K8ac_peak_14622	8.75926	PNISR_ENSG00000132424;RP11-98I9.4_ENSG00000228506
6	99968946	99969261	5Y-H4K8ac_peak_14623	5.72233	USP45_ENSG00000123552;TSTD3_ENSG00000228439
6	100016259	100016531	5Y-H4K8ac_peak_14624	8.564	CCNC_ENSG00000112237;RP1-199J3.7_ENSG00000272017
6	101849525	101849719	5Y-H4K8ac_peak_14625	8.97752	
6	101850311	101850530	5Y-H4K8ac_peak_14626	5.56912	

6	102838758	102839026	5Y-H4K8ac_peak_14627	9.93099	
6	103775068	103775273	5Y-H4K8ac_peak_14628	7.31815	
6	105307322	105307652	5Y-H4K8ac_peak_14629	8.2913	HACE1_ENSG00000085382;RP11-809N15.3_ENSG00000271099
6	105388513	105389207	5Y-H4K8ac_peak_14630	7.90751	LINC00577_ENSG00000203809
6	105584616	105585024	5Y-H4K8ac_peak_14631	10.90365	BVES_ENSG00000112276;BVES-AS1_ENSG00000203808
6	105585254	105585477	5Y-H4K8ac_peak_14632	4.84727	BVES_ENSG00000112276
6	105627462	105627797	5Y-H4K8ac_peak_14633	6.73385	POPDC3_ENSG00000132429
6	105721600	105721829	5Y-H4K8ac_peak_14634	7.38046	RP3-355L5.5_ENSG00000272102
6	106773270	106773493	5Y-H4K8ac_peak_14635	7.63144	ATG5_ENSG00000057663
6	106958199	106958425	5Y-H4K8ac_peak_14636	4.07874	
6	107349509	107349797	5Y-H4K8ac_peak_14637	5.29015	C6orf203_ENSG00000130349
6	107365069	107365411	5Y-H4K8ac_peak_14638	4.50834	
6	107366329	107366571	5Y-H4K8ac_peak_14639	5.23083	
6	107435448	107435817	5Y-H4K8ac_peak_14640	7.68492	BEND3_ENSG00000178409
6	107436009	107436569	5Y-H4K8ac_peak_14641	7.80156	BEND3_ENSG00000178409
6	107436822	107437432	5Y-H4K8ac_peak_14642	14.36519	BEND3_ENSG00000178409
6	107781087	107781304	5Y-H4K8ac_peak_14643	10.35586	PDSS2_ENSG00000164494
6	107937899	107938332	5Y-H4K8ac_peak_14644	8.43511	
6	107992337	107992595	5Y-H4K8ac_peak_14645	5.41472	
6	108033622	108033909	5Y-H4K8ac_peak_14646	8.33296	
6	108582885	108583376	5Y-H4K8ac_peak_14647	7.38046	SNX3_ENSG00000112335
6	108616118	108616386	5Y-H4K8ac_peak_14648	6.43775	LACE1_ENSG00000135537
6	109103541	109103769	5Y-H4K8ac_peak_14649	4.51076	
6	109121865	109122272	5Y-H4K8ac_peak_14650	5.89113	
6	109264679	109264931	5Y-H4K8ac_peak_14651	4.50834	
6	109415826	109416159	5Y-H4K8ac_peak_14652	8.21582	SESN1_ENSG00000080546;CEP57L1_ENSG00000183137
6	109548481	109548768	5Y-H4K8ac_peak_14653	4.84727	
6	109703209	109703962	5Y-H4K8ac_peak_14654	7.89273	CD164_ENSG00000135535;RP11-425D10.10_ENSG00000260273
6	109776431	109776768	5Y-H4K8ac_peak_14655	5.12488	
6	110012723	110012915	5Y-H4K8ac_peak_14656	7.17184	AK9_ENSG00000155085;FIG4_ENSG00000112367
6	110035053	110035592	5Y-H4K8ac_peak_14657	6.34046	
6	110500354	110500821	5Y-H4K8ac_peak_14658	7.46096	WASF1_ENSG00000112290;CDC40_ENSG00000168438
6	110679076	110679862	5Y-H4K8ac_peak_14659	12.05638	METTL24_ENSG00000053328
6	110864259	110864518	5Y-H4K8ac_peak_14660	11.57334	
6	111196577	111197018	5Y-H4K8ac_peak_14661	8.2913	AMD1_ENSG00000123505
6	111254478	111254708	5Y-H4K8ac_peak_14662	4.50834	
6	111278819	111279093	5Y-H4K8ac_peak_14663	8.2913	GTF3C6_ENSG00000155115
6	111279553	111279776	5Y-H4K8ac_peak_14664	8.62703	GTF3C6_ENSG00000155115
6	111279986	111280383	5Y-H4K8ac_peak_14665	18.77281	GTF3C6_ENSG00000155115
6	111804157	111804376	5Y-H4K8ac_peak_14666	8.21582	REV3L_ENSG00000009413;TRAF3IP2-AS1_ENSG00000231889
6	111805718	111805988	5Y-H4K8ac_peak_14667	4.84727	REV3L_ENSG00000009413
6	111875332	111875527	5Y-H4K8ac_peak_14668	7.63144	
6	111881358	111881574	5Y-H4K8ac_peak_14669	8.93923	
6	111882222	111882436	5Y-H4K8ac_peak_14670	6.77436	
6	111887312	111887624	5Y-H4K8ac_peak_14671	13.00733	
6	111887913	111888260	5Y-H4K8ac_peak_14672	11.18363	

6	111982851	111983202	5Y-H4K8ac_peak_14673	5.23083	
6	112115105	112115372	5Y-H4K8ac_peak_14674	7.60057	
6	112194018	112194409	5Y-H4K8ac_peak_14675	9.34555	FYN_ENSG00000010810
6	112575247	112575674	5Y-H4K8ac_peak_14676	7.30348	LAMA4_ENSG00000112769
6	113885768	113885972	5Y-H4K8ac_peak_14677	5.65584	
6	114053633	114053981	5Y-H4K8ac_peak_14678	10.46287	
6	114291377	114292553	5Y-H4K8ac_peak_14679	11.93212	RP3-399L15.3_ENSG00000228624
6	116422089	116422291	5Y-H4K8ac_peak_14680	8.69112	NT5DC1_ENSG00000178425
6	116990183	116990391	5Y-H4K8ac_peak_14681	4.03329	ZUFSP_ENSG00000153975
6	118973001	118973350	5Y-H4K8ac_peak_14682	7.31815	
6	119030274	119030503	5Y-H4K8ac_peak_14683	4.84727	CEP85L_ENSG00000111860
6	121655761	121656048	5Y-H4K8ac_peak_14684	5.41472	TBC1D32_ENSG00000146350
6	126111136	126111367	5Y-H4K8ac_peak_14685	7.09658	
6	126277949	126278254	5Y-H4K8ac_peak_14686	7.38046	HINT3_ENSG00000111911
6	127588167	127588392	5Y-H4K8ac_peak_14687	4.07874	RNF146_ENSG00000118518
6	127664599	127665023	5Y-H4K8ac_peak_14688	5.98695	ECHDC1_ENSG00000093144
6	131456693	131457107	5Y-H4K8ac_peak_14689	7.31815	AKAP7_ENSG00000118507
6	132833821	132834159	5Y-H4K8ac_peak_14690	5.3176	STX7_ENSG00000079950
6	133134928	133135257	5Y-H4K8ac_peak_14691	12.65187	RPS12_ENSG00000112306
6	133452675	133452869	5Y-H4K8ac_peak_14692	4.50834	
6	134273151	134273593	5Y-H4K8ac_peak_14693	16.52279	TBPL1_ENSG00000028839
6	135051195	135051406	5Y-H4K8ac_peak_14694	5.23083	
6	135376187	135376683	5Y-H4K8ac_peak_14695	7.89273	CTA-212D2.2_ENSG00000232876
6	135502008	135502257	5Y-H4K8ac_peak_14696	9.63153	MYB_ENSG00000118513
6	136433439	136433640	5Y-H4K8ac_peak_14697	4.9885	
6	136612897	136613119	5Y-H4K8ac_peak_14698	7.49746	
6	137143744	137143962	5Y-H4K8ac_peak_14699	13.33225	PEX7_ENSG00000112357
6	137242514	137243526	5Y-H4K8ac_peak_14700	17.76126	SLC35D3_ENSG00000182747
6	137244013	137244276	5Y-H4K8ac_peak_14701	5.25819	SLC35D3_ENSG00000182747
6	137244496	137244757	5Y-H4K8ac_peak_14702	9.59156	
6	137283400	137283599	5Y-H4K8ac_peak_14703	7.63144	
6	137288526	137288809	5Y-H4K8ac_peak_14704	10.89497	
6	137289214	137289675	5Y-H4K8ac_peak_14705	7.41197	
6	137292540	137292818	5Y-H4K8ac_peak_14706	12.69256	
6	137301612	137301807	5Y-H4K8ac_peak_14707	6.34046	
6	137302033	137302642	5Y-H4K8ac_peak_14708	6.34046	
6	137303378	137303813	5Y-H4K8ac_peak_14709	10.84616	
6	137309792	137309995	5Y-H4K8ac_peak_14710	7.38046	
6	137310901	137311384	5Y-H4K8ac_peak_14711	9.51254	
6	137364289	137364480	5Y-H4K8ac_peak_14712	4.51076	
6	137365251	137366182	5Y-H4K8ac_peak_14713	16.03077	IL20RA_ENSG00000016402
6	137540156	137540479	5Y-H4K8ac_peak_14714	11.19336	IFNGR1_ENSG00000027697
6	138188482	138188685	5Y-H4K8ac_peak_14715	4.84727	RP11-356I2.4_ENSG00000237499;TNFAIP3_ENSG00000118503
6	139309009	139309375	5Y-H4K8ac_peak_14716	10.69698	REPS1_ENSG00000135597
6	139309769	139310037	5Y-H4K8ac_peak_14717	7.31815	REPS1_ENSG00000135597
6	139348631	139348841	5Y-H4K8ac_peak_14718	4.84727	ABRACL_ENSG00000146386

6	142468423	142468698	5Y-H4K8ac_peak_14719	6.34046	VTA1_ENSG00000009844
6	142968836	142969031	5Y-H4K8ac_peak_14720	6.73385	
6	143247692	143247988	5Y-H4K8ac_peak_14721	10.21117	
6	143266505	143266772	5Y-H4K8ac_peak_14722	8.23578	HIVEP2_ENSG00000010818
6	143381539	143381747	5Y-H4K8ac_peak_14723	6.77436	AIG1_ENSG00000146416
6	143832438	143832852	5Y-H4K8ac_peak_14724	15.87289	FUCA2_ENSG00000001036
6	143857687	143858330	5Y-H4K8ac_peak_14725	6.64195	PHACTR2_ENSG00000112419
6	144163890	144164219	5Y-H4K8ac_peak_14726	6.50117	LTV1_ENSG00000135521
6	144164516	144164820	5Y-H4K8ac_peak_14727	8.43511	LTV1_ENSG00000135521
6	144417065	144417729	5Y-H4K8ac_peak_14728	13.64163	SF3B5_ENSG00000169976
6	146056130	146056410	5Y-H4K8ac_peak_14729	15.06486	EPM2A_ENSG00000112425;RP3-466P17.1_ENSG00000270638
6	146056613	146056988	5Y-H4K8ac_peak_14730	6.78128	EPM2A_ENSG00000112425;RP3-466P17.1_ENSG00000270638
6	146135570	146135827	5Y-H4K8ac_peak_14731	6.50117	FBXO30_ENSG00000118496;RP11-545I5.3_ENSG00000235652
6	146755322	146756029	5Y-H4K8ac_peak_14732	10.1994	
6	146864186	146864576	5Y-H4K8ac_peak_14733	13.99721	RAB32_ENSG00000118508
6	146864914	146865194	5Y-H4K8ac_peak_14734	6.1654	RAB32_ENSG00000118508
6	146972262	146972532	5Y-H4K8ac_peak_14735	7.89142	
6	149638225	149638590	5Y-H4K8ac_peak_14736	21.35466	
6	149638841	149639553	5Y-H4K8ac_peak_14737	11.57334	
6	149866799	149866990	5Y-H4K8ac_peak_14738	8.2913	PPIL4_ENSG00000131013
6	150038843	150039172	5Y-H4K8ac_peak_14739	15.80786	LATS1_ENSG00000131023
6	150067808	150068280	5Y-H4K8ac_peak_14740	5.65584	
6	151186901	151187398	5Y-H4K8ac_peak_14741	9.23159	MTHFD1L_ENSG00000120254
6	151378613	151378936	5Y-H4K8ac_peak_14742	7.12953	
6	151432675	151432874	5Y-H4K8ac_peak_14743	5.29015	
6	151562074	151562435	5Y-H4K8ac_peak_14744	13.06587	AKAP12_ENSG00000131016
6	151710861	151711329	5Y-H4K8ac_peak_14745	6.37023	
6	151711920	151712539	5Y-H4K8ac_peak_14746	21.14873	ZBTB2_ENSG00000181472
6	151771554	151771795	5Y-H4K8ac_peak_14747	8.33296	
6	153303313	153304081	5Y-H4K8ac_peak_14748	14.77644	FBXO5_ENSG00000112029;RP1-101K10.6_ENSG00000227627
6	153471968	153472161	5Y-H4K8ac_peak_14749	10.31981	
6	154360399	154361034	5Y-H4K8ac_peak_14750	11.57334	
6	154415588	154415829	5Y-H4K8ac_peak_14751	8.43511	
6	154745079	154745269	5Y-H4K8ac_peak_14752	5.29015	
6	154778558	154778811	5Y-H4K8ac_peak_14753	8.93923	
6	154830591	154830906	5Y-H4K8ac_peak_14754	5.28616	CNKSR3_ENSG00000153721
6	154862895	154863101	5Y-H4K8ac_peak_14755	5.62788	
6	155054541	155055136	5Y-H4K8ac_peak_14756	9.179	SCAF8_ENSG00000213079
6	155316889	155317290	5Y-H4K8ac_peak_14757	5.64909	
6	156188028	156188322	5Y-H4K8ac_peak_14758	5.64909	
6	157099037	157099838	5Y-H4K8ac_peak_14759	12.10416	RP11-230C9.2_ENSG00000271551;ARID1B_ENSG00000049618
6	157100140	157100767	5Y-H4K8ac_peak_14760	9.05168	MIR4466_ENSG00000271899;RP11-230C9.3_ENSG00000270487
6	157269456	157269650	5Y-H4K8ac_peak_14761	6.34046	
6	157549941	157550299	5Y-H4K8ac_peak_14762	4.8509	
6	157629673	157629923	5Y-H4K8ac_peak_14763	5.29015	
6	157695406	157695738	5Y-H4K8ac_peak_14764	6.34046	

6	157696001	157696451	5Y-H4K8ac_peak_14765	11.1169	
6	157801274	157801770	5Y-H4K8ac_peak_14766	6.03632	ZDHHC14_ENSG00000175048
6	157853928	157854142	5Y-H4K8ac_peak_14767	5.3176	
6	158070478	158070776	5Y-H4K8ac_peak_14768	10.31981	
6	158243606	158244054	5Y-H4K8ac_peak_14769	16.15586	SNX9_ENSG00000130340
6	158244356	158244933	5Y-H4K8ac_peak_14770	10.84616	SNX9_ENSG00000130340
6	158402940	158403370	5Y-H4K8ac_peak_14771	5.23083	SYNJ2_ENSG00000078269
6	158431334	158431562	5Y-H4K8ac_peak_14772	10.60003	
6	158589463	158590169	5Y-H4K8ac_peak_14773	10.36926	SERAC1_ENSG00000122335;GTF2H5_ENSG00000272047
6	158653368	158653745	5Y-H4K8ac_peak_14774	6.47245	
6	158957527	158957855	5Y-H4K8ac_peak_14775	5.449	TMEM181_ENSG00000146433
6	158958154	158958494	5Y-H4K8ac_peak_14776	6.78128	TMEM181_ENSG00000146433
6	159041732	159042057	5Y-H4K8ac_peak_14777	6.34046	TATDN2P2_ENSG00000218226
6	159042361	159042816	5Y-H4K8ac_peak_14778	8.2913	TATDN2P2_ENSG00000218226
6	159064919	159065670	5Y-H4K8ac_peak_14779	7.31102	DYNLT1_ENSG00000146425
6	159125490	159125966	5Y-H4K8ac_peak_14780	11.36844	
6	159172908	159173122	5Y-H4K8ac_peak_14781	5.03335	
6	159174274	159174525	5Y-H4K8ac_peak_14782	4.9885	
6	159176518	159176896	5Y-H4K8ac_peak_14783	8.2913	
6	159177094	159177480	5Y-H4K8ac_peak_14784	8.59175	
6	159178724	159179254	5Y-H4K8ac_peak_14785	4.07874	
6	159240556	159240767	5Y-H4K8ac_peak_14786	5.24695	EZR_ENSG00000092820
6	159525388	159525766	5Y-H4K8ac_peak_14787	9.31608	
6	160114458	160114667	5Y-H4K8ac_peak_14788	6.34046	
6	160148185	160148390	5Y-H4K8ac_peak_14789	10.72427	
6	160148749	160149009	5Y-H4K8ac_peak_14790	5.64909	
6	161412413	161412751	5Y-H4K8ac_peak_14791	8.33296	RP3-428L16.2_ENSG00000272841;MAP3K4_ENSG00000085511
6	161412970	161413202	5Y-H4K8ac_peak_14792	7.31815	RP3-428L16.2_ENSG00000272841;MAP3K4_ENSG00000085511
6	161462403	161462600	5Y-H4K8ac_peak_14793	5.81062	
6	161589246	161589577	5Y-H4K8ac_peak_14794	7.31815	
6	162043371	162043624	5Y-H4K8ac_peak_14795	5.40331	
6	163134222	163134423	5Y-H4K8ac_peak_14796	9.20645	
6	163148389	163148713	5Y-H4K8ac_peak_14797	6.43775	PARK2_ENSG00000185345;PACRG_ENSG00000112530
6	163338181	163338494	5Y-H4K8ac_peak_14798	5.28616	
6	163776094	163776350	5Y-H4K8ac_peak_14799	5.98695	
6	163834427	163834898	5Y-H4K8ac_peak_14800	7.50148	CAHM_ENSG00000270419;QKI_ENSG00000112531
6	164092265	164092550	5Y-H4K8ac_peak_14801	4.9885	
6	164134169	164134364	5Y-H4K8ac_peak_14802	6.50117	
6	164146342	164146573	5Y-H4K8ac_peak_14803	5.04571	
6	165346729	165347082	5Y-H4K8ac_peak_14804	5.28616	
6	166073951	166075466	5Y-H4K8ac_peak_14805	24.01091	
6	166314401	166314623	5Y-H4K8ac_peak_14806	7.96225	
6	166419210	166419535	5Y-H4K8ac_peak_14807	7.90751	
6	166720841	166721219	5Y-H4K8ac_peak_14808	11.1169	PRR18_ENSG00000176381
6	166756159	166756536	5Y-H4K8ac_peak_14809	14.50475	SFT2D1_ENSG00000198818
6	166817267	166817604	5Y-H4K8ac_peak_14810	7.59101	

6	166849252	166849443	5Y-H4K8ac_peak_14811	5.64909	
6	166849880	166850379	5Y-H4K8ac_peak_14812	6.82266	
6	166852633	166853194	5Y-H4K8ac_peak_14813	7.03573	
6	166854912	166855427	5Y-H4K8ac_peak_14814	6.08523	
6	166873681	166874221	5Y-H4K8ac_peak_14815	13.12848	
6	166875252	166875987	5Y-H4K8ac_peak_14816	20.07837	
6	166876280	166876504	5Y-H4K8ac_peak_14817	5.64909	
6	166881217	166881595	5Y-H4K8ac_peak_14818	5.57299	
6	166883583	166884217	5Y-H4K8ac_peak_14819	12.57157	
6	166884541	166884980	5Y-H4K8ac_peak_14820	13.00733	
6	166896268	166896477	5Y-H4K8ac_peak_14821	7.63144	
6	166903420	166904706	5Y-H4K8ac_peak_14822	9.72464	
6	166916693	166917020	5Y-H4K8ac_peak_14823	6.98394	
6	166946826	166947164	5Y-H4K8ac_peak_14824	3.91125	
6	167275718	167276006	5Y-H4K8ac_peak_14825	9.38203	
6	167276512	167276891	5Y-H4K8ac_peak_14826	7.38046	
6	167339699	167339940	5Y-H4K8ac_peak_14827	7.28311	
6	167412922	167413325	5Y-H4K8ac_peak_14828	10.4689	RP11-517H2.6_ENSG00000272980;RP1-167A14.2_ENSG00000227598;FGFR1OP_ENSG00000213066
6	167440794	167441096	5Y-H4K8ac_peak_14829	6.50117	
6	167441502	167441857	5Y-H4K8ac_peak_14830	6.91702	
6	167445295	167445801	5Y-H4K8ac_peak_14831	11.28133	
6	167528012	167528217	5Y-H4K8ac_peak_14832	9.34555	
6	167555326	167555597	5Y-H4K8ac_peak_14833	9.51785	
6	167648813	167649034	5Y-H4K8ac_peak_14834	5.65584	
6	167649270	167649648	5Y-H4K8ac_peak_14835	6.1654	
6	167691625	167691969	5Y-H4K8ac_peak_14836	10.02453	
6	167764499	167764813	5Y-H4K8ac_peak_14837	10.31981	
6	168099686	168099886	5Y-H4K8ac_peak_14838	5.12213	
6	168196672	168197192	5Y-H4K8ac_peak_14839	12.57157	AL009178.1_ENSG00000269155;C6orf123_ENSG00000146521
6	168226260	168226647	5Y-H4K8ac_peak_14840	5.98695	MLLT4-AS1_ENSG00000198221;MLLT4_ENSG00000130396
6	168396829	168397231	5Y-H4K8ac_peak_14841	4.84727	KIF25-AS1_ENSG00000229921;KIF25_ENSG00000125337
6	168435762	168435982	5Y-H4K8ac_peak_14842	8.63306	
6	168496964	168497353	5Y-H4K8ac_peak_14843	6.77436	
6	168498796	168499571	5Y-H4K8ac_peak_14844	11.1169	
6	168653862	168654060	5Y-H4K8ac_peak_14845	7.72345	
6	168956927	168957202	5Y-H4K8ac_peak_14846	4.51076	
6	169003296	169003602	5Y-H4K8ac_peak_14847	9.38203	
6	169049636	169049982	5Y-H4K8ac_peak_14848	5.98695	
6	169424423	169424614	5Y-H4K8ac_peak_14849	7.05631	
6	169613477	169613801	5Y-H4K8ac_peak_14850	16.50659	XXyac-YX65C7_A.2_ENSG00000226445
6	169614052	169614319	5Y-H4K8ac_peak_14851	6.34046	XXyac-YX65C7_A.2_ENSG00000226445
6	170102363	170102742	5Y-H4K8ac_peak_14852	14.15915	WDR27_ENSG00000184465;C6orf120_ENSG00000185127
6	170124917	170125580	5Y-H4K8ac_peak_14853	12.43358	PHF10_ENSG00000130024;RP1-266L20.2_ENSG00000232640;RP1-266L20.4_ENSG00000227704
6	170151297	170152079	5Y-H4K8ac_peak_14854	10.35586	TCTE3_ENSG00000184786;ERMARD_ENSG00000130023
6	170210541	170211038	5Y-H4K8ac_peak_14855	11.93212	RP1-182D15.2_ENSG00000236173
6	170340641	170340877	5Y-H4K8ac_peak_14856	9.52603	

6	170341242	170341599	5Y-H4K8ac_peak_14857	5.29015	
6	170403242	170403517	5Y-H4K8ac_peak_14858	6.55906	
6	170533620	170534035	5Y-H4K8ac_peak_14859	6.37023	
6	170553181	170553547	5Y-H4K8ac_peak_14860	5.03335	
6	170553817	170554260	5Y-H4K8ac_peak_14861	5.41472	
6	170615885	170616198	5Y-H4K8ac_peak_14862	12.21176	
6	170748165	170748827	5Y-H4K8ac_peak_14863	8.51022	
7	57190	57758	5Y-H4K8ac_peak_14864	5.12488	
7	87185	87582	5Y-H4K8ac_peak_14865	6.06162	
7	148962	149177	5Y-H4K8ac_peak_14866	13.92877	AC093627.9_ENSG00000242474;AC093627.10_ENSG00000240859
7	149481	150517	5Y-H4K8ac_peak_14867	24.01091	AC093627.9_ENSG00000242474;AC093627.10_ENSG00000240859
7	372345	372818	5Y-H4K8ac_peak_14868	12.2918	
7	495017	495459	5Y-H4K8ac_peak_14869	10.1994	
7	533261	534145	5Y-H4K8ac_peak_14870	16.06604	
7	706286	706486	5Y-H4K8ac_peak_14871	5.88655	
7	751332	752137	5Y-H4K8ac_peak_14872	17.04999	
7	752429	752625	5Y-H4K8ac_peak_14873	5.24695	
7	765853	766226	5Y-H4K8ac_peak_14874	16.57163	HEATR2_ENSG00000164818
7	808419	808917	5Y-H4K8ac_peak_14875	9.64738	
7	810058	810385	5Y-H4K8ac_peak_14876	10.48476	
7	818457	818712	5Y-H4K8ac_peak_14877	4.89128	
7	841672	841925	5Y-H4K8ac_peak_14878	6.08523	
7	855385	855644	5Y-H4K8ac_peak_14879	5.87725	SUN1_ENSG00000164828
7	856357	856607	5Y-H4K8ac_peak_14880	6.52343	SUN1_ENSG00000164828
7	915220	915943	5Y-H4K8ac_peak_14881	10.21117	GET4_ENSG00000239857
7	941520	941812	5Y-H4K8ac_peak_14882	8.95951	
7	949064	949367	5Y-H4K8ac_peak_14883	3.94247	
7	956131	956329	5Y-H4K8ac_peak_14884	8.73227	
7	1014854	1015107	5Y-H4K8ac_peak_14885	10.48476	COX19_ENSG00000240230
7	1067535	1067806	5Y-H4K8ac_peak_14886	4.642	AC073957.15_ENSG00000225146
7	1121237	1121565	5Y-H4K8ac_peak_14887	7.96285	AC091729.8_ENSG00000226291;GPER1_ENSG00000164850
7	1121818	1122328	5Y-H4K8ac_peak_14888	6.78128	GPER1_ENSG00000164850
7	1126180	1126452	5Y-H4K8ac_peak_14889	5.03564	
7	1178148	1178473	5Y-H4K8ac_peak_14890	4.55128	C7orf50_ENSG00000146540
7	1200139	1200652	5Y-H4K8ac_peak_14891	10.4495	ZFAND2A_ENSG00000178381;AC091729.9_ENSG00000229043
7	1215451	1215827	5Y-H4K8ac_peak_14892	6.19716	
7	1543593	1543923	5Y-H4K8ac_peak_14893	12.50545	
7	1572943	1573273	5Y-H4K8ac_peak_14894	5.34294	
7	1594921	1595165	5Y-H4K8ac_peak_14895	5.87725	
7	1609115	1610397	5Y-H4K8ac_peak_14896	24.73897	PSMG3_ENSG00000157778;PSMG3-AS1_ENSG00000230487
7	1625148	1626116	5Y-H4K8ac_peak_14897	12.50545	
7	1626388	1626895	5Y-H4K8ac_peak_14898	11.09673	
7	1678848	1679107	5Y-H4K8ac_peak_14899	7.1449	
7	1679586	1680030	5Y-H4K8ac_peak_14900	11.52237	
7	1680252	1680609	5Y-H4K8ac_peak_14901	7.50464	
7	1680856	1681107	5Y-H4K8ac_peak_14902	9.00574	

7	1681989	1682248	5Y-H4K8ac_peak_14903	10.48476	
7	1684917	1685252	5Y-H4K8ac_peak_14904	10.12457	
7	1686893	1688806	5Y-H4K8ac_peak_14905	18.60312	
7	1693862	1694093	5Y-H4K8ac_peak_14906	7.59101	
7	1703773	1704384	5Y-H4K8ac_peak_14907	7.41172	
7	1704594	1705062	5Y-H4K8ac_peak_14908	7.59101	
7	1707161	1707458	5Y-H4K8ac_peak_14909	4.04213	
7	1716175	1716387	5Y-H4K8ac_peak_14910	6.77436	
7	1753096	1753849	5Y-H4K8ac_peak_14911	3.93392	
7	1756364	1757294	5Y-H4K8ac_peak_14912	7.20869	
7	1764642	1764846	5Y-H4K8ac_peak_14913	5.88408	
7	1782849	1783204	5Y-H4K8ac_peak_14914	7.50148	AC074389.9_ENSG00000236081
7	1878848	1879216	5Y-H4K8ac_peak_14915	11.95373	AC110781.3_ENSG00000176349
7	1879477	1879833	5Y-H4K8ac_peak_14916	16.57564	
7	1893756	1893982	5Y-H4K8ac_peak_14917	8.3285	
7	1895840	1896090	5Y-H4K8ac_peak_14918	6.67704	
7	1896440	1897137	5Y-H4K8ac_peak_14919	9.02938	
7	1923803	1924016	5Y-H4K8ac_peak_14920	4.29586	
7	1939010	1939358	5Y-H4K8ac_peak_14921	4.77126	
7	1949163	1949363	5Y-H4K8ac_peak_14922	6.88247	
7	1989957	1990968	5Y-H4K8ac_peak_14923	6.08523	
7	1992165	1992355	5Y-H4K8ac_peak_14924	5.21777	
7	2041170	2041456	5Y-H4K8ac_peak_14925	4.67245	
7	2053299	2053570	5Y-H4K8ac_peak_14926	9.68742	
7	2106367	2106842	5Y-H4K8ac_peak_14927	10.56003	
7	2121209	2121493	5Y-H4K8ac_peak_14928	7.20869	
7	2142754	2142973	5Y-H4K8ac_peak_14929	6.40724	
7	2143309	2144056	5Y-H4K8ac_peak_14930	8.75963	
7	2144496	2144997	5Y-H4K8ac_peak_14931	16.73191	
7	2145209	2145541	5Y-H4K8ac_peak_14932	8.69112	
7	2146197	2146521	5Y-H4K8ac_peak_14933	7.50501	
7	2147253	2147530	5Y-H4K8ac_peak_14934	4.32488	
7	2148668	2149010	5Y-H4K8ac_peak_14935	6.80915	
7	2150776	2151047	5Y-H4K8ac_peak_14936	13.81505	
7	2160407	2160706	5Y-H4K8ac_peak_14937	6.88736	
7	2165534	2165813	5Y-H4K8ac_peak_14938	6.86362	
7	2177752	2177944	5Y-H4K8ac_peak_14939	4.18287	
7	2178190	2178684	5Y-H4K8ac_peak_14940	5.72233	
7	2191469	2192006	5Y-H4K8ac_peak_14941	9.51254	
7	2200927	2201773	5Y-H4K8ac_peak_14942	11.22005	
7	2241887	2242094	5Y-H4K8ac_peak_14943	7.09658	
7	2281395	2282429	5Y-H4K8ac_peak_14944	16.30401	FTSJ2_ENSG00000122687;NUDT1_ENSG00000106268
7	2321433	2322058	5Y-H4K8ac_peak_14945	11.4254	
7	2353532	2353732	5Y-H4K8ac_peak_14946	5.24695	
7	2393744	2395029	5Y-H4K8ac_peak_14947	13.12848	SNX8_ENSG00000106266;EIF3B_ENSG00000106263
7	2439759	2440063	5Y-H4K8ac_peak_14948	6.69752	

7	2443248	2443531	5Y-H4K8ac_peak_14949	9.89953	CHST12_ENSG00000136213
7	2491494	2492072	5Y-H4K8ac_peak_14950	8.7173	
7	2535283	2535636	5Y-H4K8ac_peak_14951	12.00715	
7	2553090	2553366	5Y-H4K8ac_peak_14952	10.12457	LFNG_ENSG00000106003
7	2553594	2554023	5Y-H4K8ac_peak_14953	6.53157	
7	2563569	2563856	5Y-H4K8ac_peak_14954	7.44077	
7	2594945	2595364	5Y-H4K8ac_peak_14955	14.89891	BRAT1_ENSG00000106009
7	2653402	2653642	5Y-H4K8ac_peak_14956	7.07275	
7	2661626	2662179	5Y-H4K8ac_peak_14957	8.62703	
7	2662784	2663250	5Y-H4K8ac_peak_14958	6.08523	
7	2670851	2671195	5Y-H4K8ac_peak_14959	5.29036	TTYH3_ENSG00000136295
7	2671678	2672068	5Y-H4K8ac_peak_14960	12.67841	TTYH3_ENSG00000136295
7	2677435	2677667	5Y-H4K8ac_peak_14961	4.642	
7	2678420	2679117	5Y-H4K8ac_peak_14962	10.48476	
7	2680344	2680855	5Y-H4K8ac_peak_14963	12.20135	
7	2683685	2684883	5Y-H4K8ac_peak_14964	6.85826	
7	2685352	2685674	5Y-H4K8ac_peak_14965	8.85352	
7	2702474	2702725	5Y-H4K8ac_peak_14966	5.67517	
7	2798102	2798401	5Y-H4K8ac_peak_14967	8.24461	
7	2903382	2903702	5Y-H4K8ac_peak_14968	9.31608	
7	2920378	2920576	5Y-H4K8ac_peak_14969	4.18845	
7	2926238	2926431	5Y-H4K8ac_peak_14970	7.89273	
7	2953264	2953578	5Y-H4K8ac_peak_14971	7.01266	
7	2961434	2961631	5Y-H4K8ac_peak_14972	7.90751	
7	2967944	2968382	5Y-H4K8ac_peak_14973	11.8592	
7	2969030	2969877	5Y-H4K8ac_peak_14974	12.50545	
7	2971897	2972777	5Y-H4K8ac_peak_14975	9.12101	
7	3036621	3036851	5Y-H4K8ac_peak_14976	5.35202	
7	3066238	3066438	5Y-H4K8ac_peak_14977	5.49775	
7	3067179	3068044	5Y-H4K8ac_peak_14978	4.92482	
7	3068841	3069090	5Y-H4K8ac_peak_14979	11.22005	
7	3073292	3073521	5Y-H4K8ac_peak_14980	4.50834	
7	3083809	3084040	5Y-H4K8ac_peak_14981	5.98695	CARD11_ENSG00000198286
7	3113384	3113740	5Y-H4K8ac_peak_14982	12.72596	
7	3179474	3179680	5Y-H4K8ac_peak_14983	5.52265	
7	3339510	3340146	5Y-H4K8ac_peak_14984	6.78128	SDK1_ENSG00000146555
7	3341554	3341752	5Y-H4K8ac_peak_14985	6.37023	SDK1_ENSG00000146555;AC073316.1_ENSG00000236708
7	4046561	4047039	5Y-H4K8ac_peak_14986	4.642	
7	4091322	4091601	5Y-H4K8ac_peak_14987	6.37023	
7	4169409	4169764	5Y-H4K8ac_peak_14988	6.78128	
7	4183156	4183572	5Y-H4K8ac_peak_14989	6.08523	
7	4183781	4184503	5Y-H4K8ac_peak_14990	19.58975	
7	4184722	4185265	5Y-H4K8ac_peak_14991	17.09777	
7	4229096	4229513	5Y-H4K8ac_peak_14992	10.44473	
7	4230004	4230888	5Y-H4K8ac_peak_14993	13.00733	
7	4280468	4280670	5Y-H4K8ac_peak_14994	9.34684	

7	4366962	4367287	5Y-H4K8ac_peak_14995	4.50834	
7	4536643	4536839	5Y-H4K8ac_peak_14996	5.78188	
7	4564032	4564432	5Y-H4K8ac_peak_14997	6.06314	
7	4680578	4680804	5Y-H4K8ac_peak_14998	8.47411	
7	4681864	4682135	5Y-H4K8ac_peak_14999	8.3285	
7	4721212	4721690	5Y-H4K8ac_peak_15000	12.10416	
7	4765104	4765524	5Y-H4K8ac_peak_15001	6.78128	
7	4813813	4814052	5Y-H4K8ac_peak_15002	6.37023	
7	4814341	4815082	5Y-H4K8ac_peak_15003	12.05638	AP5Z1_ENSG00000242802
7	4860430	4860643	5Y-H4K8ac_peak_15004	4.77126	
7	4911024	4912031	5Y-H4K8ac_peak_15005	7.64648	
7	4912392	4912753	5Y-H4K8ac_peak_15006	6.50031	
7	4913091	4913355	5Y-H4K8ac_peak_15007	4.55128	
7	4922750	4923224	5Y-H4K8ac_peak_15008	5.87725	RADIL_ENSG00000157927
7	4998399	4998839	5Y-H4K8ac_peak_15009	8.43511	MMD2_ENSG00000136297
7	5013140	5013413	5Y-H4K8ac_peak_15010	6.77436	RNF216P1_ENSG00000196204
7	5085054	5085295	5Y-H4K8ac_peak_15011	5.73251	RBAK_ENSG00000146587
7	5085553	5086135	5Y-H4K8ac_peak_15012	8.5159	RBAK_ENSG00000146587
7	5111607	5112014	5Y-H4K8ac_peak_15013	7.89273	RBAKDN_ENSG00000273313
7	5112320	5112560	5Y-H4K8ac_peak_15014	6.81557	RBAKDN_ENSG00000273313
7	5184295	5184554	5Y-H4K8ac_peak_15015	5.52265	ZNF890P_ENSG00000159904
7	5229147	5229657	5Y-H4K8ac_peak_15016	13.4304	WIPI2_ENSG00000157954
7	5302670	5302877	5Y-H4K8ac_peak_15017	5.37237	
7	5314983	5315225	5Y-H4K8ac_peak_15018	4.81081	SLC29A4_ENSG00000164638;AC093376.1_ENSG00000263808
7	5315877	5316204	5Y-H4K8ac_peak_15019	7.50501	AC093376.1_ENSG00000263808
7	5322999	5323267	5Y-H4K8ac_peak_15020	4.95697	
7	5446698	5446961	5Y-H4K8ac_peak_15021	6.95844	
7	5462781	5463333	5Y-H4K8ac_peak_15022	9.48438	
7	5463883	5464103	5Y-H4K8ac_peak_15023	10.98149	TNRC18_ENSG00000182095
7	5464628	5464987	5Y-H4K8ac_peak_15024	11.4254	TNRC18_ENSG00000182095;RP11-1275H24.2_ENSG00000272953
7	5465358	5465821	5Y-H4K8ac_peak_15025	9.00954	TNRC18_ENSG00000182095;RP11-1275H24.2_ENSG00000272953
7	5466051	5466624	5Y-H4K8ac_peak_15026	9.64145	RP11-1275H24.2_ENSG00000272953
7	5466949	5468235	5Y-H4K8ac_peak_15027	11.23789	RP11-1275H24.1_ENSG00000234432;RP11-1275H24.3_ENSG00000273084
7	5468787	5469129	5Y-H4K8ac_peak_15028	15.90402	RP11-1275H24.1_ENSG00000234432;RP11-1275H24.3_ENSG00000273084
7	5469458	5470008	5Y-H4K8ac_peak_15029	7.16882	RP11-1275H24.1_ENSG00000234432
7	5517445	5518172	5Y-H4K8ac_peak_15030	10.91993	
7	5519353	5519603	5Y-H4K8ac_peak_15031	5.54834	
7	5553003	5553760	5Y-H4K8ac_peak_15032	19.52902	FBXL18_ENSG00000155034
7	5569725	5570197	5Y-H4K8ac_peak_15033	16.23405	
7	5570459	5570907	5Y-H4K8ac_peak_15034	13.60583	
7	5578750	5579002	5Y-H4K8ac_peak_15035	4.94375	
7	5594141	5596118	5Y-H4K8ac_peak_15036	15.03212	CTB-161C1.1_ENSG00000272719
7	5601251	5601468	5Y-H4K8ac_peak_15037	4.95697	AC006483.5_ENSG00000228974
7	5601871	5602255	5Y-H4K8ac_peak_15038	7.64648	
7	5609999	5610422	5Y-H4K8ac_peak_15039	8.3285	
7	5614610	5615243	5Y-H4K8ac_peak_15040	12.91503	

7	5633732	5634621	5Y-H4K8ac_peak_15041	14.00559	
7	5660423	5660739	5Y-H4K8ac_peak_15042	10.24399	
7	5666737	5667147	5Y-H4K8ac_peak_15043	13.92877	
7	5673051	5673456	5Y-H4K8ac_peak_15044	5.85957	
7	5674293	5674632	5Y-H4K8ac_peak_15045	3.93392	
7	5821597	5821837	5Y-H4K8ac_peak_15046	8.5159	RNF216_ENSG00000011275
7	5861702	5862528	5Y-H4K8ac_peak_15047	13.30822	ZNF815P_ENSG00000235944
7	5862857	5863237	5Y-H4K8ac_peak_15048	6.4759	ZNF815P_ENSG00000235944
7	5938027	5938343	5Y-H4K8ac_peak_15049	10.1994	CCZ1_ENSG00000122674
7	6048376	6048614	5Y-H4K8ac_peak_15050	7.64648	PMS2_ENSG00000122512;AIMP2_ENSG00000106305
7	6121404	6122125	5Y-H4K8ac_peak_15051	8.31536	AC004895.4_ENSG00000231704;AC004895.1_ENSG00000264605
7	6206708	6206991	5Y-H4K8ac_peak_15052	5.64879	
7	6312413	6312676	5Y-H4K8ac_peak_15053	12.15235	CYTH3_ENSG00000008256
7	6413409	6413802	5Y-H4K8ac_peak_15054	8.07508	RAC1_ENSG00000136238
7	6414198	6414506	5Y-H4K8ac_peak_15055	8.62703	RAC1_ENSG00000136238
7	6543053	6543296	5Y-H4K8ac_peak_15056	7.87406	
7	6566026	6566244	5Y-H4K8ac_peak_15057	7.50148	
7	6576699	6576905	5Y-H4K8ac_peak_15058	8.69112	
7	6615714	6616441	5Y-H4K8ac_peak_15059	10.1994	ZDHHC4_ENSG00000136247
7	6617099	6617954	5Y-H4K8ac_peak_15060	16.0768	ZDHHC4_ENSG00000136247
7	6629237	6629478	5Y-H4K8ac_peak_15061	10.08244	AC079742.4_ENSG00000232581;C7orf26_ENSG00000146576
7	6654585	6655072	5Y-H4K8ac_peak_15062	12.26819	ZNF853_ENSG00000236609
7	6655407	6655938	5Y-H4K8ac_peak_15063	17.78344	ZNF853_ENSG00000236609
7	6676098	6676876	5Y-H4K8ac_peak_15064	26.92846	ZNF316_ENSG00000205903
7	6677245	6677519	5Y-H4K8ac_peak_15065	10.1994	ZNF316_ENSG00000205903
7	6703687	6704322	5Y-H4K8ac_peak_15066	25.62564	AC073343.13_ENSG00000228010
7	6746245	6746537	5Y-H4K8ac_peak_15067	6.80915	ZNF12_ENSG00000164631
7	6746894	6747099	5Y-H4K8ac_peak_15068	6.31818	ZNF12_ENSG00000164631
7	6768811	6769211	5Y-H4K8ac_peak_15069	8.3285	
7	6866060	6866291	5Y-H4K8ac_peak_15070	11.82621	CCZ1B_ENSG00000146574
7	6894766	6895029	5Y-H4K8ac_peak_15071	9.19793	
7	6895467	6895819	5Y-H4K8ac_peak_15072	7.50148	
7	7222303	7222943	5Y-H4K8ac_peak_15073	13.14766	
7	7606330	7607059	5Y-H4K8ac_peak_15074	14.89891	RP5-1159O4.1_ENSG00000272894;MIOS_ENSG00000164654
7	7680494	7680955	5Y-H4K8ac_peak_15075	6.31818	RPA3-AS1_ENSG00000219545
7	8008492	8008791	5Y-H4K8ac_peak_15076	11.04542	AC006042.7_ENSG00000233108
7	8254711	8254904	5Y-H4K8ac_peak_15077	5.65584	
7	8268974	8269295	5Y-H4K8ac_peak_15078	10.26997	
7	8270660	8270993	5Y-H4K8ac_peak_15079	12.69256	
7	8300753	8302324	5Y-H4K8ac_peak_15080	16.13719	ICA1_ENSG00000003147;AC007009.1_ENSG00000244239;AC007128.1_ENSG00000229970
7	8329949	8330189	5Y-H4K8ac_peak_15081	6.00382	
7	8331673	8332145	5Y-H4K8ac_peak_15082	7.20869	
7	8473619	8474257	5Y-H4K8ac_peak_15083	16.13719	NXPH1_ENSG00000122584
7	9271412	9271625	5Y-H4K8ac_peak_15084	5.29015	
7	11013615	11014283	5Y-H4K8ac_peak_15085	14.90456	PHF14_ENSG00000106443
7	11065496	11065713	5Y-H4K8ac_peak_15086	8.75926	

7	11232993	11233320	5Y-H4K8ac_peak_15087	4.6766	
7	11812951	11813152	5Y-H4K8ac_peak_15088	4.79585	
7	11871751	11872001	5Y-H4K8ac_peak_15089	6.53157	THSD7A_ENSG00000005108
7	12130159	12130876	5Y-H4K8ac_peak_15090	14.19462	
7	12131563	12131802	5Y-H4K8ac_peak_15091	7.89273	
7	12151520	12151804	5Y-H4K8ac_peak_15092	8.90774	
7	12251020	12251246	5Y-H4K8ac_peak_15093	12.10416	TMEM106B_ENSG00000106460
7	12443191	12443564	5Y-H4K8ac_peak_15094	8.3285	VWDE_ENSG00000146530
7	12726070	12726305	5Y-H4K8ac_peak_15095	5.98695	CTD-2320J21.1_ENSG00000271253;ARL4A_ENSG00000122644
7	16460616	16461202	5Y-H4K8ac_peak_15096	8.20773	ISPD_ENSG00000214960
7	16685532	16686029	5Y-H4K8ac_peak_15097	10.11191	ANKMY2_ENSG00000106524;BZW2_ENSG00000136261
7	17979711	17979949	5Y-H4K8ac_peak_15098	5.19944	SNX13_ENSG00000071189
7	18739607	18739801	5Y-H4K8ac_peak_15099	6.50117	
7	18741632	18741989	5Y-H4K8ac_peak_15100	6.76096	
7	18746564	18746757	5Y-H4K8ac_peak_15101	7.01266	
7	18868603	18868855	5Y-H4K8ac_peak_15102	5.98695	
7	19145805	19146124	5Y-H4K8ac_peak_15103	4.00285	
7	19146838	19147585	5Y-H4K8ac_peak_15104	10.77539	
7	19147997	19148263	5Y-H4K8ac_peak_15105	7.64648	
7	19152082	19152283	5Y-H4K8ac_peak_15106	7.20869	AC003986.6_ENSG00000232821
7	19156046	19156393	5Y-H4K8ac_peak_15107	4.642	TWIST1_ENSG00000122691
7	19156697	19157060	5Y-H4K8ac_peak_15108	13.60583	TWIST1_ENSG00000122691
7	19158635	19158944	5Y-H4K8ac_peak_15109	8.90223	AC003986.7_ENSG00000236536
7	20370996	20371453	5Y-H4K8ac_peak_15110	15.08122	CTA-293F17.1_ENSG00000271133;ITGB8_ENSG00000105855
7	20600321	20600512	5Y-H4K8ac_peak_15111	7.11863	
7	22373490	22373728	5Y-H4K8ac_peak_15112	4.66683	
7	22396330	22396765	5Y-H4K8ac_peak_15113	14.01193	RAPGEF5_ENSG00000136237
7	22862076	22862346	5Y-H4K8ac_peak_15114	11.29141	TOMM7_ENSG00000196683
7	23145586	23145915	5Y-H4K8ac_peak_15115	4.55128	KLHL7-AS1_ENSG00000230658;KLHL7_ENSG00000122550
7	23245692	23246040	5Y-H4K8ac_peak_15116	12.45737	AC005082.12_ENSG00000226816
7	23508338	23509072	5Y-H4K8ac_peak_15117	5.60566	
7	23509445	23509693	5Y-H4K8ac_peak_15118	6.02475	IGF2BP3_ENSG00000136231
7	23513492	23513811	5Y-H4K8ac_peak_15119	12.00715	
7	23529559	23529789	5Y-H4K8ac_peak_15120	4.97006	RPS2P32_ENSG00000232818
7	23530202	23530725	5Y-H4K8ac_peak_15121	8.82628	RPS2P32_ENSG00000232818;Y_RNA_ENSG00000200847
7	23571851	23572242	5Y-H4K8ac_peak_15122	7.68528	TRA2A_ENSG00000164548
7	23610514	23610783	5Y-H4K8ac_peak_15123	13.27281	
7	23637243	23637445	5Y-H4K8ac_peak_15124	4.42435	CCDC126_ENSG00000169193
7	23719836	23720049	5Y-H4K8ac_peak_15125	13.33225	FAM221A_ENSG00000188732;AC006026.13_ENSG00000234286
7	23944577	23944806	5Y-H4K8ac_peak_15126	8.67475	
7	23964237	23964721	5Y-H4K8ac_peak_15127	5.64909	
7	23966832	23967290	5Y-H4K8ac_peak_15128	7.50081	
7	24239015	24239210	5Y-H4K8ac_peak_15129	5.81394	
7	24250672	24250883	5Y-H4K8ac_peak_15130	7.29176	
7	24253008	24253495	5Y-H4K8ac_peak_15131	17.97359	
7	24255509	24255804	5Y-H4K8ac_peak_15132	8.28468	

7	24256043	24256421	5Y-H4K8ac_peak_15133	8.73392	
7	24257340	24257549	5Y-H4K8ac_peak_15134	4.642	
7	24266506	24266755	5Y-H4K8ac_peak_15135	9.00954	
7	24323481	24324164	5Y-H4K8ac_peak_15136	14.89891	NPY_ENSG00000122585
7	24795849	24796496	5Y-H4K8ac_peak_15137	9.51254	
7	24796760	24797522	5Y-H4K8ac_peak_15138	10.12457	
7	24962765	24963035	5Y-H4K8ac_peak_15139	6.77436	
7	25137622	25137852	5Y-H4K8ac_peak_15140	6.36454	
7	25584883	25585143	5Y-H4K8ac_peak_15141	12.21176	
7	25891880	25892489	5Y-H4K8ac_peak_15142	11.01458	
7	26118001	26118435	5Y-H4K8ac_peak_15143	14.99673	
7	26118849	26119053	5Y-H4K8ac_peak_15144	5.08308	
7	26191395	26191615	5Y-H4K8ac_peak_15145	6.98416	NFE2L3_ENSG00000050344
7	26192239	26192773	5Y-H4K8ac_peak_15146	4.0639	NFE2L3_ENSG00000050344
7	26239786	26240268	5Y-H4K8ac_peak_15147	6.19716	HNRNPA2B1_ENSG00000122566;CBX3_ENSG00000122565
7	26240719	26241175	5Y-H4K8ac_peak_15148	9.51254	HNRNPA2B1_ENSG00000122566;CBX3_ENSG00000122565
7	26241368	26241982	5Y-H4K8ac_peak_15149	14.89891	HNRNPA2B1_ENSG00000122566;CBX3_ENSG00000122565
7	26331550	26331876	5Y-H4K8ac_peak_15150	9.07085	SNX10_ENSG00000086300
7	26415794	26416581	5Y-H4K8ac_peak_15151	11.04356	AC004540.4_ENSG00000225792
7	26437642	26438032	5Y-H4K8ac_peak_15152	14.99673	AC004540.5_ENSG00000214870
7	26438299	26439046	5Y-H4K8ac_peak_15153	10.54764	AC004540.5_ENSG00000214870
7	27702296	27702557	5Y-H4K8ac_peak_15154	13.45283	HIBADH_ENSG00000106049
7	27779843	27780047	5Y-H4K8ac_peak_15155	7.93983	AC004549.6_ENSG00000229893;TAX1BP1_ENSG00000106052
7	28995938	28996233	5Y-H4K8ac_peak_15156	4.41844	
7	28996616	28996880	5Y-H4K8ac_peak_15157	7.41172	AC005013.5_ENSG00000228421
7	28997603	28998251	5Y-H4K8ac_peak_15158	13.68515	AC005013.1_ENSG00000255690;TRIL_ENSG00000176734;AC005013.5_ENSG00000228421
7	29234068	29234324	5Y-H4K8ac_peak_15159	15.17389	CPVL_ENSG00000106066
7	29525726	29526276	5Y-H4K8ac_peak_15160	7.31102	
7	29724836	29725109	5Y-H4K8ac_peak_15161	9.79526	ZNRF2P2_ENSG00000225264
7	29742435	29742676	5Y-H4K8ac_peak_15162	5.81062	
7	29810177	29810384	5Y-H4K8ac_peak_15163	5.98695	
7	30028854	30029882	5Y-H4K8ac_peak_15164	6.56228	SCRN1_ENSG00000136193;AC007285.6_ENSG00000227014
7	30265566	30265756	5Y-H4K8ac_peak_15165	7.81811	
7	30324776	30324986	5Y-H4K8ac_peak_15166	7.3565	ZNRF2_ENSG00000180233
7	30518481	30518723	5Y-H4K8ac_peak_15167	9.00954	NOD1_ENSG00000106100
7	30543961	30544370	5Y-H4K8ac_peak_15168	11.94897	
7	30544847	30545160	5Y-H4K8ac_peak_15169	11.4254	
7	30794960	30795337	5Y-H4K8ac_peak_15170	6.57751	
7	30810321	30811342	5Y-H4K8ac_peak_15171	12.99645	FAM188B_ENSG00000106125
7	30910412	30910752	5Y-H4K8ac_peak_15172	6.55841	
7	30925486	30926267	5Y-H4K8ac_peak_15173	9.56044	
7	30926769	30927095	5Y-H4K8ac_peak_15174	13.33225	
7	30927361	30927594	5Y-H4K8ac_peak_15175	6.36723	
7	30945851	30946054	5Y-H4K8ac_peak_15176	6.34891	
7	30950630	30950938	5Y-H4K8ac_peak_15177	7.43972	
7	30953349	30953868	5Y-H4K8ac_peak_15178	8.24461	

7	30954368	30954625	5Y-H4K8ac_peak_15179	5.41472	
7	30978088	30978412	5Y-H4K8ac_peak_15180	13.1133	GHRHR_ENSG00000106128
7	31068211	31068743	5Y-H4K8ac_peak_15181	7.72887	
7	31082624	31082915	5Y-H4K8ac_peak_15182	4.642	
7	31088192	31088466	5Y-H4K8ac_peak_15183	6.34245	
7	31089254	31089484	5Y-H4K8ac_peak_15184	8.39119	
7	31092200	31092606	5Y-H4K8ac_peak_15185	7.90751	ADCYAP1R1_ENSG00000078549
7	31094445	31094666	5Y-H4K8ac_peak_15186	4.47015	
7	32529670	32529867	5Y-H4K8ac_peak_15187	4.92482	
7	32535138	32535355	5Y-H4K8ac_peak_15188	7.64648	LSM5_ENSG00000106355;AVL9_ENSG00000105778
7	32767546	32768341	5Y-H4K8ac_peak_15189	16.74969	ZNRF2P1_ENSG00000237004
7	32931058	32931395	5Y-H4K8ac_peak_15190	16.73191	
7	32931837	32932435	5Y-H4K8ac_peak_15191	16.79568	
7	32982273	32982525	5Y-H4K8ac_peak_15192	7.90751	RP9P_ENSG00000205763
7	33038121	33038363	5Y-H4K8ac_peak_15193	7.87406	
7	33055723	33056041	5Y-H4K8ac_peak_15194	4.50834	
7	33148638	33148840	5Y-H4K8ac_peak_15195	6.37023	RP9_ENSG00000164610
7	33258555	33258759	5Y-H4K8ac_peak_15196	5.65584	
7	35077519	35077713	5Y-H4K8ac_peak_15197	7.41197	DPY19L1_ENSG00000173852
7	35226499	35226717	5Y-H4K8ac_peak_15198	5.70472	DPY19L2P1_ENSG00000189212
7	35293407	35293693	5Y-H4K8ac_peak_15199	8.24461	TBX20_ENSG00000164532
7	35505326	35505674	5Y-H4K8ac_peak_15200	15.82359	
7	35506291	35506510	5Y-H4K8ac_peak_15201	9.30206	
7	35734222	35734505	5Y-H4K8ac_peak_15202	4.48815	HERPUD2_ENSG00000122557;RP11-379H18.1_ENSG00000271122
7	35795630	35795837	5Y-H4K8ac_peak_15203	4.50834	
7	36192020	36192228	5Y-H4K8ac_peak_15204	5.41472	EEPD1_ENSG00000122547
7	36242556	36242998	5Y-H4K8ac_peak_15205	5.98695	
7	36428905	36429101	5Y-H4K8ac_peak_15206	6.339	KIAA0895_ENSG00000164542;ANLN_ENSG00000011426
7	36429517	36429920	5Y-H4K8ac_peak_15207	9.32639	KIAA0895_ENSG00000164542;ANLN_ENSG00000011426
7	36909689	36910134	5Y-H4K8ac_peak_15208	4.90696	
7	37371233	37371486	5Y-H4K8ac_peak_15209	9.07803	
7	37371984	37372209	5Y-H4K8ac_peak_15210	7.11863	
7	37412764	37413066	5Y-H4K8ac_peak_15211	5.41472	
7	37487498	37487823	5Y-H4K8ac_peak_15212	9.30206	
7	37488043	37488404	5Y-H4K8ac_peak_15213	6.53157	ELMO1_ENSG00000155849
7	37960413	37961075	5Y-H4K8ac_peak_15214	9.74838	
7	38409867	38410147	5Y-H4K8ac_peak_15215	7.73231	
7	39662579	39663034	5Y-H4K8ac_peak_15216	18.62331	RALA_ENSG00000006451
7	39663258	39663518	5Y-H4K8ac_peak_15217	9.36633	RALA_ENSG00000006451
7	39772354	39772792	5Y-H4K8ac_peak_15218	11.12941	LINC00265_ENSG00000188185
7	39773011	39773257	5Y-H4K8ac_peak_15219	7.60057	LINC00265_ENSG00000188185
7	39988736	39988943	5Y-H4K8ac_peak_15220	5.5192	RP11-467D6.1_ENSG00000259826;CDK13_ENSG00000065883
7	39989418	39990057	5Y-H4K8ac_peak_15221	6.31818	RP11-467D6.1_ENSG00000259826;CDK13_ENSG00000065883
7	40173749	40174132	5Y-H4K8ac_peak_15222	11.01287	SUGCT_ENSG00000175600;MPLKIP_ENSG00000168303
7	40291029	40291219	5Y-H4K8ac_peak_15223	5.65584	
7	40583551	40584023	5Y-H4K8ac_peak_15224	4.15658	

7	40585269	40585505	5Y-H4K8ac_peak_15225	4.96263	
7	40590555	40590844	5Y-H4K8ac_peak_15226	12.10416	
7	40591238	40591490	5Y-H4K8ac_peak_15227	9.79526	
7	40660146	40660360	5Y-H4K8ac_peak_15228	6.77436	
7	40660857	40661193	5Y-H4K8ac_peak_15229	7.11863	
7	40665187	40665510	5Y-H4K8ac_peak_15230	4.7605	
7	40684344	40685082	5Y-H4K8ac_peak_15231	15.65766	
7	40753694	40753886	5Y-H4K8ac_peak_15232	7.05206	
7	40763861	40764060	5Y-H4K8ac_peak_15233	7.89273	
7	41744484	41744781	5Y-H4K8ac_peak_15234	10.60083	AC005027.3_ENSG00000236310
7	42951178	42951417	5Y-H4K8ac_peak_15235	10.60083	C7orf25_ENSG00000136197
7	42971988	42972189	5Y-H4K8ac_peak_15236	8.4454	PSMA2_ENSG00000256646;PSMA2_ENSG00000106588;MRPL32_ENSG00000106591
7	43570801	43571226	5Y-H4K8ac_peak_15237	4.97086	
7	43572099	43572347	5Y-H4K8ac_peak_15238	13.92877	
7	43573157	43573619	5Y-H4K8ac_peak_15239	9.58986	
7	43576550	43576755	5Y-H4K8ac_peak_15240	7.29055	
7	43687107	43687666	5Y-H4K8ac_peak_15241	13.0168	
7	43797834	43798787	5Y-H4K8ac_peak_15242	10.4689	BLVRA_ENSG00000106605
7	43918283	43918503	5Y-H4K8ac_peak_15243	6.24511	
7	43945989	43946550	5Y-H4K8ac_peak_15244	14.6127	URGCP-MRPS24_ENSG00000270617
7	43965751	43966513	5Y-H4K8ac_peak_15245	15.90416	URGCP_ENSG00000106608;UBE2D4_ENSG00000078967
7	43975476	43975685	5Y-H4K8ac_peak_15246	7.11439	
7	43977586	43977900	5Y-H4K8ac_peak_15247	11.82624	
7	44105070	44105290	5Y-H4K8ac_peak_15248	5.36617	AC017116.11_ENSG00000239775;PGAM2_ENSG00000164708
7	44112268	44112882	5Y-H4K8ac_peak_15249	7.60057	
7	44121690	44122011	5Y-H4K8ac_peak_15250	6.98416	POLM_ENSG00000122678
7	44127467	44128408	5Y-H4K8ac_peak_15251	8.09118	
7	44143384	44143700	5Y-H4K8ac_peak_15252	16.13719	AEBP1_ENSG00000106624
7	44143917	44144576	5Y-H4K8ac_peak_15253	9.02938	AEBP1_ENSG00000106624
7	44163460	44163831	5Y-H4K8ac_peak_15254	8.24461	POLD2_ENSG00000106628
7	44184965	44185243	5Y-H4K8ac_peak_15255	6.78318	
7	44240665	44240860	5Y-H4K8ac_peak_15256	9.15007	YKT6_ENSG00000106636
7	44257583	44258084	5Y-H4K8ac_peak_15257	7.90005	
7	44258344	44258738	5Y-H4K8ac_peak_15258	4.70545	
7	44274794	44276719	5Y-H4K8ac_peak_15259	13.57316	
7	44280807	44281105	5Y-H4K8ac_peak_15260	6.10343	
7	44296985	44297254	5Y-H4K8ac_peak_15261	6.08523	
7	44303114	44303321	5Y-H4K8ac_peak_15262	7.90751	
7	44315335	44315652	5Y-H4K8ac_peak_15263	7.11863	
7	44316031	44316578	5Y-H4K8ac_peak_15264	7.50148	
7	44330880	44331096	5Y-H4K8ac_peak_15265	8.05932	
7	44360323	44360523	5Y-H4K8ac_peak_15266	4.95697	
7	44529885	44530574	5Y-H4K8ac_peak_15267	20.32089	NUDCD3_ENSG00000015676
7	44580641	44581174	5Y-H4K8ac_peak_15268	4.67245	NPC1L1_ENSG00000015520
7	44612961	44613455	5Y-H4K8ac_peak_15269	17.49408	
7	44613831	44614068	5Y-H4K8ac_peak_15270	6.37023	DDX56_ENSG00000136271

7	44621451	44621813	5Y-H4K8ac_peak_15271	9.00954	TMED4_ENSG00000158604
7	44622181	44622663	5Y-H4K8ac_peak_15272	7.87406	TMED4_ENSG00000158604
7	44788142	44788359	5Y-H4K8ac_peak_15273	13.00733	ZMIZ2_ENSG00000122515
7	44788575	44788841	5Y-H4K8ac_peak_15274	6.51488	ZMIZ2_ENSG00000122515
7	44836336	44836815	5Y-H4K8ac_peak_15275	11.9002	PPIA_ENSG00000196262
7	44887384	44887672	5Y-H4K8ac_peak_15276	8.3285	H2AFV_ENSG00000105968;AC004854.4_ENSG00000234183
7	44888427	44888665	5Y-H4K8ac_peak_15277	10.1994	H2AFV_ENSG00000105968;AC004854.4_ENSG00000234183
7	44896238	44896454	5Y-H4K8ac_peak_15278	5.69598	
7	44925375	44925806	5Y-H4K8ac_peak_15279	9.51254	PURB_ENSG00000146676;RP4-673M15.1_ENSG00000272768
7	44951622	44951878	5Y-H4K8ac_peak_15280	5.41472	
7	45025790	45026193	5Y-H4K8ac_peak_15281	14.01193	SNHG15_ENSG00000232956
7	45026516	45027035	5Y-H4K8ac_peak_15282	5.24695	SNHG15_ENSG00000232956
7	45112473	45113036	5Y-H4K8ac_peak_15283	13.2814	
7	45121618	45122173	5Y-H4K8ac_peak_15284	6.14551	
7	45128495	45128894	5Y-H4K8ac_peak_15285	8.62703	NACAD_ENSG00000136274
7	45150897	45151199	5Y-H4K8ac_peak_15286	5.72233	TBRG4_ENSG00000136270
7	45613277	45613893	5Y-H4K8ac_peak_15287	15.82359	ADCY1_ENSG00000164742
7	45614387	45614739	5Y-H4K8ac_peak_15288	5.24695	ADCY1_ENSG00000164742
7	45614940	45615277	5Y-H4K8ac_peak_15289	6.53157	
7	45618200	45618415	5Y-H4K8ac_peak_15290	4.15658	
7	45618672	45619478	5Y-H4K8ac_peak_15291	7.90751	
7	45619782	45620472	5Y-H4K8ac_peak_15292	11.69482	
7	45631992	45632303	5Y-H4K8ac_peak_15293	4.95697	
7	45632912	45633138	5Y-H4K8ac_peak_15294	6.01761	
7	45808943	45809591	5Y-H4K8ac_peak_15295	9.32595	SEPT7P2_ENSG00000214765
7	46452048	46452287	5Y-H4K8ac_peak_15296	6.76425	
7	47293644	47294092	5Y-H4K8ac_peak_15297	9.99838	
7	47342603	47342889	5Y-H4K8ac_peak_15298	4.95697	
7	47367031	47367933	5Y-H4K8ac_peak_15299	6.19716	
7	47441197	47441428	5Y-H4K8ac_peak_15300	7.01266	
7	47493640	47494143	5Y-H4K8ac_peak_15301	8.5915	
7	47494644	47494891	5Y-H4K8ac_peak_15302	7.18391	
7	47517397	47517785	5Y-H4K8ac_peak_15303	7.89273	
7	47531821	47532023	5Y-H4K8ac_peak_15304	5.23819	
7	47534615	47535194	5Y-H4K8ac_peak_15305	6.34245	
7	47535415	47535722	5Y-H4K8ac_peak_15306	9.00954	
7	47536268	47536578	5Y-H4K8ac_peak_15307	9.22492	
7	47576159	47577169	5Y-H4K8ac_peak_15308	28.75038	
7	47607691	47607992	5Y-H4K8ac_peak_15309	7.64648	
7	47617069	47617474	5Y-H4K8ac_peak_15310	7.34185	
7	47621163	47622048	5Y-H4K8ac_peak_15311	12.99645	TNS3_ENSG00000136205
7	47674592	47675280	5Y-H4K8ac_peak_15312	15.73087	
7	47675476	47676031	5Y-H4K8ac_peak_15313	10.1994	
7	47677005	47677216	5Y-H4K8ac_peak_15314	6.78128	
7	47748319	47748766	5Y-H4K8ac_peak_15315	6.19178	
7	47760142	47761085	5Y-H4K8ac_peak_15316	9.48438	

7	47761400	47761605	5Y-H4K8ac_peak_15317	6.00598	
7	47764049	47764323	5Y-H4K8ac_peak_15318	6.19716	
7	47764734	47765241	5Y-H4K8ac_peak_15319	9.02938	
7	47766716	47767120	5Y-H4K8ac_peak_15320	6.31818	
7	47797547	47797755	5Y-H4K8ac_peak_15321	7.20869	
7	47802251	47803154	5Y-H4K8ac_peak_15322	14.89891	
7	47982446	47982638	5Y-H4K8ac_peak_15323	8.00646	
7	48129025	48129239	5Y-H4K8ac_peak_15324	7.90236	UPP1_ENSG00000183696
7	50518245	50518710	5Y-H4K8ac_peak_15325	17.67055	FIGNL1_ENSG00000132436
7	50573607	50573878	5Y-H4K8ac_peak_15326	5.56894	
7	50610110	50610355	5Y-H4K8ac_peak_15327	5.89922	
7	50626449	50626943	5Y-H4K8ac_peak_15328	7.00198	
7	50635757	50636126	5Y-H4K8ac_peak_15329	9.99623	
7	51715221	51715625	5Y-H4K8ac_peak_15330	8.93923	
7	54323766	54323965	5Y-H4K8ac_peak_15331	5.23083	
7	54612473	54612668	5Y-H4K8ac_peak_15332	6.03255	
7	54614459	54614747	5Y-H4K8ac_peak_15333	7.89273	
7	54907268	54907505	5Y-H4K8ac_peak_15334	8.1667	
7	55086446	55086769	5Y-H4K8ac_peak_15335	4.642	EGFR_ENSG00000146648
7	55148948	55149289	5Y-H4K8ac_peak_15336	7.89273	
7	55201278	55202075	5Y-H4K8ac_peak_15337	7.00982	
7	55245177	55245605	5Y-H4K8ac_peak_15338	5.34199	
7	55247700	55247929	5Y-H4K8ac_peak_15339	5.5192	
7	55259874	55260342	5Y-H4K8ac_peak_15340	14.18025	
7	55261038	55261385	5Y-H4K8ac_peak_15341	10.1994	
7	55280908	55281841	5Y-H4K8ac_peak_15342	12.65187	
7	55322045	55322621	5Y-H4K8ac_peak_15343	9.86146	
7	55433206	55434274	5Y-H4K8ac_peak_15344	11.69482	LANCL2_ENSG00000132434
7	55639746	55640068	5Y-H4K8ac_peak_15345	10.1994	VOPP1_ENSG00000154978;RP11-310H4.1_ENSG00000223475
7	55640385	55640645	5Y-H4K8ac_peak_15346	8.19376	VOPP1_ENSG00000154978;RP11-310H4.1_ENSG00000223475
7	55954931	55955332	5Y-H4K8ac_peak_15347	8.62703	MRPS17_ENSG00000249773;ZNF713_ENSG00000178665
7	56032313	56032778	5Y-H4K8ac_peak_15348	26.96937	
7	56118744	56119140	5Y-H4K8ac_peak_15349	7.65114	PSPH_ENSG00000146733;CCT6A_ENSG00000146731
7	56119561	56119834	5Y-H4K8ac_peak_15350	13.92877	PSPH_ENSG00000146733;CCT6A_ENSG00000146731
7	56604917	56605601	5Y-H4K8ac_peak_15351	7.04637	RP11-760D2.5_ENSG00000233288
7	57710669	57711130	5Y-H4K8ac_peak_15352	6.62622	RP11-368M16.9_ENSG00000230796;RP11-368M16.5_ENSG00000237639
7	57829691	57829888	5Y-H4K8ac_peak_15353	4.15658	RP11-548K12.6_ENSG00000227015;RP11-548K12.7_ENSG00000232161
7	57835657	57835994	5Y-H4K8ac_peak_15354	4.79658	
7	63359991	63360366	5Y-H4K8ac_peak_15355	7.59101	RP11-340I6.7_ENSG00000236299;RP11-340I6.8_ENSG00000226581
7	63385673	63385994	5Y-H4K8ac_peak_15356	9.30505	RP11-73B2.6_ENSG00000227910
7	63386301	63386689	5Y-H4K8ac_peak_15357	7.89273	RP11-73B2.6_ENSG00000227910;AC092634.2_ENSG00000265017
7	63387209	63387696	5Y-H4K8ac_peak_15358	11.16796	RP11-73B2.6_ENSG00000227910;AC092634.2_ENSG00000265017
7	63766742	63767149	5Y-H4K8ac_peak_15359	4.642	ZNF736_ENSG00000234444
7	63924275	63924505	5Y-H4K8ac_peak_15360	8.82628	
7	64022984	64023418	5Y-H4K8ac_peak_15361	8.18236	ZNF680_ENSG00000173041
7	64035149	64035472	5Y-H4K8ac_peak_15362	9.79526	

7	64126597	64126956	5Y-H4K8ac_peak_15363	9.93099	ZNF107_ENSG00000196247
7	64253929	64254404	5Y-H4K8ac_peak_15364	6.08523	ZNF138_ENSG00000197008
7	64337984	64338345	5Y-H4K8ac_peak_15365	11.71078	
7	64362850	64363140	5Y-H4K8ac_peak_15366	7.34185	
7	64363732	64364100	5Y-H4K8ac_peak_15367	9.30505	
7	64466661	64466908	5Y-H4K8ac_peak_15368	6.19716	ZNF117_ENSG00000152926;ERV3-1_ENSG00000213462
7	64467556	64467793	5Y-H4K8ac_peak_15369	5.64909	ZNF117_ENSG00000152926;ERV3-1_ENSG00000213462
7	64541210	64541435	5Y-H4K8ac_peak_15370	8.73985	
7	64699762	64700044	5Y-H4K8ac_peak_15371	9.87097	
7	64700297	64700744	5Y-H4K8ac_peak_15372	13.00733	
7	64711374	64711925	5Y-H4K8ac_peak_15373	14.19462	
7	64712234	64712554	5Y-H4K8ac_peak_15374	7.7384	
7	64712827	64713018	5Y-H4K8ac_peak_15375	10.4689	
7	64734339	64734628	5Y-H4K8ac_peak_15376	8.69112	
7	64838091	64838306	5Y-H4K8ac_peak_15377	8.16382	ZNF92_ENSG00000146757
7	64838881	64839176	5Y-H4K8ac_peak_15378	9.36633	ZNF92_ENSG00000146757
7	65216376	65216570	5Y-H4K8ac_peak_15379	5.36617	CCT6P1_ENSG00000228409
7	65235944	65236442	5Y-H4K8ac_peak_15380	9.32595	
7	65446901	65447732	5Y-H4K8ac_peak_15381	21.45803	GUSB_ENSG00000169919
7	65579341	65580225	5Y-H4K8ac_peak_15382	8.24461	CRCP_ENSG00000241258
7	65604917	65605163	5Y-H4K8ac_peak_15383	5.12488	
7	65610657	65610864	5Y-H4K8ac_peak_15384	5.77617	
7	65617878	65618074	5Y-H4K8ac_peak_15385	4.0861	
7	65669856	65670056	5Y-H4K8ac_peak_15386	5.72233	TPST1_ENSG00000169902
7	65958741	65959128	5Y-H4K8ac_peak_15387	12.99645	GS1-124K5.3_ENSG00000223473;GS1-124K5.4_ENSG00000237310
7	66056550	66056957	5Y-H4K8ac_peak_15388	5.41472	GS1-124K5.12_ENSG00000232559
7	66057851	66058121	5Y-H4K8ac_peak_15389	6.78128	GS1-124K5.12_ENSG00000232559
7	66093154	66093544	5Y-H4K8ac_peak_15390	7.22147	KCTD7_ENSG00000243335
7	66118861	66119419	5Y-H4K8ac_peak_15391	11.88337	RP4-756H11.3_ENSG00000226824
7	66205325	66205559	5Y-H4K8ac_peak_15392	12.79198	RP11-792A8.4_ENSG00000272831
7	66309383	66309601	5Y-H4K8ac_peak_15393	7.87406	GTF2IRD1P1_ENSG00000230583
7	66310531	66311200	5Y-H4K8ac_peak_15394	9.51254	GTF2IRD1P1_ENSG00000230583
7	66386261	66386661	5Y-H4K8ac_peak_15395	9.74838	TMEM248_ENSG00000106609
7	67084933	67085142	5Y-H4K8ac_peak_15396	8.66584	
7	67688830	67689158	5Y-H4K8ac_peak_15397	9.7353	
7	68178737	68178988	5Y-H4K8ac_peak_15398	6.77436	
7	68455058	68455276	5Y-H4K8ac_peak_15399	5.10825	
7	68677042	68677268	5Y-H4K8ac_peak_15400	8.67475	
7	69289124	69289343	5Y-H4K8ac_peak_15401	8.28346	
7	69290745	69290962	5Y-H4K8ac_peak_15402	10.19948	
7	69311646	69311860	5Y-H4K8ac_peak_15403	7.38046	
7	69328646	69328993	5Y-H4K8ac_peak_15404	7.59101	
7	69331723	69331935	5Y-H4K8ac_peak_15405	8.2913	
7	70083543	70083776	5Y-H4K8ac_peak_15406	4.6468	
7	70159102	70159456	5Y-H4K8ac_peak_15407	9.31608	
7	70159680	70160087	5Y-H4K8ac_peak_15408	8.76608	

7	70228028	70228346	5Y-H4K8ac_peak_15409	8.73392	
7	72349252	72349481	5Y-H4K8ac_peak_15410	7.90751	POM121_ENSG00000196313
7	72349958	72350344	5Y-H4K8ac_peak_15411	9.51254	POM121_ENSG00000196313
7	72395307	72395763	5Y-H4K8ac_peak_15412	5.41472	RP11-313P13.5_ENSG00000272843
7	72723221	72723806	5Y-H4K8ac_peak_15413	15.44596	NSUN5_ENSG00000130305
7	72814141	72814571	5Y-H4K8ac_peak_15414	7.50148	
7	72842888	72843984	5Y-H4K8ac_peak_15415	14.86775	
7	72940755	72940969	5Y-H4K8ac_peak_15416	4.29586	
7	72971752	72972473	5Y-H4K8ac_peak_15417	22.69022	BCL7B_ENSG00000106635
7	73062280	73062526	5Y-H4K8ac_peak_15418	6.34245	
7	73097347	73097707	5Y-H4K8ac_peak_15419	11.71078	DNAJC30_ENSG00000176410;WBSCR22_ENSG00000071462
7	73152869	73153077	5Y-H4K8ac_peak_15420	5.24695	ABHD11_ENSG00000106077
7	73153378	73153714	5Y-H4K8ac_peak_15421	14.35165	ABHD11_ENSG00000106077
7	73240563	73240876	5Y-H4K8ac_peak_15422	4.8547	
7	73241984	73242241	5Y-H4K8ac_peak_15423	9.79526	
7	73247915	73248105	5Y-H4K8ac_peak_15424	5.64909	
7	73256380	73256724	5Y-H4K8ac_peak_15425	7.89273	WBSCR27_ENSG00000165171
7	73257047	73257408	5Y-H4K8ac_peak_15426	7.50148	WBSCR27_ENSG00000165171
7	73284127	73284532	5Y-H4K8ac_peak_15427	12.2918	
7	73300668	73300905	5Y-H4K8ac_peak_15428	5.87725	
7	73309340	73309697	5Y-H4K8ac_peak_15429	9.00954	
7	73333918	73334195	5Y-H4K8ac_peak_15430	7.73538	
7	73334408	73334987	5Y-H4K8ac_peak_15431	7.11863	
7	73385186	73385453	5Y-H4K8ac_peak_15432	5.24695	
7	73398093	73398572	5Y-H4K8ac_peak_15433	13.92823	
7	73399860	73400084	5Y-H4K8ac_peak_15434	4.83262	RP11-731K22.1_ENSG00000261467
7	73420846	73421053	5Y-H4K8ac_peak_15435	6.72932	
7	73436632	73437067	5Y-H4K8ac_peak_15436	5.2778	
7	73438268	73438766	5Y-H4K8ac_peak_15437	5.12488	
7	73441843	73442211	5Y-H4K8ac_peak_15438	8.62703	ELN_ENSG00000049540
7	73442588	73442973	5Y-H4K8ac_peak_15439	8.36116	ELN_ENSG00000049540
7	73447596	73447924	5Y-H4K8ac_peak_15440	12.99645	
7	73456837	73458325	5Y-H4K8ac_peak_15441	19.41527	
7	73458585	73459823	5Y-H4K8ac_peak_15442	14.18025	
7	73496775	73496980	5Y-H4K8ac_peak_15443	5.18558	LIMK1_ENSG00000106683
7	73497361	73497833	5Y-H4K8ac_peak_15444	9.74838	LIMK1_ENSG00000106683
7	73507072	73507997	5Y-H4K8ac_peak_15445	20.56946	
7	73508880	73509351	5Y-H4K8ac_peak_15446	8.21298	
7	73588085	73588427	5Y-H4K8ac_peak_15447	10.54669	EIF4H_ENSG00000106682
7	73588724	73589273	5Y-H4K8ac_peak_15448	22.9394	EIF4H_ENSG00000106682
7	73699230	73699439	5Y-H4K8ac_peak_15449	4.79585	
7	73703391	73703613	5Y-H4K8ac_peak_15450	12.65187	CLIP2_ENSG00000106665
7	73703822	73704076	5Y-H4K8ac_peak_15451	6.25061	CLIP2_ENSG00000106665
7	73721403	73721601	5Y-H4K8ac_peak_15452	5.13727	
7	73832006	73832548	5Y-H4K8ac_peak_15453	8.69112	
7	73845467	73845723	5Y-H4K8ac_peak_15454	6.46606	

7	73867726	73868172	5Y-H4K8ac_peak_15455	14.99099	GTF2IRD1_ENSG00000006704
7	73868391	73868782	5Y-H4K8ac_peak_15456	8.14883	GTF2IRD1_ENSG00000006704
7	73869054	73869451	5Y-H4K8ac_peak_15457	4.97086	GTF2IRD1_ENSG00000006704
7	73895153	73895370	5Y-H4K8ac_peak_15458	5.5192	
7	73920715	73920935	5Y-H4K8ac_peak_15459	5.60566	
7	74020779	74021553	5Y-H4K8ac_peak_15460	8.90774	RP5-1186P10.2_ENSG00000273069
7	74071369	74071719	5Y-H4K8ac_peak_15461	19.06379	GTF2I_ENSG00000077809
7	74072034	74072649	5Y-H4K8ac_peak_15462	33.23772	GTF2I_ENSG00000077809
7	74306296	74306588	5Y-H4K8ac_peak_15463	8.43511	STAG3L2_ENSG00000160828;PMS2P5_ENSG00000123965
7	74489171	74489549	5Y-H4K8ac_peak_15464	9.64145	WBSCR16_ENSG00000174374
7	74489787	74489991	5Y-H4K8ac_peak_15465	11.12941	WBSCR16_ENSG00000174374
7	75115287	75115516	5Y-H4K8ac_peak_15466	10.60083	POM121C_ENSG00000135213;AC006014.7_ENSG00000242073
7	75115806	75116084	5Y-H4K8ac_peak_15467	7.65114	POM121C_ENSG00000135213;AC006014.7_ENSG00000242073
7	75156992	75157358	5Y-H4K8ac_peak_15468	8.24461	PMS2P3_ENSG00000127957
7	75267775	75268325	5Y-H4K8ac_peak_15469	9.76277	
7	75268693	75269828	5Y-H4K8ac_peak_15470	14.26354	
7	75368123	75368322	5Y-H4K8ac_peak_15471	7.50148	HIP1_ENSG00000127946
7	75368807	75369058	5Y-H4K8ac_peak_15472	8.43999	HIP1_ENSG00000127946
7	75507129	75507351	5Y-H4K8ac_peak_15473	7.77518	
7	75676744	75677222	5Y-H4K8ac_peak_15474	8.24461	STYXL1_ENSG00000127952;MDH2_ENSG00000146701
7	75677434	75677772	5Y-H4K8ac_peak_15475	17.13377	STYXL1_ENSG00000127952;MDH2_ENSG00000146701
7	75794890	75795099	5Y-H4K8ac_peak_15476	6.08523	
7	75795549	75795796	5Y-H4K8ac_peak_15477	8.19942	
7	75796127	75796586	5Y-H4K8ac_peak_15478	11.88337	
7	75807775	75808078	5Y-H4K8ac_peak_15479	6.80915	
7	75814464	75815400	5Y-H4K8ac_peak_15480	14.68695	
7	75827619	75827811	5Y-H4K8ac_peak_15481	4.728	
7	75830484	75830829	5Y-H4K8ac_peak_15482	6.37023	SRRM3_ENSG00000177679
7	75831229	75831620	5Y-H4K8ac_peak_15483	11.22005	SRRM3_ENSG00000177679
7	75878587	75878807	5Y-H4K8ac_peak_15484	8.13965	
7	75879652	75879928	5Y-H4K8ac_peak_15485	5.21777	
7	75880682	75881196	5Y-H4K8ac_peak_15486	17.29652	
7	75910685	75911144	5Y-H4K8ac_peak_15487	6.55225	
7	75921358	75921560	5Y-H4K8ac_peak_15488	9.61965	
7	75923757	75924406	5Y-H4K8ac_peak_15489	14.6127	
7	75933262	75933479	5Y-H4K8ac_peak_15490	7.72519	
7	75936418	75937076	5Y-H4K8ac_peak_15491	9.14624	
7	75946674	75947240	5Y-H4K8ac_peak_15492	8.3285	
7	75947710	75948070	5Y-H4K8ac_peak_15493	13.86751	
7	75987687	75988627	5Y-H4K8ac_peak_15494	11.69482	YWHAG_ENSG00000170027
7	75988861	75989229	5Y-H4K8ac_peak_15495	6.86362	YWHAG_ENSG00000170027
7	76018832	76019031	5Y-H4K8ac_peak_15496	6.18374	
7	76022128	76022439	5Y-H4K8ac_peak_15497	8.27643	
7	76022723	76022992	5Y-H4K8ac_peak_15498	7.90751	
7	76026844	76027235	5Y-H4K8ac_peak_15499	11.40439	ZP3_ENSG00000188372
7	76177916	76178323	5Y-H4K8ac_peak_15500	12.10416	AC004980.7_ENSG00000205485

7	76178732	76179214	5Y-H4K8ac_peak_15501	12.00715	AC004980.7_ENSG00000205485
7	76256456	76256874	5Y-H4K8ac_peak_15502	9.15007	POMZP3_ENSG00000146707
7	77044826	77046182	5Y-H4K8ac_peak_15503	22.9394	GSAP_ENSG00000186088;RP5-899E9.1_ENSG00000273341
7	77166986	77167289	5Y-H4K8ac_peak_15504	4.48815	PTPN12_ENSG00000127947
7	77395529	77395759	5Y-H4K8ac_peak_15505	7.09444	
7	79083242	79083543	5Y-H4K8ac_peak_15506	13.12848	MAGI2_ENSG00000187391
7	81897061	81897276	5Y-H4K8ac_peak_15507	12.05638	
7	82072383	82073116	5Y-H4K8ac_peak_15508	28.78021	CACNA2D1_ENSG00000153956
7	83207573	83207781	5Y-H4K8ac_peak_15509	7.31815	
7	86274213	86274403	5Y-H4K8ac_peak_15510	5.23819	GRM3_ENSG00000198822
7	86688524	86688851	5Y-H4K8ac_peak_15511	9.00954	KIAA1324L_ENSG00000164659
7	86781898	86782507	5Y-H4K8ac_peak_15512	14.26354	AC005076.5_ENSG00000224046;DMTF1_ENSG00000135164
7	86849411	86849780	5Y-H4K8ac_peak_15513	7.89273	TMEM243_ENSG00000135185
7	86940292	86940503	5Y-H4K8ac_peak_15514	8.51022	
7	87104614	87104947	5Y-H4K8ac_peak_15515	8.26455	
7	87105170	87105374	5Y-H4K8ac_peak_15516	9.02938	
7	87505185	87505486	5Y-H4K8ac_peak_15517	7.64648	SLC25A40_ENSG00000075303;DBF4_ENSG00000006634
7	87563650	87563845	5Y-H4K8ac_peak_15518	8.91395	ADAM22_ENSG00000008277
7	87646143	87646453	5Y-H4K8ac_peak_15519	10.27264	
7	87674065	87674323	5Y-H4K8ac_peak_15520	7.21591	
7	87849065	87849273	5Y-H4K8ac_peak_15521	9.45199	AC003991.3_ENSG00000228113
7	89748301	89748518	5Y-H4K8ac_peak_15522	10.35586	DPY19L2P4_ENSG00000235436
7	89748713	89749170	5Y-H4K8ac_peak_15523	8.2913	DPY19L2P4_ENSG00000235436
7	89950633	89950831	5Y-H4K8ac_peak_15524	6.75784	
7	90225122	90225325	5Y-H4K8ac_peak_15525	4.44548	
7	90225701	90225982	5Y-H4K8ac_peak_15526	4.55128	AC002456.2_ENSG00000223969
7	90798534	90798749	5Y-H4K8ac_peak_15527	8.04943	
7	91195241	91195475	5Y-H4K8ac_peak_15528	11.28133	
7	91570321	91570554	5Y-H4K8ac_peak_15529	9.32595	AKAP9_ENSG00000127914
7	91763410	91763614	5Y-H4K8ac_peak_15530	10.31072	CTB-161K23.1_ENSG00000188693
7	91763881	91764366	5Y-H4K8ac_peak_15531	10.48695	CTB-161K23.1_ENSG00000188693
7	91808408	91809006	5Y-H4K8ac_peak_15532	9.80622	LRRD1_ENSG00000240720
7	91874771	91875702	5Y-H4K8ac_peak_15533	12.00715	KRIT1_ENSG00000001631;ANKIB1_ENSG00000001629
7	92076258	92076514	5Y-H4K8ac_peak_15534	7.8267	GATAD1_ENSG00000157259
7	92076867	92077306	5Y-H4K8ac_peak_15535	5.32952	GATAD1_ENSG00000157259
7	92219093	92219669	5Y-H4K8ac_peak_15536	9.36633	FAM133B_ENSG00000234545
7	92329133	92329323	5Y-H4K8ac_peak_15537	5.65584	
7	92400296	92400488	5Y-H4K8ac_peak_15538	5.41472	
7	92464153	92464649	5Y-H4K8ac_peak_15539	10.54764	
7	92861946	92862288	5Y-H4K8ac_peak_15540	5.85188	CCDC132_ENSG00000004766
7	93551114	93551352	5Y-H4K8ac_peak_15541	3.94241	GNG11_ENSG00000127920
7	93595139	93595406	5Y-H4K8ac_peak_15542	5.23083	
7	93997336	93997550	5Y-H4K8ac_peak_15543	5.59843	
7	94138403	94140000	5Y-H4K8ac_peak_15544	25.2532	CASD1_ENSG00000127995
7	94284882	94285453	5Y-H4K8ac_peak_15545	14.31711	SGCE_ENSG00000127990;PEG10_ENSG00000242265
7	94536707	94537423	5Y-H4K8ac_peak_15546	8.3285	PPP1R9A_ENSG00000158528

7	95064072	95065109	5Y-H4K8ac_peak_15547	12.00715	PON2_ENSG00000105854
7	95402176	95402399	5Y-H4K8ac_peak_15548	4.79585	DYNC1I1_ENSG00000158560
7	95441677	95441870	5Y-H4K8ac_peak_15549	5.65584	
7	95557774	95557982	5Y-H4K8ac_peak_15550	6.50117	
7	95950979	95951379	5Y-H4K8ac_peak_15551	12.00715	SLC25A13_ENSG00000004864
7	96338771	96339006	5Y-H4K8ac_peak_15552	8.24461	SHFM1_ENSG00000127922
7	96635274	96636508	5Y-H4K8ac_peak_15553	20.40213	DLX6_ENSG00000006377;DLX6-AS2_ENSG00000271956
7	96645739	96646994	5Y-H4K8ac_peak_15554	13.86751	
7	97557409	97558082	5Y-H4K8ac_peak_15555	8.24461	
7	97601309	97602187	5Y-H4K8ac_peak_15556	17.49408	AC004967.7_ENSG00000243554
7	97736273	97736605	5Y-H4K8ac_peak_15557	9.62747	LMTK2_ENSG00000164715
7	97840862	97841077	5Y-H4K8ac_peak_15558	7.20869	BHLHA15_ENSG00000180535
7	97907995	97908297	5Y-H4K8ac_peak_15559	7.50148	
7	97910101	97910459	5Y-H4K8ac_peak_15560	9.93116	
7	97911076	97911267	5Y-H4K8ac_peak_15561	8.1667	
7	98184181	98184371	5Y-H4K8ac_peak_15562	5.24695	
7	98188291	98188481	5Y-H4K8ac_peak_15563	6.20944	
7	98199211	98199496	5Y-H4K8ac_peak_15564	6.37023	
7	98245869	98246355	5Y-H4K8ac_peak_15565	20.32089	NPTX2_ENSG00000106236
7	98423890	98424761	5Y-H4K8ac_peak_15566	10.16277	
7	98465420	98465864	5Y-H4K8ac_peak_15567	6.53157	
7	98466979	98467212	5Y-H4K8ac_peak_15568	6.03632	
7	98467430	98468114	5Y-H4K8ac_peak_15569	11.48691	TMEM130_ENSG00000166448
7	98476163	98476552	5Y-H4K8ac_peak_15570	15.90402	TRRAP_ENSG00000196367
7	98741268	98741568	5Y-H4K8ac_peak_15571	19.35769	SMURF1_ENSG00000198742
7	98741902	98742097	5Y-H4K8ac_peak_15572	8.3285	SMURF1_ENSG00000198742
7	98922956	98923337	5Y-H4K8ac_peak_15573	6.67135	ARPC1A_ENSG00000241685
7	98923613	98923919	5Y-H4K8ac_peak_15574	10.54764	ARPC1A_ENSG00000241685
7	98990025	98990690	5Y-H4K8ac_peak_15575	10.35586	
7	99005711	99006512	5Y-H4K8ac_peak_15576	22.56714	PDAP1_ENSG00000106244;BUD31_ENSG00000106245
7	99036621	99036891	5Y-H4K8ac_peak_15577	13.68515	CPSF4_ENSG00000160917
7	99063386	99064187	5Y-H4K8ac_peak_15578	16.79568	PTCD1_ENSG00000106246;ATP5J2-PTCD1_ENSG00000248919;ATP5J2_ENSG00000241468
7	99068377	99068677	5Y-H4K8ac_peak_15579	6.08523	
7	99068914	99069156	5Y-H4K8ac_peak_15580	6.31818	
7	99069795	99070055	5Y-H4K8ac_peak_15581	9.3149	ZNF789_ENSG00000198556
7	99101773	99102068	5Y-H4K8ac_peak_15582	9.37812	ZKSCAN5_ENSG00000196652
7	99102330	99103150	5Y-H4K8ac_peak_15583	11.5813	ZKSCAN5_ENSG00000196652
7	99148517	99148817	5Y-H4K8ac_peak_15584	5.41472	
7	99149412	99149666	5Y-H4K8ac_peak_15585	11.4254	
7	99155705	99156065	5Y-H4K8ac_peak_15586	15.45397	FAM200A_ENSG00000221909;ZNF655_ENSG00000197343;GS1-259H13.10_ENSG00000272647
7	99177464	99178498	5Y-H4K8ac_peak_15587	12.51872	
7	99214201	99214402	5Y-H4K8ac_peak_15588	9.44542	ZSCAN25_ENSG00000197037
7	99214647	99214930	5Y-H4K8ac_peak_15589	13.29549	ZSCAN25_ENSG00000197037
7	99612992	99613776	5Y-H4K8ac_peak_15590	13.33225	ZKSCAN1_ENSG00000106261
7	99646983	99647268	5Y-H4K8ac_peak_15591	12.51536	ZSCAN21_ENSG00000166529
7	99678606	99679329	5Y-H4K8ac_peak_15592	11.98202	ZNF3_ENSG00000166526

7	99679904	99680159	5Y-H4K8ac_peak_15593	10.19272	ZNF3_ENSG00000166526
7	99685976	99687119	5Y-H4K8ac_peak_15594	12.99645	COPS6_ENSG00000168090
7	99698519	99698967	5Y-H4K8ac_peak_15595	24.53547	MCM7_ENSG00000166508;AP4M1_ENSG00000221838
7	99699648	99700606	5Y-H4K8ac_peak_15596	11.27006	MCM7_ENSG00000166508;AP4M1_ENSG00000221838
7	99723399	99724125	5Y-H4K8ac_peak_15597	3.93327	RP11-506M12.1_ENSG00000242798;MBLAC1_ENSG00000214309
7	99724984	99726000	5Y-H4K8ac_peak_15598	6.95285	RP11-506M12.1_ENSG00000242798;MBLAC1_ENSG00000214309
7	99745494	99746106	5Y-H4K8ac_peak_15599	12.5784	LAMTOR4_ENSG00000188186
7	99755763	99756248	5Y-H4K8ac_peak_15600	7.82121	C7orf43_ENSG00000146826
7	99774685	99774944	5Y-H4K8ac_peak_15601	11.23789	GPC2_ENSG00000213420;STAG3_ENSG00000066923
7	99870026	99870269	5Y-H4K8ac_peak_15602	7.90005	GATS_ENSG00000239521;GATS_ENSG00000160844
7	99933816	99934225	5Y-H4K8ac_peak_15603	6.66983	STAG3L5P_ENSG00000242294;STAG3L5P-PVRIG2P-PILRB_ENSG00000272752;PILRB_ENSG00000121716
7	99979221	99979482	5Y-H4K8ac_peak_15604	4.29586	
7	100025499	100025848	5Y-H4K8ac_peak_15605	7.90005	ZCWPW1_ENSG00000078487;MEPCE_ENSG00000146834
7	100027589	100028243	5Y-H4K8ac_peak_15606	13.1133	ZCWPW1_ENSG00000078487
7	100033480	100034063	5Y-H4K8ac_peak_15607	12.52787	RP11-758P17.2_ENSG00000241357;PPP1R35_ENSG00000160813;RP11-758P17.3_ENSG00000240211
7	100034458	100034916	5Y-H4K8ac_peak_15608	6.67135	PPP1R35_ENSG00000160813;RP11-758P17.3_ENSG00000240211
7	100047941	100048635	5Y-H4K8ac_peak_15609	14.50475	
7	100065313	100065554	5Y-H4K8ac_peak_15610	8.69112	
7	100081908	100082231	5Y-H4K8ac_peak_15611	7.29055	AC092849.1_ENSG00000266372;NYAP1_ENSG00000166924
7	100113477	100113703	5Y-H4K8ac_peak_15612	4.95697	
7	100167533	100168281	5Y-H4K8ac_peak_15613	19.60376	RP11-44M6.3_ENSG00000184414
7	100183439	100183695	5Y-H4K8ac_peak_15614	18.60312	LRCH4_ENSG00000077454
7	100197994	100198521	5Y-H4K8ac_peak_15615	9.13495	
7	100200056	100200641	5Y-H4K8ac_peak_15616	14.86775	PCOLCE_ENSG00000106333
7	100202115	100202345	5Y-H4K8ac_peak_15617	8.09118	PCOLCE-AS1_ENSG00000224729
7	100208977	100209468	5Y-H4K8ac_peak_15618	10.86711	MOSPD3_ENSG00000106330
7	100224459	100224826	5Y-H4K8ac_peak_15619	7.20869	
7	100230511	100231261	5Y-H4K8ac_peak_15620	19.58975	
7	100231474	100231693	5Y-H4K8ac_peak_15621	5.64909	
7	100254265	100254699	5Y-H4K8ac_peak_15622	6.37023	ACTL6B_ENSG00000077080
7	100271123	100271313	5Y-H4K8ac_peak_15623	12.19856	GNB2_ENSG00000172354
7	100271545	100271811	5Y-H4K8ac_peak_15624	7.31947	GNB2_ENSG00000172354
7	100273306	100273539	5Y-H4K8ac_peak_15625	6.79877	
7	100291138	100291594	5Y-H4K8ac_peak_15626	8.00646	
7	100292181	100292845	5Y-H4K8ac_peak_15627	11.71078	
7	100303137	100303506	5Y-H4K8ac_peak_15628	10.54669	POP7_ENSG00000172336
7	100303762	100303954	5Y-H4K8ac_peak_15629	8.09118	POP7_ENSG00000172336
7	100399553	100399986	5Y-H4K8ac_peak_15630	8.9308	
7	100471659	100472516	5Y-H4K8ac_peak_15631	13.12848	SRRT_ENSG00000087087
7	100472931	100473230	5Y-H4K8ac_peak_15632	9.79526	SRRT_ENSG00000087087
7	100486856	100487255	5Y-H4K8ac_peak_15633	10.48695	UFSP1_ENSG00000176125
7	100488165	100488840	5Y-H4K8ac_peak_15634	16.93214	UFSP1_ENSG00000176125
7	100494773	100495111	5Y-H4K8ac_peak_15635	4.36209	ACHE_ENSG00000087085
7	100797072	100797264	5Y-H4K8ac_peak_15636	7.90751	AP1S1_ENSG00000106367
7	100798041	100798337	5Y-H4K8ac_peak_15637	8.99358	AP1S1_ENSG00000106367
7	100805877	100806467	5Y-H4K8ac_peak_15638	6.19716	

7	100806981	100807320	5Y-H4K8ac_peak_15639	5.41472	
7	100809239	100809513	5Y-H4K8ac_peak_15640	8.52662	VGF_ENSG00000128564
7	100812083	100812521	5Y-H4K8ac_peak_15641	7.90751	
7	100835625	100836308	5Y-H4K8ac_peak_15642	9.38276	
7	100860968	100861227	5Y-H4K8ac_peak_15643	7.96285	PLOD3_ENSG00000106397;ZNHIT1_ENSG00000106400
7	100887763	100888842	5Y-H4K8ac_peak_15644	10.14791	
7	100925958	100926276	5Y-H4K8ac_peak_15645	8.10449	
7	100965207	100965641	5Y-H4K8ac_peak_15646	11.09673	RABL5_ENSG00000128581
7	101006955	101007286	5Y-H4K8ac_peak_15647	5.12488	COL26A1_ENSG00000160963
7	101007943	101008175	5Y-H4K8ac_peak_15648	9.51785	
7	101130067	101130421	5Y-H4K8ac_peak_15649	8.09118	
7	101130625	101131015	5Y-H4K8ac_peak_15650	4.44548	
7	101386251	101386685	5Y-H4K8ac_peak_15651	11.27089	
7	101398324	101398521	5Y-H4K8ac_peak_15652	11.27006	
7	101457608	101457886	5Y-H4K8ac_peak_15653	5.23083	
7	101458397	101458924	5Y-H4K8ac_peak_15654	7.44077	CUX1_ENSG00000257923
7	101687897	101688300	5Y-H4K8ac_peak_15655	5.98695	
7	101708214	101708524	5Y-H4K8ac_peak_15656	6.31818	
7	101754754	101754944	5Y-H4K8ac_peak_15657	7.18918	
7	101902506	101902768	5Y-H4K8ac_peak_15658	6.13919	
7	101914042	101914250	5Y-H4K8ac_peak_15659	8.36588	
7	101929330	101929974	5Y-H4K8ac_peak_15660	16.29405	SH2B2_ENSG00000160999
7	102003519	102004058	5Y-H4K8ac_peak_15661	10.31981	PRKRIP1_ENSG00000128563;RP11-163E9.2_ENSG00000239969
7	102004471	102004861	5Y-H4K8ac_peak_15662	6.80915	PRKRIP1_ENSG00000128563;RP11-163E9.2_ENSG00000239969
7	102023614	102023823	5Y-H4K8ac_peak_15663	4.8188	RP11-163E9.1_ENSG00000239486
7	102074059	102074386	5Y-H4K8ac_peak_15664	10.60083	RP11-514P8.2_ENSG00000239480;ORAI2_ENSG00000160991
7	102081453	102081649	5Y-H4K8ac_peak_15665	6.78128	
7	102082532	102082778	5Y-H4K8ac_peak_15666	5.60566	
7	102104967	102105262	5Y-H4K8ac_peak_15667	9.02938	MIR5090_ENSG00000266715;ALKBH4_ENSG00000160993;LRWD1_ENSG00000161036
7	102153354	102153735	5Y-H4K8ac_peak_15668	13.0168	
7	102172629	102172822	5Y-H4K8ac_peak_15669	10.90097	
7	102252509	102252727	5Y-H4K8ac_peak_15670	16.73191	
7	102576570	102576880	5Y-H4K8ac_peak_15671	5.98695	
7	102715661	102715978	5Y-H4K8ac_peak_15672	7.07617	FBXL13_ENSG00000161040;ARMC10_ENSG00000170632
7	102789801	102790073	5Y-H4K8ac_peak_15673	10.56003	NAPEPLD_ENSG00000161048
7	102920393	102921164	5Y-H4K8ac_peak_15674	10.60083	DPY19L2P2_ENSG00000170629
7	102937956	102938239	5Y-H4K8ac_peak_15675	13.03153	PMPCB_ENSG00000105819
7	102984880	102985191	5Y-H4K8ac_peak_15676	12.10416	DNAJC2_ENSG00000105821;PSMC2_ENSG00000161057
7	103086432	103086781	5Y-H4K8ac_peak_15677	3.94247	SLC26A5_ENSG00000170615;CTB-107G13.1_ENSG00000234715
7	103165344	103165569	5Y-H4K8ac_peak_15678	7.87048	
7	103355999	103356420	5Y-H4K8ac_peak_15679	5.98695	
7	103456534	103456750	5Y-H4K8ac_peak_15680	6.50117	
7	103629859	103630102	5Y-H4K8ac_peak_15681	11.64833	RELN_ENSG00000189056
7	103630301	103630813	5Y-H4K8ac_peak_15682	6.56228	RELN_ENSG00000189056
7	103848113	103848820	5Y-H4K8ac_peak_15683	17.04999	ORC5_ENSG00000164815
7	103968423	103970003	5Y-H4K8ac_peak_15684	11.22005	LHFPL3_ENSG00000187416

7	104580341	104580816	5Y-H4K8ac_peak_15685	6.66215	RP11-325F22.2_ENSG00000237513
7	104585212	104585468	5Y-H4K8ac_peak_15686	6.78128	
7	104624149	104624918	5Y-H4K8ac_peak_15687	7.50148	
7	104653912	104654294	5Y-H4K8ac_peak_15688	9.36633	LINC01004_ENSG00000228393;KMT2E-AS1_ENSG00000239569;KMT2E_ENSG00000005483
7	105028910	105029242	5Y-H4K8ac_peak_15689	6.10343	
7	105041886	105042139	5Y-H4K8ac_peak_15690	5.24695	
7	105162050	105162633	5Y-H4K8ac_peak_15691	9.69901	PUS7_ENSG00000091127
7	105172700	105172927	5Y-H4K8ac_peak_15692	14.77593	RINT1_ENSG00000135249
7	105252905	105253098	5Y-H4K8ac_peak_15693	7.18391	
7	105262728	105262952	5Y-H4K8ac_peak_15694	5.62696	
7	105283786	105284012	5Y-H4K8ac_peak_15695	7.418	
7	105409771	105410131	5Y-H4K8ac_peak_15696	5.38445	
7	105517270	105517501	5Y-H4K8ac_peak_15697	10.89497	ATXN7L1_ENSG00000146776;CDHR3_ENSG00000128536
7	105661058	105661304	5Y-H4K8ac_peak_15698	7.73538	
7	105698445	105699339	5Y-H4K8ac_peak_15699	11.01067	
7	105705217	105705475	5Y-H4K8ac_peak_15700	11.60058	
7	105752355	105752578	5Y-H4K8ac_peak_15701	10.1994	SYPL1_ENSG00000008282
7	105817052	105817252	5Y-H4K8ac_peak_15702	7.38046	
7	106209854	106210197	5Y-H4K8ac_peak_15703	5.67283	
7	106300974	106301506	5Y-H4K8ac_peak_15704	17.78344	CCDC71L_ENSG00000253276;AC004917.1_ENSG00000177820
7	106301732	106302113	5Y-H4K8ac_peak_15705	11.86504	CCDC71L_ENSG00000253276;AC004917.1_ENSG00000177820
7	106484145	106484428	5Y-H4K8ac_peak_15706	10.1994	
7	106744181	106744418	5Y-H4K8ac_peak_15707	5.03438	
7	107204567	107204762	5Y-H4K8ac_peak_15708	6.37023	COG5_ENSG00000164597;DUS4L_ENSG00000105865
7	107220740	107220932	5Y-H4K8ac_peak_15709	8.79399	RP4-593H12.1_ENSG00000272854;BCAP29_ENSG00000075790
7	107384237	107384469	5Y-H4K8ac_peak_15710	9.36633	AC002467.7_ENSG00000241764;CBLL1_ENSG00000105879
7	107642465	107644039	5Y-H4K8ac_peak_15711	18.78631	LAMB1_ENSG00000091136
7	107933269	107933683	5Y-H4K8ac_peak_15712	4.50834	
7	108095260	108095759	5Y-H4K8ac_peak_15713	13.03079	
7	108096275	108096813	5Y-H4K8ac_peak_15714	8.62703	NRCAM_ENSG00000091129
7	108166954	108167174	5Y-H4K8ac_peak_15715	9.79526	
7	108210422	108210878	5Y-H4K8ac_peak_15716	9.99838	PNPLA8_ENSG00000135241;THAP5_ENSG00000177683;DNAJB9_ENSG00000128590
7	110777881	110778099	5Y-H4K8ac_peak_15717	8.08688	
7	112031765	112032077	5Y-H4K8ac_peak_15718	10.90365	
7	112032525	112032855	5Y-H4K8ac_peak_15719	6.4472	
7	112060351	112060701	5Y-H4K8ac_peak_15720	6.46053	
7	112429997	112430815	5Y-H4K8ac_peak_15721	12.43028	TMEM168_ENSG00000146802
7	115850625	115851035	5Y-H4K8ac_peak_15722	8.82628	TES_ENSG00000135269
7	116501955	116502152	5Y-H4K8ac_peak_15723	7.76768	
7	116502671	116503088	5Y-H4K8ac_peak_15724	15.99783	
7	116593018	116593259	5Y-H4K8ac_peak_15725	8.11614	ST7_ENSG00000004866;ST7-OT4_ENSG00000214188
7	116593966	116594316	5Y-H4K8ac_peak_15726	8.24461	ST7-AS1_ENSG00000227199;ST7_ENSG00000004866;ST7-OT4_ENSG00000214188
7	117824296	117824584	5Y-H4K8ac_peak_15727	7.59101	NAA38_ENSG00000128534
7	122676314	122676539	5Y-H4K8ac_peak_15728	5.40331	
7	123388805	123389021	5Y-H4K8ac_peak_15729	7.21591	WASL_ENSG00000106299;RP11-390E23.6_ENSG00000272686
7	123672052	123673794	5Y-H4K8ac_peak_15730	18.08671	TMEM229A_ENSG00000234224;RP5-921G16.1_ENSG00000242593

7	123713747	123713958	5Y-H4K8ac_peak_15731	4.6766	
7	123724719	123724990	5Y-H4K8ac_peak_15732	9.89244	
7	123828092	123828323	5Y-H4K8ac_peak_15733	6.83519	
7	124497728	124498108	5Y-H4K8ac_peak_15734	4.51076	
7	125108367	125108595	5Y-H4K8ac_peak_15735	6.77436	
7	126886588	126886849	5Y-H4K8ac_peak_15736	8.49231	
7	126891098	126891327	5Y-H4K8ac_peak_15737	5.98695	
7	126891684	126892333	5Y-H4K8ac_peak_15738	7.70811	
7	126893726	126894039	5Y-H4K8ac_peak_15739	11.01067	GRM8_ENSG00000179603
7	127225415	127225634	5Y-H4K8ac_peak_15740	11.33673	
7	127227753	127228287	5Y-H4K8ac_peak_15741	17.67055	ARF5_ENSG00000004059
7	127228480	127228712	5Y-H4K8ac_peak_15742	8.46442	ARF5_ENSG00000004059
7	127291021	127291909	5Y-H4K8ac_peak_15743	10.16277	AC073934.6_ENSG00000240790;SND1_ENSG00000197157
7	127663225	127663427	5Y-H4K8ac_peak_15744	9.30206	
7	127670745	127671007	5Y-H4K8ac_peak_15745	7.50081	
7	127671222	127671772	5Y-H4K8ac_peak_15746	12.97312	LRRC4_ENSG00000128594
7	127672843	127673067	5Y-H4K8ac_peak_15747	5.60566	LRRC4_ENSG00000128594
7	127717563	127717773	5Y-H4K8ac_peak_15748	5.68578	
7	127722106	127722582	5Y-H4K8ac_peak_15749	5.41472	MIR593_ENSG00000207588
7	127724359	127725267	5Y-H4K8ac_peak_15750	17.52337	
7	127736947	127737341	5Y-H4K8ac_peak_15751	5.41472	
7	127775509	127775866	5Y-H4K8ac_peak_15752	20.47833	
7	127806650	127808178	5Y-H4K8ac_peak_15753	15.63321	
7	127899327	127899582	5Y-H4K8ac_peak_15754	4.83834	
7	127904741	127905608	5Y-H4K8ac_peak_15755	17.37977	RP11-62J1.4_ENSG00000272915
7	127906078	127906441	5Y-H4K8ac_peak_15756	7.27671	
7	127907264	127907533	5Y-H4K8ac_peak_15757	11.16796	
7	127907826	127908512	5Y-H4K8ac_peak_15758	11.4254	
7	127988536	127988849	5Y-H4K8ac_peak_15759	4.92498	
7	127989043	127989450	5Y-H4K8ac_peak_15760	5.05857	
7	127989923	127991535	5Y-H4K8ac_peak_15761	10.35586	
7	127995711	127997127	5Y-H4K8ac_peak_15762	12.29582	
7	127998223	127998470	5Y-H4K8ac_peak_15763	10.35444	
7	127999352	128000952	5Y-H4K8ac_peak_15764	18.4549	PRRT4_ENSG00000224940
7	128001262	128002938	5Y-H4K8ac_peak_15765	25.33524	PRRT4_ENSG00000224940
7	128004196	128004416	5Y-H4K8ac_peak_15766	8.62703	
7	128018745	128019318	5Y-H4K8ac_peak_15767	10.16277	
7	128020220	128021014	5Y-H4K8ac_peak_15768	10.1994	
7	128022699	128022988	5Y-H4K8ac_peak_15769	7.90236	
7	128027225	128027627	5Y-H4K8ac_peak_15770	20.56946	
7	128045508	128045887	5Y-H4K8ac_peak_15771	6.38599	
7	128086506	128086769	5Y-H4K8ac_peak_15772	5.24695	
7	128094629	128094839	5Y-H4K8ac_peak_15773	7.20869	
7	128095283	128095671	5Y-H4K8ac_peak_15774	7.8511	RP11-212P7.3_ENSG00000273184;HILPDA_ENSG00000135245;RP11-155G14.6_ENSG00000240758
7	128096040	128096308	5Y-H4K8ac_peak_15775	7.44077	RP11-212P7.3_ENSG00000273184;HILPDA_ENSG00000135245;RP11-155G14.6_ENSG00000240758
7	128116381	128116669	5Y-H4K8ac_peak_15776	7.50501	METTL2B_ENSG00000165055

7	128116896	128117428	5Y-H4K8ac_peak_15777	14.86775	METTL2B_ENSG00000165055
7	128170794	128171119	5Y-H4K8ac_peak_15778	11.29141	RP11-212P7.2_ENSG00000273270;RP11-274B21.1_ENSG00000242588
7	128171429	128172272	5Y-H4K8ac_peak_15779	13.59183	RP11-212P7.2_ENSG00000273270;RP11-274B21.1_ENSG00000242588
7	128392729	128392940	5Y-H4K8ac_peak_15780	5.98695	
7	128430830	128431291	5Y-H4K8ac_peak_15781	8.26455	CCDC136_ENSG00000128596
7	128470076	128471048	5Y-H4K8ac_peak_15782	12.40938	FLNC_ENSG00000128591
7	128471498	128472441	5Y-H4K8ac_peak_15783	7.90751	
7	128472851	128473108	5Y-H4K8ac_peak_15784	5.50568	
7	128509500	128509869	5Y-H4K8ac_peak_15785	5.64909	RP11-309L24.9_ENSG00000272899
7	128514700	128514936	5Y-H4K8ac_peak_15786	5.34199	
7	128577541	128578484	5Y-H4K8ac_peak_15787	9.79526	IRF5_ENSG00000128604
7	128694570	128695141	5Y-H4K8ac_peak_15788	17.29652	TNPO3_ENSG00000064419;TPI1P2_ENSG00000230359
7	128828459	128829310	5Y-H4K8ac_peak_15789	12.52787	SMO_ENSG00000128602
7	129251150	129251426	5Y-H4K8ac_peak_15790	11.16796	RP11-448A19.1_ENSG00000273329;NRF1_ENSG00000106459
7	129387767	129387970	5Y-H4K8ac_peak_15791	7.92818	
7	129389864	129390115	5Y-H4K8ac_peak_15792	6.20292	
7	129419753	129419947	5Y-H4K8ac_peak_15793	5.7621	
7	129464630	129464832	5Y-H4K8ac_peak_15794	6.55841	
7	129473930	129474139	5Y-H4K8ac_peak_15795	8.4454	
7	129592174	129592369	5Y-H4K8ac_peak_15796	4.77126	UBE2H_ENSG00000186591;RP11-306G20.1_ENSG00000244036
7	129690829	129691205	5Y-H4K8ac_peak_15797	15.87286	ZC3HC1_ENSG00000091732
7	129780976	129781435	5Y-H4K8ac_peak_15798	5.60566	RP11-775D22.2_ENSG00000229196
7	129793128	129793408	5Y-H4K8ac_peak_15799	5.85188	
7	129793687	129794135	5Y-H4K8ac_peak_15800	6.51488	
7	129794777	129795253	5Y-H4K8ac_peak_15801	7.44077	
7	129844977	129845183	5Y-H4K8ac_peak_15802	9.74838	
7	129845724	129846282	5Y-H4K8ac_peak_15803	6.78128	
7	130004451	130004885	5Y-H4K8ac_peak_15804	7.55879	
7	130005122	130005328	5Y-H4K8ac_peak_15805	4.51215	
7	130005760	130006096	5Y-H4K8ac_peak_15806	7.11863	
7	130013573	130013919	5Y-H4K8ac_peak_15807	13.0168	
7	130019635	130020338	5Y-H4K8ac_peak_15808	7.44077	CPA1_ENSG00000091704
7	130020623	130020845	5Y-H4K8ac_peak_15809	6.84536	CPA1_ENSG00000091704
7	130080236	130080901	5Y-H4K8ac_peak_15810	14.17466	
7	130353302	130353494	5Y-H4K8ac_peak_15811	9.02938	COPG2_ENSG00000158623
7	130794481	130795303	5Y-H4K8ac_peak_15812	19.17092	LINC-PINT_ENSG00000231721;MKLN1_ENSG00000128585
7	130921126	130921406	5Y-H4K8ac_peak_15813	6.64036	
7	131187948	131188211	5Y-H4K8ac_peak_15814	6.64195	
7	131206377	131206624	5Y-H4K8ac_peak_15815	7.35333	
7	131221999	131222198	5Y-H4K8ac_peak_15816	5.73383	
7	131222838	131223189	5Y-H4K8ac_peak_15817	9.88714	
7	131242678	131243031	5Y-H4K8ac_peak_15818	15.19225	PODXL_ENSG00000128567
7	131304485	131304892	5Y-H4K8ac_peak_15819	4.50834	
7	131305540	131305776	5Y-H4K8ac_peak_15820	7.64648	
7	131306892	131307115	5Y-H4K8ac_peak_15821	5.99504	
7	131339715	131340037	5Y-H4K8ac_peak_15822	5.98695	

7	131377141	131377518	5Y-H4K8ac_peak_15823	6.66983	
7	131593958	131594573	5Y-H4K8ac_peak_15824	9.7353	
7	131845880	131846628	5Y-H4K8ac_peak_15825	8.26455	
7	132120175	132120685	5Y-H4K8ac_peak_15826	15.93748	
7	132159829	132160069	5Y-H4K8ac_peak_15827	12.50545	
7	132160823	132161068	5Y-H4K8ac_peak_15828	5.47303	
7	132165404	132165597	5Y-H4K8ac_peak_15829	3.93411	
7	132342090	132342333	5Y-H4K8ac_peak_15830	9.30338	
7	132766412	132766763	5Y-H4K8ac_peak_15831	12.26819	CHCHD3_ENSG00000106554;U6_ENSG00000272393
7	132937897	132938088	5Y-H4K8ac_peak_15832	10.23828	EXOC4_ENSG00000131558
7	133241600	133241869	5Y-H4K8ac_peak_15833	8.43011	
7	133426743	133427057	5Y-H4K8ac_peak_15834	9.00954	
7	133428447	133428667	5Y-H4K8ac_peak_15835	11.09673	
7	133432825	133433486	5Y-H4K8ac_peak_15836	10.0861	
7	133434240	133434494	5Y-H4K8ac_peak_15837	8.80171	
7	133465302	133465570	5Y-H4K8ac_peak_15838	12.6937	
7	133465954	133466531	5Y-H4K8ac_peak_15839	13.59183	
7	133478073	133478448	5Y-H4K8ac_peak_15840	8.62044	
7	133481098	133481473	5Y-H4K8ac_peak_15841	8.09118	
7	133482457	133482764	5Y-H4K8ac_peak_15842	11.64833	
7	133491354	133491577	5Y-H4K8ac_peak_15843	5.51759	
7	133491821	133492067	5Y-H4K8ac_peak_15844	5.87725	
7	133495501	133495883	5Y-H4K8ac_peak_15845	8.3285	
7	133531119	133531595	5Y-H4K8ac_peak_15846	6.56228	
7	133544810	133545005	5Y-H4K8ac_peak_15847	5.72233	
7	133631770	133632236	5Y-H4K8ac_peak_15848	4.8887	
7	133861224	133861433	5Y-H4K8ac_peak_15849	4.03329	
7	134001252	134001559	5Y-H4K8ac_peak_15850	5.60566	SLC35B4_ENSG00000205060
7	134143542	134143783	5Y-H4K8ac_peak_15851	5.41472	AKR1B1_ENSG00000085662
7	134671586	134671935	5Y-H4K8ac_peak_15852	7.03573	AGBL3_ENSG00000146856
7	134854905	134855477	5Y-H4K8ac_peak_15853	22.69022	C7orf49_ENSG00000122783
7	134895871	134896156	5Y-H4K8ac_peak_15854	6.49458	WDR91_ENSG00000105875
7	135005764	135005964	5Y-H4K8ac_peak_15855	9.14889	
7	135006850	135007230	5Y-H4K8ac_peak_15856	8.24461	
7	135242495	135243200	5Y-H4K8ac_peak_15857	10.21117	NUP205_ENSG00000155561
7	135346375	135347024	5Y-H4K8ac_peak_15858	8.39119	C7orf73_ENSG00000243317
7	135396399	135396607	5Y-H4K8ac_peak_15859	5.64909	
7	135398907	135399712	5Y-H4K8ac_peak_15860	15.71541	
7	135486565	135486835	5Y-H4K8ac_peak_15861	6.96612	
7	135662544	135662755	5Y-H4K8ac_peak_15862	7.15925	MTPN_ENSG00000105887
7	136553814	136554392	5Y-H4K8ac_peak_15863	8.24461	CHRM2_ENSG00000181072
7	136623298	136623492	5Y-H4K8ac_peak_15864	8.2913	KRT8P51_ENSG00000223657
7	137693345	137693626	5Y-H4K8ac_peak_15865	9.59236	
7	137837922	137838204	5Y-H4K8ac_peak_15866	7.11863	
7	137838951	137839232	5Y-H4K8ac_peak_15867	12.2918	
7	137937563	137937771	5Y-H4K8ac_peak_15868	4.98439	

7	138145299	138145599	5Y-H4K8ac_peak_15869	9.68742	TRIM24_ENSG00000122779
7	138258379	138258646	5Y-H4K8ac_peak_15870	5.87382	
7	138304595	138304827	5Y-H4K8ac_peak_15871	8.2913	
7	138349105	138349453	5Y-H4K8ac_peak_15872	5.70209	
7	138365052	138365459	5Y-H4K8ac_peak_15873	4.0639	
7	138621062	138621269	5Y-H4K8ac_peak_15874	6.78318	
7	138665691	138665960	5Y-H4K8ac_peak_15875	12.52642	KIAA1549_ENSG00000122778
7	138666361	138666958	5Y-H4K8ac_peak_15876	12.26819	KIAA1549_ENSG00000122778
7	138720231	138720719	5Y-H4K8ac_peak_15877	8.39119	ZC3HAV1L_ENSG00000146858
7	138721040	138721403	5Y-H4K8ac_peak_15878	5.54834	ZC3HAV1L_ENSG00000146858
7	138793578	138794133	5Y-H4K8ac_peak_15879	10.14791	ZC3HAV1_ENSG00000105939
7	139024306	139024785	5Y-H4K8ac_peak_15880	5.87918	C7orf55_ENSG00000164898;C7orf55-LUC7L2_ENSG00000146963
7	139025626	139026398	5Y-H4K8ac_peak_15881	13.59183	C7orf55-LUC7L2_ENSG00000146963;LUC7L2_ENSG00000269955
7	139043646	139044350	5Y-H4K8ac_peak_15882	10.60083	RP11-634H22.1_ENSG00000273391
7	139044778	139044969	5Y-H4K8ac_peak_15883	7.61781	RP11-634H22.1_ENSG00000273391
7	139477974	139478510	5Y-H4K8ac_peak_15884	7.50148	HIPK2_ENSG00000064393
7	139691837	139692030	5Y-H4K8ac_peak_15885	5.23083	
7	139762685	139763052	5Y-H4K8ac_peak_15886	4.18845	PARP12_ENSG00000059378
7	139930021	139930463	5Y-H4K8ac_peak_15887	8.58926	
7	140046443	140046817	5Y-H4K8ac_peak_15888	4.15658	
7	140098701	140099076	5Y-H4K8ac_peak_15889	12.2918	
7	140340035	140341268	5Y-H4K8ac_peak_15890	12.26819	
7	140341552	140341748	5Y-H4K8ac_peak_15891	5.22219	
7	140346708	140346953	5Y-H4K8ac_peak_15892	7.09658	
7	140372797	140373677	5Y-H4K8ac_peak_15893	5.03564	DENND2A_ENSG00000146966;ADCK2_ENSG00000133597
7	140395803	140396149	5Y-H4K8ac_peak_15894	9.37778	NDUFB2-AS1_ENSG00000240889
7	140624134	140624474	5Y-H4K8ac_peak_15895	11.4254	BRAF_ENSG00000157764
7	140624925	140625143	5Y-H4K8ac_peak_15896	8.43511	BRAF_ENSG00000157764
7	140714511	140714739	5Y-H4K8ac_peak_15897	11.1169	MRPS33_ENSG00000090263
7	140714964	140715365	5Y-H4K8ac_peak_15898	10.1994	MRPS33_ENSG00000090263
7	140773827	140774600	5Y-H4K8ac_peak_15899	6.43068	TMEM178B_ENSG00000261115
7	141250494	141250863	5Y-H4K8ac_peak_15900	9.58986	RP11-744I24.2_ENSG00000261570;AGK_ENSG00000006530
7	141251281	141251497	5Y-H4K8ac_peak_15901	8.99358	RP11-744I24.2_ENSG00000261570;AGK_ENSG00000006530
7	141401433	141401832	5Y-H4K8ac_peak_15902	6.98416	KIAA1147_ENSG00000257093
7	142960621	142960846	5Y-H4K8ac_peak_15903	7.89273	
7	142984625	142985064	5Y-H4K8ac_peak_15904	6.53157	AC073342.12_ENSG00000231840;CASP2_ENSG00000106144
7	142985522	142985843	5Y-H4K8ac_peak_15905	13.33225	CASP2_ENSG00000106144
7	143042434	143042658	5Y-H4K8ac_peak_15906	7.31815	
7	143077526	143078174	5Y-H4K8ac_peak_15907	14.25523	AC093673.5_ENSG00000232533;ZYX_ENSG00000159840
7	143078380	143078980	5Y-H4K8ac_peak_15908	16.54982	AC093673.5_ENSG00000232533;ZYX_ENSG00000159840
7	143599738	143600067	5Y-H4K8ac_peak_15909	10.1994	FAM115A_ENSG00000198420
7	144533232	144533677	5Y-H4K8ac_peak_15910	7.11863	TPK1_ENSG00000196511
7	145942500	145942717	5Y-H4K8ac_peak_15911	8.33296	
7	147281776	147282093	5Y-H4K8ac_peak_15912	13.06587	
7	147501641	147501993	5Y-H4K8ac_peak_15913	6.47245	
7	148395217	148396478	5Y-H4K8ac_peak_15914	10.86648	RP5-1136G13.2_ENSG00000273314;CUL1_ENSG00000055130

7	148582005	148582671	5Y-H4K8ac_peak_15915	11.29141	EZH2_ENSG00000106462
7	148638174	148638365	5Y-H4K8ac_peak_15916	6.22669	RNY5_ENSG00000252310
7	148725095	148725498	5Y-H4K8ac_peak_15917	17.67055	PDIA4_ENSG00000155660
7	148762446	148762942	5Y-H4K8ac_peak_15918	9.37812	
7	148787504	148787708	5Y-H4K8ac_peak_15919	5.45148	ZNF786_ENSG00000197362
7	148788076	148788654	5Y-H4K8ac_peak_15920	15.2347	ZNF786_ENSG00000197362
7	148822960	148823315	5Y-H4K8ac_peak_15921	10.60083	ZNF425_ENSG00000204947;RN7SL521P_ENSG00000240877;ZNF398_ENSG00000197024
7	148823763	148824309	5Y-H4K8ac_peak_15922	8.62703	ZNF425_ENSG00000204947;RN7SL521P_ENSG00000240877;ZNF398_ENSG00000197024
7	148936871	148937135	5Y-H4K8ac_peak_15923	15.10927	ZNF212_ENSG00000170260
7	148958519	148959155	5Y-H4K8ac_peak_15924	12.72376	ZNF783_ENSG00000204946
7	148959353	148960150	5Y-H4K8ac_peak_15925	9.74838	ZNF783_ENSG00000204946
7	149119039	149119527	5Y-H4K8ac_peak_15926	10.35586	RP11-143I21.1_ENSG00000261842
7	149119842	149120152	5Y-H4K8ac_peak_15927	12.44818	RP11-143I21.1_ENSG00000261842
7	149157858	149158340	5Y-H4K8ac_peak_15928	6.56228	ZNF777_ENSG00000196453
7	149195115	149195383	5Y-H4K8ac_peak_15929	10.54764	ZNF746_ENSG00000181220
7	149321456	149321753	5Y-H4K8ac_peak_15930	11.4254	ZNF767_ENSG00000133624
7	149321961	149322298	5Y-H4K8ac_peak_15931	16.93214	ZNF767_ENSG00000133624
7	149411186	149411688	5Y-H4K8ac_peak_15932	9.51254	KRBA1_ENSG00000133619
7	149412106	149412303	5Y-H4K8ac_peak_15933	6.34245	KRBA1_ENSG00000133619
7	149439634	149439835	5Y-H4K8ac_peak_15934	9.62747	
7	149468404	149468634	5Y-H4K8ac_peak_15935	6.53157	
7	149472934	149473630	5Y-H4K8ac_peak_15936	9.79526	SSPO_ENSG00000197558
7	149474111	149474404	5Y-H4K8ac_peak_15937	6.47245	SSPO_ENSG00000197558
7	150020422	150020660	5Y-H4K8ac_peak_15938	12.56588	ACTR3C_ENSG00000106526;LRRC61_ENSG00000127399
7	150065168	150066086	5Y-H4K8ac_peak_15939	22.56714	REPIN1_ENSG00000214022;ZNF775_ENSG00000196456
7	150075737	150076045	5Y-H4K8ac_peak_15940	7.50464	
7	150101937	150102326	5Y-H4K8ac_peak_15941	16.73191	
7	150485108	150485616	5Y-H4K8ac_peak_15942	6.98416	
7	150518185	150518574	5Y-H4K8ac_peak_15943	6.37023	
7	150595893	150596262	5Y-H4K8ac_peak_15944	9.00954	
7	150597983	150598330	5Y-H4K8ac_peak_15945	7.8267	
7	150599222	150599475	5Y-H4K8ac_peak_15946	7.89273	
7	150651419	150651632	5Y-H4K8ac_peak_15947	5.87725	
7	150651967	150652858	5Y-H4K8ac_peak_15948	13.82621	
7	150653198	150653442	5Y-H4K8ac_peak_15949	7.20869	
7	150653707	150654203	5Y-H4K8ac_peak_15950	11.97508	
7	150659094	150659302	5Y-H4K8ac_peak_15951	6.92623	
7	150660433	150660629	5Y-H4K8ac_peak_15952	6.69752	
7	150661428	150661699	5Y-H4K8ac_peak_15953	11.8865	
7	150662186	150662652	5Y-H4K8ac_peak_15954	11.09899	
7	150667637	150669168	5Y-H4K8ac_peak_15955	21.95299	
7	150669430	150669685	5Y-H4K8ac_peak_15956	10.90097	
7	150671917	150673005	5Y-H4K8ac_peak_15957	11.6706	
7	150674996	150675300	5Y-H4K8ac_peak_15958	13.00733	KCNH2_ENSG00000055118
7	150676172	150676425	5Y-H4K8ac_peak_15959	5.37237	KCNH2_ENSG00000055118
7	150676842	150677105	5Y-H4K8ac_peak_15960	11.33673	

7	150677469	150677685	5Y-H4K8ac_peak_15961	4.83834	
7	150685073	150685427	5Y-H4K8ac_peak_15962	6.95844	
7	150686221	150686477	5Y-H4K8ac_peak_15963	7.61515	
7	150690362	150690565	5Y-H4K8ac_peak_15964	6.22904	
7	150691706	150692360	5Y-H4K8ac_peak_15965	6.53157	
7	150710990	150711487	5Y-H4K8ac_peak_15966	7.90005	
7	150714557	150715847	5Y-H4K8ac_peak_15967	13.89096	
7	150735713	150736292	5Y-H4K8ac_peak_15968	10.60083	
7	150755395	150755854	5Y-H4K8ac_peak_15969	8.69112	CDK5_ENSG00000164885
7	150777409	150777821	5Y-H4K8ac_peak_15970	9.79526	FASTK_ENSG00000164896
7	150778142	150779163	5Y-H4K8ac_peak_15971	6.98416	FASTK_ENSG00000164896
7	150779676	150779869	5Y-H4K8ac_peak_15972	11.01067	TMUB1_ENSG00000164897
7	150780223	150780626	5Y-H4K8ac_peak_15973	12.45737	TMUB1_ENSG00000164897
7	150782827	150783201	5Y-H4K8ac_peak_15974	6.37023	AGAP3_ENSG00000133612
7	150929042	150929448	5Y-H4K8ac_peak_15975	12.06963	CHPF2_ENSG00000033100
7	150929658	150929885	5Y-H4K8ac_peak_15976	11.22005	CHPF2_ENSG00000033100
7	150942383	150942935	5Y-H4K8ac_peak_15977	8.09118	
7	150946169	150946421	5Y-H4K8ac_peak_15978	7.11863	RP5-1070G24.2_ENSG00000243018
7	150948514	150948901	5Y-H4K8ac_peak_15979	15.17389	
7	150949128	150949925	5Y-H4K8ac_peak_15980	12.91503	
7	150974034	150974655	5Y-H4K8ac_peak_15981	14.86775	SMARCD3_ENSG00000082014
7	151104417	151104609	5Y-H4K8ac_peak_15982	4.55294	
7	151106733	151107695	5Y-H4K8ac_peak_15983	11.67272	WDR86_ENSG00000187260;WDR86-AS1_ENSG00000243836
7	151136911	151137168	5Y-H4K8ac_peak_15984	4.0639	CRYGN_ENSG00000127377
7	151217451	151217926	5Y-H4K8ac_peak_15985	10.86648	RHEB_ENSG00000106615
7	151236390	151236636	5Y-H4K8ac_peak_15986	7.61752	
7	151329038	151329237	5Y-H4K8ac_peak_15987	4.52203	
7	151329604	151329849	5Y-H4K8ac_peak_15988	9.54288	
7	151409489	151409719	5Y-H4K8ac_peak_15989	5.30014	
7	151574252	151574852	5Y-H4K8ac_peak_15990	14.75477	PRKAG2_ENSG00000106617;PRKAG2-AS1_ENSG00000239911
7	151575066	151575511	5Y-H4K8ac_peak_15991	6.55841	PRKAG2_ENSG00000106617;PRKAG2-AS1_ENSG00000239911
7	151622461	151622756	5Y-H4K8ac_peak_15992	5.23083	
7	151630050	151630411	5Y-H4K8ac_peak_15993	5.76631	
7	151722325	151722594	5Y-H4K8ac_peak_15994	5.99756	GALNT11_ENSG00000178234
7	151722921	151723162	5Y-H4K8ac_peak_15995	7.48932	GALNT11_ENSG00000178234
7	152132706	152132990	5Y-H4K8ac_peak_15996	11.19336	KMT2C_ENSG00000055609;FABP5P3_ENSG00000241735
7	152133404	152133644	5Y-H4K8ac_peak_15997	11.22005	KMT2C_ENSG00000055609;FABP5P3_ENSG00000241735
7	152161322	152162090	5Y-H4K8ac_peak_15998	16.0768	LINC01003_ENSG00000261455
7	152240944	152241145	5Y-H4K8ac_peak_15999	6.3265	
7	152311873	152312072	5Y-H4K8ac_peak_16000	6.22904	
7	152372838	152373067	5Y-H4K8ac_peak_16001	4.8547	XRCC2_ENSG00000196584
7	152433385	152433596	5Y-H4K8ac_peak_16002	6.78128	
7	152456971	152457191	5Y-H4K8ac_peak_16003	6.40778	ACTR3B_ENSG00000133627
7	152463318	152463517	5Y-H4K8ac_peak_16004	6.77436	
7	153631964	153632380	5Y-H4K8ac_peak_16005	9.87097	
7	153863315	153863536	5Y-H4K8ac_peak_16006	6.37023	

7	154001876	154002107	5Y-H4K8ac_peak_16007	16.74969	
7	154662830	154663066	5Y-H4K8ac_peak_16008	4.77126	
7	154794868	154795504	5Y-H4K8ac_peak_16009	19.99409	PAXIP1_ENSG00000157212;PAXIP1-AS1_ENSG00000273344
7	155029373	155029626	5Y-H4K8ac_peak_16010	5.0078	
7	155049360	155049894	5Y-H4K8ac_peak_16011	7.20869	
7	155088802	155089372	5Y-H4K8ac_peak_16012	17.3057	AC144652.1_ENSG00000273117;INSIG1_ENSG00000186480
7	155089762	155090019	5Y-H4K8ac_peak_16013	6.67135	AC144652.1_ENSG00000273117;INSIG1_ENSG00000186480
7	155137354	155137704	5Y-H4K8ac_peak_16014	18.10232	
7	155436342	155437841	5Y-H4K8ac_peak_16015	12.37752	AC009403.2_ENSG00000216895;RBM33_ENSG00000184863
7	156400004	156400638	5Y-H4K8ac_peak_16016	9.30206	
7	156432804	156433721	5Y-H4K8ac_peak_16017	13.33225	LINC01006_ENSG00000182648;C7orf13_ENSG00000244291;RNF32_ENSG00000105982
7	156577056	156577273	5Y-H4K8ac_peak_16018	9.13822	
7	156685081	156685293	5Y-H4K8ac_peak_16019	5.64982	LMBR1_ENSG00000105983
7	156686039	156686466	5Y-H4K8ac_peak_16020	8.69112	LMBR1_ENSG00000105983
7	156741748	156741945	5Y-H4K8ac_peak_16021	8.5915	NOM1_ENSG00000146909
7	156742479	156742935	5Y-H4K8ac_peak_16022	8.04693	NOM1_ENSG00000146909
7	157069681	157069956	5Y-H4K8ac_peak_16023	8.5915	
7	157071082	157071300	5Y-H4K8ac_peak_16024	4.0639	
7	157129155	157129514	5Y-H4K8ac_peak_16025	13.1133	
7	157190659	157190952	5Y-H4K8ac_peak_16026	4.725	
7	157257439	157257758	5Y-H4K8ac_peak_16027	5.67283	
7	157483348	157483563	5Y-H4K8ac_peak_16028	7.41197	
7	157544979	157546851	5Y-H4K8ac_peak_16029	9.88714	
7	157547064	157547375	5Y-H4K8ac_peak_16030	6.43068	
7	157698622	157698867	5Y-H4K8ac_peak_16031	5.59843	
7	157719534	157719759	5Y-H4K8ac_peak_16032	4.95697	
7	157784427	157785073	5Y-H4K8ac_peak_16033	9.51254	
7	157890602	157891352	5Y-H4K8ac_peak_16034	10.64267	
7	157903447	157903805	5Y-H4K8ac_peak_16035	21.37457	
7	157904261	157904475	5Y-H4K8ac_peak_16036	6.86362	
7	157904823	157905198	5Y-H4K8ac_peak_16037	5.23083	
7	158066389	158067511	5Y-H4K8ac_peak_16038	5.72233	
7	158221865	158222218	5Y-H4K8ac_peak_16039	7.50148	
7	158380858	158381400	5Y-H4K8ac_peak_16040	25.34984	PTPRN2_ENSG00000155093
7	158497868	158498233	5Y-H4K8ac_peak_16041	11.30548	NCAPG2_ENSG00000146918
7	158622464	158622759	5Y-H4K8ac_peak_16042	6.80915	ESYT2_ENSG00000117868
7	158648697	158648915	5Y-H4K8ac_peak_16043	9.38276	WDR60_ENSG00000126870
7	158768777	158769065	5Y-H4K8ac_peak_16044	5.57299	
7	158823250	158823783	5Y-H4K8ac_peak_16045	7.74473	
7	158896939	158897142	5Y-H4K8ac_peak_16046	3.94555	
7	158899851	158900407	5Y-H4K8ac_peak_16047	6.37023	
8	182479	183022	5Y-H4K8ac_peak_16048	10.19948	RPL23AP53_ENSG00000223508;ZNF596_ENSG00000172748;RP5-855D21.3_ENSG00000272812
8	340844	341401	5Y-H4K8ac_peak_16049	5.65584	
8	356975	357227	5Y-H4K8ac_peak_16050	9.23159	FBXO25_ENSG00000147364
8	681446	681784	5Y-H4K8ac_peak_16051	6.08523	
8	1145135	1145345	5Y-H4K8ac_peak_16052	7.37325	

8	1203151	1203342	5Y-H4K8ac_peak_16053	6.54441	
8	1287457	1287660	5Y-H4K8ac_peak_16054	8.33296	
8	1321242	1321916	5Y-H4K8ac_peak_16055	17.51434	
8	1426725	1427060	5Y-H4K8ac_peak_16056	4.728	
8	1431412	1431666	5Y-H4K8ac_peak_16057	5.51139	
8	1472227	1472489	5Y-H4K8ac_peak_16058	5.40331	
8	1474984	1475192	5Y-H4K8ac_peak_16059	8.75926	
8	1476468	1476697	5Y-H4K8ac_peak_16060	4.95697	
8	1922475	1922675	5Y-H4K8ac_peak_16061	7.50148	RP11-439C15.4_ENSG00000253764;KBTBD11_ENSG00000176595;RP11-439C15.5_ENSG00000270988
8	1949357	1949868	5Y-H4K8ac_peak_16062	10.19948	
8	6264605	6264831	5Y-H4K8ac_peak_16063	8.33296	RP11-115C21.2_ENSG00000246089;MCPH1_ENSG00000147316
8	6320465	6321128	5Y-H4K8ac_peak_16064	6.50117	
8	6322071	6322429	5Y-H4K8ac_peak_16065	9.15007	
8	6565943	6566541	5Y-H4K8ac_peak_16066	11.19336	CTD-2541M15.1_ENSG00000249898;AGPAT5_ENSG00000155189
8	7212401	7212811	5Y-H4K8ac_peak_16067	18.82931	FAM66B_ENSG00000215374
8	8243630	8243845	5Y-H4K8ac_peak_16068	5.65584	SGK223_ENSG00000182319
8	8244334	8244546	5Y-H4K8ac_peak_16069	8.33296	SGK223_ENSG00000182319
8	8267344	8267674	5Y-H4K8ac_peak_16070	6.34046	
8	8506909	8507105	5Y-H4K8ac_peak_16071	9.30505	
8	8559408	8559928	5Y-H4K8ac_peak_16072	10.79063	CLDN23_ENSG00000253958;AC087269.1_ENSG00000176305
8	9007913	9009056	5Y-H4K8ac_peak_16073	10.19948	PPP1R3B_ENSG00000173281;RP11-10A14.4_ENSG00000253426
8	9031908	9032280	5Y-H4K8ac_peak_16074	5.718	
8	9035382	9035724	5Y-H4K8ac_peak_16075	8.43511	
8	9037760	9037970	5Y-H4K8ac_peak_16076	6.17374	
8	9041409	9041661	5Y-H4K8ac_peak_16077	9.74189	
8	9048789	9048993	5Y-H4K8ac_peak_16078	4.74398	
8	9056214	9056436	5Y-H4K8ac_peak_16079	9.30505	
8	9056919	9057810	5Y-H4K8ac_peak_16080	5.64909	
8	9110683	9111030	5Y-H4K8ac_peak_16081	6.20875	
8	9763876	9764251	5Y-H4K8ac_peak_16082	4.44548	
8	9791589	9791962	5Y-H4K8ac_peak_16083	7.31815	
8	9912362	9912726	5Y-H4K8ac_peak_16084	10.69698	RP11-1E4.1_ENSG00000260093;MSRA_ENSG00000175806
8	10190919	10191489	5Y-H4K8ac_peak_16085	5.98695	
8	10235117	10235686	5Y-H4K8ac_peak_16086	5.98695	
8	10501815	10502075	5Y-H4K8ac_peak_16087	8.04493	
8	10588480	10588754	5Y-H4K8ac_peak_16088	8.73985	
8	10635166	10635546	5Y-H4K8ac_peak_16089	13.2534	
8	10851071	10851294	5Y-H4K8ac_peak_16090	4.74398	
8	10910356	10910735	5Y-H4K8ac_peak_16091	8.49231	
8	11058620	11058822	5Y-H4K8ac_peak_16092	9.01738	XKR6_ENSG00000171044
8	11059121	11059323	5Y-H4K8ac_peak_16093	14.99673	XKR6_ENSG00000171044
8	11141322	11141824	5Y-H4K8ac_peak_16094	15.06486	MTMR9_ENSG00000104643
8	11142053	11142459	5Y-H4K8ac_peak_16095	12.05638	MTMR9_ENSG00000104643
8	11269563	11269858	5Y-H4K8ac_peak_16096	13.19488	
8	11306656	11307201	5Y-H4K8ac_peak_16097	7.60057	
8	11311746	11312026	5Y-H4K8ac_peak_16098	8.43511	

8	11317890	11318290	5Y-H4K8ac_peak_16099	4.90696	
8	11318560	11318951	5Y-H4K8ac_peak_16100	10.14606	
8	11322670	11322862	5Y-H4K8ac_peak_16101	6.95844	
8	11324888	11325135	5Y-H4K8ac_peak_16102	8.69112	
8	11376044	11376518	5Y-H4K8ac_peak_16103	6.52593	
8	11450904	11451147	5Y-H4K8ac_peak_16104	5.23083	
8	11451778	11452193	5Y-H4K8ac_peak_16105	6.77436	
8	11466281	11466471	5Y-H4K8ac_peak_16106	5.10825	
8	11469606	11469934	5Y-H4K8ac_peak_16107	11.19336	
8	11470204	11470525	5Y-H4K8ac_peak_16108	9.05168	
8	11470791	11471626	5Y-H4K8ac_peak_16109	17.50045	
8	11489196	11489398	5Y-H4K8ac_peak_16110	5.41472	
8	11490167	11490373	5Y-H4K8ac_peak_16111	5.68769	
8	11498508	11498954	5Y-H4K8ac_peak_16112	8.21582	
8	11499800	11500027	5Y-H4K8ac_peak_16113	6.34046	
8	11501199	11501817	5Y-H4K8ac_peak_16114	12.20548	
8	11599340	11599746	5Y-H4K8ac_peak_16115	8.59175	
8	11599981	11601095	5Y-H4K8ac_peak_16116	14.49096	
8	11725747	11725994	5Y-H4K8ac_peak_16117	13.2534	CTSB_ENSG00000164733
8	11759932	11760372	5Y-H4K8ac_peak_16118	6.77436	
8	11807404	11807778	5Y-H4K8ac_peak_16119	12.05638	
8	11814996	11815296	5Y-H4K8ac_peak_16120	11.14154	
8	11843775	11843978	5Y-H4K8ac_peak_16121	5.65584	
8	11973441	11973727	5Y-H4K8ac_peak_16122	6.34046	FAM66D_ENSG00000255052
8	12052413	12052616	5Y-H4K8ac_peak_16123	12.71156	FAM86B1_ENSG00000186523;FAM85A_ENSG00000255495
8	12219735	12219962	5Y-H4K8ac_peak_16124	7.31815	FAM66A_ENSG00000227888
8	12293415	12293611	5Y-H4K8ac_peak_16125	6.78128	FAM86B2_ENSG00000145002
8	12611222	12611426	5Y-H4K8ac_peak_16126	11.19336	
8	12613193	12613484	5Y-H4K8ac_peak_16127	6.37023	LONRF1_ENSG00000154359
8	13133706	13133981	5Y-H4K8ac_peak_16128	8.33296	
8	15397807	15398164	5Y-H4K8ac_peak_16129	15.84891	
8	16884859	16885184	5Y-H4K8ac_peak_16130	11.57334	MICU3_ENSG00000155970
8	17014242	17014596	5Y-H4K8ac_peak_16131	7.31815	ZDHHC2_ENSG00000104219
8	17151454	17151651	5Y-H4K8ac_peak_16132	4.9885	
8	17271168	17271556	5Y-H4K8ac_peak_16133	7.58806	MTMR7_ENSG00000003987
8	17484470	17484663	5Y-H4K8ac_peak_16134	6.62622	
8	17527287	17527546	5Y-H4K8ac_peak_16135	5.59837	
8	17538173	17538434	5Y-H4K8ac_peak_16136	4.40812	MIR548V_ENSG00000265520
8	17780600	17780800	5Y-H4K8ac_peak_16137	8.46442	PCM1_ENSG00000078674
8	17941664	17942047	5Y-H4K8ac_peak_16138	9.15007	ASAH1_ENSG00000104763;CTD-2547L16.1_ENSG00000245281
8	18525408	18525627	5Y-H4K8ac_peak_16139	5.19372	
8	19050944	19051335	5Y-H4K8ac_peak_16140	8.35139	
8	20038235	20038449	5Y-H4K8ac_peak_16141	5.13364	
8	20092258	20092617	5Y-H4K8ac_peak_16142	5.03917	
8	20125909	20126141	5Y-H4K8ac_peak_16143	5.10825	
8	20126443	20126797	5Y-H4K8ac_peak_16144	8.04943	

8	20161681	20161878	5Y-H4K8ac_peak_16145	8.16031	LZTS1_ENSG00000061337
8	21867230	21867521	5Y-H4K8ac_peak_16146	7.31102	
8	21867952	21868590	5Y-H4K8ac_peak_16147	14.6127	
8	21910964	21911367	5Y-H4K8ac_peak_16148	8.90223	
8	21912397	21913020	5Y-H4K8ac_peak_16149	7.19446	
8	21913294	21914048	5Y-H4K8ac_peak_16150	8.04586	
8	21914648	21914894	5Y-H4K8ac_peak_16151	5.24695	
8	21915128	21915730	5Y-H4K8ac_peak_16152	7.59101	
8	21916184	21916462	5Y-H4K8ac_peak_16153	11.4254	
8	21923685	21924325	5Y-H4K8ac_peak_16154	9.00954	
8	21990686	21991113	5Y-H4K8ac_peak_16155	10.1994	HR_ENSG00000168453
8	22021980	22022177	5Y-H4K8ac_peak_16156	8.43511	BMP1_ENSG00000168487
8	22068817	22069686	5Y-H4K8ac_peak_16157	7.89273	
8	22070171	22070731	5Y-H4K8ac_peak_16158	7.89273	
8	22089181	22089423	5Y-H4K8ac_peak_16159	4.61816	PHYHIP_ENSG00000168490
8	22297896	22298464	5Y-H4K8ac_peak_16160	12.77513	PPP3CC_ENSG00000120910
8	22422494	22422693	5Y-H4K8ac_peak_16161	9.05168	
8	22423676	22423899	5Y-H4K8ac_peak_16162	8.26455	RP11-582J16.3_ENSG00000254230
8	22457100	22457428	5Y-H4K8ac_peak_16163	13.3385	C8orf58_ENSG00000241852
8	22461690	22461966	5Y-H4K8ac_peak_16164	10.35586	CCAR2_ENSG00000158941
8	22462356	22462619	5Y-H4K8ac_peak_16165	9.30206	CCAR2_ENSG00000158941
8	22551965	22552286	5Y-H4K8ac_peak_16166	4.15658	
8	22553143	22553542	5Y-H4K8ac_peak_16167	8.16382	
8	22722524	22723579	5Y-H4K8ac_peak_16168	15.44294	
8	22726392	22726640	5Y-H4K8ac_peak_16169	5.65584	
8	22768844	22769115	5Y-H4K8ac_peak_16170	4.79658	
8	22853286	22853775	5Y-H4K8ac_peak_16171	9.76527	
8	22925749	22926323	5Y-H4K8ac_peak_16172	5.98695	TNFRSF10B_ENSG00000120889;RP11-875O11.2_ENSG00000246130
8	23145658	23145957	5Y-H4K8ac_peak_16173	14.19462	
8	23193036	23193349	5Y-H4K8ac_peak_16174	12.44602	RP11-177H13.2_ENSG00000253837
8	23193803	23194159	5Y-H4K8ac_peak_16175	7.59101	RP11-177H13.2_ENSG00000253837
8	23224988	23225213	5Y-H4K8ac_peak_16176	6.22669	
8	23315457	23315860	5Y-H4K8ac_peak_16177	8.33296	CTC-756D1.2_ENSG00000253390;ENTPD4_ENSG00000197217
8	23385990	23386206	5Y-H4K8ac_peak_16178	7.96225	SLC25A37_ENSG00000147454
8	24799764	24800204	5Y-H4K8ac_peak_16179	9.30505	
8	24813412	24813789	5Y-H4K8ac_peak_16180	5.59843	NEFL_ENSG00000104725;CTD-2168K21.2_ENSG00000272157
8	24858122	24858500	5Y-H4K8ac_peak_16181	4.77126	
8	24858695	24859011	5Y-H4K8ac_peak_16182	4.9885	
8	24866807	24867150	5Y-H4K8ac_peak_16183	6.34046	
8	24921764	24922011	5Y-H4K8ac_peak_16184	5.8635	
8	26307193	26307558	5Y-H4K8ac_peak_16185	12.14232	
8	26429089	26429290	5Y-H4K8ac_peak_16186	5.56912	
8	26434424	26435157	5Y-H4K8ac_peak_16187	11.22005	
8	26920716	26921391	5Y-H4K8ac_peak_16188	10.31981	
8	27116305	27116582	5Y-H4K8ac_peak_16189	8.21582	STMN4_ENSG00000015592
8	27169199	27169463	5Y-H4K8ac_peak_16190	8.2913	TRIM35_ENSG00000104228;PTK2B_ENSG00000120899

8	27450031	27450292	5Y-H4K8ac_peak_16191	6.14981	
8	27491110	27491826	5Y-H4K8ac_peak_16192	8.21582	SCARA3_ENSG00000168077
8	27629854	27630104	5Y-H4K8ac_peak_16193	8.69112	CCDC25_ENSG00000147419;ESCO2_ENSG00000171320
8	27695476	27696026	5Y-H4K8ac_peak_16194	5.23083	PBK_ENSG00000168078;RP11-1149M3.2_ENSG00000253176
8	27749696	27749893	5Y-H4K8ac_peak_16195	4.77126	
8	27752094	27752336	5Y-H4K8ac_peak_16196	4.95697	
8	28190304	28190528	5Y-H4K8ac_peak_16197	10.42746	
8	28269173	28270264	5Y-H4K8ac_peak_16198	11.1169	
8	28351283	28351571	5Y-H4K8ac_peak_16199	8.2913	FZD3_ENSG00000104290
8	28558623	28558833	5Y-H4K8ac_peak_16200	7.89273	EXTL3-AS1_ENSG00000246339
8	28559237	28559618	5Y-H4K8ac_peak_16201	8.16382	EXTL3-AS1_ENSG00000246339
8	28748211	28748445	5Y-H4K8ac_peak_16202	6.78318	INTS9_ENSG00000104299;HMBOX1_ENSG00000147421
8	29208563	29208754	5Y-H4K8ac_peak_16203	7.11863	DUSP4_ENSG00000120875
8	29209375	29209761	5Y-H4K8ac_peak_16204	8.24461	RP4-676L2.1_ENSG00000260253
8	29210057	29210400	5Y-H4K8ac_peak_16205	6.18374	RP4-676L2.1_ENSG00000260253
8	29230458	29231122	5Y-H4K8ac_peak_16206	9.74189	
8	29629924	29630124	5Y-H4K8ac_peak_16207	5.85188	
8	29632226	29632420	5Y-H4K8ac_peak_16208	7.59101	
8	29655410	29655647	5Y-H4K8ac_peak_16209	4.50834	
8	29662230	29662907	5Y-H4K8ac_peak_16210	9.52603	
8	29738082	29738278	5Y-H4K8ac_peak_16211	6.53157	
8	29952246	29953089	5Y-H4K8ac_peak_16212	14.01193	LEPROTL1_ENSG00000104660
8	30013933	30014537	5Y-H4K8ac_peak_16213	9.23159	RP11-51J9.5_ENSG00000271869;DCTN6_ENSG00000104671
8	30241666	30241983	5Y-H4K8ac_peak_16214	4.95697	RBPMS-AS1_ENSG00000254109;RBPMS_ENSG00000157110
8	30242488	30242851	5Y-H4K8ac_peak_16215	7.72345	RBPMS-AS1_ENSG00000254109;RBPMS_ENSG00000157110
8	30280518	30280732	5Y-H4K8ac_peak_16216	8.79957	
8	30458732	30459011	5Y-H4K8ac_peak_16217	7.31815	
8	30601048	30601810	5Y-H4K8ac_peak_16218	9.96543	
8	30602030	30602306	5Y-H4K8ac_peak_16219	7.59101	
8	30611470	30611661	5Y-H4K8ac_peak_16220	5.40331	RP11-465K4.4_ENSG00000271081
8	30670046	30670318	5Y-H4K8ac_peak_16221	14.15915	
8	31497858	31498282	5Y-H4K8ac_peak_16222	5.59843	NRG1_ENSG00000157168
8	32504545	32504755	5Y-H4K8ac_peak_16223	4.07874	
8	33371819	33372363	5Y-H4K8ac_peak_16224	6.50117	TTI2_ENSG00000129696;SNORD13_ENSG00000239039
8	33457812	33458282	5Y-H4K8ac_peak_16225	4.95697	DUSP26_ENSG00000133878
8	35764041	35764317	5Y-H4K8ac_peak_16226	7.31102	
8	36931364	36931567	5Y-H4K8ac_peak_16227	12.49811	
8	37105094	37105333	5Y-H4K8ac_peak_16228	5.64909	
8	37105561	37105861	5Y-H4K8ac_peak_16229	7.76232	
8	37182837	37183161	5Y-H4K8ac_peak_16230	5.48291	
8	37376744	37376956	5Y-H4K8ac_peak_16231	7.07936	
8	37549046	37549267	5Y-H4K8ac_peak_16232	6.46053	
8	37593494	37593926	5Y-H4K8ac_peak_16233	9.51254	ERLIN2_ENSG00000147475
8	37641518	37641726	5Y-H4K8ac_peak_16234	6.14981	GPR124_ENSG00000020181
8	37654006	37654237	5Y-H4K8ac_peak_16235	4.642	
8	37660009	37660569	5Y-H4K8ac_peak_16236	13.12848	

8	37660975	37661470	5Y-H4K8ac_peak_16237	9.00954	
8	37683362	37684090	5Y-H4K8ac_peak_16238	6.37023	
8	37684634	37684882	5Y-H4K8ac_peak_16239	5.59843	
8	37887468	37887690	5Y-H4K8ac_peak_16240	5.70472	EIF4EBP1_ENSG00000187840
8	37888123	37888797	5Y-H4K8ac_peak_16241	6.37023	EIF4EBP1_ENSG00000187840
8	37889182	37889376	5Y-H4K8ac_peak_16242	7.60057	
8	38034404	38034673	5Y-H4K8ac_peak_16243	6.50117	LSM1_ENSG00000175324;BAG4_ENSG00000156735
8	38239460	38239724	5Y-H4K8ac_peak_16244	7.50148	WHSC1L1_ENSG00000147548;RP11-350N15.5_ENSG00000272092
8	38240048	38240374	5Y-H4K8ac_peak_16245	7.21591	WHSC1L1_ENSG00000147548;RP11-350N15.5_ENSG00000272092
8	38242978	38243184	5Y-H4K8ac_peak_16246	7.55879	LETM2_ENSG00000165046
8	38329478	38329999	5Y-H4K8ac_peak_16247	13.3959	
8	38422495	38422733	5Y-H4K8ac_peak_16248	6.24411	
8	38577565	38577794	5Y-H4K8ac_peak_16249	9.47421	
8	38591748	38591963	5Y-H4K8ac_peak_16250	8.47164	
8	38592907	38593185	5Y-H4K8ac_peak_16251	9.15007	
8	38758775	38759075	5Y-H4K8ac_peak_16252	7.53429	PLEKHA2_ENSG00000169499
8	38782020	38782217	5Y-H4K8ac_peak_16253	6.50117	
8	38793717	38794684	5Y-H4K8ac_peak_16254	13.2534	
8	38854653	38855077	5Y-H4K8ac_peak_16255	11.68317	TM2D2_ENSG00000169490;ADAM9_ENSG00000168615
8	40790777	40791096	5Y-H4K8ac_peak_16256	4.07874	
8	41324646	41325221	5Y-H4K8ac_peak_16257	6.14981	
8	41347371	41347759	5Y-H4K8ac_peak_16258	8.75926	GOLGA7_ENSG00000147533
8	41427119	41427406	5Y-H4K8ac_peak_16259	5.8635	
8	41435783	41436062	5Y-H4K8ac_peak_16260	12.82003	RP11-360L9.4_ENSG00000253133
8	41753416	41753606	5Y-H4K8ac_peak_16261	8.748	ANK1_ENSG00000029534
8	41754017	41754212	5Y-H4K8ac_peak_16262	5.8635	ANK1_ENSG00000029534
8	41754491	41754769	5Y-H4K8ac_peak_16263	6.2403	ANK1_ENSG00000029534
8	41909319	41909511	5Y-H4K8ac_peak_16264	11.53521	KAT6A_ENSG00000083168
8	41909795	41910180	5Y-H4K8ac_peak_16265	14.68407	KAT6A_ENSG00000083168
8	42009782	42010129	5Y-H4K8ac_peak_16266	12.71088	RP11-589C21.5_ENSG00000261449;AP3M2_ENSG00000070718
8	42072451	42072707	5Y-H4K8ac_peak_16267	6.47245	
8	42081513	42081790	5Y-H4K8ac_peak_16268	6.13909	
8	42396302	42397333	5Y-H4K8ac_peak_16269	10.19948	SLC20A2_ENSG00000168575;SMIM19_ENSG00000176209
8	42597031	42597253	5Y-H4K8ac_peak_16270	6.03255	
8	42698156	42698415	5Y-H4K8ac_peak_16271	9.30505	THAP1_ENSG00000131931
8	42751332	42751712	5Y-H4K8ac_peak_16272	8.35138	RNF170_ENSG00000120925;MIR4469_ENSG00000266044;HOOK3_ENSG00000168172
8	42752183	42752820	5Y-H4K8ac_peak_16273	6.77436	RNF170_ENSG00000120925;MIR4469_ENSG00000266044;HOOK3_ENSG00000168172
8	42948793	42948993	5Y-H4K8ac_peak_16274	5.87725	POMK_ENSG00000185900
8	42996183	42996695	5Y-H4K8ac_peak_16275	6.47245	HGSNAT_ENSG00000165102
8	48100369	48100727	5Y-H4K8ac_peak_16276	6.43775	RP11-1134I14.4_ENSG00000254348
8	48172722	48173343	5Y-H4K8ac_peak_16277	8.43511	SPIDR_ENSG00000164808
8	48920275	48920786	5Y-H4K8ac_peak_16278	17.56739	UBE2V2_ENSG00000169139
8	49291982	49292196	5Y-H4K8ac_peak_16279	4.84727	
8	49292547	49292992	5Y-H4K8ac_peak_16280	8.33296	
8	49342609	49342903	5Y-H4K8ac_peak_16281	8.93923	
8	49468676	49468983	5Y-H4K8ac_peak_16282	6.1654	RP11-567J20.2_ENSG00000253688

8	49494617	49494841	5Y-H4K8ac_peak_16283	7.89273	
8	49782592	49783044	5Y-H4K8ac_peak_16284	11.28133	
8	54569699	54569976	5Y-H4K8ac_peak_16285	14.00129	
8	54570972	54571791	5Y-H4K8ac_peak_16286	13.57931	
8	54792283	54793401	5Y-H4K8ac_peak_16287	11.1169	
8	54818485	54818885	5Y-H4K8ac_peak_16288	6.00388	
8	54935742	54935968	5Y-H4K8ac_peak_16289	6.79955	TCEA1_ENSG00000187735
8	55014031	55014332	5Y-H4K8ac_peak_16290	6.98118	LYPLA1_ENSG00000120992
8	56792539	56793148	5Y-H4K8ac_peak_16291	5.24695	LYN_ENSG00000254087
8	56826327	56826584	5Y-H4K8ac_peak_16292	5.65584	
8	56826778	56827248	5Y-H4K8ac_peak_16293	10.69866	
8	56828022	56828261	5Y-H4K8ac_peak_16294	6.50117	
8	56829535	56830124	5Y-H4K8ac_peak_16295	8.93923	
8	56830607	56830827	5Y-H4K8ac_peak_16296	4.50834	
8	56831754	56832038	5Y-H4K8ac_peak_16297	4.03329	
8	56835134	56835340	5Y-H4K8ac_peak_16298	9.23159	
8	56838060	56838313	5Y-H4K8ac_peak_16299	6.77436	
8	57124409	57124709	5Y-H4K8ac_peak_16300	4.48815	PLAG1_ENSG00000181690;CHCHD7_ENSG00000170791
8	59571861	59572300	5Y-H4K8ac_peak_16301	9.30505	NSMAF_ENSG00000035681
8	60031290	60031667	5Y-H4K8ac_peak_16302	10.19948	TOX_ENSG00000198846;RP11-25K19.1_ENSG00000167912
8	60032493	60032738	5Y-H4K8ac_peak_16303	7.65114	TOX_ENSG00000198846;RP11-25K19.1_ENSG00000167912
8	61564371	61564699	5Y-H4K8ac_peak_16304	6.78318	
8	61591450	61591742	5Y-H4K8ac_peak_16305	10.46287	CHD7_ENSG00000171316
8	61706339	61706547	5Y-H4K8ac_peak_16306	5.98695	
8	61822260	61822972	5Y-H4K8ac_peak_16307	25.55525	AC022182.1_ENSG00000254777
8	61918903	61919093	5Y-H4K8ac_peak_16308	5.97089	
8	61925465	61925891	5Y-H4K8ac_peak_16309	4.90471	
8	61930248	61930534	5Y-H4K8ac_peak_16310	5.65584	
8	62052078	62052389	5Y-H4K8ac_peak_16311	7.11863	
8	62200602	62200859	5Y-H4K8ac_peak_16312	7.31102	
8	62627567	62627757	5Y-H4K8ac_peak_16313	6.20875	ASPH_ENSG00000198363;MIR4470_ENSG00000264408
8	64081175	64081527	5Y-H4K8ac_peak_16314	17.63051	YTHDF3-AS1_ENSG00000270673;YTHDF3_ENSG00000185728
8	65711040	65711674	5Y-H4K8ac_peak_16315	11.19336	CYP7B1_ENSG00000172817
8	66056933	66057347	5Y-H4K8ac_peak_16316	12.49199	
8	66753153	66753681	5Y-H4K8ac_peak_16317	9.20645	PDE7A_ENSG00000205268
8	66754476	66754784	5Y-H4K8ac_peak_16318	14.89891	PDE7A_ENSG00000205268;CTD-2532N20.1_ENSG00000272192
8	67435131	67435455	5Y-H4K8ac_peak_16319	8.67475	
8	67624891	67625401	5Y-H4K8ac_peak_16320	8.24461	SGK3_ENSG00000104205
8	67838072	67838420	5Y-H4K8ac_peak_16321	8.93923	SNHG6_ENSG00000245910
8	71314603	71315012	5Y-H4K8ac_peak_16322	4.15658	
8	71315307	71316390	5Y-H4K8ac_peak_16323	9.23159	NCOA2_ENSG00000140396
8	71316600	71316851	5Y-H4K8ac_peak_16324	5.65584	NCOA2_ENSG00000140396
8	71520026	71520382	5Y-H4K8ac_peak_16325	10.5157	TRAM1_ENSG00000067167;RP11-382J12.1_ENSG00000246366
8	71581017	71581295	5Y-H4K8ac_peak_16326	6.08523	LACTB2_ENSG00000147592;XKR9_ENSG00000221947
8	71964792	71965129	5Y-H4K8ac_peak_16327	4.50834	
8	73163579	73164100	5Y-H4K8ac_peak_16328	7.76232	RP11-656G20.1_ENSG00000250979

8	74004999	74005830	5Y-H4K8ac_peak_16329	11.28133	
8	74068878	74069147	5Y-H4K8ac_peak_16330	5.65584	
8	74658848	74659052	5Y-H4K8ac_peak_16331	7.09444	STAU2_ENSG00000040341
8	74659317	74659929	5Y-H4K8ac_peak_16332	8.69112	STAU2_ENSG00000040341
8	74790395	74790686	5Y-H4K8ac_peak_16333	5.87382	RP11-463D19.2_ENSG00000258677;UBE2W_ENSG00000104343
8	74883972	74884389	5Y-H4K8ac_peak_16334	7.31815	TCEB1_ENSG00000154582;TMEM70_ENSG00000175606
8	75233278	75233756	5Y-H4K8ac_peak_16335	7.38046	JPH1_ENSG00000104369;GDAP1_ENSG00000104381
8	75262712	75262942	5Y-H4K8ac_peak_16336	4.97086	CTD-2320G14.2_ENSG00000253596
8	76236565	76236893	5Y-H4K8ac_peak_16337	7.31815	CASC9_ENSG00000249395
8	76319382	76319821	5Y-H4K8ac_peak_16338	9.00954	HNF4G_ENSG00000164749
8	77590330	77590541	5Y-H4K8ac_peak_16339	6.37023	
8	77912681	77913158	5Y-H4K8ac_peak_16340	15.82359	PEX2_ENSG00000164751
8	79428273	79428523	5Y-H4K8ac_peak_16341	16.27715	PKIA_ENSG00000171033
8	79894919	79895125	5Y-H4K8ac_peak_16342	5.65584	
8	80211724	80211916	5Y-H4K8ac_peak_16343	6.14981	
8	80524814	80525024	5Y-H4K8ac_peak_16344	5.64909	
8	80680659	80681108	5Y-H4K8ac_peak_16345	12.05638	HEY1_ENSG00000164683;RP11-27N21.3_ENSG00000272138
8	80682082	80682386	5Y-H4K8ac_peak_16346	4.50834	
8	80696035	80696404	5Y-H4K8ac_peak_16347	6.54441	
8	80739502	80740198	5Y-H4K8ac_peak_16348	6.77436	
8	81142661	81143045	5Y-H4K8ac_peak_16349	5.449	TPD52_ENSG00000076554
8	81398498	81398842	5Y-H4K8ac_peak_16350	10.89622	Y_RNA_ENSG00000251867;ZBTB10_ENSG00000205189
8	81490242	81490693	5Y-H4K8ac_peak_16351	10.19948	
8	81785632	81785856	5Y-H4K8ac_peak_16352	8.2913	
8	81786247	81786447	5Y-H4K8ac_peak_16353	11.27554	ZNF704_ENSG00000164684
8	82192865	82193534	5Y-H4K8ac_peak_16354	12.87247	RP11-363E6.3_ENSG00000254027;FABP5_ENSG00000164687
8	82633722	82633916	5Y-H4K8ac_peak_16355	5.64909	
8	86089433	86090056	5Y-H4K8ac_peak_16356	7.59101	RP11-219B4.7_ENSG00000260493;E2F5_ENSG00000133740;RP11-219B4.3_ENSG00000254208
8	87355016	87355377	5Y-H4K8ac_peak_16357	10.46287	CTD-2284J15.1_ENSG00000254231;WWP1_ENSG00000123124
8	87520441	87520700	5Y-H4K8ac_peak_16358	6.73385	
8	90770271	90770585	5Y-H4K8ac_peak_16359	4.79585	RP11-37B2.1_ENSG00000251136;RIPK2_ENSG00000104312
8	90996568	90996835	5Y-H4K8ac_peak_16360	14.30055	
8	91658116	91658586	5Y-H4K8ac_peak_16361	9.60627	RP11-68L18.1_ENSG00000246792
8	91803688	91804162	5Y-H4K8ac_peak_16362	5.23083	TMEM64_ENSG00000180694;NECAB1_ENSG00000123119
8	91997025	91997294	5Y-H4K8ac_peak_16363	4.95697	C8orf88_ENSG00000253250
8	91997645	91997839	5Y-H4K8ac_peak_16364	9.20645	C8orf88_ENSG00000253250
8	92897640	92897935	5Y-H4K8ac_peak_16365	5.40331	
8	93114239	93114561	5Y-H4K8ac_peak_16366	8.62703	RUNX1T1_ENSG00000079102
8	93115092	93115739	5Y-H4K8ac_peak_16367	13.92877	RUNX1T1_ENSG00000079102
8	94637281	94637510	5Y-H4K8ac_peak_16368	5.23083	
8	94928436	94928813	5Y-H4K8ac_peak_16369	9.15007	MIR378D2_ENSG00000264448
8	94929288	94929653	5Y-H4K8ac_peak_16370	5.59843	MIR378D2_ENSG00000264448
8	95237879	95238072	5Y-H4K8ac_peak_16371	4.95697	
8	95274328	95274527	5Y-H4K8ac_peak_16372	5.98695	GEM_ENSG00000164949
8	95835777	95835994	5Y-H4K8ac_peak_16373	4.71803	
8	95961849	95962523	5Y-H4K8ac_peak_16374	12.21176	RP11-347C18.3_ENSG00000253878;TP53INP1_ENSG00000164938

8	96036734	96036952	5Y-H4K8ac_peak_16375	5.56894	
8	97247485	97247701	5Y-H4K8ac_peak_16376	8.2589	UQCRB_ENSG00000156467;KB-1043D8.6_ENSG00000254224
8	97273516	97273728	5Y-H4K8ac_peak_16377	7.38046	MTERFD1_ENSG00000156469;PTDSS1_ENSG00000156471
8	97512172	97512444	5Y-H4K8ac_peak_16378	4.9603	
8	97537336	97537653	5Y-H4K8ac_peak_16379	6.94416	
8	97840466	97841004	5Y-H4K8ac_peak_16380	8.2589	
8	97844855	97845080	5Y-H4K8ac_peak_16381	8.2913	
8	97939419	97939875	5Y-H4K8ac_peak_16382	6.46053	
8	98289729	98290049	5Y-H4K8ac_peak_16383	5.50568	TSPYL5_ENSG00000180543
8	98305862	98306210	5Y-H4K8ac_peak_16384	7.31815	
8	98306477	98306710	5Y-H4K8ac_peak_16385	6.34046	
8	98347248	98347479	5Y-H4K8ac_peak_16386	5.67283	
8	99057376	99057698	5Y-H4K8ac_peak_16387	10.23399	RPL30_ENSG00000156482
8	99306073	99306845	5Y-H4K8ac_peak_16388	15.06486	NIPAL2_ENSG00000104361
8	99952476	99953250	5Y-H4K8ac_peak_16389	7.11863	
8	101322529	101322921	5Y-H4K8ac_peak_16390	7.09658	
8	101429220	101429465	5Y-H4K8ac_peak_16391	6.34046	
8	101506323	101506708	5Y-H4K8ac_peak_16392	9.43096	
8	101507035	101507461	5Y-H4K8ac_peak_16393	10.19948	
8	101521322	101521698	5Y-H4K8ac_peak_16394	9.43096	
8	101571642	101571973	5Y-H4K8ac_peak_16395	14.88279	ANKRD46_ENSG00000186106
8	101859626	101860471	5Y-H4K8ac_peak_16396	4.642	
8	101964537	101965127	5Y-H4K8ac_peak_16397	11.22005	YWHAZ_ENSG00000164924
8	102038138	102038559	5Y-H4K8ac_peak_16398	4.84727	
8	102064184	102064649	5Y-H4K8ac_peak_16399	9.15007	RP11-302J23.1_ENSG00000248599
8	102092597	102093290	5Y-H4K8ac_peak_16400	9.30505	
8	102138345	102138681	5Y-H4K8ac_peak_16401	11.4254	KB-1460A1.1_ENSG00000253395
8	102660765	102661169	5Y-H4K8ac_peak_16402	8.33296	
8	102669860	102670099	5Y-H4K8ac_peak_16403	4.51076	
8	102703084	102703292	5Y-H4K8ac_peak_16404	7.97699	
8	102717904	102718095	5Y-H4K8ac_peak_16405	7.97699	
8	103612838	103613085	5Y-H4K8ac_peak_16406	6.1654	
8	103668538	103669202	5Y-H4K8ac_peak_16407	13.06587	KLF10_ENSG00000155090
8	103669735	103670037	5Y-H4K8ac_peak_16408	12.24192	
8	103732857	103733221	5Y-H4K8ac_peak_16409	6.50117	
8	103818408	103818610	5Y-H4K8ac_peak_16410	5.72233	KB-1732A1.1_ENSG00000253669
8	103818993	103819773	5Y-H4K8ac_peak_16411	12.10416	
8	103822588	103822811	5Y-H4K8ac_peak_16412	5.99504	
8	103876550	103876953	5Y-H4K8ac_peak_16413	16.2188	KB-1507C5.2_ENSG00000253320
8	104152800	104152995	5Y-H4K8ac_peak_16414	4.42435	C8orf56_ENSG00000236939;BAALC_ENSG00000164929
8	104311174	104311521	5Y-H4K8ac_peak_16415	6.77436	RP11-318M2.2_ENSG00000247081;FZD6_ENSG00000164930
8	104383331	104383575	5Y-H4K8ac_peak_16416	6.77436	CTHRC1_ENSG00000164932
8	104511190	104511667	5Y-H4K8ac_peak_16417	5.23083	
8	104511921	104512137	5Y-H4K8ac_peak_16418	8.33296	RIMS2_ENSG00000176406
8	104513258	104513801	5Y-H4K8ac_peak_16419	8.3285	RP11-1C8.4_ENSG00000253477;RIMS2_ENSG00000176406
8	105424765	105425005	5Y-H4K8ac_peak_16420	4.50834	

8	105600872	105601089	5Y-H4K8ac_peak_16421	9.56187	LRP12_ENSG00000147650
8	110552047	110552616	5Y-H4K8ac_peak_16422	7.38046	EBAG9_ENSG00000147654
8	117886462	117886843	5Y-H4K8ac_peak_16423	8.2913	RAD21_ENSG00000164754;RAD21-AS1_ENSG00000253327;MIR3610_ENSG00000264875
8	119634530	119634832	5Y-H4K8ac_peak_16424	5.98695	SAMD12_ENSG00000177570
8	120868283	120868561	5Y-H4K8ac_peak_16425	5.29015	DSCC1_ENSG00000136982
8	121457444	121457787	5Y-H4K8ac_peak_16426	5.65584	MRPL13_ENSG00000172172;MTBP_ENSG00000172167
8	123256881	123257210	5Y-H4K8ac_peak_16427	6.34046	
8	123692136	123692358	5Y-H4K8ac_peak_16428	5.10825	
8	123693007	123693504	5Y-H4K8ac_peak_16429	4.55128	
8	124037586	124037851	5Y-H4K8ac_peak_16430	6.94416	
8	124055322	124055581	5Y-H4K8ac_peak_16431	4.50834	DERL1_ENSG00000136986
8	124085010	124085239	5Y-H4K8ac_peak_16432	9.51254	
8	124245400	124245601	5Y-H4K8ac_peak_16433	6.1654	
8	124253330	124253546	5Y-H4K8ac_peak_16434	8.35139	C8orf76_ENSG00000189376
8	124286309	124286591	5Y-H4K8ac_peak_16435	5.98695	
8	124720581	124720811	5Y-H4K8ac_peak_16436	6.20875	
8	124934616	124934806	5Y-H4K8ac_peak_16437	6.13909	
8	125551429	125551660	5Y-H4K8ac_peak_16438	10.1994	TATDN1_ENSG00000147687;NDUFB9_ENSG00000147684
8	125739696	125739981	5Y-H4K8ac_peak_16439	7.59101	MTSS1_ENSG00000170873
8	125740847	125741196	5Y-H4K8ac_peak_16440	11.19336	MTSS1_ENSG00000170873
8	126010125	126010454	5Y-H4K8ac_peak_16441	8.31252	RP11-6D1.3_ENSG00000253513;SQLE_ENSG00000104549
8	126440762	126440979	5Y-H4K8ac_peak_16442	8.78565	
8	126441768	126442020	5Y-H4K8ac_peak_16443	12.05638	TRIB1_ENSG00000173334
8	128806295	128806588	5Y-H4K8ac_peak_16444	9.87097	PVT1_ENSG00000249859
8	128858206	128858399	5Y-H4K8ac_peak_16445	5.04571	
8	130472208	130472430	5Y-H4K8ac_peak_16446	8.27643	
8	130951602	130951954	5Y-H4K8ac_peak_16447	7.11863	RP11-473O4.3_ENSG00000253720
8	131028168	131028516	5Y-H4K8ac_peak_16448	6.17374	FAM49B_ENSG00000153310
8	131028835	131029162	5Y-H4K8ac_peak_16449	6.37023	FAM49B_ENSG00000153310
8	131309816	131310055	5Y-H4K8ac_peak_16450	5.12213	
8	131455486	131455852	5Y-H4K8ac_peak_16451	5.72233	ASAP1_ENSG00000153317
8	131651566	131651782	5Y-H4K8ac_peak_16452	5.59843	
8	131653466	131654118	5Y-H4K8ac_peak_16453	8.21582	
8	132922577	132922775	5Y-H4K8ac_peak_16454	5.8635	
8	134038717	134038927	5Y-H4K8ac_peak_16455	8.33296	
8	134284713	134284920	5Y-H4K8ac_peak_16456	10.46287	
8	134415927	134416458	5Y-H4K8ac_peak_16457	6.50117	
8	135470499	135470785	5Y-H4K8ac_peak_16458	11.31394	
8	135673155	135673369	5Y-H4K8ac_peak_16459	4.66683	
8	135725458	135725770	5Y-H4K8ac_peak_16460	5.12488	ZFAT_ENSG00000066827
8	136469213	136469477	5Y-H4K8ac_peak_16461	12.21176	RP11-343P9.1_ENSG00000254372;KHDRBS3_ENSG00000131773
8	136737184	136737522	5Y-H4K8ac_peak_16462	9.38203	
8	139206043	139206299	5Y-H4K8ac_peak_16463	5.29015	
8	139509225	139509533	5Y-H4K8ac_peak_16464	5.99504	FAM135B_ENSG00000147724
8	139555908	139556134	5Y-H4K8ac_peak_16465	6.50117	
8	139685614	139685852	5Y-H4K8ac_peak_16466	6.72926	

8	139780230	139780611	5Y-H4K8ac_peak_16467	6.73385	
8	139828319	139828569	5Y-H4K8ac_peak_16468	4.79585	
8	139948270	139948497	5Y-H4K8ac_peak_16469	13.06587	
8	140643505	140643814	5Y-H4K8ac_peak_16470	8.93923	
8	140770039	140770254	5Y-H4K8ac_peak_16471	5.35202	
8	140801936	140802135	5Y-H4K8ac_peak_16472	8.05083	
8	140802706	140802947	5Y-H4K8ac_peak_16473	5.87725	
8	140875100	140876151	5Y-H4K8ac_peak_16474	13.2534	
8	140991905	140992182	5Y-H4K8ac_peak_16475	9.05766	
8	140993157	140993395	5Y-H4K8ac_peak_16476	4.77016	
8	141018356	141018554	5Y-H4K8ac_peak_16477	4.84727	
8	141468348	141468748	5Y-H4K8ac_peak_16478	14.99673	TRAPPC9_ENSG00000167632
8	141489218	141489485	5Y-H4K8ac_peak_16479	5.23083	
8	141490268	141490964	5Y-H4K8ac_peak_16480	7.8267	
8	141521520	141521927	5Y-H4K8ac_peak_16481	11.60058	CHRAC1_ENSG00000104472
8	141600412	141600868	5Y-H4K8ac_peak_16482	4.29586	
8	141645125	141645353	5Y-H4K8ac_peak_16483	4.55128	AGO2_ENSG00000123908
8	142010944	142011134	5Y-H4K8ac_peak_16484	7.28311	
8	142011583	142011830	5Y-H4K8ac_peak_16485	13.94477	PTK2_ENSG00000169398
8	142032025	142033019	5Y-H4K8ac_peak_16486	8.2913	
8	142094726	142094986	5Y-H4K8ac_peak_16487	4.95697	
8	142114792	142115181	5Y-H4K8ac_peak_16488	6.43775	
8	142125526	142125778	5Y-H4K8ac_peak_16489	5.39991	
8	142137813	142138009	5Y-H4K8ac_peak_16490	6.08523	
8	142174428	142174901	5Y-H4K8ac_peak_16491	10.79063	
8	142215416	142216424	5Y-H4K8ac_peak_16492	13.27281	
8	142288954	142289295	5Y-H4K8ac_peak_16493	5.12213	RP11-10J21.5_ENSG00000254197
8	142339976	142340321	5Y-H4K8ac_peak_16494	4.75045	
8	142364897	142365107	5Y-H4K8ac_peak_16495	5.23083	CTD-3064M3.3_ENSG00000261655
8	142386364	142386647	5Y-H4K8ac_peak_16496	8.79957	
8	142387762	142387965	5Y-H4K8ac_peak_16497	6.5814	
8	142401154	142401355	5Y-H4K8ac_peak_16498	4.29586	PTP4A3_ENSG00000184489
8	142401654	142402646	5Y-H4K8ac_peak_16499	12.10416	CTD-3064M3.4_ENSG00000244998;PTP4A3_ENSG00000184489
8	142409053	142409396	5Y-H4K8ac_peak_16500	6.55841	
8	142410181	142410424	5Y-H4K8ac_peak_16501	8.43511	
8	142410864	142411243	5Y-H4K8ac_peak_16502	8.43511	
8	142411456	142412655	5Y-H4K8ac_peak_16503	15.40871	
8	142422292	142422633	5Y-H4K8ac_peak_16504	6.08523	
8	142423112	142423358	5Y-H4K8ac_peak_16505	10.16277	
8	142425131	142425436	5Y-H4K8ac_peak_16506	9.22312	
8	142432408	142432603	5Y-H4K8ac_peak_16507	7.09658	
8	142517761	142517978	5Y-H4K8ac_peak_16508	8.28346	MROH5_ENSG00000226807
8	142518291	142518595	5Y-H4K8ac_peak_16509	6.77436	MROH5_ENSG00000226807
8	142867126	142867478	5Y-H4K8ac_peak_16510	7.24844	
8	142869403	142869801	5Y-H4K8ac_peak_16511	5.23083	
8	142877149	142877593	5Y-H4K8ac_peak_16512	13.61292	

8	142878397	142879240	5Y-H4K8ac_peak_16513	9.30505	
8	143001005	143001416	5Y-H4K8ac_peak_16514	11.22005	
8	143182318	143182591	5Y-H4K8ac_peak_16515	5.57299	
8	143211166	143211402	5Y-H4K8ac_peak_16516	6.03611	
8	143211811	143212066	5Y-H4K8ac_peak_16517	8.86778	
8	143330598	143331356	5Y-H4K8ac_peak_16518	14.99673	
8	143331703	143331895	5Y-H4K8ac_peak_16519	4.52203	
8	143462764	143462974	5Y-H4K8ac_peak_16520	6.77436	
8	143484726	143485216	5Y-H4K8ac_peak_16521	17.18444	TSNARE1_ENSG00000171045;RP13-467H17.1_ENSG00000261693
8	143534454	143534692	5Y-H4K8ac_peak_16522	7.89273	
8	143556223	143556443	5Y-H4K8ac_peak_16523	3.99744	
8	143556648	143556895	5Y-H4K8ac_peak_16524	7.31102	
8	143557581	143557923	5Y-H4K8ac_peak_16525	5.98695	
8	143591684	143591982	5Y-H4K8ac_peak_16526	9.05168	
8	143592412	143592869	5Y-H4K8ac_peak_16527	7.59101	
8	143609771	143610256	5Y-H4K8ac_peak_16528	4.95697	
8	143611813	143612151	5Y-H4K8ac_peak_16529	5.9726	
8	143613616	143613866	5Y-H4K8ac_peak_16530	11.22005	
8	143614298	143614739	5Y-H4K8ac_peak_16531	5.98695	
8	143630950	143631243	5Y-H4K8ac_peak_16532	4.84727	
8	143632388	143632603	5Y-H4K8ac_peak_16533	5.25503	
8	143694525	143694755	5Y-H4K8ac_peak_16534	5.03917	
8	143696401	143696618	5Y-H4K8ac_peak_16535	4.33574	ARC_ENSG00000198576
8	143697547	143698051	5Y-H4K8ac_peak_16536	6.77436	ARC_ENSG00000198576
8	143704515	143704945	5Y-H4K8ac_peak_16537	5.23083	
8	143716360	143716583	5Y-H4K8ac_peak_16538	5.24695	
8	143750929	143751384	5Y-H4K8ac_peak_16539	12.69256	PSCA_ENSG00000167653
8	143754975	143755177	5Y-H4K8ac_peak_16540	7.59101	
8	143807784	143808421	5Y-H4K8ac_peak_16541	12.11208	CTD-2292P10.4_ENSG00000253741;THEM6_ENSG00000130193;CTD-2292P10.2_ENSG00000253806
8	143820619	143820842	5Y-H4K8ac_peak_16542	6.66641	
8	143897101	143897308	5Y-H4K8ac_peak_16543	8.20773	
8	144099304	144100375	5Y-H4K8ac_peak_16544	17.8549	RP11-273G15.2_ENSG00000247317;LY6E_ENSG00000160932
8	144120729	144121391	5Y-H4K8ac_peak_16545	8.24461	C8orf31_ENSG00000177335
8	144301269	144301469	5Y-H4K8ac_peak_16546	7.38046	
8	144329192	144329504	5Y-H4K8ac_peak_16547	9.30206	ZFP41_ENSG00000181638;ZFP41_ENSG00000264668
8	144427370	144427704	5Y-H4K8ac_peak_16548	6.6946	
8	144597918	144598148	5Y-H4K8ac_peak_16549	6.64195	
8	144717798	144718746	5Y-H4K8ac_peak_16550	9.11392	ZNF623_ENSG00000183309
8	144910945	144911465	5Y-H4K8ac_peak_16551	10.48476	PUF60_ENSG00000179950
8	144922956	144923150	5Y-H4K8ac_peak_16552	4.15658	
8	144976926	144977206	5Y-H4K8ac_peak_16553	7.89273	
8	145025476	145025686	5Y-H4K8ac_peak_16554	7.90236	
8	145028413	145028632	5Y-H4K8ac_peak_16555	10.1994	
8	145103971	145104575	5Y-H4K8ac_peak_16556	6.64036	CTD-3065J16.6_ENSG00000204791
8	145132969	145133369	5Y-H4K8ac_peak_16557	11.68317	CTD-3065J16.9_ENSG00000255224;EXOSC4_ENSG00000178896
8	145150039	145150342	5Y-H4K8ac_peak_16558	10.69698	CYC1_ENSG00000179091

8	145158825	145159046	5Y-H4K8ac_peak_16559	4.0639	MAF1_ENSG00000179632
8	145330319	145330584	5Y-H4K8ac_peak_16560	13.8759	KM-PA-2_ENSG00000204775
8	145560864	145561688	5Y-H4K8ac_peak_16561	10.4689	SCRT1_ENSG00000170616
8	145562532	145562778	5Y-H4K8ac_peak_16562	8.46442	
8	145596888	145597435	5Y-H4K8ac_peak_16563	11.82621	ADCK5_ENSG00000173137
8	145635038	145635500	5Y-H4K8ac_peak_16564	7.59101	CPSF1_ENSG00000071894;GS1-393G12.14_ENSG00000272115
8	145638689	145638985	5Y-H4K8ac_peak_16565	12.81408	
8	145639212	145639597	5Y-H4K8ac_peak_16566	12.76485	
8	145654529	145654757	5Y-H4K8ac_peak_16567	4.642	VPS28_ENSG00000160948
8	145670045	145670298	5Y-H4K8ac_peak_16568	10.19948	TONSL_ENSG00000160949
8	145690529	145690723	5Y-H4K8ac_peak_16569	10.0695	CYHR1_ENSG00000187954;KIFC2_ENSG00000167702
8	145702687	145703090	5Y-H4K8ac_peak_16570	4.5541	FOXH1_ENSG00000160973;PPP1R16A_ENSG00000160972
8	145728381	145728667	5Y-H4K8ac_peak_16571	8.69112	GPT_ENSG00000167701
8	145733480	145734018	5Y-H4K8ac_peak_16572	13.68515	MFSD3_ENSG00000167700
8	145734398	145735017	5Y-H4K8ac_peak_16573	16.33454	MFSD3_ENSG00000167700
8	145752229	145752948	5Y-H4K8ac_peak_16574	7.82914	LRRC24_ENSG00000254402
8	145754149	145754433	5Y-H4K8ac_peak_16575	13.92877	C8orf82_ENSG00000213563
8	145754693	145754992	5Y-H4K8ac_peak_16576	4.96019	C8orf82_ENSG00000213563
8	145925201	145925473	5Y-H4K8ac_peak_16577	4.8509	AF186192.1_ENSG00000254533;AF186192.5_ENSG00000255085
8	146017267	146017612	5Y-H4K8ac_peak_16578	4.00285	RPL8_ENSG00000161016
8	146023700	146024134	5Y-H4K8ac_peak_16579	8.69112	ZNF517_ENSG00000197363
8	146051896	146052145	5Y-H4K8ac_peak_16580	8.93923	ZNF7_ENSG00000147789
8	146052987	146053548	5Y-H4K8ac_peak_16581	16.50659	ZNF7_ENSG00000147789
8	146078008	146078219	5Y-H4K8ac_peak_16582	6.37023	COMMD5_ENSG00000170619;AF235103.1_ENSG00000263640
8	146078630	146078853	5Y-H4K8ac_peak_16583	9.30505	COMMD5_ENSG00000170619;AF235103.1_ENSG00000263640
8	146175769	146176297	5Y-H4K8ac_peak_16584	7.90751	ZNF16_ENSG00000170631
8	146278073	146278374	5Y-H4K8ac_peak_16585	5.70472	C8orf33_ENSG00000182307
9	469719	469912	5Y-H4K8ac_peak_16586	5.87382	KANK1_ENSG00000107104
9	2620988	2621677	5Y-H4K8ac_peak_16587	7.31815	VLDLR_ENSG00000147852
9	2622624	2622973	5Y-H4K8ac_peak_16588	7.59101	VLDLR_ENSG00000147852
9	2843744	2843949	5Y-H4K8ac_peak_16589	5.65584	KIAA0020_ENSG00000080608
9	3526066	3527206	5Y-H4K8ac_peak_16590	21.10958	RFX3_ENSG00000080298;RP11-509J21.1_ENSG00000232104
9	3825878	3826096	5Y-H4K8ac_peak_16591	6.77436	
9	4792982	4793281	5Y-H4K8ac_peak_16592	10.31981	RCL1_ENSG00000120158
9	4985461	4985654	5Y-H4K8ac_peak_16593	4.29586	JAK2_ENSG00000096968
9	5304513	5304726	5Y-H4K8ac_peak_16594	7.31815	RLN2_ENSG00000107014
9	5628722	5628920	5Y-H4K8ac_peak_16595	10.54764	KIAA1432_ENSG00000107036
9	5832738	5832952	5Y-H4K8ac_peak_16596	4.95697	ERMP1_ENSG00000099219
9	6007783	6008204	5Y-H4K8ac_peak_16597	8.17203	KIAA2026_ENSG00000183354;MIR4665_ENSG00000263575
9	6015891	6016172	5Y-H4K8ac_peak_16598	15.60239	RANBP6_ENSG00000137040
9	6412470	6413012	5Y-H4K8ac_peak_16599	17.04136	UHRF2_ENSG00000147854
9	6680865	6681220	5Y-H4K8ac_peak_16600	13.10145	
9	6681445	6681765	5Y-H4K8ac_peak_16601	12.05638	
9	6681986	6682448	5Y-H4K8ac_peak_16602	6.14981	
9	6704384	6704817	5Y-H4K8ac_peak_16603	13.72401	RP11-390F4.10_ENSG00000229611
9	6925431	6925627	5Y-H4K8ac_peak_16604	6.20875	

9	7224743	7225021	5Y-H4K8ac_peak_16605	6.34046	
9	7799516	7799734	5Y-H4K8ac_peak_16606	7.89273	
9	12658496	12658760	5Y-H4K8ac_peak_16607	4.47071	
9	13278756	13279119	5Y-H4K8ac_peak_16608	6.78128	MPDZ_ENSG00000107186;RP11-272P10.2_ENSG00000234740
9	13279438	13279674	5Y-H4K8ac_peak_16609	4.29586	MPDZ_ENSG00000107186;RP11-272P10.2_ENSG00000234740
9	13431054	13431254	5Y-H4K8ac_peak_16610	9.38203	
9	14313159	14313360	5Y-H4K8ac_peak_16611	5.41472	
9	14314231	14314464	5Y-H4K8ac_peak_16612	11.20652	
9	14314827	14315375	5Y-H4K8ac_peak_16613	11.19336	
9	14315745	14316071	5Y-H4K8ac_peak_16614	12.87247	
9	14322280	14322476	5Y-H4K8ac_peak_16615	4.07874	
9	14322672	14323080	5Y-H4K8ac_peak_16616	12.71156	
9	14370645	14370874	5Y-H4K8ac_peak_16617	6.07082	
9	14374199	14374741	5Y-H4K8ac_peak_16618	6.34046	
9	14403148	14403400	5Y-H4K8ac_peak_16619	8.8236	
9	14419366	14419809	5Y-H4K8ac_peak_16620	6.66026	
9	14515141	14515897	5Y-H4K8ac_peak_16621	8.35139	
9	14517771	14517978	5Y-H4K8ac_peak_16622	6.27984	
9	14533627	14534159	5Y-H4K8ac_peak_16623	4.50834	
9	14693131	14694161	5Y-H4K8ac_peak_16624	19.17092	ZDHHC21_ENSG00000175893
9	14993481	14994026	5Y-H4K8ac_peak_16625	9.30505	RP11-54D18.2_ENSG00000215237
9	15085246	15085472	5Y-H4K8ac_peak_16626	8.35139	
9	16726927	16727162	5Y-H4K8ac_peak_16627	7.59101	RP11-62F24.2_ENSG00000234779
9	16870338	16870566	5Y-H4K8ac_peak_16628	5.40331	BNC2_ENSG00000173068
9	17059889	17060172	5Y-H4K8ac_peak_16629	4.50834	
9	17134577	17134843	5Y-H4K8ac_peak_16630	14.72057	CNTLN_ENSG00000044459
9	17135085	17135330	5Y-H4K8ac_peak_16631	10.35586	CNTLN_ENSG00000044459
9	19048255	19048475	5Y-H4K8ac_peak_16632	5.04571	RRAGA_ENSG00000155876
9	19048924	19049141	5Y-H4K8ac_peak_16633	4.15658	RRAGA_ENSG00000155876
9	19102589	19102858	5Y-H4K8ac_peak_16634	7.04637	HAUS6_ENSG00000147874
9	19127229	19127426	5Y-H4K8ac_peak_16635	11.86504	
9	19129114	19129420	5Y-H4K8ac_peak_16636	5.40331	
9	19229847	19230316	5Y-H4K8ac_peak_16637	12.21176	DENND4C_ENSG00000137145
9	19230915	19231241	5Y-H4K8ac_peak_16638	6.77436	DENND4C_ENSG00000137145
9	19408434	19408681	5Y-H4K8ac_peak_16639	4.95697	ACER2_ENSG00000177076
9	20243912	20244157	5Y-H4K8ac_peak_16640	9.51254	
9	23820842	23821423	5Y-H4K8ac_peak_16641	5.81062	
9	25677434	25678806	5Y-H4K8ac_peak_16642	7.89142	TUSC1_ENSG00000198680
9	26946864	26947056	5Y-H4K8ac_peak_16643	7.90236	PLAA_ENSG00000137055;IFT74_ENSG00000096872
9	26947263	26947469	5Y-H4K8ac_peak_16644	6.37023	PLAA_ENSG00000137055;IFT74_ENSG00000096872
9	27529413	27529815	5Y-H4K8ac_peak_16645	9.23159	MOB3B_ENSG00000120162
9	27572830	27573398	5Y-H4K8ac_peak_16646	8.84534	C9orf72_ENSG00000147894
9	29213034	29213234	5Y-H4K8ac_peak_16647	6.17374	
9	32573279	32573667	5Y-H4K8ac_peak_16648	5.65584	NDUFB6_ENSG00000165264
9	32782824	32783179	5Y-H4K8ac_peak_16649	4.95697	TMEM215_ENSG00000188133
9	33025320	33025585	5Y-H4K8ac_peak_16650	17.67751	APTX_ENSG00000137074;DNAJA1_ENSG00000086061

9	33076336	33076597	5Y-H4K8ac_peak_16651	9.65108	SMU1_ENSG00000122692
9	33159307	33159756	5Y-H4K8ac_peak_16652	5.87382	
9	33290570	33290760	5Y-H4K8ac_peak_16653	6.73385	NFX1_ENSG00000086102
9	33403191	33403411	5Y-H4K8ac_peak_16654	7.57144	AQP7_ENSG00000165269;RP11-311H10.4_ENSG00000223678
9	33510885	33511245	5Y-H4K8ac_peak_16655	13.92877	SUGT1P_ENSG00000226823
9	33511648	33511868	5Y-H4K8ac_peak_16656	6.20875	SUGT1P_ENSG00000226823
9	33523582	33523897	5Y-H4K8ac_peak_16657	9.07085	ANKRD18B_ENSG00000230453
9	33721800	33722153	5Y-H4K8ac_peak_16658	11.1169	
9	33750747	33751022	5Y-H4K8ac_peak_16659	9.01738	PRSS3_ENSG00000010438
9	33816642	33816933	5Y-H4K8ac_peak_16660	13.70457	UBE2R2_ENSG00000107341
9	33817430	33818022	5Y-H4K8ac_peak_16661	17.50049	RP11-133O22.6_ENSG00000235481;UBE2R2_ENSG00000107341
9	34048976	34049489	5Y-H4K8ac_peak_16662	9.36633	UBAP2_ENSG00000137073;RN7SKP114_ENSG00000222259
9	34178583	34178790	5Y-H4K8ac_peak_16663	5.35202	UBAP1_ENSG00000165006
9	34553949	34554697	5Y-H4K8ac_peak_16664	5.83799	
9	34555626	34555832	5Y-H4K8ac_peak_16665	7.60057	
9	34557803	34557997	5Y-H4K8ac_peak_16666	4.59665	
9	34558859	34559061	5Y-H4K8ac_peak_16667	8.20773	
9	34559271	34559491	5Y-H4K8ac_peak_16668	10.35586	
9	34559726	34560531	5Y-H4K8ac_peak_16669	7.10001	
9	34566420	34566613	5Y-H4K8ac_peak_16670	5.12488	
9	34570947	34571458	5Y-H4K8ac_peak_16671	9.51254	
9	34574312	34574505	5Y-H4K8ac_peak_16672	4.38969	
9	34577984	34578652	5Y-H4K8ac_peak_16673	15.73087	
9	34580364	34580567	5Y-H4K8ac_peak_16674	7.89273	
9	34583800	34584078	5Y-H4K8ac_peak_16675	5.21222	
9	34589911	34590142	5Y-H4K8ac_peak_16676	11.04542	CNTFR_ENSG00000122756
9	34591395	34591598	5Y-H4K8ac_peak_16677	6.50117	
9	34611678	34612018	5Y-H4K8ac_peak_16678	6.08523	RPP25L_ENSG00000164967
9	34612265	34612625	5Y-H4K8ac_peak_16679	4.50834	RPP25L_ENSG00000164967
9	34620101	34620403	5Y-H4K8ac_peak_16680	5.91107	DCTN3_ENSG00000137100
9	34637190	34637695	5Y-H4K8ac_peak_16681	10.69698	SIGMAR1_ENSG00000147955;GALT_ENSG00000213930
9	34638008	34638307	5Y-H4K8ac_peak_16682	7.58806	SIGMAR1_ENSG00000147955;GALT_ENSG00000213930
9	34651937	34652215	5Y-H4K8ac_peak_16683	5.2814	
9	34653918	34654246	5Y-H4K8ac_peak_16684	8.69895	
9	34957652	34958051	5Y-H4K8ac_peak_16685	7.31815	KIAA1045_ENSG00000122733
9	34988489	34988695	5Y-H4K8ac_peak_16686	4.59169	DNAJB5_ENSG00000137094
9	34993519	34993770	5Y-H4K8ac_peak_16687	4.50834	
9	34994663	34994884	5Y-H4K8ac_peak_16688	7.38046	
9	35095676	35096038	5Y-H4K8ac_peak_16689	9.74189	PIGO_ENSG00000165282;RP11-182N22.8_ENSG00000234181
9	35096244	35096577	5Y-H4K8ac_peak_16690	23.5028	PIGO_ENSG00000165282;RP11-182N22.8_ENSG00000234181
9	35162054	35162337	5Y-H4K8ac_peak_16691	7.31102	UNC13B_ENSG00000198722
9	35489436	35490567	5Y-H4K8ac_peak_16692	10.16277	RUSC2_ENSG00000198853
9	35617039	35617313	5Y-H4K8ac_peak_16693	6.37023	
9	35647061	35647308	5Y-H4K8ac_peak_16694	6.08523	CD72_ENSG00000137101;RP11-331F9.4_ENSG00000227933
9	35664467	35664665	5Y-H4K8ac_peak_16695	3.99744	
9	35675810	35676417	5Y-H4K8ac_peak_16696	18.10232	ARHGEF39_ENSG00000137135

9	35749408	35749599	5Y-H4K8ac_peak_16697	7.09658	GBA2_ENSG00000070610;RGP1_ENSG00000107185
9	35756544	35757395	5Y-H4K8ac_peak_16698	7.38046	MSMP_ENSG00000215183;RP11-112J3.15_ENSG00000228843
9	36023307	36023519	5Y-H4K8ac_peak_16699	5.28616	
9	36036399	36036704	5Y-H4K8ac_peak_16700	5.54013	RECK_ENSG00000122707
9	36136230	36136572	5Y-H4K8ac_peak_16701	7.89273	
9	36137269	36137468	5Y-H4K8ac_peak_16702	6.37023	
9	36148417	36148642	5Y-H4K8ac_peak_16703	5.23083	
9	36166281	36166496	5Y-H4K8ac_peak_16704	6.51488	
9	36191018	36191278	5Y-H4K8ac_peak_16705	11.09973	CLTA_ENSG00000122705
9	36258214	36258410	5Y-H4K8ac_peak_16706	5.12488	
9	36400461	36400681	5Y-H4K8ac_peak_16707	7.11863	
9	36486805	36487543	5Y-H4K8ac_peak_16708	8.21525	RNF38_ENSG00000137075
9	36766290	36766742	5Y-H4K8ac_peak_16709	8.2913	
9	36842249	36842455	5Y-H4K8ac_peak_16710	6.1654	
9	36986492	36987145	5Y-H4K8ac_peak_16711	5.98695	
9	37036526	37036788	5Y-H4K8ac_peak_16712	7.89142	
9	37037318	37038261	5Y-H4K8ac_peak_16713	5.41472	
9	37080120	37080362	5Y-H4K8ac_peak_16714	5.77617	RP11-220I1.5_ENSG00000260100;RP11-220I1.1_ENSG00000233137
9	37120649	37120864	5Y-H4K8ac_peak_16715	9.15007	ZCCHC7_ENSG00000147905
9	37415531	37415769	5Y-H4K8ac_peak_16716	7.59101	
9	37422202	37422514	5Y-H4K8ac_peak_16717	7.30348	GRHPR_ENSG00000137106
9	37464996	37465290	5Y-H4K8ac_peak_16718	10.69698	ZBTB5_ENSG00000168795
9	37466380	37466953	5Y-H4K8ac_peak_16719	10.60083	ZBTB5_ENSG00000168795
9	37482056	37482275	5Y-H4K8ac_peak_16720	9.88125	
9	37575959	37576202	5Y-H4K8ac_peak_16721	6.73385	
9	37678770	37678992	5Y-H4K8ac_peak_16722	6.94416	
9	37753872	37754188	5Y-H4K8ac_peak_16723	9.23159	TRMT10B_ENSG00000165275
9	37784696	37784914	5Y-H4K8ac_peak_16724	8.69112	
9	37904900	37905144	5Y-H4K8ac_peak_16725	9.56516	SLC25A51_ENSG00000122696
9	37937678	37937980	5Y-H4K8ac_peak_16726	8.4454	RNU7-124P_ENSG00000251745
9	37977200	37977531	5Y-H4K8ac_peak_16727	5.23083	
9	37978534	37978789	5Y-H4K8ac_peak_16728	7.35333	
9	37997203	37997482	5Y-H4K8ac_peak_16729	7.55879	
9	38019083	38019671	5Y-H4K8ac_peak_16730	7.59101	
9	38068207	38068519	5Y-H4K8ac_peak_16731	9.63153	RP11-613M10.9_ENSG00000255872;SHB_ENSG00000107338
9	38154089	38154405	5Y-H4K8ac_peak_16732	5.77617	
9	38154898	38155112	5Y-H4K8ac_peak_16733	7.59101	
9	38155669	38155967	5Y-H4K8ac_peak_16734	5.8635	
9	38214608	38214924	5Y-H4K8ac_peak_16735	9.30505	
9	38225034	38225314	5Y-H4K8ac_peak_16736	12.49199	
9	38226019	38226219	5Y-H4K8ac_peak_16737	8.17203	
9	38226434	38226826	5Y-H4K8ac_peak_16738	12.09463	
9	38228160	38228597	5Y-H4K8ac_peak_16739	8.1667	
9	38229471	38229767	5Y-H4K8ac_peak_16740	6.61077	
9	38232676	38233343	5Y-H4K8ac_peak_16741	19.72309	
9	38234603	38235577	5Y-H4K8ac_peak_16742	11.1169	

9	38284771	38285002	5Y-H4K8ac_peak_16743	7.90751	
9	38308847	38309039	5Y-H4K8ac_peak_16744	7.01266	
9	38345251	38345544	5Y-H4K8ac_peak_16745	4.07874	
9	38361509	38361825	5Y-H4K8ac_peak_16746	8.43011	
9	38372268	38372695	5Y-H4K8ac_peak_16747	10.19948	
9	38422467	38422719	5Y-H4K8ac_peak_16748	7.59101	
9	38424029	38424451	5Y-H4K8ac_peak_16749	9.02782	IGFBPL1_ENSG00000137142
9	38620806	38621649	5Y-H4K8ac_peak_16750	10.1994	ANKRD18A_ENSG00000180071;FAM201A_ENSG00000204860
9	38762555	38762877	5Y-H4K8ac_peak_16751	5.12213	
9	39078644	39079012	5Y-H4K8ac_peak_16752	9.20645	
9	41954471	41954952	5Y-H4K8ac_peak_16753	5.40265	RP11-104G3.2_ENSG00000229273
9	45356002	45356231	5Y-H4K8ac_peak_16754	16.30401	
9	45440965	45441217	5Y-H4K8ac_peak_16755	9.38203	AL354718.1_ENSG00000268578
9	66455548	66456075	5Y-H4K8ac_peak_16756	13.1986	
9	66457333	66458530	5Y-H4K8ac_peak_16757	15.41202	RP11-262H14.1_ENSG00000238113;RNA5SP283_ENSG00000202474
9	66489234	66489457	5Y-H4K8ac_peak_16758	7.11863	
9	66493232	66494136	5Y-H4K8ac_peak_16759	19.06379	RP11-262H14.5_ENSG00000229422
9	66519817	66520023	5Y-H4K8ac_peak_16760	9.02938	RP11-262H14.7_ENSG00000227582
9	66520405	66520806	5Y-H4K8ac_peak_16761	8.98784	RP11-262H14.7_ENSG00000227582
9	67339616	67339891	5Y-H4K8ac_peak_16762	7.11863	
9	68412891	68413153	5Y-H4K8ac_peak_16763	6.08471	
9	68413565	68413823	5Y-H4K8ac_peak_16764	9.87124	LINC00537_ENSG00000232815
9	71394366	71394713	5Y-H4K8ac_peak_16765	8.79957	FAM122A_ENSG00000187866
9	71736023	71736257	5Y-H4K8ac_peak_16766	8.33296	TJP2_ENSG00000119139
9	71762045	71762248	5Y-H4K8ac_peak_16767	7.76232	
9	72628049	72628259	5Y-H4K8ac_peak_16768	7.38046	
9	73034799	73034995	5Y-H4K8ac_peak_16769	6.78318	
9	73035415	73035719	5Y-H4K8ac_peak_16770	10.1994	
9	73035923	73036210	5Y-H4K8ac_peak_16771	8.21582	
9	76975019	76975323	5Y-H4K8ac_peak_16772	5.23083	
9	77567321	77567740	5Y-H4K8ac_peak_16773	17.50049	C9orf40_ENSG00000135045;RP11-197P3.4_ENSG00000203321
9	77643865	77644058	5Y-H4K8ac_peak_16774	7.17184	C9orf41_ENSG00000156017
9	79008900	79009247	5Y-H4K8ac_peak_16775	4.90696	RFK_ENSG00000135002
9	80247491	80247699	5Y-H4K8ac_peak_16776	6.34046	
9	80512466	80512730	5Y-H4K8ac_peak_16777	4.50834	
9	80604392	80604656	5Y-H4K8ac_peak_16778	6.6946	
9	80646026	80646405	5Y-H4K8ac_peak_16779	8.73985	GNAQ_ENSG00000156052
9	80646992	80647185	5Y-H4K8ac_peak_16780	5.67283	GNAQ_ENSG00000156052
9	80850540	80850823	5Y-H4K8ac_peak_16781	7.59101	CEP78_ENSG00000148019
9	80851234	80851425	5Y-H4K8ac_peak_16782	7.30348	CEP78_ENSG00000148019
9	80911557	80911754	5Y-H4K8ac_peak_16783	6.10343	PSAT1_ENSG00000135069
9	82185420	82185811	5Y-H4K8ac_peak_16784	8.2913	TLE4_ENSG00000106829
9	82846472	82847449	5Y-H4K8ac_peak_16785	5.87725	
9	84303376	84303747	5Y-H4K8ac_peak_16786	4.0639	TLE1_ENSG00000196781;RP11-154D17.1_ENSG00000233926
9	84731052	84731306	5Y-H4K8ac_peak_16787	6.20875	
9	85294511	85294869	5Y-H4K8ac_peak_16788	6.17374	

9	85573269	85573615	5Y-H4K8ac_peak_16789	9.6443	
9	85677196	85677968	5Y-H4K8ac_peak_16790	9.30505	RASEF_ENSG00000165105
9	85834364	85834559	5Y-H4K8ac_peak_16791	5.07473	RP11-439K3.1_ENSG00000229109
9	86065612	86065870	5Y-H4K8ac_peak_16792	4.50834	
9	86571072	86571933	5Y-H4K8ac_peak_16793	5.98695	C9orf64_ENSG00000165118
9	86594724	86595101	5Y-H4K8ac_peak_16794	8.04693	HNRNPK_ENSG00000165119;RMI1_ENSG00000178966
9	86595815	86596206	5Y-H4K8ac_peak_16795	7.83553	HNRNPK_ENSG00000165119;RMI1_ENSG00000178966
9	88420368	88420875	5Y-H4K8ac_peak_16796	9.52603	
9	88556204	88556578	5Y-H4K8ac_peak_16797	12.06963	NAA35_ENSG00000135040
9	88713890	88714820	5Y-H4K8ac_peak_16798	15.01219	GOLM1_ENSG00000135052
9	88968975	88969250	5Y-H4K8ac_peak_16799	4.29586	ZCCHC6_ENSG00000083223
9	88969563	88969804	5Y-H4K8ac_peak_16800	13.0168	ZCCHC6_ENSG00000083223
9	89517628	89517831	5Y-H4K8ac_peak_16801	6.83519	
9	89763409	89764045	5Y-H4K8ac_peak_16802	10.1994	C9orf170_ENSG00000204446
9	90112909	90113424	5Y-H4K8ac_peak_16803	13.14766	DAPK1_ENSG00000196730
9	90326996	90327367	5Y-H4K8ac_peak_16804	6.07082	
9	90341552	90341861	5Y-H4K8ac_peak_16805	10.84616	
9	90691206	90691626	5Y-H4K8ac_peak_16806	10.19948	
9	90692140	90692402	5Y-H4K8ac_peak_16807	7.01266	
9	91358957	91359206	5Y-H4K8ac_peak_16808	5.23083	
9	91361900	91362131	5Y-H4K8ac_peak_16809	6.2403	
9	91442512	91442707	5Y-H4K8ac_peak_16810	5.91107	
9	91503780	91503978	5Y-H4K8ac_peak_16811	7.3889	
9	91625299	91625672	5Y-H4K8ac_peak_16812	9.99623	RP11-82L18.4_ENSG00000231632
9	91849411	91849653	5Y-H4K8ac_peak_16813	5.40331	
9	91925127	91925628	5Y-H4K8ac_peak_16814	19.10058	CKS2_ENSG00000123975
9	91933025	91933647	5Y-H4K8ac_peak_16815	19.17092	SECISBP2_ENSG00000187742
9	92068326	92068567	5Y-H4K8ac_peak_16816	9.97632	
9	92112463	92112863	5Y-H4K8ac_peak_16817	6.50117	SEMA4D_ENSG00000187764;AL590233.1_ENSG00000264913
9	92113308	92113683	5Y-H4K8ac_peak_16818	5.41472	SEMA4D_ENSG00000187764;AL590233.1_ENSG00000264913
9	92152771	92152974	5Y-H4K8ac_peak_16819	5.35202	
9	92156786	92156991	5Y-H4K8ac_peak_16820	6.67017	
9	92157744	92157988	5Y-H4K8ac_peak_16821	5.35202	
9	92219023	92219227	5Y-H4K8ac_peak_16822	4.50834	GADD45G_ENSG00000130222
9	92277678	92278072	5Y-H4K8ac_peak_16823	11.08964	
9	92290302	92290932	5Y-H4K8ac_peak_16824	15.59156	
9	92292229	92292437	5Y-H4K8ac_peak_16825	5.98695	
9	92293233	92293582	5Y-H4K8ac_peak_16826	6.96612	
9	92337015	92337221	5Y-H4K8ac_peak_16827	5.87382	
9	92447341	92447552	5Y-H4K8ac_peak_16828	8.79957	
9	92447901	92448194	5Y-H4K8ac_peak_16829	9.30505	
9	92451387	92452065	5Y-H4K8ac_peak_16830	9.6443	
9	92685165	92685635	5Y-H4K8ac_peak_16831	5.65584	
9	92716087	92716732	5Y-H4K8ac_peak_16832	14.72547	
9	92717239	92717520	5Y-H4K8ac_peak_16833	6.17374	
9	92761732	92762004	5Y-H4K8ac_peak_16834	5.98695	

9	93563669	93564524	5Y-H4K8ac_peak_16835	5.72233	SYK_ENSG00000165025
9	93955567	93955931	5Y-H4K8ac_peak_16836	6.50117	
9	94183538	94183834	5Y-H4K8ac_peak_16837	10.6025	
9	94619866	94620058	5Y-H4K8ac_peak_16838	7.4047	
9	94662455	94662882	5Y-H4K8ac_peak_16839	6.37023	
9	94878142	94878344	5Y-H4K8ac_peak_16840	5.28616	SPTLC1_ENSG00000090054
9	95087974	95088195	5Y-H4K8ac_peak_16841	13.12848	NOL8_ENSG00000198000;CENPP_ENSG00000188312
9	95121347	95121658	5Y-H4K8ac_peak_16842	6.20875	
9	95123061	95123265	5Y-H4K8ac_peak_16843	5.65584	
9	95255028	95255314	5Y-H4K8ac_peak_16844	6.37023	
9	95413740	95413997	5Y-H4K8ac_peak_16845	6.77436	
9	95527532	95527752	5Y-H4K8ac_peak_16846	13.45283	BICD2_ENSG00000185963
9	95709683	95709995	5Y-H4K8ac_peak_16847	5.77617	FGD3_ENSG00000127084
9	95896477	95896687	5Y-H4K8ac_peak_16848	4.18845	NINJ1_ENSG00000131669
9	95896977	95897173	5Y-H4K8ac_peak_16849	6.22669	NINJ1_ENSG00000131669
9	95946614	95947226	5Y-H4K8ac_peak_16850	7.15925	WNK2_ENSG00000165238
9	95963497	95963851	5Y-H4K8ac_peak_16851	11.1169	
9	95965399	95965615	5Y-H4K8ac_peak_16852	6.34772	
9	95968342	95968625	5Y-H4K8ac_peak_16853	4.29586	
9	95985911	95986262	5Y-H4K8ac_peak_16854	9.77183	
9	96082368	96082615	5Y-H4K8ac_peak_16855	6.6946	
9	96108414	96108980	5Y-H4K8ac_peak_16856	8.2913	C9orf129_ENSG00000204352
9	96329067	96329567	5Y-H4K8ac_peak_16857	13.00733	AL353629.1_ENSG00000263875
9	96337666	96337972	5Y-H4K8ac_peak_16858	8.63676	PHF2_ENSG00000197724
9	96338292	96338735	5Y-H4K8ac_peak_16859	5.98695	PHF2_ENSG00000197724
9	96339309	96339718	5Y-H4K8ac_peak_16860	10.77539	PHF2_ENSG00000197724
9	96589999	96590204	5Y-H4K8ac_peak_16861	6.50117	
9	96590466	96590659	5Y-H4K8ac_peak_16862	5.59843	
9	96793141	96793394	5Y-H4K8ac_peak_16863	14.43262	PTPDC1_ENSG00000158079
9	96928671	96928938	5Y-H4K8ac_peak_16864	14.43262	RP11-2B6.3_ENSG00000269946
9	97020795	97021088	5Y-H4K8ac_peak_16865	5.64909	ZNF169_ENSG00000175787
9	97371902	97372201	5Y-H4K8ac_peak_16866	6.06162	
9	97373042	97373401	5Y-H4K8ac_peak_16867	7.28023	
9	97385585	97385949	5Y-H4K8ac_peak_16868	6.14981	
9	97466426	97466625	5Y-H4K8ac_peak_16869	6.79955	
9	97467117	97467620	5Y-H4K8ac_peak_16870	10.12234	
9	97476400	97476644	5Y-H4K8ac_peak_16871	7.31815	
9	97488339	97488663	5Y-H4K8ac_peak_16872	6.86362	C9orf3_ENSG00000148120
9	97628270	97628542	5Y-H4K8ac_peak_16873	6.59249	
9	97631247	97631457	5Y-H4K8ac_peak_16874	7.89273	
9	97689755	97690157	5Y-H4K8ac_peak_16875	7.72887	
9	97690361	97690825	5Y-H4K8ac_peak_16876	15.82359	
9	97766560	97767054	5Y-H4K8ac_peak_16877	14.55472	
9	97767517	97767757	5Y-H4K8ac_peak_16878	5.98695	
9	97853998	97854216	5Y-H4K8ac_peak_16879	7.03573	
9	97858717	97858998	5Y-H4K8ac_peak_16880	11.28133	

9	97865939	97866131	5Y-H4K8ac_peak_16881	8.24461	
9	97889690	97889949	5Y-H4K8ac_peak_16882	8.69112	
9	98079807	98080361	5Y-H4K8ac_peak_16883	6.08523	FANCC_ENSG00000158169
9	98213699	98213898	5Y-H4K8ac_peak_16884	8.21582	
9	98268520	98269109	5Y-H4K8ac_peak_16885	8.62703	RP11-435O5.5_ENSG00000271155
9	98273594	98273803	5Y-H4K8ac_peak_16886	9.05766	
9	98278658	98278850	5Y-H4K8ac_peak_16887	8.35139	PTCH1_ENSG00000185920
9	98343906	98344134	5Y-H4K8ac_peak_16888	9.63153	
9	98637974	98638198	5Y-H4K8ac_peak_16889	11.71078	LINC00476_ENSG00000175611;ERCC6L2_ENSG00000182150
9	98965759	98966018	5Y-H4K8ac_peak_16890	5.83608	
9	98981131	98981458	5Y-H4K8ac_peak_16891	7.64675	
9	99179218	99179617	5Y-H4K8ac_peak_16892	4.66683	ZNF367_ENSG00000165244
9	99179865	99180570	5Y-H4K8ac_peak_16893	8.51022	ZNF367_ENSG00000165244
9	99329276	99329494	5Y-H4K8ac_peak_16894	8.75926	
9	99381902	99382104	5Y-H4K8ac_peak_16895	10.11191	CDC14B_ENSG00000081377
9	99800898	99801090	5Y-H4K8ac_peak_16896	6.47245	CTSV_ENSG00000136943
9	99801648	99801856	5Y-H4K8ac_peak_16897	5.87382	CTSV_ENSG00000136943
9	100000122	100000548	5Y-H4K8ac_peak_16898	7.03573	RP11-498P14.5_ENSG00000203279;RP11-23J9.5_ENSG00000254876;CCDC180_ENSG00000197816;RP11-23J9.4_ENSG00000255036;RP11-23J9.6_ENSG00000254483
9	100264132	100264560	5Y-H4K8ac_peak_16899	7.60057	TMOD1_ENSG00000136842
9	100265957	100266218	5Y-H4K8ac_peak_16900	8.79957	
9	100267581	100268147	5Y-H4K8ac_peak_16901	11.35968	
9	100280102	100280342	5Y-H4K8ac_peak_16902	4.77126	RP11-244N9.6_ENSG00000228174
9	100683596	100684014	5Y-H4K8ac_peak_16903	5.23083	C9orf156_ENSG00000136932
9	100744903	100745317	5Y-H4K8ac_peak_16904	15.82713	ANP32B_ENSG00000136938
9	100745685	100747465	5Y-H4K8ac_peak_16905	12.26819	ANP32B_ENSG00000136938
9	100881153	100881607	5Y-H4K8ac_peak_16906	8.55081	TRIM14_ENSG00000106785
9	101018260	101018502	5Y-H4K8ac_peak_16907	4.90696	TBC1D2_ENSG00000095383
9	101559010	101559297	5Y-H4K8ac_peak_16908	6.37023	ANKS6_ENSG00000165138
9	101866577	101866960	5Y-H4K8ac_peak_16909	6.34046	TGFBR1_ENSG00000106799
9	101867732	101868067	5Y-H4K8ac_peak_16910	7.31102	
9	101946806	101947219	5Y-H4K8ac_peak_16911	11.93212	RN7SL794P_ENSG00000240235
9	102180621	102180922	5Y-H4K8ac_peak_16912	5.40331	
9	102227054	102227510	5Y-H4K8ac_peak_16913	9.15007	
9	102581916	102582180	5Y-H4K8ac_peak_16914	12.05638	RP11-554F20.1_ENSG00000237461
9	102669005	102669361	5Y-H4K8ac_peak_16915	5.64909	RP11-60I3.5_ENSG00000255145;STX17_ENSG00000136874
9	102861565	102861801	5Y-H4K8ac_peak_16916	6.77436	ERP44_ENSG00000023318;INVS_ENSG00000119509
9	103114895	103115179	5Y-H4K8ac_peak_16917	6.78128	TEX10_ENSG00000136891
9	103173601	103173806	5Y-H4K8ac_peak_16918	8.43511	
9	103189700	103190081	5Y-H4K8ac_peak_16919	13.54475	MSANTD3_ENSG00000066697
9	104248769	104249344	5Y-H4K8ac_peak_16920	13.4304	
9	107526539	107527003	5Y-H4K8ac_peak_16921	9.02782	NIPSNAP3B_ENSG00000165028
9	107730650	107731018	5Y-H4K8ac_peak_16922	7.80156	
9	108007001	108007684	5Y-H4K8ac_peak_16923	5.23083	SLC44A1_ENSG00000070214
9	108209840	108210202	5Y-H4K8ac_peak_16924	7.84116	FSD1L_ENSG00000106701
9	108418263	108418465	5Y-H4K8ac_peak_16925	9.15007	
9	109622928	109623221	5Y-H4K8ac_peak_16926	9.7353	

9	109625507	109625697	5Y-H4K8ac_peak_16927	6.76425	ZNF462_ENSG00000148143
9	110045681	110046005	5Y-H4K8ac_peak_16928	5.18654	RAD23B_ENSG00000119318
9	110125964	110126289	5Y-H4K8ac_peak_16929	6.46053	
9	110187355	110187648	5Y-H4K8ac_peak_16930	11.42066	
9	111929210	111929447	5Y-H4K8ac_peak_16931	9.30505	FRRS1L_ENSG00000260230
9	113017882	113018337	5Y-H4K8ac_peak_16932	5.23083	TXN_ENSG00000136810
9	113799951	113800981	5Y-H4K8ac_peak_16933	13.2534	LPAR1_ENSG00000198121
9	114245384	114245574	5Y-H4K8ac_peak_16934	6.46053	
9	114246243	114246884	5Y-H4K8ac_peak_16935	6.34046	KIAA0368_ENSG00000136813
9	114659135	114659582	5Y-H4K8ac_peak_16936	6.43775	UGCG_ENSG00000148154
9	114859921	114860120	5Y-H4K8ac_peak_16937	7.17184	
9	114937025	114938058	5Y-H4K8ac_peak_16938	15.28394	SUSD1_ENSG00000106868;RP11-165N19.2_ENSG00000225513
9	115512485	115512703	5Y-H4K8ac_peak_16939	6.73047	RP11-276E15.4_ENSG00000226609;SNX30_ENSG00000148158
9	115774081	115774422	5Y-H4K8ac_peak_16940	4.642	ZNF883_ENSG00000228623
9	116111857	116112126	5Y-H4K8ac_peak_16941	9.52603	BSPRY_ENSG00000119411
9	116139449	116139739	5Y-H4K8ac_peak_16942	7.89273	HDHD3_ENSG00000119431
9	116356643	116357301	5Y-H4K8ac_peak_16943	5.23083	
9	116413008	116413277	5Y-H4K8ac_peak_16944	6.00382	RP11-18B16.2_ENSG00000227482
9	116638671	116638979	5Y-H4K8ac_peak_16945	7.59101	ZNF618_ENSG00000157657
9	116918126	116918340	5Y-H4K8ac_peak_16946	3.88879	COL27A1_ENSG00000196739
9	117045215	117045415	5Y-H4K8ac_peak_16947	7.38046	
9	117049472	117049663	5Y-H4K8ac_peak_16948	5.70209	
9	117049978	117050252	5Y-H4K8ac_peak_16949	6.43775	
9	117157105	117157435	5Y-H4K8ac_peak_16950	5.03917	AKNA_ENSG00000106948
9	117373149	117374176	5Y-H4K8ac_peak_16951	19.17092	C9orf91_ENSG00000157693
9	117534621	117534846	5Y-H4K8ac_peak_16952	6.34046	
9	119898899	119899099	5Y-H4K8ac_peak_16953	5.03335	
9	120039244	120039471	5Y-H4K8ac_peak_16954	6.47245	RN7SKP125_ENSG00000222413
9	120044001	120044191	5Y-H4K8ac_peak_16955	4.29586	
9	120082923	120083120	5Y-H4K8ac_peak_16956	5.59843	
9	120117844	120118124	5Y-H4K8ac_peak_16957	4.14761	
9	120165309	120165540	5Y-H4K8ac_peak_16958	6.79955	
9	120165934	120166132	5Y-H4K8ac_peak_16959	8.35139	
9	120681717	120681970	5Y-H4K8ac_peak_16960	8.04493	
9	122318678	122318875	5Y-H4K8ac_peak_16961	4.29586	
9	123341849	123342251	5Y-H4K8ac_peak_16962	5.65584	CDK5RAP2_ENSG00000136861
9	123477059	123477351	5Y-H4K8ac_peak_16963	14.00559	MEGF9_ENSG00000106780
9	123555422	123555622	5Y-H4K8ac_peak_16964	4.77126	FBXW2_ENSG00000119402;RP11-27I1.4_ENSG00000214654
9	123638058	123638657	5Y-H4K8ac_peak_16965	6.53157	PHF19_ENSG00000119403
9	123638986	123639219	5Y-H4K8ac_peak_16966	9.36633	PHF19_ENSG00000119403
9	123682022	123682490	5Y-H4K8ac_peak_16967	5.44849	
9	123698486	123698775	5Y-H4K8ac_peak_16968	6.73047	
9	123837280	123837470	5Y-H4K8ac_peak_16969	11.09973	CNTRL_ENSG00000119397
9	124221700	124221894	5Y-H4K8ac_peak_16970	6.00388	
9	124270317	124270782	5Y-H4K8ac_peak_16971	7.38046	
9	124272790	124272981	5Y-H4K8ac_peak_16972	7.90236	

9	124273671	124273877	5Y-H4K8ac_peak_16973	6.50117	
9	124274167	124274358	5Y-H4K8ac_peak_16974	7.63144	
9	124289677	124290376	5Y-H4K8ac_peak_16975	9.15007	
9	124291228	124291501	5Y-H4K8ac_peak_16976	9.74189	
9	124308020	124308255	5Y-H4K8ac_peak_16977	8.43511	
9	124308553	124308962	5Y-H4K8ac_peak_16978	10.24719	
9	124327051	124327264	5Y-H4K8ac_peak_16979	5.07797	
9	124328856	124329736	5Y-H4K8ac_peak_16980	15.25183	DAB2IP_ENSG00000136848
9	124361385	124361709	5Y-H4K8ac_peak_16981	7.89142	
9	124407000	124407874	5Y-H4K8ac_peak_16982	8.79957	
9	124411837	124412111	5Y-H4K8ac_peak_16983	10.50538	
9	124461752	124462385	5Y-H4K8ac_peak_16984	5.41472	
9	124542888	124543082	5Y-H4K8ac_peak_16985	5.29015	
9	125109049	125109731	5Y-H4K8ac_peak_16986	7.49746	
9	125168488	125168961	5Y-H4K8ac_peak_16987	6.50117	
9	125590527	125590733	5Y-H4K8ac_peak_16988	8.69112	PDCL_ENSG00000136940
9	125591154	125591732	5Y-H4K8ac_peak_16989	13.60015	PDCL_ENSG00000136940
9	125667281	125667544	5Y-H4K8ac_peak_16990	6.75784	RC3H2_ENSG00000056586
9	125675706	125676053	5Y-H4K8ac_peak_16991	7.89142	ZBTB6_ENSG00000186130
9	125693428	125693778	5Y-H4K8ac_peak_16992	12.10416	ZBTB26_ENSG00000171448
9	126062728	126063199	5Y-H4K8ac_peak_16993	4.95697	
9	126106430	126106624	5Y-H4K8ac_peak_16994	4.24332	
9	126691967	126692361	5Y-H4K8ac_peak_16995	15.06486	DENND1A_ENSG00000119522
9	126692553	126693155	5Y-H4K8ac_peak_16996	9.34555	DENND1A_ENSG00000119522
9	127020895	127021185	5Y-H4K8ac_peak_16997	5.64909	
9	127082327	127082627	5Y-H4K8ac_peak_16998	10.1994	
9	127182071	127182274	5Y-H4K8ac_peak_16999	10.35586	
9	127213023	127213214	5Y-H4K8ac_peak_17000	5.98695	GPR144_ENSG00000180264
9	127239328	127240772	5Y-H4K8ac_peak_17001	9.79526	
9	127274601	127274810	5Y-H4K8ac_peak_17002	5.12488	
9	127275076	127275359	5Y-H4K8ac_peak_17003	11.09973	
9	127275801	127276050	5Y-H4K8ac_peak_17004	6.14981	
9	127539221	127540052	5Y-H4K8ac_peak_17005	8.46999	OLFML2A_ENSG00000185585
9	127623899	127624177	5Y-H4K8ac_peak_17006	11.30958	RPL35_ENSG00000136942;ARPC5L_ENSG00000136950
9	127630968	127631243	5Y-H4K8ac_peak_17007	8.58926	
9	128023444	128023794	5Y-H4K8ac_peak_17008	7.57144	GAPVD1_ENSG00000165219
9	128102412	128102614	5Y-H4K8ac_peak_17009	4.51076	
9	128170112	128171584	5Y-H4K8ac_peak_17010	15.03212	
9	128469008	128469390	5Y-H4K8ac_peak_17011	10.44054	MAPKAP1_ENSG00000119487
9	128509480	128509911	5Y-H4K8ac_peak_17012	7.31102	RP11-423C15.3_ENSG00000229582;PBX3_ENSG00000167081
9	128510114	128510315	5Y-H4K8ac_peak_17013	5.64909	PBX3_ENSG00000167081
9	128753114	128753331	5Y-H4K8ac_peak_17014	5.23083	
9	128756366	128756762	5Y-H4K8ac_peak_17015	5.97532	
9	128821404	128821645	5Y-H4K8ac_peak_17016	6.73385	
9	129088536	129088839	5Y-H4K8ac_peak_17017	11.53136	MVB12B_ENSG00000196814
9	129089471	129089683	5Y-H4K8ac_peak_17018	7.69843	MVB12B_ENSG00000196814

9	129201385	129201576	5Y-H4K8ac_peak_17019	7.35333	
9	129202375	129202608	5Y-H4K8ac_peak_17020	10.32379	
9	129268767	129269188	5Y-H4K8ac_peak_17021	5.24695	
9	129269490	129269720	5Y-H4K8ac_peak_17022	12.44818	
9	129270416	129270628	5Y-H4K8ac_peak_17023	5.41472	
9	129272694	129273504	5Y-H4K8ac_peak_17024	9.22312	
9	129275651	129277025	5Y-H4K8ac_peak_17025	10.1994	
9	129277238	129277511	5Y-H4K8ac_peak_17026	8.43511	
9	129277762	129278021	5Y-H4K8ac_peak_17027	10.69698	RP11-205K6.1_ENSG00000233721
9	129279235	129279540	5Y-H4K8ac_peak_17028	6.22669	RP11-205K6.1_ENSG00000233721
9	129282105	129282718	5Y-H4K8ac_peak_17029	6.53157	
9	129283879	129285124	5Y-H4K8ac_peak_17030	9.7353	
9	129286678	129287270	5Y-H4K8ac_peak_17031	6.95541	
9	129288695	129289375	5Y-H4K8ac_peak_17032	4.0639	RP11-205K6.2_ENSG00000226078
9	129293343	129293654	5Y-H4K8ac_peak_17033	7.79831	AL356309.1_ENSG00000221768
9	129293851	129294091	5Y-H4K8ac_peak_17034	8.73985	AL356309.1_ENSG00000221768
9	129294384	129294823	5Y-H4K8ac_peak_17035	7.89142	
9	129295186	129295764	5Y-H4K8ac_peak_17036	13.53477	
9	129296136	129296418	5Y-H4K8ac_peak_17037	18.10232	
9	129296624	129296878	5Y-H4K8ac_peak_17038	11.22005	
9	129299123	129299703	5Y-H4K8ac_peak_17039	9.37238	
9	129300500	129300709	5Y-H4K8ac_peak_17040	6.76096	
9	129309891	129310199	5Y-H4K8ac_peak_17041	7.27597	
9	129310649	129311711	5Y-H4K8ac_peak_17042	11.69482	
9	129312184	129312463	5Y-H4K8ac_peak_17043	12.10416	
9	129312739	129313846	5Y-H4K8ac_peak_17044	18.10232	
9	129319912	129320133	5Y-H4K8ac_peak_17045	8.2913	
9	129326712	129327010	5Y-H4K8ac_peak_17046	6.54441	
9	129424982	129425187	5Y-H4K8ac_peak_17047	4.84727	
9	129433272	129433980	5Y-H4K8ac_peak_17048	7.50501	
9	129434228	129434452	5Y-H4K8ac_peak_17049	5.03564	
9	129434678	129435240	5Y-H4K8ac_peak_17050	13.86507	
9	129467897	129468098	5Y-H4K8ac_peak_17051	5.64909	
9	129484496	129485659	5Y-H4K8ac_peak_17052	15.23166	
9	129485896	129486187	5Y-H4K8ac_peak_17053	13.48518	
9	129486574	129486804	5Y-H4K8ac_peak_17054	6.53157	
9	129508145	129508387	5Y-H4K8ac_peak_17055	6.87117	
9	129566796	129567101	5Y-H4K8ac_peak_17056	6.73385	ZBTB43_ENSG00000169155
9	129622643	129622869	5Y-H4K8ac_peak_17057	11.1169	ZBTB34_ENSG00000177125
9	129798521	129799046	5Y-H4K8ac_peak_17058	8.04943	
9	129885318	129885547	5Y-H4K8ac_peak_17059	6.34245	ANGPTL2_ENSG00000136859
9	129987656	129987874	5Y-H4K8ac_peak_17060	6.09071	
9	130007154	130007398	5Y-H4K8ac_peak_17061	7.04637	
9	130213213	130213622	5Y-H4K8ac_peak_17062	6.78318	RPL12_ENSG00000197958;LRSAM1_ENSG00000148356
9	130319805	130320448	5Y-H4K8ac_peak_17063	8.21582	
9	130331648	130332146	5Y-H4K8ac_peak_17064	5.41472	

9	130333275	130333894	5Y-H4K8ac_peak_17065	5.65584	
9	130370535	130371067	5Y-H4K8ac_peak_17066	4.55128	
9	130423021	130423223	5Y-H4K8ac_peak_17067	7.30348	
9	130426409	130427187	5Y-H4K8ac_peak_17068	11.62198	
9	130427615	130428462	5Y-H4K8ac_peak_17069	8.4454	
9	130429036	130429337	5Y-H4K8ac_peak_17070	5.35202	
9	130497135	130497568	5Y-H4K8ac_peak_17071	16.79568	TOR2A_ENSG00000160404
9	130515997	130516382	5Y-H4K8ac_peak_17072	6.37023	
9	130541348	130541547	5Y-H4K8ac_peak_17073	5.99504	SH2D3C_ENSG00000095370
9	130547530	130548008	5Y-H4K8ac_peak_17074	3.96434	CDK9_ENSG00000136807;MIR3960_ENSG00000266070
9	130576000	130576363	5Y-H4K8ac_peak_17075	5.23083	
9	130616464	130616973	5Y-H4K8ac_peak_17076	12.60508	ENG_ENSG00000106991
9	130617210	130617616	5Y-H4K8ac_peak_17077	5.23083	ENG_ENSG00000106991
9	130620076	130620493	5Y-H4K8ac_peak_17078	10.35586	
9	130640120	130640468	5Y-H4K8ac_peak_17079	9.34555	AK1_ENSG00000106992
9	130661421	130661637	5Y-H4K8ac_peak_17080	7.64648	
9	130666018	130666256	5Y-H4K8ac_peak_17081	4.82324	
9	130678717	130679227	5Y-H4K8ac_peak_17082	10.18964	ST6GALNAC4_ENSG00000136840
9	130699705	130700027	5Y-H4K8ac_peak_17083	5.59843	DPM2_ENSG00000136908
9	130725895	130726442	5Y-H4K8ac_peak_17084	5.03564	
9	130727059	130727286	5Y-H4K8ac_peak_17085	5.47078	
9	130728088	130728597	5Y-H4K8ac_peak_17086	6.98416	
9	130729004	130729212	5Y-H4K8ac_peak_17087	6.96612	
9	130729452	130730012	5Y-H4K8ac_peak_17088	6.77436	
9	130730369	130730578	5Y-H4K8ac_peak_17089	7.89273	
9	130732428	130732708	5Y-H4K8ac_peak_17090	4.77126	
9	130733963	130734682	5Y-H4K8ac_peak_17091	7.59101	
9	130734928	130735283	5Y-H4K8ac_peak_17092	9.89244	
9	130735518	130736034	5Y-H4K8ac_peak_17093	10.60083	
9	130736571	130737636	5Y-H4K8ac_peak_17094	10.16277	
9	130828849	130829325	5Y-H4K8ac_peak_17095	8.73392	
9	130830700	130831012	5Y-H4K8ac_peak_17096	6.73385	NAIF1_ENSG00000171169;SLC25A25_ENSG00000148339
9	130852996	130853359	5Y-H4K8ac_peak_17097	9.30505	
9	130921637	130921906	5Y-H4K8ac_peak_17098	9.15007	C9orf16_ENSG00000171159
9	130953413	130954146	5Y-H4K8ac_peak_17099	7.50148	
9	131005181	131005410	5Y-H4K8ac_peak_17100	5.91107	
9	131005629	131006111	5Y-H4K8ac_peak_17101	12.87247	MIR199B_ENSG00000207581
9	131006962	131007181	5Y-H4K8ac_peak_17102	5.96059	MIR199B_ENSG00000207581;MIR3154_ENSG00000264823
9	131007529	131008057	5Y-H4K8ac_peak_17103	18.78631	MIR199B_ENSG00000207581;MIR3154_ENSG00000264823
9	131038623	131038983	5Y-H4K8ac_peak_17104	7.38046	SWI5_ENSG00000175854;GOLGA2_ENSG00000167110
9	131102475	131102669	5Y-H4K8ac_peak_17105	6.78128	SLC27A4_ENSG00000167114
9	131124234	131124482	5Y-H4K8ac_peak_17106	6.78128	
9	131181911	131182658	5Y-H4K8ac_peak_17107	6.43775	
9	131201199	131201459	5Y-H4K8ac_peak_17108	10.35586	
9	131285517	131285767	5Y-H4K8ac_peak_17109	5.40331	
9	131419177	131419612	5Y-H4K8ac_peak_17110	9.52603	WDR34_ENSG00000119333

9	131419893	131420166	5Y-H4K8ac_peak_17111	8.69112	WDR34_ENSG00000119333
9	131450965	131451159	5Y-H4K8ac_peak_17112	9.51254	
9	131452319	131452667	5Y-H4K8ac_peak_17113	15.06486	
9	131465375	131465693	5Y-H4K8ac_peak_17114	4.0639	PKN3_ENSG00000160447
9	131486148	131486407	5Y-H4K8ac_peak_17115	11.1169	ZDHHC12_ENSG00000160446;RP11-545E17.3_ENSG00000223478
9	131534387	131534779	5Y-H4K8ac_peak_17116	9.01738	ZER1_ENSG00000160445
9	131549177	131549373	5Y-H4K8ac_peak_17117	8.73392	TBC1D13_ENSG00000107021
9	131607908	131608378	5Y-H4K8ac_peak_17118	4.84727	
9	131709465	131709799	5Y-H4K8ac_peak_17119	4.15658	DOLK_ENSG00000175283;NUP188_ENSG00000095319
9	131832416	131832739	5Y-H4K8ac_peak_17120	4.24332	
9	131901730	131902266	5Y-H4K8ac_peak_17121	7.11863	
9	131902507	131902935	5Y-H4K8ac_peak_17122	9.38276	
9	131936171	131936362	5Y-H4K8ac_peak_17123	7.72887	
9	131936627	131937121	5Y-H4K8ac_peak_17124	5.18558	RP11-247A12.8_ENSG00000268050
9	131940758	131941104	5Y-H4K8ac_peak_17125	7.30348	IER5L_ENSG00000188483
9	131986563	131987023	5Y-H4K8ac_peak_17126	4.35469	
9	132080738	132080937	5Y-H4K8ac_peak_17127	8.1667	
9	132082396	132083082	5Y-H4K8ac_peak_17128	9.01738	C9orf106_ENSG00000179082
9	132096531	132097003	5Y-H4K8ac_peak_17129	12.19856	
9	132114153	132114576	5Y-H4K8ac_peak_17130	8.14913	
9	132114798	132115432	5Y-H4K8ac_peak_17131	9.51254	
9	132154561	132154771	5Y-H4K8ac_peak_17132	4.8887	
9	132156250	132156676	5Y-H4K8ac_peak_17133	9.59236	
9	132157015	132157342	5Y-H4K8ac_peak_17134	10.60083	
9	132164711	132164905	5Y-H4K8ac_peak_17135	10.1994	
9	132170870	132171188	5Y-H4K8ac_peak_17136	4.15658	
9	132175179	132175965	5Y-H4K8ac_peak_17137	9.30206	
9	132176275	132177367	5Y-H4K8ac_peak_17138	9.74838	
9	132177690	132178044	5Y-H4K8ac_peak_17139	4.15658	
9	132200089	132200291	5Y-H4K8ac_peak_17140	5.03564	RP11-65J3.2_ENSG00000226355
9	132220625	132222466	5Y-H4K8ac_peak_17141	22.07994	
9	132222877	132223411	5Y-H4K8ac_peak_17142	13.55689	
9	132229298	132229800	5Y-H4K8ac_peak_17143	7.59101	
9	132244248	132244683	5Y-H4K8ac_peak_17144	22.9394	RP11-65J3.14_ENSG00000261334
9	132245028	132246325	5Y-H4K8ac_peak_17145	20.83544	RP11-65J3.14_ENSG00000261334;LINC00963_ENSG00000204054
9	132249838	132250701	5Y-H4K8ac_peak_17146	7.50148	
9	132252103	132252358	5Y-H4K8ac_peak_17147	5.81394	
9	132253747	132253991	5Y-H4K8ac_peak_17148	8.9562	
9	132255925	132256120	5Y-H4K8ac_peak_17149	8.05083	
9	132257395	132257645	5Y-H4K8ac_peak_17150	5.56894	
9	132258586	132258951	5Y-H4K8ac_peak_17151	8.43511	
9	132262292	132262613	5Y-H4K8ac_peak_17152	5.64909	
9	132262836	132263045	5Y-H4K8ac_peak_17153	9.15007	
9	132312806	132313085	5Y-H4K8ac_peak_17154	11.09973	
9	132319467	132320213	5Y-H4K8ac_peak_17155	10.01583	
9	132320620	132320964	5Y-H4K8ac_peak_17156	12.05638	

9	132321432	132321680	5Y-H4K8ac_peak_17157	6.77436	
9	132331224	132331425	5Y-H4K8ac_peak_17158	9.7353	
9	132370050	132370365	5Y-H4K8ac_peak_17159	5.41472	NTMT1_ENSG00000148335
9	132373106	132373443	5Y-H4K8ac_peak_17160	4.33574	
9	132382103	132382597	5Y-H4K8ac_peak_17161	11.1169	C9orf50_ENSG00000179058
9	132387866	132388262	5Y-H4K8ac_peak_17162	7.38046	
9	132388502	132388706	5Y-H4K8ac_peak_17163	9.23159	
9	132401897	132402210	5Y-H4K8ac_peak_17164	7.08802	RP11-483H20.4_ENSG00000234789
9	132404841	132405285	5Y-H4K8ac_peak_17165	13.99721	ASB6_ENSG00000148331
9	132411492	132412325	5Y-H4K8ac_peak_17166	18.33806	
9	132413175	132413791	5Y-H4K8ac_peak_17167	5.64909	
9	132438300	132438546	5Y-H4K8ac_peak_17168	5.98695	
9	132459173	132460455	5Y-H4K8ac_peak_17169	19.71555	
9	132489096	132489307	5Y-H4K8ac_peak_17170	5.51139	
9	132499837	132500538	5Y-H4K8ac_peak_17171	16.27715	
9	132514968	132515183	5Y-H4K8ac_peak_17172	7.18391	PTGES_ENSG00000148344
9	132563781	132564262	5Y-H4K8ac_peak_17173	5.8635	
9	132564864	132565093	5Y-H4K8ac_peak_17174	7.07936	TOR1B_ENSG00000136816
9	132624936	132625145	5Y-H4K8ac_peak_17175	8.04493	
9	132626317	132626580	5Y-H4K8ac_peak_17176	6.17374	
9	132647436	132647931	5Y-H4K8ac_peak_17177	12.84845	RP11-409K20.7_ENSG00000224988
9	132805791	132805986	5Y-H4K8ac_peak_17178	5.18494	FNBP1_ENSG00000187239
9	132815894	132816639	5Y-H4K8ac_peak_17179	15.40526	GPR107_ENSG00000148358
9	132962706	132962967	5Y-H4K8ac_peak_17180	11.43902	
9	133003318	133003564	5Y-H4K8ac_peak_17181	5.9856	
9	133051752	133052158	5Y-H4K8ac_peak_17182	5.65584	
9	133274822	133275112	5Y-H4K8ac_peak_17183	5.03917	AL354898.1_ENSG00000215428;RN7SL665P_ENSG00000264169
9	133281551	133281855	5Y-H4K8ac_peak_17184	5.25819	
9	133289554	133289801	5Y-H4K8ac_peak_17185	9.23159	
9	133292508	133292703	5Y-H4K8ac_peak_17186	5.15917	
9	133294254	133294732	5Y-H4K8ac_peak_17187	7.04158	
9	133295106	133295333	5Y-H4K8ac_peak_17188	7.04158	
9	133295961	133296288	5Y-H4K8ac_peak_17189	7.04158	
9	133296655	133296939	5Y-H4K8ac_peak_17190	8.5159	
9	133297663	133298287	5Y-H4K8ac_peak_17191	9.36633	
9	133300035	133300952	5Y-H4K8ac_peak_17192	10.21121	
9	133301314	133301686	5Y-H4K8ac_peak_17193	7.681	
9	133302237	133302430	5Y-H4K8ac_peak_17194	7.87406	
9	133304705	133305111	5Y-H4K8ac_peak_17195	5.99504	
9	133307371	133307652	5Y-H4K8ac_peak_17196	9.51254	
9	133308444	133309250	5Y-H4K8ac_peak_17197	13.38945	
9	133319800	133320432	5Y-H4K8ac_peak_17198	7.30348	ASS1_ENSG00000130707
9	133320914	133321409	5Y-H4K8ac_peak_17199	9.79526	ASS1_ENSG00000130707
9	133321770	133322202	5Y-H4K8ac_peak_17200	9.89244	
9	133322399	133323955	5Y-H4K8ac_peak_17201	14.39608	
9	133324217	133324504	5Y-H4K8ac_peak_17202	6.13242	snoU13_ENSG00000238298

9	133325535	133327710	5Y-H4K8ac_peak_17203	10.35586	snoU13_ENSG00000238298
9	133328580	133328784	5Y-H4K8ac_peak_17204	4.42435	
9	133329322	133329798	5Y-H4K8ac_peak_17205	9.79526	
9	133412625	133412969	5Y-H4K8ac_peak_17206	5.98695	
9	133455015	133455457	5Y-H4K8ac_peak_17207	9.11354	FUBP3_ENSG00000107164
9	133557163	133557368	5Y-H4K8ac_peak_17208	11.48691	
9	133568349	133568732	5Y-H4K8ac_peak_17209	4.50834	EXOSC2_ENSG00000130713
9	133587661	133587890	5Y-H4K8ac_peak_17210	5.91107	
9	133588250	133588635	5Y-H4K8ac_peak_17211	6.08523	ABL1_ENSG00000097007
9	133804155	133804372	5Y-H4K8ac_peak_17212	5.37588	
9	133938546	133938999	5Y-H4K8ac_peak_17213	8.14913	
9	133976476	133976672	5Y-H4K8ac_peak_17214	5.13361	
9	134000050	134000401	5Y-H4K8ac_peak_17215	7.18918	NUP214_ENSG00000126883
9	134153679	134154065	5Y-H4K8ac_peak_17216	11.60073	
9	134164724	134164982	5Y-H4K8ac_peak_17217	7.89273	PPAPDC3_ENSG00000160539
9	134193171	134193547	5Y-H4K8ac_peak_17218	6.78318	
9	134225102	134225558	5Y-H4K8ac_peak_17219	7.59101	
9	134269512	134270159	5Y-H4K8ac_peak_17220	14.31711	PRRC2B_ENSG00000130723
9	134377843	134378104	5Y-H4K8ac_peak_17221	6.37023	POMT1_ENSG00000130714
9	134378469	134378758	5Y-H4K8ac_peak_17222	6.78128	POMT1_ENSG00000130714
9	134399239	134399621	5Y-H4K8ac_peak_17223	5.29015	
9	134406826	134407424	5Y-H4K8ac_peak_17224	13.63503	UCK1_ENSG00000130717
9	134743932	134744192	5Y-H4K8ac_peak_17225	7.43638	
9	134954940	134955211	5Y-H4K8ac_peak_17226	7.63144	MED27_ENSG00000160563
9	135107124	135107511	5Y-H4K8ac_peak_17227	5.07797	
9	135281810	135282091	5Y-H4K8ac_peak_17228	6.22669	TTF1_ENSG00000125482
9	135374551	135374781	5Y-H4K8ac_peak_17229	6.35402	
9	135378317	135378683	5Y-H4K8ac_peak_17230	6.40964	
9	135381403	135381620	5Y-H4K8ac_peak_17231	4.50834	
9	135383823	135384036	5Y-H4K8ac_peak_17232	8.43511	
9	135385475	135385676	5Y-H4K8ac_peak_17233	9.66296	
9	135419353	135419645	5Y-H4K8ac_peak_17234	5.98695	
9	135420656	135421373	5Y-H4K8ac_peak_17235	7.11863	
9	135422387	135422695	5Y-H4K8ac_peak_17236	7.96285	
9	135545169	135545675	5Y-H4K8ac_peak_17237	6.37023	DDX31_ENSG00000125485;GTF3C4_ENSG00000125484
9	135709210	135709490	5Y-H4K8ac_peak_17238	4.0639	
9	135722138	135722516	5Y-H4K8ac_peak_17239	5.23083	
9	135723911	135724408	5Y-H4K8ac_peak_17240	7.38046	
9	135753845	135754195	5Y-H4K8ac_peak_17241	10.93537	AK8_ENSG00000165695;C9orf9_ENSG00000165698
9	135819608	135820011	5Y-H4K8ac_peak_17242	5.87725	TSC1_ENSG00000165699;GFI1B_ENSG00000165702
9	135905175	135905740	5Y-H4K8ac_peak_17243	9.30505	GTF3C5_ENSG00000148308
9	135906186	135906763	5Y-H4K8ac_peak_17244	10.5157	GTF3C5_ENSG00000148308
9	135989264	135989501	5Y-H4K8ac_peak_17245	4.50834	
9	135989724	135990072	5Y-H4K8ac_peak_17246	5.9856	
9	135992942	135993172	5Y-H4K8ac_peak_17247	6.4221	
9	135995229	135995626	5Y-H4K8ac_peak_17248	7.90005	

9	135996100	135996471	5Y-H4K8ac_peak_17249	6.27205	
9	136008344	136008675	5Y-H4K8ac_peak_17250	7.18391	
9	136024130	136024505	5Y-H4K8ac_peak_17251	6.54441	
9	136038620	136039071	5Y-H4K8ac_peak_17252	6.67017	RALGDS_ENSG00000160271;GBGT1_ENSG00000148288
9	136215124	136215407	5Y-H4K8ac_peak_17253	10.16277	MED22_ENSG00000148297;RPL7A_ENSG00000148303;SNORD24_ENSG00000206611
9	136242366	136242677	5Y-H4K8ac_peak_17254	10.19038	SURF4_ENSG00000148248;C9orf96_ENSG00000198870
9	136243270	136243691	5Y-H4K8ac_peak_17255	10.1994	SURF4_ENSG00000148248;C9orf96_ENSG00000198870
9	136283050	136283598	5Y-H4K8ac_peak_17256	6.80915	REXO4_ENSG00000148300
9	136313911	136314258	5Y-H4K8ac_peak_17257	17.04999	
9	136325157	136325454	5Y-H4K8ac_peak_17258	7.90751	CACFD1_ENSG00000160325
9	136334821	136335154	5Y-H4K8ac_peak_17259	5.12213	
9	136349761	136350303	5Y-H4K8ac_peak_17260	5.64988	
9	136350839	136351252	5Y-H4K8ac_peak_17261	5.98695	
9	136357972	136358311	5Y-H4K8ac_peak_17262	4.00285	
9	136359055	136359447	5Y-H4K8ac_peak_17263	5.59843	
9	136370755	136370976	5Y-H4K8ac_peak_17264	7.11863	
9	136374297	136374542	5Y-H4K8ac_peak_17265	5.24695	
9	136374744	136375068	5Y-H4K8ac_peak_17266	12.65187	
9	136377981	136378767	5Y-H4K8ac_peak_17267	14.00559	
9	136379711	136381050	5Y-H4K8ac_peak_17268	18.06205	
9	136387149	136387447	5Y-H4K8ac_peak_17269	7.00982	
9	136387661	136387977	5Y-H4K8ac_peak_17270	10.72308	
9	136391540	136393573	5Y-H4K8ac_peak_17271	11.1169	TMEM8C_ENSG00000187616
9	136398716	136399012	5Y-H4K8ac_peak_17272	10.35586	
9	136399302	136399660	5Y-H4K8ac_peak_17273	7.64648	
9	136399894	136400145	5Y-H4K8ac_peak_17274	8.54067	
9	136400831	136401051	5Y-H4K8ac_peak_17275	9.13495	
9	136457274	136457469	5Y-H4K8ac_peak_17276	4.79585	
9	136469968	136470280	5Y-H4K8ac_peak_17277	5.62406	
9	136470476	136470775	5Y-H4K8ac_peak_17278	13.72401	
9	136473992	136474472	5Y-H4K8ac_peak_17279	6.64195	
9	136479989	136480265	5Y-H4K8ac_peak_17280	8.63676	
9	136496934	136497409	5Y-H4K8ac_peak_17281	10.1994	LL09NC01-254D11.1_ENSG00000261018
9	136497702	136497986	5Y-H4K8ac_peak_17282	11.28133	LL09NC01-254D11.1_ENSG00000261018
9	136498776	136499009	5Y-H4K8ac_peak_17283	11.05199	LL09NC01-254D11.1_ENSG00000261018
9	136499212	136501131	5Y-H4K8ac_peak_17284	17.08376	LL09NC01-254D11.1_ENSG00000261018;DBH_ENSG00000123454
9	136501573	136502864	5Y-H4K8ac_peak_17285	5.72233	DBH_ENSG00000123454
9	136527292	136527558	5Y-H4K8ac_peak_17286	5.77617	
9	136544244	136544770	5Y-H4K8ac_peak_17287	10.73606	
9	136547579	136547850	5Y-H4K8ac_peak_17288	6.49786	
9	136548151	136548636	5Y-H4K8ac_peak_17289	16.01457	
9	136548927	136549400	5Y-H4K8ac_peak_17290	10.15788	
9	136549719	136549961	5Y-H4K8ac_peak_17291	6.80915	
9	136569271	136569600	5Y-H4K8ac_peak_17292	5.23083	
9	136580645	136580840	5Y-H4K8ac_peak_17293	5.41472	
9	136602201	136602448	5Y-H4K8ac_peak_17294	11.52376	

9	136603763	136603977	5Y-H4K8ac_peak_17295	4.55128	
9	136734397	136734630	5Y-H4K8ac_peak_17296	7.89142	
9	136735321	136735551	5Y-H4K8ac_peak_17297	6.95541	
9	136738605	136738806	5Y-H4K8ac_peak_17298	9.38276	
9	136740257	136740540	5Y-H4K8ac_peak_17299	10.90365	
9	136740845	136741288	5Y-H4K8ac_peak_17300	7.41197	
9	136742060	136742468	5Y-H4K8ac_peak_17301	5.18558	
9	136746513	136746777	5Y-H4K8ac_peak_17302	8.20773	
9	136747322	136747706	5Y-H4K8ac_peak_17303	6.86362	
9	136748044	136748331	5Y-H4K8ac_peak_17304	8.18236	
9	136784417	136784632	5Y-H4K8ac_peak_17305	8.39058	
9	136794106	136794364	5Y-H4K8ac_peak_17306	9.22312	
9	136809338	136809825	5Y-H4K8ac_peak_17307	4.642	
9	136820235	136820426	5Y-H4K8ac_peak_17308	9.68432	
9	136821020	136821502	5Y-H4K8ac_peak_17309	10.19948	
9	137000297	137000937	5Y-H4K8ac_peak_17310	6.80915	LL09NC01-251B2.3_ENSG00000273249;WDR5_ENSG00000196363
9	137028474	137028843	5Y-H4K8ac_peak_17311	5.89922	RNU6ATAC_ENSG00000221676
9	137030254	137030691	5Y-H4K8ac_peak_17312	11.48691	RNU6ATAC_ENSG00000221676
9	137030965	137031219	5Y-H4K8ac_peak_17313	6.15578	
9	137184204	137184701	5Y-H4K8ac_peak_17314	12.73673	
9	137184953	137186141	5Y-H4K8ac_peak_17315	18.10232	
9	137186644	137186860	5Y-H4K8ac_peak_17316	7.09658	RP11-145E17.2_ENSG00000223729
9	137212316	137212523	5Y-H4K8ac_peak_17317	6.46053	
9	137217119	137217953	5Y-H4K8ac_peak_17318	14.31711	
9	137239352	137239780	5Y-H4K8ac_peak_17319	10.6523	
9	137240907	137241554	5Y-H4K8ac_peak_17320	10.31017	
9	137242255	137242530	5Y-H4K8ac_peak_17321	6.34245	
9	137248456	137248873	5Y-H4K8ac_peak_17322	6.25061	
9	137253706	137253912	5Y-H4K8ac_peak_17323	5.64982	
9	137258958	137259606	5Y-H4K8ac_peak_17324	7.90751	
9	137334086	137334430	5Y-H4K8ac_peak_17325	6.22669	
9	137334667	137335117	5Y-H4K8ac_peak_17326	7.89273	
9	137347661	137348183	5Y-H4K8ac_peak_17327	15.77107	
9	137348568	137349032	5Y-H4K8ac_peak_17328	8.69112	
9	137409202	137409494	5Y-H4K8ac_peak_17329	7.29055	
9	137598562	137598972	5Y-H4K8ac_peak_17330	5.0078	
9	137599492	137599976	5Y-H4K8ac_peak_17331	11.19336	
9	137600478	137600882	5Y-H4K8ac_peak_17332	8.2913	
9	137966690	137967027	5Y-H4K8ac_peak_17333	7.69843	OLFM1_ENSG00000130558
9	137967377	137967660	5Y-H4K8ac_peak_17334	7.97699	OLFM1_ENSG00000130558
9	138025900	138026338	5Y-H4K8ac_peak_17335	4.50834	
9	138037574	138037864	5Y-H4K8ac_peak_17336	9.63153	
9	138038632	138040308	5Y-H4K8ac_peak_17337	14.86775	
9	138059698	138059908	5Y-H4K8ac_peak_17338	6.47245	
9	138107751	138108089	5Y-H4K8ac_peak_17339	5.23083	
9	138116294	138117202	5Y-H4K8ac_peak_17340	11.19336	

9	138142769	138142959	5Y-H4K8ac_peak_17341	5.62406	
9	138174753	138175043	5Y-H4K8ac_peak_17342	4.29586	
9	138241518	138241953	5Y-H4K8ac_peak_17343	6.54441	
9	138348789	138349025	5Y-H4K8ac_peak_17344	5.65584	
9	138370729	138371387	5Y-H4K8ac_peak_17345	9.30505	PPP1R26_ENSG00000196422
9	138392720	138392924	5Y-H4K8ac_peak_17346	6.96612	C9orf116_ENSG00000160345;MRPS2_ENSG00000122140
9	138554980	138555179	5Y-H4K8ac_peak_17347	6.50117	LCN9_ENSG00000148386
9	138594032	138594251	5Y-H4K8ac_peak_17348	10.11191	KCNT1_ENSG00000107147
9	138799740	138800244	5Y-H4K8ac_peak_17349	5.98695	CAMSAP1_ENSG00000130559;RP11-432J22.2_ENSG00000238058
9	138870796	138871553	5Y-H4K8ac_peak_17350	14.72057	
9	138884273	138885533	5Y-H4K8ac_peak_17351	11.41382	
9	138903798	138904460	5Y-H4K8ac_peak_17352	11.22005	
9	139001094	139001377	5Y-H4K8ac_peak_17353	5.34199	RP11-83N9.5_ENSG00000260193
9	139009911	139010672	5Y-H4K8ac_peak_17354	11.8513	C9orf69_ENSG00000238227
9	139010953	139011341	5Y-H4K8ac_peak_17355	6.46606	C9orf69_ENSG00000238227
9	139159308	139159694	5Y-H4K8ac_peak_17356	6.37023	
9	139219795	139220667	5Y-H4K8ac_peak_17357	17.18444	DKFZP434A062_ENSG00000267845
9	139220948	139221726	5Y-H4K8ac_peak_17358	15.84891	GPSM1_ENSG00000160360
9	139221949	139222411	5Y-H4K8ac_peak_17359	10.43045	GPSM1_ENSG00000160360
9	139240980	139241307	5Y-H4K8ac_peak_17360	6.29918	
9	139242441	139243150	5Y-H4K8ac_peak_17361	11.71078	
9	139243945	139244461	5Y-H4K8ac_peak_17362	6.60109	
9	139245524	139246209	5Y-H4K8ac_peak_17363	7.18918	
9	139254702	139255154	5Y-H4K8ac_peak_17364	5.23083	
9	139255365	139256100	5Y-H4K8ac_peak_17365	12.60556	
9	139256479	139257189	5Y-H4K8ac_peak_17366	6.78128	
9	139257907	139258127	5Y-H4K8ac_peak_17367	11.1169	DNLZ_ENSG00000213221
9	139258521	139259044	5Y-H4K8ac_peak_17368	12.65187	DNLZ_ENSG00000213221
9	139259260	139259854	5Y-H4K8ac_peak_17369	7.89273	
9	139260102	139260506	5Y-H4K8ac_peak_17370	7.89273	
9	139268241	139268865	5Y-H4K8ac_peak_17371	13.27281	CARD9_ENSG00000187796
9	139269640	139269830	5Y-H4K8ac_peak_17372	9.63153	
9	139270148	139270349	5Y-H4K8ac_peak_17373	5.98695	
9	139294266	139294544	5Y-H4K8ac_peak_17374	9.01738	
9	139294822	139295154	5Y-H4K8ac_peak_17375	6.98416	
9	139305232	139305456	5Y-H4K8ac_peak_17376	4.642	SDCCAG3_ENSG00000165689;PMPCA_ENSG00000165688
9	139333785	139334227	5Y-H4K8ac_peak_17377	6.77436	INPP5E_ENSG00000148384
9	139378473	139378686	5Y-H4K8ac_peak_17378	7.50501	C9orf163_ENSG00000196366
9	139497044	139497251	5Y-H4K8ac_peak_17379	4.50834	U3_ENSG00000252440
9	139540476	139541202	5Y-H4K8ac_peak_17380	7.89273	
9	139541616	139542004	5Y-H4K8ac_peak_17381	7.01266	
9	139550785	139551106	5Y-H4K8ac_peak_17382	7.21932	
9	139559539	139560011	5Y-H4K8ac_peak_17383	7.59101	
9	139582241	139582671	5Y-H4K8ac_peak_17384	9.51254	AGPAT2_ENSG00000169692
9	139607147	139607406	5Y-H4K8ac_peak_17385	7.38046	FAM69B_ENSG00000165716
9	139622802	139622998	5Y-H4K8ac_peak_17386	4.642	SNHG7_ENSG00000233016

9	139701764	139703070	5Y-H4K8ac_peak_17387	10.54764	CCDC183-AS1_ENSG00000228544;RABL6_ENSG00000196642
9	139760970	139761698	5Y-H4K8ac_peak_17388	23.64521	EDF1_ENSG00000107223
9	139839398	139839882	5Y-H4K8ac_peak_17389	7.01266	FBXW5_ENSG00000159069;C8G_ENSG00000176919
9	139844722	139845313	5Y-H4K8ac_peak_17390	15.44907	LCN12_ENSG00000184925
9	139845881	139846171	5Y-H4K8ac_peak_17391	8.2913	
9	139846887	139847106	5Y-H4K8ac_peak_17392	5.65584	
9	139872229	139872654	5Y-H4K8ac_peak_17393	8.21582	PTGDS_ENSG00000107317
9	139948656	139950035	5Y-H4K8ac_peak_17394	17.80031	ENTPD2_ENSG00000054179
9	139965257	139966161	5Y-H4K8ac_peak_17395	9.01738	SAPCD2_ENSG00000186193
9	139966794	139967184	5Y-H4K8ac_peak_17396	5.64988	
9	139971266	139971633	5Y-H4K8ac_peak_17397	7.59101	UAP1L1_ENSG00000197355
9	139971977	139972265	5Y-H4K8ac_peak_17398	6.1654	UAP1L1_ENSG00000197355
9	139981788	139982059	5Y-H4K8ac_peak_17399	6.37023	AL807752.1_ENSG00000268996;MAN1B1_ENSG00000177239
9	140009410	140009904	5Y-H4K8ac_peak_17400	7.54409	DPP7_ENSG00000176978
9	140024539	140024958	5Y-H4K8ac_peak_17401	7.09658	
9	140032851	140034072	5Y-H4K8ac_peak_17402	11.19336	GRIN1_ENSG00000176884
9	140053083	140053298	5Y-H4K8ac_peak_17403	4.84727	
9	140079013	140079685	5Y-H4K8ac_peak_17404	11.68317	
9	140080244	140080493	5Y-H4K8ac_peak_17405	7.38046	
9	140099745	140100333	5Y-H4K8ac_peak_17406	13.83999	TMEM203_ENSG00000187713;NDOR1_ENSG00000188566
9	140116096	140116469	5Y-H4K8ac_peak_17407	7.70703	RNF208_ENSG00000212864
9	140134905	140135157	5Y-H4K8ac_peak_17408	8.62703	TUBB4B_ENSG00000188229
9	140135820	140136021	5Y-H4K8ac_peak_17409	9.31608	TUBB4B_ENSG00000188229
9	140149215	140149528	5Y-H4K8ac_peak_17410	7.18391	NELFB_ENSG00000188986
9	140149831	140150297	5Y-H4K8ac_peak_17411	8.08337	NELFB_ENSG00000188986
9	140174920	140175322	5Y-H4K8ac_peak_17412	8.73985	
9	140175548	140175961	5Y-H4K8ac_peak_17413	8.75963	
9	140190093	140190624	5Y-H4K8ac_peak_17414	4.29586	RP13-122B23.8_ENSG00000260996
9	140197033	140197626	5Y-H4K8ac_peak_17415	7.95453	NRARP_ENSG00000198435
9	140210708	140211268	5Y-H4K8ac_peak_17416	10.35586	
9	140310749	140311119	5Y-H4K8ac_peak_17417	6.50117	
9	140317019	140317587	5Y-H4K8ac_peak_17418	13.99721	EXD3_ENSG00000187609;NOXA1_ENSG00000188747
9	140317981	140318542	5Y-H4K8ac_peak_17419	11.68317	EXD3_ENSG00000187609;NOXA1_ENSG00000188747
9	140348833	140349238	5Y-H4K8ac_peak_17420	4.29586	
9	140353307	140353714	5Y-H4K8ac_peak_17421	8.21582	NSMF_ENSG00000165802
9	140353961	140354315	5Y-H4K8ac_peak_17422	10.35586	NSMF_ENSG00000165802
9	140373620	140373904	5Y-H4K8ac_peak_17423	8.79957	
9	140375130	140375473	5Y-H4K8ac_peak_17424	8.24461	
9	140429941	140430182	5Y-H4K8ac_peak_17425	6.50117	
9	140445649	140445922	5Y-H4K8ac_peak_17426	9.56187	PNPLA7_ENSG00000130653;MRPL41_ENSG00000182154
9	140473623	140473960	5Y-H4K8ac_peak_17427	10.19948	DPH7_ENSG00000148399
9	140512914	140513315	5Y-H4K8ac_peak_17428	10.35586	C9orf37_ENSG00000203993;EHMT1_ENSG00000181090
9	140771668	140772028	5Y-H4K8ac_peak_17429	9.79526	CACNA1B_ENSG00000148408
9	140772350	140772605	5Y-H4K8ac_peak_17430	12.10416	CACNA1B_ENSG00000148408
GL	482278	482680	5Y-H4K8ac_peak_17431	4.55128	
GL	114680	115008	5Y-H4K8ac_peak_17432	6.75261	

GL	30143	30602	5Y-H4K8ac_peak_17433	16.15299	
GL	30938	31187	5Y-H4K8ac_peak_17434	18.07863	
GL	31539	31767	5Y-H4K8ac_peak_17435	18.07722	
GL	32144	32341	5Y-H4K8ac_peak_17436	9.05607	
GL	33026	33959	5Y-H4K8ac_peak_17437	5.62377	
GL	75039	75263	5Y-H4K8ac_peak_17438	6.9958	
GL	75900	76168	5Y-H4K8ac_peak_17439	11.09839	
GL	86201	86620	5Y-H4K8ac_peak_17440	8.46152	
GL	102538	102998	5Y-H4K8ac_peak_17441	6.67135	
GL	138051	138394	5Y-H4K8ac_peak_17442	19.58975	
GL	7195	7907	5Y-H4K8ac_peak_17443	7.04637	
GL	12984	13429	5Y-H4K8ac_peak_17444	4.9885	
GL	23301	23875	5Y-H4K8ac_peak_17445	8.2913	
GL	34066	34379	5Y-H4K8ac_peak_17446	9.02938	
GL	85749	86109	5Y-H4K8ac_peak_17447	33.81227	
GL	86905	87223	5Y-H4K8ac_peak_17448	25.63391	
GL	87849	88364	5Y-H4K8ac_peak_17449	7.1739	
GL	110220	111908	5Y-H4K8ac_peak_17450	14.70433	
GL	47398	47960	5Y-H4K8ac_peak_17451	9.23159	
GL	29116	29344	5Y-H4K8ac_peak_17452	7.74473	
GL	29708	30087	5Y-H4K8ac_peak_17453	10.56003	
GL	30341	30833	5Y-H4K8ac_peak_17454	39.07868	
GL	41914	42242	5Y-H4K8ac_peak_17455	9.74838	
GL	44246	44958	5Y-H4K8ac_peak_17456	7.90751	
GL	45217	45721	5Y-H4K8ac_peak_17457	12.79198	
GL	99132	99524	5Y-H4K8ac_peak_17458	7.90751	
GL	99838	100401	5Y-H4K8ac_peak_17459	37.25819	
GL	6826	7187	5Y-H4K8ac_peak_17460	12.70589	
GL	7481	7690	5Y-H4K8ac_peak_17461	10.34857	
GL	7931	8184	5Y-H4K8ac_peak_17462	8.77912	
GL	30947	31438	5Y-H4K8ac_peak_17463	10.19323	
X	64607	64915	5Y-H4K8ac_peak_17464	7.31815	
X	347254	347948	5Y-H4K8ac_peak_17465	17.56739	PPP2R3B_ENSG00000167393
X	1510679	1510961	5Y-H4K8ac_peak_17466	23.03824	SLC25A6_ENSG00000169100
X	1511339	1511764	5Y-H4K8ac_peak_17467	7.89273	SLC25A6_ENSG00000169100
X	2170939	2171259	5Y-H4K8ac_peak_17468	7.17184	
X	2418305	2418583	5Y-H4K8ac_peak_17469	8.43511	ZBED1_ENSG00000214717
X	2418817	2419100	5Y-H4K8ac_peak_17470	9.38203	ZBED1_ENSG00000214717
X	2419412	2419664	5Y-H4K8ac_peak_17471	7.31815	ZBED1_ENSG00000214717
X	2812488	2812934	5Y-H4K8ac_peak_17472	8.84534	
X	2847088	2847414	5Y-H4K8ac_peak_17473	12.71156	ARSD_ENSG00000006756
X	3733271	3733527	5Y-H4K8ac_peak_17474	11.04542	
X	3733876	3734089	5Y-H4K8ac_peak_17475	6.00747	
X	7065576	7065939	5Y-H4K8ac_peak_17476	11.09973	HDHD1_ENSG00000130021;MIR4767_ENSG00000264268
X	7895176	7895406	5Y-H4K8ac_peak_17477	10.46287	PNPLA4_ENSG00000006757
X	9157910	9158110	5Y-H4K8ac_peak_17478	4.84727	

X	9982453	9982798	5Y-H4K8ac_peak_17479	4.51076	WWC3_ENSG00000047644
X	10126546	10126833	5Y-H4K8ac_peak_17480	5.19372	
X	11129581	11129813	5Y-H4K8ac_peak_17481	4.77126	RP11-120D5.1_ENSG00000234129;HCCS_ENSG00000004961
X	13753106	13753320	5Y-H4K8ac_peak_17482	5.91107	TRAPPC2_ENSG00000196459;OFD1_ENSG00000046651
X	15693600	15694046	5Y-H4K8ac_peak_17483	11.93212	CA5BP1_ENSG00000186312
X	16730206	16730770	5Y-H4K8ac_peak_17484	10.86079	CTPS2_ENSG00000047230
X	16805142	16805364	5Y-H4K8ac_peak_17485	6.77436	TXLNG_ENSG00000086712
X	17555850	17556063	5Y-H4K8ac_peak_17486	6.79955	
X	18693167	18693778	5Y-H4K8ac_peak_17487	6.37023	PPEF1_ENSG00000086717
X	18782027	18782279	5Y-H4K8ac_peak_17488	6.47245	
X	19533053	19533258	5Y-H4K8ac_peak_17489	5.40331	MAP3K15_ENSG00000180815
X	20134613	20134924	5Y-H4K8ac_peak_17490	7.3889	MAP7D2_ENSG00000184368
X	20285387	20285620	5Y-H4K8ac_peak_17491	6.50117	RPS6KA3_ENSG00000177189
X	21857866	21858067	5Y-H4K8ac_peak_17492	4.15658	MBTPS2_ENSG00000012174
X	24167151	24168138	5Y-H4K8ac_peak_17493	17.51157	ZFX-AS1_ENSG00000234230;ZFX_ENSG00000005889
X	24168531	24168814	5Y-H4K8ac_peak_17494	12.71156	ZFX-AS1_ENSG00000234230
X	24483528	24483870	5Y-H4K8ac_peak_17495	4.79585	PDK3_ENSG00000067992
X	24712119	24712322	5Y-H4K8ac_peak_17496	5.65584	POLA1_ENSG00000101868
X	30906952	30907284	5Y-H4K8ac_peak_17497	8.33296	
X	38663963	38664194	5Y-H4K8ac_peak_17498	7.63144	MID1IP1-AS1_ENSG00000238123
X	39278393	39278629	5Y-H4K8ac_peak_17499	8.08688	
X	39378165	39378359	5Y-H4K8ac_peak_17500	6.31818	
X	39378932	39379123	5Y-H4K8ac_peak_17501	5.56912	
X	39547832	39548028	5Y-H4K8ac_peak_17502	4.77126	
X	39548316	39548606	5Y-H4K8ac_peak_17503	8.43511	
X	39574880	39575519	5Y-H4K8ac_peak_17504	10.84616	
X	39594799	39595127	5Y-H4K8ac_peak_17505	9.26157	
X	39618839	39619085	5Y-H4K8ac_peak_17506	8.35139	
X	39754989	39755349	5Y-H4K8ac_peak_17507	6.34046	
X	39867427	39867903	5Y-H4K8ac_peak_17508	8.93923	AC092198.1_ENSG00000223486
X	39868681	39868940	5Y-H4K8ac_peak_17509	10.01583	AC092198.1_ENSG00000223486
X	39872913	39873917	5Y-H4K8ac_peak_17510	12.05638	
X	39874308	39874570	5Y-H4K8ac_peak_17511	5.10825	
X	39967582	39967876	5Y-H4K8ac_peak_17512	8.2913	
X	39968671	39969224	5Y-H4K8ac_peak_17513	8.55081	
X	40005457	40005799	5Y-H4K8ac_peak_17514	7.59101	
X	40006041	40006345	5Y-H4K8ac_peak_17515	10.19948	
X	40011154	40011473	5Y-H4K8ac_peak_17516	8.28346	
X	40011734	40012024	5Y-H4K8ac_peak_17517	7.56545	
X	40012303	40012542	5Y-H4K8ac_peak_17518	5.98695	
X	40012803	40013143	5Y-H4K8ac_peak_17519	4.50834	
X	40027938	40028296	5Y-H4K8ac_peak_17520	8.90038	
X	40029445	40029668	5Y-H4K8ac_peak_17521	4.50834	
X	40035430	40035634	5Y-H4K8ac_peak_17522	9.15007	BCOR_ENSG00000183337
X	40169378	40169587	5Y-H4K8ac_peak_17523	5.64909	
X	40944121	40944366	5Y-H4K8ac_peak_17524	12.84845	USP9X_ENSG00000124486

X	41192167	41192425	5Y-H4K8ac_peak_17525	10.35586	DDX3X_ENSG00000215301
X	44731563	44731993	5Y-H4K8ac_peak_17526	7.38046	KDM6A_ENSG00000147050
X	46461778	46461991	5Y-H4K8ac_peak_17527	4.07874	
X	47053297	47053533	5Y-H4K8ac_peak_17528	8.47164	
X	47383215	47383664	5Y-H4K8ac_peak_17529	6.47245	
X	48432962	48433154	5Y-H4K8ac_peak_17530	10.19948	AC115618.1_ENSG00000204620;RBM3_ENSG00000102317
X	48768639	48769128	5Y-H4K8ac_peak_17531	5.81062	SLC35A2_ENSG00000102100
X	48769505	48769752	5Y-H4K8ac_peak_17532	4.95697	SLC35A2_ENSG00000102100
X	48814256	48815060	5Y-H4K8ac_peak_17533	8.59175	OTUD5_ENSG00000068308
X	48911270	48911484	5Y-H4K8ac_peak_17534	7.31815	CCDC120_ENSG00000147144
X	48932023	48932281	5Y-H4K8ac_peak_17535	7.11262	PRAF2_ENSG00000243279
X	48958550	48958986	5Y-H4K8ac_peak_17536	5.29015	WDR45_ENSG00000196998
X	48980324	48980518	5Y-H4K8ac_peak_17537	8.2913	GPKOW_ENSG00000068394
X	49126311	49127290	5Y-H4K8ac_peak_17538	11.09973	PPP1R3F_ENSG00000049769
X	52005126	52005427	5Y-H4K8ac_peak_17539	12.77513	
X	52964193	52964859	5Y-H4K8ac_peak_17540	9.30505	
X	53253986	53254580	5Y-H4K8ac_peak_17541	10.4495	KDM5C_ENSG00000126012
X	53461528	53461828	5Y-H4K8ac_peak_17542	7.60893	HSD17B10_ENSG00000072506
X	53468992	53469425	5Y-H4K8ac_peak_17543	5.23083	
X	54209300	54209582	5Y-H4K8ac_peak_17544	7.3889	FAM120C_ENSG00000184083
X	54384889	54385402	5Y-H4K8ac_peak_17545	5.65584	WNK3_ENSG00000196632
X	56809641	56810101	5Y-H4K8ac_peak_17546	4.50834	
X	63004360	63004835	5Y-H4K8ac_peak_17547	7.31815	ARHGEF9_ENSG00000131089
X	64754308	64754520	5Y-H4K8ac_peak_17548	5.29015	LAS1L_ENSG00000001497
X	67718367	67718969	5Y-H4K8ac_peak_17549	18.74304	YIPF6_ENSG00000181704
X	68114088	68114713	5Y-H4K8ac_peak_17550	10.19948	
X	68165781	68166112	5Y-H4K8ac_peak_17551	5.16353	
X	68399383	68399709	5Y-H4K8ac_peak_17552	6.34046	LINC00269_ENSG00000215162
X	69653938	69654305	5Y-H4K8ac_peak_17553	13.14766	
X	70339224	70339417	5Y-H4K8ac_peak_17554	4.84727	MED12_ENSG00000184634
X	70503518	70503951	5Y-H4K8ac_peak_17555	9.23159	NONO_ENSG00000147140
X	70585356	70585571	5Y-H4K8ac_peak_17556	8.17203	TAF1_ENSG00000147133
X	71261877	71262234	5Y-H4K8ac_peak_17557	5.64909	
X	71334387	71334676	5Y-H4K8ac_peak_17558	5.77946	
X	71351348	71351545	5Y-H4K8ac_peak_17559	5.98695	RGAG4_ENSG00000242732;RP11-262D11.1_ENSG00000248345
X	71400945	71401206	5Y-H4K8ac_peak_17560	8.35139	PIN4_ENSG00000102309
X	71496728	71496925	5Y-H4K8ac_peak_17561	6.73385	RPS4X_ENSG00000198034
X	71497494	71497686	5Y-H4K8ac_peak_17562	7.87048	RPS4X_ENSG00000198034
X	71526920	71527315	5Y-H4K8ac_peak_17563	7.89142	CITED1_ENSG00000125931
X	73640415	73640843	5Y-H4K8ac_peak_17564	9.38203	SLC16A2_ENSG00000147100
X	74375677	74376008	5Y-H4K8ac_peak_17565	7.38046	ABCB7_ENSG00000131269
X	74408574	74408772	5Y-H4K8ac_peak_17566	4.50834	
X	77041397	77041656	5Y-H4K8ac_peak_17567	11.9858	ATRX_ENSG00000085224
X	77155572	77155782	5Y-H4K8ac_peak_17568	6.13909	COX7B_ENSG00000131174
X	98627247	98627438	5Y-H4K8ac_peak_17569	4.71803	
X	101186646	101186917	5Y-H4K8ac_peak_17570	6.00388	ZMAT1_ENSG00000166432

X	101771380	101771582	5Y-H4K8ac_peak_17571	6.34046	TMSB15A_ENSG00000158164
X	101966851	101967124	5Y-H4K8ac_peak_17572	6.79955	GPRASP2_ENSG00000158301
X	102001023	102001337	5Y-H4K8ac_peak_17573	4.84727	
X	102863478	102863854	5Y-H4K8ac_peak_17574	9.78792	
X	103357290	103357506	5Y-H4K8ac_peak_17575	5.64909	ZCCHC18_ENSG00000166707
X	110924429	110924741	5Y-H4K8ac_peak_17576	16.79568	
X	115004497	115005222	5Y-H4K8ac_peak_17577	111.43083	
X	115084718	115084989	5Y-H4K8ac_peak_17578	6.76425	RP11-761E20.1_ENSG00000235244
X	117973173	117973365	5Y-H4K8ac_peak_17579	9.38203	RP4-562J12.2_ENSG00000203650
X	118805871	118806093	5Y-H4K8ac_peak_17580	8.00339	
X	118893140	118893353	5Y-H4K8ac_peak_17581	4.84727	SOWAHD_ENSG00000187808
X	118925140	118925518	5Y-H4K8ac_peak_17582	6.77436	RPL39_ENSG00000198918
X	119077329	119077579	5Y-H4K8ac_peak_17583	10.95042	NKAP_ENSG00000101882
X	119384909	119385125	5Y-H4K8ac_peak_17584	5.59843	ZBTB33_ENSG00000177485
X	119695085	119695288	5Y-H4K8ac_peak_17585	7.31815	
X	129040210	129040463	5Y-H4K8ac_peak_17586	7.38046	UTP14A_ENSG00000156697
X	129116063	129116291	5Y-H4K8ac_peak_17587	7.63144	BCORL1_ENSG00000085185
X	129194840	129195108	5Y-H4K8ac_peak_17588	10.69866	
X	129244235	129244504	5Y-H4K8ac_peak_17589	6.1654	ELF4_ENSG00000102034
X	129305322	129305952	5Y-H4K8ac_peak_17590	10.46287	RAB33A_ENSG00000134594
X	129402467	129402762	5Y-H4K8ac_peak_17591	9.30505	ZNF280C_ENSG00000056277
X	129853377	129853569	5Y-H4K8ac_peak_17592	4.51076	
X	130170062	130170331	5Y-H4K8ac_peak_17593	6.34046	
X	131623623	131623936	5Y-H4K8ac_peak_17594	4.84727	MBNL3_ENSG00000076770
X	132091620	132092005	5Y-H4K8ac_peak_17595	9.23159	
X	133371053	133371303	5Y-H4K8ac_peak_17596	6.50117	CCDC160_ENSG00000203952
X	133507427	133507621	5Y-H4K8ac_peak_17597	8.21582	PHF6_ENSG00000156531
X	133594374	133594576	5Y-H4K8ac_peak_17598	7.59101	HPRT1_ENSG00000165704
X	134048842	134049068	5Y-H4K8ac_peak_17599	8.2913	MOSPD1_ENSG00000101928
X	135229715	135229998	5Y-H4K8ac_peak_17600	5.87382	FHL1_ENSG00000022267
X	135824878	135825091	5Y-H4K8ac_peak_17601	6.34046	
X	151999862	152000128	5Y-H4K8ac_peak_17602	4.90696	CETN2_ENSG00000147400;NSDHL_ENSG00000147383
X	152109796	152110124	5Y-H4K8ac_peak_17603	6.06152	
X	152110438	152110667	5Y-H4K8ac_peak_17604	7.87406	
X	152742382	152742581	5Y-H4K8ac_peak_17605	7.41197	
X	152743425	152743619	5Y-H4K8ac_peak_17606	6.73385	
X	152753140	152753336	5Y-H4K8ac_peak_17607	6.50117	
X	152757053	152757474	5Y-H4K8ac_peak_17608	9.74189	
X	152791280	152791558	5Y-H4K8ac_peak_17609	10.35586	
X	152802395	152802630	5Y-H4K8ac_peak_17610	8.69112	
X	152843948	152844209	5Y-H4K8ac_peak_17611	4.29586	
X	153031552	153031779	5Y-H4K8ac_peak_17612	4.5753	U52111.14_ENSG00000232725
X	153084277	153084484	5Y-H4K8ac_peak_17613	5.77946	
X	153143955	153144204	5Y-H4K8ac_peak_17614	8.78565	
X	153145477	153145894	5Y-H4K8ac_peak_17615	6.39626	LCA10_ENSG00000196987
X	153167524	153167966	5Y-H4K8ac_peak_17616	5.59843	AVPR2_ENSG00000126895

X	153171138	153171388	5Y-H4K8ac_peak_17617	6.2403	
X	153191844	153192533	5Y-H4K8ac_peak_17618	4.77126	
X	153193076	153193592	5Y-H4K8ac_peak_17619	7.87406	
X	153236091	153236308	5Y-H4K8ac_peak_17620	8.1667	HCFC1_ENSG00000172534
X	153236785	153237563	5Y-H4K8ac_peak_17621	12.65187	HCFC1_ENSG00000172534;TMEM187_ENSG00000177854
X	153284625	153285201	5Y-H4K8ac_peak_17622	7.11863	IRAK1_ENSG00000184216;MIR718_ENSG00000211524
X	153285557	153285822	5Y-H4K8ac_peak_17623	10.1994	IRAK1_ENSG00000184216;MIR718_ENSG00000211524
X	153603405	153603627	5Y-H4K8ac_peak_17624	6.50117	FLNA_ENSG00000196924
X	153625839	153626449	5Y-H4K8ac_peak_17625	11.53136	
X	153640604	153640832	5Y-H4K8ac_peak_17626	9.23159	DNASE1L1_ENSG00000013563;TAZ_ENSG00000102125
X	154299862	154300079	5Y-H4K8ac_peak_17627	7.31815	CMC4_ENSG00000182712;BRCC3_ENSG00000185515
X	154841960	154842807	5Y-H4K8ac_peak_17628	9.38203	
Y	21154810	21155002	5Y-H4K8ac_peak_17629	8.33296	CD24P4_ENSG00000185275

G00000200913

)00116783
