# Supplementary material for: SIRT2 functions as a histone delactylase and inhibits the proliferation and migration of neuroblastoma cells
Source: Cell Discov. 2022 Jun 7;8:54. doi: 10.1038/s41421-022-00398-y (PMC9174446; doi:10.1038/s41421-022-00398-y)
Supplement: Supplementary file 2 — Supplementary Table S2 [file 41421_2022_398_MOESM2_ESM.pdf]

Table S2. The differentially expressed genes in SIRT2 KD cells vs. control SH-SY5Y cells determined by RNA-seq.

entrezgene_id	ensembl_gene_id	baseMean	og2FoldChang	lfcSE	pvalue	padj	hgnc_symbol
7105	ENSG00000000003	522.25749	0.76604117	0.11476006	5.10E-12	1.11E-10	TSPAN6
64102	ENSG00000000005	0	NA	NA	NA	NA	TNMD
8813	ENSG00000000419	669.87346	-0.01715454	0.0962409	0.85182802	0.93275912	DPM1
57147	ENSG00000000457	382.345246	0.04768479	0.13671135	0.71002147	0.86084246	SCYL3
55732	ENSG00000000460	717.684591	0.06890152	0.10507083	0.49589731	0.71459926	C1orf112
2268	ENSG00000000938	0	NA	NA	NA	NA	FGR
3075	ENSG00000000971	0.15069912	-0.01848503	0.38480575	0.84537521	NA	CFH
2519	ENSG00000001036	686.994166	-0.66179397	0.0976039	3.08E-12	6.78E-11	FUCA2
2729	ENSG00000001084	632.64089	0.44714347	0.10740671	1.43E-05	0.00013234	GCLC
4800	ENSG00000001167	2461.36269	0.16741695	0.07707723	0.026433	0.09095225	NFYA
90529	ENSG00000001460	206.91793	0.28472907	0.17363525	0.06613824	0.18395945	STPG1
57185	ENSG00000001461	464.595054	-0.00190446	0.11547201	0.98527709	0.99475332	NIPAL3
81887	ENSG00000001497	1607.83224	0.08791981	0.08160718	0.27049096	0.49374742	LAS1L
22875	ENSG00000001561	0.34114673	0.0032349	0.38339629	0.95755819	NA	ENPP4
6405	ENSG00000001617	78.285503	0.17728762	0.22853091	0.34027124	0.57056767	SEMA3F
1080	ENSG00000001626	0	NA	NA	NA	NA	CFTR
54467	ENSG00000001629	2573.48934	0.03817843	0.06133003	0.52885954	0.74053026	ANKIB1
1595	ENSG00000001630	2222.41897	-0.67130252	0.10143806	9.10E-12	1.95E-10	CYP51A1
889	ENSG00000001631	1223.94105	-0.08937193	0.08015795	0.25266766	0.47273469	KRIT1
5893	ENSG00000002016	262.810156	0.28589991	0.19817985	0.09409323	0.23820463	RAD52
NA	ENSG00000002079	0.36295163	0.02389475	0.3853924	0.609968	NA	MYH16
572	ENSG00000002330	612.615397	-0.21616969	0.11011834	0.03933105	0.12396233	BAD
51056	ENSG00000002549	679.356735	0.11657886	0.1047543	0.24682341	0.46592963	LAP3
4267	ENSG00000002586	1167.2818	0.92115202	0.08843896	3.25E-26	1.62E-24	CD99
9957	ENSG00000002587	0	NA	NA	NA	NA	HS3ST1
26	ENSG00000002726	1.59649369	-0.11790376	0.40731156	0.0839442	NA	AOC1
51384	ENSG00000002745	10.6552572	0.19920936	0.39745098	0.2505563	0.47053941	WNT16
23072	ENSG00000002746	1.39252152	0.03814872	0.38081255	0.59606634	NA	HECW1
8379	ENSG00000002822	1029.3429	0.0014247	0.09754116	0.9888484	0.99604194	MAD1L1
3927	ENSG00000002834	1878.39484	0.3237578	0.0685878	1.55E-06	1.70E-05	LASP1
29916	ENSG00000002919	516.298809	-0.0465221	0.13121143	0.70480805	0.85803894	SNX11
55365	ENSG00000002933	0.94591501	-0.05316797	0.38917212	0.25487635	NA	TMEM176A
4074	ENSG00000003056	2335.43303	0.05282188	0.0638562	0.40222362	0.63040759	M6PR
90293	ENSG00000003096	1861.29804	1.30161763	0.0711288	8.41E-76	1.56E-73	KLHL13
56603	ENSG00000003137	2.06514806	0.09626599	0.39148402	0.28647282	NA	CYP26B1
3382	ENSG00000003147	3340.4075	0.22947457	0.07951602	0.00313468	0.01572095	ICA1

79007	ENSG00000003249	1186.70189	-0.11480719	0.07573541	0.12155162	0.28808795	DBNDD1
57679	ENSG00000003393	900.572729	-0.30291598	0.1026059	0.00211427	0.01122182	ALS2
843	ENSG00000003400	1.41030328	0.01772516	0.3807661	0.76453093	NA	CASP10
8837	ENSG00000003402	1082.33082	0.20253158	0.10956437	0.05328292	0.15626505	CFLAR
7035	ENSG00000003436	3.22794941	0.1534585	0.42535728	0.04956785	0.14803945	TFPI
55471	ENSG00000003509	556.49386	0.16927111	0.10750564	0.10055555	0.25028506	NDUFAF7
10181	ENSG00000003756	2663.91774	0.03746056	0.06508077	0.55973434	0.76413192	RBM5
9108	ENSG00000003987	378.749909	-0.50434983	0.1277726	3.00E-05	0.00025761	MTMR7
6542	ENSG00000003989	231.078714	1.05003924	0.16727034	4.24E-11	8.49E-10	SLC7A2
381	ENSG00000004059	2965.6547	0.11887136	0.06964118	0.0825752	0.21637154	ARF5
23098	ENSG00000004139	2278.62106	-0.50049384	0.06531982	7.19E-15	1.94E-13	SARM1
26073	ENSG00000004142	2037.78977	-0.12830835	0.06383498	0.04130442	0.12854198	POLDIP2
23129	ENSG00000004399	191.177659	-0.50263675	0.22115283	0.00757771	0.03302038	PLXND1
204	ENSG00000004455	2486.64931	-0.16738073	0.06326123	0.00724322	0.03180029	AK2
952	ENSG00000004468	54.4478516	4.12499323	0.52036409	1.07E-16	3.24E-15	CD38
2288	ENSG00000004478	4840.55548	-0.287235	0.068453	1.94E-05	0.00017427	FKBP4
23028	ENSG00000004487	6398.13582	0.13236848	0.06948158	0.05319259	0.15609245	KDM1A
10180	ENSG00000004534	3682.08125	0.29333498	0.06320411	2.47E-06	2.61E-05	RBM6
84254	ENSG00000004660	169.405515	0.15126165	0.17164455	0.32538734	0.55337603	CAMKK1
5965	ENSG00000004700	650.447765	-0.06286207	0.13216606	0.61128309	0.79937717	RECQL
55610	ENSG00000004766	736.368084	0.28732975	0.11140861	0.0068929	0.03055299	VPS50
126393	ENSG00000004776	7.43800108	0.11324518	0.37748365	0.4146074	0.64173903	HSPB6
115703	ENSG00000004777	692.457392	0.09022453	0.10969333	0.39047961	0.61981882	ARHGAP33
4706	ENSG00000004779	1219.63778	0.12135268	0.09688213	0.19539465	0.39895336	NDUFAB1
5166	ENSG00000004799	77.5760359	1.50995432	0.32027403	1.85E-07	2.36E-06	PDK4
85413	ENSG00000004809	1.13478943	0.06737302	0.38926795	0.26350831	NA	SLC22A16
51364	ENSG00000004838	65.7268928	0.36755196	0.28135591	0.08415883	0.21946783	ZMYND10
340273	ENSG00000004846	0	NA	NA	NA	NA	ABCB5
170302	ENSG00000004848	0.79999795	-0.02985682	0.38372707	0.5462175	NA	ARX
10165	ENSG00000004864	1673.83514	-0.34923004	0.07781231	4.37E-06	4.45E-05	SLC25A13
7982	ENSG00000004866	667.359421	-0.12379352	0.12590133	0.29609241	0.52268649	ST7
93655	ENSG00000004866	667.359421	-0.12379352	0.12590133	0.29609241	0.52268649	ST7
996	ENSG00000004897	2135.41241	-0.14739812	0.08573919	0.07723727	0.20599375	CDC27
6521	ENSG00000004939	0.173124	-0.01911262	0.38486262	0.84537521	NA	SLC4A1
799	ENSG00000004948	0.51313021	0.03090336	0.38602227	0.48239305	NA	CALCR
3052	ENSG00000004961	317.39032	-0.10073179	0.13872563	0.43504534	0.66035668	HCCS
1856	ENSG00000004975	1545.559	0.19684602	0.07791115	0.00978944	0.04080306	DVL2
64063	ENSG00000005001	2.5109304	-0.00387812	0.37450917	0.96250013	NA	PRSS22

5976	ENSG00000005007	3365.29931	-0.13052159	0.07243368	0.06509765	0.18195793	UPF1
8935	ENSG00000005020	32.2554582	0.37233489	0.39987471	0.10978723	0.26670487	SKAP2
292	ENSG00000005022	4350.14093	-0.08827934	0.05750263	0.1202039	0.28547347	SLC25A5
55013	ENSG00000005059	393.621096	0.08564226	0.12486792	0.46859985	0.69133113	MCUB
3207	ENSG00000005073	0	NA	NA	NA	NA	HOXA11
5439	ENSG00000005075	1388.43905	-0.06444766	0.07622225	0.38800684	0.61697969	POLR2J
56919	ENSG00000005100	1796.76664	0.03832527	0.07730791	0.61355077	0.8012142	DHX33
4222	ENSG00000005102	0	NA	NA	NA	NA	MEOX1
221981	ENSG00000005108	1520.91559	0.97867338	0.096145	3.46E-25	1.66E-23	THSD7A
3980	ENSG00000005156	1558.11303	0.29397021	0.1230959	0.01146076	0.04643563	LIG3
79657	ENSG00000005175	518.95592	-0.07880605	0.1088444	0.44761482	0.67150685	RPAP3
6296	ENSG00000005187	3.60130902	0.16634876	0.42563653	0.06618993	0.1840258	ACSM3
81691	ENSG00000005189	270.157579	-0.02597598	0.17860065	0.86799947	0.94228001	REXO5
57019	ENSG00000005194	1260.36104	-0.18662815	0.07688412	0.01307429	0.05164121	CIAPIN1
56928	ENSG00000005206	1345.61485	0.48854109	0.07473156	2.63E-11	5.36E-10	SPPL2B
80256	ENSG00000005238	279.240084	0.32444172	0.16408204	0.02876473	0.09712258	FAM214B
51226	ENSG00000005243	52.1426431	0.52944713	0.32465189	0.02648486	0.09108332	COPZ2
5577	ENSG00000005249	1094.48274	-0.27239383	0.08280694	0.00073467	0.00451543	PRKAR2B
10943	ENSG00000005302	561.141127	0.08480974	0.10377643	0.39627237	0.62563597	MSL3
1387	ENSG00000005339	2814.58672	0.00668117	0.07119437	0.92001084	0.96633041	CREBBP
9256	ENSG00000005379	469.53262	-0.40203507	0.15117426	0.00380048	0.01846719	TSPOAP1
4353	ENSG00000005381	0	NA	NA	NA	NA	MPO
5444	ENSG00000005421	1.17149719	-0.00728038	0.37858016	0.91548856	NA	PON1
6936	ENSG00000005436	687.778007	-0.21392533	0.10783318	0.03802891	0.12085248	GCFC2
84058	ENSG00000005448	790.531531	-0.27361242	0.10999889	0.00910551	0.03836341	WDR54
54677	ENSG00000005469	209.910003	0.28706959	0.1588458	0.04691857	0.14177038	CROT
5244	ENSG00000005471	570.279066	0.56321151	0.11304508	2.07E-07	2.63E-06	ABCB4
55904	ENSG00000005483	2651.67788	-0.10370238	0.07209728	0.13952571	0.31648712	KMT2E
57414	ENSG00000005486	1676.46208	0.20511556	0.07221515	0.0038031	0.01847088	RHBDD2
30812	ENSG00000005513	0	NA	NA	NA	NA	SOX8
25998	ENSG00000005700	999.880366	-0.08831179	0.08533342	0.28837167	0.51413497	IBTK
7748	ENSG00000005801	1798.09548	-0.1051443	0.08778211	0.21788219	0.42968409	ZNF195
23077	ENSG00000005810	1940.61039	-0.12328195	0.09621441	0.18477466	0.38350847	MYCBP2
26224	ENSG00000005812	462.67383	-0.24177215	0.12667372	0.04230492	0.1311043	FBXL3
3683	ENSG00000005844	0.80812928	0.03486839	0.38584757	0.52993191	NA	ITGAL
5164	ENSG00000005882	567.558978	-0.24186873	0.12625783	0.04166108	0.12951361	PDK2
3675	ENSG00000005884	381.863516	-0.18939245	0.19487559	0.25835311	0.47964541	ITGA3
7543	ENSG00000005889	864.739688	-0.11870505	0.13652052	0.35088023	0.58113897	ZFX

3920	ENSG00000005893	513.78676	-0.07729745	0.1041893	0.43887825	0.66407447	LAMP2
3674	ENSG00000005961	59.8907763	-0.09768843	0.2896718	0.61373777	0.8013529	ITGA2B
51666	ENSG00000005981	0.16735865	0.01897177	0.38482933	0.78225479	NA	ASB4
51573	ENSG00000006007	798.266604	-0.01674402	0.09138555	0.84926828	0.93154246	GDE1
55049	ENSG00000006015	758.350083	-0.08642079	0.09722188	0.35709837	0.58712453	REX1BD
9244	ENSG00000006016	61.8489748	-0.10735919	0.24103208	0.56891147	0.76956457	CRLF1
114881	ENSG00000006025	523.561338	0.33992168	0.11809195	0.00242303	0.0126209	OSBPL7
26022	ENSG00000006042	1856.94551	0.18661654	0.06825051	0.00518415	0.02394729	TMEM98
51087	ENSG00000006047	206.305464	1.12463665	0.18041394	5.13E-11	1.02E-09	YBX2
3883	ENSG00000006059	0	NA	NA	NA	NA	KRT33A
9020	ENSG00000006062	227.612787	0.31331594	0.15168267	0.02436763	0.08536592	MAP3K14
6833	ENSG00000006071	194.272813	-0.95770047	0.23005141	3.85E-06	3.94E-05	ABCC8
10368	ENSG00000006116	0	NA	NA	NA	NA	CACNG3
54972	ENSG00000006118	5617.88941	0.14760953	0.05491687	0.00665427	0.02961432	TMEM132A
163	ENSG00000006125	6851.44074	-0.16615755	0.06468096	0.0090731	0.03823499	AP2B1
6863	ENSG00000006128	0	NA	NA	NA	NA	TAC1
10127	ENSG00000006194	670.818903	0.05420103	0.10482681	0.59158716	0.78614128	ZNF263
6376	ENSG00000006210	313.734316	-0.92999117	0.15580447	3.40E-10	6.20E-09	CX3CL1
64847	ENSG00000006282	965.032429	-0.02793854	0.08392654	0.7308703	0.8712652	SPATA20
8913	ENSG00000006283	346.832957	-2.23146606	0.1889428	2.14E-33	1.36E-31	CACNA1G
51330	ENSG00000006327	69.6588405	0.10736054	0.22587483	0.55970366	0.76413192	TNFRSF12A
1750	ENSG00000006377	327.991941	-0.04183869	0.12856686	0.72897907	0.87045369	DLX6
4293	ENSG00000006432	743.685981	-0.15787483	0.11000474	0.1330972	0.30658869	MAP3K9
5898	ENSG00000006451	2139.37006	-0.08978943	0.07294386	0.20930419	0.41755608	RALA
55971	ENSG00000006453	296.872796	0.3272981	0.13524158	0.0095479	0.03993894	BAIAP2L1
80853	ENSG00000006459	1051.59041	-0.09594022	0.0918122	0.28116231	0.50594669	KDM7A
2115	ENSG00000006468	710.906855	1.2446643	0.10144306	1.29E-35	8.86E-34	ETV1
55750	ENSG00000006530	1046.13285	-0.18202539	0.09688939	0.05153522	0.1524879	AGK
221	ENSG00000006534	32.2388291	-0.36189796	0.38778772	0.115839	0.27790154	ALDH3B1
55001	ENSG00000006555	0	NA	NA	NA	NA	TTC22
57157	ENSG00000006576	1395.52342	-0.0872433	0.07874223	0.25677851	0.47786189	PHTF2
10344	ENSG00000006606	0	NA	NA	NA	NA	CCL26
9855	ENSG00000006607	953.79531	-0.19542741	0.10777567	0.05808038	0.16712323	FARP2
10083	ENSG00000006611	1.7521339	-0.00083294	0.37612745	0.99046715	NA	USH1C
79017	ENSG00000006625	1252.62514	-0.32252039	0.08373125	7.61E-05	0.00059755	GGCT
10926	ENSG00000006634	1100.43046	-0.5345773	0.11695166	1.69E-06	1.83E-05	DBF4
6915	ENSG00000006638	56.4984033	-0.11018809	0.24881508	0.56401565	0.76665322	TBXA2R
3475	ENSG00000006652	1586.85257	-0.19040756	0.09626272	0.04054659	0.12664426	IFRD1

56891	ENSG00000006659	0	NA	NA	NA	NA	LGALS14
1352	ENSG00000006695	320.853231	0.02231887	0.13996022	0.86518029	0.94028794	COX10
9569	ENSG00000006704	1442.91874	0.24031919	0.0817108	0.00256642	0.01327412	GTF2IRD1
54623	ENSG00000006712	1073.12111	-0.10176278	0.07862424	0.18560335	0.38490337	PAF1
27072	ENSG00000006715	2041.58027	0.11662671	0.07420071	0.10918397	0.26566224	VPS41
9912	ENSG00000006740	10.2066999	0.15026294	0.38592485	0.31385641	0.54165894	ARHGAP44
60528	ENSG00000006744	1695.07673	0.01760769	0.07741072	0.81687111	0.91671007	ELAC2
85477	ENSG00000006747	0	NA	NA	NA	NA	SCIN
414	ENSG00000006756	529.497767	0.33493621	0.13206022	0.00677064	0.03007825	ARSD
8228	ENSG00000006757	37.8374833	-0.3961043	0.42278032	0.09290472	0.23587906	PNPLA4
8735	ENSG00000006788	0.16735865	0.01897177	0.38482933	0.78225479	NA	MYH13
79602	ENSG00000006831	1508.08357	-0.27301115	0.07599393	0.00023968	0.00167202	ADIPOR2
51265	ENSG00000006837	45.6210287	-0.26159406	0.2989673	0.21224603	0.42202458	CDKL3
151531	ENSG00000007001	1.18837386	0.04619176	0.38449686	0.37462067	NA	UPP2
10942	ENSG00000007038	0.15069912	-0.01848503	0.38480575	0.84537521	NA	PRSS21
57787	ENSG00000007047	1055.02244	0.00698085	0.08968583	0.93698435	0.97351594	MARK4
8842	ENSG00000007062	262.590088	4.12432046	0.21234156	1.67E-85	3.73E-83	PROM1
115098	ENSG00000007080	1486.34522	0.06576813	0.08511655	0.428576	0.65447566	CCDC124
90273	ENSG00000007129	0	NA	NA	NA	NA	CEACAM21
5048	ENSG00000007168	3828.46245	-0.24810913	0.06187423	4.62E-05	0.00038203	PAFAH1B1
4843	ENSG00000007171	82.1727675	0.72828772	0.30928283	0.00311918	0.01566633	NOS2
1770	ENSG00000007174	0.68981627	0.04028194	0.38714411	0.35752454	NA	DNAH9
9703	ENSG00000007202	4619.26443	-0.00068369	0.06072443	0.99045299	0.99676382	KIAA0100
9058	ENSG00000007216	0	NA	NA	NA	NA	SLC13A2
8522	ENSG00000007237	175.655034	-0.55772268	0.209085	0.00219307	0.01159351	GAS7
79090	ENSG00000007255	112.857627	0.55419009	0.23914195	0.00579997	0.02639156	TRAPPC6A
4145	ENSG00000007264	0.65754506	0.00334079	0.38192682	0.94271382	NA	MATK
1087	ENSG00000007306	0	NA	NA	NA	NA	CEACAM7
974	ENSG00000007312	1.11198554	-0.01893371	0.38176065	0.71997015	NA	CD79B
6329	ENSG00000007314	0	NA	NA	NA	NA	SCN4A
54879	ENSG00000007341	286.405356	-0.20466641	0.14592954	0.12829673	0.29899782	ST7L
8277	ENSG00000007350	4.46857995	0.13285024	0.40750751	0.11164106	0.2700997	TKTL1
5080	ENSG00000007372	1.08875469	-0.02005498	0.38234577	0.78769956	NA	PAX6
113000	ENSG00000007376	572.194184	-0.14616842	0.10015543	0.1298707	0.30153711	RPUSD1
64285	ENSG00000007384	28.9176656	0.48753656	0.45818108	0.05710482	0.16493806	RHBDF1
55692	ENSG00000007392	2217.0873	0.27070709	0.06616955	3.20E-05	0.00027378	LUC7L
9254	ENSG00000007402	12616.1895	1.17729298	0.04680533	9.68E-141	6.00E-138	CACNA2D2
8938	ENSG00000007516	19.4703165	0.00094998	0.33609926	0.99783311	0.99926338	BAIAP3

115939	ENSG00000007520	835.59368	0.12086141	0.09368761	0.18362927	0.38196736	TSR3
9091	ENSG00000007541	1337.6419	0.32809533	0.08294103	4.93E-05	0.00040382	PIGQ
57585	ENSG00000007545	1322.06328	0.23901557	0.12094565	0.03594517	0.11573389	CRAMP1
7005	ENSG00000007866	222.546318	0.59686754	0.18125154	0.00027374	0.00188443	TEAD3
6401	ENSG00000007908	0	NA	NA	NA	NA	SELE
55735	ENSG00000007923	1627.08409	-0.09171088	0.09341671	0.31050833	0.5382687	DNAJC11
2328	ENSG00000007933	0.173124	-0.01911262	0.38486262	0.84537521	NA	FMO3
29116	ENSG00000007944	184.143421	-0.27903237	0.18670792	0.08765723	0.22621221	MYLIP
27035	ENSG00000007952	0.15069912	-0.01848503	0.38480575	0.84537521	NA	NOX1
1870	ENSG00000007968	984.571308	-0.3057412	0.10948417	0.00345899	0.017084	E2F2
5689	ENSG00000008018	2397.67831	0.02385981	0.06487246	0.71095684	0.86118643	PSMB1
6853	ENSG00000008056	319.415649	-2.58463072	0.17651173	8.04E-50	7.90E-48	SYN1
3720	ENSG00000008083	1020.76306	0.13029263	0.08192144	0.10346484	0.25569786	JARID2
6792	ENSG00000008086	688.442088	-0.08688718	0.13537106	0.49388821	0.71263909	CDKL5
57172	ENSG00000008118	1.56173405	0.11143762	0.40628069	0.05476964	NA	CAMK1G
728642	ENSG00000008128	964.861152	0.39408212	0.08509877	1.98E-06	2.13E-05	CDK11A
65220	ENSG00000008130	1445.55597	0.18613625	0.08802207	0.02959472	0.09931651	NADK
7021	ENSG00000008196	14644.0753	0.22261758	0.05384673	2.48E-05	0.00021768	TFAP2B
83741	ENSG00000008197	8.03869294	-1.18968095	1.35853969	0.01023481	0.04220717	TFAP2D
9940	ENSG00000008226	3.94084535	-0.01276139	0.37007773	0.89710557	0.95488961	DLEC1
9265	ENSG00000008256	1015.39019	0.13692341	0.11119086	0.19876849	0.40314524	CYTH3
53616	ENSG00000008277	5360.80924	-0.29246709	0.06790854	1.17E-05	0.00010956	ADAM22
6856	ENSG00000008282	482.552543	0.15930458	0.11157429	0.13529945	0.30986276	SYPL1
1534	ENSG00000008283	9023.99082	-0.2621449	0.06460942	3.74E-05	0.000316	CYB561
9043	ENSG00000008294	5656.40565	-0.218432	0.05439874	4.90E-05	0.00040213	SPAG9
1951	ENSG00000008300	4576.13679	0.16622412	0.06552945	0.01004185	0.04160166	CELSR3
10157	ENSG00000008311	198.701469	2.11065757	0.22118727	8.73E-23	3.76E-21	AASS
55200	ENSG00000008323	7.51655365	0.23739888	0.49709332	0.00319401	0.01594194	PLEKHG6
51188	ENSG00000008324	254.22265	-0.22038718	0.14958269	0.10860018	0.26446884	SS18L2
84954	ENSG00000008382	357.78067	0.07406921	0.13759985	0.56514466	0.76729419	MPND
4257	ENSG00000008394	185.469292	2.84532145	0.20905368	2.29E-43	1.93E-41	MGST1
1407	ENSG00000008405	495.842327	0.26726681	0.11578444	0.01519367	0.0582385	CRY1
8993	ENSG00000008438	0.50753074	0.03073847	0.38601592	0.48354038	NA	PGLYRP1
4784	ENSG00000008441	1867.49772	-0.16804599	0.09271175	0.06119594	0.1739936	NFIX
6482	ENSG00000008513	104.291504	1.00835682	0.25292093	7.86E-06	7.60E-05	ST3GAL1
64386	ENSG00000008516	11.7054491	-0.00530444	0.35335029	0.96623257	0.98692619	MMP25
9235	ENSG00000008517	2.66262469	0.04717456	0.37850631	0.58109316	0.77834785	IL32
5310	ENSG00000008710	8331.79613	-0.19859562	0.07160519	0.00471474	0.02212928	PKD1

23542	ENSG00000008735	1937.36857	-1.40573386	0.07507426	2.99E-79	5.99E-77	MAPK8IP2
9862	ENSG00000008838	2045.02181	0.10124147	0.07964148	0.19325424	0.39596861	MED24
23221	ENSG00000008853	874.544836	-0.11238092	0.10436767	0.2615337	0.48353634	RHOBTB2
54497	ENSG00000008869	1520.28268	-0.26314344	0.08000601	0.00074688	0.00458199	HEATR5B
7095	ENSG00000008952	1721.83244	-0.56526574	0.08586837	1.52E-11	3.19E-10	SEC62
6224	ENSG00000008988	11401.5033	-0.15554551	0.05790148	0.00664719	0.02958946	RPS20
7812	ENSG00000009307	9660.80695	-0.25901646	0.05005525	1.71E-07	2.18E-06	CSDE1
9690	ENSG00000009335	2397.12182	-0.05177619	0.08649633	0.5382258	0.74807128	UBE3C
5980	ENSG00000009413	1327.61395	-0.41421587	0.08976599	1.99E-06	2.15E-05	REV3L
10178	ENSG00000009694	28.2087795	0.14948515	0.30794683	0.45279356	0.67610629	TENM1
5081	ENSG00000009709	0.3468566	4.37E-05	0.38336202	0.96804177	NA	PAX7
10747	ENSG00000009724	52.3147006	-0.11157856	0.25356484	0.56097396	0.7645572	MASP2
389434	ENSG00000009765	0	NA	NA	NA	NA	IYD
199870	ENSG00000009780	155.642215	0.32795198	0.20988819	0.06578041	0.18330641	FAM76A
80342	ENSG00000009790	11.0361529	-0.04349014	0.33818413	0.79044705	0.90451433	TRAF3IP3
29954	ENSG00000009830	1132.41554	0.12271702	0.08569591	0.14175137	0.32020348	POMT2
51534	ENSG00000009844	936.680841	-0.25812344	0.09445126	0.00466867	0.02194418	VTA1
51085	ENSG00000009950	75.2640447	0.51246862	0.29683969	0.02377351	0.08366474	MLXIPL
9031	ENSG00000009954	6667.82745	-0.22590004	0.05190678	1.10E-05	0.0001036	BAZ1B
10048	ENSG00000010017	998.870469	-0.03639916	0.10172164	0.70982932	0.860757	RANBP9
51513	ENSG00000010030	2.82795347	-0.19639197	0.4580382	0.00823563	0.03540516	ETV7
83932	ENSG00000010072	470.267091	0.06263712	0.15344728	0.65685771	0.82968692	SPRTN
51603	ENSG00000010165	1720.19841	-0.13625738	0.07517071	0.06418354	0.1801898	EEF1AKNMT
8798	ENSG00000010219	114.234612	0.50542363	0.23864765	0.01096007	0.04469634	DYRK4
7756	ENSG00000010244	4201.57904	0.04904323	0.06425652	0.4392923	0.66465029	ZNF207
7384	ENSG00000010256	2689.73126	0.25068561	0.08823286	0.00344866	0.0170372	UQCRC1
83930	ENSG00000010270	1262.40036	0.12158836	0.07764126	0.10896074	0.26521666	STARD3NL
928	ENSG00000010278	149.40044	5.25288661	0.34868801	1.05E-51	1.09E-49	CD9
57467	ENSG00000010282	0	NA	NA	NA	NA	HHATL
9918	ENSG00000010292	4110.32174	-0.17007203	0.07707286	0.02401966	0.08444978	NCAPD2
25900	ENSG00000010295	578.512704	0.17484976	0.11012013	0.09690668	0.24348114	IFFO1
2696	ENSG00000010310	118.868467	1.12210073	0.23369763	1.69E-07	2.17E-06	GIPR
51533	ENSG00000010318	77.7282934	0.73308741	0.28687426	0.00183722	0.00994825	PHF7
56920	ENSG00000010319	29.2174056	-0.0622347	0.29236629	0.74360875	0.87916081	SEMA3G
11188	ENSG00000010322	3331.29885	0.24352616	0.06662884	0.00020284	0.00144087	NISCH
23166	ENSG00000010327	1.07545701	0.04262835	0.38384458	0.41010704	NA	STAB1
80199	ENSG00000010361	978.176569	0.05668297	0.09391371	0.53421198	0.74539459	FUZ
6540	ENSG00000010379	2.22870597	0.02983046	0.37619956	0.7292228	NA	SLC6A13

3423	ENSG00000010404	3731.48205	-0.38789543	0.06293257	3.89E-10	7.04E-09	IDS
5646	ENSG00000010438	832.509814	-0.36236716	0.09924323	0.00015076	0.00110008	PRSS3
7752	ENSG00000010539	262.443247	-0.23853846	0.15736548	0.09551447	0.24094177	ZNF200
920	ENSG00000010610	8.79825504	0.10655184	0.36156188	0.49993898	0.71714187	CD4
10233	ENSG00000010626	133.332005	0.32812958	0.20031151	0.05745315	0.16569347	LRRC23
695	ENSG00000010671	1.40524716	0.07586116	0.39454052	0.11272121	NA	BTK
3077	ENSG00000010704	0.48617452	-0.00656019	0.38315309	0.86579112	NA	HFE
22955	ENSG00000010803	1049.30743	-0.27379393	0.09089818	0.00187191	0.01011129	SCMH1
2534	ENSG00000010810	1124.32355	0.04538007	0.11844639	0.68803988	0.84855544	FYN
3097	ENSG00000010818	634.509393	0.15850418	0.14904397	0.24803139	0.46714855	HIVEP2
2326	ENSG00000010932	0	NA	NA	NA	NA	FMO1
6924	ENSG00000011007	1491.75083	-0.2332513	0.0725738	0.00104064	0.00611377	ELOA
11313	ENSG00000011009	2714.54704	0.15261967	0.06823593	0.02296173	0.08135013	LYPLA2
1185	ENSG00000011021	1157.27746	-0.04161886	0.10208356	0.67140836	0.83825699	CLCN6
9902	ENSG00000011028	3244.27644	1.29495506	0.07326335	6.80E-71	1.12E-68	MRC2
654364	ENSG00000011052	354.547354	0.09212357	0.13969649	0.47936292	0.70064538	NME1-NME2
6534	ENSG00000011083	0.67118112	0.02020611	0.38386956	0.77887943	NA	SLC6A7
10867	ENSG00000011105	947.301839	0.0023572	0.08588227	0.98329559	0.99428951	TSPAN9
55727	ENSG00000011114	1070.92706	-0.50514764	0.08628416	1.89E-09	3.14E-08	BTBD7
9546	ENSG00000011132	372.704706	0.46182864	0.15421224	0.00113861	0.00660334	APBA3
54903	ENSG00000011143	595.318528	0.17900978	0.11292828	0.09660316	0.24301925	MKS1
51099	ENSG00000011198	426.997305	-0.09915573	0.12330491	0.39455789	0.623797	ABHD5
3730	ENSG00000011201	13.6968091	3.85857728	0.92115215	2.52E-06	2.66E-05	ANOS1
26993	ENSG00000011243	1272.87997	0.39873247	0.10101669	4.18E-05	0.00034963	AKAP8L
54799	ENSG00000011258	1881.36538	-0.13967707	0.068741	0.03869434	0.12238095	MBTD1
51096	ENSG00000011260	1066.3531	-0.13237845	0.08947154	0.12701617	0.2969598	UTP18
54476	ENSG00000011275	1824.37059	0.11260939	0.06937048	0.09888651	0.24730977	RNF216
54902	ENSG00000011295	984.108888	0.10342063	0.09209321	0.2473573	0.46632163	TTC19
5725	ENSG00000011304	10367.1765	-0.05337815	0.05605882	0.33509227	0.56494141	PTBP1
8193	ENSG00000011332	331.55015	-0.8387095	0.16460742	5.71E-08	7.77E-07	DPF1
9066	ENSG00000011347	374.678835	-2.10171226	0.18055687	1.18E-32	7.34E-31	SYT7
23395	ENSG00000011376	796.466636	0.08458272	0.09366664	0.35209404	0.5820803	LARS2
5286	ENSG00000011405	1775.43401	-0.20258361	0.09935678	0.03439178	0.11182118	PIK3C2A
5329	ENSG00000011422	315.46309	0.32764384	0.13535369	0.00955938	0.03997011	PLAUR
54443	ENSG00000011426	1792.99424	-0.24302139	0.07544058	0.00098603	0.00583788	ANLN
58525	ENSG00000011451	2425.92402	0.15835092	0.06541091	0.01396498	0.05446535	WIZ
23637	ENSG00000011454	1201.57195	0.16447892	0.09274507	0.0674058	0.18641359	RABGAP1
1634	ENSG00000011465	18.910451	5.4895013	0.96484763	8.55E-09	1.30E-07	DCN

54814	ENSG00000011478	133.077896	0.55099428	0.20096093	0.00186949	0.01010294	QPCTL
5536	ENSG00000011485	2445.33521	0.10843516	0.07393348	0.13504448	0.30949318	PPP5C
23177	ENSG00000011523	1166.22041	-0.25266513	0.08650782	0.00265221	0.01363917	CEP68
8491	ENSG00000011566	917.539105	-0.09175889	0.10231482	0.35127879	0.5814593	MAP4K3
27033	ENSG00000011590	3.19665764	-0.10741192	0.38977984	0.31545395	0.54331625	ZBTB32
7305	ENSG00000011600	0	NA	NA	NA	NA	TYROBP
57146	ENSG00000011638	35.497963	1.16978233	0.57085732	0.00254376	0.01317364	TMEM159
2556	ENSG00000011677	226.281937	0.92293884	0.18565608	9.38E-08	1.24E-06	GABRA3
672	ENSG00000012048	1321.21677	-0.07765974	0.08261547	0.33512208	0.56494367	BRCA1
2067	ENSG00000012061	1651.92364	0.18693753	0.0826295	0.02072949	0.07504982	ERCC1
933	ENSG00000012124	0	NA	NA	NA	NA	CD22
7869	ENSG00000012171	197.240408	1.45609084	0.2081277	2.07E-13	4.94E-12	SEMA3B
51360	ENSG00000012174	1462.5026	-0.23658458	0.07287083	0.00092307	0.0055312	MBTPS2
4007	ENSG00000012211	3.48272187	0.08948101	0.38390632	0.39270672	0.62191102	PRICKLE3
4057	ENSG00000012223	0	NA	NA	NA	NA	LTF
2137	ENSG00000012232	2166.50453	0.14853063	0.10701103	0.14850063	0.3308909	EXTL3
9971	ENSG00000012504	0	NA	NA	NA	NA	NR1H4
60481	ENSG00000012660	2489.4557	-0.1449389	0.07286523	0.04245605	0.13144946	ELOVL5
240	ENSG00000012779	0.18922458	0.01954053	0.38488496	0.78225479	NA	ALOX5
8284	ENSG00000012817	0	NA	NA	NA	NA	KDM5D
57658	ENSG00000012822	1015.97533	0.39488001	0.11075218	0.00019141	0.001367	CALCOCO1
55148	ENSG00000012963	1559.15555	0.08308175	0.08188447	0.29926895	0.52623381	UBR7
11183	ENSG00000012983	1218.25311	-0.19883224	0.07854929	0.00957224	0.04001543	MAP4K5
30845	ENSG00000013016	843.444627	-0.16998204	0.0992483	0.07553533	0.20246162	EHD3
5704	ENSG00000013275	2133.15911	0.05615326	0.07401605	0.43998201	0.66517856	PSMC4
23324	ENSG00000013288	359.284197	0.11603257	0.12821898	0.3371406	0.56743039	MAN2B2
57709	ENSG00000013293	405.227155	-2.64310652	0.16890838	8.10E-57	9.69E-55	SLC7A14
5010	ENSG00000013297	6.88173956	-0.32665022	0.54862883	0.05142627	0.15228317	CLDN11
51629	ENSG00000013306	1904.81202	-0.07662322	0.07473169	0.29537148	0.52197116	SLC25A39
9961	ENSG00000013364	45.092487	1.02502113	0.41419242	0.00118615	0.00685702	MVP
51667	ENSG00000013374	1767.34085	-0.06014552	0.09173854	0.49871387	0.71636861	NUB1
5238	ENSG00000013375	940.582471	0.09112253	0.10612893	0.37202734	0.60104858	PGM3
112611	ENSG00000013392	148.176601	0.06715888	0.18217338	0.67680994	0.84203356	RWDD2A
1195	ENSG00000013441	1278.21932	-0.00194464	0.08932915	0.98121543	0.99355086	CLK1
55703	ENSG00000013503	308.759557	-0.04669803	0.12840089	0.69875594	0.85472333	POLR3B
23357	ENSG00000013523	724.44843	0.29764527	0.11097077	0.00496052	0.02307542	ANGEL1
9604	ENSG00000013561	587.669674	-0.07039326	0.10537397	0.48631134	0.70622288	RNF14
1774	ENSG00000013563	282.454604	-0.12031308	0.14430913	0.3668985	0.59618256	DNASE1L1

1663	ENSG00000013573	2056.11988	0.4346915	0.0753704	3.96E-09	6.29E-08	DDX11
50865	ENSG00000013583	128.13473	-0.00946643	0.20092448	0.95457694	0.98123447	HEBP1
9052	ENSG00000013588	40.9366336	0.43791539	0.43129028	0.07408034	0.19966771	GPRC5A
10046	ENSG00000013619	77.3267178	-0.27518661	0.23770926	0.15044431	0.333984	MAMLD1
923	ENSG00000013725	25.7199483	-0.14349001	0.32546716	0.45644755	0.67989846	CD6
10460	ENSG00000013810	2819.04517	-0.03297277	0.06712349	0.61617739	0.80321678	TACC3
23376	ENSG00000014123	493.479726	0.10851455	0.11013483	0.30395869	0.53126299	UFL1
23649	ENSG00000014138	890.257201	-0.15031596	0.11224568	0.16022156	0.34797983	POLA2
23144	ENSG00000014164	324.305661	0.27249719	0.13562714	0.03128791	0.10368332	ZC3H3
823	ENSG00000014216	1530.81597	0.18065608	0.08078105	0.02202647	0.07871129	CAPN1
55	ENSG00000014257	9.28305096	6.87689503	2.67095134	2.85E-06	2.99E-05	ACP3
4190	ENSG00000014641	1815.54078	-0.16347075	0.08130937	0.03929197	0.12387615	MDH1
10463	ENSG00000014824	2214.50308	0.1028168	0.072163	0.146809	0.32800893	SLC30A9
10903	ENSG00000014914	27.1481218	0.04800993	0.33175268	0.7797047	0.89925062	MTMR11
1355	ENSG00000014919	1155.83653	-0.41846556	0.08914586	1.33E-06	1.48E-05	COX15
440193	ENSG00000015133	1269.88805	-0.48501518	0.0901976	3.15E-08	4.44E-07	CCDC88C
10138	ENSG00000015153	333.602805	-0.2985344	0.17426086	0.05452141	0.15905085	YAF2
10771	ENSG00000015171	1594.54389	0.05456395	0.0708483	0.43476096	0.66019092	ZMYND11
7454	ENSG00000015285	11.5414088	0.07150072	0.33899172	0.67508461	0.84091366	WAS
1800	ENSG00000015413	0.34746601	0.02370763	0.3853651	0.61987128	NA	DPEP1
637	ENSG00000015475	1015.78116	0.0665646	0.08606126	0.42781104	0.65371004	BID
9782	ENSG00000015479	7730.39257	-0.19458674	0.06687005	0.00314603	0.01576193	MATR3
29881	ENSG00000015520	2.90437861	0.00010425	0.37118321	0.99898054	0.99962651	NPC1L1
64132	ENSG00000015532	809.055341	0.15287691	0.09377301	0.09254319	0.2352411	XYLT2
84220	ENSG00000015568	62.9387312	-0.10648677	0.25461787	0.5783658	0.77642449	RGPD5
81551	ENSG00000015592	191.522333	-3.62439227	0.23358191	3.11E-55	3.65E-53	STMN4
23386	ENSG00000015676	3748.90688	-0.12657777	0.07720925	0.09374847	0.23753778	NUDCD3
3670	ENSG00000016082	4648.12201	0.41244516	0.05723226	3.27E-13	7.66E-12	ISL1
55349	ENSG00000016391	366.955291	-0.1019447	0.14697173	0.45119454	0.67453091	CHDH
53832	ENSG00000016402	786.8515	-0.76489294	0.10754993	2.26E-13	5.36E-12	IL20RA
1179	ENSG00000016490	0	NA	NA	NA	NA	CLCA1
22802	ENSG00000016602	0	NA	NA	NA	NA	CLCA4
55830	ENSG00000016864	928.791217	0.05350868	0.09392666	0.55769769	0.76276866	GLT8D1
27032	ENSG00000017260	1954.52406	-0.17791231	0.07467627	0.01499874	0.0577108	ATP2C1
3479	ENSG00000017427	0	NA	NA	NA	NA	IGF1
92745	ENSG00000017483	4.70139773	0.08103664	0.37258854	0.52482213	0.73753159	SLC38A5
10928	ENSG00000017797	1614.12733	-0.16574664	0.07140997	0.01801117	0.0669806	RALBP1
22902	ENSG00000018189	1703.33649	-0.18263828	0.07642938	0.01445483	0.05592508	RUFY3

1272	ENSG00000018236	3750.94004	-0.54097325	0.05999916	6.96E-20	2.58E-18	CNTN1
6556	ENSG00000018280	1.45970231	-0.00278711	0.37968125	0.96360206	NA	SLC11A1
25937	ENSG00000018408	133.336127	3.55588187	0.26401659	1.89E-42	1.52E-40	WWTR1
8540	ENSG00000018510	1790.32872	0.11151824	0.08524923	0.17969059	0.37625976	AGPS
646915	ENSG00000018607	6.70543029	0.03873209	0.35753553	0.77858291	0.89866105	ZNF806
63932	ENSG00000018610	420.650395	0.0603321	0.12139414	0.60100526	0.79181148	STEEP1
477	ENSG00000018625	0	NA	NA	NA	NA	ATP1A2
55622	ENSG00000018699	358.407692	0.0111724	0.14464802	0.93468091	0.97292421	TTC27
147948	ENSG00000018869	79.305605	0.27091943	0.2514284	0.16967843	0.36139775	ZNF582
23584	ENSG00000019102	1.59465622	0.11019222	0.40584332	0.05461339	NA	VSIG2
23187	ENSG00000019144	1472.26423	-0.24562425	0.08068144	0.00178622	0.00970655	PHLDB1
8685	ENSG00000019169	0	NA	NA	NA	NA	MARCO
1591	ENSG00000019186	0	NA	NA	NA	NA	CYP24A1
56981	ENSG00000019485	465.675879	0.05316749	0.13972138	0.68369908	0.84583087	PRDM11
57586	ENSG00000019505	245.325226	-0.99255873	0.17601811	2.24E-09	3.68E-08	SYT13
6591	ENSG00000019549	26.7019411	0.07324724	0.30097746	0.70261676	0.85699455	SNAI2
972	ENSG00000019582	14.3845777	-0.40780296	0.55216187	0.06844181	0.18843333	CD74
3082	ENSG00000019991	163.613321	2.20610796	0.22500688	5.65E-24	2.59E-22	HGF
54764	ENSG00000019995	724.945175	-0.06753079	0.10080111	0.48624882	0.70622288	ZRANB1
23154	ENSG00000020129	1671.53057	-0.01699321	0.08343175	0.8339488	0.92432089	NCDN
25960	ENSG00000020181	2765.03278	-0.3276686	0.06113257	5.41E-08	7.40E-07	ADGRA2
NA	ENSG00000020219	0.300017	-0.0229446	0.38527539	0.68644804	NA	CCT8L1P
55734	ENSG00000020256	614.613217	-0.06540489	0.11880494	0.56193405	0.76508177	ZFP64
4331	ENSG00000020426	284.720519	0.10533663	0.14710353	0.43862573	0.66394556	MNAT1
23034	ENSG00000020577	761.159021	-0.3021796	0.13179083	0.01427242	0.05540302	SAMD4A
864	ENSG00000020633	5.70743263	-0.24259961	0.46121545	0.10357036	0.25589489	RUNX3
4361	ENSG00000020922	1049.13827	0.09740381	0.13151553	0.43120124	0.65676591	MRE11
58473	ENSG00000021300	75.9357908	1.78228059	0.34422278	1.29E-08	1.93E-07	PLEKHB1
1992	ENSG00000021355	99.3855728	0.06744487	0.21782178	0.70753237	0.85903201	SERPINB1
64816	ENSG00000021461	0	NA	NA	NA	NA	CYP3A43
11136	ENSG00000021488	0.73443283	0.01971684	0.38275146	0.62605875	NA	SLC7A9
6683	ENSG00000021574	1201.31972	-0.0904456	0.09123704	0.30698147	0.53453681	SPAST
9369	ENSG00000021645	27.6295195	-4.83633151	0.86116912	6.20E-10	1.10E-08	NRXN3
114879	ENSG00000021762	114.856294	0.05947005	0.21017445	0.73661246	0.87516565	OSBPL5
9716	ENSG00000021776	1511.4132	0.04900589	0.08350316	0.54879602	0.75527976	AQR
1373	ENSG00000021826	20.9230151	0.17804587	0.3618501	0.3355233	0.5655232	CPS1
732	ENSG00000021852	0	NA	NA	NA	NA	C8B
2273	ENSG00000022267	1852.91677	0.06072276	0.10193764	0.53751928	0.74765981	FHL1

51507	ENSG00000022277	1252.09667	0.11068379	0.0760355	0.13751022	0.31319054	RTF2
2554	ENSG00000022355	0	NA	NA	NA	NA	GABRA1
55655	ENSG00000022556	0	NA	NA	NA	NA	NLRP2
57210	ENSG00000022567	488.295554	-0.01092961	0.25600475	0.95175609	0.98012235	SLC45A4
9921	ENSG00000022840	2025.58032	0.10056459	0.07174282	0.15422646	0.3394719	RNF10
55778	ENSG00000022976	310.143651	0.07306339	0.16282584	0.62104057	0.80672374	ZNF839
64429	ENSG00000023041	787.783006	-0.12459308	0.134437	0.32094623	0.54869222	ZDHHC6
57476	ENSG00000023171	399.938471	-0.15536683	0.14936534	0.25680678	0.47786969	GRAMD1B
6050	ENSG00000023191	1032.80072	0.34817659	0.089112	5.71E-05	0.00045935	RNH1
4719	ENSG00000023228	1713.91428	-0.20699958	0.08288465	0.01036207	0.04262566	NDUFS1
9821	ENSG00000023287	1258.17363	-0.11348635	0.08223727	0.15734246	0.34410503	RB1CC1
23071	ENSG00000023318	608.823486	0.36616141	0.11079197	0.00054264	0.00346505	ERP44
211	ENSG00000023330	456.04724	0.21399245	0.11637968	0.0531784	0.15607387	ALAS1
330	ENSG00000023445	7.24495109	-0.00946151	0.34907312	0.94694895	0.97815729	BIRC3
11215	ENSG00000023516	1431.84342	-0.66038884	0.07784281	5.75E-18	1.89E-16	AKAP11
51022	ENSG00000023572	273.061735	-0.01322646	0.17361864	0.93082526	0.97111825	GLRX2
6617	ENSG00000023608	416.875131	-0.02503502	0.12232944	0.82818642	0.92220943	SNAPC1
51071	ENSG00000023697	487.103379	-0.00659252	0.11623943	0.95170546	0.98012235	DERA
11171	ENSG00000023734	3151.45772	-0.07728067	0.07491765	0.29237431	0.51842938	STRAP
1244	ENSG00000023839	14.2192028	0.49821083	0.62100181	0.0494541	0.14780908	ABCC2
50619	ENSG00000023892	6.87994977	0.0490935	0.36253248	0.70587634	0.85822936	DEF6
51177	ENSG00000023902	1369.99186	0.03075625	0.07468689	0.6750668	0.84091366	PLEKHO1
2730	ENSG00000023909	212.690357	0.40753395	0.21355903	0.02473091	0.08639925	GCLM
23304	ENSG00000024048	949.467899	-0.09594187	0.09570822	0.30003933	0.52721462	UBR2
30846	ENSG00000024422	53.092055	0.989579	0.38021908	0.00090303	0.00542092	EHD2
55635	ENSG00000024526	1125.24934	-0.39382161	0.08782824	3.85E-06	3.94E-05	DEPDC1
25901	ENSG00000024862	194.965663	0.12986884	0.15542012	0.36094523	0.5908055	CCDC28A
58528	ENSG00000025039	378.757911	-0.3253925	0.14277028	0.01376447	0.05382088	RRAGD
3298	ENSG00000025156	698.190401	0.06187407	0.10073242	0.52494256	0.73755856	HSF2
51230	ENSG00000025293	1225.9768	-0.17178069	0.08106842	0.02987987	0.10005012	PHF20
8630	ENSG00000025423	48.2477944	0.32855953	0.39301209	0.14065216	0.318644	HSD17B6
10062	ENSG00000025434	60.7724877	0.19673482	0.25695218	0.31741287	0.54528282	NR1H3
1890	ENSG00000025708	145.025569	0.27733853	0.23518032	0.14530986	0.32579361	TYMP
29781	ENSG00000025770	1211.23896	-0.1219001	0.09025407	0.16461184	0.35425738	NCAPH2
10953	ENSG00000025772	750.547849	-0.104297	0.09577943	0.26006127	0.48180181	TOMM34
11231	ENSG00000025796	2433.83714	-0.09678238	0.09589119	0.29638039	0.52297409	SEC63
23633	ENSG00000025800	1620.10039	-0.05559749	0.07248477	0.43415506	0.6593919	KPNA6
7431	ENSG00000026025	8469.85449	3.3498333	0.08517701	0	0	VIM

NA	ENSG00000026036	356.667516	0.30072475	0.1278741	0.01239226	0.04952912	RTEL1-TNFRSF6B
355	ENSG00000026103	18.5279679	1.23093267	0.69485981	0.00403213	0.01943571	FAS
8635	ENSG00000026297	550.150146	0.06961996	0.10651676	0.49692135	0.71500229	RNASET2
960	ENSG00000026508	25.8808342	0.85846246	0.54341814	0.01010569	0.04175278	CD44
3755	ENSG00000026559	693.217151	0.16607156	0.107064	0.10573009	0.25948603	KCNG1
56895	ENSG00000026652	885.955599	0.27406217	0.09406429	0.00259391	0.01339143	AGPAT4
57823	ENSG00000026751	0	NA	NA	NA	NA	SLAMF7
11119	ENSG00000026950	492.615627	0.51265369	0.12104117	8.57E-06	8.25E-05	BTN3A1
4285	ENSG00000027001	94.4587391	0.22427083	0.21533698	0.21307805	0.42300907	MIPEP
5583	ENSG00000027075	162.948014	-0.34555882	0.17903344	0.02955228	0.09920766	PRKCH
3645	ENSG00000027644	519.272589	0.29901733	0.11763377	0.00742157	0.03246117	INSRR
3459	ENSG00000027697	695.04761	-0.41941217	0.10682659	4.25E-05	0.00035533	IFNGR1
11285	ENSG00000027847	307.485797	0.09827361	0.13040156	0.42339842	0.64931782	B4GALT7
9047	ENSG00000027869	0.47495241	0.00526877	0.3831615	0.96705744	NA	SH2D2A
7444	ENSG00000028116	2.19076271	-0.05054919	0.37949941	0.55745995	NA	VRK2
7133	ENSG00000028137	27.9191541	-2.07499649	0.47258061	6.45E-07	7.55E-06	TNFRSF1B
55591	ENSG00000028203	1318.03058	-0.16942357	0.08624736	0.04338806	0.13346033	VEZT
5452	ENSG00000028277	75.4965282	-1.52167472	0.32455901	1.86E-07	2.37E-06	POU2F2
65980	ENSG00000028310	1581.54658	-0.03155951	0.07170471	0.654059	0.82779603	BRD9
6642	ENSG00000028528	2681.03137	-0.1226804	0.09005364	0.16034044	0.34816179	SNX1
9519	ENSG00000028839	692.824032	0.12364313	0.09741585	0.18999412	0.39114696	TBPL1
56938	ENSG00000029153	105.056574	-0.37078678	0.25080226	0.06388585	0.17963369	ARNTL2
9774	ENSG00000029363	5197.47307	-0.44901036	0.05813827	7.79E-15	2.09E-13	BCLAF1
55334	ENSG00000029364	2089.00047	-0.33029354	0.07478013	6.42E-06	6.32E-05	SLC39A9
286	ENSG00000029534	232.83165	-2.50142383	0.190387	1.32E-40	1.02E-38	ANK1
3381	ENSG00000029559	0	NA	NA	NA	NA	IBSP
51106	ENSG00000029639	348.325539	0.2235481	0.1274929	0.06239026	0.17647886	TFB1M
9135	ENSG00000029725	1674.87222	-0.38620283	0.08317403	1.89E-06	2.04E-05	RABEP1
3149	ENSG00000029993	2064.49832	-0.0491596	0.10531926	0.62635223	0.8103044	HMGB3
23279	ENSG00000030066	3193.72611	0.1496756	0.05929931	0.01060347	0.04346546	NUP160
578	ENSG00000030110	425.275248	0.67143305	0.11884456	4.03E-09	6.39E-08	BAK1
4593	ENSG00000030304	0	NA	NA	NA	NA	MUSK
22807	ENSG00000030419	60.0242662	0.01940254	0.26822041	0.92111483	0.96677343	IKZF2
2896	ENSG00000030582	1572.41997	0.19104043	0.08291848	0.01815015	0.0673082	GRN
51306	ENSG00000031003	1072.20576	-0.21283893	0.10790911	0.03916368	0.1235306	FAM13B
57514	ENSG00000031081	1606.42758	0.29665493	0.09356052	0.00104989	0.00615537	ARHGAP31
55166	ENSG00000031691	186.421555	0.08193212	0.16008052	0.5742614	0.77352065	CENPQ
6301	ENSG00000031698	2633.22579	0.19880552	0.11673406	0.07338109	0.19821386	SARS1

8498	ENSG00000031823	1208.92272	-0.051433	0.10806814	0.61871881	0.80494303	RANBP3
5926	ENSG00000032219	640.527642	-0.17930286	0.10861938	0.08443357	0.22003967	ARID4A
7260	ENSG00000032389	960.554997	-0.08982784	0.09785361	0.34159754	0.57180671	EIPR1
10908	ENSG00000032444	1421.89642	0.24430585	0.07946142	0.00164543	0.00908077	PNPLA6
8100	ENSG00000032742	271.930969	0.07143753	0.13988759	0.58460192	0.78071566	IFT88
56052	ENSG00000033011	358.913049	0.10428565	0.13410782	0.40669324	0.6349881	ALG1
55596	ENSG00000033030	873.381434	0.18174492	0.08821878	0.03408491	0.1110783	ZCCHC8
10061	ENSG00000033050	1090.3825	0.15981712	0.08930823	0.06561717	0.18299372	ABCF2
54480	ENSG00000033100	1261.24158	0.07584269	0.09848396	0.42607144	0.6519054	CHPF2
57554	ENSG00000033122	31.2853275	2.0568268	0.54762757	9.41E-06	8.97E-05	LRRC7
2530	ENSG00000033170	612.633007	0.17403	0.10489229	0.08414267	0.21945453	FUT8
55236	ENSG00000033178	1453.32681	0.15514053	0.11146688	0.14548776	0.32597939	UBA6
9846	ENSG00000033327	2830.09033	0.36022397	0.08079775	4.92E-06	4.96E-05	GAB2
535	ENSG00000033627	1668.98167	-0.20993788	0.0745248	0.00401214	0.01935818	ATP6V0A1
8554	ENSG00000033800	1079.49384	-0.25049417	0.08976004	0.00401365	0.01936076	PIAS1
9497	ENSG00000033867	1513.2093	0.22821145	0.1042198	0.02259241	0.08029979	SLC4A7
321	ENSG00000034053	2615.42135	-0.21650365	0.07043647	0.00172037	0.00943139	APBA2
5606	ENSG00000034152	320.308101	-0.35185239	0.13828881	0.00618566	0.02778434	MAP2K3
79645	ENSG00000034239	8.88501942	0.43326397	0.66931899	0.03940651	0.12407247	EFCAB1
9168	ENSG00000034510	8127.96835	0.31711197	0.05358181	2.30E-09	3.78E-08	TMSB10
28990	ENSG00000034533	148.5496	-0.12540968	0.18425696	0.4371341	0.66229404	ASTE1
25897	ENSG00000034677	720.15043	-0.25765828	0.10234312	0.00877031	0.03724311	RNF19A
8504	ENSG00000034693	149.696782	-0.33500035	0.20950149	0.05965714	0.17039991	PEX3
11345	ENSG00000034713	1260.17264	0.02198071	0.08037378	0.77444668	0.89612867	GABARAPL2
4653	ENSG00000034971	0	NA	NA	NA	NA	MYOC
26751	ENSG00000035115	785.698638	-0.07597623	0.09339426	0.40085575	0.62929088	SH3YL1
84908	ENSG00000035141	1048.09984	-0.14368909	0.09194494	0.10689668	0.26179855	FAM136A
7414	ENSG00000035403	1389.97192	0.49198738	0.07767981	9.91E-11	1.91E-09	VCL
55789	ENSG00000035499	531.613331	0.04910119	0.11857717	0.66365561	0.83407238	DEPDC1B
23604	ENSG00000035664	29.6630582	-0.43983177	0.45454653	0.07238526	0.19603105	DAPK2
8439	ENSG00000035681	671.634632	0.03616435	0.11405676	0.74057403	0.87742184	NSMAF
159	ENSG00000035687	1129.04429	-0.13608515	0.08684938	0.10716863	0.26223799	ADSS2
26228	ENSG00000035720	0.15069912	-0.01848503	0.38480575	0.84537521	NA	STAP1
7077	ENSG00000035862	7248.44363	-0.33915255	0.05900531	5.73E-09	8.91E-08	TIMP2
5981	ENSG00000035928	1417.3543	-0.25377725	0.09199682	0.00437652	0.02083758	RFC1
55773	ENSG00000036054	361.817628	-0.22910692	0.12508143	0.05186647	0.15326642	TBC1D23
8452	ENSG00000036257	1144.55631	-0.56011711	0.09190334	3.69E-10	6.71E-09	CUL3
9172	ENSG00000036448	10.3760189	-0.15108228	0.37675964	0.34940207	0.58004303	MYOM2

5009	ENSG00000036473	0.5971745	0.04260524	0.38720333	0.38071642	NA	OTC
10858	ENSG00000036530	2.96895349	0.00545388	0.37340512	0.9304077	0.97108152	CYP46A1
26009	ENSG00000036549	1099.03905	-0.01626542	0.10683231	0.87315768	0.94441388	ZZZ3
6570	ENSG00000036565	441.228445	-0.89014645	0.17719183	7.50E-08	1.01E-06	SLC18A1
9099	ENSG00000036672	120.735039	-0.12026141	0.19425814	0.47244523	0.69457676	USP2
846	ENSG00000036828	0	NA	NA	NA	NA	CASR
27175	ENSG00000037042	288.328448	0.33928128	0.15727843	0.01809274	0.06717065	TUBG2
51121	ENSG00000037241	420.275519	0.31783034	0.11746827	0.00438526	0.02086918	RPL26L1
2324	ENSG00000037280	1.92999539	-0.05878781	0.38259371	0.46698235	NA	FLT4
54888	ENSG00000037474	2053.14281	-0.02273196	0.07714035	0.76282118	0.89062391	NSUN2
54455	ENSG00000037637	568.55345	-0.10449617	0.14776976	0.44202526	0.66680698	FBXO42
4238	ENSG00000037749	1765.67159	-0.33252348	0.08356175	4.34E-05	0.00036124	MFAP3
84245	ENSG00000037757	543.437349	0.13404492	0.10615658	0.18867036	0.38926996	MRI1
4234	ENSG00000037897	137.293091	0.25940481	0.19660762	0.12615905	0.29546661	METTL1
3224	ENSG00000037965	71.3594469	-0.48717889	0.26795523	0.02212717	0.07897159	HOXC8
175	ENSG00000038002	238.36419	0.70124016	0.17267703	1.08E-05	0.00010142	AGA
55300	ENSG00000038210	158.707765	0.10519775	0.17473068	0.50003711	0.71723078	PI4K2B
259282	ENSG00000038219	2467.28429	-0.01313631	0.07944373	0.8650446	0.94028794	BOD1L1
27430	ENSG00000038274	1222.02841	0.19929479	0.07862513	0.00952435	0.03986563	MAT2B
7092	ENSG00000038295	0	NA	NA	NA	NA	TLL1
23644	ENSG00000038358	2088.90077	0.01321923	0.08340993	0.87210283	0.94409997	EDC4
7204	ENSG00000038382	4765.39225	-0.50992188	0.0668971	1.01E-14	2.68E-13	TRIO
1462	ENSG00000038427	1322.93625	2.03440043	0.27223947	5.16E-15	1.41E-13	VCAN
23274	ENSG00000038532	1118.46831	-0.00419513	0.09158471	0.96170245	0.98442765	CLEC16A
4481	ENSG00000038945	0	NA	NA	NA	NA	MSR1
999	ENSG00000039068	15.5145344	-3.70924472	1.02745286	2.06E-05	0.00018432	CDH1
23517	ENSG00000039123	1418.9486	-0.23682704	0.12235422	0.04072666	0.12702999	MTREX
1767	ENSG00000039139	7.17529456	0.16957739	0.39961713	0.25650934	0.47758495	DNAH5
9765	ENSG00000039319	1411.90515	0.21026404	0.09848351	0.0269387	0.09230809	ZFYVE16
79567	ENSG00000039523	1297.82994	-0.1483742	0.07966458	0.05652276	0.16356646	RIPOR1
729	ENSG00000039537	54.3485977	-0.01831609	0.28451101	0.92147474	0.9670192	C6
26064	ENSG00000039560	675.980592	0.19951513	0.16620144	0.18235296	0.38010956	RAI14
11063	ENSG00000039600	0.32860161	-0.02330333	0.38532616	0.6660926	NA	SOX30
11284	ENSG00000039650	842.665174	0.15845692	0.12308518	0.17358624	0.36665079	PNKP
54831	ENSG00000039987	1.75061959	0.07016317	0.38717549	0.2951704	NA	BEST2
23035	ENSG00000040199	397.033353	0.17801562	0.12891196	0.14297753	0.32208696	PHLPP2
54908	ENSG00000040275	859.967069	0.07107113	0.13506773	0.57371879	0.7731681	SPDL1
27067	ENSG00000040341	1042.16825	-0.32218884	0.09318481	0.00035109	0.00235803	STAU2

54896	ENSG00000040487	272.423739	-0.06120715	0.1465356	0.64870005	0.82469621	SLC66A1
1497	ENSG00000040531	329.709185	-0.21980806	0.13478741	0.08050012	0.21235506	CTNS
65078	ENSG00000040608	125.252677	-0.06147952	0.21930724	0.73083998	0.8712652	RTN4R
79142	ENSG00000040633	1220.53833	0.09080002	0.08202351	0.25702707	0.47809754	PHF23
1008	ENSG00000040731	0	NA	NA	NA	NA	CDH10
3631	ENSG00000040933	1302.92573	-0.13672683	0.09894471	0.15158376	0.33557485	INPP4A
5874	ENSG00000041353	317.67437	0.01631336	0.16290091	0.91293548	0.96282372	RAB27B
5685	ENSG00000041357	2359.02582	-0.05606941	0.0784587	0.46458627	0.68740335	PSMA4
23026	ENSG00000041515	366.322875	-0.01273187	0.12170792	0.91132748	0.96225318	MYO16
55341	ENSG00000041802	847.151669	0.20105331	0.09507973	0.02882172	0.09722903	LSG1
10039	ENSG00000041880	184.383674	-0.10942047	0.17782246	0.48652855	0.7063832	PARP3
3371	ENSG00000041982	297.283894	0.71387766	0.21240627	0.00015319	0.00111613	TNC
90326	ENSG00000041988	266.349749	-0.02857548	0.14604105	0.83133676	0.92375976	THAP3
140876	ENSG00000042062	165.005993	2.53066251	0.22099629	1.39E-31	8.36E-30	RIPOR3
55775	ENSG00000042088	503.604892	0.23855535	0.12073793	0.03687491	0.118091	TDP1
84883	ENSG00000042286	230.069175	0.23119345	0.16717618	0.12426396	0.29221252	AIFM2
56918	ENSG00000042304	0	NA	NA	NA	NA	C2orf83
55812	ENSG00000042317	117.030422	0.33850787	0.22590037	0.07059475	0.19275957	SPATA7
9440	ENSG00000042429	876.850403	-0.06211092	0.09202161	0.4862786	0.70622288	MED17
54884	ENSG00000042445	534.128227	0.21545136	0.11683341	0.0523272	0.15416864	RETSAT
822	ENSG00000042493	7.20768711	0.07797067	0.36033326	0.59441471	0.7877158	CAPG
1175	ENSG00000042753	1617.79918	-0.08606046	0.06840013	0.20057622	0.40577467	AP2S1
7399	ENSG00000042781	22.553269	0.15552144	0.32171721	0.43137476	0.65692932	USH2A
11055	ENSG00000042813	0	NA	NA	NA	NA	ZPBP
7038	ENSG00000042832	3.55208756	-0.24329658	0.49733423	0.01375522	0.05379878	TG
10863	ENSG00000042980	0	NA	NA	NA	NA	ADAM28
8538	ENSG00000043039	0	NA	NA	NA	NA	BARX2
54165	ENSG00000043093	654.433142	0.00052841	0.11474138	0.99827963	0.99941443	DCUN1D1
23338	ENSG00000043143	432.808285	0.49314539	0.12468253	3.01E-05	0.00025838	JADE2
7546	ENSG00000043355	0	NA	NA	NA	NA	ZIC2
3937	ENSG00000043462	0.15069912	-0.01848503	0.38480575	0.84537521	NA	LCP2
54802	ENSG00000043514	477.165889	0.20524468	0.12286663	0.0774267	0.2064157	TRIT1
153	ENSG00000043591	0	NA	NA	NA	NA	ADRB1
2981	ENSG00000044012	0	NA	NA	NA	NA	GUCA2B
9820	ENSG00000044090	1979.29205	0.49846603	0.07525676	1.43E-11	3.00E-10	CUL7
1495	ENSG00000044115	4489.99324	0.7758064	0.06378987	1.03E-34	6.90E-33	CTNNA1
5256	ENSG00000044446	543.966628	0.01336914	0.12639445	0.91183814	0.96248504	PHKA2
54875	ENSG00000044459	589.529587	-0.28147757	0.11978429	0.01304203	0.0515446	CNTLN

2042	ENSG00000044524	0	NA	NA	NA	NA	EPHA3
3309	ENSG00000044574	13823.0698	0.91485351	0.09753077	1.10E-21	4.44E-20	HSPA5
1829	ENSG00000046604	117.099541	11.4557472	2.97607695	3.66E-18	1.23E-16	DSG2
54960	ENSG00000046647	441.4231	-0.04712925	0.11626648	0.66974029	0.83753108	GEMIN8
8481	ENSG00000046651	929.411045	0.15615809	0.10262133	0.11409319	0.27454323	OFD1
2824	ENSG00000046653	239.990315	-0.22879675	0.18002277	0.14948508	0.33230072	GPM6B
51438	ENSG00000046774	4.3388516	-0.17441401	0.42520896	0.1254722	0.29435597	MAGEC2
80243	ENSG00000046889	0	NA	NA	NA	NA	PREX2
22884	ENSG00000047056	700.412302	0.07195947	0.10088602	0.46030007	0.68340593	WDR37
64848	ENSG00000047188	1242.99685	-0.13067405	0.09334098	0.14767161	0.329636	YTHDC2
56474	ENSG00000047230	853.070435	-0.1288029	0.09546123	0.16275941	0.35164399	CTPS2
51606	ENSG00000047249	499.892785	0.17224924	0.139609	0.18397192	0.3824796	ATP6V1H
5431	ENSG00000047315	3332.8292	-0.10058553	0.05915757	0.08526993	0.22149194	POLR2B
56204	ENSG00000047346	563.325823	-0.06386028	0.13860554	0.62367763	0.80810895	FAM214A
116984	ENSG00000047365	0	NA	NA	NA	NA	ARAP2
7175	ENSG00000047410	6890.71039	0.04294894	0.06286506	0.48899462	0.70870715	TPR
1356	ENSG00000047457	1.25259581	-0.02315123	0.37948037	0.73579668	NA	CP
23247	ENSG00000047578	570.149313	0.08051996	0.13127652	0.51009664	0.72552278	KATNIP
84062	ENSG00000047579	182.549598	-0.19304858	0.16997151	0.20417587	0.41083664	DTNBP1
7504	ENSG00000047597	67.9221345	0.42858456	0.28857657	0.05016204	0.14934131	XK
57101	ENSG00000047617	0	NA	NA	NA	NA	ANO2
57102	ENSG00000047621	385.333268	-0.45336307	0.13731171	0.00041525	0.00273797	C12orf4
6322	ENSG00000047634	355.067508	0.04567733	0.12772566	0.70535526	0.85803894	SCML1
55841	ENSG00000047644	531.648357	0.30369752	0.11195586	0.00446123	0.02115964	WWC3
395	ENSG00000047648	22.2990782	2.49051417	0.67857265	1.17E-05	0.00011031	ARHGAP6
27146	ENSG00000047662	11.9001715	-0.0258292	0.33596925	0.87360464	0.94441388	FAM184B
4134	ENSG00000047849	3676.28725	0.37246058	0.0646153	4.77E-09	7.49E-08	MAP4
57120	ENSG00000047932	1243.78085	-0.33061669	0.07671743	1.03E-05	9.76E-05	GOPC
6098	ENSG00000047936	0	NA	NA	NA	NA	ROS1
57646	ENSG00000048028	681.923024	0.21054415	0.13316873	0.09113269	0.232939	USP28
9734	ENSG00000048052	5111.19952	0.02535664	0.06860307	0.70838082	0.8597973	HDAC9
26262	ENSG00000048140	877.566856	-0.16896245	0.08736816	0.04613242	0.13991007	TSPAN17
51491	ENSG00000048162	785.682552	0.15862267	0.09089862	0.07221926	0.19574701	NOP16
57545	ENSG00000048342	186.568839	-0.63971367	0.19853237	0.00030167	0.00205605	CC2D2A
50484	ENSG00000048392	431.682722	0.65776755	0.11732021	5.35E-09	8.35E-08	RRM2B
168850	ENSG00000048405	717.323522	-0.15013645	0.09605327	0.10579252	0.25954292	ZNF800
608	ENSG00000048462	0	NA	NA	NA	NA	TNFRSF17
92017	ENSG00000048471	490.028798	-0.03687734	0.12670114	0.75679907	0.88697677	SNX29

55885	ENSG00000048540	338.773899	-2.02065625	0.1422605	6.35E-47	5.78E-45	LMO3
55173	ENSG00000048544	731.850529	-0.32273684	0.09791891	0.00063011	0.00394742	MRPS10
2978	ENSG00000048545	126.778044	-0.11640657	0.18858961	0.47772453	0.69938463	GUCA1A
118142757	ENSG00000048545	126.778044	-0.11640657	0.18858961	0.47772453	0.69938463	GUCA1A
51773	ENSG00000048649	2682.44249	-0.25933561	0.06440276	4.29E-05	0.00035718	RSF1
55187	ENSG00000048707	1197.2928	0.17271755	0.09946738	0.07186766	0.19529576	VPS13D
10659	ENSG00000048740	4.64305282	-3.42189135	1.84469159	0.00243431	0.01267298	CELF2
23196	ENSG00000048828	3040.39018	0.00378216	0.06649955	0.95413383	0.98112988	FAM120A
23518	ENSG00000048991	1387.37037	-0.14893715	0.09463052	0.10411651	0.25686042	R3HDM1
1298	ENSG00000049089	372.937908	2.03736328	0.167181	2.46E-35	1.68E-33	COL9A2
4254	ENSG00000049130	552.830405	-0.5835172	0.11633404	1.62E-07	2.09E-06	KITLG
1161	ENSG00000049167	491.322508	0.11977461	0.12111974	0.29714664	0.52379972	ERCC8
11174	ENSG00000049192	144.796862	1.55531361	0.22870556	8.00E-13	1.83E-11	ADAMTS6
9563	ENSG00000049239	1639.02776	0.30326019	0.07305553	2.28E-05	0.00020244	H6PD
9341	ENSG00000049245	709.859826	0.44293968	0.10389311	9.35E-06	8.93E-05	VAMP3
8863	ENSG00000049246	403.914149	-0.04271589	0.149795	0.75546831	0.88621127	PER3
10911	ENSG00000049247	0	NA	NA	NA	NA	UTS2
3604	ENSG00000049249	0.16735865	0.01897177	0.38482933	0.78225479	NA	TNFRSF9
55040	ENSG00000049283	7.80261103	-0.09222539	0.37604395	0.44612698	0.67049158	EPN3
4052	ENSG00000049323	277.706265	2.17121798	0.16779716	1.95E-39	1.49E-37	LTBP1
5954	ENSG00000049449	2466.39628	0.43344903	0.07604673	5.91E-09	9.17E-08	RCN1
2006	ENSG00000049540	1739.92799	-0.8104184	0.09365961	9.14E-19	3.20E-17	ELN
5982	ENSG00000049541	1266.06315	-0.09420264	0.08868905	0.27359882	0.49704244	RFC2
57492	ENSG00000049618	3109.40014	0.07409259	0.06103517	0.21967007	0.43175718	ARID1B
81037	ENSG00000049656	2739.67125	0.06800191	0.07330999	0.34505615	0.57548034	CLPTM1L
23327	ENSG00000049759	515.751252	0.15535633	0.27178472	0.43597214	0.66108891	NEDD4L
50943	ENSG00000049768	8.65626858	-0.01111461	0.34600621	0.93962743	0.97474024	FOXP3
89801	ENSG00000049769	220.325144	0.36284424	0.18630626	0.02585934	0.08939699	PPP1R3F
3074	ENSG00000049860	494.944396	0.00078265	0.11012585	0.99495711	0.99852582	HEXB
79810	ENSG00000049883	442.247475	-0.20163524	0.1417367	0.12459195	0.29277559	PTCD2
340533	ENSG00000050030	41.3494138	-2.6148848	0.45907531	7.89E-10	1.38E-08	NEXMIF
51528	ENSG00000050130	762.63479	-0.33321867	0.11506198	0.00232351	0.0121696	JKAMP
27122	ENSG00000050165	1574.26778	-0.08827282	0.09253738	0.32420595	0.55212413	DKK3
7984	ENSG00000050327	0	NA	NA	NA	NA	ARHGEF5
9603	ENSG00000050344	824.524784	0.1353205	0.09612718	0.14579992	0.32633917	NFE2L3
63933	ENSG00000050393	959.542884	0.02194277	0.08570899	0.79440056	0.90579008	MCUR1
51474	ENSG00000050405	1733.90932	0.59049577	0.07370494	3.63E-16	1.07E-14	LIMA1
25875	ENSG00000050426	794.874608	-0.092741	0.10350354	0.35069845	0.58113472	LETMD1

9498	ENSG00000050438	1423.69016	-0.80659914	0.08750937	5.72E-21	2.24E-19	SLC4A8
10319	ENSG00000050555	15.6316255	-0.16730633	0.38657739	0.29109731	0.51718193	LAMC3
5733	ENSG00000050628	20.727972	-0.05062699	0.3103139	0.78374339	0.9014745	PTGER3
79931	ENSG00000050730	0.37844916	0.02407552	0.3854191	0.60038877	NA	TNIP3
5601	ENSG00000050748	727.654164	-0.21737928	0.10221246	0.02688177	0.09216075	MAPK9
91522	ENSG00000050767	68.7718736	-2.18995022	0.332403	2.95E-12	6.53E-11	COL23A1
9564	ENSG00000050820	1590.93779	-0.28977012	0.10794613	0.00498094	0.02315412	BCAR1
84067	ENSG00000051009	425.287173	-0.27226603	0.15699025	0.05647111	0.16351237	FAM160A2
9709	ENSG00000051108	1257.70256	1.2623568	0.08322306	6.30E-53	6.72E-51	HERPUD1
9454	ENSG00000051128	803.693362	1.17418888	0.09405209	1.03E-36	7.24E-35	HOMER3
5888	ENSG00000051180	429.238395	-0.51265174	0.12713477	2.04E-05	0.00018249	RAD51
10721	ENSG00000051341	843.044024	-0.52005128	0.13649756	4.97E-05	0.0004061	POLQ
5291	ENSG00000051382	339.978675	-0.60828793	0.1455248	8.12E-06	7.84E-05	PIK3CB
1535	ENSG00000051523	486.215562	0.2541938	0.11614794	0.02133233	0.07670051	CYBA
84321	ENSG00000051596	1372.3521	0.00945446	0.07852783	0.89971893	0.95564128	THOC3
23593	ENSG00000051620	348.114864	1.17607989	0.15319189	1.71E-15	4.85E-14	HEBP2
10198	ENSG00000051825	1094.39728	-0.22003463	0.10300817	0.02609773	0.09006417	MPHOSPH9
54477	ENSG00000052126	294.200214	0.52980516	0.15083629	0.00015468	0.00112579	PLEKHA5
5652	ENSG00000052344	3.30838869	0.07705588	0.38263342	0.42374354	0.64959604	PRSS8
80143	ENSG00000052723	1098.79906	0.19014138	0.13098763	0.12256885	0.28970289	SIKE1
23223	ENSG00000052749	1518.04181	0.03461558	0.07266271	0.62840361	0.81158232	RRP12
57600	ENSG00000052795	614.121929	-0.10786509	0.11867297	0.33807253	0.56829936	FNIP2
6307	ENSG00000052802	844.793952	-0.47674754	0.14126054	0.00029472	0.00201423	MSMO1
55761	ENSG00000052841	2474.21763	0.42320709	0.06153416	3.10E-12	6.83E-11	TTC17
60529	ENSG00000052850	8.50916519	0.3305188	0.52793543	0.07386363	0.19921891	ALX4
23105	ENSG00000053108	574.315292	-3.13028101	0.12864262	5.95E-132	3.11E-129	FSTL4
1112	ENSG00000053254	2155.28088	0.03795375	0.07822407	0.62130372	0.80672374	FOXN3
728464	ENSG00000053328	18.2374059	2.25419839	0.65099415	2.94E-05	0.00025379	METTL24
8574	ENSG00000053371	645.676238	-0.00474423	0.09769914	0.95915408	0.9831126	AKR7A2
51154	ENSG00000053372	1017.26832	-0.05131192	0.08993293	0.5565381	0.76208295	MRTO4
4826	ENSG00000053438	11037.036	1.26260706	0.06178067	8.30E-94	2.39E-91	NNAT
55850	ENSG00000053501	231.975472	0.03638613	0.14869009	0.79180789	0.90499209	USE1
23101	ENSG00000053524	69.2547223	-0.7343761	0.31496878	0.00317404	0.0158702	MCF2L2
83714	ENSG00000053702	69.6353952	-0.16602374	0.23191479	0.37335213	0.60236694	NRIP2
3909	ENSG00000053747	312.503741	2.10468962	0.16271141	1.66E-39	1.27E-37	LAMA3
55745	ENSG00000053770	770.959732	0.01018738	0.10422587	0.92006889	0.96634023	AP5M1
29945	ENSG00000053900	546.038579	0.09353452	0.14492293	0.48621015	0.70622288	ANAPC4
3784	ENSG00000053918	145.441919	-0.65520653	0.22573708	0.00081393	0.00494438	KCNQ1

27095	ENSG00000054116	607.884997	0.01833761	0.09953709	0.84953175	0.93154246	TRAPPC3
9967	ENSG00000054118	3181.51939	-0.13596257	0.07599422	0.06756871	0.18665906	THRAP3
29085	ENSG00000054148	990.723732	0.09831793	0.0838126	0.22943896	0.44501603	PHPT1
954	ENSG00000054179	4.11503966	-0.06800191	0.37246735	0.56170032	0.76502898	ENTPD2
4065	ENSG00000054219	0	NA	NA	NA	NA	LY75
51742	ENSG00000054267	1637.80979	0.0288795	0.0787222	0.70846604	0.8597973	ARID4B
23596	ENSG00000054277	597.150409	1.1086	0.11608224	1.53E-22	6.52E-21	OPN3
10806	ENSG00000054282	614.245702	0.00082155	0.13906285	0.99612541	0.99859366	SDCCAG8
5798	ENSG00000054356	945.936602	-2.4681967	0.09554824	2.46E-148	1.95E-145	PTPRN
55733	ENSG00000054392	739.008641	-0.50843445	0.10197489	2.49E-07	3.12E-06	HHAT
23095	ENSG00000054523	4026.26172	-0.52134904	0.06227206	1.02E-17	3.31E-16	KIF1B
2296	ENSG00000054598	256.892242	1.89006559	0.16279846	3.01E-32	1.84E-30	FOXC1
25771	ENSG00000054611	375.495141	-0.13590734	0.14183785	0.30074001	0.52797821	TBC1D22A
23224	ENSG00000054654	5054.10325	0.27169424	0.06219983	9.33E-06	8.91E-05	SYNE2
57475	ENSG00000054690	523.843909	0.12587958	0.13523773	0.31967603	0.54727882	PLEKHH1
10079	ENSG00000054793	2484.20111	-0.01613574	0.06209335	0.79223299	0.90499209	ATP9A
23626	ENSG00000054796	0	NA	NA	NA	NA	SPO11
140689	ENSG00000054803	641.347547	0.47752585	0.15007356	0.00060908	0.00383379	CBLN4
25884	ENSG00000054938	0.173124	-0.01911262	0.38486262	0.84537521	NA	CHRDL2
23201	ENSG00000054965	2875.29355	-0.1187806	0.06052335	0.04642337	0.14061674	FAM168A
84957	ENSG00000054967	432.11455	-0.14800838	0.12201797	0.19897677	0.40340274	RELT
2581	ENSG00000054983	494.169417	0.29230176	0.12406455	0.01252948	0.04992667	GALC
51602	ENSG00000055044	1658.92672	-0.14769737	0.07049784	0.03285223	0.10778817	NOP58
26099	ENSG00000055070	3458.53845	0.01074422	0.06273712	0.86364351	0.93960776	SZRD1
3757	ENSG00000055118	5733.42235	-0.36831472	0.05501652	1.29E-11	2.73E-10	KCNH2
8454	ENSG00000055130	1837.92104	0.02700972	0.07495279	0.71444204	0.86242659	CUL1
10827	ENSG00000055147	447.133747	-0.05302937	0.12376524	0.64989242	0.82529572	FAM114A2
26999	ENSG00000055163	2706.84768	-0.3922535	0.06353532	3.65E-10	6.64E-09	CYFIP2
23118	ENSG00000055208	1500.15263	-0.21649218	0.09035669	0.01346846	0.05295894	TAB2
116254	ENSG00000055211	247.374487	0.23955431	0.14923882	0.0810791	0.21357208	GINM1
5610	ENSG00000055332	2149.98215	-0.17430097	0.09876728	0.06369854	0.17918196	EIF2AK2
57602	ENSG00000055483	1198.5825	-0.16012758	0.07945665	0.03911599	0.12343904	USP36
58508	ENSG00000055609	3285.5046	-0.32330885	0.0965385	0.00051275	0.00329861	KMT2C
55283	ENSG00000055732	0	NA	NA	NA	NA	MCOLN3
114800	ENSG00000055813	0	NA	NA	NA	NA	CCDC85A
23369	ENSG00000055917	4002.26621	-0.52529538	0.06690034	1.54E-15	4.36E-14	PUM2
84545	ENSG00000055950	1023.69047	0.04046173	0.0845572	0.62440124	0.80857129	MRPL43
3700	ENSG00000055955	22.1468053	2.57918491	0.74963623	2.60E-05	0.00022721	ITIH4

3697	ENSG00000055957	0.15069912	-0.01848503	0.38480575	0.84537521	NA	ITIH1
54969	ENSG00000056050	502.160485	0.00552511	0.10918536	0.95906584	0.9831126	HPF1
51663	ENSG00000056097	2292.22207	-0.19586275	0.07115003	0.00500739	0.02324844	ZFR
55609	ENSG00000056277	418.060363	-0.02974278	0.13247234	0.81003806	0.91386075	ZNF280C
10886	ENSG00000056291	252.822748	0.31143577	0.14530723	0.02037392	0.07391569	NPFFR2
112885	ENSG00000056487	2577.12924	-1.69037074	0.06962925	2.60E-131	1.32E-128	PHF21B
7185	ENSG00000056558	17.1570115	-0.02266378	0.32195801	0.89625436	0.95469109	TRAF1
54542	ENSG00000056586	2302.98209	-0.29214422	0.08437585	0.00037329	0.00249108	RC3H2
55540	ENSG00000056736	198.716519	0.08070639	0.15932691	0.57875142	0.77656626	IL17RB
10758	ENSG00000056972	131.759239	-0.02054497	0.18526224	0.89808302	0.95533575	TRAF3IP2
8908	ENSG00000056998	120.714541	0.99964139	0.24431147	5.00E-06	5.03E-05	GYG2
131566	ENSG00000057019	1022.27233	-0.15454194	0.08388332	0.05859568	0.16816698	DCBLD2
6317	ENSG00000057149	0	NA	NA	NA	NA	SERPINB3
6646	ENSG00000057252	656.295207	0.08039376	0.1030782	0.41842591	0.6444567	SOAT1
5318	ENSG00000057294	172.566141	2.09156616	0.19751583	2.31E-27	1.19E-25	PKP2
4438	ENSG00000057468	0.34712721	0.00084574	0.3833641	0.96045798	NA	MSH4
2155	ENSG00000057593	3.41218294	-0.01743079	0.3739663	0.84118869	0.92789889	F7
2665	ENSG00000057608	3851.79871	-0.39661192	0.08393636	1.20E-06	1.34E-05	GDI2
639	ENSG00000057657	0	NA	NA	NA	NA	PRDM1
9474	ENSG00000057663	319.124097	-0.15013183	0.13726389	0.2407149	0.45879844	ATG5
57458	ENSG00000057704	74.5483529	0.20777266	0.24601357	0.28278237	0.50782063	TMCC3
57095	ENSG00000057757	580.76378	-0.14487906	0.10563856	0.15166865	0.33565042	PITHD1
57504	ENSG00000057935	1966.53165	0.09987264	0.07299832	0.16352879	0.35261507	MTA3
8975	ENSG00000058056	1019.06324	-0.05685573	0.12497295	0.62922332	0.81227084	USP13
23200	ENSG00000058063	1201.09101	0.4197021	0.09794774	9.10E-06	8.70E-05	ATP11B
3918	ENSG00000058085	0.63325157	0.0018705	0.38251164	0.96341566	NA	LAMC2
5218	ENSG00000058091	1386.81721	0.04738595	0.07878819	0.53978561	0.74874827	CDK14
29927	ENSG00000058262	4988.48445	-0.07317467	0.05963166	0.2139987	0.42415859	SEC61A1
4659	ENSG00000058272	1081.24199	0.14148447	0.12656546	0.23582753	0.45262825	PPP1R12A
5923	ENSG00000058335	2.13990379	-0.05283874	0.38368747	0.37470751	NA	RASGRF1
816	ENSG00000058404	193.750718	-1.52834595	0.27566789	1.95E-09	3.22E-08	CAMK2B
9696	ENSG00000058453	2331.72046	0.07569482	0.09311255	0.40269733	0.63083419	CROCC
55718	ENSG00000058600	1458.4059	0.11260896	0.10448913	0.26302545	0.48504013	POLR3E
493	ENSG00000058668	675.512434	0.68798791	0.11684364	9.38E-10	1.62E-08	ATP2B4
9877	ENSG00000058673	4326.67821	-0.02750275	0.07517586	0.70822852	0.85971939	ZC3H11A
55781	ENSG00000058729	320.879833	-0.31462318	0.17275704	0.03959027	0.1244606	RIOK2
54432	ENSG00000058799	225.914246	0.32934111	0.17057519	0.03148309	0.10422567	YIPF1
55706	ENSG00000058804	1248.60477	-0.63970504	0.08061661	5.75E-16	1.66E-14	NDC1

1608	ENSG00000058866	6.72394865	-0.30242382	0.5462797	0.02742102	0.09344437	DGKG
84256	ENSG00000059122	875.414504	-0.065705	0.10286586	0.50644622	0.72214266	FLYWCH1
64718	ENSG00000059145	512.835547	0.14933217	0.14247537	0.2583304	0.47964541	UNKL
6916	ENSG00000059377	40.7290682	-0.18149155	0.28992346	0.36694317	0.59618989	TBXAS1
64761	ENSG00000059378	40.8666834	0.71752104	0.40880182	0.01124651	0.04570478	PARP12
5832	ENSG00000059573	2256.46243	-0.02106102	0.08289777	0.79391939	0.90579008	ALDH18A1
6894	ENSG00000059588	1924.68711	0.18267779	0.08380235	0.02534462	0.0880241	TARBP1
5188	ENSG00000059691	456.618445	-0.03217769	0.13771487	0.80117399	0.90897872	GATB
4084	ENSG00000059728	253.412974	-0.06514051	0.16039789	0.65412597	0.82782798	MXD1
5128	ENSG00000059758	383.8963	-0.14068796	0.15060726	0.3085812	0.53621325	CDK17
548645	ENSG00000059769	178.610493	0.2925802	0.17154975	0.05686818	0.16435994	DNAJC25
6515	ENSG00000059804	0	NA	NA	NA	NA	SLC2A3
5662	ENSG00000059915	507.74821	-1.45229797	0.11925719	3.28E-35	2.24E-33	PSD
9150	ENSG00000060069	526.065785	0.15123325	0.10409805	0.13094035	0.30334616	CTDP1
8531	ENSG00000060138	0.37844916	0.02407552	0.3854191	0.60038877	NA	YBX3
55359	ENSG00000060140	0	NA	NA	NA	NA	STYK1
65125	ENSG00000060237	4585.13363	-0.22060165	0.06268238	0.00035147	0.00235899	WNK1
NA	ENSG00000060303	0.173124	-0.01911262	0.38486262	0.84537521	NA	RPS17P5
55749	ENSG00000060339	2286.06587	-0.24631444	0.09256993	0.00596838	0.0270472	CCAR1
11054	ENSG00000060491	1089.50998	-0.20993562	0.09423287	0.02086996	0.07536656	OGFR
2769	ENSG00000060558	0.40363765	0.01333376	0.38394276	0.83341597	NA	GNA15
84699	ENSG00000060566	9.90042191	-0.24332929	0.43741416	0.15366611	0.33878786	CREB3L3
55650	ENSG00000060642	193.436552	-0.13173875	0.18636275	0.41781117	0.64393487	PIGV
10076	ENSG00000060656	2550.04441	0.02678722	0.08682615	0.75223583	0.88403749	PTPRU
9410	ENSG00000060688	867.714082	0.04629124	0.09069229	0.59996125	0.79080728	SNRNP40
23504	ENSG00000060709	3431.81884	-0.23058553	0.07156748	0.00101885	0.00600176	RIMBP2
1301	ENSG00000060718	490.267549	3.83552145	0.16363982	4.57E-123	1.97E-120	COL11A1
79832	ENSG00000060749	2267.72091	-0.24607562	0.07774078	0.00119407	0.00689675	QSER1
51660	ENSG00000060762	661.628377	0.07651055	0.106243	0.45406432	0.67734106	MPC1
30	ENSG00000060971	462.187471	-0.13360574	0.12672745	0.26196478	0.48394839	ACAA1
586	ENSG00000060982	3771.07681	0.28002262	0.06724492	2.29E-05	0.00020285	BCAT1
51564	ENSG00000061273	1443.63289	-0.05170517	0.08276971	0.52144558	0.73486861	HDAC7
11178	ENSG00000061337	580.299049	0.49305881	0.10329673	7.34E-07	8.50E-06	LZTS1
93166	ENSG00000061455	9.93435802	-0.01861019	0.36635153	0.85819342	0.93665736	PRDM6
7478	ENSG00000061492	0	NA	NA	NA	NA	WNT8A
6676	ENSG00000061656	111.043499	0.14026568	0.21175571	0.42999153	0.65567843	SPAG4
10787	ENSG00000061676	3089.99289	-0.18511641	0.07424467	0.01091634	0.04456382	NCKAP1
60488	ENSG00000061794	548.854146	-0.03833955	0.11384764	0.72381839	0.86794586	MRPS35

2983	ENSG00000061918	392.457977	-0.50908701	0.13406549	5.45E-05	0.00044062	GUCY1B1
6433	ENSG00000061936	1785.84581	0.09347092	0.07786513	0.22063904	0.43304726	SFSWAP
10188	ENSG00000061938	1516.93714	-0.1876973	0.0781505	0.01401183	0.05460511	TNK2
23041	ENSG00000061987	1064.7441	0.14233839	0.10000521	0.14016105	0.31765996	MON2
1001	ENSG00000062038	35.3013425	-0.02961199	0.2943934	0.87384842	0.94444368	CDH3
416	ENSG00000062096	0.30001591	-0.02294459	0.38527538	0.68644873	NA	ARSF
65056	ENSG00000062194	1887.31669	-0.35030263	0.07085888	4.68E-07	5.61E-06	GPBP1
84649	ENSG00000062282	91.5448866	-0.12961917	0.22616063	0.47914353	0.70058277	DGAT2
7771	ENSG00000062370	274.402857	-0.47624231	0.14729938	0.00049016	0.00317078	ZNF112
1431	ENSG00000062485	3265.19471	0.02152869	0.05668939	0.69917797	0.85490515	CS
4058	ENSG00000062524	42.9879408	1.59227675	0.39125139	3.37E-06	3.48E-05	LTK
64951	ENSG00000062582	1072.00943	-0.11558578	0.0980526	0.22236573	0.43535977	MRPS24
63916	ENSG00000062598	809.283901	-0.09457013	0.09005841	0.27901068	0.50350845	ELMO2
23063	ENSG00000062650	1294.45535	-0.16102334	0.08212784	0.04437026	0.1357031	WAPL
81671	ENSG00000062716	1237.3572	-0.1790155	0.07664464	0.01695414	0.06380314	VMP1
10513	ENSG00000062725	1429.57473	-0.18467501	0.08762658	0.03002901	0.1003834	APPBP2
5424	ENSG00000062822	2232.27143	-0.00749969	0.0785706	0.92154395	0.9670192	POLD1
124925	ENSG00000063015	453.585722	-2.16857655	0.13431589	8.07E-60	1.05E-57	SEZ6
1975	ENSG00000063046	12076.6339	-0.09713412	0.04951823	0.04793827	0.14408352	EIF4B
28968	ENSG00000063127	0	NA	NA	NA	NA	SLC6A16
29998	ENSG00000063169	665.624026	0.34280128	0.12337067	0.00326929	0.01627671	BICRA
56848	ENSG00000063176	723.774509	0.24828014	0.10638004	0.01486561	0.05725758	SPHK2
6141	ENSG00000063177	9448.37519	0.06921317	0.05351248	0.1917349	0.39366867	RPL18
770	ENSG00000063180	202.76199	0.11945433	0.18480751	0.46185454	0.6848938	CA11
79763	ENSG00000063241	679.692398	0.12937843	0.09704995	0.16810285	0.35916008	ISOC2
11338	ENSG00000063244	5890.03362	0.13165085	0.06594784	0.04282162	0.13230775	U2AF2
29924	ENSG00000063245	4096.97433	-0.02830137	0.06222515	0.64415982	0.8223082	EPN1
55588	ENSG00000063322	1311.12798	0.05760742	0.1109367	0.58834762	0.78314772	MED29
57491	ENSG00000063438	12.5036808	0.23899858	0.42540577	0.18025552	0.37712427	AHRR
2928	ENSG00000063515	0.50586849	0.0145565	0.38307792	0.75000001	NA	GSC2
10838	ENSG00000063587	412.599773	-0.15056691	0.12243456	0.19334855	0.39603916	ZNF275
105373378	ENSG00000063587	412.599773	-0.15056691	0.12243456	0.19334855	0.39603916	ZNF275
8776	ENSG00000063601	811.10033	-0.08304345	0.09663909	0.37370967	0.60269874	MTMR1
2817	ENSG00000063660	824.27179	0.1544503	0.09358591	0.08848321	0.22759703	GPC1
57143	ENSG00000063761	159.585418	0.17288812	0.19108218	0.29815184	0.52487422	ADCK1
3029	ENSG00000063854	739.240893	0.13312312	0.11667688	0.23083346	0.44684775	HAGH
6047	ENSG00000063978	1011.35379	-0.49598312	0.10165466	4.18E-07	5.06E-06	RNF4
841	ENSG00000064012	10.1675833	-0.03235521	0.34797254	0.82822339	0.92220943	CASP8

22998	ENSG00000064042	2325.52929	-0.42326898	0.07456229	6.84E-09	1.05E-07	LIMCH1
55726	ENSG00000064102	493.125718	-0.17872409	0.11478636	0.10175042	0.25253045	INTS13
51768	ENSG00000064115	1668.00183	-0.49241148	0.06962307	6.20E-13	1.43E-11	TM7SF3
1747	ENSG00000064195	0	NA	NA	NA	NA	DLX3
53340	ENSG00000064199	228.620738	-0.02948383	0.22909238	0.87018362	0.94333385	SPA17
10077	ENSG00000064201	5.55217324	-0.02960274	0.3644677	0.80435302	0.91118482	TSPAN32
8839	ENSG00000064205	3.80670146	-0.07311225	0.3763259	0.5146819	0.72937777	CCN5
58524	ENSG00000064218	0	NA	NA	NA	NA	DMRT3
10402	ENSG00000064225	880.784003	-0.0345195	0.09020655	0.69279586	0.85156247	ST3GAL6
9914	ENSG00000064270	6.16967696	0.00256285	0.36810733	0.98295811	0.99428951	ATP2C2
4804	ENSG00000064300	295.342734	1.14354845	0.18175215	3.47E-11	7.00E-10	NGFR
50937	ENSG00000064309	969.549461	0.65706971	0.08430635	1.76E-15	4.96E-14	CDON
6873	ENSG00000064313	1063.17708	0.32693246	0.09823271	0.00056016	0.00356263	TAF2
28996	ENSG00000064393	3702.14826	-0.39610979	0.05791467	4.32E-12	9.42E-11	HIPK2
23534	ENSG00000064419	2307.31014	-0.18467444	0.06325192	0.00304099	0.01530912	TNPO3
NA	ENSG00000064489	43.2794601	0.80163906	0.46552951	0.00905398	0.03818204	BORCS8-MEF2B
8625	ENSG00000064490	662.603619	0.11399399	0.10222469	0.24713944	0.46606087	RFXANK
54929	ENSG00000064545	632.799407	0.09498164	0.09854716	0.31890196	0.54656314	TMEM161A
9170	ENSG00000064547	593.448608	-0.27747428	0.10441341	0.0055027	0.0252522	LPAR2
5476	ENSG00000064601	873.981775	0.29957906	0.100924	0.00203934	0.0108794	CTSA
10147	ENSG00000064607	4262.56638	-0.17992209	0.05590902	0.00112619	0.00653705	SUGP2
6558	ENSG00000064651	413.164264	-0.40895553	0.14065373	0.00177227	0.00965452	SLC12A2
28966	ENSG00000064652	263.211046	0.09446433	0.14604129	0.48479267	0.70488105	SNX24
2139	ENSG00000064655	35.8034936	1.7345305	0.4518196	7.55E-06	7.34E-05	EYA2
1265	ENSG00000064666	682.942568	2.0063517	0.11477784	1.35E-69	2.17E-67	CNN2
10347	ENSG00000064687	239.634466	0.25169205	0.15883722	0.08118562	0.21379384	ABCA7
9627	ENSG00000064692	42.5708672	-0.01984626	0.28159406	0.91571893	0.96423105	SNCAIP
11218	ENSG00000064703	618.985741	-0.2858456	0.10796073	0.00561718	0.02567852	DDX20
53339	ENSG00000064726	1302.81584	-0.23858069	0.07744354	0.00161606	0.00894855	BTBD1
55711	ENSG00000064763	177.195907	-0.27060788	0.18089603	0.08963614	0.22992799	FAR2
8537	ENSG00000064787	2.8885739	-0.13233943	0.40569864	0.17281505	0.36572272	BCAS1
5449	ENSG00000064835	0	NA	NA	NA	NA	POU1F1
1117	ENSG00000064886	0	NA	NA	NA	NA	CHI3L2
22904	ENSG00000064932	1151.08746	0.31120176	0.09062091	0.00039692	0.00262934	SBNO2
5378	ENSG00000064933	745.027745	0.02933424	0.10532969	0.77305063	0.89561741	PMS1
10362	ENSG00000064961	1508.2469	0.48680986	0.08202009	1.23E-09	2.09E-08	HMG20B
10203	ENSG00000064989	22.6358152	4.3301481	0.73774787	5.31E-10	9.51E-09	CALCRL
6882	ENSG00000064995	763.343545	-0.16926744	0.09106562	0.05495953	0.16007004	TAF11

23294	ENSG00000064999	1563.40211	0.094493	0.07173182	0.17999691	0.37673028	ANKS1A
8943	ENSG00000065000	3687.4386	-0.02758796	0.06977454	0.68616235	0.8472712	AP3D1
7629	ENSG00000065029	622.907559	0.12258614	0.11297137	0.25625759	0.47738504	ZNF76
9351	ENSG00000065054	415.78371	-0.23908752	0.11762914	0.03183314	0.10508645	SLC9A3R2
4913	ENSG00000065057	502.488962	-0.14691607	0.11073151	0.1647848	0.35443739	NTHL1
54887	ENSG00000065060	1404.63916	0.04397768	0.09720378	0.64086865	0.82008361	UHRF1BP1
2773	ENSG00000065135	2132.80987	0.10797651	0.06749087	0.10414586	0.25686895	GNAI3
3843	ENSG00000065150	4952.42333	-0.27756367	0.06862437	3.79E-05	0.00031934	IPO5
4942	ENSG00000065154	908.070296	-0.52976378	0.09041802	1.68E-09	2.82E-08	OAT
10885	ENSG00000065183	1653.83878	-0.23901763	0.09162329	0.00705336	0.03116675	WDR3
5586	ENSG00000065243	894.925505	0.35767923	0.13035554	0.00364464	0.01785624	PKN2
57418	ENSG00000065268	1122.70651	0.05319157	0.08434014	0.51831643	0.73217501	WDR18
9697	ENSG00000065308	1215.9754	-1.27178699	0.0872218	3.71E-49	3.57E-47	TRAM2
9423	ENSG00000065320	4.86237717	-0.06960162	0.3709929	0.5695269	0.77023951	NTN1
9340	ENSG00000065325	0	NA	NA	NA	NA	GLP2R
55388	ENSG00000065328	1227.23354	-0.48199773	0.07728015	1.89E-10	3.52E-09	MCM10
1606	ENSG00000065357	306.365666	0.1035651	0.15540401	0.46645318	0.68929176	DGKA
2065	ENSG00000065361	24.5879142	1.23173398	1.00989471	0.00641753	0.02870885	ERBB3
54763	ENSG00000065371	1.36392096	-0.07227669	0.39347707	0.1370696	NA	ROPN1
91526	ENSG00000065413	1381.86056	-0.23589266	0.09813003	0.01252331	0.04991209	ANKRD44
3735	ENSG00000065427	2922.70506	-0.26600955	0.06190975	1.28E-05	0.00011972	KARS1
23536	ENSG00000065457	1422.8216	0.08783152	0.09885135	0.35808922	0.5884123	ADAT1
10954	ENSG00000065485	352.600706	0.16475272	0.14257277	0.21264373	0.42247662	PDIA5
55633	ENSG00000065491	351.572959	-0.01840028	0.13293932	0.88244601	0.94823075	TBC1D22B
4710	ENSG00000065518	1049.36517	0.29256694	0.09219762	0.00105208	0.00616639	NDUFB4
23013	ENSG00000065526	3521.24226	0.03004208	0.07357104	0.67815467	0.84291038	SPEN
4638	ENSG00000065534	262.831142	0.16256215	0.19492455	0.33332972	0.56278287	MYLK
55854	ENSG00000065548	1267.40523	-0.18812996	0.07604273	0.01143352	0.04635085	ZC3H15
6416	ENSG00000065559	613.39846	-0.01518168	0.12119336	0.89358592	0.95394425	MAP2K4
55248	ENSG00000065600	876.989307	0.05866885	0.09232563	0.51691078	0.7308667	PACC1
9892	ENSG00000065609	1560.15094	-1.39664421	0.09368143	2.48E-51	2.53E-49	SNAP91
9748	ENSG00000065613	871.862951	-0.04681491	0.11998025	0.68006901	0.84377539	SLK
51167	ENSG00000065615	570.676382	-0.24631609	0.12269062	0.03349968	0.10951287	CYB5R4
1308	ENSG00000065618	11.4494889	0.26702873	0.47686744	0.08322585	0.21772162	COL17A1
119391	ENSG00000065621	13.411335	-0.00092756	0.3551063	0.99145686	0.99723721	GSTO2
55176	ENSG00000065665	360.538985	-0.26462301	0.13467112	0.03526614	0.11399155	SEC61A2
5588	ENSG00000065675	84.5938824	-0.09702974	0.2288866	0.59723508	0.78921427	PRKCQ
7089	ENSG00000065717	79.974157	0.59588755	0.30580021	0.01145822	0.04643563	TLE2

51665	ENSG00000065802	2043.30945	0.22709345	0.08230328	0.00465938	0.02191608	ASB1
83641	ENSG00000065809	1076.41621	-0.26201636	0.09156193	0.00312227	0.01567453	FAM107B
4199	ENSG00000065833	536.289859	0.00188523	0.10371536	0.98630321	0.99540871	ME1
23216	ENSG00000065882	960.958607	0.15472671	0.10280972	0.11798158	0.28184882	TBC1D1
8621	ENSG00000065883	1704.22426	-0.12483769	0.10190114	0.20252479	0.40813528	CDK13
10797	ENSG00000065911	2841.32792	0.20068249	0.10848733	0.05351149	0.15675002	MTHFD2
84679	ENSG00000065923	297.299305	0.32895373	0.15775486	0.02209493	0.07889909	SLC9A7
55810	ENSG00000065970	358.811343	0.0652617	0.1254651	0.5835797	0.77994081	FOXJ2
4904	ENSG00000065978	19961.4794	-0.26024195	0.05094519	2.48E-07	3.11E-06	YBX1
5141	ENSG00000065989	45.532229	0.4810079	0.35383614	0.0473594	0.14263366	PDE4A
5525	ENSG00000066027	317.738574	-0.13606619	0.12810299	0.25881671	0.48030339	PPP2R5A
1496	ENSG00000066032	810.834777	-0.79066117	0.10302844	3.17E-15	8.86E-14	CTNNA2
1994	ENSG00000066044	2770.39049	0.19603842	0.06670241	0.0028199	0.01437112	ELAVL1
7075	ENSG00000066056	0	NA	NA	NA	NA	TIE1
57609	ENSG00000066084	1601.08568	-0.28853912	0.08055587	0.00024	0.00167307	DIP2B
6602	ENSG00000066117	3068.74848	0.18884169	0.0730727	0.00838562	0.03592544	SMARCD1
9682	ENSG00000066135	1693.29473	0.13894396	0.09067111	0.11466058	0.27560769	KDM4A
4802	ENSG00000066136	817.789482	0.02577692	0.09062973	0.77078419	0.89494676	NFYC
84217	ENSG00000066185	7.83365596	-0.05360092	0.35653347	0.70568456	0.85810136	ZMYND12
6550	ENSG00000066230	161.112239	-0.22375812	0.17289501	0.14638876	0.32736169	SLC9A3
25791	ENSG00000066248	4.69270454	0.05675457	0.37647739	0.56328855	0.76608585	NGEF
259266	ENSG00000066279	4313.08726	-0.23062646	0.06214274	0.0001658	0.00120056	ASPM
8832	ENSG00000066294	0	NA	NA	NA	NA	CD84
64834	ENSG00000066322	498.300514	0.09741164	0.13920885	0.45327661	0.67649496	ELOVL1
6688	ENSG00000066336	0.98382358	0.0055225	0.38090519	0.92055612	NA	SPI1
30834	ENSG00000066379	308.48488	0.10873336	0.1420753	0.41050943	0.63818422	POLR1H
744	ENSG00000066382	356.471644	-1.15421454	0.14060174	2.43E-17	7.68E-16	MPPED2
51208	ENSG00000066405	14.8028961	-0.00294003	0.32927279	0.98425525	0.99457273	CLDN18
27107	ENSG00000066422	793.357869	-0.15663786	0.10495241	0.11993794	0.28511487	ZBTB11
4287	ENSG00000066427	295.385633	-0.1328854	0.16690905	0.37528349	0.60449338	ATXN3
9950	ENSG00000066455	470.381612	0.01763512	0.11173048	0.86985598	0.9432116	GOLGA5
2263	ENSG00000066468	77.1809794	1.74910838	0.33074966	8.23E-09	1.25E-07	FGFR2
55631	ENSG00000066557	522.053428	0.07096339	0.10822041	0.49499404	0.71386797	LRRC40
51015	ENSG00000066583	641.017598	0.52110061	0.09880346	5.02E-08	6.92E-07	ISOC1
2009	ENSG00000066629	793.517596	-0.8051814	0.14373099	3.78E-09	6.02E-08	EML1
60487	ENSG00000066651	327.578968	-0.06640872	0.14263069	0.614807	0.80225838	TRMT11
55623	ENSG00000066654	910.189228	-0.03314423	0.11507934	0.76160484	0.88972737	THUMPD1
91283	ENSG00000066697	540.204362	-0.00851921	0.11935972	0.93948005	0.97474024	MSANTD3

26153	ENSG00000066735	2239.80758	-0.12157283	0.08069465	0.1225077	0.28962737	KIF26A
55102	ENSG00000066739	1446.67563	-0.3596886	0.11976411	0.00150978	0.00842114	ATG2B
10565	ENSG00000066777	1260.9939	-0.11324615	0.09338854	0.21054304	0.4192266	ARFGEF1
348158	ENSG00000066813	0	NA	NA	NA	NA	ACSM2B
57623	ENSG00000066827	207.849604	-0.08003727	0.16779961	0.59473068	0.78785671	ZFAT
9650	ENSG00000066855	381.078602	0.07510212	0.15412956	0.59551648	0.78816266	MTFR1
10734	ENSG00000066923	406.246127	0.15138252	0.14956588	0.27139107	0.49479863	STAG3
2235	ENSG00000066926	739.17727	0.12237433	0.09589827	0.18757778	0.38786275	FECH
4649	ENSG00000066933	673.814876	-0.53079599	0.12471346	7.26E-06	7.09E-05	MYO9A
8653	ENSG00000067048	0	NA	NA	NA	NA	DDX3Y
5214	ENSG00000067057	2168.77891	-0.92773586	0.09907439	1.16E-21	4.69E-20	PFKP
3422	ENSG00000067064	1206.88081	-0.23155836	0.11724604	0.03733871	0.11921467	IDI1
6672	ENSG00000067066	0	NA	NA	NA	NA	SP100
1316	ENSG00000067082	428.784198	0.15500571	0.1619172	0.29076165	0.51679428	KLF6
8611	ENSG00000067113	68.4823066	0.22617885	0.24143193	0.23945233	0.45732906	PLPP1
4756	ENSG00000067141	2155.3719	0.24392126	0.09361317	0.00710103	0.0313007	NEO1
23471	ENSG00000067167	1110.57695	0.29087731	0.08751372	0.00063004	0.00394742	TRAM1
5255	ENSG00000067177	754.567948	0.18008905	0.09679536	0.0542306	0.15841212	PHKA1
7132	ENSG00000067182	21.4017542	0.03486383	0.3360017	0.83297958	0.9239848	TNFRSF1A
782	ENSG00000067191	469.224481	-0.13165328	0.14294198	0.31953204	0.54726483	CACNB1
7813	ENSG00000067208	339.924787	0.1858592	0.13913341	0.15089983	0.33458345	EVI5
9399	ENSG00000067221	240.526851	-0.31051449	0.18277907	0.05376317	0.15732467	STOML1
5315	ENSG00000067225	16335.8325	0.10169726	0.08309778	0.20552481	0.41249633	PKM
54505	ENSG00000067248	579.487487	-0.35662355	0.11372374	0.00099328	0.00587207	DHX29
30836	ENSG00000067334	1063.14761	-0.2342407	0.0867327	0.0054289	0.02496741	DNTTIP2
79091	ENSG00000067365	355.597026	0.19334079	0.12435905	0.09924115	0.24794665	METTL22
7158	ENSG00000067369	3241.27528	-0.229172	0.05847199	7.17E-05	0.0005674	TP53BP1
7216	ENSG00000067445	2126.12563	0.16496769	0.08538612	0.04735635	0.14263366	TRO
51018	ENSG00000067533	779.944177	0.24559048	0.12791192	0.04117839	0.12828422	RRP15
387	ENSG00000067560	5059.1076	0.04198217	0.06672532	0.52597028	0.73808697	RHOA
1659	ENSG00000067596	2038.38248	0.16209	0.0660245	0.01269084	0.05043796	DHX8
5382	ENSG00000067601	41.9520327	0.04226955	0.26189021	0.82769107	0.92210075	PMS2P4
5590	ENSG00000067606	825.816343	-0.0048812	0.09703224	0.95773024	0.98264776	PRKCZ
7544	ENSG00000067646	0	NA	NA	NA	NA	ZFY
55699	ENSG00000067704	2798.39186	0.03385784	0.05986441	0.56750225	0.76896903	IARS2
6857	ENSG00000067715	949.939435	0.22136445	0.08599072	0.00815682	0.03511964	SYT1
89795	ENSG00000067798	4.60351858	-0.03345196	0.37581887	0.68666263	0.84769738	NAV3
3421	ENSG00000067829	693.190618	0.28550484	0.0980937	0.00253864	0.01315056	IDH3G

79641	ENSG00000067836	509.140247	-0.09336163	0.11217582	0.38316841	0.61242739	ROGDI
57595	ENSG00000067840	2471.475	-0.56901329	0.06284328	4.60E-20	1.73E-18	PDZD4
492	ENSG00000067842	272.941164	-0.78356131	0.20262348	1.88E-05	0.00016926	ATP2B3
6093	ENSG00000067900	1289.70509	-0.19450297	0.08187687	0.01480479	0.05705655	ROCK1
865	ENSG00000067955	1261.04809	0.10157601	0.09020137	0.24686755	0.46592963	CBFB
5165	ENSG00000067992	353.288903	-0.2116745	0.20303078	0.21861688	0.43050004	PDK3
8692	ENSG00000068001	1370.18245	0.35902496	0.08632701	1.90E-05	0.00017141	HYAL2
9759	ENSG00000068024	774.733402	-0.23792775	0.09986811	0.01332301	0.05247466	HDAC4
11186	ENSG00000068028	525.094579	0.12925926	0.11212139	0.22780028	0.44270325	RASSF1
2261	ENSG00000068078	16.1006639	1.57607468	0.75253213	0.00166665	0.00918252	FGFR3
3430	ENSG00000068079	0.63756822	0.03739676	0.38672729	0.3970149	NA	IFI35
63897	ENSG00000068097	1017.82477	-0.07226636	0.12630712	0.54326264	0.75131261	HEATR6
80347	ENSG00000068120	1198.02943	0.07347805	0.08229667	0.36021419	0.59016007	COASY
79990	ENSG00000068137	775.747067	0.07337566	0.11098837	0.49035394	0.70985933	PLEKHH3
4205	ENSG00000068305	795.020067	-0.0762564	0.11758989	0.49269686	0.71179682	MEF2A
55593	ENSG00000068308	1301.70977	0.10068918	0.07452513	0.16852594	0.35979283	OTUD5
7030	ENSG00000068323	785.125103	-0.00579835	0.10620291	0.95373622	0.98083958	TFE3
4943	ENSG00000068354	366.768743	-0.08229625	0.12207731	0.4762782	0.69819414	TBC1D25
2182	ENSG00000068366	762.889038	-0.8572206	0.11946526	1.19E-13	2.89E-12	ACSL4
3632	ENSG00000068383	264.162269	0.00544941	0.14367168	0.96846335	0.98817646	INPP5A
27238	ENSG00000068394	376.06394	0.33164902	0.12942051	0.00637486	0.02854372	GPKOW
56850	ENSG00000068400	1400.59103	-0.02036236	0.07745264	0.78763991	0.90351292	GRIPAP1
24140	ENSG00000068438	683.157415	0.03293598	0.09541972	0.72235104	0.86754018	FTSJ1
55771	ENSG00000068489	2777.6694	-0.89390983	0.07352523	8.22E-35	5.53E-33	PRR11
65055	ENSG00000068615	176.737115	-0.91775351	0.18631778	1.20E-07	1.57E-06	REEP1
23250	ENSG00000068650	1393.43298	0.32198682	0.10648598	0.00160035	0.00887144	ATP11A
25885	ENSG00000068654	2290.92942	-0.12421763	0.06525646	0.05322597	0.15616729	POLR1A
9741	ENSG00000068697	2188.71858	0.10356757	0.07703335	0.17024841	0.36213659	LAPTM4A
57217	ENSG00000068724	414.547037	0.14214244	0.12904492	0.24171231	0.45967442	TTC7A
51447	ENSG00000068745	1908.54325	-0.0049599	0.06874508	0.93539045	0.97325384	IP6K2
286749	ENSG00000068781	10.3533668	0.41489235	0.64348615	0.04412839	0.13518014	STON1-GTF2A1L
55133	ENSG00000068784	552.777088	-0.49622883	0.11198091	3.67E-06	3.77E-05	SRBD1
3796	ENSG00000068796	2485.3682	-0.27668353	0.07624887	0.00020666	0.00146589	KIF2A
10235	ENSG00000068831	151.839499	-0.07287967	0.17416704	0.63763668	0.81742743	RASGRP2
23198	ENSG00000068878	2051.57876	0.01294517	0.07254034	0.85659391	0.93595581	PSME4
57560	ENSG00000068885	830.003339	-0.08196314	0.10076589	0.39852856	0.62742474	IFT80
22933	ENSG00000068903	407.567552	-1.00756519	0.13551936	1.33E-14	3.50E-13	SIRT2
27248	ENSG00000068912	699.109603	0.0903599	0.09647011	0.33345519	0.56294681	ERLEC1

5526	ENSG00000068971	441.292882	-0.04012566	0.11543972	0.71372977	0.86192204	PPP2R5B
5837	ENSG00000068976	9.22977598	0.14544713	0.37924805	0.35591545	0.58600518	PYGM
8712	ENSG00000068985	0.173124	-0.01911262	0.38486262	0.84537521	NA	PAGE1
5307	ENSG00000069011	0	NA	NA	NA	NA	PITX1
57113	ENSG00000069018	0	NA	NA	NA	NA	TRPC7
375449	ENSG00000069020	701.833859	-0.06174184	0.11703575	0.57861069	0.77656626	MAST4
221395	ENSG00000069122	0	NA	NA	NA	NA	ADGRF5
54549	ENSG00000069188	13.5669859	0.1662607	0.37584956	0.33036999	0.55911802	SDK2
8756	ENSG00000069206	0	NA	NA	NA	NA	ADAM7
55746	ENSG00000069248	1926.57973	-0.0792401	0.09314323	0.37955811	0.60845927	NUP133
64710	ENSG00000069275	15156.0847	-0.35758215	0.04777685	4.41E-14	1.12E-12	NUCKS1
55737	ENSG00000069329	1806.91633	-0.54665588	0.08267587	1.33E-11	2.80E-10	VPS35
10294	ENSG00000069345	1129.4183	-0.10545552	0.09478533	0.25009929	0.46992802	DNAJA2
602	ENSG00000069399	10.1620828	4.37330518	1.23636141	6.35E-05	0.00050743	BCL3
8514	ENSG00000069424	400.053405	-0.31514914	0.16164646	0.03119534	0.10344569	KCNAB2
10060	ENSG00000069431	45.8244256	2.72650378	0.41119329	1.70E-12	3.81E-11	ABCC9
51083	ENSG00000069482	771.510594	-0.55639801	0.14234335	2.99E-05	0.0002571	GAL
29121	ENSG00000069493	107.511817	0.32698112	0.21769564	0.07331913	0.19810043	CLEC2D
139341	ENSG00000069509	218.74431	-0.01715422	0.14823684	0.89896939	0.95556135	FUNDC1
4129	ENSG00000069535	26.0526863	0.94074456	0.51636464	0.00585121	0.02658299	MAOB
6095	ENSG00000069667	353.361378	0.01251864	0.19907701	0.94290365	0.97652077	RORA
1815	ENSG00000069696	5.84790272	-0.04701102	0.36077718	0.72388179	0.86794586	DRD4
7049	ENSG00000069702	0	NA	NA	NA	NA	TGFBR3
8399	ENSG00000069764	0.97083619	-0.06756019	0.39188254	0.1968697	NA	PLA2G10
54626	ENSG00000069812	2.41875988	0.01519793	0.37877735	0.81813737	NA	HES2
483	ENSG00000069849	1203.60499	-0.131162	0.09138512	0.13841995	0.31479274	ATP1B3
4734	ENSG00000069869	353.959514	0.39364953	0.14084823	0.00265204	0.01363917	NEDD4
9488	ENSG00000069943	106.308801	0.19961567	0.21846124	0.27089411	0.4942105	PIGB
5597	ENSG00000069956	1220.20018	-0.12011262	0.08749854	0.15784049	0.34484696	MAPK6
10681	ENSG00000069966	466.814993	0.00144193	0.11086451	0.99002031	0.99665126	GNB5
5873	ENSG00000069974	134.146443	0.13225685	0.21433932	0.45959276	0.68271342	RAB27A
27440	ENSG00000069998	1135.13869	-0.17491128	0.08129481	0.02744302	0.09350327	HDHD5
7353	ENSG00000070010	1050.4857	0.32751343	0.10814555	0.00155628	0.00866346	UFD1
4040	ENSG00000070018	1949.35838	0.20247865	0.0919208	0.02308154	0.08171625	LRP6
2984	ENSG00000070019	0.173733	0.01914545	0.38484597	0.78225479	NA	GUCY2C
6343	ENSG00000070031	0	NA	NA	NA	NA	SCT
57661	ENSG00000070047	1932.99978	0.03352335	0.0734507	0.64254706	0.82136499	PHRF1
8518	ENSG00000070061	2109.86293	-0.17483353	0.07885134	0.02313241	0.08185251	ELP1

4925	ENSG00000070081	426.744147	0.03546246	0.11582964	0.74948217	0.88205755	NUCB2
5217	ENSG00000070087	4641.88555	0.32920296	0.06479684	2.46E-07	3.09E-06	PFN2
5774	ENSG00000070159	18.0309604	0.12305305	0.37076356	0.41010226	0.63800117	PTPN3
6710	ENSG00000070182	292.155948	-0.36495655	0.13687879	0.00423721	0.02025692	SPTB
27071	ENSG00000070190	0.74967325	-0.04343647	0.38756608	0.34471339	NA	DAPP1
2255	ENSG00000070193	0	NA	NA	NA	NA	FGF10
23446	ENSG00000070214	926.553002	-0.02999628	0.10079527	0.75710417	0.88697677	SLC44A1
54916	ENSG00000070269	313.279347	0.30354657	0.16605667	0.04258701	0.13177264	TMEM260
23293	ENSG00000070366	1005.56209	0.02440568	0.11973397	0.83300404	0.9239848	SMG6
10640	ENSG00000070367	1923.35646	-0.12938628	0.08778566	0.12932841	0.30080605	EXOC5
8218	ENSG00000070371	255.14724	0.41616785	0.1779619	0.00884925	0.03748274	CLTCL1
27006	ENSG00000070388	8.76733142	0.22632753	0.43057604	0.16796082	0.35893393	FGF22
10272	ENSG00000070404	123.904378	0.30518756	0.22415515	0.09999335	0.24919835	FSTL3
9993	ENSG00000070413	1925.26211	-0.07161079	0.07240117	0.31336324	0.54126787	DGCR2
55658	ENSG00000070423	885.249375	-0.14243821	0.08918708	0.09858639	0.24665406	RNF126
4335	ENSG00000070444	762.947709	-0.24058966	0.09415053	0.00820551	0.03530631	MNT
79364	ENSG00000070476	616.414867	0.0752888	0.09993766	0.43560821	0.66073906	ZXDC
23210	ENSG00000070495	721.019327	-0.03525393	0.10835829	0.73362788	0.87316412	JMJD6
5423	ENSG00000070501	434.348914	0.06934981	0.11649066	0.53347137	0.74467543	POLB
55808	ENSG00000070526	0	NA	NA	NA	NA	ST6GALNAC1
55062	ENSG00000070540	138.655264	2.41118072	0.231852	1.86E-26	9.28E-25	WIPI1
22844	ENSG00000070601	41.7018318	0.48214785	0.40062036	0.05520879	0.16065449	FRMPD1
57704	ENSG00000070610	3015.11815	-0.04640587	0.06177783	0.44602748	0.67039282	GBA2
3340	ENSG00000070614	3402.96847	-0.06514012	0.07334536	0.364816	0.59432668	NDST1
440	ENSG00000070669	3028.52805	0.45204064	0.28480225	0.03859701	0.12224852	ASNS
10947	ENSG00000070718	660.474645	-0.26742685	0.11455371	0.0140968	0.05488235	AP3M2
1258	ENSG00000070729	0	NA	NA	NA	NA	CNGB1
10610	ENSG00000070731	4.81375467	0.1556572	0.41257938	0.15730146	0.34408611	ST6GALNAC2
1103	ENSG00000070748	6.47669384	-0.13593459	0.38354791	0.3468015	0.57746889	CHAT
26986	ENSG00000070756	15901.9091	-0.58119017	0.06363414	2.15E-20	8.20E-19	PABPC1
10420	ENSG00000070759	94.5671766	0.26357231	0.24201007	0.17303076	0.36578929	TESK2
29105	ENSG00000070761	750.167084	-0.03901886	0.09808595	0.68004629	0.84377539	CFAP20
1459	ENSG00000070770	1084.19274	-0.07006034	0.08804964	0.41305857	0.6403849	CSNK2A2
11099	ENSG00000070778	808.980955	0.28616757	0.09850973	0.00258461	0.01335035	PTPN21
8891	ENSG00000070785	349.920402	0.11676949	0.12462526	0.32339177	0.55106868	EIF2B3
815	ENSG00000070808	0.68744131	-0.03071099	0.38429993	0.55668345	NA	CAMK2A
6949	ENSG00000070814	3716.30769	-0.06870127	0.05499685	0.20672574	0.41386868	TCOF1
998	ENSG00000070831	3076.73866	-0.14677917	0.0575316	0.00976434	0.04073267	CDC42

26031	ENSG00000070882	382.533041	0.0211882	0.14053014	0.87299125	0.94439555	OSBPL3
2046	ENSG00000070886	201.986312	-1.60130775	0.21016708	1.86E-15	5.24E-14	EPHA8
6559	ENSG00000070915	1.41099212	-0.02065922	0.37959921	0.75912992	NA	SLC12A3
56852	ENSG00000070950	640.289048	0.01029006	0.1078947	0.92175849	0.96719317	RAD18
490	ENSG00000070961	817.570686	-0.2426238	0.11638915	0.02814094	0.09543867	ATP2B1
29850	ENSG00000070985	0.52978646	0.01332752	0.38290755	0.74558738	NA	TRPM5
8440	ENSG00000071051	1321.6118	-0.12067798	0.07950796	0.11965681	0.28461708	NCK2
9448	ENSG00000071054	6264.79824	-0.06823398	0.05753171	0.22645993	0.44088684	MAP4K4
11320	ENSG00000071073	256.89568	0.3047879	0.20733249	0.08366533	0.21849678	MGAT4A
6160	ENSG00000071082	9802.24134	-0.02959142	0.05869797	0.60546635	0.79536863	RPL31
9948	ENSG00000071127	2186.97424	0.01264084	0.06837141	0.8518234	0.93275912	WDR1
23161	ENSG00000071189	713.694434	-0.0228271	0.16366874	0.85973351	0.93751308	SNX13
54860	ENSG00000071203	0	NA	NA	NA	NA	MS4A12
79658	ENSG00000071205	115.141923	0.98580268	0.24924737	9.36E-06	8.93E-05	ARHGAP10
6196	ENSG00000071242	3609.60685	0.44644599	0.05511737	2.59E-16	7.71E-15	RPS6KA2
54556	ENSG00000071243	379.711828	0.13066525	0.14445686	0.32860738	0.55708539	ING3
22846	ENSG00000071246	2050.8964	0.22971517	0.07806367	0.00261172	0.01346931	VASH1
29995	ENSG00000071282	619.188481	0.14250355	0.10893239	0.17203619	0.36458	LMCD1
114049	ENSG00000071462	1511.79218	0.41199507	0.09319058	5.04E-06	5.06E-05	BUD23
6400	ENSG00000071537	1420.56722	0.29884612	0.12243976	0.00969126	0.0404533	SEL1L
9319	ENSG00000071539	1105.01941	-0.08952824	0.08832065	0.29605562	0.52267989	TRIP13
537	ENSG00000071553	2096.30511	-0.26928498	0.0782546	0.00042631	0.00280345	ATP6AP1
6929	ENSG00000071564	6132.53295	0.30831143	0.05039	6.05E-10	1.08E-08	TCF3
28951	ENSG00000071575	1261.36482	-0.67953333	0.08482264	2.79E-16	8.27E-15	TRIB2
26528	ENSG00000071626	2814.14047	-0.24280325	0.05876876	2.84E-05	0.00024625	DAZAP1
53615	ENSG00000071655	3213.30443	-0.01038585	0.06575371	0.87683389	0.94566867	MBD3
51052	ENSG00000071677	0	NA	NA	NA	NA	PRLH
6596	ENSG00000071794	1338.83224	-0.47090608	0.09344588	1.97E-07	2.51E-06	HLTF
9130	ENSG00000071859	893.292155	-0.23330411	0.09139386	0.00838973	0.03593528	FAM50A
60343	ENSG00000071889	387.311116	0.11630057	0.11961483	0.30625127	0.53396829	FAM3A
29894	ENSG00000071894	3062.10645	0.36764632	0.06239772	2.26E-09	3.71E-08	CPSF1
140469	ENSG00000071909	0.64211671	0.00270554	0.381949	0.95310032	NA	MYO3B
79901	ENSG00000071967	97.7833713	1.7619506	0.31003768	8.13E-10	1.42E-08	CYBRD1
28513	ENSG00000071991	0.29863895	-0.02292439	0.3852726	0.68753245	NA	CDH19
5134	ENSG00000071994	1872.24056	-0.11230962	0.07121147	0.10815	0.26372861	PDCD2
55117	ENSG00000072041	1368.36171	-0.28032158	0.07355256	9.97E-05	0.00075866	SLC6A15
51109	ENSG00000072042	1586.59312	0.07479663	0.07823614	0.32834517	0.55683119	RDH11
5566	ENSG00000072062	1135.31907	-0.02346437	0.10033487	0.80759653	0.91292321	PRKACA

22859	ENSG00000072071	2494.89326	-0.41806649	0.09956042	1.32E-05	0.0001233	ADGRL1
6694	ENSG00000072080	0	NA	NA	NA	NA	SPP2
87	ENSG00000072110	2303.7278	-0.50723767	0.07985425	8.34E-11	1.62E-09	ACTN1
23503	ENSG00000072121	588.369671	-0.18602269	0.12248176	0.10791665	0.2634186	ZFYVE26
27330	ENSG00000072133	1695.48937	0.41784285	0.08047376	1.06E-07	1.39E-06	RPS6KA6
22905	ENSG00000072134	1443.16292	0.1182982	0.08735735	0.16426112	0.35369445	EPN2
26469	ENSG00000072135	599.448107	0.07982308	0.11483869	0.46721197	0.69011879	PTPN18
55679	ENSG00000072163	10.0559359	0.06238189	0.35028122	0.68718588	0.84792387	LIMS2
55515	ENSG00000072182	412.841993	-0.72563404	0.14203014	6.86E-08	9.24E-07	ASIC4
10290	ENSG00000072195	1254.27088	-0.08366706	0.10945365	0.42394197	0.64969559	SPEG
84708	ENSG00000072201	28.3971416	-0.1195768	0.30314954	0.539358	0.74850934	LNX1
224	ENSG00000072210	1074.11513	-0.33037058	0.087145	9.21E-05	0.00070688	ALDH3A2
7037	ENSG00000072274	2091.27501	0.33683504	0.20297214	0.05333139	0.15638409	TFRC
6720	ENSG00000072310	2880.99457	-0.67064184	0.06354504	1.32E-26	6.65E-25	SREBF1
7224	ENSG00000072315	3.43424896	0.07901165	0.37745415	0.48768524	0.70749307	TRPC5
27125	ENSG00000072364	3255.56012	-0.19089899	0.07282126	0.00749382	0.03270508	AFF4
7321	ENSG00000072401	490.484788	0.06065207	0.10754539	0.55758289	0.76266422	UBE2D1
64398	ENSG00000072415	416.262618	-0.12431758	0.13221646	0.31530138	0.54311334	MPP5
9886	ENSG00000072422	820.790826	0.24812099	0.10917666	0.01740348	0.06513582	RHOBTB1
8243	ENSG00000072501	7661.63632	-0.69273237	0.05364369	8.40E-39	6.31E-37	SMC1A
3028	ENSG00000072506	1817.64429	-0.01529178	0.07431098	0.83290372	0.9239848	HSD17B10
2011	ENSG00000072518	1090.52336	-0.0034538	0.09736942	0.96950196	0.98853525	MARK2
3161	ENSG00000072571	1022.94365	-0.34878189	0.13965664	0.00716702	0.03152155	HMMR
55743	ENSG00000072609	1032.36645	0.14140291	0.08840891	0.10003584	0.24927294	CHFR
29953	ENSG00000072657	5.88164387	5.88308178	2.68072459	7.13E-05	0.00056456	TRHDE
8974	ENSG00000072682	255.238779	0.30893436	0.15280009	0.02735726	0.09335537	P4HA2
2213	ENSG00000072694	0	NA	NA	NA	NA	FCGR2B
4775	ENSG00000072736	1413.3291	-0.28253484	0.09958153	0.00319127	0.01593228	NFATC3
51095	ENSG00000072756	473.213469	-0.21232016	0.11549664	0.05307819	0.15584893	TRNT1
37	ENSG00000072778	2143.237	0.35946122	0.07066402	2.16E-07	2.72E-06	ACADVL
6793	ENSG00000072786	738.721236	0.1396393	0.10700843	0.17399895	0.36724886	STK10
23291	ENSG00000072803	2001.67067	-0.24099939	0.08568363	0.00381437	0.01850295	FBXW11
9744	ENSG00000072818	24.011114	0.10505754	0.34428877	0.53911066	0.74843996	ACAP1
1400	ENSG00000072832	8280.0118	-0.88483889	0.0517195	2.11E-66	3.17E-64	CRMP1
2121	ENSG00000072840	640.501481	0.78815987	0.13918001	2.79E-09	4.50E-08	EVC
51009	ENSG00000072849	737.476587	0.09606153	0.0962525	0.3030654	0.53032436	DERL2
54847	ENSG00000072858	31.5633262	0.60512385	0.74399499	0.02816306	0.09547008	SIDT1
54820	ENSG00000072864	876.734251	-0.36525629	0.09559419	7.63E-05	0.00059871	NDE1

10335	ENSG00000072952	267.365782	-0.01950353	0.14213902	0.88156584	0.94772508	IRAG1
79041	ENSG00000072954	186.838923	0.17448433	0.16527796	0.24206059	0.46022827	TMEM38A
8907	ENSG00000072958	2101.93321	-0.09533768	0.08027072	0.22341436	0.43670355	AP1M1
5817	ENSG00000073008	1276.28796	-0.48693144	0.08627439	6.86E-09	1.05E-07	PVR
7515	ENSG00000073050	649.104794	0.17588985	0.10660144	0.0851446	0.2214571	XRCC1
949	ENSG00000073060	1245.35684	-0.52195871	0.09286986	7.11E-09	1.09E-07	SCARB1
54905	ENSG00000073067	7.2489666	0.0485584	0.35520935	0.74022554	0.87736188	CYP2W1
4171	ENSG00000073111	4461.12354	-0.13124292	0.08305308	0.10461186	0.25761097	MCM2
54456	ENSG00000073146	2.31284792	-0.02179237	0.37596095	0.79044881	NA	MOV10L1
56666	ENSG00000073150	97.9047859	0.66309253	0.25586326	0.00202449	0.01081181	PANX2
83642	ENSG00000073169	672.876421	0.13690032	0.10449262	0.17284379	0.36574454	SELENOO
8626	ENSG00000073282	0	NA	NA	NA	NA	TP63
80216	ENSG00000073331	45.4628923	0.18236104	0.28592593	0.36708071	0.59632206	ALPK1
3993	ENSG00000073350	199.795585	-0.51546255	0.17025788	0.00085567	0.00516474	LLGL2
5151	ENSG00000073417	738.250233	0.84272422	0.11606111	6.54E-14	1.63E-12	PDE8A
1183	ENSG00000073464	639.559848	-0.47130097	0.12345106	5.48E-05	0.00044339	CLCN4
54475	ENSG00000073536	747.340547	-0.34524892	0.11020249	0.00103298	0.0060739	NLE1
6389	ENSG00000073578	1355.97423	-0.09659195	0.07278058	0.17605874	0.37037488	SDHA
6605	ENSG00000073584	3699.44113	0.14256846	0.06916246	0.03625272	0.11649724	SMARCE1
54752	ENSG00000073598	1.29743315	-0.01435995	0.38049881	0.81092726	NA	FNDC8
55876	ENSG00000073605	238.875138	0.5361726	0.19483417	0.00190128	0.01023367	GSDMB
5927	ENSG00000073614	2009.0231	-0.25176124	0.09256331	0.00495143	0.02304394	KDM5A
4185	ENSG00000073670	418.359885	0.28689377	0.15320232	0.04070691	0.12700504	ADAM11
5523	ENSG00000073711	179.274606	0.4391798	0.18723487	0.00800359	0.03451983	PPP2R3A
10979	ENSG00000073712	1877.97123	0.56097801	0.07431959	1.52E-14	3.97E-13	FERMT2
8647	ENSG00000073734	0	NA	NA	NA	NA	ABCB11
10170	ENSG00000073737	0	NA	NA	NA	NA	DHRS9
922	ENSG00000073754	0.14931947	-0.01844332	0.38480212	0.84537521	NA	CD5L
5743	ENSG00000073756	0	NA	NA	NA	NA	PTGS2
10644	ENSG00000073792	0	NA	NA	NA	NA	IGF2BP2
9175	ENSG00000073803	511.759872	-0.33816737	0.12025443	0.00296583	0.01499162	MAP3K13
6480	ENSG00000073849	140.890286	0.64256943	0.2031591	0.00037381	0.00249331	ST6GAL1
30009	ENSG00000073861	1.26305052	0.02098636	0.38053927	0.75775503	NA	TBX21
NA	ENSG00000073905	0	NA	NA	NA	NA	VDAC1P1
10129	ENSG00000073910	373.33258	-0.18895043	0.20823964	0.28007312	0.5047371	FRY
8301	ENSG00000073921	1436.81393	-0.24246886	0.08474634	0.0032651	0.01626735	PICALM
4905	ENSG00000073969	1366.77636	-0.34532797	0.07740862	5.01E-06	5.04E-05	NSF
2736	ENSG00000074047	277.999184	1.2503622	0.15891872	3.46E-16	1.02E-14	GLI2

23332	ENSG00000074054	3544.77656	0.32284316	0.06046341	6.18E-08	8.37E-07	CLASP1
65993	ENSG00000074071	1637.21856	-0.25484644	0.07787175	0.00081259	0.00493774	MRPS34
4854	ENSG00000074181	2481.23072	1.45190913	0.08066727	1.68E-73	2.92E-71	NOTCH3
1207	ENSG00000074201	2325.89733	-0.13897153	0.06648368	0.03327496	0.10890387	CLNS1A
5522	ENSG00000074211	444.271733	-2.12856204	0.13865017	2.29E-54	2.55E-52	PPP2R2C
8463	ENSG00000074219	1287.24791	1.39424614	0.0891584	3.80E-56	4.52E-54	TEAD2
8726	ENSG00000074266	436.573548	-0.05851712	0.11810353	0.60164142	0.79212726	EED
54825	ENSG00000074276	49.6391863	-0.26247841	0.30593026	0.21249082	0.42238326	CDHR2
6620	ENSG00000074317	67.656621	-2.19063754	0.33702745	5.64E-12	1.22E-10	SNCB
7251	ENSG00000074319	1242.34862	0.15018503	0.08848708	0.08093678	0.21334235	TSG101
55421	ENSG00000074356	1850.36482	-0.04345038	0.1175273	0.69666255	0.85365037	NCBP3
489	ENSG00000074370	239.15968	0.8435844	0.16518924	5.43E-08	7.42E-07	ATP2A3
771	ENSG00000074410	0.83239697	-0.0301577	0.3835933	0.5327641	NA	CA12
11343	ENSG00000074416	75.0840853	-0.02375429	0.26181164	0.89926753	0.95557174	MGLL
59277	ENSG00000074527	28.8331453	2.38497444	0.57428237	1.30E-06	1.45E-05	NTN4
617	ENSG00000074582	481.934813	-0.11848536	0.12420863	0.3120454	0.5397542	BCS1L
9891	ENSG00000074590	48.1388288	0.6722328	0.36714739	0.01128813	0.04583637	NUAK1
54878	ENSG00000074603	963.201603	0.03537743	0.13727773	0.78394914	0.90160286	DPP8
9187	ENSG00000074621	268.143267	0.12016184	0.18175113	0.45326336	0.67649496	SLC24A1
55205	ENSG00000074657	3692.12362	0.16604936	0.05911421	0.00446021	0.02115964	ZNF532
8578	ENSG00000074660	9.69827898	-0.10589967	0.36870791	0.46585575	0.68856963	SCARF1
3998	ENSG00000074695	2139.07164	-0.22384713	0.0748236	0.0021962	0.01160698	LMAN1
51495	ENSG00000074696	2715.21549	0.0046409	0.06154531	0.93974292	0.97474024	HACD3
26034	ENSG00000074706	27.4846939	3.54359854	0.65491169	4.26E-09	6.74E-08	IPCEF1
23140	ENSG00000074755	1502.84351	-0.08834347	0.08594216	0.29076088	0.51679428	ZZEF1
50508	ENSG00000074771	1.55151473	0.09327671	0.39636697	0.14786157	NA	NOX3
2023	ENSG00000074800	17178.491	-0.25343766	0.08214687	0.00150642	0.00841417	ENO1
6557	ENSG00000074803	10.9564616	-0.11717093	0.35262433	0.49125682	0.71060078	SLC12A1
56005	ENSG00000074842	1115.3621	0.23908568	0.07883224	0.00191688	0.01029474	MYDGF
57719	ENSG00000074855	1240.29879	0.30715859	0.08184349	0.00011973	0.00089304	ANO8
51175	ENSG00000074935	690.529284	0.63849911	0.12835693	1.76E-07	2.25E-06	TUBE1
55160	ENSG00000074964	987.489544	-0.2986516	0.10083981	0.00206595	0.01099527	ARHGEF10L
7294	ENSG00000074966	3.78628526	0.08072848	0.38067124	0.43875446	0.66399363	TXK
9671	ENSG00000075035	155.891237	-1.97640981	0.28035605	8.42E-14	2.08E-12	WSCD2
3785	ENSG00000075043	7240.76961	-0.02274764	0.06917506	0.73798802	0.8762752	KCNQ2
6865	ENSG00000075073	7.12316317	0.1915897	0.4125222	0.20945722	0.41777735	TACR2
64431	ENSG00000075089	566.469264	-0.20986239	0.11618482	0.05666652	0.16393463	ACTR6
54962	ENSG00000075131	270.040322	-0.13250811	0.15185082	0.34075913	0.57095356	TIPIN

6717	ENSG00000075142	1699.72706	0.22889989	0.09403053	0.01190287	0.04791093	SRI
8672	ENSG00000075151	2776.68063	-0.21149574	0.06430564	0.00082575	0.00500243	EIF4G3
79023	ENSG00000075188	370.647863	0.27955906	0.12594771	0.01849659	0.06829932	NUP37
10371	ENSG00000075213	154.477323	1.5185773	0.2289809	2.53E-12	5.62E-11	SEMA3A
51512	ENSG00000075218	1410.53912	-0.12328161	0.07694223	0.10137512	0.25181949	GTSE1
10512	ENSG00000075223	907.262539	0.09290556	0.09593762	0.31804683	0.54566319	SEMA3C
55020	ENSG00000075234	575.711211	-0.00054722	0.10101073	0.9929465	0.99767338	TTC38
38	ENSG00000075239	1173.50425	-0.10054524	0.09708007	0.28335143	0.50852034	ACAT1
23151	ENSG00000075240	1337.82184	-0.1468053	0.12141123	0.20101193	0.40615916	GRAMD4
9620	ENSG00000075275	71.5632308	4.08894336	1.19369178	7.34E-06	7.16E-05	CELSR1
7479	ENSG00000075290	21.0807219	-0.17533286	0.33676464	0.37250818	0.60143185	WNT8B
27332	ENSG00000075292	2433.10181	-0.12950361	0.0685878	0.05497761	0.1600992	ZNF638
55972	ENSG00000075303	705.024778	-0.21538904	0.12843021	0.07388242	0.19924252	SLC25A40
29090	ENSG00000075336	363.158909	-0.17282151	0.14026326	0.1839313	0.38247532	TIMM21
119	ENSG00000075340	2769.46776	-0.34335052	0.07414969	2.17E-06	2.33E-05	ADD2
2249	ENSG00000075388	0	NA	NA	NA	NA	FGF4
9462	ENSG00000075391	550.704963	0.23390412	0.11402048	0.03124548	0.10358167	RASAL2
9605	ENSG00000075399	241.980669	0.12609827	0.17237214	0.41311927	0.6403849	VPS9D1
7587	ENSG00000075407	1001.79985	0.16020353	0.13225615	0.19643233	0.40005705	ZNF37A
4140	ENSG00000075413	2186.18178	-0.01551579	0.06468496	0.80714571	0.91260538	MARK3
5250	ENSG00000075415	6442.49712	0.02074787	0.05208165	0.69983252	0.8552838	SLC25A3
64778	ENSG00000075420	486.739324	0.03448944	0.13504482	0.78614948	0.90280441	FNDC3B
2355	ENSG00000075426	80.9092892	0.08445769	0.22202711	0.6434075	0.82181194	FOSL2
27091	ENSG00000075429	256.058482	-1.48815794	0.23514118	1.86E-11	3.86E-10	CACNG5
27092	ENSG00000075461	18166.637	-0.31942555	0.04741632	1.08E-11	2.29E-10	CACNG4
285527	ENSG00000075539	1431.57412	-0.02126697	0.10916751	0.83758226	0.92628758	FRYL
23505	ENSG00000075568	1071.45224	0.13857269	0.10085696	0.15425616	0.3394719	TMEM131
6624	ENSG00000075618	18935.9928	0.06969678	0.05435745	0.19552902	0.39918663	FSCN1
60	ENSG00000075624	50261.31	0.16983742	0.06997571	0.01366748	0.05355773	ACTB
55034	ENSG00000075643	23.0655773	1.69896651	0.55453841	0.00013071	0.00096586	MOCOS
5337	ENSG00000075651	22.0722073	0.8956178	0.77795691	0.0130118	0.05146612	PLD1
479	ENSG00000075673	0	NA	NA	NA	NA	ATP12A
284403	ENSG00000075702	781.712185	-0.17906537	0.11316265	0.09665262	0.24306007	WDR62
1739	ENSG00000075711	1532.97204	-0.05567145	0.09017482	0.5255877	0.73806584	DLG1
7879	ENSG00000075785	3588.78967	0.06515804	0.06259843	0.28947583	0.51546747	RAB7A
55973	ENSG00000075790	444.416132	0.29273531	0.13985656	0.02435486	0.08534096	BCAP29
25956	ENSG00000075826	261.77276	0.39260061	0.17885572	0.01378334	0.05387341	SEC31B
9733	ENSG00000075856	2120.82438	0.06620814	0.08575728	0.42898057	0.65464	SART3

55843	ENSG00000075884	0	NA	NA	NA	NA	ARHGAP15
113457	ENSG00000075886	19.0218121	0.08192128	0.33350971	0.64048248	0.81992338	TUBA3D
5076	ENSG00000075891	1.92000503	-0.13335149	0.41626229	0.0343628	NA	PAX2
23016	ENSG00000075914	552.221214	-0.07200141	0.10864163	0.48826491	0.70797163	EXOSC7
22920	ENSG00000075945	1104.60573	0.15822256	0.09136446	0.074302	0.20010202	KIFAP3
23609	ENSG00000075975	966.837269	0.03247025	0.0843122	0.69370523	0.85183585	MKRN2
4175	ENSG00000076003	2505.62604	-0.50558122	0.08914171	5.63E-09	8.76E-08	MCM6
25996	ENSG00000076043	348.406837	0.3879349	0.13344889	0.00191568	0.01029165	REXO2
10179	ENSG00000076053	320.847963	-0.37024875	0.1457832	0.00596726	0.0270472	RBM7
5939	ENSG00000076067	619.270056	-0.16330586	0.11889846	0.14735283	0.32899843	RBMS2
11176	ENSG00000076108	3203.17803	0.08648435	0.08634163	0.30427195	0.53154043	BAZ2A
25930	ENSG00000076201	1474.71235	0.07836368	0.08157001	0.32579891	0.55398101	PTPN23
4292	ENSG00000076242	851.969506	-0.05534707	0.0956444	0.54956887	0.75607328	MLH1
7374	ENSG00000076248	1072.39085	-0.12228478	0.08052014	0.12019494	0.28547347	UNG
2329	ENSG00000076258	0.18922458	0.01954053	0.38488496	0.78225479	NA	FMO4
27252	ENSG00000076321	638.005901	-0.11793301	0.0986462	0.21525428	0.42582958	KLHL20
8786	ENSG00000076344	358.939169	0.9486891	0.16708937	1.83E-09	3.04E-08	RGS11
113235	ENSG00000076351	696.321209	-0.26243118	0.15537677	0.06393402	0.1796757	SLC46A1
5362	ENSG00000076356	2070.80976	-1.41962934	0.10198506	4.04E-45	3.53E-43	PLXNA2
10615	ENSG00000076382	2196.37902	-0.09150245	0.07775979	0.22860697	0.44392309	SPAG5
88455	ENSG00000076513	641.44528	0.35264031	0.10155928	0.00030691	0.00209033	ANKRD13A
7163	ENSG00000076554	1044.01618	0.00500821	0.12069489	0.9662185	0.98692619	TPD52
32	ENSG00000076555	851.620865	0.76483189	0.10996276	7.13E-13	1.64E-11	ACACB
9618	ENSG00000076604	2022.60776	0.21910183	0.08348153	0.00708536	0.03125247	TRAF4
55824	ENSG00000076641	172.216207	-0.391021	0.21476546	0.03127269	0.1036502	PAG1
55094	ENSG00000076650	219.543001	-0.25217671	0.15743271	0.07813875	0.20773003	GPATCH1
3385	ENSG00000076662	37.5273335	-0.06610111	0.28259955	0.73052933	0.87107559	ICAM3
22978	ENSG00000076685	1084.73191	-0.37842118	0.10363887	0.00014266	0.00104754	NT5C2
4162	ENSG00000076706	235.343177	-0.4275652	0.18388301	0.00866919	0.03686067	MCAM
2239	ENSG00000076716	78.9627384	3.94094723	0.36658253	6.19E-28	3.31E-26	GPC4
55796	ENSG00000076770	586.744092	0.18064885	0.11923961	0.11047518	0.26795865	MBNL3
57662	ENSG00000076826	523.632237	-0.2553772	0.15460442	0.07001275	0.19161995	CAMSAP3
5909	ENSG00000076864	700.363524	-1.73806408	0.118482	7.17E-50	7.08E-48	RAP1GAP
56949	ENSG00000076924	1267.14395	0.18761927	0.08592674	0.02496505	0.08707922	XAB2
9138	ENSG00000076928	2101.28243	0.22362009	0.08064465	0.00450237	0.02131401	ARHGEF1
100505585	ENSG00000076928	2101.28243	0.22362009	0.08064465	0.00450237	0.02131401	ARHGEF1
6813	ENSG00000076944	11.8922777	0.30283313	0.4640626	0.12121697	0.2874665	STXBP2
5609	ENSG00000076984	1164.67709	-0.10668704	0.08277333	0.18658322	0.38635899	MAP2K7

27231	ENSG00000077009	0.60792302	-0.03597367	0.38657565	0.43415757	NA	NMRK2
8527	ENSG00000077044	1594.05571	0.05057073	0.08287626	0.53262415	0.74391151	DGKD
83992	ENSG00000077063	0	NA	NA	NA	NA	CTTNBP2
51412	ENSG00000077080	643.565342	-2.18930644	0.1219224	2.46E-73	4.20E-71	ACTL6B
5915	ENSG00000077092	161.626982	0.65555693	0.20203689	0.00027774	0.00190602	RARB
7155	ENSG00000077097	6757.45083	-0.17475315	0.06013268	0.00321355	0.01602734	TOP2B
56889	ENSG00000077147	4213.0451	-0.28500209	0.07032898	3.68E-05	0.00031188	TM9SF3
4791	ENSG00000077150	360.903563	0.75460066	0.1312844	1.86E-09	3.09E-08	NFKB2
29089	ENSG00000077152	1276.1892	0.03064216	0.08315804	0.7067078	0.85864725	UBE2T
4660	ENSG00000077157	836.123297	0.00904179	0.24054857	0.96341784	0.98545957	PPP1R12B
54431	ENSG00000077232	1738.25832	0.12773393	0.10509367	0.20648373	0.41346761	DNAJC10
2975	ENSG00000077235	3262.3612	0.07542901	0.06472679	0.23700187	0.45395994	GTF3C1
3566	ENSG00000077238	5.5272736	5.40550262	2.88036782	0.00042423	0.0027916	IL4R
23032	ENSG00000077254	1651.26472	-0.08588671	0.09357266	0.34268936	0.57288144	USP33
5063	ENSG00000077264	919.732729	-0.85411805	0.08766927	3.45E-23	1.52E-21	PAK3
827	ENSG00000077274	56.892561	3.5506125	0.41096833	5.83E-19	2.07E-17	CAPN6
1641	ENSG00000077279	3161.74404	-0.27301667	0.06278061	9.99E-06	9.48E-05	DCX
6626	ENSG00000077312	1613.83028	0.12389856	0.07434452	0.08931896	0.22925243	SNRPA
9576	ENSG00000077327	88.8252869	1.21248141	0.30795011	6.96E-06	6.81E-05	SPAG6
56915	ENSG00000077348	517.538707	-0.03659178	0.10973917	0.72702171	0.86953012	EXOSC5
1781	ENSG00000077380	2289.45555	0.02159666	0.07297773	0.76406617	0.8914874	DYNC1I2
54518	ENSG00000077420	0	NA	NA	NA	NA	APBB1IP
4034	ENSG00000077454	1039.99916	0.28779446	0.12907708	0.01756394	0.06560027	LRCH4
143684	ENSG00000077458	687.767558	-0.23999535	0.11922103	0.03348918	0.10951287	FAM76B
51548	ENSG00000077463	514.400514	0.13489498	0.12336449	0.24788981	0.46697056	SIRT6
7299	ENSG00000077498	0.173733	0.01914545	0.38484597	0.78225479	NA	TYR
10714	ENSG00000077514	519.93999	-0.1365115	0.10849922	0.1886647	0.38926996	POLD3
88	ENSG00000077522	272.045988	-0.36308622	0.15048725	0.00864917	0.03678446	ACTN2
832	ENSG00000077549	1741.51636	-0.21600622	0.07198935	0.00220215	0.01162915	CAPZB
7107	ENSG00000077585	242.238945	0.65318835	0.16983326	2.95E-05	0.00025493	GPR137B
10003	ENSG00000077616	562.401555	0.13348401	0.10233741	0.17568156	0.36989516	NAALAD2
79960	ENSG00000077684	1270.35802	-0.21149273	0.10173913	0.03060273	0.10194058	JADE1
203427	ENSG00000077713	0.37844916	0.02407552	0.3854191	0.60038877	NA	SLC25A43
7319	ENSG00000077721	729.26511	0.1220651	0.09373714	0.17761164	0.37273271	UBE2A
2260	ENSG00000077782	1879.49088	0.26873517	0.10616124	0.00826729	0.03549512	FGFR1
8468	ENSG00000077800	1.24242796	0.06894829	0.39263599	0.14569156	NA	FKBP6
27127	ENSG00000077935	1.06616886	0.0030941	0.38073027	0.95600814	NA	SMC1B
2192	ENSG00000077942	5780.99085	0.04317929	0.08499428	0.60243727	0.79259054	FBLN1

8516	ENSG00000077943	0.34110933	0.02362652	0.3853534	0.62410613	NA	ITGA8
8530	ENSG00000077984	0.173733	0.01914545	0.38484597	0.78225479	NA	CST7
4133	ENSG00000078018	5136.61592	-0.61347118	0.06242053	2.66E-23	1.18E-21	MAP2
9063	ENSG00000078043	588.729109	-0.10011109	0.12780021	0.40527658	0.63362391	PIAS2
273	ENSG00000078053	0	NA	NA	NA	NA	AMPH
369	ENSG00000078061	606.455322	0.07548801	0.10268331	0.44591391	0.67028199	ARAF
56922	ENSG00000078070	454.191484	-0.09208508	0.14003431	0.47691364	0.69881585	MCCC1
27074	ENSG00000078081	13.245468	0.05880614	0.33863548	0.72660703	0.86948287	LAMP3
2191	ENSG00000078098	1.10744684	0.07128808	0.39266921	0.17137223	NA	FAP
10529	ENSG00000078114	2537.04756	-0.07944368	0.08757829	0.35073891	0.58113897	NEBL
55331	ENSG00000078124	514.028314	-0.2846236	0.13104752	0.02032113	0.07381383	ACER3
3093	ENSG00000078140	1778.81503	-0.16582709	0.10417356	0.09726133	0.24401427	UBE2K
5289	ENSG00000078142	1194.7034	-0.02469472	0.12169952	0.82941586	0.92261925	PIK3C3
55728	ENSG00000078177	1101.3846	-0.41418753	0.09455512	5.96E-06	5.92E-05	N4BP2
57103	ENSG00000078237	232.653526	-0.06188732	0.22455486	0.73297984	0.87261966	TIGAR
7289	ENSG00000078246	786.67814	0.40016795	0.09167665	6.74E-06	6.60E-05	TULP3
8871	ENSG00000078269	158.359626	1.05917566	0.21092023	5.86E-08	7.96E-07	SYNJ2
108	ENSG00000078295	0	NA	NA	NA	NA	ADCY2
5527	ENSG00000078304	2493.84733	0.15653753	0.07779306	0.03983831	0.12506204	PPP2R5C
NA	ENSG00000078319	699.171304	0.03458367	0.09907584	0.71872952	0.86509267	PMS2P1
54715	ENSG00000078328	0.80203412	-0.02961958	0.38288109	0.56594918	NA	RBFOX1
2782	ENSG00000078369	12219.6523	0.0624537	0.05863644	0.28397602	0.50913467	GNB1
3205	ENSG00000078399	0	NA	NA	NA	NA	HOXA9
1906	ENSG00000078401	2.54220909	0.04647573	0.37678438	0.61948858	NA	EDN1
8028	ENSG00000078403	1582.6614	0.0952548	0.09384298	0.29527872	0.52190021	MLLT10
55063	ENSG00000078487	41.7234678	0.1097128	0.27259945	0.57669641	0.77528586	ZCWPW1
117	ENSG00000078549	2292.49545	-1.65063655	0.06572329	2.62E-140	1.57E-137	ADCYAP1R1
26281	ENSG00000078579	0.77127108	0.03225086	0.38522181	0.47166203	NA	FGF20
27334	ENSG00000078589	0	NA	NA	NA	NA	P2RY10
9452	ENSG00000078596	59.6938139	1.29691767	0.34259439	1.24E-05	0.00011631	ITM2A
4898	ENSG00000078618	2411.00459	0.15615569	0.06820144	0.01990443	0.07267279	NRDC
7419	ENSG00000078668	1765.88737	-0.50631385	0.07554042	8.11E-12	1.74E-10	VDAC3
5108	ENSG00000078674	2799.05437	0.07605358	0.07744642	0.31636214	0.54437337	PCM1
57690	ENSG00000078687	1790.38907	-0.16814848	0.11983011	0.13603984	0.31101986	TNRC6C
9139	ENSG00000078699	4787.11665	0.62877777	0.05612697	1.15E-29	6.49E-28	CBFA2T2
1620	ENSG00000078725	216.106985	2.34063605	0.19180619	2.27E-35	1.56E-33	BRINP1
83737	ENSG00000078747	918.389391	0.05984644	0.10169163	0.54215352	0.75027258	ITCH
27039	ENSG00000078795	1.46869288	-0.03087296	0.38162923	0.6164219	NA	PKD2L2

58476	ENSG00000078804	518.154353	-0.38936012	0.11145554	0.00025216	0.00175355	TP53INP2
51150	ENSG00000078808	1966.46385	0.0196281	0.07065785	0.77815256	0.8983839	SDF4
57644	ENSG00000078814	21.4103056	0.94770837	0.60686882	0.00815893	0.03512109	MYH7B
80341	ENSG00000078898	0	NA	NA	NA	NA	BPIFB2
7161	ENSG00000078900	534.878948	-0.19471061	0.12876434	0.10697521	0.26192621	TP73
54472	ENSG00000078902	801.8087	0.10587504	0.11064094	0.3167548	0.54452953	TOLLIP
51619	ENSG00000078967	1151.28606	0.21963985	0.09109539	0.0129028	0.05115738	UBE2D4
27098	ENSG00000079101	31.3679018	2.69720218	0.56338284	6.38E-08	8.62E-07	CLUL1
862	ENSG00000079102	589.353149	-0.70642968	0.14335563	1.81E-07	2.31E-06	RUNX1T1
1015	ENSG00000079112	0.45740913	-0.00334167	0.38312944	0.92771444	NA	CDH17
9984	ENSG00000079134	746.444329	-0.0806105	0.10716005	0.43242282	0.65788827	THOC1
51661	ENSG00000079150	663.85192	0.81784993	0.12922794	4.46E-11	8.89E-10	FKBP7
114880	ENSG00000079156	175.298715	0.04991552	0.18293259	0.75730099	0.88697677	OSBPL6
6507	ENSG00000079215	0	NA	NA	NA	NA	SLC1A3
7520	ENSG00000079246	8350.29302	-0.13577782	0.0578084	0.01748525	0.06535558	XRCC5
56925	ENSG00000079257	0.18922458	0.01954053	0.38488496	0.78225479	NA	LXN
11262	ENSG00000079263	1.24279523	0.01529348	0.38130326	0.6299551	NA	SP140
8569	ENSG00000079277	310.418004	0.02816355	0.14165381	0.83178589	0.92385262	MKNK1
7145	ENSG00000079308	1462.03489	-0.20193114	0.1247585	0.08599722	0.22308902	TNS1
57455	ENSG00000079313	1030.26844	0.145774	0.09575377	0.11574729	0.27775826	REXO1
56681	ENSG00000079332	1944.59818	-0.10356389	0.07373402	0.15185085	0.33594122	SAR1A
8556	ENSG00000079335	118.710322	0.52279552	0.24626907	0.01030391	0.0424392	CDC14A
10411	ENSG00000079337	36.3832245	-0.67871792	0.44271584	0.01767728	0.06593667	RAPGEF3
634	ENSG00000079385	2.42857747	-0.0097113	0.37675014	0.89381225	NA	CEACAM1
29843	ENSG00000079387	683.237195	-0.06400163	0.12163103	0.57803803	0.77614203	SENP1
51207	ENSG00000079393	0.62731245	0.01407412	0.38259184	0.72482501	NA	DUSP13
23152	ENSG00000079432	2429.63256	-0.24014335	0.08357054	0.00316603	0.01583813	CIC
3991	ENSG00000079435	214.666859	-0.12650091	0.15471735	0.3703303	0.59943356	LIPE
2222	ENSG00000079459	2850.25893	-0.55452653	0.09761888	4.54E-09	7.18E-08	FDFT1
5050	ENSG00000079462	1341.33184	-0.16413434	0.0867012	0.05155794	0.15251371	PAFAH1B3
4983	ENSG00000079482	275.817692	0.5344764	0.15290433	0.00016034	0.00116397	OPHN1
173	ENSG00000079557	0	NA	NA	NA	NA	AFM
3835	ENSG00000079616	2178.55455	-0.04713842	0.07160641	0.50223985	0.71909062	KIF22
10590	ENSG00000079689	8.77935684	-2.00825417	1.15477614	0.00302021	0.01521609	SCGN
55604	ENSG00000079691	85.3424601	0.32891604	0.25638269	0.10242623	0.2538584	CARMIL1
5236	ENSG00000079739	1087.74346	0.08906982	0.08563398	0.28620842	0.51202661	PGM1
1653	ENSG00000079785	3293.11279	-0.13994437	0.06446957	0.02752993	0.09371891	DDX1
1785	ENSG00000079805	2426.82839	0.02088204	0.07992746	0.79107352	0.90490742	DNM2

2037	ENSG00000079819	0.67554771	-0.02885627	0.38405527	0.57775739	NA	EPB41L2
22999	ENSG00000079841	124.81114	0.1062661	0.20850917	0.5464226	0.75358156	RIMS1
26002	ENSG00000079931	0	NA	NA	NA	NA	MOXD1
8417	ENSG00000079950	225.452467	0.43489472	0.18154037	0.00716207	0.03150677	STX7
11158	ENSG00000079974	498.195792	0.2089764	0.11795001	0.0620339	0.17577159	RABL2B
9817	ENSG00000079999	1252.37091	0.21682747	0.09213742	0.01515693	0.0581426	KEAP1
55510	ENSG00000080007	0.18922458	0.01954053	0.38488496	0.78225479	NA	DDX43
5794	ENSG00000080031	47.6571095	-0.34682602	0.31178944	0.11365557	0.27365614	PTPRH
1638	ENSG00000080166	5.92361595	-0.12771682	0.38819822	0.31851905	0.54614276	DCT
51006	ENSG00000080189	1296.14199	0.10630464	0.0980157	0.26205075	0.48395112	SLC35C2
131544	ENSG00000080200	352.21187	-0.26034594	0.13832659	0.04295816	0.13257414	CRYBG3
285220	ENSG00000080224	56.4056413	-2.76518318	0.38410415	3.25E-14	8.34E-13	EPHA6
6344	ENSG00000080293	0	NA	NA	NA	NA	SCTR
5991	ENSG00000080298	864.558444	-0.16388767	0.09675555	0.07930394	0.21006544	RFX3
55183	ENSG00000080345	2312.72068	-0.44037994	0.0658535	1.07E-11	2.27E-10	RIF1
23011	ENSG00000080371	529.889392	-0.09361278	0.12567272	0.42942368	0.65511463	RAB21
8671	ENSG00000080493	0.16735865	0.01897177	0.38482933	0.78225479	NA	SLC4A4
6595	ENSG00000080503	1053.69307	0.27248052	0.10811907	0.00844374	0.03608096	SMARCA2
50700	ENSG00000080511	0	NA	NA	NA	NA	RDH8
27244	ENSG00000080546	418.588327	-0.23229148	0.12054032	0.04167028	0.1295219	SESN1
11043	ENSG00000080561	0.55147679	0.03287368	0.38623201	0.45234823	NA	MID2
139212	ENSG00000080572	0	NA	NA	NA	NA	DNAAF6
50509	ENSG00000080573	0.16735865	0.01897177	0.38482933	0.78225479	NA	COL5A3
10847	ENSG00000080603	3186.09515	-0.05947524	0.06209647	0.33198493	0.56118097	SRCAP
9933	ENSG00000080608	996.676611	-0.10524337	0.0930026	0.24003016	0.45803582	PUM3
1361	ENSG00000080618	0.92228698	-0.03223864	0.38469064	0.48158676	NA	CPB2
1136	ENSG00000080644	7049.76771	0.82187751	0.07652707	1.22E-27	6.41E-26	CHRNA3
3781	ENSG00000080709	0.173733	0.01914545	0.38484597	0.78225479	NA	KCNN2
4850	ENSG00000080802	545.951574	-0.16362509	0.11506563	0.13502554	0.30949318	CNOT4
5663	ENSG00000080815	1192.32766	0.10240808	0.10403662	0.30650528	0.53417645	PSEN1
1371	ENSG00000080819	410.717128	0.22872777	0.13412998	0.06809541	0.18779383	CPOX
56650	ENSG00000080822	1684.63004	0.19038915	0.08211437	0.01751315	0.06544753	CLDND1
5891	ENSG00000080823	67.6147588	0.25399641	0.29858256	0.22494867	0.43877008	MOK
3320	ENSG00000080824	25120.1884	-0.19853941	0.09193245	0.02578977	0.08920466	HSP90AA1
5933	ENSG00000080839	526.775021	-0.2570275	0.12186531	0.02530743	0.08793099	RBL1
22839	ENSG00000080845	1865.9736	-0.05540669	0.11382565	0.60830082	0.79713925	DLGAP4
22997	ENSG00000080854	4402.10165	-0.75769387	0.07778785	4.16E-23	1.81E-21	IGSF9B
3080	ENSG00000080910	0	NA	NA	NA	NA	CFHR2

114819	ENSG00000080947	503.971726	0.19003287	0.1798256	0.23195262	0.44861961	CROCCP3
10403	ENSG00000080986	638.009525	-0.32348612	0.11592788	0.00331322	0.01645002	NDC80
23431	ENSG00000081014	488.09659	-0.19489132	0.112105	0.06822358	0.18799387	AP4E1
54665	ENSG00000081019	556.967722	-0.22942456	0.11255098	0.03243666	0.10675902	RSBN1
260425	ENSG00000081026	1554.3248	-0.53945377	0.07876449	2.66E-12	5.88E-11	MAGI3
2920	ENSG00000081041	0	NA	NA	NA	NA	CXCL2
174	ENSG00000081051	0	NA	NA	NA	NA	AFP
1286	ENSG00000081052	3.65652751	-0.10834187	0.39460293	0.25898034	0.48036523	COL4A4
6932	ENSG00000081059	797.208293	-0.2680825	0.10978015	0.010518	0.0431597	TCF7
28962	ENSG00000081087	417.27755	-0.13340278	0.19792518	0.43031222	0.65586498	OSTM1
1005	ENSG00000081138	45.6243638	4.9569288	0.61771609	1.07E-17	3.47E-16	CDH7
50939	ENSG00000081148	5.90887707	0.00522066	0.36372828	0.9674209	0.98759962	IMPG2
57092	ENSG00000081154	2509.43919	0.01978192	0.07513815	0.78923518	0.90431711	PCNP
55218	ENSG00000081177	804.349149	-0.14184785	0.09029486	0.10544088	0.25896844	EXD2
384	ENSG00000081181	252.970692	-0.33826316	0.15567784	0.01733184	0.06491745	ARG2
4208	ENSG00000081189	247.561264	0.23461668	0.20946591	0.18457637	0.38333456	MEF2C
5788	ENSG00000081237	0.16735865	0.01897177	0.38482933	0.78225479	NA	PTPRC
779	ENSG00000081248	0	NA	NA	NA	NA	CACNA1S
5317	ENSG00000081277	0	NA	NA	NA	NA	PKP1
79876	ENSG00000081307	1065.22685	0.24557398	0.11144111	0.02094726	0.07555646	UBA5
9262	ENSG00000081320	169.208165	0.33861614	0.18842599	0.04035447	0.12622304	STK17B
8555	ENSG00000081377	801.278499	0.25055709	0.10266652	0.01114672	0.04534568	CDC14B
22869	ENSG00000081386	666.045287	0.05227552	0.13344377	0.67670597	0.84200981	ZNF510
4036	ENSG00000081479	2.58792426	0.14227898	0.41825569	0.06495332	0.1817081	LRP2
440515	ENSG00000081665	472.290723	0.08497955	0.12104678	0.45995315	0.68305646	ZNF506
65094	ENSG00000081692	489.802495	0.14226418	0.13642912	0.26448081	0.48671731	JMJD4
11266	ENSG00000081721	678.446028	-0.24576999	0.10556415	0.01512916	0.05806977	DUSP12
65985	ENSG00000081760	704.80536	-0.27928121	0.1062056	0.00602977	0.02726934	AACS
9812	ENSG00000081791	1421.86673	-0.2494726	0.07762892	0.00100602	0.00593855	DELE1
6561	ENSG00000081800	0	NA	NA	NA	NA	SLC13A1
93664	ENSG00000081803	0.173733	0.01914545	0.38484597	0.78225479	NA	CADPS2
56131	ENSG00000081818	278.346152	-3.00E-05	0.13276381	0.999822	0.9998748	PCDHB4
56142	ENSG00000081842	18.6621312	-0.03417761	0.32811157	0.84138627	0.92792546	PCDHA6
56113	ENSG00000081853	52.6094057	0.75923831	0.36181455	0.00519168	0.02397645	PCDHGA2
51668	ENSG00000081870	279.254414	-0.21377245	0.14749743	0.11513552	0.27658176	HSPB11
23239	ENSG00000081913	409.395282	-0.24122038	0.1246671	0.03997269	0.12529119	PHLPP1
5205	ENSG00000081923	7.40309601	0.13639975	0.40510001	0.16304546	0.35199367	ATP8B1
3595	ENSG00000081985	45.1877555	1.27012951	0.41661734	0.00016648	0.00120498	IL12RB2

6604	ENSG00000082014	575.290538	-0.02952218	0.12635476	0.80349602	0.91070264	SMARCD3
55100	ENSG00000082068	469.181568	0.23486136	0.14398027	0.07791935	0.20733904	WDR70
2533	ENSG00000082074	0	NA	NA	NA	NA	FYB1
58538	ENSG00000082126	1.34301837	0.02962375	0.38170127	0.62989356	NA	MPP4
55437	ENSG00000082146	508.289317	0.13605217	0.11383237	0.21048511	0.41915333	STRADB
9689	ENSG00000082153	3990.84623	-0.0548112	0.07231591	0.43771237	0.66286646	BZW1
5241	ENSG00000082175	0	NA	NA	NA	NA	PGR
114899	ENSG00000082196	209.15886	0.53552496	0.16833458	0.00048333	0.00313377	C1QTNF3
4200	ENSG00000082212	1440.67991	-0.18890954	0.08901653	0.02880376	0.09722126	ME2
55322	ENSG00000082213	604.553271	-0.24557449	0.10288056	0.01293906	0.05127042	C5orf22
905	ENSG00000082258	1404.67279	0.04949446	0.08183289	0.53646728	0.74722766	CCNT2
57579	ENSG00000082269	328.020699	0.07968105	0.1414794	0.54535192	0.75298369	FAM135A
1310	ENSG00000082293	81.5442538	2.80002626	0.38800121	1.65E-14	4.30E-13	COL19A1
23136	ENSG00000082397	0	NA	NA	NA	NA	EPB41L3
22837	ENSG00000082438	326.903072	0.52391199	0.1578409	0.00031508	0.0021408	COBLL1
1741	ENSG00000082458	449.765009	-1.47425535	0.12933759	3.40E-31	2.01E-29	DLG3
3776	ENSG00000082482	0	NA	NA	NA	NA	KCNK2
56256	ENSG00000082497	763.452034	-0.65707097	0.12867925	7.86E-08	1.05E-06	SERTAD4
7188	ENSG00000082512	1638.54081	-0.00516736	0.08024672	0.9450322	0.97756908	TRAF5
29093	ENSG00000082515	294.923405	-0.03232947	0.1507322	0.81454099	0.91592004	MRPL22
25929	ENSG00000082516	854.085247	-0.12935363	0.09353208	0.15313909	0.33796213	GEMIN5
4986	ENSG00000082556	0	NA	NA	NA	NA	OPRK1
4779	ENSG00000082641	5667.90997	0.04121511	0.0565866	0.46205601	0.68509014	NFE2L1
54437	ENSG00000082684	1279.01962	-0.46788737	0.08768236	4.12E-08	5.72E-07	SEMA5B
2932	ENSG00000082701	2076.62309	-0.14365364	0.07146395	0.04043591	0.12641796	GSK3B
3693	ENSG00000082781	1671.85588	0.22189114	0.07837996	0.00378613	0.01841552	ITGB5
23085	ENSG00000082805	1740.43733	-0.58247199	0.07548129	3.78E-15	1.05E-13	ERC1
7514	ENSG00000082898	5673.41673	-0.26252171	0.0702656	0.00014047	0.00103258	XPO1
10141	ENSG00000082929	0.29863895	-0.02292439	0.3852726	0.68753245	NA	LINC01587
11342	ENSG00000082996	503.586278	0.21332566	0.11525004	0.05201021	0.15357681	RNF13
80036	ENSG00000083067	4.00900217	4.87082168	2.83506314	0.00081999	0.0049751	TRPM3
79728	ENSG00000083093	346.145041	-0.06334658	0.1463259	0.63838651	0.81803815	PALB2
23033	ENSG00000083097	655.911158	-0.51083227	0.12773803	2.34E-05	0.00020686	DOP1A
57226	ENSG00000083099	852.076074	-0.65358358	0.09655651	3.36E-12	7.38E-11	LYRM2
594	ENSG00000083123	348.438715	-0.21807654	0.16088785	0.13414232	0.30838745	BCKDHB
7994	ENSG00000083168	2154.61674	-0.08330947	0.0700963	0.22625898	0.44057103	KAT6A
79670	ENSG00000083223	498.187992	0.07463454	0.13202721	0.54782825	0.75451702	TUT7
9706	ENSG00000083290	1214.47221	0.37485306	0.10253046	0.00014366	0.00105371	ULK2

79977	ENSG00000083307	0.5354285	-0.00914583	0.38315739	0.81662091	NA	GRHL2
3842	ENSG00000083312	5626.70964	-0.11189409	0.07089791	0.10789372	0.26339503	TNPO1
5351	ENSG00000083444	1927.75691	0.0311174	0.08731331	0.71514519	0.86271551	PLOD1
5026	ENSG00000083454	317.697229	-0.95750805	0.16155271	4.11E-10	7.40E-09	P2RX5
3682	ENSG00000083457	270.595236	-0.19663141	0.13662331	0.1222365	0.289124	ITGAE
22894	ENSG00000083520	1848.81286	-0.2368772	0.08486132	0.0041164	0.01975552	DIS3
10464	ENSG00000083535	469.502811	-0.17344043	0.11662558	0.11713086	0.28025522	PIBF1
81550	ENSG00000083544	459.795588	0.12000892	0.12126528	0.29708842	0.52379226	TDRD3
NA	ENSG00000083622	0	NA	NA	NA	NA	
26747	ENSG00000083635	255.086292	0.16770507	0.14144857	0.20176748	0.40724024	NUFIP1
23047	ENSG00000083642	1517.6978	-0.39401974	0.07406635	5.61E-08	7.64E-07	PDS5B
5019	ENSG00000083720	1128.10279	-0.50311109	0.11336129	3.47E-06	3.58E-05	OXCT1
10325	ENSG00000083750	188.42205	0.58620843	0.18833955	0.00051579	0.00331605	RRAGB
1833	ENSG00000083782	0	NA	NA	NA	NA	EPYC
1540	ENSG00000083799	563.786108	-0.23355095	0.12445764	0.04647415	0.14072758	CYLD
10998	ENSG00000083807	170.04239	0.0683144	0.16503208	0.64769515	0.82431548	SLC27A5
25799	ENSG00000083812	476.338641	-0.17398083	0.14887128	0.20290426	0.40881694	ZNF324
79891	ENSG00000083814	14.2852517	0.12233633	0.35194739	0.48124528	0.70210356	ZNF671
55659	ENSG00000083817	115.820474	-0.20125252	0.20790517	0.25166434	0.47149344	ZNF416
54807	ENSG00000083828	172.94107	-0.17311683	0.18747149	0.28953475	0.51546747	ZNF586
55663	ENSG00000083838	261.480314	0.25917266	0.16981876	0.08862683	0.22782537	ZNF446
9422	ENSG00000083844	486.379679	-0.12460212	0.12672632	0.29587153	0.52252903	ZNF264
6193	ENSG00000083845	15854.2456	-0.04577609	0.05546423	0.40610254	0.63436535	RPS5
2195	ENSG00000083857	1771.01747	1.72740459	0.09048784	1.83E-82	3.86E-80	FAT1
91746	ENSG00000083896	1736.17635	0.02446692	0.09391292	0.78730234	0.90331242	YTHDC1
25978	ENSG00000083937	263.397775	0.37757904	0.14508582	0.00493233	0.02298202	CHMP2B
64744	ENSG00000084070	635.478969	-0.42599494	0.10880815	4.32E-05	0.00035934	SMAP2
10450	ENSG00000084072	1160.49366	0.06989032	0.08299831	0.38882221	0.61802858	PPIE
10269	ENSG00000084073	850.716367	-0.36130665	0.08850086	2.61E-05	0.00022851	ZMPSTE24
56910	ENSG00000084090	2638.14275	-0.16961556	0.06673621	0.00978402	0.04078904	STARD7
84273	ENSG00000084092	657.002129	-0.13118182	0.108375	0.20612564	0.41317248	NOA1
5978	ENSG00000084093	800.435824	-0.25466073	0.09575514	0.00580242	0.02639156	REST
3034	ENSG00000084110	12.162019	-0.18229654	0.38625731	0.28421776	0.50933794	HAL
54434	ENSG00000084112	1538.75157	0.01240383	0.09974622	0.898687	0.95556135	SSH1
2950	ENSG00000084207	6206.73674	0.31534029	0.05783491	3.36E-08	4.72E-07	GSTP1
334	ENSG00000084234	6175.65934	-0.02922233	0.06139102	0.62906829	0.81212356	APLP2
57613	ENSG00000084444	515.551532	-0.00088818	0.1093887	0.99235064	0.99756121	FAM234B
6579	ENSG00000084453	138.595779	0.18515138	0.25222516	0.34150354	0.57180671	SLCO1A2

51729	ENSG00000084463	1974.44436	-0.32039033	0.07955085	3.71E-05	0.00031408	WBP11
8668	ENSG00000084623	4455.56933	-0.03696552	0.06413426	0.55824622	0.76291851	EIF3I
79570	ENSG00000084628	1234.45822	0.09797057	0.07634075	0.19053737	0.39185876	NKAIN1
1307	ENSG00000084636	471.420813	-0.2949289	0.11599641	0.00742426	0.0324658	COL16A1
200081	ENSG00000084652	2160.20057	-0.279337	0.06509402	1.32E-05	0.00012298	TXLNA
338	ENSG00000084674	4.04695161	-0.02723176	0.36707948	0.80912095	0.91334153	APOB
8648	ENSG00000084676	961.968592	-0.21271437	0.08991455	0.01469442	0.05669726	NCOA1
60509	ENSG00000084693	1469.09927	0.13740134	0.07554343	0.06348446	0.17888533	AGBL5
22979	ENSG00000084710	1160.29873	-0.39541749	0.09044721	6.57E-06	6.45E-05	EFR3B
3797	ENSG00000084731	3470.40989	-0.33990971	0.06035672	1.12E-08	1.69E-07	KIF3C
10890	ENSG00000084733	3181.88843	-0.09888916	0.07969838	0.20604638	0.41316859	RAB10
2646	ENSG00000084734	2.2499202	0.0223147	0.37776887	0.77212347	NA	GCKR
3030	ENSG00000084754	4644.78744	-0.02777482	0.05481614	0.60729944	0.79664586	HADHA
22924	ENSG00000084764	684.706263	-0.15900224	0.09668022	0.08849211	0.22759703	MAPRE3
790	ENSG00000084774	3639.97359	0.16431739	0.06246512	0.00784813	0.0339749	CAD
966	ENSG00000085063	984.944389	-0.029412	0.08528553	0.72153334	0.86709971	CD59
3732	ENSG00000085117	40.3095565	-0.5014664	0.41737	0.04944658	0.14780908	CD82
63035	ENSG00000085185	971.220055	0.22921545	0.10416107	0.02197263	0.07856136	BCORL1
546	ENSG00000085224	4998.95273	-0.18314058	0.05817525	0.00142616	0.00802923	ATRX
102157402	ENSG00000085231	273.776398	0.10736242	0.13708336	0.40203562	0.63031025	AK6
2219	ENSG00000085265	0	NA	NA	NA	NA	FCN1
55892	ENSG00000085274	443.365488	-0.17307768	0.14243728	0.18921753	0.39003384	MYNN
2122	ENSG00000085276	32.8931136	4.07656085	0.67742002	5.43E-11	1.07E-09	MECOM
9522	ENSG00000085365	1430.19138	-0.24454246	0.08017304	0.00177314	0.00965665	SCAMP1
5550	ENSG00000085377	1521.75335	-0.17649159	0.08596517	0.03479301	0.11282988	PREP
57531	ENSG00000085382	583.866131	0.23682528	0.13017514	0.05236844	0.15426725	HACE1
81929	ENSG00000085415	898.982913	-0.43044714	0.09088332	1.06E-06	1.19E-05	SEH1L
22911	ENSG00000085433	611.312439	-0.24469343	0.12000568	0.03084473	0.10243712	WDR47
57590	ENSG00000085449	664.04579	-0.00236385	0.10352723	0.98016041	0.99338199	WDFY1
5016	ENSG00000085465	15.2975615	0.1196481	0.33964961	0.51074684	0.72599857	OVGP1
29957	ENSG00000085491	1076.07664	-0.421638	0.08164155	1.21E-07	1.58E-06	SLC25A24
4216	ENSG00000085511	850.332774	0.13197148	0.13633239	0.30023426	0.52737031	MAP3K4
29992	ENSG00000085514	16.3042177	0.11022157	0.33308097	0.54823534	0.75480124	PILRA
57549	ENSG00000085552	1534.70774	0.34545067	0.07095198	7.00E-07	8.13E-06	IGSF9
5243	ENSG00000085563	2454.71962	0.08340459	0.0718108	0.23726933	0.45434065	ABCB1
7760	ENSG00000085644	606.866627	-0.10390282	0.10730134	0.31139665	0.53905904	ZNF213
231	ENSG00000085662	4142.19936	-0.34541958	0.05752157	1.19E-09	2.04E-08	AKR1B1
8895	ENSG00000085719	1549.56186	-0.14290837	0.08031632	0.06840063	0.18835094	CPNE3

54700	ENSG00000085721	1365.23432	-0.29742955	0.0793234	0.00012272	0.00091257	RRN3
2017	ENSG00000085733	3524.39453	0.18839411	0.05684029	0.00079693	0.00485749	CTTN
7481	ENSG00000085741	5.41149357	-0.19271736	0.43567528	0.10177579	0.25256182	WNT11
4528	ENSG00000085760	602.838139	-0.14262301	0.11682881	0.19876205	0.40314524	MTIF2
23259	ENSG00000085788	1510.02005	-0.09844865	0.11023018	0.34957865	0.58004855	DDHD2
22996	ENSG00000085831	86.0557512	-0.26378789	0.22501575	0.15410922	0.33936549	TTC39A
2060	ENSG00000085832	1015.83795	-0.07162453	0.11488803	0.51239474	0.7276095	EPS15
4998	ENSG00000085840	472.291088	-0.29384023	0.11117969	0.00559555	0.02559734	ORC1
4258	ENSG00000085871	69.7098674	-0.36088754	0.26839586	0.0815353	0.21429626	MGST2
10523	ENSG00000085872	2679.80005	0.04117322	0.06267374	0.50592445	0.72177288	CHERP
55054	ENSG00000085978	602.603366	-0.10892833	0.12626202	0.3595241	0.58949503	ATG16L1
55230	ENSG00000085982	827.004033	-0.03480821	0.1171201	0.75390252	0.88515954	USP40
55624	ENSG00000085998	1766.26498	0.36485848	0.07792542	1.67E-06	1.82E-05	POMGNT1
8438	ENSG00000085999	612.526345	-0.14409842	0.12059126	0.20736085	0.41455455	RAD54L
23139	ENSG00000086015	2003.31035	0.47862151	0.06668975	3.14E-13	7.38E-12	MAST2
3301	ENSG00000086061	5298.95016	0.20781663	0.09363867	0.02194003	0.07847309	DNAJA1
2683	ENSG00000086062	388.750327	0.01656741	0.12308255	0.88801805	0.95125955	B4GALT1
51510	ENSG00000086065	1335.23668	0.02418787	0.07732308	0.75058494	0.8826224	CHMP5
4799	ENSG00000086102	1075.74348	-0.07042269	0.08333669	0.38590764	0.61510241	NFX1
363	ENSG00000086159	31.3864409	0.18873974	0.34102104	0.3403152	0.57056767	AQP6
27292	ENSG00000086189	536.010387	0.01353794	0.12037739	0.90640991	0.95930719	DIMT1
51194	ENSG00000086200	433.875499	-0.11365276	0.13581775	0.36941753	0.59883728	IPO11
2346	ENSG00000086205	0	NA	NA	NA	NA	FOLH1
27102	ENSG00000086232	5631.45315	-0.02152883	0.06396853	0.73204244	0.87198139	EIF2AK1
51314	ENSG00000086288	0	NA	NA	NA	NA	NME8
54749	ENSG00000086289	575.703309	0.09657654	0.12713806	0.42119594	0.64719079	EPDR1
29887	ENSG00000086300	125.639958	-0.02620305	0.18157804	0.86883167	0.94277075	SNX10
22929	ENSG00000086475	2162.05107	-0.14820912	0.06854158	0.02779316	0.0944206	SEPHS1
10573	ENSG00000086504	1277.5272	0.00760246	0.08636215	0.92979643	0.97064482	MRPL28
3049	ENSG00000086506	71.0494068	-0.10666134	0.2470302	0.57457091	0.77361067	HBQ1
80271	ENSG00000086544	216.868175	0.29227458	0.17495445	0.06079677	0.17313162	ITPKC
4680	ENSG00000086548	0	NA	NA	NA	NA	CEACAM6
2196	ENSG00000086570	14.842522	-0.03808087	0.33531069	0.81662183	0.9166503	FAT2
55696	ENSG00000086589	925.489025	0.0397977	0.09021578	0.65059175	0.82544657	RBM22
10959	ENSG00000086598	2902.04922	0.23236965	0.08381075	0.00443041	0.02104325	TMED2
56605	ENSG00000086619	227.530237	0.65495023	0.17062442	3.07E-05	0.00026309	ERO1B
54469	ENSG00000086666	836.09206	-0.01891679	0.10223552	0.84714496	0.93052693	ZFAND6
3294	ENSG00000086696	0.34746601	0.02370763	0.3853651	0.61987128	NA	HSD17B2

55787	ENSG00000086712	978.934849	-0.17173471	0.08558148	0.03924133	0.12375585	TXLNG
5475	ENSG00000086717	214.183415	-1.74975928	0.20354871	5.72E-19	2.03E-17	PPEF1
7462	ENSG00000086730	50.731378	0.18519258	0.29594916	0.36186581	0.59157216	LAT2
10075	ENSG00000086758	8390.39368	-0.26702594	0.04996654	6.83E-08	9.21E-07	HUWE1
9183	ENSG00000086827	433.534013	-0.18065865	0.12860887	0.13480285	0.3092967	ZW10
79796	ENSG00000086848	691.929871	0.13933033	0.13014374	0.25533023	0.47623877	ALG9
4606	ENSG00000086967	0.89929436	0.05171731	0.38894682	0.25054435	NA	MYBPC2
50507	ENSG00000086991	71.3050622	0.05209877	0.22798853	0.77806055	0.8983839	NOX4
8310	ENSG00000087008	321.623377	-0.0146588	0.13698651	0.90753949	0.95963244	ACOX3
8898	ENSG00000087053	1477.53023	-0.26690221	0.08730334	0.00166331	0.00916671	MTMR2
23645	ENSG00000087074	621.30701	0.49552052	0.11952887	1.34E-05	0.00012497	PPP1R15A
51171	ENSG00000087076	128.244982	0.11888325	0.19427607	0.47956726	0.70069817	HSD17B14
7205	ENSG00000087077	27.3375461	0.1595089	0.32612701	0.41632264	0.64306408	TRIP6
43	ENSG00000087085	123.954051	-0.7519899	0.21846327	0.00010591	0.00080078	ACHE
2512	ENSG00000087086	5105.78808	0.43133249	0.05306311	2.14E-16	6.39E-15	FTL
51593	ENSG00000087087	3654.33241	0.14513001	0.06930474	0.03319171	0.10866725	SRRT
581	ENSG00000087088	794.340287	-0.02145147	0.09484352	0.8146973	0.91594007	BAX
51701	ENSG00000087095	265.879484	0.11127649	0.14238342	0.40044702	0.62894783	NLK
94005	ENSG00000087111	1338.64353	-0.55301809	0.10216546	2.10E-08	3.04E-07	PIGS
9509	ENSG00000087116	614.015409	-1.47985948	0.13623661	1.36E-28	7.47E-27	ADAMTS2
28983	ENSG00000087128	0	NA	NA	NA	NA	TMPRSS11E
56970	ENSG00000087152	1851.85181	-0.01591556	0.07162858	0.82110305	0.91847813	ATXN7L3
9489	ENSG00000087157	1323.87768	0.05798718	0.07540195	0.43329619	0.65859161	PGS1
5705	ENSG00000087191	3586.81115	-0.23740415	0.06185593	9.85E-05	0.00075201	PSMC5
51720	ENSG00000087206	504.011171	-0.26234222	0.13995207	0.04221342	0.13092298	UIMC1
1071	ENSG00000087237	0	NA	NA	NA	NA	CETP
4313	ENSG00000087245	6978.22328	-0.38171849	0.06110345	2.40E-10	4.43E-09	MMP2
4504	ENSG00000087250	0	NA	NA	NA	NA	MT3
54947	ENSG00000087253	498.680355	0.43678651	0.13871134	0.00074336	0.00456317	LPCAT2
2775	ENSG00000087258	2169.49554	-1.63680153	0.09553669	6.01E-67	9.10E-65	GNAO1
55239	ENSG00000087263	999.136572	0.09029332	0.0810341	0.25449911	0.47504585	OGFOD1
6452	ENSG00000087266	1797.49054	0.11927499	0.08011588	0.1278244	0.29835718	SH3BP2
8602	ENSG00000087269	925.482786	-0.2109677	0.09085242	0.01618691	0.06138156	NOP14
118	ENSG00000087274	4172.13359	0.04514035	0.06298106	0.4691574	0.69153662	ADD1
79944	ENSG00000087299	355.779458	0.01911054	0.12551028	0.87349224	0.94441388	L2HGDH
57544	ENSG00000087301	444.500836	0.03102149	0.14433154	0.81714597	0.91675019	TXNDC16
51637	ENSG00000087302	1756.3891	0.0289054	0.07958161	0.71149979	0.86137148	RTRAF
22795	ENSG00000087303	58.8277439	2.05492782	0.35643806	5.64E-10	1.01E-08	NID2

64395	ENSG00000087338	1098.0844	0.48753959	0.08280475	1.64E-09	2.75E-08	GMCL1
10992	ENSG00000087365	5512.09834	0.11756938	0.06285933	0.05789482	0.16678281	SF3B2
57542	ENSG00000087448	1643.83626	-0.27203176	0.07381417	0.00016743	0.00121091	KLHL42
2778	ENSG00000087460	41601.9251	0.00100361	0.06049793	0.98493264	0.99473578	GNAS
10059	ENSG00000087470	2136.16883	-0.13390945	0.06769858	0.0442528	0.13549004	DNM1L
5744	ENSG00000087494	1.78906957	0.1167159	0.40584882	0.09508355	NA	PTHLH
116154	ENSG00000087495	312.836105	-1.42525727	0.14723545	3.25E-23	1.43E-21	PHACTR3
51290	ENSG00000087502	392.151196	-0.52411503	0.14442963	9.89E-05	0.00075342	ERGIC2
7022	ENSG00000087510	0.16735865	0.01897177	0.38482933	0.78225479	NA	TFAP2C
6790	ENSG00000087586	765.992854	-0.21661423	0.1097582	0.03866306	0.122321	AURKA
57091	ENSG00000087589	0.48848646	0.010387	0.3833355	0.7981203	NA	CASS4
8544	ENSG00000087842	956.575602	-0.42027642	0.08615633	5.35E-07	6.35E-06	PIR
28971	ENSG00000087884	174.636586	0.49036324	0.18535272	0.00301368	0.0151909	AAMDC
5990	ENSG00000087903	228.849215	0.49578343	0.22168877	0.00859801	0.03661397	RFX2
339175	ENSG00000087995	929.95825	0.00476228	0.08971049	0.95796523	0.98268084	METTL2A
6820	ENSG00000088002	5.59979227	0.11821099	0.38732451	0.33507036	0.56494141	SULT2B1
29929	ENSG00000088035	239.639626	0.0894508	0.17118169	0.56036802	0.76415299	ALG6
4849	ENSG00000088038	1429.35919	0.18526915	0.09208203	0.03794946	0.12067829	CNOT3
51206	ENSG00000088053	0.51932689	-0.00272327	0.38299064	0.95315258	NA	GP6
5775	ENSG00000088179	682.707459	-0.02507769	0.16067291	0.85675315	0.93605226	PTPN4
8886	ENSG00000088205	3125.40916	-0.07282003	0.06982515	0.28901083	0.5149506	DDX18
8570	ENSG00000088247	5761.37154	-0.08600469	0.06234709	0.16194509	0.35042673	KHSRP
2767	ENSG00000088256	1871.40091	0.0774946	0.07781724	0.30953485	0.53735494	GNA11
55616	ENSG00000088280	505.216931	0.67241763	0.11629878	1.86E-09	3.08E-08	ASAP3
55741	ENSG00000088298	293.008307	0.35152248	0.14464679	0.00863682	0.03676346	EDEM2
111089941	ENSG00000088298	293.008307	0.35152248	0.14464679	0.00863682	0.03676346	EDEM2
1789	ENSG00000088305	406.318402	-0.08846948	0.11914287	0.43376533	0.65909763	DNMT3B
28954	ENSG00000088320	0.14931947	-0.01844332	0.38480212	0.84537521	NA	REM1
22974	ENSG00000088325	3194.07264	-0.21749756	0.0596917	0.00022107	0.00155479	TPX2
NA	ENSG00000088340	129.454837	1.15207406	0.25251269	5.01E-07	5.98E-06	FER1L4
81572	ENSG00000088356	413.61161	0.09268897	0.11694017	0.40570976	0.63390153	PDRG1
2036	ENSG00000088367	926.172462	-0.45141995	0.10988528	1.77E-05	0.00016089	EPB41L1
6564	ENSG00000088386	0	NA	NA	NA	NA	SLC15A1
23348	ENSG00000088387	1046.6666	-0.19220153	0.09880254	0.04351394	0.13376459	DOCK9
55608	ENSG00000088448	1667.3584	0.28815312	0.08418	0.00043887	0.00288031	ANKRD10
23483	ENSG00000088451	195.488779	-0.13159781	0.16886006	0.38452757	0.61356176	TGDS
1795	ENSG00000088538	7.8122382	0.01009596	0.35344413	0.94497249	0.97756908	DOCK3
51161	ENSG00000088543	642.735177	0.27395049	0.10224506	0.00531508	0.02450642	C3orf18

57017	ENSG00000088682	1073.38576	-0.04053269	0.08247239	0.61533974	0.80264813	COQ9
55287	ENSG00000088726	0	NA	NA	NA	NA	TMEM40
64147	ENSG00000088727	103.877597	0.06334891	0.21344823	0.72283513	0.86755463	KIF9
79822	ENSG00000088756	288.663618	1.16864547	0.14909602	5.01E-16	1.46E-14	ARHGAP28
54675	ENSG00000088766	537.53324	-0.25426736	0.1255741	0.0315222	0.10433774	CRLS1
140850	ENSG00000088782	0	NA	NA	NA	NA	DEFB127
23368	ENSG00000088808	556.152033	-0.13708538	0.12369883	0.23906976	0.45681827	PPP1R13B
8455	ENSG00000088812	1642.12496	0.03335161	0.07150133	0.63657426	0.81722218	ATRN
54498	ENSG00000088826	178.981102	0.54800388	0.18108658	0.00078469	0.00479467	SMOX
6614	ENSG00000088827	0.34051795	-0.00034736	0.38336263	0.96854535	NA	SIGLEC1
2280	ENSG00000088832	2655.48901	-0.29193314	0.07698399	0.00010472	0.00079333	FKBP1A
55968	ENSG00000088833	968.605552	0.06386157	0.10245236	0.51856212	0.73226139	NSFL1C
83959	ENSG00000088836	98.4035764	2.18656012	0.27903146	2.91E-16	8.60E-15	SLC4A11
25943	ENSG00000088854	835.019877	0.01274983	0.09487916	0.89040722	0.95232275	C20orf194
79175	ENSG00000088876	572.224343	-0.03644166	0.10678167	0.72156332	0.86709971	ZNF343
57593	ENSG00000088881	782.379161	0.26507356	0.09308599	0.00326815	0.01627514	EBF4
56265	ENSG00000088882	3532.5501	0.33963799	0.06068597	1.38E-08	2.05E-07	CPXM1
57506	ENSG00000088888	1911.32898	-0.23058027	0.06889909	0.00065566	0.00407911	MAVS
9762	ENSG00000088899	318.876858	-0.07727193	0.13447073	0.53869723	0.74837075	LZTS3
2160	ENSG00000088926	0.16735865	0.01897177	0.38482933	0.78225479	NA	F11
22803	ENSG00000088930	3186.24784	0.06333829	0.0698617	0.35738072	0.58754007	XRN2
55857	ENSG00000088970	556.649078	1.01718959	0.13423322	4.63E-15	1.27E-13	KIZ
8655	ENSG00000088986	2812.2686	-0.08043118	0.05981179	0.17304467	0.36578929	DYNLL1
54997	ENSG00000088992	32.2182841	1.92141159	0.51006847	9.57E-06	9.11E-05	TESC
27131	ENSG00000089006	1226.14358	-0.16004606	0.0842588	0.0509731	0.15127992	SNX5
6128	ENSG00000089009	23054.4307	-0.0434937	0.04576921	0.34648396	0.57723082	RPL6
55423	ENSG00000089012	0	NA	NA	NA	NA	SIRPG
8550	ENSG00000089022	1108.31483	0.20359477	0.11483394	0.06353298	0.17894581	MAPKAPK5
5027	ENSG00000089041	256.761114	-0.45597234	0.17392509	0.00339687	0.01681903	P2RX7
51575	ENSG00000089048	483.250392	-0.20105124	0.11158522	0.05882987	0.16858119	ESF1
10741	ENSG00000089050	580.024441	0.07698703	0.10444132	0.44412294	0.66905081	RBBP9
51433	ENSG00000089053	2365.73494	-0.0254029	0.07211347	0.71917381	0.86548638	ANAPC5
9962	ENSG00000089057	1123.90037	-0.18169358	0.08819317	0.03404738	0.11097421	SLC23A2
80024	ENSG00000089060	178.546697	0.72833534	0.24071	0.00045883	0.00299643	SLC8B1
29058	ENSG00000089063	1547.98995	-0.29387426	0.07393583	4.92E-05	0.00040306	TMEM230
55184	ENSG00000089091	162.845613	0.08363785	0.17505757	0.59230422	0.78672529	DZANK1
84678	ENSG00000089094	1186.94249	-0.02878862	0.09249679	0.74749637	0.88138554	KDM2B
26074	ENSG00000089101	3.29145081	0.10213872	0.39056912	0.30667213	0.53422766	CFAP61

64211	ENSG00000089116	0	NA	NA	NA	NA	LHX5
55617	ENSG00000089123	126.779518	0.13764462	0.19476525	0.41410183	0.64130672	TASP1
4938	ENSG00000089127	212.59035	-1.74912899	0.19692173	5.04E-20	1.88E-18	OAS1
10985	ENSG00000089154	7411.65121	0.21477917	0.07239911	0.00250057	0.01297707	GCN1
6175	ENSG00000089157	40712.737	-0.07057619	0.05653006	0.20606367	0.41316859	RPLP0
5829	ENSG00000089159	670.104165	0.2697192	0.10537764	0.00761419	0.0331284	PXN
23409	ENSG00000089163	25.3447074	-0.01111555	0.29935894	0.95106951	0.97996464	SIRT4
22895	ENSG00000089169	1071.94886	-3.05957045	0.1213583	9.02E-142	5.77E-139	RPH3A
55614	ENSG00000089177	747.760016	-0.44427696	0.11782252	7.27E-05	0.00057444	KIF16B
51605	ENSG00000089195	981.730704	0.20214985	0.08476444	0.01441832	0.05579472	TRMT6
1114	ENSG00000089199	5625.73052	0.27673948	0.07076962	6.73E-05	0.00053594	CHGB
5037	ENSG00000089220	3721.24647	-0.2340627	0.06231905	0.00013816	0.00101823	PEBP1
6910	ENSG00000089225	0	NA	NA	NA	NA	TBX5
8315	ENSG00000089234	518.119431	-0.09912172	0.11450745	0.36087727	0.59075159	BRAP
10961	ENSG00000089248	1870.97819	0.0773408	0.07153601	0.27142027	0.49480244	ERP29
4842	ENSG00000089250	140.733007	-1.90049167	0.27360653	1.89E-13	4.54E-12	NOS1
2521	ENSG00000089280	14969.4256	0.14797503	0.04697924	0.00150275	0.00839611	FUS
3476	ENSG00000089289	636.136294	-0.00679048	0.10731014	0.94581968	0.97800919	IGBP1
53827	ENSG00000089327	0	NA	NA	NA	NA	FXYD5
55900	ENSG00000089335	855.04795	-0.18568558	0.08822628	0.03022391	0.1009669	ZNF302
57655	ENSG00000089351	3250.26322	0.11380878	0.06049266	0.05674474	0.16411308	GRAMD1A
5349	ENSG00000089356	0.18922458	0.01954053	0.38488496	0.78225479	NA	FXYD3
9843	ENSG00000089472	6.32793369	0.059961	0.36052813	0.66780322	0.83659884	HEPH
29965	ENSG00000089486	615.144653	0.07588738	0.11201798	0.47923053	0.70061045	CDIP1
113540	ENSG00000089505	56.897814	0.15913566	0.25711234	0.4165973	0.64309452	CMTM1
23415	ENSG00000089558	80.5122893	-1.60477771	0.31129636	1.89E-08	2.75E-07	KCNH4
23193	ENSG00000089597	8233.84381	0.01914051	0.05391904	0.72051805	0.86657845	GANAB
51291	ENSG00000089639	775.105438	-0.3819732	0.10870874	0.00023988	0.00167285	GMIP
55285	ENSG00000089682	451.715148	0.10662316	0.13224513	0.39062574	0.62000118	RBM41
332	ENSG00000089685	2067.14692	-1.04473598	0.07452286	1.55E-45	1.37E-43	BIRC5
3902	ENSG00000089692	53.4228807	0.97926948	0.35407469	0.00059116	0.00373523	LAG3
8079	ENSG00000089693	3255.91609	0.07314495	0.05830181	0.20455286	0.4113032	MLF2
78990	ENSG00000089723	176.896046	0.04353749	0.16826758	0.77475554	0.89613481	OTUB2
57062	ENSG00000089737	2594.17559	-0.12730391	0.08277484	0.11465033	0.27560769	DDX24
7597	ENSG00000089775	409.052314	0.00255983	0.12390209	0.98400741	0.99453916	ZBTB25
25977	ENSG00000089818	714.241359	-0.51897032	0.10251233	1.54E-07	1.99E-06	NECAP1
393	ENSG00000089820	333.492218	0.33738432	0.14130537	0.01007611	0.04167381	ARHGAP4
170961	ENSG00000089847	12.211461	0.16837814	0.38069998	0.31964791	0.54727882	ANKRD24

55760	ENSG00000089876	477.568162	0.11221772	0.1162449	0.31096678	0.53866926	DHX32
23186	ENSG00000089902	948.939739	-0.14931142	0.08827711	0.08157778	0.21430178	RCOR1
55668	ENSG00000089916	1683.15683	0.1186947	0.07632323	0.11260659	0.27179011	GPATCH2L
8425	ENSG00000090006	1527.54642	0.45143951	0.09474603	8.63E-07	9.90E-06	LTBP4
645	ENSG00000090013	111.480064	1.07736196	0.25591091	2.81E-06	2.94E-05	BLVRB
6548	ENSG00000090020	266.901297	0.50268638	0.14563307	0.00020998	0.00148625	SLC9A1
10558	ENSG00000090054	906.221936	0.15417434	0.09553435	0.09540946	0.2407381	SPTLC1
10914	ENSG00000090060	3272.7309	-0.37142165	0.07005448	6.32E-08	8.55E-07	PAPOLA
8812	ENSG00000090061	761.032017	0.11006048	0.09663261	0.23927786	0.4571163	CCNK
57060	ENSG00000090097	3758.7523	0.0501793	0.06545251	0.43758265	0.66277119	PCBP4
5996	ENSG00000090104	0	NA	NA	NA	NA	RGS1
83719	ENSG00000090238	665.245305	-0.05258131	0.09962057	0.58394554	0.78020525	YPEL3
51650	ENSG00000090263	793.241511	-0.43679379	0.13493663	0.00054822	0.0034928	MRPS33
4708	ENSG00000090266	1329.31158	-0.03931201	0.07388361	0.58688845	0.78237765	NDUFB2
10726	ENSG00000090273	2181.45501	-0.00282518	0.07921303	0.97194544	0.98950111	NUDC
10296	ENSG00000090316	1570.85396	0.19680097	0.07219623	0.00547617	0.02514982	MAEA
3383	ENSG00000090339	0.66256418	0.02385211	0.38420072	0.58205561	NA	ICAM1
29888	ENSG00000090372	3835.65729	-0.01079498	0.06610003	0.86670713	0.94134063	STRN4
11213	ENSG00000090376	0.34110234	0.02362647	0.38535339	0.62410984	NA	IRAK3
4069	ENSG00000090382	0	NA	NA	NA	NA	LYZ
6476	ENSG00000090402	0	NA	NA	NA	NA	SI
79594	ENSG00000090432	510.562816	-0.12642945	0.11757566	0.25765933	0.47887326	MUL1
7023	ENSG00000090447	454.055317	0.34959935	0.13471825	0.00547506	0.02514982	TFAP4
10081	ENSG00000090470	685.320365	-0.22834922	0.09797252	0.01559398	0.05950848	PDCD7
51324	ENSG00000090487	1118.73488	-0.1758986	0.08127895	0.02657591	0.0913173	SPG21
26998	ENSG00000090512	0	NA	NA	NA	NA	FETUB
51726	ENSG00000090520	1583.9047	0.47559125	0.09685865	3.88E-07	4.71E-06	DNAJB11
55214	ENSG00000090530	1.2916023	-0.0017475	0.38121454	0.8540625	NA	P3H2
7066	ENSG00000090534	1.10774217	0.07660367	0.39435724	0.13775798	NA	THPO
8646	ENSG00000090539	24.9656752	1.35893396	0.56633113	0.00095343	0.00568221	CHRD
2323	ENSG00000090554	43.9032883	0.55338768	0.36619003	0.02872717	0.0970608	FLT3LG
9727	ENSG00000090565	1188.34521	0.20744834	0.11453468	0.05725361	0.16524755	RAB11FIP3
84572	ENSG00000090581	491.754742	0.24459933	0.11817242	0.02917024	0.09819645	GNPTG
10795	ENSG00000090612	591.847496	0.03080516	0.1087108	0.76853227	0.89330656	ZNF268
2802	ENSG00000090615	1642.62887	0.21365904	0.12442882	0.0689279	0.18930364	GOLGA3
8761	ENSG00000090621	3684.76772	-0.27512049	0.06738357	3.25E-05	0.00027804	PABPC4
30835	ENSG00000090659	0	NA	NA	NA	NA	CD209
79603	ENSG00000090661	334.078864	0.07195313	0.13804571	0.57724514	0.77565483	CERS4

57192	ENSG00000090674	479.109799	-0.0144912	0.1087472	0.88868954	0.95146564	MCOLN1
84196	ENSG00000090686	1988.4707	-0.19514855	0.07306243	0.00642377	0.02873028	USP48
1947	ENSG00000090776	436.352276	-0.23336968	0.11349803	0.03083398	0.10243712	EFNB1
55066	ENSG00000090857	1789.50866	-0.13758886	0.07473202	0.06015382	0.17157157	PDPR
16	ENSG00000090861	3338.22466	0.29283711	0.10449669	0.00351118	0.01731586	AARS1
2734	ENSG00000090863	3148.31885	-0.09404446	0.06742586	0.15597427	0.34213777	GLG1
24137	ENSG00000090889	1829.23689	-0.25520748	0.07984699	0.00104879	0.00615074	KIF4A
27327	ENSG00000090905	2861.2442	-0.09234472	0.08109404	0.24256933	0.46079811	TNRC6A
64857	ENSG00000090924	1617.49529	0.33086411	0.08834692	0.00011519	0.00086261	PLEKHG2
10683	ENSG00000090932	429.672711	1.14360104	0.13802042	1.30E-17	4.17E-16	DLL3
57106	ENSG00000090971	1049.88408	-0.11538073	0.09778516	0.22149515	0.43427846	NAT14
57605	ENSG00000090975	166.372564	-0.39597839	0.19150467	0.01823755	0.0674558	PITPNM2
55763	ENSG00000090989	848.63933	-0.14851791	0.10623197	0.14409975	0.32391367	EXOC1
54439	ENSG00000091009	829.769488	-0.10726314	0.10334622	0.2801695	0.50481908	RBM27
5459	ENSG00000091010	2.62351936	-0.01430628	0.37029266	0.88693064	0.95029993	POU4F3
114882	ENSG00000091039	1534.0969	0.19269783	0.07841872	0.01196865	0.04814639	OSBPL8
113878	ENSG00000091073	256.989896	0.35919057	0.18276373	0.02628559	0.09050816	DTX2
58484	ENSG00000091106	32.1608099	0.14835626	0.3079529	0.45606792	0.67958359	NLRC4
54517	ENSG00000091127	1002.55452	-0.2306384	0.10684704	0.02414558	0.08477229	PUS7
22798	ENSG00000091128	10.2512443	-0.32701741	0.50086456	0.09037644	0.23142341	LAMB4
4897	ENSG00000091129	6493.24926	-1.99646402	0.08442248	7.81E-125	3.44E-122	NRCAM
3912	ENSG00000091136	14987.1866	0.75139309	0.05029786	4.27E-51	4.35E-49	LAMB1
5172	ENSG00000091137	5.1145725	0.21780831	0.45722653	0.07344828	0.19834008	SLC26A4
1811	ENSG00000091138	0.37844916	0.02407552	0.3854191	0.60038877	NA	SLC26A3
1738	ENSG00000091140	1663.97534	-0.04376771	0.08406105	0.59292734	0.78702605	DLD
23335	ENSG00000091157	446.67366	-0.11896795	0.17365523	0.44034544	0.66517856	WDR7
9352	ENSG00000091164	1188.57673	-0.29355905	0.08409204	0.0003352	0.00226201	TXNL1
3568	ENSG00000091181	0.63841587	-0.02095331	0.38316251	0.66869642	NA	IL5RA
368	ENSG00000091262	44.1111167	0.3967814	0.37482331	0.09243266	0.23508014	ABCC6
54918	ENSG00000091317	964.764331	0.37576237	0.08613673	7.02E-06	6.87E-05	CMTM6
3655	ENSG00000091409	192.625525	1.86427439	0.58886618	7.09E-05	0.0005627	ITGA6
11069	ENSG00000091428	170.274274	0.65522603	0.24362141	0.00156125	0.00868383	RAPGEF4
51776	ENSG00000091436	774.19022	0.05788964	0.09125991	0.51416237	0.72921448	MAP3K20
23676	ENSG00000091482	0	NA	NA	NA	NA	SMPX
2271	ENSG00000091483	1549.31018	-0.02510788	0.07708248	0.73835891	0.87630444	FH
23231	ENSG00000091490	43.2495412	1.79026968	0.42052847	1.27E-06	1.41E-05	SEL1L3
7018	ENSG00000091513	26.1923303	0.20102578	0.45770739	0.0250472	0.08728903	TF
55573	ENSG00000091527	1945.0432	-0.22562937	0.07103587	0.0012063	0.00695729	CDV3

51168	ENSG00000091536	27.9750333	0.83686252	0.51158222	0.00960516	0.04013614	MYO15A
54890	ENSG00000091542	1735.90924	-0.15853793	0.08982866	0.06898013	0.18942086	ALKBH5
350	ENSG00000091583	0	NA	NA	NA	NA	APOH
22861	ENSG00000091592	1.77586445	0.11350373	0.40444609	0.10090946	NA	NLRP1
83394	ENSG00000091622	36.8650558	0.44633637	0.40162163	0.07008531	0.19173917	PITPNM3
9552	ENSG00000091640	587.151087	0.11750386	0.10264857	0.23462363	0.45154068	SPAG7
23594	ENSG00000091651	950.7034	-0.09610486	0.08972347	0.26999466	0.49316601	ORC6
79776	ENSG00000091656	1563.46446	-0.02857941	0.08195196	0.73191264	0.87198139	ZFHX4
57084	ENSG00000091664	0.16735865	0.01897177	0.38482933	0.78225479	NA	SLC17A6
1357	ENSG00000091704	14.1017182	0.6266389	0.75661373	0.02965602	0.09943814	CPA1
51530	ENSG00000091732	633.289733	-0.17937239	0.10054557	0.0638562	0.17957577	ZC3HC1
2099	ENSG00000091831	18.5037785	0.2911797	0.39474055	0.17452117	0.36815523	ESR1
26575	ENSG00000091844	349.762772	-0.3581964	0.13759351	0.00519993	0.024009	RGS17
285	ENSG00000091879	67.8093508	-0.35483489	0.2778292	0.09146504	0.23347687	ANGPT2
84336	ENSG00000091947	534.521006	0.1307369	0.12505628	0.26877988	0.49166446	TMEM101
4345	ENSG00000091972	687.163003	-0.46531438	0.10209812	2.25E-06	2.40E-05	CD200
151887	ENSG00000091986	76.4805366	3.46260159	0.40925717	5.26E-19	1.87E-17	CCDC80
1215	ENSG00000092009	0	NA	NA	NA	NA	CMA1
5720	ENSG00000092010	793.83941	0.36556298	0.0968028	9.21E-05	0.00070708	PSME1
55012	ENSG00000092020	538.378713	-0.38361536	0.10930196	0.00024277	0.00169124	PPP2R3C
54930	ENSG00000092036	416.39747	-0.01999413	0.11885279	0.85866677	0.93701934	HAUS4
84502	ENSG00000092051	2702.28621	-0.43125257	0.06523243	1.86E-11	3.85E-10	JPH4
4625	ENSG00000092054	0.32287603	0.00126362	0.38336747	0.96898071	NA	MYH7
1053	ENSG00000092067	0	NA	NA	NA	NA	CEBPE
23428	ENSG00000092068	0.3166136	0.00100275	0.38336578	0.96824525	NA	SLC7A8
55644	ENSG00000092094	499.028617	0.25397545	0.11744582	0.0227228	0.08067651	OSGEP
51310	ENSG00000092096	1778.18585	-0.43842057	0.07837681	1.06E-08	1.60E-07	SLC22A17
55072	ENSG00000092098	720.75231	0.08809168	0.12938112	0.46994468	0.6922856	RNF31
23256	ENSG00000092108	748.224877	0.08282654	0.10481445	0.41187139	0.63975013	SCFD1
55632	ENSG00000092140	968.74083	-0.03956686	0.09546937	0.66797254	0.83666483	G2E3
25831	ENSG00000092148	2591.27713	-0.64798975	0.06381678	8.64E-25	4.08E-23	HECTD1
3183	ENSG00000092199	11014.2619	-0.09963954	0.06110795	0.09615897	0.24213656	HNRNPC
57096	ENSG00000092200	2.63070417	-0.05873686	0.37950679	0.51362568	0.7287659	RPGRIP1
11198	ENSG00000092201	7276.36198	-0.0295347	0.0624724	0.63105687	0.81347335	SUPT16H
9878	ENSG00000092203	1057.17034	0.06422321	0.09116887	0.4716618	0.69399066	TOX4
8487	ENSG00000092208	115.849789	-0.00495777	0.19376964	0.97508275	0.99122005	GEMIN2
7051	ENSG00000092295	33.5015326	0.14758414	0.30532463	0.45744796	0.68095495	TGM1
26277	ENSG00000092330	466.848439	-0.10363706	0.10962417	0.32297729	0.55059885	TINF2

1618	ENSG00000092345	0	NA	NA	NA	NA	DAZL
90665	ENSG00000092377	0	NA	NA	NA	NA	TBL1Y
57556	ENSG00000092421	1171.52357	-0.46057391	0.11978958	5.18E-05	0.00042165	SEMA6A
54822	ENSG00000092439	1265.38404	-0.23542562	0.08171705	0.00312638	0.01569119	TRPM7
7301	ENSG00000092445	424.876839	0.06265595	0.1163143	0.57249568	0.7723153	TYRO3
79968	ENSG00000092470	650.524751	-0.1878856	0.1103499	0.0745274	0.20049133	WDR76
825	ENSG00000092529	15.4555439	0.46866759	0.68790857	0.04034462	0.1262121	CAPN3
8773	ENSG00000092531	495.807452	0.64912411	0.11678426	7.19E-09	1.10E-07	SNAP23
6913	ENSG00000092607	0.15069912	-0.01848503	0.38480575	0.84537521	NA	TBX15
26227	ENSG00000092621	4086.67925	0.85138955	0.07962237	1.98E-27	1.03E-25	PHGDH
1299	ENSG00000092758	70.858305	-0.28619266	0.27103074	0.16069379	0.34866195	COL9A3
7430	ENSG00000092820	2743.48916	0.07803575	0.06481975	0.22211066	0.43522721	EZR
4637	ENSG00000092841	4772.63191	0.04641563	0.05538374	0.39810042	0.62724844	MYL6
26523	ENSG00000092847	2047.05102	0.03486225	0.07398533	0.63162872	0.81371846	AGO1
27285	ENSG00000092850	72.8770416	0.06206171	0.22873361	0.73655026	0.87514416	TEKT2
63967	ENSG00000092853	1087.73625	-0.65679253	0.09684133	3.07E-12	6.76E-11	CLSPN
117584	ENSG00000092871	521.912729	0.22736374	0.13491029	0.07108569	0.19367482	RFFL
201294	ENSG00000092929	356.330608	-1.76517643	0.16750575	3.68E-27	1.89E-25	UNC13D
79157	ENSG00000092931	807.711602	0.28995077	0.11083672	0.00613593	0.02763606	MFSD11
1808	ENSG00000092964	4779.91862	-0.58152674	0.07284491	4.60E-16	1.35E-14	DPYSL2
7042	ENSG00000092969	14.002785	0.26578975	0.42725108	0.16748983	0.35846798	TGFB2
55105	ENSG00000092978	568.216099	0.04256986	0.1129478	0.69419824	0.85201936	GPATCH2
10762	ENSG00000093000	1980.3701	-0.22198268	0.0733059	0.00199642	0.01068674	NUP50
8318	ENSG00000093009	900.409443	-0.17186463	0.10213995	0.08009923	0.21143808	CDC45
1312	ENSG00000093010	1057.19898	0.47674041	0.09838428	5.36E-07	6.36E-06	COMT
51816	ENSG00000093072	9.6846022	-0.18877255	0.39062628	0.27229743	0.49567676	ADA2
NA	ENSG00000093100	163.634445	-0.12134119	0.23506504	0.51506191	0.72955207	
55350	ENSG00000093134	0	NA	NA	NA	NA	VNN3
55862	ENSG00000093144	776.460611	-0.27690046	0.09645216	0.00289192	0.01465533	ECHDC1
9209	ENSG00000093167	605.529277	0.12209088	0.09955073	0.20397409	0.41051388	LRRFIP2
9117	ENSG00000093183	1382.1905	0.04808656	0.07827336	0.53089155	0.74257279	SEC22C
9942	ENSG00000093217	229.93413	-0.47812695	0.21969274	0.0103026	0.0424392	XYLB
10013	ENSG00000094631	1245.49974	0.05337202	0.11768906	0.63471669	0.81607019	HDAC6
126370	ENSG00000094661	0	NA	NA	NA	NA	OR1I1
2568	ENSG00000094755	110.219282	0.06604363	0.20512813	0.70503455	0.85803894	GABRP
3881	ENSG00000094796	0	NA	NA	NA	NA	KRT31
990	ENSG00000094804	846.565189	-0.48393745	0.11485509	1.02E-05	9.64E-05	CDC6
139596	ENSG00000094841	192.018553	-0.04766275	0.15798443	0.73968891	0.8770894	UPRT

8697	ENSG00000094880	920.611814	-0.04289107	0.08508878	0.60461652	0.79477849	CDC23
8086	ENSG00000094914	1036.5045	0.23213276	0.08209296	0.0037419	0.01824509	AAAS
23468	ENSG00000094916	9974.70667	-0.13839723	0.06426474	0.02842603	0.09620819	CBX5
2327	ENSG00000094963	0	NA	NA	NA	NA	FMO2
51430	ENSG00000094975	1373.43903	-0.12096665	0.07543331	0.10109103	0.25133408	SUCO
4436	ENSG00000095002	2573.33608	-0.26043326	0.0732353	0.00028934	0.00197948	MSH2
4214	ENSG00000095015	2670.12307	-0.71082041	0.06763362	1.78E-26	8.93E-25	MAP3K1
1725	ENSG00000095059	2468.49078	-0.03513253	0.06350773	0.57225137	0.77229217	DHPS
29911	ENSG00000095066	670.072858	0.06319937	0.10982946	0.54838634	0.75487303	HOOK2
120400	ENSG00000095110	0	NA	NA	NA	NA	NXPE1
372	ENSG00000095139	2894.08521	-0.00950174	0.06177007	0.87549476	0.94503896	ARCN1
54566	ENSG00000095203	46.1052914	-2.14508194	0.39226232	2.99E-09	4.80E-08	EPB41L4B
55151	ENSG00000095209	192.072592	0.05138836	0.19996665	0.76572372	0.89233237	TMEM38B
5711	ENSG00000095261	673.283447	-0.24275783	0.11127202	0.02212582	0.07897159	PSMD5
5742	ENSG00000095303	16.9372067	0.51004696	0.56753505	0.05147257	0.15239282	PTGS1
23511	ENSG00000095319	2446.77666	-0.09503987	0.07911783	0.21903075	0.43093714	NUP188
1384	ENSG00000095321	535.742726	-0.04594193	0.11109091	0.6649009	0.83454529	CRAT
10044	ENSG00000095370	164.229618	-1.58642517	0.24646804	7.97E-12	1.71E-10	SH2D3C
54187	ENSG00000095380	407.364721	0.07540513	0.13195325	0.54345169	0.75149877	NANS
55357	ENSG00000095383	74.4247461	0.4589695	0.26969581	0.03083486	0.10243712	TBC1D2
25861	ENSG00000095397	104.435008	2.26640351	0.27284818	6.64E-18	2.17E-16	WHRN
5146	ENSG00000095464	4.81283863	-0.05747665	0.37155572	0.6047519	0.79485112	PDE6C
55280	ENSG00000095485	359.224066	-0.09914006	0.15956878	0.49292825	0.71202899	CWF19L1
57715	ENSG00000095539	549.379703	-0.10869713	0.11541388	0.32239947	0.54994458	SEMA4G
9044	ENSG00000095564	1469.66099	0.01131357	0.08708291	0.89405647	0.95417323	BTAF1
64376	ENSG00000095574	360.291723	0.04056864	0.1483713	0.76797362	0.89311821	IKZF5
29760	ENSG00000095585	0.31867469	0.00156311	0.38337037	0.96210221	NA	BLNK
7093	ENSG00000095587	5.02055481	-0.11774277	0.39017127	0.31279924	0.54072254	TLL2
1592	ENSG00000095596	0.50207596	0.02562368	0.38565771	0.52549886	NA	CYP26A1
56165	ENSG00000095627	0	NA	NA	NA	NA	TDRD1
10580	ENSG00000095637	305.003757	-0.68558363	0.14441044	4.78E-07	5.72E-06	SORBS1
55118	ENSG00000095713	132.551187	-2.42599133	0.27494674	6.23E-20	2.32E-18	CRTAC1
25805	ENSG00000095739	483.244301	-1.08634559	0.13363861	5.15E-17	1.59E-15	BAMBI
3589	ENSG00000095752	34.5656142	0.45248024	0.41581489	0.06754108	0.18665906	IL11
53904	ENSG00000095777	0	NA	NA	NA	NA	MYO3A
51322	ENSG00000095787	3804.55374	-0.31051109	0.06236024	4.34E-07	5.23E-06	WAC
1390	ENSG00000095794	251.929157	0.35784684	0.15094631	0.00991631	0.04119337	CREM
10101	ENSG00000095906	1793.3488	0.18495115	0.06796377	0.00567449	0.02589278	NUBP2

23430	ENSG00000095917	0	NA	NA	NA	NA	TPSD1
284422	ENSG00000095932	2.83971159	-0.09934323	0.39119734	0.2958088	0.52251089	SMIM24
3096	ENSG00000095951	485.158986	0.01020434	0.1766054	0.94959545	0.97920861	HIVEP1
54209	ENSG00000095970	0	NA	NA	NA	NA	TREM2
83795	ENSG00000095981	0	NA	NA	NA	NA	KCNK16
10321	ENSG00000096006	0	NA	NA	NA	NA	CRISP3
2289	ENSG00000096060	968.347764	-0.22920421	0.08491958	0.00553308	0.0253642	FKBP5
6732	ENSG00000096063	2074.28861	-0.33431567	0.07108426	1.63E-06	1.77E-05	SRPK1
27154	ENSG00000096070	1236.45199	0.03661019	0.08066407	0.64309577	0.82167827	BRPF3
55168	ENSG00000096080	435.718723	-0.03632647	0.12230578	0.75312635	0.88456232	MRPS18A
5225	ENSG00000096088	0.46899274	-0.02782639	0.38571851	0.56951037	NA	PGC
28978	ENSG00000096092	513.571968	-0.54872324	0.11925522	1.43E-06	1.58E-05	TMEM14A
114327	ENSG00000096093	539.60449	0.06848033	0.16580268	0.64799663	0.82447067	EFHC1
9436	ENSG00000096264	0	NA	NA	NA	NA	NCR2
3326	ENSG00000096384	29152.3147	-0.39548922	0.05691936	2.08E-12	4.66E-11	HSP90AB1
4295	ENSG00000096395	0	NA	NA	NA	NA	MLN
988	ENSG00000096401	1506.9979	-0.00703306	0.0716254	0.91628762	0.96446211	CDC5L
3710	ENSG00000096433	375.09028	1.7064069	0.14978606	3.71E-31	2.19E-29	ITPR3
7738	ENSG00000096654	259.463075	-0.08954598	0.14256488	0.49768658	0.71556647	ZNF184
1832	ENSG00000096696	0.173733	0.01914545	0.38484597	0.78225479	NA	DSP
23411	ENSG00000096717	700.088915	-0.48433516	0.11623247	1.25E-05	0.00011678	SIRT1
3189	ENSG00000096746	4600.5292	0.05252582	0.07469731	0.474264	0.69642496	HNRNPH3
80173	ENSG00000096872	251.576402	0.08183111	0.15988263	0.57438462	0.77352065	IFT74
3717	ENSG00000096968	165.563735	-0.04217816	0.18261622	0.79159287	0.90499209	JAK2
3594	ENSG00000096996	0	NA	NA	NA	NA	IL12RB1
25	ENSG00000097007	3583.37804	0.00653297	0.06154443	0.91501702	0.96403708	ABL1
11332	ENSG00000097021	2336.35937	-0.61337625	0.08269012	3.51E-14	8.97E-13	ACOT7
51100	ENSG00000097033	1003.80807	-0.31409238	0.08262862	9.57E-05	0.00073257	SH3GLB1
8317	ENSG00000097046	545.141267	-0.51714659	0.12413006	1.13E-05	0.0001064	CDC7
84144	ENSG00000097096	0.173124	-0.01911262	0.38486262	0.84537521	NA	SYDE2
5125	ENSG00000099139	0.14931947	-0.01844332	0.38480212	0.84537521	NA	PCSK5
6319	ENSG00000099194	12027.7078	-0.76889773	0.07370918	3.26E-26	1.62E-24	SCD
11018	ENSG00000099203	350.096729	0.16209693	0.14184979	0.21782665	0.42963324	TMED1
3983	ENSG00000099204	3149.29661	-0.17917573	0.06539728	0.00537202	0.02474022	ABLIM1
79956	ENSG00000099219	321.64598	0.29287379	0.13227624	0.01804643	0.06707403	ERMP1
22931	ENSG00000099246	930.216463	-0.15658336	0.12173878	0.17351102	0.36653625	RAB18
8829	ENSG00000099250	20.2095275	-0.06329453	0.33040047	0.71377919	0.86192204	NRP1
158160	ENSG00000099251	69.1685925	0.34424025	0.26036504	0.0909034	0.23256267	HSD17B7P2

56952	ENSG00000099256	104.19057	1.88235376	0.2833998	1.68E-12	3.76E-11	PRTFDC1
54873	ENSG00000099260	4.67014941	0.34438888	0.61776113	0.01905433	0.07005893	PALMD
23555	ENSG00000099282	304.580378	-0.11996644	0.12822989	0.31979679	0.54738747	TSPAN15
55506	ENSG00000099284	2487.44578	0.04443189	0.06369805	0.47953149	0.70069817	MACROH2A2
387680	ENSG00000099290	910.85465	0.03864869	0.08950624	0.65772358	0.83026251	WASHC2A
23031	ENSG00000099308	591.692436	-0.07084975	0.1102269	0.50081676	0.71803761	MAST3
7593	ENSG00000099326	1471.05783	0.19675159	0.07685886	0.00890542	0.03768041	MZF1
79629	ENSG00000099330	102.509284	0.16397341	0.21350568	0.35855714	0.5888398	OCEL1
4650	ENSG00000099331	2429.53892	0.00380308	0.07277458	0.95846048	0.98288396	MYO9B
9424	ENSG00000099337	147.564487	-0.03156065	0.18531177	0.84450506	0.92965052	KCNK6
57828	ENSG00000099338	49.1796357	1.30066454	0.41727261	0.00012743	0.00094439	CATSPERG
5714	ENSG00000099341	2604.63871	-0.07976099	0.06087893	0.18412416	0.3826844	PSMD8
54620	ENSG00000099364	2184.94438	-0.06919536	0.08182714	0.38594835	0.61510241	FBXL19
112755	ENSG00000099365	495.098172	-0.01431325	0.12737849	0.9041861	0.95807825	STX1B
80270	ENSG00000099377	54.4793218	0.87878234	0.42877315	0.00425723	0.02033792	HSD3B7
9739	ENSG00000099381	1307.28358	-0.05048842	0.08792312	0.55433811	0.76027015	SETD1A
9274	ENSG00000099385	528.38595	-0.11871339	0.10933811	0.25623784	0.47738504	BCL7C
4113	ENSG00000099399	5.21247095	-0.08390827	0.37355326	0.50333537	0.72006071	MAGEB2
1943	ENSG00000099617	465.141271	0.08739839	0.11522618	0.42701081	0.65286501	EFNA2
1153	ENSG00000099622	4889.63036	-0.13660346	0.06488642	0.03252284	0.10695396	CIRBP
513	ENSG00000099624	1193.10613	0.04880394	0.08216792	0.5428666	0.75092193	ATP5F1D
255057	ENSG00000099625	570.438944	0.02936302	0.11403561	0.7883815	0.90400376	CBARP
83259	ENSG00000099715	0	NA	NA	NA	NA	PCDH11Y
266	ENSG00000099721	0	NA	NA	NA	NA	AMELY
5616	ENSG00000099725	0	NA	NA	NA	NA	PRKY
3483	ENSG00000099769	3.70059091	0.17616579	0.42393767	0.13841044	0.31479274	IGFALS
4670	ENSG00000099783	7244.94505	0.01401712	0.07130203	0.84289926	0.92864956	HNRNPM
51257	ENSG00000099785	178.661826	0.50527422	0.19564573	0.00340963	0.0168738	MARCHF2
4713	ENSG00000099795	1216.02732	0.01003616	0.08118287	0.90008171	0.95573896	NDUFB7
9524	ENSG00000099797	1245.29758	0.06028654	0.0852682	0.46887031	0.69142165	TECR
26517	ENSG00000099800	1772.12279	0.01600822	0.07134232	0.82025582	0.91817957	TIMM13
997	ENSG00000099804	1141.55221	0.01398372	0.07987716	0.85865556	0.93701934	CDC34
4507	ENSG00000099810	1167.73084	0.05183845	0.10655188	0.61336377	0.80102272	MTAP
126353	ENSG00000099812	2.46553757	-0.07738641	0.38729787	0.33570336	NA	MISP
283638	ENSG00000099814	528.977794	0.32777646	0.10649354	0.00132107	0.00751221	CEP170B
5434	ENSG00000099817	2340.95868	0.02335563	0.06642138	0.72123843	0.86691933	POLR2E
5442	ENSG00000099821	1553.36095	0.18499288	0.09232696	0.03879572	0.12262339	POLRMT
610	ENSG00000099822	330.638438	-0.31080098	0.14845919	0.02282897	0.08099549	HCN2

53841	ENSG00000099834	3.27376587	0.03808873	0.3712284	0.72391962	0.86794586	CDHR5
113177	ENSG00000099840	38.6687979	1.09583901	0.52198789	0.00238071	0.01243635	IZUMO4
8045	ENSG00000099849	173.975149	0.16067944	0.21418575	0.3688712	0.59820516	RASSF7
4616	ENSG00000099860	6.53260176	0.20504903	0.436401	0.12728989	0.29752625	GADD45B
5064	ENSG00000099864	1583.18597	-0.08217661	0.08416446	0.31587616	0.54382011	PALM
8174	ENSG00000099866	11.4165432	0.1454486	0.37020991	0.38352304	0.61270278	MADCAM1
51214	ENSG00000099869	33.6203711	1.28217301	0.44900432	0.00031983	0.00216791	IGF2-AS
2872	ENSG00000099875	1537.86056	0.16814491	0.07781642	0.02718939	0.09298521	MKNK2
421	ENSG00000099889	953.910118	-0.57061405	0.08950023	5.98E-11	1.18E-09	ARVCF
27037	ENSG00000099899	1132.32097	0.0680048	0.0927045	0.45022876	0.67359465	TRMT2A
5902	ENSG00000099901	3697.44387	0.08632066	0.06852361	0.20066474	0.40584036	RANBP1
29801	ENSG00000099904	1425.74291	0.27050952	0.10204045	0.00583763	0.02653305	ZDHHC8
84861	ENSG00000099910	731.816878	-0.07506445	0.1288832	0.53519184	0.74618455	KLHL22
51586	ENSG00000099917	1890.27822	0.04799115	0.07306332	0.50432534	0.72088002	MED15
3053	ENSG00000099937	0.48572904	-0.01096121	0.38340473	0.81482371	NA	SERPIND1
9342	ENSG00000099940	646.956833	-0.05102255	0.12349725	0.66144995	0.8324506	SNAP29
1399	ENSG00000099942	2784.72548	-0.01603676	0.05938499	0.78402576	0.90163872	CRKL
8216	ENSG00000099949	2093.92776	0.26841182	0.06421474	2.25E-05	0.00019993	LZTR1
4320	ENSG00000099953	408.546831	0.64155715	0.15151078	5.94E-06	5.90E-05	MMP11
27443	ENSG00000099954	0	NA	NA	NA	NA	CECR2
6598	ENSG00000099956	2938.5768	0.09714974	0.06924236	0.15390475	0.3390108	SMARCB1
9127	ENSG00000099957	103.836061	-0.04754896	0.21634276	0.78873881	0.90411045	P2RX6
91319	ENSG00000099958	0.51350539	0.01481804	0.38307531	0.74411418	NA	DERL3
6545	ENSG00000099960	0.93453969	-0.05161064	0.38834234	0.35304854	NA	SLC7A4
23786	ENSG00000099968	927.943534	0.19883485	0.10834699	0.0553358	0.16097686	BCL2L13
100037417	ENSG00000099974	42.112543	-0.03397589	0.259969	0.8579906	0.936593	DDTL
1652	ENSG00000099977	823.115216	0.01037533	0.09267505	0.90936173	0.96089352	DDT
100037417	ENSG00000099977	823.115216	0.01037533	0.09267505	0.90936173	0.96089352	DDT
2953	ENSG00000099984	34.4067763	0.40183574	0.38948184	0.09165691	0.23379699	GSTT2
5008	ENSG00000099985	0.15069912	-0.01848503	0.38480575	0.84537521	NA	OSM
23523	ENSG00000099991	2272.89663	-0.01951405	0.0722826	0.78258873	0.90103067	CABIN1
83874	ENSG00000099992	118.677798	0.1155499	0.20305726	0.50583702	0.72177288	TBC1D10A
56241	ENSG00000099994	155.305859	2.93916216	0.24398793	1.63E-34	1.09E-32	SUSD2
10291	ENSG00000099995	2510.397	-0.19494511	0.10854234	0.06025153	0.17177617	SF3A1
2687	ENSG00000099998	107.223379	1.1032245	0.25584779	1.66E-06	1.81E-05	GGT5
200312	ENSG00000099999	398.343668	0.4613267	0.14847067	0.0007888	0.00481677	RNF215
23541	ENSG00000100003	202.268499	0.08554277	0.22959188	0.6428578	0.82148002	SEC14L2
266629	ENSG00000100012	0.68114595	0.00157579	0.38130168	0.9744473	NA	SEC14L3

23384	ENSG00000100014	2765.91505	0.02200025	0.06277988	0.72306853	0.86764418	SPECC1L
23759	ENSG00000100023	1674.75897	0.03489157	0.07313229	0.6275893	0.81099169	PPIL2
51733	ENSG00000100024	75.9321549	2.83794294	0.36376556	3.82E-16	1.12E-14	UPB1
29799	ENSG00000100027	783.136899	1.00778089	0.13318394	4.88E-15	1.34E-13	YPEL1
6634	ENSG00000100028	1904.9656	0.01834084	0.09750261	0.84682421	0.93052693	SNRPD3
23481	ENSG00000100029	1509.1961	-0.14748042	0.07512323	0.04456191	0.13608807	PES1
5594	ENSG00000100030	1414.51626	-0.44409874	0.09341561	9.20E-07	1.05E-05	MAPK1
2678	ENSG00000100031	14.9825898	1.17514467	0.8389459	0.00758004	0.03302328	GGT1
5625	ENSG00000100033	34.5219213	1.94878385	0.45563195	1.12E-06	1.25E-05	PRODH
9647	ENSG00000100034	1774.01162	0.01477898	0.08009728	0.85126462	0.93275912	PPM1F
339665	ENSG00000100036	177.377919	-0.38502094	0.20518129	0.02889869	0.09740774	SLC35E4
8940	ENSG00000100038	1081.19954	0.39905924	0.08710896	2.47E-06	2.61E-05	TOP3B
1417	ENSG00000100053	0.78607846	0.00108047	0.38151834	0.98756414	NA	CRYBB3
27128	ENSG00000100055	0	NA	NA	NA	NA	CYTH4
8220	ENSG00000100056	406.6422	0.07905359	0.12521447	0.50461667	0.72111066	ESS2
1416	ENSG00000100058	423.166224	0.06957884	0.1252562	0.55740872	0.76258372	CRYBB2P1
4242	ENSG00000100060	44.6750158	-0.75810566	0.36630695	0.00551304	0.02528989	MFNG
29775	ENSG00000100065	275.129998	-1.22966534	0.1513918	4.65E-17	1.44E-15	CARD10
NA	ENSG00000100068	281.483357	0.16538336	0.13976232	0.20348209	0.40973152	LRP5L
6576	ENSG00000100075	1416.21772	-0.44501676	0.09330273	8.54E-07	9.79E-06	SLC25A1
157	ENSG00000100077	4261.13671	-0.10093708	0.06746379	0.12824175	0.29899782	GRK3
50487	ENSG00000100078	0.30139824	-0.02296476	0.38527817	0.68536535	NA	PLA2G3
3957	ENSG00000100079	0	NA	NA	NA	NA	LGALS2
26088	ENSG00000100083	1976.75024	0.27113519	0.07807574	0.00038086	0.00253648	GGA1
7290	ENSG00000100084	1524.41188	-0.01155583	0.07395046	0.87284415	0.94439555	HIRA
23616	ENSG00000100092	485.694109	0.02482752	0.11238567	0.81824575	0.9171248	SH3BP1
23544	ENSG00000100095	1190.36996	-3.2469177	0.11273855	5.07E-184	6.70E-181	SEZ6L
3956	ENSG00000100097	3289.99689	0.02618789	0.06123289	0.66525325	0.83470042	LGALS1
89781	ENSG00000100099	1033.98927	0.07661381	0.10164345	0.43490556	0.66027902	HPS4
113791	ENSG00000100100	105.783623	-0.41555233	0.23248916	0.03085955	0.1024692	PIK3IP1
NA	ENSG00000100101	3.68189037	0.03069668	0.38661547	0.16772812	0.35871254	
402055	ENSG00000100104	407.796094	-0.3119122	0.16908096	0.03955703	0.12441527	SRRD
23598	ENSG00000100105	2168.10439	-8.52E-06	0.0658887	0.99917305	0.99962651	PATZ1
11078	ENSG00000100106	1511.15972	0.22407882	0.08269711	0.00545107	0.02506357	TRIOBP
24144	ENSG00000100109	653.232728	0.12396902	0.13342744	0.3229443	0.55059885	TFIP11
23464	ENSG00000100116	157.299598	0.05184984	0.21449791	0.77266584	0.89561741	GCAT
91227	ENSG00000100121	0	NA	NA	NA	NA	GGTLC2
1414	ENSG00000100122	5.87299205	-0.20985128	0.43167809	0.15274709	0.33739727	CRYBB1

129138	ENSG00000100124	683.962911	-0.17848805	0.10779106	0.08354635	0.21827226	ANKRD54
51386	ENSG00000100129	9305.55356	-0.03312376	0.0607772	0.57893284	0.77671271	EIF3L
4809	ENSG00000100138	1389.30007	-0.30372605	0.07906838	8.21E-05	0.00063781	SNU13
85377	ENSG00000100139	785.078724	0.08917533	0.09178126	0.31726496	0.54509671	MICALL1
5435	ENSG00000100142	498.235116	-0.01438286	0.12210821	0.90009993	0.95573896	POLR2F
6663	ENSG00000100146	1.95634066	0.13473642	0.41171039	0.10912239	NA	SOX10
79879	ENSG00000100147	280.048446	0.08115954	0.13943993	0.53273674	0.74395247	CCDC134
9681	ENSG00000100150	482.739791	-0.13558874	0.12698564	0.25662991	0.47767496	DEPDC5
9463	ENSG00000100151	477.95342	0.24831811	0.12735391	0.0382653	0.12144993	PICK1
23331	ENSG00000100154	2198.93643	-0.2089319	0.07783884	0.00602101	0.02723592	TTC28
23539	ENSG00000100156	24.3356508	-0.02326601	0.30351485	0.89905272	0.95557174	SLC16A8
79019	ENSG00000100162	132.858973	-0.88148067	0.24147987	3.72E-05	0.00031514	CENPM
55964	ENSG00000100167	1323.25787	-0.69847065	0.07953833	3.78E-19	1.35E-17	SEPTIN3
6523	ENSG00000100170	1.57058864	-0.00888322	0.37868862	0.89566899	NA	SLC5A1
387590	ENSG00000100181	0	NA	NA	NA	NA	TPTEP1
6527	ENSG00000100191	0.3243667	0.00127512	0.38336752	0.96809536	NA	SLC5A4
11015	ENSG00000100196	66.0257248	2.12853437	0.35197832	8.96E-11	1.73E-09	KDELR3
1565	ENSG00000100197	22.5976961	0.5094315	0.4968672	0.05325477	0.1562287	CYP2D6
107987478	ENSG00000100197	22.5976961	0.5094315	0.4968672	0.05325477	0.1562287	CYP2D6
107987479	ENSG00000100197	22.5976961	0.5094315	0.4968672	0.05325477	0.1562287	CYP2D6
10521	ENSG00000100201	6370.79696	0.00369896	0.05132618	0.97228046	0.98958828	DDX17
11144	ENSG00000100206	5.44860305	0.09355902	0.37782974	0.44690883	0.67095132	DMC1
6942	ENSG00000100207	848.481032	-0.15053105	0.11411539	0.16594655	0.35616396	TCF20
150274	ENSG00000100209	143.535453	-0.14041276	0.19747809	0.40542275	0.63375238	HSCB
25776	ENSG00000100211	166.945888	-0.0346482	0.16583631	0.81569248	0.9163678	CBY1
56993	ENSG00000100216	894.698191	-0.05528412	0.09006715	0.5270722	0.73901854	TOMM22
27156	ENSG00000100218	11.5682499	0.32847631	0.49456545	0.0974398	0.24443112	RSPH14
7494	ENSG00000100219	1601.7555	0.53520301	0.07649519	9.66E-13	2.20E-11	XBP1
51493	ENSG00000100220	1315.02953	-0.15308589	0.07836479	0.04565029	0.13867733	RTCB
9929	ENSG00000100221	763.020616	0.00364273	0.10352261	0.97178255	0.98938604	JOSD1
25793	ENSG00000100225	1652.4625	0.04326056	0.06904278	0.52454878	0.73734087	FBXO7
9567	ENSG00000100226	973.798783	-0.02312959	0.08889868	0.78837688	0.90400376	GTPBP1
84271	ENSG00000100227	1140.82876	-0.22279268	0.10519829	0.02710781	0.0927914	POLDIP3
9609	ENSG00000100228	218.39745	-0.2448649	0.20571252	0.15980176	0.3473723	RAB36
7078	ENSG00000100234	2090.01639	-1.07512039	0.07012631	5.97E-54	6.51E-52	TIMP3
9701	ENSG00000100239	1502.86839	0.15220959	0.07543147	0.03846642	0.12197623	PPP6R2
6305	ENSG00000100241	4782.61059	0.07427126	0.06423378	0.24109078	0.45895616	SBF1
25777	ENSG00000100242	1141.93077	-0.25212798	0.10919477	0.01563259	0.05962142	SUN2

1727	ENSG00000100243	1352.52794	0.26325742	0.0778352	0.0005401	0.00345019	CYB5R3
10126	ENSG00000100246	212.034733	0.40773237	0.19110104	0.01509252	0.0579852	DNAL4
25770	ENSG00000100249	0	NA	NA	NA	NA	C22orf31
55586	ENSG00000100253	4.09161237	0.18049553	0.4242033	0.14342309	0.32269382	MIOX
91289	ENSG00000100258	896.400058	0.22190136	0.10354778	0.02577157	0.0891868	LMF2
25807	ENSG00000100263	501.854954	0.37445085	0.1188844	0.00090352	0.00542222	RHBDD3
11252	ENSG00000100266	668.36082	-0.28248338	0.10600485	0.00540968	0.02488478	PACSIN2
25809	ENSG00000100271	188.776207	0.39307332	0.16577498	0.00883162	0.03742405	TTLL1
10633	ENSG00000100276	1.1233646	0.06326018	0.38839755	0.28139386	NA	RASL10A
162	ENSG00000100280	2796.8116	-0.07152593	0.07424303	0.3250967	0.55302396	AP1B1
10042	ENSG00000100281	894.139699	-0.08662655	0.08614443	0.30139122	0.52872103	HMGXB4
10043	ENSG00000100284	336.079485	0.06110641	0.14480372	0.6489847	0.82497477	TOM1
4744	ENSG00000100285	1589.37017	-1.36569484	0.08602658	8.51E-58	1.04E-55	NEFH
1120	ENSG00000100288	747.176922	0.20120708	0.10038646	0.03755584	0.1197345	CHKB
638	ENSG00000100290	1.00918704	-0.02393651	0.38044904	0.71012726	NA	BIK
3162	ENSG00000100292	38.2234353	0.22693615	0.30313095	0.27554801	0.49966845	HMOX1
27349	ENSG00000100294	140.164522	0.1293182	0.19456124	0.44004863	0.66517856	MCAT
8563	ENSG00000100296	2018.39461	0.12238857	0.06829317	0.06857718	0.18867997	THOC5
4174	ENSG00000100297	2616.55642	-0.1049419	0.06527174	0.10163092	0.25236014	MCM5
164668	ENSG00000100298	0	NA	NA	NA	NA	APOBEC3H
410	ENSG00000100299	339.609559	0.40248688	0.15365715	0.00433236	0.02065708	ARSA
706	ENSG00000100300	729.644049	0.17614613	0.10264773	0.07431676	0.2001033	TSPO
23551	ENSG00000100302	15.4084778	0.32976138	0.44610064	0.12694115	0.29688935	RASD2
23170	ENSG00000100304	1181.06437	-0.22933932	0.0806814	0.00357244	0.01758305	TTLL12
23492	ENSG00000100307	81.5233965	0.47716323	0.28546999	0.02991772	0.10009569	CBX7
5155	ENSG00000100311	0	NA	NA	NA	NA	PDGFB
49	ENSG00000100312	0.34746601	0.02370763	0.3853651	0.61987128	NA	ACR
164633	ENSG00000100314	160.512115	-2.1039316	0.61164813	2.69E-05	0.00023487	CABP7
6122	ENSG00000100316	31219.3278	-0.16574749	0.04853809	0.00055797	0.0035504	RPL3
55954	ENSG00000100319	227.264934	0.14817761	0.15172259	0.28772648	0.51340148	ZMAT5
23543	ENSG00000100320	11047.7787	0.18463865	0.04880315	0.00012586	0.00093314	RBFOX2
9145	ENSG00000100321	471.810445	-0.32830109	0.15387177	0.019546	0.07160128	SYNGR1
10454	ENSG00000100324	490.654491	0.15022807	0.11184475	0.16001891	0.34765388	TAB1
84164	ENSG00000100325	1265.30802	-0.09936365	0.08579281	0.23393314	0.45086784	ASCC2
8897	ENSG00000100330	1112.10023	0.07471845	0.11157117	0.48453963	0.70468159	MTMR3
54471	ENSG00000100335	1011.20115	-0.0964164	0.08734038	0.25640096	0.47756244	MIEF1
80832	ENSG00000100336	106.291338	1.47005911	0.28087173	1.30E-08	1.94E-07	APOL4
150379	ENSG00000100341	0	NA	NA	NA	NA	PNPLA5

8542	ENSG00000100342	0.16735865	0.01897177	0.38482933	0.78225479	NA	APOL1
80339	ENSG00000100344	22.2618301	0.12261752	0.34347558	0.49101687	0.71055979	PNPLA3
4627	ENSG00000100345	4881.64488	0.04381549	0.06276988	0.47820693	0.69986975	MYH9
8911	ENSG00000100346	1.28376817	-0.07018956	0.38938093	0.25995481	NA	CACNA1I
25813	ENSG00000100347	781.598124	-0.02796679	0.08861766	0.74494216	0.87988222	SAMM50
25828	ENSG00000100348	1400.94794	0.06363612	0.07649867	0.396397	0.62570741	TXN2
80020	ENSG00000100350	1478.88908	-0.16363785	0.07229352	0.02093307	0.07553876	FOXRED2
9402	ENSG00000100351	0.38789669	0.01214925	0.38385518	0.84879667	NA	GRAP2
8664	ENSG00000100353	3377.48615	0.01704844	0.06601821	0.79346989	0.90556117	EIF3D
23112	ENSG00000100354	674.031383	-0.16314045	0.28369998	0.41289201	0.64038252	TNRC6B
27352	ENSG00000100359	876.541306	0.21560947	0.09337886	0.01710516	0.06426176	SGSM3
11020	ENSG00000100360	814.383757	0.27394065	0.09538983	0.00295618	0.01495046	IFT27
5816	ENSG00000100362	0	NA	NA	NA	NA	PVALB
23313	ENSG00000100364	1167.60615	-0.39042384	0.08618686	3.17E-06	3.30E-05	KIAA0930
4689	ENSG00000100365	0	NA	NA	NA	NA	NCF4
1439	ENSG00000100368	0	NA	NA	NA	NA	CSF2RB
10478	ENSG00000100372	322.205311	0.06339837	0.15507095	0.65585667	0.82885511	SLC25A17
7380	ENSG00000100373	0	NA	NA	NA	NA	UPK3A
55007	ENSG00000100376	626.946557	0.12670863	0.1083384	0.22220656	0.43535977	FAM118A
79734	ENSG00000100379	624.283704	-0.43574822	0.11656841	8.59E-05	0.00066398	KCTD17
6767	ENSG00000100380	2232.76231	-0.20127573	0.08309152	0.01292309	0.05122757	ST13
3560	ENSG00000100385	1.17832196	0.05379565	0.38759742	0.29472884	NA	IL2RB
9978	ENSG00000100387	371.318198	0.09803136	0.12317146	0.4011279	0.62949876	RBX1
2033	ENSG00000100393	1309.50182	-0.04014103	0.09700255	0.65016276	0.82529572	EP300
83746	ENSG00000100395	333.96561	-0.01829754	0.13094967	0.8812178	0.94760765	L3MBTL2
150356	ENSG00000100399	6.43343641	0.12412794	0.37761356	0.39291054	0.6220352	CHADL
5905	ENSG00000100401	1936.16304	-0.03751557	0.0841269	0.64651724	0.82387449	RANGAP1
23264	ENSG00000100403	1853.30139	-0.22543172	0.07255242	0.00150798	0.00841822	ZC3H7B
84844	ENSG00000100410	403.809211	-0.10338974	0.12435718	0.37695983	0.60572472	PHF5A
50	ENSG00000100412	1165.35683	0.25260577	0.08904809	0.00347338	0.01714651	ACO2
171568	ENSG00000100413	655.281113	-0.21071332	0.13165079	0.08677017	0.22459479	POLR3H
55687	ENSG00000100416	1222.12153	0.06187434	0.08461157	0.45523538	0.67862874	TRMU
5372	ENSG00000100417	143.434808	-0.01994108	0.18984958	0.90242768	0.95713489	PMM1
27351	ENSG00000100418	396.965959	-0.3137653	0.12366811	0.00706516	0.03119804	DESI1
64781	ENSG00000100422	2872.40231	-0.38957177	0.06940556	1.10E-08	1.65E-07	CERK
23774	ENSG00000100425	1393.75019	0.14252745	0.07884806	0.06465531	0.18119939	BRD1
9889	ENSG00000100426	2475.65338	-0.10307553	0.06346742	0.09939861	0.24821504	ZBED4
23209	ENSG00000100427	0	NA	NA	NA	NA	MLC1

83933	ENSG00000100429	489.288982	0.07600757	0.11411903	0.48602144	0.70617338	HDAC10
54207	ENSG00000100433	18.8858456	-2.55056247	0.76592006	4.05E-05	0.00034013	KCNK10
63874	ENSG00000100439	173.487296	0.56873147	0.21360234	0.00219951	0.01162136	ABHD4
23351	ENSG00000100441	1285.18397	0.39992266	0.08380543	9.68E-07	1.10E-05	KHNYN
2287	ENSG00000100442	714.661409	-0.21771022	0.10634053	0.03259559	0.1071399	FKBP3
56948	ENSG00000100445	750.418345	0.06408137	0.10894112	0.53941069	0.74850934	SDR39U1
1511	ENSG00000100448	0	NA	NA	NA	NA	CTSG
2999	ENSG00000100450	0	NA	NA	NA	NA	GZMH
3002	ENSG00000100453	0	NA	NA	NA	NA	GZMB
55147	ENSG00000100461	1233.82425	-0.00544042	0.09716621	0.95257602	0.98049849	RBM23
10419	ENSG00000100462	1548.55371	0.06761508	0.0758561	0.36540226	0.5947154	PRMT5
1690	ENSG00000100473	1050.9083	0.36190041	0.08484957	1.18E-05	0.00011052	COCH
11154	ENSG00000100478	110.139612	-0.31905728	0.24240528	0.10068346	0.25054057	AP4S1
5427	ENSG00000100479	230.489429	0.01088292	0.16847731	0.94398621	0.97718285	POLE2
79609	ENSG00000100483	167.696066	-0.11195886	0.17393724	0.46944187	0.69185312	VCPKMT
6655	ENSG00000100485	785.220862	0.23247896	0.0910071	0.00843106	0.03605781	SOS2
8814	ENSG00000100490	159.274157	-0.2867005	0.29184254	0.17034959	0.36228275	CDKL1
51199	ENSG00000100503	1872.97528	0.00339074	0.09655122	0.97260543	0.98981748	NIN
5836	ENSG00000100504	111.193332	1.58550382	0.28849655	2.67E-09	4.33E-08	PYGL
114088	ENSG00000100505	226.389683	-0.19239206	0.17425335	0.21406547	0.42423923	TRIM9
5706	ENSG00000100519	1054.94708	-0.04360284	0.08820435	0.61056374	0.7987352	PSMC6
64841	ENSG00000100522	1006.22867	-0.15478992	0.08835981	0.07135954	0.19425517	GNPNAT1
80821	ENSG00000100523	995.201824	-0.04829937	0.11142679	0.64969621	0.82525848	DDHD1
1033	ENSG00000100526	280.607456	-0.41594677	0.15556086	0.00352533	0.01737703	CDKN3
10175	ENSG00000100528	1427.36034	-0.21826977	0.08325854	0.00711627	0.0313609	CNIH1
10668	ENSG00000100532	143.18825	-0.09205698	0.19450168	0.58036849	0.77774473	CGRRF1
51382	ENSG00000100554	550.619479	0.22084613	0.11232835	0.03933368	0.12396233	ATP6V1D
55195	ENSG00000100557	0.18922458	0.01954053	0.38488496	0.78225479	NA	CCDC198
26499	ENSG00000100558	2.98364397	0.10102331	0.39023597	0.30920641	0.53699712	PLEK2
5283	ENSG00000100564	395.45914	0.2059275	0.12363427	0.07787584	0.20726343	PIGH
145497	ENSG00000100565	0	NA	NA	NA	NA	LRRC74A
5684	ENSG00000100567	941.697687	-0.15139637	0.10063429	0.11824282	0.28216714	PSMA3
10490	ENSG00000100568	1504.77326	-0.06825605	0.0931788	0.44929859	0.67295604	VTI1B
26520	ENSG00000100575	284.817588	-0.30440028	0.15987871	0.03571699	0.11513073	TIMM9
2954	ENSG00000100577	326.669864	0.03641286	0.14151904	0.78304046	0.90117013	GSTZ1
9786	ENSG00000100578	681.130725	-0.2699684	0.10411449	0.00688115	0.03050772	KIAA0586
283578	ENSG00000100580	1601.88156	-0.93335748	0.07555661	7.52E-36	5.23E-34	TMED8
161394	ENSG00000100583	34.1187049	-0.41393091	0.5132831	0.07275766	0.19685148	SAMD15

10598	ENSG00000100591	2825.83342	0.0068345	0.08271672	0.9339124	0.97240128	AHSA1
23002	ENSG00000100592	435.726033	-0.20065589	0.201521	0.24198809	0.46013453	DAAM1
145501	ENSG00000100593	2.36756011	-0.07436187	0.38660919	0.35622998	NA	ISM2
9517	ENSG00000100596	1689.12505	-0.12275593	0.07141914	0.07979803	0.21086746	SPTLC2
79890	ENSG00000100599	4.90157512	0.07091501	0.37531539	0.52429693	0.73721128	RIN3
5641	ENSG00000100600	573.660437	0.19609137	0.11115658	0.06482504	0.18145157	LGMN
8846	ENSG00000100601	218.506802	-0.26041648	0.16481076	0.0793774	0.21020391	ALKBH1
22938	ENSG00000100603	1515.30075	-0.24196032	0.10387215	0.01513658	0.05808699	SNW1
1113	ENSG00000100604	19767.1564	-0.40141561	0.0744951	3.73E-08	5.20E-07	CHGA
3705	ENSG00000100605	1170.34791	-0.1217492	0.08753835	0.15209164	0.3363239	ITPK1
51635	ENSG00000100612	1239.12085	-0.08221086	0.07728579	0.27696866	0.50137393	DHRS7
5494	ENSG00000100614	1247.8008	-0.42735493	0.10174912	1.25E-05	0.00011694	PPM1A
51804	ENSG00000100625	75.0305291	1.14212467	0.37951338	0.00021844	0.00153956	SIX4
57452	ENSG00000100626	254.221116	-0.62373819	0.17515064	9.56E-05	0.00073178	GALNT16
51676	ENSG00000100628	0	NA	NA	NA	NA	ASB2
145508	ENSG00000100629	316.275967	-0.19090059	0.13954145	0.14053823	0.31847841	CEP128
2079	ENSG00000100632	3931.1153	0.11036633	0.0793854	0.15530952	0.3411582	ERH
3091	ENSG00000100644	4316.73049	0.00457093	0.06551011	0.94379598	0.97708788	HIF1A
9766	ENSG00000100647	381.713423	-0.11035159	0.12513128	0.34975508	0.58009871	SUSD6
6430	ENSG00000100650	4244.25861	0.40998424	0.07141979	4.95E-09	7.75E-08	SRSF5
6554	ENSG00000100652	0.32399419	-0.02327436	0.38532155	0.66851063	NA	SLC10A1
1983	ENSG00000100664	3855.62634	-0.30053809	0.08261143	0.00018855	0.00134898	EIF5
5267	ENSG00000100665	0	NA	NA	NA	NA	SERPINA4
6547	ENSG00000100678	1002.93459	-0.34218577	0.0947004	0.00018506	0.00132546	SLC8A3
23405	ENSG00000100697	2053.95956	0.05588882	0.06733989	0.39942091	0.6281816	DICER1
79038	ENSG00000100711	405.605514	0.36538116	0.11807995	0.00112101	0.00651078	ZFYVE21
4522	ENSG00000100714	2042.24862	-0.10189409	0.08447449	0.21566554	0.42647306	MTHFD1
8115	ENSG00000100721	0	NA	NA	NA	NA	TCL1A
79882	ENSG00000100722	1700.51238	-0.20532874	0.07608679	0.00581215	0.02642978	ZC3H14
9894	ENSG00000100726	857.976629	0.16700857	0.11931327	0.13423443	0.30845623	TELO2
22990	ENSG00000100731	1918.37631	0.08217478	0.07811767	0.28289465	0.50793031	PCNX1
623	ENSG00000100739	0.4901093	-0.00697197	0.38316072	0.85885354	NA	BDKRB1
51527	ENSG00000100744	407.619987	-0.27621053	0.11908993	0.01435708	0.05563861	GSKIP
7443	ENSG00000100749	551.460415	-0.18721829	0.10773306	0.06904451	0.18952649	VRK1
5700	ENSG00000100764	1618.42255	-0.08223681	0.09515988	0.37142337	0.60057374	PSMC1
89932	ENSG00000100767	33.55482	0.05830618	0.28304567	0.7641103	0.8914874	PAPLN
9252	ENSG00000100784	358.709979	-0.17422875	0.14030809	0.18069732	0.37770382	RPS6KA5
55671	ENSG00000100796	1717.77046	-0.11660431	0.11389622	0.28175237	0.50654372	PPP4R3A

60686	ENSG00000100802	293.310277	-0.22600031	0.16331802	0.12490495	0.29330267	C14orf93
5693	ENSG00000100804	2256.68105	-0.16189019	0.06235078	0.00843555	0.03606146	PSMB5
7528	ENSG00000100811	2633.25303	-0.16398736	0.07536631	0.02617787	0.09027793	YY1
22985	ENSG00000100813	4928.6482	0.14110344	0.06405542	0.02534686	0.0880241	ACIN1
57820	ENSG00000100814	1645.46057	0.29986512	0.07687492	6.67E-05	0.00053193	CCNB1IP1
9321	ENSG00000100815	935.11998	0.20759522	0.09862524	0.02931053	0.09858313	TRIP11
328	ENSG00000100823	3516.91172	-0.06847197	0.056341	0.21841774	0.43045674	APEX1
8106	ENSG00000100836	3842.2678	0.20828701	0.06193727	0.00065225	0.0040617	PABPN1
10278	ENSG00000100842	2000.65011	0.19083184	0.07215027	0.00702862	0.03107125	EFS
394	ENSG00000100852	890.12554	-0.08587799	0.10654073	0.39963998	0.62831531	ARHGAP5
51550	ENSG00000100865	525.842681	-0.07866902	0.11448165	0.47074354	0.69301557	CINP
10202	ENSG00000100867	92.0848023	-0.76904439	0.24755544	0.00032289	0.00218493	DHRS2
6729	ENSG00000100883	1126.81429	0.08378814	0.09011144	0.33912884	0.56932782	SRP54
9362	ENSG00000100884	0	NA	NA	NA	NA	CPNE6
57680	ENSG00000100888	2874.10989	0.00057534	0.07382552	0.99445972	0.99838482	CHD8
5106	ENSG00000100889	1379.57731	0.00737202	0.12898111	0.95300272	0.98058207	PCK2
9692	ENSG00000100890	447.578358	0.13883812	0.15364632	0.32716095	0.55553519	PRORP
80344	ENSG00000100897	1013.00565	-0.04230706	0.10157293	0.66493284	0.83454529	DCAF11
5687	ENSG00000100902	1924.62759	0.00821934	0.0770842	0.91457128	0.96388282	PSMA6
4792	ENSG00000100906	547.472187	0.65645948	0.11052266	7.55E-10	1.32E-08	NFKBIA
51016	ENSG00000100908	367.066631	-0.01763348	0.12158942	0.87759933	0.94578953	EMC9
5721	ENSG00000100911	795.154762	-0.01986207	0.10088618	0.83715617	0.92602467	PSME2
84312	ENSG00000100916	392.131137	0.08489063	0.15244364	0.54465049	0.75239282	BRMS1L
9985	ENSG00000100918	3332.1225	-0.06642662	0.05949046	0.25804219	0.47940416	REC8
10548	ENSG00000100926	715.900421	0.34190189	0.09730958	0.00027185	0.0018734	TM9SF1
10484	ENSG00000100934	701.389579	-0.04160387	0.10322933	0.67477494	0.84066152	SEC23A
51292	ENSG00000100938	1078.30851	-0.16186872	0.0943908	0.07543282	0.2022854	GMPR2
5411	ENSG00000100941	1823.94536	-0.14064416	0.10205837	0.15192797	0.33603688	PNN
5875	ENSG00000100949	335.331113	0.38162265	0.13697222	0.00283749	0.01444594	RABGGTA
4776	ENSG00000100968	830.293274	1.38288101	0.12442668	8.87E-30	5.07E-28	NFATC4
5360	ENSG00000100979	526.355453	0.6553249	0.11519046	3.33E-09	5.32E-08	PLTP
63935	ENSG00000100982	858.399675	0.52802726	0.08825636	8.20E-10	1.43E-08	PCIF1
2937	ENSG00000100983	1076.73395	-0.12602501	0.08381503	0.12287632	0.29015309	GSS
4318	ENSG00000100985	2.29631252	-0.07232408	0.38238226	0.44142351	NA	MMP9
30813	ENSG00000100987	0	NA	NA	NA	NA	VSX1
26133	ENSG00000100991	1629.52212	-0.11664261	0.07186199	0.0981255	0.24574727	TRPC4AP
5834	ENSG00000100994	1543.88049	0.16900309	0.07789885	0.02649546	0.09108817	PYGB
26090	ENSG00000100997	777.829949	-0.25467561	0.09462782	0.00536051	0.02469294	ABHD12

10544	ENSG00000101000	10.6905885	0.04459441	0.34750158	0.77244447	0.89553101	PROCR
9837	ENSG00000101003	588.963368	0.04325439	0.14300536	0.74549572	0.88008063	GINS1
22981	ENSG00000101004	1927.68606	0.43778073	0.06781304	5.25E-11	1.04E-09	NINL
958	ENSG00000101017	1.58400581	-0.06301475	0.38615781	0.37079477	NA	CD40
55245	ENSG00000101019	943.013961	0.18953864	0.09550463	0.0401935	0.12587831	UQCC1
23613	ENSG00000101040	1328.12002	-0.54916668	0.07687694	3.19E-13	7.49E-12	ZMYND8
10110	ENSG00000101049	46.3999598	0.95032949	0.38315147	0.00134481	0.00762748	SGK2
51098	ENSG00000101052	621.545053	-0.02235898	0.10303101	0.81933426	0.91761737	IFT52
4605	ENSG00000101057	3859.8554	-0.5009157	0.06142933	1.43E-16	4.29E-15	MYBL2
140902	ENSG00000101074	0	NA	NA	NA	NA	R3HDML
3172	ENSG00000101076	0	NA	NA	NA	NA	HNF4A
57446	ENSG00000101079	983.033477	-0.52255266	0.08827933	1.21E-09	2.07E-08	NDRG3
84174	ENSG00000101082	0.68805294	-0.00445254	0.3824501	0.92023421	NA	SLA2
55969	ENSG00000101084	831.372672	-0.17598694	0.0960212	0.05778598	0.16649345	RAB5IF
4773	ENSG00000101096	8.6755052	2.89315425	1.08732104	0.0004764	0.00309487	NFATC2
140730	ENSG00000101098	1501.1296	-1.18098093	0.08549584	2.27E-44	1.96E-42	RIMS4
80336	ENSG00000101104	890.614021	0.51072625	0.09236494	1.26E-08	1.88E-07	PABPC1L
6789	ENSG00000101109	1261.81821	-0.06059949	0.08403585	0.45919795	0.68252693	STK4
57167	ENSG00000101115	204.338761	0.69383716	0.21927233	0.00031699	0.0021516	SALL4
23394	ENSG00000101126	3503.11146	-0.18898416	0.060397	0.00151217	0.00842975	ADNP
5203	ENSG00000101132	531.073782	-0.15821982	0.10929841	0.12935948	0.30081651	PFDN4
55816	ENSG00000101134	0	NA	NA	NA	NA	DOK5
1477	ENSG00000101138	566.98049	-0.07433494	0.11234329	0.48750988	0.70734212	CSTF1
655	ENSG00000101144	2460.09876	-1.15120478	0.06617148	9.48E-69	1.52E-66	BMP7
8480	ENSG00000101146	917.792662	0.13058386	0.12463201	0.26760943	0.49015612	RAE1
7165	ENSG00000101150	2660.16848	0.20357223	0.06258087	0.00096851	0.00575308	TPD52L2
80331	ENSG00000101152	1935.54042	-0.2760166	0.0712729	7.85E-05	0.00061348	DNAJC5
51497	ENSG00000101158	2313.63087	0.42488584	0.08457826	2.52E-07	3.16E-06	NELFCD
1522	ENSG00000101160	176.093581	0.4495345	0.19024889	0.00742595	0.03246602	CTSZ
24148	ENSG00000101161	2262.47341	-0.02919198	0.07109463	0.67540134	0.84111426	PRPF6
81027	ENSG00000101162	11.9120719	-0.00114718	0.33907605	0.9921605	0.99756121	TUBB1
51012	ENSG00000101166	1649.7084	0.01268056	0.08126037	0.87432036	0.94470892	PRELID3B
11255	ENSG00000101180	117.540193	-3.09500243	0.26604307	2.12E-32	1.31E-30	HRH3
26164	ENSG00000101181	794.918794	-0.00073767	0.11905002	0.99320792	0.99778445	MTG2
5688	ENSG00000101182	3754.79976	-0.19114007	0.06905364	0.00483312	0.02257807	PSMA7
28231	ENSG00000101187	964.581687	0.67907865	0.11887533	2.69E-09	4.35E-08	SLCO4A1
4923	ENSG00000101188	2.05659011	-0.0754969	0.38683708	0.35762108	NA	NTSR1
55257	ENSG00000101189	563.663245	-0.06380998	0.10953283	0.54200748	0.75025664	MRGBP

10732	ENSG00000101190	325.122957	0.14255829	0.14134623	0.27746014	0.50194302	TCFL5
11083	ENSG00000101191	2786.62015	-0.09804732	0.05889472	0.09180012	0.23401187	DIDO1
54994	ENSG00000101193	1265.04992	-0.25625318	0.08408035	0.00174146	0.00952332	GID8
63910	ENSG00000101194	0	NA	NA	NA	NA	SLC17A9
79444	ENSG00000101197	0	NA	NA	NA	NA	BIRC7
128414	ENSG00000101198	0	NA	NA	NA	NA	NKAIN4
55738	ENSG00000101199	2130.13452	0.49951728	0.08622553	2.77E-09	4.48E-08	ARFGAP1
551	ENSG00000101200	0	NA	NA	NA	NA	AVP
57642	ENSG00000101203	0.173733	0.01914545	0.38484597	0.78225479	NA	COL20A1
1137	ENSG00000101204	1.05455877	-0.06787837	0.39189048	0.19208405	NA	CHRNA4
1917	ENSG00000101210	8678.99929	-1.10527231	0.0640479	1.30E-67	2.04E-65	EEF1A2
5753	ENSG00000101213	42.6174579	-0.62432515	0.38821245	0.01897746	0.06984101	PTK6
26205	ENSG00000101216	619.455837	-0.00558547	0.10166859	0.951818	0.98012235	GMEB2
54976	ENSG00000101220	756.16493	-0.16364061	0.09712515	0.08091222	0.21334235	C20orf27
25876	ENSG00000101222	6.86893078	-0.18124136	0.40501691	0.23418122	0.45103926	SPEF1
994	ENSG00000101224	1957.25075	-0.22433382	0.07904808	0.00364821	0.01786733	CDC25B
140862	ENSG00000101230	0.72521985	0.00222141	0.38233694	0.95839775	NA	ISM1
11237	ENSG00000101236	889.534588	0.0766256	0.11486332	0.48502278	0.70507415	RNF24
10139	ENSG00000101246	620.854218	0.04159607	0.11860316	0.71318743	0.86188941	ARFRP1
79133	ENSG00000101247	275.547638	0.08149072	0.15447701	0.56500323	0.76722846	NDUFAF5
80343	ENSG00000101251	0.47763262	-0.01664982	0.38407143	0.85281619	NA	SEL1L2
57761	ENSG00000101255	725.104088	0.00126626	0.14548096	0.9942729	0.9982983	TRIB3
9770	ENSG00000101265	424.458658	-0.24192107	0.11966944	0.03275869	0.10755128	RASSF2
1457	ENSG00000101266	4317.15479	0.04917586	0.06645614	0.45307933	0.67643118	CSNK2A1
113278	ENSG00000101276	0	NA	NA	NA	NA	SLC52A3
NA	ENSG00000101278	2.65860855	-0.01365037	0.37095451	0.89000117	0.95215008	RPS10P5
51378	ENSG00000101280	2.45573132	-0.00133616	0.37224682	0.98719061	NA	ANGPT4
343637	ENSG00000101282	59.9065917	-2.11899172	0.40347546	7.25E-09	1.11E-07	RSPO4
8760	ENSG00000101290	1710.33655	-0.23262677	0.08985255	0.00757345	0.03300907	CDS2
128674	ENSG00000101292	126.078917	-3.15316501	0.28521322	1.52E-29	8.55E-28	PROKR2
81502	ENSG00000101294	2042.07993	-0.08201064	0.07288668	0.25160009	0.47149344	HM13
9751	ENSG00000101298	308.336607	-0.26049048	0.1532645	0.06288744	0.17758139	SNPH
85366	ENSG00000101306	25.009287	0.26902605	0.36956685	0.20481877	0.41169887	MYLK2
10326	ENSG00000101307	0.53612635	0.03213492	0.38615517	0.46295797	NA	SIRPB1
10483	ENSG00000101310	1642.41483	0.02849869	0.08234781	0.7239489	0.86794586	SEC23B
55612	ENSG00000101311	14.7278584	3.24507432	0.84644964	1.01E-05	9.55E-05	FERMT1
54363	ENSG00000101323	0	NA	NA	NA	NA	HAO1
5173	ENSG00000101327	0	NA	NA	NA	NA	PDYN

140706	ENSG00000101331	61.5704556	-0.80446246	0.30904998	0.00134237	0.00761582	CCM2L
5332	ENSG00000101333	782.563615	-0.44585837	0.11841231	7.47E-05	0.00058802	PLCB4
10398	ENSG00000101335	0.16735865	0.01897177	0.38482933	0.78225479	NA	MYL9
3055	ENSG00000101336	0.50897541	0.01329051	0.38297807	0.76493109	NA	HCK
9777	ENSG00000101337	1751.96139	-0.16326519	0.06753278	0.01381945	0.05397201	TM9SF4
140711	ENSG00000101342	2.35382252	0.04525638	0.3823511	0.51826843	NA	TLDC2
51340	ENSG00000101343	851.741567	-0.01786222	0.09156948	0.84003159	0.92739563	CRNKL1
23509	ENSG00000101346	1334.79889	-0.03584121	0.07712394	0.63460746	0.81607019	POFUT1
25939	ENSG00000101347	400.166017	-0.44677435	0.12592482	0.00017325	0.00124854	SAMHD1
57144	ENSG00000101349	190.533084	-0.06326666	0.16177486	0.66406026	0.83431586	PAK5
9371	ENSG00000101350	1514.74329	-0.74188105	0.07794041	3.80E-22	1.58E-20	KIF3B
140699	ENSG00000101353	29.6496927	-0.04087829	0.31475825	0.81901564	0.9175195	MROH8
10528	ENSG00000101361	3180.10978	0.14431991	0.06247123	0.01916416	0.07035842	NOP56
63905	ENSG00000101363	959.178235	0.08746814	0.08765129	0.30575608	0.53334106	MANBAL
3420	ENSG00000101365	1444.01317	0.11611954	0.07870759	0.13171447	0.30446407	IDH3B
22919	ENSG00000101367	2342.3942	-0.28158527	0.06947057	3.65E-05	0.00030954	MAPRE1
182	ENSG00000101384	306.955987	1.64275649	0.19673319	5.20E-18	1.73E-16	JAG1
51654	ENSG00000101391	423.389657	0.24771788	0.1368825	0.05226429	0.15402903	CDK5RAP1
6640	ENSG00000101400	213.510665	-0.22748817	0.162131	0.12064709	0.28635462	SNTA1
5020	ENSG00000101405	0.86866502	0.03820342	0.3870704	0.32648034	NA	OXT
9675	ENSG00000101407	1274.71512	0.05504166	0.07594048	0.4601893	0.68333808	TTI1
1869	ENSG00000101412	1486.99253	-0.34811111	0.07546845	2.43E-06	2.58E-05	E2F1
58490	ENSG00000101413	766.528372	-0.00965112	0.09917633	0.91874437	0.96558446	RPRD1B
11264	ENSG00000101417	297.113047	0.012177	0.13543453	0.92444315	0.96852469	PXMP4
128866	ENSG00000101421	1131.92402	0.07485527	0.08549474	0.36948509	0.59885502	CHMP4B
671	ENSG00000101425	0	NA	NA	NA	NA	BPI
128821	ENSG00000101435	0	NA	NA	NA	NA	CST9L
140679	ENSG00000101438	243.245608	-3.1047327	0.19671936	2.84E-57	3.44E-55	SLC32A1
1471	ENSG00000101439	846.152103	0.37069361	0.09480814	5.29E-05	0.00042958	CST3
434	ENSG00000101440	0.96455001	-0.04977631	0.38563191	0.39076061	NA	ASIP
1472	ENSG00000101441	0	NA	NA	NA	NA	CST4
79913	ENSG00000101442	444.661982	0.06157159	0.11517238	0.57673406	0.77528586	ACTR5
10406	ENSG00000101443	0.76406384	0.05427165	0.38913569	0.26959566	NA	WFDC2
191	ENSG00000101444	3444.28574	-0.27010719	0.05687353	1.52E-06	1.67E-05	AHCY
26051	ENSG00000101445	1231.19524	-0.84681292	0.0900513	9.27E-22	3.78E-20	PPP1R16B
10816	ENSG00000101446	0	NA	NA	NA	NA	SPINT3
81610	ENSG00000101447	1026.22356	-0.33191239	0.08651285	8.06E-05	0.00062747	FAM83D
57119	ENSG00000101448	0	NA	NA	NA	NA	EPPIN

60625	ENSG00000101452	483.802881	-0.06608776	0.14539486	0.62276575	0.80737672	DHX35
116092	ENSG00000101457	458.364284	0.08057025	0.11698197	0.47044073	0.69270775	DNTTIP1
84557	ENSG00000101460	367.761464	0.12397481	0.12755893	0.30276407	0.52998404	MAP1LC3A
79953	ENSG00000101463	0	NA	NA	NA	NA	SYNDIG1
128869	ENSG00000101464	409.360091	0.38637155	0.12678213	0.00123197	0.00707069	PIGU
7125	ENSG00000101470	2.88306901	0.12557839	0.40164935	0.20527491	0.41224389	TNNC2
10005	ENSG00000101473	352.621157	-0.1621153	0.12620111	0.17297073	0.36578929	ACOT8
57136	ENSG00000101474	1329.13711	0.21654615	0.07803786	0.00454289	0.02146486	APMAP
56853	ENSG00000101489	960.355407	-3.14855309	0.10788495	2.10E-188	2.98E-185	CELF4
9658	ENSG00000101493	1085.00262	0.34046908	0.13084326	0.00552654	0.02534007	ZNF516
28316	ENSG00000101542	53.2051979	4.60793592	0.5100357	2.97E-20	1.13E-18	CDH20
22850	ENSG00000101544	1353.57662	-0.00987227	0.07710866	0.89549677	0.95469109	ADNP2
79863	ENSG00000101546	488.825982	0.02694609	0.10987695	0.79889621	0.90798839	RBFA
9097	ENSG00000101557	1569.27856	-0.129439	0.07882097	0.09290857	0.23587906	USP14
9218	ENSG00000101558	1299.87928	-0.44396778	0.08405499	5.95E-08	8.07E-07	VAPA
64863	ENSG00000101574	320.782927	-0.10380835	0.14987385	0.45062134	0.67397869	METTL4
9663	ENSG00000101577	630.775745	-0.17286202	0.09988786	0.07242748	0.19611863	LPIN2
23347	ENSG00000101596	2257.12996	-0.0272327	0.07374576	0.70274417	0.85699455	SMCHD1
8736	ENSG00000101605	20.2637937	1.84374535	0.65137209	0.00020174	0.00143356	MYOM1
10627	ENSG00000101608	1278.00799	0.43097282	0.08407839	1.46E-07	1.89E-06	MYL12A
79959	ENSG00000101624	277.790874	-0.19321915	0.14947288	0.15916615	0.34667433	CEP76
29906	ENSG00000101638	4.48889172	0.30012567	0.54940798	0.02331498	0.08241002	ST8SIA5
55125	ENSG00000101639	988.651994	0.03421031	0.10223003	0.72918643	0.87046879	CEP192
8731	ENSG00000101654	1962.85648	-0.1172052	0.07198045	0.09704098	0.24361546	RNMT
4092	ENSG00000101665	384.482592	0.57811827	0.14178889	1.38E-05	0.00012839	SMAD7
9388	ENSG00000101670	4.53004691	0.03286598	0.3657035	0.78300919	0.90117013	LIPG
284217	ENSG00000101680	757.539955	1.62015689	0.11255893	4.34E-48	4.04E-46	LAMA1
54941	ENSG00000101695	39.1467287	0.39309867	0.34772477	0.0904589	0.23157478	RNF125
23253	ENSG00000101745	605.497848	-0.48981143	0.13306696	8.87E-05	0.00068457	ANKRD12
8715	ENSG00000101746	590.998401	-0.81805055	0.11957071	1.40E-12	3.17E-11	NOL4
11201	ENSG00000101751	829.031053	0.13672371	0.10231682	0.16552506	0.35564031	POLI
57534	ENSG00000101752	3094.74359	-0.01903344	0.0681003	0.77588365	0.89695441	MIB1
5932	ENSG00000101773	1160.47144	-0.55102496	0.09928615	9.81E-09	1.48E-07	RBBP8
8780	ENSG00000101782	854.912913	-0.16559427	0.09276664	0.06523585	0.1822483	RIOK3
1478	ENSG00000101811	716.137123	0.11852521	0.10207614	0.22834326	0.44354125	CSTF2
286436	ENSG00000101812	0.14931947	-0.01844332	0.38480212	0.84537521	NA	H2BW2
25878	ENSG00000101825	0	NA	NA	NA	NA	MXRA5
340547	ENSG00000101842	0	NA	NA	NA	NA	VSIG1

5716	ENSG00000101843	425.552413	0.00979251	0.1130641	0.92865485	0.97011965	PSMD10
115201	ENSG00000101844	138.788729	0.07313621	0.1879634	0.65656652	0.82948802	ATG4A
412	ENSG00000101846	199.210105	-0.64005624	0.1774862	7.77E-05	0.00060859	STS
6907	ENSG00000101849	715.055291	-0.21263602	0.12042275	0.06128604	0.17419985	TBL1X
4935	ENSG00000101850	2.4308842	0.02264811	0.37477944	0.80037145	NA	GPR143
10857	ENSG00000101856	1567.46034	-0.09921803	0.07085381	0.15381151	0.33890581	PGRMC1
5422	ENSG00000101868	989.893352	-0.39941852	0.08731579	2.52E-06	2.66E-05	POLA1
4281	ENSG00000101871	219.774955	-0.5330573	0.16857686	0.00052343	0.00335645	MID1
79576	ENSG00000101882	492.910773	0.13897277	0.11774253	0.21458413	0.42492755	NKAP
158800	ENSG00000101883	0	NA	NA	NA	NA	RHOXF1
55916	ENSG00000101888	246.131176	-0.3813111	0.16154048	0.00937922	0.03936614	NXT2
2986	ENSG00000101890	0.16735865	0.01897177	0.38482933	0.78225479	NA	GUCY2F
23439	ENSG00000101892	0	NA	NA	NA	NA	ATP1B4
NA	ENSG00000101898	86.7454913	-0.0860214	0.21870102	0.63179517	0.81371846	MCTS2P
79868	ENSG00000101901	683.594193	0.06532828	0.11994232	0.56698179	0.76856463	ALG13
5634	ENSG00000101911	475.108698	0.35185238	0.11533281	0.00134608	0.00763251	PRPS2
51311	ENSG00000101916	0	NA	NA	NA	NA	TLR8
56180	ENSG00000101928	92.0405251	0.17643848	0.25036746	0.36291733	0.59271319	MOSPD1
9949	ENSG00000101935	328.012833	-0.33796918	0.13776722	0.00834752	0.03578539	AMMECR1
91851	ENSG00000101938	312.971857	0.11204225	0.14185804	0.39466537	0.62391721	CHRDL1
64743	ENSG00000101940	994.151945	0.38241082	0.10077143	8.14E-05	0.0006333	WDR13
6839	ENSG00000101945	359.44314	-0.08528874	0.12221326	0.45907009	0.68244796	SUV39H1
9506	ENSG00000101951	0	NA	NA	NA	NA	PAGE4
8406	ENSG00000101955	42.9840849	2.33517754	0.41384787	1.03E-09	1.77E-08	SRPX
2742	ENSG00000101958	1.79095171	-0.09139811	0.39520062	0.15963497	NA	GLRA2
331	ENSG00000101966	912.973659	-0.16420707	0.12324762	0.15882709	0.34620189	XIAP
10735	ENSG00000101972	2311.39563	-0.07858498	0.10038819	0.41642898	0.64306408	STAG2
286410	ENSG00000101974	1348.4952	-0.2362311	0.09351714	0.00905476	0.03818204	ATP11C
4168	ENSG00000101977	4.46198858	0.09545885	0.38466838	0.36713226	0.59635697	MCF2
2158	ENSG00000101981	0	NA	NA	NA	NA	F9
215	ENSG00000101986	313.195619	-0.21020134	0.13006024	0.08509224	0.2213782	ABCD1
28952	ENSG00000101997	256.161266	0.06024273	0.14175741	0.64854948	0.82457939	CCDC22
778	ENSG00000102001	0.95304202	-0.03273545	0.3847849	0.49375742	NA	CACNA1F
6855	ENSG00000102003	1376.07315	-1.41708922	0.08770501	8.87E-60	1.15E-57	SYP
5355	ENSG00000102007	775.176751	0.51487338	0.09477741	2.13E-08	3.08E-07	PLP2
660	ENSG00000102010	7.61767746	-0.25943301	0.46913399	0.10292499	0.25477628	BMX
51213	ENSG00000102021	0	NA	NA	NA	NA	LUZP4
5358	ENSG00000102024	822.828975	0.52038038	0.09808653	4.25E-08	5.89E-07	PLS3

8260	ENSG00000102030	1372.37541	-0.13923065	0.08304536	0.08533862	0.22164134	NAA10
5973	ENSG00000102032	33.8540671	0.35872291	0.37438024	0.11765614	0.28127459	RENBP
2000	ENSG00000102034	281.291987	0.21914657	0.14014009	0.09237032	0.23501245	ELF4
6594	ENSG00000102038	2229.98147	0.09311589	0.06723513	0.15966559	0.34722847	SMARCA1
55613	ENSG00000102043	23.2148994	0.02973861	0.31648115	0.8701664	0.94333385	MTMR8
140462	ENSG00000102048	58.5978112	-0.18770397	0.26345659	0.34175981	0.57180929	ASB9
340554	ENSG00000102053	145.330464	0.06727546	0.1849043	0.67841704	0.84291038	ZC3H12B
5931	ENSG00000102054	2188.7971	-0.02667724	0.07409095	0.71267771	0.86169546	RBBP7
80316	ENSG00000102055	0	NA	NA	NA	NA	PPP1R2C
3750	ENSG00000102057	110.176786	0.11388144	0.20778693	0.51651209	0.73056247	KCND1
5956	ENSG00000102076	0	NA	NA	NA	NA	OPN1LW
9016	ENSG00000102078	198.834616	0.22627274	0.15945959	0.11846532	0.28258066	SLC25A14
2332	ENSG00000102081	761.740246	-0.04999539	0.10324062	0.6144676	0.8019364	FMR1
11040	ENSG00000102096	187.982854	0.19443375	0.16920249	0.2005334	0.40575148	PIM2
10389	ENSG00000102098	535.378406	-0.01608737	0.10758476	0.8747425	0.94484382	SCML2
7355	ENSG00000102100	472.578149	0.15600025	0.1156445	0.15658708	0.34316665	SLC35A2
10084	ENSG00000102103	713.883311	-0.17054238	0.10244604	0.08321657	0.21772162	PQBP1
6247	ENSG00000102104	0	NA	NA	NA	NA	RS1
27344	ENSG00000102109	1275.65621	0.12021859	0.09028977	0.17061444	0.36253512	PCSK1N
2010	ENSG00000102119	862.468128	-0.08349478	0.09407135	0.35956877	0.58949503	EMD
6901	ENSG00000102125	511.572311	0.29592334	0.11473004	0.00671773	0.02986326	TAZ
282808	ENSG00000102128	6.67419048	0.02109424	0.35409178	0.88246636	0.94823075	RAB40AL
5230	ENSG00000102144	5803.02148	-0.0510183	0.08258881	0.526053	0.73808697	PGK1
2623	ENSG00000102145	0	NA	NA	NA	NA	GATA1
84061	ENSG00000102158	634.400992	1.02428972	0.13770614	1.28E-14	3.38E-13	MAGT1
6611	ENSG00000102172	1888.97308	-0.26050773	0.07764605	0.00059505	0.00375861	SMS
5251	ENSG00000102174	7.62091827	0.1474708	0.38842704	0.30963586	0.53743835	PHEX
8266	ENSG00000102178	1100.71638	-0.22827888	0.08557316	0.0060818	0.02744208	UBL4A
83692	ENSG00000102181	657.032488	0.01626461	0.11399078	0.88163999	0.94773789	CD99L2
8411	ENSG00000102189	363.518661	-0.22898818	0.13705681	0.07262105	0.19650869	EEA1
9248	ENSG00000102195	0	NA	NA	NA	NA	GPR50
6102	ENSG00000102218	263.534204	0.04853497	0.1480079	0.72327241	0.86773445	RP2
9767	ENSG00000102221	608.972063	-0.10510298	0.12242614	0.36410326	0.59381904	JADE3
5127	ENSG00000102225	2731.47218	-0.02692284	0.06320303	0.66563564	0.83494641	CDK16
8237	ENSG00000102226	3892.40302	0.00249779	0.06005729	0.96719744	0.98759962	USP11
9468	ENSG00000102230	598.524961	-0.19628127	0.10787561	0.0568798	0.16435994	PCYT1B
680	ENSG00000102239	0.70793655	0.02847024	0.38384472	0.55549389	NA	BRS3
27336	ENSG00000102241	2153.36029	0.09752014	0.06752876	0.14247097	0.3212532	HTATSF1

51442	ENSG00000102243	0	NA	NA	NA	NA	VGLL1
959	ENSG00000102245	0	NA	NA	NA	NA	CD40LG
7076	ENSG00000102265	150.459202	1.228408	0.20607472	2.48E-10	4.56E-09	TIMP1
56062	ENSG00000102271	0	NA	NA	NA	NA	KLHL4
2564	ENSG00000102287	0	NA	NA	NA	NA	GABRE
27328	ENSG00000102290	0.173124	-0.01911262	0.38486262	0.84537521	NA	PCDH11X
2245	ENSG00000102302	716.470669	-0.04572344	0.09579539	0.62131965	0.80672374	FGD1
5303	ENSG00000102309	588.86841	0.26654888	0.10655036	0.00902359	0.03811541	PIN4
64840	ENSG00000102312	278.776128	-0.43367582	0.14995658	0.00170472	0.00935593	PORCN
347365	ENSG00000102313	0.173733	0.01914545	0.38484597	0.78225479	NA	ITIH6
10916	ENSG00000102316	2463.78771	0.43242794	0.06885522	1.67E-10	3.12E-09	MAGED2
5935	ENSG00000102317	2710.61691	0.20240909	0.06681475	0.00207695	0.01104441	RBM3
11279	ENSG00000102349	0.34108768	0.02362635	0.38535337	0.62411761	NA	KLF8
27286	ENSG00000102359	0.67055544	-0.02605768	0.38455641	0.57446224	NA	SRPX2
94121	ENSG00000102362	35.8506404	1.69096942	0.62903121	0.00027321	0.00188143	SYTL4
158866	ENSG00000102383	216.458728	-0.64921886	0.17468573	4.95E-05	0.00040485	ZDHHC15
2491	ENSG00000102384	439.294672	-0.68140177	0.14277006	4.24E-07	5.12E-06	CENPI
1821	ENSG00000102385	75.4779277	-0.24977137	0.25624398	0.20687425	0.41408243	DRP2
54457	ENSG00000102387	23.486055	2.07962403	0.58891166	2.44E-05	0.00021495	TAF7L
51260	ENSG00000102390	301.715291	-0.15950641	0.14572175	0.2356418	0.45240308	PBDC1
2717	ENSG00000102393	341.413449	0.20102139	0.13236529	0.10511755	0.25849433	GLA
51566	ENSG00000102401	723.026887	0.08946246	0.09642499	0.33793967	0.56821327	ARMCX3
56271	ENSG00000102409	1193.06255	-0.10084079	0.07703352	0.18103496	0.37823647	BEX4
80183	ENSG00000102445	5.30208087	0.42016204	0.79770428	0.00938976	0.03940202	RUBCNL
259232	ENSG00000102452	25.5953003	-0.17431945	0.35677941	0.35029124	0.58064819	NALCN
2259	ENSG00000102466	428.088339	-0.95646215	0.15840032	2.10E-10	3.90E-09	FGF14
3356	ENSG00000102468	0	NA	NA	NA	NA	HTR2A
54602	ENSG00000102471	906.871324	-0.3523271	0.0961275	0.00014732	0.00107813	NDFIP2
10673	ENSG00000102524	8.06288748	0.21809965	0.42860293	0.16257573	0.35136193	TNFSF13B
22862	ENSG00000102531	1829.28161	0.28798878	0.0790609	0.00019273	0.00137548	FNDC3A
2862	ENSG00000102539	1.67394001	-0.02312954	0.37630969	0.78059233	NA	MLNR
81602	ENSG00000102543	87.3023062	-0.08671801	0.22035243	0.62987047	0.8127361	CDADC1
81617	ENSG00000102547	119.28817	-0.1809971	0.20107977	0.29127769	0.51740974	CAB39L
688	ENSG00000102554	35.755824	0.47902059	0.42416816	0.0579709	0.16694071	KLF5
8428	ENSG00000102572	1466.41438	-0.3366469	0.08532064	4.96E-05	0.00040539	STK24
54	ENSG00000102575	4.07344245	0.11812331	0.39272409	0.29278881	0.51892337	ACP5
5611	ENSG00000102580	625.546171	0.42401849	0.11825925	0.00016272	0.00118041	DNAJC3
55757	ENSG00000102595	898.882491	0.39615583	0.09454988	1.49E-05	0.00013723	UGGT2

8874	ENSG00000102606	2768.32145	0.24879778	0.08122148	0.00169337	0.00931425	ARHGEF7
2254	ENSG00000102678	0	NA	NA	NA	NA	FGF9
6445	ENSG00000102683	2.48861994	0.02683769	0.37316045	0.78221811	NA	SGCG
143	ENSG00000102699	1258.0394	0.68449711	0.08010232	3.25E-18	1.10E-16	PARP4
55578	ENSG00000102710	1550.94222	-0.18421014	0.07227843	0.00935765	0.03928391	SUPT20H
10240	ENSG00000102738	288.457907	-0.08502912	0.13365273	0.49651262	0.714811	MRPS31
10166	ENSG00000102743	880.941335	-0.32377112	0.09480233	0.00040822	0.00269522	SLC25A15
3839	ENSG00000102753	1100.01463	-0.31459133	0.09765559	0.00082847	0.0050112	KPNA3
2321	ENSG00000102755	0.18922458	0.01954053	0.38488496	0.78225479	NA	FLT1
28984	ENSG00000102760	0.99332863	0.03056551	0.38199533	0.61161126	NA	RGCC
23078	ENSG00000102763	818.33271	0.04472657	0.10170138	0.64923437	0.82505828	VWA8
160851	ENSG00000102780	754.186215	-0.32138286	0.10824957	0.0019086	0.01026723	DGKH
84056	ENSG00000102781	574.554527	0.13016808	0.11883175	0.24917976	0.46855526	KATNAL1
26512	ENSG00000102786	875.082191	-0.22090529	0.15492832	0.11826561	0.28218758	INTS6
730249	ENSG00000102794	0	NA	NA	NA	NA	ACOD1
79758	ENSG00000102796	108.226115	0.10958223	0.21871253	0.5445506	0.75235966	DHRS12
84935	ENSG00000102802	0	NA	NA	NA	NA	MEDAG
8848	ENSG00000102804	1675.84603	-0.12895989	0.07035058	0.06191399	0.17555717	TSC22D1
1203	ENSG00000102805	357.291988	-0.01815696	0.13808317	0.88691372	0.95029993	CLN5
10562	ENSG00000102837	0.53441252	0.01348423	0.38343732	0.74516574	NA	OLFM4
10232	ENSG00000102854	1.39831843	-0.02778483	0.38200337	0.62527136	NA	MSLN
23295	ENSG00000102858	1938.72521	-0.0145803	0.08027843	0.85180403	0.93275912	MGRN1
23361	ENSG00000102870	1787.40838	0.04586654	0.06870056	0.49855431	0.71624312	ZNF629
8717	ENSG00000102871	156.746972	0.24290761	0.17820971	0.12362917	0.29131232	TRADD
3299	ENSG00000102878	826.530954	0.54092346	0.12721677	7.29E-06	7.11E-05	HSF4
11151	ENSG00000102879	502.56025	-0.41569773	0.15629626	0.00364183	0.01785369	CORO1A
5595	ENSG00000102882	834.722044	-0.34540194	0.09719756	0.00022977	0.00160857	MAPK3
79153	ENSG00000102886	47.8032838	-0.03632281	0.28952577	0.84757748	0.93065124	GDPD3
79767	ENSG00000102890	80.1333897	0.27958844	0.23805532	0.14549036	0.32597939	ELMO3
84560	ENSG00000102891	0	NA	NA	NA	NA	MT4
5257	ENSG00000102893	1517.09611	-0.44941355	0.08688561	1.06E-07	1.39E-06	PHKB
57149	ENSG00000102897	378.076513	-0.22391662	0.12748172	0.06163228	0.17502196	LYRM1
10204	ENSG00000102898	2598.93222	0.03530485	0.07014995	0.60959658	0.79803651	NUTF2
9688	ENSG00000102900	1203.74082	-0.02145252	0.09132836	0.80794946	0.9131433	NUP93
80152	ENSG00000102901	1059.50045	0.12144333	0.09567051	0.18990749	0.39109037	CENPT
55815	ENSG00000102904	46.5469561	0.18906647	0.28000519	0.3493254	0.58004303	TSNAXIP1
10725	ENSG00000102908	1372.82917	-0.40400531	0.07828227	1.29E-07	1.68E-06	NFAT5
83752	ENSG00000102910	1480.28635	-0.02441964	0.08649097	0.77108333	0.8951015	LONP2

9683	ENSG00000102921	768.559921	-0.22213848	0.14259283	0.09215718	0.23465072	N4BP1
869	ENSG00000102924	170.062844	-2.12330043	0.22375916	1.55E-22	6.58E-21	CBLN1
23568	ENSG00000102931	681.248065	-0.52303807	0.09954142	5.43E-08	7.42E-07	ARL2BP
51090	ENSG00000102934	46.9777224	-0.09836965	0.28000141	0.6122798	0.80024215	PLLP
23090	ENSG00000102935	4426.90019	-1.01248655	0.06354687	5.07E-58	6.24E-56	ZNF423
6367	ENSG00000102962	0	NA	NA	NA	NA	CCL22
1723	ENSG00000102967	354.921385	0.24625203	0.14267115	0.06242233	0.17651923	DHODH
6361	ENSG00000102970	0.80356287	-0.00576102	0.38152262	0.91108175	NA	CCL17
10664	ENSG00000102974	1757.74356	-0.22126013	0.08220583	0.00574412	0.02617441	CTCF
65057	ENSG00000102977	598.114928	-0.15925771	0.10654591	0.11883673	0.28318716	ACD
5432	ENSG00000102978	1745.46748	-0.1315888	0.06941159	0.05368407	0.15715539	POLR2C
50855	ENSG00000102981	216.805903	-0.52592743	0.166087	0.0005249	0.0033637	PARD6A
55565	ENSG00000102984	326.538253	0.31922235	0.13785824	0.01287144	0.05105345	ZNF821
4324	ENSG00000102996	426.957236	-0.10644153	0.13864294	0.40903171	0.63708503	MMP15
79650	ENSG00000103005	1049.35938	-0.12223642	0.11744771	0.27292324	0.49654259	USB1
80777	ENSG00000103018	1024.19358	-0.43553421	0.10677557	2.11E-05	0.00018856	CYB5B
29070	ENSG00000103021	116.654725	0.86382335	0.24234057	5.22E-05	0.00042488	CCDC113
221191	ENSG00000103023	0	NA	NA	NA	NA	PRSS54
4832	ENSG00000103024	514.417812	-0.10849988	0.11290758	0.31373646	0.54159326	NME3
65009	ENSG00000103034	1161.56114	-1.07306586	0.10421255	8.70E-26	4.23E-24	NDRG4
5713	ENSG00000103035	1867.66183	0.08480339	0.07635496	0.25750434	0.47871871	PSMD7
79918	ENSG00000103037	1126.03538	0.04886008	0.0872294	0.56527501	0.76733419	SETD6
55238	ENSG00000103042	497.48211	0.40919707	0.12421093	0.00049153	0.00317759	SLC38A7
55697	ENSG00000103043	1155.49939	0.2173634	0.0889264	0.01197563	0.0481647	VAC14
3038	ENSG00000103044	61.3755934	-0.00243412	0.2708496	0.9879815	0.99589897	HAS3
79613	ENSG00000103047	288.963022	-0.12548823	0.13815422	0.32902372	0.55760064	TANGO6
25839	ENSG00000103051	1641.24928	-0.00066972	0.08305817	0.99212333	0.99756121	COG4
55512	ENSG00000103056	608.217601	-2.85064854	0.14160543	1.64E-91	4.47E-89	SMPD3
84138	ENSG00000103061	678.804809	0.08300581	0.10671646	0.41835618	0.6444567	SLC7A6OS
9057	ENSG00000103064	1022.72369	-0.37359331	0.11516114	0.00064654	0.00403246	SLC7A6
23659	ENSG00000103066	182.265453	0.32530058	0.19580744	0.05537548	0.16106869	PLA2G15
80004	ENSG00000103067	3.33536588	0.03598659	0.37249045	0.72501701	0.86844186	ESRP2
79152	ENSG00000103089	2.42508199	0.0272696	0.37414243	0.76773843	NA	FA2H
79726	ENSG00000103091	993.594099	-0.03032072	0.11518757	0.76391579	0.89137736	WDR59
22879	ENSG00000103111	1175.33812	0.12903523	0.08264783	0.10933108	0.26588975	MON1B
56942	ENSG00000103121	446.031248	-0.05168101	0.12436999	0.65965024	0.83126657	CMC2
8312	ENSG00000103126	848.654012	-0.04571919	0.08642032	0.58651023	0.78217133	AXIN1
54985	ENSG00000103145	264.167571	-0.07709639	0.14905581	0.572488	0.7723153	HCFC1R1

8131	ENSG00000103148	1039.49834	0.2361664	0.08691899	0.00520395	0.02401637	NPRL3
23417	ENSG00000103150	343.744796	0.04509051	0.13003388	0.71312755	0.86187687	MLYCD
4350	ENSG00000103152	522.822802	-0.16423011	0.10555157	0.10456761	0.25755703	MPG
54550	ENSG00000103154	24.6831528	-0.20913045	0.33894113	0.30367293	0.53104502	NECAB2
83693	ENSG00000103160	788.155588	-0.20777089	0.10252269	0.03492402	0.11312533	HSDL1
9013	ENSG00000103168	1941.41263	0.56608168	0.07114988	6.02E-16	1.74E-14	TAF1C
51172	ENSG00000103174	175.838293	0.52151151	0.23055737	0.00732904	0.03209895	NAGPA
58189	ENSG00000103175	0.39948737	-0.02383611	0.38540824	0.65894985	NA	WFDC1
9717	ENSG00000103184	0.81326998	-0.03812067	0.38409693	0.49180648	NA	SEC14L5
23406	ENSG00000103187	129.659037	-0.68803119	0.21521329	0.00029602	0.00202169	COTL1
9100	ENSG00000103194	1672.9629	-0.20139785	0.07046206	0.00358764	0.01764473	USP10
83716	ENSG00000103196	5.92591177	-0.0487854	0.36493407	0.6923528	0.85128158	CRISPLD2
7249	ENSG00000103197	4178.26911	0.39324987	0.07395901	5.77E-08	7.85E-07	TSC2
26048	ENSG00000103199	293.649293	0.17253876	0.14946987	0.20956712	0.4178638	ZNF500
NA	ENSG00000103200	0.15069912	-0.01848503	0.38480575	0.84537521	NA	
4833	ENSG00000103202	5414.19112	0.48510814	0.05703059	7.67E-18	2.51E-16	NME4
4363	ENSG00000103222	3113.05597	0.01322972	0.07831911	0.86458282	0.94007513	ABCC1
408050	ENSG00000103226	1509.05509	0.36858387	0.10998059	0.00045507	0.00297583	NOMO3
64788	ENSG00000103227	129.367082	0.94011659	0.25576637	2.98E-05	0.00025691	LMF1
2294	ENSG00000103241	0	NA	NA	NA	NA	FOXF1
64428	ENSG00000103245	598.635149	0.14135779	0.1060087	0.16488749	0.35458137	CIAO3
64779	ENSG00000103248	277.677442	0.04521009	0.14323689	0.73501733	0.87400323	MTHFSD
1186	ENSG00000103249	1551.98637	0.35487566	0.11168227	0.00087096	0.00525065	CLCN7
84264	ENSG00000103253	481.766743	-0.32107725	0.1189771	0.00440389	0.02093774	HAGHL
65990	ENSG00000103254	550.031759	-0.10292786	0.10944246	0.32508844	0.55302396	ANTKMT
8140	ENSG00000103257	2442.24281	0.12045461	0.2283467	0.51688463	0.7308667	SLC7A5
79006	ENSG00000103260	426.363639	-0.29397398	0.12106104	0.01016273	0.04193604	METRN
79791	ENSG00000103264	575.892449	-0.43729977	0.12857459	0.00030398	0.00207106	FBXO31
10273	ENSG00000103266	1703.19734	0.13071617	0.06843346	0.05221966	0.15396613	STUB1
9028	ENSG00000103269	40.980964	0.34462735	0.35477636	0.12785522	0.29839402	RHBDL1
4682	ENSG00000103274	319.944611	-0.12331524	0.12705202	0.30255974	0.52987991	NUBP1
7329	ENSG00000103275	2709.32127	0.09444352	0.07215936	0.18277983	0.38071934	UBE2I
7783	ENSG00000103310	0	NA	NA	NA	NA	ZP2
4210	ENSG00000103313	0.43880265	-0.02654222	0.38563237	0.5780147	NA	MEFV
1428	ENSG00000103316	30.6858606	1.66609361	0.48252735	3.55E-05	0.00030166	CRYM
29904	ENSG00000103319	898.248235	-0.01763027	0.0880033	0.83243042	0.92392457	EEF2K
6650	ENSG00000103326	1877.18994	-0.12000909	0.07448696	0.1004517	0.25008931	CAPN15
9780	ENSG00000103335	289.837051	0.34181635	0.21422313	0.05875568	0.16840693	PIEZO1

2935	ENSG00000103342	3136.39704	-0.27895837	0.06601548	1.74E-05	0.00015765	GSPT1
7727	ENSG00000103343	169.073224	0.05375901	0.17513176	0.73122174	0.87162779	ZNF174
23059	ENSG00000103351	295.634513	0.37487461	0.17054212	0.01448906	0.05602473	CLUAP1
56061	ENSG00000103353	2804.94577	-0.06932122	0.07306024	0.33325272	0.56270076	UBFD1
260429	ENSG00000103355	0.69252051	0.04040802	0.38716153	0.35603027	NA	PRSS33
124454	ENSG00000103356	779.776589	-0.12402963	0.10938695	0.23563538	0.45240308	EARS2
6923	ENSG00000103363	1768.3665	0.0555295	0.07140465	0.42908808	0.65470334	ELOB
23062	ENSG00000103365	1888.0715	-0.09911019	0.09263951	0.26929979	0.49229778	GGA2
343	ENSG00000103375	0.18922458	0.01954053	0.38488496	0.78225479	NA	AQP8
55313	ENSG00000103381	343.950513	-0.51074916	0.13873307	8.49E-05	0.00065647	CPPED1
57478	ENSG00000103404	520.118018	0.01430674	0.13226296	0.91291677	0.96282372	USP31
3163	ENSG00000103415	447.150211	-0.4537484	0.12928373	0.00019552	0.00139337	HMOX2
9093	ENSG00000103423	1003.13788	-0.06867803	0.10116684	0.48074271	0.70158112	DNAJA3
100529144	ENSG00000103426	8.98066911	0.01442512	0.36498042	0.90535714	0.95855092	CORO7-PAM16
51283	ENSG00000103429	1304.93048	-0.08874316	0.08968004	0.30823974	0.53579716	BFAR
6299	ENSG00000103449	0.34109801	0.02362643	0.38535338	0.62411213	NA	SALL1
27324	ENSG00000103460	0	NA	NA	NA	NA	TOX3
NA	ENSG00000103472	4.57064213	0.18613322	0.42683604	0.14252794	0.32128606	RRN3P2
5934	ENSG00000103479	1341.77734	0.24163395	0.1097922	0.02129601	0.07661794	RBL2
23475	ENSG00000103485	4140.83718	0.29469126	0.05378661	3.04E-08	4.29E-07	QPRT
64131	ENSG00000103489	1519.40294	0.0439461	0.07587458	0.55440068	0.76030342	XYLT1
29108	ENSG00000103490	3.95808471	0.11855277	0.39216425	0.30398098	0.53126299	PYCARD
23322	ENSG00000103494	456.668813	-0.28310178	0.12724634	0.01796475	0.06684557	RPGRIP1L
4150	ENSG00000103495	6983.48869	-0.12673078	0.05552897	0.02096223	0.075589	MAZ
6810	ENSG00000103496	342.728771	0.14085091	0.13638263	0.26796606	0.49058288	STX4
10423	ENSG00000103502	1110.67433	-0.10106718	0.07980516	0.19488313	0.39811381	CDIPT
10295	ENSG00000103507	960.429245	-0.01926961	0.0906522	0.82644076	0.92144622	BCKDK
84148	ENSG00000103510	1171.60574	0.06615195	0.08036128	0.39967621	0.62831531	KAT8
23420	ENSG00000103512	3207.27318	0.21057565	0.07849686	0.00608157	0.02744208	NOMO1
50615	ENSG00000103522	2.94641292	-0.14308076	0.4133539	0.12759414	0.29804636	IL21R
51760	ENSG00000103528	93.4967349	1.19665109	0.26704438	6.99E-07	8.13E-06	SYT17
79838	ENSG00000103534	7.16324248	-0.03589252	0.36721689	0.74227413	0.87857766	TMC5
9738	ENSG00000103540	1592.94939	-0.49478901	0.08216883	7.05E-10	1.24E-08	CCP110
57020	ENSG00000103544	777.216493	0.25203797	0.10289561	0.0108141	0.04419191	VPS35L
6530	ENSG00000103546	5275.73957	-0.10465368	0.0717609	0.13710005	0.31261521	SLC6A2
9810	ENSG00000103549	2884.66801	-0.15863908	0.07209709	0.02486927	0.0867909	RNF40
400506	ENSG00000103550	1587.17162	-0.02385874	0.09550838	0.79553101	0.90599479	KNOP1
366	ENSG00000103569	0	NA	NA	NA	NA	AQP9

79719	ENSG00000103591	780.42015	-0.15473037	0.12645408	0.19362144	0.39643441	AAGAB
64799	ENSG00000103599	49.1662352	0.9957656	0.43678653	0.00201393	0.01076702	IQCH
114294	ENSG00000103642	69.9073986	0.3237777	0.28081185	0.12270302	0.28988186	LACTB
10391	ENSG00000103647	6.95235233	-0.22163614	0.43040914	0.16546037	0.35554356	CORO2B
1445	ENSG00000103653	1820.0049	-0.28521194	0.07713368	0.0001544	0.00112413	CSK
8925	ENSG00000103657	2287.39335	-0.04168098	0.21799915	0.79169153	0.90499209	HERC1
9325	ENSG00000103671	350.395349	0.15707088	0.14451705	0.23993468	0.4579339	TRIP4
123263	ENSG00000103707	228.043826	0.05474606	0.15285529	0.69723208	0.85387893	MTFMT
51285	ENSG00000103710	7.83980473	0.04030669	0.36236383	0.75359895	0.88490786	RASL12
8120	ENSG00000103723	1523.16208	-1.53505099	0.09896011	2.18E-55	2.58E-53	AP3B2
23205	ENSG00000103740	18.8608338	2.64003515	0.71574972	7.43E-06	7.23E-05	ACSBG1
57722	ENSG00000103742	173.034221	1.10050958	0.22808768	1.53E-07	1.98E-06	IGDCC4
8766	ENSG00000103769	2517.00483	0.06105983	0.06911611	0.36972549	0.59909782	RAB11A
1512	ENSG00000103811	277.809276	0.41745833	0.17040233	0.00658082	0.02935328	CTSH
NA	ENSG00000103832	4.28220094	0.11343987	0.38594492	0.35628774	0.58627757	GOLGA8UP
64927	ENSG00000103852	137.231816	-0.31098491	0.23454699	0.10235373	0.25371038	TTC23
80381	ENSG00000103855	2547.14906	0.00995261	0.07860553	0.89886752	0.95556135	CD276
2184	ENSG00000103876	183.167702	0.07963763	0.17383896	0.60849258	0.79733788	FAH
57214	ENSG00000103888	8.45926903	0.01455865	0.34863819	0.92349587	0.96825748	CEMIP
26015	ENSG00000103932	1381.73058	0.07531968	0.09496958	0.41347837	0.64069125	RPAP1
9455	ENSG00000103942	532.587042	-0.26201489	0.12942926	0.03084399	0.10243712	HOMER2
30844	ENSG00000103966	485.934167	0.12531188	0.10987244	0.23387523	0.45084382	EHD4
25963	ENSG00000103978	826.25512	-0.12593993	0.10598751	0.21534274	0.42596211	TMEM87A
64397	ENSG00000103994	1876.3997	-0.17124234	0.08708835	0.04305124	0.13277051	ZNF106
22995	ENSG00000103995	843.455649	0.03316584	0.08931943	0.7035514	0.85751377	CEP152
79895	ENSG00000104043	3.22076333	0.01686987	0.36882402	0.87540966	0.94499851	ATP8B4
4948	ENSG00000104044	69.3778356	0.33820213	0.27303287	0.10450944	0.25747762	OCA2
56986	ENSG00000104047	376.747383	0.16439454	0.13171798	0.1830684	0.38124037	DTWD1
9333	ENSG00000104055	0	NA	NA	NA	NA	TGM5
23359	ENSG00000104059	26.6067571	1.52097581	0.53204754	0.00025513	0.00177169	FAM189A1
2553	ENSG00000104064	497.977581	-0.02371034	0.11532934	0.82850558	0.92220943	GABPB1
7082	ENSG00000104067	1608.66501	0.39924292	0.08146042	5.14E-07	6.12E-06	TJP1
90427	ENSG00000104081	476.703531	0.87180698	0.13445149	1.46E-11	3.06E-10	BMF
23312	ENSG00000104093	2235.89226	-0.37328248	0.09735982	7.08E-05	0.00056184	DMXL2
29106	ENSG00000104112	1792.1521	-1.84430021	0.07460883	4.75E-136	2.69E-133	SCG3
55192	ENSG00000104129	343.128703	-0.00646089	0.13714448	0.95867518	0.98300251	DNAJC17
8669	ENSG00000104131	1356.53326	-0.39832395	0.09225627	8.32E-06	8.03E-05	EIF3J
80208	ENSG00000104133	1504.95711	0.07148853	0.11550991	0.51701038	0.7309546	SPG11

171177	ENSG00000104140	30.2359747	-0.11605393	0.30708708	0.54871276	0.75521756	RHOV
57617	ENSG00000104142	543.924655	-0.24730267	0.10745085	0.01601509	0.06081137	VPS18
11339	ENSG00000104147	182.204259	-0.10766154	0.1656696	0.47007374	0.69237291	OIP5
7782	ENSG00000104154	239.713823	-0.23339637	0.16014463	0.10779283	0.26339391	SLC30A4
26258	ENSG00000104164	1252.82917	0.07974606	0.0876039	0.34954202	0.58004303	BLOC1S6
50804	ENSG00000104177	4356.15622	0.07864104	0.06780804	0.23922878	0.45707811	MYEF2
23678	ENSG00000104205	57.6788381	0.85706589	0.41682737	0.00421947	0.0201916	SGK3
5157	ENSG00000104213	18.0086244	-0.14277316	0.33440439	0.45089835	0.67429441	PDGFRL
79848	ENSG00000104218	445.260742	-0.03041667	0.11239699	0.77641222	0.89723881	CSPP1
51201	ENSG00000104219	104.63722	2.27553706	0.42074968	3.31E-09	5.31E-08	ZDHHC2
55290	ENSG00000104221	221.391992	-0.08872925	0.15341712	0.52989034	0.74155494	BRF2
23087	ENSG00000104228	536.377267	0.54066325	0.11823005	1.67E-06	1.81E-05	TRIM35
79752	ENSG00000104231	377.358063	-0.25310775	0.14485227	0.05811561	0.16717612	ZFAND1
6101	ENSG00000104237	0	NA	NA	NA	NA	RP1
760	ENSG00000104267	0	NA	NA	NA	NA	CA2
7976	ENSG00000104290	929.464841	-0.41963843	0.25949469	0.04127316	0.12847387	FZD3
55756	ENSG00000104299	729.032095	0.2913278	0.1068396	0.00441904	0.02099971	INTS9
8767	ENSG00000104312	247.552701	-0.05502319	0.14569214	0.68210782	0.84486322	RIPK2
2138	ENSG00000104313	2015.84856	0.32261074	0.07414888	8.88E-06	8.50E-05	EYA1
4683	ENSG00000104320	948.169285	-0.0925804	0.09567375	0.31702375	0.54483775	NBN
8989	ENSG00000104321	0.34457596	0.00783944	0.38356596	0.90071784	NA	TRPA1
10404	ENSG00000104324	3.07963713	-0.21744607	0.47081029	0.02161376	0.07746569	CPQ
1666	ENSG00000104325	344.454656	-0.2755874	0.12677024	0.02081852	0.07530345	DECR1
793	ENSG00000104327	151.402464	-2.19054077	0.24666914	3.43E-20	1.30E-18	CALB1
54928	ENSG00000104331	1580.25284	-0.11094048	0.07744487	0.14317482	0.32228142	BPNT2
6422	ENSG00000104332	377.779061	0.41458587	0.12478754	0.00043624	0.00286401	SFRP1
55353	ENSG00000104341	6485.63574	-0.09738504	0.05504231	0.07361393	0.1986805	LAPTM4B
55284	ENSG00000104343	382.962884	0.03701963	0.12693465	0.75829836	0.8876941	UBE2W
10940	ENSG00000104356	528.941448	0.0594978	0.11296476	0.58238426	0.77918294	POP1
79815	ENSG00000104361	172.971414	0.38032792	0.27807348	0.07204343	0.19555978	NIPAL2
3551	ENSG00000104365	680.684965	0.36794763	0.12102619	0.00132693	0.00753901	IKBKB
5327	ENSG00000104368	215.942327	0.00016386	0.17538809	0.99893658	0.99962651	PLAT
56704	ENSG00000104369	128.574634	0.39543546	0.23940187	0.04314362	0.13291451	JPH1
27121	ENSG00000104371	0	NA	NA	NA	NA	DKK4
6788	ENSG00000104375	190.58647	0.74113877	0.19322028	2.39E-05	0.00021102	STK3
54332	ENSG00000104381	2396.24074	-0.53982913	0.06756072	4.89E-16	1.43E-14	GDAP1
5862	ENSG00000104388	1310.00354	0.09309453	0.07638096	0.21373426	0.4238788	RAB2A
3646	ENSG00000104408	4660.54109	-0.18063782	0.0614454	0.00288172	0.01462605	EIF3E

9694	ENSG00000104412	203.909896	0.16452964	0.17000494	0.28165064	0.50647591	EMC2
54845	ENSG00000104413	0	NA	NA	NA	NA	ESRP1
8840	ENSG00000104415	0	NA	NA	NA	NA	CCN4
10397	ENSG00000104419	353.489477	0.46197159	0.18580723	0.0050867	0.02356843	NDRG1
51101	ENSG00000104427	421.326762	-0.02877555	0.13421326	0.81811881	0.9171248	ZC2HC1A
3574	ENSG00000104432	54.9596824	0.22766027	0.29482701	0.27088775	0.4942105	IL7
11075	ENSG00000104435	11987.5147	-0.51868594	0.04679702	5.58E-29	3.09E-27	STMN2
55156	ENSG00000104442	711.77921	-0.17220515	0.11873549	0.12591388	0.29508254	ARMC1
7227	ENSG00000104447	367.502097	1.82247724	0.21013736	2.27E-19	8.23E-18	TRPS1
6674	ENSG00000104450	91.0732253	-0.09493713	0.27111921	0.62221279	0.80700149	SPAG1
54108	ENSG00000104472	435.666626	0.24380681	0.11726056	0.0286976	0.09699494	CHRAC1
83988	ENSG00000104490	620.887785	-0.11818716	0.10788246	0.25056698	0.47053941	NCALD
64089	ENSG00000104497	144.522117	0.29237752	0.2070464	0.09697867	0.24355834	SNX16
2765	ENSG00000104499	0	NA	NA	NA	NA	GML
51366	ENSG00000104517	3686.98439	-0.01695471	0.08042515	0.82793891	0.9221528	UBR5
79792	ENSG00000104518	33.1969154	1.08759366	0.47920974	0.00182007	0.00986885	GSDMD
7264	ENSG00000104522	658.572384	-0.04081654	0.09799697	0.66576876	0.83494641	GFUS
65263	ENSG00000104524	181.225178	0.03598422	0.16642263	0.81153354	0.9146404	PYCR3
1936	ENSG00000104529	4992.97284	0.51877493	0.06466629	4.03E-16	1.18E-14	EEF1D
312	ENSG00000104537	0	NA	NA	NA	NA	ANXA13
6713	ENSG00000104549	1614.22143	-0.46481384	0.10494724	4.05E-06	4.13E-05	SQLE
63898	ENSG00000104611	0	NA	NA	NA	NA	SH2D4A
55174	ENSG00000104613	734.921681	0.26424595	0.11721036	0.01756072	0.06560027	INTS10
90459	ENSG00000104626	573.400735	-0.05723098	0.10406003	0.56700617	0.76856463	ERI1
23516	ENSG00000104635	1790.01282	-0.13143344	0.07174145	0.06189596	0.175546	SLC39A14
66036	ENSG00000104643	909.656443	-0.10494222	0.09666104	0.260834	0.48273755	MTMR9
23484	ENSG00000104660	1470.40931	0.06908672	0.09693249	0.46469904	0.68746767	LEPROTL1
10671	ENSG00000104671	436.475235	0.00726629	0.11549524	0.94805863	0.97876335	DCTN6
203069	ENSG00000104679	678.907202	-0.26295381	0.11114275	0.01311452	0.05177953	R3HCC1
2936	ENSG00000104687	1025.24562	0.17317218	0.0975953	0.06627961	0.18422352	GSR
8797	ENSG00000104689	68.9457208	0.74476939	0.3226792	0.00327953	0.01631544	TNFRSF10A
7993	ENSG00000104691	207.248997	-0.33787064	0.16285509	0.02192379	0.0784433	UBXN8
5516	ENSG00000104695	927.225718	0.08275853	0.0848202	0.31750104	0.54538707	PPP2CB
157697	ENSG00000104714	396.876844	0.19606977	0.12902872	0.1059864	0.25995429	ERICH1
4741	ENSG00000104722	1929.70801	-2.26945602	0.07303193	3.12E-213	6.88E-210	NEFM
7991	ENSG00000104723	1628.14017	-0.18071425	0.07128959	0.00978201	0.04078904	TUSC3
9639	ENSG00000104728	670.239587	0.11527255	0.11902517	0.30827393	0.53579981	ARHGEF10
54758	ENSG00000104731	1196.97362	0.02262777	0.08005159	0.77413983	0.89604057	KLHDC4

4173	ENSG00000104738	3845.36866	-0.57615999	0.08518026	4.31E-12	9.42E-11	MCM4
2515	ENSG00000104755	0	NA	NA	NA	NA	ADAM2
54793	ENSG00000104756	431.681369	-0.05025345	0.15446524	0.716717	0.8636823	KCTD9
2267	ENSG00000104760	1.43737226	0.06748064	0.38989989	0.25958163	NA	FGL1
427	ENSG00000104763	1056.23164	-0.04582106	0.08622286	0.58519011	0.78119847	ASAH1
665	ENSG00000104765	1059.21575	-0.0113835	0.10793322	0.91015888	0.9614798	BNIP3L
4125	ENSG00000104774	920.056432	0.27429709	0.1076167	0.00774993	0.03359374	MAN2B1
3783	ENSG00000104783	2.34131822	-0.00470251	0.37368775	0.95649651	NA	KCNN4
7288	ENSG00000104804	11.4892263	0.08562113	0.35577992	0.57643737	0.77519944	TULP2
4924	ENSG00000104805	2506.53223	0.31350206	0.07887892	4.72E-05	0.00038923	NUCB1
27294	ENSG00000104808	10.8774244	0.33619746	0.48946143	0.10185517	0.25269559	DHDH
2997	ENSG00000104812	747.840621	-0.10758129	0.12039148	0.34434485	0.5746805	GYS1
11184	ENSG00000104814	17.8915069	-0.89013459	0.8232409	0.01378764	0.05387961	MAP4K1
114336	ENSG00000104818	0.52119901	0.02576573	0.38568143	0.51723676	NA	CGB2
1891	ENSG00000104823	1458.76941	0.01410593	0.08025931	0.85811921	0.93662789	ECH1
3191	ENSG00000104824	5508.62535	0.18317593	0.08708882	0.030564	0.10186289	HNRNPL
4793	ENSG00000104825	412.546982	0.16573775	0.12744042	0.16773066	0.35871254	NFKBIB
3972	ENSG00000104826	2.9063243	0.14738227	0.41059195	0.16731958	0.35828164	LHB
1082	ENSG00000104827	0	NA	NA	NA	NA	CGB3
10382	ENSG00000104833	1233.89011	-0.15916497	0.08497793	0.05432577	0.15863564	TUBB4A
54938	ENSG00000104835	466.92116	0.32803523	0.11344587	0.00241258	0.01257963	SARS2
3743	ENSG00000104848	1.6759716	0.04924646	0.38310892	0.48408204	NA	KCNA7
6625	ENSG00000104852	4804.28419	0.0988406	0.05476723	0.0688017	0.18908793	SNRNP70
1209	ENSG00000104853	1409.15141	0.01880145	0.07748187	0.80502626	0.91147183	CLPTM1
5971	ENSG00000104856	50.6508871	0.33024846	0.31403361	0.13214088	0.30511168	RELB
11129	ENSG00000104859	1149.24016	0.20869892	0.08568923	0.01233968	0.04936867	CLASRP
64130	ENSG00000104863	52.440105	-0.09861304	0.25024689	0.60521791	0.79522617	LIN7B
284352	ENSG00000104866	524.814086	0.08585905	0.11471187	0.43324494	0.65856418	PPP1R37
2217	ENSG00000104870	515.703439	0.77045285	0.13343003	1.53E-09	2.58E-08	FCGRT
55011	ENSG00000104872	1749.58597	0.13552098	0.09266068	0.13167873	0.30441691	PIH1D1
1158	ENSG00000104879	0.4692364	-0.00817449	0.38327906	0.85817344	NA	CKM
23370	ENSG00000104880	808.621081	0.07221607	0.09007756	0.40991557	0.63781075	ARHGEF18
10848	ENSG00000104881	202.826197	0.73170769	0.18899997	2.15E-05	0.00019198	PPP1R13L
92960	ENSG00000104883	34.1935822	-0.04575721	0.2789001	0.8110582	0.91443965	PEX11G
2068	ENSG00000104884	969.196055	0.10064232	0.09155144	0.25794238	0.47930849	ERCC2
84444	ENSG00000104885	2797.7624	0.27273742	0.07525157	0.00021415	0.00151359	DOT1L
55111	ENSG00000104886	1335.61883	0.10223033	0.08027286	0.19286878	0.39542384	PLEKHJ1
57030	ENSG00000104888	18.7966319	0.43233631	0.48703714	0.07678034	0.20499556	SLC17A7

10535	ENSG00000104889	932.711102	-0.041798	0.08555475	0.61546536	0.80271053	RNASEH2A
147700	ENSG00000104892	8.36904398	0.14546085	0.3828358	0.3388902	0.56911982	KLC3
951	ENSG00000104894	14.8774689	0.76095358	0.73133547	0.02136277	0.07678212	CD37
8175	ENSG00000104897	2372.12503	0.24847196	0.06561881	0.0001192	0.00088976	SF3A2
268	ENSG00000104899	1165.12369	0.15568889	0.0994192	0.1046155	0.25761097	AMH
27120	ENSG00000104901	6.10768604	0.3220163	0.56268075	0.03648897	0.11718772	DKKL1
4066	ENSG00000104903	232.510222	-0.66826346	0.15788735	5.57E-06	5.56E-05	LYL1
4946	ENSG00000104904	5274.2933	0.11694774	0.06390395	0.06345632	0.17887414	OAZ1
55621	ENSG00000104907	688.902107	0.17356539	0.09816853	0.06706797	0.18576413	TRMT1
8677	ENSG00000104915	1634.37593	-0.00661767	0.08546803	0.93597194	0.97325384	STX10
56729	ENSG00000104918	0	NA	NA	NA	NA	RETN
2208	ENSG00000104921	0	NA	NA	NA	NA	FCER2
1760	ENSG00000104936	1696.24342	0.44588108	0.07353757	6.32E-10	1.12E-08	DMPK
10332	ENSG00000104938	0	NA	NA	NA	NA	CLEC4M
81492	ENSG00000104941	0.45209736	-0.02505403	0.38558831	0.57865948	NA	RSPH6A
79735	ENSG00000104946	683.125049	-0.10012353	0.10596208	0.32300555	0.55059971	TBC1D17
259307	ENSG00000104951	4.43855707	-0.07733217	0.3796787	0.45685967	0.68033445	IL4I1
79816	ENSG00000104953	65.9501587	0.46158438	0.31037633	0.04294624	0.13257414	TLE6
81576	ENSG00000104957	641.126674	0.50499112	0.09916026	1.39E-07	1.80E-06	CCDC130
53635	ENSG00000104960	3381.01115	0.19070848	0.0734214	0.00807852	0.03481275	PTOV1
166	ENSG00000104964	7572.22837	0.15112174	0.05684465	0.00717899	0.03156719	TLE5
4858	ENSG00000104967	1285.95731	-0.47181032	0.08387695	7.97E-09	1.21E-07	NOVA2
6449	ENSG00000104969	2531.5619	0.02700088	0.0619394	0.65926261	0.83105722	SGTA
NA	ENSG00000104970	0	NA	NA	NA	NA	KIR3DX1
10859	ENSG00000104972	0	NA	NA	NA	NA	LILRB1
81857	ENSG00000104973	1482.16975	0.2441515	0.08161463	0.00216358	0.0114529	MED25
11024	ENSG00000104974	0	NA	NA	NA	NA	LILRA1
6618	ENSG00000104976	349.29738	-0.08190476	0.13455492	0.51467772	0.72937777	SNAPC2
28974	ENSG00000104979	1032.93863	0.0056874	0.08452261	0.94555548	0.97783794	C19orf53
10469	ENSG00000104980	802.281934	0.19676497	0.11094974	0.06336256	0.17866872	TIMM44
729440	ENSG00000104983	134.258878	0.3918308	0.20673453	0.02732696	0.09328405	CCDC61
9466	ENSG00000104998	168.631144	-0.00021712	0.16564001	0.99770294	0.99926338	IL27RA
55723	ENSG00000105011	551.846834	-0.3716119	0.11560378	0.00071339	0.00439826	ASF1B
7138	ENSG00000105048	176.301571	0.3758017	0.18161437	0.01965592	0.07192429	TNNT1
51231	ENSG00000105053	381.127147	0.1271205	0.13543918	0.31574289	0.54363784	VRK3
26017	ENSG00000105058	1680.30649	0.02761528	0.06913527	0.68559748	0.8470175	FAM32A
22870	ENSG00000105063	1913.09231	-0.07089794	0.07943887	0.36105306	0.59083575	PPP6R1
84167	ENSG00000105072	276.038766	0.17974651	0.14162369	0.1715053	0.3639214	C19orf44

9441	ENSG00000105085	242.03599	-0.07971715	0.17306952	0.60482911	0.79489995	MED26
93145	ENSG00000105088	1148.90318	0.510367	0.09104106	8.11E-09	1.24E-07	OLFM2
64926	ENSG00000105122	0.173124	-0.01911262	0.38486262	0.84537521	NA	RASAL3
10270	ENSG00000105127	669.854292	0.17884859	0.10456211	0.07486177	0.20117262	AKAP8
79852	ENSG00000105131	5.91914505	-0.00847415	0.35790332	0.94665672	0.97814213	EPHX3
10994	ENSG00000105135	806.898889	-0.12800556	0.09955942	0.18216727	0.3798422	ILVBL
79744	ENSG00000105136	336.966858	0.02389821	0.15975502	0.87041698	0.94340743	ZNF419
85360	ENSG00000105137	616.404977	0.42516935	0.10576987	2.84E-05	0.00024661	SYDE1
23581	ENSG00000105141	0	NA	NA	NA	NA	CASP14
6511	ENSG00000105143	0	NA	NA	NA	NA	SLC1A6
6795	ENSG00000105146	8.3096471	0.04946678	0.36509856	0.68779288	0.84840902	AURKC
10775	ENSG00000105171	359.798068	-0.2803506	0.12452029	0.01691669	0.06370327	POP4
898	ENSG00000105173	208.551069	-0.33312728	0.16327932	0.02407477	0.08462852	CCNE1
8725	ENSG00000105176	1283.768	0.02129027	0.08038038	0.78808619	0.90384589	URI1
9141	ENSG00000105185	776.066912	-0.03515331	0.10454709	0.72545943	0.86866734	PDCD5
84079	ENSG00000105186	683.861109	-0.10191596	0.12904551	0.40187634	0.63031025	ANKRD27
6217	ENSG00000105193	17040.4796	0.03104179	0.05094491	0.53908761	0.74843996	RPS16
92609	ENSG00000105197	1072.54668	-0.00771256	0.12123221	0.94433002	0.97728379	TIMM50
29124	ENSG00000105198	0	NA	NA	NA	NA	LGALS13
2091	ENSG00000105202	2975.15108	0.04397789	0.06645975	0.50265498	0.71947729	FBL
9149	ENSG00000105204	583.396581	0.44583872	0.13486505	0.00040909	0.00270004	DYRK1B
1178	ENSG00000105205	0	NA	NA	NA	NA	CLC
79935	ENSG00000105219	6.1674186	-0.00402588	0.3592137	0.97281311	0.98992269	CCNP
2821	ENSG00000105220	5333.95789	-0.24321658	0.08274472	0.00254953	0.01319319	GPI
208	ENSG00000105221	3228.23964	0.07235997	0.06185548	0.23473681	0.45159871	AKT2
23646	ENSG00000105223	3928.1534	0.2898498	0.05715497	2.91E-07	3.61E-06	PLD3
57716	ENSG00000105227	76.0454464	0.70859592	0.28736975	0.00243053	0.01265661	PRX
51588	ENSG00000105229	753.157385	-0.01143357	0.10860604	0.91166175	0.96237542	PIAS4
9253	ENSG00000105245	863.025092	0.51513071	0.09781822	5.29E-08	7.25E-07	NUMBL
10148	ENSG00000105246	0.29863895	-0.02292439	0.3852726	0.68753245	NA	EBI3
55702	ENSG00000105248	282.22158	-0.12272592	0.13686702	0.33598696	0.56592082	YJU2
56961	ENSG00000105251	3307.77644	0.85578663	0.06160514	1.25E-44	1.09E-42	SHD
1155	ENSG00000105254	1399.05263	-0.00036994	0.08015006	0.9955125	0.99852582	TBCB
79187	ENSG00000105255	2200.26818	0.15179893	0.0696151	0.02643182	0.09095225	FSD1
5438	ENSG00000105258	561.580346	0.13169617	0.11508117	0.23064573	0.44661441	POLR2I
728361	ENSG00000105261	0.52158967	0.01056479	0.38325086	0.77480829	NA	OVOL3
25999	ENSG00000105270	1576.31537	-0.20925843	0.10003076	0.02974619	0.0997008	CLIP3
23217	ENSG00000105278	16.9632831	-0.35428426	0.46811648	0.1052119	0.25869427	ZFR2

6510	ENSG00000105281	2467.97415	0.03413123	0.06936875	0.61760831	0.8042894	SLC1A5
25865	ENSG00000105287	1128.66572	0.12600585	0.08451452	0.126306	0.29571761	PRKD2
27134	ENSG00000105289	3.36002555	0.14026115	0.40566924	0.19658011	0.4001795	TJP3
333	ENSG00000105290	2864.85499	-0.35896198	0.06430552	1.41E-08	2.08E-07	APLP1
58509	ENSG00000105298	989.684773	0.21986816	0.08597153	0.00857766	0.03655871	CACTIN
26093	ENSG00000105321	537.579893	0.31462662	0.12496865	0.0075986	0.03309688	CCDC9
11100	ENSG00000105323	6661.45029	-0.07394747	0.05638779	0.1844054	0.38305969	HNRNPUL1
51343	ENSG00000105325	1417.35399	0.03182287	0.08406538	0.69872227	0.85472333	FZR1
27113	ENSG00000105327	366.350466	0.77445183	0.13262331	1.04E-09	1.79E-08	BBC3
7040	ENSG00000105329	62.5847985	0.36830055	0.28784079	0.08601088	0.2230953	TGFB1
22898	ENSG00000105339	259.964346	-0.36786737	0.18059886	0.02146655	0.07708533	DENND3
55101	ENSG00000105341	1118.90427	0.06036415	0.09421954	0.50923921	0.72487486	DMAC2
1089	ENSG00000105352	0	NA	NA	NA	NA	CEACAM4
10226	ENSG00000105355	665.722904	0.27397541	0.09957737	0.0042911	0.02048009	PLIN3
79784	ENSG00000105357	0.173124	-0.01911262	0.38486262	0.84537521	NA	MYH14
51073	ENSG00000105364	975.285542	0.12997561	0.08466525	0.11567025	0.27763099	MRPL4
27181	ENSG00000105366	0	NA	NA	NA	NA	SIGLEC8
973	ENSG00000105369	0.97071832	0.00424683	0.38089891	0.93873791	NA	CD79A
3982	ENSG00000105370	0	NA	NA	NA	NA	LIM2
3386	ENSG00000105371	0	NA	NA	NA	NA	ICAM4
6223	ENSG00000105372	13226.9937	-0.05164857	0.0522507	0.31660239	0.54452953	RPS19
29997	ENSG00000105373	6863.74875	0.00541687	0.05366928	0.92014981	0.96637405	NOP53
4818	ENSG00000105374	0.48531136	0.01796065	0.38416958	0.79443464	NA	NKG7
7087	ENSG00000105376	7.93155467	0.17108844	0.39843268	0.26201887	0.48394839	ICAM5
2109	ENSG00000105379	653.395162	-0.24908555	0.10312994	0.01188192	0.04786922	ETFB
945	ENSG00000105383	0	NA	NA	NA	NA	CD33
1048	ENSG00000105388	0	NA	NA	NA	NA	CEACAM5
1406	ENSG00000105392	0	NA	NA	NA	NA	CRX
29086	ENSG00000105393	1603.42156	0.17019921	0.0714363	0.0152515	0.05840372	BABAM1
7297	ENSG00000105397	1927.61322	0.13962833	0.07808359	0.06776912	0.18713142	TYK2
6822	ENSG00000105398	0	NA	NA	NA	NA	SULT2A1
11140	ENSG00000105401	3825.9914	0.21984448	0.06491293	0.0005841	0.00369531	CDC37
8775	ENSG00000105402	1111.31706	0.04679656	0.09779112	0.6214621	0.80672374	NAPA
10567	ENSG00000105404	787.154812	0.33796748	0.10008544	0.00045569	0.00297789	RABAC1
478	ENSG00000105409	5023.86091	-1.99776744	0.07749345	9.81E-148	7.48E-145	ATP1A3
56917	ENSG00000105419	1693.97059	0.09070641	0.06929392	0.18351833	0.38180295	MEIS3
5802	ENSG00000105426	5352.44547	0.0215015	0.05572426	0.69750362	0.85389371	PTPRS
84518	ENSG00000105427	40.5921481	0.53493235	0.36736747	0.03360634	0.10980726	CNFN

148066	ENSG00000105428	0.33836517	0.00209965	0.3833763	0.96840955	NA	ZNRF4
1954	ENSG00000105429	4693.684	-0.03927219	0.05688714	0.48435321	0.70452718	MEGF8
10945	ENSG00000105438	1939.18483	0.00087866	0.08538239	0.99251754	0.99756121	KDELR1
9266	ENSG00000105443	2740.2806	0.41742142	0.07628025	2.27E-08	3.27E-07	CYTH2
83743	ENSG00000105447	1133.16456	-0.1193921	0.08699877	0.15852939	0.34569581	GRWD1
2906	ENSG00000105464	255.500287	0.69526066	0.18714888	4.42E-05	0.0003673	GRIN2D
23546	ENSG00000105467	1.0130207	-0.02269175	0.38298927	0.63582114	NA	SYNGR4
6320	ENSG00000105472	66.7076818	-0.20510139	0.24262037	0.28433608	0.50950396	CLEC11A
93233	ENSG00000105479	11.0160896	0.25380405	0.42823037	0.17146193	0.36389846	ODAD1
22900	ENSG00000105483	748.395622	0.68599038	0.10593947	2.28E-11	4.67E-10	CARD8
3978	ENSG00000105486	1421.28029	-0.02554016	0.08690631	0.76227228	0.890245	LIG1
946	ENSG00000105492	0	NA	NA	NA	NA	SIGLEC6
7728	ENSG00000105497	332.2538	-0.13848885	0.16423966	0.34938776	0.58004303	ZNF175
8605	ENSG00000105499	6.12424534	-0.11239136	0.3878715	0.32428329	0.55216104	PLA2G4C
NA	ENSG00000105501	0.16735865	0.01897177	0.38482933	0.78225479	NA	SIGLEC5
56344	ENSG00000105507	0	NA	NA	NA	NA	CABP5
3036	ENSG00000105509	2.3872068	0.07630564	0.38968056	0.28766667	NA	HAS1
9545	ENSG00000105514	534.311746	-0.1605977	0.119691	0.15700571	0.34378031	RAB3D
1628	ENSG00000105516	103.238891	-0.61303617	0.2978839	0.00847115	0.0361669	DBP
374882	ENSG00000105518	422.370144	0.12336977	0.12136426	0.28392489	0.50908899	TMEM205
828	ENSG00000105519	136.301186	0.35125572	0.21034713	0.04946205	0.14780908	CAPS
64748	ENSG00000105520	1142.41697	-0.15804261	0.07724692	0.03657485	0.11739928	PLPPR2
54854	ENSG00000105523	9.50195527	0.10836121	0.35721843	0.50771866	0.72359301	FAM83E
54922	ENSG00000105538	28.276517	0.40485686	0.47691892	0.08346203	0.21813816	RASIP1
51298	ENSG00000105549	0.32326427	-0.0232685	0.38532065	0.66893092	NA	THEG
26291	ENSG00000105550	1.31792497	0.07638453	0.39125895	0.21740716	NA	FGF21
587	ENSG00000105552	555.851738	0.29019314	0.10531647	0.00407382	0.01960892	BCAT2
54531	ENSG00000105556	543.214817	0.18017419	0.14978502	0.19064356	0.39199589	MIER2
57664	ENSG00000105559	165.973303	2.28597424	0.21932686	1.33E-26	6.68E-25	PLEKHA4
5518	ENSG00000105568	5019.99324	0.11745925	0.06606856	0.07124486	0.19407647	PPP2R1A
30000	ENSG00000105576	4337.85372	-0.01807746	0.0781226	0.81244142	0.91506317	TNPO2
51398	ENSG00000105583	1411.46004	0.11223968	0.08001807	0.15152313	0.33547806	WDR83OS
59284	ENSG00000105605	988.874497	-0.39516286	0.09763621	2.74E-05	0.0002386	CACNG7
2639	ENSG00000105607	382.206703	0.23880831	0.14114221	0.06810057	0.18779383	GCDH
10990	ENSG00000105609	0	NA	NA	NA	NA	LILRB5
10661	ENSG00000105610	7.72288365	0.05307281	0.3532594	0.72209843	0.86742752	KLF1
1777	ENSG00000105612	409.790904	0.49842573	0.13051179	5.18E-05	0.00042177	DNASE2
22983	ENSG00000105613	1902.3618	-0.17505133	0.08259904	0.02960757	0.09932622	MAST1

79165	ENSG00000105617	121.715251	-0.31659618	0.22884669	0.09208519	0.23452762	LENG1
26121	ENSG00000105618	1318.67914	0.04050709	0.07737368	0.59001542	0.78468333	PRPF31
29844	ENSG00000105619	272.663503	-0.03360921	0.14251999	0.79839302	0.90769144	TFPT
3718	ENSG00000105639	34.3148797	0.46484449	0.38022989	0.05898442	0.16891612	JAK3
6142	ENSG00000105640	17518.1918	-0.00381475	0.05072471	0.93844237	0.97434175	RPL18A
6528	ENSG00000105641	12.1135218	0.01094918	0.33251867	0.94995674	0.97937756	SLC5A5
3780	ENSG00000105642	335.532131	0.22688603	0.13205533	0.06665334	0.185029	KCNN1
27106	ENSG00000105643	222.850526	0.60664532	0.16632348	7.37E-05	0.00058162	ARRDC2
5296	ENSG00000105647	3987.34775	-0.29018088	0.0601742	1.01E-06	1.14E-05	PIK3R2
5864	ENSG00000105649	591.910588	-0.44099505	0.11508919	5.80E-05	0.00046612	RAB3A
5143	ENSG00000105650	119.101996	-0.36512938	0.28756066	0.08745186	0.22586501	PDE4C
51477	ENSG00000105655	1973.418	0.01482861	0.07081763	0.83269739	0.92397742	ISYNA1
8178	ENSG00000105656	327.171252	-0.25103758	0.13644156	0.0482092	0.14474434	ELL
23373	ENSG00000105662	794.948705	-0.04274821	0.09760249	0.64962979	0.82525848	CRTC1
1311	ENSG00000105664	4.0924842	-0.10791642	0.38371626	0.37757023	0.60636177	COMP
11045	ENSG00000105668	0.34624801	-0.02356563	0.38536324	0.65270996	NA	UPK1A
11316	ENSG00000105669	2404.35032	0.08524959	0.073082	0.23579379	0.45260728	COPE
54555	ENSG00000105671	1176.86625	-0.23989556	0.08778481	0.00489121	0.02280528	DDX49
2116	ENSG00000105672	9.12842975	0.04546527	0.35217803	0.75938414	0.88850157	ETV2
495	ENSG00000105675	1.75812907	0.11235116	0.40398771	0.09882788	NA	ATP4A
93436	ENSG00000105676	931.097416	-0.24690572	0.09739132	0.00857459	0.03655403	ARMC6
10430	ENSG00000105677	912.729861	0.12607079	0.08790577	0.14068989	0.318644	TMEM147
26330	ENSG00000105679	0.14931947	-0.01844332	0.38480212	0.84537521	NA	GAPDHS
NA	ENSG00000105694	0	NA	NA	NA	NA	ELOCP28
4099	ENSG00000105695	0	NA	NA	NA	NA	MAG
25789	ENSG00000105696	858.512182	-0.14035003	0.10571771	0.16554125	0.35564031	TMEM59L
57817	ENSG00000105697	0.64036599	-0.02529949	0.38298116	0.63542976	NA	HAMP
7392	ENSG00000105698	1614.70238	-0.08667765	0.08210944	0.27929346	0.50378964	USF2
51599	ENSG00000105699	19.2257146	0.07074017	0.31316609	0.70638633	0.85848115	LSR
79036	ENSG00000105700	1653.76152	0.10838297	0.07712556	0.15148382	0.33542846	KXD1
23770	ENSG00000105701	4599.58146	0.07124187	0.05745051	0.20958481	0.4178638	FKBP8
57794	ENSG00000105705	671.781914	-0.04288929	0.10803952	0.67711017	0.84229976	SUGP1
3249	ENSG00000105707	0.3390049	-0.00270765	0.38338649	0.98584234	NA	HPN
7561	ENSG00000105708	106.266289	-0.34681826	0.24368243	0.07688776	0.20525474	ZNF14
6324	ENSG00000105711	111.573617	1.34366324	0.26198235	2.45E-08	3.51E-07	SCN1B
80714	ENSG00000105717	73.9190789	0.5567518	0.30705876	0.01726867	0.06477791	PBX4
2077	ENSG00000105722	1365.99131	-0.28175311	0.07889167	0.00025449	0.00176852	ERF
2931	ENSG00000105723	1971.08021	0.0611463	0.07700987	0.41844209	0.6444567	GSK3A

57130	ENSG00000105726	2844.90307	0.23791433	0.06669975	0.00028718	0.00196671	ATP13A1
64763	ENSG00000105732	493.76052	-0.09790596	0.11127783	0.35695829	0.58708883	ZNF574
2901	ENSG00000105737	1549.65937	-0.2804262	0.08241881	0.00047692	0.0030963	GRIK5
23094	ENSG00000105738	1174.96233	0.10464739	0.22194404	0.56681975	0.76856055	SIPA1L3
7639	ENSG00000105750	298.005069	-0.47764754	0.14652348	0.00044403	0.00291031	ZNF85
23474	ENSG00000105755	207.726653	0.33360047	0.16481592	0.02518137	0.08766423	ETHE1
199731	ENSG00000105767	1101.3391	0.07096489	0.08768106	0.40588925	0.63413204	CADM4
56006	ENSG00000105771	745.324751	0.04473279	0.12703418	0.71208704	0.86159535	SMG9
23080	ENSG00000105778	2238.88917	-0.20167458	0.09253936	0.02437059	0.08536592	AVL9
154661	ENSG00000105784	88.0471043	-0.32731943	0.28750126	0.12052347	0.28609544	RUNDC3B
79846	ENSG00000105792	76.5765681	1.48600575	0.38931834	8.42E-06	8.12E-05	CFAP69
85865	ENSG00000105793	417.969134	-0.09843122	0.15090163	0.47860842	0.70031648	GTPBP10
101927446	ENSG00000105793	417.969134	-0.09843122	0.15090163	0.47860842	0.70031648	GTPBP10
10156	ENSG00000105808	1731.88834	0.02227554	0.08081368	0.76770921	0.89307241	RASA4
1021	ENSG00000105810	14529.6004	0.24600262	0.04873162	3.53E-07	4.33E-06	CDK6
9512	ENSG00000105819	2013.53138	-0.05633263	0.07723509	0.45646408	0.67989846	PMPCB
27000	ENSG00000105821	1030.82026	0.02811543	0.08523389	0.73476744	0.87375845	DNAJC2
7980	ENSG00000105825	497.584194	4.36841102	0.15437454	2.51E-177	3.12E-174	TFPI2
10282	ENSG00000105829	451.594608	0.21049623	0.12045835	0.06509028	0.18195793	BET1
10135	ENSG00000105835	962.935483	-0.10093482	0.09166716	0.25617866	0.47732763	NAMPT
221830	ENSG00000105849	528.653222	-0.1068385	0.11034141	0.31065445	0.5384279	POLR1F
5294	ENSG00000105851	0.16735865	0.01897177	0.38482933	0.78225479	NA	PIK3CG
5446	ENSG00000105852	0.36623983	0.02391775	0.38539601	0.6083452	NA	PON3
5445	ENSG00000105854	871.296713	0.62268306	0.09507443	1.68E-11	3.49E-10	PON2
3696	ENSG00000105855	159.393303	1.07806936	0.2070012	2.21E-08	3.19E-07	ITGB8
26959	ENSG00000105856	962.471965	0.24345971	0.08995562	0.00557542	0.02551703	HBP1
11062	ENSG00000105865	291.805274	-0.14497386	0.16090368	0.31959568	0.54727882	DUS4L
6671	ENSG00000105866	695.80776	-0.65673686	0.13952112	6.23E-07	7.32E-06	SP4
29062	ENSG00000105875	457.071382	0.25476164	0.16913288	0.09334088	0.23674616	WDR91
8701	ENSG00000105877	1.02288836	0.02349086	0.38188096	0.67819013	NA	DNAH11
79872	ENSG00000105879	934.151268	-0.08992508	0.09844259	0.3438519	0.57419443	CBLL1
1749	ENSG00000105880	31.103362	0.11498053	0.30300145	0.55693004	0.76235124	DLX5
136319	ENSG00000105887	6551.62104	-0.2816469	0.05856269	1.10E-06	1.24E-05	MTPN
767558	ENSG00000105887	6551.62104	-0.2816469	0.05856269	1.10E-06	1.24E-05	MTPN
256227	ENSG00000105889	2.12063112	0.11474906	0.40292562	0.14757341	NA	STEAP1B
401312	ENSG00000105889	2.12063112	0.11474906	0.40292562	0.14757341	NA	STEAP1B
5764	ENSG00000105894	420.926857	1.29431185	0.1229052	6.14E-27	3.13E-25	PTN
51678	ENSG00000105926	1954.86798	0.35721885	0.09072487	4.91E-05	0.00040275	MPP6

1687	ENSG00000105928	1549.39229	0.09000513	0.08720136	0.28953013	0.51546747	GSDME
50617	ENSG00000105929	1.01765974	0.00026751	0.38026894	0.98595981	NA	ATP6V0A4
56829	ENSG00000105939	1032.64287	0.02160381	0.08731548	0.80019123	0.90848606	ZC3HAV1
79989	ENSG00000105948	300.231924	0.13911973	0.14003854	0.28589907	0.51156544	TTC26
4967	ENSG00000105953	2211.76445	0.03074076	0.07430702	0.6742826	0.84019291	OGDH
64111	ENSG00000105954	0	NA	NA	NA	NA	NPVF
11033	ENSG00000105963	10.5864665	-0.06865039	0.35230277	0.64824209	0.82448382	ADAP1
22797	ENSG00000105967	0	NA	NA	NA	NA	TFEC
94239	ENSG00000105968	5374.65553	-0.04545136	0.06041569	0.44504591	0.66983089	H2AZ2
858	ENSG00000105971	0	NA	NA	NA	NA	CAV2
857	ENSG00000105974	0	NA	NA	NA	NA	CAV1
4233	ENSG00000105976	25.6563996	0.32533426	0.48707155	0.09129941	0.23321491	MET
140545	ENSG00000105982	69.8254586	0.51394282	0.29077715	0.02209999	0.07890295	RNF32
64327	ENSG00000105983	2374.41125	0.07778796	0.07296477	0.27781602	0.50226626	LMBR1
NA	ENSG00000105988	0	NA	NA	NA	NA	NHP2P1
7472	ENSG00000105989	0	NA	NA	NA	NA	WNT2
3198	ENSG00000105991	0.16735865	0.01897177	0.38482933	0.78225479	NA	HOXA1
10049	ENSG00000105993	4091.4782	0.22337375	0.06870104	0.00093656	0.00560354	DNAJB6
3199	ENSG00000105996	0	NA	NA	NA	NA	HOXA2
3200	ENSG00000105997	0	NA	NA	NA	NA	HOXA3
3955	ENSG00000106003	74.1568182	0.22404201	0.24659625	0.24893503	0.46831699	LFNG
3202	ENSG00000106004	0	NA	NA	NA	NA	HOXA5
3203	ENSG00000106006	0	NA	NA	NA	NA	HOXA6
221927	ENSG00000106009	1893.41047	-0.02224314	0.07456355	0.75856114	0.88794931	BRAT1
23288	ENSG00000106012	914.63776	0.0976299	0.09269495	0.27791246	0.50239483	IQCE
56311	ENSG00000106013	33.6129877	-0.0408289	0.291604	0.82879402	0.92238028	ANKRD7
7434	ENSG00000106018	2.54834583	-0.00710735	0.37452758	0.93207977	NA	VIPR2
23554	ENSG00000106025	104.078651	1.59155396	0.27702277	7.33E-10	1.28E-08	TSPAN12
6742	ENSG00000106028	2003.60717	-0.00545955	0.0747349	0.94102481	0.97559098	SSBP1
3209	ENSG00000106031	0	NA	NA	NA	NA	HOXA13
79974	ENSG00000106034	61.3698658	1.82523251	0.36045252	2.84E-08	4.04E-07	CPED1
2128	ENSG00000106038	0	NA	NA	NA	NA	EVX1
11112	ENSG00000106049	627.882631	0.2402592	0.09955922	0.01225464	0.04906802	HIBADH
8887	ENSG00000106052	1856.61863	0.01734142	0.07201402	0.8072004	0.91260538	TAX1BP1
54504	ENSG00000106066	149.831128	0.99285197	0.19997315	8.75E-08	1.16E-06	CPVL
1124	ENSG00000106069	64.0484336	-0.23409382	0.27478603	0.24697264	0.46604299	CHN2
2887	ENSG00000106070	0.16735865	0.01897177	0.38482933	0.78225479	NA	GRB10
83451	ENSG00000106077	137.546931	-0.24366751	0.20520019	0.16227169	0.35094147	ABHD11

23242	ENSG00000106078	6.93865314	0.36497182	0.64325696	0.01484918	0.0572054	COBL
55033	ENSG00000106080	717.765551	0.08338539	0.11230827	0.43783367	0.66289835	FKBP14
84725	ENSG00000106086	1825.77122	0.03478742	0.08195633	0.66464552	0.83444869	PLEKHA8
6804	ENSG00000106089	932.742486	-0.75047356	0.10745842	5.89E-13	1.36E-11	STX1A
10392	ENSG00000106100	465.570328	0.36399204	0.11327966	0.00075043	0.00459948	NOD1
2617	ENSG00000106105	4455.76178	0.45807511	0.10356447	4.38E-06	4.46E-05	GARS1
1395	ENSG00000106113	10.9264594	-3.76194275	1.09086852	5.91E-05	0.00047436	CRHR2
2051	ENSG00000106123	1.50149908	-0.04488918	0.38394934	0.46828219	NA	EPHB6
84182	ENSG00000106125	263.4895	-0.10982759	0.14531006	0.41292754	0.64038252	MINDY4
2692	ENSG00000106128	0	NA	NA	NA	NA	GHRHR
260294	ENSG00000106133	438.823972	0.47601021	0.11889869	2.63E-05	0.00022962	NSUN5P2
835	ENSG00000106144	1742.14466	-0.39512038	0.07153414	1.78E-08	2.61E-07	CASP2
51142	ENSG00000106153	6804.17905	-0.31502007	0.05782978	3.43E-08	4.81E-07	CHCHD2
6369	ENSG00000106178	0	NA	NA	NA	NA	CCL24
3315	ENSG00000106211	3064.92376	0.47021632	0.0805037	2.28E-09	3.75E-08	HSPB1
4885	ENSG00000106236	2494.81023	-0.58269978	0.06778114	2.63E-18	9.00E-17	NPTX2
11333	ENSG00000106244	5961.05308	0.13567597	0.06444027	0.0326032	0.10714717	PDAP1
8896	ENSG00000106245	1419.18352	0.0658345	0.07434966	0.36720587	0.59642771	BUD31
26024	ENSG00000106246	608.643632	0.39913382	0.10732515	0.00010521	0.00079639	PTCD1
1577	ENSG00000106258	76.5172849	-0.33708299	0.29611641	0.1164007	0.27891182	CYP3A5
7586	ENSG00000106261	6862.67187	0.02916675	0.05566923	0.59742721	0.78921427	ZKSCAN1
8662	ENSG00000106263	4377.33425	-0.08289541	0.05652303	0.13803874	0.31414184	EIF3B
29886	ENSG00000106266	684.945675	-0.22420266	0.14209904	0.08753214	0.22594959	SNX8
4521	ENSG00000106268	699.901931	0.05425036	0.0966251	0.56257795	0.76559067	NUDT1
5803	ENSG00000106278	0	NA	NA	NA	NA	PTPRZ1
6878	ENSG00000106290	1974.5265	0.30521428	0.07192426	1.53E-05	0.00014009	TAF6
8976	ENSG00000106299	1728.4581	0.17692368	0.0757586	0.01711513	0.06428705	WASL
23553	ENSG00000106302	0.173733	0.01914545	0.38484597	0.78225479	NA	HYAL4
6677	ENSG00000106304	0.18922458	0.01954053	0.38488496	0.78225479	NA	SPAM1
7965	ENSG00000106305	518.499272	-0.21934594	0.10824342	0.03400391	0.11087545	AIMP2
7036	ENSG00000106327	236.569706	-1.18410544	0.16396208	5.34E-14	1.34E-12	TFR2
29999	ENSG00000106328	10.413746	0.18760936	0.38847695	0.2787647	0.50317912	FSCN3
64598	ENSG00000106330	497.215282	0.19863346	0.10958275	0.05809227	0.16713321	MOSPD3
5078	ENSG00000106331	0.34687871	-0.00470809	0.38343604	0.96739424	NA	PAX4
5118	ENSG00000106333	9470.52215	0.63421903	0.06059604	6.08E-26	2.99E-24	PCOLCE
26261	ENSG00000106336	18.9519765	0.203596	0.37604742	0.28250959	0.50756047	FBXO24
10842	ENSG00000106341	0	NA	NA	NA	NA	PPP1R17
55131	ENSG00000106344	2356.46362	-0.22585667	0.09880188	0.01766055	0.06588665	RBM28

84132	ENSG00000106346	1112.73079	0.24320391	0.07932972	0.00169959	0.00934124	USP42
3614	ENSG00000106348	2635.58376	-0.42906174	0.08581567	2.88E-07	3.58E-06	IMPDH1
3268	ENSG00000106351	170.20902	-0.03618221	0.19263482	0.8266543	0.92144622	AGFG2
23658	ENSG00000106355	1351.11879	-0.40071517	0.09836612	2.42E-05	0.00021288	LSM5
5054	ENSG00000106366	7.48067163	-0.28288184	0.48441043	0.09480412	0.23954582	SERPINE1
1174	ENSG00000106367	2323.52018	-0.0969482	0.06638305	0.1375419	0.31322672	AP1S1
346606	ENSG00000106384	0	NA	NA	NA	NA	MOGAT3
56913	ENSG00000106392	744.991669	-0.16169959	0.11689384	0.14506009	0.32541747	C1GALT1
8985	ENSG00000106397	3565.05078	0.35706325	0.06517544	2.60E-08	3.70E-07	PLOD3
6119	ENSG00000106399	641.559704	0.0003431	0.10610849	0.99819185	0.99941443	RPA3
10467	ENSG00000106400	1494.64285	0.06497903	0.08125301	0.41361771	0.64081351	ZNHIT1
24146	ENSG00000106404	653.630158	0.50264718	0.09902635	1.53E-07	1.97E-06	CLDN15
135935	ENSG00000106410	0	NA	NA	NA	NA	NOBOX
113263	ENSG00000106415	1678.08452	0.03353935	0.08304605	0.67983078	0.84376374	GLCCI1
93408	ENSG00000106436	0	NA	NA	NA	NA	MYL10
9678	ENSG00000106443	2793.89569	-0.21996464	0.06599644	0.0007026	0.0043385	PHF14
4899	ENSG00000106459	516.462436	0.03882384	0.11324341	0.72046622	0.86656864	NRF1
54664	ENSG00000106460	1544.10496	0.07809306	0.09867896	0.41330163	0.64061753	TMEM106B
2146	ENSG00000106462	2575.32253	0.18065644	0.07341133	0.0121023	0.04856587	EZH2
95681	ENSG00000106477	901.994088	-0.25253829	0.0980718	0.00753649	0.03287688	CEP41
643641	ENSG00000106479	887.719833	0.46235803	0.10661777	6.29E-06	6.21E-05	ZNF862
6424	ENSG00000106483	3.35666611	-0.08951247	0.38319247	0.40343848	0.6314966	SFRP4
4232	ENSG00000106484	39.2259909	2.23391927	0.44382782	3.02E-08	4.26E-07	MEST
4223	ENSG00000106511	0	NA	NA	NA	NA	MEOX2
57037	ENSG00000106524	193.952889	0.44924963	0.1735153	0.00404668	0.01949455	ANKMY2
653857	ENSG00000106526	21.823934	0.04553696	0.30608242	0.80963984	0.91359357	ACTR3C
11281	ENSG00000106536	0	NA	NA	NA	NA	POU6F2
27075	ENSG00000106537	359.512133	0.36809789	0.13657821	0.00388992	0.0188234	TSPAN13
5919	ENSG00000106538	16.294079	2.23717233	0.70500027	8.46E-05	0.00065457	RARRES2
NA	ENSG00000106540	0	NA	NA	NA	NA	
10551	ENSG00000106541	0	NA	NA	NA	NA	AGR2
196	ENSG00000106546	0	NA	NA	NA	NA	AHR
54927	ENSG00000106554	1962.84039	-0.20117983	0.08009119	0.01006548	0.04165471	CHCHD3
26157	ENSG00000106560	0	NA	NA	NA	NA	GIMAP2
28959	ENSG00000106565	2.08256655	-0.06911144	0.38421728	0.41384591	NA	TMEM176B
2737	ENSG00000106571	0	NA	NA	NA	NA	GLI3
5683	ENSG00000106588	1728.20093	-0.24089602	0.08924308	0.00538576	0.02479774	PSMA2
64983	ENSG00000106591	689.678218	0.15142764	0.10170363	0.12200963	0.28884077	MRPL32

55744	ENSG00000106603	2329.84776	0.17762997	0.06191412	0.00363193	0.01780958	COA1
644	ENSG00000106605	683.802617	0.03791853	0.10589128	0.71029729	0.86089825	BLVRA
55665	ENSG00000106608	1428.41192	0.03840832	0.07598477	0.60668039	0.79627805	URGCP
55069	ENSG00000106609	2208.88894	0.01613916	0.06939685	0.81398681	0.91592004	TMEM248
64940	ENSG00000106610	331.79688	-0.18827703	0.14664618	0.16395498	0.35322761	STAG3L4
101929736	ENSG00000106610	331.79688	-0.18827703	0.14664618	0.16395498	0.35322761	STAG3L4
6009	ENSG00000106615	2054.6863	-0.26661184	0.07072685	0.00012171	0.00090576	RHEB
51422	ENSG00000106617	259.054248	0.10223945	0.16296633	0.48876924	0.7085856	PRKAG2
165	ENSG00000106624	1719.80409	-0.27820906	0.0772898	0.00022904	0.001604	AEBP1
5425	ENSG00000106628	3089.17478	-0.09987067	0.06154428	0.09980746	0.2488914	POLD2
58498	ENSG00000106631	0.46871751	-0.02821432	0.38578887	0.54668481	NA	MYL7
2645	ENSG00000106633	52.7268276	0.17582694	0.26195069	0.37351163	0.60252627	GCK
9275	ENSG00000106635	1194.68394	-0.05341565	0.07903219	0.48922503	0.70874969	BCL7B
10652	ENSG00000106636	4174.02233	-0.05686693	0.06584596	0.37980925	0.60877488	YKT6
26608	ENSG00000106638	974.366114	0.23336843	0.09019329	0.00766251	0.03329483	TBL2
168391	ENSG00000106648	0.14931947	-0.01844332	0.38480212	0.84537521	NA	GALNTL5
7461	ENSG00000106665	2008.25599	0.19325304	0.08202726	0.01575987	0.06002677	CLIP2
7458	ENSG00000106682	9799.18266	-0.06029968	0.05120503	0.23572155	0.45251238	EIF4H
3984	ENSG00000106683	1205.46614	0.08275518	0.0849281	0.31798861	0.54561048	LIMK1
55064	ENSG00000106686	78.752443	0.48231871	0.26142262	0.02131781	0.07666221	SPATA6L
6505	ENSG00000106688	77.4215168	-0.25239404	0.25649286	0.20179917	0.40725211	SLC1A1
9355	ENSG00000106689	1.43280255	-0.07694854	0.39114633	0.22102014	NA	LHX2
2218	ENSG00000106692	773.848545	-0.15191186	0.14639083	0.25982878	0.48146481	FKTN
83856	ENSG00000106701	348.327072	-0.7904933	0.14881013	1.98E-08	2.89E-07	FSD1L
79937	ENSG00000106714	187.276937	-0.99759863	0.21199164	3.06E-07	3.78E-06	CNTNAP3
10927	ENSG00000106723	2799.29449	0.03015731	0.07898495	0.70373831	0.85757322	SPIN1
54981	ENSG00000106733	2.21741499	0.06196062	0.38270153	0.45992697	NA	NMRK1
23731	ENSG00000106771	1114.97112	-0.09991631	0.09027017	0.25318337	0.47343619	TMEM245
158471	ENSG00000106772	0	NA	NA	NA	NA	PRUNE2
1955	ENSG00000106780	1008.16931	0.06115937	0.08464939	0.45941016	0.68258752	MEGF9
9830	ENSG00000106785	674.751728	0.11156094	0.0981872	0.23994236	0.4579339	TRIM14
7464	ENSG00000106789	221.766152	-0.15712799	0.15527996	0.26702669	0.48945025	CORO2A
7046	ENSG00000106799	1103.94737	-0.01533293	0.10417154	0.87707953	0.94574195	TGFBR1
10952	ENSG00000106803	732.102782	0.23001712	0.10694192	0.02469965	0.08632044	SEC61B
727	ENSG00000106804	405.477701	0.20410985	0.15349218	0.14729038	0.32889601	C5
4969	ENSG00000106809	1.03855236	-0.01321531	0.38113483	0.81116657	NA	OGN
54829	ENSG00000106819	0	NA	NA	NA	NA	ASPN
1842	ENSG00000106823	1.05688577	-0.00814344	0.38096817	0.88172235	NA	ECM2

7091	ENSG00000106829	1133.7881	0.28613864	0.09803318	0.00246887	0.01282596	TLE4
26468	ENSG00000106852	15.7476642	0.0180577	0.3462271	0.9073225	0.95963244	LHX6
22949	ENSG00000106853	51.5628826	1.54560731	0.41905078	1.51E-05	0.00013924	PTGR1
64420	ENSG00000106868	323.141496	-0.15454221	0.13652723	0.22487905	0.43874524	SUSD1
259	ENSG00000106927	1.63974568	0.10137474	0.40018473	0.12131541	NA	AMBP
80709	ENSG00000106948	380.521515	0.64172541	0.1667827	3.03E-05	0.0002597	AKNA
944	ENSG00000106952	0	NA	NA	NA	NA	TNFSF8
1759	ENSG00000106976	2971.13161	-0.46852815	0.07073369	1.54E-11	3.22E-10	DNM1
2022	ENSG00000106991	551.736778	-0.57503834	0.12905269	2.60E-06	2.74E-05	ENG
203	ENSG00000106992	508.808917	-0.26522187	0.12361985	0.02291255	0.08124326	AK1
55664	ENSG00000106993	258.733281	-0.18645246	0.13926051	0.14956112	0.33235804	CDC37L1
6019	ENSG00000107014	32.9129754	0.07084472	0.2886071	0.71569743	0.8628205	RLN2
6013	ENSG00000107018	9.22689226	0.17902032	0.38802126	0.28948123	0.51546747	RLN1
55848	ENSG00000107020	234.995409	-0.04507115	0.16055676	0.75616543	0.88670553	PLGRKT
54662	ENSG00000107021	792.777381	0.16749626	0.09491217	0.06833047	0.18818386	TBC1D13
57589	ENSG00000107036	886.383135	0.24913299	0.08697287	0.00321541	0.01603259	RIC1
23081	ENSG00000107077	389.597089	-0.00761702	0.12524882	0.94767777	0.97860443	KDM4C
81704	ENSG00000107099	0	NA	NA	NA	NA	DOCK8
23189	ENSG00000107104	0.34510075	-0.00733018	0.3835432	0.94764827	NA	KANK1
1993	ENSG00000107105	701.376317	-1.26157455	0.1149113	5.00E-29	2.79E-27	ELAVL2
23413	ENSG00000107130	3447.49955	-0.58487588	0.08052116	1.19E-13	2.89E-12	NCS1
7016	ENSG00000107140	319.154683	0.23527415	0.14101224	0.07220621	0.19574701	TESK1
57582	ENSG00000107147	158.57352	-3.29243184	0.25784126	1.02E-38	7.62E-37	KCNT1
768	ENSG00000107159	6.28898864	0.04218366	0.36254694	0.74213682	0.87846755	CA9
8939	ENSG00000107164	2256.88024	0.07179793	0.07894505	0.35314634	0.58328533	FUBP3
7306	ENSG00000107165	22.2381529	0.04053948	0.32548867	0.81843488	0.9171248	TYRP1
10488	ENSG00000107175	547.872282	-0.02014508	0.10338053	0.83835865	0.92668313	CREB3
9827	ENSG00000107185	1837.79354	0.09727183	0.07091215	0.16298174	0.35195933	RGP1
8777	ENSG00000107186	3021.9459	0.17889212	0.05682644	0.00145669	0.00817263	MPDZ
8022	ENSG00000107187	0.71866211	0.0519745	0.38872294	0.28945158	NA	LHX3
23586	ENSG00000107201	43.4592356	0.05274899	0.37398337	0.62425752	0.80849071	DDX58
8721	ENSG00000107223	2440.17609	0.09923499	0.06580228	0.12570482	0.29476228	EDF1
8395	ENSG00000107242	0	NA	NA	NA	NA	PIP5K1B
169792	ENSG00000107249	22.6836911	0.5917622	0.55612303	0.03622055	0.11645043	GLIS3
573	ENSG00000107262	1043.54182	-0.47949851	0.08473202	6.45E-09	9.96E-08	BAG1
2889	ENSG00000107263	1821.95731	-0.13955092	0.08224294	0.08232719	0.21594016	RAPGEF1
56654	ENSG00000107281	1435.15951	-0.3990929	0.0972398	2.12E-05	0.0001893	NPDC1
320	ENSG00000107282	415.152238	-2.3479612	0.15302982	2.00E-54	2.25E-52	APBA1

23064	ENSG00000107290	2567.4731	0.16776095	0.06861221	0.01289213	0.05112531	SETX
6456	ENSG00000107295	1.30147618	0.00572748	0.37796443	0.93723114	NA	SH3GL2
5730	ENSG00000107317	2.92301148	0.08636699	0.38268752	0.41807998	0.64419904	PTGDS
20	ENSG00000107331	3069.12995	-0.26343642	0.08726054	0.00187383	0.0101161	ABCA2
6461	ENSG00000107338	1341.51989	-1.18109626	0.08029803	6.19E-50	6.14E-48	SHB
54926	ENSG00000107341	2022.7277	-0.16634712	0.06985348	0.01531842	0.05859213	UBE2R2
51104	ENSG00000107362	463.053243	-0.03446764	0.11348475	0.74978614	0.88225829	ABHD17B
51010	ENSG00000107371	822.307656	-0.41654093	0.10225902	2.30E-05	0.00020377	EXOSC3
7763	ENSG00000107372	2407.54229	0.20196585	0.07051266	0.0035404	0.01744698	ZFAND5
1855	ENSG00000107404	1833.15903	0.15139768	0.07738398	0.04564895	0.13867733	DVL1
9124	ENSG00000107438	407.94237	0.45504679	0.12444486	0.00011282	0.00084628	PDLIM1
54619	ENSG00000107443	877.232334	0.18547518	0.09015751	0.03429505	0.11161645	CCNJ
1791	ENSG00000107447	0	NA	NA	NA	NA	DNTT
2625	ENSG00000107485	5312.62841	0.57860979	0.06266647	8.65E-21	3.37E-19	GATA3
26033	ENSG00000107518	516.084631	-0.46362423	0.15339114	0.00098422	0.00582939	ATRNL1
3257	ENSG00000107521	514.362674	0.13803212	0.12053055	0.22710191	0.44177223	HPS1
5264	ENSG00000107537	205.952075	0.05839088	0.15152396	0.67563347	0.84120289	PHYH
83937	ENSG00000107551	76.7871954	0.35318441	0.28111548	0.09539688	0.24073698	RASSF4
23268	ENSG00000107554	306.44503	-0.0698141	0.14292942	0.59751953	0.78921427	DNMBP
22841	ENSG00000107560	778.512569	0.13575024	0.10820387	0.19077464	0.39211202	RAB11FIP2
6387	ENSG00000107562	0.33010465	-0.00578571	0.3834757	0.96609971	NA	CXCL12
10613	ENSG00000107566	662.154562	-0.07797986	0.10289607	0.43072235	0.65613723	ERLIN1
8661	ENSG00000107581	8973.56714	-0.09567103	0.04753937	0.04243572	0.131407	EIF3A
9033	ENSG00000107593	0.32378099	-0.02327265	0.38532129	0.66863335	NA	PKD2L1
8029	ENSG00000107611	21.2815198	0.56133705	0.79124797	0.02808044	0.09528234	CUBN
1787	ENSG00000107614	208.17772	0.51347345	0.22182895	0.00662649	0.02951717	TRDMT1
79009	ENSG00000107625	1238.17985	-0.11398672	0.08200733	0.1558156	0.34200341	DDX50
5599	ENSG00000107643	1438.76007	-0.47933898	0.10624356	2.75E-06	2.89E-05	MAPK8
11196	ENSG00000107651	1180.17003	0.02067798	0.11763364	0.85435999	0.93466646	SEC23IP
11101	ENSG00000107669	1019.26629	-0.14847101	0.08472353	0.07188862	0.19532597	ATE1
54780	ENSG00000107672	807.00262	0.07700347	0.09026058	0.38049751	0.60938575	NSMCE4A
59338	ENSG00000107679	807.521782	-0.10516775	0.09199072	0.23832864	0.4558851	PLEKHA1
27143	ENSG00000107719	399.863294	-0.25514201	0.11826138	0.02281204	0.08096439	PALD1
219699	ENSG00000107731	9297.57591	0.26979997	0.04872255	2.38E-08	3.41E-07	UNC5B
64072	ENSG00000107736	90.668975	-0.62923626	0.32336892	0.01007625	0.04167381	CDH23
64115	ENSG00000107738	40.2885881	-0.05102015	0.32651263	0.76600022	0.89234011	VSIR
9806	ENSG00000107742	163.8965	-0.17021987	0.21961356	0.34609103	0.57691531	SPOCK2
10367	ENSG00000107745	1214.2823	0.10770943	0.10647459	0.29270568	0.51883147	MICU1

5532	ENSG00000107758	876.602581	-0.16493092	0.09151223	0.06293882	0.17770118	PPP3CB
54462	ENSG00000107771	741.322665	-0.33037875	0.13278436	0.00778442	0.03373589	CCSER2
657	ENSG00000107779	1093.60383	0.103474	0.13000665	0.39651527	0.62574474	BMPR1A
9562	ENSG00000107789	985.808488	-0.23481475	0.08425032	0.00419629	0.02009035	MINPP1
59	ENSG00000107796	40.4389988	0.40868304	0.35135605	0.08094966	0.21334235	ACTA2
3988	ENSG00000107798	516.295247	0.25477394	0.10584012	0.01207131	0.04849055	LIPA
3195	ENSG00000107807	71.8298336	-0.31328467	0.30275568	0.14315752	0.32228142	TLX1
56652	ENSG00000107815	1446.63357	-0.10278139	0.10309246	0.29957833	0.52654448	TWNK
84445	ENSG00000107816	1944.79336	0.30546897	0.07376009	2.36E-05	0.000209	LZTS2
81855	ENSG00000107819	1569.92053	-0.61578592	0.08758137	5.92E-13	1.37E-11	SFXN3
81621	ENSG00000107821	86.4283192	0.25390319	0.27952754	0.21609454	0.42689591	KAZALD1
6468	ENSG00000107829	660.79934	0.15167823	0.11242476	0.15729648	0.34408611	FBXW4
2253	ENSG00000107831	0.18922458	0.01954053	0.38488496	0.78225479	NA	FGF8
10360	ENSG00000107833	427.499808	0.01511653	0.128253	0.90147805	0.95673063	NPM3
80351	ENSG00000107854	1769.46967	-0.08222574	0.07274841	0.24739454	0.46634753	TNKS2
5309	ENSG00000107859	0.73627161	-0.04183898	0.38735754	0.35739953	NA	PITX3
8729	ENSG00000107862	1597.3975	0.09123227	0.12699406	0.44694016	0.67095132	GBF1
57584	ENSG00000107863	1093.58476	0.06170602	0.09238261	0.49192167	0.7110834	ARHGAP21
22849	ENSG00000107864	95.3543009	-0.49335623	0.24569939	0.01465527	0.0565733	CPEB3
79176	ENSG00000107872	394.955543	-0.77062385	0.15038146	5.71E-08	7.77E-07	FBXL15
79004	ENSG00000107874	1545.66227	0.01039103	0.07819227	0.89241528	0.9534988	CUEDC2
51684	ENSG00000107882	643.992075	-0.07149384	0.10073489	0.46120745	0.68434341	SUFU
22852	ENSG00000107890	589.865301	0.09773459	0.11577362	0.37607208	0.60513139	ANKRD26
91452	ENSG00000107897	871.991077	-0.32438189	0.11140447	0.00226247	0.01190013	ACBD5
64077	ENSG00000107902	203.043685	0.52828168	0.17718314	0.00096332	0.00573256	LHPP
23185	ENSG00000107929	1569.64397	-0.1401861	0.08098836	0.07616503	0.20371308	LARP4B
23560	ENSG00000107937	2410.69428	0.02154165	0.09766118	0.82058888	0.91840037	GTPBP4
26098	ENSG00000107938	1045.77848	0.02766785	0.09121021	0.75562563	0.88629101	EDRF1
56647	ENSG00000107949	818.162928	-0.03384891	0.09887671	0.72248824	0.86754018	BCCIP
55149	ENSG00000107951	393.896047	-0.19611838	0.16820917	0.19324588	0.39596861	MTPAP
9148	ENSG00000107954	361.392555	-1.53461156	0.15908515	3.88E-23	1.69E-21	NEURL1
9644	ENSG00000107957	1684.4346	-0.69646874	0.07924389	3.62E-19	1.30E-17	SH3PXD2A
10531	ENSG00000107959	743.950661	0.01508202	0.09389522	0.87112452	0.94384401	PITRM1
79991	ENSG00000107960	420.198708	0.01068393	0.11951318	0.92601273	0.96919669	STN1
1326	ENSG00000107968	27.053108	-0.01161037	0.31431805	0.94654738	0.97814213	MAP3K8
22943	ENSG00000107984	3027.79345	2.41502479	0.08238375	4.93E-190	7.52E-187	DKK1
253738	ENSG00000108001	65.9709196	1.91523583	0.33405691	5.76E-10	1.03E-08	EBF3
10539	ENSG00000108010	967.444931	-0.12819976	0.12340481	0.27130899	0.49473993	GLRX3

114815	ENSG00000108018	5339.48633	0.36831286	0.06755303	2.95E-08	4.17E-07	SORCS1
54906	ENSG00000108021	2500.7429	-0.07072141	0.06508419	0.26985698	0.49304379	TASOR2
7511	ENSG00000108039	1247.96376	-0.02251732	0.08840743	0.79208722	0.90499209	XPNPEP1
9126	ENSG00000108055	3323.49506	-0.22629576	0.06316096	0.00027546	0.00189427	SMC3
8036	ENSG00000108061	671.249123	-0.22390651	0.11069649	0.03404538	0.11097421	SHOC2
7019	ENSG00000108064	1145.39971	-0.53337813	0.08356692	6.56E-11	1.29E-09	TFAM
8030	ENSG00000108091	940.068653	-0.26202639	0.08582026	0.00168843	0.00929224	CCDC6
8453	ENSG00000108094	647.460282	-0.10022586	0.09787421	0.288812	0.51478125	CUL2
219771	ENSG00000108100	1343.24795	-0.12012368	0.09683171	0.19901611	0.40344126	CCNY
27338	ENSG00000108106	7284.63873	-0.32309001	0.06603478	6.87E-07	7.99E-06	UBE2S
6158	ENSG00000108107	10741.321	0.15225408	0.07810816	0.04693561	0.14178748	RPL28
57178	ENSG00000108175	8135.18476	-0.40697558	0.0655235	2.74E-10	5.03E-09	ZMIZ1
56521	ENSG00000108176	678.145263	0.03774954	0.09869435	0.69307658	0.8516694	DNAJC12
10105	ENSG00000108179	2232.83122	-0.29911412	0.07676446	6.69E-05	0.00053345	PPIF
64081	ENSG00000108187	99.8044929	-0.0501695	0.22122491	0.78012316	0.89936692	PBLD
81619	ENSG00000108219	1516.01869	0.1591982	0.08351071	0.05061551	0.15037611	TSPAN14
9211	ENSG00000108231	14.3908877	-0.00400387	0.33139697	0.97875651	0.99278156	LGI1
23232	ENSG00000108239	268.826838	-0.23544718	0.15223058	0.09107493	0.23285137	TBC1D12
1562	ENSG00000108242	1.10253973	-0.00116947	0.37984077	0.98446619	NA	CYP2C18
25984	ENSG00000108244	0	NA	NA	NA	NA	KRT23
1411	ENSG00000108255	0	NA	NA	NA	NA	CRYBA1
57532	ENSG00000108256	2429.68845	0.07836537	0.08703874	0.3555069	0.58591601	NUFIP2
28964	ENSG00000108262	2466.64162	0.23660009	0.07192665	0.00079012	0.00482338	GIT1
6143	ENSG00000108298	18930.982	-0.06947214	0.04622985	0.12960584	0.30116889	RPL19
84961	ENSG00000108306	490.845181	-0.04038784	0.11935399	0.7207976	0.86665199	FBXL20
10900	ENSG00000108309	1245.68196	-1.32289646	0.08945383	1.53E-50	1.54E-48	RUNDC3A
7343	ENSG00000108312	3003.50275	-0.00229401	0.07076586	0.97299383	0.98995885	UBTF
1440	ENSG00000108342	0.44765505	0.02632933	0.38557726	0.58574429	NA	CSF3
5709	ENSG00000108344	2955.28505	0.09876716	0.07710932	0.19107335	0.39259479	PSMD3
22794	ENSG00000108349	2076.8484	-0.48931453	0.06720988	1.38E-13	3.34E-12	CASC3
51195	ENSG00000108352	321.045307	-0.03237799	0.13355662	0.79600226	0.90627136	RAPGEFL1
8787	ENSG00000108370	233.924087	-0.82980921	0.17834306	5.40E-07	6.40E-06	RGS9
54894	ENSG00000108375	0.47596848	-0.00794879	0.38324764	0.85634226	NA	RNF43
7473	ENSG00000108379	160.702031	0.00773386	0.16842345	0.96042983	0.9839372	WNT3
101929777	ENSG00000108379	160.702031	0.00773386	0.16842345	0.96042983	0.9839372	WNT3
443	ENSG00000108381	0.15069912	-0.01848503	0.38480575	0.84537521	NA	ASPA
5889	ENSG00000108384	587.891401	-0.17379871	0.10849842	0.09303836	0.23613676	RAD51C
5414	ENSG00000108387	91.5794228	0.09392198	0.21643833	0.60150661	0.79200883	SEPTIN4

9110	ENSG00000108389	3276.4592	0.06845533	0.09568087	0.46070197	0.68379794	MTMR4
4591	ENSG00000108395	1562.82161	-0.10635381	0.07388489	0.14119894	0.31935557	TRIM37
5023	ENSG00000108405	0.55665916	-0.01733583	0.38376366	0.71051265	NA	P2RX1
79665	ENSG00000108406	2838.01604	0.22749573	0.07202889	0.00128598	0.00734213	DHX40
8688	ENSG00000108417	0	NA	NA	NA	NA	KRT37
51174	ENSG00000108423	137.330045	0.20011754	0.21607651	0.26658324	0.48900406	TUBD1
3837	ENSG00000108424	10325.9936	-0.16078699	0.06670166	0.01422532	0.05527425	KPNB1
9570	ENSG00000108433	1765.51723	-0.02067287	0.08394629	0.79940799	0.90803676	GOSR2
55163	ENSG00000108439	590.413344	-0.11658967	0.10509883	0.24779864	0.46688746	PNPO
NA	ENSG00000108442	0	NA	NA	NA	NA	TVP23BP2
6198	ENSG00000108443	1106.89647	-0.01770188	0.11048593	0.8653819	0.9403131	RPS6KB1
147166	ENSG00000108448	443.78678	0.33450376	0.13414704	0.00764344	0.03322654	TRIM16L
NA	ENSG00000108452	0.18922458	0.01954053	0.38488496	0.78225479	NA	ZNF29P
80279	ENSG00000108465	4520.2584	0.29391789	0.05686973	1.71E-07	2.19E-06	CDK5RAP3
10951	ENSG00000108468	8228.81031	-0.09739125	0.04824205	0.04174111	0.12970144	CBX1
9400	ENSG00000108469	786.913389	0.39159017	0.12756607	0.00111777	0.00649722	RECQL5
9487	ENSG00000108474	202.702783	0.33035714	0.17495837	0.03427897	0.11160073	PIGL
2584	ENSG00000108479	474.680867	0.17440999	0.11493436	0.11129726	0.26948227	GALK1
57508	ENSG00000108506	998.843274	-0.16681757	0.09840021	0.07879057	0.20920837	INTS2
23125	ENSG00000108509	697.644723	0.16846022	0.13794593	0.18995045	0.39109764	CAMTA2
9969	ENSG00000108510	3437.38504	-0.08993239	0.06881567	0.1837229	0.38212206	MED13
3216	ENSG00000108511	0	NA	NA	NA	NA	HOXB6
2027	ENSG00000108515	326.361343	0.06188744	0.16330428	0.6754775	0.84111426	ENO3
NA	ENSG00000108516	0	NA	NA	NA	NA	
5216	ENSG00000108518	12191.1614	0.11428536	0.05714587	0.04334251	0.13334089	PFN1
26001	ENSG00000108523	960.991729	0.14904936	0.085897	0.07483467	0.20112705	RNF167
8402	ENSG00000108528	1101.07829	0.03524999	0.08417867	0.66840132	0.83697256	SLC25A11
51655	ENSG00000108551	16.4158345	0.04693394	0.32301726	0.79464964	0.90579008	RASD1
1145	ENSG00000108556	3.26730219	-0.0658602	0.37454209	0.5553795	0.76129842	CHRNE
10743	ENSG00000108557	2965.64326	-0.44128445	0.0888863	3.23E-07	3.97E-06	RAI1
4927	ENSG00000108559	1134.39831	-0.21720726	0.12866742	0.07202913	0.19555978	NUP88
708	ENSG00000108561	3808.88665	-0.1125761	0.06396481	0.07400147	0.19948222	C1QBP
6532	ENSG00000108576	4.22573552	-0.00309949	0.36599784	0.97584988	0.99164422	SLC6A4
642	ENSG00000108578	886.461708	0.13938071	0.09928369	0.1461443	0.32699131	BLMH
1362	ENSG00000108582	1962.61182	0.07864286	0.10964679	0.45470488	0.67793979	CPD
9527	ENSG00000108587	1151.82578	-0.13959385	0.10430533	0.16330719	0.3522953	GOSR1
57003	ENSG00000108588	2262.14771	-0.09236173	0.07373669	0.20135556	0.40672921	CCDC47
51003	ENSG00000108590	119.818279	0.0049412	0.20035023	0.97810407	0.9925115	MED31

1819	ENSG00000108591	847.52877	0.1864955	0.10780462	0.07106634	0.19367482	DRG2
117246	ENSG00000108592	4504.28229	0.20980187	0.06079203	0.00046262	0.00301725	FTSJ3
11216	ENSG00000108599	582.932545	0.11327769	0.13258273	0.36178062	0.59153844	AKAP10
218	ENSG00000108602	8.61631266	0.11655443	0.3808104	0.38602937	0.61516648	ALDH3A1
6603	ENSG00000108604	2932.52061	0.39005254	0.06120128	1.04E-10	1.99E-09	SMARCD2
3384	ENSG00000108622	12.9722805	-0.05457326	0.33361234	0.74925336	0.88205755	ICAM2
9144	ENSG00000108639	1049.49285	0.19461707	0.089259	0.02419628	0.08493526	SYNGR2
27077	ENSG00000108641	265.201554	0.37770185	0.16730909	0.01241976	0.04959348	B9D1
55813	ENSG00000108651	808.596373	-0.0016771	0.11542062	0.98758763	0.99589897	UTP6
1655	ENSG00000108654	23976.2845	0.32059642	0.05033515	6.47E-11	1.27E-09	DDX5
64149	ENSG00000108666	645.277122	0.2620755	0.1078495	0.01109939	0.04520876	C17orf75
9267	ENSG00000108669	1632.48656	-0.42332962	0.09358628	2.97E-06	3.11E-05	CYTH1
5717	ENSG00000108671	1803.65031	-0.0809402	0.07931557	0.29619329	0.52277154	PSMD11
3959	ENSG00000108679	4039.8094	0.28796904	0.05930276	8.72E-07	9.99E-06	LGALS3BP
40	ENSG00000108684	6.50085788	-1.89151098	1.54330811	0.00644398	0.02881418	ASIC2
6354	ENSG00000108688	0	NA	NA	NA	NA	CCL7
6347	ENSG00000108691	1.72932463	0.08712096	0.39265462	0.2408329	NA	CCL2
6355	ENSG00000108700	0	NA	NA	NA	NA	CCL8
6346	ENSG00000108702	0	NA	NA	NA	NA	CCL1
5193	ENSG00000108733	126.152773	-0.25400779	0.22105906	0.16521054	0.35517682	PEX12
3882	ENSG00000108759	0	NA	NA	NA	NA	KRT32
79132	ENSG00000108771	5.73552958	0.26164585	0.48910787	0.05973815	0.17060671	DHX58
2648	ENSG00000108773	911.06328	0.43553171	0.08797001	3.57E-07	4.37E-06	KAT2A
5878	ENSG00000108774	2056.17114	0.08675624	0.07740863	0.25268908	0.47273469	RAB5C
4669	ENSG00000108784	451.67595	0.05374246	0.13098853	0.66342638	0.83395929	NAGLU
NA	ENSG00000108785	6.71529656	0.22714716	0.45536447	0.09937985	0.24819941	HSD17B1P1
3292	ENSG00000108786	60.518987	-0.85682604	0.31967339	0.00096601	0.005742	HSD17B1
6945	ENSG00000108788	1006.91265	-0.20999523	0.08222681	0.00879312	0.03730872	MLX
8506	ENSG00000108797	709.591932	-0.11381142	0.09936468	0.23474483	0.45159871	CNTNAP1
51225	ENSG00000108798	0.16735865	0.01897177	0.38482933	0.78225479	NA	ABI3
2145	ENSG00000108799	832.497208	0.17052681	0.12247292	0.14128511	0.31950382	EZH1
1748	ENSG00000108813	6.45373025	-0.09732388	0.37294575	0.46815154	0.69110148	DLX4
84687	ENSG00000108819	3315.1103	-0.15991566	0.05614373	0.00395616	0.01912995	PPP1R9B
1277	ENSG00000108821	500.009829	1.03138621	0.1304819	3.44E-16	1.02E-14	COL1A1
6442	ENSG00000108823	0	NA	NA	NA	NA	SGCA
100885850	ENSG00000108825	51.1148958	-0.08388165	0.26412374	0.66323407	0.83380582	PTGES3L-AARSD1
51264	ENSG00000108826	845.128516	-0.09686402	0.09499695	0.29185951	0.5179626	MRPL27
10493	ENSG00000108828	2347.28583	-0.22190806	0.06844758	0.00096511	0.00573974	VAT1

55379	ENSG00000108829	3695.52459	-0.07734648	0.0679348	0.24682778	0.46592963	LRRC59
8153	ENSG00000108830	457.902847	0.103395	0.12101032	0.36831197	0.59778383	RND2
239	ENSG00000108839	5.72761136	-0.07503406	0.36923274	0.55826869	0.76291851	ALOX12
10014	ENSG00000108840	1205.31894	0.05628517	0.09518981	0.54220808	0.75027258	HDAC5
8714	ENSG00000108846	5.70309598	0.18040878	0.41583713	0.19039971	0.39167661	ABCC3
51747	ENSG00000108848	8267.54233	-0.06375328	0.05477604	0.23314867	0.45004948	LUC7L3
5539	ENSG00000108849	0	NA	NA	NA	NA	PPY
4355	ENSG00000108852	555.089859	-0.40765997	0.13575054	0.00130288	0.00741802	MPP2
64750	ENSG00000108854	1197.60495	-0.06498414	0.07884379	0.39914612	0.62789874	SMURF2
1845	ENSG00000108861	566.253691	-0.20773148	0.10780275	0.04360669	0.13394304	DUSP3
786	ENSG00000108878	14.3766637	0.53844089	0.6220397	0.04480104	0.13659944	CACNG1
9343	ENSG00000108883	2698.86246	-0.12545443	0.06442647	0.04802943	0.14433562	EFTUD2
3131	ENSG00000108924	54.3366352	-0.14889515	0.27958542	0.45091929	0.67429441	HLF
9120	ENSG00000108932	3.01395098	0.10536625	0.3900913	0.31639586	0.54438418	SLC16A6
5573	ENSG00000108946	4973.60772	0.00118055	0.06122997	0.98516087	0.99475332	PRKAR1A
1949	ENSG00000108947	1094.96135	-0.39709528	0.09852701	2.94E-05	0.00025373	EFNB3
54757	ENSG00000108950	7.12161697	0.39453756	0.69813581	0.01681691	0.06339493	FAM20A
7531	ENSG00000108953	11987.1618	-0.01970281	0.05800231	0.68481689	0.84658029	YWHAE
NA	ENSG00000108958	1.5213871	0.02387902	0.37697456	0.76883479	NA	
23531	ENSG00000108960	1690.44148	-0.02409393	0.0737201	0.73818549	0.87629989	MMD
29098	ENSG00000108961	384.264307	0.02778908	0.12741828	0.81851384	0.9171248	RANGRF
1801	ENSG00000108963	653.075013	-0.02932948	0.10868729	0.77755307	0.89804709	DPH1
5608	ENSG00000108984	1017.93641	0.37674072	0.09295461	2.85E-05	0.00024718	MAP2K6
25979	ENSG00000109016	195.379037	0.01110465	0.16274481	0.94113382	0.97559098	DHRS7B
26118	ENSG00000109046	2182.82089	0.88336755	0.08049044	8.67E-29	4.76E-27	WSB1
5957	ENSG00000109047	0	NA	NA	NA	NA	RCVRN
4619	ENSG00000109061	0	NA	NA	NA	NA	MYH1
9368	ENSG00000109062	899.51349	-0.3068541	0.08901866	0.0003797	0.00252961	SLC9A3R1
4621	ENSG00000109063	54.2763923	0.22438087	0.26765418	0.26477905	0.48704028	MYH3
26151	ENSG00000109065	1115.36485	0.33340223	0.09985793	0.00053155	0.00340189	NAT9
54868	ENSG00000109066	885.523823	0.16898003	0.10263318	0.08717357	0.22540384	TMEM104
7448	ENSG00000109072	6.28865344	0.18131634	0.41351403	0.19957029	0.40423425	VTN
7126	ENSG00000109079	1203.89449	0.18871239	0.083571	0.02056533	0.07453711	TNFAIP1
90410	ENSG00000109083	439.165573	0.14852274	0.11778062	0.18477608	0.38350847	IFT20
27346	ENSG00000109084	1293.1842	-0.41006974	0.08262202	3.53E-07	4.33E-06	TMEM97
30850	ENSG00000109089	1397.88679	-0.37824975	0.07825834	7.48E-07	8.64E-06	CDR2L
5376	ENSG00000109099	359.443231	0.74169156	0.13495344	8.16E-09	1.24E-07	PMP22
8456	ENSG00000109101	0	NA	NA	NA	NA	FOXN1

9094	ENSG00000109103	673.451644	0.01655826	0.09690728	0.86066457	0.93799827	UNC119
230	ENSG00000109107	1146.05846	-0.44491001	0.12316041	0.00013554	0.00099966	ALDOC
6830	ENSG00000109111	3490.30047	-0.10881689	0.06577711	0.0928079	0.2357385	SUPT6H
83871	ENSG00000109113	2.51350838	0.02037207	0.37463075	0.81869811	NA	RAB34
57649	ENSG00000109118	1137.7263	0.19748606	0.09708096	0.03518753	0.1137931	PHF12
8929	ENSG00000109132	7176.56282	-0.46739802	0.04931456	1.19E-21	4.79E-20	PHOX2B
55161	ENSG00000109133	2424.65443	-0.03399109	0.06669605	0.60442884	0.79458443	TMEM33
2557	ENSG00000109158	0	NA	NA	NA	NA	GABRA4
2798	ENSG00000109163	29.3927446	-0.35182036	0.37457075	0.12402749	0.2918294	GNRHR
57606	ENSG00000109171	765.848127	-0.44563782	0.13941975	0.00057581	0.003651	SLAIN2
54940	ENSG00000109180	1827.96054	-0.26015171	0.08239014	0.00118806	0.00686604	OCIAD1
7365	ENSG00000109181	0	NA	NA	NA	NA	UGT2B10
80157	ENSG00000109182	0	NA	NA	NA	NA	CWH43
23142	ENSG00000109184	949.392359	-0.32264191	0.09407333	0.00038727	0.00257143	DCUN1D4
64854	ENSG00000109189	822.371296	-0.18382696	0.08887354	0.03309938	0.10843655	USP46
6783	ENSG00000109193	0	NA	NA	NA	NA	SULT1E1
54959	ENSG00000109205	0.18922458	0.01954053	0.38488496	0.78225479	NA	ODAM
26952	ENSG00000109208	0	NA	NA	NA	NA	SMR3A
26511	ENSG00000109220	160.467496	0.01870666	0.16456131	0.9010266	0.95641534	CHIC2
10874	ENSG00000109255	485.245455	-0.18365819	0.12184291	0.11074757	0.26844028	NMU
57482	ENSG00000109265	3535.21793	0.1169184	0.05923697	0.04523248	0.13772444	CRACD
8649	ENSG00000109270	691.663827	-0.21774608	0.11350704	0.0438124	0.13439055	LAMTOR3
5197	ENSG00000109272	0	NA	NA	NA	NA	PF4V1
4790	ENSG00000109320	512.210432	0.07755029	0.10904704	0.45892615	0.68235016	NFKB1
374	ENSG00000109321	0	NA	NA	NA	NA	AREG
4126	ENSG00000109323	207.156472	-0.15389967	0.15958875	0.28747896	0.513189	MANBA
7323	ENSG00000109332	4308.72507	-0.03688782	0.06306656	0.55268953	0.75905858	UBE2D3
5602	ENSG00000109339	285.136244	-0.49127708	0.16179641	0.00089201	0.00536447	MAPK10
1998	ENSG00000109381	780.767722	-0.13441479	0.10173306	0.16977466	0.36148629	ELF2
4717	ENSG00000109390	539.110481	0.10563858	0.11199375	0.32724515	0.55563061	NDUFC1
7350	ENSG00000109424	1.7341049	0.07227386	0.38511793	0.3934411	NA	UCP1
23158	ENSG00000109436	844.817758	-0.2021346	0.09922215	0.03443602	0.1119467	TBC1D9
27309	ENSG00000109445	609.292423	0.0422794	0.11212053	0.69417247	0.85201936	ZNF330
8821	ENSG00000109452	37.8229174	0.84681629	0.44042117	0.00580143	0.02639156	INPP4B
2549	ENSG00000109458	546.459055	0.04821992	0.11057272	0.6497079	0.82525848	GAB1
11275	ENSG00000109466	367.428747	-0.0490505	0.12318679	0.6733635	0.83953925	KLHL2
3558	ENSG00000109471	0.18922458	0.01954053	0.38488496	0.78225479	NA	IL2
1363	ENSG00000109472	2150.55977	0.03169082	0.06531091	0.62259211	0.80737672	CPE

6164	ENSG00000109475	6489.64829	-0.03284531	0.05395212	0.53838391	0.74816395	RPL34
7466	ENSG00000109501	582.546713	-0.03363593	0.1233326	0.77231102	0.89548539	WFS1
11199	ENSG00000109511	0	NA	NA	NA	NA	ANXA10
80273	ENSG00000109519	554.032572	-0.4173424	0.11314987	0.00011048	0.00083196	GRPEL1
54433	ENSG00000109534	441.017187	-0.02138813	0.11345569	0.84115065	0.92789889	GAR1
2483	ENSG00000109536	487.68078	0.17537597	0.12476946	0.13669769	0.31191283	FRG1
1182	ENSG00000109572	1605.83311	-0.28231623	0.10090931	0.00361685	0.01774875	CLCN3
51166	ENSG00000109576	462.975143	-0.31401218	0.12666307	0.00839239	0.03593894	AADAT
51809	ENSG00000109586	711.48856	0.12272493	0.10063736	0.20625256	0.41325498	GALNT7
1665	ENSG00000109606	3946.4074	-0.26173545	0.05953012	8.21E-06	7.93E-05	DHX15
6649	ENSG00000109610	0	NA	NA	NA	NA	SOD3
51091	ENSG00000109618	352.306816	0.37673964	0.15266074	0.0072047	0.0316592	SEPSECS
8532	ENSG00000109625	3.47262192	-0.05659922	0.376693	0.57286364	0.7725692	CPZ
23321	ENSG00000109654	1915.05351	-0.02413334	0.06986418	0.72472286	0.86844186	TRIM2
56606	ENSG00000109667	0	NA	NA	NA	NA	SLC2A9
55294	ENSG00000109670	530.156149	-0.0441355	0.14643146	0.74282513	0.87875221	FBXW7
55247	ENSG00000109674	307.262669	-0.28737984	0.13347133	0.02123851	0.07646018	NEIL3
55296	ENSG00000109680	106.08109	0.04941062	0.22607335	0.78865387	0.90411045	TBC1D19
116449	ENSG00000109684	0	NA	NA	NA	NA	CLNK
7468	ENSG00000109685	7533.0208	-0.03265264	0.05744057	0.56471395	0.76707686	NSD2
152503	ENSG00000109686	818.442177	0.01708235	0.10372643	0.86555139	0.94044576	SH3D19
57620	ENSG00000109689	427.925431	-0.36687639	0.12069152	0.0013258	0.00753476	STIM2
579	ENSG00000109705	0.51937201	-0.02557462	0.38567603	0.54680819	NA	NKX3-2
10227	ENSG00000109736	1588.27834	0.10371802	0.07582077	0.16275579	0.35164399	MFSD10
2743	ENSG00000109738	269.482588	-0.05291332	0.13783838	0.67963171	0.84363969	GLRB
683	ENSG00000109743	22.5310946	0.43578084	0.48979146	0.07337062	0.19821257	BST1
9693	ENSG00000109756	1319.76801	-0.11571453	0.09104076	0.18993384	0.39109764	RAPGEF2
3083	ENSG00000109758	25.1144172	-0.02925327	0.31817529	0.86838062	0.94253385	HGFAC
83891	ENSG00000109762	336.726861	0.16860012	0.1340834	0.1789897	0.37507273	SNX25
55805	ENSG00000109771	56.9244435	0.10013099	0.27235083	0.60939075	0.79803651	LRP2BP
55325	ENSG00000109775	342.226402	-0.11621671	0.15026433	0.39902411	0.62780637	UFSP2
51274	ENSG00000109787	777.787234	0.46405493	0.09612368	6.13E-07	7.21E-06	KLF3
51088	ENSG00000109790	768.170014	0.0416292	0.10466579	0.68021974	0.8437889	KLHL5
25854	ENSG00000109794	88.2378753	0.18159568	0.22064431	0.32052103	0.54831381	FAM149A
64151	ENSG00000109805	1723.26269	-0.40600455	0.07866679	1.27E-07	1.65E-06	NCAPG
7358	ENSG00000109814	764.925458	0.49529497	0.0996643	2.72E-07	3.38E-06	UGDH
10891	ENSG00000109819	1.38058753	0.08305142	0.39323914	0.18324153	NA	PPARGC1A
29118	ENSG00000109832	113.07469	-1.03726489	0.22970343	7.37E-07	8.52E-06	DDX25

1410	ENSG00000109846	0.88111352	0.04023644	0.38511396	0.43011874	NA	CRYAB
120237	ENSG00000109851	6.39698679	1.71864857	1.4450455	0.00671209	0.02984486	DBX1
10553	ENSG00000109854	0.75661163	0.02039878	0.38277281	0.61488206	NA	HTATIP2
1075	ENSG00000109861	1472.23436	0.41934927	0.07897028	5.58E-08	7.61E-07	CTSC
91057	ENSG00000109881	634.810491	0.10421197	0.11373853	0.33752403	0.56783488	CCDC34
7704	ENSG00000109906	5.86486181	5.85787459	2.69371741	8.22E-05	0.00063814	ZBTB16
26610	ENSG00000109911	276.192369	0.06493141	0.13443281	0.60655747	0.79616939	ELP4
8882	ENSG00000109917	1445.79406	0.01209704	0.07921174	0.87162158	0.94401928	ZPR1
23788	ENSG00000109919	1228.23704	-0.01990533	0.09582592	0.82986442	0.92286579	MTCH2
23360	ENSG00000109920	2335.50193	0.15118196	0.09179425	0.08971587	0.23005853	FNBP4
7007	ENSG00000109927	196.221605	0.00667454	0.17747865	0.96744621	0.98759962	TECTA
116804918	ENSG00000109927	196.221605	0.00667454	0.17747865	0.96744621	0.98759962	TECTA
6309	ENSG00000109929	1014.76503	-0.39374735	0.09132051	8.65E-06	8.31E-05	SC5D
56253	ENSG00000109943	0	NA	NA	NA	NA	CRTAM
79864	ENSG00000109944	249.316417	-0.15517077	0.15536329	0.27351586	0.49702826	JHY
27087	ENSG00000109956	1180.36134	-1.1765512	0.08384933	1.05E-45	9.33E-44	B3GAT1
3312	ENSG00000109971	24242.3421	0.05048398	0.15735967	0.69911079	0.85487569	HSPA8
5024	ENSG00000109991	1.16465936	0.0129262	0.38077068	0.82412664	NA	P2RX3
4013	ENSG00000110002	2.85141005	0.20572818	0.4662256	0.00617919	0.02776728	VWA5A
3338	ENSG00000110011	439.302894	0.24079158	0.11641755	0.0296084	0.09932622	DNAJC4
54414	ENSG00000110013	225.101533	0.11917485	0.15430038	0.39948503	0.62823264	SIAE
29907	ENSG00000110025	358.748659	0.03104178	0.13586427	0.80806739	0.9131433	SNX15
9404	ENSG00000110031	56.3075155	0.09458856	0.25116151	0.62277641	0.80737672	LPXN
23220	ENSG00000110042	4.28385762	-0.06837238	0.37569674	0.51802528	0.73186794	DTX4
23130	ENSG00000110046	500.653219	0.30536238	0.12983498	0.01209153	0.04853248	ATG2A
10938	ENSG00000110047	928.750974	0.13372497	0.09459129	0.14452577	0.32462259	EHD1
5007	ENSG00000110048	1013.88033	-0.18175309	0.08322043	0.02501787	0.08721718	OSBP
81622	ENSG00000110057	193.5367	0.51835565	0.19464986	0.00259364	0.01339143	UNC93B1
83480	ENSG00000110060	386.40288	-0.63848525	0.15258349	7.28E-06	7.11E-05	PUS3
28960	ENSG00000110063	632.883007	-0.13449852	0.14929448	0.32587361	0.55406054	DCPS
51111	ENSG00000110066	1035.48673	-0.20112005	0.10156243	0.03967279	0.12460156	KMT5B
55572	ENSG00000110074	780.181088	0.0849172	0.09336195	0.35008277	0.58049663	FOXRED1
55291	ENSG00000110075	2331.89512	-0.22394849	0.06636116	0.00059905	0.00377783	PPP6R3
9379	ENSG00000110076	1769.16533	-2.05590412	0.11977348	2.85E-67	4.38E-65	NRXN2
64231	ENSG00000110077	0	NA	NA	NA	NA	MS4A6A
51338	ENSG00000110079	0	NA	NA	NA	NA	MS4A4A
6484	ENSG00000110080	427.734281	-0.21786232	0.12391736	0.06202828	0.17577159	ST3GAL4
1374	ENSG00000110090	2016.07227	-0.17093179	0.07239135	0.01603998	0.06089421	CPT1A

595	ENSG00000110092	11802.8601	0.46388918	0.04840568	4.94E-22	2.03E-20	CCND1
79080	ENSG00000110104	460.223935	0.00958926	0.11474326	0.93112873	0.97127203	CCDC86
27339	ENSG00000110107	3827.62383	-0.18709649	0.0541113	0.00046662	0.00304032	PRPF19
79073	ENSG00000110108	867.341365	0.57153026	0.08965667	6.06E-11	1.19E-09	TMEM109
887	ENSG00000110148	0	NA	NA	NA	NA	CCKBR
3263	ENSG00000110169	14.3807216	0.0156211	0.32487809	0.93058727	0.97111825	HPX
10612	ENSG00000110171	510.241176	0.07266527	0.11067862	0.49346069	0.712409	TRIM3
26973	ENSG00000110172	1051.95993	-0.01000316	0.1371173	0.9363719	0.97351594	CHORDC1
2348	ENSG00000110195	9.62195929	0.15876504	0.38087816	0.33132313	0.56018597	FOLR1
25906	ENSG00000110200	404.07179	-0.13963893	0.12170931	0.22526372	0.43906825	ANAPC15
2352	ENSG00000110203	0	NA	NA	NA	NA	FOLR3
24145	ENSG00000110218	830.531119	-0.10196803	0.0962196	0.2728411	0.49643869	PANX1
9828	ENSG00000110237	1582.14066	0.02828405	0.11337194	0.79490931	0.90585603	ARHGEF17
116519	ENSG00000110243	0	NA	NA	NA	NA	APOA5
337	ENSG00000110244	0	NA	NA	NA	NA	APOA4
345	ENSG00000110245	0	NA	NA	NA	NA	APOC3
22897	ENSG00000110274	1436.87893	0.29970145	0.09728434	0.00141249	0.00797085	CEP164
50862	ENSG00000110315	604.510087	0.15036572	0.10031427	0.12016019	0.28543799	RNF141
57562	ENSG00000110318	217.483075	0.88121771	0.16482987	1.40E-08	2.06E-07	CEP126
1982	ENSG00000110321	15656.9086	-0.01975673	0.05330238	0.70733573	0.85900354	EIF4G2
3587	ENSG00000110324	26.5502945	-0.21497768	0.32882251	0.29580051	0.52251089	IL10RA
374378	ENSG00000110328	111.763786	-0.24095513	0.20094304	0.16095829	0.34896869	GALNT18
329	ENSG00000110330	811.051819	-0.16901828	0.09839598	0.07495729	0.20134749	BIRC2
9354	ENSG00000110344	1233.67794	-0.23483098	0.08373238	0.00397017	0.01917902	UBE4A
1656	ENSG00000110367	3809.47553	-0.34830445	0.07673295	3.46E-06	3.57E-05	DDX6
7379	ENSG00000110375	1.61243246	0.06586775	0.38733039	0.33307085	NA	UPK2
867	ENSG00000110395	2378.82164	-0.20747291	0.06164496	0.00063929	0.00399227	CBL
5818	ENSG00000110400	3424.53417	-1.09831713	0.0646254	1.04E-65	1.51E-63	NECTIN1
10114	ENSG00000110422	645.669401	0.22826607	0.0989464	0.01673247	0.0631366	HIPK3
25758	ENSG00000110427	492.246888	-0.02873544	0.1317981	0.81560959	0.91634261	KIAA1549L
26273	ENSG00000110429	592.599198	-0.37299958	0.10622336	0.00024885	0.00173172	FBXO3
8050	ENSG00000110435	350.393937	-0.60766164	0.13841138	3.19E-06	3.31E-05	PDHX
6506	ENSG00000110436	93.7063323	-1.6908313	0.32917952	1.89E-08	2.76E-07	SLC1A2
29099	ENSG00000110442	612.941592	-0.10667629	0.11246128	0.31905646	0.54668625	COMMD9
51296	ENSG00000110446	10.4359458	1.58793322	1.57880572	0.00456031	0.02153176	SLC15A3
921	ENSG00000110448	0.48288597	0.01075364	0.38337498	0.79448031	NA	CD5
84680	ENSG00000110455	271.829003	0.36037817	0.16692462	0.01676457	0.06323365	ACCS
4250	ENSG00000110484	0	NA	NA	NA	NA	SCGB2A2

4192	ENSG00000110492	14341.9244	0.62868041	0.04872882	1.69E-38	1.26E-36	MDK
55626	ENSG00000110497	704.983917	-0.29945028	0.10697631	0.00344117	0.01700868	AMBRA1
8567	ENSG00000110514	2177.08271	-0.34995246	0.0652744	5.08E-08	6.99E-07	MADD
114971	ENSG00000110536	687.496775	0.07067939	0.10953099	0.50072293	0.71795498	PTPMT1
79829	ENSG00000110583	1363.67728	-0.08307314	0.10284843	0.40088928	0.62929371	NAA40
833	ENSG00000110619	1837.35724	0.3683656	0.22571175	0.05064528	0.15041952	CARS1
5002	ENSG00000110628	75.3735418	0.25878205	0.24472735	0.18247278	0.3803144	SLC22A18
975	ENSG00000110651	2886.33774	0.1130447	0.06486313	0.07667895	0.20477996	CD81
54733	ENSG00000110660	610.570392	-0.03989219	0.09721403	0.67072233	0.83771678	SLC35F2
29125	ENSG00000110665	0.5141508	-0.0102321	0.38328162	0.81789405	NA	C11orf21
55531	ENSG00000110675	14.8563956	2.59521388	0.82734663	9.09E-05	0.00069919	ELMOD1
796	ENSG00000110680	0.48350556	0.01083862	0.383379	0.79316478	NA	CALCA
55553	ENSG00000110693	8.40267497	-0.04542883	0.35769744	0.73669124	0.87520684	SOX6
10944	ENSG00000110696	2712.89068	-0.00633048	0.07274783	0.93148653	0.97149202	C11orf58
9600	ENSG00000110697	742.854278	-0.24345281	0.1465415	0.07101047	0.19357622	PITPNM1
6207	ENSG00000110700	8232.7837	-0.1797297	0.06419481	0.00443563	0.02105615	RPS13
9049	ENSG00000110711	921.007428	0.21877668	0.09829404	0.0210698	0.07596307	AIP
4928	ENSG00000110713	1996.55498	-0.20080529	0.09441969	0.02757229	0.09381484	NUP98
4728	ENSG00000110717	1129.13064	0.22801851	0.08051578	0.00373366	0.01821386	NDUFS8
10312	ENSG00000110719	23.8536382	-0.04173323	0.31025238	0.81878985	0.91737015	TCIRG1
1119	ENSG00000110721	517.744602	-0.04215858	0.11538394	0.70085257	0.85575596	CHKA
23086	ENSG00000110723	237.850372	-0.72449738	0.17165996	5.03E-06	5.05E-05	EXPH5
11234	ENSG00000110756	450.923351	-0.06438196	0.12274462	0.57878454	0.77656626	HPS5
2965	ENSG00000110768	686.679346	-0.04087787	0.10220385	0.67730437	0.84233177	GTF2H1
5450	ENSG00000110777	0.50207596	0.02562368	0.38565771	0.52549886	NA	POU2AF1
84867	ENSG00000110786	14.6136215	0.19212982	0.3821038	0.28565743	0.51117916	PTPN5
7450	ENSG00000110799	3.29160881	0.22728245	0.48152555	0.01536999	0.05873277	VWF
5715	ENSG00000110801	519.827608	-0.00144811	0.1089136	0.98805324	0.99589897	PSMD9
10536	ENSG00000110811	825.336702	-0.00266572	0.0888272	0.97380885	0.99048209	P3H3
8496	ENSG00000110841	855.839492	0.04511532	0.11401696	0.67956002	0.84361793	PPFIBP1
25766	ENSG00000110844	780.653931	0.2438121	0.09169683	0.00608139	0.02744208	PRPF40B
969	ENSG00000110848	0	NA	NA	NA	NA	CD69
11108	ENSG00000110851	1084.75166	-0.05077475	0.08293854	0.53146385	0.74283399	PRDM4
9976	ENSG00000110852	0	NA	NA	NA	NA	CLEC2B
84274	ENSG00000110871	459.124522	-0.12399827	0.129089	0.30663196	0.53422766	COQ5
6404	ENSG00000110876	2.67161718	-0.02088627	0.37356275	0.81986499	0.91788244	SELPLG
23603	ENSG00000110880	2380.41965	-0.38084832	0.06969864	2.61E-08	3.73E-07	CORO1C
41	ENSG00000110881	1397.56817	0.34937787	0.08385751	1.87E-05	0.00016877	ASIC1

1610	ENSG00000110887	0.94545361	-0.06630286	0.39158611	0.20462663	NA	DAO
65981	ENSG00000110888	806.811019	0.31180871	0.1185521	0.00555675	0.02545505	CAPRIN2
441631	ENSG00000110900	1181.37849	0.38771949	0.10938419	0.00021232	0.00150118	TSPAN11
83892	ENSG00000110906	1767.5853	-0.03167316	0.0759022	0.6701198	0.83758072	KCTD10
4891	ENSG00000110911	927.383677	0.01199104	0.09471616	0.89697336	0.95487211	SLC11A2
9761	ENSG00000110917	3918.17371	0.00098569	0.0696739	0.98927223	0.99604194	MLEC
4598	ENSG00000110921	734.035878	-0.64403291	0.1216936	3.12E-08	4.40E-07	MVK
81566	ENSG00000110925	1223.32934	0.14527333	0.08516977	0.07966504	0.21062825	CSRNP2
10645	ENSG00000110931	1248.17303	-0.2594308	0.10292138	0.00864632	0.03678337	CAMKK2
51411	ENSG00000110934	0	NA	NA	NA	NA	BIN2
51561	ENSG00000110944	4.00619291	-0.03927615	0.3679199	0.73405354	0.87332827	IL23A
506	ENSG00000110955	12254.6537	-0.0396781	0.05645817	0.46097988	0.68410808	ATP5F1B
10728	ENSG00000110958	4213.61397	0.05972234	0.07927267	0.45043281	0.67378216	PTGES3
341359	ENSG00000110975	0	NA	NA	NA	NA	SYT10
605	ENSG00000110987	1799.73155	0.09832117	0.07487301	0.18069553	0.37770382	BCL7A
65117	ENSG00000111011	1642.66785	0.1969941	0.08175621	0.01352792	0.05313349	RSRC2
1594	ENSG00000111012	37.5564478	0.15471669	0.30581205	0.43762062	0.66277811	CYP27B1
4618	ENSG00000111046	0	NA	NA	NA	NA	MYF6
4617	ENSG00000111049	0	NA	NA	NA	NA	MYF5
8825	ENSG00000111052	1.92144211	0.10311487	0.39754402	0.19146554	NA	LIN7A
3875	ENSG00000111057	6.20539866	4.22933163	1.6165998	0.00041614	0.00274175	KRT18
79611	ENSG00000111058	0	NA	NA	NA	NA	ACSS3
23371	ENSG00000111077	113.683945	0.23584041	0.24378569	0.22198755	0.43502894	TNS2
2735	ENSG00000111087	14.2938164	3.84728093	0.88571466	1.39E-06	1.54E-05	GLI1
57460	ENSG00000111110	0.18922458	0.01954053	0.38488496	0.78225479	NA	PPM1H
10988	ENSG00000111142	2330.98353	-0.1755746	0.06288719	0.00459042	0.02164819	METAP2
4048	ENSG00000111144	1999.22086	-0.09704277	0.06602785	0.13538836	0.30992327	LTA4H
2004	ENSG00000111145	962.232646	0.72297377	0.1003756	1.33E-13	3.23E-12	ELK3
6539	ENSG00000111181	26.1252088	-0.01386174	0.31957348	0.93550311	0.97325384	SLC6A12
81029	ENSG00000111186	116.149179	-1.14589445	0.2346076	1.08E-07	1.42E-06	WNT5B
55110	ENSG00000111196	388.528726	-0.06186044	0.16268371	0.67406438	0.8400928	MAGOHB
59341	ENSG00000111199	42.4655853	2.7039615	0.42659893	1.65E-11	3.44E-10	TRPV4
55846	ENSG00000111203	697.709649	-0.05088096	0.11420429	0.63986107	0.81944511	ITFG2
2305	ENSG00000111206	3099.25282	-0.19644558	0.0660012	0.00249027	0.01293039	FOXM1
5554	ENSG00000111215	24.2173752	0.29276867	0.37354639	0.17927966	0.37559752	PRR4
11272	ENSG00000111215	24.2173752	0.29276867	0.37354639	0.17927966	0.37559752	PRR4
56341	ENSG00000111218	8.79792492	-3.42208217	1.27228974	0.00049525	0.00319742	PRMT8
57097	ENSG00000111224	141.32679	-0.55365379	0.25032494	0.00721883	0.03170723	PARP11

10094	ENSG00000111229	1888.37639	-0.36386245	0.07829853	1.97E-06	2.13E-05	ARPC3
51184	ENSG00000111231	397.109313	0.03938782	0.12045172	0.7335631	0.87316366	GPN3
51699	ENSG00000111237	580.52632	-0.26934226	0.10469903	0.00730954	0.03204181	VPS29
2251	ENSG00000111241	0.41622699	-0.011908	0.38380213	0.90842947	NA	FGF6
4633	ENSG00000111245	0.16735865	0.01897177	0.38482933	0.78225479	NA	MYL2
10635	ENSG00000111247	497.299307	-0.11388052	0.11598433	0.30108091	0.52835924	RAD51AP1
23316	ENSG00000111249	2231.78609	-1.64087275	0.07385442	1.75E-110	5.89E-108	CUX2
10019	ENSG00000111252	314.732362	-0.93265754	0.13868613	2.59E-12	5.75E-11	SH2B3
10566	ENSG00000111254	4.61600344	0.12881711	0.39931718	0.21869423	0.43056392	AKAP3
54682	ENSG00000111261	494.854066	0.07762804	0.10648297	0.44850531	0.67234398	MANSC1
3736	ENSG00000111262	0	NA	NA	NA	NA	KCNA1
80824	ENSG00000111266	1509.05487	0.2021548	0.08557365	0.01522328	0.05834073	DUSP16
1389	ENSG00000111269	685.977753	-0.32682549	0.11183122	0.0021645	0.0114547	CREBL2
80724	ENSG00000111271	805.824078	0.22943035	0.12152751	0.04603052	0.13970407	ACAD10
217	ENSG00000111275	1261.57088	0.17593911	0.08126273	0.02654754	0.09123563	ALDH2
1027	ENSG00000111276	1385.83813	0.29648652	0.07848381	0.00011058	0.00083228	CDKN1B
55507	ENSG00000111291	5.11030394	-0.26922247	0.5131179	0.03215629	0.10595926	GPRC5D
80018	ENSG00000111300	915.871402	0.02207142	0.12727873	0.85577116	0.93566075	NAA25
83445	ENSG00000111305	0.29863895	-0.02292439	0.3852726	0.68753245	NA	GSG1
6337	ENSG00000111319	0	NA	NA	NA	NA	SCNN1A
4055	ENSG00000111321	0	NA	NA	NA	NA	LTBR
79676	ENSG00000111325	213.38681	0.12999646	0.15585293	0.36167035	0.59140689	OGFOD2
8099	ENSG00000111328	3591.41519	-0.3132742	0.05722274	2.88E-08	4.08E-07	CDK2AP1
4940	ENSG00000111331	841.383443	-0.67526189	0.10579907	4.26E-11	8.53E-10	OAS3
4939	ENSG00000111335	0.96822761	0.01469816	0.38058074	0.80647333	NA	OAS2
420	ENSG00000111339	0.88508185	-0.02256421	0.38270569	0.65529417	NA	ART4
4256	ENSG00000111341	48.4430577	3.93603908	0.5466793	1.07E-14	2.83E-13	MGP
8437	ENSG00000111344	6.08224322	-0.39967982	0.6905099	0.0222	0.07917454	RASAL1
397	ENSG00000111348	8.52290665	-0.00405304	0.35345838	0.97421966	0.99074878	ARHGDIB
2967	ENSG00000111358	670.293816	-0.02683926	0.1232272	0.81696672	0.91671007	GTF2H3
1967	ENSG00000111361	1491.01757	-0.04813833	0.07701279	0.52275088	0.73592404	EIF2B1
57696	ENSG00000111364	1002.63086	0.00855993	0.09642197	0.92775984	0.96974634	DDX55
81539	ENSG00000111371	5879.91074	-0.67905755	0.06056619	9.00E-30	5.13E-28	SLC38A1
79785	ENSG00000111404	0	NA	NA	NA	NA	RERGL
8909	ENSG00000111405	0.33942509	0.00599294	0.38348079	0.92248644	NA	ENDOU
79794	ENSG00000111412	1421.54517	0.38419268	0.09160421	1.51E-05	0.00013924	SPRING1
7421	ENSG00000111424	1.71285337	-0.02288719	0.37784429	0.76060239	NA	VDR
11211	ENSG00000111432	0	NA	NA	NA	NA	FZD10

5985	ENSG00000111445	750.807164	-0.14219623	0.09898899	0.13628676	0.3113874	RFC5
2054	ENSG00000111450	835.219817	0.20791622	0.1066738	0.0420546	0.13057329	STX2
283383	ENSG00000111452	1.92157058	0.08055634	0.38802455	0.33354437	NA	ADGRD1
22818	ENSG00000111481	1800.69811	-0.29802313	0.08859567	0.00052578	0.00336827	COPZ1
23329	ENSG00000111490	67.4019754	-0.20322121	0.30429301	0.31674652	0.54452953	TBC1D30
55832	ENSG00000111530	3419.44204	-0.15792894	0.06919693	0.01981933	0.07246877	CAND1
55801	ENSG00000111536	0	NA	NA	NA	NA	IL26
3458	ENSG00000111537	0	NA	NA	NA	NA	IFNG
5869	ENSG00000111540	1590.11376	0.25093356	0.08229086	0.00176283	0.00961126	RAB5B
56890	ENSG00000111554	211.072788	0.23127754	0.17330416	0.1348352	0.30933515	MDM1
57122	ENSG00000111581	1738.5538	0.14988528	0.07468872	0.0406671	0.12692727	NUP107
4848	ENSG00000111596	1208.12689	-0.17056213	0.11730373	0.12553049	0.29445791	CNOT2
8914	ENSG00000111602	2941.39273	-0.19133645	0.06060372	0.00136987	0.00775635	TIMELESS
11052	ENSG00000111605	3808.57951	-0.12755102	0.07720697	0.09125901	0.23314174	CPSF6
11103	ENSG00000111615	962.686787	0.06046263	0.09127198	0.49568022	0.71444211	KRR1
51258	ENSG00000111639	1448.68338	-0.07036394	0.07967377	0.3659924	0.59518764	MRPL51
2597	ENSG00000111640	41981.5922	-0.31179498	0.04923997	1.59E-10	2.99E-09	GAPDH
4839	ENSG00000111641	1295.47275	-0.03763451	0.08180336	0.63529439	0.81649547	NOP2
1108	ENSG00000111642	9925.66245	-0.08588226	0.0547325	0.11265657	0.27179207	CHD4
84519	ENSG00000111644	29.1295179	0.11963162	0.30811459	0.53902577	0.74843996	ACRBP
23074	ENSG00000111647	362.543649	-0.16847421	0.17250278	0.27219449	0.49553485	UHRF1BP1L
50813	ENSG00000111652	953.122746	0.02882778	0.08823187	0.73785086	0.87620179	COPS7A
51147	ENSG00000111653	787.464604	0.23476921	0.11146677	0.02737401	0.09336911	ING4
2784	ENSG00000111664	209.072685	-0.18852131	0.16528472	0.20491676	0.41178452	GNB3
83461	ENSG00000111665	623.881956	0.3496305	0.10500319	0.00051996	0.0033374	CDCA3
56994	ENSG00000111666	207.450735	0.40859796	0.16758569	0.00701275	0.03102877	CHPT1
8078	ENSG00000111667	2341.54813	0.05593223	0.09990056	0.56310692	0.76599528	USP5
7167	ENSG00000111669	8608.90463	-0.31208515	0.06787942	2.86E-06	2.99E-05	TPI1
79158	ENSG00000111670	749.338788	0.10786883	0.09723673	0.25141801	0.47149344	GNPTAB
84727	ENSG00000111671	65.8295267	0.20968846	0.24919363	0.28189576	0.50671288	SPSB2
105369632	ENSG00000111671	65.8295267	0.20968846	0.24919363	0.28189576	0.50671288	SPSB2
2026	ENSG00000111674	2661.78006	-0.69921479	0.07359033	4.93E-22	2.03E-20	ENO2
1822	ENSG00000111676	4543.93138	0.03383238	0.06075363	0.57021992	0.7706786	ATN1
113246	ENSG00000111678	2729.51542	0.10837683	0.06107297	0.07231345	0.19591682	C12orf57
5777	ENSG00000111679	5.26377636	-0.02251008	0.36277216	0.84945481	0.93154246	PTPN6
10162	ENSG00000111684	240.697458	-0.35260942	0.19984103	0.04045966	0.1264723	LPCAT3
51559	ENSG00000111696	242.197957	-0.20842901	0.2150688	0.24370097	0.46210717	NT5DC3
28234	ENSG00000111700	0	NA	NA	NA	NA	SLCO1B3

339	ENSG00000111701	0	NA	NA	NA	NA	APOBEC1
79923	ENSG00000111704	1.79730873	0.04660996	0.38508775	0.41809387	NA	NANOG
64426	ENSG00000111707	1412.75509	-0.08347938	0.08304221	0.30256929	0.52987991	SUDS3
51026	ENSG00000111711	439.043704	0.26562268	0.12325521	0.02250497	0.08005423	GOLT1B
2998	ENSG00000111713	0.5972779	-0.02732172	0.38586499	0.50076333	NA	GYS2
3945	ENSG00000111716	12948.9701	-0.02382206	0.04985254	0.62218753	0.80700149	LDHB
5564	ENSG00000111725	668.264447	0.03132137	0.10978827	0.76662218	0.8924999	PRKAB1
55907	ENSG00000111726	590.588425	-0.03606751	0.10685062	0.72315344	0.86764418	CMAS
29915	ENSG00000111727	224.29375	0.28167879	0.17995584	0.07670975	0.20483464	HCFC2
6489	ENSG00000111728	142.103458	-0.47922731	0.2855609	0.02874154	0.0970608	ST8SIA1
50856	ENSG00000111729	3.70376004	0.08805402	0.38278138	0.41753251	0.64375544	CLEC4A
9847	ENSG00000111731	1225.89478	-0.31473072	0.08757205	0.00021427	0.00151393	C2CD5
57379	ENSG00000111732	0	NA	NA	NA	NA	AICDA
11021	ENSG00000111737	1252.41731	-0.18539305	0.08802957	0.03014746	0.10072846	RAB35
1911	ENSG00000111752	2025.00212	-0.07003312	0.07127535	0.31679814	0.54455685	PHC1
1337	ENSG00000111775	3892.93336	-0.22798156	0.05829421	7.44E-05	0.00058583	COX6A1
NA	ENSG00000111780	1.98388243	0.00839964	0.37501741	0.92280135	NA	
5992	ENSG00000111783	0	NA	NA	NA	NA	RFX4
55188	ENSG00000111785	504.819782	-0.14941944	0.11442916	0.17004776	0.36191941	RIC8B
8683	ENSG00000111786	3022.38086	-0.63103631	0.06078666	8.63E-26	4.21E-24	SRSF9
NA	ENSG00000111788	518.312109	0.44604543	0.11477291	4.65E-05	0.00038373	
26127	ENSG00000111790	514.147598	0.06123066	0.11118318	0.56557908	0.76741504	FGFR1OP2
3820	ENSG00000111796	0.16735865	0.01897177	0.38482933	0.78225479	NA	KLRB1
1303	ENSG00000111799	0	NA	NA	NA	NA	COL12A1
10384	ENSG00000111801	177.842693	0.14821725	0.17306758	0.33772324	0.56802552	BTN3A3
51567	ENSG00000111802	533.506077	-0.2079652	0.10985348	0.04717392	0.14221708	TDP2
2444	ENSG00000111816	29.5239557	0.06044742	0.29057571	0.75532417	0.88614698	FRK
29940	ENSG00000111817	348.605293	0.20528366	0.14447944	0.12427899	0.29221323	DSE
51389	ENSG00000111832	675.470771	-0.06311315	0.09480151	0.49205151	0.71113078	RWDD1
345895	ENSG00000111834	9.45337218	0.01490944	0.3440885	0.92529201	0.96892907	RSPH4A
4117	ENSG00000111837	10.4514753	0.21722784	0.40710845	0.21827192	0.43029759	MAK
51522	ENSG00000111843	1088.13024	0.016327	0.07975379	0.83382168	0.92431452	TMEM14C
55003	ENSG00000111845	530.179299	-0.15772416	0.11194541	0.13954785	0.31648712	PAK1IP1
2651	ENSG00000111846	312.498675	0.3253821	0.14996061	0.0183127	0.06768329	GCNT2
57150	ENSG00000111850	191.678029	-0.3233674	0.17528915	0.0381365	0.12111865	SMIM8
63914	ENSG00000111850	191.678029	-0.3233674	0.17528915	0.0381365	0.12111865	SMIM8
4739	ENSG00000111859	142.963176	0.00337826	0.25281257	0.98787041	0.99589897	NEDD9
387119	ENSG00000111860	181.804303	0.17623505	0.18151857	0.27130813	0.49473993	CEP85L

84830	ENSG00000111863	0	NA	NA	NA	NA	ADTRP
25842	ENSG00000111875	317.267318	0.24433051	0.17427927	0.11546308	0.27726792	ASF1A
254394	ENSG00000111877	273.784783	0.00902701	0.1396704	0.94450651	0.97736448	MCM9
79632	ENSG00000111879	423.593539	-0.19136229	0.1174661	0.08581768	0.22271062	FAM184A
8732	ENSG00000111880	817.046442	-0.11385018	0.10198654	0.24603455	0.46528697	RNGTT
4121	ENSG00000111885	589.252308	2.65175533	0.11487246	5.10E-119	1.98E-116	MAN1A1
2570	ENSG00000111886	24.4405494	0.5591125	0.58704782	0.03993832	0.12522429	GABRR2
57515	ENSG00000111897	2235.92398	-0.20412519	0.06595638	0.0016158	0.00894855	SERINC1
51020	ENSG00000111906	590.095193	0.08305402	0.12483076	0.48227258	0.70272483	HDDC2
7164	ENSG00000111907	0.34209262	0.02363404	0.38535456	0.62358548	NA	TPD52L1
135114	ENSG00000111911	481.176965	-0.35134169	0.1181704	0.001726	0.00945647	HINT3
135112	ENSG00000111912	440.194355	0.0190385	0.14293694	0.88649177	0.95015905	NCOA7
9750	ENSG00000111913	1.15110275	-0.00344607	0.3801718	0.97223975	NA	RIPOR2
23328	ENSG00000111961	0	NA	NA	NA	NA	SASH1
10090	ENSG00000111962	0	NA	NA	NA	NA	UST
80329	ENSG00000111981	30.5013425	0.17636042	0.37020337	0.3325414	0.56173872	ULBP1
26271	ENSG00000112029	874.136278	-0.15467251	0.0895571	0.07500852	0.20145783	FBXO5
54516	ENSG00000112031	734.579704	-0.15453004	0.10598751	0.12824164	0.29899782	MTRF1L
5467	ENSG00000112033	1070.31224	0.33954864	0.08163143	2.01E-05	0.00018029	PPARD
4988	ENSG00000112038	1574.16282	-1.96536002	0.10185379	3.26E-84	7.11E-82	OPRM1
2178	ENSG00000112039	377.005241	-0.00049125	0.12096154	0.99561306	0.99852582	FANCE
7287	ENSG00000112041	0.15069912	-0.01848503	0.38480575	0.84537521	NA	TULP1
116369	ENSG00000112053	0.52791311	0.01296299	0.38341368	0.75425252	NA	SLC26A8
1432	ENSG00000112062	1114.74744	-0.16352949	0.10375514	0.10073265	0.25062521	MAPK14
6005	ENSG00000112077	0	NA	NA	NA	NA	RHAG
222658	ENSG00000112078	3480.60352	-0.00392077	0.05812949	0.94482828	0.97754449	KCTD20
11329	ENSG00000112079	763.687359	0.02994652	0.0912247	0.73615336	0.87477001	STK38
6428	ENSG00000112081	6377.67565	-0.08509242	0.06849617	0.20743863	0.41458472	SRSF3
6648	ENSG00000112096	1001.36884	-0.2292707	0.09808348	0.01530632	0.05855714	SOD2
29074	ENSG00000112110	515.416852	-0.3613556	0.11588777	0.00103297	0.0060739	MRPL18
3605	ENSG00000112115	0	NA	NA	NA	NA	IL17A
112744	ENSG00000112116	0	NA	NA	NA	NA	IL17F
4172	ENSG00000112118	5007.08773	-0.2991758	0.05302719	1.20E-08	1.81E-07	MCM3
9025	ENSG00000112130	457.445347	-0.32679122	0.14357323	0.01380183	0.05391382	RNF8
221692	ENSG00000112137	318.592184	-1.89490268	0.14977858	7.79E-38	5.66E-36	PHACTR1
266727	ENSG00000112139	66.8409493	-2.22933472	0.43862903	1.33E-08	1.99E-07	MDGA1
22858	ENSG00000112144	712.02217	0.05279569	0.09521337	0.56849498	0.76931138	CILK1
26268	ENSG00000112146	914.224931	0.18084681	0.09251319	0.04366314	0.13403632	FBXO9

9308	ENSG00000112149	130.196442	0.25760487	0.19938968	0.13289728	0.306306	CD83
23195	ENSG00000112159	3102.87324	-0.16726575	0.06244212	0.00656146	0.02927351	MDN1
2740	ENSG00000112164	204.695471	0.03922134	0.16194038	0.79067064	0.90466583	GLP1R
55776	ENSG00000112167	211.433589	0.06958194	0.15090447	0.61815554	0.80452675	SAYSD1
653	ENSG00000112175	0	NA	NA	NA	NA	BMP5
60468	ENSG00000112182	1526.20852	-0.29951709	0.10083559	0.00187433	0.0101161	BACH2
221662	ENSG00000112183	5.08980011	3.41233259	2.0594212	0.00278906	0.01424321	RBM24
10486	ENSG00000112186	177.937152	0.81033141	0.22637563	5.55E-05	0.00044767	CAP2
79865	ENSG00000112195	0	NA	NA	NA	NA	TREML2
26036	ENSG00000112200	1088.86438	-0.12610327	0.10851502	0.22463448	0.43844053	ZNF451
9532	ENSG00000112208	349.915431	0.34134072	0.17040505	0.02576415	0.08917669	BAG2
51715	ENSG00000112210	215.259925	-0.18941208	0.15127166	0.17178923	0.36432905	RAB23
222642	ENSG00000112212	1.14950504	0.05651207	0.38624043	0.32144351	NA	TSPO2
9457	ENSG00000112214	0	NA	NA	NA	NA	FHL5
81491	ENSG00000112218	155.756819	0.32797924	0.18780791	0.04665811	0.14119844	GPR63
202559	ENSG00000112232	0	NA	NA	NA	NA	KHDRBS2
26235	ENSG00000112234	530.103006	0.23282729	0.11676115	0.03592227	0.11567893	FBXL4
892	ENSG00000112237	638.881352	-0.09241379	0.12160144	0.42271296	0.6487179	CCNC
59336	ENSG00000112238	0.47560797	-0.02854873	0.38581796	0.54111271	NA	PRDM13
1871	ENSG00000112242	1914.36769	-0.47001882	0.0743048	1.11E-10	2.11E-09	E2F3
7803	ENSG00000112245	1999.57972	-0.07049149	0.09425549	0.43885367	0.66407447	PTP4A1
6492	ENSG00000112246	0	NA	NA	NA	NA	SIM1
10973	ENSG00000112249	1171.12263	-0.1792696	0.09331949	0.04711977	0.14216185	ASCC3
154150	ENSG00000112273	0.18922458	0.01954053	0.38488496	0.78225479	NA	HDGFL1
11149	ENSG00000112276	500.093095	-0.52069373	0.11686656	3.04E-06	3.17E-05	BVES
1297	ENSG00000112280	153.964683	2.34436923	0.22144869	2.42E-27	1.25E-25	COL9A1
9439	ENSG00000112282	733.404815	0.06001911	0.09808186	0.52731839	0.73910257	MED23
8936	ENSG00000112290	2010.13418	-0.23696264	0.07912498	0.0021603	0.01143925	WASF1
2822	ENSG00000112293	227.625498	-0.3137265	0.20399746	0.0717662	0.19512696	GPLD1
7915	ENSG00000112294	636.266592	-0.66862974	0.10366046	2.80E-11	5.68E-10	ALDH5A1
202	ENSG00000112297	20.4993232	-0.69765553	0.72516613	0.0231768	0.08199496	CRYBG1
8876	ENSG00000112299	0	NA	NA	NA	NA	VNN1
8875	ENSG00000112303	0	NA	NA	NA	NA	VNN2
55856	ENSG00000112304	347.637072	0.00520147	0.12248599	0.96517903	0.98622874	ACOT13
60682	ENSG00000112305	745.443392	-0.08116644	0.09688398	0.38595816	0.61510241	SMAP1
6206	ENSG00000112306	6156.53409	-0.01400127	0.05241011	0.70483604	0.85803894	RPS12
81688	ENSG00000112308	1151.36377	0.04084743	0.0801698	0.60303848	0.79307194	C6orf62
135152	ENSG00000112309	34.7545245	0.06226363	0.27858111	0.74915917	0.88205755	B3GAT2

51053	ENSG00000112312	841.529994	-0.16089196	0.0894824	0.06392694	0.1796757	GMNN
2070	ENSG00000112319	115.596847	7.52525239	0.72756435	2.32E-24	1.08E-22	EYA4
55084	ENSG00000112320	454.767828	-1.20677858	0.16592594	3.46E-14	8.86E-13	SOBP
7101	ENSG00000112333	3.13102258	0.09710571	0.38882296	0.33051215	0.5593109	NR2E1
8724	ENSG00000112335	1727.08371	-0.04756092	0.06924414	0.48384537	0.70413645	SNX3
10246	ENSG00000112337	0.15069912	-0.01848503	0.38480575	0.84537521	NA	SLC17A2
10767	ENSG00000112339	823.842252	0.01549247	0.10574461	0.88010845	0.94683492	HBS1L
10475	ENSG00000112343	0.173733	0.01914545	0.38484597	0.78225479	NA	TRIM38
5191	ENSG00000112357	210.697515	-0.20598194	0.16772384	0.17101555	0.36315408	PEX7
9841	ENSG00000112365	321.194232	-0.23133414	0.13255336	0.06198627	0.17568679	ZBTB24
9896	ENSG00000112367	513.843149	0.07731908	0.10971661	0.46264381	0.68555181	FIG4
64065	ENSG00000112378	0	NA	NA	NA	NA	PERP
57221	ENSG00000112379	0	NA	NA	NA	NA	ARFGEF3
117247	ENSG00000112394	227.912776	0.19546514	0.18881193	0.23486093	0.45174425	SLC16A10
51696	ENSG00000112406	135.043103	0.18614919	0.20150833	0.28043603	0.5049783	HECA
57211	ENSG00000112414	0	NA	NA	NA	NA	ADGRG6
9749	ENSG00000112419	362.630757	0.2402947	0.15782362	0.09418559	0.23837761	PHACTR2
7957	ENSG00000112425	271.258658	-0.36985448	0.15111624	0.00773578	0.03354707	EPM2A
81797	ENSG00000112462	0	NA	NA	NA	NA	OR12D3
7922	ENSG00000112473	1851.47466	0.05129955	0.06703503	0.43781504	0.66289835	SLC39A7
1235	ENSG00000112486	0	NA	NA	NA	NA	CCR6
54346	ENSG00000112494	0.18922458	0.01954053	0.38488496	0.78225479	NA	UNC93A
6582	ENSG00000112499	0	NA	NA	NA	NA	SLC22A2
5252	ENSG00000112511	598.795874	0.33721192	0.11081718	0.00144239	0.00810451	PHF1
51596	ENSG00000112514	1465.45692	0.16713743	0.081695	0.03606089	0.11599352	CUTA
135138	ENSG00000112530	19.326547	0.80364546	0.74964355	0.01724173	0.06468908	PACRG
9444	ENSG00000112531	1824.5993	1.04269345	0.07712517	1.51E-42	1.22E-40	QKI
168090	ENSG00000112539	10.6885166	0.06088132	0.3488729	0.6972058	0.85387893	C6orf118
10846	ENSG00000112541	1012.29047	1.85991112	0.100471	1.37E-77	2.63E-75	PDE10A
90632	ENSG00000112541	1012.29047	1.85991112	0.100471	1.37E-77	2.63E-75	PDE10A
4188	ENSG00000112559	723.062544	0.86141651	0.10081798	2.23E-18	7.69E-17	MDFI
7942	ENSG00000112561	131.547356	-0.23606654	0.19133498	0.15532797	0.34116096	TFEB
64094	ENSG00000112562	22.3661371	0.45947977	0.46762904	0.06819399	0.18796453	SMOC2
896	ENSG00000112576	544.332697	-0.04290948	0.12957828	0.72397359	0.86794586	CCND3
705	ENSG00000112578	730.164855	-0.18585808	0.10588593	0.06686484	0.18540858	BYSL
84498	ENSG00000112584	1216.22079	0.29992628	0.08110102	0.00015148	0.00110451	FAM120B
6908	ENSG00000112592	557.013182	0.18308458	0.11873782	0.1043661	0.25722378	TBP
2979	ENSG00000112599	50.1516034	0.44066271	0.31089852	0.05245711	0.15445963	GUCA1B

5961	ENSG00000112619	0.51210169	0.01491862	0.38308811	0.74405738	NA	PRPH2
23506	ENSG00000112624	655.959856	-0.43860982	0.11499955	6.29E-05	0.00050303	BICRAL
5528	ENSG00000112640	1347.69318	-0.20889801	0.0783288	0.00634802	0.0284428	PPP2R5D
51069	ENSG00000112651	299.676488	0.27730385	0.13467652	0.02759446	0.09387416	MRPL2
5754	ENSG00000112655	4768.20151	0.0737562	0.0589343	0.20531159	0.41224389	PTK7
6722	ENSG00000112658	1465.03728	0.24363752	0.07749061	0.00130375	0.00742006	SRF
23113	ENSG00000112659	1484.21831	0.35288265	0.10879048	0.00069993	0.00432742	CUL9
10591	ENSG00000112667	938.303003	0.43124495	0.0949927	2.72E-06	2.86E-05	DNPH1
56940	ENSG00000112679	310.830676	1.60192269	0.15733956	1.91E-25	9.22E-24	DUSP22
55770	ENSG00000112685	624.570637	-0.23933758	0.12796156	0.04637313	0.14052892	EXOC2
1347	ENSG00000112695	1484.48855	-0.10261692	0.07129325	0.14258832	0.3213258	COX7A2
55754	ENSG00000112697	2161.17001	-0.38935534	0.06665572	2.86E-09	4.61E-08	TMEM30A
2762	ENSG00000112699	202.848017	-0.13717172	0.16030655	0.34500329	0.57544054	GMDS
26054	ENSG00000112701	1830.97556	-0.06142494	0.07644363	0.41218478	0.64000085	SENP6
3617	ENSG00000112706	10.6855313	0.28872376	0.49626061	0.0758828	0.20317338	IMPG1
7422	ENSG00000112715	1904.24934	-0.33599466	0.10850006	0.00120313	0.00694501	VEGFA
8899	ENSG00000112739	2525.47894	0.02562571	0.06806933	0.7081852	0.85971939	PRPF4B
7272	ENSG00000112742	793.84144	-0.37239639	0.10877264	0.00034417	0.00231737	TTK
2030	ENSG00000112759	2710.3234	-0.34453322	0.06266879	2.36E-08	3.39E-07	SLC29A1
8838	ENSG00000112761	0.3436116	0.02364564	0.38535635	0.62278312	NA	CCN6
11120	ENSG00000112763	610.720528	0.25514079	0.11050079	0.01558617	0.05949013	BTN2A1
3910	ENSG00000112769	146.828777	-0.56623951	0.27446899	0.00989951	0.04113221	LAMA4
55603	ENSG00000112773	229.935947	-1.39047527	0.16797195	1.13E-17	3.64E-16	TENT5A
53405	ENSG00000112782	0	NA	NA	NA	NA	CLIC5
57666	ENSG00000112787	2259.8788	0.18160311	0.06948868	0.00782991	0.03390342	FBRSL1
59084	ENSG00000112796	0	NA	NA	NA	NA	ENPP5
9450	ENSG00000112799	0	NA	NA	NA	NA	LY86
10279	ENSG00000112812	0.18922458	0.01954053	0.38488496	0.78225479	NA	PRSS16
4224	ENSG00000112818	0	NA	NA	NA	NA	MEP1A
9096	ENSG00000112837	16.6874347	8.11127742	2.69316361	5.60E-09	8.72E-08	TBX18
55914	ENSG00000112851	1118.93707	-0.69011202	0.0953392	1.09E-13	2.65E-12	ERBIN
56133	ENSG00000112852	116.014449	0.40465603	0.21255814	0.02556467	0.08865635	PCDHB2
23438	ENSG00000112855	1034.19134	0.28193751	0.08041819	0.00033136	0.00223764	HARS2
83594	ENSG00000112874	360.278769	-0.26586999	0.15702531	0.06284433	0.17750484	NUDT12
55722	ENSG00000112877	264.23177	0.01554055	0.14647594	0.91041813	0.96165126	CEP72
4124	ENSG00000112893	203.976244	-0.03063302	0.16511555	0.83608748	0.92527105	MAN2A1
9037	ENSG00000112902	89.605942	0.79794551	0.27896573	0.00065208	0.0040617	SEMA5A
730	ENSG00000112936	11348.6044	-1.21180138	0.05447234	1.41E-110	4.81E-108	C7

11044	ENSG00000112941	1084.29882	-0.36764112	0.09695416	8.50E-05	0.00065695	TENT4A
2690	ENSG00000112964	0	NA	NA	NA	NA	GHR
3157	ENSG00000112972	2288.7533	-0.37995775	0.20842898	0.03262908	0.10721446	HMGCS1
1611	ENSG00000112977	1576.66929	0.05002577	0.07668596	0.50605587	0.72177288	DAP
8382	ENSG00000112981	21.8909222	0.23508467	0.37696903	0.24092167	0.45885437	NME5
10902	ENSG00000112983	1327.55324	-0.09082368	0.0931361	0.31368545	0.54159326	BRD8
10112	ENSG00000112984	1817.0062	-0.04031646	0.08443566	0.62375286	0.80815364	KIF20A
23530	ENSG00000112992	2390.00083	-0.18978026	0.06959316	0.00547524	0.02514982	NNT
10884	ENSG00000112996	678.330091	-0.05032456	0.103513	0.61289453	0.80067344	MRPS30
3313	ENSG00000113013	4052.98495	-0.02609066	0.0646829	0.68154735	0.84433357	HSPA9
23107	ENSG00000113048	1278.23078	-0.41378912	0.07905326	8.54E-08	1.13E-06	MRPS27
5201	ENSG00000113068	664.551029	0.11789801	0.12073488	0.30319005	0.53049568	PFDN1
1839	ENSG00000113070	41.794568	-0.56989522	0.40070157	0.0302939	0.10114961	HBEGF
83697	ENSG00000113073	0.81228073	0.02242119	0.38334325	0.76254719	NA	SLC4A9
4015	ENSG00000113083	393.63179	0.69755713	0.12850436	1.34E-08	2.00E-07	LOX
3003	ENSG00000113088	0	NA	NA	NA	NA	GZMK
1007	ENSG00000113100	0	NA	NA	NA	NA	CDH9
10307	ENSG00000113108	357.368128	0.09493037	0.12752499	0.4303556	0.65588071	APBB3
55374	ENSG00000113119	263.594016	0.02831316	0.17268033	0.85587794	0.93567436	TMCO6
6678	ENSG00000113140	1492.72194	1.15497204	0.09695033	1.07E-33	6.89E-32	SPARC
3550	ENSG00000113141	1855.69516	0.05285185	0.08120252	0.50607775	0.72177288	IK
3156	ENSG00000113161	2813.54103	-0.28041427	0.09946997	0.00344517	0.01702418	HMGCR
10087	ENSG00000113163	760.338255	0.12753723	0.09134759	0.15052543	0.33407003	CERT1
23197	ENSG00000113194	1881.86686	-0.13520024	0.06667264	0.03925451	0.12377772	FAF2
9421	ENSG00000113196	4834.90309	0.19970942	0.05691263	0.00038836	0.00257691	HAND1
56132	ENSG00000113205	393.774894	-0.16530418	0.12885827	0.17201043	0.36458	PCDHB3
26167	ENSG00000113209	56.9542267	0.84776364	0.38614575	0.00328457	0.01633228	PCDHB5
56130	ENSG00000113211	263.424607	0.8887393	0.15390973	1.20E-09	2.05E-08	PCDHB6
56129	ENSG00000113212	42.4700176	0.25604368	0.30527318	0.2249195	0.43877008	PCDHB7
8622	ENSG00000113231	118.221549	-0.65524268	0.24728432	0.00172686	0.00945647	PDE8B
57396	ENSG00000113240	312.227087	-0.11907697	0.1279307	0.32554393	0.55359488	CLK4
56121	ENSG00000113248	265.658303	-0.19189836	0.15391099	0.17222574	0.36478687	PCDHB15
26762	ENSG00000113249	0.173733	0.01914545	0.38484597	0.78225479	NA	HAVCR1
2916	ENSG00000113262	25.2350172	0.27629749	0.38195237	0.18859997	0.38924617	GRM6
3702	ENSG00000113263	0	NA	NA	NA	NA	ITK
55819	ENSG00000113269	1387.49191	0.34975436	0.07432207	1.55E-06	1.70E-05	RNF130
54974	ENSG00000113272	455.225346	-0.1934923	0.13398096	0.12214173	0.28896853	THG1L
411	ENSG00000113273	494.126151	-0.31433795	0.12478631	0.0074375	0.0324879	ARSB

9685	ENSG00000113282	1955.10607	-0.10103749	0.0753185	0.17008753	0.36191941	CLINT1
7060	ENSG00000113296	231.664827	1.16409623	0.18419013	2.79E-11	5.68E-10	THBS4
57472	ENSG00000113300	1732.49712	-0.48520201	0.07926784	3.88E-10	7.03E-09	CNOT6
3593	ENSG00000113302	0	NA	NA	NA	NA	IL12B
79908	ENSG00000113303	0	NA	NA	NA	NA	BTNL8
7265	ENSG00000113312	656.075457	0.06433844	0.09365666	0.4792684	0.70061045	TTC1
4437	ENSG00000113318	546.423351	-0.29360868	0.10785844	0.00443139	0.02104325	MSH3
5924	ENSG00000113319	40.6883391	0.02800717	0.27020281	0.88605523	0.95015905	RASGRF2
2566	ENSG00000113327	1.25319808	-0.04068253	0.38420537	0.46803325	NA	GABRG2
900	ENSG00000113328	1609.3871	-0.2506431	0.07336759	0.0004879	0.0031582	CCNG1
10622	ENSG00000113356	147.281889	0.18024299	0.19864621	0.2905911	0.51667585	POLR3G
29102	ENSG00000113360	1630.10001	0.16051769	0.09100847	0.06920466	0.18985349	DROSHA
1004	ENSG00000113361	0	NA	NA	NA	NA	CDH6
4001	ENSG00000113368	3215.7716	-0.34960944	0.07257144	8.81E-07	1.01E-05	LMNB1
57561	ENSG00000113369	648.621295	-0.14584727	0.11374173	0.17644307	0.37102603	ARRDC3
64083	ENSG00000113384	1179.99541	-0.11043135	0.08134754	0.16432846	0.35376265	GOLPH3
10923	ENSG00000113387	1679.15256	-0.13572322	0.08510108	0.10135615	0.2518039	SUB1
4883	ENSG00000113389	1.86888861	0.06712512	0.38520622	0.39568254	NA	NPR3
83989	ENSG00000113391	1001.24028	0.12185453	0.09463216	0.18141605	0.37883323	FAM172A
28965	ENSG00000113396	16.2028081	3.05544547	0.84534311	1.96E-05	0.00017575	SLC27A6
6897	ENSG00000113407	2420.14951	0.00228926	0.08388446	0.97851949	0.99267917	TARS1
50805	ENSG00000113430	0	NA	NA	NA	NA	IRX4
4012	ENSG00000113441	552.151854	-0.23263037	0.11524043	0.0334944	0.10951287	LNPEP
5144	ENSG00000113448	304.64289	-0.03357732	0.14762694	0.80205939	0.90951407	PDE4D
5810	ENSG00000113456	745.406641	-0.24776425	0.10858385	0.0168775	0.06358709	RAD1
55299	ENSG00000113460	762.003616	0.14626068	0.09764197	0.12128951	0.28753541	BRIX1
64902	ENSG00000113492	0	NA	NA	NA	NA	AGXT2
5618	ENSG00000113494	12.8729149	2.7875109	0.94275585	0.00010634	0.00080373	PRLR
10723	ENSG00000113504	201.41572	1.84804381	0.20122164	2.60E-21	1.03E-19	SLC12A7
3565	ENSG00000113520	0	NA	NA	NA	NA	IL4
10111	ENSG00000113522	2034.80768	0.03879256	0.07706477	0.6089558	0.7978212	RAD50
3567	ENSG00000113525	0.910068	-0.05134303	0.38885868	0.26827551	NA	IL5
7903	ENSG00000113532	18.0830613	5.43550306	1.09029432	3.53E-07	4.33E-06	ST8SIA4
10007	ENSG00000113552	1146.02057	-0.08419773	0.09707394	0.3692206	0.59862194	GNPDA1
51294	ENSG00000113555	67.1511674	0.37494643	0.34213016	0.10057896	0.2503119	PCDH12
6500	ENSG00000113558	3336.85923	0.01377084	0.05934977	0.81805291	0.9171248	SKP1
9631	ENSG00000113569	1719.58497	-0.14647759	0.09699874	0.11785633	0.28165137	NUP155
5515	ENSG00000113575	2713.45316	-0.0555644	0.06710595	0.40012721	0.62854504	PPP2CA

2246	ENSG00000113578	170.44748	-1.53621607	0.23598168	5.10E-12	1.11E-10	FGF1
2908	ENSG00000113580	310.164167	0.87126083	0.14196935	1.38E-10	2.62E-09	NR3C1
56951	ENSG00000113583	639.177149	-0.30392035	0.10126752	0.00180372	0.00979359	C5orf15
23398	ENSG00000113593	478.242186	0.01397236	0.11065812	0.89541254	0.95469109	PPWD1
3977	ENSG00000113594	65.2625383	3.68117516	0.39489095	7.42E-22	3.04E-20	LIFR
373	ENSG00000113595	277.460655	-0.35644061	0.15622212	0.01242695	0.04959996	TRIM23
80006	ENSG00000113597	406.25235	0.11989827	0.12794799	0.32120717	0.54894911	TRAPPC13
735	ENSG00000113600	0	NA	NA	NA	NA	C9
10802	ENSG00000113615	664.782376	-0.28649905	0.1234944	0.0138326	0.05401276	SEC24A
79770	ENSG00000113621	600.084754	-0.37299479	0.12250629	0.00128128	0.00731947	TXNDC15
23548	ENSG00000113638	494.949369	-0.42974502	0.14533475	0.00141955	0.00799654	TTC33
5917	ENSG00000113643	1538.54628	-0.06651402	0.09935002	0.48741383	0.70726738	RARS1
23286	ENSG00000113645	543.008522	-0.4682785	0.16814792	0.0021103	0.01120673	WWC1
9555	ENSG00000113648	6446.2403	-0.01784314	0.06498339	0.77972825	0.89925062	MACROH2A1
10915	ENSG00000113649	2667.3123	-0.29956645	0.06493121	2.75E-06	2.89E-05	TCERG1
1809	ENSG00000113657	14256.3043	0.54718714	0.06136605	1.84E-19	6.68E-18	DPYSL3
4090	ENSG00000113658	1968.76669	-0.11633721	0.09494428	0.2051336	0.41205331	SMAD5
1452	ENSG00000113712	3398.70026	-0.00528834	0.06480527	0.93322288	0.97202552	CSNK1A1
22993	ENSG00000113716	2061.76038	-0.05891545	0.0689863	0.38482019	0.61378186	HMGXB3
57222	ENSG00000113719	3445.24539	-0.17511723	0.0644176	0.00557488	0.02551703	ERGIC1
5159	ENSG00000113721	2132.01116	-0.12456854	0.0873866	0.14237747	0.32117652	PDGFRB
1044	ENSG00000113722	30.3005251	-0.338375	0.37031846	0.13498306	0.30945965	CDX1
8992	ENSG00000113732	566.880119	-0.2664261	0.11525952	0.01487522	0.05727678	ATP6V0E1
662	ENSG00000113734	183.740154	-0.24586991	0.16315529	0.09518702	0.24026852	BNIP1
8614	ENSG00000113739	866.568266	0.0906492	0.14126187	0.49088652	0.7104748	STC2
80315	ENSG00000113742	606.951026	-0.56334235	0.10749989	5.30E-08	7.26E-07	CPEB4
3274	ENSG00000113749	1.4225907	-0.02835773	0.38008814	0.68061404	NA	HRH2
1627	ENSG00000113758	8727.84209	0.13623246	0.05936775	0.02019767	0.07347302	DBN1
23567	ENSG00000113761	626.046463	0.12802009	0.11752541	0.25259454	0.47264695	ZNF346
90249	ENSG00000113763	824.728401	-3.04508765	0.11964269	4.47E-144	3.06E-141	UNC5A
1962	ENSG00000113790	214.528707	0.09764155	0.19370637	0.56034499	0.76415299	EHHADH
5067	ENSG00000113805	54.2873514	1.88514523	0.47633096	3.17E-06	3.29E-05	CNTN3
10051	ENSG00000113810	5138.88785	-0.22424541	0.06592964	0.00054339	0.00346873	SMC4
58515	ENSG00000113811	561.586588	0.28562189	0.11762691	0.01052148	0.04316015	SELENOK
93973	ENSG00000113812	793.658123	0.145814	0.09819004	0.12424776	0.29220905	ACTR8
55171	ENSG00000113838	335.590973	-0.16411657	0.14838709	0.22942125	0.44501603	TBCCD1
51300	ENSG00000113845	1770.16101	0.19302911	0.07458065	0.00832743	0.03570698	TIMMDC1
51185	ENSG00000113851	603.862139	0.01478192	0.10148648	0.88077401	0.94723311	CRBN

3827	ENSG00000113889	0.49444345	-0.02942187	0.38589312	0.52715984	NA	KNG1
3273	ENSG00000113905	0	NA	NA	NA	NA	HRG
604	ENSG00000113916	1.61398434	-0.02376934	0.37985765	0.72051391	NA	BCL6
3081	ENSG00000113924	2.33009785	0.0534513	0.37981278	0.5425998	NA	HGD
10686	ENSG00000113946	0	NA	NA	NA	NA	CLDN16
84100	ENSG00000113966	453.030422	-0.43009308	0.12560299	0.0002883	0.00197305	ARL6
27031	ENSG00000113971	468.487418	0.55010357	0.17042758	0.0003957	0.00262214	NPHP3
942	ENSG00000114013	0	NA	NA	NA	NA	CD86
51421	ENSG00000114019	820.81729	0.77037607	0.09244477	1.62E-17	5.20E-16	AMOTL2
56954	ENSG00000114021	615.011348	0.08693901	0.11632813	0.43316043	0.65852706	NIT2
26355	ENSG00000114023	465.369583	-0.03399804	0.14984067	0.80420858	0.91111799	FAM162A
4968	ENSG00000114026	590.052718	-0.06025787	0.12066596	0.59780215	0.78948244	OGG1
3836	ENSG00000114030	1146.0651	-0.2804976	0.1118537	0.00850743	0.03630616	KPNA1
5096	ENSG00000114054	1225.21046	0.09881316	0.07663561	0.18870882	0.38930882	PCCB
7337	ENSG00000114062	1630.35051	-0.26892325	0.08770404	0.00158665	0.00880041	UBE3A
25852	ENSG00000114098	652.606811	-0.00340063	0.10090641	0.97129371	0.9892437	ARMC8
80321	ENSG00000114107	357.681963	-0.23490262	0.12670926	0.04864013	0.14586156	CEP70
5948	ENSG00000114113	0.29863895	-0.02292439	0.3852726	0.68753245	NA	RBP2
5947	ENSG00000114115	5269.6184	0.16929834	0.05388452	0.00149312	0.00834464	RBP1
55186	ENSG00000114120	2421.12056	0.21124536	0.06639551	0.00120504	0.00695203	SLC25A36
131890	ENSG00000114124	4.45105359	0.38890136	0.7137691	0.01138753	0.0461927	GRK7
9616	ENSG00000114125	651.43392	-0.25422365	0.11014773	0.01561908	0.05958135	RNF7
7029	ENSG00000114126	1926.59278	0.95105021	0.10513655	2.07E-20	7.94E-19	TFDP2
54464	ENSG00000114127	964.676613	-0.18220127	0.11677214	0.0998047	0.2488914	XRN1
8850	ENSG00000114166	316.812235	-0.31484827	0.13397059	0.01189073	0.04788247	KAT2B
590	ENSG00000114200	308.825779	0.6438985	0.19471849	0.00022843	0.0016003	BCHE
5276	ENSG00000114204	0	NA	NA	NA	NA	SERPINI2
11235	ENSG00000114209	503.788848	0.0044054	0.11224252	0.96817533	0.98809325	PDCD10
79782	ENSG00000114248	0.34043669	1.23E-05	0.38336209	0.96513672	NA	LRRC31
7474	ENSG00000114251	68.2509491	0.51450943	0.33367158	0.03145056	0.10413538	WNT5A
5210	ENSG00000114268	252.763888	-0.00431647	0.1538488	0.9744631	0.99089477	PFKFB4
1294	ENSG00000114270	86.5166681	0.442252	0.33773399	0.0584365	0.16783152	COL7A1
2257	ENSG00000114279	0.173124	-0.01911262	0.38486262	0.84537521	NA	FGF12
5576	ENSG00000114302	1191.59371	-0.25197658	0.12719225	0.03510904	0.11357627	PRKAR2A
3280	ENSG00000114315	32.7509636	0.14962783	0.30178459	0.4542531	0.67744995	HES1
7375	ENSG00000114316	1233.50042	-0.01219679	0.09026768	0.8886226	0.95146564	USP4
107986084	ENSG00000114316	1233.50042	-0.01219679	0.09026768	0.8886226	0.95146564	USP4
23527	ENSG00000114331	1102.00463	-0.67537516	0.08522716	5.74E-16	1.66E-14	ACAP2

1894	ENSG00000114346	1784.93654	-0.32542034	0.08762601	0.0001311	0.00096797	ECT2
2779	ENSG00000114349	0	NA	NA	NA	NA	GNAT1
2771	ENSG00000114353	1807.63024	-0.12115652	0.07288853	0.09010437	0.23086121	GNAI2
10342	ENSG00000114354	1761.34861	0.20418406	0.06631205	0.00175566	0.00958248	TFG
8287	ENSG00000114374	0	NA	NA	NA	NA	USP9Y
64595	ENSG00000114374	0	NA	NA	NA	NA	USP9Y
3373	ENSG00000114378	65.7941091	0.01040896	0.23956862	0.95731049	0.98264776	HYAL1
11334	ENSG00000114383	692.105951	-0.00764251	0.10972108	0.9411705	0.97559098	TUSC2
10641	ENSG00000114388	780.793613	0.21235806	0.10034892	0.0280578	0.0952218	NPRL2
6152	ENSG00000114391	8120.19471	-0.02676006	0.04985585	0.58695846	0.78237765	RPL24
11068	ENSG00000114395	274.338144	-0.20620091	0.14010098	0.11274313	0.27188813	CYB561D2
57415	ENSG00000114405	319.645366	-0.07226011	0.13505129	0.56647349	0.76820466	C3orf14
8087	ENSG00000114416	2925.63642	0.06434779	0.06247712	0.2969827	0.5236583	FXR1
868	ENSG00000114423	628.545657	0.34689338	0.1139392	0.0013879	0.00784722	CBLB
56987	ENSG00000114439	1331.65854	0.09554238	0.0977832	0.31236043	0.54011076	BBX
55081	ENSG00000114446	495.222485	0.3450088	0.12289362	0.00297269	0.01501864	IFT57
59345	ENSG00000114450	1074.36467	-0.29324205	0.09049943	0.00082635	0.00500297	GNB4
11148	ENSG00000114455	0.173124	-0.01911262	0.38486262	0.84537521	NA	HHLA2
84223	ENSG00000114473	108.541738	0.27159308	0.22713982	0.14504887	0.32541747	IQCG
2632	ENSG00000114480	168.996963	-0.18990224	0.16774181	0.20698498	0.41417369	GBE1
27136	ENSG00000114487	0	NA	NA	NA	NA	MORC1
7372	ENSG00000114491	929.015724	-0.08566018	0.09867442	0.36813175	0.59754021	UMPS
22916	ENSG00000114503	3616.40677	-0.064444	0.0568851	0.25166913	0.47149344	NCBP2
8723	ENSG00000114520	907.671062	0.09998894	0.08906141	0.24856873	0.46784968	SNX4
79669	ENSG00000114529	33.5954363	2.01662435	0.47922776	1.44E-06	1.59E-05	C3orf52
23150	ENSG00000114541	44.4945463	-0.12633695	0.29505762	0.51758924	0.73141449	FRMD4B
54946	ENSG00000114544	747.993422	-0.22599069	0.10427085	0.02374217	0.08361089	SLC41A3
152015	ENSG00000114547	9.40572923	-0.3132766	0.52558812	0.06198005	0.17568679	ROPN1B
5361	ENSG00000114554	3440.91632	-0.34943252	0.07989135	7.37E-06	7.18E-05	PLXNA1
523	ENSG00000114573	1305.93892	-0.51298369	0.10173926	1.72E-07	2.20E-06	ATP6V1A
80325	ENSG00000114626	276.171408	0.34090255	0.16005072	0.019131	0.07027571	ABTB1
50512	ENSG00000114631	3034.8558	-0.10842794	0.05880924	0.06155507	0.17488938	PODXL2
7348	ENSG00000114638	0.18922458	0.01954053	0.38488496	0.78225479	NA	UPK1B
10675	ENSG00000114646	105.342697	-0.33789525	0.21309633	0.06059201	0.17264764	CSPG5
23276	ENSG00000114648	685.976057	-0.09582535	0.10430286	0.33741044	0.56778823	KLHL18
22937	ENSG00000114650	2475.44922	0.08373193	0.06853672	0.21444441	0.42477806	SCAP
79825	ENSG00000114654	1.04143875	0.05900488	0.39038104	0.19798031	NA	EFCC1
57501	ENSG00000114656	25.2705923	0.13270733	0.31133327	0.49934093	0.71682702	CFAP92

79858	ENSG00000114670	64.1471507	0.11418229	0.24152554	0.5476458	0.75435339	NEK11
11222	ENSG00000114686	1981.95576	-0.15543766	0.07154187	0.02678521	0.09189323	MRPL3
57088	ENSG00000114698	0	NA	NA	NA	NA	PLSCR4
51409	ENSG00000114735	870.720418	0.26106877	0.11827546	0.01998861	0.07291303	HEMK1
1154	ENSG00000114737	194.960333	0.77494705	0.17480519	1.75E-06	1.90E-05	CISH
7867	ENSG00000114738	427.996922	0.13430789	0.114882	0.22036459	0.43277467	MAPKAPK3
93	ENSG00000114739	1537.93961	-0.1089098	0.07182015	0.12231897	0.28928458	ACVR2B
57599	ENSG00000114742	1262.49133	0.13655289	0.08816906	0.11128937	0.26948227	WDR48
51122	ENSG00000114744	544.970115	-0.08376193	0.12745046	0.48486409	0.70489511	COMMD2
64689	ENSG00000114745	659.172769	-0.16782774	0.11479263	0.12440277	0.29243495	GORASP1
51555	ENSG00000114757	118.630286	-1.81780733	0.30499196	1.55E-10	2.92E-09	PEX5L
9136	ENSG00000114767	477.308815	0.02284552	0.10816004	0.82662665	0.92144622	RRP9
10057	ENSG00000114770	2113.31158	0.41867462	0.07604694	1.89E-08	2.75E-07	ABCC5
13	ENSG00000114771	0	NA	NA	NA	NA	AADAC
84836	ENSG00000114779	491.668449	0.00891936	0.11719052	0.93691046	0.97351594	ABHD14B
10289	ENSG00000114784	630.882687	-0.22193788	0.10460004	0.02700083	0.09245712	EIF1B
100526760	ENSG00000114786	88.6384314	-0.00615837	0.278199	0.97226429	0.98958828	ABHD14A-ACY1
26084	ENSG00000114790	121.991486	0.54838835	0.22275562	0.00407993	0.01963274	ARHGEF26
54800	ENSG00000114796	1351.46209	0.02908481	0.11812704	0.79403555	0.90579008	KLHL24
23007	ENSG00000114805	126.960851	0.2370349	0.20141784	0.16956734	0.3612387	PLCH1
7433	ENSG00000114812	11.2541091	-0.18846025	0.41176714	0.20235944	0.40788492	VIPR1
25981	ENSG00000114841	189.959823	0.1989836	0.35412955	0.31439709	0.5421676	DNAH1
6747	ENSG00000114850	1513.49616	0.10991526	0.07487726	0.13451842	0.3089299	SSR3
92999	ENSG00000114853	436.039125	-0.15550398	0.14543642	0.24662414	0.46578072	ZBTB47
7134	ENSG00000114854	2.06076984	0.05901115	0.38222193	0.47725525	NA	TNNC1
4820	ENSG00000114857	4237.20364	0.06489516	0.0726765	0.36363975	0.59353748	NKTR
1181	ENSG00000114859	246.519936	0.13446475	0.18898981	0.40985125	0.63781072	CLCN2
27086	ENSG00000114861	1297.71254	-0.57226327	0.0849266	5.21E-12	1.13E-10	FOXP1
1981	ENSG00000114867	9763.57151	-0.09842059	0.05608321	0.07536286	0.20221788	EIF4G1
28972	ENSG00000114902	1129.21194	0.14841208	0.08865548	0.08521127	0.22148098	SPCS1
6787	ENSG00000114904	584.906273	0.48462213	0.11009385	4.39E-06	4.46E-05	NEK4
6508	ENSG00000114923	606.491858	0.34892972	0.1117474	0.00106708	0.00624141	SLC4A3
54891	ENSG00000114933	512.272057	-0.01667303	0.11128146	0.87442715	0.94470892	INO80D
1933	ENSG00000114942	5601.2476	-0.36254057	0.05403196	1.17E-11	2.48E-10	EEF1B2
8745	ENSG00000114948	459.131481	-0.43461377	0.17202053	0.004957	0.02306446	ADAM23
1716	ENSG00000114956	1144.7836	0.01269631	0.08049639	0.87174078	0.94401928	DGUOK
55233	ENSG00000114978	1546.64979	-0.024542	0.08009982	0.75326954	0.88464062	MOB1A
55683	ENSG00000114982	1944.97231	-0.10557652	0.06786357	0.11362423	0.27361388	KANSL3

81562	ENSG00000114988	289.579161	-0.06105158	0.13178692	0.62102614	0.80672374	LMAN2L
6242	ENSG00000114993	518.460699	0.23745716	0.12884742	0.04982129	0.14857274	RTKN
150465	ENSG00000114999	1810.89303	-0.05516787	0.07365803	0.44483952	0.66962186	TTL
3552	ENSG00000115008	0	NA	NA	NA	NA	IL1A
6364	ENSG00000115009	0	NA	NA	NA	NA	CCL20
200576	ENSG00000115020	1821.9356	-0.10496609	0.07223908	0.1383309	0.31466234	PIKFYVE
30818	ENSG00000115041	30.3645276	0.14120033	0.31857821	0.47025942	0.69253479	KCNIP3
51011	ENSG00000115042	249.891133	0.62436175	0.16611375	4.49E-05	0.00037287	FAHD2A
4691	ENSG00000115053	13102.9952	-0.28199878	0.04764895	2.41E-09	3.93E-08	NCL
10120	ENSG00000115073	801.805035	0.04776944	0.09912559	0.61901393	0.80522138	ACTR1B
80255	ENSG00000115084	968.990031	0.12105301	0.10547364	0.23258451	0.44940365	SLC35F5
7535	ENSG00000115085	11.6164891	0.14638797	0.3604583	0.4051589	0.63353977	ZAP70
10096	ENSG00000115091	2037.60006	-0.12828698	0.08404725	0.11704905	0.28012704	ACTR3
55240	ENSG00000115107	210.716688	-0.25330336	0.1567426	0.07573931	0.20287132	STEAP3
57669	ENSG00000115109	943.182552	0.18564576	0.1078998	0.07249958	0.19628706	EPB41L5
29842	ENSG00000115112	38.2518756	2.53235516	0.59433638	8.83E-07	1.01E-05	TFCP2L1
51639	ENSG00000115128	1775.26267	-0.05491585	0.07260594	0.44018945	0.66517856	SF3B6
9540	ENSG00000115129	412.334106	0.30581956	0.1256938	0.00979672	0.04082482	TP53I3
51277	ENSG00000115137	279.161436	0.12427472	0.18296146	0.44058654	0.66549197	DNAJC27
5443	ENSG00000115138	0.69153686	0.01680424	0.38318935	0.66879163	NA	POMC
10254	ENSG00000115145	350.055951	0.00390131	0.14308412	0.97769181	0.99244848	STAM2
9381	ENSG00000115155	2.88571501	-0.05246782	0.38387485	0.30767401	0.53516122	OTOF
2820	ENSG00000115159	681.456796	0.3384716	0.14241873	0.01044717	0.04290453	GPD2
1058	ENSG00000115163	604.737691	-0.22735081	0.10916219	0.02932384	0.09860761	CENPA
9595	ENSG00000115165	0.173124	-0.01911262	0.38486262	0.84537521	NA	CYTIP
90	ENSG00000115170	467.343992	-0.25970607	0.11781309	0.02011883	0.07323992	ACVR1
85461	ENSG00000115183	2009.08016	0.39193752	0.07232745	3.31E-08	4.65E-07	TANC1
7781	ENSG00000115194	588.986664	-2.53533635	0.13362791	1.78E-81	3.68E-79	SLC30A3
4358	ENSG00000115204	1722.8654	0.02212509	0.06775016	0.74091734	0.87763281	MPV17
2976	ENSG00000115207	2753.35684	0.18380426	0.07555306	0.01297636	0.05137715	GTF3C2
8890	ENSG00000115211	864.339595	-0.02550655	0.08829215	0.76598541	0.89234011	EIF2B4
29959	ENSG00000115216	2028.84018	0.13375096	0.06657242	0.04130966	0.12854198	NRBP1
3694	ENSG00000115221	0	NA	NA	NA	NA	ITGB6
64838	ENSG00000115226	601.323019	0.89818306	0.10872823	2.31E-17	7.33E-16	FNDC4
3676	ENSG00000115232	217.293814	-0.02479276	0.1578414	0.86203021	0.9387792	ITGA4
10213	ENSG00000115233	1201.11734	0.08820808	0.09644969	0.34536256	0.57584615	PSMD14
9784	ENSG00000115234	2429.13101	0.14719639	0.08351747	0.07075746	0.1930817	SNX17
51130	ENSG00000115239	423.552814	-0.11809992	0.11482505	0.27980359	0.50438881	ASB3

100302652	ENSG00000115239	423.552814	-0.11809992	0.11482505	0.27980359	0.50438881	ASB3
5496	ENSG00000115241	6405.13818	-0.42793488	0.05508204	3.99E-15	1.10E-13	PPM1G
5136	ENSG00000115252	3.91110082	0.05357879	0.38220507	0.37094004	0.60017919	PDE1A
92840	ENSG00000115255	104.823853	0.79696232	0.23314657	0.00010528	0.00079661	REEP6
54760	ENSG00000115257	117.137936	0.78616173	0.27115753	0.00058671	0.00370948	PCSK4
2641	ENSG00000115263	0	NA	NA	NA	NA	GCG
10297	ENSG00000115266	5823.33164	-0.69421892	0.05794477	1.09E-33	7.03E-32	APC2
64135	ENSG00000115267	11.5726527	7.36195021	2.68153296	3.99E-07	4.84E-06	IFIH1
6209	ENSG00000115268	9123.66852	-0.05117488	0.05719259	0.36532989	0.5947154	RPS15
25801	ENSG00000115271	180.296703	1.02213663	0.19556456	2.06E-08	2.99E-07	GCA
83444	ENSG00000115274	493.535195	0.31408857	0.13311032	0.01167774	0.04712905	INO80B
7841	ENSG00000115275	1579.47671	0.16579073	0.07410217	0.02291519	0.08124326	MOGS
64427	ENSG00000115282	813.489933	0.1178289	0.10701306	0.2516195	0.47149344	TTC31
374291	ENSG00000115286	1274.38196	0.0485762	0.08108712	0.54019507	0.748907	NDUFS7
84759	ENSG00000115289	260.396601	0.25261522	0.15114504	0.06878499	0.1890682	PCGF1
2888	ENSG00000115290	61.805761	0.31675031	0.27898718	0.13036596	0.30247464	GRB14
79745	ENSG00000115295	798.078734	0.31167659	0.14465733	0.01971992	0.07214518	CLIP4
3196	ENSG00000115297	971.305181	0.04226503	0.09519573	0.6475299	0.82430661	TLX2
6711	ENSG00000115306	11074.1077	-0.43040143	0.05812398	3.52E-14	8.98E-13	SPTBN1
550	ENSG00000115307	1714.83322	-0.02201321	0.07204637	0.75508847	0.88608005	AUP1
57142	ENSG00000115310	3890.53653	0.07092171	0.06108419	0.23983573	0.45788499	RTN4
27429	ENSG00000115317	591.343067	-0.00211231	0.11425705	0.98361769	0.99439363	HTRA2
84695	ENSG00000115318	591.941938	-0.25077412	0.11124467	0.01785452	0.06652275	LOXL3
1796	ENSG00000115325	217.40121	0.0212454	0.16815915	0.88946431	0.95181279	DOK1
2591	ENSG00000115339	2.68293064	0.05288393	0.37759984	0.57139928	0.77166993	GALNT3
56655	ENSG00000115350	318.162224	-0.3325101	0.13402305	0.0079546	0.03433094	POLE4
6869	ENSG00000115353	1.19478899	0.0442412	0.38337837	0.4894765	NA	TACR1
55704	ENSG00000115355	2746.50598	-0.17129157	0.07846091	0.02738836	0.09338117	CCDC88A
33	ENSG00000115361	22.5693318	2.64790836	0.59693204	4.41E-07	5.30E-06	ACADL
84141	ENSG00000115363	23.0638542	0.22393499	0.38193965	0.24711009	0.46606087	EVA1A
9801	ENSG00000115364	1024.08124	-0.36219954	0.0961078	9.49E-05	0.00072665	MRPL19
10314	ENSG00000115365	2426.35681	0.00241678	0.06440647	0.97027689	0.98888633	LANCL1
84128	ENSG00000115368	1119.46975	-0.05094892	0.09605207	0.5822557	0.77911602	WDR75
2202	ENSG00000115380	0	NA	NA	NA	NA	EFEMP1
5967	ENSG00000115386	0	NA	NA	NA	NA	REG1A
55120	ENSG00000115392	578.030476	0.10978106	0.10270165	0.26737157	0.48990136	FANCL
2335	ENSG00000115414	2128.04616	2.38331323	0.12652112	2.18E-80	4.46E-78	FN1
6772	ENSG00000115415	977.2243	0.01884332	0.09694069	0.84160344	0.92800104	STAT1

2744	ENSG00000115419	2118.00517	-0.40213816	0.06617882	6.56E-10	1.16E-08	GLS
64895	ENSG00000115421	489.458713	-0.43771617	0.12523461	0.00021688	0.0015302	PAPOLG
1768	ENSG00000115423	0	NA	NA	NA	NA	DNAH6
55825	ENSG00000115425	169.12219	-0.11095505	0.18270323	0.48915347	0.70874969	PECR
25972	ENSG00000115446	402.842756	-0.01093198	0.12676071	0.94664493	0.97814213	UNC50
3485	ENSG00000115457	5193.30347	0.44313013	0.05962518	5.17E-14	1.30E-12	IGFBP2
84173	ENSG00000115459	293.181961	0.18818153	0.15658307	0.18772772	0.38805147	ELMOD3
3488	ENSG00000115461	297.611845	1.43683161	0.18963907	2.92E-15	8.17E-14	IGFBP5
9736	ENSG00000115464	3901.7672	-0.14151916	0.07436676	0.05210528	0.15376598	USP34
80303	ENSG00000115468	70.4573876	2.34994391	0.34598255	7.15E-13	1.64E-11	EFHD1
3769	ENSG00000115474	2.11692692	0.11279713	0.40262084	0.14011401	NA	KCNJ13
10575	ENSG00000115484	2997.24705	-0.05076929	0.07604883	0.4951444	0.71392913	CCT4
2677	ENSG00000115486	718.757202	0.15090205	0.11202026	0.15836812	0.34554311	GGCX
4759	ENSG00000115488	0	NA	NA	NA	NA	NEU2
23301	ENSG00000115504	1117.84173	-0.5239274	0.14083187	6.91E-05	0.00054937	EHBP1
5013	ENSG00000115507	22.243587	2.48429488	0.59951843	2.00E-06	2.15E-05	OTX1
10190	ENSG00000115514	337.852042	-0.17400352	0.12881094	0.15053357	0.33407003	TXNDC9
80219	ENSG00000115520	164.887245	0.21460677	0.19090552	0.19635149	0.40005705	COQ10B
10578	ENSG00000115523	1.46586965	0.02917769	0.37938916	0.68577375	NA	GNLY
23451	ENSG00000115524	9148.72937	0.02455738	0.07676482	0.74437843	0.87963225	SF3B1
8869	ENSG00000115525	470.459212	-0.89570907	0.14070627	2.92E-11	5.91E-10	ST3GAL5
9486	ENSG00000115526	571.670494	-0.1986068	0.11610357	0.07184565	0.19526459	CHST10
79031	ENSG00000115539	353.946129	-0.00657305	0.12582185	0.95428559	0.98112988	PDCL3
25843	ENSG00000115540	790.785254	-0.03860957	0.12621524	0.74494519	0.87988222	MOB4
3336	ENSG00000115541	2044.4852	-0.15820681	0.10076086	0.10292328	0.25477628	HSPE1
55818	ENSG00000115548	745.434832	0.07943783	0.26210187	0.68381845	0.84592581	KDM3A
84812	ENSG00000115556	1043.06513	0.02843066	0.09815036	0.7652998	0.89210036	PLCD4
51652	ENSG00000115561	1267.58592	0.07310282	0.08525257	0.37951308	0.60845927	CHMP3
7701	ENSG00000115568	1474.05873	-0.03650926	0.08190756	0.64755868	0.82430661	ZNF142
7850	ENSG00000115590	0	NA	NA	NA	NA	IL1R2
53632	ENSG00000115592	0	NA	NA	NA	NA	PRKAG3
150572	ENSG00000115593	0	NA	NA	NA	NA	SMYD1
3554	ENSG00000115594	0	NA	NA	NA	NA	IL1R1
7475	ENSG00000115596	242.2688	-0.95296458	0.18629462	4.21E-08	5.83E-07	WNT6
8808	ENSG00000115598	0	NA	NA	NA	NA	IL1RL2
9173	ENSG00000115602	0	NA	NA	NA	NA	IL1RL1
8809	ENSG00000115604	0	NA	NA	NA	NA	IL18R1
8807	ENSG00000115607	0	NA	NA	NA	NA	IL18RAP

6549	ENSG00000115616	0.14931947	-0.01844332	0.38480212	0.84537521	NA	SLC9A2
2274	ENSG00000115641	168.581636	-0.31956712	0.17430768	0.03966444	0.12459508	FHL2
79083	ENSG00000115648	6.52603067	-0.04205545	0.36226814	0.73768134	0.87612079	MLPH
27013	ENSG00000115649	1071.26253	-0.28936134	0.09572924	0.00174518	0.00954102	CNPPD1
80146	ENSG00000115652	397.828715	-0.01357849	0.11676407	0.90170929	0.95678213	UXS1
10058	ENSG00000115657	961.693664	0.242917	0.09865145	0.01068198	0.04376016	ABCB6
8576	ENSG00000115661	412.854792	-0.04960397	0.12410204	0.67168521	0.83828612	STK16
60482	ENSG00000115665	0.96513545	0.01588278	0.3807244	0.771561	NA	SLC5A7
3069	ENSG00000115677	10029.9349	-0.12036206	0.05397651	0.02413332	0.08475925	HDLBP
5510	ENSG00000115685	673.558637	-0.22256308	0.09991373	0.02066286	0.07486324	PPP1R7
23178	ENSG00000115687	764.669141	-0.35701217	0.1366388	0.00502453	0.0233101	PASK
10494	ENSG00000115694	2258.66039	0.03773661	0.06360258	0.54752199	0.75431125	STK25
7173	ENSG00000115705	7.04333111	0.05111882	0.35391593	0.72919311	0.87046879	TPO
5624	ENSG00000115718	4.08331077	0.05914903	0.37658576	0.55505242	0.7609868	PROC
3398	ENSG00000115738	367.150738	-0.31872409	0.12607344	0.00718823	0.0316008	ID2
9014	ENSG00000115750	516.641452	0.15489882	0.11836161	0.16826	0.35937974	TAF1B
3241	ENSG00000115756	1921.20108	-0.90742491	0.07632941	2.13E-33	1.35E-31	HPCAL1
4953	ENSG00000115758	3960.80574	-0.22484981	0.05484624	3.38E-05	0.00028767	ODC1
57448	ENSG00000115760	5252.63477	-0.13560148	0.08617405	0.10438048	0.25722378	BIRC6
79954	ENSG00000115761	864.528412	-0.16270394	0.088487	0.05823621	0.16740168	NOL10
55041	ENSG00000115762	1847.92908	0.07024903	0.07207847	0.32195606	0.54956623	PLEKHB2
26003	ENSG00000115806	1306.7081	0.02785877	0.08021007	0.72304706	0.86764418	GORASP2
6801	ENSG00000115808	1560.34993	-0.12436888	0.08015978	0.11213088	0.2710046	STRN
10153	ENSG00000115816	2294.92407	-0.3474522	0.07886195	6.40E-06	6.31E-05	CEBPZ
23683	ENSG00000115825	4074.47711	0.08821425	0.05581478	0.11026302	0.2676313	PRKD3
80067	ENSG00000115827	744.262771	-0.00311596	0.13129337	0.97828708	0.99254491	DCAF17
25797	ENSG00000115828	2.19291843	0.01463026	0.37334638	0.87431724	NA	QPCT
22930	ENSG00000115839	954.59661	-0.19108031	0.08831071	0.02577914	0.08919746	RAB3GAP1
8604	ENSG00000115840	239.59036	0.12882855	0.16033765	0.37641151	0.6053339	SLC25A12
151393	ENSG00000115841	92.659429	-0.32260797	0.24549694	0.09980147	0.2488914	RMDN2
1746	ENSG00000115844	0.53116992	0.01535836	0.38361063	0.72195714	NA	DLX2
3938	ENSG00000115850	0.50565632	0.00490052	0.38258351	0.87118475	NA	LCT
1615	ENSG00000115866	1379.58959	-0.08798256	0.09882729	0.35569448	0.58599659	DARS1
6432	ENSG00000115875	4504.811	0.04316518	0.07454608	0.55575596	0.76147778	SRSF7
6382	ENSG00000115884	2878.14544	-0.20414936	0.07387629	0.00498307	0.02315859	SDC1
5334	ENSG00000115896	137.542839	-1.81845557	0.24418213	6.15E-15	1.67E-13	PLCL1
6509	ENSG00000115902	710.308797	-0.1062713	0.14495878	0.43246869	0.65788827	SLC1A4
6654	ENSG00000115904	2172.88341	-0.0637884	0.07039443	0.35159823	0.58169688	SOS1

8942	ENSG00000115919	0	NA	NA	NA	NA	KYNU
NA	ENSG00000115934	0	NA	NA	NA	NA	
7456	ENSG00000115935	1174.63698	0.56262078	0.106347	4.02E-08	5.60E-07	WIPF1
4999	ENSG00000115942	1059.15002	-0.34832781	0.10860522	0.00079199	0.00483331	ORC2
9167	ENSG00000115944	1743.34924	-0.06678478	0.07136494	0.33955453	0.56999424	COX7A2L
56902	ENSG00000115946	334.361464	-0.31540728	0.1533264	0.02454945	0.08588629	PNO1
5000	ENSG00000115947	777.982001	-0.11611902	0.10753311	0.25983084	0.48146481	ORC4
5341	ENSG00000115956	0.77422295	-0.02883461	0.38364886	0.55985386	NA	PLEK
390	ENSG00000115963	1090.4215	0.16714921	0.07847738	0.02948182	0.09905492	RND3
1386	ENSG00000115966	933.472385	-0.16436449	0.08630843	0.05017215	0.14934131	ATF2
63892	ENSG00000115970	1149.78082	0.01280441	0.09542605	0.89497122	0.95469109	THADA
22848	ENSG00000115977	886.060486	-0.35042314	0.15146801	0.01163468	0.04697436	AAK1
66008	ENSG00000115993	1292.69303	-0.1346342	0.08149634	0.09051069	0.23167746	TRAK2
54980	ENSG00000115998	134.934174	-0.39224381	0.21129555	0.02939791	0.09878974	C2orf42
7072	ENSG00000116001	3145.27124	0.04302376	0.06840278	0.52332981	0.73621666	TIA1
51449	ENSG00000116005	1108.17747	-0.31014134	0.10890913	0.00286553	0.01457746	PCYOX1
84634	ENSG00000116014	5.06881374	-0.14223211	0.40008954	0.23588881	0.45270207	KISS1R
2034	ENSG00000116016	2121.97338	-0.35568318	0.09284325	7.55E-05	0.00059356	EPAS1
1820	ENSG00000116017	720.105903	0.61280239	0.1247426	2.58E-07	3.22E-06	ARID3A
7341	ENSG00000116030	2371.61591	-0.22541422	0.06738066	0.00066619	0.00413813	SUMO1
50489	ENSG00000116031	27.8991691	0.12803755	0.31104194	0.51359038	0.7287659	CD207
116444	ENSG00000116032	4.31020164	0.16696513	0.41835102	0.15265777	0.33723751	GRIN3B
25806	ENSG00000116035	144.118283	-0.13918204	0.18504779	0.3897317	0.61887921	VAX2
525	ENSG00000116039	83.786324	0.2689405	0.23271466	0.1557914	0.34198954	ATP6V1B1
4780	ENSG00000116044	681.117288	0.24071228	0.1123973	0.02475019	0.08645137	NFE2L2
2956	ENSG00000116062	3338.46718	-0.34872293	0.06184105	1.06E-08	1.60E-07	MSH6
65977	ENSG00000116095	260.654743	-0.03889079	0.20948073	0.83418368	0.92433422	PLEKHA3
6697	ENSG00000116096	296.463517	-0.46769725	0.14145762	0.00038946	0.00258253	SPR
2043	ENSG00000116106	0	NA	NA	NA	NA	EPHA4
117583	ENSG00000116117	140.151917	0.96879934	0.24826967	1.08E-05	0.00010216	PARD3B
10056	ENSG00000116120	694.45934	-0.22403599	0.10490987	0.02592972	0.08959345	FARSB
7840	ENSG00000116127	1478.24908	-0.12361145	0.08219189	0.12499366	0.29343866	ALMS1
607	ENSG00000116128	1663.42992	-0.00038064	0.09912421	0.99581209	0.99852582	BCL9
5396	ENSG00000116132	0	NA	NA	NA	NA	PRRX1
1718	ENSG00000116133	5774.71055	-0.65133727	0.20622943	0.00036145	0.00241863	DHCR24
23341	ENSG00000116138	404.194185	-0.25384788	0.17595005	0.10397555	0.25670421	DNAJC16
4139	ENSG00000116141	653.054945	0.13716576	0.13158607	0.26749718	0.490041	MARK1
7143	ENSG00000116147	235.474983	-1.55321521	0.17327021	2.44E-20	9.30E-19	TNR

79906	ENSG00000116151	197.878985	0.45122635	0.1794951	0.00494472	0.02302891	MORN1
2882	ENSG00000116157	725.285329	0.20094505	0.10968287	0.05542907	0.16113007	GPX7
27101	ENSG00000116161	2442.54474	0.0098395	0.09060836	0.91191818	0.96251831	CACYBP
6342	ENSG00000116171	656.984452	0.11187462	0.09805862	0.2380379	0.45546069	SCP2
25823	ENSG00000116176	0	NA	NA	NA	NA	TPSG1
60676	ENSG00000116183	7.07430099	0.84067236	1.62026462	0.01536085	0.05872047	PAPPA2
55103	ENSG00000116191	966.762075	0.15930608	0.23614697	0.39902156	0.62780637	RALGPS2
9068	ENSG00000116194	5.93565993	0.36472652	0.64414349	0.0192299	0.07054757	ANGPTL1
9731	ENSG00000116198	791.952741	0.10613783	0.14135753	0.41873258	0.64465387	CEP104
9917	ENSG00000116199	2662.11144	0.02864804	0.07132354	0.68354377	0.84579696	FAM20B
127428	ENSG00000116205	746.302155	0.2393304	0.11641655	0.03055333	0.10184445	TCEANC2
9528	ENSG00000116209	1866.13279	-0.27272442	0.06961224	6.57E-05	0.00052388	TMEM59
115353	ENSG00000116212	440.08428	0.14978283	0.11394084	0.1686769	0.36003762	LRRC42
49856	ENSG00000116213	1790.24119	0.27386984	0.08542041	0.00098243	0.00582528	WRAP73
7827	ENSG00000116218	0	NA	NA	NA	NA	NPHS2
51253	ENSG00000116221	1117.2577	-0.24749357	0.08230031	0.00201348	0.01076702	MRPL37
23463	ENSG00000116237	2017.89527	-0.16991379	0.06692161	0.00945181	0.03961214	ICMT
6146	ENSG00000116251	8102.97219	-0.22271346	0.04890964	4.32E-06	4.40E-05	RPL22
26038	ENSG00000116254	228.31602	-1.8175197	0.18834083	3.08E-23	1.36E-21	CHD5
5768	ENSG00000116260	1755.18281	0.05807217	0.07268861	0.41643585	0.64306408	QSOX1
6814	ENSG00000116266	358.997992	0.36149525	0.13653282	0.00456559	0.0215516	STXBP3
148479	ENSG00000116273	691.991827	0.03903131	0.09192171	0.66256512	0.83345752	PHF13
54206	ENSG00000116285	258.095816	-0.21776672	0.15253742	0.11852845	0.28264477	ERRFI1
11315	ENSG00000116288	2777.86301	-0.30425215	0.06200624	6.32E-07	7.41E-06	PARK7
57535	ENSG00000116299	73.7672625	-2.97661684	0.35587765	2.81E-18	9.58E-17	ELAPOR1
4985	ENSG00000116329	1032.97891	0.58584698	0.0828243	4.86E-13	1.13E-11	OPRD1
271	ENSG00000116337	1635.76561	0.36013781	0.10749332	0.00046887	0.00305098	AMPD2
6429	ENSG00000116350	2377.84123	-0.20237403	0.07382244	0.00512506	0.02372403	SRSF4
51102	ENSG00000116353	392.963177	0.11249663	0.14766062	0.40960883	0.63763351	MECR
3749	ENSG00000116396	169.412493	-0.15514009	0.19631464	0.35674287	0.58678316	KCNC4
80267	ENSG00000116406	1626.0932	0.10690654	0.07354603	0.13497333	0.30945965	EDEM3
79084	ENSG00000116455	891.901249	-0.14976347	0.09282935	0.09444755	0.23891609	WDR77
515	ENSG00000116459	1731.13275	-0.38470541	0.07288981	7.29E-08	9.77E-07	ATP5PB
5906	ENSG00000116473	540.218744	-0.35705968	0.12884655	0.00316908	0.01584938	RAP1A
3065	ENSG00000116478	1269.58291	0.0499251	0.07573646	0.5021114	0.71895859	HDAC1
829	ENSG00000116489	2005.21792	-0.15558086	0.07118005	0.02593594	0.08959933	CAPZA1
64766	ENSG00000116497	753.58033	0.25719082	0.10988227	0.01432037	0.05554485	S100PBP
127544	ENSG00000116514	253.01925	0.02057656	0.14532614	0.87859272	0.94627217	RNF19B

10067	ENSG00000116521	1835.22006	0.14157521	0.07081637	0.04187416	0.13005373	SCAMP3
55223	ENSG00000116525	135.53945	0.06146906	0.21597382	0.73240385	0.87210781	TRIM62
55870	ENSG00000116539	3122.24497	-0.17499503	0.06500986	0.00625068	0.02803834	ASH1L
58512	ENSG00000116544	253.384056	-1.20919695	0.16692143	4.53E-14	1.15E-12	DLGAP3
6421	ENSG00000116560	14077.1817	-0.01009259	0.05059548	0.83594459	0.92522644	SFPQ
58480	ENSG00000116574	1080.43279	-0.97378058	0.08612241	1.73E-30	1.02E-28	RHOU
54856	ENSG00000116580	2684.08519	-0.02655477	0.07358254	0.7117685	0.86153908	GON4L
9181	ENSG00000116584	2315.82455	-0.17787319	0.07525788	0.01576005	0.06002677	ARHGEF2
28956	ENSG00000116586	973.023753	0.07765098	0.08344382	0.34073805	0.57095356	LAMTOR2
4209	ENSG00000116604	1052.81652	0.33511087	0.09905087	0.00044773	0.00293169	MEF2D
85440	ENSG00000116641	2413.94306	-0.07863067	0.1933864	0.63715702	0.81722218	DOCK7
6723	ENSG00000116649	1361.7876	-0.066485	0.08320499	0.41239295	0.64005916	SRM
NA	ENSG00000116652	21.7585068	0.09945458	0.31505196	0.60194327	0.79229168	DLEU2L
26232	ENSG00000116661	93.9491671	-1.02653948	0.26819443	1.46E-05	0.00013477	FBXO2
26270	ENSG00000116663	44.8100822	0.03926774	0.26893354	0.8402818	0.92746542	FBXO6
81563	ENSG00000116667	1130.17587	-0.10435045	0.13313797	0.40189154	0.63031025	C1orf21
54823	ENSG00000116668	203.515653	0.0660859	0.16413338	0.65695413	0.82971354	SWT1
10459	ENSG00000116670	938.793093	-0.26647012	0.08836349	0.00189155	0.01019518	MAD2L2
9829	ENSG00000116675	1704.15804	-0.64343563	0.0766631	1.29E-17	4.15E-16	DNAJC6
3953	ENSG00000116678	948.120613	0.87734566	0.10749162	5.45E-17	1.68E-15	LEPR
10625	ENSG00000116679	4349.23303	0.16574111	0.05250527	0.00143451	0.00806709	IVNS1ABP
90231	ENSG00000116685	1217.34199	-0.26224181	0.08035211	0.0008238	0.0049921	KIAA2013
9927	ENSG00000116688	2413.28544	-0.17083958	0.06808686	0.01070928	0.04385391	MFN2
10216	ENSG00000116690	3.86660865	0.18140287	0.42876625	0.12034927	0.28578451	PRG4
60672	ENSG00000116691	456.68976	0.34695714	0.13240028	0.00514743	0.02381089	MIIP
9887	ENSG00000116698	2880.78544	-0.23310828	0.061283	0.00011404	0.00085444	SMG7
4688	ENSG00000116701	0.65250675	0.01174478	0.38266871	0.92947371	NA	NCF2
5132	ENSG00000116703	0.91776575	-0.06294814	0.3908818	0.22039491	NA	PDC
23169	ENSG00000116704	441.880607	0.15146722	0.11488269	0.16676928	0.35754295	SLC35D1
5321	ENSG00000116711	0	NA	NA	NA	NA	PLA2G4A
1647	ENSG00000116717	597.408272	-0.40801887	0.10372593	4.26E-05	0.00035568	GADD45A
65121	ENSG00000116721	0	NA	NA	NA	NA	PRAMEF1
390999	ENSG00000116726	0	NA	NA	NA	NA	PRAMEF12
79971	ENSG00000116729	1206.57872	0.85498358	0.10593883	1.19E-16	3.60E-15	WLS
7799	ENSG00000116731	1160.13867	-0.04952152	0.08676956	0.55737965	0.76258372	PRDM2
5997	ENSG00000116741	191.510724	0.20916056	0.1684152	0.166671	0.35740949	RGS2
6121	ENSG00000116745	0	NA	NA	NA	NA	RPE65
6738	ENSG00000116747	1023.30966	0.02072894	0.09907868	0.81020076	0.91399233	RO60

270	ENSG00000116748	0.173733	0.01914545	0.38484597	0.78225479	NA	AMPD1
51377	ENSG00000116750	1339.40856	-0.21276675	0.07925901	0.00598941	0.02711771	UCHL5
10286	ENSG00000116752	576.666639	-0.21473733	0.10283718	0.02974943	0.0997008	BCAS2
9295	ENSG00000116754	4894.93137	-0.04786549	0.05742045	0.39869929	0.62756224	SRSF11
1491	ENSG00000116761	105.516545	0.26632249	0.2422398	0.16891113	0.36034672	CTH
79814	ENSG00000116771	31.9363874	-0.17167422	0.30251516	0.39400347	0.62317897	AGMAT
56944	ENSG00000116774	35.4664215	0.47714716	0.37104752	0.05252273	0.15462992	OLFML3
51086	ENSG00000116783	6.86514723	0.47277056	0.84834498	0.01627398	0.06162927	TNNI3K
10878	ENSG00000116785	0.94407361	0.00100349	0.3809701	0.9850961	NA	CFHR3
23207	ENSG00000116786	1626.00539	0.04660439	0.06919557	0.49373017	0.7125217	PLEKHM2
1429	ENSG00000116791	455.485514	0.0521614	0.11364427	0.63168213	0.81371846	CRYZ
10745	ENSG00000116793	748.673034	-0.09172139	0.09494221	0.31797398	0.54561048	PHTF1
7709	ENSG00000116809	905.642256	0.2335839	0.09632387	0.01207781	0.04849701	ZBTB17
965	ENSG00000116815	87.3082484	0.33907044	0.23586351	0.07813797	0.20773003	CD58
339488	ENSG00000116819	52.9286865	0.89918643	0.44939668	0.00439348	0.02089824	TFAP2E
914	ENSG00000116824	0	NA	NA	NA	NA	CD2
8458	ENSG00000116830	844.561881	-0.39315299	0.14481159	0.00331563	0.01645788	TTF2
2494	ENSG00000116833	0	NA	NA	NA	NA	NR5A2
23046	ENSG00000116852	1161.8608	-2.07174586	0.08626866	1.31E-128	6.33E-126	KIF21B
252839	ENSG00000116857	2159.39877	0.21760381	0.07261922	0.00225372	0.01185729	TMEM9
54936	ENSG00000116863	901.078327	-0.11556607	0.08971473	0.18252791	0.3803144	ADPRS
55700	ENSG00000116871	3147.85894	-0.0467604	0.07739917	0.53660605	0.74729267	MAP7D1
10352	ENSG00000116874	164.684888	-0.22557175	0.19051983	0.17290172	0.36578929	WARS2
51179	ENSG00000116882	0	NA	NA	NA	NA	HAO2
127700	ENSG00000116885	89.3029836	0.61165022	0.30080211	0.00903377	0.03811782	OSCP1
64960	ENSG00000116898	691.607154	-0.07047391	0.10296001	0.47654253	0.69841458	MRPS15
149371	ENSG00000116903	347.070317	-0.34283444	0.12767599	0.00430621	0.02054721	EXOC8
8443	ENSG00000116906	1381.54638	-0.02262223	0.07884628	0.76896654	0.89364433	GNPAT
7257	ENSG00000116918	1121.48569	-0.16773144	0.0885151	0.05102342	0.15140663	TSNAX
54955	ENSG00000116922	601.947629	0.1109712	0.10384473	0.2666803	0.48908911	C1orf109
64121	ENSG00000116954	307.101022	-0.18539512	0.14015241	0.15412585	0.33936549	RRAGC
4811	ENSG00000116962	2617.3088	0.37434578	0.07004101	5.24E-08	7.18E-07	NID1
3964	ENSG00000116977	1133.2305	-0.30749104	0.09729609	0.00104378	0.00612859	LGALS8
84618	ENSG00000116981	1137.94895	-0.53253059	0.09907622	2.75E-08	3.92E-07	NT5C1A
51440	ENSG00000116983	424.179618	-1.91404675	0.13105604	2.01E-49	1.95E-47	HPCAL4
4548	ENSG00000116984	5238.79752	-0.63339181	0.07806227	1.37E-16	4.13E-15	MTR
656	ENSG00000116985	305.644201	-0.52022978	0.14325858	0.00010004	0.00076105	BMP8B
4610	ENSG00000116990	339.68885	0.98554029	0.14187521	5.13E-13	1.19E-11	MYCL

57568	ENSG00000116991	1123.80243	0.31087823	0.09764489	0.00096796	0.0057515	SIPA1L2
57829	ENSG00000116996	0	NA	NA	NA	NA	ZP4
6018	ENSG00000117000	708.238689	-0.22567945	0.09848239	0.0174423	0.0652444	RLF
8564	ENSG00000117009	4.77255431	0.15790144	0.40842911	0.1984291	0.4027039	KMO
127396	ENSG00000117010	87.0295829	0.27617957	0.259427	0.16880013	0.36022314	ZNF684
9132	ENSG00000117013	40.3907877	0.68023006	0.38811509	0.01264638	0.05031164	KCNQ4
9783	ENSG00000117016	2176.44444	0.14809689	0.11731927	0.18472533	0.38350847	RIMS3
10000	ENSG00000117020	4007.76123	-0.09639775	0.08274298	0.23229091	0.44909196	AKT3
2117	ENSG00000117036	1065.96187	-0.08965834	0.09037312	0.30669622	0.53422766	ETV3
34	ENSG00000117054	1028.62048	-0.06111457	0.086086	0.46547221	0.68820095	ACADM
81849	ENSG00000117069	230.680646	-0.19588425	0.16820385	0.19424866	0.39721723	ST6GALNAC5
6504	ENSG00000117090	0	NA	NA	NA	NA	SLAMF1
962	ENSG00000117091	0	NA	NA	NA	NA	CD48
23266	ENSG00000117114	8181.47226	0.09540355	0.05274987	0.06806829	0.1877746	ADGRL2
11240	ENSG00000117115	14.7607837	0.15330137	0.35587361	0.39809274	0.62724844	PADI2
6390	ENSG00000117118	627.845151	-0.20779307	0.10490004	0.03889196	0.12286424	SDHB
4237	ENSG00000117122	2945.26667	0.12579556	0.06319864	0.0436125	0.13394304	MFAP2
80135	ENSG00000117133	703.953372	0.11304512	0.0974118	0.23006714	0.44597284	RPF1
10765	ENSG00000117139	4026.57681	0.04281855	0.05919218	0.46533375	0.6880988	KDM5B
6675	ENSG00000117143	859.896297	-0.03080259	0.09731539	0.74269145	0.87875221	UAP1
81569	ENSG00000117148	0.80488441	-0.03027885	0.38378171	0.54468417	NA	ACTL8
1486	ENSG00000117151	131.599568	0.11485232	0.18708384	0.48321128	0.70357492	CTBS
5999	ENSG00000117152	4155.43711	-0.05187465	0.20043415	0.75949781	0.88850157	RGS4
59349	ENSG00000117153	1052.02533	0.12613673	0.0871531	0.13733434	0.31289771	KLHL12
84966	ENSG00000117154	18.102735	-0.88226944	0.74016309	0.01373962	0.05375547	IGSF21
117178	ENSG00000117155	745.62063	-0.25444477	0.09803025	0.00708129	0.03124842	SSX2IP
54680	ENSG00000117174	358.599518	0.03717979	0.12143965	0.74831509	0.8819396	ZNHIT6
26279	ENSG00000117215	0.14931947	-0.01844332	0.38480212	0.84537521	NA	PLA2G2D
5929	ENSG00000117222	936.638451	-0.24853334	0.09704171	0.00794499	0.03429692	RBBP5
2635	ENSG00000117226	0.37844916	0.02407552	0.3854191	0.60038877	NA	GBP3
2633	ENSG00000117228	0.35150941	0.00330166	0.383397	0.93716717	NA	GBP1
100861548	ENSG00000117242	265.487654	0.40171315	0.16890049	0.00843435	0.03606146	PINK1-AS
57576	ENSG00000117245	62.5700404	-0.09212054	0.26185659	0.63230257	0.81396853	KIF17
653519	ENSG00000117262	477.602232	-0.02264676	0.13743177	0.8587849	0.93709671	GPR89A
5129	ENSG00000117266	394.900357	-0.34919934	0.1409029	0.00760641	0.03312361	CDK18
8934	ENSG00000117280	423.732507	0.11797225	0.12446848	0.31887844	0.54656314	RAB29
11126	ENSG00000117281	31.6389228	0.05617504	0.2922496	0.77068289	0.89494676	CD160
1889	ENSG00000117298	2061.43493	0.26583132	0.0651623	3.40E-05	0.00028989	ECE1

3155	ENSG00000117305	283.09655	0.05612776	0.15926374	0.69922659	0.85491191	HMGCL
2582	ENSG00000117308	309.971708	-0.03884466	0.14727366	0.77419438	0.89604057	GALE
3399	ENSG00000117318	227.740411	0.21645196	0.15811808	0.13274907	0.3060355	ID3
1380	ENSG00000117322	0	NA	NA	NA	NA	CR2
4179	ENSG00000117335	2824.48911	0.13136649	0.07043542	0.0577825	0.16649345	CD46
9129	ENSG00000117360	1549.75198	-0.02965488	0.07317051	0.67880253	0.84319072	PRPF3
51107	ENSG00000117362	3802.72613	0.08189753	0.06001194	0.1659137	0.35616336	APH1A
64175	ENSG00000117385	864.069494	0.12327696	0.10832688	0.23441804	0.45139906	P3H1
6513	ENSG00000117394	913.035833	-0.19779961	0.14754842	0.14505761	0.32541747	SLC2A1
10969	ENSG00000117395	1418.44618	0.1794606	0.0954302	0.05194278	0.15342337	EBNA1BP2
991	ENSG00000117399	1431.72719	-0.15851145	0.07168837	0.02420135	0.08493803	CDC20
4352	ENSG00000117400	2.08940217	0.09002326	0.39422673	0.20926	NA	MPL
9048	ENSG00000117407	10.6394081	0.06024034	0.35255752	0.69058923	0.85006144	ARTN
9670	ENSG00000117408	866.424687	-0.15049607	0.0983985	0.11280992	0.27198308	IPO13
533	ENSG00000117410	1764.60849	-0.36131889	0.07518014	9.10E-07	1.04E-05	ATP6V0B
8704	ENSG00000117411	1817.53059	-0.01227667	0.07726923	0.86984972	0.9432116	B4GALT2
79033	ENSG00000117419	903.429941	0.10678558	0.10560886	0.29297352	0.51909756	ERI3
8643	ENSG00000117425	56.4565753	1.15365236	0.36270207	0.00013017	0.00096259	PTCH2
10327	ENSG00000117448	1195.72154	0.12275239	0.08322486	0.13068285	0.30292648	AKR1A1
5052	ENSG00000117450	3419.33515	-0.13269548	0.07496445	0.07066729	0.19290575	PRDX1
8503	ENSG00000117461	1858.05555	-0.50679855	0.07772489	2.75E-11	5.59E-10	PIK3R3
10103	ENSG00000117472	1.30910033	0.07207082	0.39349794	0.12888999	NA	TSPAN1
8548	ENSG00000117475	629.536262	0.15896139	0.1125373	0.13915463	0.31601249	BLZF1
57821	ENSG00000117477	209.838291	0.20501254	0.16226605	0.16309865	0.35207021	CCDC181
10560	ENSG00000117479	615.495664	0.00629858	0.10114964	0.94936968	0.97912847	SLC19A2
2166	ENSG00000117480	126.987561	-0.39187802	0.23523248	0.04225126	0.13101983	FAAH
387338	ENSG00000117481	632.510834	-0.03748647	0.11092166	0.72248189	0.86754018	NSUN4
50999	ENSG00000117500	889.697806	0.34794923	0.10848073	0.00080209	0.00488371	TMED5
80133	ENSG00000117501	0	NA	NA	NA	NA	MROH9
1810	ENSG00000117505	1689.39478	-0.09938502	0.06958291	0.14601274	0.32674178	DR1
388714	ENSG00000117507	0	NA	NA	NA	NA	FMO6P
1266	ENSG00000117519	4250.65355	0.08058869	0.06574789	0.21411083	0.42428673	CNN3
23215	ENSG00000117523	8455.64627	-0.04056187	0.04923993	0.40569283	0.63390153	PRRC2C
2152	ENSG00000117525	44.4653492	0.57998027	0.36430226	0.02294686	0.08131196	F3
5825	ENSG00000117528	1227.63364	-0.27885319	0.09506842	0.00239484	0.01250357	ABCD3
8674	ENSG00000117533	502.998416	0.18465306	0.13732141	0.1489178	0.33152468	VAMP4
51611	ENSG00000117543	411.465641	-0.01741174	0.13758358	0.89112588	0.95273684	DPH5
356	ENSG00000117560	0	NA	NA	NA	NA	FASLG

58155	ENSG00000117569	916.713083	0.10830721	0.12614882	0.36330767	0.59305778	PTBP2
7292	ENSG00000117586	23.3375293	0.35849965	0.41359646	0.11869375	0.28297093	TNFSF4
9588	ENSG00000117592	2684.03983	0.07980875	0.07172293	0.25765988	0.47887326	PRDX6
55157	ENSG00000117593	1423.89128	-0.36917454	0.08026051	2.57E-06	2.71E-05	DARS2
3290	ENSG00000117594	0	NA	NA	NA	NA	HSD11B1
3664	ENSG00000117595	71.8570453	-2.04343026	0.32330533	1.57E-11	3.27E-10	IRF6
27042	ENSG00000117597	905.13477	-0.28258571	0.08989419	0.001186	0.00685702	UTP25
163404	ENSG00000117598	457.770438	-1.5896545	0.12892935	5.09E-36	3.56E-34	PLPPR5
9890	ENSG00000117600	595.833541	-0.27866817	0.10258627	0.00468304	0.0219961	PLPPR4
462	ENSG00000117601	1.5998353	0.05265818	0.38313675	0.43549321	NA	SERPINC1
11123	ENSG00000117602	548.917826	0.66271113	0.12598122	3.61E-08	5.05E-07	RCAN3
25949	ENSG00000117614	818.852994	0.05545564	0.08910606	0.52283158	0.73594472	SYF2
57035	ENSG00000117616	1234.29372	0.19153851	0.09025902	0.02882487	0.09722903	RSRP1
23443	ENSG00000117620	316.510515	0.00280386	0.14391283	0.98310788	0.99428951	SLC35A3
55758	ENSG00000117625	1343.73326	-0.16180689	0.08715655	0.05602862	0.16256329	RCOR3
3925	ENSG00000117632	12178.8983	-0.10026622	0.05872464	0.08379077	0.21870922	STMN1
56181	ENSG00000117640	1046.13813	-0.12232978	0.08099843	0.12356752	0.2912855	MTFR1L
57134	ENSG00000117643	76.4826564	-0.08576176	0.2280016	0.64031202	0.81986385	MAN1C1
4751	ENSG00000117650	999.990323	-0.04428387	0.08932975	0.60906313	0.79787471	NEK2
6195	ENSG00000117676	377.332392	-0.32565615	0.19474581	0.05346599	0.15663988	RPS6KA1
79947	ENSG00000117682	411.465395	0.0584088	0.13979665	0.65463649	0.82810431	DHDDS
29937	ENSG00000117691	757.202679	-0.20885801	0.1082133	0.04350904	0.13376459	NENF
25936	ENSG00000117697	1213.45731	0.00335837	0.08407214	0.9686693	0.98824492	NSL1
5629	ENSG00000117707	6367.06336	-0.14325728	0.07339077	0.04645377	0.14068735	PROX1
8289	ENSG00000117713	5849.93855	-0.14609065	0.05769664	0.01035083	0.04258823	ARID1A
1063	ENSG00000117724	12210.8841	-0.5221726	0.05571459	2.56E-21	1.02E-19	CENPF
6118	ENSG00000117748	1171.58807	-0.20280931	0.08177481	0.01102607	0.04494703	RPA2
5511	ENSG00000117751	1074.48506	-0.03261867	0.07904802	0.67264921	0.83906698	PPP1R8
23673	ENSG00000117758	634.959186	0.12819773	0.10581361	0.20731501	0.41450466	STX12
54996	ENSG00000117791	206.126019	0.19590097	0.1824831	0.22234967	0.43535977	MTARC2
200010	ENSG00000117834	12.1006409	0.20295362	0.39715666	0.24685852	0.46592963	SLC5A9
114883	ENSG00000117859	1337.5727	0.05105868	0.07649101	0.49706348	0.71500229	OSBPL9
51060	ENSG00000117862	1033.9433	0.23440306	0.08293236	0.00373836	0.01823233	TXNDC12
57488	ENSG00000117868	1381.26584	0.08531068	0.07458023	0.24377735	0.4621195	ESYT2
10849	ENSG00000117877	160.291751	-0.15503977	0.18074485	0.33000996	0.55861736	POLR1G
23184	ENSG00000117899	772.895902	0.03874295	0.10502162	0.70223232	0.85686744	MESD
5955	ENSG00000117906	2848.04673	0.14197034	0.06279023	0.02172199	0.07780545	RCN2
1143	ENSG00000117971	1056.48315	0.31317099	0.08662827	0.00020154	0.00143262	CHRNB4

727897	ENSG00000117983	1.71976341	0.04259761	0.38190092	0.52927044	NA	MUC5B
1509	ENSG00000117984	1874.09081	0.33587724	0.07438988	3.95E-06	4.04E-05	CTSD
78989	ENSG00000118004	1760.92027	-0.41727706	0.08252382	2.15E-07	2.72E-06	COLEC11
10274	ENSG00000118007	1281.79922	-0.1802534	0.08378877	0.02719255	0.09298521	STAG1
51146	ENSG00000118017	2.0831918	-0.11313163	0.39983459	0.18933273	NA	A4GNT
6794	ENSG00000118046	1142.17186	0.29589398	0.0832942	0.00026628	0.00183949	STK11
4297	ENSG00000118058	3758.88564	-0.01937376	0.10927814	0.85154427	0.93275912	KMT2A
11181	ENSG00000118094	0.52977536	0.01340197	0.38291275	0.74469277	NA	TREH
56912	ENSG00000118096	369.787026	0.09177612	0.12762532	0.44626954	0.67062583	IFT46
4317	ENSG00000118113	0	NA	NA	NA	NA	MMP8
335	ENSG00000118137	0.15069912	-0.01848503	0.38480575	0.84537521	NA	APOA1
84215	ENSG00000118156	0.9809279	-0.03757886	0.38418971	0.45799278	NA	ZNF541
6543	ENSG00000118160	531.794755	-1.24553982	0.15311076	3.85E-17	1.20E-15	SLC8A2
11133	ENSG00000118162	430.331262	-0.03227758	0.12824832	0.78610961	0.90280441	KPTN
6230	ENSG00000118181	12170.5058	0.01388902	0.05307886	0.79214932	0.90499209	RPS25
9928	ENSG00000118193	1361.10994	-0.74743635	0.08605792	7.99E-19	2.80E-17	KIF14
7139	ENSG00000118194	3.46996475	0.08669282	0.37933056	0.45219727	0.67536839	TNNT2
83479	ENSG00000118197	523.192853	0.0866014	0.11078514	0.41450101	0.6417034	DDX59
23271	ENSG00000118200	2130.51903	-0.29234981	0.0880814	0.00062812	0.0039399	CAMSAP2
22926	ENSG00000118217	1044.96226	0.03063731	0.09973442	0.75127645	0.88327851	ATF6
1421	ENSG00000118231	0	NA	NA	NA	NA	CRYGD
55686	ENSG00000118242	135.089757	-0.99724988	0.23613478	2.88E-06	3.02E-05	MREG
7141	ENSG00000118245	0	NA	NA	NA	NA	TNP1
22868	ENSG00000118246	644.203182	-0.2997945	0.10154567	0.00213548	0.01131926	FASTKD2
8828	ENSG00000118257	28.2956297	0.90711927	1.24989208	0.01149878	0.04653421	NRP2
1385	ENSG00000118260	1493.595	-0.03052634	0.0936356	0.73564888	0.87443975	CREB1
8609	ENSG00000118263	2513.22908	0.71827367	0.07282605	1.40E-23	6.37E-22	KLF7
7276	ENSG00000118271	0	NA	NA	NA	NA	TTR
9331	ENSG00000118276	249.095848	1.14234803	0.19157673	2.78E-10	5.11E-09	B4GALT6
79630	ENSG00000118292	8.07093576	-0.04255829	0.35076292	0.77372893	0.89600234	C1orf54
23632	ENSG00000118298	35.3453528	0.31130532	0.34686623	0.15992606	0.34752825	CA14
55259	ENSG00000118307	4.98595501	0.08530186	0.37551028	0.48679249	0.70661128	CFAP94
4033	ENSG00000118308	1.00882577	0.05620494	0.38978218	0.2164917	NA	IRAG2
23120	ENSG00000118322	0	NA	NA	NA	NA	ATP10B
9789	ENSG00000118363	1280.14211	-0.26459929	0.11082245	0.01229522	0.04922057	SPCS2
57558	ENSG00000118369	438.938421	0.17034251	0.11813066	0.12904245	0.30038744	USP35
6785	ENSG00000118402	153.681664	-0.15858005	0.18360078	0.32453558	0.55221149	ELOVL4
27145	ENSG00000118407	8.83544506	0.19212918	0.39996461	0.24764921	0.46665024	FILIP1

9994	ENSG00000118412	1039.25119	-0.32690945	0.10334942	0.0009817	0.00582268	CASP8AP2
9324	ENSG00000118418	1131.85843	0.06449321	0.08183884	0.42031792	0.64619198	HMGN3
90025	ENSG00000118420	101.393494	0.03595837	0.20334652	0.83596821	0.92522644	UBE3D
1268	ENSG00000118432	25.5564887	1.25366561	0.56547333	0.00170423	0.00935593	CNR1
81833	ENSG00000118434	0	NA	NA	NA	NA	SPACA1
81573	ENSG00000118454	474.068088	0.28222161	0.11903821	0.01241377	0.0495851	ANKRD13C
84251	ENSG00000118473	1011.88256	-0.1575433	0.08242746	0.05002189	0.14903652	SGIP1
23469	ENSG00000118482	1383.14319	-0.33600924	0.0734753	3.03E-06	3.16E-05	PHF3
153918	ENSG00000118491	0	NA	NA	NA	NA	ZC2HC1B
79747	ENSG00000118492	0.15069912	-0.01848503	0.38480575	0.84537521	NA	ADGB
5325	ENSG00000118495	483.082271	3.29302662	0.17029041	1.18E-84	2.60E-82	PLAGL1
84085	ENSG00000118496	542.343008	0.03450786	0.10254647	0.72845955	0.87026999	FBXO30
7128	ENSG00000118503	6.06966877	0.38127119	0.66060299	0.02079352	0.07522674	TNFAIP3
9465	ENSG00000118507	28.4774373	-0.24327817	0.33607264	0.24615553	0.46542702	AKAP7
10981	ENSG00000118508	292.385766	-0.28602853	0.14231724	0.02990879	0.1000827	RAB32
4602	ENSG00000118513	212.155475	-0.14335279	0.15495207	0.31061927	0.53841396	MYB
64577	ENSG00000118514	7.47283966	0.07755272	0.37807924	0.46438262	0.68715328	ALDH8A1
6446	ENSG00000118515	62.1602575	-1.69664286	0.36206315	1.67E-07	2.15E-06	SGK1
81847	ENSG00000118518	699.578014	-0.01617383	0.09856481	0.86428049	0.93988845	RNF146
383	ENSG00000118520	0.50018037	-0.01271546	0.38349606	0.78722594	NA	ARG1
1490	ENSG00000118523	0	NA	NA	NA	NA	CCN2
6943	ENSG00000118526	0	NA	NA	NA	NA	TCF21
83449	ENSG00000118557	44.2208298	-0.2391899	0.33726672	0.24905285	0.4684498	PMFBP1
26234	ENSG00000118564	581.436396	0.08564652	0.1038957	0.39213102	0.62144576	FBXL5
80306	ENSG00000118579	1407.19641	-0.18937415	0.24233214	0.3201229	0.54785114	MED28
9194	ENSG00000118596	0.34746601	0.02370763	0.3853651	0.61987128	NA	SLC16A7
10329	ENSG00000118600	202.149759	-0.10784743	0.16980492	0.47768908	0.69938463	RXYLT1
80264	ENSG00000118620	280.210168	-0.34327345	0.15446427	0.01504954	0.05786489	ZNF430
8673	ENSG00000118640	2.68458397	0.04363934	0.37392591	0.66718362	0.83613941	VAMP8
64858	ENSG00000118655	353.340188	-0.37980652	0.12709244	0.00150853	0.00841879	DCLRE1B
103910	ENSG00000118680	2140.37942	0.17834787	0.06734405	0.00714187	0.03144527	MYL12B
2309	ENSG00000118689	2998.09001	-0.23177357	0.06924988	0.00061501	0.00386377	FOXO3
84071	ENSG00000118690	62.4534746	0.29763841	0.33501766	0.17208836	0.36461546	ARMC2
2691	ENSG00000118702	0.16735865	0.01897177	0.38482933	0.78225479	NA	GHRH
6185	ENSG00000118705	4579.29095	0.1188609	0.06236097	0.05341405	0.15653395	RPN2
60436	ENSG00000118707	1581.74068	0.3847437	0.0812734	1.23E-06	1.37E-05	TGIF2
845	ENSG00000118729	0	NA	NA	NA	NA	CASQ2
118427	ENSG00000118733	71.4058738	-1.85492029	0.33433016	1.99E-09	3.29E-08	OLFM3

5311	ENSG00000118762	984.157051	0.10405655	0.0919184	0.2437588	0.4621195	PKD2
9429	ENSG00000118777	0	NA	NA	NA	NA	ABCG2
6696	ENSG00000118785	10.1856972	0.0867964	0.35867772	0.57147915	0.77166993	SPP1
8987	ENSG00000118804	266.895131	0.45237744	0.15840542	0.0018362	0.00994544	STBD1
10983	ENSG00000118816	5050.22306	-0.47586968	0.06527842	1.35E-13	3.27E-12	CCNI
5918	ENSG00000118849	25.1218368	0.01641665	0.31697274	0.92908296	0.97031141	RARRES1
64747	ENSG00000118855	421.585209	0.19645334	0.1219997	0.08878573	0.22817472	MFSD1
25782	ENSG00000118873	2109.35413	0.06492463	0.08201617	0.41841631	0.6444567	RAB3GAP2
196483	ENSG00000118894	265.682056	0.00507627	0.14831858	0.97344207	0.99020176	EEF2KMT
5493	ENSG00000118898	23.8756172	0.1954757	0.35354188	0.31783028	0.54556488	PPL
29855	ENSG00000118900	1577.04748	-0.13018795	0.07578103	0.07929247	0.21006311	UBN1
NA	ENSG00000118903	0	NA	NA	NA	NA	BTF3P11
11278	ENSG00000118922	1655.23327	-0.47242664	0.07192215	2.22E-11	4.56E-10	KLF12
7347	ENSG00000118939	185.095068	-0.13770858	0.18503798	0.39565892	0.62508942	UCHL3
27253	ENSG00000118946	78.4617515	-0.86896151	0.30207162	0.00050849	0.00327544	PCDH17
64342	ENSG00000118960	496.243609	0.23067748	0.13340505	0.06449843	0.18086909	HS1BP3
60526	ENSG00000118961	1112.60331	-0.14936855	0.0961789	0.10784067	0.26339391	LDAH
57539	ENSG00000118965	1906.64976	-0.11228192	0.07704804	0.1367471	0.31191797	WDR35
894	ENSG00000118971	0	NA	NA	NA	NA	CCND2
8074	ENSG00000118972	1.51652797	0.08755393	0.39515627	0.16226803	NA	FGF23
NA	ENSG00000118976	0.51314949	-0.01008878	0.38327516	0.82010522	NA	OTUD4P1
22936	ENSG00000118985	26.7637766	0.64227296	0.57388767	0.02816026	0.09547008	ELL2
NA	ENSG00000118990	0	NA	NA	NA	NA	GLRXP3
56171	ENSG00000118997	30.0017969	0.14944207	0.36149475	0.3866789	0.61575643	DNAH7
57404	ENSG00000119004	210.795646	0.09263825	0.18927678	0.57401995	0.77333896	CYP20A1
4709	ENSG00000119013	476.490748	0.05103944	0.10710818	0.62045731	0.80630595	NDUFB3
9330	ENSG00000119041	1212.72093	-0.07401102	0.0825048	0.35823288	0.58854144	GTF3C3
23314	ENSG00000119042	5.32276645	-0.01658887	0.36812462	0.87198072	0.94401928	SATB2
7320	ENSG00000119048	582.891131	-0.32782716	0.10804636	0.00150893	0.00841879	UBE2B
140803	ENSG00000119121	1.14358999	-0.06092496	0.38759591	0.30748337	NA	TRPM6
9615	ENSG00000119125	0	NA	NA	NA	NA	GDA
687	ENSG00000119138	150.013578	-0.04830731	0.18741275	0.76831341	0.89324733	KLF9
9414	ENSG00000119139	1249.08432	-0.66366334	0.10680473	1.28E-10	2.44E-09	TJP2
84417	ENSG00000119147	12.9856435	-0.0703494	0.34472693	0.66465688	0.83444869	ECRG4
9270	ENSG00000119185	1357.78869	-0.03221011	0.07429899	0.65763353	0.83025448	ITGB1BP1
51692	ENSG00000119203	1999.92211	-0.21773335	0.08755266	0.01042824	0.04284454	CPSF3
80235	ENSG00000119227	103.506647	0.54512738	0.22881788	0.0051148	0.02368761	PIGZ
205564	ENSG00000119231	1005.05313	-0.11829041	0.09074223	0.17899136	0.37507273	SENP5

80212	ENSG00000119242	899.174012	0.07357696	0.09124988	0.40694172	0.6352261	CCDC92
84886	ENSG00000119280	1178.30973	-0.09304497	0.07659891	0.21452385	0.42489301	C1orf198
440730	ENSG00000119283	460.861188	-3.44213763	0.16378147	3.85E-99	1.18E-96	TRIM67
55127	ENSG00000119285	3547.13784	-0.17267216	0.06489539	0.00686867	0.0304592	HEATR1
9991	ENSG00000119314	1134.158	-0.06678198	0.08933719	0.44124824	0.6661181	PTBP3
5887	ENSG00000119318	4124.16381	-0.07726643	0.05968523	0.18958233	0.39058291	RAD23B
23307	ENSG00000119321	775.159515	-0.1573623	0.15726369	0.27081138	0.49415045	FKBP15
8727	ENSG00000119326	655.33568	-0.21459559	0.117366	0.05398059	0.15777474	CTNNAL1
54942	ENSG00000119328	261.844873	0.13329988	0.14099586	0.30935952	0.53712343	ABITRAM
89891	ENSG00000119333	2239.87656	-0.02777183	0.06259257	0.65248681	0.82643886	DYNC2I2
6418	ENSG00000119335	9064.78308	-0.10603985	0.05202327	0.03957231	0.12444359	SET
5524	ENSG00000119383	2037.08629	0.37569436	0.0952423	4.53E-05	0.00037551	PTPA
2733	ENSG00000119392	738.077863	0.02648915	0.09456117	0.77405113	0.89604057	GLE1
51552	ENSG00000119396	1281.67452	-0.01942292	0.07632412	0.79475775	0.90579008	RAB14
11064	ENSG00000119397	1150.29806	0.08209728	0.08029156	0.2959121	0.52255416	CNTRL
22954	ENSG00000119401	372.260964	0.22311131	0.13667765	0.08001795	0.21130787	TRIM32
26190	ENSG00000119402	2280.63498	-0.14571877	0.06581972	0.02451093	0.08581205	FBXW2
26147	ENSG00000119403	1452.21754	-0.14328341	0.09182869	0.10771202	0.26327541	PHF19
10783	ENSG00000119408	607.221785	-0.41227806	0.10829358	7.01E-05	0.00055703	NEK6
54836	ENSG00000119411	11.9147899	0.21490661	0.39589079	0.24080304	0.45879844	BSPRY
5537	ENSG00000119414	1060.607	-0.13423229	0.08163527	0.09199781	0.23436524	PPP6C
4702	ENSG00000119421	475.547103	-0.13309577	0.11144522	0.21109557	0.42012066	NDUFA8
81932	ENSG00000119431	170.000124	-0.06191086	0.17456192	0.68840756	0.84869236	HDHD3
NA	ENSG00000119440	0	NA	NA	NA	NA	LCN1P1
92400	ENSG00000119446	714.415444	-0.17791425	0.15595382	0.2100778	0.41859431	RBM18
57864	ENSG00000119457	0.76951065	-0.0295156	0.38376407	0.55723895	NA	SLC46A2
84263	ENSG00000119471	395.429011	-0.17913302	0.1356174	0.1565632	0.34316665	HSDL2
79109	ENSG00000119487	1906.9369	0.13466494	0.08056894	0.08724718	0.22550599	MAPKAP1
8013	ENSG00000119508	8.79956554	0.0704797	0.35349804	0.6477358	0.82431548	NR4A3
27130	ENSG00000119509	557.822217	-0.15014907	0.13307958	0.22761251	0.44251172	INVS
79695	ENSG00000119514	52.7799483	1.45822543	0.36134047	4.06E-06	4.14E-05	GALNT12
57706	ENSG00000119522	901.089579	-0.16083382	0.09209314	0.07138425	0.19425517	DENND1A
85365	ENSG00000119523	312.776666	-0.07845831	0.1311425	0.52341435	0.73623706	ALG2
1441	ENSG00000119535	17.1629894	0.4016671	0.51418447	0.07964383	0.21062825	CSF3R
2531	ENSG00000119537	676.343998	-0.0146182	0.11819342	0.89556003	0.95469109	KDSR
9525	ENSG00000119541	769.067141	-0.00955296	0.09078261	0.91302994	0.96283789	VPS4B
9480	ENSG00000119547	1232.18786	-0.35384847	0.08558707	2.13E-05	0.00019042	ONECUT2
148223	ENSG00000119559	523.755935	0.01878928	0.12433253	0.87406451	0.94457431	C19orf25

84878	ENSG00000119574	458.483199	0.13740459	0.12861398	0.25843281	0.47972557	ZBTB45
56252	ENSG00000119596	2988.0222	0.0602499	0.07746581	0.43143561	0.65697157	YLPM1
26094	ENSG00000119599	124.067877	-0.0316927	0.23907919	0.86386228	0.93969119	DCAF4
283571	ENSG00000119608	6.27161519	-0.21318625	0.43110152	0.15804768	0.34503367	PROX2
338917	ENSG00000119614	1.10518223	0.06168576	0.39096434	0.18060216	NA	VSX2
51077	ENSG00000119616	782.766796	-0.06764932	0.09773209	0.47353822	0.69551963	FCF1
5228	ENSG00000119630	180.220787	0.51552459	0.19026523	0.0022933	0.01203679	PGF
83982	ENSG00000119632	158.71175	0.30142448	0.17565986	0.05414148	0.15819211	IFI27L2
80127	ENSG00000119636	34.3702415	0.32346573	0.37677137	0.14565719	0.32616688	BBOF1
91754	ENSG00000119638	3148.12047	0.20760951	0.07489458	0.00466156	0.02192085	NEK9
97	ENSG00000119640	246.903468	0.38597694	0.17528215	0.01385698	0.0540973	ACYP1
112752	ENSG00000119650	230.202221	0.39569445	0.15758433	0.00599951	0.0271538	IFT43
10577	ENSG00000119655	445.335416	1.02823833	0.14160265	4.82E-14	1.22E-12	NPC2
NA	ENSG00000119660	0	NA	NA	NA	NA	DPPA5P4
83544	ENSG00000119661	290.234891	0.15721725	0.19190421	0.34619142	0.57693723	DNAL1
64207	ENSG00000119669	1974.84038	0.45686064	0.06974055	2.64E-11	5.39E-10	IRF2BPL
10965	ENSG00000119673	630.494637	0.16581212	0.1067654	0.10547789	0.25899521	ACOT2
4053	ENSG00000119681	406.666245	1.0085939	0.14286896	2.20E-13	5.22E-12	LTBP2
9870	ENSG00000119682	1776.04399	0.17023587	0.07966454	0.02882046	0.09722903	AREL1
27030	ENSG00000119684	735.1674	-0.24980419	0.10679465	0.01454194	0.05619637	MLH3
23093	ENSG00000119685	626.998202	-0.44723893	0.17448585	0.00429112	0.02048009	TTLL5
55640	ENSG00000119686	1.44444657	0.05314176	0.38625921	0.34924497	NA	FLVCR2
5826	ENSG00000119688	1244.27669	0.20329581	0.08091233	0.01008034	0.04167381	ABCD4
1743	ENSG00000119689	2349.37608	0.03166836	0.06581234	0.62561762	0.8095124	DLST
57718	ENSG00000119698	1.32916261	0.07178669	0.39161775	0.20808645	NA	PPP4R4
7043	ENSG00000119699	183.736846	2.22314105	0.20004145	7.19E-30	4.16E-28	TGFB3
79696	ENSG00000119703	45.2292044	-0.09043825	0.28656638	0.63876824	0.81845605	ZC2HC1C
81892	ENSG00000119705	739.647164	-0.02871532	0.09247223	0.74809237	0.88173637	SLIRP
58517	ENSG00000119707	3873.76305	-0.14786763	0.0750718	0.04426679	0.13549029	RBM25
4329	ENSG00000119711	389.147313	0.68383404	0.18481118	4.76E-05	0.00039207	ALDH6A1
8111	ENSG00000119714	275.516557	-0.96730524	0.1576443	1.16E-10	2.21E-09	GPR68
2103	ENSG00000119715	5.40908485	0.24903411	0.4983603	0.02030971	0.07378587	ESRRB
8892	ENSG00000119718	834.735002	-0.22322153	0.09249998	0.01267552	0.05040737	EIF2B2
55051	ENSG00000119720	632.002897	-0.23702192	0.12284561	0.0408619	0.12740848	NRDE2
51004	ENSG00000119723	297.798564	-0.01366487	0.14385289	0.91731772	0.96503439	COQ6
57862	ENSG00000119725	860.153373	0.00814319	0.0871141	0.9243351	0.96852	ZNF410
23433	ENSG00000119729	1621.21499	-0.60153463	0.10189148	1.06E-09	1.82E-08	RHOQ
10936	ENSG00000119737	101.140924	0.10050207	0.20528266	0.56424873	0.76680562	GPR75

9913	ENSG00000119760	1890.00755	-0.04050163	0.06685344	0.53773163	0.74776213	SUPT7L
114818	ENSG00000119771	317.466345	-0.90649997	0.14630031	8.69E-11	1.69E-09	KLHL29
1788	ENSG00000119772	5662.32045	-0.18941097	0.05329689	0.00032843	0.00222014	DNMT3A
54867	ENSG00000119777	1178.16003	0.1147464	0.08952981	0.18753722	0.38786275	TMEM214
54454	ENSG00000119778	894.308237	0.02317594	0.08592154	0.78269387	0.90105949	ATAD2B
2281	ENSG00000119782	416.757909	-0.17069577	0.11877288	0.1292797	0.30076329	FKBP1B
64225	ENSG00000119787	1357.73636	-0.12529187	0.08995917	0.15132812	0.33531348	ATL2
51646	ENSG00000119801	733.864792	0.18842342	0.09446338	0.03935644	0.12400059	YPEL5
25940	ENSG00000119812	908.350775	0.09621537	0.26217024	0.62192353	0.80695268	FAM98A
84272	ENSG00000119820	1506.91759	-0.10065376	0.119261	0.36979295	0.59912004	YIPF4
54812	ENSG00000119844	678.350224	-0.041627	0.11221339	0.6972585	0.85387893	AFTPH
29094	ENSG00000119862	491.537335	-0.48861236	0.13085134	7.39E-05	0.00058248	LGALSL
25927	ENSG00000119865	1447.10622	-0.53606188	0.07235006	4.58E-14	1.16E-12	CNRIP1
53335	ENSG00000119866	345.67095	0.70459498	0.16008983	2.36E-06	2.51E-05	BCL11A
9419	ENSG00000119878	583.131038	-0.29999352	0.1153534	0.00615757	0.02770185	CRIPT
4072	ENSG00000119888	22.7366734	0.13119191	0.31983415	0.49837324	0.71619429	EPCAM
26503	ENSG00000119899	215.795316	0.25874415	0.16493123	0.08224978	0.21582272	SLC17A5
79627	ENSG00000119900	603.001247	0.72112348	0.1075206	4.43E-12	9.66E-11	OGFRL1
55719	ENSG00000119906	868.397566	-0.07070569	0.0917529	0.42639203	0.65221241	SLF2
3416	ENSG00000119912	805.762907	-0.21617111	0.09251655	0.01579982	0.0601436	IDE
6975	ENSG00000119913	0	NA	NA	NA	NA	TECTB
83401	ENSG00000119915	12.9494607	-0.00154592	0.33153744	0.99057595	0.99679705	ELOVL3
3437	ENSG00000119917	0.3223625	-0.02325318	0.38531854	0.66968133	NA	IFIT3
159296	ENSG00000119919	0	NA	NA	NA	NA	NKX2-3
3433	ENSG00000119922	10.7294149	-2.68734937	1.27507052	0.0008578	0.00517603	IFIT2
57678	ENSG00000119927	742.913839	-0.0326519	0.122647	0.77772655	0.89814288	GPAM
51076	ENSG00000119929	202.139733	-0.18370298	0.15644119	0.19725891	0.40121304	CUTC
5507	ENSG00000119938	62.9620956	-0.62910418	0.30091029	0.00765801	0.03328254	PPP1R3C
84795	ENSG00000119943	24.3766692	-0.1095554	0.33914092	0.53597666	0.74664913	PYROXD2
26507	ENSG00000119946	471.467008	-0.43786701	0.14670471	0.00123238	0.00707069	CNNM1
4601	ENSG00000119950	759.953398	0.06511741	0.09906896	0.49762847	0.71553478	MXI1
10285	ENSG00000119953	659.319027	0.01235185	0.11032989	0.90787943	0.95978728	SMNDC1
80007	ENSG00000119965	301.565388	-0.1778334	0.1378677	0.16566898	0.35583766	C10orf88
3070	ENSG00000119969	1001.61205	0.04672515	0.12498701	0.69347587	0.85183585	HELLS
2834	ENSG00000119973	173.185912	-2.63776156	0.22556672	6.18E-33	3.87E-31	PRLHR
26123	ENSG00000119977	671.176329	0.26104723	0.09967317	0.00654051	0.02918662	TCTN3
404636	ENSG00000119979	554.396248	-0.39510462	0.11179466	0.00021447	0.00151478	DENND10
60370	ENSG00000119986	57.1551777	-0.00547188	0.25226017	0.97545214	0.99139237	AVPI1

55717	ENSG00000120008	1487.21575	0.24283317	0.08346304	0.00282788	0.01440808	WDR11
79591	ENSG00000120029	508.001221	-0.26936629	0.11269288	0.01207479	0.0484947	ARMH3
30819	ENSG00000120049	60.089505	-0.24115998	0.26424331	0.22822978	0.44340764	KCNIP2
159686	ENSG00000120051	9.91435712	0.11711156	0.3753945	0.40628363	0.63454832	CFAP58
2805	ENSG00000120053	529.41739	-0.22496265	0.11140202	0.03415518	0.11127072	GOT1
1369	ENSG00000120054	0	NA	NA	NA	NA	CPN1
79946	ENSG00000120055	16.5611201	-0.0275759	0.32709173	0.87169699	0.94401928	C10orf95
6425	ENSG00000120057	57.7363954	-5.37450602	0.6261458	3.30E-18	1.12E-16	SFRP5
10672	ENSG00000120063	2740.08663	-0.5154045	0.06658757	3.85E-15	1.07E-13	GNA13
3218	ENSG00000120068	0	NA	NA	NA	NA	HOXB8
284058	ENSG00000120071	2062.44241	0.12225601	0.06725501	0.06483746	0.18146058	KANSL1
3215	ENSG00000120075	0	NA	NA	NA	NA	HOXB5
1394	ENSG00000120088	93.5110989	-0.88036747	0.29428492	0.0003725	0.00248666	CRHR1
104909134	ENSG00000120088	93.5110989	-0.88036747	0.29428492	0.0003725	0.00248666	CRHR1
3213	ENSG00000120093	0.173124	-0.01911262	0.38486262	0.84537521	NA	HOXB3
3211	ENSG00000120094	0	NA	NA	NA	NA	HOXB1
1843	ENSG00000120129	857.343908	1.46950082	0.09975396	3.58E-50	3.57E-48	DUSP1
79646	ENSG00000120137	6763.20309	-0.20524492	0.05832454	0.00036582	0.00244454	PANK3
4488	ENSG00000120149	21.0434459	0.23184447	0.37653472	0.24548053	0.46454384	MSX2
7010	ENSG00000120156	0	NA	NA	NA	NA	TEK
10171	ENSG00000120158	433.847976	-0.07699634	0.11230171	0.4725845	0.69470213	RCL1
79886	ENSG00000120159	540.631999	0.03979337	0.11508011	0.71791384	0.8643386	CAAP1
54586	ENSG00000120160	0	NA	NA	NA	NA	EQTN
79817	ENSG00000120162	69.7848138	-0.0205554	0.24853819	0.91226129	0.96260718	MOB3B
11172	ENSG00000120210	0.33997242	0.00281064	0.38338782	0.96162062	NA	INSL6
3641	ENSG00000120211	0	NA	NA	NA	NA	INSL4
2315	ENSG00000120215	5.7289566	-0.25973048	0.48181162	0.07259529	0.19646576	MLANA
29126	ENSG00000120217	20.335057	-0.01875484	0.34910199	0.89462025	0.95456012	CD274
3443	ENSG00000120235	0	NA	NA	NA	NA	IFNA6
3445	ENSG00000120242	0	NA	NA	NA	NA	IFNA8
2891	ENSG00000120251	459.250026	-1.84056563	0.12877631	1.79E-47	1.65E-45	GRIA2
348995	ENSG00000120253	960.836171	-0.14039688	0.08683075	0.09639355	0.24263487	NUP43
25902	ENSG00000120254	786.115544	0.24299246	0.14783698	0.07432264	0.2001033	MTHFD1L
84918	ENSG00000120256	515.215183	0.15908704	0.11152627	0.13546429	0.3099898	LRP11
80129	ENSG00000120262	0	NA	NA	NA	NA	CCDC170
5110	ENSG00000120265	1052.31092	-0.0136195	0.08906876	0.87370972	0.94441388	PCMT1
57480	ENSG00000120278	0	NA	NA	NA	NA	PLEKHG1
80177	ENSG00000120279	0.64236694	-0.01780933	0.38349976	0.68678394	NA	MYCT1

80231	ENSG00000120280	0	NA	NA	NA	NA	TASL
4115	ENSG00000120289	0	NA	NA	NA	NA	MAGEB4
84418	ENSG00000120306	160.84937	0.19246093	0.1847011	0.23533555	0.45219979	CYSTM1
54853	ENSG00000120314	1023.41179	0.04074081	0.08104558	0.60740778	0.7967057	WDR55
64411	ENSG00000120318	2379.06882	-0.40966167	0.06413246	8.78E-11	1.70E-09	ARAP3
56128	ENSG00000120322	92.1367951	0.15439673	0.21104516	0.3839572	0.61309544	PCDHB8
56126	ENSG00000120324	282.207207	0.58595814	0.1469309	2.00E-05	0.00017934	PCDHB10
56122	ENSG00000120327	405.780244	0.27068115	0.12263434	0.0194878	0.07145408	PCDHB14
56124	ENSG00000120328	167.096544	0.60960253	0.19082337	0.00037387	0.00249331	PCDHB12
83884	ENSG00000120329	0	NA	NA	NA	NA	SLC25A2
63923	ENSG00000120332	0	NA	NA	NA	NA	TNN
63931	ENSG00000120333	504.070988	-0.20545082	0.12025192	0.07108284	0.19367482	MRPS14
91687	ENSG00000120334	318.608209	-0.05845216	0.14191938	0.6553748	0.82851001	CENPL
8995	ENSG00000120337	0	NA	NA	NA	NA	TNFSF18
89866	ENSG00000120341	37.215499	0.228893	0.36391082	0.25803351	0.47940416	SEC16B
111240474	ENSG00000120341	37.215499	0.228893	0.36391082	0.25803351	0.47940416	SEC16B
92344	ENSG00000120370	201.712828	-0.06196111	0.15863981	0.66710623	0.83613156	GORAB
2853	ENSG00000120436	0	NA	NA	NA	NA	GPR31
39	ENSG00000120437	1374.67827	-0.29673139	0.11891045	0.0084037	0.03597182	ACAT2
6950	ENSG00000120438	4294.80409	-0.16627618	0.07298735	0.02009946	0.07320204	TCP1
83887	ENSG00000120440	35.3070264	0.08386398	0.29661234	0.66545432	0.83481591	TTLL2
399979	ENSG00000120451	2037.5408	-0.18712652	0.09732365	0.0462175	0.14012153	SNX19
3762	ENSG00000120457	17.3650061	-0.1379713	0.34524466	0.44838062	0.67234398	KCNJ5
79684	ENSG00000120458	550.13967	0.08344655	0.11044726	0.43061915	0.65606363	MSANTD2
63970	ENSG00000120471	1.63396956	0.06161887	0.38411344	0.42832792	NA	TP53AIP1
56159	ENSG00000120498	0.34624801	-0.02356563	0.38536324	0.65270996	NA	TEX11
407	ENSG00000120500	1.45944124	0.07028406	0.38875353	0.30037243	NA	ARR3
51248	ENSG00000120509	571.581987	0.16544791	0.10612832	0.10437294	0.25722378	PDZD11
84068	ENSG00000120519	118.682612	0.23240874	0.2232719	0.20625115	0.41325498	SLC10A7
84955	ENSG00000120526	653.096444	-0.18752884	0.11010583	0.0745429	0.20050585	NUDCD1
56943	ENSG00000120533	1211.00101	-0.02705449	0.08072863	0.73142849	0.87166875	ENY2
84930	ENSG00000120539	931.633376	-0.25550982	0.08720575	0.00256651	0.01327412	MASTL
56243	ENSG00000120549	148.379528	2.62333394	0.24129862	8.49E-29	4.68E-27	KIAA1217
NA	ENSG00000120555	2.39624697	-0.04839539	0.377767	0.58892803	NA	SEPTIN7P9
84569	ENSG00000120563	0	NA	NA	NA	NA	LYZL1
84898	ENSG00000120594	119.720045	-0.25425581	0.29890465	0.22233592	0.43535977	PLXDC2
80314	ENSG00000120616	828.1884	0.05603617	0.09290604	0.53471352	0.74572704	EPC1
440073	ENSG00000120645	371.262426	-4.75530407	0.21128816	4.91E-113	1.71E-110	IQSEC3

84318	ENSG00000120647	498.811904	-0.17981982	0.13026822	0.14093831	0.31898686	CCDC77
6883	ENSG00000120656	423.27743	-0.04450115	0.11421278	0.68237534	0.8449992	TAF12
55068	ENSG00000120658	174.876746	-0.60166448	0.18541886	0.00031691	0.0021516	ENOX1
8600	ENSG00000120659	0	NA	NA	NA	NA	TNFSF11
9617	ENSG00000120662	192.601811	-0.20174927	0.18258448	0.20849578	0.4162363	MTRF1
NA	ENSG00000120664	11.7655225	0.09655536	0.3495569	0.56433994	0.76683134	SPART-AS1
54937	ENSG00000120669	28.5070653	0.30151217	0.37846802	0.16902965	0.36039348	SOHLH2
29103	ENSG00000120675	249.20731	0.37146513	0.16186376	0.01150006	0.04653421	DNAJC15
80209	ENSG00000120685	1419.8766	-0.12958998	0.08471802	0.1160893	0.27839903	PROSER1
51569	ENSG00000120686	785.482465	-0.03728861	0.10389224	0.70883951	0.86019792	UFM1
11193	ENSG00000120688	317.537635	0.06856774	0.12842378	0.57178542	0.77185678	WBP4
1997	ENSG00000120690	321.741567	0.36696264	0.13572379	0.00379998	0.01846719	ELF1
4093	ENSG00000120693	1117.01092	-0.30215963	0.08179442	0.00015061	0.0010994	SMAD9
10808	ENSG00000120694	3490.84661	-0.10691638	0.10890956	0.30469532	0.53198909	HSPH1
84078	ENSG00000120696	374.795041	-0.12501542	0.1356454	0.32325903	0.55093712	KBTBD7
29880	ENSG00000120697	462.944904	-0.05160088	0.11294109	0.63185219	0.81371846	ALG5
11340	ENSG00000120699	760.125469	0.10729422	0.09268248	0.23019674	0.44613689	EXOSC8
2107	ENSG00000120705	2806.21252	-0.2631622	0.06619744	5.29E-05	0.00042958	ETF1
7045	ENSG00000120708	0.16735865	0.01897177	0.38482933	0.78225479	NA	TGFBI
51307	ENSG00000120709	1410.50167	-0.09312466	0.07352204	0.19626676	0.39997732	FAM53C
100128966	ENSG00000120709	1410.50167	-0.09312466	0.07352204	0.19626676	0.39997732	FAM53C
64374	ENSG00000120725	333.050348	-0.01207624	0.14285797	0.92667372	0.96932621	SIL1
51247	ENSG00000120727	2734.24196	0.16969027	0.05898303	0.00359296	0.01765775	PAIP2
9499	ENSG00000120729	0.64135813	0.03718182	0.38666599	0.41086295	NA	MYOT
51780	ENSG00000120733	2745.08494	-0.11374129	0.06241727	0.06453206	0.18093782	KDM3B
1958	ENSG00000120738	4996.43647	4.06310543	0.41368801	4.85E-24	2.24E-22	EGR1
27230	ENSG00000120742	2434.74932	0.32413002	0.06821109	1.33E-06	1.48E-05	SERP1
5357	ENSG00000120756	288.880579	-0.12465289	0.16976512	0.41034583	0.63815925	PLS1
22835	ENSG00000120784	496.624608	-0.38404221	0.1652565	0.01008447	0.04167381	ZFP30
7181	ENSG00000120798	576.21415	0.03062685	0.13077995	0.80445749	0.91118482	NR2C1
27340	ENSG00000120800	963.348902	0.06319517	0.08804338	0.46110034	0.68423565	UTP20
7112	ENSG00000120802	4038.54265	-0.36493955	0.06543573	1.44E-08	2.12E-07	TMPO
400	ENSG00000120805	438.847293	0.04493661	0.12514456	0.70513367	0.85803894	ARL1
83468	ENSG00000120820	49.2276728	0.64859104	0.36002737	0.0128457	0.05099213	GLT8D2
80298	ENSG00000120832	362.394939	0.08458065	0.13486535	0.50463208	0.72111066	MTERF2
8835	ENSG00000120833	421.357819	-0.26256633	0.13527989	0.03731991	0.11917383	SOCS2
4801	ENSG00000120837	709.092025	-0.29319442	0.100317	0.00245167	0.01274996	NFYB
51019	ENSG00000120860	173.431773	0.10492392	0.1888564	0.52457492	0.73734087	WASHC3

317	ENSG00000120868	767.920285	-0.16832327	0.09600625	0.06953469	0.19052178	APAF1
1846	ENSG00000120875	3403.40835	-0.44602661	0.06915428	5.00E-11	9.92E-10	DUSP4
1191	ENSG00000120885	21.9108667	-0.48370572	0.67104545	0.03937525	0.12401014	CLU
8795	ENSG00000120889	1810.28868	0.13976935	0.07597222	0.06052385	0.17247818	TNFRSF10B
10174	ENSG00000120896	785.559624	0.257622	0.12949812	0.03399772	0.11087545	SORBS3
2185	ENSG00000120899	180.616252	0.15673729	0.23933717	0.40825751	0.63640649	PTK2B
1135	ENSG00000120903	3.78621096	-0.08248107	0.38584972	0.3416745	0.57180671	CHRNA2
148	ENSG00000120907	0.45187964	0.00140723	0.38312334	0.99378719	NA	ADRA1A
5533	ENSG00000120910	196.713757	-0.0881236	0.16498465	0.55287427	0.75919187	PPP3CC
64236	ENSG00000120913	215.305627	-0.02158905	0.14921476	0.87425426	0.94470892	PDLIM2
2053	ENSG00000120915	387.329003	0.29710364	0.15906709	0.03994049	0.12522429	EPHX2
81790	ENSG00000120925	476.578082	-0.01343496	0.1092176	0.89831298	0.95552914	RNF170
4879	ENSG00000120937	0.62258031	-0.04505531	0.38756585	0.37427725	NA	NPPB
29914	ENSG00000120942	431.434997	-0.19323706	0.14645419	0.15187436	0.33595576	UBIAD1
23435	ENSG00000120948	4199.18554	-0.01926511	0.08577117	0.81697301	0.91671007	TARDBP
943	ENSG00000120949	0.173124	-0.01911262	0.38486262	0.84537521	NA	TNFRSF8
65122	ENSG00000120952	0	NA	NA	NA	NA	PRAMEF2
51123	ENSG00000120963	664.942646	-0.83015697	0.13436379	1.09E-10	2.09E-09	ZNF706
10434	ENSG00000120992	1176.55143	-0.11924934	0.09669469	0.20177276	0.40724024	LYPLA1
83690	ENSG00000121005	1053.39887	0.84018148	0.10362377	9.11E-17	2.77E-15	CRISPLD1
10987	ENSG00000121022	955.115551	-0.01342789	0.08991834	0.87716611	0.94574195	COPS5
157506	ENSG00000121039	94.9529099	-0.09618639	0.26494297	0.61837474	0.80469863	RDH10
8288	ENSG00000121053	2.50967355	-0.122475	0.4052678	0.14170571	NA	EPX
8165	ENSG00000121057	2384.41053	-0.31319067	0.06892581	3.72E-06	3.82E-05	AKAP1
8161	ENSG00000121058	608.426203	-0.31462872	0.09981326	0.00106014	0.00620629	COIL
7706	ENSG00000121060	1326.24236	0.10441238	0.09801549	0.27066736	0.49398241	TRIM25
59342	ENSG00000121064	575.479855	0.51800504	0.11850939	4.60E-06	4.66E-05	SCPEP1
8405	ENSG00000121067	963.04294	-0.06566276	0.11153392	0.53661232	0.74729267	SPOP
6909	ENSG00000121068	3060.57016	0.68133843	0.06010567	2.24E-30	1.30E-28	TBX2
10237	ENSG00000121073	1010.25867	0.32981606	0.08780832	0.00011093	0.00083399	SLC35B1
9496	ENSG00000121075	24.0685901	2.13646531	0.63581549	3.82E-05	0.00032221	TBX4
NA	ENSG00000121089	0.53000235	0.01362607	0.38292808	0.74194811	NA	NACA3P
56155	ENSG00000121101	21.372039	-0.03448964	0.30460577	0.85216373	0.93300196	TEX14
81558	ENSG00000121104	259.882569	0.60253702	0.16894701	0.00010048	0.00076377	FAM117A
23397	ENSG00000121152	1296.86791	-0.04802993	0.09955691	0.61634942	0.80326549	NCAPH
9227	ENSG00000121207	0	NA	NA	NA	NA	LRAT
23240	ENSG00000121210	573.168491	-0.14188223	0.10927311	0.1738051	0.36691776	TMEM131L
84057	ENSG00000121211	183.241121	-0.23082174	0.17431388	0.13630411	0.3113874	MND1

117854	ENSG00000121236	0	NA	NA	NA	NA	TRIM6
85320	ENSG00000121270	13.7729283	0.06656442	0.33824625	0.69629209	0.85340714	ABCC11
64282	ENSG00000121274	314.151634	-0.16153298	0.1504482	0.24157656	0.45960686	TENT4B
113	ENSG00000121281	994.014068	0.00927499	0.09700272	0.92214937	0.96738383	ADCY7
84902	ENSG00000121289	615.698644	0.33300729	0.14328507	0.01225164	0.04906593	CEP89
57616	ENSG00000121297	777.221587	0.03597399	0.09502495	0.69669584	0.85365037	TSHZ3
55268	ENSG00000121310	195.38927	0.08064184	0.20585664	0.64423778	0.82234302	ECHDC2
50836	ENSG00000121314	0.81915356	-0.00712032	0.38011689	0.90685717	NA	TAS2R8
79887	ENSG00000121316	9.85508768	0.46702557	0.75040772	0.02625165	0.09046946	PLBD1
50839	ENSG00000121318	24.5793946	0.19775705	0.33182453	0.33080652	0.55961812	TAS2R10
653247	ENSG00000121335	0.66900215	0.02598789	0.38362153	0.58754139	NA	PRB2
79912	ENSG00000121350	368.624639	0.22113381	0.164323	0.13619886	0.31128106	PYROXD1
3375	ENSG00000121351	0	NA	NA	NA	NA	IAPP
3764	ENSG00000121361	363.932469	1.30671331	0.13438417	2.38E-23	1.06E-21	KCNJ8
50837	ENSG00000121377	0	NA	NA	NA	NA	TAS2R7
79370	ENSG00000121380	0.78914922	-0.00349613	0.38198127	0.90760115	NA	BCL2L14
50835	ENSG00000121381	1.19489416	0.05598592	0.38741728	0.28969146	NA	TAS2R9
NA	ENSG00000121388	0	NA	NA	NA	NA	
55269	ENSG00000121390	1087.8728	-0.08252417	0.08732678	0.33146765	0.5603543	PSPC1
256051	ENSG00000121406	260.359865	-0.02811003	0.14288333	0.83113175	0.92369221	ZNF549
1	ENSG00000121410	135.013648	-0.05091677	0.1889804	0.75577098	0.88640901	A1BG
65982	ENSG00000121413	726.216728	0.1128204	0.12313631	0.33623829	0.56617316	ZSCAN18
10520	ENSG00000121417	285.749092	0.03914377	0.16219839	0.79096137	0.9048941	ZNF211
23024	ENSG00000121440	351.054683	3.4968401	0.16778293	2.65E-97	7.97E-95	PDZRN3
353299	ENSG00000121446	0	NA	NA	NA	NA	RGSL1
89884	ENSG00000121454	260.7436	-0.03399061	0.13552398	0.78970095	0.9043268	LHX4
6045	ENSG00000121481	583.748372	-0.02333246	0.11094947	0.82436059	0.92028551	RNF2
81627	ENSG00000121486	501.554348	-0.39276086	0.1366578	0.00207306	0.01102966	TRMT1L
26984	ENSG00000121542	247.127037	-0.28605567	0.1735399	0.06363522	0.17913205	SEC22A
1475	ENSG00000121552	0.18922458	0.01954053	0.38488496	0.78225479	NA	CSTA
55211	ENSG00000121570	0	NA	NA	NA	NA	DPPA4
64091	ENSG00000121577	1118.3943	0.42512404	0.10210211	1.53E-05	0.00014069	POPDC2
8702	ENSG00000121578	560.53179	0.50266114	0.1345428	7.11E-05	0.00056398	B4GALT4
80218	ENSG00000121579	2365.7545	-0.03384136	0.06580827	0.59952066	0.79057618	NAA50
941	ENSG00000121594	0.69493202	0.03154416	0.38626309	0.41391645	NA	CD80
81930	ENSG00000121621	404.866942	-0.27055708	0.1197984	0.01707618	0.06418935	KIF18A
2703	ENSG00000121634	0	NA	NA	NA	NA	GJA8
51029	ENSG00000121644	1773.70147	-0.07466425	0.07512318	0.3102543	0.53796162	DESI2

9479	ENSG00000121653	1300.43883	-1.35171049	0.08138178	5.42E-63	7.52E-61	MAPK8IP1
1408	ENSG00000121671	220.809296	0.0524849	0.20446976	0.76314986	0.89085039	CRY2
9409	ENSG00000121680	346.575178	0.1264764	0.14301249	0.33965455	0.57006566	PEX16
91614	ENSG00000121690	94.3578889	0.08324015	0.20947578	0.63706331	0.81722218	DEPDC7
847	ENSG00000121691	628.166417	0.26540097	0.09707607	0.0046198	0.0217557	CAT
29990	ENSG00000121716	900.209515	0.03030085	0.10231703	0.75968549	0.88853152	PILRB
7750	ENSG00000121741	3018.42368	-0.18803466	0.07806143	0.01371934	0.05368874	ZMYM2
10804	ENSG00000121742	0	NA	NA	NA	NA	GJB6
2700	ENSG00000121743	0	NA	NA	NA	NA	GJA3
64786	ENSG00000121749	598.651502	-0.00420197	0.10235506	0.96520179	0.98622874	TBC1D15
576	ENSG00000121753	2544.80636	-0.46319966	0.07228772	6.58E-11	1.29E-09	ADGRB2
3061	ENSG00000121764	10.6594486	0.1253313	0.3713007	0.41730994	0.64361234	HCRTR1
51538	ENSG00000121766	449.003466	-0.34775695	0.11460506	0.00142923	0.00804233	ZCCHC17
2170	ENSG00000121769	7.71635917	-0.14514783	0.38606559	0.32114097	0.54888325	FABP3
10657	ENSG00000121774	6244.13451	0.03957766	0.05778642	0.48910365	0.70874969	KHDRBS1
55116	ENSG00000121775	403.391051	0.05694853	0.13006616	0.6426587	0.82136499	TMEM39B
9034	ENSG00000121797	0	NA	NA	NA	NA	CCRL2
729230	ENSG00000121807	0	NA	NA	NA	NA	CCR2
84265	ENSG00000121851	362.547481	-0.10893454	0.12608132	0.35940124	0.58949503	POLR3GL
2693	ENSG00000121853	0	NA	NA	NA	NA	GHSR
8743	ENSG00000121858	0	NA	NA	NA	NA	TNFSF10
51193	ENSG00000121864	661.983566	-0.31539624	0.10521087	0.00176287	0.00961126	ZNF639
22865	ENSG00000121871	1.54953355	-0.02066408	0.38105813	0.71588314	NA	SLITRK3
5290	ENSG00000121879	837.048329	-0.23569634	0.11969492	0.03743886	0.11945755	PIK3CA
23244	ENSG00000121892	2732.09001	-0.61013706	0.06464004	1.18E-21	4.74E-20	PDS5A
80008	ENSG00000121895	0.173733	0.01914545	0.38484597	0.78225479	NA	TMEM156
11019	ENSG00000121897	205.694614	0.08087927	0.16784111	0.59429936	0.7877158	LIAS
119587	ENSG00000121898	0	NA	NA	NA	NA	CPXM2
113452	ENSG00000121900	175.430686	-0.35174437	0.17711465	0.02553485	0.08857763	TMEM54
7579	ENSG00000121903	371.057318	-0.16910659	0.12267424	0.14451324	0.32462259	ZSCAN20
114784	ENSG00000121904	100.687104	-3.08814704	0.31635684	6.19E-24	2.83E-22	CSMD2
3208	ENSG00000121905	16.0329994	-0.18219079	0.38678918	0.28064421	0.50530731	HPCA
55791	ENSG00000121931	400.807097	-0.5364186	0.14721928	9.05E-05	0.00069675	LRIF1
57413	ENSG00000121933	0	NA	NA	NA	NA	TMIGD3
23155	ENSG00000121940	1038.95682	-0.05948867	0.09274718	0.50786766	0.72375336	CLCC1
29899	ENSG00000121957	1289.36491	-0.26235213	0.07867725	0.00063782	0.00398436	GPSM2
79712	ENSG00000121964	371.385403	-0.18368444	0.17908175	0.24367574	0.4621035	GTDC1
7852	ENSG00000121966	456.809835	-4.13856234	0.22878369	2.24E-74	4.01E-72	CXCR4

84083	ENSG00000121988	274.362723	-0.34067212	0.18581174	0.03678146	0.11789082	ZRANB3
92	ENSG00000121989	245.942358	-0.28635728	0.15580591	0.04376443	0.13428491	ACVR2A
51426	ENSG00000122008	566.250565	-0.31094138	0.11922909	0.00592555	0.02689612	POLK
22987	ENSG00000122012	1953.27118	-1.09977393	0.08267928	2.61E-41	2.02E-39	SV2C
2322	ENSG00000122025	0	NA	NA	NA	NA	FLT3
6144	ENSG00000122026	10820.5641	-0.24662728	0.06384724	7.88E-05	0.0006149	RPL21
219402	ENSG00000122033	226.150264	-0.1078615	0.14622179	0.42394943	0.64969559	MTIF3
2971	ENSG00000122034	1288.35767	-0.12343752	0.08938985	0.15505571	0.34067612	GTF3A
387496	ENSG00000122035	9.51668623	0.13957286	0.39290979	0.27675286	0.50116619	RASL11A
5412	ENSG00000122042	387.622989	-0.25686369	0.11887692	0.02257895	0.08026633	UBL3
NA	ENSG00000122043	0	NA	NA	NA	NA	LINC00544
84248	ENSG00000122068	1261.52606	-0.36528511	0.08394996	7.84E-06	7.60E-05	FYTTD1
130916	ENSG00000122085	902.473688	-0.15722479	0.10077909	0.10504057	0.2584011	MTERF4
7512	ENSG00000122121	72.3866459	0.53597711	0.28458071	0.01632554	0.06176559	XPNPEP2
54440	ENSG00000122122	0.36233267	0.0008537	0.38336412	0.96773832	NA	SASH3
4952	ENSG00000122126	1731.27921	-0.18165388	0.06727199	0.00600011	0.0271538	OCRL
5047	ENSG00000122133	0	NA	NA	NA	NA	PAEP
29991	ENSG00000122136	0.15069912	-0.01848503	0.38480575	0.84537521	NA	OBP2A
51116	ENSG00000122140	989.39985	-0.17678877	0.10357595	0.07545362	0.20229727	MRPS2
50945	ENSG00000122145	20.2770022	-0.38835454	0.45383964	0.09610874	0.24207151	TBX22
2331	ENSG00000122176	0.173124	-0.01911262	0.38486262	0.84537521	NA	FMOD
4656	ENSG00000122180	0.16735865	0.01897177	0.38482933	0.78225479	NA	MYOG
54900	ENSG00000122188	0.79333025	-0.04367995	0.38521218	0.44950511	NA	LAX1
5340	ENSG00000122194	1.25830373	-0.04225424	0.38176262	0.54677263	NA	PLG
57179	ENSG00000122203	1977.61345	-0.12260751	0.07161678	0.08105569	0.21356507	KIAA1191
1314	ENSG00000122218	5175.89918	-0.19875446	0.06322257	0.00141397	0.00797179	COPA
51744	ENSG00000122223	0	NA	NA	NA	NA	CD244
4063	ENSG00000122224	0	NA	NA	NA	NA	LY9
9956	ENSG00000122254	15.9181193	-0.67712142	0.72485277	0.02722522	0.0930328	HS3ST2
5930	ENSG00000122257	1636.06246	-0.06136985	0.08651922	0.46567544	0.68839883	RBBP6
29066	ENSG00000122299	1205.77966	-0.15782731	0.11199079	0.13935185	0.31622334	ZC3H7A
5620	ENSG00000122304	0.16735865	0.01897177	0.38482933	0.78225479	NA	PRM2
84947	ENSG00000122335	261.073183	-0.06703928	0.1478385	0.62227736	0.80703248	SERAC1
311	ENSG00000122359	702.778102	0.53190057	0.12438316	6.71E-06	6.57E-05	ANXA11
11155	ENSG00000122367	16.0050718	-0.19324956	0.40496531	0.21901268	0.43093714	LDB3
94233	ENSG00000122375	0	NA	NA	NA	NA	OPN4
54537	ENSG00000122376	812.137477	-0.48024755	0.09788019	3.87E-07	4.70E-06	SHLD2
84293	ENSG00000122378	2018.65487	-0.17151941	0.06701813	0.00897076	0.03792452	PRXL2A

7755	ENSG00000122386	367.875733	0.0837556	0.12973722	0.49320355	0.71236151	ZNF205
79903	ENSG00000122390	1370.05937	0.24091431	0.08746817	0.00459416	0.0216598	NAA60
6125	ENSG00000122406	15027.6684	-0.02985483	0.04863188	0.53391951	0.74519614	RPL5
57489	ENSG00000122417	485.651734	0.45926236	0.11451311	2.68E-05	0.00023401	ODF2L
5737	ENSG00000122420	37.0820644	-0.17066989	0.31491483	0.39423997	0.62344341	PTGFR
100505741	ENSG00000122432	6.1176465	0.27580614	0.5083537	0.04427337	0.13549029	SPATA1
54482	ENSG00000122435	725.743569	-0.06099765	0.10639188	0.5495316	0.75607328	TRMT13
127495	ENSG00000122477	59.392765	0.14431208	0.25395078	0.45780944	0.68133969	LRRC39
25950	ENSG00000122481	239.670299	0.06033494	0.15290435	0.66798241	0.83666483	RWDD3
84146	ENSG00000122482	961.848606	-0.11323269	0.08452083	0.16904344	0.36039348	ZNF644
343099	ENSG00000122483	292.910795	0.19062902	0.16047531	0.19041632	0.39167661	CCDC18
79871	ENSG00000122484	568.631105	-0.28889342	0.11896636	0.01037045	0.04264242	RPAP2
80148	ENSG00000122490	711.756199	-0.18169197	0.09559513	0.04916564	0.14714833	SLC66A2
27241	ENSG00000122507	241.771491	0.02734727	0.18012432	0.86464168	0.94007513	BBS9
5395	ENSG00000122512	1336.27866	-0.12235624	0.07314429	0.08810234	0.22680037	PMS2
83637	ENSG00000122515	2774.50752	0.37792066	0.08028739	1.43E-06	1.58E-05	ZMIZ2
654231	ENSG00000122543	0.688222	0.04020838	0.3871341	0.3583845	NA	OCM
989	ENSG00000122545	4628.06854	-0.1411079	0.05507677	0.01006083	0.04165471	SEPTIN7
80820	ENSG00000122547	171.580587	0.29156469	0.17593115	0.06240278	0.17648913	EEPD1
9808	ENSG00000122548	0.9749599	-0.01781151	0.38087417	0.76347278	NA	KIAA0087
55975	ENSG00000122550	954.246067	-0.38367793	0.09977683	6.53E-05	0.00052142	KLHL7
64224	ENSG00000122557	557.07968	0.0487919	0.10980796	0.64332229	0.82181194	HERPUD2
11335	ENSG00000122565	6228.22173	-0.16329007	0.06575968	0.01162402	0.04696	CBX3
3181	ENSG00000122566	28454.1571	-0.11787959	0.070044	0.08522106	0.22148098	HNRNPA2B1
644150	ENSG00000122574	91.1378045	1.158497	0.27537419	2.60E-06	2.74E-05	WIPF3
30010	ENSG00000122584	53.8445284	-1.02747806	0.43993987	0.00145748	0.00817306	NXPH1
4852	ENSG00000122585	2553.66933	0.58803574	0.11237583	5.17E-08	7.10E-07	NPY
84668	ENSG00000122591	49.3319628	-0.24538137	0.37742253	0.22117297	0.43373822	FAM126A
3204	ENSG00000122592	0	NA	NA	NA	NA	HOXA7
3624	ENSG00000122641	1512.92148	-0.32492417	0.07696811	1.57E-05	0.00014374	INHBA
11328	ENSG00000122642	2084.47256	0.24171855	0.06776615	0.00028537	0.00195569	FKBP9
51251	ENSG00000122643	715.734902	-0.29101078	0.1244367	0.01317555	0.05200193	NT5C3A
10124	ENSG00000122644	230.913367	0.56273355	0.15987772	0.000136	0.00100271	ARL4A
51622	ENSG00000122674	834.116678	0.25564888	0.102371	0.00940639	0.03945512	CCZ1
27434	ENSG00000122678	715.673308	0.43183824	0.11086159	4.65E-05	0.00038373	POLM
10268	ENSG00000122679	7.26292058	-0.08689183	0.37069475	0.50571252	0.72177288	RAMP3
29960	ENSG00000122687	1347.32529	-0.35459098	0.08225646	9.76E-06	9.28E-05	MRM2
7291	ENSG00000122691	5415.23184	0.50784349	0.06494723	2.12E-15	5.98E-14	TWIST1

55234	ENSG00000122692	1105.15502	0.0050579	0.08259734	0.9497545	0.97932173	SMU1
152007	ENSG00000122694	349.991958	0.10208939	0.14300213	0.44191389	0.66678618	GLIPR2
92014	ENSG00000122696	332.839033	-0.11427816	0.12645501	0.33840044	0.56877877	SLC25A51
1211	ENSG00000122705	2262.36463	-0.22088765	0.06823523	0.0009843	0.00582939	CLTA
8434	ENSG00000122707	380.194117	0.25680181	0.12882705	0.03389544	0.11060618	RECK
27290	ENSG00000122711	0.55908305	0.01280346	0.38331087	0.73663191	NA	SPINK4
138474	ENSG00000122728	1.01693633	0.0226591	0.38210562	0.67438295	NA	TAF1L
48	ENSG00000122729	456.375309	-0.11054425	0.10974336	0.29230419	0.51835137	ACO1
23349	ENSG00000122733	6.65235695	0.15036975	0.40305039	0.21968664	0.43175718	PHF24
27019	ENSG00000122735	8.37748049	0.39788262	0.66158237	0.02524701	0.0877849	DNAI1
79269	ENSG00000122741	1128.18868	-0.167775	0.08588854	0.04470039	0.13639738	DCAF10
1271	ENSG00000122756	2829.0782	-1.12879228	0.06439298	1.01E-69	1.64E-67	CNTFR
57670	ENSG00000122778	3752.42359	0.09429586	0.05442143	0.08007321	0.21142567	KIAA1549
8805	ENSG00000122779	2837.61908	-0.03576185	0.07017566	0.61807895	0.80452675	TRIM24
78996	ENSG00000122783	863.315635	-0.1127651	0.08499159	0.17169747	0.36421231	CYREN
800	ENSG00000122786	3621.20393	0.14840946	0.06544391	0.02153527	0.07724818	CALD1
6718	ENSG00000122787	0	NA	NA	NA	NA	AKR1D1
170685	ENSG00000122824	288.594996	-0.03033121	0.13940766	0.81436515	0.91592004	NUDT10
653509	ENSG00000122852	0.173733	0.01914545	0.38484597	0.78225479	NA	SFTPA1
50674	ENSG00000122859	0.15069912	-0.01848503	0.38480575	0.84537521	NA	NEUROG3
5328	ENSG00000122861	28.8920033	-0.06555672	0.29361361	0.73140627	0.87166875	PLAU
5552	ENSG00000122862	0.29863895	-0.02292439	0.3852726	0.68753245	NA	SRGN
9469	ENSG00000122863	912.611218	-0.34541639	0.09125697	9.36E-05	0.00071719	CHST3
80114	ENSG00000122870	365.787799	-0.08886826	0.13192032	0.47235034	0.69454019	BICC1
NA	ENSG00000122872	0	NA	NA	NA	NA	ARL4AP1
55847	ENSG00000122873	312.594921	-0.29600217	0.1614569	0.04229272	0.13108695	CISD1
1959	ENSG00000122877	5.06394307	0.23488832	0.45880991	0.09772823	0.24490691	EGR2
11319	ENSG00000122882	545.972093	0.05048029	0.11622522	0.64842344	0.82455589	ECD
5033	ENSG00000122884	682.008031	0.49055643	0.12878209	5.55E-05	0.00044773	P4HA1
8034	ENSG00000122912	271.134997	0.03974506	0.16207024	0.78778411	0.90355167	SLC25A16
11130	ENSG00000122952	1278.524	-0.11500454	0.09657162	0.21750697	0.42908802	ZWINT
9559	ENSG00000122958	802.817034	-0.01671034	0.13479805	0.8859267	0.95015905	VPS26A
9904	ENSG00000122965	821.434725	0.14691319	0.10507052	0.14569635	0.32618095	RBM19
11113	ENSG00000122966	1388.19269	-0.39997966	0.0851813	1.41E-06	1.56E-05	CIT
28981	ENSG00000122970	884.704267	0.15107331	0.11352874	0.16324533	0.35223353	IFT81
35	ENSG00000122971	99.0569615	0.44916084	0.24553622	0.02524274	0.0877849	ACADS
84329	ENSG00000122986	15.3434913	0.54827014	0.66858417	0.04122772	0.12836755	HVCN1
NA	ENSG00000123009	0	NA	NA	NA	NA	NME2P1

79039	ENSG00000123064	1265.57622	-0.14717229	0.08696154	0.08167261	0.21450683	DDX54
23389	ENSG00000123066	1746.93735	-0.10080336	0.07344781	0.16157197	0.34999912	MED13L
1031	ENSG00000123080	901.635574	0.62804465	0.09290468	3.90E-12	8.53E-11	CDKN2C
26994	ENSG00000123091	788.80925	-0.04131773	0.10034026	0.66895887	0.83720078	RNF11
11228	ENSG00000123094	771.287311	0.44172076	0.11474772	5.45E-05	0.00044062	RASSF8
79365	ENSG00000123095	6.49663573	-0.25099086	0.50029873	0.0181695	0.0673422	BHLHE41
8082	ENSG00000123096	221.247834	0.38924106	0.23649427	0.04462406	0.13624829	SSPN
3709	ENSG00000123104	697.936797	0.28314648	0.12157226	0.01375611	0.05379878	ITPR2
55297	ENSG00000123106	486.484824	0.05255455	0.14332459	0.693515	0.85183585	CCDC91
64168	ENSG00000123119	81.2840671	-0.24623719	0.23123812	0.18948168	0.39045835	NECAB1
11059	ENSG00000123124	399.499712	0.55316101	0.14647619	5.17E-05	0.00042123	WWP1
23597	ENSG00000123130	495.306436	-0.06541914	0.12341368	0.57466733	0.77361067	ACOT9
10549	ENSG00000123131	867.417387	0.17608044	0.08840441	0.04046848	0.12647995	PRDX4
10212	ENSG00000123136	1785.50307	-0.16313585	0.07284099	0.0218877	0.07835654	DDX39A
5585	ENSG00000123143	4566.56153	-0.34951281	0.05371804	4.77E-11	9.48E-10	PKN1
79002	ENSG00000123144	3008.43225	-0.05229311	0.06349343	0.40244553	0.63061911	TRIR
976	ENSG00000123146	434.452793	0.36503278	0.12928256	0.00266498	0.01369417	ADGRE5
84292	ENSG00000123154	265.227834	0.01724356	0.14073777	0.89638983	0.95469109	WDR83
10755	ENSG00000123159	946.058872	0.05744058	0.09155139	0.51877451	0.73239566	GIPC1
139741	ENSG00000123165	0	NA	NA	NA	NA	ACTRT1
83446	ENSG00000123171	0	NA	NA	NA	NA	CCDC70
57213	ENSG00000123178	203.893814	0.18171125	0.1594755	0.2108908	0.41983479	SPRYD7
84650	ENSG00000123179	714.285031	-0.00956097	0.09915416	0.91946083	0.96585497	EBPL
540	ENSG00000123191	364.01599	0.13143991	0.12423153	0.26355304	0.48550532	ATP7B
23091	ENSG00000123200	3033.67298	-0.10964381	0.0613101	0.06965211	0.19079082	ZC3H13
2974	ENSG00000123201	0.14931947	-0.01844332	0.38480212	0.84537521	NA	GUCY1B2
57486	ENSG00000123213	988.797933	0.0610368	0.08339239	0.45420961	0.67744995	NLN
64105	ENSG00000123219	478.481038	0.05806591	0.11412631	0.59487642	0.78787011	CENPK
10133	ENSG00000123240	401.018861	0.13740195	0.13166139	0.26625829	0.48853828	OPTN
80760	ENSG00000123243	1.84834672	0.05202777	0.38288463	0.42373663	NA	ITIH5
466	ENSG00000123268	273.903881	-0.21330089	0.13708557	0.09459504	0.23913934	ATF1
10102	ENSG00000123297	467.704787	-0.03638102	0.11528392	0.74059002	0.87742184	TSFM
58158	ENSG00000123307	0	NA	NA	NA	NA	NEUROD4
64333	ENSG00000123329	91.2256454	2.74148619	0.36834245	5.86E-15	1.59E-13	ARHGAP9
3071	ENSG00000123338	0	NA	NA	NA	NA	NCKAP1L
4327	ENSG00000123342	2.20517753	0.09136999	0.39125184	0.28032328	NA	MMP19
5204	ENSG00000123349	2445.61687	-0.14059725	0.06041482	0.01833276	0.06774481	PFDN5
65244	ENSG00000123352	1069.52111	0.09361166	0.0944798	0.30686238	0.53442325	SPATS2

29095	ENSG00000123353	268.776301	-0.02852819	0.15906446	0.84259095	0.928413	ORMDL2
3164	ENSG00000123358	605.758161	0.74946385	0.14473096	4.52E-08	6.24E-07	NR4A1
5153	ENSG00000123360	0	NA	NA	NA	NA	PDE1B
3229	ENSG00000123364	0.49101099	-0.01135408	0.38292455	0.81858369	NA	HOXC13
1017	ENSG00000123374	1297.10383	0.04305683	0.07859259	0.57709511	0.77557535	CDK2
4035	ENSG00000123384	6078.20363	0.46213735	0.05136511	1.21E-19	4.41E-18	LRP1
3227	ENSG00000123388	0.52119901	0.02576573	0.38568143	0.51723676	NA	HOXC11
60673	ENSG00000123395	765.30798	0.19396083	0.09381265	0.03275774	0.10755128	ATG101
4778	ENSG00000123405	0	NA	NA	NA	NA	NFE2
3228	ENSG00000123407	0	NA	NA	NA	NA	HOXC12
64375	ENSG00000123411	393.486505	0.03679168	0.12695519	0.76012684	0.88889051	IKZF4
23583	ENSG00000123415	779.345202	-0.21209676	0.10977909	0.04295963	0.13257414	SMUG1
10376	ENSG00000123416	38290.9744	-0.23948412	0.05579124	1.39E-05	0.00012849	TUBA1B
25895	ENSG00000123427	152.720712	0.31076856	0.18201896	0.05326295	0.15622958	EEF1AKMT3
55709	ENSG00000123444	470.567428	-0.19876411	0.12893049	0.1005283	0.25024862	KBTBD4
NA	ENSG00000123447	0	NA	NA	NA	NA	TYRL
1757	ENSG00000123453	454.095949	0.12046257	0.1625576	0.41342896	0.64066701	SARDH
1621	ENSG00000123454	55435.6891	0.98744618	0.0528108	7.82E-79	1.55E-76	DBH
64756	ENSG00000123472	1168.9884	-0.08341373	0.08787658	0.32906117	0.55761363	ATPAF1
6491	ENSG00000123473	734.157335	-0.43444921	0.11292074	5.60E-05	0.00045174	STIL
55355	ENSG00000123485	843.327856	-0.21606261	0.1082419	0.03687606	0.118091	HJURP
3598	ENSG00000123496	8.43539783	0.1394119	0.37222604	0.38943009	0.61864785	IL13RA2
1300	ENSG00000123500	2.96305376	-0.00127599	0.36949505	0.98847317	0.99604194	COL10A1
262	ENSG00000123505	2000.08139	-0.4534459	0.09582347	1.00E-06	1.13E-05	AMD1
29078	ENSG00000123545	404.155157	-1.04785456	0.16725615	4.34E-11	8.68E-10	NDUFAF4
85015	ENSG00000123552	368.065418	0.04199725	0.14746376	0.7588259	0.88815444	USP45
5354	ENSG00000123560	3.52274042	0.26961314	0.51602203	0.02382069	0.08379476	PLP1
6906	ENSG00000123561	0	NA	NA	NA	NA	SERPINA7
9643	ENSG00000123562	3920.386	-0.11709633	0.08433994	0.15059806	0.33417577	MORF4L2
158983	ENSG00000123569	0	NA	NA	NA	NA	H2BW1
51209	ENSG00000123570	112.785088	-0.46794394	0.23365323	0.0163002	0.06169325	RAB9B
203447	ENSG00000123572	0.70622758	0.00412423	0.38184091	0.9305563	NA	NRK
139231	ENSG00000123575	757.155308	-0.17574313	0.10599856	0.08356624	0.21829549	FAM199X
80712	ENSG00000123576	0	NA	NA	NA	NA	ESX1
4108	ENSG00000123584	0	NA	NA	NA	NA	MAGEA9
92552	ENSG00000123594	0	NA	NA	NA	NA	ATXN3L
9367	ENSG00000123595	151.738696	0.24534333	0.18310819	0.12781277	0.29835718	RAB9A
79828	ENSG00000123600	402.090705	-0.12148606	0.1391581	0.34730234	0.57801178	METTL8

79809	ENSG00000123607	816.439565	0.03264686	0.11958538	0.77460223	0.89613481	TTC21B
9111	ENSG00000123609	0.15069912	-0.01848503	0.38480575	0.84537521	NA	NMI
7130	ENSG00000123610	182.741623	-0.09741593	0.1655413	0.5129569	0.72818152	TNFAIP6
130399	ENSG00000123612	8.05533593	-0.00707557	0.36421241	0.94824595	0.97876335	ACVR1C
29994	ENSG00000123636	795.399797	0.07669732	0.12951316	0.53096514	0.74257279	BAZ2B
206358	ENSG00000123643	413.688076	0.08369465	0.11607976	0.44987507	0.67340229	SLC36A1
9926	ENSG00000123684	2991.19514	-0.26449447	0.06359676	2.40E-05	0.00021136	LPGAT1
55509	ENSG00000123685	11.7145671	1.45390006	0.87384172	0.00410766	0.01972792	BATF3
50486	ENSG00000123689	23.9688854	-0.27606664	0.35676414	0.19913241	0.40359455	G0S2
3759	ENSG00000123700	7.08512819	3.62934626	1.35682515	0.00072554	0.00446349	KCNJ2
57826	ENSG00000123728	585.097648	-0.26723397	0.11380725	0.01362008	0.05341418	RAP2C
5393	ENSG00000123737	842.795156	0.16520947	0.10209304	0.09291305	0.23587906	EXOSC9
81579	ENSG00000123739	650.245827	-0.08626001	0.10750568	0.40223436	0.63040759	PLA2G12A
80776	ENSG00000123810	53.2590572	-0.11363319	0.25513215	0.55464043	0.76057964	B9D2
79934	ENSG00000123815	673.537731	0.16680818	0.10533637	0.09908733	0.24762471	COQ8B
5208	ENSG00000123836	226.989207	-0.08408044	0.20088023	0.62166257	0.80692086	PFKFB2
722	ENSG00000123838	0	NA	NA	NA	NA	C4BPA
725	ENSG00000123843	0	NA	NA	NA	NA	C4BPB
NA	ENSG00000123870	61.6532559	-0.22344078	0.33235036	0.27446787	0.49825626	ZNF137P
23682	ENSG00000123892	12.9250034	2.65865249	0.84856758	0.00010512	0.00079605	RAB38
10888	ENSG00000123901	1.46441736	0.02130092	0.37800852	0.77960751	NA	GPR83
27161	ENSG00000123908	3322.1343	-0.07718879	0.06804123	0.24785532	0.46694991	AGO2
10608	ENSG00000123933	1901.80471	0.0236223	0.08308401	0.77193773	0.89538192	MXD4
NA	ENSG00000123965	10.8967658	-0.01540309	0.33757854	0.92248496	0.96749493	PMS2P5
1164	ENSG00000123975	1193.81371	0.17937559	0.09849488	0.05923833	0.16954535	CKS2
164781	ENSG00000123977	0	NA	NA	NA	NA	DAW1
2181	ENSG00000123983	1750.8466	-0.56217945	0.07488944	2.06E-14	5.34E-13	ACSL3
79586	ENSG00000123989	1220.83793	-0.57532352	0.08890311	3.06E-11	6.18E-10	CHPF
23549	ENSG00000123992	475.706267	0.17613492	0.12386849	0.13233561	0.30543724	DNPEP
3623	ENSG00000123999	8.04318878	0.07164053	0.3656025	0.59566534	0.78816266	INHA
116255	ENSG00000124003	5.23483775	0.16880368	0.40828061	0.21789575	0.42968409	MOGAT1
23363	ENSG00000124006	2624.08663	0.06936702	0.06187506	0.25779934	0.47908756	OBSL1
79843	ENSG00000124019	2.81137807	0.22351985	0.48220921	0.00514577	0.02380881	FAM124B
6560	ENSG00000124067	616.964769	0.52773803	0.13927871	5.29E-05	0.00042979	SLC12A4
84080	ENSG00000124074	424.824752	0.02069018	0.11269993	0.84876086	0.93128058	ENKD1
4159	ENSG00000124089	0.37844916	0.02407552	0.3854191	0.60038877	NA	MC3R
140687	ENSG00000124091	1.84581566	0.02980015	0.37601244	0.73423608	NA	GCNT7
140690	ENSG00000124092	0	NA	NA	NA	NA	CTCFL

NA	ENSG00000124097	1.68525324	0.03526429	0.37865048	0.65853274	NA	HMGB1P1
116151	ENSG00000124098	2611.39881	0.07444729	0.06838574	0.26874802	0.49165152	FAM210B
5266	ENSG00000124102	0	NA	NA	NA	NA	PI3
200232	ENSG00000124103	0	NA	NA	NA	NA	FAM209A
90203	ENSG00000124104	428.173197	-0.36297756	0.13944179	0.0050778	0.02353273	SNX21
6590	ENSG00000124107	0	NA	NA	NA	NA	SLPI
140686	ENSG00000124116	0.65185969	0.01369813	0.38247831	0.70831419	NA	WFDC3
79183	ENSG00000124120	819.817877	-0.44545884	0.10110449	4.85E-06	4.90E-05	TTPAL
57580	ENSG00000124126	505.058007	0.20615661	0.10659797	0.04372711	0.13419116	PREX1
3787	ENSG00000124134	12.1820403	-1.522812	0.84528364	0.00316549	0.01583813	KCNS1
57468	ENSG00000124140	78.888045	-0.42103924	0.26592951	0.04363551	0.13397223	SLC12A5
343578	ENSG00000124143	2.63783925	-0.02348306	0.37526965	0.78357384	0.9014745	ARHGAP40
6385	ENSG00000124145	285.580738	1.20805264	0.16140916	7.61E-15	2.04E-13	SDC4
8202	ENSG00000124151	657.738991	-0.17042447	0.1004862	0.07813958	0.20773003	NCOA3
51604	ENSG00000124155	1782.20114	0.10680013	0.06747003	0.10813771	0.26372861	PIGT
6407	ENSG00000124157	0	NA	NA	NA	NA	SEMG2
8785	ENSG00000124159	0.16735865	0.01897177	0.38482933	0.78225479	NA	MATN4
57727	ENSG00000124160	1134.25418	-0.2011679	0.09120014	0.02285265	0.08105052	NCOA5
9217	ENSG00000124164	1546.72737	-0.07844031	0.10935623	0.45264543	0.67593598	VAPB
84612	ENSG00000124171	78.1818345	-0.0340734	0.22760812	0.85161306	0.93275912	PARD6B
514	ENSG00000124172	2966.42782	-0.09644319	0.06721301	0.14619551	0.32701731	ATP5F1E
84181	ENSG00000124177	2434.25573	0.30177147	0.07932483	9.81E-05	0.00074952	CHD6
5335	ENSG00000124181	6281.18725	-0.17598421	0.05397065	0.00097001	0.00576025	PLCG1
84969	ENSG00000124191	1459.70047	0.03557333	0.08178806	0.65685384	0.82968692	TOX2
6431	ENSG00000124193	5701.90035	-0.00631512	0.05315665	0.90391767	0.95803483	SRSF6
78997	ENSG00000124194	610.482415	-2.5495838	0.13294473	3.46E-83	7.39E-81	GDAP1L1
149699	ENSG00000124196	0	NA	NA	NA	NA	GTSF1L
10564	ENSG00000124198	1006.50004	-0.10939763	0.08498876	0.1862925	0.38596943	ARFGEF2
57169	ENSG00000124201	483.165995	-0.09265025	0.10640381	0.364017	0.59381904	ZNFX1
128611	ENSG00000124203	0.35660095	0.02381648	0.38538097	0.6140575	NA	ZNF831
1908	ENSG00000124205	9.16108682	4.12434866	1.30089863	0.00019524	0.00139235	EDN3
1434	ENSG00000124207	2781.33811	-0.34055481	0.0875397	6.15E-05	0.00049262	CSE1L
387522	ENSG00000124208	23.8278064	-0.04767157	0.38517263	0.41225321	0.64004254	PEDS1-UBE2V1
57403	ENSG00000124209	713.995291	0.0238281	0.10741564	0.81818984	0.9171248	RAB22A
5740	ENSG00000124212	176.184888	0.03661409	0.19224105	0.82733723	0.92189669	PTGIS
6780	ENSG00000124214	2762.29703	-0.19880276	0.06271686	0.00129397	0.00737925	STAU1
60437	ENSG00000124215	7.68492129	0.04619994	0.3619717	0.72149326	0.86709971	CDH26
6615	ENSG00000124216	55.1922062	1.08463423	0.33020707	0.00010186	0.0007737	SNAI1

27304	ENSG00000124217	334.133374	-0.22527303	0.14471956	0.09167439	0.23381152	MOCS3
8675	ENSG00000124222	2131.46323	0.18949908	0.06931484	0.00540855	0.02488478	STX16
NA	ENSG00000124224	202.910519	0.51904713	0.19510626	0.00260912	0.01345957	PPP4R1L
56937	ENSG00000124225	1198.59762	-0.78469441	0.08234515	3.14E-22	1.31E-20	PMEPA1
55905	ENSG00000124226	1182.88272	0.0827649	0.08253848	0.30501982	0.53238261	RNF114
140731	ENSG00000124227	0	NA	NA	NA	NA	ANKRD60
55661	ENSG00000124228	1516.662	0.16191234	0.07025558	0.01909847	0.07019518	DDX27
11317	ENSG00000124232	0	NA	NA	NA	NA	RBPJL
6406	ENSG00000124233	0	NA	NA	NA	NA	SEMG1
128602	ENSG00000124237	0	NA	NA	NA	NA	C20orf85
55653	ENSG00000124243	115.394696	-0.03717637	0.21375866	0.83274741	0.92398124	BCAS4
60598	ENSG00000124249	26.3955039	-0.07402903	0.29551296	0.69892862	0.8548268	KCNK15
27296	ENSG00000124251	15.3957272	-0.07430531	0.33365904	0.66895109	0.83720078	TP53TG5
5105	ENSG00000124253	0.173733	0.01914545	0.38484597	0.78225479	NA	PCK1
81030	ENSG00000124256	0	NA	NA	NA	NA	ZBP1
140825	ENSG00000124257	68.3802414	0.29229471	0.25722198	0.14526907	0.32577579	NEURL2
4109	ENSG00000124260	8.04563358	-0.93437286	2.24210584	0.01005368	0.04164195	MAGEA10
4552	ENSG00000124275	326.895727	-0.1199131	0.17842461	0.44591996	0.67028199	MTRR
79072	ENSG00000124279	152.063375	-0.47429841	0.2353495	0.01529827	0.05854681	FASTKD3
5184	ENSG00000124299	1187.04598	-0.00368416	0.08467844	0.96399512	0.9857573	PEPD
64377	ENSG00000124302	81.7422249	-0.63571903	0.34607206	0.01233825	0.04936867	CHST8
23096	ENSG00000124313	125.341359	0.5576402	0.20646424	0.00205975	0.01097354	IQSEC2
6845	ENSG00000124333	741.502823	-0.13368765	0.09240384	0.13421258	0.30845623	VAMP7
3581	ENSG00000124334	1.84080832	0.02124037	0.37829173	0.77649847	NA	IL9R
7499	ENSG00000124343	0	NA	NA	NA	NA	XG
100132596	ENSG00000124343	0	NA	NA	NA	NA	XG
10617	ENSG00000124356	1420.31869	0.00172443	0.07891597	0.98265553	0.99428951	STAMBP
55577	ENSG00000124357	595.587312	0.25065181	0.10733877	0.0147542	0.05690577	NAGK
84693	ENSG00000124370	72.4548769	0.23822029	0.25124641	0.22513984	0.43895189	MCEE
400961	ENSG00000124374	232.06362	0.00121269	0.14659613	0.99518692	0.99852582	PAIP2B
11017	ENSG00000124380	527.491101	-0.39705354	0.11924314	0.00044719	0.0029291	SNRNP27
10199	ENSG00000124383	656.579426	-0.18836212	0.10887091	0.07027492	0.19207251	MPHOSPH10
27189	ENSG00000124391	0.51556888	0.03113434	0.38605466	0.47762515	NA	IL17C
NA	ENSG00000124399	0	NA	NA	NA	NA	NDUFB4P12
10396	ENSG00000124406	2017.32468	-0.22868491	0.0910347	0.00952092	0.03985969	ATP8A1
23326	ENSG00000124422	10468.7945	-0.12138273	0.04866807	0.01187889	0.04786922	USP22
79983	ENSG00000124429	2.73765988	0.0789101	0.38126945	0.44663934	0.67085506	POF1B
64344	ENSG00000124440	467.003251	3.35228864	0.48304986	1.88E-13	4.51E-12	HIF3A

79177	ENSG00000124444	182.97946	-0.03759274	0.17566357	0.8088941	0.91334153	ZNF576
56269	ENSG00000124449	0	NA	NA	NA	NA	IRGC
7596	ENSG00000124459	694.672715	-0.2349045	0.10765948	0.02250767	0.08005423	ZNF45
27076	ENSG00000124466	3.35807478	0.10359202	0.3902883	0.31423552	0.54212505	LYPD3
440533	ENSG00000124467	0	NA	NA	NA	NA	PSG8
1088	ENSG00000124469	0	NA	NA	NA	NA	CEACAM8
4693	ENSG00000124479	2.68546129	0.13276317	0.41139336	0.09225466	0.23483865	NDP
8239	ENSG00000124486	2923.56111	-0.1933707	0.06775163	0.00374605	0.01825637	USP9X
7180	ENSG00000124490	0	NA	NA	NA	NA	CRISP2
2162	ENSG00000124491	0	NA	NA	NA	NA	F13A1
2914	ENSG00000124493	2.38246573	-0.14361402	0.41496076	0.09906832	NA	GRM4
55809	ENSG00000124496	721.871213	-0.15609429	0.09297409	0.08298581	0.21726558	TRERF1
29993	ENSG00000124507	37.1674571	-0.9015694	0.45067728	0.00439065	0.02088982	PACSIN1
10385	ENSG00000124508	450.769962	0.1374312	0.11802876	0.22087011	0.43326706	BTN2A2
23408	ENSG00000124523	283.079508	-0.03045312	0.15862725	0.83257752	0.92392457	SIRT5
57380	ENSG00000124532	668.618528	-0.09241921	0.11491927	0.39821578	0.62727695	MRS2
56897	ENSG00000124535	1211.90486	-0.20825888	0.07750804	0.00598084	0.02709036	WRNIP1
88745	ENSG00000124541	862.851039	-0.29957031	0.09375997	0.00095254	0.00567859	RRP36
54718	ENSG00000124549	110.44635	0.43312801	0.24886571	0.03162344	0.10460523	BTN2A3P
696	ENSG00000124557	16.5698707	-0.3544768	0.45844721	0.11036535	0.2677814	BTN1A1
6631	ENSG00000124562	1498.85546	-0.17404815	0.08324669	0.0319255	0.10535628	SNRPC
10786	ENSG00000124564	0.29863895	-0.02292439	0.3852726	0.68753245	NA	SLC17A3
6568	ENSG00000124568	0	NA	NA	NA	NA	SLC17A1
5269	ENSG00000124570	1326.70571	-0.10265351	0.08602681	0.22046275	0.43280124	SERPINB6
57510	ENSG00000124571	2818.40019	0.00995903	0.07297062	0.89037588	0.95232275	XPO5
89845	ENSG00000124574	644.370679	0.3011675	0.11511681	0.00595668	0.02700795	ABCC10
3007	ENSG00000124575	1.78985855	-0.03402492	0.37658058	0.69541495	NA	H1-3
5190	ENSG00000124587	673.959337	0.12020366	0.10905201	0.2499815	0.46975118	PEX6
4835	ENSG00000124588	103.178768	0.36109406	0.22305474	0.05258135	0.15473361	NQO2
NA	ENSG00000124593	304.08001	0.41813427	0.15445449	0.00318516	0.01591777	
221443	ENSG00000124596	438.63712	-0.15678503	0.11741205	0.15977532	0.34735289	OARD1
222643	ENSG00000124602	35.0979517	0.64586689	0.44633083	0.02159271	0.07741223	UNC5CL
57505	ENSG00000124608	1129.24192	0.00147933	0.09065325	0.98740439	0.9958875	AARS2
3024	ENSG00000124610	0	NA	NA	NA	NA	H1-1
346157	ENSG00000124613	207.067703	0.26865744	0.18585008	0.09942458	0.24822342	ZNF391
6204	ENSG00000124614	8308.75052	-0.09584436	0.0562675	0.08518418	0.2214571	RPS10
4337	ENSG00000124615	274.495763	-0.2569289	0.1403296	0.04838921	0.14519692	MOCS1
8970	ENSG00000124635	16.1255624	-0.19016856	0.35872489	0.32374629	0.55153069	H2BC11

9477	ENSG00000124641	437.013864	0.09359506	0.12648215	0.43350255	0.65875388	MED20
26212	ENSG00000124657	0.65897915	0.00113328	0.38189975	0.97955736	NA	OR2B6
6903	ENSG00000124659	360.779738	0.03389136	0.11924075	0.76635335	0.8924999	TBCC
25803	ENSG00000124664	4.06533206	0.09590584	0.37986199	0.42883852	0.65462623	SPDEF
6954	ENSG00000124678	0.30139824	-0.02296476	0.38527817	0.68536535	NA	TCP11
9587	ENSG00000124688	401.222093	-0.27005682	0.12244904	0.01946463	0.07138231	MAD2L1BP
10930	ENSG00000124701	3.57997077	0.10929309	0.38779649	0.34150039	0.57180671	APOBEC2
116138	ENSG00000124702	3536.93288	-0.01341981	0.05751391	0.80687808	0.91256903	KLHDC3
27232	ENSG00000124713	23.250565	0.45451499	0.46369676	0.07043792	0.192412	GNMT
107080644	ENSG00000124713	23.250565	0.45451499	0.46369676	0.07043792	0.192412	GNMT
1769	ENSG00000124721	2.92903275	0.13075912	0.41300137	0.06974479	0.19093923	DNAH8
54210	ENSG00000124731	0	NA	NA	NA	NA	TREM1
4201	ENSG00000124733	1029.89182	-0.10558969	0.09285066	0.24030961	0.45826053	MEA1
401265	ENSG00000124743	11.9416035	-8.96E-06	0.33683567	0.9978509	0.99926338	KLHL31
81578	ENSG00000124749	0.16735865	0.01897177	0.38482933	0.78225479	NA	COL21A1
1026	ENSG00000124762	726.915665	0.86846088	0.10398599	1.12E-17	3.63E-16	CDKN1A
6659	ENSG00000124766	11033.4505	0.3090284	0.04726132	4.31E-11	8.61E-10	SOX4
2739	ENSG00000124767	2446.84408	0.15627506	0.07954362	0.04456118	0.13608807	GLO1
57699	ENSG00000124772	4.10910488	-0.19247522	0.43216481	0.12335197	0.29097102	CPNE5
89822	ENSG00000124780	0.34746601	0.02370763	0.3853651	0.61987128	NA	KCNK17
6239	ENSG00000124782	244.537069	1.89183531	0.19864458	1.06E-22	4.54E-21	RREB1
6745	ENSG00000124783	1102.50738	-0.0642419	0.10235354	0.51403177	0.72918564	SSR1
83732	ENSG00000124784	915.337039	0.00343079	0.0879862	0.96892461	0.98835292	RIOK1
51299	ENSG00000124785	158.258629	0.05010065	0.1799661	0.75403211	0.8852593	NRN1
51000	ENSG00000124786	210.395732	0.0441866	0.16392806	0.76426364	0.89148768	SLC35B3
10799	ENSG00000124787	77.018627	0.06396273	0.25524673	0.74008096	0.87734262	RPP40
6310	ENSG00000124788	119.920023	1.44272068	0.38508828	1.15E-05	0.00010831	ATXN1
9972	ENSG00000124789	2158.45009	-0.24638924	0.06680038	0.00017566	0.00126363	NUP153
7913	ENSG00000124795	3858.2574	-0.35866294	0.06278747	6.71E-09	1.03E-07	DEK
9521	ENSG00000124802	301.000847	-0.28855896	0.15166231	0.03784997	0.12049799	EEF1E1
167	ENSG00000124812	0	NA	NA	NA	NA	CRISP1
860	ENSG00000124813	0.72074055	-0.03017113	0.38526582	0.62254501	NA	RUNX2
221391	ENSG00000124818	0	NA	NA	NA	NA	OPN5
9247	ENSG00000124827	0.99556546	0.02989887	0.38202549	0.61540303	NA	GCM2
9208	ENSG00000124831	1331.80945	-0.2213546	0.09350155	0.0144077	0.05578625	LRRFIP1
93463	ENSG00000124835	1.03885373	0.07430075	0.39365123	0.15225457	NA	
64284	ENSG00000124839	2.16125305	0.00030064	0.3742673	0.99861695	NA	RAB17
6372	ENSG00000124875	0	NA	NA	NA	NA	CXCL6

2069	ENSG00000124882	0.15069912	-0.01848503	0.38480575	0.84537521	NA	EREG
84767	ENSG00000124900	0	NA	NA	NA	NA	TRIM51
26070	ENSG00000124915	0.15069912	-0.01848503	0.38480575	0.84537521	NA	MYRF-AS1
745	ENSG00000124920	287.860345	1.01710708	0.14747941	6.89E-13	1.59E-11	MYRF
10647	ENSG00000124935	0	NA	NA	NA	NA	SCGB1D2
4246	ENSG00000124939	0	NA	NA	NA	NA	SCGB2A1
79026	ENSG00000124942	685.366807	3.55891435	0.13924581	2.98E-145	2.11E-142	AHNAK
55831	ENSG00000125037	571.905876	0.00838207	0.10852342	0.93701757	0.97351594	EMC3
51066	ENSG00000125046	102.142908	-0.82855352	0.27549673	0.00038497	0.00255785	SSUH2
7471	ENSG00000125084	0	NA	NA	NA	NA	WNT1
54436	ENSG00000125089	204.373156	-1.14539667	0.24596815	3.14E-07	3.88E-06	SH3TC1
23019	ENSG00000125107	4620.25619	-0.1142016	0.0950153	0.21389804	0.42407686	CNOT1
26231	ENSG00000125122	29.1902449	0.11072843	0.31109228	0.56814408	0.76919502	LRRC29
583	ENSG00000125124	767.840515	0.20385362	0.09872445	0.03235801	0.10653394	BBS2
4495	ENSG00000125144	0.53474877	0.0320676	0.38614822	0.46394167	NA	MT1G
4502	ENSG00000125148	176.995179	0.86741039	0.19604797	1.49E-06	1.64E-05	MT2A
80262	ENSG00000125149	413.92738	-0.10849338	0.12451097	0.35590825	0.58600518	C16orf70
2806	ENSG00000125166	2328.33714	-0.21712155	0.07126602	0.00189919	0.01022801	GOT2
55715	ENSG00000125170	3042.91025	-0.91061545	0.06133386	1.15E-50	1.17E-48	DOK4
9271	ENSG00000125207	258.517386	0.40900769	0.15688752	0.00440136	0.02093073	PIWIL1
2841	ENSG00000125245	0.47262548	-0.02840375	0.38580526	0.54352804	NA	GPR18
171425	ENSG00000125246	99.3246645	0.60613858	0.24365978	0.0031579	0.01580543	CLYBL
84899	ENSG00000125247	1167.36342	-0.00201415	0.08605749	0.97953667	0.99317507	TMTC4
5911	ENSG00000125249	1156.80197	-0.47460592	0.08417727	7.40E-09	1.13E-07	RAP2A
6555	ENSG00000125255	0	NA	NA	NA	NA	SLC10A2
10257	ENSG00000125257	633.807716	-0.71986446	0.10595032	2.45E-12	5.44E-11	ABCC4
1948	ENSG00000125266	2814.47463	1.43469151	0.07429346	4.19E-84	9.03E-82	EFNB2
11166	ENSG00000125285	0.81937543	-0.04649727	0.38803309	0.31234927	NA	SOX21
9375	ENSG00000125304	2077.24121	0.0898535	0.0639248	0.1541342	0.33936549	TM9SF2
78995	ENSG00000125319	140.425827	-0.32470198	0.20405344	0.06266585	0.1771069	HROB
3834	ENSG00000125337	10.6896127	-0.03844252	0.34462752	0.80414018	0.91109252	KIF25
3659	ENSG00000125347	67.1691869	1.25245958	0.44549291	0.00032439	0.00219428	IRF1
65109	ENSG00000125351	752.039936	-0.09453899	0.09346476	0.2963931	0.52297409	UPF3B
7737	ENSG00000125352	193.66401	-0.11237199	0.15750831	0.43318733	0.65852706	RNF113A
23157	ENSG00000125354	4077.46391	-0.15953409	0.06018146	0.00724681	0.03180197	SEPTIN6
55026	ENSG00000125355	3.9047523	0.14452693	0.41055969	0.15989758	0.34750443	TMEM255A
4694	ENSG00000125356	622.704936	-0.20101993	0.1014795	0.03943057	0.12409621	NDUFA1
265	ENSG00000125363	0	NA	NA	NA	NA	AMELX

27109	ENSG00000125375	124.94955	-0.32895238	0.23136009	0.08191912	0.21504039	DMAC2L
652	ENSG00000125378	1.70717773	0.09098026	0.39488702	0.18213239	NA	BMP4
5732	ENSG00000125384	390.367692	7.44255427	0.39907672	1.13E-76	2.16E-74	PTGER2
NA	ENSG00000125385	0	NA	NA	NA	NA	RPL32P21
8603	ENSG00000125386	976.067992	-0.09372316	0.08295676	0.24654133	0.46577702	FAM193A
2868	ENSG00000125388	168.802669	-0.00721049	0.17055678	0.96118847	0.98419142	GRK4
6662	ENSG00000125398	65.4796472	1.42206037	0.30523759	2.61E-07	3.26E-06	SOX9
64518	ENSG00000125409	5.32952314	0.10197884	0.37873508	0.42568165	0.65149487	TEKT3
4620	ENSG00000125414	0	NA	NA	NA	NA	MYH2
9953	ENSG00000125430	0.16735865	0.01897177	0.38482933	0.78225479	NA	HS3ST3B1
399512	ENSG00000125434	128.250619	0.50686734	0.21851736	0.00677659	0.03009552	SLC25A35
51081	ENSG00000125445	1323.69377	-0.08854894	0.07414639	0.22310703	0.43623174	MRPS7
23163	ENSG00000125447	1513.75516	-0.00991768	0.08219118	0.90106901	0.95641534	GGA3
79637	ENSG00000125449	330.552874	0.09654944	0.13162456	0.43515355	0.66040284	ARMC7
79902	ENSG00000125450	948.335268	0.10762408	0.1119228	0.31493063	0.5427103	NUP85
60386	ENSG00000125454	287.459813	-0.11360032	0.1383574	0.37731408	0.6061395	SLC25A19
57409	ENSG00000125457	379.618903	-0.10195669	0.13887161	0.43003118	0.65568848	MIF4GD
30833	ENSG00000125458	884.190383	0.17875288	0.09179159	0.04464461	0.13629004	NT5C
55154	ENSG00000125459	1035.25	0.22334936	0.09611377	0.016207	0.06143205	MSTO1
10485	ENSG00000125462	492.32162	-0.33192921	0.12504891	0.00485716	0.02266901	MIR9-1HG
7270	ENSG00000125482	627.544307	-0.13564026	0.11041781	0.1981906	0.4024257	TTF1
9329	ENSG00000125484	1876.83317	-0.32783817	0.07180617	3.21E-06	3.34E-05	GTF3C4
64794	ENSG00000125485	526.094315	0.04865898	0.12500676	0.68125899	0.84428624	DDX31
56751	ENSG00000125492	0	NA	NA	NA	NA	BARHL1
3802	ENSG00000125498	0	NA	NA	NA	NA	KIR2DL1
54776	ENSG00000125503	1073.20122	0.09546881	0.09786081	0.31333838	0.54126787	PPP1R12C
79143	ENSG00000125505	989.979256	0.22666575	0.10299811	0.02202608	0.07871129	MBOAT7
6725	ENSG00000125508	8.85594361	0.04999735	0.35082729	0.74290881	0.87875221	SRMS
4987	ENSG00000125510	171.154617	0.29094938	0.18856102	0.07775724	0.20704652	OPRL1
NA	ENSG00000125514	0	NA	NA	NA	NA	LINC00029
56731	ENSG00000125520	857.62006	0.28861449	0.09981776	0.0026833	0.01378119	SLC2A4RG
2832	ENSG00000125522	0.30139824	-0.02296476	0.38527817	0.68536535	NA	NPBWR2
79025	ENSG00000125531	86.0089347	0.68096469	0.28129898	0.00299567	0.01510396	FNDC11
128408	ENSG00000125533	0	NA	NA	NA	NA	BHLHE23
79144	ENSG00000125534	2097.09139	0.14012052	0.07186189	0.04708386	0.14207511	PPDPF
3553	ENSG00000125538	0.16735865	0.01897177	0.38482933	0.78225479	NA	IL1B
5342	ENSG00000125551	92.5746393	0.05962149	0.21977337	0.74270655	0.87875221	PLGLB2
27178	ENSG00000125571	0	NA	NA	NA	NA	IL37

84269	ENSG00000125611	257.106172	0.15361369	0.15976481	0.29026212	0.51620656	CHCHD5
7849	ENSG00000125618	0.33656195	-0.00108945	0.38336593	0.99872987	NA	PAX8
51141	ENSG00000125629	398.175153	0.09314469	0.13580206	0.46396277	0.68678459	INSIG2
84172	ENSG00000125630	2154.74509	-0.08982315	0.10806201	0.38525847	0.61433274	POLR1B
NA	ENSG00000125631	0.50604797	0.01474136	0.3830919	0.74769424	NA	HTR5BP
54520	ENSG00000125633	1706.34852	0.14591019	0.09551181	0.11449257	0.27537066	CCDC93
23550	ENSG00000125637	10.3488325	0.20472821	0.41644236	0.19060034	0.39194762	PSD4
79085	ENSG00000125648	1098.60067	-0.17339226	0.0975327	0.06539321	0.18250124	SLC25A23
5623	ENSG00000125650	79.2761926	0.2887925	0.24950849	0.1433231	0.32257859	PSPN
2962	ENSG00000125651	2279.35934	0.01389924	0.07195274	0.84481522	0.92965052	GTF2F1
84266	ENSG00000125652	374.561046	-0.12515418	0.12196852	0.27810662	0.50258055	ALKBH7
8192	ENSG00000125656	1089.85734	-0.07650301	0.08645204	0.36377654	0.59354103	CLPP
8744	ENSG00000125657	2.64513933	0.06786231	0.3824027	0.45144478	0.67474853	TNFSF9
2892	ENSG00000125675	16.910799	1.14629082	1.00045294	0.00882253	0.03740949	GRIA3
57187	ENSG00000125676	2296.42043	0.12731342	0.07967532	0.10128975	0.25167045	THOC2
5469	ENSG00000125686	2232.53622	-0.16537906	0.07400881	0.02254739	0.0801685	MED1
9349	ENSG00000125691	15831.5886	-0.00368887	0.04939516	0.93940098	0.97474024	RPL23
NA	ENSG00000125695	0	NA	NA	NA	NA	
84938	ENSG00000125703	207.081796	-0.02650834	0.16714537	0.85896315	0.93718813	ATG4C
970	ENSG00000125726	0	NA	NA	NA	NA	CD70
718	ENSG00000125730	0	NA	NA	NA	NA	C3
10045	ENSG00000125731	7.93118069	0.85811982	1.7482914	0.01332497	0.05247466	SH2D3A
9322	ENSG00000125733	452.130311	0.41969031	0.14902704	0.00228661	0.01200804	TRIP10
56927	ENSG00000125734	545.934372	0.54924424	0.10820345	1.33E-07	1.74E-06	GPR108
8740	ENSG00000125735	0	NA	NA	NA	NA	TNFSF14
2354	ENSG00000125740	275.814582	6.0876067	0.56667602	2.34E-28	1.27E-26	FOSB
80207	ENSG00000125741	582.012627	-0.0156512	0.12957864	0.89613788	0.95469109	OPA3
6633	ENSG00000125743	3784.02271	-0.03965122	0.05815641	0.53437303	0.74551445	SNRPD2
6253	ENSG00000125744	938.253978	-1.08061806	0.08957802	1.97E-34	1.30E-32	RTN2
24139	ENSG00000125746	322.352115	0.06843284	0.13944208	0.59875663	0.79005929	EML2
7408	ENSG00000125753	466.905661	0.66355596	0.16644581	1.60E-05	0.00014659	VASP
8189	ENSG00000125755	2688.39969	0.06465996	0.06176585	0.29136622	0.51742125	SYMPK
56261	ENSG00000125772	312.553269	0.19337508	0.14974124	0.16038593	0.34822247	GPCPD1
27111	ENSG00000125775	23.7268173	0.16799171	0.32788327	0.39660281	0.6258107	SDCBP2
80025	ENSG00000125779	859.932332	-0.17417123	0.08991053	0.04519261	0.13762417	PANK2
7053	ENSG00000125780	0.33471731	0.02354391	0.38534154	0.62842204	NA	TGM3
2797	ENSG00000125787	2.96826605	-0.00830598	0.37188832	0.92811932	0.96985158	GNRH2
81623	ENSG00000125788	0	NA	NA	NA	NA	DEFB126

3170	ENSG00000125798	0	NA	NA	NA	NA	FOXA2
284800	ENSG00000125804	12.5026294	0.0619887	0.33856036	0.71328965	0.86190557	FAM182A
22918	ENSG00000125810	0	NA	NA	NA	NA	CD93
64412	ENSG00000125812	447.512453	-0.11599504	0.19096667	0.48271232	0.70315803	GZF1
5075	ENSG00000125813	0	NA	NA	NA	NA	PAX1
63908	ENSG00000125814	573.357561	-0.59083069	0.11654612	1.20E-07	1.57E-06	NAPB
10047	ENSG00000125815	0	NA	NA	NA	NA	CST8
644524	ENSG00000125816	0	NA	NA	NA	NA	NKX2-4
1059	ENSG00000125817	1939.14134	-0.4812186	0.0667106	2.30E-13	5.44E-12	CENPB
9491	ENSG00000125818	1273.78253	0.0357574	0.07548263	0.6295973	0.8124371	PSMF1
4821	ENSG00000125820	0	NA	NA	NA	NA	NKX2-2
92675	ENSG00000125821	824.21087	0.11834346	0.09399395	0.19399155	0.39683151	DTD1
128817	ENSG00000125823	0	NA	NA	NA	NA	CSTL1
10616	ENSG00000125826	1274.20558	0.08579165	0.08790999	0.31622748	0.54428326	RBCK1
56255	ENSG00000125827	1740.34121	-0.26016771	0.07287909	0.0002695	0.00185783	TMX4
140880	ENSG00000125831	0	NA	NA	NA	NA	CST11
140901	ENSG00000125834	890.082305	-0.04873559	0.08773083	0.56325154	0.76608585	STK35
6628	ENSG00000125835	5016.33246	0.11392433	0.07015756	0.09862105	0.24670807	SNRPB
80023	ENSG00000125841	1302.26265	-0.14557493	0.0817118	0.06911833	0.18966912	NRSN2
55317	ENSG00000125843	185.038829	-0.15326365	0.18824814	0.34951813	0.58004303	AP5S1
6238	ENSG00000125844	806.295017	0.2071236	0.10471615	0.03937822	0.12401014	RRBP1
650	ENSG00000125845	7.3953478	4.55754405	1.5356558	0.00014651	0.00107297	BMP2
7692	ENSG00000125846	415.23004	0.00939256	0.12946974	0.93950689	0.97474024	ZNF133
23767	ENSG00000125848	480.810901	0.0896319	0.13297916	0.4757038	0.69776455	FLRT3
58495	ENSG00000125850	0	NA	NA	NA	NA	OVOL2
5126	ENSG00000125851	0	NA	NA	NA	NA	PCSK2
64096	ENSG00000125861	0	NA	NA	NA	NA	GFRA4
8195	ENSG00000125863	777.486674	-0.01119283	0.09527299	0.90279654	0.95739939	MKKS
631	ENSG00000125864	7.47984673	0.14942377	0.3863313	0.32443085	0.55221149	BFSP1
11034	ENSG00000125868	1349.82371	-0.23402113	0.08095996	0.00304234	0.015312	DSTN
24141	ENSG00000125869	2202.55792	-0.53618431	0.06910661	3.15E-15	8.80E-14	LAMP5
6629	ENSG00000125870	2393.23647	-0.95801466	0.06314533	7.94E-53	8.43E-51	SNRPB2
92667	ENSG00000125871	642.159726	0.00324678	0.1097219	0.97696665	0.99216924	MGME1
164312	ENSG00000125872	0.49492041	0.01127072	0.38337735	0.78451667	NA	LRRN4
128637	ENSG00000125875	1041.73863	-0.11604882	0.08595855	0.16556297	0.35564848	TBC1D20
3704	ENSG00000125877	985.94097	0.01926586	0.08461913	0.81614608	0.91637704	ITPA
6939	ENSG00000125878	82.0242856	-0.76436512	0.28014914	0.00103324	0.0060739	TCF15
56914	ENSG00000125879	211.169707	-3.55504655	0.23077455	1.59E-54	1.80E-52	OTOR

84515	ENSG00000125885	1347.09337	-0.00895423	0.08708916	0.91602071	0.96435405	MCM8
140836	ENSG00000125888	0	NA	NA	NA	NA	BANF2
55321	ENSG00000125895	71.694115	0.55317398	0.29350886	0.01523449	0.05837164	TMEM74B
83541	ENSG00000125898	333.957025	-0.29823928	0.14871764	0.02909606	0.09799113	FAM110A
NA	ENSG00000125899	0	NA	NA	NA	NA	LINC02871
128646	ENSG00000125900	0	NA	NA	NA	NA	SIRPD
64949	ENSG00000125901	1543.70336	0.01807411	0.07569922	0.8083632	0.91317199	MRPS26
140881	ENSG00000125903	0	NA	NA	NA	NA	DEFB129
8698	ENSG00000125910	0	NA	NA	NA	NA	S1PR4
56926	ENSG00000125912	2112.64871	-0.0964729	0.0864257	0.25120394	0.47115582	NCLN
4435	ENSG00000125931	83.3370381	0.83251136	0.27908194	0.00041879	0.00275672	CITED1
10236	ENSG00000125944	6836.9131	0.01903186	0.06290917	0.85031786	0.93221564	HNRNPR
80818	ENSG00000125945	309.513274	-0.04619211	0.12878499	0.70252706	0.85694048	ZNF436
4149	ENSG00000125952	999.792286	-0.18166176	0.12802937	0.13091599	0.30332515	MAX
100529261	ENSG00000125954	21.588102	0.93042163	0.714715	0.01212891	0.04864315	CHURC1-FNTB
64860	ENSG00000125962	171.361427	-0.20871467	0.18579917	0.19894094	0.4033713	ARMCX5
8200	ENSG00000125965	3.89796405	0.14288996	0.4058409	0.20148012	0.40689796	GDF5
10893	ENSG00000125966	901.203606	-3.02498867	0.11083662	3.18E-165	3.71E-162	MMP24
63941	ENSG00000125967	306.614823	0.03844558	0.15713152	0.7896001	0.9043268	NECAB3
3397	ENSG00000125968	213.547702	-0.08969505	0.1657471	0.54766664	0.75435339	ID1
22913	ENSG00000125970	3746.57006	0.10555094	0.06523378	0.09593679	0.24179191	RALY
83658	ENSG00000125971	1475.74448	0.0324845	0.07892821	0.66959636	0.83747033	DYNLRB1
140873	ENSG00000125975	0.16735865	0.01897177	0.38482933	0.78225479	NA	C20orf173
8894	ENSG00000125977	1799.83781	0.05246254	0.1014693	0.59290773	0.78702605	EIF2S2
51614	ENSG00000125991	3364.05073	0.22943927	0.06060785	0.0001244	0.00092302	ERGIC3
140823	ENSG00000125995	915.368001	-0.03726138	0.09352625	0.68068224	0.84396798	ROMO1
NA	ENSG00000125997	0.60279648	-0.02752712	0.38588313	0.49730371	NA	BPIFB9P
128876	ENSG00000125998	0.5080512	0.03076426	0.38601843	0.48315201	NA	FAM83C
92747	ENSG00000125999	0	NA	NA	NA	NA	BPIFB1
11190	ENSG00000126001	2150.28656	0.00048346	0.07725331	0.99643069	0.99873692	CEP250
5326	ENSG00000126003	1030.68688	0.23026964	0.0826819	0.00429109	0.02048009	PLAGL2
101410538	ENSG00000126005	234.948488	-0.42527942	0.15868349	0.00334827	0.01660326	MMP24OS
2925	ENSG00000126010	4.54894044	-0.1477689	0.40253738	0.23219598	0.44897628	GRPR
8242	ENSG00000126012	4680.26162	0.03077105	0.08132212	0.70019481	0.85541355	KDM5C
154796	ENSG00000126016	316.688162	-0.05762382	0.13504315	0.64807021	0.82447067	AMOT
11070	ENSG00000126062	1285.41518	0.00663263	0.09794345	0.94513263	0.97760447	TMEM115
5690	ENSG00000126067	1636.31147	0.14888372	0.08019224	0.05749109	0.16576416	PSMB2
192669	ENSG00000126070	1190.24013	-0.09552091	0.09999647	0.32145223	0.54914291	AGO3

7389	ENSG00000126088	843.565844	0.15249512	0.08784445	0.0743479	0.20011698	UROD
6487	ENSG00000126091	467.772555	0.30065653	0.12577335	0.01113386	0.04530267	ST3GAL3
79639	ENSG00000126106	119.830618	0.21867465	0.24993316	0.26244566	0.48440989	TMEM53
79654	ENSG00000126107	602.980003	0.31709584	0.11838202	0.00474847	0.02224549	HECTD3
3831	ENSG00000126214	3306.20155	-0.18223827	0.05870731	0.00166118	0.00915749	KLC1
7517	ENSG00000126215	616.366109	-0.23246717	0.11932188	0.0397485	0.12479986	XRCC3
10426	ENSG00000126216	1002.11709	0.01882815	0.08893014	0.8285197	0.92220943	TUBGCP3
23263	ENSG00000126217	1496.34168	-0.15757606	0.08871244	0.06734886	0.18634601	MCF2L
2159	ENSG00000126218	442.480383	0.44013894	0.12301343	0.00015888	0.00115419	F10
55795	ENSG00000126226	1214.70964	0.05106843	0.09232173	0.56963087	0.77027512	PCID2
8858	ENSG00000126231	4.24308067	-0.05793452	0.36834836	0.63494677	0.81626022	PROZ
57152	ENSG00000126233	0	NA	NA	NA	NA	SLURP1
79414	ENSG00000126243	439.020782	0.00858695	0.14857948	0.95140878	0.98008882	LRFN3
79713	ENSG00000126246	55.6792994	-0.11475507	0.28247296	0.55658175	0.76208295	IGFLR1
826	ENSG00000126247	1995.53094	0.30455076	0.06874726	6.50E-06	6.39E-05	CAPNS1
84306	ENSG00000126249	155.597408	-0.31069793	0.17811243	0.0492454	0.14732036	PDCD2L
2866	ENSG00000126251	0	NA	NA	NA	NA	GPR42
79171	ENSG00000126254	1046.20742	0.23306225	0.09193839	0.00889128	0.03763666	RBM42
84063	ENSG00000126259	108.611361	-0.47050604	0.2577739	0.02308729	0.08171822	KIRREL2
10054	ENSG00000126261	3876.14931	-0.09952435	0.05753998	0.07989536	0.21106844	UBA2
2867	ENSG00000126262	0	NA	NA	NA	NA	FFAR2
10870	ENSG00000126264	0.82391088	-0.00162191	0.38069451	0.9775606	NA	HCST
2864	ENSG00000126266	0	NA	NA	NA	NA	FFAR1
1340	ENSG00000126267	1567.05576	-0.00135779	0.06943927	0.98318241	0.99428951	COX6B1
8689	ENSG00000126337	0	NA	NA	NA	NA	KRT36
7067	ENSG00000126351	1192.47998	-0.07307199	0.08361962	0.36996014	0.59928226	THRA
1236	ENSG00000126353	2.02974961	0.13306556	0.41465152	0.05731135	NA	CCR7
9572	ENSG00000126368	79.9324967	0.25174672	0.23777111	0.18833918	0.38882927	NR1D1
83786	ENSG00000126391	610.380983	0.34633568	0.10682007	0.00071513	0.00440625	FRMD8
25824	ENSG00000126432	3805.51385	0.00300309	0.06103421	0.96411037	0.98577354	PRDX5
83596	ENSG00000126453	295.858439	0.9822099	0.15748597	6.08E-11	1.20E-09	BCL2L12
3661	ENSG00000126456	526.786538	-0.11978478	0.13717789	0.34824704	0.5790012	IRF3
3276	ENSG00000126457	5519.1107	0.00170271	0.07436012	0.98254929	0.99428951	PRMT1
6237	ENSG00000126458	87.7089273	1.37351875	0.27669104	5.93E-08	8.05E-07	RRAS
5639	ENSG00000126460	5.43388866	-0.14749742	0.40725319	0.17826838	0.37383412	PRRG2
58506	ENSG00000126461	2550.42549	0.14966668	0.06153385	0.01371986	0.05368874	SCAF1
57479	ENSG00000126464	1631.65521	0.02168369	0.06861082	0.74899385	0.88205755	PRR12
60385	ENSG00000126467	0	NA	NA	NA	NA	TSKS

23769	ENSG00000126500	583.344631	0.11279799	0.11371193	0.29910592	0.52612826	FLRT1
435	ENSG00000126522	409.556046	0.40409585	0.14272122	0.00230755	0.01210196	ASL
51119	ENSG00000126524	847.771244	-0.11165895	0.08563485	0.18087808	0.37795923	SBDS
1446	ENSG00000126545	0	NA	NA	NA	NA	CSN1S1
6779	ENSG00000126549	0	NA	NA	NA	NA	STATH
3346	ENSG00000126550	0	NA	NA	NA	NA	HTN1
6776	ENSG00000126561	9.96039781	-0.35801693	0.56022413	0.06109579	0.17383343	STAT5A
65266	ENSG00000126562	6.35899059	1.0993745	2.0743207	0.01049204	0.04307991	WNK4
8678	ENSG00000126581	782.451862	-0.0202593	0.09075069	0.82203544	0.91867195	BECN1
5582	ENSG00000126583	109.503755	0.76029013	0.29148601	0.00145164	0.00814957	PRKCG
10131	ENSG00000126602	2882.939	-0.11067813	0.06720833	0.09350416	0.23701577	TRAP1
84662	ENSG00000126603	505.710065	-0.10574078	0.1105486	0.31700874	0.54483775	GLIS2
84081	ENSG00000126653	419.983517	-0.18513671	0.11383709	0.08767115	0.22621869	NSRP1
22826	ENSG00000126698	1687.81676	-0.10128443	0.06857665	0.13299096	0.30644076	DNAJC8
27245	ENSG00000126705	1091.88321	0.12513291	0.08299201	0.1221562	0.28896853	AHDC1
2537	ENSG00000126709	218.263498	0.96712892	0.19287994	7.03E-08	9.45E-07	IFI6
117154	ENSG00000126733	0	NA	NA	NA	NA	DACH2
171017	ENSG00000126746	2032.28271	0.21706051	0.06829092	0.00121499	0.00699723	ZNF384
10436	ENSG00000126749	938.206141	-0.05608163	0.08848286	0.5141418	0.72921448	EMG1
6756	ENSG00000126752	0	NA	NA	NA	NA	SSX1
8409	ENSG00000126756	453.338642	-0.06036032	0.11960544	0.59447476	0.7877158	UXT
5199	ENSG00000126759	19.9289739	0.25741648	0.43072869	0.15698057	0.34378031	CFP
2002	ENSG00000126767	681.132049	-0.1622013	0.09765833	0.08518955	0.2214571	ELK1
10245	ENSG00000126768	681.943814	0.18964032	0.09659913	0.04221179	0.13092298	TIMM17B
64430	ENSG00000126773	2311.33945	-0.08969272	0.07913889	0.24618791	0.46544388	PCNX4
22863	ENSG00000126775	367.713189	0.27009518	0.12347517	0.02054116	0.07447673	ATG14
3895	ENSG00000126777	3813.35328	-0.2167232	0.07130963	0.00194039	0.01041314	KTN1
6495	ENSG00000126778	2.73291672	0.17395879	0.44179679	0.00968968	0.0404533	SIX1
57381	ENSG00000126785	0.48241915	0.02941591	0.3858847	0.50463983	NA	RHOJ
9787	ENSG00000126787	1151.71787	-0.25341852	0.10683165	0.01319116	0.05204067	DLGAP5
112849	ENSG00000126790	183.742516	0.2126437	0.16331939	0.14970244	0.33259758	L3HYPDH
3306	ENSG00000126803	271.735471	0.03493122	0.15753586	0.80900489	0.91334153	HSPA2
22890	ENSG00000126804	710.714207	-0.28880357	0.11754845	0.0096008	0.04012637	ZBTB1
57570	ENSG00000126814	553.758308	-0.36437527	0.13779136	0.0045268	0.02139394	TRMT5
81537	ENSG00000126821	269.974325	-0.3613943	0.1490464	0.00845368	0.03610787	SGPP1
26030	ENSG00000126822	984.652921	-0.37667628	0.10662844	0.0002268	0.00159055	PLEKHG3
5858	ENSG00000126838	1.13347067	-0.04696378	0.38740561	0.30427083	NA	PZP
11105	ENSG00000126856	0.46723714	-0.01002979	0.38291395	0.84210182	NA	PRDM7

55288	ENSG00000126858	972.017619	0.00677917	0.10081868	0.94504914	0.97756908	RHOT1
2123	ENSG00000126860	2.30886449	-0.03548369	0.37720263	0.67492142	NA	EVI2A
4974	ENSG00000126861	3.12727196	0.06361256	0.3803864	0.49540157	0.71414424	OMG
101927057	ENSG00000126861	3.12727196	0.06361256	0.3803864	0.49540157	0.71414424	OMG
55112	ENSG00000126870	812.160667	0.5277448	0.09026392	1.87E-09	3.10E-08	DYNC2I1
83543	ENSG00000126878	431.076448	2.0274143	0.1355225	1.07E-51	1.10E-49	AIF1L
286336	ENSG00000126882	231.936617	0.13374583	0.14895821	0.32997135	0.55861736	FAM78A
8021	ENSG00000126883	1722.03632	-0.07235602	0.07843007	0.34542121	0.57589553	NUP214
30848	ENSG00000126890	2.02163866	-0.03460677	0.38110749	0.6058908	NA	CTAG2
554	ENSG00000126895	15.194229	-0.11904183	0.34095378	0.50793074	0.7237561	AVPR2
8273	ENSG00000126903	196.232939	0.05699192	0.16476243	0.70360215	0.85751377	SLC10A3
5605	ENSG00000126934	1813.26613	0.01761941	0.06859916	0.7946998	0.90579008	MAP2K2
3188	ENSG00000126945	2062.93777	0.02703515	0.08678982	0.74998043	0.8824202	HNRNPH2
51309	ENSG00000126947	272.817238	-0.25813095	0.14164477	0.04906569	0.14693784	ARMCX1
59353	ENSG00000126950	238.842731	-0.14040387	0.15150592	0.31191232	0.53958168	TMEM35A
55998	ENSG00000126952	0.51380477	0.01507728	0.38309512	0.74089101	NA	NXF5
1678	ENSG00000126953	294.148434	-0.23552032	0.13537162	0.06194251	0.17561295	TIMM8A
55906	ENSG00000126970	655.803184	-0.13975467	0.1073779	0.17396081	0.36720742	ZC4H2
821	ENSG00000127022	9602.36548	-0.08212286	0.05947403	0.16166617	0.3500826	CANX
54973	ENSG00000127054	2175.481	-0.02117421	0.07177389	0.76326506	0.89093245	INTS11
6003	ENSG00000127074	0.98605025	0.00607633	0.38006497	0.92025621	NA	RGS13
64768	ENSG00000127080	489.499699	0.07340224	0.14586082	0.5870773	0.78237765	IPPK
83744	ENSG00000127081	140.937703	-0.30424337	0.188952	0.06527801	0.18230796	ZNF484
4958	ENSG00000127083	0.99480918	0.02209923	0.38284268	0.6506095	NA	OMD
89846	ENSG00000127084	27.2571144	0.32304112	0.36671536	0.1495052	0.33230822	FGD3
59269	ENSG00000127124	329.035206	-1.22726671	0.14000584	1.99E-19	7.25E-18	HIVEP3
79717	ENSG00000127125	283.523135	-0.63458092	0.14802641	4.72E-06	4.79E-05	PPCS
1907	ENSG00000127129	1.44450725	0.01025023	0.37826463	0.88335199	NA	EDN2
64919	ENSG00000127152	0	NA	NA	NA	NA	BCL11B
1350	ENSG00000127184	2625.95467	-0.14068664	0.06303968	0.02335012	0.08247576	COX7C
7186	ENSG00000127191	503.787105	-0.20917822	0.11285194	0.05166899	0.15279665	TRAF2
79575	ENSG00000127220	616.881533	0.08482844	0.09806438	0.37139083	0.60057374	ABHD8
5648	ENSG00000127241	0	NA	NA	NA	NA	MASP1
84239	ENSG00000127249	10.0404169	0.50077101	0.98497124	0.00722046	0.03170737	ATP13A4
57110	ENSG00000127252	31.2252004	-0.0325132	0.29494496	0.86259232	0.93899493	PLAAT1
92797	ENSG00000127311	67.0842107	0.41526849	0.2830938	0.0543402	0.15863564	HELB
5908	ENSG00000127314	1204.52822	-0.12684166	0.08222827	0.1137319	0.27377941	RAP1B
50616	ENSG00000127318	0	NA	NA	NA	NA	IL22

7103	ENSG00000127324	4.7073598	0.05454146	0.36877643	0.64727712	0.82430661	TSPAN8
144453	ENSG00000127325	0.173124	-0.01911262	0.38486262	0.84537521	NA	BEST3
117177	ENSG00000127328	482.830127	-0.32383896	0.15325384	0.02085988	0.07535879	RAB3IP
5787	ENSG00000127329	31.049319	2.58175763	0.50422946	1.87E-08	2.73E-07	PTPRB
8445	ENSG00000127334	2311.7898	0.21845641	0.07146456	0.00183782	0.00994882	DYRK2
8089	ENSG00000127337	405.310293	-0.10277516	0.1669662	0.49195216	0.7110834	YEATS4
50831	ENSG00000127362	9.21392174	-0.03007097	0.34461762	0.84489979	0.92965052	TAS2R3
50832	ENSG00000127364	94.7685875	-0.04781038	0.20794863	0.78350783	0.90146111	TAS2R4
54429	ENSG00000127366	30.9821222	-0.04856459	0.28463395	0.79942302	0.90803676	TAS2R5
155051	ENSG00000127377	0.70027077	0.00203698	0.38203102	0.85183428	NA	CRYGN
65999	ENSG00000127399	559.676285	0.50138762	0.11274185	3.40E-06	3.51E-05	LRRC61
56302	ENSG00000127412	0.32112006	-0.00294009	0.38339209	0.99470998	NA	TRPV5
3425	ENSG00000127415	215.232277	0.81144065	0.21289397	2.26E-05	0.00020133	IDUA
53834	ENSG00000127418	17.3403769	3.11420161	0.71085536	8.72E-07	9.99E-06	FGFRL1
84286	ENSG00000127419	496.309024	0.05413504	0.11434185	0.62073945	0.80656694	TMEM175
79000	ENSG00000127423	101.878052	-0.42648506	0.24673265	0.03301139	0.10818399	AUNIP
5300	ENSG00000127445	1358.18184	-0.10166166	0.08188151	0.20294689	0.40886131	PIN1
54850	ENSG00000127452	484.157288	0.40044537	0.11823163	0.00036403	0.00243422	FBXL12
23065	ENSG00000127463	1451.31318	-0.30569787	0.07310257	1.97E-05	0.00017724	EMC1
5322	ENSG00000127472	0	NA	NA	NA	NA	PLA2G5
23352	ENSG00000127481	8047.02624	0.22001727	0.05474479	4.84E-05	0.00039787	UBR4
50809	ENSG00000127483	4864.08573	-0.0051865	0.06468017	0.93407174	0.97250122	HP1BP3
30817	ENSG00000127507	5.63941949	0.03154468	0.35752549	0.81842791	0.9171248	ADGRE2
23309	ENSG00000127511	1708.15326	0.2142061	0.09407545	0.01862008	0.0687042	SIN3B
390892	ENSG00000127515	0	NA	NA	NA	NA	OR7A10
79939	ENSG00000127526	1214.48917	-0.06082334	0.11168854	0.56788646	0.76918607	SLC35E1
58513	ENSG00000127527	794.534741	0.02085737	0.10228542	0.83362187	0.92422788	EPS15L1
10365	ENSG00000127528	0	NA	NA	NA	NA	KLF2
26658	ENSG00000127529	0	NA	NA	NA	NA	OR7C2
26664	ENSG00000127530	0.39935548	-0.01092807	0.38376568	0.92097753	NA	OR7C1
9002	ENSG00000127533	2.54387008	-0.08257224	0.38562861	0.37317167	NA	F2RL3
10975	ENSG00000127540	1806.19343	-0.03064821	0.06981881	0.65481265	0.82814932	UQCR11
2671	ENSG00000127554	564.581488	0.06976037	0.11414205	0.52264419	0.73587826	GFER
9143	ENSG00000127561	249.577372	-0.47768319	0.1771738	0.00267755	0.01375518	SYNGR3
9088	ENSG00000127564	1033.71224	-0.18284583	0.08759053	0.03178856	0.10503632	PKMYT1
117166	ENSG00000127578	26.9948854	-0.00433677	0.31265554	0.97877053	0.99278156	WFIKKN1
84219	ENSG00000127580	411.859849	0.12526966	0.11856667	0.26760504	0.49015612	WDR24
146330	ENSG00000127585	1456.89159	-0.25904418	0.08318675	0.0013895	0.00785178	FBXL16

63922	ENSG00000127586	1301.4162	0.31720069	0.09386803	0.00047708	0.0030963	CHTF18
51764	ENSG00000127588	5.67775627	-0.1088758	0.37587297	0.4288738	0.65462623	GNG13
NA	ENSG00000127589	1.67866563	0.04002483	0.37799829	0.63856675	NA	TUBBP1
23499	ENSG00000127603	6714.41138	-0.12846558	0.05241652	0.01334793	0.05255466	MACF1
6597	ENSG00000127616	10345.2533	0.08393891	0.05626683	0.13248879	0.30568409	SMARCA4
23030	ENSG00000127663	1555.38825	-0.11738326	0.08061635	0.13569502	0.31033882	KDM4B
148022	ENSG00000127666	375.88941	0.22897009	0.12390404	0.05033907	0.14971174	TICAM1
84190	ENSG00000127720	40.5521515	0.33692951	0.33704655	0.13137981	0.30404473	METTL25
27190	ENSG00000127743	0.32819249	-0.02330795	0.38532666	0.66610365	NA	IL17B
83460	ENSG00000127774	420.434391	-0.00236974	0.11698177	0.98209237	0.99406728	EMC6
8388	ENSG00000127780	0	NA	NA	NA	NA	OR1E2
79066	ENSG00000127804	764.902115	-0.07197768	0.12101192	0.53099194	0.74257279	METTL16
7277	ENSG00000127824	114.618478	-1.78645107	0.28078471	1.32E-11	2.79E-10	TUBA4A
7429	ENSG00000127831	1.54309679	0.09696304	0.39791336	0.14281108	NA	VIL1
14	ENSG00000127837	1718.54356	-0.0929326	0.08638151	0.26871787	0.49164169	AAMP
25953	ENSG00000127838	335.135951	-0.02178182	0.13720101	0.86628077	0.94103211	PNKD
55504	ENSG00000127863	65.826058	-1.381296	0.31626802	1.01E-06	1.14E-05	TNFRSF19
6049	ENSG00000127870	344.400953	0.00083509	0.12977359	0.99548097	0.99852582	RNF6
1892	ENSG00000127884	1078.35124	0.12885748	0.08765379	0.13109172	0.30355506	ECHS1
90485	ENSG00000127903	151.606199	-0.31477763	0.19117152	0.05883601	0.16858119	ZNF835
10142	ENSG00000127914	4007.77002	-0.06311227	0.06273454	0.30741356	0.53486671	AKAP9
2791	ENSG00000127920	1015.29956	0.99859026	0.09307686	1.05E-27	5.52E-26	GNG11
7979	ENSG00000127922	1541.89753	-0.15576193	0.08853917	0.07005326	0.19167792	SEM1
2792	ENSG00000127928	0.18922458	0.01954053	0.38488496	0.78225479	NA	GNGT1
3092	ENSG00000127946	2663.97196	-0.53159659	0.06484258	8.98E-17	2.74E-15	HIP1
5782	ENSG00000127947	1751.90521	-0.57631977	0.09882607	1.75E-09	2.93E-08	PTPN12
5447	ENSG00000127948	1175.83812	-0.03891909	0.08306377	0.63062248	0.81317757	POR
10875	ENSG00000127951	1.90436144	0.1018786	0.39747466	0.19055868	NA	FGL2
51657	ENSG00000127952	427.807057	0.01447254	0.12427493	0.90302379	0.95751119	STYXL1
79689	ENSG00000127954	9.39448695	-0.08896486	0.35272056	0.57740871	0.77582208	STEAP4
2770	ENSG00000127955	843.838425	-0.1690507	0.0963711	0.06934697	0.19013872	GNAI1
5387	ENSG00000127957	234.290973	0.25090955	0.17715482	0.11069665	0.26835552	PMS2P3
5189	ENSG00000127980	1215.39357	0.09680146	0.09397341	0.28824468	0.5139923	PEX1
7978	ENSG00000127989	366.041093	-0.19973147	0.15175248	0.15050339	0.33407003	MTERF1
8910	ENSG00000127990	918.195391	0.07411376	0.117987	0.50987426	0.72545712	SGCE
84060	ENSG00000127993	413.541853	0.10903583	0.12674264	0.36231005	0.59197696	RBM48
64921	ENSG00000127995	1793.70137	-0.03637368	0.07291489	0.6103089	0.7985846	CASD1
163131	ENSG00000128000	855.961886	-0.20159633	0.1207683	0.07770509	0.20696321	ZNF780B

57622	ENSG00000128011	550.820133	0.01168385	0.10411212	0.90820425	0.96007952	LRFN1
7538	ENSG00000128016	84.169258	0.47726581	0.24616008	0.01815837	0.06731907	ZFP36
79644	ENSG00000128039	277.548926	-0.05785641	0.14545017	0.66527811	0.83470042	SRD5A3
6691	ENSG00000128040	6.79436239	-0.17531088	0.40755419	0.2223711	0.43535977	SPINK2
65997	ENSG00000128045	1460.66895	0.11117974	0.07727424	0.14197328	0.32055876	RASL11B
10606	ENSG00000128050	4591.0648	-0.33689493	0.0826389	2.85E-05	0.00024718	PAICS
3791	ENSG00000128052	3.23404702	-0.10774035	0.39135893	0.29203943	0.51811341	KDR
5471	ENSG00000128059	1158.96101	-0.29053753	0.09779723	0.00203551	0.01086189	PPAT
85378	ENSG00000128159	1599.92767	0.20094095	0.07069685	0.00379916	0.01846719	TUBGCP6
79924	ENSG00000128165	304.202371	1.30881077	0.58845622	0.00148635	0.0083162	ADM2
85359	ENSG00000128185	599.561724	-0.03578251	0.10098608	0.71046383	0.86099206	DGCR6L
54487	ENSG00000128191	1293.39785	0.00731681	0.07441445	0.92073768	0.96670947	DGCR8
57168	ENSG00000128203	215.668128	-0.1817416	0.15328456	0.19431974	0.39729014	ASPHD2
29802	ENSG00000128218	2.3162771	0.0100306	0.37449946	0.91025463	NA	VPREB3
23753	ENSG00000128228	612.252769	0.65331417	0.11959546	1.22E-08	1.84E-07	SDF2L1
9514	ENSG00000128242	28.6918141	-1.09487994	0.47755823	0.00170945	0.0093767	GAL3ST1
7533	ENSG00000128245	1973.97282	-0.43958141	0.06507717	6.85E-12	1.48E-10	YWHAH
5988	ENSG00000128250	0.94612289	0.04084733	0.38743659	0.29648824	NA	RFPL1
10739	ENSG00000128253	3.53486295	0.08316779	0.38031072	0.44940755	0.672975	RFPL2
NA	ENSG00000128254	15.5918957	0.04222092	0.32819205	0.80960828	0.91359357	C22orf24
29774	ENSG00000128262	73.2592246	2.23894044	0.32176848	2.46E-13	5.81E-12	POM121L9P
2781	ENSG00000128266	913.196898	-0.37595308	0.10433897	0.00017548	0.00126278	GNAZ
4248	ENSG00000128268	546.47479	-0.50389698	0.13269849	5.51E-05	0.00044492	MGAT3
135	ENSG00000128271	841.118169	1.49972041	0.10622241	2.41E-46	2.17E-44	ADORA2A
468	ENSG00000128272	5371.33571	0.45993973	0.22103311	0.01396245	0.05446535	ATF4
53947	ENSG00000128274	0	NA	NA	NA	NA	A4GALT
10738	ENSG00000128276	0	NA	NA	NA	NA	RFPL3
11135	ENSG00000128283	163.740933	0.45547716	0.2234138	0.01588001	0.06038154	CDC42EP1
80833	ENSG00000128284	5.94936346	3.49013628	2.02886205	0.00207986	0.01105395	APOL3
2847	ENSG00000128285	0	NA	NA	NA	NA	MCHR1
8459	ENSG00000128294	257.060136	0.14182712	0.16637761	0.34430098	0.57465563	TPST2
80115	ENSG00000128298	59.4439203	0.42137107	0.29420919	0.05572862	0.16188226	BAIAP2L2
4357	ENSG00000128309	2664.5767	0.06298999	0.07037183	0.36318792	0.59291108	MPST
8484	ENSG00000128310	0.31775907	0.00054329	0.38336346	0.97186541	NA	GALR3
7263	ENSG00000128311	243.459064	0.12016446	0.15381632	0.39400993	0.62317897	TST
80831	ENSG00000128313	0.15069912	-0.01848503	0.38480575	0.84537521	NA	APOL5
3543	ENSG00000128322	0	NA	NA	NA	NA	IGLL1
23780	ENSG00000128335	178.638631	0.02350184	0.17081182	0.87906636	0.94641763	APOL2

5880	ENSG00000128340	23.8950371	-1.84606025	0.58606939	8.98E-05	0.00069156	RAC2
3976	ENSG00000128342	53.2289888	0.09205915	0.2588269	0.63511407	0.8163695	LIF
84645	ENSG00000128346	7.0434226	0.01426718	0.35451074	0.92038605	0.96646868	C22orf23
200315	ENSG00000128383	0	NA	NA	NA	NA	APOBEC3A
100913187	ENSG00000128383	0	NA	NA	NA	NA	APOBEC3A
200316	ENSG00000128394	34.3167508	0.15639655	0.29424662	0.43515159	0.66040284	APOBEC3F
26150	ENSG00000128408	0.18922458	0.01954053	0.38488496	0.78225479	NA	RIBC2
3872	ENSG00000128422	0.15069912	-0.01848503	0.38480575	0.84537521	NA	KRT17
NA	ENSG00000128438	0	NA	NA	NA	NA	TBC1D27P
51234	ENSG00000128463	1549.59332	0.17375751	0.08140047	0.02882566	0.09722903	EMC4
7732	ENSG00000128482	468.071815	-0.25628956	0.12706515	0.03180522	0.10504671	RNF112
92521	ENSG00000128487	348.285102	-0.20084097	0.13007283	0.09942699	0.24822342	SPECC1
51200	ENSG00000128510	0.14931947	-0.01844332	0.38480212	0.84537521	NA	CPA4
9732	ENSG00000128512	1230.86094	-0.3831536	0.10521636	0.0001463	0.00107182	DOCK4
25913	ENSG00000128513	732.148409	-0.2051206	0.14747459	0.13056308	0.30279036	POT1
50833	ENSG00000128519	0	NA	NA	NA	NA	TAS2R16
9296	ENSG00000128524	1394.63153	-0.09400889	0.07585915	0.20564996	0.41264935	ATP6V1F
51691	ENSG00000128534	1636.59805	0.32492145	0.10321139	0.00104718	0.00614315	LSM8
222256	ENSG00000128536	200.270565	0.59669407	0.20860961	0.00111778	0.00649722	CDHR3
79706	ENSG00000128563	1322.32002	0.30376429	0.08101926	0.00012169	0.00090576	PRKRIP1
7425	ENSG00000128564	8353.61222	-2.37288604	0.07074854	7.38E-248	2.93E-244	VGF
5420	ENSG00000128567	2054.4006	-0.49548516	0.07204926	2.50E-12	5.55E-11	PODXL
93986	ENSG00000128573	0	NA	NA	NA	NA	FOXP2
57464	ENSG00000128578	121.550624	-0.47473933	0.28598923	0.02975984	0.09971884	STRIP2
64792	ENSG00000128581	657.217883	0.09509767	0.10080489	0.32831657	0.55683028	IFT22
4289	ENSG00000128585	2267.27172	-0.21025166	0.09481467	0.02175868	0.07792279	MKLN1
4189	ENSG00000128590	440.549245	0.20445599	0.11456023	0.06096447	0.17355935	DNAJB9
2318	ENSG00000128591	1336.30023	1.20827135	0.08345654	1.83E-48	1.71E-46	FLNC
64101	ENSG00000128594	594.963161	-1.8021641	0.12626648	2.42E-47	2.22E-45	LRRC4
813	ENSG00000128595	4062.28185	-0.20302974	0.0715613	0.00380956	0.01848412	CALU
64753	ENSG00000128596	1612.6278	-0.10242717	0.07497554	0.16317492	0.35215821	CCDC136
6608	ENSG00000128602	1955.68639	0.92325746	0.07070479	9.21E-40	7.08E-38	SMO
3663	ENSG00000128604	52.4314596	0.15992155	0.29062107	0.42436991	0.65010417	IRF5
10234	ENSG00000128606	17.1974746	0.41348375	0.50365881	0.0802994	0.2118819	LRRC17
23008	ENSG00000128607	1176.4456	0.02475263	0.09237708	0.78319553	0.90125859	KLHDC10
4698	ENSG00000128609	1293.09977	-0.35389862	0.07819716	3.63E-06	3.73E-05	NDUFA5
389549	ENSG00000128610	0	NA	NA	NA	NA	FEZF1
611	ENSG00000128617	2.80006029	0.08385933	0.38552359	0.37913273	0.6081327	OPN1SW

6183	ENSG00000128626	419.595665	-0.16396064	0.11407151	0.13058941	0.30281602	MRPS12
4430	ENSG00000128641	4062.77879	-0.23373629	0.0631143	0.00016826	0.00121614	MYO1B
3231	ENSG00000128645	20.6497146	0.96642897	0.5937044	0.00762048	0.0331485	HOXD1
3232	ENSG00000128652	494.130279	-0.1105511	0.10766478	0.28387134	0.50903899	HOXD3
10651	ENSG00000128654	959.040636	-0.12394709	0.08976717	0.15481456	0.34030142	MTX2
50940	ENSG00000128655	70.6262444	0.33974983	0.29966419	0.11612882	0.27839903	PDE11A
1123	ENSG00000128656	1320.87575	0.16808528	0.08218678	0.03609784	0.11607474	CHN1
2571	ENSG00000128683	2.0300085	-0.03540306	0.38000666	0.62346039	NA	GAD1
NA	ENSG00000128692	0.99185888	0.03322072	0.38235116	0.58147144	NA	EIF2S2P4
64172	ENSG00000128694	269.088136	-0.13285755	0.15287577	0.34219063	0.57224058	OSGEPL1
94101	ENSG00000128699	790.969435	-0.11765762	0.10948549	0.26095676	0.48276874	ORMDL1
8520	ENSG00000128708	772.076279	-0.09721847	0.11447757	0.3726527	0.60153198	HAT1
3235	ENSG00000128709	290.693056	-0.13994875	0.15075956	0.31124171	0.53888083	HOXD9
3236	ENSG00000128710	20.8463829	0.28435332	0.37873138	0.18660756	0.38635899	HOXD10
3237	ENSG00000128713	8.32933386	0.27460476	0.46901014	0.11218566	0.27107099	HOXD11
3239	ENSG00000128714	42.8218363	2.97002638	0.43215765	4.53E-13	1.06E-11	HOXD13
8924	ENSG00000128731	3780.2272	-0.06060219	0.08676272	0.47185208	0.69421175	HERC2
6638	ENSG00000128739	1873.37677	-0.22134069	0.07535599	0.0026902	0.01380587	SNRPN
56984	ENSG00000128789	417.999246	-0.07321154	0.12037621	0.52070281	0.7341869	PSMG2
57045	ENSG00000128791	402.985173	0.51041422	0.12047527	8.62E-06	8.28E-05	TWSG1
58504	ENSG00000128805	110.189759	0.00629623	0.20717172	0.97274508	0.98990882	ARHGAP22
57705	ENSG00000128815	2.9398666	0.00865473	0.3778817	0.93607339	0.97330832	WDFY4
440275	ENSG00000128829	1324.45008	0.09729699	0.07838926	0.20496608	0.41184191	EIF2AK4
55930	ENSG00000128833	411.789839	0.60991307	0.13258582	1.21E-06	1.36E-05	MYO5C
84952	ENSG00000128849	381.842659	0.23415203	0.11992783	0.03953389	0.12436219	CGNL1
29767	ENSG00000128872	1407.08955	-1.19474135	0.0835952	2.60E-47	2.38E-45	TMOD2
146057	ENSG00000128881	1147.01365	-0.13955592	0.10081545	0.15031331	0.33373053	TTBK2
80237	ENSG00000128886	13.3121281	0.08417503	0.34030595	0.62390921	0.80825065	ELL3
90416	ENSG00000128891	297.30599	-0.17641517	0.18969603	0.28259487	0.50762175	CCDC32
54617	ENSG00000128908	808.310192	-0.03952445	0.10975359	0.70606689	0.85825065	INO80
79664	ENSG00000128915	1258.19412	0.07151907	0.12245671	0.53827952	0.74807128	ICE2
54567	ENSG00000128917	2.19397701	0.051928	0.3774275	0.58379402	NA	DLL4
8854	ENSG00000128918	26.6030683	1.72586216	0.66004361	0.00038099	0.0025365	ALDH1A2
54629	ENSG00000128923	493.919212	0.14965646	0.11247697	0.16368348	0.35275692	MINDY2
3712	ENSG00000128928	1206.10077	-0.12831461	0.0975483	0.17295267	0.36578929	IVD
90417	ENSG00000128944	1089.58764	-0.11112608	0.09083543	0.20725643	0.41447107	KNSTRN
1854	ENSG00000128951	2418.6224	-0.46457263	0.07677574	6.36E-10	1.13E-08	DUT
79094	ENSG00000128965	495.041616	0.36774051	0.51291038	0.08748695	0.22591971	CHAC1

54982	ENSG00000128973	830.463229	-0.1988795	0.09482616	0.02961764	0.09932622	CLN6
10776	ENSG00000128989	4726.25565	-0.04680784	0.07099351	0.50157542	0.71850227	ARPP19
54832	ENSG00000129003	2402.8753	-0.42032586	0.06984581	8.73E-10	1.52E-08	VPS13C
91860	ENSG00000129007	78.6998659	0.20881214	0.22963839	0.26389151	0.4860837	CALML4
3671	ENSG00000129009	185.229672	-0.8737119	0.21305407	5.86E-06	5.83E-05	ISLR
56906	ENSG00000129028	207.625098	-0.26281457	0.17497825	0.09133532	0.23324658	THAP10
4016	ENSG00000129038	3.19668163	-0.0101024	0.37131323	0.91661836	0.96470788	LOXL1
51554	ENSG00000129048	5.5126972	0.06850852	0.3665477	0.60376954	0.79398076	ACKR4
25847	ENSG00000129055	635.384833	-0.14503684	0.10087581	0.13544688	0.30998745	ANAPC13
8930	ENSG00000129071	466.699189	-0.0710835	0.13466464	0.57173682	0.77185678	MBD4
1315	ENSG00000129083	2195.64349	0.00087625	0.07849071	0.99125063	0.9971497	COPB1
5682	ENSG00000129084	1516.55043	-0.02914168	0.08113897	0.71159941	0.86140448	PSMA1
25870	ENSG00000129103	1822.72861	0.0106935	0.07744118	0.88877031	0.95146564	SUMF2
23022	ENSG00000129116	1663.53611	0.71311192	0.09031787	6.72E-16	1.93E-14	PALLD
60559	ENSG00000129128	1763.12447	0.62468557	0.08485178	5.21E-14	1.31E-12	SPCS3
8424	ENSG00000129151	0	NA	NA	NA	NA	BBOX1
4654	ENSG00000129152	0.15069912	-0.01848503	0.38480575	0.84537521	NA	MYOD1
26297	ENSG00000129158	146.369489	-0.00068998	0.22026542	0.99533071	0.99852582	SERGEF
3746	ENSG00000129159	582.971214	-1.77992018	0.18214438	7.75E-24	3.53E-22	KCNC1
7166	ENSG00000129167	13.9582171	-0.05477161	0.32929621	0.75227848	0.88403749	TPH1
8048	ENSG00000129170	0	NA	NA	NA	NA	CSRP3
79733	ENSG00000129173	287.532554	-0.39274927	0.14494496	0.00340939	0.0168738	E2F8
1635	ENSG00000129187	861.015921	-0.01304427	0.0889756	0.87955496	0.94660386	DCTD
6665	ENSG00000129194	22.6469198	0.32615316	0.41483593	0.14000503	0.3173426	SOX15
54478	ENSG00000129195	1102.48467	0.02341469	0.09078389	0.79150487	0.90499209	PIMREG
84268	ENSG00000129197	1729.24454	0.1875915	0.06754303	0.00475135	0.02225371	RPAIN
9098	ENSG00000129204	12.6900417	-0.46790336	0.62828802	0.05106547	0.15148609	USP6
6462	ENSG00000129214	2.89629976	-0.1091912	0.39272404	0.27949609	0.50401767	SHBG
5338	ENSG00000129219	456.516564	0.1326135	0.11593718	0.23002258	0.44593003	PLD2
23746	ENSG00000129221	0.173124	-0.01911262	0.38486262	0.84537521	NA	AIPL1
968	ENSG00000129226	2.92510069	0.07505733	0.38025096	0.46827439	0.69115934	CD68
84817	ENSG00000129235	632.092603	-0.10629054	0.10337447	0.28492534	0.51028332	TXNDC17
482	ENSG00000129244	111.767364	0.1841466	0.19721352	0.27830585	0.50270347	ATP1B2
9513	ENSG00000129245	1525.83921	0.16731531	0.07650858	0.02552815	0.08857615	FXR2
10749	ENSG00000129250	647.414179	1.06908376	0.18745288	1.32E-09	2.24E-08	KIF1C
9526	ENSG00000129255	873.84351	0.0382071	0.08519622	0.65073195	0.8254613	MPDU1
51105	ENSG00000129292	1537.43311	0.05488046	0.07630082	0.46338422	0.68623892	PHF20L1
23639	ENSG00000129295	35.9084375	0.49458821	0.39236468	0.04969038	0.14833843	LRRC6

904	ENSG00000129315	1570.40424	0.11170371	0.09067608	0.20531055	0.41224389	CCNT1
83448	ENSG00000129317	634.016424	-0.52967797	0.12110541	4.29E-06	4.37E-05	PUS7L
65095	ENSG00000129347	979.531341	0.12737055	0.08560026	0.126802	0.29662228	KRI1
3609	ENSG00000129351	18488.2578	-0.0619924	0.0499976	0.2107875	0.41967125	ILF3
57153	ENSG00000129353	1324.13962	0.2785292	0.08194781	0.00049136	0.00317751	SLC44A2
10053	ENSG00000129354	0.173733	0.01914545	0.38484597	0.78225479	NA	AP1M2
1032	ENSG00000129355	196.402857	-0.41899212	0.17337134	0.00713891	0.03143974	CDKN2D
57509	ENSG00000129422	430.253659	-2.80158418	0.21196211	2.51E-41	1.95E-39	MTUS1
43847	ENSG00000129437	2.93585794	0.15933053	0.42483867	0.07600293	0.20344011	KLK14
27180	ENSG00000129450	0	NA	NA	NA	NA	SIGLEC9
5655	ENSG00000129451	0.14931947	-0.01844332	0.38480212	0.84537521	NA	KLK10
11202	ENSG00000129455	0	NA	NA	NA	NA	KLK8
25983	ENSG00000129460	596.524513	0.19321811	0.11260111	0.07206589	0.19555978	NGDN
11035	ENSG00000129465	0.37844916	0.02407552	0.3854191	0.60038877	NA	RIPK3
196883	ENSG00000129467	3.95266142	0.15932285	0.41332864	0.163683	0.35275692	ADCY4
84932	ENSG00000129472	672.520509	-0.0259638	0.12448933	0.82500268	0.92054353	RAB2B
599	ENSG00000129473	855.652264	-0.08946845	0.08958515	0.30369137	0.53104502	BCL2L2
84962	ENSG00000129474	327.324767	1.44272899	0.17565626	1.63E-17	5.22E-16	AJUBA
112487	ENSG00000129480	242.707733	0.03349175	0.14733221	0.80659409	0.91241386	DTD2
10038	ENSG00000129484	606.64096	0.05524543	0.10838667	0.5958899	0.78826859	PARP2
25938	ENSG00000129493	501.350934	0.39845718	0.35156928	0.08707871	0.22523253	HEATR5A
3169	ENSG00000129514	0	NA	NA	NA	NA	FOXA1
58533	ENSG00000129515	1239.14503	-0.19669513	0.09450459	0.03133516	0.10378792	SNX6
55837	ENSG00000129518	296.194122	-0.07569149	0.1311151	0.53812339	0.7480639	EAPP
112399	ENSG00000129521	54.4059118	-0.04865545	0.27749014	0.79861029	0.90773005	EGLN3
55320	ENSG00000129534	531.483754	-0.40530863	0.13238256	0.00108637	0.00633741	MIS18BP1
4901	ENSG00000129535	29.4961466	-0.06463893	0.29313217	0.73462654	0.87374168	NRL
6035	ENSG00000129538	0	NA	NA	NA	NA	RNASE1
4738	ENSG00000129559	2140.25472	0.0130315	0.06776775	0.84601255	0.93016841	NEDD8
1603	ENSG00000129562	1194.73698	0.18590174	0.08279559	0.02131698	0.07666221	DAD1
7011	ENSG00000129566	489.154691	0.63097789	0.16555213	3.57E-05	0.00030326	TEP1
64097	ENSG00000129595	82.5745967	1.017115	0.40495907	0.00102231	0.00602039	EPB41L4A
1036	ENSG00000129596	147.501343	-0.44237529	0.22075393	0.01756145	0.06560027	CDO1
7905	ENSG00000129625	762.348174	-0.05189734	0.1002078	0.59082326	0.78554834	REEP5
81533	ENSG00000129636	789.946235	-0.36855015	0.09432339	5.33E-05	0.00043214	ITFG1
84074	ENSG00000129646	197.487532	0.65809931	0.1739943	3.76E-05	0.00031779	QRICH2
2302	ENSG00000129654	5.24381335	0.14653226	0.39843869	0.26155918	0.4835377	FOXJ1
6397	ENSG00000129657	2776.88861	-0.08278759	0.06574271	0.20100227	0.40615916	SEC14L1

79651	ENSG00000129667	449.003207	-0.23127865	0.13545373	0.06678716	0.18529677	RHBDF2
15	ENSG00000129673	2.3986071	-0.0640059	0.38134128	0.47713288	NA	AANAT
9459	ENSG00000129675	255.368648	0.96617203	0.18226736	1.58E-08	2.32E-07	ARHGEF6
79649	ENSG00000129680	416.781593	0.43366926	0.13171717	0.00046073	0.00300783	MAP7D3
2258	ENSG00000129682	3.68571695	0.07129875	0.37694014	0.51513836	0.72960772	FGF13
9070	ENSG00000129691	740.014684	-0.11608084	0.10026838	0.22920834	0.44474264	ASH2L
80185	ENSG00000129696	298.604864	0.14030601	0.15224746	0.31547395	0.54331625	TTI2
417	ENSG00000129744	0.173124	-0.01911262	0.38486262	0.84537521	NA	ART1
57053	ENSG00000129749	32.8968237	-0.20342475	0.30459439	0.32091768	0.54869068	CHRNA10
1028	ENSG00000129757	854.449049	0.55412838	0.09982257	9.74E-09	1.47E-07	CDKN1C
151648	ENSG00000129810	380.542201	-0.40911599	0.13249853	0.00099312	0.00587207	SGO1
100101116	ENSG00000129816	0	NA	NA	NA	NA	TTTY1B
6192	ENSG00000129824	0	NA	NA	NA	NA	RPS4Y1
353513	ENSG00000129862	0	NA	NA	NA	NA	VCY1B
9084	ENSG00000129864	0	NA	NA	NA	NA	VCY
203611	ENSG00000129873	0	NA	NA	NA	NA	CDY2B
1013	ENSG00000129910	8.9664042	0.05526419	0.34717641	0.7269549	0.86953012	CDH15
83855	ENSG00000129911	645.94772	-0.00833595	0.11783349	0.93995266	0.97488948	KLF16
58986	ENSG00000129925	989.072157	-0.15397652	0.1005124	0.11146437	0.2697881	PGAP6
83475	ENSG00000129932	322.711775	-0.11506497	0.13064669	0.34779894	0.57854707	DOHH
23383	ENSG00000129933	2255.6835	0.24130367	0.07057536	0.00049864	0.00321621	MAU2
25759	ENSG00000129946	756.252438	-0.06995095	0.09364786	0.44008043	0.66517856	SHC2
79948	ENSG00000129951	994.016917	0.60392448	0.10299924	1.36E-09	2.30E-08	PLPPR3
723961	ENSG00000129965	0	NA	NA	NA	NA	INS-IGF2
81926	ENSG00000129968	2144.40251	0.11954082	0.06912497	0.07873028	0.20910425	ABHD17A
3929	ENSG00000129988	3.24199459	-0.05937409	0.37538658	0.57308914	0.7727401	LBP
6861	ENSG00000129990	115.501083	-1.06404506	0.23902172	9.44E-07	1.07E-05	SYT5
7137	ENSG00000129991	61.7410611	-0.86644032	0.30951623	0.00067642	0.00419604	TNNI3
863	ENSG00000129993	2.00176869	0.10034808	0.3965826	0.18626772	NA	CBFA2T3
2593	ENSG00000130005	806.38658	0.04998863	0.08945056	0.56614644	0.76789354	GAMT
8226	ENSG00000130021	249.536851	-0.05850011	0.15564451	0.67901123	0.84320727	PUDP
55780	ENSG00000130023	312.417729	0.18480162	0.16975762	0.22497221	0.43877008	ERMARD
55274	ENSG00000130024	1646.70226	0.10115255	0.08726564	0.23381392	0.45077791	PHF10
79057	ENSG00000130032	0	NA	NA	NA	NA	PRRG3
26290	ENSG00000130035	0.87316756	0.03844629	0.38477768	0.44621224	NA	GALNT8
3741	ENSG00000130037	0	NA	NA	NA	NA	KCNA5
84766	ENSG00000130038	158.159075	-1.1505171	0.23795502	1.27E-07	1.65E-06	CRACR2A
158046	ENSG00000130045	0.84471752	-0.00134595	0.38065581	0.98151753	NA	NXNL2

9754	ENSG00000130052	26.5263011	2.04228546	0.57064462	1.85E-05	0.00016703	STARD8
27112	ENSG00000130054	172.647789	-2.40311804	0.20260617	1.34E-33	8.57E-32	FAM155B
54857	ENSG00000130055	2.62831827	0.02983857	0.37421123	0.7525931	0.88425267	GDPD2
6303	ENSG00000130066	100.997754	0.92874425	0.24467462	1.96E-05	0.00017632	SAT1
54552	ENSG00000130119	1084.97481	-0.42879581	0.11330274	7.28E-05	0.00057491	GNL3L
23677	ENSG00000130147	701.390283	-0.26683197	0.10441304	0.00771796	0.03347713	SH3BP4
158747	ENSG00000130150	245.844996	0.1282917	0.14419426	0.33708369	0.56738274	MOSPD2
57572	ENSG00000130158	943.362357	0.81263426	0.12497306	1.44E-11	3.01E-10	DOCK6
51295	ENSG00000130159	869.273012	-0.1306907	0.10076159	0.17795132	0.37336655	ECSIT
3949	ENSG00000130164	1405.9045	-0.00660002	0.10665641	0.94680981	0.97815729	LDLR
84337	ENSG00000130165	944.905403	-0.06944037	0.08350648	0.39344664	0.62246703	ELOF1
26526	ENSG00000130167	0	NA	NA	NA	NA	TSPAN16
55908	ENSG00000130173	0.173733	0.01914545	0.38484597	0.78225479	NA	ANGPTL8
5589	ENSG00000130175	6659.15882	0.47122232	0.05491886	4.28E-18	1.44E-16	PRKCSH
1264	ENSG00000130176	6.31226382	-0.03509484	0.35803612	0.79212413	0.90499209	CNN1
8881	ENSG00000130177	1347.86102	0.18196244	0.07969651	0.01949575	0.0714584	CDC16
84891	ENSG00000130182	2.87409863	0.04953616	0.37570674	0.61163186	0.79965575	ZSCAN10
51337	ENSG00000130193	937.70229	-0.59199917	0.09612366	2.24E-10	4.15E-09	THEM6
5819	ENSG00000130202	73.0003452	2.09179494	0.31074786	9.58E-13	2.19E-11	NECTIN2
348	ENSG00000130203	109.393018	1.67594196	0.26203852	1.17E-11	2.49E-10	APOE
10452	ENSG00000130204	1428.75176	0.00856704	0.09201258	0.92436761	0.96852	TOMM40
341	ENSG00000130208	11.3133074	-0.02122711	0.33839197	0.89336438	0.95389529	APOC1
10912	ENSG00000130222	354.298149	1.07910153	0.13735721	4.89E-16	1.43E-14	GADD45G
57631	ENSG00000130224	448.217292	-0.09399368	0.12114559	0.41261614	0.64014381	LRCH2
1804	ENSG00000130226	251.776486	-0.96892734	0.16914121	1.34E-09	2.27E-08	DPP6
23039	ENSG00000130227	2930.41174	-0.29085555	0.09454743	0.00136493	0.00773058	XPO7
59272	ENSG00000130234	2.68881683	-0.06166396	0.3839297	0.4242875	0.65002811	ACE2
147965	ENSG00000130244	230.557255	-0.25062093	0.17561631	0.10785839	0.26339391	FAM98C
9667	ENSG00000130254	1825.39999	-0.11715213	0.07674884	0.11880807	0.28318716	SAFB2
25873	ENSG00000130255	8593.99103	-0.04710887	0.05874481	0.41652294	0.64309452	RPL36
148229	ENSG00000130270	71.872144	1.26393333	0.35128015	2.53E-05	0.00022207	ATP8B3
2657	ENSG00000130283	209.438067	-0.25910586	0.16586165	0.08254114	0.21637154	GDF1
1463	ENSG00000130287	533.979634	-2.52023294	0.14004483	1.21E-73	2.13E-71	NCAN
547	ENSG00000130294	12778.0325	-0.13651502	0.06415758	0.03022942	0.10096828	KIF1A
84705	ENSG00000130299	1110.97836	0.20512791	0.08678005	0.01512675	0.05806977	GTPBP3
83483	ENSG00000130300	0.18922458	0.01954053	0.38488496	0.78225479	NA	PLVAP
684	ENSG00000130303	23.3162049	0.04925309	0.30047807	0.79650192	0.90658744	BST2
376497	ENSG00000130304	221.540497	0.20254412	0.16784211	0.17966455	0.37624495	SLC27A1

55695	ENSG00000130305	1266.28035	0.07002809	0.07802579	0.35978014	0.58972554	NSUN5
83878	ENSG00000130307	2.72140812	0.02938956	0.37710053	0.70987369	0.860757	USHBP1
79709	ENSG00000130309	1391.22023	-0.23728672	0.07891688	0.00207614	0.01104305	COLGALT1
79016	ENSG00000130311	1367.04117	0.20624848	0.09635	0.02671537	0.0917012	DDA1
64981	ENSG00000130312	666.144438	0.06211009	0.1010783	0.5247915	0.73753159	MRPL34
25796	ENSG00000130313	876.844126	0.2654404	0.09459672	0.00371586	0.01815382	PGLS
51690	ENSG00000130332	1410.66988	0.08373137	0.07896788	0.27910825	0.50354712	LSM7
56995	ENSG00000130338	1431.21226	-0.4853112	0.07609806	7.33E-11	1.43E-09	TULP4
51429	ENSG00000130340	991.427415	0.37816861	0.108516	0.000273	0.00188069	SNX9
84816	ENSG00000130347	117.165414	0.19101632	0.21303223	0.28464813	0.50987892	RTN4IP1
55278	ENSG00000130348	406.400171	-0.04791134	0.12209467	0.67792991	0.84273994	QRSL1
51250	ENSG00000130349	216.55262	-0.17201919	0.15248257	0.217538	0.42910658	MTRES1
83861	ENSG00000130363	216.523542	0.2969164	0.18673546	0.0697384	0.19093923	RSPH3
4142	ENSG00000130368	0.3404686	-0.00018999	0.38336229	0.96705355	NA	MAS1
81616	ENSG00000130377	6.98792366	-0.05993475	0.37776879	0.52983032	0.74152324	ACSBG2
4298	ENSG00000130382	2796.20323	-0.1901706	0.06188445	0.00181907	0.00986616	MLLT1
2527	ENSG00000130383	3.70259504	0.16040904	0.42862625	0.05123602	0.15188997	FUT5
9210	ENSG00000130385	0.37844916	0.02407552	0.3854191	0.60038877	NA	BMP15
4301	ENSG00000130396	3309.53745	-0.01740808	0.06682396	0.78557582	0.90248778	AFDN
81	ENSG00000130402	3256.80739	0.3647484	0.0817146	4.74E-06	4.79E-05	ACTN4
65975	ENSG00000130413	89.533396	-0.39383608	0.30659482	0.07552673	0.20246162	STK33
4705	ENSG00000130414	1534.86492	0.05716178	0.09169355	0.52233627	0.73570578	NDUFA10
2056	ENSG00000130427	2.32318451	0.05666066	0.38047005	0.5194166	NA	EPO
10095	ENSG00000130429	691.565841	0.21734399	0.13309229	0.08100276	0.21345396	ARPC1B
59285	ENSG00000130433	0.51559444	0.03113559	0.38605479	0.4776066	NA	CACNG6
57688	ENSG00000130449	985.694427	0.2125248	0.0891293	0.01409178	0.05487356	ZSWIM6
23149	ENSG00000130475	216.462022	-0.24724442	0.16285133	0.0928688	0.23585726	FCHO1
23025	ENSG00000130477	2422.38778	-2.0726662	0.07826923	1.07E-155	1.06E-152	UNC13A
55201	ENSG00000130479	961.386854	-0.37516628	0.08994049	1.70E-05	0.00015521	MAP1S
113730	ENSG00000130487	1.46908626	0.07041286	0.38853882	0.30889259	NA	KLHDC7B
7837	ENSG00000130508	8842.33745	-0.03592214	0.05588776	0.51533949	0.72968723	PXDN
170463	ENSG00000130511	1290.92386	-0.21496063	0.07787706	0.00472147	0.0221399	SSBP4
9518	ENSG00000130513	177.74883	1.28677979	0.2092708	7.33E-11	1.43E-09	GDF15
54858	ENSG00000130517	666.025236	-0.24170119	0.11631106	0.02867259	0.09694344	PGPEP1
80726	ENSG00000130518	97.1407451	0.83519961	0.25252721	0.00014216	0.00104419	IQCN
25804	ENSG00000130520	3339.81815	-0.38098257	0.06495861	2.54E-09	4.12E-08	LSM4
3727	ENSG00000130522	2119.83296	0.53201333	0.07243943	7.71E-14	1.91E-12	JUND
3270	ENSG00000130528	1.22646606	-0.04062268	0.383066	0.52860218	NA	HRC

54795	ENSG00000130529	100.985189	1.19035172	0.25509425	2.94E-07	3.65E-06	TRPM4
81061	ENSG00000130538	0	NA	NA	NA	NA	OR11H1
25830	ENSG00000130540	227.85108	-0.63555107	0.15947454	1.73E-05	0.00015733	SULT4A1
79230	ENSG00000130544	262.242173	-0.12938608	0.15381743	0.35699214	0.58709582	ZNF557
92359	ENSG00000130545	1.15402791	0.00195627	0.37937999	0.97603885	NA	CRB3
10439	ENSG00000130558	825.650436	-1.53073771	0.10534272	6.69E-49	6.35E-47	OLFM1
157922	ENSG00000130559	2948.0177	-0.12646854	0.06729283	0.05609748	0.16266792	CAMSAP1
10422	ENSG00000130560	1141.56888	0.05556478	0.08057546	0.48073053	0.70158112	UBAC1
6295	ENSG00000130561	0	NA	NA	NA	NA	SAG
140685	ENSG00000130584	117.627181	0.46953451	0.21773159	0.01146241	0.04643563	ZBTB46
85441	ENSG00000130589	646.731884	0.40812578	0.14269672	0.00209828	0.01114589	HELZ2
140700	ENSG00000130590	281.487209	0.29935335	0.15643378	0.0358634	0.11552685	SAMD10
4046	ENSG00000130592	30.1553752	0.38284978	0.44707761	0.09714267	0.24380906	LSP1
7140	ENSG00000130595	1.37398733	0.06160358	0.38548417	0.38797179	NA	TNNT3
7136	ENSG00000130598	3.73780599	-0.13747442	0.40488075	0.19241838	0.39466357	TNNI2
283120	ENSG00000130600	79266.1821	0.73983328	0.20088395	4.51E-05	0.0003744	H19
102724852	ENSG00000130600	79266.1821	0.73983328	0.20088395	4.51E-05	0.0003744	H19
22952	ENSG00000130612	0.50366145	0.03047707	0.3859845	0.48834145	NA	CYP2G1P
1289	ENSG00000130635	75.8244717	0.09202072	0.24118364	0.62688181	0.81066736	COL5A1
25814	ENSG00000130638	2630.69175	-0.30539621	0.06871611	6.06E-06	6.00E-05	ATXN10
10844	ENSG00000130640	1884.93577	-0.00310172	0.06721759	0.96190361	0.98458276	TUBGCP2
50632	ENSG00000130643	175.223835	-1.25807966	0.18628886	1.44E-12	3.25E-11	CALY
1571	ENSG00000130649	1.45913899	0.02244385	0.37819378	0.76749814	NA	CYP2E1
375775	ENSG00000130653	76.2496153	0.31314892	0.28355064	0.13565766	0.31030495	PNPLA7
3050	ENSG00000130656	0.70129728	-0.01817036	0.38274402	0.68636655	NA	HBZ
10298	ENSG00000130669	1375.88907	0.14924802	0.0810577	0.05942691	0.16984009	PAK4
3110	ENSG00000130675	2.41247224	0.02032553	0.37457639	0.82136493	NA	MNX1
26152	ENSG00000130684	902.11159	0.11636634	0.08964257	0.18190446	0.37957585	ZNF337
64793	ENSG00000130695	989.755461	-0.14648691	0.09122336	0.09765784	0.24479236	CEP85
6874	ENSG00000130699	619.241225	-0.08231649	0.1051572	0.41252735	0.64014381	TAF4
140628	ENSG00000130700	0.9244433	-0.06525595	0.39134464	0.2113244	NA	GATA5
140893	ENSG00000130701	0	NA	NA	NA	NA	RBBP8NL
3911	ENSG00000130702	6243.94941	0.9096038	0.08133015	7.79E-30	4.49E-28	LAMA5
9885	ENSG00000130703	723.491032	-0.13652273	0.09902088	0.15264126	0.33723751	OSBPL2
11047	ENSG00000130706	1674.87752	0.0655781	0.09113269	0.45896991	0.68235016	ADRM1
445	ENSG00000130707	151.15029	1.12764175	0.22656972	7.02E-08	9.45E-07	ASS1
59335	ENSG00000130711	6.81283592	0.09076327	0.36634972	0.52606524	0.73808697	PRDM12
23404	ENSG00000130713	1049.08075	-0.18964715	0.0922758	0.03369466	0.11004519	EXOSC2

10585	ENSG00000130714	891.568554	0.33579565	0.1388871	0.00934352	0.03923288	POMT1
83549	ENSG00000130717	913.165586	-0.31125764	0.08427871	0.00014807	0.00108285	UCK1
84929	ENSG00000130720	0	NA	NA	NA	NA	FIBCD1
27243	ENSG00000130724	1697.63058	0.26980788	0.0695729	7.85E-05	0.00061348	CHMP2A
9040	ENSG00000130725	1621.98677	-0.01210373	0.08770365	0.8864681	0.95015905	UBE2M
10155	ENSG00000130726	8361.21022	-0.05258386	0.06175122	0.39078811	0.62011007	TRIM28
84326	ENSG00000130731	914.460145	0.17292464	0.08584	0.03857887	0.12221056	METTL26
78992	ENSG00000130733	592.062377	-0.11152689	0.09970231	0.24441583	0.46302016	YIPF2
84971	ENSG00000130734	608.451684	0.14866799	0.13085003	0.22646561	0.44088684	ATG4D
1968	ENSG00000130741	4716.26221	-0.04579747	0.05791354	0.42345589	0.64935576	EIF2S3
54958	ENSG00000130748	453.648128	-0.12270909	0.12312888	0.29149606	0.51742702	TMEM160
23211	ENSG00000130749	2123.86926	-0.12721597	0.07878709	0.10016065	0.24951784	ZC3H4
4861	ENSG00000130751	27.2845974	0.0408432	0.30303244	0.82889488	0.92244079	NPAS1
9535	ENSG00000130755	0.89984857	0.06462883	0.3912593	0.20169955	NA	GMFG
4294	ENSG00000130758	824.350284	0.36549276	0.10417638	0.00025829	0.00179112	MAP3K10
27237	ENSG00000130762	79.7984478	-0.19052271	0.24186133	0.31714784	0.54496909	ARHGEF16
57470	ENSG00000130764	1616.51025	-0.17041183	0.07549591	0.02109442	0.07603803	LRRC47
83667	ENSG00000130766	440.647231	0.89905316	0.18683255	2.22E-07	2.80E-06	SESN2
27293	ENSG00000130768	44.5866703	-0.11119932	0.26718161	0.56766273	0.76899226	SMPDL3B
93974	ENSG00000130770	1254.08739	-0.0745386	0.09438053	0.41438495	0.641645	ATP5IF1
54797	ENSG00000130772	155.736949	-0.3025448	0.17738157	0.05464445	0.15938634	MED18
9473	ENSG00000130775	113.823395	0.18996059	0.19733259	0.26343452	0.48542909	THEMIS2
6249	ENSG00000130779	747.21408	0.03203126	0.09762657	0.73525412	0.87418001	CLIP1
84660	ENSG00000130783	12.2106881	-0.02759061	0.33308731	0.86750931	0.94194356	CCDC62
9026	ENSG00000130787	1490.77562	0.11833315	0.09831824	0.21301458	0.42295929	HIP1R
57693	ENSG00000130803	813.120315	-0.09291929	0.09249722	0.30014129	0.52733399	ZNF317
56342	ENSG00000130810	1138.36296	0.06122324	0.08736793	0.47229137	0.69450495	PPAN
8666	ENSG00000130811	4201.16203	0.17594899	0.05887556	0.00246876	0.01282596	EIF3G
83854	ENSG00000130812	6.49097312	-0.26749405	0.48243703	0.08343851	0.21810542	ANGPTL6
55337	ENSG00000130813	200.595539	0.42144829	0.1921241	0.01249209	0.04980768	SHFL
1786	ENSG00000130816	5923.93995	-0.29310159	0.0717209	3.07E-05	0.00026337	DNMT1
79088	ENSG00000130818	393.753152	0.05174863	0.1409453	0.69398053	0.8520157	ZNF426
6535	ENSG00000130821	2068.0062	-0.25445424	0.07856682	0.00091145	0.00546812	SLC6A8
139728	ENSG00000130822	121.351667	-0.99081502	0.23398772	2.81E-06	2.94E-05	PNCK
1736	ENSG00000130826	1971.74544	-0.1181924	0.0721941	0.09518056	0.24026852	DKC1
55558	ENSG00000130827	1537.69719	-0.17752055	0.11428559	0.10221045	0.25341857	PLXNA3
1852	ENSG00000130829	15.2597148	0.0346429	0.33769233	0.83544252	0.92495422	DUSP9
4354	ENSG00000130830	69.1016232	0.2587564	0.26590452	0.20088035	0.40614145	MPP1

55422	ENSG00000130844	867.60856	-0.09699847	0.09355993	0.28408135	0.50919216	ZNF331
7776	ENSG00000130856	530.350892	-0.07985035	0.15128749	0.56462569	0.7670095	ZNF236
56301	ENSG00000130876	8.47098171	0.17082426	0.39316404	0.28152868	0.50630249	SLC7A10
4037	ENSG00000130881	1819.26811	0.00293872	0.07907463	0.97044231	0.98888633	LRP3
91574	ENSG00000130921	524.736288	0.00243757	0.11176791	0.98274217	0.99428951	C12orf65
25926	ENSG00000130935	1441.41441	-0.20051296	0.08851456	0.01962478	0.07182359	NOL11
10277	ENSG00000130939	1786.2523	-0.02280419	0.06986516	0.73933997	0.87688337	UBE4B
54897	ENSG00000130940	162.678753	0.39432595	0.20013232	0.02294082	0.08130509	CASZ1
10343	ENSG00000130943	56.6846857	0.47774679	0.30573347	0.03623262	0.11647038	PKDREJ
3293	ENSG00000130948	0	NA	NA	NA	NA	HSD17B3
54754	ENSG00000130950	0	NA	NA	NA	NA	NUTM2F
22927	ENSG00000130956	280.419323	0.09118532	0.13881918	0.4817205	0.70240082	HABP4
8789	ENSG00000130957	0.33471731	0.02354391	0.38534154	0.62842204	NA	FBP2
11046	ENSG00000130958	310.085806	0.33470964	0.13934532	0.00980336	0.04084389	SLC35D2
5638	ENSG00000130962	11.2239492	0.34881739	0.56275679	0.04393348	0.13469946	PRRG1
7317	ENSG00000130985	6658.8233	-0.13758676	0.05319552	0.00898195	0.03796375	UBA1
9104	ENSG00000130988	3.96726212	0.10786403	0.38729746	0.34166581	0.57180671	RGN
353497	ENSG00000130997	8.26959685	0.12891373	0.39005439	0.29843896	0.52528647	POLN
246126	ENSG00000131002	0	NA	NA	NA	NA	TXLNGY
425057	ENSG00000131007	0	NA	NA	NA	NA	TTTY9B
85313	ENSG00000131013	456.844425	-0.0423109	0.11401284	0.6978161	0.85403149	PPIL4
80328	ENSG00000131015	3.80949374	0.0077451	0.36472169	0.94902706	0.97902958	ULBP2
9590	ENSG00000131016	3279.47946	-0.43670437	0.05822945	3.11E-14	8.00E-13	AKAP12
23345	ENSG00000131018	944.316293	0.25006496	0.14450534	0.06114724	0.17392993	SYNE1
79465	ENSG00000131019	4.82876159	-0.01494573	0.36209287	0.90151609	0.95673063	ULBP3
9113	ENSG00000131023	1221.69372	-0.09167424	0.09100146	0.29949508	0.52648412	LATS1
54869	ENSG00000131037	161.223196	-0.05351545	0.18814583	0.74427847	0.87963225	EPS8L1
10288	ENSG00000131042	0	NA	NA	NA	NA	LILRB2
25980	ENSG00000131043	857.66333	-0.14060886	0.09126137	0.11208008	0.27094782	AAR2
164395	ENSG00000131044	52.787547	0.11009264	0.25186406	0.56767443	0.76899226	TTLL9
140683	ENSG00000131050	0	NA	NA	NA	NA	BPIFA2
9584	ENSG00000131051	4391.18728	0.08174987	0.05388857	0.12554791	0.29446395	RBM39
84701	ENSG00000131055	27.6857854	0.43329661	0.42586918	0.07768207	0.20692968	COX4I2
128861	ENSG00000131059	0	NA	NA	NA	NA	BPIFA3
84905	ENSG00000131061	184.594648	0.09540683	0.16076152	0.51431738	0.72927793	ZNF341
2686	ENSG00000131067	1045.44414	-0.26332122	0.08989535	0.00251948	0.01306199	GGT7
117285	ENSG00000131068	0	NA	NA	NA	NA	DEFB118
55902	ENSG00000131069	383.151539	-0.10197755	0.12835621	0.39829377	0.62727695	ACSS2

60401	ENSG00000131080	136.130581	-0.7438403	0.22407948	0.00016748	0.00121091	EDA2R
23229	ENSG00000131089	462.669581	-0.50916723	0.13507009	5.97E-05	0.0004791	ARHGEF9
10882	ENSG00000131094	245.979429	-0.00632879	0.14945107	0.96225476	0.98476398	C1QL1
2670	ENSG00000131095	3.59970135	-0.0844431	0.3847686	0.38701749	0.61602672	GFAP
5697	ENSG00000131096	0.173733	0.01914545	0.38484597	0.78225479	NA	PYY
51751	ENSG00000131097	0	NA	NA	NA	NA	HIGD1B
529	ENSG00000131100	1015.63818	0.05194657	0.09324863	0.56644346	0.76820466	ATP6V1E1
7770	ENSG00000131115	619.618197	-0.21110328	0.10660242	0.03867458	0.12233795	ZNF227
126299	ENSG00000131116	982.703219	-0.31254296	0.08663386	0.00020498	0.00145503	ZNF428
83639	ENSG00000131126	0	NA	NA	NA	NA	TEX101
7700	ENSG00000131127	576.499167	-0.39472093	0.12245465	0.00065555	0.00407911	ZNF141
6370	ENSG00000131142	0.173733	0.01914545	0.38484597	0.78225479	NA	CCL25
1327	ENSG00000131143	4542.78392	0.02106602	0.06003936	0.7243491	0.86820892	COX4I1
10328	ENSG00000131148	506.473768	0.11655639	0.10856839	0.26307611	0.48504013	EMC8
23199	ENSG00000131149	8850.72756	-0.52134572	0.04882703	4.81E-27	2.46E-25	GSE1
NA	ENSG00000131152	1.56359704	0.06153354	0.39132085	0.13522809	NA	
51659	ENSG00000131153	627.021705	-0.31398768	0.10108406	0.00123757	0.00709227	GINS2
5119	ENSG00000131165	2087.99249	0.17976979	0.08322408	0.02676042	0.09183996	CHMP1A
6451	ENSG00000131171	689.937087	0.07872982	0.09688249	0.40231687	0.63048711	SH3BGRL
1349	ENSG00000131174	1422.22439	-0.00652801	0.0812788	0.93375897	0.97237716	COX7B
6569	ENSG00000131183	0	NA	NA	NA	NA	SLC34A1
2161	ENSG00000131187	348.607963	-0.43705652	0.13507122	0.00055002	0.00350317	F12
80758	ENSG00000131188	394.20198	-0.54491983	0.12273633	3.10E-06	3.23E-05	PRR7
4772	ENSG00000131196	0	NA	NA	NA	NA	NFATC1
3620	ENSG00000131203	0.29863895	-0.02292439	0.3852726	0.68753245	NA	IDO1
81025	ENSG00000131233	1.21613103	0.05045324	0.38902488	0.20730064	NA	GJA9
10487	ENSG00000131236	3799.97427	0.00645142	0.06831401	0.92434215	0.96852	CAP1
5538	ENSG00000131238	1838.88997	0.1118752	0.07379151	0.1228194	0.2900532	PPT1
84440	ENSG00000131242	1647.45586	-1.30569452	0.10528658	2.33E-36	1.64E-34	RAB11FIP4
51132	ENSG00000131263	1663.61067	-0.31193566	0.06966292	5.08E-06	5.09E-05	RLIM
1046	ENSG00000131264	0	NA	NA	NA	NA	CDX4
22	ENSG00000131269	667.011638	0.09515485	0.12325546	0.41560605	0.64249293	ABCB7
7187	ENSG00000131323	1086.54022	0.02212517	0.10426056	0.82658476	0.92144622	TRAF3
93323	ENSG00000131351	251.268378	-0.30667828	0.14884216	0.0250829	0.08736739	HAUS8
84658	ENSG00000131355	0	NA	NA	NA	NA	ADGRE3
64432	ENSG00000131368	1903.79521	0.08536392	0.07284279	0.23321364	0.45004948	MRPS25
9467	ENSG00000131370	537.57024	0.09380598	0.11140177	0.37918146	0.60816169	SH3BP5
26061	ENSG00000131373	217.297872	0.1071329	0.16820506	0.48053139	0.70158112	HACL1

9779	ENSG00000131374	2273.28759	-0.19455267	0.09101378	0.02738731	0.09338117	TBC1D5
23473	ENSG00000131375	887.057309	-0.11241882	0.11088736	0.28793501	0.51368788	CAPN7
23180	ENSG00000131378	2601.88199	-0.21638638	0.06654038	0.00094295	0.00563331	RFTN1
84077	ENSG00000131379	0	NA	NA	NA	NA	C3orf20
64145	ENSG00000131381	1078.36768	-0.03371252	0.07818845	0.65906061	0.83094576	RBSN
117248	ENSG00000131386	4.53908786	0.19692395	0.4509815	0.03552401	0.11461967	GALNT15
6533	ENSG00000131389	1255.62563	-0.1268352	0.09917459	0.18464916	0.38340548	SLC6A6
3748	ENSG00000131398	433.275446	-1.31162134	0.12872947	2.14E-25	1.03E-23	KCNC3
9476	ENSG00000131400	27.0073833	0.05837605	0.29727582	0.76211574	0.89011458	NAPSA
256236	ENSG00000131401	0.32404836	-0.00108855	0.38336652	0.99066559	NA	NAPSB
7376	ENSG00000131408	903.005495	0.47544879	0.11791422	2.31E-05	0.00020494	NR1H2
94030	ENSG00000131409	1648.50563	-0.18193993	0.07348646	0.0114638	0.04643563	LRRC4B
8572	ENSG00000131435	846.57548	0.09719595	0.11923365	0.3918082	0.62114503	PDLIM4
11127	ENSG00000131437	869.201103	-0.58826195	0.1409542	8.84E-06	8.48E-05	KIF3A
4245	ENSG00000131446	1162.60015	0.14878324	0.08143554	0.06138105	0.17444492	MGAT1
9945	ENSG00000131459	67.9292915	-0.19060872	0.27271364	0.33853435	0.56881111	GFPT2
7283	ENSG00000131462	1446.70783	-0.05035578	0.07542058	0.49561513	0.71440019	TUBG1
10197	ENSG00000131467	2824.76613	-0.24226357	0.07442311	0.00088523	0.00532696	PSME3
6155	ENSG00000131469	9922.86196	-0.22066539	0.04851388	4.37E-06	4.45E-05	RPL27
29893	ENSG00000131470	629.546314	0.0601363	0.10558304	0.55408319	0.76002561	PSMC3IP
8639	ENSG00000131471	9.09686134	0.48515344	0.75250304	0.03313449	0.10851572	AOC3
47	ENSG00000131473	8471.81551	-0.07152383	0.06525972	0.26570659	0.48802762	ACLY
84313	ENSG00000131475	805.199285	0.18726089	0.0879909	0.02860606	0.09673498	VPS25
10266	ENSG00000131477	81.9367422	-0.12120182	0.22525888	0.50759419	0.72355808	RAMP2
314	ENSG00000131480	19.7871494	0.30441486	0.40246937	0.16007605	0.34773993	AOC2
2538	ENSG00000131482	0	NA	NA	NA	NA	G6PC1
NA	ENSG00000131484	1.55991453	-0.0934555	0.39621208	0.1649301	NA	
4695	ENSG00000131495	807.287269	-0.01802584	0.10436618	0.85653751	0.93595581	NDUFA2
54882	ENSG00000131503	2438.63582	-0.11737931	0.07463424	0.10842262	0.26429596	ANKHD1
1729	ENSG00000131504	2317.72327	0.11266167	0.08376024	0.1682346	0.35936418	DIAPH1
80762	ENSG00000131507	1920.16186	-0.09071692	0.08524959	0.27709274	0.50146127	NDFIP1
7322	ENSG00000131508	1621.27851	-0.40475172	0.10383802	4.96E-05	0.00040539	UBE2D2
441543	ENSG00000131548	0	NA	NA	NA	NA	TTTY6B
60412	ENSG00000131558	2611.21809	-0.04995645	0.06169445	0.41152689	0.63926507	EXOC4
116983	ENSG00000131584	2265.31998	0.21275685	0.08835391	0.01317872	0.05200193	ACAP3
54991	ENSG00000131591	588.444128	0.1863235	0.10891601	0.07393177	0.19932142	C1orf159
55107	ENSG00000131620	0.356286	0.02381422	0.38538061	0.6142166	NA	ANO1
8500	ENSG00000131626	1207.7045	-0.02993402	0.09226237	0.74532498	0.87994031	PPFIA1

79652	ENSG00000131634	31.9782647	-0.2777083	0.3320212	0.19754297	0.40156333	TMEM204
79412	ENSG00000131650	63.3957838	-0.8824916	0.31821774	0.00070108	0.00433315	KREMEN2
79228	ENSG00000131652	599.772004	0.08720823	0.10392542	0.38343148	0.61266407	THOC6
84231	ENSG00000131653	3201.93291	-0.01301723	0.06096261	0.828516	0.92220943	TRAF7
56033	ENSG00000131668	0	NA	NA	NA	NA	BARX1
4814	ENSG00000131669	259.009823	-0.17494489	0.1470278	0.19638148	0.40005705	NINJ1
765	ENSG00000131686	0	NA	NA	NA	NA	CA6
261734	ENSG00000131697	539.40084	-0.1151539	0.12148632	0.31648424	0.54448902	NPHP4
4131	ENSG00000131711	11589.2376	-1.04496932	0.06075331	3.41E-67	5.21E-65	MAP1B
84528	ENSG00000131721	0	NA	NA	NA	NA	RHOXF2
3597	ENSG00000131724	121.719875	1.86150766	0.23562071	2.09E-16	6.26E-15	IL13RA1
54521	ENSG00000131725	242.085224	0.04010347	0.14575054	0.7672741	0.89280791	WDR44
1160	ENSG00000131730	9.05666656	-0.07181636	0.3550682	0.63267005	0.81417935	CKMT2
84240	ENSG00000131732	409.721732	0.3211729	0.12342204	0.00593154	0.02691097	ZCCHC9
3885	ENSG00000131737	0	NA	NA	NA	NA	KRT34
3884	ENSG00000131738	0	NA	NA	NA	NA	KRT33B
84951	ENSG00000131746	0.80763644	0.03653866	0.38486192	0.46699599	NA	TNS4
7153	ENSG00000131747	10879.7774	-0.28228548	0.04737423	1.74E-09	2.90E-08	TOP2A
10948	ENSG00000131748	1003.25352	0.09019301	0.08938751	0.30047819	0.52765864	STARD3
5914	ENSG00000131759	995.761342	0.21749768	0.08744461	0.01050686	0.04312292	RARA
84152	ENSG00000131771	21.9190303	0.12961971	0.32980235	0.49334144	0.71236493	PPP1R1B
10656	ENSG00000131773	261.814132	0.21134411	0.16852401	0.16227505	0.35094147	KHDRBS3
9557	ENSG00000131778	1453.04294	-0.0919025	0.07969189	0.23770089	0.45499138	CHD1L
8799	ENSG00000131779	355.469208	-0.1893965	0.13293832	0.12782339	0.29835718	PEX11B
2330	ENSG00000131781	65.7195449	0.06452762	0.23391542	0.7301949	0.87103576	FMO5
10401	ENSG00000131788	860.95666	0.53384453	0.09652383	1.15E-08	1.73E-07	PIAS3
5565	ENSG00000131791	1138.65904	-0.01468819	0.07953738	0.85273094	0.93348077	PRKAB2
100132341	ENSG00000131797	476.024007	0.13132047	0.11784076	0.24144522	0.45949862	CLUHP3
2488	ENSG00000131808	0	NA	NA	NA	NA	FSHB
5160	ENSG00000131828	1078.01557	-0.00217155	0.08769107	0.97893503	0.99288138	PDHA1
10742	ENSG00000131831	41.3699895	-0.00268865	0.27917568	0.98701817	0.99565021	RAI2
64087	ENSG00000131844	1574.52487	-0.14352952	0.08034071	0.06724716	0.18615649	MCCC2
57343	ENSG00000131845	251.751328	-0.09796258	0.15032961	0.47772482	0.69938463	ZNF304
79149	ENSG00000131848	88.4203518	-0.05106021	0.24848502	0.78574444	0.90256888	ZSCAN5A
7691	ENSG00000131849	160.713756	-0.1010627	0.17212176	0.5103812	0.72572757	ZNF132
57663	ENSG00000131864	0	NA	NA	NA	NA	USP29
55829	ENSG00000131871	738.125752	0.39518004	0.09430778	1.49E-05	0.00013707	SELENOS
22856	ENSG00000131873	1380.74456	0.11426857	0.07268984	0.10937518	0.26596439	CHSY1

6627	ENSG00000131876	717.532509	-0.12632533	0.1076599	0.22040097	0.43277467	SNRPA1
NA	ENSG00000131885	0	NA	NA	NA	NA	KRT17P1
3996	ENSG00000131899	2117.16614	0.20801381	0.06546291	0.00125305	0.00717474	LLGL1
8431	ENSG00000131910	1.01348691	0.0309244	0.38200515	0.60807314	NA	NR0B2
79727	ENSG00000131914	0.81896772	-0.00791533	0.38149477	0.87919575	NA	LIN28A
55145	ENSG00000131931	180.051291	-0.02021576	0.17145665	0.89403682	0.95417323	THAP1
85415	ENSG00000131941	182.653341	-0.18682495	0.21209521	0.29156967	0.51751138	RHPN2
83636	ENSG00000131943	647.988392	-0.11266645	0.09844782	0.23550077	0.45230739	C19orf12
91442	ENSG00000131944	80.5503936	0.06759322	0.22926338	0.71517551	0.86271551	FAAP24
341883	ENSG00000131951	3.76615309	-0.01007961	0.37698167	0.88615346	0.95015905	LRRC9
55860	ENSG00000131966	625.081701	-0.01370119	0.09988346	0.88623908	0.95015905	ACTR10
145447	ENSG00000131969	5.95805383	0.3668254	0.67005705	0.01164237	0.04699584	ABHD12B
2643	ENSG00000131979	2572.03685	0.07541056	0.06843574	0.26304051	0.48504013	GCH1
3958	ENSG00000131981	109.519635	0.14463983	0.20816254	0.41082287	0.63847247	LGALS3
NA	ENSG00000131982	0	NA	NA	NA	NA	UBE2L2
79883	ENSG00000132000	38.7137392	-0.78279047	0.46217267	0.00997362	0.04137946	PODNL1
3337	ENSG00000132002	1510.88464	0.22900499	0.10988653	0.02934858	0.09864065	DNAJB1
65249	ENSG00000132003	969.742607	0.20716191	0.08836418	0.01584906	0.0603035	ZSWIM4
84261	ENSG00000132004	347.127675	0.36390916	0.13082988	0.00304899	0.01534161	FBXW9
5989	ENSG00000132005	536.202306	-0.16133906	0.12420037	0.16833144	0.35949363	RFX1
7568	ENSG00000132010	61.4558057	0.2495826	0.28924423	0.23000167	0.44593003	ZNF20
79173	ENSG00000132016	228.895719	-0.11524491	0.1691003	0.44630712	0.67062583	BRME1
90379	ENSG00000132017	795.10219	0.00251507	0.10504217	0.9811125	0.99355086	DCAF15
54862	ENSG00000132024	922.215853	0.03023369	0.10146497	0.7581655	0.88767331	CC2D1A
83546	ENSG00000132026	64.5619567	-0.18113528	0.25975538	0.35516708	0.58555051	RTBDN
4148	ENSG00000132031	112.345655	-0.34453416	0.2159435	0.05804576	0.16707209	MATN3
6737	ENSG00000132109	66.981002	0.26336144	0.27592336	0.19969399	0.40440224	TRIM21
54558	ENSG00000132122	230.962941	0.38101271	0.18864133	0.02152829	0.07723713	SPATA6
10489	ENSG00000132128	2225.8871	0.07929391	0.06812612	0.23725113	0.45434065	LRRC41
10693	ENSG00000132141	8.93044771	0.35086313	0.53379977	0.0769183	0.20530862	CCT6B
22907	ENSG00000132153	3240.7178	0.02180533	0.06256146	0.72494821	0.86844186	DHX30
5894	ENSG00000132155	2732.27077	0.10189395	0.07065816	0.14245983	0.3212532	RAF1
6538	ENSG00000132164	487.284079	-0.26488434	0.12604292	0.02550933	0.08852635	SLC6A11
5468	ENSG00000132170	10.906083	0.25013724	0.47456332	0.07539134	0.20223962	PPARG
23225	ENSG00000132182	6106.49795	-0.49441272	0.05743561	3.06E-18	1.04E-16	NUP210
84824	ENSG00000132185	0	NA	NA	NA	NA	FCRLA
51478	ENSG00000132196	385.240843	-0.05884952	0.14098364	0.65255307	0.82647002	HSD17B7
55556	ENSG00000132199	477.134833	0.22441027	0.14626448	0.09594925	0.24179261	ENOSF1

NA	ENSG00000132204	8.50184539	-1.58315225	1.23141817	0.00653572	0.02917839	LINC00470
84034	ENSG00000132205	147.23681	0.14734201	0.19523926	0.38172831	0.61081455	EMILIN2
548593	ENSG00000132207	1501.10878	0.29282131	0.07577866	7.86E-05	0.00061391	SLX1A
23647	ENSG00000132254	1533.98098	0.03803035	0.07766224	0.61769105	0.80429156	ARFIP2
85363	ENSG00000132256	29.5923731	5.38422323	0.9698527	6.92E-10	1.22E-08	TRIM5
1262	ENSG00000132259	1.24099725	0.08646518	0.39720667	0.10582374	NA	CNGA4
10346	ENSG00000132274	0.34110387	0.02362648	0.38535339	0.62410903	NA	TRIM22
23378	ENSG00000132275	479.580273	-0.45713338	0.14503269	0.00068237	0.00422805	RRP8
26515	ENSG00000132286	873.71573	-0.14652839	0.11223759	0.17086426	0.36291049	TIMM10B
23167	ENSG00000132294	1064.65033	0.00741265	0.09243928	0.94034732	0.97517789	EFR3A
10086	ENSG00000132297	0.54618237	0.03261825	0.38620504	0.45601618	NA	HHLA1
55037	ENSG00000132300	1487.67955	0.11192679	0.10179788	0.25423355	0.47477349	PTCD3
10989	ENSG00000132305	2045.45807	-0.02737785	0.07213111	0.69834554	0.85455335	IMMT
51318	ENSG00000132313	668.180188	-0.33200888	0.10512977	0.00098941	0.00585271	MRPL35
79781	ENSG00000132321	0.33471731	0.02354391	0.38534154	0.62842204	NA	IQCA1
80895	ENSG00000132323	500.813415	0.09183217	0.11764451	0.41259094	0.64014381	ILKAP
8864	ENSG00000132326	97.1995002	-0.29605068	0.22194328	0.10786671	0.26339391	PER2
10267	ENSG00000132329	1471.39227	0.36324481	0.08897337	2.62E-05	0.00022926	RAMP1
51540	ENSG00000132330	1147.56593	0.13215108	0.08430403	0.10811018	0.26372572	SCLY
5791	ENSG00000132334	24.0411772	0.71499798	0.95650101	0.01919588	0.07046185	PTPRE
5901	ENSG00000132341	6954.33991	-0.27907489	0.05727063	7.63E-07	8.80E-06	RAN
5562	ENSG00000132356	931.264755	-0.22538259	0.10832865	0.02954922	0.09920766	PRKAA1
84674	ENSG00000132357	1.31194181	0.02135235	0.379559	0.77048313	NA	CARD6
23108	ENSG00000132359	2504.23782	1.29059226	0.06546383	1.63E-87	3.77E-85	RAP1GAP2
23277	ENSG00000132361	1760.83573	-0.00270629	0.0715378	0.96745331	0.98759962	CLUH
51763	ENSG00000132376	448.919359	-0.04254015	0.15383753	0.76155657	0.88972737	INPP5K
10514	ENSG00000132382	2778.78763	0.09978604	0.06728594	0.1327013	0.30599649	MYBBP1A
6117	ENSG00000132383	2108.61164	-0.22118719	0.09017898	0.0113942	0.04621034	RPA1
5176	ENSG00000132386	625.483781	2.875874	0.12017836	8.28E-128	3.82E-125	SERPINF1
7326	ENSG00000132388	1482.43806	0.22097135	0.08132675	0.00536011	0.02469294	UBE2G1
60678	ENSG00000132394	614.423314	0.11963011	0.10278005	0.22675109	0.44131269	EEFSEC
57533	ENSG00000132405	2243.39711	-0.17574564	0.06289969	0.00457629	0.0215918	TBC1D14
85013	ENSG00000132406	427.572973	0.13892877	0.13350429	0.26696175	0.48937642	TMEM128
51805	ENSG00000132423	118.473439	-0.00595171	0.19540147	0.9704444	0.98888633	COQ3
25957	ENSG00000132424	5356.32168	0.18726384	0.05242039	0.00030904	0.00210413	PNISR
64208	ENSG00000132429	306.356906	-0.66165496	0.14915377	2.23E-06	2.38E-05	POPDC3
23480	ENSG00000132432	1179.0293	-0.10054666	0.08446582	0.2217876	0.43480888	SEC61G
55915	ENSG00000132434	458.396178	-0.48294398	0.12509479	4.53E-05	0.00037565	LANCL2

63979	ENSG00000132436	1102.11624	-0.25241566	0.08493344	0.0022494	0.01184083	FIGNL1
1644	ENSG00000132437	1700.948	-1.61525412	0.08853616	1.86E-75	3.41E-73	DDC
53940	ENSG00000132446	0	NA	NA	NA	NA	FTHL17
2926	ENSG00000132463	1934.64962	-0.18677039	0.07870194	0.01514064	0.05809133	GRSF1
10117	ENSG00000132464	0	NA	NA	NA	NA	ENAM
3512	ENSG00000132465	0	NA	NA	NA	NA	JCHAIN
26057	ENSG00000132466	3859.10999	-0.20141286	0.05531215	0.00021724	0.00153162	ANKRD17
57050	ENSG00000132467	853.897145	0.00806621	0.10288355	0.93675127	0.97351594	UTP3
3691	ENSG00000132470	40.8515741	0.00603457	0.28218191	0.97667042	0.99207153	ITGB4
23558	ENSG00000132471	2076.07091	-0.01282282	0.06704263	0.84515387	0.92965052	WBP2
3021	ENSG00000132475	14152.2091	0.28137565	0.05073313	2.16E-08	3.12E-07	H3-3B
85451	ENSG00000132478	1703.47279	0.01992104	0.07921415	0.79802658	0.9074513	UNK
91107	ENSG00000132481	39.7208835	-0.18256947	0.28580647	0.36461606	0.59423075	TRIM47
9406	ENSG00000132485	2404.84547	0.09130796	0.07834962	0.23406122	0.45093611	ZRANB2
1984	ENSG00000132507	8553.64109	-0.18647062	0.05952892	0.00147628	0.00826683	EIF5A
23135	ENSG00000132510	2380.66821	0.21046625	0.08847436	0.0143316	0.05556751	KDM6B
10462	ENSG00000132514	0	NA	NA	NA	NA	CLEC10A
55065	ENSG00000132517	1.47380488	0.02867712	0.3815687	0.64111572	NA	SLC52A1
3000	ENSG00000132518	2.20425671	0.0901798	0.38881274	0.32703552	NA	GUCY2D
2874	ENSG00000132522	1235.43597	-0.13442753	0.0806428	0.08776809	0.22632101	GPS2
54739	ENSG00000132530	0	NA	NA	NA	NA	XAF1
1742	ENSG00000132535	921.424771	-0.16211801	0.09839384	0.08744279	0.22586501	DLG4
10247	ENSG00000132541	141.55961	-0.99794608	0.21369732	3.74E-07	4.56E-06	RIDA
157680	ENSG00000132549	1310.04197	0.10443886	0.08920431	0.22874275	0.44405638	VPS13B
26166	ENSG00000132554	0.76994472	-0.03644129	0.38642285	0.53879405	NA	RGS22
4147	ENSG00000132561	24.7958343	0.48205298	0.45413678	0.06014417	0.17157108	MATN2
51308	ENSG00000132563	1224.55472	-0.44972679	0.08689319	1.03E-07	1.36E-06	REEP2
84105	ENSG00000132570	257.635586	-0.65041914	0.1747307	4.72E-05	0.00038923	PCBD2
6388	ENSG00000132581	550.24919	0.18170575	0.1066871	0.07579147	0.20295623	SDF2
2319	ENSG00000132589	3510.08478	0.32937775	0.06884556	1.11E-06	1.25E-05	FLOT2
26284	ENSG00000132591	915.360659	-0.02206928	0.09436062	0.84659883	0.93050362	ERAL1
54496	ENSG00000132600	689.700011	-0.08417354	0.10230925	0.39245301	0.62172726	PRMT7
51388	ENSG00000132603	897.361967	0.00200391	0.09414848	0.98336436	0.99428951	NIP7
7014	ENSG00000132604	842.235828	-0.47159358	0.11016091	7.82E-06	7.59E-05	TERF2
27183	ENSG00000132612	2144.15051	-0.05666638	0.06662996	0.38734605	0.61635625	VPS4A
92154	ENSG00000132613	1941.43165	-0.18099507	0.09042915	0.03898132	0.12307279	MTSS2
116835	ENSG00000132622	31.9438631	-0.32242755	0.36450782	0.14891885	0.33152468	HSPA12B
63926	ENSG00000132623	202.769397	0.27975871	0.16574477	0.06117569	0.17396096	ANKEF1

140856	ENSG00000132631	0	NA	NA	NA	NA	SCP2D1
64773	ENSG00000132635	1168.92565	0.40783404	0.08009043	1.86E-07	2.37E-06	PCED1A
6616	ENSG00000132639	1170.15003	-1.54602786	0.09691944	2.39E-58	3.01E-56	SNAP25
22903	ENSG00000132640	1295.90603	0.54847802	0.08405636	2.39E-11	4.89E-10	BTBD3
5111	ENSG00000132646	3092.3559	-0.21856895	0.08260057	0.00663296	0.02953564	PCNA
29107	ENSG00000132661	306.641997	-0.18233326	0.13294177	0.14235923	0.32117652	NXT1
10621	ENSG00000132664	271.494921	0.11901101	0.14727759	0.3818139	0.61090223	POLR3F
54453	ENSG00000132669	45.8643257	-0.20805958	0.30104119	0.30745723	0.53489578	RIN2
5786	ENSG00000132670	1761.17924	0.07042379	0.07110869	0.31384074	0.54165894	PTPRA
6754	ENSG00000132671	0	NA	NA	NA	NA	SSTR4
7818	ENSG00000132676	2117.2518	-0.06815435	0.06263314	0.26999845	0.49316601	DAP3
57127	ENSG00000132677	2.69629369	0.08815649	0.38725108	0.34751618	0.57819389	RHBG
22889	ENSG00000132680	2541.72473	0.0695402	0.08687486	0.41630305	0.64306408	KHDC4
480	ENSG00000132681	0.3400998	0.00219723	0.38337765	0.96670829	NA	ATP1A4
10763	ENSG00000132688	7312.74703	-0.61177823	0.06096199	3.15E-24	1.45E-22	NES
63827	ENSG00000132692	110.872641	-0.58725763	0.23217405	0.00298825	0.01507802	BCAN
1401	ENSG00000132693	0	NA	NA	NA	NA	CRP
9826	ENSG00000132694	1939.40012	0.03876404	0.0958967	0.67791978	0.84273994	ARHGEF11
57111	ENSG00000132698	3.16349965	0.21630871	0.46764594	0.03600843	0.11586234	RAB25
60484	ENSG00000132702	13.6365786	0.35320898	0.53905315	0.0736608	0.19875293	HAPLN2
325	ENSG00000132703	0	NA	NA	NA	NA	APCS
79368	ENSG00000132704	0	NA	NA	NA	NA	FCRL2
50717	ENSG00000132716	2366.27666	0.45338678	0.07080194	7.06E-11	1.38E-09	DCAF8
23208	ENSG00000132718	3195.52583	-1.09403958	0.06354061	2.31E-67	3.62E-65	SYT11
3508	ENSG00000132740	561.505582	-0.06380166	0.10742392	0.53537096	0.74627695	IGHMBP2
91703	ENSG00000132744	0.34624801	-0.02356563	0.38536324	0.65270996	NA	ACY3
222	ENSG00000132746	0	NA	NA	NA	NA	ALDH3B2
9633	ENSG00000132749	239.94464	0.28368276	0.17135946	0.06428697	0.18040361	TESMIN
25974	ENSG00000132763	285.861848	0.02919328	0.13743533	0.82099103	0.91847813	MMACHC
1802	ENSG00000132768	1288.32042	-0.15407585	0.08966175	0.07659566	0.20464017	DPH2
114034	ENSG00000132773	339.318347	-0.20617347	0.1309764	0.0927589	0.23566865	TOE1
4678	ENSG00000132780	6392.5325	0.07158135	0.05277155	0.17167695	0.36420774	NASP
4595	ENSG00000132781	567.850194	0.34575603	0.12075558	0.00250636	0.01300029	MUTYH
56259	ENSG00000132792	826.408275	-0.07403443	0.09085899	0.4010612	0.62946398	CTNNBL1
64900	ENSG00000132793	8.80066804	2.20069613	1.08384469	0.00154515	0.0086039	LPIN3
140831	ENSG00000132801	100.975586	-0.20371652	0.21321945	0.25337921	0.47375776	ZSWIM3
55544	ENSG00000132819	896.140855	-0.26880552	0.0907959	0.0022417	0.01180969	RBM38
128434	ENSG00000132821	507.699159	-1.47954301	0.15508834	1.09E-22	4.67E-21	VSTM2L

51526	ENSG00000132823	393.593258	-0.0574134	0.13126005	0.64076771	0.82008361	OSER1
10955	ENSG00000132824	1053.79748	-0.08416165	0.08394515	0.30348778	0.53085409	SERINC3
5509	ENSG00000132825	96.2828541	0.4976174	0.23494643	0.01139853	0.04621842	PPP1R3D
NA	ENSG00000132832	3.61664045	0.2221978	0.46636813	0.05193223	0.15341504	
29958	ENSG00000132837	23.1249388	0.55593428	0.54505645	0.04109681	0.12808053	DMGDH
23743	ENSG00000132840	0	NA	NA	NA	NA	BHMT2
8546	ENSG00000132842	778.042907	0.30071181	0.09548013	0.00111802	0.00649722	AP3B1
84327	ENSG00000132846	819.866833	0.59799866	0.12900179	1.05E-06	1.19E-05	ZBED3
10207	ENSG00000132849	239.356119	0.39609695	0.19982881	0.02216014	0.07906081	PATJ
163782	ENSG00000132854	64.1835187	-3.06541001	0.39960434	1.11E-15	3.16E-14	KANK4
27329	ENSG00000132855	1.8526157	-0.09795016	0.39820621	0.13508234	NA	ANGPTL3
6860	ENSG00000132872	6862.50392	-1.10465088	0.0768358	9.07E-48	8.41E-46	SYT4
8170	ENSG00000132874	1.13134361	0.06144845	0.39097037	0.17672426	NA	SLC14A2
93611	ENSG00000132879	541.507927	-0.18602081	0.11049992	0.07757199	0.2066702	FBXO44
79363	ENSG00000132881	112.181582	0.30464525	0.27224129	0.13917929	0.31601249	CPLANE2
842	ENSG00000132906	196.375697	0.29734298	0.1678974	0.0490758	0.14693914	CASP9
56923	ENSG00000132911	0	NA	NA	NA	NA	NMUR2
51164	ENSG00000132912	1966.94826	-0.43362077	0.07238245	1.02E-09	1.75E-08	DCTN4
5145	ENSG00000132915	23.9484474	-0.07977111	0.30909093	0.67151599	0.83826144	PDE6A
51761	ENSG00000132932	2.13104563	0.14381209	0.42231578	0.02204775	NA	ATP8A2
23281	ENSG00000132938	11.9058067	-0.4350607	0.63047575	0.04413114	0.13518014	MTUS2
9205	ENSG00000132950	226.973789	-0.19865621	0.17451841	0.19999762	0.40493447	ZMYM5
10208	ENSG00000132952	583.593768	-0.21011887	0.12556856	0.07560179	0.20255766	USPL1
64328	ENSG00000132953	1414.90403	0.01816363	0.0914264	0.83868006	0.92674595	XPO4
93492	ENSG00000132958	1.50517803	0.0221318	0.37949143	0.74905281	NA	TPTE2
51371	ENSG00000132963	1146.13741	-0.13541462	0.08982987	0.1204917	0.28605424	POMP
1024	ENSG00000132964	533.552437	-0.02616425	0.11010935	0.80307202	0.9104022	CDK8
241	ENSG00000132965	0.3518339	-0.00497886	0.38344636	0.98710913	NA	ALOX5AP
NA	ENSG00000132967	74.4631695	0.62986545	0.37107351	0.01620477	0.06143205	HMGB1P5
10810	ENSG00000132970	1667.85243	0.08274291	0.07921328	0.28501085	0.51037097	WASF3
56163	ENSG00000132972	0.15069912	-0.01848503	0.38480575	0.84537521	NA	RNF17
2835	ENSG00000132975	12.7384737	-3.42098154	1.00659286	5.46E-05	0.00044198	GPR12
1131	ENSG00000133019	6683.83418	-0.20371563	0.06021217	0.00060313	0.00380116	CHRM3
4626	ENSG00000133020	0	NA	NA	NA	NA	MYH8
4628	ENSG00000133026	9553.57816	0.26669887	0.0677756	6.30E-05	0.00050369	MYH10
10400	ENSG00000133027	471.681291	0.12096531	0.12755841	0.31462197	0.5422726	PEMT
6341	ENSG00000133028	1264.48964	0.10725028	0.08454859	0.19296823	0.39558686	SCO1
23164	ENSG00000133030	8392.03769	-0.18952254	0.0778307	0.012737	0.05060114	MPRIP

1116	ENSG00000133048	0.33471731	0.02354391	0.38534154	0.62842204	NA	CHI3L1
4608	ENSG00000133055	0.173124	-0.01911262	0.38486262	0.84537521	NA	MYBPH
5287	ENSG00000133056	757.48408	-0.15160377	0.09628002	0.1031591	0.2551347	PIK3C2B
25778	ENSG00000133059	872.04995	0.07684543	0.09373709	0.39834742	0.62727695	DSTYK
1118	ENSG00000133063	0.43485182	0.02575927	0.3855378	0.59020656	NA	CHIT1
254428	ENSG00000133065	1112.76551	0.03242704	0.09093171	0.67957166	0.84361793	SLC41A1
59352	ENSG00000133067	2.86813471	-0.02327039	0.37383332	0.79923071	0.90801909	LGR6
9911	ENSG00000133069	583.677436	0.07731431	0.12198379	0.50403011	0.72050994	TMCC2
9201	ENSG00000133083	445.941105	-0.58845656	0.12612269	9.22E-07	1.05E-05	DCLK1
8900	ENSG00000133101	69.9043033	-0.04494744	0.23053196	0.8063145	0.9122093	CCNA1
57511	ENSG00000133103	384.473664	0.25562495	0.16298054	0.0825658	0.21637154	COG6
23111	ENSG00000133104	580.55397	0.09010548	0.11493216	0.41117876	0.63882432	SPART
122042	ENSG00000133105	2.2094203	0.0994326	0.39771759	0.17149161	NA	RXFP2
94240	ENSG00000133106	0.14931947	-0.01844332	0.38480212	0.84537521	NA	EPSTI1
7223	ENSG00000133107	0	NA	NA	NA	NA	TRPC4
10631	ENSG00000133110	0	NA	NA	NA	NA	POSTN
5994	ENSG00000133111	298.886878	-0.28897334	0.13574461	0.02241961	0.07981428	RFXAP
7178	ENSG00000133112	24016.9141	-0.1503269	0.0475074	0.00140514	0.00793566	TPT1
55425	ENSG00000133114	660.052009	-0.12934533	0.10216962	0.18820177	0.38858602	GPALPP1
161003	ENSG00000133115	0	NA	NA	NA	NA	STOML3
9365	ENSG00000133116	1.7228245	-0.07172359	0.38739277	0.33555723	NA	KL
5983	ENSG00000133119	1369.1854	-0.44861847	0.07603147	1.69E-09	2.82E-08	RFC3
90627	ENSG00000133121	509.474292	-0.93265542	0.12611668	2.07E-14	5.36E-13	STARD13
8471	ENSG00000133124	0	NA	NA	NA	NA	IRS4
79710	ENSG00000133131	230.01971	-0.0580824	0.14852422	0.6723101	0.83874944	MORC4
84707	ENSG00000133134	608.566992	-0.54872392	0.10164134	2.32E-08	3.33E-07	BEX2
79589	ENSG00000133135	0	NA	NA	NA	NA	RNF128
NA	ENSG00000133136	0	NA	NA	NA	NA	GNG5P2
54885	ENSG00000133138	5.93739281	-0.00257001	0.354916	0.98316743	0.99428951	TBC1D8B
79921	ENSG00000133142	879.742477	-0.20261206	0.10488048	0.0439943	0.13484427	TCEAL4
55859	ENSG00000133169	2094.83998	-0.80037734	0.07891951	6.93E-25	3.29E-23	BEX1
84923	ENSG00000133193	1003.47106	0.17787088	0.09430022	0.05154152	0.1524879	FAM104A
201266	ENSG00000133195	283.744903	-0.20778772	0.15161402	0.13435146	0.30858343	SLC39A11
2048	ENSG00000133216	2116.75652	-0.30173507	0.08271932	0.00017972	0.00129127	EPHB2
10250	ENSG00000133226	1957.76685	-0.04616241	0.08014381	0.55161964	0.75806149	SRRM1
55643	ENSG00000133243	3809.82755	0.13047715	0.05616371	0.0188381	0.06937959	BTBD2
84106	ENSG00000133246	1.86974123	0.12639289	0.4100604	0.08150668	NA	PRAM1
84787	ENSG00000133247	753.718145	0.26205542	0.10197267	0.00753241	0.03286629	KMT5C

84330	ENSG00000133250	265.362814	0.46079526	0.1673412	0.00239959	0.01252178	ZNF414
5158	ENSG00000133256	112.244349	0.04034948	0.20748738	0.81894748	0.91749495	PDE6B
23640	ENSG00000133265	1437.06703	0.12591738	0.09070539	0.15300401	0.33777674	HSPBP1
1455	ENSG00000133275	2497.54403	0.20308351	0.0622375	0.00093524	0.00559735	CSNK1G2
84250	ENSG00000133302	674.637524	0.33031062	0.11031802	0.00170936	0.0093767	SLF1
55748	ENSG00000133313	810.808223	0.12411448	0.10664633	0.22555938	0.43951541	CNDP2
28992	ENSG00000133315	763.173203	0.10626768	0.10816217	0.3051415	0.53254815	MACROD1
54663	ENSG00000133316	853.569176	0.25219708	0.0896922	0.00376329	0.01832689	WDR74
85329	ENSG00000133317	0	NA	NA	NA	NA	LGALS12
10313	ENSG00000133318	3317.13383	-0.58451356	0.06369921	1.42E-20	5.49E-19	RTN3
5920	ENSG00000133321	0	NA	NA	NA	NA	PLAAT4
54979	ENSG00000133328	0.31814294	0.00109006	0.38336631	0.9666328	NA	PLAAT2
4629	ENSG00000133392	14.3323178	-0.1299677	0.35798262	0.43782377	0.66289835	MYH11
123811	ENSG00000133393	497.478167	-0.17077609	0.12296586	0.14148272	0.31988799	CEP20
84246	ENSG00000133398	598.349701	0.29433638	0.1097321	0.00500826	0.02324844	MED10
23037	ENSG00000133401	184.290378	-0.00864476	0.1802802	0.95540953	0.9817074	PDZD2
22880	ENSG00000133422	1234.55873	-0.04304991	0.11513204	0.69413137	0.85201936	MORC2
9215	ENSG00000133424	169.45521	0.22489845	0.18804937	0.17127734	0.36363215	LARGE1
653689	ENSG00000133433	239.166513	0.24554227	0.16955062	0.10637205	0.26080345	GSTT2B
84700	ENSG00000133454	28.3388274	-0.17163107	0.31039016	0.3926813	0.62191102	MYO18B
66035	ENSG00000133460	453.686547	-0.12532793	0.12429183	0.28523548	0.51070046	SLC2A11
114904	ENSG00000133466	489.323747	0.5237942	0.12118741	5.63E-06	5.62E-05	C1QTNF6
728441	ENSG00000133475	1.78740445	0.07744508	0.38968192	0.28656823	NA	GGT2
113828	ENSG00000133477	0	NA	NA	NA	NA	FAM83F
284904	ENSG00000133488	0	NA	NA	NA	NA	SEC14L4
NA	ENSG00000133519	185.678089	-0.30585908	0.17275746	0.04720581	0.1422916	ZDHHC8P1
474344	ENSG00000133561	0	NA	NA	NA	NA	GIMAP6
55303	ENSG00000133574	0	NA	NA	NA	NA	GIMAP4
90956	ENSG00000133597	459.700111	0.06072094	0.11936471	0.59323339	0.78711401	ADCK2
23608	ENSG00000133606	1951.93216	0.14785404	0.08357857	0.06969078	0.19085983	MKRN1
116988	ENSG00000133612	3440.18166	-0.13615602	0.0630829	0.02850053	0.09642745	AGAP3
84626	ENSG00000133619	501.827338	0.15157281	0.1367363	0.23502242	0.45186105	KRBA1
79970	ENSG00000133624	571.507277	0.21027349	0.13066207	0.08618201	0.22346533	ZNF767P
57180	ENSG00000133627	832.291495	-0.14381748	0.09800219	0.1283094	0.29899782	ACTR3B
4922	ENSG00000133636	14.8391575	-0.00974549	0.32824546	0.95285176	0.98058207	NTS
694	ENSG00000133639	1249.7922	-0.00421976	0.07893791	0.95573041	0.9818048	BTG1
84125	ENSG00000133640	93.0006033	0.28449522	0.27322938	0.16534564	0.35537401	LRRIQ1
91298	ENSG00000133641	685.201374	-0.3669532	0.11329283	0.00067473	0.00418724	C12orf29

79572	ENSG00000133657	2434.56607	-0.58823019	0.09832872	6.73E-10	1.19E-08	ATP13A3
6441	ENSG00000133661	11.8470388	0.15101753	0.37391305	0.35622176	0.58626626	SFTPD
84332	ENSG00000133665	23.1957258	0.05019177	0.33531591	0.76623498	0.89247205	DYDC2
80195	ENSG00000133678	238.401648	-0.19682302	0.16939134	0.1938021	0.39665339	TMEM254
83857	ENSG00000133687	168.538843	0.62168322	0.2155421	0.00097744	0.00579914	TMTC1
3845	ENSG00000133703	1459.37756	0.01371894	0.0746853	0.85274221	0.93348077	KRAS
10526	ENSG00000133704	1678.7958	-0.04804994	0.07021652	0.48523371	0.70532909	IPO8
51520	ENSG00000133706	6723.44369	-0.02333705	0.06060107	0.68928648	0.84914274	LARS1
11005	ENSG00000133710	0.18922458	0.01954053	0.38488496	0.78225479	NA	SPINK5
3612	ENSG00000133731	672.822094	-0.18056398	0.10456555	0.07203154	0.19555978	IMPA1
85444	ENSG00000133739	197.736547	0.19216053	0.16047377	0.18722655	0.38733835	LRRCC1
1875	ENSG00000133740	315.992666	0.01412416	0.12951801	0.90874672	0.96049944	E2F5
759	ENSG00000133742	0	NA	NA	NA	NA	CA1
29080	ENSG00000133773	361.413752	0.08105035	0.12512809	0.49373731	0.7125217	CCDC59
23075	ENSG00000133789	707.062924	-0.04460342	0.09643521	0.63043425	0.81309339	SWAP70
406	ENSG00000133794	421.618981	-0.59524921	0.14540901	1.24E-05	0.00011579	ARNTL
10894	ENSG00000133800	3.17594966	0.21551357	0.46807088	0.03198224	0.10552598	LYVE1
272	ENSG00000133805	92.5917105	-0.22916381	0.23417179	0.22422888	0.43795047	AMPD3
81846	ENSG00000133812	1442.8789	-0.04433055	0.07906503	0.56461248	0.7670095	SBF2
9645	ENSG00000133816	71.2035137	-0.51608018	0.31277773	0.02628569	0.09050816	MICAL2
84953	ENSG00000133816	71.2035137	-0.51608018	0.31277773	0.02628569	0.09050816	MICAL2
22800	ENSG00000133818	308.194578	-0.20440221	0.14084735	0.11756763	0.28111968	RRAS2
3295	ENSG00000133835	2094.47712	0.15853327	0.08387131	0.05255405	0.15469917	HSD17B4
196441	ENSG00000133858	1420.35153	-0.03570403	0.07738232	0.63703794	0.81722218	ZFC3H1
56154	ENSG00000133863	686.905644	-1.00142063	0.12132476	1.94E-17	6.17E-16	TEX15
51669	ENSG00000133872	3006.29307	-0.00886968	0.06389443	0.88752037	0.95077776	SARAF
79845	ENSG00000133874	441.988512	0.25591846	0.11715124	0.02150448	0.07717959	RNF122
78986	ENSG00000133878	775.691985	-0.34186299	0.10480929	0.00067209	0.00417219	DUSP26
5977	ENSG00000133884	1086.5642	0.14680344	0.10083971	0.13113662	0.30358816	DPF2
4221	ENSG00000133895	1582.14557	0.15183387	0.07748321	0.04529943	0.1378648	MEN1
11161	ENSG00000133935	509.505053	0.06179816	0.1126814	0.56584791	0.76767113	ERG28
145258	ENSG00000133937	49.313173	0.73979054	0.35692669	0.00578028	0.02631501	GSC
80017	ENSG00000133943	356.275399	-0.19122557	0.13380414	0.12616901	0.29546661	DGLUCY
57578	ENSG00000133958	1044.21236	-2.15686028	0.10449925	7.29E-96	2.13E-93	UNC79
8650	ENSG00000133961	1144.71197	-0.47274572	0.11013052	7.36E-06	7.17E-05	NUMB
79820	ENSG00000133962	0	NA	NA	NA	NA	CATSPERB
55237	ENSG00000133980	0	NA	NA	NA	NA	VRTN
51241	ENSG00000133983	596.408165	0.00937195	0.10786468	0.93068082	0.97111825	COX16

23508	ENSG00000133985	212.405487	0.26658119	0.1595225	0.06585416	0.18345221	TTC9
10001	ENSG00000133997	536.076123	0.05672793	0.10918776	0.58849362	0.78323697	MED6
1965	ENSG00000134001	1390.0038	-0.30241774	0.11523009	0.00575842	0.02622752	EIF2S1
8748	ENSG00000134007	0.33471731	0.02354391	0.38534154	0.62842204	NA	ADAM20
4017	ENSG00000134013	175.869765	0.92059751	0.24228745	1.93E-05	0.00017329	LOXL2
55140	ENSG00000134014	733.400892	-0.19006414	0.09813146	0.04451937	0.13604654	ELP3
157310	ENSG00000134020	0.69644283	0.04118148	0.38726566	0.34952825	NA	PEBP4
27299	ENSG00000134028	0	NA	NA	NA	NA	ADAMDEC1
9811	ENSG00000134030	1922.75255	-0.18689866	0.07108576	0.00736319	0.03222714	CTIF
83876	ENSG00000134042	5.72905871	0.15084192	0.40010722	0.25028437	0.47023123	MRO
8932	ENSG00000134046	526.188837	-0.04952002	0.14010721	0.70229443	0.85686744	MBD2
51124	ENSG00000134049	764.341859	-0.07243068	0.0994665	0.44811537	0.67210531	IER3IP1
92259	ENSG00000134056	285.005329	-0.2274068	0.14659186	0.09184164	0.23405757	MRPS36
891	ENSG00000134057	2072.3222	-0.18972432	0.08221824	0.01784413	0.06649655	CCNB1
1022	ENSG00000134058	252.934484	0.21820375	0.15232537	0.11803298	0.2818698	CDK7
4064	ENSG00000134061	0	NA	NA	NA	NA	CD180
3656	ENSG00000134070	14.8428807	-0.16278462	0.35624705	0.37689245	0.60570261	IRAK2
8536	ENSG00000134072	263.045699	-0.44884536	0.15463112	0.00157982	0.00876745	CAMK1
25917	ENSG00000134077	1075.89069	-0.34915665	0.09083542	7.34E-05	0.00057947	THUMPD3
7428	ENSG00000134086	2079.6928	-0.20220719	0.07103949	0.00371797	0.01815964	VHL
8553	ENSG00000134107	24.7408314	-0.16421447	0.3421694	0.39173935	0.62114503	BHLHE40
55207	ENSG00000134108	860.570234	-0.202482	0.09788864	0.03179678	0.10503632	ARL8B
9695	ENSG00000134109	629.125047	0.44334802	0.1532736	0.00164692	0.00908647	EDEM1
27255	ENSG00000134115	48.3180063	1.2408909	0.39809913	0.00013888	0.001022	CNTN6
10752	ENSG00000134121	15.2917251	0.11419109	0.38651867	0.3053564	0.5327826	CHL1
4212	ENSG00000134138	3865.13024	-0.24423028	0.07471519	0.00083943	0.00507291	MEIS2
89978	ENSG00000134146	168.494973	-0.29143116	0.1924021	0.08091908	0.21334235	DPH6
79768	ENSG00000134152	540.893753	0.39578778	0.13584038	0.00183472	0.00994017	KATNBL1
56851	ENSG00000134153	506.077392	-0.16084515	0.11030985	0.12681201	0.29662228	EMC7
4308	ENSG00000134160	0	NA	NA	NA	NA	TRPM1
2780	ENSG00000134183	1.86981588	0.01826221	0.37632415	0.83263004	NA	GNAT2
2944	ENSG00000134184	0.173733	0.01914545	0.38484597	0.78225479	NA	GSTM1
55119	ENSG00000134186	2295.92231	-0.04728251	0.07349723	0.51148945	0.72672234	PRPF38B
83998	ENSG00000134193	0	NA	NA	NA	NA	REG4
10100	ENSG00000134198	265.111147	-0.02983424	0.14369905	0.82181571	0.91847813	TSPAN2
7252	ENSG00000134200	0.52119901	0.02576573	0.38568143	0.51723676	NA	TSHB
2949	ENSG00000134201	2.14208696	0.08259543	0.39492863	0.11757026	NA	GSTM5
2947	ENSG00000134202	653.86164	0.07100179	0.1073133	0.49120391	0.71060078	GSTM3

148281	ENSG00000134207	0	NA	NA	NA	NA	SYT6
10451	ENSG00000134215	164.137192	-0.36450929	0.2051354	0.03793944	0.12066663	VAV3
27159	ENSG00000134216	0	NA	NA	NA	NA	CHIA
84722	ENSG00000134222	677.28814	-0.20864783	0.11370915	0.0531025	0.15587419	PSRC1
3158	ENSG00000134240	0	NA	NA	NA	NA	HMGCS2
26191	ENSG00000134242	0.84907133	-0.04552917	0.38787827	0.31635245	NA	PTPN22
6272	ENSG00000134243	1744.31909	-0.25155936	0.07464671	0.00057446	0.00364477	SORT1
7482	ENSG00000134245	98.5776044	0.10434933	0.22202457	0.56747898	0.76896903	WNT2B
5738	ENSG00000134247	1708.95272	0.37441213	0.08419914	5.05E-06	5.07E-05	PTGFRN
10542	ENSG00000134248	964.231264	0.28173653	0.09574185	0.00232135	0.01216291	LAMTOR5
11085	ENSG00000134249	0	NA	NA	NA	NA	ADAM30
4853	ENSG00000134250	2088.28031	0.37083423	0.07039045	8.08E-08	1.08E-06	NOTCH2
80263	ENSG00000134253	194.852556	0.4819453	0.17693573	0.00244121	0.01270225	TRIM45
10390	ENSG00000134255	410.213806	-0.09736447	0.12143738	0.39648167	0.62574149	CEPT1
9398	ENSG00000134256	28.5362845	0.86266017	0.641398	0.0128708	0.05105345	CD101
79679	ENSG00000134258	1.27075225	0.06925222	0.38969475	0.23537421	NA	VTCN1
4803	ENSG00000134259	16.1488062	-0.78169589	0.69381993	0.01957	0.07166922	NGF
10717	ENSG00000134262	305.530327	-0.09788801	0.14956795	0.47601678	0.69806881	AP4B1
8774	ENSG00000134265	531.670598	0.11201741	0.11549455	0.30933408	0.53712343	NAPG
56907	ENSG00000134278	535.756381	-0.58365209	0.13826195	7.25E-06	7.08E-05	SPIRE1
51535	ENSG00000134283	1302.68599	0.06523197	0.08046045	0.4030226	0.63099498	PPHLN1
51303	ENSG00000134285	413.775484	0.80378002	0.12655949	3.98E-11	8.00E-10	FKBP11
377	ENSG00000134287	3230.25757	-0.18130485	0.07182869	0.01006261	0.04165471	ARF3
79022	ENSG00000134291	1741.10563	-0.07087966	0.08397427	0.38639815	0.61555639	TMEM106C
54407	ENSG00000134294	4097.43031	0.14244247	0.06475126	0.02560499	0.08874965	SLC38A2
51054	ENSG00000134297	104.310177	0.15041918	0.2414662	0.4289058	0.65462623	PLEKHA8P1
10971	ENSG00000134308	11769.1522	-0.03307593	0.05084601	0.5105092	0.72579739	YWHAQ
57498	ENSG00000134313	3955.53025	-0.14458588	0.0543418	0.00714492	0.03144527	KIDINS220
29841	ENSG00000134317	98.3672081	-0.07426437	0.21936702	0.67843838	0.84291038	GRHL1
9475	ENSG00000134318	1513.49026	-0.28421132	0.08282966	0.00042735	0.00280936	ROCK2
91543	ENSG00000134321	11.7536946	0.41109276	0.58261195	0.05908601	0.16915821	RSAD2
4613	ENSG00000134323	299.795102	2.08715904	0.18166457	9.83E-32	5.91E-30	MYCN
23175	ENSG00000134324	1945.75419	-0.37103179	0.07500296	4.33E-07	5.22E-06	LPIN1
129607	ENSG00000134326	15.5463065	0.24331092	0.40080459	0.20724318	0.41447107	CMPK2
285148	ENSG00000134330	830.417689	-0.0906035	0.11074252	0.39193379	0.62123244	IAH1
3939	ENSG00000134333	5797.13024	-0.43804674	0.18595045	0.00789112	0.03411845	LDHA
6289	ENSG00000134339	0	NA	NA	NA	NA	SAA2
63982	ENSG00000134343	26.0933054	1.09531305	0.91953115	0.00660545	0.0294499	ANO3

3572	ENSG00000134352	1147.43004	0.08325041	0.09723148	0.37844978	0.60738098	IL6ST
10468	ENSG00000134363	0	NA	NA	NA	NA	FST
10877	ENSG00000134365	0.74962837	0.04901693	0.38810411	0.32628015	NA	CFHR4
89796	ENSG00000134369	1454.90205	-1.87530389	0.09438128	5.36E-89	1.31E-86	NAV1
79577	ENSG00000134371	1013.3258	0.03055974	0.09405972	0.73788203	0.87620179	CDC73
10440	ENSG00000134375	1187.94288	-0.17659361	0.08089367	0.02527453	0.0878468	TIMM17A
23418	ENSG00000134376	136.408867	0.10862088	0.1928635	0.51639386	0.73049941	CRB1
81494	ENSG00000134389	0	NA	NA	NA	NA	CFHR5
10595	ENSG00000134398	0.46848334	-0.00803935	0.38327402	0.86029833	NA	ERN2
6210	ENSG00000134419	11180.2314	-0.08105411	0.05369427	0.1258031	0.29492303	RPS15A
30062	ENSG00000134438	18.7532335	2.79953216	0.61000232	2.57E-07	3.22E-06	RAX
4677	ENSG00000134440	3498.50049	0.29566076	0.07469925	5.31E-05	0.00043059	NARS1
2922	ENSG00000134443	0	NA	NA	NA	NA	GRP
57614	ENSG00000134444	581.748552	-0.23248458	0.10151164	0.01721887	0.06461552	RELCH
84893	ENSG00000134452	1335.46709	0.06903299	0.08050524	0.38084496	0.60984374	FBH1
84991	ENSG00000134453	1349.02521	-0.14449722	0.07818614	0.05872205	0.16833485	RBM17
3559	ENSG00000134460	0	NA	NA	NA	NA	IL2RA
54522	ENSG00000134461	198.203587	0.01027699	0.15387952	0.94472468	0.97752994	ANKRD16
79746	ENSG00000134463	127.169573	-0.1058444	0.19361321	0.52611192	0.73808941	ECHDC3
3601	ENSG00000134470	0	NA	NA	NA	NA	IL15RA
902	ENSG00000134480	443.139639	-0.14074738	0.14317628	0.28904425	0.51496389	CCNH
59340	ENSG00000134489	1.57145922	-0.01069123	0.37965719	0.85894273	NA	HRH4
85019	ENSG00000134490	233.817945	0.04940164	0.15878999	0.73354295	0.87316366	TMEM241
284252	ENSG00000134504	659.840072	0.23502277	0.09977722	0.01447083	0.05597607	KCTD1
91768	ENSG00000134508	357.962423	-1.13994918	0.15033982	3.67E-15	1.02E-13	CABLES1
1794	ENSG00000134516	0.37844916	0.02407552	0.3854191	0.60038877	NA	DOCK2
2012	ENSG00000134531	0.94504246	0.01121999	0.38197933	0.74133166	NA	EMP1
6660	ENSG00000134532	70.0984686	1.14952031	0.33680017	5.61E-05	0.00045241	SOX5
85004	ENSG00000134533	82.3789253	3.14772869	0.31681432	2.31E-24	1.08E-22	RERG
10599	ENSG00000134538	0.90005598	-0.05128821	0.38884012	0.27034634	NA	SLCO1B1
3824	ENSG00000134539	0	NA	NA	NA	NA	KLRD1
3821	ENSG00000134545	0	NA	NA	NA	NA	KLRC1
80763	ENSG00000134548	1.07380051	-0.04718186	0.38502688	0.42270356	NA	SPX
5555	ENSG00000134551	12.128728	0.14786015	0.36094331	0.40218715	0.63040759	PRH2
4038	ENSG00000134569	1105.48611	4.45135427	0.26924038	5.82E-63	8.02E-61	LRP4
4607	ENSG00000134571	0	NA	NA	NA	NA	MYBPC3
1643	ENSG00000134574	523.106783	0.41024944	0.12272936	0.00041243	0.00272117	DDB2
53	ENSG00000134575	580.140289	-0.11151841	0.1281263	0.35457318	0.58505754	ACP2

83844	ENSG00000134588	0.14931947	-0.01844332	0.38480212	0.84537521	NA	USP26
8933	ENSG00000134590	1574.5485	0.32512374	0.07827285	2.13E-05	0.00019052	RTL8C
9363	ENSG00000134594	213.079329	-0.47089803	0.17572979	0.00288708	0.01464203	RAB33A
6658	ENSG00000134595	0	NA	NA	NA	NA	SOX3
51634	ENSG00000134597	557.487773	-0.11605616	0.10170182	0.23417397	0.45103926	RBMX2
51765	ENSG00000134602	654.969767	0.38506018	0.10305547	0.00010235	0.00077653	STK26
219595	ENSG00000134612	0	NA	NA	NA	NA	FOLH1B
143689	ENSG00000134627	9.39210416	0.13408953	0.36954795	0.40788562	0.63609894	PIWIL4
4544	ENSG00000134640	0	NA	NA	NA	NA	MTNR1B
9698	ENSG00000134644	3220.86012	0.1450988	0.06060849	0.01530067	0.05854681	PUM1
90853	ENSG00000134668	3.34131511	0.02281896	0.37319787	0.80095763	0.90883611	SPOCD1
8565	ENSG00000134684	2605.79062	0.35812776	0.11065692	0.00070871	0.00437352	YARS1
1912	ENSG00000134686	2400.66169	0.22501897	0.06703951	0.00064528	0.00402714	PHC2
55143	ENSG00000134690	953.821602	-0.24765085	0.09335142	0.00608879	0.02746741	CDCA8
29889	ENSG00000134697	1467.73871	0.02415319	0.08305753	0.76654619	0.8924999	GNL2
192670	ENSG00000134698	860.263035	0.45087046	0.14494565	0.00080598	0.00490361	AGO4
51361	ENSG00000134709	552.044293	-0.00541723	0.14748655	0.967539	0.98759962	HOOK1
1573	ENSG00000134716	132.825615	0.1223055	0.20186442	0.48054236	0.70158112	CYP2J2
91408	ENSG00000134717	1465.76677	-0.30869155	0.08500054	0.00019257	0.00137484	BTF3L4
23318	ENSG00000134744	1657.0629	-0.0762239	0.11039998	0.46880505	0.69137681	TUT4
84950	ENSG00000134748	1407.52301	-0.10796995	0.07433242	0.13873304	0.31543588	PRPF38A
1824	ENSG00000134755	0	NA	NA	NA	NA	DSC2
1830	ENSG00000134757	0	NA	NA	NA	NA	DSG3
51444	ENSG00000134758	732.578227	-0.16148627	0.09322158	0.07282369	0.19700327	RNF138
55250	ENSG00000134759	1964.66937	-0.06732083	0.07330703	0.34903817	0.57983064	ELP2
1828	ENSG00000134760	0.89438146	0.01649853	0.38218118	0.70664981	NA	DSG1
1825	ENSG00000134762	0.15069912	-0.01848503	0.38480575	0.84537521	NA	DSC3
1823	ENSG00000134765	0	NA	NA	NA	NA	DSC1
1837	ENSG00000134769	435.326964	0.14953665	0.14267333	0.25862495	0.48003733	DTNA
80206	ENSG00000134775	1199.61145	-0.01945677	0.08337481	0.81046003	0.91412888	FHOD3
25941	ENSG00000134779	2530.20562	-0.18171929	0.0692533	0.00755056	0.03292376	TPGS2
747	ENSG00000134780	595.752736	0.01460867	0.10259749	0.88382694	0.94897201	DAGLA
29015	ENSG00000134802	232.756047	-1.48888245	0.18060875	1.42E-17	4.57E-16	SLC43A3
26519	ENSG00000134809	373.763944	-0.0334628	0.11833214	0.76547863	0.89220398	TIMM10
2694	ENSG00000134812	0	NA	NA	NA	NA	CBLIF
9704	ENSG00000134815	907.217255	0.29392627	0.0887505	0.00064578	0.00402903	DHX34
187	ENSG00000134817	5.05798726	-5.38468738	2.71171378	0.0002209	0.00155415	APLNR
9415	ENSG00000134824	9133.67809	-0.37677841	0.08211649	2.50E-06	2.65E-05	FADS2

746	ENSG00000134825	1001.05384	0.1103476	0.1075684	0.2855041	0.51095086	TMEM258
6947	ENSG00000134827	0	NA	NA	NA	NA	TCN1
27202	ENSG00000134830	6.26179199	0.07394434	0.37695379	0.48943804	0.70894726	C5AR2
55858	ENSG00000134851	1106.45966	0.23221041	0.08291905	0.00407376	0.01960892	TMEM165
9575	ENSG00000134852	856.905223	-0.52498117	0.14561729	0.00010801	0.00081513	CLOCK
5156	ENSG00000134853	5413.89268	-0.58539508	0.07561948	2.89E-15	8.12E-14	PDGFRA
87769	ENSG00000134864	71.1035964	-0.07546808	0.26322969	0.6940797	0.85201936	GGACT
1284	ENSG00000134871	4221.69532	0.22072993	0.05668279	8.15E-05	0.0006333	COL4A2
9071	ENSG00000134873	3.9441789	-0.22225355	0.45787195	0.08154923	0.21429626	CLDN10
22873	ENSG00000134874	2082.92764	-0.18521832	0.08724635	0.02889688	0.09740774	DZIP1
337867	ENSG00000134882	1093.48702	0.08218097	0.08761217	0.33559643	0.56555111	UBAC2
55082	ENSG00000134884	4690.37241	0.30560324	0.05442581	1.37E-08	2.03E-07	ARGLU1
54841	ENSG00000134897	1302.90266	-0.20665587	0.07774871	0.00652683	0.02915178	BIVM
2073	ENSG00000134899	1128.7562	0.16243324	0.09531164	0.07823257	0.20794939	ERCC5
7174	ENSG00000134900	1792.19138	-0.11201443	0.08164514	0.15965939	0.34722847	TPP2
79070	ENSG00000134901	599.337403	0.08602198	0.10234351	0.38339359	0.61266407	POGLUT2
79587	ENSG00000134905	1000.10783	-0.0338198	0.09399805	0.75044365	0.88259197	CARS2
9743	ENSG00000134909	1082.52522	-0.22986774	0.08982148	0.00837083	0.03587741	ARHGAP32
3703	ENSG00000134910	2189.3331	0.29367466	0.0869421	0.0005094	0.00328028	STT3A
11095	ENSG00000134917	0	NA	NA	NA	NA	ADAMTS8
56	ENSG00000134940	7.74286318	-0.37701518	0.58984567	0.05186163	0.15326642	ACRV1
2113	ENSG00000134954	1421.57476	-1.66139485	0.07618178	1.56E-106	5.09E-104	ETS1
219855	ENSG00000134955	39.5466272	0.3536522	0.33655945	0.11744233	0.28096658	SLC37A2
152831	ENSG00000134962	7.51819657	-0.07220524	0.3611115	0.6112979	0.79937717	KLB
51014	ENSG00000134970	1208.28112	-0.05433153	0.0846647	0.50991317	0.72545712	TMED7
324	ENSG00000134982	1233.44295	-0.50961946	0.09838684	8.52E-08	1.13E-06	APC
9315	ENSG00000134986	6631.0302	0.31815759	0.05058864	2.14E-10	3.97E-09	NREP
134430	ENSG00000134987	1196.72735	-0.08886046	0.08923092	0.30642537	0.53411107	WDR36
26578	ENSG00000134996	1.82026699	-0.07142347	0.38789106	0.32246275	NA	OSTF1
55312	ENSG00000135002	272.236189	-0.30565926	0.14851347	0.02522645	0.08774419	RFK
29979	ENSG00000135018	3136.26364	-0.06842502	0.06532073	0.28826574	0.5139923	UBQLN1
60560	ENSG00000135040	759.688978	-0.16467286	0.1459621	0.22047995	0.43280124	NAA35
55071	ENSG00000135045	309.849118	-0.01047996	0.13230664	0.93181152	0.9715756	C9orf40
301	ENSG00000135046	0	NA	NA	NA	NA	ANXA1
1514	ENSG00000135047	556.992958	0.21560926	0.10382834	0.03070111	0.10216479	CTSL
23670	ENSG00000135048	11.6821917	0.03407082	0.35188952	0.81357324	0.91581805	CEMIP2
23287	ENSG00000135049	748.889694	-0.00909451	0.09143501	0.91773523	0.9652015	AGTPBP1
51280	ENSG00000135052	2554.07107	-0.12747138	0.07005335	0.06394607	0.1796757	GOLM1

9413	ENSG00000135063	0.15069912	-0.01848503	0.38480575	0.84537521	NA	FAM189A2
29968	ENSG00000135069	2721.76105	0.06794635	0.10346405	0.49630763	0.714811	PSAT1
81689	ENSG00000135070	616.49397	-0.23477736	0.10592221	0.02067583	0.07489655	ISCA1
8728	ENSG00000135074	310.682872	-0.1893053	0.1417346	0.14953811	0.33234414	ADAM19
84868	ENSG00000135077	3.23825709	-0.02796289	0.37194222	0.77935458	0.89913327	HAVCR2
79616	ENSG00000135083	603.788844	-0.22169847	0.10466178	0.02720979	0.0930121	CCNJL
51347	ENSG00000135090	517.422802	0.02491402	0.11134801	0.8160477	0.9163678	TAOK3
84749	ENSG00000135093	441.232306	0.21744515	0.15373657	0.12168507	0.28819766	USP30
10993	ENSG00000135094	1.08372236	0.02404129	0.3820154	0.66253461	NA	SDS
4440	ENSG00000135097	1044.58001	0.38971478	0.11250621	0.0002837	0.00194493	MSI1
6927	ENSG00000135100	2.55400699	-0.14020803	0.41423226	0.09497602	NA	HNF1A
23014	ENSG00000135108	1546.85567	0.12938441	0.07152363	0.0654339	0.18258912	FBXO21
6926	ENSG00000135111	393.407248	0.05338044	0.11733071	0.63626929	0.81722218	TBX3
8638	ENSG00000135114	3.05805924	0.15915208	0.42267596	0.09146245	0.23347687	OASL
8739	ENSG00000135116	201.844411	4.08300446	0.23516817	1.62E-68	2.57E-66	HRK
84900	ENSG00000135119	388.51545	0.00335063	0.14018858	0.9807898	0.99355086	RNFT2
5025	ENSG00000135124	218.577779	0.73511059	0.21405911	0.00011098	0.00083399	P2RX4
92558	ENSG00000135127	159.555315	-1.86044092	0.22635731	1.23E-17	3.96E-16	BICDL1
1840	ENSG00000135144	1080.64143	-0.36573093	0.08866814	2.13E-05	0.00019042	DTX1
10906	ENSG00000135148	746.083782	-0.17308097	0.09799087	0.06696214	0.18552445	TRAFD1
9988	ENSG00000135164	3556.50437	0.04713234	0.06472322	0.46077269	0.68385174	DMTF1
4951	ENSG00000135175	0	NA	NA	NA	NA	OCM2
79161	ENSG00000135185	256.883749	0.10763681	0.15985576	0.45922641	0.68252693	TMEM243
57639	ENSG00000135205	114.930719	0.91457815	0.2449192	2.50E-05	0.00021943	CCDC146
85025	ENSG00000135211	196.202009	-0.00178442	0.16310323	0.98889098	0.99604194	TMEM60
948	ENSG00000135218	5.33545897	-0.05903543	0.37215521	0.59376495	0.78750562	CD36
79799	ENSG00000135220	0	NA	NA	NA	NA	UGT2A3
1447	ENSG00000135222	0	NA	NA	NA	NA	CSN2
54490	ENSG00000135226	0	NA	NA	NA	NA	UGT2B28
50640	ENSG00000135241	707.689675	0.0280769	0.12314114	0.81229544	0.91499949	PNPLA8
29923	ENSG00000135245	644.173443	0.23236218	0.11853507	0.03884382	0.12275587	HILPDA
84691	ENSG00000135248	0	NA	NA	NA	NA	FAM71F1
60561	ENSG00000135249	718.59237	-0.15087161	0.10166226	0.12268251	0.28988186	RINT1
6733	ENSG00000135250	1632.05697	-0.28787003	0.0876815	0.00072066	0.0044362	SRPK2
375616	ENSG00000135253	41.7130235	0.38166398	0.3784806	0.10275137	0.2544735	KCP
26136	ENSG00000135269	2414.12876	0.63307246	0.06613107	2.98E-22	1.25E-20	TES
29969	ENSG00000135272	0.18922458	0.01954053	0.38488496	0.78225479	NA	MDFIC
25821	ENSG00000135297	270.102639	0.01554939	0.1457822	0.9093106	0.96089067	MTO1

577	ENSG00000135298	522.964393	-1.59584239	0.14968361	1.18E-27	6.22E-26	ADGRB3
22881	ENSG00000135299	404.408469	-0.70289642	0.14820926	4.60E-07	5.52E-06	ANKRD6
3351	ENSG00000135312	0	NA	NA	NA	NA	HTR1B
80759	ENSG00000135314	48.4380258	-0.067728	0.25245591	0.72204163	0.86742752	KHDC1
22832	ENSG00000135315	387.787858	0.05738026	0.13928941	0.65918909	0.83105493	CEP162
10492	ENSG00000135316	5628.82296	-0.39831255	0.0620171	6.79E-11	1.33E-09	SYNCRIP
57231	ENSG00000135317	1722.16101	0.05027559	0.10720452	0.62752628	0.81099169	SNX14
4907	ENSG00000135318	52.0425309	3.101905	0.42112598	1.09E-14	2.88E-13	NT5E
112609	ENSG00000135324	219.81082	-0.73954699	0.19270495	2.41E-05	0.00021241	MRAP2
2045	ENSG00000135333	0	NA	NA	NA	NA	EPHA7
55122	ENSG00000135334	992.772979	0.2688933	0.08587488	0.00129646	0.00738916	AKIRIN2
23595	ENSG00000135336	560.206604	-0.23578149	0.10928606	0.02377529	0.08366474	ORC3
167691	ENSG00000135338	174.378184	-0.41766295	0.18530146	0.0108232	0.04421998	LCA5
6885	ENSG00000135341	1385.13852	-0.0819792	0.07536468	0.26744676	0.48999388	MAP3K7
1081	ENSG00000135346	0	NA	NA	NA	NA	CGA
84694	ENSG00000135355	0	NA	NA	NA	NA	GJA10
79899	ENSG00000135362	16.0971495	0.07314347	0.35208409	0.63470693	0.81607019	PRR5L
4005	ENSG00000135363	8.02938913	0.36009271	0.56659786	0.05726964	0.16526977	LMO2
51317	ENSG00000135365	2036.54575	0.17312083	0.06980003	0.01163409	0.04697436	PHF21A
55226	ENSG00000135372	1386.18099	0.12557853	0.07854703	0.10232436	0.25366929	NAT10
26298	ENSG00000135373	0	NA	NA	NA	NA	EHF
2001	ENSG00000135374	0	NA	NA	NA	NA	ELF5
79056	ENSG00000135378	0	NA	NA	NA	NA	PRRG4
4076	ENSG00000135387	7376.67273	-0.37932662	0.07194135	7.60E-08	1.02E-06	CAPRIN1
517	ENSG00000135390	3321.7681	0.02281839	0.05646297	0.68334566	0.84571005	ATP5MC2
85406	ENSG00000135392	1326.73555	-0.22735969	0.07602954	0.00223955	0.01180149	DNAJC14
967	ENSG00000135404	3429.86802	0.14782703	0.06615844	0.02069776	0.07496232	CD63
5630	ENSG00000135406	14.0251819	0.84668528	0.7817434	0.01644824	0.06219424	PRPH
10677	ENSG00000135407	28.5926156	0.16956395	0.34111563	0.37860076	0.607525	AVIL
269	ENSG00000135409	0.7148979	0.02945505	0.38490835	0.51061702	NA	AMHR2
90070	ENSG00000135413	0	NA	NA	NA	NA	LACRT
10220	ENSG00000135414	1313.6738	-0.15065375	0.08021378	0.05441337	0.15876841	GDF11
27165	ENSG00000135423	57.1552192	-0.12705571	0.27473894	0.5161772	0.73029706	GLS2
3679	ENSG00000135424	99.4570264	0.67329658	0.33896521	0.00827695	0.03552891	ITGA7
9840	ENSG00000135426	0	NA	NA	NA	NA	TESPA1
84070	ENSG00000135436	44.3992719	-0.01059171	0.2575054	0.95442574	0.98112988	FAM186B
5959	ENSG00000135437	25.3514526	0.08504067	0.30669226	0.65747967	0.83021873	RDH5
116986	ENSG00000135439	1324.84878	-0.78227251	0.07956156	1.62E-23	7.33E-22	AGAP2

2647	ENSG00000135441	550.616734	0.04209222	0.108918	0.68777443	0.84840902	BLOC1S1
3891	ENSG00000135443	0	NA	NA	NA	NA	KRT85
1019	ENSG00000135446	3936.6835	0.04775974	0.05886147	0.41546101	0.64244323	CDK4
5502	ENSG00000135447	0	NA	NA	NA	NA	PPP1R1A
10024	ENSG00000135451	961.135456	0.05379109	0.10657594	0.60009804	0.79083488	TROAP
6302	ENSG00000135452	523.305381	0.14595191	0.14875816	0.28772737	0.51340148	TSPAN31
2583	ENSG00000135454	1706.12538	-0.52644834	0.08056405	2.36E-11	4.84E-10	B4GALNT1
7024	ENSG00000135457	983.005971	-0.07067763	0.0940359	0.43740716	0.66264578	TFCP2
93058	ENSG00000135469	501.894889	-0.12589116	0.10608453	0.21587765	0.42667359	COQ10A
23017	ENSG00000135472	387.348285	-1.18488086	0.14126358	5.23E-18	1.73E-16	FAIM2
9924	ENSG00000135473	1110.88387	0.47399124	0.10219927	1.52E-06	1.66E-05	PAN2
9700	ENSG00000135476	1837.77316	-0.01900788	0.07780142	0.80144081	0.90902045	ESPL1
85349	ENSG00000135477	0	NA	NA	NA	NA	KRT87P
3855	ENSG00000135480	0.99626146	0.0153023	0.38222224	0.75501554	NA	KRT7
84872	ENSG00000135482	349.631131	0.07721996	0.13760312	0.54684054	0.75400072	ZC3H10
3178	ENSG00000135486	43950.1918	0.03306932	0.04261039	0.43488692	0.66027902	HNRNPA1
NA	ENSG00000135502	329.188955	0.83859474	0.15870501	2.15E-08	3.11E-07	SLC26A10
91	ENSG00000135503	1170.47253	-0.12484634	0.09412594	0.17080823	0.36283417	ACVR1B
10956	ENSG00000135506	2419.86689	0.08678323	0.06424144	0.17038148	0.36230289	OS9
4284	ENSG00000135517	0.91194749	-0.03735534	0.3845512	0.46467032	NA	MIP
23416	ENSG00000135519	20.0525048	0.10692812	0.31993957	0.57342249	0.7729263	KCNH3
84946	ENSG00000135521	597.855391	-0.08120069	0.11543611	0.45996141	0.68305646	LTV1
9053	ENSG00000135525	353.980543	-1.32095039	0.13867852	1.56E-22	6.60E-21	MAP7
8763	ENSG00000135535	1654.69383	0.37805842	0.07322946	1.39E-07	1.80E-06	CD164
246269	ENSG00000135537	103.331422	0.58962703	0.25245968	0.00487367	0.02274074	AFG1L
57224	ENSG00000135540	0.34746601	0.02370763	0.3853651	0.61987128	NA	NHSL1
54806	ENSG00000135541	827.455265	0.1696891	0.11868777	0.13201462	0.30496778	AHI1
23493	ENSG00000135547	0	NA	NA	NA	NA	HEY2
5570	ENSG00000135549	2.54084671	0.12073835	0.39994038	0.20834867	NA	PKIB
9038	ENSG00000135569	0	NA	NA	NA	NA	TAAR5
4829	ENSG00000135577	0.14931947	-0.01844332	0.38480212	0.84537521	NA	NMBR
6610	ENSG00000135587	139.293188	0.34409058	0.22354799	0.064332	0.18047892	SMPD2
64780	ENSG00000135596	2614.21135	0.08957412	0.06609227	0.16899017	0.36039348	MICAL1
85021	ENSG00000135597	1002.59543	0.38668751	0.09629304	3.24E-05	0.00027676	REPS1
8676	ENSG00000135604	0	NA	NA	NA	NA	STX11
7006	ENSG00000135605	9.55560461	1.88229859	1.2099621	0.00333944	0.01656776	TEC
84279	ENSG00000135617	177.035102	-0.268886	0.17690801	0.08645526	0.22396418	PRADC1
10505	ENSG00000135622	578.152399	0.13777652	0.11772657	0.21841543	0.43045674	SEMA4F

10574	ENSG00000135624	5548.60492	-0.36324795	0.05843364	3.04E-10	5.56E-09	CCT7
1961	ENSG00000135625	2.2990923	0.0500886	0.37980647	0.55697654	NA	EGR4
26056	ENSG00000135631	453.686136	-0.11070403	0.14206764	0.39987249	0.62834391	RAB11FIP5
10322	ENSG00000135632	554.323164	0.35145036	0.1390156	0.00661154	0.02947042	SMYD5
8291	ENSG00000135636	6.42108751	-0.02210732	0.37204283	0.79846515	0.90769144	DYSF
84865	ENSG00000135637	469.139018	0.3893426	0.12799454	0.00123494	0.00708129	CCDC142
2016	ENSG00000135638	0.69464464	-0.03798381	0.38678913	0.40441551	NA	EMX1
27345	ENSG00000135643	151.556747	0.20658714	0.18030438	0.19477138	0.39796747	KCNMB4
9958	ENSG00000135655	494.223574	-0.21747164	0.14433291	0.10247302	0.25394264	USP15
2799	ENSG00000135677	1457.99361	0.19200201	0.08029359	0.01435835	0.05563861	GNS
1368	ENSG00000135678	0.63890143	-0.01817255	0.38287285	0.68787973	NA	CPM
4193	ENSG00000135679	1661.88481	-0.26230721	0.07857618	0.00062911	0.00394488	MDM2
79786	ENSG00000135686	505.697651	0.28227941	0.14676908	0.03702967	0.11843762	KLHL36
53630	ENSG00000135697	0	NA	NA	NA	NA	BCO1
10200	ENSG00000135698	205.95796	0.00657125	0.1540593	0.96162506	0.98439922	MPHOSPH6
23563	ENSG00000135702	4.70852485	0.11133268	0.39416269	0.27327327	0.49676945	CHST5
9764	ENSG00000135709	274.814413	-0.41664853	0.14790751	0.00229055	0.01202554	KIAA0513
1783	ENSG00000135720	3369.68554	0.26577437	0.0667697	5.19E-05	0.00042223	DYNC1LI2
55336	ENSG00000135722	178.656548	0.65229367	0.21849113	0.00065016	0.00405376	FBXL8
29109	ENSG00000135723	973.828554	-0.09041859	0.09531134	0.32646169	0.55472764	FHOD1
92922	ENSG00000135736	283.465128	0.77347486	0.16251284	3.70E-07	4.51E-06	CCDC102A
6553	ENSG00000135740	294.661608	0.00674854	0.15253035	0.96376187	0.98562039	SLC9A5
183	ENSG00000135744	33.5179136	0.07401296	0.28269972	0.70453254	0.85803894	AGT
NA	ENSG00000135747	19.4295342	-0.15672007	0.34303827	0.40786056	0.63609894	ZNF670-ZNF695
80003	ENSG00000135749	1226.87846	-0.02506984	0.10997787	0.81063058	0.9141961	PCNX2
3775	ENSG00000135750	511.486181	-1.22090947	0.14489129	3.66E-18	1.23E-16	KCNK1
9816	ENSG00000135763	1153.55887	-0.07287659	0.08827233	0.39673229	0.6258107	URB2
54583	ENSG00000135766	790.445254	-0.46013985	0.11819456	4.27E-05	0.0003563	EGLN1
10753	ENSG00000135773	0.84529519	0.02200162	0.3832441	0.76339043	NA	CAPN9
22796	ENSG00000135775	850.278633	-0.15243107	0.09497873	0.09702687	0.24361087	COG2
23456	ENSG00000135776	498.67024	-0.15462546	0.110967	0.14437939	0.32440399	ABCB10
84284	ENSG00000135778	1114.31034	-0.14732376	0.08361243	0.07054095	0.19264038	NTPCR
27097	ENSG00000135801	916.89916	-0.14394027	0.08931641	0.09691384	0.24348114	TAF5L
2752	ENSG00000135821	2948.11752	0.33911481	0.06461299	9.78E-08	1.29E-06	GLUL
10228	ENSG00000135823	1347.76452	-0.3727668	0.0785566	1.20E-06	1.34E-05	STX6
85397	ENSG00000135824	8.05069043	-0.14897025	0.3906545	0.30146077	0.52877568	RGS8
6041	ENSG00000135828	0	NA	NA	NA	NA	RNASEL
1660	ENSG00000135829	8472.31585	-0.03010269	0.06312053	0.64416905	0.8223082	DHX9

57710	ENSG00000135835	1300.50941	-1.32668449	0.11520205	9.66E-32	5.83E-30	KIAA1614
9857	ENSG00000135837	1704.99605	-0.36533025	0.10387343	0.00024778	0.00172486	CEP350
80896	ENSG00000135838	169.614355	0.89230034	0.21023441	3.12E-06	3.24E-05	NPL
116496	ENSG00000135842	124.673299	1.69271254	0.261175	7.35E-12	1.58E-10	NIBAN1
5279	ENSG00000135845	478.982339	-0.35659943	0.11545659	0.0011605	0.00672438	PIGC
3915	ENSG00000135862	4459.55197	0.26727859	0.05825262	3.37E-06	3.48E-05	LAMC1
149041	ENSG00000135870	675.743743	-0.38162747	0.12877396	0.0016214	0.00897563	RC3H1
9290	ENSG00000135898	0	NA	NA	NA	NA	GPR55
3431	ENSG00000135899	52.2343379	4.64837966	0.55741106	8.79E-18	2.87E-16	SP110
65080	ENSG00000135900	461.297795	-0.16545025	0.11981236	0.1450449	0.32541747	MRPL44
1144	ENSG00000135902	15.4524416	1.39675649	0.82705669	0.00414172	0.01986263	CHRND
5077	ENSG00000135903	0	NA	NA	NA	NA	PAX3
55619	ENSG00000135905	752.479278	-0.02903391	0.10514655	0.773102	0.89561741	DOCK10
9654	ENSG00000135912	798.385811	-0.34613917	0.11627167	0.0017244	0.00945088	TTLL4
57695	ENSG00000135913	580.321087	-0.37131536	0.13967328	0.00423544	0.02025333	USP37
3357	ENSG00000135914	5.46797749	-0.12808905	0.39055038	0.31330329	0.54126787	HTR2B
81618	ENSG00000135916	6985.56426	-0.0219671	0.05443762	0.68618718	0.8472712	ITM2C
80704	ENSG00000135917	0	NA	NA	NA	NA	SLC19A3
5270	ENSG00000135919	364.175701	1.06871343	0.13037832	3.01E-17	9.51E-16	SERPINE2
3300	ENSG00000135924	820.793491	0.04199897	0.09034598	0.63308336	0.8146565	DNAJB2
80326	ENSG00000135925	13.5056203	-1.3508571	0.84623722	0.00471857	0.02213638	WNT10A
64114	ENSG00000135926	2.59326384	0.0573287	0.38148694	0.4816714	0.70240082	TMBIM1
1593	ENSG00000135929	10.5114477	0.05049561	0.34073961	0.75934691	0.88850157	CYP27A1
9470	ENSG00000135930	1589.35099	-0.09321626	0.1001916	0.33391644	0.56367754	EIF4E2
80210	ENSG00000135931	610.653492	0.38272258	0.1292337	0.00163792	0.00904935	ARMC9
51719	ENSG00000135932	856.491317	-0.20983562	0.1029486	0.03392493	0.11068419	CAB39
1329	ENSG00000135940	1584.84159	0.08824781	0.08166234	0.26887631	0.49179552	COX5B
51455	ENSG00000135945	804.303483	-0.02430327	0.12468591	0.83581399	0.92515898	REV1
80705	ENSG00000135951	92.68385	0.66616502	0.29028122	0.00420789	0.02014102	TSGA10
84804	ENSG00000135953	449.092885	0.0592786	0.13329855	0.64035677	0.81986824	MFSD9
55654	ENSG00000135956	993.33222	-0.01589927	0.08516114	0.84685226	0.93052693	TMEM127
10913	ENSG00000135960	1.52691924	-0.00089333	0.37956646	0.9876641	NA	EDAR
9392	ENSG00000135966	959.904998	-0.2075049	0.09327101	0.02151712	0.07721099	TGFBRAP1
9648	ENSG00000135968	818.039671	0.34842251	0.10945618	0.00087194	0.00525421	GCC2
64965	ENSG00000135972	396.249344	0.25326511	0.13817854	0.0488842	0.14644564	MRPS9
11250	ENSG00000135973	0.79620076	-0.00721012	0.38151312	0.88962119	NA	GPR45
79074	ENSG00000135974	535.755188	-0.14358354	0.1216273	0.21200793	0.42163567	C2orf49
375248	ENSG00000135976	449.536024	0.08975039	0.15012464	0.51650062	0.73056247	ANKRD36

26122	ENSG00000135999	703.679126	-0.2316063	0.10202612	0.01820804	0.0674095	EPC2
50649	ENSG00000136002	522.118712	1.34E-05	0.11898696	0.99911734	0.99962651	ARHGEF4
23479	ENSG00000136003	822.835289	-0.0138151	0.08969382	0.87347957	0.94441388	ISCU
160428	ENSG00000136010	586.907613	0.61792421	0.15859233	2.67E-05	0.00023334	ALDH1L2
55576	ENSG00000136011	3.1532859	-0.09825307	0.39957152	0.09516866	0.24026852	STAB2
84101	ENSG00000136014	281.847113	0.11591559	0.17256313	0.45162389	0.67491849	USP44
55681	ENSG00000136021	1171.92264	-0.12506974	0.08751125	0.1415004	0.3198915	SCYL2
10970	ENSG00000136026	1328.0599	0.08119794	0.07672965	0.28036896	0.5049178	CKAP4
10154	ENSG00000136040	143.202841	-1.5042337	0.23796728	1.94E-11	3.99E-10	PLXNC1
55198	ENSG00000136044	1477.60575	0.47334665	0.0799218	1.39E-09	2.34E-08	APPL2
11137	ENSG00000136045	872.583764	-0.19210555	0.09528036	0.03688262	0.118091	PWP1
55332	ENSG00000136048	34.5402496	4.72405174	0.66680439	1.59E-13	3.84E-12	DRAM1
23325	ENSG00000136051	1063.69356	0.11865635	0.10252945	0.22974051	0.44547026	WASHC4
84102	ENSG00000136052	85.9345724	-0.14879262	0.27272617	0.44857285	0.67234398	SLC41A2
50853	ENSG00000136059	163.714094	0.45348871	0.21316687	0.01299725	0.05142908	VILL
2317	ENSG00000136068	3331.65219	-0.08748161	0.0619346	0.15115984	0.33504762	FLNB
4752	ENSG00000136098	359.92346	0.29191429	0.13104308	0.01747559	0.06534407	NEK3
5100	ENSG00000136099	15.7114922	-0.16357174	0.35445154	0.37613685	0.60518654	PCDH8
51028	ENSG00000136100	1122.39819	0.06921167	0.09932137	0.47120996	0.69358302	VPS36
79621	ENSG00000136104	748.30194	-0.25612527	0.11839825	0.02251122	0.08005423	RNASEH2B
26586	ENSG00000136108	2248.58404	-0.34288963	0.07929817	9.47E-06	9.02E-05	CKAP2
11061	ENSG00000136110	0	NA	NA	NA	NA	CNMD
9882	ENSG00000136111	565.276447	-0.25847158	0.12090646	0.02376076	0.08364327	TBC1D4
55901	ENSG00000136114	59.71724	-0.03161587	0.24715465	0.86621983	0.94101743	THSD1
79866	ENSG00000136122	293.241943	0.11548402	0.13441344	0.35851581	0.58882066	BORA
23143	ENSG00000136141	887.915051	0.12251303	0.08800477	0.15256369	0.33710476	LRCH1
8803	ENSG00000136143	684.615646	-0.04582875	0.11768345	0.68134618	0.84428882	SUCLA2
55213	ENSG00000136144	832.643646	0.07336833	0.09318768	0.41744917	0.64370875	RCBTB1
29079	ENSG00000136146	665.418414	0.01774573	0.10736995	0.86425258	0.93988845	MED4
51131	ENSG00000136147	2.23786561	-0.07590221	0.38736235	0.34319693	NA	PHF11
NA	ENSG00000136149	2.07725048	-0.10268584	0.39703388	0.20207701	NA	RPL13AP25
83548	ENSG00000136152	688.308551	0.15819871	0.0986493	0.09674804	0.24318777	COG3
4008	ENSG00000136153	962.319575	0.03810011	0.10300545	0.70090899	0.85575596	LMO7
8796	ENSG00000136155	0	NA	NA	NA	NA	SCEL
9445	ENSG00000136156	2281.53616	0.02393611	0.06956767	0.72725859	0.86953012	ITM2B
10253	ENSG00000136158	2619.73898	0.54714992	0.0714584	6.85E-15	1.85E-13	SPRY2
55270	ENSG00000136159	491.602086	-0.37043195	0.11703835	0.00085931	0.00518354	NUDT15
1910	ENSG00000136160	12.8605937	1.87670316	0.80940713	0.00090175	0.00541651	EDNRB

1102	ENSG00000136161	185.178509	-0.56282429	0.2092216	0.00205228	0.01093959	RCBTB2
3936	ENSG00000136167	41.9729054	0.08212027	0.26341337	0.67292467	0.83933156	LCP1
83852	ENSG00000136169	246.450424	0.08275434	0.16127074	0.57260925	0.77240737	SETDB2
9805	ENSG00000136193	6717.9516	-0.32913907	0.05312836	3.76E-10	6.82E-09	SCRN1
79020	ENSG00000136197	213.833414	-0.06856272	0.16339467	0.64193592	0.82088358	C7orf25
64759	ENSG00000136205	3416.50271	-0.8776012	0.07516727	2.80E-32	1.71E-30	TNS3
285955	ENSG00000136206	25.0834685	0.33799673	0.38831131	0.13566457	0.31030495	SPDYE1
55501	ENSG00000136213	1482.01675	-0.00779982	0.09522395	0.92719154	0.96953959	CHST12
10643	ENSG00000136231	2062.22687	0.08495222	0.06935788	0.21371673	0.4238788	IGF2BP3
10457	ENSG00000136235	2.54920605	0.0224789	0.37686318	0.77173087	NA	GPNMB
9771	ENSG00000136237	503.263619	0.70491609	0.11735203	4.42E-10	7.95E-09	RAPGEF5
5879	ENSG00000136238	5847.35436	-0.07536461	0.06435909	0.19660786	0.40019493	RAC1
11014	ENSG00000136240	3093.50755	-0.16315719	0.07267292	0.02215001	0.07903887	KDELR2
11097	ENSG00000136243	889.247574	-0.02541366	0.09930273	0.79018173	0.90434126	NUP42
3569	ENSG00000136244	0	NA	NA	NA	NA	IL6
55146	ENSG00000136247	1114.47995	0.03489082	0.10862419	0.73868404	0.87647218	ZDHHC4
313	ENSG00000136250	1.71748819	-0.06485623	0.38712854	0.33809145	NA	AOAH
28969	ENSG00000136261	1590.3298	-0.11921955	0.07519617	0.10560418	0.25927323	BZW2
1607	ENSG00000136267	22.7452794	4.29587059	0.78876868	4.98E-09	7.79E-08	DGKB
9238	ENSG00000136270	2010.22698	0.03855746	0.07743858	0.61181593	0.7998437	TBRG4
54606	ENSG00000136271	2361.5661	-0.03475754	0.06439179	0.5821862	0.77907556	DDX56
3364	ENSG00000136273	487.532951	-0.40770556	0.14317524	0.00213482	0.01131877	HUS1
23148	ENSG00000136274	1465.02321	-0.76451753	0.09354066	6.12E-17	1.88E-15	NACAD
80099	ENSG00000136275	0	NA	NA	NA	NA	C7orf69
28988	ENSG00000136279	4159.96013	-0.00285188	0.05839726	0.95919305	0.9831126	DBNL
83605	ENSG00000136280	1180.55026	0.01053158	0.07691744	0.88948211	0.95181279	CCM2
64005	ENSG00000136286	18.8640418	0.04474401	0.33239563	0.7947184	0.90579008	MYO1G
80727	ENSG00000136295	12191.3388	-0.18882547	0.05446238	0.00045175	0.00295601	TTYH3
221938	ENSG00000136297	31.3048476	1.36534327	0.5195731	0.00053491	0.00341898	MMD2
27141	ENSG00000136305	5.73219115	-0.10882225	0.40032792	0.14558572	0.32613386	CIDEB
NA	ENSG00000136315	0	NA	NA	NA	NA	
91875	ENSG00000136319	536.252632	0.05347188	0.15513928	0.707405	0.85903201	TTC5
26257	ENSG00000136327	0	NA	NA	NA	NA	NKX2-8
7080	ENSG00000136352	0	NA	NA	NA	NA	NKX2-1
85446	ENSG00000136367	600.26467	0.20564598	0.1062637	0.0436355	0.13397223	ZFHX2
10588	ENSG00000136371	68.0426917	-0.40235783	0.29143271	0.06466739	0.18119939	MTHFS
11173	ENSG00000136378	2479.19359	0.89966455	0.0658078	2.50E-43	2.07E-41	ADAMTS7
58489	ENSG00000136379	184.661192	-0.21091493	0.1678881	0.16135053	0.34958987	ABHD17C

3658	ENSG00000136381	2876.94108	-0.10334031	0.07203859	0.14373874	0.32322075	IREB2
57538	ENSG00000136383	55.0480872	0.09576336	0.38576577	0.37141351	0.60057374	ALPK3
53346	ENSG00000136404	2.91224807	0.00505477	0.3745015	0.95358494	0.98082457	TM6SF1
10518	ENSG00000136425	669.845165	-0.04697848	0.098341	0.62030883	0.80621861	CIB2
10241	ENSG00000136436	599.778615	0.31832926	0.11676919	0.00413128	0.01982214	CALCOCO2
55316	ENSG00000136444	1464.22839	0.20719741	0.0853276	0.01285764	0.05102931	RSAD1
4836	ENSG00000136448	2184.8563	0.09387211	0.07516618	0.20322798	0.40930371	NMT1
84073	ENSG00000136449	111.606325	0.4141581	0.27674062	0.05216395	0.15389334	MYCBPAP
6426	ENSG00000136450	11444.4215	0.04719528	0.06677194	0.47126923	0.69361881	SRSF1
7716	ENSG00000136451	2260.76358	-0.08820756	0.0661656	0.17520274	0.36912205	VEZF1
1101	ENSG00000136457	13.2386537	0.64058607	0.91522878	0.02561858	0.08878125	CHAD
51204	ENSG00000136463	591.190986	-0.02179054	0.10819292	0.83258931	0.92392457	TACO1
55852	ENSG00000136478	1173.22747	-0.1557201	0.07917257	0.04408758	0.13508843	TEX2
10238	ENSG00000136485	11047.7436	-0.05863877	0.04929292	0.23023337	0.4461643	DCAF7
2689	ENSG00000136487	0	NA	NA	NA	NA	GH2
1442	ENSG00000136488	0	NA	NA	NA	NA	CSH1
80774	ENSG00000136490	3588.18327	0.38069728	0.07021216	3.35E-08	4.69E-07	LIMD2
83990	ENSG00000136492	1435.0552	-0.3820954	0.08263042	2.09E-06	2.24E-05	BRIP1
11143	ENSG00000136504	1290.80994	-0.06111873	0.07678578	0.41601606	0.64286616	KAT7
64108	ENSG00000136514	0	NA	NA	NA	NA	RTP4
86	ENSG00000136518	1432.39388	0.13967196	0.0891021	0.10682334	0.26165125	ACTL6A
4711	ENSG00000136521	1065.57135	-0.06557923	0.09245006	0.46416311	0.68693094	NDUFB5
57129	ENSG00000136522	583.193834	0.03737253	0.11010721	0.72408563	0.86797529	MRPL47
6434	ENSG00000136527	4688.23015	-0.08434586	0.06776764	0.20635374	0.41333377	TRA2B
6326	ENSG00000136531	503.444153	-0.28607413	0.12881455	0.01798762	0.0669056	SCN2A
10716	ENSG00000136535	6.687336	0.40551956	0.70734586	0.02030573	0.07378491	TBR1
64844	ENSG00000136536	1371.84368	-0.1431787	0.09736132	0.12761612	0.29804636	MARCHF7
57471	ENSG00000136541	0	NA	NA	NA	NA	ERMN
11227	ENSG00000136542	0	NA	NA	NA	NA	GALNT5
6332	ENSG00000136546	2.72431821	0.05656952	0.37693513	0.56829906	0.76920816	SCN7A
10010	ENSG00000136560	412.223266	0.01618641	0.12663016	0.89470766	0.95456012	TANK
640	ENSG00000136573	48.1307517	-0.57444772	0.37056406	0.02502219	0.08721718	BLK
2626	ENSG00000136574	387.367747	-0.41468934	0.15834141	0.00410233	0.01971184	GATA4
6498	ENSG00000136603	929.939955	0.13581525	0.10556342	0.18117223	0.37844358	SKIL
2058	ENSG00000136628	4335.8086	-0.0290862	0.07102193	0.67602787	0.84148271	EPRS1
3142	ENSG00000136630	43.5558498	0.97088273	0.38134514	0.0011343	0.00658025	HLX
11311	ENSG00000136631	1703.10946	-0.08535743	0.07429844	0.23710283	0.45410948	VPS45
3586	ENSG00000136634	0	NA	NA	NA	NA	IL10

51133	ENSG00000136636	2224.65984	0.20561829	0.0663113	0.00162874	0.00901119	KCTD3
26750	ENSG00000136643	1047.83424	-0.3000209	0.10106786	0.00202611	0.01081755	RPS6KC1
150472	ENSG00000136682	474.566969	0.02947767	0.11021745	0.78086325	0.89982732	CBWD2
56300	ENSG00000136688	0	NA	NA	NA	NA	IL36G
3557	ENSG00000136689	0	NA	NA	NA	NA	IL1RN
27179	ENSG00000136694	0	NA	NA	NA	NA	IL36A
26525	ENSG00000136695	0	NA	NA	NA	NA	IL36RN
27177	ENSG00000136696	0	NA	NA	NA	NA	IL36B
84639	ENSG00000136697	0	NA	NA	NA	NA	IL1F10
55997	ENSG00000136698	0	NA	NA	NA	NA	CFC1
55627	ENSG00000136699	2221.04839	0.13992225	0.07466741	0.05604963	0.16260047	SMPD4
55339	ENSG00000136709	2045.68421	-0.44164385	0.10104344	5.72E-06	5.69E-05	WDR33
84317	ENSG00000136710	454.25845	0.03889624	0.11825759	0.73049771	0.87107559	CCDC115
79595	ENSG00000136715	839.575714	-0.18268723	0.0892693	0.03500018	0.11331652	SAP130
274	ENSG00000136717	2212.76325	-0.56194253	0.06795639	4.56E-17	1.42E-15	BIN1
92856	ENSG00000136718	1334.05612	-0.01173863	0.08692443	0.88900971	0.95165386	IMP4
9394	ENSG00000136720	1681.17913	-0.84248585	0.07330297	2.55E-31	1.51E-29	HS6ST1
56886	ENSG00000136731	3845.37303	-0.09118034	0.07864291	0.23558356	0.45240308	UGGT1
2995	ENSG00000136732	0	NA	NA	NA	NA	GYPC
8027	ENSG00000136738	575.692409	-0.07784471	0.09923811	0.41605441	0.64287535	STAM
2572	ENSG00000136750	0	NA	NA	NA	NA	GAD2
10006	ENSG00000136754	540.631959	0.01309923	0.10650066	0.89940131	0.95557174	ABI1
10730	ENSG00000136758	3447.71752	-0.05203374	0.06021727	0.3794728	0.60845927	YME1L1
64215	ENSG00000136770	335.287401	0.54174478	0.16568576	0.0003524	0.00236443	DNAJC1
25934	ENSG00000136783	206.937482	0.18676943	0.161425	0.20210605	0.4076641	NIPSNAP3A
56262	ENSG00000136802	2233.99519	-0.08130204	0.0671297	0.21859308	0.43050004	LRRC8A
1025	ENSG00000136807	1071.31348	-0.20143469	0.09325215	0.02557773	0.08867067	CDK9
7295	ENSG00000136810	1176.5322	0.25197833	0.08207731	0.00163462	0.00903399	TXN
4957	ENSG00000136811	1521.73537	-0.1454552	0.09313148	0.10659219	0.26121406	ODF2
23392	ENSG00000136813	1863.18583	0.16992643	0.06810209	0.01118184	0.04546992	ECPAS
27348	ENSG00000136816	708.137704	-0.16198335	0.124288	0.16692502	0.35783819	TOR1B
51759	ENSG00000136819	1069.31402	-0.2188848	0.08727117	0.00985662	0.0409823	C9orf78
10592	ENSG00000136824	1722.24801	-0.36773666	0.07655189	9.06E-07	1.03E-05	SMC2
9314	ENSG00000136826	0.173733	0.01914545	0.38484597	0.78225479	NA	KLF4
1861	ENSG00000136827	1149.82874	-0.13510597	0.09090987	0.12547108	0.29435597	TOR1A
9649	ENSG00000136828	793.165529	-0.0883813	0.13345092	0.47953025	0.70069817	RALGPS1
64855	ENSG00000136830	921.688424	-0.07060728	0.09052665	0.42188911	0.64785449	NIBAN2
347168	ENSG00000136834	0.70195379	0.02750895	0.38372988	0.5667643	NA	OR1J1

286362	ENSG00000136839	0	NA	NA	NA	NA	OR13C9
27090	ENSG00000136840	251.624992	-0.24333087	0.1597611	0.09281015	0.2357385	ST6GALNAC4
7111	ENSG00000136842	1694.91302	-0.48152983	0.07217396	1.08E-11	2.29E-10	TMOD1
153090	ENSG00000136848	2678.05867	-0.20118859	0.06276044	0.00112546	0.00653472	DAB2IP
6812	ENSG00000136854	3001.85712	-0.19367753	0.0698483	0.00475685	0.02227424	STXBP1
29988	ENSG00000136856	217.680506	0.0959217	0.16160027	0.51499204	0.72955207	SLC2A8
23452	ENSG00000136859	403.470963	-0.34762235	0.1609531	0.01715903	0.06443973	ANGPTL2
55755	ENSG00000136861	2875.64079	-0.19284464	0.07759901	0.01101076	0.04489384	CDK5RAP2
7539	ENSG00000136866	187.539261	-0.26579533	0.16165514	0.06930486	0.19007581	ZFP37
1318	ENSG00000136867	32.2459783	0.66715745	0.44707162	0.0198291	0.07247778	SLC31A2
1317	ENSG00000136868	728.328926	0.22618875	0.09935151	0.01817985	0.06735733	SLC31A1
7099	ENSG00000136869	3.00969588	-0.07122255	0.39016502	0.22322201	0.43637055	TLR4
7743	ENSG00000136870	503.637734	-0.16015484	0.11428881	0.14112474	0.31929662	ZNF189
229	ENSG00000136872	2.92530196	0.00854652	0.36869571	0.93746541	0.97366414	ALDOB
55014	ENSG00000136874	814.164946	-0.05694639	0.10091285	0.55733516	0.76258372	STX17
9128	ENSG00000136875	1228.49802	0.06054404	0.0798071	0.43839481	0.66364665	PRPF4
2356	ENSG00000136877	981.311293	0.21554573	0.09859528	0.02341102	0.08263201	FPGS
10868	ENSG00000136878	831.016952	-0.10351732	0.12337734	0.3744246	0.60355736	USP20
570	ENSG00000136881	1.11474076	0.07782957	0.39460367	0.13653044	NA	BAAT
113220	ENSG00000136883	3.86977168	-0.0279629	0.36995526	0.78665886	0.90294962	KIF12
9550	ENSG00000136888	1475.38139	0.08712581	0.07107742	0.21250361	0.42238326	ATP6V1G1
54881	ENSG00000136891	1026.41554	-0.29123911	0.08525027	0.00044226	0.00289969	TEX10
84253	ENSG00000136895	302.060153	0.16216521	0.16011878	0.26498147	0.48718221	GARNL3
54534	ENSG00000136897	424.665054	-0.16748248	0.12369695	0.15162291	0.33558862	MRPL50
8818	ENSG00000136908	1210.58348	-0.035197	0.07879086	0.64856661	0.82457939	DPM2
401551	ENSG00000136918	0	NA	NA	NA	NA	WDR38
158427	ENSG00000136925	633.147926	-0.13186222	0.1312977	0.28379232	0.50898929	TSTD2
9568	ENSG00000136928	0.3237694	-0.02327256	0.38532127	0.66864002	NA	GABBR2
55363	ENSG00000136929	0.3565367	0.02381602	0.3853809	0.61408995	NA	HEMGN
5695	ENSG00000136930	1982.90634	0.02793473	0.07185336	0.69241575	0.85130623	PSMB7
2516	ENSG00000136931	2.0441195	0.05224442	0.38112862	0.52099165	NA	NR5A1
51531	ENSG00000136932	111.469708	-0.22378709	0.21113773	0.2072353	0.41447107	TRMO
10244	ENSG00000136933	402.855372	0.26756089	0.12818089	0.02639102	0.09083932	RABEPK
2800	ENSG00000136935	609.624148	0.12455022	0.11069003	0.23981624	0.45788499	GOLGA1
7507	ENSG00000136936	228.235939	0.07442888	0.14665739	0.58162157	0.77868762	XPA
4686	ENSG00000136937	1973.14829	-0.18014052	0.08444711	0.02837671	0.09607405	NCBP1
10541	ENSG00000136938	3086.37795	-0.13504286	0.06313959	0.02996123	0.10020747	ANP32B
254973	ENSG00000136939	0	NA	NA	NA	NA	OR1L4

5082	ENSG00000136940	660.916522	-0.2007957	0.09683946	0.03175044	0.10495312	PDCL
11224	ENSG00000136942	8772.2788	-0.08033186	0.05197185	0.11950101	0.28438287	RPL35
1515	ENSG00000136943	191.410429	-0.04555864	0.15637542	0.74880577	0.88205755	CTSV
4010	ENSG00000136944	25.2840827	4.70954976	0.76765554	1.91E-10	3.57E-09	LMX1B
81873	ENSG00000136950	867.163955	-0.08058816	0.08965412	0.35435921	0.58485041	ARPC5L
5168	ENSG00000136960	2.97946291	0.2394008	0.49856423	0.00393216	0.01902316	ENPP2
79075	ENSG00000136982	370.292289	-0.23637541	0.1317343	0.05515057	0.16050862	DSCC1
79139	ENSG00000136986	1389.42078	-0.46514525	0.08198815	6.19E-09	9.59E-08	DERL1
4609	ENSG00000136997	1875.44982	-0.29046996	0.06749035	1.17E-05	0.00010998	MYC
4856	ENSG00000136999	0	NA	NA	NA	NA	CCN3
90865	ENSG00000137033	0	NA	NA	NA	NA	IL33
90871	ENSG00000137038	740.0952	0.02214861	0.1013278	0.82181095	0.91847813	DMAC1
26953	ENSG00000137040	734.551107	-0.02531783	0.09883022	0.78958133	0.9043268	RANBP6
64425	ENSG00000137054	837.272231	-0.29151577	0.09363875	0.00125755	0.00719638	POLR1E
9373	ENSG00000137055	1013.11458	-0.13647832	0.08684843	0.1059198	0.25982306	PLAA
3590	ENSG00000137070	301.434908	0.38665357	0.16808766	0.01083055	0.04424092	IL11RA
55833	ENSG00000137073	1174.68663	0.01027482	0.08354304	0.89888376	0.95556135	UBAP2
54840	ENSG00000137074	489.644984	-0.09255373	0.12548907	0.4338897	0.65914024	APTX
152006	ENSG00000137075	737.318082	0.32492812	0.10097656	0.00082823	0.0050112	RNF38
7094	ENSG00000137076	5916.20065	0.17644383	0.06195521	0.00385874	0.01869078	TLN1
6366	ENSG00000137077	0	NA	NA	NA	NA	CCL21
27240	ENSG00000137078	0.36303624	0.02389534	0.38539249	0.60992614	NA	SIT1
3452	ENSG00000137080	0	NA	NA	NA	NA	IFNA21
1761	ENSG00000137090	0	NA	NA	NA	NA	DMRT1
25822	ENSG00000137094	588.080984	-0.44431855	0.1128921	3.78E-05	0.00031924	DNAJB5
26206	ENSG00000137098	6.18277647	0.18607886	0.41878212	0.17673614	0.37152417	SPAG8
11258	ENSG00000137100	736.105936	-0.29156234	0.10026001	0.00266278	0.01368641	DCTN3
971	ENSG00000137101	15.3814777	0.54047411	0.63382057	0.04404221	0.13497025	CD72
51754	ENSG00000137103	421.866207	0.41846235	0.16172918	0.00448007	0.02123379	TMEM8B
9380	ENSG00000137106	1385.80109	-0.00693471	0.07543128	0.92418207	0.96852	GRHPR
219	ENSG00000137124	747.496909	-0.61697889	0.11065243	7.00E-09	1.07E-07	ALDH1B1
84681	ENSG00000137133	308.044559	0.30108877	0.16078805	0.03915637	0.12352715	HINT2
84904	ENSG00000137135	330.109742	0.39043035	0.14430435	0.00350047	0.01727597	ARHGEF39
347252	ENSG00000137142	13300.4726	-0.02276531	0.04683254	0.62455096	0.80860493	IGFBPL1
55667	ENSG00000137145	853.374391	0.01706006	0.11670696	0.87929327	0.94652699	DENND4C
6194	ENSG00000137154	23910.4138	-0.18255294	0.0486177	0.00014965	0.00109357	RPS6
10695	ENSG00000137161	1126.26726	0.0582826	0.08118512	0.46198766	0.68504	CNPY3
116113	ENSG00000137166	710.065485	0.34082941	0.1038288	0.00063038	0.00394783	FOXP4

51645	ENSG00000137168	833.508834	-0.17425631	0.09081208	0.0478384	0.14387052	PPIL1
89953	ENSG00000137171	510.925492	0.21052904	0.11487743	0.05425614	0.15846339	KLC4
63971	ENSG00000137177	567.605913	0.27730542	0.11194375	0.00942201	0.03949561	KIF13A
7746	ENSG00000137185	569.041262	0.2116677	0.11061462	0.04525923	0.13776361	ZSCAN9
5292	ENSG00000137193	415.895488	-0.65916633	0.12312268	2.19E-08	3.15E-07	PIM1
2766	ENSG00000137198	19.6701992	0.38593751	0.45332561	0.09897412	0.24743531	GMPR
23070	ENSG00000137200	1267.1114	-0.13749853	0.08816173	0.10845988	0.26431057	CMTR1
7020	ENSG00000137203	1.36525802	-0.09760918	0.4006911	0.08918952	NA	TFAP2A
10864	ENSG00000137204	1.07664962	-0.02090245	0.38201282	0.5848267	NA	SLC22A7
25844	ENSG00000137207	1799.96908	0.08212258	0.07156889	0.24303658	0.46142064	YIPF3
81853	ENSG00000137210	678.686533	-0.18285347	0.09823293	0.05363654	0.15706995	TMEM14B
55362	ENSG00000137216	861.948529	0.11954433	0.14916809	0.38463379	0.6136819	TMEM63B
10817	ENSG00000137218	272.481428	-0.15466107	0.14036559	0.23524955	0.45208747	FRS3
93643	ENSG00000137221	897.655388	-0.03696484	0.08689189	0.66146499	0.8324506	TJAP1
11131	ENSG00000137225	0	NA	NA	NA	NA	CAPN11
27283	ENSG00000137251	0	NA	NA	NA	NA	TINAG
3062	ENSG00000137252	0.84180466	-0.0320569	0.38394834	0.51982775	NA	HCRTR2
9856	ENSG00000137261	55.7437362	-0.28006965	0.29736213	0.18507435	0.38396685	KIAA0319
3662	ENSG00000137265	0.18922458	0.01954053	0.38488496	0.78225479	NA	IRF4
63027	ENSG00000137266	64.5064706	0.56397921	0.29364285	0.01369109	0.05359848	SLC22A23
7280	ENSG00000137267	1312.36539	-0.74983772	0.08846702	4.91E-18	1.64E-16	TUBB2A
55227	ENSG00000137269	41.1469585	1.12301872	0.39795438	0.00041853	0.00275595	LRRC1
8521	ENSG00000137270	1.83591866	0.08463931	0.39231667	0.24009992	NA	GCM1
2295	ENSG00000137273	0	NA	NA	NA	NA	FOXF2
670	ENSG00000137274	478.478514	0.10343501	0.11529989	0.34718274	0.57794986	BPHL
8737	ENSG00000137275	530.80389	0.12435618	0.11593225	0.26038413	0.48228775	RIPK1
347733	ENSG00000137285	11902.3076	-0.50285018	0.05496309	3.50E-20	1.33E-18	TUBB2B
84300	ENSG00000137288	660.462633	-0.03998583	0.10815979	0.69909924	0.85487569	UQCC2
3159	ENSG00000137309	5951.55614	-0.28426829	0.06306002	4.66E-06	4.72E-05	HMGA1
6941	ENSG00000137310	552.266647	-0.10494725	0.10510319	0.29743633	0.52412619	TCF19
10211	ENSG00000137312	2286.81796	-0.07475517	0.06461657	0.24020481	0.45820056	FLOT1
8870	ENSG00000137331	1.47656161	-0.0565733	0.38506853	0.4071063	NA	IER3
9656	ENSG00000137337	2272.57512	0.02463036	0.07262088	0.73052919	0.87107559	MDC1
84547	ENSG00000137338	340.43	-0.0705586	0.12884246	0.56016469	0.76415299	PGBD1
79969	ENSG00000137343	1032.51101	0.44361434	0.0904031	4.34E-07	5.23E-06	ATAT1
7172	ENSG00000137364	346.18004	-0.19687873	0.13518997	0.11857291	0.2827168	TPMT
1208	ENSG00000137392	0	NA	NA	NA	NA	CLPS
255488	ENSG00000137393	19.8277968	1.19808485	0.64911497	0.00372138	0.01816736	RNF144B

11270	ENSG00000137404	346.173731	-0.00191268	0.12380186	0.98632762	0.99540871	NRM
23787	ENSG00000137409	2196.36782	0.10979097	0.08250463	0.17306774	0.36578929	MTCH1
57176	ENSG00000137411	1023.62704	0.16168665	0.08446473	0.04956228	0.14803945	VARS2
129685	ENSG00000137413	552.954437	0.20045108	0.12827466	0.09650079	0.24281239	TAF8
51439	ENSG00000137414	1104.98562	-0.35058254	0.08530047	2.40E-05	0.00021136	FAM8A1
347744	ENSG00000137434	19.5239493	0.34470414	0.44642236	0.11919895	0.28385039	C6orf52
9982	ENSG00000137440	0	NA	NA	NA	NA	FGFBP1
83888	ENSG00000137441	0.173733	0.01914545	0.38484597	0.78225479	NA	FGFBP2
132864	ENSG00000137449	123.841805	0.6181575	0.24026161	0.00243605	0.01267872	CPEB2
85462	ENSG00000137460	43.2071579	0.44271389	0.35963758	0.06478961	0.181378	FHDC1
7097	ENSG00000137462	21.4740889	1.59865626	0.56327792	0.00025598	0.00177701	TLR2
84709	ENSG00000137463	14.9480423	-0.44007834	0.53788263	0.06821039	0.18798362	MGARP
83894	ENSG00000137473	2.28648112	-0.0407789	0.38016519	0.57532206	NA	TTC29
4647	ENSG00000137474	60.5978013	-0.7809553	0.32140559	0.0022105	0.01167014	MYO7A
9873	ENSG00000137478	700.051821	-0.33664837	0.09752933	0.00034488	0.00231946	FCHSD2
408	ENSG00000137486	1235.34493	-0.79731123	0.08920494	8.11E-20	2.98E-18	ARRB1
11309	ENSG00000137491	2.48640729	0.06649655	0.38308505	0.43977902	NA	SLCO2B1
5612	ENSG00000137492	1302.54983	-0.51872731	0.08112025	6.08E-11	1.20E-09	THAP12
338699	ENSG00000137494	170.115672	0.46847476	0.19195445	0.00568848	0.02594468	ANKRD42
10068	ENSG00000137496	389.873481	0.2214169	0.13649536	0.08173042	0.21460185	IL18BP
4926	ENSG00000137497	12864.3344	0.20884122	0.05192067	4.89E-05	0.00040132	NUMA1
60492	ENSG00000137500	996.191145	-0.10482504	0.09268581	0.24307103	0.4614419	CCDC90B
54843	ENSG00000137501	17.033588	0.07440245	0.3334658	0.66786003	0.83661718	SYTL2
27314	ENSG00000137502	420.966405	-0.05241925	0.13050077	0.6667407	0.83582576	RAB30
58487	ENSG00000137504	2918.76811	0.2168216	0.07032393	0.0016907	0.0093021	CREBZF
2615	ENSG00000137507	34.0373183	-0.63529015	0.42393237	0.0212754	0.07656521	LRRC32
5547	ENSG00000137509	882.059986	0.22291382	0.08658204	0.00812137	0.0349822	PRCP
79731	ENSG00000137513	483.940987	0.11715418	0.12643081	0.32590343	0.55406376	NARS2
55298	ENSG00000137522	503.380304	0.24299153	0.12749672	0.04267815	0.1320147	RNF121
29088	ENSG00000137547	602.367594	-0.18060814	0.13907588	0.1619458	0.35042673	MRPL15
51050	ENSG00000137558	655.03579	1.34149456	0.11558144	3.73E-32	2.26E-30	PI15
7274	ENSG00000137561	0	NA	NA	NA	NA	TTPA
8836	ENSG00000137563	763.44571	0.28493751	0.09425306	0.00177763	0.00967311	GGH
81796	ENSG00000137571	4.61069147	0.04307198	0.36800791	0.7093134	0.86050983	SLCO5A1
23213	ENSG00000137573	0	NA	NA	NA	NA	SULF1
96764	ENSG00000137574	944.581771	-0.00113384	0.08750558	0.98861912	0.99604194	TGS1
6386	ENSG00000137575	1268.67016	0.60165399	0.07918926	9.31E-15	2.48E-13	SDCBP
4750	ENSG00000137601	385.540056	0.02509127	0.12188241	0.82903259	0.9225423	NEK1

55601	ENSG00000137628	72.7399537	0.25920482	0.25742043	0.19240347	0.39466357	DDX60
54827	ENSG00000137634	0	NA	NA	NA	NA	NXPE4
6653	ENSG00000137642	696.16855	-0.7072943	0.10010707	3.69E-13	8.64E-12	SORL1
56649	ENSG00000137648	0.18922458	0.01954053	0.38488496	0.78225479	NA	TMPRSS4
84811	ENSG00000137656	420.326522	-0.04780528	0.11831795	0.67027489	0.83763251	BUD13
7225	ENSG00000137672	0	NA	NA	NA	NA	TRPC6
4316	ENSG00000137673	0	NA	NA	NA	NA	MMP7
9313	ENSG00000137674	0	NA	NA	NA	NA	MMP20
64066	ENSG00000137675	0	NA	NA	NA	NA	MMP27
85016	ENSG00000137691	0.16735865	0.01897177	0.38482933	0.78225479	NA	CFAP300
84259	ENSG00000137692	1077.69075	0.05052001	0.096215	0.58827285	0.78310073	DCUN1D5
10413	ENSG00000137693	38.8316798	6.26994944	1.01388237	4.07E-10	7.33E-09	YAP1
23650	ENSG00000137699	17.1038828	0.09659913	0.34054198	0.57949566	0.77706744	TRIM29
2542	ENSG00000137700	961.097972	0.19931283	0.10231853	0.04294875	0.13257414	SLC37A4
54766	ENSG00000137707	0	NA	NA	NA	NA	BTG4
25833	ENSG00000137709	1.96755609	0.13267835	0.41631926	0.02925138	NA	POU2F3
5962	ENSG00000137710	1869.12628	-0.23580861	0.08323106	0.00361264	0.01773691	RDX
5519	ENSG00000137713	866.670118	0.43378903	0.0991512	5.83E-06	5.80E-05	PPP2R1B
2230	ENSG00000137714	271.571944	-0.39759475	0.15430895	0.0049493	0.02304052	FDX1
64776	ENSG00000137720	224.387926	0.19215437	0.169131	0.20544734	0.41239126	C11orf1
53826	ENSG00000137726	2076.01554	-0.02301857	0.0796011	0.76624844	0.89247205	FXYD6
57569	ENSG00000137727	2.190634	0.12153585	0.40578099	0.08841696	NA	ARHGAP20
486	ENSG00000137731	2.5954829	0.1533084	0.42210407	0.0719571	0.19545849	FXYD2
4322	ENSG00000137745	0	NA	NA	NA	NA	MMP13
84000	ENSG00000137747	2.10157625	0.05706524	0.38531284	0.3993012	NA	TMPRSS13
834	ENSG00000137752	0	NA	NA	NA	NA	CASP1
838	ENSG00000137757	0	NA	NA	NA	NA	CASP5
91801	ENSG00000137760	247.275301	0.13601041	0.14823753	0.31967814	0.54727882	ALKBH8
5607	ENSG00000137764	360.98644	0.24069595	0.13192275	0.05137801	0.15218103	MAP2K5
440279	ENSG00000137766	0	NA	NA	NA	NA	UNC13C
58472	ENSG00000137767	16.7890462	2.99653078	0.71932277	2.17E-06	2.33E-05	SQOR
51496	ENSG00000137770	1315.43609	-0.20119621	0.09425477	0.02737541	0.09336911	CTDSPL2
79811	ENSG00000137776	2216.05444	0.13793024	0.07064991	0.04685049	0.14165097	SLTM
7057	ENSG00000137801	85.9130024	1.61959712	0.29386503	2.37E-09	3.87E-08	THBS1
23005	ENSG00000137802	1046.70031	-0.0542549	0.09647448	0.56018502	0.76415299	MAPKBP1
51203	ENSG00000137804	2366.46052	-0.19443404	0.07691776	0.00971198	0.04053128	NUSAP1
51103	ENSG00000137806	210.540434	0.00373256	0.18380673	0.98303792	0.99428951	NDUFAF1
9493	ENSG00000137807	1124.63954	0.02806606	0.0964069	0.76441637	0.89148768	KIF23

NA	ENSG00000137808	0	NA	NA	NA	NA	
22801	ENSG00000137809	31.7852283	0.00538758	0.33246781	0.97606419	0.99170422	ITGA11
57082	ENSG00000137812	1434.17567	-0.52648944	0.11058425	6.94E-07	8.07E-06	KNL1
55142	ENSG00000137814	452.286471	0.01395689	0.12549641	0.90785058	0.95978728	HAUS2
23168	ENSG00000137815	1809.94371	0.10665635	0.07136391	0.12834547	0.29903961	RTF1
56965	ENSG00000137817	1546.74416	-0.84424818	0.07641252	3.97E-29	2.22E-27	PARP6
6176	ENSG00000137818	21183.7608	-0.07750311	0.04902451	0.11067503	0.26834208	RPLP1
54852	ENSG00000137819	33.2063829	0.39298761	0.39178325	0.09665928	0.24306007	PAQR5
54839	ENSG00000137821	499.876431	-0.20827581	0.12556639	0.07806333	0.20763862	LRRC49
27229	ENSG00000137822	1562.81438	-0.10408822	0.09488472	0.25661152	0.47767496	TUBGCP4
55177	ENSG00000137824	454.130013	-0.23672021	0.12158554	0.03937642	0.12401014	RMDN3
3706	ENSG00000137825	9.02027366	-0.03246432	0.3548686	0.8130131	0.91544721	ITPKA
55075	ENSG00000137831	459.410121	0.3007954	0.13438551	0.01660308	0.06268392	UACA
4091	ENSG00000137834	46.0207022	2.7624316	0.40935022	1.15E-12	2.61E-11	SMAD6
5330	ENSG00000137841	72.2448503	-0.15131549	0.26362559	0.43865949	0.663946	PLCB2
80021	ENSG00000137842	285.418923	0.19546132	0.13795375	0.12818693	0.29899232	TMEM62
56924	ENSG00000137843	94.8147683	-0.32483574	0.25463665	0.10410681	0.25686042	PAK6
106821730	ENSG00000137843	94.8147683	-0.32483574	0.25463665	0.10410681	0.25686042	PAK6
102	ENSG00000137845	1241.42955	-0.06985353	0.09620242	0.45238419	0.67559671	ADAM10
53905	ENSG00000137857	156.938478	0.71455744	0.20097951	7.81E-05	0.00061115	DUOX1
9153	ENSG00000137860	3.64663931	-0.10012268	0.38631401	0.35831613	0.58854144	SLC28A2
64220	ENSG00000137868	3699.86852	-0.79478998	0.07099806	8.37E-30	4.80E-28	STRA6
1588	ENSG00000137869	0	NA	NA	NA	NA	CYP19A1
54816	ENSG00000137871	924.667197	-0.2562513	0.10706145	0.01236782	0.04945132	ZNF280D
80031	ENSG00000137872	436.128408	-0.12030723	0.13792316	0.34841071	0.57911259	SEMA6D
10017	ENSG00000137875	1.5998228	-0.11145782	0.40606854	0.06065909	NA	BCL2L10
51187	ENSG00000137876	2514.61034	0.22821174	0.06937975	0.0008146	0.00494694	RSL24D1
51332	ENSG00000137877	16.0490697	0.0548401	0.33728114	0.74086143	0.87763281	SPTBN5
145781	ENSG00000137878	52.118168	-0.74685599	0.34157268	0.0043551	0.02075553	GCOM1
2644	ENSG00000137880	17.2632774	-1.46330909	0.70564007	0.00193691	0.01039728	GCHFR
8412	ENSG00000137936	127.17502	0.68862965	0.21517477	0.00029188	0.00199549	BCAR3
723788	ENSG00000137936	127.17502	0.68862965	0.21517477	0.00029188	0.00199549	BCAR3
79739	ENSG00000137941	924.908563	-0.04453436	0.10968898	0.67070945	0.83771678	TTLL7
54874	ENSG00000137942	2921.39841	0.15476168	0.05855182	0.00746982	0.03261469	FNBP1L
56267	ENSG00000137944	618.384675	0.11536713	0.10263377	0.24352296	0.46198062	KYAT3
2959	ENSG00000137947	329.809434	0.01386991	0.1268672	0.90890913	0.96056875	GTF2B
676	ENSG00000137948	0	NA	NA	NA	NA	BRDT
5876	ENSG00000137955	1215.28486	-0.17651189	0.08657152	0.03599916	0.11585132	RABGGTB

10964	ENSG00000137959	0.53686373	0.03973037	0.3868486	0.41316805	NA	IFI44L
54810	ENSG00000137960	1.32426889	-0.05755939	0.38758647	0.29338197	NA	GIPC2
9411	ENSG00000137962	232.740447	0.48210175	0.16297439	0.00120843	0.00696553	ARHGAP29
10561	ENSG00000137965	4.44451348	5.1958381	2.76366752	0.00042505	0.00279607	IFI44
204962	ENSG00000137968	654.821985	0.34530566	0.10890307	0.00092099	0.00552039	SLC44A5
NA	ENSG00000137970	4.46731687	0.05974695	0.37653066	0.56350619	0.76627833	RPL7P9
9635	ENSG00000137975	0	NA	NA	NA	NA	CLCA2
58511	ENSG00000137976	0.32361704	-0.00016112	0.38336248	0.98180736	NA	DNASE2B
1629	ENSG00000137992	571.402412	-0.11592541	0.13068164	0.34405458	0.57438933	DBT
8634	ENSG00000137996	566.765984	0.05518611	0.12546724	0.64254425	0.82136499	RTCA
26160	ENSG00000138002	1092.88632	0.48845691	0.10781748	2.38E-06	2.53E-05	IFT172
85465	ENSG00000138018	2233.7795	-0.30871212	0.08593638	0.00022073	0.00155346	SELENOI
10669	ENSG00000138028	932.207286	-0.27934491	0.08640953	0.0008799	0.00529648	CGREF1
3032	ENSG00000138029	1479.85799	0.03422494	0.07821698	0.65636363	0.8292845	HADHB
3795	ENSG00000138030	394.334822	0.06781372	0.13820179	0.59971017	0.79066681	KHK
109	ENSG00000138031	878.3366	-0.51341046	0.09463589	2.20E-08	3.18E-07	ADCY3
5495	ENSG00000138032	1086.14923	0.02774786	0.08001402	0.72356695	0.86792244	PPM1B
87178	ENSG00000138035	749.85036	0.18169155	0.09812897	0.05512731	0.16046446	PNPT1
51626	ENSG00000138036	350.142364	0.32675929	0.14416294	0.01432241	0.05554485	DYNC2LI1
3973	ENSG00000138039	0.18922458	0.01954053	0.38488496	0.78225479	NA	LHCGR
80745	ENSG00000138050	424.158518	0.10651281	0.14162868	0.41856143	0.64459045	THUMPD2
1545	ENSG00000138061	141.136325	0.07501803	0.18432243	0.64685167	0.82421635	CYP1B1
391365	ENSG00000138068	0	NA	NA	NA	NA	SULT6B1
5861	ENSG00000138069	1621.34838	-0.07924793	0.08561575	0.34167514	0.57180671	RAB1A
10097	ENSG00000138071	4710.26485	-0.22051042	0.05634877	7.97E-05	0.00062123	ACTR2
10113	ENSG00000138073	1296.89464	0.27808197	0.08922885	0.00132148	0.00751235	PREB
8884	ENSG00000138074	1516.82629	-0.03326044	0.08312043	0.68112819	0.84419446	SLC5A6
64240	ENSG00000138075	2.62288524	0.02075514	0.37245614	0.83218655	0.92392457	ABCG5
9581	ENSG00000138078	2168.62101	-0.22734387	0.0771297	0.00257199	0.01329555	PREPL
6519	ENSG00000138079	1.55097574	0.0929806	0.39470205	0.21059753	NA	SLC3A1
11117	ENSG00000138080	7215.26682	-0.18793854	0.055512	0.00061302	0.00385495	EMILIN1
80204	ENSG00000138081	2466.41915	-0.12778891	0.07334215	0.07558395	0.20253722	FBXO11
6496	ENSG00000138083	2617.93489	0.14395663	0.07941664	0.06369262	0.17918196	SIX3
51374	ENSG00000138085	1480.9932	0.08274678	0.07735588	0.27463695	0.49847198	ATRAID
79172	ENSG00000138092	1789.60489	-0.2515907	0.07242298	0.00038466	0.00255669	CENPO
10128	ENSG00000138095	5643.08266	-0.20844666	0.0540974	9.03E-05	0.00069501	LRPPRC
57159	ENSG00000138100	60.5822755	-1.98483356	0.34027046	3.64E-10	6.63E-09	TRIM54
1838	ENSG00000138101	656.744739	-0.00307039	0.10029841	0.97385757	0.99048209	DTNB

10121	ENSG00000138107	3107.95307	-0.06697938	0.06514287	0.29641349	0.52297409	ACTR1A
1559	ENSG00000138109	0	NA	NA	NA	NA	CYP2C9
79847	ENSG00000138111	437.107103	-0.50141021	0.14777185	0.00025499	0.00177133	MFSD13A
1558	ENSG00000138115	0.18922458	0.01954053	0.38488496	0.78225479	NA	CYP2C8
26509	ENSG00000138119	2.6240105	0.15683239	0.42689945	0.03864407	0.12228832	MYOF
84171	ENSG00000138131	79.6659816	-0.06658663	0.21994084	0.71118755	0.86120368	LOXL4
57559	ENSG00000138134	216.59416	0.03452163	0.16147214	0.8149092	0.91598064	STAMBPL1
9023	ENSG00000138135	0	NA	NA	NA	NA	CH25H
10660	ENSG00000138136	0.33817773	0.00200115	0.38337497	0.96934855	NA	LBX1
84896	ENSG00000138138	1660.00112	-0.18443279	0.07449752	0.01132947	0.04598543	ATAD1
118663	ENSG00000138152	0.173733	0.01914545	0.38484597	0.78225479	NA	BTBD16
3832	ENSG00000138160	2238.94734	-0.40349932	0.0670896	9.53E-10	1.64E-08	KIF11
50624	ENSG00000138161	9.46963836	0.10230064	0.36058982	0.51467683	0.72937777	CUZD1
10579	ENSG00000138162	1523.68904	-0.58538537	0.08919614	1.67E-11	3.47E-10	TACC2
1847	ENSG00000138166	51.7698117	0.1485277	0.25527642	0.44590491	0.67028199	DUSP5
51063	ENSG00000138172	77.6932023	-0.0257596	0.22674859	0.88672169	0.95023138	CALHM2
403	ENSG00000138175	793.362228	-0.17771988	0.08959431	0.04050567	0.1265563	ARL3
55165	ENSG00000138180	699.418186	-0.42467581	0.09631652	5.07E-06	5.08E-05	CEP55
9585	ENSG00000138182	876.304143	-0.20322375	0.09831031	0.03176963	0.10499908	KIF20B
953	ENSG00000138185	1002.34267	0.19118223	0.10029099	0.04814509	0.1446175	ENTPD1
54536	ENSG00000138190	392.039288	-0.23908103	0.12589134	0.04358442	0.13391897	EXOC6
51196	ENSG00000138193	174.061657	-0.31262029	0.27204599	0.12755432	0.29800727	PLCE1
5950	ENSG00000138207	5.4180837	0.16063624	0.40421716	0.23643391	0.45322211	RBP4
51163	ENSG00000138231	620.142342	-0.03430094	0.11197201	0.74653232	0.8807279	DBR1
23317	ENSG00000138246	1455.32964	0.05565395	0.07831841	0.46820035	0.69110148	DNAJC13
53836	ENSG00000138271	0	NA	NA	NA	NA	GPR87
310	ENSG00000138279	742.02519	0.11379065	0.10658037	0.26649223	0.48892231	ANXA7
317662	ENSG00000138286	826.410198	0.17032362	0.09453969	0.06281034	0.17744542	FAM149B1
51008	ENSG00000138303	390.601097	-0.22356118	0.12654864	0.06022211	0.17171698	ASCC1
84647	ENSG00000138308	0.83861444	-0.01118817	0.38091392	0.84358121	NA	PLA2G12B
22891	ENSG00000138311	102.25309	-1.27439245	0.24592817	1.98E-08	2.89E-07	ZNF365
170392	ENSG00000138315	0.31802559	0.00095554	0.38336542	0.9679205	NA	OIT3
140766	ENSG00000138316	58.5328156	-0.51661718	0.30358713	0.02453887	0.08587956	ADAMTS14
6229	ENSG00000138326	12965.1539	-0.30252525	0.04930418	5.89E-10	1.05E-08	RPS24
80312	ENSG00000138336	504.881898	-0.15217906	0.11699528	0.17081004	0.36283417	TET1
1763	ENSG00000138346	766.142464	-0.28324779	0.12657942	0.01731212	0.06489096	DNA2
84665	ENSG00000138347	0	NA	NA	NA	NA	MYPN
316	ENSG00000138356	46.5162578	-0.90901824	0.38526863	0.00200836	0.0107459	AOX1

471	ENSG00000138363	1641.3653	0.1500289	0.09250317	0.09442921	0.23890276	ATIC
50485	ENSG00000138375	518.146425	0.18797697	0.12208746	0.10383208	0.25644573	SMARCAL1
580	ENSG00000138376	378.281634	0.33441936	0.14989808	0.01524068	0.05837356	BARD1
6775	ENSG00000138378	21.5574355	0.24622015	0.37251846	0.23085632	0.44684775	STAT4
2660	ENSG00000138379	3.48831158	0.06783578	0.37515113	0.54622353	0.75345287	MSTN
79800	ENSG00000138380	271.914405	0.06401474	0.17005763	0.67560685	0.84120289	CARF
54529	ENSG00000138381	658.264664	-0.47169234	0.11496673	1.71E-05	0.00015545	ASNSD1
29081	ENSG00000138382	462.340481	0.2006977	0.11019854	0.05680066	0.16421638	METTL5
6741	ENSG00000138385	2186.62087	-0.13066349	0.07795335	0.08650042	0.22401363	SSB
4664	ENSG00000138386	1274.96011	-0.08401987	0.0896192	0.33480044	0.56474327	NAB1
65061	ENSG00000138395	6.15818008	-0.02539268	0.36060022	0.8398335	0.92728013	CDK15
9360	ENSG00000138398	1376.90361	-0.37276905	0.09050348	2.16E-05	0.00019281	PPIG
79675	ENSG00000138399	457.284475	-0.23880364	0.13592826	0.05934719	0.16966113	FASTKD1
130752	ENSG00000138400	14.5754375	0.2448064	0.40712114	0.20117134	0.40643985	MDH1B
57520	ENSG00000138411	345.34902	-0.00935624	0.12412238	0.93560977	0.97325384	HECW2
3417	ENSG00000138413	1147.80504	-0.00414275	0.09565623	0.96344916	0.98545957	IDH1
29789	ENSG00000138430	1686.52682	-0.13823963	0.0773431	0.06737197	0.18634601	OLA1
9541	ENSG00000138433	389.161744	0.10695416	0.12036148	0.34951452	0.58004303	CIR1
6744	ENSG00000138434	1045.0858	-0.19552875	0.09089954	0.02636669	0.09077131	ITPRID2
1134	ENSG00000138435	5.71943676	0.00711825	0.35787174	0.95896419	0.9831126	CHRNA1
150864	ENSG00000138439	639.876377	-0.23181522	0.11026659	0.02764733	0.09400566	FAM117B
55759	ENSG00000138442	1136.62298	-0.17271937	0.13869394	0.18001023	0.37673028	WDR12
10152	ENSG00000138443	3489.12813	-0.09058313	0.06274783	0.14307072	0.32222162	ABI2
3685	ENSG00000138448	1393.6415	-0.16586597	0.11835938	0.13933645	0.31622334	ITGAV
30061	ENSG00000138449	196.646546	0.43203387	0.16711255	0.00432396	0.02062195	SLC40A1
55032	ENSG00000138459	627.025982	-0.55463632	0.12820622	4.98E-06	5.02E-05	SLC35A5
84925	ENSG00000138463	217.239003	-0.08494665	0.17433002	0.58305071	0.77960143	SLC49A4
57337	ENSG00000138468	612.868962	-0.03111954	0.11077457	0.76695641	0.89261979	SENP7
9626	ENSG00000138472	0	NA	NA	NA	NA	GUCA1C
84692	ENSG00000138483	0.69837401	0.02518644	0.38341849	0.57787734	NA	CCDC54
10063	ENSG00000138495	389.159566	0.04787691	0.13900599	0.71266079	0.86169546	COX17
83666	ENSG00000138496	346.195857	0.1288254	0.13030873	0.29325511	0.5194341	PARP9
55329	ENSG00000138587	162.223315	-0.07419949	0.17645108	0.63483985	0.81617564	MNS1
9101	ENSG00000138592	999.87625	-0.00189567	0.0843202	0.98119686	0.99355086	USP8
9728	ENSG00000138593	1134.05779	0.27134361	0.0993479	0.00458041	0.02160614	SECISBP2L
29766	ENSG00000138594	993.386437	-0.00592666	0.10809896	0.95310764	0.98058207	TMOD3
112268148	ENSG00000138594	993.386437	-0.00592666	0.10809896	0.95310764	0.98058207	TMOD3
84888	ENSG00000138600	629.339619	-0.37458678	0.10455691	0.00018945	0.00135494	SPPL2A

26035	ENSG00000138604	287.060367	0.02720279	0.13880766	0.83460722	0.92451691	GLCE
90525	ENSG00000138606	1158.74173	0.12888956	0.07969019	0.09836321	0.24621828	SHF
83464	ENSG00000138613	160.915608	0.41488353	0.22979988	0.02985577	0.09998962	APH1B
81556	ENSG00000138614	871.874358	-0.05464453	0.12791042	0.64972467	0.82525848	INTS14
8483	ENSG00000138615	14.2307896	0.02802963	0.32527855	0.87472557	0.94484382	CILP
54956	ENSG00000138617	214.623359	0.14704864	0.1502114	0.28732437	0.51306915	PARP16
60490	ENSG00000138621	143.59762	-0.17572147	0.24541749	0.35801489	0.58833889	PPCDC
10021	ENSG00000138622	14.3298217	-2.42878105	0.78276487	0.00010654	0.00080489	HCN4
8482	ENSG00000138623	122.73509	-0.10932281	0.19335476	0.51218196	0.72744578	SEMA7A
84993	ENSG00000138629	1389.08566	0.14060446	0.08921611	0.10533683	0.25877695	UBL7
83478	ENSG00000138639	31.2261993	1.55834569	0.59908492	0.00037674	0.00251078	ARHGAP24
10144	ENSG00000138640	112.481191	0.45377837	0.22914427	0.0181903	0.06735733	FAM13A
8916	ENSG00000138641	109.93599	-0.04785113	0.20978198	0.7841312	0.9017077	HERC3
55008	ENSG00000138642	105.603856	0.05535934	0.22445329	0.76276053	0.89060552	HERC6
51191	ENSG00000138646	212.873064	0.75532766	0.18651948	9.92E-06	9.42E-05	HERC5
57575	ENSG00000138650	68.18483	-0.62803885	0.33334438	0.01160936	0.04691032	PCDH10
64579	ENSG00000138653	0	NA	NA	NA	NA	NDST4
55345	ENSG00000138658	878.046894	0.00367984	0.08779144	0.96660694	0.98715611	ZGRF1
55435	ENSG00000138660	848.741889	0.03354915	0.09503099	0.71639407	0.86350293	AP1AR
51138	ENSG00000138663	532.199023	-0.17097566	0.12218731	0.13876635	0.31543588	COPS4
3184	ENSG00000138668	8045.7671	-0.23995966	0.06278153	0.00010392	0.00078783	HNRNPD
5593	ENSG00000138669	0	NA	NA	NA	NA	PRKG2
153020	ENSG00000138670	0	NA	NA	NA	NA	RASGEF1B
22872	ENSG00000138674	2796.41657	0.03703028	0.08177653	0.64379343	0.82204027	SEC31A
2250	ENSG00000138675	18.7920263	0.12124225	0.33415629	0.51421058	0.72923073	FGF5
84803	ENSG00000138678	0	NA	NA	NA	NA	GPAT3
59067	ENSG00000138684	0	NA	NA	NA	NA	IL21
2247	ENSG00000138685	363.372493	-0.15970788	0.13098037	0.19627919	0.39997732	FGF2
55212	ENSG00000138686	284.031858	0.10426364	0.15278343	0.45718751	0.6806183	BBS7
84162	ENSG00000138688	1704.76659	0.27012662	0.07426473	0.00020439	0.00145135	KIAA1109
658	ENSG00000138696	970.591513	-0.53311301	0.09273229	3.24E-09	5.20E-08	BMPR1B
5910	ENSG00000138698	811.25595	-0.15115535	0.09140101	0.08797932	0.2266307	RAP1GDS1
55132	ENSG00000138709	326.531553	0.04359553	0.14034058	0.73971909	0.8770894	LARP1B
22915	ENSG00000138722	1.03111363	-0.00079013	0.37916723	0.99025885	NA	MMRN1
8654	ENSG00000138735	360.560849	1.21486482	0.16407123	1.26E-14	3.32E-13	PDE5A
11107	ENSG00000138738	257.025376	0.45835268	0.17819051	0.00408602	0.01964299	PRDM5
7222	ENSG00000138741	1.48147409	0.10022246	0.4018286	0.07221301	NA	TRPC3
27163	ENSG00000138744	55.7183287	0.33304111	0.29777899	0.12206058	0.28886671	NAAA

53371	ENSG00000138750	531.945492	-0.15645432	0.11629326	0.15693526	0.34373985	NUP54
4283	ENSG00000138755	0	NA	NA	NA	NA	CXCL9
55589	ENSG00000138756	430.022698	0.33728207	0.15785583	0.01900533	0.06992015	BMP2K
9908	ENSG00000138757	4097.77092	-0.18113742	0.06367007	0.00385151	0.01866943	G3BP2
55752	ENSG00000138758	3788.6936	-0.18828987	0.06496297	0.0032482	0.01618796	SEPTIN11
80144	ENSG00000138759	4671.28705	-1.11946122	0.05406592	3.71E-96	1.10E-93	FRAS1
950	ENSG00000138760	4949.77799	0.18917201	0.05466658	0.00047709	0.0030963	SCARB2
901	ENSG00000138764	1148.74058	0.21284139	0.10230024	0.03058915	0.10191246	CCNG2
246175	ENSG00000138767	509.193204	-0.24088705	0.15871719	0.09329202	0.23668972	CNOT6L
8615	ENSG00000138768	1538.21833	0.04626145	0.08918314	0.59447927	0.7877158	USO1
8999	ENSG00000138769	84.606132	0.11211632	0.25234499	0.5600472	0.76415299	CDKL2
57619	ENSG00000138771	1945.91495	-0.12333821	0.08772166	0.1479938	0.33012187	SHROOM3
306	ENSG00000138772	2.07802796	0.07411435	0.3851653	0.39405882	NA	ANXA3
27068	ENSG00000138777	579.998048	-0.022072	0.10744003	0.82844735	0.92220943	PPA2
1062	ENSG00000138778	1557.88498	-0.49341709	0.07031594	9.31E-13	2.13E-11	CENPE
79807	ENSG00000138780	555.766156	-0.21475915	0.13088297	0.07977804	0.21084412	GSTCD
57117	ENSG00000138785	356.699081	-0.04798627	0.13941235	0.71049204	0.86099206	INTS12
2028	ENSG00000138792	0.16735865	0.01897177	0.38482933	0.78225479	NA	ENPEP
839	ENSG00000138794	232.147211	0.46994415	0.2061468	0.0084696	0.0361669	CASP6
51176	ENSG00000138795	11.7798426	0.30055951	0.55293956	0.00721557	0.03169994	LEF1
3033	ENSG00000138796	1286.44456	-0.29012879	0.09212087	0.00113961	0.00660722	HADH
1950	ENSG00000138798	0	NA	NA	NA	NA	EGF
9061	ENSG00000138801	1902.93936	0.36268142	0.07137238	2.26E-07	2.85E-06	PAPSS1
10427	ENSG00000138802	836.925885	-0.1439763	0.08802462	0.09234415	0.2349928	SEC24B
84103	ENSG00000138813	0	NA	NA	NA	NA	C4orf17
5530	ENSG00000138814	748.854892	-0.20496645	0.09628578	0.02746577	0.09356471	PPP3CA
64116	ENSG00000138821	320.993934	0.94700413	0.13564633	4.30E-13	1.00E-11	SLC39A8
4547	ENSG00000138823	4.88393185	-0.00428062	0.36238045	0.96964561	0.98858077	MTTP
2201	ENSG00000138829	0	NA	NA	NA	NA	FBN2
23162	ENSG00000138834	3885.23377	0.30602911	0.06977712	7.94E-06	7.68E-05	MAPK8IP3
5998	ENSG00000138835	120.708033	0.07130846	0.19184481	0.6692627	0.83726414	RGS3
83606	ENSG00000138867	1250.87756	0.24441971	0.09851574	0.01008401	0.04167381	GUCD1
164714	ENSG00000138892	0	NA	NA	NA	NA	TTLL8
91445	ENSG00000138942	736.242862	-0.03162853	0.10215224	0.74716291	0.88120985	RNF185
85352	ENSG00000138944	0.173124	-0.01911262	0.38486262	0.84537521	NA	SHISAL1
64098	ENSG00000138964	0.51937201	-0.02557462	0.38567603	0.54680819	NA	PARVG
283358	ENSG00000139044	218.785465	-0.33718571	0.17598571	0.03124679	0.10358167	B4GALNT3
5149	ENSG00000139053	0.14931947	-0.01844332	0.38480212	0.84537521	NA	PDE6H

121506	ENSG00000139055	0	NA	NA	NA	NA	ERP27
2120	ENSG00000139083	295.949988	0.62022189	0.14916266	8.69E-06	8.34E-05	ETV6
23710	ENSG00000139112	145.432853	0.36730938	0.19480939	0.03050706	0.10172437	GABARAPL1
55605	ENSG00000139116	8378.59343	-0.03192756	0.05671484	0.56813822	0.76919502	KIF21A
144402	ENSG00000139117	5.8342597	0.07695163	0.36878857	0.55722586	0.76258372	CPNE8
51067	ENSG00000139131	296.304138	0.07419154	0.13024363	0.54524254	0.75294856	YARS2
121512	ENSG00000139132	870.97427	-0.04554902	0.09662333	0.62535228	0.80924616	FGD4
84920	ENSG00000139133	149.575532	-0.02414026	0.18275779	0.87940182	0.94652699	ALG10
5288	ENSG00000139144	0	NA	NA	NA	NA	PIK3C2G
58516	ENSG00000139146	2950.15106	-0.09512785	0.0855925	0.25354179	0.47391189	SINHCAF
89869	ENSG00000139151	0	NA	NA	NA	NA	PLCZ1
121536	ENSG00000139154	510.234173	0.23214291	0.14358667	0.08048201	0.21233551	AEBP2
53919	ENSG00000139155	18.6034227	1.34996225	0.83843315	0.00366989	0.0179602	SLCO1C1
254013	ENSG00000139160	35.3660611	-0.19069187	0.30138037	0.34862438	0.57928875	ETFBKMT
55500	ENSG00000139163	1222.96323	-0.36746258	0.07734037	1.18E-06	1.32E-05	ETNK1
85437	ENSG00000139168	682.271518	-0.00787092	0.10445285	0.93670129	0.97351594	ZCRB1
84216	ENSG00000139173	209.239633	0.05089531	0.15945725	0.72701743	0.86953012	TMEM117
144165	ENSG00000139174	213.12015	-0.39256365	0.18240881	0.01511278	0.05803579	PRICKLE1
51279	ENSG00000139178	145.327226	1.42638547	0.2574979	2.38E-09	3.89E-08	C1RL
4704	ENSG00000139180	723.438896	0.05934517	0.13792302	0.65122226	0.82578612	NDUFA9
9746	ENSG00000139182	1240.82906	0.00831613	0.09161448	0.92735474	0.96962941	CLSTN3
10219	ENSG00000139187	71.6660616	-0.25496889	0.29396579	0.21852646	0.43050004	KLRG1
6843	ENSG00000139190	165.537678	0.11291435	0.22847017	0.5407122	0.74929575	VAMP1
55080	ENSG00000139192	57.8848764	0.4733119	0.33334168	0.04532784	0.13793011	TAPBPL
939	ENSG00000139193	2.20332748	0.11338524	0.40304365	0.13794575	NA	CD27
83758	ENSG00000139194	23.1982672	0.40510933	0.42450731	0.0911164	0.23292738	RBP5
5830	ENSG00000139197	822.003608	-0.26601502	0.10041369	0.00588721	0.02673434	PEX5
196500	ENSG00000139200	951.03358	-0.331998	0.08991007	0.00013966	0.00102739	PIANP
55089	ENSG00000139209	0	NA	NA	NA	NA	SLC38A4
347902	ENSG00000139211	0	NA	NA	NA	NA	AMIGO2
9169	ENSG00000139218	2066.39592	-0.15465832	0.09543109	0.0935335	0.23705987	SCAF11
1280	ENSG00000139219	49.5433631	3.41016316	1.19760693	9.08E-05	0.00069833	COL2A1
8499	ENSG00000139220	0	NA	NA	NA	NA	PPFIA2
23519	ENSG00000139223	0	NA	NA	NA	NA	ANP32D
84298	ENSG00000139233	611.817033	-0.1629894	0.1276465	0.17461756	0.36831942	LLPH
NA	ENSG00000139239	6.99554107	-0.02296309	0.35676504	0.86356335	0.93960776	RPL14P1
121227	ENSG00000139263	1.04366226	0.02700427	0.38148494	0.65366058	NA	LRIG3
92979	ENSG00000139266	707.358792	0.13455098	0.09658281	0.14985571	0.33287611	MARCHF9

83729	ENSG00000139269	182.605731	4.35661836	1.0104815	6.14E-07	7.22E-06	INHBE
11010	ENSG00000139278	47.3285057	-0.1882209	0.27147848	0.34560663	0.57615625	GLIPR1
121278	ENSG00000139287	0.37039575	-0.01090982	0.38376479	0.90497973	NA	TPH2
22822	ENSG00000139289	616.734235	1.1406246	0.10635276	9.24E-28	4.89E-26	PHLDA1
55266	ENSG00000139291	496.584653	-0.28280897	0.12863766	0.01911983	0.07024768	TMEM19
8549	ENSG00000139292	1054.91122	2.60244181	0.11126163	2.75E-122	1.16E-119	LGR5
374462	ENSG00000139304	0	NA	NA	NA	NA	PTPRQ
1848	ENSG00000139318	2622.53785	0.25930185	0.06775398	9.89E-05	0.00075342	DUSP6
282809	ENSG00000139323	384.934598	0.42253376	0.16249954	0.00427005	0.02039427	POC1B
160418	ENSG00000139324	730.68256	-0.03681378	0.11195431	0.73008569	0.8709773	TMTC3
4060	ENSG00000139329	203.183862	2.37715626	0.56841393	1.31E-06	1.45E-05	LUM
11081	ENSG00000139330	0	NA	NA	NA	NA	KERA
6636	ENSG00000139343	961.297236	0.17805018	0.08382007	0.02950214	0.09908963	SNRPF
144193	ENSG00000139344	30.3226471	1.60008256	0.49590175	7.21E-05	0.00057039	AMDHD1
121441	ENSG00000139350	651.897405	0.37156463	0.10937152	0.00038383	0.00255203	NEDD1
50511	ENSG00000139351	3.91880041	-0.01940386	0.37098661	0.84544236	0.92979914	SYCP3
429	ENSG00000139352	392.54863	5.69404374	0.24344933	2.87E-121	1.19E-118	ASCL1
283431	ENSG00000139354	529.357236	-0.43463844	0.12452667	0.00022227	0.00156208	GAS2L3
114795	ENSG00000139364	0	NA	NA	NA	NA	TMEM132B
121260	ENSG00000139370	332.799644	-0.0483407	0.12679313	0.68543421	0.84697401	SLC15A4
6996	ENSG00000139372	1403.073	-0.10911619	0.07327183	0.12936323	0.30081651	TDG
84934	ENSG00000139405	402.708381	-0.35555208	0.13937686	0.00607729	0.02744045	RITA1
113675	ENSG00000139410	17.4050934	0.02881302	0.32630733	0.87020646	0.94333385	SDSL
326625	ENSG00000139428	854.085699	-0.93673629	0.09954988	7.44E-22	3.04E-20	MMAB
51228	ENSG00000139433	476.274364	0.01640952	0.11162829	0.87887044	0.94636578	GLTP
9815	ENSG00000139436	1183.93332	-0.00705139	0.08250965	0.92954097	0.97060391	GIT2
84260	ENSG00000139437	665.68382	0.00686336	0.13001639	0.95477797	0.98139026	TCHP
84915	ENSG00000139438	2625.52636	-1.91851422	0.07069022	2.29E-163	2.52E-160	FAM222A
121643	ENSG00000139445	526.496286	-1.00479332	0.13613975	2.04E-14	5.30E-13	FOXN4
9818	ENSG00000139496	1686.6239	-0.09558033	0.08227284	0.23357774	0.4504454	NUP58
9107	ENSG00000139505	790.23015	-0.45215404	0.09948636	2.48E-06	2.62E-05	MTMR6
283537	ENSG00000139508	27.8024198	0.28938279	0.35290125	0.18494816	0.38378105	SLC46A3
6541	ENSG00000139514	4295.27353	-0.05652278	0.1073012	0.58236432	0.77918294	SLC7A1
3651	ENSG00000139515	0	NA	NA	NA	NA	PDX1
222484	ENSG00000139517	167.246335	-0.14615568	0.17896685	0.35489924	0.58535214	LNX2
6821	ENSG00000139531	359.371787	-0.02173644	0.12554725	0.8537127	0.93418233	SUOX
85478	ENSG00000139537	20.3882352	0.1227822	0.32669394	0.51586671	0.73002854	CCDC65
283375	ENSG00000139540	3.84285716	0.06485967	0.38033218	0.48424835	0.7045162	SLC39A5

6895	ENSG00000139546	478.963251	0.25540801	0.1129517	0.01769259	0.06596898	TARBP2
8608	ENSG00000139547	9.07287509	0.78181	1.30780556	0.01739428	0.0651137	RDH16
50846	ENSG00000139549	7.20658093	0.11453821	0.36968141	0.4491266	0.6728081	DHH
94	ENSG00000139567	82.9245198	0.73266778	0.28947803	0.00190001	0.01022965	ACVRL1
53831	ENSG00000139572	5.00381307	0.24621288	0.46960432	0.08784316	0.22642697	GPR84
8620	ENSG00000139574	23.3777089	0.09741093	0.30810874	0.61276608	0.80066379	NPFF
79035	ENSG00000139579	1131.85449	-0.27846475	0.09177088	0.0017277	0.00945849	NABP2
90634	ENSG00000139597	186.385377	0.24288115	0.16797526	0.10784253	0.26339391	N4BP2L1
1990	ENSG00000139610	0	NA	NA	NA	NA	CELA1
6601	ENSG00000139613	3934.30539	-0.08201097	0.07169774	0.24377059	0.4621195	SMARCC2
675	ENSG00000139618	788.276534	-0.37002741	0.3125089	0.09447787	0.23893446	BRCA2
54934	ENSG00000139620	540.558461	0.00637045	0.14484105	0.96669252	0.98719269	KANSL2
91012	ENSG00000139624	558.174292	-0.2352171	0.1255927	0.04662339	0.14111488	CERS5
7786	ENSG00000139625	853.067696	-0.12714165	0.09214123	0.15454731	0.33997324	MAP3K12
3695	ENSG00000139626	5.25062743	0.09518494	0.38724135	0.33781133	0.56812552	ITGB7
11226	ENSG00000139629	337.556441	0.04887362	0.14238365	0.7124963	0.86169546	GALNT6
51380	ENSG00000139631	305.240268	0.59871489	0.15277421	2.55E-05	0.00022392	CSAD
55716	ENSG00000139636	954.860269	0.5003851	0.10325306	4.97E-07	5.94E-06	LMBR1L
60314	ENSG00000139637	798.300935	-0.02825033	0.09017978	0.74644638	0.8807279	MYG1
23344	ENSG00000139641	1699.96089	0.04999345	0.07890933	0.51793554	0.73179327	ESYT1
7009	ENSG00000139644	4296.11525	-0.05431277	0.07187683	0.44034553	0.66517856	TMBIM6
283373	ENSG00000139645	3028.20094	0.49310504	0.06488497	1.24E-14	3.28E-13	ANKRD52
112802	ENSG00000139648	0	NA	NA	NA	NA	KRT71
283337	ENSG00000139651	1096.65113	0.07044595	0.09031626	0.42283541	0.64881495	ZNF740
79024	ENSG00000139656	0.173124	-0.01911262	0.38486262	0.84537521	NA	SMIM2
115825	ENSG00000139668	1083.93368	0.31990627	0.10354792	0.0012961	0.00738916	WDFY2
144983	ENSG00000139675	10.2145771	6.72E-06	0.38287261	0.98969382	0.99637314	HNRNPA1L2
10161	ENSG00000139679	0.65371431	0.004689	0.38251884	0.91176106	NA	LPAR6
2098	ENSG00000139684	1120.92332	-0.06719868	0.08035929	0.39262059	0.6218739	ESD
5925	ENSG00000139687	1588.61794	-0.0134073	0.11809906	0.90395222	0.95803483	RB1
55206	ENSG00000139697	1523.2871	-0.22312069	0.08149975	0.00500017	0.02322176	SBNO1
283385	ENSG00000139714	74.7120504	0.12364597	0.22373779	0.49979187	0.7169827	MORN3
23067	ENSG00000139718	976.704914	0.11320527	0.08831225	0.1879919	0.38838712	SETD1B
65082	ENSG00000139719	644.57038	0.13828609	0.12629124	0.2458925	0.46515134	VPS33A
79720	ENSG00000139722	1098.02345	0.00026845	0.08142578	0.99700965	0.99912473	VPS37B
54509	ENSG00000139725	184.877147	0.08217883	0.19233834	0.62134769	0.80672374	RHOF
8562	ENSG00000139726	1706.2333	-0.19312071	0.07757489	0.01088131	0.04443912	DENR
81624	ENSG00000139734	487.137855	-0.15173773	0.11740391	0.17347504	0.36653299	DIAPH3

122060	ENSG00000139737	705.47068	-0.1307971	0.09828933	0.16780956	0.35875832	SLAIN1
64062	ENSG00000139746	1997.28739	-0.18055088	0.06710727	0.00621207	0.02788959	RBM26
84530	ENSG00000139767	1495.65467	-1.03768046	0.07748984	8.99E-42	7.08E-40	SRRM4
196541	ENSG00000139780	0	NA	NA	NA	NA	METTL21C
10150	ENSG00000139793	357.187572	0.31266686	0.13668619	0.01411892	0.05493616	MBNL2
140432	ENSG00000139797	0.47311071	-0.03505208	0.38628403	0.496036	NA	RNF113B
85416	ENSG00000139800	0	NA	NA	NA	NA	ZIC5
84945	ENSG00000139826	452.099368	0.06111883	0.1162168	0.58190287	0.778894	ABHD13
55647	ENSG00000139832	55.4724014	1.28175757	0.38756898	7.48E-05	0.0005886	RAB20
79774	ENSG00000139835	95.8794468	0.27157191	0.22835169	0.14687811	0.32812316	GRTP1
8451	ENSG00000139842	1706.40158	0.12604322	0.0842475	0.12500724	0.29343866	CUL4A
319089	ENSG00000139865	0	NA	NA	NA	NA	TTC6
6751	ENSG00000139874	0	NA	NA	NA	NA	SSTR1
64403	ENSG00000139880	1043.92452	0.03243826	0.08747189	0.7037806	0.85757322	CDH24
161253	ENSG00000139890	58.2214561	0.28604724	0.28746901	0.17295116	0.36578929	REM2
643866	ENSG00000139899	9.98634996	0.20958729	0.41095645	0.21367233	0.4238788	CBLN3
283629	ENSG00000139908	57.0393826	-0.04141639	0.2414865	0.82422546	0.92018644	TSSK4
4857	ENSG00000139910	254.187564	0.06740175	0.17775788	0.66983868	0.83753108	NOVA1
161247	ENSG00000139914	8.01419383	0.02198525	0.34905742	0.88396934	0.94897201	FITM1
161357	ENSG00000139915	0	NA	NA	NA	NA	MDGA2
81542	ENSG00000139921	858.143545	0.27900717	0.08983599	0.00137148	0.00776322	TMX1
122786	ENSG00000139926	466.124517	2.27175943	0.12715885	1.65E-72	2.80E-70	FRMD6
57161	ENSG00000139946	0	NA	NA	NA	NA	PELI2
6252	ENSG00000139970	3075.22048	-0.09396211	0.06122916	0.11963683	0.28460369	RTN1
145407	ENSG00000139971	51.7902248	-0.06716283	0.27835338	0.72671836	0.86949966	ARMH4
83851	ENSG00000139973	671.600189	-0.62304664	0.1424927	3.29E-06	3.41E-05	SYT16
145389	ENSG00000139974	74.0626954	-0.0640755	0.27510027	0.7382891	0.87630444	SLC38A6
122830	ENSG00000139977	550.164239	-0.15233708	0.12493641	0.1958168	0.39944263	NAA30
8747	ENSG00000139985	16.0009477	0.87847271	0.71150099	0.01424663	0.0553462	ADAM21
145226	ENSG00000139988	3.83916817	0.0682149	0.37539545	0.54181998	0.75017788	RDH12
8816	ENSG00000139990	1468.78421	0.12928004	0.07699836	0.08467035	0.22056982	DCAF5
376267	ENSG00000139998	723.086612	-0.11885813	0.11040771	0.25989252	0.48153414	RAB15
112840	ENSG00000140006	303.949321	-0.39170739	0.15448046	0.00562416	0.02570451	WDR89
2100	ENSG00000140009	33.5835089	0.28123845	0.3235757	0.19215549	0.39428741	ESR2
27133	ENSG00000140015	1.20625659	-0.00221032	0.38074056	0.82204911	NA	KCNH5
85439	ENSG00000140022	72.3422256	-0.60399013	0.32978827	0.01352976	0.05313349	STON2
90141	ENSG00000140025	99.3882478	0.07178817	0.21647504	0.68978894	0.84944526	EFCAB11
8477	ENSG00000140030	0.18922458	0.01954053	0.38488496	0.78225479	NA	GPR65

145482	ENSG00000140043	443.489922	0.05071412	0.14192428	0.70216083	0.85686252	PTGR2
122953	ENSG00000140044	93.1128306	1.74392485	0.33593266	1.51E-08	2.22E-07	JDP2
122481	ENSG00000140057	106.31364	-0.48941639	0.24093145	0.0138632	0.05410254	AK7
90050	ENSG00000140067	0	NA	NA	NA	NA	FAM181A
123041	ENSG00000140090	0	NA	NA	NA	NA	SLC24A4
10516	ENSG00000140092	0	NA	NA	NA	NA	FBLN5
51156	ENSG00000140093	0	NA	NA	NA	NA	SERPINA10
122616	ENSG00000140104	241.337038	0.25351318	0.16812259	0.09344502	0.23695669	CLBA1
7453	ENSG00000140105	1520.5333	0.49944137	0.0895886	9.98E-09	1.51E-07	WARS1
283600	ENSG00000140107	1.32137066	0.07872657	0.39199322	0.20799964	NA	SLC25A47
91833	ENSG00000140153	435.389945	0.00987935	0.14162015	0.94133309	0.97559098	WDR20
81614	ENSG00000140157	1143.0522	-0.06710905	0.09450713	0.46149455	0.68465911	NIPA2
9990	ENSG00000140199	693.374829	0.53098194	0.10572884	1.86E-07	2.36E-06	SLC12A6
90527	ENSG00000140254	19.7398548	2.029775	0.61119115	4.69E-05	0.00038722	DUOXA1
4236	ENSG00000140259	880.190034	-0.04795719	0.08424607	0.55886669	0.7635226	MFAP1
6938	ENSG00000140262	5023.36941	0.16816038	0.0606667	0.00497389	0.02313219	TCF12
6652	ENSG00000140263	1770.98514	-0.03789672	0.07696483	0.61424886	0.80178045	SORD
10169	ENSG00000140264	2894.02233	-0.09501526	0.07342188	0.18689748	0.38676207	SERF2
146050	ENSG00000140265	1307.45579	-0.17208907	0.08183788	0.03108539	0.10311556	ZSCAN29
405753	ENSG00000140274	2.31747265	0.04034072	0.38318097	0.51567308	NA	DUOXA2
50506	ENSG00000140279	20.129113	3.98950056	0.86903106	2.06E-07	2.61E-06	DUOX2
256586	ENSG00000140280	180.250173	-0.63352383	0.1869483	0.00017506	0.0012602	LYSMD2
11001	ENSG00000140284	89.8575504	-0.58441087	0.26483028	0.00684187	0.03035391	SLC27A2
2252	ENSG00000140285	2.82266031	0.14049663	0.41953941	0.03314373	0.10852807	FGF7
3067	ENSG00000140287	0.34746601	0.02370763	0.3853651	0.61987128	NA	HDC
9245	ENSG00000140297	0.85078897	0.02111374	0.38250161	0.58859804	NA	GCNT3
663	ENSG00000140299	1478.68725	0.06536531	0.08161414	0.41304773	0.6403849	BNIP2
2958	ENSG00000140307	1039.95609	-0.09116898	0.09272665	0.31012023	0.53778389	GTF2A2
6727	ENSG00000140319	3999.44554	0.03533861	0.0626531	0.56789504	0.76918607	SRP14
22893	ENSG00000140320	973.665498	-0.11792622	0.09615174	0.20439591	0.41102928	BAHD1
85455	ENSG00000140323	621.163097	-0.54655744	0.40200725	0.03495199	0.11318725	DISP2
146059	ENSG00000140326	730.79557	0.10608992	0.13347538	0.39674632	0.6258107	CDAN1
7090	ENSG00000140332	1707.73706	-0.25352827	0.08773182	0.00291782	0.01477528	TLE3
8125	ENSG00000140350	4215.42258	-0.04811361	0.07301897	0.50132302	0.71841948	ANP32A
54939	ENSG00000140365	1012.75927	0.14844222	0.08250585	0.06527289	0.18230796	COMMD4
92912	ENSG00000140367	432.964237	0.15572954	0.11896072	0.16812874	0.35917673	UBE2Q2
9051	ENSG00000140368	19.0743375	-0.7465933	0.75575015	0.02027957	0.07370335	PSTPIP1
2108	ENSG00000140374	1133.69515	-0.17424859	0.08679189	0.03893	0.12293032	ETFA

597	ENSG00000140379	0	NA	NA	NA	NA	BCL2A1
10363	ENSG00000140382	1378.54965	0.09682082	0.10428763	0.33472351	0.5647036	HMG20A
49855	ENSG00000140386	459.870553	-0.16004824	0.11362243	0.13926293	0.31613004	SCAPER
10099	ENSG00000140391	3708.31539	-0.11677098	0.05588936	0.03449058	0.11208731	TSPAN3
80349	ENSG00000140395	607.52164	0.01318736	0.10089804	0.89308679	0.95380535	WDR61
10499	ENSG00000140396	759.421766	-0.46084444	0.11255174	1.83E-05	0.00016575	NCOA2
79661	ENSG00000140398	367.378013	0.35596297	0.13582564	0.00498931	0.0231822	NEIL1
4123	ENSG00000140400	1906.6537	0.58821087	0.09738464	4.88E-10	8.77E-09	MAN2C1
55466	ENSG00000140403	22.9873857	0.12153114	0.31422979	0.53094912	0.74257279	DNAJA4
59274	ENSG00000140406	1321.61276	0.02913502	0.07379509	0.69066298	0.85009948	TLNRD1
7168	ENSG00000140416	835.375646	-0.27102392	0.09649219	0.00360022	0.01768467	TPM1
3480	ENSG00000140443	4465.11566	0.60605155	0.09878065	2.37E-10	4.37E-09	IGF1R
91947	ENSG00000140450	252.447811	-0.00772692	0.14589557	0.95332139	0.98070602	ARRDC4
80119	ENSG00000140451	560.357108	0.18765076	0.12978775	0.12363154	0.29131232	PIF1
9960	ENSG00000140455	681.304639	0.09791902	0.10179559	0.31868877	0.54633934	USP3
1583	ENSG00000140459	2.88689944	0.11337458	0.39656328	0.2404944	0.45856886	CYP11A1
585	ENSG00000140463	434.675304	-0.07943945	0.12650755	0.50498372	0.72130144	BBS4
5371	ENSG00000140464	546.370228	0.01536982	0.11959795	0.89273105	0.95357124	PML
1543	ENSG00000140465	4.29660944	0.03541119	0.37409103	0.71600209	0.86313534	CYP1A1
170691	ENSG00000140470	154.828724	-1.15807004	0.21986324	1.32E-08	1.97E-07	ADAMTS17
55180	ENSG00000140471	242.22997	0.08299259	0.15589702	0.56119623	0.76470695	LINS1
25989	ENSG00000140474	1290.37894	-0.04153223	0.07440296	0.56860797	0.76931138	ULK3
653643	ENSG00000140478	0.44795842	-0.02501848	0.38558245	0.5807882	NA	GOLGA6D
5046	ENSG00000140479	410.17157	-0.24866798	0.14507113	0.06319079	0.17823504	PCSK6
80125	ENSG00000140481	171.7156	-0.43677431	0.19354479	0.01001842	0.04153062	CCDC33
60677	ENSG00000140488	114.493527	-1.51641233	0.25158415	1.31E-10	2.48E-09	CELF6
10066	ENSG00000140497	609.069971	0.41046462	0.11646841	0.00021908	0.00154323	SCAMP2
1544	ENSG00000140505	0	NA	NA	NA	NA	CYP1A2
79748	ENSG00000140506	0.95001248	-0.02968431	0.38428612	0.53539752	NA	LMAN1L
145864	ENSG00000140511	134.801866	-0.3698471	0.19731293	0.03072852	0.10220515	HAPLN3
51458	ENSG00000140519	5.52006437	-0.10053988	0.37616033	0.4422496	0.66698837	RHCG
5428	ENSG00000140521	1515.02571	0.04445189	0.07888685	0.56541261	0.76735248	POLG
6017	ENSG00000140522	0.38611928	-0.01155562	0.38381297	0.89621533	NA	RLBP1
55215	ENSG00000140525	3067.59321	-0.24969419	0.06760498	0.00017077	0.0012334	FANCI
11057	ENSG00000140526	3567.20437	-0.8393718	0.06268044	1.21E-41	9.49E-40	ABHD2
56964	ENSG00000140527	6.36305267	0.40518322	0.65813483	0.03653112	0.11729688	WDR93
90381	ENSG00000140534	971.618998	-0.22429342	0.0965173	0.01604357	0.06089619	TICRR
4916	ENSG00000140538	11.6310775	-4.49269962	1.54406589	4.18E-05	0.00035016	NTRK3

55070	ENSG00000140543	264.994312	-0.20817081	0.15735726	0.14579064	0.32633917	DET1
4240	ENSG00000140545	1312.31747	0.56536059	0.0816721	1.49E-12	3.36E-11	MFGE8
374655	ENSG00000140548	709.106172	0.24137781	0.10262929	0.01441107	0.05578843	ZNF710
55898	ENSG00000140553	1870.30865	-0.1767476	0.07482012	0.01585095	0.0603035	UNC45A
8128	ENSG00000140557	636.558739	-0.47631547	0.13567398	0.00018005	0.00129289	ST8SIA2
55784	ENSG00000140563	91.406055	3.6872195	0.8014348	1.45E-07	1.87E-06	MCTP2
5045	ENSG00000140564	688.266271	0.09544539	0.09551528	0.30242459	0.52987991	FURIN
8826	ENSG00000140575	2030.00146	0.15373131	0.10989858	0.14466648	0.32485387	IQGAP1
64784	ENSG00000140577	1266.46677	0.13194259	0.10055524	0.17377256	0.36689354	CRTC3
79631	ENSG00000140598	616.095287	0.05915977	0.1025258	0.54985877	0.75633105	EFL1
6457	ENSG00000140600	0.66106017	-0.03839137	0.38686595	0.40270318	NA	SH3GL3
23478	ENSG00000140612	2515.54942	-0.04220769	0.06173682	0.48803427	0.70784413	SEC11A
124404	ENSG00000140623	0.50207596	0.02562368	0.38565771	0.52549886	NA	SEPTIN12
84656	ENSG00000140632	1822.86106	-0.10671054	0.09839148	0.26090467	0.48276874	GLYR1
5373	ENSG00000140650	1170.83426	-0.17854774	0.08608737	0.03296215	0.10807232	PMM2
6524	ENSG00000140675	17.3586598	-0.00660956	0.32073412	0.96832142	0.98809325	SLC5A2
3687	ENSG00000140678	0	NA	NA	NA	NA	ITGAX
7041	ENSG00000140682	480.905512	0.43374133	0.119892	0.00013854	0.00102064	TGFB1I1
64755	ENSG00000140688	1677.43598	0.32486343	0.0807778	3.78E-05	0.00031891	RUSF1
79798	ENSG00000140691	309.617274	-0.15798809	0.13738395	0.21677046	0.42789035	ARMC5
5073	ENSG00000140694	485.773845	-0.05766332	0.12104752	0.61464436	0.80211433	PARN
79068	ENSG00000140718	1162.277	-0.13114428	0.07961897	0.09249237	0.2351722	FTO
7385	ENSG00000140740	2030.24747	0.0666929	0.069126	0.32757175	0.55599483	UQCRC2
1039	ENSG00000140743	414.050006	-0.06509849	0.1298047	0.59291544	0.78702605	CDR2
10261	ENSG00000140749	10.9104554	0.1813837	0.37894606	0.30729252	0.5347968	IGSF6
55114	ENSG00000140750	998.367182	-0.20580963	0.09091306	0.01949763	0.0714584	ARHGAP17
91807	ENSG00000140795	16.8469977	0.08737793	0.34467213	0.60288537	0.79296792	MYLK3
94160	ENSG00000140798	0	NA	NA	NA	NA	ABCC12
85407	ENSG00000140807	389.298962	-1.48151375	0.21402062	3.10E-13	7.30E-12	NKD1
9785	ENSG00000140829	2339.47513	0.04150888	0.07644991	0.58077143	0.77801412	DHX38
54957	ENSG00000140830	410.257167	-0.25561035	0.13160061	0.03793401	0.12066663	TXNL4B
91862	ENSG00000140832	1.51835697	0.07660256	0.39245408	0.18001508	NA	MARVELD3
10164	ENSG00000140835	0.44946387	-0.00197002	0.38312958	0.95034056	NA	CHST4
463	ENSG00000140836	3080.93298	0.01873987	0.07771556	0.80625791	0.9122093	ZFHX3
497190	ENSG00000140839	3.18473798	-0.03917056	0.37109716	0.71032784	0.86089825	CLEC18B
221184	ENSG00000140848	603.431673	0.05901324	0.10112267	0.54708119	0.75418804	CPNE2
84166	ENSG00000140853	44.5700766	0.28914116	0.3553337	0.18377164	0.38218336	NLRC5
10300	ENSG00000140854	792.248703	-0.12028505	0.08768085	0.15896819	0.34635723	KATNB1

3801	ENSG00000140859	575.849799	-0.38831529	0.10547704	0.00012431	0.00092267	KIFC3
170692	ENSG00000140873	0	NA	NA	NA	NA	ADAMTS18
283927	ENSG00000140876	66.5673918	-0.22914955	0.2639857	0.25113804	0.47110579	NUDT7
2653	ENSG00000140905	810.792539	-0.08189217	0.09276899	0.36220539	0.59197696	GCSH
123920	ENSG00000140931	768.332783	0.75668821	0.10456298	9.56E-14	2.35E-12	CMTM3
146225	ENSG00000140932	1.64458291	0.03297455	0.37882525	0.66717033	NA	CMTM2
1009	ENSG00000140937	416.899778	1.47424398	0.16535648	3.54E-20	1.34E-18	CDH11
8996	ENSG00000140939	517.552771	0.17702576	0.10669988	0.08372215	0.21858763	NOL3
81631	ENSG00000140941	846.988161	-0.31164607	0.09228124	0.00048524	0.00314507	MAP1LC3B
8720	ENSG00000140943	3038.56862	0.10892618	0.06248084	0.07726078	0.20602875	MBTPS1
1012	ENSG00000140945	0	NA	NA	NA	NA	CDH13
23174	ENSG00000140948	3059.94084	0.25501439	0.06269352	3.73E-05	0.00031548	ZCCHC14
57707	ENSG00000140950	267.785441	-0.23334377	0.15789193	0.104043	0.25677486	MEAK7
161931	ENSG00000140955	0.14931947	-0.01844332	0.38480212	0.84537521	NA	ADAD2
29948	ENSG00000140961	30.3997066	1.24523183	0.70413968	0.00368035	0.01800358	OSGIN1
3394	ENSG00000140968	0	NA	NA	NA	NA	IRF8
89941	ENSG00000140983	3729.26735	0.27089449	0.06076694	6.18E-06	6.11E-05	RHOT2
6123	ENSG00000140986	2.08236117	-0.05495263	0.38030324	0.52611089	NA	RPL3L
54925	ENSG00000140987	318.221877	0.0339583	0.1393832	0.7942466	0.90579008	ZSCAN32
6187	ENSG00000140988	50884.6115	-0.17292826	0.0510998	0.00058288	0.0036888	RPS2
4716	ENSG00000140990	1175.28154	0.07348478	0.08718586	0.3873973	0.61635625	NDUFB10
5170	ENSG00000140992	1652.71276	-0.22412682	0.0788448	0.00359977	0.01768467	PDPK1
91151	ENSG00000140993	110.563833	-0.40878846	0.23762571	0.03584206	0.11549476	TIGD7
54849	ENSG00000140995	1053.63933	-0.34894736	0.11235549	0.00111478	0.00648789	DEF8
22980	ENSG00000141002	1285.66023	-0.07811244	0.126161	0.51016956	0.72557448	TCF25
2588	ENSG00000141012	348.584777	0.35037297	0.13644357	0.00592959	0.02690828	GALNS
2622	ENSG00000141013	602.487458	0.25758313	0.1174015	0.02083057	0.07532259	GAS8
55090	ENSG00000141026	309.222269	0.03750488	0.1318744	0.76341538	0.89095066	MED9
9611	ENSG00000141027	3230.49336	-0.08089447	0.10375714	0.41520595	0.64226462	NCOR1
NA	ENSG00000141028	2.05226808	-0.11285233	0.39986263	0.18982868	NA	CDRT15P1
8533	ENSG00000141030	988.492684	-0.360739	0.08891053	2.91E-05	0.00025116	COPS3
79018	ENSG00000141034	351.116822	0.03551854	0.1292423	0.77134879	0.89515442	GID4
57336	ENSG00000141040	241.749368	0.0084805	0.18726468	0.96001511	0.98361391	ZNF287
93649	ENSG00000141052	0	NA	NA	NA	NA	MYOCD
8844	ENSG00000141068	741.319048	0.31360049	0.09648883	0.00076124	0.00465713	KSR1
84916	ENSG00000141076	1228.46498	-0.01593897	0.08323101	0.8445867	0.92965052	UTP4
57610	ENSG00000141084	806.136712	0.24962937	0.1016987	0.01073837	0.04395239	RANBP10
1506	ENSG00000141086	39.9164406	0.30067076	0.368673	0.16768316	0.35871254	CTRL

64180	ENSG00000141096	0	NA	NA	NA	NA	DPEP3
81577	ENSG00000141098	900.07567	0.03330862	0.09312393	0.71307557	0.86187687	GFOD2
28987	ENSG00000141101	773.805802	-0.76153944	0.10320719	3.29E-14	8.42E-13	NOB1
5636	ENSG00000141127	691.977635	-0.38175726	0.09837053	5.71E-05	0.00045935	PRPSAP2
146862	ENSG00000141161	0	NA	NA	NA	NA	UNC45B
58488	ENSG00000141179	454.947399	0.69111114	0.12489232	7.49E-09	1.14E-07	PCTP
26689	ENSG00000141194	0.50778071	0.01499268	0.38310604	0.74337842	NA	OR4D1
10040	ENSG00000141198	1210.41922	0.45487813	0.07760478	2.12E-09	3.49E-08	TOM1L1
84643	ENSG00000141200	0	NA	NA	NA	NA	KIF2B
55028	ENSG00000141219	1021.0432	-0.18350223	0.0816809	0.02163361	0.07750287	C17orf80
10140	ENSG00000141232	529.298508	0.35848835	0.10899634	0.00058606	0.00370654	TOB1
55275	ENSG00000141252	1767.51354	0.09605362	0.08328275	0.23759253	0.45482784	VPS53
84690	ENSG00000141255	0	NA	NA	NA	NA	SPATA22
9905	ENSG00000141258	2722.01413	0.14172342	0.06595915	0.02908096	0.09795693	SGSM2
9520	ENSG00000141279	2901.75666	0.05254682	0.10013932	0.58781966	0.78276005	NPEPPS
8631	ENSG00000141293	0	NA	NA	NA	NA	SKAP1
90506	ENSG00000141294	26.9936992	0.01433325	0.28995344	0.94190472	0.97594973	LRRC46
90507	ENSG00000141295	842.073981	0.2906864	0.10231596	0.00313027	0.01570675	SCRN2
85464	ENSG00000141298	2507.13078	-0.14347536	0.06975963	0.03628037	0.11654835	SSH2
162494	ENSG00000141314	154.22144	0.20843985	0.2153822	0.24625854	0.46553305	RHBDL3
124912	ENSG00000141316	0.173124	-0.01911262	0.38486262	0.84537521	NA	SPACA3
22901	ENSG00000141337	22.2321592	2.36037937	0.64407497	1.25E-05	0.00011694	ARSG
10351	ENSG00000141338	0.18922458	0.01954053	0.38488496	0.78225479	NA	ABCA8
92579	ENSG00000141349	1176.14453	-0.08500259	0.08457619	0.30305374	0.53032436	G6PC3
1213	ENSG00000141367	9781.6921	0.0363381	0.07214194	0.60869389	0.79754899	CLTC
124773	ENSG00000141371	0.3245007	0.0013895	0.38336851	0.96700238	NA	C17orf64
54828	ENSG00000141376	445.577494	0.39987928	0.153911	0.00461863	0.02175536	BCAS3
51651	ENSG00000141378	943.855547	-0.02522806	0.08950342	0.76655412	0.8924999	PTRH2
6760	ENSG00000141380	2088.56058	0.15275949	0.06383057	0.0151884	0.05822953	SS18
6875	ENSG00000141384	232.683527	0.06384873	0.1435983	0.63252776	0.81415276	TAF4B
10939	ENSG00000141385	1144.09488	-0.34567687	0.08868049	5.91E-05	0.00047457	AFG3L2
10650	ENSG00000141391	235.812954	-0.01472278	0.14622269	0.91224669	0.96260718	PRELID3A
3613	ENSG00000141401	0.55789229	-0.03299273	0.38624378	0.47434443	NA	IMPA2
2774	ENSG00000141404	32.8859578	0.57310532	0.4517833	0.03459306	0.11230997	GNAL
25800	ENSG00000141424	3389.35537	0.32453684	0.06906077	1.72E-06	1.86E-05	SLC39A6
55197	ENSG00000141425	3242.07957	0.12640254	0.06529743	0.04967364	0.14831075	RPRD1A
83608	ENSG00000141428	266.178443	-0.00848173	0.1441507	0.94878416	0.9789317	C18orf21
2589	ENSG00000141429	1236.02948	0.07906341	0.08644739	0.34845998	0.57911259	GALNT1

80816	ENSG00000141431	395.895265	-0.03801325	0.15017286	0.78185628	0.90044863	ASXL3
116	ENSG00000141433	0	NA	NA	NA	NA	ADCYAP1
4225	ENSG00000141434	0.173733	0.01914545	0.38484597	0.78225479	NA	MEP1B
147407	ENSG00000141437	0.8241027	-0.05695426	0.38962115	0.26311886	NA	SLC25A52
64762	ENSG00000141441	734.986777	-0.16289043	0.28434097	0.41209682	0.63994998	GAREM1
114799	ENSG00000141446	506.403036	-0.40180355	0.11228538	0.00017482	0.00125893	ESCO1
114876	ENSG00000141447	672.717855	0.1436695	0.11737002	0.19811887	0.40237519	OSBPL1A
2627	ENSG00000141448	0	NA	NA	NA	NA	GATA6
80000	ENSG00000141449	1.4925153	-0.08233195	0.39311264	0.19374394	NA	GREB1L
29919	ENSG00000141452	374.006048	0.40244581	0.1688796	0.00826064	0.03548193	RMC1
27043	ENSG00000141456	1592.42347	0.35306021	0.07505598	1.55E-06	1.70E-05	PELP1
4864	ENSG00000141458	658.545077	0.08042835	0.09590269	0.38679351	0.6158895	NPC1
6563	ENSG00000141469	0	NA	NA	NA	NA	SLC14A1
409	ENSG00000141480	1088.78776	-0.38093182	0.08827859	8.87E-06	8.49E-05	ARRB2
284111	ENSG00000141485	0.16735865	0.01897177	0.38482933	0.78225479	NA	SLC13A5
84225	ENSG00000141497	0	NA	NA	NA	NA	ZMYND15
55135	ENSG00000141499	492.393554	-0.06257163	0.11523273	0.56822145	0.76919502	WRAP53
50488	ENSG00000141503	1743.35092	0.05781664	0.08224444	0.47218673	0.69445401	MINK1
112483	ENSG00000141504	534.033131	0.02600403	0.10675822	0.80072387	0.90877553	SAT2
432	ENSG00000141505	82.4651119	-0.07560883	0.2294714	0.68078949	0.84396798	ASGR1
23533	ENSG00000141506	0	NA	NA	NA	NA	PIK3R5
7157	ENSG00000141510	2168.36823	0.44626007	0.09387993	9.34E-07	1.06E-05	TP53
55036	ENSG00000141519	507.193466	0.51573741	0.11964993	6.09E-06	6.03E-05	CCDC40
396	ENSG00000141522	10016.9989	-0.1020551	0.05401459	0.05618664	0.16287885	ARHGDIA
11322	ENSG00000141524	280.948285	-0.1552776	0.18114863	0.33000404	0.55861736	TMC6
9123	ENSG00000141526	58.2130859	0.02960338	0.2490949	0.8777046	0.94578953	SLC16A3
79092	ENSG00000141527	23.1270478	0.344379	0.40025254	0.12920509	0.30069547	CARD14
94015	ENSG00000141540	599.832879	0.30686275	0.10923601	0.00329883	0.01639909	TTYH2
10966	ENSG00000141542	319.232189	-0.01102486	0.15420399	0.93593963	0.97325384	RAB40B
9775	ENSG00000141543	1526.57883	-0.12993799	0.07559164	0.07919361	0.2099675	EIF4A3
1453	ENSG00000141551	4159.54363	0.22580526	0.06438153	0.00036867	0.00246278	CSNK1D
51529	ENSG00000141552	2221.05456	0.06893787	0.0715343	0.32703856	0.55537491	ANAPC11
6904	ENSG00000141556	3338.92473	0.02806011	0.08445874	0.73444545	0.87363737	TBCD
79672	ENSG00000141560	880.430328	-0.04957309	0.09653991	0.59491341	0.78787011	FN3KRP
26502	ENSG00000141562	2344.29747	0.14205004	0.06933031	0.03966211	0.12459508	NARF
57521	ENSG00000141564	1468.75162	0.09940682	0.08377263	0.22408432	0.43775431	RPTOR
3607	ENSG00000141568	3620.70636	-0.19790956	0.06427845	0.0017645	0.00961747	FOXK2
201292	ENSG00000141569	804.789348	-1.64E-05	0.10275382	0.99879963	0.99957022	TRIM65

57332	ENSG00000141570	738.951347	0.13007892	0.12270921	0.26293033	0.48497991	CBX8
6398	ENSG00000141574	6.87698546	0.12430072	0.38112291	0.37224357	0.60116503	SECTM1
114804	ENSG00000141576	1981.54583	-0.1697872	0.09514262	0.06447294	0.18082317	RNF157
22994	ENSG00000141577	1374.51016	0.0933453	0.07694621	0.21595305	0.42670138	CEP131
79755	ENSG00000141579	10.0609654	-0.13649305	0.3741684	0.38479408	0.61378186	ZNF750
56270	ENSG00000141580	2529.04748	0.15178493	0.0637599	0.01573341	0.05995981	WDR45B
8535	ENSG00000141582	546.451717	0.18873117	0.10839829	0.06900019	0.18944974	CBX4
494470	ENSG00000141622	2196.28797	0.20629381	0.07240734	0.00368107	0.01800358	RNF165
54808	ENSG00000141627	1285.45961	-0.23978215	0.09897762	0.01188286	0.04786922	DYM
5596	ENSG00000141639	314.603359	-0.12874917	0.14349075	0.33207585	0.56128684	MAPK4
55520	ENSG00000141642	192.432501	-0.50214772	0.19965992	0.00410477	0.01971879	ELAC1
4152	ENSG00000141644	1742.9236	0.1017485	0.08092343	0.19848137	0.40276877	MBD1
4089	ENSG00000141646	1728.69127	-0.35509373	0.07529958	1.44E-06	1.59E-05	SMAD4
8792	ENSG00000141655	96.9709183	1.44814894	0.4596553	8.68E-05	0.00067039	TNFRSF11A
54877	ENSG00000141664	376.294318	0.23100714	0.12464346	0.04958665	0.14807331	ZCCHC2
201456	ENSG00000141665	38.2408614	0.43688815	0.40262622	0.07463628	0.2007026	FBXO15
147381	ENSG00000141668	1120.54188	-1.01218549	0.09487584	1.88E-27	9.79E-26	CBLN2
5366	ENSG00000141682	600.822066	0.64226412	0.13729253	7.28E-07	8.44E-06	PMAIP1
10609	ENSG00000141696	1010.19927	0.06643474	0.09485328	0.47028837	0.69253479	P3H4
115024	ENSG00000141698	1462.11295	-0.20041601	0.07523153	0.00649726	0.02903278	NT5C3B
162427	ENSG00000141699	799.308747	0.09753315	0.09842864	0.30636923	0.53408008	RETREG3
2064	ENSG00000141736	211.742003	0.07403267	0.15450736	0.60174121	0.79216856	ERBB2
2886	ENSG00000141738	0	NA	NA	NA	NA	GRB7
84299	ENSG00000141741	380.336667	-0.05986425	0.12298621	0.60624377	0.79607018	MIEN1
5409	ENSG00000141744	93.7842189	0.0246994	0.21548608	0.89147614	0.95288397	PNMT
390790	ENSG00000141748	7.09632263	0.03931015	0.38621094	0.34100063	0.57126172	ARL5C
342667	ENSG00000141750	123.45179	-0.29985112	0.20674486	0.08829274	0.2271726	STAC2
3487	ENSG00000141753	2966.97999	0.50606754	0.06332922	5.43E-16	1.58E-14	IGFBP4
60681	ENSG00000141756	3419.8021	0.29216412	0.05773125	2.98E-07	3.69E-06	FKBP10
10907	ENSG00000141759	1883.39339	-0.01698539	0.08070882	0.82878591	0.92238028	TXNL4A
773	ENSG00000141837	0.41052975	0.01218565	0.38382487	0.86311641	NA	CACNA1A
113230	ENSG00000141854	70.2391152	-0.07911868	0.23188186	0.66851784	0.83701843	MISP3
90378	ENSG00000141858	1437.6605	-0.14368539	0.11153872	0.17680761	0.37159566	SAMD1
23476	ENSG00000141867	1859.84562	-0.0564888	0.10031462	0.55816571	0.76291851	BRD4
29985	ENSG00000141873	670.953416	-0.66671467	0.09875359	3.74E-12	8.20E-11	SLC39A3
4782	ENSG00000141905	2066.15497	-0.10190894	0.0852788	0.21968693	0.43175718	NFIC
91978	ENSG00000141933	495.20601	-0.10937763	0.10813789	0.29088264	0.51693941	TPGS1
8612	ENSG00000141934	841.204718	-0.49290749	0.09095233	2.42E-08	3.46E-07	PLPP2

114026	ENSG00000141946	0	NA	NA	NA	NA	ZIM3
63977	ENSG00000141956	461.714165	0.1752311	0.15129606	0.20682437	0.41402436	PRDM15
5211	ENSG00000141959	1607.45944	0.24239017	0.07954666	0.00181295	0.00983566	PFKL
55527	ENSG00000141965	768.462046	0.03948339	0.09259297	0.66209784	0.83297534	FEM1A
7409	ENSG00000141968	0	NA	NA	NA	NA	VAV1
93343	ENSG00000141971	823.307142	0.02484585	0.09972256	0.79761669	0.90722439	MVB12A
117286	ENSG00000141977	0.173124	-0.01911262	0.38486262	0.84537521	NA	CIB3
NA	ENSG00000141979	0.71952772	0.02578377	0.38343699	0.56827864	NA	
6455	ENSG00000141985	2403.77647	0.0738113	0.07687142	0.32785355	0.55637794	SH3GL1
56931	ENSG00000141994	743.342841	0.23383897	0.12179328	0.04228862	0.13108695	DUS3L
91039	ENSG00000142002	1176.64306	0.26744934	0.11242576	0.01256431	0.05005538	DPP9
63946	ENSG00000142025	0	NA	NA	NA	NA	DMRTC2
90324	ENSG00000142039	810.116692	0.04007282	0.0907217	0.6500905	0.82529572	CCDC97
641649	ENSG00000142046	72.8506335	0.88138345	0.29618433	0.00038518	0.0025584	TMEM91
57677	ENSG00000142065	387.169379	-0.25941589	0.12707034	0.02988787	0.10005012	ZFP14
23410	ENSG00000142082	315.389372	-0.05259906	0.14420777	0.69294079	0.85163509	SIRT3
10410	ENSG00000142089	9.5869203	0.09121288	0.3571328	0.55855909	0.76326279	IFITM3
80162	ENSG00000142102	96.5455013	0.66734369	0.31200352	0.00597615	0.02707622	PGGHG
30811	ENSG00000142149	639.314911	-0.29599418	0.0980041	0.00173882	0.00951147	HUNK
1291	ENSG00000142156	1958.52587	-0.07546631	0.09053026	0.39005315	0.61929053	COL6A1
8389	ENSG00000142163	0	NA	NA	NA	NA	OR1E3
3454	ENSG00000142166	551.732662	-0.06505299	0.11552425	0.55407379	0.76002561	IFNAR1
6647	ENSG00000142168	2340.22796	0.19964897	0.07185791	0.00460803	0.0217106	SOD1
1292	ENSG00000142173	1702.64173	0.44143995	0.08434331	7.89E-08	1.05E-06	COL6A2
150094	ENSG00000142178	1.57407922	-0.0688141	0.38651449	0.3618503	NA	SIK1
29947	ENSG00000142182	0	NA	NA	NA	NA	DNMT3L
7226	ENSG00000142185	3.09057399	-0.00825692	0.37207899	0.92677428	0.96932899	TRPM2
57410	ENSG00000142186	974.240649	0.07695341	0.08470932	0.35197482	0.58206363	SCYL1
757	ENSG00000142188	407.914319	0.63133817	0.15914714	1.92E-05	0.00017267	TMEM50B
351	ENSG00000142192	14619.86	-0.37819781	0.05335401	7.72E-13	1.77E-11	APP
9980	ENSG00000142197	394.94251	-0.02009522	0.1178952	0.85702668	0.93609679	DOP1B
9875	ENSG00000142207	2344.42791	-0.17993545	0.06521335	0.00505926	0.02345774	URB1
207	ENSG00000142208	3894.34496	-0.10577136	0.05876231	0.06823389	0.18799616	AKT1
29949	ENSG00000142224	0	NA	NA	NA	NA	IL19
2014	ENSG00000142227	9.24958289	0.41873652	0.5996117	0.05745822	0.16569347	EMP3
10055	ENSG00000142230	2760.16524	-0.2846417	0.07261055	6.28E-05	0.0005026	SAE1
126147	ENSG00000142233	7.47337169	0.9933058	1.68018652	0.01331449	0.05245423	NTN5
114783	ENSG00000142235	367.86484	-0.36606706	0.13532613	0.00377321	0.01836688	LMTK3

79760	ENSG00000142252	234.636022	0.08787312	0.14505752	0.51292017	0.72818148	GEMIN7
23624	ENSG00000142273	0	NA	NA	NA	NA	CBLC
126374	ENSG00000142279	20.9497	-0.14394703	0.38151398	0.32889473	0.5575249	WTIP
81794	ENSG00000142303	664.67577	0.05948079	0.09701566	0.52686962	0.73878671	ADAMTS10
6531	ENSG00000142319	0.68509521	-0.0057442	0.38217916	0.84869912	NA	SLC6A3
57140	ENSG00000142327	656.880674	0.34562587	0.13103378	0.00485162	0.02265384	RNPEPL1
11132	ENSG00000142330	903.009396	0.32926403	0.08560812	7.76E-05	0.00060795	CAPN10
4542	ENSG00000142347	14.6281857	1.68264561	1.42457764	0.00359147	0.01765651	MYO1F
NA	ENSG00000142396	1145.28188	-0.02551282	0.07989526	0.74470564	0.87982849	ERVK3-1
91662	ENSG00000142405	0	NA	NA	NA	NA	NLRP12
59283	ENSG00000142408	731.131873	-0.35779175	0.10059148	0.00021994	0.00154849	CACNG8
126208	ENSG00000142409	808.220389	-0.15057497	0.10962845	0.15083779	0.33448325	ZNF787
90580	ENSG00000142444	274.560192	-0.02607874	0.14749262	0.84712521	0.93052693	TIMM29
84467	ENSG00000142449	7.57004741	-0.0582363	0.37242857	0.58157324	0.77868762	FBN3
10498	ENSG00000142453	1785.28278	0.22560988	0.07768472	0.00297829	0.01503914	CARM1
115704	ENSG00000142459	489.200381	-0.10831082	0.11087473	0.30668251	0.53422766	EVI5L
9032	ENSG00000142484	0.35689629	0.02381861	0.3853813	0.61390838	NA	TM4SF5
55244	ENSG00000142494	131.526964	0.54593847	0.20793026	0.00264349	0.0136049	SLC47A1
5694	ENSG00000142507	1165.37026	0.23608283	0.08590366	0.00473822	0.02221322	PSMB6
2854	ENSG00000142511	0	NA	NA	NA	NA	GPR32
89790	ENSG00000142512	12.8922647	0.20602755	0.39281975	0.25071541	0.47063996	SIGLEC10
93650	ENSG00000142513	0.18922458	0.01954053	0.38488496	0.78225479	NA	ACP4
354	ENSG00000142515	0	NA	NA	NA	NA	KLK3
25888	ENSG00000142528	649.908893	-0.18476572	0.13005253	0.12979013	0.30146269	ZNF473
112703	ENSG00000142530	13.6638871	0.02752636	0.3309756	0.8734897	0.94441388	FAM71E1
6205	ENSG00000142534	26507.6506	0.19133998	0.04782441	6.00E-05	0.0004809	RPS11
113091	ENSG00000142538	0	NA	NA	NA	NA	PTH2
NA	ENSG00000142539	0.173124	-0.01911262	0.38486262	0.84537521	NA	
23521	ENSG00000142541	48529.7486	-0.00168968	0.05504	0.99048381	0.99676382	RPL13A
90353	ENSG00000142544	248.736692	-0.04063574	0.14426729	0.76019273	0.88889389	CTU1
51070	ENSG00000142546	991.712263	0.18755602	0.0879486	0.02828562	0.09583107	NOSIP
402665	ENSG00000142549	1799.80022	-0.21357697	0.06745107	0.00127427	0.00728575	IGLON5
57333	ENSG00000142552	225.246899	0.2804304	0.15860033	0.05195814	0.15344589	RCN3
80110	ENSG00000142556	253.140166	-0.5049103	0.16688429	0.00089491	0.00538032	ZNF614
6518	ENSG00000142583	0.15069912	-0.01848503	0.38480575	0.84537521	NA	SLC2A5
473	ENSG00000142599	3555.69845	-0.18185706	0.07360132	0.0119294	0.04800797	RERE
79258	ENSG00000142606	25.984368	-1.33719976	0.63905709	0.00166069	0.00915735	MMEL1
85452	ENSG00000142609	7.51919866	-0.09090965	0.38723333	0.30753485	0.53498391	CFAP74

63976	ENSG00000142611	0.14931947	-0.01844332	0.38480212	0.84537521	NA	PRDM16
63036	ENSG00000142615	0.37844916	0.02407552	0.3854191	0.60038877	NA	CELA2A
51702	ENSG00000142619	0.18922458	0.01954053	0.38488496	0.78225479	NA	PADI3
114827	ENSG00000142621	56.9081009	-0.02403759	0.2398634	0.89658678	0.95469109	FHAD1
29943	ENSG00000142623	0.173733	0.01914545	0.38484597	0.78225479	NA	PADI1
1969	ENSG00000142627	684.824784	-0.38238544	0.12741302	0.0014293	0.00804233	EPHA2
128272	ENSG00000142632	152.757694	0.14560222	0.20673934	0.40301132	0.63099498	ARHGEF19
79180	ENSG00000142634	488.814737	-0.07449113	0.12168507	0.51759386	0.73141449	EFHD2
5195	ENSG00000142655	470.123124	0.1917893	0.12683941	0.108484	0.26431568	PEX14
5226	ENSG00000142657	3512.40759	-0.45087532	0.07243694	2.23E-10	4.12E-09	PGD
127294	ENSG00000142661	1.13933553	0.02148567	0.38071517	0.70032822	NA	MYOM3
83442	ENSG00000142669	703.857581	0.04464288	0.10053009	0.64617948	0.82370422	SH3BGRL3
10256	ENSG00000142675	41.9532046	0.64483897	0.39237846	0.01678441	0.06328444	CNKSR1
6135	ENSG00000142676	13611.7097	-0.12928958	0.05032107	0.00939764	0.03942678	RPL11
58985	ENSG00000142677	0.173733	0.01914545	0.38484597	0.78225479	NA	IL22RA1
51042	ENSG00000142684	284.412618	-0.42778331	0.15107676	0.00211603	0.01122516	ZNF593
127703	ENSG00000142686	632.195932	-0.56717923	0.11844422	5.38E-07	6.38E-06	C1orf216
79932	ENSG00000142687	1947.26409	0.23825247	0.08976537	0.00627659	0.0281482	KIAA0319L
55194	ENSG00000142694	89.9961264	0.02698849	0.21124658	0.87849003	0.9462643	EVA1B
84970	ENSG00000142698	0.173124	-0.01911262	0.38486262	0.84537521	NA	C1orf94
63950	ENSG00000142700	6.19049428	-0.03207846	0.36250371	0.79685999	0.90662359	DMRTA2
10733	ENSG00000142731	824.464748	0.11603515	0.12317893	0.31975629	0.54736538	PLK4
9064	ENSG00000142733	150.293561	1.00715207	0.20530215	1.16E-07	1.52E-06	MAP3K6
8547	ENSG00000142748	0.34624801	-0.02356563	0.38536324	0.65270996	NA	FCN3
54707	ENSG00000142751	838.618687	-0.22048609	0.08843067	0.01013921	0.04185642	GPN2
84958	ENSG00000142765	44.2956294	0.82952912	0.37443564	0.00338892	0.01678388	SYTL1
23038	ENSG00000142784	1503.79206	0.35046828	0.07320791	1.04E-06	1.17E-05	WDTC1
10136	ENSG00000142789	0	NA	NA	NA	NA	CELA3A
84224	ENSG00000142794	505.554191	-0.29767342	0.11396116	0.0060501	0.02733735	NBPF3
3339	ENSG00000142798	840.599413	0.42704547	0.11513501	9.84E-05	0.00075114	HSPG2
23421	ENSG00000142856	490.017591	-0.0103429	0.10734379	0.91875186	0.96558446	ITGB3BP
26135	ENSG00000142864	9248.26989	-0.24179693	0.04881695	5.79E-07	6.84E-06	SERBP1
8915	ENSG00000142867	159.390237	0.04157673	0.18422212	0.79835498	0.90769144	BCL10
3491	ENSG00000142871	226.363812	2.36294304	0.17424062	4.90E-43	4.04E-41	CCN1
5567	ENSG00000142875	1131.74708	-0.53348953	0.09013592	1.18E-09	2.01E-08	PRKACB
10026	ENSG00000142892	326.014555	-0.57793619	0.14201974	1.43E-05	0.00013246	PIGK
64129	ENSG00000142910	0.40000988	-0.01273014	0.38391013	0.87965238	NA	TINAGL1
113451	ENSG00000142920	151.878134	-0.34133545	0.23403454	0.07340707	0.19825705	AZIN2

6202	ENSG00000142937	18269.0076	-0.05697059	0.04610604	0.21246015	0.42238326	RPS8
11004	ENSG00000142945	1601.48724	-0.20148451	0.08198662	0.01170599	0.04721425	KIF2C
5792	ENSG00000142949	3821.11869	0.01469807	0.05501908	0.78993583	0.9043268	PTPRF
266675	ENSG00000142959	2.79924884	0.06907865	0.38044503	0.48173225	0.70240082	BEST4
148932	ENSG00000142961	177.435522	0.19340714	0.16719859	0.19881303	0.40318637	MOB3C
1580	ENSG00000142973	0	NA	NA	NA	NA	CYP4B1
199964	ENSG00000143001	0.66566499	-0.03804996	0.38684084	0.40282748	NA	TMEM61
63948	ENSG00000143006	0	NA	NA	NA	NA	DMRTB1
8543	ENSG00000143013	179.828985	0.78789045	0.17862432	1.90E-06	2.05E-05	LMO4
284612	ENSG00000143028	0	NA	NA	NA	NA	SYPL2
343472	ENSG00000143032	0	NA	NA	NA	NA	BARHL2
22823	ENSG00000143033	1079.13654	0.19040215	0.09419817	0.03683087	0.11801107	MTF2
126969	ENSG00000143036	178.214761	1.62295769	0.23171707	1.89E-13	4.54E-12	SLC44A3
3321	ENSG00000143061	4855.78473	0.08164216	0.05802904	0.15486281	0.34030142	IGSF3
90874	ENSG00000143067	674.091946	-0.31790977	0.11515931	0.00368932	0.01803301	ZNF697
55917	ENSG00000143079	723.6113	-0.00428337	0.12456082	0.96879256	0.98831985	CTTNBP2NL
85369	ENSG00000143093	1262.38188	-0.25033065	0.08312757	0.00198429	0.01062858	STRIP1
3744	ENSG00000143105	0	NA	NA	NA	NA	KCNA10
5686	ENSG00000143106	774.915913	0.0975052	0.10127727	0.31849106	0.54614197	PSMA5
163479	ENSG00000143107	0.50207596	0.02562368	0.38565771	0.52549886	NA	FNDC7
128346	ENSG00000143110	10.438724	0.6325385	0.9825162	0.02337374	0.08251513	C1orf162
963	ENSG00000143119	0	NA	NA	NA	NA	CD53
84432	ENSG00000143125	0.16735865	0.01897177	0.38482933	0.78225479	NA	PROK1
1952	ENSG00000143126	2718.41471	-0.40999021	0.06558903	2.13E-10	3.96E-09	CELSR2
8515	ENSG00000143127	7.41793499	0.07480052	0.36383096	0.58797476	0.7828366	ITGA10
23432	ENSG00000143147	1905.7714	0.62757356	0.06944545	4.69E-20	1.76E-18	GPR161
223	ENSG00000143149	1095.92857	0.04829422	0.08832952	0.57481767	0.77370302	ALDH9A1
481	ENSG00000143153	5382.6407	-0.21050403	0.05718262	0.00020736	0.00147034	ATP1B1
261726	ENSG00000143155	1719.27315	-0.36294743	0.07442584	6.37E-07	7.46E-06	TIPRL
29922	ENSG00000143156	538.843996	-0.15314118	0.10619205	0.13242852	0.30558086	NME7
57645	ENSG00000143157	3118.4887	-0.06032561	0.05905645	0.30084301	0.52811231	POGK
25874	ENSG00000143158	523.585282	0.03571652	0.1069923	0.72906177	0.87045369	MPC2
8804	ENSG00000143162	374.224038	-0.0921467	0.12776743	0.44334186	0.66838164	CREG1
55827	ENSG00000143164	604.846732	0.01186163	0.1088601	0.910482	0.96166753	DCAF6
10223	ENSG00000143167	0	NA	NA	NA	NA	GPA33
6258	ENSG00000143171	0	NA	NA	NA	NA	RXRG
9095	ENSG00000143178	58.0747115	0.31007375	0.28510295	0.1412967	0.31950382	TBX19
7371	ENSG00000143179	2610.92759	0.04245526	0.07156426	0.54658986	0.75371048	UCK2

54499	ENSG00000143183	2333.98677	-0.17349319	0.06663412	0.00812767	0.03500174	TMCO1
6375	ENSG00000143184	0.30139824	-0.02296476	0.38527817	0.68536535	NA	XCL1
6846	ENSG00000143185	0	NA	NA	NA	NA	XCL2
5451	ENSG00000143190	1588.80113	-0.12078322	0.08987784	0.16599267	0.35622439	POU2F1
84944	ENSG00000143194	0.14931947	-0.01844332	0.38480212	0.84537521	NA	MAEL
387597	ENSG00000143195	0.64639521	0.03389546	0.38619789	0.52618369	NA	ILDR2
1805	ENSG00000143196	156.186749	-0.01305689	0.17938019	0.93319607	0.97202552	DPT
4259	ENSG00000143198	1096.6806	0.10454467	0.08823299	0.22360178	0.43698378	MGST3
55811	ENSG00000143199	3.17560158	0.16355094	0.42387406	0.10301964	0.25491514	ADCY10
64326	ENSG00000143207	828.83205	-0.11559967	0.09484347	0.20779696	0.41513359	COP1
81607	ENSG00000143217	5.16933527	-0.05232195	0.36771097	0.66067125	0.83186632	NECTIN4
51506	ENSG00000143222	1176.88396	-0.06106837	0.07780712	0.41992512	0.64583833	UFC1
5498	ENSG00000143224	574.154682	0.2285629	0.13236069	0.06504594	0.18187757	PPOX
2212	ENSG00000143226	0	NA	NA	NA	NA	FCGR2A
83540	ENSG00000143228	1238.62175	-0.03246947	0.07812281	0.67056343	0.83767652	NUF2
8490	ENSG00000143248	51470.0919	0.17195814	0.08934584	0.03987803	0.12512313	RGS5
6391	ENSG00000143252	1447.56393	0.23414976	0.08313966	0.00385834	0.01869078	SDHC
5202	ENSG00000143256	2012.8498	-0.02119633	0.07315194	0.76653153	0.8924999	PFDN2
9970	ENSG00000143257	10.8461734	-0.06715748	0.35437887	0.65373499	0.82754433	NR1I3
27005	ENSG00000143258	1054.54934	0.31405149	0.08429903	0.00013077	0.0009659	USP21
2165	ENSG00000143278	0.32782306	0.00309619	0.38339363	0.95064653	NA	F13B
5546	ENSG00000143294	2302.26406	-0.12354185	0.06401798	0.05015316	0.14933795	PRCC
83416	ENSG00000143297	0	NA	NA	NA	NA	FCRL5
51093	ENSG00000143303	532.587958	0.08968432	0.12436386	0.44671116	0.67086136	RRNAD1
79590	ENSG00000143314	989.027516	-0.02606869	0.09627865	0.77884972	0.89886014	MRPL24
93183	ENSG00000143315	1152.44505	-0.20680722	0.08677119	0.0141558	0.05504726	PIGM
844	ENSG00000143318	0.50207596	0.02562368	0.38565771	0.52549886	NA	CASQ1
81875	ENSG00000143319	1985.77461	-0.19872135	0.08649144	0.01809887	0.06718084	ISG20L2
1382	ENSG00000143320	6488.42405	0.9865814	0.06228369	2.34E-57	2.85E-55	CRABP2
3068	ENSG00000143321	9042.01103	0.0807512	0.06086453	0.17303443	0.36578929	HDGF
27	ENSG00000143322	1834.27839	-0.02660714	0.06926099	0.69598946	0.8531799	ABL2
9213	ENSG00000143324	2600.42178	0.06284042	0.07628078	0.40114682	0.62949876	XPR1
6004	ENSG00000143333	261.07749	0.3453465	0.150058	0.01238091	0.04949371	RGS16
26092	ENSG00000143337	878.913698	-0.23247733	0.08832083	0.00668709	0.0297404	TOR1AIP1
148753	ENSG00000143340	2021.91447	0.06989033	0.06848709	0.30028851	0.52741891	FAM163A
83872	ENSG00000143341	24.9541458	8.24859988	3.17401811	9.57E-06	9.11E-05	HMCN1
23179	ENSG00000143344	435.124002	0.21046191	0.11260357	0.05017688	0.14934131	RGL1
127018	ENSG00000143353	227.87672	-0.41809673	0.17173817	0.00677756	0.03009552	LYPLAL1

56956	ENSG00000143355	89.8429281	2.24754895	0.27642492	3.05E-17	9.62E-16	LHX9
58497	ENSG00000143363	876.515349	0.02820071	0.09505551	0.7602547	0.88889389	PRUNE1
6097	ENSG00000143365	5.79784067	-0.27716794	0.50122577	0.0618566	0.17549479	RORC
7286	ENSG00000143367	282.839748	-0.20640769	0.15018209	0.13400268	0.30828075	TUFT1
10262	ENSG00000143368	1964.44865	0.15603568	0.0771863	0.0389267	0.12293032	SF3B4
1893	ENSG00000143369	27.7702002	0.14521065	0.30983461	0.46367091	0.68652759	ECM1
57592	ENSG00000143373	1314.39997	0.06784509	0.08348623	0.40492929	0.63328055	ZNF687
80222	ENSG00000143374	937.707642	-0.10449602	0.10109873	0.28299886	0.50802546	TARS2
57530	ENSG00000143375	301.889294	-0.62313522	0.17687498	0.00010995	0.00082852	CGN
81609	ENSG00000143376	1101.44313	-0.15653283	0.1154287	0.15381482	0.33890581	SNX27
9869	ENSG00000143379	2087.14096	-0.02978833	0.06793505	0.65535567	0.82851001	SETDB1
54507	ENSG00000143382	42.1663919	0.29893126	0.30359819	0.16390471	0.35319533	ADAMTSL4
4170	ENSG00000143384	6147.84541	0.12316545	0.049938	0.01287107	0.05105345	MCL1
574406	ENSG00000143384	6147.84541	0.12316545	0.049938	0.01287107	0.05105345	MCL1
1513	ENSG00000143387	283.41284	0.2960372	0.1367883	0.02024408	0.07360136	CTSK
5993	ENSG00000143390	1830.38896	0.1294384	0.07673893	0.0852602	0.22149194	RFX5
5298	ENSG00000143393	1410.55897	0.00266979	0.07965442	0.9733691	0.99018833	PI4KB
8394	ENSG00000143398	1825.84779	-0.3610312	0.08615523	1.56E-05	0.00014338	PIP5K1A
81611	ENSG00000143401	3691.55923	-0.31687127	0.08429679	0.00011203	0.00084127	ANP32E
55793	ENSG00000143409	505.549588	-0.17822834	0.13068684	0.14560957	0.32613386	MINDY1
8416	ENSG00000143412	29.1014017	0.05192114	0.29138266	0.78853655	0.90410108	ANXA9
8991	ENSG00000143416	8.58480815	2.06853959	1.10496608	0.00236576	0.0123713	SELENBP1
29956	ENSG00000143418	3712.9618	-0.02597303	0.05730885	0.64686859	0.82421635	CERS2
2029	ENSG00000143420	2913.78902	-0.41690963	0.07195403	3.51E-09	5.61E-08	ENSA
645166	ENSG00000143429	75.5426187	0.08617336	0.246082	0.65199428	0.82607871	LSP1P4
10500	ENSG00000143434	717.936864	0.42227673	0.14087734	0.00127661	0.00729491	SEMA6C
65005	ENSG00000143436	1229.02802	-0.0547598	0.08168094	0.49205662	0.71113078	MRPL9
405	ENSG00000143437	1280.5412	-0.12756473	0.08660762	0.12980585	0.30146269	ARNT
23126	ENSG00000143442	4061.05271	-0.10916255	0.0868947	0.1964393	0.40005705	POGZ
54964	ENSG00000143443	178.190901	-0.08637404	0.16531508	0.5616252	0.76502898	C1orf56
51686	ENSG00000143450	53.4580843	-0.11881548	0.30771132	0.53737443	0.74759591	OAZ3
84072	ENSG00000143452	0	NA	NA	NA	NA	HORMAD1
55204	ENSG00000143457	555.200811	-0.14364628	0.11429494	0.18684835	0.38675756	GOLPH3L
126626	ENSG00000143458	202.004747	-0.12661255	0.18931107	0.4401926	0.66517856	GABPB2
255928	ENSG00000143469	983.031044	-1.64498555	0.09017979	2.02E-75	3.67E-73	SYT14
3756	ENSG00000143473	2696.02239	-0.77295357	0.06355991	1.03E-34	6.90E-33	KCNH1
51514	ENSG00000143476	1677.87408	-0.42346563	0.08640902	4.63E-07	5.56E-06	DTL
8444	ENSG00000143479	486.449785	-0.12574979	0.11340616	0.24476128	0.46347387	DYRK3

1939	ENSG00000143486	720.208235	0.15963556	0.09969276	0.0969814	0.24355834	EIF2D
25896	ENSG00000143493	955.270352	-0.33431295	0.08439202	4.70E-05	0.00038746	INTS7
79805	ENSG00000143494	667.72615	-0.27355859	0.12080883	0.01654987	0.0625666	VASH2
9015	ENSG00000143498	434.637067	0.20453031	0.12914503	0.09201807	0.23438676	TAF1A
56950	ENSG00000143499	1176.09973	-0.63740041	0.07839301	1.19E-16	3.59E-15	SMYD2
55061	ENSG00000143502	10.3879538	-0.16492361	0.38883044	0.29323138	0.5194341	SUSD4
11221	ENSG00000143507	49.9381495	0.48145164	0.34219035	0.04440185	0.13577876	DUSP10
79802	ENSG00000143512	3.53460626	0.09307175	0.38311702	0.40294866	0.63099498	HHIPL2
7159	ENSG00000143514	2311.44378	0.21163427	0.06651136	0.0012298	0.00706407	TP53BP2
57198	ENSG00000143515	2336.14789	0.35945406	0.08487602	1.36E-05	0.00012637	ATP8B2
388698	ENSG00000143520	0	NA	NA	NA	NA	FLG2
49860	ENSG00000143536	0	NA	NA	NA	NA	CRNN
8751	ENSG00000143537	1481.25519	-0.4518985	0.07664028	1.70E-09	2.85E-08	ADAM15
10899	ENSG00000143543	1795.0692	-0.23090295	0.07206155	0.00107434	0.00627831	JTB
5872	ENSG00000143545	1029.59292	-0.00476804	0.08788273	0.94917738	0.97907226	RAB13
6279	ENSG00000143546	0	NA	NA	NA	NA	S100A8
7170	ENSG00000143549	7968.88778	0.04636447	0.0791165	0.5507833	0.75727932	TPM3
91181	ENSG00000143552	1.05937298	0.02341631	0.38189215	0.67321425	NA	NUP210L
23557	ENSG00000143553	786.479623	0.01299976	0.08745391	0.87938543	0.94652699	SNAPIN
11000	ENSG00000143554	323.580502	0.03391061	0.13773489	0.79296096	0.90530242	SLC27A3
6278	ENSG00000143556	0	NA	NA	NA	NA	S100A7
9898	ENSG00000143569	6549.78825	0.11750296	0.07000863	0.08795914	0.22660814	UBAP2L
27173	ENSG00000143570	2638.69066	0.10652704	0.06231216	0.08378385	0.21870922	SLC39A1
10456	ENSG00000143575	1361.1722	0.1895087	0.08026211	0.01564478	0.05964814	HAX1
148327	ENSG00000143578	483.090078	0.49465018	0.1281301	4.45E-05	0.00036924	CREB3L4
1944	ENSG00000143590	289.587296	0.05876698	0.13335304	0.63829141	0.81801574	EFNA3
89872	ENSG00000143595	42.6756677	-0.71882233	0.47166881	0.01517941	0.0582063	AQP10
3782	ENSG00000143603	27.1460338	0.13202401	0.31759666	0.49463913	0.71350347	KCNN3
25912	ENSG00000143612	5222.73332	-0.14982847	0.05501099	0.0060517	0.02733735	C1orf43
57459	ENSG00000143614	1581.77935	-0.15127189	0.08541121	0.06878307	0.1890682	GATAD2B
3608	ENSG00000143621	5600.22396	0.03899084	0.07163826	0.58159135	0.77868762	ILF2
6016	ENSG00000143622	599.482812	0.39423898	0.10904217	0.00015816	0.00114939	RIT1
65123	ENSG00000143624	2600.64918	0.32202129	0.06663699	9.42E-07	1.07E-05	INTS3
5313	ENSG00000143627	1.3203214	0.02918128	0.38055347	0.66485404	NA	PKLR
57657	ENSG00000143630	826.517471	-0.58260055	0.11409094	1.01E-07	1.33E-06	HCN3
2312	ENSG00000143631	1.13319958	-0.05891097	0.38707981	0.31404748	NA	FLG
58	ENSG00000143632	6.08696193	0.12388535	0.37979457	0.38408763	0.613205	ACTA1
128061	ENSG00000143633	344.91883	-0.18918458	0.13282916	0.12765058	0.29808895	C1orf131

2590	ENSG00000143641	3036.43634	0.06315595	0.05908454	0.27943092	0.50394595	GALNT2
79573	ENSG00000143643	664.222096	-0.04400832	0.10457985	0.65974121	0.8312756	TTC13
51097	ENSG00000143653	778.299835	0.17345549	0.09296166	0.05439769	0.15875997	SCCPDH
1130	ENSG00000143669	432.792744	-0.39092699	0.19054608	0.01916017	0.0703568	LYST
84451	ENSG00000143674	386.317175	-0.18445694	0.1224623	0.1107994	0.26850619	MAP3K21
9859	ENSG00000143702	8195.75229	-0.23425907	0.05728799	3.36E-05	0.00028697	CEP170
645455	ENSG00000143702	8195.75229	-0.23425907	0.05728799	3.36E-05	0.00028697	CEP170
52	ENSG00000143727	2215.77006	-0.08118528	0.07106547	0.24485185	0.46353633	ACP1
116841	ENSG00000143740	810.178956	-0.11783465	0.10144526	0.2271208	0.44177223	SNAP47
6726	ENSG00000143742	5422.6254	-0.59491569	0.05620564	1.10E-26	5.57E-25	SRP9
4931	ENSG00000143748	675.260175	-0.19014663	0.10308707	0.05476224	0.15963604	NVL
163859	ENSG00000143751	747.764635	0.11298757	0.09795693	0.23304001	0.44988944	SDE2
8560	ENSG00000143753	988.516592	-0.10488339	0.08700207	0.21506032	0.42553074	DEGS1
23219	ENSG00000143756	1115.4723	0.127476	0.09587872	0.16954863	0.36123762	FBXO28
375	ENSG00000143761	6928.98939	0.10191069	0.05650189	0.06832737	0.18818386	ARF1
7044	ENSG00000143768	2.48345687	0.02305836	0.37301364	0.81113485	NA	LEFTY2
29097	ENSG00000143771	1147.01991	0.26970079	0.09570348	0.00354701	0.01747521	CNIH4
3707	ENSG00000143772	129.065054	2.45204296	0.23074222	1.64E-27	8.61E-26	ITPKB
2987	ENSG00000143774	3058.35953	0.14302544	0.06880331	0.03453833	0.11218738	GUK1
8476	ENSG00000143776	2208.32366	-0.31392912	0.06365324	5.33E-07	6.33E-06	CDC42BPA
149111	ENSG00000143786	61.7528212	0.12390909	0.2483471	0.51994282	0.73349514	CNIH3
79169	ENSG00000143793	813.466581	0.29279302	0.09360466	0.00122787	0.00705909	C1orf35
129642	ENSG00000143797	2123.81289	-0.17683531	0.07096842	0.01112083	0.04526821	MBOAT2
142	ENSG00000143799	11192.4889	0.02560961	0.06785549	0.7007811	0.85575596	PARP1
5664	ENSG00000143801	349.293124	0.01629796	0.14930178	0.90762418	0.95967084	PSEN2
29920	ENSG00000143811	1298.8656	0.05800258	0.08991231	0.50762121	0.72355808	PYCR2
3930	ENSG00000143815	2548.17119	-0.22141117	0.07069168	0.00142449	0.00802213	LBR
7483	ENSG00000143816	0.34109985	0.02362645	0.38535338	0.62411116	NA	WNT9A
2052	ENSG00000143819	147.201229	-0.10626503	0.17458217	0.49343575	0.712409	EPHX1
5972	ENSG00000143839	0	NA	NA	NA	NA	REN
9580	ENSG00000143842	238.286744	0.66328053	0.16022904	8.44E-06	8.13E-05	SOX13
55224	ENSG00000143845	360.665483	-0.2418462	0.15636879	0.08898023	0.22858576	ETNK2
8497	ENSG00000143847	438.053055	0.22774962	0.15527002	0.10795158	0.26343905	PPFIA4
22874	ENSG00000143850	2326.33721	-1.20287478	0.20943598	8.97E-10	1.55E-08	PLEKHA6
5778	ENSG00000143851	2.19864757	-0.04660297	0.38202989	0.50275204	NA	PTPN7
127833	ENSG00000143858	53.2612417	0.74013343	0.3758712	0.00706168	0.0311896	SYT2
127829	ENSG00000143862	1310.26585	-0.09216084	0.08441725	0.26238349	0.4843402	ARL8A
130497	ENSG00000143867	0.37844916	0.02407552	0.3854191	0.60038877	NA	OSR1

151449	ENSG00000143869	77.7719032	-0.07522024	0.22678555	0.68078945	0.84396798	GDF7
10130	ENSG00000143870	8959.62071	0.33772228	0.06241878	4.06E-08	5.65E-07	PDIA6
388	ENSG00000143878	1830.99057	0.69768776	0.07268199	1.97E-22	8.29E-21	RHOB
245973	ENSG00000143882	97.5960881	0.18994008	0.21773152	0.29409804	0.52027686	ATP6V1C2
92906	ENSG00000143889	1450.28812	0.1477011	0.09771745	0.11787689	0.28166658	HNRNPLL
130589	ENSG00000143891	245.362718	0.64716622	0.16294912	1.80E-05	0.00016326	GALM
79823	ENSG00000143919	223.828644	-0.36990531	0.15943193	0.01080249	0.04417638	CAMKMT
64241	ENSG00000143921	2.41982694	-0.04113889	0.37818899	0.62263279	NA	ABCG8
27436	ENSG00000143924	5256.87091	0.20173272	0.06222795	0.00100791	0.00594792	EML4
805	ENSG00000143933	19277.067	-0.31409099	0.06627204	1.43E-06	1.58E-05	CALM2
494143	ENSG00000143942	63.4155464	-0.25303675	0.25230561	0.19857634	0.40288134	CHAC2
6233	ENSG00000143947	8682.01983	-0.20853077	0.05398759	9.42E-05	0.00072173	RPS27A
51057	ENSG00000143951	140.611142	0.17337204	0.19483319	0.30258098	0.52987991	WDPCP
51542	ENSG00000143952	921.505888	0.09857614	0.09144946	0.26723713	0.48970026	VPS54
130120	ENSG00000143954	0	NA	NA	NA	NA	REG3G
55252	ENSG00000143970	1643.12835	-0.03547053	0.22680086	0.84400625	0.92948958	ASXL2
54465	ENSG00000143971	756.402007	-0.01233733	0.09033329	0.88715014	0.95048379	ETAA1
6637	ENSG00000143977	1349.2107	-0.15952888	0.08402766	0.05126003	0.15192695	SNRPG
84696	ENSG00000143994	47.2926964	0.27143394	0.32741876	0.20632821	0.41333377	ABHD1
4211	ENSG00000143995	1853.24071	-0.01769088	0.07746099	0.81450333	0.91592004	MEIS1
653192	ENSG00000144010	0.18922458	0.01954053	0.38488496	0.78225479	NA	TRIM43B
129868	ENSG00000144015	0.16735865	0.01897177	0.38482933	0.78225479	NA	TRIM43
9391	ENSG00000144021	3215.17076	-0.07224358	0.06564319	0.2660857	0.48831193	CIAO1
84874	ENSG00000144026	586.703237	0.07413692	0.12099291	0.51901937	0.73254209	ZNF514
23020	ENSG00000144028	15225.4922	-0.08850637	0.05931861	0.14540096	0.32596101	SNRNP200
64969	ENSG00000144029	847.439368	0.22711499	0.09741285	0.01574706	0.06000031	MRPS5
79998	ENSG00000144031	9.5784895	0.07896009	0.37560613	0.4774515	0.69924247	ANKRD53
51002	ENSG00000144034	518.282628	-0.03144837	0.11282495	0.76954392	0.89405696	TPRKB
9027	ENSG00000144035	0.64813526	-0.02080678	0.38311595	0.66495951	NA	NAT8
23233	ENSG00000144036	452.528659	0.17601481	0.13165164	0.15407222	0.339342	EXOC6B
94097	ENSG00000144040	233.58798	-0.19221936	0.17831313	0.22240031	0.43535977	SFXN5
113419	ENSG00000144043	1422.13705	-0.49377769	0.10007008	3.19E-07	3.94E-06	TEX261
165545	ENSG00000144045	85.889545	0.09426058	0.21884441	0.60255257	0.79259054	DQX1
8446	ENSG00000144048	227.063222	-0.137286	0.16049908	0.34526666	0.57573462	DUSP11
84620	ENSG00000144057	549.83981	0.03088165	0.13168312	0.80383068	0.91089792	ST6GAL2
4867	ENSG00000144061	86.1109387	1.15158874	0.31915514	2.70E-05	0.0002355	NPHP1
7851	ENSG00000144063	0.35662409	0.02381665	0.38538099	0.61404581	NA	MALL
55258	ENSG00000144115	0	NA	NA	NA	NA	THNSL2

5899	ENSG00000144118	516.94811	-0.36200473	0.11608909	0.00102982	0.00605918	RALB
165257	ENSG00000144119	0.30002146	-0.02294464	0.38527539	0.68644526	NA	C1QL2
80775	ENSG00000144120	367.929221	-0.13044527	0.13508808	0.3010902	0.52835924	TMEM177
284958	ENSG00000144130	0.33471731	0.02354391	0.38534154	0.62842204	NA	NT5DC4
11159	ENSG00000144134	197.894643	0.3350541	0.17909927	0.03503878	0.11340607	RABL2A
6574	ENSG00000144136	1738.07064	-0.245648	0.08025265	0.00170395	0.00935593	SLC20A1
129804	ENSG00000144152	321.007945	0.02003744	0.14173282	0.88031091	0.94694038	FBLN7
NA	ENSG00000144158	0.16735865	0.01897177	0.38482933	0.78225479	NA	SNRPA1P1
84524	ENSG00000144161	546.884083	-0.38854148	0.10673739	0.00014293	0.00104908	ZC3H8
51601	ENSG00000144182	90.9637322	-0.0330953	0.23286483	0.85652909	0.93595581	LIPT1
NA	ENSG00000144188	0	NA	NA	NA	NA	TRIM43CP
1261	ENSG00000144191	0.173124	-0.01911262	0.38486262	0.84537521	NA	CNGA3
151313	ENSG00000144199	173.517592	0.3937629	0.1882416	0.0175679	0.06560268	FAHD2B
129530	ENSG00000144214	13.1255416	0.34758128	0.48527479	0.10315981	0.2551347	LYG1
3899	ENSG00000144218	573.467257	-0.84154695	0.14036665	3.22E-10	5.90E-09	AFF3
23190	ENSG00000144224	2043.38731	0.02961507	0.08773752	0.72961666	0.87071245	UBXN4
11249	ENSG00000144227	23.6776015	-0.43111412	0.44044902	0.07881929	0.20925662	NXPH2
339745	ENSG00000144228	501.72865	0.03010124	0.13540701	0.81315116	0.91549876	SPOPL
80731	ENSG00000144229	7.36281147	-3.16336109	1.42683827	0.00122494	0.00704636	THSD7B
2840	ENSG00000144230	0.43109345	0.01211155	0.38375314	0.85851826	NA	GPR17
5433	ENSG00000144231	1732.63084	0.01853487	0.08577654	0.82529018	0.92066374	POLR2D
83607	ENSG00000144233	1069.89543	0.00187058	0.08117501	0.98123163	0.99355086	AMMECR1L
114805	ENSG00000144278	1753.09293	-0.44015699	0.07656007	4.24E-09	6.72E-08	GALNT13
8502	ENSG00000144283	878.551789	-0.24121852	0.09384734	0.00785485	0.03399657	PKP4
6323	ENSG00000144285	17.6961962	-0.2418107	0.40917783	0.1933741	0.3960506	SCN1A
57282	ENSG00000144290	0.18922458	0.01954053	0.38488496	0.78225479	NA	SLC4A10
79634	ENSG00000144306	639.550399	-0.13922256	0.10329906	0.16052235	0.34848051	SCRN3
80856	ENSG00000144320	997.067941	0.13183188	0.22618928	0.47517492	0.69740127	LNPK
151126	ENSG00000144331	8.97568024	1.22590508	1.79408303	0.01012774	0.04183108	ZNF385B
23671	ENSG00000144339	29.9643349	-0.86924205	0.46323192	0.00617984	0.02776728	TMEFF2
83879	ENSG00000144354	1181.10499	-0.41208785	0.08646969	9.60E-07	1.09E-05	CDCA7
1745	ENSG00000144355	2.24254199	0.12333057	0.40405214	0.16588926	NA	DLX1
130507	ENSG00000144357	1192.58015	-0.22624433	0.09654496	0.01511305	0.05803579	UBR3
493911	ENSG00000144362	85.9962915	-0.12953712	0.23019013	0.48285582	0.7032638	PHOSPHO2
51454	ENSG00000144366	1420.87151	0.52519289	0.07714057	3.75E-12	8.22E-11	GULP1
165215	ENSG00000144369	697.791471	-0.73343236	0.09676552	7.61E-15	2.04E-13	FAM171B
3329	ENSG00000144381	9196.72804	-0.29934575	0.07503146	4.56E-05	0.00037768	HSPD1
284992	ENSG00000144395	272.980885	0.09992833	0.16342376	0.49968976	0.71690947	CCDC150

151194	ENSG00000144401	397.639364	0.16224603	0.11537162	0.13992568	0.31723522	METTL21A
285175	ENSG00000144406	867.168057	-1.7917926	0.11058278	2.91E-60	3.85E-58	UNC80
5746	ENSG00000144407	144.073225	0.43556982	0.19784128	0.01168039	0.04713015	PTH2R
130749	ENSG00000144410	0.173124	-0.01911262	0.38486262	0.84537521	NA	CPO
65065	ENSG00000144426	308.788499	0.10027333	0.17678876	0.52313753	0.73618679	NBEAL1
151050	ENSG00000144445	331.164123	0.04654458	0.13710455	0.71691831	0.8637162	KANSL1L
79582	ENSG00000144451	245.582825	-0.06954996	0.14113308	0.59502281	0.78790447	SPAG16
26154	ENSG00000144452	103.375637	0.99027957	0.31196479	0.00016665	0.00120582	ABCA12
285362	ENSG00000144455	208.163225	-0.42356769	0.1942506	0.01258336	0.05011116	SUMF1
100130207	ENSG00000144455	208.163225	-0.42356769	0.1942506	0.01258336	0.05011116	SUMF1
57624	ENSG00000144460	105.1985	0.01752741	0.19422922	0.91913456	0.96566562	NYAP2
84236	ENSG00000144468	434.604346	0.18776434	0.15509747	0.18496544	0.38378105	RHBDD1
57007	ENSG00000144476	0	NA	NA	NA	NA	ACKR3
79054	ENSG00000144481	1.20624581	0.01767401	0.38002972	0.78258358	NA	TRPM8
55502	ENSG00000144485	2419.48099	1.34023828	0.06621552	4.16E-92	1.15E-89	HES6
339768	ENSG00000144488	25.1759544	-0.37634474	0.40210625	0.10753203	0.26296505	ESPNL
51281	ENSG00000144504	272.701956	0.51750343	0.17138216	0.00086675	0.00522686	ANKMY1
64708	ENSG00000144524	996.70736	0.03351079	0.08296016	0.67987151	0.84376374	COPS7B
129563	ENSG00000144535	922.51443	0.14527523	0.08761663	0.08842087	0.22747277	DIS3L2
151835	ENSG00000144550	13.3983749	-0.01816825	0.33879418	0.9072083	0.95963244	CPNE9
2177	ENSG00000144554	1312.80282	0.09346178	0.09639492	0.31619717	0.54427831	FANCD2
132001	ENSG00000144559	154.58678	0.08508886	0.18762826	0.60429862	0.79446588	TAMM41
9686	ENSG00000144560	2253.23706	0.03786446	0.06643861	0.56305041	0.76597094	VGLL4
5868	ENSG00000144566	852.794687	0.05802815	0.09128695	0.51327278	0.72852568	RAB5A
79137	ENSG00000144567	1731.8415	-0.21581912	0.07407758	0.00292551	0.01481044	RETREG2
58190	ENSG00000144579	879.721018	0.37575937	0.092129	2.56E-05	0.00022476	CTDSP1
9125	ENSG00000144580	1857.7906	-0.17937984	0.07985861	0.02144441	0.07701976	CNOT9
57574	ENSG00000144583	274.623622	-2.47079849	0.17556685	3.33E-46	2.96E-44	MARCHF4
114790	ENSG00000144589	541.382149	0.16364496	0.12242067	0.15775449	0.34477297	STK11IP
29926	ENSG00000144591	505.852045	0.22347555	0.11771633	0.04558008	0.13856846	GMPPA
80852	ENSG00000144596	289.230607	-0.74052918	0.16640792	1.73E-06	1.87E-05	GRIP2
85403	ENSG00000144597	1219.27976	-0.36644264	0.08906623	2.28E-05	0.00020284	EAF1
152330	ENSG00000144619	1377.84281	-0.70861248	0.07571913	1.90E-21	7.60E-20	CNTN4
51143	ENSG00000144635	831.656445	0.09956045	0.09839069	0.2951503	0.52176619	DYNC1LI1
27303	ENSG00000144642	1398.69634	-0.16738123	0.08133411	0.03490208	0.11308412	RBMS3
339896	ENSG00000144644	0	NA	NA	NA	NA	GADL1
114884	ENSG00000144645	58.0050825	0.06419612	0.29278451	0.73924667	0.87688337	OSBPL10
84892	ENSG00000144647	516.635417	0.01354378	0.10423525	0.89345723	0.95389529	POMGNT2

1238	ENSG00000144648	7.16305007	-0.15257672	0.39261031	0.28754265	0.51323739	ACKR2
729085	ENSG00000144649	12.4065413	3.7354242	0.97990607	1.57E-05	0.00014359	GASK1A
64651	ENSG00000144655	60.3491501	0.17590004	0.26366853	0.37464125	0.60375943	CSRNP1
54977	ENSG00000144659	434.229427	-0.12053989	0.11323629	0.26458649	0.48686662	SLC25A38
3680	ENSG00000144668	1460.72751	-0.79144719	0.08340364	4.50E-22	1.86E-20	ITGA9
9389	ENSG00000144671	2.59809514	0.05155757	0.3770724	0.58997084	0.78467664	SLC22A14
2803	ENSG00000144674	1671.86682	-0.07831112	0.10087361	0.42008718	0.6460375	GOLGA4
10217	ENSG00000144677	760.282112	0.31840444	0.09484068	0.00051651	0.0033196	CTDSPL
6769	ENSG00000144681	948.070503	1.53438332	0.09369961	2.40E-61	3.26E-59	STAC
9922	ENSG00000144711	1433.27568	-0.44794467	0.07544172	1.34E-09	2.27E-08	IQSEC1
23066	ENSG00000144712	405.820337	0.09421239	0.13731636	0.46295963	0.68581491	CAND2
6161	ENSG00000144713	11558.7562	-0.16505732	0.04835788	0.00057707	0.00365668	RPL32
5793	ENSG00000144724	410.646533	0.32749016	0.13103373	0.00768892	0.03337303	PTPRG
54756	ENSG00000144730	11756.3676	-0.50777844	0.04603348	8.10E-30	4.66E-28	IL17RD
55164	ENSG00000144736	691.092506	-0.1199087	0.10731807	0.24353845	0.46198062	SHQ1
115286	ENSG00000144741	264.86607	0.08971131	0.14587526	0.50808559	0.72385596	SLC25A26
9039	ENSG00000144744	922.40159	0.04879374	0.09105697	0.58193275	0.778894	UBA3
10550	ENSG00000144746	804.416001	-0.15061499	0.09442991	0.09921498	0.2479125	ARL6IP5
7110	ENSG00000144747	646.655826	-0.27520018	0.13171983	0.02544406	0.08833073	TMF1
26018	ENSG00000144749	147.665497	0.90000741	0.23910552	2.32E-05	0.00020518	LRIG1
57408	ENSG00000144771	0	NA	NA	NA	NA	LRTM1
NA	ENSG00000144785	248.27796	-0.05506702	0.14548683	0.68072032	0.84396798	
8994	ENSG00000144791	508.194619	0.09926455	0.15496533	0.48433254	0.70452718	LIMD1
285349	ENSG00000144792	257.71153	-0.15375218	0.16129621	0.29093461	0.51698545	ZNF660
64332	ENSG00000144802	54.9857979	0.04013505	0.26801669	0.83652784	0.92562282	NFKBIZ
1295	ENSG00000144810	0.64342128	0.02056942	0.38306882	0.64913344	NA	COL8A1
91775	ENSG00000144815	1202.99139	0.15938501	0.08786235	0.06230457	0.17636219	NXPE3
84873	ENSG00000144820	0	NA	NA	NA	NA	ADGRG7
22989	ENSG00000144821	68.8506636	1.44727388	0.31779639	3.99E-07	4.83E-06	MYH15
90102	ENSG00000144824	326.225946	1.71553542	0.16336832	6.83E-27	3.47E-25	PHLDB2
55347	ENSG00000144827	430.681498	0.02165098	0.12468242	0.85615958	0.93593069	ABHD10
29114	ENSG00000144834	1361.79896	-0.12649119	0.07962743	0.10393088	0.25665779	TAGLN3
51365	ENSG00000144837	0.65294698	0.0259575	0.38366461	0.58982774	NA	PLA1A
285282	ENSG00000144840	247.484099	0.20274507	0.15219472	0.1461572	0.32699131	RABL3
141	ENSG00000144843	71.621897	0.08768226	0.23035867	0.63676855	0.81722218	ADPRH
152404	ENSG00000144847	34.0242698	3.29167551	0.5115899	9.88E-12	2.11E-10	IGSF11
64422	ENSG00000144848	660.639516	-0.11308393	0.11614212	0.3061773	0.53388623	ATG3
8856	ENSG00000144852	2.87561771	0.1172767	0.39582607	0.25587329	0.47698262	NR1I2

91653	ENSG00000144857	808.200194	0.09557263	0.09841907	0.31517185	0.54297665	BOC
58477	ENSG00000144867	802.386312	-0.29508281	0.11887917	0.00895068	0.03784773	SRPRB
66000	ENSG00000144868	820.679181	2.04368233	0.12032305	5.57E-66	8.25E-64	TMEM108
185	ENSG00000144891	590.016045	-0.50945086	0.11219788	2.11E-06	2.26E-05	AGTR1
116931	ENSG00000144893	101.011776	-0.4098627	0.27664598	0.05285211	0.15530003	MED12L
83939	ENSG00000144895	1683.73597	-0.11258167	0.07653362	0.13276455	0.30603562	EIF2A
10840	ENSG00000144908	22.8358975	-0.01999383	0.31084123	0.91117312	0.9621926	ALDH1L1
114885	ENSG00000144909	477.592579	-0.22649623	0.11478024	0.03803442	0.12085248	OSBPL11
7220	ENSG00000144935	211.501928	0.16447987	0.2341198	0.3810952	0.61009673	TRPC1
57552	ENSG00000144959	61.0187084	-0.01291037	0.25784004	0.94430719	0.97728379	NCEH1
83893	ENSG00000144962	0	NA	NA	NA	NA	SPATA16
653333	ENSG00000145002	0.49694214	0.01153459	0.38339044	0.7804583	NA	FAM86B2
4026	ENSG00000145012	7.60344169	3.14240183	1.25016804	0.00071839	0.00442363	LPP
93109	ENSG00000145014	470.897925	0.00848269	0.11167994	0.93763816	0.97366414	TMEM44
9711	ENSG00000145016	750.071684	-0.19994589	0.10016475	0.03805655	0.12090343	RUBCN
275	ENSG00000145020	338.829559	0.53446298	0.15365974	0.00017187	0.00124018	AMT
6988	ENSG00000145022	214.785244	-0.48500114	0.17139161	0.00176199	0.00961126	TCTA
84276	ENSG00000145029	335.26582	0.20008075	0.1400904	0.12361533	0.29131232	NICN1
90226	ENSG00000145040	1.08175734	0.04913157	0.38642238	0.34484369	NA	UCN2
9730	ENSG00000145041	1263.55815	-0.03385239	0.07970178	0.66275334	0.83353574	DCAF1
7873	ENSG00000145050	1764.61819	0.67043931	0.09225673	9.53E-14	2.34E-12	MANF
NA	ENSG00000145063	0.76401627	-0.02749518	0.38342601	0.57911717	NA	
NA	ENSG00000145075	1.05489384	-0.04391844	0.38790839	0.27834512	NA	CCDC39
9515	ENSG00000145087	399.019462	-1.02480837	0.17902448	1.22E-09	2.08E-08	STXBP5L
55840	ENSG00000145088	22.1397012	0.02042242	0.31631019	0.91215596	0.96260718	EAF2
286676	ENSG00000145103	1.68406501	0.12404622	0.41177607	0.04108289	NA	ILDR1
116211	ENSG00000145107	1.71004271	-0.06683772	0.39132443	0.18656897	NA	TM4SF19
4585	ENSG00000145113	5.28852537	-0.06361994	0.37053243	0.59354046	0.78731315	MUC4
9353	ENSG00000145147	98.0893646	0.01623902	0.20843357	0.92751466	0.96969441	SLIT2
8893	ENSG00000145191	1482.57952	0.1350191	0.07093281	0.05269173	0.1548976	EIF2B5
197	ENSG00000145192	2.72757994	0.04535152	0.38135755	0.54533093	0.75298369	AHSG
9718	ENSG00000145194	84.1839394	1.28057129	0.29488395	1.21E-06	1.35E-05	ECE2
90113	ENSG00000145198	156.089103	0.13107284	0.20117657	0.44573993	0.67028199	VWA5B2
1609	ENSG00000145214	297.402871	0.35486453	0.1460695	0.00858339	0.03656741	DGKQ
81608	ENSG00000145216	1088.75233	-0.0499851	0.08168755	0.53054354	0.74231198	FIP1L1
10861	ENSG00000145217	63.2944328	0.11927424	0.31164204	0.53586225	0.74657844	SLC26A1
55646	ENSG00000145220	382.265843	-0.05687584	0.11852645	0.61307021	0.80076208	LYAR
1060	ENSG00000145241	413.697275	-0.18634508	0.11647103	0.09218379	0.23468836	CENPC

2044	ENSG00000145242	0.14931947	-0.01844332	0.38480212	0.84537521	NA	EPHA5
10699	ENSG00000145244	21.313988	3.18671044	0.69403896	2.87E-07	3.57E-06	CORIN
57205	ENSG00000145246	631.401508	0.97722113	0.12573659	1.07E-15	3.06E-14	ATP10D
132299	ENSG00000145247	874.525608	-0.022651	0.08537523	0.78465538	0.9020424	OCIAD2
201780	ENSG00000145248	547.020945	0.22725835	0.1104414	0.03132823	0.1037823	SLC10A4
345274	ENSG00000145283	0.34746601	0.02370763	0.3853651	0.61987128	NA	SLC10A6
79966	ENSG00000145284	372.739237	-0.21101187	0.12266431	0.06854463	0.18864273	SCD5
51316	ENSG00000145287	0.55356856	0.03297301	0.38624244	0.45094446	NA	PLAC8
58478	ENSG00000145293	917.586377	-0.32593115	0.09666747	0.00047252	0.00307169	ENOPH1
85438	ENSG00000145309	0	NA	NA	NA	NA	CABS1
2638	ENSG00000145321	0	NA	NA	NA	NA	GC
93587	ENSG00000145331	148.736342	0.02484515	0.21686537	0.8911897	0.95275372	TRMT10A
57563	ENSG00000145332	688.777358	-0.37923794	0.16116067	0.00950657	0.03980799	KLHL8
6622	ENSG00000145335	240.471817	-0.34477844	0.2251226	0.06423991	0.18029704	SNCA
100996939	ENSG00000145337	841.862377	0.07457141	0.08798784	0.38431539	0.61337119	PYURF
93627	ENSG00000145348	599.029126	0.1570086	0.1059638	0.12269271	0.28988186	TBCK
817	ENSG00000145349	518.824952	-0.02075756	0.11787574	0.85200857	0.93288356	CAMK2D
493856	ENSG00000145354	722.143973	-0.36004126	0.11547613	0.00104251	0.00612296	CISD2
115265	ENSG00000145358	7.09404882	2.49294987	1.28168608	0.00214142	0.01134469	DDIT4L
287	ENSG00000145362	3981.96901	0.39342798	0.07603422	1.26E-07	1.64E-06	ANK2
92610	ENSG00000145365	424.567233	-0.05922922	0.13161176	0.63075297	0.81329297	TIFA
166378	ENSG00000145375	308.09186	-0.03168742	0.13960289	0.8065107	0.91237156	SPATA5
2169	ENSG00000145384	0	NA	NA	NA	NA	FABP2
890	ENSG00000145386	1289.66566	-0.17414537	0.08786433	0.04135968	0.12865727	CCNA2
57721	ENSG00000145388	666.644244	-0.33796537	0.14242873	0.01029971	0.0424392	METTL14
54532	ENSG00000145390	290.626266	0.25025768	0.15609711	0.07886948	0.20933386	USP53
80854	ENSG00000145391	1198.16051	-0.71064989	0.08165509	7.53E-19	2.65E-17	SETD7
92345	ENSG00000145414	229.586049	0.22666572	0.17358176	0.14299103	0.32208696	NAF1
55016	ENSG00000145416	125.771088	-0.2171867	0.208304	0.21672712	0.42784737	MARCHF1
6423	ENSG00000145423	0.49887772	0.01178183	0.38340295	0.77665762	NA	SFRP2
6189	ENSG00000145425	18331.709	-0.15211358	0.05019904	0.00221852	0.01170933	RPS3A
285533	ENSG00000145428	198.483728	0.15921985	0.15906872	0.27190847	0.49528683	RNF175
56034	ENSG00000145431	30.8858792	2.03313438	0.4864665	1.80E-06	1.95E-05	PDGFC
84869	ENSG00000145439	663.482966	-0.05100908	0.10014336	0.59692623	0.78900843	CBR4
8001	ENSG00000145451	10.7495481	-0.4504519	0.65210421	0.04609532	0.13985441	GLRA3
285440	ENSG00000145476	538.50324	-0.45360027	0.13234646	0.00026426	0.00182871	CYP4V2
83853	ENSG00000145491	1.03870336	0.05888287	0.39035467	0.19883368	NA	ROPN1L
4726	ENSG00000145494	764.217134	0.13893141	0.09736022	0.13998502	0.3173335	NDUFS6

10299	ENSG00000145495	5578.59489	0.04658448	0.06973896	0.49707795	0.71500229	MARCHF6
85409	ENSG00000145506	15.9334067	0.34953792	0.46436483	0.10921242	0.26569888	NKD2
1016	ENSG00000145526	2.99675661	0.11055188	0.39261616	0.2803424	0.5049178	CDH18
170690	ENSG00000145536	3.26176294	-0.03466558	0.37354106	0.7211028	0.86691933	ADAMTS16
6715	ENSG00000145545	664.895624	-0.22834338	0.12551843	0.05335301	0.15642438	SRD5A1
4651	ENSG00000145555	807.453954	1.4894105	0.09885091	2.32E-52	2.43E-50	MYO10
54491	ENSG00000145569	586.713731	-1.01390987	0.10807863	8.66E-22	3.53E-20	OTULINL
6167	ENSG00000145592	16968.4493	-0.07329403	0.05506785	0.17816787	0.37366284	RPL37
6502	ENSG00000145604	2865.13966	0.33517467	0.08747241	8.01E-05	0.00062357	SKP2
9180	ENSG00000145623	1.27964908	0.05379502	0.38671458	0.30663808	NA	OSMR
133688	ENSG00000145626	0	NA	NA	NA	NA	UGT3A1
10769	ENSG00000145632	90.4627641	1.01729948	0.27313647	2.14E-05	0.00019081	PLK2
100132916	ENSG00000145642	0.52119901	0.02576573	0.38568143	0.51723676	NA	SHISAL2B
3001	ENSG00000145649	0	NA	NA	NA	NA	GZMA
5295	ENSG00000145675	1618.70644	-1.38712934	0.08953169	3.45E-55	4.01E-53	PIK3R1
1404	ENSG00000145681	0	NA	NA	NA	NA	HAPLN1
10184	ENSG00000145685	826.368604	-1.51231121	0.12831259	3.66E-33	2.30E-31	LHFPL2
23635	ENSG00000145687	784.037554	0.5927026	0.09562858	1.78E-10	3.32E-09	SSBP2
635	ENSG00000145692	3.02473945	-0.05360609	0.37780344	0.56024586	0.76415299	BHMT
256006	ENSG00000145700	32.1075008	-0.08543281	0.29179236	0.65806035	0.83031782	ANKRD31
10788	ENSG00000145703	267.897125	0.65875888	0.16428339	1.48E-05	0.00013614	IQGAP2
1393	ENSG00000145708	0	NA	NA	NA	NA	CRHBP
5921	ENSG00000145715	1081.66287	-0.21869213	0.09235428	0.01441547	0.05579457	RASA1
167410	ENSG00000145721	0.45004261	-0.02731475	0.38571429	0.56165931	NA	LIX1
54826	ENSG00000145723	106.133917	-0.74428585	0.24743693	0.00046968	0.00305525	GIN1
23262	ENSG00000145725	1231.26944	-0.27080603	0.10824997	0.0088727	0.03756603	PPIP5K2
5066	ENSG00000145730	1226.44715	0.37793999	0.07908803	1.00E-06	1.13E-05	PAM
55814	ENSG00000145734	1732.13689	-0.1408311	0.07819042	0.06535928	0.18243221	BDP1
2966	ENSG00000145736	574.035811	-0.17023018	0.11965655	0.13326582	0.30691105	GTF2H2
728340	ENSG00000145736	574.035811	-0.17023018	0.11965655	0.13326582	0.30691105	GTF2H2
64924	ENSG00000145740	1153.62503	-0.1382732	0.08980466	0.11258913	0.27178103	SLC30A5
689	ENSG00000145741	8528.07228	0.0280306	0.05101667	0.57935538	0.77706744	BTF3
64839	ENSG00000145743	564.020133	0.01881729	0.10170587	0.84898367	0.93137282	FBXL17
83890	ENSG00000145757	8.85955804	0.02473621	0.3506018	0.86688206	0.94147909	SPATA9
85480	ENSG00000145777	37.055434	1.11246771	0.41769598	0.0006579	0.00409046	TSLP
25816	ENSG00000145779	131.951296	0.65080234	0.21065315	0.00046718	0.00304196	TNFAIP8
56929	ENSG00000145780	339.929243	-0.08185149	0.1310556	0.5054822	0.7217017	FEM1C
51397	ENSG00000145781	288.519274	-0.25637381	0.14562749	0.05628185	0.16308337	COMMD10

9140	ENSG00000145782	967.257239	-0.4035593	0.10255469	4.30E-05	0.00035752	ATG12
84466	ENSG00000145794	6.44804491	0.22552132	0.44332065	0.13544764	0.30998745	MEGF10
171019	ENSG00000145808	256.092541	3.44116673	0.22999369	2.55E-52	2.66E-50	ADAMTS19
81555	ENSG00000145817	658.18634	0.04679943	0.10447908	0.64223756	0.82116344	YIPF5
23092	ENSG00000145819	366.659327	-0.26503924	0.1912248	0.1100579	0.26723153	ARHGAP26
9547	ENSG00000145824	0	NA	NA	NA	NA	CXCL14
3950	ENSG00000145826	0.87431337	0.0632895	0.39095804	0.21028855	NA	LECT2
153328	ENSG00000145832	0	NA	NA	NA	NA	SLC25A48
9879	ENSG00000145833	2822.38882	0.04614312	0.09589626	0.62013016	0.80605425	DDX46
NA	ENSG00000145835	0	NA	NA	NA	NA	CDKN2AIPNLP2
3578	ENSG00000145839	0	NA	NA	NA	NA	IL9
91937	ENSG00000145850	0	NA	NA	NA	NA	TIMD4
153830	ENSG00000145860	2513.54096	-0.12954615	0.07920947	0.09452913	0.23903363	RNF145
114898	ENSG00000145861	18.5277379	-0.06893614	0.31763211	0.70662292	0.85861856	C1QTNF2
2559	ENSG00000145863	0	NA	NA	NA	NA	GABRA6
2561	ENSG00000145864	0	NA	NA	NA	NA	GABRB2
81545	ENSG00000145868	1073.26837	0.12008779	0.09634078	0.19782988	0.40199972	FBXO38
84651	ENSG00000145879	1.27833632	0.07076668	0.39314745	0.13508016	NA	SPINK7
78991	ENSG00000145882	715.986145	-0.29929547	0.09988428	0.00185284	0.01002735	PCYOX1L
2741	ENSG00000145888	0.173124	-0.01911262	0.38486262	0.84537521	NA	GLRA1
10318	ENSG00000145901	924.992111	-0.15997271	0.09921414	0.09407289	0.23818352	TNIP1
10146	ENSG00000145907	3639.57371	-0.06287319	0.0766817	0.40192774	0.63031025	G3BP1
91975	ENSG00000145908	1198.58214	-0.20254123	0.08736551	0.01698335	0.06390093	ZNF300
23138	ENSG00000145911	105.264956	-0.52700392	0.257844	0.01195483	0.04810055	N4BP3
55651	ENSG00000145912	2396.48467	0.0136531	0.06355253	0.83010913	0.92296908	NHP2
64777	ENSG00000145916	1188.51566	-0.04288386	0.0833749	0.59809169	0.78965456	RMND5B
91272	ENSG00000145919	962.794017	0.12311837	0.10065825	0.20507942	0.41202791	BOD1
10814	ENSG00000145920	1870.02123	-2.84357207	0.1141209	1.43E-138	8.35E-136	CPLX2
57451	ENSG00000145934	36.8102061	-0.5977953	0.69355094	0.02739342	0.09338238	TENM2
3779	ENSG00000145936	0	NA	NA	NA	NA	KCNMB1
26240	ENSG00000145945	284.714461	-0.20655278	0.16728447	0.16893069	0.36034672	FAM50B
340156	ENSG00000145949	23.8270171	-0.00405097	0.29757871	0.98113785	0.99355086	MYLK4
222826	ENSG00000145975	0.34624801	-0.02356563	0.38536324	0.65270996	NA	FAM217A
51256	ENSG00000145979	308.972718	0.16435446	0.14261379	0.21396314	0.42415859	TBC1D7
107080638	ENSG00000145979	308.972718	0.16435446	0.14261379	0.21396314	0.42415859	TBC1D7
10667	ENSG00000145982	152.316738	0.15907952	0.19597518	0.3466588	0.57745009	FARS2
54438	ENSG00000145990	131.534217	-0.33623123	0.20063069	0.05147866	0.15239282	GFOD1
54901	ENSG00000145996	349.641208	-0.56611159	0.14571655	3.17E-05	0.00027126	CDKAL1

54660	ENSG00000146001	19.951733	0.06835963	0.3102924	0.7171101	0.86381491	PCDHB18P
84249	ENSG00000146005	94.5962292	0.25758435	0.22014541	0.15916391	0.34667433	PSD2
26045	ENSG00000146006	251.64582	-1.48247658	0.17765993	5.82E-18	1.91E-16	LRRTM2
153527	ENSG00000146007	813.387229	-0.10086201	0.09268446	0.26139442	0.48341399	ZMAT2
2676	ENSG00000146013	1050.35347	-1.51108618	0.09784283	6.83E-55	7.83E-53	GFRA3
26249	ENSG00000146021	268.056859	-0.21512785	0.15846828	0.13462317	0.30902739	KLHL3
51473	ENSG00000146038	4.23778789	-0.06262202	0.38116691	0.46986683	0.69227372	DCDC2
10050	ENSG00000146039	0	NA	NA	NA	NA	SLC17A4
255626	ENSG00000146047	0	NA	NA	NA	NA	H2BC1
353219	ENSG00000146049	0	NA	NA	NA	NA	KAAG1
81786	ENSG00000146054	58.2773039	-0.53337933	0.34341973	0.02873551	0.0970608	TRIM7
90933	ENSG00000146063	1172.82018	0.27298062	0.08155868	0.00060401	0.00380554	TRIM41
192286	ENSG00000146066	754.07463	0.15131327	0.09755047	0.10846858	0.26431057	HIGD2A
54540	ENSG00000146067	1300.20234	0.19679986	0.07678571	0.00882907	0.03742122	FAM193B
7941	ENSG00000146070	0	NA	NA	NA	NA	PLA2G7
27242	ENSG00000146072	138.82966	2.17531757	0.21768596	1.22E-24	5.72E-23	TNFRSF21
22838	ENSG00000146083	3324.37298	0.43475978	0.07195484	7.45E-10	1.30E-08	RNF44
4594	ENSG00000146085	534.763625	0.22165268	0.10729348	0.03106276	0.10305772	MMUT
255426	ENSG00000146090	11.4942165	0.21938383	0.40758294	0.21686449	0.42803338	RASGEF1C
79930	ENSG00000146094	116.92076	0.14229394	0.22631959	0.43990742	0.66517856	DOK3
29777	ENSG00000146109	538.496386	0.10287249	0.1085957	0.32355722	0.55130327	ABT1
170954	ENSG00000146112	1468.84943	0.08590509	0.0775809	0.25726931	0.47832659	PPP1R18
107987457	ENSG00000146112	1468.84943	0.08590509	0.0775809	0.25726931	0.47832659	PPP1R18
23500	ENSG00000146122	342.127673	2.23366975	0.1460186	5.55E-54	6.09E-52	DAAM2
5558	ENSG00000146143	343.426569	0.06323852	0.12666205	0.59942069	0.79057618	PRIM2
90523	ENSG00000146147	0	NA	NA	NA	NA	MLIP
54511	ENSG00000146151	14.7707925	-0.15483039	0.36017632	0.38659151	0.61566669	HMGCLL1
51557	ENSG00000146166	0	NA	NA	NA	NA	LGSN
221472	ENSG00000146192	0.18922458	0.01954053	0.38488496	0.78225479	NA	FGD2
222663	ENSG00000146197	15.8696393	-0.01409529	0.33521593	0.92973471	0.97064482	SCUBE3
50636	ENSG00000146205	137.71335	-0.24746308	0.18601278	0.12856351	0.29941308	ANO7
401262	ENSG00000146215	0.18922458	0.01954053	0.38488496	0.78225479	NA	CRIP3
84630	ENSG00000146216	1320.06876	-1.38584275	0.08159525	1.01E-65	1.49E-63	TTBK1
202500	ENSG00000146221	7.63320458	0.22850669	0.45982141	0.08384127	0.21881225	TCTE1
285855	ENSG00000146223	3843.85958	-0.14600193	0.08218214	0.06849159	0.18852292	RPL7L1
4794	ENSG00000146232	169.482432	-0.16841025	0.17251575	0.27319934	0.49676945	NFKBIE
51302	ENSG00000146233	0	NA	NA	NA	NA	CYP39A1
7162	ENSG00000146242	452.359212	0.69207486	0.14281878	2.95E-07	3.66E-06	TPBG

134728	ENSG00000146243	328.26349	-0.16740763	0.14571568	0.2125563	0.42238752	IRAK1BP1
55023	ENSG00000146247	3301.56078	-0.15485694	0.06501628	0.01555081	0.05936659	PHIP
167681	ENSG00000146250	52.716925	0.91218699	0.35422715	0.00116856	0.00676713	PRSS35
253714	ENSG00000146263	865.011652	-0.26946313	0.1187552	0.01656405	0.06259633	MMS22L
84553	ENSG00000146267	850.4075	-0.54500409	0.11207122	3.96E-07	4.80E-06	FAXC
2569	ENSG00000146276	0.15069912	-0.01848503	0.38480575	0.84537521	NA	GABRR1
10957	ENSG00000146278	523.66627	0.4836851	0.11216502	6.63E-06	6.50E-05	PNRC1
135293	ENSG00000146281	1239.00016	0.02149082	0.0808932	0.78457703	0.90201126	PM20D2
57038	ENSG00000146282	748.72841	0.11392153	0.10838706	0.27320938	0.49676945	RARS2
256380	ENSG00000146285	27.9151292	-1.44696329	0.61362816	0.00090243	0.00541894	SCML4
221322	ENSG00000146350	264.944819	0.25246869	0.16972	0.0971948	0.24387819	TBC1D32
134829	ENSG00000146352	286.88909	0.98344861	0.50987664	0.00423952	0.02026308	CLVS2
2830	ENSG00000146360	0.14931947	-0.01844332	0.38480212	0.84537521	NA	GPR6
154214	ENSG00000146373	0.30139824	-0.02296476	0.38527817	0.68536535	NA	RNF217
84870	ENSG00000146374	0	NA	NA	NA	NA	RSPO3
93663	ENSG00000146376	0	NA	NA	NA	NA	ARHGAP18
9287	ENSG00000146378	0	NA	NA	NA	NA	TAAR2
319100	ENSG00000146383	0	NA	NA	NA	NA	TAAR6
83551	ENSG00000146385	0	NA	NA	NA	NA	TAAR8
58527	ENSG00000146386	54.9363137	0.58573297	0.33883828	0.0181162	0.06720935	ABRACL
134864	ENSG00000146399	0	NA	NA	NA	NA	TAAR1
116843	ENSG00000146409	0	NA	NA	NA	NA	SLC18B1
113115	ENSG00000146410	85.8013756	-0.13879811	0.2230207	0.44571246	0.67028199	MTFR2
154091	ENSG00000146411	66.6173003	1.92511634	0.35242651	3.29E-09	5.27E-08	SLC2A12
257218	ENSG00000146414	986.925656	-0.20571367	0.0857201	0.01367456	0.05356431	SHPRH
51390	ENSG00000146416	556.985012	-0.28244817	0.10383456	0.00451431	0.02134509	AIG1
6993	ENSG00000146425	1103.78705	-0.29289186	0.08722613	0.00054742	0.00348997	DYNLT1
26230	ENSG00000146426	360.843218	0.49482434	0.142407	0.00019669	0.00140068	TIAM2
57583	ENSG00000146433	2003.72789	-0.41885109	0.06793535	3.54E-10	6.45E-09	TMEM181
154197	ENSG00000146453	0.56790858	-0.00208417	0.3828124	0.94123501	NA	PNLDC1
9589	ENSG00000146457	1500.72685	-0.19799773	0.07886222	0.01017159	0.04196387	WTAP
9202	ENSG00000146463	2284.33028	-0.11803422	0.07960932	0.12959207	0.30116889	ZMYM4
7432	ENSG00000146469	8.22627928	-1.57316732	1.35811245	0.00726534	0.03187622	VIP
79624	ENSG00000146476	273.769169	-0.05621778	0.1473777	0.67849187	0.84291038	ARMT1
6581	ENSG00000146477	0	NA	NA	NA	NA	SLC22A3
26238	ENSG00000146521	0	NA	NA	NA	NA	LINC01558
221806	ENSG00000146530	183.215622	1.61367476	0.28609104	8.90E-10	1.54E-08	VWDE
2768	ENSG00000146535	1884.21808	-0.13511138	0.0695274	0.04790193	0.14399612	GNA12

84310	ENSG00000146540	1405.69718	0.19407798	0.07700851	0.01001166	0.04151127	C7orf50
221935	ENSG00000146555	1554.06194	0.15578091	0.09033321	0.07576334	0.20290829	SDK1
NA	ENSG00000146556	411.209417	0.29084484	0.12315742	0.01244578	0.04965299	WASH2P
221960	ENSG00000146574	2170.36407	0.16533499	0.07009087	0.01636521	0.06190386	CCZ1B
79034	ENSG00000146576	923.136496	0.20318549	0.11220005	0.05776416	0.16647892	C7orf26
57786	ENSG00000146587	1013.57853	-0.36826184	0.08698006	1.31E-05	0.00012266	RBAK
9586	ENSG00000146592	244.94446	-0.48631936	0.21088337	0.00734252	0.03214379	CREB5
222894	ENSG00000146618	0	NA	NA	NA	NA	FERD3L
1956	ENSG00000146648	669.317008	-1.80566815	0.11183665	9.38E-60	1.21E-57	EGFR
84847	ENSG00000146666	5.41117012	0.02763548	0.36077058	0.83179331	0.92385262	LINC00525
113130	ENSG00000146670	1584.85369	-0.44421201	0.07128125	2.11E-10	3.93E-09	CDCA5
3486	ENSG00000146674	34.3735421	2.72506004	0.50177585	3.49E-09	5.58E-08	IGFBP3
5814	ENSG00000146676	2794.47703	0.07834934	0.06672069	0.23430081	0.45122578	PURB
NA	ENSG00000146677	0.47107016	-0.00696797	0.38321369	0.8716432	NA	RPL32P18
3484	ENSG00000146678	0	NA	NA	NA	NA	IGFBP1
136853	ENSG00000146700	61.4055851	1.59337892	0.36128457	7.44E-07	8.60E-06	SSC4D
4191	ENSG00000146701	5234.60294	-0.03287008	0.06629119	0.61604727	0.80315271	MDH2
22932	ENSG00000146707	595.894387	0.18260976	0.10352047	0.06657717	0.18484342	POMZP3
541473	ENSG00000146722	12.3793694	0.08628856	0.35597064	0.58076084	0.77801412	
2631	ENSG00000146729	1264.65213	-0.05339194	0.07832408	0.48564024	0.7057649	NIPSNAP2
908	ENSG00000146731	6389.4327	-0.21365183	0.07059508	0.00204033	0.01088178	CCT6A
5723	ENSG00000146733	839.643551	0.5286667	0.11932931	3.39E-06	3.50E-05	PSPH
135892	ENSG00000146755	0.66943461	0.03059546	0.38616409	0.42784935	NA	TRIM50
168374	ENSG00000146757	616.87195	-0.39233424	0.10753792	0.00014007	0.00103004	ZNF92
222255	ENSG00000146776	238.222784	-0.20385308	0.19215277	0.21943079	0.43146748	ATXN7L1
64418	ENSG00000146802	726.081073	-0.06057819	0.13203083	0.62537108	0.80924616	TMEM168
142685	ENSG00000146809	0.33471731	0.02354391	0.38534154	0.62842204	NA	ASB15
55262	ENSG00000146826	637.591508	0.19451446	0.10323632	0.04994406	0.14887172	TRAPPC14
56996	ENSG00000146828	1029.28729	0.30918673	0.10590461	0.00232162	0.01216291	SLC12A9
64599	ENSG00000146830	3707.48636	0.25969369	0.05827631	6.38E-06	6.29E-05	GIGYF1
89122	ENSG00000146833	315.762072	-0.03617715	0.12932195	0.76540357	0.89216889	TRIM4
56257	ENSG00000146834	1256.70518	-0.1195422	0.07893271	0.12126452	0.28753541	MEPCE
7455	ENSG00000146839	0.16735865	0.01897177	0.38482933	0.78225479	NA	ZAN
84928	ENSG00000146842	1139.91804	0.07741341	0.10280619	0.43505656	0.66035668	TMEM209
340351	ENSG00000146856	41.2652512	0.10361184	0.32362385	0.57849924	0.77655107	AGBL3
346673	ENSG00000146857	0	NA	NA	NA	NA	STRA8
92092	ENSG00000146858	434.000893	0.45196466	0.11757679	5.42E-05	0.00043898	ZC3HAV1L
55281	ENSG00000146859	387.928877	-0.02291551	0.12661283	0.8465081	0.93045544	TMEM140

11011	ENSG00000146872	1737.0424	0.00137043	0.08138282	0.98698533	0.99565021	TLK2
2041	ENSG00000146904	1.00332036	0.0055579	0.37925585	0.93202442	NA	EPHA1
64434	ENSG00000146909	1332.44175	0.38852009	0.09722672	3.50E-05	0.00029733	NOM1
285888	ENSG00000146910	0	NA	NA	NA	NA	CNPY1
54892	ENSG00000146918	2201.09691	-0.0181691	0.09493679	0.84232678	0.92837824	NCAPG2
136371	ENSG00000146926	0	NA	NA	NA	NA	ASB10
57502	ENSG00000146938	267.842873	-0.96459757	0.16393752	5.38E-10	9.62E-09	NLGN4X
357	ENSG00000146950	130.455665	-0.32909887	0.20045263	0.05635621	0.16322734	SHROOM2
401409	ENSG00000146955	3.18109652	-0.07835945	0.38243106	0.42838612	0.65428642	RAB19
51631	ENSG00000146963	2184.04668	-0.09454141	0.09578128	0.30725268	0.53477438	LUC7L2
27147	ENSG00000146966	700.25135	0.14732729	0.12398848	0.2088139	0.41674558	DENND2A
57393	ENSG00000147003	11.5735335	0.00456542	0.33952227	0.9793716	0.99317507	CLTRN
30011	ENSG00000147010	359.196565	-0.79196403	0.16760962	4.07E-07	4.92E-06	SH3KBP1
83604	ENSG00000147027	0	NA	NA	NA	NA	TMEM47
347404	ENSG00000147036	0.53262177	0.02083861	0.384376	0.75767191	NA	LANCL3
94122	ENSG00000147041	1.52401327	0.07848063	0.38951113	0.29612947	NA	SYTL5
8573	ENSG00000147044	818.084278	-0.1098054	0.09431828	0.22905295	0.4444846	CASK
7403	ENSG00000147050	651.201108	0.15724134	0.10068992	0.10526287	0.25874017	KDM6A
54466	ENSG00000147059	17.0518024	-0.14526405	0.33905317	0.43994788	0.66517856	SPIN2A
4478	ENSG00000147065	2255.30626	-0.1032831	0.08238936	0.19882941	0.40318637	MSN
8852	ENSG00000147081	0	NA	NA	NA	NA	AKAP4
85417	ENSG00000147082	28.7002441	-0.25343944	0.38518757	0.20376807	0.41022409	CCNB3
55869	ENSG00000147099	820.154709	-0.12724241	0.09795137	0.17814502	0.37365442	HDAC8
6567	ENSG00000147100	436.205255	0.22838733	0.1234932	0.05024553	0.1495008	SLC16A2
79742	ENSG00000147113	0	NA	NA	NA	NA	DIPK2B
7712	ENSG00000147117	33.2685892	-0.13302079	0.30536919	0.4978381	0.71568059	ZNF157
7569	ENSG00000147118	242.998691	-0.20891704	0.14964251	0.12829383	0.29899782	ZNF182
56548	ENSG00000147119	254.31546	1.01532919	0.15351704	4.91E-12	1.07E-10	CHST7
55634	ENSG00000147121	222.873972	0.02215601	0.1564735	0.87781091	0.94578953	KRBOX4
54539	ENSG00000147123	1782.79394	0.08288247	0.07525419	0.26178893	0.48374648	NDUFB11
7592	ENSG00000147124	368.287259	-0.23722461	0.12969237	0.05119366	0.15179828	ZNF41
347517	ENSG00000147127	4.99639845	0.02515755	0.36407444	0.83610178	0.92527105	RAB41
9203	ENSG00000147130	1716.31454	0.06659075	0.07471427	0.36389378	0.5936726	ZMYM3
6872	ENSG00000147133	1727.00859	-0.09848119	0.09116181	0.26548181	0.48783542	TAF1
84636	ENSG00000147138	0.16735865	0.01897177	0.38482933	0.78225479	NA	GPR174
4841	ENSG00000147140	17095.2703	0.09220809	0.05639778	0.07102627	0.19359269	NONO
90060	ENSG00000147144	364.186232	0.17326317	0.12397523	0.13912349	0.31599427	CCDC120
2846	ENSG00000147145	0	NA	NA	NA	NA	LPAR4

10682	ENSG00000147155	743.942675	-0.58167444	0.0998021	1.76E-09	2.93E-08	EBP
158835	ENSG00000147160	0	NA	NA	NA	NA	AWAT2
8473	ENSG00000147162	3813.80779	0.25270629	0.06554022	8.98E-05	0.00069156	OGT
29934	ENSG00000147164	632.372143	0.07959324	0.11742123	0.47688242	0.69881585	SNX12
26548	ENSG00000147166	13.7024586	-0.0560391	0.33909193	0.73183949	0.87194905	ITGB1BP2
3561	ENSG00000147168	0	NA	NA	NA	NA	IL2RG
93953	ENSG00000147174	15.9376848	0.08713231	0.34185744	0.610165	0.79847461	GCNA
7552	ENSG00000147180	1575.83555	-0.16220843	0.07714854	0.03160063	0.1045799	ZNF711
53336	ENSG00000147183	0	NA	NA	NA	NA	CPXCR1
1730	ENSG00000147202	199.440521	0.23995838	0.16649825	0.1095746	0.26631876	DIAPH2
56000	ENSG00000147206	0.30139824	-0.02296476	0.38527817	0.68536535	NA	NXF3
92129	ENSG00000147223	0	NA	NA	NA	NA	RIPPLY1
5631	ENSG00000147224	895.314564	0.03699631	0.08884715	0.67162318	0.83826144	PRPS1
55086	ENSG00000147231	1.91120367	-0.05237235	0.38174001	0.50334617	NA	RADX
84443	ENSG00000147234	1.60863909	0.10434938	0.40355579	0.06090712	NA	FRMPD3
3358	ENSG00000147246	5.28802511	0.07981759	0.37430743	0.51240726	0.7276095	HTR2C
139818	ENSG00000147251	474.334831	-0.40287633	0.1145711	0.00022524	0.00158071	DOCK11
3547	ENSG00000147255	436.233945	0.56179098	0.14377767	2.99E-05	0.00025716	IGSF1
158763	ENSG00000147256	415.615818	0.04493366	0.14593095	0.7403205	0.87741689	ARHGAP36
2719	ENSG00000147257	399.020059	3.0754557	0.15443639	1.89E-89	4.74E-87	GPC3
139760	ENSG00000147262	0	NA	NA	NA	NA	GPR119
27316	ENSG00000147274	8899.90398	-0.00805391	0.05958512	0.89049445	0.95232275	RBMX
79648	ENSG00000147316	468.62581	-0.3968272	0.16231124	0.00706859	0.03119928	MCPH1
9258	ENSG00000147324	903.09399	-0.28912504	0.26264458	0.15143623	0.33539121	MFHAS1
26260	ENSG00000147364	1047.29492	-0.24179356	0.08805129	0.00467922	0.02198336	FBXO25
89885	ENSG00000147378	0	NA	NA	NA	NA	FATE1
4103	ENSG00000147381	0	NA	NA	NA	NA	MAGEA4
50814	ENSG00000147383	385.874497	-0.20307301	0.13047646	0.09701131	0.24360263	NSDHL
7739	ENSG00000147394	168.354531	1.2334445	0.21275173	6.67E-10	1.18E-08	ZNF185
1069	ENSG00000147400	349.802045	0.05604546	0.13165884	0.65120444	0.82578612	CETN2
6134	ENSG00000147403	23295.5986	0.08596745	0.04801155	0.07077067	0.1930817	RPL10
55790	ENSG00000147408	1.14036012	0.07722112	0.39253222	0.20972802	NA	CSGALNACT1
526	ENSG00000147416	2868.61525	-0.4679965	0.0642599	1.45E-13	3.50E-12	ATP6V1B2
55246	ENSG00000147419	1137.53042	-0.00839588	0.09287598	0.92484159	0.96868601	CCDC25
79618	ENSG00000147421	950.703432	0.30556085	0.09760108	0.00117443	0.00679719	HMBOX1
1142	ENSG00000147432	0	NA	NA	NA	NA	CHRNB3
8973	ENSG00000147434	1.39259898	-0.00432957	0.37918364	0.8730313	NA	CHRNA6
2796	ENSG00000147437	22.5105389	0.22595058	0.35068699	0.27160925	0.49501447	GNRH1

55909	ENSG00000147439	241.467411	-0.00032455	0.14758229	0.99653371	0.99873692	BIN3
9046	ENSG00000147443	0.15069912	-0.01848503	0.38480575	0.84537521	NA	DOK2
51312	ENSG00000147454	998.541148	0.04212669	0.08804004	0.62885088	0.81194855	SLC25A37
91782	ENSG00000147457	1251.09611	-0.1067098	0.10305393	0.28141632	0.50624272	CHMP7
80005	ENSG00000147459	149.677828	1.46450598	0.22507414	7.07E-12	1.52E-10	DOCK5
6770	ENSG00000147465	39.3031151	0.02296167	0.27974331	0.90637045	0.95930719	STAR
11212	ENSG00000147471	558.660268	-0.02787068	0.11106927	0.79089076	0.90486551	PLPBP
11160	ENSG00000147475	852.066477	-0.05215129	0.10268496	0.59651365	0.78867326	ERLIN2
54212	ENSG00000147481	1.62372068	-0.10818151	0.4046584	0.06530737	NA	SNTG1
137902	ENSG00000147485	103.51425	1.17911785	0.24582526	1.58E-07	2.04E-06	PXDNL
9705	ENSG00000147488	0.34746601	0.02370763	0.3853651	0.61987128	NA	ST18
8601	ENSG00000147509	21.8076496	0.48486266	0.48754361	0.05929202	0.1695767	RGS20
6867	ENSG00000147526	402.977045	-0.31838167	0.1378739	0.01359014	0.05333894	TACC1
51125	ENSG00000147533	748.885403	0.02614087	0.10410185	0.79525828	0.90586169	GOLGA7
84513	ENSG00000147535	537.781827	0.36669286	0.11838224	0.00110823	0.00645735	PLPP5
84296	ENSG00000147536	479.604593	0.23924846	0.16923125	0.11495828	0.27628979	GINS4
54904	ENSG00000147548	3512.30673	-0.36611597	0.09836897	0.0001126	0.00084495	NSD3
85479	ENSG00000147570	0	NA	NA	NA	NA	DNAJC5B
1392	ENSG00000147571	0	NA	NA	NA	NA	CRH
84675	ENSG00000147573	0	NA	NA	NA	NA	TRIM55
137872	ENSG00000147576	16.6243915	0.36950826	0.48577263	0.09667385	0.24306007	ADHFE1
28957	ENSG00000147586	219.743292	-0.27996455	0.15476634	0.04752134	0.14306879	MRPS28
5375	ENSG00000147588	0	NA	NA	NA	NA	PMP2
51110	ENSG00000147592	111.846198	0.15390158	0.21071599	0.38499435	0.61396093	LACTB2
63978	ENSG00000147596	0	NA	NA	NA	NA	PRDM14
7013	ENSG00000147601	775.919886	-0.42014165	0.09566483	5.58E-06	5.57E-05	TERF1
6129	ENSG00000147604	16678.158	-0.13360118	0.05474046	0.01358814	0.05333894	RPL7
115111	ENSG00000147606	0.49850962	-0.02963375	0.38591404	0.52353366	NA	SLC26A7
85481	ENSG00000147613	0	NA	NA	NA	NA	PSKH2
245972	ENSG00000147614	0	NA	NA	NA	NA	ATP6V0D2
55638	ENSG00000147642	80.8943957	0.07971758	0.24639602	0.67611749	0.84154147	SYBU
1807	ENSG00000147647	0.89961696	-0.05075436	0.38821636	0.35902327	NA	DPYS
92140	ENSG00000147649	2577.06345	-0.47484308	0.07298728	3.33E-11	6.73E-10	MTDH
29967	ENSG00000147650	593.077573	-0.14615952	0.12573936	0.2172086	0.42858464	LRP12
9166	ENSG00000147654	262.964352	-0.28607667	0.16235316	0.05133884	0.15209235	EBAG9
340419	ENSG00000147655	0.173124	-0.01911262	0.38486262	0.84537521	NA	RSPO2
5440	ENSG00000147669	679.15173	0.24389564	0.09773292	0.0097212	0.04056123	POLR2K
114569	ENSG00000147676	0	NA	NA	NA	NA	MAL2

8667	ENSG00000147677	4009.10779	0.01050908	0.05587017	0.84988123	0.93178844	EIF3H
84294	ENSG00000147679	494.093892	0.24302814	0.14196009	0.06477481	0.181378	UTP23
4715	ENSG00000147684	2138.5999	-0.04993574	0.06272074	0.41878672	0.6446754	NDUFB9
83940	ENSG00000147687	420.067331	-0.12180191	0.11817909	0.27768459	0.50221169	TATDN1
84985	ENSG00000147689	1.40798341	-0.03526476	0.38166771	0.58231557	NA	FAM83A
56169	ENSG00000147697	0	NA	NA	NA	NA	GSDMC
51059	ENSG00000147724	70.2167031	-2.87059636	0.3336563	5.87E-19	2.08E-17	FAM135B
246122	ENSG00000147753	0	NA	NA	NA	NA	TTTY7
100101120	ENSG00000147761	0	NA	NA	NA	NA	TTTY7B
7553	ENSG00000147789	597.335417	-0.05631704	0.11413105	0.60488372	0.79491906	ZNF7
80728	ENSG00000147799	475.244185	0.10144704	0.1107108	0.3387678	0.5690106	ARHGAP39
55630	ENSG00000147804	247.442785	0.20896261	0.14552661	0.12015112	0.28543799	SLC39A4
93100	ENSG00000147813	73.2203921	-0.02280735	0.22352752	0.89885932	0.95556135	NAPRT
7436	ENSG00000147852	254.238965	0.10101825	0.16875911	0.50599819	0.72177288	VLDLR
50808	ENSG00000147853	1155.01955	-0.18642994	0.09041921	0.03344033	0.109409	AK3
115426	ENSG00000147854	1072.07724	0.06818746	0.08918706	0.43229256	0.65787255	UHRF2
4781	ENSG00000147862	4081.24638	-0.8888183	0.06501776	1.86E-43	1.57E-41	NFIB
9350	ENSG00000147869	6.06266688	0.12606195	0.38072511	0.37630693	0.60526384	CER1
123	ENSG00000147872	955.86879	0.19268922	0.08691572	0.02271093	0.08064878	PLIN2
3442	ENSG00000147873	0.31716937	0.00152043	0.38337001	0.96328461	NA	IFNA5
54801	ENSG00000147874	1895.62216	-0.2312271	0.08706594	0.00624372	0.02801342	HAUS6
1030	ENSG00000147883	7.46355748	0.14158984	0.39254217	0.284643	0.50987892	CDKN2B
3449	ENSG00000147885	0	NA	NA	NA	NA	IFNA16
1029	ENSG00000147889	340.182156	-0.09569161	0.13217485	0.43957424	0.66481822	CDKN2A
203228	ENSG00000147894	530.997305	0.12467275	0.10424235	0.2138241	0.42397262	C9orf72
56832	ENSG00000147896	0	NA	NA	NA	NA	IFNK
84186	ENSG00000147905	350.898646	-0.15974642	0.15226048	0.25163376	0.47149344	ZCCHC7
26267	ENSG00000147912	883.395982	0.02748749	0.10996978	0.79503764	0.90585603	FBXO10
10280	ENSG00000147955	1794.27351	-0.22818942	0.07912305	0.00314094	0.01574579	SIGMAR1
220869	ENSG00000147996	556.794527	0.08337389	0.10860288	0.42397221	0.64969559	CBWD5
84131	ENSG00000148019	1502.67799	0.07586109	0.08320409	0.35070191	0.58113472	CEP78
4915	ENSG00000148053	26.2655232	-0.07398231	0.30133396	0.6969259	0.85375952	NTRK2
414328	ENSG00000148057	47.0718473	-0.00715604	0.25975326	0.96855239	0.98817646	IDNK
53358	ENSG00000148082	1263.46059	-0.4891272	0.08914321	1.68E-08	2.45E-07	SHC3
549	ENSG00000148090	215.894883	0.34312175	0.15819881	0.01733204	0.06491745	AUH
84641	ENSG00000148110	789.290912	0.06966734	0.09510472	0.44997573	0.67340229	MFSD14B
84909	ENSG00000148120	314.255543	0.25273467	0.15043915	0.0672202	0.18610782	AOPEP
54886	ENSG00000148123	0.16735865	0.01897177	0.38482933	0.78225479	NA	PLPPR1

138804	ENSG00000148136	0	NA	NA	NA	NA	OR13C4
58499	ENSG00000148143	1458.17258	-0.06686251	0.07508362	0.36304346	0.59281472	ZNF462
58493	ENSG00000148153	695.704673	-0.49461975	0.13769179	0.00012781	0.00094689	INIP
7357	ENSG00000148154	463.81408	0.11134072	0.13084258	0.36538344	0.5947154	UGCG
10880	ENSG00000148156	0.18922458	0.01954053	0.38488496	0.78225479	NA	ACTL7B
401548	ENSG00000148158	445.091271	-0.22757238	0.11917793	0.04389212	0.13459347	SNX30
2040	ENSG00000148175	230.981618	-0.06427241	0.14568626	0.63267152	0.81417935	STOM
2934	ENSG00000148180	468.649281	0.44917652	0.13908986	0.00054421	0.00347173	GSN
92399	ENSG00000148187	776.929303	-0.09101869	0.0903566	0.29865138	0.52551632	MRRF
2649	ENSG00000148200	65.3029835	0.46143684	0.37324387	0.05830063	0.16751071	NR6A1
286204	ENSG00000148204	2.25754674	0.03989187	0.37727946	0.65195724	NA	CRB2
392391	ENSG00000148215	0	NA	NA	NA	NA	OR5C1
210	ENSG00000148218	560.109714	0.3218156	0.1042355	0.00130161	0.00741428	ALAD
23245	ENSG00000148219	1751.91056	-0.34016171	0.07213897	1.51E-06	1.66E-05	ASTN2
114987	ENSG00000148225	88.0290417	0.02005488	0.23001599	0.91481211	0.9640318	WDR31
54107	ENSG00000148229	1514.80948	-0.45513384	0.07519	6.40E-10	1.13E-08	POLE3
6836	ENSG00000148248	3250.04061	-0.12408415	0.08593189	0.13780052	0.31363569	SURF4
26301	ENSG00000148288	69.6230581	-0.75015886	0.37973399	0.00650022	0.0290395	GBGT1
6834	ENSG00000148290	856.169801	0.10044403	0.08792249	0.24089889	0.45885437	SURF1
6835	ENSG00000148291	418.216813	-0.11549523	0.14183776	0.37958167	0.60845927	SURF2
6838	ENSG00000148296	1357.28544	-0.04738702	0.07468008	0.51710766	0.73098795	SURF6
6837	ENSG00000148297	1163.97541	0.05437931	0.10948128	0.60523766	0.79522617	MED22
57109	ENSG00000148300	965.529247	0.02282006	0.08578603	0.78562835	0.90248778	REXO4
6130	ENSG00000148303	24397.6652	-0.0045151	0.05135793	0.92525516	0.96892907	RPL7A
9328	ENSG00000148308	1300.17439	0.16758974	0.08372008	0.03991954	0.12519819	GTF3C5
140459	ENSG00000148331	1546.82847	-0.11556856	0.07891857	0.13405808	0.30828734	ASB6
80142	ENSG00000148334	1206.16116	-0.22225762	0.08046769	0.00467205	0.02195486	PTGES2
28989	ENSG00000148335	684.99757	-0.0131925	0.10374313	0.89404855	0.95417323	NTMT1
25792	ENSG00000148337	2519.03309	-0.08119486	0.0639954	0.19785033	0.40199972	CIZ1
114789	ENSG00000148339	767.81794	-0.38051397	0.10037802	8.29E-05	0.0006434	SLC25A25
56904	ENSG00000148341	1459.17377	0.18845217	0.07937054	0.0150917	0.0579852	SH3GLB2
84895	ENSG00000148343	633.287347	0.19096751	0.11470103	0.08063292	0.21267709	MIGA2
9536	ENSG00000148344	106.938928	-0.17172101	0.20941976	0.32928522	0.55780556	PTGES
3934	ENSG00000148346	0	NA	NA	NA	NA	LCN2
90678	ENSG00000148356	633.184291	0.20884777	0.10934511	0.04585697	0.13926256	LRSAM1
256158	ENSG00000148357	248.861723	0.73507925	0.18578759	1.52E-05	0.00013962	HMCN2
57720	ENSG00000148358	2249.86056	-0.00173951	0.07103885	0.9793839	0.99317507	GPR107
286257	ENSG00000148362	536.691694	0.1064322	0.10670027	0.29920581	0.52616941	PAXX

91734	ENSG00000148377	0.34624801	-0.02356563	0.38536324	0.65270996	NA	IDI2
56623	ENSG00000148384	622.629873	0.46281309	0.1111341	1.36E-05	0.00012616	INPP5E
392399	ENSG00000148386	0	NA	NA	NA	NA	LCN9
9919	ENSG00000148396	2960.85352	-0.1087985	0.06401495	0.08393871	0.21898015	SEC16A
92715	ENSG00000148399	905.164267	0.23454201	0.0910985	0.00793617	0.03427672	DPH7
4851	ENSG00000148400	150.176326	2.2837787	0.28302058	4.08E-17	1.27E-15	NOTCH1
774	ENSG00000148408	1268.54985	-2.03360792	0.09014189	6.91E-114	2.49E-111	CACNA1B
138151	ENSG00000148411	114.141467	0.46432826	0.23676636	0.01806868	0.067119	NACC2
254427	ENSG00000148426	39.9769396	3.23158147	0.45057086	5.32E-14	1.33E-12	PROSER2
9712	ENSG00000148429	725.539931	0.19791781	0.09208179	0.02660607	0.09138928	USP6NL
23412	ENSG00000148444	678.214576	0.02107377	0.09347369	0.81699937	0.91671007	COMMD3
22921	ENSG00000148450	926.989718	0.02386826	0.09210912	0.79349898	0.90556117	MSRB2
23590	ENSG00000148459	269.359636	-0.41694009	0.15296847	0.00302384	0.01523049	PDSS1
221061	ENSG00000148468	1744.97365	0.12556148	0.07065854	0.07060402	0.19275957	FAM171A1
80013	ENSG00000148481	468.757353	-0.31589996	0.13162759	0.01035013	0.04258823	MINDY3
221074	ENSG00000148482	0	NA	NA	NA	NA	SLC39A12
653567	ENSG00000148483	1.12281109	-0.04714939	0.38566392	0.3665206	NA	TMEM236
6251	ENSG00000148484	505.278246	0.57150169	0.11307075	1.41E-07	1.82E-06	RSU1
338596	ENSG00000148488	1.43982294	0.00381868	0.38005775	0.94901222	NA	ST8SIA6
56288	ENSG00000148498	1180.14916	-0.0190385	0.08221967	0.81127605	0.91456796	PARD3
91074	ENSG00000148513	0.33471731	0.02354391	0.38534154	0.62842204	NA	ANKRD30A
6935	ENSG00000148516	1377.77953	0.01496987	0.08123653	0.84746646	0.93065124	ZEB1
220965	ENSG00000148541	179.306472	-0.44341728	0.18684555	0.00731976	0.03207953	FAM13C
29982	ENSG00000148572	264.74368	-0.18676886	0.14542076	0.16398397	0.35325103	NRBF2
29974	ENSG00000148584	0.68892055	0.00490802	0.38130194	0.92401061	NA	A1CF
92211	ENSG00000148600	208.628343	-1.06890144	0.21210955	5.13E-08	7.05E-07	CDHR1
26103	ENSG00000148602	0	NA	NA	NA	NA	LRIT1
5995	ENSG00000148604	0	NA	NA	NA	NA	RGR
11128	ENSG00000148606	1652.13894	-0.28080017	0.06968015	4.01E-05	0.00033665	POLR3A
26091	ENSG00000148634	793.509968	0.15487803	0.10608014	0.12830985	0.29899782	HERC4
83938	ENSG00000148655	20.2736789	0.04465153	0.33045135	0.79462509	0.90579008	LRMDA
818	ENSG00000148660	1397.1048	0.07352167	0.07431453	0.31425842	0.54212505	CAMK2G
10974	ENSG00000148671	0	NA	NA	NA	NA	ADIRF
2746	ENSG00000148672	1764.86136	-0.27611381	0.082276	0.00057022	0.00362019	GLUD1
27063	ENSG00000148677	0	NA	NA	NA	NA	ANKRD1
3363	ENSG00000148680	0.16735865	0.01897177	0.38482933	0.78225479	NA	HTR7
10556	ENSG00000148688	320.13674	-0.06164517	0.12783884	0.60796527	0.79695264	RPP30
118924	ENSG00000148690	542.50492	0.10600862	0.14507947	0.4358257	0.66096789	FRA10AC1

120	ENSG00000148700	1075.3548	0.2859432	0.1135504	0.00821843	0.03534655	ADD3
3026	ENSG00000148702	0	NA	NA	NA	NA	HABP2
11023	ENSG00000148704	0	NA	NA	NA	NA	VAX1
54788	ENSG00000148719	633.430761	0.18283516	0.11604462	0.097453	0.24443331	DNAJB12
1979	ENSG00000148730	6548.47209	-0.11101675	0.0612034	0.06603835	0.18375052	EIF4EBP2
64106	ENSG00000148734	3.00414779	0.01684629	0.36924773	0.87523251	0.94499851	NPFFR1
79949	ENSG00000148735	0.16735865	0.01897177	0.38482933	0.78225479	NA	PLEKHS1
6934	ENSG00000148737	237.011836	0.90853375	0.18654762	1.58E-07	2.04E-06	TCF7L2
4288	ENSG00000148773	10512.8548	-0.75635392	0.05396845	2.65E-45	2.33E-43	MKI67
1586	ENSG00000148795	0	NA	NA	NA	NA	CYP17A1
9118	ENSG00000148798	3873.40384	-2.7089968	0.06014563	0	0	INA
282969	ENSG00000148803	120.99544	0.08960865	0.19010208	0.58882411	0.78351083	FUOM
80313	ENSG00000148814	182.582899	0.4691387	0.17786543	0.00328235	0.01632536	LRRC27
92170	ENSG00000148824	1279.21283	-0.02258668	0.08388437	0.78172697	0.90040424	MTG1
84504	ENSG00000148826	0.30002379	-0.02294466	0.38527539	0.6864438	NA	NKX6-2
196743	ENSG00000148832	44.4893293	0.11980286	0.27098293	0.5416374	0.750006	PAOX
9446	ENSG00000148834	605.248622	-0.05769438	0.10723194	0.57288499	0.7725692	GSTO1
6877	ENSG00000148835	310.864598	-0.22602005	0.13314152	0.06922163	0.18987379	TAF5
23082	ENSG00000148840	1683.9368	-0.03319545	0.07109183	0.63019025	0.81297489	PPRC1
85450	ENSG00000148841	392.882742	0.25054118	0.12766788	0.03699519	0.11835094	ITPRIP
54805	ENSG00000148842	608.03869	-0.55801293	0.12430587	2.32E-06	2.47E-05	CNNM2
22984	ENSG00000148843	3815.15944	-0.05934859	0.06058242	0.31926573	0.5469361	PDCD11
8038	ENSG00000148848	2564.99262	-1.93601746	0.0791536	2.84E-133	1.53E-130	ADAM12
6001	ENSG00000148908	72.4553689	0.13597625	0.25376313	0.48350003	0.70373712	RGS10
84280	ENSG00000148925	523.16077	-0.14244804	0.10987507	0.17482253	0.36867335	BTBD10
133	ENSG00000148926	52.9508049	-0.57790757	0.399238	0.02775815	0.09431781	ADM
2620	ENSG00000148935	81.2374504	0.78828621	0.28819582	0.00096365	0.00573279	GAS2
159963	ENSG00000148942	0.33471731	0.02354391	0.38534154	0.62842204	NA	SLC5A12
55327	ENSG00000148943	518.019265	-0.51052297	0.11047958	1.45E-06	1.60E-05	LIN7C
57689	ENSG00000148948	10.9342917	0.1027082	0.35736179	0.52164463	0.73494218	LRRC4C
196294	ENSG00000148950	124.988643	-0.39878826	0.20730872	0.02488861	0.08684313	IMMP1L
6291	ENSG00000148965	0	NA	NA	NA	NA	SAA4
27315	ENSG00000148985	542.84796	0.15259907	0.10453568	0.12893964	0.30021857	PGAP2
64852	ENSG00000149016	769.138924	0.05439249	0.1062264	0.5948493	0.78787011	TUT1
7356	ENSG00000149021	0	NA	NA	NA	NA	SCGB1A1
90019	ENSG00000149043	17.8641785	-0.50776693	0.62119969	0.04558651	0.13856846	SYT8
7761	ENSG00000149050	88.6395329	0.06072086	0.25758793	0.75286397	0.8843065	ZNF214
7762	ENSG00000149054	0.78122969	0.00103505	0.38149724	0.98289957	NA	ZNF215

51144	ENSG00000149084	815.704551	-0.26866314	0.09778099	0.00437054	0.0208191	HSD17B12
51074	ENSG00000149089	303.998664	0.14515128	0.13774601	0.25881506	0.48030339	APIP
25891	ENSG00000149090	2.03544701	0.03351314	0.37964023	0.57428394	NA	PAMR1
8525	ENSG00000149091	1367.17884	-0.30980715	0.08824804	0.0002978	0.00203244	DGKZ
10480	ENSG00000149100	2533.82095	0.05046245	0.06417876	0.42559098	0.65142147	EIF3M
85456	ENSG00000149115	958.806484	-0.13952779	0.10757248	0.17572149	0.36993999	TNKS1BP1
10249	ENSG00000149124	1.23759164	0.06131861	0.39085027	0.17525616	NA	GLYAT
710	ENSG00000149131	244.87328	1.80962876	0.17027123	1.66E-27	8.64E-26	SERPING1
338674	ENSG00000149133	0	NA	NA	NA	NA	OR5F1
6749	ENSG00000149136	6828.43902	-0.17651389	0.05411571	0.00096607	0.005742	SSRP1
8501	ENSG00000149150	193.621859	0.01574753	0.16931431	0.91878396	0.96558446	SLC43A1
5795	ENSG00000149177	284.009768	0.23205635	0.16608347	0.12106754	0.28721511	PTPRJ
79096	ENSG00000149179	748.978004	0.50726348	0.10666531	7.63E-07	8.80E-06	C11orf49
84364	ENSG00000149182	1230.25489	-0.0493025	0.07613769	0.50818231	0.72394177	ARFGAP2
10658	ENSG00000149187	3810.15125	-0.11267434	0.06475272	0.07698188	0.2054507	CELF1
51501	ENSG00000149196	561.737929	-0.03162861	0.10251384	0.74612309	0.8804068	HIKESHI
60494	ENSG00000149201	18.4141217	0.36878002	0.44269676	0.10850528	0.2643325	CCDC81
143686	ENSG00000149212	692.850799	-0.22136164	0.11740098	0.04689316	0.14175837	SESN3
23052	ENSG00000149218	106.21832	-0.46835211	0.22420008	0.01341046	0.0527799	ENDOD1
79780	ENSG00000149231	981.746675	-0.37929532	0.1090603	0.00027481	0.00189053	CCDC82
283212	ENSG00000149243	94.5346901	0.37847337	0.23893076	0.05217845	0.1539026	KLHL35
26011	ENSG00000149256	5028.42669	0.26270838	0.08567475	0.00162673	0.00900259	TENM4
871	ENSG00000149257	2584.77616	0.76216039	0.06722038	1.82E-30	1.06E-28	SERPINH1
726	ENSG00000149260	616.475378	-0.05967824	0.11418308	0.58284747	0.77951315	CAPN5
92105	ENSG00000149262	759.313159	0.22074379	0.09851143	0.02017837	0.07342972	INTS4
5058	ENSG00000149269	706.433433	0.12997858	0.10825054	0.21031095	0.41893265	PAK1
6188	ENSG00000149273	23137.6585	-0.20944104	0.04912619	1.69E-05	0.00015358	RPS3
85463	ENSG00000149289	906.847917	0.83409468	0.10417176	2.10E-16	6.28E-15	ZC3H12C
54970	ENSG00000149292	232.233933	0.04236591	0.15219674	0.7642974	0.89148768	TTC12
4684	ENSG00000149294	3401.7132	-1.43748432	0.07035648	4.36E-93	1.24E-90	NCAM1
1813	ENSG00000149295	794.33448	-2.51194411	0.10820529	2.18E-120	8.84E-118	DRD2
91894	ENSG00000149300	0.34746601	0.02370763	0.3853651	0.61987128	NA	C11orf52
9177	ENSG00000149305	2.34842905	0.15776707	0.42873771	0.03308935	NA	HTR3B
4863	ENSG00000149308	596.523116	-0.21563122	0.10708553	0.03546267	0.11446692	NPAT
472	ENSG00000149311	2588.94007	0.08507896	0.07301984	0.23537319	0.45219979	ATM
60496	ENSG00000149313	1587.13352	-0.34128552	0.08230342	2.08E-05	0.00018659	AASDHPPT
89944	ENSG00000149328	660.06708	0.02158976	0.09622175	0.81754274	0.91685309	GLB1L2
128710	ENSG00000149346	327.545853	-0.14672671	0.13820352	0.25402046	0.47451017	SLX4IP

55004	ENSG00000149357	1196.11286	0.11964905	0.08722218	0.15893415	0.3463211	LAMTOR1
283208	ENSG00000149380	1.17947757	0.01850914	0.38013115	0.77237192	NA	P4HA3
2900	ENSG00000149403	171.504736	-2.55408642	0.20654858	2.82E-36	1.98E-34	GRIK4
6768	ENSG00000149418	0.94185167	-0.02000688	0.38110359	0.73516374	NA	ST14
10525	ENSG00000149428	6233.95169	0.3971878	0.10056769	4.25E-05	0.00035498	HYOU1
92086	ENSG00000149435	0	NA	NA	NA	NA	GGTLC1
NA	ENSG00000149443	0	NA	NA	NA	NA	SCP2D1-AS1
80332	ENSG00000149451	0.173733	0.01914545	0.38484597	0.78225479	NA	ADAM33
9376	ENSG00000149452	0.173733	0.01914545	0.38484597	0.78225479	NA	SLC22A8
57325	ENSG00000149474	444.231565	-0.03787253	0.14050462	0.77080899	0.89494676	KAT14
26007	ENSG00000149476	1284.64518	0.24465713	0.08075051	0.0019101	0.01027001	TKFC
9219	ENSG00000149480	2829.03533	0.04935703	0.06155504	0.41702552	0.64347381	MTA2
51524	ENSG00000149483	439.224076	-0.23580105	0.12108013	0.03942546	0.12409621	TMEM138
3992	ENSG00000149485	3857.92629	-0.38238401	0.06958339	2.05E-08	2.98E-07	FADS1
117532	ENSG00000149488	6.95584252	0.19085767	0.41982949	0.18209352	0.37980817	TMC2
6094	ENSG00000149489	32.9452239	-0.22383258	0.36207073	0.26221441	0.48416323	ROM1
256364	ENSG00000149499	860.601191	0.05714263	0.09400905	0.53096763	0.74257279	EML3
3619	ENSG00000149503	2273.74309	-0.23779112	0.06708895	0.00031232	0.00212474	INCENP
22917	ENSG00000149506	0.173733	0.01914545	0.38484597	0.78225479	NA	ZP1
219990	ENSG00000149507	0	NA	NA	NA	NA	OOSP2
932	ENSG00000149516	0	NA	NA	NA	NA	MS4A3
9651	ENSG00000149527	376.88182	-1.96604668	0.15495087	3.87E-38	2.84E-36	PLCH2
NA	ENSG00000149531	110.123315	-0.02974112	0.19765599	0.86034925	0.93787522	FRG1BP
79869	ENSG00000149532	2576.56041	-0.05646385	0.07556641	0.44410473	0.66905081	CPSF7
2206	ENSG00000149534	0	NA	NA	NA	NA	MS4A2
26229	ENSG00000149541	935.776021	0.24792288	0.10646378	0.01511446	0.05803579	B3GAT3
9538	ENSG00000149547	1964.90261	-0.21304413	0.06625529	0.00107611	0.00628684	EI24
80071	ENSG00000149548	164.679269	0.18172465	0.18072131	0.25529814	0.47622369	CCDC15
1111	ENSG00000149554	1068.16648	-0.28718578	0.08676165	0.00065692	0.0040857	CHEK1
9638	ENSG00000149557	730.929749	-1.39033799	0.10431313	1.49E-41	1.16E-39	FEZ1
105369550	ENSG00000149557	730.929749	-1.39033799	0.10431313	1.49E-41	1.16E-39	FEZ1
90952	ENSG00000149564	32.9163073	0.2872443	0.33649164	0.18638905	0.38610218	ESAM
84623	ENSG00000149571	246.209805	-0.37724237	0.15576045	0.0080762	0.03481275	KIRREL3
10205	ENSG00000149573	0	NA	NA	NA	NA	MPZL2
6327	ENSG00000149575	18.5791846	0.14621626	0.33746109	0.44032175	0.66517856	SCN2B
51092	ENSG00000149577	403.899749	0.27208653	0.14382371	0.04078426	0.12718637	SIDT2
84866	ENSG00000149582	454.557365	0.11045837	0.11502722	0.31421498	0.54212505	TMEM25
6876	ENSG00000149591	54.3193354	0.92321926	0.36597761	0.00122922	0.00706277	TAGLN

57158	ENSG00000149596	3.13556017	-0.0820709	0.38527045	0.3672852	0.59650772	JPH2
128853	ENSG00000149599	137.62461	-1.01145652	0.22278816	6.78E-07	7.90E-06	DUSP15
149951	ENSG00000149600	506.673921	-0.05298081	0.10584487	0.60179257	0.79216856	COMMD7
128864	ENSG00000149609	13.73156	0.01573984	0.3291393	0.92865134	0.97011965	C20orf144
85449	ENSG00000149633	101.753725	0.9125145	0.25668692	4.94E-05	0.00040406	KIAA1755
128497	ENSG00000149634	32.6054919	0.32985968	0.34844328	0.141556	0.3199443	SPATA25
128506	ENSG00000149635	0	NA	NA	NA	NA	OCSTAMP
79980	ENSG00000149636	472.713153	-0.06552019	0.11287591	0.54298178	0.75099868	DSN1
140710	ENSG00000149639	4781.16163	-0.15670332	0.06455669	0.01365173	0.05351717	SOGA1
140894	ENSG00000149646	30.9115366	0.09237365	0.30097951	0.63352889	0.81495647	CNBD2
391253	ENSG00000149651	0	NA	NA	NA	NA	SPINT4
64405	ENSG00000149654	9.80686136	-4.19422789	1.22181032	0.00010721	0.00080972	CDH22
NA	ENSG00000149656	0.51086009	-0.01927154	0.38352315	0.70504665	NA	LINC00266-1
149986	ENSG00000149657	1698.58404	-0.09494148	0.07148058	0.17604032	0.37037488	LSM14B
54915	ENSG00000149658	1071.98548	-0.16277345	0.08126484	0.04010216	0.12563191	YTHDF1
81928	ENSG00000149679	513.011205	0.2810504	0.13984994	0.03062186	0.10197567	CABLES2
220064	ENSG00000149716	447.496178	-0.08176781	0.11998331	0.4721051	0.69438543	LTO1
170589	ENSG00000149735	0	NA	NA	NA	NA	GPHA2
114571	ENSG00000149742	0	NA	NA	NA	NA	SLC22A9
83707	ENSG00000149743	442.94398	0.15885038	0.11681536	0.15386018	0.33895025	TRPT1
84304	ENSG00000149761	426.352121	0.22586289	0.14172219	0.0856067	0.22219218	NUDT22
83706	ENSG00000149781	8.66698231	-0.06947315	0.36178602	0.60805761	0.79695264	FERMT3
5331	ENSG00000149782	1288.65493	-0.12148537	0.08400436	0.13775812	0.31361113	PLCB3
740	ENSG00000149792	938.774626	-0.20187054	0.11313406	0.06069469	0.17286574	MRPL49
10435	ENSG00000149798	75.9470851	0.29515716	0.29703375	0.16579837	0.35599996	CDC42EP2
2197	ENSG00000149806	4588.20042	0.07973433	0.05585251	0.14937576	0.33213215	FAU
7108	ENSG00000149809	821.267887	-0.19454686	0.10370731	0.05074372	0.15068933	TM7SF2
738	ENSG00000149823	2471.98153	-0.09798057	0.07891063	0.20397394	0.41051388	VPS51
6911	ENSG00000149922	69.1112025	0.28277279	0.26184266	0.16133332	0.34958987	TBX6
5531	ENSG00000149923	2805.85129	0.1298118	0.06840222	0.05377541	0.15733729	PPP4C
226	ENSG00000149925	6229.78811	-0.21199243	0.07623715	0.00454984	0.02148745	ALDOA
83723	ENSG00000149926	1634.90176	-0.7065131	0.08281192	3.35E-18	1.13E-16	TLCD3B
8448	ENSG00000149927	185.253303	0.29401302	0.16645472	0.05007306	0.1491442	DOC2A
8479	ENSG00000149929	917.297877	-0.31300014	0.09335934	0.00052372	0.00335719	HIRIP3
9344	ENSG00000149930	1549.49726	-0.06146513	0.07741253	0.41668246	0.64309452	TAOK2
124446	ENSG00000149932	638.744193	0.2332072	0.10874762	0.02498256	0.08712497	TMEM219
8091	ENSG00000149948	0	NA	NA	NA	NA	HMGA2
4314	ENSG00000149968	0	NA	NA	NA	NA	MMP3

22866	ENSG00000149970	188.766581	-0.25747284	0.25720129	0.19350306	0.39626941	CNKSR2
53942	ENSG00000149972	9.62175875	-1.71143157	0.97444379	0.00298081	0.01504431	CNTN5
51348	ENSG00000150045	0.16735865	0.01897177	0.38482933	0.78225479	NA	KLRF1
51267	ENSG00000150048	0	NA	NA	NA	NA	CLEC1A
283078	ENSG00000150051	0	NA	NA	NA	NA	MKX
143098	ENSG00000150054	0.34113261	0.0236267	0.38535342	0.6240938	NA	MPP7
3688	ENSG00000150093	8399.74718	0.14991124	0.04790113	0.00160561	0.00889812	ITGB1
NA	ENSG00000150175	0	NA	NA	NA	NA	FRMPD2B
53828	ENSG00000150201	0	NA	NA	NA	NA	FXYD4
79097	ENSG00000150244	0	NA	NA	NA	NA	TRIM48
390157	ENSG00000150261	0	NA	NA	NA	NA	OR8K1
390162	ENSG00000150269	0	NA	NA	NA	NA	OR5M9
65217	ENSG00000150275	3.97022521	0.02968007	0.37056272	0.77464727	0.89613481	PCDH15
NA	ENSG00000150276	0	NA	NA	NA	NA	PPIAP26
1489	ENSG00000150281	36.4981409	0.49563597	0.37845247	0.04696181	0.14179583	CTF1
51503	ENSG00000150316	519.667729	-0.0346621	0.1175703	0.75581559	0.88640901	CWC15
2209	ENSG00000150337	0	NA	NA	NA	NA	FCGR1A
84159	ENSG00000150347	720.325581	-0.878844	0.10264118	1.81E-18	6.25E-17	ARID5B
57626	ENSG00000150361	0.173733	0.01914545	0.38484597	0.78225479	NA	KLHL1
1006	ENSG00000150394	3.21230678	0.06076682	0.38143753	0.44542981	0.67025615	CDH8
55208	ENSG00000150401	595.235897	-0.10143639	0.13958535	0.43621865	0.6612819	DCUN1D2
55002	ENSG00000150403	1099.33353	0.01995599	0.08850197	0.81748419	0.91685309	TMCO3
219854	ENSG00000150433	437.671538	0.10404891	0.11521714	0.34387995	0.57419443	TMEM218
114609	ENSG00000150455	275.523825	-0.14356486	0.16024845	0.32290678	0.55059885	TIRAP
221143	ENSG00000150456	60.9830237	-0.19137472	0.26006148	0.33060116	0.55938713	EEF1AKMT1
26524	ENSG00000150457	0	NA	NA	NA	NA	LATS2
10284	ENSG00000150459	1016.88807	0.03605168	0.0941198	0.69343428	0.85183585	SAP18
23284	ENSG00000150471	113.538179	-0.27804014	0.25655953	0.16093542	0.34896869	ADGRL3
57536	ENSG00000150477	243.566481	-0.02645455	0.15151777	0.84823822	0.93095493	KIAA1328
220108	ENSG00000150510	213.376425	0.12769109	0.15888483	0.37755917	0.60636177	FAM124A
4253	ENSG00000150527	150.2633	0.02068229	0.18511665	0.89852637	0.95556135	MIA2
3176	ENSG00000150540	0.46100927	0.00464453	0.38317317	0.96931797	NA	HNMT
116372	ENSG00000150551	8.27530393	0.28133188	0.4872365	0.08722692	0.22548301	LYPD1
130576	ENSG00000150556	6.39079761	-0.05161616	0.3589517	0.70659649	0.85861856	LYPD6B
27250	ENSG00000150593	1327.0627	0.22389631	0.08908346	0.00964702	0.04029407	PDCD4
150	ENSG00000150594	0.82167337	0.01148781	0.38170777	0.82204659	NA	ADRA2A
2823	ENSG00000150625	24.3978374	4.82530832	1.00121837	1.52E-08	2.23E-07	GPM6A
116966	ENSG00000150627	628.642236	0.26894762	0.12265799	0.02036824	0.07391569	WDR17

132851	ENSG00000150628	3.86742618	0.01005886	0.36545111	0.93251717	0.97195497	SPATA4
7424	ENSG00000150630	0	NA	NA	NA	NA	VEGFC
79839	ENSG00000150636	63.0144628	-0.27054218	0.26443078	0.17986338	0.37654209	CCDC102B
10666	ENSG00000150637	9.13821423	-3.260779	1.13517386	0.00021533	0.00151978	CD226
84735	ENSG00000150656	0.37844916	0.02407552	0.3854191	0.60038877	NA	CNDP1
161835	ENSG00000150667	18.7228239	-0.19692987	0.35530971	0.31373463	0.54159326	FSIP1
1740	ENSG00000150672	43.8240852	1.1758565	0.42643256	0.00046166	0.00301222	DLG2
220047	ENSG00000150676	0	NA	NA	NA	NA	CCDC83
64407	ENSG00000150681	0	NA	NA	NA	NA	RGS18
11098	ENSG00000150687	30.9840434	0.41641523	0.45622892	0.08150516	0.21429626	PRSS23
54545	ENSG00000150712	526.717251	0.19721816	0.11794667	0.07828905	0.20807163	MTMR12
151242	ENSG00000150722	3.54436604	0.01851122	0.36752451	0.86893157	0.94277602	PPP1R1C
NA	ENSG00000150732	0	NA	NA	NA	NA	
341032	ENSG00000150750	0	NA	NA	NA	NA	C11orf53
22948	ENSG00000150753	7396.5569	-0.15817557	0.06683815	0.01764576	0.06585625	CCT5
134145	ENSG00000150756	243.271132	-0.12104311	0.15778134	0.39833981	0.62727695	ATPSCKMT
1793	ENSG00000150760	1873.25905	0.20979604	0.06719308	0.00150683	0.00841417	DOCK1
85458	ENSG00000150764	855.214867	-0.11507988	0.09325814	0.20233871	0.40788457	DIXDC1
1737	ENSG00000150768	1103.26953	-0.03474542	0.0835424	0.66934053	0.83727912	DLAT
120379	ENSG00000150773	19.2326377	0.30497897	0.42995678	0.14620201	0.32701731	PIH1D2
55216	ENSG00000150776	1084.91566	-0.21048668	0.10465042	0.0359948	0.11585132	NKAPD1
26521	ENSG00000150779	948.706418	-0.19255667	0.09135372	0.02954089	0.09920298	TIMM8B
3606	ENSG00000150782	0.34112095	0.02362661	0.38535341	0.62409997	NA	IL18
56158	ENSG00000150783	0.82706411	-8.73E-06	0.38068677	0.99954138	NA	TEX12
5805	ENSG00000150787	343.489692	-0.13240581	0.12400188	0.25812032	0.47950441	PTS
5305	ENSG00000150867	378.748828	0.30928393	0.15145264	0.02599538	0.08974218	PIP4K2A
130813	ENSG00000150873	13.9508087	0.23462387	0.41582978	0.19070572	0.39208305	C2orf50
341640	ENSG00000150893	64.1987158	4.85970637	0.4835342	2.37E-24	1.10E-22	FREM2
2308	ENSG00000150907	61.1380131	2.65200836	0.35287607	4.11E-15	1.13E-13	FOXO1
51232	ENSG00000150938	0.78623954	-0.00672434	0.38158221	0.89598968	NA	CRIM1
9871	ENSG00000150961	1304.99227	0.63805262	0.08299176	3.69E-15	1.02E-13	SEC24D
23457	ENSG00000150967	93.8334739	0.15538072	0.24901038	0.41974526	0.64566181	ABCB9
196383	ENSG00000150977	119.302312	0.69805889	0.21740998	0.00027555	0.00189427	RILPL2
57647	ENSG00000150990	619.799095	0.13774305	0.10203265	0.16095784	0.34896869	DHX37
7316	ENSG00000150991	15311.6131	-0.15459967	0.04701257	0.00091463	0.00548556	UBC
3708	ENSG00000150995	2900.36365	-0.01608563	0.06707129	0.80718517	0.91260538	ITPR1
84076	ENSG00000151005	0.14931947	-0.01844332	0.38480212	0.84537521	NA	TKTL2
339105	ENSG00000151006	241.257434	0.6145476	0.21958267	0.00128269	0.00732544	PRSS53

23657	ENSG00000151012	124.786585	2.92460947	0.81721611	1.39E-05	0.00012872	SLC7A11
25819	ENSG00000151014	184.326484	-0.47241238	0.18565987	0.00419245	0.02007682	NOCT
219670	ENSG00000151023	3.54673989	0.06974561	0.37815918	0.50737287	0.72330803	ENKUR
57512	ENSG00000151025	18.7655004	-2.9716497	0.78525201	8.58E-06	8.26E-05	GPR158
119180	ENSG00000151033	0	NA	NA	NA	NA	LYZL2
93589	ENSG00000151062	2.29108476	-0.01937071	0.37847798	0.76547426	NA	CACNA2D4
196513	ENSG00000151065	307.744815	0.10729219	0.13088691	0.38243767	0.61165364	DCP1B
775	ENSG00000151067	605.661902	-1.10901539	0.17067154	7.33E-12	1.58E-10	CACNA1C
100874369	ENSG00000151067	605.661902	-1.10901539	0.17067154	7.33E-12	1.58E-10	CACNA1C
3742	ENSG00000151079	0	NA	NA	NA	NA	KCNA6
7068	ENSG00000151090	30.0394022	-0.39843209	0.38455172	0.09149615	0.23350704	THRB
55768	ENSG00000151092	375.437441	0.1204411	0.1237202	0.30373479	0.53107413	NGLY1
54995	ENSG00000151093	149.806604	-0.41684488	0.19190038	0.01327324	0.05232288	OXSM
55293	ENSG00000151116	200.439161	0.08206708	0.18698501	0.6164172	0.80326549	UEVLD
144110	ENSG00000151117	108.812968	0.41924332	0.21816133	0.02334409	0.08246914	TMEM86A
121053	ENSG00000151131	499.471753	-0.06888144	0.11917205	0.5422026	0.75027258	C12orf45
90488	ENSG00000151135	925.893191	-0.025382	0.08826982	0.76673014	0.89256152	TMEM263
121551	ENSG00000151136	653.261822	-1.29966612	0.10944903	1.56E-33	9.98E-32	BTBD11
89910	ENSG00000151148	1432.78817	0.10736739	0.08038892	0.17196293	0.36454157	UBE3B
288	ENSG00000151150	295.293845	-1.42007417	0.17463099	3.57E-17	1.12E-15	ANK3
253430	ENSG00000151151	388.057697	-0.55446019	0.1236793	2.44E-06	2.59E-05	IPMK
144715	ENSG00000151164	11.2833665	0.16785902	0.39777547	0.25960726	0.48121669	RAD9B
196463	ENSG00000151176	758.665327	-0.08545411	0.09739533	0.36378321	0.59354103	PLBD2
9231	ENSG00000151208	3985.12963	0.20495899	0.06313635	0.00098328	0.00582682	DLG5
4143	ENSG00000151224	7.74968025	0.43438856	0.66233025	0.04277939	0.13222358	MAT1A
114134	ENSG00000151229	294.230216	0.06950132	0.1672981	0.64443374	0.822478	SLC2A13
283464	ENSG00000151233	803.387328	0.07529914	0.10767747	0.46673528	0.68965725	GXYLT1
5756	ENSG00000151239	1057.49435	-0.00815952	0.10402275	0.93412926	0.97251008	TWF1
22982	ENSG00000151240	1020.6598	-0.07121714	0.08319061	0.38023078	0.60925365	DIP2C
1977	ENSG00000151247	1334.72969	-0.2471891	0.11088455	0.01944564	0.07132585	EIF4E
9223	ENSG00000151276	2360.7473	0.05035929	0.07911369	0.51592224	0.73004045	MAGI1
93081	ENSG00000151287	245.251941	-0.08272441	0.14640477	0.54008621	0.748907	TEX30
1456	ENSG00000151292	868.867749	-0.07331021	0.08787522	0.39077053	0.62011007	CSNK1G3
NA	ENSG00000151303	2.54833488	0.13235516	0.41702184	0.01999829	NA	
153443	ENSG00000151304	152.147098	-0.01156179	0.17507389	0.93969533	0.97474024	SRFBP1
9472	ENSG00000151320	380.154328	-0.73196487	0.17606236	6.41E-06	6.32E-05	AKAP6
64067	ENSG00000151322	15.2685081	1.45778782	0.78925623	0.00288418	0.01463366	NPAS3
283635	ENSG00000151327	544.3645	0.03865558	0.10720314	0.7082285	0.85971939	FAM177A1

51562	ENSG00000151332	235.882861	0.20036259	0.15158412	0.14913793	0.33171487	MBIP
145282	ENSG00000151338	0.6040165	-0.03154773	0.38596638	0.58767911	NA	MIPOL1
2132	ENSG00000151348	1200.10883	0.01544967	0.09096184	0.86255773	0.93899493	EXT2
129787	ENSG00000151353	816.340014	-0.0839049	0.10785528	0.41640023	0.64306408	TMEM18
55821	ENSG00000151360	3.96826624	-0.04589152	0.36957332	0.6820025	0.84478552	ALLC
65987	ENSG00000151364	5.62700283	0.05161765	0.36216265	0.69844814	0.85459212	KCTD14
7069	ENSG00000151365	0	NA	NA	NA	NA	THRSP
4718	ENSG00000151366	1426.25848	0.08598316	0.08563864	0.3029537	0.53022242	NDUFC2
10873	ENSG00000151376	241.658898	0.00601229	0.17452705	0.97039688	0.98888633	ME3
343930	ENSG00000151379	0	NA	NA	NA	NA	MSGN1
81792	ENSG00000151388	61.7879994	0.95342765	0.45119997	0.00279151	0.01425209	ADAMTS12
80224	ENSG00000151413	190.179937	-0.21140453	0.18394298	0.18986048	0.39103414	NUBPL
140609	ENSG00000151414	652.5797	0.54193133	0.10840431	2.02E-07	2.56E-06	NEK7
127124	ENSG00000151418	0.16735865	0.01897177	0.38482933	0.78225479	NA	ATP6V1G3
2241	ENSG00000151422	960.801092	-0.28157347	0.09889728	0.00311984	0.01566633	FER
63894	ENSG00000151445	587.86549	-0.09014959	0.10837621	0.38418125	0.61330512	VIPAS39
57182	ENSG00000151458	1093.21694	-0.05538481	0.08479715	0.50319927	0.72004858	ANKRD50
26019	ENSG00000151461	853.8865	0.100553	0.09065576	0.25386062	0.47439002	UPF2
8872	ENSG00000151465	1697.78205	0.02822967	0.07153847	0.68829476	0.8486324	CDC123
132320	ENSG00000151466	354.464282	-0.25855488	0.13087314	0.03475029	0.11270978	SCLT1
83643	ENSG00000151468	66.1118222	0.29183692	0.26444475	0.15081999	0.33448181	CCDC3
132321	ENSG00000151470	100.509235	0.01276543	0.23147108	0.94652587	0.97814213	C4orf33
55691	ENSG00000151474	903.106133	-0.56802001	0.1183735	5.08E-07	6.06E-06	FRMD4A
83447	ENSG00000151475	0	NA	NA	NA	NA	SLC25A31
5800	ENSG00000151490	487.312368	0.21122103	0.11994407	0.06308583	0.17804949	PTPRO
2059	ENSG00000151491	181.960232	0.43640585	0.19400605	0.01031584	0.04247068	EPS8
27034	ENSG00000151498	631.423117	-0.10790772	0.09953579	0.26080448	0.48273755	ACAD8
29087	ENSG00000151500	520.723651	0.16596178	0.13422447	0.18690901	0.38676207	THYN1
112936	ENSG00000151502	2392.88607	-0.38422172	0.06512342	2.03E-09	3.35E-08	VPS26B
23310	ENSG00000151503	2470.46485	-0.37463124	0.08964372	1.64E-05	0.0001499	NCAPD3
143187	ENSG00000151532	493.356439	-0.4889677	0.11611546	1.02E-05	9.64E-05	VTI1A
5860	ENSG00000151552	793.232585	0.16933859	0.09042942	0.05388654	0.15759272	QDPR
57700	ENSG00000151553	484.621669	-0.05097294	0.13951973	0.69357557	0.85183585	FAM160B1
121601	ENSG00000151572	1.53769215	0.11260496	0.40669265	0.05481322	NA	ANO4
374618	ENSG00000151575	140.824795	0.18074645	0.1823721	0.26024418	0.48209565	TEX9
79691	ENSG00000151576	560.697549	-0.04718129	0.14086591	0.7162296	0.86335715	QTRT2
1814	ENSG00000151577	0.34624801	-0.02356563	0.38536324	0.65270996	NA	DRD3
166785	ENSG00000151611	93.6189627	0.08391046	0.21442549	0.6393569	0.8190109	MMAA

152485	ENSG00000151612	837.109805	-0.10464891	0.11263808	0.33002046	0.55861736	ZNF827
5458	ENSG00000151615	43.443629	-2.48719404	0.45544778	3.13E-09	5.03E-08	POU4F2
1909	ENSG00000151617	124.680464	1.81443918	0.23284589	5.11E-16	1.48E-14	EDNRA
4306	ENSG00000151623	14.2639712	2.53760751	1.05045036	0.00050736	0.00326926	NR3C2
NA	ENSG00000151631	0	NA	NA	NA	NA	AKR1C6P
1646	ENSG00000151632	2.85994077	-0.01180014	0.37293934	0.89625569	0.95469109	AKR1C2
10570	ENSG00000151640	2252.36716	0.07968075	0.06417942	0.20812899	0.41567135	DPYSL4
27287	ENSG00000151650	0	NA	NA	NA	NA	VENTX
101	ENSG00000151651	20.6681421	1.57045965	0.63078615	0.00063447	0.00396757	ADAM8
3698	ENSG00000151655	0.95429709	0.0026938	0.38167118	0.95671165	NA	ITIH2
22944	ENSG00000151657	291.941728	0.01868133	0.16529455	0.9014782	0.95673063	KIN
5281	ENSG00000151665	490.87608	-0.11250167	0.11576759	0.30715461	0.5346975	PIGF
150709	ENSG00000151687	42.1664356	-0.02178334	0.28739123	0.90719047	0.95963244	ANKAR
3628	ENSG00000151689	200.735043	0.28614293	0.16921675	0.05955476	0.17018096	INPP1
54842	ENSG00000151690	628.203601	-0.13366609	0.10756108	0.19316744	0.39591344	MFSD6
9781	ENSG00000151692	2272.19296	0.09765469	0.07209198	0.16809075	0.35916008	RNF144A
8853	ENSG00000151693	2601.66634	0.13025326	0.06616027	0.04534567	0.1379632	ASAP2
6868	ENSG00000151694	1613.70957	-0.01972344	0.08199113	0.80586073	0.91210433	ADAM17
2313	ENSG00000151702	5.13982123	2.37764317	1.7364849	0.00674745	0.02998195	FLI1
3758	ENSG00000151704	0.36009051	0.02384153	0.38538488	0.61230064	NA	KCNJ1
120224	ENSG00000151715	151.152408	-0.66638198	0.20138236	0.00021158	0.00149653	TMEM45B
80014	ENSG00000151718	840.962534	0.4979271	0.09493121	6.31E-08	8.55E-07	WWC2
79682	ENSG00000151725	1275.44261	-0.44090938	0.08662167	1.69E-07	2.17E-06	CENPU
2180	ENSG00000151726	1266.11564	-0.42543128	0.09042711	1.24E-06	1.39E-05	ACSL1
291	ENSG00000151729	907.81997	-0.32800155	0.08830204	0.00012992	0.00096106	SLC25A4
196394	ENSG00000151743	154.186753	-0.33981682	0.18294422	0.03529445	0.11406449	AMN1
636	ENSG00000151746	1125.32895	-0.10307878	0.09125161	0.2438901	0.4622449	BICD1
60485	ENSG00000151748	418.493376	0.2040817	0.13347826	0.10207811	0.25315371	SAV1
160857	ENSG00000151773	91.9236531	-0.03925016	0.21606105	0.82475082	0.92041041	CCDC122
387923	ENSG00000151778	157.207275	-0.60232283	0.19128234	0.00044156	0.00289698	SERP2
51594	ENSG00000151779	1163.10664	0.24556699	0.09123181	0.00549811	0.02523887	NBAS
79750	ENSG00000151789	322.597755	0.08299541	0.1900652	0.616327	0.80326549	ZNF385D
6999	ENSG00000151790	0	NA	NA	NA	NA	TDO2
60558	ENSG00000151806	745.980851	0.18792096	0.09680667	0.0447907	0.13658891	GUF1
341880	ENSG00000151812	0.173733	0.01914545	0.38484597	0.78225479	NA	SLC35F4
2555	ENSG00000151834	0	NA	NA	NA	NA	GABRA2
26278	ENSG00000151835	3012.18248	-0.61693612	0.06943023	1.75E-19	6.36E-18	SACS
729665	ENSG00000151838	0.16735865	0.01897177	0.38482933	0.78225479	NA	CCDC175

5042	ENSG00000151846	0	NA	NA	NA	NA	PABPC3
55835	ENSG00000151849	890.576781	0.15452848	0.1145105	0.15704549	0.34380314	CENPJ
26272	ENSG00000151876	68.2138353	0.18762143	0.27683445	0.3515535	0.58167138	FBXO4
64417	ENSG00000151881	102.217712	0.3618687	0.32044907	0.10476413	0.25791293	TMEM267
56477	ENSG00000151882	0	NA	NA	NA	NA	CCL28
79668	ENSG00000151883	647.60286	0.04585015	0.10026304	0.63639617	0.81722218	PARP8
2674	ENSG00000151892	34.5026604	0.54747131	0.45128092	0.04066919	0.12692727	GFRA1
143384	ENSG00000151893	1362.02754	-0.0545239	0.08080479	0.48718119	0.70702032	CACUL1
667	ENSG00000151914	5724.095	0.28866969	0.06857832	1.83E-05	0.00016581	DST
221336	ENSG00000151917	106.093206	0.03269966	0.23432414	0.86187344	0.93871152	BEND6
7073	ENSG00000151923	1844.3605	-0.10943603	0.07892887	0.15586211	0.34203123	TIAL1
9531	ENSG00000151929	304.371164	0.06798305	0.14715496	0.61774348	0.80430703	BAG3
144423	ENSG00000151948	5.68429783	1.59813158	1.72513822	0.00797517	0.03441221	GLT1D1
121256	ENSG00000151952	0	NA	NA	NA	NA	TMEM132D
166863	ENSG00000151962	0	NA	NA	NA	NA	RBM46
NA	ENSG00000151963	0.173733	0.01914545	0.38484597	0.78225479	NA	ZNF37CP
29970	ENSG00000151967	1882.76643	0.51513765	0.08309505	2.18E-10	4.05E-09	SCHIP1
84539	ENSG00000152034	0	NA	NA	NA	NA	MCHR2
23704	ENSG00000152049	0.55157499	0.01184175	0.38326009	0.75076895	NA	KCNE4
130340	ENSG00000152056	95.1864567	0.14901441	0.21957608	0.41030527	0.63815925	AP1S3
9910	ENSG00000152061	418.306379	-0.17274519	0.15338735	0.21861557	0.43050004	RABGAP1L
91409	ENSG00000152076	188.571013	0.53358152	0.19675862	0.00214136	0.01134469	CCDC74B
148534	ENSG00000152078	192.248734	-0.1294197	0.16931118	0.39308045	0.62215525	TLCD4
80097	ENSG00000152082	2911.01149	0.01322421	0.06556614	0.84047006	0.92760529	MZT2B
112714	ENSG00000152086	9.31314226	0.03801839	0.34324449	0.81252298	0.91507108	TUBA3E
460	ENSG00000152092	242.942924	-1.35847589	0.17217785	2.77E-16	8.20E-15	ASTN1
653275	ENSG00000152093	0	NA	NA	NA	NA	CFC1B
130074	ENSG00000152102	5653.819	0.04785081	0.04949745	0.32995163	0.55861736	FAM168B
5784	ENSG00000152104	235.381172	3.10269057	0.19089245	1.65E-60	2.20E-58	PTPN14
150776	ENSG00000152117	580.297242	0.35974084	0.10300272	0.00027987	0.00191934	SMPD4BP
4249	ENSG00000152127	4783.7224	-0.37280456	0.05855483	1.04E-10	2.00E-09	MGAT5
81615	ENSG00000152128	20.0202864	-0.27087578	0.39098143	0.1927605	0.3952427	TMEM163
253635	ENSG00000152133	669.088315	-0.29395071	0.10728838	0.00417955	0.02002468	GPATCH11
26353	ENSG00000152137	29.1107337	1.30284536	0.47814331	0.00045416	0.00297084	HSPB8
79833	ENSG00000152147	629.091071	-0.35527608	0.13070899	0.00375302	0.01828136	GEMIN6
130733	ENSG00000152154	53.4589305	0.0172993	0.25601981	0.92955871	0.97060391	TMEM178A
5457	ENSG00000152192	42.3554163	1.82673606	0.39670138	2.78E-07	3.46E-06	POU4F1
79596	ENSG00000152193	419.128581	-0.46024368	0.11793455	4.13E-05	0.00034578	OBI1

57105	ENSG00000152207	3.09894292	0.19911197	0.46106089	0.005882	0.02671679	CYSLTR2
2895	ENSG00000152208	4.48488173	-0.05289128	0.37660303	0.56958776	0.77026932	GRID2
115761	ENSG00000152213	0.18922458	0.01954053	0.38488496	0.78225479	NA	ARL11
6014	ENSG00000152214	183.975293	-3.24108829	0.2571336	1.38E-37	9.95E-36	RIT2
26040	ENSG00000152217	623.243151	0.04159265	0.11748859	0.71100623	0.86118643	SETBP1
120534	ENSG00000152219	520.484799	-0.4565219	0.12157574	7.57E-05	0.00059484	ARL14EP
57724	ENSG00000152223	822.555514	0.21006095	0.10867141	0.04333369	0.13334089	EPG5
9050	ENSG00000152229	103.407947	0.27542747	0.24189856	0.15486802	0.34030142	PSTPIP2
498	ENSG00000152234	8255.29185	-0.11745257	0.05111426	0.02033136	0.0738375	ATP5F1A
115106	ENSG00000152240	574.108467	-0.07107364	0.10786954	0.49025524	0.70981553	HAUS1
147339	ENSG00000152242	638.211665	-0.1289926	0.10755267	0.21044269	0.41911091	C18orf25
57405	ENSG00000152253	366.418335	-0.19382669	0.15635319	0.17351353	0.36653625	SPC25
57818	ENSG00000152254	1.83907284	-0.09192542	0.39288796	0.22828405	NA	G6PC2
5163	ENSG00000152256	502.431469	0.28660185	0.18870417	0.082495	0.21632312	PDK1
5741	ENSG00000152266	0	NA	NA	NA	NA	PTH
5140	ENSG00000152270	993.501326	0.45048086	0.10908201	1.65E-05	0.00015067	PDE3B
83439	ENSG00000152284	283.218259	1.32839781	0.15043175	1.01E-19	3.70E-18	TCF7L1
10618	ENSG00000152291	2996.64583	-0.2271973	0.06460091	0.00035368	0.00237143	TGOLN2
284948	ENSG00000152292	4.71412	0.01781107	0.36244919	0.8865118	0.95015905	SH2D6
56659	ENSG00000152315	0.81358106	-0.00736337	0.3808088	0.89651778	NA	KCNK13
127933	ENSG00000152332	1717.05637	-0.29592609	0.09255466	0.0009504	0.00567269	UHMK1
83734	ENSG00000152348	225.166528	0.01391122	0.17514919	0.93016492	0.97093028	ATG10
134359	ENSG00000152359	352.561419	0.01820612	0.13532396	0.88660267	0.95020511	POC5
6695	ENSG00000152377	5075.12994	-2.27921588	0.07684656	1.13E-194	2.25E-191	SPOCK1
167555	ENSG00000152380	30.0050296	0.14051459	0.3028057	0.4788699	0.70038424	FAM151B
117143	ENSG00000152382	526.210867	-0.09553638	0.11596268	0.38606619	0.61517574	TADA1
2977	ENSG00000152402	2.22571534	-0.03058065	0.37471713	0.73958904	NA	GUCY1A2
143884	ENSG00000152404	193.127114	0.091635	0.15989826	0.52634299	0.73825698	CWF19L2
133746	ENSG00000152409	655.968033	-0.16985272	0.09756316	0.07125267	0.19407647	JMY
9456	ENSG00000152413	328.165862	0.08495442	0.19397384	0.61286459	0.80067344	HOMER1
7518	ENSG00000152422	141.576495	-0.48966225	0.20715497	0.00640377	0.02865375	XRCC4
66037	ENSG00000152430	1.5016155	0.04242929	0.3822091	0.53699502	NA	BOLL
284306	ENSG00000152433	61.407801	-0.30151381	0.29797458	0.15723164	0.34400923	ZNF547
374928	ENSG00000152439	169.901622	0.44023557	0.20829108	0.0139772	0.05450228	ZNF773
284309	ENSG00000152443	578.4712	0.181612	0.116816	0.10196723	0.25294197	ZNF776
10172	ENSG00000152454	148.085638	0.04809618	0.17332095	0.7571855	0.88697677	ZNF256
79723	ENSG00000152455	521.212683	-0.54673442	0.14098582	3.46E-05	0.00029399	SUV39H2
64421	ENSG00000152457	362.292408	-0.50300702	0.16956938	0.00106949	0.00625183	DCLRE1C

55301	ENSG00000152463	0.173124	-0.01911262	0.38486262	0.84537521	NA	OLAH
10557	ENSG00000152464	180.529945	-0.20012871	0.17653398	0.20092173	0.40615916	RPP38
9397	ENSG00000152465	350.660001	0.11146557	0.14776541	0.41402335	0.64129366	NMT2
284312	ENSG00000152467	31.5955776	-0.15476051	0.30946217	0.43542039	0.66055517	ZSCAN1
116412	ENSG00000152475	66.5743725	0.3805402	0.26428938	0.06578335	0.18330641	ZNF837
219333	ENSG00000152484	747.112834	-0.28844774	0.09666773	0.00196453	0.01053412	USP12
152137	ENSG00000152492	1413.11087	0.11331391	0.0876759	0.18353099	0.38180295	CCDC50
814	ENSG00000152495	2680.85821	0.20837593	0.08317172	0.00996434	0.04135825	CAMK4
55521	ENSG00000152503	400.524163	-0.09239652	0.11876032	0.41219409	0.64000085	TRIM36
678	ENSG00000152518	933.973462	0.48166712	0.08537081	7.14E-09	1.09E-07	ZFP36L2
255967	ENSG00000152520	596.865541	-0.04346339	0.10310698	0.65810403	0.83032012	PAN3
130271	ENSG00000152527	169.057735	1.65972839	0.23783638	2.05E-13	4.89E-12	PLEKHH2
5213	ENSG00000152556	2750.1506	-0.10176611	0.07468217	0.16418674	0.35357266	PFKM
114908	ENSG00000152558	2733.21472	-0.0662926	0.08056154	0.39930139	0.62809321	TMEM123
2893	ENSG00000152578	175.130663	-0.70224324	0.26143116	0.00131893	0.00750219	GRIA4
285313	ENSG00000152580	0.173733	0.01914545	0.38484597	0.78225479	NA	IGSF10
79925	ENSG00000152582	45.1201083	0.74256456	0.4626859	0.01236528	0.04945116	SPEF2
8404	ENSG00000152583	6.49238021	0.08286509	0.37961664	0.42319763	0.64917034	SPARCL1
1834	ENSG00000152591	0.85900754	0.04865624	0.38843866	0.27179449	NA	DSPP
1758	ENSG00000152592	0.37844916	0.02407552	0.3854191	0.60038877	NA	DMP1
56955	ENSG00000152595	0	NA	NA	NA	NA	MEPE
4154	ENSG00000152601	1171.61917	0.25836649	0.07793353	0.00067678	0.00419604	MBNL1
133690	ENSG00000152611	0	NA	NA	NA	NA	CAPSL
133686	ENSG00000152620	544.450331	0.28677911	0.13764775	0.02520427	0.08768238	NADK2
23171	ENSG00000152642	580.097609	-0.30173088	0.10321631	0.00232426	0.0121703	GPD1L
2697	ENSG00000152661	57.8179949	6.70993273	0.84467998	4.99E-15	1.37E-13	GJA1
10309	ENSG00000152669	0.15069912	-0.01848503	0.38480575	0.84537521	NA	CCNO
54514	ENSG00000152670	0.34058028	0.00242265	0.38338108	0.96455862	NA	DDX4
165530	ENSG00000152672	32.7457456	0.23920866	0.34508972	0.2535572	0.47391189	CLEC4F
55676	ENSG00000152683	1144.79981	0.0577092	0.08910694	0.50579784	0.72177288	SLC30A6
53918	ENSG00000152684	435.171479	-0.04578896	0.14163597	0.72675972	0.86949966	PELO
25780	ENSG00000152689	1.10600457	0.04824209	0.38612995	0.33928934	NA	RASGRP3
51128	ENSG00000152700	445.82993	-0.2320982	0.16037581	0.10999432	0.26710984	SAR1B
347732	ENSG00000152705	9.71262777	0.15898587	0.38192542	0.32921681	0.55775099	CATSPER3
160897	ENSG00000152749	481.832901	-0.20572605	0.10916551	0.04860781	0.14580805	GPR180
200132	ENSG00000152760	0	NA	NA	NA	NA	DYNLT5
79819	ENSG00000152763	19.363678	0.1195935	0.34438403	0.50150048	0.71844678	DNAI4
118932	ENSG00000152766	0	NA	NA	NA	NA	ANKRD22

10160	ENSG00000152767	3441.40824	-7.50E-05	0.09353093	0.99881449	0.99957022	FARP1
24138	ENSG00000152778	284.912673	-0.7126277	0.15568178	9.91E-07	1.12E-05	IFIT5
387700	ENSG00000152779	3.5042049	-0.11197774	0.39396866	0.27210415	0.49550677	SLC16A12
53354	ENSG00000152782	395.276087	-0.27964997	0.13509148	0.02649265	0.09108817	PANK1
56978	ENSG00000152784	287.928718	0.32928495	0.15963853	0.02331904	0.08241002	PRDM8
651	ENSG00000152785	0	NA	NA	NA	NA	BMP3
9987	ENSG00000152795	6879.68117	0.21525378	0.07680272	0.0041899	0.02006944	HNRNPDL
3087	ENSG00000152804	33.8305926	-0.01635105	0.29269434	0.92984251	0.97064482	HHEX
7402	ENSG00000152818	93.8904251	-0.1074452	0.23381833	0.562639	0.7656036	UTRN
2911	ENSG00000152822	18.426401	-0.38030531	0.52553957	0.07788049	0.20726343	GRM1
5796	ENSG00000152894	4.5035961	5.13595977	2.80435387	0.00052059	0.00334038	PTPRK
9453	ENSG00000152904	944.83393	0.04493367	0.08455515	0.58688615	0.78237765	GGPS1
85445	ENSG00000152910	4.77519633	0.15945732	0.40407582	0.23284036	0.4497666	CNTNAP4
51351	ENSG00000152926	3199.05991	0.62605925	0.07566941	3.78E-17	1.18E-15	ZNF117
109504726	ENSG00000152926	3199.05991	0.62605925	0.07566941	3.78E-17	1.18E-15	ZNF117
25859	ENSG00000152931	18.9638994	1.77925774	0.626918	0.00022717	0.00159256	PART1
115827	ENSG00000152932	897.828208	-1.31793673	0.16973051	7.09E-16	2.03E-14	RAB3C
160492	ENSG00000152936	72.036727	-1.33838215	0.31175158	1.43E-06	1.58E-05	LMNTD1
153562	ENSG00000152939	0.49552866	-0.01729184	0.38338507	0.74005398	NA	MARVELD2
5884	ENSG00000152942	817.586609	0.03095566	0.11553143	0.77968823	0.89925062	RAD17
9412	ENSG00000152944	433.006324	-0.04706743	0.13905783	0.71544077	0.86274919	MED21
5352	ENSG00000152952	1.47022721	0.00275908	0.37913342	0.89081549	NA	PLOD2
55351	ENSG00000152953	1.50394928	0.02043922	0.37890699	0.77464689	NA	STK32B
140767	ENSG00000152954	475.263251	-1.6384859	0.12676115	2.58E-39	1.96E-37	NRSN1
152789	ENSG00000152969	209.817797	-1.55975262	0.17191805	9.53E-21	3.71E-19	JAKMIP1
7545	ENSG00000152977	418.404973	0.35654093	0.1284077	0.003156	0.01580388	ZIC1
166647	ENSG00000152990	2092.03878	0.38669524	0.08907157	7.80E-06	7.57E-05	ADGRA3
1360	ENSG00000153002	37.5185997	0.3188303	0.34522757	0.15142196	0.33539121	CPB1
285672	ENSG00000153006	495.242642	-0.02789952	0.14420312	0.83341438	0.92419724	SREK1IP1
55203	ENSG00000153012	0	NA	NA	NA	NA	LGI2
10283	ENSG00000153015	381.295404	0.00561566	0.12888605	0.96408896	0.98577354	CWC27
3140	ENSG00000153029	4.10298545	0.08366457	0.38565803	0.37577107	0.60489233	MR1
6728	ENSG00000153037	630.120225	-0.03293353	0.10505801	0.74247786	0.87870936	SRP19
64946	ENSG00000153044	875.5901	0.124991	0.10514097	0.21632846	0.42723041	CENPH
9425	ENSG00000153046	480.197092	0.14235834	0.1318859	0.25078379	0.47069581	CDYL
23589	ENSG00000153048	2376.2907	-0.05226921	0.08257104	0.51578814	0.73002247	CARHSP1
146279	ENSG00000153060	32.154996	-0.02295696	0.28605511	0.90282183	0.95739939	TEKT5
55024	ENSG00000153064	0	NA	NA	NA	NA	BANK1

51061	ENSG00000153066	342.315981	0.46182681	0.13707466	0.00031888	0.00216365	TXNDC11
1601	ENSG00000153071	0	NA	NA	NA	NA	DAB2
112267931	ENSG00000153071	0	NA	NA	NA	NA	DAB2
130013	ENSG00000153086	4.23372703	-0.09011032	0.38490387	0.38079931	0.60981987	ACMSD
55289	ENSG00000153093	43.166748	0.12591529	0.27398235	0.52379947	0.73661622	ACOXL
10018	ENSG00000153094	1918.88667	0.12424256	0.08217119	0.1215202	0.28804788	BCL2L11
64682	ENSG00000153107	1600.74874	-0.08696789	0.09085224	0.32453487	0.55221149	ANAPC1
831	ENSG00000153113	1007.56576	0.5056576	0.08818957	3.88E-09	6.16E-08	CAST
60592	ENSG00000153130	721.471313	-0.28600522	0.13912374	0.02682958	0.09202955	SCOC
1047	ENSG00000153132	795.5065	-0.07102611	0.09496975	0.43960434	0.66481822	CLGN
1070	ENSG00000153140	507.364758	-0.2557242	0.13424473	0.04127482	0.12847387	CETN3
8467	ENSG00000153147	3792.85329	-0.11316773	0.06196923	0.06367444	0.17916619	SMARCA5
221711	ENSG00000153157	223.569649	0.27153824	0.17041612	0.07528911	0.20204732	SYCP2L
654	ENSG00000153162	0.173733	0.01914545	0.38484597	0.78225479	NA	BMP6
653489	ENSG00000153165	10.8845418	0.01907129	0.33826423	0.90847576	0.9602642	RGPD3
283349	ENSG00000153179	678.999016	-0.0990967	0.11057885	0.34842114	0.57911259	RASSF3
3192	ENSG00000153187	19288.1974	-0.06243625	0.05849448	0.27964111	0.50414172	HNRNPU
5903	ENSG00000153201	2669.03668	-0.16300258	0.06783815	0.01450804	0.05608721	RANBP2
25909	ENSG00000153207	3304.37776	-0.0193936	0.08703505	0.81809179	0.9171248	AHCTF1
10461	ENSG00000153208	324.481427	0.36424924	0.1505908	0.00850562	0.03630616	MERTK
84910	ENSG00000153214	237.78131	0.20553359	0.16509444	0.16771937	0.35871254	TMEM87B
343170	ENSG00000153230	0	NA	NA	NA	NA	OR14K1
5801	ENSG00000153233	53.3060906	0.22455763	0.27457193	0.2686655	0.49159121	PTPRR
4929	ENSG00000153234	1.40774761	0.09918123	0.40153846	0.07494634	NA	NR4A2
130940	ENSG00000153237	3.2868938	0.11267332	0.39782648	0.20614898	0.41317262	CCDC148
22925	ENSG00000153246	0	NA	NA	NA	NA	PLA2R1
5937	ENSG00000153250	1065.00325	0.38577397	0.08783857	6.20E-06	6.12E-05	RBMS1
6328	ENSG00000153253	948.546945	-0.48165178	0.08882433	2.46E-08	3.52E-07	SCN3A
55079	ENSG00000153266	0	NA	NA	NA	NA	FEZF2
10225	ENSG00000153283	0.18922458	0.01954053	0.38488496	0.78225479	NA	CD96
9481	ENSG00000153291	15.6538075	-0.00964401	0.35136435	0.94212499	0.97606984	SLC25A27
266977	ENSG00000153292	0	NA	NA	NA	NA	ADGRF1
221393	ENSG00000153294	0	NA	NA	NA	NA	ADGRF4
79981	ENSG00000153303	20.2094069	-1.27492972	0.66202634	0.00286877	0.01459023	FRMD1
51571	ENSG00000153310	1573.18476	-0.02223023	0.08433345	0.78630273	0.90287569	CYRIB
50807	ENSG00000153317	3785.73849	0.24254132	0.05543951	9.64E-06	9.17E-05	ASAP1
22878	ENSG00000153339	707.198393	-0.24001189	0.10521432	0.01728435	0.06482447	TRAPPC8
153643	ENSG00000153347	0.18922458	0.01954053	0.38488496	0.78225479	NA	FAM81B

NA	ENSG00000153363	231.060984	-0.25333429	0.20547485	0.14658609	0.32769217	LINC00467
125476	ENSG00000153391	213.323124	-0.06545814	0.15739091	0.64754602	0.82430661	INO80C
79888	ENSG00000153395	2026.35117	-0.05756097	0.09179673	0.51710434	0.73098795	LPCAT1
153478	ENSG00000153404	1322.55172	0.63421799	0.08328342	7.44E-15	2.00E-13	PLEKHG4B
57407	ENSG00000153406	752.919472	0.02778113	0.09774682	0.76974241	0.89412702	NMRAL1
124402	ENSG00000153443	460.694292	0.07077109	0.11658633	0.52470766	0.73747521	UBALD1
146556	ENSG00000153446	1.14331157	-0.03331457	0.38177304	0.59882389	NA	C16orf89
26175	ENSG00000153485	204.857204	-0.3867033	0.16820312	0.01080362	0.04417638	TMEM251
3621	ENSG00000153487	514.920206	0.06665057	0.11870157	0.55546601	0.7613434	ING1
121793	ENSG00000153495	0.51610347	0.03116049	0.38605724	0.47723758	NA	TEX29
122258	ENSG00000153498	0	NA	NA	NA	NA	SPACA7
113622	ENSG00000153531	204.449508	-0.38350691	0.16932898	0.01187985	0.04786922	ADPRHL1
101928841	ENSG00000153531	204.449508	-0.38350691	0.16932898	0.01187985	0.04786922	ADPRHL1
112616	ENSG00000153551	462.673629	0.27317458	0.12074377	0.01684464	0.06347534	CMTM7
25827	ENSG00000153558	188.300161	0.03456151	0.1642315	0.81710568	0.91675019	FBXL2
7342	ENSG00000153560	1901.21295	-0.01072432	0.06661965	0.86945383	0.94307645	UBP1
64795	ENSG00000153561	1613.68155	-0.16026019	0.07141672	0.02220809	0.07918916	RMND5A
925	ENSG00000153563	75.5635511	-0.27000976	0.24643936	0.16589917	0.35616336	CD8A
22934	ENSG00000153574	550.684296	-0.16643149	0.10977655	0.11218568	0.27107099	RPIA
100132565	ENSG00000153684	0	NA	NA	NA	NA	GOLGA8F
5789	ENSG00000153707	311.204702	1.88098284	0.15544634	7.80E-35	5.26E-33	PTPRD
286343	ENSG00000153714	62.0423782	0.54535055	0.29449126	0.01682128	0.06339936	LURAP1L
154043	ENSG00000153721	904.321283	0.51932555	0.09057231	3.75E-09	5.97E-08	CNKSR3
2960	ENSG00000153767	342.887746	0.25314764	0.13039829	0.03846801	0.12197623	GTF2E1
10428	ENSG00000153774	1000.73734	-0.17781865	0.08349079	0.02887369	0.09737446	CFDP1
90316	ENSG00000153779	14.6606986	0.39329554	0.49662526	0.08804854	0.22670443	TGIF2LX
55625	ENSG00000153786	1213.95741	0.0498164	0.08767105	0.56033635	0.76415299	ZDHHC7
339145	ENSG00000153789	3.25594014	-0.09457828	0.38571813	0.35989876	0.58982252	CIBAR2
136895	ENSG00000153790	95.1252596	0.93112244	0.27741939	9.87E-05	0.00075301	C7orf31
9407	ENSG00000153802	0.50765625	0.01580256	0.38317427	0.73437663	NA	TMPRSS11D
221895	ENSG00000153814	422.575724	-0.66612932	0.12711987	3.97E-08	5.54E-07	JAZF1
80790	ENSG00000153815	1625.13066	-0.13427796	0.07981477	0.08495624	0.22105335	CMIP
80309	ENSG00000153820	0	NA	NA	NA	NA	SPHKAP
3773	ENSG00000153822	0	NA	NA	NA	NA	KCNJ16
55022	ENSG00000153823	0	NA	NA	NA	NA	PID1
9320	ENSG00000153827	4141.76946	0.01859987	0.07044964	0.78913167	0.90431308	TRIP12
130888	ENSG00000153832	51.3033795	0.49615	0.36854246	0.04504724	0.13726573	FBXO36
1054	ENSG00000153879	1272.50481	-0.21930772	0.13409412	0.07975615	0.21084103	CEBPG

79047	ENSG00000153885	2314.37495	0.16361678	0.06716739	0.0133062	0.05243199	KCTD15
148103	ENSG00000153896	210.150763	0.14646566	0.16486009	0.32530508	0.55328357	ZNF599
255231	ENSG00000153898	0.18922458	0.01954053	0.38488496	0.78225479	NA	MCOLN2
163175	ENSG00000153902	10.8800846	0.91457968	1.24824271	0.01427615	0.05540667	LGI4
23576	ENSG00000153904	3672.01731	-0.85983278	0.05610983	9.39E-54	1.02E-51	DDAH1
140890	ENSG00000153914	2296.80887	-0.10624956	0.07052446	0.12482169	0.29314184	SREK1
1105	ENSG00000153922	1281.84933	-0.03440979	0.11082206	0.74433256	0.87963225	CHD1
9629	ENSG00000153923	0	NA	NA	NA	NA	CLCA3P
162282	ENSG00000153930	0.3411051	0.02362649	0.38535339	0.62410837	NA	ANKFN1
8526	ENSG00000153933	358.75566	0.01921011	0.121403	0.86842333	0.94253385	DGKE
9653	ENSG00000153936	1004.12673	-0.32612414	0.1006563	0.00075393	0.00461949	HS2ST1
124540	ENSG00000153944	6806.53892	0.14180834	0.07052756	0.04068126	0.12694498	MSI2
781	ENSG00000153956	2685.53885	0.67244011	0.07327167	1.13E-20	4.39E-19	CACNA2D1
221302	ENSG00000153975	265.962128	0.2019746	0.14903769	0.14085183	0.31892169	ZUP1
9955	ENSG00000153976	0.3000162	-0.02294459	0.38527538	0.68644854	NA	HS3ST3A1
284161	ENSG00000153982	191.527308	-0.17064216	0.18723792	0.29541256	0.52199725	GDPD1
116150	ENSG00000153989	1419.31758	-0.0810162	0.08798418	0.34351999	0.57383485	NUS1
223117	ENSG00000153993	15.1463558	0.30088617	0.42265879	0.15458874	0.33998898	SEMA3D
5529	ENSG00000154001	1414.04401	-0.13183198	0.09355267	0.14549131	0.32597939	PPP2R5E
127247	ENSG00000154007	0	NA	NA	NA	NA	ASB17
10750	ENSG00000154016	4.69570198	-0.18428473	0.43197956	0.10110552	0.25133862	GRAP
125206	ENSG00000154025	1.3617202	-0.01344143	0.38019884	0.82593296	NA	SLC5A10
26289	ENSG00000154027	29.5967475	0.27071347	0.37902162	0.19583561	0.39944263	AK5
26256	ENSG00000154040	214.142584	-0.0932489	0.15052287	0.49809341	0.71599575	CABYR
55364	ENSG00000154059	717.00525	-0.12975745	0.13197164	0.29366546	0.51992885	IMPACT
147463	ENSG00000154065	123.104545	2.21360995	0.23481247	3.10E-22	1.29E-20	ANKRD29
135154	ENSG00000154079	117.678902	0.34894883	0.2299157	0.0655154	0.18276512	SDHAF4
83539	ENSG00000154080	53.517635	0.72004073	0.36246827	0.00702677	0.03107002	CHST9
7070	ENSG00000154096	1999.56299	-0.8259818	0.06826472	1.92E-34	1.27E-32	THY1
123872	ENSG00000154099	11.585145	0.03180038	0.34218655	0.84225381	0.92836346	DNAAF1
404550	ENSG00000154102	15.6146019	0.08479843	0.32808906	0.6409946	0.82014987	C16orf74
219899	ENSG00000154114	527.539202	0.01489884	0.11319726	0.89160991	0.95294622	TBCEL
57338	ENSG00000154118	1088.09907	-1.14553142	0.08837961	2.32E-39	1.76E-37	JPH3
56172	ENSG00000154122	873.284188	-0.16499418	0.14082439	0.20598398	0.4130923	ANKH
90268	ENSG00000154124	850.592501	-0.2299478	0.11558653	0.03627586	0.11654835	OTULIN
84959	ENSG00000154127	22.2214461	-1.39107394	0.68083732	0.0018972	0.01022282	UBASH3B
54538	ENSG00000154133	1.62328417	0.10558046	0.40389383	0.06055386	NA	ROBO4
64221	ENSG00000154134	248.734038	0.37469819	0.17277696	0.0155986	0.05951467	ROBO3

116337	ENSG00000154143	0.16735865	0.01897177	0.38482933	0.78225479	NA	PANX3
84897	ENSG00000154144	646.708208	0.21631112	0.09580603	0.01949979	0.0714584	TBRG1
4900	ENSG00000154146	239.353804	-0.62365458	0.16694402	4.91E-05	0.00040257	NRGN
54463	ENSG00000154153	101.631589	-0.15602764	0.21641799	0.3842216	0.61332018	RETREG1
1010	ENSG00000154162	0.15069912	-0.01848503	0.38480575	0.84537521	NA	CDH12
2838	ENSG00000154165	0.173124	-0.01911262	0.38486262	0.84537521	NA	GPR15
9868	ENSG00000154174	2457.25191	-0.22578282	0.0672256	0.00063453	0.00396757	TOMM70
25890	ENSG00000154175	13.9692711	0.49001724	0.61819312	0.04918329	0.14717895	ABI3BP
284	ENSG00000154188	58.5307633	-0.11860369	0.25572937	0.53740992	0.74759591	ANGPT1
163720	ENSG00000154198	0	NA	NA	NA	NA	CYP4Z2P
26207	ENSG00000154217	1229.18818	-0.0742624	0.07938732	0.33506869	0.56494141	PITPNC1
200014	ENSG00000154222	629.260469	0.19710604	0.10546473	0.05151274	0.1524482	CC2D1B
204219	ENSG00000154227	0.18922458	0.01954053	0.38488496	0.78225479	NA	CERS3
5578	ENSG00000154229	3779.11618	1.61234295	0.06289476	4.76E-146	3.50E-143	PRKCA
79705	ENSG00000154237	927.393482	-0.07403442	0.10156198	0.44878416	0.67234598	LRRK1
201134	ENSG00000154240	191.883427	-0.00060262	0.18963077	0.99567662	0.99852582	CEP112
64090	ENSG00000154252	8.13749556	0.14147907	0.38183556	0.35109854	0.58132031	GAL3ST2
10350	ENSG00000154258	0	NA	NA	NA	NA	ABCA9
23460	ENSG00000154262	0	NA	NA	NA	NA	ABCA6
10349	ENSG00000154263	0	NA	NA	NA	NA	ABCA10
23461	ENSG00000154265	158.389753	1.51089891	0.24947336	1.04E-10	2.00E-09	ABCA5
5169	ENSG00000154269	0.62270811	0.03703505	0.3867132	0.3983139	NA	ENPP3
55286	ENSG00000154274	1.8203124	0.04654439	0.38022014	0.56539705	NA	C4orf19
7345	ENSG00000154277	9682.52632	0.39484563	0.06082316	4.70E-11	9.34E-10	UCHL1
375056	ENSG00000154305	2085.09455	0.50581179	0.09169905	1.37E-08	2.03E-07	MIA3
84976	ENSG00000154309	280.470777	-0.07521049	0.13868004	0.5595112	0.76413192	DISP1
23043	ENSG00000154310	938.065121	-1.15766732	0.11851297	1.67E-23	7.55E-22	TNIK
157739	ENSG00000154316	7.8809995	-0.00380169	0.35217359	0.97638771	0.99184704	TDH
83648	ENSG00000154319	1314.48601	-1.62011091	0.08953173	2.78E-74	4.93E-72	FAM167A
252969	ENSG00000154328	346.092694	-0.29695742	0.13718107	0.02008468	0.07316932	NEIL2
5239	ENSG00000154330	0	NA	NA	NA	NA	PGM5
89780	ENSG00000154342	0.35667843	0.02381704	0.38538106	0.61401837	NA	WNT3A
84033	ENSG00000154358	4609.68497	0.10649032	0.09391788	0.24240513	0.46064214	OBSCN
91694	ENSG00000154359	191.44204	0.56388892	0.18610182	0.00073196	0.00450016	LONRF1
81559	ENSG00000154370	1416.71678	0.16121336	0.08177341	0.04340729	0.13349876	TRIM11
55740	ENSG00000154380	5110.93876	-0.31757843	0.07930713	4.09E-05	0.00034276	ENAH
5506	ENSG00000154415	0	NA	NA	NA	NA	PPP1R3A
126731	ENSG00000154429	1124.21955	-0.37054087	0.0822499	3.80E-06	3.90E-05	CCSAP

136991	ENSG00000154438	0	NA	NA	NA	NA	ASZ1
57630	ENSG00000154447	332.790623	0.0236721	0.13939464	0.85646237	0.93595581	SH3RF1
115362	ENSG00000154451	2.41491726	-0.13218935	0.40972206	0.11394106	NA	GBP5
9184	ENSG00000154473	3080.51521	0.0521786	0.06668173	0.4288224	0.65462623	BUB3
2849	ENSG00000154478	0.173733	0.01914545	0.38484597	0.78225479	NA	GPR26
129881	ENSG00000154479	3.0016387	0.04198037	0.37492165	0.66725212	0.83616392	CCDC173
118856	ENSG00000154485	15.7565101	-0.05431097	0.32452159	0.75873248	0.88809749	MMP21
118611	ENSG00000154493	0.79261673	-0.0452504	0.38782936	0.32594236	NA	C10orf90
388650	ENSG00000154511	150.74338	-0.22506601	0.18081174	0.15782404	0.34484696	DIPK1A
518	ENSG00000154518	1871.61172	0.13594662	0.08556948	0.10300196	0.25491514	ATP5MC3
728577	ENSG00000154529	13.7700498	-0.33413581	0.47290277	0.11013688	0.26735784	CNTNAP3B
728239	ENSG00000154545	72.0057152	-0.27039925	0.49757892	0.06361559	0.1791022	MAGED4
135295	ENSG00000154548	336.167124	-0.10600851	0.14822747	0.43681703	0.6619272	SRSF12
27295	ENSG00000154553	368.078549	1.51890124	0.18122115	3.87E-18	1.30E-16	PDLIM3
8470	ENSG00000154556	558.751079	0.54764374	0.10842143	1.53E-07	1.97E-06	SORBS2
6921	ENSG00000154582	828.373304	-0.10618431	0.10666455	0.2985395	0.5253702	ELOC
23643	ENSG00000154589	0	NA	NA	NA	NA	LY96
NA	ENSG00000154608	28.5482779	-0.03106711	0.29633185	0.86827343	0.94247428	CEP170P1
143471	ENSG00000154611	0	NA	NA	NA	NA	PSMA8
9087	ENSG00000154620	0	NA	NA	NA	NA	TMSB4Y
1525	ENSG00000154639	439.612564	0.17289531	0.13654855	0.17504491	0.36892512	CXADR
10950	ENSG00000154640	419.76188	0.34745231	0.14216863	0.00842951	0.03605781	BTG3
54149	ENSG00000154642	283.424278	0.64119733	0.17989503	8.97E-05	0.00069156	C21orf91
140578	ENSG00000154645	0.98248796	-0.02104553	0.38024242	0.74333687	NA	CHODL
5651	ENSG00000154646	0	NA	NA	NA	NA	TMPRSS15
4685	ENSG00000154654	89.6299859	0.4124038	0.29625926	0.0601901	0.17165037	NCAM2
91133	ENSG00000154655	200.949092	0.69621144	0.169399	8.86E-06	8.49E-05	L3MBTL4
5137	ENSG00000154678	0	NA	NA	NA	NA	PDE1C
27342	ENSG00000154710	779.01583	0.1126992	0.09309087	0.21207451	0.4217258	RABGEF1
54148	ENSG00000154719	253.466962	-0.06518861	0.1555128	0.64572154	0.82359463	MRPL39
58494	ENSG00000154721	311.138981	0.05899183	0.15567202	0.67907085	0.84320727	JAM2
522	ENSG00000154723	1730.25738	-0.19361503	0.07105497	0.00548567	0.0251876	ATP5PF
2551	ENSG00000154727	960.124964	-0.18498852	0.09028505	0.03459158	0.11230997	GABPA
9510	ENSG00000154734	660.289265	1.14533728	0.11983472	1.39E-22	5.93E-21	ADAMTS1
11096	ENSG00000154736	95.2567617	1.02038361	0.29153537	5.02E-05	0.00040965	ADAMTS5
80746	ENSG00000154743	322.634168	0.03057203	0.13736033	0.81269547	0.91516078	TSEN2
146857	ENSG00000154760	0	NA	NA	NA	NA	SLFN13
7476	ENSG00000154764	5.88239617	-3.75127301	1.66297228	0.00095529	0.00569157	WNT7A

7508	ENSG00000154767	1284.40125	0.16535583	0.07924926	0.03285258	0.10778817	XPC
51244	ENSG00000154781	443.651136	0.32135469	0.121463	0.00517914	0.02393528	CCDC174
152273	ENSG00000154783	8.50806756	-0.39398195	0.62265816	0.04232171	0.13113584	FGD5
201163	ENSG00000154803	471.07173	0.20569694	0.11912068	0.06872193	0.18899959	FLCN
285381	ENSG00000154813	486.314226	0.10560549	0.12301894	0.36514002	0.59463005	DPH3
92106	ENSG00000154814	268.504996	0.12808543	0.13885572	0.32273603	0.55037672	OXNAD1
23228	ENSG00000154822	431.904953	-0.326502	0.11920559	0.00384407	0.01863793	PLCL2
30827	ENSG00000154832	1401.82034	0.12878446	0.08730754	0.12967677	0.30129841	CXXC1
220134	ENSG00000154839	297.634745	-0.28813386	0.13907538	0.02567419	0.08892737	SKA1
9989	ENSG00000154845	1266.78636	-0.12698748	0.08831144	0.1388766	0.31565034	PPP4R1
147495	ENSG00000154856	53.8146661	0.19302466	0.30411649	0.34163949	0.57180671	APCDD1
63895	ENSG00000154864	0	NA	NA	NA	NA	PIEZO2
284047	ENSG00000154874	12.6196644	2.2988497	1.26844044	0.00156088	0.00868383	CCDC144B
65258	ENSG00000154889	205.732309	-0.32290757	0.21399046	0.07291948	0.19718174	MPPE1
NA	ENSG00000154898	41.4245895	0.08659263	0.29394081	0.65507681	0.82823884	CCDC144CP
124739	ENSG00000154914	0	NA	NA	NA	NA	USP43
51560	ENSG00000154917	804.544123	-0.06999307	0.11992928	0.53666462	0.74729267	RAB6B
146956	ENSG00000154920	781.26572	0.06575321	0.12528122	0.57951057	0.77706744	EME1
2047	ENSG00000154928	2.11109298	0.06576683	0.38390138	0.42383853	NA	EPHB1
84532	ENSG00000154930	0	NA	NA	NA	NA	ACSS1
91369	ENSG00000154945	1966.64536	-0.01730797	0.06620462	0.78996439	0.9043268	ANKRD40
7566	ENSG00000154957	83.1197074	-0.0671189	0.22750688	0.71339186	0.86190557	ZNF18
56934	ENSG00000154975	1.05385652	0.04033127	0.38247713	0.5346697	NA	CA10
81552	ENSG00000154978	1032.48443	0.0855332	0.08288853	0.29077493	0.51679428	VOPP1
346288	ENSG00000154997	0	NA	NA	NA	NA	SEPTIN14
139322	ENSG00000155008	424.897394	-0.12647	0.12310883	0.27679787	0.50120195	APOOL
27123	ENSG00000155011	79.0768814	3.23421623	0.35254724	3.16E-21	1.24E-19	DKK2
113612	ENSG00000155016	390.683476	0.04041952	0.11574302	0.71500464	0.86271551	CYP2U1
222967	ENSG00000155026	6.07411942	0.35384898	0.59507559	0.03825009	0.12144053	RSPH10B
80028	ENSG00000155034	1564.50129	-0.11537223	0.07124065	0.09897175	0.24743531	FBXL18
129684	ENSG00000155052	823.574436	-1.69510761	0.10633822	2.54E-58	3.15E-56	CNTNAP5
150696	ENSG00000155066	44.7355485	0.76148836	0.38390992	0.00648879	0.0290015	PROM2
NA	ENSG00000155070	1.91433091	0.04719228	0.38000272	0.56430486	NA	UNC93B2
221264	ENSG00000155085	125.536856	-0.10417943	0.20771083	0.55036063	0.75685555	AK9
4956	ENSG00000155087	0	NA	NA	NA	NA	ODF1
7071	ENSG00000155090	648.833952	0.06060329	0.11301706	0.57543462	0.7743233	KLF10
5799	ENSG00000155093	1491.82853	-1.47966728	0.10179028	5.87E-49	5.62E-47	PTPRN2
51582	ENSG00000155096	4659.29378	0.28405567	0.06387262	6.32E-06	6.24E-05	AZIN1

528	ENSG00000155097	672.494571	-0.31006027	0.15345243	0.02717338	0.09295171	ATP6V1C1
55529	ENSG00000155099	227.467672	0.0930537	0.14911311	0.49889591	0.71652634	PIP4P2
51633	ENSG00000155100	436.129801	0.03087497	0.13345522	0.80611801	0.9121533	OTUD6B
23097	ENSG00000155111	1395.71639	-0.31596852	0.08052892	5.75E-05	0.00046237	CDK19
112495	ENSG00000155115	737.536229	-0.13965008	0.09574745	0.13136546	0.30404473	GTF3C6
158219	ENSG00000155158	103.576596	0.35725364	0.27735454	0.08948172	0.22960648	TTC39B
55326	ENSG00000155189	1730.5469	-0.46338428	0.07843346	1.54E-09	2.59E-08	AGPAT5
64210	ENSG00000155229	1728.19364	0.03690843	0.07101427	0.59801617	0.78960741	MMS19
79544	ENSG00000155249	0	NA	NA	NA	NA	OR4K1
55361	ENSG00000155252	805.542819	-0.1819079	0.08880031	0.03438431	0.11181524	PI4K2A
83742	ENSG00000155254	602.534534	-0.21680093	0.11337068	0.04417658	0.13529846	MARVELD1
118813	ENSG00000155256	896.186635	-0.39588611	0.0980996	2.78E-05	0.00024158	ZFYVE27
401647	ENSG00000155265	420.622981	-2.32613925	0.15811573	2.78E-50	2.79E-48	GOLGA7B
27201	ENSG00000155269	2.77736763	-0.09708752	0.39307471	0.25923527	0.48076535	GPR78
152992	ENSG00000155275	225.885284	-0.12477954	0.17319799	0.41788466	0.64399811	TRMT44
81894	ENSG00000155287	295.37435	0.0849869	0.13430357	0.50013972	0.7173261	SLC25A28
6782	ENSG00000155304	1151.19563	-0.05887378	0.08331312	0.47006186	0.69237291	HSPA13
64092	ENSG00000155307	0	NA	NA	NA	NA	SAMSN1
388813	ENSG00000155307	0	NA	NA	NA	NA	SAMSN1
29761	ENSG00000155313	453.702375	0.55211006	0.12624794	4.14E-06	4.22E-05	USP25
65983	ENSG00000155324	31.6085666	4.0212928	0.56913974	1.78E-13	4.29E-12	GRAMD2B
54819	ENSG00000155329	280.930549	-0.06051874	0.14967433	0.65935845	0.83105722	ZCCHC10
388272	ENSG00000155330	354.020191	-0.40815063	0.22049048	0.02753815	0.0937308	C16orf87
4343	ENSG00000155363	889.029877	0.59554417	0.10517239	4.57E-09	7.22E-08	MOV10
389	ENSG00000155366	609.864388	0.56964744	0.11194599	1.18E-07	1.54E-06	RHOC
333926	ENSG00000155367	7.22668368	-0.28969642	0.48712013	0.09500048	0.23991343	PPM1J
1622	ENSG00000155368	1278.71225	-0.54487118	0.09289272	1.55E-09	2.61E-08	DBI
6566	ENSG00000155380	2095.953	-0.16101868	0.11769657	0.14945236	0.33226522	SLC16A1
55027	ENSG00000155393	399.401996	-0.10098494	0.13463545	0.42215188	0.64814241	HEATR3
378108	ENSG00000155428	6.36863609	-0.00411486	0.36959259	0.96527777	0.98625559	TRIM74
84365	ENSG00000155438	1037.47714	-0.44342819	0.10672003	1.48E-05	0.00013627	NIFK
5018	ENSG00000155463	1510.60145	-0.21527516	0.07465465	0.00322671	0.0160849	OXA1L
9056	ENSG00000155465	1.70353084	0.02357298	0.3793468	0.73471499	NA	SLC7A7
9947	ENSG00000155495	22.4901132	-1.70250223	0.59156932	0.00022166	0.0015584	MAGEC1
23367	ENSG00000155506	7568.55166	-0.09698352	0.06205033	0.12416411	0.29208041	LARP1
9337	ENSG00000155508	1610.83452	-0.00511237	0.07126708	0.94115176	0.97559098	CNOT8
2890	ENSG00000155511	0	NA	NA	NA	NA	GRIA1
136332	ENSG00000155530	159.251917	-0.05463096	0.17556356	0.72557814	0.86866734	LRGUK

133383	ENSG00000155542	184.57155	-0.27203782	0.17256514	0.07728232	0.20605848	SETD9
166968	ENSG00000155545	1152.76607	-0.41378255	0.10856324	6.81E-05	0.00054247	MIER3
23165	ENSG00000155561	4706.01324	0.0038128	0.06193726	0.95091377	0.97985505	NUP205
342357	ENSG00000155592	605.064578	-0.1658142	0.10819992	0.10928691	0.26581493	ZKSCAN2
138241	ENSG00000155621	236.52332	0.2141061	0.14833987	0.11694557	0.27994692	C9orf85
9502	ENSG00000155622	0	NA	NA	NA	NA	XAGE2
118788	ENSG00000155629	155.201582	-0.66771024	0.1923419	0.00011838	0.0008846	PIK3AP1
129831	ENSG00000155636	220.763187	-0.25708532	0.15649031	0.07128033	0.19409851	RBM45
7273	ENSG00000155657	319.092835	0.07466398	0.129645	0.54125064	0.74967974	TTN
11326	ENSG00000155659	0	NA	NA	NA	NA	VSIG4
9601	ENSG00000155660	5107.60898	0.83452722	0.0752242	2.47E-29	1.39E-27	PDIA4
79831	ENSG00000155666	85.0853404	0.13922729	0.21788691	0.43875789	0.66399363	KDM8
255762	ENSG00000155714	1.19233976	0.01955149	0.38005787	0.76257316	NA	PDZD9
146183	ENSG00000155719	5.26384778	5.7040874	2.67821432	0.00011092	0.00083399	OTOA
130535	ENSG00000155729	279.213542	0.09493533	0.14299584	0.47473234	0.69695794	KCTD18
285172	ENSG00000155744	369.79401	-0.13221337	0.1570767	0.35417695	0.58474417	FAM126B
130540	ENSG00000155749	13.5127988	-0.10751881	0.34655261	0.52953693	0.7412695	FLACC1
151254	ENSG00000155754	3.12667316	0.11262166	0.39157062	0.30262446	0.52987991	C2CD6
65062	ENSG00000155755	1624.47555	-0.0600042	0.0929643	0.50498124	0.72130144	TMEM237
8324	ENSG00000155760	1157.67541	0.06349571	0.08198478	0.42737026	0.65313711	FZD7
200162	ENSG00000155761	0.6645966	-0.02196995	0.38320164	0.65254366	NA	SPAG17
64798	ENSG00000155792	12.003612	0.02461826	0.33567957	0.88358435	0.94890373	DEPTOR
56776	ENSG00000155816	646.63559	-0.85288943	0.17994027	3.33E-07	4.09E-06	FMN2
56254	ENSG00000155827	1664.79123	-0.23744968	0.09425946	0.00914415	0.03850987	RNF20
1539	ENSG00000155833	0	NA	NA	NA	NA	CYLC2
133522	ENSG00000155846	103.05789	0.04286615	0.20115705	0.80398257	0.911018	PPARGC1B
9844	ENSG00000155849	1063.08095	-0.49823635	0.12418663	2.31E-05	0.00020482	ELMO1
1836	ENSG00000155850	823.264386	-0.35381145	0.09225527	7.48E-05	0.0005886	SLC26A2
134353	ENSG00000155858	503.557362	-0.74858303	0.11938606	7.53E-11	1.46E-09	LSM11
9443	ENSG00000155868	217.70634	-0.10184408	0.15211157	0.46485977	0.68755165	MED7
158297	ENSG00000155875	0.34045604	-0.0001331	0.38336221	0.9665143	NA	SAXO1
10670	ENSG00000155876	896.038108	-0.06724584	0.09058808	0.44431621	0.66924033	RRAGA
25769	ENSG00000155886	16.9855572	3.71272072	0.8632569	1.66E-06	1.81E-05	SLC24A2
287015	ENSG00000155890	0	NA	NA	NA	NA	TRIM42
92370	ENSG00000155893	425.836083	0.40978464	0.14844743	0.00278009	0.01420474	PXYLP1
114	ENSG00000155897	1.14109546	-0.00031907	0.37936945	0.99567772	NA	ADCY8
5922	ENSG00000155903	475.302408	0.04048496	0.13088674	0.74297263	0.8787753	RASA2
55005	ENSG00000155906	592.924729	-0.03993217	0.09809216	0.67298823	0.83933156	RMND1

154064	ENSG00000155918	0	NA	NA	NA	NA	RAET1L
6503	ENSG00000155926	20.8472452	0.02673244	0.30982985	0.88643837	0.95015905	SLA
51643	ENSG00000155957	374.470373	0.40038954	0.16141499	0.00639249	0.02861621	TMBIM4
7411	ENSG00000155959	961.75353	-0.31968324	0.09705085	0.00063656	0.00397899	VBP1
116442	ENSG00000155961	343.220372	-0.19124748	0.16416972	0.19641303	0.40005705	RAB39B
1193	ENSG00000155962	17.9421013	-0.01420973	0.32967489	0.93083446	0.97111825	CLIC2
2334	ENSG00000155966	0	NA	NA	NA	NA	AFF2
286097	ENSG00000155970	173.272668	-0.29549914	0.1950248	0.07953369	0.2105454	MICU3
23426	ENSG00000155974	30.1442535	5.45687222	0.89251076	1.92E-11	3.97E-10	GRIP1
137492	ENSG00000155975	506.815749	0.45765232	0.14750053	0.00081606	0.00495428	VPS37A
3798	ENSG00000155980	1139.2903	-0.79949811	0.1060973	8.93E-15	2.38E-13	KIF5A
10	ENSG00000156006	0.14931947	-0.01844332	0.38480212	0.84537521	NA	NAT2
4107	ENSG00000156009	0.72009102	0.02303266	0.38385915	0.59437427	NA	MAGEA8
23362	ENSG00000156011	1534.66316	-0.54022396	0.07621932	4.92E-13	1.14E-11	PSD3
138199	ENSG00000156017	171.497044	0.01307992	0.17272247	0.93364208	0.97236005	CARNMT1
90550	ENSG00000156026	388.251326	-0.33733004	0.14393598	0.01128177	0.04581993	MCU
91748	ENSG00000156030	703.378419	-0.1326398	0.10949635	0.20513137	0.41205331	MIDEAS
118491	ENSG00000156042	11.7175948	0.84522044	1.05391811	0.01691797	0.06370327	CFAP70
9630	ENSG00000156049	5.04594042	0.13864263	0.39642109	0.27311123	0.49676945	GNA14
145483	ENSG00000156050	109.175701	-0.10468949	0.20708673	0.54749177	0.75431125	FAM161B
2776	ENSG00000156052	1862.75177	-0.51817117	0.08196222	9.81E-11	1.89E-09	GNAQ
11197	ENSG00000156076	0	NA	NA	NA	NA	WIF1
7363	ENSG00000156096	0	NA	NA	NA	NA	UGT2B4
83873	ENSG00000156097	4.90139228	0.02987664	0.37315812	0.74485436	0.87988222	GPR61
4325	ENSG00000156103	1418.84251	0.03166744	0.07461867	0.66582086	0.83495897	MMP16
132	ENSG00000156110	394.538064	-0.07228643	0.12116626	0.52661462	0.73858575	ADK
3778	ENSG00000156113	360.493914	-3.31347362	0.16804776	1.45E-87	3.46E-85	KCNMA1
10538	ENSG00000156127	0	NA	NA	NA	NA	BATF
1633	ENSG00000156136	710.772421	-0.10783578	0.10125122	0.26764047	0.49016772	DCK
9508	ENSG00000156140	531.708786	3.67125981	0.14943697	1.88E-134	1.04E-131	ADAMTS3
257	ENSG00000156150	0.30139824	-0.02296476	0.38527817	0.68536535	NA	ALX3
286148	ENSG00000156162	1139.42162	0.15611503	0.10930044	0.13597896	0.3109165	DPY19L4
137682	ENSG00000156170	250.722709	-0.168041	0.14688095	0.21420426	0.42438929	NDUFAF6
128338	ENSG00000156171	394.924834	0.01999243	0.16618366	0.89515627	0.95469109	DRAM2
157657	ENSG00000156172	89.7444624	-0.08636033	0.2174178	0.62957428	0.8124371	C8orf37
5470	ENSG00000156194	1.31607109	0.05440781	0.38675805	0.29196184	NA	PPEF2
161502	ENSG00000156206	1.32737373	-0.01667605	0.37940508	0.80257198	NA	CFAP161
57188	ENSG00000156218	0	NA	NA	NA	NA	ADAMTSL3

419	ENSG00000156219	0.38962757	0.02407781	0.38542126	0.60160411	NA	ART3
9154	ENSG00000156222	0.3348156	0.00657317	0.38350531	0.91414739	NA	SLC28A1
123720	ENSG00000156232	185.565394	0.05063786	0.16248513	0.73199014	0.87198139	WHAMM
10563	ENSG00000156234	0.66471966	0.00545819	0.38195458	0.90769313	NA	CXCL13
29104	ENSG00000156239	622.008214	0.28532011	0.10346681	0.00408596	0.01964299	N6AMT1
10069	ENSG00000156253	393.240174	-0.26625378	0.11984426	0.01891914	0.06963927	RWDD2B
10600	ENSG00000156256	846.897867	-0.18819845	0.08905834	0.02948998	0.09906554	USP16
10694	ENSG00000156261	3710.294	-0.18999689	0.07398794	0.00875392	0.03719011	CCT8
56911	ENSG00000156265	0.78300971	-0.00623337	0.38219998	0.89370184	NA	MAP3K7CL
84779	ENSG00000156269	0.84544779	0.03692112	0.38467463	0.45953839	NA	NAA11
571	ENSG00000156273	469.891855	0.30176453	0.15970766	0.03737237	0.1192962	BACH1
100379661	ENSG00000156273	469.891855	0.30176453	0.15970766	0.03737237	0.1192962	BACH1
26285	ENSG00000156282	0	NA	NA	NA	NA	CLDN17
9073	ENSG00000156284	0	NA	NA	NA	NA	CLDN8
7102	ENSG00000156298	372.783614	-0.24346987	0.12087989	0.0332283	0.10876911	TSPAN7
7074	ENSG00000156299	1109.87917	-0.47467525	0.08930733	4.54E-08	6.26E-07	TIAM1
57466	ENSG00000156304	1458.39751	0.07823356	0.07676715	0.29872899	0.52556382	SCAF4
6103	ENSG00000156313	190.650799	0.23066877	0.2060725	0.18757745	0.38786275	RPGR
23552	ENSG00000156345	253.586265	0.23751086	0.18100312	0.13711745	0.31261897	CDK20
84108	ENSG00000156374	252.878102	-0.22263697	0.15442479	0.1142699	0.27493509	PCGF6
122416	ENSG00000156381	692.938321	0.39742321	0.11491105	0.00027661	0.00190026	ANKRD9
119392	ENSG00000156384	95.0482288	-0.43398914	0.2365177	0.02628055	0.09050816	SFR1
22986	ENSG00000156395	20.3293309	0.01608191	0.30800566	0.93262447	0.97195551	SORCS3
118980	ENSG00000156398	323.790795	-0.34092864	0.14116234	0.00922135	0.03877735	SFXN2
9556	ENSG00000156411	978.318973	-0.15350213	0.09209223	0.08542127	0.22179793	ATP5MJ
2528	ENSG00000156413	0.96707992	-0.02489566	0.38141133	0.67795312	NA	FUT6
122402	ENSG00000156414	2.39771539	-0.11938069	0.40812908	0.08115854	NA	TDRD9
8817	ENSG00000156427	1.62132929	-0.01706455	0.37715472	0.82603318	NA	FGF18
5097	ENSG00000156453	86.6989924	-2.60895589	0.3012279	2.48E-19	8.93E-18	PCDH1
153769	ENSG00000156463	0.29863895	-0.02292439	0.3852726	0.68753245	NA	SH3RF2
392255	ENSG00000156466	6.75839875	3.35479759	1.49705083	0.00165928	0.00915212	GDF6
7381	ENSG00000156467	2025.72449	-0.22935766	0.07553921	0.00189802	0.01022448	UQCRB
51001	ENSG00000156469	213.787935	-0.26522894	0.16697341	0.07716714	0.20583438	MTERF3
9791	ENSG00000156471	1072.21158	-0.57861932	0.09039349	4.99E-11	9.91E-10	PTDSS1
5521	ENSG00000156475	78.2415419	-2.67171794	0.34834978	6.43E-16	1.85E-14	PPP2R2B
6156	ENSG00000156482	11540.4707	0.01207558	0.05496461	0.82512081	0.92061618	RPL30
3788	ENSG00000156486	2.85743744	-0.15519212	0.42205375	0.08770992	0.22627844	KCNS2
159091	ENSG00000156500	106.802045	0.16036656	0.20225475	0.35307476	0.58322456	PABIR3

6832	ENSG00000156502	477.413092	-0.13950159	0.12031072	0.22077629	0.43316867	SUPV3L1
159090	ENSG00000156504	857.369026	-0.19656203	0.10033763	0.04178533	0.12979818	PABIR2
1915	ENSG00000156508	178792.759	-0.07639208	0.04300203	0.06802283	0.18770138	EEF1A1
286151	ENSG00000156509	59.2658865	-0.12097123	0.24574716	0.52497008	0.73755856	FBXO43
80201	ENSG00000156510	0	NA	NA	NA	NA	HKDC1
3098	ENSG00000156515	3059.69461	-0.37696976	0.07823156	8.18E-07	9.41E-06	HK1
219743	ENSG00000156521	369.442265	0.13644336	0.12651617	0.2529992	0.47325073	TYSND1
84295	ENSG00000156531	1807.61791	0.08276452	0.0909554	0.34987407	0.58019905	PHF6
135228	ENSG00000156535	0	NA	NA	NA	NA	CD109
57497	ENSG00000156564	593.243258	-0.98446734	0.11511618	1.66E-18	5.77E-17	LRFN2
4838	ENSG00000156574	48.4662995	0.5911428	0.35452698	0.01982848	0.07247778	NODAL
10394	ENSG00000156575	0	NA	NA	NA	NA	PRG3
9246	ENSG00000156587	628.076887	-0.03372413	0.09887929	0.72338081	0.86781203	UBE2L6
25921	ENSG00000156599	1224.05149	-0.16246035	0.08595217	0.05204438	0.15365484	ZDHHC5
219541	ENSG00000156603	314.918902	-0.11309155	0.14021826	0.38446763	0.61356176	MED19
60685	ENSG00000156639	1496.56084	-0.00922152	0.09125545	0.9174079	0.96505604	ZFAND3
27020	ENSG00000156642	630.030354	-0.13169936	0.10490314	0.19090286	0.39232575	NPTN
23522	ENSG00000156650	1528.9751	0.66121275	0.09559514	1.22E-12	2.77E-11	KAT6B
142891	ENSG00000156671	801.89291	-0.16598098	0.09261117	0.06364763	0.17914159	SAMD8
80223	ENSG00000156675	485.170862	0.10427486	0.13460449	0.40847912	0.63652585	RAB11FIP1
137970	ENSG00000156687	675.436565	-1.02741753	0.10773427	1.97E-22	8.29E-21	UNC5D
219970	ENSG00000156689	28.6456605	0.40200168	0.41717845	0.09214472	0.23464913	GLYATL2
10813	ENSG00000156697	1184.93813	-0.22985836	0.07702428	0.00227532	0.01195508	UTP14A
9131	ENSG00000156709	623.035381	-0.04568947	0.11886558	0.68590917	0.8472443	AIFM1
5603	ENSG00000156711	88.1201528	0.15429803	0.25225391	0.42581527	0.65161395	MAPK13
9530	ENSG00000156735	433.158138	-0.56111706	0.12858608	4.13E-06	4.21E-05	BAG4
931	ENSG00000156738	0	NA	NA	NA	NA	MS4A1
NA	ENSG00000156755	0	NA	NA	NA	NA	IGKV1OR-2
93594	ENSG00000156787	438.161795	0.10794506	0.11439762	0.32296321	0.55059885	TBC1D31
55093	ENSG00000156795	331.408546	-0.27115564	0.16424936	0.06683868	0.18536194	NTAQ1
29028	ENSG00000156802	1944.54807	-0.52589469	0.08143439	4.04E-11	8.09E-10	ATAD2
114907	ENSG00000156804	55.4598951	0.32012939	0.28744085	0.13148961	0.30415692	FBXO32
286053	ENSG00000156831	201.567939	0.14050403	0.15931657	0.33261505	0.5618153	NSMCE2
115509	ENSG00000156853	350.911623	-0.21840491	0.1318439	0.07641256	0.20422871	ZNF689
78994	ENSG00000156858	757.579653	-0.04397793	0.08886801	0.61032955	0.7985846	PRR14
64319	ENSG00000156860	1482.6937	0.22549926	0.09799375	0.01709332	0.06424162	FBRS
391059	ENSG00000156869	3.35340384	0.23327832	0.48522958	0.016598	0.06267687	FRRS1
5261	ENSG00000156873	394.17112	0.04310258	0.11826714	0.70249912	0.85694048	PHKG2

64645	ENSG00000156875	1178.25024	-0.10463225	0.08037256	0.18251677	0.3803144	MFSD14A
163786	ENSG00000156876	573.825616	-0.35563672	0.11337972	0.00099529	0.00588222	SASS6
1339	ENSG00000156885	0.18922458	0.01954053	0.38488496	0.78225479	NA	COX6A2
3681	ENSG00000156886	0	NA	NA	NA	NA	ITGAD
139378	ENSG00000156920	2.89084641	0.05809901	0.37652958	0.56859274	0.76931138	ADGRG4
7547	ENSG00000156925	0.5945563	0.0208396	0.38402101	0.64002282	NA	ZIC3
115416	ENSG00000156928	521.500912	0.15532548	0.10516568	0.12417834	0.29208041	MALSU1
23355	ENSG00000156931	672.188283	-0.3320442	0.13235284	0.00732792	0.03209895	VPS8
2585	ENSG00000156958	176.905975	0.33956049	0.16962126	0.02599518	0.08974218	GALK2
375323	ENSG00000156959	1273.36702	-1.3363792	0.13783105	2.74E-23	1.21E-21	LHFPL4
93010	ENSG00000156966	17.1079343	0.04441448	0.32293279	0.80535564	0.91174071	B3GNT7
255027	ENSG00000156968	196.76542	-0.6905737	0.19512555	8.61E-05	0.00066525	MPV17L
701	ENSG00000156970	1936.34932	-0.00810773	0.08682818	0.92234504	0.96745047	BUB1B
5147	ENSG00000156973	348.210035	-0.16974686	0.13199156	0.16975692	0.36148629	PDE6D
1974	ENSG00000156976	6456.18115	-0.11203035	0.05661515	0.04524991	0.13775638	EIF4A2
7862	ENSG00000156983	830.871145	-0.00500077	0.08614076	0.95146194	0.98008882	BRPF1
285367	ENSG00000156990	392.862349	0.13130224	0.12303174	0.25962417	0.48121669	RPUSD3
6750	ENSG00000157005	0	NA	NA	NA	NA	SST
9797	ENSG00000157014	1084.79462	-0.15502224	0.09573553	0.09376995	0.23753778	TATDN2
51738	ENSG00000157017	2.68766026	-0.05748061	0.38428716	0.38116309	0.61011123	GHRL
6396	ENSG00000157020	1375.09464	-0.01858641	0.08165823	0.81510845	0.91599551	SEC13
NA	ENSG00000157021	0	NA	NA	NA	NA	CIBAR1P1
9941	ENSG00000157036	95.1356879	0.47151974	0.24939417	0.02035225	0.07388629	EXOG
123803	ENSG00000157045	553.241842	0.24138868	0.10826294	0.01984422	0.07251969	NTAN1
81626	ENSG00000157060	0	NA	NA	NA	NA	SHCBP1L
23057	ENSG00000157064	1518.34726	-3.23922558	0.10948834	3.22E-194	5.81E-191	NMNAT2
9372	ENSG00000157077	752.024109	-0.06577358	0.096958	0.48169466	0.70240082	ZFYVE9
491	ENSG00000157087	296.204686	-0.40688578	0.16343783	0.00607671	0.02744045	ATP2B2
131375	ENSG00000157093	0	NA	NA	NA	NA	LYZL4
6529	ENSG00000157103	14.9689014	0.0250597	0.33783916	0.8791094	0.94641763	SLC6A1
23049	ENSG00000157106	3692.64027	0.03410308	0.0657969	0.59874956	0.79005929	SMG1
115548	ENSG00000157107	309.381581	-0.0129378	0.12841448	0.91354056	0.96320586	FCHO2
11030	ENSG00000157110	357.730159	1.63042698	0.14702906	1.09E-29	6.18E-28	RBPMS
134285	ENSG00000157111	1.74784129	0.11187679	0.40303439	0.10602727	NA	TMEM171
131377	ENSG00000157119	0	NA	NA	NA	NA	KLHL40
731	ENSG00000157131	0	NA	NA	NA	NA	C8A
7079	ENSG00000157150	0	NA	NA	NA	NA	TIMP4
6854	ENSG00000157152	256.215291	-0.27559441	0.14578958	0.04032578	0.1262121	SYN2

3084	ENSG00000157168	108.23448	-1.01408724	0.25125487	6.17E-06	6.10E-05	NRG1
54953	ENSG00000157181	762.853441	0.16645349	0.10983042	0.11322893	0.27296015	ODR4
1376	ENSG00000157184	529.85696	-0.15052829	0.10747576	0.14347904	0.32277095	CPT2
55707	ENSG00000157191	979.756064	0.15216107	0.09327901	0.09244371	0.23508014	NECAP2
7804	ENSG00000157193	2201.98847	-0.60930984	0.08444656	1.59E-13	3.84E-12	LRP8
200008	ENSG00000157211	0.94964547	0.00243572	0.38167557	0.96079652	NA	CDCP2
22976	ENSG00000157212	720.060133	0.07155116	0.10503944	0.47941438	0.70066897	PAXIP1
261729	ENSG00000157214	286.440889	-0.08516514	0.14525588	0.52528084	0.73786263	STEAP2
23648	ENSG00000157216	1786.68093	-0.08800935	0.09106608	0.31896107	0.54659755	SSBP3
3361	ENSG00000157219	0	NA	NA	NA	NA	HTR5A
9069	ENSG00000157224	1289.48032	-0.21876557	0.08887291	0.011186	0.04547752	CLDN12
4323	ENSG00000157227	27.4208563	1.61166446	0.69052813	0.0008118	0.00493444	MMP14
8321	ENSG00000157240	521.669648	0.04280082	0.10689218	0.67756861	0.84255476	FZD1
57798	ENSG00000157259	1717.7799	0.08627437	0.07420695	0.23619254	0.4530211	GATAD1
203328	ENSG00000157303	1.37292794	-0.01486915	0.37910267	0.82626189	NA	SUSD3
NA	ENSG00000157306	69.8109345	0.12937851	0.24203616	0.4970045	0.71500229	ZFHX2-AS1
146456	ENSG00000157315	10.0412497	-0.00330494	0.34139322	0.98094119	0.99355086	TMED6
348174	ENSG00000157322	2.72068317	0.09291158	0.39207274	0.27024399	0.49343279	CLEC18A
10901	ENSG00000157326	309.486256	0.30691129	0.1374767	0.01655879	0.06258837	DHRS4
728635	ENSG00000157326	309.486256	0.30691129	0.1374767	0.01655879	0.06258837	DHRS4
93190	ENSG00000157330	0	NA	NA	NA	NA	C1orf158
283971	ENSG00000157335	0.90740109	-0.05159104	0.38889269	0.2677938	NA	CLEC18C
221481	ENSG00000157343	44.0126169	0.57436978	0.35832259	0.02327083	0.08226894	ARMC12
11269	ENSG00000157349	584.388708	-0.04363795	0.1142402	0.68813292	0.84857639	DDX19B
6483	ENSG00000157350	1212.84537	-0.2164036	0.10747833	0.03537315	0.11420719	ST3GAL2
197258	ENSG00000157353	379.892773	0.5167324	0.15113021	0.00022519	0.00158071	FCSK
146433	ENSG00000157368	2.69357229	-0.13043345	0.40445666	0.18070386	0.37770382	IL34
115817	ENSG00000157379	169.021926	0.57972829	0.19309509	0.00076152	0.00465737	DHRS1
776	ENSG00000157388	400.428853	-0.69319754	0.17043424	1.05E-05	9.88E-05	CACNA1D
415	ENSG00000157399	0.87772087	0.02512699	0.38268914	0.63058073	NA	ARSL
3815	ENSG00000157404	222.404596	-0.51899985	0.16221307	0.00047885	0.00310675	KIT
54768	ENSG00000157423	7.22835248	0.28888677	0.50138363	0.07283628	0.19701047	HYDIN
132949	ENSG00000157426	303.791824	-0.05272401	0.13233844	0.67042299	0.83767652	AASDH
7567	ENSG00000157429	59.5646889	-0.16491675	0.25833158	0.39874054	0.62756224	ZNF19
55799	ENSG00000157445	5.28697407	-0.20022535	0.44111494	0.09986665	0.24900772	CACNA2D3
54778	ENSG00000157450	878.082357	0.28536017	0.123323	0.01429017	0.05545021	RNF111
9133	ENSG00000157456	1279.08251	-0.16088709	0.09897985	0.09185732	0.23406746	CCNB2
145773	ENSG00000157470	34.1840393	-0.5578361	0.44826318	0.03774335	0.12021642	FAM81A

4643	ENSG00000157483	585.470031	1.80276025	0.15494576	1.80E-32	1.11E-30	MYO1E
26060	ENSG00000157500	1499.2086	-0.25165886	0.07809898	0.00097075	0.0057629	APPL1
139221	ENSG00000157502	0	NA	NA	NA	NA	PWWP3B
134265	ENSG00000157510	131.106075	1.06017373	0.2276937	3.74E-07	4.55E-06	AFAP1L1
1831	ENSG00000157514	504.121916	-0.0351689	0.12670469	0.76706991	0.89269515	TSC22D3
10311	ENSG00000157538	1086.36315	0.02359339	0.09200965	0.79254532	0.90514041	VPS26C
1859	ENSG00000157540	1108.97349	-0.14892873	0.08398892	0.06876291	0.1890682	DYRK1A
3763	ENSG00000157542	0	NA	NA	NA	NA	KCNJ6
3772	ENSG00000157551	0.173733	0.01914545	0.38484597	0.78225479	NA	KCNJ15
2078	ENSG00000157554	0.14931947	-0.01844332	0.38480212	0.84537521	NA	ERG
2114	ENSG00000157557	367.645462	-0.63358529	0.14400897	2.82E-06	2.96E-05	ETS2
90139	ENSG00000157570	3060.1594	-0.26135488	0.06295205	2.50E-05	0.00021943	TSPAN18
150082	ENSG00000157578	13.6640242	0.2595643	0.41119099	0.18694021	0.3867863	LCA5L
347734	ENSG00000157593	1420.01962	0.12863132	0.07309496	0.0730366	0.19747154	SLC35B2
84187	ENSG00000157600	1492.88514	0.09271275	0.09426485	0.31079109	0.53855384	TMEM164
4599	ENSG00000157601	0.37837616	0.0239029	0.38539626	0.61496056	NA	MX1
90993	ENSG00000157613	5.15925134	0.16597691	0.42123603	0.10101325	0.25117219	CREB3L1
25966	ENSG00000157617	209.886925	-0.13414311	0.23174199	0.46877093	0.69137681	C2CD2
257397	ENSG00000157625	754.150862	-0.17163411	0.11528578	0.11746617	0.2809883	TAB3
124565	ENSG00000157637	3384.26285	0.00103485	0.05998672	0.98657306	0.99555514	SLC38A10
257169	ENSG00000157653	8.67125087	-0.00547558	0.35367253	0.96611081	0.98690343	C9orf43
445815	ENSG00000157654	64.8881017	1.65650924	0.4139666	3.35E-06	3.46E-05	PALM2AKAP2
114991	ENSG00000157657	2660.83489	0.00997921	0.06323013	0.87290803	0.94439555	ZNF618
9162	ENSG00000157680	746.774617	-1.90824982	0.15601733	9.99E-36	6.91E-34	DGKI
203197	ENSG00000157693	439.032765	-0.61035926	0.16824977	7.65E-05	0.00059993	TMEM268
136306	ENSG00000157703	1.59523611	-0.00081364	0.38000234	0.98804209	NA	SVOPL
79856	ENSG00000157734	119.265143	-0.64570917	0.25811384	0.00263642	0.01357553	SNX22
254048	ENSG00000157741	1937.31684	0.00590292	0.06572638	0.92797869	0.96982181	UBN2
673	ENSG00000157764	1114.32664	-0.17113255	0.12966576	0.16226946	0.35094147	BRAF
10568	ENSG00000157765	0	NA	NA	NA	NA	SLC34A2
176	ENSG00000157766	0	NA	NA	NA	NA	ACAN
84262	ENSG00000157778	785.670483	-0.13451155	0.0947597	0.14210753	0.32078887	PSMG3
9478	ENSG00000157782	17.9390635	0.0482747	0.31828157	0.79155162	0.90499209	CABP1
57728	ENSG00000157796	589.94221	0.38565174	0.13476282	0.00222937	0.01175723	WDR19
84255	ENSG00000157800	1544.12894	0.08878093	0.08380594	0.27778332	0.5022529	SLC37A3
10239	ENSG00000157823	835.595883	0.07050315	0.11020408	0.50489247	0.72130144	AP3S2
114793	ENSG00000157827	2347.4924	0.15043489	0.07255633	0.03467291	0.112514	FMNL2
150946	ENSG00000157833	1039.13303	-0.69094421	0.09658668	2.01E-13	4.81E-12	GAREM2

121665	ENSG00000157837	955.413923	-0.22372385	0.10383831	0.02476636	0.08649264	SPPL3
56896	ENSG00000157851	8759.00006	-0.2381955	0.05843852	3.33E-05	0.00028417	DPYSL5
92749	ENSG00000157856	9.58222565	0.03316562	0.34179201	0.83801966	0.92636004	DRC1
9364	ENSG00000157869	209.657874	-0.02620225	0.1642694	0.85834667	0.93677309	RAB28
127281	ENSG00000157870	875.048868	-0.03227568	0.10898436	0.75653105	0.88693352	PRXL2B
8764	ENSG00000157873	5.02754008	0.06792205	0.36969591	0.58476494	0.78078479	TNFRSF14
55229	ENSG00000157881	637.848893	0.18561816	0.103971	0.06308911	0.17804949	PANK4
130106	ENSG00000157884	0	NA	NA	NA	NA	CIB4
84465	ENSG00000157890	39.221154	-0.6990937	0.49540924	0.01789378	0.06663148	MEGF11
64897	ENSG00000157895	383.450927	-0.16229573	0.13444272	0.19603374	0.39974691	C12orf43
5192	ENSG00000157911	421.918509	0.13299602	0.15157101	0.33976611	0.57015645	PEX10
11079	ENSG00000157916	1753.62763	-0.17870182	0.07860098	0.0198189	0.07246877	RER1
55698	ENSG00000157927	1444.86375	0.00189604	0.09498189	0.98474203	0.99473578	RADIL
6497	ENSG00000157933	1590.51162	-0.13032948	0.07565863	0.0784314	0.20835248	SKI
26100	ENSG00000157954	2003.78439	0.33024037	0.06689734	5.16E-07	6.14E-06	WIPI2
NA	ENSG00000157965	0	NA	NA	NA	NA	SSX8P
26119	ENSG00000157978	132.739427	1.20524587	0.2142546	1.85E-09	3.07E-08	LDLRAP1
116987	ENSG00000157985	2615.36396	-0.77773054	0.07120228	1.77E-28	9.63E-27	AGAP1
200634	ENSG00000157992	12.1445202	0.11090939	0.35261486	0.51083351	0.72605035	KRTCAP3
100310846	ENSG00000157999	40.3716779	-0.27131671	0.32795739	0.20570058	0.41269082	ANKRD61
5051	ENSG00000158006	244.112866	-0.35319697	0.17654582	0.02454798	0.08588629	PAFAH2
2134	ENSG00000158008	35.0846837	0.05364348	0.28680864	0.78135072	0.90003181	EXTL1
7780	ENSG00000158014	0.33842187	0.00212859	0.38337671	0.96813356	NA	SLC30A2
9577	ENSG00000158019	449.907829	-0.04649482	0.11144993	0.66208089	0.83297534	BABAM2
84676	ENSG00000158022	0.33471731	0.02354391	0.38534154	0.62842204	NA	TRIM63
144406	ENSG00000158023	6.73627554	-0.10259586	0.37098819	0.46759674	0.69041597	CFAP251
63875	ENSG00000158042	718.758564	0.08712051	0.11451762	0.42506488	0.65086728	MRPL17
1844	ENSG00000158050	6.91893002	1.72041513	1.78934948	0.00653367	0.02917579	DUSP2
57822	ENSG00000158055	0	NA	NA	NA	NA	GRHL3
91544	ENSG00000158062	325.343704	0.52470853	0.1350459	3.63E-05	0.00030857	UBXN11
338323	ENSG00000158077	2.18914891	0.02372344	0.3752586	0.78847317	NA	NLRP14
138639	ENSG00000158079	526.732606	0.03453769	0.10741121	0.73864039	0.87647218	PTPDC1
79623	ENSG00000158089	1068.24971	-1.74333921	0.09497717	2.20E-76	4.16E-74	GALNT14
4690	ENSG00000158092	278.882465	0.0091144	0.14639473	0.94626087	0.97814213	NCK1
3242	ENSG00000158104	1.34399714	-0.04455947	0.38505332	0.40971879	NA	HPD
114822	ENSG00000158106	456.465831	0.50030039	0.11522273	5.63E-06	5.62E-05	RHPN1
127262	ENSG00000158109	604.09066	0.15164435	0.10175044	0.12184969	0.2885531	TPRG1L
254050	ENSG00000158113	11.0710017	1.81874697	1.07751174	0.00263503	0.01357191	LRRC43

195827	ENSG00000158122	120.007329	0.07907181	0.19256451	0.63659818	0.81722218	PRXL2C
7498	ENSG00000158125	0	NA	NA	NA	NA	XDH
55113	ENSG00000158156	9.73403924	-0.11621248	0.36286838	0.46406371	0.68683506	XKR8
26504	ENSG00000158158	509.019618	-0.03081616	0.11627761	0.78014469	0.89936692	CNNM4
2140	ENSG00000158161	981.212244	0.09645945	0.09870632	0.31221413	0.53995191	EYA3
199221	ENSG00000158163	227.485648	-0.22806935	0.16770002	0.12925631	0.30074413	DZIP1L
11013	ENSG00000158164	886.556291	-0.03327513	0.08996685	0.70295703	0.85716076	TMSB15A
2176	ENSG00000158169	705.764371	-0.1304115	0.11334361	0.22733464	0.44210148	FANCC
22808	ENSG00000158186	1150.25319	0.15237522	0.11205116	0.1546997	0.3401953	MRAS
10163	ENSG00000158195	2337.94384	0.21480667	0.07160191	0.00223534	0.01178559	WASF2
171586	ENSG00000158201	139.421437	-0.46423884	0.22111867	0.01320234	0.05207442	ABHD3
83850	ENSG00000158220	55.1496268	0.10964837	0.2623608	0.57235314	0.7723153	ESYT3
55179	ENSG00000158234	164.265681	-0.35447811	0.17746934	0.02471827	0.0863703	FAIM
115572	ENSG00000158246	27.0607011	0.11312244	0.31473902	0.55737404	0.76258372	TENT5B
64084	ENSG00000158258	477.155704	-1.05722044	0.14165693	9.97E-15	2.65E-13	CLSTN2
81035	ENSG00000158270	3.70175244	0.1636535	0.41980341	0.13632616	0.3113874	COLEC12
388591	ENSG00000158286	397.988651	-0.19524057	0.11956968	0.08473205	0.22069796	RNF207
8450	ENSG00000158290	1320.35846	-0.17546634	0.08167426	0.02760374	0.09388964	CUL4B
387509	ENSG00000158292	942.122763	-0.48509154	0.09808697	3.12E-07	3.85E-06	GPR153
64849	ENSG00000158296	7.21886994	0.1387266	0.38058261	0.35915647	0.58949503	SLC13A3
114928	ENSG00000158301	719.360531	-0.33439862	0.09922439	0.00046808	0.00304681	GPRASP2
100528062	ENSG00000158301	719.360531	-0.33439862	0.09922439	0.00046808	0.00304681	GPRASP2
54933	ENSG00000158315	3.03264136	0.02722745	0.37400475	0.77281805	0.89561741	RHBDL2
26053	ENSG00000158321	3270.41939	0.16755272	0.10092755	0.08547618	0.22188243	AUTS2
57477	ENSG00000158352	0	NA	NA	NA	NA	SHROOM4
3017	ENSG00000158373	101.282007	-1.01795828	0.2653031	1.41E-05	0.00013044	H2BC5
995	ENSG00000158402	216.960796	0.24109005	0.15246417	0.08431278	0.21975376	CDC25C
8365	ENSG00000158406	13.6226322	0.10376258	0.35713381	0.51504001	0.72955207	H4C8
129531	ENSG00000158411	293.322932	0.35291408	0.1650644	0.01808395	0.06715058	MITD1
9669	ENSG00000158417	2767.24244	-0.15324233	0.07036042	0.02660186	0.09138928	EIF5B
158787	ENSG00000158423	6.53970011	0.05806779	0.35765066	0.68737086	0.84809937	RIBC1
286527	ENSG00000158427	272.370184	-0.16603479	0.14924199	0.2258767	0.4400858	TMSB15B
375307	ENSG00000158428	0.16735865	0.01897177	0.38482933	0.78225479	NA	CATIP
55571	ENSG00000158435	716.611149	-0.10500336	0.09773439	0.26546081	0.48783542	CNOT11
3745	ENSG00000158445	685.685421	-1.41734297	0.42562033	5.52E-05	0.00044565	KCNB1
340348	ENSG00000158457	463.222991	-0.25141382	0.12323644	0.03062354	0.10197567	TSPAN33
9542	ENSG00000158458	171.230416	-1.00082613	0.1862419	9.71E-09	1.47E-07	NRG2
23382	ENSG00000158467	415.018291	-0.02230924	0.13042796	0.85470797	0.93485893	AHCYL2

9334	ENSG00000158470	2228.23424	-0.42913149	0.08489072	2.09E-07	2.65E-06	B4GALT5
912	ENSG00000158473	0	NA	NA	NA	NA	CD1D
909	ENSG00000158477	0	NA	NA	NA	NA	CD1A
9825	ENSG00000158480	314.99649	0.10642185	0.12761919	0.37691555	0.60570261	SPATA2
911	ENSG00000158481	0	NA	NA	NA	NA	CD1C
NA	ENSG00000158482	3.48875602	0.21776802	0.46252764	0.05540657	0.16108826	SNX29P1
NA	ENSG00000158483	142.534276	0.03821878	0.18415665	0.81429838	0.91592004	FAM86C1P
910	ENSG00000158485	0	NA	NA	NA	NA	CD1B
55567	ENSG00000158486	0.15069912	-0.01848503	0.38480575	0.84537521	NA	DNAH3
913	ENSG00000158488	0	NA	NA	NA	NA	CD1E
57824	ENSG00000158497	0	NA	NA	NA	NA	HMHB1
1358	ENSG00000158516	0.173733	0.01914545	0.38484597	0.78225479	NA	CPA2
653361	ENSG00000158517	0	NA	NA	NA	NA	NCF1
93979	ENSG00000158525	0.35666205	0.02381692	0.38538104	0.61402664	NA	CPA5
90121	ENSG00000158526	794.220465	-0.13872124	0.09517328	0.13700105	0.31242538	TSR2
55607	ENSG00000158528	1030.17832	-0.18647493	0.08521254	0.02452561	0.08584828	PPP1R9A
124245	ENSG00000158545	1868.54421	0.0031227	0.06744493	0.96320843	0.98545957	ZC3H18
130617	ENSG00000158552	297.046316	-0.24479817	0.14819129	0.07237429	0.19602811	ZFAND2B
94026	ENSG00000158553	0.173733	0.01914545	0.38484597	0.78225479	NA	POM121L2
81544	ENSG00000158555	164.855308	-0.40988306	0.19258363	0.01501205	0.05773195	GDPD5
1780	ENSG00000158560	618.468543	0.83222515	0.13402839	9.51E-11	1.84E-09	DYNC1I1
5207	ENSG00000158571	5.52082752	-0.05796023	0.36579346	0.64995644	0.82529572	PFKFB1
212	ENSG00000158578	0.18922458	0.01954053	0.38488496	0.78225479	NA	ALAS2
222068	ENSG00000158604	2951.85353	0.09392828	0.06084798	0.11802582	0.2818698	TMED4
84919	ENSG00000158615	2251.50908	0.01043541	0.08271014	0.89802947	0.95533001	PPP1R15B
26958	ENSG00000158623	1254.16645	0.15091941	0.07598572	0.04269142	0.1320147	COPG2
56946	ENSG00000158636	576.195705	-0.10836746	0.1249092	0.35744814	0.58755353	EMSY
90737	ENSG00000158639	2.65170264	-0.12804767	0.40776715	0.12871668	0.29973461	PAGE5
137964	ENSG00000158669	2136.3798	-0.10135364	0.06500351	0.11332698	0.27313013	GPAT4
168507	ENSG00000158683	19.3075279	-0.33759122	0.42046651	0.13032477	0.30241443	PKD1L1
9753	ENSG00000158691	761.386641	-0.0167281	0.10320606	0.86526131	0.94028794	ZSCAN12
8407	ENSG00000158710	2533.79463	-0.29626677	0.06344905	2.11E-06	2.26E-05	TAGLN2
2005	ENSG00000158711	758.275897	0.13599271	0.10578864	0.18088321	0.37795923	ELK4
56833	ENSG00000158714	0.15069912	-0.01848503	0.38480575	0.84537521	NA	SLAMF8
85414	ENSG00000158715	240.468418	0.27275165	0.15389293	0.05267907	0.1548976	SLC45A3
54935	ENSG00000158716	298.711701	0.18289273	0.14074671	0.16196016	0.35042673	DUSP23
115992	ENSG00000158717	405.199955	-0.02410901	0.15260856	0.86219032	0.9388505	RNF166
NA	ENSG00000158731	0	NA	NA	NA	NA	OR10J6P

4681	ENSG00000158747	1325.7552	-0.45907094	0.08812229	8.40E-08	1.12E-06	NBL1
100532736	ENSG00000158747	1325.7552	-0.45907094	0.08812229	8.40E-08	1.12E-06	NBL1
3362	ENSG00000158748	160.011474	-0.15033257	0.17331204	0.330046	0.55861736	HTR6
142683	ENSG00000158764	0	NA	NA	NA	NA	ITLN2
50848	ENSG00000158769	366.063042	0.29870323	0.20938666	0.09240056	0.23505924	F11R
7391	ENSG00000158773	1491.67769	0.05889983	0.07392393	0.41724899	0.64356838	USF1
64600	ENSG00000158786	0	NA	NA	NA	NA	PLA2G2F
124044	ENSG00000158792	202.913556	0.00012853	0.15301819	0.99987375	0.9998748	SPATA2L
4817	ENSG00000158793	493.862179	0.13917899	0.11735025	0.21253287	0.42238326	NIT1
9191	ENSG00000158796	1124.1486	0.21348486	0.09059124	0.01516183	0.05815016	DEDD
92822	ENSG00000158805	1310.77216	0.23230993	0.09178279	0.00904851	0.03817189	ZNF276
10361	ENSG00000158806	4.20028012	0.05983987	0.37310779	0.58799551	0.7828366	NPM2
1896	ENSG00000158813	3.13451338	-0.08430713	0.38471501	0.38845229	0.61758902	EDA
8822	ENSG00000158815	17.4367339	0.29384154	0.41787335	0.15888548	0.3462911	FGF17
127731	ENSG00000158816	3.53736892	-0.30162202	0.55940331	0.0171737	0.06448264	VWA5B1
978	ENSG00000158825	0	NA	NA	NA	NA	CDA
65018	ENSG00000158828	513.636128	0.03225156	0.1074257	0.75526273	0.88614698	PINK1
8703	ENSG00000158850	1407.83175	-0.00111231	0.08145957	0.98820828	0.99593885	B4GALT3
2039	ENSG00000158856	445.121941	-0.58433421	0.12751369	1.40E-06	1.55E-05	DMTN
9507	ENSG00000158859	873.441837	0.0602861	0.14843466	0.66449428	0.83444869	ADAMTS4
64760	ENSG00000158863	1095.35387	0.3480861	0.09348331	0.0001195	0.00089166	FAM160B2
4720	ENSG00000158864	3082.504	0.05089251	0.05880875	0.38124991	0.61019669	NDUFS2
115584	ENSG00000158865	5.08154885	0.24710898	0.47978046	0.06204978	0.17579147	SLC5A11
2207	ENSG00000158869	1.80000047	0.02197139	0.3752095	0.80305595	NA	FCER1G
336	ENSG00000158874	0	NA	NA	NA	NA	APOA2
84134	ENSG00000158882	539.904817	-0.09605994	0.12612759	0.41881155	0.6446754	TOMM40L
4359	ENSG00000158887	23.7636868	-0.65021981	0.50778871	0.02568772	0.0889587	MPZ
90199	ENSG00000158901	0	NA	NA	NA	NA	WFDC8
57805	ENSG00000158941	4198.38537	0.05996704	0.09227438	0.50444285	0.72099605	CCAR2
7484	ENSG00000158955	0	NA	NA	NA	NA	WNT9B
57685	ENSG00000158966	3032.79018	-0.71620054	0.05948157	5.05E-34	3.30E-32	CACHD1
56990	ENSG00000158985	397.616907	0.12786833	0.12568417	0.28149047	0.50630249	CDC42SE2
51735	ENSG00000158987	617.406924	-0.27365814	0.11858072	0.01352726	0.05313349	RAPGEF6
2035	ENSG00000159023	3219.74375	0.04136727	0.06774511	0.5347504	0.74572704	EPB41
54069	ENSG00000159055	384.732218	-0.05144756	0.12358387	0.65932212	0.83105722	MIS18A
79053	ENSG00000159063	817.49536	0.07608972	0.09125725	0.38858098	0.61774412	ALG8
54461	ENSG00000159069	1670.16497	0.09208888	0.07372429	0.20327181	0.40934965	FBXW5
56683	ENSG00000159079	1074.66485	0.14697898	0.09035392	0.0943841	0.23881909	CFAP298

8867	ENSG00000159082	526.117603	-0.97315349	0.15277643	2.02E-11	4.15E-10	SYNJ1
94104	ENSG00000159086	1656.57749	0.03951373	0.07043391	0.56878538	0.76944648	PAXBP1
3455	ENSG00000159110	287.587423	0.07156412	0.14169677	0.58643174	0.78212395	IFNAR2
124995	ENSG00000159111	1502.22884	-0.00034704	0.09195985	0.99473379	0.99852582	MRPL10
3460	ENSG00000159128	1044.9163	0.25325615	0.082424	0.00163959	0.00905606	IFNGR2
2618	ENSG00000159131	3094.34318	0.12711341	0.07314082	0.07663662	0.20469446	GART
6651	ENSG00000159140	6793.93047	-0.4511384	0.07777094	2.97E-09	4.78E-08	SON
29980	ENSG00000159147	484.998926	-0.09349837	0.11885718	0.40699808	0.63526409	DONSON
9900	ENSG00000159164	2562.31951	-0.47028592	0.07136534	1.93E-11	3.98E-10	SV2A
3898	ENSG00000159166	0	NA	NA	NA	NA	LAD1
6781	ENSG00000159167	0.173124	-0.01911262	0.38486262	0.84537521	NA	STC1
7135	ENSG00000159173	0.93703287	-0.03548087	0.38531853	0.45338	NA	TNNI1
1465	ENSG00000159176	540.276867	0.3983824	0.10773842	0.00011386	0.00085339	CSRP1
84366	ENSG00000159182	0	NA	NA	NA	NA	PRAC1
10481	ENSG00000159184	0	NA	NA	NA	NA	HOXB13
NA	ENSG00000159186	0	NA	NA	NA	NA	ATP5POP1
714	ENSG00000159189	0	NA	NA	NA	NA	C1QC
9992	ENSG00000159197	1.31524186	0.00929914	0.37896592	0.89090372	NA	KCNE2
516	ENSG00000159199	686.200927	-0.13778849	0.12238752	0.23347657	0.45042543	ATP5MC1
1827	ENSG00000159200	260.351749	-0.26880999	0.15279585	0.0543471	0.15863564	RCAN1
65264	ENSG00000159202	2162.55686	-0.0234656	0.06439499	0.71012919	0.86086784	UBE2Z
148523	ENSG00000159208	8.92430247	0.07876939	0.35949942	0.59564827	0.78816266	CIART
11267	ENSG00000159210	1553.61415	-0.18692906	0.08888576	0.03026209	0.10106039	SNF8
54102	ENSG00000159212	0.18922458	0.01954053	0.38488496	0.78225479	NA	CLIC6
149473	ENSG00000159214	173.81325	-0.04838275	0.17611829	0.75616699	0.88670553	CCDC24
861	ENSG00000159216	14.3472486	0.126314	0.35238508	0.46880107	0.69137681	RUNX1
100506403	ENSG00000159216	14.3472486	0.126314	0.35238508	0.46880107	0.69137681	RUNX1
10642	ENSG00000159217	11.839335	-2.37821327	1.18783491	0.00082786	0.0050106	IGF2BP1
2695	ENSG00000159224	0	NA	NA	NA	NA	GIP
873	ENSG00000159228	791.832836	-0.00578998	0.09624389	0.94867521	0.97892111	CBR1
874	ENSG00000159231	30.599798	-0.31731534	0.35196228	0.15324613	0.33804794	CBR3
NA	ENSG00000159239	2.92030553	0.08565032	0.38788142	0.34168301	0.57180671	
NA	ENSG00000159247	6.79858016	-0.19142116	0.41216588	0.20771436	0.41501037	TUBBP5
57369	ENSG00000159248	4.7729524	-0.18098626	0.41970547	0.16954555	0.36123762	GJD2
70	ENSG00000159251	0	NA	NA	NA	NA	ACTC1
23515	ENSG00000159256	1012.72113	0.06876055	0.08524351	0.41076173	0.63847247	MORC3
8208	ENSG00000159259	513.044943	-0.38313535	0.12414979	0.00107901	0.00630193	CHAF1B
23562	ENSG00000159261	0.30139824	-0.02296476	0.38527817	0.68536535	NA	CLDN14

6493	ENSG00000159263	5.71390944	-3.02120843	1.51101076	0.00272389	0.01396431	SIM2
3141	ENSG00000159267	753.290229	0.02175541	0.13030387	0.85896176	0.93718813	HLCS
342096	ENSG00000159289	2.91469743	0.17698053	0.4360494	0.06277151	0.17738025	GOLGA6A
80274	ENSG00000159307	47.7977227	-1.97329486	0.49479221	1.99E-06	2.14E-05	SCUBE1
201176	ENSG00000159314	75.3579481	0.38276091	0.26850784	0.06587365	0.18348074	ARHGAP27
83440	ENSG00000159322	831.653151	-0.13636046	0.1021157	0.16436387	0.35380049	ADPGK
5763	ENSG00000159335	6091.35722	-0.06022479	0.06548152	0.35118538	0.58135318	PTMS
283748	ENSG00000159337	0	NA	NA	NA	NA	PLA2G4D
23569	ENSG00000159339	0	NA	NA	NA	NA	PADI4
51094	ENSG00000159346	1702.55296	0.2699649	0.07118908	0.00011202	0.00084127	ADIPOR1
51706	ENSG00000159348	398.969093	-0.21555523	0.12454625	0.06600554	0.18369334	CYB5R1
5710	ENSG00000159352	4445.54991	0.00586654	0.05811125	0.91928355	0.96577102	PSMD4
23400	ENSG00000159363	1301.93703	-0.16291439	0.0826121	0.04301183	0.13271456	ATP13A2
130951	ENSG00000159374	0.71437841	-0.01573492	0.38252123	0.79790796	NA	M1AP
5692	ENSG00000159377	5038.74943	0.03204622	0.06136603	0.59740001	0.78921427	PSMB4
79190	ENSG00000159387	62.4383822	0.64073462	0.32762288	0.00983992	0.04094361	IRX6
7832	ENSG00000159388	545.346378	1.73054371	0.11337573	1.05E-53	1.13E-51	BTG2
221223	ENSG00000159398	0.54443584	0.03032242	0.38588423	0.55227227	NA	CES5A
3099	ENSG00000159399	1504.01117	0.20896412	0.11061216	0.04819064	0.14471721	HK2
715	ENSG00000159403	18.7127237	0.19157855	0.37501865	0.29544804	0.52201345	C1R
11189	ENSG00000159409	1948.47176	-1.56276695	0.07343471	1.36E-101	4.21E-99	CELF3
8659	ENSG00000159423	291.553626	0.11302889	0.15186118	0.42184652	0.64783923	ALDH4A1
57519	ENSG00000159433	1495.51787	0.6131873	0.09541187	3.80E-11	7.64E-10	STARD9
117145	ENSG00000159445	668.660568	0.17645922	0.09517758	0.05538683	0.16107809	THEM4
7062	ENSG00000159450	0	NA	NA	NA	NA	TCHH
26239	ENSG00000159455	0	NA	NA	NA	NA	LCE2B
197131	ENSG00000159459	874.674653	-0.45650194	0.1116027	1.88E-05	0.00016962	UBR1
267	ENSG00000159461	656.391049	-0.21211494	0.1107455	0.04474245	0.13648374	AMFR
112950	ENSG00000159479	433.934101	-0.16910343	0.11904316	0.13403849	0.30828734	MED8
116179	ENSG00000159495	0	NA	NA	NA	NA	TGM7
266747	ENSG00000159496	11.4016277	0.26323285	0.49855302	0.03571722	0.11513073	RGL4
6706	ENSG00000159516	0	NA	NA	NA	NA	SPRR2G
114771	ENSG00000159527	0	NA	NA	NA	NA	PGLYRP3
64843	ENSG00000159556	188.655445	-0.08850038	0.15871263	0.53942472	0.74850934	ISL2
89970	ENSG00000159579	665.813843	0.23183642	0.11259535	0.03080577	0.10237631	RSPRY1
149483	ENSG00000159588	12.0867291	0.10193288	0.35416602	0.53379421	0.74507367	CCDC17
60313	ENSG00000159592	1332.57452	-0.12274219	0.09749252	0.19212847	0.39427275	GPBP1L1
8883	ENSG00000159593	1380.74256	-0.07425737	0.07814776	0.33123145	0.56014592	NAE1

51249	ENSG00000159596	447.52945	-0.05803866	0.11583419	0.59837554	0.78991172	TMEM69
221188	ENSG00000159618	0	NA	NA	NA	NA	ADGRG5
84229	ENSG00000159625	13.0276999	0.0686593	0.35200883	0.65425027	0.82790673	DRC7
1636	ENSG00000159640	457.824699	0.15663848	0.12249301	0.17684722	0.37163689	ACE
374739	ENSG00000159648	7.31828275	-0.01803717	0.35542525	0.89341791	0.95389529	TEPP
131669	ENSG00000159650	0.5290841	0.03179438	0.38612054	0.46790964	NA	UROC1
9813	ENSG00000159658	636.976757	0.41312371	0.12213945	0.00035593	0.00238487	EFCAB14
10417	ENSG00000159674	10.7774803	-0.08474818	0.34942173	0.59980948	0.79066681	SPON2
84303	ENSG00000159685	276.402288	0.03747407	0.14397606	0.77877449	0.8988299	CHCHD6
1487	ENSG00000159692	3029.24509	0.10145843	0.06921358	0.13628125	0.3113874	CTBP1
55282	ENSG00000159708	20.7136545	0.47721908	0.49747536	0.06159438	0.17497602	LRRC36
NA	ENSG00000159712	2.29168666	-0.03643855	0.37489887	0.69788913	NA	ANKRD18CP
51673	ENSG00000159713	208.944217	-0.21229173	0.16754084	0.15796422	0.34496534	TPPP3
29800	ENSG00000159714	179.439167	0.72583507	0.19547032	4.10E-05	0.00034399	ZDHHC1
9114	ENSG00000159720	878.367148	-0.27655114	0.11563294	0.0118319	0.04771238	ATP6V0D1
181	ENSG00000159723	0.64500811	-0.03770254	0.386784	0.41114084	NA	AGRP
57732	ENSG00000159733	29.5905768	-0.09560861	0.31853516	0.60573056	0.79555768	ZFYVE28
146206	ENSG00000159753	443.963619	-1.84036631	0.15881204	2.51E-32	1.54E-30	CARMIL2
388284	ENSG00000159761	83.9533237	0.37389605	0.24875958	0.06111215	0.17385505	C16orf86
5304	ENSG00000159763	0	NA	NA	NA	NA	PIP
9715	ENSG00000159784	6.43557884	-0.14305166	0.38938032	0.30996018	0.53774299	FAM131B
6002	ENSG00000159788	1397.57912	0.20128617	0.12660575	0.09147256	0.23347687	RGS12
5681	ENSG00000159792	1281.80786	0.27953189	0.093004	0.00191245	0.01027725	PSKH1
7791	ENSG00000159840	864.955764	0.57578914	0.09330073	2.21E-10	4.09E-09	ZYX
29	ENSG00000159842	3980.08014	-0.13480262	0.05457594	0.01251943	0.04990667	ABR
NA	ENSG00000159860	0	NA	NA	NA	NA	TCAF2P1
284348	ENSG00000159871	4.47221864	-0.11921702	0.38888796	0.32452883	0.55221149	LYPD5
150275	ENSG00000159873	741.054466	0.03906404	0.0999686	0.68652718	0.84763827	CCDC117
7773	ENSG00000159882	208.769231	-0.14767354	0.15833537	0.30472322	0.53198909	ZNF230
203260	ENSG00000159884	115.845871	0.2718356	0.20555408	0.12121291	0.2874665	CCDC107
7673	ENSG00000159885	110.197622	0.08357508	0.22393826	0.64821514	0.82448382	ZNF222
4882	ENSG00000159899	173.347989	0.16253164	0.22112194	0.37216709	0.60109044	NPR2
NA	ENSG00000159904	1.67970679	0.03180163	0.37856483	0.68439553	NA	ZNF890P
7638	ENSG00000159905	101.15811	-0.2467136	0.25346212	0.20975142	0.41815395	ZNF221
353355	ENSG00000159915	156.135276	-0.03429417	0.18951494	0.83252198	0.92392457	ZNF233
9310	ENSG00000159917	354.235635	0.09701468	0.13693445	0.44871506	0.67234398	ZNF235
10020	ENSG00000159921	985.330268	-0.22270409	0.08679009	0.00830676	0.03563374	GNE
115650	ENSG00000159958	40.3665593	0.30987356	0.32598999	0.15720892	0.34399746	TNFRSF13C

8392	ENSG00000159961	0	NA	NA	NA	NA	OR3A3
2909	ENSG00000160007	4285.67442	-0.39534359	0.07294816	3.23E-08	4.54E-07	ARHGAP35
5739	ENSG00000160013	186.100261	0.3179084	0.19323458	0.0586293	0.1682196	PTGIR
808	ENSG00000160014	7786.80381	-0.24539752	0.05973682	2.96E-05	0.00025552	CALM3
1676	ENSG00000160049	2227.34592	-0.19821322	0.07051888	0.0041788	0.02002468	DFFA
79140	ENSG00000160050	334.80692	-0.42167865	0.14910394	0.00217905	0.01152249	CCDC28B
55721	ENSG00000160051	106.69828	0.03590314	0.20743207	0.83851043	0.92674595	IQCC
56063	ENSG00000160055	142.014508	0.26018226	0.20884901	0.14114772	0.31929662	TMEM234
55108	ENSG00000160058	1417.88808	0.06893041	0.07405129	0.34292341	0.57311341	BSDC1
653121	ENSG00000160062	163.069846	-0.19049124	0.18070743	0.23094683	0.44697932	ZBTB8A
83858	ENSG00000160072	1154.12791	0.21493368	0.08583669	0.01013096	0.04183108	ATAD3B
29101	ENSG00000160075	1414.52757	-0.08403793	0.07633812	0.2608123	0.48273755	SSU72
118424	ENSG00000160087	1426.43945	-0.12261161	0.08470013	0.13721239	0.31276353	UBE2J2
149076	ENSG00000160094	1266.54487	0.01327574	0.08736365	0.87768945	0.94578953	ZNF362
252995	ENSG00000160097	54.1001293	-1.75775326	0.39054084	4.26E-07	5.14E-06	FNDC5
27151	ENSG00000160111	11.0804674	0.59842107	0.97461971	0.02083524	0.07532272	CPAMD8
2063	ENSG00000160113	1713.67424	-0.14639641	0.07282684	0.04033285	0.1262121	NR2F6
126549	ENSG00000160117	515.869009	-0.52305939	0.10990537	7.14E-07	8.29E-06	ANKLE1
131076	ENSG00000160124	194.799588	-0.0799693	0.18239269	0.61722259	0.80396906	MIX23
203547	ENSG00000160131	791.505567	0.26102135	0.10023837	0.00685985	0.03042688	VMA21
8997	ENSG00000160145	2045.55958	-0.36008382	0.09802273	0.00013878	0.00102164	KALRN
148113	ENSG00000160161	54.0941054	0.08789454	0.24634591	0.64610004	0.82370422	CILP2
645332	ENSG00000160172	104.180687	0.00235472	0.20953012	0.99074459	0.99682401	FAM86C2P
9619	ENSG00000160179	85.3525977	-1.81073966	0.33692319	4.89E-09	7.68E-08	ABCG1
7033	ENSG00000160180	0	NA	NA	NA	NA	TFF3
7032	ENSG00000160181	0	NA	NA	NA	NA	TFF2
7031	ENSG00000160182	0	NA	NA	NA	NA	TFF1
64699	ENSG00000160183	0	NA	NA	NA	NA	TMPRSS3
53347	ENSG00000160185	0	NA	NA	NA	NA	UBASH3A
89765	ENSG00000160188	5.64931037	-0.06610175	0.36570368	0.61232238	0.80024215	RSPH1
54020	ENSG00000160190	88.9501421	-0.25624712	0.25085499	0.19106443	0.39259479	SLC37A1
5152	ENSG00000160191	525.333731	-0.40107114	0.12209634	0.00051744	0.0033245	PDE9A
10785	ENSG00000160193	499.1987	-0.0975298	0.11648224	0.3784008	0.60737234	WDR4
4731	ENSG00000160194	536.311154	-0.08832008	0.12684589	0.45934122	0.68256874	NDUFV3
5316	ENSG00000160199	562.23866	0.0817839	0.14422119	0.54122354	0.74967974	PKNOX1
875	ENSG00000160200	3383.79123	0.1198158	0.05828341	0.03752814	0.11968467	CBS
7307	ENSG00000160201	1435.05198	-0.24723251	0.2326483	0.18811696	0.38853222	U2AF1
1409	ENSG00000160202	0	NA	NA	NA	NA	CRYAA

11077	ENSG00000160207	14.7367698	0.36863761	0.47830976	0.09961135	0.24855856	HSF2BP
23076	ENSG00000160208	1907.52953	-0.15673961	0.07049852	0.02352835	0.08298336	RRP1B
8566	ENSG00000160209	1338.00413	-0.02293807	0.09554111	0.80342052	0.91070264	PDXK
105372824	ENSG00000160209	1338.00413	-0.02293807	0.09554111	0.80342052	0.91070264	PDXK
2539	ENSG00000160211	1014.02894	0.07419862	0.08891937	0.39237269	0.62167977	G6PD
1476	ENSG00000160213	575.968481	-0.10254781	0.10133262	0.29138499	0.51742125	CSTB
8568	ENSG00000160214	702.796682	0.0303064	0.11445622	0.78235227	0.90086297	RRP1
56894	ENSG00000160216	2295.32885	0.11319463	0.07329448	0.11512568	0.27658176	AGPAT3
7109	ENSG00000160218	1217.18382	-0.08340161	0.09539661	0.36595773	0.59518764	TRAPPC10
139716	ENSG00000160219	0	NA	NA	NA	NA	GAB3
8209	ENSG00000160221	133.645631	-0.0838785	0.18722533	0.60777869	0.79695264	GATD3A
23308	ENSG00000160223	0	NA	NA	NA	NA	ICOSLG
326	ENSG00000160224	0.60687039	0.03538224	0.38650176	0.41848568	NA	AIRE
755	ENSG00000160226	387.70931	0.33957708	0.12426045	0.00379757	0.01846659	CFAP410
7617	ENSG00000160229	194.519639	-0.12977369	0.1728571	0.39954239	0.62826628	ZNF66
81543	ENSG00000160233	240.655458	-0.00368047	0.15944842	0.97857704	0.9926868	LRRC3
3689	ENSG00000160255	0.8936208	-0.02377628	0.38232713	0.71099375	NA	ITGB2
85395	ENSG00000160256	419.242327	-0.08468081	0.11867408	0.45211452	0.67536839	FAM207A
5900	ENSG00000160271	6052.50801	0.1648705	0.05630013	0.00279775	0.01428023	RALGDS
10841	ENSG00000160282	4.5108189	-0.06038661	0.36752376	0.62739405	0.81091192	FTCD
84221	ENSG00000160284	156.483948	0.17543576	0.18060738	0.271661	0.49506331	SPATC1L
4047	ENSG00000160285	3176.81201	-0.14053689	0.09237455	0.11606822	0.27838412	LSS
7410	ENSG00000160293	1241.53625	-0.66780328	0.08695037	4.05E-15	1.12E-13	VAV2
8888	ENSG00000160294	3109.05491	0.12941246	0.06409204	0.04040925	0.12635452	MCM3AP
54058	ENSG00000160298	1027.40214	0.34264932	0.09605519	0.00022243	0.00156268	C21orf58
5116	ENSG00000160299	2182.72775	0.19420919	0.08246948	0.01578823	0.060111	PCNT
23181	ENSG00000160305	1445.57794	0.09339667	0.07808822	0.22195499	0.43502894	DIP2A
6285	ENSG00000160307	6.49096972	-0.14296852	0.38946892	0.31177613	0.53952362	S100B
3275	ENSG00000160310	1716.14842	0.02495663	0.07053148	0.71952715	0.86580657	PRMT2
125875	ENSG00000160318	3.36051433	-0.02625494	0.37211443	0.78877202	0.90411045	CLDND2
7757	ENSG00000160321	136.180962	-0.89131515	0.26322983	9.18E-05	0.00070511	ZNF208
11093	ENSG00000160323	564.532031	0.79225384	0.1227806	2.09E-11	4.29E-10	ADAMTS13
11094	ENSG00000160325	754.723805	0.34291972	0.10448467	0.0006273	0.00393597	CACFD1
11182	ENSG00000160326	485.939937	-0.32259298	0.13085168	0.00851363	0.03632481	SLC2A6
388561	ENSG00000160336	311.686603	0.15184415	0.14216968	0.24993876	0.46971535	ZNF761
110116772	ENSG00000160336	311.686603	0.15184415	0.14216968	0.24993876	0.46971535	ZNF761
2220	ENSG00000160339	0	NA	NA	NA	NA	FCN2
138162	ENSG00000160345	37.7893717	0.81069308	0.44730459	0.00791795	0.03421739	C9orf116

3933	ENSG00000160349	0	NA	NA	NA	NA	LCN1
148206	ENSG00000160352	1125.28826	-0.37636669	0.09471551	3.95E-05	0.00033274	ZNF714
26086	ENSG00000160360	2663.12004	-0.3098862	0.06833514	3.89E-06	3.99E-05	GPSM1
126526	ENSG00000160392	387.646087	-0.01192705	0.12648885	0.9197282	0.96608468	C19orf47
147746	ENSG00000160396	3.38466066	0.06503111	0.37432309	0.56269276	0.7656036	HIPK4
286207	ENSG00000160401	34.8933453	0.10152007	0.30742365	0.59617226	0.78832911	CFAP157
27433	ENSG00000160404	322.187713	0.19404588	0.14295876	0.14247514	0.3212532	TOR2A
30815	ENSG00000160408	612.409493	0.10454851	0.10724597	0.30986569	0.53771847	ST6GALNAC6
92799	ENSG00000160410	866.96294	0.31984161	0.09364357	0.00041624	0.00274175	SHKBP1
112724	ENSG00000160439	374.787296	-0.07136675	0.14714383	0.59799386	0.78960741	RDH13
10444	ENSG00000160445	824.323679	0.18640298	0.09404634	0.04065337	0.12691785	ZER1
84885	ENSG00000160446	366.040621	0.25463633	0.12987348	0.03668522	0.11767741	ZDHHC12
29941	ENSG00000160447	526.005527	-0.06675154	0.1088921	0.52179415	0.73509876	PKN3
57731	ENSG00000160460	637.10812	-1.77641433	0.11797969	2.26E-52	2.39E-50	SPTBN4
84446	ENSG00000160469	1687.97264	-0.13609108	0.07308601	0.05752058	0.16582509	BRSK1
125965	ENSG00000160471	4.07397354	-0.0972237	0.38393493	0.37450016	0.60363012	COX6B2
147744	ENSG00000160472	0.31807308	0.00101207	0.38336578	0.96737941	NA	TMEM190
147945	ENSG00000160505	0.52119901	0.02576573	0.38568143	0.51723676	NA	NLRP4
84814	ENSG00000160539	317.233174	-0.09865015	0.12891045	0.4155157	0.64244323	PLPP7
57551	ENSG00000160551	3402.01972	-0.11143727	0.0618169	0.0672863	0.18623888	TAOK1
9442	ENSG00000160563	322.590216	0.20893871	0.13437948	0.09588155	0.24171409	MED27
162989	ENSG00000160570	426.277269	0.15388203	0.11424977	0.1579364	0.34494256	DEDD2
23387	ENSG00000160584	860.612977	-0.11150191	0.08869301	0.19390553	0.39677039	SIK3
196264	ENSG00000160588	6.16348434	-0.31593454	0.55428393	0.0348688	0.1130018	MPZL3
120425	ENSG00000160593	0	NA	NA	NA	NA	JAML
284086	ENSG00000160602	76.7555261	-0.06866281	0.25024119	0.7165568	0.86359415	NEK8
116238	ENSG00000160606	45.2672398	-0.13354181	0.28491752	0.49852307	0.71624312	TLCD1
9159	ENSG00000160613	1892.12918	0.09108283	0.08525018	0.273558	0.49704244	PCSK7
6294	ENSG00000160633	3099.82554	-0.10249194	0.07140138	0.14348991	0.32277095	SAFB
917	ENSG00000160654	0	NA	NA	NA	NA	CD3G
6271	ENSG00000160678	14.9241741	0.12254502	0.33744052	0.50590128	0.72177288	S100A1
26097	ENSG00000160679	2690.16605	-0.09236316	0.0590651	0.11324469	0.27296498	CHTOP
643	ENSG00000160683	0	NA	NA	NA	NA	CXCR5
51043	ENSG00000160685	860.041277	0.40300771	0.09000139	3.98E-06	4.07E-05	ZBTB7B
80308	ENSG00000160688	1189.16778	0.19913823	0.08124036	0.01203995	0.04838417	FLAD1
6464	ENSG00000160691	9586.08119	0.4412578	0.06184655	4.66E-13	1.09E-11	SHC1
55823	ENSG00000160695	734.79319	-0.02828636	0.09857084	0.76557184	0.89220778	VPS11
79671	ENSG00000160703	353.653868	-0.11771331	0.142613	0.37253004	0.60143185	NLRX1

103	ENSG00000160710	7676.69872	-0.04675698	0.05299555	0.37215199	0.60109044	ADAR
3570	ENSG00000160712	6.26731095	0.01702473	0.37690542	0.80850702	0.91317199	IL6R
55585	ENSG00000160714	2481.60063	-0.31369307	0.06653049	1.62E-06	1.77E-05	UBE2Q1
1141	ENSG00000160716	3080.75167	-0.81722714	0.06746652	1.66E-34	1.10E-32	CHRNB2
200186	ENSG00000160741	904.081213	0.18558017	0.08814553	0.03033688	0.10125905	CRTC2
55129	ENSG00000160746	272.902754	-0.08607981	0.14429345	0.51853378	0.73226139	ANO10
2224	ENSG00000160752	3252.75353	-0.43567679	0.08425502	1.11E-07	1.46E-06	FDPS
23623	ENSG00000160753	1083.73143	0.04512062	0.08559581	0.58877526	0.78350668	RUSC1
2630	ENSG00000160766	317.911978	0.06235718	0.1453605	0.64394799	0.82218471	GBAP1
10712	ENSG00000160767	2019.20766	0.11722453	0.07609887	0.11634649	0.27881559	FAM189B
79957	ENSG00000160781	309.56672	1.06868069	0.14607315	3.18E-14	8.17E-13	PAQR6
11243	ENSG00000160783	839.858068	0.20575542	0.08726112	0.01500074	0.0577108	PMF1
9673	ENSG00000160785	1257.00936	-0.13737517	0.08781769	0.10737285	0.26264053	SLC25A44
4000	ENSG00000160789	1883.85391	0.57151416	0.09104147	1.13E-10	2.15E-09	LMNA
1234	ENSG00000160791	0	NA	NA	NA	NA	CCR5
23218	ENSG00000160796	1521.24087	0.52805228	0.10239815	9.23E-08	1.22E-06	NBEAL2
151903	ENSG00000160799	474.575775	-0.05330602	0.12832648	0.65837039	0.83060337	CCDC12
5745	ENSG00000160801	13.5329399	-0.36029491	0.52186776	0.07994083	0.21113234	PTH1R
56893	ENSG00000160803	3542.85539	0.16868312	0.05806013	0.00327662	0.01630909	UBQLN4
4634	ENSG00000160808	3.50049941	0.11690241	0.39743939	0.22526591	0.43906825	MYL3
221908	ENSG00000160813	977.666367	-0.0554852	0.10282662	0.5746694	0.77361067	PPP1R35
54865	ENSG00000160818	1624.74894	-0.18901998	0.09376711	0.03717938	0.11883984	GPATCH4
149499	ENSG00000160838	3.16564577	0.02620411	0.37201488	0.79443001	0.90579008	LRRC71
115352	ENSG00000160856	0	NA	NA	NA	NA	FCRL3
563	ENSG00000160862	3.28301578	-0.17049018	0.42574685	0.10970541	0.26657138	AZGP1
2264	ENSG00000160867	197.878715	-0.1003218	0.15635774	0.48136179	0.7022219	FGFR4
1576	ENSG00000160868	0.18922458	0.01954053	0.38488496	0.78225479	NA	CYP3A4
1551	ENSG00000160870	0.80657935	-0.01800035	0.38258291	0.78501635	NA	CYP3A7
112939	ENSG00000160877	1740.40738	-0.05186891	0.08115193	0.51244446	0.72761026	NACC1
1584	ENSG00000160882	0	NA	NA	NA	NA	CYP11B1
3101	ENSG00000160883	2.39291242	-0.14839312	0.42043244	0.0573652	NA	HK3
54742	ENSG00000160886	0.33471731	0.02354391	0.38534154	0.62842204	NA	LY6K
9592	ENSG00000160888	4119.64145	1.40499392	0.07029792	6.37E-90	1.64E-87	IER2
84124	ENSG00000160908	728.063276	0.07603178	0.09643699	0.4157269	0.64256946	ZNF394
10898	ENSG00000160917	1457.13813	0.06726212	0.07204268	0.34203586	0.57202996	CPSF4
4061	ENSG00000160932	895.468332	-0.46863472	0.08996462	8.23E-08	1.10E-06	LY6E
51160	ENSG00000160948	1067.80928	0.0930513	0.08356178	0.25412487	0.47465989	VPS28
4796	ENSG00000160949	1380.95305	-0.28022727	0.0734836	9.88E-05	0.00075342	TONSL

5731	ENSG00000160951	41.9492801	-0.00677873	0.27735524	0.96979239	0.98858357	PTGER1
84939	ENSG00000160953	2003.583	0.32216737	0.06834566	1.61E-06	1.76E-05	PWWP3A
9401	ENSG00000160957	1638.37829	-0.17236325	0.07723411	0.02246542	0.07996302	RECQL4
9684	ENSG00000160959	822.326571	0.0920161	0.10477576	0.36161968	0.59140575	LRRC14
84449	ENSG00000160961	554.987953	0.18789464	0.1209076	0.10098914	0.25116589	ZNF333
136227	ENSG00000160963	150.674824	0.1764048	0.18279524	0.27326831	0.49676945	COL26A1
84988	ENSG00000160972	923.579149	0.16639164	0.08986918	0.05647959	0.16351308	PPP1R16A
8928	ENSG00000160973	3.42630868	-0.00161702	0.36851843	0.98601818	0.99524831	FOXH1
80228	ENSG00000160991	3828.42885	-0.20809801	0.05860348	0.00032021	0.00216972	ORAI2
54784	ENSG00000160993	447.148383	-0.09419075	0.12392879	0.42109653	0.64708816	ALKBH4
126402	ENSG00000160994	0	NA	NA	NA	NA	CCDC105
10603	ENSG00000160999	328.891258	-0.36654343	0.13866044	0.00451347	0.02134509	SH2B2
51149	ENSG00000161010	888.505954	0.27611763	0.10966875	0.00840698	0.03597811	MRNIP
8878	ENSG00000161011	1407.14321	0.16574813	0.10066322	0.08761432	0.22613087	SQSTM1
11282	ENSG00000161013	1680.53956	0.06573092	0.08852666	0.44588093	0.67028199	MGAT4B
6132	ENSG00000161016	29172.7682	-0.06345771	0.04658092	0.16729347	0.35828164	RPL8
9794	ENSG00000161021	1491.16769	-0.06619767	0.12611476	0.57580663	0.7744963	MAML1
114770	ENSG00000161031	0	NA	NA	NA	NA	PGLYRP2
222229	ENSG00000161036	1576.39915	-0.0146562	0.08012757	0.8565181	0.93595581	LRWD1
222235	ENSG00000161040	10.2925309	0.13072192	0.3796058	0.37596336	0.60500552	FBXL13
222236	ENSG00000161048	441.852174	-0.34315662	0.14301527	0.00953974	0.03991321	NAPEPLD
92304	ENSG00000161055	0.173124	-0.01911262	0.38486262	0.84537521	NA	SCGB3A1
5701	ENSG00000161057	1495.93208	0.00860395	0.121759	0.94492034	0.97756908	PSMC2
60680	ENSG00000161082	794.819449	-0.6535394	0.15241923	4.45E-06	4.51E-05	CELF5
126321	ENSG00000161091	923.272096	0.16028731	0.09664456	0.08629315	0.22368122	MFSD12
102725072	ENSG00000161103	10.0835096	0.28641281	0.5186988	0.03453315	0.11218738	
NA	ENSG00000161132	0.47253961	-0.00769265	0.38324381	0.86091375	NA	
373856	ENSG00000161133	0	NA	NA	NA	NA	USP41
113691	ENSG00000161149	192.208888	-0.35195659	0.16464392	0.01806213	0.067119	TUBA3FP
150223	ENSG00000161179	555.081119	-0.18229068	0.10894909	0.07985526	0.2109906	YDJC
164592	ENSG00000161180	7.37950909	0.17989093	0.40709066	0.22607046	0.44033358	CCDC116
1857	ENSG00000161202	4282.10213	0.26427609	0.05778819	3.41E-06	3.51E-05	DVL3
1173	ENSG00000161203	4287.80655	-0.23765435	0.05366325	7.56E-06	7.34E-05	AP2M1
55324	ENSG00000161204	727.903071	-0.00124465	0.11653216	0.9885318	0.99604194	ABCF3
5130	ENSG00000161217	894.758949	0.10016534	0.09968539	0.29831426	0.52511356	PCYT1A
126433	ENSG00000161243	246.797214	0.12015985	0.21462838	0.501409	0.71841948	FBXO27
93099	ENSG00000161249	0	NA	NA	NA	NA	DMKN
199746	ENSG00000161265	153.174348	0.05816958	0.18061709	0.71645602	0.86352515	U2AF1L4

622	ENSG00000161267	530.845076	0.00131999	0.1149729	0.99126933	0.9971497	BDH1
4868	ENSG00000161270	3.28454602	0.01890749	0.37740587	0.79380374	0.90579008	NPHS1
199745	ENSG00000161277	114.783913	-0.21946791	0.2202453	0.22777914	0.44270325	THAP8
1346	ENSG00000161281	0.69493202	0.03154416	0.38626309	0.41391645	NA	COX7A1
84911	ENSG00000161298	395.175987	-0.43044171	0.14208109	0.00111385	0.00648629	ZNF382
115399	ENSG00000161328	59.1249395	0.36393557	0.29380625	0.0930156	0.2361092	LRRC56
57125	ENSG00000161381	40.2861237	0.79395764	0.59023959	0.0145254	0.05614337	PLXDC1
93210	ENSG00000161395	271.937562	0.43925088	0.15217684	0.00173226	0.00947824	PGAP3
22806	ENSG00000161405	0	NA	NA	NA	NA	IKZF3
2905	ENSG00000161509	19.9771784	0.56766257	0.55909715	0.04105735	0.12797767	GRIN2C
2232	ENSG00000161513	344.849728	0.17867792	0.13589985	0.15872207	0.34601096	FDXR
29115	ENSG00000161526	1601.50312	0.14971411	0.09625531	0.10785359	0.26339391	SAP30BP
51	ENSG00000161533	750.892404	-0.01847915	0.1014894	0.8496324	0.9315671	ACOX1
5635	ENSG00000161542	1222.78233	0.09456469	0.07814989	0.21664419	0.42772621	PRPSAP1
114757	ENSG00000161544	1280.52549	-0.86923824	0.09103076	2.18E-22	9.14E-21	CYGB
6427	ENSG00000161547	9329.5588	0.14340656	0.05318345	0.00678021	0.03010054	SRSF2
84765	ENSG00000161551	180.89008	-0.01857013	0.21096375	0.91434414	0.96378282	ZNF577
55260	ENSG00000161558	249.079023	0.64210446	0.16374167	2.25E-05	0.00019993	TMEM143
317719	ENSG00000161594	0.89486476	-0.03580092	0.38549223	0.44826384	NA	KLHL10
147872	ENSG00000161609	0	NA	NA	NA	NA	KASH5
3060	ENSG00000161610	0	NA	NA	NA	NA	HCRT
126133	ENSG00000161618	554.656889	-0.19505205	0.11308151	0.07018247	0.1919256	ALDH16A1
117159	ENSG00000161634	0	NA	NA	NA	NA	DCD
3678	ENSG00000161638	3.51330291	-0.0584799	0.38185938	0.46913186	0.69153662	ITGA5
114132	ENSG00000161640	0	NA	NA	NA	NA	SIGLEC11
25946	ENSG00000161642	721.990841	0.3158978	0.10520534	0.00174721	0.00954158	ZNF385A
400709	ENSG00000161643	3.36460558	-0.0129914	0.36843743	0.90195347	0.95687554	SIGLEC16
4356	ENSG00000161647	303.506096	-0.67898526	0.1473527	9.51E-07	1.08E-05	MPP3
146894	ENSG00000161649	0	NA	NA	NA	NA	CD300LG
126123	ENSG00000161652	0	NA	NA	NA	NA	IZUMO2
162417	ENSG00000161653	7.44165696	0.02683749	0.35624135	0.84704966	0.93052693	NAGS
124801	ENSG00000161654	724.171914	-0.26472807	0.10254734	0.00720415	0.0316592	LSM12
92591	ENSG00000161664	55.3533675	0.32750928	0.28778326	0.1234786	0.29112482	ASB16
284361	ENSG00000161671	3005.40692	-0.05487793	0.05798361	0.33793547	0.56821327	EMC10
126119	ENSG00000161677	254.208893	0.53661114	0.15616275	0.00019888	0.0014158	JOSD2
50944	ENSG00000161681	1082.35819	-2.33579582	0.15153167	5.15E-55	5.94E-53	SHANK1
284069	ENSG00000161682	868.511912	-0.12898327	0.09489908	0.15949409	0.34700764	FAM171A2
80174	ENSG00000161692	543.181304	0.11130205	0.11665084	0.31668785	0.54452953	DBF4B

113026	ENSG00000161714	191.829195	0.04313135	0.24100294	0.81989479	0.91788244	PLCD3
91010	ENSG00000161791	943.448968	-0.04941685	0.08648614	0.5569314	0.76235124	FMNL3
362	ENSG00000161798	6.86751148	0.19713175	0.41882748	0.19028783	0.39162973	AQP5
29127	ENSG00000161800	2541.36808	-0.23579703	0.06044675	7.64E-05	0.00059936	RACGAP1
125962	ENSG00000161807	0	NA	NA	NA	NA	OR7G1
113251	ENSG00000161813	1366.31227	-0.27421822	0.11239208	0.01041377	0.04280283	LARP4
160622	ENSG00000161835	57.361454	0.0586027	0.29224665	0.76134842	0.88963735	TAMALIN
125950	ENSG00000161847	1597.0023	0.11727874	0.07678066	0.11892536	0.28328481	RAVER1
3890	ENSG00000161849	0	NA	NA	NA	NA	KRT84
3888	ENSG00000161850	0	NA	NA	NA	NA	KRT82
256126	ENSG00000161860	18.9960999	0.01636775	0.34176387	0.91862394	0.96558446	SYCE2
147841	ENSG00000161888	583.724349	0.00211672	0.11761218	0.9857226	0.99508557	SPC24
117283	ENSG00000161896	0.32239579	-0.02325345	0.38531858	0.66966207	NA	IP6K3
221496	ENSG00000161904	1336.28971	0.06584985	0.08413165	0.42217462	0.64814241	LEMD2
246	ENSG00000161905	0.173124	-0.01911262	0.38486262	0.84537521	NA	ALOX15
340205	ENSG00000161911	0	NA	NA	NA	NA	TREML1
221442	ENSG00000161912	14.5523736	0.12086394	0.34289514	0.50233738	0.7191395	ADCY10P1
115950	ENSG00000161914	244.794873	0.53826328	0.16274549	0.00031323	0.00212898	ZNF653
400569	ENSG00000161920	112.367391	0.28644509	0.21172153	0.1091824	0.26566224	MED11
58191	ENSG00000161921	6.87625574	0.2937282	0.51462206	0.060145	0.17157108	CXCL16
388325	ENSG00000161929	2.39776484	0.05639652	0.38069187	0.5126686	NA	SCIMP
NA	ENSG00000161939	43.3854256	0.14476068	0.31311439	0.46303524	0.68582451	RNASEK-C17orf49
255877	ENSG00000161940	6.22776568	-0.02000596	0.35514309	0.88309541	0.94857242	BCL6B
433	ENSG00000161944	0	NA	NA	NA	NA	ASGR2
8741	ENSG00000161955	54.6406021	0.10730597	0.29007552	0.58447559	0.7806123	TNFSF13
26168	ENSG00000161956	1648.25111	0.02160799	0.0693619	0.75263986	0.88425267	SENP3
2256	ENSG00000161958	220.450337	0.21828527	0.17298511	0.1582569	0.3453764	FGF11
1973	ENSG00000161960	18406.9446	-0.09451516	0.05815894	0.09997674	0.24918825	EIF4A1
6154	ENSG00000161970	13502.7898	-0.10761748	0.05283575	0.03759456	0.11981944	RPL26
146849	ENSG00000161973	0	NA	NA	NA	NA	CCDC42
51728	ENSG00000161980	456.405946	-0.0548809	0.11755375	0.62295785	0.80754549	POLR3K
79622	ENSG00000161981	493.53283	0.04830037	0.12154514	0.67602333	0.84148271	SNRNP25
146325	ENSG00000161992	2.30080806	0.02731772	0.3762151	0.75141889	NA	PRR35
197335	ENSG00000161996	1358.76898	0.47071092	0.07786879	6.62E-10	1.17E-08	WDR90
339123	ENSG00000161999	1236.79334	0.16246122	0.0909775	0.06588785	0.18349451	JMJD8
124093	ENSG00000162004	244.291013	0.21312823	0.18001635	0.1803305	0.37720159	CCDC78
NA	ENSG00000162006	0	NA	NA	NA	NA	MSLNL
6755	ENSG00000162009	0	NA	NA	NA	NA	SSTR5

90864	ENSG00000162032	673.758071	0.35322235	0.11048931	0.00082203	0.0049857	SPSB3
254528	ENSG00000162039	14.2910806	-0.23296875	0.40321429	0.21277851	0.42263393	MEIOB
64711	ENSG00000162040	0.29863895	-0.02292439	0.3852726	0.68753245	NA	HS3ST6
80178	ENSG00000162062	718.523539	0.09127797	0.10153291	0.35140808	0.58156116	TEDC2
899	ENSG00000162063	1128.01901	-0.50102833	0.08525079	1.66E-09	2.78E-08	CCNF
57465	ENSG00000162065	1217.35571	-0.13726783	0.07770714	0.07080321	0.19311736	TBC1D24
51005	ENSG00000162066	522.428028	0.19463506	0.11700055	0.0800895	0.21143808	AMDHD2
4917	ENSG00000162068	206.818508	-0.11266086	0.15340268	0.42197645	0.64793845	NTN3
146439	ENSG00000162069	0	NA	NA	NA	NA	BICDL2
124222	ENSG00000162073	1271.84159	-0.75797737	0.09260612	5.67E-17	1.74E-15	PAQR4
114984	ENSG00000162076	272.19473	-0.0245778	0.14958365	0.85730209	0.93625109	FLYWCH2
124220	ENSG00000162078	0	NA	NA	NA	NA	ZG16B
7627	ENSG00000162086	405.177954	-0.29022102	0.13908655	0.02461934	0.08611458	ZNF75A
115	ENSG00000162104	726.006066	-0.63520428	0.10396167	2.68E-10	4.93E-09	ADCY9
22941	ENSG00000162105	263.564169	-2.72340387	0.1814619	2.99E-52	3.11E-50	SHANK2
81570	ENSG00000162129	1421.62695	-0.08188416	0.0838602	0.3165391	0.54452953	CLPB
10825	ENSG00000162139	670.367842	-0.22706507	0.13527637	0.07207324	0.19555978	NEU3
220002	ENSG00000162144	894.170827	0.34705851	0.09900517	0.00027584	0.00189564	CYB561A3
220004	ENSG00000162148	32.4489353	0.57390886	0.53577381	0.03900475	0.12312718	PPP1R32
80150	ENSG00000162174	570.973268	-0.1080778	0.12024997	0.34255802	0.5727584	ASRGL1
2785	ENSG00000162188	9.51935208	-0.09281921	0.3593418	0.54541291	0.75298369	GNG3
51035	ENSG00000162191	2377.84118	0.14953155	0.06587784	0.02116596	0.07622857	UBXN1
79081	ENSG00000162194	182.50798	0.0573532	0.17119045	0.71186167	0.86154673	LBHD1
283237	ENSG00000162222	284.171798	-0.0025463	0.14417468	0.98374816	0.99447485	TTC9C
10629	ENSG00000162227	367.855173	-0.08063576	0.16519402	0.587506	0.78256674	TAF6L
10482	ENSG00000162231	1167.44141	0.22125772	0.09881654	0.02025942	0.07364363	NXF1
6811	ENSG00000162236	450.941016	0.26127332	0.11627754	0.01821844	0.06741028	STX5
283130	ENSG00000162241	169.136252	0.54272008	0.19303281	0.0015631	0.00869165	SLC25A45
6159	ENSG00000162244	16652.407	-0.04920276	0.0488517	0.30711656	0.53467818	RPL29
3699	ENSG00000162267	0	NA	NA	NA	NA	ITIH3
84447	ENSG00000162298	1182.60099	0.33683087	0.09790795	0.00036451	0.00243659	SYVN1
7542	ENSG00000162300	289.180097	0.29304802	0.15442049	0.03795527	0.12067829	ZFPL1
8986	ENSG00000162302	295.5288	0.64904405	0.19375819	0.00019045	0.00136163	RPS6KA4
4041	ENSG00000162337	2823.8517	-0.05716403	0.06171013	0.34970464	0.58009871	LRP5
219931	ENSG00000162341	332.676088	0.10340683	0.13559224	0.41402876	0.64129366	TPCN2
9965	ENSG00000162344	39.7394393	0.63804841	0.3832638	0.01692736	0.06372655	FGF19
284541	ENSG00000162365	0	NA	NA	NA	NA	CYP4A22
10158	ENSG00000162366	0	NA	NA	NA	NA	PDZK1IP1

6886	ENSG00000162367	0	NA	NA	NA	NA	TAL1
51727	ENSG00000162368	941.54762	-0.20529589	0.09666133	0.02729948	0.09320627	CMPK1
79656	ENSG00000162373	181.929691	-0.15373907	0.17320456	0.31864448	0.54631061	BEND5
1996	ENSG00000162374	3175.99639	-1.26277919	0.0627298	3.98E-91	1.07E-88	ELAVL4
65260	ENSG00000162377	739.627183	-0.3025216	0.10485308	0.00260915	0.01345957	COA7
79699	ENSG00000162378	1041.01853	-0.38641202	0.08619626	4.04E-06	4.13E-05	ZYG11B
6512	ENSG00000162383	0.96756134	0.02449976	0.38147848	0.67780217	NA	SLC1A7
54987	ENSG00000162384	621.653194	0.11334512	0.10100943	0.24466192	0.46339612	CZIB
4116	ENSG00000162385	567.293229	0.45933983	0.1055371	5.99E-06	5.94E-05	MAGOH
26027	ENSG00000162390	321.06173	0.05807595	0.14910944	0.67342525	0.83953925	ACOT11
338094	ENSG00000162391	0.51364586	0.01490651	0.38308195	0.74300609	NA	FAM151A
25973	ENSG00000162396	106.380867	-0.05773133	0.20824354	0.74051497	0.87742184	PARS2
163747	ENSG00000162398	0	NA	NA	NA	NA	LEXM
7809	ENSG00000162399	0.50207596	0.02562368	0.38565771	0.52549886	NA	BSND
23358	ENSG00000162402	2372.13813	0.06387023	0.12204557	0.58254517	0.77929311	USP24
8613	ENSG00000162407	2591.09758	-0.69057512	0.06945389	6.65E-24	3.04E-22	PLPP3
79707	ENSG00000162408	456.477265	-0.43884464	0.16637146	0.00357022	0.01757647	NOL9
5563	ENSG00000162409	884.901653	-0.39684566	0.08680649	2.59E-06	2.73E-05	PRKAA2
9903	ENSG00000162413	471.98262	-0.09863194	0.13341777	0.42769343	0.65358066	KLHL21
57643	ENSG00000162415	395.259321	-0.48860461	0.12706247	4.76E-05	0.00039207	ZSWIM5
10691	ENSG00000162419	424.20645	-0.28913588	0.12618267	0.01483534	0.05716317	GMEB1
50651	ENSG00000162426	80.3717353	-0.38439851	0.29906306	0.07895157	0.20949568	SLC45A1
57190	ENSG00000162430	3308.04414	-0.17062316	0.06515026	0.00786206	0.03402033	SELENON
205	ENSG00000162433	376.34134	0.1914723	0.1859131	0.23995367	0.4579339	AK4
3716	ENSG00000162434	2482.28069	-0.44545384	0.07220615	3.27E-10	5.98E-09	JAK1
55225	ENSG00000162437	1669.09375	-0.04220091	0.07538272	0.56744167	0.76896903	RAVER2
11330	ENSG00000162438	2.80162734	-0.01483476	0.37616812	0.84515806	0.92965052	CTRC
84328	ENSG00000162441	845.4091	-0.35459344	0.10364227	0.00036335	0.0024305	LZIC
116362	ENSG00000162444	5.60388216	0.57573156	1.38685265	0.01025959	0.04230054	RBP7
148930	ENSG00000162456	0	NA	NA	NA	NA	KNCN
54751	ENSG00000162458	2099.10153	0.46320559	0.07946061	2.49E-09	4.06E-08	FBLIM1
388595	ENSG00000162460	0.67240293	0.02556207	0.38355592	0.59055641	NA	TMEM82
284723	ENSG00000162461	85.5719436	0.37580054	0.28052315	0.07627938	0.20395995	SLC25A34
22977	ENSG00000162482	26.4656297	0.21866462	0.3355163	0.28993615	0.51590433	AKR7A3
374946	ENSG00000162490	80.8601923	-0.80025257	0.27248212	0.00051337	0.00330154	DRAXIN
10630	ENSG00000162493	0	NA	NA	NA	NA	PDPN
126755	ENSG00000162494	0	NA	NA	NA	NA	LRRC38
9249	ENSG00000162496	41.2006269	2.04547748	0.4272004	1.10E-07	1.44E-06	DHRS3

4146	ENSG00000162510	5.91789012	-0.13600623	0.39613792	0.26500355	0.48718221	MATN1
7805	ENSG00000162511	0.64286122	-0.04587562	0.38767119	0.36615328	NA	LAPTM5
9672	ENSG00000162512	1430.85064	0.02733848	0.07953575	0.72601841	0.86899144	SDC3
553115	ENSG00000162517	827.565537	0.24046619	0.08861353	0.00512895	0.02373651	PEF1
81493	ENSG00000162520	18.052452	-0.03332615	0.32476529	0.84835833	0.93099338	SYNC
5928	ENSG00000162521	5550.12153	-0.12387974	0.05610324	0.0228239	0.08099199	RBBP4
57648	ENSG00000162522	856.328986	-0.08941723	0.12537602	0.44832083	0.67234398	KIAA1522
81629	ENSG00000162526	51.9031474	0.28965873	0.30862091	0.17679588	0.37159566	TSSK3
255104	ENSG00000162542	65.4912099	1.45182861	0.3667106	5.52E-06	5.51E-05	TMCO4
127733	ENSG00000162543	13.3295657	2.77303391	0.87561126	7.99E-05	0.00062227	UBXN10
55450	ENSG00000162545	658.807448	-0.49078034	0.10591679	1.45E-06	1.59E-05	CAMK2N1
249	ENSG00000162551	118.377604	-0.46455314	0.31601794	0.04277137	0.13221938	ALPL
54361	ENSG00000162552	12.3255888	-1.46636562	0.86258921	0.00380148	0.01846752	WNT4
254173	ENSG00000162571	0	NA	NA	NA	NA	TTLL10
6339	ENSG00000162572	138.736894	0.14670659	0.35430493	0.4171477	0.64356223	SCNN1D
54587	ENSG00000162576	139.355201	0.29487955	0.26669008	0.14716828	0.32869736	MXRA8
199990	ENSG00000162585	1157.45014	0.04716214	0.08686683	0.57829939	0.77638786	FAAP20
1953	ENSG00000162591	599.320396	1.08198875	0.15640344	5.25E-13	1.22E-11	MEGF6
148870	ENSG00000162592	1.90316167	0.10396118	0.39730915	0.20083214	NA	CCDC27
149233	ENSG00000162594	0	NA	NA	NA	NA	IL23R
9077	ENSG00000162595	22.3909994	-0.34015426	0.39874543	0.13203682	0.30496778	DIRAS3
127795	ENSG00000162598	0	NA	NA	NA	NA	C1orf87
4774	ENSG00000162599	653.202607	0.73696432	0.10829476	2.16E-12	4.82E-11	NFIA
115209	ENSG00000162600	130.097135	-0.16381971	0.19589623	0.33077325	0.55960955	OMA1
114803	ENSG00000162601	1530.99996	0.20472766	0.12894728	0.0913191	0.2332352	MYSM1
83941	ENSG00000162604	352.927155	-0.08938356	0.12376206	0.44349464	0.66851038	TM2D1
7398	ENSG00000162607	1609.64602	-0.519902	0.08206799	8.87E-11	1.72E-09	USP1
8880	ENSG00000162613	4621.87785	0.04148209	0.0730225	0.56355464	0.76627833	FUBP1
91624	ENSG00000162614	28.6085747	1.56976117	0.52364851	0.00015369	0.00111935	NEXN
11080	ENSG00000162616	195.613893	0.30777948	0.1752251	0.04871498	0.14601982	DNAJB4
64123	ENSG00000162618	0.16735865	0.01897177	0.38482933	0.78225479	NA	ADGRL4
127255	ENSG00000162620	33.472964	-0.26930068	0.35935565	0.2042199	0.41088053	LRRIQ3
105378803	ENSG00000162621	0.14931947	-0.01844332	0.38480212	0.84537521	NA	LRRC53
127253	ENSG00000162623	469.04904	-0.37436897	0.11318076	0.00051977	0.00333728	TYW3
431707	ENSG00000162624	1.99137393	0.13373244	0.4169259	0.02804919	NA	LHX8
51375	ENSG00000162627	666.065408	-0.01321253	0.11199518	0.90093828	0.95641534	SNX7
8707	ENSG00000162630	26.4581776	2.45504057	0.52075397	1.52E-07	1.97E-06	B3GALT2
22854	ENSG00000162631	16.0107721	0.04462095	0.32141468	0.80526883	0.91169445	NTNG1

284611	ENSG00000162636	600.259791	-0.3154394	0.10850621	0.00235394	0.012316	FAM102B
113802	ENSG00000162639	0	NA	NA	NA	NA	HENMT1
254268	ENSG00000162641	10.4484345	0.00383372	0.36061284	0.9781671	0.99252469	AKNAD1
148423	ENSG00000162642	529.99873	0.07376044	0.12266251	0.5259912	0.73808697	C1orf52
126820	ENSG00000162643	0	NA	NA	NA	NA	DNAI3
2634	ENSG00000162645	1.3405357	0.06490519	0.38782687	0.32006197	NA	GBP2
127002	ENSG00000162650	326.159164	-0.0067168	0.13451137	0.95649458	0.9821879	ATXN7L2
115361	ENSG00000162654	1.44393847	-0.02208191	0.37948064	0.74740963	NA	GBP4
284695	ENSG00000162664	1383.57415	0.04475409	0.0764093	0.55077691	0.75727932	ZNF326
164045	ENSG00000162669	132.065724	0.05479074	0.20978521	0.75699188	0.88697677	HFM1
339479	ENSG00000162670	0.80195464	0.05170262	0.38861068	0.28387399	NA	BRINP3
2672	ENSG00000162676	2.16643338	0.09140788	0.39133367	0.28672559	NA	GFI1
NA	ENSG00000162685	0	NA	NA	NA	NA	LSP1P3
343450	ENSG00000162687	741.737596	0.32567701	0.09243392	0.00027463	0.00188992	KCNT2
178	ENSG00000162688	891.181089	0.02823624	0.09642328	0.76306122	0.89079933	AGL
7412	ENSG00000162692	0	NA	NA	NA	NA	VCAM1
2135	ENSG00000162694	1232.87667	-0.09373078	0.08301697	0.24655249	0.46577702	EXTL2
148867	ENSG00000162695	572.012899	-0.15859783	0.1094187	0.12835793	0.29903961	SLC30A7
NA	ENSG00000162699	0	NA	NA	NA	NA	DNAJA1P5
23528	ENSG00000162702	1041.43358	-0.22183646	0.08525216	0.00748751	0.03268473	ZNF281
10092	ENSG00000162704	2571.48347	-0.1190581	0.06260055	0.05367782	0.15715539	ARPC5
57863	ENSG00000162706	50.2456642	-3.77803608	0.46955937	7.22E-17	2.21E-15	CADM3
114548	ENSG00000162711	0.51805479	-0.0147833	0.38308519	0.75798034	NA	NLRP3
84838	ENSG00000162714	2447.74201	-0.03708988	0.06992519	0.58889761	0.78351083	ZNF496
25893	ENSG00000162722	0	NA	NA	NA	NA	TRIM58
89886	ENSG00000162723	0.173124	-0.01911262	0.38486262	0.84537521	NA	SLAMF9
127059	ENSG00000162727	0	NA	NA	NA	NA	OR2M5
3765	ENSG00000162728	193.393514	-3.51794757	0.2227838	3.24E-57	3.90E-55	KCNJ9
93185	ENSG00000162729	1039.05247	-0.00528316	0.0846201	0.94823301	0.97876335	IGSF8
4921	ENSG00000162733	5160.87656	0.21937034	0.18406308	0.17554782	0.36969203	DDR2
8682	ENSG00000162734	2932.64192	-0.53270897	0.06140709	1.50E-18	5.23E-17	PEA15
5824	ENSG00000162735	1490.1104	0.11938394	0.11153582	0.26320838	0.48523083	PEX19
23385	ENSG00000162736	1882.20656	0.06550996	0.08846817	0.44843412	0.67234398	NCSTN
57216	ENSG00000162738	2711.66218	0.02519322	0.06047496	0.67511347	0.84091366	VANGL2
114836	ENSG00000162739	0	NA	NA	NA	NA	SLAMF6
25903	ENSG00000162745	32.4292096	1.03365312	0.47974099	0.00254819	0.01318971	OLFML2B
127943	ENSG00000162746	2.92173933	-0.09572563	0.38611335	0.36366137	0.59353748	FCRLB
2215	ENSG00000162747	0	NA	NA	NA	NA	FCGR3B

284525	ENSG00000162753	0.91378936	0.05235943	0.3890638	0.24527608	NA	SLC9C2
126823	ENSG00000162755	15.2346664	0.0485743	0.32518679	0.7855697	0.90248778	KLHDC9
148304	ENSG00000162757	350.90066	-0.35489836	0.13743246	0.00560053	0.02561423	C1orf74
4009	ENSG00000162761	0	NA	NA	NA	NA	LMX1A
440699	ENSG00000162763	0	NA	NA	NA	NA	LRRC52
28982	ENSG00000162769	1195.04641	-0.0958557	0.11868722	0.39442272	0.623633	FLVCR1
149647	ENSG00000162771	0.14931947	-0.01844332	0.38480212	0.84537521	NA	FAM71A
467	ENSG00000162772	350.308713	1.9673877	0.21157657	9.97E-22	4.05E-20	ATF3
64783	ENSG00000162775	460.465999	-0.33810297	0.12161666	0.00327805	0.01631212	RBM15
79961	ENSG00000162777	1.14317494	-0.04424769	0.38409198	0.40081813	NA	DENND2D
126859	ENSG00000162779	2.3905836	0.08296977	0.38702321	0.35554863	NA	AXDND1
163589	ENSG00000162782	6.54835896	2.46671601	1.46033191	0.00342289	0.01693099	TDRD5
51278	ENSG00000162783	874.165409	0.06160678	0.09162169	0.48923019	0.70874969	IER5
25992	ENSG00000162804	69.7706395	0.04277099	0.22391992	0.81588453	0.9163678	SNED1
10380	ENSG00000162813	639.270552	0.14052169	0.11122604	0.1863955	0.38610218	BPNT1
128153	ENSG00000162814	36.1467709	0.49427914	0.43423068	0.05393025	0.15767405	SPATA17
79762	ENSG00000162817	347.320389	0.9106851	0.14750024	1.02E-10	1.97E-09	C1orf115
148362	ENSG00000162819	812.706524	-0.22089621	0.10264119	0.02506622	0.08732798	BROX
100288142	ENSG00000162825	1876.88947	-0.29592473	0.0688102	1.19E-05	0.00011146	NBPF20
51205	ENSG00000162836	324.404023	0.26132242	0.15239101	0.06109041	0.17383343	ACP6
NA	ENSG00000162840	0	NA	NA	NA	NA	MT2P1
128025	ENSG00000162843	0.16735865	0.01897177	0.38482933	0.78225479	NA	WDR64
55083	ENSG00000162849	4666.07869	-0.89852573	0.08331679	6.43E-28	3.43E-26	KIF26B
64216	ENSG00000162851	635.690803	-0.04402934	0.09570979	0.63396857	0.81531966	TFB2M
163882	ENSG00000162852	1411.47595	0.01900067	0.09604246	0.83895408	0.92674595	CNST
129285	ENSG00000162869	250.339261	0.34858941	0.18141376	0.03005551	0.10045507	PPP1R21
55220	ENSG00000162873	13.8814635	0.28623551	0.4519845	0.13180818	0.30460971	KLHDC8A
148811	ENSG00000162877	6.34564116	-0.05041258	0.36314585	0.69548322	0.85296328	PM20D1
91461	ENSG00000162878	427.026652	0.47005998	0.12830268	0.00010406	0.00078856	PKDCC
165140	ENSG00000162881	3.11635141	-0.02422893	0.36881879	0.82174031	0.91847813	OXER1
23498	ENSG00000162882	1.29380723	0.03216309	0.38112497	0.62867729	NA	HAAO
148789	ENSG00000162885	1743.17697	-0.21828172	0.07143486	0.00182742	0.00990601	B3GALNT2
NA	ENSG00000162888	0	NA	NA	NA	NA	C1orf147
9261	ENSG00000162889	1125.91862	0.5951578	0.08296337	2.28E-13	5.41E-12	MAPKAPK2
50604	ENSG00000162891	0	NA	NA	NA	NA	IL20
11009	ENSG00000162892	0.15069912	-0.01848503	0.38480575	0.84537521	NA	IL24
9214	ENSG00000162894	4.62311937	-0.27483891	0.52420677	0.01367263	0.05356431	FCMR
5284	ENSG00000162896	0	NA	NA	NA	NA	PIGR

83953	ENSG00000162897	0	NA	NA	NA	NA	FCAMR
824	ENSG00000162909	2982.75895	0.12737458	0.06505601	0.04673155	0.14137754	CAPN2
128308	ENSG00000162910	556.266017	0.13299001	0.11314702	0.21871085	0.43056392	MRPL55
574407	ENSG00000162913	62.8121386	0.52481661	0.35111296	0.03229055	0.10634868	OBSCN-AS1
80232	ENSG00000162923	3894.57926	-0.17280762	0.06404039	0.00614498	0.02766124	WDR26
5966	ENSG00000162924	371.482068	0.03998673	0.12974652	0.74407846	0.8795065	REL
150962	ENSG00000162927	116.12764	-0.06961665	0.22650063	0.70269282	0.85699455	PUS10
5194	ENSG00000162928	446.325678	-0.02320443	0.12115935	0.83908087	0.92674595	PEX13
84542	ENSG00000162929	405.809195	0.16120756	0.15428551	0.25313597	0.47339218	KIAA1841
51127	ENSG00000162931	189.472256	0.71125996	0.17483144	1.02E-05	9.64E-05	TRIM17
130132	ENSG00000162944	19.2033821	2.91566273	0.91588677	4.45E-05	0.00036941	RFTN2
27185	ENSG00000162946	311.241844	-0.42339494	0.15827021	0.00341941	0.01691795	DISC1
150596	ENSG00000162947	0	NA	NA	NA	NA	LINC01931
92291	ENSG00000162949	22.9718157	-0.44740092	0.50227256	0.0701808	0.1919256	CAPN13
347730	ENSG00000162951	16.5280501	1.26466179	0.76314832	0.0044846	0.02124509	LRRTM1
51072	ENSG00000162959	750.29172	-0.19258533	0.1080331	0.06222017	0.17614842	MEMO1
84661	ENSG00000162961	683.155104	0.03024328	0.10104233	0.75725869	0.88697677	DPY30
129450	ENSG00000162971	340.099876	0.01777183	0.12644624	0.88188328	0.94786089	TYW5
79568	ENSG00000162972	181.401396	0.1144984	0.1648345	0.44208686	0.66680698	MAIP1
3754	ENSG00000162975	2.87043955	-0.04588572	0.37600384	0.62872183	0.81183476	KCNF1
130814	ENSG00000162976	71.6366403	0.46677272	0.31425531	0.04236429	0.13122678	SLC66A3
26225	ENSG00000162980	1123.76084	0.10654661	0.09049878	0.22601307	0.44026503	ARL5A
151354	ENSG00000162981	240.251814	0.4392347	0.1783278	0.0059224	0.02688797	LRATD1
3760	ENSG00000162989	45.4379967	-0.90073601	0.39994055	0.00262959	0.01354741	KCNJ3
4760	ENSG00000162992	130.970491	0.23027758	0.19986329	0.17945555	0.37588663	NEUROD1
130162	ENSG00000162994	196.037646	0.31967996	0.18986573	0.05412767	0.15818132	CLHC1
344405	ENSG00000162997	65.8610631	0.14427515	0.25049728	0.45467694	0.67793979	PRORSD1P
2487	ENSG00000162998	302.688012	0.38386079	0.13802781	0.00287386	0.01460438	FRZB
142679	ENSG00000162999	87.702068	-0.66631651	0.30068873	0.00506247	0.02346713	DUSP19
112942	ENSG00000163001	409.98464	0.14390189	0.12076689	0.20923687	0.41746374	CFAP36
129401	ENSG00000163002	321.223942	-0.32757627	0.14135767	0.01240764	0.04957059	NUP35
165055	ENSG00000163006	388.10475	-0.14882418	0.12354465	0.20076099	0.40598289	CCDC138
151112	ENSG00000163012	1.98379226	-0.07503058	0.38670646	0.35311588	NA	ZSWIM2
150726	ENSG00000163013	961.503419	-0.36777066	0.08855407	1.88E-05	0.00016926	FBXO41
200420	ENSG00000163016	10.0507794	0.12167893	0.36537285	0.44557406	0.67028199	ALMS1P1
72	ENSG00000163017	0.37282482	-0.01055631	0.38373802	0.9081619	NA	ACTG2
80304	ENSG00000163026	887.836933	-0.19576176	0.11199889	0.06674892	0.1852425	WDCP
79677	ENSG00000163029	1604.4946	-0.04609597	0.07681794	0.53955236	0.74863409	SMC6

7447	ENSG00000163032	141.573097	2.08758841	0.24627431	1.51E-18	5.27E-17	VSNL1
90557	ENSG00000163040	280.177705	0.14739387	0.15808971	0.3060482	0.53375494	CCDC74A
3020	ENSG00000163041	12438.1888	-0.00015524	0.0499021	0.99281031	0.99758706	H3-3A
554226	ENSG00000163046	7.10424242	0.16787258	0.40272035	0.24468436	0.46339612	ANKRD30BL
56997	ENSG00000163050	997.579021	-0.13313194	0.09873194	0.16198405	0.35042673	COQ8A
151473	ENSG00000163053	173.534612	-0.50849538	0.22496253	0.00762995	0.03318243	SLC16A14
150483	ENSG00000163060	0.29863895	-0.02292439	0.3852726	0.68753245	NA	TEKT4
2019	ENSG00000163064	0	NA	NA	NA	NA	EN1
6443	ENSG00000163069	1257.53093	-0.31594248	0.07770772	3.17E-05	0.00027109	SGCB
132671	ENSG00000163071	41.4563213	0.16488476	0.33339078	0.39708637	0.62614796	SPATA18
115677	ENSG00000163072	19.7290535	-0.02241569	0.32339221	0.89607548	0.95469109	NOSTRIN
200373	ENSG00000163075	0.3885105	0.0240703	0.38542007	0.6015462	NA	CFAP221
151278	ENSG00000163081	0	NA	NA	NA	NA	CCDC140
130367	ENSG00000163082	50.0586851	0.17867616	0.26730672	0.36944396	0.59883728	SGPP2
3625	ENSG00000163083	0.33471731	0.02354391	0.38534154	0.62842204	NA	INHBB
129446	ENSG00000163092	0	NA	NA	NA	NA	XIRP2
129880	ENSG00000163093	159.949275	0.37576742	0.19382046	0.02621608	0.09036257	BBS5
112401	ENSG00000163098	0	NA	NA	NA	NA	BIRC8
56916	ENSG00000163104	2348.21828	-0.17897993	0.0674208	0.0069307	0.03070682	SMARCAD1
27306	ENSG00000163106	0	NA	NA	NA	NA	HPGDS
10611	ENSG00000163110	1867.42125	0.27246071	0.0945884	0.00288941	0.01464636	PDLIM5
5161	ENSG00000163114	0	NA	NA	NA	NA	PDHA2
285555	ENSG00000163116	1.20108203	-0.00601984	0.37851058	0.92996282	NA	STPG2
93082	ENSG00000163121	0.173733	0.01914545	0.38484597	0.78225479	NA	NEURL3
23248	ENSG00000163125	1801.72574	-0.31234203	0.07611947	2.72E-05	0.00023675	RPRD2
200539	ENSG00000163126	111.865698	0.03527078	0.20654172	0.84089104	0.92777657	ANKRD23
1520	ENSG00000163131	4.42880755	0.15153924	0.40688703	0.20554127	0.41249633	CTSS
4487	ENSG00000163132	6.81236598	0.03088644	0.3568635	0.82087749	0.91847813	MSX1
133015	ENSG00000163138	323.140649	0.18513132	0.14183327	0.15959051	0.34717936	PACRGL
149428	ENSG00000163141	27.1894859	-1.46684594	0.67441653	0.00123681	0.00708992	BNIPL
114905	ENSG00000163145	0.64397944	0.03581381	0.38644566	0.46226837	NA	C1QTNF7
79626	ENSG00000163154	5.68146329	0.05089546	0.36051023	0.70925236	0.86048841	TNFAIP8L2
388695	ENSG00000163155	474.204549	0.02600217	0.11613958	0.81521344	0.91605316	LYSMD1
79005	ENSG00000163156	458.118701	0.18605435	0.11315164	0.08487392	0.22086812	SCNM1
100534012	ENSG00000163156	458.118701	0.18605435	0.11315164	0.08487392	0.22086812	SCNM1
29765	ENSG00000163157	9.2425594	0.21862463	0.41249217	0.20823678	0.4158149	TMOD4
6944	ENSG00000163159	1718.87005	0.08055096	0.06862957	0.23303474	0.44988944	VPS72
2071	ENSG00000163161	1854.42567	0.15233599	0.07030498	0.02750236	0.09365718	ERCC3

284996	ENSG00000163162	398.479359	0.22225457	0.11976009	0.05037129	0.14978512	RNF149
55677	ENSG00000163166	1100.19345	-0.02531868	0.08579847	0.76123672	0.88962039	IWS1
388962	ENSG00000163170	201.61628	0.06716933	0.15730437	0.64625907	0.82370422	BOLA3
10602	ENSG00000163171	1606.2504	0.00610723	0.07869926	0.93760629	0.97366414	CDC42EP3
6282	ENSG00000163191	87.609111	10.9843322	2.93735994	1.11E-16	3.37E-15	S100A11
84648	ENSG00000163202	0	NA	NA	NA	NA	LCE3D
4184	ENSG00000163206	0	NA	NA	NA	NA	SMCP
3713	ENSG00000163207	0	NA	NA	NA	NA	IVL
6707	ENSG00000163209	0	NA	NA	NA	NA	SPRR3
90957	ENSG00000163214	1249.64821	-0.02057486	0.09633362	0.83944854	0.92700985	DHX57
6703	ENSG00000163216	0	NA	NA	NA	NA	SPRR2D
27302	ENSG00000163217	0	NA	NA	NA	NA	BMP10
57115	ENSG00000163218	0	NA	NA	NA	NA	PGLYRP4
9938	ENSG00000163219	14.0709136	0.14893854	0.35227003	0.41503386	0.64209855	ARHGAP25
6280	ENSG00000163220	0	NA	NA	NA	NA	S100A9
6283	ENSG00000163221	0	NA	NA	NA	NA	S100A12
7039	ENSG00000163235	5.42758061	-0.02278587	0.36669359	0.83403599	0.92432089	TGFA
126668	ENSG00000163239	2.0793011	0.09478261	0.39228211	0.27095834	NA	TDRD10
151195	ENSG00000163249	337.291403	0.54864062	0.15097182	9.16E-05	0.00070379	CCNYL1
7855	ENSG00000163251	46.211518	-0.48494519	0.34301774	0.04334069	0.13334089	FZD5
1420	ENSG00000163254	0.52119901	0.02576573	0.38568143	0.51723676	NA	CRYGC
54876	ENSG00000163257	1411.20106	0.14516349	0.08558335	0.08171224	0.2145825	DCAF16
388701	ENSG00000163263	0.16735865	0.01897177	0.38482933	0.78225479	NA	C1orf189
4880	ENSG00000163273	0.33468635	-0.02324288	0.38531994	0.67674993	NA	NPPC
132789	ENSG00000163281	488.769171	0.18886678	0.11146989	0.07614012	0.2036974	GNPDA2
250	ENSG00000163283	0.33471731	0.02354391	0.38534154	0.62842204	NA	ALPP
2565	ENSG00000163285	0	NA	NA	NA	NA	GABRG1
251	ENSG00000163286	0	NA	NA	NA	NA	ALPG
2560	ENSG00000163288	2.71263968	0.20765234	0.46761039	0.00653964	0.02918662	GABRB1
152559	ENSG00000163291	932.342087	-0.15595904	0.09095812	0.0770427	0.20558039	PAQR3
152519	ENSG00000163293	19.5410369	0.05779417	0.32000561	0.75218127	0.88402798	NIPAL1
248	ENSG00000163295	0	NA	NA	NA	NA	ALPI
118429	ENSG00000163297	1077.81585	0.4555108	0.08443818	3.14E-08	4.42E-07	ANTXR2
113510	ENSG00000163312	190.879778	-0.20041573	0.18053053	0.20740468	0.41455862	HELQ
51023	ENSG00000163319	280.880124	0.14931248	0.14265929	0.25687536	0.47790765	MRPS18C
8545	ENSG00000163320	2463.67413	-0.25999503	0.08005928	0.00087208	0.00525421	CGGBP1
84142	ENSG00000163322	150.45336	-0.07835121	0.1800377	0.62133339	0.80672374	ABRAXAS1
151556	ENSG00000163328	377.433973	1.71218802	0.13706683	6.88E-37	4.91E-35	GPR155

92196	ENSG00000163331	0.50363226	0.01236985	0.38343365	0.76764028	NA	DAPL1
10654	ENSG00000163344	810.519117	0.10616527	0.09365357	0.24285167	0.4611137	PMVK
57326	ENSG00000163346	1149.44046	1.06640248	0.07946343	5.93E-42	4.71E-40	PBXIP1
9076	ENSG00000163347	0	NA	NA	NA	NA	CLDN1
90780	ENSG00000163348	2323.34178	-0.16549089	0.06349814	0.00825754	0.03547631	PYGO2
204851	ENSG00000163349	1284.77312	0.03784238	0.07674629	0.61596783	0.80313338	HIPK1
55891	ENSG00000163352	2.71037926	0.16420915	0.43054495	0.03724782	0.11900108	LENEP
127579	ENSG00000163354	16.1014659	0.41040612	0.55596531	0.06523828	0.1822483	DCST2
149095	ENSG00000163357	3.97696662	0.21296212	0.45117373	0.08584397	0.22274971	DCST1
1293	ENSG00000163359	9.3882341	-2.67370013	1.22614028	0.00073538	0.00451842	COL6A3
55765	ENSG00000163362	207.68932	0.34212956	0.17357366	0.02754787	0.09374783	INAVA
NA	ENSG00000163364	163.164504	-0.08068206	0.18452775	0.61742675	0.80410573	LINC01116
55249	ENSG00000163374	1352.68393	-0.15562123	0.08639441	0.06402565	0.17979744	YY1AP1
84541	ENSG00000163376	86.0719604	0.21385971	0.26087667	0.28068238	0.50533016	KBTBD8
151647	ENSG00000163377	8.58941824	-0.23455646	0.43120796	0.16703996	0.35796855	TAFA4
285203	ENSG00000163378	166.194514	-0.06700287	0.19788604	0.69101134	0.85031724	EOGT
56203	ENSG00000163380	1.17084403	0.02420981	0.37961559	0.72694653	NA	LMOD3
128240	ENSG00000163382	1982.21204	0.28329697	0.06678002	1.60E-05	0.00014598	NAXE
56983	ENSG00000163389	893.296908	0.02861992	0.08656611	0.73519233	0.87415892	POGLUT1
55356	ENSG00000163393	102.395437	0.1535761	0.23353734	0.41264109	0.64014381	SLC22A15
886	ENSG00000163394	1.04448589	0.07458384	0.39372702	0.15090449	NA	CCKAR
91156	ENSG00000163395	0.173733	0.01914545	0.38484597	0.78225479	NA	IGFN1
476	ENSG00000163399	3379.58665	0.28393721	0.05798344	7.09E-07	8.23E-06	ATP1A1
6565	ENSG00000163406	34.9507478	-0.10646899	0.34119086	0.5387595	0.74837139	SLC15A2
317649	ENSG00000163412	373.187573	0.18208713	0.12760127	0.12980826	0.30146269	EIF4E3
60675	ENSG00000163421	5.10823466	0.14387413	0.39310122	0.29400301	0.52021105	PROK2
152405	ENSG00000163424	0	NA	NA	NA	NA	TEX55
116064	ENSG00000163428	1377.03263	-0.15547029	0.08174373	0.05128432	0.15197622	LRRC58
11167	ENSG00000163430	1696.12125	1.21651086	0.1079066	1.81E-30	1.06E-28	FSTL1
25802	ENSG00000163431	20.6221793	0.30487235	0.4028423	0.15935046	0.34688536	LMOD1
1999	ENSG00000163435	11.9853519	3.48695872	0.97154564	1.65E-05	0.00015064	ELF3
132954	ENSG00000163440	1.56028331	-0.0253943	0.37981867	0.71068851	NA	PDCL2
92703	ENSG00000163444	2001.79987	0.17486447	0.07996127	0.02519038	0.08767516	TMEM183A
92691	ENSG00000163449	570.209168	-0.35388426	0.1058465	0.00048758	0.0031582	TMEM169
3490	ENSG00000163453	2.12875089	-0.07186301	0.38481615	0.40358949	NA	IGFBP7
80128	ENSG00000163462	1072.40085	0.0552525	0.0858419	0.51123066	0.72640667	TRIM46
200185	ENSG00000163463	730.97631	0.31528853	0.10850952	0.00238315	0.01244585	KRTCAP2
3577	ENSG00000163464	0	NA	NA	NA	NA	CXCR1

10109	ENSG00000163466	2853.2754	-0.17260524	0.06663144	0.00843764	0.03606263	ARPC2
128229	ENSG00000163467	3.40145801	-0.14654707	0.40731725	0.1920392	0.39417108	TSACC
7203	ENSG00000163468	8310.36984	-0.16415603	0.06219754	0.00743469	0.03248276	CCT3
84283	ENSG00000163472	262.239396	-0.02812339	0.17608627	0.85583673	0.93567436	TMEM79
6746	ENSG00000163479	7551.93836	0.17599841	0.04960791	0.00035611	0.00238527	SSR2
64320	ENSG00000163481	420.120135	-0.46427305	0.12478003	8.46E-05	0.00065457	RNF25
27148	ENSG00000163482	902.415571	0.07132184	0.11627317	0.52025278	0.73376094	STK36
134	ENSG00000163485	94.5580735	-1.18604856	0.37697437	0.00012359	0.00091838	ADORA1
152110	ENSG00000163491	5.73812682	-0.02817473	0.36344994	0.80943877	0.91359357	NEK10
285025	ENSG00000163492	0	NA	NA	NA	NA	CCDC141
54738	ENSG00000163497	1331.35367	-0.94068373	0.08678975	3.24E-28	1.75E-26	FEV
1412	ENSG00000163499	14.6181531	-0.07425561	0.33299207	0.6690143	0.83720681	CRYBA2
3549	ENSG00000163501	5.16872557	0.26836849	0.49925494	0.05595352	0.16239288	IHH
57650	ENSG00000163507	1139.31932	-0.10433285	0.10218706	0.28866662	0.51460846	CIP2A
8320	ENSG00000163508	0	NA	NA	NA	NA	EOMES
57703	ENSG00000163510	730.402522	-0.37871613	0.10098336	9.76E-05	0.00074641	CWC22
64343	ENSG00000163512	796.972312	-0.135226	0.09806808	0.15292556	0.33767871	AZI2
7048	ENSG00000163513	139.694195	0.38252743	0.19759966	0.0258314	0.08931597	TGFBR2
84666	ENSG00000163515	0	NA	NA	NA	NA	RETNLB
55139	ENSG00000163516	739.393912	0.01965801	0.09827514	0.83691979	0.92591806	ANKZF1
79885	ENSG00000163517	209.944628	0.09542314	0.17320019	0.53764311	0.7477106	HDAC11
83417	ENSG00000163518	0	NA	NA	NA	NA	FCRL4
50852	ENSG00000163519	0.15069912	-0.01848503	0.38480575	0.84537521	NA	TRAT1
2199	ENSG00000163520	546.331334	-0.17519094	0.12697487	0.14233952	0.32117652	FBLN2
79411	ENSG00000163521	95.4908893	1.15843033	0.24314745	1.94E-07	2.46E-06	GLB1L
201595	ENSG00000163527	1994.6363	0.02829681	0.09324585	0.75524703	0.88614698	STT3B
131474	ENSG00000163528	281.030303	-0.60567316	0.14528462	8.64E-06	8.30E-05	CHCHD4
151871	ENSG00000163530	0	NA	NA	NA	NA	DPPA2
23114	ENSG00000163531	1063.2287	-1.2923657	0.09355256	2.86E-44	2.46E-42	NFASC
115350	ENSG00000163534	0	NA	NA	NA	NA	FCRL1
151246	ENSG00000163535	699.497332	-0.44752624	0.10241817	5.67E-06	5.65E-05	SGO2
5274	ENSG00000163536	191.985679	0.29055317	0.1772246	0.06505057	0.18187757	SERPINI1
23122	ENSG00000163539	2804.39296	-0.4176851	0.07549298	1.60E-08	2.34E-07	CLASP2
8802	ENSG00000163541	673.430034	-0.01328671	0.11168709	0.89977873	0.95565364	SUCLG1
81788	ENSG00000163545	8.96153288	0.1041716	0.3621522	0.50387535	0.72045005	NUAK2
6708	ENSG00000163554	0	NA	NA	NA	NA	SPTA1
5584	ENSG00000163558	1413.28547	-0.11340423	0.10449689	0.25829831	0.47964541	PRKCI
4332	ENSG00000163563	0	NA	NA	NA	NA	MNDA

149628	ENSG00000163564	0	NA	NA	NA	NA	PYHIN1
3428	ENSG00000163565	0	NA	NA	NA	NA	IFI16
9447	ENSG00000163568	0	NA	NA	NA	NA	AIM2
151651	ENSG00000163576	2.701327	0.15674546	0.42682817	0.0468292	0.14160818	EFHB
56648	ENSG00000163577	97.8959436	0.11461647	0.29956727	0.55558813	0.7613563	EIF5A2
6514	ENSG00000163581	0	NA	NA	NA	NA	SLC2A2
200916	ENSG00000163584	316.360333	-0.06865439	0.14364466	0.60542014	0.79536059	RPL22L1
2168	ENSG00000163586	0	NA	NA	NA	NA	FABP1
151742	ENSG00000163590	1252.81993	-0.19502325	0.07741033	0.01000259	0.04148231	PPM1L
130026	ENSG00000163596	274.560146	-0.08780447	0.15342613	0.53112391	0.74260031	ICA1L
100507246	ENSG00000163597	3355.3833	-0.62376528	0.06331369	1.96E-23	8.80E-22	SNHG16
1493	ENSG00000163599	0	NA	NA	NA	NA	CTLA4
29851	ENSG00000163600	0	NA	NA	NA	NA	ICOS
23429	ENSG00000163602	1464.52944	0.36192133	0.07361272	5.31E-07	6.31E-06	RYBP
151987	ENSG00000163605	1371.45073	-0.12512738	0.08150763	0.11576447	0.27775826	PPP4R2
131450	ENSG00000163606	1.1902675	-0.02624359	0.3824067	0.61976493	NA	CD200R1
29083	ENSG00000163607	369.858482	0.04800822	0.11955494	0.67336125	0.83953925	GTPBP8
25871	ENSG00000163608	1104.01103	-0.26435269	0.09160307	0.00288888	0.01464636	NEPRO
152185	ENSG00000163611	383.00383	0.63396591	0.14499674	3.24E-06	3.36E-05	SPICE1
NA	ENSG00000163612	0	NA	NA	NA	NA	FAM86KP
57577	ENSG00000163617	136.579437	0.87512571	0.21848932	9.01E-06	8.62E-05	CCDC191
8618	ENSG00000163618	13.0348989	-0.0788741	0.34036895	0.64135904	0.82041028	CADPS
4825	ENSG00000163623	0	NA	NA	NA	NA	NKX6-1
1040	ENSG00000163624	2.58875482	-0.0077415	0.37403501	0.92886973	0.97019086	CDS1
23001	ENSG00000163625	1558.24183	-0.04039342	0.11590003	0.71346145	0.86190557	WDFY3
285521	ENSG00000163626	444.06727	0.26286423	0.11655724	0.01764957	0.06585808	COX18
5783	ENSG00000163629	882.933926	0.54756237	0.08982571	3.88E-10	7.03E-09	PTPN13
132204	ENSG00000163630	8.35579812	2.00179557	1.60535936	0.00479779	0.02243415	SYNPR
213	ENSG00000163631	1.40427668	0.06662381	0.39038066	0.21491866	NA	ALB
132200	ENSG00000163632	1.74956889	0.10306946	0.39945445	0.12203887	NA	C3orf49
132989	ENSG00000163633	5.36401112	0.05891279	0.36628931	0.64587241	0.82368764	C4orf36
80145	ENSG00000163634	599.428966	-0.03181293	0.09845264	0.73742831	0.87597758	THOC7
6314	ENSG00000163635	524.250583	0.14795164	0.14177176	0.26179365	0.48374648	ATXN7
9861	ENSG00000163636	1126.91822	0.10106502	0.08498729	0.22297005	0.43606299	PSMD6
166336	ENSG00000163637	10.5326744	0.17526649	0.40231057	0.233922	0.45086784	PRICKLE2
56999	ENSG00000163638	9905.27793	-0.35278298	0.05385637	3.77E-11	7.58E-10	ADAMTS9
152926	ENSG00000163644	429.27703	-0.4257959	0.13800523	0.00094146	0.00562947	PPM1K
131831	ENSG00000163645	4.25050557	0.00091418	0.36315867	0.99585839	0.99852582	ERICH6

7401	ENSG00000163646	0	NA	NA	NA	NA	CLRN1
8833	ENSG00000163655	1749.94615	-0.42307842	0.08062498	7.62E-08	1.02E-06	GMPS
25976	ENSG00000163659	188.434282	-0.08728764	0.17825451	0.57950594	0.77706744	TIPARP
57018	ENSG00000163660	1827.16348	0.25853652	0.07252101	0.00027706	0.00190266	CCNL1
5806	ENSG00000163661	22.080159	0.45923781	0.47056358	0.06855584	0.18864741	PTX3
8820	ENSG00000163666	26.9029056	0.43285977	0.41055796	0.07713679	0.20578109	HESX1
85443	ENSG00000163673	30.6416156	3.31654233	0.57286813	5.09E-10	9.13E-09	DCLK3
7871	ENSG00000163681	750.801554	-0.25713626	0.10708221	0.01212035	0.04861862	SLMAP
6133	ENSG00000163682	8668.08523	-0.1772415	0.0584261	0.00212876	0.01128963	RPL9
201895	ENSG00000163683	449.592535	0.05893502	0.1521677	0.67399883	0.84006388	SMIM14
11102	ENSG00000163684	495.530824	-0.13346818	0.15555661	0.34617051	0.57693723	RPP14
57406	ENSG00000163686	123.010858	-0.17462886	0.18889513	0.28821682	0.5139923	ABHD6
1776	ENSG00000163687	0	NA	NA	NA	NA	DNASE1L3
200844	ENSG00000163689	139.678154	-0.10015084	0.20091737	0.55725822	0.76258372	CFAP20DC
54502	ENSG00000163694	1.02328604	0.05874709	0.39031919	0.20106626	NA	RBM47
323	ENSG00000163697	980.695569	-0.40432209	0.11318476	0.00017802	0.00127966	APBB2
132014	ENSG00000163701	39.7660688	0.58222334	0.41371844	0.02821858	0.09562026	IL17RE
84818	ENSG00000163702	237.663058	1.03560868	0.17910663	9.06E-10	1.57E-08	IL17RC
78987	ENSG00000163703	1046.6015	0.36234245	0.10241541	0.00023413	0.00163501	CRELD1
285368	ENSG00000163704	222.475052	-0.10233161	0.16479456	0.49116343	0.71060078	PRRT3
115795	ENSG00000163705	0.15069912	-0.01848503	0.38480575	0.84537521	NA	FANCD2OS
26577	ENSG00000163710	105.377647	0.47334923	0.23331683	0.01506656	0.05791677	PCOLCE2
23350	ENSG00000163714	2849.4426	-0.00855438	0.07981176	0.91243976	0.96260718	U2SURP
64419	ENSG00000163719	650.550259	0.18762959	0.10037091	0.05231598	0.15415849	MTMR14
151613	ENSG00000163728	1312.86166	0.55441104	0.08727687	7.31E-11	1.43E-09	TTC14
2921	ENSG00000163734	0	NA	NA	NA	NA	CXCL3
6374	ENSG00000163735	0	NA	NA	NA	NA	CXCL5
5473	ENSG00000163736	0	NA	NA	NA	NA	PPBP
5196	ENSG00000163737	0	NA	NA	NA	NA	PF4
441024	ENSG00000163738	261.367868	-0.13477537	0.13912602	0.29725273	0.52389572	MTHFD2L
2919	ENSG00000163739	0	NA	NA	NA	NA	CXCL1
25898	ENSG00000163743	469.348848	-0.39714204	0.11811269	0.00039841	0.00263835	RCHY1
57047	ENSG00000163746	0	NA	NA	NA	NA	PLSCR2
339965	ENSG00000163749	4.59341369	-0.00530033	0.36411561	0.96117265	0.98419142	CCDC158
1359	ENSG00000163751	1.66388074	0.03581179	0.37899126	0.64944584	NA	CPA3
2992	ENSG00000163754	215.349066	0.1259479	0.15985447	0.38753859	0.61648218	GYG1
84343	ENSG00000163755	513.754934	-0.07225984	0.10886445	0.48794505	0.70776647	HPS3
116441	ENSG00000163762	0.173124	-0.01911262	0.38486262	0.84537521	NA	TM4SF18

11073	ENSG00000163781	2462.57842	-0.08380666	0.0726533	0.23971954	0.45775127	TOPBP1
6259	ENSG00000163785	1268.65698	0.20704794	0.08526137	0.01269054	0.05043796	RYK
54861	ENSG00000163788	605.601644	-0.16609778	0.10956915	0.11279375	0.27197715	SNRK
150921	ENSG00000163792	0	NA	NA	NA	NA	TCF23
285126	ENSG00000163793	56.5320828	-1.7093179	0.38663308	6.16E-07	7.24E-06	DNAJC5G
7349	ENSG00000163794	32.6288283	-0.07457027	0.27897379	0.69973826	0.8552838	UCN
130557	ENSG00000163795	1469.45247	0.24040721	0.07099656	0.00056025	0.00356263	ZNF513
22950	ENSG00000163798	771.627389	0.03265016	0.10204443	0.74094504	0.87763281	SLC4A1AP
151056	ENSG00000163803	11.4678376	-0.07947665	0.3699268	0.52140831	0.73486829	PLB1
245711	ENSG00000163806	12.0830339	-0.03763281	0.34826438	0.80079663	0.90877553	SPDYA
57456	ENSG00000163807	1038.83945	-0.21599563	0.24695357	0.2639571	0.48609939	KIAA1143
56992	ENSG00000163808	1081.07157	-0.32749193	0.10659045	0.00133116	0.00756092	KIF15
7047	ENSG00000163810	2.85863137	-0.14639347	0.41125319	0.15551888	0.34146686	TGM4
23160	ENSG00000163811	1530.89778	-0.17865593	0.10486662	0.07569712	0.2027857	WDR43
51304	ENSG00000163812	1536.42355	-0.2279705	0.12132859	0.04696272	0.14179583	ZDHHC3
64866	ENSG00000163814	0.44427848	0.01506333	0.38397328	0.81042032	NA	CDCP1
7123	ENSG00000163815	4.76223654	0.05812573	0.3723885	0.60944639	0.79803651	CLEC3B
54716	ENSG00000163817	0.14931947	-0.01844332	0.38480212	0.84537521	NA	SLC6A20
54585	ENSG00000163818	343.439965	0.3064799	0.12940517	0.01163235	0.04697436	LZTFL1
79443	ENSG00000163820	435.715904	0.95261226	0.14178747	2.60E-12	5.77E-11	FYCO1
1230	ENSG00000163823	0	NA	NA	NA	NA	CCR1
83597	ENSG00000163825	0.173733	0.01914545	0.38484597	0.78225479	NA	RTP3
79442	ENSG00000163827	32.150259	0.0006676	0.28955498	0.99905009	0.99962651	LRRC2
54859	ENSG00000163832	684.957222	-0.11541503	0.11949446	0.30861991	0.53621325	ELP6
51725	ENSG00000163833	0	NA	NA	NA	NA	FBXO40
151636	ENSG00000163840	484.58259	0.00247453	0.12498778	0.98499241	0.99473578	DTX3L
7707	ENSG00000163848	888.454685	-0.21547226	0.09252636	0.01612566	0.06118439	ZNF148
349565	ENSG00000163864	11.3716482	-0.53412382	0.71041328	0.03852992	0.1221335	NMNAT3
113444	ENSG00000163866	487.035837	0.02265582	0.11270816	0.83423746	0.92434219	SMIM12
9204	ENSG00000163867	332.248608	-0.08118111	0.13737537	0.52489789	0.73755856	ZMYM6
131601	ENSG00000163870	378.985922	0.13219103	0.12644095	0.26825055	0.49101309	TPRA1
55689	ENSG00000163872	3230.42976	0.06684343	0.06253731	0.27802261	0.50254816	YEATS2
2899	ENSG00000163873	40.8086422	-4.28828655	0.54640609	6.19E-16	1.79E-14	GRIK3
80149	ENSG00000163874	36.6019046	0.68899972	0.39962201	0.01296106	0.05133707	ZC3H12A
64769	ENSG00000163875	911.209264	-0.13115176	0.09103241	0.13724964	0.31281251	MEAF6
79753	ENSG00000163877	475.913878	-0.07054279	0.17545587	0.64901712	0.82497477	SNIP1
7802	ENSG00000163879	372.935734	0.25270946	0.16206398	0.08484819	0.22086345	DNALI1
5437	ENSG00000163882	1107.20735	-0.03170404	0.08188159	0.69258871	0.85146611	POLR2H

28999	ENSG00000163884	239.230971	0.07142868	0.14944395	0.6057104	0.79555768	KLF15
348807	ENSG00000163885	0.15069912	-0.01848503	0.38480575	0.84537521	NA	CFAP100
94032	ENSG00000163888	204.75336	-1.46694757	0.20307379	4.12E-14	1.05E-12	CAMK2N2
200879	ENSG00000163898	1.80341675	0.08745899	0.39546778	0.14318956	NA	LIPH
90407	ENSG00000163900	308.440484	0.36460367	0.16462519	0.01440118	0.05577188	TMEM41A
6184	ENSG00000163902	3133.7349	0.19660903	0.08266063	0.01474848	0.0568948	RPN1
59343	ENSG00000163904	858.89801	0.24764856	0.09879718	0.00931846	0.03914424	SENP2
26508	ENSG00000163909	0.86893111	0.01664743	0.38116958	0.76840041	NA	HEYL
55764	ENSG00000163913	590.777603	0.07553207	0.12530652	0.52433527	0.73721294	IFT122
6010	ENSG00000163914	0.37844916	0.02407552	0.3854191	0.60038877	NA	RHO
646600	ENSG00000163915	0	NA	NA	NA	NA	IGF2BP2-AS1
5984	ENSG00000163918	725.991845	-0.09321938	0.10132298	0.33909698	0.5693225	RFC4
116832	ENSG00000163923	365.083601	-0.15851378	0.12170505	0.16860527	0.35992344	RPL39L
8314	ENSG00000163930	2393.26123	0.1197381	0.06188447	0.04998779	0.1489573	BAP1
7086	ENSG00000163931	4363.6994	-0.54798342	0.0642558	5.22E-18	1.73E-16	TKT
5580	ENSG00000163932	235.592521	0.00738884	0.14599621	0.95742677	0.98264776	PRKCD
91869	ENSG00000163933	616.83858	-0.20448779	0.10876631	0.04884159	0.1463551	RFT1
51460	ENSG00000163935	298.373871	-0.22457131	0.16506209	0.13053084	0.30275099	SFMBT1
26354	ENSG00000163938	2017.43906	-0.1926651	0.07662071	0.0101834	0.04200386	GNL3
55193	ENSG00000163939	2866.19554	-0.05945471	0.07994348	0.44136194	0.66615602	PBRM1
57654	ENSG00000163945	1207.55314	0.26788356	0.12398185	0.02114269	0.07618153	UVSSA
23272	ENSG00000163946	2123.93948	-0.15561741	0.08614983	0.06310616	0.17807227	TASOR
50650	ENSG00000163947	128.791817	0.02458843	0.20523623	0.88861782	0.95146564	ARHGEF3
7884	ENSG00000163950	1097.30472	-0.32388755	0.08852104	0.00016289	0.00118118	SLBP
4043	ENSG00000163956	1330.94636	0.29075273	0.08829728	0.00070679	0.00436302	LRPAP1
131540	ENSG00000163958	11.4195	0.2628518	0.43275087	0.16291191	0.35193514	ZDHHC19
200931	ENSG00000163959	10.7155833	0.19448188	0.40038856	0.24380904	0.46213541	SLC51A
26043	ENSG00000163960	2234.88571	-0.12914658	0.0789968	0.09099586	0.23271133	UBXN7
165918	ENSG00000163961	711.833947	-0.33970406	0.10215112	0.00054014	0.00345019	RNF168
54965	ENSG00000163964	534.451611	-0.06802843	0.1545147	0.62276869	0.80737672	PIGX
4241	ENSG00000163975	359.039574	0.76042378	0.14314643	2.16E-08	3.12E-07	MELTF
133060	ENSG00000163982	0	NA	NA	NA	NA	OTOP1
6286	ENSG00000163993	0.69017422	0.03903171	0.3869417	0.37258575	NA	S100P
84448	ENSG00000163995	104.525257	-1.28329631	0.24414457	1.33E-08	1.98E-07	ABLIM2
64789	ENSG00000164002	216.239719	-0.03808378	0.14825602	0.77969021	0.89925062	EXO5
149461	ENSG00000164007	1.14146079	0.06770743	0.38954926	0.25074476	NA	CLDN19
79078	ENSG00000164008	211.880299	-0.33099036	0.22163725	0.07235843	0.19601191	C1orf50
114625	ENSG00000164010	147.951813	-0.06130064	0.21218423	0.72724912	0.86953012	ERMAP

51058	ENSG00000164011	265.541884	-0.15718393	0.15095392	0.25597024	0.47707369	ZNF691
9255	ENSG00000164022	1215.04669	-0.05019541	0.07826859	0.51176596	0.72695902	AIMP1
166929	ENSG00000164023	1.06425219	0.03847341	0.38467702	0.47350963	NA	SGMS2
23173	ENSG00000164024	1053.97649	-0.06080594	0.08247791	0.4495938	0.67315229	METAP1
79982	ENSG00000164031	887.559386	0.69371576	0.12812235	1.40E-08	2.07E-07	DNAJB14
3015	ENSG00000164032	7526.31036	0.02361045	0.0536894	0.65782421	0.83028388	H2AZ1
51705	ENSG00000164035	0	NA	NA	NA	NA	EMCN
150159	ENSG00000164037	4.26473574	0.01670639	0.36944539	0.87377324	0.94441388	SLC9B1
133308	ENSG00000164038	675.373075	-0.30044439	0.1299611	0.0136186	0.05341418	SLC9B2
56898	ENSG00000164039	589.877711	0.44455443	0.13379792	0.00039501	0.00261844	BDH2
10424	ENSG00000164040	770.014164	-0.20880663	0.10114977	0.03186319	0.10516813	PGRMC2
993	ENSG00000164045	1076.57444	-0.1861355	0.09445332	0.04143663	0.12885621	CDC25A
820	ENSG00000164047	0	NA	NA	NA	NA	CAMP
51385	ENSG00000164048	692.54264	-0.12675992	0.11507114	0.24725955	0.46618165	ZNF589
285231	ENSG00000164049	5.51262989	0.01693555	0.36033481	0.89668148	0.95469109	FBXW12
5364	ENSG00000164050	2846.97158	0.53627767	0.06827151	1.48E-15	4.22E-14	PLXNB1
79714	ENSG00000164051	455.872928	-0.28327566	0.12832085	0.01874874	0.0691018	CCDC51
84126	ENSG00000164053	458.206541	0.00703174	0.11071583	0.94799564	0.97876335	ATRIP
51246	ENSG00000164054	190.692626	0.47260352	0.21303423	0.00985731	0.0409823	SHISA5
10252	ENSG00000164056	60.4465313	0.46070134	0.31328393	0.04470036	0.13639738	SPRY1
8927	ENSG00000164061	1688.85755	-1.55115614	0.07191999	3.09E-104	9.88E-102	BSN
327	ENSG00000164062	1669.73757	0.04340901	0.0801103	0.57775201	0.77605301	APEH
27152	ENSG00000164066	458.694472	0.22916521	0.13520472	0.06945746	0.19038905	INTU
63891	ENSG00000164068	1053.03718	0.18316491	0.08947664	0.03512984	0.11362505	RNF123
22824	ENSG00000164070	858.977165	-0.16315786	0.1057341	0.10764145	0.26314781	HSPA4L
256471	ENSG00000164073	227.13419	-0.19001771	0.15658552	0.18214017	0.37982563	MFSD8
80167	ENSG00000164074	217.312366	0.11174099	0.16613404	0.45611568	0.67958359	ABHD18
79012	ENSG00000164076	1180.83584	-1.0012436	0.08540863	1.34E-32	8.30E-31	CAMKV
84315	ENSG00000164077	312.430314	-0.15020346	0.14276488	0.25550518	0.47650319	MON1A
4486	ENSG00000164078	26.2977633	1.90502778	0.55940568	3.16E-05	0.00027078	MST1R
23132	ENSG00000164080	1396.83127	-0.08483579	0.08257736	0.29219353	0.51825408	RAD54L2
51368	ENSG00000164081	418.6777	0.03243091	0.13688529	0.80056806	0.90868418	TEX264
2912	ENSG00000164082	108.275722	-2.2939638	0.28086884	2.23E-17	7.07E-16	GRM2
1849	ENSG00000164086	1459.79656	-1.03333909	0.08174786	1.63E-37	1.17E-35	DUSP7
25886	ENSG00000164087	376.432147	-0.00658956	0.12236512	0.95379728	0.98083958	POC1A
132160	ENSG00000164088	392.27733	0.36809776	0.1274781	0.00216754	0.01146772	PPM1M
64850	ENSG00000164089	0	NA	NA	NA	NA	ETNPPL
80335	ENSG00000164091	5208.64438	-0.4227484	0.05082934	4.58E-17	1.42E-15	WDR82

5308	ENSG00000164093	0	NA	NA	NA	NA	PITX2
401152	ENSG00000164096	807.660851	-0.30403267	0.10994296	0.00378171	0.01839941	C4orf3
8492	ENSG00000164099	2659.26438	1.77949476	0.07697985	2.59E-119	1.03E-116	PRSS12
9348	ENSG00000164100	24.5066338	0.04364533	0.29707032	0.8191578	0.91752333	NDST3
3148	ENSG00000164104	6679.11935	0.30326492	0.05693379	8.02E-08	1.07E-06	HMGB2
8819	ENSG00000164105	371.661773	0.63157367	0.12600464	1.47E-07	1.91E-06	SAP30
11341	ENSG00000164106	6.61451978	0.19750107	0.41715601	0.19421204	0.39719274	SCRG1
9464	ENSG00000164107	9005.21868	1.40823789	0.05865674	2.63E-128	1.24E-125	HAND2
4085	ENSG00000164109	1840.78263	-0.38737882	0.10189553	7.73E-05	0.0006058	MAD2L1
308	ENSG00000164111	1629.42043	0.46381972	0.09212131	2.13E-07	2.69E-06	ANXA5
132332	ENSG00000164112	0	NA	NA	NA	NA	SMIM43
132612	ENSG00000164113	0	NA	NA	NA	NA	ADAD1
79884	ENSG00000164114	739.241986	-0.14184661	0.11871311	0.20808408	0.41566533	MAP9
2982	ENSG00000164116	580.137337	0.16334883	0.10380677	0.1017487	0.25253045	GUCY1A1
26269	ENSG00000164117	404.038572	1.37971513	0.12423266	1.09E-29	6.19E-28	FBXO8
80817	ENSG00000164118	1850.58205	1.72098378	0.08671149	9.22E-89	2.23E-86	CEP44
3248	ENSG00000164120	10.1526405	1.31928375	1.08072169	0.00679973	0.03018044	HPGD
140458	ENSG00000164122	0	NA	NA	NA	NA	ASB5
152940	ENSG00000164123	0.48561729	0.00575914	0.38311112	0.86236059	NA	C4orf45
55314	ENSG00000164124	0.14931947	-0.01844332	0.38480212	0.84537521	NA	TMEM144
51313	ENSG00000164125	3.21187715	-0.00436165	0.37392956	0.95528669	0.98165887	GASK1B
4886	ENSG00000164128	0	NA	NA	NA	NA	NPY1R
4889	ENSG00000164129	0	NA	NA	NA	NA	NPY5R
80155	ENSG00000164134	1517.32728	-0.30560762	0.10025108	0.00146813	0.00823048	NAA15
3600	ENSG00000164136	3.88484992	-0.08239994	0.38129917	0.43134589	0.65692932	IL15
729830	ENSG00000164142	55.4066488	1.93312451	0.41243949	1.64E-07	2.11E-06	FAM160A1
27236	ENSG00000164144	557.278171	-0.01703965	0.104994	0.86376142	0.93963299	ARFIP1
23379	ENSG00000164151	1743.10404	0.16023006	0.07315108	0.0255375	0.08857763	ICE1
64399	ENSG00000164161	4.05310157	0.01641231	0.37384447	0.85224697	0.9330416	HHIP
10393	ENSG00000164162	133.048417	0.09453142	0.17914043	0.5519326	0.75833399	ANAPC10
6059	ENSG00000164163	1641.71932	-0.16524076	0.07382486	0.0221842	0.07913243	ABCE1
54726	ENSG00000164164	1239.53545	-0.03463034	0.08609324	0.67918897	0.84330115	OTUD4
11157	ENSG00000164167	261.118125	0.01444853	0.17568523	0.92770146	0.9697364	LSM6
55751	ENSG00000164168	983.317752	-0.12741755	0.09083173	0.1479138	0.33006521	TMEM184C
90826	ENSG00000164169	283.639045	0.14884719	0.13671089	0.24364703	0.4620932	PRMT9
3673	ENSG00000164171	388.861315	0.3977526	0.13010752	0.0011455	0.00663939	ITGA2
4338	ENSG00000164172	412.658843	-0.38767648	0.1524104	0.00559052	0.02558025	MOCS2
51151	ENSG00000164175	16.9462416	0.088072	0.35006779	0.58890429	0.78351083	SLC45A2

10085	ENSG00000164176	149.715972	-2.06677594	0.23104672	2.70E-20	1.03E-18	EDIL3
153396	ENSG00000164180	730.583679	0.05988328	0.11093689	0.57364656	0.77312328	TMEM161B
79993	ENSG00000164181	109.977107	0.35963162	0.24326409	0.06690519	0.18544027	ELOVL7
91942	ENSG00000164182	304.757804	0.09746079	0.13516321	0.44122412	0.6661181	NDUFAF2
133923	ENSG00000164185	3.46770211	0.0504828	0.37194771	0.64718192	0.82430661	ZNF474
92255	ENSG00000164187	639.489953	-0.21392398	0.11496726	0.0503386	0.14971174	LMBRD2
202151	ENSG00000164188	1.70553796	-0.06664453	0.38677239	0.35221034	NA	RANBP3L
25836	ENSG00000164190	1913.67103	-0.13055053	0.07566125	0.07803936	0.20760269	NIPBL
285671	ENSG00000164197	0.92335854	-0.01445502	0.38045929	0.78669419	NA	RNF180
84059	ENSG00000164199	812.96524	0.36343376	0.11832444	0.00120844	0.00696553	ADGRV1
91137	ENSG00000164209	1339.30174	-0.32717208	0.08035251	2.97E-05	0.00025568	SLC25A46
134429	ENSG00000164211	2519.72573	0.27772263	0.06882793	4.00E-05	0.00033601	STARD4
5229	ENSG00000164219	524.060122	-0.33042414	0.11874018	0.00330483	0.0164248	PGGT1B
2151	ENSG00000164220	1.01344486	0.00310693	0.37996385	0.95929643	NA	F2RL2
153733	ENSG00000164221	452.95321	-0.07963516	0.12454827	0.49748328	0.71542971	CCDC112
651746	ENSG00000164236	8.85339891	0.3276223	0.52550247	0.07345781	0.19834008	ANKRD33B
134147	ENSG00000164237	470.339962	-0.11985976	0.14113302	0.35954121	0.58949503	CMBL
401207	ENSG00000164241	197.127447	-0.09924787	0.20480843	0.56547068	0.76736924	C5orf63
133619	ENSG00000164244	991.460498	0.07662027	0.09448651	0.40306164	0.6310063	PRRC1
2150	ENSG00000164251	38.088838	-0.91312331	0.43543073	0.00363037	0.01780629	F2RL1
55109	ENSG00000164252	1058.90144	-0.04851319	0.08277915	0.54709043	0.75418804	AGGF1
55255	ENSG00000164253	791.021939	-0.1881716	0.11991167	0.09771458	0.24490364	WDR41
56979	ENSG00000164256	0	NA	NA	NA	NA	PRDM9
4724	ENSG00000164258	547.214803	0.17324878	0.10844572	0.09544388	0.24079433	NDUFS4
117156	ENSG00000164265	0	NA	NA	NA	NA	SCGB3A2
6690	ENSG00000164266	0	NA	NA	NA	NA	SPINK1
3360	ENSG00000164270	1.72801861	0.02900077	0.37859304	0.7041367	NA	HTR4
11082	ENSG00000164283	0	NA	NA	NA	NA	ESM1
134266	ENSG00000164284	845.535747	-0.33739179	0.12420676	0.00397989	0.0192177	GRPEL2
166979	ENSG00000164287	0.173733	0.01914545	0.38484597	0.78225479	NA	CDC20B
153642	ENSG00000164291	152.183757	0.31591673	0.19442948	0.06116231	0.17394786	ARSK
22836	ENSG00000164292	6413.61489	-0.10483937	0.06551808	0.10402066	0.25677486	RHOBTB3
493869	ENSG00000164294	7.50195193	6.47062241	2.65366252	1.05E-05	9.92E-05	GPX8
81789	ENSG00000164296	140.451409	-0.23606486	0.18563451	0.14647737	0.32752294	TIGD6
84654	ENSG00000164299	0.83570807	-0.02152789	0.3825715	0.6114649	NA	SPZ1
256987	ENSG00000164300	489.100832	-0.52005983	0.11772486	3.64E-06	3.75E-05	SERINC5
133121	ENSG00000164303	58.6833823	-0.07286521	0.2671112	0.70461527	0.85803894	ENPP6
285782	ENSG00000164304	3.36740333	0.03974674	0.37141738	0.71211898	0.86159535	CAGE1

836	ENSG00000164305	627.791139	-0.32560991	0.10705152	0.00148328	0.00830132	CASP3
201973	ENSG00000164306	312.218816	0.01795999	0.16420536	0.9050055	0.95843435	PRIMPOL
51752	ENSG00000164307	232.548195	0.18657282	0.15827009	0.19504697	0.39840749	ERAP1
64167	ENSG00000164308	6.6318146	0.13998287	0.39331509	0.28701979	0.51287694	ERAP2
202333	ENSG00000164309	23.6729538	0.31764191	0.43586792	0.13060894	0.30282593	CMYA5
133584	ENSG00000164318	40.4544751	2.78754718	0.44027163	1.67E-11	3.47E-10	EGFLAM
57587	ENSG00000164323	1467.62986	-0.14829036	0.07361063	0.03987383	0.12512313	CFAP97
134288	ENSG00000164325	0	NA	NA	NA	NA	TMEM174
9607	ENSG00000164326	1.47727575	-0.0052354	0.37817175	0.94015928	NA	CARTPT
253260	ENSG00000164327	564.526618	-0.24230474	0.12388347	0.03798349	0.12074866	RICTOR
167153	ENSG00000164329	611.11806	-0.10606689	0.12423228	0.36583653	0.59508049	TENT2
1879	ENSG00000164330	102.960735	-0.03412058	0.21545932	0.84691561	0.93052693	EBF1
57763	ENSG00000164331	247.758775	0.00917332	0.16366107	0.9518051	0.98012235	ANKRA2
134510	ENSG00000164332	521.056474	-0.2405026	0.11140365	0.02345933	0.08277309	UBLCP1
340069	ENSG00000164334	0.64235609	-0.00831672	0.38275593	0.80555485	NA	FAM170A
84135	ENSG00000164338	301.160605	0.17278143	0.16769789	0.25201693	0.47192221	UTP15
7098	ENSG00000164342	0.16735865	0.01897177	0.38482933	0.78225479	NA	TLR3
3818	ENSG00000164344	1.94806512	0.04888178	0.38314495	0.46185431	NA	KLKB1
10412	ENSG00000164346	2308.89729	0.04498342	0.06351208	0.47299504	0.69507604	NSA2
84340	ENSG00000164347	917.788337	-0.2845871	0.09561264	0.00205222	0.01093959	GFM2
7015	ENSG00000164362	401.303272	0.32246029	0.162665	0.02849847	0.09642745	TERT
348932	ENSG00000164363	0	NA	NA	NA	NA	SLC6A18
133957	ENSG00000164366	576.776097	-0.11372723	0.11407947	0.29479693	0.52123439	CCDC127
94234	ENSG00000164379	0	NA	NA	NA	NA	FOXQ1
154386	ENSG00000164385	0.3404787	-0.00022855	0.38336236	0.96741903	NA	LINC01600
222611	ENSG00000164393	0	NA	NA	NA	NA	ADGRF2
23305	ENSG00000164398	61.5074176	-0.5795502	0.37776467	0.02430432	0.08519395	ACSL6
3562	ENSG00000164399	0	NA	NA	NA	NA	IL3
1437	ENSG00000164400	0	NA	NA	NA	NA	CSF2
23176	ENSG00000164402	1837.89912	0.20318367	0.06813451	0.00242271	0.0126209	SEPTIN8
134549	ENSG00000164403	105.783575	-0.49539618	0.31731056	0.03246962	0.10683204	SHROOM1
2661	ENSG00000164404	38.2874449	-0.0820614	0.27473551	0.67181957	0.83832583	GDF9
27089	ENSG00000164405	2138.53575	-0.27190331	0.07955917	0.0004617	0.00301222	UQCRQ
116842	ENSG00000164406	25.6487	0.22235983	0.33746937	0.28223913	0.50716641	LEAP2
375519	ENSG00000164411	0	NA	NA	NA	NA	GJB7
10559	ENSG00000164414	384.091304	-0.11416143	0.13474586	0.36460972	0.59423075	SLC35A1
2898	ENSG00000164418	414.952843	-1.03781518	0.15263569	1.26E-12	2.86E-11	GRIK2
115004	ENSG00000164430	2.40442519	0.11907397	0.400059	0.20330447	NA	CGAS

2173	ENSG00000164434	0	NA	NA	NA	NA	FABP7
30012	ENSG00000164438	172.65799	-0.15558409	0.16710287	0.29993601	0.52707976	TLX3
167838	ENSG00000164440	0	NA	NA	NA	NA	TXLNB
10370	ENSG00000164442	1.94965725	-0.0183244	0.37692774	0.81313113	NA	CITED2
221301	ENSG00000164451	0	NA	NA	NA	NA	CALHM4
6862	ENSG00000164458	0.88131531	-0.0007412	0.38066796	0.98790266	NA	TBXT
153222	ENSG00000164463	173.494779	0.57433389	0.20111741	0.00122362	0.00704108	CREBRF
285761	ENSG00000164465	7.74077074	0.41173504	0.66908574	0.0355782	0.11473855	DCBLD1
94081	ENSG00000164466	1732.53166	-0.22449808	0.09059266	0.01052246	0.04316015	SFXN1
154075	ENSG00000164483	0	NA	NA	NA	NA	SAMD3
114801	ENSG00000164484	0	NA	NA	NA	NA	TMEM200A
116379	ENSG00000164485	0	NA	NA	NA	NA	IL22RA2
168002	ENSG00000164488	0	NA	NA	NA	NA	DACT2
57107	ENSG00000164494	254.682024	-0.18032694	0.14269655	0.17210805	0.36461546	PDSS2
100130988	ENSG00000164500	0	NA	NA	NA	NA	SPATA48
134957	ENSG00000164506	575.373405	-0.53929793	0.11307112	6.40E-07	7.49E-06	STXBP5
221613	ENSG00000164508	0	NA	NA	NA	NA	H2AC1
133396	ENSG00000164509	2.40403862	0.05389603	0.38337884	0.4344984	NA	IL31RA
79722	ENSG00000164512	0.55145755	0.04045112	0.38693546	0.4047702	NA	ANKRD55
135250	ENSG00000164520	0	NA	NA	NA	NA	RAET1E
221476	ENSG00000164530	0	NA	NA	NA	NA	PI16
57057	ENSG00000164532	72.0742764	0.42417036	0.27339111	0.04587381	0.13929237	TBX20
221955	ENSG00000164535	464.985138	0.05079088	0.15098376	0.71553732	0.86274919	DAGLB
23366	ENSG00000164542	538.157434	-0.55197867	0.17003771	0.00034424	0.00231737	KIAA0895
9263	ENSG00000164543	802.084895	0.01133034	0.0933471	0.90107437	0.95641534	STK17A
29896	ENSG00000164548	2346.22693	-0.08810689	0.06602516	0.1751849	0.36912205	TRA2A
340286	ENSG00000164556	0.18922458	0.01954053	0.38488496	0.78225479	NA	FAM183BP
55568	ENSG00000164574	457.302672	-0.49275925	0.1679806	0.00122823	0.00705909	GALNT10
79685	ENSG00000164576	822.536208	-0.06629765	0.10790787	0.52109636	0.73457437	SAP30L
6208	ENSG00000164587	15697.2975	0.12998251	0.05120748	0.01041843	0.04281309	RPS14
348980	ENSG00000164588	0	NA	NA	NA	NA	HCN1
91977	ENSG00000164591	399.209047	0.0488624	0.13206465	0.69512823	0.85273877	MYOZ3
10466	ENSG00000164597	1012.19305	0.38991096	0.11829061	0.00052172	0.00334658	COG5
63974	ENSG00000164600	0	NA	NA	NA	NA	NEUROD6
154743	ENSG00000164603	326.151965	0.09293093	0.13203841	0.45427145	0.67744995	BMT2
54329	ENSG00000164604	299.902008	-0.88040966	0.20148157	1.72E-06	1.86E-05	GPR85
10569	ENSG00000164609	969.505647	0.56849113	0.13550913	8.59E-06	8.27E-05	SLU7
6100	ENSG00000164610	558.802148	0.12071021	0.10864359	0.24657541	0.46577702	RP9

9232	ENSG00000164611	1023.02813	-0.0361883	0.08441146	0.65986745	0.83138187	PTTG1
819	ENSG00000164615	740.524326	-0.29974873	0.10420412	0.00271791	0.01394086	CAMLG
NA	ENSG00000164616	22.6327393	3.74076545	0.66832471	1.96E-09	3.24E-08	FBXL21P
168667	ENSG00000164619	2.7437711	0.17469171	0.43225651	0.07964386	0.21062825	BMPER
285613	ENSG00000164620	506.087242	-0.67411612	0.14392073	6.70E-07	7.82E-06	RELL2
9597	ENSG00000164621	0.46777338	-0.00944564	0.38288077	0.84957036	NA	SMAD5-AS1
8645	ENSG00000164626	84.2540828	0.5124552	0.25174517	0.01300851	0.05146338	KCNK5
221458	ENSG00000164627	20.6845616	0.31235763	0.44227393	0.13557496	0.31017149	KIF6
7559	ENSG00000164631	958.814625	-0.112753	0.12610047	0.34294367	0.57311341	ZNF12
222962	ENSG00000164638	2754.11854	0.84958138	0.07385806	2.04E-31	1.22E-29	SLC29A4
219557	ENSG00000164645	0	NA	NA	NA	NA	TEX47
26872	ENSG00000164647	31.9576771	0.22433614	0.31918385	0.28247129	0.50753763	STEAP1
55536	ENSG00000164649	480.180401	0.98511476	0.12350381	2.03E-16	6.09E-15	CDCA7L
221833	ENSG00000164651	0.33873386	0.0022816	0.38337893	0.96667412	NA	SP8
54468	ENSG00000164654	1156.66705	-0.27420425	0.09472164	0.00273899	0.01402364	MIOS
222223	ENSG00000164659	1533.61498	-0.08285067	0.08516676	0.31778481	0.54556488	ELAPOR2
25862	ENSG00000164663	908.247209	0.01807003	0.11572784	0.87807345	0.94596966	USP49
285905	ENSG00000164669	44.4734434	0.05145671	0.26393237	0.7913146	0.90493295	INTS4P1
94120	ENSG00000164674	16.0248557	0.04566666	0.32778301	0.79578419	0.90615541	SYTL3
154865	ENSG00000164675	6.56782281	0.57864161	1.04844847	0.02014532	0.0733229	IQUB
23462	ENSG00000164683	549.230581	-0.67952932	0.12993682	4.07E-08	5.66E-07	HEY1
619279	ENSG00000164684	8266.54137	0.12769971	0.06353171	0.04171079	0.12962752	ZNF704
2171	ENSG00000164687	466.992782	0.09939399	0.11698524	0.37260208	0.6014992	FABP5
6469	ENSG00000164690	1.89628344	0.07740229	0.38977458	0.28312786	NA	SHH
117289	ENSG00000164691	2.86014515	0.06221439	0.38227659	0.46021993	0.68333808	TAGAP
1278	ENSG00000164692	11.9322	-0.08472336	0.36365001	0.54547838	0.75301218	COL1A2
84624	ENSG00000164694	0.16735865	0.01897177	0.38482933	0.78225479	NA	FNDC1
92421	ENSG00000164695	0	NA	NA	NA	NA	CHMP4C
26266	ENSG00000164707	349.398022	0.32799686	0.13478622	0.00920554	0.03872729	SLC13A4
5224	ENSG00000164708	74.1789648	0.29494721	0.25512698	0.13973117	0.31686665	PGAM2
25798	ENSG00000164713	644.088798	0.1071095	0.10624162	0.29400839	0.52021105	BRI3
22853	ENSG00000164715	2014.19542	0.06914	0.06799849	0.30101521	0.52832111	LMTK2
146861	ENSG00000164729	0	NA	NA	NA	NA	SLC35G3
1508	ENSG00000164733	2119.78492	-0.14055323	0.06589054	0.03014157	0.10072575	CTSB
64321	ENSG00000164736	0	NA	NA	NA	NA	SOX17
NA	ENSG00000164740	0	NA	NA	NA	NA	SEPTIN7P5
10395	ENSG00000164741	54.1580132	-0.08502012	0.30119581	0.65399654	0.82779603	DLC1
107	ENSG00000164742	6996.93194	-0.80363473	0.04985056	3.43E-59	4.37E-57	ADCY1

157773	ENSG00000164743	22.4798284	0.40322427	0.47740718	0.08805085	0.22670443	C8orf48
256979	ENSG00000164744	0	NA	NA	NA	NA	SUN3
136288	ENSG00000164746	0.83104738	0.01171515	0.38170116	0.81896071	NA	C7orf57
3174	ENSG00000164749	46.5145765	1.07420316	0.39865189	0.00063067	0.00394838	HNF4G
5828	ENSG00000164751	446.533568	-0.13736873	0.12760648	0.25209979	0.47202308	PEX2
5885	ENSG00000164754	5198.55433	-0.0320375	0.05489737	0.54815944	0.75477016	RAD21
169026	ENSG00000164756	0	NA	NA	NA	NA	SLC30A8
90390	ENSG00000164758	228.804388	-0.10207929	0.15727931	0.47561741	0.69768942	MED30
4982	ENSG00000164761	0	NA	NA	NA	NA	TNFRSF11B
157869	ENSG00000164764	66.6745606	-0.57024808	0.31128958	0.01584205	0.06029278	SBSPON
5260	ENSG00000164776	55.0218854	0.0974207	0.2842871	0.61786026	0.80440629	PHKG1
2020	ENSG00000164778	1.373176	-0.09355926	0.39942046	0.09419964	NA	EN2
27012	ENSG00000164794	0	NA	NA	NA	NA	KCNV1
114788	ENSG00000164796	0	NA	NA	NA	NA	CSMD3
23514	ENSG00000164808	874.89097	0.18176398	0.11909865	0.10569369	0.25942878	SPIDR
5001	ENSG00000164815	479.568272	-0.36016758	0.17106828	0.0186773	0.06888969	ORC5
1670	ENSG00000164816	0	NA	NA	NA	NA	DEFA5
54919	ENSG00000164818	2140.1192	-0.04605396	0.07445346	0.52714586	0.73905402	DNAAF5
1669	ENSG00000164821	0	NA	NA	NA	NA	DEFA4
1671	ENSG00000164822	0	NA	NA	NA	NA	DEFA6
734	ENSG00000164823	373.31555	-0.39102482	0.15038898	0.0047196	0.02213638	OSGIN2
1672	ENSG00000164825	0	NA	NA	NA	NA	DEFB1
23353	ENSG00000164828	4339.85952	0.03815329	0.05778599	0.5049113	0.72130144	SUN1
55074	ENSG00000164830	84.2714474	0.18285136	0.23807885	0.33611133	0.56608225	OXR1
157753	ENSG00000164841	9.91612148	0.12399879	0.35977519	0.45896196	0.68235016	TMEM74
NA	ENSG00000164845	2.21043564	0.06358325	0.38489367	0.40928205	NA	FAM86FP
115330	ENSG00000164849	31.8523968	-1.06103126	0.50730898	0.00273734	0.01401878	GPR146
2852	ENSG00000164850	45.0471998	-0.43758328	0.33680698	0.06105984	0.17378098	GPER1
340260	ENSG00000164853	0	NA	NA	NA	NA	UNCX
202915	ENSG00000164855	11.5833128	-0.05681496	0.34327631	0.72271694	0.86754018	TMEM184A
4846	ENSG00000164867	21.3330576	0.26684595	0.39801487	0.19208283	0.39421985	NOS3
10407	ENSG00000164871	0	NA	NA	NA	NA	SPAG11B
79778	ENSG00000164877	130.22863	0.46585221	0.2799201	0.03171353	0.10486607	MICALL2
761	ENSG00000164879	0.14931947	-0.01844332	0.38480212	0.84537521	NA	CA3
26173	ENSG00000164880	8820.44384	0.14215501	0.05331978	0.00709968	0.0313007	INTS1
1020	ENSG00000164885	919.584573	0.09313484	0.09110829	0.29288202	0.51900494	CDK5
6522	ENSG00000164889	5454.38291	0.22816924	0.05650476	4.39E-05	0.00036462	SLC4A2
157724	ENSG00000164893	0	NA	NA	NA	NA	SLC7A13

10922	ENSG00000164896	1355.14287	0.10609667	0.07484901	0.14843126	0.33077346	FASTK
83590	ENSG00000164897	1043.21519	-0.04810047	0.08808876	0.57404047	0.77333896	TMUB1
154791	ENSG00000164898	295.764627	-0.08407434	0.14702104	0.53464111	0.74572704	FMC1
2636	ENSG00000164900	0.70129453	0.02714705	0.38368153	0.56969794	NA	GBX1
51808	ENSG00000164902	768.770156	-0.39735636	0.14310071	0.00274615	0.01405297	PHAX
501	ENSG00000164904	1191.63213	0.35209181	0.09576223	0.00014209	0.00104413	ALDH7A1
221937	ENSG00000164916	1705.9571	-0.17357989	0.0769872	0.02116649	0.07622857	FOXK1
1345	ENSG00000164919	1436.70927	0.08961881	0.08613021	0.2854523	0.51095035	COX6C
116039	ENSG00000164920	1.28097681	0.09129719	0.39863959	0.09641927	NA	OSR2
7534	ENSG00000164924	9125.33152	-0.04270425	0.05796218	0.45555093	0.67899711	YWHAZ
79870	ENSG00000164929	153.803382	1.01531982	0.30436923	8.78E-05	0.00067797	BAALC
8323	ENSG00000164930	333.510819	-0.06605203	0.13185652	0.59271113	0.78702605	FZD6
115908	ENSG00000164932	82.6139721	0.60520115	0.26661535	0.00554684	0.02541556	CTHRC1
81034	ENSG00000164933	511.021044	-0.05721452	0.106703	0.57570559	0.7744963	SLC25A32
25879	ENSG00000164934	1486.41796	-0.12347738	0.07291952	0.08397198	0.21903814	DCAF13
81501	ENSG00000164935	0	NA	NA	NA	NA	DCSTAMP
94241	ENSG00000164938	883.527625	0.35475519	0.09812622	0.00017976	0.00129127	TP53INP1
55656	ENSG00000164941	876.677853	0.18957782	0.15327738	0.17660926	0.37133614	INTS8
25962	ENSG00000164944	1574.44096	-0.16011193	0.087578	0.05997656	0.17116441	VIRMA
158326	ENSG00000164946	1166.35774	0.46562947	0.12105882	5.14E-05	0.0004192	FREM1
2669	ENSG00000164949	121.452398	-0.93497181	0.23018974	6.38E-06	6.29E-05	GEM
54704	ENSG00000164951	421.228568	-0.14645161	0.13539776	0.24645709	0.46573089	PDP1
91147	ENSG00000164953	293.540792	0.34812967	0.18800907	0.03474778	0.11270978	TMEM67
9897	ENSG00000164961	840.791875	-0.12966754	0.12321109	0.2660687	0.48831193	WASHC5
138716	ENSG00000164967	104.037522	-0.42854357	0.25084792	0.03413418	0.11122057	RPP25L
203259	ENSG00000164970	948.584678	-0.92940278	0.11065063	6.35E-18	2.08E-16	FAM219A
84688	ENSG00000164972	4.65871149	-0.0183832	0.3713867	0.843707	0.92928146	C9orf24
6619	ENSG00000164975	1445.83473	0.38680726	0.08462079	2.69E-06	2.83E-05	SNAPC3
57462	ENSG00000164976	799.423406	-2.30703986	0.10089022	7.82E-117	2.93E-114	MYORG
318	ENSG00000164978	205.126392	-0.12359727	0.15815551	0.39228852	0.62159606	NUDT2
157378	ENSG00000164983	519.424694	-0.83150669	0.11257141	2.68E-14	6.91E-13	TMEM65
11168	ENSG00000164985	4355.95094	-0.1965172	0.06367665	0.00174692	0.00954158	PSIP1
203238	ENSG00000164989	105.185728	-0.02079999	0.21130826	0.90467063	0.95823316	CCDC171
51271	ENSG00000165006	658.39316	-0.21902029	0.09630354	0.01849187	0.06829463	UBAP1
54769	ENSG00000165023	20.7159513	0.03061663	0.30832441	0.87061022	0.94350971	DIRAS2
6850	ENSG00000165025	1048.00787	0.21205699	0.07997593	0.00663361	0.02953564	SYK
55335	ENSG00000165028	202.462226	0.40756941	0.20286977	0.02010696	0.07321016	NIPSNAP3B
19	ENSG00000165029	17.4524807	0.40310948	0.48426196	0.08743552	0.22586501	ABCA1

4783	ENSG00000165030	192.647532	-0.30851099	0.18033251	0.05299004	0.15563616	NFIL3
137994	ENSG00000165046	81.7770544	-0.2851271	0.25450924	0.15064865	0.33423321	LETM2
55798	ENSG00000165055	1472.09936	-0.0470477	0.09566379	0.61056542	0.7987352	METTL2B
5568	ENSG00000165059	0	NA	NA	NA	NA	PRKACG
2395	ENSG00000165060	153.160041	-0.34444599	0.19067101	0.03864656	0.12228832	FXN
79698	ENSG00000165061	45.7074162	-0.13902259	0.29809628	0.48059761	0.70158112	ZMAT4
157848	ENSG00000165066	0.32374618	-0.02327237	0.38532124	0.66865339	NA	NKX6-3
137835	ENSG00000165071	0.173733	0.01914545	0.38484597	0.78225479	NA	TMEM71
256691	ENSG00000165072	1.79120473	0.02878021	0.3815672	0.62134372	NA	MAMDC2
136242	ENSG00000165076	1.38173259	-0.02647048	0.38186702	0.64145721	NA	PRSS37
57094	ENSG00000165078	0	NA	NA	NA	NA	CPA6
116328	ENSG00000165084	0	NA	NA	NA	NA	C8orf34
117531	ENSG00000165091	0	NA	NA	NA	NA	TMC1
216	ENSG00000165092	0	NA	NA	NA	NA	ALDH1A1
221656	ENSG00000165097	631.946829	0.10571877	0.10373086	0.28989097	0.51587019	KDM1B
138050	ENSG00000165102	777.329298	0.13619107	0.10648931	0.18232216	0.38008529	HGSNAT
158158	ENSG00000165105	380.801609	-0.70973723	0.13328703	2.26E-08	3.25E-07	RASEF
80318	ENSG00000165113	196.667001	0.18098435	0.17173345	0.23884842	0.45652723	GKAP1
55582	ENSG00000165115	216.28517	0.08565827	0.17105394	0.57672648	0.77528586	KIF27
84267	ENSG00000165118	449.903468	0.02316463	0.11363747	0.83193824	0.92391014	C9orf64
3190	ENSG00000165119	15712.2025	-0.02434375	0.05469925	0.65219481	0.8261929	HNRNPK
136263	ENSG00000165120	0.29863895	-0.02292439	0.3852726	0.68753245	NA	SSMEM1
NA	ENSG00000165121	110.33797	0.2707977	0.21333196	0.13145993	0.30412372	
79987	ENSG00000165124	3.64214623	0.22878965	0.4718851	0.04939177	0.14766919	SVEP1
55503	ENSG00000165125	0	NA	NA	NA	NA	TRPV6
135927	ENSG00000165131	0	NA	NA	NA	NA	LLCFC1
203286	ENSG00000165138	938.759768	0.30505198	0.08883437	0.00040461	0.00267402	ANKS6
2203	ENSG00000165140	35.262048	1.76208904	0.44899933	5.49E-06	5.49E-05	FBP1
84302	ENSG00000165152	891.73292	-0.52564853	0.09220851	4.42E-09	7.00E-08	PGAP4
11244	ENSG00000165156	1176.29375	-0.28283049	0.10571501	0.0052307	0.0241342	ZHX1
286464	ENSG00000165164	0.18922458	0.01954053	0.38488496	0.78225479	NA	CFAP47
1536	ENSG00000165168	16.0917065	2.61847523	0.71049925	1.34E-05	0.00012498	CYBB
6990	ENSG00000165169	140.504406	0.21606113	0.19157113	0.19583482	0.39944263	DYNLT3
155368	ENSG00000165171	15.4443141	0.09626966	0.33491696	0.59204475	0.7865913	METTL27
58526	ENSG00000165175	1391.50989	0.11266014	0.09221459	0.20824279	0.4158149	MID1IP1
654817	ENSG00000165178	0	NA	NA	NA	NA	NCF1C
158401	ENSG00000165181	0.4819103	-0.00539734	0.38311991	0.88361852	NA	SHOC1
254158	ENSG00000165182	0.89037701	-0.04737719	0.38810027	0.30457487	NA	CXorf58

158405	ENSG00000165185	1517.97012	0.01121532	0.07765661	0.88483011	0.94964311	KIAA1958
139411	ENSG00000165186	1.5402434	-0.10893911	0.40503843	0.06530338	NA	PTCHD1
138065	ENSG00000165188	0.62659733	0.0368787	0.3866636	0.4036524	NA	RNF183
140456	ENSG00000165192	0.173733	0.01914545	0.38484597	0.78225479	NA	ASB11
57526	ENSG00000165194	7.81696745	0.09037238	0.35952523	0.5547956	0.76068726	PCDH19
5277	ENSG00000165195	315.148349	-0.13766193	0.14518711	0.30400346	0.53126299	PIGA
2277	ENSG00000165197	25.0482183	-0.08265314	0.31149469	0.65856183	0.83073135	VEGFD
158131	ENSG00000165202	0.18922458	0.01954053	0.38488496	0.78225479	NA	OR1Q1
392392	ENSG00000165204	0	NA	NA	NA	NA	OR1K1
55342	ENSG00000165209	977.833527	-0.22426449	0.09403939	0.01360998	0.0533957	STRBP
1365	ENSG00000165215	12.6449211	0.13050313	0.35872231	0.44524583	0.67004935	CLDN3
26130	ENSG00000165219	1351.25515	-0.46856226	0.08031386	2.35E-09	3.85E-08	GAPVD1
84270	ENSG00000165233	348.165471	-0.09526364	0.15381065	0.49704328	0.71500229	CARD19
65268	ENSG00000165238	2412.52731	-0.30739387	0.09504379	0.0008115	0.00493414	WNK2
538	ENSG00000165240	798.660948	0.52865658	0.09741124	2.10E-08	3.05E-07	ATP7A
195828	ENSG00000165244	468.468355	-0.50538205	0.11372202	3.38E-06	3.49E-05	ZNF367
22829	ENSG00000165246	0	NA	NA	NA	NA	NLGN4Y
139324	ENSG00000165259	225.346524	-0.32121009	0.18435609	0.0476618	0.14344814	HDX
4712	ENSG00000165264	485.372556	0.11123625	0.11353297	0.30520031	0.53260394	NDUFB6
364	ENSG00000165269	0.37844916	0.02407552	0.3854191	0.60038877	NA	AQP7
112267859	ENSG00000165269	0.37844916	0.02407552	0.3854191	0.60038877	NA	AQP7
65083	ENSG00000165271	1398.51253	0.1421368	0.07786482	0.06215155	0.17598767	NOL6
360	ENSG00000165272	95.4788297	0.46648311	0.24457881	0.02000757	0.07295538	AQP3
158234	ENSG00000165275	369.507474	0.09772868	0.12695612	0.41544539	0.64244323	TRMT10B
7415	ENSG00000165280	5765.96118	0.07409245	0.05957797	0.23883392	0.45652723	VCP
84720	ENSG00000165282	843.935829	0.12136087	0.10788144	0.24107649	0.45895616	PIGO
30968	ENSG00000165283	2244.89283	-0.12887172	0.06427106	0.04177411	0.12978364	STOML2
254065	ENSG00000165288	1145.88971	-0.24200092	0.08170329	0.00237332	0.01240429	BRWD3
26050	ENSG00000165300	254.439862	0.14754165	0.15691871	0.30283591	0.53006303	SLITRK5
9833	ENSG00000165304	1103.412	-0.10347981	0.0892384	0.23257151	0.44940365	MELK
219681	ENSG00000165309	6.50278616	-0.06882259	0.36862118	0.57667014	0.77528586	ARMC3
220213	ENSG00000165312	65.3695128	0.19162595	0.24627784	0.32166085	0.54929861	OTUD1
94134	ENSG00000165322	457.148032	0.31276956	0.13337995	0.01213543	0.04865943	ARHGAP12
120114	ENSG00000165323	492.649375	-1.26219828	0.14960741	2.97E-18	1.01E-16	FAT3
159989	ENSG00000165325	1.71514281	0.03366814	0.37907901	0.65213618	NA	DEUP1
143279	ENSG00000165338	457.621234	-0.34782076	0.13103597	0.00460288	0.02169148	HECTD2
84889	ENSG00000165349	8.55055255	4.02212687	1.24279198	0.00017772	0.001278	SLC7A3
254170	ENSG00000165355	147.108441	0.17486957	0.19758899	0.30348734	0.53085409	FBXO33

203522	ENSG00000165359	302.324472	-0.00709402	0.13088083	0.95308308	0.98058207	INTS6L
83550	ENSG00000165370	0	NA	NA	NA	NA	GPR101
9075	ENSG00000165376	1.17613771	0.05312853	0.38675507	0.31748969	NA	CLDN2
145581	ENSG00000165379	0.34179133	0.00186157	0.38337287	0.94760546	NA	LRFN5
474354	ENSG00000165383	0	NA	NA	NA	NA	LRRC18
171546	ENSG00000165389	856.575601	-0.11714474	0.08973712	0.17872825	0.3746401	SPTSSA
7486	ENSG00000165392	843.608187	0.07578563	0.1549013	0.59374519	0.78750562	WRN
220972	ENSG00000165406	522.119947	0.04135165	0.15219689	0.76835419	0.89324733	MARCHF8
7253	ENSG00000165409	1.37821731	0.04902936	0.38392253	0.4591067	NA	TSHR
1073	ENSG00000165410	508.358537	-1.59594897	0.13968438	2.23E-31	1.33E-29	CFL2
10910	ENSG00000165416	698.464769	-0.26615227	0.10866564	0.0103485	0.04258823	SUGT1
2957	ENSG00000165417	1397.85772	-0.24903554	0.10419889	0.01268154	0.05042118	GTF2A1
219654	ENSG00000165424	557.736948	0.82834159	0.11141559	1.91E-14	4.97E-13	ZCCHC24
283209	ENSG00000165434	334.703141	-0.09097578	0.13802286	0.47986611	0.70107095	PGM2L1
84457	ENSG00000165443	973.383916	-1.76179164	0.08810408	4.43E-90	1.16E-87	PHYHIPL
220963	ENSG00000165449	325.360432	0.02780763	0.13189055	0.82323533	0.91945578	SLC16A9
2350	ENSG00000165457	0.173733	0.01914545	0.38484597	0.78225479	NA	FOLR2
3636	ENSG00000165458	2448.02166	0.19772467	0.0681216	0.00315445	0.01580013	INPPL1
401	ENSG00000165462	9030.15705	0.45755716	0.06286274	1.78E-13	4.28E-12	PHOX2A
4153	ENSG00000165471	0.35648909	0.02381568	0.38538084	0.614114	NA	MBL2
2706	ENSG00000165474	0	NA	NA	NA	NA	GJB2
51084	ENSG00000165475	81.7222712	0.77267111	0.26158481	0.00052681	0.00337378	CRYL1
221035	ENSG00000165476	804.809004	0.29120247	0.09168614	0.00104675	0.00614243	REEP3
220296	ENSG00000165478	0.173124	-0.01911262	0.38486262	0.84537521	NA	HEPACAM
221150	ENSG00000165480	512.224791	-0.2475952	0.1264812	0.03704704	0.11847409	SKA3
221154	ENSG00000165487	537.624513	0.08094156	0.10657114	0.42947781	0.65514684	MICU2
220042	ENSG00000165490	522.294102	-0.06364118	0.14364814	0.63155265	0.81371846	DDIAS
51585	ENSG00000165494	1406.15709	-0.23827533	0.1056881	0.01859793	0.06863521	PCF11
63876	ENSG00000165495	12.594192	-0.02989504	0.34398939	0.84468787	0.92965052	PKNOX2
140801	ENSG00000165496	0	NA	NA	NA	NA	RPL10L
122769	ENSG00000165501	257.516704	-0.44575593	0.14998392	0.00128778	0.00735028	LRR1
6166	ENSG00000165502	1010.14342	0.06018433	0.09413504	0.51038694	0.72572757	RPL36AL
55172	ENSG00000165506	669.430769	-0.07837378	0.10133272	0.42171547	0.64775467	DNAAF2
11067	ENSG00000165507	7.08193596	0.63431637	1.22756783	0.01803893	0.06707127	DEPP1
139081	ENSG00000165509	0	NA	NA	NA	NA	MAGEC3
220979	ENSG00000165511	18.2507471	-0.20303138	0.36342346	0.29685991	0.52357548	ZNF22-AS1
7570	ENSG00000165512	947.108207	0.00279966	0.09931026	0.97776278	0.99246974	ZNF22
23588	ENSG00000165516	900.799767	0.22514372	0.08988776	0.00985629	0.0409823	KLHDC2

161436	ENSG00000165521	1839.75261	-0.0737864	0.07530299	0.31565477	0.5435333	EML5
9147	ENSG00000165525	1161.12649	-0.00230587	0.08420024	0.9768543	0.99214102	NEMF
84881	ENSG00000165526	724.487193	-0.25996579	0.10903865	0.01256695	0.05005587	RPUSD4
382	ENSG00000165527	1488.83759	-0.22827754	0.07679521	0.0023644	0.01236742	ARF6
123016	ENSG00000165533	1645.12038	0.46116087	0.08058691	4.75E-09	7.47E-08	TTC8
57156	ENSG00000165548	404.031986	-0.91848684	0.15445787	3.88E-10	7.03E-09	TMEM63C
58157	ENSG00000165553	0.92922319	-0.05058936	0.38876113	0.26831827	NA	NGB
122945	ENSG00000165555	11.0534601	0.01590901	0.33470265	0.92583719	0.9691565	NOXRED1
1045	ENSG00000165556	0	NA	NA	NA	NA	CDX2
219287	ENSG00000165566	0	NA	NA	NA	NA	AMER2
83592	ENSG00000165568	136.47361	0.03555263	0.20538224	0.83904315	0.92674595	AKR1E2
89890	ENSG00000165572	688.03291	-0.43858266	0.09961499	5.02E-06	5.05E-05	KBTBD6
6758	ENSG00000165583	0	NA	NA	NA	NA	SSX5
10214	ENSG00000165584	0	NA	NA	NA	NA	SSX3
5015	ENSG00000165588	0	NA	NA	NA	NA	OTX2
158584	ENSG00000165591	3.99449654	0.17055395	0.4180074	0.16239323	0.35108224	FAAH2
644168	ENSG00000165606	830.02522	-0.01321934	0.08604476	0.87168532	0.94401928	DRGX
11164	ENSG00000165609	1512.73033	0.02267245	0.09399129	0.80482892	0.91135243	NUDT5
51339	ENSG00000165617	114.129219	-1.78945128	0.29736698	1.19E-10	2.26E-09	DACT1
27199	ENSG00000165621	6.88460709	0.03430981	0.35467868	0.81065786	0.9141961	OXGR1
221044	ENSG00000165623	0.173733	0.01914545	0.38484597	0.78225479	NA	UCMA
222389	ENSG00000165626	372.145977	0.21811148	0.15483544	0.12296438	0.29029191	BEND7
509	ENSG00000165629	1887.80565	-0.09194996	0.07006477	0.18158325	0.37906267	ATP5F1C
8559	ENSG00000165630	415.892492	-0.13471795	0.12243566	0.24445483	0.46304981	PRPF18
83860	ENSG00000165632	370.148534	-0.274081	0.12458131	0.01960942	0.07178059	TAF3
196740	ENSG00000165633	725.177341	-0.1953422	0.0925937	0.02933436	0.09860958	VSTM4
7417	ENSG00000165637	2119.35711	-0.11987226	0.06397389	0.05717444	0.16506708	VDAC2
402381	ENSG00000165643	0.44795842	-0.02501848	0.38558245	0.5807882	NA	SOHLH1
118881	ENSG00000165644	124.835039	-0.57123648	0.22199228	0.00278787	0.01424083	COMTD1
6571	ENSG00000165646	30.266315	-1.72001326	0.49440381	3.11E-05	0.00026655	SLC18A2
118987	ENSG00000165650	1102.82444	-0.23072456	0.08932596	0.00773912	0.03355421	PDZD8
84858	ENSG00000165655	772.470789	-0.80886875	0.10138206	2.76E-16	8.18E-15	ZNF503
23172	ENSG00000165660	500.387331	-0.11977151	0.11372796	0.26919996	0.49225135	ABRAXAS2
169714	ENSG00000165661	1199.74112	-0.30491955	0.08235047	0.00014495	0.00106236	QSOX2
63877	ENSG00000165669	471.52031	-0.11784887	0.11296628	0.27435854	0.49810335	FAM204A
64324	ENSG00000165671	3529.82785	-0.2222504	0.09969735	0.02057604	0.0745623	NSD1
10935	ENSG00000165672	2084.64083	-0.48316834	0.06739188	3.19E-13	7.49E-12	PRDX3
10495	ENSG00000165675	239.515747	-0.06221871	0.19262716	0.70749544	0.85903201	ENOX2

27069	ENSG00000165678	1564.76486	-0.07800327	0.07003394	0.25687337	0.47790765	GHITM
51266	ENSG00000165682	0	NA	NA	NA	NA	CLEC1B
6621	ENSG00000165684	1250.8276	0.2055674	0.08050614	0.00891882	0.03772908	SNAPC4
120939	ENSG00000165685	0	NA	NA	NA	NA	TMEM52B
23203	ENSG00000165688	1513.91457	-0.20677687	0.09009661	0.01792417	0.06671962	PMPCA
10807	ENSG00000165689	1198.4798	-0.0617582	0.09833968	0.51466792	0.72937777	ENTR1
90167	ENSG00000165694	2.79364949	-0.03632127	0.37485393	0.70035793	0.85550433	FRMD7
158067	ENSG00000165695	9.74422171	0.17715836	0.39605679	0.26708999	0.48947584	AK8
11092	ENSG00000165698	107.019156	0.304906	0.21066967	0.08750045	0.22592516	SPACA9
7248	ENSG00000165699	1393.94127	0.09387856	0.08179431	0.24020553	0.45820056	TSC1
8328	ENSG00000165702	0.47217664	-0.00720291	0.38322146	0.86798003	NA	GFI1B
3251	ENSG00000165704	529.380678	-0.20668305	0.14352496	0.11908704	0.28363587	HPRT1
118426	ENSG00000165714	361.219984	0.10078186	0.16117138	0.49133891	0.71066297	BORCS5
138311	ENSG00000165716	1316.12058	-0.14755867	0.08147457	0.06314403	0.17813182	DIPK1B
116225	ENSG00000165724	590.918101	-0.17882466	0.10190943	0.06806219	0.1877746	ZMYND19
219736	ENSG00000165730	83.1110943	1.08438945	0.2656025	4.77E-06	4.82E-05	STOX1
5979	ENSG00000165731	5787.03069	1.41794732	0.09206685	1.14E-54	1.29E-52	RET
9188	ENSG00000165732	5506.14861	-0.21713765	0.05291158	3.40E-05	0.00028989	DDX21
9790	ENSG00000165733	1809.70409	-0.00572326	0.0706262	0.93357333	0.97233948	BMS1
282974	ENSG00000165752	420.90348	-0.28125523	0.11873666	0.01242087	0.04959348	STK32C
57608	ENSG00000165757	56.874746	-0.22382112	0.26630875	0.26348184	0.48542909	JCAD
390431	ENSG00000165762	0	NA	NA	NA	NA	OR4K2
65991	ENSG00000165775	1116.10387	-0.13074866	0.09469892	0.15349278	0.33847913	FUNDC2
90809	ENSG00000165782	523.406873	0.23547979	0.11161124	0.0271193	0.09279871	PIP4P1
64745	ENSG00000165792	1013.69313	0.07537964	0.09188451	0.39874242	0.62756224	METTL17
29986	ENSG00000165794	0	NA	NA	NA	NA	SLC39A2
57447	ENSG00000165795	326.051571	0.67489738	0.1554309	3.33E-06	3.45E-05	NDRG2
84659	ENSG00000165799	0.16735865	0.01897177	0.38482933	0.78225479	NA	RNASE7
55701	ENSG00000165801	1652.96838	-0.12732352	0.08407567	0.12004946	0.28527742	ARHGEF40
26012	ENSG00000165802	2771.97664	0.0470259	0.06677535	0.47870269	0.7003511	NSMF
51222	ENSG00000165804	1555.69108	0.22087279	0.08251787	0.00604757	0.02733735	ZNF219
160419	ENSG00000165805	2.20660589	-0.12127001	0.41080128	0.03673019	NA	C12orf50
840	ENSG00000165806	248.056994	0.32127468	0.18437074	0.04756486	0.14317808	CASP7
145376	ENSG00000165807	1.80139273	-0.03908088	0.38020193	0.59126722	NA	PPP1R36
153579	ENSG00000165810	0	NA	NA	NA	NA	BTNL9
55088	ENSG00000165813	350.132215	0.23548881	0.14657301	0.08111614	0.21363921	CCDC186
340706	ENSG00000165816	0.61751279	0.03585022	0.38655501	0.41235257	NA	VWA2
56339	ENSG00000165819	1970.33342	0.34555013	0.07047723	5.88E-07	6.94E-06	METTL3

6297	ENSG00000165821	1103.62318	-0.24370629	0.08273144	0.00250603	0.01300029	SALL2
118471	ENSG00000165828	4.98636661	-0.06829719	0.37020129	0.58031487	0.77774473	PRAP1
142940	ENSG00000165832	494.873464	-0.09265131	0.10719869	0.36673598	0.59606123	TRUB1
220081	ENSG00000165837	5.77840572	0.10246568	0.37389772	0.45598613	0.67958359	ERICH6B
1557	ENSG00000165841	0.45209736	-0.02505403	0.38558831	0.57865948	NA	CYP2C19
53349	ENSG00000165861	470.839946	-0.09026992	0.12611622	0.44727406	0.67119864	ZFYVE1
143379	ENSG00000165863	2.19471467	0.00837943	0.37467418	0.9242075	NA	C10orf82
259217	ENSG00000165868	1544.72148	-1.13179115	0.08257965	1.05E-43	8.97E-42	HSPA12A
NA	ENSG00000165874	19.8866609	0.04988449	0.34720481	0.75047009	0.88259197	SHLD2P1
10023	ENSG00000165879	82.448636	0.44558648	0.2527594	0.02907597	0.09795675	FRAT1
80019	ENSG00000165886	112.232366	0.24112495	0.22060455	0.18779626	0.38811227	UBTD1
26287	ENSG00000165887	0.48001468	-0.0354033	0.38631979	0.49116481	NA	ANKRD2
144455	ENSG00000165891	479.3957	0.46954547	0.133981	0.00019129	0.00136667	E2F7
143872	ENSG00000165895	28.888656	6.60530171	1.06851193	4.49E-10	8.07E-09	ARHGAP42
122961	ENSG00000165898	283.814336	-0.08582915	0.13624242	0.49946657	0.71682702	ISCA2
283310	ENSG00000165899	0	NA	NA	NA	NA	OTOGL
120071	ENSG00000165905	135.593508	1.40588496	0.24816378	1.17E-09	2.00E-08	LARGE2
29763	ENSG00000165912	492.193843	1.12822633	0.11506759	1.27E-23	5.78E-22	PACSIN3
145567	ENSG00000165914	573.773947	-0.30036227	0.11101152	0.00449698	0.02129357	TTC7B
91252	ENSG00000165915	928.441429	-0.23006098	0.09577133	0.01264993	0.05031567	SLC39A13
5702	ENSG00000165916	1788.65053	-0.15221699	0.08833841	0.07601573	0.20344694	PSMC3
5913	ENSG00000165917	0.83112686	0.02089355	0.38085822	0.73074749	NA	RAPSN
79841	ENSG00000165923	20.4697377	0.05515624	0.31843626	0.76414489	0.8914874	AGBL2
123036	ENSG00000165929	0	NA	NA	NA	NA	TC2N
53981	ENSG00000165934	1339.23936	-0.25389997	0.09236238	0.0045098	0.02133899	CPSF2
341346	ENSG00000165935	0.36029818	0.02384302	0.38538512	0.61219644	NA	SMCO2
64112	ENSG00000165943	955.889332	-0.38703688	0.08841776	6.56E-06	6.44E-05	MOAP1
122509	ENSG00000165948	147.362209	0.24098899	0.20944842	0.17306146	0.36578929	IFI27L1
3429	ENSG00000165949	41.2621299	1.05495374	0.40362309	0.00082242	0.0049857	IFI27
145264	ENSG00000165953	0	NA	NA	NA	NA	SERPINA12
79789	ENSG00000165959	179.341362	0.0128027	0.21335852	0.94420183	0.97728379	CLMN
29951	ENSG00000165966	3.79951255	0.08689175	0.38289048	0.41256035	0.64014381	PDZRN4
9152	ENSG00000165970	19.0675828	-1.32286959	0.65742718	0.00241963	0.01260981	SLC6A5
120935	ENSG00000165972	18.8357248	0.02676577	0.31908751	0.88361877	0.94890373	CCDC38
4745	ENSG00000165973	796.331629	-0.41628542	0.1060464	4.28E-05	0.00035718	NELL1
9317	ENSG00000165983	12.1090045	1.5536218	0.88121621	0.00326577	0.01626735	PTER
389941	ENSG00000165985	13.4284147	3.10135971	0.9902274	7.50E-05	0.00058997	C1QL3
783	ENSG00000165995	151.63063	0.4680142	0.19404457	0.00616647	0.02772326	CACNB2

9200	ENSG00000165996	263.200279	0.24836721	0.16008516	0.08736055	0.2257402	HACD1
221079	ENSG00000165997	1837.12218	0.06644474	0.07527889	0.36859482	0.59798654	ARL5B
56935	ENSG00000166002	190.814288	0.73267221	0.22043251	0.00016529	0.0011976	SMCO4
85459	ENSG00000166004	919.17644	-0.26633255	0.09369346	0.00320751	0.01600127	CEP295
3747	ENSG00000166006	0	NA	NA	NA	NA	KCNC2
NA	ENSG00000166007	0	NA	NA	NA	NA	TRIM51HP
79101	ENSG00000166012	2034.13437	-0.13001973	0.07129693	0.06320065	0.17823752	TAF1D
NA	ENSG00000166013	0	NA	NA	NA	NA	TRIM53BP
25841	ENSG00000166016	199.633758	-1.6614008	0.18632088	3.65E-20	1.38E-18	ABTB2
27291	ENSG00000166024	339.88938	0.01545274	0.12661325	0.89872126	0.95556135	R3HCC1L
154810	ENSG00000166025	4330.27158	-0.12386062	0.05484453	0.02239702	0.07975033	AMOTL1
5654	ENSG00000166033	66.6457416	1.85726214	0.32942661	1.10E-09	1.88E-08	HTRA1
3990	ENSG00000166035	0	NA	NA	NA	NA	LIPC
9702	ENSG00000166037	1032.56959	-0.2123608	0.09037787	0.01534658	0.05867723	CEP57
255394	ENSG00000166046	53.66854	0.08467835	0.27673186	0.66459289	0.83444869	TCP11L2
139135	ENSG00000166049	0	NA	NA	NA	NA	PASD1
161742	ENSG00000166068	879.770018	0.01887959	0.09711488	0.84156181	0.92800104	SPRED1
145942	ENSG00000166069	0	NA	NA	NA	NA	TMCO5A
11245	ENSG00000166073	918.126618	-0.73079414	0.09664142	8.77E-15	2.34E-13	GPR176
83700	ENSG00000166086	1738.51858	-0.16153849	0.07269733	0.02343372	0.08269745	JAM3
64806	ENSG00000166090	0	NA	NA	NA	NA	IL25
116173	ENSG00000166091	0	NA	NA	NA	NA	CMTM5
NA	ENSG00000166104	0	NA	NA	NA	NA	
112937	ENSG00000166105	213.865815	-2.76015251	0.20422028	7.23E-43	5.88E-41	GLB1L3
170689	ENSG00000166106	0.53038805	0.03940474	0.38680981	0.41702279	NA	ADAMTS15
55530	ENSG00000166111	274.652086	-3.10936608	0.21472318	6.25E-49	5.96E-47	SVOP
219938	ENSG00000166118	0	NA	NA	NA	NA	SPATA19
84706	ENSG00000166123	1933.62728	0.37067895	0.11482527	0.00070185	0.00433655	GPT2
81693	ENSG00000166126	1.12588503	-0.0459897	0.38306303	0.49565157	NA	AMN
51762	ENSG00000166128	409.582957	-0.27064582	0.12450459	0.0209494	0.07555646	RAB8B
121457	ENSG00000166130	356.988058	-0.04244138	0.13055109	0.72855313	0.87026999	IKBIP
27079	ENSG00000166133	216.254845	0.30399987	0.20147235	0.07841384	0.20834747	RPUSD2
55662	ENSG00000166135	2003.15918	-0.20551843	0.06710138	0.0018335	0.00993627	HIF1AN
4714	ENSG00000166136	1532.76589	-0.05576679	0.08424681	0.4969279	0.71500229	NDUFB8
84936	ENSG00000166140	322.110496	0.14436812	0.1477952	0.28938843	0.51546747	ZFYVE19
54866	ENSG00000166143	0.173124	-0.01911262	0.38486262	0.84537521	NA	PPP1R14D
6692	ENSG00000166145	66.3851875	0.61876501	0.31330676	0.01006639	0.04165471	SPINT1
2200	ENSG00000166147	1226.54979	-0.43356232	0.08964495	6.29E-07	7.39E-06	FBN1

552	ENSG00000166148	0	NA	NA	NA	NA	AVPR1A
123970	ENSG00000166152	0	NA	NA	NA	NA	C16orf78
120863	ENSG00000166153	34.5411496	0.11325831	0.30030392	0.56170248	0.76502898	DEPDC4
654429	ENSG00000166159	4.89637704	-0.01015455	0.36732039	0.92218399	0.96738383	LRTM2
728458	ENSG00000166160	0	NA	NA	NA	NA	OPN1MW2
29117	ENSG00000166164	1891.10149	0.01857323	0.0778394	0.80821393	0.91317199	BRD7
1152	ENSG00000166165	8312.94075	-0.51159225	0.06178454	4.77E-17	1.48E-15	CKB
115708	ENSG00000166166	467.40254	-0.07905896	0.12960822	0.51612361	0.73027332	TRMT61A
8945	ENSG00000166167	763.07839	-0.33371614	0.11490533	0.00225195	0.01185112	BTRC
27343	ENSG00000166169	525.635847	0.10885576	0.12422877	0.3544769	0.58494733	POLL
9529	ENSG00000166170	763.960708	-0.28965574	0.09762513	0.00209228	0.01111697	BAG5
25911	ENSG00000166171	253.312244	0.37751472	0.1487638	0.00593761	0.02693239	DPCD
55323	ENSG00000166173	274.367615	-0.12890118	0.17718464	0.41100563	0.63865537	LARP6
8539	ENSG00000166181	1844.52111	0.09978047	0.07778813	0.19039699	0.39167661	API5
374569	ENSG00000166183	1.65534621	0.07420061	0.38963652	0.27786859	NA	ASPG
57567	ENSG00000166188	360.783454	-0.04605036	0.16493336	0.7572656	0.88697677	ZNF319
79803	ENSG00000166189	336.693065	-0.10974566	0.12815665	0.36249474	0.59216926	HPS6
123228	ENSG00000166192	46.8731832	-0.38217815	0.35112078	0.09627348	0.2423634	SENP8
9221	ENSG00000166197	4656.7939	-0.32388075	0.05313576	7.20E-10	1.26E-08	NOLC1
221120	ENSG00000166199	222.989379	0.02209675	0.16542174	0.88380884	0.94897201	ALKBH3
9318	ENSG00000166200	1239.88703	-0.49208372	0.08211144	8.45E-10	1.47E-08	COPS2
2562	ENSG00000166206	1273.49138	-3.19875738	0.09909673	5.68E-230	1.61E-226	GABRB3
121599	ENSG00000166211	0	NA	NA	NA	NA	SPIC
219793	ENSG00000166220	0	NA	NA	NA	NA	TBATA
8879	ENSG00000166224	1563.05563	0.14722525	0.07098408	0.03433822	0.11170355	SGPL1
10818	ENSG00000166225	812.607213	0.11609134	0.12393654	0.32207293	0.54965002	FRS2
10576	ENSG00000166226	4320.7004	-0.04320803	0.0740999	0.54912941	0.75563376	CCT2
5092	ENSG00000166228	931.538811	-0.3055369	0.09967355	0.00144735	0.00813008	PCBD1
25820	ENSG00000166233	1321.63518	-0.17217595	0.08892811	0.0461012	0.13985441	ARIH1
146562	ENSG00000166246	15.8127491	-0.3954845	0.53813401	0.07320075	0.19780754	C16orf71
79827	ENSG00000166250	2229.50361	-0.3054968	0.06540751	2.00E-06	2.16E-05	CLMP
55800	ENSG00000166257	780.372459	-2.08127205	0.10391672	2.09E-90	5.53E-88	SCN3B
1353	ENSG00000166260	1274.72555	-0.08074352	0.08512416	0.32974834	0.55849471	COX11
7753	ENSG00000166261	493.830715	-0.04770488	0.11239198	0.65615073	0.82907086	ZNF202
196951	ENSG00000166262	72.3486432	0.00924345	0.25507708	0.9630764	0.98545957	FAM227B
252983	ENSG00000166263	531.524771	-0.17808303	0.26966677	0.36887308	0.59820516	STXBP4
116159	ENSG00000166265	0.15069912	-0.01848503	0.38480575	0.84537521	NA	CYYR1
8065	ENSG00000166266	1023.91743	-0.1708566	0.1119662	0.10963278	0.26642754	CUL5

196446	ENSG00000166268	0	NA	NA	NA	NA	MYRFL
54838	ENSG00000166272	596.309083	-0.02653613	0.10550291	0.79215147	0.90499209	WBP1L
102724307	ENSG00000166272	596.309083	-0.02653613	0.10550291	0.79215147	0.90499209	WBP1L
119032	ENSG00000166275	313.566476	-0.00994678	0.14662151	0.94037819	0.97517789	BORCS7
717	ENSG00000166278	0.70025675	0.016725	0.38289955	0.74186697	NA	C2
79156	ENSG00000166289	50.9916888	-0.03301562	0.26161403	0.86199492	0.9387792	PLEKHF1
55273	ENSG00000166292	326.330483	2.43469839	0.17884846	2.43E-43	2.03E-41	TMEM100
119504	ENSG00000166295	921.295829	-0.21709428	0.09183849	0.01465659	0.0565733	ANAPC16
6609	ENSG00000166311	302.246883	-0.04528374	0.18014766	0.77452658	0.89613481	SMPD1
322	ENSG00000166313	1463.57308	-0.10480948	0.074568	0.15180757	0.33588293	APBB1
79933	ENSG00000166317	25.0300082	0.03359504	0.31663831	0.85341538	0.93396002	SYNPO2L
25961	ENSG00000166321	89.1001574	0.11627185	0.22100471	0.52255063	0.73580254	NUDT13
160140	ENSG00000166323	10.3770335	-0.05440945	0.35087517	0.71511769	0.86271551	C11orf65
54765	ENSG00000166326	2995.6089	0.1054351	0.10557007	0.29916786	0.52614931	TRIM44
101927581	ENSG00000166329	1.86498491	0.07560952	0.38709497	0.35244729	NA	CCDC182
3611	ENSG00000166333	1105.91226	0.04231734	0.07726476	0.5757679	0.7744963	ILK
6881	ENSG00000166337	1429.44304	0.02907167	0.07798182	0.70356393	0.85751377	TAF10
1200	ENSG00000166340	820.179927	0.13857838	0.12021623	0.22437235	0.43811368	TPP1
8642	ENSG00000166341	6105.67825	-0.25183336	0.05442528	2.90E-06	3.04E-05	DCHS1
81832	ENSG00000166342	0	NA	NA	NA	NA	NETO1
118490	ENSG00000166343	47.1682303	0.1594494	0.27979861	0.42361891	0.64955555	MSS51
1528	ENSG00000166347	489.798007	-0.00992536	0.11400314	0.92647303	0.96932621	CYB5A
159195	ENSG00000166348	591.174615	-0.23540753	0.11536598	0.03172671	0.10489217	USP54
5896	ENSG00000166349	78.2591022	-0.80255939	0.34302795	0.00261323	0.01347361	RAG1
317754	ENSG00000166351	0.18922458	0.01954053	0.38488496	0.78225479	NA	POTED
119710	ENSG00000166352	621.264058	-0.43509073	0.10487111	1.57E-05	0.00014387	IFTAP
126248	ENSG00000166359	14.4210309	0.14506648	0.3586186	0.41229933	0.64005916	WDR88
144124	ENSG00000166363	0	NA	NA	NA	NA	OR10A5
120776	ENSG00000166368	0	NA	NA	NA	NA	OR2D2
374868	ENSG00000166377	378.747709	0.06747588	0.13843375	0.60251936	0.79259054	ATP9B
8495	ENSG00000166387	400.337795	-1.03748761	0.20283143	3.47E-08	4.86E-07	PPFIBP2
80168	ENSG00000166391	0	NA	NA	NA	NA	MOGAT2
51700	ENSG00000166394	16.5923783	0.0583635	0.34733345	0.70565146	0.85810136	CYB5R2
8710	ENSG00000166396	0	NA	NA	NA	NA	SERPINB7
9710	ENSG00000166398	821.699917	0.16672689	0.0902585	0.0573078	0.16533181	GARRE1
5271	ENSG00000166401	14.0487968	0.17764796	0.37952812	0.30739485	0.53486671	SERPINB8
7275	ENSG00000166402	4387.75904	-0.46797157	0.05677641	3.48E-17	1.09E-15	TUB
79608	ENSG00000166405	522.50336	-0.48725048	0.12294733	2.90E-05	0.00025076	RIC3

4004	ENSG00000166407	554.811193	-0.8325246	0.11426462	5.65E-14	1.41E-12	LMO1
NA	ENSG00000166408	0	NA	NA	NA	NA	OR5P1P
3419	ENSG00000166411	537.219685	-0.31460118	0.11196845	0.00318977	0.01593228	IDH3A
256764	ENSG00000166415	0	NA	NA	NA	NA	WDR72
1381	ENSG00000166426	480.688212	0.36606369	0.12568837	0.00201635	0.01077441	CRABP1
122618	ENSG00000166428	0.71563014	0.02307282	0.38312712	0.59572404	NA	PLD4
84460	ENSG00000166432	395.265776	0.35627477	0.14232355	0.00699088	0.03094578	ZMAT1
143570	ENSG00000166435	346.376775	-0.26091132	0.15316948	0.06232829	0.17640417	XRRA1
9866	ENSG00000166436	532.793906	-0.08156215	0.14655971	0.54424633	0.75220113	TRIM66
254225	ENSG00000166439	636.658573	0.03558064	0.10419101	0.72498318	0.86844186	RNF169
6157	ENSG00000166441	17129.6918	-0.06467749	0.04458162	0.13875648	0.31543588	RPL27A
6764	ENSG00000166444	910.392535	0.17082324	0.09116678	0.05352961	0.15677997	DENND2B
124359	ENSG00000166446	1.26304592	0.07023556	0.39299654	0.13809485	NA	CDYL2
222865	ENSG00000166448	693.700599	-0.4104831	0.10759948	6.86E-05	0.00054557	TMEM130
283659	ENSG00000166450	939.345641	-0.24405702	0.11069443	0.0208425	0.07533523	PRTG
55839	ENSG00000166451	493.219757	-0.51285997	0.14230019	0.00011221	0.00084232	CENPN
56672	ENSG00000166452	223.467972	0.02037476	0.17044894	0.89540763	0.95469109	AKIP1
23300	ENSG00000166454	1550.9223	-0.10763056	0.07815504	0.16342313	0.35246384	ATMIN
123775	ENSG00000166455	8.15458342	0.05578857	0.3661037	0.65935209	0.83105722	C16orf46
440026	ENSG00000166471	792.725469	0.07520923	0.11062898	0.4782274	0.69986975	TMEM41B
114780	ENSG00000166473	0.173124	-0.01911262	0.38486262	0.84537521	NA	PKD1L2
123169	ENSG00000166477	715.998175	-0.72477565	0.09851746	4.16E-14	1.06E-12	LEO1
7702	ENSG00000166478	431.779585	-0.19366342	0.13827377	0.13163549	0.30439464	ZNF143
54495	ENSG00000166479	1205.91458	-0.01389098	0.08503582	0.86091141	0.9380757	TMX3
4239	ENSG00000166482	8152.64825	1.22702558	0.06614207	8.84E-78	1.72E-75	MFAP4
7465	ENSG00000166483	1031.65292	-0.37756083	0.11875323	0.0007973	0.00485824	WEE1
5598	ENSG00000166484	863.352353	0.25172929	0.10560732	0.01293245	0.05125444	MAPK7
NA	ENSG00000166492	0.3180506	0.00098569	0.38336561	0.96763183	NA	FAM86GP
5579	ENSG00000166501	0	NA	NA	NA	NA	PRKCB
50810	ENSG00000166503	2193.05806	-0.37767575	0.06759284	1.31E-08	1.96E-07	HDGFL3
8509	ENSG00000166507	437.757323	0.0921751	0.15487469	0.51587702	0.73002854	NDST2
4176	ENSG00000166508	11033.8124	0.06718476	0.05582165	0.22453184	0.43832645	MCM7
10143	ENSG00000166509	0	NA	NA	NA	NA	CLEC3A
80323	ENSG00000166510	45.0100406	0.0419051	0.27216492	0.82978234	0.922859	CCDC68
26253	ENSG00000166523	0	NA	NA	NA	NA	CLEC4E
7551	ENSG00000166526	1895.12402	0.2543176	0.07024077	0.00022642	0.00158845	ZNF3
338339	ENSG00000166527	0	NA	NA	NA	NA	CLEC4D
7589	ENSG00000166529	413.178469	0.16686109	0.12282983	0.15129641	0.33531286	ZSCAN21

NA	ENSG00000166530	0	NA	NA	NA	NA	HSBP1P2
57494	ENSG00000166532	1605.39756	0.04897609	0.08138094	0.53850176	0.74827532	RIMKLB
144568	ENSG00000166535	0.18922458	0.01954053	0.38488496	0.78225479	NA	A2ML1
146227	ENSG00000166546	3.55471473	0.0556904	0.37584806	0.58717306	0.78237765	BEAN1
7084	ENSG00000166548	245.429391	0.33192895	0.17021709	0.02979707	0.09982672	TK2
23423	ENSG00000166557	1153.13003	-0.10536872	0.08503435	0.20368599	0.41010046	TMED3
146167	ENSG00000166558	0	NA	NA	NA	NA	SLC38A8
90701	ENSG00000166562	516.918661	-0.03860176	0.10703139	0.70606626	0.85825065	SEC11C
339302	ENSG00000166569	0	NA	NA	NA	NA	CPLX4
2587	ENSG00000166573	0	NA	NA	NA	NA	GALR1
65084	ENSG00000166575	399.169347	-0.11594434	0.15968497	0.4228416	0.64881495	TMEM135
115811	ENSG00000166578	31.2082929	1.15127648	0.52280118	0.00196268	0.01052991	IQCD
81565	ENSG00000166579	457.699619	0.11560636	0.11459464	0.29062274	0.51667585	NDEL1
201161	ENSG00000166582	2420.02969	0.00816539	0.08100681	0.91909427	0.96566562	CENPV
1014	ENSG00000166589	0.15069912	-0.01848503	0.38480575	0.84537521	NA	CDH16
6236	ENSG00000166592	4.45275555	0.24292628	0.47686876	0.06465931	0.18119939	RRAD
51647	ENSG00000166595	1473.3152	0.13884048	0.08321764	0.0872656	0.22552422	CIAO2B
146845	ENSG00000166596	0	NA	NA	NA	NA	CFAP52
7184	ENSG00000166598	15958.0263	0.55140103	0.08400955	1.84E-11	3.82E-10	HSP90B1
4160	ENSG00000166603	8.93865668	0.0440981	0.3496991	0.77229561	0.89548539	MC4R
10904	ENSG00000166619	1207.56552	0.04271386	0.0817534	0.59345679	0.7872548	BLCAP
89777	ENSG00000166634	0	NA	NA	NA	NA	SERPINB12
89832	ENSG00000166664	206.982309	0.5081114	0.2168905	0.00636525	0.02851342	CHRFAM7A
80063	ENSG00000166669	189.660004	0.41858672	0.2031254	0.01718653	0.06451859	ATF7IP2
4319	ENSG00000166670	0	NA	NA	NA	NA	MMP10
780776	ENSG00000166676	330.711417	-0.03022752	0.15841064	0.83251997	0.92392457	TVP23A
27018	ENSG00000166681	3020.12422	-0.07551187	0.06223623	0.21808761	0.42997696	BEX3
80975	ENSG00000166682	22.6051055	1.2723861	0.97728158	0.00621331	0.02788959	TMPRSS5
9382	ENSG00000166685	937.37038	-0.00012106	0.08990039	0.99786794	0.99926338	COG1
144100	ENSG00000166689	341.985636	0.5602419	0.14286903	2.83E-05	0.00024624	PLEKHA7
NA	ENSG00000166693	0	NA	NA	NA	NA	OR5M13P
80095	ENSG00000166704	345.832087	0.13962897	0.16128165	0.33993135	0.57028903	ZNF606
644353	ENSG00000166707	169.857484	0.20825105	0.1893297	0.20696273	0.41417369	ZCCHC18
567	ENSG00000166710	1562.99793	0.07528788	0.06846828	0.26402253	0.48609939	B2M
9640	ENSG00000166716	1444.09345	-0.23162933	0.07408596	0.00140579	0.00793707	ZNF592
113201	ENSG00000166734	1627.31538	0.03440755	0.07605041	0.6449507	0.82283284	GOLM2
3359	ENSG00000166736	0	NA	NA	NA	NA	HTR3A
4837	ENSG00000166741	0.173733	0.01914545	0.38484597	0.78225479	NA	NNMT

116285	ENSG00000166743	0	NA	NA	NA	NA	ACSM1
164	ENSG00000166747	1374.71809	-0.19732301	0.08454529	0.01656941	0.06260468	AP1G1
162394	ENSG00000166750	0	NA	NA	NA	NA	SLFN5
117155	ENSG00000166762	91.3833054	-0.49189637	0.296006	0.02816464	0.09547008	CATSPER2
554225	ENSG00000166763	12.1021893	1.8145793	0.87846417	0.00163061	0.00901906	STRCP1
NA	ENSG00000166770	384.741464	0.07254564	0.12105567	0.52847328	0.74019836	ZNF667-AS1
89927	ENSG00000166780	840.186506	-0.52467737	0.09244777	5.12E-09	8.00E-08	BMERB1
9665	ENSG00000166783	1909.05541	0.18472011	0.10998467	0.07924437	0.20999175	MARF1
6290	ENSG00000166787	0	NA	NA	NA	NA	SAA3P
113174	ENSG00000166788	636.61494	-0.22895907	0.10824939	0.02700081	0.09245712	SAAL1
219539	ENSG00000166793	85.0944552	0.24331229	0.24537857	0.21039291	0.41905384	YPEL4
5479	ENSG00000166794	5278.50592	0.39399397	0.06342828	2.90E-10	5.32E-09	PPIB
3948	ENSG00000166796	0.93312285	-0.0220967	0.38190803	0.60757747	NA	LDHC
84191	ENSG00000166797	468.543911	-0.15989019	0.11763169	0.15218612	0.33649534	CIAO2A
160287	ENSG00000166800	8.67624426	-0.02868348	0.34715586	0.84845944	0.93105283	LDHAL6A
63901	ENSG00000166801	176.139606	3.96196011	0.2921695	1.82E-43	1.54E-41	FAM111A
9768	ENSG00000166803	739.404132	-0.41047931	0.09297366	5.16E-06	5.16E-05	PCLAF
374654	ENSG00000166813	591.79113	0.36401596	0.10908738	0.00048784	0.0031582	KIF7
197257	ENSG00000166816	4.18586365	0.24872558	0.49109393	0.03964415	0.12457077	LDHD
5346	ENSG00000166819	10.3089048	0.15606641	0.38121582	0.33416203	0.56389725	PLIN1
8800	ENSG00000166821	218.056939	0.17270216	0.17813145	0.27411797	0.49775768	PEX11A
124491	ENSG00000166822	586.842113	-0.18285804	0.12632656	0.12398367	0.29179342	TMEM170A
55897	ENSG00000166823	57.4374589	0.46324134	0.32442015	0.04693852	0.14178748	MESP1
290	ENSG00000166825	154.679631	-0.21483335	0.22960957	0.24821981	0.46741467	ANPEP
6340	ENSG00000166828	0	NA	NA	NA	NA	SCNN1G
348093	ENSG00000166831	240.026894	1.44286606	0.16544785	2.40E-19	8.69E-18	RBPMS2
89797	ENSG00000166833	8005.1776	0.53492526	0.06525171	9.11E-17	2.77E-15	NAV2
348094	ENSG00000166839	278.180559	-0.13589653	0.16941211	0.37004171	0.59932029	ANKDD1A
92292	ENSG00000166840	16.186122	0.05887029	0.33981254	0.72549773	0.86866734	GLYATL1
162681	ENSG00000166845	424.412588	-0.52923843	0.14411946	8.31E-05	0.0006449	C18orf54
84516	ENSG00000166847	4115.88445	-0.1385592	0.05956776	0.01848707	0.0682896	DCTN5
54386	ENSG00000166848	2227.94113	-0.49304064	0.07181128	2.72E-12	6.02E-11	TERF2IP
5347	ENSG00000166851	1761.89544	-0.12687541	0.08046415	0.10526969	0.25874017	PLK1
10845	ENSG00000166855	762.336017	0.25131085	0.10466949	0.01235156	0.04940623	CLPX
11318	ENSG00000166856	9.60212874	0.26052813	0.44808361	0.14190367	0.32043805	GPR182
9880	ENSG00000166860	871.330102	0.01078201	0.09029448	0.9029863	0.95751119	ZBTB39
10369	ENSG00000166862	136.668927	-3.07454366	0.25776233	6.80E-34	4.41E-32	CACNG2
6866	ENSG00000166863	20.7669879	-0.34482436	0.42403836	0.12470802	0.29301363	TAC3

4640	ENSG00000166866	1.2465507	-0.03277431	0.38265371	0.57241099	NA	MYO1A
63928	ENSG00000166869	0	NA	NA	NA	NA	CHP2
23306	ENSG00000166881	1352.05965	-0.43419614	0.0781878	1.35E-08	2.01E-07	NEMP1
219983	ENSG00000166884	0	NA	NA	NA	NA	OR4D6
4665	ENSG00000166886	309.78288	-0.0941335	0.1389457	0.46643927	0.68929176	NAB2
23339	ENSG00000166887	2239.92182	0.00047049	0.07151217	0.99548404	0.99852582	VPS39
6778	ENSG00000166888	4.11486524	0.12158304	0.38837952	0.33289188	0.56223503	STAT6
219988	ENSG00000166889	941.687105	0.26172946	0.08619628	0.00179965	0.00977684	PATL1
91419	ENSG00000166896	122.236116	-0.04175814	0.20233182	0.80692774	0.91256903	ATP23
114794	ENSG00000166897	43.1419485	-3.05925136	0.45067211	8.16E-13	1.87E-11	ELFN2
6809	ENSG00000166900	459.741296	-0.57424823	0.13120488	3.71E-06	3.81E-05	STX3
54948	ENSG00000166902	414.955154	-0.08945691	0.12898178	0.46063314	0.68374693	MRPL16
79837	ENSG00000166908	653.012439	0.22325376	0.11845237	0.04703473	0.14194844	PIP4K2C
54893	ENSG00000166912	400.814852	0.02336439	0.12018451	0.8386201	0.92674595	MTMR10
7529	ENSG00000166913	4320.7218	-0.2341623	0.06939738	0.00058895	0.00372244	YWHAB
101927671	ENSG00000166917	0	NA	NA	NA	NA	MIR202HG
84419	ENSG00000166920	1.3020088	-0.01851317	0.3776269	0.80753936	NA	C15orf48
6447	ENSG00000166922	166.775217	1.67354776	0.20582652	3.19E-17	1.00E-15	SCG5
26585	ENSG00000166923	1.96510275	-0.06220858	0.38811123	0.30407606	NA	GREM1
222950	ENSG00000166924	652.226081	-0.22491597	0.10637681	0.0272669	0.09311107	NYAP1
81628	ENSG00000166925	610.291673	0.15567691	0.10273952	0.11548358	0.27727493	TSC22D4
245802	ENSG00000166926	0	NA	NA	NA	NA	MS4A6E
58475	ENSG00000166927	0	NA	NA	NA	NA	MS4A7
84689	ENSG00000166928	0	NA	NA	NA	NA	MS4A14
64232	ENSG00000166930	0	NA	NA	NA	NA	MS4A5
115752	ENSG00000166938	812.67053	-0.06605762	0.10320243	0.50479261	0.72128811	DIS3L
23582	ENSG00000166946	752.070874	-0.01759859	0.09917267	0.85331796	0.93390491	CCNDBP1
2038	ENSG00000166947	0.18922458	0.01954053	0.38488496	0.78225479	NA	EPB42
343641	ENSG00000166948	0	NA	NA	NA	NA	TGM6
4088	ENSG00000166949	897.475418	0.16663236	0.10738434	0.10564561	0.25934285	SMAD3
83661	ENSG00000166959	0	NA	NA	NA	NA	MS4A8
374864	ENSG00000166960	0.74020022	-0.00504882	0.382087	0.94748844	NA	CCDC178
219995	ENSG00000166961	0	NA	NA	NA	NA	MS4A15
4130	ENSG00000166963	1460.48387	-0.19896897	0.07659484	0.00793686	0.03427672	MAP1A
91433	ENSG00000166965	924.06723	0.0008441	0.09398337	0.98030779	0.99338199	RCCD1
64400	ENSG00000166971	330.249143	0.03655046	0.14264888	0.78363126	0.9014745	AKTIP
10982	ENSG00000166974	958.132106	-0.13223048	0.09110039	0.13268171	0.30598688	MAPRE2
59271	ENSG00000166979	45.20187	-0.03051817	0.26751348	0.87197901	0.94401928	EVA1C

NA	ENSG00000166984	0.47855198	-0.0099778	0.38335877	0.83031632	NA	TCP10L2
4141	ENSG00000166986	4533.885	0.4938922	0.07315873	6.16E-12	1.33E-10	MARS1
114785	ENSG00000166987	1096.67389	0.61797626	0.11061397	6.55E-09	1.01E-07	MBD6
245812	ENSG00000166997	312.143346	0.28340277	0.13807851	0.02774395	0.0942857	CNPY4
2923	ENSG00000167004	5960.25857	0.32361104	0.08002901	3.45E-05	0.00029372	PDIA3
11051	ENSG00000167005	4170.5484	-0.20839676	0.07348288	0.00380578	0.01847935	NUDT21
375607	ENSG00000167011	215.143167	-0.345306	0.1649546	0.0203621	0.07390853	NAT16
145645	ENSG00000167014	0	NA	NA	NA	NA	TERB2
4824	ENSG00000167034	0	NA	NA	NA	NA	NKX3-1
129049	ENSG00000167037	308.039137	-0.05771454	0.14679756	0.66923522	0.83726414	SGSM1
NA	ENSG00000167046	13.1854438	0.17001326	0.36364226	0.35366978	0.58401019	
150290	ENSG00000167065	159.745282	0.20573907	0.17860487	0.19380836	0.39665339	DUSP18
7008	ENSG00000167074	105.430104	0.0532155	0.20831475	0.76265902	0.89054624	TEF
150365	ENSG00000167077	0.56622391	0.04121299	0.38703405	0.39547994	NA	MEI1
124872	ENSG00000167080	0	NA	NA	NA	NA	B4GALNT2
5090	ENSG00000167081	1305.16483	-0.02712542	0.08512834	0.74340003	0.87901881	PBX3
2793	ENSG00000167083	0	NA	NA	NA	NA	GNGT2
5245	ENSG00000167085	3563.00125	0.01505942	0.06430477	0.81224764	0.91499949	PHB
6632	ENSG00000167088	1741.72001	0.03675181	0.10226181	0.71030767	0.86089825	SNRPD1
158248	ENSG00000167094	4.41770387	0.12116064	0.38822201	0.33415525	0.56389725	TTC16
140732	ENSG00000167098	0	NA	NA	NA	NA	SUN5
201191	ENSG00000167100	1003.71944	-0.28200197	0.09556289	0.00223855	0.01179939	SAMD14
138429	ENSG00000167103	87.7239145	0.10394809	0.21728725	0.56426427	0.76680562	PIP5KL1
128859	ENSG00000167104	0	NA	NA	NA	NA	BPIFB6
162461	ENSG00000167105	0.37844916	0.02407552	0.3854191	0.60038877	NA	TMEM92
399665	ENSG00000167106	549.170372	-0.18119688	0.28177491	0.36536731	0.5947154	FAM102A
80221	ENSG00000167107	361.053151	1.58334082	0.13254597	5.76E-34	3.76E-32	ACSF2
2801	ENSG00000167110	1481.25715	0.10287622	0.08647347	0.22264205	0.43570904	GOLGA2
26995	ENSG00000167112	898.432498	-0.36029555	0.09484081	8.45E-05	0.00065457	TRUB2
51117	ENSG00000167113	705.305155	0.12673415	0.09401785	0.16428287	0.35370289	COQ4
10999	ENSG00000167114	716.356601	0.10929723	0.09519502	0.23598553	0.45280389	SLC27A4
55018	ENSG00000167117	0	NA	NA	NA	NA	ANKRD40CL
81605	ENSG00000167118	1107.08697	0.12273998	0.08823619	0.15283714	0.3375586	URM1
51148	ENSG00000167123	426.357875	0.08082658	0.11808011	0.47242139	0.69457676	CERCAM
57171	ENSG00000167130	579.753896	-0.15401735	0.10444379	0.12345648	0.29110727	DOLPP1
388389	ENSG00000167131	33.3659611	0.27228805	0.34333463	0.20638429	0.41335193	CCDC103
2021	ENSG00000167136	598.653851	-0.1864428	0.10978906	0.07474749	0.20094718	ENDOG
161514	ENSG00000167139	0	NA	NA	NA	NA	TBC1D21

51450	ENSG00000167157	162.409592	1.05136201	0.19113977	4.59E-09	7.24E-08	PRRX2
54578	ENSG00000167165	0	NA	NA	NA	NA	UGT1A6
56905	ENSG00000167173	389.562545	-0.20163986	0.13398365	0.10703391	0.26200528	C15orf39
57611	ENSG00000167178	5615.98338	-1.16350155	0.06933716	3.46E-64	4.94E-62	ISLR2
6668	ENSG00000167182	552.720662	-0.09608611	0.13458248	0.44464256	0.66947775	SP2
79170	ENSG00000167183	2.80742508	0.02826377	0.37109534	0.78616464	0.90280441	PRR15L
10229	ENSG00000167186	355.742255	0.07249214	0.1251613	0.54068124	0.74929575	COQ7
51704	ENSG00000167191	223.600623	0.4376306	0.16398102	0.00334568	0.01659458	GPRC5B
1398	ENSG00000167193	2073.05951	-0.19386825	0.06977432	0.0046626	0.02192085	CRK
146378	ENSG00000167194	1.85940607	0.07739212	0.38730093	0.34878705	NA	C16orf92
653641	ENSG00000167195	0.75689831	0.03380966	0.38651369	0.3822658	NA	GOLGA6C
26263	ENSG00000167196	1336.33502	0.3068937	0.07941579	7.38E-05	0.00058207	FBXO22
23102	ENSG00000167202	1426.17031	0.05675323	0.09270232	0.52944173	0.74118854	TBC1D2B
64127	ENSG00000167207	8.81506738	-0.17640691	0.41637547	0.16895749	0.36036515	NOD2
124460	ENSG00000167208	1.03647493	0.02178715	0.38186455	0.69088788	NA	SNX20
125336	ENSG00000167210	0.4596103	0.00434526	0.38316676	0.97273885	NA	LOXHD1
83473	ENSG00000167216	210.735076	0.55791753	0.20305279	0.00178091	0.00968567	KATNAL2
84064	ENSG00000167220	765.761963	-0.28625	0.09447289	0.00171404	0.00939925	HDHD2
7644	ENSG00000167232	856.632897	-0.11470545	0.13488661	0.36229566	0.59197696	ZNF91
3481	ENSG00000167244	9561.09702	0.82534899	0.4439738	0.00699465	0.03095557	IGF2
257160	ENSG00000167257	674.555559	-0.10501052	0.11964896	0.35444885	0.58494733	RNF214
51755	ENSG00000167258	1310.66028	0.00211193	0.09313103	0.98218241	0.99408818	CDK12
64174	ENSG00000167261	0	NA	NA	NA	NA	DPEP2
54920	ENSG00000167264	215.843501	0.03543373	0.16883921	0.81804689	0.9171248	DUS2
51367	ENSG00000167272	269.577802	0.31039543	0.14357873	0.01957177	0.07166922	POP5
64772	ENSG00000167280	1046.99818	0.31139606	0.13002787	0.01067302	0.04373248	ENGASE
146713	ENSG00000167281	12.9459316	0.32303099	0.47374097	0.11342714	0.27329189	RBFOX3
10632	ENSG00000167283	1517.62566	-0.00503877	0.07974645	0.94636868	0.97814213	ATP5MG
915	ENSG00000167286	0	NA	NA	NA	NA	CD3D
125058	ENSG00000167291	6415.9684	-0.05566458	0.05051876	0.26589391	0.48827746	TBC1D16
146705	ENSG00000167302	661.216025	0.15316522	0.1103525	0.14669865	0.32786991	TEPSIN
4645	ENSG00000167306	22.5800395	-0.10817627	0.32928833	0.55631108	0.76192268	MYO5B
116969	ENSG00000167311	2.93956936	0.03212106	0.37215353	0.75056771	0.8826224	ART5
10449	ENSG00000167315	1311.56762	-0.1290284	0.08514888	0.1196293	0.28460369	ACAA2
6786	ENSG00000167323	589.209848	0.47046458	0.1421309	0.00038195	0.00254208	STIM1
6240	ENSG00000167325	2685.8703	-0.36140526	0.07879466	2.66E-06	2.80E-05	RRM1
81285	ENSG00000167332	0	NA	NA	NA	NA	OR51E2
55128	ENSG00000167333	182.046515	0.46984263	0.2082585	0.00899397	0.03800482	TRIM68

56547	ENSG00000167346	0	NA	NA	NA	NA	MMP26
282763	ENSG00000167355	0.16735865	0.01897177	0.38482933	0.78225479	NA	OR51B5
390063	ENSG00000167359	0	NA	NA	NA	NA	OR51I1
390061	ENSG00000167360	0	NA	NA	NA	NA	OR51Q1
64122	ENSG00000167363	455.380293	0.22667604	0.13460565	0.0713979	0.19425868	FN3K
112476	ENSG00000167371	378.519382	0.0637186	0.12540311	0.59154125	0.78613293	PRRT2
7571	ENSG00000167377	401.968963	0.01898357	0.12856337	0.8764589	0.94556523	ZNF23
126298	ENSG00000167378	1026.94303	-0.08352581	0.10115949	0.39128347	0.62079681	IRGQ
7769	ENSG00000167380	566.720675	0.01976322	0.14364959	0.88316682	0.94857242	ZNF226
7733	ENSG00000167384	238.934047	-0.42854664	0.15911869	0.00319052	0.01593228	ZNF180
NA	ENSG00000167390	0	NA	NA	NA	NA	POM121L3P
28227	ENSG00000167393	762.058597	0.36634667	0.09583505	7.60E-05	0.00059681	PPP2R3B
79759	ENSG00000167394	438.571459	0.11682279	0.1219155	0.31225752	0.53997989	ZNF668
9726	ENSG00000167395	1141.75321	0.00275432	0.07910264	0.97202389	0.9895302	ZNF646
79001	ENSG00000167397	1339.17606	-0.18339618	0.07470254	0.0121864	0.04883422	VKORC1
94235	ENSG00000167414	144.181977	0.17325242	0.18784451	0.29149532	0.51742702	GNG8
4025	ENSG00000167419	0.14931947	-0.01844332	0.38480212	0.84537521	NA	LPO
762	ENSG00000167434	0.29863895	-0.02292439	0.3852726	0.68753245	NA	CA4
55181	ENSG00000167447	684.417632	-0.1072363	0.10228121	0.27575853	0.49982179	SMG8
148231	ENSG00000167459	0	NA	NA	NA	NA	LINC00905
7171	ENSG00000167460	9826.22524	0.05356668	0.05294017	0.30997084	0.53774299	TPM4
4218	ENSG00000167461	830.008981	-0.15478207	0.11578161	0.15854265	0.34569581	RAB8A
2879	ENSG00000167468	1624.7812	-0.61673391	0.08288375	2.87E-14	7.41E-13	GPX4
90007	ENSG00000167470	2506.14231	0.14628665	0.06337513	0.01921158	0.07049338	MIDN
126306	ENSG00000167476	363.422359	2.42566377	0.15673696	3.43E-55	4.01E-53	JSRP1
105372240	ENSG00000167476	363.422359	2.42566377	0.15673696	3.43E-55	4.01E-53	JSRP1
199786	ENSG00000167483	3.27433269	-0.03245497	0.37055502	0.75770342	0.88730562	NIBAN3
55295	ENSG00000167487	302.750423	-0.12860951	0.15563572	0.365395	0.5947154	KLHL26
54815	ENSG00000167491	2235.81339	-0.22776236	0.07910279	0.00317943	0.01589315	GATAD2A
NA	ENSG00000167494	0	NA	NA	NA	NA	NOS2P2
4597	ENSG00000167508	996.075114	-0.3132242	0.12336887	0.00713283	0.03141992	MVD
81620	ENSG00000167513	1026.22229	-0.32491004	0.09938259	0.00068523	0.00424446	CDT1
51693	ENSG00000167515	487.204639	-0.37949679	0.16273106	0.01003	0.04156122	TRAPPC2L
29123	ENSG00000167522	5748.11431	0.26687301	0.07373697	0.00022271	0.00156412	ANKRD11
124045	ENSG00000167523	148.118163	0.14338736	0.18397867	0.37535631	0.60449338	SPATA33
124923	ENSG00000167524	297.161692	-0.03845163	0.13871388	0.76496796	0.89181833	RSKR
147011	ENSG00000167525	132.940662	0.8065229	0.27718492	0.00054807	0.0034928	PROCA1
6137	ENSG00000167526	31376.2646	0.06951121	0.05197643	0.16368042	0.35275692	RPL13

121274	ENSG00000167528	320.421626	0.41554607	0.16052659	0.00446965	0.02118943	ZNF641
3906	ENSG00000167531	0	NA	NA	NA	NA	LALBA
784	ENSG00000167535	1130.26827	0.3258852	0.08260553	5.18E-05	0.00042165	CACNB3
147015	ENSG00000167536	534.973497	-0.50960469	0.10616177	6.05E-07	7.13E-06	DHRS13
90313	ENSG00000167543	626.832644	0.83597991	0.13807321	2.41E-10	4.44E-09	TP53I13
8085	ENSG00000167548	5312.12187	0.14910945	0.05163508	0.00355856	0.01752774	KMT2D
84940	ENSG00000167549	2016.89125	1.32013348	0.07999081	3.33E-62	4.55E-60	CORO6
121268	ENSG00000167550	72.4263769	-0.06056058	0.2282001	0.74167471	0.87812748	RHEBL1
7846	ENSG00000167552	26365.6829	-0.1795062	0.06237414	0.00350469	0.01729247	TUBA1A
84790	ENSG00000167553	78.6283242	0.37435382	0.24823575	0.06044461	0.17230188	TUBA1C
162963	ENSG00000167554	102.939818	0.36929927	0.25018307	0.06421776	0.18026038	ZNF610
84436	ENSG00000167555	675.513611	-0.08614158	0.11425502	0.42856824	0.65447566	ZNF528
55762	ENSG00000167562	171.564197	-0.25901632	0.18343334	0.10795085	0.26343905	ZNF701
29946	ENSG00000167565	135.099302	0.49515859	0.20755475	0.005957	0.02700795	SERTAD3
57701	ENSG00000167566	1602.26676	-0.05668625	0.07236814	0.42474736	0.65048148	NCKAP5L
53916	ENSG00000167578	178.682582	0.17353484	0.17035331	0.2557736	0.47684158	RAB4B
359	ENSG00000167580	1.90249889	0.13691703	0.41831211	0.02867139	NA	AQP2
2819	ENSG00000167588	5.92426203	0.04972271	0.3677097	0.67514686	0.84091366	GPD1
148137	ENSG00000167595	465.530214	0.24014714	0.12303547	0.03864141	0.12228832	PROSER3
29785	ENSG00000167600	0	NA	NA	NA	NA	CYP2S1
558	ENSG00000167601	0.94162468	-0.03816095	0.38462091	0.45455056	NA	AXL
84807	ENSG00000167604	63.8531182	0.05145438	0.25496744	0.78936639	0.90431711	NFKBID
147798	ENSG00000167608	3.50212295	-0.04815289	0.37681384	0.59057269	0.78531918	TMC4
341405	ENSG00000167612	10.8405603	1.9106033	0.93423556	0.00157444	0.00874246	ANKRD33
3903	ENSG00000167613	0	NA	NA	NA	NA	LAIR1
57348	ENSG00000167614	7.10007211	-0.15939202	0.39122303	0.28975255	0.51571638	TTYH1
114823	ENSG00000167615	5170.77604	0.32359052	0.05474353	2.38E-09	3.89E-08	LENG8
148170	ENSG00000167617	0.89228056	0.03000497	0.3843321	0.48640127	NA	CDC42EP5
3904	ENSG00000167618	0	NA	NA	NA	NA	LAIR2
284339	ENSG00000167619	540.097735	-1.26198504	0.1991413	2.05E-11	4.21E-10	TMEM145
116115	ENSG00000167625	310.673747	-0.02394496	0.13008773	0.84408579	0.92948958	ZNF526
83696	ENSG00000167632	642.874246	0.29245042	0.10103709	0.0026265	0.01353851	TRAPPC9
3811	ENSG00000167633	0	NA	NA	NA	NA	KIR3DL1
199713	ENSG00000167634	0	NA	NA	NA	NA	NLRP7
7705	ENSG00000167635	2538.01683	-0.18957776	0.07716637	0.01198064	0.04817509	ZNF146
284349	ENSG00000167637	267.139934	-0.11528557	0.14529248	0.39001364	0.61927737	ZNF283
94274	ENSG00000167641	145.134601	0.99689649	0.22091755	7.90E-07	9.11E-06	PPP1R14A
10653	ENSG00000167642	28.931429	-0.07593251	0.34184504	0.64281684	0.82148002	SPINT2

64073	ENSG00000167644	0	NA	NA	NA	NA	C19orf33
90522	ENSG00000167645	762.989422	0.03132204	0.09162396	0.72726728	0.86953012	YIF1B
352909	ENSG00000167646	32.3937833	-0.18893911	0.31507666	0.35094222	0.5811749	DNAAF3
8000	ENSG00000167653	1.95089704	-0.03571095	0.37771029	0.6695401	NA	PSCA
85300	ENSG00000167654	1348.51056	-1.44971169	0.10641128	2.42E-43	2.03E-41	ATCAY
8581	ENSG00000167656	0	NA	NA	NA	NA	LY6D
1613	ENSG00000167657	819.611089	-0.09879065	0.1018681	0.31341156	0.54126787	DAPK3
1938	ENSG00000167658	65029.2794	-0.31002491	0.05652678	2.71E-08	3.86E-07	EEF2
126259	ENSG00000167664	266.777837	0.41150978	0.15013337	0.0029702	0.01500987	TMIGD2
10036	ENSG00000167670	1598.97874	-0.05724975	0.08551918	0.49157502	0.71084901	CHAF1A
80700	ENSG00000167671	1640.39118	-0.18574527	0.09820737	0.04971273	0.14834779	UBXN6
84717	ENSG00000167674	2114.21011	-0.12528434	0.06860627	0.06314522	0.17813182	HDGFL2
729359	ENSG00000167676	142.438698	0.85214846	0.21081012	8.35E-06	8.06E-05	PLIN4
10501	ENSG00000167680	80.6769494	-0.19663526	0.23383364	0.29527412	0.52190021	SEMA6B
55311	ENSG00000167685	931.207021	0.03889929	0.09649323	0.67777114	0.84270098	ZNF444
64359	ENSG00000167693	633.121151	-0.14716166	0.11246777	0.16901822	0.36039348	NXN
79850	ENSG00000167695	526.437897	-0.04716173	0.14902969	0.72661421	0.86948287	TLCD3A
51031	ENSG00000167699	996.527194	-0.08205989	0.09243604	0.35960979	0.58949503	GLOD4
113655	ENSG00000167700	601.458066	0.23106163	0.10865211	0.02628082	0.09050816	MFSD3
2875	ENSG00000167701	44.9316678	0.36541533	0.33374279	0.10665995	0.26134783	GPT
90990	ENSG00000167702	845.065838	0.30344852	0.09056458	0.00055021	0.00350326	KIFC2
124935	ENSG00000167703	249.331362	-0.56418242	0.16258387	0.0001601	0.00116264	SLC43A2
83547	ENSG00000167705	9.57264379	0.13052781	0.37824722	0.37620145	0.60519502	RILP
5345	ENSG00000167711	4.84422154	0.03304591	0.36448237	0.78963702	0.9043268	SERPINF2
124997	ENSG00000167716	672.24794	0.08730795	0.12103988	0.44667512	0.67085802	WDR81
63826	ENSG00000167720	296.951801	0.36751844	0.1447681	0.00605077	0.02733735	SRR
55720	ENSG00000167721	2529.92366	-0.07556233	0.06855118	0.26259039	0.48463194	TSR1
162514	ENSG00000167723	1.81407516	-0.11258364	0.40381775	0.10889324	NA	TRPV3
374875	ENSG00000167733	352.688697	0.44256961	0.13647332	0.00053228	0.00340548	HSD11B1L
124936	ENSG00000167740	258.042276	-0.24106519	0.16181883	0.09904472	0.24754941	CYB5D2
124975	ENSG00000167741	0	NA	NA	NA	NA	GGT6
84798	ENSG00000167747	1506.92765	-0.45869085	0.08046447	5.38E-09	8.40E-08	C19orf48
3816	ENSG00000167748	0.15069912	-0.01848503	0.38480575	0.84537521	NA	KLK1
9622	ENSG00000167749	0	NA	NA	NA	NA	KLK4
3817	ENSG00000167751	0.76318994	-0.02061218	0.38210442	0.69450557	NA	KLK2
25818	ENSG00000167754	0	NA	NA	NA	NA	KLK5
5653	ENSG00000167755	0	NA	NA	NA	NA	KLK6
11012	ENSG00000167757	0	NA	NA	NA	NA	KLK11

26085	ENSG00000167759	0	NA	NA	NA	NA	KLK13
55769	ENSG00000167766	652.408786	0.2105951	0.10228376	0.03232194	0.10643442	ZNF83
144501	ENSG00000167767	32.4318195	0.1632381	0.30258851	0.41724247	0.64356838	KRT80
3848	ENSG00000167768	0	NA	NA	NA	NA	KRT1
125981	ENSG00000167769	0	NA	NA	NA	NA	ACER1
55611	ENSG00000167770	2258.37506	0.16975439	0.06563733	0.00863976	0.0367681	OTUB1
283248	ENSG00000167771	1750.682	0.30956027	0.07257472	1.36E-05	0.00012602	RCOR2
51129	ENSG00000167772	7.27007445	0.13632395	0.38000199	0.36545862	0.59471759	ANGPTL4
NA	ENSG00000167774	199.858191	0.07546615	0.23554428	0.68707265	0.84783688	
51293	ENSG00000167775	915.937564	0.0718527	0.09156474	0.41956435	0.64543356	CD320
84926	ENSG00000167778	240.333236	0.02171075	0.15823285	0.8816733	0.94773789	SPRYD3
3489	ENSG00000167779	36.7632594	0.05465994	0.27847903	0.77763621	0.89809083	IGFBP6
8435	ENSG00000167780	0.35668925	0.02381712	0.38538107	0.61401291	NA	SOAT2
148156	ENSG00000167785	678.220227	0.1507569	0.11714829	0.17586823	0.37013108	ZNF558
51475	ENSG00000167791	0	NA	NA	NA	NA	CABP2
4723	ENSG00000167792	2069.01931	0.21234043	0.06943935	0.00185933	0.010057	NDUFV1
10263	ENSG00000167797	627.007661	0.46166339	0.1050812	4.92E-06	4.96E-05	CDK2AP2
388503	ENSG00000167798	0	NA	NA	NA	NA	C3P1
254552	ENSG00000167799	57.5468415	0.95812927	0.3220253	0.00033739	0.00227446	NUDT8
347853	ENSG00000167800	0.18922458	0.01954053	0.38488496	0.78225479	NA	TBX10
NA	ENSG00000167807	19.145921	0.06042791	0.32184232	0.74304433	0.87880772	
7001	ENSG00000167815	4608.57407	-0.09609985	0.05982456	0.10183066	0.25266637	PRDX2
81168	ENSG00000167822	0	NA	NA	NA	NA	OR8J3
10798	ENSG00000167825	0	NA	NA	NA	NA	OR5I1
7775	ENSG00000167840	303.076675	-0.06088712	0.1439977	0.6470744	0.82430661	ZNF232
79003	ENSG00000167842	660.994742	-0.04021833	0.10090011	0.67832122	0.84291038	MIS12
10871	ENSG00000167850	0	NA	NA	NA	NA	CD300C
11314	ENSG00000167851	0.37844916	0.02407552	0.3854191	0.60038877	NA	CD300A
83659	ENSG00000167858	0	NA	NA	NA	NA	TEKT1
283987	ENSG00000167861	1764.95477	-0.23258264	0.11353716	0.03111488	0.10319613	HID1
3396	ENSG00000167862	429.499747	-0.12157483	0.11710893	0.27466945	0.49848536	MRPL58
10476	ENSG00000167863	1511.89699	-0.12190973	0.07359867	0.09177746	0.23401187	ATP5PD
92162	ENSG00000167874	21.2401478	0.07472903	0.31740247	0.68814241	0.84857639	TMEM88
2125	ENSG00000167880	5.4003877	0.17229613	0.42265891	0.13729951	0.31285428	EVPL
6730	ENSG00000167881	1981.88493	0.03364264	0.06679409	0.60959597	0.79803651	SRP68
146664	ENSG00000167889	595.694659	-1.09465958	0.11212485	1.89E-23	8.50E-22	MGAT5B
147138	ENSG00000167895	5.05574237	-0.00319067	0.37170622	0.96929331	0.98853525	TMC8
7083	ENSG00000167900	1144.94678	-0.32614046	0.08421051	6.90E-05	0.00054874	TK1

137695	ENSG00000167904	301.412379	0.05448014	0.14705372	0.68910566	0.8490152	TMEM68
1581	ENSG00000167910	0	NA	NA	NA	NA	CYP7A1
100505501	ENSG00000167912	108.856334	-0.00182592	0.19545674	0.98814502	0.99592568	
284110	ENSG00000167914	0.16735865	0.01897177	0.38482933	0.78225479	NA	GSDMA
192666	ENSG00000167916	0	NA	NA	NA	NA	KRT24
147184	ENSG00000167920	24.571699	-1.45294383	0.63683393	0.00108883	0.00634992	TMEM99
84514	ENSG00000167925	355.95757	0.12402765	0.12815029	0.30460835	0.53198909	GHDC
83986	ENSG00000167930	2051.25733	0.08031754	0.07559994	0.27889274	0.5033414	FAM234A
50964	ENSG00000167941	0	NA	NA	NA	NA	SOST
90850	ENSG00000167962	1945.49209	-0.09642422	0.07067583	0.1645803	0.35422793	ZNF598
25837	ENSG00000167964	232.967325	-0.11981691	0.15441537	0.39549572	0.62498088	RAB26
64223	ENSG00000167965	1030.37328	-0.02490194	0.08359207	0.76117757	0.88962039	MLST8
1877	ENSG00000167967	714.543687	0.37706256	0.09694369	5.62E-05	0.00045276	E4F1
1775	ENSG00000167968	41.568589	0.17286895	0.29717462	0.39170523	0.62114503	DNASE1L2
1632	ENSG00000167969	696.695418	0.20156066	0.10737215	0.05012876	0.1492877	ECI1
57524	ENSG00000167971	471.760722	0.25731141	0.17691367	0.10114543	0.25140633	CASKIN1
21	ENSG00000167972	3501.8039	-0.31146802	0.06127306	2.50E-07	3.13E-06	ABCA3
54442	ENSG00000167977	858.674748	-0.11622921	0.12029217	0.30776571	0.53524471	KCTD5
23524	ENSG00000167978	18705.3409	-0.06964811	0.06293827	0.26130885	0.4833008	SRRM2
146434	ENSG00000167981	109.470787	-0.06445372	0.20595573	0.7099076	0.860757	ZNF597
197358	ENSG00000167984	18.2360684	-0.06564362	0.32248806	0.71535783	0.86274919	NLRC3
54949	ENSG00000167985	414.552187	0.08865648	0.13980186	0.49631435	0.714811	SDHAF2
1642	ENSG00000167986	7257.56575	-0.0652346	0.05619283	0.24025506	0.45820056	DDB1
55048	ENSG00000167987	565.242059	-0.04241798	0.1377451	0.74022986	0.87736188	VPS37C
220001	ENSG00000167992	242.093362	-0.30927647	0.16520993	0.03792298	0.12066567	VWCE
5866	ENSG00000167994	106.304273	0.14956453	0.20161473	0.38542555	0.6145004	RAB3IL1
7439	ENSG00000167995	58.9809717	0.11028274	0.27317139	0.57467107	0.77361067	BEST1
2495	ENSG00000167996	13907.4443	0.24925901	0.05136304	9.16E-07	1.05E-05	FTH1
26580	ENSG00000168000	520.963674	0.13036895	0.12370324	0.2655273	0.48787384	BSCL2
5436	ENSG00000168002	1300.57709	-0.08496583	0.08429639	0.30094859	0.52825091	POLR2G
6520	ENSG00000168003	1411.88939	0.52982876	0.09575658	1.16E-08	1.75E-07	SLC3A2
117245	ENSG00000168004	0	NA	NA	NA	NA	PLAAT5
144097	ENSG00000168005	1429.77029	0.17370687	0.08294155	0.0317941	0.10503632	SPINDOC
89849	ENSG00000168010	463.023312	0.37997254	0.1525012	0.00667815	0.02971397	ATG16L2
26005	ENSG00000168014	1200.64953	-0.3907496	0.07800041	2.98E-07	3.69E-06	C2CD3
9881	ENSG00000168016	9.05639305	0.03300338	0.35579703	0.8103458	0.914052	TRANK1
199223	ENSG00000168026	89.3959611	0.27126341	0.24397821	0.16234701	0.35102057	TTC21A
3921	ENSG00000168028	21203.3365	-0.20825645	0.04900469	1.78E-05	0.00016176	RPSA

956	ENSG00000168032	1.37675766	0.02715541	0.38025224	0.68672835	NA	ENTPD3
1499	ENSG00000168036	4585.42883	0.15816254	0.08814503	0.06514662	0.18206915	CTNNB1
54986	ENSG00000168038	94.8523529	-0.09223038	0.21069711	0.59974969	0.79066681	ULK4
8772	ENSG00000168040	438.712329	-0.18143869	0.11208417	0.08968926	0.23002002	FADD
4054	ENSG00000168056	2732.88401	0.30252273	0.0722916	1.98E-05	0.00017734	LTBP3
10004	ENSG00000168060	2.87521398	0.13072423	0.4036099	0.19165273	0.39354068	NAALADL1
29901	ENSG00000168061	354.673278	-0.19523232	0.13626406	0.12393817	0.29179226	SAC3D1
116071	ENSG00000168062	1.60253421	0.08104892	0.39121639	0.26020817	NA	BATF2
55867	ENSG00000168065	0.16735865	0.01897177	0.38482933	0.78225479	NA	SLC22A11
7536	ENSG00000168066	5487.78247	0.00054879	0.05698849	0.99316563	0.99778445	SF1
5871	ENSG00000168067	438.95639	0.08971855	0.15508104	0.52777993	0.73958565	MAP4K2
283129	ENSG00000168070	0	NA	NA	NA	NA	MAJIN
283234	ENSG00000168071	10.4222872	0.1892069	0.40521703	0.2261415	0.44042873	CCDC88B
51435	ENSG00000168077	160.928758	0.73812856	0.19970195	4.27E-05	0.0003563	SCARA3
55872	ENSG00000168078	719.438265	0.00875535	0.09416437	0.92153181	0.9670192	PBK
286133	ENSG00000168079	0.50832795	0.03077797	0.38601977	0.48294583	NA	SCARA5
5368	ENSG00000168081	0	NA	NA	NA	NA	PNOC
10980	ENSG00000168090	2326.22901	0.06728712	0.08822745	0.43379521	0.65909763	COPS6
5049	ENSG00000168092	2361.9719	-0.15580848	0.07448316	0.0326403	0.10723051	PAFAH1B2
124401	ENSG00000168096	772.69248	0.27711825	0.0960038	0.00280508	0.01431399	ANKS3
84309	ENSG00000168101	511.935171	-0.21185537	0.10847434	0.04105559	0.12797767	NUDT16L1
57691	ENSG00000168116	473.678974	-0.41180357	0.11896166	0.00026555	0.00183572	KIAA1586
5867	ENSG00000168118	987.773681	-0.13425213	0.08242193	0.09613011	0.24209462	RAB4A
NA	ENSG00000168122	0	NA	NA	NA	NA	ZNF355P
4992	ENSG00000168124	4.64970397	0.02716374	0.36502312	0.82125186	0.91847813	OR1F1
NA	ENSG00000168126	0.45209736	-0.02505403	0.38558831	0.57865948	NA	OR2W6P
NA	ENSG00000168129	0	NA	NA	NA	NA	DHFRP3
81697	ENSG00000168131	0	NA	NA	NA	NA	OR2B2
3761	ENSG00000168135	11.5876665	0.04703806	0.3366934	0.7801464	0.89936692	KCNJ4
55209	ENSG00000168137	4318.22695	0.13828063	0.06327361	0.02667956	0.09159412	SETD5
114990	ENSG00000168140	332.318708	-0.91438615	0.14160092	1.59E-11	3.32E-10	VASN
222584	ENSG00000168143	0	NA	NA	NA	NA	FAM83B
8290	ENSG00000168148	0.4877242	-0.00640555	0.38314294	0.86753788	NA	H3-4
79725	ENSG00000168152	116.863432	-0.14791101	0.22064054	0.4141001	0.64130672	THAP9
4993	ENSG00000168158	0.15069912	-0.01848503	0.38480575	0.84537521	NA	OR2C1
149603	ENSG00000168159	2380.45996	0.23496701	0.06866511	0.00049694	0.00320628	RNF187
84376	ENSG00000168172	1555.96272	-0.52747512	0.11437698	1.43E-06	1.58E-05	HOOK3
93487	ENSG00000168175	1952.43058	0.03346414	0.07468077	0.64838642	0.82455589	MAPK1IP1L

54541	ENSG00000168209	1721.18331	0.56686452	0.11940438	6.73E-07	7.85E-06	DDIT4
3516	ENSG00000168214	1696.45818	-0.06996872	0.07769013	0.35709755	0.58712453	RBPJ
55788	ENSG00000168216	303.37	0.12155652	0.13464091	0.3350785	0.56494141	LMBRD1
29063	ENSG00000168228	189.232077	0.21364396	0.17420631	0.16891308	0.36034672	ZCCHC4
5729	ENSG00000168229	0.75689831	0.03380966	0.38651369	0.3822658	NA	PTGDR
125488	ENSG00000168234	349.473378	-0.17605988	0.13080161	0.15092755	0.33460752	TTC39C
132158	ENSG00000168237	235.663699	-0.31807194	0.18485633	0.0503207	0.14970202	GLYCTK
2786	ENSG00000168243	6390.49018	-0.58690074	0.0646741	3.60E-20	1.36E-18	GNG4
92181	ENSG00000168246	1377.79274	0.0941733	0.08732106	0.26834662	0.49109833	UBTD2
NA	ENSG00000168255	504.45199	0.02755089	0.14018026	0.83404801	0.92432089	POLR2J3
28511	ENSG00000168256	2221.25752	0.07437291	0.06917603	0.2752744	0.49935486	NKIRAS2
7266	ENSG00000168259	1700.13719	-0.1145359	0.08182724	0.15145009	0.33539121	DNAJC7
169522	ENSG00000168263	4.51682814	0.08037776	0.37232638	0.52558506	0.73806584	KCNV2
359948	ENSG00000168264	2601.19714	-0.28338528	0.07038801	4.06E-05	0.00034085	IRF2BP2
256297	ENSG00000168267	0.14931947	-0.01844332	0.38480212	0.84537521	NA	PTF1A
64943	ENSG00000168268	7860.09182	0.20278676	0.06095362	0.00074629	0.0045798	NT5DC2
2299	ENSG00000168269	0	NA	NA	NA	NA	FOXI1
440957	ENSG00000168273	112.691352	0.07126118	0.22171465	0.69583514	0.8531799	SMIM4
388753	ENSG00000168275	315.341695	-0.18750038	0.13424755	0.13495586	0.30945965	COA6
3800	ENSG00000168280	4571.81814	-0.92596227	0.07285794	8.09E-38	5.86E-36	KIF5C
4247	ENSG00000168282	553.307654	-0.37105883	0.10608823	0.00026365	0.00182576	MGAT2
648	ENSG00000168283	1507.40692	0.04044091	0.08812494	0.63766756	0.81742743	BMI1
57215	ENSG00000168286	1206.67452	-0.04143148	0.08027676	0.59706843	0.78909124	THAP11
27249	ENSG00000168288	1053.16591	-0.27514389	0.09572059	0.00290108	0.01469803	MMADHC
5162	ENSG00000168291	896.908116	-0.30445972	0.08590277	0.00026778	0.00184791	PDHB
54899	ENSG00000168297	228.558545	-0.26864252	0.15375877	0.05584861	0.16214719	PXK
3008	ENSG00000168298	2.01685815	0.06174029	0.381431	0.48420728	NA	H1-4
115294	ENSG00000168300	630.687425	0.24934448	0.10979777	0.01748489	0.06535558	PCMTD1
200845	ENSG00000168301	179.228609	-0.25167413	0.17878117	0.11137225	0.26959803	KCTD6
136647	ENSG00000168303	642.67488	0.05727071	0.11234269	0.59442107	0.7877158	MPLKIP
8309	ENSG00000168306	0.15069912	-0.01848503	0.38480575	0.84537521	NA	ACOX2
11170	ENSG00000168309	0.58687465	-0.02575243	0.38410181	0.63655746	NA	FAM107A
3660	ENSG00000168310	257.913087	0.15309696	0.14522705	0.25460548	0.47519969	IRF2
4336	ENSG00000168314	1.56061342	-0.02098207	0.3801929	0.73489371	NA	MOBP
1524	ENSG00000168329	0	NA	NA	NA	NA	CX3CR1
492307	ENSG00000168333	0	NA	NA	NA	NA	PPDPFL
165904	ENSG00000168334	0.58994281	0.02421937	0.383846	0.64003656	NA	XIRP1
84684	ENSG00000168348	4053.57029	1.71565295	0.07083693	1.04E-130	5.14E-128	INSM2

123099	ENSG00000168350	16.2273942	-0.27137929	0.41862935	0.17045761	0.36235712	DEGS2
11280	ENSG00000168356	14.7669358	0.6651124	0.73803656	0.02820633	0.09559509	SCN11A
732275	ENSG00000168367	0	NA	NA	NA	NA	LINC00917
378	ENSG00000168374	2571.60518	0.0290212	0.07209399	0.68238797	0.8449992	ARF4
4735	ENSG00000168385	6587.45554	0.00501827	0.06596397	0.93941283	0.97474024	SEPTIN2
11259	ENSG00000168386	37.9699368	2.10741559	0.42553064	4.56E-08	6.29E-07	FILIP1L
84879	ENSG00000168389	186.679011	-0.18822887	0.20789251	0.28262886	0.50763685	MFSD2A
1841	ENSG00000168393	1013.98748	-0.20174336	0.08133863	0.01095039	0.04467526	DTYMK
6890	ENSG00000168394	2.8433397	0.22865042	0.4871942	0.00480407	0.02245825	TAP1
84289	ENSG00000168395	1143.85059	0.03464131	0.10996087	0.74316751	0.87884863	ING5
23192	ENSG00000168397	1503.54024	0.24546391	0.07881959	0.00143576	0.0080718	ATG4B
624	ENSG00000168398	30.9903809	-0.73454359	0.46314984	0.01379786	0.05390894	BDKRB2
197259	ENSG00000168404	38.358814	1.43018586	0.43180839	6.44E-05	0.00051436	MLKL
NA	ENSG00000168405	27.2624534	1.18532167	0.54693572	0.00199495	0.01068279	CMAHP
55159	ENSG00000168411	1491.9132	-0.24591604	0.09511687	0.00743428	0.03248276	RFWD3
4543	ENSG00000168412	6.84163408	1.94781098	1.35084729	0.00482492	0.02254772	MTNR1A
93107	ENSG00000168418	0.16735865	0.01897177	0.38482933	0.78225479	NA	KCNG4
399	ENSG00000168421	0	NA	NA	NA	NA	RHOH
377007	ENSG00000168427	1.00931534	0.00167771	0.37995553	0.97801625	NA	KLHL30
91949	ENSG00000168434	324.837546	0.26295323	0.13276549	0.03434434	0.11170355	COG7
51362	ENSG00000168438	607.509532	-0.02490953	0.1018407	0.79899707	0.90801363	CDC40
10963	ENSG00000168439	4270.0072	-0.06592353	0.08826193	0.44207267	0.66680698	STIP1
6338	ENSG00000168447	0	NA	NA	NA	NA	SCNN1B
55806	ENSG00000168453	160.323909	-2.4637599	0.72909373	2.64E-05	0.00023038	HR
84203	ENSG00000168454	0.70013361	0.02121893	0.38367343	0.60571412	NA	TXNDC2
11031	ENSG00000168461	229.458011	-0.31559767	0.16939588	0.03788516	0.1205713	RAB31
80346	ENSG00000168476	704.943734	-0.01480692	0.09690304	0.87356235	0.94441388	REEP4
7148	ENSG00000168477	11.515266	-0.27036021	0.51643642	0.01543022	0.05895159	TNXB
203190	ENSG00000168481	26.8666841	-1.66914712	0.5938506	0.00026709	0.00184444	LGI3
6440	ENSG00000168484	2.07205005	-0.03838997	0.38020194	0.57449899	NA	SFTPC
649	ENSG00000168487	711.615527	0.15162055	0.10989056	0.14907082	0.33163994	BMP1
11273	ENSG00000168488	4877.03235	0.43565072	0.06137274	6.23E-13	1.44E-11	ATXN2L
9796	ENSG00000168490	237.585901	-0.89715932	0.18987135	3.32E-07	4.08E-06	PHYHIP
256309	ENSG00000168491	18.5089661	1.15201947	0.74263298	0.00567731	0.02589969	CCDC110
661	ENSG00000168495	2434.6086	-0.27194088	0.07649719	0.00027743	0.0019046	POLR3D
2237	ENSG00000168496	2513.30313	-0.41263456	0.06195529	1.42E-11	2.98E-10	FEN1
8436	ENSG00000168497	0.173733	0.01914545	0.38484597	0.78225479	NA	CAVIN2
23255	ENSG00000168502	491.291707	0.06372829	0.13156737	0.60700068	0.79648767	MTCL1

2637	ENSG00000168505	0.48176888	0.00243862	0.38304173	0.89348694	NA	GBX2
148738	ENSG00000168509	0.173733	0.01914545	0.38484597	0.78225479	NA	HJV
10648	ENSG00000168515	0	NA	NA	NA	NA	SCGB1D1
124790	ENSG00000168517	76.1098792	0.03091586	0.24915578	0.87125525	0.94385192	HEXIM2
2339	ENSG00000168522	1176.35255	-0.58829511	0.09171517	4.40E-11	8.78E-10	FNTA
347735	ENSG00000168528	933.847079	0.57759938	0.10267274	5.94E-09	9.21E-08	SERINC2
4632	ENSG00000168530	0	NA	NA	NA	NA	MYL1
60684	ENSG00000168538	1014.23232	0.09976308	0.13529046	0.43043867	0.65595693	TRAPPC11
1128	ENSG00000168539	2.79472673	-0.0373209	0.37764807	0.6095755	0.79803651	CHRM1
1281	ENSG00000168542	463.992435	6.96888398	0.49083983	1.56E-46	1.42E-44	COL3A1
2675	ENSG00000168546	5.43274031	-0.14723662	0.39949235	0.24229685	0.4605892	GFRA2
3622	ENSG00000168556	130.007809	0.40811946	0.20997197	0.02309034	0.08171822	ING2
55602	ENSG00000168564	548.537672	-0.10618844	0.10412414	0.2873408	0.51306915	CDKN2AIP
154007	ENSG00000168566	395.819998	0.24242246	0.12539908	0.04033973	0.1262121	SNRNP48
79064	ENSG00000168569	412.038843	0.12387864	0.13210722	0.31782819	0.54556488	TMEM223
6575	ENSG00000168575	731.896517	-0.19875535	0.12046829	0.08138687	0.21415324	SLC20A2
1418	ENSG00000168582	0	NA	NA	NA	NA	CRYGA
83657	ENSG00000168589	0.34746601	0.02370763	0.3853651	0.61987128	NA	DYNLRB2
79089	ENSG00000168591	586.078149	0.11815772	0.10804743	0.25428036	0.47479259	TMUB2
11086	ENSG00000168594	1.75978561	-0.05439265	0.38342907	0.46148969	NA	ADAM29
6774	ENSG00000168610	1235.07659	0.17206331	0.08733837	0.04283108	0.13230775	STAT3
90204	ENSG00000168612	194.440801	-0.04218372	0.16208352	0.77306344	0.89561741	ZSWIM1
8754	ENSG00000168615	729.625608	-0.13065833	0.09997483	0.17520158	0.36912205	ADAM9
8749	ENSG00000168619	0.173124	-0.01911262	0.38486262	0.84537521	NA	ADAM18
2668	ENSG00000168621	0	NA	NA	NA	NA	GDNF
NA	ENSG00000168630	0	NA	NA	NA	NA	SPINT5P
135656	ENSG00000168631	0	NA	NA	NA	NA	MUCL3
164237	ENSG00000168634	0	NA	NA	NA	NA	WFDC13
8313	ENSG00000168646	282.793558	-0.20377002	0.15618036	0.15162381	0.33558862	AXIN2
4725	ENSG00000168653	3156.24468	0.04501536	0.07462736	0.53941629	0.74850934	NDUFS5
200403	ENSG00000168658	0	NA	NA	NA	NA	VWA3B
90075	ENSG00000168661	190.233626	-0.15601209	0.17798326	0.32091055	0.54869068	ZNF30
167127	ENSG00000168671	11.1924977	-0.06723187	0.35483403	0.64532734	0.82320416	UGT3A2
157638	ENSG00000168672	0.86430786	0.03673462	0.38459125	0.45892896	NA	LRATD2
753	ENSG00000168675	966.072292	-0.5359648	0.09453336	5.12E-09	8.00E-08	LDLRAD4
146212	ENSG00000168676	109.225717	0.84834188	0.2424734	7.08E-05	0.00056222	KCTD19
9122	ENSG00000168679	17.5010063	0.10249244	0.32780223	0.57951036	0.77706744	SLC16A4
3575	ENSG00000168685	0	NA	NA	NA	NA	IL7R

29100	ENSG00000168701	439.303752	0.10283068	0.12220287	0.37489089	0.60411267	TMEM208
53353	ENSG00000168702	12.2180446	0.07608161	0.35466717	0.61697262	0.80383089	LRP1B
128488	ENSG00000168703	0	NA	NA	NA	NA	WFDC12
10768	ENSG00000168710	2074.9398	0.06799438	0.0760709	0.36226659	0.59197696	AHCYL1
134218	ENSG00000168724	634.456243	0.01605692	0.1070084	0.87677874	0.94566867	DNAJC21
11142	ENSG00000168734	596.277948	0.29549286	0.117047	0.00788458	0.03410292	PKIG
255743	ENSG00000168743	4.56993529	0.22345952	0.47244814	0.01432296	0.05554485	NPNT
140834	ENSG00000168746	0	NA	NA	NA	NA	LINC01620
101927242	ENSG00000168746	0	NA	NA	NA	NA	LINC01620
766	ENSG00000168748	1.15648962	0.03374533	0.38179791	0.59354971	NA	CA7
51252	ENSG00000168754	0.173124	-0.01911262	0.38486262	0.84537521	NA	FAM178B
64591	ENSG00000168757	0	NA	NA	NA	NA	TSPY2
54910	ENSG00000168758	1807.57523	0.28118765	0.08067749	0.00035566	0.00238387	SEMA4C
26505	ENSG00000168763	989.81821	0.11230412	0.08379929	0.17026261	0.36213659	CNNM3
2948	ENSG00000168765	225.522134	0.87281666	0.16266393	1.26E-08	1.89E-07	GSTM4
54790	ENSG00000168769	933.20701	-0.00829584	0.09267642	0.92600632	0.96919669	TET2
80319	ENSG00000168772	453.155237	-0.11870682	0.11720194	0.28652009	0.51226085	CXXC4
79867	ENSG00000168778	165.470273	0.81638012	0.18914673	2.70E-06	2.84E-05	TCTN2
6474	ENSG00000168779	1301.94728	1.45487665	0.090245	1.57E-59	2.02E-57	SHOX2
9677	ENSG00000168781	719.961053	-0.60732165	0.18337624	0.00024195	0.00168611	PPIP5K1
10098	ENSG00000168785	637.441137	-0.37894968	0.10236931	0.0001177	0.00088019	TSPAN5
116236	ENSG00000168792	127.088314	0.85962215	0.2259631	2.14E-05	0.00019061	ABHD15
9925	ENSG00000168795	1086.04599	-0.02241082	0.08516023	0.78706482	0.90324885	ZBTB5
54921	ENSG00000168802	1465.07403	-0.18157599	0.10807577	0.0790833	0.20976104	CHTF8
113455421	ENSG00000168802	1465.07403	-0.18157599	0.10807577	0.0790833	0.20976104	CHTF8
161823	ENSG00000168803	315.349077	0.06499523	0.14953466	0.6378976	0.81760985	ADAL
9836	ENSG00000168806	631.684819	-0.54100044	0.10703654	1.49E-07	1.93E-06	LCMT2
6645	ENSG00000168807	636.4291	0.15619008	0.14767683	0.25147491	0.47149344	SNTB2
3592	ENSG00000168811	19.732868	0.32691188	0.42107015	0.13817725	0.31440929	IL12A
22847	ENSG00000168813	964.481305	-0.11853337	0.09126066	0.18042932	0.37732873	ZNF507
53407	ENSG00000168818	454.344922	-0.1771459	0.11702997	0.11100374	0.26893566	STX18
27065	ENSG00000168824	1462.23224	-1.70543645	0.08656211	1.53E-87	3.62E-85	NSG1
166793	ENSG00000168826	185.378948	0.9746664	0.20614272	2.90E-07	3.60E-06	ZBTB49
85476	ENSG00000168827	1137.88323	0.21645071	0.09628961	0.02001907	0.07297048	GFM1
392309	ENSG00000168828	0	NA	NA	NA	NA	OR13J1
3354	ENSG00000168830	145.640777	-1.39372384	0.2259268	5.86E-11	1.16E-09	HTR1E
56884	ENSG00000168843	404.952492	-1.31037927	0.13546177	3.73E-23	1.63E-21	FSTL5
NA	ENSG00000168852	14.4180301	0.1227247	0.34840429	0.48812449	0.70792323	TPTE2P5

55308	ENSG00000168872	1417.58166	0.18905132	0.07841099	0.01367915	0.05357172	DDX19A
84913	ENSG00000168874	40.2975651	0.07081705	0.28782151	0.71460876	0.86245136	ATOH8
8403	ENSG00000168875	5.95559713	-0.10000687	0.37345347	0.46227548	0.6851832	SOX14
54851	ENSG00000168876	182.79467	0.22729988	0.1709169	0.13674383	0.31191797	ANKRD49
6439	ENSG00000168878	0.64021618	0.02085573	0.38403002	0.77165456	NA	SFTPB
10713	ENSG00000168883	1221.89476	-0.05866875	0.07886152	0.44686194	0.67093544	USP39
79155	ENSG00000168884	506.05146	0.03095088	0.11173111	0.77286986	0.89561741	TNIP2
388969	ENSG00000168887	1137.5556	0.21835533	0.09414031	0.01658713	0.06264775	C2orf68
129303	ENSG00000168890	93.7211341	0.22510651	0.21762942	0.21455532	0.4249129	TMEM150A
51255	ENSG00000168894	348.074214	0.09273499	0.12925346	0.44649745	0.6707435	RNF181
10791	ENSG00000168899	44.9541133	0.75386981	0.40337543	0.00826281	0.03548357	VAMP5
10917	ENSG00000168903	0	NA	NA	NA	NA	BTNL3
123355	ENSG00000168904	259.509066	-0.20786627	0.15646838	0.14428807	0.32427223	LRRC28
4144	ENSG00000168906	13070.0074	-0.68242584	0.21303086	0.00028744	0.00196781	MAT2A
255189	ENSG00000168907	0.18922458	0.01954053	0.38488496	0.78225479	NA	PLA2G4F
375704	ENSG00000168913	157.469023	-0.77615262	0.20159658	2.13E-05	0.00019042	ENHO
57507	ENSG00000168916	1402.82247	-0.03817342	0.08058982	0.6273754	0.81091192	ZNF608
80723	ENSG00000168917	283.434019	-0.74191392	0.1463673	8.27E-08	1.10E-06	SLC35G2
3635	ENSG00000168918	12.3103653	-0.00528734	0.34581047	0.96964626	0.98858077	INPP5D
3954	ENSG00000168924	1926.23154	-0.05048989	0.07684831	0.50171899	0.71856283	LETM1
1504	ENSG00000168925	0.36166929	0.00714652	0.38353093	0.918697	NA	CTRB1
440387	ENSG00000168928	0.173733	0.01914545	0.38484597	0.78225479	NA	CTRB2
57093	ENSG00000168930	0	NA	NA	NA	NA	TRIM49
92305	ENSG00000168936	989.410119	0.48995431	0.11049468	3.75E-06	3.85E-05	TMEM129
5480	ENSG00000168938	236.75505	1.46543611	0.17255441	1.78E-18	6.19E-17	PPIC
10251	ENSG00000168939	50.3418084	0.22696637	0.28039276	0.26674841	0.48915731	SPRY3
153241	ENSG00000168944	654.954237	-0.23860522	0.10024665	0.01340571	0.05277169	CEP120
29091	ENSG00000168952	0.15069912	-0.01848503	0.38480575	0.84537521	NA	STXBP6
79853	ENSG00000168955	0.99819238	0.00770367	0.37932145	0.90586153	NA	TM4SF20
56947	ENSG00000168958	989.727447	-0.02924539	0.08521253	0.72369913	0.86794586	MFF
2915	ENSG00000168959	1.39377048	-0.01541691	0.38025183	0.79378053	NA	GRM5
3965	ENSG00000168961	22.0788684	0.22977381	0.36791414	0.25498198	0.47574957	LGALS9
NA	ENSG00000168967	0	NA	NA	NA	NA	PMCHL1
8681	ENSG00000168970	346.688251	-0.02002576	0.14083189	0.87736248	0.94576863	JMJD7-PLA2G4B
NA	ENSG00000168992	0	NA	NA	NA	NA	OR7E102P
10815	ENSG00000168993	683.368527	-2.15800703	0.16024678	1.32E-42	1.07E-40	CPLX1
221749	ENSG00000168994	11.5413155	0.27425041	0.45965975	0.12700945	0.2969598	PXDC1
27036	ENSG00000168995	0	NA	NA	NA	NA	SIGLEC7

23620	ENSG00000169006	0	NA	NA	NA	NA	NTSR2
1876	ENSG00000169016	1094.57103	0.12349952	0.09012904	0.15851058	0.34569581	E2F6
10116	ENSG00000169018	1620.97157	-0.30539284	0.07581015	3.80E-05	0.00032056	FEM1B
54951	ENSG00000169019	112.915298	0.09483678	0.20333918	0.58353691	0.77994081	COMMD8
521	ENSG00000169020	752.03865	-0.17376715	0.10414024	0.08209643	0.21544882	ATP5ME
7386	ENSG00000169021	954.178672	-0.14059409	0.08209143	0.07911929	0.20982153	UQCRFS1
84179	ENSG00000169026	6.95593021	0.18387837	0.41091973	0.21315525	0.42306924	SLC49A3
1285	ENSG00000169031	0.88851901	0.02796225	0.38351453	0.64030088	NA	COL4A3
5604	ENSG00000169032	535.382115	-0.1239871	0.13960314	0.34478129	0.57516697	MAP2K1
5650	ENSG00000169035	0	NA	NA	NA	NA	KLK7
5370	ENSG00000169040	0	NA	NA	NA	NA	PMCHL2
3187	ENSG00000169045	14186.9775	0.01512785	0.05791833	0.65947864	0.8311559	HNRNPH1
3667	ENSG00000169047	292.647965	-0.46416864	0.18943202	0.00551474	0.0252918	IRS1
4204	ENSG00000169057	1999.20199	-0.04268438	0.09609619	0.64472649	0.82270219	MECP2
51481	ENSG00000169059	0	NA	NA	NA	NA	VCX3A
65110	ENSG00000169062	1384.0694	-0.00569683	0.08750926	0.94581164	0.97800919	UPF3A
79740	ENSG00000169064	0.16735865	0.01897177	0.38482933	0.78225479	NA	ZBBX
345651	ENSG00000169067	0	NA	NA	NA	NA	ACTBL2
4920	ENSG00000169071	611.31879	-0.37406201	0.11461549	0.00060558	0.00381422	ROR2
NA	ENSG00000169075	0.32361177	0.00180997	0.38337295	0.963754	NA	BRD7P3
367	ENSG00000169083	0	NA	NA	NA	NA	AR
207063	ENSG00000169084	504.191765	0.25034313	0.11613945	0.02332998	0.08243399	DHRSX
254778	ENSG00000169085	0.52119901	0.02576573	0.38568143	0.51723676	NA	VXN
79663	ENSG00000169087	217.369908	0.17207612	0.15473616	0.22416679	0.43787232	HSPBAP1
8623	ENSG00000169093	291.816986	0.13703728	0.14574152	0.30954786	0.53735494	ASMTL
293	ENSG00000169100	21608.8393	0.10206171	0.05609298	0.06593809	0.1835829	SLC25A6
113189	ENSG00000169105	631.99791	0.32825158	0.10771422	0.00145699	0.00817263	CHST14
25849	ENSG00000169116	0	NA	NA	NA	NA	PARM1
53944	ENSG00000169118	996.424451	-0.06644929	0.08781636	0.4363344	0.66138547	CSNK1G1
90362	ENSG00000169122	219.024376	0.01255774	0.1478742	0.92892781	0.97020045	FAM110B
55130	ENSG00000169126	0.14931947	-0.01844332	0.38480212	0.84537521	NA	ODAD2
84632	ENSG00000169129	43.5474237	0.06565478	0.30920212	0.72775975	0.86981806	AFAP1L2
6940	ENSG00000169131	389.693301	0.05517539	0.13336699	0.65999773	0.83146071	ZNF354A
22809	ENSG00000169136	254.543312	-0.05883101	0.15448401	0.67648856	0.84184489	ATF5
7336	ENSG00000169139	1234.0358	-0.24009718	0.11114825	0.02353147	0.08298336	UBE2V2
137362	ENSG00000169154	0	NA	NA	NA	NA	GOT1L1
23099	ENSG00000169155	500.986569	0.26621373	0.11613449	0.01588363	0.06038154	ZBTB43
NA	ENSG00000169164	0	NA	NA	NA	NA	

126129	ENSG00000169169	2547.00349	-0.10444712	0.06096636	0.08253899	0.21637154	CPT1C
255738	ENSG00000169174	6.59502432	2.58542503	1.2917935	0.00187386	0.0101161	PCSK9
23214	ENSG00000169180	2926.22964	-0.32677245	0.08030741	2.99E-05	0.00025716	XPO6
146395	ENSG00000169181	3.257549	-0.00834531	0.36931265	0.933761	0.97237716	GSG1L
4330	ENSG00000169184	112.575834	1.06395644	0.26587031	6.61E-06	6.48E-05	MN1
27301	ENSG00000169188	464.158335	0.06223659	0.13027681	0.61231508	0.80024215	APEX2
197370	ENSG00000169189	608.762765	-0.0030437	0.09720622	0.97102842	0.98911805	NSMCE1
90693	ENSG00000169193	172.870319	-0.11393195	0.17396594	0.46152602	0.68465911	CCDC126
3596	ENSG00000169194	1.87494329	0.1010284	0.40282411	0.05228604	NA	IL13
440353	ENSG00000169203	774.752107	0.20615317	0.11209286	0.0538383	0.15749807	NPIPB12
26533	ENSG00000169208	0.16735865	0.01897177	0.38482933	0.78225479	NA	OR10G3
5865	ENSG00000169213	245.339412	0.06178728	0.14700645	0.65017843	0.82529572	RAB3B
343169	ENSG00000169214	0	NA	NA	NA	NA	OR6F1
10421	ENSG00000169217	2553.30466	-0.13919654	0.07009656	0.04317363	0.1329863	CD2BP2
284654	ENSG00000169218	1.84967541	-0.13051236	0.41476694	0.037638	NA	RSPO1
10636	ENSG00000169220	216.104117	0.16980943	0.17088551	0.26705474	0.48945645	RGS14
26000	ENSG00000169221	1416.49127	-0.03302727	0.07585266	0.65615275	0.82907086	TBC1D10B
10960	ENSG00000169223	1879.34775	0.23331918	0.06831667	0.00051135	0.00329066	LMAN2
148823	ENSG00000169224	0	NA	NA	NA	NA	GCSAML
53917	ENSG00000169228	701.102992	-0.06065513	0.10455646	0.54578374	0.75327648	RAB24
27166	ENSG00000169230	3808.52123	-0.03328356	0.06305188	0.59539404	0.78816266	PRELID1
7059	ENSG00000169231	1194.48011	0.279776	0.08230288	0.00048839	0.00316034	THBS3
11238	ENSG00000169239	99.9994337	0.27588821	0.24806369	0.1590778	0.34655798	CA5B
55974	ENSG00000169241	464.714799	0.06880103	0.10926185	0.51185294	0.72703053	SLC50A1
1942	ENSG00000169242	437.460902	-0.20054334	0.11890609	0.07515232	0.20173481	EFNA1
3627	ENSG00000169245	0	NA	NA	NA	NA	CXCL10
23117	ENSG00000169246	660.994367	0.20421872	0.11304289	0.0581325	0.16720045	NPIPB3
79628	ENSG00000169247	1.33990564	-0.04870425	0.38792374	0.38932232	NA	SH3TC2
6373	ENSG00000169248	0.15069912	-0.01848503	0.38480575	0.84537521	NA	CXCL11
8233	ENSG00000169249	220.56668	0.00925777	0.15644882	0.94948692	0.97919849	ZRSR2
51068	ENSG00000169251	865.335125	-0.22586972	0.09663794	0.01543487	0.058958	NMD3
154	ENSG00000169252	5.23279836	-0.02349839	0.36608261	0.83703455	0.92594176	ADRB2
NA	ENSG00000169253	0	NA	NA	NA	NA	
8706	ENSG00000169255	171.484643	2.27892911	0.23990138	1.01E-22	4.34E-21	B3GALNT1
114787	ENSG00000169258	1849.28632	-0.98391403	0.07680341	2.01E-38	1.50E-36	GPRIN1
8988	ENSG00000169271	0	NA	NA	NA	NA	HSPB3
7881	ENSG00000169282	35.4148637	0.16297786	0.2886731	0.41763569	0.64376444	KCNAB1
65008	ENSG00000169288	241.986751	-0.24289142	0.15622278	0.08777798	0.22632101	MRPL1

126669	ENSG00000169291	2.94205353	0.01787123	0.37248821	0.85439067	0.93466646	SHE
190	ENSG00000169297	0	NA	NA	NA	NA	NR0B1
55276	ENSG00000169299	373.87724	0.13185535	0.12485976	0.26121772	0.48317728	PGM2
202374	ENSG00000169302	25.1416858	-0.00083021	0.30814183	0.99433083	0.99830594	STK32A
11141	ENSG00000169306	0	NA	NA	NA	NA	IL1RAPL1
64805	ENSG00000169313	1.06970021	0.06073329	0.3907427	0.18811887	NA	P2RY12
150248	ENSG00000169314	3.88048685	0.03585436	0.36656339	0.76198721	0.89001685	C22orf15
NA	ENSG00000169325	0	NA	NA	NA	NA	GUSBP12
390445	ENSG00000169327	0	NA	NA	NA	NA	OR5AU1
23251	ENSG00000169330	129.197138	0.36618528	0.20964945	0.04038808	0.12630824	MINAR1
204474	ENSG00000169340	0	NA	NA	NA	NA	PDILT
7369	ENSG00000169344	0	NA	NA	NA	NA	UMOD
2813	ENSG00000169347	0	NA	NA	NA	NA	GP2
9197	ENSG00000169359	523.034209	0.11259958	0.11309927	0.29765909	0.5243318	SLC33A1
10073	ENSG00000169371	280.831677	-0.10150975	0.13881439	0.43181711	0.65745157	SNUPN
8738	ENSG00000169372	158.510172	-0.46685272	0.21806356	0.01189097	0.04788247	CRADD
25942	ENSG00000169375	1608.25054	-0.14262094	0.08392056	0.08094548	0.21334235	SIN3A
200894	ENSG00000169379	244.713798	0.15847416	0.15117328	0.25393265	0.47447954	ARL13B
6036	ENSG00000169385	0	NA	NA	NA	NA	RNASE2
64100	ENSG00000169393	0	NA	NA	NA	NA	ELSPBP1
6037	ENSG00000169397	0	NA	NA	NA	NA	RNASE3
5747	ENSG00000169398	2575.57792	0.03002085	0.08285532	0.71136084	0.86130838	PTK2
728194	ENSG00000169402	3.29690753	0.11728821	0.40498978	0.12696663	0.29691395	RSPH10B2
5724	ENSG00000169403	6.81615427	0.10291758	0.37200312	0.46630224	0.68917137	PTAFR
5780	ENSG00000169410	1299.37574	-0.28080207	0.08255723	0.00048104	0.0031199	PTPN9
6039	ENSG00000169413	0	NA	NA	NA	NA	RNASE6
4881	ENSG00000169418	2.4167718	0.01717039	0.37595718	0.8354662	NA	NPR1
51305	ENSG00000169427	6.90702877	-0.01696567	0.35192698	0.90385478	0.95803392	KCNK9
3576	ENSG00000169429	0	NA	NA	NA	NA	CXCL8
6335	ENSG00000169432	429.656812	2.6253469	0.14361612	7.54E-76	1.41E-73	SCN9A
166824	ENSG00000169435	0	NA	NA	NA	NA	RASSF6
169044	ENSG00000169436	1.17522906	-0.03111975	0.38143648	0.62258339	NA	COL22A1
6383	ENSG00000169439	1722.08994	-1.58320859	0.11217474	2.30E-46	2.07E-44	SDC2
1043	ENSG00000169442	0	NA	NA	NA	NA	CD52
93380	ENSG00000169446	619.998881	-0.42879745	0.12423284	0.00026189	0.00181487	MMGT1
6699	ENSG00000169469	0	NA	NA	NA	NA	SPRR1B
6698	ENSG00000169474	0	NA	NA	NA	NA	SPRR1A
122740	ENSG00000169484	0	NA	NA	NA	NA	OR4K14

81127	ENSG00000169488	0	NA	NA	NA	NA	OR4K15
83877	ENSG00000169490	289.648514	-0.04045195	0.14945611	0.76775893	0.89307241	TM2D2
203100	ENSG00000169495	0.31809377	0.00103576	0.38336594	0.96715256	NA	HTRA4
59339	ENSG00000169499	326.805791	0.4503031	0.19559933	0.00858109	0.03656547	PLEKHA2
25932	ENSG00000169504	3145.78088	0.36999576	0.09379632	4.60E-05	0.00038084	CLIC4
151258	ENSG00000169507	0	NA	NA	NA	NA	SLC38A11
1880	ENSG00000169508	0.32429952	-0.02327681	0.38532192	0.66833494	NA	GPR183
54544	ENSG00000169509	0	NA	NA	NA	NA	CRCT1
83987	ENSG00000169515	5.77651257	0.16908771	0.40844079	0.21735659	0.428834	CCDC8
196074	ENSG00000169519	307.80849	0.01771454	0.14908207	0.8985883	0.95556135	METTL15
129025	ENSG00000169548	0.3225948	-0.02325506	0.38531882	0.66954696	NA	ZNF280A
143662	ENSG00000169550	0.85361557	0.03055138	0.38453456	0.49607004	NA	MUC15
54967	ENSG00000169551	5.42832209	0.05774386	0.36665698	0.64848578	0.82457939	CT55
9839	ENSG00000169554	1166.42194	-0.07589059	0.12148715	0.50913425	0.72482947	ZEB2
2705	ENSG00000169562	0	NA	NA	NA	NA	GJB1
5093	ENSG00000169564	4292.59474	0.01731157	0.05868203	0.76587061	0.89234011	PCBP1
3094	ENSG00000169567	3178.33582	-0.05340858	0.06224301	0.38390724	0.61306501	HINT1
285605	ENSG00000169570	130.716478	0.34309924	0.2149445	0.05828858	0.16750367	DTWD2
7441	ENSG00000169575	0	NA	NA	NA	NA	VPREB1
9022	ENSG00000169583	0.70498675	0.05128	0.38860286	0.29567183	NA	CLIC3
283899	ENSG00000169592	1377.21237	-0.1604111	0.1032589	0.1057774	0.25953792	INO80E
646	ENSG00000169594	0.97492369	0.00557836	0.38007023	0.92670034	NA	BNC1
1677	ENSG00000169598	300.26987	0.01815434	0.15653027	0.90002656	0.95573896	DFFB
27247	ENSG00000169599	238.80385	-0.25110203	0.14839898	0.06570905	0.18315081	NFU1
84168	ENSG00000169604	2090.68199	0.90453341	0.0721893	8.56E-37	6.09E-35	ANTXR1
56287	ENSG00000169605	0	NA	NA	NA	NA	GKN1
150468	ENSG00000169607	725.18605	-0.37760274	0.11340402	0.00046714	0.00304196	CKAP2L
123207	ENSG00000169609	746.359196	0.24221025	0.2883244	0.24315411	0.46155545	C15orf40
83640	ENSG00000169612	420.000335	-0.13946056	0.11374466	0.19825815	0.40248048	RAMAC
10887	ENSG00000169618	1.70753617	0.11354004	0.40738421	0.04738105	NA	PROKR1
200558	ENSG00000169621	71.4550092	0.62194196	0.29458745	0.00754773	0.03291867	APLF
552900	ENSG00000169627	698.330479	-0.00471658	0.10172982	0.96066404	0.98407795	BOLA2B
654483	ENSG00000169627	698.330479	-0.00471658	0.10172982	0.96066404	0.98407795	BOLA2B
727851	ENSG00000169629	100.547723	-0.21458137	0.23420325	0.25436902	0.47484771	RGPD8
23119	ENSG00000169635	692.897957	0.61067653	0.09803078	1.39E-10	2.62E-09	HIC2
7798	ENSG00000169641	987.121227	0.01115436	0.10423656	0.91261876	0.96264321	LUZP1
284004	ENSG00000169660	728.879134	0.22694604	0.09960137	0.01808069	0.06715058	HEXD
NA	ENSG00000169662	1.13919152	0.01776856	0.38013248	0.78070577	NA	BCRP6

NA	ENSG00000169668	42.3483586	0.06900453	0.2667603	0.72286016	0.86755463	BCRP2
1816	ENSG00000169676	0	NA	NA	NA	NA	DRD5
699	ENSG00000169679	1207.70933	-0.18342137	0.09280571	0.04118156	0.12828422	BUB1
83985	ENSG00000169682	980.394971	0.13243846	0.0983079	0.16368021	0.35275692	SPNS1
201255	ENSG00000169683	814.473204	0.35339561	0.10224994	0.00032525	0.00219936	LRRC45
1138	ENSG00000169684	989.157297	-0.01381107	0.09277972	0.8772424	0.94574195	CHRNA5
4490	ENSG00000169688	0	NA	NA	NA	NA	MT1B
201254	ENSG00000169689	950.809773	0.0920807	0.08989172	0.2922232	0.51825408	CENPX
10555	ENSG00000169692	579.897154	0.12890735	0.10179744	0.18891387	0.38965081	AGPAT2
79058	ENSG00000169696	769.670561	0.08578912	0.09284007	0.34117207	0.57145242	ASPSCR1
2815	ENSG00000169704	0	NA	NA	NA	NA	GP9
2194	ENSG00000169710	20167.9208	-0.42822698	0.0692989	9.86E-10	1.70E-08	FASN
7555	ENSG00000169714	5475.61881	-0.05480724	0.05338409	0.29638241	0.52297409	CNBP
4493	ENSG00000169715	1.29633656	-0.02000461	0.37876945	0.77925424	NA	MT1E
140625	ENSG00000169717	0	NA	NA	NA	NA	ACTRT2
64118	ENSG00000169718	2289.16358	0.30363084	0.07576468	4.22E-05	0.00035303	DUS1L
2873	ENSG00000169727	4108.14277	0.07443553	0.05401145	0.16337392	0.35239601	GPS1
5986	ENSG00000169733	1078.69796	0.1940799	0.10483423	0.05385331	0.15751876	RFNG
51181	ENSG00000169738	885.085509	-0.32221493	0.08622256	0.00012097	0.00090156	DCXR
7580	ENSG00000169740	384.814694	-0.07965891	0.12362978	0.49523725	0.7139953	ZNF32
9079	ENSG00000169744	201.381685	0.20431309	0.18102451	0.20161864	0.40709482	LDB2
5881	ENSG00000169750	1592.80283	-0.29956832	0.07323886	2.86E-05	0.00024757	RAC3
145957	ENSG00000169752	11.4602532	0.1182751	0.35470063	0.4854981	0.70561001	NRG4
3987	ENSG00000169756	1128.73807	0.27521491	0.10085558	0.00457454	0.0215887	LIMS1
123591	ENSG00000169758	49.5145025	0.56365293	0.35625385	0.02491867	0.08693273	TMEM266
22871	ENSG00000169760	79.7558712	-0.09042378	0.24489009	0.63206277	0.81381825	NLGN1
202018	ENSG00000169762	432.925049	0.06445646	0.13966104	0.62005652	0.8060491	TAPT1
NA	ENSG00000169763	0	NA	NA	NA	NA	PRYP3
7360	ENSG00000169764	380.86374	-0.02651091	0.11863799	0.81341477	0.91569162	UGP2
50834	ENSG00000169777	0	NA	NA	NA	NA	TAS2R1
84894	ENSG00000169783	252.751379	-1.886323	0.22410538	2.29E-18	7.86E-17	LINGO1
9081	ENSG00000169789	0	NA	NA	NA	NA	PRY
159163	ENSG00000169800	0	NA	NA	NA	NA	RBMY1F
442862	ENSG00000169807	0	NA	NA	NA	NA	PRY2
NA	ENSG00000169811	0	NA	NA	NA	NA	RBMY1HP
3185	ENSG00000169813	4702.37203	0.03322659	0.0594658	0.57248249	0.7723153	HNRNPF
686	ENSG00000169814	441.465563	0.22173248	0.13277281	0.07444389	0.20030974	BTD
55454	ENSG00000169826	940.448364	0.25749095	0.08504035	0.00186442	0.01008177	CSGALNACT2

6870	ENSG00000169836	0.16735865	0.01897177	0.38482933	0.78225479	NA	TACR3
219409	ENSG00000169840	0	NA	NA	NA	NA	GSX1
NA	ENSG00000169849	0	NA	NA	NA	NA	TSPY14P
5099	ENSG00000169851	7.82589174	0.06814153	0.37290092	0.54854093	0.75503345	PCDH7
6091	ENSG00000169855	1001.62983	-0.07528395	0.14810137	0.57996075	0.77743886	ROBO1
3175	ENSG00000169856	0.49687332	-0.02955746	0.38590719	0.52474722	NA	ONECUT1
57099	ENSG00000169857	195.95589	-0.13501569	0.15879285	0.34920586	0.57998769	AVEN
5028	ENSG00000169860	3.01298285	0.18759872	0.44383995	0.05337541	0.15645711	P2RY1
1501	ENSG00000169862	0	NA	NA	NA	NA	CTNND2
81844	ENSG00000169871	11.3585229	0.11059551	0.35459271	0.50826572	0.7239622	TRIM56
140453	ENSG00000169876	0	NA	NA	NA	NA	MUC17
51327	ENSG00000169877	0	NA	NA	NA	NA	AHSP
7480	ENSG00000169884	0.69723612	0.03065898	0.38448208	0.58082614	NA	WNT10B
163688	ENSG00000169885	6.53402955	-0.03045907	0.35986537	0.81370149	0.91591045	CALML6
9185	ENSG00000169891	48.7365805	-0.21074422	0.28166297	0.29965101	0.52662556	REPS2
4584	ENSG00000169894	0.68684686	0.00320496	0.38127506	0.94988598	NA	MUC3A
94056	ENSG00000169895	1114.44307	-0.20933005	0.09727934	0.02566232	0.08890176	SYAP1
3684	ENSG00000169896	0	NA	NA	NA	NA	ITGAM
260434	ENSG00000169900	0	NA	NA	NA	NA	PYDC1
8460	ENSG00000169902	559.39648	0.50611427	0.11005352	1.65E-06	1.80E-05	TPST1
7104	ENSG00000169903	0	NA	NA	NA	NA	TM4SF4
163590	ENSG00000169905	2262.74478	-0.084054	0.07089769	0.22720249	0.4418878	TOR1AIP2
795	ENSG00000169906	0	NA	NA	NA	NA	S100G
4071	ENSG00000169908	0.34746601	0.02370763	0.3853651	0.61987128	NA	TM4SF1
23252	ENSG00000169914	664.431119	0.10702565	0.12628446	0.37019659	0.59943356	OTUD3
161725	ENSG00000169918	252.786475	-0.52607689	0.15188354	0.00018462	0.0013233	OTUD7A
2990	ENSG00000169919	1112.92759	0.0532934	0.08319463	0.51237168	0.7276095	GUSB
8019	ENSG00000169925	2713.42236	0.23320105	0.080796	0.003103	0.01559038	BRD3
51621	ENSG00000169926	2147.84476	-0.17454301	0.07520594	0.01770657	0.06600871	KLF13
9758	ENSG00000169933	0	NA	NA	NA	NA	FRMPD4
23414	ENSG00000169946	26.121443	3.75699376	0.74539666	2.42E-08	3.46E-07	ZFPM2
92595	ENSG00000169951	407.702036	0.19640202	0.11938588	0.08336134	0.21800589	ZNF764
159119	ENSG00000169953	0	NA	NA	NA	NA	HSFY2
65988	ENSG00000169955	445.214915	-0.0465216	0.14789563	0.73271663	0.87239916	ZNF747
79724	ENSG00000169957	1315.66771	-0.01025882	0.09414413	0.90965122	0.96107209	ZNF768
83756	ENSG00000169962	9.25751943	0.10740134	0.35779217	0.50839272	0.72403357	TAS1R3
131616	ENSG00000169964	159.317602	-0.06624102	0.1689854	0.66146428	0.8324506	TMEM42
10746	ENSG00000169967	942.243284	-0.04974501	0.09349991	0.58214356	0.77907104	MAP3K2

126789	ENSG00000169972	259.802826	-0.1791477	0.13972454	0.16732883	0.35828164	PUSL1
83443	ENSG00000169976	1086.53545	0.39535216	0.09742401	2.65E-05	0.00023154	SF3B5
7584	ENSG00000169981	304.980794	0.10162711	0.1448063	0.44870988	0.67234398	ZNF35
201798	ENSG00000169989	35.8675381	0.22188486	0.32540219	0.28636446	0.51225386	TIGD4
126917	ENSG00000169991	126.726728	-0.71598274	0.24296421	0.00059716	0.00376716	IFFO2
57555	ENSG00000169992	3191.43242	-0.22227366	0.0716507	0.00156789	0.00871099	NLGN2
4648	ENSG00000169994	0.15069912	-0.01848503	0.38480575	0.84537521	NA	MYO7B
1107	ENSG00000170004	4013.71651	0.08716638	0.06643534	0.18335843	0.38156401	CHD3
201799	ENSG00000170006	39.5874761	3.15723937	0.6190836	1.24E-08	1.86E-07	TMEM154
25924	ENSG00000170011	173.511122	0.01326916	0.17398644	0.93299559	0.97202552	MYRIP
214	ENSG00000170017	3175.75883	0.55594491	0.05876278	1.08E-21	4.40E-20	ALCAM
7532	ENSG00000170027	10941.0307	-0.27675726	0.0469854	2.84E-09	4.57E-08	YWHAG
10477	ENSG00000170035	1491.01472	-0.03844674	0.07216347	0.58751671	0.78256674	UBE2E3
116840	ENSG00000170037	1014.85622	0.10797781	0.09945044	0.26093812	0.48276874	CNTROB
58485	ENSG00000170043	1066.4037	0.21119953	0.08025937	0.00706143	0.0311896	TRAPPC1
131368	ENSG00000170044	1.53473883	0.02335072	0.37723536	0.76964634	NA	ZPLD1
9196	ENSG00000170049	432.926489	0.27285526	0.12602054	0.02154473	0.07726816	KCNAB3
327657	ENSG00000170054	0	NA	NA	NA	NA	SERPINA9
285596	ENSG00000170074	0.83002665	0.02308492	0.38355603	0.61702622	NA	FAM153A
9283	ENSG00000170075	4.59426043	0.10330928	0.38855657	0.31444033	0.54217557	GPR37L1
375484	ENSG00000170085	705.408885	-0.05801656	0.10681108	0.57085622	0.77109827	SIMC1
201931	ENSG00000170088	403.079904	-0.323453	0.13714252	0.01131976	0.04595542	TMEM192
NA	ENSG00000170089	261.953525	-0.2766912	0.14907175	0.04330741	0.13329491	
51617	ENSG00000170091	3676.24809	-3.60652643	0.08080996	0	0	NSG2
442590	ENSG00000170092	22.1720714	-0.21669939	0.34814443	0.28643364	0.51225526	SPDYE5
866	ENSG00000170099	0	NA	NA	NA	NA	SERPINA6
197320	ENSG00000170100	968.956706	0.20202294	0.12703139	0.09119322	0.23300366	ZNF778
123606	ENSG00000170113	1439.25411	-0.24225795	0.08831973	0.00471781	0.02213638	NIPA1
2298	ENSG00000170122	31.1991243	0.15944877	0.2969006	0.42787725	0.65375395	FOXD4
2848	ENSG00000170128	2.75418029	-0.1088411	0.39392267	0.26974893	0.49289177	GPR25
7324	ENSG00000170142	1609.93466	-0.23306497	0.07860527	0.00240135	0.01252769	UBE2E1
220988	ENSG00000170144	11732.2763	0.02156966	0.07701866	0.77618697	0.89709598	HNRNPA3
23235	ENSG00000170145	1209.51707	0.63749969	0.0872855	7.80E-14	1.93E-12	SIK2
57484	ENSG00000170153	4892.26885	-0.3516516	0.07717077	3.10E-06	3.23E-05	RNF150
9720	ENSG00000170160	26.6654547	-0.24766194	0.40554249	0.19373399	0.39662392	CCDC144A
554249	ENSG00000170161	157.920818	0.12442403	0.17180023	0.41864524	0.64464626	
245806	ENSG00000170162	0	NA	NA	NA	NA	VGLL2
3233	ENSG00000170166	110.895706	-0.06068292	0.19738483	0.71903702	0.86537424	HOXD4

1140	ENSG00000170175	347.920122	0.28780182	0.12878623	0.01742958	0.06522123	CHRNB1
3238	ENSG00000170178	0.52119901	0.02576573	0.38568143	0.51723676	NA	HOXD12
2993	ENSG00000170180	0	NA	NA	NA	NA	GYPA
84640	ENSG00000170185	661.141091	-0.16722699	0.10559617	0.09850287	0.24653676	USP38
9121	ENSG00000170190	0	NA	NA	NA	NA	SLC16A5
140838	ENSG00000170191	193.963764	-0.38167523	0.17138143	0.01320587	0.052078	NANP
255239	ENSG00000170209	3.6548343	0.03573914	0.36980932	0.74612701	0.8804068	ANKK1
147	ENSG00000170214	0	NA	NA	NA	NA	ADRA1B
56985	ENSG00000170222	151.869628	-0.13606017	0.18373097	0.39837145	0.62727695	ADPRM
2172	ENSG00000170231	13.5403007	-1.40015846	0.89714664	0.0048841	0.02278406	FABP6
114825	ENSG00000170234	435.142426	-0.25429442	0.12263336	0.02799325	0.09501899	PWWP2A
373509	ENSG00000170236	14.7368343	0.16727824	0.36264676	0.35936017	0.58949503	USP50
55031	ENSG00000170242	1699.19162	0.34740635	0.09480423	0.00015101	0.00110145	USP47
10015	ENSG00000170248	2151.15264	-0.20057578	0.08252442	0.0126427	0.0503071	PDCD6IP
259249	ENSG00000170255	0	NA	NA	NA	NA	MRGPRX1
7988	ENSG00000170260	475.065549	0.11023644	0.11145054	0.30140297	0.52872103	ZNF212
56246	ENSG00000170262	0	NA	NA	NA	NA	MRAP
84140	ENSG00000170264	337.301988	-0.28227405	0.13349542	0.02364843	0.08332657	FAM161A
8427	ENSG00000170265	1270.03515	-0.04956355	0.10354645	0.6181204	0.80452675	ZNF282
2720	ENSG00000170266	884.735583	0.30935376	0.11816633	0.00578906	0.02634892	GLB1
84520	ENSG00000170270	237.122116	-0.40514312	0.15641712	0.00464057	0.02184314	GON7
10826	ENSG00000170271	270.786797	0.46180177	0.16095985	0.00169839	0.00933926	FAXDC2
10491	ENSG00000170275	1833.20069	0.19146552	0.07907075	0.01323237	0.05217216	CRTAP
3316	ENSG00000170276	0.173124	-0.01911262	0.38486262	0.84537521	NA	HSPB2
202865	ENSG00000170279	0	NA	NA	NA	NA	C7orf33
54714	ENSG00000170289	0.18922458	0.01954053	0.38488496	0.78225479	NA	CNGB3
6588	ENSG00000170290	0.66943461	0.03059546	0.38616409	0.42784935	NA	SLN
23587	ENSG00000170291	830.810254	0.22543688	0.08801797	0.00840132	0.03596938	ELP5
152189	ENSG00000170293	75.8223806	0.28357767	0.26234835	0.16031886	0.34815303	CMTM8
11337	ENSG00000170296	3235.76641	0.09277744	0.06562615	0.15069872	0.33428696	GABARAP
284194	ENSG00000170298	0	NA	NA	NA	NA	LGALS9B
9482	ENSG00000170310	217.878986	0.1189251	0.15733837	0.40883335	0.63702612	STX8
983	ENSG00000170312	1832.92836	-0.37250499	0.07387225	2.64E-07	3.29E-06	CDK1
7314	ENSG00000170315	12350.8112	-0.10094091	0.04640433	0.02836205	0.0960408	UBB
4798	ENSG00000170322	1287.76843	0.11452285	0.07892985	0.13818795	0.31440929	NFRKB
2167	ENSG00000170323	0	NA	NA	NA	NA	FABP4
143162	ENSG00000170324	4.9949858	0.19906643	0.4458268	0.07597106	0.20338223	FRMPD2
56980	ENSG00000170325	392.513088	-0.14477981	0.12848008	0.23059236	0.44655466	PRDM10

10678	ENSG00000170340	304.614223	-0.14850641	0.13320168	0.23320337	0.45004948	B3GNT2
2353	ENSG00000170345	927.495378	2.61893509	0.2914582	1.54E-20	5.94E-19	FOS
10972	ENSG00000170348	3406.03136	0.25683411	0.07611086	0.00057878	0.00366632	TMED10
401428	ENSG00000170356	0	NA	NA	NA	NA	OR2A20P
6419	ENSG00000170364	428.93572	-0.21890776	0.12259035	0.05865467	0.16825136	SETMAR
4086	ENSG00000170365	297.485371	-0.09071956	0.1787617	0.56488828	0.76715609	SMAD1
1473	ENSG00000170367	0	NA	NA	NA	NA	CST5
1470	ENSG00000170369	0	NA	NA	NA	NA	CST2
2018	ENSG00000170370	0	NA	NA	NA	NA	EMX2
1469	ENSG00000170373	0	NA	NA	NA	NA	CST1
121340	ENSG00000170374	3.50947625	-0.0761553	0.37940605	0.46969062	0.69206549	SP7
285966	ENSG00000170379	0.35628637	0.02381422	0.38538061	0.61421642	NA	TCAF2
9723	ENSG00000170381	0.75016689	-0.00543912	0.38183361	0.8995577	NA	SEMA3E
10446	ENSG00000170382	109.085332	-0.20726015	0.22268887	0.25672147	0.47780055	LRRN2
7779	ENSG00000170385	573.829622	-0.67184148	0.11290804	6.62E-10	1.17E-08	SLC30A1
166614	ENSG00000170390	753.578605	-0.29133631	0.10856343	0.00497667	0.02313971	DCLK2
91752	ENSG00000170396	232.672743	-0.72006807	0.16349616	2.26E-06	2.41E-05	ZNF804A
NA	ENSG00000170409	0	NA	NA	NA	NA	
55890	ENSG00000170412	10.4091984	3.55844558	1.1749026	0.00017106	0.00123504	GPRC5C
130827	ENSG00000170417	71.7372274	0.09580265	0.23540181	0.60933286	0.79803651	TMEM182
222008	ENSG00000170419	19.1455709	-0.31041756	0.42728611	0.13895263	0.31571462	VSTM2A
3856	ENSG00000170421	0	NA	NA	NA	NA	KRT8
196374	ENSG00000170423	0	NA	NA	NA	NA	KRT78
136	ENSG00000170425	30.0239335	-0.05546562	0.28620175	0.77161474	0.89527792	ADORA2B
121214	ENSG00000170426	0	NA	NA	NA	NA	SDR9C7
4255	ENSG00000170430	283.218713	0.1148444	0.14162279	0.3829023	0.61227467	MGMT
196410	ENSG00000170439	0	NA	NA	NA	NA	METTL7B
3892	ENSG00000170442	0	NA	NA	NA	NA	KRT86
3035	ENSG00000170445	2403.9034	0.01362874	0.06312154	0.8273322	0.92189669	HARS1
152518	ENSG00000170448	549.676088	0.0786919	0.10692964	0.44405167	0.66905081	NFXL1
9119	ENSG00000170454	0	NA	NA	NA	NA	KRT75
160518	ENSG00000170456	793.237341	-0.178674	0.13521826	0.15679513	0.34358464	DENND5B
929	ENSG00000170458	20.5041188	1.15438401	0.61333435	0.00375117	0.01827681	CD14
202052	ENSG00000170464	591.239359	0.00672421	0.13201461	0.95986677	0.98360159	DNAJC18
286887	ENSG00000170465	0	NA	NA	NA	NA	KRT6C
79697	ENSG00000170468	430.057133	-0.13767952	0.11303879	0.20152368	0.40694451	RIOX1
202051	ENSG00000170469	42.0820082	0.09452936	0.26446239	0.62700701	0.81067573	SPATA24
57148	ENSG00000170471	2605.10351	-0.04415677	0.06012107	0.45702633	0.68052466	RALGAPB

84305	ENSG00000170473	419.034116	-0.0495058	0.11608343	0.65333484	0.82730171	PYM1
51237	ENSG00000170476	0.8230849	-0.00547373	0.38077018	0.92289514	NA	MZB1
3851	ENSG00000170477	0	NA	NA	NA	NA	KRT4
9963	ENSG00000170482	7.90429217	0.20158279	0.4104864	0.21561415	0.42641394	SLC23A1
121391	ENSG00000170484	0	NA	NA	NA	NA	KRT74
4862	ENSG00000170485	29.8829652	-0.07919062	0.3217465	0.66007363	0.83146071	NPAS2
140807	ENSG00000170486	0	NA	NA	NA	NA	KRT72
3814	ENSG00000170498	1.67792723	0.06154521	0.38500862	0.39937632	NA	KISS1
164832	ENSG00000170500	183.558136	0.0345242	0.16630439	0.8191077	0.91752333	LONRF2
53343	ENSG00000170502	418.315325	-0.06552342	0.13184395	0.59566295	0.78816266	NUDT9
345275	ENSG00000170509	14.9677522	0.26379989	0.4019622	0.19332036	0.3960223	HSD17B13
5036	ENSG00000170515	3821.64546	-0.05284576	0.07156394	0.45167445	0.67494322	PA2G4
170712	ENSG00000170516	0	NA	NA	NA	NA	COX7B2
79071	ENSG00000170522	1463.10695	-0.33319258	0.1162638	0.00254562	0.01317983	ELOVL6
3889	ENSG00000170523	0	NA	NA	NA	NA	KRT83
5209	ENSG00000170525	572.714701	0.00710597	0.14177984	0.95882133	0.98310156	PFKFB3
79905	ENSG00000170537	100.076654	0.18712621	0.22784434	0.31318053	0.54124579	TMC7
23204	ENSG00000170540	5351.04922	-0.286684	0.0518263	2.29E-08	3.29E-07	ARL6IP1
5272	ENSG00000170542	8.22735833	-0.18585081	0.39656173	0.25960343	0.48121669	SERPINB9
57228	ENSG00000170545	26.1705334	-0.10986598	0.32587276	0.55370101	0.75980255	SMAGP
79192	ENSG00000170549	0	NA	NA	NA	NA	IRX1
1000	ENSG00000170558	3106.0314	1.0272181	0.06135669	9.13E-64	1.29E-61	CDH2
153572	ENSG00000170561	0	NA	NA	NA	NA	IRX2
133418	ENSG00000170571	616.416884	0.11252573	0.0988461	0.23882392	0.45652723	EMB
10736	ENSG00000170577	29.3309238	0.23940226	0.33016879	0.25554405	0.47650319	SIX2
9229	ENSG00000170579	0.71501162	-0.04064198	0.38714626	0.37606669	NA	DLGAP1
6773	ENSG00000170581	800.652759	0.30527244	0.10003939	0.00152594	0.0085017	STAT2
134492	ENSG00000170584	548.04027	-0.09536623	0.12975567	0.43335586	0.65863186	NUDCD2
26145	ENSG00000170604	1312.62008	0.02096173	0.08788657	0.80722337	0.91260538	IRF2BP1
441639	ENSG00000170605	0	NA	NA	NA	NA	OR9K2
3308	ENSG00000170606	2893.27686	-0.35657451	0.08035705	5.44E-06	5.44E-05	HSPA4
3171	ENSG00000170608	5.30711147	0.00098151	0.35887432	0.99597878	0.99859366	FOXA3
153745	ENSG00000170613	0	NA	NA	NA	NA	FAM71B
375611	ENSG00000170615	9.68397929	1.2953348	1.36035894	0.00880275	0.03734159	SLC26A5
28991	ENSG00000170619	565.564101	0.04189975	0.09933045	0.66326227	0.83380582	COMMD5
6444	ENSG00000170624	74.3437157	0.00622106	0.25513455	0.97575468	0.99159826	SGCD
121355	ENSG00000170627	23.151985	-1.95412553	0.61637529	7.90E-05	0.00061601	GTSF1
349152	ENSG00000170629	362.628805	-0.23624593	0.13779251	0.06531185	0.18235113	DPY19L2P2

7564	ENSG00000170631	153.071349	-0.00373758	0.18731935	0.98206668	0.99406728	ZNF16
83787	ENSG00000170632	939.880534	-0.22185436	0.10278484	0.02465063	0.08617946	ARMC10
80196	ENSG00000170633	603.033588	0.03296072	0.11521855	0.7650925	0.89191112	RNF34
98	ENSG00000170634	118.453314	0.03742741	0.18755837	0.82051252	0.91836671	ACYP2
80305	ENSG00000170638	1441.9526	-0.09245122	0.08051135	0.23902785	0.45678218	TRABD
11016	ENSG00000170653	925.095631	0.07515401	0.09602376	0.4201837	0.64613584	ATF7
100271927	ENSG00000170667	1141.41506	0.1197955	0.09802019	0.20620369	0.41324052	RASA4B
9306	ENSG00000170677	652.692117	0.52780444	0.1089639	4.62E-07	5.55E-06	SOCS6
347273	ENSG00000170681	153.006944	-0.13703721	0.17498064	0.37810239	0.60702282	CAVIN4
26496	ENSG00000170683	0	NA	NA	NA	NA	OR10A3
162979	ENSG00000170684	19.7357671	0.42543104	0.49083921	0.07837909	0.20828302	ZNF296
NA	ENSG00000170688	0	NA	NA	NA	NA	OR5E1P
3219	ENSG00000170689	0	NA	NA	NA	NA	HOXB9
284076	ENSG00000170703	0	NA	NA	NA	NA	TTLL6
5429	ENSG00000170734	626.244774	0.0362626	0.27034457	0.8532285	0.93385853	POLH
143425	ENSG00000170743	19.4946776	2.46743912	0.62493743	4.71E-06	4.77E-05	SYT9
3790	ENSG00000170745	4.19961508	0.28997988	0.55691392	0.00620984	0.02788666	KCNS3
27288	ENSG00000170748	0.16735865	0.01897177	0.38482933	0.78225479	NA	RBMXL2
3799	ENSG00000170759	4809.14291	-0.24712449	0.05450513	4.53E-06	4.60E-05	KIF5B
2861	ENSG00000170775	211.131079	1.81428456	0.18072118	8.09E-25	3.83E-23	GPR37
11214	ENSG00000170776	2129.27642	0.21519275	0.08300226	0.00782716	0.03389892	AKAP13
89882	ENSG00000170777	0	NA	NA	NA	NA	TPD52L3
55038	ENSG00000170779	1006.24629	-0.41691439	0.08417224	3.69E-07	4.50E-06	CDCA4
283297	ENSG00000170782	0	NA	NA	NA	NA	OR10A4
195814	ENSG00000170786	0	NA	NA	NA	NA	SDR16C5
143241	ENSG00000170788	0.85833586	0.04613547	0.38538553	0.40603712	NA	DYDC1
341276	ENSG00000170790	0	NA	NA	NA	NA	OR10A2
79145	ENSG00000170791	100.262932	-0.07431098	0.23488783	0.68894778	0.84898907	CHCHD7
94031	ENSG00000170801	2.27953673	-0.15767178	0.43003068	0.01919798	NA	HTRA3
3344	ENSG00000170802	474.22979	-0.41720219	0.11547745	0.00014742	0.00107846	FOXN2
442721	ENSG00000170807	0.50818559	0.03077092	0.38601908	0.48305186	NA	LMOD2
8419	ENSG00000170819	0	NA	NA	NA	NA	BFSP2
2492	ENSG00000170820	0	NA	NA	NA	NA	FSHR
NA	ENSG00000170827	2.0221701	0.03281618	0.3767489	0.70355011	NA	CELP
84669	ENSG00000170832	1277.81613	-0.2029806	0.08905857	0.01884299	0.06938475	USP32
1056	ENSG00000170835	41.7098787	0.25562132	0.31230241	0.22747008	0.44227817	CEL
8493	ENSG00000170836	876.377947	-0.12922933	0.08509028	0.11881708	0.28318716	PPM1D
2850	ENSG00000170837	375.013675	-0.25905564	0.123219	0.0259129	0.08955568	GPR27

NA	ENSG00000170846	457.383903	-0.13837176	0.12705668	0.24576918	0.46496238	
5675	ENSG00000170848	0	NA	NA	NA	NA	PSG6
25948	ENSG00000170852	1475.54325	-0.2140699	0.08006192	0.00615923	0.02770185	KBTBD2
84864	ENSG00000170854	527.300537	-0.21417458	0.12913092	0.0770512	0.20558039	RIOX2
51499	ENSG00000170855	333.746306	-0.20349095	0.13528094	0.10693762	0.2618665	TRIAP1
79166	ENSG00000170858	0	NA	NA	NA	NA	LILRP2
27258	ENSG00000170860	665.977471	-0.33991535	0.10443393	0.00069512	0.00430169	LSM3
9778	ENSG00000170871	998.91637	0.01432969	0.10257442	0.88583718	0.95015905	KIAA0232
9788	ENSG00000170873	401.863248	-0.54049312	0.14929824	9.78E-05	0.00074748	MTSS1
79188	ENSG00000170876	1484.91216	-0.39600421	0.07635267	1.14E-07	1.50E-06	TMEM43
11236	ENSG00000170881	522.159125	-0.11070522	0.10555967	0.2739229	0.49754008	RNF139
6203	ENSG00000170889	10831.0395	-0.1112736	0.0490427	0.0220835	0.07887246	RPS9
5319	ENSG00000170890	0.36265744	0.00119746	0.38336636	0.9645027	NA	PLA2G1B
54360	ENSG00000170891	115.675067	-0.00409768	0.20128044	0.98020387	0.99338199	CYTL1
79042	ENSG00000170892	899.206127	-0.25500112	0.09210905	0.00416501	0.01996949	TSEN34
7200	ENSG00000170893	1.87644317	-0.06322292	0.38708455	0.33781037	NA	TRH
2941	ENSG00000170899	774.480054	-0.5543777	0.09866614	6.58E-09	1.01E-07	GSTA4
84437	ENSG00000170903	850.25664	-0.1308926	0.10708804	0.20215925	0.40772996	MSANTD4
4696	ENSG00000170906	510.68271	0.09871706	0.11504966	0.36861771	0.59798654	NDUFA3
126014	ENSG00000170909	1.2677063	0.03906889	0.38264176	0.53563598	NA	OSCAR
85315	ENSG00000170915	605.44267	-0.49863329	0.11136239	2.94E-06	3.07E-05	PAQR8
11162	ENSG00000170917	220.939489	-0.00672792	0.16747897	0.96319047	0.98545957	NUDT6
NA	ENSG00000170919	479.158149	0.58629626	0.13330817	3.34E-06	3.46E-05	TPT1-AS1
390883	ENSG00000170920	0	NA	NA	NA	NA	OR7G3
26115	ENSG00000170921	2657.5517	-0.05796517	0.0998356	0.54601168	0.75333225	TANC2
390882	ENSG00000170923	0	NA	NA	NA	NA	OR7G2
56156	ENSG00000170925	0	NA	NA	NA	NA	TEX13B
5314	ENSG00000170927	40.8107718	-0.13108846	0.28647204	0.50683525	0.72264544	PKHD1
125963	ENSG00000170929	0	NA	NA	NA	NA	OR1M1
392517	ENSG00000170935	0	NA	NA	NA	NA	NCBP2L
120526	ENSG00000170946	343.196436	0.06734579	0.15402231	0.63544285	0.8165276	DNAJC24
85509	ENSG00000170948	0	NA	NA	NA	NA	MBD3L1
90338	ENSG00000170949	553.320797	0.10563911	0.12727724	0.37865384	0.60756103	ZNF160
5232	ENSG00000170950	0	NA	NA	NA	NA	PGK2
219858	ENSG00000170953	0	NA	NA	NA	NA	OR8B12
55786	ENSG00000170954	183.697425	-0.04070591	0.17189737	0.78992985	0.9043268	ZNF415
112464	ENSG00000170955	0	NA	NA	NA	NA	CAVIN3
1084	ENSG00000170956	0	NA	NA	NA	NA	CEACAM3

341019	ENSG00000170959	1.3736986	0.03881598	0.38199749	0.56108184	NA	DCDC1
3037	ENSG00000170961	0.35651483	0.02381586	0.38538087	0.614101	NA	HAS2
80310	ENSG00000170962	219.651156	0.78406635	0.16791573	5.67E-07	6.71E-06	PDGFD
10761	ENSG00000170965	3.29634248	-0.01844288	0.36953426	0.85934224	0.9374775	PLAC1
414301	ENSG00000170967	0.18922458	0.01954053	0.38488496	0.78225479	NA	DDI1
NA	ENSG00000170983	0.47455697	-0.02849777	0.38581349	0.54195944	NA	LINC00208
1901	ENSG00000170989	5.34543424	3.74593527	1.70641036	0.00106185	0.00621448	S1PR1
90161	ENSG00000171004	2483.50465	0.81337677	0.07836478	5.83E-26	2.88E-24	HS6ST2
219875	ENSG00000171014	0	NA	NA	NA	NA	OR4D5
26108	ENSG00000171016	571.766813	-0.08988502	0.11919475	0.42643646	0.65221241	PYGO1
80131	ENSG00000171017	0.16735865	0.01897177	0.38482933	0.78225479	NA	LRRC8E
5569	ENSG00000171033	659.725302	-0.28261935	0.11071675	0.00743975	0.03249057	PKIA
286046	ENSG00000171044	147.749066	-0.21504409	0.24917681	0.26781428	0.49039551	XKR6
203062	ENSG00000171045	280.37473	-0.03471011	0.18029097	0.82535306	0.92066374	TSNARE1
2358	ENSG00000171049	0	NA	NA	NA	NA	FPR2
2357	ENSG00000171051	0	NA	NA	NA	NA	FPR1
160065	ENSG00000171053	0	NA	NA	NA	NA	PATE1
347468	ENSG00000171054	0.48994503	-0.00693475	0.38315951	0.85942445	NA	OR13H1
9637	ENSG00000171055	526.376855	0.06176695	0.10682596	0.54789953	0.75451702	FEZ2
83595	ENSG00000171056	291.798051	-1.4986547	0.16123557	1.24E-21	4.96E-20	SOX7
203076	ENSG00000171060	0	NA	NA	NA	NA	C8orf74
53838	ENSG00000171067	626.247559	-0.15354008	0.1007123	0.11337248	0.2732066	C11orf24
100125556	ENSG00000171084	82.772385	-0.07126738	0.2236463	0.69437933	0.85208349	FAM86JP
238	ENSG00000171094	5182.08604	-1.05705027	0.05799607	4.14E-75	7.46E-73	ALK
883	ENSG00000171097	251.705928	0.10954893	0.15749851	0.44633257	0.67062583	KYAT1
4534	ENSG00000171100	89.9338077	0.3416254	0.30005154	0.11373437	0.27377941	MTM1
284367	ENSG00000171101	0	NA	NA	NA	NA	SIGLEC17P
29989	ENSG00000171102	0	NA	NA	NA	NA	OBP2B
55006	ENSG00000171103	480.573884	0.09223721	0.11531309	0.40199568	0.63031025	TRMT61B
3643	ENSG00000171105	530.989676	0.10719305	0.28793948	0.58478927	0.78078479	INSR
55669	ENSG00000171109	1098.48276	0.1300738	0.09276247	0.14834412	0.33065355	MFN1
155038	ENSG00000171115	0	NA	NA	NA	NA	GIMAP8
100506164	ENSG00000171116	1.13976687	-0.06245039	0.39108221	0.18895555	NA	HSFX1
4902	ENSG00000171119	101.697659	-0.07235435	0.20590453	0.67632148	0.84170926	NRTN
27094	ENSG00000171121	60.3891718	-0.21130972	0.2630185	0.2873601	0.51306915	KCNMB3
2525	ENSG00000171124	1.8243984	-0.04306502	0.38052105	0.56737642	NA	FUT3
170850	ENSG00000171126	33.0356508	0.97445919	0.45610026	0.00297902	0.01503914	KCNG3
155066	ENSG00000171130	2152.91888	-0.25015585	0.07531637	0.00068636	0.00425014	ATP6V0E2

5581	ENSG00000171132	328.191775	-0.21875661	0.14284362	0.09757699	0.24466031	PRKCE
26248	ENSG00000171133	0	NA	NA	NA	NA	OR2K2
84522	ENSG00000171135	689.119232	-0.15949508	0.09973559	0.09696608	0.24355834	JAGN1
117579	ENSG00000171136	0.173733	0.01914545	0.38484597	0.78225479	NA	RLN3
10474	ENSG00000171148	1797.95468	0.18799424	0.07428712	0.00980928	0.04085998	TADA3
9655	ENSG00000171150	718.857381	-0.04240653	0.10435646	0.67184379	0.83832583	SOCS5
29071	ENSG00000171155	210.36947	0.16637404	0.15503972	0.239442	0.45732906	C1GALT1C1
79095	ENSG00000171159	632.91868	0.28671708	0.09941426	0.00275866	0.01410612	C9orf16
118812	ENSG00000171160	521.78199	0.21090469	0.11909298	0.06181336	0.17539718	MORN4
79894	ENSG00000171161	897.663058	-0.23144737	0.09242225	0.00950141	0.0397948	ZNF672
55657	ENSG00000171163	936.425377	0.21813157	0.09155509	0.01398347	0.05451602	ZNF692
203245	ENSG00000171169	254.098811	-0.0638286	0.14725738	0.63793349	0.81760985	NAIF1
64080	ENSG00000171174	24.0977835	0.23894127	0.36002671	0.24825817	0.46743944	RBKS
26245	ENSG00000171180	0.173733	0.01914545	0.38484597	0.78225479	NA	OR2M4
2897	ENSG00000171189	0.16735865	0.01897177	0.38482933	0.78225479	NA	GRIK1
4589	ENSG00000171195	0	NA	NA	NA	NA	MUC7
58503	ENSG00000171199	0	NA	NA	NA	NA	OPRPN
10879	ENSG00000171201	0	NA	NA	NA	NA	SMR3B
84233	ENSG00000171202	193.988057	-0.03031366	0.15739613	0.83409339	0.92432089	TMEM126A
55863	ENSG00000171204	332.105923	0.05770862	0.14455847	0.66766279	0.83652856	TMEM126B
81603	ENSG00000171206	913.095726	-0.27351522	0.10650078	0.00729314	0.03198408	TRIM8
81831	ENSG00000171208	1464.77985	-0.61089561	0.09832457	1.49E-10	2.81E-09	NETO2
1448	ENSG00000171209	0	NA	NA	NA	NA	CSN3
49861	ENSG00000171217	16.6310814	0.03864302	0.31940366	0.83189176	0.92391014	CLDN20
55561	ENSG00000171219	236.174961	0.20706421	0.14906378	0.13112451	0.30358816	CDC42BPG
51282	ENSG00000171222	990.467289	-0.03736652	0.08490722	0.65118513	0.82578612	SCAND1
3726	ENSG00000171223	1722.72581	0.28268669	0.07454772	0.00010781	0.00081388	JUNB
219738	ENSG00000171224	291.265337	-0.08021824	0.13958224	0.53624231	0.74696675	FAM241B
140738	ENSG00000171227	2.07889136	-0.02173606	0.37788953	0.77487526	NA	TMEM37
7364	ENSG00000171234	0	NA	NA	NA	NA	UGT2B7
116844	ENSG00000171236	3.39388008	-0.15255637	0.41398273	0.15138717	0.33539121	LRG1
79801	ENSG00000171241	396.228911	-0.12384823	0.12639469	0.29763108	0.52432966	SHCBP1
25928	ENSG00000171243	13.1276958	0.0803825	0.33825461	0.64202453	0.82094397	SOSTDC1
4884	ENSG00000171246	0.61831094	-0.03593288	0.38658432	0.43088585	NA	NPTX1
283742	ENSG00000171262	1187.22842	-0.10356771	0.11000461	0.32441062	0.55221149	FAM98B
90594	ENSG00000171291	302.904868	-0.02519732	0.16183575	0.86247977	0.93899493	ZNF439
126070	ENSG00000171295	195.642081	0.00011928	0.169127	0.99829641	0.99941443	ZNF440
2548	ENSG00000171298	2330.19486	0.21506421	0.07941127	0.00556809	0.02549524	GAA

124583	ENSG00000171302	1598.31272	-0.08432143	0.1086561	0.4167852	0.64320305	CANT1
3777	ENSG00000171303	419.16107	-2.59829379	0.15465835	1.41E-64	2.04E-62	KCNK3
84287	ENSG00000171307	539.153326	-0.0456374	0.10741075	0.6570982	0.82978985	ZDHHC16
50515	ENSG00000171310	269.159255	-0.58298297	0.20521005	0.00120358	0.00694559	CHST11
51013	ENSG00000171311	330.33166	0.08098611	0.13197421	0.51355427	0.7287659	EXOSC1
5223	ENSG00000171314	4467.71393	-0.42687307	0.08113296	6.98E-08	9.39E-07	PGAM1
55636	ENSG00000171316	3126.00829	0.14878534	0.20856425	0.39912163	0.62789874	CHD7
157570	ENSG00000171320	378.59052	-0.19601571	0.16319312	0.1820596	0.37980817	ESCO2
3880	ENSG00000171345	0.92180311	-0.05028412	0.38870729	0.27104919	NA	KRT19
3866	ENSG00000171346	0	NA	NA	NA	NA	KRT15
541468	ENSG00000171357	37.6424885	0.10335307	0.28967398	0.5992	0.79043404	LURAP1
8687	ENSG00000171360	0	NA	NA	NA	NA	KRT38
1184	ENSG00000171365	526.102273	-0.38290112	0.12234646	0.00093111	0.00557431	CLCN5
11076	ENSG00000171368	307.572064	-0.63702012	0.1363872	8.04E-07	9.26E-06	TPPP
3752	ENSG00000171385	2.85046178	-0.01451295	0.37335799	0.87297343	0.94439555	KCND3
8862	ENSG00000171388	127.980964	1.73263792	0.2387161	3.07E-14	7.91E-13	APLN
84616	ENSG00000171396	0	NA	NA	NA	NA	KRTAP4-4
3860	ENSG00000171401	0	NA	NA	NA	NA	KRT13
170626	ENSG00000171402	0	NA	NA	NA	NA	XAGE3
3857	ENSG00000171403	0	NA	NA	NA	NA	KRT9
170627	ENSG00000171405	0	NA	NA	NA	NA	XAGE5
27115	ENSG00000171408	90.7161746	1.30446928	0.27354682	1.67E-07	2.15E-06	PDE7B
64979	ENSG00000171421	248.782216	-0.13808579	0.14808085	0.31103806	0.53866926	MRPL36
51545	ENSG00000171425	373.507409	-0.13647315	0.12684643	0.25308921	0.47334935	ZNF581
9	ENSG00000171428	28.3168071	-0.05181381	0.29834794	0.78336521	0.90135811	NAT1
54474	ENSG00000171431	0	NA	NA	NA	NA	KRT20
392465	ENSG00000171433	0	NA	NA	NA	NA	GLOD5
283455	ENSG00000171435	114.080587	0.35941553	0.21023777	0.04477316	0.13655643	KSR2
147807	ENSG00000171443	87.8279157	-0.20785745	0.2433988	0.27917465	0.50362112	ZNF524
4163	ENSG00000171444	970.395578	-0.05103405	0.09703594	0.58591439	0.7817695	MCC
342574	ENSG00000171446	0	NA	NA	NA	NA	KRT27
57684	ENSG00000171448	415.604773	-0.09451416	0.12073598	0.40818519	0.63640649	ZBTB26
8941	ENSG00000171450	754.396459	-1.63912016	0.10021106	3.13E-61	4.19E-59	CDK5R2
92126	ENSG00000171451	518.204329	-0.23743023	0.11293933	0.02724779	0.09307784	DSEL
9533	ENSG00000171453	444.312172	0.24358065	0.12536691	0.03946358	0.12418039	POLR1C
171023	ENSG00000171456	3492.24361	0.01081107	0.08892783	0.90158237	0.9567498	ASXL1
392390	ENSG00000171459	0	NA	NA	NA	NA	OR1L6
65989	ENSG00000171462	57.2038104	-0.40003533	0.30210303	0.07021027	0.1919487	DLK2

54811	ENSG00000171466	832.736276	-0.19773021	0.11145128	0.06281246	0.17744542	ZNF562
24149	ENSG00000171467	1244.68112	-0.23114183	0.07959562	0.00293094	0.01483038	ZNF318
93134	ENSG00000171469	738.880543	0.06215466	0.0984568	0.51426655	0.72925798	ZNF561
147179	ENSG00000171475	981.571764	0.23896684	0.13173515	0.0528785	0.15535456	WIPF2
84525	ENSG00000171476	2.34923132	0.08052168	0.38684956	0.36184912	NA	HOPX
729201	ENSG00000171478	0.173733	0.01914545	0.38484597	0.78225479	NA	SPACA5B
26735	ENSG00000171481	0	NA	NA	NA	NA	OR1L3
NA	ENSG00000171483	0.15069912	-0.01848503	0.38480575	0.84537521	NA	SSX6P
126206	ENSG00000171487	0	NA	NA	NA	NA	NLRP5
84230	ENSG00000171488	459.923502	-0.91791042	0.12202033	8.16E-15	2.18E-13	LRRC8C
389852	ENSG00000171489	0	NA	NA	NA	NA	SPACA5
26156	ENSG00000171490	2747.05217	-0.36099189	0.06321093	6.69E-09	1.03E-07	RSL1D1
55144	ENSG00000171492	613.733486	-0.19516818	0.12128135	0.08876628	0.22815429	LRRC8D
133558	ENSG00000171495	0.93555031	-0.01646483	0.38151541	0.76418188	NA	MROH2B
138881	ENSG00000171496	0	NA	NA	NA	NA	OR1L8
5481	ENSG00000171497	1015.12945	0.1068678	0.09250202	0.23291429	0.44986562	PPID
138882	ENSG00000171501	0	NA	NA	NA	NA	OR1N2
255631	ENSG00000171502	1.92307328	0.13013286	0.41147964	0.0782998	NA	COL24A1
2110	ENSG00000171503	474.65502	0.12201297	0.14528398	0.36447188	0.594135	ETFDH
138883	ENSG00000171505	0	NA	NA	NA	NA	OR1N1
59350	ENSG00000171509	0.14931947	-0.01844332	0.38480212	0.84537521	NA	RXFP1
23566	ENSG00000171517	79.5843338	0.58025519	0.27224432	0.00828623	0.03555628	LPAR3
5734	ENSG00000171522	0.34624801	-0.02356563	0.38536324	0.65270996	NA	PTGER4
6902	ENSG00000171530	1644.68168	0.09618797	0.0810359	0.2246998	0.4385072	TBCA
4761	ENSG00000171532	3.0009515	0.07981591	0.38325908	0.4130919	0.6403849	NEUROD2
4135	ENSG00000171533	1524.09456	-1.5577278	0.07978558	5.45E-86	1.23E-83	MAP6
23440	ENSG00000171540	0.49167029	-0.00731448	0.3831721	0.85359874	NA	OTP
9427	ENSG00000171551	74.9264526	4.58489632	0.45847334	2.47E-24	1.15E-22	ECEL1
598	ENSG00000171552	868.13671	-0.07335706	0.08635454	0.37906576	0.60807444	BCL2L1
2266	ENSG00000171557	0	NA	NA	NA	NA	FGG
2243	ENSG00000171560	0	NA	NA	NA	NA	FGA
341152	ENSG00000171561	0.173733	0.01914545	0.38484597	0.78225479	NA	OR2AT4
2244	ENSG00000171564	0	NA	NA	NA	NA	FGB
5356	ENSG00000171566	984.964447	-0.03383522	0.102137	0.73026986	0.87106623	PLRG1
NA	ENSG00000171570	7.47175324	0.19460226	0.4137992	0.20868925	0.4165387	RAB4B-EGLN2
201514	ENSG00000171574	195.076392	-0.02586344	0.16161543	0.86070391	0.93799827	ZNF584
1826	ENSG00000171587	0	NA	NA	NA	NA	DSCAM
64446	ENSG00000171595	0	NA	NA	NA	NA	DNAI2

10316	ENSG00000171596	12.8065149	0.11571055	0.354941	0.49326726	0.71236151	NMUR1
22883	ENSG00000171603	3953.46809	-0.20428347	0.06275589	0.00095579	0.00569287	CLSTN1
51523	ENSG00000171604	2127.38352	-0.13388924	0.07337356	0.06215452	0.17598767	CXXC5
10782	ENSG00000171606	244.828715	-0.42402377	0.15174053	0.00239671	0.01251004	ZNF274
5293	ENSG00000171608	133.617658	0.54004773	0.21873774	0.00408291	0.01964231	PIK3CD
171558	ENSG00000171611	0	NA	NA	NA	NA	PTCRA
84275	ENSG00000171612	431.957938	-0.40521098	0.11968239	0.00035856	0.00240088	SLC25A33
8507	ENSG00000171617	1168.2968	0.09543553	0.11301737	0.37727301	0.60612976	ENC1
80176	ENSG00000171621	41.2320921	-0.42059498	0.34071214	0.07074434	0.1930817	SPSB1
5031	ENSG00000171631	19.7641453	0.01441495	0.32565714	0.93582419	0.97325384	P2RY6
2186	ENSG00000171634	5242.89871	-0.18494388	0.05968238	0.00156475	0.00869842	BPTF
170591	ENSG00000171643	0	NA	NA	NA	NA	S100Z
284307	ENSG00000171649	162.107403	0.34248069	0.202761	0.04930831	0.14748635	ZIK1
27197	ENSG00000171657	0.32279419	-0.02325667	0.38531906	0.66943166	NA	GPR82
344887	ENSG00000171658	0	NA	NA	NA	NA	NMRAL2P
2857	ENSG00000171659	0.48406658	-0.00538943	0.38311431	0.88309449	NA	GPR34
220070	ENSG00000171671	0	NA	NA	NA	NA	SHANK2-AS3
57449	ENSG00000171680	1601.48298	0.37526112	0.10402391	0.00017323	0.00124854	PLEKHG5
55729	ENSG00000171681	1424.77885	-0.4089201	0.11156747	0.00012346	0.00091773	ATF7IP
198437	ENSG00000171695	10.052071	0.13555194	0.37705154	0.37768658	0.60649953	LKAAEAR1
10287	ENSG00000171700	297.62989	0.17296524	0.14151482	0.18802713	0.38838712	RGS19
6919	ENSG00000171703	630.05897	0.34373132	0.11311047	0.00143384	0.0080656	TCEA2
1673	ENSG00000171711	0	NA	NA	NA	NA	DEFB4A
100289462	ENSG00000171711	0	NA	NA	NA	NA	DEFB4A
203859	ENSG00000171714	638.202699	-0.60876702	0.14992437	1.35E-05	0.00012586	ANO5
102723370	ENSG00000171714	638.202699	-0.60876702	0.14992437	1.35E-05	0.00012586	ANO5
8841	ENSG00000171720	1241.70782	0.07485484	0.08479437	0.36560944	0.59480864	HDAC3
284680	ENSG00000171722	63.161059	-2.26422477	0.35831882	1.46E-11	3.06E-10	SPATA46
10243	ENSG00000171723	448.895033	-0.48301998	0.12076927	2.56E-05	0.00022476	GPHN
57687	ENSG00000171724	181.712789	-1.15795777	0.20334189	1.30E-09	2.20E-08	VAT1L
55092	ENSG00000171729	457.113838	-0.01722493	0.12193309	0.880591	0.94713457	TMEM51
23261	ENSG00000171735	838.505028	-0.58699431	0.10067699	1.72E-09	2.87E-08	CAMTA1
3960	ENSG00000171747	2.57822357	-0.0279604	0.37702859	0.7211761	0.86691933	LGALS4
151827	ENSG00000171757	204.151107	-0.38427708	0.18976262	0.02086041	0.07535879	LRRC34
5053	ENSG00000171759	2.61596974	0.17637302	0.44264197	0.01111043	0.04524446	PAH
79029	ENSG00000171763	340.112352	0.04762192	0.14143445	0.71784837	0.8643386	SPATA5L1
2628	ENSG00000171766	205.740952	0.48177667	0.17881157	0.00268659	0.01379448	GATM
93426	ENSG00000171772	0.85676824	-0.01016063	0.38083338	0.8579291	NA	SYCE1

115861	ENSG00000171773	0	NA	NA	NA	NA	NXNL1
115727	ENSG00000171777	0.30139824	-0.02296476	0.38527817	0.68536535	NA	RASGRP4
4807	ENSG00000171786	14.8621908	0.31541116	0.43391054	0.13951623	0.31648712	NHLH1
200172	ENSG00000171790	29.0458599	0.12777393	0.2986228	0.51839502	0.73221886	SLFNL1
596	ENSG00000171791	926.287796	-0.81020172	0.09114766	1.14E-19	4.17E-18	BCL2
83695	ENSG00000171792	610.676351	-0.28334992	0.13177339	0.02137016	0.07679476	RHNO1
1503	ENSG00000171793	1792.03756	0.16239872	0.09634048	0.08130633	0.21402647	CTPS1
8433	ENSG00000171794	0	NA	NA	NA	NA	UTF1
85442	ENSG00000171798	59.258124	0.21317004	0.2661521	0.28646484	0.51225526	KNDC1
83889	ENSG00000171804	0.29863895	-0.02292439	0.3852726	0.68753245	NA	WDR87
92342	ENSG00000171806	150.667122	-0.42370582	0.19969717	0.01464343	0.05655552	METTL18
54777	ENSG00000171811	71.6098725	0.46278589	0.29639928	0.03826369	0.12144993	CFAP46
1296	ENSG00000171812	124.981805	0.05408461	0.20013408	0.75235249	0.8840721	COL8A2
170394	ENSG00000171813	404.395889	-0.39622248	0.12148517	0.00057125	0.00362556	PWWP2B
29930	ENSG00000171815	2.30262893	0.17361184	0.4406657	0.01298917	NA	PCDHB1
163255	ENSG00000171817	55.7709356	0.14009824	0.26437292	0.47555034	0.69768942	ZNF540
10218	ENSG00000171819	0.14931947	-0.01844332	0.38480212	0.84537521	NA	ANGPTL7
144699	ENSG00000171823	400.341114	0.09347492	0.12178811	0.41866266	0.64464626	FBXL14
5394	ENSG00000171824	1888.99561	0.12701622	0.07957918	0.10267681	0.2543206	EXOSC10
148268	ENSG00000171827	335.331771	-0.64642842	0.14210453	1.37E-06	1.53E-05	ZNF570
4815	ENSG00000171840	0.29863895	-0.02292439	0.3852726	0.68753245	NA	NINJ2
4300	ENSG00000171843	357.818931	-0.181115	0.1477829	0.18265734	0.38050416	MLLT3
55138	ENSG00000171847	3.29187655	0.07510127	0.38533167	0.35932192	0.58949503	FAM90A1
6241	ENSG00000171848	3549.32203	-0.27815801	0.10232023	0.00465637	0.02190715	RRM2
51112	ENSG00000171853	1725.51072	0.17193919	0.07539208	0.01994719	0.07281548	TRAPPC12
3456	ENSG00000171855	0	NA	NA	NA	NA	IFNB1
6227	ENSG00000171858	8616.26043	0.00412752	0.05519175	0.94051678	0.97527063	RPS21
719	ENSG00000171860	2.95601563	-0.11153525	0.40053597	0.14889178	0.33152468	C3AR1
55178	ENSG00000171861	302.29297	-0.16532116	0.15204076	0.23465589	0.45155897	MRM3
5728	ENSG00000171862	1953.60805	-0.56049324	0.11186217	1.78E-07	2.27E-06	PTEN
6201	ENSG00000171863	19662.929	-0.02192682	0.04669575	0.63099533	0.81344688	RPS7
23627	ENSG00000171864	0	NA	NA	NA	NA	PRND
246243	ENSG00000171865	2191.00047	0.1859844	0.07103894	0.00767178	0.03332779	RNASEH1
5621	ENSG00000171867	214.302672	-1.09819835	0.18063984	1.38E-10	2.62E-09	PRNP
128209	ENSG00000171872	0	NA	NA	NA	NA	KLF17
146	ENSG00000171873	0.16735865	0.01897177	0.38482933	0.78225479	NA	ADRA1D
84978	ENSG00000171877	390.688653	-0.72093815	0.15057908	3.57E-07	4.37E-06	FRMD5
361	ENSG00000171885	3.95056174	-0.04357075	0.3709579	0.6814371	0.84429601	AQP4

554202	ENSG00000171889	0	NA	NA	NA	NA	MIR31HG
57834	ENSG00000171903	0	NA	NA	NA	NA	CYP4F11
83660	ENSG00000171914	1419.12171	0.13541623	0.12145441	0.23887804	0.45653986	TLN2
654346	ENSG00000171916	0	NA	NA	NA	NA	LGALS9C
51030	ENSG00000171928	535.421588	-0.05466627	0.1099461	0.60236827	0.79259054	TVP23B
10517	ENSG00000171931	2.5882608	0.16635213	0.42766217	0.08340137	0.21803706	FBXW10
26532	ENSG00000171936	0	NA	NA	NA	NA	OR10H3
7764	ENSG00000171940	523.976576	0.64784289	0.13730628	6.09E-07	7.17E-06	ZNF217
26538	ENSG00000171942	0	NA	NA	NA	NA	OR10H2
653464	ENSG00000171943	432.529927	-0.3505104	0.12451824	0.0028161	0.01435545	SRGAP2C
390054	ENSG00000171944	0	NA	NA	NA	NA	OR52A5
7857	ENSG00000171951	342.925517	-1.4373638	0.14320866	9.58E-25	4.51E-23	SCG2
91647	ENSG00000171953	336.500918	-0.08777581	0.12515564	0.45709255	0.680528	ATPAF2
126410	ENSG00000171954	0	NA	NA	NA	NA	CYP4F22
27023	ENSG00000171956	0	NA	NA	NA	NA	FOXB1
10465	ENSG00000171960	979.880901	-0.07549782	0.08854045	0.37985655	0.60880151	PPIH
83450	ENSG00000171962	150.592761	0.09426875	0.19385792	0.57433272	0.77352065	DRC3
126295	ENSG00000171970	64.1934265	-0.10877719	0.25238273	0.57029384	0.7706786	ZNF57
149840	ENSG00000171984	46.3254907	-0.06298307	0.2609744	0.74211071	0.87846755	SHLD1
143501	ENSG00000171987	0	NA	NA	NA	NA	C11orf40
221037	ENSG00000171988	1974.43185	0.09353192	0.11221032	0.38386325	0.61304411	JMJD1C
92483	ENSG00000171989	0.70177513	0.02815439	0.38381803	0.55959831	NA	LDHAL6B
11346	ENSG00000171992	2603.32219	-0.76757124	0.06541461	1.79E-32	1.11E-30	SYNPO
NA	ENSG00000171999	0	NA	NA	NA	NA	OR52P2P
80032	ENSG00000172000	104.715725	-0.49840283	0.25618366	0.01626256	0.06159779	ZNF556
4118	ENSG00000172005	0	NA	NA	NA	NA	MAL
115196	ENSG00000172006	169.905875	-0.01747829	0.16221515	0.90432338	0.9581211	ZNF554
83452	ENSG00000172007	246.571619	0.15714349	0.15818406	0.27559954	0.49971621	RAB33B
7064	ENSG00000172009	1873.1541	0.09850892	0.08475069	0.23353818	0.45043378	THOP1
NA	ENSG00000172014	15.1255468	0.0925997	0.33636116	0.60224757	0.79255716	ANKRD20A4P
5068	ENSG00000172016	0	NA	NA	NA	NA	REG3A
2596	ENSG00000172020	543.044175	-1.55652537	0.11620732	5.37E-42	4.28E-40	GAP43
5968	ENSG00000172023	0	NA	NA	NA	NA	REG1B
253152	ENSG00000172031	36.3915727	0.1352225	0.28482043	0.49647762	0.714811	EPHX4
3913	ENSG00000172037	1694.02835	0.13003604	0.09574517	0.16077323	0.34879615	LAMB2
10869	ENSG00000172046	2456.93788	0.0271	0.06674928	0.68078018	0.84396798	USP19
5859	ENSG00000172053	4226.33008	0.20561191	0.07541665	0.00539106	0.02481554	QARS1
94103	ENSG00000172057	770.022555	0.24544111	0.09431701	0.00714481	0.03144527	ORMDL3

8293	ENSG00000172058	360.954275	0.04361224	0.18957887	0.79320881	0.9054599	SERF1A
8462	ENSG00000172059	197.062192	-0.33045902	0.1644095	0.02609646	0.09006417	KLF11
131578	ENSG00000172061	0	NA	NA	NA	NA	LRRC15
6606	ENSG00000172062	1039.22618	-0.38182154	0.09463468	3.00E-05	0.00025785	SMN1
9451	ENSG00000172071	842.421174	0.53807593	0.09406311	3.86E-09	6.14E-08	EIF2AK3
200523	ENSG00000172073	0.33895101	0.00238255	0.38338048	0.96571076	NA	TEX37
126308	ENSG00000172081	552.435997	-0.036625	0.12144601	0.74947183	0.88205755	MOB3A
51315	ENSG00000172086	346.156124	-0.01943214	0.12712009	0.86864702	0.94267349	KRCC1
10201	ENSG00000172113	496.992878	-0.10809086	0.12903632	0.37250693	0.60143185	NME6
54205	ENSG00000172115	2370.62012	-0.51265552	0.08359395	3.33E-10	6.08E-09	CYCS
926	ENSG00000172116	0	NA	NA	NA	NA	CD8B
55106	ENSG00000172123	0	NA	NA	NA	NA	SLFN12
794	ENSG00000172137	0	NA	NA	NA	NA	CALB2
285335	ENSG00000172139	0	NA	NA	NA	NA	SLC9C1
8383	ENSG00000172146	0	NA	NA	NA	NA	OR1A1
NA	ENSG00000172148	0	NA	NA	NA	NA	OR7A2P
26189	ENSG00000172150	0	NA	NA	NA	NA	OR1A2
120586	ENSG00000172154	0	NA	NA	NA	NA	OR8I2
353134	ENSG00000172155	0	NA	NA	NA	NA	LCE1D
6356	ENSG00000172156	0	NA	NA	NA	NA	CCL11
257019	ENSG00000172159	119.161014	-1.4050869	0.25329864	2.27E-09	3.73E-08	FRMD3
6641	ENSG00000172164	20.4206881	2.13651124	0.63435713	4.06E-05	0.00034088	SNTB1
27085	ENSG00000172167	338.134329	0.11245866	0.13058037	0.35982774	0.58975484	MTBP
79736	ENSG00000172171	100.627827	-0.30528513	0.22807808	0.10325087	0.25526319	TEFM
28998	ENSG00000172172	363.848654	0.14874045	0.13735316	0.24610954	0.46538442	MRPL13
10892	ENSG00000172175	460.270467	0.13344777	0.12616675	0.26268392	0.48471437	MALT1
5617	ENSG00000172179	0.67639953	-0.00104602	0.38188924	0.9880707	NA	PRL
3669	ENSG00000172183	16.8228166	-0.01961518	0.34704511	0.889494	0.95181279	ISG20
NA	ENSG00000172186	0	NA	NA	NA	NA	HMGN1P35
219429	ENSG00000172188	0	NA	NA	NA	NA	OR4C11
154141	ENSG00000172197	269.912577	0.87820442	0.1586165	4.91E-09	7.69E-08	MBOAT1
219417	ENSG00000172199	0	NA	NA	NA	NA	OR8U1
3400	ENSG00000172201	15.2390727	0.35556084	0.48479268	0.101164	0.25142098	ID4
119764	ENSG00000172208	0	NA	NA	NA	NA	OR4X2
2845	ENSG00000172209	29.0942494	1.46536138	0.7146591	0.00170426	0.00935593	GPR22
10663	ENSG00000172215	3.47099464	0.01355262	0.36773504	0.90348704	0.95785538	CXCR6
1051	ENSG00000172216	226.177126	0.48886494	0.45949714	0.05792877	0.1668564	CEBPB
566	ENSG00000172232	10.0282826	0.43219562	0.64400011	0.04446555	0.13591073	AZU1

7177	ENSG00000172236	0	NA	NA	NA	NA	TPSAB1
474	ENSG00000172238	1.37372765	0.03116858	0.37977055	0.665074	NA	ATOH1
10605	ENSG00000172239	1965.79285	0.12446381	0.07950923	0.10950568	0.26621648	PAIP1
64581	ENSG00000172243	0	NA	NA	NA	NA	CLEC7A
375444	ENSG00000172244	207.080718	-0.21687268	0.19500616	0.19675106	0.40043787	C5orf34
114900	ENSG00000172247	60.5534006	0.18708057	0.26104811	0.34381534	0.57418319	C1QTNF4
94009	ENSG00000172250	4.0452977	-0.18037811	0.42691959	0.12357611	0.2912855	SERHL
257194	ENSG00000172260	208.410673	-2.00292794	0.19141595	9.75E-27	4.95E-25	NEGR1
7690	ENSG00000172262	695.526697	0.01412002	0.09560056	0.87903551	0.94641763	ZNF131
140733	ENSG00000172264	87.70858	0.2963022	0.2647344	0.14436164	0.32440083	MACROD2
1798	ENSG00000172269	975.131398	0.37741573	0.10474862	0.00017449	0.00125705	DPAGT1
682	ENSG00000172270	8014.21055	0.12521965	0.05595837	0.02408481	0.08463382	BSG
25988	ENSG00000172273	478.609482	0.17238459	0.10863316	0.09762338	0.24473689	HINFP
NA	ENSG00000172283	0	NA	NA	NA	NA	PRYP4
9085	ENSG00000172288	0	NA	NA	NA	NA	CDY1
390201	ENSG00000172289	0	NA	NA	NA	NA	OR10V1
253782	ENSG00000172292	3125.76533	-0.37615966	0.06290099	1.27E-09	2.16E-08	CERS6
NA	ENSG00000172294	0	NA	NA	NA	NA	CSPG4P4Y
55304	ENSG00000172296	0	NA	NA	NA	NA	SPTLC3
NA	ENSG00000172297	0	NA	NA	NA	NA	GOLGA2P3Y
55352	ENSG00000172301	391.437589	-0.36597092	0.12001793	0.00128941	0.00735746	COPRS
112858	ENSG00000172315	407.892875	-0.28940496	0.11659394	0.00893909	0.03780678	TP53RK
8708	ENSG00000172318	51.2415604	-0.66602701	0.33194508	0.00817585	0.0351863	B3GALT1
219982	ENSG00000172320	0	NA	NA	NA	NA	OR5A1
160364	ENSG00000172322	0	NA	NA	NA	NA	CLEC12A
219981	ENSG00000172324	0	NA	NA	NA	NA	OR5A2
669	ENSG00000172331	509.629	0.05363039	0.12150777	0.64266454	0.82136499	BPGM
10248	ENSG00000172336	786.443095	-0.19938538	0.09008896	0.02221393	0.07919574	POP7
199857	ENSG00000172339	124.996089	-0.09200708	0.35534349	0.55716098	0.76256028	ALG14
8801	ENSG00000172340	373.559499	0.53812094	0.12700978	7.91E-06	7.65E-05	SUCLG2
NA	ENSG00000172342	0	NA	NA	NA	NA	CSPG4P3Y
80765	ENSG00000172345	53.9746848	0.18588751	0.31215411	0.35825779	0.58854144	STARD5
27254	ENSG00000172346	81.5559032	-0.24015253	0.22682521	0.19572402	0.39935567	CSDC2
10231	ENSG00000172348	0	NA	NA	NA	NA	RCAN2
3603	ENSG00000172349	5.39595836	0.22582536	0.46225505	0.06570791	0.18315081	IL16
64137	ENSG00000172350	46.3367579	0.63666288	0.39488042	0.01790484	0.06666016	ABCG4
253175	ENSG00000172352	0	NA	NA	NA	NA	CDY1B
2783	ENSG00000172354	5221.91239	-0.12535231	0.05709061	0.02644027	0.09096149	GNB2

220136	ENSG00000172361	13.2840674	0.28094344	0.43093775	0.15851085	0.34569581	CFAP53
390191	ENSG00000172362	0	NA	NA	NA	NA	OR5B12
390190	ENSG00000172365	0	NA	NA	NA	NA	OR5B2
84331	ENSG00000172366	449.159389	-0.2564791	0.13544633	0.0421069	0.13069475	MCRIP2
79849	ENSG00000172367	0.30139824	-0.02296476	0.38527817	0.68536535	NA	PDZD3
9854	ENSG00000172375	585.3053	0.07578071	0.10254087	0.44355137	0.66854511	C2CD2L
219954	ENSG00000172377	0	NA	NA	NA	NA	OR9I1
9915	ENSG00000172379	1816.62644	-0.09641217	0.08022726	0.21859274	0.43050004	ARNT2
55970	ENSG00000172380	1207.19986	0.06656568	0.07960801	0.39315537	0.62217456	GNG12
83886	ENSG00000172382	173.601398	1.03251415	0.22184528	3.81E-07	4.63E-06	PRSS27
51778	ENSG00000172399	10.301981	1.73933444	0.97081261	0.00288469	0.01463366	MYOZ2
171024	ENSG00000172403	3937.61208	0.61072641	0.05704543	3.02E-27	1.55E-25	SYNPO2
150353	ENSG00000172404	4.48924974	0.28052433	0.51469847	0.04671602	0.14135211	DNAJB7
10978	ENSG00000172409	241.235435	-0.13599692	0.14957242	0.32200375	0.54960035	CLP1
10022	ENSG00000172410	0.16735865	0.01897177	0.38482933	0.78225479	NA	INSL5
146779	ENSG00000172421	0	NA	NA	NA	NA	EFCAB3
143941	ENSG00000172425	17.9661746	-0.02520142	0.31757349	0.88647	0.95015905	TTC36
221421	ENSG00000172426	15.2062442	0.13922598	0.34371785	0.45213255	0.67536839	RSPH9
150678	ENSG00000172428	417.431076	0.002379	0.11672495	0.98416267	0.99453916	COPS9
54676	ENSG00000172432	1459.47081	-0.05087283	0.0901351	0.56079621	0.76437187	GTPBP2
55277	ENSG00000172456	64.4940333	-0.81157077	0.30896228	0.00121712	0.00700743	FGGY
283189	ENSG00000172457	0	NA	NA	NA	NA	OR9G4
53342	ENSG00000172458	76.1793902	-0.96274499	0.30977436	0.00021718	0.00153162	IL17D
219493	ENSG00000172459	0	NA	NA	NA	NA	OR5AR1
124221	ENSG00000172460	0	NA	NA	NA	NA	PRSS30P
10690	ENSG00000172461	465.6709	-0.37538557	0.12936478	0.00200947	0.01074897	FUT9
338675	ENSG00000172464	0	NA	NA	NA	NA	OR5AP2
9338	ENSG00000172465	144.504103	-0.09483494	0.17606298	0.54350962	0.75149877	TCEAL1
7572	ENSG00000172466	2615.14411	-0.24414836	0.0630192	8.39E-05	0.00064987	ZNF24
86614	ENSG00000172468	0	NA	NA	NA	NA	HSFY1
79694	ENSG00000172469	405.224806	0.01564316	0.12977321	0.89889484	0.95556135	MANEA
142684	ENSG00000172476	55.2378656	0.02120891	0.24694939	0.91248756	0.96260718	RAB40A
79919	ENSG00000172478	0	NA	NA	NA	NA	MAB21L4
189	ENSG00000172482	0	NA	NA	NA	NA	AGXT
219477	ENSG00000172487	0	NA	NA	NA	NA	OR8J1
390154	ENSG00000172489	0	NA	NA	NA	NA	OR5T3
4299	ENSG00000172493	788.796244	-0.13856131	0.10845845	0.18147421	0.37887491	AFF1
134526	ENSG00000172497	0.36240779	0.00094427	0.38336464	0.96688599	NA	ACOT12

9158	ENSG00000172500	1206.24246	0.00961854	0.07725663	0.89971504	0.95564128	FIBP
57571	ENSG00000172508	17.3609886	0.5102489	0.63636377	0.04213557	0.13072239	CARNS1
284433	ENSG00000172519	0	NA	NA	NA	NA	OR10H5
54971	ENSG00000172530	458.950671	0.15631567	0.12095898	0.17276945	0.36566523	BANP
5499	ENSG00000172531	2813.05143	-0.1548693	0.06158689	0.01073154	0.04393598	PPP1CA
3054	ENSG00000172534	5352.28426	-0.02370377	0.06379516	0.73274494	0.87239916	HCFC1
170370	ENSG00000172538	0	NA	NA	NA	NA	FAM170B
1521	ENSG00000172543	0.29863895	-0.02292439	0.3852726	0.68753245	NA	CTSW
348938	ENSG00000172548	139.768394	0.71828595	0.20440736	8.97E-05	0.00069156	NIPAL4
118430	ENSG00000172551	0	NA	NA	NA	NA	MUCL1
54221	ENSG00000172554	9.02195092	4.1466744	1.32573523	7.18E-05	0.00056816	SNTG2
408263	ENSG00000172568	0.30139824	-0.02296476	0.38527817	0.68536535	NA	FNDC9
5139	ENSG00000172572	3604.16999	1.29947669	0.07552859	2.56E-67	3.97E-65	PDE3A
10125	ENSG00000172575	139.587435	-0.07822945	0.18102318	0.62325737	0.8078282	RASGRP1
89857	ENSG00000172578	0	NA	NA	NA	NA	KLHL6
118487	ENSG00000172586	448.615182	-0.34796351	0.1250031	0.00315705	0.01580516	CHCHD1
122704	ENSG00000172590	821.092939	0.05570466	0.0928173	0.53681991	0.74734999	MRPL52
10924	ENSG00000172594	0	NA	NA	NA	NA	SMPDL3A
27289	ENSG00000172602	32.5787561	0.33082091	0.37371492	0.14236717	0.32117652	RND1
5883	ENSG00000172613	373.421877	0.19255865	0.14162205	0.14275076	0.32165532	RAD9A
30008	ENSG00000172638	447.171516	0.36101528	0.12878797	0.00287784	0.01461386	EFEMP2
121275	ENSG00000172640	3.08338977	0.09510866	0.38516411	0.37680598	0.60570261	OR10AD1
729092	ENSG00000172650	154.312881	0.29525466	0.22035844	0.10702325	0.26200528	AGAP5
253725	ENSG00000172661	867.520848	0.15776678	0.09133677	0.07509501	0.20160826	WASHC2C
80194	ENSG00000172663	281.771099	0.79224471	0.17055648	6.09E-07	7.17E-06	TMEM134
64393	ENSG00000172667	1141.69091	0.1251132	0.08864999	0.14669226	0.32786991	ZMAT3
93550	ENSG00000172671	161.944445	-0.01566897	0.19679982	0.9246627	0.96862095	ZFAND4
387357	ENSG00000172673	0	NA	NA	NA	NA	THEMIS
4342	ENSG00000172680	0	NA	NA	NA	NA	MOS
148203	ENSG00000172687	546.259974	-0.09528306	0.12021099	0.40294055	0.63099498	ZNF738
341116	ENSG00000172689	0	NA	NA	NA	NA	MS4A10
91607	ENSG00000172716	0.32334531	0.00163257	0.38337096	0.96545193	NA	SLFN11
161142	ENSG00000172717	0.35361408	0.0039586	0.38341258	0.93089565	NA	FAM71D
6363	ENSG00000172724	0	NA	NA	NA	NA	CCL19
57175	ENSG00000172725	1192.98626	0.12888733	0.0834008	0.11349355	0.27336554	CORO1B
84750	ENSG00000172728	165.067078	0.1472793	0.17674803	0.3479	0.57861815	FUT10
55222	ENSG00000172731	672.45122	0.16662256	0.0973193	0.07642107	0.20422871	LRRC20
80198	ENSG00000172732	925.862125	0.36318036	0.09770531	0.00011753	0.00087928	MUS81

29942	ENSG00000172733	187.123476	-0.00091139	0.15707826	0.99390824	0.99814114	PURG
221468	ENSG00000172738	8.12619093	0.11687337	0.38743757	0.33861847	0.56890427	TMEM217
390199	ENSG00000172742	0.29863895	-0.02292439	0.3852726	0.68753245	NA	OR4D9
NA	ENSG00000172746	0.173124	-0.01911262	0.38486262	0.84537521	NA	RPL12P13
169270	ENSG00000172748	183.045825	0.15675279	0.19056113	0.34365631	0.57400025	ZNF596
256076	ENSG00000172752	0.16735865	0.01897177	0.38482933	0.78225479	NA	COL6A5
1072	ENSG00000172757	17996.4755	0.04105983	0.04816907	0.39146623	0.62088815	CFL1
23023	ENSG00000172765	435.550662	0.12111767	0.12954152	0.32053143	0.54831381	TMCC1
79612	ENSG00000172766	804.175179	0.00739657	0.10650363	0.94336663	0.97689827	NAA16
441608	ENSG00000172769	0	NA	NA	NA	NA	OR5B3
90288	ENSG00000172771	4.95306072	0.04635511	0.36504001	0.71280271	0.86173942	EFCAB12
81341	ENSG00000172772	0	NA	NA	NA	NA	OR10W1
80011	ENSG00000172775	1509.96302	-0.01383947	0.07566174	0.85130655	0.93275912	PSME3IP1
339122	ENSG00000172780	60.5350603	1.39246937	0.33479869	2.67E-06	2.81E-05	RAB43
283985	ENSG00000172782	6.1119881	-0.05032637	0.36522035	0.68613149	0.8472712	FADS6
55871	ENSG00000172785	431.272381	-0.02748879	0.11314877	0.79853056	0.90769144	CBWD1
3222	ENSG00000172789	131.583363	-0.59725324	0.23447493	0.00274454	0.01404842	HOXC5
326624	ENSG00000172794	1.30199031	-0.08404756	0.39649408	0.11552913	NA	RAB37
167227	ENSG00000172795	1926.22128	0.02250076	0.10363978	0.82272283	0.91918127	DCP2
NA	ENSG00000172799	0	NA	NA	NA	NA	ZBTB8OSP2
254122	ENSG00000172803	143.912883	0.22872861	0.18647452	0.16109501	0.34915229	SNX32
6169	ENSG00000172809	9033.85189	-0.13299796	0.05493485	0.01564561	0.05964814	RPL38
9420	ENSG00000172817	246.232395	-0.02913342	0.15712991	0.83787223	0.92628758	CYP7B1
5017	ENSG00000172818	0.37844916	0.02407552	0.3854191	0.60038877	NA	OVOL1
5916	ENSG00000172819	886.974119	0.40100685	0.08876425	3.31E-06	3.43E-05	RARG
283848	ENSG00000172824	96.5527907	0.2893324	0.22772166	0.12194078	0.28873433	CES4A
23491	ENSG00000172828	40.9436801	0.9879794	0.42743183	0.00187718	0.01012594	CES3
54961	ENSG00000172830	256.137612	0.18552912	0.15074346	0.18032922	0.37720159	SSH3
8824	ENSG00000172831	817.295026	0.15468381	0.1191719	0.17177561	0.36432905	CES2
57546	ENSG00000172840	565.863951	-0.01362096	0.1513797	0.91168578	0.96237542	PDP2
6670	ENSG00000172845	1996.22077	-0.1570005	0.06779602	0.01828135	0.06760522	SP3
3849	ENSG00000172867	0	NA	NA	NA	NA	KRT2
1657	ENSG00000172869	815.008121	-0.43007092	0.12771633	0.00034912	0.00234635	DMXL1
254042	ENSG00000172878	196.128049	-0.00684744	0.17328376	0.96342409	0.98545957	METAP1D
285268	ENSG00000172888	1117.18726	0.02584606	0.08150412	0.74581406	0.8802926	ZNF621
51162	ENSG00000172889	477.999809	-0.16593578	0.11393509	0.12621792	0.29554626	EGFL7
55191	ENSG00000172890	644.794708	0.5534381	0.11523169	5.27E-07	6.27E-06	NADSYN1
1717	ENSG00000172893	2027.59417	-0.66758228	0.08897977	1.60E-14	4.19E-13	DHCR7

399923	ENSG00000172900	0	NA	NA	NA	NA	ACTE1P
206338	ENSG00000172901	9.31475304	0.0590432	0.3643685	0.64402801	0.82223396	LVRN
NA	ENSG00000172912	0	NA	NA	NA	NA	COX6B1P3
26960	ENSG00000172915	3096.40872	0.52981409	0.05963499	2.43E-19	8.78E-18	NBEA
84153	ENSG00000172922	1284.02081	-0.13337976	0.07450885	0.06770654	0.18698467	RNASEH2C
26579	ENSG00000172927	1.08299052	0.03431385	0.38421053	0.57393189	NA	MYEOV
338692	ENSG00000172932	867.789023	0.15263208	0.09078693	0.0833026	0.21789368	ANKRD13D
116535	ENSG00000172935	1.78698867	-0.0040706	0.37779845	0.95394306	NA	MRGPRF
4615	ENSG00000172936	769.41061	0.08399494	0.09181794	0.3474427	0.57819389	MYD88
116512	ENSG00000172938	0.49044334	0.01388611	0.38307155	0.76199047	NA	MRGPRD
9943	ENSG00000172939	1517.96541	0.00633168	0.0847551	0.9394831	0.97474024	OXSR1
9390	ENSG00000172940	1.37620723	0.00697379	0.37804349	0.92248536	NA	SLC22A13
23133	ENSG00000172943	1120.42421	-0.12665986	0.0847799	0.12547187	0.29435597	PHF8
253558	ENSG00000172954	682.230172	-0.42756445	0.1349716	0.00071493	0.00440625	LCLAT1
130	ENSG00000172955	4.59289199	0.1432414	0.40265933	0.23067844	0.44663416	ADH6
541471	ENSG00000172965	52.1884114	-0.77585133	0.37354431	0.00517427	0.02392393	MIR4435-2HG
150165	ENSG00000172967	0	NA	NA	NA	NA	XKR3
100288801	ENSG00000172969	0.84210987	0.01615443	0.38118446	0.77437227	NA	FRG2C
NA	ENSG00000172971	1.54598581	0.09392177	0.39689612	0.14890243	NA	UNC93B3
NA	ENSG00000172974	2.6369726	-0.20165643	0.46175246	0.00878606	0.03728674	VDAC2P5
10524	ENSG00000172977	635.73658	0.05900082	0.10178921	0.54827885	0.75480124	KAT5
344558	ENSG00000172985	44.5855955	-0.07283601	0.25996874	0.7041044	0.85774517	SH3RF3
727936	ENSG00000172986	72.5520704	2.05139103	0.30380653	9.99E-13	2.28E-11	GXYLT2
60495	ENSG00000172987	11.0496166	7.31307848	2.66398423	3.68E-07	4.50E-06	HPSE2
79877	ENSG00000172992	1233.98632	0.12517338	0.09267187	0.16406869	0.35339516	DCAKD
10777	ENSG00000172995	11.9847789	-0.08982225	0.35934523	0.54618061	0.75345287	ARPP21
93624	ENSG00000173011	485.484342	0.00229331	0.1434622	0.98745689	0.99588983	TADA2B
257236	ENSG00000173013	6.34400257	0.21774352	0.43814425	0.14155115	0.3199443	CCDC96
156	ENSG00000173020	1501.69032	-0.1961131	0.07109985	0.00493434	0.02298598	GRK2
5970	ENSG00000173039	1451.37746	-0.08795868	0.08054756	0.26344715	0.48542909	RELA
132884	ENSG00000173040	51.3746475	-0.08479279	0.2576998	0.65875846	0.83073135	EVC2
340252	ENSG00000173041	531.194794	0.16710411	0.12543903	0.15825033	0.3453764	ZNF680
283450	ENSG00000173064	4159.7452	-0.04567721	0.06376753	0.4670939	0.69008437	HECTD4
55731	ENSG00000173065	606.941008	0.16212557	0.10132088	0.09668498	0.24306007	FAM222B
54796	ENSG00000173068	35.9050392	-0.30382836	0.41046983	0.15133049	0.33531348	BNC2
NA	ENSG00000173077	0.94820793	-0.01598938	0.38145775	0.77103862	NA	DELEC1
339403	ENSG00000173080	3.85861295	0.14283707	0.40568929	0.20222942	0.40778604	RXFP4
10855	ENSG00000173083	0	NA	NA	NA	NA	HPSE

27235	ENSG00000173085	121.926149	0.03831113	0.20210748	0.82409885	0.92009686	COQ2
160762	ENSG00000173093	0	NA	NA	NA	NA	CCDC63
3310	ENSG00000173110	0	NA	NA	NA	NA	HSPA6
51504	ENSG00000173113	3153.42723	-0.03957748	0.06254516	0.5213781	0.73486829	TRMT112
54674	ENSG00000173114	403.954394	1.57885376	0.14400068	5.13E-29	2.85E-27	LRRN3
22992	ENSG00000173120	1817.18544	0.11944993	0.08663526	0.15691254	0.34373985	KDM2A
142827	ENSG00000173124	0	NA	NA	NA	NA	ACSM6
203054	ENSG00000173137	171.352177	0.20388128	0.18116909	0.20224872	0.40778604	ADCK5
78988	ENSG00000173141	643.137685	-0.12256858	0.09987243	0.20286784	0.40878509	MRPL57
64318	ENSG00000173145	806.673192	-0.47714737	0.09564507	2.56E-07	3.20E-06	NOC3L
2101	ENSG00000173153	812.98635	-0.06880129	0.10221692	0.48338343	0.70370318	ESRRA
29984	ENSG00000173156	0	NA	NA	NA	NA	RHOD
80070	ENSG00000173157	37.644992	0.25334764	0.31011519	0.23138603	0.44769835	ADAMTS20
150684	ENSG00000173163	136.205017	0.04880888	0.18553935	0.76470981	0.8916054	COMMD1
65059	ENSG00000173166	688.564088	-0.33691056	0.11978464	0.00297824	0.01503914	RAPH1
4580	ENSG00000173171	828.451482	0.14646896	0.10143732	0.1340677	0.30828734	MTX1
111	ENSG00000173175	183.834542	-0.47799769	0.19708789	0.00562863	0.02571904	ADCY5
54625	ENSG00000173193	287.198605	0.50994112	0.15409833	0.00033626	0.00226841	PARP14
10800	ENSG00000173198	0.33471731	0.02354391	0.38534154	0.62842204	NA	CYSLTR1
165631	ENSG00000173200	0.14931947	-0.01844332	0.38480212	0.84537521	NA	PARP15
1163	ENSG00000173207	2772.48252	0.02708844	0.0613269	0.65503526	0.82823884	CKS1B
225	ENSG00000173208	709.09776	0.247824	0.1051477	0.01404708	0.05473174	ABCD2
130872	ENSG00000173209	1454.48198	0.31377065	0.0863292	0.00018504	0.00132546	AHSA2P
22885	ENSG00000173210	21.1518038	0.4157182	0.45959747	0.08517653	0.2214571	ABLIM3
126868	ENSG00000173212	3.03057773	0.09276338	0.38544275	0.37630055	0.60526384	MAB21L3
260334	ENSG00000173213	0.87518259	-0.02812402	0.38332521	0.55074935	NA	TUBB8B
91749	ENSG00000173214	469.493442	-0.16892399	0.15374681	0.22864697	0.44394937	MFSD4B
81839	ENSG00000173218	445.096979	0.31569106	0.11894101	0.00512219	0.02371628	VANGL1
2745	ENSG00000173221	51.5303908	-0.25671334	0.28509028	0.21375541	0.4238788	GLRX
9657	ENSG00000173226	473.171384	0.36044578	0.12415812	0.00211567	0.01122516	IQCB1
91683	ENSG00000173227	1.53927943	0.02803555	0.38109342	0.65873963	NA	SYT12
2804	ENSG00000173230	1907.52814	-0.04191634	0.08596317	0.615819	0.8030134	GOLGB1
NA	ENSG00000173231	0	NA	NA	NA	NA	TERF1P1
254439	ENSG00000173237	0	NA	NA	NA	NA	C11orf86
340654	ENSG00000173239	0	NA	NA	NA	NA	LIPM
134391	ENSG00000173250	0	NA	NA	NA	NA	GPR151
10655	ENSG00000173253	0	NA	NA	NA	NA	DMRT2
158399	ENSG00000173258	333.340359	-0.1419734	0.15639469	0.31809683	0.54567516	ZNF483

153770	ENSG00000173261	16.2589978	0.05065891	0.32854927	0.7736427	0.8959822	PLAC8L1
144195	ENSG00000173262	0	NA	NA	NA	NA	SLC2A14
56834	ENSG00000173264	572.836683	0.15737088	0.12164236	0.17193662	0.36452474	GPR137
6623	ENSG00000173267	0.173733	0.01914545	0.38484597	0.78225479	NA	SNCG
79812	ENSG00000173269	1.4613793	-0.06232146	0.38500179	0.40217421	NA	MMRN2
653784	ENSG00000173272	837.149445	-0.15370986	0.08632573	0.06719949	0.18607646	MZT2A
8658	ENSG00000173273	2970.13628	-0.15902615	0.06230501	0.00948751	0.03974498	TNKS
203523	ENSG00000173275	159.141029	0.02465899	0.17150017	0.87366133	0.94441388	ZNF449
49854	ENSG00000173276	551.446363	-0.46939394	0.12130635	4.58E-05	0.00037907	ZBTB21
79660	ENSG00000173281	1218.79387	-0.40719256	0.08537651	9.55E-07	1.09E-05	PPP1R3B
391109	ENSG00000173285	0	NA	NA	NA	NA	OR10K1
286042	ENSG00000173295	142.637573	0.38787527	0.19619774	0.02323865	0.08218445	FAM86B3P
344561	ENSG00000173302	0.74831009	-0.04237872	0.38743592	0.3510469	NA	GPR148
56977	ENSG00000173320	1450.30554	0.24038797	0.10608878	0.01821414	0.06741028	STOX2
4296	ENSG00000173327	1039.22008	0.21805874	0.08917111	0.01158338	0.04681489	MAP3K11
10221	ENSG00000173334	246.289707	-0.04083296	0.16330782	0.78122745	0.90003181	TRIB1
128822	ENSG00000173335	0	NA	NA	NA	NA	CST9
10089	ENSG00000173338	7.00761611	0.0952657	0.3700009	0.49464406	0.71350347	KCNK7
84826	ENSG00000173349	54.9052306	-0.01399833	0.38367646	0.69005226	0.84966405	SFT2D3
NA	ENSG00000173357	0	NA	NA	NA	NA	
NA	ENSG00000173366	5.51001601	0.24021459	0.47413587	0.06082522	0.17318776	
713	ENSG00000173369	0	NA	NA	NA	NA	C1QB
712	ENSG00000173372	0	NA	NA	NA	NA	C1QA
79625	ENSG00000173376	65.1572149	1.82321487	0.3310653	2.49E-09	4.06E-08	NDNF
132141	ENSG00000173389	0.37844916	0.02407552	0.3854191	0.60038877	NA	IQCF1
4973	ENSG00000173391	0	NA	NA	NA	NA	OLR1
256710	ENSG00000173401	4.6483808	0.00608348	0.36287963	0.96135857	0.98422798	GLIPR1L1
1605	ENSG00000173402	2246.68062	-0.13733007	0.06626873	0.03520976	0.11384642	DAG1
3642	ENSG00000173404	1.53892252	0.04892919	0.38152842	0.52454881	NA	INSM1
1600	ENSG00000173406	25.394526	-3.00639689	0.64488049	1.76E-07	2.25E-06	DAB1
64801	ENSG00000173409	421.986335	-0.04955694	0.12678072	0.67789499	0.84273994	ARV1
51126	ENSG00000173418	683.512073	-0.07405616	0.09294903	0.40973612	0.63778163	NAA20
339834	ENSG00000173421	1.09660671	0.03490317	0.38340376	0.52786228	NA	IHO1
122665	ENSG00000173431	0	NA	NA	NA	NA	RNASE8
6288	ENSG00000173432	0	NA	NA	NA	NA	SAA1
440574	ENSG00000173436	666.916446	-0.25671964	0.1302298	0.03523801	0.11391919	MICOS10
254102	ENSG00000173442	824.197775	1.44126244	0.11377624	7.34E-38	5.35E-36	EHBP1L1
83591	ENSG00000173451	324.283578	0.07363066	0.13438584	0.55824278	0.76291851	THAP2

256130	ENSG00000173452	0.50634052	0.03067938	0.38601017	0.48443161	NA	TMEM196
79102	ENSG00000173456	1077.52259	-0.05708613	0.07796014	0.457056	0.68052466	RNF26
26472	ENSG00000173457	2745.48148	0.02410691	0.06444431	0.70530484	0.85803894	PPP1R14B
122651	ENSG00000173464	0	NA	NA	NA	NA	RNASE11
10534	ENSG00000173465	400.843313	-0.00120124	0.12784389	0.99106209	0.99704234	ZNRD2
155465	ENSG00000173467	0	NA	NA	NA	NA	AGR3
6599	ENSG00000173473	6850.15966	0.16973483	0.05524234	0.00191248	0.01027725	SMARCC1
147687	ENSG00000173480	399.735216	-0.20675886	0.14066584	0.1126622	0.27179207	ZNF417
5797	ENSG00000173482	509.59629	-1.37829252	0.14415346	9.63E-23	4.14E-21	PTPRM
2286	ENSG00000173486	484.564506	0.06360256	0.11370406	0.55871597	0.76342457	FKBP2
7423	ENSG00000173511	828.457558	0.14487587	0.10413693	0.14800575	0.33012187	VEGFB
79834	ENSG00000173517	1035.85016	0.22295387	0.11937843	0.04877259	0.14617043	PEAK1
8793	ENSG00000173530	0.173733	0.01914545	0.38484597	0.78225479	NA	TNFRSF10D
4485	ENSG00000173531	263.816129	0.877307	0.19525256	1.05E-06	1.18E-05	MST1
8794	ENSG00000173535	0.44795842	-0.02501848	0.38558245	0.5807882	NA	TNFRSF10C
29925	ENSG00000173540	867.138815	0.22154859	0.10094496	0.02265601	0.08051141	GMPPB
92597	ENSG00000173542	1232.54862	-0.25764419	0.08371866	0.00157642	0.00875104	MOB1B
90441	ENSG00000173545	513.314364	-0.0203314	0.11063472	0.8469648	0.93052693	ZNF622
1464	ENSG00000173546	77.2891716	0.25638786	0.24691596	0.18882468	0.38950735	CSPG4
257364	ENSG00000173548	1025.88579	0.22137238	0.08450011	0.00715632	0.03148844	SNX33
339778	ENSG00000173557	15.6116354	-0.56046615	0.64095222	0.039883	0.12512313	FAM166C
64859	ENSG00000173559	47.1707612	-0.33519397	0.31673225	0.12672374	0.29648573	NABP1
165082	ENSG00000173567	13.2380406	0.03799265	0.334483	0.82333928	0.91945578	ADGRF3
126204	ENSG00000173572	0	NA	NA	NA	NA	NLRP13
1106	ENSG00000173575	2836.08718	0.10352498	0.08260904	0.19918748	0.40366492	CHD2
2829	ENSG00000173578	1.03543838	0.02903081	0.38173321	0.63000381	NA	XCR1
29903	ENSG00000173581	573.835455	-0.03244221	0.11360385	0.76445748	0.89148768	CCDC106
10803	ENSG00000173585	0.173733	0.01914545	0.38484597	0.78225479	NA	CCR9
51134	ENSG00000173588	294.514658	0.30645777	0.15517078	0.03057977	0.10189834	CEP83
27284	ENSG00000173597	0.50854511	0.03078872	0.38602082	0.48278419	NA	SULT1B1
11163	ENSG00000173598	1454.25714	0.30428108	0.08110241	0.00012026	0.00089661	NUDT4
5091	ENSG00000173599	991.277983	0.01572589	0.0854007	0.85143798	0.93275912	PC
10941	ENSG00000173610	0	NA	NA	NA	NA	UGT2A1
286205	ENSG00000173611	507.428652	-0.19301958	0.11895512	0.08681738	0.22465852	SCAI
222545	ENSG00000173612	0	NA	NA	NA	NA	GPRC6A
64802	ENSG00000173614	191.582347	0.17544258	0.18440116	0.27903868	0.50351318	NMNAT1
78999	ENSG00000173621	809.972551	-0.41498465	0.10256889	2.60E-05	0.00022751	LRFN4
100128327	ENSG00000173626	0	NA	NA	NA	NA	TRAPPC3L

403314	ENSG00000173627	0.16735865	0.01897177	0.38482933	0.78225479	NA	APOBEC4
6573	ENSG00000173638	1192.92682	-0.21542237	0.08984795	0.01343933	0.05286207	SLC19A1
27129	ENSG00000173641	89.139932	-1.08478882	0.29532572	2.37E-05	0.00020971	HSPB7
9986	ENSG00000173653	412.472314	-0.01114984	0.11342001	0.91637747	0.96450552	RCE1
7388	ENSG00000173660	5146.50585	-0.08401407	0.05460859	0.11969438	0.28467232	UQCRH
80835	ENSG00000173662	1.19363074	-0.00731775	0.37939456	0.90922581	NA	TAS1R1
390992	ENSG00000173673	0.31646653	0.00081478	0.38336468	0.9700455	NA	HES3
1964	ENSG00000173674	2473.79619	-0.13762596	0.06772378	0.03877614	0.12260057	EIF1AX
100310812	ENSG00000173678	6.43363564	0.0312741	0.38672691	0.11258499	0.27178103	SPDYE2B
26737	ENSG00000173679	0	NA	NA	NA	NA	OR1L1
256643	ENSG00000173681	253.631495	-0.17819385	0.16808439	0.23655851	0.45337334	BCLAF3
5707	ENSG00000173692	2059.08397	-0.01176204	0.0951562	0.89750959	0.95508423	PSMD1
10149	ENSG00000173698	22.8577788	0.25112942	0.35654103	0.23330171	0.45017563	ADGRG2
130560	ENSG00000173699	0	NA	NA	NA	NA	SPATA3
56667	ENSG00000173702	0.52119901	0.02576573	0.38568143	0.51723676	NA	MUC13
26032	ENSG00000173705	31.0289255	1.5014769	0.50875703	0.00019518	0.00139235	SUSD5
57493	ENSG00000173706	236.866546	1.2939322	0.17494266	1.30E-14	3.43E-13	HEG1
124857	ENSG00000173714	4.03003492	-0.05584076	0.37532679	0.58405284	0.78020525	WFIKKN2
79703	ENSG00000173715	245.871032	0.29994512	0.16541011	0.0445237	0.13604654	C11orf80
9804	ENSG00000173726	10106.1511	-0.24972486	0.04721458	9.11E-08	1.21E-06	TOMM20
NA	ENSG00000173727	19.453169	-0.16705312	0.34123378	0.386521	0.61565323	
200159	ENSG00000173728	1.91344469	-0.0070394	0.3760225	0.92942498	NA	C1orf100
3267	ENSG00000173744	1146.4842	-0.1541323	0.08815627	0.0721622	0.1956941	AGFG1
6777	ENSG00000173757	1742.49418	0.25024477	0.08197571	0.00174654	0.00954158	STAT5B
924	ENSG00000173762	1.29934018	0.09125185	0.39877176	0.09270252	NA	CD7
375337	ENSG00000173769	0	NA	NA	NA	NA	TOPAZ1
1267	ENSG00000173786	1943.92455	0.09517705	0.07222296	0.17972853	0.37629949	CNP
3728	ENSG00000173801	1462.3583	0.65896204	0.09256336	2.85E-13	6.72E-12	JUP
9001	ENSG00000173805	4.84920484	0.0396741	0.36348282	0.75482418	0.8858747	HAP1
91646	ENSG00000173809	7.17529182	-0.03087355	0.36188919	0.80142529	0.90902045	TDRD12
NA	ENSG00000173810	0	NA	NA	NA	NA	PPIAP7
100874114	ENSG00000173811	4.14839482	0.1287227	0.3942593	0.28445715	0.50967487	CCDC13-AS1
10209	ENSG00000173812	7093.66333	0.20505255	0.06411078	0.0011793	0.00682338	EIF1
284131	ENSG00000173818	468.012302	0.54291864	0.12546016	5.17E-06	5.17E-05	ENDOV
57674	ENSG00000173821	3490.51085	0.28985583	0.08904485	0.00081976	0.0049751	RNF213
220359	ENSG00000173825	75.7949162	0.19426028	0.2335348	0.3024423	0.52987991	TIGD3
81033	ENSG00000173826	97.8600851	-2.18656428	0.25941257	2.41E-18	8.27E-17	KCNH6
162333	ENSG00000173838	1.12816751	-0.02393095	0.38168695	0.67645623	NA	MARCHF10

1263	ENSG00000173846	165.283217	0.21177572	0.17894094	0.18145785	0.37887491	PLK3
10276	ENSG00000173848	943.47472	-0.28924513	0.09828947	0.00226431	0.0119067	NET1
23333	ENSG00000173852	1297.16522	-0.00257404	0.09097715	0.97540999	0.99139237	DPY19L1
NA	ENSG00000173862	1.03867714	-0.0343278	0.38474401	0.45647693	NA	
NA	ENSG00000173867	31.5855053	0.02900002	0.31450021	0.87491305	0.94487915	
162466	ENSG00000173868	0.51937201	-0.02557462	0.38567603	0.54680819	NA	PHOSPHO1
163049	ENSG00000173875	773.370756	0.08507487	0.13876973	0.5109983	0.72623254	ZNF791
80012	ENSG00000173889	802.082483	-0.01134266	0.09431853	0.89880937	0.95556135	PHC3
26996	ENSG00000173890	18.3429212	-0.13212974	0.3324401	0.48192153	0.70262515	GPR160
84733	ENSG00000173894	4269.08078	0.15357779	0.05483686	0.00465328	0.02189778	CBX2
6712	ENSG00000173898	4793.18964	-0.02762808	0.09003256	0.75148146	0.88344203	SPTBN2
27333	ENSG00000173905	2036.3951	0.02334712	0.06993384	0.73553832	0.87436071	GOLIM4
162605	ENSG00000173908	0	NA	NA	NA	NA	KRT28
83759	ENSG00000173914	690.828509	0.03146984	0.09615075	0.73610162	0.87476826	RBM4B
84833	ENSG00000173915	1037.35569	-0.20885556	0.08380926	0.0104364	0.04286918	ATP5MK
3212	ENSG00000173917	0.15069912	-0.01848503	0.38480575	0.84537521	NA	HOXB2
114897	ENSG00000173918	163.499377	-0.93436301	0.20400038	6.30E-07	7.39E-06	C1QTNF1
115123	ENSG00000173926	60.6316742	-1.04773692	0.3665157	0.00038314	0.00254912	MARCHF3
126074	ENSG00000173928	43.3299626	0.01229418	0.25654576	0.95034777	0.97957708	SWSAP1
353189	ENSG00000173930	55.2730184	0.10321949	0.25899053	0.5959692	0.78826859	SLCO4C1
5936	ENSG00000173933	2647.66129	-0.24697828	0.07800943	0.00119351	0.00689552	RBM4
128344	ENSG00000173947	0	NA	NA	NA	NA	PIFO
152002	ENSG00000173950	790.826531	0.03133351	0.09523918	0.73545064	0.87432359	XXYLT1
NA	ENSG00000173954	0	NA	NA	NA	NA	SNURFL
165324	ENSG00000173960	865.365644	0.05610159	0.08823472	0.51415243	0.72921448	UBXN2A
NA	ENSG00000173966	0	NA	NA	NA	NA	
84839	ENSG00000173976	0	NA	NA	NA	NA	RAX2
220416	ENSG00000173988	29.1743014	0.23439677	0.34096136	0.26097252	0.48276874	LRRC63
8557	ENSG00000173991	23.3041162	0.05920391	0.30303805	0.7562025	0.88670553	TCAP
9973	ENSG00000173992	276.77501	0.21525118	0.15526842	0.12900096	0.30032609	CCS
375387	ENSG00000174004	45.3833886	1.09256958	0.40260715	0.00057475	0.00364546	NRROS
84984	ENSG00000174007	83.3230411	0.16734278	0.22094421	0.35891683	0.58923542	CEP19
80311	ENSG00000174010	286.785069	-0.37447609	0.13870738	0.0037336	0.01821386	KLHL15
200933	ENSG00000174013	1161.47419	-0.42358617	0.08564067	3.75E-07	4.57E-06	FBXO45
220082	ENSG00000174015	0	NA	NA	NA	NA	CBY2
169966	ENSG00000174016	0	NA	NA	NA	NA	TENT5D
2787	ENSG00000174021	1336.18427	0.30788144	0.08264191	0.00013202	0.00097444	GNG5
NA	ENSG00000174028	55.1751419	-0.2777742	0.30055743	0.18907498	0.38986152	FAM3C2P

253512	ENSG00000174032	218.67651	0.55114382	0.18356353	0.0008262	0.00500297	SLC25A30
138724	ENSG00000174038	3.49022519	0.04834291	0.3792808	0.55959225	0.76413192	C9orf131
947	ENSG00000174059	6.92931541	0.04957304	0.36180734	0.70499509	0.85803894	CD34
8722	ENSG00000174080	605.358415	0.48814439	0.11827567	1.47E-05	0.00013594	CTSF
253827	ENSG00000174099	79.0265256	7.22893452	0.83679429	1.84E-17	5.86E-16	MSRB3
23592	ENSG00000174106	538.229601	0.25325503	0.12092564	0.02691082	0.09224441	LEMD3
283951	ENSG00000174109	318.753615	0.04328015	0.1310062	0.72708853	0.86953012	C16orf91
81793	ENSG00000174123	0.14931947	-0.01844332	0.38480212	0.84537521	NA	TLR10
7096	ENSG00000174125	7.6155168	0.1028715	0.37057426	0.46879507	0.69137681	TLR1
10333	ENSG00000174130	2.22155685	0.0613082	0.38165025	0.48097066	NA	TLR6
345757	ENSG00000174132	183.386744	0.46665431	0.17790774	0.00346373	0.01710316	FAM174A
285704	ENSG00000174136	1004.24228	-0.28138206	0.08248373	0.00046333	0.00302086	RGMB
152877	ENSG00000174137	103.711103	0.07903021	0.22472555	0.66650437	0.83560485	FAM53A
57495	ENSG00000174145	0	NA	NA	NA	NA	NWD2
284613	ENSG00000174151	255.442716	-0.00221192	0.16968945	0.98711278	0.99567688	CYB561D1
2940	ENSG00000174156	0	NA	NA	NA	NA	GSTA3
254359	ENSG00000174165	428.624349	-0.149356	0.12571641	0.20700334	0.41417369	ZDHHC24
NA	ENSG00000174171	2.54269252	-0.17296645	0.43485956	0.04234011	NA	
54931	ENSG00000174173	557.350517	-0.09795566	0.10312423	0.32328807	0.55093929	TRMT10C
6403	ENSG00000174175	0	NA	NA	NA	NA	SELP
348180	ENSG00000174177	324.139701	0.06978781	0.1316721	0.57340749	0.7729263	CTU2
23269	ENSG00000174197	2464.71239	-0.18688398	0.07810436	0.01419998	0.05519743	MGA
144577	ENSG00000174206	134.535725	-0.33368831	0.22626483	0.074199	0.19988632	C12orf66
392509	ENSG00000174225	0.70148749	0.00128865	0.38181396	0.97734568	NA	ARL13A
169166	ENSG00000174226	0.68271199	0.04027334	0.38711563	0.36252232	NA	SNX31
54872	ENSG00000174227	1178.02682	0.11504278	0.08711427	0.17504403	0.36892512	PIGG
10594	ENSG00000174231	11195.7811	-0.1261609	0.04822291	0.00831812	0.03567479	PRPF8
112	ENSG00000174233	2154.65697	0.02705083	0.06768236	0.68537161	0.8469494	ADCY6
387849	ENSG00000174236	7.49560803	0.20100517	0.41697988	0.19726982	0.40121304	REP15
5306	ENSG00000174238	1268.08161	-0.11313472	0.10824808	0.27484973	0.4987213	PITPNA
9416	ENSG00000174243	2310.6222	-0.08374463	0.10025373	0.38528975	0.61433325	DDX23
7634	ENSG00000174255	0.69197381	-0.00049013	0.38183012	0.99325142	NA	ZNF80
741	ENSG00000174276	220.84773	-0.06997984	0.15930368	0.62792558	0.81117654	ZNHIT2
344191	ENSG00000174279	3.00685641	0.23092792	0.4836506	0.01744236	0.0652444	EVX2
57659	ENSG00000174282	1018.46376	0.15810753	0.09069901	0.07255914	0.19642152	ZBTB4
8711	ENSG00000174292	60.2648844	0.13921265	0.24516385	0.46752744	0.69041597	TNK1
23051	ENSG00000174306	1052.69621	-0.05310363	0.10878481	0.6096291	0.79803651	ZHX3
23612	ENSG00000174307	525.024673	0.59742667	0.13264282	1.96E-06	2.11E-05	PHLDA3

NA	ENSG00000174325	0	NA	NA	NA	NA	DIRC1
162515	ENSG00000174326	4.15658738	0.04487263	0.36812958	0.70548522	0.85803894	SLC16A11
201232	ENSG00000174327	51.0276253	0.16866779	0.27958062	0.40085468	0.62929088	SLC16A13
148979	ENSG00000174332	11.0980439	-1.77998122	0.94778518	0.002463	0.01280216	GLIS1
134083	ENSG00000174339	0	NA	NA	NA	NA	OR2Y1
55584	ENSG00000174343	0	NA	NA	NA	NA	CHRNA9
127435	ENSG00000174348	15.0421397	0.23817275	0.39084471	0.22594115	0.44016814	PODN
378108	ENSG00000174353	766.913428	0.08276135	0.09101597	0.34962177	0.58007157	STAG3L3
340024	ENSG00000174358	0	NA	NA	NA	NA	SLC6A19
128439	ENSG00000174365	228.817742	0.29432759	0.15331523	0.03598936	0.11585132	SNHG11
NA	ENSG00000174370	17.0210227	-0.25376593	0.39758797	0.20095091	0.40615916	C11orf45
9156	ENSG00000174371	1598.34578	-0.5899588	0.0853102	1.44E-12	3.25E-11	EXO1
253959	ENSG00000174373	1324.83827	0.28352682	0.07896186	0.00023748	0.00165785	RALGAPA1
NA	ENSG00000174384	65.6321635	-0.03779666	0.23120083	0.83666564	0.9256885	PMS2P6
NA	ENSG00000174403	14.78654	0.18459528	0.38054412	0.29618461	0.52277154	MIR1-1HG-AS1
3981	ENSG00000174405	451.952561	-0.08702939	0.1162155	0.4486704	0.67234398	LIG4
NA	ENSG00000174407	1.22135173	-0.05687993	0.38754548	0.30779291	NA	MIR1-1HG
NA	ENSG00000174408	0	NA	NA	NA	NA	PPIAP24
7201	ENSG00000174417	7.79982031	0.18128896	0.40833187	0.2199424	0.43209275	TRHR
NA	ENSG00000174418	0	NA	NA	NA	NA	RPL35P9
389524	ENSG00000174428	600.718882	0.22001646	0.10679663	0.0316171	0.10460523	GTF2IRD2B
137735	ENSG00000174429	0	NA	NA	NA	NA	ABRA
488	ENSG00000174437	7347.54522	0.02634354	0.06087803	0.71192197	0.86156715	ATP2A2
55055	ENSG00000174442	982.641896	-0.44260029	0.09343567	1.01E-06	1.14E-05	ZWILCH
6124	ENSG00000174444	37387.6133	0.03474432	0.05254466	0.50797337	0.7237561	RPL4
10302	ENSG00000174446	149.846792	-0.10276247	0.17088273	0.50106451	0.71823711	SNAPC5
147323	ENSG00000174448	2.11408623	0.06968243	0.38387942	0.41946714	NA	STARD6
283685	ENSG00000174450	0	NA	NA	NA	NA	GOLGA6L2
402117	ENSG00000174453	0	NA	NA	NA	NA	VWC2L
400073	ENSG00000174456	320.040548	0.01750319	0.14225292	0.89446148	0.95445145	C12orf76
170261	ENSG00000174460	137.81006	-0.46306035	0.21228785	0.0110632	0.04508913	ZCCHC12
26047	ENSG00000174469	290.283875	-0.50855709	0.2145485	0.00573135	0.0261222	CNTNAP2
442117	ENSG00000174473	13.8020884	-0.05422016	0.3326429	0.75035897	0.88256592	GALNTL6
158038	ENSG00000174482	116.101847	-0.3718705	0.21305207	0.03952726	0.12436105	LINGO2
582	ENSG00000174483	496.376364	0.31366437	0.11124429	0.00313303	0.01571666	BBS1
10260	ENSG00000174485	541.795414	-0.50727053	0.13339894	5.35E-05	0.00043351	DENND4A
NA	ENSG00000174495	0	NA	NA	NA	NA	PPIAP80
9543	ENSG00000174498	118.648978	0.58820366	0.27601942	0.00787775	0.03408079	IGDCC3

257144	ENSG00000174500	4.85700299	0.23318518	0.47153804	0.05939059	0.16976075	GCSAM
400986	ENSG00000174501	342.596887	-0.23845692	0.16704423	0.11148562	0.26980661	ANKRD36C
115019	ENSG00000174502	0.53035984	0.03940331	0.38680964	0.41703988	NA	SLC26A9
148808	ENSG00000174514	69.1607622	-0.79037467	0.39238596	0.00547202	0.0251482	MFSD4A
246330	ENSG00000174516	232.299849	-0.24713404	0.1555842	0.08151169	0.21429626	PELI3
148014	ENSG00000174521	408.032895	-5.2097965	0.21867539	6.14E-126	2.77E-123	TTC9B
283446	ENSG00000174527	17.3233516	-0.2368008	0.43357279	0.15321877	0.33804794	MYO1H
388730	ENSG00000174529	96.6594998	-0.32555701	0.23740491	0.0909967	0.23271133	TMEM81
65003	ENSG00000174547	809.828783	0.02496129	0.11146663	0.81590993	0.9163678	MRPL11
55554	ENSG00000174562	0	NA	NA	NA	NA	KLK15
53833	ENSG00000174564	32.8113182	0.53635409	0.43707432	0.04212281	0.13070323	IL20RB
127845	ENSG00000174567	16.3792258	0.05937741	0.32694077	0.73929783	0.87688337	GOLT1A
NA	ENSG00000174572	0.18922458	0.01954053	0.38488496	0.78225479	NA	RPL10P2
79647	ENSG00000174574	1224.43924	-0.22001848	0.08929937	0.01108665	0.04516613	AKIRIN1
266743	ENSG00000174576	5.0039016	0.18351023	0.42171989	0.16469952	0.3543692	NPAS4
55167	ENSG00000174579	763.066776	-0.26743706	0.1539538	0.05716576	0.16506603	MSL2
162968	ENSG00000174586	227.608571	0.24848206	0.16114947	0.0888969	0.22841422	ZNF497
133022	ENSG00000174599	168.604158	-0.10580182	0.17308746	0.4921454	0.71115547	TRAM1L1
1240	ENSG00000174600	13.9026916	-2.04498438	0.87245047	0.00083413	0.00504237	CMKLR1
90806	ENSG00000174606	1072.59794	-0.16749899	0.11240063	0.11808202	0.28192478	ANGEL2
7368	ENSG00000174607	151.680865	-0.50741735	0.2051717	0.004516	0.02134799	UGT8
339855	ENSG00000174611	11.728127	-0.07779876	0.34447181	0.63721911	0.81722218	KY
124152	ENSG00000174628	87.3747096	0.11821333	0.22389453	0.52217018	0.73552406	IQCK
6578	ENSG00000174640	1.38677075	0.00988362	0.37925781	0.87965884	NA	SLCO2A1
10781	ENSG00000174652	776.635534	-0.0146622	0.09942552	0.87766485	0.94578953	ZNF266
125958	ENSG00000174667	0	NA	NA	NA	NA	OR7D4
3177	ENSG00000174669	582.329704	0.18632816	0.10734124	0.07016484	0.1919256	SLC29A2
9024	ENSG00000174672	1079.66193	-1.46422628	0.09537192	2.68E-54	2.98E-52	BRSK2
NA	ENSG00000174677	0	NA	NA	NA	NA	VN1R6P
NA	ENSG00000174678	0	NA	NA	NA	NA	FAM47DP
NA	ENSG00000174680	3.61628275	0.15565515	0.4233615	0.07468097	0.20079555	GRIK1-AS1
11041	ENSG00000174684	806.925089	-0.08921798	0.08808914	0.29732158	0.52397052	B4GAT1
153339	ENSG00000174695	1702.13623	-0.38247887	0.08852978	8.60E-06	8.27E-05	TMEM167A
3952	ENSG00000174697	0.84934393	0.04950338	0.38855591	0.26958967	NA	LEP
285590	ENSG00000174705	2436.52228	0.28034185	0.0643762	9.99E-06	9.48E-05	SH3PXD2B
NA	ENSG00000174715	0	NA	NA	NA	NA	PPIAP72
55196	ENSG00000174718	411.038618	-0.18831933	0.13749377	0.1406916	0.318644	RESF1
51574	ENSG00000174720	858.337441	-0.00275677	0.10003084	0.97639943	0.99184704	LARP7

143282	ENSG00000174721	147.58555	-0.64444805	0.20541827	0.00040642	0.00268511	FGFBP3
9975	ENSG00000174738	1006.67113	-0.27672121	0.09790464	0.00336067	0.01665642	NR1D2
140886	ENSG00000174740	1.07741191	0.02469562	0.38188171	0.66691784	NA	PABPC5
25855	ENSG00000174744	509.766041	0.00693222	0.11889859	0.95201299	0.98012235	BRMS1
6138	ENSG00000174748	24083.2592	0.03489426	0.05231326	0.50128496	0.71841948	RPL15
132720	ENSG00000174749	149.840608	-0.16622946	0.17936924	0.29377139	0.52006999	FAM241A
3265	ENSG00000174775	760.117673	-0.29623487	0.09853657	0.00180338	0.00979359	HRAS
151790	ENSG00000174776	0.69493202	0.03154416	0.38626309	0.41391645	NA	WDR49
6731	ENSG00000174780	2801.07398	-0.22771625	0.06638992	0.00047438	0.00308276	SRP72
126006	ENSG00000174788	0.34746601	0.02370763	0.3853651	0.61987128	NA	PCP2
9610	ENSG00000174791	78.0723206	-0.53270769	0.34151094	0.02868804	0.09697916	RIN1
152816	ENSG00000174792	1.2186262	0.011649	0.38062608	0.84212455	NA	ODAPH
152815	ENSG00000174796	267.186811	-0.04841205	0.16068224	0.73970114	0.8770894	THAP6
9662	ENSG00000174799	538.996492	0.01692037	0.11007506	0.87362981	0.94441388	CEP135
8322	ENSG00000174804	70.9202892	0.30537668	0.25296121	0.12559202	0.29453259	FZD4
57124	ENSG00000174807	315.650888	0.07388874	0.15252001	0.59868332	0.79005929	CD248
685	ENSG00000174808	0	NA	NA	NA	NA	BTC
5174	ENSG00000174827	386.77279	-4.3638571	0.19174236	1.31E-115	4.81E-113	PDZK1
2015	ENSG00000174837	0	NA	NA	NA	NA	ADGRE1
201627	ENSG00000174839	817.972827	0.07069773	0.10947294	0.50100227	0.71823519	DENND6A
201626	ENSG00000174840	758.493689	0.00278346	0.10940481	0.97981829	0.99328572	PDE12
11146	ENSG00000174842	196.451478	0.00099683	0.1635795	0.99576313	0.99852582	GLMN
201625	ENSG00000174844	1.93322063	0.01430367	0.3808549	0.65351262	NA	DNAH12
10897	ENSG00000174851	820.452811	-0.02266276	0.10586416	0.82244577	0.91897523	YIF1A
254263	ENSG00000174871	744.792277	-0.10166497	0.09371529	0.26956078	0.4927127	CNIH2
277	ENSG00000174876	0	NA	NA	NA	NA	AMY1B
171389	ENSG00000174885	0	NA	NA	NA	NA	NLRP6
126328	ENSG00000174886	1812.97756	-0.03077189	0.06905571	0.64456569	0.8225499	NDUFA11
51319	ENSG00000174891	674.799164	0.35316317	0.0978244	0.0001834	0.0013155	RSRC1
257062	ENSG00000174898	0.86773468	-0.03757764	0.38469315	0.46173226	NA	CATSPERD
152078	ENSG00000174899	33.1478635	1.09179822	0.46427516	0.00151507	0.00844355	SLC66A1L
81876	ENSG00000174903	2337.1451	-0.00390134	0.06748694	0.95283717	0.98058207	RAB1B
NA	ENSG00000174912	3.20296728	0.23380462	0.48097459	0.03649122	0.11718772	METTL15P1
390174	ENSG00000174914	0	NA	NA	NA	NA	OR9G1
81490	ENSG00000174915	699.021698	0.06995227	0.10040344	0.47088911	0.69316219	PTDSS2
125988	ENSG00000174917	475.786846	0.05960979	0.11313207	0.58167473	0.77870625	MICOS13
285315	ENSG00000174928	64.9463171	-0.16010978	0.24134208	0.39956975	0.62826628	C3orf33
NA	ENSG00000174930	0	NA	NA	NA	NA	VN2R1P

219482	ENSG00000174937	0	NA	NA	NA	NA	OR5M3
26470	ENSG00000174938	2104.18632	-2.09367136	0.0779	2.76E-160	2.88E-157	SEZ6L2
253982	ENSG00000174939	657.528455	-2.67469834	0.13399581	6.88E-90	1.75E-87	ASPHD1
253980	ENSG00000174943	625.622598	0.53055065	0.13881427	4.83E-05	0.00039707	KCTD13
9934	ENSG00000174944	0.40091344	-0.01266455	0.3839044	0.88153981	NA	P2RY14
155185	ENSG00000174945	8.35457453	-0.15392319	0.39661869	0.25945029	0.48111914	AMZ1
29909	ENSG00000174946	0	NA	NA	NA	NA	GPR171
344758	ENSG00000174948	0.16735865	0.01897177	0.38482933	0.78225479	NA	GPR149
388611	ENSG00000174950	0.15069912	-0.01848503	0.38480575	0.84537521	NA	CD164L2
2523	ENSG00000174951	27.0325603	0.06326843	0.29964486	0.7416499	0.87812748	FUT1
170506	ENSG00000174953	1873.8311	0.0428377	0.08249824	0.59567309	0.78816266	DHX36
282775	ENSG00000174957	0	NA	NA	NA	NA	OR5J2
84107	ENSG00000174963	142.499491	0.34969488	0.25187067	0.08020532	0.21166182	ZIC4
282770	ENSG00000174970	0	NA	NA	NA	NA	OR10AG1
NA	ENSG00000174977	0	NA	NA	NA	NA	
219431	ENSG00000174982	0	NA	NA	NA	NA	OR4S2
26259	ENSG00000174989	1650.71525	0.62911652	0.07524657	1.79E-17	5.70E-16	FBXW8
763	ENSG00000174990	0	NA	NA	NA	NA	CA5A
653808	ENSG00000174992	0	NA	NA	NA	NA	ZG16
64837	ENSG00000174996	1449.14552	0.11967954	0.07425172	0.09932044	0.24808862	KLC2
6580	ENSG00000175003	0.32459315	0.00146341	0.3833692	0.96629595	NA	SLC22A1
387718	ENSG00000175018	0	NA	NA	NA	NA	TEX36
1488	ENSG00000175029	2857.04934	0.0600494	0.06745806	0.36548842	0.59471759	CTBP2
9435	ENSG00000175040	2.22879901	-0.06657069	0.3821461	0.45926665	NA	CHST2
79683	ENSG00000175048	207.698422	0.37720424	0.18307182	0.019893	0.07265777	ZDHHC14
545	ENSG00000175054	863.292066	-0.32378655	0.11601496	0.00330708	0.01642774	ATR
125144	ENSG00000175061	6279.98307	-0.15044662	0.05131696	0.00307024	0.01543288	SNHG29
11065	ENSG00000175063	1527.91879	-0.11505362	0.08942512	0.18533159	0.38437996	UBE2C
147409	ENSG00000175065	0	NA	NA	NA	NA	DSG4
256356	ENSG00000175066	552.055511	0.14898345	0.13997171	0.25250639	0.47252657	GK5
80124	ENSG00000175073	409.623632	-0.00143054	0.12137259	0.98915511	0.99604194	VCPIP1
132112	ENSG00000175077	0	NA	NA	NA	NA	RTP1
1674	ENSG00000175084	0.36303109	0.0238953	0.38539249	0.60992868	NA	DES
149420	ENSG00000175087	421.633107	-0.24608694	0.1689353	0.10350172	0.25575714	PDIK1L
92369	ENSG00000175093	211.691316	-0.72599105	0.17392473	6.17E-06	6.11E-05	SPSB4
5897	ENSG00000175097	412.146536	-5.81958757	0.25391788	9.59E-118	3.66E-115	RAG2
7189	ENSG00000175104	859.167889	-0.87742024	0.1076856	5.94E-17	1.82E-15	TRAF6
55279	ENSG00000175105	200.137375	0.06622968	0.15986455	0.64966838	0.82525848	ZNF654

201158	ENSG00000175106	106.699942	0.09629167	0.20787375	0.58354725	0.77994081	TVP23C
56945	ENSG00000175110	441.510668	0.15848837	0.1164077	0.15286038	0.33757234	MRPS22
55690	ENSG00000175115	1458.17105	0.38016798	0.0861183	5.70E-06	5.67E-05	PACS1
149708	ENSG00000175121	0	NA	NA	NA	NA	WFDC5
65108	ENSG00000175130	10554.7279	-0.14848315	0.04665711	0.0013318	0.00756237	MARCKSL1
80851	ENSG00000175137	1233.18118	-0.06648781	0.07761001	0.38106366	0.61009547	SH3BP5L
26696	ENSG00000175143	0	NA	NA	NA	NA	OR2T1
200197	ENSG00000175147	450.197706	0.48627616	0.15827557	0.00082249	0.0049857	TMEM51-AS1
388403	ENSG00000175155	660.81639	-1.005454	0.11952308	5.23E-18	1.73E-16	YPEL2
253559	ENSG00000175161	180.959636	0.3967208	0.23894177	0.04320971	0.13305613	CADM2
28	ENSG00000175164	0	NA	NA	NA	NA	ABO
5708	ENSG00000175166	3699.13876	-0.02149291	0.07584431	0.77148204	0.89520433	PSMD2
NA	ENSG00000175170	23.3839078	0.05998249	0.31438522	0.74693576	0.88109908	FAM182B
22843	ENSG00000175175	1391.85264	-0.37207139	0.09206336	3.03E-05	0.0002598	PPM1E
131408	ENSG00000175182	965.372829	-0.20502212	0.08699301	0.0152855	0.05851134	FAM131A
1466	ENSG00000175183	574.896512	0.97438198	0.12700314	2.39E-15	6.71E-14	CSRP2
3626	ENSG00000175189	9.75951403	0.39626736	0.56747614	0.06581353	0.18336477	INHBC
55486	ENSG00000175193	605.771916	-0.15255008	0.11395217	0.15926859	0.34685937	PARL
1649	ENSG00000175197	184.241881	0.92471445	0.29719411	0.00023017	0.0016108	DDIT3
5095	ENSG00000175198	310.762731	0.16180604	0.16874574	0.28698444	0.51285995	PCCA
123346	ENSG00000175202	0.173733	0.01914545	0.38484597	0.78225479	NA	HIGD2B
10540	ENSG00000175203	2134.31819	0.30000086	0.09216093	0.00078281	0.00478467	DCTN2
4878	ENSG00000175206	123.589801	-2.07816213	0.26858001	6.70E-16	1.93E-14	NPPA
79797	ENSG00000175213	256.090369	0.07533672	0.14857214	0.58309002	0.77960145	ZNF408
10106	ENSG00000175215	3313.91439	0.24660314	0.07103449	0.00040404	0.00267119	CTDSP2
9793	ENSG00000175216	6002.79563	-0.31726604	0.07674402	2.32E-05	0.00020552	CKAP5
392	ENSG00000175220	1293.7054	-0.07433811	0.07874613	0.33426466	0.56397745	ARHGAP1
10025	ENSG00000175221	1195.32097	-0.02023622	0.07514643	0.78292393	0.90115512	MED16
9776	ENSG00000175224	1286.63334	-0.08135811	0.10077106	0.40177707	0.63028837	ATG13
89792	ENSG00000175229	370.084461	0.24213989	0.1435386	0.06812855	0.18781025	GAL3ST3
148345	ENSG00000175262	3.82802516	-0.07984808	0.38492317	0.37096761	0.60017919	C1orf127
8534	ENSG00000175264	59.1293702	-3.8007809	0.458265	5.69E-18	1.88E-16	CHST1
23015	ENSG00000175265	3610.09106	0.21002674	0.06110539	0.0004944	0.00319403	GOLGA8A
146177	ENSG00000175267	7.5104178	0.13020489	0.39836262	0.21891558	0.43081814	VWA3A
9537	ENSG00000175274	1459.39348	-0.36189293	0.10227804	0.00023165	0.00161997	TP53I11
378708	ENSG00000175279	179.713524	0.13394828	0.16256203	0.36419818	0.59387751	CENPS
22845	ENSG00000175283	219.760974	-0.09874976	0.15394958	0.48279084	0.70322078	DOLK
254295	ENSG00000175287	0.14931947	-0.01844332	0.38480212	0.84537521	NA	PHYHD1

117144	ENSG00000175294	0.86172829	0.03799542	0.38472774	0.45126332	NA	CATSPER1
NA	ENSG00000175302	0	NA	NA	NA	NA	ANKRD30BP1
9134	ENSG00000175305	206.225099	-0.28863118	0.20032921	0.09251938	0.23521072	CCNE2
85007	ENSG00000175309	457.545493	0.26899067	0.11726813	0.0158553	0.06030849	PHYKPL
257629	ENSG00000175311	0	NA	NA	NA	NA	ANKS4B
1474	ENSG00000175315	0.84909805	0.02962074	0.38439909	0.50670033	NA	CST6
196996	ENSG00000175318	12.0776643	0.13811326	0.3876213	0.31506781	0.54289729	GRAMD2A
NA	ENSG00000175319	0	NA	NA	NA	NA	NF1P5
162655	ENSG00000175322	480.263549	0.20969859	0.12118657	0.06767137	0.18691355	ZNF519
27257	ENSG00000175324	621.274714	-0.22802483	0.0983289	0.01614111	0.06123127	LSM1
5626	ENSG00000175325	0.18922458	0.01954053	0.38488496	0.78225479	NA	PROP1
91464	ENSG00000175329	1.45662398	-0.02534541	0.38091292	0.68693583	NA	ISX
8815	ENSG00000175334	2142.01451	0.03229663	0.07077821	0.64145361	0.82042546	BANF1
319	ENSG00000175336	0.4964018	-0.01055555	0.38285916	0.82391731	NA	APOF
1139	ENSG00000175344	1589.53485	0.2760389	0.08068891	0.00045529	0.00297629	CHRNA7
56674	ENSG00000175348	301.836787	-0.22825523	0.13338052	0.06691366	0.18544027	TMEM9B
56675	ENSG00000175352	108.481661	0.21802475	0.21287247	0.22198586	0.43502894	NRIP3
5771	ENSG00000175354	423.930849	-0.06302185	0.11991581	0.57912809	0.7768696	PTPN2
57758	ENSG00000175356	32.1768887	0.22692176	0.38148109	0.24150568	0.45956961	SCUBE2
84285	ENSG00000175376	515.949696	-0.04884172	0.10399955	0.62476134	0.80877365	EIF1AD
4087	ENSG00000175387	1536.43956	-0.30165292	0.07773391	7.14E-05	0.00056533	SMAD2
8665	ENSG00000175390	5204.46157	0.14601517	0.05337132	0.00576555	0.02625394	EIF3F
219749	ENSG00000175395	474.352291	-0.17561152	0.11224732	0.10099806	0.25116589	ZNF25
121130	ENSG00000175398	0	NA	NA	NA	NA	OR10P1
285598	ENSG00000175414	2603.37955	-0.28727988	0.20139621	0.09591809	0.24177549	ARL10
1212	ENSG00000175416	538.502902	-0.50435629	0.13547556	7.43E-05	0.00058524	CLTB
5122	ENSG00000175426	5.49100605	-3.67398681	1.73254651	0.00135097	0.00765587	PCSK1
4023	ENSG00000175445	3.31003049	0.21663377	0.47617538	0.00451231	0.02134509	LPL
317671	ENSG00000175449	49.0021121	-0.25213759	0.36478278	0.22229066	0.43535977	RFESD
64770	ENSG00000175455	3347.76527	-0.06351879	0.07723507	0.40041816	0.62894783	CCDC14
374403	ENSG00000175463	23.4324705	0.50787491	0.5269882	0.05309689	0.15587419	TBC1D10C
9092	ENSG00000175467	1882.95198	0.2356442	0.09452834	0.0099552	0.04132895	SART1
55844	ENSG00000175470	776.858614	-0.10197087	0.09810329	0.28150658	0.50630249	PPP2R2D
79772	ENSG00000175471	79.9944846	0.2593207	0.24968967	0.18628228	0.38596943	MCTP1
57804	ENSG00000175482	94.9787764	0.49584815	0.25667723	0.01705974	0.06413972	POLD4
120787	ENSG00000175485	0.36244485	0.00098593	0.38336489	0.96649389	NA	OR52W1
126364	ENSG00000175489	0	NA	NA	NA	NA	LRRC25
57628	ENSG00000175497	21.5056274	2.07009092	0.57229121	1.60E-05	0.00014659	DPP10

23529	ENSG00000175505	11.5601592	0.14893412	0.37067034	0.37275248	0.60164409	CLCF1
NA	ENSG00000175509	0.55365942	0.03297732	0.38624289	0.45088369	NA	ACRP1
254187	ENSG00000175513	0.16735865	0.01897177	0.38482933	0.78225479	NA	TSGA10IP
390212	ENSG00000175514	0	NA	NA	NA	NA	GPR152
143630	ENSG00000175518	0	NA	NA	NA	NA	UBQLNL
50613	ENSG00000175520	0	NA	NA	NA	NA	UBQLN3
5406	ENSG00000175535	0	NA	NA	NA	NA	PNLIP
387787	ENSG00000175536	74.7192834	-0.06392536	0.23809773	0.73126233	0.87162779	LIPT2
10008	ENSG00000175538	0	NA	NA	NA	NA	KCNE3
57010	ENSG00000175544	1.35294443	0.00802665	0.37866486	0.90485094	NA	CABP4
144245	ENSG00000175548	616.130215	0.06993985	0.14283868	0.60151154	0.79200883	ALG10B
10589	ENSG00000175550	1083.42408	0.09480102	0.08941515	0.2757897	0.49983261	DRAP1
79836	ENSG00000175556	0.49037226	0.01381349	0.38306631	0.76290694	NA	LONRF3
7352	ENSG00000175564	62.0252933	0.14750843	0.25800156	0.45045598	0.67378216	UCP3
7351	ENSG00000175567	583.507772	0.82520138	0.14841503	4.60E-09	7.25E-08	UCP2
83638	ENSG00000175573	365.983922	0.21300331	0.13023657	0.08148936	0.21429626	C11orf68
80227	ENSG00000175575	518.520428	0.16375559	0.10957287	0.11847308	0.28258066	PAAF1
51642	ENSG00000175581	400.269392	-0.15993985	0.13379531	0.20051873	0.40575148	MRPL48
5870	ENSG00000175582	1766.26052	-0.26372123	0.08237135	0.00101604	0.00598698	RAB6A
5029	ENSG00000175591	14.4848203	0.05358756	0.34467665	0.73783943	0.87620179	P2RY2
8061	ENSG00000175592	13.2455086	0.42368682	0.59102611	0.05850006	0.16796543	FOSL1
2072	ENSG00000175595	438.843018	-0.20570968	0.14451669	0.12311737	0.29054555	ERCC4
79783	ENSG00000175600	51.434978	0.2210259	0.27387633	0.27568503	0.49980793	SUGCT
11007	ENSG00000175602	579.968377	-0.75908249	0.10595806	1.62E-13	3.90E-12	CCDC85B
NA	ENSG00000175604	0.33471731	0.02354391	0.38534154	0.62842204	NA	
54968	ENSG00000175606	354.198421	-0.08747059	0.15069697	0.527172	0.73905402	TMEM70
100128782	ENSG00000175611	96.2714561	0.17271655	0.24043616	0.36732809	0.59652854	LINC00476
119765	ENSG00000175619	0	NA	NA	NA	NA	OR4B1
6199	ENSG00000175634	853.627858	0.18024583	0.09173827	0.04279977	0.13226595	RPS6KB2
116028	ENSG00000175643	450.437786	-0.2056785	0.12081633	0.07205199	0.19555978	RMI2
5619	ENSG00000175646	0	NA	NA	NA	NA	PRM1
NA	ENSG00000175658	0	NA	NA	NA	NA	DRD5P2
146691	ENSG00000175662	722.731915	-0.05552225	0.10949362	0.59579259	0.7882452	TOM1L2
122046	ENSG00000175664	0	NA	NA	NA	NA	TEX26
100132979	ENSG00000175676	0	NA	NA	NA	NA	GOLGA8DP
58492	ENSG00000175691	144.633377	-0.24384805	0.20846106	0.16054401	0.3484894	ZNF77
165829	ENSG00000175697	41.3917297	1.27527663	0.40357334	0.00012408	0.00092131	GPR156
256369	ENSG00000175699	0	NA	NA	NA	NA	CCDC197

205251	ENSG00000175701	123.490693	0.13346144	0.20750216	0.44559059	0.67028199	MTLN
126695	ENSG00000175707	0.173733	0.01914545	0.38484597	0.78225479	NA	KDF1
146712	ENSG00000175711	207.948093	0.19225591	0.16150721	0.18953252	0.39052085	B3GNTL1
139804	ENSG00000175718	0.34624801	-0.02356563	0.38536324	0.65270996	NA	RBMXL3
22877	ENSG00000175727	1812.40708	-0.22824763	0.10110459	0.01885411	0.06941278	MLXIP
283171	ENSG00000175728	0	NA	NA	NA	NA	LINC02873
NA	ENSG00000175730	0.83487678	0.03685432	0.38459835	0.46326182	NA	BAK1P1
NA	ENSG00000175741	0.5211355	0.01049697	0.38324774	0.77582193	NA	RWDD4P2
7025	ENSG00000175745	2245.47569	1.1929386	0.07088348	1.71E-64	2.45E-62	NR2F1
400360	ENSG00000175746	0	NA	NA	NA	NA	C15orf54
NA	ENSG00000175749	0.14931947	-0.01844332	0.38480212	0.84537521	NA	EIF3KP1
54998	ENSG00000175756	1301.965	-0.16643929	0.07705386	0.02725927	0.09310102	AURKAIP1
158135	ENSG00000175764	316.125599	-0.02259211	0.13978503	0.86114388	0.93812292	TTLL11
253314	ENSG00000175766	0.14931947	-0.01844332	0.38480212	0.84537521	NA	EIF4E1B
401505	ENSG00000175768	1200.80271	-0.06378922	0.07888216	0.40804962	0.63630468	TOMM5
NA	ENSG00000175772	14.3715132	-0.39778844	0.55940718	0.06736435	0.18634601	LINC01106
NA	ENSG00000175773	0.53947826	-0.00667326	0.38308251	0.99653506	NA	ZBTB44-DT
400359	ENSG00000175779	0.15069912	-0.01848503	0.38480575	0.84537521	NA	LINC02694
55508	ENSG00000175782	518.897543	-0.32340508	0.11788298	0.00381908	0.0185213	SLC35E3
145270	ENSG00000175785	514.816304	-1.36569216	0.12249555	6.52E-30	3.78E-28	PRIMA1
169841	ENSG00000175787	167.330465	-0.34086462	0.18186821	0.0338949	0.11060618	ZNF169
8607	ENSG00000175792	2116.86092	-0.12147296	0.06730819	0.06650092	0.18468341	RUVBL1
2810	ENSG00000175793	2.63792138	0.08366915	0.38952631	0.30837174	0.53592286	SFN
NA	ENSG00000175800	0	NA	NA	NA	NA	OR52B3P
4482	ENSG00000175806	83.3837072	0.15740835	0.2253115	0.39280037	0.62197758	MSRA
158506	ENSG00000175809	0	NA	NA	NA	NA	CBLL2
643677	ENSG00000175820	13.4395951	0.07283579	0.33473152	0.67685692	0.84203921	CCDC168
23399	ENSG00000175826	2243.72521	-0.08744935	0.06751104	0.18772525	0.38805147	CTDNEP1
2118	ENSG00000175832	1429.28015	0.29817199	0.07265698	2.84E-05	0.00024625	ETV4
NA	ENSG00000175841	0	NA	NA	NA	NA	FAM172BP
375757	ENSG00000175854	147.857053	0.35361156	0.19622306	0.03792697	0.12066567	SWI5
202309	ENSG00000175857	0	NA	NA	NA	NA	GAPT
10458	ENSG00000175866	533.741527	0.37083241	0.1252835	0.00170077	0.0093446	BAIAP2
797	ENSG00000175868	55.1385881	-0.34830339	0.28162993	0.09946692	0.24826063	CALCB
200407	ENSG00000175874	1.55971086	-0.03247983	0.38287115	0.46128998	NA	CREG2
135886	ENSG00000175877	0.33889064	0.0023549	0.38338005	0.96597464	NA	TMEM270
3234	ENSG00000175879	369.174002	-0.22220596	0.15097829	0.10801155	0.26355297	HOXD8
NA	ENSG00000175886	1.02247641	0.03650131	0.38191249	0.57430161	NA	RPL7AP66

340481	ENSG00000175893	1452.38766	-0.05575109	0.0881073	0.51481876	0.72951962	ZDHHC21
54084	ENSG00000175894	107.05391	-0.87729281	0.36228791	0.00180662	0.00980395	TSPEAR
79666	ENSG00000175895	134.768725	0.09170224	0.18283039	0.56969332	0.77030706	PLEKHF2
2	ENSG00000175899	2.87869126	-0.01565114	0.37620961	0.84077918	0.92773074	A2M
379	ENSG00000175906	173.918671	0.62704901	0.18867482	0.00022763	0.00159523	ARL4D
285489	ENSG00000175920	5.76785446	-0.0782688	0.36934625	0.54219846	0.75027258	DOK7
57633	ENSG00000175928	534.122833	0.00370828	0.11687195	0.97824103	0.99254491	LRRN1
63893	ENSG00000175931	2678.02522	-0.15456201	0.07324077	0.03141566	0.10403717	UBE2O
93129	ENSG00000175938	155.748753	0.02996956	0.17584737	0.84927077	0.93154246	ORAI3
340359	ENSG00000175946	0	NA	NA	NA	NA	KLHL38
NA	ENSG00000175967	0.30139824	-0.02296476	0.38527817	0.68536535	NA	LINC02880
84747	ENSG00000175970	1506.38765	-0.08103635	0.08923909	0.35018774	0.58060182	UNC119B
163259	ENSG00000175984	0.34669998	0.02366913	0.38535997	0.62115904	NA	DENND2C
400224	ENSG00000175985	39.5467083	-0.43486034	0.37463378	0.0709599	0.19346495	PLEKHD1
NA	ENSG00000176007	0	NA	NA	NA	NA	FAM220BP
56676	ENSG00000176009	0.66235055	-0.0195514	0.38294208	0.6679054	NA	ASCL3
84617	ENSG00000176014	391.147795	0.53364833	0.14142419	5.56E-05	0.00044851	TUBB6
116068	ENSG00000176018	335.216414	-0.09962452	0.16928131	0.51070288	0.72599857	LYSMD3
386724	ENSG00000176020	155.385306	0.21119652	0.20058912	0.2194896	0.43154035	AMIGO3
126792	ENSG00000176022	986.902355	-0.14230403	0.08848204	0.0975805	0.24466031	B3GALT6
79898	ENSG00000176024	76.631781	-0.30242726	0.28634356	0.14842529	0.33077346	ZNF613
56673	ENSG00000176029	0.89389376	0.01361547	0.38205178	0.71945716	NA	C11orf16
344805	ENSG00000176040	0.18922458	0.01954053	0.38488496	0.78225479	NA	TMPRSS7
NA	ENSG00000176043	0	NA	NA	NA	NA	
26471	ENSG00000176046	0.14931947	-0.01844332	0.38480212	0.84537521	NA	NUPR1
9832	ENSG00000176049	785.325659	-0.42971466	0.09730281	4.84E-06	4.89E-05	JAKMIP2
NA	ENSG00000176054	6.28092594	0.077268	0.37092126	0.54597196	0.75333225	RPL23P2
153364	ENSG00000176055	391.194337	-0.16502215	0.11761771	0.13934402	0.31622334	MBLAC2
286262	ENSG00000176058	351.026319	-0.18082539	0.13702335	0.15642316	0.34292104	TPRN
23630	ENSG00000176076	3.67451544	0.03958305	0.36872319	0.72972144	0.87078512	KCNE5
257101	ENSG00000176083	0	NA	NA	NA	NA	ZNF683
113829	ENSG00000176087	1955.46255	0.10879598	0.0678294	0.10319122	0.25514879	SLC35A4
55057	ENSG00000176092	45.0417082	-0.09111132	0.34215893	0.58709212	0.78237765	CRYBG2
9807	ENSG00000176095	1606.00581	0.00311832	0.08690573	0.97175945	0.98938604	IP6K1
8636	ENSG00000176101	877.822097	-0.20087555	0.09197302	0.02411101	0.08469589	SSNA1
1479	ENSG00000176102	734.346278	-0.14830111	0.1028099	0.13358428	0.30743129	CSTF3
7525	ENSG00000176105	614.673868	0.44987204	0.10360569	6.47E-06	6.36E-05	YES1
79643	ENSG00000176108	493.429939	0.04064785	0.10842774	0.69677491	0.85365037	CHMP6

NA	ENSG00000176115	0	NA	NA	NA	NA	AQP7P4
10301	ENSG00000176124	145.444262	-0.06729628	0.18295943	0.67416971	0.84017129	DLEU1
100874074	ENSG00000176124	145.444262	-0.06729628	0.18295943	0.67416971	0.84017129	DLEU1
402682	ENSG00000176125	82.6446373	0.043327	0.22787871	0.81511596	0.91599551	UFSP1
NA	ENSG00000176134	0.5972779	-0.02732172	0.38586499	0.50076333	NA	
4161	ENSG00000176136	0	NA	NA	NA	NA	MC5R
55254	ENSG00000176142	552.89655	1.07587632	0.14505319	1.38E-14	3.62E-13	TMEM39A
55346	ENSG00000176148	565.797055	-0.12381946	0.10408145	0.21479135	0.42508333	TCP11L1
2877	ENSG00000176153	2.55742763	-0.07445035	0.38759497	0.33070897	NA	GPX2
284001	ENSG00000176155	1704.20078	-0.07191994	0.08308834	0.37454244	0.60364923	CCDC57
124535	ENSG00000176160	0.30139824	-0.02296476	0.38527817	0.68536535	NA	HSF5
2290	ENSG00000176165	0	NA	NA	NA	NA	FOXG1
8877	ENSG00000176170	36.1772329	2.43111005	0.44338224	2.68E-09	4.35E-08	SPHK1
664	ENSG00000176171	1230.43746	-0.10202519	0.11412709	0.34796092	0.57864958	BNIP3
150350	ENSG00000176177	0	NA	NA	NA	NA	ENTHD1
339344	ENSG00000176182	217.641868	0.19918414	0.1538373	0.1573448	0.34410503	MYPOP
NA	ENSG00000176183	0	NA	NA	NA	NA	
1149	ENSG00000176194	0	NA	NA	NA	NA	CIDEA
390442	ENSG00000176198	0	NA	NA	NA	NA	OR11H4
219986	ENSG00000176200	0	NA	NA	NA	NA	OR4D11
80059	ENSG00000176204	192.111171	-1.21666469	0.19289202	2.81E-11	5.70E-10	LRRTM4
79915	ENSG00000176208	605.033864	-0.20384783	0.10973408	0.05190381	0.15335394	ATAD5
114926	ENSG00000176209	425.641967	-0.02553547	0.14184013	0.84531069	0.92970586	SMIM19
122748	ENSG00000176219	0	NA	NA	NA	NA	OR11H6
342908	ENSG00000176222	53.7205254	-0.0072297	0.2692825	0.96791623	0.98788302	ZNF404
25914	ENSG00000176225	748.620601	0.13424845	0.10883672	0.19754325	0.40156333	RTTN
390436	ENSG00000176230	0	NA	NA	NA	NA	OR4K17
126541	ENSG00000176231	0	NA	NA	NA	NA	OR10H4
NA	ENSG00000176232	0	NA	NA	NA	NA	
221060	ENSG00000176236	2.54082223	-0.05947596	0.37825898	0.53946919	NA	RPP38-DT
390058	ENSG00000176239	0	NA	NA	NA	NA	OR51B6
NA	ENSG00000176243	0	NA	NA	NA	NA	CDV3P1
414149	ENSG00000176244	254.475961	-0.22823711	0.1492017	0.09599113	0.24186741	ACBD7
122742	ENSG00000176246	0	NA	NA	NA	NA	OR4L1
29882	ENSG00000176248	1051.05133	0.03343174	0.09309938	0.71203495	0.86159535	ANAPC2
390433	ENSG00000176253	0	NA	NA	NA	NA	OR4K13
127540	ENSG00000176256	0	NA	NA	NA	NA	HMGB4
339487	ENSG00000176261	295.855338	-0.22405998	0.15632207	0.11525045	0.27682433	ZBTB8OS

NA	ENSG00000176268	8.70616966	0.18634112	0.40649485	0.23110528	0.44719874	CYCSP34
441308	ENSG00000176269	0.65871511	0.02516691	0.38354497	0.59695666	NA	OR4F21
159371	ENSG00000176273	285.110353	0.07111989	0.1535367	0.61410248	0.80172357	SLC35G1
79317	ENSG00000176281	0	NA	NA	NA	NA	OR4K5
NA	ENSG00000176289	0	NA	NA	NA	NA	IDSP1
283617	ENSG00000176290	0	NA	NA	NA	NA	OR4K3
7694	ENSG00000176293	51.592909	0.09679065	0.26337768	0.61988765	0.80593519	ZNF135
390429	ENSG00000176294	0	NA	NA	NA	NA	OR4N2
441670	ENSG00000176299	0	NA	NA	NA	NA	OR4M1
283150	ENSG00000176302	0.16735865	0.01897177	0.38482933	0.78225479	NA	FOXR1
NA	ENSG00000176312	0	NA	NA	NA	NA	OR4H12P
NA	ENSG00000176318	0	NA	NA	NA	NA	FOXN3P1
NA	ENSG00000176320	0	NA	NA	NA	NA	
1351	ENSG00000176340	3745.9193	0.00973052	0.0619707	0.87454868	0.94478878	COX8A
NA	ENSG00000176343	0.46658771	-0.00768756	0.38326124	0.86582814	NA	RPL37AP8
NA	ENSG00000176349	22.5694439	-1.49919455	0.60288538	0.00071196	0.00439081	
255061	ENSG00000176358	0	NA	NA	NA	NA	TAC4
54993	ENSG00000176371	592.535571	0.12052311	0.12082233	0.29311444	0.51927766	ZSCAN2
NA	ENSG00000176378	0.83404269	-0.00965768	0.38153684	0.85309719	NA	PFN1P10
285800	ENSG00000176381	58.8311416	-0.1095846	0.26015999	0.57028862	0.7706786	PRR18
79369	ENSG00000176383	53.7438299	1.38017594	0.57075533	0.00073821	0.00453296	B3GNT4
246184	ENSG00000176386	407.69078	-0.00856314	0.12042302	0.93902548	0.97474024	CDC26
3291	ENSG00000176387	44.9881432	0.07635793	0.25829621	0.69307976	0.8516694	HSD11B2
51379	ENSG00000176390	463.526352	0.11595936	0.11175707	0.27812609	0.50258055	CRLF3
6051	ENSG00000176393	1454.00084	0.27589434	0.08004655	0.00041464	0.00273488	RNPEP
163126	ENSG00000176396	324.790252	-0.3158957	0.1350001	0.01215251	0.04871807	EID2
63951	ENSG00000176399	0	NA	NA	NA	NA	DMRTA1
126272	ENSG00000176401	82.0332386	-0.18794877	0.2447385	0.32699604	0.55535024	EID2B
349149	ENSG00000176402	1.00807697	0.00468448	0.37999709	0.93862016	NA	GJC3
9699	ENSG00000176406	354.477245	-1.85279531	0.14589215	5.03E-38	3.68E-36	RIMS2
56888	ENSG00000176407	863.202088	-0.22852058	0.09080878	0.00925322	0.03889487	KCMF1
84277	ENSG00000176410	219.164454	-0.08345928	0.15030403	0.54481029	0.75256117	DNAJC30
283377	ENSG00000176422	237.3548	0.06262913	0.1522944	0.65478229	0.82814932	SPRYD4
155382	ENSG00000176428	296.52136	0.13365895	0.13388954	0.28667883	0.51245229	VPS37D
161198	ENSG00000176435	0	NA	NA	NA	NA	CLEC14A
161176	ENSG00000176438	807.09916	-0.40121777	0.11158881	0.00016534	0.0011976	SYNE3
1196	ENSG00000176444	2128.24397	0.11935044	0.064636	0.06104746	0.17377066	CLK2
254531	ENSG00000176454	648.949948	0.17122138	0.10242439	0.08228987	0.21587082	LPCAT4

28232	ENSG00000176463	179.562297	-0.51046513	0.22943227	0.00828691	0.03555628	SLCO3A1
284346	ENSG00000176472	16.3084999	0.27727427	0.42270645	0.1634634	0.35251239	ZNF575
79446	ENSG00000176473	197.821213	-0.13207723	0.16630051	0.37690586	0.60570261	WDR25
112869	ENSG00000176476	150.137727	-0.27170304	0.18478794	0.09386723	0.23775385	SGF29
11145	ENSG00000176485	182.455994	-0.33097132	0.1908842	0.04681082	0.14159576	PLAAT3
148252	ENSG00000176490	1489.52987	-0.54417626	0.11354694	5.56E-07	6.59E-06	DIRAS1
390195	ENSG00000176495	0	NA	NA	NA	NA	OR5AN1
392133	ENSG00000176510	0	NA	NA	NA	NA	OR10AC1
NA	ENSG00000176515	0	NA	NA	NA	NA	
653583	ENSG00000176531	228.258895	-0.28982561	0.19611372	0.08649327	0.22401363	PHLDB3
222171	ENSG00000176532	0.8079061	-0.00822484	0.3808485	0.88438785	NA	PRR15
2788	ENSG00000176533	330.391078	0.7514349	0.1525209	1.70E-07	2.17E-06	GNG7
79346	ENSG00000176540	0	NA	NA	NA	NA	OR4C5
205717	ENSG00000176542	643.301865	-0.07320089	0.12157419	0.52447778	0.73730881	USF3
256144	ENSG00000176547	0	NA	NA	NA	NA	OR4C3
256148	ENSG00000176555	0	NA	NA	NA	NA	OR4S1
124817	ENSG00000176563	19.2925267	-0.16858943	0.35289096	0.37024114	0.59943356	CNTD1
138009	ENSG00000176566	0.173733	0.01914545	0.38484597	0.78225479	NA	DCAF4L2
390113	ENSG00000176567	0	NA	NA	NA	NA	OR4X1
168975	ENSG00000176571	0	NA	NA	NA	NA	CNBD1
375940	ENSG00000176584	0	NA	NA	NA	NA	DMBT1L1
100128398	ENSG00000176593	248.677764	-0.08748516	0.18479962	0.587176	0.78237765	
9920	ENSG00000176595	778.021049	0.31266394	0.10485383	0.0018829	0.01015404	KBTBD11
84002	ENSG00000176597	594.023637	0.35774051	0.10847882	0.00056997	0.0036198	B3GNT5
80122	ENSG00000176601	0	NA	NA	NA	NA	MAP3K19
283598	ENSG00000176605	0	NA	NA	NA	NA	C14orf177
84823	ENSG00000176619	6707.91687	-0.00182554	0.06212126	0.88595766	0.95015905	LMNB2
51115	ENSG00000176623	611.897368	0.19555643	0.10861724	0.05999783	0.17120049	RMDN1
51320	ENSG00000176624	1632.67339	-0.06170416	0.07449712	0.39782403	0.62701213	MEX3C
150280	ENSG00000176635	0	NA	NA	NA	NA	HORMAD2
220441	ENSG00000176641	1139.79409	0.58801649	0.0856477	2.10E-12	4.68E-11	RNF152
NA	ENSG00000176654	0	NA	NA	NA	NA	NANOGP1
4642	ENSG00000176658	342.417237	-0.4525475	0.14179262	0.00060653	0.00381897	MYO1D
284756	ENSG00000176659	0	NA	NA	NA	NA	C20orf197
2300	ENSG00000176678	0	NA	NA	NA	NA	FOXL1
90655	ENSG00000176679	0	NA	NA	NA	NA	TGIF2LY
9884	ENSG00000176681	133.90541	0.34862794	0.20060533	0.04395771	0.13475294	LRRC37A
2303	ENSG00000176692	0.35686611	0.02381839	0.38538127	0.61392362	NA	FOXC2

81099	ENSG00000176695	0.30139824	-0.02296476	0.38527817	0.68536535	NA	OR4F17
627	ENSG00000176697	24.4547915	3.43616925	0.63693089	4.59E-09	7.24E-08	BDNF
NA	ENSG00000176700	334.754704	-0.05863268	0.31208087	0.74908799	0.88205755	SCAND2P
79635	ENSG00000176714	160.182043	0.09505595	0.1904545	0.56574777	0.76758775	CCDC121
197322	ENSG00000176715	829.953097	0.15300695	0.10572133	0.13175825	0.30452978	ACSF3
NA	ENSG00000176716	0	NA	NA	NA	NA	OR10AB1P
666	ENSG00000176720	70.9896025	-0.19191798	0.24812535	0.32040736	0.54819611	BOK
283933	ENSG00000176723	56.429291	0.11867992	0.27777165	0.54795788	0.754545	ZNF843
83869	ENSG00000176728	0	NA	NA	NA	NA	TTTY14
401466	ENSG00000176731	455.127631	0.04424224	0.11460498	0.68679015	0.84769917	RBIS
375189	ENSG00000176732	24.0580812	0.30424106	0.38173686	0.16521809	0.35517682	PFN4
283111	ENSG00000176742	0	NA	NA	NA	NA	OR51V1
158809	ENSG00000176746	2.28252939	-0.04545442	0.38037891	0.56303576	NA	MAGEB6
283110	ENSG00000176748	0	NA	NA	NA	NA	OR52Z1
8851	ENSG00000176749	1159.60435	-0.80141518	0.08472276	5.54E-22	2.27E-20	CDK5R1
NA	ENSG00000176752	0	NA	NA	NA	NA	OR51A1P
644809	ENSG00000176753	2.88483091	0.0695805	0.37839631	0.50512054	0.72144493	C15orf56
NA	ENSG00000176754	0	NA	NA	NA	NA	LINC00303
NA	ENSG00000176761	9.74065866	0.01037137	0.34471301	0.94835238	0.97876335	ZNF285B
256536	ENSG00000176769	0.3000209	-0.02294463	0.38527539	0.68644561	NA	TCERG1L
344148	ENSG00000176771	157.158516	1.11587599	0.21941136	3.99E-08	5.56E-07	NCKAP5
286514	ENSG00000176774	0	NA	NA	NA	NA	MAGEB18
140596	ENSG00000176782	0	NA	NA	NA	NA	DEFB104A
80230	ENSG00000176783	936.991424	0.24312651	0.0832577	0.0027313	0.01399146	RUFY1
119678	ENSG00000176787	0	NA	NA	NA	NA	OR52E2
10409	ENSG00000176788	1467.83015	-0.3861137	0.07924331	6.07E-07	7.15E-06	BASP1
55894	ENSG00000176797	0	NA	NA	NA	NA	DEFB103A
414325	ENSG00000176797	0	NA	NA	NA	NA	DEFB103A
119682	ENSG00000176798	0	NA	NA	NA	NA	OR51L1
374819	ENSG00000176809	576.012169	0.20874965	0.15545079	0.14103467	0.31912955	LRRC37A3
NA	ENSG00000176812	0	NA	NA	NA	NA	METTL1P1
360132	ENSG00000176826	0.16735865	0.01897177	0.38482933	0.78225479	NA	FKBP9P1
54621	ENSG00000176834	521.526171	0.17170593	0.13265296	0.16724028	0.35828164	VSIG10
284424	ENSG00000176840	10.0467854	-0.37217834	0.54736312	0.07029092	0.19208977	MIR7-3HG
10265	ENSG00000176842	181.144196	-0.21080453	0.16720824	0.1601757	0.34791832	IRX5
284207	ENSG00000176845	151.556128	-0.04396288	0.19136538	0.78980622	0.9043268	METRNL
157769	ENSG00000176853	1098.91869	-0.0822035	0.09703513	0.38043916	0.60938575	FAM91A1
NA	ENSG00000176855	0	NA	NA	NA	NA	KRT18P28

NA	ENSG00000176857	0.54648734	0.03263277	0.38620655	0.45580981	NA	GJA1P1
NA	ENSG00000176868	8.65900385	0.39058596	0.64788645	0.03344927	0.10942019	
55884	ENSG00000176871	1727.75474	-0.35756534	0.07143751	3.34E-07	4.10E-06	WSB2
NA	ENSG00000176882	0	NA	NA	NA	NA	
2902	ENSG00000176884	952.679746	-4.21189377	0.13144577	5.63E-227	1.40E-223	GRIN1
6664	ENSG00000176887	6480.31137	0.64338861	0.05257313	5.68E-35	3.85E-33	SOX11
7298	ENSG00000176890	3232.68996	-0.19694744	0.06447646	0.00191729	0.01029474	TYMS
81282	ENSG00000176893	0	NA	NA	NA	NA	OR51G2
5827	ENSG00000176894	261.496851	-0.44908313	0.15320896	0.00144905	0.0081373	PXMP2
119687	ENSG00000176895	0	NA	NA	NA	NA	OR51A7
170082	ENSG00000176896	45.3573948	-0.19151664	0.29604438	0.34641902	0.57719207	TCEANC
401665	ENSG00000176900	0	NA	NA	NA	NA	OR51T1
9240	ENSG00000176903	1568.10326	-0.24408022	0.07220258	0.00056395	0.00358502	PNMA1
401663	ENSG00000176904	0	NA	NA	NA	NA	OR51H1
56892	ENSG00000176907	0	NA	NA	NA	NA	TCIM
284358	ENSG00000176909	41.6678267	0.46483845	0.41232682	0.0619012	0.175546	MAMSTR
NA	ENSG00000176912	39.4301531	-0.02281546	0.27589075	0.90377425	0.95800026	TYMSOS
23141	ENSG00000176915	1903.25711	0.14508813	0.07790712	0.05699194	0.16463601	ANKLE2
733	ENSG00000176919	2.43328913	-0.0570374	0.37746952	0.5593085	NA	C8G
2524	ENSG00000176920	37.6659502	0.20946199	0.38754613	0.24762797	0.46665024	FUT2
119692	ENSG00000176922	0	NA	NA	NA	NA	OR51S1
NA	ENSG00000176923	0	NA	NA	NA	NA	OR7A15P
119694	ENSG00000176925	0	NA	NA	NA	NA	OR51F2
374786	ENSG00000176927	12.9439131	0.14734118	0.37496014	0.36163985	0.59140575	EFCAB5
51301	ENSG00000176928	4.07060528	0.32066555	0.59079402	0.01527047	0.05846508	GCNT4
NA	ENSG00000176933	11.4575629	-0.06783142	0.34795652	0.6686006	0.83706921	TOB2P1
200958	ENSG00000176945	54.4547559	0.38719498	0.32429826	0.08678144	0.22459479	MUC20
51078	ENSG00000176946	912.67351	-0.04883606	0.08634676	0.56100958	0.7645572	THAP4
NA	ENSG00000176951	0	NA	NA	NA	NA	OR51N1P
84901	ENSG00000176953	1421.19052	-0.06608377	0.07673383	0.3756306	0.60471528	NFATC2IP
4062	ENSG00000176956	0	NA	NA	NA	NA	LY6H
NA	ENSG00000176970	0	NA	NA	NA	NA	RPL7L1P11
387758	ENSG00000176971	0	NA	NA	NA	NA	FIBIN
23625	ENSG00000176973	1126.83045	-0.19211619	0.09881491	0.04368976	0.13409727	FAM89B
6470	ENSG00000176974	449.964824	-0.08206369	0.11767789	0.46271403	0.6855676	SHMT1
29952	ENSG00000176978	1637.04306	-0.12155777	0.07189784	0.0848575	0.22086345	DPP7
166655	ENSG00000176979	0	NA	NA	NA	NA	TRIM60
107984399	ENSG00000176984	1.85647236	0.02509684	0.37544933	0.77544964	NA	

9632	ENSG00000176986	2812.8122	0.02370897	0.0782524	0.75806132	0.8876261	SEC24C
158521	ENSG00000176988	0.18922458	0.01954053	0.38488496	0.78225479	NA	FMR1NB
140775	ENSG00000176994	1333.71668	-0.05883785	0.07986748	0.45397308	0.67730681	SMCR8
NA	ENSG00000176998	0.173124	-0.01911262	0.38486262	0.84537521	NA	HCG4
4524	ENSG00000177000	1262.81584	0.2495337	0.0938531	0.00600671	0.02717743	MTHFR
503618	ENSG00000177023	0	NA	NA	NA	NA	DEFB104B
147685	ENSG00000177025	4.01393402	0.0736889	0.37435921	0.53249637	0.7437975	C19orf18
10522	ENSG00000177030	1372.26944	-0.24581859	0.08022256	0.00168552	0.00927879	DEAF1
345778	ENSG00000177034	1277.25592	0.01790034	0.07880115	0.81632689	0.9165257	MTX3
283232	ENSG00000177042	219.63907	0.11060369	0.15752823	0.44182156	0.66669757	TMEM80
147912	ENSG00000177045	471.61984	2.43641023	0.13718297	7.93E-72	1.33E-69	SIX5
3467	ENSG00000177047	0.14931947	-0.01844332	0.38480212	0.84537521	NA	IFNW1
23403	ENSG00000177051	641.601916	0.12678533	0.113272	0.24082095	0.45879844	FBXO46
54503	ENSG00000177054	718.833545	-0.21375702	0.09899999	0.02520009	0.08768238	ZDHHC13
153129	ENSG00000177058	295.376671	0.20950518	0.16166081	0.15200779	0.33617597	SLC38A9
340485	ENSG00000177076	57.6576559	0.20893587	0.27422762	0.3017185	0.52913363	ACER2
84942	ENSG00000177082	956.584469	0.19237292	0.09287731	0.03250601	0.10692141	WDR73
5426	ENSG00000177084	3163.16045	-0.01578743	0.07650416	0.83225443	0.92392457	POLE
150368	ENSG00000177096	257.936623	0.18867211	0.14968207	0.17050409	0.36238863	PHETA2
6330	ENSG00000177098	31.1090983	0.85824602	0.49075871	0.00769827	0.03339901	SCN4B
57453	ENSG00000177103	33.5582165	-0.27776971	0.34068709	0.19760648	0.4016507	DSCAML1
391	ENSG00000177105	372.924376	0.06778681	0.13243538	0.58721701	0.78237765	RHOG
64787	ENSG00000177106	52.4574056	-0.1163546	0.2751284	0.55148969	0.75793541	EPS8L2
283576	ENSG00000177108	245.095978	-2.38111014	0.1753444	4.00E-43	3.31E-41	ZDHHC22
100129827	ENSG00000177112	1.80708766	-0.0356991	0.37677633	0.68136958	NA	IRAG1-AS1
196527	ENSG00000177119	1771.47421	-0.0424325	0.07011855	0.53758271	0.747679	ANO6
403341	ENSG00000177125	783.784376	-0.29826439	0.10163794	0.00226807	0.01192329	ZBTB34
440556	ENSG00000177133	0	NA	NA	NA	NA	PRDM16-DT
171483	ENSG00000177138	0	NA	NA	NA	NA	FAM9B
1068	ENSG00000177143	0	NA	NA	NA	NA	CETN1
440672	ENSG00000177144	82.2124532	-0.05678066	0.23522426	0.75889836	0.88818685	NUDT4B
125228	ENSG00000177150	314.539921	-0.2959458	0.16180954	0.04310027	0.13284284	FAM210A
403244	ENSG00000177151	0	NA	NA	NA	NA	OR2T35
6888	ENSG00000177156	1290.38618	-0.16703532	0.08114001	0.0349056	0.11308412	TALDO1
8408	ENSG00000177169	1728.06963	0.3799313	0.10724964	0.00021912	0.00154323	ULK1
NA	ENSG00000177173	6.09728451	0.13480198	0.38935174	0.31911892	0.54674606	NAP1L4P1
127066	ENSG00000177174	0	NA	NA	NA	NA	OR14C36
284716	ENSG00000177181	472.533069	-0.77017584	0.14864529	4.07E-08	5.65E-07	RIMKLA

157807	ENSG00000177182	65.6694504	-0.8227494	0.44666119	0.00647234	0.02893446	CLVS1
391196	ENSG00000177186	0	NA	NA	NA	NA	OR2M7
6197	ENSG00000177189	864.120331	-0.08266523	0.10680271	0.41871036	0.64465387	RPS6KA3
374907	ENSG00000177191	7.36240267	0.10148517	0.37379349	0.45992131	0.68305646	B3GNT8
80324	ENSG00000177192	540.505555	-0.04152546	0.11192868	0.69192357	0.85101966	PUS1
NA	ENSG00000177197	0	NA	NA	NA	NA	PCNPP5
80205	ENSG00000177200	1970.95217	-0.36510279	0.07939137	2.45E-06	2.60E-05	CHD9
127064	ENSG00000177201	0	NA	NA	NA	NA	OR2T12
171169	ENSG00000177202	2.03263474	0.06810864	0.38235125	0.45283665	NA	SPACA4
391195	ENSG00000177212	0	NA	NA	NA	NA	OR2T33
347862	ENSG00000177225	1401.25178	0.21987866	0.07555464	0.00295567	0.01495046	GATD1
NA	ENSG00000177233	0	NA	NA	NA	NA	OR2M1P
404216	ENSG00000177234	0.15069912	-0.01848503	0.38480575	0.84537521	NA	LINC01561
493829	ENSG00000177238	0.14931947	-0.01844332	0.38480212	0.84537521	NA	TRIM72
11253	ENSG00000177239	1402.91117	0.08235844	0.08499623	0.32074116	0.54862527	MAN1B1
55894	ENSG00000177243	0	NA	NA	NA	NA	DEFB103B
1673	ENSG00000177257	0	NA	NA	NA	NA	DEFB4B
100289462	ENSG00000177257	0	NA	NA	NA	NA	DEFB4B
NA	ENSG00000177261	0	NA	NA	NA	NA	ENSAP3
3738	ENSG00000177272	68.2447376	-4.52573839	0.4689857	7.08E-23	3.06E-21	KCNA3
127608	ENSG00000177275	0	NA	NA	NA	NA	OR2AJ1
8325	ENSG00000177283	23.0488539	1.35457361	0.62076908	0.00167158	0.00920716	FZD8
219770	ENSG00000177291	1.14391802	-0.00477282	0.38048857	0.93391025	NA	GJD4
162517	ENSG00000177294	0	NA	NA	NA	NA	FBXO39
53842	ENSG00000177300	0	NA	NA	NA	NA	CLDN22
3737	ENSG00000177301	1.32085411	-0.01339762	0.38120608	0.64923384	NA	KCNA2
7156	ENSG00000177302	2073.18505	-0.29537943	0.08096892	0.00018299	0.00131301	TOP3A
57513	ENSG00000177303	762.737416	0.13225878	0.09891642	0.16630021	0.35680721	CASKIN2
NA	ENSG00000177306	0	NA	NA	NA	NA	OR7E125P
253461	ENSG00000177311	656.936961	-0.39439734	0.10831529	0.00014298	0.00104908	ZBTB38
139105	ENSG00000177324	0.16735865	0.01897177	0.38482933	0.78225479	NA	BEND2
286122	ENSG00000177335	35.6889534	-1.61377851	0.45929877	2.83E-05	0.00024558	C8orf31
NA	ENSG00000177337	60.6322512	0.36163397	0.28007184	0.08805365	0.22670443	DLGAP1-AS1
NA	ENSG00000177338	0	NA	NA	NA	NA	LINC00469
79857	ENSG00000177340	4.73809702	0.03781085	0.37057026	0.72906625	0.87045369	
645683	ENSG00000177350	0.32472341	0.00156183	0.38337017	0.96535514	NA	RPL13AP3
64925	ENSG00000177352	540.457806	-0.18294578	0.12299966	0.11528417	0.2768718	CCDC71
118461	ENSG00000177354	2.42144075	0.08769662	0.38783799	0.34084348	NA	C10orf71

NA	ENSG00000177359	19.2801758	0.00442035	0.34196163	0.97971498	0.9932825	
221091	ENSG00000177363	6.57073337	0.26922097	0.48077034	0.09025504	0.23114238	LRRN4CL
124871	ENSG00000177369	0	NA	NA	NA	NA	
29928	ENSG00000177370	334.040669	0.0986485	0.12459998	0.40297845	0.63099498	TIMM22
3090	ENSG00000177374	150.393755	-1.03850765	0.20957356	8.39E-08	1.12E-06	HIC1
8541	ENSG00000177380	672.495239	-0.28818886	0.10315209	0.00361406	0.01773946	PPFIA3
64110	ENSG00000177383	1859.77247	-0.10020556	0.06654132	0.12586724	0.29503851	MAGEF1
89766	ENSG00000177398	0.14931947	-0.01844332	0.38480212	0.84537521	NA	UMODL1
NA	ENSG00000177400	0	NA	NA	NA	NA	OR7E8P
100049716	ENSG00000177406	0.32256794	-0.02325484	0.38531879	0.66956249	NA	NINJ2-AS1
219285	ENSG00000177409	2.84477125	0.19288227	0.45200142	0.02371077	0.08352659	SAMD9L
441951	ENSG00000177410	1420.06795	-0.02897811	0.08478464	0.72508475	0.86844186	ZFAS1
148581	ENSG00000177414	0	NA	NA	NA	NA	UBE2U
NA	ENSG00000177418	0.15069912	-0.01848503	0.38480575	0.84537521	NA	
5074	ENSG00000177425	0.173733	0.01914545	0.38484597	0.78225479	NA	PAWR
7050	ENSG00000177426	386.604549	0.6851492	0.14538417	5.68E-07	6.72E-06	TGIF1
125170	ENSG00000177427	228.515918	-0.02854077	0.15048519	0.83566081	0.92509265	MIEF2
266812	ENSG00000177432	398.766977	-0.09610869	0.11928434	0.39560405	0.62505251	NAP1L5
NA	ENSG00000177447	0	NA	NA	NA	NA	CBX3P1
NA	ENSG00000177452	0	NA	NA	NA	NA	
167359	ENSG00000177453	2.48472207	-0.08395096	0.39013164	0.23334115	NA	NIM1K
930	ENSG00000177455	0.49179166	0.02819073	0.38571158	0.57316017	NA	CD19
203111	ENSG00000177459	9.64689267	3.75194209	1.11230822	7.85E-05	0.0006137	ERICH5
343172	ENSG00000177462	0	NA	NA	NA	NA	OR2T8
7182	ENSG00000177463	3484.13496	-0.07548395	0.06512178	0.23695126	0.45390682	NR2C2
2828	ENSG00000177464	0.14931947	-0.01844332	0.38480212	0.84537521	NA	GPR4
122970	ENSG00000177465	28.8827229	-0.25996358	0.33762856	0.22236513	0.43535977	ACOT4
167826	ENSG00000177468	0	NA	NA	NA	NA	OLIG3
284119	ENSG00000177469	25.2812529	2.97220508	0.58676036	2.93E-08	4.15E-07	CAVIN1
81469	ENSG00000177476	0	NA	NA	NA	NA	OR2G3
10425	ENSG00000177479	2388.86662	0.03018109	0.06493227	0.63697288	0.81722218	ARIH2
375316	ENSG00000177483	15.9115939	0.35555615	0.45344655	0.1122794	0.27126441	RBM44
10009	ENSG00000177485	700.086381	-0.13901363	0.09689572	0.1374416	0.31307018	ZBTB33
81470	ENSG00000177489	0	NA	NA	NA	NA	OR2G2
79413	ENSG00000177494	0	NA	NA	NA	NA	ZBED2
51480	ENSG00000177504	0	NA	NA	NA	NA	VCX2
79191	ENSG00000177508	506.61005	-0.36678414	0.11649859	0.00091788	0.0055034	IRX3
51046	ENSG00000177511	1647.09059	-2.40737672	0.09251207	1.77E-150	1.59E-147	ST8SIA3

56475	ENSG00000177519	89.5924147	1.9746695	0.26784198	1.19E-14	3.14E-13	RPRM
127623	ENSG00000177535	0	NA	NA	NA	NA	OR2B11
79751	ENSG00000177542	1318.55728	0.05279339	0.08812255	0.5373679	0.74759591	SLC25A22
79874	ENSG00000177548	286.708156	0.09431805	0.13924935	0.46688128	0.68982161	RABEP2
4808	ENSG00000177551	5.86674893	0.15501412	0.40341508	0.23403282	0.45093611	NHLH2
102724659	ENSG00000177553	0	NA	NA	NA	NA	C1orf167-AS1
475	ENSG00000177556	273.852098	0.32643878	0.14731719	0.01628658	0.0616652	ATOX1
148109	ENSG00000177558	0	NA	NA	NA	NA	FAM187B
79718	ENSG00000177565	2224.73087	0.24848625	0.0736161	0.00057689	0.00365668	TBL1XR1
401474	ENSG00000177570	2.7509509	-0.03777846	0.37858287	0.62733486	0.81091192	SAMD12
9332	ENSG00000177575	0	NA	NA	NA	NA	CD163
497661	ENSG00000177576	543.799057	0.29142053	0.29772977	0.17068981	0.36265644	C18orf32
NA	ENSG00000177586	0	NA	NA	NA	NA	OR7E149P
NA	ENSG00000177590	0	NA	NA	NA	NA	GIMAP3P
55367	ENSG00000177595	971.183866	0.46990927	0.0935261	2.20E-07	2.77E-06	PIDD1
100288208	ENSG00000177596	0.173124	-0.01911262	0.38486262	0.84537521	NA	
126069	ENSG00000177599	88.5315782	0.05166081	0.22240693	0.77738394	0.8979563	ZNF491
6181	ENSG00000177600	15990.9288	0.07325675	0.05488973	0.17701627	0.37183726	RPLP2
83903	ENSG00000177602	246.752081	-0.45152953	0.16845919	0.00306225	0.01540108	HASPIN
3725	ENSG00000177606	3279.88248	1.38357637	0.07450324	5.31E-78	1.04E-75	JUN
23283	ENSG00000177613	1217.33717	0.04318291	0.08450639	0.60001934	0.79083131	CSTF2T
79605	ENSG00000177614	493.514419	-2.76185956	0.14544326	9.69E-82	2.02E-79	PGBD5
121273	ENSG00000177627	0	NA	NA	NA	NA	C12orf54
2629	ENSG00000177628	817.570956	-0.24269225	0.10617111	0.01702719	0.06404159	GBA
255082	ENSG00000177640	58.9788998	0.54260438	0.32951354	0.02423791	0.08501712	CASC2
28976	ENSG00000177646	947.09921	0.09538828	0.09638521	0.30672934	0.53423845	ACAD9
23765	ENSG00000177663	8.02665796	-0.00969145	0.36754441	0.92180792	0.96719389	IL17RA
57104	ENSG00000177666	915.478539	0.159695	0.09828273	0.09257981	0.23530405	PNPLA2
619373	ENSG00000177669	5.6979679	0.00199759	0.35614405	0.99017707	0.99673744	MBOAT4
165100	ENSG00000177673	0	NA	NA	NA	NA	TEX44
57085	ENSG00000177674	289.802193	0.29459467	0.14030534	0.02375331	0.08363188	AGTRAP
283316	ENSG00000177675	66.8901971	-1.62773687	0.32670054	4.01E-08	5.58E-07	CD163L1
222183	ENSG00000177679	1867.87004	-1.28886019	0.07742423	3.11E-63	4.37E-61	SRRM3
168451	ENSG00000177683	788.042842	-0.18287504	0.10379918	0.06641621	0.18449981	THAP5
245928	ENSG00000177684	0	NA	NA	NA	NA	DEFB114
283229	ENSG00000177685	56.8845131	0.07179637	0.24336508	0.70838359	0.8597973	CRACR2B
387082	ENSG00000177688	1.80509735	0.0043994	0.37717304	0.9549297	NA	SUMO4
139422	ENSG00000177689	0	NA	NA	NA	NA	MAGEB10

54943	ENSG00000177692	26.1388594	0.13141477	0.31455766	0.501362	0.71841948	DNAJC28
26682	ENSG00000177693	0	NA	NA	NA	NA	OR4F4
254827	ENSG00000177694	9.15259905	0.04900351	0.35798506	0.72122151	0.86691933	NAALADL2
977	ENSG00000177697	2117.06785	0.15598968	0.07123685	0.02579022	0.08920466	CD151
100129540	ENSG00000177699	0.9574744	0.00409411	0.3809187	0.94080245	NA	
5441	ENSG00000177700	674.707739	0.05837515	0.09406311	0.52273823	0.73592404	POLR2L
56975	ENSG00000177706	1.06813846	0.07576372	0.39404622	0.14540818	NA	FAM20C
25945	ENSG00000177707	251.834745	1.68023228	0.20103718	4.63E-18	1.55E-16	NECTIN3
83650	ENSG00000177710	3.35071816	0.07061938	0.37555213	0.53199747	0.74324553	SLC35G5
389289	ENSG00000177721	4.93245147	0.28499973	0.51628438	0.04833253	0.14507071	ANXA2R
NA	ENSG00000177725	3.03394831	-0.22087344	0.47324085	0.02207339	0.07885057	
9772	ENSG00000177728	2875.79175	0.14561814	0.10154569	0.13634278	0.31138953	TMEM94
2314	ENSG00000177731	1106.95657	0.23729946	0.10373725	0.01730595	0.06488097	FLII
6666	ENSG00000177732	2918.07477	0.02061789	0.05965568	0.72701665	0.86953012	SOX12
10949	ENSG00000177733	8256.38798	0.00072717	0.05122317	0.9892657	0.99604194	HNRNPA0
NA	ENSG00000177736	0.16735865	0.01897177	0.38482933	0.78225479	NA	FXNP2
648987	ENSG00000177738	19.8758208	-0.07767644	0.32611901	0.66404877	0.83431586	
285525	ENSG00000177752	2.51737687	-0.05619384	0.37866156	0.54684104	NA	YIPF7
400728	ENSG00000177757	15.1733586	-0.34710794	0.46055711	0.1126542	0.27179207	FAM87B
NA	ENSG00000177770	0	NA	NA	NA	NA	CDKN2AIPNLP1
NA	ENSG00000177776	0	NA	NA	NA	NA	RPS2P2
105373159	ENSG00000177788	421.933511	-0.26861197	0.12257823	0.02033703	0.07384457	
58529	ENSG00000177791	8.93885014	0.00854525	0.34491854	0.95739484	0.98264776	MYOZ1
NA	ENSG00000177800	1.02477112	0.00735648	0.38089199	0.89488303	NA	TMEM78
NA	ENSG00000177803	0	NA	NA	NA	NA	YWHAQP4
3766	ENSG00000177807	5.39696165	0.10409247	0.38911836	0.30224945	0.52973755	KCNJ10
NA	ENSG00000177822	136.106673	-0.06075003	0.19094874	0.71267991	0.86169546	TENM3-AS1
66005	ENSG00000177830	1379.22897	0.09536966	0.07491265	0.19426405	0.39721723	CHID1
56127	ENSG00000177839	259.040779	0.50964086	0.18750948	0.00224722	0.01183251	PCDHB9
253639	ENSG00000177842	290.129056	-0.31909032	0.16338885	0.03077338	0.10232007	ZNF620
9849	ENSG00000177853	1064.73753	-0.00351107	0.09737215	0.96931908	0.98853525	ZNF518A
8269	ENSG00000177854	108.14198	-0.05425207	0.23073934	0.76765436	0.89306101	TMEM187
NA	ENSG00000177855	0.55699435	0.01254336	0.38329678	0.74045158	NA	CACYBPP2
374969	ENSG00000177868	273.242375	-0.18074631	0.1428207	0.17144071	0.36389846	SVBP
285267	ENSG00000177873	147.980965	-0.34008698	0.21646074	0.06140846	0.17449782	ZNF619
387856	ENSG00000177875	96.2873142	0.19647711	0.21182467	0.26967216	0.4928325	CCDC184
1176	ENSG00000177879	588.543771	0.0673004	0.09848877	0.48014094	0.70142083	AP3S1
2885	ENSG00000177885	3317.36699	-0.12350747	0.06503281	0.05343676	0.15657737	GRB2

360023	ENSG00000177888	624.041524	0.01325716	0.10650255	0.89781383	0.95520304	ZBTB41
7334	ENSG00000177889	2684.5713	-0.22535936	0.0735021	0.00175088	0.00955901	UBE2N
645961	ENSG00000177910	0	NA	NA	NA	NA	SPATA31C2
151188	ENSG00000177917	639.229563	-0.09811164	0.1005297	0.3111162	0.53874253	ARL6IP6
30832	ENSG00000177932	752.778999	-0.36012456	0.10094283	0.00020918	0.00148165	ZNF354C
93661	ENSG00000177938	0	NA	NA	NA	NA	CAPZA3
158056	ENSG00000177943	229.886693	0.18596131	0.32951096	0.35627344	0.58627757	MAMDC4
92806	ENSG00000177946	287.517387	0.01471538	0.14484049	0.91390919	0.96354333	CENPBD1
113746	ENSG00000177947	0	NA	NA	NA	NA	ODF3
51272	ENSG00000177951	839.026956	-0.13448645	0.09672617	0.15005936	0.33324129	BET1L
6232	ENSG00000177954	15199.7056	0.01375877	0.05332236	0.79527716	0.90586169	RPS27
60626	ENSG00000177963	2070.53393	-0.05806299	0.07282927	0.41642195	0.64306408	RIC8A
55272	ENSG00000177971	1115.65763	-0.13272001	0.07985427	0.09059952	0.23187492	IMP3
140461	ENSG00000177981	397.96259	-0.10563637	0.13165516	0.39180109	0.62114503	ASB8
389812	ENSG00000177984	21.976351	0.22715623	0.36405008	0.26200282	0.48394839	LCN15
440836	ENSG00000177989	147.67754	1.3535063	0.25022898	5.45E-09	8.49E-08	ODF3B
283417	ENSG00000177990	369.737752	-0.5533726	0.14598421	4.81E-05	0.00039523	DPY19L2
286234	ENSG00000177992	0.56767373	0.02627943	0.38574813	0.49686938	NA	SPATA31E1
100874123	ENSG00000177993	0	NA	NA	NA	NA	ZNRF3-AS1
129852	ENSG00000177994	1.2900775	0.05530874	0.38725416	0.29245582	NA	C2orf73
285601	ENSG00000178015	25.0505794	0.10081365	0.31834132	0.59502711	0.78790447	GPR150
388951	ENSG00000178021	0.173733	0.01914545	0.38484597	0.78225479	NA	TSPYL6
388886	ENSG00000178026	324.155259	0.10722901	0.15064848	0.44088439	0.66584047	LRRC75B
55929	ENSG00000178028	696.846485	0.04509587	0.1189142	0.69001327	0.84966405	DMAP1
92949	ENSG00000178031	4.46867511	-0.00495475	0.37143599	0.95505417	0.98157246	ADAMTSL1
254228	ENSG00000178033	0.52980934	0.01391988	0.3829495	0.73845638	NA	CALHM5
3615	ENSG00000178035	3804.00475	-0.20198138	0.05698296	0.00033247	0.00224436	IMPDH2
259173	ENSG00000178038	33.6413268	-0.45647764	0.38733692	0.06302228	0.1779115	ALS2CL
4291	ENSG00000178053	249.43568	0.18466391	0.15407508	0.19042906	0.39167661	MLF1
339906	ENSG00000178055	0.99047471	-0.01444432	0.38065914	0.80629562	NA	PRSS42P
25915	ENSG00000178057	861.65808	0.28310332	0.09482742	0.00201896	0.01078516	NDUFAF3
205327	ENSG00000178074	332.571802	-0.06814183	0.12461085	0.56191381	0.76508177	C2orf69
54762	ENSG00000178075	100.192459	0.22776724	0.28721169	0.26499409	0.48718221	GRAMD1C
55620	ENSG00000178078	32.9163827	0.26267142	0.32221209	0.22067076	0.43304726	STAP2
89837	ENSG00000178081	37.0258738	0.18161583	0.30639753	0.37289157	0.60181964	ULK4P3
NA	ENSG00000178082	0.15069912	-0.01848503	0.38480575	0.84537521	NA	TWF1P1
170572	ENSG00000178084	0	NA	NA	NA	NA	HTR3C
83983	ENSG00000178093	46.3112331	0.22030681	0.28769334	0.28408508	0.50919216	TSSK6

51027	ENSG00000178096	225.38797	-0.07679046	0.15740327	0.59182154	0.78640007	BOLA1
9659	ENSG00000178104	606.560892	-0.51431019	0.14291317	0.00011362	0.00085193	PDE4DIP
1662	ENSG00000178105	569.771021	-0.00170071	0.10622644	0.98518542	0.99475332	DDX10
NA	ENSG00000178107	0	NA	NA	NA	NA	
727909	ENSG00000178115	17.811858	-0.15017575	0.35386073	0.40539643	0.63375238	GOLGA8Q
728047	ENSG00000178115	17.811858	-0.15017575	0.35386073	0.40539643	0.63375238	GOLGA8Q
286187	ENSG00000178125	0.61572015	0.03581773	0.38655464	0.41248855	NA	PPP1R42
4729	ENSG00000178127	849.445464	0.04230764	0.09453417	0.6449546	0.82283284	NDUFV2
NA	ENSG00000178130	0	NA	NA	NA	NA	FAM27E5
NA	ENSG00000178146	0	NA	NA	NA	NA	GK4P
55152	ENSG00000178149	583.926125	0.18695161	0.11014704	0.07616651	0.20371308	DALRD3
163071	ENSG00000178150	84.9688136	-0.36995689	0.29013503	0.08517278	0.2214571	ZNF114
NA	ENSG00000178162	0.30001968	-0.02294462	0.38527539	0.68644637	NA	FAR2P2
85460	ENSG00000178163	345.653942	0.09294919	0.1431088	0.48436244	0.70452718	ZNF518B
205147	ENSG00000178171	301.080897	-2.99041783	0.19567966	1.97E-54	2.22E-52	AMER3
404203	ENSG00000178172	0	NA	NA	NA	NA	SPINK6
167465	ENSG00000178175	0	NA	NA	NA	NA	ZNF366
254251	ENSG00000178177	708.67627	0.07847516	0.11658765	0.48039207	0.70158112	LCORL
84552	ENSG00000178184	362.464798	0.43956712	0.13072346	0.00035126	0.00235839	PARD6G
285676	ENSG00000178187	83.7212085	0.27249372	0.23716384	0.1547672	0.34030142	ZNF454
25970	ENSG00000178188	1906.05307	0.1775946	0.07644537	0.0176867	0.06595943	SH2B1
340152	ENSG00000178199	3.00970201	0.06521518	0.38488569	0.31772081	0.54552842	ZC3H12D
57191	ENSG00000178201	23.8000168	0.07637734	0.30463056	0.68971921	0.84943947	VN1R1
143888	ENSG00000178202	882.547625	0.1879248	0.12035511	0.09952916	0.24838472	POGLUT3
5339	ENSG00000178209	4494.05147	-0.15787711	0.05554513	0.00404034	0.01947059	PLEC
387694	ENSG00000178217	1.15754537	0.01898254	0.38117088	0.74436376	NA	SH2D4B
285498	ENSG00000178222	67.6988493	0.31387269	0.30653173	0.14653471	0.32761423	RNF212
146547	ENSG00000178226	1.83570253	0.02716197	0.37757679	0.73354996	NA	PRSS36
125919	ENSG00000178229	135.850066	0.15854057	0.19031618	0.3389905	0.56924008	ZNF543
441151	ENSG00000178233	654.385093	-0.73472218	0.10080006	6.80E-14	1.70E-12	TMEM151B
63917	ENSG00000178234	1220.90531	0.08394954	0.07601121	0.26143312	0.4834405	GALNT11
114798	ENSG00000178235	0	NA	NA	NA	NA	SLITRK1
NA	ENSG00000178243	0	NA	NA	NA	NA	C9orf62
388882	ENSG00000178248	0	NA	NA	NA	NA	FAM230I
11180	ENSG00000178252	5923.21604	-0.04359711	0.05393973	0.40834571	0.63646625	WDR6
58531	ENSG00000178257	0	NA	NA	NA	NA	PRM3
7142	ENSG00000178279	0	NA	NA	NA	NA	TNP2
653423	ENSG00000178287	0	NA	NA	NA	NA	SPAG11A

348654	ENSG00000178295	1953.9692	-0.27458683	0.07196624	9.93E-05	0.00075606	GEN1
360200	ENSG00000178297	3.62242559	0.011204	0.37398944	0.89921944	0.95557174	TMPRSS9
282679	ENSG00000178301	31.1660451	0.00425374	0.28225597	0.98401284	0.99453916	AQP11
8834	ENSG00000178307	406.091682	-0.15586596	0.1186422	0.16592832	0.35616336	TMEM11
117608	ENSG00000178338	267.58527	0.1787644	0.13986398	0.16922679	0.36066812	ZNF354B
26251	ENSG00000178342	3.62822109	0.09387814	0.38708198	0.35469227	0.58520537	KCNG2
152573	ENSG00000178343	37.0273374	2.65777414	0.45646857	3.98E-10	7.20E-09	SHISA3
120775	ENSG00000178358	0	NA	NA	NA	NA	OR2D3
810	ENSG00000178363	0	NA	NA	NA	NA	CALML3
51806	ENSG00000178372	0	NA	NA	NA	NA	CALML5
90637	ENSG00000178381	153.021007	0.07248617	0.190775	0.66292665	0.83370086	ZFAND2A
389072	ENSG00000178385	557.478782	0.02717492	0.1227291	0.8161483	0.91637704	PLEKHM3
7766	ENSG00000178386	157.686027	0.16873993	0.20479371	0.33226364	0.56141294	ZNF223
3350	ENSG00000178394	0	NA	NA	NA	NA	HTR1A
164127	ENSG00000178395	0	NA	NA	NA	NA	CCDC185
84792	ENSG00000178397	462.718483	-0.24229023	0.14151921	0.06467101	0.18119939	FAM220A
79962	ENSG00000178401	25.8097226	2.51503656	0.53093399	1.33E-07	1.73E-06	DNAJC22
63973	ENSG00000178403	4832.88079	0.72273079	0.0590019	3.87E-35	2.63E-33	NEUROG2
100653515	ENSG00000178404	3.9586743	-0.01482645	0.36579177	0.89575342	0.95469109	CEP295NL
57673	ENSG00000178409	410.011449	-0.16271595	0.1187466	0.14827001	0.33052552	BEND3
284241	ENSG00000178412	0.79971516	-0.00577684	0.38215497	0.90182286	NA	
221294	ENSG00000178425	310.678291	0.34931916	0.14391017	0.00870668	0.03700527	NT5DC1
NA	ENSG00000178429	8.13463755	-0.03591301	0.34816673	0.81096556	0.91443142	RPS3AP5
NA	ENSG00000178440	347.72363	0.28963725	0.13734364	0.02364979	0.08332657	TIMM23B-AGAP6
2731	ENSG00000178445	921.526129	-0.04904717	0.14212078	0.70897894	0.8603145	GLDC
84987	ENSG00000178449	217.891213	0.07396535	0.17312549	0.63330126	0.81478111	COX14
246705	ENSG00000178457	0	NA	NA	NA	NA	LINC00314
NA	ENSG00000178458	1.02058948	-0.02089271	0.38105229	0.72518351	NA	H3P16
157777	ENSG00000178460	11.998771	-0.14410901	0.36571766	0.39632473	0.62564311	MCMDC2
79861	ENSG00000178462	0	NA	NA	NA	NA	TUBAL3
NA	ENSG00000178464	1.10850786	-0.02630484	0.38212841	0.64133864	NA	RPL10P16
54681	ENSG00000178467	2.02986455	0.00455933	0.37747651	0.94929687	NA	P4HTM
114131	ENSG00000178473	0	NA	NA	NA	NA	UCN3
196403	ENSG00000178498	1483.80602	0.15981212	0.07789947	0.03603043	0.11591433	DTX3
55175	ENSG00000178502	353.436117	-0.3780579	0.14023487	0.00374567	0.01825637	KLHL11
NA	ENSG00000178503	0	NA	NA	NA	NA	NECAP1P1
258	ENSG00000178522	0	NA	NA	NA	NA	AMBN
404217	ENSG00000178531	1741.0163	0.27929322	0.07595966	0.0001719	0.00124018	CTXN1

788	ENSG00000178537	327.552836	-0.08887706	0.12872159	0.46275813	0.6855676	SLC25A20
767	ENSG00000178538	14.4124011	0.19458446	0.40923703	0.19534759	0.39889833	CA8
NA	ENSG00000178556	0	NA	NA	NA	NA	CKS1BP6
940	ENSG00000178562	0	NA	NA	NA	NA	CD28
9852	ENSG00000178567	1219.58778	-0.23766852	0.09326953	0.00844692	0.03608673	EPM2AIP1
2066	ENSG00000178568	19.0197734	0.22346855	0.37948092	0.25415176	0.47466543	ERBB4
4094	ENSG00000178573	5.80681331	-0.13203792	0.394185	0.2709755	0.49431353	MAF
56998	ENSG00000178585	783.613576	0.23271545	0.09703105	0.01299583	0.05142908	CTNNBIP1
150681	ENSG00000178586	0	NA	NA	NA	NA	OR6B3
245938	ENSG00000178591	0	NA	NA	NA	NA	DEFB125
NA	ENSG00000178596	0	NA	NA	NA	NA	GAPDHP29
768239	ENSG00000178597	0	NA	NA	NA	NA	PSAPL1
150677	ENSG00000178602	0	NA	NA	NA	NA	OTOS
8225	ENSG00000178605	907.194906	-0.03227419	0.09229158	0.71253599	0.86169546	GTPBP6
2081	ENSG00000178607	438.586292	0.16004513	0.14648791	0.23598751	0.45280389	ERN1
2859	ENSG00000178623	20.8811978	0.27035331	0.44415168	0.14250728	0.32128606	GPR35
NA	ENSG00000178631	11.1330842	0.09712437	0.3502562	0.56049104	0.7641659	ACTG1P1
NA	ENSG00000178636	0	NA	NA	NA	NA	
282966	ENSG00000178645	2.77882287	0.04409403	0.37491078	0.6554463	0.8285476	C10orf53
NA	ENSG00000178654	0	NA	NA	NA	NA	PPIAP33
NA	ENSG00000178660	0.81287042	0.0158478	0.38192767	0.7585084	NA	ARMC10P1
80034	ENSG00000178662	685.688279	-0.47750757	0.13168209	0.00011522	0.00086261	CSRNP3
349075	ENSG00000178665	303.673093	-0.39662059	0.17101086	0.0098593	0.0409823	ZNF713
84875	ENSG00000178685	16.4922579	-0.13769662	0.36261043	0.41342912	0.64066701	PARP10
284254	ENSG00000178690	0	NA	NA	NA	NA	DYNAP
23512	ENSG00000178691	1458.52906	-0.31678833	0.08142427	6.42E-05	0.00051305	SUZ12
63899	ENSG00000178694	299.504689	-0.25978412	0.17176334	0.09011034	0.23086121	NSUN3
115207	ENSG00000178695	727.36454	-1.38422368	0.10117289	1.25E-43	1.07E-41	KCTD12
200895	ENSG00000178700	261.412173	-0.00488548	0.14951975	0.97061608	0.9889696	DHFR2
NA	ENSG00000178715	0.99877021	0.00589672	0.37927292	0.92785106	NA	
54913	ENSG00000178718	750.968163	-1.12892707	0.11091136	2.79E-25	1.34E-23	RPP25
2907	ENSG00000178719	1834.31787	-0.21139755	0.07248744	0.00292716	0.014815	GRINA
285668	ENSG00000178722	0.50544106	0.03058872	0.38599731	0.48636788	NA	C5orf64
NA	ENSG00000178723	0	NA	NA	NA	NA	GLULP4
7056	ENSG00000178726	0	NA	NA	NA	NA	THBD
2814	ENSG00000178732	1.67230964	-0.02201813	0.37758524	0.77598962	NA	GP5
729420	ENSG00000178734	0	NA	NA	NA	NA	LMO7DN
9377	ENSG00000178741	1205.93733	-0.08974061	0.0774458	0.23620434	0.4530211	COX5A

415117	ENSG00000178750	0.64944402	-0.03790105	0.3868082	0.40861375	NA	STX19
151176	ENSG00000178752	159.363113	0.60521133	0.19076745	0.00040715	0.00268901	ERFE
57184	ENSG00000178761	1321.46143	0.36058016	0.08534605	1.41E-05	0.00013058	FAM219B
NA	ENSG00000178762	0	NA	NA	NA	NA	H2BC2P
22882	ENSG00000178764	95.709881	0.2493036	0.22397487	0.1769986	0.37183726	ZHX2
1370	ENSG00000178772	0	NA	NA	NA	NA	CPN2
27132	ENSG00000178773	0.14931947	-0.01844332	0.38480212	0.84537521	NA	CPNE7
389336	ENSG00000178776	0	NA	NA	NA	NA	C5orf46
124599	ENSG00000178789	0	NA	NA	NA	NA	CD300LB
220032	ENSG00000178795	0.45209736	-0.02505403	0.38558831	0.57865948	NA	GDPD4
284485	ENSG00000178796	17.6792732	-1.87449337	0.74150695	0.00043536	0.00285919	RIIAD1
4351	ENSG00000178802	614.142434	0.07230608	0.12742272	0.54718425	0.75424436	MPI
646023	ENSG00000178803	59.4252054	1.22287889	0.33795407	2.57E-05	0.00022491	ADORA2A-AS1
132243	ENSG00000178804	0	NA	NA	NA	NA	H1-8
375593	ENSG00000178809	77.6077269	1.35220552	0.33928722	4.93E-06	4.97E-05	TRIM73
26873	ENSG00000178814	4.16176063	-0.05688562	0.37759508	0.55313247	0.75940406	OPLAH
339456	ENSG00000178821	18.9688713	0.15265624	0.36312525	0.3803023	0.60931904	TMEM52
135932	ENSG00000178826	13.8066179	0.19653608	0.3929892	0.25891419	0.48036523	TMEM139
54546	ENSG00000178828	0	NA	NA	NA	NA	RNF186
100286922	ENSG00000178836	0	NA	NA	NA	NA	
124989	ENSG00000178852	38.0833892	-0.45748603	0.42355368	0.06478582	0.181378	EFCAB13
9242	ENSG00000178860	0	NA	NA	NA	NA	MSC
81575	ENSG00000178878	342.544949	0.87680656	0.16340565	1.25E-08	1.88E-07	APOLD1
144347	ENSG00000178882	50.2192123	0.29356156	0.33953213	0.17666644	0.37141698	RFLNA
100533183	ENSG00000178882	50.2192123	0.29356156	0.33953213	0.17666644	0.37141698	RFLNA
54512	ENSG00000178896	804.376664	-0.11354536	0.1032947	0.2522505	0.47222371	EXOSC4
147991	ENSG00000178904	1646.86979	-0.20246679	0.08477176	0.0141025	0.05489379	DPY19L3
6879	ENSG00000178913	2144.98048	-0.14862783	0.06490948	0.02003335	0.07300914	TAF7
285346	ENSG00000178917	206.794026	-0.07330227	0.16701682	0.62505544	0.80894324	ZNF852
2304	ENSG00000178919	0	NA	NA	NA	NA	FOXE1
5198	ENSG00000178921	1609.91528	-0.21474545	0.08388559	0.00870921	0.03700808	PFAS
81888	ENSG00000178922	269.014884	0.20104167	0.13897299	0.1199985	0.28519048	HYI
79415	ENSG00000178927	1615.49228	0.06776012	0.0837221	0.4075869	0.63598301	CYBC1
284355	ENSG00000178928	0	NA	NA	NA	NA	TPRX1
3963	ENSG00000178934	0	NA	NA	NA	NA	LGALS7B
653499	ENSG00000178934	0	NA	NA	NA	NA	LGALS7B
79818	ENSG00000178935	122.989779	-0.09772392	0.23637468	0.59945525	0.79057618	ZNF552
399668	ENSG00000178947	51.1277134	1.92212867	0.36382455	8.43E-09	1.28E-07	SMIM10L2A

2580	ENSG00000178950	1757.38279	0.25039367	0.0854147	0.00258406	0.01335035	GAK
51341	ENSG00000178951	615.990795	-0.14552209	0.1093421	0.16254746	0.35133911	ZBTB7A
7284	ENSG00000178952	5608.60468	0.00307405	0.0584192	0.95829477	0.98281563	TUFM
127254	ENSG00000178965	82.4621485	-4.14240759	0.45025211	1.72E-21	6.88E-20	ERICH3
80010	ENSG00000178966	537.731556	-0.50446282	0.11135197	2.27E-06	2.42E-05	RMI1
80169	ENSG00000178971	598.205937	0.14460162	0.10714656	0.15930126	0.34688536	CTC1
55030	ENSG00000178974	531.715302	0.00412126	0.10387165	0.9682412	0.98809325	FBXO34
284029	ENSG00000178977	11.5001909	-0.19950976	0.40754473	0.22280644	0.43585878	LINC00324
6415	ENSG00000178980	1171.34182	-0.06602582	0.08825161	0.44099805	0.66591075	SELENOW
27335	ENSG00000178982	2078.607	0.03629485	0.07173068	0.60718196	0.79658855	EIF3K
114932	ENSG00000178988	1465.90644	-0.34644477	0.07977445	8.61E-06	8.28E-05	MRFAP1L1
112574	ENSG00000178996	315.368381	-0.25398243	0.14160814	0.05277947	0.15510956	SNX18
161829	ENSG00000178997	2.35970785	-0.015353	0.37955794	0.82841235	NA	EXD1
9212	ENSG00000178999	1062.47032	0.19504698	0.08576775	0.01950908	0.07147926	AURKB
80834	ENSG00000179002	0	NA	NA	NA	NA	TAS1R2
317761	ENSG00000179008	37.251711	-0.17361667	0.32207078	0.38267328	0.61198112	C14orf39
93621	ENSG00000179010	5287.97478	-0.14548128	0.05495673	0.00743109	0.03248133	MRFAP1
285237	ENSG00000179021	301.966148	0.01501105	0.13120161	0.90403942	0.95807607	C3orf38
127707	ENSG00000179023	0.74951642	-0.04342943	0.38756505	0.34479115	NA	KLHDC7A
84314	ENSG00000179029	167.976762	0.14162906	0.17315358	0.36049997	0.5904664	TMEM107
NA	ENSG00000179031	0.50207596	0.02562368	0.38565771	0.52549886	NA	NSA2P3
NA	ENSG00000179038	0.51331499	0.01065258	0.38327774	0.77572498	NA	
23212	ENSG00000179041	798.013271	-0.04973847	0.10154533	0.61005244	0.79837997	RRS1
283849	ENSG00000179044	13.074799	-0.30330918	0.46177226	0.12202431	0.28884077	EXOC3L1
205860	ENSG00000179046	0	NA	NA	NA	NA	TRIML2
55920	ENSG00000179051	7879.45498	-0.13436141	0.05221272	0.00933564	0.0392081	RCC2
286365	ENSG00000179055	0	NA	NA	NA	NA	OR13D1
283284	ENSG00000179057	13.9376939	0.02319388	0.35867137	0.85985286	0.93754016	IGSF22
375759	ENSG00000179058	11.2339888	0.23797935	0.42017416	0.18948251	0.39045835	C9orf50
132625	ENSG00000179059	0	NA	NA	NA	NA	ZFP42
NA	ENSG00000179066	2.34946023	0.12116545	0.40584433	0.1273233	NA	
220388	ENSG00000179071	14.4512556	-0.31428922	0.43420106	0.13900572	0.31576294	CCDC89
9288	ENSG00000179073	0	NA	NA	NA	NA	TAAR3P
NA	ENSG00000179082	2.70042498	0.06189462	0.37958819	0.51518812	0.72962613	C9orf106
286499	ENSG00000179083	1.27253463	0.00909548	0.38021527	0.8813383	NA	FAM133A
54344	ENSG00000179085	435.743132	0.11815744	0.11244445	0.27179039	0.49516265	DPM3
374470	ENSG00000179088	0	NA	NA	NA	NA	C12orf42
1537	ENSG00000179091	1769.73695	0.07003656	0.07593157	0.34722372	0.57794986	CYC1

5187	ENSG00000179094	564.422331	0.22155136	0.16870503	0.14316518	0.32228142	PER1
3355	ENSG00000179097	0	NA	NA	NA	NA	HTR1F
NA	ENSG00000179101	0.15069912	-0.01848503	0.38480575	0.84537521	NA	H3P47
160335	ENSG00000179104	1281.59639	0.01526997	0.07401726	0.83412506	0.92432089	TMTC2
84667	ENSG00000179111	56.4329878	2.05893123	0.36261721	8.22E-10	1.43E-08	HES7
2193	ENSG00000179115	3345.21397	0.06690827	0.07376539	0.35576255	0.58599659	FARSA
144108	ENSG00000179119	656.23932	0.22431801	0.10009713	0.02000195	0.07294827	SPTY2D1
NA	ENSG00000179131	0.6511535	0.00367376	0.38250135	0.93040605	NA	
256815	ENSG00000179133	1.5351185	0.04633005	0.38477194	0.41931537	NA	C10orf67
55095	ENSG00000179134	2208.11035	0.06768027	0.07604445	0.36434047	0.59405941	SAMD4B
284034	ENSG00000179136	0	NA	NA	NA	NA	LINC00670
NA	ENSG00000179141	0.53290632	0.01988167	0.38349612	0.67961517	NA	MTUS2-AS1
1585	ENSG00000179142	0	NA	NA	NA	NA	CYP11B2
168537	ENSG00000179144	0	NA	NA	NA	NA	GIMAP7
59344	ENSG00000179148	11.254009	-0.2813108	0.45112703	0.13650941	0.31158286	ALOXE3
80153	ENSG00000179151	1410.2616	0.04560574	0.08091417	0.56302375	0.76597094	EDC3
285343	ENSG00000179152	583.456601	0.03166483	0.11523643	0.77405505	0.89604057	TCAIM
NA	ENSG00000179157	0	NA	NA	NA	NA	RPS2P28
2517	ENSG00000179163	196.522354	0.28486217	0.16846175	0.05994198	0.17111821	FUCA1
222659	ENSG00000179165	0.36242552	0.00096442	0.38336476	0.96669627	NA	PXT1
199720	ENSG00000179168	28.2343118	0.06050883	0.30467315	0.74937042	0.88205755	GGN
NA	ENSG00000179170	0	NA	NA	NA	NA	OR7E97P
343069	ENSG00000179172	0	NA	NA	NA	NA	HNRNPCL1
128218	ENSG00000179178	0	NA	NA	NA	NA	TMEM125
144348	ENSG00000179195	5997.23696	-0.15267676	0.05162702	0.00285498	0.01452751	ZNF664
284369	ENSG00000179213	0	NA	NA	NA	NA	SIGLECL1
811	ENSG00000179218	12294.0813	0.46733936	0.08772421	4.44E-08	6.14E-07	CALR
NA	ENSG00000179219	1.12402921	0.01595474	0.38002808	0.80244378	NA	LINC00311
9500	ENSG00000179222	7550.86403	-0.08741783	0.07065281	0.20763481	0.41489321	MAGED1
100506127	ENSG00000179240	726.416163	0.15913266	0.11034485	0.13155471	0.30427203	GVQW3
143458	ENSG00000179241	1526.45758	-1.36549354	0.08560313	2.42E-58	3.02E-56	LDLRAD3
1002	ENSG00000179242	9.55487673	-0.19943457	0.40467819	0.2316801	0.44822361	CDH4
100128310	ENSG00000179253	0	NA	NA	NA	NA	
440087	ENSG00000179256	1.63855702	-0.03819293	0.38062094	0.59839428	NA	SMCO3
5886	ENSG00000179262	1894.56579	0.15274661	0.07637416	0.04110732	0.12809316	RAD23A
388939	ENSG00000179270	2.89148372	-0.079196	0.38455459	0.39900513	0.62780637	PCARE
90480	ENSG00000179271	1403.45061	0.13590058	0.07207769	0.05486614	0.15989192	GADD45GIP1
NA	ENSG00000179277	68.345089	0.59025778	0.37456741	0.02292811	0.08127455	MEIS3P1

199699	ENSG00000179284	473.324187	-0.65797757	0.12596832	4.38E-08	6.06E-07	DAND5
256472	ENSG00000179292	265.050419	-1.75638498	0.16423192	8.45E-28	4.48E-26	TMEM151A
5781	ENSG00000179295	3835.19586	-0.26856873	0.0753589	0.00026815	0.00184983	PTPN11
79730	ENSG00000179299	162.373971	0.13565824	0.20450825	0.43494322	0.66028567	NSUN7
203430	ENSG00000179300	0	NA	NA	NA	NA	RTL3
727866	ENSG00000179304	340.5227	-0.18055851	0.18084205	0.25692251	0.47795055	FAM156B
23302	ENSG00000179314	2.90451731	-0.03512095	0.37367039	0.71732891	0.86394741	WSCD1
54734	ENSG00000179331	495.989912	-0.99608536	0.11393182	3.11E-19	1.12E-17	RAB39A
1198	ENSG00000179335	1394.05994	0.23118073	0.08348394	0.00448237	0.0212396	CLK3
NA	ENSG00000179342	0.66399629	0.0038693	0.3819207	0.93405804	NA	
3119	ENSG00000179344	0	NA	NA	NA	NA	HLA-DQB1
2624	ENSG00000179348	19449.3206	0.23476393	0.06334832	0.00016978	0.0012267	GATA2
10620	ENSG00000179361	792.023112	0.55658972	0.09345276	8.83E-10	1.53E-08	ARID3B
NA	ENSG00000179362	0.67183493	0.0229586	0.38322735	0.6051825	NA	HMGN2P46
203562	ENSG00000179363	3.35785905	0.12451246	0.39617323	0.2651833	0.48737717	TMEM31
23241	ENSG00000179364	2191.65402	0.21935349	0.0635224	0.00045714	0.0029864	PACS2
NA	ENSG00000179381	0	NA	NA	NA	NA	OR4K11P
255520	ENSG00000179387	1424.34005	-0.36922683	0.08017991	2.38E-06	2.53E-05	ELMOD2
1960	ENSG00000179388	12.4128668	2.16250147	0.8722672	0.00053464	0.00341837	EGR3
257044	ENSG00000179397	7.69749471	0.20514492	0.42672746	0.15761862	0.34451398	CATSPERE
2262	ENSG00000179399	21.501492	-0.01632313	0.32123629	0.92425248	0.96852	GPC5
64856	ENSG00000179403	177.651169	-0.26563356	0.18976407	0.10749002	0.26289472	VWA1
285908	ENSG00000179406	523.825486	-0.1711253	0.10704599	0.09501007	0.23991343	LINC00174
165721	ENSG00000179407	4.87236174	0.00617433	0.35949169	0.96346684	0.98545957	DNAJB8
50628	ENSG00000179409	909.030725	-0.27932431	0.10159203	0.0042341	0.02025179	GEMIN4
101060301	ENSG00000179412	0	NA	NA	NA	NA	HNRNPCL4
NA	ENSG00000179420	0.18922458	0.01954053	0.38488496	0.78225479	NA	OR6W1P
541472	ENSG00000179428	0	NA	NA	NA	NA	IL6-AS1
24147	ENSG00000179431	798.647224	-1.62689418	0.12112825	3.02E-42	2.41E-40	FJX1
NA	ENSG00000179443	0	NA	NA	NA	NA	OR13C6P
100130264	ENSG00000179447	0	NA	NA	NA	NA	SLC24A3-AS1
100287948	ENSG00000179452	0	NA	NA	NA	NA	LINC01699
54813	ENSG00000179454	297.54259	0.08740362	0.15189045	0.53330772	0.74452219	KLHL28
7681	ENSG00000179455	250.055144	0.41219287	0.15845071	0.00443623	0.02105615	MKRN3
10472	ENSG00000179456	1433.13736	1.02984975	0.07977821	5.56E-39	4.19E-37	ZBTB18
NA	ENSG00000179460	0	NA	NA	NA	NA	EEF1A1P27
135924	ENSG00000179468	0	NA	NA	NA	NA	OR9A2
122525	ENSG00000179476	17.7043895	-0.10880737	0.32490075	0.55963034	0.76413192	C14orf28

242	ENSG00000179477	14.6827427	-0.24104684	0.41117629	0.19604523	0.39974691	ALOX12B
246213	ENSG00000179520	0	NA	NA	NA	NA	SLC17A8
645212	ENSG00000179523	180.533377	0.20517275	0.16879917	0.17593999	0.37020356	EIF3J-DT
81858	ENSG00000179526	638.66546	0.2202354	0.10301036	0.02615003	0.0902133	SHARPIN
85474	ENSG00000179528	72.8635169	1.87843063	0.37583559	3.00E-08	4.24E-07	LBX2
144132	ENSG00000179532	610.059391	0.63758882	0.12463206	8.49E-08	1.13E-06	DNHD1
139065	ENSG00000179542	0	NA	NA	NA	NA	SLITRK4
3352	ENSG00000179546	0.54607335	0.03261306	0.38620451	0.45608999	NA	HTR1D
79571	ENSG00000179562	846.802083	-0.15794458	0.10471597	0.11611584	0.27839903	GCC1
132228	ENSG00000179564	0.82952557	-0.01007874	0.38156424	0.84666063	NA	LSMEM2
NA	ENSG00000179571	0	NA	NA	NA	NA	NBPF17P
NA	ENSG00000179577	0	NA	NA	NA	NA	
146310	ENSG00000179580	0.78820934	-0.02799992	0.38348471	0.55826532	NA	RNF151
4261	ENSG00000179583	47.2976088	-0.52064332	0.37757367	0.03752118	0.11968171	CIITA
161882	ENSG00000179588	594.123098	-0.48357852	0.13042441	8.30E-05	0.00064426	ZFPM1
247	ENSG00000179593	0	NA	NA	NA	NA	ALOX15B
201164	ENSG00000179598	285.199802	-0.30273737	0.14855502	0.0266795	0.09159412	PLD6
122876	ENSG00000179600	0	NA	NA	NA	NA	GPHB5
2918	ENSG00000179603	287.770769	-1.97191662	0.26492043	3.57E-15	9.93E-14	GRM8
23580	ENSG00000179604	991.078853	0.5692286	0.09885061	2.78E-09	4.49E-08	CDC42EP4
NA	ENSG00000179611	0.69605637	0.02462452	0.3833564	0.58388227	NA	DGKZP1
121129	ENSG00000179615	0	NA	NA	NA	NA	OR2AP1
341418	ENSG00000179626	0	NA	NA	NA	NA	OR6C4
100128927	ENSG00000179627	152.36081	0.23353367	0.19253967	0.16233526	0.35102057	ZBTB42
144811	ENSG00000179630	69.2353143	0.17401127	0.27862059	0.3848	0.61378186	LACC1
84232	ENSG00000179632	2065.75267	0.08411744	0.07808197	0.27144303	0.49480244	MAF1
122664	ENSG00000179636	0	NA	NA	NA	NA	TPPP2
2205	ENSG00000179639	0	NA	NA	NA	NA	FCER1A
388394	ENSG00000179673	0.90361723	0.03142195	0.38455169	0.48209261	NA	RPRML
80117	ENSG00000179674	0	NA	NA	NA	NA	ARL14
221241	ENSG00000179676	0	NA	NA	NA	NA	LINC00305
341416	ENSG00000179695	0	NA	NA	NA	NA	OR6C2
340390	ENSG00000179698	49.4527267	0.28287897	0.30957721	0.18610884	0.38570975	WDR97
126205	ENSG00000179709	0	NA	NA	NA	NA	NLRP8
91523	ENSG00000179715	0	NA	NA	NA	NA	PCED1B
729614	ENSG00000179743	195.686389	0.12449152	0.16150106	0.39646172	0.62574149	
9582	ENSG00000179750	0	NA	NA	NA	NA	APOBEC3B
342898	ENSG00000179751	0	NA	NA	NA	NA	SYCN

NA	ENSG00000179755	0	NA	NA	NA	NA	
51268	ENSG00000179761	58.244991	0.17757284	0.27623858	0.37639541	0.6053339	PIPOX
158381	ENSG00000179766	0.34034124	0.00295036	0.38339048	0.96028375	NA	ATP8B5P
2307	ENSG00000179772	0	NA	NA	NA	NA	FOXS1
220202	ENSG00000179774	5.74044398	0.1420166	0.39389395	0.29023627	0.51620656	ATOH7
1003	ENSG00000179776	0.18922458	0.01954053	0.38488496	0.78225479	NA	CDH5
116135	ENSG00000179796	1.88087679	0.13537473	0.41739912	0.03033852	NA	LRRC3B
NA	ENSG00000179799	0.16735865	0.01897177	0.38482933	0.78225479	NA	OR7E22P
144809	ENSG00000179813	0	NA	NA	NA	NA	FAM216B
117196	ENSG00000179817	0	NA	NA	NA	NA	MRGPRX4
400960	ENSG00000179818	449.892744	0.14393821	0.12953145	0.23749604	0.45473088	PCBP1-AS1
91663	ENSG00000179820	932.859474	0.07107029	0.09475616	0.43933117	0.66465842	MYADM
117195	ENSG00000179826	0	NA	NA	NA	NA	MRGPRX3
727957	ENSG00000179832	590.214703	-0.01613226	0.09918332	0.86509302	0.94028794	MROH1
9792	ENSG00000179833	545.513217	0.41252415	0.1684047	0.00662216	0.02950451	SERTAD2
644997	ENSG00000179840	0	NA	NA	NA	NA	PIK3CD-AS1
9495	ENSG00000179841	114.952985	-0.52963338	0.23556933	0.00738621	0.0323208	AKAP5
284353	ENSG00000179846	21.7445841	-1.19596755	0.56437732	0.00220057	0.01162386	NKPD1
126326	ENSG00000179855	15.4075157	0.11611457	0.34369753	0.51440759	0.72931984	GIPC3
284023	ENSG00000179859	124.764377	0.03822938	0.22052117	0.83360608	0.92422788	RNF227
163732	ENSG00000179862	31.6353214	0.28784165	0.34299984	0.18688979	0.38676207	CITED4
154664	ENSG00000179869	0	NA	NA	NA	NA	ABCA13
204801	ENSG00000179873	0	NA	NA	NA	NA	NLRP11
84948	ENSG00000179886	526.2292	-0.09827178	0.10301758	0.32081648	0.54867706	TIGD5
23042	ENSG00000179889	1409.48356	-0.29102525	0.09143997	0.00101086	0.00596003	PDXDC1
NA	ENSG00000179899	491.841297	0.06423514	0.12060421	0.57559259	0.7744446	PHC1P1
127003	ENSG00000179902	0.64103849	0.00333272	0.38196039	0.94259469	NA	C1orf194
7710	ENSG00000179909	18.3044164	-0.24276811	0.37571657	0.23281334	0.4497582	ZNF154
22864	ENSG00000179912	1639.14087	0.16962576	0.21677145	0.34797722	0.57864958	R3HDM2
10331	ENSG00000179913	3.93265368	-0.09593285	0.3845475	0.38116576	0.61011123	B3GNT3
55600	ENSG00000179914	0	NA	NA	NA	NA	ITLN1
9378	ENSG00000179915	335.179935	-2.53722638	0.23209789	2.79E-29	1.57E-27	NRXN1
22928	ENSG00000179918	704.169253	-0.34180547	0.09710692	0.0002642	0.00182871	SEPHS2
121364	ENSG00000179919	0	NA	NA	NA	NA	OR10A7
151306	ENSG00000179921	9.8742744	-0.1470023	0.37447196	0.36673989	0.59606123	GPBAR1
147808	ENSG00000179922	183.103075	0.0680656	0.15923041	0.63965902	0.81929212	ZNF784
127665	ENSG00000179930	11.9308662	0.04457722	0.33529941	0.79184917	0.90499209	ZNF648
55017	ENSG00000179933	420.218065	0.02408445	0.11985336	0.83332744	0.92415967	C14orf119

1237	ENSG00000179934	0	NA	NA	NA	NA	CCR8
NA	ENSG00000179935	3.38017786	-0.05173074	0.3728049	0.63499887	0.81627431	LINC00652
653073	ENSG00000179938	2.43438211	0.12857222	0.41291282	0.05511344	NA	GOLGA8J
79738	ENSG00000179941	558.932668	0.00644261	0.11049964	0.95276591	0.98058207	BBS10
84922	ENSG00000179943	585.51575	0.0652645	0.11743929	0.55994272	0.76415299	FIZ1
22827	ENSG00000179950	2590.73393	0.11191533	0.06388052	0.07556958	0.20252608	PUF60
284297	ENSG00000179954	25.3155096	0.10250823	0.31093769	0.59454709	0.78775306	SSC5D
79077	ENSG00000179958	763.843985	-0.44007011	0.09390789	1.30E-06	1.45E-05	DCTPP1
51333	ENSG00000179965	216.796361	-0.0714475	0.15896812	0.62056133	0.80638832	ZNF771
NA	ENSG00000179967	1.15669714	0.01660958	0.37989121	0.7970795	NA	PPP1R14BP3
NA	ENSG00000179978	651.283275	0.45418347	0.11462153	3.33E-05	0.00028437	NAIPP2
10194	ENSG00000179981	571.580633	0.28736203	0.10504279	0.00434735	0.02072355	TSHZ1
118672	ENSG00000179988	88.3200295	0.07210176	0.21894215	0.69045471	0.85000133	PSTK
441251	ENSG00000179994	0.173733	0.01914545	0.38484597	0.78225479	NA	SPDYE7P
NA	ENSG00000179997	0	NA	NA	NA	NA	RPA3P1
122809	ENSG00000180008	1220.58791	-0.13627833	0.1026029	0.16709532	0.35804851	SOCS4
284273	ENSG00000180011	763.34358	0.08297409	0.13214732	0.50330423	0.72006071	ZADH2
NA	ENSG00000180015	23.6220553	-0.19890674	0.34196536	0.32110317	0.54886593	
8387	ENSG00000180016	0	NA	NA	NA	NA	OR1E1
NA	ENSG00000180019	0.34624801	-0.02356563	0.38536324	0.65270996	NA	
197407	ENSG00000180035	1078.71749	0.25976032	0.09523052	0.00477681	0.02234661	ZNF48
NA	ENSG00000180042	0	NA	NA	NA	NA	OR1R1P
284418	ENSG00000180043	0	NA	NA	NA	NA	FAM71E2
401097	ENSG00000180044	8.00148977	-0.22481678	0.4337138	0.15692102	0.34373985	C3orf80
137814	ENSG00000180053	0	NA	NA	NA	NA	NKX2-6
284417	ENSG00000180061	0.33471731	0.02354391	0.38534154	0.62842204	NA	TMEM150B
NA	ENSG00000180066	0.14931947	-0.01844332	0.38480212	0.84537521	NA	LINC02870
NA	ENSG00000180068	0	NA	NA	NA	NA	OR3A4P
253650	ENSG00000180071	284.590223	-0.74640904	0.17264058	2.94E-06	3.08E-05	ANKRD18A
259239	ENSG00000180083	0	NA	NA	NA	NA	WFDC11
255043	ENSG00000180089	68.9736613	0.09992781	0.23528227	0.59462239	0.78780021	TMEM86B
4994	ENSG00000180090	0	NA	NA	NA	NA	OR3A1
1731	ENSG00000180096	42.8669524	-0.0586965	0.26715798	0.75960455	0.88850157	SEPTIN1
54952	ENSG00000180098	318.635882	0.07278233	0.15711603	0.61263538	0.80056246	TRNAU1AP
11336	ENSG00000180104	985.074731	-0.00409414	0.08136009	0.95817692	0.98274556	EXOC3
NA	ENSG00000180105	0	NA	NA	NA	NA	ACER2P1
221400	ENSG00000180113	0.34746601	0.02370763	0.3853651	0.61987128	NA	TDRD6
283461	ENSG00000180116	1.40567573	-0.04465103	0.38346077	0.48196492	NA	C12orf40

122011	ENSG00000180138	0	NA	NA	NA	NA	CSNK1A1L
NA	ENSG00000180139	3.3182044	-0.01220471	0.37152256	0.8969254	0.95487211	ACTA2-AS1
NA	ENSG00000180150	0	NA	NA	NA	NA	HMGN2P9
NA	ENSG00000180152	0	NA	NA	NA	NA	XIAPP3
66004	ENSG00000180155	98.9214791	0.81732916	0.40423669	0.00482549	0.02254772	LYNX1
NA	ENSG00000180172	0	NA	NA	NA	NA	RPS12P23
7054	ENSG00000180176	3.3145817	-0.09817112	0.38473276	0.37592694	0.60500552	TH
NA	ENSG00000180178	0	NA	NA	NA	NA	FAR2P1
9282	ENSG00000180182	1567.43842	0.12942215	0.1001616	0.18065114	0.37770382	MED14
81889	ENSG00000180185	472.846964	0.01321433	0.1305284	0.91532986	0.96406776	FAHD1
NA	ENSG00000180189	3.3529607	-0.00565818	0.36674214	0.9577635	0.98264776	HMGB1P14
157695	ENSG00000180190	359.833354	-0.01460011	0.12602701	0.89722466	0.95488961	TDRP
1104	ENSG00000180198	1655.01184	-0.07401262	0.07090484	0.28755781	0.51323739	RCC1
259240	ENSG00000180205	0	NA	NA	NA	NA	WFDC9
29895	ENSG00000180209	1.12172923	-0.06172621	0.39092167	0.19353557	NA	MYLPF
2147	ENSG00000180210	0.51122455	0.00365648	0.38254769	0.88431555	NA	F2
NA	ENSG00000180211	0.34624801	-0.02356563	0.38536324	0.65270996	NA	
196472	ENSG00000180219	0.173124	-0.01911262	0.38486262	0.84537521	NA	FAM71C
NA	ENSG00000180221	0.3415213	0.00281377	0.38338784	0.96082379	NA	TPT1P10
8575	ENSG00000180228	1220.16625	0.40592436	0.08610775	1.26E-06	1.41E-05	PRKRA
283755	ENSG00000180229	1547.21601	0.2993656	0.08551793	0.00032055	0.0021709	HERC2P3
NA	ENSG00000180230	0	NA	NA	NA	NA	NACAP2
223082	ENSG00000180233	372.934192	-0.0701957	0.12663967	0.55783199	0.76280883	ZNRF2
10692	ENSG00000180245	4.4787083	0.19286042	0.43480143	0.11162823	0.2700997	RRH
389015	ENSG00000180251	0.32398763	-0.02327431	0.38532154	0.6685144	NA	SLC9A4
125893	ENSG00000180257	151.809504	-0.06457057	0.19771618	0.70193493	0.85663955	ZNF816
149830	ENSG00000180259	0	NA	NA	NA	NA	PRNT
55785	ENSG00000180263	488.475947	0.12707776	0.11770892	0.2565701	0.47765326	FGD6
347088	ENSG00000180264	8.74215519	-0.06860179	0.35309262	0.65174572	0.82602754	ADGRD2
124274	ENSG00000180269	0.47363805	-0.0284531	0.38580958	0.54270361	NA	GPR139
284365	ENSG00000180279	0.94190373	-0.00195309	0.38007536	0.97438512	NA	LINC01869
NA	ENSG00000180284	0	NA	NA	NA	NA	CNEP1R1P1
200150	ENSG00000180287	1075.5625	-0.33213768	0.09209488	0.0001954	0.00139298	PLD5
4947	ENSG00000180304	2343.38975	0.19104498	0.07763182	0.01189634	0.04789434	OAZ2
140832	ENSG00000180305	0	NA	NA	NA	NA	WFDC10A
285848	ENSG00000180316	2.68070602	0.0797623	0.38451956	0.39832451	0.62727695	PNPLA1
8092	ENSG00000180318	49.2713853	0.5102025	0.33546522	0.03372254	0.11011441	ALX1
124808	ENSG00000180329	560.884881	-0.06210764	0.10486403	0.53740104	0.74759591	CCDC43

386618	ENSG00000180332	0	NA	NA	NA	NA	KCTD4
284071	ENSG00000180336	194.177523	0.07150606	0.21614629	0.69053611	0.85004879	MEIOC
2535	ENSG00000180340	653.182278	0.91352665	0.10239316	7.30E-20	2.70E-18	FZD2
166815	ENSG00000180346	176.648586	0.16733352	0.17076077	0.27408087	0.49773588	TIGD2
223075	ENSG00000180347	0	NA	NA	NA	NA	ITPRID1
3059	ENSG00000180353	1.37636305	-0.02475218	0.38030679	0.70427314	NA	HCLS1
222166	ENSG00000180354	1194.05878	-0.27078503	0.09181963	0.00232006	0.01216116	MTURN
23060	ENSG00000180357	2949.30181	-0.14651361	0.05871738	0.01150023	0.04653421	ZNF609
5062	ENSG00000180370	2425.34129	-0.10549187	0.0801524	0.17799386	0.37341632	PAK2
285331	ENSG00000180376	397.645146	-0.1399905	0.17746565	0.37212355	0.60109044	CCDC66
245937	ENSG00000180383	0	NA	NA	NA	NA	DEFB124
NA	ENSG00000180385	140.436615	-0.1733669	0.20572159	0.31823033	0.54576013	EMC3-AS1
100505724	ENSG00000180386	0	NA	NA	NA	NA	KRTAP9-7
NA	ENSG00000180389	0	NA	NA	NA	NA	ATP5F1EP2
90411	ENSG00000180398	2464.93948	-0.17520711	0.06759784	0.0083726	0.03587741	MCFD2
NA	ENSG00000180409	0	NA	NA	NA	NA	OR10AA1P
283860	ENSG00000180422	24.9669085	0.93061251	0.61981879	0.00948103	0.0397262	LINC00304
283254	ENSG00000180423	65.040768	0.05306474	0.2478162	0.78054972	0.89965693	HARBI1
245936	ENSG00000180424	0	NA	NA	NA	NA	DEFB123
54494	ENSG00000180425	127.895747	0.04371315	0.19435932	0.79580906	0.90615541	C11orf71
1582	ENSG00000180432	0.92826579	0.0036884	0.38036159	0.94948299	NA	CYP8B1
128371	ENSG00000180433	0	NA	NA	NA	NA	OR6K6
NA	ENSG00000180437	0	NA	NA	NA	NA	OR6K4P
348825	ENSG00000180438	0.173733	0.01914545	0.38484597	0.78225479	NA	TPRXL
400120	ENSG00000180440	66.2521795	1.12672742	0.3119105	2.96E-05	0.00025541	SERTM1
2619	ENSG00000180447	122.109095	0.83564818	0.2383453	7.04E-05	0.00055875	GAS1
23526	ENSG00000180448	115.609763	-0.15042103	0.21839219	0.40396894	0.63202774	ARHGAP45
NA	ENSG00000180458	25.252641	0.02910502	0.2940009	0.88023668	0.94691188	
219960	ENSG00000180475	0	NA	NA	NA	NA	OR10Q1
51276	ENSG00000180479	97.9194221	0.05506128	0.2127816	0.75730767	0.88697677	ZNF571
144321	ENSG00000180481	1.22161593	0.08649353	0.3971884	0.10725541	NA	GLIPR1L2
245932	ENSG00000180483	0	NA	NA	NA	NA	DEFB119
374986	ENSG00000180488	502.065946	-0.02532836	0.10953615	0.80839639	0.91317199	MIGA1
3753	ENSG00000180509	0.47260901	-0.01593465	0.38332663	0.76502704	NA	KCNE1
NA	ENSG00000180525	1.38665865	-0.02485663	0.37808655	0.743302	NA	DIP2C-AS1
8204	ENSG00000180530	0.16735865	0.01897177	0.38482933	0.78225479	NA	NRIP1
201516	ENSG00000180532	0	NA	NA	NA	NA	ZSCAN4
168620	ENSG00000180535	50.5888911	0.05669178	0.25493304	0.76878913	0.8934905	BHLHA15

221687	ENSG00000180537	420.980636	0.2523717	0.14995958	0.0670066	0.18562007	RNF182
NA	ENSG00000180539	10.2417841	-0.09672766	0.37461972	0.4495663	0.67315229	C9orf139
85453	ENSG00000180543	2088.63914	-0.31366649	0.06985195	4.73E-06	4.79E-05	TSPYL5
2529	ENSG00000180549	1.33189532	0.00355429	0.37788968	0.96130846	NA	FUT7
8334	ENSG00000180573	104.280308	-1.23978232	0.2538739	9.88E-08	1.30E-06	H2AC6
255308	ENSG00000180574	6.31684192	-0.01986001	0.35592302	0.88255498	0.94827461	EIF2S3B
NA	ENSG00000180581	0	NA	NA	NA	NA	SRP9P1
387640	ENSG00000180592	465.518325	0.35593015	0.11851671	0.0015583	0.00867227	SKIDA1
8347	ENSG00000180596	8.36139519	-0.47752063	0.76233643	0.03071123	0.10216479	H2BC4
NA	ENSG00000180610	1.17723217	0.01418799	0.3797094	0.82630723	NA	ZBTB12BP
151963	ENSG00000180611	49.8297485	0.18024391	0.27228123	0.36767759	0.59703579	MB21D2
170825	ENSG00000180613	1.11954738	-0.05801677	0.39009015	0.21465513	NA	GSX2
6752	ENSG00000180616	158.007519	0.53363439	0.20366672	0.00281211	0.01433879	SSTR2
84622	ENSG00000180626	543.754834	0.06413344	0.11184347	0.54960125	0.75607328	ZNF594
84333	ENSG00000180628	155.909149	0.30694077	0.19043031	0.06478429	0.181378	PCGF5
146802	ENSG00000180638	2.25589684	-0.00964389	0.372044	0.91882671	NA	SLC47A2
5551	ENSG00000180644	0.66468534	0.02522022	0.38353444	0.5953632	NA	PRF1
79541	ENSG00000180658	0	NA	NA	NA	NA	OR2A4
4081	ENSG00000180660	3253.87191	1.09579909	0.0624558	8.89E-70	1.46E-67	MAB21L1
NA	ENSG00000180662	0	NA	NA	NA	NA	RPL21P8
NA	ENSG00000180663	0	NA	NA	NA	NA	VN1R3
55432	ENSG00000180667	743.853941	0.07839043	0.09095583	0.37557142	0.60466908	YOD1
NA	ENSG00000180673	0	NA	NA	NA	NA	EXOC5P1
169200	ENSG00000180694	607.837034	-1.2175623	0.11846806	9.31E-26	4.52E-24	TMEM64
152065	ENSG00000180697	0.37844916	0.02407552	0.3854191	0.60038877	NA	C3orf22
391107	ENSG00000180708	0	NA	NA	NA	NA	OR10K2
NA	ENSG00000180712	5.29754517	-0.16528602	0.41339048	0.16846839	0.35970865	LINC02363
NA	ENSG00000180714	0	NA	NA	NA	NA	OR5AZ1P
1132	ENSG00000180720	8.28806334	-0.30948627	0.51528045	0.07222117	0.19574701	CHRM4
NA	ENSG00000180723	0	NA	NA	NA	NA	OR51A9P
387914	ENSG00000180730	1.24840401	0.03481126	0.38284651	0.54911218	NA	SHISA2
53637	ENSG00000180739	0.53606622	0.0137271	0.38291855	0.73918762	NA	S1PR5
119467	ENSG00000180745	0	NA	NA	NA	NA	CLRN3
NA	ENSG00000180747	687.615873	0.16659939	0.11013326	0.11356485	0.27350408	SMG1P3
80045	ENSG00000180758	184.612004	-0.25211927	0.39555863	0.20485339	0.41169887	GPR157
NA	ENSG00000180764	21.1220891	0.45814606	0.46833382	0.06891262	0.18928789	PIPSL
166012	ENSG00000180767	24.6241461	-0.12790199	0.33549556	0.48960481	0.70913707	CHST13
404201	ENSG00000180769	34.419185	-0.12417324	0.30053327	0.52546073	0.73801083	WDFY3-AS2

NA	ENSG00000180770	0	NA	NA	NA	NA	OR7E129P
186	ENSG00000180772	0	NA	NA	NA	NA	AGTR2
120103	ENSG00000180773	1038.54171	0.25060441	0.11181147	0.01881925	0.06934881	SLC36A4
253832	ENSG00000180776	624.531024	0.03097953	0.12124579	0.78886371	0.90411045	ZDHHC20
374860	ENSG00000180777	0	NA	NA	NA	NA	ANKRD30B
143503	ENSG00000180785	0	NA	NA	NA	NA	OR51E1
124961	ENSG00000180787	580.977625	-0.01202293	0.10911501	0.90716344	0.95963244	ZFP3
79642	ENSG00000180801	66.863363	3.0551786	0.44650985	4.17E-13	9.75E-12	ARSJ
NA	ENSG00000180803	0.18922458	0.01954053	0.38488496	0.78225479	NA	
3225	ENSG00000180806	117.410496	-0.56838695	0.21565108	0.00237735	0.01242206	HOXC9
389840	ENSG00000180815	39.0212482	0.93373907	0.40741468	0.00227506	0.01195508	MAP3K15
5464	ENSG00000180817	2297.41951	-0.2823732	0.07405103	9.89E-05	0.00075342	PPA1
3226	ENSG00000180818	7.84022109	-0.03772491	0.35494966	0.78526861	0.90233576	HOXC10
389362	ENSG00000180822	383.605681	0.10408722	0.14075753	0.42729194	0.65306772	PSMG4
27319	ENSG00000180828	0	NA	NA	NA	NA	BHLHE22
79929	ENSG00000180834	79.1665653	-0.14023766	0.26201564	0.47075458	0.69301557	MAP6D1
255193	ENSG00000180846	2.99092271	0.09847653	0.3913117	0.29650048	0.52308105	CSNK1G2-AS1
10224	ENSG00000180855	143.439139	-0.07584806	0.17986591	0.63389313	0.81527549	ZNF443
NA	ENSG00000180861	0	NA	NA	NA	NA	LINC01559
NA	ENSG00000180867	288.00526	-0.04156297	0.14027159	0.74927816	0.88205755	PDIA3P1
439927	ENSG00000180869	0.34624801	-0.02356563	0.38536324	0.65270996	NA	LINC01555
3579	ENSG00000180871	0	NA	NA	NA	NA	CXCR2
245915	ENSG00000180872	0	NA	NA	NA	NA	DEFB112
64388	ENSG00000180875	8.76611368	3.53259639	1.14982269	0.00019115	0.00136617	GREM2
160298	ENSG00000180878	2.49885388	0.042267	0.37624632	0.64988007	NA	C11orf42
6748	ENSG00000180879	1473.75234	0.05247547	0.07599566	0.48214794	0.70272402	SSR4
84698	ENSG00000180881	101.393379	0.109006	0.22613656	0.55372908	0.75980255	CAPS2
126375	ENSG00000180884	423.249074	-0.04701673	0.11567668	0.6688301	0.83720078	ZNF792
404093	ENSG00000180891	660.287167	0.14873899	0.13311783	0.23297507	0.44988944	CUEDC1
23513	ENSG00000180900	3081.86958	0.30349745	0.0751345	3.68E-05	0.00031188	SCRIB
23510	ENSG00000180901	1194.70123	-0.00782492	0.08100749	0.92083533	0.96670947	KCTD2
728294	ENSG00000180902	466.113112	0.27750323	0.11457717	0.0109534	0.04467835	D2HGDH
NA	ENSG00000180909	0	NA	NA	NA	NA	OR52B1P
83866	ENSG00000180910	0	NA	NA	NA	NA	TTTY11
NA	ENSG00000180913	0	NA	NA	NA	NA	OR56B3P
5021	ENSG00000180914	104.909575	-0.21192737	0.20833381	0.22804625	0.44313786	OXTR
55783	ENSG00000180917	499.644819	-0.00201297	0.10703976	0.98300951	0.99428951	CMTR2
196335	ENSG00000180919	0	NA	NA	NA	NA	OR56B4

286077	ENSG00000180921	14.332492	0.07004287	0.33683133	0.6841648	0.84603775	FAM83H
NA	ENSG00000180926	0	NA	NA	NA	NA	OR7E18P
118442	ENSG00000180929	39.2110754	0.31021248	0.34377309	0.16062777	0.34855682	GPR62
120796	ENSG00000180934	0	NA	NA	NA	NA	OR56A1
137209	ENSG00000180938	97.5133237	-0.30984965	0.23846105	0.10786156	0.26339391	ZNF572
NA	ENSG00000180953	68.1776296	-0.08227454	0.2567261	0.66711131	0.83613156	ST20
23760	ENSG00000180957	2383.81611	-0.10321647	0.08336737	0.20411283	0.41075144	PITPNB
90843	ENSG00000180964	703.240524	-0.28863777	0.10301899	0.00351605	0.01733557	TCEAL8
390081	ENSG00000180974	0	NA	NA	NA	NA	OR52E4
255252	ENSG00000180979	390.566071	0.38276258	0.1385257	0.00301579	0.01519768	LRRC57
390077	ENSG00000180988	0	NA	NA	NA	NA	OR52N2
64928	ENSG00000180992	464.278271	-0.0870748	0.1139048	0.42258941	0.64862864	MRPL14
283554	ENSG00000180998	340.167967	-0.01433098	0.15148344	0.91697361	0.96477469	GPR137C
92346	ENSG00000180999	0	NA	NA	NA	NA	C1orf105
79473	ENSG00000181001	0	NA	NA	NA	NA	OR52N1
166379	ENSG00000181004	60.1615785	0.07768878	0.25046015	0.68570941	0.84710303	BBS12
284406	ENSG00000181007	339.265949	-0.1123818	0.14455516	0.40011481	0.62854504	ZFP82
390075	ENSG00000181009	0	NA	NA	NA	NA	OR52N5
286006	ENSG00000181016	7.74939829	0.23658436	0.45945652	0.08890194	0.22841422	LSMEM1
NA	ENSG00000181017	0	NA	NA	NA	NA	OR56B2P
1728	ENSG00000181019	300.731008	1.90312067	0.1741797	5.95E-29	3.29E-27	NQO1
387748	ENSG00000181023	0	NA	NA	NA	NA	OR56B1
64782	ENSG00000181026	992.393848	0.12716296	0.08168115	0.11179174	0.27034959	AEN
79147	ENSG00000181027	865.023287	0.02074685	0.12532894	0.86443231	0.94000203	FKRP
126003	ENSG00000181029	586.517554	0.01855085	0.10667727	0.85762677	0.93650256	TRAPPC5
9501	ENSG00000181031	85.8101536	0.18758484	0.26839573	0.34485112	0.5752351	RPH3AL
284439	ENSG00000181035	161.647071	0.09288901	0.19672272	0.58418538	0.78032976	SLC25A42
343413	ENSG00000181036	1.1727418	0.05198808	0.38669146	0.35522169	NA	FCRL6
124512	ENSG00000181038	509.802798	0.11823531	0.11406184	0.27754163	0.50204466	METTL23
284129	ENSG00000181045	572.678715	0.30140022	0.11196994	0.00476221	0.02228879	SLC26A11
25994	ENSG00000181061	880.822966	-0.00909171	0.09350429	0.91670572	0.96473288	HIGD1A
1129	ENSG00000181072	212.005103	-1.33473864	0.20855651	1.26E-11	2.65E-10	CHRM2
390072	ENSG00000181074	0	NA	NA	NA	NA	OR52N4
225689	ENSG00000181085	218.026975	0.3945329	0.17801258	0.01306547	0.05162695	MAPK15
79813	ENSG00000181090	2061.19762	-0.01085312	0.07409079	0.88063465	0.94713457	EHMT1
9370	ENSG00000181092	0	NA	NA	NA	NA	ADIPOQ
NA	ENSG00000181097	11.0186777	0.02202053	0.34035234	0.89257988	0.95351371	LINC02878
NA	ENSG00000181101	0	NA	NA	NA	NA	ENTR1P2

2149	ENSG00000181104	1619.22178	0.27591009	0.0789128	0.00034488	0.00231946	F2R
NA	ENSG00000181109	0	NA	NA	NA	NA	OR52P1P
NA	ENSG00000181123	0.14931947	-0.01844332	0.38480212	0.84537521	NA	
352962	ENSG00000181126	0	NA	NA	NA	NA	HLA-V
286075	ENSG00000181135	269.394862	0.09742254	0.13759851	0.44858008	0.67234398	ZNF707
101928160	ENSG00000181135	269.394862	0.09742254	0.13759851	0.44858008	0.67234398	ZNF707
94025	ENSG00000181143	0	NA	NA	NA	NA	MUC16
4869	ENSG00000181163	11970.9025	-0.10231861	0.05696795	0.06969655	0.19085983	NPM1
439941	ENSG00000181171	0	NA	NA	NA	NA	FER1L6-AS1
64219	ENSG00000181191	1032.86235	-0.27988698	0.08581821	0.00079437	0.00484634	PJA1
55526	ENSG00000181192	1929.69234	-0.11211636	0.07679355	0.13552273	0.31008775	DHTKD1
5179	ENSG00000181195	0	NA	NA	NA	NA	PENK
NA	ENSG00000181201	8.73575637	-0.02108797	0.34935677	0.8844318	0.94936574	H2BU2P
100127950	ENSG00000181211	0	NA	NA	NA	NA	HECW1-IT1
26492	ENSG00000181214	0	NA	NA	NA	NA	OR8G2P
389197	ENSG00000181215	22.1002061	-1.23093023	0.61981995	0.00283225	0.01442665	C4orf50
92815	ENSG00000181218	438.88889	-0.40241204	0.12887641	0.00090054	0.00541088	H2AW
155061	ENSG00000181220	791.759867	-0.00759922	0.10166858	0.93720983	0.97354401	ZNF746
5430	ENSG00000181222	6309.58508	-0.09550821	0.05746419	0.09235075	0.2349928	POLR2A
NA	ENSG00000181227	4.7765237	-0.05816224	0.38148147	0.49348094	0.712409	DLSTP1
92293	ENSG00000181234	0	NA	NA	NA	NA	TMEM132C
284427	ENSG00000181240	2.85705093	0.06205243	0.38062176	0.49876961	0.71639681	SLC25A41
NA	ENSG00000181260	0	NA	NA	NA	NA	MTHFD2P7
219902	ENSG00000181264	577.25941	-0.19557501	0.11219064	0.06696273	0.18552445	TLCD5
390181	ENSG00000181273	0	NA	NA	NA	NA	OR5AK2
23401	ENSG00000181274	258.087195	0.37305798	0.1506413	0.00712073	0.0313736	FRAT2
NA	ENSG00000181282	0	NA	NA	NA	NA	OR5AK3P
284114	ENSG00000181284	131.493821	0.81144959	0.21226632	2.22E-05	0.00019748	TMEM102
124842	ENSG00000181291	0.84989367	-0.00687349	0.38140222	0.8954221	NA	TMEM132E
NA	ENSG00000181296	0	NA	NA	NA	NA	OR5G1P
79692	ENSG00000181315	866.505341	-0.06716498	0.10084063	0.48928258	0.70877384	ZNF322
347736	ENSG00000181322	19.3258722	-0.12189316	0.33250939	0.51163283	0.72687381	NME9
374768	ENSG00000181323	0	NA	NA	NA	NA	SPEM1
NA	ENSG00000181325	0	NA	NA	NA	NA	OR9G3P
341208	ENSG00000181333	5.13375642	0.08367849	0.37402108	0.50490337	0.72130144	HEPHL1
388341	ENSG00000181350	107.670477	0.13845062	0.19779131	0.41661824	0.64309452	LRRC75A
NA	ENSG00000181355	0	NA	NA	NA	NA	OFCC1
NA	ENSG00000181358	0	NA	NA	NA	NA	CTAGE10P

NA	ENSG00000181359	0.173733	0.01914545	0.38484597	0.78225479	NA	HSP90AA6P
219484	ENSG00000181371	0	NA	NA	NA	NA	OR5M8
6357	ENSG00000181374	0	NA	NA	NA	NA	CCL13
255101	ENSG00000181378	41.344527	0.1617374	0.28208492	0.41936435	0.64519864	CFAP65
91351	ENSG00000181381	151.43721	1.01019871	0.25400768	7.83E-06	7.59E-05	DDX60L
163183	ENSG00000181392	5.77539532	0.07773831	0.36718175	0.56028589	0.76415299	SYNE4
79701	ENSG00000181396	1384.12168	-0.0099912	0.07804262	0.89528601	0.95469109	OGFOD3
100287171	ENSG00000181404	411.562682	0.2297367	0.1278522	0.05608157	0.16266792	WASHC1
2837	ENSG00000181408	8.62510678	0.0281355	0.34541093	0.85737522	0.93627941	UTS2R
9625	ENSG00000181409	1238.1999	-0.27534756	0.09945203	0.00401489	0.01936202	AATK
23109	ENSG00000181418	95.007888	-0.67819907	0.24849742	0.00130027	0.0074088	DDN
55511	ENSG00000181433	0	NA	NA	NA	NA	SAGE1
168544	ENSG00000181444	54.9595238	0.06523081	0.25614352	0.73546303	0.87432359	ZNF467
6657	ENSG00000181449	68.0509386	4.46922308	0.45674691	2.30E-23	1.03E-21	SOX2
339500	ENSG00000181450	519.30085	-0.1238104	0.12426435	0.29042642	0.51645247	ZNF678
55076	ENSG00000181458	209.184167	-0.52470294	0.17648786	0.00100236	0.00592224	TMEM45A
5912	ENSG00000181467	306.213339	0.56456752	0.14016913	1.81E-05	0.00016349	RAP2B
57621	ENSG00000181472	298.15669	-0.3143921	0.13702848	0.01377024	0.05383283	ZBTB2
84282	ENSG00000181481	0	NA	NA	NA	NA	RNF135
219874	ENSG00000181499	0	NA	NA	NA	NA	OR6T1
NA	ENSG00000181511	0	NA	NA	NA	NA	GLRX5P1
79777	ENSG00000181513	72.9160482	-0.09324249	0.23806567	0.61870526	0.80494303	ACBD4
NA	ENSG00000181514	0	NA	NA	NA	NA	
338662	ENSG00000181518	0	NA	NA	NA	NA	OR8D4
6448	ENSG00000181523	831.755972	0.15234726	0.14353655	0.25154023	0.47149344	SGSH
NA	ENSG00000181524	4.98735765	0.03294239	0.36924084	0.76715463	0.89274138	RPL24P4
10586	ENSG00000181541	30.5128033	7.04804454	1.3304378	2.37E-09	3.88E-08	MAB21L2
2187	ENSG00000181544	182.357067	-0.3066106	0.18934452	0.0635114	0.17891043	FANCB
64184	ENSG00000181552	0	NA	NA	NA	NA	EDDM3B
29072	ENSG00000181555	2277.54618	-0.12668091	0.08054528	0.10720817	0.2623024	SETD2
10876	ENSG00000181562	0	NA	NA	NA	NA	EDDM3A
221416	ENSG00000181577	0.9842078	-0.01042811	0.38036263	0.86059005	NA	C6orf223
259236	ENSG00000181585	67.3509605	-0.82460502	0.31090921	0.00111693	0.00649658	TMIE
399664	ENSG00000181588	1290.95477	-0.17003755	0.07825823	0.02618738	0.09029505	MEX3D
390066	ENSG00000181609	0	NA	NA	NA	NA	OR52D1
51649	ENSG00000181610	1229.76516	0.06955838	0.07757759	0.36022371	0.59016007	MRPS23
390067	ENSG00000181616	0	NA	NA	NA	NA	OR52H1
260436	ENSG00000181617	0	NA	NA	NA	NA	FDCSP

64582	ENSG00000181619	185.925787	-0.06542462	0.16192719	0.65489372	0.82814932	GPR135
79008	ENSG00000181625	31.3138021	0.73082647	0.5894244	0.02071923	0.07502636	SLX1B
342850	ENSG00000181626	1.50834298	0.05574805	0.38471721	0.41390417	NA	ANKRD62
53829	ENSG00000181631	0.36291009	0.02389445	0.38539235	0.60998858	NA	P2RY13
9966	ENSG00000181634	0	NA	NA	NA	NA	TNFSF15
286128	ENSG00000181638	656.937882	0.0402812	0.12994064	0.74344586	0.87902062	ZFP41
7262	ENSG00000181649	0.46756584	-0.00823939	0.38328691	0.85770315	NA	PHLDA2
285973	ENSG00000181652	178.24959	-0.6798449	0.18348205	4.75E-05	0.00039098	ATG9B
54112	ENSG00000181656	1.38849842	-0.07830509	0.39181065	0.21714084	NA	GPR88
284459	ENSG00000181666	1097.53844	0.07121386	0.10335869	0.47499518	0.69724063	ZNF875
5324	ENSG00000181690	3.76136559	-0.05441754	0.37329282	0.60953064	0.79803651	PLAG1
219469	ENSG00000181693	0	NA	NA	NA	NA	OR8H1
390155	ENSG00000181698	0	NA	NA	NA	NA	OR5T1
286451	ENSG00000181704	1456.28472	0.09255932	0.08702034	0.27489686	0.49876121	YIPF6
NA	ENSG00000181705	0	NA	NA	NA	NA	
219464	ENSG00000181718	0	NA	NA	NA	NA	OR5T2
26137	ENSG00000181722	320.326352	-0.05743686	0.32040765	0.74383398	0.87932229	ZBTB20
284383	ENSG00000181733	0	NA	NA	NA	NA	OR2Z1
NA	ENSG00000181741	0.15069912	-0.01848503	0.38480575	0.84537521	NA	FDX1P1
205428	ENSG00000181744	5.54732603	0.02224828	0.35748316	0.87019674	0.94333385	DIPK2A
90355	ENSG00000181751	488.531097	-0.00459831	0.11560343	0.96574976	0.98663622	MACIR
219453	ENSG00000181752	0	NA	NA	NA	NA	OR8K5
57463	ENSG00000181754	231.65756	0.25631233	0.16877932	0.0906502	0.23197471	AMIGO1
390152	ENSG00000181761	0	NA	NA	NA	NA	OR8H3
390151	ENSG00000181767	0	NA	NA	NA	NA	OR8H2
2827	ENSG00000181773	92.3138266	0.02925579	0.20849728	0.86888362	0.94277555	GPR3
169693	ENSG00000181778	0	NA	NA	NA	NA	TMEM252
NA	ENSG00000181780	0	NA	NA	NA	NA	OR5J1P
284451	ENSG00000181781	1.68375986	0.02490047	0.37966438	0.71417112	NA	ODF3L2
219447	ENSG00000181785	0	NA	NA	NA	NA	OR5AS1
284382	ENSG00000181786	0	NA	NA	NA	NA	ACTL9
6478	ENSG00000181788	478.319269	-0.07972163	0.11020239	0.44932705	0.67295604	SIAH2
22820	ENSG00000181789	2108.35671	0.11915378	0.07256872	0.09463614	0.23921278	COPG1
575	ENSG00000181790	319.766484	-2.34859364	0.48306668	5.23E-08	7.18E-07	ADGRB1
151477	ENSG00000181798	28.1981045	-0.00815845	0.28832172	0.9642134	0.98582808	LINC00471
414196	ENSG00000181800	0	NA	NA	NA	NA	CELF2-AS1
341799	ENSG00000181803	0	NA	NA	NA	NA	OR6S1
285195	ENSG00000181804	0	NA	NA	NA	NA	SLC9A9

84967	ENSG00000181817	346.159379	-0.19659095	0.12902625	0.10423021	0.2570084	LSM10
NA	ENSG00000181819	2.17927467	-0.00446207	0.37459335	0.95729961	NA	KCTD9P2
768211	ENSG00000181826	89.5597205	0.64937982	0.27310055	0.0036445	0.01785624	RELL1
64864	ENSG00000181827	1780.78048	-0.10903446	0.06885754	0.10707903	0.26208338	RFX7
55343	ENSG00000181830	332.083829	-0.02353203	0.13028445	0.84772562	0.93071088	SLC35C1
NA	ENSG00000181837	0	NA	NA	NA	NA	OR5D17P
201633	ENSG00000181847	0.66695587	-0.02130958	0.38247053	0.65852154	NA	TIGIT
10193	ENSG00000181852	1167.15139	-0.00343373	0.0819198	0.96505341	0.98622874	RNF41
6517	ENSG00000181856	54.1381088	0.40095563	0.30946114	0.07307715	0.19752734	SLC2A4
94033	ENSG00000181867	0	NA	NA	NA	NA	FTMT
200205	ENSG00000181873	292.164632	-0.66769775	0.17048432	2.09E-05	0.00018709	IBA57
1366	ENSG00000181885	6.01955361	0.20245197	0.43969465	0.10004879	0.24927389	CLDN7
79673	ENSG00000181894	217.353203	-0.0363944	0.15738697	0.7989291	0.90798839	ZNF329
94039	ENSG00000181896	199.337755	-0.51769209	0.20337758	0.00359182	0.01765651	ZNF101
219432	ENSG00000181903	0	NA	NA	NA	NA	OR4C6
134553	ENSG00000181904	1717.0416	-0.4448621	0.08215641	2.88E-08	4.08E-07	C5orf24
100996455	ENSG00000181908	12.511603	1.41677314	0.91705511	0.00485301	0.022655	LINC02724
84890	ENSG00000181915	1523.46028	-0.15484425	0.07683059	0.03941052	0.12407247	ADO
51287	ENSG00000181924	946.382278	0.08104793	0.08290552	0.31704394	0.54483775	COA4
81300	ENSG00000181927	0	NA	NA	NA	NA	OR4P4
5571	ENSG00000181929	734.139098	0.09279928	0.09741278	0.32493768	0.55284827	PRKAG1
64785	ENSG00000181938	306.369817	-0.35735224	0.14022296	0.00609193	0.02747164	GINS3
81309	ENSG00000181939	0	NA	NA	NA	NA	OR4C15
NA	ENSG00000181943	0	NA	NA	NA	NA	OR4A21P
NA	ENSG00000181950	0	NA	NA	NA	NA	OR4A13P
81328	ENSG00000181958	0	NA	NA	NA	NA	OR4A15
81327	ENSG00000181961	0	NA	NA	NA	NA	OR4A16
119774	ENSG00000181963	0	NA	NA	NA	NA	OR52K2
4762	ENSG00000181965	0	NA	NA	NA	NA	NEUROG1
NA	ENSG00000181977	0	NA	NA	NA	NA	PYY3
91050	ENSG00000181982	0.62151788	-0.03202751	0.38600238	0.58906207	NA	CCDC149
729786	ENSG00000181984	0.14931947	-0.01844332	0.38480212	0.84537521	NA	GOLGA8CP
64963	ENSG00000181991	553.921746	-0.14774655	0.11370252	0.17237504	0.36498622	MRPS11
283197	ENSG00000181995	0	NA	NA	NA	NA	LINC00301
NA	ENSG00000181997	0	NA	NA	NA	NA	AQP7P2
NA	ENSG00000182000	0	NA	NA	NA	NA	PPIAP73
6635	ENSG00000182004	2565.94832	-0.06401591	0.07045915	0.35382225	0.58420717	SNRPE
219790	ENSG00000182010	1031.64382	-0.5174003	0.09057982	4.22E-09	6.69E-08	RTKN2

55228	ENSG00000182013	1490.04436	-0.09587527	0.10111267	0.3241597	0.55209275	PNMA8A
100996643	ENSG00000182021	0	NA	NA	NA	NA	
51363	ENSG00000182022	4.27808858	-0.15119338	0.40685125	0.20205708	0.40760676	CHST15
149685	ENSG00000182035	0	NA	NA	NA	NA	ADIG
124590	ENSG00000182040	37.5426893	-0.07006912	0.28496308	0.71559479	0.86274919	USH1G
7221	ENSG00000182048	0	NA	NA	NA	NA	TRPC2
25834	ENSG00000182050	5.33362753	0.12342893	0.3980785	0.24015226	0.45820056	MGAT4C
283116	ENSG00000182053	0	NA	NA	NA	NA	TRIM49B
3418	ENSG00000182054	3691.76718	0.11464076	0.0605282	0.05506531	0.16031256	IDH2
NA	ENSG00000182057	6.91463229	-0.12570449	0.39268441	0.27357822	0.49704244	OGFRP1
23538	ENSG00000182070	0	NA	NA	NA	NA	OR52A1
374308	ENSG00000182077	0.18922458	0.01954053	0.38488496	0.78225479	NA	PTCHD3
389090	ENSG00000182083	0	NA	NA	NA	NA	OR6B2
91304	ENSG00000182087	3248.51821	0.14511279	0.05987227	0.01411371	0.05492662	TMEM259
7485	ENSG00000182093	724.346543	-0.18353512	0.09378729	0.04307011	0.13277051	GET1
84629	ENSG00000182095	3425.73376	0.12276687	0.11326114	0.25079261	0.47069581	TNRC18
220382	ENSG00000182103	97.6466835	0.34417726	0.23920538	0.07633536	0.20405465	FAM181B
161291	ENSG00000182107	11.4869414	0.08417693	0.34864321	0.60783764	0.79695264	TMEM30B
28955	ENSG00000182108	553.316365	0.13908587	0.10908645	0.18321121	0.38141767	DEXI
441234	ENSG00000182111	0	NA	NA	NA	NA	ZNF716
55505	ENSG00000182117	1127.70837	-0.06844401	0.07900693	0.37620313	0.60519502	NOP10
375061	ENSG00000182118	359.503341	-0.4916544	0.1278803	4.75E-05	0.00039098	FAM89A
30820	ENSG00000182132	18.0714717	-0.06676313	0.3220748	0.71102744	0.86118643	KCNIP1
11022	ENSG00000182134	831.340508	-0.17795952	0.09129015	0.04427241	0.13549029	TDRKH
7562	ENSG00000182141	303.623023	0.13605624	0.13516613	0.28194841	0.5067358	ZNF708
9798	ENSG00000182149	2013.0859	0.19186416	0.08008603	0.01421085	0.05522884	IST1
375748	ENSG00000182150	925.235786	-0.25881415	0.09193419	0.00364505	0.01785624	ERCC6L2
64975	ENSG00000182154	590.069026	-0.21780913	0.10006551	0.02374313	0.08361089	MRPL41
339221	ENSG00000182156	0	NA	NA	NA	NA	ENPP7
64764	ENSG00000182158	2471.66241	1.07320361	0.0941473	5.43E-31	3.20E-29	CREB3L2
286530	ENSG00000182162	0	NA	NA	NA	NA	P2RY8
NA	ENSG00000182165	173.423334	0.36371719	0.18392605	0.02531119	0.08793099	TP53TG1
8633	ENSG00000182168	7605.56612	0.43773103	0.05192813	1.71E-17	5.45E-16	UNC5C
386746	ENSG00000182170	0	NA	NA	NA	NA	MRGPRG
283989	ENSG00000182173	842.331359	-0.31749773	0.08794749	0.00020066	0.00142693	TSEN54
56963	ENSG00000182175	174.81107	1.17104116	0.20102712	6.01E-10	1.07E-08	RGMA
401036	ENSG00000182177	0	NA	NA	NA	NA	ASB18
7318	ENSG00000182179	42.9432151	0.75707205	0.37383547	0.00604117	0.02731466	UBA7

51021	ENSG00000182180	1556.37419	-0.03553084	0.07685629	0.63632642	0.81722218	MRPS16
348378	ENSG00000182183	3.95628535	-0.01376179	0.36756407	0.89639252	0.95469109	SHISAL2A
5890	ENSG00000182185	59.227752	0.01488809	0.2673024	0.9400679	0.97495803	RAD51B
1419	ENSG00000182187	0	NA	NA	NA	NA	CRYGB
23641	ENSG00000182195	289.783368	0.01790221	0.18449971	0.91316447	0.96291167	LDOC1
51329	ENSG00000182196	1573.59852	-0.40746217	0.08324023	5.10E-07	6.09E-06	ARL6IP4
2131	ENSG00000182197	361.99283	0.26287274	0.14011602	0.04320215	0.13305349	EXT1
6472	ENSG00000182199	3508.57273	-0.09656275	0.07123311	0.16730985	0.35828164	SHMT2
81532	ENSG00000182208	595.266848	-0.10406058	0.10042403	0.2808741	0.50556476	MOB2
84439	ENSG00000182218	54.2051084	-0.02334005	0.24348518	0.89998849	0.95573896	HHIPL1
10159	ENSG00000182220	968.436711	-0.14400477	0.1034821	0.14754421	0.32938867	ATP6AP2
326340	ENSG00000182223	25.8384814	0.37844988	0.40154415	0.10658236	0.26121406	ZAR1
124637	ENSG00000182224	242.188592	-0.22542615	0.1631519	0.12607954	0.29536168	CYB5D1
202134	ENSG00000182230	0	NA	NA	NA	NA	FAM153B
25825	ENSG00000182240	0	NA	NA	NA	NA	BACE2
7325	ENSG00000182247	377.647697	-0.128183	0.1268838	0.28346436	0.508585	UBE2E2
23336	ENSG00000182253	292.982484	0.03439019	0.13512596	0.78666755	0.90294962	SYNM
3739	ENSG00000182255	0.99919462	0.02216778	0.38208598	0.68033314	NA	KCNA4
2567	ENSG00000182256	13.3646285	-0.15982661	0.37145202	0.3508548	0.58113897	GABRG3
NA	ENSG00000182257	7.70011931	-0.07737177	0.35624483	0.60898207	0.7978212	PRR34
338322	ENSG00000182261	0	NA	NA	NA	NA	NLRP10
55137	ENSG00000182263	368.221708	0.24303665	0.17796393	0.12323659	0.29076157	FIGN
284359	ENSG00000182264	4.67687247	-0.08607206	0.37630398	0.47460818	0.69683765	IZUMO1
388364	ENSG00000182271	0	NA	NA	NA	NA	TMIGD1
338707	ENSG00000182272	3182.09222	0.31211196	0.07101316	7.48E-06	7.28E-05	B4GALNT4
8905	ENSG00000182287	2012.21003	-0.81546793	0.07525838	4.39E-28	2.36E-26	AP1S2
65265	ENSG00000182307	1227.47903	0.13144819	0.0904871	0.13511832	0.30959088	C8orf33
285429	ENSG00000182308	20.7800974	0.50008736	0.50334609	0.05558713	0.16154221	DCAF4L1
147650	ENSG00000182310	81.6803017	0.27718408	0.24663974	0.15636644	0.34283458	SPACA6
653657	ENSG00000182315	0	NA	NA	NA	NA	MBD3L3
342945	ENSG00000182318	244.372207	-0.28056858	0.16666548	0.06121702	0.17402861	ZSCAN22
3770	ENSG00000182324	103.563117	0.24969986	0.21566039	0.1667416	0.35752225	KCNJ14
26233	ENSG00000182325	664.551777	0.13025514	0.10355643	0.19125343	0.3929241	FBXL6
716	ENSG00000182326	6.70878014	0.528381	0.9755501	0.01819055	0.06735733	C1S
388323	ENSG00000182327	10.280095	-0.03120427	0.33980783	0.84490639	0.92965052	GLTPD2
100652824	ENSG00000182329	1.39341735	-0.00640538	0.37787071	0.9280557	NA	KIAA2012
391002	ENSG00000182330	0	NA	NA	NA	NA	PRAMEF8
8513	ENSG00000182333	0	NA	NA	NA	NA	LIPF

120066	ENSG00000182334	0	NA	NA	NA	NA	OR5P3
267012	ENSG00000182346	0	NA	NA	NA	NA	DAOA
NA	ENSG00000182347	0.14931947	-0.01844332	0.38480212	0.84537521	NA	PDSS1P1
219578	ENSG00000182348	0	NA	NA	NA	NA	ZNF804B
NA	ENSG00000182351	0	NA	NA	NA	NA	CRIP1P4
NA	ENSG00000182352	0	NA	NA	NA	NA	C17orf77
143879	ENSG00000182359	54.3976142	-0.84701693	0.47366698	0.00672625	0.02989443	KBTBD3
54059	ENSG00000182362	146.066889	0.22511645	0.18308936	0.16248612	0.35124479	YBEY
NA	ENSG00000182365	0	NA	NA	NA	NA	OR5F2P
157693	ENSG00000182366	7.17890443	0.15193715	0.38867343	0.30769073	0.53516122	FAM87A
2055	ENSG00000182372	462.080764	0.02137378	0.12088127	0.85366545	0.93418215	CLN8
339059	ENSG00000182376	12.6812496	0.14866636	0.35815437	0.40552085	0.6337561	
55344	ENSG00000182378	1141.14497	-0.06632543	0.10865796	0.52323437	0.73618679	PLCXD1
11247	ENSG00000182379	54.3502495	0.07301886	0.25761036	0.70596662	0.85825065	NXPH4
NA	ENSG00000182383	0	NA	NA	NA	NA	RPL27AP5
785	ENSG00000182389	285.602162	0.03954366	0.14720283	0.77213602	0.89539725	CACNB4
282618	ENSG00000182393	0	NA	NA	NA	NA	IFNL1
196968	ENSG00000182397	13.4340685	0.04878237	0.33814619	0.77093628	0.89504218	DNM1P46
122553	ENSG00000182400	327.481473	-0.16283478	0.16686533	0.2770056	0.50139504	TRAPPC6B
161779	ENSG00000182405	237.504823	0.15630158	0.14521926	0.2435525	0.46198062	PGBD4
9426	ENSG00000182415	0	NA	NA	NA	NA	CDY2A
55666	ENSG00000182446	3259.61263	0.17480209	0.06738616	0.00825622	0.03547631	NPLOC4
131149	ENSG00000182447	0	NA	NA	NA	NA	OTOL1
50801	ENSG00000182450	15.0354422	-0.58731483	0.6667727	0.03557196	0.11473707	KCNK4
400629	ENSG00000182459	4.65008364	0.04356094	0.36530198	0.72614363	0.86907701	TEX19
128553	ENSG00000182463	301.407617	-1.97828066	0.20031661	3.07E-24	1.42E-22	TSHZ2
147968	ENSG00000182472	24.2407431	0.88999648	0.58072849	0.00984174	0.04094361	CAPN12
23265	ENSG00000182473	4844.61964	0.08950502	0.05961226	0.12947082	0.30096087	EXOC7
NA	ENSG00000182477	0	NA	NA	NA	NA	OR2B8P
3838	ENSG00000182481	5279.43138	-0.14091831	0.08262018	0.07959502	0.21058343	KPNA2
NA	ENSG00000182484	761.114637	0.17055558	0.09573108	0.06549937	0.18274611	WASH6P
654816	ENSG00000182487	0.16735865	0.01897177	0.38482933	0.78225479	NA	NCF1B
402415	ENSG00000182489	9.83489309	0.22716885	0.41747802	0.19573259	0.39935567	XKRX
633	ENSG00000182492	1.58966381	-0.03842805	0.38062286	0.58822104	NA	BGN
79598	ENSG00000182504	805.357981	0.00025833	0.08814226	0.99832375	0.99941443	CEP97
340596	ENSG00000182508	0.30001698	-0.0229446	0.38527539	0.68644806	NA	LHFPL1
2242	ENSG00000182511	10.1700202	-0.0741799	0.36562157	0.57507665	0.77394659	FES
51218	ENSG00000182512	798.741952	-0.24443326	0.08956011	0.0048921	0.02280528	GLRX5

90736	ENSG00000182518	225.624207	-0.02525673	0.16003577	0.86108849	0.93812292	FAM104B
387332	ENSG00000182521	0.173733	0.01914545	0.38484597	0.78225479	NA	TBPL2
NA	ENSG00000182531	0.34746601	0.02370763	0.3853651	0.61987128	NA	OR7E115P
859	ENSG00000182533	0.14931947	-0.01844332	0.38480212	0.84537521	NA	CAV3
439921	ENSG00000182534	2412.18614	-0.51794971	0.07050798	7.79E-14	1.93E-12	MXRA7
3985	ENSG00000182541	895.990305	-0.50354385	0.1088796	1.45E-06	1.59E-05	LIMK2
84975	ENSG00000182544	339.009834	-0.01326855	0.13784566	0.91673936	0.96473288	MFSD5
338879	ENSG00000182545	2.95933702	0.01496225	0.3700817	0.88649058	0.95015905	RNASE10
55256	ENSG00000182551	1292.294	-0.10899725	0.08240002	0.175047	0.36892512	ADI1
201965	ENSG00000182552	659.400821	-0.13792341	0.10960195	0.18813948	0.38853827	RWDD4
201305	ENSG00000182557	0	NA	NA	NA	NA	SPNS3
NA	ENSG00000182565	0	NA	NA	NA	NA	OR4C2P
339390	ENSG00000182566	0	NA	NA	NA	NA	CLEC4G
6304	ENSG00000182568	2198.09648	0.64269725	0.11230529	2.82E-09	4.55E-08	SATB1
NA	ENSG00000182574	0	NA	NA	NA	NA	CSGALNACT2P1
11248	ENSG00000182575	0.83842976	-0.00906171	0.38082224	0.87298405	NA	NXPH3
1436	ENSG00000182578	1.62959926	-0.07203796	0.39059462	0.24848065	NA	CSF1R
2049	ENSG00000182580	251.126465	-0.87621469	0.20115883	1.90E-06	2.05E-05	EPHB3
26609	ENSG00000182583	0.173124	-0.01911262	0.38486262	0.84537521	NA	VCX
255324	ENSG00000182585	0.72092638	0.02647699	0.38352105	0.56127935	NA	EPGN
114042	ENSG00000182586	1.5275847	0.07309172	0.38803268	0.32338244	NA	LINC00334
337880	ENSG00000182591	0	NA	NA	NA	NA	KRTAP11-1
389084	ENSG00000182600	40.2047055	-0.66211481	0.44464713	0.01910202	0.07019524	SNORC
9951	ENSG00000182601	0	NA	NA	NA	NA	HS3ST4
22906	ENSG00000182606	1786.36708	-0.19695246	0.07094072	0.0046376	0.02183436	TRAK1
83882	ENSG00000182612	48.121086	1.02249708	0.39742704	0.00095983	0.00571346	TSPAN10
285659	ENSG00000182613	0	NA	NA	NA	NA	OR2V2
23236	ENSG00000182621	220.247798	-0.0306117	0.15562833	0.82854753	0.92220943	PLCB1
NA	ENSG00000182625	0	NA	NA	NA	NA	
348235	ENSG00000182628	1730.77793	-0.99955528	0.0799092	9.43E-37	6.68E-35	SKA2
51289	ENSG00000182631	0.6228022	-0.04506827	0.38756783	0.3741287	NA	RXFP3
414194	ENSG00000182632	431.969655	0.33023656	0.12150813	0.00407399	0.01960892	CCNYL2
390265	ENSG00000182634	0	NA	NA	NA	NA	OR10G7
4692	ENSG00000182636	122.324227	2.45360424	0.26520853	1.55E-21	6.21E-20	NDN
374355	ENSG00000182645	0	NA	NA	NA	NA	CCDC172
100506380	ENSG00000182648	175.206685	0.23700954	0.23712804	0.21311567	0.42303301	LINC01006
NA	ENSG00000182652	0	NA	NA	NA	NA	OR4Q3
50863	ENSG00000182667	3.92580813	0.20624412	0.46746261	0.00395862	0.0191359	NTM

7267	ENSG00000182670	6270.72896	-0.53569988	0.06081708	5.25E-19	1.87E-17	TTC3
9312	ENSG00000182674	0	NA	NA	NA	NA	KCNB2
116729	ENSG00000182676	1.68915012	0.09460189	0.39444981	0.22138417	NA	PPP1R27
283870	ENSG00000182685	68.1956213	0.02218538	0.24130194	0.90750757	0.95963244	BRICD5
8811	ENSG00000182687	1.89698084	0.04621535	0.38011388	0.56749294	NA	GALR2
389075	ENSG00000182698	0	NA	NA	NA	NA	RESP18
492311	ENSG00000182700	84.046996	-0.20477609	0.22922057	0.27111488	0.49452232	IGIP
25987	ENSG00000182704	778.304601	0.37735652	0.0923568	2.47E-05	0.00021739	TSKU
NA	ENSG00000182707	0	NA	NA	NA	NA	FXYD6P3
100272147	ENSG00000182712	74.7021254	-0.13793868	0.22704024	0.45345703	0.67663865	CMC4
302	ENSG00000182718	1091.19499	0.95920695	0.1107401	6.51E-19	2.29E-17	ANXA2
NA	ENSG00000182722	0.36237696	0.00090817	0.38336443	0.96722566	NA	SEPHS1P1
9628	ENSG00000182732	0.173733	0.01914545	0.38484597	0.78225479	NA	RGS6
3214	ENSG00000182742	0	NA	NA	NA	NA	HOXB4
340146	ENSG00000182747	749.365496	-1.26765064	0.11927204	2.22E-27	1.15E-25	SLC35D3
164091	ENSG00000182749	534.606489	0.09780473	0.11035771	0.3552001	0.58555629	PAQR7
5069	ENSG00000182752	68.6738558	0.04364174	0.26779302	0.82179087	0.91847813	PAPPA
389692	ENSG00000182759	0.74659737	-0.03303578	0.38644226	0.4112877	NA	MAFA
51335	ENSG00000182768	2987.97264	-0.02431481	0.05855425	0.67377985	0.83989647	NGRN
2894	ENSG00000182771	264.391328	-1.69983788	0.15889652	7.95E-28	4.23E-26	GRID1
6218	ENSG00000182774	13759.354	-0.1388492	0.0514415	0.00639476	0.0286199	RPS17
NA	ENSG00000182776	0	NA	NA	NA	NA	
338442	ENSG00000182782	0.61725667	-0.01616397	0.38340632	0.71508012	NA	HCAR2
343563	ENSG00000182783	0	NA	NA	NA	NA	OR2T29
55231	ENSG00000182791	6.2090406	0.04011627	0.35746265	0.77304752	0.89561741	CCDC87
221357	ENSG00000182793	0	NA	NA	NA	NA	GSTA5
79098	ENSG00000182795	4.11138321	0.27884768	0.51886602	0.03654239	0.11731408	C1orf116
440104	ENSG00000182796	542.378911	0.40737386	0.11057039	0.00011728	0.00087769	TMEM198B
645864	ENSG00000182798	0.64893849	-0.03787929	0.38680562	0.40888116	NA	MAGEB17
1397	ENSG00000182809	2193.94354	-0.52046323	0.06795965	7.16E-15	1.93E-13	CRIP2
55794	ENSG00000182810	222.853385	0.22504724	0.16727384	0.13487664	0.30939444	DDX28
NA	ENSG00000182814	0	NA	NA	NA	NA	FUNDC2P2
337959	ENSG00000182816	0	NA	NA	NA	NA	KRTAP13-2
642643	ENSG00000182824	0.33471731	0.02354391	0.38534154	0.62842204	NA	FAM230E
64746	ENSG00000182827	1393.86311	-0.13595276	0.07262066	0.05636595	0.16323171	ACBD3
29035	ENSG00000182831	1894.57746	-0.27359139	0.07028962	7.28E-05	0.00057491	C16orf72
345557	ENSG00000182836	3215.9047	-1.19766158	0.06759487	2.32E-71	3.87E-69	PLCXD3
91695	ENSG00000182841	228.792835	0.27652857	0.15114884	0.04634684	0.14047072	RRP7BP

284013	ENSG00000182853	3.15406849	-0.02567856	0.3716976	0.79673682	0.90658744	VMO1
390649	ENSG00000182854	0.14931947	-0.01844332	0.38480212	0.84537521	NA	OR4F15
79087	ENSG00000182858	590.945071	0.27328623	0.14419561	0.04033895	0.1262121	ALG12
3932	ENSG00000182866	0	NA	NA	NA	NA	LCK
50614	ENSG00000182870	0	NA	NA	NA	NA	GALNT9
80781	ENSG00000182871	1291.08811	-0.00151768	0.08451586	0.98495952	0.99473578	COL18A1
8241	ENSG00000182872	2207.04903	-0.08133003	0.06805043	0.22427772	0.43800275	RBM10
100506504	ENSG00000182873	58.5663039	0.29860578	0.32099988	0.16960171	0.36127312	PRKCZ-AS1
222487	ENSG00000182885	0	NA	NA	NA	NA	ADGRG3
NA	ENSG00000182888	0.14931947	-0.01844332	0.38480212	0.84537521	NA	RPS27AP20
2747	ENSG00000182890	0.34111665	0.02362658	0.3853534	0.62410225	NA	GLUD2
339168	ENSG00000182896	0	NA	NA	NA	NA	TMEM95
126637	ENSG00000182898	0	NA	NA	NA	NA	TCHHL1
6165	ENSG00000182899	6276.01539	0.01254535	0.05224009	0.92453796	0.96857287	RPL35A
6000	ENSG00000182901	178.687389	-1.74892312	0.19755763	6.50E-20	2.41E-18	RGS7
83733	ENSG00000182902	3.75382409	0.09097394	0.38110037	0.42614263	0.65196405	SLC25A18
170960	ENSG00000182903	668.600993	0.18013489	0.09861928	0.05844691	0.16783712	ZNF721
NA	ENSG00000182912	250.158656	-0.18242409	0.32875837	0.36072598	0.59064153	TSPEAR-AS2
56849	ENSG00000182916	419.494361	-0.27437792	0.11895457	0.01489813	0.05734884	TCEAL7
28970	ENSG00000182919	469.126672	-0.02204004	0.14252773	0.86663204	0.94131062	C11orf54
NA	ENSG00000182921	0	NA	NA	NA	NA	CCDC75P1
80254	ENSG00000182923	487.373047	-0.13809158	0.10930784	0.18653346	0.38634762	CEP63
280664	ENSG00000182931	0	NA	NA	NA	NA	WFDC10B
6734	ENSG00000182934	2406.61691	0.23387133	0.06920794	0.00058277	0.0036888	SRPRA
347741	ENSG00000182938	0	NA	NA	NA	NA	OTOP3
2130	ENSG00000182944	8113.9076	0.07945057	0.05635693	0.1544292	0.33975109	EWSR1
161753	ENSG00000182950	1.80988999	0.0855585	0.39189238	0.25050022	NA	ODF3L1
10473	ENSG00000182952	1330.48353	-0.15352355	0.08049677	0.05081352	0.15087402	HMGN4
221178	ENSG00000182957	339.106348	0.30086306	0.14646932	0.02591427	0.08955568	SPATA13
10052	ENSG00000182963	1653.73281	0.1171334	0.09821602	0.21718093	0.42857268	GJC1
NA	ENSG00000182965	0	NA	NA	NA	NA	NPM1P14
6656	ENSG00000182968	0	NA	NA	NA	NA	SOX1
25904	ENSG00000182973	774.036403	-0.07695428	0.10941419	0.46211261	0.68512286	CNOT10
NA	ENSG00000182974	0	NA	NA	NA	NA	OR4M2B
9112	ENSG00000182979	5988.28778	0.27457211	0.05779718	1.51E-06	1.66E-05	MTA1
389114	ENSG00000182983	300.31674	0.04226168	0.13029861	0.72955123	0.87068674	ZNF662
23705	ENSG00000182985	3686.35791	-0.54360001	0.06641659	9.83E-17	2.99E-15	CADM1
162967	ENSG00000182986	1296.86085	-0.13257804	0.0865134	0.11508591	0.27652954	ZNF320

144608	ENSG00000182993	39.0566859	0.17576946	0.32309272	0.38047231	0.60938575	C12orf60
5831	ENSG00000183010	3447.90184	0.50039068	0.08183751	3.99E-10	7.21E-09	PYCR1
84316	ENSG00000183011	699.259752	-0.04687215	0.09690647	0.61622537	0.80322655	NAA38
124976	ENSG00000183018	46.6823325	-0.42365113	0.39366423	0.07843673	0.20835248	SPNS2
199675	ENSG00000183019	0.14931947	-0.01844332	0.38480212	0.84537521	NA	MCEMP1
161	ENSG00000183020	2222.3679	-0.17690089	0.0834091	0.02931159	0.09858313	AP2A2
NA	ENSG00000183022	0	NA	NA	NA	NA	TPM3P8
6546	ENSG00000183023	97.9832383	-0.94117601	0.29011116	0.00013872	0.00102161	SLC8A1
8390	ENSG00000183024	0	NA	NA	NA	NA	OR1G1
89874	ENSG00000183032	0	NA	NA	NA	NA	SLC25A21
92736	ENSG00000183034	0	NA	NA	NA	NA	OTOP2
1538	ENSG00000183035	0	NA	NA	NA	NA	CYLC1
5121	ENSG00000183036	0.173124	-0.01911262	0.38486262	0.84537521	NA	PCP4
18	ENSG00000183044	1417.46512	-0.66242276	0.08268382	2.91E-16	8.60E-15	ABAT
1468	ENSG00000183048	785.754966	0.05179308	0.08975674	0.55345964	0.75964298	SLC25A10
57118	ENSG00000183049	708.124266	-0.17458133	0.11522053	0.1111431	0.26924042	CAMK1D
729540	ENSG00000183054	860.462356	-0.15649157	0.10548123	0.12164525	0.28819117	RGPD6
NA	ENSG00000183055	0	NA	NA	NA	NA	FAM133CP
145748	ENSG00000183060	202.17867	-0.16619834	0.17096535	0.27605083	0.50016881	LYSMD4
164684	ENSG00000183066	9.59951775	-0.06689328	0.35110151	0.6632172	0.83380582	WBP2NL
150084	ENSG00000183067	0.9298696	0.00937803	0.38200548	0.84585339	NA	IGSF5
1482	ENSG00000183072	0	NA	NA	NA	NA	NKX2-5
125061	ENSG00000183077	453.925712	0.0980781	0.113376	0.36549594	0.59471759	AFMID
2621	ENSG00000183087	1118.17219	-0.35891313	0.08602655	1.78E-05	0.00016151	GAS6
166752	ENSG00000183090	0.173124	-0.01911262	0.38486262	0.84537521	NA	FREM3
4703	ENSG00000183091	21.7846093	-0.0730346	0.30794126	0.69458437	0.85228239	NEB
57596	ENSG00000183092	6200.48128	0.62930014	0.06441651	4.41E-23	1.92E-21	BEGAIN
10082	ENSG00000183098	1037.19608	-0.14145602	0.13079458	0.24828008	0.46743944	GPC6
389337	ENSG00000183111	258.33703	0.41181784	0.16171726	0.00515068	0.02382041	ARHGEF37
163933	ENSG00000183114	3.18077887	0.24723647	0.50048853	0.01436346	0.05564753	FAM43B
64478	ENSG00000183117	0	NA	NA	NA	NA	CSMD1
NA	ENSG00000183122	0	NA	NA	NA	NA	OR2A3P
119395	ENSG00000183128	0.45049191	-0.02733669	0.38571609	0.56128806	NA	CALHM3
11251	ENSG00000183134	4.61036505	0.07344575	0.37378476	0.53723911	0.74759591	PTGDR2
285753	ENSG00000183137	515.678155	-0.40938753	0.13850346	0.00151035	0.00842196	CEP57L1
53820	ENSG00000183145	5.34379603	0.19593501	0.4322084	0.13363379	0.3075034	RIPPLY3
NA	ENSG00000183146	0	NA	NA	NA	NA	PRORY
NA	ENSG00000183148	1.49319474	-0.05783425	0.39048926	0.16515854	NA	ANKRD20A2P

2842	ENSG00000183150	87.5812986	-1.4166223	0.33891376	2.05E-06	2.20E-05	GPR19
125111	ENSG00000183153	57.8579662	0.27587726	0.31206127	0.19712719	0.40108743	GJD3
102723701	ENSG00000183154	2.08694033	0.08914678	0.39094022	0.28568966	NA	
5877	ENSG00000183155	364.855272	-0.41059359	0.12560703	0.00053355	0.00341254	RABIF
338773	ENSG00000183160	1464.34697	-0.49174017	0.07258829	5.10E-12	1.11E-10	TMEM119
2188	ENSG00000183161	615.902127	0.05566112	0.11094945	0.60091472	0.79174845	FANCF
83698	ENSG00000183166	0.74084089	0.04259458	0.38746842	0.33153098	NA	CALN1
NA	ENSG00000183169	0	NA	NA	NA	NA	POM121L1P
NA	ENSG00000183171	0.50081678	-0.02974107	0.38592371	0.52183155	NA	
91689	ENSG00000183172	179.920641	-0.36119079	0.18582018	0.02722423	0.0930328	SMDT1
200959	ENSG00000183185	0	NA	NA	NA	NA	GABRR3
126567	ENSG00000183186	259.087312	0.08201372	0.14166329	0.53416779	0.74539459	C2CD4C
4166	ENSG00000183196	6.50725897	0.0001403	0.35792586	0.99835658	0.99941443	CHST6
NA	ENSG00000183199	0.37844916	0.02407552	0.3854191	0.60038877	NA	HSP90AB3P
388468	ENSG00000183206	0	NA	NA	NA	NA	POTEC
10856	ENSG00000183207	3267.49111	-0.0769629	0.06279436	0.21461133	0.42493901	RUVBL2
390637	ENSG00000183208	50.944386	-0.28314974	0.2985247	0.18121066	0.378484	GDPGP1
29119	ENSG00000183230	1.95964608	-0.09244964	0.39567729	0.18279773	NA	CTNNA3
NA	ENSG00000183239	0.62281892	-0.04506767	0.38756765	0.37413999	NA	
NA	ENSG00000183242	2.32339574	0.16804855	0.43703502	0.01376837	NA	WT1-AS
150221	ENSG00000183246	1.27594059	0.04712006	0.38669214	0.32115792	NA	RIMBP3C
80164	ENSG00000183248	1421.96355	0.29190567	0.09679058	0.00178339	0.00969646	PRR36
NA	ENSG00000183249	0	NA	NA	NA	NA	NF1P3
84536	ENSG00000183250	143.566392	-0.39579906	0.20651129	0.02547023	0.08840612	LINC01547
79339	ENSG00000183251	0	NA	NA	NA	NA	OR51B4
754	ENSG00000183255	3824.58761	0.03757725	0.05343429	0.47877357	0.70035842	PTTG1IP
51428	ENSG00000183258	1397.32833	0.1660517	0.08421041	0.04306489	0.13277051	DDX41
140701	ENSG00000183260	0	NA	NA	NA	NA	ABHD16B
390079	ENSG00000183269	0	NA	NA	NA	NA	OR52E8
160777	ENSG00000183273	0.16735865	0.01897177	0.38482933	0.78225479	NA	CCDC60
5343	ENSG00000183281	66.8019665	0.02636458	0.2320165	0.88813123	0.95127807	PLGLB1
9802	ENSG00000183283	1409.60354	0.1063095	0.08276416	0.18836657	0.38884536	DAZAP2
147372	ENSG00000183287	173.623839	-1.31987244	0.21432024	6.63E-11	1.30E-09	CCBE1
9403	ENSG00000183291	1368.1843	-0.15215822	0.07965795	0.05046875	0.14998501	SELENOF
150527	ENSG00000183292	0	NA	NA	NA	NA	PRSS40A
646743	ENSG00000183292	0	NA	NA	NA	NA	PRSS40A
NA	ENSG00000183298	1.13550476	-0.03439825	0.38119686	0.60753377	NA	RPSAP19
120065	ENSG00000183303	0	NA	NA	NA	NA	OR5P2

171482	ENSG00000183304	0	NA	NA	NA	NA	FAM9A
266740	ENSG00000183305	881.252936	-0.51348747	0.11562946	3.34E-06	3.46E-05	MAGEA2B
27439	ENSG00000183307	238.799174	-1.32439814	0.16156031	2.34E-17	7.39E-16	TMEM121B
NA	ENSG00000183308	1.96235268	-0.04398916	0.37897764	0.59876214	NA	
9831	ENSG00000183309	1128.71671	-0.12237347	0.08476308	0.13830036	0.31462897	ZNF623
127068	ENSG00000183310	0	NA	NA	NA	NA	OR2T34
338751	ENSG00000183313	0	NA	NA	NA	NA	OR52L1
284656	ENSG00000183317	142.735155	-1.55226283	0.97925863	0.00332039	0.01647737	EPHA10
388333	ENSG00000183318	0	NA	NA	NA	NA	SPDYE4
202243	ENSG00000183323	299.760637	-0.25291979	0.1491481	0.06487628	0.18151817	CCDC125
283677	ENSG00000183324	0	NA	NA	NA	NA	REC114
552900	ENSG00000183336	477.37078	-0.00147351	0.13222623	0.98931465	0.99604194	BOLA2
54880	ENSG00000183337	2913.81868	-0.01233759	0.08238089	0.87688773	0.94566867	BCOR
8690	ENSG00000183340	256.046049	0.20147875	0.15844579	0.16300904	0.35195933	JRKL
219621	ENSG00000183346	50.9948343	0.00089343	0.25461116	0.9978654	0.99926338	CABCOCO1
163351	ENSG00000183347	0.49008205	-0.00828035	0.38275294	0.84705846	NA	GBP6
158358	ENSG00000183354	613.373373	-0.13922118	0.11202212	0.19159747	0.39346794	KIAA2026
341277	ENSG00000183378	0	NA	NA	NA	NA	OVCH2
646658	ENSG00000183379	0.14931947	-0.01844332	0.38480212	0.84537521	NA	SYNDIG1L
2275	ENSG00000183386	216.839551	-0.33024555	0.15925611	0.02249359	0.08003652	FHL3
120793	ENSG00000183389	0	NA	NA	NA	NA	OR56A4
5367	ENSG00000183395	0.34746601	0.02370763	0.3853651	0.61987128	NA	PMCH
440955	ENSG00000183396	0.173733	0.01914545	0.38484597	0.78225479	NA	TMEM89
100128569	ENSG00000183397	10.6974654	0.19708328	0.39555933	0.25552079	0.47650319	C19orf71
126075	ENSG00000183401	56.3465642	0.33277442	0.28726674	0.11767454	0.28128469	CCDC159
54101	ENSG00000183421	0.66333729	0.00158865	0.38130712	0.97431639	NA	RIPK4
345193	ENSG00000183423	3.11784857	0.14673233	0.41199684	0.1522651	0.33663244	LRIT3
9284	ENSG00000183426	2178.91567	0.13164073	0.07050357	0.05743543	0.16567591	NPIPA1
642799	ENSG00000183426	2178.91567	0.13164073	0.07050357	0.05743543	0.16567591	NPIPA1
10946	ENSG00000183431	2064.72768	-0.25082029	0.08516101	0.00246207	0.01280072	SF3A3
NA	ENSG00000183432	0	NA	NA	NA	NA	ZBTB8OSP1
51270	ENSG00000183434	0.29863895	-0.02292439	0.3852726	0.68753245	NA	TFDP3
391712	ENSG00000183439	0.70350977	-0.04037975	0.3871535	0.37490811	NA	TRIM61
NA	ENSG00000183444	41.8999704	0.05755485	0.26242981	0.76684628	0.89261979	OR7E38P
2903	ENSG00000183454	0	NA	NA	NA	NA	GRIN2A
NA	ENSG00000183458	1707.62447	0.42273476	0.08104768	9.26E-08	1.23E-06	PKD1P3
646625	ENSG00000183463	0	NA	NA	NA	NA	URAD
286023	ENSG00000183470	0	NA	NA	NA	NA	

728340	ENSG00000183474	1070.69753	0.09265866	0.091351	0.29662484	0.52325392	GTF2H2C
140460	ENSG00000183475	357.105754	-0.22577618	0.13459501	0.07221512	0.19574701	ASB7
646892	ENSG00000183476	21.2075591	0.18275889	0.334205	0.36048215	0.5904664	SH2D7
11219	ENSG00000183479	8.32235357	-0.06549389	0.36669979	0.61050156	0.7987352	TREX2
29933	ENSG00000183484	0.67813863	0.00201297	0.38127911	0.96796511	NA	GPR132
4600	ENSG00000183486	0	NA	NA	NA	NA	MX2
57634	ENSG00000183495	3821.61799	-0.02216248	0.05937145	0.70459866	0.85803894	EP400
84206	ENSG00000183496	1909.00365	0.32876382	0.06795378	8.53E-07	9.79E-06	MEX3B
NA	ENSG00000183506	1086.86217	0.15130766	0.15032468	0.27278616	0.49638425	PI4KAP2
54855	ENSG00000183508	0.48674198	0.01127224	0.38339997	0.7864547	NA	TENT5C
493753	ENSG00000183513	689.34976	0.0287561	0.10294749	0.77280314	0.89561741	COA5
NA	ENSG00000183514	0	NA	NA	NA	NA	TDGF1P2
51118	ENSG00000183520	842.189234	-0.08066967	0.09850859	0.39595411	0.62540637	UTP11
8624	ENSG00000183527	265.838524	-0.28309486	0.1567402	0.04714193	0.14220708	PSMG1
253143	ENSG00000183530	1800.18372	-0.22808034	0.08068785	0.00372133	0.01816736	PRR14L
378832	ENSG00000183535	1.70346212	-0.0263742	0.37665076	0.7499803	NA	COL18A1-AS1
8302	ENSG00000183542	0	NA	NA	NA	NA	KLRC4
54988	ENSG00000183549	0	NA	NA	NA	NA	ACSM5
399814	ENSG00000183559	0	NA	NA	NA	NA	C10orf120
390243	ENSG00000183560	0	NA	NA	NA	NA	IZUMO1R
NA	ENSG00000183562	5.63414418	-0.12360921	0.38785469	0.33222153	0.56138959	
317716	ENSG00000183566	0	NA	NA	NA	NA	BPIFA4P
253190	ENSG00000183569	26.2186865	0.07221953	0.31236504	0.700407	0.8555116	SERHL2
54039	ENSG00000183570	905.062567	-0.95520975	0.13613263	3.06E-13	7.23E-12	PCBP3
145814	ENSG00000183571	0.4479964	0.02634439	0.38557832	0.58562477	NA	PGPEP1L
84193	ENSG00000183576	606.650351	-0.23638342	0.10518962	0.01907146	0.07010892	SETD3
388121	ENSG00000183578	1.62877765	0.04686899	0.3828543	0.50010504	NA	TNFAIP8L3
84133	ENSG00000183579	451.18227	0.07232069	0.11112043	0.49712954	0.71502465	ZNRF3
23194	ENSG00000183580	799.15552	0.56792935	0.11949725	6.53E-07	7.64E-06	FBXL7
NA	ENSG00000183586	0	NA	NA	NA	NA	HMGN3P1
128989	ENSG00000183597	288.814562	-0.23805204	0.16469849	0.10851755	0.2643325	TANGO2
653604	ENSG00000183598	4.56898049	-0.09629338	0.38440524	0.38151357	0.61056943	H3C13
595101	ENSG00000183604	542.704554	0.11340265	0.12231395	0.32803069	0.55648815	SMG1P5
119559	ENSG00000183605	388.155232	-0.13972306	0.12562703	0.23795844	0.45535257	SFXN4
200504	ENSG00000183607	0	NA	NA	NA	NA	GKN2
84734	ENSG00000183615	9.49116109	0.54746156	0.80730728	0.03168835	0.10480027	FAM167B
116541	ENSG00000183617	203.530296	-0.10782347	0.15339013	0.44203063	0.66680698	MRPL54
220929	ENSG00000183621	97.4255286	0.06571326	0.20348937	0.70503767	0.85803894	ZNF438

56941	ENSG00000183624	638.867134	-0.22392539	0.1056091	0.02694508	0.09231401	HMCES
1232	ENSG00000183625	0	NA	NA	NA	NA	CCR3
105377067	ENSG00000183625	0	NA	NA	NA	NA	CCR3
8214	ENSG00000183628	100.826678	0.55621206	0.25213645	0.00756369	0.03297377	DGCR6
283768	ENSG00000183629	0	NA	NA	NA	NA	GOLGA8G
100130613	ENSG00000183631	0	NA	NA	NA	NA	PRR32
24150	ENSG00000183632	16.1842529	-0.24492703	0.39041187	0.218635	0.43050004	TP53TG3
102723655	ENSG00000183632	16.1842529	-0.24492703	0.39041187	0.218635	0.43050004	TP53TG3
102723713	ENSG00000183632	16.1842529	-0.24492703	0.39041187	0.218635	0.43050004	TP53TG3
102724101	ENSG00000183632	16.1842529	-0.24492703	0.39041187	0.218635	0.43050004	TP53TG3
102724127	ENSG00000183632	16.1842529	-0.24492703	0.39041187	0.218635	0.43050004	TP53TG3
94137	ENSG00000183638	5.61302993	0.1493574	0.39942894	0.25712386	0.47814578	RP1L1
337879	ENSG00000183640	0	NA	NA	NA	NA	KRTAP8-1
145858	ENSG00000183643	0.60279648	-0.02752712	0.38588313	0.49730371	NA	C15orf32
399949	ENSG00000183644	0.50207596	0.02562368	0.38565771	0.52549886	NA	HOATZ
348327	ENSG00000183647	223.712586	-0.01264424	0.16380931	0.93172742	0.9715756	ZNF530
4707	ENSG00000183648	335.903068	-0.28513905	0.12920994	0.01869577	0.06893219	NDUFB1
441061	ENSG00000183654	836.940151	-1.17085801	0.09787517	6.14E-34	3.99E-32	MARCHF11
64410	ENSG00000183655	602.648348	-0.09406624	0.10773814	0.36113867	0.59092711	KLHL25
407738	ENSG00000183662	0.16735865	0.01897177	0.38482933	0.78225479	NA	TAFA1
NA	ENSG00000183663	0	NA	NA	NA	NA	GAPDHP30
55039	ENSG00000183665	289.98739	0.20048785	0.15370822	0.15381039	0.33890581	TRMT12
728411	ENSG00000183666	284.8359	0.16691142	0.15790118	0.24559542	0.46467796	GUSBP1
5678	ENSG00000183668	0	NA	NA	NA	NA	PSG9
2825	ENSG00000183671	13.6967807	0.04221513	0.34937051	0.7796528	0.89925062	GPR1
NA	ENSG00000183674	0	NA	NA	NA	NA	LINC00518
353500	ENSG00000183682	130.331849	-0.22868713	0.19493095	0.17411914	0.36742436	BMP8A
10189	ENSG00000183684	2385.95601	0.26001227	0.08755041	0.00224266	0.01181162	ALYREF
359845	ENSG00000183688	1653.66952	0.33919367	0.07659365	5.98E-06	5.93E-05	RFLNB
80258	ENSG00000183690	13.7750127	0.71964844	0.77071215	0.02414305	0.08477229	EFHC2
9241	ENSG00000183691	0	NA	NA	NA	NA	NOG
117194	ENSG00000183695	0	NA	NA	NA	NA	MRGPRX2
7378	ENSG00000183696	82.6515048	0.01241706	0.22603636	0.94729638	0.97843954	UPP1
NA	ENSG00000183704	0	NA	NA	NA	NA	SLC9B1P1
283694	ENSG00000183706	0	NA	NA	NA	NA	OR4N4
282616	ENSG00000183709	0	NA	NA	NA	NA	IFNL2
4978	ENSG00000183715	1.18815028	0.06717315	0.39222996	0.15327856	NA	OPCML
84851	ENSG00000183718	656.182941	0.00700202	0.1119826	0.94893184	0.97900074	TRIM52

10186	ENSG00000183722	126.66632	0.0204	0.19132454	0.90349323	0.95785538	LHFPL6
146223	ENSG00000183723	1066.72043	0.20333415	0.08461731	0.01363161	0.05344884	CMTM4
23585	ENSG00000183726	1011.94453	0.0290556	0.08455879	0.72528511	0.86857346	TMEM50A
344018	ENSG00000183733	1.02999244	0.03390006	0.3815362	0.60064137	NA	FIGLA
430	ENSG00000183734	0	NA	NA	NA	NA	ASCL2
29110	ENSG00000183735	528.001095	-0.18126435	0.11099386	0.08685592	0.22472894	TBK1
23466	ENSG00000183741	1014.91667	-0.24866338	0.08538821	0.00274685	0.01405297	CBX6
346389	ENSG00000183742	0.36122628	-0.00938616	0.38365907	0.92306001	NA	MACC1
123876	ENSG00000183747	0	NA	NA	NA	NA	ACSM2A
10607	ENSG00000183751	981.934489	0.19749443	0.09670863	0.03456711	0.11226248	TBL3
9083	ENSG00000183753	0	NA	NA	NA	NA	BPY2
390928	ENSG00000183760	0	NA	NA	NA	NA	ACP7
83999	ENSG00000183762	709.281892	0.5825542	0.11737135	2.16E-07	2.73E-06	KREMEN1
10293	ENSG00000183763	302.701009	-0.04207861	0.14215569	0.74887582	0.88205755	TRAIP
11200	ENSG00000183765	283.094731	0.00029895	0.1496135	0.99923868	0.99963176	CHEK2
668	ENSG00000183770	0	NA	NA	NA	NA	FOXL2
150209	ENSG00000183773	39.9955936	0.1891163	0.30378423	0.35434173	0.58485041	AIFM3
57528	ENSG00000183775	25.1264103	-0.06896403	0.30254519	0.71536661	0.86274919	KCTD16
10317	ENSG00000183778	75.5655546	-0.07770025	0.29181203	0.6840146	0.84597592	B3GALT5
80139	ENSG00000183779	190.57416	-1.41074038	0.19794753	9.11E-14	2.24E-12	ZNF703
148641	ENSG00000183780	392.628697	-1.82970776	0.13489259	5.07E-43	4.16E-41	SLC35F3
386617	ENSG00000183783	0	NA	NA	NA	NA	KCTD8
NA	ENSG00000183784	0	NA	NA	NA	NA	DOCK8-AS1
51807	ENSG00000183785	38.2014817	-0.1922599	0.32512115	0.3404433	0.57071357	TUBA8
100288332	ENSG00000183793	24.3014879	-0.1862385	0.40354043	0.23496489	0.45186105	NPIPA5
90187	ENSG00000183798	56.3057157	-0.49273219	0.3204256	0.03505683	0.11342589	EMILIN3
283298	ENSG00000183801	0.44808424	0.02634827	0.38557859	0.58559402	NA	OLFML1
221303	ENSG00000183807	32.5547868	2.36937783	0.47570963	3.97E-08	5.54E-07	FAM162B
389677	ENSG00000183808	1362.28683	-0.03092918	0.09195066	0.72808996	0.87004185	RBM12B
1233	ENSG00000183813	0	NA	NA	NA	NA	CCR4
286826	ENSG00000183814	448.991986	-0.11691845	0.11720607	0.29393792	0.52021105	LIN9
107985578	ENSG00000183822	0	NA	NA	NA	NA	NCF4-AS1
114781	ENSG00000183826	432.235308	-0.17104354	0.13363928	0.1704172	0.36231003	BTBD9
256281	ENSG00000183828	94.9110855	-0.05992958	0.21205522	0.73396271	0.87327664	NUDT14
339416	ENSG00000183831	116.495445	0.08893599	0.18999031	0.59190992	0.78646482	ANKRD45
89876	ENSG00000183833	143.417404	0.38667709	0.22488095	0.03933805	0.12396233	CFAP91
29944	ENSG00000183837	56.0348515	-0.87040695	0.3393478	0.00129319	0.00737693	PNMA3
2863	ENSG00000183840	6.00777454	1.98895326	1.91822416	0.00704353	0.03113025	GPR39

54097	ENSG00000183844	0	NA	NA	NA	NA	FAM3B
100129543	ENSG00000183850	166.932057	-0.2055263	0.18282898	0.20083115	0.40608337	ZNF730
55243	ENSG00000183853	0	NA	NA	NA	NA	KIRREL1
128239	ENSG00000183856	2726.38364	0.2348702	0.06033777	7.97E-05	0.00062137	IQGAP3
1260	ENSG00000183862	0	NA	NA	NA	NA	CNGA2
10766	ENSG00000183864	471.910589	0.07714513	0.12317642	0.5093292	0.72495095	TOB2
6331	ENSG00000183873	145.925391	-0.72211182	0.19605779	4.65E-05	0.00038373	SCN5A
340075	ENSG00000183876	0	NA	NA	NA	NA	ARSI
7404	ENSG00000183878	0	NA	NA	NA	NA	UTY
149563	ENSG00000183888	0	NA	NA	NA	NA	SRARP
NA	ENSG00000183889	3359.91575	-0.20828063	0.1002947	0.03080348	0.10237631	
130502	ENSG00000183891	111.84851	0.25079921	0.2114047	0.15982258	0.3473795	TTC32
219527	ENSG00000183908	0.14931947	-0.01844332	0.38480212	0.84537521	NA	LRRC55
NA	ENSG00000183909	0	NA	NA	NA	NA	OR4G2P
NA	ENSG00000183911	0.173733	0.01914545	0.38484597	0.78225479	NA	RPL21P132
146754	ENSG00000183914	11.8334534	0.16545937	0.4066389	0.19676758	0.40043787	DNAH2
4068	ENSG00000183918	0	NA	NA	NA	NA	SH2D1A
100288072	ENSG00000183921	0.18922458	0.01954053	0.38488496	0.78225479	NA	SDR42E2
NA	ENSG00000183929	90.1944052	-0.15734297	0.22407801	0.38959918	0.61876783	DUSP5P1
93164	ENSG00000183935	43.4496271	-0.16832391	0.28530918	0.4006072	0.6290998	HTR7P1
NA	ENSG00000183938	0	NA	NA	NA	NA	TRBV21OR9-2
5613	ENSG00000183943	1193.03794	0.0690996	0.09266628	0.44344604	0.66848792	PRKX
387893	ENSG00000183955	1115.92505	-0.2151225	0.10282345	0.02932941	0.09860958	KMT5A
131096	ENSG00000183960	501.342653	-0.31865283	0.1116691	0.00276331	0.01412629	KCNH8
6525	ENSG00000183963	632.082081	0.2999592	0.09979838	0.00180475	0.00979648	SMTN
283869	ENSG00000183971	121.220949	-0.10280374	0.19728241	0.54299779	0.75099868	NPW
151649	ENSG00000183977	5.55463117	0.0602902	0.36496604	0.64604706	0.82370422	PP2D1
28958	ENSG00000183978	407.471486	0.07619686	0.11699642	0.49466937	0.71350347	COA3
256933	ENSG00000183979	25.4317907	0.35271198	0.38398128	0.12408994	0.29194169	NPB
NA	ENSG00000183981	0	NA	NA	NA	NA	MAGEA13P
256435	ENSG00000184005	353.177909	-0.10213322	0.15095229	0.46045089	0.68357869	ST6GALNAC3
8073	ENSG00000184007	2536.76397	-0.32936649	0.06225721	7.89E-08	1.05E-06	PTP4A2
71	ENSG00000184009	35005.2675	0.09164841	0.05152944	0.07229325	0.19588883	ACTG1
7113	ENSG00000184012	0	NA	NA	NA	NA	TMPRSS2
23258	ENSG00000184014	2035.28055	-0.0216186	0.09772543	0.81729081	0.91682968	DENND5A
127069	ENSG00000184022	0.16735865	0.01897177	0.38482933	0.78225479	NA	OR2T10
10281	ENSG00000184029	0	NA	NA	NA	NA	DSCR4
337976	ENSG00000184032	0	NA	NA	NA	NA	KRTAP20-2

1485	ENSG00000184033	0.34746601	0.02370763	0.3853651	0.61987128	NA	CTAG1B
56616	ENSG00000184047	1243.15823	-0.03334015	0.09257128	0.70979727	0.860757	DIABLO
NA	ENSG00000184055	0	NA	NA	NA	NA	OR7E87P
26276	ENSG00000184056	188.012496	0.19306063	0.18531844	0.23517847	0.45199466	VPS33B
6899	ENSG00000184058	4.59591499	0.00181957	0.36722786	0.98902418	0.99604194	TBX1
55803	ENSG00000184060	9.71966644	-0.06891831	0.36813944	0.57786718	0.77608611	ADAP2
NA	ENSG00000184068	43.6268152	-0.07432663	0.27610491	0.70019738	0.85541355	SREBF2-AS1
29796	ENSG00000184076	795.10979	-0.08241164	0.09100843	0.35070053	0.58113472	UQCR10
54954	ENSG00000184083	435.818461	0.06766321	0.12396187	0.56485266	0.76715609	FAM120C
NA	ENSG00000184084	0	NA	NA	NA	NA	
NA	ENSG00000184100	0.67935032	0.0029718	0.38128818	0.95334318	NA	BRD7P2
NA	ENSG00000184106	0	NA	NA	NA	NA	TREML3P
339976	ENSG00000184108	0	NA	NA	NA	NA	TRIML1
8663	ENSG00000184110	18975.8737	-0.07107432	0.0510278	0.15933313	0.34688536	EIF3C
NA	ENSG00000184111	0	NA	NA	NA	NA	RPL11P4
7122	ENSG00000184113	13.0200556	0.04455294	0.33436326	0.7942049	0.90579008	CLDN5
NA	ENSG00000184115	0	NA	NA	NA	NA	
8508	ENSG00000184117	3907.22328	0.04429369	0.05809635	0.44242593	0.66720358	NIPSNAP1
NA	ENSG00000184139	0	NA	NA	NA	NA	RPL7AP28
390648	ENSG00000184140	0	NA	NA	NA	NA	OR4F6
6900	ENSG00000184144	159.393348	-2.87591259	0.28020175	6.25E-26	3.07E-24	CNTN2
163778	ENSG00000184148	0	NA	NA	NA	NA	SPRR4
220074	ENSG00000184154	182.932525	0.25718273	0.17026628	0.09179522	0.23401187	LRTOMT
127385	ENSG00000184155	0	NA	NA	NA	NA	OR10J5
3786	ENSG00000184156	453.84702	0.04591886	0.11152275	0.66779697	0.83659884	KCNQ3
152	ENSG00000184160	292.145786	-0.97091501	0.14892451	9.68E-12	2.07E-10	ADRA2C
126382	ENSG00000184162	207.619321	-0.20574215	0.17357231	0.18304418	0.38122995	NR2C2AP
388581	ENSG00000184163	6.21831818	0.20294806	0.42503995	0.17209795	0.36461546	C1QTNF12
79174	ENSG00000184164	1185.59647	0.82247605	0.09018945	1.39E-20	5.38E-19	CRELD2
4991	ENSG00000184166	0	NA	NA	NA	NA	OR1D2
152579	ENSG00000184178	273.097461	-0.20151032	0.14808276	0.13892828	0.31571462	SCFD2
140739	ENSG00000184182	649.498047	0.09855795	0.11166106	0.35586468	0.58600518	UBE2F
3768	ENSG00000184185	16.3435109	-0.13809988	0.34481884	0.44985768	0.67340229	KCNJ12
NA	ENSG00000184188	0.33471731	0.02354391	0.38534154	0.62842204	NA	
54328	ENSG00000184194	1404.6745	0.14032003	0.0839047	0.08642527	0.22393591	GPR173
5504	ENSG00000184203	587.061225	-0.23236807	0.13135043	0.05868202	0.16825136	PPP1R2
64061	ENSG00000184205	584.028612	0.10438592	0.11624601	0.34618175	0.57693723	TSPYL2
643707	ENSG00000184206	82.0495501	0.36951517	0.26032763	0.07076949	0.1930817	GOLGA6L4

283871	ENSG00000184207	722.05622	-0.22700484	0.10524498	0.02434137	0.08530875	PGP
79640	ENSG00000184208	540.121829	0.25813076	0.10596717	0.01107912	0.04514474	C22orf46
11066	ENSG00000184209	350.458852	0.24670947	0.13768159	0.05433834	0.15863564	SNRNP35
347516	ENSG00000184210	0	NA	NA	NA	NA	DGAT2L6
3654	ENSG00000184216	3075.13251	0.03633327	0.06278929	0.55894165	0.7635226	IRAK1
84319	ENSG00000184220	503.653533	-0.12659944	0.14255068	0.33723494	0.56754103	CMSS1
116448	ENSG00000184221	0	NA	NA	NA	NA	OLIG1
100505621	ENSG00000184224	11.7123317	0.17978398	0.38688393	0.28943388	0.51546747	C11orf72
5101	ENSG00000184226	179.55354	-1.51955517	0.28875995	9.28E-09	1.41E-07	PCDH9
641371	ENSG00000184227	111.443143	0.03629501	0.19689286	0.83149917	0.9237845	ACOT1
220323	ENSG00000184232	454.642888	-0.27319859	0.11958113	0.01577337	0.06006594	OAF
220	ENSG00000184254	1010.96357	0.86279711	0.12524498	8.49E-13	1.94E-11	ALDH1A3
8338	ENSG00000184260	44.8612721	-0.0103628	0.25846629	0.95551307	0.9817278	H2AC20
56660	ENSG00000184261	49.9536157	-0.38371738	0.33915063	0.09368448	0.23741219	KCNK12
317772	ENSG00000184270	1.54581079	0.03668979	0.38135524	0.58959859	NA	H2AC21
5463	ENSG00000184271	258.331553	0.01675698	0.16119063	0.9100288	0.96139357	POU6F1
NA	ENSG00000184274	0.36301683	0.0238952	0.38539247	0.60993574	NA	LINC00315
245911	ENSG00000184276	0.14931947	-0.01844332	0.38480212	0.84537521	NA	DEFB108B
80213	ENSG00000184277	551.904922	-0.29028838	0.12162075	0.01154986	0.04670794	TM2D3
10078	ENSG00000184281	705.467645	0.05916767	0.10076223	0.54351088	0.75149877	TSSC4
4070	ENSG00000184292	0	NA	NA	NA	NA	TACSTD2
160365	ENSG00000184293	0	NA	NA	NA	NA	CLECL1
4990	ENSG00000184302	122.591414	-0.48337463	0.23785409	0.01438484	0.05571951	SIX6
NA	ENSG00000184303	0	NA	NA	NA	NA	DRD5P1
5587	ENSG00000184304	3.05149192	0.19920663	0.44688349	0.06947046	0.19039838	PRKD1
401145	ENSG00000184305	48.5963035	-0.81547834	0.37824871	0.00380938	0.01848412	CCSER1
254887	ENSG00000184307	264.96224	-0.0199554	0.13910153	0.87663024	0.94566867	ZDHHC23
374977	ENSG00000184313	39.6542778	0.38445967	0.39841183	0.10170968	0.25249251	MROH7
NA	ENSG00000184319	261.260526	-0.02688116	0.15280958	0.84685882	0.93052693	RPL23AP82
79470	ENSG00000184321	0	NA	NA	NA	NA	OR51J1
338324	ENSG00000184330	0	NA	NA	NA	NA	S100A7A
26576	ENSG00000184343	29.6986025	0.18927905	0.3422335	0.33849084	0.56881111	SRPK3
9573	ENSG00000184344	0	NA	NA	NA	NA	GDF3
389123	ENSG00000184345	0	NA	NA	NA	NA	IQCF2
6586	ENSG00000184347	12980.3199	0.02277674	0.05287328	0.6641661	0.83431586	SLIT3
1946	ENSG00000184349	592.804258	0.72412801	0.17842056	9.90E-06	9.41E-05	EFNA5
116534	ENSG00000184350	21.3618245	-0.28997091	0.39545759	0.17363332	0.3667112	MRGPRE
337882	ENSG00000184351	0	NA	NA	NA	NA	KRTAP19-1

3009	ENSG00000184357	2.30186897	-0.04535901	0.3761815	0.63075496	NA	H1-5
124783	ENSG00000184361	0.53636214	0.01011868	0.38269945	0.78318813	NA	SPATA32
11187	ENSG00000184363	8.47080239	-0.41950536	0.65833226	0.04013296	0.12570856	PKP3
256714	ENSG00000184368	390.924221	-0.45660401	0.14376637	0.00062934	0.00394505	MAP7D2
1435	ENSG00000184371	34.1983067	1.19436467	0.60900225	0.00259547	0.01339596	CSF1
10584	ENSG00000184374	0	NA	NA	NA	NA	COLEC10
84517	ENSG00000184378	27.4991528	0.04433165	0.29445711	0.81801588	0.9171248	ACTRT3
8398	ENSG00000184381	145.843255	0.60677866	0.20867924	0.00094097	0.00562823	PLA2G6
84441	ENSG00000184384	106.587466	-1.17569501	0.24857503	2.21E-07	2.79E-06	MAML2
150147	ENSG00000184385	0	NA	NA	NA	NA	UMODL1-AS1
645974	ENSG00000184388	0.30139824	-0.02296476	0.38527817	0.68536535	NA	PABPC1L2B
127550	ENSG00000184389	0.91126447	0.06383254	0.39112218	0.20226405	NA	A3GALT2
390437	ENSG00000184394	0	NA	NA	NA	NA	OR4N5
26039	ENSG00000184402	1302.91603	-0.10784683	0.1000177	0.26292987	0.48497991	SS18L1
3751	ENSG00000184408	0.35653705	0.02381602	0.3853809	0.61408977	NA	KCND2
NA	ENSG00000184414	1.37425861	-0.0186538	0.38077972	0.75583429	NA	IRS3P
NA	ENSG00000184423	0	NA	NA	NA	NA	RPL23AP38
116447	ENSG00000184428	669.284017	0.05095941	0.09564657	0.58294159	0.77951315	TOP1MT
9276	ENSG00000184432	2442.90757	-0.07075992	0.07101524	0.31027696	0.53796162	COPB2
64922	ENSG00000184434	0.4990887	0.01258375	0.38346215	0.76502333	NA	LRRC19
80764	ENSG00000184436	527.303755	-0.28740552	0.11570132	0.00878399	0.03728592	THAP7
NA	ENSG00000184441	90.1645305	0.00409555	0.21479513	0.98678625	0.99565021	
9735	ENSG00000184445	2140.93762	-0.03318274	0.07872645	0.66573967	0.83494641	KNTC1
2826	ENSG00000184451	57.130177	-0.10165589	0.25437626	0.59569048	0.78816266	CCR10
400746	ENSG00000184454	0	NA	NA	NA	NA	NCMAP
254240	ENSG00000184459	0	NA	NA	NA	NA	BPIFC
253769	ENSG00000184465	1859.77795	0.23836318	0.07245135	0.00079596	0.00485434	WDR27
10587	ENSG00000184470	407.845579	0.00916757	0.12626148	0.93971713	0.97474024	TXNRD2
390664	ENSG00000184471	0	NA	NA	NA	NA	C1QTNF8
390083	ENSG00000184478	0	NA	NA	NA	NA	OR56A3
4303	ENSG00000184481	349.309463	-0.17474881	0.164094	0.23678134	0.45366893	FOXO4
5454	ENSG00000184486	753.335003	-1.25098719	0.1016366	8.51E-36	5.90E-34	POU3F2
11156	ENSG00000184489	410.099589	0.26193001	0.13703866	0.04023984	0.12600355	PTP4A3
200350	ENSG00000184492	5.8573231	0.18765166	0.4159029	0.1956001	0.39920852	FOXD4L1
348013	ENSG00000184497	62.5917727	1.66012474	0.36011555	2.61E-07	3.26E-06	TMEM255B
5627	ENSG00000184500	520.060001	0.33728209	0.10972767	0.00130301	0.00741802	PROS1
2520	ENSG00000184502	0	NA	NA	NA	NA	GAST
256646	ENSG00000184507	0.9593479	-0.01907145	0.3809965	0.74718274	NA	NUTM1

374659	ENSG00000184508	236.429843	-0.34344991	0.16427629	0.02075104	0.07510044	HDDC3
340542	ENSG00000184515	1.27492684	0.03450414	0.38293094	0.5471287	NA	BEX5
162239	ENSG00000184517	374.358775	-0.21858839	0.14026336	0.09261466	0.23536248	ZFP1
NA	ENSG00000184523	3.96947903	0.18006847	0.43466482	0.08182239	0.21481489	PTGER4P2
51286	ENSG00000184524	431.371413	-0.77825243	0.14656695	2.08E-08	3.02E-07	CEND1
352999	ENSG00000184530	1.35830311	0.00386659	0.37877003	0.95501466	NA	C6orf58
201140	ENSG00000184544	0	NA	NA	NA	NA	DHRS7C
1850	ENSG00000184545	519.558894	-0.87271244	0.11266378	1.56E-15	4.43E-14	DUSP8
9021	ENSG00000184557	137.37167	0.50012483	0.21533641	0.00690861	0.03061578	SOCS3
201243	ENSG00000184560	0.173124	-0.01911262	0.38486262	0.84537521	NA	SPEM2
84189	ENSG00000184564	474.453411	1.67712755	0.121458	1.89E-44	1.64E-42	SLITRK6
NA	ENSG00000184566	0	NA	NA	NA	NA	
440822	ENSG00000184571	0	NA	NA	NA	NA	PIWIL3
57121	ENSG00000184574	5.62594584	-0.00068712	0.35586821	0.9940063	0.99818216	LPAR5
11260	ENSG00000184575	4157.69294	0.01813693	0.06173765	0.76864653	0.8933771	XPOT
340061	ENSG00000184584	13.377469	-0.13968245	0.35913115	0.4166808	0.64309452	STING1
5142	ENSG00000184588	1292.45247	0.01875686	0.09120672	0.83345448	0.92419724	PDE4B
284467	ENSG00000184599	0.83752982	0.01555468	0.38115542	0.7824946	NA	TAFA3
400258	ENSG00000184601	9.01464274	-0.14629586	0.37813579	0.35488174	0.58535214	C14orf180
8303	ENSG00000184602	725.536913	0.21914574	0.09648456	0.0187112	0.06897625	SNN
83656	ENSG00000184608	1.1513741	-0.00401811	0.37950367	0.94927846	NA	FAM167A-AS1
90134	ENSG00000184611	51.4866377	-1.16607565	0.35045913	7.79E-05	0.00060943	KCNH7
NA	ENSG00000184612	0.71350263	0.0420622	0.38737624	0.34118409	NA	RPL7P26
4753	ENSG00000184613	99.489288	1.25081799	0.26018273	1.35E-07	1.75E-06	NELL2
NA	ENSG00000184616	0.51567365	0.03113947	0.38605517	0.47754913	NA	SPDYE12P
NA	ENSG00000184617	2.76198497	0.00756053	0.37373066	0.93500735	0.97306034	ZNF840P
124751	ENSG00000184619	852.768494	0.29689244	0.09351766	0.00103556	0.00608576	KRBA2
NA	ENSG00000184624	0	NA	NA	NA	NA	ZNF72P
9968	ENSG00000184634	2022.78771	0.15437401	0.09058232	0.07921441	0.20996844	MED12
81931	ENSG00000184635	237.618736	-0.33937723	0.15531071	0.016768	0.06323456	ZNF93
10801	ENSG00000184640	9865.67133	-0.23913979	0.05976063	4.91E-05	0.00040257	SEPTIN9
203074	ENSG00000184647	0	NA	NA	NA	NA	PRSS55
146852	ENSG00000184650	0	NA	NA	NA	NA	ODF4
349334	ENSG00000184659	7.50019949	0.25858226	0.45966431	0.12047028	0.28603762	FOXD4L4
157313	ENSG00000184661	487.066211	-0.54693151	0.12149823	2.29E-06	2.44E-05	CDCA2
NA	ENSG00000184669	11.2742338	-0.06455166	0.34525792	0.68697821	0.84777704	OR7E14P
138046	ENSG00000184672	0.54592432	0.03260597	0.38620377	0.45619093	NA	RALYL
139285	ENSG00000184675	400.574117	0.01953092	0.11689241	0.8606184	0.93799827	AMER1

9923	ENSG00000184677	1488.93641	-0.23097703	0.07894376	0.00272808	0.01398219	ZBTB40
8349	ENSG00000184678	147.674066	0.04306285	0.17652338	0.78487831	0.90209627	H2BC21
9074	ENSG00000184697	10.1693576	-0.24546042	0.43725618	0.15431249	0.33953197	CLDN6
390059	ENSG00000184698	0	NA	NA	NA	NA	OR51M1
5413	ENSG00000184702	1745.80015	-0.08252941	0.09140151	0.35112002	0.58132031	SEPTIN5
56478	ENSG00000184708	724.270413	0.13809796	0.10191363	0.15963469	0.34722847	EIF4ENIF1
389816	ENSG00000184709	10.2568001	-2.86047035	1.01403745	0.00029665	0.0020253	LRRC26
619189	ENSG00000184716	1.14012245	-0.03623364	0.38219595	0.56803734	NA	SERINC4
55328	ENSG00000184719	0.71148248	0.00456663	0.3818398	0.92335791	NA	RNLS
337966	ENSG00000184724	0	NA	NA	NA	NA	KRTAP6-1
55911	ENSG00000184730	1.48490281	-0.00830353	0.37725882	0.91165979	NA	APOBR
642273	ENSG00000184731	0.51618059	0.03116426	0.38605761	0.47718172	NA	FAM110C
168400	ENSG00000184735	0.51384646	-0.00893201	0.38272151	0.83199937	NA	DDX53
NA	ENSG00000184741	0	NA	NA	NA	NA	OR5AQ1P
25923	ENSG00000184743	622.552165	0.23385393	0.10788815	0.02358091	0.08312815	ATL3
55967	ENSG00000184752	516.624794	0.06398156	0.10743455	0.53562284	0.74647226	NDUFA12
NA	ENSG00000184761	0	NA	NA	NA	NA	PRSS40B
641515	ENSG00000184774	2.11634394	0.05690135	0.37899318	0.54361741	NA	MGAT4EP
644538	ENSG00000184785	1.43460945	0.05921468	0.38615675	0.36893512	NA	SMIM10
6991	ENSG00000184786	45.0833911	-0.15859181	0.26895794	0.42172616	0.64775467	DYNLT2
7327	ENSG00000184787	5701.44118	0.17701834	0.06005494	0.00287257	0.01460438	UBE2G2
340562	ENSG00000184788	31.1553601	-0.04098428	0.28544505	0.82939691	0.92261925	SATL1
NA	ENSG00000184789	0	NA	NA	NA	NA	OR4C10P
23762	ENSG00000184792	310.34526	-0.47417382	0.16932499	0.00197188	0.01057066	OSBP2
NA	ENSG00000184795	0.14931947	-0.01844332	0.38480212	0.84537521	NA	UNC93B5
NA	ENSG00000184809	12.4730782	0.50296726	0.77973674	0.03066522	0.10208021	B3GALT5-AS1
286753	ENSG00000184811	0.173733	0.01914545	0.38484597	0.78225479	NA	TRARG1
389151	ENSG00000184814	0	NA	NA	NA	NA	PRR23B
201501	ENSG00000184828	126.973011	-2.18281079	0.24744817	6.83E-20	2.53E-18	ZBTB7C
79135	ENSG00000184831	216.490802	-0.30114764	0.16818093	0.04621633	0.14012153	APOO
51334	ENSG00000184838	67.0792203	0.88139015	0.31332204	0.00061292	0.00385495	PRR16
54732	ENSG00000184840	1406.06908	0.20683921	0.07510985	0.00493031	0.022978	TMED9
NA	ENSG00000184844	0.14931947	-0.01844332	0.38480212	0.84537521	NA	CYCSP45
1812	ENSG00000184845	0	NA	NA	NA	NA	DRD1
54143	ENSG00000184856	0	NA	NA	NA	NA	LINC00308
25880	ENSG00000184857	253.992065	-0.05784206	0.14534685	0.66621492	0.83537685	TMEM186
93517	ENSG00000184860	0	NA	NA	NA	NA	SDR42E1
155435	ENSG00000184863	2513.35098	0.15305236	0.08137978	0.05414731	0.15819211	RBM33

9823	ENSG00000184867	904.648283	-0.07560555	0.09129383	0.3932147	0.62221883	ARMCX2
90135	ENSG00000184887	275.770945	-0.21769351	0.14471213	0.10314917	0.2551347	BTBD6
6736	ENSG00000184895	0	NA	NA	NA	NA	SRY
8971	ENSG00000184897	4930.70881	-0.1047148	0.07285366	0.14278817	0.32170307	H1-10
375287	ENSG00000184898	139.202552	0.3184646	0.20222714	0.06689689	0.18544027	RBM43
6612	ENSG00000184900	2200.18901	-0.23166151	0.06536452	0.00031914	0.00216466	SUMO3
83943	ENSG00000184903	204.137626	0.11853357	0.15996643	0.41580153	0.64263476	IMMP2L
140597	ENSG00000184905	139.444792	-0.57265496	0.21446543	0.00211169	0.01121109	TCEAL2
101929583	ENSG00000184906	0	NA	NA	NA	NA	
1188	ENSG00000184908	6.27169319	0.31189137	0.56086025	0.02677068	0.09185925	CLCNKB
63947	ENSG00000184911	1.19666822	-0.0414823	0.38309698	0.50942755	NA	DMRTC1B
728656	ENSG00000184911	1.19666822	-0.0414823	0.38309698	0.50942755	NA	DMRTC1B
3714	ENSG00000184916	369.699271	-1.95640496	0.20705043	1.68E-22	7.10E-21	JAG2
752	ENSG00000184922	201.528357	-0.03473515	0.18393474	0.82819151	0.92220943	FMNL1
728118	ENSG00000184923	102.21806	0.11791756	0.21076928	0.50568463	0.72177288	NUTM2A
391356	ENSG00000184924	577.992404	0.05873617	0.10678113	0.56756801	0.76899226	PTRHD1
286256	ENSG00000184925	9.03797507	-0.04281605	0.34828584	0.77603845	0.89702883	LCN12
8590	ENSG00000184933	0	NA	NA	NA	NA	OR6A2
7490	ENSG00000184937	4.47748108	5.26076808	2.7375239	0.00035063	0.00235573	WT1
146198	ENSG00000184939	1225.69728	0.04108719	0.09964754	0.67017712	0.83758072	ZFP90
375318	ENSG00000184945	0	NA	NA	NA	NA	AQP12A
646851	ENSG00000184949	171.545492	0.39170876	0.2907501	0.07024338	0.19201277	FAM227A
390327	ENSG00000184954	0	NA	NA	NA	NA	OR6C70
4588	ENSG00000184956	6.20369479	0.02150508	0.35757858	0.87488755	0.94487915	MUC6
NA	ENSG00000184961	0	NA	NA	NA	NA	RPL7AP49
79050	ENSG00000184967	438.346426	0.06538223	0.11751679	0.5596695	0.76413192	NOC4L
11274	ENSG00000184979	131.748891	1.56709155	0.2213309	1.18E-13	2.88E-12	USP18
4700	ENSG00000184983	366.601492	-0.10595847	0.12732997	0.3766837	0.60567343	NDUFA6
1133	ENSG00000184984	3.56860837	-0.05907882	0.37590939	0.56685215	0.76856055	CHRM5
57537	ENSG00000184985	49.0363914	0.19684127	0.30695787	0.33484171	0.56474327	SORCS2
80757	ENSG00000184986	128.260741	-0.39246328	0.22507756	0.03663248	0.11754623	TMEM121
113277	ENSG00000184988	0.4953803	0.01133127	0.38338033	0.78358522	NA	TMEM106A
10572	ENSG00000184990	910.699564	0.11408733	0.09632978	0.22147438	0.43427846	SIVA1
83868	ENSG00000184991	0	NA	NA	NA	NA	TTTY13
140707	ENSG00000184992	1028.68559	-0.34311365	0.12151052	0.00280779	0.01432239	BRI3BP
338376	ENSG00000184995	0	NA	NA	NA	NA	IFNE
387775	ENSG00000184999	0.173733	0.01914545	0.38484597	0.78225479	NA	SLC22A10
8694	ENSG00000185000	895.206931	-0.28297689	0.10143078	0.00370695	0.01811477	DGAT1

222546	ENSG00000185002	0.173733	0.01914545	0.38484597	0.78225479	NA	RFX6
6092	ENSG00000185008	30.0403479	0.74160356	0.59086171	0.01859725	0.06863521	ROBO2
26985	ENSG00000185009	1513.96524	-0.15200119	0.07040225	0.02786626	0.09465274	AP3M1
2157	ENSG00000185010	34.3821576	0.07396313	0.29602682	0.70225447	0.85686744	F8
93034	ENSG00000185013	1.8047487	-0.07260544	0.38611241	0.36056885	NA	NT5C1B
377677	ENSG00000185015	14.0202798	1.05851162	2.42316457	0.00695732	0.03081786	CA13
22888	ENSG00000185019	233.05984	0.14992659	0.15018132	0.27806809	0.50258055	UBOX5
23764	ENSG00000185022	97.1099057	0.3789454	0.23712148	0.05089665	0.15109824	MAFF
2972	ENSG00000185024	1386.40714	-0.24066869	0.10187997	0.01390632	0.05425789	BRF1
389257	ENSG00000185028	2.13839055	0.03444476	0.37885719	0.66170889	NA	LRRC14B
NA	ENSG00000185031	2.2609842	-0.05448502	0.38086488	0.51586056	NA	SLC2A3P2
10509	ENSG00000185033	618.530548	0.0739042	0.14308055	0.57801162	0.77614203	SEMA4B
NA	ENSG00000185037	0.173124	-0.01911262	0.38486262	0.84537521	NA	ZNF733P
339766	ENSG00000185038	0.16735865	0.01897177	0.38482933	0.78225479	NA	MROH2A
285955	ENSG00000185040	15.0107804	-0.01823109	0.3247951	0.91512655	0.96403708	SPDYE16
102723555	ENSG00000185040	15.0107804	-0.01823109	0.3247951	0.91512655	0.96403708	SPDYE16
10519	ENSG00000185043	195.144565	0.31871033	0.16828296	0.03530394	0.11407657	CIB1
56899	ENSG00000185046	28.3534607	0.36987607	0.40413685	0.11133985	0.26955249	ANKS1B
7469	ENSG00000185049	944.551671	0.05666827	0.08472572	0.49331041	0.71236493	NELFA
57419	ENSG00000185052	0	NA	NA	NA	NA	SLC24A3
137868	ENSG00000185053	0	NA	NA	NA	NA	SGCZ
100130771	ENSG00000185055	8.82384784	-0.45334981	0.67796909	0.0428333	0.13230775	EFCAB10
133491	ENSG00000185056	0	NA	NA	NA	NA	C5orf47
NA	ENSG00000185065	35.3591223	0.12375	0.296775	0.53074928	0.74249512	
51350	ENSG00000185069	0	NA	NA	NA	NA	KRT76
23768	ENSG00000185070	0	NA	NA	NA	NA	FLRT2
NA	ENSG00000185074	0	NA	NA	NA	NA	OR7E31P
NA	ENSG00000185078	0	NA	NA	NA	NA	RPL9P14
80789	ENSG00000185085	623.451788	-0.18722274	0.11662676	0.09105094	0.23282002	INTS5
51065	ENSG00000185088	616.523669	0.44354429	0.11835959	8.15E-05	0.00063367	RPS27L
149175	ENSG00000185090	1697.94856	-1.19743996	0.08235274	7.23E-49	6.83E-47	MANEAL
122622	ENSG00000185100	138.586873	0.25221541	0.20469678	0.14824623	0.33050962	ADSS1
338440	ENSG00000185101	4.41560161	0.0886088	0.3809813	0.4232507	0.64919165	ANO9
11124	ENSG00000185104	913.2714	-0.14497566	0.09559243	0.11667257	0.27939454	FAF1
255275	ENSG00000185105	29.0944599	-0.0299087	0.28846102	0.87461175	0.94480547	MYADML2
131583	ENSG00000185112	88.9090385	-0.30781696	0.24851212	0.11818705	0.28210313	FAM43A
56160	ENSG00000185115	396.485912	0.23832863	0.13201506	0.05392799	0.15767405	NSMCE3
3297	ENSG00000185122	1655.78715	0.05752903	0.09098754	0.51579914	0.73002247	HSF1

387263	ENSG00000185127	631.288799	0.0485589	0.09672487	0.60466941	0.79479535	C6orf120
5813	ENSG00000185129	689.702996	-0.03780296	0.10242223	0.70090701	0.85575596	PURA
8340	ENSG00000185130	1.9458894	-0.04901211	0.38348595	0.47203446	NA	H2BC13
27124	ENSG00000185133	232.888103	-0.12140456	0.16036319	0.40338658	0.63146519	INPP5J
4887	ENSG00000185149	77.8650269	-0.17083771	0.23180228	0.35959066	0.58949503	NPY2R
83881	ENSG00000185155	7.92556308	0.08300061	0.36188303	0.56981108	0.77039833	MIXL1
162387	ENSG00000185156	0	NA	NA	NA	NA	MFSD6L
114659	ENSG00000185158	205.097826	0.26416575	0.15608885	0.06358884	0.17907774	LRRC37B
317781	ENSG00000185163	716.784358	0.28343333	0.12886514	0.01914596	0.07031764	DDX51
283820	ENSG00000185164	2212.21279	0.14012881	0.09734033	0.13646875	0.31153759	NOMO2
102723728	ENSG00000185164	2212.21279	0.14012881	0.09734033	0.13646875	0.31153759	NOMO2
NA	ENSG00000185168	27.7763353	-0.56611258	0.50712392	0.03870961	0.12240974	LINC00482
653437	ENSG00000185176	0.93243087	0.01447017	0.38162763	0.80425453	NA	AQP12B
90827	ENSG00000185177	0	NA	NA	NA	NA	ZNF479
390535	ENSG00000185182	0	NA	NA	NA	NA	GOLGA8EP
NA	ENSG00000185186	1.01749276	0.05370793	0.38930842	0.22860634	NA	LINC00313
59307	ENSG00000185187	201.910913	0.21012647	0.17069256	0.16911228	0.36046278	SIGIRR
340371	ENSG00000185189	676.315182	0.75365252	0.09921816	6.50E-15	1.76E-13	NRBP2
400668	ENSG00000185198	0.34624801	-0.02356563	0.38536324	0.65270996	NA	PRSS57
10581	ENSG00000185201	19.2602839	3.25960097	0.78872339	2.35E-06	2.50E-05	IFITM2
NA	ENSG00000185203	3.23861798	-0.19903463	0.45031535	0.05556704	0.16150749	WASIR1
7127	ENSG00000185215	204.677464	-0.70698487	0.19917833	7.78E-05	0.00060901	TNFAIP2
353274	ENSG00000185219	1782.53829	-0.15300191	0.07007534	0.02616576	0.09025186	ZNF445
267002	ENSG00000185220	144.768293	-0.1134581	0.17906541	0.47308923	0.69516297	PGBD2
NA	ENSG00000185221	0	NA	NA	NA	NA	GAPDHP24
51186	ENSG00000185222	580.699443	0.26878532	0.10395266	0.00708838	0.03125883	TCEAL9
4158	ENSG00000185231	0	NA	NA	NA	NA	MC2R
9230	ENSG00000185236	2209.70032	0.2045623	0.06920239	0.00262758	0.01354055	RAB11B
10196	ENSG00000185238	967.415107	0.27789995	0.09287298	0.00199676	0.01068674	PRMT3
2811	ENSG00000185245	25.8790055	0.70348269	0.51261263	0.02010104	0.07320204	GP1BA
55015	ENSG00000185246	504.603999	0.12204746	0.10872516	0.24159476	0.45960686	PRPF39
4110	ENSG00000185247	0	NA	NA	NA	NA	MAGEA11
285755	ENSG00000185250	44.8994184	0.61224032	0.51217843	0.03102152	0.10295552	PPIL6
7625	ENSG00000185252	1291.26407	0.20695773	0.07935645	0.00761329	0.0331284	ZNF74
285600	ENSG00000185261	39.0825019	0.11192371	0.28243894	0.56933536	0.77008546	KIAA0825
283991	ENSG00000185262	761.654459	-0.050364	0.12985985	0.67990176	0.84376374	UBALD2
339669	ENSG00000185264	0	NA	NA	NA	NA	TEX33
441549	ENSG00000185267	19.3801977	0.44963905	0.52607776	0.06632367	0.18429434	CDNF

147111	ENSG00000185269	45.056738	-1.60278273	0.43751182	1.57E-05	0.00014401	NOTUM
123103	ENSG00000185271	0.33471731	0.02354391	0.38534154	0.62842204	NA	KLHL33
54033	ENSG00000185272	0.173733	0.01914545	0.38484597	0.78225479	NA	RBM11
64409	ENSG00000185274	0	NA	NA	NA	NA	GALNT17
NA	ENSG00000185275	0.14931947	-0.01844332	0.38480212	0.84537521	NA	CD24P4
84614	ENSG00000185278	1375.8642	0.01393217	0.08061881	0.8597029	0.93751308	ZBTB37
389493	ENSG00000185290	0	NA	NA	NA	NA	NUPR2
3563	ENSG00000185291	1.48550844	-0.00791088	0.37724569	0.91578172	NA	IL3RA
162540	ENSG00000185294	1.07548859	-0.07448626	0.39363112	0.16128604	NA	SPPL2C
NA	ENSG00000185296	0	NA	NA	NA	NA	
339230	ENSG00000185298	1543.84495	-0.00825895	0.08005925	0.91519207	0.96403708	CCDC137
729238	ENSG00000185303	0.3439991	0.02364859	0.3853568	0.62257909	NA	SFTPA2
729857	ENSG00000185304	461.89828	-0.09875814	0.1156569	0.36970316	0.59909782	RGPD2
54622	ENSG00000185305	232.359316	-0.64069508	0.25691375	0.00265737	0.01366216	ARL15
115749	ENSG00000185306	0.18922458	0.01954053	0.38488496	0.78225479	NA	C12orf56
6336	ENSG00000185313	0	NA	NA	NA	NA	SCN10A
NA	ENSG00000185319	0	NA	NA	NA	NA	SSXP4
8558	ENSG00000185324	1237.17356	0.22620331	0.08081859	0.00414001	0.01985924	CDK10
284186	ENSG00000185332	16.0889605	0.23404292	0.38543589	0.23254684	0.44940365	TMEM105
8651	ENSG00000185338	15.2603331	-0.2703479	0.41104986	0.18017587	0.37700128	SOCS1
6948	ENSG00000185339	53.668979	0.03327561	0.25146986	0.8627382	0.93907741	TCN2
10634	ENSG00000185340	373.005909	-0.05813141	0.13959516	0.65367331	0.82751905	GAS2L1
23545	ENSG00000185344	1073.90479	0.05816664	0.0815853	0.46551373	0.68821106	ATP6V0A2
5071	ENSG00000185345	69.0674787	1.33023739	0.32731087	3.62E-06	3.73E-05	PRKN
283643	ENSG00000185347	416.95994	-0.22116822	0.13698898	0.08262542	0.21646485	TEDC1
266722	ENSG00000185352	753.227377	-3.94756715	0.15531304	6.46E-144	4.27E-141	HS6ST3
9146	ENSG00000185359	3560.73636	0.14750039	0.08885154	0.08778973	0.22632101	HGS
126282	ENSG00000185361	361.905952	-0.15148367	0.13740984	0.23631832	0.45308809	TNFAIP8L1
26693	ENSG00000185372	0	NA	NA	NA	NA	OR2V1
5892	ENSG00000185379	381.917745	-0.12228523	0.11958314	0.27937914	0.50389837	RAD51D
26333	ENSG00000185385	0	NA	NA	NA	NA	OR7A17
5600	ENSG00000185386	857.754062	-0.61830185	0.09856352	1.00E-10	1.93E-09	MAPK11
NA	ENSG00000185390	0	NA	NA	NA	NA	FGF7P2
93349	ENSG00000185404	25.8341823	-1.17160837	0.58468298	0.0027716	0.014165	SP140L
51263	ENSG00000185414	1168.98739	-0.41231041	0.12017968	0.00029548	0.00201868	MRPL30
123283	ENSG00000185418	326.689902	-0.24866228	0.12990191	0.04119971	0.12832059	TARS3
64754	ENSG00000185420	470.692478	0.07770377	0.11166693	0.46754116	0.69041597	SMYD3
25840	ENSG00000185432	723.680362	0.93157111	0.13706236	1.54E-12	3.47E-11	METTL7A

54072	ENSG00000185433	0	NA	NA	NA	NA	LINC00158
163702	ENSG00000185436	88.1072329	0.74712452	0.27866723	0.00123293	0.0070718	IFNLR1
6450	ENSG00000185437	44.5511888	-0.06176245	0.28598861	0.74714129	0.88120985	SH3BGR
400451	ENSG00000185442	173.434523	-0.92745788	0.20462703	7.97E-07	9.18E-06	FAM174B
158724	ENSG00000185448	0	NA	NA	NA	NA	FAM47A
374920	ENSG00000185453	379.203549	-0.08046664	0.12505531	0.49526227	0.7139953	ZSWIM9
402569	ENSG00000185467	0.16735865	0.01897177	0.38482933	0.78225479	NA	KPNA7
374395	ENSG00000185475	339.377018	-0.10683342	0.13380536	0.39345873	0.62246703	TMEM179B
285513	ENSG00000185477	94.9159404	0.43358507	0.25040053	0.03207913	0.10577528	GPRIN3
3854	ENSG00000185479	0	NA	NA	NA	NA	KRT6B
55010	ENSG00000185480	397.453293	-0.64337275	0.12685517	1.04E-07	1.37E-06	PARPBP
246329	ENSG00000185482	22.2697336	-0.03625128	0.31367854	0.84080274	0.92773074	STAC3
4919	ENSG00000185483	584.85229	0.18392195	0.11371839	0.08994833	0.23053894	ROR1
255812	ENSG00000185485	490.71557	0.49844325	0.12405527	2.28E-05	0.00020244	SDHAP1
NA	ENSG00000185495	108.255408	0.18233424	0.21205418	0.30575712	0.53334106	SEPTIN7P13
4582	ENSG00000185499	45.5174046	0.31465804	0.3326658	0.15303964	0.33781781	MUC1
80233	ENSG00000185504	1707.22483	-0.01032643	0.08158405	0.89603919	0.95469109	FAAP100
3665	ENSG00000185507	182.703771	0.69130178	0.18450121	3.99E-05	0.00033586	IRF7
26013	ENSG00000185513	629.26258	0.55798514	0.12004943	1.11E-06	1.25E-05	L3MBTL1
79184	ENSG00000185515	368.789041	-0.26701004	0.12852584	0.02687703	0.09216043	BRCC3
9899	ENSG00000185518	0.34746601	0.02370763	0.3853651	0.61987128	NA	SV2B
348487	ENSG00000185519	97.8016112	-0.2105663	0.21403158	0.23872307	0.45646355	FAM131C
256329	ENSG00000185522	16.8491188	0.3570003	0.49579901	0.09470757	0.23933234	LMNTD2
149643	ENSG00000185523	1.15092985	0.01828074	0.37930053	0.78918978	NA	SPATA45
5148	ENSG00000185527	0.32599697	-0.02328245	0.38532298	0.66758559	NA	PDE6G
5592	ENSG00000185532	242.686794	0.90483884	0.16820077	1.15E-08	1.73E-07	PRKG1
7026	ENSG00000185551	1.71977115	0.111473	0.40324436	0.10891299	NA	NR2F2
8788	ENSG00000185559	45139.4219	0.66226724	0.06303163	2.17E-26	1.08E-24	DLK1
727910	ENSG00000185561	38.7211128	0.98294353	0.4044011	0.00149304	0.00834464	TLCD2
4045	ENSG00000185565	1440.59178	-0.24312569	0.07624453	0.00111183	0.00647645	LSAMP
113146	ENSG00000185567	156.064936	-0.070087	0.22672747	0.69977522	0.8552838	AHNAK2
169611	ENSG00000185585	15.3884041	-0.13295626	0.34771076	0.45844823	0.68183005	OLFML2A
6667	ENSG00000185591	2287.17592	0.16732269	0.06663817	0.01074259	0.04395401	SP1
145946	ENSG00000185594	0	NA	NA	NA	NA	SPATA8
NA	ENSG00000185596	871.111462	0.18125506	0.10146042	0.06361126	0.1791022	WASH3P
NA	ENSG00000185607	0.64878957	0.00236416	0.38248516	0.95453482	NA	ACTBP7
64976	ENSG00000185608	287.221412	-0.13783699	0.1481351	0.31184465	0.53954807	MRPL40
440097	ENSG00000185610	0.173124	-0.01911262	0.38486262	0.84537521	NA	DBX2

389119	ENSG00000185614	125.499162	-1.0083741	0.25925724	1.17E-05	0.00010998	INKA1
64714	ENSG00000185615	123.28823	0.6697616	0.21450327	0.00040014	0.00264888	PDIA2
10336	ENSG00000185619	2453.42921	0.21913483	0.06728571	0.00092631	0.00554888	PCGF3
89782	ENSG00000185621	154.606895	0.36428463	0.22478358	0.05158965	0.15258477	LMLN
5034	ENSG00000185624	8348.96359	0.2557326	0.05210657	7.35E-07	8.52E-06	P4HB
5719	ENSG00000185627	1622.79039	-0.05669513	0.07206695	0.42226283	0.64822768	PSMD13
5087	ENSG00000185630	3761.16543	-1.28067018	0.06430864	2.94E-89	7.30E-87	PBX1
NA	ENSG00000185631	0.35735973	0.02382194	0.38538182	0.61367454	NA	RPL14P6
56901	ENSG00000185633	77.7162175	-0.85730406	0.33922514	0.00145299	0.00815481	NDUFA4L2
399694	ENSG00000185634	26.4233697	-0.18544297	0.34637157	0.33744401	0.56779656	SHC4
NA	ENSG00000185638	0	NA	NA	NA	NA	FTH1P14
338785	ENSG00000185640	0	NA	NA	NA	NA	KRT79
NA	ENSG00000185641	0.32562574	0.00212452	0.38337695	0.95997109	NA	
677	ENSG00000185650	111.995097	0.75423422	0.28896552	0.0014693	0.00823474	ZFP36L1
7332	ENSG00000185651	1691.02245	-0.10393678	0.07510743	0.15778462	0.34480085	UBE2L3
4908	ENSG00000185652	0.35668631	0.0238171	0.38538106	0.6140144	NA	NTF3
54014	ENSG00000185658	2247.9003	-0.00443643	0.06571874	0.94476495	0.97752994	BRWD1
644994	ENSG00000185662	0	NA	NA	NA	NA	SMIM23
6490	ENSG00000185664	78.2085079	0.11881748	0.24022526	0.52823142	0.73996408	PMEL
8224	ENSG00000185666	331.873266	-3.03717077	0.54324275	8.77E-10	1.52E-08	SYN3
5453	ENSG00000185668	0	NA	NA	NA	NA	POU3F1
333929	ENSG00000185669	6.07182568	-0.01397691	0.35497095	0.91743558	0.96505604	SNAI3
79842	ENSG00000185670	65.5639782	-0.19738216	0.27198014	0.32238474	0.54994458	ZBTB3
254773	ENSG00000185674	1.28826906	-0.01473589	0.37948775	0.82255434	NA	LYG2
254956	ENSG00000185681	0.34746601	0.02370763	0.3853651	0.61987128	NA	MORN5
NA	ENSG00000185684	569.634842	0.4265808	0.10710733	3.30E-05	0.00028159	EP400P1
23532	ENSG00000185686	8506.16818	0.11744979	0.04986492	0.01761077	0.06573804	PRAME
404220	ENSG00000185689	6.10922617	0.03511164	0.36278597	0.78281653	0.90108377	C6orf201
4603	ENSG00000185697	298.926067	-0.26208415	0.13879786	0.0420897	0.13066181	MYBL1
100101118	ENSG00000185700	0	NA	NA	NA	NA	TTTY8B
NA	ENSG00000185701	0	NA	NA	NA	NA	OR5M5P
NA	ENSG00000185710	22.3622181	-0.07603636	0.39271078	0.18313695	0.38130308	SMG1P4
730094	ENSG00000185716	521.370773	0.11717983	0.11101362	0.27011913	0.49334102	MOSMO
4733	ENSG00000185721	776.957261	-0.16828718	0.10770262	0.10264647	0.25430894	DRG1
51479	ENSG00000185722	2123.56841	-0.05237096	0.07090143	0.45173293	0.67497976	ANKFY1
253943	ENSG00000185728	1383.2439	0.03709009	0.12149723	0.74847497	0.88197086	YTHDF3
79943	ENSG00000185730	670.99254	-0.00759061	0.11084328	0.94178659	0.97592427	ZNF696
105	ENSG00000185736	334.035633	-0.27687621	0.16241876	0.05884197	0.16858119	ADARB2

10718	ENSG00000185737	46.2201847	-1.65466579	0.38863882	1.38E-06	1.54E-05	NRG3
6345	ENSG00000185739	0	NA	NA	NA	NA	SRL
399947	ENSG00000185742	1.57252297	-0.03734208	0.37994987	0.61925401	NA	C11orf87
3434	ENSG00000185745	20.9111975	0.3004639	0.39191926	0.16736212	0.35828164	IFIT1
159013	ENSG00000185753	321.319957	0.27452931	0.1317393	0.02611391	0.09010434	CXorf38
100132463	ENSG00000185758	0	NA	NA	NA	NA	CLDN24
56479	ENSG00000185760	270.118701	-0.25332059	0.14778728	0.06251074	0.17671887	KCNQ5
339366	ENSG00000185761	2.26042715	0.10704501	0.40457972	0.04544843	NA	ADAMTSL5
80333	ENSG00000185774	1.07582017	-0.01671425	0.38215846	0.74013154	NA	KCNIP4
389730	ENSG00000185775	0	NA	NA	NA	NA	SPATA31A6
10933	ENSG00000185787	4848.24447	0.02605187	0.06416789	0.68113237	0.84419446	MORF4L1
338321	ENSG00000185792	0	NA	NA	NA	NA	NLRP9
348793	ENSG00000185798	137.698483	0.00941946	0.17823979	0.95365795	0.98083958	WDR53
1762	ENSG00000185800	1532.50529	0.41764324	0.0790693	6.53E-08	8.82E-07	DMWD
79581	ENSG00000185803	1089.18458	0.00540826	0.08519706	0.94935677	0.97912847	SLC52A2
51227	ENSG00000185808	203.41391	-0.23847258	0.16204905	0.10333321	0.25540436	PIGP
10320	ENSG00000185811	63.3613344	5.04785615	0.58814524	5.64E-20	2.10E-18	IKZF1
5833	ENSG00000185813	857.406329	-0.2771544	0.11729287	0.01274467	0.05062147	PCYT2
339983	ENSG00000185818	1222.77007	0.03064353	0.07655118	0.67582731	0.84136185	NAT8L
390326	ENSG00000185821	0	NA	NA	NA	NA	OR6C76
23742	ENSG00000185823	0	NA	NA	NA	NA	NPAP1
10134	ENSG00000185825	1768.38083	-0.27687057	0.07951374	0.00036089	0.00241568	BCAP31
51326	ENSG00000185829	539.381177	0.07345063	0.15669337	0.60811825	0.79695264	ARL17A
NA	ENSG00000185834	0.31679374	0.0011946	0.38336714	0.96640738	NA	RPL12P4
100130717	ENSG00000185837	4.06052668	-0.0476876	0.37045582	0.67053186	0.83767652	HDHD5-AS1
54584	ENSG00000185838	254.004554	0.10032977	0.14186299	0.447107	0.67106427	GNB1L
NA	ENSG00000185839	0.16735865	0.01897177	0.38482933	0.78225479	NA	
127602	ENSG00000185842	481.556644	-0.10186286	0.12698556	0.3942052	0.62343812	DNAH14
100131138	ENSG00000185847	7.45233305	-0.15201105	0.40770027	0.19411963	0.39704468	LINC01405
339512	ENSG00000185860	0.56660955	0.00913976	0.38298795	0.79124371	NA	CCDC190
2124	ENSG00000185862	3.39793369	0.03084916	0.3705591	0.77176037	0.89531784	EVI2B
100505993	ENSG00000185863	0	NA	NA	NA	NA	TMEM210
440345	ENSG00000185864	382.69427	0.51807465	0.16234505	0.00049411	0.00319318	NPIPB4
374899	ENSG00000185869	247.187148	-0.20527929	0.16054724	0.15801763	0.34501645	ZNF829
132724	ENSG00000185873	0	NA	NA	NA	NA	TMPRSS11B
79896	ENSG00000185875	172.472517	-0.23199826	0.21441956	0.19556581	0.39920852	THNSL1
140691	ENSG00000185880	582.671416	0.18134397	0.13658454	0.15510052	0.34073683	TRIM69
527	ENSG00000185883	2350.54646	-0.03449433	0.07250177	0.62696737	0.81067573	ATP6V0C

8519	ENSG00000185885	0.16735865	0.01897177	0.38482933	0.78225479	NA	IFITM1
339501	ENSG00000185888	0	NA	NA	NA	NA	PRSS38
3916	ENSG00000185896	1892.06774	-0.07873883	0.07936756	0.30999423	0.53774299	LAMP1
2865	ENSG00000185897	0	NA	NA	NA	NA	FFAR3
338398	ENSG00000185899	0	NA	NA	NA	NA	TAS2R60
84197	ENSG00000185900	1295.98698	-0.66684134	0.08845659	1.22E-14	3.23E-13	POMK
NA	ENSG00000185903	2.04435331	-0.0198972	0.37775949	0.79329593	NA	OR11N1P
84856	ENSG00000185904	935.919208	0.03126826	0.09288307	0.72938064	0.87058789	LINC00839
283897	ENSG00000185905	0	NA	NA	NA	NA	C16orf54
200942	ENSG00000185909	856.03825	-0.52712379	0.09967635	4.52E-08	6.24E-07	KLHDC8B
257240	ENSG00000185915	4.72386909	-0.10021948	0.38431256	0.38102639	0.61008503	KLHL34
54093	ENSG00000185917	509.978352	0.24007178	0.14365296	0.0707868	0.19309914	SETD4
5727	ENSG00000185920	944.880237	0.09221033	0.11171363	0.38690034	0.61601017	PTCH1
146760	ENSG00000185924	2.29083902	-0.07191458	0.38227526	0.44397118	NA	RTN4RL1
119749	ENSG00000185926	0	NA	NA	NA	NA	OR4C46
255022	ENSG00000185933	1.02846556	0.02192143	0.38179602	0.69190042	NA	CALHM1
439915	ENSG00000185940	2.17244002	0.07612488	0.38508202	0.39421107	NA	KRTAP5-5
286183	ENSG00000185942	2.71208092	0.07089374	0.37875443	0.49693892	0.71500229	NKAIN3
55599	ENSG00000185946	352.739121	0.10053934	0.13302721	0.42074557	0.64669911	RNPC3
10308	ENSG00000185947	110.183576	-0.33929369	0.21225553	0.0589304	0.16878577	ZNF267
8660	ENSG00000185950	5522.17919	0.62243625	0.05171891	7.68E-34	4.96E-32	IRS2
402573	ENSG00000185955	3.82930458	0.02707166	0.36674564	0.81483542	0.91598064	C7orf61
121006	ENSG00000185958	3.70062887	0.13742642	0.41161885	0.10473015	0.25786128	FAM186A
6473	ENSG00000185960	0	NA	NA	NA	NA	SHOX
353142	ENSG00000185962	0	NA	NA	NA	NA	LCE3A
23299	ENSG00000185963	1568.31614	-0.19004896	0.08258294	0.01821621	0.06741028	BICD2
353145	ENSG00000185966	0	NA	NA	NA	NA	LCE3E
881	ENSG00000185972	1.57535977	-0.03615585	0.38067973	0.61058017	NA	CCIN
55217	ENSG00000185973	416.073259	-0.04712913	0.13053858	0.7001969	0.85541355	TMLHE
6011	ENSG00000185974	13.383329	0.04566492	0.34338433	0.77491423	0.89613481	GRK1
245939	ENSG00000185982	0	NA	NA	NA	NA	DEFB128
84631	ENSG00000185985	0	NA	NA	NA	NA	SLITRK2
NA	ENSG00000185986	385.533452	0.00944983	0.12388598	0.93692737	0.97351594	SDHAP3
126520	ENSG00000185988	1.38696581	0.01023845	0.37995175	0.87077323	NA	PLK5
22821	ENSG00000185989	322.252002	-0.47557968	0.13886768	0.00025003	0.00173934	RASA3
84859	ENSG00000186001	1156.04773	-0.20872588	0.0934138	0.02084784	0.07534082	LRCH3
93273	ENSG00000186007	0	NA	NA	NA	NA	LEMD1
NA	ENSG00000186008	0	NA	NA	NA	NA	RPS4XP21

496	ENSG00000186009	0.76972557	-0.04329735	0.38756885	0.340737	NA	ATP4B
51079	ENSG00000186010	3171.16699	0.10798286	0.05976691	0.06743878	0.18646699	NDUFA13
84924	ENSG00000186017	340.084723	-0.03894389	0.16588238	0.79323581	0.9054599	ZNF566
NA	ENSG00000186019	171.435653	-0.57667197	0.21785639	0.00221925	0.0117101	
57711	ENSG00000186020	625.918443	-0.04285665	0.12411015	0.71420526	0.8622791	ZNF529
342909	ENSG00000186026	268.56155	-0.06325553	0.15703361	0.65803877	0.83031782	ZNF284
285242	ENSG00000186038	0	NA	NA	NA	NA	HTR3E
220107	ENSG00000186047	0.4988949	-0.01359831	0.38305388	0.7848689	NA	DLEU7
103689915	ENSG00000186047	0.4988949	-0.01359831	0.38305388	0.7848689	NA	DLEU7
319101	ENSG00000186049	0	NA	NA	NA	NA	KRT73
6887	ENSG00000186051	8.20094784	0.12843713	0.37258243	0.40774839	0.63603571	TAL2
100129196	ENSG00000186056	16.1270499	0.01829203	0.3410132	0.90967605	0.96107209	MATN1-AS1
64853	ENSG00000186063	1313.68191	0.20698429	0.0797577	0.00790789	0.03418139	AIDA
84529	ENSG00000186073	281.778307	-0.77888613	0.1783695	2.28E-06	2.43E-05	CDIN1
146722	ENSG00000186074	0	NA	NA	NA	NA	CD300LF
124626	ENSG00000186075	0	NA	NA	NA	NA	ZPBP2
NA	ENSG00000186076	1.19952539	-0.06487716	0.39166475	0.17292621	NA	
3852	ENSG00000186081	0	NA	NA	NA	NA	KRT5
NA	ENSG00000186082	0.16735865	0.01897177	0.38482933	0.78225479	NA	KRT18P14
653149	ENSG00000186086	242.028032	0.56306741	0.16102687	0.00014703	0.00107641	NBPF6
54103	ENSG00000186088	261.537611	0.36609569	0.15611328	0.01037035	0.04264242	GSAP
200909	ENSG00000186090	0	NA	NA	NA	NA	HTR3D
79501	ENSG00000186092	0	NA	NA	NA	NA	OR4F5
84871	ENSG00000186094	13.353661	0.21667355	0.39691314	0.23443629	0.45139906	AGBL4
503582	ENSG00000186103	0.33471731	0.02354391	0.38534154	0.62842204	NA	ARGFX
120227	ENSG00000186104	289.993507	-0.08508086	0.1577542	0.55290138	0.75919187	CYP2R1
100130733	ENSG00000186105	2.80995163	0.12639883	0.40287689	0.18766131	0.38799505	LRRC70
157567	ENSG00000186106	429.407846	-0.53296853	0.13376014	2.36E-05	0.00020827	ANKRD46
23396	ENSG00000186111	1111.47298	-0.11355452	0.09953298	0.23650263	0.45331004	PIP5K1C
219436	ENSG00000186113	0	NA	NA	NA	NA	OR5D14
8529	ENSG00000186115	0	NA	NA	NA	NA	CYP4F2
374973	ENSG00000186118	2.45093186	0.08100945	0.38370706	0.41039592	NA	TEX38
219438	ENSG00000186119	0	NA	NA	NA	NA	OR5D18
NA	ENSG00000186124	0	NA	NA	NA	NA	OR9M1P
10773	ENSG00000186130	499.671686	-0.39618009	0.12137121	0.00056645	0.00359974	ZBTB6
130355	ENSG00000186132	81.1117838	-0.09013648	0.22149189	0.61813643	0.80452675	C2orf76
353164	ENSG00000186136	1.01332516	0.02939345	0.38193292	0.62120384	NA	TAS2R42
10623	ENSG00000186141	986.397048	-0.05088745	0.08218017	0.52686749	0.73878671	POLR3C

339779	ENSG00000186143	0	NA	NA	NA	NA	PRR30
644414	ENSG00000186146	0	NA	NA	NA	NA	DEFB131A
NA	ENSG00000186148	0	NA	NA	NA	NA	
164153	ENSG00000186150	1.52613919	-0.05270386	0.38448056	0.42811681	NA	UBL4B
NA	ENSG00000186152	0	NA	NA	NA	NA	LILRP1
51741	ENSG00000186153	359.154759	-0.06354111	0.16215182	0.66410598	0.83431586	WWOX
199974	ENSG00000186160	0	NA	NA	NA	NA	CYP4Z1
NA	ENSG00000186162	123.02061	-0.07526247	0.19441394	0.65268631	0.82653324	CIDECP1
207147	ENSG00000186163	0	NA	NA	NA	NA	
338657	ENSG00000186166	272.580467	0.17521546	0.15441166	0.21471491	0.42507156	CENATAC
283149	ENSG00000186174	680.295408	-0.00216107	0.1375363	0.9852641	0.99475332	BCL9L
51082	ENSG00000186184	1257.01395	-0.16825996	0.07517939	0.02228614	0.07942465	POLR1D
146909	ENSG00000186185	1810.8973	0.04814703	0.07129496	0.49239608	0.71141409	KIF18B
84937	ENSG00000186187	1109.56443	0.0904994	0.09858862	0.34241339	0.57256482	ZNRF1
338557	ENSG00000186188	0	NA	NA	NA	NA	FFAR4
359710	ENSG00000186190	0	NA	NA	NA	NA	BPIFB3
149954	ENSG00000186191	0	NA	NA	NA	NA	BPIFB4
89958	ENSG00000186193	2235.40542	-0.68711936	0.06997755	2.28E-23	1.02E-21	SAPCD2
128178	ENSG00000186197	10.5354018	1.0164805	2.12504542	0.01158151	0.04681489	EDARADD
123264	ENSG00000186198	0.47437202	-0.00764478	0.3832367	0.86108696	NA	SLC51B
66002	ENSG00000186204	0	NA	NA	NA	NA	CYP4F12
64757	ENSG00000186205	702.169735	-0.40220083	0.1302269	0.0010153	0.00598444	MTARC1
254910	ENSG00000186207	0	NA	NA	NA	NA	LCE5A
345079	ENSG00000186212	0.16735865	0.01897177	0.38482933	0.78225479	NA	SOWAHB
55330	ENSG00000186222	368.9723	-0.19082875	0.13017112	0.118003	0.28186606	BLOC1S4
353135	ENSG00000186226	0	NA	NA	NA	NA	LCE1E
388567	ENSG00000186230	296.497594	0.11236864	0.13493489	0.37349154	0.60252627	ZNF749
114792	ENSG00000186231	55.7207741	-0.10238358	0.26767185	0.59706469	0.78909124	KLHL32
NA	ENSG00000186234	0.14931947	-0.01844332	0.38480212	0.84537521	NA	FAM86MP
151174	ENSG00000186235	0.6463683	0.00271477	0.38249497	0.94800508	NA	LINC02610
NA	ENSG00000186244	0	NA	NA	NA	NA	
57496	ENSG00000186260	1240.38901	-0.37539246	0.09478768	4.17E-05	0.00034889	MRTFB
151888	ENSG00000186265	0.67290387	0.0274814	0.38380982	0.57165405	NA	BTLA
NA	ENSG00000186268	0	NA	NA	NA	NA	OR10D4P
7565	ENSG00000186272	138.886739	0.22310319	0.19919116	0.19186887	0.39390298	ZNF17
55693	ENSG00000186280	74.6636524	0.17412792	0.25081604	0.37033461	0.59943356	KDM4D
150763	ENSG00000186281	1.79378296	0.00907106	0.37784869	0.90233359	NA	GPAT2
64222	ENSG00000186283	725.911237	0.2811149	0.1379694	0.02888642	0.09740083	TOR3A

340529	ENSG00000186288	0.173733	0.01914545	0.38484597	0.78225479	NA	PABPC1L2A
2558	ENSG00000186297	0.14931947	-0.01844332	0.38480212	0.84537521	NA	GABRA5
5501	ENSG00000186298	3057.70181	-0.15567963	0.07231445	0.02835591	0.09603639	PPP1CC
148254	ENSG00000186300	271.656777	-0.03877729	0.15238569	0.78057935	0.89965693	ZNF555
NA	ENSG00000186301	34.011486	1.56709731	0.50171753	0.00011093	0.00083399	MST1P2
128360	ENSG00000186306	0	NA	NA	NA	NA	OR10T2
4675	ENSG00000186310	88.370793	-0.81234751	0.26217725	0.00030088	0.00205204	NAP1L3
340591	ENSG00000186312	259.258147	-0.46786385	0.16259125	0.00161444	0.00894457	CA5BP1
153768	ENSG00000186314	162.114506	-0.25093899	0.21162596	0.15846312	0.34567436	PRELID2
23621	ENSG00000186318	2360.025	-0.22074362	0.06884842	0.00109848	0.00640428	BACE1
388531	ENSG00000186326	1.73633161	-0.08056338	0.39348829	0.17401971	NA	RGS9BP
NA	ENSG00000186328	0	NA	NA	NA	NA	
389177	ENSG00000186329	0	NA	NA	NA	NA	TMEM212
285641	ENSG00000186334	0.173733	0.01914545	0.38484597	0.78225479	NA	SLC36A3
153201	ENSG00000186335	0.16735865	0.01897177	0.38482933	0.78225479	NA	SLC36A2
7058	ENSG00000186340	0.66295541	-0.03787121	0.38676331	0.41297456	NA	THBS2
6256	ENSG00000186350	855.616271	-0.56288168	0.09033588	1.56E-10	2.92E-09	RXRA
353322	ENSG00000186352	69.7945178	0.40483608	0.30826158	0.07035063	0.19222647	ANKRD37
200035	ENSG00000186364	80.2931966	-0.0394906	0.22607199	0.8278144	0.92210075	NUDT17
100127206	ENSG00000186367	2.96509162	0.23875254	0.49784971	0.00399119	0.01926649	MINAR2
NA	ENSG00000186369	7.97774504	-0.05531809	0.36642138	0.64995088	0.82529572	LINC00643
7626	ENSG00000186376	328.328804	0.00603817	0.13757564	0.96366272	0.98556979	ZNF75D
260293	ENSG00000186377	0	NA	NA	NA	NA	CYP4X1
353288	ENSG00000186393	0	NA	NA	NA	NA	KRT26
3858	ENSG00000186395	207.16851	-0.39834855	0.18325277	0.01412214	0.05493788	KRT10
101059918	ENSG00000186399	42.4979129	0.22406933	0.3112507	0.28191012	0.50671288	GOLGA8R
342510	ENSG00000186407	0.30001757	-0.0229446	0.38527539	0.68644769	NA	CD300E
728621	ENSG00000186409	70.8113313	-0.17768837	0.26073523	0.36462526	0.59423075	CCDC30
55922	ENSG00000186416	496.72453	-0.19893782	0.10713315	0.05239277	0.15429309	NKRF
342035	ENSG00000186417	0.45209736	-0.02505403	0.38558831	0.57865948	NA	GLDN
2204	ENSG00000186431	0.16735865	0.01897177	0.38482933	0.78225479	NA	FCAR
3840	ENSG00000186432	927.382454	-0.81649126	0.10688491	3.98E-15	1.10E-13	KPNA4
10345	ENSG00000186439	0	NA	NA	NA	NA	TRDN
128366	ENSG00000186440	0	NA	NA	NA	NA	OR6P1
3850	ENSG00000186442	0	NA	NA	NA	NA	KRT3
115560	ENSG00000186446	127.485711	-0.35601334	0.20411786	0.04198394	0.1303743	ZNF501
10168	ENSG00000186448	998.321287	-0.15369137	0.1018217	0.1161924	0.27851362	ZNF197
110354863	ENSG00000186448	998.321287	-0.15369137	0.1018217	0.1161924	0.27851362	ZNF197

353324	ENSG00000186451	5.48314062	0.03604082	0.3607403	0.78501124	0.90214452	SPATA12
283471	ENSG00000186452	0.29863895	-0.02292439	0.3852726	0.68753245	NA	TMPRSS12
653140	ENSG00000186453	13.7261209	0.56998755	0.67311445	0.03768955	0.12006434	FAM228A
400830	ENSG00000186458	0	NA	NA	NA	NA	DEFB132
4674	ENSG00000186462	0	NA	NA	NA	NA	NAP1L2
NA	ENSG00000186466	0	NA	NA	NA	NA	AQP7P1
6228	ENSG00000186468	14272.0525	-0.12893917	0.0554864	0.01868839	0.06891779	RPS23
54331	ENSG00000186469	1077.83888	0.8228366	0.09450778	5.90E-19	2.08E-17	GNG2
11118	ENSG00000186470	564.073303	0.93890128	0.14456618	1.19E-11	2.51E-10	BTN3A2
158798	ENSG00000186471	0	NA	NA	NA	NA	AKAP14
27445	ENSG00000186472	2192.13244	0.63271467	0.06448949	2.45E-23	1.09E-21	PCLO
43849	ENSG00000186474	0	NA	NA	NA	NA	KLK12
401190	ENSG00000186479	0	NA	NA	NA	NA	RGS7BP
3638	ENSG00000186480	1586.78974	-0.20341605	0.12141062	0.07617804	0.20371644	INSIG1
NA	ENSG00000186481	0.46052845	-0.02780411	0.38575284	0.5537291	NA	ANKRD20A5P
23040	ENSG00000186487	1420.70072	-0.83550274	0.09824595	3.22E-18	1.10E-16	MYT1L
153571	ENSG00000186493	0	NA	NA	NA	NA	C5orf38
252884	ENSG00000186496	48.3320433	0.41665335	0.31567158	0.06631822	0.18429434	ZNF396
84065	ENSG00000186501	882.256106	0.16436259	0.09207657	0.06567765	0.18311476	TMEM222
NA	ENSG00000186508	0	NA	NA	NA	NA	OR9I2P
219956	ENSG00000186509	0	NA	NA	NA	NA	OR9Q1
1187	ENSG00000186510	18.2783153	0.07706258	0.33308321	0.66040822	0.83169348	CLCNKA
219957	ENSG00000186513	0	NA	NA	NA	NA	OR9Q2
257106	ENSG00000186517	10.3468901	0.20619582	0.3970425	0.24482071	0.4635216	ARHGAP30
151011	ENSG00000186522	865.345586	0.42759594	0.0906762	1.19E-06	1.34E-05	SEPTIN10
85002	ENSG00000186523	100.017046	0.58503071	0.24347765	0.00422534	0.0202148	FAM86B1
11283	ENSG00000186526	0	NA	NA	NA	NA	CYP4F8
4051	ENSG00000186529	0.77034759	-0.0147326	0.3819582	0.77486312	NA	CYP4F3
114826	ENSG00000186532	596.581273	0.02361112	0.10719465	0.81956068	0.91771554	SMYD4
NA	ENSG00000186543	0	NA	NA	NA	NA	CROCCP5
245908	ENSG00000186562	0	NA	NA	NA	NA	DEFB105A
2306	ENSG00000186564	0.51937201	-0.02557462	0.38567603	0.54680819	NA	FOXD2
23131	ENSG00000186566	2026.29109	-0.14085018	0.0789294	0.0678217	0.18722451	GPATCH8
56971	ENSG00000186567	15.3887747	0.38524397	0.50597252	0.08640836	0.22393591	CEACAM19
245910	ENSG00000186572	0	NA	NA	NA	NA	DEFB107A
503614	ENSG00000186572	0	NA	NA	NA	NA	DEFB107A
4771	ENSG00000186575	771.677045	-0.19718159	0.09716687	0.03537222	0.11420719	NF2
221491	ENSG00000186577	196.740763	-0.26912975	0.17686126	0.08618733	0.22346533	SMIM29

245909	ENSG00000186579	0	NA	NA	NA	NA	DEFB106A
375686	ENSG00000186583	0.85196166	-0.01053786	0.3808599	0.85264278	NA	SPATC1
7328	ENSG00000186591	2945.29801	0.24083157	0.06719266	0.00026729	0.00184516	UBE2H
84981	ENSG00000186594	41.1665137	1.33714804	0.46016138	0.00024372	0.00169721	MIR22HG
245908	ENSG00000186599	0	NA	NA	NA	NA	DEFB105B
504180	ENSG00000186599	0	NA	NA	NA	NA	DEFB105B
84842	ENSG00000186603	38.9065801	0.56536984	0.39226812	0.03036804	0.10131192	HPDL
100129075	ENSG00000186615	57.623058	0.15637625	0.2734179	0.43163694	0.65722769	KTN1-AS1
11104	ENSG00000186625	286.85903	0.09501153	0.15331402	0.49897034	0.71658137	KATNA1
123722	ENSG00000186628	4.27302986	0.05268248	0.37707547	0.58630981	0.78206176	FSD2
116985	ENSG00000186635	1681.95777	0.01812982	0.09453727	0.84406641	0.92948958	ARAP1
347240	ENSG00000186638	315.286575	-0.13346193	0.14610329	0.32208826	0.54965002	KIF24
5138	ENSG00000186642	238.438106	-1.28363723	0.23052972	2.01E-09	3.32E-08	PDE2A
643862	ENSG00000186645	4.20381132	-0.03110807	0.37119558	0.76266488	0.89054624	SPDYE17
100101268	ENSG00000186645	4.20381132	-0.03110807	0.37119558	0.76266488	0.89054624	SPDYE17
102723849	ENSG00000186645	4.20381132	-0.03110807	0.37119558	0.76266488	0.89054624	SPDYE17
90668	ENSG00000186648	591.426961	-0.67485917	0.11351097	6.78E-10	1.19E-08	CARMIL3
5553	ENSG00000186652	0	NA	NA	NA	NA	PRG2
55615	ENSG00000186654	58.3317858	0.03725265	0.24755959	0.84529532	0.92970586	PRR5
80829	ENSG00000186660	1902.35327	-0.16197794	0.07146878	0.02088288	0.07538507	ZFP91
284018	ENSG00000186665	475.118767	-0.29889984	0.11470927	0.00614225	0.02765826	C17orf58
144233	ENSG00000186666	260.923503	-0.21073906	0.15928299	0.14481984	0.32509907	BCDIN3D
139599	ENSG00000186675	4.38811121	-0.03293176	0.36801134	0.76965618	0.89407919	MAGEE2
NA	ENSG00000186676	0.50207596	0.02562368	0.38565771	0.52549886	NA	EEF1GP1
NA	ENSG00000186678	0	NA	NA	NA	NA	
339761	ENSG00000186684	62.6280985	0.81187596	0.33287175	0.00196448	0.01053412	CYP27C1
90624	ENSG00000186687	678.288686	-0.21487612	0.15376062	0.12496375	0.29340601	LYRM7
NA	ENSG00000186704	2.9043025	0.11130381	0.39280042	0.28793966	0.51368788	DTX2P1
387885	ENSG00000186710	6.18567993	-0.11815879	0.37882825	0.39591579	0.62539564	CFAP73
493860	ENSG00000186714	0	NA	NA	NA	NA	CCDC73
11223	ENSG00000186715	20.0591453	0.75798981	0.73035407	0.02129778	0.07661794	MST1L
613	ENSG00000186716	1912.53912	-0.20387419	0.09782781	0.03065189	0.10205296	BCR
26539	ENSG00000186723	0	NA	NA	NA	NA	OR10H1
758	ENSG00000186732	21.3574941	-0.19842601	0.3862775	0.25836545	0.47964541	MPPED1
NA	ENSG00000186743	0	NA	NA	NA	NA	TPI1P3
25794	ENSG00000186765	5.12274503	-0.01747448	0.36231898	0.88584004	0.95015905	FSCN2
399823	ENSG00000186766	0	NA	NA	NA	NA	FOXI2
139886	ENSG00000186767	405.365644	-0.31469946	0.13615225	0.01317609	0.05200193	SPIN4

654254	ENSG00000186777	62.8146109	-0.73678401	0.35426543	0.00561359	0.02566803	ZNF732
474343	ENSG00000186787	124.295165	-0.00425045	0.18789013	0.97798819	0.99249544	SPIN2B
389762	ENSG00000186788	0	NA	NA	NA	NA	SPATA31D3
2301	ENSG00000186790	3.23040893	0.03882731	0.37742807	0.65684059	0.82968692	FOXE3
8372	ENSG00000186792	129.177798	-0.06652147	0.18093382	0.67584596	0.84136185	HYAL3
338567	ENSG00000186795	0	NA	NA	NA	NA	KCNK18
3446	ENSG00000186803	0.173733	0.01914545	0.38484597	0.78225479	NA	IFNA10
147645	ENSG00000186806	71.6180565	-0.29643327	0.25841459	0.13953246	0.31648712	VSIG10L
2833	ENSG00000186810	0	NA	NA	NA	NA	CXCR3
84307	ENSG00000186812	1769.82942	0.07268955	0.08133384	0.36086499	0.59075159	ZNF397
100101467	ENSG00000186814	828.439005	-0.05506232	0.11036967	0.60150558	0.79200883	ZSCAN30
53373	ENSG00000186815	854.363751	0.41085334	0.1284439	0.0006767	0.00419604	TPCN1
11006	ENSG00000186818	0	NA	NA	NA	NA	LILRB4
408029	ENSG00000186825	0.32450141	0.00139008	0.38336851	0.9669968	NA	CDRT15P3
7293	ENSG00000186827	0	NA	NA	NA	NA	TNFRSF4
NA	ENSG00000186831	0.173733	0.01914545	0.38484597	0.78225479	NA	KRT17P2
3868	ENSG00000186832	0	NA	NA	NA	NA	KRT16
10614	ENSG00000186834	643.103088	0.11193561	0.10197261	0.25492888	0.47571378	HEXIM1
348303	ENSG00000186838	10.9116456	0.1256982	0.3665329	0.42837653	0.65428642	SELENOV
353131	ENSG00000186844	0	NA	NA	NA	NA	LCE1A
3861	ENSG00000186847	0	NA	NA	NA	NA	KRT14
129293	ENSG00000186854	1.10382659	-0.02405627	0.38060958	0.69120644	NA	TRABD2A
105374836	ENSG00000186854	1.10382659	-0.02405627	0.38060958	0.69120644	NA	TRABD2A
83902	ENSG00000186860	0	NA	NA	NA	NA	KRTAP17-1
79955	ENSG00000186862	205.985526	-0.55100876	0.16454552	0.00025815	0.00179082	PDZD7
23275	ENSG00000186866	721.940635	0.20234659	0.1238295	0.08354503	0.21827226	POFUT2
84109	ENSG00000186867	0.15069912	-0.01848503	0.38480575	0.84537521	NA	QRFPR
4137	ENSG00000186868	1298.31481	-1.37699016	0.08503332	5.09E-60	6.68E-58	MAPT
54821	ENSG00000186871	389.99423	-0.28447645	0.12832836	0.01812215	0.06721694	ERCC6L
138805	ENSG00000186881	0	NA	NA	NA	NA	OR13F1
NA	ENSG00000186886	0	NA	NA	NA	NA	OR5D3P
200728	ENSG00000186889	65.4852636	-0.26730825	0.25187953	0.17505348	0.36892512	TMEM17
8784	ENSG00000186891	0.37844916	0.02407552	0.3854191	0.60038877	NA	TNFRSF18
2248	ENSG00000186895	0	NA	NA	NA	NA	FGF3
338761	ENSG00000186897	89.7310998	0.45140609	0.27236589	0.03445823	0.11200054	C1QL4
349667	ENSG00000186907	73.5022455	-0.01747245	0.24402624	0.92506783	0.96882104	RTN4RL2
23390	ENSG00000186908	1407.28836	0.18155316	0.08079209	0.02149598	0.07716307	ZDHHC17
256394	ENSG00000186910	0	NA	NA	NA	NA	SERPINA11

5030	ENSG00000186912	8.65798831	0.6872621	1.17182842	0.01977566	0.07233574	P2RY4
55893	ENSG00000186918	1421.60753	0.3969186	0.10612034	9.68E-05	0.00074059	ZNF395
353174	ENSG00000186919	9.45190671	-0.09643965	0.36307195	0.51874893	0.73239566	ZACN
337979	ENSG00000186924	0	NA	NA	NA	NA	KRTAP22-1
337973	ENSG00000186925	0	NA	NA	NA	NA	KRTAP19-6
337967	ENSG00000186930	0	NA	NA	NA	NA	KRTAP6-2
NA	ENSG00000186940	0	NA	NA	NA	NA	CHCHD2P9
138802	ENSG00000186943	0	NA	NA	NA	NA	OR13C8
5465	ENSG00000186951	517.916366	-0.25805951	0.14506544	0.05337995	0.15645711	PPARA
642987	ENSG00000186952	16.3238789	0.03951127	0.33159745	0.81774761	0.91703101	TMEM232
387978	ENSG00000186960	0	NA	NA	NA	NA	LINC01551
337969	ENSG00000186965	0	NA	NA	NA	NA	KRTAP19-2
337971	ENSG00000186967	0	NA	NA	NA	NA	KRTAP19-4
254950	ENSG00000186970	0	NA	NA	NA	NA	KRTAP15-1
284827	ENSG00000186971	0	NA	NA	NA	NA	KRTAP13-4
440585	ENSG00000186973	0	NA	NA	NA	NA	FAM183A
64800	ENSG00000186976	13.1864624	1.04905771	0.87110031	0.0098989	0.04113221	EFCAB6
337972	ENSG00000186977	0	NA	NA	NA	NA	KRTAP19-5
337963	ENSG00000186980	0	NA	NA	NA	NA	KRTAP23-1
256949	ENSG00000186994	1.48093186	0.06638933	0.38841369	0.24651703	NA	KANK3
129080	ENSG00000186998	157.384111	-0.03825749	0.20073499	0.8215678	0.91847813	EMID1
10881	ENSG00000187003	0.72077386	0.05211251	0.38874868	0.28818718	NA	ACTL7A
337977	ENSG00000187005	0	NA	NA	NA	NA	KRTAP21-1
6007	ENSG00000187010	2.80418962	-0.02113247	0.37434995	0.81333278	0.91565127	RHD
NA	ENSG00000187012	0	NA	NA	NA	NA	LINC00207
388407	ENSG00000187013	21.1369187	0.42014729	0.46764812	0.08307384	0.21743865	LINC02875
83715	ENSG00000187017	5.66734383	0.08161964	0.37954179	0.44428847	0.66924033	ESPN
5407	ENSG00000187021	0.78963195	0.0447097	0.38778241	0.30924938	NA	PNLIPRP1
138428	ENSG00000187024	129.998251	0.04030214	0.2090358	0.81985149	0.91788244	PTRH1
337978	ENSG00000187026	0	NA	NA	NA	NA	KRTAP21-2
344658	ENSG00000187033	0.173124	-0.01911262	0.38486262	0.84537521	NA	SAMD7
353345	ENSG00000187037	0	NA	NA	NA	NA	GPR141
164656	ENSG00000187045	9.53535932	0.13249891	0.37011143	0.4084802	0.63652585	TMPRSS6
1579	ENSG00000187048	0	NA	NA	NA	NA	CYP4A11
51259	ENSG00000187049	277.845683	-0.10310154	0.14661201	0.44567123	0.67028199	TMEM216
91582	ENSG00000187051	493.50925	-0.10632683	0.10931879	0.30936035	0.53712343	RPS19BP1
339967	ENSG00000187054	1.71253596	0.03169261	0.3799317	0.65977011	NA	TMPRSS11A
100130348	ENSG00000187066	13.0144815	-0.04430693	0.34701807	0.77247451	0.89553101	TMEM262

285382	ENSG00000187068	62.6694408	-0.30434955	0.26756699	0.13647042	0.31153759	C3orf70
7003	ENSG00000187079	2579.14235	0.15002864	0.09094726	0.08929931	0.22925243	TEAD1
391191	ENSG00000187080	0.15069912	-0.01848503	0.38480575	0.84537521	NA	OR2AK2
503841	ENSG00000187082	0	NA	NA	NA	NA	DEFB106B
5333	ENSG00000187091	163.914752	0.66990316	0.19061332	0.00010191	0.00077375	PLCD1
885	ENSG00000187094	0	NA	NA	NA	NA	CCK
957	ENSG00000187097	338.597341	0.26146694	0.17160393	0.08854161	0.22766532	ENTPD5
4286	ENSG00000187098	98.6040078	1.22595997	0.36509986	5.98E-05	0.00048	MITF
NA	ENSG00000187103	0	NA	NA	NA	NA	FUNDC2P3
399671	ENSG00000187105	12.5717164	-0.19384824	0.42347838	0.14887106	0.33152468	HEATR4
4673	ENSG00000187109	8809.29183	-0.12818735	0.06739479	0.05293234	0.15548973	NAP1L1
353514	ENSG00000187116	0	NA	NA	NA	NA	LILRA5
152100	ENSG00000187118	406.463511	0.22294198	0.13368542	0.07456803	0.20054626	CMC1
6585	ENSG00000187122	2188.36514	-2.36746114	0.07990765	4.88E-194	8.07E-191	SLIT1
130574	ENSG00000187123	251.222158	0.18002509	0.16372606	0.22399409	0.43762114	LYPD6
1645	ENSG00000187134	41.4053194	-0.00221632	0.26110431	0.98922993	0.99604194	AKR1C1
342865	ENSG00000187135	0	NA	NA	NA	NA	VSTM2B
27022	ENSG00000187140	0.80827351	0.0274469	0.38417302	0.5339441	NA	FOXD3
374955	ENSG00000187144	0.32373548	-0.02327229	0.38532123	0.66865955	NA	SPATA21
55182	ENSG00000187147	1570.30424	-0.03713435	0.07227201	0.59977493	0.79066681	RNF220
253935	ENSG00000187151	0	NA	NA	NA	NA	ANGPTL5
57698	ENSG00000187164	720.060749	-0.01550065	0.09704814	0.86809665	0.94233395	SHTN1
341567	ENSG00000187166	0.3164871	0.00084348	0.38336483	0.96977064	NA	H1-7
199834	ENSG00000187170	0	NA	NA	NA	NA	LCE4A
NA	ENSG00000187172	0	NA	NA	NA	NA	BAGE2
353139	ENSG00000187173	0	NA	NA	NA	NA	LCE2A
353332	ENSG00000187175	0	NA	NA	NA	NA	KRTAP12-1
353140	ENSG00000187180	0	NA	NA	NA	NA	LCE2C
388282	ENSG00000187185	0	NA	NA	NA	NA	
730098	ENSG00000187186	60.4956779	-0.03191833	0.24801335	0.86485919	0.94026008	
339327	ENSG00000187187	179.094901	-0.04340544	0.19389793	0.79462833	0.90579008	ZNF546
23270	ENSG00000187189	1927.06718	-0.10403291	0.09375705	0.25166307	0.47149344	TSPYL4
57054	ENSG00000187191	0	NA	NA	NA	NA	DAZ3
4501	ENSG00000187193	73.2154307	0.50004443	0.27596331	0.02142917	0.07697895	MT1X
2650	ENSG00000187210	0.33471731	0.02354391	0.38534154	0.62842204	NA	GCNT1
353141	ENSG00000187223	0	NA	NA	NA	NA	LCE2D
NA	ENSG00000187229	0	NA	NA	NA	NA	
91404	ENSG00000187231	1192.64581	-0.1143958	0.11194736	0.28414617	0.50925565	SESTD1

353143	ENSG00000187238	0	NA	NA	NA	NA	LCE3B
23048	ENSG00000187239	899.310639	-0.55898199	0.11765788	6.64E-07	7.75E-06	FNBP1
79659	ENSG00000187240	416.364232	0.62634974	0.1567612	1.72E-05	0.00015613	DYNC2H1
3859	ENSG00000187242	0	NA	NA	NA	NA	KRT12
81557	ENSG00000187243	1789.22848	0.05197849	0.09925316	0.58862079	0.78335367	MAGED4B
4059	ENSG00000187244	4.61042887	0.32826967	0.59474043	0.01710025	0.06425549	BCAM
222194	ENSG00000187257	941.815393	-0.35984319	0.09772812	0.00013341	0.00098435	RSBN1L
387129	ENSG00000187258	0	NA	NA	NA	NA	NPSR1
349136	ENSG00000187260	723.088566	0.17025075	0.09329015	0.05964912	0.17039991	WDR86
2057	ENSG00000187266	455.057203	0.19416674	0.11283434	0.07131001	0.19415268	EPOR
171484	ENSG00000187268	0	NA	NA	NA	NA	FAM9C
83901	ENSG00000187272	0	NA	NA	NA	NA	KRTAP9-8
63924	ENSG00000187288	0.173733	0.01914545	0.38484597	0.78225479	NA	CIDEC
1630	ENSG00000187323	180.057789	-1.65480759	0.20902025	1.85E-16	5.55E-15	DCC
51616	ENSG00000187325	728.58987	-0.26018401	0.09474594	0.0044148	0.0209846	TAF9B
56123	ENSG00000187372	182.500069	0.6538422	0.17865785	6.15E-05	0.00049262	PCDHB13
9863	ENSG00000187391	425.186061	-0.20525231	0.13735696	0.10833221	0.26410801	MAGI2
338645	ENSG00000187398	13.2746931	0.14771686	0.35752414	0.4097764	0.63779429	LUZP2
375612	ENSG00000187416	99.3878923	-2.61907924	0.2951907	4.82E-20	1.81E-18	LHFPL3
11261	ENSG00000187446	869.377843	0.29545419	0.09724548	0.00164388	0.0090747	CHP1
NA	ENSG00000187472	0	NA	NA	NA	NA	
2359	ENSG00000187474	0	NA	NA	NA	NA	FPR3
3010	ENSG00000187475	0	NA	NA	NA	NA	H1-6
387763	ENSG00000187479	2099.93155	1.11744461	0.0727657	3.80E-54	4.19E-52	C11orf96
NA	ENSG00000187481	0	NA	NA	NA	NA	HSD3BP1
388007	ENSG00000187483	0	NA	NA	NA	NA	SERPINA13P
3767	ENSG00000187486	111.316202	-0.42145841	0.2189423	0.02272761	0.08067916	KCNJ11
389118	ENSG00000187492	0.38566494	0.00705828	0.38352517	0.90845506	NA	CDHR4
1282	ENSG00000187498	2462.60831	0.9047211	0.07780492	4.77E-32	2.88E-30	COL4A1
NA	ENSG00000187504	0.51435463	0.01620069	0.3831885	0.72798278	NA	RPL7P48
440107	ENSG00000187510	0	NA	NA	NA	NA	PLEKHG7
2701	ENSG00000187513	1.69878526	-0.07085894	0.3870089	0.34489856	NA	GJA4
5757	ENSG00000187514	31910.0334	-0.0987373	0.0526453	0.05815819	0.16725009	PTMA
25763	ENSG00000187516	0	NA	NA	NA	NA	H2AP
51182	ENSG00000187522	312.619888	-0.14237114	0.14260115	0.28088913	0.50556476	HSPA14
344905	ENSG00000187527	0.56767373	0.02627943	0.38574813	0.49686938	NA	ATP13A5
51547	ENSG00000187531	665.479814	0.12219065	0.11168778	0.25246184	0.47248778	SIRT7
401137	ENSG00000187533	0	NA	NA	NA	NA	PRR27

NA	ENSG00000187534	0.14931947	-0.01844332	0.38480212	0.84537521	NA	PRR13P5
9742	ENSG00000187535	870.550948	-0.02359514	0.08903796	0.78455533	0.90201126	IFT140
NA	ENSG00000187536	0	NA	NA	NA	NA	TPM3P7
404785	ENSG00000187537	7.40334748	-0.18639137	0.41576655	0.19231563	0.39457518	POTEG
343071	ENSG00000187545	0	NA	NA	NA	NA	PRAMEF10
392636	ENSG00000187546	0.34687852	0.00028476	0.3833622	0.96576209	NA	AGMO
646643	ENSG00000187550	4.63712248	-0.05432129	0.36712428	0.66009766	0.83146071	SBK2
340665	ENSG00000187553	0.36295525	0.02389477	0.3853924	0.60996621	NA	CYP26C1
7100	ENSG00000187554	22.4097577	0.16629482	0.38304071	0.30707644	0.53465526	TLR5
7874	ENSG00000187555	2699.42483	-0.34183649	0.06187024	2.06E-08	2.99E-07	USP7
342977	ENSG00000187556	23.753633	-0.3318693	0.43375282	0.12474075	0.29302114	NANOS3
286380	ENSG00000187559	2.76380768	0.10343029	0.39981911	0.1417232	0.32019418	FOXD4L3
378884	ENSG00000187566	0	NA	NA	NA	NA	NHLRC1
359787	ENSG00000187569	0	NA	NA	NA	NA	DPPA3
341947	ENSG00000187581	0.63433561	0.00268403	0.3825192	0.94833541	NA	COX8C
84069	ENSG00000187583	2.76114498	0.00247887	0.37524666	0.97742384	0.99232877	PLEKHN1
NA	ENSG00000187589	0	NA	NA	NA	NA	TERF1P2
201181	ENSG00000187595	50.8190373	1.37445241	0.40327814	4.92E-05	0.00040282	ZNF385C
101805491	ENSG00000187600	0.16735865	0.01897177	0.38482933	0.78225479	NA	LINC02583
28986	ENSG00000187601	285.214796	0.10259657	0.13912495	0.42958719	0.6552631	MAGEH1
200424	ENSG00000187605	2592.9195	0.17132374	0.05996269	0.00380701	0.01848083	TET3
57335	ENSG00000187607	1394.09174	-0.39518742	0.09240288	1.01E-05	9.56E-05	ZNF286A
9636	ENSG00000187608	15.6407638	-0.13797871	0.34687115	0.44789712	0.67182875	ISG15
54932	ENSG00000187609	186.192083	0.96756335	0.18268962	1.61E-08	2.36E-07	EXD3
390148	ENSG00000187612	0	NA	NA	NA	NA	OR5W2
389827	ENSG00000187616	1.02319012	0.00696526	0.38088185	0.9004305	NA	MYMK
NA	ENSG00000187621	0	NA	NA	NA	NA	TCL6
400566	ENSG00000187624	28.5210004	-0.05011119	0.30393749	0.78770479	0.90351292	C17orf97
387032	ENSG00000187626	200.305341	0.03584743	0.15521793	0.80140488	0.90902045	ZKSCAN4
400966	ENSG00000187627	118.860418	1.06271309	0.28220953	1.72E-05	0.00015645	RGPD1
317749	ENSG00000187630	155.713964	0.40135848	0.18999097	0.01622917	0.06149481	DHRS4L2
148398	ENSG00000187634	345.717047	-0.32402795	0.15660662	0.0230607	0.08165702	SAMD11
84808	ENSG00000187642	3.59646748	0.01543991	0.36895294	0.88611428	0.95015905	PERM1
400673	ENSG00000187650	161.992422	0.24506282	0.1863797	0.13336592	0.30706508	VMAC
NA	ENSG00000187653	0	NA	NA	NA	NA	TMSB4XP8
NA	ENSG00000187657	0	NA	NA	NA	NA	TSPY13P
100190949	ENSG00000187658	0	NA	NA	NA	NA	C5orf52
404037	ENSG00000187664	54.8566452	0.11964289	0.40439259	0.15323914	0.33804794	HAPLN4

26059	ENSG00000187672	162.014242	-0.24775425	0.18611788	0.1282424	0.29899782	ERC2
145173	ENSG00000187676	327.049033	0.1910632	0.12942314	0.11633236	0.27881542	B3GLCT
81848	ENSG00000187678	810.85265	-0.15132296	0.0952568	0.1003899	0.24996681	SPRY4
3266	ENSG00000187682	1.94795485	0.13737587	0.41535593	0.06556439	NA	ERAS
NA	ENSG00000187686	3.62409428	0.01224251	0.37236046	0.89770352	0.9551369	KRT18P59
51393	ENSG00000187688	8.75692912	0.11305815	0.375051	0.42092329	0.64692215	TRPV2
401138	ENSG00000187689	0	NA	NA	NA	NA	AMTN
340602	ENSG00000187690	10.8410997	0.12205298	0.35650632	0.47163847	0.69399066	EZHIP
84281	ENSG00000187699	76.1093231	0.21212996	0.23060908	0.25759425	0.47884098	C2orf88
403239	ENSG00000187701	0	NA	NA	NA	NA	OR2T27
94107	ENSG00000187713	792.199989	-0.05304332	0.09259076	0.5532349	0.75941303	TMEM203
6572	ENSG00000187714	493.436339	-1.00080332	0.12960442	1.55E-15	4.40E-14	SLC18A3
166348	ENSG00000187715	1.0860262	-0.03571045	0.38482539	0.43320026	NA	KBTBD12
79875	ENSG00000187720	97.7754247	-1.81138762	0.27557731	3.17E-12	6.96E-11	THSD4
NA	ENSG00000187721	0	NA	NA	NA	NA	GTF2IP3
374407	ENSG00000187726	8.69944145	0.08954564	0.37291166	0.49015761	0.70978228	DNAJB13
2563	ENSG00000187730	49.5137396	-0.81067592	0.40005328	0.00517641	0.02392821	GABRD
278	ENSG00000187733	0	NA	NA	NA	NA	AMY1C
6917	ENSG00000187735	1634.56867	-0.43855393	0.07231173	6.32E-10	1.12E-08	TCEA1
79840	ENSG00000187736	603.881301	-0.16584028	0.12975386	0.17302411	0.36578929	NHEJ1
2175	ENSG00000187741	1493.33075	-0.20724022	0.08802961	0.01532138	0.05859217	FANCA
79048	ENSG00000187742	1684.04873	-0.29043608	0.08825601	0.00069898	0.00432419	SECISBP2
340980	ENSG00000187747	0	NA	NA	NA	NA	OR52B6
389766	ENSG00000187753	1.00770394	0.03798146	0.38215804	0.55883654	NA	C9orf153
280658	ENSG00000187754	0	NA	NA	NA	NA	SSX7
124	ENSG00000187758	0	NA	NA	NA	NA	ADH1A
NA	ENSG00000187762	0	NA	NA	NA	NA	HSPE1P11
NA	ENSG00000187763	0	NA	NA	NA	NA	OR2B7P
10507	ENSG00000187764	930.69582	-0.24819148	0.0925015	0.005561	0.02546867	SEMA4D
386681	ENSG00000187766	0	NA	NA	NA	NA	KRTAP10-8
389421	ENSG00000187772	366.02265	-0.29883213	0.12873072	0.01341934	0.05280437	LIN28B
125704	ENSG00000187773	0	NA	NA	NA	NA	DIPK1C
8632	ENSG00000187775	38.6836298	0.30765956	0.41301797	0.14912043	0.33171312	DNAH17
10445	ENSG00000187778	1113.19844	0.11297709	0.08083605	0.15229491	0.33666083	MCRS1
643236	ENSG00000187783	0.37844916	0.02407552	0.3854191	0.60038877	NA	TMEM72
57697	ENSG00000187790	154.242325	0.37118866	0.22785972	0.04921763	0.14725949	FANCM
100129969	ENSG00000187791	0.173124	-0.01911262	0.38486262	0.84537521	NA	FAM205C
7621	ENSG00000187792	358.527547	0.1654457	0.12278351	0.15457288	0.33998898	ZNF70

64170	ENSG00000187796	752.451805	0.0708074	0.11023911	0.50234649	0.7191395	CARD9
375033	ENSG00000187800	160.11904	-0.32886547	0.25802593	0.1019812	0.252945	PEAR1
65243	ENSG00000187801	230.190052	0.0314719	0.15958325	0.82997697	0.92292025	ZFP69B
338949	ENSG00000187806	0	NA	NA	NA	NA	TMEM202
347454	ENSG00000187808	4.68917404	0.04165224	0.36986844	0.71030811	0.86089825	SOWAHD
NA	ENSG00000187812	0	NA	NA	NA	NA	
339559	ENSG00000187815	168.599148	-0.2282113	0.17463994	0.14162724	0.3200324	ZFP69
391723	ENSG00000187821	0	NA	NA	NA	NA	HELT
340595	ENSG00000187823	0.173733	0.01914545	0.38484597	0.78225479	NA	RTL4
388335	ENSG00000187824	83.5122995	-0.03300836	0.23804187	0.85809148	0.93662789	TMEM220
388960	ENSG00000187833	0	NA	NA	NA	NA	C2orf78
3006	ENSG00000187837	83.1612413	-0.49726406	0.25768907	0.01701426	0.06400509	H1-2
57048	ENSG00000187838	585.684295	0.54401109	0.11050049	2.97E-07	3.67E-06	PLSCR3
1978	ENSG00000187840	1891.78963	-0.16889042	0.0863557	0.04434117	0.13565472	EIF4EBP1
NA	ENSG00000187847	0	NA	NA	NA	NA	OR7E25P
22953	ENSG00000187848	0.3435056	0.02364483	0.38535622	0.62283903	NA	P2RX2
121549	ENSG00000187855	0.173124	-0.01911262	0.38486262	0.84537521	NA	ASCL4
390323	ENSG00000187857	0	NA	NA	NA	NA	OR6C75
550631	ENSG00000187860	145.628109	0.21232346	0.18470807	0.19027079	0.39162973	CCDC157
164118	ENSG00000187862	3.73744928	-0.01984875	0.36576482	0.86149043	0.93844892	TTC24
116224	ENSG00000187866	303.513514	-0.05740975	0.13374465	0.64613081	0.82370422	PABIR1
342979	ENSG00000187867	249.067848	0.51204298	0.15363252	0.00031315	0.00212898	PALM3
NA	ENSG00000187870	0	NA	NA	NA	NA	RNFT1P3
389400	ENSG00000187871	0	NA	NA	NA	NA	GFRAL
199920	ENSG00000187889	2.76715221	0.03837586	0.37799264	0.63135826	0.81365039	FYB2
392459	ENSG00000187893	0	NA	NA	NA	NA	CXXC1P1
NA	ENSG00000187900	0	NA	NA	NA	NA	OR5H7P
729956	ENSG00000187902	78.0895738	-1.74279365	0.29041826	1.43E-10	2.70E-09	SHISA7
NA	ENSG00000187904	0.45209736	-0.02505403	0.38558831	0.57865948	NA	
400891	ENSG00000187905	2.84418523	-0.0011301	0.3708319	0.98924211	0.99604194	LRRC74B
1755	ENSG00000187908	1.03458168	0.0506224	0.38552444	0.35142596	NA	DMBT1
388512	ENSG00000187912	0	NA	NA	NA	NA	CLEC17A
390064	ENSG00000187918	0	NA	NA	NA	NA	OR51I2
414332	ENSG00000187922	1.06573756	0.04978228	0.38642528	0.3445109	NA	LCN10
401944	ENSG00000187942	25.4595071	0.05598461	0.3300006	0.74783226	0.88161596	LDLRAD2
401027	ENSG00000187944	6.95647971	0.03158882	0.35719271	0.81914092	0.91752333	C2orf66
341350	ENSG00000187950	0.173733	0.01914545	0.38484597	0.78225479	NA	OVCH1
100288637	ENSG00000187951	492.929071	0.13796217	0.1359025	0.27728367	0.50167437	

NA	ENSG00000187952	0.32427719	0.00119291	0.38336686	0.96888079	NA	HS6ST1P1
NA	ENSG00000187953	410.922911	0.12294973	0.11593138	0.26590894	0.48827746	PMS2CL
50626	ENSG00000187954	1104.2157	0.06485009	0.09054689	0.46167586	0.68473119	CYHR1
7373	ENSG00000187955	1.42887348	0.10356603	0.40306616	0.0690672	NA	COL14A1
92737	ENSG00000187957	1.55831389	-0.09505766	0.39662707	0.16243725	NA	DNER
642843	ENSG00000187959	0.37844916	0.02407552	0.3854191	0.60038877	NA	CPSF4L
339451	ENSG00000187961	739.821805	0.02393993	0.09226987	0.79000968	0.9043268	KLHL17
389874	ENSG00000187969	0	NA	NA	NA	NA	ZCCHC13
NA	ENSG00000187979	0.56767373	0.02627943	0.38574813	0.49686938	NA	FAM230D
391013	ENSG00000187980	0	NA	NA	NA	NA	PLA2G2C
NA	ENSG00000187984	2.29286552	0.04980121	0.38371094	0.46617227	NA	ANKRD19P
222696	ENSG00000187987	181.909219	0.64093898	0.18684887	0.00015015	0.00109643	ZSCAN23
NA	ENSG00000187988	0.34746601	0.02370763	0.3853651	0.61987128	NA	KCTD9P3
126432	ENSG00000187994	123.520675	0.59569643	0.27246289	0.00683912	0.03034851	RINL
100141515	ENSG00000187997	0	NA	NA	NA	NA	C17orf99
NA	ENSG00000187999	0.16735865	0.01897177	0.38482933	0.78225479	NA	HNRNPA1P61
162998	ENSG00000188000	0.35704327	0.02381967	0.38538147	0.61383419	NA	OR7D2
285386	ENSG00000188001	0.173124	-0.01911262	0.38486262	0.84537521	NA	TPRG1
NA	ENSG00000188002	183.336213	0.02635569	0.15912362	0.85678977	0.93605226	
284677	ENSG00000188004	12.47467	-0.1370155	0.36471567	0.41191416	0.63976648	SNHG28
729967	ENSG00000188010	248.063206	0.03252636	0.14398692	0.80869227	0.91327725	MORN2
285093	ENSG00000188011	3.12131613	0.04457994	0.37786446	0.61502268	0.80239693	RTP5
NA	ENSG00000188013	20.6368497	0.44099669	0.5109431	0.07206484	0.19555978	MEIS3P2
6274	ENSG00000188015	0.33471731	0.02354391	0.38534154	0.62842204	NA	S100A3
29978	ENSG00000188021	1319.34999	-0.02962943	0.07612204	0.69111895	0.85035049	UBQLN2
353116	ENSG00000188026	429.505785	0.41642903	0.12856297	0.00058261	0.0036888	RILPL1
392360	ENSG00000188029	0	NA	NA	NA	NA	CTSL3P
646457	ENSG00000188032	0	NA	NA	NA	NA	C19orf67
57474	ENSG00000188033	167.562452	0.20919124	0.19073498	0.20723858	0.41447107	ZNF490
1180	ENSG00000188037	0	NA	NA	NA	NA	CLCN1
123904	ENSG00000188038	1.98674645	0.09856895	0.3998048	0.10747501	NA	NRN1L
284434	ENSG00000188039	0.34117358	0.02362701	0.38535347	0.62407208	NA	NWD1
10123	ENSG00000188042	560.993558	-0.21272783	0.11023086	0.04295368	0.13257414	ARL4C
168433	ENSG00000188050	0	NA	NA	NA	NA	RNF133
100130519	ENSG00000188051	11.7560591	0.39316763	0.60153495	0.05219045	0.15390289	TMEM221
285852	ENSG00000188056	0	NA	NA	NA	NA	TREML4
115273	ENSG00000188060	28.1191833	0.12488936	0.29853919	0.52725373	0.73906416	RAB42
7477	ENSG00000188064	0.15069912	-0.01848503	0.38480575	0.84537521	NA	WNT7B

65998	ENSG00000188070	2096.82517	-0.16247466	0.06776203	0.01476231	0.05692599	C11orf95
147199	ENSG00000188076	0	NA	NA	NA	NA	SCGB1C1
NA	ENSG00000188078	0	NA	NA	NA	NA	
NA	ENSG00000188086	0.48437198	0.00977975	0.38330854	0.807476	NA	PRSS45P
123745	ENSG00000188089	0	NA	NA	NA	NA	PLA2G4E
51463	ENSG00000188092	259.347232	0.44930145	0.16603811	0.00287544	0.01460542	GPR89B
145873	ENSG00000188095	2.62408805	-0.04650319	0.37637428	0.62147004	0.80672374	MESP2
643161	ENSG00000188100	0	NA	NA	NA	NA	FAM25A
NA	ENSG00000188101	0	NA	NA	NA	NA	ALOX15P2
346007	ENSG00000188107	2.01109226	0.05326836	0.38301945	0.46516719	NA	EYS
647024	ENSG00000188112	0.83757895	-0.03117678	0.3837275	0.52283675	NA	C6orf132
1617	ENSG00000188120	0	NA	NA	NA	NA	DAZ1
338755	ENSG00000188124	0	NA	NA	NA	NA	OR2AG2
6300	ENSG00000188130	641.724295	-0.88898547	0.10112682	2.42E-19	8.73E-18	MAPK12
401498	ENSG00000188133	50.2420772	-0.73456406	0.41147092	0.01002228	0.04153792	TMEM215
441457	ENSG00000188152	21.236359	0.00787571	0.32377478	0.96633677	0.98698181	NUTM2G
1287	ENSG00000188153	0.9406116	0.06504731	0.39124699	0.20236004	NA	COL4A5
386674	ENSG00000188155	0	NA	NA	NA	NA	KRTAP10-6
375790	ENSG00000188157	2014.08388	-0.4607871	0.06826684	6.66E-12	1.44E-10	AGRN
4810	ENSG00000188158	1005.12526	-0.01897438	0.10407603	0.84870214	0.93126766	NHS
340990	ENSG00000188162	0.14931947	-0.01844332	0.38480212	0.84537521	NA	OTOG
401565	ENSG00000188163	6.52873372	-0.03127133	0.35612579	0.81829659	0.9171248	FAM166A
643853	ENSG00000188167	58.5056222	-0.64030095	0.31131649	0.00793372	0.03427672	TMPPE
199777	ENSG00000188171	138.243206	0.06057303	0.1846119	0.70960481	0.86070548	ZNF626
253012	ENSG00000188175	1.97246452	-0.01170005	0.37459631	0.89203485	NA	HEPACAM2
342527	ENSG00000188176	0.29863895	-0.02292439	0.3852726	0.68753245	NA	SMTNL2
376940	ENSG00000188177	530.889368	-0.08423275	0.15223502	0.54563566	0.7531245	ZC3H6
NA	ENSG00000188185	542.551556	0.00373299	0.10735686	0.9720923	0.98954906	LINC00265
389541	ENSG00000188186	1052.57103	0.04776804	0.08846252	0.57969488	0.77720951	LAMTOR4
5575	ENSG00000188191	2440.87846	-0.0643508	0.06908571	0.34318515	0.57337207	PRKAR1B
729262	ENSG00000188199	53.2737858	0.37360241	0.30783105	0.09116438	0.23298963	NUTM2B
374383	ENSG00000188211	710.245663	-0.72048928	0.10946481	1.03E-11	2.19E-10	NCR3LG1
123879	ENSG00000188215	243.704068	0.06369874	0.15785669	0.65876493	0.83073135	DCUN1D3
445582	ENSG00000188219	0.93488885	-0.01830083	0.38087361	0.75923893	NA	POTEE
NA	ENSG00000188223	9.45392371	-0.02795971	0.36904291	0.79460484	0.90579008	
390927	ENSG00000188227	534.926869	0.0247701	0.12348466	0.83332678	0.92415967	ZNF793
10383	ENSG00000188229	5726.92823	-0.10588917	0.05978663	0.07257187	0.19642919	TUBB4B
119016	ENSG00000188234	419.258937	-0.09557853	0.14822632	0.48319471	0.70357492	AGAP4

25845	ENSG00000188242	187.804745	0.08466666	0.16233857	0.56362397	0.76627833	
170622	ENSG00000188243	654.672943	-0.25584791	0.10168579	0.00886055	0.03752258	COMMD6
5320	ENSG00000188257	0	NA	NA	NA	NA	PLA2G2A
400935	ENSG00000188263	0.80833901	-0.00835916	0.38085242	0.88250937	NA	IL17REL
123688	ENSG00000188266	40.7523706	0.717657	0.399991	0.01050244	0.04311366	HYKK
26659	ENSG00000188269	0	NA	NA	NA	NA	OR7A5
643338	ENSG00000188277	85.9114398	-0.18989805	0.22606338	0.30363611	0.531042	C15orf62
NA	ENSG00000188280	0	NA	NA	NA	NA	FAM230G
285180	ENSG00000188282	4.05995382	0.29892364	0.56234355	0.01466769	0.05660512	RUFY4
163087	ENSG00000188283	204.919683	-0.10326782	0.17143962	0.4995901	0.71690059	ZNF383
57801	ENSG00000188290	128.727018	-2.18617726	0.2327514	4.14E-22	1.72E-20	HES4
374918	ENSG00000188293	0	NA	NA	NA	NA	IGFL1
79862	ENSG00000188295	416.861066	0.11671757	0.12729627	0.33094074	0.55979745	ZNF669
374872	ENSG00000188305	2.51616042	-0.00324804	0.37121721	0.9720491	NA	PEAK3
344657	ENSG00000188306	3.40165696	0.0346672	0.3710835	0.74389224	0.87933877	LRRIQ4
401541	ENSG00000188312	399.585351	-0.4936886	0.14141144	0.00018228	0.00130843	CENPP
5359	ENSG00000188313	30.1465739	3.08150422	0.60429933	1.35E-08	2.01E-07	PLSCR1
NA	ENSG00000188314	0	NA	NA	NA	NA	OR7D1P
375341	ENSG00000188315	221.194293	0.08701528	0.20989721	0.62170774	0.8069267	C3orf62
387712	ENSG00000188316	2.39501451	0.05440127	0.38412491	0.43353401	NA	ENO4
84527	ENSG00000188321	387.501186	-0.0564411	0.12287768	0.6258894	0.80978006	ZNF559
388228	ENSG00000188322	3825.59496	-0.08961901	0.06120934	0.13773939	0.31360448	SBK1
283365	ENSG00000188324	0	NA	NA	NA	NA	OR6C6
100131137	ENSG00000188334	0	NA	NA	NA	NA	BSPH1
10991	ENSG00000188338	37.6868208	0.22276744	0.33206411	0.28096157	0.50564928	SLC38A3
81442	ENSG00000188340	0	NA	NA	NA	NA	OR6N2
2963	ENSG00000188342	513.72323	0.14103254	0.11461467	0.19691587	0.40069856	GTF2F2
137392	ENSG00000188343	271.121271	-0.5955133	0.15659302	4.08E-05	0.00034254	CIBAR1
54914	ENSG00000188352	876.129948	-0.10316359	0.08911299	0.23174705	0.44830942	FOCAD
NA	ENSG00000188365	1.48022204	-0.04721801	0.38436184	0.44515493	NA	
284338	ENSG00000188368	20.9231813	0.09356134	0.31413475	0.62278696	0.80737672	PRR19
7784	ENSG00000188372	104.509726	1.00281399	0.26456355	1.79E-05	0.0001619	ZP3
387695	ENSG00000188373	0	NA	NA	NA	NA	C10orf99
440093	ENSG00000188375	0.37844916	0.02407552	0.3854191	0.60038877	NA	H3-5
3440	ENSG00000188379	0	NA	NA	NA	NA	IFNA2
NA	ENSG00000188383	2.36346797	0.10828532	0.39758601	0.21310758	NA	GPAT2P2
282973	ENSG00000188385	79.8459509	-0.36129596	0.26395101	0.07812622	0.20773003	JAKMIP3
5535	ENSG00000188386	0	NA	NA	NA	NA	PPP3R2

100133220	ENSG00000188388	8.08522151	0.00496618	0.35059841	0.97480445	0.99114028	GOLGA6L3
5133	ENSG00000188389	1.38237915	0.0317034	0.37963765	0.66358205	NA	PDCD1
387836	ENSG00000188393	0	NA	NA	NA	NA	CLEC2A
2844	ENSG00000188394	0.15069912	-0.01848503	0.38480575	0.84537521	NA	GPR21
343521	ENSG00000188396	0.68304084	-0.01111829	0.38155519	0.83537345	NA	DYNLT4
NA	ENSG00000188399	0	NA	NA	NA	NA	ANKRD36P1
NA	ENSG00000188403	0	NA	NA	NA	NA	IGHV1OR15-9
6402	ENSG00000188404	0	NA	NA	NA	NA	SELL
347541	ENSG00000188408	0	NA	NA	NA	NA	MAGEB5
1121	ENSG00000188419	687.681952	-0.2023677	0.11237926	0.05885393	0.16859111	CHM
339345	ENSG00000188425	1.00245096	-0.02492772	0.38055569	0.69887298	NA	NANOS2
63915	ENSG00000188428	322.33704	-0.02449834	0.13345579	0.84426509	0.92958651	BLOC1S5
NA	ENSG00000188438	0	NA	NA	NA	NA	DEFB108F
NA	ENSG00000188439	0	NA	NA	NA	NA	OR4P1P
NA	ENSG00000188451	0.50211935	0.01195435	0.38340609	0.77280437	NA	SRP72P2
375298	ENSG00000188452	17.9997604	0.06012679	0.31928402	0.74273847	0.87875221	CERKL
NA	ENSG00000188459	0.33856635	0.00220071	0.38337774	0.96744578	NA	WASF4P
NA	ENSG00000188460	0	NA	NA	NA	NA	ACTBP11
283652	ENSG00000188467	1.96593827	-0.04694562	0.38168285	0.52680511	NA	SLC24A5
389792	ENSG00000188483	163.734387	0.31938872	0.18579248	0.05052935	0.15014263	IER5L
3014	ENSG00000188486	4143.97036	-0.09840044	0.05777265	0.08474179	0.22069796	H2AX
387755	ENSG00000188487	15.1211704	0.71732885	0.69582304	0.0240821	0.08463382	INSC
5104	ENSG00000188488	149.57733	-2.1437361	0.23341287	2.57E-21	1.02E-19	SERPINA5
284325	ENSG00000188493	322.88195	0.15144644	0.15138641	0.27602751	0.50016881	C19orf54
197021	ENSG00000188501	10.6645192	-0.21826874	0.41627814	0.19656354	0.4001795	LCTL
342897	ENSG00000188505	6.50332862	-0.07576329	0.36842795	0.56207334	0.76518802	NCCRP1
388533	ENSG00000188508	0	NA	NA	NA	NA	KRTDAP
348645	ENSG00000188511	31.4747708	-2.07835842	0.49531893	1.63E-06	1.78E-05	C22orf34
NA	ENSG00000188512	0	NA	NA	NA	NA	
84570	ENSG00000188517	0	NA	NA	NA	NA	COL25A1
644815	ENSG00000188522	174.816988	0.29845773	0.17259401	0.05307009	0.15584823	FAM83G
389799	ENSG00000188523	11.8145361	0.1409379	0.35640975	0.42706059	0.65286501	CFAP77
NA	ENSG00000188525	0.36294433	0.02389469	0.38539239	0.60997161	NA	
10772	ENSG00000188529	4357.57525	0.04684816	0.06664184	0.47561052	0.69768942	SRSF10
3040	ENSG00000188536	0.30139824	-0.02296476	0.38527817	0.68536535	NA	HBA2
285193	ENSG00000188542	123.087393	0.20812732	0.21571834	0.24749664	0.46645136	DUSP28
388115	ENSG00000188549	318.702348	0.22741913	0.19673436	0.17999939	0.37673028	CCDC9B
4077	ENSG00000188554	1769.51138	-0.06788312	0.07774671	0.37199599	0.60104858	NBR1

391211	ENSG00000188558	0	NA	NA	NA	NA	OR2G6
57186	ENSG00000188559	835.914914	-0.12315604	0.08697878	0.14545968	0.32597939	RALGAPA2
27158	ENSG00000188566	964.590685	0.33495758	0.09875101	0.00043321	0.00284601	NDOR1
345630	ENSG00000188573	778.318411	-0.83515338	0.13612614	1.46E-10	2.75E-09	FBLL1
154215	ENSG00000188580	0	NA	NA	NA	NA	NKAIN2
81851	ENSG00000188581	0	NA	NA	NA	NA	KRTAP1-1
344838	ENSG00000188582	28.7677677	1.01442088	0.50319146	0.00357709	0.01760154	PAQR9
400797	ENSG00000188585	0	NA	NA	NA	NA	CLEC20A
144535	ENSG00000188596	3.99120327	0.2270698	0.46785555	0.05832015	0.16752161	CFAP54
NA	ENSG00000188599	268.288429	0.00292948	0.13906025	0.98197725	0.99406451	NPIPP1
1201	ENSG00000188603	468.634514	0.07326398	0.11168235	0.49351559	0.712409	CLN3
653820	ENSG00000188610	301.495986	-0.4298222	0.15130264	0.002034	0.01085677	FAM72B
56624	ENSG00000188611	148.006976	0.52389124	0.29891076	0.0213394	0.07671201	ASAH2
6613	ENSG00000188612	5806.38188	-0.23542121	0.05791347	3.84E-05	0.00032333	SUMO2
340719	ENSG00000188613	1117.45078	0.74261734	0.08301841	8.06E-20	2.97E-18	NANOS1
340784	ENSG00000188620	0	NA	NA	NA	NA	HMX3
388555	ENSG00000188624	0	NA	NA	NA	NA	IGFL3
653720	ENSG00000188626	10.315969	0.04635457	0.34065556	0.77698878	0.89765663	GOLGA8M
7730	ENSG00000188629	46.2028634	-0.33775871	0.30915683	0.12206948	0.28886671	ZNF177
84247	ENSG00000188636	2159.91674	0.08780872	0.07249851	0.21589115	0.42667359	RTL6
1806	ENSG00000188641	956.86507	-0.24078765	0.09273854	0.00729866	0.03200117	DPYD
140576	ENSG00000188643	1.11896681	-0.01229829	0.37984734	0.84604941	NA	S100A16
NA	ENSG00000188646	0	NA	NA	NA	NA	
375743	ENSG00000188647	2089.08553	-0.36517541	0.08189177	4.75E-06	4.80E-05	PTAR1
387707	ENSG00000188649	4.20506458	-0.09927049	0.38739421	0.33587099	0.56585025	CC2D2B
390443	ENSG00000188655	0	NA	NA	NA	NA	RNASE9
NA	ENSG00000188656	0	NA	NA	NA	NA	TSPY7P
283726	ENSG00000188659	25.91281	0.42718547	0.48584539	0.07746427	0.20648812	SAXO2
NA	ENSG00000188660	1.87161035	-0.02319256	0.37843127	0.75321352	NA	LINC00319
NA	ENSG00000188662	0	NA	NA	NA	NA	H1-9P
NA	ENSG00000188668	0	NA	NA	NA	NA	OR7E90P
6006	ENSG00000188672	10.0833596	-0.03767367	0.35044145	0.7976658	0.90722825	RHCE
389073	ENSG00000188674	0	NA	NA	NA	NA	C2orf80
169355	ENSG00000188676	0	NA	NA	NA	NA	IDO2
29780	ENSG00000188677	398.747572	-0.05783013	0.12431238	0.62210098	0.80696197	PARVB
100132288	ENSG00000188681	312.200194	0.33553472	0.14173522	0.01073998	0.04395239	TEKT4P2
100233156	ENSG00000188681	312.200194	0.33553472	0.14173522	0.01073998	0.04395239	TEKT4P2
57835	ENSG00000188687	76.5130619	0.64422127	0.36647499	0.01357843	0.0533141	SLC4A5

7390	ENSG00000188690	819.768402	0.01958349	0.0912016	0.82566425	0.92091178	UROS
390084	ENSG00000188691	0	NA	NA	NA	NA	OR56A5
613126	ENSG00000188693	8.7777055	-0.01897026	0.35423911	0.88974242	0.95202525	CYP51A1-AS1
643803	ENSG00000188694	0	NA	NA	NA	NA	KRTAP24-1
51114	ENSG00000188706	746.776726	-0.16339133	0.09177096	0.06612103	0.18393735	ZDHHC9
113763	ENSG00000188707	384.943558	0.54243854	0.1276598	7.39E-06	7.20E-05	ZBED6CL
347148	ENSG00000188710	8.98511539	-0.29087737	0.52205335	0.04434761	0.13565472	QRFP
NA	ENSG00000188712	0	NA	NA	NA	NA	OR13D3P
338599	ENSG00000188716	0	NA	NA	NA	NA	DUSP29
643155	ENSG00000188725	1178.21433	0.07508762	0.10388071	0.45315926	0.67644871	SMIM15
344901	ENSG00000188729	0	NA	NA	NA	NA	OSTN
375567	ENSG00000188730	0	NA	NA	NA	NA	VWC2
340277	ENSG00000188732	642.95557	-0.17665727	0.10212583	0.07203665	0.19555978	FAM221A
144404	ENSG00000188735	964.728111	0.28513142	0.10726699	0.00550356	0.0252522	TMEM120B
401024	ENSG00000188738	24.3465881	-0.03577885	0.30607313	0.84608295	0.93019427	FSIP2
23029	ENSG00000188739	899.338063	-0.53303452	0.10743284	2.49E-07	3.13E-06	RBM34
10811	ENSG00000188747	18.8213837	1.87312455	0.61523381	0.00012936	0.00095766	NOXA1
NA	ENSG00000188755	0.173124	-0.01911262	0.38486262	0.84537521	NA	TBC1D3P2
130612	ENSG00000188760	626.38942	-1.00851968	0.13851727	4.17E-14	1.06E-12	TMEM198
440603	ENSG00000188761	16.4081812	0.07939731	0.36396394	0.54205223	0.75026624	BCL2L15
8326	ENSG00000188763	66.6976943	-0.03415297	0.22987593	0.85169438	0.93275912	FZD9
NA	ENSG00000188765	0	NA	NA	NA	NA	TMSB4XP2
399473	ENSG00000188766	318.325193	-0.0483687	0.14182219	0.71342369	0.86190557	SPRED3
26254	ENSG00000188770	0	NA	NA	NA	NA	OPTC
349633	ENSG00000188771	0.14931947	-0.01844332	0.38480212	0.84537521	NA	PLET1
155	ENSG00000188778	0	NA	NA	NA	NA	ADRB3
390598	ENSG00000188779	71.1995907	0.57269308	0.287639	0.01150221	0.04653421	SKOR1
378807	ENSG00000188782	0.18922458	0.01954053	0.38488496	0.78225479	NA	CATSPER4
5549	ENSG00000188783	1.41277326	0.08182413	0.39286045	0.18490829	NA	PRELP
30814	ENSG00000188784	0	NA	NA	NA	NA	PLA2G2E
147694	ENSG00000188785	411.985131	-0.08889042	0.15145061	0.52238261	0.73571881	ZNF548
4520	ENSG00000188786	561.960936	-0.24038378	0.15341853	0.08525566	0.22149194	MTF1
127391	ENSG00000188800	0	NA	NA	NA	NA	TMCO2
NA	ENSG00000188801	0.30139824	-0.02296476	0.38527817	0.68536535	NA	ZNF322P1
388336	ENSG00000188803	0	NA	NA	NA	NA	SHISA6
199953	ENSG00000188807	1171.0333	-0.28854182	0.09005868	0.0009489	0.00566542	TMEM201
387921	ENSG00000188811	530.935021	-0.02784954	0.1105749	0.79124641	0.90493295	NHLRC3
3167	ENSG00000188816	2.77966984	0.20924479	0.46923597	0.0060925	0.02747164	HMX2

132203	ENSG00000188817	0	NA	NA	NA	NA	SNTN
79844	ENSG00000188818	96.9443703	-0.80858592	0.2893005	0.00075578	0.00462944	ZDHHC11
441168	ENSG00000188820	1.40002076	-0.02075353	0.37955003	0.75889573	NA	CALHM6
1269	ENSG00000188822	0.9967882	0.00664525	0.38006109	0.91293995	NA	CNR2
NA	ENSG00000188825	102.787097	0.00107753	0.20531024	0.99612738	0.99859366	LINC00910
84464	ENSG00000188827	630.458234	0.09302495	0.09975861	0.33481226	0.56474327	SLX4
NA	ENSG00000188828	9.31050253	0.18364839	0.39703782	0.26187173	0.48384571	GLRA4
NA	ENSG00000188831	0	NA	NA	NA	NA	DPPA3P2
377841	ENSG00000188833	9.757961	0.35123166	0.52410806	0.08207441	0.21541955	ENTPD8
9045	ENSG00000188846	10455.0667	-0.04480762	0.04816831	0.34932119	0.58004303	RPL14
389206	ENSG00000188848	1799.57883	-0.40152163	0.07052257	6.56E-09	1.01E-07	BEND4
NA	ENSG00000188850	0	NA	NA	NA	NA	
NA	ENSG00000188856	0.66261589	-0.02369954	0.38272313	0.63558227	NA	RPSAP47
149297	ENSG00000188859	67.7597592	1.08049086	0.29925474	3.18E-05	0.00027234	FAM78B
147837	ENSG00000188868	31.7439915	0.08038904	0.29061445	0.67905824	0.84320727	ZNF563
342125	ENSG00000188869	6.33149251	0.02624361	0.35650228	0.84945288	0.93154246	TMC3
NA	ENSG00000188873	0.15069912	-0.01848503	0.38480575	0.84537521	NA	RPL10AP2
340441	ENSG00000188877	0	NA	NA	NA	NA	POTEA
85302	ENSG00000188878	372.634461	0.18887498	0.13392788	0.13163841	0.30439464	FBF1
346689	ENSG00000188883	19.1646025	-0.17259837	0.34001795	0.37542854	0.60453716	KLRG2
431705	ENSG00000188886	0.18922458	0.01954053	0.38488496	0.78225479	NA	ASTL
339287	ENSG00000188895	2184.82679	-0.21765439	0.06659111	0.00089613	0.00538601	MSL1
400499	ENSG00000188897	27.2057953	-0.23858806	0.39219095	0.21860011	0.43050004	
120892	ENSG00000188906	109.389211	1.08439396	0.26687576	5.11E-06	5.12E-05	LRRK2
390259	ENSG00000188909	3.35861435	-0.15949122	0.42157949	0.10671699	0.26142302	BSX
2707	ENSG00000188910	0.36303115	0.0238953	0.38539249	0.60992866	NA	GJB3
642938	ENSG00000188916	1.18374889	-0.02672335	0.38111485	0.66921013	NA	INSYN2A
79979	ENSG00000188917	212.094616	0.07054544	0.15640851	0.622069	0.80696197	TRMT2B
401494	ENSG00000188921	62.4983342	0.40577981	0.28244837	0.05916543	0.16936116	HACD4
257177	ENSG00000188931	21.1274391	0.01531404	0.30735046	0.93590267	0.97325384	CFAP126
NA	ENSG00000188933	8.69922755	0.00825658	0.3481437	0.95761361	0.98264776	USP32P1
60506	ENSG00000188937	0	NA	NA	NA	NA	NYX
158293	ENSG00000188938	969.169309	0.00615292	0.09332249	0.94669423	0.97814213	FAM120AOS
257313	ENSG00000188958	0.30139824	-0.02296476	0.38527817	0.68536535	NA	UTS2B
401546	ENSG00000188959	0	NA	NA	NA	NA	C9orf152
26155	ENSG00000188976	4629.14409	-0.05913262	0.06379095	0.34708219	0.57783927	NOC2L
345222	ENSG00000188981	5.03352153	0.03721576	0.36362572	0.76780868	0.89307241	MSANTD1
126767	ENSG00000188984	0	NA	NA	NA	NA	AADACL3

NA	ENSG00000188985	67.9254194	-0.14149625	0.41416335	0.11245043	0.27161145	DHFRP1
25920	ENSG00000188986	1738.53803	-0.03847211	0.06973807	0.57431556	0.77352065	NELFB
729025	ENSG00000188991	0	NA	NA	NA	NA	SLC15A5
149998	ENSG00000188992	3.09577389	0.02362963	0.37212595	0.81205077	0.91493482	LIPI
339977	ENSG00000188993	6.0194751	0.09246411	0.37364764	0.48311718	0.70354116	LRRC66
23036	ENSG00000188994	1894.18285	-0.08708086	0.0954033	0.34467437	0.57503696	ZNF292
135458	ENSG00000188996	2.02332216	0.03500669	0.37654975	0.69096363	NA	HUS1B
283219	ENSG00000188997	215.729401	-0.24115854	0.18462922	0.13537305	0.30992327	KCTD21
374897	ENSG00000189001	0.53238261	-0.01818742	0.38336655	0.72877183	NA	SBSN
NA	ENSG00000189002	0	NA	NA	NA	NA	PROS2P
134637	ENSG00000189007	339.952496	-0.13693	0.13683919	0.28292059	0.50793092	ADAT2
3805	ENSG00000189013	0	NA	NA	NA	NA	KIR2DL4
NA	ENSG00000189014	11.6837505	0.00936588	0.3464606	0.95297519	0.98058207	SHLD2P3
139604	ENSG00000189023	0	NA	NA	NA	NA	MAGEB16
391104	ENSG00000189030	0.5063993	0.01486836	0.38310111	0.74606538	NA	VHLL
63904	ENSG00000189037	0	NA	NA	NA	NA	DUSP21
163081	ENSG00000189042	197.33139	-0.00165968	0.18417365	0.99066755	0.99679705	ZNF567
4697	ENSG00000189043	2556.84013	0.03805456	0.06242713	0.53723912	0.74759591	NDUFA4
728780	ENSG00000189045	23.994756	0.84030801	0.63955755	0.01415423	0.05504726	ANKDD1B
121642	ENSG00000189046	610.44218	0.38303789	0.10178961	9.27E-05	0.00071088	ALKBH2
51136	ENSG00000189050	216.335368	-0.22547205	0.15335622	0.10764655	0.26314781	RNFT1
643904	ENSG00000189051	1.32702544	0.00669838	0.37907339	0.92036681	NA	RNF222
93659	ENSG00000189052	0.15069912	-0.01848503	0.38480575	0.84537521	NA	CGB5
5649	ENSG00000189056	2471.5358	-0.86379455	0.08488477	4.26E-25	2.03E-23	RELN
374393	ENSG00000189057	420.280982	-0.86768999	0.14785103	6.85E-10	1.21E-08	FAM111B
347	ENSG00000189058	0	NA	NA	NA	NA	APOD
3005	ENSG00000189060	7673.74942	-0.17887034	0.08749764	0.03533451	0.11413817	H1-0
645037	ENSG00000189064	0.34047096	-0.00019945	0.38336231	0.96714319	NA	GAGE2A
729447	ENSG00000189064	0.34047096	-0.00019945	0.38336231	0.96714319	NA	GAGE2A
100101629	ENSG00000189064	0.34047096	-0.00019945	0.38336231	0.96714319	NA	GAGE2A
9516	ENSG00000189067	973.038757	-0.48568054	0.10499743	1.52E-06	1.66E-05	LITAF
284415	ENSG00000189068	0	NA	NA	NA	NA	VSTM1
83862	ENSG00000189077	328.861523	0.01139485	0.14849985	0.93477224	0.97292421	TMEM120A
196528	ENSG00000189079	1580.26271	-0.06973844	0.08302039	0.38905944	0.61827567	ARID2
NA	ENSG00000189089	2.45617409	0.06276569	0.38019947	0.50054333	NA	RIMKLBP1
100133093	ENSG00000189090	0	NA	NA	NA	NA	FAM25G
23450	ENSG00000189091	5582.32177	-0.18723228	0.05799915	0.00108298	0.00631952	SF3B3
345062	ENSG00000189099	0	NA	NA	NA	NA	PRSS48

26280	ENSG00000189108	0	NA	NA	NA	NA	IL1RAPL2
388552	ENSG00000189114	114.024209	-0.38792971	0.21596726	0.03386754	0.1105697	BLOC1S3
80320	ENSG00000189120	2.10272856	0.14110165	0.41697607	0.0638867	NA	SP6
340120	ENSG00000189127	9.58732186	2.47455378	1.01198682	0.00066524	0.00413424	ANKRD34B
219348	ENSG00000189129	19.3864316	1.0693438	0.64773085	0.00618046	0.02776728	PLAC9
170062	ENSG00000189132	0	NA	NA	NA	NA	FAM47B
222698	ENSG00000189134	0	NA	NA	NA	NA	NKAPL
388165	ENSG00000189136	92.8174414	0.38476704	0.29633276	0.07773294	0.2070096	UBE2Q2P1
84075	ENSG00000189139	0	NA	NA	NA	NA	FSCB
1364	ENSG00000189143	87.0016942	0.38895429	0.28013645	0.06780918	0.18721599	CLDN4
126231	ENSG00000189144	108.472649	-0.04487141	0.20559436	0.79493827	0.90585603	ZNF573
NA	ENSG00000189145	0	NA	NA	NA	NA	RPL32P36
NA	ENSG00000189149	6.73257459	-0.06922771	0.36056644	0.6212714	0.80672374	CRYM-AS1
400581	ENSG00000189152	0.4721408	-0.0076152	0.38324107	0.86212392	NA	GRAPL
100129583	ENSG00000189157	66.7724351	-0.05377902	0.24301547	0.77395323	0.89604057	FAM47E
51155	ENSG00000189159	3336.44928	0.02056661	0.06339475	0.74370499	0.87922221	JPT1
84503	ENSG00000189164	154.516216	-0.48346075	0.19737871	0.00518415	0.02394729	ZNF527
NA	ENSG00000189166	0.16735865	0.01897177	0.38482933	0.78225479	NA	TNRC18P3
646799	ENSG00000189167	0.47250969	-0.00768687	0.38324361	0.86100405	NA	ZAR1L
386685	ENSG00000189169	0	NA	NA	NA	NA	KRTAP10-12
6284	ENSG00000189171	22.6208288	0.04035508	0.30390001	0.83164352	0.92378968	S100A13
7581	ENSG00000189180	1254.46866	-0.26238337	0.09148315	0.00306938	0.01543248	ZNF33A
401994	ENSG00000189181	0	NA	NA	NA	NA	OR14I1
374454	ENSG00000189182	0	NA	NA	NA	NA	KRT77
54510	ENSG00000189184	854.549029	-0.08387878	0.10509917	0.40519125	0.63354042	PCDH18
347442	ENSG00000189186	0	NA	NA	NA	NA	DCAF8L2
162966	ENSG00000189190	179.569096	0.36085493	0.19757044	0.03503927	0.11340607	ZNF600
284697	ENSG00000189195	108.13369	-0.27320656	0.21176586	0.12518432	0.29381959	BTBD8
100287879	ENSG00000189196	0	NA	NA	NA	NA	LINC00994
NA	ENSG00000189212	174.578214	-0.7453385	0.18298965	9.06E-06	8.67E-05	DPY19L2P1
4128	ENSG00000189221	263.872752	0.88138098	0.17500343	7.23E-08	9.70E-07	MAOA
654433	ENSG00000189223	0.34746601	0.02370763	0.3853651	0.61987128	NA	PAX8-AS1
145853	ENSG00000189227	272.478075	0.13621449	0.14672702	0.31519466	0.54297665	C15orf61
NA	ENSG00000189229	11.2335194	0.03552379	0.3370907	0.83163749	0.92378968	
389643	ENSG00000189233	0.16735865	0.01897177	0.38482933	0.78225479	NA	NUGGC
100507206	ENSG00000189238	0	NA	NA	NA	NA	LINC00943
7259	ENSG00000189241	1176.57817	-0.11470028	0.08063781	0.14522865	0.32572196	TSPYL1
139067	ENSG00000189252	0	NA	NA	NA	NA	SPANXN3

642446	ENSG00000189253	0	NA	NA	NA	NA	TRIM64B
55629	ENSG00000189266	2792.84977	0.16939257	0.06108273	0.00494953	0.02304052	PNRC2
51233	ENSG00000189269	7.21049358	0.14752279	0.38231911	0.34302181	0.57318007	DRICH1
399827	ENSG00000189275	0.29863895	-0.02292439	0.3852726	0.68753245	NA	LINC01164
2709	ENSG00000189280	0.69493202	0.03154416	0.38626309	0.41391645	NA	GJB5
2272	ENSG00000189283	105.572749	0.3944641	0.23711538	0.04269185	0.1320147	FHIT
285016	ENSG00000189292	2.52739347	0.02258703	0.37271808	0.81436162	NA	ALKAL2
NA	ENSG00000189295	0	NA	NA	NA	NA	NKRD62P1-PARP4P3
80317	ENSG00000189298	210.712833	0.00831251	0.15291658	0.95377282	0.98083958	ZKSCAN3
139628	ENSG00000189299	246.578014	-0.24134432	0.15002028	0.07954881	0.2105454	FOXR2
27341	ENSG00000189306	697.854968	-0.06172982	0.11033731	0.5581971	0.76291851	RRP7A
132660	ENSG00000189308	430.269743	-0.12369055	0.12214225	0.2836642	0.50881632	LIN54
441239	ENSG00000189316	3.21305998	-0.09297622	0.38344365	0.39775403	0.62695161	
9679	ENSG00000189319	1268.06821	-0.04906499	0.09067823	0.576633	0.77528586	FAM53B
389558	ENSG00000189320	2.14863989	-0.06068254	0.38033313	0.50365621	NA	FAM180A
389384	ENSG00000189325	0	NA	NA	NA	NA	BNIP5
441525	ENSG00000189326	0	NA	NA	NA	NA	SPANXN4
494141	ENSG00000189332	0	NA	NA	NA	NA	SLC25A51P4
57402	ENSG00000189334	0	NA	NA	NA	NA	S100A14
23254	ENSG00000189337	273.021141	0.40582791	0.18937131	0.01476539	0.05692682	KAZN
728661	ENSG00000189339	4967.96791	0.08795575	0.05694445	0.11843518	0.28255821	SLC35E2B
NA	ENSG00000189343	18.331099	-0.00973004	0.31619369	0.95504367	0.98157246	RPS2P46
646508	ENSG00000189348	0	NA	NA	NA	NA	FAM90A27P
165186	ENSG00000189350	77.9812223	-0.30674432	0.26917643	0.13251753	0.30570716	TOGARAM2
389761	ENSG00000189357	0	NA	NA	NA	NA	SPATA31D4
100131211	ENSG00000189362	276.570098	-0.40611236	0.39195623	0.08722414	0.22548301	NEMP2
200810	ENSG00000189366	9.11356466	0.3692222	0.57465702	0.05830791	0.16751071	ALG1L
9729	ENSG00000189367	735.827033	-1.67425056	0.15594785	4.89E-28	2.62E-26	KIAA0408
23708	ENSG00000189369	327.037146	-0.31284178	0.12896035	0.00976904	0.04074368	GSPT2
NA	ENSG00000189372	0	NA	NA	NA	NA	
254272	ENSG00000189375	0	NA	NA	NA	NA	TBC1D28
84933	ENSG00000189376	409.175524	-0.16991786	0.12107355	0.13778624	0.31363569	C8orf76
284340	ENSG00000189377	0	NA	NA	NA	NA	CXCL17
NA	ENSG00000189398	6.22266874	-0.09567934	0.37694956	0.44997836	0.67340229	OR7E12P
139562	ENSG00000189401	0.18922458	0.01954053	0.38488496	0.78225479	NA	OTUD6A
3146	ENSG00000189403	12218.3021	-0.14878696	0.05791617	0.00789161	0.03411845	HMGB1
8510	ENSG00000189409	11.0349952	1.34509327	1.1599925	0.00708441	0.03125247	MMP23B
400745	ENSG00000189410	32.2296199	-0.46586462	0.41379876	0.06236105	0.17644655	SH2D5

NA	ENSG00000189419	17.2923491	0.03644138	0.31988281	0.84064362	0.92770994	SPATA41
139735	ENSG00000189420	158.020322	-0.28929993	0.19212849	0.08294603	0.21719009	ZFP92
NA	ENSG00000189423	121.561373	0.03941578	0.22719602	0.83091417	0.92360004	USP32P3
9437	ENSG00000189430	0	NA	NA	NA	NA	NCR1
644943	ENSG00000189431	12.2987401	-0.86874117	0.87847267	0.01676262	0.06323365	RASSF10
127534	ENSG00000189433	0.36304042	0.02389537	0.3853925	0.60992407	NA	GJB4
NA	ENSG00000194297	0	NA	NA	NA	NA	RNU1-75P
574452	ENSG00000194717	0	NA	NA	NA	NA	MIR494
NA	ENSG00000195024	0	NA	NA	NA	NA	RNU1-15P
NA	ENSG00000195401	0	NA	NA	NA	NA	
284521	ENSG00000196071	0	NA	NA	NA	NA	OR2L13
282991	ENSG00000196072	402.79382	-0.46915645	0.15095208	0.00075659	0.00463152	BLOC1S2
10388	ENSG00000196074	148.312695	0.05120714	0.22914246	0.78234013	0.90086297	SYCP2
440519	ENSG00000196081	177.570079	-0.23405423	0.1714829	0.12636113	0.29581177	ZNF724
3556	ENSG00000196083	0.53092972	0.01187066	0.38331447	0.7553138	NA	IL1RAP
NA	ENSG00000196085	0	NA	NA	NA	NA	SMIM7P1
11122	ENSG00000196090	18.9382718	-3.82218398	0.88113228	1.41E-06	1.56E-05	PTPRT
4604	ENSG00000196091	0	NA	NA	NA	NA	MYBPC1
5079	ENSG00000196092	743.998386	-2.70726864	0.13881837	4.50E-86	1.03E-83	PAX5
NA	ENSG00000196096	2.39598147	0.09416575	0.39613267	0.17933514	NA	SPAG16-DT
403278	ENSG00000196098	0	NA	NA	NA	NA	OR5K4
390261	ENSG00000196099	0	NA	NA	NA	NA	OR6M1
50859	ENSG00000196104	23.4238734	2.24390558	0.55517664	2.99E-06	3.12E-05	SPOCK3
163223	ENSG00000196109	2.50636778	-0.08976281	0.38853179	0.32721887	NA	ZNF676
374879	ENSG00000196110	127.66978	-0.04179207	0.17994194	0.79160515	0.90499209	ZNF699
NA	ENSG00000196114	0.44795842	-0.02501848	0.38558245	0.5807882	NA	IFITM3P3
NA	ENSG00000196115	0	NA	NA	NA	NA	ADAM5
23424	ENSG00000196116	164.636951	0.36355214	0.19495938	0.03222852	0.10617966	TDRD7
90835	ENSG00000196118	132.049657	0.44170203	0.20498542	0.01276411	0.05068852	CCDC189
390275	ENSG00000196119	0	NA	NA	NA	NA	OR8A1
653319	ENSG00000196123	1666.31447	0.1186638	0.07581716	0.11047892	0.26795865	KIAA0895L
3123	ENSG00000196126	0	NA	NA	NA	NA	HLA-DRB1
317701	ENSG00000196131	0	NA	NA	NA	NA	VN1R2
4661	ENSG00000196132	551.144667	-1.05471734	0.12654051	9.40E-18	3.06E-16	MYT1
12	ENSG00000196136	0.67266818	0.00970948	0.38267945	0.82290888	NA	SERPINA3
8644	ENSG00000196139	9.25808076	0.17740356	0.39108001	0.28176494	0.50654372	AKR1C3
26010	ENSG00000196141	323.091597	0.29815063	0.1546721	0.03495455	0.11318725	SPATS2L
NA	ENSG00000196143	1.35190218	0.0514076	0.38444859	0.43248709	NA	OR11H13P

58500	ENSG00000196150	418.777426	-0.05629207	0.13763374	0.65870274	0.83073135	ZNF250
151525	ENSG00000196151	148.308688	-0.14064972	0.18411396	0.38344001	0.61266407	WDSUB1
7633	ENSG00000196152	144.268313	-0.02380682	0.18358965	0.88153077	0.94772508	ZNF79
6275	ENSG00000196154	33.6763122	-0.61526456	0.43767349	0.02599119	0.08974218	S100A4
25894	ENSG00000196155	1576.67656	0.12361644	0.1033748	0.21422675	0.42438929	PLEKHG4
85290	ENSG00000196156	0	NA	NA	NA	NA	KRTAP4-3
79633	ENSG00000196159	92.3464074	-0.12625831	0.2372758	0.50068333	0.71795498	FAT4
NA	ENSG00000196166	0.34624801	-0.02356563	0.38536324	0.65270996	NA	C8orf86
399948	ENSG00000196167	0.33471731	0.02354391	0.38534154	0.62842204	NA	COLCA1
124602	ENSG00000196169	17.0749825	2.64465164	0.72907906	1.26E-05	0.00011776	KIF19
81448	ENSG00000196171	0	NA	NA	NA	NA	OR6K2
148213	ENSG00000196172	467.72776	-0.5264011	0.14305015	8.01E-05	0.00062369	ZNF681
36	ENSG00000196177	435.734031	0.17657379	0.12449681	0.13326808	0.30691105	ACADSB
83931	ENSG00000196182	1541.8801	0.42820794	0.07080234	7.33E-10	1.28E-08	STK40
NA	ENSG00000196183	1.15217991	-0.00364109	0.37949693	0.95399678	NA	RPS2P4
26476	ENSG00000196184	0	NA	NA	NA	NA	OR10J1
9725	ENSG00000196187	405.982053	2.56768667	0.17622416	1.50E-49	1.46E-47	TMEM63A
1510	ENSG00000196188	1.48390247	0.03434546	0.38072464	0.60904738	NA	CTSE
64218	ENSG00000196189	47.6171664	0.14868192	0.26444684	0.45189761	0.67512414	SEMA4A
646962	ENSG00000196196	0	NA	NA	NA	NA	HRCT1
54737	ENSG00000196199	1011.11028	-0.161719	0.08951353	0.06253185	0.17675338	MPHOSPH8
441191	ENSG00000196204	585.146994	-0.16234017	0.11826961	0.1479759	0.33012187	RNF216P1
NA	ENSG00000196205	131.992795	-0.2063957	0.20876065	0.24086673	0.45883782	EEF1A1P5
9687	ENSG00000196208	3878.44446	-0.02578421	0.0838437	0.75189262	0.88379345	GREB1
284759	ENSG00000196209	0	NA	NA	NA	NA	SIRPB2
90321	ENSG00000196214	300.38659	-0.18778897	0.19434319	0.26150256	0.48352384	ZNF766
6261	ENSG00000196218	85.1101413	0.18715703	0.43214443	0.11648127	0.27907117	RYR1
9901	ENSG00000196220	389.266332	0.10167822	0.1934855	0.54497	0.75267698	SRGAP3
387266	ENSG00000196224	0	NA	NA	NA	NA	KRTAP5-3
63939	ENSG00000196227	591.044626	-1.05170947	0.12505395	4.96E-18	1.65E-16	FAM217B
442038	ENSG00000196228	0	NA	NA	NA	NA	SULT1C3
203068	ENSG00000196230	43349.6101	-0.12195643	0.05418859	0.02231296	0.07950595	TUBB
84458	ENSG00000196233	1189.68921	-0.1552557	0.27759546	0.43259929	0.65796603	LCOR
6829	ENSG00000196235	3294.39986	0.18619802	0.06571827	0.00402137	0.01938859	SUPT5H
63929	ENSG00000196236	228.110194	-0.07562049	0.1488446	0.58057757	0.77797237	XPNPEP3
401992	ENSG00000196240	0	NA	NA	NA	NA	OR2T2
81472	ENSG00000196242	0	NA	NA	NA	NA	OR2C3
148824	ENSG00000196242	0	NA	NA	NA	NA	OR2C3

439916	ENSG00000196243	0	NA	NA	NA	NA	LINC00615
51427	ENSG00000196247	1110.27823	-0.41310221	0.08336062	3.68E-07	4.49E-06	ZNF107
219873	ENSG00000196248	0	NA	NA	NA	NA	OR10S1
389376	ENSG00000196260	0	NA	NA	NA	NA	SFTA2
5478	ENSG00000196262	23909.4215	-0.21153754	0.05669612	0.00015224	0.00110963	PPIA
57573	ENSG00000196263	208.336067	-0.0810928	0.1848066	0.61525282	0.80259162	ZNF471
441911	ENSG00000196266	0	NA	NA	NA	NA	OR10J3
162962	ENSG00000196267	108.117164	0.16854623	0.23697298	0.37205028	0.60104858	ZNF836
284443	ENSG00000196268	306.279794	-0.00565959	0.14226665	0.96481114	0.98622874	ZNF493
283601	ENSG00000196273	3.91222055	-0.21861656	0.45852439	0.07150544	0.19452459	LINC00523
NA	ENSG00000196274	0.15069912	-0.01848503	0.38480575	0.84537521	NA	
84163	ENSG00000196275	505.566565	0.32165718	0.10822973	0.00190306	0.01024051	GTF2IRD2
2917	ENSG00000196277	359.759849	-0.03196735	0.17370314	0.83475818	0.92454439	GRM7
8464	ENSG00000196284	155.901856	0.04308292	0.17642318	0.78479039	0.90204747	SUPT3H
441925	ENSG00000196289	0	NA	NA	NA	NA	BECN2
60491	ENSG00000196290	566.688524	-0.12996925	0.1041363	0.1932479	0.39596861	NIF3L1
NA	ENSG00000196295	1020.31406	0.28832961	0.09467226	0.00163468	0.00903399	GARS1-DT
487	ENSG00000196296	59.5332015	0.01630584	0.29981345	0.9326843	0.97195551	ATP2A1
NA	ENSG00000196301	0	NA	NA	NA	NA	HLA-DRB9
NA	ENSG00000196302	102.113297	0.27688617	0.24350778	0.15426808	0.3394719	GUSBP15
3376	ENSG00000196305	4775.72618	0.3043026	0.08748578	0.00034434	0.00231737	IARS1
84278	ENSG00000196312	1377.66564	0.05775346	0.13580779	0.65026996	0.82529572	MFSD14C
9883	ENSG00000196313	4923.29598	-0.01025384	0.05430165	0.82745919	0.92198082	POM121
29068	ENSG00000196323	1081.36377	0.01054453	0.11095205	0.92194985	0.96729164	ZBTB44
55340	ENSG00000196329	0	NA	NA	NA	NA	GIMAP5
56164	ENSG00000196335	10.8145382	0.1185405	0.3729554	0.42026553	0.64619198	STK31
94027	ENSG00000196337	3.04438347	0.10604707	0.39288286	0.27331103	0.49679256	CGB7
54413	ENSG00000196338	247.75333	-0.39669862	0.1671434	0.00857472	0.03655403	NLGN3
283159	ENSG00000196341	0	NA	NA	NA	NA	OR8D1
131	ENSG00000196344	0	NA	NA	NA	NA	ADH7
55888	ENSG00000196345	524.021272	-0.0936727	0.12672474	0.43265701	0.65797295	ZKSCAN7
100287226	ENSG00000196350	0.64926934	-0.03789353	0.38680731	0.40870612	NA	ZNF729
1604	ENSG00000196352	0	NA	NA	NA	NA	CD55
131034	ENSG00000196353	87.0350409	-1.77064365	0.29389569	1.14E-10	2.18E-09	CPNE4
147929	ENSG00000196357	62.5638395	-0.08572131	0.23949152	0.64739925	0.82430661	ZNF565
84628	ENSG00000196358	88.2717509	0.18056675	0.30576785	0.37155572	0.60060191	NTNG2
1995	ENSG00000196361	4137.86925	-0.91181377	0.07115285	2.13E-38	1.58E-36	ELAVL3
11091	ENSG00000196363	1551.41516	-0.05039243	0.07092693	0.46915147	0.69153662	WDR5

NA	ENSG00000196364	0	NA	NA	NA	NA	PRSS29P
9361	ENSG00000196365	3395.37522	0.07495663	0.05837695	0.1939954	0.39683151	LONP1
158055	ENSG00000196366	16.1655484	-0.23554834	0.38467828	0.23354098	0.45043378	C9orf163
8295	ENSG00000196367	6536.3733	-0.10984828	0.05334393	0.03738228	0.1192962	TRRAP
55190	ENSG00000196368	420.96925	-0.11897136	0.11737611	0.28660607	0.5123684	NUDT11
647135	ENSG00000196369	1030.7164	-0.43115728	0.09437741	2.35E-06	2.50E-05	SRGAP2B
2526	ENSG00000196371	76.3628149	0.07318664	0.23422045	0.69573265	0.85316371	FUT4
79754	ENSG00000196372	909.470214	-0.25701658	0.08669413	0.00227927	0.01197266	ASB13
222553	ENSG00000196376	1.34775073	-0.04155115	0.38271622	0.52040837	NA	SLC35F1
80778	ENSG00000196378	87.0817792	-0.16643457	0.24475449	0.38304418	0.61240159	ZNF34
163115	ENSG00000196381	100.838208	-0.12956583	0.26302005	0.50395191	0.72045005	ZNF781
NA	ENSG00000196383	0	NA	NA	NA	NA	OR4Q2
7699	ENSG00000196387	294.211099	-0.01475674	0.15975699	0.91783927	0.9652015	ZNF140
388324	ENSG00000196388	7.45147874	0.07682923	0.35953537	0.60251596	0.79259054	INCA1
NA	ENSG00000196390	0	NA	NA	NA	NA	
342132	ENSG00000196391	236.010063	-0.57925059	0.16961076	0.00018443	0.00132241	ZNF774
NA	ENSG00000196395	0	NA	NA	NA	NA	
5770	ENSG00000196396	1088.54858	-0.04415827	0.08129391	0.57776351	0.77605301	PTPN1
NA	ENSG00000196403	0	NA	NA	NA	NA	OR10D1P
51466	ENSG00000196405	2708.25843	-0.05416944	0.06726804	0.41286784	0.64038252	EVL
64648	ENSG00000196406	0	NA	NA	NA	NA	SPANXD
284486	ENSG00000196407	0.36325155	0.02389685	0.38539273	0.60981965	NA	THEM5
124056	ENSG00000196408	2.36430715	0.11586212	0.4003901	0.18719163	NA	NOXO1
2050	ENSG00000196411	1448.1558	2.42016174	0.092731	1.94E-151	1.83E-148	EPHB4
5657	ENSG00000196415	0	NA	NA	NA	NA	PRTN3
91661	ENSG00000196417	230.402875	0.04060891	0.17275905	0.7935071	0.90556117	ZNF765
7678	ENSG00000196418	379.128489	-0.22293282	0.13072946	0.06876954	0.1890682	ZNF124
2547	ENSG00000196419	2834.7753	-0.12569762	0.09319542	0.16366814	0.35275692	XRCC6
6276	ENSG00000196420	0	NA	NA	NA	NA	S100A5
284739	ENSG00000196421	66.5641943	0.24992968	0.24781666	0.20054431	0.40575148	C20orf204
9858	ENSG00000196422	1639.89707	0.02246613	0.07876259	0.77285543	0.89561741	PPP1R26
148545	ENSG00000196427	146.999886	0.93034518	0.26556294	5.81E-05	0.0004672	NBPF4
9819	ENSG00000196428	475.197898	-0.06905767	0.13174013	0.57583644	0.7744963	TSC22D2
1413	ENSG00000196431	0	NA	NA	NA	NA	CRYBA4
438	ENSG00000196433	0.173733	0.01914545	0.38484597	0.78225479	NA	ASMT
440348	ENSG00000196436	0.49335459	-0.01684786	0.38404234	0.84749744	NA	NPIPB15
148266	ENSG00000196437	268.751324	-0.19111421	0.15578466	0.17806632	0.37352884	ZNF569
100131755	ENSG00000196440	683.949022	-0.02171721	0.10133559	0.82240217	0.91897523	ARMCX4

79693	ENSG00000196449	693.485474	-0.02166566	0.09710289	0.81671509	0.9166503	YRDC
27153	ENSG00000196453	497.535325	-0.28728971	0.10878948	0.00571359	0.02604725	ZNF777
30849	ENSG00000196455	647.888832	-0.15613415	0.12018477	0.17008462	0.36191941	PIK3R4
285971	ENSG00000196456	504.30229	0.22783055	0.12790721	0.05823272	0.16740168	ZNF775
100289635	ENSG00000196458	819.490411	-0.26887036	0.11339524	0.01263355	0.05028078	ZNF605
6399	ENSG00000196459	466.488702	-0.48884488	0.14564478	0.0003014	0.00205487	TRAPPC2
731220	ENSG00000196460	1.39562213	-0.05561223	0.38872542	0.26074571	NA	RFX8
140465	ENSG00000196465	2128.24818	0.06126339	0.0729094	0.39246534	0.62172726	MYL6B
90576	ENSG00000196466	96.0645502	-0.20670867	0.24138192	0.27957459	0.5040676	ZNF799
8823	ENSG00000196468	0	NA	NA	NA	NA	FGF16
6477	ENSG00000196470	624.803207	0.04096653	0.10861052	0.6949118	0.85252598	SIAH1
NA	ENSG00000196472	0	NA	NA	NA	NA	
2712	ENSG00000196475	0	NA	NA	NA	NA	GK2
140680	ENSG00000196476	331.153876	0.20919969	0.12819435	0.08276211	0.2167657	C20orf96
2104	ENSG00000196482	2067.68979	0.12301824	0.07756604	0.10541859	0.25894574	ESRRG
79711	ENSG00000196497	1783.46503	-0.05718824	0.07502605	0.43553463	0.66067796	IPO4
9612	ENSG00000196498	5171.34423	0.05777238	0.06976555	0.39982262	0.62831531	NCOR2
6817	ENSG00000196502	9.40902836	0.13652097	0.39550988	0.26348719	0.48542909	SULT1A1
132946	ENSG00000196503	0.173124	-0.01911262	0.38486262	0.84537521	NA	ARL9
55660	ENSG00000196504	2677.9917	0.03008446	0.07279717	0.679789	0.84376374	PRPF40A
54834	ENSG00000196505	338.083423	-0.39148634	0.1393622	0.00252566	0.01309017	GDAP2
85012	ENSG00000196507	272.524198	-0.21782192	0.14572424	0.10478547	0.25792917	TCEAL3
51434	ENSG00000196510	1094.73484	-0.08742164	0.08800962	0.30644084	0.53411107	ANAPC7
27010	ENSG00000196511	84.6064932	0.04381831	0.21730065	0.80841452	0.91317199	TPK1
6536	ENSG00000196517	269.85889	1.82362041	0.25694449	8.88E-14	2.19E-12	SLC6A9
60312	ENSG00000196526	6738.74318	-0.16410678	0.05036719	0.00100291	0.0059237	AFAP1
4666	ENSG00000196531	10875.5626	0.02262594	0.05004724	0.66305581	0.83375759	NACA
NA	ENSG00000196534	0	NA	NA	NA	NA	OR9K1P
399687	ENSG00000196535	1295.86121	-0.24546579	0.07955992	0.00156993	0.00871989	MYO18A
343173	ENSG00000196539	0	NA	NA	NA	NA	OR2T3
165679	ENSG00000196542	189.069947	0.3845637	0.17960482	0.01610653	0.06112347	SPTSSB
54785	ENSG00000196544	186.836319	-0.02539557	0.16049693	0.8607181	0.93799827	BORCS6
4122	ENSG00000196547	1892.96651	-0.1153528	0.07072896	0.09607139	0.24200817	MAN2A2
4311	ENSG00000196549	34.0103746	-0.43514484	0.41170752	0.07347026	0.19834672	MME
729533	ENSG00000196550	349.493146	-0.48154252	0.1314754	0.00010062	0.00076454	FAM72A
440184	ENSG00000196553	0.173733	0.01914545	0.38484597	0.78225479	NA	CCDC196
8912	ENSG00000196557	0	NA	NA	NA	NA	CACNA1H
55959	ENSG00000196562	1878.155	-2.08816806	0.08079744	1.74E-148	1.43E-145	SULF2

NA	ENSG00000196564	0	NA	NA	NA	NA	BLOC1S6P1
3048	ENSG00000196565	0	NA	NA	NA	NA	HBG2
NA	ENSG00000196566	0	NA	NA	NA	NA	
3908	ENSG00000196569	0	NA	NA	NA	NA	LAMA2
345456	ENSG00000196570	0.34057487	-0.00047727	0.38336304	0.96977705	NA	PFN3
23654	ENSG00000196576	4635.92879	-0.19397138	0.07496299	0.00823972	0.0354151	PLXNB2
81050	ENSG00000196578	0	NA	NA	NA	NA	OR5AC2
55966	ENSG00000196581	22.0036629	-2.83770735	0.77892699	1.32E-05	0.0001233	AJAP1
7516	ENSG00000196584	1626.9507	-0.08908419	0.07694113	0.23682556	0.45370983	XRCC2
4646	ENSG00000196586	1279.1522	0.24342045	0.07758977	0.00133691	0.00758921	MYO6
57591	ENSG00000196588	336.549875	0.07896742	0.15765897	0.58403211	0.78020525	MRTFA
729458	ENSG00000196589	0	NA	NA	NA	NA	MBD3L2B
3066	ENSG00000196591	5043.8138	0.05431184	0.05829903	0.34673388	0.57745322	HDAC2
NA	ENSG00000196593	1.3933945	-0.06015007	0.38588951	0.37463236	NA	ANKRD20A19P
158431	ENSG00000196597	106.163383	-0.11293636	0.237261	0.54605197	0.75333225	ZNF782
387601	ENSG00000196600	0.33471731	0.02354391	0.38534154	0.62842204	NA	SLC22A25
728378	ENSG00000196604	0.18922458	0.01954053	0.38488496	0.78225479	NA	POTEF
162993	ENSG00000196605	98.0294702	0.501885	0.2507982	0.01448116	0.05600512	ZNF846
100505555	ENSG00000196605	98.0294702	0.501885	0.2507982	0.01448116	0.05600512	ZNF846
4312	ENSG00000196611	0	NA	NA	NA	NA	MMP1
125	ENSG00000196616	0.70829826	0.02419826	0.38327581	0.58592423	NA	ADH1B
7366	ENSG00000196620	0	NA	NA	NA	NA	UGT2B15
6925	ENSG00000196628	12673.6232	-0.23149599	0.05427509	1.44E-05	0.00013269	TCF4
65267	ENSG00000196632	226.874103	-0.18727436	0.1946628	0.26332894	0.48536289	WNK3
106182249	ENSG00000196634	0	NA	NA	NA	NA	LUADT1
57001	ENSG00000196636	347.73718	0.04111755	0.12214733	0.72735449	0.86958199	SDHAF3
3269	ENSG00000196639	0.18922458	0.01954053	0.38488496	0.78225479	NA	HRH1
55684	ENSG00000196642	3813.06809	-0.04686428	0.06012665	0.42720098	0.652979	RABL6
7695	ENSG00000196646	176.286977	-0.19197268	0.18995395	0.24432691	0.4628959	ZNF136
23660	ENSG00000196652	887.570344	0.03348194	0.0961438	0.71979427	0.86597043	ZKSCAN5
91392	ENSG00000196653	170.787235	-0.12204991	0.16635285	0.41468974	0.64180766	ZNF502
51399	ENSG00000196655	775.701737	0.09635126	0.09239067	0.28284692	0.50789057	TRAPPC4
NA	ENSG00000196656	1370.06871	-0.04749416	0.0797156	0.54188457	0.75019119	
150737	ENSG00000196659	249.837971	0.34587524	0.15297547	0.01369139	0.05359848	TTC30B
55532	ENSG00000196660	91.6883939	0.46549705	0.27530636	0.03078117	0.10232882	SLC30A10
9895	ENSG00000196663	764.203704	0.5998391	0.11565882	6.40E-08	8.65E-07	TECPR2
51284	ENSG00000196664	0	NA	NA	NA	NA	TLR7
399888	ENSG00000196666	2.78108551	0.02531872	0.37163223	0.80360691	0.91070264	FAM180B

100287569	ENSG00000196668	6.99384931	0.24974389	0.46546914	0.09176182	0.23400444	LINC00173
643836	ENSG00000196670	903.909129	0.02779117	0.09670644	0.76742749	0.89288602	ZFP62
112479	ENSG00000196678	612.386357	-0.16979288	0.10515417	0.09230647	0.2349404	ERI2
54543	ENSG00000196683	1089.7402	0.04836994	0.096805	0.60643051	0.79607018	TOMM7
84941	ENSG00000196684	0	NA	NA	NA	NA	HSH2D
7442	ENSG00000196689	228.689719	0.04336387	0.14547332	0.74837314	0.88195564	TRPV1
7582	ENSG00000196693	1057.23808	-0.39588973	0.09139595	7.84E-06	7.60E-05	ZNF33B
283970	ENSG00000196696	165.217127	0.62944199	0.20957392	0.00065111	0.00405715	
57473	ENSG00000196700	1895.81508	0.31030904	0.07074741	7.88E-06	7.62E-05	ZNF512B
51321	ENSG00000196704	1111.3987	-0.24073012	0.09329275	0.00763786	0.03320955	AMZ2
170959	ENSG00000196705	923.829995	0.11299517	0.09717296	0.22926937	0.44481755	ZNF431
389658	ENSG00000196711	0	NA	NA	NA	NA	ALKAL1
4763	ENSG00000196712	2617.00127	-0.09467004	0.08632533	0.25955991	0.48121669	NF1
154807	ENSG00000196715	1723.13746	-0.2680727	0.07945447	0.00054698	0.0034883	VKORC1L1
147686	ENSG00000196724	8.79941896	0.24434016	0.48248185	0.04211862	0.13070323	ZNF418
1612	ENSG00000196730	1373.55278	0.01845611	0.07497267	0.80280408	0.91020247	DAPK1
353132	ENSG00000196734	0.173124	-0.01911262	0.38486262	0.84537521	NA	LCE1B
3117	ENSG00000196735	0	NA	NA	NA	NA	HLA-DQA1
85301	ENSG00000196739	174.993325	-1.347993	0.19556559	4.79E-13	1.12E-11	COL27A1
NA	ENSG00000196741	44.5197113	0.18449943	0.30067819	0.36500909	0.59446563	LINC01560
2760	ENSG00000196743	407.377164	0.38139733	0.11996556	0.00080135	0.0048814	GM2A
8329	ENSG00000196747	5.80107887	-0.2241277	0.44636492	0.1210442	0.28719408	H2AC13
389383	ENSG00000196748	0.49160492	-0.00761637	0.38272042	0.85526143	NA	CLPSL2
6273	ENSG00000196754	0.63354864	0.00212699	0.38251372	0.95865884	NA	S100A2
388796	ENSG00000196756	701.04475	0.07233896	0.09454954	0.43027608	0.65586029	SNHG17
90592	ENSG00000196757	288.667931	0.02342592	0.14103968	0.85937013	0.9374775	ZNF700
401040	ENSG00000196758	0	NA	NA	NA	NA	
5456	ENSG00000196767	0	NA	NA	NA	NA	POU3F4
284532	ENSG00000196772	0	NA	NA	NA	NA	OR14A16
961	ENSG00000196776	1178.39384	-0.17022181	0.10150381	0.08123392	0.21389263	CD47
390036	ENSG00000196778	0	NA	NA	NA	NA	OR52K1
7088	ENSG00000196781	1916.39821	0.10650347	0.08292867	0.18845464	0.3889867	TLE1
55534	ENSG00000196782	2375.50094	-0.32085327	0.06763894	1.38E-06	1.53E-05	MAML3
8969	ENSG00000196787	57.3099036	-0.8628971	0.32533907	0.00105258	0.00616751	H2AC11
29966	ENSG00000196792	526.187295	-0.10555157	0.13421524	0.40012109	0.62854504	STRN3
8187	ENSG00000196793	311.079221	-0.53790812	0.18627497	0.00123232	0.00707069	ZNF239
NA	ENSG00000196796	0.34746601	0.02370763	0.3853651	0.61987128	NA	NPIPB10P
408187	ENSG00000196800	0	NA	NA	NA	NA	SPINK14

6701	ENSG00000196805	0	NA	NA	NA	NA	SPRR2B
NA	ENSG00000196810	504.390882	-0.08950089	0.13205646	0.46911991	0.69153662	CTBP1-DT
1146	ENSG00000196811	0.14931947	-0.01844332	0.38480212	0.84537521	NA	CHRNG
80345	ENSG00000196812	245.630462	-0.01888709	0.1393011	0.88375368	0.94897201	ZSCAN16
89853	ENSG00000196814	1013.14032	-0.09133961	0.09384559	0.31509387	0.54289729	MVB12B
64771	ENSG00000196821	1684.4389	0.04176307	0.07761064	0.5834721	0.77994081	ILRUN
NA	ENSG00000196826	0	NA	NA	NA	NA	
390439	ENSG00000196832	0.62178598	-0.04501091	0.38755918	0.37478114	NA	OR11G2
653269	ENSG00000196834	0	NA	NA	NA	NA	POTEI
100	ENSG00000196839	146.963754	0.3407159	0.18895748	0.03969602	0.12465479	ADA
10865	ENSG00000196843	90.0176547	0.45241765	0.25438571	0.02747182	0.09356925	ARID5A
399967	ENSG00000196844	0.173733	0.01914545	0.38484597	0.78225479	NA	PATE2
160760	ENSG00000196850	568.298632	0.06511165	0.10207354	0.50896708	0.72469548	PPTC7
390792	ENSG00000196859	0	NA	NA	NA	NA	KRT39
387990	ENSG00000196860	1.1264234	-0.00654307	0.38052282	0.90997405	NA	TOMM20L
285190	ENSG00000196862	17.7085264	0.19300388	0.366484	0.31325864	0.54126787	RGPD4
374354	ENSG00000196865	1186.30094	-0.18679092	0.07715929	0.01330163	0.05242437	NHLRC2
3013	ENSG00000196866	0.7534956	-0.03327829	0.38646938	0.40782637	NA	H2AC7
140612	ENSG00000196867	254.258062	0.01098868	0.18991977	0.94836984	0.97876335	ZFP28
343990	ENSG00000196872	52.3697297	2.01735431	0.3754165	4.75E-09	7.47E-08	CRACDL
445571	ENSG00000196873	384.723517	0.11067859	0.12677079	0.35506675	0.58548241	CBWD3
6334	ENSG00000196876	821.717781	-0.76279953	0.10284844	2.38E-14	6.15E-13	SCN8A
3914	ENSG00000196878	1.65839172	-0.05277493	0.38343898	0.46101376	NA	LAMB3
128312	ENSG00000196890	57.8471066	-0.32942402	0.28120276	0.11697163	0.27997551	H2BU1
NA	ENSG00000196893	0	NA	NA	NA	NA	
389320	ENSG00000196900	0	NA	NA	NA	NA	TEX43
3841	ENSG00000196911	495.74099	0.17420228	0.13296925	0.16197632	0.35042673	KPNA5
57730	ENSG00000196912	325.036199	0.24500774	0.14350751	0.06502771	0.18187757	ANKRD36B
23365	ENSG00000196914	4117.05242	-0.36263979	0.06013293	9.61E-10	1.65E-08	ARHGEF12
27198	ENSG00000196917	0	NA	NA	NA	NA	HCAR1
286101	ENSG00000196922	848.703656	-0.2527223	0.11019665	0.01630014	0.06169325	ZNF252P
9260	ENSG00000196923	866.664539	-0.0074821	0.09149676	0.93209725	0.97175748	PDLIM7
2316	ENSG00000196924	13880.1579	0.62516256	0.04860418	2.22E-38	1.64E-36	FLNA
219623	ENSG00000196932	29.8430221	-0.81549272	0.50407685	0.01061309	0.04349591	TMEM26
NA	ENSG00000196933	0.14931947	-0.01844332	0.38480212	0.84537521	NA	RPS26P11
57522	ENSG00000196935	862.0233	0.58582932	0.10851885	2.09E-08	3.03E-07	SRGAP1
10447	ENSG00000196937	400.889548	-0.41883903	0.14642454	0.00197394	0.01057888	FAM3C
161424	ENSG00000196943	1353.96025	-0.03948142	0.07573177	0.594254	0.7877158	NOP9

127074	ENSG00000196944	0	NA	NA	NA	NA	OR2T4
440077	ENSG00000196946	0.34746601	0.02370763	0.3853651	0.61987128	NA	ZNF705A
57181	ENSG00000196950	1430.97038	-0.19699511	0.0940021	0.03041106	0.10142132	SLC39A10
100129858	ENSG00000196951	71.0171262	-0.20976845	0.29931471	0.3049759	0.5323528	SCOC-AS1
837	ENSG00000196954	0.5897162	-0.03106455	0.38592535	0.59281556	NA	CASP4
160	ENSG00000196961	1545.56114	0.09481175	0.09605873	0.30860746	0.53621325	AP2A1
199704	ENSG00000196967	313.182644	-0.11019882	0.19206094	0.50579949	0.72177288	ZNF585A
170384	ENSG00000196968	294.820592	-0.24462205	0.1496398	0.07481832	0.20111035	FUT11
55999	ENSG00000196970	0.52119901	0.02576573	0.38568143	0.51723676	NA	NXF4
644596	ENSG00000196972	7.77442052	-0.00248133	0.35435911	0.98345415	0.99432963	SMIM10L2B
307	ENSG00000196975	276.586207	0.93903915	0.15171873	8.83E-11	1.71E-09	ANXA4
8270	ENSG00000196976	371.02623	0.01774594	0.12299459	0.87991032	0.94676614	LAGE3
401554	ENSG00000196979	0.71683569	0.02258975	0.38381088	0.59908846	NA	GPRACR
54554	ENSG00000196981	317.2767	0.09858333	0.14361015	0.45972263	0.6828552	WDR5B
642968	ENSG00000196990	1881.63497	-0.40140431	0.06872665	2.77E-09	4.48E-08	FAM163B
100507607	ENSG00000196993	17.3961523	0.53163167	0.62854707	0.04544641	0.13822733	NPIPB9
11152	ENSG00000196998	740.803379	0.24571246	0.09942123	0.01029578	0.04243214	WDR45
51108	ENSG00000197006	2649.1153	-0.07005578	0.06066716	0.24171594	0.45967442	METTL9
7697	ENSG00000197008	601.650048	-0.06840122	0.09967232	0.4765575	0.69841458	ZNF138
353088	ENSG00000197013	219.647235	-0.54116354	0.18784182	0.00123018	0.00706421	ZNF429
388566	ENSG00000197016	360.914998	-0.07860025	0.12446591	0.50342765	0.72011569	ZNF470
29950	ENSG00000197019	58.7901742	0.39499343	0.29273936	0.0703821	0.192286	SERTAD1
163227	ENSG00000197020	339.993637	-0.41757057	0.1406309	0.00141298	0.00797085	ZNF100
541578	ENSG00000197021	286.026246	-0.01914437	0.15348693	0.89054657	0.95232275	EOLA2
NA	ENSG00000197023	0	NA	NA	NA	NA	OR51A6P
57541	ENSG00000197024	683.668388	-0.16217567	0.11374786	0.13330117	0.30695163	ZNF398
221785	ENSG00000197037	940.194347	-0.05981155	0.08926782	0.49028791	0.70981553	ZSCAN25
NA	ENSG00000197038	0	NA	NA	NA	NA	RBMY1A3P
309	ENSG00000197043	1899.64312	-0.17802166	0.07321175	0.01310486	0.05175169	ANXA6
126068	ENSG00000197044	147.259228	-0.35050963	0.20597333	0.04640921	0.14061674	ZNF441
2764	ENSG00000197045	1724.38522	-0.55671136	0.08531854	2.33E-11	4.77E-10	GMFB
284266	ENSG00000197046	0	NA	NA	NA	NA	SIGLEC15
147923	ENSG00000197050	318.208584	-0.51862549	0.1512186	0.00021136	0.00149549	ZNF420
284390	ENSG00000197054	33.6465798	0.14516409	0.33504265	0.44285131	0.66774358	ZNF763
79830	ENSG00000197056	379.061925	-0.17271751	0.12531589	0.14367292	0.32310935	ZMYM1
401124	ENSG00000197057	0	NA	NA	NA	NA	DTHD1
8364	ENSG00000197061	2.34312805	-0.06514319	0.38272177	0.45196845	NA	H4C3
7741	ENSG00000197062	283.664302	0.11865589	0.20290455	0.49221059	0.71119788	ZSCAN26

4097	ENSG00000197063	1757.72805	0.14992341	0.06760354	0.02421031	0.08495446	MAFG
NA	ENSG00000197067	0	NA	NA	NA	NA	OR2T32P
92714	ENSG00000197070	448.34799	0.48055327	0.12589541	5.52E-05	0.00044598	ARRDC1
85379	ENSG00000197077	724.216707	0.30433309	0.09878197	0.00138913	0.00785178	KIAA1671
3886	ENSG00000197079	0	NA	NA	NA	NA	KRT35
3482	ENSG00000197081	4425.94249	0.03737642	0.05907842	0.52341853	0.73623706	IGF2R
NA	ENSG00000197083	0.34656964	-0.02344504	0.38534777	0.6613171	NA	ZNF300P1
353133	ENSG00000197084	0	NA	NA	NA	NA	LCE1C
404744	ENSG00000197085	0	NA	NA	NA	NA	NPSR1-AS1
NA	ENSG00000197092	0	NA	NA	NA	NA	GOLGA6L16P
79690	ENSG00000197093	276.148286	0.64373038	0.15882037	1.30E-05	0.00012108	GAL3ST4
NA	ENSG00000197099	0	NA	NA	NA	NA	
1778	ENSG00000197102	16304.2961	0.24731051	0.05719719	1.21E-05	0.00011374	DYNC1H1
388662	ENSG00000197106	223.856853	-2.17554786	0.21300005	1.08E-25	5.25E-24	SLC6A17
282617	ENSG00000197110	0	NA	NA	NA	NA	IFNL3
5094	ENSG00000197111	10324.5624	-0.01240309	0.04836596	0.79400393	0.90579008	PCBP2
84619	ENSG00000197114	643.361119	0.36564775	0.10011823	0.00014984	0.00109459	ZGPAT
123096	ENSG00000197119	3942.63193	0.49948938	0.06430198	3.26E-15	9.08E-14	SLC25A29
80055	ENSG00000197121	2204.09832	-0.5239867	0.07893834	1.19E-11	2.52E-10	PGAP1
6714	ENSG00000197122	4961.47782	0.14716129	0.06104504	0.0146113	0.05644239	SRC
168417	ENSG00000197123	6.04430401	-0.22293922	0.44048227	0.13984647	0.31709188	ZNF679
91120	ENSG00000197124	221.694227	0.00071111	0.15392241	0.99707574	0.99913206	ZNF682
26493	ENSG00000197125	0	NA	NA	NA	NA	OR8B8
400720	ENSG00000197128	230.257688	-0.15110177	0.19247924	0.36304321	0.59281472	ZNF772
113835	ENSG00000197134	89.7711906	-0.35127437	0.31011348	0.10763513	0.26314781	ZNF257
399909	ENSG00000197136	2303.7076	0.28629606	0.06802795	1.86E-05	0.00016751	PCNX3
203102	ENSG00000197140	25.8599949	-0.28375934	0.43352038	0.14543853	0.32597939	ADAM32
51703	ENSG00000197142	0.80612149	-0.00727628	0.38082098	0.89768298	NA	ACSL5
23507	ENSG00000197147	1000.29155	-0.50136929	0.10340365	4.83E-07	5.78E-06	LRRC8B
NA	ENSG00000197149	7.27574921	-0.03718276	0.35098642	0.79955832	0.90807929	
11194	ENSG00000197150	975.047591	0.0661081	0.10130164	0.49914318	0.71669126	ABCB8
8356	ENSG00000197153	2.86198723	-0.09319768	0.39089866	0.29387064	0.52019927	H3C12
27044	ENSG00000197157	4929.87644	-0.03037919	0.05241223	0.55880347	0.76349156	SND1
NA	ENSG00000197161	0	NA	NA	NA	NA	OR4C4P
146540	ENSG00000197162	654.490539	0.1872325	0.11115166	0.0777937	0.2071028	ZNF785
6799	ENSG00000197165	0	NA	NA	NA	NA	SULT1A2
341676	ENSG00000197168	18.1734469	1.71117313	0.64921616	0.00044216	0.00289969	NEK5
5718	ENSG00000197170	1441.41913	-0.15515204	0.08339204	0.05616579	0.16284223	PSMD12

101929006	ENSG00000197171	0	NA	NA	NA	NA	
4105	ENSG00000197172	1042.39153	0.43741387	0.08329978	7.30E-08	9.78E-07	MAGEA6
100129345	ENSG00000197176	0	NA	NA	NA	NA	LINC02291
84435	ENSG00000197177	0.34624801	-0.02356563	0.38536324	0.65270996	NA	ADGRA1
158960	ENSG00000197180	23.2587319	-0.07526297	0.30411356	0.69116641	0.85035049	
55124	ENSG00000197181	4.8365582	0.18989055	0.4261557	0.14923729	0.33189865	PIWIL2
400931	ENSG00000197182	163.668495	-0.52871712	0.22413829	0.00566996	0.02588997	MIRLET7BHG
140688	ENSG00000197183	1527.69138	-0.02605702	0.08393712	0.74954938	0.88208433	NOL4L
NA	ENSG00000197185	0	NA	NA	NA	NA	SSXP1
375791	ENSG00000197191	44.3664394	0.63926119	0.44627866	0.0229844	0.0814159	CYSRT1
6583	ENSG00000197208	5.17591352	0.04943108	0.36997343	0.66266934	0.83353574	SLC22A4
NA	ENSG00000197210	5.96875521	3.9574274	1.7321715	0.00088832	0.00534395	
342933	ENSG00000197213	0.173733	0.01914545	0.38484597	0.78225479	NA	ZSCAN5B
NA	ENSG00000197214	0.15069912	-0.01848503	0.38480575	0.84537521	NA	TCEA1P1
9583	ENSG00000197217	1973.084	0.05221903	0.06913687	0.44307299	0.66802706	ENTPD4
10438	ENSG00000197223	299.578972	-0.014502	0.13306772	0.90665272	0.95941063	C1D
23061	ENSG00000197226	2322.04984	0.0942743	0.07223162	0.18437429	0.38303517	TBC1D9B
26740	ENSG00000197233	0	NA	NA	NA	NA	OR1J2
8363	ENSG00000197238	45.6077213	-0.00348571	0.38068474	0.93948196	0.97474024	H4C11
155184	ENSG00000197241	0	NA	NA	NA	NA	SLC2A7
79927	ENSG00000197245	21.2723428	-0.32867539	0.42379746	0.13418601	0.30845214	FAM110D
5265	ENSG00000197249	0	NA	NA	NA	NA	SERPINA1
401253	ENSG00000197251	0.98055222	0.00600475	0.3793004	0.92649228	NA	LINC00336
64499	ENSG00000197253	0	NA	NA	NA	NA	TPSB2
NA	ENSG00000197254	0	NA	NA	NA	NA	
25959	ENSG00000197256	1696.02236	-0.01948077	0.10791526	0.84898576	0.93137282	KANK2
NA	ENSG00000197258	4.05024091	-0.15409509	0.40744512	0.20525323	0.41224389	EIF4BP6
135398	ENSG00000197261	0.96944804	0.0519479	0.38613807	0.35679999	NA	C6orf141
2961	ENSG00000197265	560.256188	-0.01422945	0.10144186	0.88281591	0.94840089	GTF2E2
246778	ENSG00000197272	0.15069912	-0.01848503	0.38480575	0.84537521	NA	IL27
2980	ENSG00000197273	0	NA	NA	NA	NA	GUCA2A
25788	ENSG00000197275	281.325852	-0.41033533	0.14685181	0.00249606	0.01295705	RAD54B
7718	ENSG00000197279	12.1106976	0.18213193	0.37549447	0.31365838	0.54159326	ZNF165
8831	ENSG00000197283	1126.4483	-0.17086721	0.09206709	0.05527155	0.16081355	SYNGAP1
100190938	ENSG00000197291	116.025822	-0.66782756	0.22509097	0.0006529	0.00406446	RAMP2-AS1
128486	ENSG00000197296	298.716911	-0.19908156	0.1561647	0.16158574	0.34999912	FITM2
641	ENSG00000197299	768.599347	-0.31738467	0.0908525	0.00031239	0.00212474	BLM
100129940	ENSG00000197301	0	NA	NA	NA	NA	HMGA2-AS1

124411	ENSG00000197302	432.83212	-0.2217969	0.14978183	0.10636518	0.26080345	ZNF720
399717	ENSG00000197308	458.071944	0.47860273	0.14254422	0.00031567	0.00214407	GATA3-AS1
26497	ENSG00000197309	0	NA	NA	NA	NA	OR10D3
84301	ENSG00000197312	1308.36146	-0.06665671	0.07994193	0.39346568	0.62246703	DDI2
NA	ENSG00000197320	0.173733	0.01914545	0.38484597	0.78225479	NA	
6840	ENSG00000197321	594.648025	1.48890611	0.12629567	3.62E-33	2.28E-31	SVIL
400591	ENSG00000197322	0	NA	NA	NA	NA	C17orf102
51592	ENSG00000197323	1569.71625	-0.19654371	0.08579676	0.01852182	0.06837978	TRIM33
26020	ENSG00000197324	1152.26715	0.24268944	0.07884038	0.00163153	0.00902162	LRP10
57162	ENSG00000197329	825.994168	0.12098396	0.10373668	0.22556179	0.43951541	PELI1
NA	ENSG00000197332	81.1217835	-0.18484148	0.26419126	0.34908329	0.57985703	
NA	ENSG00000197334	0	NA	NA	NA	NA	
79027	ENSG00000197343	2573.62988	-0.13666701	0.0746725	0.06177106	0.17534507	ZNF655
219927	ENSG00000197345	596.584415	0.00339608	0.10384875	0.97435153	0.9908321	MRPL21
137797	ENSG00000197353	0	NA	NA	NA	NA	LYPD2
91373	ENSG00000197355	486.264863	-0.68687124	0.12005615	2.53E-09	4.11E-08	UAP1L1
NA	ENSG00000197358	0	NA	NA	NA	NA	BNIP3P1
148198	ENSG00000197360	0.9480641	-0.06797803	0.39193571	0.19890261	NA	ZNF98
283807	ENSG00000197361	6.181953	0.1254054	0.39022368	0.30174486	0.52913363	FBXL22
136051	ENSG00000197362	451.736699	-0.06639258	0.10936338	0.5257921	0.73808697	ZNF786
340385	ENSG00000197363	373.163577	0.03929999	0.14218264	0.76693459	0.89261979	ZNF517
NA	ENSG00000197364	0	NA	NA	NA	NA	S100A7L2
171392	ENSG00000197372	277.812618	0.0411826	0.15398177	0.77134431	0.89515442	ZNF675
6584	ENSG00000197375	299.026848	0.02381252	0.15965664	0.87074913	0.94350971	SLC22A5
NA	ENSG00000197376	0.173124	-0.01911262	0.38486262	0.84537521	NA	
147906	ENSG00000197380	799.482017	-0.10312115	0.08939325	0.23486603	0.45174425	DACT3
104	ENSG00000197381	897.147649	-1.2678691	0.12309852	6.30E-26	3.09E-24	ADARB1
344787	ENSG00000197385	3.28994562	0.05411987	0.37752635	0.5580412	0.76291851	ZNF860
3064	ENSG00000197386	2830.77042	-0.10315261	0.08294433	0.2018715	0.40731522	HTT
128372	ENSG00000197403	0	NA	NA	NA	NA	OR6N1
728	ENSG00000197405	0.34109461	0.02362641	0.38535338	0.62411393	NA	C5AR1
1735	ENSG00000197406	120.020797	4.61464382	0.34363317	8.61E-42	6.81E-40	DIO3
1555	ENSG00000197408	0	NA	NA	NA	NA	CYP2B6
8351	ENSG00000197409	1.02142397	-0.00016943	0.38155016	0.99724909	NA	H3C4
54798	ENSG00000197410	0	NA	NA	NA	NA	DCHS2
79674	ENSG00000197415	1.78145877	0.0057804	0.37797608	0.93629417	NA	VEPH1
646486	ENSG00000197416	0	NA	NA	NA	NA	FABP12
23729	ENSG00000197417	511.491632	-0.07791243	0.11102778	0.46272645	0.6855676	SHPK

NA	ENSG00000197421	11.2878594	5.35995768	1.43906756	9.11E-06	8.70E-05	GGT3P
390038	ENSG00000197428	0	NA	NA	NA	NA	OR51D1
3652	ENSG00000197429	261.140833	-0.14713241	0.15492294	0.29800731	0.52474847	IPP
93377	ENSG00000197430	0	NA	NA	NA	NA	OPALIN
441933	ENSG00000197437	0	NA	NA	NA	NA	OR13G1
4217	ENSG00000197442	278.849992	-0.81247014	0.15129085	1.40E-08	2.07E-07	MAP3K5
55753	ENSG00000197444	1360.94846	-0.96714853	0.08861596	1.37E-28	7.49E-27	OGDHL
1572	ENSG00000197446	0.18922458	0.01954053	0.38488496	0.78225479	NA	CYP2F1
373156	ENSG00000197448	922.048516	0.42154791	0.08539657	4.01E-07	4.86E-06	GSTK1
3182	ENSG00000197451	5452.59265	-0.2180465	0.06053452	0.00026017	0.00180356	HNRNPAB
81466	ENSG00000197454	0.173733	0.01914545	0.38484597	0.78225479	NA	OR2L5
50861	ENSG00000197457	3395.72038	-0.80691959	0.06789635	2.67E-33	1.69E-31	STMN3
5154	ENSG00000197461	2.05258534	-0.03692088	0.3759929	0.68397674	NA	PDGFA
NA	ENSG00000197462	0.173733	0.01914545	0.38484597	0.78225479	NA	
2996	ENSG00000197465	1.88832085	0.11020293	0.39945066	0.18654202	NA	GYPE
1305	ENSG00000197467	1.93619754	0.11337097	0.40415247	0.11947396	NA	COL13A1
NA	ENSG00000197468	0	NA	NA	NA	NA	OR7E85BP
6693	ENSG00000197471	2.40960067	0.09609494	0.39451071	0.21269724	NA	SPN
57116	ENSG00000197472	275.897166	-0.17339594	0.14206028	0.18779353	0.38811227	ZNF695
1587	ENSG00000197475	0	NA	NA	NA	NA	ADAM3A
NA	ENSG00000197476	0	NA	NA	NA	NA	
56125	ENSG00000197479	39.5402771	0.89335352	0.51009964	0.00706719	0.03119928	PCDHB11
89887	ENSG00000197483	200.351265	0.03190233	0.16394617	0.83075089	0.92347029	ZNF628
85569	ENSG00000197487	0	NA	NA	NA	NA	GALP
81031	ENSG00000197496	118.857386	0.53823904	0.2499805	0.00903179	0.03811757	SLC2A10
79788	ENSG00000197497	82.011314	-0.10948774	0.23702841	0.5580583	0.76291851	ZNF665
84154	ENSG00000197498	576.071889	-0.18624537	0.12449912	0.11265205	0.27179207	RPF2
144360	ENSG00000197503	0	NA	NA	NA	NA	LINC00477
64078	ENSG00000197506	0	NA	NA	NA	NA	SLC28A3
400823	ENSG00000197520	0	NA	NA	NA	NA	FAM177B
142678	ENSG00000197530	921.948999	0.59856671	0.12476972	4.74E-07	5.67E-06	MIB2
391112	ENSG00000197532	0	NA	NA	NA	NA	OR6Y1
4644	ENSG00000197535	1238.31225	-0.4576993	0.10534038	5.90E-06	5.86E-05	MYO5A
NA	ENSG00000197536	37.9864218	0.6716406	0.48721065	0.02088266	0.07538507	IRF1-AS1
3004	ENSG00000197540	0.16735865	0.01897177	0.38482933	0.78225479	NA	GZMM
10533	ENSG00000197548	329.838722	-0.07019317	0.14548753	0.60217629	0.79251592	ATG7
649179	ENSG00000197549	0	NA	NA	NA	NA	PRAMENP
NA	ENSG00000197550	2.66353136	-0.08606225	0.38657027	0.36104782	0.59083575	

26037	ENSG00000197555	3790.0146	-0.33202245	0.06092802	3.25E-08	4.57E-07	SIPA1L1
92104	ENSG00000197557	217.513815	0.16782021	0.15745197	0.24252028	0.46074905	TTC30A
NA	ENSG00000197558	110.904496	0.37817968	0.26872739	0.06810366	0.18779383	SSPOP
1991	ENSG00000197561	0	NA	NA	NA	NA	ELANE
57799	ENSG00000197562	505.64391	-0.1218279	0.11423945	0.26288649	0.48497991	RAB40C
23556	ENSG00000197563	613.737238	-0.01616729	0.11426099	0.88206072	0.9479489	PIGN
1288	ENSG00000197565	0.77735646	-0.00320859	0.38207188	0.93334478	NA	COL4A6
57547	ENSG00000197566	116.323563	-0.32073988	0.20480315	0.06682611	0.185353	ZNF624
11147	ENSG00000197568	72.205216	0.1342231	0.24114689	0.48040415	0.70158112	HHLA3
NA	ENSG00000197575	0	NA	NA	NA	NA	RPS17P2
3201	ENSG00000197576	0	NA	NA	NA	NA	HOXA4
10210	ENSG00000197579	503.168137	-0.16713298	0.10814867	0.10623096	0.26052192	TOPORS
83875	ENSG00000197580	30.7120308	0.64943474	0.49543805	0.02465764	0.08618877	BCO2
NA	ENSG00000197582	0.32248618	-0.02325418	0.38531869	0.66960978	NA	GPX1P1
10242	ENSG00000197584	2.41415358	0.1045837	0.39692663	0.2065756	NA	KCNMB2
NA	ENSG00000197585	0.5456005	0.02195801	0.38368909	0.65343716	NA	
955	ENSG00000197586	967.100798	0.0723541	0.10212356	0.46300224	0.68582451	ENTPD6
127343	ENSG00000197587	0	NA	NA	NA	NA	DMBX1
606293	ENSG00000197588	0	NA	NA	NA	NA	KLKP1
391189	ENSG00000197591	0	NA	NA	NA	NA	OR11L1
5167	ENSG00000197594	0	NA	NA	NA	NA	ENPP1
400165	ENSG00000197595	0	NA	NA	NA	NA	ATP11AUN
645811	ENSG00000197599	6.90956304	0.03491695	0.36654866	0.76099078	0.88953379	CCDC154
84188	ENSG00000197601	1536.555	0.11323365	0.08066434	0.15115922	0.33504762	FAR1
65250	ENSG00000197603	641.073671	-0.07190649	0.12262002	0.52957785	0.74127448	CPLANE1
284371	ENSG00000197608	291.875089	0.06825769	0.15318559	0.62795022	0.81117654	ZNF841
8076	ENSG00000197614	0	NA	NA	NA	NA	MFAP5
4624	ENSG00000197616	0	NA	NA	NA	NA	MYH6
NA	ENSG00000197617	0.36293605	0.02389464	0.38539238	0.60997572	NA	VN1R5
284370	ENSG00000197619	156.647051	-0.04771493	0.20248503	0.78002075	0.89936692	ZNF615
91966	ENSG00000197620	152.502532	-0.08979874	0.17562673	0.56428236	0.76680562	EOLA1
56882	ENSG00000197622	2563.3211	-0.31767926	0.06831046	2.21E-06	2.36E-05	CDC42SE1
NA	ENSG00000197627	0	NA	NA	NA	NA	UBE2Q2P12
219972	ENSG00000197629	0	NA	NA	NA	NA	MPEG1
5055	ENSG00000197632	0	NA	NA	NA	NA	SERPINB2
1803	ENSG00000197635	0.48453952	0.02955208	0.38589984	0.50215184	NA	DPP4
5275	ENSG00000197641	0	NA	NA	NA	NA	SERPINB13
NA	ENSG00000197644	0	NA	NA	NA	NA	

80380	ENSG00000197646	0.66053232	0.02589183	0.38363366	0.5899582	NA	PDCD1LG2
163059	ENSG00000197647	46.9820195	-0.1492263	0.27307232	0.45039194	0.67378216	ZNF433
196477	ENSG00000197651	0	NA	NA	NA	NA	CCER1
196385	ENSG00000197653	31.4802143	-0.03053573	0.30481328	0.86754711	0.94194356	DNAH10
283238	ENSG00000197658	0	NA	NA	NA	NA	SLC22A24
NA	ENSG00000197665	0.15069912	-0.01848503	0.38480575	0.84537521	NA	
NA	ENSG00000197670	5.18153268	0.06244514	0.36432679	0.64010825	0.81965584	
NA	ENSG00000197674	0	NA	NA	NA	NA	OR51C1P
388818	ENSG00000197683	0	NA	NA	NA	NA	KRTAP26-1
NA	ENSG00000197692	0.14931947	-0.01844332	0.38480212	0.84537521	NA	CBX3P7
6709	ENSG00000197694	9661.70154	-0.26963825	0.0679409	5.31E-05	0.00043086	SPTAN1
4828	ENSG00000197696	222.678989	0.19046923	0.16086439	0.1919759	0.39408192	NMB
55742	ENSG00000197702	426.513646	0.07693961	0.11646549	0.49013852	0.70978228	PARVA
57565	ENSG00000197705	150.221193	1.59222645	0.21271804	5.56E-15	1.52E-13	KLHL14
254783	ENSG00000197706	0	NA	NA	NA	NA	OR6C74
92689	ENSG00000197712	158.310782	1.49230652	0.26738888	1.77E-09	2.94E-08	FAM114A1
6120	ENSG00000197713	793.900292	-0.11553453	0.09969443	0.22932896	0.44488965	RPE
10794	ENSG00000197714	54.3411729	0.56988767	0.33568954	0.02022755	0.07355471	ZNF460
1379	ENSG00000197721	0	NA	NA	NA	NA	CR1L
5253	ENSG00000197724	1437.25357	0.08130736	0.08085021	0.30392283	0.53126299	PHF2
6231	ENSG00000197728	4250.44369	0.00641397	0.07119763	0.92812426	0.96985158	RPS26
283579	ENSG00000197734	0.16735865	0.01897177	0.38482933	0.78225479	NA	C14orf178
NA	ENSG00000197744	1.56482097	-0.00544317	0.37739752	0.94054094	NA	PTMAP2
404552	ENSG00000197745	0	NA	NA	NA	NA	SCGB1D4
5660	ENSG00000197746	9212.6643	-0.16884805	0.04750647	0.00033726	0.0022744	PSAP
6281	ENSG00000197747	0.173733	0.01914545	0.38484597	0.78225479	NA	S100A10
80217	ENSG00000197748	32.7317276	-0.11486388	0.32071246	0.54263495	0.75070618	CFAP43
222662	ENSG00000197753	101.618311	-0.24956232	0.22442859	0.17632324	0.37081334	LHFPL5
6168	ENSG00000197756	13517.2458	-0.10791917	0.05796531	0.05868445	0.16825136	RPL37A
3223	ENSG00000197757	239.809744	-0.51228849	0.15662954	0.00038328	0.00254917	HOXC6
114112	ENSG00000197763	185.273422	0.27878307	0.18437241	0.08538219	0.22172547	TXNRD3
1675	ENSG00000197766	8.26095679	-0.01238383	0.34958164	0.93072139	0.97111825	CFD
441476	ENSG00000197768	2.422914	0.06671136	0.38173898	0.46613122	NA	STPG3
440738	ENSG00000197769	0.60003487	-0.03562272	0.38653687	0.4387237	NA	MAP1LC3C
79892	ENSG00000197771	1577.40208	-0.15539608	0.07958048	0.04561225	0.13860424	MCMBP
197342	ENSG00000197774	881.848574	0.98106644	0.14671609	3.01E-12	6.65E-11	EME2
122773	ENSG00000197776	49.9256516	-0.18722119	0.3006367	0.35526156	0.58560895	KLHDC1
347344	ENSG00000197779	607.433046	-0.07119995	0.15041008	0.60802789	0.79695264	ZNF81

6884	ENSG00000197780	286.425296	-0.3718552	0.14179797	0.00471114	0.02211763	TAF13
284323	ENSG00000197782	482.934208	-0.62636547	0.13807379	1.52E-06	1.66E-05	ZNF780A
55210	ENSG00000197785	1206.47886	0.09377074	0.08712116	0.2692763	0.49229778	ATAD3A
219965	ENSG00000197786	0	NA	NA	NA	NA	OR5B17
119772	ENSG00000197790	0	NA	NA	NA	NA	OR52M1
NA	ENSG00000197794	0	NA	NA	NA	NA	IGKV7-3
79607	ENSG00000197798	318.873372	0.00985078	0.12767821	0.93576347	0.97325384	FAM118B
92283	ENSG00000197808	161.522447	-0.29370479	0.23517789	0.12250244	0.28962737	ZNF461
NA	ENSG00000197813	3.52794107	0.23358666	0.47921639	0.03878491	0.12260878	
NA	ENSG00000197815	23.053928	-0.21795546	0.34391883	0.2864886	0.51225526	
100499483	ENSG00000197816	68.4578716	0.48289101	0.30170052	0.03312957	0.10851572	CCDC180
23315	ENSG00000197818	444.343719	0.22087301	0.11743832	0.04772913	0.14360721	SLC9A8
100506658	ENSG00000197822	384.801861	0.5845177	0.14318431	1.33E-05	0.00012333	OCLN
255119	ENSG00000197826	0.31710024	-0.00155351	0.38337104	0.99273608	NA	CFAP299
121504	ENSG00000197837	24.555644	0.41199688	0.46667602	0.08455161	0.22028941	H4-16
1553	ENSG00000197838	0	NA	NA	NA	NA	CYP2A13
339318	ENSG00000197841	288.690337	-0.14391274	0.13685976	0.25899531	0.48036523	ZNF181
NA	ENSG00000197847	16.9931649	1.89879433	0.77354018	0.00049472	0.00319506	SLC22A20P
26494	ENSG00000197849	0	NA	NA	NA	NA	OR8G1
55924	ENSG00000197852	410.718624	0.26851365	0.15079068	0.0521826	0.1539026	INKA2
51710	ENSG00000197857	134.359941	0.28417491	0.19965704	0.09820194	0.24587665	ZNF44
8733	ENSG00000197858	1998.94071	0.13272226	0.07140525	0.05850863	0.16796572	GPAA1
9719	ENSG00000197859	1452.87456	0.57548122	0.07754121	3.86E-14	9.84E-13	ADAMTSL2
54557	ENSG00000197860	353.177321	-0.40360114	0.13338544	0.00124069	0.00710808	SGTB
388536	ENSG00000197863	141.969345	-0.25325653	0.22696299	0.17222474	0.36478687	ZNF790
NA	ENSG00000197866	0	NA	NA	NA	NA	OR5M12P
5544	ENSG00000197870	0	NA	NA	NA	NA	PRB3
81553	ENSG00000197872	99.9289679	0.71776249	0.29866622	0.0028332	0.01442779	CYRIA
4641	ENSG00000197879	1505.2315	0.82280562	0.10877811	6.99E-15	1.89E-13	MYO1C
259283	ENSG00000197880	0	NA	NA	NA	NA	MDS2
NA	ENSG00000197882	0.16735865	0.01897177	0.38482933	0.78225479	NA	OR7E13P
28512	ENSG00000197885	477.904934	-0.43719135	0.13879978	0.00073584	0.00451981	NKIRAS1
219958	ENSG00000197887	0	NA	NA	NA	NA	OR1S2
7367	ENSG00000197888	0	NA	NA	NA	NA	UGT2B17
644890	ENSG00000197889	9.01466693	0.04725023	0.35436255	0.74585403	0.8802926	MEIG1
116085	ENSG00000197891	0.51520883	0.03111672	0.38605293	0.47788656	NA	SLC22A12
23303	ENSG00000197892	911.497709	0.13197447	0.10434863	0.18862512	0.38925758	KIF13B
4892	ENSG00000197893	0.18922458	0.01954053	0.38488496	0.78225479	NA	NRAP

128	ENSG00000197894	2418.84874	0.15931906	0.09676339	0.08860668	0.2278031	ADH5
9356	ENSG00000197901	0	NA	NA	NA	NA	SLC22A6
85236	ENSG00000197903	175.607414	-0.52248475	0.17917326	0.00120077	0.0069334	H2BC12
7004	ENSG00000197905	325.803088	-0.68290658	0.13987847	2.48E-07	3.11E-06	TEAD4
6687	ENSG00000197912	2334.57712	0.11467407	0.07986131	0.1421905	0.32093965	SPG7
388697	ENSG00000197915	2.40279159	-0.10350488	0.39420512	0.25412705	NA	HRNR
3439	ENSG00000197919	0	NA	NA	NA	NA	IFNA1
388585	ENSG00000197921	0.14931947	-0.01844332	0.38480212	0.84537521	NA	HES5
NA	ENSG00000197927	1.57908417	-0.0019652	0.37827053	0.97640547	NA	NBEAP2
342926	ENSG00000197928	395.166623	-0.33170314	0.12892598	0.0061457	0.02766124	ZNF677
30001	ENSG00000197930	1448.7654	0.78589174	0.08464808	3.26E-21	1.28E-19	ERO1A
55552	ENSG00000197933	103.24668	-0.24422207	0.25568702	0.21589598	0.42667359	ZNF823
100996571	ENSG00000197934	3.51221084	-0.11707323	0.39525633	0.247172	0.46606087	CYYR1-AS1
282890	ENSG00000197935	0.44956412	-0.02729138	0.38571238	0.56205533	NA	ZNF311
84671	ENSG00000197937	314.4331	0.23975209	0.16378419	0.10499462	0.25832009	ZNF347
79310	ENSG00000197938	0	NA	NA	NA	NA	OR5H2
5336	ENSG00000197943	0	NA	NA	NA	NA	PLCG2
89848	ENSG00000197948	505.65873	0.62775779	0.11526413	1.44E-08	2.13E-07	FCHSD1
58491	ENSG00000197951	559.27917	0.14094807	0.10586453	0.16575256	0.35597865	ZNF71
344752	ENSG00000197953	0	NA	NA	NA	NA	AADACL2
6277	ENSG00000197956	531.020088	0.80326142	0.11108407	9.02E-14	2.22E-12	S100A6
6136	ENSG00000197958	13253.3119	-0.35966398	0.0540761	2.00E-11	4.12E-10	RPL12
26052	ENSG00000197959	139.30292	-1.21908669	0.22132189	3.40E-09	5.43E-08	DNM3
7675	ENSG00000197961	1108.61813	0.14812225	0.11360604	0.17191331	0.36451957	ZNF121
9019	ENSG00000197965	2795.07067	-0.05487424	0.07136474	0.43340385	0.65865433	MPZL1
23230	ENSG00000197969	1728.11678	-0.11108345	0.10657747	0.2768325	0.50121841	VPS13A
4155	ENSG00000197971	1.18477697	0.01659746	0.37998305	0.7952965	NA	MBP
8227	ENSG00000197976	1744.49181	0.42559266	0.07059324	7.98E-10	1.39E-08	AKAP17A
54898	ENSG00000197977	354.683714	-0.42583224	0.14367762	0.00140093	0.00791414	ELOVL2
440295	ENSG00000197978	1199.94044	0.38510562	0.08687245	5.12E-06	5.12E-05	GOLGA6L9
389170	ENSG00000197980	7.7966061	0.18304268	0.40367865	0.2340562	0.45093611	LEKR1
127687	ENSG00000197982	852.070992	0.11328571	0.08838198	0.18817996	0.38858143	C1orf122
NA	ENSG00000197984	0	NA	NA	NA	NA	OR51A8P
85028	ENSG00000197989	390.881949	-0.0840445	0.12788055	0.48228668	0.70272483	SNHG12
NA	ENSG00000197990	0	NA	NA	NA	NA	ZNF734P
NA	ENSG00000197991	0	NA	NA	NA	NA	
283420	ENSG00000197992	0	NA	NA	NA	NA	CLEC9A
3792	ENSG00000197993	8.72398493	0.37162457	0.59911942	0.03987261	0.12512313	KEL

55035	ENSG00000198000	843.058288	-0.17482993	0.1245004	0.13672993	0.31191797	NOL8
51135	ENSG00000198001	203.242689	0.0656126	0.17079965	0.66917919	0.83726414	IRAK4
115948	ENSG00000198003	43.2869664	0.08014055	0.27665954	0.68230886	0.8449992	ODAD3
9228	ENSG00000198010	7.18519105	-0.22499853	0.44765485	0.11270084	0.27181918	DLGAP2
28977	ENSG00000198015	1542.98232	0.10349084	0.08326467	0.20315575	0.40924055	MRPL42
57089	ENSG00000198018	534.181383	0.08868026	0.11624186	0.42396113	0.64969559	ENTPD7
NA	ENSG00000198019	0.33471731	0.02354391	0.38534154	0.62842204	NA	FCGR1B
30014	ENSG00000198021	0	NA	NA	NA	NA	SPANXA1
NA	ENSG00000198022	0	NA	NA	NA	NA	SAGE2P
63925	ENSG00000198026	624.545987	0.06545583	0.10535211	0.51864955	0.73233273	ZNF335
147741	ENSG00000198028	3.72966254	-0.29129736	0.55510892	0.0092831	0.03900397	ZNF560
7278	ENSG00000198033	0.14931947	-0.01844332	0.38480212	0.84537521	NA	TUBA3C
6191	ENSG00000198034	24610.8826	-0.1354961	0.0499134	0.00610459	0.02751988	RPS4X
10793	ENSG00000198039	416.295424	-0.25478592	0.12457198	0.02999679	0.10030952	ZNF273
7637	ENSG00000198040	1733.96685	0.08277134	0.08841377	0.33623085	0.56617316	ZNF84
84549	ENSG00000198042	757.821249	-0.11404267	0.11630909	0.30212149	0.52960685	MAK16
63934	ENSG00000198046	300.76475	-0.05124205	0.16828609	0.73381297	0.87327664	ZNF667
553	ENSG00000198049	0.50207596	0.02562368	0.38565771	0.52549886	NA	AVPR1B
140885	ENSG00000198053	86.5485941	0.05545036	0.21299579	0.7547103	0.88583526	SIRPA
84677	ENSG00000198054	0	NA	NA	NA	NA	DSCR8
2870	ENSG00000198055	1292.30543	-0.33214605	0.07743124	1.13E-05	0.0001061	GRK6
5557	ENSG00000198056	663.928004	-0.36748261	0.09674386	8.33E-05	0.000646	PRIM1
54708	ENSG00000198060	1044.83291	-0.35408435	0.10094898	0.00026571	0.0018362	MARCHF5
23784	ENSG00000198062	19.9494421	0.01739909	0.30995005	0.92661059	0.96932621	POTEH
613037	ENSG00000198064	94.7066051	0.52708638	0.35987288	0.03348344	0.10951287	NPIPB13
57016	ENSG00000198074	0	NA	NA	NA	NA	AKR1B10
27233	ENSG00000198075	1037.34853	0.60933669	0.08841695	1.65E-12	3.71E-11	SULT1C4
1549	ENSG00000198077	0.37859049	-0.02359453	0.38537326	0.6731922	NA	CYP2A7
7541	ENSG00000198081	287.507088	-0.16402098	0.1480713	0.22841269	0.44363268	ZBTB14
81870	ENSG00000198083	0	NA	NA	NA	NA	KRTAP9-9
23607	ENSG00000198087	345.096189	0.2903903	0.13954573	0.02506718	0.08732798	CD2AP
54830	ENSG00000198088	103.023071	0.34663071	0.22691639	0.06533813	0.18239884	NUP62CL
9814	ENSG00000198089	675.357182	0.1845609	0.11091967	0.08158374	0.21430178	SFI1
81871	ENSG00000198090	0	NA	NA	NA	NA	KRTAP4-6
389208	ENSG00000198092	0	NA	NA	NA	NA	TMPRSS11F
65251	ENSG00000198093	220.291904	-0.05306868	0.16935817	0.72558241	0.86866734	ZNF649
127	ENSG00000198099	0.18922458	0.01954053	0.38488496	0.78225479	NA	ADH4
254879	ENSG00000198104	0	NA	NA	NA	NA	OR2T6

57209	ENSG00000198105	620.669499	-0.13873028	0.14542977	0.29934093	0.52631373	ZNF248
NA	ENSG00000198106	26.6206484	-0.24132744	0.34368085	0.24873349	0.46802661	
337876	ENSG00000198108	0	NA	NA	NA	NA	CHSY3
54863	ENSG00000198113	17.745418	-0.05959338	0.32059001	0.74081553	0.87763281	TOR4A
1902	ENSG00000198121	68.706676	1.09672265	0.36314852	0.00023218	0.00162314	LPAR1
4151	ENSG00000198125	3.07656568	-0.04292809	0.37214507	0.68673128	0.84769738	MB
391192	ENSG00000198128	0	NA	NA	NA	NA	OR2L3
245910	ENSG00000198129	0	NA	NA	NA	NA	DEFB107B
503614	ENSG00000198129	0	NA	NA	NA	NA	DEFB107B
26275	ENSG00000198130	464.049455	-0.03585776	0.1088205	0.73032898	0.87106623	HIBCH
27300	ENSG00000198131	12.7350944	-0.18792661	0.37404388	0.30429618	0.53154043	ZNF544
161145	ENSG00000198133	64.5508939	0.15495602	0.25869707	0.42901689	0.65464506	TMEM229B
NA	ENSG00000198134	0	NA	NA	NA	NA	PTMAP9
65124	ENSG00000198142	299.581826	-0.06465402	0.1382088	0.61577148	0.80301072	SOWAHC
54989	ENSG00000198146	1311.11966	-0.54200065	0.09603517	5.83E-09	9.05E-08	ZNF770
NA	ENSG00000198153	23.8830399	-0.46116156	0.45480111	0.06733667	0.18634601	ZNF849P
642280	ENSG00000198155	111.191622	-0.31103636	0.23333328	0.10170346	0.25249251	ZNF876P
728741	ENSG00000198156	9.9070415	-0.00433003	0.35921754	0.97043429	0.98888633	NPIPB6
79366	ENSG00000198157	51.545056	2.76058648	0.36603991	2.80E-15	7.86E-14	HMGN5
57708	ENSG00000198160	698.683819	0.02666763	0.10071872	0.78476489	0.90204747	MIER1
10905	ENSG00000198162	1314.52378	-0.06763047	0.08693517	0.42371608	0.64959604	MAN1A2
258010	ENSG00000198168	521.025529	-0.16982701	0.11703579	0.12603236	0.29528602	SVIP
90987	ENSG00000198169	489.424921	0.26605683	0.11838891	0.01785906	0.06652719	ZNF251
65992	ENSG00000198171	662.66659	0.00335731	0.10827421	0.97572746	0.99159826	DDRGK1
442444	ENSG00000198173	0	NA	NA	NA	NA	FAM47C
7027	ENSG00000198176	1931.42738	-0.31416043	0.06593546	1.27E-06	1.41E-05	TFDP1
170482	ENSG00000198178	0	NA	NA	NA	NA	CLEC4C
84775	ENSG00000198182	335.993007	-0.01065633	0.13429385	0.9316831	0.9715756	ZNF607
51297	ENSG00000198183	0	NA	NA	NA	NA	BPIFA1
55713	ENSG00000198185	662.208164	-0.38703649	0.11428006	0.00037432	0.00249543	ZNF334
51170	ENSG00000198189	805.15581	-0.08119193	0.09258968	0.3655235	0.59471759	HSD17B11
23334	ENSG00000198198	1654.24756	0.20896962	0.08367259	0.01030753	0.04244527	SZT2
6819	ENSG00000198203	0.66943461	0.03059546	0.38616409	0.42784935	NA	SULT1C2
7789	ENSG00000198205	130.832255	0.21295082	0.19812387	0.21109792	0.42012066	ZXDA
83694	ENSG00000198208	195.693195	-1.20577111	0.23679594	3.31E-08	4.65E-07	RPS6KL1
NA	ENSG00000198211	0	NA	NA	NA	NA	
777	ENSG00000198216	0.32360239	0.00418348	0.38342001	0.93761449	NA	CACNA1E
NA	ENSG00000198217	0	NA	NA	NA	NA	OR51H2P

54870	ENSG00000198218	1997.16814	0.04864874	0.09857527	0.61074655	0.79891947	QRICH1
NA	ENSG00000198221	33.8950485	0.33739955	0.41059653	0.13064872	0.30288275	AFDN-DT
1438	ENSG00000198223	0	NA	NA	NA	NA	CSF2RA
NA	ENSG00000198225	0.49663606	-0.01036196	0.3828476	0.8263336	NA	FKBP1C
11325	ENSG00000198231	4978.72775	-0.02791363	0.07046209	0.68558718	0.8470175	DDX42
NA	ENSG00000198237	208.932401	0.25825855	0.18792834	0.11613056	0.27839903	GUSBP13
6147	ENSG00000198242	6013.89043	-0.13409497	0.06119267	0.02385859	0.08389835	RPL23A
55315	ENSG00000198246	220.277784	-0.19078079	0.15919255	0.18658871	0.38635899	SLC29A3
NA	ENSG00000198251	0	NA	NA	NA	NA	CYP2A7P2
6815	ENSG00000198252	293.6808	0.24521817	0.13563704	0.0526168	0.154792	STYX
59286	ENSG00000198258	1498.39515	0.07283462	0.0718385	0.30218661	0.52967421	UBL5
NA	ENSG00000198261	0	NA	NA	NA	NA	OR5BB1P
9931	ENSG00000198265	2666.11023	-0.27231783	0.07656516	0.00027914	0.001915	HELZ
89894	ENSG00000198270	171.607413	0.01696985	0.18802183	0.91714786	0.96490686	TMEM116
85289	ENSG00000198271	0	NA	NA	NA	NA	KRTAP4-5
54963	ENSG00000198276	1051.12198	0.0504869	0.0905932	0.56702853	0.76856463	UCKL1
NA	ENSG00000198277	0	NA	NA	NA	NA	
219968	ENSG00000198283	0.18922458	0.01954053	0.38488496	0.78225479	NA	OR5B21
255330	ENSG00000198284	0	NA	NA	NA	NA	NUP210P1
84433	ENSG00000198286	42.0597646	-0.39439646	0.40679024	0.09415193	0.23832283	CARD11
220992	ENSG00000198298	151.212527	-0.25458859	0.19554681	0.1316562	0.30440031	ZNF485
5178	ENSG00000198300	624.752108	-0.56200741	0.11773042	5.86E-07	6.92E-06	PEG3
55153	ENSG00000198301	1011.43093	-0.00366763	0.09433461	0.96717516	0.98759962	SDAD1
NA	ENSG00000198312	0.14931947	-0.01844332	0.38480212	0.84537521	NA	BMS1P9
7745	ENSG00000198315	2203.27703	0.03444118	0.0700558	0.6176623	0.80429156	ZKSCAN8
144717	ENSG00000198324	459.702047	-0.01011115	0.14071549	0.93774064	0.97366414	PHETA1
100288797	ENSG00000198326	0.18922458	0.01954053	0.38488496	0.78225479	NA	TMEM239
219844	ENSG00000198331	101.417896	-0.1781836	0.20704166	0.30801698	0.53554973	HYLS1
4635	ENSG00000198336	0.61294449	-0.03619595	0.38660045	0.43128479	NA	MYL4
79973	ENSG00000198342	23.8896358	-0.33603911	0.43669672	0.1220294	0.28884077	ZNF442
126017	ENSG00000198346	177.671882	-0.02500685	0.17615352	0.87238412	0.94419841	ZNF813
3221	ENSG00000198353	540.639701	-0.35415667	0.11439526	0.00113055	0.00656041	HOXC4
340578	ENSG00000198354	0.14931947	-0.01844332	0.38480212	0.84537521	NA	DCAF12L2
415116	ENSG00000198355	1255.32322	-0.64387626	0.0899442	2.22E-13	5.26E-12	PIM3
439	ENSG00000198356	1050.18104	0.32011829	0.10159503	0.00105362	0.00617176	GET3
101928372	ENSG00000198358	0	NA	NA	NA	NA	
444	ENSG00000198363	1043.11008	-0.0922503	0.08917565	0.28691794	0.51278731	ASPH
NA	ENSG00000198367	0	NA	NA	NA	NA	OR7A11P

200734	ENSG00000198369	1693.82808	-0.12326254	0.06893787	0.06871478	0.18899959	SPRED2
11060	ENSG00000198373	752.05824	-0.39852333	0.0961389	1.78E-05	0.00016106	WWP2
2673	ENSG00000198380	713.420784	-0.38417313	0.12103484	0.00079617	0.00485434	GFPT1
7405	ENSG00000198382	398.564253	-0.32508591	0.13366097	0.0092337	0.03882105	UVRAG
140258	ENSG00000198390	0	NA	NA	NA	NA	KRTAP13-1
7574	ENSG00000198393	435.29259	0.23462928	0.16136448	0.10845671	0.26431057	ZNF26
131920	ENSG00000198398	0	NA	NA	NA	NA	TMEM207
50618	ENSG00000198399	751.482202	0.03225326	0.09194238	0.71852928	0.86497305	ITSN2
4914	ENSG00000198400	315.261049	0.01509602	0.14622378	0.91223623	0.96260718	NTRK1
NA	ENSG00000198406	164.382057	0.32394917	0.25263337	0.10506744	0.25840943	BZW1P2
10724	ENSG00000198408	3035.00274	-0.04585223	0.0729367	0.52320596	0.73618679	OGA
NA	ENSG00000198414	0.16735865	0.01897177	0.38482933	0.78225479	NA	TATDN2P1
NA	ENSG00000198416	91.5710988	-0.25316377	0.23098501	0.17769314	0.37286428	ZNF658B
4494	ENSG00000198417	12.8421094	0.23727538	0.41022801	0.20227179	0.4077911	MT1F
9747	ENSG00000198420	5835.12369	-0.08961587	0.06175639	0.14096257	0.31900278	TCAF1
7620	ENSG00000198429	158.079043	-0.15642738	0.17564266	0.31552547	0.54335781	ZNF69
7296	ENSG00000198431	3283.96486	0.2046819	0.07071154	0.00320678	0.01600127	TXNRD1
441478	ENSG00000198435	349.898121	-0.05669339	0.1342288	0.65167818	0.82602754	NRARP
147949	ENSG00000198440	99.0274618	0.06034964	0.20269455	0.72713841	0.86953012	ZNF583
85285	ENSG00000198443	0	NA	NA	NA	NA	KRTAP4-1
150160	ENSG00000198445	0	NA	NA	NA	NA	CCT8L2
NA	ENSG00000198452	0	NA	NA	NA	NA	OR14L1P
374900	ENSG00000198453	312.124317	-0.09155523	0.14515934	0.49408138	0.71286267	ZNF568
NA	ENSG00000198454	0.16735865	0.01897177	0.38482933	0.78225479	NA	LINC02692
158586	ENSG00000198455	336.61456	0.07080263	0.13368063	0.57152685	0.77168182	ZXDB
147657	ENSG00000198464	467.964414	0.18706864	0.13853908	0.14722493	0.32878688	ZNF480
84914	ENSG00000198466	911.900913	0.0128667	0.09675625	0.89150364	0.95288397	ZNF587
7169	ENSG00000198467	165.877597	0.71664637	0.22054558	0.00023107	0.00161651	TPM2
642946	ENSG00000198468	114.625787	-0.23550874	0.21009114	0.18325032	0.38145907	FLVCR1-DT
344892	ENSG00000198471	0	NA	NA	NA	NA	RTP2
83699	ENSG00000198478	859.610262	-0.90338898	0.10826404	1.12E-17	3.63E-16	SH3BGRL2
388558	ENSG00000198482	234.093874	-0.10790757	0.15985352	0.45935775	0.68256874	ZNF808
148741	ENSG00000198483	0	NA	NA	NA	NA	ANKRD35
192134	ENSG00000198488	0.173124	-0.01911262	0.38486262	0.84537521	NA	B3GNT6
101929106	ENSG00000198491	0	NA	NA	NA	NA	
51441	ENSG00000198492	2092.46723	-0.02939835	0.07967756	0.70537789	0.85803894	YTHDF2
10230	ENSG00000198496	60.5020031	0.19508587	0.2547942	0.32039236	0.54819611	NBR2
55319	ENSG00000198498	420.941921	0.12218688	0.11505538	0.26560782	0.48795885	TMA16

3127	ENSG00000198502	0	NA	NA	NA	NA	HLA-DRB5
51062	ENSG00000198513	399.974539	-0.57935217	0.12629539	1.39E-06	1.54E-05	ATL1
1259	ENSG00000198515	1.3093995	-0.01594918	0.37938199	0.81069756	NA	CNGA1
7975	ENSG00000198517	372.928653	-0.58239517	0.1946797	0.00076357	0.00466845	MAFK
339541	ENSG00000198520	28.8736237	0.1623618	0.32053111	0.41343043	0.64066701	ARMH1
7594	ENSG00000198521	884.977049	-0.40530286	0.0901914	3.67E-06	3.77E-05	ZNF43
11321	ENSG00000198522	1049.08286	0.01461195	0.0817732	0.85469762	0.93485893	GPN1
5350	ENSG00000198523	0.173733	0.01914545	0.38484597	0.78225479	NA	PLN
NA	ENSG00000198526	0	NA	NA	NA	NA	PABPC1P2
145741	ENSG00000198535	1.71937656	-0.06004075	0.38610862	0.36968488	NA	C2CD4A
7576	ENSG00000198538	306.370688	-0.11780645	0.13809501	0.35929375	0.58949503	ZNF28
9358	ENSG00000198542	0	NA	NA	NA	NA	ITGBL1
118472	ENSG00000198546	519.116298	0.04581735	0.10677278	0.65571037	0.82872301	ZNF511
284805	ENSG00000198547	0.16735865	0.01897177	0.38482933	0.78225479	NA	C20orf203
199692	ENSG00000198551	390.736144	-0.13193063	0.13637067	0.29951745	0.52648412	ZNF627
283518	ENSG00000198553	16.8401497	0.10891023	0.33092346	0.55612465	0.76171992	KCNRG
11169	ENSG00000198554	893.711287	0.00717941	0.10060205	0.94134317	0.97559098	WDHD1
NA	ENSG00000198555	1.19523293	-0.00843647	0.38136715	0.87247901	NA	
285989	ENSG00000198556	504.553281	0.14908582	0.14774904	0.27418939	0.49784182	ZNF789
1500	ENSG00000198561	2061.84129	0.00114213	0.08786126	0.99023674	0.99673744	CTNND1
7919	ENSG00000198563	8747.86023	0.21084763	0.05044152	2.46E-05	0.00021609	DDX39B
142680	ENSG00000198569	19.6418446	0.26448499	0.37261992	0.21020321	0.41876008	SLC34A3
343035	ENSG00000198570	1222.62289	-2.48122558	0.0955352	8.01E-150	6.91E-147	RD3
64663	ENSG00000198573	0	NA	NA	NA	NA	SPANXC
117157	ENSG00000198574	0	NA	NA	NA	NA	SH2D1B
23237	ENSG00000198576	393.832363	0.23326066	0.16853354	0.12313039	0.29054555	ARC
NA	ENSG00000198580	6.01350941	-0.05787295	0.36792637	0.63680514	0.81722218	
131870	ENSG00000198585	1323.76538	0.05373801	0.075284	0.46722155	0.69011879	NUDT16
9874	ENSG00000198586	922.779718	0.03782058	0.10152628	0.70019233	0.85541355	TLK1
987	ENSG00000198589	1440.19502	-0.01962834	0.08173127	0.80603962	0.9121533	LRBA
NA	ENSG00000198590	21.1878033	0.9711524	0.61303427	0.00798947	0.03446641	C3orf35
9745	ENSG00000198597	1002.97042	0.63590881	0.10144906	1.01E-10	1.94E-09	ZNF536
4326	ENSG00000198598	181.071728	0.21221092	0.17030857	0.1639553	0.35322761	MMP17
391194	ENSG00000198601	0	NA	NA	NA	NA	OR2M2
11177	ENSG00000198604	2012.25032	0.33314875	0.08867425	0.00010924	0.00082342	BAZ1A
1109	ENSG00000198610	0	NA	NA	NA	NA	AKR1C4
10920	ENSG00000198612	1172.1204	-0.20385569	0.08878149	0.01804281	0.06707314	COPS8
NA	ENSG00000198618	1.81768097	-0.07487825	0.38913232	0.29849152	NA	PPIAP22

26112	ENSG00000198624	13.17697	0.20269175	0.37878616	0.28036615	0.5049178	CCDC69
4194	ENSG00000198625	2275.23389	-0.1814957	0.10268471	0.06562502	0.18299372	MDM4
6262	ENSG00000198626	430.081686	-3.24041039	0.21504379	2.50E-53	2.68E-51	RYR2
147658	ENSG00000198633	2.05190235	0.0763992	0.38703754	0.34516056	NA	ZNF534
55958	ENSG00000198642	1169.42374	-0.15001895	0.08117903	0.05806001	0.16708886	KLHL9
131177	ENSG00000198643	0.15069912	-0.01848503	0.38480575	0.84537521	NA	FAM3D
23054	ENSG00000198646	2012.9477	-0.39258057	0.07873305	3.33E-07	4.10E-06	NCOA6
27347	ENSG00000198648	773.918721	-0.30935616	0.09349698	0.00062322	0.00391285	STK39
6898	ENSG00000198650	0.15069912	-0.01848503	0.38480575	0.84537521	NA	TAT
NA	ENSG00000198658	3.23180956	0.07005272	0.37964132	0.48893868	0.70868961	ABHD17AP1
221477	ENSG00000198663	2493.27337	-0.1502889	0.06329744	0.01596194	0.06062114	C6orf89
801	ENSG00000198668	6427.56432	-0.70605738	0.06040927	3.34E-32	2.03E-30	CALM1
4018	ENSG00000198670	0	NA	NA	NA	NA	LPA
NA	ENSG00000198671	0	NA	NA	NA	NA	
338811	ENSG00000198673	6.39570601	0.25527823	0.48296039	0.05649915	0.16353915	TAFA2
79490	ENSG00000198674	0	NA	NA	NA	NA	OR10G6
9652	ENSG00000198677	2849.86639	-0.2135295	0.0658722	0.00098322	0.00582682	TTC37
NA	ENSG00000198678	0	NA	NA	NA	NA	OR5BS1P
286319	ENSG00000198680	334.887738	0.22272148	0.14241298	0.09153281	0.23357054	TUSC1
4100	ENSG00000198681	0.15069912	-0.01848503	0.38480575	0.84537521	NA	MAGEA1
9060	ENSG00000198682	57.5197515	0.35065108	0.29158367	0.10309614	0.25507263	PAPSS2
23434	ENSG00000198685	0.68219046	0.04060292	0.38717358	0.35756446	NA	LINC01565
10479	ENSG00000198689	422.712602	-0.26334839	0.13488096	0.03607249	0.11601202	SLC9A6
22909	ENSG00000198690	1291.24806	0.00022067	0.0965905	0.99912696	0.99962651	FAN1
24	ENSG00000198691	0.747933	0.01552071	0.38292809	0.7287086	NA	ABCA4
9086	ENSG00000198692	0	NA	NA	NA	NA	EIF1AY
4541	ENSG00000198695	9202.72782	-0.02403816	0.09646349	0.79672647	0.90658744	MT-ND6
55705	ENSG00000198700	5452.62862	-0.11933186	0.07525591	0.1053298	0.25877695	IPO9
NA	ENSG00000198703	0	NA	NA	NA	NA	OR10R3P
257202	ENSG00000198704	0	NA	NA	NA	NA	GPX6
80184	ENSG00000198707	1008.39569	0.14130658	0.11122836	0.18412829	0.3826844	CEP290
619518	ENSG00000198711	56.6295732	0.05858466	0.24349905	0.75732794	0.88697677	SSBP3-AS1
4513	ENSG00000198712	98080.0834	0.43628689	0.07477176	1.76E-09	2.93E-08	MT-CO2
112770	ENSG00000198715	693.886299	0.22420604	0.10787489	0.02989032	0.10005012	GLMP
23116	ENSG00000198718	220.297447	-0.02620079	0.18365707	0.86957233	0.94311021	TOGARAM1
28514	ENSG00000198719	62.2257167	3.28474291	0.37725529	2.77E-19	9.95E-18	DLL1
124930	ENSG00000198720	2476.38341	0.25668876	0.06436143	5.12E-05	0.00041735	ANKRD13B
10455	ENSG00000198721	553.420737	0.22505178	0.11972456	0.04736243	0.14263366	ECI2

10497	ENSG00000198722	1321.82054	-0.02318705	0.07396085	0.74711021	0.88120985	UNC13B
374877	ENSG00000198723	37.7988345	0.13194192	0.28842252	0.50629792	0.72198309	TEX45
4519	ENSG00000198727	57491.1905	-0.16247671	0.06028146	0.00631659	0.02830836	MT-CYB
8861	ENSG00000198728	3378.67561	0.31830041	0.05762432	2.23E-08	3.21E-07	LDB1
81706	ENSG00000198729	0	NA	NA	NA	NA	PPP1R14C
9646	ENSG00000198730	2344.62192	0.04286331	0.07508117	0.56143209	0.76492328	CTR9
64093	ENSG00000198732	43.0363606	0.08911869	0.28637925	0.64748925	0.82430661	SMOC1
2153	ENSG00000198734	0	NA	NA	NA	NA	F5
51734	ENSG00000198736	294.305987	-0.19270404	0.13301436	0.12119155	0.2874665	MSRB1
NA	ENSG00000198738	0	NA	NA	NA	NA	SMIM11P1
347731	ENSG00000198739	0	NA	NA	NA	NA	LRRTM3
22834	ENSG00000198740	828.478201	-0.27634918	0.103333	0.00532848	0.02455678	ZNF652
57154	ENSG00000198742	1036.01847	-0.12585837	0.08146128	0.11344919	0.27329189	SMURF1
6526	ENSG00000198743	637.701612	-0.14244775	0.10553531	0.15945464	0.34700764	SLC5A3
NA	ENSG00000198744	2.8638861	0.0603525	0.37583909	0.56704884	0.76856463	MTCO3P12
63906	ENSG00000198746	227.918208	0.16686533	0.18851955	0.31009672	0.53778389	GPATCH3
9578	ENSG00000198752	3254.9315	-0.06169795	0.08516678	0.45671299	0.68021813	CDC42BPB
5365	ENSG00000198753	371.114115	-0.95409588	0.15371297	7.37E-11	1.43E-09	PLXNB3
64064	ENSG00000198754	41.8092442	-0.79718251	0.43532147	0.00779238	0.03376301	OXCT2
4736	ENSG00000198755	11673.3115	-0.11924827	0.05325076	0.02364198	0.08332657	RPL10A
23127	ENSG00000198756	201.966325	0.21285477	0.16569903	0.15423132	0.3394719	COLGALT2
79574	ENSG00000198758	0	NA	NA	NA	NA	EPS8L3
25975	ENSG00000198759	0.15069912	-0.01848503	0.38480575	0.84537521	NA	EGFL6
4536	ENSG00000198763	72947.0136	-0.27663529	0.09010233	0.00177498	0.00966134	MT-ND2
6847	ENSG00000198765	0	NA	NA	NA	NA	SYCP1
164284	ENSG00000198768	0.69958169	0.02707939	0.38367853	0.57143862	NA	APCDD1L
92241	ENSG00000198771	0.14931947	-0.01844332	0.38480212	0.84537521	NA	RCSD1
9182	ENSG00000198774	2.63218231	0.11061433	0.39563201	0.24820236	0.46741467	RASSF9
26049	ENSG00000198780	351.067587	-0.80140384	0.13537856	5.90E-10	1.05E-08	FAM169A
91603	ENSG00000198783	376.726368	0.26305345	0.12657513	0.0273481	0.09334015	ZNF830
116443	ENSG00000198785	15.3716447	-0.10391541	0.33476068	0.56373653	0.76632633	GRIN3A
4540	ENSG00000198786	51500.145	-0.08368088	0.07474662	0.25819409	0.47950675	MT-ND5
NA	ENSG00000198787	0	NA	NA	NA	NA	OR7E103P
4583	ENSG00000198788	0	NA	NA	NA	NA	MUC2
29883	ENSG00000198791	2075.92493	-0.38257163	0.07803807	5.18E-07	6.16E-06	CNOT7
25829	ENSG00000198792	524.073847	-0.16791953	0.11039575	0.1112917	0.26948227	TMEM184B
2475	ENSG00000198793	2567.9306	-0.02842896	0.0844503	0.72923896	0.87047115	MTOR
192683	ENSG00000198794	2215.46485	-0.65094197	0.06814999	3.42E-22	1.42E-20	SCAMP5

25925	ENSG00000198795	453.689888	0.38456198	0.14601957	0.00444344	0.02108533	ZNF521
115701	ENSG00000198796	5.61411006	5.86194866	2.66817996	7.17E-05	0.00056753	ALPK2
57795	ENSG00000198797	53.214555	-3.2226162	0.45289673	7.59E-14	1.88E-12	BRINP2
4114	ENSG00000198798	0	NA	NA	NA	NA	MAGEB3
9860	ENSG00000198799	541.16988	-0.30050379	0.11897287	0.00768071	0.0333547	LRIG2
4512	ENSG00000198804	265616.416	0.13758536	0.04548416	0.00234593	0.01227733	MT-CO1
4860	ENSG00000198805	863.62433	-0.32851166	0.08788843	0.00011843	0.00088464	PNP
5083	ENSG00000198807	0	NA	NA	NA	NA	PAX9
376132	ENSG00000198812	0	NA	NA	NA	NA	LRRC10
2710	ENSG00000198814	201.076396	0.05830863	0.15939262	0.68879707	0.8489088	GK
22887	ENSG00000198815	1751.15486	-0.11609742	0.08732053	0.17144436	0.36389846	FOXJ3
140467	ENSG00000198816	1564.19847	0.10993127	0.07435155	0.13307696	0.30657768	ZNF358
113402	ENSG00000198818	456.337563	1.02695159	0.12063249	2.22E-18	7.65E-17	SFT2D1
919	ENSG00000198821	5.51154977	-0.08749626	0.37200881	0.50165816	0.71856283	CD247
2913	ENSG00000198822	9.16615361	-0.01948325	0.34759274	0.89470679	0.95456012	GRM3
283489	ENSG00000198824	956.737779	-0.45676475	0.10129085	2.85E-06	2.99E-05	CHAMP1
22876	ENSG00000198825	2742.29961	-0.19938626	0.07174722	0.00459507	0.0216598	INPP5F
9824	ENSG00000198826	2254.914	-0.59135681	0.12120107	3.16E-07	3.89E-06	ARHGAP11A
56670	ENSG00000198829	0	NA	NA	NA	NA	SUCNR1
3151	ENSG00000198830	9112.33729	-0.46333062	0.05136866	8.51E-20	3.13E-18	HMGN2
140606	ENSG00000198832	250.777898	0.18615459	0.15509778	0.18897766	0.38974187	SELENOM
51465	ENSG00000198833	1084.14498	-0.1785153	0.08004856	0.02239762	0.07975033	UBE2J1
57165	ENSG00000198835	27.869716	0.0370979	0.29031898	0.84807176	0.93095493	GJC2
4976	ENSG00000198836	1840.15507	-0.54835869	0.09724563	5.92E-09	9.18E-08	OPA1
9909	ENSG00000198837	2555.70492	0.08990513	0.07646714	0.23041296	0.44633794	DENND4B
6263	ENSG00000198838	30.8325622	-0.25059848	0.33962225	0.23339245	0.45030693	RYR3
11179	ENSG00000198839	452.356912	-0.35317032	0.11991093	0.00186986	0.01010294	ZNF277
4537	ENSG00000198840	10161.8723	-0.10582789	0.08734311	0.21296258	0.42289836	MT-ND3
112970	ENSG00000198841	230.746342	0.11889168	0.15320921	0.39765051	0.62683824	KTI12
92235	ENSG00000198842	0	NA	NA	NA	NA	STYXL2
51714	ENSG00000198843	716.682826	-0.25452419	0.13055541	0.03715676	0.11878666	SELENOT
22899	ENSG00000198844	0.16735865	0.01897177	0.38482933	0.78225479	NA	ARHGEF15
9760	ENSG00000198846	938.451958	0.05090143	0.08283305	0.53135538	0.7427669	TOX
1066	ENSG00000198848	0	NA	NA	NA	NA	CES1
916	ENSG00000198851	0	NA	NA	NA	NA	CD3E
9853	ENSG00000198853	892.678664	-0.05579578	0.10701445	0.58540616	0.78132918	RUSC2
100129271	ENSG00000198854	0	NA	NA	NA	NA	C1orf68
11153	ENSG00000198855	72.3927849	0.21051123	0.24049101	0.27170719	0.495102	FICD

58505	ENSG00000198856	924.732818	0.15373391	0.08600668	0.06646284	0.18460348	OSTC
NA	ENSG00000198857	1.50088203	-0.00795158	0.37605697	0.92070917	NA	HSD3BP5
91300	ENSG00000198858	621.630992	0.29063411	0.10124823	0.00285015	0.01450666	R3HDM4
116461	ENSG00000198860	898.444873	0.18245219	0.09042951	0.03765416	0.11997086	TSEN15
26046	ENSG00000198862	1337.45313	-0.30115298	0.08912385	0.00049596	0.003201	LTN1
146923	ENSG00000198863	433.485232	-0.1436491	0.11858668	0.20164965	0.40711601	RUNDC1
100129792	ENSG00000198865	354.215428	-0.08528275	0.14680044	0.52800861	0.73970419	CCDC152
NA	ENSG00000198868	0.32417112	-0.02327578	0.38532176	0.66840881	NA	MTND4LP30
169436	ENSG00000198870	29.7380799	-0.34590324	0.37014878	0.12766441	0.29808895	STKLD1
2869	ENSG00000198873	349.780582	-0.44998182	0.14145626	0.00063772	0.00398436	GRK5
55253	ENSG00000198874	566.458803	-0.02963058	0.10496638	0.76696016	0.89261979	TYW1
25853	ENSG00000198876	1677.16778	0.14945163	0.07095561	0.03202811	0.10562461	DCAF12
57713	ENSG00000198879	878.743796	-2.18249145	0.10206281	1.29E-102	4.07E-100	SFMBT2
142689	ENSG00000198881	2.69770731	0.13763045	0.40995838	0.14386669	0.3234718	ASB12
114824	ENSG00000198883	0	NA	NA	NA	NA	PNMA5
150771	ENSG00000198885	501.323514	-0.15938317	0.12582015	0.17882173	0.37479645	ITPRIPL1
4538	ENSG00000198886	104890.627	0.0843407	0.05504274	0.10788698	0.26339503	MT-ND4
23137	ENSG00000198887	1059.4413	-0.26188455	0.11217805	0.01426298	0.05539728	SMC5
4535	ENSG00000198888	45730.9262	-0.20497941	0.07851728	0.00768492	0.03336297	MT-ND1
139170	ENSG00000198889	0	NA	NA	NA	NA	DCAF12L1
55170	ENSG00000198890	1358.03713	-0.21783373	0.08984974	0.01243607	0.04962421	PRMT6
149345	ENSG00000198892	161.548455	0.06105297	0.17386987	0.69483418	0.85248347	SHISA4
85457	ENSG00000198894	597.595184	-0.23910587	0.12654997	0.04456473	0.13608807	CIPC
830	ENSG00000198898	1385.28356	-0.3206051	0.1112783	0.00251957	0.01306199	CAPZA2
4508	ENSG00000198899	57837.5034	0.05842909	0.07199323	0.40909552	0.63710637	MT-ATP6
7150	ENSG00000198900	2714.06263	-0.15423253	0.07576685	0.03753467	0.11968626	TOP1
9055	ENSG00000198901	3574.19917	-0.19308766	0.07958586	0.01294215	0.05127242	PRC1
80823	ENSG00000198908	204.162652	-0.62982475	0.22966541	0.00143782	0.00808111	BHLHB9
4215	ENSG00000198909	1317.9889	-0.17503486	0.10116645	0.07213562	0.19564875	MAP3K3
3897	ENSG00000198910	4384.36524	-2.59167258	0.07836554	3.54E-241	1.17E-237	L1CAM
6721	ENSG00000198911	4762.58512	-0.40384699	0.07291835	1.62E-08	2.37E-07	SREBF2
339448	ENSG00000198912	551.801202	0.03509184	0.11120521	0.74256255	0.87870936	C1orf174
5455	ENSG00000198914	260.263113	4.90564279	0.24849302	1.59E-87	3.71E-85	POU3F3
221002	ENSG00000198915	1.00287065	-6.68E-05	0.37920934	0.9991813	NA	RASGEF1A
51490	ENSG00000198917	1067.21663	0.11400414	0.09922489	0.23460022	0.45153942	SPOUT1
6170	ENSG00000198918	10130.3335	-0.08102999	0.05988478	0.17008284	0.36191941	RPL39
9666	ENSG00000198919	1028.35495	-0.13218797	0.09909899	0.1660398	0.35628699	DZIP3
9851	ENSG00000198920	570.051545	-0.16620697	0.13079838	0.17478702	0.36863767	KIAA0753

NA	ENSG00000198923	0	NA	NA	NA	NA	
9937	ENSG00000198924	611.740924	-0.47854731	0.12620449	5.73E-05	0.00046135	DCLRE1A
79065	ENSG00000198925	1197.06118	0.06798105	0.08307931	0.4012791	0.62960674	ATG9A
9722	ENSG00000198929	270.50543	-1.2712507	0.15682517	5.19E-17	1.60E-15	NOS1AP
158511	ENSG00000198930	424.150097	-0.38353618	0.12447478	0.00110254	0.00642609	CSAG1
353	ENSG00000198931	800.591211	0.13466852	0.09618434	0.14795746	0.33012187	APRT
9737	ENSG00000198932	402.050694	-0.37845329	0.12522466	0.00134794	0.00764088	GPRASP1
9755	ENSG00000198933	1091.10619	0.1705198	0.08254336	0.03423663	0.11149947	TBKBP1
57692	ENSG00000198934	148.444315	0.17601697	0.1801914	0.26907585	0.49211513	MAGEE1
154467	ENSG00000198937	568.906753	-0.36130532	0.103042	0.0002624	0.00181772	CCDC167
4514	ENSG00000198938	98239.8613	-0.09426941	0.06653311	0.15323606	0.33804794	MT-CO3
80108	ENSG00000198939	14.6130443	0.22213159	0.39955956	0.22950554	0.44510165	ZFP2
134548	ENSG00000198944	14.232951	-0.06736075	0.33273245	0.69753303	0.85389371	SOWAHA
84456	ENSG00000198945	168.766827	0.17105031	0.17602308	0.27470456	0.49850348	L3MBTL3
1756	ENSG00000198947	552.615257	1.48368943	0.1563498	1.93E-22	8.14E-21	DMD
9848	ENSG00000198948	219.869689	-0.26492217	0.24895932	0.1756084	0.36978038	MFAP3L
101928198	ENSG00000198948	219.869689	-0.26492217	0.24895932	0.1756084	0.36978038	MFAP3L
4668	ENSG00000198951	312.491905	0.01648413	0.13402681	0.89668482	0.95469109	NAGA
23381	ENSG00000198952	3653.8172	0.20319539	0.05776548	0.00037079	0.00247606	SMG5
26128	ENSG00000198954	513.611153	-0.15897169	0.11538498	0.14716089	0.32869736	KIFBP
7052	ENSG00000198959	306.996327	2.87852525	0.20484535	2.80E-46	2.50E-44	TGM2
54470	ENSG00000198960	415.435884	0.12059364	0.1160734	0.27571295	0.49980793	ARMCX6
9867	ENSG00000198961	1669.2489	-0.43793381	0.07928566	1.59E-08	2.34E-07	PJA2
6096	ENSG00000198963	1167.3719	-0.65926036	0.09073067	9.58E-14	2.35E-12	RORB
259230	ENSG00000198964	171.726866	-0.22634687	0.18189595	0.15708985	0.34385069	SGMS1
343406	ENSG00000198965	0	NA	NA	NA	NA	OR10R2
128368	ENSG00000198967	0	NA	NA	NA	NA	OR10Z1
406887	ENSG00000198972	0	NA	NA	NA	NA	MIRLET7E
494324	ENSG00000198973	0	NA	NA	NA	NA	MIR375
407034	ENSG00000198974	0	NA	NA	NA	NA	MIR30E
406882	ENSG00000198975	0	NA	NA	NA	NA	MIRLET7A2
554210	ENSG00000198976	0	NA	NA	NA	NA	MIR429
494329	ENSG00000198982	0	NA	NA	NA	NA	MIR380
554213	ENSG00000198983	0	NA	NA	NA	NA	MIR449A
442910	ENSG00000198984	0	NA	NA	NA	NA	MIR345
406951	ENSG00000198987	0	NA	NA	NA	NA	MIR16-2
442908	ENSG00000198995	0	NA	NA	NA	NA	MIR340
406901	ENSG00000198997	0	NA	NA	NA	NA	MIR107

554212	ENSG00000199001	0	NA	NA	NA	NA	MIR448
442915	ENSG00000199005	0	NA	NA	NA	NA	MIR370
574433	ENSG00000199012	0	NA	NA	NA	NA	MIR412
494326	ENSG00000199015	0	NA	NA	NA	NA	MIR377
406904	ENSG00000199017	0	NA	NA	NA	NA	MIR1-1
494330	ENSG00000199020	0	NA	NA	NA	NA	MIR381
442907	ENSG00000199023	0	NA	NA	NA	NA	MIR339
406896	ENSG00000199024	0	NA	NA	NA	NA	MIR103A2
442914	ENSG00000199025	0	NA	NA	NA	NA	MIR369
406885	ENSG00000199030	0	NA	NA	NA	NA	MIRLET7C
442916	ENSG00000199031	0	NA	NA	NA	NA	MIR371A
494337	ENSG00000199032	0	NA	NA	NA	NA	MIR425
406895	ENSG00000199035	0	NA	NA	NA	NA	MIR103A1
407002	ENSG00000199036	0	NA	NA	NA	NA	MIR219A1
406992	ENSG00000199038	0	NA	NA	NA	NA	MIR210
442904	ENSG00000199043	0	NA	NA	NA	NA	MIR335
494327	ENSG00000199047	0	NA	NA	NA	NA	MIR378A
494323	ENSG00000199051	0	NA	NA	NA	NA	MIR361
442898	ENSG00000199053	0	NA	NA	NA	NA	MIR324
442891	ENSG00000199059	0	NA	NA	NA	NA	MIR135B
406894	ENSG00000199065	0	NA	NA	NA	NA	MIR101-2
442897	ENSG00000199069	0	NA	NA	NA	NA	MIR323A
406888	ENSG00000199072	0	NA	NA	NA	NA	MIRLET7F1
407015	ENSG00000199075	0	NA	NA	NA	NA	MIR26A1
406918	ENSG00000199077	0	NA	NA	NA	NA	MIR129-2
442890	ENSG00000199080	0	NA	NA	NA	NA	MIR133B
442909	ENSG00000199082	0	NA	NA	NA	NA	MIR342
406940	ENSG00000199085	0	NA	NA	NA	NA	MIR148A
494328	ENSG00000199088	0	NA	NA	NA	NA	MIR379
442900	ENSG00000199090	0	NA	NA	NA	NA	MIR326
574434	ENSG00000199092	0	NA	NA	NA	NA	MIR410
407032	ENSG00000199094	0	NA	NA	NA	NA	MIR30C2
442917	ENSG00000199095	0	NA	NA	NA	NA	MIR372
442895	ENSG00000199102	0	NA	NA	NA	NA	MIR302C
442911	ENSG00000199104	0	NA	NA	NA	NA	MIR346
574413	ENSG00000199107	0	NA	NA	NA	NA	MIR409
693121	ENSG00000199109	0	NA	NA	NA	NA	MIR411
407017	ENSG00000199121	0	NA	NA	NA	NA	MIR26B

442892	ENSG00000199122	0	NA	NA	NA	NA	MIR148B
494332	ENSG00000199127	0	NA	NA	NA	NA	MIR383
100126355	ENSG00000199130	0	NA	NA	NA	NA	MIR365A
554214	ENSG00000199132	0	NA	NA	NA	NA	MIR450A1
406886	ENSG00000199133	0	NA	NA	NA	NA	MIRLET7D
406893	ENSG00000199135	0	NA	NA	NA	NA	MIR101-1
442918	ENSG00000199143	0	NA	NA	NA	NA	MIR373
442896	ENSG00000199145	0	NA	NA	NA	NA	MIR302D
406890	ENSG00000199150	0	NA	NA	NA	NA	MIRLET7G
442905	ENSG00000199151	0	NA	NA	NA	NA	MIR337
407033	ENSG00000199153	0	NA	NA	NA	NA	MIR30D
494334	ENSG00000199156	0	NA	NA	NA	NA	MIR422A
406990	ENSG00000199157	0	NA	NA	NA	NA	MIR208A
407053	ENSG00000199158	0	NA	NA	NA	NA	MIR96
406913	ENSG00000199161	0	NA	NA	NA	NA	MIR126
406881	ENSG00000199165	0	NA	NA	NA	NA	MIRLET7A1
442919	ENSG00000199168	0	NA	NA	NA	NA	MIR374A
442912	ENSG00000199169	0	NA	NA	NA	NA	MIR367
442903	ENSG00000199172	0	NA	NA	NA	NA	MIR331
407035	ENSG00000199177	0	NA	NA	NA	NA	MIR31
406891	ENSG00000199179	0	NA	NA	NA	NA	MIRLET7I
NA	ENSG00000199196	0	NA	NA	NA	NA	
NA	ENSG00000199197	0	NA	NA	NA	NA	RN7SKP72
NA	ENSG00000199200	0	NA	NA	NA	NA	
NA	ENSG00000199201	0	NA	NA	NA	NA	
NA	ENSG00000199202	0	NA	NA	NA	NA	RNA5SP289
NA	ENSG00000199203	0	NA	NA	NA	NA	
NA	ENSG00000199204	0	NA	NA	NA	NA	
26766	ENSG00000199212	0	NA	NA	NA	NA	RNU105C
NA	ENSG00000199217	0	NA	NA	NA	NA	RNU6-1123P
NA	ENSG00000199218	0	NA	NA	NA	NA	RN7SKP184
NA	ENSG00000199219	0	NA	NA	NA	NA	RNU6-500P
NA	ENSG00000199220	0	NA	NA	NA	NA	
NA	ENSG00000199222	0	NA	NA	NA	NA	
NA	ENSG00000199223	0	NA	NA	NA	NA	
NA	ENSG00000199224	0	NA	NA	NA	NA	
NA	ENSG00000199226	0	NA	NA	NA	NA	RNU6-50P
NA	ENSG00000199231	0	NA	NA	NA	NA	

NA	ENSG00000199237	0	NA	NA	NA	NA	RNU6-834P
NA	ENSG00000199240	0	NA	NA	NA	NA	RNA5SP46
NA	ENSG00000199241	0	NA	NA	NA	NA	
NA	ENSG00000199245	0	NA	NA	NA	NA	
NA	ENSG00000199246	0	NA	NA	NA	NA	RNU6-896P
NA	ENSG00000199248	0	NA	NA	NA	NA	RNU6-28P
NA	ENSG00000199251	0	NA	NA	NA	NA	RNU6-664P
NA	ENSG00000199260	0	NA	NA	NA	NA	RNU6-874P
NA	ENSG00000199263	0	NA	NA	NA	NA	
NA	ENSG00000199266	0	NA	NA	NA	NA	SNORA60
NA	ENSG00000199272	0	NA	NA	NA	NA	RNU6-349P
NA	ENSG00000199273	0	NA	NA	NA	NA	
NA	ENSG00000199276	0	NA	NA	NA	NA	RNA5SP26
NA	ENSG00000199279	0	NA	NA	NA	NA	RNU6-661P
NA	ENSG00000199282	0	NA	NA	NA	NA	
NA	ENSG00000199283	0	NA	NA	NA	NA	RNU1-58P
NA	ENSG00000199285	0	NA	NA	NA	NA	
NA	ENSG00000199286	0	NA	NA	NA	NA	RNU6-1094P
NA	ENSG00000199289	0	NA	NA	NA	NA	RNU6-502P
NA	ENSG00000199290	0	NA	NA	NA	NA	
NA	ENSG00000199291	0	NA	NA	NA	NA	
NA	ENSG00000199293	0	NA	NA	NA	NA	SNORA21
NA	ENSG00000199295	0	NA	NA	NA	NA	RNU6-1312P
NA	ENSG00000199299	0	NA	NA	NA	NA	RNA5SP430
NA	ENSG00000199301	0	NA	NA	NA	NA	RNU6-208P
NA	ENSG00000199303	0	NA	NA	NA	NA	
NA	ENSG00000199306	0	NA	NA	NA	NA	RNU6-858P
NA	ENSG00000199308	0	NA	NA	NA	NA	RNU6-222P
100033808	ENSG00000199311	0	NA	NA	NA	NA	SNORD115-34
NA	ENSG00000199313	0	NA	NA	NA	NA	RNU4-82P
NA	ENSG00000199315	0	NA	NA	NA	NA	RNA5SP347
NA	ENSG00000199318	0	NA	NA	NA	NA	RNA5SP52
NA	ENSG00000199319	0.173124	-0.01911262	0.38486262	0.84537521	NA	RN7SKP25
NA	ENSG00000199321	0	NA	NA	NA	NA	
NA	ENSG00000199322	0	NA	NA	NA	NA	RNA5SP183
NA	ENSG00000199325	0	NA	NA	NA	NA	RNU4-39P
NA	ENSG00000199326	0	NA	NA	NA	NA	RNU6-1298P
NA	ENSG00000199327	0	NA	NA	NA	NA	RNU6-230P

NA	ENSG00000199331	0	NA	NA	NA	NA	
NA	ENSG00000199332	0	NA	NA	NA	NA	
NA	ENSG00000199335	0	NA	NA	NA	NA	RNU6-204P
NA	ENSG00000199347	0	NA	NA	NA	NA	RNU5E-1
NA	ENSG00000199348	0	NA	NA	NA	NA	RNU6-766P
NA	ENSG00000199349	0	NA	NA	NA	NA	
NA	ENSG00000199350	0	NA	NA	NA	NA	RNA5SP432
100169751	ENSG00000199352	0.15069912	-0.01848503	0.38480575	0.84537521	NA	RNA5S1
NA	ENSG00000199354	0	NA	NA	NA	NA	RNA5SP273
NA	ENSG00000199357	0	NA	NA	NA	NA	
NA	ENSG00000199360	0	NA	NA	NA	NA	RNU6-1115P
NA	ENSG00000199361	0	NA	NA	NA	NA	RNU1-150P
NA	ENSG00000199362	0	NA	NA	NA	NA	
NA	ENSG00000199363	0	NA	NA	NA	NA	SNORA63E
NA	ENSG00000199364	0	NA	NA	NA	NA	RNA5SP327
NA	ENSG00000199366	0	NA	NA	NA	NA	
NA	ENSG00000199368	0	NA	NA	NA	NA	RNU6-1084P
NA	ENSG00000199370	0	NA	NA	NA	NA	
NA	ENSG00000199373	0	NA	NA	NA	NA	RNA5SP453
26828	ENSG00000199377	0	NA	NA	NA	NA	RNU5F-1
NA	ENSG00000199378	0	NA	NA	NA	NA	
NA	ENSG00000199381	0	NA	NA	NA	NA	RNU6-79P
NA	ENSG00000199385	0	NA	NA	NA	NA	RNU6-369P
767583	ENSG00000199390	0	NA	NA	NA	NA	SNORD114-7
NA	ENSG00000199392	0	NA	NA	NA	NA	
NA	ENSG00000199394	0	NA	NA	NA	NA	RNU6-600P
NA	ENSG00000199395	0	NA	NA	NA	NA	RNA5SP93
NA	ENSG00000199398	0	NA	NA	NA	NA	
NA	ENSG00000199400	0	NA	NA	NA	NA	RNY4P19
NA	ENSG00000199402	0	NA	NA	NA	NA	RNA5SP308
109617001	ENSG00000199405	0	NA	NA	NA	NA	SNORA1B
NA	ENSG00000199407	0	NA	NA	NA	NA	RNA5SP301
NA	ENSG00000199409	0	NA	NA	NA	NA	
NA	ENSG00000199410	0	NA	NA	NA	NA	
NA	ENSG00000199411	0	NA	NA	NA	NA	
NA	ENSG00000199415	0.14931947	-0.01844332	0.38480212	0.84537521	NA	RNA5SP370
NA	ENSG00000199420	0	NA	NA	NA	NA	RN7SKP170
NA	ENSG00000199422	0	NA	NA	NA	NA	VTRNA3-1P

NA	ENSG00000199424	0	NA	NA	NA	NA	
NA	ENSG00000199426	0	NA	NA	NA	NA	RNU1-108P
692053	ENSG00000199436	0	NA	NA	NA	NA	SNORD9
NA	ENSG00000199440	0	NA	NA	NA	NA	
NA	ENSG00000199442	0	NA	NA	NA	NA	
NA	ENSG00000199444	0	NA	NA	NA	NA	
NA	ENSG00000199446	0	NA	NA	NA	NA	RNU6-543P
NA	ENSG00000199448	0	NA	NA	NA	NA	RNU6-845P
NA	ENSG00000199450	0	NA	NA	NA	NA	RNA5SP276
NA	ENSG00000199454	0	NA	NA	NA	NA	RNA5SP388
NA	ENSG00000199455	0	NA	NA	NA	NA	RNA5SP191
NA	ENSG00000199458	0	NA	NA	NA	NA	RNU4-35P
NA	ENSG00000199459	0	NA	NA	NA	NA	
NA	ENSG00000199460	0	NA	NA	NA	NA	RNU6-1216P
NA	ENSG00000199461	0	NA	NA	NA	NA	
NA	ENSG00000199466	0	NA	NA	NA	NA	
NA	ENSG00000199468	0	NA	NA	NA	NA	RNU6-556P
NA	ENSG00000199469	0	NA	NA	NA	NA	RNU1-62P
NA	ENSG00000199470	0	NA	NA	NA	NA	
NA	ENSG00000199471	0	NA	NA	NA	NA	
NA	ENSG00000199472	0	NA	NA	NA	NA	
NA	ENSG00000199473	0	NA	NA	NA	NA	
NA	ENSG00000199474	0	NA	NA	NA	NA	
NA	ENSG00000199475	0	NA	NA	NA	NA	RN7SKP104
NA	ENSG00000199476	0	NA	NA	NA	NA	
677814	ENSG00000199477	0	NA	NA	NA	NA	SNORA31
NA	ENSG00000199480	0	NA	NA	NA	NA	RNA5SP389
NA	ENSG00000199482	0	NA	NA	NA	NA	RNU6-633P
NA	ENSG00000199483	0	NA	NA	NA	NA	RNU4-15P
NA	ENSG00000199487	0	NA	NA	NA	NA	
NA	ENSG00000199488	0	NA	NA	NA	NA	RNU1-70P
100033801	ENSG00000199489	0	NA	NA	NA	NA	SNORD115-25
NA	ENSG00000199490	0	NA	NA	NA	NA	
NA	ENSG00000199492	0	NA	NA	NA	NA	RNU6-1313P
NA	ENSG00000199497	0.173733	0.01914545	0.38484597	0.78225479	NA	RNU1-94P
NA	ENSG00000199506	0	NA	NA	NA	NA	RNU6-247P
NA	ENSG00000199508	0	NA	NA	NA	NA	RNA5SP110
NA	ENSG00000199509	0	NA	NA	NA	NA	RNA5SP477

NA	ENSG00000199512	0	NA	NA	NA	NA	RNU6-212P
NA	ENSG00000199514	0	NA	NA	NA	NA	RNU6-235P
NA	ENSG00000199515	0	NA	NA	NA	NA	
NA	ENSG00000199516	0	NA	NA	NA	NA	
NA	ENSG00000199520	0	NA	NA	NA	NA	RNU6-386P
NA	ENSG00000199523	0	NA	NA	NA	NA	RNA5SP226
NA	ENSG00000199525	0	NA	NA	NA	NA	RNA5SP295
NA	ENSG00000199529	0	NA	NA	NA	NA	RNU6-462P
NA	ENSG00000199530	0	NA	NA	NA	NA	
NA	ENSG00000199535	0	NA	NA	NA	NA	RNA5SP305
NA	ENSG00000199536	0	NA	NA	NA	NA	RNU6-315P
NA	ENSG00000199540	0	NA	NA	NA	NA	
NA	ENSG00000199545	0	NA	NA	NA	NA	RNA5SP195
NA	ENSG00000199546	0	NA	NA	NA	NA	
NA	ENSG00000199550	0	NA	NA	NA	NA	
NA	ENSG00000199551	0	NA	NA	NA	NA	RNU6-545P
NA	ENSG00000199552	0	NA	NA	NA	NA	
NA	ENSG00000199556	0	NA	NA	NA	NA	RNA5SP361
NA	ENSG00000199562	0	NA	NA	NA	NA	RNU6-37P
NA	ENSG00000199564	0	NA	NA	NA	NA	RNA5SP502
NA	ENSG00000199565	0	NA	NA	NA	NA	
NA	ENSG00000199566	0	NA	NA	NA	NA	
NA	ENSG00000199567	0	NA	NA	NA	NA	
26831	ENSG00000199568	0	NA	NA	NA	NA	RNU5A-1
NA	ENSG00000199570	0	NA	NA	NA	NA	RNU6-228P
NA	ENSG00000199571	0	NA	NA	NA	NA	
NA	ENSG00000199572	0	NA	NA	NA	NA	RNA5SP174
595100	ENSG00000199574	0	NA	NA	NA	NA	SNORD18C
767577	ENSG00000199575	0	NA	NA	NA	NA	SNORD114-1
NA	ENSG00000199577	0	NA	NA	NA	NA	RNU6-822P
NA	ENSG00000199580	0	NA	NA	NA	NA	
NA	ENSG00000199584	0	NA	NA	NA	NA	
NA	ENSG00000199585	0	NA	NA	NA	NA	RNA5SP119
NA	ENSG00000199591	0	NA	NA	NA	NA	
NA	ENSG00000199592	0	NA	NA	NA	NA	RNA5SP321
767592	ENSG00000199593	0	NA	NA	NA	NA	SNORD114-14
NA	ENSG00000199594	0	NA	NA	NA	NA	RNU6-1301P
NA	ENSG00000199595	0	NA	NA	NA	NA	

NA	ENSG00000199598	0	NA	NA	NA	NA	RNU6-859P
NA	ENSG00000199601	0	NA	NA	NA	NA	RNU6-562P
NA	ENSG00000199603	0	NA	NA	NA	NA	RNU6-951P
NA	ENSG00000199605	0	NA	NA	NA	NA	RNY4P24
NA	ENSG00000199609	0	NA	NA	NA	NA	RNA5SP124
NA	ENSG00000199620	0	NA	NA	NA	NA	RNA5SP258
NA	ENSG00000199622	0	NA	NA	NA	NA	RN7SKP20
NA	ENSG00000199626	0	NA	NA	NA	NA	RNU6-989P
NA	ENSG00000199627	0	NA	NA	NA	NA	RNU6-1010P
NA	ENSG00000199629	0	NA	NA	NA	NA	RNU1-14P
NA	ENSG00000199630	0	NA	NA	NA	NA	
26818	ENSG00000199631	0	NA	NA	NA	NA	SNORD33
NA	ENSG00000199633	0	NA	NA	NA	NA	
NA	ENSG00000199634	0	NA	NA	NA	NA	RNU4-79P
NA	ENSG00000199635	0	NA	NA	NA	NA	
NA	ENSG00000199636	0	NA	NA	NA	NA	
NA	ENSG00000199638	0	NA	NA	NA	NA	RNA5SP319
NA	ENSG00000199640	0	NA	NA	NA	NA	RN7SKP294
NA	ENSG00000199643	0	NA	NA	NA	NA	RNU4-90P
NA	ENSG00000199645	0	NA	NA	NA	NA	RNU6-1330P
NA	ENSG00000199646	0	NA	NA	NA	NA	RNU6-1272P
NA	ENSG00000199652	0	NA	NA	NA	NA	RNU1-35P
NA	ENSG00000199664	0	NA	NA	NA	NA	RNU6-1266P
109616984	ENSG00000199666	0	NA	NA	NA	NA	SNORD3G
NA	ENSG00000199667	0	NA	NA	NA	NA	
NA	ENSG00000199668	0	NA	NA	NA	NA	
NA	ENSG00000199672	0	NA	NA	NA	NA	RNU4-21P
NA	ENSG00000199673	0	NA	NA	NA	NA	SNORD16
NA	ENSG00000199674	0	NA	NA	NA	NA	RNU6-862P
NA	ENSG00000199676	0	NA	NA	NA	NA	
NA	ENSG00000199677	0	NA	NA	NA	NA	
NA	ENSG00000199683	0.15069912	-0.01848503	0.38480575	0.84537521	NA	RN7SKP185
NA	ENSG00000199687	0	NA	NA	NA	NA	RNU1-38P
NA	ENSG00000199691	2.78253675	0.12034343	0.39932476	0.21966157	0.43175718	RN7SKP173
NA	ENSG00000199695	0	NA	NA	NA	NA	RNU6-1128P
NA	ENSG00000199697	0	NA	NA	NA	NA	RNU6-446P
NA	ENSG00000199698	0	NA	NA	NA	NA	
NA	ENSG00000199700	0	NA	NA	NA	NA	RNU6-223P

NA	ENSG00000199701	0	NA	NA	NA	NA	
NA	ENSG00000199702	0	NA	NA	NA	NA	RNU6-170P
NA	ENSG00000199705	0	NA	NA	NA	NA	
NA	ENSG00000199709	0	NA	NA	NA	NA	RNU4-23P
NA	ENSG00000199710	0	NA	NA	NA	NA	
NA	ENSG00000199711	0	NA	NA	NA	NA	
100033437	ENSG00000199712	0	NA	NA	NA	NA	SNORD115-2
NA	ENSG00000199713	0	NA	NA	NA	NA	
NA	ENSG00000199715	0	NA	NA	NA	NA	
NA	ENSG00000199716	0	NA	NA	NA	NA	
NA	ENSG00000199719	3.25044116	0.07247012	0.3798361	0.47935997	0.70064538	RN7SKP74
NA	ENSG00000199728	0	NA	NA	NA	NA	RNU6-655P
NA	ENSG00000199730	0	NA	NA	NA	NA	RN7SKP95
NA	ENSG00000199731	0	NA	NA	NA	NA	RNU6-1079P
NA	ENSG00000199732	0	NA	NA	NA	NA	
NA	ENSG00000199733	0	NA	NA	NA	NA	RNA5SP307
NA	ENSG00000199735	0	NA	NA	NA	NA	RNU6-227P
NA	ENSG00000199739	0	NA	NA	NA	NA	RNU6-552P
NA	ENSG00000199740	0	NA	NA	NA	NA	
26815	ENSG00000199744	0	NA	NA	NA	NA	SNORD36A
NA	ENSG00000199751	0	NA	NA	NA	NA	
NA	ENSG00000199753	0	NA	NA	NA	NA	SNORD104
NA	ENSG00000199755	0	NA	NA	NA	NA	
NA	ENSG00000199756	0	NA	NA	NA	NA	
NA	ENSG00000199762	0	NA	NA	NA	NA	
NA	ENSG00000199764	0	NA	NA	NA	NA	
NA	ENSG00000199765	0	NA	NA	NA	NA	RNU6-363P
NA	ENSG00000199767	0	NA	NA	NA	NA	RNU6-78P
NA	ENSG00000199771	0	NA	NA	NA	NA	RNA5SP426
NA	ENSG00000199773	0	NA	NA	NA	NA	RNU1-76P
NA	ENSG00000199780	0	NA	NA	NA	NA	
NA	ENSG00000199781	0	NA	NA	NA	NA	
100033446	ENSG00000199782	0	NA	NA	NA	NA	SNORD115-9
NA	ENSG00000199783	0	NA	NA	NA	NA	
NA	ENSG00000199784	0	NA	NA	NA	NA	RNU6-1287P
619565	ENSG00000199785	0	NA	NA	NA	NA	SNORA52
NA	ENSG00000199786	0	NA	NA	NA	NA	RNA5SP188
109616959	ENSG00000199787	0	NA	NA	NA	NA	SNORA80C

NA	ENSG00000199788	0	NA	NA	NA	NA	RNY3P2
NA	ENSG00000199790	0	NA	NA	NA	NA	RNU6-836P
NA	ENSG00000199791	0	NA	NA	NA	NA	RNU6-451P
NA	ENSG00000199792	0	NA	NA	NA	NA	RNU6-1233P
NA	ENSG00000199796	0	NA	NA	NA	NA	RNU6-924P
NA	ENSG00000199797	0	NA	NA	NA	NA	
767581	ENSG00000199798	0	NA	NA	NA	NA	SNORD114-5
NA	ENSG00000199801	0	NA	NA	NA	NA	
NA	ENSG00000199803	0	NA	NA	NA	NA	RNU6-1159P
NA	ENSG00000199804	0	NA	NA	NA	NA	RNA5SP383
NA	ENSG00000199805	0	NA	NA	NA	NA	RNU1-134P
NA	ENSG00000199806	0	NA	NA	NA	NA	RNA5SP491
NA	ENSG00000199809	0	NA	NA	NA	NA	RNA5SP374
NA	ENSG00000199812	0	NA	NA	NA	NA	RNU6-428P
NA	ENSG00000199814	0	NA	NA	NA	NA	RNU6-1260P
NA	ENSG00000199815	0	NA	NA	NA	NA	
NA	ENSG00000199824	0	NA	NA	NA	NA	RNU6-199P
NA	ENSG00000199827	0	NA	NA	NA	NA	RNU6-1290P
NA	ENSG00000199831	0	NA	NA	NA	NA	RN7SKP291
NA	ENSG00000199832	0	NA	NA	NA	NA	
100033603	ENSG00000199833	0	NA	NA	NA	NA	SNORD115-21
NA	ENSG00000199836	0	NA	NA	NA	NA	RNU1-47P
NA	ENSG00000199839	0	NA	NA	NA	NA	RNA5SP150
NA	ENSG00000199840	0	NA	NA	NA	NA	
NA	ENSG00000199843	0	NA	NA	NA	NA	RNA5SP358
NA	ENSG00000199845	0	NA	NA	NA	NA	RNA5SP375
NA	ENSG00000199846	0	NA	NA	NA	NA	RNU1-72P
NA	ENSG00000199849	0	NA	NA	NA	NA	RNU5F-6P
NA	ENSG00000199851	0	NA	NA	NA	NA	
NA	ENSG00000199855	0	NA	NA	NA	NA	RNU6-490P
NA	ENSG00000199856	0	NA	NA	NA	NA	
NA	ENSG00000199857	0	NA	NA	NA	NA	
NA	ENSG00000199858	0	NA	NA	NA	NA	RNU6-1120P
NA	ENSG00000199859	0	NA	NA	NA	NA	RNU6-582P
NA	ENSG00000199862	0	NA	NA	NA	NA	
NA	ENSG00000199865	0	NA	NA	NA	NA	RNU6-495P
NA	ENSG00000199866	0	NA	NA	NA	NA	
NA	ENSG00000199867	0	NA	NA	NA	NA	

NA	ENSG00000199872	0	NA	NA	NA	NA	RNU6-942P
NA	ENSG00000199874	0	NA	NA	NA	NA	RNA5SP452
NA	ENSG00000199875	0	NA	NA	NA	NA	
NA	ENSG00000199878	0	NA	NA	NA	NA	RN7SKP149
NA	ENSG00000199879	0	NA	NA	NA	NA	RNVU1-22
NA	ENSG00000199880	0	NA	NA	NA	NA	RNU6-888P
NA	ENSG00000199881	0	NA	NA	NA	NA	
NA	ENSG00000199883	0	NA	NA	NA	NA	RN7SKP90
NA	ENSG00000199884	0	NA	NA	NA	NA	
NA	ENSG00000199885	0	NA	NA	NA	NA	RNU6-330P
NA	ENSG00000199886	0	NA	NA	NA	NA	RNU6-249P
NA	ENSG00000199890	0	NA	NA	NA	NA	
NA	ENSG00000199892	0	NA	NA	NA	NA	RNU1-17P
NA	ENSG00000199894	0	NA	NA	NA	NA	
NA	ENSG00000199895	0	NA	NA	NA	NA	
NA	ENSG00000199899	0	NA	NA	NA	NA	
NA	ENSG00000199901	0	NA	NA	NA	NA	
NA	ENSG00000199903	0	NA	NA	NA	NA	RNU6-1273P
NA	ENSG00000199905	0	NA	NA	NA	NA	RNU6-492P
NA	ENSG00000199906	0	NA	NA	NA	NA	RNU5B-2P
NA	ENSG00000199911	0	NA	NA	NA	NA	
NA	ENSG00000199912	0	NA	NA	NA	NA	
767594	ENSG00000199914	0	NA	NA	NA	NA	SNORD114-16
NA	ENSG00000199920	0	NA	NA	NA	NA	RNU4-44P
NA	ENSG00000199921	0	NA	NA	NA	NA	RNU6-161P
NA	ENSG00000199924	0	NA	NA	NA	NA	RNU6-1252P
NA	ENSG00000199927	0	NA	NA	NA	NA	
NA	ENSG00000199929	0	NA	NA	NA	NA	RNA5SP405
NA	ENSG00000199932	0	NA	NA	NA	NA	RNU1-18P
NA	ENSG00000199933	0	NA	NA	NA	NA	RNY1P16
NA	ENSG00000199934	0	NA	NA	NA	NA	
NA	ENSG00000199936	0	NA	NA	NA	NA	
NA	ENSG00000199938	0	NA	NA	NA	NA	
NA	ENSG00000199940	0	NA	NA	NA	NA	RN7SKP75
767597	ENSG00000199942	0	NA	NA	NA	NA	SNORD114-19
NA	ENSG00000199944	0	NA	NA	NA	NA	RNU6-653P
NA	ENSG00000199949	0	NA	NA	NA	NA	
NA	ENSG00000199953	0	NA	NA	NA	NA	RNA5SP443

NA	ENSG00000199959	0	NA	NA	NA	NA	
100033451	ENSG00000199960	0	NA	NA	NA	NA	SNORD115-14
677849	ENSG00000199961	0	NA	NA	NA	NA	SNORD1B
NA	ENSG00000199962	0	NA	NA	NA	NA	
NA	ENSG00000199963	0	NA	NA	NA	NA	RNU6-605P
NA	ENSG00000199964	0	NA	NA	NA	NA	
100033440	ENSG00000199970	0	NA	NA	NA	NA	SNORD115-3
NA	ENSG00000199971	0	NA	NA	NA	NA	RNU6-797P
NA	ENSG00000199975	0	NA	NA	NA	NA	RN7SKP243
NA	ENSG00000199977	0	NA	NA	NA	NA	
NA	ENSG00000199979	0	NA	NA	NA	NA	
NA	ENSG00000199985	0	NA	NA	NA	NA	RNA5SP264
NA	ENSG00000199986	0	NA	NA	NA	NA	RNU6-486P
56664	ENSG00000199990	0	NA	NA	NA	NA	VTRNA1-1
NA	ENSG00000199994	0	NA	NA	NA	NA	RNA5SP145
NA	ENSG00000200003	0	NA	NA	NA	NA	RNU6-986P
NA	ENSG00000200008	0	NA	NA	NA	NA	
NA	ENSG00000200011	0	NA	NA	NA	NA	
NA	ENSG00000200013	0	NA	NA	NA	NA	RNU6-623P
NA	ENSG00000200021	0	NA	NA	NA	NA	RNA5SP448
NA	ENSG00000200024	0	NA	NA	NA	NA	
NA	ENSG00000200026	0	NA	NA	NA	NA	
NA	ENSG00000200028	0	NA	NA	NA	NA	RNA5SP98
NA	ENSG00000200029	0	NA	NA	NA	NA	RNU6-642P
NA	ENSG00000200033	0	NA	NA	NA	NA	RNU6-403P
NA	ENSG00000200034	0	NA	NA	NA	NA	RNU4-73P
NA	ENSG00000200036	0	NA	NA	NA	NA	RNA5SP65
NA	ENSG00000200040	0	NA	NA	NA	NA	
NA	ENSG00000200041	0	NA	NA	NA	NA	
NA	ENSG00000200047	0	NA	NA	NA	NA	RN7SKP115
NA	ENSG00000200048	0	NA	NA	NA	NA	RNU6-812P
NA	ENSG00000200049	0	NA	NA	NA	NA	
NA	ENSG00000200051	0	NA	NA	NA	NA	
NA	ENSG00000200052	0	NA	NA	NA	NA	
NA	ENSG00000200058	0	NA	NA	NA	NA	RNA5SP218
NA	ENSG00000200059	0	NA	NA	NA	NA	
NA	ENSG00000200060	0	NA	NA	NA	NA	
NA	ENSG00000200062	0	NA	NA	NA	NA	RNU4-58P

NA	ENSG00000200063	0	NA	NA	NA	NA	
NA	ENSG00000200064	0	NA	NA	NA	NA	RNY4P9
NA	ENSG00000200065	0	NA	NA	NA	NA	
NA	ENSG00000200070	0	NA	NA	NA	NA	RNU4-80P
NA	ENSG00000200072	0	NA	NA	NA	NA	
NA	ENSG00000200075	0	NA	NA	NA	NA	
NA	ENSG00000200079	0	NA	NA	NA	NA	RNA5SP326
NA	ENSG00000200084	0	NA	NA	NA	NA	SNORD68
NA	ENSG00000200085	0	NA	NA	NA	NA	
NA	ENSG00000200086	0	NA	NA	NA	NA	RNU6-433P
26768	ENSG00000200087	2.46684816	-0.00368137	0.37209828	0.96765836	NA	SNORA73B
767612	ENSG00000200089	0	NA	NA	NA	NA	SNORD114-31
NA	ENSG00000200090	0	NA	NA	NA	NA	
NA	ENSG00000200091	0	NA	NA	NA	NA	RN7SKP163
NA	ENSG00000200095	0	NA	NA	NA	NA	RNU6-181P
NA	ENSG00000200097	0	NA	NA	NA	NA	RNU6-1167P
NA	ENSG00000200101	0	NA	NA	NA	NA	RNU6-508P
NA	ENSG00000200102	0	NA	NA	NA	NA	RNU6-252P
NA	ENSG00000200105	0	NA	NA	NA	NA	RNU6-251P
NA	ENSG00000200106	0	NA	NA	NA	NA	RNU6-984P
NA	ENSG00000200107	0	NA	NA	NA	NA	RNU6-1249P
NA	ENSG00000200108	0	NA	NA	NA	NA	RNU6-774P
NA	ENSG00000200112	0	NA	NA	NA	NA	
NA	ENSG00000200113	0	NA	NA	NA	NA	
NA	ENSG00000200114	0	NA	NA	NA	NA	RNA5SP123
NA	ENSG00000200118	0	NA	NA	NA	NA	
NA	ENSG00000200120	0	NA	NA	NA	NA	
NA	ENSG00000200121	0	NA	NA	NA	NA	
NA	ENSG00000200131	0	NA	NA	NA	NA	RN7SKP77
NA	ENSG00000200132	0	NA	NA	NA	NA	RNU6-1021P
NA	ENSG00000200135	0	NA	NA	NA	NA	
NA	ENSG00000200138	0	NA	NA	NA	NA	RNY1P10
NA	ENSG00000200139	0	NA	NA	NA	NA	RNU6-778P
NA	ENSG00000200142	0	NA	NA	NA	NA	
NA	ENSG00000200146	0	NA	NA	NA	NA	RNU6-544P
NA	ENSG00000200150	0	NA	NA	NA	NA	
NA	ENSG00000200151	0	NA	NA	NA	NA	RN7SKP231
NA	ENSG00000200152	0	NA	NA	NA	NA	RNU6-216P

NA	ENSG00000200153	0	NA	NA	NA	NA	RNU6-23P
26832	ENSG00000200156	0	NA	NA	NA	NA	RNU5B-1
NA	ENSG00000200161	0	NA	NA	NA	NA	RN7SKP145
NA	ENSG00000200162	0	NA	NA	NA	NA	
NA	ENSG00000200164	0	NA	NA	NA	NA	
NA	ENSG00000200168	0	NA	NA	NA	NA	RNA5SP350
NA	ENSG00000200169	0	NA	NA	NA	NA	RNU5D-1
NA	ENSG00000200170	0	NA	NA	NA	NA	
NA	ENSG00000200171	0	NA	NA	NA	NA	
NA	ENSG00000200174	0	NA	NA	NA	NA	
NA	ENSG00000200175	0	NA	NA	NA	NA	RNU6-1309P
NA	ENSG00000200176	0	NA	NA	NA	NA	RNU1-19P
NA	ENSG00000200179	0	NA	NA	NA	NA	
NA	ENSG00000200183	0	NA	NA	NA	NA	RNU6-238P
NA	ENSG00000200184	0	NA	NA	NA	NA	RNU1-20P
NA	ENSG00000200189	0	NA	NA	NA	NA	RNU6-832P
NA	ENSG00000200191	0	NA	NA	NA	NA	
NA	ENSG00000200197	0	NA	NA	NA	NA	RNU1-21P
NA	ENSG00000200198	0	NA	NA	NA	NA	RN7SKP211
NA	ENSG00000200201	0	NA	NA	NA	NA	
NA	ENSG00000200204	0	NA	NA	NA	NA	RNU1-22P
NA	ENSG00000200206	0	NA	NA	NA	NA	
NA	ENSG00000200208	0	NA	NA	NA	NA	
NA	ENSG00000200209	0	NA	NA	NA	NA	
NA	ENSG00000200211	0	NA	NA	NA	NA	RNY4P27
NA	ENSG00000200213	0	NA	NA	NA	NA	RNU6-992P
767566	ENSG00000200215	0	NA	NA	NA	NA	SNORD113-6
NA	ENSG00000200216	0	NA	NA	NA	NA	RNU6-745P
NA	ENSG00000200217	0	NA	NA	NA	NA	RNU6-839P
NA	ENSG00000200218	0	NA	NA	NA	NA	RNU6-697P
NA	ENSG00000200220	0	NA	NA	NA	NA	RNU6-179P
NA	ENSG00000200222	0	NA	NA	NA	NA	
NA	ENSG00000200224	0	NA	NA	NA	NA	RNU6-626P
NA	ENSG00000200225	0	NA	NA	NA	NA	RNA5SP382
NA	ENSG00000200227	0	NA	NA	NA	NA	RNA5SP197
NA	ENSG00000200231	0	NA	NA	NA	NA	RNU1-23P
NA	ENSG00000200235	0	NA	NA	NA	NA	
NA	ENSG00000200237	0	NA	NA	NA	NA	

NA	ENSG00000200238	0	NA	NA	NA	NA	RNA5SP133
NA	ENSG00000200241	0	NA	NA	NA	NA	
NA	ENSG00000200243	0	NA	NA	NA	NA	RN7SKP79
NA	ENSG00000200246	0	NA	NA	NA	NA	RNA5SP263
NA	ENSG00000200247	0	NA	NA	NA	NA	RNU6-254P
NA	ENSG00000200248	0	NA	NA	NA	NA	RNA5SP214
NA	ENSG00000200250	0	NA	NA	NA	NA	RNU6-1147P
NA	ENSG00000200252	0	NA	NA	NA	NA	
NA	ENSG00000200253	0	NA	NA	NA	NA	RNU6-529P
NA	ENSG00000200254	0	NA	NA	NA	NA	RNU6-536P
NA	ENSG00000200256	0	NA	NA	NA	NA	
NA	ENSG00000200257	0	NA	NA	NA	NA	RNU6-97P
26816	ENSG00000200259	0	NA	NA	NA	NA	SNORD35A
NA	ENSG00000200261	0	NA	NA	NA	NA	
NA	ENSG00000200262	0	NA	NA	NA	NA	
NA	ENSG00000200267	0	NA	NA	NA	NA	RNU6-66P
NA	ENSG00000200269	0	NA	NA	NA	NA	RNU6-838P
NA	ENSG00000200274	0	NA	NA	NA	NA	RNU4-32P
NA	ENSG00000200275	0	NA	NA	NA	NA	RNA5SP199
NA	ENSG00000200278	0	NA	NA	NA	NA	RNA5SP352
767588	ENSG00000200279	0	NA	NA	NA	NA	SNORD114-10
NA	ENSG00000200281	0	NA	NA	NA	NA	RNU6-624P
NA	ENSG00000200283	0	NA	NA	NA	NA	
NA	ENSG00000200287	0	NA	NA	NA	NA	
NA	ENSG00000200288	0	NA	NA	NA	NA	
NA	ENSG00000200291	0	NA	NA	NA	NA	
NA	ENSG00000200293	0.15069912	-0.01848503	0.38480575	0.84537521	NA	RNA5SP393
NA	ENSG00000200294	0	NA	NA	NA	NA	
NA	ENSG00000200295	0	NA	NA	NA	NA	RNU6-527P
NA	ENSG00000200296	0	NA	NA	NA	NA	RNU1-83P
NA	ENSG00000200298	0	NA	NA	NA	NA	
NA	ENSG00000200301	0	NA	NA	NA	NA	RNA5SP483
NA	ENSG00000200303	0	NA	NA	NA	NA	RNU6-940P
NA	ENSG00000200304	0	NA	NA	NA	NA	RNU6-1255P
NA	ENSG00000200305	0	NA	NA	NA	NA	
NA	ENSG00000200309	0	NA	NA	NA	NA	
NA	ENSG00000200310	0	NA	NA	NA	NA	RNA5SP215
NA	ENSG00000200312	0	NA	NA	NA	NA	RN7SKP255

NA	ENSG00000200313	0	NA	NA	NA	NA	RNA5-8SP7
NA	ENSG00000200314	0	NA	NA	NA	NA	
NA	ENSG00000200318	0	NA	NA	NA	NA	SNORD3P1
NA	ENSG00000200320	0	NA	NA	NA	NA	SNORA63
NA	ENSG00000200325	0	NA	NA	NA	NA	
NA	ENSG00000200326	0	NA	NA	NA	NA	RNA5SP391
NA	ENSG00000200327	0	NA	NA	NA	NA	RNA5SP62
NA	ENSG00000200331	0	NA	NA	NA	NA	RNU6-183P
NA	ENSG00000200332	0	NA	NA	NA	NA	
NA	ENSG00000200334	0	NA	NA	NA	NA	
NA	ENSG00000200336	0	NA	NA	NA	NA	RNA5SP333
NA	ENSG00000200338	0	NA	NA	NA	NA	RNU1-49P
NA	ENSG00000200340	0	NA	NA	NA	NA	RNU1-105P
NA	ENSG00000200344	0	NA	NA	NA	NA	
NA	ENSG00000200345	0	NA	NA	NA	NA	RNU6-485P
NA	ENSG00000200350	0	NA	NA	NA	NA	RNU6-1285P
NA	ENSG00000200351	0	NA	NA	NA	NA	
NA	ENSG00000200354	0	NA	NA	NA	NA	SNORA71D
NA	ENSG00000200355	0	NA	NA	NA	NA	
NA	ENSG00000200356	0	NA	NA	NA	NA	RNU6-833P
NA	ENSG00000200360	0	NA	NA	NA	NA	RNU6-792P
NA	ENSG00000200361	0	NA	NA	NA	NA	
NA	ENSG00000200366	0	NA	NA	NA	NA	RNU6-511P
767568	ENSG00000200367	0	NA	NA	NA	NA	SNORD113-8
NA	ENSG00000200369	0	NA	NA	NA	NA	RNU6-666P
NA	ENSG00000200372	0	NA	NA	NA	NA	RNU5E-8P
NA	ENSG00000200376	0	NA	NA	NA	NA	RNU5E-10P
NA	ENSG00000200377	0	NA	NA	NA	NA	
NA	ENSG00000200378	0	NA	NA	NA	NA	RNU5B-4P
NA	ENSG00000200379	0	NA	NA	NA	NA	RN7SKP45
NA	ENSG00000200385	0	NA	NA	NA	NA	
NA	ENSG00000200388	0	NA	NA	NA	NA	RNU6-618P
NA	ENSG00000200389	0	NA	NA	NA	NA	RNU6-509P
NA	ENSG00000200390	0	NA	NA	NA	NA	
NA	ENSG00000200391	0	NA	NA	NA	NA	
NA	ENSG00000200393	0	NA	NA	NA	NA	RNU6-698P
100124536	ENSG00000200394	0	NA	NA	NA	NA	SNORA38B
NA	ENSG00000200397	0	NA	NA	NA	NA	

NA	ENSG00000200398	0	NA	NA	NA	NA	SNORD115-24
NA	ENSG00000200403	0	NA	NA	NA	NA	RNU6-1099P
767603	ENSG00000200406	0	NA	NA	NA	NA	SNORD114-23
NA	ENSG00000200407	0	NA	NA	NA	NA	RNU6-1169P
NA	ENSG00000200408	0	NA	NA	NA	NA	RNA5SP74
NA	ENSG00000200411	0	NA	NA	NA	NA	RNA5SP346
767606	ENSG00000200413	0	NA	NA	NA	NA	SNORD114-26
NA	ENSG00000200418	0	NA	NA	NA	NA	SNORA63B
NA	ENSG00000200419	0	NA	NA	NA	NA	
NA	ENSG00000200421	0	NA	NA	NA	NA	
NA	ENSG00000200422	0	NA	NA	NA	NA	
NA	ENSG00000200424	0	NA	NA	NA	NA	RNU6-1155P
NA	ENSG00000200427	0	NA	NA	NA	NA	
NA	ENSG00000200428	0	NA	NA	NA	NA	
NA	ENSG00000200431	0	NA	NA	NA	NA	RNU4-81P
NA	ENSG00000200432	0	NA	NA	NA	NA	
NA	ENSG00000200434	0	NA	NA	NA	NA	RNA5-8SP2
NA	ENSG00000200436	0	NA	NA	NA	NA	
NA	ENSG00000200437	0	NA	NA	NA	NA	RNU6-799P
NA	ENSG00000200443	0	NA	NA	NA	NA	RNU6-1066P
NA	ENSG00000200444	0	NA	NA	NA	NA	RNU6-1339P
NA	ENSG00000200446	0	NA	NA	NA	NA	RNU6-1085P
NA	ENSG00000200448	0	NA	NA	NA	NA	
NA	ENSG00000200455	0	NA	NA	NA	NA	RNU6-1112P
NA	ENSG00000200456	0	NA	NA	NA	NA	RNU6-1097P
NA	ENSG00000200462	0	NA	NA	NA	NA	RNU6-727P
727676	ENSG00000200463	0	NA	NA	NA	NA	SNORD118
NA	ENSG00000200468	0	NA	NA	NA	NA	RNA5SP403
NA	ENSG00000200469	0	NA	NA	NA	NA	RNU6-112P
NA	ENSG00000200471	0	NA	NA	NA	NA	RNU6-1125P
NA	ENSG00000200472	0.33471731	0.02354391	0.38534154	0.62842204	NA	RN7SKP44
NA	ENSG00000200473	0	NA	NA	NA	NA	RNA5SP507
NA	ENSG00000200475	0	NA	NA	NA	NA	RN7SKP162
100033815	ENSG00000200478	0	NA	NA	NA	NA	SNORD115-41
767609	ENSG00000200480	0	NA	NA	NA	NA	SNORD114-28
NA	ENSG00000200483	0	NA	NA	NA	NA	RNU6-1017P
NA	ENSG00000200484	0	NA	NA	NA	NA	RNU6-1244P
NA	ENSG00000200485	0	NA	NA	NA	NA	

100033448	ENSG00000200486	0	NA	NA	NA	NA	SNORD115-11
NA	ENSG00000200487	0	NA	NA	NA	NA	RNA5SP322
NA	ENSG00000200488	0	NA	NA	NA	NA	RN7SKP203
NA	ENSG00000200492	0	NA	NA	NA	NA	
NA	ENSG00000200494	0	NA	NA	NA	NA	
NA	ENSG00000200495	0	NA	NA	NA	NA	RNU6-1205P
NA	ENSG00000200496	0	NA	NA	NA	NA	
NA	ENSG00000200502	0	NA	NA	NA	NA	
100033442	ENSG00000200503	0	NA	NA	NA	NA	SNORD115-5
NA	ENSG00000200506	0	NA	NA	NA	NA	
NA	ENSG00000200508	0	NA	NA	NA	NA	
NA	ENSG00000200516	0	NA	NA	NA	NA	RNA5SP378
NA	ENSG00000200520	0	NA	NA	NA	NA	RNU6-1214P
NA	ENSG00000200521	0	NA	NA	NA	NA	
NA	ENSG00000200522	0	NA	NA	NA	NA	RNU6-957P
NA	ENSG00000200525	0	NA	NA	NA	NA	RNU6-1209P
NA	ENSG00000200526	0	NA	NA	NA	NA	RNY4P14
NA	ENSG00000200527	0	NA	NA	NA	NA	RNA5SP457
NA	ENSG00000200528	0	NA	NA	NA	NA	RNU6-1331P
NA	ENSG00000200530	0	NA	NA	NA	NA	SNORD35B
594839	ENSG00000200534	0	NA	NA	NA	NA	SNORA33
NA	ENSG00000200536	0	NA	NA	NA	NA	
NA	ENSG00000200537	0	NA	NA	NA	NA	RNY4P6
NA	ENSG00000200538	0	NA	NA	NA	NA	
NA	ENSG00000200544	0	NA	NA	NA	NA	
NA	ENSG00000200545	0	NA	NA	NA	NA	
NA	ENSG00000200547	0	NA	NA	NA	NA	
NA	ENSG00000200550	0	NA	NA	NA	NA	RNU6-137P
NA	ENSG00000200553	0	NA	NA	NA	NA	
NA	ENSG00000200554	0	NA	NA	NA	NA	RNU6-1020P
NA	ENSG00000200555	0	NA	NA	NA	NA	RNU6-525P
NA	ENSG00000200556	0	NA	NA	NA	NA	RNU6-103P
NA	ENSG00000200558	0	NA	NA	NA	NA	RNA5SP429
NA	ENSG00000200560	0	NA	NA	NA	NA	RNU6-288P
NA	ENSG00000200563	0	NA	NA	NA	NA	RNU6-640P
100033813	ENSG00000200564	0	NA	NA	NA	NA	SNORD115-39
NA	ENSG00000200566	0	NA	NA	NA	NA	RNU5F-7P
NA	ENSG00000200570	0	NA	NA	NA	NA	RNU6-829P

NA	ENSG00000200571	0	NA	NA	NA	NA	RNU6-1284P
NA	ENSG00000200572	0	NA	NA	NA	NA	
NA	ENSG00000200575	0	NA	NA	NA	NA	RNU6-414P
NA	ENSG00000200579	0	NA	NA	NA	NA	
NA	ENSG00000200587	0	NA	NA	NA	NA	RNA5SP514
NA	ENSG00000200591	0	NA	NA	NA	NA	
100033807	ENSG00000200593	0	NA	NA	NA	NA	SNORD115-33
NA	ENSG00000200594	0	NA	NA	NA	NA	RNU6-824P
NA	ENSG00000200597	0	NA	NA	NA	NA	RNVU1-33
NA	ENSG00000200600	0	NA	NA	NA	NA	
NA	ENSG00000200601	0	NA	NA	NA	NA	RNA5SP50
NA	ENSG00000200605	0	NA	NA	NA	NA	
767589	ENSG00000200608	0	NA	NA	NA	NA	SNORD114-11
NA	ENSG00000200610	0	NA	NA	NA	NA	
767605	ENSG00000200612	0	NA	NA	NA	NA	SNORD114-25
NA	ENSG00000200613	0	NA	NA	NA	NA	RNA5SP349
NA	ENSG00000200615	0	NA	NA	NA	NA	
NA	ENSG00000200616	0	NA	NA	NA	NA	
NA	ENSG00000200619	0	NA	NA	NA	NA	RNA5SP447
NA	ENSG00000200620	0	NA	NA	NA	NA	
NA	ENSG00000200622	0	NA	NA	NA	NA	RNU6-1059P
595098	ENSG00000200623	0	NA	NA	NA	NA	SNORD18A
NA	ENSG00000200626	0	NA	NA	NA	NA	RNA5SP325
NA	ENSG00000200629	0	NA	NA	NA	NA	RNY1P11
NA	ENSG00000200630	0	NA	NA	NA	NA	
767567	ENSG00000200632	0	NA	NA	NA	NA	SNORD113-7
NA	ENSG00000200635	0	NA	NA	NA	NA	
767608	ENSG00000200636	0	NA	NA	NA	NA	SNORD114-27
NA	ENSG00000200637	0	NA	NA	NA	NA	RNU5F-3P
100033811	ENSG00000200638	0	NA	NA	NA	NA	SNORD115-37
NA	ENSG00000200645	0	NA	NA	NA	NA	RNU6-1210P
NA	ENSG00000200646	0	NA	NA	NA	NA	
NA	ENSG00000200648	0	NA	NA	NA	NA	RNU6-226P
NA	ENSG00000200650	0	NA	NA	NA	NA	RNA5SP114
NA	ENSG00000200651	0	NA	NA	NA	NA	
NA	ENSG00000200652	0	NA	NA	NA	NA	
NA	ENSG00000200653	0.16735865	0.01897177	0.38482933	0.78225479	NA	RNU4-92P
677795	ENSG00000200656	0	NA	NA	NA	NA	SNORA5B

100033422	ENSG00000200661	0	NA	NA	NA	NA	SNORD116-10
NA	ENSG00000200664	0	NA	NA	NA	NA	
NA	ENSG00000200665	0	NA	NA	NA	NA	RNU6-1188P
NA	ENSG00000200670	0	NA	NA	NA	NA	RNU6-912P
NA	ENSG00000200673	0	NA	NA	NA	NA	RN7SKP174
NA	ENSG00000200674	0.66980615	-0.02102309	0.3830816	0.65829298	NA	RN7SKP160
NA	ENSG00000200677	0	NA	NA	NA	NA	
100033441	ENSG00000200680	0	NA	NA	NA	NA	SNORD115-4
NA	ENSG00000200681	0	NA	NA	NA	NA	RNU6-263P
NA	ENSG00000200683	0	NA	NA	NA	NA	RNU6-379P
NA	ENSG00000200685	0	NA	NA	NA	NA	
NA	ENSG00000200686	0	NA	NA	NA	NA	RNY3P3
NA	ENSG00000200687	0	NA	NA	NA	NA	RNA5SP335
NA	ENSG00000200688	0	NA	NA	NA	NA	
NA	ENSG00000200693	0.34758969	-0.00123046	0.38336747	0.98009982	NA	
NA	ENSG00000200701	0	NA	NA	NA	NA	RNU6-674P
NA	ENSG00000200702	0	NA	NA	NA	NA	
NA	ENSG00000200703	0	NA	NA	NA	NA	RNU6-873P
NA	ENSG00000200706	0	NA	NA	NA	NA	
NA	ENSG00000200708	0	NA	NA	NA	NA	RN7SKP93
NA	ENSG00000200709	0	NA	NA	NA	NA	RNA5SP27
NA	ENSG00000200711	0	NA	NA	NA	NA	RNA5SP28
NA	ENSG00000200713	0	NA	NA	NA	NA	RNU6-682P
NA	ENSG00000200714	0	NA	NA	NA	NA	
NA	ENSG00000200718	0	NA	NA	NA	NA	RN7SKP103
NA	ENSG00000200719	0	NA	NA	NA	NA	RNA5SP260
NA	ENSG00000200720	0	NA	NA	NA	NA	RNU6-931P
100033445	ENSG00000200726	0	NA	NA	NA	NA	SNORD115-8
NA	ENSG00000200728	0	NA	NA	NA	NA	RNU6-271P
NA	ENSG00000200731	0	NA	NA	NA	NA	RNU1-124P
NA	ENSG00000200732	0	NA	NA	NA	NA	RNU6-194P
NA	ENSG00000200733	0	NA	NA	NA	NA	
NA	ENSG00000200737	0	NA	NA	NA	NA	
NA	ENSG00000200738	0	NA	NA	NA	NA	RNA5SP472
NA	ENSG00000200741	0	NA	NA	NA	NA	RNA5SP161
NA	ENSG00000200742	0	NA	NA	NA	NA	
NA	ENSG00000200745	0	NA	NA	NA	NA	RNU1-31P
NA	ENSG00000200750	0	NA	NA	NA	NA	

NA	ENSG00000200752	0	NA	NA	NA	NA	
NA	ENSG00000200753	0	NA	NA	NA	NA	
NA	ENSG00000200754	0	NA	NA	NA	NA	
NA	ENSG00000200755	0	NA	NA	NA	NA	RNA5SP68
NA	ENSG00000200756	0	NA	NA	NA	NA	RNU6-236P
100033454	ENSG00000200757	0	NA	NA	NA	NA	SNORD115-16
NA	ENSG00000200759	0	NA	NA	NA	NA	RNU6-884P
NA	ENSG00000200761	0	NA	NA	NA	NA	RN7SKP113
NA	ENSG00000200763	0	NA	NA	NA	NA	RNU6-961P
NA	ENSG00000200764	0	NA	NA	NA	NA	
NA	ENSG00000200769	0.15069912	-0.01848503	0.38480575	0.84537521	NA	
NA	ENSG00000200774	0	NA	NA	NA	NA	RNU6-478P
NA	ENSG00000200779	0	NA	NA	NA	NA	RNU6-105P
NA	ENSG00000200783	0	NA	NA	NA	NA	RN7SKP180
319103	ENSG00000200785	0	NA	NA	NA	NA	SNORD8
NA	ENSG00000200786	0	NA	NA	NA	NA	RNA5SP234
NA	ENSG00000200788	0	NA	NA	NA	NA	
NA	ENSG00000200789	0	NA	NA	NA	NA	RNU1-65P
677846	ENSG00000200792	0.16735865	0.01897177	0.38482933	0.78225479	NA	SNORA80A
NA	ENSG00000200794	0	NA	NA	NA	NA	RN7SKP250
26835	ENSG00000200795	0	NA	NA	NA	NA	RNU4-1
NA	ENSG00000200796	0	NA	NA	NA	NA	RNU6-753P
NA	ENSG00000200799	0	NA	NA	NA	NA	RNU6-770P
NA	ENSG00000200800	0	NA	NA	NA	NA	RNU1-130P
100036565	ENSG00000200801	0	NA	NA	NA	NA	SNORD115-28
NA	ENSG00000200806	0	NA	NA	NA	NA	RNA5SP275
NA	ENSG00000200807	0	NA	NA	NA	NA	RNU1-32P
100033443	ENSG00000200812	0	NA	NA	NA	NA	SNORD115-6
NA	ENSG00000200814	0	NA	NA	NA	NA	RNU6-595P
NA	ENSG00000200815	0	NA	NA	NA	NA	RNU6-1091P
677820	ENSG00000200816	0	NA	NA	NA	NA	SNORA38
NA	ENSG00000200817	0	NA	NA	NA	NA	RNU6-899P
NA	ENSG00000200818	0	NA	NA	NA	NA	RNU6-1204P
NA	ENSG00000200822	0	NA	NA	NA	NA	
767578	ENSG00000200823	0	NA	NA	NA	NA	SNORD114-2
NA	ENSG00000200827	0	NA	NA	NA	NA	RNU6-324P
NA	ENSG00000200829	0	NA	NA	NA	NA	
NA	ENSG00000200830	0	NA	NA	NA	NA	RN7SKP134

NA	ENSG00000200831	0	NA	NA	NA	NA	SNORD36B
NA	ENSG00000200832	0	NA	NA	NA	NA	SNORD114-4
NA	ENSG00000200834	0	NA	NA	NA	NA	
NA	ENSG00000200839	0	NA	NA	NA	NA	RNA5SP48
NA	ENSG00000200840	0	NA	NA	NA	NA	RNU6-82P
NA	ENSG00000200842	0	NA	NA	NA	NA	
NA	ENSG00000200847	0.173733	0.01914545	0.38484597	0.78225479	NA	
NA	ENSG00000200849	0	NA	NA	NA	NA	
NA	ENSG00000200852	0	NA	NA	NA	NA	RNA5SP499
NA	ENSG00000200855	0	NA	NA	NA	NA	
NA	ENSG00000200857	0	NA	NA	NA	NA	
NA	ENSG00000200860	0	NA	NA	NA	NA	
NA	ENSG00000200867	0	NA	NA	NA	NA	RN7SKP36
NA	ENSG00000200869	0	NA	NA	NA	NA	RNU6-457P
NA	ENSG00000200871	0	NA	NA	NA	NA	RNU6-810P
NA	ENSG00000200872	0	NA	NA	NA	NA	RNA5SP456
NA	ENSG00000200873	0	NA	NA	NA	NA	RNA5SP399
NA	ENSG00000200874	0	NA	NA	NA	NA	
NA	ENSG00000200875	0	NA	NA	NA	NA	RNU6-340P
NA	ENSG00000200877	0	NA	NA	NA	NA	RNU6-560P
85391	ENSG00000200879	0	NA	NA	NA	NA	SNORD14E
NA	ENSG00000200882	0	NA	NA	NA	NA	RNU6-681P
NA	ENSG00000200883	0	NA	NA	NA	NA	
NA	ENSG00000200884	0	NA	NA	NA	NA	
NA	ENSG00000200885	0	NA	NA	NA	NA	RNU1-146P
NA	ENSG00000200887	0	NA	NA	NA	NA	RNU6-598P
NA	ENSG00000200888	0	NA	NA	NA	NA	
NA	ENSG00000200889	0	NA	NA	NA	NA	RNU4-13P
NA	ENSG00000200890	0	NA	NA	NA	NA	RNA5SP99
NA	ENSG00000200891	0	NA	NA	NA	NA	
NA	ENSG00000200893	0	NA	NA	NA	NA	RNU6-944P
NA	ENSG00000200895	0	NA	NA	NA	NA	RN7SKP245
NA	ENSG00000200897	0	NA	NA	NA	NA	
NA	ENSG00000200898	0	NA	NA	NA	NA	RNU6-1243P
NA	ENSG00000200902	0	NA	NA	NA	NA	RNU6-627P
NA	ENSG00000200903	0	NA	NA	NA	NA	RNU1-42P
NA	ENSG00000200906	0	NA	NA	NA	NA	RNU6-854P
94161	ENSG00000200913	0	NA	NA	NA	NA	SNORD46

NA	ENSG00000200914	0	NA	NA	NA	NA	RNA5SP435
NA	ENSG00000200917	0	NA	NA	NA	NA	RNU6-553P
NA	ENSG00000200922	0	NA	NA	NA	NA	
NA	ENSG00000200924	0	NA	NA	NA	NA	RNU6-1048P
NA	ENSG00000200926	0	NA	NA	NA	NA	RNU6-960P
NA	ENSG00000200935	0	NA	NA	NA	NA	RNU6-1090P
NA	ENSG00000200941	0	NA	NA	NA	NA	RNU6-694P
NA	ENSG00000200942	0	NA	NA	NA	NA	RNU6-501P
100033806	ENSG00000200949	0	NA	NA	NA	NA	SNORD115-32
NA	ENSG00000200953	0	NA	NA	NA	NA	
NA	ENSG00000200957	0	NA	NA	NA	NA	RNU6-801P
26821	ENSG00000200959	0	NA	NA	NA	NA	SNORA74A
NA	ENSG00000200963	0	NA	NA	NA	NA	RNU6-289P
NA	ENSG00000200966	1.32469153	-0.01993956	0.37965136	0.76529411	NA	RN7SKP87
NA	ENSG00000200969	0	NA	NA	NA	NA	
NA	ENSG00000200972	0	NA	NA	NA	NA	RNU5A-8P
NA	ENSG00000200974	0	NA	NA	NA	NA	RNU4-87P
NA	ENSG00000200975	0	NA	NA	NA	NA	RNU1-7P
NA	ENSG00000200976	0	NA	NA	NA	NA	
NA	ENSG00000200982	0	NA	NA	NA	NA	
619562	ENSG00000200983	0	NA	NA	NA	NA	SNORA3A
NA	ENSG00000200985	0	NA	NA	NA	NA	RNA5SP493
NA	ENSG00000200986	0	NA	NA	NA	NA	RNU6-819P
100033804	ENSG00000200987	0	NA	NA	NA	NA	SNORD115-30
NA	ENSG00000200991	0	NA	NA	NA	NA	
NA	ENSG00000200997	0	NA	NA	NA	NA	RNVU1-34
NA	ENSG00000200998	0	NA	NA	NA	NA	
NA	ENSG00000200999	0	NA	NA	NA	NA	
NA	ENSG00000201000	0	NA	NA	NA	NA	RNA5SP516
NA	ENSG00000201001	0	NA	NA	NA	NA	RNU6-270P
NA	ENSG00000201003	0	NA	NA	NA	NA	
NA	ENSG00000201006	0	NA	NA	NA	NA	
NA	ENSG00000201009	0	NA	NA	NA	NA	
NA	ENSG00000201010	0	NA	NA	NA	NA	RN7SKP224
NA	ENSG00000201012	0	NA	NA	NA	NA	
NA	ENSG00000201013	0	NA	NA	NA	NA	
NA	ENSG00000201014	0	NA	NA	NA	NA	RNA5SP232
NA	ENSG00000201015	0	NA	NA	NA	NA	RNU6-280P

NA	ENSG00000201016	0	NA	NA	NA	NA	RNU6-374P
NA	ENSG00000201021	0	NA	NA	NA	NA	RNU6-743P
NA	ENSG00000201023	0	NA	NA	NA	NA	RNY3P11
109617014	ENSG00000201025	0	NA	NA	NA	NA	SNORD74B
NA	ENSG00000201026	0	NA	NA	NA	NA	
NA	ENSG00000201027	0	NA	NA	NA	NA	RN7SKP107
NA	ENSG00000201028	0	NA	NA	NA	NA	RNU6-151P
NA	ENSG00000201031	0	NA	NA	NA	NA	
NA	ENSG00000201032	0	NA	NA	NA	NA	RNU6-704P
NA	ENSG00000201033	0	NA	NA	NA	NA	RNU1-96P
NA	ENSG00000201034	0	NA	NA	NA	NA	
NA	ENSG00000201035	0	NA	NA	NA	NA	RNA5SP469
NA	ENSG00000201036	0	NA	NA	NA	NA	
NA	ENSG00000201041	0	NA	NA	NA	NA	RNA5SP242
NA	ENSG00000201042	0	NA	NA	NA	NA	
NA	ENSG00000201044	0	NA	NA	NA	NA	RNU6-268P
NA	ENSG00000201047	0	NA	NA	NA	NA	
NA	ENSG00000201048	0	NA	NA	NA	NA	
NA	ENSG00000201050	0	NA	NA	NA	NA	RNU6-668P
NA	ENSG00000201059	0	NA	NA	NA	NA	RNA5SP336
NA	ENSG00000201065	0	NA	NA	NA	NA	RNU6-461P
NA	ENSG00000201066	0	NA	NA	NA	NA	RN7SKP280
NA	ENSG00000201070	0	NA	NA	NA	NA	RNU4-84P
NA	ENSG00000201071	0	NA	NA	NA	NA	
NA	ENSG00000201074	0	NA	NA	NA	NA	
NA	ENSG00000201075	0	NA	NA	NA	NA	
NA	ENSG00000201076	0	NA	NA	NA	NA	RNU4-51P
NA	ENSG00000201077	0	NA	NA	NA	NA	RNU6-188P
NA	ENSG00000201078	0	NA	NA	NA	NA	RN7SKP214
NA	ENSG00000201085	0	NA	NA	NA	NA	RNU6-173P
NA	ENSG00000201086	0	NA	NA	NA	NA	RNA5SP394
NA	ENSG00000201088	0	NA	NA	NA	NA	
NA	ENSG00000201095	0	NA	NA	NA	NA	RNU6-266P
NA	ENSG00000201096	0	NA	NA	NA	NA	RNA5SP387
NA	ENSG00000201097	0	NA	NA	NA	NA	RNU6-366P
6084	ENSG00000201098	0	NA	NA	NA	NA	RNY1
NA	ENSG00000201102	0	NA	NA	NA	NA	
NA	ENSG00000201109	0	NA	NA	NA	NA	RNA5SP245

NA	ENSG00000201113	0	NA	NA	NA	NA	RNU6-647P
NA	ENSG00000201114	0	NA	NA	NA	NA	
NA	ENSG00000201118	0	NA	NA	NA	NA	
NA	ENSG00000201119	0	NA	NA	NA	NA	RNU1-33P
NA	ENSG00000201121	0	NA	NA	NA	NA	RNY1P12
109617023	ENSG00000201129	0	NA	NA	NA	NA	SNORA58B
NA	ENSG00000201133	0	NA	NA	NA	NA	
NA	ENSG00000201134	0	NA	NA	NA	NA	
NA	ENSG00000201135	0	NA	NA	NA	NA	RNU6-777P
NA	ENSG00000201136	0	NA	NA	NA	NA	RNU6-353P
NA	ENSG00000201140	0	NA	NA	NA	NA	RN7SKP128
NA	ENSG00000201142	0	NA	NA	NA	NA	RNU1-151P
100033816	ENSG00000201143	0	NA	NA	NA	NA	SNORD115-42
NA	ENSG00000201145	0	NA	NA	NA	NA	RNA5SP175
NA	ENSG00000201148	0	NA	NA	NA	NA	RNA5SP42
NA	ENSG00000201151	0	NA	NA	NA	NA	
NA	ENSG00000201153	0	NA	NA	NA	NA	RNU6-371P
NA	ENSG00000201155	0	NA	NA	NA	NA	RNU1-24P
NA	ENSG00000201157	0	NA	NA	NA	NA	
NA	ENSG00000201160	0	NA	NA	NA	NA	
NA	ENSG00000201161	0	NA	NA	NA	NA	RN7SKP10
NA	ENSG00000201162	0	NA	NA	NA	NA	RNU6-454P
NA	ENSG00000201164	0	NA	NA	NA	NA	RNU4-36P
NA	ENSG00000201165	0	NA	NA	NA	NA	RNU6-1229P
NA	ENSG00000201168	0	NA	NA	NA	NA	RNA5SP368
NA	ENSG00000201170	0	NA	NA	NA	NA	RNU1-132P
NA	ENSG00000201176	0	NA	NA	NA	NA	RNU6-853P
NA	ENSG00000201178	0	NA	NA	NA	NA	
NA	ENSG00000201179	0	NA	NA	NA	NA	RNU6-1322P
NA	ENSG00000201180	0	NA	NA	NA	NA	RNU6-115P
NA	ENSG00000201182	0	NA	NA	NA	NA	RNU6-670P
101954272	ENSG00000201183	0	NA	NA	NA	NA	RNVU1-3
NA	ENSG00000201184	0	NA	NA	NA	NA	RNU4-68P
NA	ENSG00000201185	0	NA	NA	NA	NA	RNA5SP202
NA	ENSG00000201186	0	NA	NA	NA	NA	RNU4-33P
NA	ENSG00000201196	0	NA	NA	NA	NA	
NA	ENSG00000201198	0	NA	NA	NA	NA	RNU6-879P
NA	ENSG00000201201	0.92036559	-0.05022512	0.38869686	0.2715874	NA	RN7SKP118

NA	ENSG00000201207	0	NA	NA	NA	NA	
NA	ENSG00000201208	0	NA	NA	NA	NA	
NA	ENSG00000201209	0	NA	NA	NA	NA	
NA	ENSG00000201210	0	NA	NA	NA	NA	RNA5SP139
NA	ENSG00000201216	0	NA	NA	NA	NA	
NA	ENSG00000201217	0	NA	NA	NA	NA	
NA	ENSG00000201218	0	NA	NA	NA	NA	
NA	ENSG00000201221	0	NA	NA	NA	NA	RNU4-40P
NA	ENSG00000201223	0	NA	NA	NA	NA	RNU6-1305P
NA	ENSG00000201228	0	NA	NA	NA	NA	
109616971	ENSG00000201229	0	NA	NA	NA	NA	SNORA63D
NA	ENSG00000201231	0	NA	NA	NA	NA	RNU4-50P
NA	ENSG00000201239	0	NA	NA	NA	NA	
767585	ENSG00000201240	0	NA	NA	NA	NA	SNORD114-9
NA	ENSG00000201241	0	NA	NA	NA	NA	RNU6-978P
NA	ENSG00000201242	0	NA	NA	NA	NA	RNY1P1
NA	ENSG00000201243	0	NA	NA	NA	NA	RNU6-654P
NA	ENSG00000201245	0	NA	NA	NA	NA	
767591	ENSG00000201247	0	NA	NA	NA	NA	SNORD114-13
NA	ENSG00000201253	0	NA	NA	NA	NA	RNU4-10P
NA	ENSG00000201255	0	NA	NA	NA	NA	RNU6-990P
NA	ENSG00000201260	0	NA	NA	NA	NA	RNU6-1075P
767582	ENSG00000201263	0	NA	NA	NA	NA	SNORD114-6
114655	ENSG00000201264	0	NA	NA	NA	NA	SNORD73B
NA	ENSG00000201270	0	NA	NA	NA	NA	RNU6-755P
NA	ENSG00000201271	0	NA	NA	NA	NA	RNU1-112P
NA	ENSG00000201273	0	NA	NA	NA	NA	RNU6-776P
NA	ENSG00000201274	0	NA	NA	NA	NA	RNA5SP192
NA	ENSG00000201277	0	NA	NA	NA	NA	
NA	ENSG00000201279	0	NA	NA	NA	NA	
NA	ENSG00000201282	0	NA	NA	NA	NA	
NA	ENSG00000201285	0	NA	NA	NA	NA	RNA5SP524
NA	ENSG00000201287	0	NA	NA	NA	NA	RN7SKP171
NA	ENSG00000201288	0	NA	NA	NA	NA	RNU6-1047P
NA	ENSG00000201289	0	NA	NA	NA	NA	RN7SKP76
NA	ENSG00000201291	0	NA	NA	NA	NA	RNU1-34P
NA	ENSG00000201292	0	NA	NA	NA	NA	RNU6-153P
NA	ENSG00000201294	0	NA	NA	NA	NA	RNU6-1019P

NA	ENSG00000201296	0	NA	NA	NA	NA	RNU4-41P
NA	ENSG00000201297	0	NA	NA	NA	NA	
NA	ENSG00000201298	0	NA	NA	NA	NA	RNU6-1164P
100036564	ENSG00000201300	0	NA	NA	NA	NA	SNORD115-27
NA	ENSG00000201301	0	NA	NA	NA	NA	RNA5SP130
26783	ENSG00000201302	0	NA	NA	NA	NA	SNORA65
NA	ENSG00000201308	0	NA	NA	NA	NA	RNU6-512P
NA	ENSG00000201309	0	NA	NA	NA	NA	
NA	ENSG00000201311	0	NA	NA	NA	NA	
NA	ENSG00000201312	0	NA	NA	NA	NA	RNA5SP72
NA	ENSG00000201314	0	NA	NA	NA	NA	
NA	ENSG00000201315	0	NA	NA	NA	NA	RN7SKP227
NA	ENSG00000201316	0	NA	NA	NA	NA	
NA	ENSG00000201317	0	NA	NA	NA	NA	RNU4-59P
767611	ENSG00000201318	0	NA	NA	NA	NA	SNORD114-30
100169760	ENSG00000201321	0	NA	NA	NA	NA	RNA5S9
NA	ENSG00000201324	0	NA	NA	NA	NA	RNU6-361P
NA	ENSG00000201325	0	NA	NA	NA	NA	RNA5SP142
100033799	ENSG00000201326	0	NA	NA	NA	NA	SNORD115-22
NA	ENSG00000201329	0	NA	NA	NA	NA	
692092	ENSG00000201330	0	NA	NA	NA	NA	SNORD32B
100033800	ENSG00000201331	0	NA	NA	NA	NA	SNORD115-23
NA	ENSG00000201339	0	NA	NA	NA	NA	
NA	ENSG00000201340	0	NA	NA	NA	NA	
NA	ENSG00000201341	0	NA	NA	NA	NA	RNU6-421P
NA	ENSG00000201342	0	NA	NA	NA	NA	RNU4-38P
NA	ENSG00000201343	0	NA	NA	NA	NA	
NA	ENSG00000201346	0	NA	NA	NA	NA	
NA	ENSG00000201347	0	NA	NA	NA	NA	RNA5SP69
26767	ENSG00000201348	0	NA	NA	NA	NA	RNU105B
NA	ENSG00000201351	0	NA	NA	NA	NA	
NA	ENSG00000201354	0	NA	NA	NA	NA	
NA	ENSG00000201356	0	NA	NA	NA	NA	RNA5SP500
NA	ENSG00000201358	0	NA	NA	NA	NA	RN7SKP193
NA	ENSG00000201361	0	NA	NA	NA	NA	RNA5SP433
NA	ENSG00000201363	0	NA	NA	NA	NA	
NA	ENSG00000201364	0	NA	NA	NA	NA	RN7SKP37
NA	ENSG00000201365	0	NA	NA	NA	NA	

NA	ENSG00000201367	0	NA	NA	NA	NA	RNU6-522P
NA	ENSG00000201368	0	NA	NA	NA	NA	
NA	ENSG00000201370	0	NA	NA	NA	NA	
NA	ENSG00000201371	0	NA	NA	NA	NA	
NA	ENSG00000201372	0	NA	NA	NA	NA	RNU6-1251P
NA	ENSG00000201376	0	NA	NA	NA	NA	
NA	ENSG00000201377	0	NA	NA	NA	NA	RNY4P23
NA	ENSG00000201379	0	NA	NA	NA	NA	RNU4-76P
NA	ENSG00000201382	0	NA	NA	NA	NA	RNU6-558P
NA	ENSG00000201384	0	NA	NA	NA	NA	
NA	ENSG00000201386	0	NA	NA	NA	NA	RNU6-1163P
109616972	ENSG00000201388	0	NA	NA	NA	NA	SNORA68B
NA	ENSG00000201390	0	NA	NA	NA	NA	RNU6-1141P
NA	ENSG00000201392	0	NA	NA	NA	NA	RNU6-1078P
NA	ENSG00000201393	0	NA	NA	NA	NA	
NA	ENSG00000201394	0	NA	NA	NA	NA	RNA5SP140
NA	ENSG00000201395	0	NA	NA	NA	NA	RN7SKP257
NA	ENSG00000201398	0	NA	NA	NA	NA	
NA	ENSG00000201403	0	NA	NA	NA	NA	SNORD14B
NA	ENSG00000201405	0	NA	NA	NA	NA	
NA	ENSG00000201407	0	NA	NA	NA	NA	
NA	ENSG00000201410	0	NA	NA	NA	NA	
NA	ENSG00000201412	0	NA	NA	NA	NA	
NA	ENSG00000201413	0	NA	NA	NA	NA	RNA5SP141
NA	ENSG00000201415	0	NA	NA	NA	NA	RNA5SP204
NA	ENSG00000201420	0	NA	NA	NA	NA	RNA5SP512
NA	ENSG00000201421	0	NA	NA	NA	NA	
NA	ENSG00000201423	0	NA	NA	NA	NA	RN7SKP246
NA	ENSG00000201426	0	NA	NA	NA	NA	
NA	ENSG00000201428	0.18922458	0.01954053	0.38488496	0.78225479	NA	RN7SKP71
NA	ENSG00000201431	0	NA	NA	NA	NA	RNU6-1277P
NA	ENSG00000201432	0	NA	NA	NA	NA	
NA	ENSG00000201433	0	NA	NA	NA	NA	RNU6-335P
NA	ENSG00000201435	0	NA	NA	NA	NA	RNU4-24P
NA	ENSG00000201436	0	NA	NA	NA	NA	RNU6-660P
NA	ENSG00000201439	0	NA	NA	NA	NA	RNU4-67P
NA	ENSG00000201440	0	NA	NA	NA	NA	RNA5SP515
NA	ENSG00000201441	0	NA	NA	NA	NA	RNU6-646P

NA	ENSG00000201442	0	NA	NA	NA	NA	
NA	ENSG00000201443	0	NA	NA	NA	NA	RNU6-309P
NA	ENSG00000201444	0	NA	NA	NA	NA	RNU6-1082P
NA	ENSG00000201447	0	NA	NA	NA	NA	RNA5SP509
109616970	ENSG00000201448	0	NA	NA	NA	NA	SNORA63C
NA	ENSG00000201451	0	NA	NA	NA	NA	
NA	ENSG00000201452	0	NA	NA	NA	NA	RNU6-1317P
677834	ENSG00000201457	0	NA	NA	NA	NA	SNORA55
NA	ENSG00000201458	0	NA	NA	NA	NA	RNU4-4P
NA	ENSG00000201464	0	NA	NA	NA	NA	
NA	ENSG00000201465	0	NA	NA	NA	NA	
NA	ENSG00000201466	0	NA	NA	NA	NA	
NA	ENSG00000201467	0	NA	NA	NA	NA	
NA	ENSG00000201469	0	NA	NA	NA	NA	RNA5SP379
NA	ENSG00000201470	0	NA	NA	NA	NA	RNY4P7
NA	ENSG00000201474	0	NA	NA	NA	NA	RNU6-164P
NA	ENSG00000201476	0	NA	NA	NA	NA	RNA5SP353
100033455	ENSG00000201482	0	NA	NA	NA	NA	SNORD115-17
NA	ENSG00000201483	0	NA	NA	NA	NA	
26804	ENSG00000201487	0	NA	NA	NA	NA	SNORD45B
NA	ENSG00000201489	0	NA	NA	NA	NA	
NA	ENSG00000201491	0	NA	NA	NA	NA	RNU4-75P
NA	ENSG00000201492	0	NA	NA	NA	NA	RNA5SP78
NA	ENSG00000201493	0	NA	NA	NA	NA	RNU1-141P
NA	ENSG00000201496	0	NA	NA	NA	NA	RN7SKP275
NA	ENSG00000201498	0	NA	NA	NA	NA	
NA	ENSG00000201499	0	NA	NA	NA	NA	RNU6-312P
NA	ENSG00000201500	0	NA	NA	NA	NA	
NA	ENSG00000201501	0	NA	NA	NA	NA	
NA	ENSG00000201502	0	NA	NA	NA	NA	
NA	ENSG00000201510	0	NA	NA	NA	NA	RN7SKP217
NA	ENSG00000201511	0	NA	NA	NA	NA	
NA	ENSG00000201512	0	NA	NA	NA	NA	SNORA71C
NA	ENSG00000201516	0	NA	NA	NA	NA	
NA	ENSG00000201517	0	NA	NA	NA	NA	RNU6-707P
NA	ENSG00000201518	0	NA	NA	NA	NA	RNA5SP513
NA	ENSG00000201519	0	NA	NA	NA	NA	RNU6-645P
NA	ENSG00000201523	0	NA	NA	NA	NA	RNA5SP205

NA	ENSG00000201524	0	NA	NA	NA	NA	RNU6-450P
NA	ENSG00000201527	0	NA	NA	NA	NA	RNA5SP478
NA	ENSG00000201529	0	NA	NA	NA	NA	
NA	ENSG00000201532	0	NA	NA	NA	NA	RNA5SP194
NA	ENSG00000201533	0	NA	NA	NA	NA	RN7SKP237
NA	ENSG00000201535	0	NA	NA	NA	NA	
NA	ENSG00000201541	0	NA	NA	NA	NA	
NA	ENSG00000201542	0	NA	NA	NA	NA	
692157	ENSG00000201544	0	NA	NA	NA	NA	SNORA16B
NA	ENSG00000201545	0	NA	NA	NA	NA	RNU4-85P
NA	ENSG00000201547	0	NA	NA	NA	NA	
NA	ENSG00000201548	0	NA	NA	NA	NA	
NA	ENSG00000201549	0	NA	NA	NA	NA	
NA	ENSG00000201550	0	NA	NA	NA	NA	RNU6-726P
NA	ENSG00000201554	0	NA	NA	NA	NA	
NA	ENSG00000201555	0	NA	NA	NA	NA	
767593	ENSG00000201557	0	NA	NA	NA	NA	SNORD114-15
101954276	ENSG00000201558	0.85016097	0.06074473	0.3904457	0.22300825	NA	RNVU1-6
NA	ENSG00000201560	0	NA	NA	NA	NA	RNU6-973P
NA	ENSG00000201563	0	NA	NA	NA	NA	
NA	ENSG00000201564	0	NA	NA	NA	NA	RN7SKP50
NA	ENSG00000201565	0	NA	NA	NA	NA	
NA	ENSG00000201566	0	NA	NA	NA	NA	
NA	ENSG00000201567	0	NA	NA	NA	NA	RNA5SP510
767595	ENSG00000201569	0	NA	NA	NA	NA	SNORD114-17
NA	ENSG00000201570	0	NA	NA	NA	NA	RNU4-56P
NA	ENSG00000201573	0	NA	NA	NA	NA	
NA	ENSG00000201574	0	NA	NA	NA	NA	RNU1-93P
NA	ENSG00000201579	0	NA	NA	NA	NA	RNU6-343P
NA	ENSG00000201581	0	NA	NA	NA	NA	RN7SKP78
NA	ENSG00000201583	0	NA	NA	NA	NA	RN7SKP191
NA	ENSG00000201584	0	NA	NA	NA	NA	
NA	ENSG00000201586	0	NA	NA	NA	NA	RNU6-593P
NA	ENSG00000201591	0	NA	NA	NA	NA	RNU6-99P
NA	ENSG00000201592	0	NA	NA	NA	NA	
NA	ENSG00000201594	0	NA	NA	NA	NA	RNA5SP517
NA	ENSG00000201595	0	NA	NA	NA	NA	RNA5SP132
NA	ENSG00000201596	0	NA	NA	NA	NA	

NA	ENSG00000201598	0	NA	NA	NA	NA	RNU6-219P
NA	ENSG00000201600	0	NA	NA	NA	NA	RN7SKP124
NA	ENSG00000201602	0	NA	NA	NA	NA	
NA	ENSG00000201604	0	NA	NA	NA	NA	RNU6-740P
NA	ENSG00000201607	0	NA	NA	NA	NA	RNU4-16P
NA	ENSG00000201608	0	NA	NA	NA	NA	RNU4-42P
NA	ENSG00000201609	0	NA	NA	NA	NA	RNU4-28P
NA	ENSG00000201610	0	NA	NA	NA	NA	RNA5SP84
NA	ENSG00000201612	0	NA	NA	NA	NA	RN7SKP15
NA	ENSG00000201613	0	NA	NA	NA	NA	RNU6-200P
NA	ENSG00000201616	0	NA	NA	NA	NA	RNU1-91P
NA	ENSG00000201618	0	NA	NA	NA	NA	RNA5SP505
NA	ENSG00000201619	0	NA	NA	NA	NA	
NA	ENSG00000201620	0	NA	NA	NA	NA	RNA5SP51
NA	ENSG00000201622	0	NA	NA	NA	NA	RNU6-621P
NA	ENSG00000201623	0	NA	NA	NA	NA	RNU6-923P
NA	ENSG00000201624	0	NA	NA	NA	NA	
NA	ENSG00000201627	0	NA	NA	NA	NA	
NA	ENSG00000201628	0	NA	NA	NA	NA	RNU4-7P
NA	ENSG00000201633	0	NA	NA	NA	NA	
100033822	ENSG00000201634	0	NA	NA	NA	NA	SNORD115-48
NA	ENSG00000201635	0	NA	NA	NA	NA	
NA	ENSG00000201638	0	NA	NA	NA	NA	RNY4P16
NA	ENSG00000201640	0	NA	NA	NA	NA	RN7SKP28
NA	ENSG00000201641	0	NA	NA	NA	NA	RNU6-1187P
NA	ENSG00000201642	0	NA	NA	NA	NA	RNU6-865P
677801	ENSG00000201643	0	NA	NA	NA	NA	SNORA14A
NA	ENSG00000201644	0	NA	NA	NA	NA	
NA	ENSG00000201648	0	NA	NA	NA	NA	RNU4-91P
NA	ENSG00000201649	0	NA	NA	NA	NA	RNY4P34
101954275	ENSG00000201654	0	NA	NA	NA	NA	RNU6-7
NA	ENSG00000201658	0	NA	NA	NA	NA	RNU6-283P
NA	ENSG00000201659	0	NA	NA	NA	NA	RNU12-2P
NA	ENSG00000201660	0	NA	NA	NA	NA	
NA	ENSG00000201662	0	NA	NA	NA	NA	RNU6-60P
NA	ENSG00000201663	0	NA	NA	NA	NA	
NA	ENSG00000201665	0	NA	NA	NA	NA	RN7SKP6
NA	ENSG00000201666	0	NA	NA	NA	NA	

NA	ENSG00000201668	0	NA	NA	NA	NA	
NA	ENSG00000201671	0	NA	NA	NA	NA	RNA5SP90
767564	ENSG00000201672	0	NA	NA	NA	NA	SNORD113-4
NA	ENSG00000201674	0	NA	NA	NA	NA	
26819	ENSG00000201675	0	NA	NA	NA	NA	SNORD32A
NA	ENSG00000201676	0	NA	NA	NA	NA	
100033453	ENSG00000201679	0	NA	NA	NA	NA	SNORD115-15
NA	ENSG00000201680	0	NA	NA	NA	NA	
NA	ENSG00000201683	0	NA	NA	NA	NA	
NA	ENSG00000201684	0	NA	NA	NA	NA	RN7SKP239
NA	ENSG00000201687	0	NA	NA	NA	NA	RNU6-1107P
767610	ENSG00000201689	0	NA	NA	NA	NA	SNORD114-29
NA	ENSG00000201690	0	NA	NA	NA	NA	RNY1P2
NA	ENSG00000201699	0	NA	NA	NA	NA	RNVU1-24
767563	ENSG00000201700	0	NA	NA	NA	NA	SNORD113-3
NA	ENSG00000201701	0	NA	NA	NA	NA	
NA	ENSG00000201704	0	NA	NA	NA	NA	RNA5SP396
NA	ENSG00000201707	0	NA	NA	NA	NA	RNU6-818P
NA	ENSG00000201708	0	NA	NA	NA	NA	RNA5SP359
NA	ENSG00000201709	0	NA	NA	NA	NA	RNU6-686P
NA	ENSG00000201710	0	NA	NA	NA	NA	
NA	ENSG00000201711	0	NA	NA	NA	NA	RNU6-1303P
NA	ENSG00000201713	0	NA	NA	NA	NA	RNA5SP125
NA	ENSG00000201715	0	NA	NA	NA	NA	RN7SKP133
NA	ENSG00000201723	0	NA	NA	NA	NA	
NA	ENSG00000201724	0	NA	NA	NA	NA	
NA	ENSG00000201725	0	NA	NA	NA	NA	RNU6-304P
NA	ENSG00000201727	0	NA	NA	NA	NA	RNA5SP173
NA	ENSG00000201728	0	NA	NA	NA	NA	RNA5SP479
NA	ENSG00000201733	0	NA	NA	NA	NA	
NA	ENSG00000201736	0	NA	NA	NA	NA	RNA5SP160
NA	ENSG00000201737	0	NA	NA	NA	NA	RNU1-133P
NA	ENSG00000201741	0	NA	NA	NA	NA	
NA	ENSG00000201742	0	NA	NA	NA	NA	RNU6-563P
NA	ENSG00000201744	0	NA	NA	NA	NA	RNU6-34P
NA	ENSG00000201746	0	NA	NA	NA	NA	RNU6-828P
NA	ENSG00000201747	0	NA	NA	NA	NA	RNU6-534P
NA	ENSG00000201749	0	NA	NA	NA	NA	

NA	ENSG00000201752	0	NA	NA	NA	NA	RNU6-119P
26797	ENSG00000201754	0	NA	NA	NA	NA	SNORD52
NA	ENSG00000201756	0	NA	NA	NA	NA	
NA	ENSG00000201758	0	NA	NA	NA	NA	RN7SKP55
NA	ENSG00000201761	0	NA	NA	NA	NA	RNU6-336P
NA	ENSG00000201763	0	NA	NA	NA	NA	RNA5SP267
NA	ENSG00000201766	0	NA	NA	NA	NA	RNA5SP302
NA	ENSG00000201770	0	NA	NA	NA	NA	RNU6-384P
677796	ENSG00000201772	0	NA	NA	NA	NA	SNORA5C
NA	ENSG00000201774	0	NA	NA	NA	NA	
NA	ENSG00000201775	0	NA	NA	NA	NA	RNU6-1325P
NA	ENSG00000201778	0	NA	NA	NA	NA	
NA	ENSG00000201780	0	NA	NA	NA	NA	RNU6-275P
NA	ENSG00000201782	0	NA	NA	NA	NA	RN7SKP226
692233	ENSG00000201785	0	NA	NA	NA	NA	SNORD117
NA	ENSG00000201786	0	NA	NA	NA	NA	
NA	ENSG00000201788	0	NA	NA	NA	NA	
NA	ENSG00000201790	0	NA	NA	NA	NA	RNA5SP189
NA	ENSG00000201791	0	NA	NA	NA	NA	
NA	ENSG00000201793	0.74659737	-0.03303578	0.38644226	0.4112877	NA	RN7SKP9
NA	ENSG00000201794	0	NA	NA	NA	NA	RN7SKP130
NA	ENSG00000201796	0	NA	NA	NA	NA	RNU6-392P
NA	ENSG00000201800	0	NA	NA	NA	NA	
NA	ENSG00000201801	0	NA	NA	NA	NA	RNU5E-4P
NA	ENSG00000201805	0	NA	NA	NA	NA	RNU6-1180P
NA	ENSG00000201806	0.14931947	-0.01844332	0.38480212	0.84537521	NA	RNU4-8P
NA	ENSG00000201807	0	NA	NA	NA	NA	
NA	ENSG00000201809	0	NA	NA	NA	NA	
NA	ENSG00000201810	0	NA	NA	NA	NA	
NA	ENSG00000201811	0	NA	NA	NA	NA	
NA	ENSG00000201812	0	NA	NA	NA	NA	RNA5SP488
NA	ENSG00000201813	0	NA	NA	NA	NA	RNU6-915P
NA	ENSG00000201815	0	NA	NA	NA	NA	RNU6-526P
NA	ENSG00000201816	0	NA	NA	NA	NA	
NA	ENSG00000201818	0	NA	NA	NA	NA	RNY4P17
NA	ENSG00000201820	0	NA	NA	NA	NA	
NA	ENSG00000201821	0	NA	NA	NA	NA	RNU4-9P
NA	ENSG00000201822	0	NA	NA	NA	NA	RNA5SP149

26801	ENSG00000201823	0	NA	NA	NA	NA	SNORD48
NA	ENSG00000201825	0	NA	NA	NA	NA	RNU6-1131P
NA	ENSG00000201826	0	NA	NA	NA	NA	RNU6-867P
NA	ENSG00000201827	0	NA	NA	NA	NA	
NA	ENSG00000201830	0	NA	NA	NA	NA	
338433	ENSG00000201831	0	NA	NA	NA	NA	SNORD115-1
767579	ENSG00000201839	0	NA	NA	NA	NA	SNORD114-3
NA	ENSG00000201843	0	NA	NA	NA	NA	
NA	ENSG00000201846	0	NA	NA	NA	NA	RNA5SP166
109616978	ENSG00000201847	0	NA	NA	NA	NA	SNORD31B
NA	ENSG00000201850	0	NA	NA	NA	NA	
NA	ENSG00000201852	0	NA	NA	NA	NA	RNU6-702P
NA	ENSG00000201856	0	NA	NA	NA	NA	RNA5SP331
NA	ENSG00000201860	0	NA	NA	NA	NA	
NA	ENSG00000201861	0	NA	NA	NA	NA	RNA5SP298
NA	ENSG00000201863	0	NA	NA	NA	NA	
NA	ENSG00000201867	0	NA	NA	NA	NA	
NA	ENSG00000201868	0	NA	NA	NA	NA	
NA	ENSG00000201869	0	NA	NA	NA	NA	RNU6-294P
NA	ENSG00000201875	0	NA	NA	NA	NA	RN7SKP178
NA	ENSG00000201876	0	NA	NA	NA	NA	RNA5SP97
NA	ENSG00000201881	0	NA	NA	NA	NA	
NA	ENSG00000201882	0	NA	NA	NA	NA	
NA	ENSG00000201884	0	NA	NA	NA	NA	
NA	ENSG00000201892	0	NA	NA	NA	NA	
NA	ENSG00000201894	0	NA	NA	NA	NA	RNU6-967P
NA	ENSG00000201896	0	NA	NA	NA	NA	RN7SKP153
NA	ENSG00000201898	0	NA	NA	NA	NA	
767604	ENSG00000201899	0	NA	NA	NA	NA	SNORD114-24
NA	ENSG00000201900	0	NA	NA	NA	NA	RNY1P13
NA	ENSG00000201901	0	NA	NA	NA	NA	RN7SKP48
100033812	ENSG00000201907	0	NA	NA	NA	NA	SNORD115-38
NA	ENSG00000201909	0	NA	NA	NA	NA	RNU6-590P
NA	ENSG00000201910	0	NA	NA	NA	NA	RNU1-140P
NA	ENSG00000201912	0	NA	NA	NA	NA	RN7SKP189
NA	ENSG00000201913	0	NA	NA	NA	NA	
NA	ENSG00000201916	0	NA	NA	NA	NA	
NA	ENSG00000201919	0	NA	NA	NA	NA	RNU6-1022P

NA	ENSG00000201920	0	NA	NA	NA	NA	RNA5SP442
NA	ENSG00000201922	0	NA	NA	NA	NA	RNU1-43P
NA	ENSG00000201923	0	NA	NA	NA	NA	RNA5SP485
NA	ENSG00000201931	0	NA	NA	NA	NA	RNA5SP172
NA	ENSG00000201933	0	NA	NA	NA	NA	
NA	ENSG00000201938	0	NA	NA	NA	NA	
NA	ENSG00000201939	0	NA	NA	NA	NA	RNA5SP224
NA	ENSG00000201942	0	NA	NA	NA	NA	RNA5SP363
100033447	ENSG00000201943	0	NA	NA	NA	NA	SNORD115-10
NA	ENSG00000201944	0	NA	NA	NA	NA	
NA	ENSG00000201945	0	NA	NA	NA	NA	
767569	ENSG00000201950	0	NA	NA	NA	NA	SNORD113-9
NA	ENSG00000201954	0	NA	NA	NA	NA	RNU6-673P
NA	ENSG00000201955	0	NA	NA	NA	NA	RNY3P1
NA	ENSG00000201957	0	NA	NA	NA	NA	
NA	ENSG00000201962	0	NA	NA	NA	NA	RNA5SP126
NA	ENSG00000201965	0	NA	NA	NA	NA	
NA	ENSG00000201966	0	NA	NA	NA	NA	RNA5-8SP4
NA	ENSG00000201967	0	NA	NA	NA	NA	RN7SKP22
NA	ENSG00000201968	0	NA	NA	NA	NA	RNA5SP138
100033460	ENSG00000201969	0	NA	NA	NA	NA	SNORD115-20
NA	ENSG00000201980	0	NA	NA	NA	NA	
NA	ENSG00000201984	0	NA	NA	NA	NA	
NA	ENSG00000201987	0	NA	NA	NA	NA	
NA	ENSG00000201988	0	NA	NA	NA	NA	
NA	ENSG00000201990	0	NA	NA	NA	NA	
100033809	ENSG00000201992	0	NA	NA	NA	NA	SNORD115-35
677808	ENSG00000201998	0.173733	0.01914545	0.38484597	0.78225479	NA	SNORA23
NA	ENSG00000201999	0	NA	NA	NA	NA	RNA5SP428
NA	ENSG00000202000	0	NA	NA	NA	NA	RNU1-36P
NA	ENSG00000202001	0	NA	NA	NA	NA	
NA	ENSG00000202008	0	NA	NA	NA	NA	
NA	ENSG00000202014	0	NA	NA	NA	NA	
NA	ENSG00000202016	0	NA	NA	NA	NA	RNU6-619P
NA	ENSG00000202017	0	NA	NA	NA	NA	RNU6-806P
NA	ENSG00000202019	0	NA	NA	NA	NA	
NA	ENSG00000202023	0	NA	NA	NA	NA	
NA	ENSG00000202024	0	NA	NA	NA	NA	RNU6-934P

NA	ENSG00000202025	0	NA	NA	NA	NA	RNU6-1240P
NA	ENSG00000202026	0	NA	NA	NA	NA	RNU6-1134P
NA	ENSG00000202027	0	NA	NA	NA	NA	
NA	ENSG00000202029	0	NA	NA	NA	NA	RNU6-580P
94162	ENSG00000202031	0	NA	NA	NA	NA	SNORD38A
NA	ENSG00000202034	0	NA	NA	NA	NA	RNU6-399P
NA	ENSG00000202035	0	NA	NA	NA	NA	
NA	ENSG00000202039	0	NA	NA	NA	NA	RNU6-215P
NA	ENSG00000202041	0	NA	NA	NA	NA	
NA	ENSG00000202044	0	NA	NA	NA	NA	RNA5SP223
NA	ENSG00000202046	0	NA	NA	NA	NA	
NA	ENSG00000202047	0	NA	NA	NA	NA	RNA5SP324
767598	ENSG00000202048	0	NA	NA	NA	NA	SNORD114-20
NA	ENSG00000202050	0	NA	NA	NA	NA	RNU6-391P
NA	ENSG00000202051	0	NA	NA	NA	NA	RNU6-382P
NA	ENSG00000202054	0	NA	NA	NA	NA	RNA5SP152
NA	ENSG00000202056	0	NA	NA	NA	NA	RNA5SP19
NA	ENSG00000202058	2.00237687	0.04693819	0.38082646	0.54798347	NA	RN7SKP80
NA	ENSG00000202059	0	NA	NA	NA	NA	
NA	ENSG00000202060	0	NA	NA	NA	NA	RNA5SP455
NA	ENSG00000202063	0	NA	NA	NA	NA	RNA5SP79
NA	ENSG00000202069	0	NA	NA	NA	NA	RNU4-66P
NA	ENSG00000202070	0	NA	NA	NA	NA	RNU6-1175P
NA	ENSG00000202071	0	NA	NA	NA	NA	
NA	ENSG00000202074	0	NA	NA	NA	NA	RNU6-715P
NA	ENSG00000202078	0	NA	NA	NA	NA	
NA	ENSG00000202079	0	NA	NA	NA	NA	
NA	ENSG00000202081	0	NA	NA	NA	NA	RNU6-1280P
NA	ENSG00000202082	0	NA	NA	NA	NA	RNU6-290P
NA	ENSG00000202089	0	NA	NA	NA	NA	RNU6-1306P
NA	ENSG00000202092	0	NA	NA	NA	NA	RNA5SP190
NA	ENSG00000202093	0	NA	NA	NA	NA	SNORD58C
NA	ENSG00000202095	0	NA	NA	NA	NA	RNU6-1172P
NA	ENSG00000202099	0	NA	NA	NA	NA	RNU6-234P
NA	ENSG00000202100	0	NA	NA	NA	NA	
NA	ENSG00000202103	0	NA	NA	NA	NA	RNU4-12P
56663	ENSG00000202111	0	NA	NA	NA	NA	VTRNA1-2
NA	ENSG00000202112	0	NA	NA	NA	NA	RNU6-690P

NA	ENSG00000202119	0	NA	NA	NA	NA	RNU6-302P
NA	ENSG00000202124	0	NA	NA	NA	NA	RNY4P3
NA	ENSG00000202125	0	NA	NA	NA	NA	RNU1-100P
NA	ENSG00000202137	0	NA	NA	NA	NA	
NA	ENSG00000202141	0	NA	NA	NA	NA	
767596	ENSG00000202142	0	NA	NA	NA	NA	SNORD114-18
NA	ENSG00000202144	0	NA	NA	NA	NA	
NA	ENSG00000202146	0	NA	NA	NA	NA	
NA	ENSG00000202147	0	NA	NA	NA	NA	RNA5SP329
NA	ENSG00000202150	0	NA	NA	NA	NA	RNU6-407P
NA	ENSG00000202151	0	NA	NA	NA	NA	RNY4P28
NA	ENSG00000202157	0	NA	NA	NA	NA	RNU4-11P
NA	ENSG00000202159	0	NA	NA	NA	NA	RNU6-742P
NA	ENSG00000202160	0	NA	NA	NA	NA	RNU5E-7P
NA	ENSG00000202164	0	NA	NA	NA	NA	RNA5SP117
NA	ENSG00000202167	0	NA	NA	NA	NA	RNU1-114P
NA	ENSG00000202169	0	NA	NA	NA	NA	
NA	ENSG00000202172	0	NA	NA	NA	NA	RNU6-1327P
NA	ENSG00000202174	0	NA	NA	NA	NA	RNA5-8SP5
NA	ENSG00000202175	0	NA	NA	NA	NA	RNA5SP128
NA	ENSG00000202177	0	NA	NA	NA	NA	
NA	ENSG00000202182	0	NA	NA	NA	NA	
NA	ENSG00000202183	0	NA	NA	NA	NA	
NA	ENSG00000202184	0	NA	NA	NA	NA	RNU6-1283P
NA	ENSG00000202186	0	NA	NA	NA	NA	RNU6-497P
NA	ENSG00000202187	0	NA	NA	NA	NA	RNA5SP355
100033805	ENSG00000202188	0	NA	NA	NA	NA	SNORD115-31
109616992	ENSG00000202189	0	NA	NA	NA	NA	SNORA30B
NA	ENSG00000202190	0	NA	NA	NA	NA	
767561	ENSG00000202191	0	NA	NA	NA	NA	SNORD113-1
NA	ENSG00000202193	0	NA	NA	NA	NA	RNA5SP427
NA	ENSG00000202195	0	NA	NA	NA	NA	
NA	ENSG00000202198	0	NA	NA	NA	NA	
NA	ENSG00000202199	0	NA	NA	NA	NA	RNU1-115P
NA	ENSG00000202200	0	NA	NA	NA	NA	
NA	ENSG00000202205	0	NA	NA	NA	NA	RNU6-1034P
NA	ENSG00000202206	0	NA	NA	NA	NA	RNU6-169P
NA	ENSG00000202211	0	NA	NA	NA	NA	

NA	ENSG00000202215	0	NA	NA	NA	NA	RNU1-51P
NA	ENSG00000202216	0	NA	NA	NA	NA	
NA	ENSG00000202217	0	NA	NA	NA	NA	RN7SKP47
NA	ENSG00000202222	0	NA	NA	NA	NA	
NA	ENSG00000202224	0	NA	NA	NA	NA	
NA	ENSG00000202225	0	NA	NA	NA	NA	RNA5SP240
NA	ENSG00000202227	0	NA	NA	NA	NA	RNU6-282P
NA	ENSG00000202229	0	NA	NA	NA	NA	RNU6-1138P
NA	ENSG00000202231	0	NA	NA	NA	NA	
NA	ENSG00000202233	0	NA	NA	NA	NA	
NA	ENSG00000202237	0	NA	NA	NA	NA	RNU6-53P
NA	ENSG00000202239	0	NA	NA	NA	NA	RNU6-396P
NA	ENSG00000202240	0	NA	NA	NA	NA	RNU6-737P
NA	ENSG00000202241	0	NA	NA	NA	NA	RN7SKP186
NA	ENSG00000202242	0	NA	NA	NA	NA	RNU6-1276P
NA	ENSG00000202245	0	NA	NA	NA	NA	RNU6-728P
NA	ENSG00000202249	0	NA	NA	NA	NA	RNU5E-5P
NA	ENSG00000202251	0	NA	NA	NA	NA	
85389	ENSG00000202252	0	NA	NA	NA	NA	SNORD14C
NA	ENSG00000202254	0	NA	NA	NA	NA	
NA	ENSG00000202255	0	NA	NA	NA	NA	
NA	ENSG00000202259	0	NA	NA	NA	NA	RNU6-1318P
NA	ENSG00000202260	0	NA	NA	NA	NA	RN7SKP69
100033818	ENSG00000202261	0	NA	NA	NA	NA	SNORD115-44
NA	ENSG00000202263	0	NA	NA	NA	NA	RNA5SP22
NA	ENSG00000202264	0	NA	NA	NA	NA	RNA5SP77
NA	ENSG00000202265	0	NA	NA	NA	NA	RNU6-596P
NA	ENSG00000202268	0	NA	NA	NA	NA	
NA	ENSG00000202269	0	NA	NA	NA	NA	
767590	ENSG00000202270	0	NA	NA	NA	NA	SNORD114-12
NA	ENSG00000202272	0	NA	NA	NA	NA	
NA	ENSG00000202273	0	NA	NA	NA	NA	
NA	ENSG00000202275	0	NA	NA	NA	NA	
NA	ENSG00000202276	0	NA	NA	NA	NA	
NA	ENSG00000202279	0	NA	NA	NA	NA	
NA	ENSG00000202281	0	NA	NA	NA	NA	RNA5SP235
NA	ENSG00000202283	0	NA	NA	NA	NA	
NA	ENSG00000202285	0	NA	NA	NA	NA	RNU6-117P

NA	ENSG00000202290	0	NA	NA	NA	NA	RNA5SP37
767600	ENSG00000202293	0	NA	NA	NA	NA	SNORD114-22
NA	ENSG00000202296	0	NA	NA	NA	NA	RNU6-1335P
NA	ENSG00000202297	0	NA	NA	NA	NA	RNY3P12
NA	ENSG00000202300	0	NA	NA	NA	NA	RNU6-487P
NA	ENSG00000202304	0	NA	NA	NA	NA	RNU6-1130P
NA	ENSG00000202306	0	NA	NA	NA	NA	
NA	ENSG00000202308	0	NA	NA	NA	NA	RNU6-996P
NA	ENSG00000202309	0	NA	NA	NA	NA	
NA	ENSG00000202310	0	NA	NA	NA	NA	
NA	ENSG00000202313	0	NA	NA	NA	NA	RNU1-64P
692075	ENSG00000202314	0	NA	NA	NA	NA	SNORD6
NA	ENSG00000202317	0	NA	NA	NA	NA	RNU1-84P
NA	ENSG00000202318	0	NA	NA	NA	NA	
NA	ENSG00000202322	0	NA	NA	NA	NA	RNA5SP131
NA	ENSG00000202324	0	NA	NA	NA	NA	RNA5SP366
NA	ENSG00000202329	0	NA	NA	NA	NA	RNU6-609P
NA	ENSG00000202331	0	NA	NA	NA	NA	RNA5SP167
NA	ENSG00000202332	0	NA	NA	NA	NA	
NA	ENSG00000202334	0	NA	NA	NA	NA	RNA5SP238
NA	ENSG00000202335	0	NA	NA	NA	NA	
NA	ENSG00000202336	0	NA	NA	NA	NA	RNU6-359P
NA	ENSG00000202339	0	NA	NA	NA	NA	RNU4-45P
NA	ENSG00000202341	0	NA	NA	NA	NA	RNU6-1051P
NA	ENSG00000202343	0	NA	NA	NA	NA	
NA	ENSG00000202344	0	NA	NA	NA	NA	RN7SKP208
NA	ENSG00000202345	0	NA	NA	NA	NA	
NA	ENSG00000202347	0	NA	NA	NA	NA	RNU1-16P
NA	ENSG00000202350	0	NA	NA	NA	NA	RNU6-326P
NA	ENSG00000202351	0	NA	NA	NA	NA	RN7SKP204
6085	ENSG00000202354	0	NA	NA	NA	NA	RNY3
NA	ENSG00000202356	0	NA	NA	NA	NA	RNA5SP277
NA	ENSG00000202357	0	NA	NA	NA	NA	
NA	ENSG00000202358	0	NA	NA	NA	NA	RNU6-652P
NA	ENSG00000202360	0	NA	NA	NA	NA	RN7SKP249
NA	ENSG00000202361	0	NA	NA	NA	NA	
NA	ENSG00000202363	0	NA	NA	NA	NA	SNORA62
NA	ENSG00000202368	0	NA	NA	NA	NA	

NA	ENSG00000202374	0	NA	NA	NA	NA	
109623459	ENSG00000202377	0	NA	NA	NA	NA	SNORA25B
NA	ENSG00000202379	0	NA	NA	NA	NA	
NA	ENSG00000202380	0	NA	NA	NA	NA	RNU1-55P
NA	ENSG00000202382	0	NA	NA	NA	NA	
NA	ENSG00000202383	0	NA	NA	NA	NA	RNA5SP279
NA	ENSG00000202385	0	NA	NA	NA	NA	
NA	ENSG00000202386	0	NA	NA	NA	NA	RNA5SP211
NA	ENSG00000202388	0	NA	NA	NA	NA	
NA	ENSG00000202389	0	NA	NA	NA	NA	
NA	ENSG00000202392	0	NA	NA	NA	NA	RN7SKP292
NA	ENSG00000202395	0	NA	NA	NA	NA	RN7SKP1
NA	ENSG00000202398	0	NA	NA	NA	NA	RNU6-61P
NA	ENSG00000202399	0	NA	NA	NA	NA	
25826	ENSG00000202400	0	NA	NA	NA	NA	SNORD82
NA	ENSG00000202402	0	NA	NA	NA	NA	
NA	ENSG00000202406	0.18922458	0.01954053	0.38488496	0.78225479	NA	RN7SKP187
NA	ENSG00000202407	0	NA	NA	NA	NA	
NA	ENSG00000202408	0	NA	NA	NA	NA	RNVU1-21
NA	ENSG00000202410	0	NA	NA	NA	NA	
NA	ENSG00000202411	0	NA	NA	NA	NA	RNA5SP259
NA	ENSG00000202412	0	NA	NA	NA	NA	RNY3P13
NA	ENSG00000202414	0	NA	NA	NA	NA	
NA	ENSG00000202415	0	NA	NA	NA	NA	RN7SKP269
NA	ENSG00000202417	0	NA	NA	NA	NA	
NA	ENSG00000202422	0	NA	NA	NA	NA	RNA5SP30
NA	ENSG00000202423	0	NA	NA	NA	NA	RNU6-827P
NA	ENSG00000202427	0	NA	NA	NA	NA	RNU6-744P
NA	ENSG00000202428	0	NA	NA	NA	NA	RNU6-108P
NA	ENSG00000202429	0	NA	NA	NA	NA	RNU4-48P
NA	ENSG00000202430	0	NA	NA	NA	NA	RNA5SP88
NA	ENSG00000202431	0	NA	NA	NA	NA	RNU6-438P
NA	ENSG00000202433	0	NA	NA	NA	NA	RNU6-54P
NA	ENSG00000202434	0	NA	NA	NA	NA	
NA	ENSG00000202438	0	NA	NA	NA	NA	
NA	ENSG00000202440	0	NA	NA	NA	NA	
NA	ENSG00000202441	0.14931947	-0.01844332	0.38480212	0.84537521	NA	RNY4P10
NA	ENSG00000202444	0	NA	NA	NA	NA	RNU5E-6P

NA	ENSG00000202445	0	NA	NA	NA	NA	RNU6-669P
NA	ENSG00000202449	0	NA	NA	NA	NA	
NA	ENSG00000202459	0	NA	NA	NA	NA	
NA	ENSG00000202461	0	NA	NA	NA	NA	
NA	ENSG00000202468	0	NA	NA	NA	NA	RNU4-17P
NA	ENSG00000202469	0	NA	NA	NA	NA	
NA	ENSG00000202470	0	NA	NA	NA	NA	
NA	ENSG00000202471	0	NA	NA	NA	NA	RNU4-20P
NA	ENSG00000202472	0	NA	NA	NA	NA	RNA5SP248
NA	ENSG00000202473	0	NA	NA	NA	NA	RN7SKP81
NA	ENSG00000202474	0	NA	NA	NA	NA	RNA5SP283
NA	ENSG00000202476	0	NA	NA	NA	NA	
NA	ENSG00000202478	0	NA	NA	NA	NA	RNU6-81P
NA	ENSG00000202479	0	NA	NA	NA	NA	
NA	ENSG00000202485	0	NA	NA	NA	NA	RNU6-499P
NA	ENSG00000202490	0	NA	NA	NA	NA	RNU6-597P
NA	ENSG00000202491	0	NA	NA	NA	NA	RNU6-716P
NA	ENSG00000202495	0	NA	NA	NA	NA	
NA	ENSG00000202497	0	NA	NA	NA	NA	RNU6-898P
NA	ENSG00000202498	0	NA	NA	NA	NA	
NA	ENSG00000202502	0	NA	NA	NA	NA	RNA5SP151
26817	ENSG00000202503	0	NA	NA	NA	NA	SNORD34
NA	ENSG00000202508	0	NA	NA	NA	NA	
NA	ENSG00000202512	0	NA	NA	NA	NA	RN7SKP230
NA	ENSG00000202513	0	NA	NA	NA	NA	RNU6-805P
NA	ENSG00000202514	0	NA	NA	NA	NA	
56662	ENSG00000202515	0	NA	NA	NA	NA	VTRNA1-3
NA	ENSG00000202517	0	NA	NA	NA	NA	
NA	ENSG00000202522	0	NA	NA	NA	NA	
NA	ENSG00000202523	0	NA	NA	NA	NA	
NA	ENSG00000202528	0	NA	NA	NA	NA	RNU6-650P
595099	ENSG00000202529	0	NA	NA	NA	NA	SNORD18B
NA	ENSG00000202532	0	NA	NA	NA	NA	RNU6-395P
NA	ENSG00000202533	0	NA	NA	NA	NA	
NA	ENSG00000202534	0	NA	NA	NA	NA	RNU6-1329P
NA	ENSG00000202536	0	NA	NA	NA	NA	
NA	ENSG00000202537	0	NA	NA	NA	NA	
26834	ENSG00000202538	0	NA	NA	NA	NA	RNU4-2

NA	ENSG00000202542	0	NA	NA	NA	NA	
664612	ENSG00000202560	0	NA	NA	NA	NA	MIR539
693122	ENSG00000202566	0	NA	NA	NA	NA	MIR421
574447	ENSG00000202569	0	NA	NA	NA	NA	MIR146B
693167	ENSG00000202601	0	NA	NA	NA	NA	MIR582
574441	ENSG00000202609	0	NA	NA	NA	NA	MIR488
NA	ENSG00000203258	0.16735865	0.01897177	0.38482933	0.78225479	NA	
NA	ENSG00000203262	0	NA	NA	NA	NA	
NA	ENSG00000203266	0	NA	NA	NA	NA	
NA	ENSG00000203279	9.76262612	0.10495272	0.35665667	0.51884778	0.73240412	
100128531	ENSG00000203280	0	NA	NA	NA	NA	KIAA1671-AS1
NA	ENSG00000203286	0.69020615	0.00613976	0.38192084	0.89704592	NA	
109729141	ENSG00000203288	45.3216208	-0.5015411	0.34355352	0.03749383	0.1196137	TDRKH-AS1
NA	ENSG00000203307	0.89608518	0.04469613	0.38496371	0.40125479	NA	FAM78B-AS1
NA	ENSG00000203321	0.84255051	0.03666442	0.38463912	0.46277423	NA	CARNMT1-AS1
NA	ENSG00000203325	0.15069912	-0.01848503	0.38480575	0.84537521	NA	
170958	ENSG00000203326	236.07309	0.12555669	0.1846215	0.43813695	0.66330691	ZNF525
NA	ENSG00000203327	7.39300655	-0.01633107	0.35189342	0.90734664	0.95963244	
NA	ENSG00000203334	0	NA	NA	NA	NA	
NA	ENSG00000203335	0	NA	NA	NA	NA	
105378716	ENSG00000203356	0.34109944	0.02362644	0.38535338	0.62411138	NA	LINC01562
NA	ENSG00000203362	8.37344345	-0.18057821	0.39659584	0.26202488	0.48394839	POLH-AS1
NA	ENSG00000203363	0.34746601	0.02370763	0.3853651	0.61987128	NA	GPR160P1
NA	ENSG00000203364	0	NA	NA	NA	NA	
NA	ENSG00000203387	0	NA	NA	NA	NA	SNF8P1
NA	ENSG00000203392	44.9908564	0.23871617	0.29099456	0.2496883	0.46933358	
NA	ENSG00000203395	0	NA	NA	NA	NA	
NA	ENSG00000203396	0	NA	NA	NA	NA	
NA	ENSG00000203397	0.8282343	0.01216318	0.38173245	0.81198921	NA	SHISA5P2
NA	ENSG00000203402	0	NA	NA	NA	NA	
NA	ENSG00000203408	0	NA	NA	NA	NA	OR6C71P
NA	ENSG00000203411	0	NA	NA	NA	NA	
NA	ENSG00000203413	0	NA	NA	NA	NA	ACTBP6
NA	ENSG00000203414	0.14931947	-0.01844332	0.38480212	0.84537521	NA	BTBD7P1
NA	ENSG00000203416	0	NA	NA	NA	NA	FAM32BP
NA	ENSG00000203434	0	NA	NA	NA	NA	
NA	ENSG00000203435	2.35011189	0.04085406	0.37434019	0.6788896	NA	E2F3P2
NA	ENSG00000203436	0	NA	NA	NA	NA	

NA	ENSG00000203437	0.34113746	0.02362674	0.38535343	0.62409123	NA	
106478991	ENSG00000203441	4.4585776	0.05560565	0.3719583	0.62490469	0.80885366	LINC00449
NA	ENSG00000203446	0.6208875	-0.01840553	0.38295181	0.69702053	NA	SUGCT-AS1
NA	ENSG00000203462	0	NA	NA	NA	NA	
NA	ENSG00000203469	0.36296777	0.00145226	0.38336853	0.96210322	NA	
NA	ENSG00000203472	0.15069912	-0.01848503	0.38480575	0.84537521	NA	
64423	ENSG00000203485	913.899191	-0.28333865	0.09260445	0.00156595	0.00870266	INF2
NA	ENSG00000203489	0.15069912	-0.01848503	0.38480575	0.84537521	NA	HMGB1P39
NA	ENSG00000203492	0	NA	NA	NA	NA	
NA	ENSG00000203496	0	NA	NA	NA	NA	
NA	ENSG00000203497	52.4502943	0.09688736	0.26665788	0.61973266	0.80578648	PDCD4-AS1
NA	ENSG00000203498	0	NA	NA	NA	NA	
642574	ENSG00000203499	4.37456158	-0.10521568	0.38561538	0.35501021	0.58543783	IQANK1
NA	ENSG00000203506	25.9426369	-0.06017761	0.30977092	0.74523612	0.87993124	RBMS3-AS2
NA	ENSG00000203520	0.16735865	0.01897177	0.38482933	0.78225479	NA	
NA	ENSG00000203523	0	NA	NA	NA	NA	TAS2R2P
101927447	ENSG00000203527	0	NA	NA	NA	NA	SCUBE1-AS1
NA	ENSG00000203531	0.50756658	0.03074024	0.38601609	0.48351361	NA	
NA	ENSG00000203546	14.9053587	-0.39105716	0.53009971	0.07977857	0.21084412	
NA	ENSG00000203560	0	NA	NA	NA	NA	OR52J1P
NA	ENSG00000203565	0	NA	NA	NA	NA	
NA	ENSG00000203573	0	NA	NA	NA	NA	
26184	ENSG00000203581	1.36135297	0.03410779	0.37996684	0.64030339	NA	OR1F2P
100507175	ENSG00000203585	0	NA	NA	NA	NA	LINC02408
NA	ENSG00000203588	0.48819159	-0.0065213	0.38314647	0.86576387	NA	IGBP1-AS1
NA	ENSG00000203593	0	NA	NA	NA	NA	
101978719	ENSG00000203601	0	NA	NA	NA	NA	LINC00970
NA	ENSG00000203605	0	NA	NA	NA	NA	
NA	ENSG00000203616	1.22892552	-0.03221908	0.3826028	0.57854601	NA	RHOT1P2
2812	ENSG00000203618	221.886881	0.06256602	0.15756611	0.66460608	0.83444869	GP1BB
NA	ENSG00000203620	1.85354485	0.02141132	0.37798452	0.77884469	NA	
NA	ENSG00000203635	1.0366024	0.02185682	0.3818712	0.68993517	NA	
NA	ENSG00000203643	0	NA	NA	NA	NA	
NA	ENSG00000203644	24.7422266	-0.42079078	0.41967021	0.08321426	0.21772162	
100820709	ENSG00000203645	0.173124	-0.01911262	0.38486262	0.84537521	NA	LINC00501
NA	ENSG00000203647	0	NA	NA	NA	NA	
NA	ENSG00000203648	0	NA	NA	NA	NA	
101928287	ENSG00000203650	22.4725383	-0.30040432	0.39231876	0.16640834	0.356962	LINC01285

401993	ENSG00000203661	0	NA	NA	NA	NA	OR2T5
26246	ENSG00000203663	1.18857405	-0.00828753	0.38052827	0.88628064	NA	OR2L2
441932	ENSG00000203664	0	NA	NA	NA	NA	OR2W5P
84288	ENSG00000203666	195.862871	-0.13702468	0.17696772	0.3816738	0.6107766	EFCAB2
116228	ENSG00000203667	1009.18932	-0.25018633	0.0921967	0.00502507	0.0233101	COX20
1122	ENSG00000203668	2191.72575	-0.4311898	0.09239803	1.48E-06	1.62E-05	CHML
23596	ENSG00000203668	2191.72575	-0.4311898	0.09239803	1.48E-06	1.62E-05	CHML
574432	ENSG00000203684	2.64512127	0.04106861	0.37552362	0.66537299	0.83476667	IBA57-DT
375057	ENSG00000203685	9.79637966	0.04399185	0.34166208	0.78711755	0.90325712	STUM
441178	ENSG00000203688	2.82342767	-0.0657187	0.37918655	0.50335292	0.72006071	LINC02487
6953	ENSG00000203690	0.173733	0.01914545	0.38484597	0.78225479	NA	TCP10L3
388743	ENSG00000203697	0.34706974	-0.02334524	0.38533535	0.6762196	NA	CAPN8
128387	ENSG00000203705	361.749859	0.30466423	0.15663538	0.03284271	0.10778817	TATDN3
NA	ENSG00000203706	56.9840651	-0.17260172	0.25439798	0.37505	0.60431998	SERTAD4-AS1
NA	ENSG00000203709	63.0231255	-0.23395991	0.25106276	0.2319402	0.44861961	MIR29B2CHG
1378	ENSG00000203710	0	NA	NA	NA	NA	CR1
100130967	ENSG00000203711	10.1782314	0.04772016	0.3523614	0.74925648	0.88205755	C6orf99
554279	ENSG00000203721	0.66329621	-0.0236254	0.38271394	0.63618766	NA	LINC00862
353091	ENSG00000203722	1.13250251	0.06450809	0.38888264	0.27168088	NA	RAET1G
388722	ENSG00000203724	44.4299226	0.70298238	0.37612455	0.00967959	0.04042159	C1orf53
389432	ENSG00000203727	0	NA	NA	NA	NA	SAMD5
388719	ENSG00000203729	0	NA	NA	NA	NA	LINC00272
127670	ENSG00000203730	1.03528245	0.02217544	0.38190372	0.68565319	NA	TEDDM1
100126572	ENSG00000203733	0	NA	NA	NA	NA	GJE1
345930	ENSG00000203734	2.11418982	0.00383731	0.37533968	0.96487873	NA	ECT2L
9293	ENSG00000203737	0	NA	NA	NA	NA	GPR52
101928673	ENSG00000203739	43.8958869	0.3962983	0.34149283	0.08652708	0.22405342	PRDX6-AS1
149281	ENSG00000203740	0.33971012	0.00270698	0.38338593	0.96261173	NA	METTL11B
2214	ENSG00000203747	0	NA	NA	NA	NA	FCGR3A
253582	ENSG00000203756	0	NA	NA	NA	NA	TMEM244
391114	ENSG00000203757	0	NA	NA	NA	NA	OR6K3
NA	ENSG00000203758	0	NA	NA	NA	NA	OR10T1P
387103	ENSG00000203760	289.675973	0.23650806	0.15262889	0.09074888	0.23219728	CENPW
NA	ENSG00000203761	131.772535	0.31766083	0.19609863	0.06161581	0.17501182	MSTO2P
503542	ENSG00000203772	42.2334349	-0.80465532	0.42121893	0.00661942	0.02949895	SPRN
619208	ENSG00000203778	135.079538	0.09494336	0.20077528	0.58074732	0.77801412	FAM229B
92565	ENSG00000203780	5.06454	0.1776593	0.42669533	0.10669164	0.26139321	FANK1
NA	ENSG00000203781	0	NA	NA	NA	NA	S100A7P1

4014	ENSG00000203782	0	NA	NA	NA	NA	LORICRIN
574414	ENSG00000203783	0	NA	NA	NA	NA	PRR9
149018	ENSG00000203784	0	NA	NA	NA	NA	LELP1
6704	ENSG00000203785	0	NA	NA	NA	NA	SPRR2E
448834	ENSG00000203786	0	NA	NA	NA	NA	KPRP
399818	ENSG00000203791	257.676594	-0.29780193	0.15450733	0.03470936	0.11261384	EEF1AKMT2
118670	ENSG00000203795	0	NA	NA	NA	NA	FAM24A
8528	ENSG00000203797	2.0504227	-0.04896227	0.37926474	0.56923272	NA	DDO
NA	ENSG00000203799	1.84799025	0.04674527	0.38174718	0.52705153	NA	CCDC162P
387111	ENSG00000203801	1.48460441	-0.0566891	0.38529305	0.39601673	NA	LINC00222
NA	ENSG00000203804	9.12769133	-0.05245096	0.35925196	0.7017986	0.85652585	ADAMTSL4-AS1
196051	ENSG00000203805	4.43935481	-0.16943806	0.42821148	0.08336747	0.21800589	PLPP4
154442	ENSG00000203808	149.231411	-0.48363486	0.20308501	0.00623909	0.02799899	BVES-AS1
100113403	ENSG00000203809	17.6024994	-0.23789997	0.41013355	0.19783965	0.40199972	LIN28B-AS1
126961	ENSG00000203811	83.4568125	-1.24845961	0.26845749	3.07E-07	3.79E-06	H3C14
8337	ENSG00000203812	19.6318295	0.03209741	0.38686882	NA	NA	H2AC18
440689	ENSG00000203814	30.8221836	-0.15812268	0.3148089	0.42450412	0.65018652	H2BC18
NA	ENSG00000203825	0	NA	NA	NA	NA	
119548	ENSG00000203837	0	NA	NA	NA	NA	PNLIPRP3
NA	ENSG00000203855	0	NA	NA	NA	NA	HSD3BP4
3283	ENSG00000203857	0	NA	NA	NA	NA	HSD3B1
NA	ENSG00000203858	0	NA	NA	NA	NA	HSD3BP2
3284	ENSG00000203859	0	NA	NA	NA	NA	HSD3B2
388667	ENSG00000203864	0	NA	NA	NA	NA	LINC02868
84852	ENSG00000203865	111.946112	0.04857957	0.22915873	0.78840611	0.90400376	ATP1A1-AS1
282996	ENSG00000203867	1051.384	-0.00472065	0.0817706	0.95345871	0.98074558	RBM20
100132963	ENSG00000203870	0	NA	NA	NA	NA	SMIM9
206412	ENSG00000203872	23.0733747	0.09091674	0.30743964	0.63566384	0.81670577	C6orf163
NA	ENSG00000203875	3164.98281	-0.04021408	0.07133843	0.56541965	0.76735248	SNHG5
100505933	ENSG00000203876	4.90780552	0.03203307	0.36157291	0.804592	0.91124017	ADD3-AS1
134701	ENSG00000203877	207.96704	-0.73719051	0.19172432	2.35E-05	0.00020759	RIPPLY2
149620	ENSG00000203878	0	NA	NA	NA	NA	CHIAP2
2664	ENSG00000203879	5936.05558	-0.24504538	0.05081793	1.11E-06	1.25E-05	GDI1
55251	ENSG00000203880	1841.33204	-0.04794613	0.08036842	0.54100696	0.74955154	PCMTD2
54345	ENSG00000203883	0.70234143	0.00341976	0.38183495	0.94213787	NA	SOX18
54923	ENSG00000203896	252.749206	0.22761334	0.14840473	0.09563154	0.24117576	LIME1
642864	ENSG00000203897	0	NA	NA	NA	NA	SPATA42
100130587	ENSG00000203900	0.34051569	-0.00034141	0.38336262	0.96848892	NA	

441161	ENSG00000203907	0	NA	NA	NA	NA	OOEP
154288	ENSG00000203908	0	NA	NA	NA	NA	KHDC3L
340168	ENSG00000203909	0	NA	NA	NA	NA	DPPA5
388649	ENSG00000203910	0.18922458	0.01954053	0.38488496	0.78225479	NA	C1orf146
NA	ENSG00000203914	0.16735865	0.01897177	0.38482933	0.78225479	NA	HSP90B3P
494118	ENSG00000203923	0	NA	NA	NA	NA	SPANXN1
728712	ENSG00000203926	0	NA	NA	NA	NA	SPANXA2
NA	ENSG00000203930	706.619838	-0.17355218	0.15268113	0.21359997	0.42378234	LINC00632
347487	ENSG00000203933	0	NA	NA	NA	NA	CXorf66
414157	ENSG00000203942	0.18922458	0.01954053	0.38488496	0.78225479	NA	C10orf62
148418	ENSG00000203943	50.6671663	0.31620114	0.30715542	0.14451103	0.32462259	SAMD13
26071	ENSG00000203950	993.182835	0.34371622	0.08343131	2.36E-05	0.00020827	RTL8A
347475	ENSG00000203952	3.98718959	0.20647611	0.44781699	0.08708461	0.22523253	CCDC160
400757	ENSG00000203963	0	NA	NA	NA	NA	C1orf141
84455	ENSG00000203965	180.480856	0.07797186	0.17534993	0.61795139	0.80447215	EFCAB7
245913	ENSG00000203970	0	NA	NA	NA	NA	DEFB110
NA	ENSG00000203971	0	NA	NA	NA	NA	
389396	ENSG00000203972	0.16735865	0.01897177	0.38482933	0.78225479	NA	GLYATL3
388633	ENSG00000203985	0	NA	NA	NA	NA	LDLRAD1
100133077	ENSG00000203987	1.61125817	-0.10583626	0.40142466	0.12672416	NA	
727940	ENSG00000203989	0	NA	NA	NA	NA	RHOXF2B
85026	ENSG00000203993	315.702611	0.15004096	0.13860543	0.24595616	0.46518306	ARRDC1-AS1
440590	ENSG00000203995	174.44121	-0.15648226	0.16848275	0.30017673	0.52733399	ZYG11A
284751	ENSG00000203999	275.309881	0.50514763	0.1606872	0.00061445	0.00386236	LINC01270
138307	ENSG00000204001	0	NA	NA	NA	NA	LCN8
NA	ENSG00000204003	0	NA	NA	NA	NA	
284546	ENSG00000204006	0	NA	NA	NA	NA	C1orf185
360203	ENSG00000204007	0	NA	NA	NA	NA	GLT6D1
439996	ENSG00000204010	0.15069912	-0.01848503	0.38480575	0.84537521	NA	IFIT1B
NA	ENSG00000204011	0	NA	NA	NA	NA	COL5A1-AS1
203413	ENSG00000204019	0	NA	NA	NA	NA	CT83
643418	ENSG00000204020	0	NA	NA	NA	NA	LIPN
643414	ENSG00000204021	0	NA	NA	NA	NA	LIPK
142910	ENSG00000204022	0	NA	NA	NA	NA	LIPJ
100329135	ENSG00000204025	1.08255373	0.05607251	0.38976327	0.21070257	NA	TRPC5OS
NA	ENSG00000204031	0	NA	NA	NA	NA	LCN1P2
340745	ENSG00000204033	1.61614074	-0.09757152	0.3974854	0.15210627	NA	LRIT2
109729184	ENSG00000204044	46.7613863	-0.43262566	0.3515118	0.06689509	0.18544027	SLC12A5-AS1

NA	ENSG00000204049	1.22788028	0.01823213	0.38022092	0.77283183	NA	
221424	ENSG00000204052	44.1925979	0.10681445	0.28273765	0.58744379	0.78256674	LRRC73
NA	ENSG00000204053	0	NA	NA	NA	NA	MYCLP1
NA	ENSG00000204054	302.690865	0.08986846	0.14324362	0.49967844	0.71690947	LINC00963
NA	ENSG00000204055	1.25686791	-0.05755156	0.3858471	0.38486497	NA	
100132074	ENSG00000204060	89.2050536	-0.34727907	0.23189698	0.06800623	0.18768167	FOXO6
340543	ENSG00000204065	293.232678	-0.29035951	0.13997042	0.02541514	0.08824579	TCEAL5
90196	ENSG00000204070	313.1477	-0.06457944	0.14593503	0.63127252	0.8136456	SYS1
158931	ENSG00000204071	2.41939602	-0.05224609	0.37827401	0.56952389	NA	TCEAL6
NA	ENSG00000204072	0	NA	NA	NA	NA	ARMCX7P
3633	ENSG00000204084	386.645625	0.57735019	0.13370475	4.86E-06	4.90E-05	INPP5B
29935	ENSG00000204086	1.62561171	0.02838365	0.37940571	0.69677524	NA	RPA4
732253	ENSG00000204091	109.773133	-1.02636359	0.23961128	2.16E-06	2.31E-05	TDRG1
NA	ENSG00000204092	31.3610613	-1.3352491	0.47471636	0.0003431	0.0023114	LINC00951
129807	ENSG00000204099	15.629894	-0.41008674	0.52810896	0.07365839	0.19875293	NEU4
9935	ENSG00000204103	57.6762759	1.31880337	0.34639637	1.15E-05	0.00010836	MAFB
26146	ENSG00000204104	340.017718	0.36655501	0.19263002	0.02927361	0.09848879	TRAF3IP1
NA	ENSG00000204110	0.49034168	-0.01094288	0.3829019	0.82405874	NA	LINC02520
NA	ENSG00000204113	0.32444695	0.00134473	0.38336811	0.96743017	NA	BMP2KL
53344	ENSG00000204116	175.789763	0.08864327	0.18151438	0.58036024	0.77774473	CHIC1
100287792	ENSG00000204117	0.18922458	0.01954053	0.38488496	0.78225479	NA	
645996	ENSG00000204118	0	NA	NA	NA	NA	NAP1L6P
26058	ENSG00000204120	1596.00386	-0.41297682	0.09476323	6.62E-06	6.49E-05	GIGYF2
NA	ENSG00000204121	0	NA	NA	NA	NA	ECEL1P1
257407	ENSG00000204128	175.508947	-0.03175912	0.18518626	0.84317419	0.9288493	C2orf72
55680	ENSG00000204130	442.885167	-0.54688969	0.11678517	9.70E-07	1.10E-05	RUFY2
340527	ENSG00000204131	1359.39403	0.08927849	0.08551231	0.28382867	0.50900848	NHSL2
392490	ENSG00000204131	1359.39403	0.08927849	0.08551231	0.28382867	0.50900848	NHSL2
2681	ENSG00000204136	164.982164	-0.94115262	0.19888333	3.04E-07	3.76E-06	GGTA1
65979	ENSG00000204138	1695.72051	-0.02884711	0.07632328	0.69908829	0.85487569	PHACTR4
340204	ENSG00000204140	0	NA	NA	NA	NA	CLPSL1
653308	ENSG00000204147	567.524269	-0.24592667	0.15316118	0.0788437	0.20929342	ASAH2B
58483	ENSG00000204148	0	NA	NA	NA	NA	LINC00474
414189	ENSG00000204149	641.904687	0.19824816	0.10657662	0.05238997	0.15429309	AGAP6
100652748	ENSG00000204152	389.432129	0.04997866	0.12211244	0.66676483	0.83582576	TIMM23B
84243	ENSG00000204160	731.08727	-0.06619089	0.10925839	0.5252708	0.73786263	ZDHHC18
170371	ENSG00000204161	0	NA	NA	NA	NA	TMEM273
158830	ENSG00000204165	0	NA	NA	NA	NA	CXorf65

642517	ENSG00000204172	428.060713	0.24414701	0.12016461	0.03200493	0.10558327	AGAP9
652972	ENSG00000204173	1.16601517	-0.00703431	0.38037911	0.90452519	NA	LRRC37A5P
5540	ENSG00000204174	0.69675292	0.02298921	0.3839153	0.59834713	NA	NPY4R
9721	ENSG00000204175	30.4689553	0.9975471	0.53232719	0.00477064	0.02232299	GPRIN2
83849	ENSG00000204176	18.5927666	-0.21163999	0.40078715	0.22275921	0.4358511	SYT15
399761	ENSG00000204177	321.192274	-0.00742598	0.13454349	0.95198159	0.98012235	BMS1P1
55219	ENSG00000204178	895.208482	-0.37214397	0.09273456	3.37E-05	0.0002871	MACO1
26095	ENSG00000204179	27.8719757	-0.29838105	0.37139479	0.17089085	0.36292812	PTPN20
NA	ENSG00000204183	0.173124	-0.01911262	0.38486262	0.84537521	NA	GDF5-AS1
57683	ENSG00000204186	815.44751	-0.32001898	0.10699615	0.0017859	0.00970655	ZDBF2
449520	ENSG00000204188	0.85183244	0.0162954	0.38188389	0.75356296	NA	GGNBP1
255220	ENSG00000204193	0	NA	NA	NA	NA	TXNDC8
NA	ENSG00000204194	3.42688125	0.04309508	0.37971794	0.59479757	0.78787011	RPL12P1
158833	ENSG00000204195	0	NA	NA	NA	NA	AWAT1
NA	ENSG00000204196	0.34624801	-0.02356563	0.38536324	0.65270996	NA	RPL12P16
1616	ENSG00000204209	2441.87321	-0.09765878	0.06443045	0.12385404	0.29169785	DAXX
113523636	ENSG00000204209	2441.87321	-0.09765878	0.06443045	0.12385404	0.29169785	DAXX
659	ENSG00000204217	2159.77044	-0.32382462	0.06624302	6.65E-07	7.77E-06	BMPR2
6920	ENSG00000204219	9.31560075	1.18512121	1.50598859	0.00920855	0.03873173	TCEA3
10471	ENSG00000204220	945.53148	-0.00179095	0.09024317	0.98296934	0.99428951	PFDN6
6015	ENSG00000204227	985.891521	0.1705309	0.08311263	0.03549333	0.11453931	RING1
7923	ENSG00000204228	84.6740845	0.58692328	0.28178151	0.00903086	0.03811757	HSD17B8
6257	ENSG00000204231	1163.20793	-0.00085181	0.08035027	0.99092967	0.99695967	RXRB
339229	ENSG00000204237	746.148645	0.09758588	0.11260296	0.36491484	0.59440972	OXLD1
100128239	ENSG00000204241	25.2862957	-0.19864687	0.33359245	0.32447029	0.55221149	LINC02731
138803	ENSG00000204246	0	NA	NA	NA	NA	OR13C3
1302	ENSG00000204248	103.275525	0.47871977	0.29684228	0.03236291	0.10653394	COL11A2
414319	ENSG00000204250	0	NA	NA	NA	NA	LINC00587
3111	ENSG00000204252	0.32291469	0.00129779	0.38336776	0.96865395	NA	HLA-DOA
NA	ENSG00000204253	1.63660251	0.02613906	0.37923633	0.71809995	NA	HNRNPCP2
6046	ENSG00000204256	5229.15646	0.2109022	0.05751253	0.00020905	0.00148124	BRD2
3108	ENSG00000204257	11.0027819	0.7454281	0.94116254	0.02114575	0.07618153	HLA-DMA
100507463	ENSG00000204261	3.20331856	0.03455801	0.3734787	0.72254315	0.86754018	PSMB8-AS1
1290	ENSG00000204262	66.9243645	0.45156273	0.30779198	0.04659612	0.14105386	COL5A2
5696	ENSG00000204264	37.6057281	0.17118559	0.29156214	0.39700275	0.62606586	PSMB8
6891	ENSG00000204267	160.043391	0.34620043	0.21413853	0.05585251	0.16214719	TAP2
169981	ENSG00000204271	560.790883	0.19558422	0.11192201	0.06710028	0.18582768	SPIN3
550643	ENSG00000204272	419.415152	0.12984945	0.12546947	0.27315818	0.49676945	NBDY

100996291	ENSG00000204277	0.64084514	0.00289197	0.38136785	0.95418768	NA	LINC01993
283999	ENSG00000204278	8.61055269	0.15252667	0.38196971	0.33417143	0.56389725	TMEM235
139793	ENSG00000204279	0.30001564	-0.02294458	0.38527538	0.68644889	NA	PAGE3
400624	ENSG00000204283	6.14101164	-0.04375929	0.37202451	0.66442369	0.83444869	LINC01973
3122	ENSG00000204287	0	NA	NA	NA	NA	HLA-DRA
56244	ENSG00000204290	0	NA	NA	NA	NA	BTNL2
1306	ENSG00000204291	0	NA	NA	NA	NA	COL15A1
10665	ENSG00000204296	0	NA	NA	NA	NA	TSBP1
338661	ENSG00000204300	0	NA	NA	NA	NA	TMEM225
4855	ENSG00000204301	226.546945	-0.33094984	0.17776191	0.03588639	0.11558215	NOTCH4
5089	ENSG00000204304	1769.04627	0.25678932	0.07961771	0.00095803	0.00570447	PBX2
177	ENSG00000204305	85.4713429	0.19479307	0.22944875	0.29583492	0.52251089	AGER
6048	ENSG00000204308	890.667048	0.01204224	0.09000105	0.89108194	0.95273684	RNF5
10554	ENSG00000204310	2465.60218	-0.0623665	0.06608486	0.33757044	0.56786481	AGPAT1
494513	ENSG00000204311	40.8338211	0.52249464	0.39415055	0.04122499	0.12836755	PJVK
80863	ENSG00000204314	100.049057	0.60089311	0.24654686	0.00368458	0.0180143	PRRT1
100507547	ENSG00000204314	100.049057	0.60089311	0.24654686	0.00368458	0.0180143	PRRT1
63943	ENSG00000204315	93.2126766	-0.15734834	0.20798309	0.36921427	0.59862194	FKBPL
64978	ENSG00000204316	985.949724	0.06701155	0.0966045	0.47398901	0.69607269	MRPL38
643008	ENSG00000204323	14.1185683	0.02204598	0.3331282	0.89663196	0.95469109	SMIM5
285141	ENSG00000204334	1.020046	0.05850572	0.38757233	0.313004	NA	ERICH2
389058	ENSG00000204335	0	NA	NA	NA	NA	SP5
NA	ENSG00000204338	1.75524888	-0.01958176	0.37682227	0.80619051	NA	CYP21A1P
8859	ENSG00000204344	357.072769	0.29588605	0.13134348	0.01623374	0.06150035	STK19
100131439	ENSG00000204345	0	NA	NA	NA	NA	CD300LD
388419	ENSG00000204347	0.62399897	-0.03619255	0.38661507	0.4273305	NA	BTBD17
1797	ENSG00000204348	511.597788	0.13877791	0.1099726	0.18744972	0.38775961	DXO
6499	ENSG00000204351	1492.87787	0.28881885	0.08052266	0.0002377	0.00165882	SKIV2L
NA	ENSG00000204352	43.0585812	0.53982087	0.35846757	0.03036443	0.10131192	C9orf129
7936	ENSG00000204356	1305.42385	0.19897401	0.07614952	0.00760844	0.03312519	NELFE
120406	ENSG00000204361	0.18922458	0.01954053	0.38488496	0.78225479	NA	NXPE2
400743	ENSG00000204362	0	NA	NA	NA	NA	LINC02783
494197	ENSG00000204363	0	NA	NA	NA	NA	SPANXN5
221527	ENSG00000204366	1131.25474	-0.01206027	0.09274683	0.89262147	0.95351371	ZBTB12
NA	ENSG00000204368	0	NA	NA	NA	NA	
6392	ENSG00000204370	745.835332	-0.00229616	0.0975629	0.98005752	0.99338199	SDHD
10919	ENSG00000204371	2908.70712	0.09517687	0.06177118	0.11822478	0.28215801	EHMT2
653220	ENSG00000204379	353.813255	-1.09741853	0.1440367	3.02E-15	8.44E-14	XAGE1A

100129029	ENSG00000204380	11.2961098	0.03199383	0.33998257	0.84467964	0.92965052	PKP4-AS1
143903	ENSG00000204381	217.455872	1.67433881	0.19843692	2.36E-18	8.11E-17	LAYN
653067	ENSG00000204382	1.60690846	0.04707782	0.38111878	0.54091693	NA	XAGE1B
80736	ENSG00000204385	1.47948131	0.03726333	0.38295883	0.50725379	NA	SLC44A4
4758	ENSG00000204386	444.949237	-0.36896483	0.13508717	0.00342832	0.01695359	NEU1
50854	ENSG00000204387	1733.15093	0.1175847	0.07763375	0.12160199	0.2881729	SNHG32
3304	ENSG00000204388	1484.45397	0.49886804	0.26920471	0.01988978	0.07265777	HSPA1B
3303	ENSG00000204389	1840.74147	0.56381923	0.23275768	0.00431272	0.02057329	HSPA1A
3305	ENSG00000204390	54.9302597	0.23665846	0.27405554	0.24506402	0.46389372	HSPA1L
57819	ENSG00000204392	675.062708	0.06729285	0.10485522	0.50541641	0.72165971	LSM2
149950	ENSG00000204393	0	NA	NA	NA	NA	NOL4L-DT
7407	ENSG00000204394	3540.45801	0.21847594	0.06786781	0.00102694	0.00604406	VARS1
80737	ENSG00000204396	10.0723146	0.15412082	0.38539783	0.31241551	0.54015893	VWA7
114769	ENSG00000204397	0	NA	NA	NA	NA	CARD16
NA	ENSG00000204398	0	NA	NA	NA	NA	
NA	ENSG00000204399	0	NA	NA	NA	NA	
100506742	ENSG00000204403	0	NA	NA	NA	NA	CASP12
55777	ENSG00000204406	520.902178	-0.15946741	0.30650285	0.42319721	0.64917034	MBD5
4439	ENSG00000204410	758.084608	0.49458131	0.09947964	2.68E-07	3.34E-06	MSH5
1444	ENSG00000204414	0	NA	NA	NA	NA	CSHL1
80739	ENSG00000204420	6.03638886	0.16253691	0.40393116	0.23408221	0.45093611	MPIG6B
80740	ENSG00000204421	1.4729201	0.06935505	0.39038739	0.20951455	NA	LY6G6C
NA	ENSG00000204422	0	NA	NA	NA	NA	
259215	ENSG00000204424	0	NA	NA	NA	NA	LY6G6F
7920	ENSG00000204427	535.510558	0.28332001	0.10836201	0.0062856	0.02818224	ABHD16A
80741	ENSG00000204428	27.5209044	0.20689134	0.32615356	0.31433845	0.5421676	LY6G5C
NA	ENSG00000204429	0.18922458	0.01954053	0.38488496	0.78225479	NA	
NA	ENSG00000204434	0.47049773	-0.02830157	0.38579648	0.54521756	NA	POTEKP
1460	ENSG00000204435	1881.12656	0.31335607	0.08226473	9.34E-05	0.00071649	CSNK2B
NA	ENSG00000204437	0	NA	NA	NA	NA	CTSLP6
7918	ENSG00000204438	502.854767	-0.07256581	0.13971767	0.57597881	0.77463528	GPANK1
57827	ENSG00000204439	390.209298	0.14207193	0.13124214	0.24910769	0.46846413	C6orf47
728215	ENSG00000204442	866.619538	-0.08271821	0.08585307	0.32162551	0.54929628	FAM155A
55937	ENSG00000204444	36.0486289	0.21716454	0.30163236	0.29438434	0.52061418	APOM
401535	ENSG00000204446	0.5972779	-0.02732172	0.38586499	0.50076333	NA	LINC02872
642612	ENSG00000204449	0	NA	NA	NA	NA	TRIM49C
120146	ENSG00000204450	0	NA	NA	NA	NA	TRIM64
NA	ENSG00000204455	0	NA	NA	NA	NA	TRIM51BP

NA	ENSG00000204456	0	NA	NA	NA	NA	
151121	ENSG00000204460	0.18922458	0.01954053	0.38488496	0.78225479	NA	LINC01854
7917	ENSG00000204463	6371.21001	0.17535989	0.06155512	0.0038843	0.01880082	BAG6
727684	ENSG00000204464	0	NA	NA	NA	NA	C1orf195
7916	ENSG00000204469	10714.3544	0.16222107	0.04743602	0.00056783	0.00360737	PRRC2A
NA	ENSG00000204471	0	NA	NA	NA	NA	TBC1D3P4
199	ENSG00000204472	0	NA	NA	NA	NA	AIF1
259197	ENSG00000204475	0	NA	NA	NA	NA	NCR3
645425	ENSG00000204478	0	NA	NA	NA	NA	PRAMEF20
391004	ENSG00000204479	0	NA	NA	NA	NA	PRAMEF17
645414	ENSG00000204480	0	NA	NA	NA	NA	PRAMEF19
729528	ENSG00000204481	0	NA	NA	NA	NA	PRAMEF14
7940	ENSG00000204482	1.21358277	-0.00117298	0.38068136	0.91953419	NA	LST1
4795	ENSG00000204498	646.118882	-0.03082098	0.12315017	0.79194643	0.90499209	NFKBIL1
653619	ENSG00000204501	0	NA	NA	NA	NA	PRAMEF15
343070	ENSG00000204505	0	NA	NA	NA	NA	PRAMEF9
441871	ENSG00000204510	0	NA	NA	NA	NA	PRAMEF7
401250	ENSG00000204511	0.18922458	0.01954053	0.38488496	0.78225479	NA	MCCD1
730051	ENSG00000204514	399.177505	-0.36821692	0.19938851	0.03250756	0.10692141	ZNF814
4277	ENSG00000204516	0	NA	NA	NA	NA	MICB
343066	ENSG00000204518	0	NA	NA	NA	NA	AADACL4
90233	ENSG00000204519	401.141732	-0.47215068	0.16003195	0.00124441	0.00712732	ZNF551
100507436	ENSG00000204520	0	NA	NA	NA	NA	MICA
390980	ENSG00000204524	232.670435	-0.03853072	0.15312428	0.7827046	0.90105949	ZNF805
3107	ENSG00000204525	509.037216	1.19443164	0.11672778	1.56E-25	7.55E-24	HLA-C
NA	ENSG00000204528	0	NA	NA	NA	NA	PSORS1C3
NA	ENSG00000204529	0	NA	NA	NA	NA	GUCY2EP
5460	ENSG00000204531	2.68213873	0.04115475	0.376934	0.64810709	0.82447067	POU5F1
649137	ENSG00000204532	0	NA	NA	NA	NA	ZSCAN5C
54535	ENSG00000204536	396.325561	0.29592165	0.14224996	0.02478676	0.08654862	CCHCR1
170680	ENSG00000204538	0	NA	NA	NA	NA	PSORS1C2
1041	ENSG00000204539	0	NA	NA	NA	NA	CDSN
170679	ENSG00000204540	0	NA	NA	NA	NA	PSORS1C1
29113	ENSG00000204542	0	NA	NA	NA	NA	C6orf15
394263	ENSG00000204544	0	NA	NA	NA	NA	MUC21
245935	ENSG00000204547	0	NA	NA	NA	NA	DEFB122
245934	ENSG00000204548	0	NA	NA	NA	NA	DEFB121
NA	ENSG00000204555	0	NA	NA	NA	NA	CFTRP3

NA	ENSG00000204556	3.6981796	0.10333135	0.39059326	0.31180589	0.53952807	
8449	ENSG00000204560	1041.44261	-0.03840697	0.09769803	0.68339148	0.845714	DHX16
221545	ENSG00000204564	173.338585	-0.18128114	0.16741752	0.2276885	0.44257273	C6orf136
28973	ENSG00000204568	566.329695	-0.38792741	0.1406752	0.00299386	0.01509866	MRPS18B
5514	ENSG00000204569	1923.98578	-0.61392404	0.08373132	6.96E-14	1.73E-12	PPP1R10
440051	ENSG00000204571	0	NA	NA	NA	NA	KRTAP5-11
387273	ENSG00000204572	0.14931947	-0.01844332	0.38480212	0.84537521	NA	KRTAP5-10
23	ENSG00000204574	2563.25362	-0.03278613	0.06766732	0.62195926	0.80695268	ABCF1
80742	ENSG00000204576	1132.46923	-0.15730434	0.07975082	0.04342904	0.13354497	PRR3
11025	ENSG00000204577	1.56561859	-0.02688328	0.37794866	0.73059694	NA	LILRB3
102725035	ENSG00000204577	1.56561859	-0.02688328	0.37794866	0.73059694	NA	LILRB3
107987425	ENSG00000204577	1.56561859	-0.02688328	0.37794866	0.73059694	NA	LILRB3
107987462	ENSG00000204577	1.56561859	-0.02688328	0.37794866	0.73059694	NA	LILRB3
780	ENSG00000204580	605.68724	0.78194665	0.16139352	2.33E-07	2.93E-06	DDR1
400997	ENSG00000204581	6.70143712	0.1882635	0.4096735	0.21917825	0.43105634	ACOXL-AS1
100507055	ENSG00000204583	0	NA	NA	NA	NA	LRCOL1
NA	ENSG00000204584	2.3213679	0.0229678	0.37691629	0.7787354	NA	
440894	ENSG00000204588	3.15051756	-0.04147031	0.37685431	0.64479319	0.82273439	LINC01123
2794	ENSG00000204590	2163.22276	-0.33373952	0.07877426	1.44E-05	0.0001328	GNL1
3133	ENSG00000204592	112.867827	2.62485841	0.27358241	6.09E-23	2.64E-21	HLA-E
503834	ENSG00000204595	0	NA	NA	NA	NA	DPRX
56658	ENSG00000204599	321.595599	-0.07802698	0.17555434	0.61599197	0.80313338	TRIM39
116437	ENSG00000204603	0.49101175	-0.02928285	0.38588267	0.52914013	NA	LINC01257
90333	ENSG00000204604	288.978781	0.14124516	0.1350598	0.26327431	0.48530728	ZNF468
89870	ENSG00000204610	0	NA	NA	NA	NA	TRIM15
90317	ENSG00000204611	337.971397	-0.01048297	0.13905943	0.93438237	0.97267151	ZNF616
442425	ENSG00000204612	0	NA	NA	NA	NA	FOXB2
10107	ENSG00000204613	0.16735865	0.01897177	0.38482933	0.78225479	NA	TRIM10
135644	ENSG00000204614	0.18922458	0.01954053	0.38488496	0.78225479	NA	TRIM40
11074	ENSG00000204616	0.16735865	0.01897177	0.38482933	0.78225479	NA	TRIM31
80352	ENSG00000204618	43.1399357	0.59298959	0.36716154	0.02139258	0.07686143	RNF39
6992	ENSG00000204619	687.271808	-0.34875443	0.10256472	0.00040094	0.00265244	PPP1R11
NA	ENSG00000204620	11.4561692	0.08345113	0.34227619	0.62459025	0.80860493	
NA	ENSG00000204622	4.52429247	-0.03395698	0.36577002	0.77163571	0.89527792	HLA-J
80862	ENSG00000204623	313.767055	0.07200613	0.14087364	0.58349335	0.77994081	ZNRD1ASP
57540	ENSG00000204624	846.415246	-2.02552679	0.11884004	2.54E-66	3.79E-64	DISP3
10255	ENSG00000204625	0.173124	-0.01911262	0.38486262	0.84537521	NA	HCG9
10399	ENSG00000204628	28128.9094	0.05249021	0.05458372	0.33324081	0.56270076	RACK1

3135	ENSG00000204632	0.14931947	-0.01844332	0.38480212	0.84537521	NA	HLA-G
11138	ENSG00000204634	556.71771	1.19121229	0.12270712	3.07E-23	1.36E-21	TBC1D8
NA	ENSG00000204637	0	NA	NA	NA	NA	ARPP19P2
129521	ENSG00000204640	0	NA	NA	NA	NA	NMS
3134	ENSG00000204642	0.91404668	-0.00896902	0.3812081	0.86891107	NA	HLA-F
346171	ENSG00000204644	0	NA	NA	NA	NA	ZFP57
280660	ENSG00000204648	0	NA	NA	NA	NA	SSX9P
147081	ENSG00000204650	1009.93247	-0.10476	0.08665001	0.21373079	0.4238788	LINC02210
NA	ENSG00000204652	0	NA	NA	NA	NA	RPS26P8
554235	ENSG00000204653	138.21949	-1.70376183	0.24127235	1.19E-13	2.89E-12	ASPDH
4340	ENSG00000204655	0.15069912	-0.01848503	0.38480575	0.84537521	NA	MOG
7932	ENSG00000204657	2.13817174	0.06313732	0.38200839	0.47386831	NA	OR2H2
NA	ENSG00000204658	0	NA	NA	NA	NA	CST9LP2
646019	ENSG00000204659	2.63491305	0.08611121	0.39268695	0.23666649	0.45349268	CBY3
285679	ENSG00000204661	0.7961785	-0.0042968	0.38212318	0.92706881	NA	C5orf60
NA	ENSG00000204662	0	NA	NA	NA	NA	CST9LP1
164380	ENSG00000204663	0	NA	NA	NA	NA	CST13P
400710	ENSG00000204666	0.8566524	0.04855441	0.38842135	0.27272764	NA	
138240	ENSG00000204669	0	NA	NA	NA	NA	C9orf57
NA	ENSG00000204670	0	NA	NA	NA	NA	IGKV1OR2-3
386653	ENSG00000204671	0	NA	NA	NA	NA	IL31
84335	ENSG00000204673	1453.21489	-0.0678032	0.08017877	0.38658148	0.61566669	AKT1S1
653316	ENSG00000204677	3.92878899	-0.10959775	0.38811972	0.33555136	0.5655232	FAM153CP
2550	ENSG00000204681	770.717538	0.62110545	0.12050745	7.05E-08	9.47E-07	GABBR1
399726	ENSG00000204682	347.007603	0.82177902	0.13216385	9.04E-11	1.75E-09	MIR1915HG
284788	ENSG00000204684	0	NA	NA	NA	NA	
NA	ENSG00000204685	160.318572	-0.02912105	0.17320515	0.84937371	0.93154246	STARD7-AS1
116511	ENSG00000204687	0	NA	NA	NA	NA	MAS1L
26716	ENSG00000204688	0	NA	NA	NA	NA	OR2H1
26531	ENSG00000204694	0	NA	NA	NA	NA	OR11A1
442191	ENSG00000204695	0	NA	NA	NA	NA	OR14J1
NA	ENSG00000204697	0	NA	NA	NA	NA	OR2U1P
NA	ENSG00000204699	0	NA	NA	NA	NA	UBTFL3
26707	ENSG00000204700	0	NA	NA	NA	NA	OR2J2
442186	ENSG00000204701	0	NA	NA	NA	NA	OR2J3
442185	ENSG00000204702	0	NA	NA	NA	NA	OR2J1
442184	ENSG00000204703	0	NA	NA	NA	NA	OR2B3
26692	ENSG00000204704	0	NA	NA	NA	NA	OR2W1

NA	ENSG00000204705	0	NA	NA	NA	NA	UBTFL5
NA	ENSG00000204706	5.7859449	0.04847669	0.36203485	0.71536693	0.86274919	MAMDC2-AS1
NA	ENSG00000204709	0.45209736	-0.02505403	0.38558831	0.57865948	NA	LINC01556
387778	ENSG00000204710	0	NA	NA	NA	NA	SPDYC
138255	ENSG00000204711	0	NA	NA	NA	NA	C9orf135
5987	ENSG00000204713	2477.71545	-0.09670379	0.06393463	0.124731	0.29302114	TRIM27
NA	ENSG00000204718	0	NA	NA	NA	NA	CNN2P12
340895	ENSG00000204740	0.56767373	0.02627943	0.38574813	0.49686938	NA	MALRD1
NA	ENSG00000204745	20.4013739	0.20295917	0.36563841	0.29694732	0.5236583	
NA	ENSG00000204754	0	NA	NA	NA	NA	LINC01951
100268168	ENSG00000204758	2.87227254	-0.00715993	0.37239544	0.93769195	0.97366414	
64901	ENSG00000204764	224.145589	0.0671173	0.18377896	0.67839613	0.84291038	RANBP17
100131897	ENSG00000204767	1.62332779	-0.10774728	0.40471665	0.06293501	NA	INSYN2B
NA	ENSG00000204776	0	NA	NA	NA	NA	IGKV1OR-3
NA	ENSG00000204778	17.5187644	-0.05644151	0.34199931	0.72763603	0.86981375	CBWD4P
653427	ENSG00000204779	3.97332606	-0.00830487	0.36775332	0.93723126	0.97354401	FOXD4L5
NA	ENSG00000204780	0	NA	NA	NA	NA	IGKV1OR9-1
NA	ENSG00000204787	0	NA	NA	NA	NA	REG1CP
NA	ENSG00000204789	117.863517	0.34646526	0.22066108	0.06068362	0.17285902	ZNF204P
NA	ENSG00000204790	0.16735865	0.01897177	0.38482933	0.78225479	NA	
NA	ENSG00000204791	31.0774475	-0.71786568	0.47586191	0.01631915	0.0617532	SMPD5
NA	ENSG00000204792	68.0364568	-0.05833747	0.22577707	0.74883912	0.88205755	LINC01291
NA	ENSG00000204801	0	NA	NA	NA	NA	FGF7P7
NA	ENSG00000204802	92.608758	0.00481816	0.26640035	0.98198943	0.99406451	
NA	ENSG00000204814	0.15069912	-0.01848503	0.38480575	0.84537521	NA	
83538	ENSG00000204815	85.1947479	0.48838916	0.26855734	0.02201333	0.07869268	ODAD4
NA	ENSG00000204816	0	NA	NA	NA	NA	FGF7P5
NA	ENSG00000204818	0	NA	NA	NA	NA	ADGRF5P2
116540	ENSG00000204822	746.933638	0.12024345	0.09995384	0.21278681	0.42263393	MRPL53
100128098	ENSG00000204832	0	NA	NA	NA	NA	ST8SIA6-AS1
NA	ENSG00000204837	24.8475453	-0.14528455	0.32690854	0.45147614	0.67474853	FGF7P3
642475	ENSG00000204839	57.6015709	0.21567992	0.27243615	0.28549738	0.51095086	MROH6
6311	ENSG00000204842	1346.89733	0.15796011	0.08510944	0.05668334	0.16395938	ATXN2
1639	ENSG00000204843	5161.35735	-0.18422868	0.05573094	0.00065072	0.00405601	DCTN1
647060	ENSG00000204849	0	NA	NA	NA	NA	SPATA31A1
NA	ENSG00000204850	14.3811596	-0.00808861	0.35740352	0.94835044	0.97876335	
57469	ENSG00000204851	146.314609	-0.0203309	0.1934233	0.90203287	0.95687554	PNMA8B
79600	ENSG00000204852	682.060466	0.10173053	0.10856916	0.3285659	0.55706267	TCTN1

29902	ENSG00000204856	542.277406	-0.10566613	0.11324811	0.32789361	0.55639832	FAM216A
3104	ENSG00000204859	431.638899	0.41073223	0.15219312	0.00336529	0.01667518	ZBTB48
NA	ENSG00000204860	138.867279	-0.60033652	0.22848816	0.00217136	0.01148486	FAM201A
147920	ENSG00000204866	0	NA	NA	NA	NA	IGFL2
444882	ENSG00000204869	0.173124	-0.01911262	0.38486262	0.84537521	NA	IGFL4
51471	ENSG00000204872	0.18922458	0.01954053	0.38488496	0.78225479	NA	NAT8B
83900	ENSG00000204873	0	NA	NA	NA	NA	KRTAP9-3
NA	ENSG00000204876	0	NA	NA	NA	NA	
728224	ENSG00000204880	0	NA	NA	NA	NA	KRTAP4-8
2843	ENSG00000204882	0.95047514	-0.05304929	0.38916907	0.25367556	NA	GPR20
728255	ENSG00000204887	0	NA	NA	NA	NA	KRTAP1-4
125115	ENSG00000204889	0	NA	NA	NA	NA	KRT40
NA	ENSG00000204894	0	NA	NA	NA	NA	
147183	ENSG00000204897	0	NA	NA	NA	NA	KRT25
440145	ENSG00000204899	773.677476	-0.26720857	0.09853713	0.00483665	0.02258926	MZT1
724087	ENSG00000204904	1.85127649	0.04458732	0.38120659	0.55237706	NA	LINC01545
643394	ENSG00000204909	0.14931947	-0.01844332	0.38480212	0.84537521	NA	SPINK9
100505591	ENSG00000204913	0.52985576	0.01340518	0.38291283	0.74463734	NA	LRRC3C
NA	ENSG00000204915	0	NA	NA	NA	NA	MFFP3
122183	ENSG00000204919	0	NA	NA	NA	NA	PRR20A
7711	ENSG00000204920	148.460559	0.06127998	0.18823355	0.70959047	0.86070548	ZNF155
790955	ENSG00000204922	363.965795	-0.058443	0.12479217	0.61962459	0.80569877	UQCC3
554251	ENSG00000204923	105.410898	0.03427237	0.19863792	0.84218074	0.92836346	FBXO48
643226	ENSG00000204928	0	NA	NA	NA	NA	GRXCR2
101927533	ENSG00000204929	4.24264568	0.00902674	0.36428499	0.94125734	0.97559098	
392307	ENSG00000204930	0.72631173	0.01461167	0.38232703	0.78672146	NA	FAM221B
NA	ENSG00000204933	0	NA	NA	NA	NA	CD177P1
401431	ENSG00000204934	204.14043	0.16126695	0.15716041	0.26063129	0.48260891	ATP6V0E2-AS1
57126	ENSG00000204936	0	NA	NA	NA	NA	CD177
5673	ENSG00000204941	0	NA	NA	NA	NA	PSG5
100289678	ENSG00000204946	892.523704	0.15724374	0.0888347	0.06864793	0.18884842	ZNF783
155054	ENSG00000204947	117.404186	0.01583064	0.20070454	0.92668345	0.96932621	ZNF425
100131726	ENSG00000204949	0.16735865	0.01897177	0.38482933	0.78225479	NA	FAM83A-AS1
390205	ENSG00000204950	11.8108903	-0.17556904	0.38610559	0.29603013	0.52267989	LRRC10B
494188	ENSG00000204952	0	NA	NA	NA	NA	FBXO47
728568	ENSG00000204954	284.107948	0.09677296	0.14931154	0.48211109	0.70272402	C12orf73
56114	ENSG00000204956	41.7915446	0.08330248	0.26654125	0.66922612	0.83726414	PCDHGA1
NA	ENSG00000204959	0	NA	NA	NA	NA	ARHGEF34P

338436	ENSG00000204960	0.14931947	-0.01844332	0.38480212	0.84537521	NA	BLACE
9752	ENSG00000204961	8.16419195	-0.39674454	0.67122915	0.02715946	0.09292009	PCDHA9
56140	ENSG00000204962	124.332498	-0.66150805	0.22370989	0.00068725	0.00425427	PCDHA8
56141	ENSG00000204963	21.4381297	-0.06255987	0.31030263	0.73423591	0.87349286	PCDHA7
56143	ENSG00000204965	24.0710221	-0.0662624	0.3092015	0.7227812	0.86755463	PCDHA5
56144	ENSG00000204967	157.816204	0.06810323	0.17635517	0.66489656	0.83454529	PCDHA4
56146	ENSG00000204969	142.529158	0.09491364	0.17555038	0.54413233	0.75209596	PCDHA2
56147	ENSG00000204970	27.1219304	-0.00485553	0.29877411	0.97755497	0.99237749	PCDHA1
NA	ENSG00000204971	0	NA	NA	NA	NA	
10206	ENSG00000204977	833.966628	-0.25786625	0.11450271	0.01788791	0.06662215	TRIM13
100170765	ENSG00000204978	0	NA	NA	NA	NA	ERICH4
503497	ENSG00000204979	0	NA	NA	NA	NA	MS4A13
NA	ENSG00000204982	0	NA	NA	NA	NA	PRSS3P4
5644	ENSG00000204983	0	NA	NA	NA	NA	PRSS1
NA	ENSG00000204989	0	NA	NA	NA	NA	OR4D8P
NA	ENSG00000204990	0.173124	-0.01911262	0.38486262	0.84537521	NA	
84501	ENSG00000204991	589.830083	-0.46838224	0.10833797	6.55E-06	6.44E-05	SPIRE2
NA	ENSG00000204993	0	NA	NA	NA	NA	CRTC1P1
441376	ENSG00000205002	2.97792685	0.23933132	0.49848727	0.00393856	0.01904948	AARD
400558	ENSG00000205015	0.30139824	-0.02296476	0.38527817	0.68536535	NA	LINC02138
NA	ENSG00000205018	0	NA	NA	NA	NA	
390748	ENSG00000205022	0	NA	NA	NA	NA	PABPN1L
NA	ENSG00000205025	0	NA	NA	NA	NA	OR5G5P
390144	ENSG00000205029	0	NA	NA	NA	NA	OR5D16
26338	ENSG00000205030	0	NA	NA	NA	NA	OR5L2
440040	ENSG00000205035	0	NA	NA	NA	NA	
400553	ENSG00000205037	82.5402522	0.09918332	0.30413629	0.60517217	0.79522617	
93035	ENSG00000205038	0.14931947	-0.01844332	0.38480212	0.84537521	NA	PKHD1L1
NA	ENSG00000205041	60.0910708	-0.04596738	0.24091209	0.80554853	0.91190704	
NA	ENSG00000205044	0	NA	NA	NA	NA	
100506736	ENSG00000205045	0	NA	NA	NA	NA	
400952	ENSG00000205054	3.38415093	-0.09283552	0.39384954	0.13144232	0.30411845	LINC01121
NA	ENSG00000205056	0	NA	NA	NA	NA	LINC02397
574016	ENSG00000205057	0	NA	NA	NA	NA	CLLU1-AS1
84912	ENSG00000205060	3308.45786	-0.00756255	0.06114193	0.89719639	0.95488961	SLC35B4
3963	ENSG00000205076	0	NA	NA	NA	NA	LGALS7
653499	ENSG00000205076	0	NA	NA	NA	NA	LGALS7
100130958	ENSG00000205078	29.7349183	0.40894427	0.38637765	0.08745439	0.22586501	SYCE1L

79583	ENSG00000205084	264.968205	-0.0254054	0.14736224	0.85080365	0.93249058	TMEM231
346653	ENSG00000205085	15.3614627	-0.06783374	0.34239723	0.67535129	0.84111426	FAM71F2
400950	ENSG00000205086	33.7346619	0.0385041	0.29045153	0.84226523	0.92836346	C2orf91
645121	ENSG00000205089	4.59024297	-0.05001064	0.36583992	0.68674597	0.84769738	CCNI2
339453	ENSG00000205090	125.275887	-0.03037416	0.19941674	0.85660714	0.93595581	TMEM240
448831	ENSG00000205097	2.8287159	0.0945773	0.39288114	0.2597402	0.48138679	FRG2
NA	ENSG00000205100	0	NA	NA	NA	NA	HSP90AA4P
NA	ENSG00000205105	0	NA	NA	NA	NA	COX17P1
374387	ENSG00000205106	0	NA	NA	NA	NA	LINC02716
259308	ENSG00000205108	3.53478474	0.02338491	0.36781749	0.83515282	0.92475394	FAM205A
344387	ENSG00000205111	35.4564556	-0.14069823	0.31051843	0.47210043	0.69438543	CDKL4
643965	ENSG00000205116	10.729126	-0.03430632	0.3374612	0.8329667	0.9239848	TMEM88B
390110	ENSG00000205126	2.10594681	0.0990863	0.3996738	0.10167793	NA	ACCSL
441054	ENSG00000205129	8.59712268	0.2105712	0.4200688	0.19033532	0.39167661	C4orf47
286144	ENSG00000205133	360.09082	0.00757778	0.13320632	0.95223619	0.98019954	TRIQK
644096	ENSG00000205138	301.018547	-0.14810399	0.13903476	0.25180496	0.47165884	SDHAF1
138715	ENSG00000205143	3.19611991	0.02110297	0.36953972	0.84364689	0.92926684	ARID3C
55851	ENSG00000205155	443.884627	0.07771727	0.13214063	0.53158875	0.74286115	PSENEN
NA	ENSG00000205174	0	NA	NA	NA	NA	C7orf66
NA	ENSG00000205176	0	NA	NA	NA	NA	REXO1L1P
100131378	ENSG00000205177	1.49027968	-0.00723017	0.37722093	0.92291141	NA	C11orf91
149837	ENSG00000205181	45.4486501	-2.18828654	0.40681264	4.68E-09	7.36E-08	LINC00654
NA	ENSG00000205184	0	NA	NA	NA	NA	SLC10A5P1
646480	ENSG00000205186	0	NA	NA	NA	NA	FABP9
65986	ENSG00000205189	847.781415	-0.00567366	0.10295684	0.95300749	0.98058207	ZBTB10
201725	ENSG00000205208	1028.64596	0.11429228	0.09236044	0.20241915	0.40796383	C4orf46
284402	ENSG00000205209	50.842098	0.53362916	0.51847831	0.04429736	0.13554282	SCGB2B2
339184	ENSG00000205212	48.5823395	-0.90259929	0.45523877	0.00442473	0.02102172	CCDC144NL
55366	ENSG00000205213	441.066501	0.33166189	0.13826022	0.00997053	0.04137529	LGR4
NA	ENSG00000205215	0	NA	NA	NA	NA	KRT17P7
5699	ENSG00000205220	152.998434	-0.24546957	0.18182247	0.12535141	0.29417696	PSMB10
5212	ENSG00000205221	0	NA	NA	NA	NA	VIT
100506376	ENSG00000205231	0	NA	NA	NA	NA	TTLL10-AS1
NA	ENSG00000205236	85.5467879	0.91972194	0.36783369	0.00137966	0.00780393	
441273	ENSG00000205238	146.297597	0.01919313	0.1764057	0.90374374	0.95800026	SPDYE2
NA	ENSG00000205240	0	NA	NA	NA	NA	OR7E36P
1874	ENSG00000205250	2183.73184	0.30186518	0.0697758	1.05E-05	9.94E-05	E2F4
NA	ENSG00000205266	0	NA	NA	NA	NA	KRT17P5

NA	ENSG00000205267	0	NA	NA	NA	NA	DGAT2L7P
5150	ENSG00000205268	1201.59341	-0.05923456	0.08915613	0.49326721	0.71236151	PDE7A
100113407	ENSG00000205269	882.04586	-0.25160063	0.09415745	0.00569375	0.02596278	TMEM170B
NA	ENSG00000205274	0.18922458	0.01954053	0.38488496	0.78225479	NA	TRBV20OR9-2
10071	ENSG00000205277	2.89809442	-0.25405354	0.51690153	0.00080222	0.00488371	MUC12
613212	ENSG00000205279	0	NA	NA	NA	NA	CTXN3
100505477	ENSG00000205293	0.14931947	-0.01844332	0.38480212	0.84537521	NA	LINC01602
NA	ENSG00000205300	0	NA	NA	NA	NA	
152586	ENSG00000205301	0	NA	NA	NA	NA	MGAT4D
6643	ENSG00000205302	674.76614	-0.22125865	0.10395501	0.0265013	0.09109249	SNX2
100316904	ENSG00000205307	21.2919997	0.01069653	0.30850417	0.95570663	0.9818048	SAP25
56953	ENSG00000205309	306.998286	0.21635878	0.17051162	0.1558865	0.34204692	NT5M
NA	ENSG00000205312	0	NA	NA	NA	NA	KRT17P4
NA	ENSG00000205318	1.0121396	0.03785925	0.38213069	0.56024448	NA	GCNT2P1
84324	ENSG00000205323	802.700177	-0.11318985	0.09258425	0.20715537	0.41443608	SARNP
NA	ENSG00000205325	0	NA	NA	NA	NA	
403284	ENSG00000205327	0	NA	NA	NA	NA	OR6C68
403282	ENSG00000205328	0	NA	NA	NA	NA	OR6C65
254786	ENSG00000205329	0	NA	NA	NA	NA	OR6C3
390321	ENSG00000205330	0	NA	NA	NA	NA	OR6C1
NA	ENSG00000205331	0	NA	NA	NA	NA	OR6C72P
NA	ENSG00000205333	0	NA	NA	NA	NA	GOLGA2P1
100129995	ENSG00000205334	2.11376986	-0.08086298	0.38714667	0.34982452	NA	LINC01460
9289	ENSG00000205336	40.3597794	-0.55613756	0.43993157	0.03677426	0.11788679	ADGRG1
10527	ENSG00000205339	6883.37071	-0.37052294	0.05029918	1.03E-13	2.53E-12	IPO7
54458	ENSG00000205352	400.75805	0.00444685	0.11996022	0.9698136	0.98858357	PRR13
25851	ENSG00000205356	1525.75031	0.39183132	0.13263797	0.00159833	0.00886272	TECPR1
4496	ENSG00000205358	0	NA	NA	NA	NA	MT1H
133482	ENSG00000205359	0	NA	NA	NA	NA	SLCO6A1
NA	ENSG00000205360	0	NA	NA	NA	NA	MT1CP
NA	ENSG00000205361	0	NA	NA	NA	NA	MT1DP
4489	ENSG00000205362	0.173124	-0.01911262	0.38486262	0.84537521	NA	MT1A
388135	ENSG00000205363	75.2910582	-1.41785741	0.42016304	4.23E-05	0.00035365	INSYN1
4499	ENSG00000205364	0	NA	NA	NA	NA	MT1M
126536	ENSG00000205396	0	NA	NA	NA	NA	LINC00661
3426	ENSG00000205403	0	NA	NA	NA	NA	CFI
390078	ENSG00000205409	0	NA	NA	NA	NA	OR52E6
NA	ENSG00000205412	0	NA	NA	NA	NA	HNRNPA1P20

54809	ENSG00000205413	33.8784539	-0.56290899	0.44560246	0.03688558	0.118091	SAMD9
NA	ENSG00000205414	0.16735865	0.01897177	0.38482933	0.78225479	NA	
3853	ENSG00000205420	0	NA	NA	NA	NA	KRT6A
255919	ENSG00000205423	261.497396	0.23449114	0.14174968	0.0744498	0.20030974	CNEP1R1
NA	ENSG00000205424	2.97842005	-0.06656431	0.37712515	0.52634059	0.73825698	PCBP3-AS1
3887	ENSG00000205426	0	NA	NA	NA	NA	KRT81
NA	ENSG00000205433	0	NA	NA	NA	NA	SNRPGP6
91828	ENSG00000205436	0.16735865	0.01897177	0.38482933	0.78225479	NA	EXOC3L4
386683	ENSG00000205439	0	NA	NA	NA	NA	KRTAP12-3
100129669	ENSG00000205442	0	NA	NA	NA	NA	IZUMO3
497048	ENSG00000205444	0	NA	NA	NA	NA	
386679	ENSG00000205445	0	NA	NA	NA	NA	KRTAP10-2
NA	ENSG00000205449	0	NA	NA	NA	NA	NBPF22P
NA	ENSG00000205452	0	NA	NA	NA	NA	
729264	ENSG00000205456	12.0571254	-0.103549	0.35029095	0.53526286	0.74623113	TP53TG3D
102723655	ENSG00000205456	12.0571254	-0.103549	0.35029095	0.53526286	0.74623113	TP53TG3D
102723713	ENSG00000205456	12.0571254	-0.103549	0.35029095	0.53526286	0.74623113	TP53TG3D
102724101	ENSG00000205456	12.0571254	-0.103549	0.35029095	0.53526286	0.74623113	TP53TG3D
102724127	ENSG00000205456	12.0571254	-0.103549	0.35029095	0.53526286	0.74623113	TP53TG3D
653550	ENSG00000205457	0	NA	NA	NA	NA	TP53TG3C
102723655	ENSG00000205457	0	NA	NA	NA	NA	TP53TG3C
102723713	ENSG00000205457	0	NA	NA	NA	NA	TP53TG3C
102724101	ENSG00000205457	0	NA	NA	NA	NA	TP53TG3C
102724127	ENSG00000205457	0	NA	NA	NA	NA	TP53TG3C
92270	ENSG00000205464	96.2089794	0.04836806	0.21939749	0.79121402	0.90493295	ATP6AP1L
317762	ENSG00000205476	1789.40605	-0.1117641	0.08600779	0.18161617	0.37909151	CCDC85C
100505767	ENSG00000205482	20.3211031	0.03735304	0.31122463	0.84123575	0.92789889	SPDYE18
100133091	ENSG00000205485	298.311702	-0.08882873	0.18761168	0.58585864	0.7817695	
NA	ENSG00000205488	0	NA	NA	NA	NA	CALML3-AS1
NA	ENSG00000205494	0	NA	NA	NA	NA	OR52A4P
119679	ENSG00000205495	0	NA	NA	NA	NA	OR52J3
401667	ENSG00000205496	0	NA	NA	NA	NA	OR51A2
401666	ENSG00000205497	0	NA	NA	NA	NA	OR51A4
100129724	ENSG00000205500	2.06379365	0.03001433	0.38128642	0.64968935	NA	MAPRE3-AS1
388125	ENSG00000205502	4.68532141	-0.04055094	0.36568531	0.73727224	0.87584464	C2CD4B
57139	ENSG00000205517	358.017323	0.41625487	0.13073248	0.00071148	0.00438925	RGL3
4676	ENSG00000205531	2633.73349	-0.10470429	0.06894323	0.12264903	0.28985787	NAP1L4
NA	ENSG00000205534	105.3034	-0.06392977	0.38453056	0.41495128	0.64205213	SMG1P2

NA	ENSG00000205537	0	NA	NA	NA	NA	
7114	ENSG00000205542	2702.13783	0.61315204	0.06229393	2.08E-23	9.32E-22	TMSB4X
254863	ENSG00000205544	402.760891	0.12565221	0.11818073	0.26401762	0.48609939	TMEM256
NA	ENSG00000205549	0.75047983	-0.04347263	0.38757139	0.34431439	NA	C9orf92
100144603	ENSG00000205559	94.7683719	0.14073264	0.21904269	0.43743306	0.66264578	CHKB-DT
1375	ENSG00000205560	639.583464	0.386645	0.11659064	0.00048684	0.00315442	CPT1B
NA	ENSG00000205562	0	NA	NA	NA	NA	
6606	ENSG00000205571	1156.55922	-0.05630142	0.11882571	0.61733928	0.8040446	SMN2
6607	ENSG00000205571	1156.55922	-0.05630142	0.11882571	0.61733928	0.8040446	SMN2
8293	ENSG00000205572	597.691192	-0.37798365	0.41507501	0.1043697	0.25722378	SERF1B
728492	ENSG00000205572	597.691192	-0.37798365	0.41507501	0.1043697	0.25722378	SERF1B
NA	ENSG00000205578	25.2433216	-0.03787716	0.29837526	0.83962337	0.92709972	POM121B
NA	ENSG00000205579	0	NA	NA	NA	NA	DYNLL1P1
3150	ENSG00000205581	7352.35954	0.17371977	0.0499451	0.0004461	0.00292292	HMGN1
NA	ENSG00000205583	220.68313	0.2745394	0.15432603	0.05182951	0.15320285	STAG3L1
NA	ENSG00000205584	0.31177432	0.00117239	0.38336728	0.96390626	NA	
283463	ENSG00000205592	8.77580797	0.04647282	0.37296822	0.64804289	0.82447067	MUC19
414918	ENSG00000205593	227.664407	0.14359854	0.17152853	0.34947591	0.58004303	DENND6B
NA	ENSG00000205596	0	NA	NA	NA	NA	
728689	ENSG00000205609	147.730103	-0.08947205	0.36135968	0.54508205	0.75277933	EIF3CL
107985433	ENSG00000205611	14.2610525	-0.22967585	0.41064299	0.1964001	0.40005705	LINC01597
NA	ENSG00000205622	0.51891447	0.00905376	0.38272017	0.8223472	NA	
NA	ENSG00000205625	0	NA	NA	NA	NA	
401337	ENSG00000205628	0	NA	NA	NA	NA	LINC01446
51451	ENSG00000205629	436.8227	0.22011335	0.11735477	0.04835926	0.14512897	LCMT1
100128946	ENSG00000205632	0	NA	NA	NA	NA	LINC01310
400932	ENSG00000205634	6.64558863	-0.02502363	0.35351372	0.85776398	0.93653665	LINC00898
100113404	ENSG00000205636	0	NA	NA	NA	NA	LINC00583
388931	ENSG00000205639	3.03917239	0.00606451	0.37214502	0.95126853	0.98008882	MFSD2B
425054	ENSG00000205642	0.8622178	0.03413631	0.38407784	0.48192307	NA	VCX3B
150383	ENSG00000205643	152.646085	0.23057768	0.20408212	0.18516071	0.38410586	CDPF1
NA	ENSG00000205644	0.79037561	-0.00857012	0.38089464	0.87942776	NA	
3347	ENSG00000205649	0	NA	NA	NA	NA	HTN3
NA	ENSG00000205653	0	NA	NA	NA	NA	
91750	ENSG00000205659	379.72568	0.24875892	0.1399959	0.05580588	0.16205922	LIN52
347527	ENSG00000205667	0	NA	NA	NA	NA	ARSH
641372	ENSG00000205669	0.14931947	-0.01844332	0.38480212	0.84537521	NA	ACOT6
54065	ENSG00000205670	150.654459	0.07637777	0.37605803	0.51455261	0.72937777	SMIM11A

253017	ENSG00000205678	0	NA	NA	NA	NA	TECRL
NA	ENSG00000205682	0	NA	NA	NA	NA	
8110	ENSG00000205683	87.6363297	-0.98318382	0.26817236	2.87E-05	0.00024886	DPF3
100287284	ENSG00000205693	1.67548751	0.03326149	0.37897726	0.66693942	NA	MANSC4
NA	ENSG00000205695	0	NA	NA	NA	NA	
642394	ENSG00000205696	0.67850399	0.00447323	0.38246378	0.91645901	NA	ADARB2-AS1
NA	ENSG00000205702	58.9422821	0.53269532	0.38858674	0.03665825	0.1176099	CYP2D7
NA	ENSG00000205704	37.5683937	-0.31148288	0.32409423	0.15480398	0.34030142	LINC00634
144363	ENSG00000205707	146.580451	-0.36820267	0.20494608	0.03624373	0.11648721	ETFRF1
100130311	ENSG00000205710	17.4412041	0.17663219	0.34995778	0.36032352	0.59027484	C17orf107
NA	ENSG00000205716	0	NA	NA	NA	NA	FAM183DP
653656	ENSG00000205718	0.16735865	0.01897177	0.38482933	0.78225479	NA	MBD3L4
6453	ENSG00000205726	1138.82018	-0.51089886	0.14277682	0.00012391	0.00092038	ITSN1
162073	ENSG00000205730	522.976569	0.10214629	0.12485919	0.38719699	0.61623231	ITPRIPL2
107984285	ENSG00000205740	34.493796	-0.14070665	0.296164	0.47724791	0.69909912	
79958	ENSG00000205744	21.5361354	0.75185128	0.59236165	0.0190402	0.07001995	DENND1C
NA	ENSG00000205745	0	NA	NA	NA	NA	
NA	ENSG00000205746	1302.75495	0.33350625	0.10539179	0.00097395	0.00578019	PKD1P4
NA	ENSG00000205754	0	NA	NA	NA	NA	SLCO1B7
64109	ENSG00000205755	0	NA	NA	NA	NA	CRLF2
9946	ENSG00000205758	510.483772	-0.11875617	0.10700034	0.24662432	0.46578072	CRYZL1
441212	ENSG00000205763	221.590473	0.05218015	0.14597252	0.70016144	0.85541355	RP9P
285636	ENSG00000205765	995.366497	-0.34553402	0.09806808	0.00025677	0.00178187	C5orf51
NA	ENSG00000205767	0	NA	NA	NA	NA	
440278	ENSG00000205771	18.9732673	0.15462382	0.3526526	0.40098923	0.62940082	CATSPER2P1
2543	ENSG00000205777	0	NA	NA	NA	NA	GAGE1
645432	ENSG00000205784	0	NA	NA	NA	NA	ARRDC5
100128682	ENSG00000205786	0	NA	NA	NA	NA	LINC01531
100131094	ENSG00000205790	16.1155037	0.63480477	0.71640398	0.03102158	0.10295552	DPP9-AS1
503693	ENSG00000205791	16.6304075	-0.0797781	0.32273811	0.66147095	0.8324506	LOH12CR2
344967	ENSG00000205794	0	NA	NA	NA	NA	
192668	ENSG00000205795	4.3071191	0.07394161	0.38546495	0.35176131	0.58182113	CYS1
403313	ENSG00000205808	388.697089	-0.14842706	0.12440633	0.20609677	0.41317248	PLPP6
3822	ENSG00000205809	0	NA	NA	NA	NA	KLRC2
3823	ENSG00000205810	0	NA	NA	NA	NA	KLRC3
NA	ENSG00000205822	4.35767379	0.10863637	0.39026431	0.30657264	0.53422766	TPTE2P6
NA	ENSG00000205830	0	NA	NA	NA	NA	
342346	ENSG00000205832	2.98950586	0.0808532	0.38385722	0.4103647	0.63815925	C16orf96

647309	ENSG00000205835	0	NA	NA	NA	NA	GMNC
400941	ENSG00000205837	0	NA	NA	NA	NA	LINC00487
153657	ENSG00000205838	7.91895163	0.07363526	0.37200658	0.53998168	0.74887232	TTC23L
93978	ENSG00000205846	0	NA	NA	NA	NA	CLEC6A
79315	ENSG00000205847	0	NA	NA	NA	NA	OR7E91P
10737	ENSG00000205853	11.7180319	0.14151209	0.37538129	0.37025326	0.59943356	RFPL3S
150297	ENSG00000205856	0	NA	NA	NA	NA	C22orf42
360030	ENSG00000205857	0	NA	NA	NA	NA	NANOGNB
100506049	ENSG00000205858	0	NA	NA	NA	NA	LRRC72
542767	ENSG00000205861	1.03670879	-0.00919083	0.38057052	0.87137982	NA	PCOTH
387911	ENSG00000205863	0	NA	NA	NA	NA	C1QTNF9B
440023	ENSG00000205864	0	NA	NA	NA	NA	KRTAP5-6
100132464	ENSG00000205865	0	NA	NA	NA	NA	FAM99B
387742	ENSG00000205866	0	NA	NA	NA	NA	FAM99A
440021	ENSG00000205867	0	NA	NA	NA	NA	KRTAP5-2
387264	ENSG00000205869	16.2337564	-1.07425047	0.77937507	0.00864706	0.03678337	KRTAP5-1
NA	ENSG00000205871	0.80331222	-0.00593024	0.38078992	0.91660053	NA	RPS3AP47
NA	ENSG00000205879	1.76652586	0.08916685	0.39615261	0.13993453	NA	FAM90A2P
613211	ENSG00000205882	0	NA	NA	NA	NA	DEFB134
613209	ENSG00000205883	0	NA	NA	NA	NA	DEFB135
613210	ENSG00000205884	0	NA	NA	NA	NA	DEFB136
283314	ENSG00000205885	64.5655088	0.14093297	0.28159867	0.47724066	0.69909912	C1RL-AS1
100128770	ENSG00000205890	0.32425478	0.00117146	0.3833667	0.96908568	NA	
NA	ENSG00000205897	4.30642117	0.06816772	0.37203838	0.57086146	0.77109827	OR7E136P
NA	ENSG00000205898	3.63509304	0.0415513	0.37317168	0.68698142	0.84777704	
727857	ENSG00000205899	0	NA	NA	NA	NA	BHLHA9
100131017	ENSG00000205903	3739.96162	-0.1257336	0.07110481	0.07163198	0.19481541	ZNF316
100128788	ENSG00000205913	18.3640462	0.08299413	0.32636105	0.65032989	0.82529572	SRRM2-AS1
57135	ENSG00000205916	0	NA	NA	NA	NA	DAZ4
NA	ENSG00000205918	438.491441	0.24805166	0.11616574	0.02484512	0.08672186	PDPK2P
390874	ENSG00000205922	0	NA	NA	NA	NA	ONECUT3
752014	ENSG00000205923	0	NA	NA	NA	NA	CEMP1
10215	ENSG00000205927	0	NA	NA	NA	NA	OLIG2
56245	ENSG00000205929	2.93323207	0.02951638	0.37253508	0.76556241	0.89220778	C21orf62
NA	ENSG00000205930	58.9223842	0.15798274	0.27761627	0.42705633	0.65286501	C21orf62-AS1
NA	ENSG00000205936	0	NA	NA	NA	NA	PPP1R12BP2
10921	ENSG00000205937	4494.67392	0.00490575	0.06530172	0.94136662	0.97559098	RNPS1
NA	ENSG00000205940	8.74768383	-0.21723323	0.41934544	0.19310932	0.3958352	HSP90AB2P

57055	ENSG00000205944	0	NA	NA	NA	NA	DAZ2
391622	ENSG00000205946	0.16735865	0.01897177	0.38482933	0.78225479	NA	USP17L6P
NA	ENSG00000205955	0.84232392	0.01659916	0.38121629	0.76831796	NA	HSP90AA5P
NA	ENSG00000205959	3.78418819	0.15247952	0.41436074	0.14855843	0.33098252	
NA	ENSG00000205971	0.47363781	-0.02845309	0.38580958	0.5427038	NA	
NA	ENSG00000205976	0.55138487	0.01181637	0.3832588	0.75114255	NA	
57523	ENSG00000205978	1268.26474	0.29131687	0.08731261	0.00059664	0.00376622	NYNRIN
131118	ENSG00000205981	469.963562	0.16530319	0.12686383	0.16694706	0.35784677	DNAJC19
387733	ENSG00000206013	0.173733	0.01914545	0.38484597	0.78225479	NA	IFITM5
NA	ENSG00000206014	0	NA	NA	NA	NA	OR7E161P
284274	ENSG00000206026	0	NA	NA	NA	NA	SMIM21
NA	ENSG00000206028	16.5482393	-0.16770941	0.36613777	0.35167274	0.58172313	
641517	ENSG00000206034	0	NA	NA	NA	NA	DEFB109B
NA	ENSG00000206042	0	NA	NA	NA	NA	DEFA7P
644041	ENSG00000206043	0	NA	NA	NA	NA	C18orf63
1667	ENSG00000206047	0	NA	NA	NA	NA	DEFA1
220164	ENSG00000206052	818.813428	-0.05583527	0.24756399	0.76604786	0.89234321	DOK6
90861	ENSG00000206053	2932.18821	-0.2108632	0.06317703	0.00070216	0.00433711	JPT2
NA	ENSG00000206062	0.173733	0.01914545	0.38484597	0.78225479	NA	
NA	ENSG00000206066	0	NA	NA	NA	NA	IGLL3P
255349	ENSG00000206069	0.6906061	0.01674122	0.38323095	0.65806079	NA	TMEM211
89778	ENSG00000206072	0	NA	NA	NA	NA	SERPINB11
6318	ENSG00000206073	0	NA	NA	NA	NA	SERPINB4
5268	ENSG00000206075	0	NA	NA	NA	NA	SERPINB5
653082	ENSG00000206077	368.167322	-0.71622905	0.13572855	2.86E-08	4.06E-07	ZDHHC11B
NA	ENSG00000206090	0	NA	NA	NA	NA	
728299	ENSG00000206102	0	NA	NA	NA	NA	KRTAP19-8
337985	ENSG00000206104	0	NA	NA	NA	NA	KRTAP20-3
100151643	ENSG00000206105	0	NA	NA	NA	NA	KRTAP20-4
100288287	ENSG00000206106	0	NA	NA	NA	NA	KRTAP22-2
643812	ENSG00000206107	0	NA	NA	NA	NA	KRTAP27-1
402160	ENSG00000206113	1.58898392	0.04313051	0.38063193	0.567313	NA	CFAP99
NA	ENSG00000206120	21.7032862	0.49603614	0.49795074	0.05661805	0.16381831	EGFEM1P
728047	ENSG00000206127	121.094345	0.67143829	0.27297844	0.00271	0.01390391	GOLGA8O
642484	ENSG00000206129	1.72373679	-0.04431607	0.38505299	0.41859324	NA	
645426	ENSG00000206140	20.7660514	1.45770285	0.61783334	0.00098884	0.00585105	TMEM191C
100996335	ENSG00000206142	0.45808616	0.00126518	0.38310474	0.98724547	NA	FAM230H
NA	ENSG00000206144	0.49322054	0.0149268	0.38314382	0.74765203	NA	MAPK6P1

NA	ENSG00000206145	0.33471731	0.02354391	0.38534154	0.62842204	NA	P2RX6P
NA	ENSG00000206147	0.173124	-0.01911262	0.38486262	0.84537521	NA	RPL23AP57
440248	ENSG00000206149	909.407173	0.02379931	0.09849492	0.80409218	0.91109017	HERC2P9
440163	ENSG00000206150	0.18922458	0.01954053	0.38488496	0.78225479	NA	RNASE13
NA	ENSG00000206159	0	NA	NA	NA	NA	GYG2P1
NA	ENSG00000206168	0.52457699	0.01099992	0.38327131	0.76830712	NA	
3039	ENSG00000206172	0.34746601	0.02370763	0.3853651	0.61987128	NA	HBA1
3042	ENSG00000206177	0	NA	NA	NA	NA	HBM
NA	ENSG00000206178	0.173733	0.01914545	0.38484597	0.78225479	NA	HBZP1
51224	ENSG00000206181	1.13761627	-0.00537635	0.37954972	0.93228938	NA	ELOA2
100128714	ENSG00000206187	0	NA	NA	NA	NA	LINC02346
57194	ENSG00000206190	0	NA	NA	NA	NA	ATP10A
NA	ENSG00000206192	0	NA	NA	NA	NA	ANKRD20A9P
503637	ENSG00000206195	1640.92773	-0.12284731	0.07433628	0.09160676	0.23372918	DUXAP8
389161	ENSG00000206199	0	NA	NA	NA	NA	ANKUB1
23617	ENSG00000206203	0	NA	NA	NA	NA	TSSK2
NA	ENSG00000206228	0.64047942	0.00308824	0.38195794	0.94667208	NA	HNRNPA1P4
NA	ENSG00000206249	0	NA	NA	NA	NA	
729627	ENSG00000206260	0	NA	NA	NA	NA	PRR23A
401089	ENSG00000206262	0	NA	NA	NA	NA	FOXL2NB
10866	ENSG00000206337	0	NA	NA	NA	NA	HCP5
NA	ENSG00000206341	22.7566104	0.04638782	0.30915092	0.8044972	0.91118482	HLA-H
253018	ENSG00000206344	10.0210693	-0.24922237	0.43493046	0.15634921	0.34283458	HCG27
100652833	ENSG00000206356	0	NA	NA	NA	NA	
131873	ENSG00000206384	0.173733	0.01914545	0.38484597	0.78225479	NA	COL6A6
NA	ENSG00000206417	32.0093521	-0.00979981	0.28012798	0.95761906	0.98264776	H1-10-AS1
201475	ENSG00000206418	381.910483	0.21448384	0.13281176	0.08448316	0.22013999	RAB12
339291	ENSG00000206422	0	NA	NA	NA	NA	LRRC30
645369	ENSG00000206432	83.8974756	2.0895972	0.35193606	1.51E-10	2.85E-09	TMEM200C
NA	ENSG00000206448	0.14931947	-0.01844332	0.38480212	0.84537521	NA	PPIAP30
442194	ENSG00000206474	0	NA	NA	NA	NA	OR10C1
3105	ENSG00000206503	4970.10161	0.21330246	0.0582596	0.00020624	0.00146348	HLA-A
201562	ENSG00000206527	638.637055	-0.50289636	0.10529803	7.01E-07	8.14E-06	HACD2
55779	ENSG00000206530	756.151421	-0.07819608	0.11515913	0.47548327	0.6976474	CFAP44
344807	ENSG00000206531	0	NA	NA	NA	NA	CD200R1L
348801	ENSG00000206535	84.2233853	0.50554403	0.25697966	0.01536738	0.05873277	LNP1
403277	ENSG00000206536	0	NA	NA	NA	NA	OR5K3
389136	ENSG00000206538	0	NA	NA	NA	NA	VGLL3

NA	ENSG00000206549	7.10266875	0.09224829	0.36731412	0.51762968	0.73141449	
100506275	ENSG00000206552	0	NA	NA	NA	NA	KRBOX1-AS1
131405	ENSG00000206557	55.6493955	0.78698326	0.32353542	0.00222522	0.01173844	TRIM71
152098	ENSG00000206559	18.9874476	0.05544334	0.32634164	0.75500688	0.8860367	ZCWPW2
23243	ENSG00000206560	951.87656	0.0814757	0.1049345	0.42038576	0.6462065	ANKRD28
8292	ENSG00000206561	141.389627	0.04046695	0.18627235	0.80502414	0.91147183	COLQ
131965	ENSG00000206562	402.53255	-0.33967371	0.1409755	0.00941714	0.03948351	METTL6
NA	ENSG00000206567	153.081606	-0.03434828	0.19049629	0.83252322	0.92392457	
440944	ENSG00000206573	360.071032	-0.28235105	0.12940155	0.01996498	0.07286703	THUMPD3-AS1
114786	ENSG00000206579	0.29863895	-0.02292439	0.3852726	0.68753245	NA	XKR4
NA	ENSG00000206582	0	NA	NA	NA	NA	
NA	ENSG00000206583	0	NA	NA	NA	NA	RNU6-1292P
26864	ENSG00000206585	0	NA	NA	NA	NA	RNVU1-7
NA	ENSG00000206587	0	NA	NA	NA	NA	RNU6-46P
NA	ENSG00000206590	0	NA	NA	NA	NA	RNU6-132P
NA	ENSG00000206592	0	NA	NA	NA	NA	
NA	ENSG00000206593	0	NA	NA	NA	NA	RNU6-47P
NA	ENSG00000206595	0	NA	NA	NA	NA	RNU6-877P
692158	ENSG00000206597	0	NA	NA	NA	NA	SNORA57
NA	ENSG00000206598	0	NA	NA	NA	NA	RNU6-1286P
NA	ENSG00000206599	0	NA	NA	NA	NA	RNU6-841P
NA	ENSG00000206600	0	NA	NA	NA	NA	RNU6-25P
NA	ENSG00000206601	0	NA	NA	NA	NA	RNU6-431P
26791	ENSG00000206602	0	NA	NA	NA	NA	SNORD58A
109616964	ENSG00000206603	0	NA	NA	NA	NA	SNORA22B
NA	ENSG00000206604	0	NA	NA	NA	NA	RNU6-425P
NA	ENSG00000206605	0	NA	NA	NA	NA	RNU6-946P
NA	ENSG00000206606	0	NA	NA	NA	NA	RNU6-1296P
100033423	ENSG00000206609	0	NA	NA	NA	NA	SNORD116-11
26820	ENSG00000206611	0	NA	NA	NA	NA	SNORD24
677793	ENSG00000206612	0	NA	NA	NA	NA	SNORA2A
NA	ENSG00000206613	0	NA	NA	NA	NA	RNU6-1336P
NA	ENSG00000206614	0	NA	NA	NA	NA	RNU6-477P
NA	ENSG00000206615	0	NA	NA	NA	NA	RNU6-338P
NA	ENSG00000206616	0	NA	NA	NA	NA	RNU6-741P
NA	ENSG00000206617	0	NA	NA	NA	NA	RNY1P5
NA	ENSG00000206618	0	NA	NA	NA	NA	RNU6-1264P
692085	ENSG00000206620	0	NA	NA	NA	NA	SNORD45C

NA	ENSG00000206621	0	NA	NA	NA	NA	SNORD116-14
26779	ENSG00000206622	0	NA	NA	NA	NA	SNORA69
NA	ENSG00000206623	0	NA	NA	NA	NA	RNU6-979P
NA	ENSG00000206624	0	NA	NA	NA	NA	RNU1-39P
NA	ENSG00000206627	0	NA	NA	NA	NA	RNU6-969P
NA	ENSG00000206629	0	NA	NA	NA	NA	RNU1-63P
26788	ENSG00000206630	0	NA	NA	NA	NA	SNORD60
NA	ENSG00000206631	0	NA	NA	NA	NA	RNU6-657P
100302743	ENSG00000206633	0	NA	NA	NA	NA	SNORA80B
677807	ENSG00000206634	0	NA	NA	NA	NA	SNORA22
NA	ENSG00000206635	0	NA	NA	NA	NA	RNU6-1062P
NA	ENSG00000206636	0	NA	NA	NA	NA	
109616993	ENSG00000206637	0	NA	NA	NA	NA	SNORA70H
NA	ENSG00000206638	0	NA	NA	NA	NA	RNU6-672P
NA	ENSG00000206639	0	NA	NA	NA	NA	
NA	ENSG00000206640	0	NA	NA	NA	NA	
NA	ENSG00000206641	0	NA	NA	NA	NA	RNU6-449P
NA	ENSG00000206644	0	NA	NA	NA	NA	RNU6-1279P
NA	ENSG00000206645	0	NA	NA	NA	NA	
NA	ENSG00000206646	0	NA	NA	NA	NA	
NA	ENSG00000206647	0.15069912	-0.01848503	0.38480575	0.84537521	NA	
NA	ENSG00000206649	0	NA	NA	NA	NA	
100379132	ENSG00000206650	0	NA	NA	NA	NA	SNORA70G
NA	ENSG00000206651	0	NA	NA	NA	NA	
26871	ENSG00000206652	0	NA	NA	NA	NA	RNU1-1
NA	ENSG00000206654	0	NA	NA	NA	NA	RNU6-608P
NA	ENSG00000206656	0	NA	NA	NA	NA	SNORD116-17
NA	ENSG00000206658	0	NA	NA	NA	NA	RNU6-1039P
NA	ENSG00000206659	0	NA	NA	NA	NA	
NA	ENSG00000206660	0	NA	NA	NA	NA	
NA	ENSG00000206661	0	NA	NA	NA	NA	
NA	ENSG00000206662	0	NA	NA	NA	NA	
NA	ENSG00000206663	0	NA	NA	NA	NA	
NA	ENSG00000206665	0	NA	NA	NA	NA	RNU6-573P
NA	ENSG00000206669	0	NA	NA	NA	NA	
NA	ENSG00000206671	0	NA	NA	NA	NA	RNU6-471P
NA	ENSG00000206672	0	NA	NA	NA	NA	
NA	ENSG00000206674	0	NA	NA	NA	NA	RNU6-945P

NA	ENSG00000206675	0	NA	NA	NA	NA	RNU6-32P
NA	ENSG00000206676	0	NA	NA	NA	NA	
NA	ENSG00000206677	0	NA	NA	NA	NA	
NA	ENSG00000206678	0	NA	NA	NA	NA	RNU6-144P
NA	ENSG00000206679	0	NA	NA	NA	NA	
6083	ENSG00000206680	0	NA	NA	NA	NA	SNORD21
NA	ENSG00000206681	0	NA	NA	NA	NA	RNU6-730P
NA	ENSG00000206682	0	NA	NA	NA	NA	
NA	ENSG00000206684	0	NA	NA	NA	NA	RNU6-157P
NA	ENSG00000206685	0	NA	NA	NA	NA	RNU6-1189P
NA	ENSG00000206686	0	NA	NA	NA	NA	RNU6-1036P
NA	ENSG00000206687	0	NA	NA	NA	NA	RNU1-109P
NA	ENSG00000206688	0	NA	NA	NA	NA	SNORD116-18
NA	ENSG00000206690	0	NA	NA	NA	NA	
NA	ENSG00000206692	0	NA	NA	NA	NA	
677835	ENSG00000206693	0	NA	NA	NA	NA	SNORA56
NA	ENSG00000206695	0	NA	NA	NA	NA	RNU6-442P
NA	ENSG00000206697	0	NA	NA	NA	NA	RNY1P8
NA	ENSG00000206698	0.15069912	-0.01848503	0.38480575	0.84537521	NA	RNU1-73P
NA	ENSG00000206699	0	NA	NA	NA	NA	
NA	ENSG00000206700	0	NA	NA	NA	NA	RNU6-723P
NA	ENSG00000206701	0	NA	NA	NA	NA	RNU6-1040P
NA	ENSG00000206702	0	NA	NA	NA	NA	RNU1-11P
NA	ENSG00000206703	0	NA	NA	NA	NA	RNU6-128P
NA	ENSG00000206704	0	NA	NA	NA	NA	
NA	ENSG00000206705	0	NA	NA	NA	NA	
NA	ENSG00000206708	0	NA	NA	NA	NA	RNU6-1227P
NA	ENSG00000206709	0	NA	NA	NA	NA	RNU6-1080P
NA	ENSG00000206710	0	NA	NA	NA	NA	RNU6-147P
NA	ENSG00000206711	0	NA	NA	NA	NA	
NA	ENSG00000206712	0	NA	NA	NA	NA	RNU6-26P
NA	ENSG00000206713	0	NA	NA	NA	NA	
NA	ENSG00000206714	0	NA	NA	NA	NA	
NA	ENSG00000206715	0	NA	NA	NA	NA	RNU6-444P
NA	ENSG00000206716	0	NA	NA	NA	NA	RNU6-133P
NA	ENSG00000206717	0	NA	NA	NA	NA	
NA	ENSG00000206718	0	NA	NA	NA	NA	RNU6-546P
NA	ENSG00000206719	0	NA	NA	NA	NA	

NA	ENSG00000206721	0	NA	NA	NA	NA	
NA	ENSG00000206722	0	NA	NA	NA	NA	RNU6-1074P
NA	ENSG00000206723	0	NA	NA	NA	NA	RNU6-1056P
NA	ENSG00000206724	0	NA	NA	NA	NA	RNU6-756P
NA	ENSG00000206725	0	NA	NA	NA	NA	RNU6-1027P
NA	ENSG00000206726	0	NA	NA	NA	NA	RNU6-259P
NA	ENSG00000206728	0	NA	NA	NA	NA	
NA	ENSG00000206729	0	NA	NA	NA	NA	RNU6-1126P
NA	ENSG00000206730	0	NA	NA	NA	NA	RNU6-468P
NA	ENSG00000206731	0	NA	NA	NA	NA	
NA	ENSG00000206732	0	NA	NA	NA	NA	RNU6-936P
NA	ENSG00000206733	0	NA	NA	NA	NA	RNU6-641P
NA	ENSG00000206734	0	NA	NA	NA	NA	
NA	ENSG00000206738	0	NA	NA	NA	NA	
NA	ENSG00000206739	0	NA	NA	NA	NA	
NA	ENSG00000206741	0	NA	NA	NA	NA	
NA	ENSG00000206743	0	NA	NA	NA	NA	RNU6-484P
NA	ENSG00000206744	0	NA	NA	NA	NA	RNU6-620P
NA	ENSG00000206745	0	NA	NA	NA	NA	RNU6-643P
NA	ENSG00000206746	0	NA	NA	NA	NA	RNU6-142P
NA	ENSG00000206747	0	NA	NA	NA	NA	RNU6-245P
NA	ENSG00000206749	0	NA	NA	NA	NA	RNU6-1083P
NA	ENSG00000206751	0	NA	NA	NA	NA	
NA	ENSG00000206752	0	NA	NA	NA	NA	RNU6-1323P
594837	ENSG00000206754	0	NA	NA	NA	NA	SNORD101
677813	ENSG00000206755	0	NA	NA	NA	NA	SNORA30
NA	ENSG00000206756	0	NA	NA	NA	NA	
NA	ENSG00000206758	0	NA	NA	NA	NA	RNU6-317P
NA	ENSG00000206759	0	NA	NA	NA	NA	RNU6-787P
574040	ENSG00000206760	0	NA	NA	NA	NA	SNORA6
NA	ENSG00000206762	0	NA	NA	NA	NA	RNU6-418P
NA	ENSG00000206763	0	NA	NA	NA	NA	RNU6-10P
NA	ENSG00000206764	0	NA	NA	NA	NA	RNU6-152P
NA	ENSG00000206765	0	NA	NA	NA	NA	RNU6-203P
NA	ENSG00000206766	0	NA	NA	NA	NA	RNU6-435P
NA	ENSG00000206767	0	NA	NA	NA	NA	RNU6-949P
NA	ENSG00000206768	0	NA	NA	NA	NA	
NA	ENSG00000206769	0	NA	NA	NA	NA	RNU6-116P

NA	ENSG00000206770	0	NA	NA	NA	NA	
NA	ENSG00000206772	0	NA	NA	NA	NA	RNU6-44P
NA	ENSG00000206774	0	NA	NA	NA	NA	RNU6-211P
26812	ENSG00000206775	0	NA	NA	NA	NA	SNORD37
NA	ENSG00000206776	0	NA	NA	NA	NA	
NA	ENSG00000206777	0	NA	NA	NA	NA	RNU6-637P
NA	ENSG00000206778	0	NA	NA	NA	NA	RNU6-213P
NA	ENSG00000206779	0	NA	NA	NA	NA	
109616994	ENSG00000206780	0	NA	NA	NA	NA	SNORA75B
NA	ENSG00000206781	0	NA	NA	NA	NA	
NA	ENSG00000206782	0	NA	NA	NA	NA	RNU6-224P
NA	ENSG00000206784	0	NA	NA	NA	NA	
NA	ENSG00000206786	0	NA	NA	NA	NA	RNU6-701P
NA	ENSG00000206787	0	NA	NA	NA	NA	RNU6-76P
NA	ENSG00000206788	0	NA	NA	NA	NA	RNU6-629P
NA	ENSG00000206790	0	NA	NA	NA	NA	
NA	ENSG00000206791	0	NA	NA	NA	NA	RNU1-129P
NA	ENSG00000206792	0	NA	NA	NA	NA	
NA	ENSG00000206795	0	NA	NA	NA	NA	
NA	ENSG00000206796	0	NA	NA	NA	NA	RNU6-811P
NA	ENSG00000206797	0	NA	NA	NA	NA	
692063	ENSG00000206799	0	NA	NA	NA	NA	SNORA32
NA	ENSG00000206800	0	NA	NA	NA	NA	RNY3P7
NA	ENSG00000206801	0	NA	NA	NA	NA	RNU6-639P
NA	ENSG00000206802	0	NA	NA	NA	NA	RNU6-926P
NA	ENSG00000206803	0	NA	NA	NA	NA	RNU6-968P
NA	ENSG00000206805	0	NA	NA	NA	NA	
NA	ENSG00000206806	0	NA	NA	NA	NA	
NA	ENSG00000206807	0	NA	NA	NA	NA	RNU6-815P
NA	ENSG00000206808	0	NA	NA	NA	NA	
574042	ENSG00000206811	0	NA	NA	NA	NA	SNORA10
NA	ENSG00000206812	0	NA	NA	NA	NA	RNU6-38P
NA	ENSG00000206813	0	NA	NA	NA	NA	
NA	ENSG00000206814	0	NA	NA	NA	NA	
NA	ENSG00000206815	0	NA	NA	NA	NA	RNU6-483P
NA	ENSG00000206816	0	NA	NA	NA	NA	
NA	ENSG00000206817	0	NA	NA	NA	NA	
NA	ENSG00000206818	0	NA	NA	NA	NA	RNU6-849P

NA	ENSG00000206819	0	NA	NA	NA	NA	RNU6-260P
NA	ENSG00000206820	0	NA	NA	NA	NA	RNU1-138P
NA	ENSG00000206822	0	NA	NA	NA	NA	
NA	ENSG00000206824	0	NA	NA	NA	NA	
NA	ENSG00000206826	0	NA	NA	NA	NA	RNU6-974P
NA	ENSG00000206827	0	NA	NA	NA	NA	RNU6-1032P
NA	ENSG00000206828	0	NA	NA	NA	NA	RNVU1-30
NA	ENSG00000206832	0	NA	NA	NA	NA	RNU6V
NA	ENSG00000206833	0	NA	NA	NA	NA	RNU6-20P
677792	ENSG00000206834	0	NA	NA	NA	NA	SNORA1
NA	ENSG00000206835	0	NA	NA	NA	NA	RNU1-74P
NA	ENSG00000206836	0	NA	NA	NA	NA	RNU6-1029P
654319	ENSG00000206838	0	NA	NA	NA	NA	SNORA5A
NA	ENSG00000206839	0	NA	NA	NA	NA	RNU6-746P
NA	ENSG00000206840	0	NA	NA	NA	NA	
NA	ENSG00000206841	0	NA	NA	NA	NA	RNU6-409P
NA	ENSG00000206842	0	NA	NA	NA	NA	RNU6-413P
NA	ENSG00000206843	0	NA	NA	NA	NA	RNU6-206P
NA	ENSG00000206844	0	NA	NA	NA	NA	
NA	ENSG00000206845	0	NA	NA	NA	NA	RNU6-209P
NA	ENSG00000206846	0	NA	NA	NA	NA	
NA	ENSG00000206847	0	NA	NA	NA	NA	
NA	ENSG00000206848	0	NA	NA	NA	NA	RNU6-890P
NA	ENSG00000206849	0	NA	NA	NA	NA	
NA	ENSG00000206850	0	NA	NA	NA	NA	
NA	ENSG00000206852	0	NA	NA	NA	NA	RNU6-895P
NA	ENSG00000206853	0	NA	NA	NA	NA	
NA	ENSG00000206854	0	NA	NA	NA	NA	RNY3P5
NA	ENSG00000206855	0	NA	NA	NA	NA	RNU6-571P
NA	ENSG00000206857	0	NA	NA	NA	NA	RNU6-214P
NA	ENSG00000206858	0	NA	NA	NA	NA	
NA	ENSG00000206859	0	NA	NA	NA	NA	RNU6-767P
NA	ENSG00000206862	0	NA	NA	NA	NA	
NA	ENSG00000206863	0	NA	NA	NA	NA	RNU5A-6P
NA	ENSG00000206864	0	NA	NA	NA	NA	RNU6-207P
NA	ENSG00000206865	0	NA	NA	NA	NA	
NA	ENSG00000206866	0	NA	NA	NA	NA	RNU6-187P
NA	ENSG00000206867	0	NA	NA	NA	NA	RNU6-356P

100337591	ENSG00000206869	0	NA	NA	NA	NA	SNORA70F
NA	ENSG00000206870	0	NA	NA	NA	NA	RNU6-398P
NA	ENSG00000206871	0	NA	NA	NA	NA	RNU6-533P
NA	ENSG00000206875	0	NA	NA	NA	NA	RNU6-761P
NA	ENSG00000206877	0	NA	NA	NA	NA	RNU6-1103P
NA	ENSG00000206878	0.173733	0.01914545	0.38484597	0.78225479	NA	
NA	ENSG00000206880	0	NA	NA	NA	NA	RNU6-1310P
NA	ENSG00000206881	0	NA	NA	NA	NA	RNU6-190P
NA	ENSG00000206882	0	NA	NA	NA	NA	RNA5SP87
654321	ENSG00000206885	0	NA	NA	NA	NA	SNORA75
NA	ENSG00000206886	0	NA	NA	NA	NA	
NA	ENSG00000206887	0	NA	NA	NA	NA	RNU6-1008P
NA	ENSG00000206888	0	NA	NA	NA	NA	RNU6-48P
NA	ENSG00000206889	0	NA	NA	NA	NA	RNU6-1200P
NA	ENSG00000206891	0	NA	NA	NA	NA	RNU6-217P
NA	ENSG00000206892	0	NA	NA	NA	NA	RNU6-42P
NA	ENSG00000206895	0	NA	NA	NA	NA	
NA	ENSG00000206896	0	NA	NA	NA	NA	RNU6-1124P
109616977	ENSG00000206897	0	NA	NA	NA	NA	SNORA9B
NA	ENSG00000206898	0	NA	NA	NA	NA	
NA	ENSG00000206899	0	NA	NA	NA	NA	RNU6-36P
NA	ENSG00000206900	0	NA	NA	NA	NA	RNU6-98P
NA	ENSG00000206901	0	NA	NA	NA	NA	
109617003	ENSG00000206903	0	NA	NA	NA	NA	SNORA24B
NA	ENSG00000206905	0	NA	NA	NA	NA	
NA	ENSG00000206906	0	NA	NA	NA	NA	RNU6-458P
NA	ENSG00000206907	0	NA	NA	NA	NA	RNU6-1013P
NA	ENSG00000206908	0	NA	NA	NA	NA	RNU1-136P
109616973	ENSG00000206909	0	NA	NA	NA	NA	SNORA70J
677812	ENSG00000206910	0	NA	NA	NA	NA	SNORA29
NA	ENSG00000206911	0	NA	NA	NA	NA	
NA	ENSG00000206912	0	NA	NA	NA	NA	
NA	ENSG00000206913	0	NA	NA	NA	NA	
NA	ENSG00000206914	0	NA	NA	NA	NA	
NA	ENSG00000206915	0	NA	NA	NA	NA	RNU6-439P
NA	ENSG00000206917	0	NA	NA	NA	NA	RNU1-52P
NA	ENSG00000206918	0	NA	NA	NA	NA	RNU6-1181P
NA	ENSG00000206920	0	NA	NA	NA	NA	RNY1P6

NA	ENSG00000206921	0	NA	NA	NA	NA	RNU6-481P
NA	ENSG00000206922	0	NA	NA	NA	NA	RNU6-80P
NA	ENSG00000206923	0	NA	NA	NA	NA	RNU6-432P
NA	ENSG00000206924	0	NA	NA	NA	NA	RNU6-689P
NA	ENSG00000206925	0	NA	NA	NA	NA	
NA	ENSG00000206926	0	NA	NA	NA	NA	RNU6-1024P
NA	ENSG00000206929	0	NA	NA	NA	NA	
NA	ENSG00000206931	0	NA	NA	NA	NA	RNU6-1042P
NA	ENSG00000206935	0	NA	NA	NA	NA	RNU6-514P
NA	ENSG00000206936	0.18922458	0.01954053	0.38488496	0.78225479	NA	RNU4-52P
100124537	ENSG00000206937	0	NA	NA	NA	NA	SNORA70B
NA	ENSG00000206938	0	NA	NA	NA	NA	RNU4-31P
NA	ENSG00000206939	0	NA	NA	NA	NA	RNU6-281P
6079	ENSG00000206941	0	NA	NA	NA	NA	SNORD15A
NA	ENSG00000206944	0	NA	NA	NA	NA	RNU6-708P
109616963	ENSG00000206947	0	NA	NA	NA	NA	SNORA20B
677817	ENSG00000206948	0	NA	NA	NA	NA	SNORA36A
NA	ENSG00000206949	0	NA	NA	NA	NA	RNU6-1324P
NA	ENSG00000206950	0	NA	NA	NA	NA	
NA	ENSG00000206951	0	NA	NA	NA	NA	
NA	ENSG00000206952	0	NA	NA	NA	NA	SNORA50A
NA	ENSG00000206954	0	NA	NA	NA	NA	RNU6-1201P
NA	ENSG00000206957	0	NA	NA	NA	NA	
NA	ENSG00000206958	0	NA	NA	NA	NA	
NA	ENSG00000206959	0	NA	NA	NA	NA	
NA	ENSG00000206960	0	NA	NA	NA	NA	RNU6-793P
NA	ENSG00000206961	0	NA	NA	NA	NA	
NA	ENSG00000206962	0	NA	NA	NA	NA	RNU6-74P
NA	ENSG00000206963	0	NA	NA	NA	NA	RNU6-675P
NA	ENSG00000206964	0	NA	NA	NA	NA	
NA	ENSG00000206965	0	NA	NA	NA	NA	RNU6-5P
NA	ENSG00000206967	0	NA	NA	NA	NA	
NA	ENSG00000206969	0	NA	NA	NA	NA	RNU6-1316P
NA	ENSG00000206970	0	NA	NA	NA	NA	RNU6-474P
NA	ENSG00000206972	0	NA	NA	NA	NA	RNU6-17P
NA	ENSG00000206973	0	NA	NA	NA	NA	RNU6-1145P
NA	ENSG00000206974	0	NA	NA	NA	NA	RNU6-1144P
NA	ENSG00000206976	0	NA	NA	NA	NA	

NA	ENSG00000206977	0	NA	NA	NA	NA	
NA	ENSG00000206978	0	NA	NA	NA	NA	
26787	ENSG00000206979	0	NA	NA	NA	NA	SNORD61
NA	ENSG00000206980	0	NA	NA	NA	NA	RNU6-662P
NA	ENSG00000206981	0	NA	NA	NA	NA	RNU6-40P
NA	ENSG00000206982	0	NA	NA	NA	NA	
NA	ENSG00000206983	0	NA	NA	NA	NA	RNU6-49P
NA	ENSG00000206985	0	NA	NA	NA	NA	RNU6-198P
NA	ENSG00000206987	0	NA	NA	NA	NA	
26785	ENSG00000206989	0	NA	NA	NA	NA	SNORD63
NA	ENSG00000206990	0	NA	NA	NA	NA	
NA	ENSG00000206991	0	NA	NA	NA	NA	RNU6-610P
NA	ENSG00000206992	0	NA	NA	NA	NA	RNU6-574P
NA	ENSG00000206995	0	NA	NA	NA	NA	
NA	ENSG00000206996	0	NA	NA	NA	NA	RNU6-842P
NA	ENSG00000206997	0	NA	NA	NA	NA	RNU6-524P
NA	ENSG00000206998	0	NA	NA	NA	NA	
NA	ENSG00000206999	0	NA	NA	NA	NA	RNU6-622P
NA	ENSG00000207000	0	NA	NA	NA	NA	RNU6-820P
NA	ENSG00000207001	0	NA	NA	NA	NA	SNORD116-2
NA	ENSG00000207002	0	NA	NA	NA	NA	
NA	ENSG00000207003	0	NA	NA	NA	NA	RNU6-611P
NA	ENSG00000207004	0	NA	NA	NA	NA	RNU6-301P
NA	ENSG00000207007	0	NA	NA	NA	NA	RNU6-891P
677833	ENSG00000207008	0	NA	NA	NA	NA	SNORA54
NA	ENSG00000207009	0	NA	NA	NA	NA	
NA	ENSG00000207010	0	NA	NA	NA	NA	RNU6-318P
NA	ENSG00000207011	0	NA	NA	NA	NA	RNY4P13
NA	ENSG00000207012	0	NA	NA	NA	NA	RNU6-72P
NA	ENSG00000207013	0	NA	NA	NA	NA	RNU6-804P
NA	ENSG00000207014	0	NA	NA	NA	NA	SNORD116-3
100124535	ENSG00000207016	0	NA	NA	NA	NA	SNORA36C
NA	ENSG00000207019	0	NA	NA	NA	NA	RNU6-167P
NA	ENSG00000207020	0	NA	NA	NA	NA	
NA	ENSG00000207021	0	NA	NA	NA	NA	
NA	ENSG00000207022	0.14931947	-0.01844332	0.38480212	0.84537521	NA	
NA	ENSG00000207023	0	NA	NA	NA	NA	RNU6-975P
NA	ENSG00000207024	0	NA	NA	NA	NA	

NA	ENSG00000207025	0	NA	NA	NA	NA	
NA	ENSG00000207026	0	NA	NA	NA	NA	RNU6-472P
NA	ENSG00000207027	0	NA	NA	NA	NA	
NA	ENSG00000207029	0	NA	NA	NA	NA	RNU6-43P
26789	ENSG00000207031	0	NA	NA	NA	NA	SNORD59A
NA	ENSG00000207032	0	NA	NA	NA	NA	
NA	ENSG00000207033	0	NA	NA	NA	NA	RNU6-154P
NA	ENSG00000207034	0	NA	NA	NA	NA	
NA	ENSG00000207036	0	NA	NA	NA	NA	RNY3P14
NA	ENSG00000207037	0	NA	NA	NA	NA	RNU6-339P
NA	ENSG00000207039	0	NA	NA	NA	NA	
NA	ENSG00000207042	0	NA	NA	NA	NA	RNU6-196P
NA	ENSG00000207044	0	NA	NA	NA	NA	RNU6-1226P
NA	ENSG00000207045	0	NA	NA	NA	NA	RNU6-532P
NA	ENSG00000207046	0	NA	NA	NA	NA	RNU6-886P
NA	ENSG00000207047	0	NA	NA	NA	NA	SNORD51
NA	ENSG00000207049	0	NA	NA	NA	NA	
619499	ENSG00000207051	0	NA	NA	NA	NA	SNORA27
NA	ENSG00000207052	0	NA	NA	NA	NA	RNU6-378P
NA	ENSG00000207053	0	NA	NA	NA	NA	RNU6-937P
NA	ENSG00000207056	0	NA	NA	NA	NA	RNU1-8P
NA	ENSG00000207058	0	NA	NA	NA	NA	RNU6-784P
NA	ENSG00000207060	0	NA	NA	NA	NA	RNU6-480P
NA	ENSG00000207061	0	NA	NA	NA	NA	
109616962	ENSG00000207062	0	NA	NA	NA	NA	SNORA15B-1
NA	ENSG00000207063	0	NA	NA	NA	NA	SNORD116-1
NA	ENSG00000207065	0	NA	NA	NA	NA	RNU5A-2P
26775	ENSG00000207067	0	NA	NA	NA	NA	SNORA72
NA	ENSG00000207068	0	NA	NA	NA	NA	RNU6-463P
NA	ENSG00000207069	0	NA	NA	NA	NA	
NA	ENSG00000207071	0	NA	NA	NA	NA	RNU6-1207P
NA	ENSG00000207072	0	NA	NA	NA	NA	RNU6-39P
NA	ENSG00000207073	0	NA	NA	NA	NA	
NA	ENSG00000207075	0	NA	NA	NA	NA	
NA	ENSG00000207076	0	NA	NA	NA	NA	RNU6-1113P
NA	ENSG00000207077	0	NA	NA	NA	NA	RNU6-1119P
NA	ENSG00000207080	0	NA	NA	NA	NA	
NA	ENSG00000207081	0	NA	NA	NA	NA	RNU6-616P

NA	ENSG00000207082	0	NA	NA	NA	NA	RNU6-171P
NA	ENSG00000207083	0	NA	NA	NA	NA	RNU6-22P
NA	ENSG00000207084	0	NA	NA	NA	NA	
NA	ENSG00000207086	0	NA	NA	NA	NA	
NA	ENSG00000207087	0	NA	NA	NA	NA	RNU6-242P
677797	ENSG00000207088	0	NA	NA	NA	NA	SNORA7B
NA	ENSG00000207089	0	NA	NA	NA	NA	RNU6-921P
NA	ENSG00000207090	0	NA	NA	NA	NA	RNU6-517P
NA	ENSG00000207091	0	NA	NA	NA	NA	
NA	ENSG00000207092	0	NA	NA	NA	NA	
NA	ENSG00000207093	0	NA	NA	NA	NA	SNORD116-8
NA	ENSG00000207094	0	NA	NA	NA	NA	
NA	ENSG00000207095	0	NA	NA	NA	NA	RNU6-424P
NA	ENSG00000207097	0	NA	NA	NA	NA	RNU6-614P
NA	ENSG00000207098	0	NA	NA	NA	NA	
NA	ENSG00000207099	0	NA	NA	NA	NA	RNU6-166P
NA	ENSG00000207100	0	NA	NA	NA	NA	
NA	ENSG00000207101	0	NA	NA	NA	NA	
NA	ENSG00000207104	0	NA	NA	NA	NA	RNU6-434P
NA	ENSG00000207105	0	NA	NA	NA	NA	
NA	ENSG00000207108	0	NA	NA	NA	NA	
109617024	ENSG00000207109	0	NA	NA	NA	NA	SNORD38C
NA	ENSG00000207110	0	NA	NA	NA	NA	RNVU1-32
684959	ENSG00000207112	0	NA	NA	NA	NA	SNORA25
NA	ENSG00000207113	0	NA	NA	NA	NA	RNU6-16P
NA	ENSG00000207114	0	NA	NA	NA	NA	RNU6-348P
NA	ENSG00000207115	0	NA	NA	NA	NA	RNU6-364P
NA	ENSG00000207116	0	NA	NA	NA	NA	RNU6-31P
NA	ENSG00000207117	0	NA	NA	NA	NA	
85390	ENSG00000207118	0	NA	NA	NA	NA	SNORD14D
NA	ENSG00000207119	0	NA	NA	NA	NA	
NA	ENSG00000207121	0	NA	NA	NA	NA	RNU6-1230P
NA	ENSG00000207122	0	NA	NA	NA	NA	RNU6-591P
NA	ENSG00000207123	0	NA	NA	NA	NA	
NA	ENSG00000207124	0	NA	NA	NA	NA	RNU6-163P
NA	ENSG00000207127	0	NA	NA	NA	NA	
NA	ENSG00000207128	0	NA	NA	NA	NA	RNU6-729P
NA	ENSG00000207129	0	NA	NA	NA	NA	RNA5SP187

NA	ENSG00000207130	0	NA	NA	NA	NA	
NA	ENSG00000207131	0	NA	NA	NA	NA	
NA	ENSG00000207132	0	NA	NA	NA	NA	
NA	ENSG00000207134	0	NA	NA	NA	NA	RNU6-106P
NA	ENSG00000207135	0	NA	NA	NA	NA	RNU6-452P
NA	ENSG00000207136	0	NA	NA	NA	NA	RNU6-769P
NA	ENSG00000207137	0	NA	NA	NA	NA	SNORD116-13
NA	ENSG00000207138	0	NA	NA	NA	NA	RNU6-869P
NA	ENSG00000207139	0	NA	NA	NA	NA	
NA	ENSG00000207142	0	NA	NA	NA	NA	
NA	ENSG00000207144	0	NA	NA	NA	NA	RNU6-1297P
677805	ENSG00000207145	0	NA	NA	NA	NA	SNORA18
NA	ENSG00000207146	0	NA	NA	NA	NA	
NA	ENSG00000207147	0	NA	NA	NA	NA	
NA	ENSG00000207148	0	NA	NA	NA	NA	
NA	ENSG00000207149	0	NA	NA	NA	NA	RNU6-465P
NA	ENSG00000207150	0	NA	NA	NA	NA	RNU6-185P
NA	ENSG00000207151	0	NA	NA	NA	NA	
NA	ENSG00000207153	0	NA	NA	NA	NA	RNU6-933P
NA	ENSG00000207154	0	NA	NA	NA	NA	RNU1-46P
NA	ENSG00000207155	0	NA	NA	NA	NA	RNY1P14
NA	ENSG00000207156	0	NA	NA	NA	NA	RNU6-505P
NA	ENSG00000207157	0	NA	NA	NA	NA	RNY3P4
NA	ENSG00000207158	0	NA	NA	NA	NA	RNU6-929P
NA	ENSG00000207160	0	NA	NA	NA	NA	RNU6-1289P
NA	ENSG00000207161	0	NA	NA	NA	NA	
NA	ENSG00000207162	0	NA	NA	NA	NA	RNU6-549P
NA	ENSG00000207163	0	NA	NA	NA	NA	RNU6-905P
NA	ENSG00000207164	0	NA	NA	NA	NA	
26778	ENSG00000207165	0	NA	NA	NA	NA	SNORA70
26780	ENSG00000207166	0	NA	NA	NA	NA	SNORA68
NA	ENSG00000207167	0	NA	NA	NA	NA	RNU6-1340P
677803	ENSG00000207168	0	NA	NA	NA	NA	SNORA15
NA	ENSG00000207169	0	NA	NA	NA	NA	RNU6-24P
NA	ENSG00000207170	0	NA	NA	NA	NA	RNU6-1215P
NA	ENSG00000207171	0	NA	NA	NA	NA	
NA	ENSG00000207172	0	NA	NA	NA	NA	RNU6-1162P
NA	ENSG00000207173	0	NA	NA	NA	NA	

NA	ENSG00000207174	0	NA	NA	NA	NA	SNORD116-15
NA	ENSG00000207175	0	NA	NA	NA	NA	RNU1-67P
NA	ENSG00000207176	0	NA	NA	NA	NA	
NA	ENSG00000207177	0	NA	NA	NA	NA	
NA	ENSG00000207178	0	NA	NA	NA	NA	RNU6-1122P
NA	ENSG00000207180	0	NA	NA	NA	NA	RNU6-411P
677802	ENSG00000207181	0	NA	NA	NA	NA	SNORA14B
NA	ENSG00000207182	0	NA	NA	NA	NA	RNU1-44P
NA	ENSG00000207183	0	NA	NA	NA	NA	RNU6-843P
NA	ENSG00000207185	0	NA	NA	NA	NA	RNU6-1157P
NA	ENSG00000207186	0	NA	NA	NA	NA	RNA5SP122
109616960	ENSG00000207187	0	NA	NA	NA	NA	SNORA10B
NA	ENSG00000207189	0	NA	NA	NA	NA	
NA	ENSG00000207190	0	NA	NA	NA	NA	RNU6-809P
NA	ENSG00000207191	0	NA	NA	NA	NA	SNORD116-5
NA	ENSG00000207192	0	NA	NA	NA	NA	RNU6-649P
NA	ENSG00000207193	0	NA	NA	NA	NA	
NA	ENSG00000207194	0	NA	NA	NA	NA	RNU6-1026P
NA	ENSG00000207195	0	NA	NA	NA	NA	
NA	ENSG00000207196	0	NA	NA	NA	NA	
NA	ENSG00000207197	0	NA	NA	NA	NA	SNORD116-12
NA	ENSG00000207198	0	NA	NA	NA	NA	RNU6-1195P
109616979	ENSG00000207199	0	NA	NA	NA	NA	
NA	ENSG00000207200	0	NA	NA	NA	NA	RNU6-45P
NA	ENSG00000207201	0	NA	NA	NA	NA	RNU1-148P
NA	ENSG00000207202	0	NA	NA	NA	NA	RNU6-800P
NA	ENSG00000207203	0	NA	NA	NA	NA	RNU6-71P
NA	ENSG00000207204	0	NA	NA	NA	NA	RNU6-393P
101954267	ENSG00000207205	0.16735865	0.01897177	0.38482933	0.78225479	NA	RNVU1-15
NA	ENSG00000207206	0	NA	NA	NA	NA	RNU6-1035P
NA	ENSG00000207207	0	NA	NA	NA	NA	
NA	ENSG00000207208	0	NA	NA	NA	NA	RNU6-790P
NA	ENSG00000207209	0	NA	NA	NA	NA	
NA	ENSG00000207210	0	NA	NA	NA	NA	
NA	ENSG00000207214	0	NA	NA	NA	NA	RNU6-102P
109617016	ENSG00000207215	0	NA	NA	NA	NA	SNORD3H
109616999	ENSG00000207217	0.173124	-0.01911262	0.38486262	0.84537521	NA	SNORA80D
NA	ENSG00000207218	0	NA	NA	NA	NA	

NA	ENSG00000207220	0	NA	NA	NA	NA	RNU1-57P
100379250	ENSG00000207221	0	NA	NA	NA	NA	SNORA70E
NA	ENSG00000207222	0	NA	NA	NA	NA	RNU6-456P
NA	ENSG00000207223	0	NA	NA	NA	NA	
NA	ENSG00000207225	0	NA	NA	NA	NA	RNU6-692P
NA	ENSG00000207227	0	NA	NA	NA	NA	RNU6-900P
NA	ENSG00000207229	0	NA	NA	NA	NA	
NA	ENSG00000207231	0	NA	NA	NA	NA	
677819	ENSG00000207233	0	NA	NA	NA	NA	SNORA37
NA	ENSG00000207234	0	NA	NA	NA	NA	RNU6-125P
NA	ENSG00000207235	0	NA	NA	NA	NA	
NA	ENSG00000207237	0	NA	NA	NA	NA	RNU6-110P
NA	ENSG00000207240	0	NA	NA	NA	NA	
26805	ENSG00000207241	0	NA	NA	NA	NA	SNORD45A
NA	ENSG00000207242	0	NA	NA	NA	NA	RNU6-1065P
NA	ENSG00000207243	0	NA	NA	NA	NA	
NA	ENSG00000207244	0	NA	NA	NA	NA	
NA	ENSG00000207245	0	NA	NA	NA	NA	SNORD116-29
NA	ENSG00000207247	0	NA	NA	NA	NA	
NA	ENSG00000207248	0	NA	NA	NA	NA	RNU6-1005P
NA	ENSG00000207249	0	NA	NA	NA	NA	
NA	ENSG00000207251	0	NA	NA	NA	NA	RNU6-342P
NA	ENSG00000207252	0	NA	NA	NA	NA	
NA	ENSG00000207255	0	NA	NA	NA	NA	RNU6-1117P
NA	ENSG00000207256	0	NA	NA	NA	NA	RNU6-880P
NA	ENSG00000207258	0	NA	NA	NA	NA	
NA	ENSG00000207260	0	NA	NA	NA	NA	RNU6-35P
NA	ENSG00000207261	0	NA	NA	NA	NA	RNU6-738P
NA	ENSG00000207263	0	NA	NA	NA	NA	SNORD116-16
NA	ENSG00000207264	0	NA	NA	NA	NA	RNU6-15P
NA	ENSG00000207265	0	NA	NA	NA	NA	
NA	ENSG00000207266	0	NA	NA	NA	NA	RNU6-1241P
NA	ENSG00000207267	0	NA	NA	NA	NA	RNU6-1081P
100124538	ENSG00000207268	0	NA	NA	NA	NA	SNORA70C
NA	ENSG00000207269	0	NA	NA	NA	NA	RN7SKP62
NA	ENSG00000207270	0	NA	NA	NA	NA	RNU6-601P
NA	ENSG00000207271	0	NA	NA	NA	NA	
109617008	ENSG00000207274	0	NA	NA	NA	NA	SNORA70I

NA	ENSG00000207275	0	NA	NA	NA	NA	RNU6-441P
NA	ENSG00000207276	0	NA	NA	NA	NA	RNU6-1160P
NA	ENSG00000207277	0	NA	NA	NA	NA	RNA5SP284
NA	ENSG00000207278	0	NA	NA	NA	NA	RNU6-831P
NA	ENSG00000207279	0	NA	NA	NA	NA	SNORD116-24
6082	ENSG00000207280	0	NA	NA	NA	NA	SNORD20
NA	ENSG00000207281	0	NA	NA	NA	NA	
NA	ENSG00000207282	0	NA	NA	NA	NA	
NA	ENSG00000207283	0	NA	NA	NA	NA	
NA	ENSG00000207286	0	NA	NA	NA	NA	
NA	ENSG00000207287	0	NA	NA	NA	NA	RNU6-1274P
NA	ENSG00000207290	0	NA	NA	NA	NA	
NA	ENSG00000207291	0	NA	NA	NA	NA	RNU6-30P
NA	ENSG00000207292	0	NA	NA	NA	NA	
NA	ENSG00000207293	0	NA	NA	NA	NA	
NA	ENSG00000207295	0	NA	NA	NA	NA	RNU6-851P
NA	ENSG00000207296	0	NA	NA	NA	NA	RNU6-140P
692076	ENSG00000207297	0	NA	NA	NA	NA	SNORD7
NA	ENSG00000207298	0	NA	NA	NA	NA	RNU6-83P
NA	ENSG00000207299	0	NA	NA	NA	NA	
NA	ENSG00000207300	0	NA	NA	NA	NA	
NA	ENSG00000207302	0	NA	NA	NA	NA	
NA	ENSG00000207303	0	NA	NA	NA	NA	
654320	ENSG00000207304	0	NA	NA	NA	NA	SNORA8
NA	ENSG00000207305	0	NA	NA	NA	NA	
NA	ENSG00000207306	0	NA	NA	NA	NA	RNU6-1152P
NA	ENSG00000207307	0	NA	NA	NA	NA	RNU6-145P
NA	ENSG00000207308	0	NA	NA	NA	NA	RNU6-878P
NA	ENSG00000207309	0	NA	NA	NA	NA	RNU4-70P
NA	ENSG00000207310	0	NA	NA	NA	NA	RNU6-1127P
NA	ENSG00000207312	0	NA	NA	NA	NA	RNU6-429P
677794	ENSG00000207313	0	NA	NA	NA	NA	SNORA2B
NA	ENSG00000207316	0	NA	NA	NA	NA	
NA	ENSG00000207317	0	NA	NA	NA	NA	
NA	ENSG00000207318	0	NA	NA	NA	NA	RNU6-1184P
NA	ENSG00000207319	0	NA	NA	NA	NA	
NA	ENSG00000207320	0	NA	NA	NA	NA	
NA	ENSG00000207321	0	NA	NA	NA	NA	RNU6-160P

NA	ENSG00000207322	0	NA	NA	NA	NA	RNU1-89P
NA	ENSG00000207323	0	NA	NA	NA	NA	RNU6-901P
NA	ENSG00000207325	0	NA	NA	NA	NA	RNY1P4
NA	ENSG00000207326	0	NA	NA	NA	NA	
NA	ENSG00000207327	0	NA	NA	NA	NA	RNU6-883P
NA	ENSG00000207328	0	NA	NA	NA	NA	RNU6-636P
NA	ENSG00000207329	0	NA	NA	NA	NA	
NA	ENSG00000207330	0	NA	NA	NA	NA	RNU6-73P
NA	ENSG00000207331	0	NA	NA	NA	NA	RNU6-1263P
NA	ENSG00000207332	0	NA	NA	NA	NA	RNU6-146P
NA	ENSG00000207333	0	NA	NA	NA	NA	RNU6-680P
NA	ENSG00000207336	0	NA	NA	NA	NA	RNU6-658P
NA	ENSG00000207338	0	NA	NA	NA	NA	RNU6-296P
101954273	ENSG00000207340	0	NA	NA	NA	NA	RNVU1-1
NA	ENSG00000207341	0	NA	NA	NA	NA	RNA5SP64
NA	ENSG00000207342	0	NA	NA	NA	NA	
NA	ENSG00000207343	0	NA	NA	NA	NA	RNU6-225P
109616965	ENSG00000207344	0	NA	NA	NA	NA	SNORA22C
NA	ENSG00000207345	0	NA	NA	NA	NA	RNU6-1222P
NA	ENSG00000207347	0	NA	NA	NA	NA	RNU6-306P
101954269	ENSG00000207349	0	NA	NA	NA	NA	RNVU1-17
NA	ENSG00000207351	0	NA	NA	NA	NA	
NA	ENSG00000207352	0	NA	NA	NA	NA	RNU6-540P
NA	ENSG00000207355	0	NA	NA	NA	NA	RNU6-1257P
NA	ENSG00000207356	0	NA	NA	NA	NA	
NA	ENSG00000207359	0	NA	NA	NA	NA	RNU6-925P
NA	ENSG00000207360	0	NA	NA	NA	NA	RNU6-14P
NA	ENSG00000207361	0	NA	NA	NA	NA	RNU6-178P
NA	ENSG00000207362	0	NA	NA	NA	NA	RNU6-422P
NA	ENSG00000207363	0	NA	NA	NA	NA	
NA	ENSG00000207364	0	NA	NA	NA	NA	RNU6-939P
NA	ENSG00000207365	0	NA	NA	NA	NA	
NA	ENSG00000207366	0	NA	NA	NA	NA	RNU6-297P
NA	ENSG00000207367	0	NA	NA	NA	NA	RNU6-29P
NA	ENSG00000207368	0	NA	NA	NA	NA	
NA	ENSG00000207369	0	NA	NA	NA	NA	RNU6-665P
NA	ENSG00000207370	0	NA	NA	NA	NA	
NA	ENSG00000207371	0	NA	NA	NA	NA	

NA	ENSG00000207375	0	NA	NA	NA	NA	SNORD116-23
NA	ENSG00000207378	0	NA	NA	NA	NA	RNU6-748P
NA	ENSG00000207379	0	NA	NA	NA	NA	
NA	ENSG00000207380	0	NA	NA	NA	NA	
NA	ENSG00000207381	0	NA	NA	NA	NA	RNU6-950P
NA	ENSG00000207382	0	NA	NA	NA	NA	
NA	ENSG00000207383	0	NA	NA	NA	NA	
NA	ENSG00000207384	0	NA	NA	NA	NA	
NA	ENSG00000207385	0	NA	NA	NA	NA	RNU6-310P
NA	ENSG00000207386	0	NA	NA	NA	NA	
NA	ENSG00000207387	0	NA	NA	NA	NA	
NA	ENSG00000207391	0	NA	NA	NA	NA	
677806	ENSG00000207392	0	NA	NA	NA	NA	SNORA20
NA	ENSG00000207393	0	NA	NA	NA	NA	RNU6-136P
NA	ENSG00000207394	0	NA	NA	NA	NA	RNU6-1060P
NA	ENSG00000207395	0	NA	NA	NA	NA	
NA	ENSG00000207397	0	NA	NA	NA	NA	RNU6-1179P
NA	ENSG00000207399	0	NA	NA	NA	NA	RNU6-1011P
NA	ENSG00000207401	0	NA	NA	NA	NA	
NA	ENSG00000207402	0	NA	NA	NA	NA	RNU6-959P
NA	ENSG00000207403	0	NA	NA	NA	NA	
NA	ENSG00000207404	0	NA	NA	NA	NA	
26784	ENSG00000207405	0	NA	NA	NA	NA	SNORA64
619569	ENSG00000207406	0	NA	NA	NA	NA	SNORA41
NA	ENSG00000207407	0	NA	NA	NA	NA	
NA	ENSG00000207408	0	NA	NA	NA	NA	
NA	ENSG00000207410	0	NA	NA	NA	NA	
NA	ENSG00000207411	0	NA	NA	NA	NA	
NA	ENSG00000207412	0	NA	NA	NA	NA	RNU6-455P
NA	ENSG00000207414	0	NA	NA	NA	NA	RNU6-768P
NA	ENSG00000207415	0	NA	NA	NA	NA	RNU6-1151P
NA	ENSG00000207416	0	NA	NA	NA	NA	
NA	ENSG00000207417	0	NA	NA	NA	NA	
NA	ENSG00000207419	0	NA	NA	NA	NA	
NA	ENSG00000207420	0	NA	NA	NA	NA	
NA	ENSG00000207421	0	NA	NA	NA	NA	SNORD38B
NA	ENSG00000207422	0	NA	NA	NA	NA	RNU6-813P
NA	ENSG00000207425	0	NA	NA	NA	NA	

NA	ENSG00000207426	0	NA	NA	NA	NA	
NA	ENSG00000207428	0	NA	NA	NA	NA	RNU6-95P
NA	ENSG00000207431	0	NA	NA	NA	NA	RNU6-906P
NA	ENSG00000207432	0	NA	NA	NA	NA	
NA	ENSG00000207433	0	NA	NA	NA	NA	RNU6-246P
NA	ENSG00000207434	0	NA	NA	NA	NA	RNU6-1072P
NA	ENSG00000207435	0	NA	NA	NA	NA	RNU6-114P
NA	ENSG00000207438	0	NA	NA	NA	NA	
NA	ENSG00000207439	0	NA	NA	NA	NA	
NA	ENSG00000207440	0	NA	NA	NA	NA	RNU6-541P
NA	ENSG00000207441	0	NA	NA	NA	NA	RNU6-21P
NA	ENSG00000207442	0	NA	NA	NA	NA	SNORD116-6
NA	ENSG00000207443	0	NA	NA	NA	NA	RNU6-417P
319139	ENSG00000207444	0	NA	NA	NA	NA	SNORD56B
114599	ENSG00000207445	0	NA	NA	NA	NA	SNORD15B
NA	ENSG00000207448	0	NA	NA	NA	NA	RNU6-520P
NA	ENSG00000207449	0	NA	NA	NA	NA	RNU6-19P
NA	ENSG00000207450	0	NA	NA	NA	NA	
NA	ENSG00000207451	0	NA	NA	NA	NA	RNU6-291P
NA	ENSG00000207452	0	NA	NA	NA	NA	RNU6-606P
NA	ENSG00000207453	0	NA	NA	NA	NA	RNU6-535P
NA	ENSG00000207454	0	NA	NA	NA	NA	RNU6-1104P
NA	ENSG00000207455	0	NA	NA	NA	NA	RNU6-331P
NA	ENSG00000207456	0	NA	NA	NA	NA	RNU6-997P
NA	ENSG00000207457	0	NA	NA	NA	NA	RNU6-476P
NA	ENSG00000207458	0	NA	NA	NA	NA	RNY3P9
NA	ENSG00000207459	0	NA	NA	NA	NA	RNU6-1311P
NA	ENSG00000207461	0	NA	NA	NA	NA	RNU6-253P
NA	ENSG00000207462	0	NA	NA	NA	NA	RNU6-376P
NA	ENSG00000207466	0	NA	NA	NA	NA	
NA	ENSG00000207467	0	NA	NA	NA	NA	
641451	ENSG00000207468	0	NA	NA	NA	NA	SNORA19
NA	ENSG00000207472	0	NA	NA	NA	NA	RNU6-41P
NA	ENSG00000207473	0	NA	NA	NA	NA	
NA	ENSG00000207474	0	NA	NA	NA	NA	RNY1P7
677823	ENSG00000207475	0	NA	NA	NA	NA	SNORA80E
NA	ENSG00000207476	0	NA	NA	NA	NA	RNU6-286P
NA	ENSG00000207480	0	NA	NA	NA	NA	

NA	ENSG00000207481	0	NA	NA	NA	NA	
NA	ENSG00000207483	0	NA	NA	NA	NA	RNU6-1067P
NA	ENSG00000207484	0	NA	NA	NA	NA	
NA	ENSG00000207486	0	NA	NA	NA	NA	RNU6-1044P
NA	ENSG00000207488	0	NA	NA	NA	NA	
NA	ENSG00000207490	0	NA	NA	NA	NA	RNU6-987P
NA	ENSG00000207492	0	NA	NA	NA	NA	RNU6-713P
677827	ENSG00000207493	0	NA	NA	NA	NA	SNORA46
NA	ENSG00000207494	0	NA	NA	NA	NA	
NA	ENSG00000207495	0	NA	NA	NA	NA	RNY3P10
619563	ENSG00000207496	0	NA	NA	NA	NA	SNORA7A
NA	ENSG00000207497	0	NA	NA	NA	NA	
NA	ENSG00000207499	0	NA	NA	NA	NA	
26771	ENSG00000207500	0	NA	NA	NA	NA	SNORD102
101954266	ENSG00000207501	0	NA	NA	NA	NA	RNVU1-14
NA	ENSG00000207502	0	NA	NA	NA	NA	
NA	ENSG00000207503	0	NA	NA	NA	NA	
NA	ENSG00000207504	0	NA	NA	NA	NA	RNU6-1031P
NA	ENSG00000207505	0	NA	NA	NA	NA	RNU6-1105P
NA	ENSG00000207508	0	NA	NA	NA	NA	RNU6-1237P
NA	ENSG00000207512	0	NA	NA	NA	NA	
NA	ENSG00000207515	0	NA	NA	NA	NA	RNU6-555P
109617005	ENSG00000207516	0	NA	NA	NA	NA	SNORA41B
NA	ENSG00000207518	0	NA	NA	NA	NA	RNU6-59P
26782	ENSG00000207523	0	NA	NA	NA	NA	SNORA66
NA	ENSG00000207524	0	NA	NA	NA	NA	RNU6-33P
NA	ENSG00000207525	0	NA	NA	NA	NA	
693129	ENSG00000207546	0	NA	NA	NA	NA	MIR548C
407014	ENSG00000207547	0	NA	NA	NA	NA	MIR25
406999	ENSG00000207548	0	NA	NA	NA	NA	MIR217
574481	ENSG00000207549	0	NA	NA	NA	NA	MIR521-2
407056	ENSG00000207550	0	NA	NA	NA	NA	MIR99B
693193	ENSG00000207551	0	NA	NA	NA	NA	MIR608
693218	ENSG00000207552	0	NA	NA	NA	NA	MIR633
693232	ENSG00000207554	0	NA	NA	NA	NA	MIR647
693163	ENSG00000207559	0	NA	NA	NA	NA	MIR578
693220	ENSG00000207561	0	NA	NA	NA	NA	MIR635
407042	ENSG00000207562	0	NA	NA	NA	NA	MIR34C

407011	ENSG00000207563	0	NA	NA	NA	NA	MIR23B
406986	ENSG00000207568	0	NA	NA	NA	NA	MIR203A
574034	ENSG00000207569	0	NA	NA	NA	NA	MIR433
693200	ENSG00000207571	0	NA	NA	NA	NA	MIR615
693134	ENSG00000207573	0	NA	NA	NA	NA	MIR550A2
724031	ENSG00000207574	0	NA	NA	NA	NA	MIR661
693234	ENSG00000207575	0	NA	NA	NA	NA	MIR649
693172	ENSG00000207577	0	NA	NA	NA	NA	MIR587
693168	ENSG00000207578	0	NA	NA	NA	NA	MIR583
724032	ENSG00000207579	0	NA	NA	NA	NA	MIR662
574468	ENSG00000207580	0	NA	NA	NA	NA	MIR526B
406978	ENSG00000207581	0	NA	NA	NA	NA	MIR199B
407030	ENSG00000207582	0	NA	NA	NA	NA	MIR30B
693191	ENSG00000207583	0	NA	NA	NA	NA	MIR606
574457	ENSG00000207585	0	NA	NA	NA	NA	MIR181D
406926	ENSG00000207586	0	NA	NA	NA	NA	MIR135A2
664613	ENSG00000207587	0	NA	NA	NA	NA	MIR544A
693178	ENSG00000207588	0	NA	NA	NA	NA	MIR593
574513	ENSG00000207589	0	NA	NA	NA	NA	MIR508
406997	ENSG00000207590	0	NA	NA	NA	NA	MIR215
574467	ENSG00000207594	0	NA	NA	NA	NA	MIR520A
406954	ENSG00000207595	0	NA	NA	NA	NA	MIR181A2
574443	ENSG00000207597	0	NA	NA	NA	NA	MIR490
406909	ENSG00000207598	0	NA	NA	NA	NA	MIR124-3
574461	ENSG00000207599	0	NA	NA	NA	NA	MIR520E
693183	ENSG00000207600	0	NA	NA	NA	NA	MIR598
406989	ENSG00000207604	0	NA	NA	NA	NA	MIR206
406966	ENSG00000207605	0	NA	NA	NA	NA	MIR191
693139	ENSG00000207606	0	NA	NA	NA	NA	MIR554
406983	ENSG00000207607	0	NA	NA	NA	NA	MIR200A
406914	ENSG00000207608	0	NA	NA	NA	NA	MIR127
574444	ENSG00000207609	0	NA	NA	NA	NA	MIR491
406941	ENSG00000207611	0	NA	NA	NA	NA	MIR149
693189	ENSG00000207612	0	NA	NA	NA	NA	MIR604
406957	ENSG00000207613	0	NA	NA	NA	NA	MIR181C
406968	ENSG00000207614	0	NA	NA	NA	NA	MIR193A
574462	ENSG00000207616	0	NA	NA	NA	NA	MIR515-1
100422842	ENSG00000207617	0	NA	NA	NA	NA	MIR3074

693170	ENSG00000207619	0	NA	NA	NA	NA	MIR585
574499	ENSG00000207620	0	NA	NA	NA	NA	MIR516A2
693204	ENSG00000207622	0	NA	NA	NA	NA	MIR619
406969	ENSG00000207624	0	NA	NA	NA	NA	MIR194-1
406916	ENSG00000207625	0	NA	NA	NA	NA	MIR128-2
693147	ENSG00000207626	0	NA	NA	NA	NA	MIR562
693166	ENSG00000207627	0	NA	NA	NA	NA	MIR581
723779	ENSG00000207628	0	NA	NA	NA	NA	MIR651
574475	ENSG00000207629	0	NA	NA	NA	NA	MIR526A1
407045	ENSG00000207630	0	NA	NA	NA	NA	MIR7-3
693226	ENSG00000207631	0	NA	NA	NA	NA	MIR641
693173	ENSG00000207632	0	NA	NA	NA	NA	MIR588
574508	ENSG00000207633	0	NA	NA	NA	NA	MIR505
574494	ENSG00000207634	0	NA	NA	NA	NA	MIR521-1
574501	ENSG00000207635	0	NA	NA	NA	NA	MIR499A
693180	ENSG00000207637	0	NA	NA	NA	NA	MIR595
407055	ENSG00000207638	0	NA	NA	NA	NA	MIR99A
574455	ENSG00000207639	0	NA	NA	NA	NA	MIR193B
574515	ENSG00000207641	0	NA	NA	NA	NA	MIR510
693156	ENSG00000207642	0	NA	NA	NA	NA	MIR571
574459	ENSG00000207644	0	NA	NA	NA	NA	MIR512-2
574458	ENSG00000207645	0	NA	NA	NA	NA	MIR512-1
724025	ENSG00000207646	0	NA	NA	NA	NA	MIR655
406944	ENSG00000207647	0	NA	NA	NA	NA	MIR153-1
406930	ENSG00000207649	0	NA	NA	NA	NA	MIR138-2
693155	ENSG00000207650	0	NA	NA	NA	NA	MIR570
407020	ENSG00000207651	0	NA	NA	NA	NA	MIR28
693206	ENSG00000207652	0	NA	NA	NA	NA	MIR621
693143	ENSG00000207653	0	NA	NA	NA	NA	MIR558
406915	ENSG00000207654	0	NA	NA	NA	NA	MIR128-1
574442	ENSG00000207656	0	NA	NA	NA	NA	MIR489
100500895	ENSG00000207688	0	NA	NA	NA	NA	MIR548AA2
693126	ENSG00000207689	0	NA	NA	NA	NA	MIR548A2
406959	ENSG00000207691	0	NA	NA	NA	NA	MIR183
693177	ENSG00000207692	0	NA	NA	NA	NA	MIR592
693187	ENSG00000207693	0	NA	NA	NA	NA	MIR602
406960	ENSG00000207695	0	NA	NA	NA	NA	MIR184
724029	ENSG00000207696	0	NA	NA	NA	NA	MIR659

693158	ENSG00000207697	0	NA	NA	NA	NA	MIR573
407036	ENSG00000207698	0	NA	NA	NA	NA	MIR32
574491	ENSG00000207699	0	NA	NA	NA	NA	MIR518A2
693182	ENSG00000207701	0	NA	NA	NA	NA	MIR597
406993	ENSG00000207702	0	NA	NA	NA	NA	MIR211
407044	ENSG00000207703	0	NA	NA	NA	NA	MIR7-2
100500863	ENSG00000207704	0	NA	NA	NA	NA	MIR548AA1
406917	ENSG00000207705	0	NA	NA	NA	NA	MIR129-1
574472	ENSG00000207706	0	NA	NA	NA	NA	MIR518F
406933	ENSG00000207708	0	NA	NA	NA	NA	MIR141
574470	ENSG00000207711	0	NA	NA	NA	NA	MIR525
693212	ENSG00000207712	0	NA	NA	NA	NA	MIR627
406985	ENSG00000207713	0	NA	NA	NA	NA	MIR200C
693169	ENSG00000207714	0	NA	NA	NA	NA	MIR584
693157	ENSG00000207716	0	NA	NA	NA	NA	MIR572
693136	ENSG00000207717	0	NA	NA	NA	NA	MIR551B
693208	ENSG00000207719	0	NA	NA	NA	NA	MIR623
406962	ENSG00000207721	0.173733	0.01914545	0.38484597	0.78225479	NA	MIR186
574473	ENSG00000207722	0	NA	NA	NA	NA	MIR520B
407007	ENSG00000207725	0	NA	NA	NA	NA	MIR222
619556	ENSG00000207726	0	NA	NA	NA	NA	MIR455
693123	ENSG00000207728	0	NA	NA	NA	NA	MIR449B
693141	ENSG00000207729	0	NA	NA	NA	NA	MIR556
406984	ENSG00000207730	0	NA	NA	NA	NA	MIR200B
574511	ENSG00000207731	0	NA	NA	NA	NA	MIR506
407000	ENSG00000207732	0	NA	NA	NA	NA	MIR218-1
574479	ENSG00000207734	0	NA	NA	NA	NA	MIR517A
574482	ENSG00000207735	0	NA	NA	NA	NA	MIR520D
724027	ENSG00000207736	0	NA	NA	NA	NA	MIR657
406956	ENSG00000207737	0	NA	NA	NA	NA	MIR181B2
574476	ENSG00000207738	0	NA	NA	NA	NA	MIR520C
407001	ENSG00000207739	0	NA	NA	NA	NA	MIR218-2
693175	ENSG00000207741	0	NA	NA	NA	NA	MIR590
619555	ENSG00000207742	0	NA	NA	NA	NA	MIR487A
574453	ENSG00000207743	0	NA	NA	NA	NA	MIR495
406903	ENSG00000207744	0	NA	NA	NA	NA	MIR10B
693160	ENSG00000207746	0	NA	NA	NA	NA	MIR575
407023	ENSG00000207749	0	NA	NA	NA	NA	MIR299

693138	ENSG00000207750	0	NA	NA	NA	NA	MIR553
NA	ENSG00000207751	17.2169031	0.09170739	0.32486456	0.61999763	0.80602536	
406976	ENSG00000207752	0	NA	NA	NA	NA	MIR199A1
664616	ENSG00000207754	0	NA	NA	NA	NA	MIR487B
574505	ENSG00000207755	0	NA	NA	NA	NA	MIR450A2
693165	ENSG00000207756	0	NA	NA	NA	NA	MIR580
407050	ENSG00000207757	0	NA	NA	NA	NA	MIR93
693124	ENSG00000207758	0	NA	NA	NA	NA	MIR532
406995	ENSG00000207759	0	NA	NA	NA	NA	MIR181A1
574408	ENSG00000207761	0	NA	NA	NA	NA	MIR329-1
574409	ENSG00000207762	0	NA	NA	NA	NA	MIR329-2
693202	ENSG00000207763	0	NA	NA	NA	NA	MIR617
693211	ENSG00000207766	0	NA	NA	NA	NA	MIR626
574498	ENSG00000207767	0	NA	NA	NA	NA	MIR516A1
406964	ENSG00000207768	0	NA	NA	NA	NA	MIR188
693171	ENSG00000207769	0	NA	NA	NA	NA	MIR586
693133	ENSG00000207771	0	NA	NA	NA	NA	MIR550A1
693227	ENSG00000207773	0	NA	NA	NA	NA	MIR642A
693125	ENSG00000207775	0	NA	NA	NA	NA	MIR548A1
693135	ENSG00000207776	0	NA	NA	NA	NA	MIR551A
100616357	ENSG00000207778	0	NA	NA	NA	NA	MIR3591
406949	ENSG00000207779	0	NA	NA	NA	NA	MIR15B
693233	ENSG00000207780	0	NA	NA	NA	NA	MIR648
693210	ENSG00000207781	0	NA	NA	NA	NA	MIR625
406942	ENSG00000207782	0	NA	NA	NA	NA	MIR150
664617	ENSG00000207784	0	NA	NA	NA	NA	MIR542
574502	ENSG00000207785	0	NA	NA	NA	NA	MIR500A
574466	ENSG00000207788	0	NA	NA	NA	NA	MIR519C
407016	ENSG00000207789	0	NA	NA	NA	NA	MIR26A2
406963	ENSG00000207797	0	NA	NA	NA	NA	MIR187
406998	ENSG00000207798	0	NA	NA	NA	NA	MIR216A
574484	ENSG00000207799	0	NA	NA	NA	NA	MIR520G
574507	ENSG00000207800	0	NA	NA	NA	NA	MIR504
693231	ENSG00000207802	0	NA	NA	NA	NA	MIR646
574488	ENSG00000207803	0	NA	NA	NA	NA	MIR518A1
693184	ENSG00000207804	0	NA	NA	NA	NA	MIR599
619552	ENSG00000207805	0	NA	NA	NA	NA	MIR483
407052	ENSG00000207807	0	NA	NA	NA	NA	MIR95

407018	ENSG00000207808	0	NA	NA	NA	NA	MIR27A
574463	ENSG00000207810	0	NA	NA	NA	NA	MIR519E
407041	ENSG00000207811	0	NA	NA	NA	NA	MIR34B
693190	ENSG00000207813	0	NA	NA	NA	NA	MIR605
406939	ENSG00000207814	0	NA	NA	NA	NA	MIR147A
693148	ENSG00000207815	0	NA	NA	NA	NA	MIR563
406908	ENSG00000207816	0	NA	NA	NA	NA	MIR124-2
406898	ENSG00000207818	0	NA	NA	NA	NA	MIR105-2
664614	ENSG00000207820	0	NA	NA	NA	NA	MIR545
693225	ENSG00000207821	0	NA	NA	NA	NA	MIR640
574469	ENSG00000207825	0	NA	NA	NA	NA	MIR519B
693181	ENSG00000207826	0	NA	NA	NA	NA	MIR596
407029	ENSG00000207827	0	NA	NA	NA	NA	MIR30A
574483	ENSG00000207837	0	NA	NA	NA	NA	MIR517B
574492	ENSG00000207838	0	NA	NA	NA	NA	MIR517C
693120	ENSG00000207839	0	NA	NA	NA	NA	MIR33B
574493	ENSG00000207861	0	NA	NA	NA	NA	MIR520H
574474	ENSG00000207862	0	NA	NA	NA	NA	MIR518B
406912	ENSG00000207863	0	NA	NA	NA	NA	MIR125B2
407019	ENSG00000207864	0	NA	NA	NA	NA	MIR27B
574517	ENSG00000207866	0	NA	NA	NA	NA	MIR514A2
574516	ENSG00000207868	0	NA	NA	NA	NA	MIR514A1
574460	ENSG00000207869	0	NA	NA	NA	NA	MIR498
407006	ENSG00000207870	0	NA	NA	NA	NA	MIR221
100313822	ENSG00000207871	0	NA	NA	NA	NA	MIR513B
574509	ENSG00000207873	0	NA	NA	NA	NA	MIR513A1
693195	ENSG00000207874	0	NA	NA	NA	NA	MIR610
NA	ENSG00000207922	0	NA	NA	NA	NA	
693144	ENSG00000207923	0	NA	NA	NA	NA	MIR559
406973	ENSG00000207924	0	NA	NA	NA	NA	MIR196A2
574485	ENSG00000207925	0	NA	NA	NA	NA	MIR516B2
406925	ENSG00000207926	0	NA	NA	NA	NA	MIR135A1
407028	ENSG00000207927	0	NA	NA	NA	NA	MIR302A
693188	ENSG00000207930	0	NA	NA	NA	NA	MIR603
693162	ENSG00000207931	0	NA	NA	NA	NA	MIR577
407039	ENSG00000207932	0	NA	NA	NA	NA	MIR33A
407046	ENSG00000207933	0	NA	NA	NA	NA	MIR9-1
724024	ENSG00000207934	0	NA	NA	NA	NA	MIR654

406987	ENSG00000207935	0	NA	NA	NA	NA	MIR204
574445	ENSG00000207938	0	NA	NA	NA	NA	MIR511
693152	ENSG00000207940	0	NA	NA	NA	NA	MIR567
693137	ENSG00000207941	0	NA	NA	NA	NA	MIR552
406927	ENSG00000207942	0	NA	NA	NA	NA	MIR136
693219	ENSG00000207943	0	NA	NA	NA	NA	MIR634
693159	ENSG00000207944	0	NA	NA	NA	NA	MIR574
574490	ENSG00000207946	0	NA	NA	NA	NA	MIR516B1
406943	ENSG00000207947	0	NA	NA	NA	NA	MIR152
442901	ENSG00000207948	0	NA	NA	NA	NA	MIR328
693146	ENSG00000207951	0	NA	NA	NA	NA	MIR561
693209	ENSG00000207952	0	NA	NA	NA	NA	MIR624
406929	ENSG00000207954	0	NA	NA	NA	NA	MIR138-1
NA	ENSG00000207955	1.13471309	0.01599169	0.38001474	0.8021103	NA	
693164	ENSG00000207956	0	NA	NA	NA	NA	MIR579
406897	ENSG00000207957	0	NA	NA	NA	NA	MIR105-1
724026	ENSG00000207959	0	NA	NA	NA	NA	MIR656
406945	ENSG00000207960	0	NA	NA	NA	NA	MIR153-2
574454	ENSG00000207961	0	NA	NA	NA	NA	MIR496
407031	ENSG00000207962	0	NA	NA	NA	NA	MIR30C1
693154	ENSG00000207963	0	NA	NA	NA	NA	MIR569
693214	ENSG00000207965	0	NA	NA	NA	NA	MIR629
693205	ENSG00000207967	0	NA	NA	NA	NA	MIR620
574512	ENSG00000207969	0	NA	NA	NA	NA	MIR507
724030	ENSG00000207970	0	NA	NA	NA	NA	MIR660
406911	ENSG00000207971	0	NA	NA	NA	NA	MIR125B1
693223	ENSG00000207972	0	NA	NA	NA	NA	MIR638
693174	ENSG00000207973	0	NA	NA	NA	NA	MIR589
693142	ENSG00000207974	0	NA	NA	NA	NA	MIR557
406955	ENSG00000207975	0	NA	NA	NA	NA	MIR181B1
693192	ENSG00000207976	0	NA	NA	NA	NA	MIR607
406946	ENSG00000207978	0	NA	NA	NA	NA	MIR154
574497	ENSG00000207979	0	NA	NA	NA	NA	MIR527
407010	ENSG00000207980	0	NA	NA	NA	NA	MIR23A
574480	ENSG00000207981	0	NA	NA	NA	NA	MIR519D
693128	ENSG00000207982	0	NA	NA	NA	NA	MIR548B
693198	ENSG00000207983	0	NA	NA	NA	NA	MIR613
574510	ENSG00000207984	0	NA	NA	NA	NA	MIR513A2

574487	ENSG00000207987	0	NA	NA	NA	NA	MIR518E
693161	ENSG00000207988	0	NA	NA	NA	NA	MIR576
574450	ENSG00000207989	0	NA	NA	NA	NA	MIR493
406958	ENSG00000207990	0	NA	NA	NA	NA	MIR182
693186	ENSG00000207991	0	NA	NA	NA	NA	MIR601
574496	ENSG00000207992	0	NA	NA	NA	NA	MIR519A1
406924	ENSG00000207993	0	NA	NA	NA	NA	MIR134
406892	ENSG00000207994	0	NA	NA	NA	NA	MIR100
442899	ENSG00000207995	0	NA	NA	NA	NA	MIR325
407027	ENSG00000207996	0	NA	NA	NA	NA	MIR301A
693229	ENSG00000207997	0	NA	NA	NA	NA	MIR644A
574514	ENSG00000208000	0	NA	NA	NA	NA	MIR509-1
574038	ENSG00000208001	0	NA	NA	NA	NA	MIR431
693228	ENSG00000208002	0	NA	NA	NA	NA	MIR643
693132	ENSG00000208003	0	NA	NA	NA	NA	MIR549A
574410	ENSG00000208004	0	NA	NA	NA	NA	MIR323B
574506	ENSG00000208005	0	NA	NA	NA	NA	MIR503
406950	ENSG00000208006	0	NA	NA	NA	NA	MIR16-1
406910	ENSG00000208008	0	NA	NA	NA	NA	MIR125A
406919	ENSG00000208009	0	NA	NA	NA	NA	MIR130A
406889	ENSG00000208012	0	NA	NA	NA	NA	MIRLET7F2
724022	ENSG00000208013	0	NA	NA	NA	NA	MIR652
724023	ENSG00000208014	0	NA	NA	NA	NA	MIR653
574030	ENSG00000208015	0	NA	NA	NA	NA	MIR362
406932	ENSG00000208017	0	NA	NA	NA	NA	MIR140
693230	ENSG00000208018	0	NA	NA	NA	NA	MIR645
693203	ENSG00000208022	0	NA	NA	NA	NA	MIR618
406961	ENSG00000208023	0	NA	NA	NA	NA	MIR185
406977	ENSG00000208024	0	NA	NA	NA	NA	MIR199A2
693176	ENSG00000208025	0	NA	NA	NA	NA	MIR591
574436	ENSG00000208027	0	NA	NA	NA	NA	MIR485
693201	ENSG00000208028	0	NA	NA	NA	NA	MIR616
693127	ENSG00000208032	0	NA	NA	NA	NA	MIR548A3
693194	ENSG00000208033	0	NA	NA	NA	NA	MIR609
406900	ENSG00000208036	0	NA	NA	NA	NA	MIR106B
NA	ENSG00000208037	0	NA	NA	NA	NA	MIR320A
109616967	ENSG00000208308	0	NA	NA	NA	NA	SNORA40B
692225	ENSG00000208772	0	NA	NA	NA	NA	SNORD94

8944	ENSG00000208797	0	NA	NA	NA	NA	SNORD73A
677816	ENSG00000208839	0	NA	NA	NA	NA	SNORA35
692226	ENSG00000208883	0	NA	NA	NA	NA	SNORD96B
677829	ENSG00000208892	0	NA	NA	NA	NA	SNORA49
NA	ENSG00000209042	0	NA	NA	NA	NA	SNORD12C
NA	ENSG00000209082	0	NA	NA	NA	NA	MT-TL1
116938	ENSG00000209480	0	NA	NA	NA	NA	SNORD83B
116937	ENSG00000209482	0.15069912	-0.01848503	0.38480575	0.84537521	NA	SNORD83A
652965	ENSG00000209582	0	NA	NA	NA	NA	SNORA48
692229	ENSG00000209645	0	NA	NA	NA	NA	SNORD105
26810	ENSG00000209702	0	NA	NA	NA	NA	SNORD41
NA	ENSG00000210049	0.34746601	0.02370763	0.3853651	0.61987128	NA	MT-TF
NA	ENSG00000210077	0	NA	NA	NA	NA	MT-TV
NA	ENSG00000210082	125227.312	0.48733973	0.09429628	9.85E-08	1.30E-06	MT-RNR2
NA	ENSG00000210100	0	NA	NA	NA	NA	MT-TI
NA	ENSG00000210107	0.47237597	-0.03501478	0.38628028	0.496551	NA	MT-TQ
NA	ENSG00000210112	0	NA	NA	NA	NA	MT-TM
NA	ENSG00000210117	0	NA	NA	NA	NA	MT-TW
NA	ENSG00000210127	2.31913423	0.04038395	0.37525791	0.67277406	NA	MT-TA
NA	ENSG00000210135	0.4920739	0.01062491	0.38334076	0.79320141	NA	MT-TN
NA	ENSG00000210140	0.68208768	0.00452981	0.38130786	0.92966277	NA	MT-TC
NA	ENSG00000210144	0.15069912	-0.01848503	0.38480575	0.84537521	NA	MT-TY
113219467	ENSG00000210151	0.32443212	0.00133214	0.383368	0.96755054	NA	MT-TS1
NA	ENSG00000210154	0	NA	NA	NA	NA	MT-TD
NA	ENSG00000210156	0	NA	NA	NA	NA	MT-TK
NA	ENSG00000210164	0	NA	NA	NA	NA	MT-TG
NA	ENSG00000210174	0	NA	NA	NA	NA	MT-TR
NA	ENSG00000210176	0	NA	NA	NA	NA	MT-TH
NA	ENSG00000210181	0	NA	NA	NA	NA	RNU6ATAC4P
NA	ENSG00000210184	0	NA	NA	NA	NA	MT-TS2
NA	ENSG00000210191	0	NA	NA	NA	NA	MT-TL2
NA	ENSG00000210194	0	NA	NA	NA	NA	MT-TE
NA	ENSG00000210195	0	NA	NA	NA	NA	MT-TT
NA	ENSG00000210196	0	NA	NA	NA	NA	MT-TP
NA	ENSG00000210678	0	NA	NA	NA	NA	RNU6ATAC2P
NA	ENSG00000210709	0	NA	NA	NA	NA	RNU6ATAC3P
406972	ENSG00000210741	0	NA	NA	NA	NA	MIR196A1
NA	ENSG00000210825	0	NA	NA	NA	NA	SNORA40

NA	ENSG00000210839	0	NA	NA	NA	NA	RNU6ATAC5P
NA	ENSG00000210841	0	NA	NA	NA	NA	RNU6ATAC26P
406965	ENSG00000211137	0	NA	NA	NA	NA	MIR190A
2878	ENSG00000211445	163.401311	-1.20905838	0.23966548	4.34E-08	6.01E-07	GPX3
1734	ENSG00000211448	7.6221866	0.00124153	0.36115535	0.9945766	0.99845164	DIO2
280636	ENSG00000211450	992.006301	-0.27555063	0.0914747	0.00186572	0.01008609	SELENOH
114814	ENSG00000211451	18.7678091	0.01639362	0.31817013	0.9293525	0.97049075	GNRHR2
1733	ENSG00000211452	1.28850183	0.02995256	0.38153639	0.56886766	NA	DIO1
NA	ENSG00000211454	25.8700232	0.05485562	0.31674917	0.7659564	0.89234011	AKR7L
23012	ENSG00000211455	754.97295	0.12307075	0.10832977	0.23624575	0.45303651	STK38L
22908	ENSG00000211456	1005.86115	0.3098123	0.09145039	0.00047135	0.00306508	SACM1L
NA	ENSG00000211459	17086.4398	0.22349174	0.05098906	9.64E-06	9.17E-05	MT-RNR1
7247	ENSG00000211460	1339.47789	0.02782407	0.08166179	0.72744444	0.86963712	TSN
NA	ENSG00000211491	0	NA	NA	NA	NA	MIR320D1
NA	ENSG00000211513	0	NA	NA	NA	NA	MIR320E
768216	ENSG00000211514	0	NA	NA	NA	NA	MIR454
100126319	ENSG00000211520	0	NA	NA	NA	NA	MIR216B
574486	ENSG00000211532	0	NA	NA	NA	NA	MIR526A2
574503	ENSG00000211538	0	NA	NA	NA	NA	MIR501
NA	ENSG00000211543	0	NA	NA	NA	NA	MIR320B1
100422915	ENSG00000211563	0	NA	NA	NA	NA	MIR3065
100313777	ENSG00000211568	0	NA	NA	NA	NA	MIR670
768222	ENSG00000211574	0	NA	NA	NA	NA	MIR770
100126348	ENSG00000211575	0	NA	NA	NA	NA	MIR760
768218	ENSG00000211578	0	NA	NA	NA	NA	MIR766
100313778	ENSG00000211579	0	NA	NA	NA	NA	MIR759
768217	ENSG00000211580	0	NA	NA	NA	NA	MIR769
768220	ENSG00000211581	0	NA	NA	NA	NA	MIR765
768212	ENSG00000211582	0	NA	NA	NA	NA	MIR758
768215	ENSG00000211583	0	NA	NA	NA	NA	MIR767
55652	ENSG00000211584	255.763616	-0.19400138	0.16243066	0.18628149	0.38596943	SLC48A1
768219	ENSG00000211590	0	NA	NA	NA	NA	MIR802
100313837	ENSG00000211591	0	NA	NA	NA	NA	MIR762
NA	ENSG00000211592	0	NA	NA	NA	NA	IGKC
NA	ENSG00000211593	0	NA	NA	NA	NA	IGKJ5
NA	ENSG00000211594	0	NA	NA	NA	NA	IGKJ4
NA	ENSG00000211595	0	NA	NA	NA	NA	IGKJ3
NA	ENSG00000211596	0	NA	NA	NA	NA	IGKJ2

NA	ENSG00000211597	0	NA	NA	NA	NA	IGKJ1
NA	ENSG00000211598	0	NA	NA	NA	NA	IGKV4-1
NA	ENSG00000211599	0	NA	NA	NA	NA	IGKV5-2
NA	ENSG00000211611	0	NA	NA	NA	NA	IGKV6-21
NA	ENSG00000211623	0	NA	NA	NA	NA	IGKV2D-26
NA	ENSG00000211625	0	NA	NA	NA	NA	IGKV3D-20
NA	ENSG00000211626	0	NA	NA	NA	NA	IGKV6D-41
NA	ENSG00000211632	0	NA	NA	NA	NA	IGKV3D-11
NA	ENSG00000211633	0	NA	NA	NA	NA	IGKV1D-42
NA	ENSG00000211637	0	NA	NA	NA	NA	IGLV4-69
NA	ENSG00000211638	0	NA	NA	NA	NA	IGLV8-61
NA	ENSG00000211639	0	NA	NA	NA	NA	IGLV4-60
NA	ENSG00000211640	0	NA	NA	NA	NA	IGLV6-57
NA	ENSG00000211641	0	NA	NA	NA	NA	IGLV11-55
NA	ENSG00000211642	0	NA	NA	NA	NA	IGLV10-54
NA	ENSG00000211643	0	NA	NA	NA	NA	IGLV5-52
NA	ENSG00000211644	0	NA	NA	NA	NA	IGLV1-51
NA	ENSG00000211645	0.29863895	-0.02292439	0.3852726	0.68753245	NA	IGLV1-50
NA	ENSG00000211647	0	NA	NA	NA	NA	IGLV5-48
NA	ENSG00000211648	0	NA	NA	NA	NA	IGLV1-47
NA	ENSG00000211649	0	NA	NA	NA	NA	IGLV7-46
NA	ENSG00000211650	0	NA	NA	NA	NA	IGLV5-45
NA	ENSG00000211651	0	NA	NA	NA	NA	IGLV1-44
NA	ENSG00000211652	0	NA	NA	NA	NA	IGLV7-43
NA	ENSG00000211653	0	NA	NA	NA	NA	IGLV1-40
NA	ENSG00000211654	0	NA	NA	NA	NA	IGLV5-37
NA	ENSG00000211655	0	NA	NA	NA	NA	IGLV1-36
NA	ENSG00000211656	0	NA	NA	NA	NA	IGLV2-33
NA	ENSG00000211657	0	NA	NA	NA	NA	IGLV3-32
NA	ENSG00000211658	0	NA	NA	NA	NA	IGLV3-27
NA	ENSG00000211659	0	NA	NA	NA	NA	IGLV3-25
NA	ENSG00000211660	0	NA	NA	NA	NA	IGLV2-23
NA	ENSG00000211661	0	NA	NA	NA	NA	IGLV3-22
NA	ENSG00000211662	0	NA	NA	NA	NA	IGLV3-21
NA	ENSG00000211663	0	NA	NA	NA	NA	IGLV3-19
NA	ENSG00000211664	0	NA	NA	NA	NA	IGLV2-18
NA	ENSG00000211665	0	NA	NA	NA	NA	IGLV3-16
NA	ENSG00000211666	0	NA	NA	NA	NA	IGLV2-14

NA	ENSG00000211667	0	NA	NA	NA	NA	IGLV3-12
NA	ENSG00000211668	0	NA	NA	NA	NA	IGLV2-11
NA	ENSG00000211669	0	NA	NA	NA	NA	IGLV3-10
NA	ENSG00000211670	0	NA	NA	NA	NA	IGLV3-9
NA	ENSG00000211672	0	NA	NA	NA	NA	IGLV4-3
NA	ENSG00000211673	0	NA	NA	NA	NA	IGLV3-1
NA	ENSG00000211674	0	NA	NA	NA	NA	IGLJ1
NA	ENSG00000211675	0	NA	NA	NA	NA	IGLC1
NA	ENSG00000211676	0	NA	NA	NA	NA	IGLJ2
NA	ENSG00000211677	0	NA	NA	NA	NA	IGLC2
NA	ENSG00000211678	0	NA	NA	NA	NA	IGLJ3
NA	ENSG00000211679	0	NA	NA	NA	NA	IGLC3
NA	ENSG00000211680	0	NA	NA	NA	NA	IGLJ4
NA	ENSG00000211681	0	NA	NA	NA	NA	IGLJ5
NA	ENSG00000211682	0	NA	NA	NA	NA	IGLJ6
NA	ENSG00000211683	3.33260119	-0.01399309	0.36746709	0.89738075	0.95499834	
NA	ENSG00000211684	0	NA	NA	NA	NA	IGLJ7
NA	ENSG00000211685	0	NA	NA	NA	NA	IGLC7
NA	ENSG00000211687	0	NA	NA	NA	NA	TRGJ2
NA	ENSG00000211688	0	NA	NA	NA	NA	TRGJP2
445347	ENSG00000211689	0	NA	NA	NA	NA	TRGC1
NA	ENSG00000211691	0	NA	NA	NA	NA	TRGJP
NA	ENSG00000211692	0	NA	NA	NA	NA	TRGJP1
NA	ENSG00000211693	0	NA	NA	NA	NA	TRGV11
NA	ENSG00000211694	0	NA	NA	NA	NA	TRGV10
NA	ENSG00000211695	0	NA	NA	NA	NA	TRGV9
NA	ENSG00000211696	0	NA	NA	NA	NA	TRGV8
NA	ENSG00000211697	0	NA	NA	NA	NA	TRGV5
NA	ENSG00000211698	0	NA	NA	NA	NA	TRGV4
NA	ENSG00000211699	0.14931947	-0.01844332	0.38480212	0.84537521	NA	TRGV3
NA	ENSG00000211701	0	NA	NA	NA	NA	TRGV1
NA	ENSG00000211706	0	NA	NA	NA	NA	TRBV6-1
NA	ENSG00000211707	0	NA	NA	NA	NA	TRBV7-1
NA	ENSG00000211710	0	NA	NA	NA	NA	TRBV4-1
NA	ENSG00000211713	0	NA	NA	NA	NA	TRBV6-4
NA	ENSG00000211714	0	NA	NA	NA	NA	TRBV7-3
NA	ENSG00000211715	0	NA	NA	NA	NA	TRBV5-3
NA	ENSG00000211716	0	NA	NA	NA	NA	TRBV9

NA	ENSG00000211717	0	NA	NA	NA	NA	TRBV10-1
NA	ENSG00000211720	0	NA	NA	NA	NA	TRBV11-1
NA	ENSG00000211721	0	NA	NA	NA	NA	TRBV6-5
NA	ENSG00000211724	0	NA	NA	NA	NA	TRBV6-6
NA	ENSG00000211725	0	NA	NA	NA	NA	TRBV5-5
NA	ENSG00000211727	0	NA	NA	NA	NA	TRBV7-6
NA	ENSG00000211728	0	NA	NA	NA	NA	TRBV5-6
NA	ENSG00000211731	0	NA	NA	NA	NA	TRBV5-7
NA	ENSG00000211734	0	NA	NA	NA	NA	TRBV5-1
NA	ENSG00000211739	0	NA	NA	NA	NA	TRBV12-2
NA	ENSG00000211745	0	NA	NA	NA	NA	TRBV4-2
NA	ENSG00000211746	0	NA	NA	NA	NA	TRBV19
NA	ENSG00000211747	0	NA	NA	NA	NA	TRBV20-1
NA	ENSG00000211749	0.173124	-0.01911262	0.38486262	0.84537521	NA	TRBV23-1
NA	ENSG00000211750	0	NA	NA	NA	NA	TRBV24-1
NA	ENSG00000211751	0	NA	NA	NA	NA	TRBC1
NA	ENSG00000211752	0	NA	NA	NA	NA	TRBV27
NA	ENSG00000211753	0	NA	NA	NA	NA	TRBV28
NA	ENSG00000211764	0	NA	NA	NA	NA	TRBJ2-1
NA	ENSG00000211765	0	NA	NA	NA	NA	TRBJ2-2
NA	ENSG00000211766	0	NA	NA	NA	NA	TRBJ2-2P
NA	ENSG00000211767	0	NA	NA	NA	NA	TRBJ2-3
NA	ENSG00000211768	0	NA	NA	NA	NA	TRBJ2-4
NA	ENSG00000211769	0	NA	NA	NA	NA	TRBJ2-5
NA	ENSG00000211770	0	NA	NA	NA	NA	TRBJ2-6
NA	ENSG00000211771	0	NA	NA	NA	NA	TRBJ2-7
NA	ENSG00000211772	0	NA	NA	NA	NA	TRBC2
NA	ENSG00000211776	0	NA	NA	NA	NA	TRAV2
NA	ENSG00000211777	0	NA	NA	NA	NA	TRAV3
NA	ENSG00000211778	0	NA	NA	NA	NA	TRAV4
NA	ENSG00000211779	0	NA	NA	NA	NA	TRAV5
NA	ENSG00000211780	0	NA	NA	NA	NA	TRAV6
NA	ENSG00000211781	0	NA	NA	NA	NA	TRAV7
NA	ENSG00000211782	0	NA	NA	NA	NA	TRAV8-1
NA	ENSG00000211783	0	NA	NA	NA	NA	TRAV9-1
NA	ENSG00000211784	0	NA	NA	NA	NA	TRAV10
NA	ENSG00000211785	0	NA	NA	NA	NA	TRAV12-1
NA	ENSG00000211786	0	NA	NA	NA	NA	TRAV8-2

NA	ENSG00000211787	0	NA	NA	NA	NA	TRAV8-3
NA	ENSG00000211788	0	NA	NA	NA	NA	TRAV13-1
NA	ENSG00000211789	0	NA	NA	NA	NA	TRAV12-2
NA	ENSG00000211790	0	NA	NA	NA	NA	TRAV8-4
NA	ENSG00000211791	0	NA	NA	NA	NA	TRAV13-2
NA	ENSG00000211792	0	NA	NA	NA	NA	TRAV14DV4
NA	ENSG00000211793	0	NA	NA	NA	NA	TRAV9-2
NA	ENSG00000211794	0	NA	NA	NA	NA	TRAV12-3
NA	ENSG00000211795	0	NA	NA	NA	NA	TRAV8-6
NA	ENSG00000211796	0	NA	NA	NA	NA	TRAV16
NA	ENSG00000211797	0	NA	NA	NA	NA	TRAV17
NA	ENSG00000211798	0	NA	NA	NA	NA	TRAV18
NA	ENSG00000211799	0	NA	NA	NA	NA	TRAV19
NA	ENSG00000211800	0	NA	NA	NA	NA	TRAV20
NA	ENSG00000211801	0	NA	NA	NA	NA	TRAV21
NA	ENSG00000211802	0	NA	NA	NA	NA	TRAV22
NA	ENSG00000211803	0	NA	NA	NA	NA	TRAV23DV6
NA	ENSG00000211804	0	NA	NA	NA	NA	TRDV1
NA	ENSG00000211805	0	NA	NA	NA	NA	TRAV24
NA	ENSG00000211806	0	NA	NA	NA	NA	TRAV25
NA	ENSG00000211807	0	NA	NA	NA	NA	TRAV26-1
NA	ENSG00000211808	0	NA	NA	NA	NA	TRAV8-7
NA	ENSG00000211809	0	NA	NA	NA	NA	TRAV27
NA	ENSG00000211810	0	NA	NA	NA	NA	TRAV29DV5
NA	ENSG00000211812	0	NA	NA	NA	NA	TRAV26-2
NA	ENSG00000211813	0	NA	NA	NA	NA	TRAV34
NA	ENSG00000211814	0	NA	NA	NA	NA	TRAV35
NA	ENSG00000211815	0	NA	NA	NA	NA	TRAV36DV7
NA	ENSG00000211816	0	NA	NA	NA	NA	TRAV38-1
NA	ENSG00000211817	0	NA	NA	NA	NA	TRAV38-2DV8
NA	ENSG00000211818	0	NA	NA	NA	NA	TRAV39
NA	ENSG00000211819	0	NA	NA	NA	NA	TRAV40
NA	ENSG00000211820	0	NA	NA	NA	NA	TRAV41
NA	ENSG00000211821	0	NA	NA	NA	NA	TRDV2
NA	ENSG00000211825	0	NA	NA	NA	NA	TRDJ1
NA	ENSG00000211826	0	NA	NA	NA	NA	TRDJ4
NA	ENSG00000211827	0	NA	NA	NA	NA	TRDJ2
NA	ENSG00000211828	0	NA	NA	NA	NA	TRDJ3

NA	ENSG00000211829	0	NA	NA	NA	NA	TRDC
NA	ENSG00000211831	0	NA	NA	NA	NA	TRAJ61
NA	ENSG00000211832	0	NA	NA	NA	NA	TRAJ59
NA	ENSG00000211833	0	NA	NA	NA	NA	TRAJ58
NA	ENSG00000211834	0	NA	NA	NA	NA	TRAJ57
NA	ENSG00000211835	0	NA	NA	NA	NA	TRAJ56
NA	ENSG00000211836	0	NA	NA	NA	NA	TRAJ54
NA	ENSG00000211837	0	NA	NA	NA	NA	TRAJ53
NA	ENSG00000211838	0	NA	NA	NA	NA	TRAJ52
NA	ENSG00000211839	0	NA	NA	NA	NA	TRAJ50
NA	ENSG00000211840	0	NA	NA	NA	NA	TRAJ49
NA	ENSG00000211841	0	NA	NA	NA	NA	TRAJ48
NA	ENSG00000211842	0	NA	NA	NA	NA	TRAJ47
NA	ENSG00000211843	0	NA	NA	NA	NA	TRAJ46
NA	ENSG00000211844	0	NA	NA	NA	NA	TRAJ45
NA	ENSG00000211845	0	NA	NA	NA	NA	TRAJ44
NA	ENSG00000211846	0	NA	NA	NA	NA	TRAJ43
NA	ENSG00000211847	0	NA	NA	NA	NA	TRAJ42
NA	ENSG00000211848	0	NA	NA	NA	NA	TRAJ41
NA	ENSG00000211849	0	NA	NA	NA	NA	TRAJ40
NA	ENSG00000211850	0	NA	NA	NA	NA	TRAJ39
NA	ENSG00000211851	0	NA	NA	NA	NA	TRAJ38
NA	ENSG00000211854	0	NA	NA	NA	NA	TRAJ35
NA	ENSG00000211855	0	NA	NA	NA	NA	TRAJ34
NA	ENSG00000211856	0	NA	NA	NA	NA	TRAJ33
NA	ENSG00000211857	0	NA	NA	NA	NA	TRAJ32
NA	ENSG00000211858	0	NA	NA	NA	NA	TRAJ31
NA	ENSG00000211859	0	NA	NA	NA	NA	TRAJ30
NA	ENSG00000211860	0	NA	NA	NA	NA	TRAJ29
NA	ENSG00000211861	0	NA	NA	NA	NA	TRAJ28
NA	ENSG00000211862	0	NA	NA	NA	NA	TRAJ27
NA	ENSG00000211863	0	NA	NA	NA	NA	TRAJ26
NA	ENSG00000211864	0	NA	NA	NA	NA	TRAJ25
NA	ENSG00000211865	0	NA	NA	NA	NA	TRAJ24
NA	ENSG00000211866	0	NA	NA	NA	NA	TRAJ23
NA	ENSG00000211867	0	NA	NA	NA	NA	TRAJ22
NA	ENSG00000211868	0	NA	NA	NA	NA	TRAJ21
NA	ENSG00000211869	0	NA	NA	NA	NA	TRAJ20

NA	ENSG00000211870	0	NA	NA	NA	NA	TRAJ19
NA	ENSG00000211871	0	NA	NA	NA	NA	TRAJ18
NA	ENSG00000211872	0	NA	NA	NA	NA	TRAJ17
NA	ENSG00000211873	0	NA	NA	NA	NA	TRAJ16
NA	ENSG00000211875	0	NA	NA	NA	NA	TRAJ14
NA	ENSG00000211876	0	NA	NA	NA	NA	TRAJ13
NA	ENSG00000211877	0	NA	NA	NA	NA	TRAJ12
NA	ENSG00000211878	0	NA	NA	NA	NA	TRAJ11
NA	ENSG00000211879	0	NA	NA	NA	NA	TRAJ10
NA	ENSG00000211880	0	NA	NA	NA	NA	TRAJ9
NA	ENSG00000211881	0	NA	NA	NA	NA	TRAJ8
NA	ENSG00000211882	0	NA	NA	NA	NA	TRAJ7
NA	ENSG00000211883	0	NA	NA	NA	NA	TRAJ6
NA	ENSG00000211884	0	NA	NA	NA	NA	TRAJ5
NA	ENSG00000211885	0	NA	NA	NA	NA	TRAJ4
NA	ENSG00000211886	0	NA	NA	NA	NA	TRAJ3
NA	ENSG00000211887	0	NA	NA	NA	NA	TRAJ2
NA	ENSG00000211888	0	NA	NA	NA	NA	TRAJ1
NA	ENSG00000211890	0	NA	NA	NA	NA	IGHA2
NA	ENSG00000211891	0	NA	NA	NA	NA	IGHE
NA	ENSG00000211892	0	NA	NA	NA	NA	IGHG4
NA	ENSG00000211893	0	NA	NA	NA	NA	IGHG2
NA	ENSG00000211895	0.14931947	-0.01844332	0.38480212	0.84537521	NA	IGHA1
NA	ENSG00000211896	0.3244501	0.0013474	0.38336813	0.9674047	NA	IGHG1
NA	ENSG00000211897	0	NA	NA	NA	NA	IGHG3
NA	ENSG00000211898	0	NA	NA	NA	NA	IGHD
NA	ENSG00000211899	0	NA	NA	NA	NA	IGHM
NA	ENSG00000211900	0	NA	NA	NA	NA	IGHJ6
NA	ENSG00000211904	0	NA	NA	NA	NA	IGHJ2
NA	ENSG00000211905	0	NA	NA	NA	NA	IGHJ1
NA	ENSG00000211907	0	NA	NA	NA	NA	IGHD1-26
NA	ENSG00000211909	0	NA	NA	NA	NA	IGHD5-24
NA	ENSG00000211911	0	NA	NA	NA	NA	IGHD3-22
NA	ENSG00000211912	0	NA	NA	NA	NA	IGHD2-21
NA	ENSG00000211914	0	NA	NA	NA	NA	IGHD6-19
NA	ENSG00000211915	0	NA	NA	NA	NA	IGHD5-18
NA	ENSG00000211917	0	NA	NA	NA	NA	IGHD3-16
NA	ENSG00000211918	0	NA	NA	NA	NA	IGHD2-15

NA	ENSG00000211920	0	NA	NA	NA	NA	IGHD6-13
NA	ENSG00000211921	0	NA	NA	NA	NA	IGHD5-12
NA	ENSG00000211923	0	NA	NA	NA	NA	IGHD3-10
NA	ENSG00000211924	0	NA	NA	NA	NA	IGHD3-9
NA	ENSG00000211925	0	NA	NA	NA	NA	IGHD2-8
NA	ENSG00000211930	0	NA	NA	NA	NA	IGHD3-3
NA	ENSG00000211931	0	NA	NA	NA	NA	IGHD2-2
NA	ENSG00000211933	0	NA	NA	NA	NA	IGHV6-1
NA	ENSG00000211934	0	NA	NA	NA	NA	IGHV1-2
NA	ENSG00000211935	0	NA	NA	NA	NA	IGHV1-3
NA	ENSG00000211937	0	NA	NA	NA	NA	IGHV2-5
NA	ENSG00000211938	0	NA	NA	NA	NA	IGHV3-7
NA	ENSG00000211941	0	NA	NA	NA	NA	IGHV3-11
NA	ENSG00000211942	0	NA	NA	NA	NA	IGHV3-13
NA	ENSG00000211943	0	NA	NA	NA	NA	IGHV3-15
NA	ENSG00000211944	0	NA	NA	NA	NA	IGHV3-16
NA	ENSG00000211945	0	NA	NA	NA	NA	IGHV1-18
NA	ENSG00000211946	0	NA	NA	NA	NA	IGHV3-20
NA	ENSG00000211947	0	NA	NA	NA	NA	IGHV3-21
NA	ENSG00000211949	0	NA	NA	NA	NA	IGHV3-23
NA	ENSG00000211950	0	NA	NA	NA	NA	IGHV1-24
NA	ENSG00000211951	0	NA	NA	NA	NA	IGHV2-26
NA	ENSG00000211952	0	NA	NA	NA	NA	IGHV4-28
NA	ENSG00000211955	1.45179463	0.01900771	0.37885436	0.78970841	NA	IGHV3-33
NA	ENSG00000211956	12.338686	0.15511534	0.37499464	0.35427843	0.58481438	IGHV4-34
NA	ENSG00000211957	0.76437655	-0.02307021	0.38373951	0.60708111	NA	IGHV3-35
NA	ENSG00000211958	2.27613262	0.05802668	0.37919282	0.53243445	NA	IGHV3-38
NA	ENSG00000211959	0.15069912	-0.01848503	0.38480575	0.84537521	NA	IGHV4-39
NA	ENSG00000211961	1.18566459	0.01896899	0.38015591	0.76681443	NA	IGHV1-45
NA	ENSG00000211962	5.97503313	0.14812238	0.39526151	0.28037698	0.5049178	IGHV1-46
NA	ENSG00000211964	0	NA	NA	NA	NA	IGHV3-48
NA	ENSG00000211965	0	NA	NA	NA	NA	IGHV3-49
NA	ENSG00000211966	0	NA	NA	NA	NA	IGHV5-51
NA	ENSG00000211967	0	NA	NA	NA	NA	IGHV3-53
NA	ENSG00000211968	0	NA	NA	NA	NA	IGHV1-58
NA	ENSG00000211970	0	NA	NA	NA	NA	IGHV4-61
NA	ENSG00000211972	0	NA	NA	NA	NA	IGHV3-66
NA	ENSG00000211973	0	NA	NA	NA	NA	IGHV1-69

NA	ENSG00000211974	0	NA	NA	NA	NA	IGHV2-70D
NA	ENSG00000211976	0	NA	NA	NA	NA	IGHV3-73
NA	ENSG00000211978	0	NA	NA	NA	NA	IGHV5-78
NA	ENSG00000211979	0	NA	NA	NA	NA	IGHV7-81
100500887	ENSG00000211991	0	NA	NA	NA	NA	MIR676
100126333	ENSG00000211997	0	NA	NA	NA	NA	MIR708
100126301	ENSG00000212013	0	NA	NA	NA	NA	MIR509-2
100126337	ENSG00000212014	0	NA	NA	NA	NA	MIR509-3
100422884	ENSG00000212017	0	NA	NA	NA	NA	MIR548U
100616354	ENSG00000212024	0	NA	NA	NA	NA	MIR550A3
100126317	ENSG00000212027	0	NA	NA	NA	NA	MIR374B
102465854	ENSG00000212036	0	NA	NA	NA	NA	MIR548BA
100126335	ENSG00000212040	0	NA	NA	NA	NA	MIR543
NA	ENSG00000212051	0	NA	NA	NA	NA	MIR320C2
100313838	ENSG00000212100	0	NA	NA	NA	NA	MIR764
100126318	ENSG00000212102	0	NA	NA	NA	NA	MIR301B
83942	ENSG00000212122	1.83880827	0.09057265	0.39681034	0.13153654	NA	TSSK1B
163154	ENSG00000212123	40.4799834	0.15194566	0.28111467	0.44654984	0.67077141	PRR22
259294	ENSG00000212124	6.68558122	0.38481437	0.65033043	0.03001163	0.1003422	TAS2R19
NA	ENSG00000212125	6.11039886	0.14715573	0.39049783	0.30697385	0.53453681	TAS2R15P
259296	ENSG00000212126	1.51884284	0.02451317	0.37925517	0.73143546	NA	TAS2R50
50840	ENSG00000212127	31.424637	-0.330237	0.3539733	0.14087851	0.31892169	TAS2R14
106707243	ENSG00000212127	31.424637	-0.330237	0.3539733	0.14087851	0.31892169	TAS2R14
50838	ENSG00000212128	1.96005034	0.11217192	0.40103568	0.16668453	NA	TAS2R13
NA	ENSG00000212133	0	NA	NA	NA	NA	RNU6-1168P
NA	ENSG00000212134	0	NA	NA	NA	NA	
692108	ENSG00000212135	0	NA	NA	NA	NA	SNORD67
NA	ENSG00000212136	0	NA	NA	NA	NA	RNU6-696P
NA	ENSG00000212138	0	NA	NA	NA	NA	RNA5SP372
NA	ENSG00000212140	0	NA	NA	NA	NA	RNU6-1320P
NA	ENSG00000212144	0	NA	NA	NA	NA	
NA	ENSG00000212145	0	NA	NA	NA	NA	
NA	ENSG00000212146	0	NA	NA	NA	NA	RNU6-910P
NA	ENSG00000212147	0	NA	NA	NA	NA	RNU6-1106P
NA	ENSG00000212149	0	NA	NA	NA	NA	
NA	ENSG00000212153	0	NA	NA	NA	NA	RNU1-82P
NA	ENSG00000212156	0	NA	NA	NA	NA	RNU6-576P
NA	ENSG00000212157	0	NA	NA	NA	NA	RNU6-1319P

692107	ENSG00000212158	0	NA	NA	NA	NA	SNORD66
NA	ENSG00000212160	0	NA	NA	NA	NA	RNU6-205P
NA	ENSG00000212161	0	NA	NA	NA	NA	
NA	ENSG00000212163	0	NA	NA	NA	NA	SNORD91A
NA	ENSG00000212165	0	NA	NA	NA	NA	
NA	ENSG00000212167	0	NA	NA	NA	NA	RNU6-763P
NA	ENSG00000212168	0	NA	NA	NA	NA	
NA	ENSG00000212170	0	NA	NA	NA	NA	RNU1-77P
NA	ENSG00000212171	0	NA	NA	NA	NA	RNA5SP86
NA	ENSG00000212172	0	NA	NA	NA	NA	RNU1-149P
NA	ENSG00000212175	0.18922458	0.01954053	0.38488496	0.78225479	NA	
NA	ENSG00000212176	0	NA	NA	NA	NA	RNA5SP207
NA	ENSG00000212181	0	NA	NA	NA	NA	
NA	ENSG00000212182	0	NA	NA	NA	NA	
NA	ENSG00000212184	0	NA	NA	NA	NA	RNU6-440P
NA	ENSG00000212186	0	NA	NA	NA	NA	RNU6-258P
NA	ENSG00000212187	0	NA	NA	NA	NA	
NA	ENSG00000212189	0	NA	NA	NA	NA	RNU6-328P
NA	ENSG00000212190	0	NA	NA	NA	NA	RNU6-298P
NA	ENSG00000212191	0	NA	NA	NA	NA	
NA	ENSG00000212195	0	NA	NA	NA	NA	
NA	ENSG00000212199	0	NA	NA	NA	NA	RNU6-127P
NA	ENSG00000212204	0	NA	NA	NA	NA	RNA5SP91
NA	ENSG00000212205	0	NA	NA	NA	NA	
NA	ENSG00000212206	0	NA	NA	NA	NA	
NA	ENSG00000212207	0	NA	NA	NA	NA	RNU6-1321P
NA	ENSG00000212211	0	NA	NA	NA	NA	
109616969	ENSG00000212214	0	NA	NA	NA	NA	SNORA48B
NA	ENSG00000212215	0	NA	NA	NA	NA	RNU6-913P
NA	ENSG00000212219	0	NA	NA	NA	NA	RNU6-604P
NA	ENSG00000212221	0	NA	NA	NA	NA	RNU6-220P
NA	ENSG00000212224	0	NA	NA	NA	NA	
NA	ENSG00000212226	0	NA	NA	NA	NA	RNU6-907P
NA	ENSG00000212228	0	NA	NA	NA	NA	
NA	ENSG00000212229	0	NA	NA	NA	NA	
692086	ENSG00000212232	2.05322451	-0.0234758	0.37561835	0.78397318	NA	SNORD17
NA	ENSG00000212237	0	NA	NA	NA	NA	RNA5SP18
NA	ENSG00000212238	0	NA	NA	NA	NA	RNA5SP244

NA	ENSG00000212240	0	NA	NA	NA	NA	RNU6-930P
NA	ENSG00000212241	0	NA	NA	NA	NA	
NA	ENSG00000212242	0	NA	NA	NA	NA	RNA5SP219
NA	ENSG00000212246	0	NA	NA	NA	NA	RNU6-360P
NA	ENSG00000212247	0	NA	NA	NA	NA	RNU6-278P
NA	ENSG00000212248	0	NA	NA	NA	NA	RNU6-750P
NA	ENSG00000212249	0	NA	NA	NA	NA	
NA	ENSG00000212251	0	NA	NA	NA	NA	RNA5SP376
NA	ENSG00000212257	0	NA	NA	NA	NA	RNU6-1176P
NA	ENSG00000212258	0	NA	NA	NA	NA	RNA5SP176
NA	ENSG00000212259	0	NA	NA	NA	NA	RNU6-308P
NA	ENSG00000212260	0	NA	NA	NA	NA	RNU6-724P
109616981	ENSG00000212264	0	NA	NA	NA	NA	SNORD65C
NA	ENSG00000212265	0	NA	NA	NA	NA	RNA5SP185
NA	ENSG00000212266	0	NA	NA	NA	NA	
NA	ENSG00000212269	0	NA	NA	NA	NA	RNU6-788P
NA	ENSG00000212270	0	NA	NA	NA	NA	
NA	ENSG00000212273	0	NA	NA	NA	NA	
NA	ENSG00000212276	0	NA	NA	NA	NA	RNA5SP292
NA	ENSG00000212278	0	NA	NA	NA	NA	
NA	ENSG00000212279	0	NA	NA	NA	NA	
NA	ENSG00000212280	0	NA	NA	NA	NA	RNA5SP256
NA	ENSG00000212282	0	NA	NA	NA	NA	RNU6-578P
692205	ENSG00000212283	0	NA	NA	NA	NA	SNORD89
NA	ENSG00000212289	0	NA	NA	NA	NA	RNA5SP339
NA	ENSG00000212292	0	NA	NA	NA	NA	RNU6-239P
NA	ENSG00000212293	0	NA	NA	NA	NA	
109617011	ENSG00000212295	0	NA	NA	NA	NA	SNORD28B
619564	ENSG00000212296	0	NA	NA	NA	NA	SNORD72
NA	ENSG00000212297	0	NA	NA	NA	NA	RNU6-821P
NA	ENSG00000212298	0	NA	NA	NA	NA	RNU6-1009P
NA	ENSG00000212302	0	NA	NA	NA	NA	
NA	ENSG00000212303	0	NA	NA	NA	NA	RNU6-1154P
692057	ENSG00000212304	0	NA	NA	NA	NA	SNORD12
NA	ENSG00000212305	0	NA	NA	NA	NA	RNU6-679P
NA	ENSG00000212306	0	NA	NA	NA	NA	
NA	ENSG00000212308	0	NA	NA	NA	NA	RNA5SP23
NA	ENSG00000212309	0	NA	NA	NA	NA	SNORD70B

NA	ENSG00000212312	0	NA	NA	NA	NA	RNA5SP109
NA	ENSG00000212314	0	NA	NA	NA	NA	RNU6-1307P
NA	ENSG00000212316	0	NA	NA	NA	NA	RNU6-1228P
NA	ENSG00000212319	0	NA	NA	NA	NA	
NA	ENSG00000212321	0	NA	NA	NA	NA	
NA	ENSG00000212324	0	NA	NA	NA	NA	RNU6-129P
NA	ENSG00000212325	0	NA	NA	NA	NA	
NA	ENSG00000212327	0	NA	NA	NA	NA	RNU6-882P
NA	ENSG00000212329	0	NA	NA	NA	NA	RNU6-316P
NA	ENSG00000212330	0	NA	NA	NA	NA	RNU6-244P
NA	ENSG00000212331	0	NA	NA	NA	NA	RNA5SP297
NA	ENSG00000212332	0	NA	NA	NA	NA	RNU6-780P
NA	ENSG00000212333	0	NA	NA	NA	NA	RNA5SP213
NA	ENSG00000212335	0	NA	NA	NA	NA	
NA	ENSG00000212336	0	NA	NA	NA	NA	RNA5SP210
NA	ENSG00000212338	0	NA	NA	NA	NA	
NA	ENSG00000212340	0	NA	NA	NA	NA	RNU6-739P
NA	ENSG00000212342	0	NA	NA	NA	NA	
NA	ENSG00000212344	0	NA	NA	NA	NA	RNU6-823P
NA	ENSG00000212345	0	NA	NA	NA	NA	RNU6-700P
NA	ENSG00000212347	0	NA	NA	NA	NA	
NA	ENSG00000212348	0	NA	NA	NA	NA	RNU6-1300P
NA	ENSG00000212354	0	NA	NA	NA	NA	RNU6-1242P
NA	ENSG00000212358	0	NA	NA	NA	NA	RNU6-837P
NA	ENSG00000212359	0	NA	NA	NA	NA	RNU6-550P
NA	ENSG00000212360	0	NA	NA	NA	NA	RNU6-1177P
NA	ENSG00000212363	0	NA	NA	NA	NA	
NA	ENSG00000212365	0	NA	NA	NA	NA	RNA5SP332
NA	ENSG00000212366	0	NA	NA	NA	NA	RNU6-1246P
NA	ENSG00000212368	0	NA	NA	NA	NA	RNU6-1000P
NA	ENSG00000212370	0	NA	NA	NA	NA	RNU6-482P
NA	ENSG00000212371	0.69465532	0.00250623	0.38183794	0.95712618	NA	
NA	ENSG00000212373	0	NA	NA	NA	NA	RNA5SP171
NA	ENSG00000212374	0	NA	NA	NA	NA	RNU6-401P
NA	ENSG00000212377	0	NA	NA	NA	NA	
NA	ENSG00000212378	0	NA	NA	NA	NA	
NA	ENSG00000212379	0	NA	NA	NA	NA	RNU6-269P
NA	ENSG00000212380	0	NA	NA	NA	NA	SNORD115-45

NA	ENSG00000212382	0	NA	NA	NA	NA	RNU6-159P
NA	ENSG00000212383	0	NA	NA	NA	NA	
767562	ENSG00000212384	0	NA	NA	NA	NA	SNORD113-2
NA	ENSG00000212385	0	NA	NA	NA	NA	RNU6-817P
NA	ENSG00000212387	0	NA	NA	NA	NA	RNU6-1055P
NA	ENSG00000212388	0	NA	NA	NA	NA	RNU6-796P
NA	ENSG00000212389	0	NA	NA	NA	NA	RNU6-1275P
NA	ENSG00000212391	0	NA	NA	NA	NA	
NA	ENSG00000212392	0	NA	NA	NA	NA	
NA	ENSG00000212395	0	NA	NA	NA	NA	
NA	ENSG00000212396	0	NA	NA	NA	NA	RNA5SP323
NA	ENSG00000212397	0	NA	NA	NA	NA	
NA	ENSG00000212398	0	NA	NA	NA	NA	RNU6-1248P
677841	ENSG00000212402	0	NA	NA	NA	NA	SNORA74B
NA	ENSG00000212404	0	NA	NA	NA	NA	
NA	ENSG00000212407	0	NA	NA	NA	NA	RNU6-663P
NA	ENSG00000212409	0	NA	NA	NA	NA	RNY4P18
NA	ENSG00000212410	0	NA	NA	NA	NA	RNU6-932P
NA	ENSG00000212411	0	NA	NA	NA	NA	
NA	ENSG00000212413	0	NA	NA	NA	NA	RNU11-3P
109616982	ENSG00000212414	0	NA	NA	NA	NA	SNORD77B
NA	ENSG00000212415	0	NA	NA	NA	NA	
NA	ENSG00000212418	0	NA	NA	NA	NA	RNY4P36
NA	ENSG00000212420	0	NA	NA	NA	NA	RNU6-1111P
NA	ENSG00000212421	0	NA	NA	NA	NA	
NA	ENSG00000212422	0	NA	NA	NA	NA	
NA	ENSG00000212424	0	NA	NA	NA	NA	RNU1-119P
NA	ENSG00000212425	0	NA	NA	NA	NA	RNA5SP105
NA	ENSG00000212428	0	NA	NA	NA	NA	
NA	ENSG00000212429	0	NA	NA	NA	NA	RNU11-6P
NA	ENSG00000212432	0	NA	NA	NA	NA	
NA	ENSG00000212433	0	NA	NA	NA	NA	RNA5SP252
NA	ENSG00000212434	0	NA	NA	NA	NA	
NA	ENSG00000212440	0	NA	NA	NA	NA	
NA	ENSG00000212441	0	NA	NA	NA	NA	RNU6-1269P
NA	ENSG00000212442	0	NA	NA	NA	NA	RNU6-243P
677832	ENSG00000212443	0.36234858	0.00087365	0.38336423	0.96755054	NA	SNORA53
NA	ENSG00000212445	0	NA	NA	NA	NA	

NA	ENSG00000212446	0	NA	NA	NA	NA	RNU6-131P
692206	ENSG00000212447	0	NA	NA	NA	NA	SNORD90
NA	ENSG00000212448	0	NA	NA	NA	NA	
NA	ENSG00000212451	0	NA	NA	NA	NA	RNU11-4P
692109	ENSG00000212452	0	NA	NA	NA	NA	SNORD69
NA	ENSG00000212454	0	NA	NA	NA	NA	RNA5SP286
NA	ENSG00000212455	0	NA	NA	NA	NA	
NA	ENSG00000212457	0	NA	NA	NA	NA	RNU6-644P
NA	ENSG00000212458	0	NA	NA	NA	NA	
NA	ENSG00000212459	0	NA	NA	NA	NA	RNU6-695P
NA	ENSG00000212460	0	NA	NA	NA	NA	RNU6-460P
NA	ENSG00000212461	0	NA	NA	NA	NA	
677800	ENSG00000212464	0	NA	NA	NA	NA	SNORA12
NA	ENSG00000212466	0	NA	NA	NA	NA	RNU6-952P
NA	ENSG00000212468	0	NA	NA	NA	NA	RNU6-754P
NA	ENSG00000212469	0	NA	NA	NA	NA	RNU6-1158P
NA	ENSG00000212473	0	NA	NA	NA	NA	RNU1-101P
NA	ENSG00000212475	0	NA	NA	NA	NA	RNU6-400P
NA	ENSG00000212479	0	NA	NA	NA	NA	
NA	ENSG00000212482	0	NA	NA	NA	NA	RNU6-530P
NA	ENSG00000212485	0	NA	NA	NA	NA	RNU6-1197P
NA	ENSG00000212489	0	NA	NA	NA	NA	RNU6-1109P
NA	ENSG00000212490	0	NA	NA	NA	NA	
NA	ENSG00000212493	0	NA	NA	NA	NA	SNORD19
NA	ENSG00000212495	0	NA	NA	NA	NA	RNU6-332P
NA	ENSG00000212496	0	NA	NA	NA	NA	RNU6-1093P
NA	ENSG00000212497	0	NA	NA	NA	NA	RNA5SP465
692201	ENSG00000212498	0	NA	NA	NA	NA	SNORD86
NA	ENSG00000212499	0	NA	NA	NA	NA	RNA5SP300
NA	ENSG00000212505	0	NA	NA	NA	NA	RNA5SP299
NA	ENSG00000212510	0	NA	NA	NA	NA	RNU6-972P
NA	ENSG00000212511	0	NA	NA	NA	NA	
NA	ENSG00000212512	0	NA	NA	NA	NA	
NA	ENSG00000212517	0	NA	NA	NA	NA	
NA	ENSG00000212518	0	NA	NA	NA	NA	RNU11-5P
NA	ENSG00000212520	0	NA	NA	NA	NA	RNU6-1250P
NA	ENSG00000212521	0	NA	NA	NA	NA	RNU6-918P
NA	ENSG00000212525	0	NA	NA	NA	NA	RNA5SP212

NA	ENSG00000212526	0	NA	NA	NA	NA	RNU6-466P
NA	ENSG00000212527	0	NA	NA	NA	NA	RNA5SP63
NA	ENSG00000212528	0	NA	NA	NA	NA	SNORD115-47
NA	ENSG00000212529	0	NA	NA	NA	NA	
NA	ENSG00000212532	0	NA	NA	NA	NA	
NA	ENSG00000212533	0	NA	NA	NA	NA	
692110	ENSG00000212534	0	NA	NA	NA	NA	SNORD70
NA	ENSG00000212535	0	NA	NA	NA	NA	RNU6-808P
NA	ENSG00000212536	0	NA	NA	NA	NA	RNA5SP474
NA	ENSG00000212538	0	NA	NA	NA	NA	
NA	ENSG00000212539	0	NA	NA	NA	NA	
NA	ENSG00000212541	0	NA	NA	NA	NA	RNU6-510P
NA	ENSG00000212542	0	NA	NA	NA	NA	RNA5SP496
NA	ENSG00000212545	0	NA	NA	NA	NA	RNU6-337P
NA	ENSG00000212546	0	NA	NA	NA	NA	RNU6-995P
NA	ENSG00000212549	0	NA	NA	NA	NA	RNA5SP354
NA	ENSG00000212550	0	NA	NA	NA	NA	RNU1-78P
NA	ENSG00000212551	0	NA	NA	NA	NA	
NA	ENSG00000212553	0	NA	NA	NA	NA	
NA	ENSG00000212555	0	NA	NA	NA	NA	RNU6-192P
NA	ENSG00000212556	0	NA	NA	NA	NA	
NA	ENSG00000212558	0	NA	NA	NA	NA	
NA	ENSG00000212559	0	NA	NA	NA	NA	RNA5SP35
NA	ENSG00000212560	0	NA	NA	NA	NA	RNU6-630P
NA	ENSG00000212561	0	NA	NA	NA	NA	RNU6-381P
NA	ENSG00000212564	0	NA	NA	NA	NA	RNU6-1326P
NA	ENSG00000212565	0	NA	NA	NA	NA	
NA	ENSG00000212567	0	NA	NA	NA	NA	
NA	ENSG00000212568	0	NA	NA	NA	NA	RNU6-1254P
NA	ENSG00000212569	0	NA	NA	NA	NA	
NA	ENSG00000212571	0	NA	NA	NA	NA	RNA5SP482
NA	ENSG00000212572	0	NA	NA	NA	NA	RNU6-903P
NA	ENSG00000212576	0	NA	NA	NA	NA	RNA5SP467
NA	ENSG00000212579	0	NA	NA	NA	NA	
NA	ENSG00000212580	0	NA	NA	NA	NA	
NA	ENSG00000212581	0	NA	NA	NA	NA	
NA	ENSG00000212584	0	NA	NA	NA	NA	RNU6-587P
NA	ENSG00000212586	0	NA	NA	NA	NA	

109616968	ENSG00000212587	0	NA	NA	NA	NA	SNORA40C
677810	ENSG00000212588	0	NA	NA	NA	NA	SNORA26
NA	ENSG00000212589	0	NA	NA	NA	NA	
NA	ENSG00000212590	0	NA	NA	NA	NA	
NA	ENSG00000212593	0	NA	NA	NA	NA	
NA	ENSG00000212594	0	NA	NA	NA	NA	
NA	ENSG00000212595	0	NA	NA	NA	NA	RNA5SP525
NA	ENSG00000212597	0	NA	NA	NA	NA	RNU6-876P
NA	ENSG00000212598	0	NA	NA	NA	NA	
NA	ENSG00000212599	0	NA	NA	NA	NA	RNU6-279P
NA	ENSG00000212601	0	NA	NA	NA	NA	RNA5SP53
NA	ENSG00000212604	0	NA	NA	NA	NA	
NA	ENSG00000212605	0	NA	NA	NA	NA	RNU1-56P
677826	ENSG00000212607	0.18922458	0.01954053	0.38488496	0.78225479	NA	SNORA3B
NA	ENSG00000212608	0	NA	NA	NA	NA	
NA	ENSG00000212609	0	NA	NA	NA	NA	RNU1-139P
NA	ENSG00000212610	0	NA	NA	NA	NA	
NA	ENSG00000212611	0	NA	NA	NA	NA	
NA	ENSG00000212612	0	NA	NA	NA	NA	RNU6-1239P
NA	ENSG00000212615	0	NA	NA	NA	NA	
NA	ENSG00000212618	0	NA	NA	NA	NA	
NA	ENSG00000212620	0	NA	NA	NA	NA	
NA	ENSG00000212623	0	NA	NA	NA	NA	RNU6-493P
NA	ENSG00000212624	0	NA	NA	NA	NA	
NA	ENSG00000212625	0	NA	NA	NA	NA	RNA5SP397
NA	ENSG00000212626	0	NA	NA	NA	NA	
NA	ENSG00000212628	0	NA	NA	NA	NA	RNA5SP241
NA	ENSG00000212643	27.2680034	-0.00961403	0.38325266	0.78938444	0.90431711	ZRSR2P1
100505753	ENSG00000212657	0	NA	NA	NA	NA	KRTAP16-1
100533177	ENSG00000212658	0	NA	NA	NA	NA	KRTAP29-1
100507608	ENSG00000212659	0	NA	NA	NA	NA	KRTAP9-6
NA	ENSG00000212663	0	NA	NA	NA	NA	
NA	ENSG00000212664	2.74670479	-0.05154414	0.37512204	0.61128703	0.79937717	
338799	ENSG00000212694	330.975236	0.19306495	0.14038367	0.13897693	0.31573369	LINC01089
NA	ENSG00000212695	0.173733	0.01914545	0.38484597	0.78225479	NA	
64693	ENSG00000212710	0	NA	NA	NA	NA	CTAGE1
NA	ENSG00000212712	2.43988	0.01019985	0.37445534	0.90859895	NA	
NA	ENSG00000212717	0	NA	NA	NA	NA	DEFB117

339263	ENSG00000212719	276.0483	0.02761054	0.14635759	0.83916943	0.92675321	LINC02693
653240	ENSG00000212721	0	NA	NA	NA	NA	KRTAP4-11
100132386	ENSG00000212722	0	NA	NA	NA	NA	KRTAP4-9
730755	ENSG00000212724	0	NA	NA	NA	NA	KRTAP2-3
81872	ENSG00000212725	0	NA	NA	NA	NA	KRTAP2-1
NA	ENSG00000212743	0.52978722	0.01376682	0.38293852	0.74030017	NA	LINC02656
441518	ENSG00000212747	522.807144	-0.44467526	0.110906	2.77E-05	0.00024084	RTL8B
283673	ENSG00000212766	8.03575157	-0.15174294	0.38350356	0.33132022	0.56018597	EWSAT1
NA	ENSG00000212769	0	NA	NA	NA	NA	HMGN2P8
NA	ENSG00000212789	0	NA	NA	NA	NA	ST13P5
NA	ENSG00000212802	1.70399282	0.03597974	0.37805124	0.66278317	NA	RPL15P3
402317	ENSG00000212807	0	NA	NA	NA	NA	OR2A42
NA	ENSG00000212829	0.173124	-0.01911262	0.38486262	0.84537521	NA	RPS26P3
NA	ENSG00000212856	0	NA	NA	NA	NA	TTTY2B
727800	ENSG00000212864	266.037082	-0.08844006	0.14534084	0.50973135	0.72536724	RNF208
85293	ENSG00000212899	0	NA	NA	NA	NA	KRTAP3-3
83897	ENSG00000212900	0	NA	NA	NA	NA	KRTAP3-2
83896	ENSG00000212901	0	NA	NA	NA	NA	KRTAP3-1
4539	ENSG00000212907	10526.4715	-0.10706663	0.05631185	0.05019831	0.14938269	MT-ND4L
54627	ENSG00000212916	128.826283	-0.78849626	0.25030087	0.00026518	0.0018338	MAP10
NA	ENSG00000212930	0	NA	NA	NA	NA	
NA	ENSG00000212932	0	NA	NA	NA	NA	RPL23AP4
386684	ENSG00000212933	0	NA	NA	NA	NA	KRTAP12-4
386682	ENSG00000212935	0	NA	NA	NA	NA	KRTAP10-3
337968	ENSG00000212938	0	NA	NA	NA	NA	KRTAP6-3
NA	ENSG00000212939	0	NA	NA	NA	NA	
NA	ENSG00000212951	0.3235666	0.0017811	0.38337261	0.96403034	NA	
NA	ENSG00000212952	7.69959972	0.08198605	0.36649586	0.5555989	0.7613563	
NA	ENSG00000212961	0.34746601	0.02370763	0.3853651	0.61987128	NA	HNRNPA1P40
339803	ENSG00000212978	135.575299	0.38560933	0.21494349	0.03426203	0.11156386	
NA	ENSG00000212989	0	NA	NA	NA	NA	
5462	ENSG00000212993	2.83576885	0.10165852	0.39197368	0.28032836	0.5049178	POU5F1B
NA	ENSG00000212994	0.63986519	-0.02081523	0.38314397	0.66991272	NA	RPS26P6
NA	ENSG00000213003	0	NA	NA	NA	NA	BTF3P12
NA	ENSG00000213005	0	NA	NA	NA	NA	PTTG3P
NA	ENSG00000213013	0	NA	NA	NA	NA	RPS15AP36
NA	ENSG00000213014	0.66089049	0.00409269	0.38193143	0.93028673	NA	VN2R17P
51157	ENSG00000213015	893.335246	0.20498519	0.09497708	0.02573774	0.08910082	ZNF580

NA	ENSG00000213016	0	NA	NA	NA	NA	
NA	ENSG00000213018	2.08468623	0.06532423	0.38272907	0.45113518	NA	PABPN1P1
81856	ENSG00000213020	230.46799	-0.1648196	0.17435176	0.28719247	0.51295451	ZNF611
284366	ENSG00000213022	0	NA	NA	NA	NA	KLK9
84258	ENSG00000213023	259.137832	-0.66863736	0.15902605	6.12E-06	6.06E-05	SYT3
23636	ENSG00000213024	2734.78533	-0.04464442	0.06511439	0.47527153	0.69749147	NUP62
NA	ENSG00000213025	0.46427351	-0.0076353	0.38326478	0.86721182	NA	COX20P1
NA	ENSG00000213026	0	NA	NA	NA	NA	CFL1P4
NA	ENSG00000213028	1.54062275	-0.00754278	0.37624286	0.92369286	NA	
94115	ENSG00000213030	0.173733	0.01914545	0.38484597	0.78225479	NA	CGB8
NA	ENSG00000213032	0	NA	NA	NA	NA	NDUFA3P3
NA	ENSG00000213033	4.28484873	0.10275379	0.38657999	0.35947133	0.58949503	AURKAP1
NA	ENSG00000213035	0	NA	NA	NA	NA	RPL23AP80
NA	ENSG00000213036	0	NA	NA	NA	NA	
NA	ENSG00000213041	0.173733	0.01914545	0.38484597	0.78225479	NA	
NA	ENSG00000213045	0	NA	NA	NA	NA	
NA	ENSG00000213046	0.173733	0.01914545	0.38484597	0.78225479	NA	EEF1A1P32
163486	ENSG00000213047	231.884795	0.6672605	0.199729	0.00018788	0.00134469	DENND1B
NA	ENSG00000213048	0	NA	NA	NA	NA	OR5S1P
NA	ENSG00000213049	0.46746036	-0.00785167	0.38326714	0.86324876	NA	HNRNPA1P34
NA	ENSG00000213050	0	NA	NA	NA	NA	TPM3P1
NA	ENSG00000213051	0	NA	NA	NA	NA	RPL5P5
NA	ENSG00000213055	0	NA	NA	NA	NA	EEF1B2P7
400798	ENSG00000213057	54.0619982	0.42259532	0.30889785	0.06050212	0.17244104	C1orf220
NA	ENSG00000213058	0	NA	NA	NA	NA	
NA	ENSG00000213060	1.02576835	0.0052515	0.37921714	0.93582806	NA	
NA	ENSG00000213061	0.67997283	0.02431813	0.38336266	0.58986099	NA	PFN1P11
NA	ENSG00000213062	83.4149193	-0.23705511	0.22944012	0.20432864	0.41096092	
NA	ENSG00000213063	0	NA	NA	NA	NA	RPL29P7
375035	ENSG00000213064	1519.48211	-0.0919173	0.10297962	0.35308015	0.58322456	SFT2D2
NA	ENSG00000213065	0	NA	NA	NA	NA	
11116	ENSG00000213066	879.948845	-0.71077197	0.08817594	1.77E-16	5.32E-15	CEP43
NA	ENSG00000213067	0.14931947	-0.01844332	0.38480212	0.84537521	NA	
NA	ENSG00000213068	0	NA	NA	NA	NA	
NA	ENSG00000213069	0	NA	NA	NA	NA	KRT8P40
NA	ENSG00000213070	0	NA	NA	NA	NA	HMGB3P6
80350	ENSG00000213071	0.50207596	0.02562368	0.38565771	0.52549886	NA	LPAL2
NA	ENSG00000213073	0	NA	NA	NA	NA	CHP1P2

641311	ENSG00000213075	0	NA	NA	NA	NA	RPL31P11
NA	ENSG00000213076	0	NA	NA	NA	NA	
NA	ENSG00000213078	0.68982227	0.04028222	0.38714415	0.35752131	NA	
22828	ENSG00000213079	1467.63894	0.09589241	0.07459092	0.19019311	0.39151601	SCAF8
NA	ENSG00000213080	0.14931947	-0.01844332	0.38480212	0.84537521	NA	
NA	ENSG00000213081	0.32247871	-0.02325412	0.38531868	0.66961411	NA	ARPC1BP1
NA	ENSG00000213082	0	NA	NA	NA	NA	PPP1R14BP2
NA	ENSG00000213083	0	NA	NA	NA	NA	VPS26CP1
25790	ENSG00000213085	1.84915154	-0.10390519	0.39767812	0.19008535	NA	CFAP45
NA	ENSG00000213087	0	NA	NA	NA	NA	
2532	ENSG00000213088	3.75057221	-0.30159846	0.56969761	0.00877203	0.03724311	ACKR1
NA	ENSG00000213089	0.18922458	0.01954053	0.38488496	0.78225479	NA	PDCL3P5
NA	ENSG00000213090	2.73444438	0.13160455	0.40791356	0.14485784	0.32514759	SCYL2P1
NA	ENSG00000213091	0	NA	NA	NA	NA	PHBP1
9534	ENSG00000213096	642.576847	0.21996872	0.11451565	0.04358053	0.13391897	ZNF254
NA	ENSG00000213100	0	NA	NA	NA	NA	KRT18P68
NA	ENSG00000213104	0.45209736	-0.02505403	0.38558831	0.57865948	NA	NPM1P46
NA	ENSG00000213107	0	NA	NA	NA	NA	AHCYP5
NA	ENSG00000213108	0	NA	NA	NA	NA	BTF3L4P3
NA	ENSG00000213109	14.127704	-0.95766086	0.87764462	0.01279097	0.05078505	
NA	ENSG00000213110	0	NA	NA	NA	NA	RNF11P1
NA	ENSG00000213111	0	NA	NA	NA	NA	COX5BP2
NA	ENSG00000213113	0	NA	NA	NA	NA	TUBB8P8
NA	ENSG00000213115	0	NA	NA	NA	NA	
NA	ENSG00000213117	0	NA	NA	NA	NA	MEMO1P2
NA	ENSG00000213118	0	NA	NA	NA	NA	CHCHD2P4
NA	ENSG00000213120	0	NA	NA	NA	NA	LIN28AP1
NA	ENSG00000213121	1.7099929	0.05301345	0.38363269	0.45777663	NA	
NA	ENSG00000213122	0	NA	NA	NA	NA	RPL23AP46
255758	ENSG00000213123	208.507629	0.14654917	0.16760538	0.33212583	0.5613235	DYNLT2B
NA	ENSG00000213126	0	NA	NA	NA	NA	TXNL4AP1
NA	ENSG00000213128	0	NA	NA	NA	NA	RPL32P31
NA	ENSG00000213130	0	NA	NA	NA	NA	EEF1DP5
NA	ENSG00000213131	19.1605197	-0.15642237	0.33734166	0.41426623	0.64151123	YWHAZP4
NA	ENSG00000213133	0.34746601	0.02370763	0.3853651	0.61987128	NA	PPP1R14BP5
NA	ENSG00000213137	0	NA	NA	NA	NA	ARF1P2
1427	ENSG00000213139	31.0211557	-0.07313628	0.28696571	0.70413788	0.85774517	CRYGS
NA	ENSG00000213140	0	NA	NA	NA	NA	ELK2AP

NA	ENSG00000213144	1.44264563	-0.00314235	0.37834837	0.96325604	NA	
1396	ENSG00000213145	11.3318131	-0.09849807	0.34987652	0.55040397	0.75686268	CRIP1
NA	ENSG00000213147	4.87444824	0.03350749	0.38697799	0.12399749	0.29179342	RPL23AP60
NA	ENSG00000213148	0	NA	NA	NA	NA	JPT1P1
NA	ENSG00000213149	0.8593066	0.01676755	0.38119569	0.76654753	NA	CNN2P9
NA	ENSG00000213150	0	NA	NA	NA	NA	
NA	ENSG00000213152	1.15592065	0.0352035	0.38305248	0.54219027	NA	RPL7AP60
NA	ENSG00000213153	0	NA	NA	NA	NA	
NA	ENSG00000213155	0	NA	NA	NA	NA	
NA	ENSG00000213157	0	NA	NA	NA	NA	RPL32P10
NA	ENSG00000213158	0	NA	NA	NA	NA	GAPDHP36
NA	ENSG00000213159	0	NA	NA	NA	NA	CEND1P1
151230	ENSG00000213160	1830.60004	-0.27660308	0.07524392	0.00017235	0.00124295	KLHL23
100526832	ENSG00000213160	1830.60004	-0.27660308	0.07524392	0.00017235	0.00124295	KLHL23
NA	ENSG00000213167	0	NA	NA	NA	NA	
NA	ENSG00000213169	0	NA	NA	NA	NA	
NA	ENSG00000213170	0	NA	NA	NA	NA	SAR1AP2
339398	ENSG00000213171	6.84545883	-4.03956835	1.66068599	0.00059691	0.00376677	LINGO4
NA	ENSG00000213172	0	NA	NA	NA	NA	
NA	ENSG00000213174	0	NA	NA	NA	NA	
NA	ENSG00000213176	0	NA	NA	NA	NA	RPL13P6
NA	ENSG00000213177	0	NA	NA	NA	NA	PRDX2P4
NA	ENSG00000213178	357.700735	-0.47111755	0.17999874	0.0034347	0.01698093	RPL22P1
NA	ENSG00000213179	0	NA	NA	NA	NA	RPL17P41
NA	ENSG00000213180	0	NA	NA	NA	NA	RPL36AP48
NA	ENSG00000213181	0	NA	NA	NA	NA	OR10N1P
NA	ENSG00000213182	0	NA	NA	NA	NA	OR10D5P
NA	ENSG00000213183	0.16735865	0.01897177	0.38482933	0.78225479	NA	RPS7P8
NA	ENSG00000213184	0	NA	NA	NA	NA	
196792	ENSG00000213185	35.3017019	0.02525348	0.27272354	0.89766079	0.9551369	FAM24B
286827	ENSG00000213186	192.341465	-0.19323273	0.16237869	0.18793018	0.38830813	TRIM59
NA	ENSG00000213187	0	NA	NA	NA	NA	COPS5P1
NA	ENSG00000213188	3.9410326	0.20719752	0.44481934	0.09945117	0.24825255	YBX1P4
NA	ENSG00000213189	84.3499718	-0.27947272	0.28590134	0.17959559	0.37614023	BTF3L4P2
10962	ENSG00000213190	4882.74612	-0.33268468	0.07935951	1.75E-05	0.0001591	MLLT11
NA	ENSG00000213194	0	NA	NA	NA	NA	CDK7P1
NA	ENSG00000213197	0	NA	NA	NA	NA	NUDCP1
9311	ENSG00000213199	149.927656	0.37918234	0.23330178	0.04811681	0.14455444	ASIC3

NA	ENSG00000213201	0	NA	NA	NA	NA	FABP5P10
170575	ENSG00000213203	0	NA	NA	NA	NA	GIMAP1
NA	ENSG00000213204	5.90451971	0.03213549	0.36230751	0.80192346	0.90941191	
NA	ENSG00000213205	0	NA	NA	NA	NA	STRADBP1
NA	ENSG00000213209	0	NA	NA	NA	NA	
NA	ENSG00000213210	0	NA	NA	NA	NA	RPL32P17
NA	ENSG00000213211	0	NA	NA	NA	NA	KRT18P64
NA	ENSG00000213212	3.33913556	0.08038533	0.38259757	0.42838089	0.65428642	NCLP1
84960	ENSG00000213213	45.4045536	0.35855508	0.3779914	0.11851502	0.28264477	CCDC183
445328	ENSG00000213214	0.16735865	0.01897177	0.38482933	0.78225479	NA	ARHGEF35
26211	ENSG00000213215	0.49540215	0.00752424	0.38315145	0.82277631	NA	OR2F1
NA	ENSG00000213216	0.173124	-0.01911262	0.38486262	0.84537521	NA	
1443	ENSG00000213218	0	NA	NA	NA	NA	CSH2
728489	ENSG00000213221	260.819764	0.04513018	0.15951478	0.75695702	0.88697677	DNLZ
NA	ENSG00000213222	40.1761839	-0.16350274	0.29720126	0.41457774	0.64173903	TOMM40P4
NA	ENSG00000213225	0	NA	NA	NA	NA	NOC2LP1
NA	ENSG00000213226	0.50767003	0.03074537	0.38601659	0.48343636	NA	
NA	ENSG00000213228	3.05222448	-0.1443781	0.41869935	0.06466674	0.18119939	RPL12P38
9623	ENSG00000213231	0	NA	NA	NA	NA	TCL1B
NA	ENSG00000213232	0	NA	NA	NA	NA	PPP1R2P10
NA	ENSG00000213233	0	NA	NA	NA	NA	RPL23AP62
NA	ENSG00000213234	0.173124	-0.01911262	0.38486262	0.84537521	NA	ST13P10
NA	ENSG00000213235	0.51414327	-0.01023103	0.38328157	0.81791057	NA	EEF1A1P16
NA	ENSG00000213236	0	NA	NA	NA	NA	YWHAZP2
NA	ENSG00000213237	0.173733	0.01914545	0.38484597	0.78225479	NA	PTMAP10
NA	ENSG00000213238	0	NA	NA	NA	NA	
NA	ENSG00000213239	0	NA	NA	NA	NA	NPM1P32
NA	ENSG00000213244	33.0493188	-0.03978877	0.28174972	0.83456196	0.92451691	H3P4
6827	ENSG00000213246	1560.76905	-0.08763068	0.08960316	0.31090621	0.53864136	SUPT4H1
NA	ENSG00000213247	0	NA	NA	NA	NA	
NA	ENSG00000213250	0.14931947	-0.01844332	0.38480212	0.84537521	NA	RBMS2P1
NA	ENSG00000213252	0	NA	NA	NA	NA	
NA	ENSG00000213253	0.51528081	0.01588328	0.38315771	0.73078657	NA	RPL12P42
NA	ENSG00000213260	0	NA	NA	NA	NA	YWHAZP5
NA	ENSG00000213261	0.16735865	0.01897177	0.38482933	0.78225479	NA	EEF1B2P6
NA	ENSG00000213262	0	NA	NA	NA	NA	VDAC2P3
NA	ENSG00000213264	0	NA	NA	NA	NA	NIP7P2
114960	ENSG00000213265	0	NA	NA	NA	NA	TSGA13

NA	ENSG00000213269	0	NA	NA	NA	NA	RPS6P26
NA	ENSG00000213270	0	NA	NA	NA	NA	RPL6P25
NA	ENSG00000213272	0.49759159	-0.029592	0.38591039	0.52418284	NA	RPL7AP9
NA	ENSG00000213275	0	NA	NA	NA	NA	IFITM9P
NA	ENSG00000213277	0.44795842	-0.02501848	0.38558245	0.5807882	NA	MARCKSL1P1
NA	ENSG00000213279	0.32233294	-0.02325294	0.3853185	0.66969844	NA	
NA	ENSG00000213280	0	NA	NA	NA	NA	
4893	ENSG00000213281	1479.32735	0.07640423	0.08046305	0.3310047	0.55985791	NRAS
NA	ENSG00000213285	0	NA	NA	NA	NA	RPL23AP45
NA	ENSG00000213287	0	NA	NA	NA	NA	
NA	ENSG00000213290	1.28175491	-0.0186554	0.3786825	0.79358113	NA	PGK1P2
NA	ENSG00000213291	0	NA	NA	NA	NA	
NA	ENSG00000213293	0	NA	NA	NA	NA	
NA	ENSG00000213295	0	NA	NA	NA	NA	RPL22P10
NA	ENSG00000213296	0	NA	NA	NA	NA	
NA	ENSG00000213297	57.205732	-0.01537657	0.37554971	0.8475428	0.93065124	ZNF625-ZNF20
NA	ENSG00000213300	4.38569108	0.08047679	0.37244908	0.52444023	0.73730826	HNRNPA3P6
NA	ENSG00000213301	0	NA	NA	NA	NA	HMGB3P11
NA	ENSG00000213302	0	NA	NA	NA	NA	
NA	ENSG00000213303	1.50835317	0.01953588	0.37782723	0.79729578	NA	
NA	ENSG00000213304	5.01699043	0.01167659	0.36185982	0.92699572	0.96945838	
NA	ENSG00000213305	0	NA	NA	NA	NA	HNRNPCP6
NA	ENSG00000213307	1.83645378	0.01976025	0.37651416	0.80975585	NA	RPL18P11
NA	ENSG00000213309	0	NA	NA	NA	NA	RPL9P18
NA	ENSG00000213310	0	NA	NA	NA	NA	CYCSP19
NA	ENSG00000213312	0	NA	NA	NA	NA	RPL7AP34
NA	ENSG00000213315	0.14931947	-0.01844332	0.38480212	0.84537521	NA	
4056	ENSG00000213316	3.19837301	0.01718981	0.37086867	0.86655906	0.94128288	LTC4S
NA	ENSG00000213318	0	NA	NA	NA	NA	
NA	ENSG00000213326	1.43675806	-0.00343999	0.37859179	0.95918045	NA	RPS7P11
NA	ENSG00000213328	0	NA	NA	NA	NA	
NA	ENSG00000213331	0.173733	0.01914545	0.38484597	0.78225479	NA	
NA	ENSG00000213332	0	NA	NA	NA	NA	SLC25A5P6
NA	ENSG00000213333	0.35668774	0.02381711	0.38538107	0.61401367	NA	NPM1P50
NA	ENSG00000213335	0	NA	NA	NA	NA	CLNS1AP1
51239	ENSG00000213337	304.233303	0.00522962	0.13273644	0.96752504	0.98759962	ANKRD39
81890	ENSG00000213339	599.150016	0.22704933	0.09954015	0.01794866	0.06679822	QTRT1
1147	ENSG00000213341	753.492862	-0.27674443	0.10126711	0.00446891	0.02118943	CHUK

NA	ENSG00000213343	0	NA	NA	NA	NA	RPL21P18
NA	ENSG00000213344	1.66244426	0.01289225	0.37556555	0.87856525	NA	PCNPP3
83463	ENSG00000213347	535.893469	0.38220672	0.10913524	0.00025307	0.00175924	MXD3
NA	ENSG00000213352	0	NA	NA	NA	NA	GAPDHP44
NA	ENSG00000213355	0	NA	NA	NA	NA	CNN2P8
NA	ENSG00000213358	0	NA	NA	NA	NA	
NA	ENSG00000213361	0	NA	NA	NA	NA	
NA	ENSG00000213362	0.34118024	0.02362706	0.38535348	0.62406855	NA	FTH1P12
NA	ENSG00000213363	0	NA	NA	NA	NA	RPS3P6
NA	ENSG00000213365	1.83663988	-0.00612791	0.37611102	0.93791053	NA	
2946	ENSG00000213366	464.681261	0.67100197	0.12576736	2.36E-08	3.38E-07	GSTM2
NA	ENSG00000213368	0	NA	NA	NA	NA	ST13P11
NA	ENSG00000213370	0	NA	NA	NA	NA	RANP6
NA	ENSG00000213371	0.18922458	0.01954053	0.38488496	0.78225479	NA	NAP1L1P3
NA	ENSG00000213373	0	NA	NA	NA	NA	LINC00671
NA	ENSG00000213376	0	NA	NA	NA	NA	GAPDHP71
84342	ENSG00000213380	1449.29705	0.00151827	0.07172511	0.98302729	0.99428951	COG8
NA	ENSG00000213383	0	NA	NA	NA	NA	
NA	ENSG00000213384	0	NA	NA	NA	NA	EIF4E2P1
NA	ENSG00000213385	19.4496388	0.0573526	0.31458567	0.7577429	0.88730562	
NA	ENSG00000213386	1.33860123	0.06340102	0.38594526	0.37761587	NA	
84986	ENSG00000213390	788.356784	-0.24582697	0.09375318	0.00660438	0.0294499	ARHGAP19
NA	ENSG00000213393	0	NA	NA	NA	NA	
NA	ENSG00000213394	1.16082574	0.01846621	0.38015171	0.77267721	NA	RPSAP46
55559	ENSG00000213397	359.753191	-0.23371762	0.13208068	0.0580037	0.16698875	HAUS7
3931	ENSG00000213398	90.2332081	0.57980471	0.2785394	0.00919094	0.03869005	LCAT
NA	ENSG00000213399	1.49127727	-0.03418512	0.37950117	0.64838336	NA	RPS2P17
NA	ENSG00000213400	0	NA	NA	NA	NA	RPL12P18
4111	ENSG00000213401	1274.302	-0.601275	0.07822952	4.63E-15	1.27E-13	MAGEA12
5790	ENSG00000213402	6.35520851	0.03886443	0.38591773	0.3669693	0.59618989	PTPRCAP
NA	ENSG00000213403	0	NA	NA	NA	NA	CISD1P1
NA	ENSG00000213406	1.82697718	0.04566138	0.38024497	0.56907949	NA	ANXA2P1
NA	ENSG00000213409	0.34624801	-0.02356563	0.38536324	0.65270996	NA	C1QBPP2
NA	ENSG00000213411	2.02046179	0.00697498	0.37414321	0.93823868	NA	RBM22P2
NA	ENSG00000213412	0.55276188	0.03293474	0.38623842	0.45148476	NA	HNRNPA1P33
79037	ENSG00000213413	15.3475149	-0.10613981	0.33899042	0.54986487	0.75633105	PVRIG
NA	ENSG00000213414	0	NA	NA	NA	NA	
83755	ENSG00000213416	0	NA	NA	NA	NA	KRTAP4-12

85294	ENSG00000213417	0	NA	NA	NA	NA	KRTAP2-4
221914	ENSG00000213420	2154.78016	0.19506281	0.07650907	0.00919279	0.03869005	GPC2
NA	ENSG00000213421	0	NA	NA	NA	NA	
NA	ENSG00000213423	0	NA	NA	NA	NA	RBMX2P2
125113	ENSG00000213424	0.30001683	-0.0229446	0.38527539	0.68644815	NA	KRT222
NA	ENSG00000213430	0.173124	-0.01911262	0.38486262	0.84537521	NA	HSPD1P1
NA	ENSG00000213431	0	NA	NA	NA	NA	
NA	ENSG00000213432	0	NA	NA	NA	NA	RPL17P34
NA	ENSG00000213433	1.51456752	-0.05449132	0.3829632	0.47123388	NA	RPLP1P6
NA	ENSG00000213434	0.29863895	-0.02292439	0.3852726	0.68753245	NA	VTI1BP2
NA	ENSG00000213435	0	NA	NA	NA	NA	ATP6V0CP3
NA	ENSG00000213438	0	NA	NA	NA	NA	YBX2P1
NA	ENSG00000213439	0	NA	NA	NA	NA	OR5AC1
NA	ENSG00000213440	0	NA	NA	NA	NA	H2AZP1
NA	ENSG00000213442	1.15084481	-0.03612456	0.38126044	0.59448301	NA	RPL18AP3
NA	ENSG00000213443	18.8744719	0.01866423	0.3111201	0.92080637	0.96670947	NPM1P7
6494	ENSG00000213445	766.829874	0.2448997	0.09720562	0.00905842	0.03818933	SIPA1
NA	ENSG00000213448	0	NA	NA	NA	NA	RPS23P2
NA	ENSG00000213449	0	NA	NA	NA	NA	GAPDHP28
NA	ENSG00000213450	0	NA	NA	NA	NA	VDAC1P7
NA	ENSG00000213451	0	NA	NA	NA	NA	OR6C69P
NA	ENSG00000213452	0	NA	NA	NA	NA	AKR1B1P2
NA	ENSG00000213453	1.47032194	0.02276794	0.37812784	0.7648725	NA	FTH1P3
NA	ENSG00000213455	0	NA	NA	NA	NA	PPIAP82
NA	ENSG00000213461	0	NA	NA	NA	NA	RPL32P15
2086	ENSG00000213462	1774.71355	0.81017884	0.07959395	4.78E-25	2.28E-23	ERV3-1
55333	ENSG00000213463	1674.81883	-0.15529409	0.08189308	0.05176718	0.15304141	SYNJ2BP
402	ENSG00000213465	812.752187	0.20957563	0.10187119	0.03257196	0.10707998	ARL2
NA	ENSG00000213467	0	NA	NA	NA	NA	HMGB1P37
286467	ENSG00000213468	0.78598915	-0.05618249	0.38943957	0.27411323	NA	FIRRE
NA	ENSG00000213470	0	NA	NA	NA	NA	
440307	ENSG00000213471	3.93761092	0.0597632	0.37134175	0.60935878	0.79803651	TTLL13P
[truncated: 464,292 more chars]
