# Supplementary material for: SIRT2 functions as a histone delactylase and inhibits the proliferation and migration of neuroblastoma cells
Source: Cell Discov. 2022 Jun 7;8:54. doi: 10.1038/s41421-022-00398-y (PMC9174446; doi:10.1038/s41421-022-00398-y)
Supplement: Supplementary file 6 — Supplementary Table S6 [file 41421_2022_398_MOESM6_ESM.pdf]

Table S6. ChIP-seq peaks and annotated genes by anti-H4K8ac antibody in SIRT2 KD SH-SY5Y cells.

chr	start	end	peakid	-LOG(pvalue)	gene
1	713595	714000	KD2-H4K8ac_peak_1	11.60333	RP11-206L10.2_ENSG00000228327;RP11-206L10.9_ENSG00000237491
1	714251	714773	KD2-H4K8ac_peak_2	10.0746	RP11-206L10.2_ENSG00000228327;RP11-206L10.9_ENSG00000237491
1	762378	762862	KD2-H4K8ac_peak_3	10.19949	LINC00115_ENSG00000225880;LINC01128_ENSG00000228794
1	805148	805560	KD2-H4K8ac_peak_4	5.57044	
1	839303	839916	KD2-H4K8ac_peak_5	8.30974	RP11-54O7.16_ENSG00000272438
1	871520	871850	KD2-H4K8ac_peak_6	6.76298	
1	876150	876988	KD2-H4K8ac_peak_7	10.43145	
1	877768	878366	KD2-H4K8ac_peak_8	9.00811	
1	894246	894551	KD2-H4K8ac_peak_9	12.57143	NOC2L_ENSG00000188976
1	896028	896648	KD2-H4K8ac_peak_10	8.94469	KLHL17_ENSG00000187961
1	934711	935086	KD2-H4K8ac_peak_11	5.14125	HES4_ENSG00000188290
1	935854	936067	KD2-H4K8ac_peak_12	6.05809	HES4_ENSG00000188290
1	936793	937282	KD2-H4K8ac_peak_13	9.94337	
1	939652	941668	KD2-H4K8ac_peak_14	11.05467	
1	955043	955306	KD2-H4K8ac_peak_15	7.35686	AGRN_ENSG00000188157
1	955777	956105	KD2-H4K8ac_peak_16	5.14125	AGRN_ENSG00000188157
1	956811	957073	KD2-H4K8ac_peak_17	4.9025	
1	1014734	1015939	KD2-H4K8ac_peak_18	5.9696	
1	1016387	1016646	KD2-H4K8ac_peak_19	8.4939	
1	1051214	1051441	KD2-H4K8ac_peak_20	12.84642	C1orf159_ENSG00000131591
1	1051976	1052186	KD2-H4K8ac_peak_21	5.54647	C1orf159_ENSG00000131591
1	1057734	1058100	KD2-H4K8ac_peak_22	6.25869	
1	1079754	1080581	KD2-H4K8ac_peak_23	12.93209	
1	1092348	1092561	KD2-H4K8ac_peak_24	10.78136	
1	1092931	1093620	KD2-H4K8ac_peak_25	13.73635	
1	1167701	1168107	KD2-H4K8ac_peak_26	13.51987	SDF4_ENSG00000078808;B3GALT6_ENSG00000176022
1	1208647	1209220	KD2-H4K8ac_peak_27	14.68449	UBE2J2_ENSG00000160087
1	1209437	1210022	KD2-H4K8ac_peak_28	20.13709	UBE2J2_ENSG00000160087;RP5-902P8.10_ENSG00000230415
1	1239173	1241575	KD2-H4K8ac_peak_29	15.65094	
1	1242838	1243127	KD2-H4K8ac_peak_30	11.43863	PUSL1_ENSG00000169972
1	1243500	1243757	KD2-H4K8ac_peak_31	14.68449	PUSL1_ENSG00000169972
1	1259745	1259988	KD2-H4K8ac_peak_32	15.22643	CPSF3L_ENSG00000127054
1	1260232	1260544	KD2-H4K8ac_peak_33	14.63458	CPSF3L_ENSG00000127054
1	1284082	1284430	KD2-H4K8ac_peak_34	19.43192	DVL1_ENSG00000107404
1	1285001	1285316	KD2-H4K8ac_peak_35	10.19949	DVL1_ENSG00000107404
1	1290664	1291071	KD2-H4K8ac_peak_36	5.54647	
1	1294914	1295827	KD2-H4K8ac_peak_37	11.7608	
1	1309938	1310554	KD2-H4K8ac_peak_38	12.46522	AURKAIP1_ENSG00000175756
1	1311010	1311933	KD2-H4K8ac_peak_39	12.57143	AURKAIP1_ENSG00000175756
1	1334187	1334673	KD2-H4K8ac_peak_40	12.65397	CCNL2_ENSG00000221978;RP4-758J18.2_ENSG00000224870
1	1335007	1335511	KD2-H4K8ac_peak_41	13.13969	CCNL2_ENSG00000221978;RP4-758J18.2_ENSG00000224870
1	1342145	1342607	KD2-H4K8ac_peak_42	9.36516	MRPL20_ENSG00000242485
1	1342810	1343128	KD2-H4K8ac_peak_43	8.85813	MRPL20_ENSG00000242485
1	1355736	1355982	KD2-H4K8ac_peak_44	8.55271	RP4-758J18.7_ENSG00000225905

1	1362964	1363310	KD2-H4K8ac_peak_45	24.37317	
1	1365709	1366349	KD2-H4K8ac_peak_46	10.50156	
1	1366560	1366777	KD2-H4K8ac_peak_47	5.09296	
1	1370603	1370937	KD2-H4K8ac_peak_48	10.96455	RP4-758J18.10_ENSG00000225285;VWA1_ENSG00000179403
1	1390762	1391267	KD2-H4K8ac_peak_49	4.54838	
1	1397252	1398466	KD2-H4K8ac_peak_50	8.79106	
1	1440052	1440777	KD2-H4K8ac_peak_51	6.65673	
1	1446829	1447296	KD2-H4K8ac_peak_52	12.93209	ATAD3A_ENSG00000197785
1	1475610	1475837	KD2-H4K8ac_peak_53	5.32933	TMEM240_ENSG00000205090
1	1476095	1476493	KD2-H4K8ac_peak_54	10.96455	TMEM240_ENSG00000205090
1	1509684	1510664	KD2-H4K8ac_peak_55	17.16047	SSU72_ENSG00000160075;AL645728.1_ENSG00000215014
1	1534985	1535333	KD2-H4K8ac_peak_56	6.30229	C1orf233_ENSG00000228594
1	1535823	1536131	KD2-H4K8ac_peak_57	4.91674	C1orf233_ENSG00000228594
1	1550844	1551458	KD2-H4K8ac_peak_58	28.03233	RP11-345P4.9_ENSG00000272106;MIB2_ENSG00000197530
1	1590273	1590702	KD2-H4K8ac_peak_59	15.22643	CDK11B_ENSG00000248333;RP11-345P4.10_ENSG00000272004
1	1591202	1591572	KD2-H4K8ac_peak_60	9.15329	CDK11B_ENSG00000248333;RP11-345P4.10_ENSG00000272004
1	1623764	1624333	KD2-H4K8ac_peak_61	15.9336	SLC35E2B_ENSG00000189339
1	1709424	1709742	KD2-H4K8ac_peak_62	7.18615	
1	1710031	1710700	KD2-H4K8ac_peak_63	9.67696	
1	1821425	1822800	KD2-H4K8ac_peak_64	13.12288	GNB1_ENSG00000078369;RP1-140A9.1_ENSG00000231050
1	1823025	1823566	KD2-H4K8ac_peak_65	12.84642	GNB1_ENSG00000078369;RP1-140A9.1_ENSG00000231050
1	1851199	1851536	KD2-H4K8ac_peak_66	13.80019	TMEM52_ENSG00000178821
1	1875039	1875588	KD2-H4K8ac_peak_67	16.09057	
1	1891329	1891887	KD2-H4K8ac_peak_68	8.74596	
1	1963269	1963562	KD2-H4K8ac_peak_69	8.30974	
1	1963774	1964142	KD2-H4K8ac_peak_70	13.80019	
1	1975846	1976440	KD2-H4K8ac_peak_71	15.05364	
1	1981062	1981651	KD2-H4K8ac_peak_72	9.20193	RP11-547D24.3_ENSG00000226969;PRKCZ_ENSG00000067606
1	2004801	2005501	KD2-H4K8ac_peak_73	4.67342	
1	2126019	2127577	KD2-H4K8ac_peak_74	14.90957	
1	2135841	2136079	KD2-H4K8ac_peak_75	7.82935	
1	2136853	2137746	KD2-H4K8ac_peak_76	7.26089	
1	2143560	2145049	KD2-H4K8ac_peak_77	13.72842	C1orf86_ENSG00000162585;RP11-181G12.5_ENSG00000243558;RP11-181G12.4_ENSG00000234396;AL590822.1_ENSG00000203301
1	2145832	2146250	KD2-H4K8ac_peak_78	4.211	AL590822.1_ENSG00000203301
1	2158131	2159091	KD2-H4K8ac_peak_79	13.12288	
1	2201780	2202186	KD2-H4K8ac_peak_80	4.52514	
1	2231767	2231979	KD2-H4K8ac_peak_81	5.14125	
1	2245322	2245520	KD2-H4K8ac_peak_82	6.45083	
1	2245792	2247340	KD2-H4K8ac_peak_83	17.8868	
1	2258216	2258411	KD2-H4K8ac_peak_84	5.30799	RP4-713A8.1_ENSG00000272161;AL589739.1_ENSG00000269753
1	2323308	2323730	KD2-H4K8ac_peak_85	12.23	MORN1_ENSG00000116151;RER1_ENSG00000157916
1	2343501	2343956	KD2-H4K8ac_peak_86	14.17488	
1	2344231	2344868	KD2-H4K8ac_peak_87	10.29842	PEX10_ENSG00000157911
1	2403845	2404145	KD2-H4K8ac_peak_88	3.90565	
1	2405169	2406352	KD2-H4K8ac_peak_89	14.66631	
1	2406572	2407428	KD2-H4K8ac_peak_90	12.36441	

1	2411509	2412190	KD2-H4K8ac_peak_91	10.88013	
1	2412635	2413233	KD2-H4K8ac_peak_92	8.10216	
1	2413696	2414241	KD2-H4K8ac_peak_93	6.99815	
1	2436462	2436805	KD2-H4K8ac_peak_94	9.65086	
1	2457240	2457498	KD2-H4K8ac_peak_95	6.25869	PANK4_ENSG00000157881
1	2471863	2472161	KD2-H4K8ac_peak_96	6.35295	
1	2474150	2476613	KD2-H4K8ac_peak_97	11.3923	
1	2476827	2477122	KD2-H4K8ac_peak_98	6.76298	RP3-395M20.12_ENSG00000272449
1	2478024	2478221	KD2-H4K8ac_peak_99	7.07117	RP3-395M20.12_ENSG00000272449
1	2518579	2518845	KD2-H4K8ac_peak_100	11.2938	FAM213B_ENSG00000157870
1	2573931	2574577	KD2-H4K8ac_peak_101	13.56049	
1	3073511	3073743	KD2-H4K8ac_peak_102	5.90414	
1	3086794	3087037	KD2-H4K8ac_peak_103	11.86082	
1	3087345	3087615	KD2-H4K8ac_peak_104	5.01002	
1	3370675	3370970	KD2-H4K8ac_peak_105	9.16531	ARHGEF16_ENSG00000130762
1	3371377	3371922	KD2-H4K8ac_peak_106	8.12706	ARHGEF16_ENSG00000130762
1	3376571	3376775	KD2-H4K8ac_peak_107	7.44324	
1	3433074	3434493	KD2-H4K8ac_peak_108	11.43863	
1	3447506	3448008	KD2-H4K8ac_peak_109	14.49045	
1	3455970	3456229	KD2-H4K8ac_peak_110	4.44748	
1	3456930	3457125	KD2-H4K8ac_peak_111	5.88845	
1	3467595	3468431	KD2-H4K8ac_peak_112	6.75424	
1	3468697	3468989	KD2-H4K8ac_peak_113	6.30229	
1	3469839	3470389	KD2-H4K8ac_peak_114	6.66692	
1	3489929	3490218	KD2-H4K8ac_peak_115	6.67092	
1	3515582	3516185	KD2-H4K8ac_peak_116	10.0089	
1	3541077	3541296	KD2-H4K8ac_peak_117	11.50346	RP11-46F15.2_ENSG00000238260;TPRG1L_ENSG00000158109
1	3566068	3566543	KD2-H4K8ac_peak_118	14.56955	
1	3567977	3569354	KD2-H4K8ac_peak_119	17.70837	WRAP73_ENSG00000116213;TP73_ENSG00000078900
1	3613732	3614267	KD2-H4K8ac_peak_120	10.42398	
1	3627883	3628353	KD2-H4K8ac_peak_121	5.34636	
1	3628977	3629815	KD2-H4K8ac_peak_122	13.81781	
1	3632744	3633950	KD2-H4K8ac_peak_123	8.20434	
1	3634246	3634574	KD2-H4K8ac_peak_124	7.49599	
1	3635450	3635832	KD2-H4K8ac_peak_125	8.82366	
1	3636216	3637124	KD2-H4K8ac_peak_126	11.06636	
1	3663404	3663882	KD2-H4K8ac_peak_127	18.25771	TP73-AS1_ENSG00000227372
1	3688721	3688985	KD2-H4K8ac_peak_128	6.31838	SMIM1_ENSG00000235169
1	3692756	3692980	KD2-H4K8ac_peak_129	9.95342	
1	3712251	3712934	KD2-H4K8ac_peak_130	4.37809	LRRC47_ENSG00000130764
1	3713304	3714081	KD2-H4K8ac_peak_131	10.86378	LRRC47_ENSG00000130764
1	3737266	3737537	KD2-H4K8ac_peak_132	4.56666	
1	3816439	3816732	KD2-H4K8ac_peak_133	7.36799	C1orf174_ENSG00000198912;LINC01134_ENSG00000236423
1	3817541	3817850	KD2-H4K8ac_peak_134	19.28826	C1orf174_ENSG00000198912;LINC01134_ENSG00000236423
1	4786971	4787440	KD2-H4K8ac_peak_135	8.02571	
1	5483616	5484034	KD2-H4K8ac_peak_136	6.13909	

1	5484400	5484778	KD2-H4K8ac_peak_137	8.55552	
1	5524341	5524538	KD2-H4K8ac_peak_138	8.02571	
1	6052074	6052439	KD2-H4K8ac_peak_139	7.13615	NPHP4_ENSG00000131697;KCNAB2_ENSG00000069424
1	6094300	6094668	KD2-H4K8ac_peak_140	10.19949	
1	6259072	6259618	KD2-H4K8ac_peak_141	17.76565	
1	6260075	6260717	KD2-H4K8ac_peak_142	10.19949	
1	6268414	6268653	KD2-H4K8ac_peak_143	9.34989	RPL22_ENSG00000116251
1	6269204	6270058	KD2-H4K8ac_peak_144	7.81657	RPL22_ENSG00000116251
1	6295605	6295961	KD2-H4K8ac_peak_145	11.86082	ICMT_ENSG00000116237;LINC00337_ENSG00000225077
1	6296204	6296535	KD2-H4K8ac_peak_146	9.11031	ICMT_ENSG00000116237;LINC00337_ENSG00000225077
1	6320705	6320943	KD2-H4K8ac_peak_147	8.30974	GPR153_ENSG00000158292
1	6321352	6321559	KD2-H4K8ac_peak_148	6.66692	GPR153_ENSG00000158292
1	6329878	6330450	KD2-H4K8ac_peak_149	5.82799	
1	6389690	6390125	KD2-H4K8ac_peak_150	9.32235	
1	6419115	6419662	KD2-H4K8ac_peak_151	5.61067	
1	6420241	6420530	KD2-H4K8ac_peak_152	8.58069	
1	6454080	6454288	KD2-H4K8ac_peak_153	6.13909	RP1-202O8.3_ENSG00000271746;ACOT7_ENSG00000097021
1	6544848	6546594	KD2-H4K8ac_peak_154	8.16906	
1	6550261	6551325	KD2-H4K8ac_peak_155	8.10216	
1	6557016	6557395	KD2-H4K8ac_peak_156	13.04385	
1	6566607	6566838	KD2-H4K8ac_peak_157	6.0525	
1	6567410	6567640	KD2-H4K8ac_peak_158	9.46843	
1	6567990	6568730	KD2-H4K8ac_peak_159	12.08081	
1	6569906	6570195	KD2-H4K8ac_peak_160	9.90914	
1	6570440	6571056	KD2-H4K8ac_peak_161	8.58725	
1	6571327	6571876	KD2-H4K8ac_peak_162	12.14909	
1	6572086	6572836	KD2-H4K8ac_peak_163	7.07117	
1	6577847	6578272	KD2-H4K8ac_peak_164	6.66692	
1	6579404	6580061	KD2-H4K8ac_peak_165	4.67342	PLEKHG5_ENSG00000171680
1	6614109	6614527	KD2-H4K8ac_peak_166	12.08081	NOL9_ENSG00000162408;TAS1R1_ENSG00000173662
1	6614952	6615655	KD2-H4K8ac_peak_167	11.50346	NOL9_ENSG00000162408;TAS1R1_ENSG00000173662
1	6639373	6639852	KD2-H4K8ac_peak_168	8.4716	ZBTB48_ENSG00000204859
1	6663084	6663458	KD2-H4K8ac_peak_169	5.54647	
1	6673210	6673451	KD2-H4K8ac_peak_170	7.40111	PHF13_ENSG00000116273
1	6673857	6674106	KD2-H4K8ac_peak_171	7.98621	KLHL21_ENSG00000162413;PHF13_ENSG00000116273
1	6674453	6674645	KD2-H4K8ac_peak_172	8.49564	KLHL21_ENSG00000162413;PHF13_ENSG00000116273
1	6685272	6685740	KD2-H4K8ac_peak_173	13.09607	THAP3_ENSG00000041988
1	6761157	6761809	KD2-H4K8ac_peak_174	12.06346	DNAJC11_ENSG00000007923
1	6990881	6991076	KD2-H4K8ac_peak_175	5.57044	
1	7020360	7020561	KD2-H4K8ac_peak_176	9.08816	
1	7325059	7325707	KD2-H4K8ac_peak_177	9.36516	
1	7326694	7326997	KD2-H4K8ac_peak_178	6.76298	
1	7351179	7351691	KD2-H4K8ac_peak_179	4.68932	
1	7394297	7394597	KD2-H4K8ac_peak_180	7.73418	
1	7396129	7396418	KD2-H4K8ac_peak_181	11.03212	
1	7401206	7401974	KD2-H4K8ac_peak_182	7.95563	

1	7439631	7439975	KD2-H4K8ac_peak_183	8.17902	
1	7446009	7446285	KD2-H4K8ac_peak_184	5.98124	
1	7448435	7449913	KD2-H4K8ac_peak_185	7.07117	RP3-453P22.2_ENSG00000237728
1	7513751	7514072	KD2-H4K8ac_peak_186	3.75035	
1	7517843	7518059	KD2-H4K8ac_peak_187	7.01494	
1	7531536	7531782	KD2-H4K8ac_peak_188	4.54006	
1	7536459	7537330	KD2-H4K8ac_peak_189	10.05606	
1	7537654	7538221	KD2-H4K8ac_peak_190	13.81781	
1	7538922	7539316	KD2-H4K8ac_peak_191	13.73574	
1	7560962	7561497	KD2-H4K8ac_peak_192	9.11031	
1	7568910	7569149	KD2-H4K8ac_peak_193	12.06346	
1	7584802	7585247	KD2-H4K8ac_peak_194	9.85259	
1	7594544	7594918	KD2-H4K8ac_peak_195	9.08816	
1	7597099	7597689	KD2-H4K8ac_peak_196	5.88845	
1	7616551	7616810	KD2-H4K8ac_peak_197	4.8888	
1	7617682	7618053	KD2-H4K8ac_peak_198	8.85813	
1	7618388	7618811	KD2-H4K8ac_peak_199	14.71663	
1	7619017	7619393	KD2-H4K8ac_peak_200	9.11891	
1	7620212	7620415	KD2-H4K8ac_peak_201	6.72624	
1	7621060	7621477	KD2-H4K8ac_peak_202	10.19723	
1	7642106	7642414	KD2-H4K8ac_peak_203	7.42204	
1	7645604	7647504	KD2-H4K8ac_peak_204	17.76565	
1	7647736	7647948	KD2-H4K8ac_peak_205	7.76329	
1	7688856	7689110	KD2-H4K8ac_peak_206	3.83717	
1	7690807	7691802	KD2-H4K8ac_peak_207	9.07715	
1	7692713	7693611	KD2-H4K8ac_peak_208	6.95649	
1	7706943	7707203	KD2-H4K8ac_peak_209	11.79585	
1	7727525	7728376	KD2-H4K8ac_peak_210	15.61791	
1	7728863	7730931	KD2-H4K8ac_peak_211	22.93827	
1	7732012	7732476	KD2-H4K8ac_peak_212	4.06758	
1	7733363	7733608	KD2-H4K8ac_peak_213	7.146	
1	7739800	7741576	KD2-H4K8ac_peak_214	19.66144	
1	7764265	7764558	KD2-H4K8ac_peak_215	12.30509	
1	7764811	7765634	KD2-H4K8ac_peak_216	6.0073	
1	7813235	7813470	KD2-H4K8ac_peak_217	4.09222	
1	7831388	7831678	KD2-H4K8ac_peak_218	12.99417	VAMP3_ENSG00000049245
1	8013641	8014708	KD2-H4K8ac_peak_219	18.5703	PARK7_ENSG00000116288
1	8085727	8086277	KD2-H4K8ac_peak_220	9.72244	ERRFI1_ENSG00000116285;RP11-431K24.1_ENSG00000238290
1	8086490	8087036	KD2-H4K8ac_peak_221	18.94636	ERRFI1_ENSG00000116285;RP11-431K24.1_ENSG00000238290
1	8229375	8229753	KD2-H4K8ac_peak_222	7.1181	
1	8230189	8230698	KD2-H4K8ac_peak_223	9.402	
1	8377717	8378641	KD2-H4K8ac_peak_224	15.80947	SLC45A1_ENSG00000162426
1	8483481	8483738	KD2-H4K8ac_peak_225	6.84261	RP5-1115A15.1_ENSG00000232912
1	8762892	8763324	KD2-H4K8ac_peak_226	14.66631	
1	8877031	8877605	KD2-H4K8ac_peak_227	6.66692	RERE_ENSG00000142599
1	8877987	8878670	KD2-H4K8ac_peak_228	11.81257	RERE_ENSG00000142599

1	8882454	8882659	KD2-H4K8ac_peak_229	6.40683	
1	8882903	8883274	KD2-H4K8ac_peak_230	6.07178	
1	8938344	8938707	KD2-H4K8ac_peak_231	7.36799	ENO1_ENSG00000074800;ENO1-IT1_ENSG00000236269;ENO1-AS1_ENSG00000230679
1	8938919	8939239	KD2-H4K8ac_peak_232	11.00372	ENO1_ENSG00000074800;ENO1-IT1_ENSG00000236269;ENO1-AS1_ENSG00000230679
1	9189525	9189893	KD2-H4K8ac_peak_233	5.9696	GPR157_ENSG00000180758
1	9242062	9243234	KD2-H4K8ac_peak_234	8.73935	RP3-510D11.2_ENSG00000234546
1	9294865	9295363	KD2-H4K8ac_peak_235	15.22643	H6PD_ENSG00000049239
1	9488601	9488928	KD2-H4K8ac_peak_236	9.08816	
1	9554992	9555747	KD2-H4K8ac_peak_237	12.99417	
1	9598806	9599186	KD2-H4K8ac_peak_238	10.08707	SLC25A33_ENSG00000171612
1	9663906	9664205	KD2-H4K8ac_peak_239	10.16666	
1	9664502	9664821	KD2-H4K8ac_peak_240	10.09705	
1	9665592	9666061	KD2-H4K8ac_peak_241	12.43831	
1	9672804	9673205	KD2-H4K8ac_peak_242	4.54006	
1	9686704	9687793	KD2-H4K8ac_peak_243	18.86697	
1	9747289	9747848	KD2-H4K8ac_peak_244	6.99815	RP11-558F24.4_ENSG00000231789
1	9749162	9749474	KD2-H4K8ac_peak_245	10.08707	
1	9749677	9750181	KD2-H4K8ac_peak_246	5.82869	
1	9777567	9777825	KD2-H4K8ac_peak_247	5.88845	
1	9784918	9785186	KD2-H4K8ac_peak_248	6.56211	
1	9883444	9883982	KD2-H4K8ac_peak_249	12.08081	CLSTN1_ENSG00000171603
1	9884293	9884849	KD2-H4K8ac_peak_250	13.95627	CLSTN1_ENSG00000171603
1	9970940	9971132	KD2-H4K8ac_peak_251	6.00226	CTNNBIP1_ENSG00000178585
1	10002819	10003377	KD2-H4K8ac_peak_252	6.83936	LZIC_ENSG00000162441;RP11-84A14.4_ENSG00000228150;NMNAT1_ENSG00000173614
1	10003711	10004266	KD2-H4K8ac_peak_253	7.13615	LZIC_ENSG00000162441;RP11-84A14.4_ENSG00000228150;NMNAT1_ENSG00000173614
1	10057326	10057544	KD2-H4K8ac_peak_254	10.21502	RBP7_ENSG00000162444
1	10057829	10058108	KD2-H4K8ac_peak_255	8.17902	RBP7_ENSG00000162444
1	10092008	10092650	KD2-H4K8ac_peak_256	11.85362	UBE4B_ENSG00000130939
1	10093097	10093441	KD2-H4K8ac_peak_257	4.54006	UBE4B_ENSG00000130939
1	10269931	10270374	KD2-H4K8ac_peak_258	18.80827	KIF1B_ENSG00000054523
1	10270701	10271640	KD2-H4K8ac_peak_259	5.54647	KIF1B_ENSG00000054523
1	10399620	10400011	KD2-H4K8ac_peak_260	10.0692	
1	10400496	10400703	KD2-H4K8ac_peak_261	4.06758	
1	10490221	10490664	KD2-H4K8ac_peak_262	9.73431	RP4-736L20.3_ENSG00000271989;APITD1_ENSG00000175279;APITD1-CORT_ENSG00000251503
1	10490923	10491303	KD2-H4K8ac_peak_263	5.14125	RP4-736L20.3_ENSG00000271989;APITD1_ENSG00000175279;APITD1-CORT_ENSG00000251503
1	10533319	10533586	KD2-H4K8ac_peak_264	6.33462	DFFA_ENSG00000160049;PEX14_ENSG00000142655
1	10534377	10534771	KD2-H4K8ac_peak_265	8.23274	
1	10685858	10686057	KD2-H4K8ac_peak_266	9.96798	
1	10686378	10686683	KD2-H4K8ac_peak_267	6.4562	
1	10687004	10687262	KD2-H4K8ac_peak_268	7.1733	
1	10694887	10695330	KD2-H4K8ac_peak_269	6.99815	
1	10696083	10696530	KD2-H4K8ac_peak_270	5.61067	
1	10700110	10700304	KD2-H4K8ac_peak_271	10.4923	RP4-734G22.3_ENSG00000272078
1	10700648	10701698	KD2-H4K8ac_peak_272	7.76329	
1	10707572	10707892	KD2-H4K8ac_peak_273	4.211	
1	10723720	10724060	KD2-H4K8ac_peak_274	6.48713	

1	10731633	10731951	KD2-H4K8ac_peak_275	5.51783	
1	10739304	10739637	KD2-H4K8ac_peak_276	5.30799	
1	10741456	10741980	KD2-H4K8ac_peak_277	5.01002	
1	10745664	10745968	KD2-H4K8ac_peak_278	12.84642	
1	10753936	10754300	KD2-H4K8ac_peak_279	7.27716	
1	10926876	10927090	KD2-H4K8ac_peak_280	6.05809	
1	10927415	10927779	KD2-H4K8ac_peak_281	21.75906	
1	10948443	10948650	KD2-H4K8ac_peak_282	6.89708	
1	10948962	10949238	KD2-H4K8ac_peak_283	4.24345	
1	10950109	10951021	KD2-H4K8ac_peak_284	5.54647	
1	10958270	10958865	KD2-H4K8ac_peak_285	10.78136	
1	10959842	10960450	KD2-H4K8ac_peak_286	6.99815	
1	10960672	10962819	KD2-H4K8ac_peak_287	16.89963	
1	10963107	10963302	KD2-H4K8ac_peak_288	4.26323	
1	10963578	10965122	KD2-H4K8ac_peak_289	29.1888	
1	10966063	10966810	KD2-H4K8ac_peak_290	25.82946	
1	10969004	10969237	KD2-H4K8ac_peak_291	8.39745	
1	10969538	10969805	KD2-H4K8ac_peak_292	8.49564	
1	10970084	10970950	KD2-H4K8ac_peak_293	17.70837	
1	10971165	10971791	KD2-H4K8ac_peak_294	9.95119	
1	11001100	11001396	KD2-H4K8ac_peak_295	5.70596	
1	11001775	11002224	KD2-H4K8ac_peak_296	14.84212	
1	11003516	11004006	KD2-H4K8ac_peak_297	7.1181	
1	11004799	11006232	KD2-H4K8ac_peak_298	13.6476	
1	11006563	11006786	KD2-H4K8ac_peak_299	8.4716	
1	11007823	11008298	KD2-H4K8ac_peak_300	8.30974	
1	11020557	11020781	KD2-H4K8ac_peak_301	7.13615	
1	11072519	11073078	KD2-H4K8ac_peak_302	9.20193	TARDBP_ENSG00000120948
1	11113101	11113441	KD2-H4K8ac_peak_303	5.53033	
1	11119684	11119916	KD2-H4K8ac_peak_304	9.11876	SRM_ENSG00000116649
1	11159410	11160715	KD2-H4K8ac_peak_305	10.19949	EXOSC10_ENSG00000171824;RP4-635E18.6_ENSG00000230337
1	11322175	11322548	KD2-H4K8ac_peak_306	9.95119	MTOR_ENSG00000198793
1	11323216	11323719	KD2-H4K8ac_peak_307	12.65964	MTOR_ENSG00000198793
1	11333284	11333788	KD2-H4K8ac_peak_308	18.5703	UBIAD1_ENSG00000120942
1	11361552	11361796	KD2-H4K8ac_peak_309	6.0073	
1	11372132	11372462	KD2-H4K8ac_peak_310	4.98318	
1	11398310	11398569	KD2-H4K8ac_peak_311	10.81728	
1	11400086	11400604	KD2-H4K8ac_peak_312	10.96455	
1	11405426	11405646	KD2-H4K8ac_peak_313	6.08655	
1	11436756	11436951	KD2-H4K8ac_peak_314	6.39197	
1	11513974	11514235	KD2-H4K8ac_peak_315	4.73543	
1	11518636	11519025	KD2-H4K8ac_peak_316	4.8888	
1	11519245	11519594	KD2-H4K8ac_peak_317	8.36432	
1	11538969	11539554	KD2-H4K8ac_peak_318	10.44656	PTCHD2_ENSG00000204624
1	11623391	11623723	KD2-H4K8ac_peak_319	11.86082	
1	11710828	11711052	KD2-H4K8ac_peak_320	6.0605	

1	11741280	11741548	KD2-H4K8ac_peak_321	9.80695	
1	11750783	11751225	KD2-H4K8ac_peak_322	6.04107	MAD2L2_ENSG00000116670;DRAXIN_ENSG00000162490
1	11761190	11761423	KD2-H4K8ac_peak_323	5.30799	
1	11779465	11779998	KD2-H4K8ac_peak_324	4.06758	
1	11796248	11796645	KD2-H4K8ac_peak_325	6.65673	AGTRAP_ENSG00000177674
1	11824330	11824685	KD2-H4K8ac_peak_326	7.36799	
1	11828216	11828441	KD2-H4K8ac_peak_327	9.11031	
1	11865313	11865939	KD2-H4K8ac_peak_328	13.13969	CLCN6_ENSG00000011021
1	11901539	11901833	KD2-H4K8ac_peak_329	6.4562	NPPA-AS1_ENSG00000242349
1	11907155	11907404	KD2-H4K8ac_peak_330	6.66692	NPPA_ENSG00000175206
1	11907627	11907824	KD2-H4K8ac_peak_331	5.61067	NPPA_ENSG00000175206
1	11908499	11908737	KD2-H4K8ac_peak_332	9.15069	NPPA_ENSG00000175206
1	11939873	11940522	KD2-H4K8ac_peak_333	10.7186	
1	11941525	11941977	KD2-H4K8ac_peak_334	10.65724	
1	11943137	11943730	KD2-H4K8ac_peak_335	8.10216	
1	11968710	11969012	KD2-H4K8ac_peak_336	6.40297	RNU5E-1_ENSG00000199347;RNU5E-4P_ENSG00000201801
1	11985805	11986290	KD2-H4K8ac_peak_337	5.88845	KIAA2013_ENSG00000116685
1	11986821	11987034	KD2-H4K8ac_peak_338	5.88845	KIAA2013_ENSG00000116685
1	11991088	11991454	KD2-H4K8ac_peak_339	8.02571	
1	11994088	11994544	KD2-H4K8ac_peak_340	10.53377	PLOD1_ENSG00000083444
1	11994778	11995036	KD2-H4K8ac_peak_341	10.65724	PLOD1_ENSG00000083444
1	11995629	11995831	KD2-H4K8ac_peak_342	7.48822	
1	11998485	11998768	KD2-H4K8ac_peak_343	4.76709	
1	12004665	12005386	KD2-H4K8ac_peak_344	7.76104	
1	12011065	12011261	KD2-H4K8ac_peak_345	4.42765	
1	12040329	12040933	KD2-H4K8ac_peak_346	9.34195	MFN2_ENSG00000116688
1	12079910	12080127	KD2-H4K8ac_peak_347	7.07117	MIIP_ENSG00000116691
1	12114103	12114913	KD2-H4K8ac_peak_348	10.96455	
1	12167146	12167398	KD2-H4K8ac_peak_349	5.29352	
1	12215060	12215393	KD2-H4K8ac_peak_350	6.24114	
1	12243956	12246085	KD2-H4K8ac_peak_351	19.33018	
1	12265209	12265426	KD2-H4K8ac_peak_352	6.99815	
1	12289604	12289894	KD2-H4K8ac_peak_353	5.54647	VPS13D_ENSG00000048707
1	12290180	12290474	KD2-H4K8ac_peak_354	13.27022	VPS13D_ENSG00000048707
1	12496277	12496600	KD2-H4K8ac_peak_355	4.59928	
1	12533404	12533985	KD2-H4K8ac_peak_356	7.1181	
1	12537760	12538369	KD2-H4K8ac_peak_357	5.54647	
1	12538687	12539446	KD2-H4K8ac_peak_358	9.20193	
1	12541029	12541254	KD2-H4K8ac_peak_359	4.42765	
1	12580797	12581039	KD2-H4K8ac_peak_360	10.21502	
1	12655025	12655328	KD2-H4K8ac_peak_361	15.61313	
1	12656104	12656401	KD2-H4K8ac_peak_362	17.10757	
1	12678087	12678578	KD2-H4K8ac_peak_363	11.89502	DHRS3_ENSG00000162496;RP11-474O21.5_ENSG00000272482
1	14026181	14027230	KD2-H4K8ac_peak_364	12.42761	PRDM2_ENSG00000116731
1	14075275	14075623	KD2-H4K8ac_peak_365	6.25869	
1	15250304	15250509	KD2-H4K8ac_peak_366	6.99815	

1	15250739	15250936	KD2-H4K8ac_peak_367	8.02571	
1	15251202	15251561	KD2-H4K8ac_peak_368	7.47462	
1	15270333	15270553	KD2-H4K8ac_peak_369	5.57044	
1	15270867	15271638	KD2-H4K8ac_peak_370	14.275	
1	15272243	15272798	KD2-H4K8ac_peak_371	10.29842	
1	15273252	15273981	KD2-H4K8ac_peak_372	7.58939	
1	15276163	15276436	KD2-H4K8ac_peak_373	5.66317	
1	15295040	15295246	KD2-H4K8ac_peak_374	5.14125	
1	15466733	15467094	KD2-H4K8ac_peak_375	8.55271	
1	15467569	15467808	KD2-H4K8ac_peak_376	8.74596	
1	15477601	15477867	KD2-H4K8ac_peak_377	8.86787	
1	15478554	15478882	KD2-H4K8ac_peak_378	9.89383	TMEM51-AS1_ENSG00000175147;TMEM51_ENSG00000171729
1	15479319	15480029	KD2-H4K8ac_peak_379	9.07715	TMEM51-AS1_ENSG00000175147;TMEM51_ENSG00000171729
1	15505281	15505692	KD2-H4K8ac_peak_380	5.20908	
1	15506520	15506712	KD2-H4K8ac_peak_381	8.30974	
1	15512380	15512630	KD2-H4K8ac_peak_382	6.00226	
1	15513287	15513539	KD2-H4K8ac_peak_383	8.30974	
1	15514255	15514500	KD2-H4K8ac_peak_384	5.14125	
1	15514803	15515098	KD2-H4K8ac_peak_385	6.98292	
1	15533576	15533798	KD2-H4K8ac_peak_386	3.75035	
1	15573733	15574086	KD2-H4K8ac_peak_387	7.69287	FHAD1_ENSG00000142621
1	15851108	15851326	KD2-H4K8ac_peak_388	14.15075	
1	15853385	15853654	KD2-H4K8ac_peak_389	5.81222	CASP9_ENSG00000132906;DNAJC16_ENSG00000116138
1	15910703	15910945	KD2-H4K8ac_peak_390	3.7063	AGMAT_ENSG00000116771
1	15911467	15911895	KD2-H4K8ac_peak_391	9.34989	AGMAT_ENSG00000116771
1	15930254	15930809	KD2-H4K8ac_peak_392	5.66705	RP4-680D5.2_ENSG00000237301;CHCHD2P6_ENSG00000235084
1	15944055	15944418	KD2-H4K8ac_peak_393	7.76329	DDI2_ENSG00000197312
1	16010031	16010797	KD2-H4K8ac_peak_394	27.43676	RP4-680D5.9_ENSG00000271742;PLEKHM2_ENSG00000116786;AL121992.1_ENSG00000264048
1	16011101	16011554	KD2-H4K8ac_peak_395	5.30799	PLEKHM2_ENSG00000116786;AL121992.1_ENSG00000264048
1	16064843	16065130	KD2-H4K8ac_peak_396	6.99815	
1	16065918	16066122	KD2-H4K8ac_peak_397	5.40356	
1	16075594	16075957	KD2-H4K8ac_peak_398	14.66631	RP11-169K16.4_ENSG00000224459
1	16083806	16084166	KD2-H4K8ac_peak_399	7.36799	FBLIM1_ENSG00000162458
1	16085396	16086100	KD2-H4K8ac_peak_400	14.66631	
1	16159754	16159969	KD2-H4K8ac_peak_401	7.27716	
1	16160993	16162238	KD2-H4K8ac_peak_402	21.57439	
1	16176098	16176588	KD2-H4K8ac_peak_403	6.92624	
1	16302343	16302831	KD2-H4K8ac_peak_404	30.77376	ZBTB17_ENSG00000116809
1	16303332	16303619	KD2-H4K8ac_peak_405	4.77079	ZBTB17_ENSG00000116809
1	16339430	16340137	KD2-H4K8ac_peak_406	23.66011	
1	16340471	16340845	KD2-H4K8ac_peak_407	5.02474	
1	16342283	16342505	KD2-H4K8ac_peak_408	4.85088	
1	16343918	16344969	KD2-H4K8ac_peak_409	9.07715	CLCNKA_ENSG00000186510
1	16399600	16400355	KD2-H4K8ac_peak_410	14.56955	FAM131C_ENSG00000185519
1	16405642	16406121	KD2-H4K8ac_peak_411	5.30799	
1	16414329	16414567	KD2-H4K8ac_peak_412	3.75035	

1	16468070	16468433	KD2-H4K8ac_peak_413	6.40683	
1	16481414	16481790	KD2-H4K8ac_peak_414	8.20667	EPHA2_ENSG00000142627;RP11-276H7.2_ENSG00000227959
1	16482114	16482327	KD2-H4K8ac_peak_415	5.72327	EPHA2_ENSG00000142627;RP11-276H7.2_ENSG00000227959
1	16493572	16494466	KD2-H4K8ac_peak_416	13.12608	
1	16500765	16501178	KD2-H4K8ac_peak_417	11.90231	
1	16501490	16502592	KD2-H4K8ac_peak_418	9.95119	
1	16502854	16503525	KD2-H4K8ac_peak_419	13.73635	
1	16532124	16532976	KD2-H4K8ac_peak_420	5.75757	
1	16533673	16534211	KD2-H4K8ac_peak_421	8.55271	
1	16535151	16535448	KD2-H4K8ac_peak_422	4.82973	
1	16542988	16543190	KD2-H4K8ac_peak_423	4.56666	
1	16563324	16564335	KD2-H4K8ac_peak_424	17.75427	RSG1_ENSG00000132881
1	16574648	16575329	KD2-H4K8ac_peak_425	5.14125	
1	16678537	16678825	KD2-H4K8ac_peak_426	3.86308	FBXO42_ENSG00000037637;SZRD1_ENSG00000055070
1	16692557	16693210	KD2-H4K8ac_peak_427	7.76329	
1	16693718	16694227	KD2-H4K8ac_peak_428	19.33018	
1	16766749	16767037	KD2-H4K8ac_peak_429	11.05467	NECAP2_ENSG00000157191
1	16767307	16767580	KD2-H4K8ac_peak_430	8.58069	NECAP2_ENSG00000157191
1	16825086	16825694	KD2-H4K8ac_peak_431	13.73635	CROCCP3_ENSG00000080947
1	16825909	16826377	KD2-H4K8ac_peak_432	11.79585	CROCCP3_ENSG00000080947
1	16840779	16840987	KD2-H4K8ac_peak_433	11.05467	RNU1-1_ENSG00000206652;RP4-798A10.7_ENSG00000261135
1	16887509	16888074	KD2-H4K8ac_peak_434	12.96622	
1	16939393	16939614	KD2-H4K8ac_peak_435	9.13332	NBPF1_ENSG00000219481
1	16939810	16940553	KD2-H4K8ac_peak_436	39.28297	NBPF1_ENSG00000219481
1	16970587	16972468	KD2-H4K8ac_peak_437	41.21368	CROCCP2_ENSG00000215908;MST1P2_ENSG00000186301
1	17026283	17026646	KD2-H4K8ac_peak_438	5.00796	
1	17030393	17030707	KD2-H4K8ac_peak_439	6.77963	
1	17034273	17034720	KD2-H4K8ac_peak_440	16.42728	
1	17090733	17091150	KD2-H4K8ac_peak_441	8.17557	
1	17222957	17223555	KD2-H4K8ac_peak_442	16.67374	RNU1-2_ENSG00000207005
1	17229485	17230038	KD2-H4K8ac_peak_443	6.2827	
1	17230553	17230838	KD2-H4K8ac_peak_444	5.59859	RP11-108M9.6_ENSG00000272426
1	17231116	17231673	KD2-H4K8ac_peak_445	12.45682	RP11-108M9.6_ENSG00000272426
1	17232522	17233042	KD2-H4K8ac_peak_446	7.86315	
1	17240133	17240347	KD2-H4K8ac_peak_447	11.19666	
1	17240719	17241412	KD2-H4K8ac_peak_448	33.50961	
1	17247689	17248966	KD2-H4K8ac_peak_449	14.43794	
1	17286718	17288089	KD2-H4K8ac_peak_450	15.53798	
1	17294049	17294333	KD2-H4K8ac_peak_451	5.30799	
1	17304619	17305204	KD2-H4K8ac_peak_452	10.05606	RP1-37C10.3_ENSG00000226526
1	17305525	17306637	KD2-H4K8ac_peak_453	15.13261	MFAP2_ENSG00000117122;RP1-37C10.3_ENSG00000226526
1	17306861	17307634	KD2-H4K8ac_peak_454	15.9754	MFAP2_ENSG00000117122
1	17380901	17381346	KD2-H4K8ac_peak_455	8.4716	SDHB_ENSG00000117118
1	17446140	17446592	KD2-H4K8ac_peak_456	6.66692	PADI2_ENSG00000117115
1	17764560	17765042	KD2-H4K8ac_peak_457	15.09202	
1	17765261	17765776	KD2-H4K8ac_peak_458	5.13615	RCC2_ENSG00000179051

1	17828813	17829257	KD2-H4K8ac_peak_459	6.56926	
1	17865648	17866715	KD2-H4K8ac_peak_460	25.10776	ARHGEF10L_ENSG00000074964
1	17867029	17867605	KD2-H4K8ac_peak_461	6.97919	ARHGEF10L_ENSG00000074964
1	17913783	17914330	KD2-H4K8ac_peak_462	6.20982	
1	17914902	17915262	KD2-H4K8ac_peak_463	9.1707	
1	17915768	17915967	KD2-H4K8ac_peak_464	4.62142	
1	17916684	17917006	KD2-H4K8ac_peak_465	5.08628	
1	17918239	17918601	KD2-H4K8ac_peak_466	4.98072	
1	17919786	17920484	KD2-H4K8ac_peak_467	13.58431	
1	17920918	17921302	KD2-H4K8ac_peak_468	6.98517	
1	17921868	17922293	KD2-H4K8ac_peak_469	7.36799	
1	17922881	17923624	KD2-H4K8ac_peak_470	7.45501	
1	17923868	17924344	KD2-H4K8ac_peak_471	8.30974	
1	17929088	17929638	KD2-H4K8ac_peak_472	10.05606	
1	17998386	17998615	KD2-H4K8ac_peak_473	8.26129	
1	18027378	18027625	KD2-H4K8ac_peak_474	6.83936	
1	18098987	18099239	KD2-H4K8ac_peak_475	5.57044	
1	18099646	18099839	KD2-H4K8ac_peak_476	5.51783	
1	18101949	18102185	KD2-H4K8ac_peak_477	4.66652	
1	18104275	18104581	KD2-H4K8ac_peak_478	4.66652	
1	18461064	18461356	KD2-H4K8ac_peak_479	9.36516	
1	18469220	18469664	KD2-H4K8ac_peak_480	4.06758	
1	18576110	18576472	KD2-H4K8ac_peak_481	6.16888	
1	19122197	19122397	KD2-H4K8ac_peak_482	4.42765	
1	19228796	19229211	KD2-H4K8ac_peak_483	6.65673	ALDH4A1_ENSG00000159423
1	19229513	19229723	KD2-H4K8ac_peak_484	7.74292	ALDH4A1_ENSG00000159423
1	19283452	19283786	KD2-H4K8ac_peak_485	5.14125	IFFO2_ENSG00000169991
1	19536333	19536719	KD2-H4K8ac_peak_486	9.11891	UBR4_ENSG00000127481;RP1-43E13.2_ENSG00000230424
1	19577821	19578805	KD2-H4K8ac_peak_487	25.8903	EMC1_ENSG00000127463;MRTO4_ENSG00000053372
1	19638189	19638536	KD2-H4K8ac_peak_488	7.07117	AKR7A2_ENSG00000053371;PQLC2_ENSG00000040487
1	19664946	19665327	KD2-H4K8ac_peak_489	9.73431	
1	19666260	19666557	KD2-H4K8ac_peak_490	6.7498	
1	19696100	19696372	KD2-H4K8ac_peak_491	10.01478	
1	19698162	19698558	KD2-H4K8ac_peak_492	8.52881	
1	19716861	19717239	KD2-H4K8ac_peak_493	4.44527	
1	19718231	19718467	KD2-H4K8ac_peak_494	7.37678	
1	19722355	19722736	KD2-H4K8ac_peak_495	6.25869	
1	19772407	19772618	KD2-H4K8ac_peak_496	5.88845	
1	19774060	19774771	KD2-H4K8ac_peak_497	6.65673	
1	19776013	19776503	KD2-H4K8ac_peak_498	5.14125	
1	19779823	19780067	KD2-H4K8ac_peak_499	5.45396	
1	19781488	19781682	KD2-H4K8ac_peak_500	4.91674	
1	19811472	19811981	KD2-H4K8ac_peak_501	10.29842	CAPZB_ENSG00000077549
1	19812262	19813031	KD2-H4K8ac_peak_502	7.96322	CAPZB_ENSG00000077549
1	19922771	19923130	KD2-H4K8ac_peak_503	6.40683	RP5-1056L3.1_ENSG00000235185;MINOS1-NBL1_ENSG00000270136;MINOS1_ENSG00000173436
1	19923522	19923979	KD2-H4K8ac_peak_504	13.13965	RP5-1056L3.1_ENSG00000235185;MINOS1-NBL1_ENSG00000270136;MINOS1_ENSG00000173436

1	19970326	19970651	KD2-H4K8ac_peak_505	11.66477	
1	19970898	19972301	KD2-H4K8ac_peak_506	9.63487	
1	19991096	19991781	KD2-H4K8ac_peak_507	11.22681	HTR6_ENSG00000158748
1	20125824	20126730	KD2-H4K8ac_peak_508	11.50346	TMCO4_ENSG00000162542
1	20208280	20208626	KD2-H4K8ac_peak_509	13.73635	OTUD3_ENSG00000169914
1	20208953	20209415	KD2-H4K8ac_peak_510	10.43145	OTUD3_ENSG00000169914
1	20478281	20478651	KD2-H4K8ac_peak_511	3.87368	
1	20539674	20539948	KD2-H4K8ac_peak_512	5.88845	
1	20634882	20635233	KD2-H4K8ac_peak_513	9.16531	
1	20767200	20767726	KD2-H4K8ac_peak_514	15.06766	
1	20799461	20799797	KD2-H4K8ac_peak_515	10.25088	
1	20801199	20801450	KD2-H4K8ac_peak_516	7.13615	
1	20802445	20803387	KD2-H4K8ac_peak_517	10.05606	
1	20803602	20804493	KD2-H4K8ac_peak_518	17.19888	
1	20810581	20810831	KD2-H4K8ac_peak_519	6.35843	
1	20812804	20813248	KD2-H4K8ac_peak_520	11.18097	CAMK2N1_ENSG00000162545
1	20820594	20820817	KD2-H4K8ac_peak_521	5.9696	
1	20834243	20835238	KD2-H4K8ac_peak_522	8.55271	MUL1_ENSG00000090432
1	20940506	20940720	KD2-H4K8ac_peak_523	6.66692	
1	20987499	20987826	KD2-H4K8ac_peak_524	7.28628	DDOST_ENSG00000244038
1	20988303	20988913	KD2-H4K8ac_peak_525	9.65086	DDOST_ENSG00000244038
1	21023256	21023741	KD2-H4K8ac_peak_526	4.52039	
1	21043837	21044722	KD2-H4K8ac_peak_527	10.87093	KIF17_ENSG00000117245
1	21112902	21113122	KD2-H4K8ac_peak_528	4.42765	HP1BP3_ENSG00000127483
1	21113391	21114330	KD2-H4K8ac_peak_529	13.73635	HP1BP3_ENSG00000127483
1	21350510	21350993	KD2-H4K8ac_peak_530	8.00457	
1	21502275	21502545	KD2-H4K8ac_peak_531	10.1591	EIF4G3_ENSG00000075151;AL031005.1_ENSG00000266867
1	21503608	21504175	KD2-H4K8ac_peak_532	21.60954	EIF4G3_ENSG00000075151;AL031005.1_ENSG00000266867
1	21546630	21547441	KD2-H4K8ac_peak_533	4.37809	
1	21581485	21581752	KD2-H4K8ac_peak_534	8.85813	
1	21588568	21588820	KD2-H4K8ac_peak_535	5.14125	
1	21605777	21606109	KD2-H4K8ac_peak_536	4.211	
1	21606557	21607365	KD2-H4K8ac_peak_537	10.16972	
1	21607568	21607788	KD2-H4K8ac_peak_538	4.75875	
1	21609815	21610145	KD2-H4K8ac_peak_539	13.09607	
1	21610392	21610807	KD2-H4K8ac_peak_540	16.64426	
1	21611029	21611423	KD2-H4K8ac_peak_541	10.53377	
1	21615735	21616304	KD2-H4K8ac_peak_542	10.33736	
1	21616638	21617172	KD2-H4K8ac_peak_543	12.40345	
1	21628618	21628849	KD2-H4K8ac_peak_544	6.16747	
1	21629259	21629845	KD2-H4K8ac_peak_545	5.87879	
1	21632251	21632472	KD2-H4K8ac_peak_546	3.97079	
1	21633318	21633593	KD2-H4K8ac_peak_547	4.82973	
1	21633791	21634139	KD2-H4K8ac_peak_548	4.68059	
1	21634583	21635324	KD2-H4K8ac_peak_549	11.58876	
1	21636422	21637271	KD2-H4K8ac_peak_550	12.2507	

1	21640732	21641210	KD2-H4K8ac_peak_551	5.82859	
1	21646018	21646341	KD2-H4K8ac_peak_552	10.0692	
1	21648002	21648256	KD2-H4K8ac_peak_553	7.13615	
1	21651233	21651433	KD2-H4K8ac_peak_554	4.42765	
1	21653407	21653613	KD2-H4K8ac_peak_555	4.56666	
1	21687726	21688000	KD2-H4K8ac_peak_556	7.44366	
1	21762973	21763655	KD2-H4K8ac_peak_557	14.66631	
1	21763850	21764242	KD2-H4K8ac_peak_558	6.30229	
1	21767088	21767296	KD2-H4K8ac_peak_559	3.75035	NBPF3_ENSG00000142794
1	21835276	21835641	KD2-H4K8ac_peak_560	5.88845	ALPL_ENSG00000162551
1	21835871	21836195	KD2-H4K8ac_peak_561	11.43863	ALPL_ENSG00000162551
1	21901183	21901448	KD2-H4K8ac_peak_562	4.44748	
1	21948523	21949020	KD2-H4K8ac_peak_563	6.25869	
1	21949527	21949747	KD2-H4K8ac_peak_564	8.04448	
1	21950556	21951421	KD2-H4K8ac_peak_565	7.49599	
1	21952565	21952993	KD2-H4K8ac_peak_566	6.05809	
1	21953459	21953758	KD2-H4K8ac_peak_567	9.43437	
1	21958480	21958706	KD2-H4K8ac_peak_568	5.14125	
1	21977692	21978109	KD2-H4K8ac_peak_569	6.25869	
1	21995336	21995815	KD2-H4K8ac_peak_570	8.4716	RAP1GAP_ENSG00000076864
1	22109183	22109564	KD2-H4K8ac_peak_571	11.05467	USP48_ENSG00000090686
1	22109819	22110041	KD2-H4K8ac_peak_572	9.36516	USP48_ENSG00000090686
1	22110404	22110792	KD2-H4K8ac_peak_573	4.42765	USP48_ENSG00000090686
1	22140900	22141481	KD2-H4K8ac_peak_574	9.20193	
1	22263307	22263506	KD2-H4K8ac_peak_575	5.57044	HSPG2_ENSG00000142798
1	22264014	22264335	KD2-H4K8ac_peak_576	10.96455	HSPG2_ENSG00000142798
1	22350634	22351401	KD2-H4K8ac_peak_577	15.22643	RP1-224A6.3_ENSG00000228397;LINC00339_ENSG00000218510
1	22352033	22352599	KD2-H4K8ac_peak_578	7.76329	RP1-224A6.3_ENSG00000228397;LINC00339_ENSG00000218510
1	22378471	22378947	KD2-H4K8ac_peak_579	9.6247	CDC42_ENSG00000070831
1	22379282	22379694	KD2-H4K8ac_peak_580	9.20193	CDC42_ENSG00000070831
1	22641915	22642229	KD2-H4K8ac_peak_581	6.65673	
1	22644379	22644649	KD2-H4K8ac_peak_582	6.92624	
1	22644865	22645061	KD2-H4K8ac_peak_583	11.05467	
1	22645411	22645648	KD2-H4K8ac_peak_584	8.4716	
1	22653031	22653250	KD2-H4K8ac_peak_585	8.55271	
1	22654965	22655780	KD2-H4K8ac_peak_586	11.51672	
1	22668674	22669740	KD2-H4K8ac_peak_587	8.79106	
1	22676613	22677377	KD2-H4K8ac_peak_588	12.00273	
1	22677650	22678447	KD2-H4K8ac_peak_589	10.34425	
1	22689459	22689857	KD2-H4K8ac_peak_590	10.77651	
1	22691866	22692242	KD2-H4K8ac_peak_591	10.06887	
1	22755589	22755811	KD2-H4K8ac_peak_592	5.835	
1	22773999	22774356	KD2-H4K8ac_peak_593	4.42765	
1	22777516	22778256	KD2-H4K8ac_peak_594	14.56955	ZBTB40_ENSG00000184677
1	22798234	22798443	KD2-H4K8ac_peak_595	6.56211	
1	22897224	22897449	KD2-H4K8ac_peak_596	4.42765	

1	22908526	22908758	KD2-H4K8ac_peak_597	9.65086	
1	22909990	22910415	KD2-H4K8ac_peak_598	4.06758	
1	22910649	22910864	KD2-H4K8ac_peak_599	8.30114	
1	23036230	23036743	KD2-H4K8ac_peak_600	7.27716	EPHB2_ENSG00000133216
1	23036995	23037261	KD2-H4K8ac_peak_601	21.00776	EPHB2_ENSG00000133216
1	23116378	23116792	KD2-H4K8ac_peak_602	4.8888	
1	23120734	23120946	KD2-H4K8ac_peak_603	4.86324	
1	23141901	23142367	KD2-H4K8ac_peak_604	7.95934	
1	23143022	23143246	KD2-H4K8ac_peak_605	7.47462	
1	23153582	23153975	KD2-H4K8ac_peak_606	15.13633	
1	23346054	23346525	KD2-H4K8ac_peak_607	8.4716	KDM1A_ENSG00000004487
1	23543488	23543885	KD2-H4K8ac_peak_608	5.45396	
1	23670183	23670766	KD2-H4K8ac_peak_609	12.88108	HNRNPR_ENSG00000125944
1	23671222	23671683	KD2-H4K8ac_peak_610	11.22681	HNRNPR_ENSG00000125944
1	23695099	23695752	KD2-H4K8ac_peak_611	12.99417	ZNF436_ENSG00000125945;C1orf213_ENSG00000249087;Y_RNA_ENSG00000201405
1	23810205	23810624	KD2-H4K8ac_peak_612	8.17902	ASAP3_ENSG00000088280
1	23823806	23824313	KD2-H4K8ac_peak_613	10.05606	
1	23856956	23857723	KD2-H4K8ac_peak_614	11.92978	E2F2_ENSG00000007968
1	23858008	23858227	KD2-H4K8ac_peak_615	9.95119	E2F2_ENSG00000007968
1	23858788	23859444	KD2-H4K8ac_peak_616	7.45501	
1	23880335	23881342	KD2-H4K8ac_peak_617	26.77453	
1	23881632	23882087	KD2-H4K8ac_peak_618	4.06758	
1	23884657	23885063	KD2-H4K8ac_peak_619	8.52559	
1	23894243	23894575	KD2-H4K8ac_peak_620	8.10216	
1	23894797	23895903	KD2-H4K8ac_peak_621	9.95759	
1	23899444	23899669	KD2-H4K8ac_peak_622	5.88845	
1	23907787	23908097	KD2-H4K8ac_peak_623	6.8836	MDS2_ENSG00000197880
1	23924749	23925106	KD2-H4K8ac_peak_624	7.07117	
1	23946053	23946409	KD2-H4K8ac_peak_625	7.07117	
1	23961001	23961556	KD2-H4K8ac_peak_626	19.94588	
1	24018348	24018661	KD2-H4K8ac_peak_627	13.92465	RPL11_ENSG00000142676
1	24069230	24070509	KD2-H4K8ac_peak_628	14.48084	TCEB3_ENSG00000011007
1	24104941	24105326	KD2-H4K8ac_peak_629	11.43353	RP5-886K2.3_ENSG00000236810;PITHD1_ENSG00000057757
1	24151472	24151907	KD2-H4K8ac_peak_630	22.05243	
1	24164577	24164784	KD2-H4K8ac_peak_631	5.25071	HMGCL_ENSG00000117305
1	24194964	24195367	KD2-H4K8ac_peak_632	7.13615	FUCA1_ENSG00000179163
1	24231833	24232186	KD2-H4K8ac_peak_633	4.42765	
1	24437786	24437980	KD2-H4K8ac_peak_634	8.10216	MYOM3_ENSG00000142661
1	24513448	24513829	KD2-H4K8ac_peak_635	20.59319	IFNLR1_ENSG00000185436
1	24514032	24514295	KD2-H4K8ac_peak_636	13.09607	IFNLR1_ENSG00000185436
1	24522343	24522584	KD2-H4K8ac_peak_637	4.37709	
1	24667898	24668170	KD2-H4K8ac_peak_638	5.23853	
1	24740407	24740932	KD2-H4K8ac_peak_639	13.09607	
1	24741695	24742032	KD2-H4K8ac_peak_640	6.66692	NIPAL3_ENSG00000001461
1	24828915	24829243	KD2-H4K8ac_peak_641	6.65673	RCAN3_ENSG00000117602
1	24878492	24878713	KD2-H4K8ac_peak_642	8.00457	

1	24886056	24886277	KD2-H4K8ac_peak_643	7.76329	
1	24969101	24969327	KD2-H4K8ac_peak_644	10.53377	
1	25020059	25020733	KD2-H4K8ac_peak_645	4.86324	
1	25057047	25057298	KD2-H4K8ac_peak_646	11.89502	
1	25061886	25062143	KD2-H4K8ac_peak_647	6.72334	
1	25062763	25063226	KD2-H4K8ac_peak_648	5.88845	
1	25197472	25197710	KD2-H4K8ac_peak_649	5.71749	
1	25240051	25241442	KD2-H4K8ac_peak_650	9.6247	
1	25381659	25381910	KD2-H4K8ac_peak_651	4.42765	
1	25391494	25392495	KD2-H4K8ac_peak_652	5.9696	
1	25558610	25559408	KD2-H4K8ac_peak_653	11.43353	SYF2_ENSG00000117614
1	25565490	25565872	KD2-H4K8ac_peak_654	6.98292	
1	25573621	25573885	KD2-H4K8ac_peak_655	15.57998	RP3-465N24.6_ENSG00000272432
1	25574146	25574545	KD2-H4K8ac_peak_656	11.3394	RP3-465N24.6_ENSG00000272432
1	25664854	25665310	KD2-H4K8ac_peak_657	17.43705	C1orf63_ENSG00000117616;RP11-335G20.7_ENSG00000259984;TMEM50A_ENSG00000183726
1	25756384	25756677	KD2-H4K8ac_peak_658	8.30974	RHCE_ENSG00000188672;TMEM57_ENSG00000204178
1	25757509	25757781	KD2-H4K8ac_peak_659	6.84261	RHCE_ENSG00000188672;TMEM57_ENSG00000204178
1	25928550	25928762	KD2-H4K8ac_peak_660	6.30229	
1	25942499	25942960	KD2-H4K8ac_peak_661	16.12525	MAN1C1_ENSG00000117643
1	25985653	25985847	KD2-H4K8ac_peak_662	4.42765	RP1-187B23.1_ENSG00000233478
1	26017122	26017314	KD2-H4K8ac_peak_663	6.40683	
1	26053777	26054285	KD2-H4K8ac_peak_664	7.98152	
1	26091739	26092210	KD2-H4K8ac_peak_665	3.72574	
1	26097473	26097671	KD2-H4K8ac_peak_666	8.79106	
1	26098356	26098631	KD2-H4K8ac_peak_667	8.65175	
1	26125870	26126063	KD2-H4K8ac_peak_668	6.80488	SEPN1_ENSG00000162430
1	26126257	26126515	KD2-H4K8ac_peak_669	8.86099	SEPN1_ENSG00000162430
1	26145254	26145797	KD2-H4K8ac_peak_670	7.94441	RP1-317E23.3_ENSG00000228172;MTFR1L_ENSG00000117640
1	26146035	26146983	KD2-H4K8ac_peak_671	9.20193	RP1-317E23.3_ENSG00000228172;MTFR1L_ENSG00000117640;AL020996.1_ENSG00000223474
1	26185700	26185900	KD2-H4K8ac_peak_672	5.54647	AUNIP_ENSG00000127423;RP1-125I3.2_ENSG00000236528
1	26201221	26202863	KD2-H4K8ac_peak_673	12.66412	
1	26232391	26232793	KD2-H4K8ac_peak_674	6.99815	STMN1_ENSG00000117632;MIR3917_ENSG00000264021
1	26233063	26233935	KD2-H4K8ac_peak_675	20.53952	STMN1_ENSG00000117632;MIR3917_ENSG00000264021
1	26254669	26254862	KD2-H4K8ac_peak_676	4.86324	
1	26323939	26324466	KD2-H4K8ac_peak_677	14.98671	PAFAH2_ENSG00000158006
1	26324927	26325624	KD2-H4K8ac_peak_678	7.69294	PAFAH2_ENSG00000158006
1	26348367	26348634	KD2-H4K8ac_peak_679	6.60096	
1	26361610	26361825	KD2-H4K8ac_peak_680	6.80488	
1	26437193	26438112	KD2-H4K8ac_peak_681	17.8868	PDIK1L_ENSG00000175087
1	26482762	26483384	KD2-H4K8ac_peak_682	12.74171	
1	26487768	26488316	KD2-H4K8ac_peak_683	5.9696	
1	26490667	26491483	KD2-H4K8ac_peak_684	10.43145	
1	26495662	26496245	KD2-H4K8ac_peak_685	7.36799	ZNF593_ENSG00000142684
1	26496493	26496807	KD2-H4K8ac_peak_686	10.19949	ZNF593_ENSG00000142684
1	26559708	26560302	KD2-H4K8ac_peak_687	7.74292	CEP85_ENSG00000130695
1	26617632	26617845	KD2-H4K8ac_peak_688	8.26945	

1	26632897	26633141	KD2-H4K8ac_peak_689	7.70289	
1	26633380	26633720	KD2-H4K8ac_peak_690	8.45238	
1	26647914	26648148	KD2-H4K8ac_peak_691	4.86324	
1	26662978	26663330	KD2-H4K8ac_peak_692	6.25869	
1	26686519	26686738	KD2-H4K8ac_peak_693	5.14125	
1	26735875	26736296	KD2-H4K8ac_peak_694	6.16888	LIN28A_ENSG00000131914
1	26758359	26758568	KD2-H4K8ac_peak_695	9.34989	DHDDS_ENSG00000117682
1	26798337	26798681	KD2-H4K8ac_peak_696	5.75757	HMGN2_ENSG00000198830
1	26799022	26799491	KD2-H4K8ac_peak_697	16.57619	HMGN2_ENSG00000198830
1	26826401	26827315	KD2-H4K8ac_peak_698	8.4716	
1	26827570	26828040	KD2-H4K8ac_peak_699	11.89502	
1	26855698	26856088	KD2-H4K8ac_peak_700	16.32216	RPS6KA1_ENSG00000117676
1	26947140	26947396	KD2-H4K8ac_peak_701	13.31016	
1	26947619	26948322	KD2-H4K8ac_peak_702	7.47462	
1	27022180	27022394	KD2-H4K8ac_peak_703	15.20126	ARID1A_ENSG00000117713
1	27022813	27024113	KD2-H4K8ac_peak_704	16.67374	ARID1A_ENSG00000117713
1	27113105	27113428	KD2-H4K8ac_peak_705	9.96941	PIGV_ENSG00000060642
1	27113836	27114214	KD2-H4K8ac_peak_706	8.4716	PIGV_ENSG00000060642
1	27114451	27115069	KD2-H4K8ac_peak_707	6.66359	PIGV_ENSG00000060642
1	27136753	27136982	KD2-H4K8ac_peak_708	10.0746	
1	27152077	27153042	KD2-H4K8ac_peak_709	8.4716	ZDHHC18_ENSG00000204160
1	27153369	27153609	KD2-H4K8ac_peak_710	5.61377	ZDHHC18_ENSG00000204160
1	27191044	27191475	KD2-H4K8ac_peak_711	3.97079	
1	27191904	27192144	KD2-H4K8ac_peak_712	7.0754	
1	27192423	27192798	KD2-H4K8ac_peak_713	9.402	
1	27216379	27216748	KD2-H4K8ac_peak_714	16.61426	GPN2_ENSG00000142751
1	27216973	27217414	KD2-H4K8ac_peak_715	4.91674	GPN2_ENSG00000142751
1	27226372	27227624	KD2-H4K8ac_peak_716	13.34404	GPATCH3_ENSG00000198746;NUDC_ENSG00000090273
1	27240421	27240917	KD2-H4K8ac_peak_717	6.05809	NR0B2_ENSG00000131910
1	27338965	27339216	KD2-H4K8ac_peak_718	5.75757	FAM46B_ENSG00000158246
1	27339828	27340057	KD2-H4K8ac_peak_719	7.33266	FAM46B_ENSG00000158246
1	27397274	27397733	KD2-H4K8ac_peak_720	6.94255	
1	27425612	27425850	KD2-H4K8ac_peak_721	12.16946	
1	27426157	27426924	KD2-H4K8ac_peak_722	10.27185	
1	27481206	27481576	KD2-H4K8ac_peak_723	14.8083	
1	27560376	27560786	KD2-H4K8ac_peak_724	7.36799	RP11-40H20.4_ENSG00000224311;WDTC1_ENSG00000142784
1	27561122	27561574	KD2-H4K8ac_peak_725	16.73922	RP11-40H20.4_ENSG00000224311;WDTC1_ENSG00000142784
1	27624928	27625135	KD2-H4K8ac_peak_726	7.47462	
1	27625412	27626246	KD2-H4K8ac_peak_727	9.58924	
1	27626593	27627872	KD2-H4K8ac_peak_728	16.25886	
1	27644517	27645050	KD2-H4K8ac_peak_729	10.0692	
1	27648798	27649400	KD2-H4K8ac_peak_730	10.66719	TMEM222_ENSG00000186501
1	27675717	27676924	KD2-H4K8ac_peak_731	25.35008	
1	27683788	27684122	KD2-H4K8ac_peak_732	6.05809	
1	27692519	27693439	KD2-H4K8ac_peak_733	11.05467	MAP3K6_ENSG00000142733
1	27718495	27718823	KD2-H4K8ac_peak_734	6.25869	GPR3_ENSG00000181773

1	27719165	27719474	KD2-H4K8ac_peak_735	9.63487	GPR3_ENSG00000181773
1	27725005	27725201	KD2-H4K8ac_peak_736	6.05809	
1	27816102	27816637	KD2-H4K8ac_peak_737	13.73635	WASF2_ENSG00000158195
1	27816979	27817508	KD2-H4K8ac_peak_738	11.70505	WASF2_ENSG00000158195
1	27830555	27830838	KD2-H4K8ac_peak_739	8.02571	
1	27831162	27831591	KD2-H4K8ac_peak_740	17.25154	
1	27845675	27846147	KD2-H4K8ac_peak_741	6.48713	
1	27849207	27849983	KD2-H4K8ac_peak_742	9.08816	
1	27853861	27854953	KD2-H4K8ac_peak_743	8.85813	
1	27862409	27862662	KD2-H4K8ac_peak_744	10.08707	
1	27887573	27888012	KD2-H4K8ac_peak_745	7.36799	
1	27888332	27888710	KD2-H4K8ac_peak_746	9.57101	
1	27986088	27986424	KD2-H4K8ac_peak_747	6.97919	
1	27988883	27989679	KD2-H4K8ac_peak_748	8.82427	RP11-288L9.1_ENSG00000233975
1	27998803	27999173	KD2-H4K8ac_peak_749	7.1181	IFI6_ENSG00000126709
1	28051420	28052011	KD2-H4K8ac_peak_750	10.86378	FAM76A_ENSG00000009780
1	28156683	28157123	KD2-H4K8ac_peak_751	10.46874	PPP1R8_ENSG00000117751
1	28157407	28157960	KD2-H4K8ac_peak_752	7.07117	PPP1R8_ENSG00000117751
1	28198531	28198740	KD2-H4K8ac_peak_753	6.48242	THEMIS2_ENSG00000130775
1	28240720	28241037	KD2-H4K8ac_peak_754	10.19949	RPA2_ENSG00000117748
1	28241732	28242022	KD2-H4K8ac_peak_755	7.40111	RPA2_ENSG00000117748
1	28414695	28415076	KD2-H4K8ac_peak_756	7.67504	EYA3_ENSG00000158161
1	28431611	28431847	KD2-H4K8ac_peak_757	7.9076	
1	28500353	28500869	KD2-H4K8ac_peak_758	15.09202	
1	28559231	28559473	KD2-H4K8ac_peak_759	12.30509	DNAJC8_ENSG00000126698
1	28562091	28562392	KD2-H4K8ac_peak_760	6.66692	ATPIF1_ENSG00000130770
1	28575573	28575888	KD2-H4K8ac_peak_761	10.25088	
1	28654635	28655350	KD2-H4K8ac_peak_762	10.05606	MED18_ENSG00000130772
1	28695040	28695351	KD2-H4K8ac_peak_763	4.06758	PHACTR4_ENSG00000204138
1	28695633	28696071	KD2-H4K8ac_peak_764	5.54647	PHACTR4_ENSG00000204138
1	28696333	28696818	KD2-H4K8ac_peak_765	14.83804	PHACTR4_ENSG00000204138
1	28831918	28832218	KD2-H4K8ac_peak_766	6.65673	RCC1_ENSG00000180198;SNHG3_ENSG00000242125
1	28833027	28833249	KD2-H4K8ac_peak_767	4.77079	RCC1_ENSG00000180198;SNHG3_ENSG00000242125
1	28834226	28834429	KD2-H4K8ac_peak_768	6.83936	SNORA73B_ENSG00000200087
1	28879648	28880081	KD2-H4K8ac_peak_769	14.98936	TRNAU1AP_ENSG00000180098
1	28908089	28908291	KD2-H4K8ac_peak_770	9.63487	
1	28908547	28908867	KD2-H4K8ac_peak_771	6.67092	SNHG12_ENSG00000197989
1	28918832	28919129	KD2-H4K8ac_peak_772	5.45396	RAB42_ENSG00000188060
1	28919387	28919603	KD2-H4K8ac_peak_773	6.99815	RAB42_ENSG00000188060
1	28969801	28970648	KD2-H4K8ac_peak_774	10.86378	TAF12_ENSG00000120656;RP11-442N24__B.1_ENSG00000229388
1	28974004	28974380	KD2-H4K8ac_peak_775	8.10216	RNU11_ENSG00000270103
1	28975546	28976127	KD2-H4K8ac_peak_776	8.17902	RNU11_ENSG00000270103
1	28994011	28994996	KD2-H4K8ac_peak_777	8.17902	GMEB1_ENSG00000162419
1	28995597	28996261	KD2-H4K8ac_peak_778	10.19949	GMEB1_ENSG00000162419
1	29062772	29063211	KD2-H4K8ac_peak_779	12.82393	YTHDF2_ENSG00000198492
1	29063528	29064104	KD2-H4K8ac_peak_780	21.11201	YTHDF2_ENSG00000198492

1	29111926	29112139	KD2-H4K8ac_peak_781	6.4095	
1	29138110	29138388	KD2-H4K8ac_peak_782	6.30229	OPRD1_ENSG00000116329
1	29138584	29139604	KD2-H4K8ac_peak_783	12.57865	OPRD1_ENSG00000116329
1	29142119	29142363	KD2-H4K8ac_peak_784	6.65673	
1	29148861	29149078	KD2-H4K8ac_peak_785	5.14125	
1	29149957	29150192	KD2-H4K8ac_peak_786	6.0073	
1	29151375	29151589	KD2-H4K8ac_peak_787	5.47943	
1	29157668	29158379	KD2-H4K8ac_peak_788	10.08707	
1	29158818	29159281	KD2-H4K8ac_peak_789	6.0073	
1	29161361	29161764	KD2-H4K8ac_peak_790	11.92978	
1	29162191	29162465	KD2-H4K8ac_peak_791	7.36799	
1	29163098	29163456	KD2-H4K8ac_peak_792	4.64388	
1	29213727	29214419	KD2-H4K8ac_peak_793	6.66692	EPB41_ENSG00000159023
1	29240547	29240797	KD2-H4K8ac_peak_794	6.66692	
1	29448165	29448443	KD2-H4K8ac_peak_795	4.54006	
1	29450891	29451755	KD2-H4K8ac_peak_796	6.66359	TMEM200B_ENSG00000253304
1	29507726	29508391	KD2-H4K8ac_peak_797	26.15943	SRSF4_ENSG00000116350
1	29509090	29509309	KD2-H4K8ac_peak_798	8.59579	SRSF4_ENSG00000116350
1	29557170	29557375	KD2-H4K8ac_peak_799	9.63487	MECR_ENSG00000116353
1	29563156	29563393	KD2-H4K8ac_peak_800	10.41994	PTPRU_ENSG00000060656
1	29564785	29565107	KD2-H4K8ac_peak_801	10.50587	
1	29709634	29710096	KD2-H4K8ac_peak_802	6.51023	
1	29710385	29710580	KD2-H4K8ac_peak_803	4.01115	
1	29710954	29711177	KD2-H4K8ac_peak_804	6.40683	
1	29721031	29721623	KD2-H4K8ac_peak_805	5.88845	
1	29754084	29754338	KD2-H4K8ac_peak_806	9.32235	
1	29763485	29763737	KD2-H4K8ac_peak_807	4.01115	
1	29768504	29769165	KD2-H4K8ac_peak_808	6.94954	
1	29776217	29776563	KD2-H4K8ac_peak_809	9.66321	
1	29785299	29785501	KD2-H4K8ac_peak_810	6.20982	
1	29788625	29788902	KD2-H4K8ac_peak_811	6.40683	
1	29794403	29794654	KD2-H4K8ac_peak_812	9.19305	
1	29794931	29795402	KD2-H4K8ac_peak_813	13.1258	
1	29795743	29796701	KD2-H4K8ac_peak_814	18.41184	
1	29796933	29798345	KD2-H4K8ac_peak_815	29.57597	
1	29798762	29799157	KD2-H4K8ac_peak_816	8.79106	
1	29800027	29800797	KD2-H4K8ac_peak_817	13.81781	
1	29801393	29802311	KD2-H4K8ac_peak_818	11.50346	
1	29803945	29804302	KD2-H4K8ac_peak_819	5.47943	
1	29804924	29805122	KD2-H4K8ac_peak_820	4.42765	
1	29816921	29817288	KD2-H4K8ac_peak_821	9.06703	
1	29841104	29841312	KD2-H4K8ac_peak_822	7.79898	
1	30029726	30030151	KD2-H4K8ac_peak_823	3.7063	
1	30065821	30066084	KD2-H4K8ac_peak_824	5.82799	
1	30104831	30105493	KD2-H4K8ac_peak_825	10.05606	
1	30105971	30106446	KD2-H4K8ac_peak_826	6.99815	

1	30107561	30108014	KD2-H4K8ac_peak_827	6.97919	
1	30119018	30119436	KD2-H4K8ac_peak_828	6.44277	
1	30124276	30124606	KD2-H4K8ac_peak_829	7.67504	
1	30157835	30158037	KD2-H4K8ac_peak_830	8.4084	
1	30161715	30162816	KD2-H4K8ac_peak_831	9.72244	
1	30171062	30171457	KD2-H4K8ac_peak_832	5.30799	
1	30180558	30181636	KD2-H4K8ac_peak_833	14.68449	RP4-656G21.1_ENSG00000228176
1	30181851	30183003	KD2-H4K8ac_peak_834	9.34195	RP4-656G21.1_ENSG00000228176
1	30228372	30228620	KD2-H4K8ac_peak_835	6.0605	
1	30239921	30240285	KD2-H4K8ac_peak_836	5.43522	
1	30274447	30274847	KD2-H4K8ac_peak_837	7.76329	
1	30558252	30558709	KD2-H4K8ac_peak_838	5.13893	
1	30559133	30559342	KD2-H4K8ac_peak_839	11.60333	
1	30562771	30563016	KD2-H4K8ac_peak_840	6.30229	
1	30604867	30605223	KD2-H4K8ac_peak_841	5.14125	
1	30605533	30605830	KD2-H4K8ac_peak_842	11.89502	
1	30606290	30606634	KD2-H4K8ac_peak_843	6.99815	
1	30852105	30853282	KD2-H4K8ac_peak_844	8.55271	
1	31123919	31124144	KD2-H4K8ac_peak_845	6.1846	
1	31164980	31165389	KD2-H4K8ac_peak_846	4.42765	
1	31190596	31190792	KD2-H4K8ac_peak_847	7.167	MATN1-AS1_ENSG00000186056
1	31191677	31191942	KD2-H4K8ac_peak_848	8.23274	MATN1-AS1_ENSG00000186056
1	31221390	31221729	KD2-H4K8ac_peak_849	4.211	
1	31255419	31255839	KD2-H4K8ac_peak_850	8.59579	
1	31296903	31297293	KD2-H4K8ac_peak_851	12.88108	RP1-65J11.1_ENSG00000223382
1	31361032	31361435	KD2-H4K8ac_peak_852	8.10216	
1	31381834	31382279	KD2-H4K8ac_peak_853	14.8083	SDC3_ENSG00000162512
1	31386034	31386263	KD2-H4K8ac_peak_854	6.2781	
1	31627557	31627763	KD2-H4K8ac_peak_855	24.48793	
1	31635191	31635651	KD2-H4K8ac_peak_856	11.05407	
1	31711964	31713011	KD2-H4K8ac_peak_857	7.07117	NKAIN1_ENSG00000084628
1	31769291	31769572	KD2-H4K8ac_peak_858	9.90914	SNRNP40_ENSG00000060688;ZCCHC17_ENSG00000121766
1	31770030	31770324	KD2-H4K8ac_peak_859	5.20998	SNRNP40_ENSG00000060688;ZCCHC17_ENSG00000121766
1	31846001	31846270	KD2-H4K8ac_peak_860	6.83936	
1	31886263	31887120	KD2-H4K8ac_peak_861	15.01306	
1	32014130	32014501	KD2-H4K8ac_peak_862	10.46264	
1	32040566	32041077	KD2-H4K8ac_peak_863	6.24747	RP11-73M7.1_ENSG00000229167
1	32079577	32080082	KD2-H4K8ac_peak_864	10.0692	
1	32083037	32083253	KD2-H4K8ac_peak_865	5.04237	HCRTR1_ENSG00000121764
1	32083457	32083856	KD2-H4K8ac_peak_866	12.06346	HCRTR1_ENSG00000121764
1	32091788	32094509	KD2-H4K8ac_peak_867	19.9503	
1	32095521	32095748	KD2-H4K8ac_peak_868	10.30284	
1	32109769	32110453	KD2-H4K8ac_peak_869	14.12659	PEF1_ENSG00000162517;RP11-73M7.6_ENSG00000235790;RP11-73M7.9_ENSG00000264078
1	32110950	32111233	KD2-H4K8ac_peak_870	5.54647	PEF1_ENSG00000162517;RP11-73M7.9_ENSG00000264078
1	32166481	32166735	KD2-H4K8ac_peak_871	7.1181	
1	32167691	32168125	KD2-H4K8ac_peak_872	5.5051	

1	32169389	32170195	KD2-H4K8ac_peak_873	7.76329	COL16A1_ENSG00000084636
1	32199281	32199540	KD2-H4K8ac_peak_874	6.9556	
1	32226285	32226754	KD2-H4K8ac_peak_875	8.69797	
1	32234874	32235077	KD2-H4K8ac_peak_876	9.34195	
1	32235558	32236618	KD2-H4K8ac_peak_877	10.19949	
1	32241395	32241620	KD2-H4K8ac_peak_878	4.98072	
1	32242199	32242598	KD2-H4K8ac_peak_879	4.67342	
1	32246457	32248090	KD2-H4K8ac_peak_880	16.57619	
1	32248448	32248903	KD2-H4K8ac_peak_881	3.90565	
1	32249361	32249674	KD2-H4K8ac_peak_882	4.54006	
1	32250596	32251326	KD2-H4K8ac_peak_883	6.25869	
1	32253626	32254098	KD2-H4K8ac_peak_884	4.67342	RP11-84A19.3_ENSG00000254545
1	32279512	32280319	KD2-H4K8ac_peak_885	11.03331	
1	32403574	32403854	KD2-H4K8ac_peak_886	7.08474	
1	32404151	32404719	KD2-H4K8ac_peak_887	15.13261	
1	32420331	32420645	KD2-H4K8ac_peak_888	6.78668	
1	32478177	32478521	KD2-H4K8ac_peak_889	5.14125	KHDRBS1_ENSG00000121774
1	32478730	32479060	KD2-H4K8ac_peak_890	11.03212	KHDRBS1_ENSG00000121774
1	32479499	32479958	KD2-H4K8ac_peak_891	19.28826	KHDRBS1_ENSG00000121774
1	32538108	32538338	KD2-H4K8ac_peak_892	9.01262	RP11-277A4.4_ENSG00000203325;TMEM39B_ENSG00000121775
1	32538600	32538795	KD2-H4K8ac_peak_893	8.1235	RP11-277A4.4_ENSG00000203325;TMEM39B_ENSG00000121775
1	32670887	32671642	KD2-H4K8ac_peak_894	8.52559	RP4-622L5.7_ENSG00000224066;IQCC_ENSG00000160051
1	32681478	32681701	KD2-H4K8ac_peak_895	4.77079	
1	32687516	32687835	KD2-H4K8ac_peak_896	6.85193	TMEM234_ENSG00000160055;EIF3I_ENSG00000084623
1	32688161	32688453	KD2-H4K8ac_peak_897	6.13909	TMEM234_ENSG00000160055;EIF3I_ENSG00000084623
1	32705526	32706645	KD2-H4K8ac_peak_898	10.19949	MTMR9LP_ENSG00000220785
1	32757693	32758006	KD2-H4K8ac_peak_899	12.56039	HDAC1_ENSG00000116478
1	32801062	32801631	KD2-H4K8ac_peak_900	15.08752	MARCKSL1_ENSG00000175130
1	32817310	32817526	KD2-H4K8ac_peak_901	5.75757	TSSK3_ENSG00000162526
1	32827876	32828080	KD2-H4K8ac_peak_902	4.76709	
1	32859626	32859919	KD2-H4K8ac_peak_903	5.53033	BSDC1_ENSG00000160058
1	32892500	32892785	KD2-H4K8ac_peak_904	5.54647	LRRC37A12P_ENSG00000229259
1	32960371	32960707	KD2-H4K8ac_peak_905	7.94441	
1	32966148	32966695	KD2-H4K8ac_peak_906	6.99815	
1	32966943	32967136	KD2-H4K8ac_peak_907	6.66692	
1	33004794	33005047	KD2-H4K8ac_peak_908	9.32235	ZBTB8A_ENSG00000160062
1	33116277	33116486	KD2-H4K8ac_peak_909	7.36799	ZBTB8OS_ENSG00000176261;RBBP4_ENSG00000162521
1	33116812	33117414	KD2-H4K8ac_peak_910	28.26254	ZBTB8OS_ENSG00000176261;RBBP4_ENSG00000162521
1	33168394	33168860	KD2-H4K8ac_peak_911	9.26408	SYNC_ENSG00000162520
1	33177260	33177557	KD2-H4K8ac_peak_912	6.00226	
1	33178753	33179015	KD2-H4K8ac_peak_913	6.40683	
1	33191074	33191277	KD2-H4K8ac_peak_914	7.11925	RP11-114B7.6_ENSG00000224409
1	33206803	33207248	KD2-H4K8ac_peak_915	5.59859	KIAA1522_ENSG00000162522
1	33207573	33208084	KD2-H4K8ac_peak_916	10.43145	KIAA1522_ENSG00000162522
1	33231272	33231498	KD2-H4K8ac_peak_917	9.20193	
1	33231936	33232153	KD2-H4K8ac_peak_918	6.24114	

1	33282582	33282894	KD2-H4K8ac_peak_919	19.88671	YARS_ENSG00000134684;S100PBP_ENSG00000116497
1	33283339	33284083	KD2-H4K8ac_peak_920	14.43794	YARS_ENSG00000134684;S100PBP_ENSG00000116497
1	33342284	33342521	KD2-H4K8ac_peak_921	4.56666	
1	33358831	33359156	KD2-H4K8ac_peak_922	10.96455	
1	33366602	33367373	KD2-H4K8ac_peak_923	6.84261	TMEM54_ENSG00000121900
1	33430206	33430440	KD2-H4K8ac_peak_924	11.05467	RNF19B_ENSG00000116514
1	33438488	33439319	KD2-H4K8ac_peak_925	12.84642	FKSG48_ENSG00000267885
1	33502056	33502433	KD2-H4K8ac_peak_926	9.31907	
1	33503150	33503472	KD2-H4K8ac_peak_927	10.8368	
1	33592383	33592796	KD2-H4K8ac_peak_928	7.47462	
1	33619755	33619956	KD2-H4K8ac_peak_929	7.993	
1	33626530	33627635	KD2-H4K8ac_peak_930	9.36516	
1	33720578	33721092	KD2-H4K8ac_peak_931	18.05111	
1	33721343	33722266	KD2-H4K8ac_peak_932	15.61313	ZNF362_ENSG00000160094
1	33815008	33815385	KD2-H4K8ac_peak_933	12.00273	RP11-415J8.5_ENSG00000233246
1	33815697	33816240	KD2-H4K8ac_peak_934	16.8435	RP11-415J8.5_ENSG00000233246
1	33829148	33829520	KD2-H4K8ac_peak_935	15.20126	
1	33938326	33938782	KD2-H4K8ac_peak_936	4.8485	ZSCAN20_ENSG00000121903
1	34284588	34284865	KD2-H4K8ac_peak_937	5.14125	
1	34285842	34286053	KD2-H4K8ac_peak_938	6.61376	
1	34522687	34523251	KD2-H4K8ac_peak_939	9.19305	
1	35162941	35163162	KD2-H4K8ac_peak_940	6.2781	
1	35324798	35325257	KD2-H4K8ac_peak_941	7.76329	SMIM12_ENSG00000163866
1	35326028	35326425	KD2-H4K8ac_peak_942	7.44366	SMIM12_ENSG00000163866
1	35450583	35451282	KD2-H4K8ac_peak_943	7.28628	RP11-244H3.1_ENSG00000241014;ZMYM6NB_ENSG00000243749
1	35496895	35497533	KD2-H4K8ac_peak_944	11.89502	RP11-244H3.4_ENSG00000271741;ZMYM6_ENSG00000163867
1	35528790	35529041	KD2-H4K8ac_peak_945	7.51935	
1	35544552	35544754	KD2-H4K8ac_peak_946	5.53033	
1	35657855	35658703	KD2-H4K8ac_peak_947	12.96152	SFPQ_ENSG00000116560
1	35659433	35659635	KD2-H4K8ac_peak_948	7.70289	SFPQ_ENSG00000116560
1	35734376	35734803	KD2-H4K8ac_peak_949	17.55491	ZMYM4_ENSG00000146463
1	36022841	36023257	KD2-H4K8ac_peak_950	10.28547	KIAA0319L_ENSG00000142687;NCDN_ENSG00000020129
1	36023878	36024156	KD2-H4K8ac_peak_951	4.52039	KIAA0319L_ENSG00000142687;NCDN_ENSG00000020129
1	36038946	36039141	KD2-H4K8ac_peak_952	7.44324	TFAP2E_ENSG00000116819
1	36108105	36108401	KD2-H4K8ac_peak_953	9.9363	PSMB2_ENSG00000126067
1	36172899	36174528	KD2-H4K8ac_peak_954	20.97251	
1	36184075	36184942	KD2-H4K8ac_peak_955	14.8083	C1orf216_ENSG00000142686
1	36233630	36233922	KD2-H4K8ac_peak_956	4.42765	
1	36234855	36235378	KD2-H4K8ac_peak_957	10.86378	CLSPN_ENSG00000092853
1	36272829	36274380	KD2-H4K8ac_peak_958	15.82362	AGO4_ENSG00000134698
1	36348348	36348552	KD2-H4K8ac_peak_959	7.69287	
1	36348878	36349574	KD2-H4K8ac_peak_960	8.82366	
1	36396267	36396625	KD2-H4K8ac_peak_961	9.63487	RP4-789D17.5_ENSG00000271914;AGO3_ENSG00000126070
1	36396921	36397267	KD2-H4K8ac_peak_962	11.24702	AGO3_ENSG00000126070
1	36550477	36550676	KD2-H4K8ac_peak_963	5.30799	TEKT2_ENSG00000092850
1	36553966	36555010	KD2-H4K8ac_peak_964	15.2334	ADPRHL2_ENSG00000116863

1	36564961	36565198	KD2-H4K8ac_peak_965	5.51783	
1	36580374	36581148	KD2-H4K8ac_peak_966	10.19949	
1	36590487	36591337	KD2-H4K8ac_peak_967	8.79106	COL8A2_ENSG00000171812
1	36614795	36614995	KD2-H4K8ac_peak_968	8.02571	TRAPPC3_ENSG00000054116
1	36615904	36616496	KD2-H4K8ac_peak_969	9.36516	TRAPPC3_ENSG00000054116
1	36620839	36622602	KD2-H4K8ac_peak_970	23.14382	MAP7D1_ENSG00000116871
1	36653372	36653823	KD2-H4K8ac_peak_971	11.2938	
1	36668874	36669318	KD2-H4K8ac_peak_972	6.99815	
1	36688872	36689098	KD2-H4K8ac_peak_973	7.79898	THRAP3_ENSG00000054118
1	36771287	36771618	KD2-H4K8ac_peak_974	4.80593	SH3D21_ENSG00000214193
1	36772182	36772384	KD2-H4K8ac_peak_975	3.90565	SH3D21_ENSG00000214193
1	36816896	36817252	KD2-H4K8ac_peak_976	5.20908	
1	36839839	36840087	KD2-H4K8ac_peak_977	7.25989	
1	36852302	36852643	KD2-H4K8ac_peak_978	4.68806	STK40_ENSG00000196182
1	36863128	36863477	KD2-H4K8ac_peak_979	6.25869	LSM10_ENSG00000181817
1	36929764	36930350	KD2-H4K8ac_peak_980	9.95119	MRPS15_ENSG00000116898
1	36941161	36941685	KD2-H4K8ac_peak_981	6.66692	
1	36947320	36947595	KD2-H4K8ac_peak_982	7.76569	
1	36948162	36948811	KD2-H4K8ac_peak_983	9.65086	CSF3R_ENSG00000119535
1	37233911	37234142	KD2-H4K8ac_peak_984	5.40356	
1	37253339	37253534	KD2-H4K8ac_peak_985	9.06703	
1	37322769	37323239	KD2-H4K8ac_peak_986	11.56469	
1	37331108	37331907	KD2-H4K8ac_peak_987	12.46264	
1	37939781	37940293	KD2-H4K8ac_peak_988	9.11031	LINC01137_ENSG00000233621;ZC3H12A_ENSG00000163874
1	37942811	37943263	KD2-H4K8ac_peak_989	6.98292	
1	37980090	37980282	KD2-H4K8ac_peak_990	6.0605	MEAF6_ENSG00000163875
1	37980545	37980928	KD2-H4K8ac_peak_991	6.76298	MEAF6_ENSG00000163875
1	38019479	38020323	KD2-H4K8ac_peak_992	16.8435	SNIP1_ENSG00000163877
1	38155793	38156069	KD2-H4K8ac_peak_993	9.1707	
1	38156421	38156638	KD2-H4K8ac_peak_994	10.46264	
1	38258922	38259953	KD2-H4K8ac_peak_995	14.275	MANEAL_ENSG00000185090
1	38273112	38273812	KD2-H4K8ac_peak_996	7.28628	YRDC_ENSG00000196449;C1orf122_ENSG00000197982
1	38274080	38274698	KD2-H4K8ac_peak_997	7.26089	YRDC_ENSG00000196449
1	38325460	38325900	KD2-H4K8ac_peak_998	16.57619	MTF1_ENSG00000188786;RP11-109P14.10_ENSG00000230955
1	38397166	38397385	KD2-H4K8ac_peak_999	12.06346	
1	38412849	38413441	KD2-H4K8ac_peak_1000	9.55735	INPP5B_ENSG00000204084
1	38456156	38456418	KD2-H4K8ac_peak_1001	5.88845	SF3A3_ENSG00000183431;RNU6-510P_ENSG00000212541
1	38461524	38461760	KD2-H4K8ac_peak_1002	5.00214	
1	38465221	38465518	KD2-H4K8ac_peak_1003	5.88845	
1	38470866	38471188	KD2-H4K8ac_peak_1004	14.15075	FHL3_ENSG00000183386
1	38477803	38478071	KD2-H4K8ac_peak_1005	12.68903	
1	38478390	38478939	KD2-H4K8ac_peak_1006	18.29344	
1	38495327	38495613	KD2-H4K8ac_peak_1007	4.98072	
1	39456461	39456692	KD2-H4K8ac_peak_1008	10.46264	AKIRIN1_ENSG00000174574
1	39491145	39491440	KD2-H4K8ac_peak_1009	7.76329	NDUFS5_ENSG00000168653
1	39492174	39492605	KD2-H4K8ac_peak_1010	6.83936	NDUFS5_ENSG00000168653

1	39546621	39546832	KD2-H4K8ac_peak_1011	8.25022	MACF1_ENSG00000127603
1	39555768	39556058	KD2-H4K8ac_peak_1012	5.04237	
1	39570406	39572030	KD2-H4K8ac_peak_1013	8.85813	
1	39873784	39874285	KD2-H4K8ac_peak_1014	9.06703	
1	39874949	39875259	KD2-H4K8ac_peak_1015	11.90231	KIAA0754_ENSG00000255103
1	40024943	40025255	KD2-H4K8ac_peak_1016	13.21293	PPIEL_ENSG00000243970
1	40025579	40025913	KD2-H4K8ac_peak_1017	4.211	
1	40041716	40042379	KD2-H4K8ac_peak_1018	8.86099	PABPC4_ENSG00000090621
1	40090654	40090864	KD2-H4K8ac_peak_1019	7.07512	
1	40091859	40092472	KD2-H4K8ac_peak_1020	6.66692	
1	40112307	40112575	KD2-H4K8ac_peak_1021	5.71749	
1	40120637	40121055	KD2-H4K8ac_peak_1022	5.28072	
1	40122451	40124167	KD2-H4K8ac_peak_1023	14.00658	
1	40156776	40157063	KD2-H4K8ac_peak_1024	9.08816	HPCAL4_ENSG00000116983;PPIE_ENSG00000084072
1	40157437	40158279	KD2-H4K8ac_peak_1025	7.40314	HPCAL4_ENSG00000116983;PPIE_ENSG00000084072
1	40204624	40204994	KD2-H4K8ac_peak_1026	5.9696	
1	40254758	40255201	KD2-H4K8ac_peak_1027	7.07117	BMP8B_ENSG00000116985;RP1-118J21.25_ENSG00000261798
1	40292396	40292674	KD2-H4K8ac_peak_1028	4.73064	
1	40348821	40349144	KD2-H4K8ac_peak_1029	16.32216	TRIT1_ENSG00000043514
1	40349364	40349657	KD2-H4K8ac_peak_1030	11.02863	TRIT1_ENSG00000043514
1	40367237	40367496	KD2-H4K8ac_peak_1031	8.30974	MYCL_ENSG00000116990
1	40367772	40368120	KD2-H4K8ac_peak_1032	8.55271	MYCL_ENSG00000116990
1	40506473	40506789	KD2-H4K8ac_peak_1033	9.36516	CAP1_ENSG00000131236
1	40562712	40563519	KD2-H4K8ac_peak_1034	10.19949	PPT1_ENSG00000131238
1	40626350	40626733	KD2-H4K8ac_peak_1035	4.77079	RLF_ENSG00000117000
1	40723125	40723579	KD2-H4K8ac_peak_1036	9.1707	RP1-39G22.7_ENSG00000259943;ZMPSTE24_ENSG00000084073
1	40723998	40724458	KD2-H4K8ac_peak_1037	8.52559	RP1-39G22.7_ENSG00000259943;ZMPSTE24_ENSG00000084073
1	40780556	40782361	KD2-H4K8ac_peak_1038	15.81711	
1	40915403	40916924	KD2-H4K8ac_peak_1039	14.98936	ZFP69B_ENSG00000187801
1	40974060	40974889	KD2-H4K8ac_peak_1040	12.84642	RP11-656D10.3_ENSG00000238287;EXO5_ENSG00000164002
1	40996772	40997065	KD2-H4K8ac_peak_1041	9.72244	ZNF684_ENSG00000117010
1	41120572	41120833	KD2-H4K8ac_peak_1042	9.15329	
1	41128576	41129063	KD2-H4K8ac_peak_1043	5.53033	
1	41134425	41134656	KD2-H4K8ac_peak_1044	11.27037	
1	41157392	41158235	KD2-H4K8ac_peak_1045	12.57865	NFYC-AS1_ENSG00000272145;NFYC_ENSG00000066136
1	41249411	41249729	KD2-H4K8ac_peak_1046	7.40111	KCNQ4_ENSG00000117013
1	41264685	41264890	KD2-H4K8ac_peak_1047	5.57044	
1	41268290	41268524	KD2-H4K8ac_peak_1048	8.37231	
1	41349793	41350369	KD2-H4K8ac_peak_1049	9.11031	
1	41414285	41414551	KD2-H4K8ac_peak_1050	10.86378	
1	41444516	41446171	KD2-H4K8ac_peak_1051	12.82393	CTPS1_ENSG00000171793
1	41707101	41707786	KD2-H4K8ac_peak_1052	20.58734	SCMH1_ENSG00000010803;RP11-399E6.1_ENSG00000235358
1	41707980	41708523	KD2-H4K8ac_peak_1053	20.18584	SCMH1_ENSG00000010803;RP11-399E6.1_ENSG00000235358
1	41823180	41823480	KD2-H4K8ac_peak_1054	5.42867	
1	41831591	41832710	KD2-H4K8ac_peak_1055	20.90492	
1	41847438	41848673	KD2-H4K8ac_peak_1056	12.84642	

1	41897530	41898020	KD2-H4K8ac_peak_1057	13.12608	
1	41898687	41899198	KD2-H4K8ac_peak_1058	8.82366	
1	41908189	41908691	KD2-H4K8ac_peak_1059	18.29344	
1	41921105	41921572	KD2-H4K8ac_peak_1060	12.3187	
1	41940369	41940693	KD2-H4K8ac_peak_1061	8.4716	
1	41948124	41948413	KD2-H4K8ac_peak_1062	7.96322	
1	41967250	41967481	KD2-H4K8ac_peak_1063	5.30799	
1	41968369	41968863	KD2-H4K8ac_peak_1064	5.75757	
1	41970504	41970753	KD2-H4K8ac_peak_1065	5.18553	
1	41972780	41973224	KD2-H4K8ac_peak_1066	5.82799	
1	41981979	41982613	KD2-H4K8ac_peak_1067	22.39508	
1	41987440	41987736	KD2-H4K8ac_peak_1068	5.14125	
1	42078573	42078875	KD2-H4K8ac_peak_1069	6.25869	
1	42128161	42128409	KD2-H4K8ac_peak_1070	4.211	
1	42178414	42178611	KD2-H4K8ac_peak_1071	4.54006	
1	42179404	42179612	KD2-H4K8ac_peak_1072	7.98621	
1	42181784	42182224	KD2-H4K8ac_peak_1073	5.59859	
1	42199252	42199448	KD2-H4K8ac_peak_1074	5.15777	
1	42500970	42501259	KD2-H4K8ac_peak_1075	13.81781	HIVEP3_ENSG00000127124
1	42501856	42502098	KD2-H4K8ac_peak_1076	12.42761	HIVEP3_ENSG00000127124
1	42800254	42800834	KD2-H4K8ac_peak_1077	8.55271	FOXJ3_ENSG00000198815
1	42801110	42801635	KD2-H4K8ac_peak_1078	13.80319	FOXJ3_ENSG00000198815
1	42845849	42846266	KD2-H4K8ac_peak_1079	9.6247	RIMKLA_ENSG00000177181
1	42846535	42847052	KD2-H4K8ac_peak_1080	8.52559	RIMKLA_ENSG00000177181
1	42920730	42921087	KD2-H4K8ac_peak_1081	5.57044	ZMYND12_ENSG00000066185;PPCS_ENSG00000127125
1	42921422	42922610	KD2-H4K8ac_peak_1082	13.48341	ZMYND12_ENSG00000066185;PPCS_ENSG00000127125
1	43124139	43124685	KD2-H4K8ac_peak_1083	14.45402	PPIH_ENSG00000171960
1	43147419	43147777	KD2-H4K8ac_peak_1084	5.14125	YBX1_ENSG00000065978
1	43148150	43149036	KD2-H4K8ac_peak_1085	12.3187	YBX1_ENSG00000065978
1	43222340	43222721	KD2-H4K8ac_peak_1086	7.12754	
1	43233013	43233217	KD2-H4K8ac_peak_1087	8.07129	LEPRE1_ENSG00000117385;C1orf50_ENSG00000164008
1	43281983	43282604	KD2-H4K8ac_peak_1088	7.40111	CCDC23_ENSG00000177868;ERMAP_ENSG00000164010
1	43312340	43312742	KD2-H4K8ac_peak_1089	21.75906	RP11-342M1.3_ENSG00000228192;ZNF691_ENSG00000164011
1	43390155	43390507	KD2-H4K8ac_peak_1090	15.59949	
1	43390719	43391068	KD2-H4K8ac_peak_1091	8.1233	
1	43419072	43419386	KD2-H4K8ac_peak_1092	4.98072	
1	43425238	43425518	KD2-H4K8ac_peak_1093	5.45396	SLC2A1_ENSG00000117394;SLC2A1-AS1_ENSG00000227533
1	43450199	43450492	KD2-H4K8ac_peak_1094	6.99815	
1	43450870	43451146	KD2-H4K8ac_peak_1095	3.75035	
1	43459402	43459601	KD2-H4K8ac_peak_1096	7.0983	
1	43471547	43472337	KD2-H4K8ac_peak_1097	16.32216	
1	43524227	43524812	KD2-H4K8ac_peak_1098	7.47462	
1	43527344	43527557	KD2-H4K8ac_peak_1099	6.16888	
1	43533683	43534431	KD2-H4K8ac_peak_1100	12.57297	
1	43534671	43534941	KD2-H4K8ac_peak_1101	8.10216	
1	43637690	43637936	KD2-H4K8ac_peak_1102	8.4716	WDR65_ENSG00000243710

1	43814234	43815252	KD2-H4K8ac_peak_1103	17.70837	
1	43824110	43824309	KD2-H4K8ac_peak_1104	5.30799	RP1-92O14.3_ENSG00000234694;CDC20_ENSG00000117399
1	43832841	43833175	KD2-H4K8ac_peak_1105	4.67342	ELOVL1_ENSG00000066322
1	43833398	43833641	KD2-H4K8ac_peak_1106	14.15075	ELOVL1_ENSG00000066322
1	43833899	43834190	KD2-H4K8ac_peak_1107	6.25869	ELOVL1_ENSG00000066322
1	43855064	43855402	KD2-H4K8ac_peak_1108	5.9696	MED8_ENSG00000159479;SZT2_ENSG00000198198
1	43855601	43855871	KD2-H4K8ac_peak_1109	15.52826	MED8_ENSG00000159479;SZT2_ENSG00000198198
1	43919126	43920268	KD2-H4K8ac_peak_1110	10.05606	HYI_ENSG00000178922;HYI-AS1_ENSG00000229348
1	43996692	43996885	KD2-H4K8ac_peak_1111	5.61067	
1	44031202	44031437	KD2-H4K8ac_peak_1112	5.82799	
1	44116216	44116502	KD2-H4K8ac_peak_1113	5.88845	KDM4A_ENSG00000066135
1	44173268	44173498	KD2-H4K8ac_peak_1114	11.00004	KDM4A-AS1_ENSG00000236200
1	44300142	44300395	KD2-H4K8ac_peak_1115	4.20044	
1	44378413	44378702	KD2-H4K8ac_peak_1116	9.11031	
1	44401502	44402054	KD2-H4K8ac_peak_1117	12.70983	
1	44412352	44412566	KD2-H4K8ac_peak_1118	15.58442	RP11-7O11.3_ENSG00000237950;IPO13_ENSG00000117408
1	44435760	44436194	KD2-H4K8ac_peak_1119	8.85813	DPH2_ENSG00000132768
1	44439956	44440475	KD2-H4K8ac_peak_1120	18.68066	ATP6V0B_ENSG00000117410
1	44440696	44440919	KD2-H4K8ac_peak_1121	6.90231	ATP6V0B_ENSG00000117410
1	44445113	44445638	KD2-H4K8ac_peak_1122	13.80019	B4GALT2_ENSG00000117411
1	44456941	44458252	KD2-H4K8ac_peak_1123	15.66774	CCDC24_ENSG00000159214
1	44461178	44461881	KD2-H4K8ac_peak_1124	12.17278	
1	44462350	44463012	KD2-H4K8ac_peak_1125	10.53377	
1	44495437	44496511	KD2-H4K8ac_peak_1126	16.8435	SLC6A9_ENSG00000196517
1	44496950	44497572	KD2-H4K8ac_peak_1127	6.90231	SLC6A9_ENSG00000196517
1	44501902	44502763	KD2-H4K8ac_peak_1128	15.77481	RP5-1198O20.4_ENSG00000230615
1	44513226	44513465	KD2-H4K8ac_peak_1129	10.99365	
1	44678410	44678785	KD2-H4K8ac_peak_1130	7.47462	DMAP1_ENSG00000178028
1	44679131	44679564	KD2-H4K8ac_peak_1131	13.3695	DMAP1_ENSG00000178028
1	44821268	44821923	KD2-H4K8ac_peak_1132	10.39154	ERI3_ENSG00000117419
1	45097056	45098313	KD2-H4K8ac_peak_1133	16.04168	
1	45241350	45241651	KD2-H4K8ac_peak_1134	5.9696	RP11-269F19.2_ENSG00000225721;RPS8_ENSG00000142937;SNORD55_ENSG00000264294;SNORD46_ENSG00000200913
1	45251990	45252279	KD2-H4K8ac_peak_1135	8.1233	
1	45265236	45265470	KD2-H4K8ac_peak_1136	11.03331	PLK3_ENSG00000173846
1	45265664	45265898	KD2-H4K8ac_peak_1137	14.66631	PLK3_ENSG00000173846
1	45266094	45266356	KD2-H4K8ac_peak_1138	8.52559	PLK3_ENSG00000173846
1	45273700	45274144	KD2-H4K8ac_peak_1139	8.14467	TCTEX1D4_ENSG00000188396;BTBD19_ENSG00000222009
1	45278734	45279831	KD2-H4K8ac_peak_1140	22.65017	
1	45280083	45280828	KD2-H4K8ac_peak_1141	7.47462	
1	45285742	45286069	KD2-H4K8ac_peak_1142	4.211	RNU5E-6P_ENSG00000202444
1	45308595	45308865	KD2-H4K8ac_peak_1143	9.16531	PTCH2_ENSG00000117425;RP5-882O7.1_ENSG00000226499
1	45411908	45412241	KD2-H4K8ac_peak_1144	8.23274	
1	45414568	45414897	KD2-H4K8ac_peak_1145	7.29632	
1	45452470	45452743	KD2-H4K8ac_peak_1146	6.40297	EIF2B3_ENSG00000070785
1	45671780	45672152	KD2-H4K8ac_peak_1147	7.07117	
1	45769585	45770377	KD2-H4K8ac_peak_1148	11.24702	

1	45792257	45793142	KD2-H4K8ac_peak_1149	14.00658	HPDL_ENSG00000186603
1	45805862	45806063	KD2-H4K8ac_peak_1150	5.59859	MUTYH_ENSG00000132781;TOE1_ENSG00000132773
1	45966130	45966396	KD2-H4K8ac_peak_1151	4.42765	CCDC163P_ENSG00000236624;MMACHC_ENSG00000132763
1	45987242	45987494	KD2-H4K8ac_peak_1152	8.55271	
1	46016191	46016954	KD2-H4K8ac_peak_1153	11.86082	AKR1A1_ENSG00000117448
1	46049794	46050099	KD2-H4K8ac_peak_1154	9.06369	NASP_ENSG00000132780
1	46152422	46152683	KD2-H4K8ac_peak_1155	8.86099	TMEM69_ENSG00000159596
1	46153450	46153730	KD2-H4K8ac_peak_1156	6.05809	GPBP1L1_ENSG00000159592;TMEM69_ENSG00000159596
1	46154667	46154885	KD2-H4K8ac_peak_1157	8.12706	GPBP1L1_ENSG00000159592
1	46201560	46201787	KD2-H4K8ac_peak_1158	6.40683	
1	46215482	46216312	KD2-H4K8ac_peak_1159	16.71655	IPP_ENSG00000197429
1	46216706	46217371	KD2-H4K8ac_peak_1160	12.26331	IPP_ENSG00000197429
1	46268164	46268700	KD2-H4K8ac_peak_1161	8.1718	
1	46598387	46598583	KD2-H4K8ac_peak_1162	12.30509	
1	46598914	46599380	KD2-H4K8ac_peak_1163	7.76104	RP4-533D7.5_ENSG00000227857
1	46663782	46664776	KD2-H4K8ac_peak_1164	11.3394	
1	46668758	46669281	KD2-H4K8ac_peak_1165	14.56955	LURAP1_ENSG00000171357
1	46712644	46712983	KD2-H4K8ac_peak_1166	5.43522	RAD54L_ENSG00000085999
1	46766921	46768936	KD2-H4K8ac_peak_1167	15.66774	LRRC41_ENSG00000132128;UQCRH_ENSG00000173660
1	46805316	46805658	KD2-H4K8ac_peak_1168	9.48012	NSUN4_ENSG00000117481
1	46805899	46806812	KD2-H4K8ac_peak_1169	16.72302	NSUN4_ENSG00000117481
1	46871944	46872160	KD2-H4K8ac_peak_1170	7.47462	
1	47082333	47082681	KD2-H4K8ac_peak_1171	5.9696	MKNK1_ENSG00000079277;MOB3C_ENSG00000142961
1	47082962	47083329	KD2-H4K8ac_peak_1172	6.66692	MKNK1_ENSG00000079277;MOB3C_ENSG00000142961
1	47133335	47134014	KD2-H4K8ac_peak_1173	6.99815	TEX38_ENSG00000186118
1	47134267	47134956	KD2-H4K8ac_peak_1174	7.13615	TEX38_ENSG00000186118
1	47184010	47185144	KD2-H4K8ac_peak_1175	5.30799	EFCAB14_ENSG00000159658
1	47780409	47781300	KD2-H4K8ac_peak_1176	9.31907	STIL_ENSG00000123473
1	47799547	47799853	KD2-H4K8ac_peak_1177	18.29344	CMPK1_ENSG00000162368
1	47800086	47800399	KD2-H4K8ac_peak_1178	13.44374	CMPK1_ENSG00000162368
1	47973982	47974415	KD2-H4K8ac_peak_1179	5.13615	
1	48104184	48104454	KD2-H4K8ac_peak_1180	4.66652	
1	48154001	48154198	KD2-H4K8ac_peak_1181	5.20908	RP11-543D5.1_ENSG00000227947
1	48313983	48314239	KD2-H4K8ac_peak_1182	5.20998	
1	48315244	48315461	KD2-H4K8ac_peak_1183	5.88845	
1	48382002	48382277	KD2-H4K8ac_peak_1184	6.40683	
1	48937384	48937749	KD2-H4K8ac_peak_1185	5.88845	SPATA6_ENSG00000132122
1	48938145	48938572	KD2-H4K8ac_peak_1186	8.5781	SPATA6_ENSG00000132122
1	49242924	49243599	KD2-H4K8ac_peak_1187	6.40683	BEND5_ENSG00000162373
1	49353932	49354264	KD2-H4K8ac_peak_1188	7.29632	
1	49383768	49384235	KD2-H4K8ac_peak_1189	6.56211	
1	49514308	49514584	KD2-H4K8ac_peak_1190	5.20908	RP11-141A19.1_ENSG00000229846
1	50513788	50514015	KD2-H4K8ac_peak_1191	9.95759	ELAVL4_ENSG00000162374
1	50571326	50571567	KD2-H4K8ac_peak_1192	5.25071	
1	50574020	50574362	KD2-H4K8ac_peak_1193	6.16888	
1	50575220	50575745	KD2-H4K8ac_peak_1194	5.25071	

1	50576051	50576461	KD2-H4K8ac_peak_1195	12.42547	
1	50717182	50717472	KD2-H4K8ac_peak_1196	9.51625	
1	50730600	50731531	KD2-H4K8ac_peak_1197	12.68903	
1	50737393	50737758	KD2-H4K8ac_peak_1198	5.32397	
1	50739432	50739712	KD2-H4K8ac_peak_1199	5.13615	
1	50741565	50742140	KD2-H4K8ac_peak_1200	14.66631	
1	50742543	50742966	KD2-H4K8ac_peak_1201	9.36516	
1	50743541	50743771	KD2-H4K8ac_peak_1202	7.13615	
1	51433924	51434148	KD2-H4K8ac_peak_1203	9.20193	
1	51434658	51435209	KD2-H4K8ac_peak_1204	4.86324	
1	51442632	51444017	KD2-H4K8ac_peak_1205	23.27293	
1	51762542	51763102	KD2-H4K8ac_peak_1206	11.90231	
1	51791620	51791928	KD2-H4K8ac_peak_1207	6.44277	
1	51795858	51796852	KD2-H4K8ac_peak_1208	20.78681	RP11-275F13.1_ENSG00000261664
1	51797134	51797367	KD2-H4K8ac_peak_1209	7.36799	
1	51887977	51888383	KD2-H4K8ac_peak_1210	4.37708	
1	52081854	52082255	KD2-H4K8ac_peak_1211	5.54647	
1	52194895	52195246	KD2-H4K8ac_peak_1212	11.70505	
1	52195545	52195809	KD2-H4K8ac_peak_1213	10.96455	
1	52343659	52344357	KD2-H4K8ac_peak_1214	7.36799	NRD1_ENSG00000078618
1	52344968	52345761	KD2-H4K8ac_peak_1215	15.83877	NRD1_ENSG00000078618
1	52499080	52499434	KD2-H4K8ac_peak_1216	8.26945	KTI12_ENSG00000198841;RP11-91A18.4_ENSG00000223390
1	52521996	52522615	KD2-H4K8ac_peak_1217	23.22963	TXNDC12_ENSG00000117862;BTF3L4_ENSG00000134717
1	52607258	52607459	KD2-H4K8ac_peak_1218	5.51783	ZFYVE9_ENSG00000157077
1	52607832	52608337	KD2-H4K8ac_peak_1219	5.4779	ZFYVE9_ENSG00000157077
1	52831447	52831764	KD2-H4K8ac_peak_1220	11.80283	CC2D1B_ENSG00000154222
1	52834119	52834612	KD2-H4K8ac_peak_1221	5.9696	PLA2G12AP1_ENSG00000224680
1	53018353	53018706	KD2-H4K8ac_peak_1222	12.14738	ZCCHC11_ENSG00000134744
1	53019280	53019694	KD2-H4K8ac_peak_1223	19.33018	RP11-25O10.2_ENSG00000272371;ZCCHC11_ENSG00000134744
1	53067113	53067487	KD2-H4K8ac_peak_1224	5.54647	GPX7_ENSG00000116157
1	53067777	53068602	KD2-H4K8ac_peak_1225	16.71655	GPX7_ENSG00000116157
1	53098602	53099360	KD2-H4K8ac_peak_1226	11.22681	FAM159A_ENSG00000182183
1	53128803	53129067	KD2-H4K8ac_peak_1227	4.77079	
1	53163371	53164448	KD2-H4K8ac_peak_1228	10.43145	COA7_ENSG00000162377
1	53192194	53192509	KD2-H4K8ac_peak_1229	14.74047	ZYG11B_ENSG00000162378
1	53308581	53309014	KD2-H4K8ac_peak_1230	6.0605	ZYG11A_ENSG00000203995
1	53527304	53527662	KD2-H4K8ac_peak_1231	8.30974	PODN_ENSG00000174348
1	53527861	53528480	KD2-H4K8ac_peak_1232	7.07117	PODN_ENSG00000174348
1	53587204	53587620	KD2-H4K8ac_peak_1233	5.82799	
1	53661799	53662015	KD2-H4K8ac_peak_1234	10.96455	CPT2_ENSG00000157184
1	53686082	53686599	KD2-H4K8ac_peak_1235	9.11891	C1orf123_ENSG00000162384;RP5-1024G6.7_ENSG00000259818
1	53703909	53705087	KD2-H4K8ac_peak_1236	5.75757	MAGOH_ENSG00000162385;RP5-1024G6.5_ENSG00000226754
1	53780071	53780550	KD2-H4K8ac_peak_1237	6.18308	
1	53781487	53781752	KD2-H4K8ac_peak_1238	7.20567	
1	53782092	53782459	KD2-H4K8ac_peak_1239	8.36432	
1	53782899	53784042	KD2-H4K8ac_peak_1240	10.59979	

1	53784912	53785329	KD2-H4K8ac_peak_1241	5.54647	
1	53786358	53786606	KD2-H4K8ac_peak_1242	5.70596	
1	53791324	53791845	KD2-H4K8ac_peak_1243	8.30974	
1	53793323	53793687	KD2-H4K8ac_peak_1244	7.79741	LRP8_ENSG00000157193;RP4-784A16.5_ENSG00000225675
1	53793893	53794371	KD2-H4K8ac_peak_1245	8.98093	LRP8_ENSG00000157193;RP4-784A16.5_ENSG00000225675
1	53794695	53795184	KD2-H4K8ac_peak_1246	7.47462	LRP8_ENSG00000157193;RP4-784A16.5_ENSG00000225675
1	53833292	53833564	KD2-H4K8ac_peak_1247	8.85813	RP11-117D22.2_ENSG00000230138
1	53834317	53834712	KD2-H4K8ac_peak_1248	9.61895	RP11-117D22.2_ENSG00000230138
1	53835891	53836174	KD2-H4K8ac_peak_1249	5.08628	
1	53874928	53875133	KD2-H4K8ac_peak_1250	8.23274	
1	53928517	53928856	KD2-H4K8ac_peak_1251	5.20908	
1	53976937	53977298	KD2-H4K8ac_peak_1252	8.55271	
1	53978376	53978876	KD2-H4K8ac_peak_1253	11.22681	
1	53991630	53993376	KD2-H4K8ac_peak_1254	11.11797	
1	54009520	54009719	KD2-H4K8ac_peak_1255	4.42765	
1	54048809	54049318	KD2-H4K8ac_peak_1256	11.47476	
1	54049599	54050276	KD2-H4K8ac_peak_1257	6.2781	
1	54051389	54051581	KD2-H4K8ac_peak_1258	7.27716	
1	54055717	54056015	KD2-H4K8ac_peak_1259	7.09076	
1	54134895	54135384	KD2-H4K8ac_peak_1260	4.86324	
1	54150725	54151054	KD2-H4K8ac_peak_1261	5.29555	
1	54303504	54304093	KD2-H4K8ac_peak_1262	9.63487	NDC1_ENSG00000058804
1	54411716	54411941	KD2-H4K8ac_peak_1263	14.66631	HSPB11_ENSG00000081870;LRRC42_ENSG00000116212
1	54494085	54494516	KD2-H4K8ac_peak_1264	7.12754	RP4-758J24.5_ENSG00000272091
1	54519356	54519647	KD2-H4K8ac_peak_1265	8.10216	TMEM59_ENSG00000116209;TCEANC2_ENSG00000116205;MIR4781_ENSG00000264208
1	54586923	54587264	KD2-H4K8ac_peak_1266	8.30974	
1	54601506	54601720	KD2-H4K8ac_peak_1267	6.50927	
1	54665890	54666215	KD2-H4K8ac_peak_1268	13.21979	RP11-446E24.4_ENSG00000256407;CYB5RL_ENSG00000215883
1	54694325	54694663	KD2-H4K8ac_peak_1269	8.83582	
1	54752305	54752584	KD2-H4K8ac_peak_1270	4.86324	
1	54762929	54763428	KD2-H4K8ac_peak_1271	12.17789	
1	54763650	54764109	KD2-H4K8ac_peak_1272	6.30229	
1	54775649	54775971	KD2-H4K8ac_peak_1273	8.33649	
1	54792177	54792791	KD2-H4K8ac_peak_1274	6.25869	
1	54793772	54794018	KD2-H4K8ac_peak_1275	7.43041	
1	54821407	54821734	KD2-H4K8ac_peak_1276	5.80056	
1	54871010	54871388	KD2-H4K8ac_peak_1277	6.66692	
1	54872905	54873260	KD2-H4K8ac_peak_1278	4.42765	
1	54940355	54941706	KD2-H4K8ac_peak_1279	7.07117	
1	54942248	54942588	KD2-H4K8ac_peak_1280	6.66692	
1	54946035	54946412	KD2-H4K8ac_peak_1281	5.88845	
1	54952516	54952748	KD2-H4K8ac_peak_1282	7.52168	
1	54953344	54953982	KD2-H4K8ac_peak_1283	14.43704	
1	54954519	54954784	KD2-H4K8ac_peak_1284	10.43145	
1	54956640	54956861	KD2-H4K8ac_peak_1285	5.77265	
1	54964519	54965707	KD2-H4K8ac_peak_1286	11.89502	

1	55007532	55008268	KD2-H4K8ac_peak_1287	15.50901	ACOT11_ENSG00000162390
1	55009523	55009725	KD2-H4K8ac_peak_1288	8.55271	
1	55024427	55024673	KD2-H4K8ac_peak_1289	4.77079	
1	55180625	55181384	KD2-H4K8ac_peak_1290	12.00273	TTC4_ENSG00000243725
1	55181599	55181848	KD2-H4K8ac_peak_1291	5.75757	TTC4_ENSG00000243725
1	55229677	55230126	KD2-H4K8ac_peak_1292	6.25869	PARS2_ENSG00000162396
1	55258044	55258337	KD2-H4K8ac_peak_1293	6.85193	RP11-67L3.2_ENSG00000237453
1	55276103	55276514	KD2-H4K8ac_peak_1294	4.01115	
1	55352265	55352617	KD2-H4K8ac_peak_1295	5.29555	DHCR24_ENSG00000116133;RP11-67L3.5_ENSG00000242396;RP11-67L3.4_ENSG00000233203
1	55353162	55353709	KD2-H4K8ac_peak_1296	5.01002	DHCR24_ENSG00000116133;RP11-67L3.5_ENSG00000242396;RP11-67L3.4_ENSG00000233203
1	55415964	55416387	KD2-H4K8ac_peak_1297	7.36799	
1	55416760	55416968	KD2-H4K8ac_peak_1298	5.88845	
1	55446253	55446656	KD2-H4K8ac_peak_1299	8.59579	TMEM61_ENSG00000143001
1	55453981	55454217	KD2-H4K8ac_peak_1300	4.67342	
1	55487974	55488324	KD2-H4K8ac_peak_1301	10.05606	
1	55514991	55515372	KD2-H4K8ac_peak_1302	5.88845	
1	55680119	55681561	KD2-H4K8ac_peak_1303	18.37277	USP24_ENSG00000162402
1	55750923	55751389	KD2-H4K8ac_peak_1304	10.21502	
1	55775828	55776261	KD2-H4K8ac_peak_1305	7.38933	
1	55777503	55777951	KD2-H4K8ac_peak_1306	4.77079	
1	55844164	55844534	KD2-H4K8ac_peak_1307	5.57044	
1	55988162	55988377	KD2-H4K8ac_peak_1308	5.04237	
1	56020456	56021440	KD2-H4K8ac_peak_1309	5.9696	
1	56022402	56022609	KD2-H4K8ac_peak_1310	6.56926	
1	56024179	56024676	KD2-H4K8ac_peak_1311	8.30974	
1	56106609	56106990	KD2-H4K8ac_peak_1312	10.46264	
1	56108851	56109161	KD2-H4K8ac_peak_1313	4.28372	
1	56152145	56152739	KD2-H4K8ac_peak_1314	7.27716	
1	56156555	56157321	KD2-H4K8ac_peak_1315	8.1233	
1	56159063	56159300	KD2-H4K8ac_peak_1316	5.43522	
1	56160324	56160574	KD2-H4K8ac_peak_1317	5.14125	
1	56161143	56161377	KD2-H4K8ac_peak_1318	4.95402	
1	56232301	56232610	KD2-H4K8ac_peak_1319	6.40683	
1	56233074	56233694	KD2-H4K8ac_peak_1320	5.20908	
1	56234856	56235207	KD2-H4K8ac_peak_1321	4.77079	
1	56235519	56235973	KD2-H4K8ac_peak_1322	5.25071	
1	56383345	56383602	KD2-H4K8ac_peak_1323	4.37708	
1	56404232	56404782	KD2-H4K8ac_peak_1324	4.77079	PIGQP1_ENSG00000227104
1	56559399	56559624	KD2-H4K8ac_peak_1325	6.09229	
1	56826670	56826870	KD2-H4K8ac_peak_1326	9.23578	
1	56838966	56839298	KD2-H4K8ac_peak_1327	8.1233	
1	56841078	56841298	KD2-H4K8ac_peak_1328	4.77079	
1	56842450	56842709	KD2-H4K8ac_peak_1329	7.70289	AC119674.1_ENSG00000223307
1	56843380	56843829	KD2-H4K8ac_peak_1330	9.11031	AC119674.1_ENSG00000223307
1	56917729	56917946	KD2-H4K8ac_peak_1331	5.82799	
1	56964232	56964570	KD2-H4K8ac_peak_1332	6.25869	

1	57043277	57043536	KD2-H4K8ac_peak_1333	7.27716	
1	57043800	57045299	KD2-H4K8ac_peak_1334	10.05606	
1	57110363	57111277	KD2-H4K8ac_peak_1335	10.72656	PPAP2B_ENSG00000162407;PRKAA2_ENSG00000162409
1	57285030	57285233	KD2-H4K8ac_peak_1336	7.96322	C1orf168_ENSG00000187889
1	58373923	58374187	KD2-H4K8ac_peak_1337	5.20908	
1	58898214	58898707	KD2-H4K8ac_peak_1338	4.211	
1	59011806	59012183	KD2-H4K8ac_peak_1339	14.42504	DAB1_ENSG00000173406;OMA1_ENSG00000162600
1	59165474	59165682	KD2-H4K8ac_peak_1340	8.4716	MYSM1_ENSG00000162601
1	59238801	59239192	KD2-H4K8ac_peak_1341	6.40683	
1	59250072	59250515	KD2-H4K8ac_peak_1342	10.44656	JUN_ENSG00000177606;LINC01135_ENSG00000234807
1	59281037	59281233	KD2-H4K8ac_peak_1343	7.70289	
1	59281802	59282431	KD2-H4K8ac_peak_1344	16.32216	
1	59364656	59364925	KD2-H4K8ac_peak_1345	9.10868	
1	59369298	59370148	KD2-H4K8ac_peak_1346	17.8868	
1	59762711	59763440	KD2-H4K8ac_peak_1347	5.86783	FGGY_ENSG00000172456
1	59913052	59913277	KD2-H4K8ac_peak_1348	7.63551	
1	60019950	60020146	KD2-H4K8ac_peak_1349	4.3289	
1	60280684	60281046	KD2-H4K8ac_peak_1350	8.16906	HOOK1_ENSG00000134709
1	60392184	60392462	KD2-H4K8ac_peak_1351	10.19949	CYP2J2_ENSG00000134716
1	61337680	61337883	KD2-H4K8ac_peak_1352	7.12754	
1	61352772	61353004	KD2-H4K8ac_peak_1353	9.34989	
1	61370283	61370540	KD2-H4K8ac_peak_1354	5.57044	
1	61400801	61401081	KD2-H4K8ac_peak_1355	15.88083	
1	61415453	61415688	KD2-H4K8ac_peak_1356	6.79289	
1	61455497	61455715	KD2-H4K8ac_peak_1357	8.00457	
1	61515926	61516774	KD2-H4K8ac_peak_1358	12.00597	
1	61519759	61520084	KD2-H4K8ac_peak_1359	8.30974	
1	61522546	61522759	KD2-H4K8ac_peak_1360	6.14257	
1	61523339	61523538	KD2-H4K8ac_peak_1361	6.99815	
1	61542345	61542649	KD2-H4K8ac_peak_1362	9.1707	
1	61545236	61545493	KD2-H4K8ac_peak_1363	5.90098	
1	61548794	61549562	KD2-H4K8ac_peak_1364	9.36516	AC096534.1_ENSG00000263380
1	61828916	61829156	KD2-H4K8ac_peak_1365	6.24747	
1	61919344	61919712	KD2-H4K8ac_peak_1366	8.37043	
1	62190392	62191097	KD2-H4K8ac_peak_1367	9.95119	TM2D1_ENSG00000162604
1	62191833	62192068	KD2-H4K8ac_peak_1368	6.2781	TM2D1_ENSG00000162604
1	62207960	62208849	KD2-H4K8ac_peak_1369	18.80827	RP11-430G17.3_ENSG00000271200;INADL_ENSG00000132849
1	62737217	62737713	KD2-H4K8ac_peak_1370	7.11824	
1	62738121	62738335	KD2-H4K8ac_peak_1371	6.0073	
1	62738599	62739024	KD2-H4K8ac_peak_1372	6.85193	
1	62784388	62784809	KD2-H4K8ac_peak_1373	12.00273	KANK4_ENSG00000132854
1	62792913	62793703	KD2-H4K8ac_peak_1374	7.49599	
1	62850761	62851017	KD2-H4K8ac_peak_1375	8.00457	
1	62902470	62903304	KD2-H4K8ac_peak_1376	7.28628	USP1_ENSG00000162607
1	63153296	63154023	KD2-H4K8ac_peak_1377	12.93209	DOCK7_ENSG00000116641;RP11-230B22.1_ENSG00000235545
1	63248657	63249224	KD2-H4K8ac_peak_1378	8.1233	ATG4C_ENSG00000125703

1	63249549	63249744	KD2-H4K8ac_peak_1379	8.02571	ATG4C_ENSG00000125703
1	63832522	63833139	KD2-H4K8ac_peak_1380	12.84642	ALG6_ENSG00000088035
1	63833362	63833758	KD2-H4K8ac_peak_1381	21.75906	ALG6_ENSG00000088035
1	64058768	64059015	KD2-H4K8ac_peak_1382	6.25869	ITGB3BP_ENSG00000142856;PGM1_ENSG00000079739
1	64140425	64141354	KD2-H4K8ac_peak_1383	11.70505	
1	64239748	64239973	KD2-H4K8ac_peak_1384	6.40683	ROR1_ENSG00000185483
1	64240458	64240736	KD2-H4K8ac_peak_1385	4.211	ROR1_ENSG00000185483
1	64283389	64283646	KD2-H4K8ac_peak_1386	14.10697	
1	64503866	64504121	KD2-H4K8ac_peak_1387	3.92226	
1	64543137	64543364	KD2-H4K8ac_peak_1388	8.66387	
1	64575142	64575480	KD2-H4K8ac_peak_1389	4.66652	
1	64636758	64637321	KD2-H4K8ac_peak_1390	8.55271	RP11-24J23.2_ENSG00000223949
1	64637595	64637826	KD2-H4K8ac_peak_1391	4.35346	RP11-24J23.2_ENSG00000223949
1	64638778	64639632	KD2-H4K8ac_peak_1392	9.63776	
1	64653769	64654076	KD2-H4K8ac_peak_1393	4.24345	
1	64712306	64712743	KD2-H4K8ac_peak_1394	11.15509	
1	64735116	64735464	KD2-H4K8ac_peak_1395	4.98318	
1	64747097	64747536	KD2-H4K8ac_peak_1396	5.9696	
1	64802202	64802889	KD2-H4K8ac_peak_1397	7.79558	
1	64804041	64804681	KD2-H4K8ac_peak_1398	7.40633	
1	64935258	64935639	KD2-H4K8ac_peak_1399	6.66692	CACHD1_ENSG00000158966
1	64935867	64936312	KD2-H4K8ac_peak_1400	7.44324	CACHD1_ENSG00000158966
1	64971040	64971254	KD2-H4K8ac_peak_1401	10.94087	
1	64971601	64972013	KD2-H4K8ac_peak_1402	12.82393	
1	65062092	65062834	KD2-H4K8ac_peak_1403	5.14125	
1	65066391	65066694	KD2-H4K8ac_peak_1404	6.00226	
1	65088801	65089060	KD2-H4K8ac_peak_1405	5.75452	
1	65210318	65211452	KD2-H4K8ac_peak_1406	11.90231	RAVER2_ENSG00000162437
1	65259809	65260011	KD2-H4K8ac_peak_1407	5.25071	
1	65341438	65341979	KD2-H4K8ac_peak_1408	6.99815	
1	65431597	65432136	KD2-H4K8ac_peak_1409	6.40683	JAK1_ENSG00000162434
1	65432464	65432704	KD2-H4K8ac_peak_1410	6.24747	JAK1_ENSG00000162434
1	65532238	65533224	KD2-H4K8ac_peak_1411	7.49599	RP4-535B20.1_ENSG00000231485
1	65533564	65534066	KD2-H4K8ac_peak_1412	17.43705	RP4-535B20.1_ENSG00000231485
1	65613611	65614769	KD2-H4K8ac_peak_1413	15.59774	AK4_ENSG00000162433
1	65731426	65731839	KD2-H4K8ac_peak_1414	12.81138	
1	65886414	65886784	KD2-H4K8ac_peak_1415	11.80283	LEPR_ENSG00000116678;LEPROT_ENSG00000213625
1	65991029	65991740	KD2-H4K8ac_peak_1416	12.88108	
1	66429025	66429217	KD2-H4K8ac_peak_1417	6.40683	
1	66709329	66709554	KD2-H4K8ac_peak_1418	4.62507	
1	66737809	66738164	KD2-H4K8ac_peak_1419	12.3187	
1	66797836	66798138	KD2-H4K8ac_peak_1420	9.11031	
1	66911532	66911827	KD2-H4K8ac_peak_1421	6.0073	
1	66916985	66917398	KD2-H4K8ac_peak_1422	9.11031	
1	66955682	66956109	KD2-H4K8ac_peak_1423	10.21502	
1	66957308	66957517	KD2-H4K8ac_peak_1424	5.14125	

1	66959838	66960198	KD2-H4K8ac_peak_1425	6.79289	
1	66971165	66971587	KD2-H4K8ac_peak_1426	4.3015	
1	66999283	66999492	KD2-H4K8ac_peak_1427	5.82799	SGIP1_ENSG00000118473
1	67390616	67390859	KD2-H4K8ac_peak_1428	10.05606	WDR78_ENSG00000152763;MIER1_ENSG00000198160
1	67391066	67391657	KD2-H4K8ac_peak_1429	8.28994	WDR78_ENSG00000152763;MIER1_ENSG00000198160
1	67519412	67519944	KD2-H4K8ac_peak_1430	9.44322	SLC35D1_ENSG00000116704
1	67773294	67773978	KD2-H4K8ac_peak_1431	4.86324	IL12RB2_ENSG00000081985
1	67895399	67896043	KD2-H4K8ac_peak_1432	15.05364	SERBP1_ENSG00000142864
1	67896336	67896927	KD2-H4K8ac_peak_1433	15.22643	SERBP1_ENSG00000142864
1	68074943	68075512	KD2-H4K8ac_peak_1434	7.95934	
1	68140932	68141350	KD2-H4K8ac_peak_1435	9.11031	
1	68142134	68142327	KD2-H4K8ac_peak_1436	5.57044	
1	68150157	68150416	KD2-H4K8ac_peak_1437	5.57044	GADD45A_ENSG00000116717
1	68151040	68152391	KD2-H4K8ac_peak_1438	10.05606	GADD45A_ENSG00000116717
1	68232363	68232576	KD2-H4K8ac_peak_1439	7.91974	
1	68298337	68298733	KD2-H4K8ac_peak_1440	11.89502	GNG12_ENSG00000172380;GNG12-AS1_ENSG00000232284
1	68298979	68299375	KD2-H4K8ac_peak_1441	5.13615	GNG12_ENSG00000172380;GNG12-AS1_ENSG00000232284
1	68403592	68403834	KD2-H4K8ac_peak_1442	4.37708	
1	68404787	68405520	KD2-H4K8ac_peak_1443	9.59147	
1	68405923	68406184	KD2-H4K8ac_peak_1444	7.27716	
1	68411296	68411603	KD2-H4K8ac_peak_1445	4.35346	
1	68697796	68698160	KD2-H4K8ac_peak_1446	7.47462	WLS_ENSG00000116729
1	70671443	70671695	KD2-H4K8ac_peak_1447	6.25869	LRRC40_ENSG00000066557;SRSF11_ENSG00000116754
1	70819913	70820844	KD2-H4K8ac_peak_1448	11.89502	ANKRD13C_ENSG00000118454;HHLA3_ENSG00000197568
1	70948352	70948557	KD2-H4K8ac_peak_1449	6.9809	
1	71546363	71546893	KD2-H4K8ac_peak_1450	10.0692	ZRANB2_ENSG00000132485;ZRANB2-AS2_ENSG00000229956
1	71547450	71547741	KD2-H4K8ac_peak_1451	15.7388	ZRANB2_ENSG00000132485;ZRANB2-AS2_ENSG00000229956
1	72252595	72252789	KD2-H4K8ac_peak_1452	5.43522	
1	72749545	72749798	KD2-H4K8ac_peak_1453	6.16888	
1	75081560	75081810	KD2-H4K8ac_peak_1454	4.89656	
1	75197973	75198174	KD2-H4K8ac_peak_1455	8.00457	CRYZ_ENSG00000116791;TYW3_ENSG00000162623
1	75198928	75199253	KD2-H4K8ac_peak_1456	8.94469	CRYZ_ENSG00000116791;TYW3_ENSG00000162623
1	76081447	76081797	KD2-H4K8ac_peak_1457	6.99815	
1	76082011	76082527	KD2-H4K8ac_peak_1458	16.09057	
1	76190424	76190704	KD2-H4K8ac_peak_1459	11.1952	RP4-682C21.5_ENSG00000178193;ACADM_ENSG00000117054
1	76540029	76540266	KD2-H4K8ac_peak_1460	11.30637	ST6GALNAC3_ENSG00000184005
1	76745045	76745263	KD2-H4K8ac_peak_1461	6.16888	
1	76803337	76803533	KD2-H4K8ac_peak_1462	7.45501	
1	77230619	77231213	KD2-H4K8ac_peak_1463	10.96455	
1	77333269	77334091	KD2-H4K8ac_peak_1464	10.88859	ST6GALNAC5_ENSG00000117069
1	77334450	77334659	KD2-H4K8ac_peak_1465	5.61067	
1	77684889	77685082	KD2-H4K8ac_peak_1466	10.58496	PIGK_ENSG00000142892
1	77747721	77747926	KD2-H4K8ac_peak_1467	6.13909	AK5_ENSG00000154027
1	78004982	78005298	KD2-H4K8ac_peak_1468	6.83936	
1	78149273	78149645	KD2-H4K8ac_peak_1469	7.36799	ZZZ3_ENSG00000036549
1	78225099	78225406	KD2-H4K8ac_peak_1470	3.82059	USP33_ENSG00000077254

1	78244504	78244991	KD2-H4K8ac_peak_1471	6.40683	FAM73A_ENSG00000180488;RNA5SP21_ENSG00000222849
1	78444274	78444581	KD2-H4K8ac_peak_1472	8.4716	FUBP1_ENSG00000162613;DNAJB4_ENSG00000162616;GIPC2_ENSG00000137960
1	78445552	78445756	KD2-H4K8ac_peak_1473	5.25071	FUBP1_ENSG00000162613;DNAJB4_ENSG00000162616;GIPC2_ENSG00000137960
1	78511736	78511940	KD2-H4K8ac_peak_1474	4.8485	
1	81358620	81358859	KD2-H4K8ac_peak_1475	5.73839	
1	81697017	81697301	KD2-H4K8ac_peak_1476	9.11031	
1	81700102	81700300	KD2-H4K8ac_peak_1477	5.25071	
1	81704524	81704870	KD2-H4K8ac_peak_1478	6.16888	
1	82266656	82266849	KD2-H4K8ac_peak_1479	7.76329	
1	82267216	82267498	KD2-H4K8ac_peak_1480	5.38581	
1	82268318	82269091	KD2-H4K8ac_peak_1481	8.4716	
1	83981857	83982141	KD2-H4K8ac_peak_1482	7.12754	
1	84157814	84158225	KD2-H4K8ac_peak_1483	5.20998	
1	84160002	84160353	KD2-H4K8ac_peak_1484	7.62364	
1	84162979	84163193	KD2-H4K8ac_peak_1485	5.18447	
1	84227673	84227902	KD2-H4K8ac_peak_1486	6.16888	
1	84464428	84465112	KD2-H4K8ac_peak_1487	17.43705	TTLL7_ENSG00000137941
1	84543060	84543598	KD2-H4K8ac_peak_1488	14.66631	RP11-486G15.2_ENSG00000271576;PRKACB_ENSG00000142875
1	84763394	84763596	KD2-H4K8ac_peak_1489	5.25071	SAMD13_ENSG00000203943
1	84764155	84764959	KD2-H4K8ac_peak_1490	15.22643	SAMD13_ENSG00000203943
1	84819112	84819326	KD2-H4K8ac_peak_1491	6.66692	
1	84944136	84944787	KD2-H4K8ac_peak_1492	11.30637	RPF1_ENSG00000117133
1	85313405	85314129	KD2-H4K8ac_peak_1493	8.30974	
1	85358882	85359331	KD2-H4K8ac_peak_1494	6.66692	LPAR3_ENSG00000171517
1	85741798	85742226	KD2-H4K8ac_peak_1495	11.22681	BCL10_ENSG00000142867;RP11-131L23.1_ENSG00000223653
1	85930502	85931345	KD2-H4K8ac_peak_1496	7.92775	
1	86042006	86043108	KD2-H4K8ac_peak_1497	5.01002	DDAH1_ENSG00000153904
1	86046970	86047204	KD2-H4K8ac_peak_1498	8.30974	CYR61_ENSG00000142871
1	86174231	86174895	KD2-H4K8ac_peak_1499	12.84642	ZNHIT6_ENSG00000117174
1	86861016	86861661	KD2-H4K8ac_peak_1500	7.13615	ODF2L_ENSG00000122417
1	86862320	86862540	KD2-H4K8ac_peak_1501	5.14125	ODF2L_ENSG00000122417
1	86862779	86863063	KD2-H4K8ac_peak_1502	6.16888	ODF2L_ENSG00000122417
1	87170063	87170901	KD2-H4K8ac_peak_1503	9.20193	RP4-612B15.3_ENSG00000261737
1	87259624	87259857	KD2-H4K8ac_peak_1504	4.35346	
1	87380417	87380763	KD2-H4K8ac_peak_1505	4.67342	SEP15_ENSG00000183291;HS2ST1_ENSG00000153936
1	87793729	87794002	KD2-H4K8ac_peak_1506	9.36516	LMO4_ENSG00000143013
1	87797024	87797246	KD2-H4K8ac_peak_1507	5.45396	
1	87800942	87801324	KD2-H4K8ac_peak_1508	8.85813	
1	89150008	89150704	KD2-H4K8ac_peak_1509	13.70753	RP11-76N22.2_ENSG00000237505;PKN2_ENSG00000065243
1	89458035	89458290	KD2-H4K8ac_peak_1510	8.4716	CCBL2_ENSG00000137944;RBMXL1_ENSG00000213516
1	89829648	89829947	KD2-H4K8ac_peak_1511	4.42765	GBP6_ENSG00000183347
1	89989444	89990269	KD2-H4K8ac_peak_1512	12.84642	LRRC8B_ENSG00000197147
1	90098205	90098936	KD2-H4K8ac_peak_1513	8.85813	RP5-1007M22.2_ENSG00000231999;LRRC8C_ENSG00000171488;RP11-302M6.4_ENSG00000271949
1	90166679	90166948	KD2-H4K8ac_peak_1514	5.14125	
1	90223516	90223776	KD2-H4K8ac_peak_1515	8.26945	
1	90228764	90229224	KD2-H4K8ac_peak_1516	6.99815	

1	90286703	90286979	KD2-H4K8ac_peak_1517	17.09929	RP5-943J3.2_ENSG00000272931;LRRC8D_ENSG00000171492
1	90459853	90460194	KD2-H4K8ac_peak_1518	3.75035	GEMIN8P4_ENSG00000228175;ZNF326_ENSG00000162664
1	90460755	90461268	KD2-H4K8ac_peak_1519	7.36799	GEMIN8P4_ENSG00000228175;ZNF326_ENSG00000162664
1	91316348	91317000	KD2-H4K8ac_peak_1520	9.31907	RP4-665J23.1_ENSG00000233593;RP4-665J23.2_ENSG00000225446
1	91486718	91486963	KD2-H4K8ac_peak_1521	6.25869	ZNF644_ENSG00000122482
1	91487331	91488201	KD2-H4K8ac_peak_1522	7.76104	ZNF644_ENSG00000122482
1	91966442	91966912	KD2-H4K8ac_peak_1523	14.48922	CDC7_ENSG00000097046
1	92495532	92495904	KD2-H4K8ac_peak_1524	4.84794	EPHX4_ENSG00000172031
1	92545909	92546377	KD2-H4K8ac_peak_1525	7.07117	BTBD8_ENSG00000189195
1	92764666	92764997	KD2-H4K8ac_peak_1526	5.20998	RPAP2_ENSG00000122484
1	93250900	93251537	KD2-H4K8ac_peak_1527	9.11031	
1	93426472	93426704	KD2-H4K8ac_peak_1528	9.1707	FAM69A_ENSG00000154511
1	93427455	93427725	KD2-H4K8ac_peak_1529	8.55271	FAM69A_ENSG00000154511
1	93544443	93544635	KD2-H4K8ac_peak_1530	10.72656	MTF2_ENSG00000143033
1	93544910	93545127	KD2-H4K8ac_peak_1531	10.63441	MTF2_ENSG00000143033
1	93645509	93645841	KD2-H4K8ac_peak_1532	10.7186	TMED5_ENSG00000117500;CCDC18_ENSG00000122483
1	93811001	93812096	KD2-H4K8ac_peak_1533	7.36799	RP4-717I23.3_ENSG00000223745;DR1_ENSG00000117505
1	93913257	93915041	KD2-H4K8ac_peak_1534	16.32216	FNBP1L_ENSG00000137942
1	94041012	94041259	KD2-H4K8ac_peak_1535	3.82059	
1	94043257	94043827	KD2-H4K8ac_peak_1536	8.55271	
1	94044622	94044815	KD2-H4K8ac_peak_1537	8.59579	
1	94055412	94055663	KD2-H4K8ac_peak_1538	3.91903	
1	94075055	94075785	KD2-H4K8ac_peak_1539	9.70057	
1	94079396	94079592	KD2-H4K8ac_peak_1540	10.21366	
1	94080892	94081444	KD2-H4K8ac_peak_1541	6.66692	
1	94082405	94082631	KD2-H4K8ac_peak_1542	5.9696	
1	94082850	94083082	KD2-H4K8ac_peak_1543	7.81657	
1	94083579	94083824	KD2-H4K8ac_peak_1544	5.82799	
1	94086239	94086526	KD2-H4K8ac_peak_1545	4.12757	
1	94092212	94092457	KD2-H4K8ac_peak_1546	5.25071	
1	94146663	94147058	KD2-H4K8ac_peak_1547	5.14125	
1	94147420	94147647	KD2-H4K8ac_peak_1548	7.81657	
1	94174434	94174780	KD2-H4K8ac_peak_1549	5.88845	
1	94205833	94206096	KD2-H4K8ac_peak_1550	7.69287	
1	94293434	94293718	KD2-H4K8ac_peak_1551	9.90914	
1	94293995	94294586	KD2-H4K8ac_peak_1552	9.31907	
1	94311184	94311419	KD2-H4K8ac_peak_1553	6.45018	MIR760_ENSG00000211575
1	94312036	94312411	KD2-H4K8ac_peak_1554	11.86082	BCAR3_ENSG00000137936;MIR760_ENSG00000211575;RP4-561L24.3_ENSG00000260464
1	94313374	94313613	KD2-H4K8ac_peak_1555	6.25869	BCAR3_ENSG00000137936;MIR760_ENSG00000211575;RP4-561L24.3_ENSG00000260464
1	94345457	94345945	KD2-H4K8ac_peak_1556	13.39763	DNTTIP2_ENSG00000067334
1	94374555	94374864	KD2-H4K8ac_peak_1557	5.9696	GCLM_ENSG00000023909
1	94509867	94510122	KD2-H4K8ac_peak_1558	8.59301	
1	94510321	94510589	KD2-H4K8ac_peak_1559	8.30974	
1	94566894	94567272	KD2-H4K8ac_peak_1560	9.96798	
1	94582905	94583492	KD2-H4K8ac_peak_1561	7.27716	
1	94712869	94713237	KD2-H4K8ac_peak_1562	4.211	RP11-148B18.1_ENSG00000231363

1	94746774	94746989	KD2-H4K8ac_peak_1563	4.65111	
1	94786643	94786857	KD2-H4K8ac_peak_1564	4.9025	
1	94884018	94884244	KD2-H4K8ac_peak_1565	8.79106	ABCD3_ENSG00000117528
1	95006927	95008022	KD2-H4K8ac_peak_1566	13.81781	F3_ENSG00000117525
1	95220803	95221097	KD2-H4K8ac_peak_1567	12.46946	
1	95222301	95222601	KD2-H4K8ac_peak_1568	8.10216	
1	95223499	95223770	KD2-H4K8ac_peak_1569	7.22457	
1	95285414	95286274	KD2-H4K8ac_peak_1570	10.05606	LINC01057_ENSG00000224081;SLC44A3_ENSG00000143036
1	95320723	95321060	KD2-H4K8ac_peak_1571	4.77079	RP11-465K1.2_ENSG00000237416
1	95350136	95350332	KD2-H4K8ac_peak_1572	6.13909	
1	95391818	95392560	KD2-H4K8ac_peak_1573	6.80488	CNN3_ENSG00000117519;RP4-639F20.1_ENSG00000235501
1	95392882	95393331	KD2-H4K8ac_peak_1574	15.76765	CNN3_ENSG00000117519;RP4-639F20.1_ENSG00000235501
1	95413748	95413981	KD2-H4K8ac_peak_1575	7.83656	
1	95414821	95415188	KD2-H4K8ac_peak_1576	5.93446	
1	95418076	95418356	KD2-H4K8ac_peak_1577	7.59643	
1	95421305	95421509	KD2-H4K8ac_peak_1578	5.57044	
1	95582897	95583465	KD2-H4K8ac_peak_1579	6.84261	TMEM56_ENSG00000152078;TMEM56-RWDD3_ENSG00000271092
1	95699221	95699591	KD2-H4K8ac_peak_1580	10.17341	RP11-57H12.3_ENSG00000226026;RWDD3_ENSG00000122481
1	95776695	95777188	KD2-H4K8ac_peak_1581	5.15777	
1	95789874	95790169	KD2-H4K8ac_peak_1582	7.12754	
1	95790399	95790962	KD2-H4K8ac_peak_1583	7.12754	
1	95791838	95792090	KD2-H4K8ac_peak_1584	6.07178	
1	95846585	95846800	KD2-H4K8ac_peak_1585	8.41122	RP11-14O19.2_ENSG00000237954
1	96038199	96038470	KD2-H4K8ac_peak_1586	4.44748	
1	96041535	96041831	KD2-H4K8ac_peak_1587	4.59928	
1	96069408	96069688	KD2-H4K8ac_peak_1588	5.57044	
1	96071567	96071965	KD2-H4K8ac_peak_1589	7.27716	
1	96108112	96108720	KD2-H4K8ac_peak_1590	5.71749	
1	96119158	96119351	KD2-H4K8ac_peak_1591	4.42765	
1	96120944	96121352	KD2-H4K8ac_peak_1592	6.40683	
1	96371029	96371258	KD2-H4K8ac_peak_1593	7.47462	
1	96416916	96417155	KD2-H4K8ac_peak_1594	8.00457	
1	96449228	96449439	KD2-H4K8ac_peak_1595	4.37708	
1	96451020	96451413	KD2-H4K8ac_peak_1596	10.21502	
1	96526387	96526649	KD2-H4K8ac_peak_1597	7.12754	
1	97186699	97186953	KD2-H4K8ac_peak_1598	4.20044	PTBP2_ENSG00000117569
1	97187367	97187994	KD2-H4K8ac_peak_1599	11.66477	PTBP2_ENSG00000117569
1	97432536	97432769	KD2-H4K8ac_peak_1600	4.77079	
1	97434263	97434459	KD2-H4K8ac_peak_1601	8.74596	
1	97462823	97463279	KD2-H4K8ac_peak_1602	9.02371	
1	97464281	97464680	KD2-H4K8ac_peak_1603	20.88898	
1	98514886	98515136	KD2-H4K8ac_peak_1604	5.54647	MIR137HG_ENSG00000225206
1	98518961	98520777	KD2-H4K8ac_peak_1605	15.58442	
1	98920285	98920534	KD2-H4K8ac_peak_1606	6.64891	
1	99126857	99127668	KD2-H4K8ac_peak_1607	11.89502	SNX7_ENSG00000162627
1	99388319	99388526	KD2-H4K8ac_peak_1608	10.21502	

1	99389949	99390204	KD2-H4K8ac_peak_1609	10.21502	
1	99469550	99470423	KD2-H4K8ac_peak_1610	15.9336	LPPR5_ENSG00000117598;RP5-896L10.1_ENSG00000232825
1	100111376	1E+08	KD2-H4K8ac_peak_1611	5.57044	PALMD_ENSG00000099260
1	100314992	1E+08	KD2-H4K8ac_peak_1612	7.57336	
1	100409388	1E+08	KD2-H4K8ac_peak_1613	7.22457	
1	100434983	1E+08	KD2-H4K8ac_peak_1614	8.55271	RP5-884G6.2_ENSG00000228084;SLC35A3_ENSG00000117620
1	100435650	1E+08	KD2-H4K8ac_peak_1615	6.40297	RP5-884G6.2_ENSG00000228084;SLC35A3_ENSG00000117620
1	100503746	1.01E+08	KD2-H4K8ac_peak_1616	5.98692	HIAT1_ENSG00000156875
1	100598140	1.01E+08	KD2-H4K8ac_peak_1617	6.20982	SASS6_ENSG00000156876;TRMT13_ENSG00000122435
1	100714965	1.01E+08	KD2-H4K8ac_peak_1618	11.30637	DBT_ENSG00000137992;RP11-305E17.4_ENSG00000230287
1	100715577	1.01E+08	KD2-H4K8ac_peak_1619	6.16888	DBT_ENSG00000137992;RP11-305E17.4_ENSG00000230287
1	100731835	1.01E+08	KD2-H4K8ac_peak_1620	11.43863	RP11-305E17.6_ENSG00000224616;RTCA_ENSG00000137996
1	100816586	1.01E+08	KD2-H4K8ac_peak_1621	6.04107	
1	101360575	1.01E+08	KD2-H4K8ac_peak_1622	8.30974	EXTL2_ENSG00000162694;RP4-549L20.3_ENSG00000273204;SLC30A7_ENSG00000162695
1	101361745	1.01E+08	KD2-H4K8ac_peak_1623	5.14125	EXTL2_ENSG00000162694;SLC30A7_ENSG00000162695
1	101491467	1.01E+08	KD2-H4K8ac_peak_1624	5.14125	DPH5_ENSG00000117543;RP11-421L21.3_ENSG00000233184
1	103574360	1.04E+08	KD2-H4K8ac_peak_1625	4.42765	COL11A1_ENSG00000060718
1	105680687	1.06E+08	KD2-H4K8ac_peak_1626	11.03331	
1	107598853	1.08E+08	KD2-H4K8ac_peak_1627	24.42768	PRMT6_ENSG00000198890
1	107600260	1.08E+08	KD2-H4K8ac_peak_1628	4.8485	PRMT6_ENSG00000198890
1	107682649	1.08E+08	KD2-H4K8ac_peak_1629	6.83936	NTNG1_ENSG00000162631
1	108507153	1.09E+08	KD2-H4K8ac_peak_1630	10.2048	VAV3_ENSG00000134215;VAV3-AS1_ENSG00000230489
1	108507913	1.09E+08	KD2-H4K8ac_peak_1631	8.55271	VAV3_ENSG00000134215;VAV3-AS1_ENSG00000230489
1	108742275	1.09E+08	KD2-H4K8ac_peak_1632	11.05467	SLC25A24_ENSG00000085491;RP11-483I13.5_ENSG00000260879
1	108743088	1.09E+08	KD2-H4K8ac_peak_1633	11.24702	SLC25A24_ENSG00000085491;RP11-483I13.5_ENSG00000260879
1	109234458	1.09E+08	KD2-H4K8ac_peak_1634	8.5092	PRPF38B_ENSG00000134186
1	109288840	1.09E+08	KD2-H4K8ac_peak_1635	7.47462	STXBP3_ENSG00000116266
1	109289348	1.09E+08	KD2-H4K8ac_peak_1636	7.47462	STXBP3_ENSG00000116266
1	109358104	1.09E+08	KD2-H4K8ac_peak_1637	6.16888	
1	109359134	1.09E+08	KD2-H4K8ac_peak_1638	7.27716	
1	109360288	1.09E+08	KD2-H4K8ac_peak_1639	6.94255	
1	109372794	1.09E+08	KD2-H4K8ac_peak_1640	6.0605	
1	109506335	1.1E+08	KD2-H4K8ac_peak_1641	6.8836	AKNAD1_ENSG00000162641;CLCC1_ENSG00000121940
1	109619178	1.1E+08	KD2-H4K8ac_peak_1642	9.06703	TAF13_ENSG00000197780
1	109642861	1.1E+08	KD2-H4K8ac_peak_1643	6.40297	SCARNA2_ENSG00000270066
1	109756656	1.1E+08	KD2-H4K8ac_peak_1644	4.98072	SARS_ENSG00000031698
1	109791585	1.1E+08	KD2-H4K8ac_peak_1645	5.75757	CELSR2_ENSG00000143126
1	109792383	1.1E+08	KD2-H4K8ac_peak_1646	5.32397	CELSR2_ENSG00000143126
1	109795957	1.1E+08	KD2-H4K8ac_peak_1647	9.6589	
1	109825475	1.1E+08	KD2-H4K8ac_peak_1648	5.59859	PSRC1_ENSG00000134222
1	109825999	1.1E+08	KD2-H4K8ac_peak_1649	6.67538	PSRC1_ENSG00000134222
1	109940059	1.1E+08	KD2-H4K8ac_peak_1650	11.05407	SORT1_ENSG00000134243
1	109968726	1.1E+08	KD2-H4K8ac_peak_1651	4.06758	PSMA5_ENSG00000143106
1	110025855	1.1E+08	KD2-H4K8ac_peak_1652	9.11031	ATXN7L2_ENSG00000162650
1	110026607	1.1E+08	KD2-H4K8ac_peak_1653	23.27293	ATXN7L2_ENSG00000162650
1	110036398	1.1E+08	KD2-H4K8ac_peak_1654	5.13615	CYB561D1_ENSG00000174151

1	110051829	1.1E+08	KD2-H4K8ac_peak_1655	8.55271	AMIGO1_ENSG00000181754
1	110072482	1.1E+08	KD2-H4K8ac_peak_1656	5.82799	
1	110073388	1.1E+08	KD2-H4K8ac_peak_1657	6.60096	
1	110074786	1.1E+08	KD2-H4K8ac_peak_1658	18.82114	
1	110089349	1.1E+08	KD2-H4K8ac_peak_1659	5.61067	
1	110090633	1.1E+08	KD2-H4K8ac_peak_1660	6.99815	GNAI3_ENSG00000065135
1	110161160	1.1E+08	KD2-H4K8ac_peak_1661	7.22457	
1	110173943	1.1E+08	KD2-H4K8ac_peak_1662	9.63487	
1	110174680	1.1E+08	KD2-H4K8ac_peak_1663	7.86151	
1	110175528	1.1E+08	KD2-H4K8ac_peak_1664	10.98959	
1	110198694	1.1E+08	KD2-H4K8ac_peak_1665	7.49599	GSTM4_ENSG00000168765
1	110211013	1.1E+08	KD2-H4K8ac_peak_1666	8.17902	GSTM2_ENSG00000213366
1	110282307	1.1E+08	KD2-H4K8ac_peak_1667	3.90565	
1	110283055	1.1E+08	KD2-H4K8ac_peak_1668	7.44324	GSTM3_ENSG00000134202
1	110453509	1.1E+08	KD2-H4K8ac_peak_1669	8.94469	CSF1_ENSG00000184371
1	110526624	1.11E+08	KD2-H4K8ac_peak_1670	5.9696	AHCYL1_ENSG00000168710
1	110527448	1.11E+08	KD2-H4K8ac_peak_1671	15.039	AHCYL1_ENSG00000168710
1	110577292	1.11E+08	KD2-H4K8ac_peak_1672	20.5798	
1	110593087	1.11E+08	KD2-H4K8ac_peak_1673	5.14125	
1	110601453	1.11E+08	KD2-H4K8ac_peak_1674	7.67504	RP4-773N10.4_ENSG00000258634
1	110602228	1.11E+08	KD2-H4K8ac_peak_1675	14.69247	
1	110602756	1.11E+08	KD2-H4K8ac_peak_1676	7.47462	
1	110605600	1.11E+08	KD2-H4K8ac_peak_1677	4.42765	
1	110637323	1.11E+08	KD2-H4K8ac_peak_1678	6.20982	
1	110640855	1.11E+08	KD2-H4K8ac_peak_1679	7.06358	
1	110649128	1.11E+08	KD2-H4K8ac_peak_1680	12.00273	
1	110714476	1.11E+08	KD2-H4K8ac_peak_1681	8.55271	RP5-1028L10.1_ENSG00000227091
1	110752311	1.11E+08	KD2-H4K8ac_peak_1682	7.27716	KCNC4-AS1_ENSG00000224965
1	110753334	1.11E+08	KD2-H4K8ac_peak_1683	6.97919	KCNC4-AS1_ENSG00000224965;KCNC4_ENSG00000116396
1	110778094	1.11E+08	KD2-H4K8ac_peak_1684	10.43145	
1	110880932	1.11E+08	KD2-H4K8ac_peak_1685	6.8713	RP5-1074L1.1_ENSG00000227963;RBM15_ENSG00000162775
1	110882097	1.11E+08	KD2-H4K8ac_peak_1686	14.27912	RP5-1074L1.1_ENSG00000227963;RBM15_ENSG00000162775
1	110949621	1.11E+08	KD2-H4K8ac_peak_1687	11.03331	LAMTOR5_ENSG00000134248
1	111505851	1.12E+08	KD2-H4K8ac_peak_1688	7.76329	LRIF1_ENSG00000121931;RP11-96K19.5_ENSG00000273010
1	111682446	1.12E+08	KD2-H4K8ac_peak_1689	9.36516	DRAM2_ENSG00000156171;CEPT1_ENSG00000134255
1	111682932	1.12E+08	KD2-H4K8ac_peak_1690	9.08816	DRAM2_ENSG00000156171;CEPT1_ENSG00000134255
1	111888977	1.12E+08	KD2-H4K8ac_peak_1691	9.95119	PIFO_ENSG00000173947
1	111992143	1.12E+08	KD2-H4K8ac_peak_1692	12.42547	WDR77_ENSG00000116455;ATP5F1_ENSG00000116459
1	112050597	1.12E+08	KD2-H4K8ac_peak_1693	8.10216	
1	112281215	1.12E+08	KD2-H4K8ac_peak_1694	9.08816	FAM212B-AS1_ENSG00000227811
1	112282751	1.12E+08	KD2-H4K8ac_peak_1695	12.42761	FAM212B-AS1_ENSG00000227811
1	112298310	1.12E+08	KD2-H4K8ac_peak_1696	7.36799	FAM212B_ENSG00000197852;DDX20_ENSG00000064703
1	112438261	1.12E+08	KD2-H4K8ac_peak_1697	11.70505	
1	112933720	1.13E+08	KD2-H4K8ac_peak_1698	9.63487	
1	112938002	1.13E+08	KD2-H4K8ac_peak_1699	10.30284	CTTNBP2NL_ENSG00000143079
1	112938837	1.13E+08	KD2-H4K8ac_peak_1700	16.61426	CTTNBP2NL_ENSG00000143079

1	113051401	1.13E+08	KD2-H4K8ac_peak_1701	5.29555	
1	113161446	1.13E+08	KD2-H4K8ac_peak_1702	17.42214	CAPZA1_ENSG00000116489
1	113162493	1.13E+08	KD2-H4K8ac_peak_1703	10.30284	CAPZA1_ENSG00000116489;ST7L_ENSG00000007341
1	113217051	1.13E+08	KD2-H4K8ac_peak_1704	5.75757	
1	113218023	1.13E+08	KD2-H4K8ac_peak_1705	7.76329	
1	113249320	1.13E+08	KD2-H4K8ac_peak_1706	9.1707	RHOC_ENSG00000155366
1	113392182	1.13E+08	KD2-H4K8ac_peak_1707	10.7186	RP3-522D1.1_ENSG00000224167;RP11-426L16.8_ENSG00000215866
1	113499083	1.13E+08	KD2-H4K8ac_peak_1708	4.67342	SLC16A1_ENSG00000155380;SLC16A1-AS1_ENSG00000226419
1	113615880	1.14E+08	KD2-H4K8ac_peak_1709	8.82366	RP11-31F15.2_ENSG00000238198;LRIG2_ENSG00000198799
1	113683472	1.14E+08	KD2-H4K8ac_peak_1710	9.20193	
1	113688154	1.14E+08	KD2-H4K8ac_peak_1711	6.66359	
1	113690501	1.14E+08	KD2-H4K8ac_peak_1712	13.81781	
1	113691945	1.14E+08	KD2-H4K8ac_peak_1713	5.54647	
1	113696233	1.14E+08	KD2-H4K8ac_peak_1714	4.16871	
1	113766651	1.14E+08	KD2-H4K8ac_peak_1715	4.12757	
1	113825621	1.14E+08	KD2-H4K8ac_peak_1716	6.66692	
1	113933167	1.14E+08	KD2-H4K8ac_peak_1717	11.05467	MAGI3_ENSG00000081026
1	114301709	1.14E+08	KD2-H4K8ac_peak_1718	12.23	PHTF1_ENSG00000116793
1	114354382	1.14E+08	KD2-H4K8ac_peak_1719	12.84642	RSBN1_ENSG00000081019;RP5-1073O3.2_ENSG00000231128
1	114356016	1.14E+08	KD2-H4K8ac_peak_1720	12.03366	RSBN1_ENSG00000081019
1	114447270	1.14E+08	KD2-H4K8ac_peak_1721	3.97079	AP4B1_ENSG00000134262;DCLRE1B_ENSG00000118655
1	114448082	1.14E+08	KD2-H4K8ac_peak_1722	5.38581	AP4B1_ENSG00000134262;DCLRE1B_ENSG00000118655
1	114471550	1.14E+08	KD2-H4K8ac_peak_1723	6.66692	RP5-1073O3.7_ENSG00000235527;HIPK1_ENSG00000163349
1	114472459	1.14E+08	KD2-H4K8ac_peak_1724	6.40683	RP5-1073O3.7_ENSG00000235527;HIPK1_ENSG00000163349
1	114521644	1.15E+08	KD2-H4K8ac_peak_1725	6.54442	OLFML3_ENSG00000116774
1	114888574	1.15E+08	KD2-H4K8ac_peak_1726	14.06758	
1	115053159	1.15E+08	KD2-H4K8ac_peak_1727	6.40874	TRIM33_ENSG00000197323
1	115054032	1.15E+08	KD2-H4K8ac_peak_1728	9.6589	TRIM33_ENSG00000197323
1	115124496	1.15E+08	KD2-H4K8ac_peak_1729	17.83665	BCAS2_ENSG00000116752
1	115258658	1.15E+08	KD2-H4K8ac_peak_1730	5.88845	NRAS_ENSG00000213281
1	115300276	1.15E+08	KD2-H4K8ac_peak_1731	27.58135	CSDE1_ENSG00000009307
1	115300857	1.15E+08	KD2-H4K8ac_peak_1732	8.1233	CSDE1_ENSG00000009307
1	115322960	1.15E+08	KD2-H4K8ac_peak_1733	4.37809	SIKE1_ENSG00000052723
1	115681830	1.16E+08	KD2-H4K8ac_peak_1734	6.15357	
1	115845264	1.16E+08	KD2-H4K8ac_peak_1735	11.30637	
1	115860233	1.16E+08	KD2-H4K8ac_peak_1736	5.57044	
1	115860981	1.16E+08	KD2-H4K8ac_peak_1737	9.1707	
1	115880103	1.16E+08	KD2-H4K8ac_peak_1738	7.76329	NGF_ENSG00000134259
1	116135185	1.16E+08	KD2-H4K8ac_peak_1739	4.37708	
1	116184644	1.16E+08	KD2-H4K8ac_peak_1740	8.53092	
1	116370964	1.16E+08	KD2-H4K8ac_peak_1741	7.69287	
1	116371912	1.16E+08	KD2-H4K8ac_peak_1742	5.14125	
1	116473290	1.16E+08	KD2-H4K8ac_peak_1743	6.86699	
1	116518589	1.17E+08	KD2-H4K8ac_peak_1744	11.24702	SLC22A15_ENSG00000163393
1	116694357	1.17E+08	KD2-H4K8ac_peak_1745	4.54006	
1	116694967	1.17E+08	KD2-H4K8ac_peak_1746	4.30033	

1	116915392	1.17E+08	KD2-H4K8ac_peak_1747	7.50083	ATP1A1_ENSG00000163399
1	116960977	1.17E+08	KD2-H4K8ac_peak_1748	11.09936	ATP1A1OS_ENSG00000203865
1	116961948	1.17E+08	KD2-H4K8ac_peak_1749	7.96194	ATP1A1OS_ENSG00000203865
1	117027533	1.17E+08	KD2-H4K8ac_peak_1750	10.77651	
1	117113905	1.17E+08	KD2-H4K8ac_peak_1751	8.10216	CD58_ENSG00000116815
1	117189358	1.17E+08	KD2-H4K8ac_peak_1752	5.28072	
1	117222356	1.17E+08	KD2-H4K8ac_peak_1753	6.0073	
1	117228073	1.17E+08	KD2-H4K8ac_peak_1754	9.06703	
1	117230589	1.17E+08	KD2-H4K8ac_peak_1755	10.05606	
1	117231743	1.17E+08	KD2-H4K8ac_peak_1756	6.30229	
1	117233980	1.17E+08	KD2-H4K8ac_peak_1757	9.44423	
1	117235894	1.17E+08	KD2-H4K8ac_peak_1758	10.05606	C1orf137_ENSG00000203864
1	117239389	1.17E+08	KD2-H4K8ac_peak_1759	10.68431	
1	117240483	1.17E+08	KD2-H4K8ac_peak_1760	10.0692	
1	117249948	1.17E+08	KD2-H4K8ac_peak_1761	8.05667	
1	117250646	1.17E+08	KD2-H4K8ac_peak_1762	9.1707	
1	117279509	1.17E+08	KD2-H4K8ac_peak_1763	3.75035	
1	117367338	1.17E+08	KD2-H4K8ac_peak_1764	11.05407	
1	117368888	1.17E+08	KD2-H4K8ac_peak_1765	13.56049	
1	117390256	1.17E+08	KD2-H4K8ac_peak_1766	7.67496	
1	117396420	1.17E+08	KD2-H4K8ac_peak_1767	6.1228	
1	117397985	1.17E+08	KD2-H4K8ac_peak_1768	12.25733	
1	117421100	1.17E+08	KD2-H4K8ac_peak_1769	13.92307	
1	117452167	1.17E+08	KD2-H4K8ac_peak_1770	6.66359	RP4-753F5.1_ENSG00000272715;PTGFRN_ENSG00000134247
1	117452801	1.17E+08	KD2-H4K8ac_peak_1771	9.11891	RP4-753F5.1_ENSG00000272715;PTGFRN_ENSG00000134247
1	117601917	1.18E+08	KD2-H4K8ac_peak_1772	6.0073	RP11-27K13.3_ENSG00000236137;TTF2_ENSG00000116830
1	117664558	1.18E+08	KD2-H4K8ac_peak_1773	13.21979	TRIM45_ENSG00000134253
1	117909106	1.18E+08	KD2-H4K8ac_peak_1774	10.4923	MAN1A2_ENSG00000198162
1	118108338	1.18E+08	KD2-H4K8ac_peak_1775	5.26591	
1	118147916	1.18E+08	KD2-H4K8ac_peak_1776	6.40683	AL157902.3_ENSG00000236866;FAM46C_ENSG00000183508
1	118472465	1.18E+08	KD2-H4K8ac_peak_1777	8.79106	GDAP2_ENSG00000196505;WDR3_ENSG00000065183
1	119683041	1.2E+08	KD2-H4K8ac_peak_1778	8.30974	WARS2_ENSG00000116874;RP11-418J17.1_ENSG00000231365
1	120190531	1.2E+08	KD2-H4K8ac_peak_1779	13.31016	ZNF697_ENSG00000143067
1	120190992	1.2E+08	KD2-H4K8ac_peak_1780	21.11201	ZNF697_ENSG00000143067
1	120254981	1.2E+08	KD2-H4K8ac_peak_1781	11.24702	
1	120611665	1.21E+08	KD2-H4K8ac_peak_1782	6.98405	NOTCH2_ENSG00000134250
1	120836617	1.21E+08	KD2-H4K8ac_peak_1783	11.05407	FAM72B_ENSG00000188610
1	142617952	1.43E+08	KD2-H4K8ac_peak_1784	28.25849	RP11-417J8.3_ENSG00000230880
1	143275549	1.43E+08	KD2-H4K8ac_peak_1785	8.86099	
1	143467269	1.43E+08	KD2-H4K8ac_peak_1786	17.43705	RP11-435B5.4_ENSG00000185044
1	143467987	1.43E+08	KD2-H4K8ac_peak_1787	25.8903	RP11-435B5.4_ENSG00000185044
1	143743386	1.44E+08	KD2-H4K8ac_peak_1788	11.05155	
1	144519938	1.45E+08	KD2-H4K8ac_peak_1789	12.82393	
1	144593764	1.45E+08	KD2-H4K8ac_peak_1790	17.42905	RP11-640M9.2_ENSG00000225241
1	144931670	1.45E+08	KD2-H4K8ac_peak_1791	15.39448	
1	145209073	1.45E+08	KD2-H4K8ac_peak_1792	4.37809	NOTCH2NL_ENSG00000213240;RP11-458D21.5_ENSG00000255168

1	145381628	1.45E+08	KD2-H4K8ac_peak_1793	7.87536	RP11-458D21.1_ENSG00000233396;RNVU1-6_ENSG00000201558
1	145400054	1.45E+08	KD2-H4K8ac_peak_1794	4.85088	
1	145437517	1.45E+08	KD2-H4K8ac_peak_1795	11.55418	TXNIP_ENSG00000117289
1	145473481	1.45E+08	KD2-H4K8ac_peak_1796	4.27127	
1	145477259	1.45E+08	KD2-H4K8ac_peak_1797	7.57336	LIX1L_ENSG00000152022
1	145516722	1.46E+08	KD2-H4K8ac_peak_1798	6.99815	GNRHR2_ENSG00000211451;PEX11B_ENSG00000131779
1	145542662	1.46E+08	KD2-H4K8ac_peak_1799	10.17486	RP11-315I20.3_ENSG00000244619
1	145575319	1.46E+08	KD2-H4K8ac_peak_1800	9.34989	PIAS3_ENSG00000131788
1	145576036	1.46E+08	KD2-H4K8ac_peak_1801	13.73635	PIAS3_ENSG00000131788
1	145588901	1.46E+08	KD2-H4K8ac_peak_1802	14.68449	NUDT17_ENSG00000186364
1	145610578	1.46E+08	KD2-H4K8ac_peak_1803	16.57619	POLR3C_ENSG00000186141;RNF115_ENSG00000121848
1	145713477	1.46E+08	KD2-H4K8ac_peak_1804	5.85291	
1	145717359	1.46E+08	KD2-H4K8ac_peak_1805	4.90805	
1	145727045	1.46E+08	KD2-H4K8ac_peak_1806	6.24114	PDZK1_ENSG00000174827
1	145826638	1.46E+08	KD2-H4K8ac_peak_1807	29.57597	GPR89A_ENSG00000117262;WI2-925H4.1_ENSG00000235702
1	146009284	1.46E+08	KD2-H4K8ac_peak_1808	11.00004	
1	146643509	1.47E+08	KD2-H4K8ac_peak_1809	7.57336	PRKAB2_ENSG00000131791;RP11-337C18.8_ENSG00000237188;RP11-337C18.10_ENSG00000273071
1	146644347	1.47E+08	KD2-H4K8ac_peak_1810	14.84212	PRKAB2_ENSG00000131791;RP11-337C18.8_ENSG00000237188;RP11-337C18.10_ENSG00000273071
1	146645129	1.47E+08	KD2-H4K8ac_peak_1811	8.90012	RP11-337C18.8_ENSG00000237188;RP11-337C18.10_ENSG00000273071
1	146714379	1.47E+08	KD2-H4K8ac_peak_1812	19.63735	FMO5_ENSG00000131781;CHD1L_ENSG00000131778
1	147012293	1.47E+08	KD2-H4K8ac_peak_1813	7.94441	BCL9_ENSG00000116128
1	147013437	1.47E+08	KD2-H4K8ac_peak_1814	10.19949	BCL9_ENSG00000116128
1	147070854	1.47E+08	KD2-H4K8ac_peak_1815	5.75757	
1	147072005	1.47E+08	KD2-H4K8ac_peak_1816	8.85813	
1	147141737	1.47E+08	KD2-H4K8ac_peak_1817	12.08081	ACP6_ENSG00000162836
1	147142782	1.47E+08	KD2-H4K8ac_peak_1818	12.57143	ACP6_ENSG00000162836
1	147400585	1.47E+08	KD2-H4K8ac_peak_1819	9.26408	GPR89B_ENSG00000188092
1	148193386	1.48E+08	KD2-H4K8ac_peak_1820	4.42765	RP11-289I10.2_ENSG00000223612
1	148246712	1.48E+08	KD2-H4K8ac_peak_1821	10.94087	
1	148247317	1.48E+08	KD2-H4K8ac_peak_1822	15.50901	RP11-89F3.2_ENSG00000236140
1	148526870	1.49E+08	KD2-H4K8ac_peak_1823	5.25071	
1	148532175	1.49E+08	KD2-H4K8ac_peak_1824	10.19949	
1	148556077	1.49E+08	KD2-H4K8ac_peak_1825	17.88235	NBPF15_ENSG00000243452
1	148598513	1.49E+08	KD2-H4K8ac_peak_1826	8.4716	RP11-666A1.5_ENSG00000237253;RP11-666A1.3_ENSG00000226675
1	148927929	1.49E+08	KD2-H4K8ac_peak_1827	6.56646	RP11-14N7.2_ENSG00000232527
1	148928433	1.49E+08	KD2-H4K8ac_peak_1828	12.42526	RP11-14N7.2_ENSG00000232527
1	149222384	1.49E+08	KD2-H4K8ac_peak_1829	15.44609	
1	149223205	1.49E+08	KD2-H4K8ac_peak_1830	8.32007	RNVU1-18_ENSG00000206737
1	149298774	1.49E+08	KD2-H4K8ac_peak_1831	7.76104	
1	149784624	1.5E+08	KD2-H4K8ac_peak_1832	8.85813	HIST2H2BF_ENSG00000203814;HIST2H3D_ENSG00000183598
1	149857793	1.5E+08	KD2-H4K8ac_peak_1833	4.79789	HIST2H2BE_ENSG00000184678;HIST2H2AC_ENSG00000184260
1	149858570	1.5E+08	KD2-H4K8ac_peak_1834	6.40874	HIST2H2BE_ENSG00000184678;HIST2H2AC_ENSG00000184260;HIST2H2AB_ENSG00000184270;BOLA1_ENSG00000178096
1	149860314	1.5E+08	KD2-H4K8ac_peak_1835	4.66592	HIST2H2AB_ENSG00000184270;BOLA1_ENSG00000178096
1	149871197	1.5E+08	KD2-H4K8ac_peak_1836	26.38304	
1	149888858	1.5E+08	KD2-H4K8ac_peak_1837	11.05467	SV2A_ENSG00000159164
1	149899838	1.5E+08	KD2-H4K8ac_peak_1838	12.08081	SF3B4_ENSG00000143368

1	149982151	1.5E+08	KD2-H4K8ac_peak_1839	9.63487	OTUD7B_ENSG00000163113
1	149982815	1.5E+08	KD2-H4K8ac_peak_1840	10.29842	OTUD7B_ENSG00000163113
1	150039066	1.5E+08	KD2-H4K8ac_peak_1841	10.53377	VPS45_ENSG00000136631
1	150039496	1.5E+08	KD2-H4K8ac_peak_1842	12.93209	VPS45_ENSG00000136631
1	150121594	1.5E+08	KD2-H4K8ac_peak_1843	14.85549	PLEKHO1_ENSG00000023902
1	150185400	1.5E+08	KD2-H4K8ac_peak_1844	6.65673	
1	150186755	1.5E+08	KD2-H4K8ac_peak_1845	5.82799	
1	150207390	1.5E+08	KD2-H4K8ac_peak_1846	14.27217	ANP32E_ENSG00000143401;RNU2-17P_ENSG00000222222
1	150209609	1.5E+08	KD2-H4K8ac_peak_1847	4.67342	RNU2-17P_ENSG00000222222
1	150228878	1.5E+08	KD2-H4K8ac_peak_1848	5.88845	RP4-790G17.7_ENSG00000271845;CA14_ENSG00000118298
1	150229278	1.5E+08	KD2-H4K8ac_peak_1849	4.84206	RP4-790G17.7_ENSG00000271845;CA14_ENSG00000118298
1	150240979	1.5E+08	KD2-H4K8ac_peak_1850	13.83097	APH1A_ENSG00000117362;C1orf54_ENSG00000118292
1	150241698	1.5E+08	KD2-H4K8ac_peak_1851	14.8083	APH1A_ENSG00000117362
1	150254036	1.5E+08	KD2-H4K8ac_peak_1852	3.90565	C1orf51_ENSG00000159208
1	150264660	1.5E+08	KD2-H4K8ac_peak_1853	7.40314	
1	150266010	1.5E+08	KD2-H4K8ac_peak_1854	8.16906	MRPS21_ENSG00000187145
1	150293210	1.5E+08	KD2-H4K8ac_peak_1855	12.08081	PRPF3_ENSG00000117360
1	150335038	1.5E+08	KD2-H4K8ac_peak_1856	7.36799	RPRD2_ENSG00000163125
1	150336181	1.5E+08	KD2-H4K8ac_peak_1857	17.70837	RPRD2_ENSG00000163125
1	150338160	1.5E+08	KD2-H4K8ac_peak_1858	4.91674	
1	150418381	1.5E+08	KD2-H4K8ac_peak_1859	5.97274	
1	150458211	1.5E+08	KD2-H4K8ac_peak_1860	5.04761	
1	150459489	1.5E+08	KD2-H4K8ac_peak_1861	9.58924	TARS2_ENSG00000143374
1	150459922	1.5E+08	KD2-H4K8ac_peak_1862	20.42458	TARS2_ENSG00000143374
1	150487653	1.5E+08	KD2-H4K8ac_peak_1863	7.49599	LINC00568_ENSG00000228126
1	150488248	1.5E+08	KD2-H4K8ac_peak_1864	23.28866	LINC00568_ENSG00000228126
1	150504364	1.51E+08	KD2-H4K8ac_peak_1865	6.25869	
1	150521155	1.51E+08	KD2-H4K8ac_peak_1866	7.33452	ADAMTSL4_ENSG00000143382;AL356356.1_ENSG00000225996
1	150533617	1.51E+08	KD2-H4K8ac_peak_1867	11.8457	ADAMTSL4-AS1_ENSG00000203804
1	150534650	1.51E+08	KD2-H4K8ac_peak_1868	10.12072	ADAMTSL4-AS1_ENSG00000203804
1	150551359	1.51E+08	KD2-H4K8ac_peak_1869	11.44887	MCL1_ENSG00000143384
1	150552356	1.51E+08	KD2-H4K8ac_peak_1870	10.43145	MCL1_ENSG00000143384
1	150594278	1.51E+08	KD2-H4K8ac_peak_1871	4.38478	
1	150601791	1.51E+08	KD2-H4K8ac_peak_1872	8.48413	ENSA_ENSG00000143420
1	150602449	1.51E+08	KD2-H4K8ac_peak_1873	10.20046	ENSA_ENSG00000143420
1	150670242	1.51E+08	KD2-H4K8ac_peak_1874	6.30229	GOLPH3L_ENSG00000143457
1	150685148	1.51E+08	KD2-H4K8ac_peak_1875	5.25071	
1	150736828	1.51E+08	KD2-H4K8ac_peak_1876	9.51625	
1	150738028	1.51E+08	KD2-H4K8ac_peak_1877	5.9696	CTSS_ENSG00000163131
1	150848730	1.51E+08	KD2-H4K8ac_peak_1878	7.98621	ARNT_ENSG00000143437
1	150849436	1.51E+08	KD2-H4K8ac_peak_1879	13.81781	ARNT_ENSG00000143437
1	150851656	1.51E+08	KD2-H4K8ac_peak_1880	8.26565	
1	150898037	1.51E+08	KD2-H4K8ac_peak_1881	13.72769	SETDB1_ENSG00000143379
1	150898875	1.51E+08	KD2-H4K8ac_peak_1882	8.83582	SETDB1_ENSG00000143379
1	150946448	1.51E+08	KD2-H4K8ac_peak_1883	17.04347	CERS2_ENSG00000143418;RP11-316M1.3_ENSG00000231073
1	150951798	1.51E+08	KD2-H4K8ac_peak_1884	7.36799	

1	150978943	1.51E+08	KD2-H4K8ac_peak_1885	8.04448	
1	150981001	1.51E+08	KD2-H4K8ac_peak_1886	11.24702	FAM63A_ENSG00000143409;PRUNE_ENSG00000143363
1	151019613	1.51E+08	KD2-H4K8ac_peak_1887	11.05155	C1orf56_ENSG00000143443
1	151031289	1.51E+08	KD2-H4K8ac_peak_1888	15.66774	
1	151042765	1.51E+08	KD2-H4K8ac_peak_1889	20.53631	CDC42SE1_ENSG00000197622;GABPB2_ENSG00000143458
1	151118814	1.51E+08	KD2-H4K8ac_peak_1890	5.19622	SEMA6C_ENSG00000143434
1	151137513	1.51E+08	KD2-H4K8ac_peak_1891	6.20982	LYSMD1_ENSG00000163155
1	151138052	1.51E+08	KD2-H4K8ac_peak_1892	10.80014	LYSMD1_ENSG00000163155
1	151138671	1.51E+08	KD2-H4K8ac_peak_1893	7.93948	LYSMD1_ENSG00000163155
1	151162399	1.51E+08	KD2-H4K8ac_peak_1894	16.49944	
1	151167979	1.51E+08	KD2-H4K8ac_peak_1895	5.88845	VPS72_ENSG00000163159
1	151226583	1.51E+08	KD2-H4K8ac_peak_1896	18.25771	PSMD4_ENSG00000159352
1	151253510	1.51E+08	KD2-H4K8ac_peak_1897	7.49599	RP11-126K1.2_ENSG00000232671;ZNF687_ENSG00000143373
1	151254034	1.51E+08	KD2-H4K8ac_peak_1898	6.01128	RP11-126K1.2_ENSG00000232671;ZNF687_ENSG00000143373
1	151254786	1.51E+08	KD2-H4K8ac_peak_1899	6.15163	RP11-126K1.2_ENSG00000232671;ZNF687_ENSG00000143373
1	151300597	1.51E+08	KD2-H4K8ac_peak_1900	14.27217	PI4KB_ENSG00000143393;RP11-126K1.9_ENSG00000273481
1	151319393	1.51E+08	KD2-H4K8ac_peak_1901	10.09333	RFX5_ENSG00000143390;RP11-126K1.6_ENSG00000237976
1	151371388	1.51E+08	KD2-H4K8ac_peak_1902	11.60333	PSMB4_ENSG00000159377
1	151372068	1.51E+08	KD2-H4K8ac_peak_1903	3.86308	PSMB4_ENSG00000159377
1	151432170	1.51E+08	KD2-H4K8ac_peak_1904	15.01306	POGZ_ENSG00000143442
1	151483328	1.51E+08	KD2-H4K8ac_peak_1905	6.0605	CGN_ENSG00000143375
1	151580404	1.52E+08	KD2-H4K8ac_peak_1906	7.50083	
1	151584610	1.52E+08	KD2-H4K8ac_peak_1907	8.36079	RP11-404E16.1_ENSG00000250734;SNX27_ENSG00000143376
1	151700333	1.52E+08	KD2-H4K8ac_peak_1908	6.65673	
1	151735379	1.52E+08	KD2-H4K8ac_peak_1909	19.33282	MRPL9_ENSG00000143436;OAZ3_ENSG00000143450
1	151762688	1.52E+08	KD2-H4K8ac_peak_1910	10.29842	TDRKH_ENSG00000182134;RP11-98D18.9_ENSG00000203288
1	151763584	1.52E+08	KD2-H4K8ac_peak_1911	11.43863	TDRKH_ENSG00000182134;RP11-98D18.9_ENSG00000203288
1	151820414	1.52E+08	KD2-H4K8ac_peak_1912	5.01002	
1	151882156	1.52E+08	KD2-H4K8ac_peak_1913	24.3087	THEM4_ENSG00000159445
1	152020018	1.52E+08	KD2-H4K8ac_peak_1914	10.74343	S100A11_ENSG00000163191
1	152020469	1.52E+08	KD2-H4K8ac_peak_1915	13.50024	S100A11_ENSG00000163191
1	152161365	1.52E+08	KD2-H4K8ac_peak_1916	4.8485	
1	153508112	1.54E+08	KD2-H4K8ac_peak_1917	5.75757	S100A6_ENSG00000197956
1	153508649	1.54E+08	KD2-H4K8ac_peak_1918	13.80019	S100A6_ENSG00000197956
1	153518184	1.54E+08	KD2-H4K8ac_peak_1919	9.48511	
1	153519048	1.54E+08	KD2-H4K8ac_peak_1920	4.06758	
1	153538035	1.54E+08	KD2-H4K8ac_peak_1921	8.85813	
1	153538930	1.54E+08	KD2-H4K8ac_peak_1922	10.43145	S100A2_ENSG00000196754
1	153540749	1.54E+08	KD2-H4K8ac_peak_1923	10.05606	S100A2_ENSG00000196754
1	153541326	1.54E+08	KD2-H4K8ac_peak_1924	7.40314	S100A2_ENSG00000196754
1	153559160	1.54E+08	KD2-H4K8ac_peak_1925	9.11891	
1	153571055	1.54E+08	KD2-H4K8ac_peak_1926	5.13893	
1	153606607	1.54E+08	KD2-H4K8ac_peak_1927	13.65567	S100A13_ENSG00000189171;RP1-178F15.5_ENSG00000271853;RP1-178F15.4_ENSG00000272030;CHTOP_ENSG00000160679
1	153630370	1.54E+08	KD2-H4K8ac_peak_1928	12.93209	SNAPIN_ENSG00000143553
1	153630892	1.54E+08	KD2-H4K8ac_peak_1929	17.43561	SNAPIN_ENSG00000143553
1	153642936	1.54E+08	KD2-H4K8ac_peak_1930	8.18084	ILF2_ENSG00000143621

1	153644361	1.54E+08	KD2-H4K8ac_peak_1931	9.96798	ILF2_ENSG00000143621
1	153669691	1.54E+08	KD2-H4K8ac_peak_1932	10.43145	
1	153683462	1.54E+08	KD2-H4K8ac_peak_1933	3.76742	
1	153698949	1.54E+08	KD2-H4K8ac_peak_1934	7.61941	Y_RNA_ENSG00000199565
1	153699608	1.54E+08	KD2-H4K8ac_peak_1935	11.80283	INTS3_ENSG00000143624;Y_RNA_ENSG00000199565
1	153700571	1.54E+08	KD2-H4K8ac_peak_1936	21.2844	INTS3_ENSG00000143624
1	153747353	1.54E+08	KD2-H4K8ac_peak_1937	9.34195	SLC27A3_ENSG00000143554
1	153748817	1.54E+08	KD2-H4K8ac_peak_1938	7.45629	
1	153755338	1.54E+08	KD2-H4K8ac_peak_1939	5.6845	
1	153852999	1.54E+08	KD2-H4K8ac_peak_1940	9.06369	
1	153853835	1.54E+08	KD2-H4K8ac_peak_1941	6.40425	
1	153895849	1.54E+08	KD2-H4K8ac_peak_1942	15.13116	GATAD2B_ENSG00000143614
1	153918178	1.54E+08	KD2-H4K8ac_peak_1943	11.80283	DENND4B_ENSG00000198837
1	153930456	1.54E+08	KD2-H4K8ac_peak_1944	10.73392	CRTC2_ENSG00000160741
1	153935545	1.54E+08	KD2-H4K8ac_peak_1945	13.75159	
1	153936375	1.54E+08	KD2-H4K8ac_peak_1946	11.08187	
1	153940924	1.54E+08	KD2-H4K8ac_peak_1947	14.17488	SLC39A1_ENSG00000143570;CREB3L4_ENSG00000143578
1	153949805	1.54E+08	KD2-H4K8ac_peak_1948	12.70959	JTB_ENSG00000143543;RP11-422P24.11_ENSG00000272654
1	153958483	1.54E+08	KD2-H4K8ac_peak_1949	5.01002	RAB13_ENSG00000143545
1	153959726	1.54E+08	KD2-H4K8ac_peak_1950	7.76329	RAB13_ENSG00000143545
1	153962552	1.54E+08	KD2-H4K8ac_peak_1951	15.40891	RPS27_ENSG00000177954
1	153963269	1.54E+08	KD2-H4K8ac_peak_1952	16.57619	RPS27_ENSG00000177954
1	154155191	1.54E+08	KD2-H4K8ac_peak_1953	10.7186	
1	154155848	1.54E+08	KD2-H4K8ac_peak_1954	12.57865	
1	154192755	1.54E+08	KD2-H4K8ac_peak_1955	14.99434	C1orf43_ENSG00000143612;UBAP2L_ENSG00000143569
1	154193397	1.54E+08	KD2-H4K8ac_peak_1956	23.72814	C1orf43_ENSG00000143612;UBAP2L_ENSG00000143569
1	154244701	1.54E+08	KD2-H4K8ac_peak_1957	12.93209	HAX1_ENSG00000143575
1	154296722	1.54E+08	KD2-H4K8ac_peak_1958	15.9754	ATP8B2_ENSG00000143515
1	154324680	1.54E+08	KD2-H4K8ac_peak_1959	4.44527	
1	154325397	1.54E+08	KD2-H4K8ac_peak_1960	6.66359	
1	154405104	1.54E+08	KD2-H4K8ac_peak_1961	6.80057	
1	154454326	1.54E+08	KD2-H4K8ac_peak_1962	3.75035	
1	154465760	1.54E+08	KD2-H4K8ac_peak_1963	10.08874	
1	154530606	1.55E+08	KD2-H4K8ac_peak_1964	12.13241	UBE2Q1_ENSG00000160714
1	154531512	1.55E+08	KD2-H4K8ac_peak_1965	31.44317	UBE2Q1_ENSG00000160714
1	154540459	1.55E+08	KD2-H4K8ac_peak_1966	6.84261	CHRNB2_ENSG00000160716
1	154580404	1.55E+08	KD2-H4K8ac_peak_1967	12.00597	
1	154599927	1.55E+08	KD2-H4K8ac_peak_1968	17.43561	ADAR_ENSG00000160710
1	154719543	1.55E+08	KD2-H4K8ac_peak_1969	4.86324	
1	154720305	1.55E+08	KD2-H4K8ac_peak_1970	11.76541	
1	154733101	1.55E+08	KD2-H4K8ac_peak_1971	6.78247	
1	154805839	1.55E+08	KD2-H4K8ac_peak_1972	6.65673	
1	154810038	1.55E+08	KD2-H4K8ac_peak_1973	5.1628	
1	154813247	1.55E+08	KD2-H4K8ac_peak_1974	7.74292	
1	154814766	1.55E+08	KD2-H4K8ac_peak_1975	10.43145	
1	154819477	1.55E+08	KD2-H4K8ac_peak_1976	8.84853	

1	154908732	1.55E+08	KD2-H4K8ac_peak_1977	9.95759	PMVK_ENSG00000163344;RP11-307C12.13_ENSG00000270361
1	154909670	1.55E+08	KD2-H4K8ac_peak_1978	5.3157	PMVK_ENSG00000163344;RP11-307C12.13_ENSG00000270361
1	154914086	1.55E+08	KD2-H4K8ac_peak_1979	5.81188	
1	154928285	1.55E+08	KD2-H4K8ac_peak_1980	7.95563	PBXIP1_ENSG00000163346
1	154933821	1.55E+08	KD2-H4K8ac_peak_1981	28.03233	RP11-307C12.12_ENSG00000271380
1	154941053	1.55E+08	KD2-H4K8ac_peak_1982	7.4653	
1	154941631	1.55E+08	KD2-H4K8ac_peak_1983	4.67342	
1	154942553	1.55E+08	KD2-H4K8ac_peak_1984	9.95119	
1	154943363	1.55E+08	KD2-H4K8ac_peak_1985	8.36432	
1	154946452	1.55E+08	KD2-H4K8ac_peak_1986	7.49599	SHC1_ENSG00000160691;CKS1B_ENSG00000173207
1	154948039	1.55E+08	KD2-H4K8ac_peak_1987	7.07117	CKS1B_ENSG00000173207;MIR4258_ENSG00000264349
1	154956330	1.55E+08	KD2-H4K8ac_peak_1988	6.66359	FLAD1_ENSG00000160688
1	154973437	1.55E+08	KD2-H4K8ac_peak_1989	8.81558	
1	154975342	1.55E+08	KD2-H4K8ac_peak_1990	18.59584	ZBTB7B_ENSG00000160685
1	154989407	1.55E+08	KD2-H4K8ac_peak_1991	22.03039	
1	154990361	1.55E+08	KD2-H4K8ac_peak_1992	5.1707	
1	155022315	1.55E+08	KD2-H4K8ac_peak_1993	5.12995	ADAM15_ENSG00000143537
1	155023836	1.55E+08	KD2-H4K8ac_peak_1994	7.43893	ADAM15_ENSG00000143537
1	155036302	1.55E+08	KD2-H4K8ac_peak_1995	10.45683	EFNA4_ENSG00000243364;EFNA3_ENSG00000143590;EFNA3_ENSG00000251246
1	155042980	1.55E+08	KD2-H4K8ac_peak_1996	16.86242	
1	155051374	1.55E+08	KD2-H4K8ac_peak_1997	5.85291	
1	155052741	1.55E+08	KD2-H4K8ac_peak_1998	6.31838	
1	155056540	1.55E+08	KD2-H4K8ac_peak_1999	7.92775	
1	155066099	1.55E+08	KD2-H4K8ac_peak_2000	7.90806	
1	155098484	1.55E+08	KD2-H4K8ac_peak_2001	4.67342	EFNA1_ENSG00000169242
1	155099602	1.55E+08	KD2-H4K8ac_peak_2002	30.17017	EFNA1_ENSG00000169242
1	155101526	1.55E+08	KD2-H4K8ac_peak_2003	5.13615	
1	155102150	1.55E+08	KD2-H4K8ac_peak_2004	7.70067	
1	155107536	1.55E+08	KD2-H4K8ac_peak_2005	13.38658	SLC50A1_ENSG00000169241
1	155111695	1.55E+08	KD2-H4K8ac_peak_2006	7.42046	
1	155112406	1.55E+08	KD2-H4K8ac_peak_2007	4.76255	DPM3_ENSG00000179085
1	155113074	1.55E+08	KD2-H4K8ac_peak_2008	14.59156	DPM3_ENSG00000179085
1	155139382	1.55E+08	KD2-H4K8ac_peak_2009	8.4716	
1	155145160	1.55E+08	KD2-H4K8ac_peak_2010	16.54484	KRTCAP2_ENSG00000163463;TRIM46_ENSG00000163462
1	155146386	1.55E+08	KD2-H4K8ac_peak_2011	21.61343	KRTCAP2_ENSG00000163463;TRIM46_ENSG00000163462
1	155162860	1.55E+08	KD2-H4K8ac_peak_2012	12.13241	MUC1_ENSG00000185499;MIR92B_ENSG00000271748
1	155176864	1.55E+08	KD2-H4K8ac_peak_2013	10.55956	MTX1_ENSG00000173171
1	155177850	1.55E+08	KD2-H4K8ac_peak_2014	21.61343	THBS3_ENSG00000169231;MTX1_ENSG00000173171
1	155188979	1.55E+08	KD2-H4K8ac_peak_2015	17.10757	
1	155196748	1.55E+08	KD2-H4K8ac_peak_2016	9.36516	GBAP1_ENSG00000160766
1	155197577	1.55E+08	KD2-H4K8ac_peak_2017	19.16083	GBAP1_ENSG00000160766
1	155213399	1.55E+08	KD2-H4K8ac_peak_2018	4.68059	GBA_ENSG00000177628
1	155214755	1.55E+08	KD2-H4K8ac_peak_2019	12.14337	GBA_ENSG00000177628
1	155220265	1.55E+08	KD2-H4K8ac_peak_2020	13.65567	
1	155223355	1.55E+08	KD2-H4K8ac_peak_2021	8.35576	
1	155224283	1.55E+08	KD2-H4K8ac_peak_2022	19.25648	FAM189B_ENSG00000160767

1	155225883	1.55E+08	KD2-H4K8ac_peak_2023	7.07117	FAM189B_ENSG00000160767
1	155231548	1.55E+08	KD2-H4K8ac_peak_2024	10.43145	SCAMP3_ENSG00000116521
1	155232312	1.55E+08	KD2-H4K8ac_peak_2025	21.11201	SCAMP3_ENSG00000116521
1	155242792	1.55E+08	KD2-H4K8ac_peak_2026	9.48511	
1	155247304	1.55E+08	KD2-H4K8ac_peak_2027	6.40297	CLK2_ENSG00000176444;HCN3_ENSG00000143630
1	155277504	1.55E+08	KD2-H4K8ac_peak_2028	9.68182	FDPS_ENSG00000160752
1	155278788	1.55E+08	KD2-H4K8ac_peak_2029	6.66359	FDPS_ENSG00000160752
1	155292541	1.55E+08	KD2-H4K8ac_peak_2030	7.07117	RUSC1-AS1_ENSG00000225855
1	155293774	1.55E+08	KD2-H4K8ac_peak_2031	12.48406	RUSC1-AS1_ENSG00000225855
1	155531921	1.56E+08	KD2-H4K8ac_peak_2032	17.19888	ASH1L-AS1_ENSG00000235919;ASH1L_ENSG00000116539
1	155532769	1.56E+08	KD2-H4K8ac_peak_2033	7.49599	ASH1L-AS1_ENSG00000235919;ASH1L_ENSG00000116539
1	155579162	1.56E+08	KD2-H4K8ac_peak_2034	8.52559	RP11-29H23.4_ENSG00000232519;MSTO1_ENSG00000125459
1	155621214	1.56E+08	KD2-H4K8ac_peak_2035	5.54647	
1	155657879	1.56E+08	KD2-H4K8ac_peak_2036	31.8111	YY1AP1_ENSG00000163374;DAP3_ENSG00000132676
1	155826575	1.56E+08	KD2-H4K8ac_peak_2037	8.4716	
1	155827219	1.56E+08	KD2-H4K8ac_peak_2038	11.15405	
1	155830042	1.56E+08	KD2-H4K8ac_peak_2039	10.66719	SYT11_ENSG00000132718;GON4L_ENSG00000116580
1	155903879	1.56E+08	KD2-H4K8ac_peak_2040	19.28826	KIAA0907_ENSG00000132680
1	155904552	1.56E+08	KD2-H4K8ac_peak_2041	9.63487	KIAA0907_ENSG00000132680
1	155910311	1.56E+08	KD2-H4K8ac_peak_2042	11.66477	RXFP4_ENSG00000173080
1	155947456	1.56E+08	KD2-H4K8ac_peak_2043	9.95119	RP11-336K24.12_ENSG00000273002
1	155948138	1.56E+08	KD2-H4K8ac_peak_2044	14.03283	RP11-336K24.12_ENSG00000273002
1	155990247	1.56E+08	KD2-H4K8ac_peak_2045	10.21366	SSR2_ENSG00000163479
1	155990879	1.56E+08	KD2-H4K8ac_peak_2046	6.99815	SSR2_ENSG00000163479
1	155995816	1.56E+08	KD2-H4K8ac_peak_2047	10.19949	
1	156022904	1.56E+08	KD2-H4K8ac_peak_2048	10.19949	UBQLN4_ENSG00000160803
1	156023654	1.56E+08	KD2-H4K8ac_peak_2049	23.33626	UBQLN4_ENSG00000160803;LAMTOR2_ENSG00000116586
1	156024639	1.56E+08	KD2-H4K8ac_peak_2050	13.3695	LAMTOR2_ENSG00000116586
1	156039893	1.56E+08	KD2-H4K8ac_peak_2051	7.92775	
1	156051204	1.56E+08	KD2-H4K8ac_peak_2052	14.39182	MEX3A_ENSG00000254726;LMNA_ENSG00000160789
1	156051999	1.56E+08	KD2-H4K8ac_peak_2053	11.18097	MEX3A_ENSG00000254726;LMNA_ENSG00000160789
1	156073570	1.56E+08	KD2-H4K8ac_peak_2054	5.26288	
1	156074710	1.56E+08	KD2-H4K8ac_peak_2055	5.49478	
1	156075232	1.56E+08	KD2-H4K8ac_peak_2056	9.48511	
1	156075944	1.56E+08	KD2-H4K8ac_peak_2057	13.27022	
1	156082226	1.56E+08	KD2-H4K8ac_peak_2058	6.99194	
1	156082900	1.56E+08	KD2-H4K8ac_peak_2059	6.04107	
1	156083757	1.56E+08	KD2-H4K8ac_peak_2060	26.00444	
1	156092511	1.56E+08	KD2-H4K8ac_peak_2061	8.88428	
1	156095843	1.56E+08	KD2-H4K8ac_peak_2062	11.80283	
1	156116087	1.56E+08	KD2-H4K8ac_peak_2063	8.10216	SEMA4A_ENSG00000196189
1	156130605	1.56E+08	KD2-H4K8ac_peak_2064	10.43145	
1	156162974	1.56E+08	KD2-H4K8ac_peak_2065	11.28831	SNORA26_ENSG00000252236;SLC25A44_ENSG00000160785
1	156182288	1.56E+08	KD2-H4K8ac_peak_2066	5.32713	PMF1_ENSG00000160783
1	156182812	1.56E+08	KD2-H4K8ac_peak_2067	10.76532	PMF1_ENSG00000160783
1	156220758	1.56E+08	KD2-H4K8ac_peak_2068	10.92268	

1	156221692	1.56E+08	KD2-H4K8ac_peak_2069	8.10216	
1	156252178	1.56E+08	KD2-H4K8ac_peak_2070	15.96909	SMG5_ENSG00000198952;TMEM79_ENSG00000163472
1	156252927	1.56E+08	KD2-H4K8ac_peak_2071	3.90565	SMG5_ENSG00000198952;TMEM79_ENSG00000163472
1	156261391	1.56E+08	KD2-H4K8ac_peak_2072	7.36799	
1	156265164	1.56E+08	KD2-H4K8ac_peak_2073	9.26408	C1orf85_ENSG00000198715
1	156265822	1.56E+08	KD2-H4K8ac_peak_2074	8.76221	C1orf85_ENSG00000198715
1	156307662	1.56E+08	KD2-H4K8ac_peak_2075	9.26408	TSACC_ENSG00000163467
1	156308392	1.56E+08	KD2-H4K8ac_peak_2076	5.75757	
1	156315023	1.56E+08	KD2-H4K8ac_peak_2077	8.93255	
1	156337905	1.56E+08	KD2-H4K8ac_peak_2078	8.85813	CCT3_ENSG00000163468;RHBG_ENSG00000132677
1	156353026	1.56E+08	KD2-H4K8ac_peak_2079	5.41891	
1	156390027	1.56E+08	KD2-H4K8ac_peak_2080	5.32713	MIR9-1_ENSG00000207933
1	156390549	1.56E+08	KD2-H4K8ac_peak_2081	16.44604	MIR9-1_ENSG00000207933
1	156405850	1.56E+08	KD2-H4K8ac_peak_2082	9.402	
1	156425862	1.56E+08	KD2-H4K8ac_peak_2083	8.16906	RP11-98G7.1_ENSG00000236947
1	156426579	1.56E+08	KD2-H4K8ac_peak_2084	22.07161	RP11-98G7.1_ENSG00000236947
1	156470236	1.56E+08	KD2-H4K8ac_peak_2085	6.52179	MEF2D_ENSG00000116604
1	156470870	1.56E+08	KD2-H4K8ac_peak_2086	23.24123	MEF2D_ENSG00000116604
1	156472445	1.56E+08	KD2-H4K8ac_peak_2087	5.26743	
1	156473026	1.56E+08	KD2-H4K8ac_peak_2088	5.90519	
1	156473919	1.56E+08	KD2-H4K8ac_peak_2089	9.89383	
1	156474536	1.56E+08	KD2-H4K8ac_peak_2090	20.26332	
1	156475462	1.56E+08	KD2-H4K8ac_peak_2091	8.36432	
1	156542560	1.57E+08	KD2-H4K8ac_peak_2092	4.34409	IQGAP3_ENSG00000183856
1	156561137	1.57E+08	KD2-H4K8ac_peak_2093	9.26408	APOA1BP_ENSG00000163382
1	156570936	1.57E+08	KD2-H4K8ac_peak_2094	7.28628	GPATCH4_ENSG00000160818
1	156611744	1.57E+08	KD2-H4K8ac_peak_2095	8.85813	RP11-284F21.9_ENSG00000272068;BCAN_ENSG00000132692
1	156626527	1.57E+08	KD2-H4K8ac_peak_2096	5.72657	
1	156627715	1.57E+08	KD2-H4K8ac_peak_2097	6.55628	
1	156629503	1.57E+08	KD2-H4K8ac_peak_2098	10.73134	
1	156630325	1.57E+08	KD2-H4K8ac_peak_2099	8.34752	RP11-284F21.7_ENSG00000229953
1	156631214	1.57E+08	KD2-H4K8ac_peak_2100	14.66631	RP11-284F21.7_ENSG00000229953
1	156637798	1.57E+08	KD2-H4K8ac_peak_2101	5.13615	
1	156645968	1.57E+08	KD2-H4K8ac_peak_2102	10.54778	NES_ENSG00000132688
1	156659679	1.57E+08	KD2-H4K8ac_peak_2103	16.31471	
1	156660804	1.57E+08	KD2-H4K8ac_peak_2104	7.59794	RP11-66D17.3_ENSG00000237588
1	156661802	1.57E+08	KD2-H4K8ac_peak_2105	10.5887	RP11-66D17.3_ENSG00000237588
1	156662683	1.57E+08	KD2-H4K8ac_peak_2106	14.66259	RP11-66D17.3_ENSG00000237588
1	156663450	1.57E+08	KD2-H4K8ac_peak_2107	9.048	
1	156674713	1.57E+08	KD2-H4K8ac_peak_2108	8.16906	CRABP2_ENSG00000143320
1	156675505	1.57E+08	KD2-H4K8ac_peak_2109	6.66359	CRABP2_ENSG00000143320
1	156676153	1.57E+08	KD2-H4K8ac_peak_2110	8.36432	CRABP2_ENSG00000143320
1	156677355	1.57E+08	KD2-H4K8ac_peak_2111	7.44247	
1	156678433	1.57E+08	KD2-H4K8ac_peak_2112	7.08571	
1	156679050	1.57E+08	KD2-H4K8ac_peak_2113	5.96384	
1	156679942	1.57E+08	KD2-H4K8ac_peak_2114	18.33759	

1	156681380	1.57E+08	KD2-H4K8ac_peak_2115	10.78621	RP11-66D17.5_ENSG00000223356
1	156698743	1.57E+08	KD2-H4K8ac_peak_2116	22.28045	ISG20L2_ENSG00000143319;RRNAD1_ENSG00000143303
1	156710454	1.57E+08	KD2-H4K8ac_peak_2117	11.50346	MRPL24_ENSG00000143314
1	156716632	1.57E+08	KD2-H4K8ac_peak_2118	9.26408	
1	156717110	1.57E+08	KD2-H4K8ac_peak_2119	11.24702	
1	156718166	1.57E+08	KD2-H4K8ac_peak_2120	8.58069	
1	156719963	1.57E+08	KD2-H4K8ac_peak_2121	5.73924	PRCC_ENSG00000143294
1	156720590	1.57E+08	KD2-H4K8ac_peak_2122	17.65594	PRCC_ENSG00000143294
1	156721789	1.57E+08	KD2-H4K8ac_peak_2123	22.03039	
1	156730147	1.57E+08	KD2-H4K8ac_peak_2124	4.211	
1	156736159	1.57E+08	KD2-H4K8ac_peak_2125	8.43115	HDGF_ENSG00000143321
1	156737351	1.57E+08	KD2-H4K8ac_peak_2126	20.85474	HDGF_ENSG00000143321
1	156744130	1.57E+08	KD2-H4K8ac_peak_2127	4.67342	
1	156770962	1.57E+08	KD2-H4K8ac_peak_2128	8.45455	
1	156771669	1.57E+08	KD2-H4K8ac_peak_2129	9.63487	
1	156822552	1.57E+08	KD2-H4K8ac_peak_2130	7.7553	
1	156828177	1.57E+08	KD2-H4K8ac_peak_2131	6.84261	INSRR_ENSG00000027644
1	156828658	1.57E+08	KD2-H4K8ac_peak_2132	13.20642	INSRR_ENSG00000027644
1	156830294	1.57E+08	KD2-H4K8ac_peak_2133	12.94604	
1	156859648	1.57E+08	KD2-H4K8ac_peak_2134	7.82254	
1	156864838	1.57E+08	KD2-H4K8ac_peak_2135	9.36516	
1	156883144	1.57E+08	KD2-H4K8ac_peak_2136	13.34725	
1	156884408	1.57E+08	KD2-H4K8ac_peak_2137	9.6589	
1	156897325	1.57E+08	KD2-H4K8ac_peak_2138	16.822	
1	157015060	1.57E+08	KD2-H4K8ac_peak_2139	11.80283	ARHGEF11_ENSG00000132694
1	157015795	1.57E+08	KD2-H4K8ac_peak_2140	9.89383	ARHGEF11_ENSG00000132694
1	157087428	1.57E+08	KD2-H4K8ac_peak_2141	5.08628	
1	157107786	1.57E+08	KD2-H4K8ac_peak_2142	23.57681	ETV3_ENSG00000117036
1	157133688	1.57E+08	KD2-H4K8ac_peak_2143	7.51054	
1	158119144	1.58E+08	KD2-H4K8ac_peak_2144	4.42765	
1	158119870	1.58E+08	KD2-H4K8ac_peak_2145	6.31143	
1	159750799	1.6E+08	KD2-H4K8ac_peak_2146	9.6589	DUSP23_ENSG00000158716
1	159824229	1.6E+08	KD2-H4K8ac_peak_2147	9.6247	C1orf204_ENSG00000188004
1	159824790	1.6E+08	KD2-H4K8ac_peak_2148	6.36375	C1orf204_ENSG00000188004
1	159892295	1.6E+08	KD2-H4K8ac_peak_2149	4.86324	
1	159894476	1.6E+08	KD2-H4K8ac_peak_2150	7.28628	
1	159915738	1.6E+08	KD2-H4K8ac_peak_2151	6.52179	IGSF9_ENSG00000085552
1	160001908	1.6E+08	KD2-H4K8ac_peak_2152	5.66705	PIGM_ENSG00000143315
1	160067866	1.6E+08	KD2-H4K8ac_peak_2153	6.04107	IGSF8_ENSG00000162729
1	160068947	1.6E+08	KD2-H4K8ac_peak_2154	6.84261	IGSF8_ENSG00000162729
1	160071105	1.6E+08	KD2-H4K8ac_peak_2155	7.07117	
1	160163268	1.6E+08	KD2-H4K8ac_peak_2156	12.79505	
1	160174533	1.6E+08	KD2-H4K8ac_peak_2157	15.9754	PEA15_ENSG00000162734;AL121987.1_ENSG00000265381
1	160231704	1.6E+08	KD2-H4K8ac_peak_2158	25.44388	RP11-574F21.2_ENSG00000228606
1	160232490	1.6E+08	KD2-H4K8ac_peak_2159	11.43863	RP11-574F21.2_ENSG00000228606
1	160254659	1.6E+08	KD2-H4K8ac_peak_2160	12.57297	DCAF8_ENSG00000132716;DCAF8_ENSG00000258465

1	160312637	1.6E+08	KD2-H4K8ac_peak_2161	19.63735	COPA_ENSG00000122218;NCSTN_ENSG00000162736
1	160332196	1.6E+08	KD2-H4K8ac_peak_2162	6.84261	
1	160346079	1.6E+08	KD2-H4K8ac_peak_2163	7.03656	
1	160369561	1.6E+08	KD2-H4K8ac_peak_2164	16.86242	
1	160588863	1.61E+08	KD2-H4K8ac_peak_2165	6.0073	
1	161008284	1.61E+08	KD2-H4K8ac_peak_2166	6.24114	RP11-544M22.13_ENSG00000270149;TSTD1_ENSG00000215845
1	161015183	1.61E+08	KD2-H4K8ac_peak_2167	19.28826	USF1_ENSG00000158773
1	161015892	1.61E+08	KD2-H4K8ac_peak_2168	11.92978	USF1_ENSG00000158773
1	161068267	1.61E+08	KD2-H4K8ac_peak_2169	4.27244	KLHDC9_ENSG00000162755
1	161087393	1.61E+08	KD2-H4K8ac_peak_2170	19.28826	PFDN2_ENSG00000143256;NIT1_ENSG00000158793
1	161101927	1.61E+08	KD2-H4K8ac_peak_2171	10.43145	DEDD_ENSG00000158796
1	161102407	1.61E+08	KD2-H4K8ac_peak_2172	14.66631	DEDD_ENSG00000158796
1	161103672	1.61E+08	KD2-H4K8ac_peak_2173	4.53319	
1	161122500	1.61E+08	KD2-H4K8ac_peak_2174	6.27719	UFC1_ENSG00000143222
1	161123705	1.61E+08	KD2-H4K8ac_peak_2175	16.67374	
1	161128956	1.61E+08	KD2-H4K8ac_peak_2176	10.35678	RP11-297K8.2_ENSG00000224985;USP21_ENSG00000143258
1	161146726	1.61E+08	KD2-H4K8ac_peak_2177	7.40314	B4GALT3_ENSG00000158850
1	161147391	1.61E+08	KD2-H4K8ac_peak_2178	8.30974	B4GALT3_ENSG00000158850
1	161166092	1.61E+08	KD2-H4K8ac_peak_2179	16.89142	NDUFS2_ENSG00000158864
1	161167261	1.61E+08	KD2-H4K8ac_peak_2180	16.25682	NDUFS2_ENSG00000158864;ADAMTS4_ENSG00000158859
1	161170157	1.61E+08	KD2-H4K8ac_peak_2181	8.21852	
1	161170627	1.61E+08	KD2-H4K8ac_peak_2182	9.69846	
1	161171315	1.61E+08	KD2-H4K8ac_peak_2183	7.43893	
1	161172218	1.61E+08	KD2-H4K8ac_peak_2184	13.1923	
1	161195043	1.61E+08	KD2-H4K8ac_peak_2185	9.89383	TOMM40L_ENSG00000158882
1	161195883	1.61E+08	KD2-H4K8ac_peak_2186	10.45683	TOMM40L_ENSG00000158882;MIR5187_ENSG00000263548
1	161275771	1.61E+08	KD2-H4K8ac_peak_2187	16.95399	
1	161283486	1.61E+08	KD2-H4K8ac_peak_2188	15.58442	SDHC_ENSG00000143252
1	161359019	1.61E+08	KD2-H4K8ac_peak_2189	9.36516	
1	161368257	1.61E+08	KD2-H4K8ac_peak_2190	7.76329	
1	161368875	1.61E+08	KD2-H4K8ac_peak_2191	18.53991	
1	161369768	1.61E+08	KD2-H4K8ac_peak_2192	6.84261	
1	161410255	1.61E+08	KD2-H4K8ac_peak_2193	7.73264	
1	161696496	1.62E+08	KD2-H4K8ac_peak_2194	8.85813	
1	161697040	1.62E+08	KD2-H4K8ac_peak_2195	12.79505	
1	161719610	1.62E+08	KD2-H4K8ac_peak_2196	11.08187	DUSP12_ENSG00000081721
1	161993431	1.62E+08	KD2-H4K8ac_peak_2197	4.91674	OLFML2B_ENSG00000162745
1	162039169	1.62E+08	KD2-H4K8ac_peak_2198	11.90231	NOS1AP_ENSG00000198929
1	162087273	1.62E+08	KD2-H4K8ac_peak_2199	7.90806	
1	162137049	1.62E+08	KD2-H4K8ac_peak_2200	4.67342	
1	162466965	1.62E+08	KD2-H4K8ac_peak_2201	6.66359	UHMK1_ENSG00000152332
1	162467619	1.62E+08	KD2-H4K8ac_peak_2202	24.23147	UHMK1_ENSG00000152332
1	162530654	1.63E+08	KD2-H4K8ac_peak_2203	24.27967	RP11-359K18.3_ENSG00000259788;UAP1_ENSG00000117143
1	162531331	1.63E+08	KD2-H4K8ac_peak_2204	9.95119	RP11-359K18.3_ENSG00000259788;UAP1_ENSG00000117143
1	162792168	1.63E+08	KD2-H4K8ac_peak_2205	5.85291	
1	162849968	1.63E+08	KD2-H4K8ac_peak_2206	6.7498	

1	162851102	1.63E+08	KD2-H4K8ac_peak_2207	17.65594	
1	162852485	1.63E+08	KD2-H4K8ac_peak_2208	6.16883	
1	162854170	1.63E+08	KD2-H4K8ac_peak_2209	8.55271	
1	162855559	1.63E+08	KD2-H4K8ac_peak_2210	8.08678	
1	162857037	1.63E+08	KD2-H4K8ac_peak_2211	11.09936	
1	162857673	1.63E+08	KD2-H4K8ac_peak_2212	18.37277	
1	162858454	1.63E+08	KD2-H4K8ac_peak_2213	9.95759	
1	162859405	1.63E+08	KD2-H4K8ac_peak_2214	7.44324	
1	162860498	1.63E+08	KD2-H4K8ac_peak_2215	13.81781	
1	162862046	1.63E+08	KD2-H4K8ac_peak_2216	5.30799	
1	162887080	1.63E+08	KD2-H4K8ac_peak_2217	19.28826	
1	162888506	1.63E+08	KD2-H4K8ac_peak_2218	14.99434	
1	162892633	1.63E+08	KD2-H4K8ac_peak_2219	5.96443	
1	162897213	1.63E+08	KD2-H4K8ac_peak_2220	10.21366	
1	163019112	1.63E+08	KD2-H4K8ac_peak_2221	12.00273	
1	163088900	1.63E+08	KD2-H4K8ac_peak_2222	9.72244	
1	163089845	1.63E+08	KD2-H4K8ac_peak_2223	5.61377	
1	163092016	1.63E+08	KD2-H4K8ac_peak_2224	7.36799	
1	163117795	1.63E+08	KD2-H4K8ac_peak_2225	12.95073	
1	163141402	1.63E+08	KD2-H4K8ac_peak_2226	4.70206	
1	163144963	1.63E+08	KD2-H4K8ac_peak_2227	7.81699	
1	163155808	1.63E+08	KD2-H4K8ac_peak_2228	4.50891	
1	163166625	1.63E+08	KD2-H4K8ac_peak_2229	9.90914	
1	163380245	1.63E+08	KD2-H4K8ac_peak_2230	9.96798	
1	163405380	1.63E+08	KD2-H4K8ac_peak_2231	6.55628	
1	163406395	1.63E+08	KD2-H4K8ac_peak_2232	10.05606	
1	163407599	1.63E+08	KD2-H4K8ac_peak_2233	7.27716	
1	163408407	1.63E+08	KD2-H4K8ac_peak_2234	10.05606	
1	163409139	1.63E+08	KD2-H4K8ac_peak_2235	8.52559	
1	163410237	1.63E+08	KD2-H4K8ac_peak_2236	7.73418	
1	163414374	1.63E+08	KD2-H4K8ac_peak_2237	5.1313	
1	163634129	1.64E+08	KD2-H4K8ac_peak_2238	4.44527	
1	164556920	1.65E+08	KD2-H4K8ac_peak_2239	10.65982	
1	164558011	1.65E+08	KD2-H4K8ac_peak_2240	10.73134	
1	164614421	1.65E+08	KD2-H4K8ac_peak_2241	6.34074	
1	164655018	1.65E+08	KD2-H4K8ac_peak_2242	4.32338	
1	164680660	1.65E+08	KD2-H4K8ac_peak_2243	14.56495	
1	164681719	1.65E+08	KD2-H4K8ac_peak_2244	6.20982	
1	164723261	1.65E+08	KD2-H4K8ac_peak_2245	8.30974	
1	165567713	1.66E+08	KD2-H4K8ac_peak_2246	8.10216	RP11-306I1.2_ENSG00000236206
1	165599719	1.66E+08	KD2-H4K8ac_peak_2247	20.42458	MGST3_ENSG00000143198
1	165667355	1.66E+08	KD2-H4K8ac_peak_2248	10.7186	ALDH9A1_ENSG00000143149;RP11-466F5.6_ENSG00000215838
1	165668078	1.66E+08	KD2-H4K8ac_peak_2249	9.55262	ALDH9A1_ENSG00000143149;RP11-466F5.6_ENSG00000215838
1	165737709	1.66E+08	KD2-H4K8ac_peak_2250	10.53377	RP11-466F5.8_ENSG00000224358
1	165738351	1.66E+08	KD2-H4K8ac_peak_2251	9.89383	RP11-466F5.8_ENSG00000224358
1	165796854	1.66E+08	KD2-H4K8ac_peak_2252	16.89963	TMCO1_ENSG00000143183;UCK2_ENSG00000143179

1	166135654	1.66E+08	KD2-H4K8ac_peak_2253	15.81711	FAM78B_ENSG00000188859
1	166320409	1.66E+08	KD2-H4K8ac_peak_2254	5.82799	
1	166808224	1.67E+08	KD2-H4K8ac_peak_2255	9.20193	POGK_ENSG00000143157
1	166844957	1.67E+08	KD2-H4K8ac_peak_2256	19.28826	TADA1_ENSG00000152382
1	167028248	1.67E+08	KD2-H4K8ac_peak_2257	5.20908	
1	167090607	1.67E+08	KD2-H4K8ac_peak_2258	11.02863	
1	167106784	1.67E+08	KD2-H4K8ac_peak_2259	6.27931	
1	167189069	1.67E+08	KD2-H4K8ac_peak_2260	23.04547	RP11-277B15.3_ENSG00000272205;POU2F1_ENSG00000143190
1	167190307	1.67E+08	KD2-H4K8ac_peak_2261	17.27647	RP11-277B15.3_ENSG00000272205;POU2F1_ENSG00000143190
1	167424031	1.67E+08	KD2-H4K8ac_peak_2262	22.62254	
1	167652222	1.68E+08	KD2-H4K8ac_peak_2263	6.66692	
1	167683692	1.68E+08	KD2-H4K8ac_peak_2264	14.03283	
1	167690339	1.68E+08	KD2-H4K8ac_peak_2265	5.13615	MPZL1_ENSG00000197965
1	167691286	1.68E+08	KD2-H4K8ac_peak_2266	7.92775	MPZL1_ENSG00000197965
1	167789224	1.68E+08	KD2-H4K8ac_peak_2267	8.23274	RP1-313L4.3_ENSG00000232194;RP1-313L4.4_ENSG00000250762
1	167905537	1.68E+08	KD2-H4K8ac_peak_2268	7.49599	MPC2_ENSG00000143158;DCAF6_ENSG00000143164
1	167905991	1.68E+08	KD2-H4K8ac_peak_2269	7.07117	MPC2_ENSG00000143158;DCAF6_ENSG00000143164
1	168105674	1.68E+08	KD2-H4K8ac_peak_2270	8.36432	GPR161_ENSG00000143147
1	168114537	1.68E+08	KD2-H4K8ac_peak_2271	8.59579	
1	168147619	1.68E+08	KD2-H4K8ac_peak_2272	5.4779	TIPRL_ENSG00000143155
1	168148207	1.68E+08	KD2-H4K8ac_peak_2273	16.49944	TIPRL_ENSG00000143155
1	168194468	1.68E+08	KD2-H4K8ac_peak_2274	8.38692	SFT2D2_ENSG00000213064
1	168195286	1.68E+08	KD2-H4K8ac_peak_2275	8.63033	SFT2D2_ENSG00000213064
1	168356443	1.68E+08	KD2-H4K8ac_peak_2276	9.9363	
1	168408728	1.68E+08	KD2-H4K8ac_peak_2277	9.36516	
1	168436029	1.68E+08	KD2-H4K8ac_peak_2278	6.85193	
1	168440404	1.68E+08	KD2-H4K8ac_peak_2279	7.27716	
1	168582479	1.69E+08	KD2-H4K8ac_peak_2280	5.01002	
1	168607678	1.69E+08	KD2-H4K8ac_peak_2281	6.61376	
1	168658902	1.69E+08	KD2-H4K8ac_peak_2282	16.61426	
1	168660247	1.69E+08	KD2-H4K8ac_peak_2283	9.90914	
1	168664995	1.69E+08	KD2-H4K8ac_peak_2284	5.9696	
1	168706651	1.69E+08	KD2-H4K8ac_peak_2285	5.88845	
1	168725134	1.69E+08	KD2-H4K8ac_peak_2286	5.14125	
1	168909744	1.69E+08	KD2-H4K8ac_peak_2287	9.40857	
1	169055191	1.69E+08	KD2-H4K8ac_peak_2288	5.82799	LINC00970_ENSG00000203601
1	169057003	1.69E+08	KD2-H4K8ac_peak_2289	5.18553	LINC00970_ENSG00000203601
1	169074199	1.69E+08	KD2-H4K8ac_peak_2290	21.09086	RP5-1018K9.1_ENSG00000237707;ATP1B1_ENSG00000143153
1	169076007	1.69E+08	KD2-H4K8ac_peak_2291	8.25809	
1	169306293	1.69E+08	KD2-H4K8ac_peak_2292	4.37815	
1	169396818	1.69E+08	KD2-H4K8ac_peak_2293	8.30974	
1	169397862	1.69E+08	KD2-H4K8ac_peak_2294	6.51023	
1	169763309	1.7E+08	KD2-H4K8ac_peak_2295	17.67328	METTL18_ENSG00000171806
1	169862423	1.7E+08	KD2-H4K8ac_peak_2296	10.35678	SCYL3_ENSG00000000457
1	169882489	1.7E+08	KD2-H4K8ac_peak_2297	4.62507	
1	170043441	1.7E+08	KD2-H4K8ac_peak_2298	8.2413	

1	170500270	1.71E+08	KD2-H4K8ac_peak_2299	9.1707	GORAB_ENSG00000120370
1	170500993	1.71E+08	KD2-H4K8ac_peak_2300	7.76329	RP11-576I22.2_ENSG00000231407;GORAB_ENSG00000120370
1	171342773	1.71E+08	KD2-H4K8ac_peak_2301	6.16888	
1	171454820	1.71E+08	KD2-H4K8ac_peak_2302	6.84261	PRRC2C_ENSG00000117523
1	171614294	1.72E+08	KD2-H4K8ac_peak_2303	9.67696	
1	171710763	1.72E+08	KD2-H4K8ac_peak_2304	10.29842	VAMP4_ENSG00000117533
1	171711342	1.72E+08	KD2-H4K8ac_peak_2305	6.8713	VAMP4_ENSG00000117533
1	171749879	1.72E+08	KD2-H4K8ac_peak_2306	11.08187	METTL13_ENSG00000010165
1	171810241	1.72E+08	KD2-H4K8ac_peak_2307	5.01002	DNM3_ENSG00000197959
1	171811594	1.72E+08	KD2-H4K8ac_peak_2308	4.49003	DNM3_ENSG00000197959
1	171868114	1.72E+08	KD2-H4K8ac_peak_2309	7.47462	
1	171974596	1.72E+08	KD2-H4K8ac_peak_2310	5.73839	
1	172113512	1.72E+08	KD2-H4K8ac_peak_2311	11.50346	DNM3OS_ENSG00000230630;MIR199A2_ENSG00000208024
1	172133463	1.72E+08	KD2-H4K8ac_peak_2312	4.54006	
1	172291373	1.72E+08	KD2-H4K8ac_peak_2313	5.14125	
1	172327185	1.72E+08	KD2-H4K8ac_peak_2314	7.38933	
1	172412827	1.72E+08	KD2-H4K8ac_peak_2315	21.09086	PIGC_ENSG00000135845
1	172419288	1.72E+08	KD2-H4K8ac_peak_2316	16.49944	
1	172466535	1.72E+08	KD2-H4K8ac_peak_2317	4.211	
1	172501205	1.73E+08	KD2-H4K8ac_peak_2318	7.58939	SUCO_ENSG00000094975
1	172502261	1.73E+08	KD2-H4K8ac_peak_2319	11.04256	SUCO_ENSG00000094975
1	173445866	1.73E+08	KD2-H4K8ac_peak_2320	6.65673	PRDX6_ENSG00000117592
1	173446708	1.73E+08	KD2-H4K8ac_peak_2321	9.87655	PRDX6_ENSG00000117592
1	173638666	1.74E+08	KD2-H4K8ac_peak_2322	3.82059	ANKRD45_ENSG00000183831
1	173683331	1.74E+08	KD2-H4K8ac_peak_2323	17.8868	KLHL20_ENSG00000076321
1	173793151	1.74E+08	KD2-H4K8ac_peak_2324	4.44527	CENPL_ENSG00000120334;DARS2_ENSG00000117593
1	173793885	1.74E+08	KD2-H4K8ac_peak_2325	8.55271	CENPL_ENSG00000120334;DARS2_ENSG00000117593
1	173837458	1.74E+08	KD2-H4K8ac_peak_2326	14.43794	GAS5_ENSG00000234741;ZBTB37_ENSG00000185278
1	173991142	1.74E+08	KD2-H4K8ac_peak_2327	9.65086	RC3H1_ENSG00000135870;RP11-160H22.3_ENSG00000224977
1	173991665	1.74E+08	KD2-H4K8ac_peak_2328	11.92978	RC3H1_ENSG00000135870;RP11-160H22.3_ENSG00000224977
1	174127904	1.74E+08	KD2-H4K8ac_peak_2329	9.96798	RP11-160H22.5_ENSG00000227373;RABGAP1L_ENSG00000152061
1	174128663	1.74E+08	KD2-H4K8ac_peak_2330	15.66774	RP11-160H22.5_ENSG00000227373;RABGAP1L_ENSG00000152061
1	174967855	1.75E+08	KD2-H4K8ac_peak_2331	12.69561	CACYBP_ENSG00000116161
1	174969141	1.75E+08	KD2-H4K8ac_peak_2332	11.50346	CACYBP_ENSG00000116161
1	174992290	1.75E+08	KD2-H4K8ac_peak_2333	11.66477	MRPS14_ENSG00000120333;Y_RNA_ENSG00000206659
1	174998253	1.75E+08	KD2-H4K8ac_peak_2334	7.8711	
1	175161600	1.75E+08	KD2-H4K8ac_peak_2335	14.00658	KIAA0040_ENSG00000235750
1	175482599	1.75E+08	KD2-H4K8ac_peak_2336	6.35843	
1	175484471	1.75E+08	KD2-H4K8ac_peak_2337	7.14562	
1	175687551	1.76E+08	KD2-H4K8ac_peak_2338	7.13615	
1	175850621	1.76E+08	KD2-H4K8ac_peak_2339	5.61067	
1	175856688	1.76E+08	KD2-H4K8ac_peak_2340	6.05809	
1	176175878	1.76E+08	KD2-H4K8ac_peak_2341	13.95627	RFWD2_ENSG00000143207;RP11-195C7.1_ENSG00000236021
1	176685858	1.77E+08	KD2-H4K8ac_peak_2342	3.75035	
1	177133621	1.77E+08	KD2-H4K8ac_peak_2343	6.66692	ASTN1_ENSG00000152092
1	177150501	1.77E+08	KD2-H4K8ac_peak_2344	6.05809	

1	178501672	1.79E+08	KD2-H4K8ac_peak_2345	14.69247	
1	178510436	1.79E+08	KD2-H4K8ac_peak_2346	18.37277	C1orf220_ENSG00000213057;C1ORF220_ENSG00000184909
1	178693832	1.79E+08	KD2-H4K8ac_peak_2347	19.67275	RALGPS2_ENSG00000116191
1	178694709	1.79E+08	KD2-H4K8ac_peak_2348	11.92978	RP11-428K3.1_ENSG00000273062;RALGPS2_ENSG00000116191
1	178954774	1.79E+08	KD2-H4K8ac_peak_2349	6.16888	
1	178971536	1.79E+08	KD2-H4K8ac_peak_2350	6.99194	
1	178994531	1.79E+08	KD2-H4K8ac_peak_2351	9.36516	FAM20B_ENSG00000116199
1	178995170	1.79E+08	KD2-H4K8ac_peak_2352	15.40925	FAM20B_ENSG00000116199
1	179005048	1.79E+08	KD2-H4K8ac_peak_2353	9.26823	RP11-177A2.5_ENSG00000232750
1	179007225	1.79E+08	KD2-H4K8ac_peak_2354	6.25869	
1	179050673	1.79E+08	KD2-H4K8ac_peak_2355	6.99815	TOR3A_ENSG00000186283
1	179051151	1.79E+08	KD2-H4K8ac_peak_2356	17.65612	TOR3A_ENSG00000186283
1	179060329	1.79E+08	KD2-H4K8ac_peak_2357	11.31076	
1	179198399	1.79E+08	KD2-H4K8ac_peak_2358	11.50346	ABL2_ENSG00000143322
1	179199058	1.79E+08	KD2-H4K8ac_peak_2359	11.24702	ABL2_ENSG00000143322
1	179262510	1.79E+08	KD2-H4K8ac_peak_2360	12.84642	SOAT1_ENSG00000057252
1	179262975	1.79E+08	KD2-H4K8ac_peak_2361	13.13969	SOAT1_ENSG00000057252
1	179334464	1.79E+08	KD2-H4K8ac_peak_2362	7.62396	AXDND1_ENSG00000162779
1	179555343	1.8E+08	KD2-H4K8ac_peak_2363	9.26408	RP11-545A16.3_ENSG00000227141
1	179560630	1.8E+08	KD2-H4K8ac_peak_2364	12.30509	TDRD5_ENSG00000162782
1	179711750	1.8E+08	KD2-H4K8ac_peak_2365	28.49742	FAM163A_ENSG00000143340
1	179715725	1.8E+08	KD2-H4K8ac_peak_2366	6.49399	
1	179723842	1.8E+08	KD2-H4K8ac_peak_2367	8.79106	
1	179729646	1.8E+08	KD2-H4K8ac_peak_2368	5.30799	
1	179731545	1.8E+08	KD2-H4K8ac_peak_2369	8.40646	
1	179735037	1.8E+08	KD2-H4K8ac_peak_2370	9.11876	
1	179749598	1.8E+08	KD2-H4K8ac_peak_2371	6.25869	
1	179750650	1.8E+08	KD2-H4K8ac_peak_2372	10.87173	
1	179751532	1.8E+08	KD2-H4K8ac_peak_2373	34.98954	
1	179753202	1.8E+08	KD2-H4K8ac_peak_2374	10.19949	
1	179755092	1.8E+08	KD2-H4K8ac_peak_2375	17.10757	
1	179756754	1.8E+08	KD2-H4K8ac_peak_2376	17.46525	
1	179757854	1.8E+08	KD2-H4K8ac_peak_2377	17.67328	
1	179758389	1.8E+08	KD2-H4K8ac_peak_2378	8.7497	
1	179764067	1.8E+08	KD2-H4K8ac_peak_2379	7.74292	
1	179766085	1.8E+08	KD2-H4K8ac_peak_2380	5.13476	
1	179786430	1.8E+08	KD2-H4K8ac_peak_2381	5.54647	RP11-12M5.3_ENSG00000229407
1	179850957	1.8E+08	KD2-H4K8ac_peak_2382	20.90514	RP11-533E19.7_ENSG00000272906;TOR1AIP1_ENSG00000143337
1	179922392	1.8E+08	KD2-H4K8ac_peak_2383	7.76329	RP11-533E19.5_ENSG00000260360
1	179922928	1.8E+08	KD2-H4K8ac_peak_2384	6.25869	RP11-533E19.5_ENSG00000260360;CEP350_ENSG00000135837
1	179923980	1.8E+08	KD2-H4K8ac_peak_2385	10.09705	RP11-533E19.5_ENSG00000260360;CEP350_ENSG00000135837
1	180123251	1.8E+08	KD2-H4K8ac_peak_2386	3.82395	QSOX1_ENSG00000116260
1	180124251	1.8E+08	KD2-H4K8ac_peak_2387	10.21366	QSOX1_ENSG00000116260
1	180180819	1.8E+08	KD2-H4K8ac_peak_2388	6.66692	
1	180471194	1.8E+08	KD2-H4K8ac_peak_2389	10.13704	ACBD6_ENSG00000135847
1	180564272	1.81E+08	KD2-H4K8ac_peak_2390	6.30229	

1	180893346	1.81E+08	KD2-H4K8ac_peak_2391	6.66359	
1	180894606	1.81E+08	KD2-H4K8ac_peak_2392	26.75898	
1	180895975	1.81E+08	KD2-H4K8ac_peak_2393	23.97836	
1	180898037	1.81E+08	KD2-H4K8ac_peak_2394	12.49757	
1	180898780	1.81E+08	KD2-H4K8ac_peak_2395	12.08081	
1	180900267	1.81E+08	KD2-H4K8ac_peak_2396	8.16906	
1	180902386	1.81E+08	KD2-H4K8ac_peak_2397	7.20567	
1	180991457	1.81E+08	KD2-H4K8ac_peak_2398	9.63487	STX6_ENSG00000135823
1	181057344	1.81E+08	KD2-H4K8ac_peak_2399	11.09936	IER5_ENSG00000162783
1	181058082	1.81E+08	KD2-H4K8ac_peak_2400	4.70206	IER5_ENSG00000162783
1	181058499	1.81E+08	KD2-H4K8ac_peak_2401	8.85813	IER5_ENSG00000162783
1	181073938	1.81E+08	KD2-H4K8ac_peak_2402	31.14568	
1	181074801	1.81E+08	KD2-H4K8ac_peak_2403	13.3695	
1	181082363	1.81E+08	KD2-H4K8ac_peak_2404	6.65673	
1	181097669	1.81E+08	KD2-H4K8ac_peak_2405	19.28826	
1	181103957	1.81E+08	KD2-H4K8ac_peak_2406	11.92978	
1	181121457	1.81E+08	KD2-H4K8ac_peak_2407	4.49003	
1	181122065	1.81E+08	KD2-H4K8ac_peak_2408	8.21852	
1	181135355	1.81E+08	KD2-H4K8ac_peak_2409	9.16531	
1	181149402	1.81E+08	KD2-H4K8ac_peak_2410	4.54729	
1	181998983	1.82E+08	KD2-H4K8ac_peak_2411	4.27244	
1	182030697	1.82E+08	KD2-H4K8ac_peak_2412	4.211	ZNF648_ENSG00000179930
1	182054354	1.82E+08	KD2-H4K8ac_peak_2413	6.67092	
1	182112915	1.82E+08	KD2-H4K8ac_peak_2414	9.07715	
1	182198096	1.82E+08	KD2-H4K8ac_peak_2415	8.55271	
1	182269310	1.82E+08	KD2-H4K8ac_peak_2416	6.66692	
1	182360210	1.82E+08	KD2-H4K8ac_peak_2417	12.13241	
1	182361158	1.82E+08	KD2-H4K8ac_peak_2418	22.92963	
1	182570728	1.83E+08	KD2-H4K8ac_peak_2419	9.08816	
1	182573184	1.83E+08	KD2-H4K8ac_peak_2420	6.40297	RGS16_ENSG00000143333
1	182583745	1.83E+08	KD2-H4K8ac_peak_2421	13.97093	RP11-317P15.4_ENSG00000261504
1	182585305	1.83E+08	KD2-H4K8ac_peak_2422	8.16906	RP11-317P15.4_ENSG00000261504
1	182598926	1.83E+08	KD2-H4K8ac_peak_2423	6.48071	
1	182602729	1.83E+08	KD2-H4K8ac_peak_2424	6.7498	
1	182669110	1.83E+08	KD2-H4K8ac_peak_2425	5.98071	
1	182758477	1.83E+08	KD2-H4K8ac_peak_2426	12.08081	NPL_ENSG00000135838
1	182808076	1.83E+08	KD2-H4K8ac_peak_2427	8.79106	DHX9_ENSG00000135829
1	182808517	1.83E+08	KD2-H4K8ac_peak_2428	12.08081	DHX9_ENSG00000135829
1	182991647	1.83E+08	KD2-H4K8ac_peak_2429	28.03233	LAMC1_ENSG00000135862
1	183285406	1.83E+08	KD2-H4K8ac_peak_2430	5.405	
1	183440439	1.83E+08	KD2-H4K8ac_peak_2431	29.92854	SMG7-AS1_ENSG00000232860;SMG7_ENSG00000116698
1	183441642	1.83E+08	KD2-H4K8ac_peak_2432	15.66774	SMG7-AS1_ENSG00000232860;SMG7_ENSG00000116698
1	183578314	1.84E+08	KD2-H4K8ac_peak_2433	10.02431	
1	183604327	1.84E+08	KD2-H4K8ac_peak_2434	10.43145	ARPC5_ENSG00000162704
1	183774071	1.84E+08	KD2-H4K8ac_peak_2435	4.44527	
1	183813029	1.84E+08	KD2-H4K8ac_peak_2436	8.30974	

1	184006038	1.84E+08	KD2-H4K8ac_peak_2437	7.57336	COLGALT2_ENSG00000198756
1	184020245	1.84E+08	KD2-H4K8ac_peak_2438	7.40373	TSEN15_ENSG00000198860
1	184020882	1.84E+08	KD2-H4K8ac_peak_2439	6.08439	TSEN15_ENSG00000198860
1	184355145	1.84E+08	KD2-H4K8ac_peak_2440	7.76104	RP11-382D12.2_ENSG00000271387;C1orf21_ENSG00000116667
1	184356266	1.84E+08	KD2-H4K8ac_peak_2441	8.2413	RP11-382D12.2_ENSG00000271387;C1orf21_ENSG00000116667
1	184591811	1.85E+08	KD2-H4K8ac_peak_2442	6.66692	
1	184653790	1.85E+08	KD2-H4K8ac_peak_2443	6.40683	
1	184723385	1.85E+08	KD2-H4K8ac_peak_2444	6.40874	EDEM3_ENSG00000116406
1	184942752	1.85E+08	KD2-H4K8ac_peak_2445	8.16906	FAM129A_ENSG00000135842
1	185014115	1.85E+08	KD2-H4K8ac_peak_2446	10.19949	RNF2_ENSG00000121481
1	185014707	1.85E+08	KD2-H4K8ac_peak_2447	8.4716	RNF2_ENSG00000121481
1	185125407	1.85E+08	KD2-H4K8ac_peak_2448	26.47675	TRMT1L_ENSG00000121486;SWT1_ENSG00000116668
1	185126391	1.85E+08	KD2-H4K8ac_peak_2449	8.86099	TRMT1L_ENSG00000121486;SWT1_ENSG00000116668
1	185226097	1.85E+08	KD2-H4K8ac_peak_2450	16.25886	Y_RNA_ENSG00000206640
1	185226561	1.85E+08	KD2-H4K8ac_peak_2451	16.71655	Y_RNA_ENSG00000206640
1	185285516	1.85E+08	KD2-H4K8ac_peak_2452	21.11201	IVNS1ABP_ENSG00000116679;GS1-279B7.2_ENSG00000273004
1	185286694	1.85E+08	KD2-H4K8ac_peak_2453	6.8713	IVNS1ABP_ENSG00000116679;GS1-279B7.2_ENSG00000273004
1	185703516	1.86E+08	KD2-H4K8ac_peak_2454	8.26945	HMCN1_ENSG00000143341
1	186343961	1.86E+08	KD2-H4K8ac_peak_2455	12.23	TPR_ENSG00000047410;C1orf27_ENSG00000157181
1	186344618	1.86E+08	KD2-H4K8ac_peak_2456	12.30509	TPR_ENSG00000047410;C1orf27_ENSG00000157181
1	187246280	1.87E+08	KD2-H4K8ac_peak_2457	5.57044	
1	187518090	1.88E+08	KD2-H4K8ac_peak_2458	5.75452	
1	188355721	1.88E+08	KD2-H4K8ac_peak_2459	16.94317	
1	190256208	1.9E+08	KD2-H4K8ac_peak_2460	4.37708	
1	192777690	1.93E+08	KD2-H4K8ac_peak_2461	9.69846	RGS2_ENSG00000116741
1	192803659	1.93E+08	KD2-H4K8ac_peak_2462	5.96828	
1	192816998	1.93E+08	KD2-H4K8ac_peak_2463	7.13615	
1	193028144	1.93E+08	KD2-H4K8ac_peak_2464	7.73264	UCHL5_ENSG00000116750;TROVE2_ENSG00000116747
1	193028825	1.93E+08	KD2-H4K8ac_peak_2465	8.16906	UCHL5_ENSG00000116750;TROVE2_ENSG00000116747
1	193090596	1.93E+08	KD2-H4K8ac_peak_2466	9.90914	CDC73_ENSG00000134371
1	193091207	1.93E+08	KD2-H4K8ac_peak_2467	11.66477	CDC73_ENSG00000134371
1	196317826	1.96E+08	KD2-H4K8ac_peak_2468	6.37652	
1	196577069	1.97E+08	KD2-H4K8ac_peak_2469	5.58324	KCNT2_ENSG00000162687
1	196577665	1.97E+08	KD2-H4K8ac_peak_2470	9.26408	KCNT2_ENSG00000162687
1	196866446	1.97E+08	KD2-H4K8ac_peak_2471	7.76329	
1	197116539	1.97E+08	KD2-H4K8ac_peak_2472	7.82935	ASPM_ENSG00000066279
1	197170133	1.97E+08	KD2-H4K8ac_peak_2473	14.43794	ZBTB41_ENSG00000177888;CRB1_ENSG00000134376
1	197406275	1.97E+08	KD2-H4K8ac_peak_2474	6.47635	
1	197409812	1.97E+08	KD2-H4K8ac_peak_2475	6.75638	
1	197416205	1.97E+08	KD2-H4K8ac_peak_2476	5.86783	RP11-75C23.1_ENSG00000230260
1	197653980	1.98E+08	KD2-H4K8ac_peak_2477	11.92978	Y_RNA_ENSG00000207139
1	197743904	1.98E+08	KD2-H4K8ac_peak_2478	14.84673	DENND1B_ENSG00000213047
1	197870912	1.98E+08	KD2-H4K8ac_peak_2479	20.48577	C1orf53_ENSG00000203724
1	198126346	1.98E+08	KD2-H4K8ac_peak_2480	10.52259	NEK7_ENSG00000151414
1	198127903	1.98E+08	KD2-H4K8ac_peak_2481	4.86324	
1	198174262	1.98E+08	KD2-H4K8ac_peak_2482	5.43522	

1	198619620	1.99E+08	KD2-H4K8ac_peak_2483	3.75035	
1	198627051	1.99E+08	KD2-H4K8ac_peak_2484	5.88845	
1	198872242	1.99E+08	KD2-H4K8ac_peak_2485	4.66652	
1	199053466	1.99E+08	KD2-H4K8ac_peak_2486	5.14125	
1	199321537	1.99E+08	KD2-H4K8ac_peak_2487	5.3778	
1	199562647	2E+08	KD2-H4K8ac_peak_2488	4.37709	
1	199585189	2E+08	KD2-H4K8ac_peak_2489	11.05407	AL450244.1_ENSG00000263805
1	199588223	2E+08	KD2-H4K8ac_peak_2490	5.91443	
1	200271398	2E+08	KD2-H4K8ac_peak_2491	9.65086	
1	200332469	2E+08	KD2-H4K8ac_peak_2492	5.88845	
1	200378738	2E+08	KD2-H4K8ac_peak_2493	19.72564	ZNF281_ENSG00000162702
1	200379339	2E+08	KD2-H4K8ac_peak_2494	6.40297	ZNF281_ENSG00000162702
1	200433985	2E+08	KD2-H4K8ac_peak_2495	5.88845	
1	200442007	2E+08	KD2-H4K8ac_peak_2496	6.1228	RP11-469A15.2_ENSG00000230623
1	200442706	2E+08	KD2-H4K8ac_peak_2497	6.66692	RP11-469A15.2_ENSG00000230623
1	200589554	2.01E+08	KD2-H4K8ac_peak_2498	11.04256	KIF14_ENSG00000118193
1	200638616	2.01E+08	KD2-H4K8ac_peak_2499	28.66603	DDX59_ENSG00000118197;RP11-92G12.3_ENSG00000260088
1	200708140	2.01E+08	KD2-H4K8ac_peak_2500	12.84642	CAMSAP2_ENSG00000118200
1	200860134	2.01E+08	KD2-H4K8ac_peak_2501	21.57439	C1orf106_ENSG00000163362
1	201083074	2.01E+08	KD2-H4K8ac_peak_2502	24.80313	
1	201123015	2.01E+08	KD2-H4K8ac_peak_2503	11.3923	
1	201123784	2.01E+08	KD2-H4K8ac_peak_2504	32.88903	
1	201140206	2.01E+08	KD2-H4K8ac_peak_2505	13.87818	TMEM9_ENSG00000116857
1	201238277	2.01E+08	KD2-H4K8ac_peak_2506	4.03256	
1	201405481	2.01E+08	KD2-H4K8ac_peak_2507	5.02474	
1	201419812	2.01E+08	KD2-H4K8ac_peak_2508	13.87818	
1	201437295	2.01E+08	KD2-H4K8ac_peak_2509	19.35756	PHLDA3_ENSG00000174307
1	201451413	2.01E+08	KD2-H4K8ac_peak_2510	16.07478	
1	201475930	2.01E+08	KD2-H4K8ac_peak_2511	8.36432	RP11-134G8.7_ENSG00000224536
1	201508407	2.02E+08	KD2-H4K8ac_peak_2512	7.43893	
1	201528522	2.02E+08	KD2-H4K8ac_peak_2513	9.08816	
1	201617897	2.02E+08	KD2-H4K8ac_peak_2514	3.83717	
1	201619162	2.02E+08	KD2-H4K8ac_peak_2515	7.90806	
1	201664989	2.02E+08	KD2-H4K8ac_peak_2516	6.62457	
1	201708699	2.02E+08	KD2-H4K8ac_peak_2517	9.41619	
1	201798331	2.02E+08	KD2-H4K8ac_peak_2518	14.27217	IPO9-AS1_ENSG00000231871;IPO9_ENSG00000198700
1	201857288	2.02E+08	KD2-H4K8ac_peak_2519	13.3695	SHISA4_ENSG00000198892
1	201915174	2.02E+08	KD2-H4K8ac_peak_2520	6.36375	LMOD1_ENSG00000163431
1	201923924	2.02E+08	KD2-H4K8ac_peak_2521	9.96798	TIMM17A_ENSG00000134375
1	201924710	2.02E+08	KD2-H4K8ac_peak_2522	9.357	TIMM17A_ENSG00000134375
1	201951443	2.02E+08	KD2-H4K8ac_peak_2523	18.41034	RNPEP_ENSG00000176393
1	202092051	2.02E+08	KD2-H4K8ac_peak_2524	6.99194	GPR37L1_ENSG00000170075
1	202113113	2.02E+08	KD2-H4K8ac_peak_2525	11.379	ARL8A_ENSG00000143862
1	202113918	2.02E+08	KD2-H4K8ac_peak_2526	19.3691	ARL8A_ENSG00000143862
1	202124178	2.02E+08	KD2-H4K8ac_peak_2527	3.97079	
1	202132190	2.02E+08	KD2-H4K8ac_peak_2528	5.9696	

1	202136670	2.02E+08	KD2-H4K8ac_peak_2529	10.32481	PTPRVP_ENSG00000243323
1	202137661	2.02E+08	KD2-H4K8ac_peak_2530	6.59798	PTPRVP_ENSG00000243323
1	202183166	2.02E+08	KD2-H4K8ac_peak_2531	10.43145	
1	202206035	2.02E+08	KD2-H4K8ac_peak_2532	12.43831	
1	202273052	2.02E+08	KD2-H4K8ac_peak_2533	7.49599	
1	202310787	2.02E+08	KD2-H4K8ac_peak_2534	11.05467	UBE2T_ENSG00000077152
1	202316716	2.02E+08	KD2-H4K8ac_peak_2535	7.87496	PPP1R12B_ENSG00000077157
1	202317892	2.02E+08	KD2-H4K8ac_peak_2536	12.13241	PPP1R12B_ENSG00000077157
1	202544233	2.03E+08	KD2-H4K8ac_peak_2537	11.43863	
1	202592454	2.03E+08	KD2-H4K8ac_peak_2538	8.01338	
1	202680876	2.03E+08	KD2-H4K8ac_peak_2539	13.09338	
1	202681968	2.03E+08	KD2-H4K8ac_peak_2540	6.04107	
1	202776044	2.03E+08	KD2-H4K8ac_peak_2541	15.84211	
1	202780092	2.03E+08	KD2-H4K8ac_peak_2542	8.0287	RP11-480I12.10_ENSG00000260021;PCAT6_ENSG00000228288
1	202829679	2.03E+08	KD2-H4K8ac_peak_2543	6.99815	RP11-480I12.5_ENSG00000214796;RP11-480I12.7_ENSG00000234996
1	202831092	2.03E+08	KD2-H4K8ac_peak_2544	7.70067	RP11-480I12.5_ENSG00000214796;RP11-480I12.7_ENSG00000234996
1	202857656	2.03E+08	KD2-H4K8ac_peak_2545	12.57865	RABIF_ENSG00000183155
1	202895937	2.03E+08	KD2-H4K8ac_peak_2546	13.94213	
1	202896920	2.03E+08	KD2-H4K8ac_peak_2547	8.39745	KLHL12_ENSG00000117153
1	202926920	2.03E+08	KD2-H4K8ac_peak_2548	13.12288	ADIPOR1_ENSG00000159346
1	202935982	2.03E+08	KD2-H4K8ac_peak_2549	6.99815	CYB5R1_ENSG00000159348
1	202936663	2.03E+08	KD2-H4K8ac_peak_2550	4.8485	CYB5R1_ENSG00000159348
1	202975495	2.03E+08	KD2-H4K8ac_peak_2551	9.65086	TMEM183A_ENSG00000163444
1	202976561	2.03E+08	KD2-H4K8ac_peak_2552	10.66719	TMEM183A_ENSG00000163444
1	202995727	2.03E+08	KD2-H4K8ac_peak_2553	10.43145	PPFIA4_ENSG00000143847
1	203020807	2.03E+08	KD2-H4K8ac_peak_2554	5.01002	
1	203037961	2.03E+08	KD2-H4K8ac_peak_2555	7.28827	
1	203096503	2.03E+08	KD2-H4K8ac_peak_2556	15.58442	RP11-335O13.7_ENSG00000234775
1	203110251	2.03E+08	KD2-H4K8ac_peak_2557	5.02474	
1	203150865	2.03E+08	KD2-H4K8ac_peak_2558	6.62541	
1	203236725	2.03E+08	KD2-H4K8ac_peak_2559	4.33139	
1	203239738	2.03E+08	KD2-H4K8ac_peak_2560	7.51054	
1	203242516	2.03E+08	KD2-H4K8ac_peak_2561	10.31695	CHIT1_ENSG00000133063
1	203247010	2.03E+08	KD2-H4K8ac_peak_2562	7.90806	
1	203247478	2.03E+08	KD2-H4K8ac_peak_2563	6.52179	
1	203248047	2.03E+08	KD2-H4K8ac_peak_2564	7.18615	
1	203256756	2.03E+08	KD2-H4K8ac_peak_2565	5.5896	RP11-134P9.3_ENSG00000231507
1	203258473	2.03E+08	KD2-H4K8ac_peak_2566	6.60096	
1	203259427	2.03E+08	KD2-H4K8ac_peak_2567	14.66631	
1	203274126	2.03E+08	KD2-H4K8ac_peak_2568	14.68449	LINC01136_ENSG00000233791;BTG2_ENSG00000159388
1	203274697	2.03E+08	KD2-H4K8ac_peak_2569	6.8713	LINC01136_ENSG00000233791;BTG2_ENSG00000159388
1	203295972	2.03E+08	KD2-H4K8ac_peak_2570	9.95759	
1	203296763	2.03E+08	KD2-H4K8ac_peak_2571	21.09086	
1	203406949	2.03E+08	KD2-H4K8ac_peak_2572	4.06758	
1	203475958	2.03E+08	KD2-H4K8ac_peak_2573	13.42721	
1	203524028	2.04E+08	KD2-H4K8ac_peak_2574	8.16906	

1	203596159	2.04E+08	KD2-H4K8ac_peak_2575	8.45455	ATP2B4_ENSG00000058668
1	203619449	2.04E+08	KD2-H4K8ac_peak_2576	7.07117	
1	203738131	2.04E+08	KD2-H4K8ac_peak_2577	6.99815	
1	203738980	2.04E+08	KD2-H4K8ac_peak_2578	7.81699	
1	203764754	2.04E+08	KD2-H4K8ac_peak_2579	5.54647	ZC3H11A_ENSG00000058673;ZBED6_ENSG00000257315
1	203829875	2.04E+08	KD2-H4K8ac_peak_2580	17.19888	SNRPE_ENSG00000182004
1	203830811	2.04E+08	KD2-H4K8ac_peak_2581	14.80472	SNRPE_ENSG00000182004
1	204042341	2.04E+08	KD2-H4K8ac_peak_2582	4.91674	SOX13_ENSG00000143842
1	204096060	2.04E+08	KD2-H4K8ac_peak_2583	10.77651	
1	204098579	2.04E+08	KD2-H4K8ac_peak_2584	12.93209	
1	204100472	2.04E+08	KD2-H4K8ac_peak_2585	6.35843	RP11-74C13.4_ENSG00000261065
1	204121400	2.04E+08	KD2-H4K8ac_peak_2586	12.10889	ETNK2_ENSG00000143845
1	204182867	2.04E+08	KD2-H4K8ac_peak_2587	7.70067	GOLT1A_ENSG00000174567
1	204255955	2.04E+08	KD2-H4K8ac_peak_2588	5.71781	
1	204256781	2.04E+08	KD2-H4K8ac_peak_2589	4.44527	
1	204261484	2.04E+08	KD2-H4K8ac_peak_2590	13.12288	
1	204265568	2.04E+08	KD2-H4K8ac_peak_2591	8.55271	
1	204301220	2.04E+08	KD2-H4K8ac_peak_2592	8.56442	
1	204302358	2.04E+08	KD2-H4K8ac_peak_2593	3.90565	
1	204318091	2.04E+08	KD2-H4K8ac_peak_2594	7.02255	
1	204319345	2.04E+08	KD2-H4K8ac_peak_2595	8.4716	
1	204320396	2.04E+08	KD2-H4K8ac_peak_2596	20.46575	
1	204329186	2.04E+08	KD2-H4K8ac_peak_2597	5.59859	
1	204332183	2.04E+08	KD2-H4K8ac_peak_2598	28.04957	
1	204380719	2.04E+08	KD2-H4K8ac_peak_2599	8.18084	PPP1R15B_ENSG00000158615
1	204381274	2.04E+08	KD2-H4K8ac_peak_2600	12.82393	PPP1R15B_ENSG00000158615
1	204463103	2.04E+08	KD2-H4K8ac_peak_2601	7.11824	PIK3C2B_ENSG00000133056
1	204476528	2.04E+08	KD2-H4K8ac_peak_2602	11.92978	
1	204484678	2.04E+08	KD2-H4K8ac_peak_2603	21.2844	MDM4_ENSG00000198625
1	204553853	2.05E+08	KD2-H4K8ac_peak_2604	4.86324	
1	204599660	2.05E+08	KD2-H4K8ac_peak_2605	14.66259	
1	204606469	2.05E+08	KD2-H4K8ac_peak_2606	9.26408	
1	204607657	2.05E+08	KD2-H4K8ac_peak_2607	9.41619	
1	204654171	2.05E+08	KD2-H4K8ac_peak_2608	9.20193	LRRN2_ENSG00000170382
1	205030476	2.05E+08	KD2-H4K8ac_peak_2609	13.77586	AL583832.1_ENSG00000251696
1	205091617	2.05E+08	KD2-H4K8ac_peak_2610	8.93255	RBBP5_ENSG00000117222
1	205179940	2.05E+08	KD2-H4K8ac_peak_2611	15.82743	DSTYK_ENSG00000133059
1	205196696	2.05E+08	KD2-H4K8ac_peak_2612	12.14909	TMCC2_ENSG00000133069
1	205218647	2.05E+08	KD2-H4K8ac_peak_2613	9.89383	
1	205253782	2.05E+08	KD2-H4K8ac_peak_2614	5.96384	
1	205312835	2.05E+08	KD2-H4K8ac_peak_2615	7.89329	
1	205320936	2.05E+08	KD2-H4K8ac_peak_2616	10.73134	SNRPGP10_ENSG00000235363
1	205473820	2.05E+08	KD2-H4K8ac_peak_2617	16.46127	CDK18_ENSG00000117266
1	205477940	2.05E+08	KD2-H4K8ac_peak_2618	5.81222	
1	205497841	2.05E+08	KD2-H4K8ac_peak_2619	19.81292	
1	205503103	2.06E+08	KD2-H4K8ac_peak_2620	6.85193	

1	205512267	2.06E+08	KD2-H4K8ac_peak_2621	7.72045	
1	205600215	2.06E+08	KD2-H4K8ac_peak_2622	19.77698	ELK4_ENSG00000158711
1	205601288	2.06E+08	KD2-H4K8ac_peak_2623	7.49599	ELK4_ENSG00000158711
1	205648878	2.06E+08	KD2-H4K8ac_peak_2624	10.05606	SLC45A3_ENSG00000158715
1	205649768	2.06E+08	KD2-H4K8ac_peak_2625	9.01262	SLC45A3_ENSG00000158715
1	205718890	2.06E+08	KD2-H4K8ac_peak_2626	9.6247	NUCKS1_ENSG00000069275
1	205719655	2.06E+08	KD2-H4K8ac_peak_2627	9.34989	NUCKS1_ENSG00000069275
1	205720294	2.06E+08	KD2-H4K8ac_peak_2628	6.67081	NUCKS1_ENSG00000069275
1	205744025	2.06E+08	KD2-H4K8ac_peak_2629	13.12288	RAB7L1_ENSG00000117280
1	205861785	2.06E+08	KD2-H4K8ac_peak_2630	5.54647	
1	206223337	2.06E+08	KD2-H4K8ac_peak_2631	6.0073	RP11-38J22.3_ENSG00000226780;AVPR1B_ENSG00000198049
1	206235463	2.06E+08	KD2-H4K8ac_peak_2632	15.61313	
1	206247257	2.06E+08	KD2-H4K8ac_peak_2633	10.78136	
1	206309763	2.06E+08	KD2-H4K8ac_peak_2634	8.02571	
1	206707629	2.07E+08	KD2-H4K8ac_peak_2635	4.75875	
1	206730620	2.07E+08	KD2-H4K8ac_peak_2636	10.18408	
1	206785365	2.07E+08	KD2-H4K8ac_peak_2637	16.87414	EIF2D_ENSG00000143486
1	206786085	2.07E+08	KD2-H4K8ac_peak_2638	5.59859	EIF2D_ENSG00000143486
1	206808176	2.07E+08	KD2-H4K8ac_peak_2639	7.40373	RP11-343H5.6_ENSG00000237605;DYRK3_ENSG00000143479
1	206858361	2.07E+08	KD2-H4K8ac_peak_2640	12.69561	MAPKAPK2_ENSG00000162889
1	207223742	2.07E+08	KD2-H4K8ac_peak_2641	17.66411	PFKFB2_ENSG00000123836
1	207226391	2.07E+08	KD2-H4K8ac_peak_2642	9.20193	YOD1_ENSG00000180667
1	207924779	2.08E+08	KD2-H4K8ac_peak_2643	11.24702	CD46_ENSG00000117335
1	208041905	2.08E+08	KD2-H4K8ac_peak_2644	28.04957	C1orf132_ENSG00000203709
1	208058780	2.08E+08	KD2-H4K8ac_peak_2645	6.55628	
1	208084093	2.08E+08	KD2-H4K8ac_peak_2646	9.01262	CD34_ENSG00000174059
1	208136080	2.08E+08	KD2-H4K8ac_peak_2647	22.82182	
1	208137245	2.08E+08	KD2-H4K8ac_peak_2648	6.29608	
1	208232240	2.08E+08	KD2-H4K8ac_peak_2649	9.48511	
1	208233936	2.08E+08	KD2-H4K8ac_peak_2650	6.66359	
1	208257472	2.08E+08	KD2-H4K8ac_peak_2651	11.04256	
1	208332832	2.08E+08	KD2-H4K8ac_peak_2652	10.74343	
1	208336708	2.08E+08	KD2-H4K8ac_peak_2653	10.84871	
1	208352932	2.08E+08	KD2-H4K8ac_peak_2654	5.4919	
1	208583616	2.09E+08	KD2-H4K8ac_peak_2655	7.146	
1	208618893	2.09E+08	KD2-H4K8ac_peak_2656	6.25869	
1	208673226	2.09E+08	KD2-H4K8ac_peak_2657	9.51625	
1	208674213	2.09E+08	KD2-H4K8ac_peak_2658	8.7325	
1	208683550	2.09E+08	KD2-H4K8ac_peak_2659	6.83936	
1	208700120	2.09E+08	KD2-H4K8ac_peak_2660	6.95649	
1	208706999	2.09E+08	KD2-H4K8ac_peak_2661	5.88845	
1	208731303	2.09E+08	KD2-H4K8ac_peak_2662	4.38478	
1	208824942	2.09E+08	KD2-H4K8ac_peak_2663	4.211	
1	208921856	2.09E+08	KD2-H4K8ac_peak_2664	6.40683	
1	208930034	2.09E+08	KD2-H4K8ac_peak_2665	5.95537	
1	209065119	2.09E+08	KD2-H4K8ac_peak_2666	6.4095	

1	209148785	2.09E+08	KD2-H4K8ac_peak_2667	6.60096	
1	209237169	2.09E+08	KD2-H4K8ac_peak_2668	5.53033	
1	209279498	2.09E+08	KD2-H4K8ac_peak_2669	6.76298	
1	209380550	2.09E+08	KD2-H4K8ac_peak_2670	14.68449	
1	209384340	2.09E+08	KD2-H4K8ac_peak_2671	5.82859	
1	209393728	2.09E+08	KD2-H4K8ac_peak_2672	10.44656	
1	209395298	2.09E+08	KD2-H4K8ac_peak_2673	10.72656	
1	209437710	2.09E+08	KD2-H4K8ac_peak_2674	6.2781	
1	209475848	2.09E+08	KD2-H4K8ac_peak_2675	5.13615	
1	209477095	2.09E+08	KD2-H4K8ac_peak_2676	6.05809	
1	209734989	2.1E+08	KD2-H4K8ac_peak_2677	6.0073	
1	209814916	2.1E+08	KD2-H4K8ac_peak_2678	6.98302	
1	209958585	2.1E+08	KD2-H4K8ac_peak_2679	5.30799	C1orf74_ENSG00000162757
1	209975429	2.1E+08	KD2-H4K8ac_peak_2680	7.9265	
1	210000667	2.1E+08	KD2-H4K8ac_peak_2681	10.73134	DIEXF_ENSG00000117597
1	210001449	2.1E+08	KD2-H4K8ac_peak_2682	5.08628	DIEXF_ENSG00000117597
1	210111068	2.1E+08	KD2-H4K8ac_peak_2683	13.87818	SYT14_ENSG00000143469
1	210166898	2.1E+08	KD2-H4K8ac_peak_2684	4.42765	
1	210406049	2.1E+08	KD2-H4K8ac_peak_2685	21.23512	SERTAD4-AS1_ENSG00000203706;SERTAD4_ENSG00000082497
1	210424575	2.1E+08	KD2-H4K8ac_peak_2686	12.93862	
1	210430914	2.1E+08	KD2-H4K8ac_peak_2687	5.54647	
1	210435402	2.1E+08	KD2-H4K8ac_peak_2688	7.146	
1	210445731	2.1E+08	KD2-H4K8ac_peak_2689	8.79106	
1	210464109	2.1E+08	KD2-H4K8ac_peak_2690	9.36908	
1	210465486	2.1E+08	KD2-H4K8ac_peak_2691	23.89229	
1	210467797	2.1E+08	KD2-H4K8ac_peak_2692	8.80432	
1	210468971	2.1E+08	KD2-H4K8ac_peak_2693	6.04107	
1	210484700	2.1E+08	KD2-H4K8ac_peak_2694	14.66631	
1	210501825	2.11E+08	KD2-H4K8ac_peak_2695	23.8784	HHAT_ENSG00000054392
1	210637603	2.11E+08	KD2-H4K8ac_peak_2696	6.99815	
1	210639794	2.11E+08	KD2-H4K8ac_peak_2697	7.167	
1	210708936	2.11E+08	KD2-H4K8ac_peak_2698	6.65673	
1	210710478	2.11E+08	KD2-H4K8ac_peak_2699	6.20282	
1	210743625	2.11E+08	KD2-H4K8ac_peak_2700	8.10216	
1	210744421	2.11E+08	KD2-H4K8ac_peak_2701	4.76255	
1	210745790	2.11E+08	KD2-H4K8ac_peak_2702	9.71513	
1	210746625	2.11E+08	KD2-H4K8ac_peak_2703	6.0605	
1	210757959	2.11E+08	KD2-H4K8ac_peak_2704	8.52559	
1	210758698	2.11E+08	KD2-H4K8ac_peak_2705	5.01002	
1	210819050	2.11E+08	KD2-H4K8ac_peak_2706	12.81138	
1	210837023	2.11E+08	KD2-H4K8ac_peak_2707	7.25989	
1	210838013	2.11E+08	KD2-H4K8ac_peak_2708	7.76329	
1	210878148	2.11E+08	KD2-H4K8ac_peak_2709	5.40356	
1	210895052	2.11E+08	KD2-H4K8ac_peak_2710	11.24702	
1	211090242	2.11E+08	KD2-H4K8ac_peak_2711	7.76569	
1	211306568	2.11E+08	KD2-H4K8ac_peak_2712	11.86355	KCNH1_ENSG00000143473;KCNH1-IT1_ENSG00000234233

1	211307843	2.11E+08	KD2-H4K8ac_peak_2713	3.90565	KCNH1_ENSG00000143473
1	211431959	2.11E+08	KD2-H4K8ac_peak_2714	7.90191	RCOR3_ENSG00000117625
1	211432780	2.11E+08	KD2-H4K8ac_peak_2715	17.43561	
1	211499268	2.12E+08	KD2-H4K8ac_peak_2716	19.54082	TRAF5_ENSG00000082512
1	211505215	2.12E+08	KD2-H4K8ac_peak_2717	6.85508	
1	211555584	2.12E+08	KD2-H4K8ac_peak_2718	7.81657	LINC00467_ENSG00000153363
1	211556228	2.12E+08	KD2-H4K8ac_peak_2719	7.11824	LINC00467_ENSG00000153363
1	211658653	2.12E+08	KD2-H4K8ac_peak_2720	7.87496	
1	211663009	2.12E+08	KD2-H4K8ac_peak_2721	15.81243	RD3_ENSG00000198570;RP11-359E8.3_ENSG00000223649
1	211665559	2.12E+08	KD2-H4K8ac_peak_2722	9.95119	RD3_ENSG00000198570;RP11-359E8.3_ENSG00000223649
1	211666537	2.12E+08	KD2-H4K8ac_peak_2723	11.91259	RD3_ENSG00000198570;RP11-359E8.3_ENSG00000223649
1	211667320	2.12E+08	KD2-H4K8ac_peak_2724	8.4716	
1	211687622	2.12E+08	KD2-H4K8ac_peak_2725	8.53092	
1	211688489	2.12E+08	KD2-H4K8ac_peak_2726	15.82743	
1	211752017	2.12E+08	KD2-H4K8ac_peak_2727	8.35267	SLC30A1_ENSG00000170385
1	211784618	2.12E+08	KD2-H4K8ac_peak_2728	7.96322	
1	211790031	2.12E+08	KD2-H4K8ac_peak_2729	7.81657	
1	211803735	2.12E+08	KD2-H4K8ac_peak_2730	4.06758	
1	211816377	2.12E+08	KD2-H4K8ac_peak_2731	10.43145	RP11-354K1.2_ENSG00000228792
1	211822578	2.12E+08	KD2-H4K8ac_peak_2732	6.53538	
1	211849342	2.12E+08	KD2-H4K8ac_peak_2733	4.211	NEK2_ENSG00000117650;RP11-122M14.1_ENSG00000231057
1	211854528	2.12E+08	KD2-H4K8ac_peak_2734	5.54647	
1	211889412	2.12E+08	KD2-H4K8ac_peak_2735	4.76709	
1	212003497	2.12E+08	KD2-H4K8ac_peak_2736	19.63735	LPGAT1_ENSG00000123684;RP11-552D8.1_ENSG00000229258
1	212062850	2.12E+08	KD2-H4K8ac_peak_2737	6.8713	
1	212458156	2.12E+08	KD2-H4K8ac_peak_2738	10.6194	PPP2R5A_ENSG00000066027
1	212458871	2.12E+08	KD2-H4K8ac_peak_2739	10.54778	PPP2R5A_ENSG00000066027
1	212588391	2.13E+08	KD2-H4K8ac_peak_2740	6.40874	TMEM206_ENSG00000065600
1	212588783	2.13E+08	KD2-H4K8ac_peak_2741	6.66359	TMEM206_ENSG00000065600
1	212605456	2.13E+08	KD2-H4K8ac_peak_2742	5.14125	NENF_ENSG00000117691
1	212606311	2.13E+08	KD2-H4K8ac_peak_2743	12.93209	NENF_ENSG00000117691
1	212692030	2.13E+08	KD2-H4K8ac_peak_2744	9.18237	
1	212731420	2.13E+08	KD2-H4K8ac_peak_2745	24.75834	RP11-61J19.4_ENSG00000260805
1	212732585	2.13E+08	KD2-H4K8ac_peak_2746	6.8713	
1	212739724	2.13E+08	KD2-H4K8ac_peak_2747	12.57297	
1	212780192	2.13E+08	KD2-H4K8ac_peak_2748	4.54006	
1	212780817	2.13E+08	KD2-H4K8ac_peak_2749	3.88385	
1	212781326	2.13E+08	KD2-H4K8ac_peak_2750	8.58725	
1	212808907	2.13E+08	KD2-H4K8ac_peak_2751	12.10889	
1	212810440	2.13E+08	KD2-H4K8ac_peak_2752	8.7577	
1	212812693	2.13E+08	KD2-H4K8ac_peak_2753	6.42941	
1	212906539	2.13E+08	KD2-H4K8ac_peak_2754	5.57044	
1	212964685	2.13E+08	KD2-H4K8ac_peak_2755	15.82362	NSL1_ENSG00000117697;TATDN3_ENSG00000203705
1	212965285	2.13E+08	KD2-H4K8ac_peak_2756	13.80019	NSL1_ENSG00000117697;TATDN3_ENSG00000203705
1	213031051	2.13E+08	KD2-H4K8ac_peak_2757	8.04448	FLVCR1-AS1_ENSG00000198468;FLVCR1_ENSG00000162769
1	213031673	2.13E+08	KD2-H4K8ac_peak_2758	16.95399	FLVCR1-AS1_ENSG00000198468;FLVCR1_ENSG00000162769

1	213090087	2.13E+08	KD2-H4K8ac_peak_2759	7.47462	
1	213101971	2.13E+08	KD2-H4K8ac_peak_2760	10.41994	
1	213123464	2.13E+08	KD2-H4K8ac_peak_2761	10.87093	VASH2_ENSG00000143494
1	213124018	2.13E+08	KD2-H4K8ac_peak_2762	4.33139	VASH2_ENSG00000143494
1	213188488	2.13E+08	KD2-H4K8ac_peak_2763	13.65567	ANGEL2_ENSG00000174606
1	213189348	2.13E+08	KD2-H4K8ac_peak_2764	16.71291	ANGEL2_ENSG00000174606
1	213223491	2.13E+08	KD2-H4K8ac_peak_2765	5.14125	RPS6KC1_ENSG00000136643
1	213224041	2.13E+08	KD2-H4K8ac_peak_2766	5.75757	RPS6KC1_ENSG00000136643
1	213224714	2.13E+08	KD2-H4K8ac_peak_2767	9.71513	RPS6KC1_ENSG00000136643
1	214155953	2.14E+08	KD2-H4K8ac_peak_2768	20.18584	PROX1_ENSG00000117707
1	214162919	2.14E+08	KD2-H4K8ac_peak_2769	5.59859	
1	214179701	2.14E+08	KD2-H4K8ac_peak_2770	6.47962	
1	214181459	2.14E+08	KD2-H4K8ac_peak_2771	7.48958	
1	214182263	2.14E+08	KD2-H4K8ac_peak_2772	9.33795	
1	214192341	2.14E+08	KD2-H4K8ac_peak_2773	10.17486	
1	214193631	2.14E+08	KD2-H4K8ac_peak_2774	4.70206	
1	214281343	2.14E+08	KD2-H4K8ac_peak_2775	4.44748	
1	214316493	2.14E+08	KD2-H4K8ac_peak_2776	5.88845	
1	214327557	2.14E+08	KD2-H4K8ac_peak_2777	9.36516	
1	214329929	2.14E+08	KD2-H4K8ac_peak_2778	10.96455	
1	214453244	2.14E+08	KD2-H4K8ac_peak_2779	5.88845	
1	214453988	2.14E+08	KD2-H4K8ac_peak_2780	27.46477	SMYD2_ENSG00000143499
1	214724558	2.15E+08	KD2-H4K8ac_peak_2781	12.93209	PTPN14_ENSG00000152104
1	214776596	2.15E+08	KD2-H4K8ac_peak_2782	18.37277	CENPF_ENSG00000117724
1	215740100	2.16E+08	KD2-H4K8ac_peak_2783	16.18205	KCTD3_ENSG00000136636
1	216105867	2.16E+08	KD2-H4K8ac_peak_2784	4.70388	
1	216621944	2.17E+08	KD2-H4K8ac_peak_2785	8.49564	
1	216674093	2.17E+08	KD2-H4K8ac_peak_2786	4.57371	
1	216675820	2.17E+08	KD2-H4K8ac_peak_2787	7.35686	
1	216691693	2.17E+08	KD2-H4K8ac_peak_2788	6.99815	
1	216704537	2.17E+08	KD2-H4K8ac_peak_2789	6.66692	
1	216705131	2.17E+08	KD2-H4K8ac_peak_2790	6.72624	
1	216705649	2.17E+08	KD2-H4K8ac_peak_2791	6.90231	
1	216707881	2.17E+08	KD2-H4K8ac_peak_2792	7.67504	
1	216749334	2.17E+08	KD2-H4K8ac_peak_2793	9.08816	
1	216754691	2.17E+08	KD2-H4K8ac_peak_2794	9.1707	
1	216755768	2.17E+08	KD2-H4K8ac_peak_2795	5.13615	
1	216756860	2.17E+08	KD2-H4K8ac_peak_2796	5.73839	
1	216769238	2.17E+08	KD2-H4K8ac_peak_2797	8.23274	
1	216773179	2.17E+08	KD2-H4K8ac_peak_2798	9.65086	
1	216774329	2.17E+08	KD2-H4K8ac_peak_2799	8.0287	
1	217099216	2.17E+08	KD2-H4K8ac_peak_2800	5.405	
1	217262309	2.17E+08	KD2-H4K8ac_peak_2801	6.66692	
1	217262934	2.17E+08	KD2-H4K8ac_peak_2802	19.28826	
1	217580856	2.18E+08	KD2-H4K8ac_peak_2803	4.211	
1	217804123	2.18E+08	KD2-H4K8ac_peak_2804	23.5321	GPATCH2_ENSG00000092978;SPATA17_ENSG00000162814

1	217847880	2.18E+08	KD2-H4K8ac_peak_2805	7.76335	
1	217976326	2.18E+08	KD2-H4K8ac_peak_2806	5.96828	
1	218338084	2.18E+08	KD2-H4K8ac_peak_2807	12.93209	
1	218457871	2.18E+08	KD2-H4K8ac_peak_2808	16.49944	RRP15_ENSG00000067533
1	218458706	2.18E+08	KD2-H4K8ac_peak_2809	8.10216	RRP15_ENSG00000067533
1	218518623	2.19E+08	KD2-H4K8ac_peak_2810	9.402	RP11-224O19.2_ENSG00000232480;TGFB2_ENSG00000092969
1	218545371	2.19E+08	KD2-H4K8ac_peak_2811	4.62142	
1	218636579	2.19E+08	KD2-H4K8ac_peak_2812	4.67342	
1	219347255	2.19E+08	KD2-H4K8ac_peak_2813	12.84642	RP11-135J2.4_ENSG00000228063;LYPLAL1_ENSG00000143353
1	220101244	2.2E+08	KD2-H4K8ac_peak_2814	9.20193	
1	220219457	2.2E+08	KD2-H4K8ac_peak_2815	8.58725	EPRS_ENSG00000136628
1	220220376	2.2E+08	KD2-H4K8ac_peak_2816	12.57865	EPRS_ENSG00000136628
1	220445339	2.2E+08	KD2-H4K8ac_peak_2817	16.57619	RAB3GAP2_ENSG00000118873
1	220543404	2.21E+08	KD2-H4K8ac_peak_2818	5.45396	
1	220701541	2.21E+08	KD2-H4K8ac_peak_2819	12.15474	MARK1_ENSG00000116141
1	220863702	2.21E+08	KD2-H4K8ac_peak_2820	13.65567	C1orf115_ENSG00000162817
1	220906151	2.21E+08	KD2-H4K8ac_peak_2821	6.45063	
1	220921600	2.21E+08	KD2-H4K8ac_peak_2822	9.26408	MARC2_ENSG00000117791
1	220959508	2.21E+08	KD2-H4K8ac_peak_2823	7.11824	MARC1_ENSG00000186205
1	220960256	2.21E+08	KD2-H4K8ac_peak_2824	14.85549	MARC1_ENSG00000186205
1	221052938	2.21E+08	KD2-H4K8ac_peak_2825	10.7186	HLA-AS1_ENSG00000257551
1	221140840	2.21E+08	KD2-H4K8ac_peak_2826	5.54647	
1	221437015	2.21E+08	KD2-H4K8ac_peak_2827	5.07901	
1	221915894	2.22E+08	KD2-H4K8ac_peak_2828	15.59774	DUSP10_ENSG00000143507
1	222006551	2.22E+08	KD2-H4K8ac_peak_2829	7.70289	
1	222221411	2.22E+08	KD2-H4K8ac_peak_2830	6.69844	
1	222623258	2.23E+08	KD2-H4K8ac_peak_2831	5.92967	
1	222639559	2.23E+08	KD2-H4K8ac_peak_2832	7.99241	
1	222762720	2.23E+08	KD2-H4K8ac_peak_2833	10.46874	TAF1A_ENSG00000143498;RP11-378J18.3_ENSG00000225265
1	222790686	2.23E+08	KD2-H4K8ac_peak_2834	7.49599	MIA3_ENSG00000154305
1	222816654	2.23E+08	KD2-H4K8ac_peak_2835	12.13241	
1	222885833	2.23E+08	KD2-H4K8ac_peak_2836	9.95119	AIDA_ENSG00000186063;BROX_ENSG00000162819
1	222886896	2.23E+08	KD2-H4K8ac_peak_2837	6.36375	AIDA_ENSG00000186063
1	222987595	2.23E+08	KD2-H4K8ac_peak_2838	8.16906	RP11-452F19.3_ENSG00000228106;DISP1_ENSG00000154309
1	222988420	2.23E+08	KD2-H4K8ac_peak_2839	17.67328	RP11-452F19.3_ENSG00000228106;DISP1_ENSG00000154309
1	223195709	2.23E+08	KD2-H4K8ac_peak_2840	6.84261	
1	223263249	2.23E+08	KD2-H4K8ac_peak_2841	6.85193	
1	223302252	2.23E+08	KD2-H4K8ac_peak_2842	6.41015	
1	223316116	2.23E+08	KD2-H4K8ac_peak_2843	4.89305	TLR5_ENSG00000187554;RP11-239E10.2_ENSG00000236846
1	223317205	2.23E+08	KD2-H4K8ac_peak_2844	5.13615	TLR5_ENSG00000187554;RP11-239E10.2_ENSG00000236846
1	223886336	2.24E+08	KD2-H4K8ac_peak_2845	9.36516	
1	223899856	2.24E+08	KD2-H4K8ac_peak_2846	16.04168	
1	223914586	2.24E+08	KD2-H4K8ac_peak_2847	16.3889	
1	224020450	2.24E+08	KD2-H4K8ac_peak_2848	7.47462	
1	224033064	2.24E+08	KD2-H4K8ac_peak_2849	14.80472	TP53BP2_ENSG00000143514
1	224033917	2.24E+08	KD2-H4K8ac_peak_2850	21.2844	TP53BP2_ENSG00000143514

1	224180576	2.24E+08	KD2-H4K8ac_peak_2851	7.73264	
1	224301498	2.24E+08	KD2-H4K8ac_peak_2852	36.65606	FBXO28_ENSG00000143756
1	224364174	2.24E+08	KD2-H4K8ac_peak_2853	3.97079	RP11-365O16.1_ENSG00000236773;DEGS1_ENSG00000143753
1	224371424	2.24E+08	KD2-H4K8ac_peak_2854	9.95119	
1	224517522	2.25E+08	KD2-H4K8ac_peak_2855	19.42408	NVL_ENSG00000143748
1	224528655	2.25E+08	KD2-H4K8ac_peak_2856	9.11031	
1	224543849	2.25E+08	KD2-H4K8ac_peak_2857	8.43115	CNIH4_ENSG00000143771
1	224544673	2.25E+08	KD2-H4K8ac_peak_2858	11.3923	CNIH4_ENSG00000143771
1	224622527	2.25E+08	KD2-H4K8ac_peak_2859	26.38304	CNIH3_ENSG00000143786
1	224688482	2.25E+08	KD2-H4K8ac_peak_2860	5.54647	
1	224690843	2.25E+08	KD2-H4K8ac_peak_2861	14.63458	
1	224921388	2.25E+08	KD2-H4K8ac_peak_2862	6.40297	
1	225116995	2.25E+08	KD2-H4K8ac_peak_2863	22.62254	
1	225615417	2.26E+08	KD2-H4K8ac_peak_2864	6.99194	LBR_ENSG00000143815
1	225615974	2.26E+08	KD2-H4K8ac_peak_2865	11.03268	LBR_ENSG00000143815
1	225647843	2.26E+08	KD2-H4K8ac_peak_2866	6.07398	
1	225649787	2.26E+08	KD2-H4K8ac_peak_2867	7.92775	
1	225650662	2.26E+08	KD2-H4K8ac_peak_2868	4.81427	
1	225662920	2.26E+08	KD2-H4K8ac_peak_2869	7.90806	
1	225797462	2.26E+08	KD2-H4K8ac_peak_2870	8.93112	
1	225839854	2.26E+08	KD2-H4K8ac_peak_2871	28.7663	ENAH_ENSG00000154380
1	225949590	2.26E+08	KD2-H4K8ac_peak_2872	5.20998	
1	225950299	2.26E+08	KD2-H4K8ac_peak_2873	6.66692	
1	225965722	2.26E+08	KD2-H4K8ac_peak_2874	3.82395	SRP9_ENSG00000143742
1	226069027	2.26E+08	KD2-H4K8ac_peak_2875	4.50477	TMEM63A_ENSG00000196187
1	226069631	2.26E+08	KD2-H4K8ac_peak_2876	6.0605	TMEM63A_ENSG00000196187
1	226070210	2.26E+08	KD2-H4K8ac_peak_2877	26.62585	TMEM63A_ENSG00000196187
1	226086808	2.26E+08	KD2-H4K8ac_peak_2878	4.211	
1	226111634	2.26E+08	KD2-H4K8ac_peak_2879	11.85249	RP4-559A3.7_ENSG00000255835;PYCR2_ENSG00000143811
1	226112303	2.26E+08	KD2-H4K8ac_peak_2880	17.43561	RP4-559A3.7_ENSG00000255835;PYCR2_ENSG00000143811
1	226115960	2.26E+08	KD2-H4K8ac_peak_2881	6.34074	
1	226128387	2.26E+08	KD2-H4K8ac_peak_2882	6.84261	LEFTY2_ENSG00000143768
1	226186642	2.26E+08	KD2-H4K8ac_peak_2883	9.91109	SDE2_ENSG00000143751
1	226187378	2.26E+08	KD2-H4K8ac_peak_2884	14.17488	SDE2_ENSG00000143751
1	226249312	2.26E+08	KD2-H4K8ac_peak_2885	19.35989	RP11-396C23.4_ENSG00000272562;H3F3A_ENSG00000163041
1	226250511	2.26E+08	KD2-H4K8ac_peak_2886	16.43913	RP11-396C23.4_ENSG00000272562;H3F3A_ENSG00000163041
1	226271054	2.26E+08	KD2-H4K8ac_peak_2887	21.04966	RP11-396C23.2_ENSG00000225518
1	226297275	2.26E+08	KD2-H4K8ac_peak_2888	8.10216	
1	226297842	2.26E+08	KD2-H4K8ac_peak_2889	25.67347	
1	226308908	2.26E+08	KD2-H4K8ac_peak_2890	34.51095	
1	226315624	2.26E+08	KD2-H4K8ac_peak_2891	8.2413	RP11-396C23.3_ENSG00000270598
1	226323089	2.26E+08	KD2-H4K8ac_peak_2892	6.40297	
1	226373860	2.26E+08	KD2-H4K8ac_peak_2893	27.48654	ACBD3_ENSG00000182827
1	226411068	2.26E+08	KD2-H4K8ac_peak_2894	12.66412	MIXL1_ENSG00000185155
1	226496079	2.26E+08	KD2-H4K8ac_peak_2895	18.40451	LIN9_ENSG00000183814
1	226497064	2.26E+08	KD2-H4K8ac_peak_2896	16.04168	LIN9_ENSG00000183814

1	226595236	2.27E+08	KD2-H4K8ac_peak_2897	10.43145	PARP1_ENSG00000143799
1	226596078	2.27E+08	KD2-H4K8ac_peak_2898	4.33139	PARP1_ENSG00000143799
1	226843008	2.27E+08	KD2-H4K8ac_peak_2899	5.54647	
1	226878036	2.27E+08	KD2-H4K8ac_peak_2900	7.78826	
1	226879102	2.27E+08	KD2-H4K8ac_peak_2901	4.68541	
1	226890891	2.27E+08	KD2-H4K8ac_peak_2902	20.48577	
1	226925266	2.27E+08	KD2-H4K8ac_peak_2903	8.4716	
1	226926422	2.27E+08	KD2-H4K8ac_peak_2904	9.18237	ITPKB_ENSG00000143772
1	227057884	2.27E+08	KD2-H4K8ac_peak_2905	19.3524	PSEN2_ENSG00000143801
1	227127026	2.27E+08	KD2-H4K8ac_peak_2906	12.66165	
1	227161900	2.27E+08	KD2-H4K8ac_peak_2907	8.10216	
1	227505459	2.28E+08	KD2-H4K8ac_peak_2908	6.31838	CDC42BPA_ENSG00000143776
1	227619404	2.28E+08	KD2-H4K8ac_peak_2909	6.66692	
1	227729365	2.28E+08	KD2-H4K8ac_peak_2910	20.30085	
1	227730558	2.28E+08	KD2-H4K8ac_peak_2911	7.76104	
1	227750660	2.28E+08	KD2-H4K8ac_peak_2912	13.99847	ZNF678_ENSG00000181450
1	227751246	2.28E+08	KD2-H4K8ac_peak_2913	10.7186	ZNF678_ENSG00000181450
1	227916399	2.28E+08	KD2-H4K8ac_peak_2914	8.30974	SNAP47_ENSG00000143740
1	227922596	2.28E+08	KD2-H4K8ac_peak_2915	7.73264	JMJD4_ENSG00000081692
1	227923211	2.28E+08	KD2-H4K8ac_peak_2916	17.66411	JMJD4_ENSG00000081692
1	227980850	2.28E+08	KD2-H4K8ac_peak_2917	9.63487	
1	228074311	2.28E+08	KD2-H4K8ac_peak_2918	6.40874	
1	228248037	2.28E+08	KD2-H4K8ac_peak_2919	4.44527	
1	228269362	2.28E+08	KD2-H4K8ac_peak_2920	16.07478	ARF1_ENSG00000143761
1	228270635	2.28E+08	KD2-H4K8ac_peak_2921	6.52179	ARF1_ENSG00000143761
1	228291236	2.28E+08	KD2-H4K8ac_peak_2922	12.14337	
1	228296626	2.28E+08	KD2-H4K8ac_peak_2923	19.39602	MRPL55_ENSG00000162910
1	228297201	2.28E+08	KD2-H4K8ac_peak_2924	19.55721	MRPL55_ENSG00000162910
1	228327114	2.28E+08	KD2-H4K8ac_peak_2925	16.21671	GUK1_ENSG00000143774
1	228328102	2.28E+08	KD2-H4K8ac_peak_2926	5.61306	GUK1_ENSG00000143774
1	228337463	2.28E+08	KD2-H4K8ac_peak_2927	9.07715	GJC2_ENSG00000198835
1	228339011	2.28E+08	KD2-H4K8ac_peak_2928	4.211	
1	228352893	2.28E+08	KD2-H4K8ac_peak_2929	14.63458	IBA57-AS1_ENSG00000203684;IBA57_ENSG00000181873
1	228353444	2.28E+08	KD2-H4K8ac_peak_2930	27.01413	IBA57-AS1_ENSG00000203684;IBA57_ENSG00000181873
1	228354031	2.28E+08	KD2-H4K8ac_peak_2931	10.74343	IBA57-AS1_ENSG00000203684;IBA57_ENSG00000181873
1	228400392	2.28E+08	KD2-H4K8ac_peak_2932	8.55271	C1orf145_ENSG00000162913
1	228565869	2.29E+08	KD2-H4K8ac_peak_2933	8.84009	
1	228566366	2.29E+08	KD2-H4K8ac_peak_2934	8.18084	
1	228603661	2.29E+08	KD2-H4K8ac_peak_2935	9.36665	TRIM17_ENSG00000162931
1	228604883	2.29E+08	KD2-H4K8ac_peak_2936	9.14807	TRIM17_ENSG00000162931
1	228644860	2.29E+08	KD2-H4K8ac_peak_2937	3.97079	HIST3H2A_ENSG00000181218;HIST3H2BB_ENSG00000196890
1	228647411	2.29E+08	KD2-H4K8ac_peak_2938	8.11396	
1	228651805	2.29E+08	KD2-H4K8ac_peak_2939	6.80488	HIST3H2BA_ENSG00000181201
1	228653310	2.29E+08	KD2-H4K8ac_peak_2940	5.54647	HIST3H2BA_ENSG00000181201
1	228674039	2.29E+08	KD2-H4K8ac_peak_2941	11.7608	RNF187_ENSG00000168159
1	228674791	2.29E+08	KD2-H4K8ac_peak_2942	13.40544	RNF187_ENSG00000168159

1	228688944	2.29E+08	KD2-H4K8ac_peak_2943	7.40314	
1	228782712	2.29E+08	KD2-H4K8ac_peak_2944	8.79106	RNA5S17_ENSG00000200370;RNA5SP18_ENSG00000212237
1	228784056	2.29E+08	KD2-H4K8ac_peak_2945	9.34195	RNA5SP18_ENSG00000212237
1	228870783	2.29E+08	KD2-H4K8ac_peak_2946	11.24702	RHOU_ENSG00000116574
1	228999350	2.29E+08	KD2-H4K8ac_peak_2947	7.70067	
1	229000321	2.29E+08	KD2-H4K8ac_peak_2948	4.67342	
1	229002054	2.29E+08	KD2-H4K8ac_peak_2949	11.14749	
1	229003705	2.29E+08	KD2-H4K8ac_peak_2950	8.38045	
1	229004418	2.29E+08	KD2-H4K8ac_peak_2951	10.44656	
1	229007939	2.29E+08	KD2-H4K8ac_peak_2952	7.90806	
1	229008510	2.29E+08	KD2-H4K8ac_peak_2953	5.5478	
1	229035346	2.29E+08	KD2-H4K8ac_peak_2954	12.46522	
1	229123554	2.29E+08	KD2-H4K8ac_peak_2955	12.78054	
1	229126131	2.29E+08	KD2-H4K8ac_peak_2956	12.14909	
1	229164133	2.29E+08	KD2-H4K8ac_peak_2957	9.74423	
1	229193920	2.29E+08	KD2-H4K8ac_peak_2958	17.8737	
1	229195204	2.29E+08	KD2-H4K8ac_peak_2959	8.82006	
1	229249642	2.29E+08	KD2-H4K8ac_peak_2960	5.72327	
1	229250585	2.29E+08	KD2-H4K8ac_peak_2961	11.90231	
1	229284060	2.29E+08	KD2-H4K8ac_peak_2962	3.86308	
1	229389803	2.29E+08	KD2-H4K8ac_peak_2963	6.66692	
1	229406343	2.29E+08	KD2-H4K8ac_peak_2964	19.21269	RP5-1061H20.4_ENSG00000177788;RP5-1061H20.3_ENSG00000240524;RAB4A_ENSG00000168118
1	229478501	2.29E+08	KD2-H4K8ac_peak_2965	14.57762	CCSAP_ENSG00000154429
1	229545045	2.3E+08	KD2-H4K8ac_peak_2966	4.91796	
1	229545664	2.3E+08	KD2-H4K8ac_peak_2967	9.34195	RN7SKP276_ENSG00000252051
1	229546671	2.3E+08	KD2-H4K8ac_peak_2968	5.48272	RN7SKP276_ENSG00000252051
1	229547426	2.3E+08	KD2-H4K8ac_peak_2969	10.43145	RN7SKP276_ENSG00000252051
1	229643831	2.3E+08	KD2-H4K8ac_peak_2970	7.57336	NUP133_ENSG00000069248;RP4-613A2.1_ENSG00000223635
1	229644580	2.3E+08	KD2-H4K8ac_peak_2971	10.77651	NUP133_ENSG00000069248;RP4-613A2.1_ENSG00000223635
1	229694101	2.3E+08	KD2-H4K8ac_peak_2972	8.0287	ABCB10_ENSG00000135776
1	229715106	2.3E+08	KD2-H4K8ac_peak_2973	12.84642	
1	229762066	2.3E+08	KD2-H4K8ac_peak_2974	7.28628	TAF5L_ENSG00000135801;URB2_ENSG00000135763
1	229827696	2.3E+08	KD2-H4K8ac_peak_2975	7.36799	
1	229828794	2.3E+08	KD2-H4K8ac_peak_2976	10.17341	
1	229829469	2.3E+08	KD2-H4K8ac_peak_2977	15.66774	
1	229831710	2.3E+08	KD2-H4K8ac_peak_2978	9.20193	
1	230018479	2.3E+08	KD2-H4K8ac_peak_2979	6.17833	
1	230029510	2.3E+08	KD2-H4K8ac_peak_2980	7.14562	
1	230078285	2.3E+08	KD2-H4K8ac_peak_2981	5.72657	
1	230079440	2.3E+08	KD2-H4K8ac_peak_2982	9.20193	
1	230080046	2.3E+08	KD2-H4K8ac_peak_2983	3.86308	
1	230202223	2.3E+08	KD2-H4K8ac_peak_2984	4.211	
1	230401022	2.3E+08	KD2-H4K8ac_peak_2985	4.56666	
1	230404353	2.3E+08	KD2-H4K8ac_peak_2986	6.98244	RP5-956O18.2_ENSG00000227006
1	230405397	2.3E+08	KD2-H4K8ac_peak_2987	8.10216	
1	230406881	2.3E+08	KD2-H4K8ac_peak_2988	5.94405	

1	230417034	2.3E+08	KD2-H4K8ac_peak_2989	8.1761	RP5-956O18.3_ENSG00000224407
1	230435165	2.3E+08	KD2-H4K8ac_peak_2990	6.99815	
1	230451089	2.3E+08	KD2-H4K8ac_peak_2991	5.30494	AL691479.1_ENSG00000203257
1	230777617	2.31E+08	KD2-H4K8ac_peak_2992	5.59859	COG2_ENSG00000135775
1	230778269	2.31E+08	KD2-H4K8ac_peak_2993	12.00597	COG2_ENSG00000135775
1	230879267	2.31E+08	KD2-H4K8ac_peak_2994	8.58725	
1	231004562	2.31E+08	KD2-H4K8ac_peak_2995	15.2334	C1orf198_ENSG00000119280
1	231114150	2.31E+08	KD2-H4K8ac_peak_2996	5.94405	TTC13_ENSG00000143643;ARV1_ENSG00000173409
1	231175056	2.31E+08	KD2-H4K8ac_peak_2997	10.49196	FAM89A_ENSG00000182118
1	231194792	2.31E+08	KD2-H4K8ac_peak_2998	7.36799	
1	231243741	2.31E+08	KD2-H4K8ac_peak_2999	6.25869	
1	231245232	2.31E+08	KD2-H4K8ac_peak_3000	9.1707	
1	231297056	2.31E+08	KD2-H4K8ac_peak_3001	6.0605	TRIM67_ENSG00000119283
1	231297943	2.31E+08	KD2-H4K8ac_peak_3002	7.11824	TRIM67_ENSG00000119283
1	231335515	2.31E+08	KD2-H4K8ac_peak_3003	8.79106	
1	231346937	2.31E+08	KD2-H4K8ac_peak_3004	12.30509	
1	231376536	2.31E+08	KD2-H4K8ac_peak_3005	14.39182	C1orf131_ENSG00000143633;GNPAT_ENSG00000116906
1	231474051	2.31E+08	KD2-H4K8ac_peak_3006	7.08655	EXOC8_ENSG00000116903
1	231557689	2.32E+08	KD2-H4K8ac_peak_3007	10.78621	
1	231558213	2.32E+08	KD2-H4K8ac_peak_3008	11.04256	
1	231663240	2.32E+08	KD2-H4K8ac_peak_3009	12.84642	RP11-295G20.2_ENSG00000233461;TSNAX-DISC1_ENSG00000270106;TSNAX_ENSG00000116918
1	231664473	2.32E+08	KD2-H4K8ac_peak_3010	6.40297	RP11-295G20.2_ENSG00000233461;TSNAX-DISC1_ENSG00000270106;TSNAX_ENSG00000116918
1	231762591	2.32E+08	KD2-H4K8ac_peak_3011	8.16906	DISC1_ENSG00000162946
1	231830362	2.32E+08	KD2-H4K8ac_peak_3012	9.048	
1	231942352	2.32E+08	KD2-H4K8ac_peak_3013	6.13909	
1	232354785	2.32E+08	KD2-H4K8ac_peak_3014	7.47462	
1	232363061	2.32E+08	KD2-H4K8ac_peak_3015	5.61377	
1	232727145	2.33E+08	KD2-H4K8ac_peak_3016	7.9042	
1	232735051	2.33E+08	KD2-H4K8ac_peak_3017	4.54006	
1	232755398	2.33E+08	KD2-H4K8ac_peak_3018	5.14125	
1	232766238	2.33E+08	KD2-H4K8ac_peak_3019	7.49599	
1	232940597	2.33E+08	KD2-H4K8ac_peak_3020	8.0287	MAP10_ENSG00000212916
1	233007084	2.33E+08	KD2-H4K8ac_peak_3021	13.95627	
1	233009168	2.33E+08	KD2-H4K8ac_peak_3022	6.25869	
1	233029114	2.33E+08	KD2-H4K8ac_peak_3023	6.84261	
1	233078645	2.33E+08	KD2-H4K8ac_peak_3024	6.56926	
1	233085830	2.33E+08	KD2-H4K8ac_peak_3025	8.3261	NTPCR_ENSG00000135778
1	233086364	2.33E+08	KD2-H4K8ac_peak_3026	10.24981	NTPCR_ENSG00000135778
1	233431049	2.33E+08	KD2-H4K8ac_peak_3027	10.76532	PCNXL2_ENSG00000135749;RP5-862P8.3_ENSG00000251508
1	233463522	2.33E+08	KD2-H4K8ac_peak_3028	9.78882	MLK4_ENSG00000143674
1	233582870	2.34E+08	KD2-H4K8ac_peak_3029	9.1707	
1	233584878	2.34E+08	KD2-H4K8ac_peak_3030	5.87879	RNU4-77P_ENSG00000252501
1	233590797	2.34E+08	KD2-H4K8ac_peak_3031	5.09296	
1	233704834	2.34E+08	KD2-H4K8ac_peak_3032	6.40683	
1	233723887	2.34E+08	KD2-H4K8ac_peak_3033	5.88845	
1	233749346	2.34E+08	KD2-H4K8ac_peak_3034	10.7186	KCNK1_ENSG00000135750

1	233951723	2.34E+08	KD2-H4K8ac_peak_3035	6.60096	
1	234040497	2.34E+08	KD2-H4K8ac_peak_3036	10.17486	SLC35F3_ENSG00000183780
1	234417719	2.34E+08	KD2-H4K8ac_peak_3037	8.79106	
1	234508768	2.35E+08	KD2-H4K8ac_peak_3038	14.99434	RP5-827C21.6_ENSG00000273367;RP5-827C21.4_ENSG00000231663;COA6_ENSG00000168275
1	234613793	2.35E+08	KD2-H4K8ac_peak_3039	22.08065	TARBP1_ENSG00000059588
1	234634601	2.35E+08	KD2-H4K8ac_peak_3040	8.85813	
1	234645158	2.35E+08	KD2-H4K8ac_peak_3041	6.99815	
1	234645701	2.35E+08	KD2-H4K8ac_peak_3042	5.01002	
1	234666949	2.35E+08	KD2-H4K8ac_peak_3043	15.61313	RP5-855F14.1_ENSG00000231768
1	234669149	2.35E+08	KD2-H4K8ac_peak_3044	5.9696	
1	234670188	2.35E+08	KD2-H4K8ac_peak_3045	11.50346	
1	234671440	2.35E+08	KD2-H4K8ac_peak_3046	8.53092	
1	234675961	2.35E+08	KD2-H4K8ac_peak_3047	4.37809	
1	234677957	2.35E+08	KD2-H4K8ac_peak_3048	23.04547	
1	234678566	2.35E+08	KD2-H4K8ac_peak_3049	9.20193	
1	234679374	2.35E+08	KD2-H4K8ac_peak_3050	8.10216	
1	234680565	2.35E+08	KD2-H4K8ac_peak_3051	7.92775	
1	234681588	2.35E+08	KD2-H4K8ac_peak_3052	13.50626	
1	234682966	2.35E+08	KD2-H4K8ac_peak_3053	6.52841	
1	234685412	2.35E+08	KD2-H4K8ac_peak_3054	6.17833	
1	234686837	2.35E+08	KD2-H4K8ac_peak_3055	5.66705	
1	234688702	2.35E+08	KD2-H4K8ac_peak_3056	8.85813	
1	234689629	2.35E+08	KD2-H4K8ac_peak_3057	13.02811	
1	234693881	2.35E+08	KD2-H4K8ac_peak_3058	4.37809	
1	234694634	2.35E+08	KD2-H4K8ac_peak_3059	7.69287	
1	234695493	2.35E+08	KD2-H4K8ac_peak_3060	6.16883	
1	234697295	2.35E+08	KD2-H4K8ac_peak_3061	12.74171	
1	234698879	2.35E+08	KD2-H4K8ac_peak_3062	10.65724	
1	234706373	2.35E+08	KD2-H4K8ac_peak_3063	9.48012	
1	234707188	2.35E+08	KD2-H4K8ac_peak_3064	10.54073	
1	234707701	2.35E+08	KD2-H4K8ac_peak_3065	5.9696	
1	234708293	2.35E+08	KD2-H4K8ac_peak_3066	6.99815	
1	234708900	2.35E+08	KD2-H4K8ac_peak_3067	6.78242	
1	234736028	2.35E+08	KD2-H4K8ac_peak_3068	9.36516	
1	234744187	2.35E+08	KD2-H4K8ac_peak_3069	6.2827	IRF2BP2_ENSG00000168264
1	234746132	2.35E+08	KD2-H4K8ac_peak_3070	12.93209	IRF2BP2_ENSG00000168264
1	234747614	2.35E+08	KD2-H4K8ac_peak_3071	8.21523	
1	234748723	2.35E+08	KD2-H4K8ac_peak_3072	12.82393	
1	234811562	2.35E+08	KD2-H4K8ac_peak_3073	15.9608	
1	234858140	2.35E+08	KD2-H4K8ac_peak_3074	9.20193	
1	234858903	2.35E+08	KD2-H4K8ac_peak_3075	9.97685	LINC01132_ENSG00000227630
1	234860372	2.35E+08	KD2-H4K8ac_peak_3076	17.9614	LINC01132_ENSG00000227630
1	234882646	2.35E+08	KD2-H4K8ac_peak_3077	8.23274	
1	234905802	2.35E+08	KD2-H4K8ac_peak_3078	14.03725	
1	235003175	2.35E+08	KD2-H4K8ac_peak_3079	6.99815	
1	235093581	2.35E+08	KD2-H4K8ac_peak_3080	5.4779	RP11-443B7.2_ENSG00000237520

1	235104614	2.35E+08	KD2-H4K8ac_peak_3081	8.48181	RP11-443B7.1_ENSG00000238005
1	235105520	2.35E+08	KD2-H4K8ac_peak_3082	16.68991	RP11-443B7.1_ENSG00000238005
1	235106558	2.35E+08	KD2-H4K8ac_peak_3083	10.45683	RP11-443B7.1_ENSG00000238005
1	235133301	2.35E+08	KD2-H4K8ac_peak_3084	7.84589	
1	235184155	2.35E+08	KD2-H4K8ac_peak_3085	7.28827	
1	235246768	2.35E+08	KD2-H4K8ac_peak_3086	7.49599	
1	235291717	2.35E+08	KD2-H4K8ac_peak_3087	11.7608	TOMM20_ENSG00000173726;SNORA14B_ENSG00000207181
1	235292439	2.35E+08	KD2-H4K8ac_peak_3088	12.36441	TOMM20_ENSG00000173726
1	235491038	2.35E+08	KD2-H4K8ac_peak_3089	13.95627	ARID4B_ENSG00000054267;GGPS1_ENSG00000152904
1	235493429	2.35E+08	KD2-H4K8ac_peak_3090	14.1139	
1	235668146	2.36E+08	KD2-H4K8ac_peak_3091	20.30085	B3GALNT2_ENSG00000162885
1	235711761	2.36E+08	KD2-H4K8ac_peak_3092	11.55418	
1	235713516	2.36E+08	KD2-H4K8ac_peak_3093	11.04256	
1	235714259	2.36E+08	KD2-H4K8ac_peak_3094	6.84261	
1	235715427	2.36E+08	KD2-H4K8ac_peak_3095	9.20193	
1	235716988	2.36E+08	KD2-H4K8ac_peak_3096	9.63487	
1	235812226	2.36E+08	KD2-H4K8ac_peak_3097	10.53377	GNG4_ENSG00000168243
1	235813645	2.36E+08	KD2-H4K8ac_peak_3098	19.71728	GNG4_ENSG00000168243
1	236029924	2.36E+08	KD2-H4K8ac_peak_3099	7.07117	
1	236030546	2.36E+08	KD2-H4K8ac_peak_3100	6.20982	
1	236081401	2.36E+08	KD2-H4K8ac_peak_3101	7.49599	
1	236082137	2.36E+08	KD2-H4K8ac_peak_3102	7.93664	
1	236134766	2.36E+08	KD2-H4K8ac_peak_3103	8.55271	RP4-580N22.2_ENSG00000229291
1	236135374	2.36E+08	KD2-H4K8ac_peak_3104	11.09936	RP4-580N22.2_ENSG00000229291
1	236227017	2.36E+08	KD2-H4K8ac_peak_3105	16.53429	NID1_ENSG00000116962
1	236263169	2.36E+08	KD2-H4K8ac_peak_3106	4.8888	
1	236292137	2.36E+08	KD2-H4K8ac_peak_3107	5.1628	
1	236304735	2.36E+08	KD2-H4K8ac_peak_3108	11.89502	GPR137B_ENSG00000077585
1	236305797	2.36E+08	KD2-H4K8ac_peak_3109	7.07117	GPR137B_ENSG00000077585
1	236558606	2.37E+08	KD2-H4K8ac_peak_3110	10.29842	
1	236686557	2.37E+08	KD2-H4K8ac_peak_3111	20.96279	LGALS8-AS1_ENSG00000223776
1	236767939	2.37E+08	KD2-H4K8ac_peak_3112	12.82393	HEATR1_ENSG00000119285
1	236849344	2.37E+08	KD2-H4K8ac_peak_3113	23.33626	ACTN2_ENSG00000077522
1	236958004	2.37E+08	KD2-H4K8ac_peak_3114	6.35843	MTR_ENSG00000116984
1	236958682	2.37E+08	KD2-H4K8ac_peak_3115	11.3923	MTR_ENSG00000116984
1	237056241	2.37E+08	KD2-H4K8ac_peak_3116	6.99815	
1	237057534	2.37E+08	KD2-H4K8ac_peak_3117	8.58725	
1	237058520	2.37E+08	KD2-H4K8ac_peak_3118	16.62311	
1	237176214	2.37E+08	KD2-H4K8ac_peak_3119	4.77079	
1	237205405	2.37E+08	KD2-H4K8ac_peak_3120	5.98692	RYR2_ENSG00000198626
1	237362514	2.37E+08	KD2-H4K8ac_peak_3121	5.54647	
1	237875326	2.38E+08	KD2-H4K8ac_peak_3122	11.75669	
1	237963017	2.38E+08	KD2-H4K8ac_peak_3123	9.50015	
1	237997733	2.38E+08	KD2-H4K8ac_peak_3124	3.71956	
1	238027837	2.38E+08	KD2-H4K8ac_peak_3125	9.57101	
1	238182059	2.38E+08	KD2-H4K8ac_peak_3126	5.75452	

1	238331077	2.38E+08	KD2-H4K8ac_peak_3127	3.75035	
1	238434502	2.38E+08	KD2-H4K8ac_peak_3128	8.17902	
1	238580505	2.39E+08	KD2-H4K8ac_peak_3129	5.13341	
1	238623164	2.39E+08	KD2-H4K8ac_peak_3130	3.75035	
1	238627884	2.39E+08	KD2-H4K8ac_peak_3131	6.83936	
1	238629816	2.39E+08	KD2-H4K8ac_peak_3132	7.65815	
1	238634781	2.39E+08	KD2-H4K8ac_peak_3133	5.29555	
1	238642439	2.39E+08	KD2-H4K8ac_peak_3134	4.77079	
1	238648717	2.39E+08	KD2-H4K8ac_peak_3135	4.98072	LINC01139_ENSG00000215808;RP11-177F15.1_ENSG00000231877
1	238864425	2.39E+08	KD2-H4K8ac_peak_3136	6.00243	
1	239351425	2.39E+08	KD2-H4K8ac_peak_3137	6.47962	
1	239429615	2.39E+08	KD2-H4K8ac_peak_3138	7.94441	
1	239520420	2.4E+08	KD2-H4K8ac_peak_3139	10.05606	
1	239521851	2.4E+08	KD2-H4K8ac_peak_3140	9.11031	
1	239549484	2.4E+08	KD2-H4K8ac_peak_3141	5.9696	CHRM3_ENSG00000133019
1	239550019	2.4E+08	KD2-H4K8ac_peak_3142	8.16906	CHRM3_ENSG00000133019
1	239573865	2.4E+08	KD2-H4K8ac_peak_3143	4.46868	
1	239587187	2.4E+08	KD2-H4K8ac_peak_3144	5.57044	
1	239634963	2.4E+08	KD2-H4K8ac_peak_3145	5.14125	
1	239688449	2.4E+08	KD2-H4K8ac_peak_3146	5.42867	
1	239796379	2.4E+08	KD2-H4K8ac_peak_3147	5.30799	
1	239993772	2.4E+08	KD2-H4K8ac_peak_3148	10.96455	
1	239995485	2.4E+08	KD2-H4K8ac_peak_3149	14.88282	
1	239996347	2.4E+08	KD2-H4K8ac_peak_3150	11.79585	
1	239999258	2.4E+08	KD2-H4K8ac_peak_3151	7.76329	
1	239999864	2.4E+08	KD2-H4K8ac_peak_3152	9.11031	
1	240001612	2.4E+08	KD2-H4K8ac_peak_3153	6.30626	
1	240002338	2.4E+08	KD2-H4K8ac_peak_3154	6.66692	
1	240004222	2.4E+08	KD2-H4K8ac_peak_3155	5.82799	
1	240005508	2.4E+08	KD2-H4K8ac_peak_3156	5.59859	
1	240006101	2.4E+08	KD2-H4K8ac_peak_3157	6.36375	
1	240008651	2.4E+08	KD2-H4K8ac_peak_3158	5.08628	
1	240010930	2.4E+08	KD2-H4K8ac_peak_3159	6.30626	
1	240031167	2.4E+08	KD2-H4K8ac_peak_3160	8.16906	
1	240032290	2.4E+08	KD2-H4K8ac_peak_3161	10.20046	
1	240033800	2.4E+08	KD2-H4K8ac_peak_3162	6.67092	
1	240035386	2.4E+08	KD2-H4K8ac_peak_3163	4.86324	
1	240037019	2.4E+08	KD2-H4K8ac_peak_3164	7.42964	
1	240038048	2.4E+08	KD2-H4K8ac_peak_3165	6.66692	
1	240042186	2.4E+08	KD2-H4K8ac_peak_3166	11.8457	
1	240044081	2.4E+08	KD2-H4K8ac_peak_3167	6.66359	
1	240051578	2.4E+08	KD2-H4K8ac_peak_3168	8.23274	
1	240052184	2.4E+08	KD2-H4K8ac_peak_3169	11.41236	
1	240054264	2.4E+08	KD2-H4K8ac_peak_3170	13.38928	
1	240056682	2.4E+08	KD2-H4K8ac_peak_3171	6.25869	
1	240057970	2.4E+08	KD2-H4K8ac_peak_3172	10.29842	

1	240059138	2.4E+08	KD2-H4K8ac_peak_3173	12.73749	
1	240060338	2.4E+08	KD2-H4K8ac_peak_3174	12.93253	
1	240060899	2.4E+08	KD2-H4K8ac_peak_3175	6.60096	
1	240160803	2.4E+08	KD2-H4K8ac_peak_3176	7.36799	
1	240627970	2.41E+08	KD2-H4K8ac_peak_3177	6.86812	
1	241136913	2.41E+08	KD2-H4K8ac_peak_3178	6.25869	
1	241520103	2.42E+08	KD2-H4K8ac_peak_3179	9.36516	RGS7_ENSG00000182901
1	241587151	2.42E+08	KD2-H4K8ac_peak_3180	24.00712	RP11-527D7.1_ENSG00000225554
1	241803151	2.42E+08	KD2-H4K8ac_peak_3181	18.37277	
1	241803819	2.42E+08	KD2-H4K8ac_peak_3182	15.61313	
1	242010393	2.42E+08	KD2-H4K8ac_peak_3183	5.20908	EXO1_ENSG00000174371
1	242011226	2.42E+08	KD2-H4K8ac_peak_3184	12.85233	EXO1_ENSG00000174371
1	242134229	2.42E+08	KD2-H4K8ac_peak_3185	6.36375	
1	242135425	2.42E+08	KD2-H4K8ac_peak_3186	9.36516	
1	242137235	2.42E+08	KD2-H4K8ac_peak_3187	6.03353	
1	242294910	2.42E+08	KD2-H4K8ac_peak_3188	5.95537	
1	242588975	2.43E+08	KD2-H4K8ac_peak_3189	6.20982	
1	242589459	2.43E+08	KD2-H4K8ac_peak_3190	5.59859	
1	242591351	2.43E+08	KD2-H4K8ac_peak_3191	9.96798	
1	242592163	2.43E+08	KD2-H4K8ac_peak_3192	10.96455	
1	242597104	2.43E+08	KD2-H4K8ac_peak_3193	6.33462	
1	242612425	2.43E+08	KD2-H4K8ac_peak_3194	9.63487	
1	242623063	2.43E+08	KD2-H4K8ac_peak_3195	8.59301	
1	242687085	2.43E+08	KD2-H4K8ac_peak_3196	6.20982	PLD5_ENSG00000180287
1	242688474	2.43E+08	KD2-H4K8ac_peak_3197	15.84211	PLD5_ENSG00000180287
1	242937638	2.43E+08	KD2-H4K8ac_peak_3198	5.57044	
1	243417683	2.43E+08	KD2-H4K8ac_peak_3199	6.30229	CEP170_ENSG00000143702
1	243418167	2.43E+08	KD2-H4K8ac_peak_3200	6.52179	CEP170_ENSG00000143702;SDCCAG8_ENSG00000054282
1	243419407	2.43E+08	KD2-H4K8ac_peak_3201	12.65012	CEP170_ENSG00000143702;SDCCAG8_ENSG00000054282
1	243584588	2.44E+08	KD2-H4K8ac_peak_3202	4.67342	
1	243585023	2.44E+08	KD2-H4K8ac_peak_3203	7.67504	
1	243645700	2.44E+08	KD2-H4K8ac_peak_3204	9.20193	
1	243647156	2.44E+08	KD2-H4K8ac_peak_3205	4.67342	
1	243654103	2.44E+08	KD2-H4K8ac_peak_3206	4.33139	
1	243656087	2.44E+08	KD2-H4K8ac_peak_3207	7.92932	
1	243658441	2.44E+08	KD2-H4K8ac_peak_3208	9.39486	
1	243659900	2.44E+08	KD2-H4K8ac_peak_3209	20.0792	
1	244012882	2.44E+08	KD2-H4K8ac_peak_3210	5.75757	
1	244013787	2.44E+08	KD2-H4K8ac_peak_3211	24.3087	AKT3_ENSG00000117020
1	244046257	2.44E+08	KD2-H4K8ac_peak_3212	6.9809	
1	244080170	2.44E+08	KD2-H4K8ac_peak_3213	18.24798	RP11-278H7.1_ENSG00000226828
1	244211064	2.44E+08	KD2-H4K8ac_peak_3214	8.4716	
1	244212159	2.44E+08	KD2-H4K8ac_peak_3215	21.55474	
1	244212757	2.44E+08	KD2-H4K8ac_peak_3216	12.60983	
1	244318461	2.44E+08	KD2-H4K8ac_peak_3217	10.96455	
1	244394440	2.44E+08	KD2-H4K8ac_peak_3218	14.92369	

1	244419484	2.44E+08	KD2-H4K8ac_peak_3219	5.72327	
1	244435875	2.44E+08	KD2-H4K8ac_peak_3220	7.40314	
1	244614393	2.45E+08	KD2-H4K8ac_peak_3221	21.2844	ADSS_ENSG00000035687
1	244615921	2.45E+08	KD2-H4K8ac_peak_3222	6.66692	ADSS_ENSG00000035687
1	244624003	2.45E+08	KD2-H4K8ac_peak_3223	5.94405	
1	244624464	2.45E+08	KD2-H4K8ac_peak_3224	22.03039	
1	244816507	2.45E+08	KD2-H4K8ac_peak_3225	11.8457	DESI2_ENSG00000121644
1	244996619	2.45E+08	KD2-H4K8ac_peak_3226	18.3598	
1	244997736	2.45E+08	KD2-H4K8ac_peak_3227	10.46874	COX20_ENSG00000203667
1	245026809	2.45E+08	KD2-H4K8ac_peak_3228	16.81258	HNRNPU_ENSG00000153187;RP11-11N7.4_ENSG00000273175
1	245028074	2.45E+08	KD2-H4K8ac_peak_3229	9.18237	HNRNPU_ENSG00000153187;RP11-11N7.4_ENSG00000273175
1	245127645	2.45E+08	KD2-H4K8ac_peak_3230	7.47462	
1	245131655	2.45E+08	KD2-H4K8ac_peak_3231	12.05219	EFCAB2_ENSG00000203666
1	245133062	2.45E+08	KD2-H4K8ac_peak_3232	30.41045	RP11-156E8.1_ENSG00000272195;EFCAB2_ENSG00000203666
1	245135587	2.45E+08	KD2-H4K8ac_peak_3233	6.65673	
1	245316578	2.45E+08	KD2-H4K8ac_peak_3234	17.51891	KIF26B_ENSG00000162849
1	245318802	2.45E+08	KD2-H4K8ac_peak_3235	11.86355	KIF26B_ENSG00000162849
1	245320079	2.45E+08	KD2-H4K8ac_peak_3236	14.30326	
1	245365720	2.45E+08	KD2-H4K8ac_peak_3237	7.44324	
1	245398022	2.45E+08	KD2-H4K8ac_peak_3238	6.66692	RP11-62I21.1_ENSG00000232192
1	245403237	2.45E+08	KD2-H4K8ac_peak_3239	4.67342	
1	245431378	2.45E+08	KD2-H4K8ac_peak_3240	4.77079	
1	245473981	2.45E+08	KD2-H4K8ac_peak_3241	8.46569	
1	245501971	2.46E+08	KD2-H4K8ac_peak_3242	6.24114	
1	245508234	2.46E+08	KD2-H4K8ac_peak_3243	4.211	
1	245534350	2.46E+08	KD2-H4K8ac_peak_3244	6.55245	AL359983.1_ENSG00000221165
1	245593062	2.46E+08	KD2-H4K8ac_peak_3245	3.67045	
1	245613234	2.46E+08	KD2-H4K8ac_peak_3246	7.47462	
1	245735550	2.46E+08	KD2-H4K8ac_peak_3247	8.30974	
1	245751682	2.46E+08	KD2-H4K8ac_peak_3248	7.53715	
1	245753388	2.46E+08	KD2-H4K8ac_peak_3249	12.82393	
1	245768565	2.46E+08	KD2-H4K8ac_peak_3250	5.3157	
1	245769231	2.46E+08	KD2-H4K8ac_peak_3251	7.44247	
1	245771337	2.46E+08	KD2-H4K8ac_peak_3252	5.18791	
1	245777684	2.46E+08	KD2-H4K8ac_peak_3253	4.44527	RP11-522M21.2_ENSG00000238224
1	245846743	2.46E+08	KD2-H4K8ac_peak_3254	7.28628	
1	245847880	2.46E+08	KD2-H4K8ac_peak_3255	6.86985	
1	245848445	2.46E+08	KD2-H4K8ac_peak_3256	4.06758	
1	245914941	2.46E+08	KD2-H4K8ac_peak_3257	10.21366	
1	245916779	2.46E+08	KD2-H4K8ac_peak_3258	5.20908	
1	245953678	2.46E+08	KD2-H4K8ac_peak_3259	6.66359	
1	245956726	2.46E+08	KD2-H4K8ac_peak_3260	4.86324	
1	245971630	2.46E+08	KD2-H4K8ac_peak_3261	5.405	
1	245973634	2.46E+08	KD2-H4K8ac_peak_3262	4.67342	
1	246032990	2.46E+08	KD2-H4K8ac_peak_3263	5.81222	
1	246033468	2.46E+08	KD2-H4K8ac_peak_3264	4.44527	

1	246034490	2.46E+08	KD2-H4K8ac_peak_3265	10.19949	
1	246037001	2.46E+08	KD2-H4K8ac_peak_3266	9.57101	
1	246037707	2.46E+08	KD2-H4K8ac_peak_3267	11.8696	
1	246039189	2.46E+08	KD2-H4K8ac_peak_3268	11.24702	
1	246040142	2.46E+08	KD2-H4K8ac_peak_3269	4.86324	
1	246158216	2.46E+08	KD2-H4K8ac_peak_3270	8.23274	
1	246159021	2.46E+08	KD2-H4K8ac_peak_3271	5.88845	
1	246198793	2.46E+08	KD2-H4K8ac_peak_3272	6.30229	
1	246447416	2.46E+08	KD2-H4K8ac_peak_3273	5.49367	
1	246670080	2.47E+08	KD2-H4K8ac_peak_3274	5.30799	SMYD3_ENSG00000185420
1	246670716	2.47E+08	KD2-H4K8ac_peak_3275	9.44423	SMYD3_ENSG00000185420
1	246673579	2.47E+08	KD2-H4K8ac_peak_3276	6.25869	
1	246822154	2.47E+08	KD2-H4K8ac_peak_3277	3.65228	
1	246853003	2.47E+08	KD2-H4K8ac_peak_3278	18.14936	RP11-439E19.7_ENSG00000235021
1	246862060	2.47E+08	KD2-H4K8ac_peak_3279	7.07117	
1	246887039	2.47E+08	KD2-H4K8ac_peak_3280	13.10368	SCCPDH_ENSG00000143653
1	246956783	2.47E+08	KD2-H4K8ac_peak_3281	8.30114	
1	246958249	2.47E+08	KD2-H4K8ac_peak_3282	6.45083	
1	247043609	2.47E+08	KD2-H4K8ac_peak_3283	5.57044	
1	247094878	2.47E+08	KD2-H4K8ac_peak_3284	14.39182	AHCTF1_ENSG00000153207
1	247095712	2.47E+08	KD2-H4K8ac_peak_3285	6.99194	AHCTF1_ENSG00000153207
1	247170910	2.47E+08	KD2-H4K8ac_peak_3286	12.08081	ZNF695_ENSG00000197472
1	247171784	2.47E+08	KD2-H4K8ac_peak_3287	9.95119	ZNF695_ENSG00000197472
1	247241595	2.47E+08	KD2-H4K8ac_peak_3288	10.52259	ZNF670_ENSG00000135747
1	247267099	2.47E+08	KD2-H4K8ac_peak_3289	18.64825	ZNF669_ENSG00000188295
1	247274336	2.47E+08	KD2-H4K8ac_peak_3290	18.48145	C1orf229_ENSG00000221953
1	247494459	2.47E+08	KD2-H4K8ac_peak_3291	12.92667	ZNF496_ENSG00000162714
1	247495389	2.47E+08	KD2-H4K8ac_peak_3292	21.9832	ZNF496_ENSG00000162714
1	249119624	2.49E+08	KD2-H4K8ac_peak_3293	5.14125	SH3BP5L_ENSG00000175137;MIR3124_ENSG00000264500
1	249120539	2.49E+08	KD2-H4K8ac_peak_3294	9.36516	SH3BP5L_ENSG00000175137;MIR3124_ENSG00000264500
1	249131634	2.49E+08	KD2-H4K8ac_peak_3295	6.61376	ZNF672_ENSG00000171161
1	249132581	2.49E+08	KD2-H4K8ac_peak_3296	17.83665	ZNF672_ENSG00000171161
1	249152169	2.49E+08	KD2-H4K8ac_peak_3297	9.95119	ZNF692_ENSG00000171163;AL672294.1_ENSG00000227237
1	249153385	2.49E+08	KD2-H4K8ac_peak_3298	13.86993	ZNF692_ENSG00000171163;AL672294.1_ENSG00000227237
1	249156940	2.49E+08	KD2-H4K8ac_peak_3299	7.76329	
1	249157627	2.49E+08	KD2-H4K8ac_peak_3300	22.5974	
1	249166804	2.49E+08	KD2-H4K8ac_peak_3301	7.01964	
1	249167411	2.49E+08	KD2-H4K8ac_peak_3302	3.97079	
1	249200346	2.49E+08	KD2-H4K8ac_peak_3303	12.82393	PGBD2_ENSG00000185220
1	249201009	2.49E+08	KD2-H4K8ac_peak_3304	10.0692	PGBD2_ENSG00000185220
10	122194	122514	KD2-H4K8ac_peak_3305	5.9696	
10	180567	181997	KD2-H4K8ac_peak_3306	14.66631	ZMYND11_ENSG00000015171
10	352710	352983	KD2-H4K8ac_peak_3307	5.18447	
10	353731	353927	KD2-H4K8ac_peak_3308	8.30094	
10	414609	414972	KD2-H4K8ac_peak_3309	6.45083	
10	735232	735607	KD2-H4K8ac_peak_3310	7.70289	DIP2C_ENSG00000151240

10	736178	736620	KD2-H4K8ac_peak_3311	5.57044	DIP2C_ENSG00000151240
10	830762	831343	KD2-H4K8ac_peak_3312	7.70289	
10	977094	977609	KD2-H4K8ac_peak_3313	11.79585	LARP4B_ENSG00000107929
10	1016267	1016518	KD2-H4K8ac_peak_3314	6.83936	
10	1017655	1017895	KD2-H4K8ac_peak_3315	7.61026	
10	1033697	1034187	KD2-H4K8ac_peak_3316	8.10216	AL359878.1_ENSG00000205740;GTPBP4_ENSG00000107937
10	1034449	1034840	KD2-H4K8ac_peak_3317	17.09315	AL359878.1_ENSG00000205740;GTPBP4_ENSG00000107937
10	1095000	1095334	KD2-H4K8ac_peak_3318	13.81781	IDI1_ENSG00000067064;WDR37_ENSG00000047056
10	1101847	1102048	KD2-H4K8ac_peak_3319	4.54838	
10	1102243	1102602	KD2-H4K8ac_peak_3320	14.8083	
10	1102827	1103154	KD2-H4K8ac_peak_3321	12.93209	
10	1312587	1312890	KD2-H4K8ac_peak_3322	10.0692	
10	1313284	1313628	KD2-H4K8ac_peak_3323	8.28234	
10	1313918	1314174	KD2-H4K8ac_peak_3324	10.44656	
10	1314650	1315205	KD2-H4K8ac_peak_3325	16.32216	
10	1315520	1316486	KD2-H4K8ac_peak_3326	22.00006	
10	1316767	1317717	KD2-H4K8ac_peak_3327	9.11031	
10	1330724	1331252	KD2-H4K8ac_peak_3328	5.34946	
10	1331646	1332141	KD2-H4K8ac_peak_3329	16.11042	
10	1332480	1332853	KD2-H4K8ac_peak_3330	10.05606	
10	1333288	1333923	KD2-H4K8ac_peak_3331	12.06346	
10	1334145	1335090	KD2-H4K8ac_peak_3332	13.56049	
10	1336247	1337295	KD2-H4K8ac_peak_3333	11.79585	
10	1398427	1398706	KD2-H4K8ac_peak_3334	6.66692	
10	1402799	1403001	KD2-H4K8ac_peak_3335	5.88845	RP11-398B16.2_ENSG00000233052
10	1443950	1444936	KD2-H4K8ac_peak_3336	4.86324	
10	1692641	1693120	KD2-H4K8ac_peak_3337	6.85193	
10	1693484	1694228	KD2-H4K8ac_peak_3338	7.36799	
10	1694909	1695548	KD2-H4K8ac_peak_3339	6.25869	
10	2267817	2268230	KD2-H4K8ac_peak_3340	8.1233	
10	2653846	2654317	KD2-H4K8ac_peak_3341	5.15777	
10	3110141	3110744	KD2-H4K8ac_peak_3342	7.40314	
10	3115483	3115688	KD2-H4K8ac_peak_3343	8.83749	
10	3438315	3438564	KD2-H4K8ac_peak_3344	10.0692	
10	3440416	3441220	KD2-H4K8ac_peak_3345	8.2413	
10	3441912	3442114	KD2-H4K8ac_peak_3346	4.52514	
10	3827837	3828165	KD2-H4K8ac_peak_3347	6.25869	KLF6_ENSG00000067082
10	3929708	3930153	KD2-H4K8ac_peak_3348	9.15069	
10	4857288	4857539	KD2-H4K8ac_peak_3349	6.25869	
10	4867777	4868143	KD2-H4K8ac_peak_3350	14.66631	
10	4868450	4869261	KD2-H4K8ac_peak_3351	19.59051	
10	5625941	5626134	KD2-H4K8ac_peak_3352	4.44748	
10	5632564	5633083	KD2-H4K8ac_peak_3353	5.51783	
10	5633409	5633806	KD2-H4K8ac_peak_3354	5.14125	
10	5637196	5637498	KD2-H4K8ac_peak_3355	4.77079	RP13-463N16.6_ENSG00000242147
10	5637747	5637958	KD2-H4K8ac_peak_3356	4.06758	RP13-463N16.6_ENSG00000242147

10	5641778	5641999	KD2-H4K8ac_peak_3357	4.01115	
10	5665056	5665314	KD2-H4K8ac_peak_3358	9.92477	
10	5708047	5708458	KD2-H4K8ac_peak_3359	9.18237	ASB13_ENSG00000196372
10	5726095	5726515	KD2-H4K8ac_peak_3360	7.12754	FAM208B_ENSG00000108021
10	5726861	5727252	KD2-H4K8ac_peak_3361	11.92978	FAM208B_ENSG00000108021
10	5734467	5734836	KD2-H4K8ac_peak_3362	8.79106	
10	5855639	5856219	KD2-H4K8ac_peak_3363	13.09607	RP11-318E3.9_ENSG00000272764
10	5873178	5873372	KD2-H4K8ac_peak_3364	5.73839	
10	5918947	5919183	KD2-H4K8ac_peak_3365	6.14257	
10	5931193	5931709	KD2-H4K8ac_peak_3366	16.07006	ANKRD16_ENSG00000134461;FBXO18_ENSG00000134452
10	5932305	5932508	KD2-H4K8ac_peak_3367	7.96322	ANKRD16_ENSG00000134461;FBXO18_ENSG00000134452
10	6131048	6131608	KD2-H4K8ac_peak_3368	16.57619	RBM17_ENSG00000134453
10	6186601	6186953	KD2-H4K8ac_peak_3369	4.42765	PFKFB3_ENSG00000170525
10	6244926	6245274	KD2-H4K8ac_peak_3370	6.67092	RP11-414H17.5_ENSG00000213994
10	6245550	6245910	KD2-H4K8ac_peak_3371	5.20908	RP11-414H17.5_ENSG00000213994
10	6253389	6254059	KD2-H4K8ac_peak_3372	10.20046	
10	6622376	6622859	KD2-H4K8ac_peak_3373	7.47462	PRKCQ_ENSG00000065675;PRKCQ-AS1_ENSG00000237943
10	7289121	7289392	KD2-H4K8ac_peak_3374	6.00243	
10	7291001	7291304	KD2-H4K8ac_peak_3375	4.70206	
10	7293733	7293931	KD2-H4K8ac_peak_3376	6.99815	
10	7301267	7301613	KD2-H4K8ac_peak_3377	8.30974	
10	7305926	7306214	KD2-H4K8ac_peak_3378	5.45396	
10	7308200	7308452	KD2-H4K8ac_peak_3379	4.42765	
10	7319985	7320378	KD2-H4K8ac_peak_3380	5.7068	
10	7452797	7453396	KD2-H4K8ac_peak_3381	10.35678	SFMBT2_ENSG00000198879
10	7486571	7486774	KD2-H4K8ac_peak_3382	5.29352	
10	7830211	7830466	KD2-H4K8ac_peak_3383	10.17486	KIN_ENSG00000151657;ATP5C1_ENSG00000165629
10	7860510	7860751	KD2-H4K8ac_peak_3384	6.99815	TAF3_ENSG00000165632
10	8086784	8087115	KD2-H4K8ac_peak_3385	6.99815	
10	8089055	8089323	KD2-H4K8ac_peak_3386	8.39745	
10	8089589	8089852	KD2-H4K8ac_peak_3387	3.75035	
10	8090074	8090684	KD2-H4K8ac_peak_3388	10.87093	
10	8091071	8091344	KD2-H4K8ac_peak_3389	8.83582	
10	8091568	8094033	KD2-H4K8ac_peak_3390	13.31016	RP11-379F12.4_ENSG00000232638
10	8095693	8096190	KD2-H4K8ac_peak_3391	7.54952	GATA3-AS1_ENSG00000197308;RP11-379F12.3_ENSG00000243350;GATA3_ENSG00000107485
10	8097007	8097335	KD2-H4K8ac_peak_3392	8.5092	
10	8097609	8098327	KD2-H4K8ac_peak_3393	15.81711	
10	8099323	8099542	KD2-H4K8ac_peak_3394	4.8888	
10	8100124	8101177	KD2-H4K8ac_peak_3395	14.56955	
10	8102289	8102481	KD2-H4K8ac_peak_3396	7.1181	
10	8103517	8104201	KD2-H4K8ac_peak_3397	8.30974	
10	8138582	8138892	KD2-H4K8ac_peak_3398	5.29352	
10	8154184	8154532	KD2-H4K8ac_peak_3399	15.22643	
10	8158126	8158702	KD2-H4K8ac_peak_3400	11.03331	
10	8159307	8160101	KD2-H4K8ac_peak_3401	11.89502	
10	8222768	8223002	KD2-H4K8ac_peak_3402	5.18447	

10	8286658	8287319	KD2-H4K8ac_peak_3403	12.80176	
10	8340824	8341256	KD2-H4K8ac_peak_3404	5.30799	RP5-1119O21.2_ENSG00000226861
10	8342264	8343085	KD2-H4K8ac_peak_3405	8.17902	
10	8374417	8374734	KD2-H4K8ac_peak_3406	6.61376	
10	8486504	8486763	KD2-H4K8ac_peak_3407	4.77079	
10	8683640	8683940	KD2-H4K8ac_peak_3408	5.04237	
10	8781396	8781628	KD2-H4K8ac_peak_3409	8.30974	
10	9280121	9280334	KD2-H4K8ac_peak_3410	8.1233	
10	9280613	9280810	KD2-H4K8ac_peak_3411	6.40683	
10	11653700	11654193	KD2-H4K8ac_peak_3412	14.3374	USP6NL_ENSG00000148429;RP11-138I18.1_ENSG00000271360
10	11784605	11784837	KD2-H4K8ac_peak_3413	6.24114	ECHDC3_ENSG00000134463
10	11865451	11865736	KD2-H4K8ac_peak_3414	6.97919	PROSER2_ENSG00000148426
10	12084303	12084732	KD2-H4K8ac_peak_3415	5.30799	UPF2_ENSG00000151461
10	12095853	12096248	KD2-H4K8ac_peak_3416	7.47462	
10	12110333	12110552	KD2-H4K8ac_peak_3417	6.13909	DHTKD1_ENSG00000181192
10	12111042	12111240	KD2-H4K8ac_peak_3418	8.55271	DHTKD1_ENSG00000181192
10	12171219	12171420	KD2-H4K8ac_peak_3419	8.59579	SEC61A2_ENSG00000065665
10	12238026	12238549	KD2-H4K8ac_peak_3420	9.73431	NUDT5_ENSG00000165609;CDC123_ENSG00000151465
10	12306445	12306883	KD2-H4K8ac_peak_3421	7.13615	
10	12390701	12390948	KD2-H4K8ac_peak_3422	5.1628	CAMK1D_ENSG00000183049
10	12391705	12392171	KD2-H4K8ac_peak_3423	6.65673	CAMK1D_ENSG00000183049
10	12882068	12882327	KD2-H4K8ac_peak_3424	6.0073	
10	13141023	13141239	KD2-H4K8ac_peak_3425	4.42765	CCDC3_ENSG00000151468;OPTN_ENSG00000123240
10	13141551	13141912	KD2-H4K8ac_peak_3426	6.8836	CCDC3_ENSG00000151468;OPTN_ENSG00000123240
10	13142245	13142752	KD2-H4K8ac_peak_3427	9.15329	CCDC3_ENSG00000151468;OPTN_ENSG00000123240
10	13341683	13342501	KD2-H4K8ac_peak_3428	8.02571	
10	13344149	13344931	KD2-H4K8ac_peak_3429	10.46264	PHYH_ENSG00000107537
10	13388987	13390054	KD2-H4K8ac_peak_3430	15.61313	SEPHS1_ENSG00000086475
10	13390437	13390903	KD2-H4K8ac_peak_3431	14.84212	SEPHS1_ENSG00000086475
10	13392030	13392471	KD2-H4K8ac_peak_3432	5.45396	
10	13481932	13482336	KD2-H4K8ac_peak_3433	5.14125	
10	13571234	13571454	KD2-H4K8ac_peak_3434	8.5781	BEND7_ENSG00000165626;RP11-214D15.2_ENSG00000227175
10	13628968	13629293	KD2-H4K8ac_peak_3435	10.65724	PRPF18_ENSG00000165630
10	13875984	13876218	KD2-H4K8ac_peak_3436	6.47962	
10	13933098	13933346	KD2-H4K8ac_peak_3437	7.27716	
10	13933584	13933790	KD2-H4K8ac_peak_3438	8.55271	
10	13934099	13934304	KD2-H4K8ac_peak_3439	13.13969	
10	14051734	14052124	KD2-H4K8ac_peak_3440	7.71478	
10	14130897	14131397	KD2-H4K8ac_peak_3441	14.74047	
10	14879578	14879959	KD2-H4K8ac_peak_3442	15.39543	CDNF_ENSG00000185267;HSPA14_ENSG00000187522
10	14880334	14881085	KD2-H4K8ac_peak_3443	14.8083	CDNF_ENSG00000185267;HSPA14_ENSG00000187522
10	14920216	14920582	KD2-H4K8ac_peak_3444	14.45402	RP11-398C13.6_ENSG00000272853;SUV39H2_ENSG00000152455
10	14920965	14921576	KD2-H4K8ac_peak_3445	7.146	RP11-398C13.6_ENSG00000272853;SUV39H2_ENSG00000152455
10	15000699	15001172	KD2-H4K8ac_peak_3446	4.67342	MEIG1_ENSG00000197889
10	15001392	15002009	KD2-H4K8ac_peak_3447	9.34195	MEIG1_ENSG00000197889
10	15002530	15002826	KD2-H4K8ac_peak_3448	11.90231	

10	15139151	15139632	KD2-H4K8ac_peak_3449	7.95563	C10orf111_ENSG00000176236;RPP38_ENSG00000152464
10	15211268	15211493	KD2-H4K8ac_peak_3450	4.67342	NMT2_ENSG00000152465
10	15412396	15413037	KD2-H4K8ac_peak_3451	15.9336	FAM171A1_ENSG00000148468
10	15413458	15413668	KD2-H4K8ac_peak_3452	8.17902	FAM171A1_ENSG00000148468
10	15901783	15902413	KD2-H4K8ac_peak_3453	6.25869	FAM188A_ENSG00000148481
10	16562638	16563312	KD2-H4K8ac_peak_3454	6.36375	C1QL3_ENSG00000165985
10	16985000	16985253	KD2-H4K8ac_peak_3455	6.16888	
10	17071848	17072140	KD2-H4K8ac_peak_3456	6.47635	
10	17243485	17243697	KD2-H4K8ac_peak_3457	9.20193	TRDMT1_ENSG00000107614
10	17270276	17271073	KD2-H4K8ac_peak_3458	19.1048	VIM-AS1_ENSG00000229124;VIM_ENSG00000026025
10	17659571	17659969	KD2-H4K8ac_peak_3459	7.1181	PTPLA_ENSG00000165996
10	17685958	17686157	KD2-H4K8ac_peak_3460	7.69287	RP11-390B4.5_ENSG00000260589;STAM_ENSG00000136738
10	18429437	18429870	KD2-H4K8ac_peak_3461	7.76329	CACNB2_ENSG00000165995
10	18491895	18492236	KD2-H4K8ac_peak_3462	10.21502	
10	18492554	18492757	KD2-H4K8ac_peak_3463	4.01115	
10	18916456	18917070	KD2-H4K8ac_peak_3464	11.15509	
10	18947656	18948093	KD2-H4K8ac_peak_3465	6.40683	ARL5B-AS1_ENSG00000152487;ARL5B_ENSG00000165997
10	18948393	18948851	KD2-H4K8ac_peak_3466	13.80019	ARL5B-AS1_ENSG00000152487;ARL5B_ENSG00000165997
10	20104683	20105053	KD2-H4K8ac_peak_3467	7.95563	PLXDC2_ENSG00000120594
10	21150136	21150424	KD2-H4K8ac_peak_3468	5.25071	
10	21179486	21179779	KD2-H4K8ac_peak_3469	6.99815	
10	21246822	21247073	KD2-H4K8ac_peak_3470	5.58333	
10	21352861	21353061	KD2-H4K8ac_peak_3471	5.3778	
10	21389184	21389413	KD2-H4K8ac_peak_3472	4.06758	
10	21462860	21463761	KD2-H4K8ac_peak_3473	4.86324	NEBL_ENSG00000078114;NEBL-AS1_ENSG00000231920
10	21651735	21652002	KD2-H4K8ac_peak_3474	5.58333	
10	21785299	21785528	KD2-H4K8ac_peak_3475	7.92775	CASC10_ENSG00000204682;MIR1915_ENSG00000222071
10	21785805	21786028	KD2-H4K8ac_peak_3476	9.34195	CASC10_ENSG00000204682;MIR1915_ENSG00000222071
10	21805296	21806556	KD2-H4K8ac_peak_3477	10.21366	
10	21808323	21808674	KD2-H4K8ac_peak_3478	5.57044	
10	21813873	21814465	KD2-H4K8ac_peak_3479	10.86378	SKIDA1_ENSG00000180592
10	21814724	21815510	KD2-H4K8ac_peak_3480	7.76329	SKIDA1_ENSG00000180592
10	21815949	21816569	KD2-H4K8ac_peak_3481	12.82393	
10	21822482	21822919	KD2-H4K8ac_peak_3482	15.5861	MLLT10_ENSG00000078403
10	21823620	21823897	KD2-H4K8ac_peak_3483	6.66692	MLLT10_ENSG00000078403
10	21874752	21874979	KD2-H4K8ac_peak_3484	6.10699	
10	22518165	22518388	KD2-H4K8ac_peak_3485	10.0692	
10	22541233	22542020	KD2-H4K8ac_peak_3486	11.05467	
10	22604875	22605590	KD2-H4K8ac_peak_3487	15.59774	COMMD3_ENSG00000148444;COMMD3-BMI1_ENSG00000269897
10	22610189	22610720	KD2-H4K8ac_peak_3488	14.66631	BMI1_ENSG00000168283
10	22624971	22625957	KD2-H4K8ac_peak_3489	11.05467	
10	22629416	22630343	KD2-H4K8ac_peak_3490	25.34026	RP11-573G6.10_ENSG00000272366
10	22634141	22634692	KD2-H4K8ac_peak_3491	9.63487	SPAG6_ENSG00000077327
10	22764820	22765383	KD2-H4K8ac_peak_3492	5.54647	
10	23003043	23004220	KD2-H4K8ac_peak_3493	27.46046	PIP4K2A_ENSG00000150867
10	23183688	23183976	KD2-H4K8ac_peak_3494	7.12754	

10	23384500	23385073	KD2-H4K8ac_peak_3495	16.8435	MSRB2_ENSG00000148450
10	23400327	23400540	KD2-H4K8ac_peak_3496	6.00226	
10	23632565	23632966	KD2-H4K8ac_peak_3497	7.12754	C10orf67_ENSG00000179133;RP11-371A19.2_ENSG00000224215
10	23727246	23727785	KD2-H4K8ac_peak_3498	16.30236	OTUD1_ENSG00000165312
10	24404468	24404692	KD2-H4K8ac_peak_3499	6.16888	
10	24408472	24408745	KD2-H4K8ac_peak_3500	6.16888	
10	24497954	24498181	KD2-H4K8ac_peak_3501	11.43863	
10	24543902	24544576	KD2-H4K8ac_peak_3502	16.61426	
10	25011555	25012486	KD2-H4K8ac_peak_3503	19.94588	ARHGAP21_ENSG00000107863
10	25012740	25012979	KD2-H4K8ac_peak_3504	7.1181	ARHGAP21_ENSG00000107863
10	25241003	25242080	KD2-H4K8ac_peak_3505	14.66631	PRTFDC1_ENSG00000099256;RP11-165A20.3_ENSG00000273107
10	26932234	26932436	KD2-H4K8ac_peak_3506	5.25071	LINC00202-2_ENSG00000231976;RP13-16H11.7_ENSG00000234296
10	26985798	26986531	KD2-H4K8ac_peak_3507	7.76104	PDSS1_ENSG00000148459
10	27388787	27389370	KD2-H4K8ac_peak_3508	15.61313	ANKRD26_ENSG00000107890
10	27443018	27443261	KD2-H4K8ac_peak_3509	7.76329	YME1L1_ENSG00000136758;MASTL_ENSG00000120539
10	27443756	27444056	KD2-H4K8ac_peak_3510	13.73635	YME1L1_ENSG00000136758;MASTL_ENSG00000120539
10	27528658	27528893	KD2-H4K8ac_peak_3511	6.78242	
10	27619601	27619830	KD2-H4K8ac_peak_3512	11.70505	
10	27634377	27634720	KD2-H4K8ac_peak_3513	7.01964	
10	27637394	27637595	KD2-H4K8ac_peak_3514	5.01262	
10	27639079	27639291	KD2-H4K8ac_peak_3515	7.27716	FAM210CP_ENSG00000213770
10	27792461	27792955	KD2-H4K8ac_peak_3516	6.25869	RAB18_ENSG00000099246
10	27793443	27793708	KD2-H4K8ac_peak_3517	9.6247	RAB18_ENSG00000099246
10	28759704	28759954	KD2-H4K8ac_peak_3518	9.02573	
10	28821224	28821950	KD2-H4K8ac_peak_3519	17.83665	WAC-AS1_ENSG00000254635;WAC_ENSG00000095787
10	28822197	28822473	KD2-H4K8ac_peak_3520	8.74596	WAC-AS1_ENSG00000254635;WAC_ENSG00000095787
10	28822687	28823044	KD2-H4K8ac_peak_3521	18.91876	
10	28957659	28958154	KD2-H4K8ac_peak_3522	5.82799	RNU6-1067P_ENSG00000207483
10	28965832	28966150	KD2-H4K8ac_peak_3523	7.36799	BAMBI_ENSG00000095739
10	29095593	29096093	KD2-H4K8ac_peak_3524	9.06703	RP11-478H13.4_ENSG00000271220
10	29097689	29098063	KD2-H4K8ac_peak_3525	5.3778	
10	29346146	29346365	KD2-H4K8ac_peak_3526	7.27716	
10	29390071	29390306	KD2-H4K8ac_peak_3527	4.67342	
10	29401130	29401355	KD2-H4K8ac_peak_3528	7.94441	
10	29402629	29402906	KD2-H4K8ac_peak_3529	6.2781	
10	29403312	29403578	KD2-H4K8ac_peak_3530	5.64449	
10	29489219	29489693	KD2-H4K8ac_peak_3531	5.14125	
10	29555588	29555943	KD2-H4K8ac_peak_3532	4.66652	
10	29607403	29607740	KD2-H4K8ac_peak_3533	18.09077	
10	29608063	29608313	KD2-H4K8ac_peak_3534	4.211	
10	29608526	29608721	KD2-H4K8ac_peak_3535	4.01115	
10	29698205	29698789	KD2-H4K8ac_peak_3536	6.99194	PTCHD3P1_ENSG00000224597
10	29907400	29907903	KD2-H4K8ac_peak_3537	6.31143	
10	29937724	29938018	KD2-H4K8ac_peak_3538	4.3015	
10	30024012	30024815	KD2-H4K8ac_peak_3539	9.63487	SVIL_ENSG00000197321
10	30128707	30129143	KD2-H4K8ac_peak_3540	5.54647	

10	30347727	30348478	KD2-H4K8ac_peak_3541	11.15509	
10	30637784	30638183	KD2-H4K8ac_peak_3542	7.47462	
10	30638480	30638780	KD2-H4K8ac_peak_3543	11.89502	
10	30721981	30722719	KD2-H4K8ac_peak_3544	10.7186	MAP3K8_ENSG00000107968
10	30990399	30991342	KD2-H4K8ac_peak_3545	9.19305	
10	30994454	30994992	KD2-H4K8ac_peak_3546	6.34383	
10	31009075	31009440	KD2-H4K8ac_peak_3547	6.40683	
10	31037603	31037804	KD2-H4K8ac_peak_3548	5.14125	
10	31073810	31074289	KD2-H4K8ac_peak_3549	15.61313	
10	31319966	31320797	KD2-H4K8ac_peak_3550	11.30637	ZNF438_ENSG00000183621
10	31321463	31321937	KD2-H4K8ac_peak_3551	13.57066	ZNF438_ENSG00000183621
10	31421943	31422658	KD2-H4K8ac_peak_3552	9.1707	
10	31423322	31423747	KD2-H4K8ac_peak_3553	11.92978	
10	31446698	31446947	KD2-H4K8ac_peak_3554	9.11031	
10	31607572	31607918	KD2-H4K8ac_peak_3555	7.38799	ZEB1_ENSG00000148516
10	31608144	31608643	KD2-H4K8ac_peak_3556	11.43353	ZEB1-AS1_ENSG00000237036;ZEB1_ENSG00000148516
10	31608999	31609377	KD2-H4K8ac_peak_3557	8.83582	ZEB1-AS1_ENSG00000237036
10	31871912	31872138	KD2-H4K8ac_peak_3558	10.46874	
10	31873377	31873825	KD2-H4K8ac_peak_3559	17.70837	
10	31891777	31892402	KD2-H4K8ac_peak_3560	10.66719	
10	31892806	31893084	KD2-H4K8ac_peak_3561	12.00597	RP11-472N13.2_ENSG00000223834
10	31937728	31937944	KD2-H4K8ac_peak_3562	7.47462	
10	31938511	31938819	KD2-H4K8ac_peak_3563	8.02571	
10	32038256	32039593	KD2-H4K8ac_peak_3564	18.41184	
10	32063534	32064063	KD2-H4K8ac_peak_3565	4.42765	
10	32217396	32217670	KD2-H4K8ac_peak_3566	12.74171	ARHGAP12_ENSG00000165322
10	32344516	32345260	KD2-H4K8ac_peak_3567	9.08816	KIF5B_ENSG00000170759;Y_RNA_ENSG00000206660
10	32635390	32636580	KD2-H4K8ac_peak_3568	43.23227	RP11-135A24.4_ENSG00000233825;AL391839.1_ENSG00000222309;RP11-135A24.2_ENSG00000229327
10	32733994	32734235	KD2-H4K8ac_peak_3569	7.12754	CCDC7_ENSG00000216937
10	32735177	32735655	KD2-H4K8ac_peak_3570	7.96322	CCDC7_ENSG00000216937
10	33246369	33247141	KD2-H4K8ac_peak_3571	9.15329	RP11-462L8.1_ENSG00000229656
10	33247411	33247901	KD2-H4K8ac_peak_3572	10.96455	RP11-462L8.1_ENSG00000229656
10	33269344	33269543	KD2-H4K8ac_peak_3573	8.85813	
10	33270067	33270292	KD2-H4K8ac_peak_3574	6.65673	
10	33294722	33295029	KD2-H4K8ac_peak_3575	6.27931	ITGB1_ENSG00000150093
10	33624300	33624502	KD2-H4K8ac_peak_3576	4.54037	NRP1_ENSG00000099250
10	35081973	35082324	KD2-H4K8ac_peak_3577	7.51054	
10	35086014	35086277	KD2-H4K8ac_peak_3578	4.73064	
10	35103483	35104175	KD2-H4K8ac_peak_3579	12.69604	PARD3_ENSG00000148498;PARD3-AS1_ENSG00000226386
10	35104631	35104881	KD2-H4K8ac_peak_3580	12.82393	PARD3_ENSG00000148498;PARD3-AS1_ENSG00000226386
10	35414840	35415502	KD2-H4K8ac_peak_3581	5.75757	RP11-297A16.2_ENSG00000230534;CREM_ENSG00000095794
10	35416288	35416672	KD2-H4K8ac_peak_3582	9.59814	RP11-297A16.2_ENSG00000230534;CREM_ENSG00000095794
10	35507126	35507343	KD2-H4K8ac_peak_3583	6.16888	
10	35624825	35625587	KD2-H4K8ac_peak_3584	15.81711	RP11-324I22.4_ENSG00000271335
10	36799501	36799775	KD2-H4K8ac_peak_3585	6.90458	
10	38146680	38146992	KD2-H4K8ac_peak_3586	18.22876	ZNF248_ENSG00000198105;RP11-162G10.5_ENSG00000236514

10	38265223	38265482	KD2-H4K8ac_peak_3587	10.72313	ZNF25_ENSG00000175395;RP11-393J16.4_ENSG00000226447
10	38299255	38299890	KD2-H4K8ac_peak_3588	12.93209	ZNF33A_ENSG00000189180
10	38382387	38383078	KD2-H4K8ac_peak_3589	5.14125	ZNF37A_ENSG00000075407
10	38383456	38383902	KD2-H4K8ac_peak_3590	7.40111	ZNF37A_ENSG00000075407
10	38691267	38691723	KD2-H4K8ac_peak_3591	13.09607	SEPT7P9_ENSG00000120555
10	38692262	38692775	KD2-H4K8ac_peak_3592	11.30637	SEPT7P9_ENSG00000120555
10	42863083	42863396	KD2-H4K8ac_peak_3593	5.82799	RP11-313J2.1_ENSG00000215146
10	42970471	42970815	KD2-H4K8ac_peak_3594	11.05407	LINC00839_ENSG00000185904
10	43133136	43133334	KD2-H4K8ac_peak_3595	5.43522	ZNF33B_ENSG00000272373;ZNF33B_ENSG00000196693
10	43133739	43134303	KD2-H4K8ac_peak_3596	22.28045	ZNF33B_ENSG00000272373;ZNF33B_ENSG00000196693
10	43186904	43187119	KD2-H4K8ac_peak_3597	8.30974	AL022344.4_ENSG00000233515
10	43187407	43187821	KD2-H4K8ac_peak_3598	6.56211	AL022344.4_ENSG00000233515
10	43277649	43278127	KD2-H4K8ac_peak_3599	7.44247	BMS1_ENSG00000165733
10	43361861	43362195	KD2-H4K8ac_peak_3600	13.35993	
10	43362436	43362628	KD2-H4K8ac_peak_3601	5.13615	
10	43366106	43366750	KD2-H4K8ac_peak_3602	15.61313	RP11-124O11.1_ENSG00000234944
10	43367158	43368124	KD2-H4K8ac_peak_3603	12.57865	RP11-124O11.1_ENSG00000234944
10	43368419	43368878	KD2-H4K8ac_peak_3604	10.08707	
10	43370331	43371306	KD2-H4K8ac_peak_3605	10.96455	
10	43372025	43372241	KD2-H4K8ac_peak_3606	9.58924	
10	43429013	43430153	KD2-H4K8ac_peak_3607	10.96455	
10	43435037	43435660	KD2-H4K8ac_peak_3608	9.63487	
10	43446905	43447709	KD2-H4K8ac_peak_3609	15.53798	
10	43448310	43448558	KD2-H4K8ac_peak_3610	6.66692	
10	43456906	43458593	KD2-H4K8ac_peak_3611	12.08081	
10	43464461	43466017	KD2-H4K8ac_peak_3612	16.72302	
10	43466344	43467466	KD2-H4K8ac_peak_3613	17.53289	
10	43469363	43470115	KD2-H4K8ac_peak_3614	6.99194	
10	43470333	43470688	KD2-H4K8ac_peak_3615	9.89383	
10	43475431	43476261	KD2-H4K8ac_peak_3616	12.30509	RP11-124O11.2_ENSG00000229630
10	43476482	43476972	KD2-H4K8ac_peak_3617	8.1235	RP11-124O11.2_ENSG00000229630
10	43477206	43477856	KD2-H4K8ac_peak_3618	10.40701	RP11-124O11.2_ENSG00000229630
10	43478398	43478686	KD2-H4K8ac_peak_3619	5.18937	
10	43484919	43485324	KD2-H4K8ac_peak_3620	8.55581	
10	43486229	43486885	KD2-H4K8ac_peak_3621	9.20193	
10	43487787	43488386	KD2-H4K8ac_peak_3622	6.36375	
10	43488653	43488862	KD2-H4K8ac_peak_3623	4.67342	
10	43534077	43534272	KD2-H4K8ac_peak_3624	6.90231	
10	43535177	43535851	KD2-H4K8ac_peak_3625	9.73431	
10	43538792	43540182	KD2-H4K8ac_peak_3626	6.40297	
10	43540750	43541065	KD2-H4K8ac_peak_3627	5.47227	
10	43542272	43542523	KD2-H4K8ac_peak_3628	7.25739	
10	43550140	43550416	KD2-H4K8ac_peak_3629	6.40297	
10	43551106	43551568	KD2-H4K8ac_peak_3630	11.92978	
10	43552080	43552446	KD2-H4K8ac_peak_3631	12.93209	
10	43552941	43555098	KD2-H4K8ac_peak_3632	11.92978	

10	43555843	43556087	KD2-H4K8ac_peak_3633	5.75757	
10	43564115	43564568	KD2-H4K8ac_peak_3634	7.99241	
10	43564766	43565401	KD2-H4K8ac_peak_3635	10.19949	
10	43571756	43572296	KD2-H4K8ac_peak_3636	10.19949	RET_ENSG00000165731
10	43572643	43572879	KD2-H4K8ac_peak_3637	7.09622	RET_ENSG00000165731
10	43573111	43573393	KD2-H4K8ac_peak_3638	5.405	RET_ENSG00000165731
10	43573731	43574385	KD2-H4K8ac_peak_3639	3.90565	
10	43596824	43597055	KD2-H4K8ac_peak_3640	5.14125	
10	43633237	43634156	KD2-H4K8ac_peak_3641	17.46525	RP11-351D16.3_ENSG00000273008;CSGALNACT2_ENSG00000169826
10	43857362	43857595	KD2-H4K8ac_peak_3642	6.2463	
10	43857977	43858573	KD2-H4K8ac_peak_3643	22.28045	
10	43891973	43892179	KD2-H4K8ac_peak_3644	6.40297	
10	43902857	43903065	KD2-H4K8ac_peak_3645	8.23274	
10	43904443	43904635	KD2-H4K8ac_peak_3646	7.47462	HNRNPF_ENSG00000169813
10	43904917	43905273	KD2-H4K8ac_peak_3647	7.40314	HNRNPF_ENSG00000169813
10	43915486	43916537	KD2-H4K8ac_peak_3648	14.66631	RP11-517P14.2_ENSG00000230555
10	43931969	43932479	KD2-H4K8ac_peak_3649	10.19949	ZNF487_ENSG00000243660
10	43950555	43950864	KD2-H4K8ac_peak_3650	12.88108	
10	43951388	43952122	KD2-H4K8ac_peak_3651	8.28234	
10	44069418	44070088	KD2-H4K8ac_peak_3652	9.36516	ZNF239_ENSG00000196793
10	44070342	44070563	KD2-H4K8ac_peak_3653	6.16888	ZNF239_ENSG00000196793
10	44101502	44102684	KD2-H4K8ac_peak_3654	12.74171	CAP1P2_ENSG00000232004;ZNF485_ENSG00000198298
10	44143810	44144772	KD2-H4K8ac_peak_3655	21.06321	ZNF32_ENSG00000169740
10	44185016	44185942	KD2-H4K8ac_peak_3656	12.81138	
10	44374074	44374268	KD2-H4K8ac_peak_3657	9.35804	
10	44374496	44374830	KD2-H4K8ac_peak_3658	7.45501	
10	44506405	44506633	KD2-H4K8ac_peak_3659	6.16888	
10	44756023	44756280	KD2-H4K8ac_peak_3660	6.97919	RP11-20J15.2_ENSG00000237590
10	44756490	44756689	KD2-H4K8ac_peak_3661	9.6589	RP11-20J15.2_ENSG00000237590
10	44806145	44806404	KD2-H4K8ac_peak_3662	11.75669	
10	45455271	45455555	KD2-H4K8ac_peak_3663	8.02571	TMEM72-AS1_ENSG00000224812;RP11-285G1.14_ENSG00000273363;RASSF4_ENSG00000107551
10	45469883	45470771	KD2-H4K8ac_peak_3664	16.66847	
10	45474068	45474269	KD2-H4K8ac_peak_3665	8.26945	C10orf10_ENSG00000165507
10	45495941	45496169	KD2-H4K8ac_peak_3666	9.89126	C10orf25_ENSG00000165511;ZNF22_ENSG00000165512
10	45496420	45497325	KD2-H4K8ac_peak_3667	15.53798	C10orf25_ENSG00000165511;ZNF22_ENSG00000165512
10	45914595	45915337	KD2-H4K8ac_peak_3668	10.0692	
10	46090702	46091148	KD2-H4K8ac_peak_3669	6.13909	MARCH8_ENSG00000165406
10	46167722	46168075	KD2-H4K8ac_peak_3670	11.92978	ZFAND4_ENSG00000172671
10	46168428	46168972	KD2-H4K8ac_peak_3671	15.22643	ZFAND4_ENSG00000172671
10	46222169	46222934	KD2-H4K8ac_peak_3672	17.42905	FAM21FP_ENSG00000237840;RP11-671E7.1_ENSG00000228702;FAM21C_ENSG00000172661
10	46951521	46952026	KD2-H4K8ac_peak_3673	6.66359	RP11-38L15.2_ENSG00000229227;RP11-38L15.3_ENSG00000231187
10	46962130	46962667	KD2-H4K8ac_peak_3674	6.20982	
10	46974970	46975800	KD2-H4K8ac_peak_3675	5.16726	
10	46992638	46993804	KD2-H4K8ac_peak_3676	32.02706	GPRIN2_ENSG00000204175
10	47056776	47057159	KD2-H4K8ac_peak_3677	15.20126	
10	47057791	47058207	KD2-H4K8ac_peak_3678	11.8675	

10	47107825	47108024	KD2-H4K8ac_peak_3679	5.32397	
10	47108304	47109188	KD2-H4K8ac_peak_3680	11.92978	
10	47619138	47619948	KD2-H4K8ac_peak_3681	9.1707	
10	47653765	47654305	KD2-H4K8ac_peak_3682	6.52179	
10	47655664	47656420	KD2-H4K8ac_peak_3683	8.82366	RP11-292F22.7_ENSG00000259942
10	47769170	47769406	KD2-H4K8ac_peak_3684	15.9336	
10	48354450	48354671	KD2-H4K8ac_peak_3685	8.2413	ZNF488_ENSG00000165388
10	48451807	48452554	KD2-H4K8ac_peak_3686	10.63441	
10	48579497	48579736	KD2-H4K8ac_peak_3687	4.9025	
10	49514777	49514969	KD2-H4K8ac_peak_3688	8.4716	MAPK8_ENSG00000107643
10	49515213	49515409	KD2-H4K8ac_peak_3689	3.97079	MAPK8_ENSG00000107643
10	49664374	49664615	KD2-H4K8ac_peak_3690	7.76104	
10	49672974	49673181	KD2-H4K8ac_peak_3691	7.47462	
10	49678477	49678830	KD2-H4K8ac_peak_3692	5.59859	
10	49753767	49754090	KD2-H4K8ac_peak_3693	5.53095	RP11-541M12.3_ENSG00000231906
10	49788120	49788651	KD2-H4K8ac_peak_3694	5.30799	
10	49812803	49813234	KD2-H4K8ac_peak_3695	7.26089	
10	49863995	49864253	KD2-H4K8ac_peak_3696	10.21366	ARHGAP22_ENSG00000128805
10	49864851	49865053	KD2-H4K8ac_peak_3697	5.30799	ARHGAP22_ENSG00000128805
10	50011906	50012329	KD2-H4K8ac_peak_3698	5.61377	
10	50012810	50014074	KD2-H4K8ac_peak_3699	12.42547	
10	50209843	50210122	KD2-H4K8ac_peak_3700	11.1952	
10	50289267	50289474	KD2-H4K8ac_peak_3701	5.45396	
10	50323733	50323978	KD2-H4K8ac_peak_3702	9.11031	VSTM4_ENSG00000165633
10	50358767	50358982	KD2-H4K8ac_peak_3703	4.39858	
10	50480765	50481127	KD2-H4K8ac_peak_3704	5.45396	
10	50506669	50506885	KD2-H4K8ac_peak_3705	4.211	C10orf71-AS1_ENSG00000236208;C10orf71_ENSG00000177354
10	50507202	50508059	KD2-H4K8ac_peak_3706	10.43145	C10orf71-AS1_ENSG00000236208;C10orf71_ENSG00000177354
10	50508664	50509253	KD2-H4K8ac_peak_3707	12.74171	
10	50568278	50569545	KD2-H4K8ac_peak_3708	11.90231	
10	50569934	50570145	KD2-H4K8ac_peak_3709	8.79106	
10	50602854	50604029	KD2-H4K8ac_peak_3710	9.57101	DRGX_ENSG00000165606
10	50604366	50604673	KD2-H4K8ac_peak_3711	9.97896	DRGX_ENSG00000165606
10	50605583	50605936	KD2-H4K8ac_peak_3712	5.4779	
10	50813396	50813931	KD2-H4K8ac_peak_3713	8.30974	
10	50818580	50818805	KD2-H4K8ac_peak_3714	3.97079	SLC18A3_ENSG00000187714
10	50824853	50825168	KD2-H4K8ac_peak_3715	6.16747	
10	50830098	50830389	KD2-H4K8ac_peak_3716	10.46264	
10	50836787	50837093	KD2-H4K8ac_peak_3717	5.72327	
10	50838097	50838565	KD2-H4K8ac_peak_3718	7.07512	
10	50841387	50841673	KD2-H4K8ac_peak_3719	9.16531	
10	50871046	50871522	KD2-H4K8ac_peak_3720	4.70206	
10	50874255	50874603	KD2-H4K8ac_peak_3721	7.93362	
10	50887695	50888056	KD2-H4K8ac_peak_3722	26.40805	C10orf53_ENSG00000178645
10	50969891	50970334	KD2-H4K8ac_peak_3723	24.30324	OGDHL_ENSG00000197444
10	51489255	51489763	KD2-H4K8ac_peak_3724	5.66705	

10	51497867	51498448	KD2-H4K8ac_peak_3725	6.40297	
10	51498876	51499379	KD2-H4K8ac_peak_3726	6.05809	
10	51499744	51500472	KD2-H4K8ac_peak_3727	6.99815	
10	51561930	51562133	KD2-H4K8ac_peak_3728	6.16888	
10	51623658	51623861	KD2-H4K8ac_peak_3729	13.57066	TIMM23_ENSG00000138297;PARGP1_ENSG00000214982
10	51827242	51828204	KD2-H4K8ac_peak_3730	15.9336	RP11-324H6.5_ENSG00000235618;FAM21A_ENSG00000099290
10	52384015	52384254	KD2-H4K8ac_peak_3731	6.65673	SGMS1_ENSG00000198964;RP11-50E11.3_ENSG00000226200
10	52384462	52385313	KD2-H4K8ac_peak_3732	11.05467	SGMS1_ENSG00000198964;RP11-50E11.3_ENSG00000226200
10	52499168	52499761	KD2-H4K8ac_peak_3733	9.95342	ASAH2B_ENSG00000204147
10	52751466	52752354	KD2-H4K8ac_peak_3734	10.46264	PRKG1_ENSG00000185532
10	52752808	52753001	KD2-H4K8ac_peak_3735	7.27716	
10	54068435	54068732	KD2-H4K8ac_peak_3736	11.05467	
10	54069678	54069902	KD2-H4K8ac_peak_3737	5.54647	
10	54072881	54073336	KD2-H4K8ac_peak_3738	10.08707	PRKG1-AS1_ENSG00000236671;DKK1_ENSG00000107984
10	54299483	54299965	KD2-H4K8ac_peak_3739	6.40683	
10	54301638	54302103	KD2-H4K8ac_peak_3740	11.92978	
10	54356786	54357169	KD2-H4K8ac_peak_3741	5.01002	
10	54360958	54361158	KD2-H4K8ac_peak_3742	13.57066	
10	54379025	54379753	KD2-H4K8ac_peak_3743	10.05606	
10	54380161	54380462	KD2-H4K8ac_peak_3744	4.67342	
10	54405313	54405879	KD2-H4K8ac_peak_3745	7.27716	
10	55458697	55458952	KD2-H4K8ac_peak_3746	6.16888	
10	57278494	57278727	KD2-H4K8ac_peak_3747	10.0692	
10	57390593	57391031	KD2-H4K8ac_peak_3748	10.02417	
10	59242804	59243090	KD2-H4K8ac_peak_3749	7.59643	
10	60026910	60027611	KD2-H4K8ac_peak_3750	10.05606	IPMK_ENSG00000151151
10	60027974	60028219	KD2-H4K8ac_peak_3751	5.58333	IPMK_ENSG00000151151;CISD1_ENSG00000122873
10	60029029	60029248	KD2-H4K8ac_peak_3752	11.92978	CISD1_ENSG00000122873
10	60094546	60095436	KD2-H4K8ac_peak_3753	11.03331	UBE2D1_ENSG00000072401
10	60145272	60145660	KD2-H4K8ac_peak_3754	7.40314	TFAM_ENSG00000108064
10	60272923	60273239	KD2-H4K8ac_peak_3755	4.67342	BICC1_ENSG00000122870
10	60412976	60413388	KD2-H4K8ac_peak_3756	8.97719	
10	60936510	60936924	KD2-H4K8ac_peak_3757	13.81781	PHYHIPL_ENSG00000165443
10	61043207	61043430	KD2-H4K8ac_peak_3758	8.4939	
10	61468683	61470011	KD2-H4K8ac_peak_3759	12.57865	
10	61665717	61666810	KD2-H4K8ac_peak_3760	7.76104	CCDC6_ENSG00000108091
10	62492697	62493548	KD2-H4K8ac_peak_3761	23.43329	ANK3_ENSG00000151150;RP11-131N11.4_ENSG00000254271
10	62537150	62537548	KD2-H4K8ac_peak_3762	5.25071	CDK1_ENSG00000170312
10	62538261	62538559	KD2-H4K8ac_peak_3763	14.69247	CDK1_ENSG00000170312
10	62702664	62702940	KD2-H4K8ac_peak_3764	5.18447	
10	62703381	62704872	KD2-H4K8ac_peak_3765	9.26408	
10	62760699	62761150	KD2-H4K8ac_peak_3766	5.54647	RHOBTB1_ENSG00000072422
10	62761348	62761647	KD2-H4K8ac_peak_3767	15.61313	RHOBTB1_ENSG00000072422
10	63213021	63213260	KD2-H4K8ac_peak_3768	4.42765	TMEM26_ENSG00000196932;RP11-809M12.1_ENSG00000237233
10	63661046	63661240	KD2-H4K8ac_peak_3769	7.96194	ARID5B_ENSG00000150347
10	63663071	63663407	KD2-H4K8ac_peak_3770	12.42547	

10	63798797	63799021	KD2-H4K8ac_peak_3771	5.04237	
10	64028007	64028339	KD2-H4K8ac_peak_3772	5.54647	RTKN2_ENSG00000182010
10	64133554	64134434	KD2-H4K8ac_peak_3773	14.66631	ZNF365_ENSG00000138311
10	64564565	64565147	KD2-H4K8ac_peak_3774	15.82362	RP11-436D10.3_ENSG00000238280;ADO_ENSG00000181915
10	65030227	65030438	KD2-H4K8ac_peak_3775	5.88845	
10	65224996	65226023	KD2-H4K8ac_peak_3776	17.17257	JMJD1C_ENSG00000171988;JMJD1C-AS1_ENSG00000272767
10	65281239	65282111	KD2-H4K8ac_peak_3777	10.43145	REEP3_ENSG00000165476
10	65386444	65386658	KD2-H4K8ac_peak_3778	4.92515	
10	65387027	65387368	KD2-H4K8ac_peak_3779	7.59643	
10	65388803	65389050	KD2-H4K8ac_peak_3780	8.79557	
10	65389564	65389822	KD2-H4K8ac_peak_3781	5.13615	
10	65390515	65390853	KD2-H4K8ac_peak_3782	14.69247	
10	65391107	65391607	KD2-H4K8ac_peak_3783	7.40633	
10	65432418	65432750	KD2-H4K8ac_peak_3784	5.57044	
10	65460027	65460315	KD2-H4K8ac_peak_3785	5.93446	
10	65471246	65471595	KD2-H4K8ac_peak_3786	7.36799	
10	65472366	65473118	KD2-H4K8ac_peak_3787	13.07235	
10	65473543	65473894	KD2-H4K8ac_peak_3788	9.76452	
10	65477790	65478321	KD2-H4K8ac_peak_3789	7.22457	
10	65480447	65480853	KD2-H4K8ac_peak_3790	6.15357	
10	65481805	65482233	KD2-H4K8ac_peak_3791	5.57044	
10	65555908	65556175	KD2-H4K8ac_peak_3792	7.22457	
10	65609208	65609458	KD2-H4K8ac_peak_3793	6.72021	
10	66287650	66288227	KD2-H4K8ac_peak_3794	11.70505	
10	67330016	67330565	KD2-H4K8ac_peak_3795	7.12754	RP11-222A11.1_ENSG00000228065
10	69523909	69524167	KD2-H4K8ac_peak_3796	13.86286	
10	69609461	69610107	KD2-H4K8ac_peak_3797	13.81781	RP11-57G10.8_ENSG00000272892
10	69643720	69644140	KD2-H4K8ac_peak_3798	4.8888	SIRT1_ENSG00000096717
10	69644608	69645041	KD2-H4K8ac_peak_3799	11.86082	SIRT1_ENSG00000096717
10	69834653	69834933	KD2-H4K8ac_peak_3800	8.30974	HERC4_ENSG00000148634
10	69991444	69991816	KD2-H4K8ac_peak_3801	5.9696	ATOH7_ENSG00000179774
10	70088695	70088955	KD2-H4K8ac_peak_3802	5.29555	
10	70091054	70091644	KD2-H4K8ac_peak_3803	6.20982	HNRNPH3_ENSG00000096746
10	70091868	70092709	KD2-H4K8ac_peak_3804	18.80827	PBLD_ENSG00000108187;HNRNPH3_ENSG00000096746
10	70166280	70167385	KD2-H4K8ac_peak_3805	19.43192	RUFY2_ENSG00000204130
10	70230970	70232252	KD2-H4K8ac_peak_3806	14.23242	DNA2_ENSG00000138346
10	70286866	70287244	KD2-H4K8ac_peak_3807	7.07117	SLC25A16_ENSG00000122912
10	70287488	70287970	KD2-H4K8ac_peak_3808	12.81138	SLC25A16_ENSG00000122912
10	70320661	70320906	KD2-H4K8ac_peak_3809	9.91109	TET1_ENSG00000138336
10	70358511	70358768	KD2-H4K8ac_peak_3810	10.0692	
10	70480116	70480383	KD2-H4K8ac_peak_3811	18.08496	CCAR1_ENSG00000060339
10	70481009	70481515	KD2-H4K8ac_peak_3812	16.33377	CCAR1_ENSG00000060339
10	70586462	70587475	KD2-H4K8ac_peak_3813	5.82799	STOX1_ENSG00000165730
10	70660136	70660829	KD2-H4K8ac_peak_3814	13.01932	DDX50_ENSG00000107625
10	70661180	70661412	KD2-H4K8ac_peak_3815	4.84794	DDX50_ENSG00000107625
10	70715169	70716654	KD2-H4K8ac_peak_3816	11.92978	DDX21_ENSG00000165732

10	70748564	70749052	KD2-H4K8ac_peak_3817	16.63953	KIAA1279_ENSG00000198954
10	70883327	70883639	KD2-H4K8ac_peak_3818	6.25869	VPS26A_ENSG00000122958
10	70884095	70884371	KD2-H4K8ac_peak_3819	7.47462	VPS26A_ENSG00000122958
10	70940040	70940340	KD2-H4K8ac_peak_3820	15.58442	SUPV3L1_ENSG00000156502
10	71077847	71078391	KD2-H4K8ac_peak_3821	24.91145	
10	71094784	71094984	KD2-H4K8ac_peak_3822	4.20044	
10	71168750	71169107	KD2-H4K8ac_peak_3823	23.27293	
10	71211275	71211929	KD2-H4K8ac_peak_3824	7.98621	TSPAN15_ENSG00000099282
10	71266942	71267156	KD2-H4K8ac_peak_3825	6.47635	
10	71390066	71390601	KD2-H4K8ac_peak_3826	8.16906	C10orf35_ENSG00000171224
10	71445995	71446349	KD2-H4K8ac_peak_3827	6.25869	
10	71516716	71517949	KD2-H4K8ac_peak_3828	8.4716	
10	71518986	71519428	KD2-H4K8ac_peak_3829	6.30626	
10	71626827	71627362	KD2-H4K8ac_peak_3830	10.19949	
10	71721004	71721204	KD2-H4K8ac_peak_3831	3.75035	
10	71734955	71735362	KD2-H4K8ac_peak_3832	5.16439	
10	71812117	71813878	KD2-H4K8ac_peak_3833	12.82393	H2AFY2_ENSG00000099284
10	71891796	71892767	KD2-H4K8ac_peak_3834	18.33557	AIFM2_ENSG00000042286
10	71905110	71905397	KD2-H4K8ac_peak_3835	5.53033	
10	71905604	71905898	KD2-H4K8ac_peak_3836	6.20982	TYSND1_ENSG00000156521
10	71930794	71931079	KD2-H4K8ac_peak_3837	14.42504	SAR1A_ENSG00000079332
10	71992751	71993051	KD2-H4K8ac_peak_3838	6.25869	PPA1_ENSG00000180817
10	71993336	71993541	KD2-H4K8ac_peak_3839	9.89126	PPA1_ENSG00000180817
10	72123322	72123573	KD2-H4K8ac_peak_3840	9.34989	
10	72141713	72142319	KD2-H4K8ac_peak_3841	11.3394	LRRC20_ENSG00000172731
10	72142723	72142917	KD2-H4K8ac_peak_3842	5.92591	LRRC20_ENSG00000172731
10	72163958	72164200	KD2-H4K8ac_peak_3843	10.77651	EIF4EBP2_ENSG00000148730
10	72218016	72218783	KD2-H4K8ac_peak_3844	7.76104	
10	72237861	72238611	KD2-H4K8ac_peak_3845	8.55271	PALD1_ENSG00000107719
10	72259237	72259484	KD2-H4K8ac_peak_3846	4.54838	
10	72439117	72439316	KD2-H4K8ac_peak_3847	7.1181	
10	72444503	72444980	KD2-H4K8ac_peak_3848	9.11031	
10	72445695	72445946	KD2-H4K8ac_peak_3849	5.7068	
10	72452877	72453087	KD2-H4K8ac_peak_3850	4.86324	
10	72455205	72455783	KD2-H4K8ac_peak_3851	5.54647	
10	72458192	72458386	KD2-H4K8ac_peak_3852	6.66692	
10	72520200	72520544	KD2-H4K8ac_peak_3853	6.64337	
10	72575716	72576272	KD2-H4K8ac_peak_3854	22.36554	SGPL1_ENSG00000166224
10	72621574	72621774	KD2-H4K8ac_peak_3855	4.24345	
10	72647567	72648575	KD2-H4K8ac_peak_3856	6.84261	PCBD1_ENSG00000166228
10	72685069	72685388	KD2-H4K8ac_peak_3857	5.51783	
10	72795207	72795558	KD2-H4K8ac_peak_3858	4.30033	
10	72796589	72796797	KD2-H4K8ac_peak_3859	6.33462	
10	72800886	72801511	KD2-H4K8ac_peak_3860	9.3973	
10	72802469	72802691	KD2-H4K8ac_peak_3861	7.96322	
10	72805665	72806161	KD2-H4K8ac_peak_3862	6.0073	

10	72834517	72834865	KD2-H4K8ac_peak_3863	4.68806	
10	72845546	72845741	KD2-H4K8ac_peak_3864	4.86824	
10	72971827	72972029	KD2-H4K8ac_peak_3865	4.82973	UNC5B_ENSG00000107731
10	72972502	72973016	KD2-H4K8ac_peak_3866	6.99815	UNC5B_ENSG00000107731
10	72982609	72983159	KD2-H4K8ac_peak_3867	9.66321	
10	72989047	72989444	KD2-H4K8ac_peak_3868	4.86324	
10	72991492	72991704	KD2-H4K8ac_peak_3869	4.86324	
10	72992198	72992493	KD2-H4K8ac_peak_3870	7.76329	
10	72992742	72994677	KD2-H4K8ac_peak_3871	9.63487	
10	72995041	72995816	KD2-H4K8ac_peak_3872	29.69376	
10	72996031	72997846	KD2-H4K8ac_peak_3873	9.89383	
10	72998302	72998564	KD2-H4K8ac_peak_3874	4.50891	
10	72999347	72999779	KD2-H4K8ac_peak_3875	15.17328	
10	73000039	73001142	KD2-H4K8ac_peak_3876	16.57619	
10	73001561	73002784	KD2-H4K8ac_peak_3877	18.11807	
10	73003737	73004144	KD2-H4K8ac_peak_3878	10.7186	
10	73004366	73004579	KD2-H4K8ac_peak_3879	11.83301	
10	73005086	73006252	KD2-H4K8ac_peak_3880	14.52423	
10	73009384	73010071	KD2-H4K8ac_peak_3881	20.5798	
10	73012045	73013721	KD2-H4K8ac_peak_3882	8.88082	
10	73014050	73014667	KD2-H4K8ac_peak_3883	11.92978	
10	73015650	73017742	KD2-H4K8ac_peak_3884	22.20916	
10	73018308	73019046	KD2-H4K8ac_peak_3885	8.55271	
10	73019765	73020730	KD2-H4K8ac_peak_3886	7.20567	
10	73024956	73028280	KD2-H4K8ac_peak_3887	12.82393	
10	73029621	73029837	KD2-H4K8ac_peak_3888	5.54647	
10	73030622	73030879	KD2-H4K8ac_peak_3889	7.20567	
10	73031194	73031700	KD2-H4K8ac_peak_3890	6.40297	
10	73034803	73035193	KD2-H4K8ac_peak_3891	4.96644	
10	73036177	73036546	KD2-H4K8ac_peak_3892	6.99815	
10	73036941	73039031	KD2-H4K8ac_peak_3893	11.89502	
10	73039406	73040001	KD2-H4K8ac_peak_3894	9.65086	
10	73078297	73078664	KD2-H4K8ac_peak_3895	5.30799	SLC29A3_ENSG00000198246
10	73082647	73082903	KD2-H4K8ac_peak_3896	5.14125	
10	73136658	73137122	KD2-H4K8ac_peak_3897	10.77651	
10	73156200	73156426	KD2-H4K8ac_peak_3898	8.30974	CDH23_ENSG00000107736
10	73398549	73398754	KD2-H4K8ac_peak_3899	4.8402	
10	73418235	73418434	KD2-H4K8ac_peak_3900	6.19483	
10	73473426	73473627	KD2-H4K8ac_peak_3901	4.76709	
10	73484736	73485494	KD2-H4K8ac_peak_3902	8.10216	
10	73485933	73487560	KD2-H4K8ac_peak_3903	8.85813	
10	73488496	73489028	KD2-H4K8ac_peak_3904	5.59859	
10	73497283	73497559	KD2-H4K8ac_peak_3905	5.53095	C10orf105_ENSG00000214688
10	73508636	73509257	KD2-H4K8ac_peak_3906	9.11031	
10	73524886	73525110	KD2-H4K8ac_peak_3907	7.11925	
10	73610525	73611007	KD2-H4K8ac_peak_3908	12.84642	PSAP_ENSG00000197746

10	73611252	73611784	KD2-H4K8ac_peak_3909	10.46264	PSAP_ENSG00000197746
10	73619972	73620580	KD2-H4K8ac_peak_3910	7.146	
10	73625517	73625795	KD2-H4K8ac_peak_3911	3.91903	
10	73640772	73641412	KD2-H4K8ac_peak_3912	12.81138	
10	73648384	73648839	KD2-H4K8ac_peak_3913	13.72842	
10	73723600	73724934	KD2-H4K8ac_peak_3914	18.7894	CHST3_ENSG00000122863
10	73725196	73725604	KD2-H4K8ac_peak_3915	3.75035	
10	73758794	73759220	KD2-H4K8ac_peak_3916	9.59905	
10	73769014	73770044	KD2-H4K8ac_peak_3917	15.20126	
10	73804087	73804753	KD2-H4K8ac_peak_3918	7.76104	
10	73847280	73848059	KD2-H4K8ac_peak_3919	7.36799	SPOCK2_ENSG00000107742
10	73992464	73992941	KD2-H4K8ac_peak_3920	7.11925	
10	73993865	73994128	KD2-H4K8ac_peak_3921	3.74127	
10	74014306	74015310	KD2-H4K8ac_peak_3922	15.61313	
10	74020138	74021342	KD2-H4K8ac_peak_3923	23.33626	
10	74032193	74033348	KD2-H4K8ac_peak_3924	10.7186	DDIT4_ENSG00000168209
10	74057409	74057797	KD2-H4K8ac_peak_3925	13.95627	
10	74068069	74068695	KD2-H4K8ac_peak_3926	8.4716	
10	74070204	74070719	KD2-H4K8ac_peak_3927	12.2118	
10	74074765	74075290	KD2-H4K8ac_peak_3928	14.8083	
10	74076019	74076651	KD2-H4K8ac_peak_3929	6.82618	
10	74079182	74080540	KD2-H4K8ac_peak_3930	15.61313	
10	74080838	74081535	KD2-H4K8ac_peak_3931	15.57998	
10	74081787	74082307	KD2-H4K8ac_peak_3932	16.54162	
10	74082983	74083313	KD2-H4K8ac_peak_3933	6.55628	
10	74084663	74085483	KD2-H4K8ac_peak_3934	12.76142	
10	74087758	74088734	KD2-H4K8ac_peak_3935	9.048	
10	74090921	74091698	KD2-H4K8ac_peak_3936	15.49029	
10	74092273	74092472	KD2-H4K8ac_peak_3937	10.52915	
10	74093009	74093698	KD2-H4K8ac_peak_3938	10.87173	
10	74094418	74095978	KD2-H4K8ac_peak_3939	14.39182	
10	74113739	74114041	KD2-H4K8ac_peak_3940	8.5092	DNAJB12_ENSG00000148719
10	74114266	74114674	KD2-H4K8ac_peak_3941	5.54647	DNAJB12_ENSG00000148719
10	74114962	74115206	KD2-H4K8ac_peak_3942	4.62507	DNAJB12_ENSG00000148719
10	74115413	74115608	KD2-H4K8ac_peak_3943	6.47635	DNAJB12_ENSG00000148719
10	74370805	74371215	KD2-H4K8ac_peak_3944	4.211	
10	74385449	74385686	KD2-H4K8ac_peak_3945	6.56211	MICU1_ENSG00000107745
10	74386177	74386697	KD2-H4K8ac_peak_3946	8.78496	MICU1_ENSG00000107745
10	74452364	74452667	KD2-H4K8ac_peak_3947	5.57044	MCU_ENSG00000156026
10	74713421	74713639	KD2-H4K8ac_peak_3948	4.211	PLA2G12B_ENSG00000138308
10	74852583	74852782	KD2-H4K8ac_peak_3949	6.24747	
10	74927522	74927724	KD2-H4K8ac_peak_3950	4.8888	FAM149B1_ENSG00000138286
10	75012137	75012344	KD2-H4K8ac_peak_3951	11.28765	MRPS16_ENSG00000182180;RP11-152N13.5_ENSG00000227540
10	75118152	75118366	KD2-H4K8ac_peak_3952	7.36799	TTC18_ENSG00000156042
10	75255309	75255558	KD2-H4K8ac_peak_3953	5.88845	PPP3CB_ENSG00000107758;RP11-137L10.6_ENSG00000221817
10	75385880	75386375	KD2-H4K8ac_peak_3954	13.09607	USP54_ENSG00000166348;RP11-464F9.20_ENSG00000268584

10	75406483	75407176	KD2-H4K8ac_peak_3955	6.25869	
10	75407528	75408099	KD2-H4K8ac_peak_3956	14.66631	
10	75425234	75425442	KD2-H4K8ac_peak_3957	7.27716	
10	75489986	75490596	KD2-H4K8ac_peak_3958	14.8083	RP11-464F9.1_ENSG00000242288;RP11-574K11.28_ENSG00000271816;BMS1P4_ENSG00000242338;DUSP8P5_ENSG00000235316
10	75504221	75504504	KD2-H4K8ac_peak_3959	4.67342	SEC24C_ENSG00000176986
10	75531424	75532525	KD2-H4K8ac_peak_3960	13.83097	AC022400.2_ENSG00000267964;FUT11_ENSG00000196968
10	75541864	75542219	KD2-H4K8ac_peak_3961	14.66631	CHCHD1_ENSG00000172586
10	75545409	75545795	KD2-H4K8ac_peak_3962	6.13909	ZSWIM8_ENSG00000214655
10	75571293	75571486	KD2-H4K8ac_peak_3963	7.07117	RP11-574K11.31_ENSG00000272916;NDST2_ENSG00000166507
10	75634527	75635067	KD2-H4K8ac_peak_3964	12.82393	CAMK2G_ENSG00000148660
10	75758496	75758689	KD2-H4K8ac_peak_3965	4.98072	VCL_ENSG00000035403
10	75910234	75910481	KD2-H4K8ac_peak_3966	5.57044	AP3M1_ENSG00000185009;ADK_ENSG00000156110
10	75935368	75935585	KD2-H4K8ac_peak_3967	4.24756	
10	76345699	76345892	KD2-H4K8ac_peak_3968	7.47462	
10	76346367	76346596	KD2-H4K8ac_peak_3969	8.37043	
10	76584071	76585574	KD2-H4K8ac_peak_3970	19.55721	KAT6B_ENSG00000156650
10	76613471	76613840	KD2-H4K8ac_peak_3971	6.40683	
10	76620628	76620830	KD2-H4K8ac_peak_3972	4.65111	
10	76728577	76728769	KD2-H4K8ac_peak_3973	6.40683	
10	76821585	76822086	KD2-H4K8ac_peak_3974	5.88845	
10	76830228	76830888	KD2-H4K8ac_peak_3975	8.02571	
10	76831124	76831422	KD2-H4K8ac_peak_3976	9.36516	
10	76859097	76859424	KD2-H4K8ac_peak_3977	6.37852	SAMD8_ENSG00000156671
10	76859968	76860366	KD2-H4K8ac_peak_3978	4.37809	SAMD8_ENSG00000156671
10	76870614	76870853	KD2-H4K8ac_peak_3979	7.98621	
10	76969910	76971002	KD2-H4K8ac_peak_3980	11.3923	VDAC2_ENSG00000165637
10	76994325	76994740	KD2-H4K8ac_peak_3981	5.54647	
10	76995005	76995500	KD2-H4K8ac_peak_3982	13.70605	COMTD1_ENSG00000165644
10	76997989	76998669	KD2-H4K8ac_peak_3983	6.56211	
10	77022542	77022771	KD2-H4K8ac_peak_3984	5.57044	
10	77053571	77054281	KD2-H4K8ac_peak_3985	12.06346	
10	77054872	77055177	KD2-H4K8ac_peak_3986	13.73635	
10	77162587	77162943	KD2-H4K8ac_peak_3987	8.16906	ZNF503_ENSG00000165655
10	77164154	77164706	KD2-H4K8ac_peak_3988	14.68449	
10	77165049	77165433	KD2-H4K8ac_peak_3989	6.05809	
10	77169047	77169288	KD2-H4K8ac_peak_3990	13.95627	RP11-399K21.13_ENSG00000273248;RP11-399K21.14_ENSG00000272692
10	77169551	77169836	KD2-H4K8ac_peak_3991	6.04107	RP11-399K21.13_ENSG00000273248;RP11-399K21.14_ENSG00000272692
10	77191407	77191655	KD2-H4K8ac_peak_3992	12.42547	RP11-399K21.10_ENSG00000236842
10	77266629	77268063	KD2-H4K8ac_peak_3993	10.25088	
10	77771213	77771540	KD2-H4K8ac_peak_3994	5.25071	
10	77871882	77872371	KD2-H4K8ac_peak_3995	11.05467	
10	77978105	77978797	KD2-H4K8ac_peak_3996	9.15329	
10	78190934	78191156	KD2-H4K8ac_peak_3997	9.97423	
10	78312034	78312308	KD2-H4K8ac_peak_3998	11.86082	
10	78313649	78314125	KD2-H4K8ac_peak_3999	9.89126	
10	78696408	78696718	KD2-H4K8ac_peak_4000	16.32216	

10	78697808	78698339	KD2-H4K8ac_peak_4001	4.42498	
10	78801229	78801579	KD2-H4K8ac_peak_4002	9.11031	
10	78849930	78850349	KD2-H4K8ac_peak_4003	6.40683	
10	78851240	78851520	KD2-H4K8ac_peak_4004	7.27716	
10	78864578	78865134	KD2-H4K8ac_peak_4005	10.0692	
10	78866091	78866283	KD2-H4K8ac_peak_4006	5.34946	
10	78866614	78866807	KD2-H4K8ac_peak_4007	6.99815	
10	79473724	79474003	KD2-H4K8ac_peak_4008	3.72574	
10	79606693	79607137	KD2-H4K8ac_peak_4009	7.76329	
10	79632147	79632458	KD2-H4K8ac_peak_4010	5.20998	
10	79637217	79637883	KD2-H4K8ac_peak_4011	16.19273	
10	79638101	79638776	KD2-H4K8ac_peak_4012	13.73635	
10	79639004	79639452	KD2-H4K8ac_peak_4013	7.1181	
10	79685769	79687329	KD2-H4K8ac_peak_4014	13.92465	DLG5_ENSG00000151208;DLG5-AS1_ENSG00000233871
10	79788889	79789245	KD2-H4K8ac_peak_4015	5.14125	POLR3A_ENSG00000148606
10	79790688	79790901	KD2-H4K8ac_peak_4016	6.16888	
10	79793696	79794093	KD2-H4K8ac_peak_4017	9.11031	RPS24_ENSG00000138326
10	79807388	79807746	KD2-H4K8ac_peak_4018	11.43353	
10	79819663	79820072	KD2-H4K8ac_peak_4019	4.77079	
10	79845439	79845811	KD2-H4K8ac_peak_4020	8.37231	
10	79899041	79899469	KD2-H4K8ac_peak_4021	6.65673	
10	79954915	79955391	KD2-H4K8ac_peak_4022	6.65673	
10	79963203	79963713	KD2-H4K8ac_peak_4023	5.88845	
10	79964231	79964621	KD2-H4K8ac_peak_4024	7.47462	
10	79964920	79965124	KD2-H4K8ac_peak_4025	5.30799	
10	79970964	79971240	KD2-H4K8ac_peak_4026	5.51783	
10	79971662	79971914	KD2-H4K8ac_peak_4027	9.63487	
10	79972257	79973016	KD2-H4K8ac_peak_4028	13.12288	
10	80048662	80049476	KD2-H4K8ac_peak_4029	6.1846	
10	80050835	80051187	KD2-H4K8ac_peak_4030	6.16747	
10	80052181	80053175	KD2-H4K8ac_peak_4031	8.10216	RP11-90J7.2_ENSG00000229543
10	80053704	80054176	KD2-H4K8ac_peak_4032	12.17278	RP11-90J7.2_ENSG00000229543
10	80055397	80055782	KD2-H4K8ac_peak_4033	6.99815	
10	80061368	80061874	KD2-H4K8ac_peak_4034	5.57044	
10	80062224	80062958	KD2-H4K8ac_peak_4035	22.21705	
10	80063470	80064057	KD2-H4K8ac_peak_4036	9.36516	
10	80064321	80064544	KD2-H4K8ac_peak_4037	6.24114	
10	80076316	80077122	KD2-H4K8ac_peak_4038	8.59579	
10	80161541	80162261	KD2-H4K8ac_peak_4039	7.95934	
10	80166442	80167617	KD2-H4K8ac_peak_4040	12.93209	
10	80167964	80168785	KD2-H4K8ac_peak_4041	10.19949	
10	80169092	80169367	KD2-H4K8ac_peak_4042	6.53538	
10	80177857	80178052	KD2-H4K8ac_peak_4043	7.0983	
10	80180485	80181056	KD2-H4K8ac_peak_4044	7.47462	
10	80193573	80193950	KD2-H4K8ac_peak_4045	8.23274	
10	80194160	80195327	KD2-H4K8ac_peak_4046	8.86099	

10	80195811	80196379	KD2-H4K8ac_peak_4047	8.4716
10	80201780	80202021	KD2-H4K8ac_peak_4048	9.73431
10	80202263	80202491	KD2-H4K8ac_peak_4049	8.30974
10	80202914	80203305	KD2-H4K8ac_peak_4050	18.57315
10	80224559	80225631	KD2-H4K8ac_peak_4051	9.1707
10	80230533	80231542	KD2-H4K8ac_peak_4052	12.37731
10	80270294	80270677	KD2-H4K8ac_peak_4053	9.15069
10	80284585	80284854	KD2-H4K8ac_peak_4054	5.14125
10	80302282	80302495	KD2-H4K8ac_peak_4055	5.29352
10	80339571	80339774	KD2-H4K8ac_peak_4056	8.17902
10	80343989	80344671	KD2-H4K8ac_peak_4057	5.61377
10	80394706	80394902	KD2-H4K8ac_peak_4058	5.45396
10	80416123	80416660	KD2-H4K8ac_peak_4059	15.10495
10	80444255	80444480	KD2-H4K8ac_peak_4060	4.06758
10	80444961	80445155	KD2-H4K8ac_peak_4061	5.54647
10	80448275	80448530	KD2-H4K8ac_peak_4062	9.1707
10	80448890	80449408	KD2-H4K8ac_peak_4063	8.5092
10	80463394	80463742	KD2-H4K8ac_peak_4064	4.16871
10	80485837	80486270	KD2-H4K8ac_peak_4065	6.1846
10	80488697	80488893	KD2-H4K8ac_peak_4066	6.36319
10	80502233	80502919	KD2-H4K8ac_peak_4067	20.99972
10	80503693	80505109	KD2-H4K8ac_peak_4068	17.05477
10	80505740	80506433	KD2-H4K8ac_peak_4069	11.50346
10	80514943	80515250	KD2-H4K8ac_peak_4070	5.73016
10	80516518	80516780	KD2-H4K8ac_peak_4071	6.97919
10	80531907	80532156	KD2-H4K8ac_peak_4072	4.211
10	80561958	80562627	KD2-H4K8ac_peak_4073	9.1707
10	80562845	80563091	KD2-H4K8ac_peak_4074	9.96798
10	80563404	80563754	KD2-H4K8ac_peak_4075	9.36516
10	80564273	80564590	KD2-H4K8ac_peak_4076	11.00004
10	80567177	80567501	KD2-H4K8ac_peak_4077	6.32754
10	80621101	80621303	KD2-H4K8ac_peak_4078	8.20667
10	80621498	80621885	KD2-H4K8ac_peak_4079	5.9696
10	80622796	80623025	KD2-H4K8ac_peak_4080	6.82618
10	80655315	80655530	KD2-H4K8ac_peak_4081	5.14125
10	80665607	80665895	KD2-H4K8ac_peak_4082	6.66692
10	80704275	80704615	KD2-H4K8ac_peak_4083	7.40111
10	80722065	80722318	KD2-H4K8ac_peak_4084	5.88845
10	80723697	80723910	KD2-H4K8ac_peak_4085	7.74292
10	80724237	80724690	KD2-H4K8ac_peak_4086	7.78826
10	80724959	80725490	KD2-H4K8ac_peak_4087	12.82393
10	80725754	80726052	KD2-H4K8ac_peak_4088	8.20667
10	80732100	80734176	KD2-H4K8ac_peak_4089	18.94636
10	80756328	80757138	KD2-H4K8ac_peak_4090	6.83936
10	80768687	80769177	KD2-H4K8ac_peak_4091	8.17902
10	80774497	80774699	KD2-H4K8ac_peak_4092	6.42097

10	80778037	80778358	KD2-H4K8ac_peak_4093	6.40297	
10	80782737	80783077	KD2-H4K8ac_peak_4094	5.08628	
10	80784401	80785084	KD2-H4K8ac_peak_4095	15.59774	
10	80785445	80786472	KD2-H4K8ac_peak_4096	8.55271	
10	80806966	80808942	KD2-H4K8ac_peak_4097	8.16906	
10	80826351	80826955	KD2-H4K8ac_peak_4098	15.81711	ZMIZ1-AS1_ENSG00000224596
10	80827202	80827548	KD2-H4K8ac_peak_4099	12.84642	ZMIZ1-AS1_ENSG00000224596
10	80827789	80828572	KD2-H4K8ac_peak_4100	11.05467	ZMIZ1-AS1_ENSG00000224596;ZMIZ1_ENSG00000108175
10	80828842	80829350	KD2-H4K8ac_peak_4101	8.23274	ZMIZ1_ENSG00000108175
10	80889062	80889835	KD2-H4K8ac_peak_4102	8.23274	
10	80891894	80892610	KD2-H4K8ac_peak_4103	7.95934	
10	80908897	80909203	KD2-H4K8ac_peak_4104	4.98072	
10	80919995	80920197	KD2-H4K8ac_peak_4105	11.3394	
10	80936990	80937481	KD2-H4K8ac_peak_4106	12.93209	
10	80937771	80938337	KD2-H4K8ac_peak_4107	17.55491	
10	80939084	80939357	KD2-H4K8ac_peak_4108	6.98244	
10	80939787	80940275	KD2-H4K8ac_peak_4109	8.82366	
10	80954312	80954842	KD2-H4K8ac_peak_4110	5.61067	
10	80957042	80957269	KD2-H4K8ac_peak_4111	3.97079	
10	80962560	80962977	KD2-H4K8ac_peak_4112	6.66359	
10	80963994	80964354	KD2-H4K8ac_peak_4113	9.95119	
10	80978876	80979288	KD2-H4K8ac_peak_4114	7.27281	
10	80979865	80980506	KD2-H4K8ac_peak_4115	15.38707	
10	80980957	80981377	KD2-H4K8ac_peak_4116	7.44324	
10	80981620	80983165	KD2-H4K8ac_peak_4117	12.11734	
10	80983536	80984844	KD2-H4K8ac_peak_4118	13.73635	
10	80988113	80988557	KD2-H4K8ac_peak_4119	9.30297	
10	80998787	80999103	KD2-H4K8ac_peak_4120	5.6746	
10	81002067	81003180	KD2-H4K8ac_peak_4121	14.60723	
10	81003389	81003974	KD2-H4K8ac_peak_4122	13.66685	
10	81006883	81007365	KD2-H4K8ac_peak_4123	6.97919	
10	81013946	81014240	KD2-H4K8ac_peak_4124	4.54729	
10	81018023	81018399	KD2-H4K8ac_peak_4125	6.05809	
10	81019481	81019698	KD2-H4K8ac_peak_4126	5.1685	
10	81020792	81021025	KD2-H4K8ac_peak_4127	11.89502	
10	81021612	81021854	KD2-H4K8ac_peak_4128	7.1181	
10	81023473	81024176	KD2-H4K8ac_peak_4129	5.29555	
10	81025617	81025864	KD2-H4K8ac_peak_4130	5.09296	
10	81026322	81026974	KD2-H4K8ac_peak_4131	6.25869	
10	81028137	81029051	KD2-H4K8ac_peak_4132	11.0821	
10	81029286	81029803	KD2-H4K8ac_peak_4133	7.70067	
10	81030161	81030670	KD2-H4K8ac_peak_4134	9.90914	
10	81031095	81031697	KD2-H4K8ac_peak_4135	9.90914	
10	81032174	81033164	KD2-H4K8ac_peak_4136	11.58876	
10	81033478	81033970	KD2-H4K8ac_peak_4137	5.4779	
10	81034424	81036143	KD2-H4K8ac_peak_4138	13.91698	

10	81106345	81106882	KD2-H4K8ac_peak_4139	9.16531	PPIF_ENSG00000108179
10	81107308	81107908	KD2-H4K8ac_peak_4140	7.36799	PPIF_ENSG00000108179
10	81156979	81157205	KD2-H4K8ac_peak_4141	6.65673	
10	81157576	81157846	KD2-H4K8ac_peak_4142	6.87784	
10	81159294	81159815	KD2-H4K8ac_peak_4143	9.74423	
10	81160664	81161402	KD2-H4K8ac_peak_4144	7.76329	
10	81184855	81185165	KD2-H4K8ac_peak_4145	3.87414	
10	81663680	81664971	KD2-H4K8ac_peak_4146	8.5092	MBL1P_ENSG00000242600
10	81741208	81742015	KD2-H4K8ac_peak_4147	11.5744	SFTPD_ENSG00000133661;ZNRF2P3_ENSG00000235924;RP11-479O17.7_ENSG00000225100
10	81782483	81782675	KD2-H4K8ac_peak_4148	6.16888	
10	81837789	81838831	KD2-H4K8ac_peak_4149	20.47312	TMEM254-AS1_ENSG00000230091;TMEM254_ENSG00000133678
10	81892162	81892464	KD2-H4K8ac_peak_4150	7.73264	PLAC9_ENSG00000189129
10	81964701	81965742	KD2-H4K8ac_peak_4151	6.65673	ANXA11_ENSG00000122359
10	82117036	82117596	KD2-H4K8ac_peak_4152	20.67885	DYDC1_ENSG00000170788
10	82168361	82168806	KD2-H4K8ac_peak_4153	10.96455	FAM213A_ENSG00000122378
10	82203733	82204100	KD2-H4K8ac_peak_4154	6.8836	
10	82213205	82213552	KD2-H4K8ac_peak_4155	6.83936	TSPAN14_ENSG00000108219
10	82214054	82214551	KD2-H4K8ac_peak_4156	9.81419	TSPAN14_ENSG00000108219
10	82219051	82219664	KD2-H4K8ac_peak_4157	9.11031	
10	82224743	82225344	KD2-H4K8ac_peak_4158	10.05606	
10	84836327	84836584	KD2-H4K8ac_peak_4159	6.16888	
10	85340415	85340654	KD2-H4K8ac_peak_4160	5.08628	
10	85899338	85899651	KD2-H4K8ac_peak_4161	8.85813	GHITM_ENSG00000165678;RP11-338I21.1_ENSG00000271933
10	86088470	86088850	KD2-H4K8ac_peak_4162	7.0754	CCSER2_ENSG00000107771
10	88004757	88005166	KD2-H4K8ac_peak_4163	13.78165	
10	88005384	88006308	KD2-H4K8ac_peak_4164	4.211	
10	88013386	88013803	KD2-H4K8ac_peak_4165	5.54647	
10	88024485	88024898	KD2-H4K8ac_peak_4166	4.33139	MIR346_ENSG00000199104
10	88126273	88126503	KD2-H4K8ac_peak_4167	6.0073	GRID1_ENSG00000182771
10	88126715	88127446	KD2-H4K8ac_peak_4168	7.36799	GRID1_ENSG00000182771
10	88136676	88136944	KD2-H4K8ac_peak_4169	7.40314	
10	88159901	88160481	KD2-H4K8ac_peak_4170	6.40297	
10	88281084	88281456	KD2-H4K8ac_peak_4171	5.59859	WAPAL_ENSG00000062650;RP11-77P6.2_ENSG00000227896
10	88281766	88282155	KD2-H4K8ac_peak_4172	10.44656	WAPAL_ENSG00000062650;RP11-77P6.2_ENSG00000227896
10	88406275	88406508	KD2-H4K8ac_peak_4173	6.40683	
10	88425602	88425818	KD2-H4K8ac_peak_4174	5.92591	
10	88434047	88434279	KD2-H4K8ac_peak_4175	6.8108	
10	88441113	88441317	KD2-H4K8ac_peak_4176	6.40683	
10	88470372	88472156	KD2-H4K8ac_peak_4177	16.54484	
10	88515620	88515981	KD2-H4K8ac_peak_4178	8.86099	RP11-359E3.4_ENSG00000272631;BMPR1A_ENSG00000107779
10	88516417	88516825	KD2-H4K8ac_peak_4179	7.25989	RP11-359E3.4_ENSG00000272631;BMPR1A_ENSG00000107779
10	88777477	88777850	KD2-H4K8ac_peak_4180	9.63487	
10	88779611	88779838	KD2-H4K8ac_peak_4181	4.59928	FAM25A_ENSG00000188100
10	88854184	88854615	KD2-H4K8ac_peak_4182	7.47462	FAM35A_ENSG00000122376
10	88854983	88855317	KD2-H4K8ac_peak_4183	10.96455	
10	89101943	89102266	KD2-H4K8ac_peak_4184	10.96455	NUTM2A-AS1_ENSG00000223482;LINC00863_ENSG00000224914

10	89102551	89102920	KD2-H4K8ac_peak_4185	7.70289	NUTM2A-AS1_ENSG00000223482;LINC00863_ENSG00000224914
10	89264148	89264497	KD2-H4K8ac_peak_4186	8.55271	MINPP1_ENSG00000107789
10	89419449	89420089	KD2-H4K8ac_peak_4187	12.10284	RP11-57C13.3_ENSG00000196566;PAPSS2_ENSG00000198682
10	89577526	89577912	KD2-H4K8ac_peak_4188	7.49599	CFL1P1_ENSG00000223820
10	89578402	89578915	KD2-H4K8ac_peak_4189	5.54647	CFL1P1_ENSG00000223820
10	89621454	89622032	KD2-H4K8ac_peak_4190	11.22681	PTEN_ENSG00000171862
10	89622769	89623133	KD2-H4K8ac_peak_4191	7.1181	KLLN_ENSG00000227268;PTEN_ENSG00000171862
10	89623407	89623737	KD2-H4K8ac_peak_4192	10.29842	KLLN_ENSG00000227268;PTEN_ENSG00000171862
10	89623963	89624187	KD2-H4K8ac_peak_4193	6.67092	KLLN_ENSG00000227268
10	89824713	89825323	KD2-H4K8ac_peak_4194	4.86324	
10	90002553	90002802	KD2-H4K8ac_peak_4195	6.37652	
10	90145295	90145569	KD2-H4K8ac_peak_4196	12.06346	
10	90639660	90640728	KD2-H4K8ac_peak_4197	14.56955	STAMBPL1_ENSG00000138134
10	90750724	90750933	KD2-H4K8ac_peak_4198	6.67538	ACTA2_ENSG00000107796;FAS_ENSG00000026103
10	91011259	91011538	KD2-H4K8ac_peak_4199	10.19949	
10	91044481	91044831	KD2-H4K8ac_peak_4200	7.11925	RP11-149I23.3_ENSG00000232110
10	91174035	91174343	KD2-H4K8ac_peak_4201	6.97919	LIPA_ENSG00000107798;IFIT5_ENSG00000152778
10	91174676	91175067	KD2-H4K8ac_peak_4202	5.38581	LIPA_ENSG00000107798;IFIT5_ENSG00000152778
10	91404453	91405037	KD2-H4K8ac_peak_4203	20.18584	PANK1_ENSG00000152782;RP11-80H5.5_ENSG00000249962;RP11-80H5.2_ENSG00000232936
10	91460835	91461104	KD2-H4K8ac_peak_4204	12.25733	KIF20B_ENSG00000138182
10	91461427	91461655	KD2-H4K8ac_peak_4205	8.74596	KIF20B_ENSG00000138182
10	92631772	92631977	KD2-H4K8ac_peak_4206	7.36799	RPP30_ENSG00000148688
10	92794381	92794584	KD2-H4K8ac_peak_4207	6.16888	
10	92980549	92980774	KD2-H4K8ac_peak_4208	8.55271	PCGF5_ENSG00000180628
10	93058350	93058691	KD2-H4K8ac_peak_4209	7.22457	
10	93169055	93170651	KD2-H4K8ac_peak_4210	11.89502	HECTD2_ENSG00000165338
10	93349104	93349331	KD2-H4K8ac_peak_4211	4.47369	
10	93351921	93352136	KD2-H4K8ac_peak_4212	3.75035	
10	93431796	93432429	KD2-H4K8ac_peak_4213	6.25869	
10	93441197	93441501	KD2-H4K8ac_peak_4214	5.71749	
10	93558283	93558863	KD2-H4K8ac_peak_4215	7.13615	TNKS2-AS1_ENSG00000228701;TNKS2_ENSG00000107854
10	93646868	93647772	KD2-H4K8ac_peak_4216	16.61426	
10	93667956	93668635	KD2-H4K8ac_peak_4217	11.79585	FGFBP3_ENSG00000174721;RP11-402D21.2_ENSG00000272817
10	94050982	94051464	KD2-H4K8ac_peak_4218	14.98671	CPEB3_ENSG00000107864;MARCH5_ENSG00000198060
10	94333286	94333720	KD2-H4K8ac_peak_4219	7.0754	IDE_ENSG00000119912
10	94333977	94334200	KD2-H4K8ac_peak_4220	9.65086	IDE_ENSG00000119912
10	94350916	94351352	KD2-H4K8ac_peak_4221	5.54647	
10	94404172	94404372	KD2-H4K8ac_peak_4222	5.14125	
10	94448750	94448946	KD2-H4K8ac_peak_4223	6.13909	HHEX_ENSG00000152804
10	94449703	94450064	KD2-H4K8ac_peak_4224	6.83936	
10	94603309	94603597	KD2-H4K8ac_peak_4225	7.13615	
10	94607952	94608961	KD2-H4K8ac_peak_4226	12.84642	
10	95255002	95255195	KD2-H4K8ac_peak_4227	9.15329	
10	95302651	95302879	KD2-H4K8ac_peak_4228	9.02371	
10	95390764	95391010	KD2-H4K8ac_peak_4229	6.64891	
10	95461622	95461868	KD2-H4K8ac_peak_4230	4.98318	FRA10AC1_ENSG00000148690

10	95609194	95609433	KD2-H4K8ac_peak_4231	5.73839	
10	95653286	95654097	KD2-H4K8ac_peak_4232	15.81711	SLC35G1_ENSG00000176273
10	95753333	95753945	KD2-H4K8ac_peak_4233	10.96455	PLCE1_ENSG00000138193
10	96162386	96162601	KD2-H4K8ac_peak_4234	6.0073	TBC1D12_ENSG00000108239
10	96304411	96304606	KD2-H4K8ac_peak_4235	11.2938	HELLS_ENSG00000119969
10	96943121	96944085	KD2-H4K8ac_peak_4236	37.89986	PAWRP1_ENSG00000225533
10	97050308	97051219	KD2-H4K8ac_peak_4237	12.06346	PDLIM1_ENSG00000107438
10	97453356	97453642	KD2-H4K8ac_peak_4238	8.74596	TCTN3_ENSG00000119977
10	97667299	97667594	KD2-H4K8ac_peak_4239	6.0605	C10orf131_ENSG00000173088
10	97802798	97804143	KD2-H4K8ac_peak_4240	23.52296	CCNJ_ENSG00000107443
10	97849495	97850590	KD2-H4K8ac_peak_4241	10.05606	ENTPD1-AS1_ENSG00000226688
10	97884437	97884799	KD2-H4K8ac_peak_4242	14.74047	
10	97889208	97890535	KD2-H4K8ac_peak_4243	13.87818	ZNF518A_ENSG00000177853
10	98134909	98135420	KD2-H4K8ac_peak_4244	10.0692	
10	98201005	98201389	KD2-H4K8ac_peak_4245	4.77079	
10	98346129	98347224	KD2-H4K8ac_peak_4246	14.4458	TM9SF3_ENSG00000077147
10	98479633	98480867	KD2-H4K8ac_peak_4247	21.11201	PIK3AP1_ENSG00000155629
10	98591558	98592620	KD2-H4K8ac_peak_4248	21.3105	LCOR_ENSG00000196233
10	98834787	98835002	KD2-H4K8ac_peak_4249	7.79898	
10	98899706	98900568	KD2-H4K8ac_peak_4250	9.51625	
10	98944886	98945851	KD2-H4K8ac_peak_4251	12.5605	SLIT1_ENSG00000187122
10	98956016	98956438	KD2-H4K8ac_peak_4252	12.00273	
10	99052021	99052216	KD2-H4K8ac_peak_4253	8.53092	ARHGAP19-SLIT1_ENSG00000269891;ARHGAP19_ENSG00000213390
10	99094557	99095029	KD2-H4K8ac_peak_4254	7.28628	FRAT2_ENSG00000181274;RP11-452K12.4_ENSG00000225850
10	99161346	99161564	KD2-H4K8ac_peak_4255	9.06369	RRP12_ENSG00000052749;RP11-452K12.7_ENSG00000231970
10	99166354	99166858	KD2-H4K8ac_peak_4256	7.98152	
10	99186097	99186379	KD2-H4K8ac_peak_4257	11.24702	AL355490.1_ENSG00000224474;PGAM1_ENSG00000171314
10	99205454	99205693	KD2-H4K8ac_peak_4258	10.46264	EXOSC1_ENSG00000171311;ZDHHC16_ENSG00000171307
10	99205970	99206311	KD2-H4K8ac_peak_4259	13.81781	EXOSC1_ENSG00000171311;ZDHHC16_ENSG00000171307
10	99309652	99310075	KD2-H4K8ac_peak_4260	6.8836	
10	99332327	99332661	KD2-H4K8ac_peak_4261	4.19765	ANKRD2_ENSG00000165887
10	99392938	99393184	KD2-H4K8ac_peak_4262	9.63487	MORN4_ENSG00000171160
10	99418824	99419052	KD2-H4K8ac_peak_4263	6.8836	
10	99419906	99420370	KD2-H4K8ac_peak_4264	7.27716	
10	99446780	99447353	KD2-H4K8ac_peak_4265	13.51255	AVPI1_ENSG00000119986
10	99461383	99461731	KD2-H4K8ac_peak_4266	3.75035	
10	99472944	99474618	KD2-H4K8ac_peak_4267	19.38373	MARVELD1_ENSG00000155254
10	99496380	99496748	KD2-H4K8ac_peak_4268	11.89502	ZFYVE27_ENSG00000155256
10	99496981	99497296	KD2-H4K8ac_peak_4269	7.40314	ZFYVE27_ENSG00000155256
10	99540786	99541069	KD2-H4K8ac_peak_4270	8.02571	
10	99609446	99609784	KD2-H4K8ac_peak_4271	4.86324	LINC00866_ENSG00000227356;GOLGA7B_ENSG00000155265
10	99649017	99649437	KD2-H4K8ac_peak_4272	12.25441	
10	99674329	99675092	KD2-H4K8ac_peak_4273	10.05606	
10	99790554	99791123	KD2-H4K8ac_peak_4274	8.17902	CRTAC1_ENSG00000095713
10	99894582	99895141	KD2-H4K8ac_peak_4275	14.8083	R3HCC1L_ENSG00000166024
10	100027613	1E+08	KD2-H4K8ac_peak_4276	9.36516	LOXL4_ENSG00000138131

10	100055280	1E+08	KD2-H4K8ac_peak_4277	9.73431	
10	100068688	1E+08	KD2-H4K8ac_peak_4278	8.59579	
10	100069565	1E+08	KD2-H4K8ac_peak_4279	5.54647	
10	100081203	1E+08	KD2-H4K8ac_peak_4280	8.23274	
10	100174607	1E+08	KD2-H4K8ac_peak_4281	10.53377	PYROXD2_ENSG00000119943
10	100206076	1E+08	KD2-H4K8ac_peak_4282	11.92978	HPS1_ENSG00000107521
10	100227634	1E+08	KD2-H4K8ac_peak_4283	6.40297	
10	100766775	1.01E+08	KD2-H4K8ac_peak_4284	6.66692	
10	100767641	1.01E+08	KD2-H4K8ac_peak_4285	5.25071	
10	101088877	1.01E+08	KD2-H4K8ac_peak_4286	17.17257	CNNM1_ENSG00000119946
10	101190035	1.01E+08	KD2-H4K8ac_peak_4287	8.23274	GOT1_ENSG00000120053;RP11-441O15.3_ENSG00000224934
10	101190715	1.01E+08	KD2-H4K8ac_peak_4288	11.86082	GOT1_ENSG00000120053;RP11-441O15.3_ENSG00000224934
10	101380626	1.01E+08	KD2-H4K8ac_peak_4289	9.20193	SLC25A28_ENSG00000155287;RP11-85A1.3_ENSG00000260475
10	101419241	1.01E+08	KD2-H4K8ac_peak_4290	5.82799	ENTPD7_ENSG00000198018
10	101491521	1.01E+08	KD2-H4K8ac_peak_4291	15.9336	COX15_ENSG00000014919
10	101492258	1.01E+08	KD2-H4K8ac_peak_4292	7.12754	COX15_ENSG00000014919
10	101945450	1.02E+08	KD2-H4K8ac_peak_4293	6.99815	
10	101988875	1.02E+08	KD2-H4K8ac_peak_4294	17.14886	CHUK_ENSG00000213341;RP11-316M21.6_ENSG00000227492
10	102027026	1.02E+08	KD2-H4K8ac_peak_4295	16.23223	CWF19L1_ENSG00000095485
10	102046018	1.02E+08	KD2-H4K8ac_peak_4296	10.96455	BLOC1S2_ENSG00000196072
10	102106375	1.02E+08	KD2-H4K8ac_peak_4297	6.8713	RP11-34D15.2_ENSG00000231188;SCD_ENSG00000099194
10	102107006	1.02E+08	KD2-H4K8ac_peak_4298	7.95563	RP11-34D15.2_ENSG00000231188;SCD_ENSG00000099194
10	102133413	1.02E+08	KD2-H4K8ac_peak_4299	10.73134	LINC00263_ENSG00000235823
10	102133982	1.02E+08	KD2-H4K8ac_peak_4300	8.09303	LINC00263_ENSG00000235823
10	102289600	1.02E+08	KD2-H4K8ac_peak_4301	7.13615	SEC31B_ENSG00000075826;NDUFB8_ENSG00000255339;NDUFB8_ENSG00000166136;HIF1AN_ENSG00000166135
10	102321947	1.02E+08	KD2-H4K8ac_peak_4302	7.1181	
10	102381131	1.02E+08	KD2-H4K8ac_peak_4303	5.88845	
10	102414560	1.02E+08	KD2-H4K8ac_peak_4304	13.80019	
10	102445215	1.02E+08	KD2-H4K8ac_peak_4305	5.60968	
10	102446960	1.02E+08	KD2-H4K8ac_peak_4306	5.57044	
10	102448093	1.02E+08	KD2-H4K8ac_peak_4307	7.47462	
10	102469288	1.02E+08	KD2-H4K8ac_peak_4308	9.63487	
10	102472857	1.02E+08	KD2-H4K8ac_peak_4309	11.05467	
10	102473578	1.02E+08	KD2-H4K8ac_peak_4310	21.1285	
10	102527108	1.03E+08	KD2-H4K8ac_peak_4311	8.84031	
10	102548049	1.03E+08	KD2-H4K8ac_peak_4312	7.07117	
10	102551420	1.03E+08	KD2-H4K8ac_peak_4313	8.55552	
10	102565786	1.03E+08	KD2-H4K8ac_peak_4314	4.75875	
10	102568192	1.03E+08	KD2-H4K8ac_peak_4315	7.47462	
10	102672787	1.03E+08	KD2-H4K8ac_peak_4316	16.49944	RP11-179B2.2_ENSG00000272572;FAM178A_ENSG00000119906
10	102728619	1.03E+08	KD2-H4K8ac_peak_4317	6.66359	SEMA4G_ENSG00000095539
10	102746997	1.03E+08	KD2-H4K8ac_peak_4318	8.28234	MRPL43_ENSG00000055950;C10orf2_ENSG00000107815
10	102756325	1.03E+08	KD2-H4K8ac_peak_4319	6.0605	LZTS2_ENSG00000107816
10	102757740	1.03E+08	KD2-H4K8ac_peak_4320	7.51054	
10	102759089	1.03E+08	KD2-H4K8ac_peak_4321	9.26408	
10	102772389	1.03E+08	KD2-H4K8ac_peak_4322	9.402	

10	102778474	1.03E+08	KD2-H4K8ac_peak_4323	11.44456	
10	102790576	1.03E+08	KD2-H4K8ac_peak_4324	9.36516	PDZD7_ENSG00000186862;SFXN3_ENSG00000107819
10	102802149	1.03E+08	KD2-H4K8ac_peak_4325	12.82393	
10	102820544	1.03E+08	KD2-H4K8ac_peak_4326	13.83097	RP11-108L7.15_ENSG00000273162;KAZALD1_ENSG00000107821
10	102821041	1.03E+08	KD2-H4K8ac_peak_4327	5.4779	RP11-108L7.15_ENSG00000273162;KAZALD1_ENSG00000107821
10	102826060	1.03E+08	KD2-H4K8ac_peak_4328	6.40683	
10	102827053	1.03E+08	KD2-H4K8ac_peak_4329	4.67342	
10	102827494	1.03E+08	KD2-H4K8ac_peak_4330	6.2781	
10	102873104	1.03E+08	KD2-H4K8ac_peak_4331	4.42765	
10	102882991	1.03E+08	KD2-H4K8ac_peak_4332	9.6589	HUG1_ENSG00000269259
10	102891298	1.03E+08	KD2-H4K8ac_peak_4333	6.40297	TLX1NB_ENSG00000236311
10	102901812	1.03E+08	KD2-H4K8ac_peak_4334	10.43145	
10	102975625	1.03E+08	KD2-H4K8ac_peak_4335	6.19483	
10	103051060	1.03E+08	KD2-H4K8ac_peak_4336	13.22091	
10	103052558	1.03E+08	KD2-H4K8ac_peak_4337	5.75757	
10	103070231	1.03E+08	KD2-H4K8ac_peak_4338	8.85813	RP11-107I14.2_ENSG00000225208
10	103070855	1.03E+08	KD2-H4K8ac_peak_4339	6.25869	RP11-107I14.2_ENSG00000225208
10	103113202	1.03E+08	KD2-H4K8ac_peak_4340	9.36665	BTRC_ENSG00000166167
10	103113986	1.03E+08	KD2-H4K8ac_peak_4341	10.08707	BTRC_ENSG00000166167
10	103318431	1.03E+08	KD2-H4K8ac_peak_4342	6.0605	
10	103319502	1.03E+08	KD2-H4K8ac_peak_4343	4.39858	
10	103320498	1.03E+08	KD2-H4K8ac_peak_4344	4.211	
10	103326361	1.03E+08	KD2-H4K8ac_peak_4345	12.08081	
10	103329434	1.03E+08	KD2-H4K8ac_peak_4346	15.22356	DPCD_ENSG00000166171
10	103347229	1.03E+08	KD2-H4K8ac_peak_4347	7.74292	POLL_ENSG00000166169
10	103454203	1.03E+08	KD2-H4K8ac_peak_4348	9.26408	FBXW4_ENSG00000107829
10	103542816	1.04E+08	KD2-H4K8ac_peak_4349	5.30799	NPM3_ENSG00000107833
10	103577564	1.04E+08	KD2-H4K8ac_peak_4350	6.25869	MGEA5_ENSG00000198408;KCNIP2-AS1_ENSG00000226009
10	103578840	1.04E+08	KD2-H4K8ac_peak_4351	8.17902	MGEA5_ENSG00000198408;KCNIP2-AS1_ENSG00000226009
10	103591008	1.04E+08	KD2-H4K8ac_peak_4352	5.9696	
10	103815560	1.04E+08	KD2-H4K8ac_peak_4353	7.49599	C10orf76_ENSG00000120029
10	103816450	1.04E+08	KD2-H4K8ac_peak_4354	7.76329	C10orf76_ENSG00000120029
10	103825083	1.04E+08	KD2-H4K8ac_peak_4355	12.96152	HPS6_ENSG00000166189
10	103874089	1.04E+08	KD2-H4K8ac_peak_4356	10.43601	
10	103874885	1.04E+08	KD2-H4K8ac_peak_4357	9.58942	
10	103876819	1.04E+08	KD2-H4K8ac_peak_4358	11.05467	
10	103877452	1.04E+08	KD2-H4K8ac_peak_4359	7.98621	
10	103878390	1.04E+08	KD2-H4K8ac_peak_4360	5.27406	
10	103879678	1.04E+08	KD2-H4K8ac_peak_4361	8.65175	LDB1_ENSG00000198728
10	103880481	1.04E+08	KD2-H4K8ac_peak_4362	8.43115	LDB1_ENSG00000198728
10	103881063	1.04E+08	KD2-H4K8ac_peak_4363	10.90543	LDB1_ENSG00000198728
10	103892167	1.04E+08	KD2-H4K8ac_peak_4364	6.75488	PPRC1_ENSG00000148840
10	103892886	1.04E+08	KD2-H4K8ac_peak_4365	8.4716	PPRC1_ENSG00000148840
10	103911360	1.04E+08	KD2-H4K8ac_peak_4366	11.81678	NOLC1_ENSG00000166197
10	103912256	1.04E+08	KD2-H4K8ac_peak_4367	6.85193	NOLC1_ENSG00000166197
10	103929586	1.04E+08	KD2-H4K8ac_peak_4368	6.99815	

10	103986103	1.04E+08	KD2-H4K8ac_peak_4369	10.19949	ELOVL3_ENSG00000119915
10	104004317	1.04E+08	KD2-H4K8ac_peak_4370	6.99815	GBF1_ENSG00000107862
10	104005349	1.04E+08	KD2-H4K8ac_peak_4371	6.66692	GBF1_ENSG00000107862
10	104169158	1.04E+08	KD2-H4K8ac_peak_4372	6.90231	
10	104179694	1.04E+08	KD2-H4K8ac_peak_4373	4.68059	FBXL15_ENSG00000107872
10	104180535	1.04E+08	KD2-H4K8ac_peak_4374	8.23274	PSD_ENSG00000059915
10	104181009	1.04E+08	KD2-H4K8ac_peak_4375	4.37809	PSD_ENSG00000059915
10	104191999	1.04E+08	KD2-H4K8ac_peak_4376	11.55418	CUEDC2_ENSG00000107874
10	104192715	1.04E+08	KD2-H4K8ac_peak_4377	6.30229	CUEDC2_ENSG00000107874
10	104209058	1.04E+08	KD2-H4K8ac_peak_4378	9.1707	RP11-18I14.10_ENSG00000269609
10	104209929	1.04E+08	KD2-H4K8ac_peak_4379	9.34195	RP11-18I14.10_ENSG00000269609
10	104210460	1.04E+08	KD2-H4K8ac_peak_4380	7.36799	RP11-18I14.10_ENSG00000269609;C10orf95_ENSG00000120055
10	104220585	1.04E+08	KD2-H4K8ac_peak_4381	19.72117	TMEM180_ENSG00000138111
10	104263770	1.04E+08	KD2-H4K8ac_peak_4382	11.89502	SUFU_ENSG00000107882
10	104387272	1.04E+08	KD2-H4K8ac_peak_4383	14.43794	
10	104388358	1.04E+08	KD2-H4K8ac_peak_4384	9.36665	
10	104388836	1.04E+08	KD2-H4K8ac_peak_4385	11.66477	
10	104401628	1.04E+08	KD2-H4K8ac_peak_4386	6.99815	
10	104402104	1.04E+08	KD2-H4K8ac_peak_4387	25.44327	RP11-47A8.5_ENSG00000272933
10	104403500	1.04E+08	KD2-H4K8ac_peak_4388	12.99417	RP11-47A8.5_ENSG00000272933;TRIM8_ENSG00000171206
10	104404927	1.04E+08	KD2-H4K8ac_peak_4389	5.14125	TRIM8_ENSG00000171206
10	104420711	1.04E+08	KD2-H4K8ac_peak_4390	4.89305	
10	104433890	1.04E+08	KD2-H4K8ac_peak_4391	7.01658	
10	104434835	1.04E+08	KD2-H4K8ac_peak_4392	5.14125	
10	104473461	1.04E+08	KD2-H4K8ac_peak_4393	10.29842	ARL3_ENSG00000138175;SFXN2_ENSG00000156398
10	104502520	1.05E+08	KD2-H4K8ac_peak_4394	7.36933	WBP1L_ENSG00000166272
10	104535960	1.05E+08	KD2-H4K8ac_peak_4395	4.42765	
10	104613634	1.05E+08	KD2-H4K8ac_peak_4396	13.73635	C10orf32_ENSG00000166275;C10orf32-ASMT_ENSG00000270316
10	104629008	1.05E+08	KD2-H4K8ac_peak_4397	8.79106	AS3MT_ENSG00000214435;RNU6-372P_ENSG00000201080
10	104678334	1.05E+08	KD2-H4K8ac_peak_4398	6.99194	CNNM2_ENSG00000148842
10	104953250	1.05E+08	KD2-H4K8ac_peak_4399	5.13615	NT5C2_ENSG00000076685
10	105036215	1.05E+08	KD2-H4K8ac_peak_4400	5.14125	INA_ENSG00000148798
10	105036983	1.05E+08	KD2-H4K8ac_peak_4401	6.83936	INA_ENSG00000148798
10	105111420	1.05E+08	KD2-H4K8ac_peak_4402	10.96455	PCGF6_ENSG00000156374
10	105126838	1.05E+08	KD2-H4K8ac_peak_4403	10.29344	TAF5_ENSG00000148835
10	105155875	1.05E+08	KD2-H4K8ac_peak_4404	4.06758	USMG5_ENSG00000173915;PDCD11_ENSG00000148843
10	105240291	1.05E+08	KD2-H4K8ac_peak_4405	4.42765	
10	105253654	1.05E+08	KD2-H4K8ac_peak_4406	5.01002	NEURL1_ENSG00000107954
10	105262616	1.05E+08	KD2-H4K8ac_peak_4407	9.16531	
10	105377117	1.05E+08	KD2-H4K8ac_peak_4408	7.47462	
10	105377751	1.05E+08	KD2-H4K8ac_peak_4409	16.79595	
10	105378970	1.05E+08	KD2-H4K8ac_peak_4410	5.81222	
10	105475911	1.05E+08	KD2-H4K8ac_peak_4411	6.04107	
10	105482831	1.05E+08	KD2-H4K8ac_peak_4412	4.19765	
10	105486804	1.05E+08	KD2-H4K8ac_peak_4413	14.80472	
10	105489564	1.05E+08	KD2-H4K8ac_peak_4414	7.146	

10	105492259	1.05E+08	KD2-H4K8ac_peak_4415	13.81781	
10	105532623	1.06E+08	KD2-H4K8ac_peak_4416	8.34752	
10	105554162	1.06E+08	KD2-H4K8ac_peak_4417	7.01658	
10	105566045	1.06E+08	KD2-H4K8ac_peak_4418	5.47943	
10	105572142	1.06E+08	KD2-H4K8ac_peak_4419	4.47369	
10	105583307	1.06E+08	KD2-H4K8ac_peak_4420	8.17557	
10	105614337	1.06E+08	KD2-H4K8ac_peak_4421	4.20044	SH3PXD2A_ENSG00000107957
10	105614744	1.06E+08	KD2-H4K8ac_peak_4422	11.89502	SH3PXD2A_ENSG00000107957
10	105667594	1.06E+08	KD2-H4K8ac_peak_4423	6.90231	
10	105677119	1.06E+08	KD2-H4K8ac_peak_4424	8.79106	OBFC1_ENSG00000107960
10	105725799	1.06E+08	KD2-H4K8ac_peak_4425	8.26945	SLK_ENSG00000065613
10	105726377	1.06E+08	KD2-H4K8ac_peak_4426	4.211	SLK_ENSG00000065613
10	105880968	1.06E+08	KD2-H4K8ac_peak_4427	12.00597	SFR1_ENSG00000156384
10	105991816	1.06E+08	KD2-H4K8ac_peak_4428	14.43704	WDR96_ENSG00000197748
10	106014197	1.06E+08	KD2-H4K8ac_peak_4429	8.85813	
10	106028507	1.06E+08	KD2-H4K8ac_peak_4430	10.02417	MIR4482-1_ENSG00000266852;GSTO2_ENSG00000065621
10	106059283	1.06E+08	KD2-H4K8ac_peak_4431	8.51832	
10	106059971	1.06E+08	KD2-H4K8ac_peak_4432	6.85193	
10	106060998	1.06E+08	KD2-H4K8ac_peak_4433	3.7063	
10	106088693	1.06E+08	KD2-H4K8ac_peak_4434	7.94441	
10	106097976	1.06E+08	KD2-H4K8ac_peak_4435	7.36799	ITPRIP_ENSG00000148841
10	106656209	1.07E+08	KD2-H4K8ac_peak_4436	6.0073	
10	108467377	1.08E+08	KD2-H4K8ac_peak_4437	6.56211	
10	108475225	1.08E+08	KD2-H4K8ac_peak_4438	8.30974	
10	108475984	1.08E+08	KD2-H4K8ac_peak_4439	4.86824	
10	108476451	1.08E+08	KD2-H4K8ac_peak_4440	5.14125	
10	108477110	1.08E+08	KD2-H4K8ac_peak_4441	5.16439	
10	108483981	1.08E+08	KD2-H4K8ac_peak_4442	7.87794	
10	108486488	1.08E+08	KD2-H4K8ac_peak_4443	6.40683	
10	108574813	1.09E+08	KD2-H4K8ac_peak_4444	11.30637	
10	108576950	1.09E+08	KD2-H4K8ac_peak_4445	4.77079	
10	108665956	1.09E+08	KD2-H4K8ac_peak_4446	7.12754	
10	108720415	1.09E+08	KD2-H4K8ac_peak_4447	8.79557	
10	108755695	1.09E+08	KD2-H4K8ac_peak_4448	7.01964	
10	108759705	1.09E+08	KD2-H4K8ac_peak_4449	5.15777	
10	108793485	1.09E+08	KD2-H4K8ac_peak_4450	6.83936	
10	108802309	1.09E+08	KD2-H4K8ac_peak_4451	4.77079	
10	108820248	1.09E+08	KD2-H4K8ac_peak_4452	6.51023	
10	108831902	1.09E+08	KD2-H4K8ac_peak_4453	6.16888	
10	108862093	1.09E+08	KD2-H4K8ac_peak_4454	4.98318	
10	108923806	1.09E+08	KD2-H4K8ac_peak_4455	9.95119	SORCS1_ENSG00000108018
10	109304715	1.09E+08	KD2-H4K8ac_peak_4456	8.17902	
10	109305612	1.09E+08	KD2-H4K8ac_peak_4457	8.30974	
10	109674313	1.1E+08	KD2-H4K8ac_peak_4458	15.69572	
10	111652768	1.12E+08	KD2-H4K8ac_peak_4459	6.8836	
10	111682937	1.12E+08	KD2-H4K8ac_peak_4460	13.70753	XPNPEP1_ENSG00000108039

10	111737207	1.12E+08	KD2-H4K8ac_peak_4461	8.79557	
10	111766897	1.12E+08	KD2-H4K8ac_peak_4462	21.09577	
10	111768078	1.12E+08	KD2-H4K8ac_peak_4463	13.44374	
10	111912146	1.12E+08	KD2-H4K8ac_peak_4464	4.24345	
10	111970105	1.12E+08	KD2-H4K8ac_peak_4465	6.65673	
10	112064255	1.12E+08	KD2-H4K8ac_peak_4466	9.31907	SMNDC1_ENSG00000119953
10	112065610	1.12E+08	KD2-H4K8ac_peak_4467	4.81242	SMNDC1_ENSG00000119953
10	112219951	1.12E+08	KD2-H4K8ac_peak_4468	6.30229	
10	112257249	1.12E+08	KD2-H4K8ac_peak_4469	7.76329	DUSP5_ENSG00000138166
10	112326149	1.12E+08	KD2-H4K8ac_peak_4470	8.6308	SMC3_ENSG00000108055
10	112327597	1.12E+08	KD2-H4K8ac_peak_4471	7.43893	SMC3_ENSG00000108055
10	112403727	1.12E+08	KD2-H4K8ac_peak_4472	9.26408	RBM20_ENSG00000203867
10	112540540	1.13E+08	KD2-H4K8ac_peak_4473	6.25869	
10	112541234	1.13E+08	KD2-H4K8ac_peak_4474	6.66692	
10	112545800	1.13E+08	KD2-H4K8ac_peak_4475	5.34636	
10	112547870	1.13E+08	KD2-H4K8ac_peak_4476	9.57101	
10	112549230	1.13E+08	KD2-H4K8ac_peak_4477	6.66692	
10	112551370	1.13E+08	KD2-H4K8ac_peak_4478	7.27716	
10	112552845	1.13E+08	KD2-H4K8ac_peak_4479	6.40683	
10	112554621	1.13E+08	KD2-H4K8ac_peak_4480	6.8836	
10	112619011	1.13E+08	KD2-H4K8ac_peak_4481	8.1233	
10	112620648	1.13E+08	KD2-H4K8ac_peak_4482	6.64891	
10	112630350	1.13E+08	KD2-H4K8ac_peak_4483	8.74596	RP11-313D6.3_ENSG00000232470;PDCD4_ENSG00000150593
10	112631025	1.13E+08	KD2-H4K8ac_peak_4484	8.28234	PDCD4-AS1_ENSG00000203497;PDCD4_ENSG00000150593
10	112631743	1.13E+08	KD2-H4K8ac_peak_4485	19.43192	PDCD4-AS1_ENSG00000203497;PDCD4_ENSG00000150593
10	112678427	1.13E+08	KD2-H4K8ac_peak_4486	14.06758	BBIP1_ENSG00000214413;SHOC2_ENSG00000108061
10	112898961	1.13E+08	KD2-H4K8ac_peak_4487	5.25071	
10	113943089	1.14E+08	KD2-H4K8ac_peak_4488	7.47462	
10	113970285	1.14E+08	KD2-H4K8ac_peak_4489	6.66692	
10	113974659	1.14E+08	KD2-H4K8ac_peak_4490	14.87555	GPAM_ENSG00000119927
10	113975330	1.14E+08	KD2-H4K8ac_peak_4491	13.81781	GPAM_ENSG00000119927
10	114206221	1.14E+08	KD2-H4K8ac_peak_4492	13.13969	ZDHHC6_ENSG00000023041;VTI1A_ENSG00000151532
10	114207029	1.14E+08	KD2-H4K8ac_peak_4493	14.84212	ZDHHC6_ENSG00000023041;VTI1A_ENSG00000151532
10	114711169	1.15E+08	KD2-H4K8ac_peak_4494	12.42547	RP11-57H14.2_ENSG00000233547
10	114711660	1.15E+08	KD2-H4K8ac_peak_4495	12.42547	RP11-57H14.2_ENSG00000233547
10	115214245	1.15E+08	KD2-H4K8ac_peak_4496	10.17698	
10	115310281	1.15E+08	KD2-H4K8ac_peak_4497	4.49837	HABP2_ENSG00000148702
10	115439268	1.15E+08	KD2-H4K8ac_peak_4498	11.02863	CASP7_ENSG00000165806
10	115613687	1.16E+08	KD2-H4K8ac_peak_4499	14.8083	DCLRE1A_ENSG00000198924;NHLRC2_ENSG00000196865
10	115933555	1.16E+08	KD2-H4K8ac_peak_4500	4.84794	C10orf118_ENSG00000165813;MIR2110_ENSG00000238742
10	115934149	1.16E+08	KD2-H4K8ac_peak_4501	5.1628	C10orf118_ENSG00000165813;MIR2110_ENSG00000238742
10	116070174	1.16E+08	KD2-H4K8ac_peak_4502	10.21502	
10	116126497	1.16E+08	KD2-H4K8ac_peak_4503	12.96672	
10	116444208	1.16E+08	KD2-H4K8ac_peak_4504	4.70388	ABLIM1_ENSG00000099204
10	116527269	1.17E+08	KD2-H4K8ac_peak_4505	11.15509	
10	116581232	1.17E+08	KD2-H4K8ac_peak_4506	11.05467	FAM160B1_ENSG00000151553

10	116852276	1.17E+08	KD2-H4K8ac_peak_4507	9.048	ATRNL1_ENSG00000107518
10	116853827	1.17E+08	KD2-H4K8ac_peak_4508	5.73839	ATRNL1_ENSG00000107518
10	118475377	1.18E+08	KD2-H4K8ac_peak_4509	4.77079	
10	118481192	1.18E+08	KD2-H4K8ac_peak_4510	4.77079	
10	118481718	1.18E+08	KD2-H4K8ac_peak_4511	7.47462	
10	118500747	1.19E+08	KD2-H4K8ac_peak_4512	7.47462	HSPA12A_ENSG00000165868
10	118501422	1.19E+08	KD2-H4K8ac_peak_4513	8.2413	HSPA12A_ENSG00000165868
10	118502317	1.19E+08	KD2-H4K8ac_peak_4514	8.55271	HSPA12A_ENSG00000165868
10	118608056	1.19E+08	KD2-H4K8ac_peak_4515	4.06758	RP11-539I5.1_ENSG00000225302;ENO4_ENSG00000188316
10	118651125	1.19E+08	KD2-H4K8ac_peak_4516	4.01115	
10	118764140	1.19E+08	KD2-H4K8ac_peak_4517	13.9234	
10	118975899	1.19E+08	KD2-H4K8ac_peak_4518	6.56926	
10	118976452	1.19E+08	KD2-H4K8ac_peak_4519	6.84261	
10	119134675	1.19E+08	KD2-H4K8ac_peak_4520	8.34752	PDZD8_ENSG00000165650
10	119135213	1.19E+08	KD2-H4K8ac_peak_4521	10.30284	PDZD8_ENSG00000165650
10	119262066	1.19E+08	KD2-H4K8ac_peak_4522	15.52826	
10	119805768	1.2E+08	KD2-H4K8ac_peak_4523	14.43704	RAB11FIP2_ENSG00000107560;CASC2_ENSG00000177640
10	119867574	1.2E+08	KD2-H4K8ac_peak_4524	4.59928	
10	120101465	1.2E+08	KD2-H4K8ac_peak_4525	14.84212	FAM204A_ENSG00000165669
10	120102014	1.2E+08	KD2-H4K8ac_peak_4526	6.66692	FAM204A_ENSG00000165669
10	120129443	1.2E+08	KD2-H4K8ac_peak_4527	7.12754	
10	120369559	1.2E+08	KD2-H4K8ac_peak_4528	6.99815	
10	120370752	1.2E+08	KD2-H4K8ac_peak_4529	4.42765	
10	120427751	1.2E+08	KD2-H4K8ac_peak_4530	4.86324	
10	120428489	1.2E+08	KD2-H4K8ac_peak_4531	4.86324	
10	120429547	1.2E+08	KD2-H4K8ac_peak_4532	11.0722	
10	120513916	1.21E+08	KD2-H4K8ac_peak_4533	6.99815	CACUL1_ENSG00000151893
10	120577972	1.21E+08	KD2-H4K8ac_peak_4534	4.42765	
10	120591329	1.21E+08	KD2-H4K8ac_peak_4535	8.83582	
10	120667652	1.21E+08	KD2-H4K8ac_peak_4536	10.65724	
10	120669268	1.21E+08	KD2-H4K8ac_peak_4537	10.0089	
10	120718838	1.21E+08	KD2-H4K8ac_peak_4538	6.0073	
10	120782452	1.21E+08	KD2-H4K8ac_peak_4539	9.6261	
10	120783172	1.21E+08	KD2-H4K8ac_peak_4540	6.0073	
10	120784624	1.21E+08	KD2-H4K8ac_peak_4541	7.73132	
10	120839779	1.21E+08	KD2-H4K8ac_peak_4542	13.81781	EIF3A_ENSG00000107581
10	120840493	1.21E+08	KD2-H4K8ac_peak_4543	8.02571	EIF3A_ENSG00000107581
10	120863015	1.21E+08	KD2-H4K8ac_peak_4544	6.56926	FAM45A_ENSG00000119979
10	120863664	1.21E+08	KD2-H4K8ac_peak_4545	12.06346	FAM45A_ENSG00000119979
10	120924728	1.21E+08	KD2-H4K8ac_peak_4546	15.47237	SFXN4_ENSG00000183605
10	120937949	1.21E+08	KD2-H4K8ac_peak_4547	25.6154	PRDX3_ENSG00000165672
10	120966450	1.21E+08	KD2-H4K8ac_peak_4548	12.57297	GRK5_ENSG00000198873
10	121130742	1.21E+08	KD2-H4K8ac_peak_4549	3.80759	
10	121301832	1.21E+08	KD2-H4K8ac_peak_4550	17.17257	RGS10_ENSG00000148908
10	121355728	1.21E+08	KD2-H4K8ac_peak_4551	7.59643	TIAL1_ENSG00000151923
10	121356216	1.21E+08	KD2-H4K8ac_peak_4552	10.86378	TIAL1_ENSG00000151923

10	121368189	1.21E+08	KD2-H4K8ac_peak_4553	10.43925	
10	121410243	1.21E+08	KD2-H4K8ac_peak_4554	10.19949	BAG3_ENSG00000151929
10	121485780	1.21E+08	KD2-H4K8ac_peak_4555	5.08628	INPP5F_ENSG00000198825
10	121577769	1.22E+08	KD2-H4K8ac_peak_4556	11.04694	
10	121631740	1.22E+08	KD2-H4K8ac_peak_4557	11.92978	
10	121632663	1.22E+08	KD2-H4K8ac_peak_4558	11.82518	
10	122610419	1.23E+08	KD2-H4K8ac_peak_4559	7.13615	WDR11-AS1_ENSG00000227165;WDR11_ENSG00000120008
10	122610900	1.23E+08	KD2-H4K8ac_peak_4560	12.84642	WDR11-AS1_ENSG00000227165;WDR11_ENSG00000120008
10	122683457	1.23E+08	KD2-H4K8ac_peak_4561	8.1233	
10	122739018	1.23E+08	KD2-H4K8ac_peak_4562	7.70289	RP11-95I16.2_ENSG00000227307
10	122739487	1.23E+08	KD2-H4K8ac_peak_4563	6.98292	RP11-95I16.2_ENSG00000227307
10	122848801	1.23E+08	KD2-H4K8ac_peak_4564	5.75452	
10	123357586	1.23E+08	KD2-H4K8ac_peak_4565	8.1235	FGFR2_ENSG00000066468
10	123358118	1.23E+08	KD2-H4K8ac_peak_4566	18.75732	FGFR2_ENSG00000066468
10	123368350	1.23E+08	KD2-H4K8ac_peak_4567	8.20938	
10	123427228	1.23E+08	KD2-H4K8ac_peak_4568	6.04107	
10	123429590	1.23E+08	KD2-H4K8ac_peak_4569	6.67474	
10	123468343	1.23E+08	KD2-H4K8ac_peak_4570	4.20044	
10	123687074	1.24E+08	KD2-H4K8ac_peak_4571	8.4716	ATE1_ENSG00000107669;ATE1-AS1_ENSG00000226864
10	123687749	1.24E+08	KD2-H4K8ac_peak_4572	11.58876	ATE1_ENSG00000107669;ATE1-AS1_ENSG00000226864
10	123734374	1.24E+08	KD2-H4K8ac_peak_4573	8.16906	NSMCE4A_ENSG00000107672
10	123872391	1.24E+08	KD2-H4K8ac_peak_4574	19.6416	
10	123898250	1.24E+08	KD2-H4K8ac_peak_4575	7.79741	
10	123899604	1.24E+08	KD2-H4K8ac_peak_4576	6.40683	
10	123900253	1.24E+08	KD2-H4K8ac_peak_4577	19.30653	
10	123907815	1.24E+08	KD2-H4K8ac_peak_4578	4.86324	
10	123914197	1.24E+08	KD2-H4K8ac_peak_4579	8.90871	
10	123922070	1.24E+08	KD2-H4K8ac_peak_4580	17.70837	
10	123989886	1.24E+08	KD2-H4K8ac_peak_4581	9.1707	
10	123993020	1.24E+08	KD2-H4K8ac_peak_4582	7.76329	
10	123994626	1.24E+08	KD2-H4K8ac_peak_4583	9.1707	
10	124058522	1.24E+08	KD2-H4K8ac_peak_4584	5.59859	
10	124134264	1.24E+08	KD2-H4K8ac_peak_4585	10.08707	PLEKHA1_ENSG00000107679
10	124263547	1.24E+08	KD2-H4K8ac_peak_4586	6.0073	
10	124713454	1.25E+08	KD2-H4K8ac_peak_4587	10.19949	C10orf88_ENSG00000119965;PSTK_ENSG00000179988
10	124739763	1.25E+08	KD2-H4K8ac_peak_4588	12.84642	
10	124908101	1.25E+08	KD2-H4K8ac_peak_4589	5.40356	HMX2_ENSG00000188816
10	124913949	1.25E+08	KD2-H4K8ac_peak_4590	13.31016	BUB3_ENSG00000154473
10	125108136	1.25E+08	KD2-H4K8ac_peak_4591	6.33462	
10	125111013	1.25E+08	KD2-H4K8ac_peak_4592	4.86324	
10	125114738	1.25E+08	KD2-H4K8ac_peak_4593	4.76709	
10	125117766	1.25E+08	KD2-H4K8ac_peak_4594	5.51783	
10	125503815	1.26E+08	KD2-H4K8ac_peak_4595	7.27716	
10	125972699	1.26E+08	KD2-H4K8ac_peak_4596	5.9696	
10	125983807	1.26E+08	KD2-H4K8ac_peak_4597	3.75035	
10	126042680	1.26E+08	KD2-H4K8ac_peak_4598	12.99417	

10	126077165	1.26E+08	KD2-H4K8ac_peak_4599	6.0073	
10	126106940	1.26E+08	KD2-H4K8ac_peak_4600	4.06758	OAT_ENSG00000065154
10	126107686	1.26E+08	KD2-H4K8ac_peak_4601	14.74047	OAT_ENSG00000065154
10	126149972	1.26E+08	KD2-H4K8ac_peak_4602	10.08707	LHPP_ENSG00000107902
10	126212156	1.26E+08	KD2-H4K8ac_peak_4603	6.97919	
10	126214015	1.26E+08	KD2-H4K8ac_peak_4604	15.81711	
10	126218054	1.26E+08	KD2-H4K8ac_peak_4605	6.98517	
10	126218945	1.26E+08	KD2-H4K8ac_peak_4606	10.05606	
10	126225011	1.26E+08	KD2-H4K8ac_peak_4607	5.88845	
10	126301610	1.26E+08	KD2-H4K8ac_peak_4608	5.86783	
10	126336129	1.26E+08	KD2-H4K8ac_peak_4609	13.70753	
10	126355968	1.26E+08	KD2-H4K8ac_peak_4610	12.0857	
10	126357154	1.26E+08	KD2-H4K8ac_peak_4611	5.75452	
10	126359855	1.26E+08	KD2-H4K8ac_peak_4612	12.65397	
10	126360695	1.26E+08	KD2-H4K8ac_peak_4613	9.33795	
10	126431908	1.26E+08	KD2-H4K8ac_peak_4614	11.43353	FAM53B_ENSG00000189319
10	126479812	1.26E+08	KD2-H4K8ac_peak_4615	7.49599	RP11-12J10.3_ENSG00000258539;METTL10_ENSG00000203791
10	126480999	1.26E+08	KD2-H4K8ac_peak_4616	4.86324	RP11-12J10.3_ENSG00000258539;METTL10_ENSG00000203791
10	126489342	1.26E+08	KD2-H4K8ac_peak_4617	6.40683	FAM175B_ENSG00000165660
10	126490441	1.26E+08	KD2-H4K8ac_peak_4618	10.19949	FAM175B_ENSG00000165660
10	126605530	1.27E+08	KD2-H4K8ac_peak_4619	4.86324	RP11-298J20.4_ENSG00000249456
10	126689174	1.27E+08	KD2-H4K8ac_peak_4620	3.97079	
10	126696680	1.27E+08	KD2-H4K8ac_peak_4621	4.42765	
10	126703181	1.27E+08	KD2-H4K8ac_peak_4622	9.73431	
10	126711033	1.27E+08	KD2-H4K8ac_peak_4623	12.93209	
10	126714691	1.27E+08	KD2-H4K8ac_peak_4624	6.99815	
10	126745300	1.27E+08	KD2-H4K8ac_peak_4625	3.97079	
10	126847511	1.27E+08	KD2-H4K8ac_peak_4626	9.11031	
10	126848304	1.27E+08	KD2-H4K8ac_peak_4627	10.05606	CTBP2_ENSG00000175029
10	126849391	1.27E+08	KD2-H4K8ac_peak_4628	16.11042	CTBP2_ENSG00000175029
10	127202032	1.27E+08	KD2-H4K8ac_peak_4629	6.65673	
10	127202611	1.27E+08	KD2-H4K8ac_peak_4630	5.29555	
10	127407409	1.27E+08	KD2-H4K8ac_peak_4631	10.96455	RP11-383C5.4_ENSG00000224023;RP11-383C5.5_ENSG00000234134;EDRF1_ENSG00000107938
10	127408324	1.27E+08	KD2-H4K8ac_peak_4632	10.35678	RP11-383C5.4_ENSG00000224023;RP11-383C5.5_ENSG00000234134;EDRF1_ENSG00000107938
10	127511108	1.28E+08	KD2-H4K8ac_peak_4633	10.63441	UROS_ENSG00000188690;BCCIP_ENSG00000107949
10	127584292	1.28E+08	KD2-H4K8ac_peak_4634	9.34195	DHX32_ENSG00000089876;FANK1_ENSG00000203780
10	127746064	1.28E+08	KD2-H4K8ac_peak_4635	13.80019	
10	127751288	1.28E+08	KD2-H4K8ac_peak_4636	5.97274	
10	127924137	1.28E+08	KD2-H4K8ac_peak_4637	8.17902	
10	127976473	1.28E+08	KD2-H4K8ac_peak_4638	5.14125	
10	128068496	1.28E+08	KD2-H4K8ac_peak_4639	4.57371	
10	128076433	1.28E+08	KD2-H4K8ac_peak_4640	20.88898	ADAM12_ENSG00000148848
10	128084513	1.28E+08	KD2-H4K8ac_peak_4641	8.17902	
10	128086758	1.28E+08	KD2-H4K8ac_peak_4642	7.71478	
10	128593333	1.29E+08	KD2-H4K8ac_peak_4643	10.44656	DOCK1_ENSG00000150760
10	128594563	1.29E+08	KD2-H4K8ac_peak_4644	7.69287	DOCK1_ENSG00000150760

10	128606280	1.29E+08	KD2-H4K8ac_peak_4645	9.15329	
10	129013471	1.29E+08	KD2-H4K8ac_peak_4646	6.16888	
10	129860633	1.3E+08	KD2-H4K8ac_peak_4647	5.90098	
10	129924112	1.3E+08	KD2-H4K8ac_peak_4648	11.05407	MKI67_ENSG00000148773
10	129947855	1.3E+08	KD2-H4K8ac_peak_4649	17.50963	
10	129948503	1.3E+08	KD2-H4K8ac_peak_4650	7.146	
10	130008737	1.3E+08	KD2-H4K8ac_peak_4651	13.92307	
10	130338744	1.3E+08	KD2-H4K8ac_peak_4652	11.89502	
10	130377199	1.3E+08	KD2-H4K8ac_peak_4653	5.88845	
10	130539433	1.31E+08	KD2-H4K8ac_peak_4654	7.12754	
10	131264737	1.31E+08	KD2-H4K8ac_peak_4655	25.8903	MGMT_ENSG00000170430
10	131265520	1.31E+08	KD2-H4K8ac_peak_4656	13.87818	MGMT_ENSG00000170430
10	131668326	1.32E+08	KD2-H4K8ac_peak_4657	12.74171	
10	131687353	1.32E+08	KD2-H4K8ac_peak_4658	6.66692	
10	131733897	1.32E+08	KD2-H4K8ac_peak_4659	6.40683	
10	131749948	1.32E+08	KD2-H4K8ac_peak_4660	5.20998	
10	131908359	1.32E+08	KD2-H4K8ac_peak_4661	10.43145	LINC00959_ENSG00000237489
10	131909608	1.32E+08	KD2-H4K8ac_peak_4662	8.34752	LINC00959_ENSG00000237489
10	131933891	1.32E+08	KD2-H4K8ac_peak_4663	6.84261	
10	131934714	1.32E+08	KD2-H4K8ac_peak_4664	14.57259	
10	131988737	1.32E+08	KD2-H4K8ac_peak_4665	9.6247	
10	133775510	1.34E+08	KD2-H4K8ac_peak_4666	7.40111	
10	133776905	1.34E+08	KD2-H4K8ac_peak_4667	6.66692	
10	133794946	1.34E+08	KD2-H4K8ac_peak_4668	15.22643	BNIP3_ENSG00000176171
10	133795656	1.34E+08	KD2-H4K8ac_peak_4669	22.21705	BNIP3_ENSG00000176171
10	133946156	1.34E+08	KD2-H4K8ac_peak_4670	7.44366	
10	133956165	1.34E+08	KD2-H4K8ac_peak_4671	11.60333	
10	133989864	1.34E+08	KD2-H4K8ac_peak_4672	4.44748	
10	133999804	1.34E+08	KD2-H4K8ac_peak_4673	4.67342	RP11-140A10.3_ENSG00000235010;DPYSL4_ENSG00000151640
10	134036406	1.34E+08	KD2-H4K8ac_peak_4674	9.11031	
10	134051040	1.34E+08	KD2-H4K8ac_peak_4675	9.95759	
10	134052507	1.34E+08	KD2-H4K8ac_peak_4676	10.45683	
10	134054246	1.34E+08	KD2-H4K8ac_peak_4677	8.2413	
10	134078049	1.34E+08	KD2-H4K8ac_peak_4678	7.05051	
10	134078734	1.34E+08	KD2-H4K8ac_peak_4679	10.81728	
10	134106558	1.34E+08	KD2-H4K8ac_peak_4680	10.7186	
10	134110523	1.34E+08	KD2-H4K8ac_peak_4681	6.85193	
10	134121772	1.34E+08	KD2-H4K8ac_peak_4682	5.61067	
10	134144953	1.34E+08	KD2-H4K8ac_peak_4683	7.76329	STK32C_ENSG00000165752;LRRC27_ENSG00000148814
10	134145802	1.34E+08	KD2-H4K8ac_peak_4684	11.03331	STK32C_ENSG00000165752;LRRC27_ENSG00000148814
10	134201280	1.34E+08	KD2-H4K8ac_peak_4685	5.57044	
10	134209542	1.34E+08	KD2-H4K8ac_peak_4686	13.81781	PWWP2B_ENSG00000171813
10	134210759	1.34E+08	KD2-H4K8ac_peak_4687	9.0912	PWWP2B_ENSG00000171813
10	134231951	1.34E+08	KD2-H4K8ac_peak_4688	5.57044	RP11-432J24.2_ENSG00000231705
10	134260847	1.34E+08	KD2-H4K8ac_peak_4689	12.3187	
10	134262997	1.34E+08	KD2-H4K8ac_peak_4690	6.2781	

10	134266857	1.34E+08	KD2-H4K8ac_peak_4691	13.9234	
10	134330415	1.34E+08	KD2-H4K8ac_peak_4692	20.58734	RP11-432J24.5_ENSG00000226900
10	134350266	1.34E+08	KD2-H4K8ac_peak_4693	8.52559	INPP5A_ENSG00000068383
10	134419724	1.34E+08	KD2-H4K8ac_peak_4694	6.25869	
10	134650158	1.35E+08	KD2-H4K8ac_peak_4695	6.15357	
10	134755730	1.35E+08	KD2-H4K8ac_peak_4696	11.66477	TTC40_ENSG00000171811
10	134800921	1.35E+08	KD2-H4K8ac_peak_4697	7.05051	
10	134801862	1.35E+08	KD2-H4K8ac_peak_4698	20.88898	
10	135074833	1.35E+08	KD2-H4K8ac_peak_4699	23.64558	
10	135122121	1.35E+08	KD2-H4K8ac_peak_4700	15.96909	ZNF511_ENSG00000198546
10	135132041	1.35E+08	KD2-H4K8ac_peak_4701	20.67885	
10	135137050	1.35E+08	KD2-H4K8ac_peak_4702	6.5996	RP11-122K13.14_ENSG00000270235
10	135138468	1.35E+08	KD2-H4K8ac_peak_4703	4.91674	RP11-122K13.14_ENSG00000270235
10	135139033	1.35E+08	KD2-H4K8ac_peak_4704	11.28831	RP11-122K13.14_ENSG00000270235
10	135170897	1.35E+08	KD2-H4K8ac_peak_4705	8.4716	FUOM_ENSG00000148803
10	135192826	1.35E+08	KD2-H4K8ac_peak_4706	9.402	PAOX_ENSG00000148832;AL360181.1_ENSG00000268882
10	135207742	1.35E+08	KD2-H4K8ac_peak_4707	10.7186	MTG1_ENSG00000148824
10	135333591	1.35E+08	KD2-H4K8ac_peak_4708	7.47462	CYP2E1_ENSG00000130649
11	206959	207295	KD2-H4K8ac_peak_4709	9.34989	BET1L_ENSG00000177951;RIC8A_ENSG00000177963
11	207743	207969	KD2-H4K8ac_peak_4710	10.77651	BET1L_ENSG00000177951;RIC8A_ENSG00000177963
11	208227	208437	KD2-H4K8ac_peak_4711	5.30799	BET1L_ENSG00000177951;RIC8A_ENSG00000177963
11	236065	236271	KD2-H4K8ac_peak_4712	4.67342	SIRT3_ENSG00000142082;PSMD13_ENSG00000185627
11	236590	237362	KD2-H4K8ac_peak_4713	21.75906	SIRT3_ENSG00000142082;PSMD13_ENSG00000185627
11	288616	289315	KD2-H4K8ac_peak_4714	12.42761	ATHL1_ENSG00000142102
11	355524	356066	KD2-H4K8ac_peak_4715	13.88933	
11	364234	364538	KD2-H4K8ac_peak_4716	10.96455	
11	396634	397255	KD2-H4K8ac_peak_4717	16.15695	
11	406658	407115	KD2-H4K8ac_peak_4718	14.74047	
11	407561	407788	KD2-H4K8ac_peak_4719	10.21502	
11	441869	442101	KD2-H4K8ac_peak_4720	6.05995	ANO9_ENSG00000185101
11	447738	448052	KD2-H4K8ac_peak_4721	7.76329	PTDSS2_ENSG00000174915
11	448272	448619	KD2-H4K8ac_peak_4722	7.59794	PTDSS2_ENSG00000174915
11	450618	450828	KD2-H4K8ac_peak_4723	7.69287	
11	506510	506746	KD2-H4K8ac_peak_4724	11.43863	RNH1_ENSG00000023191
11	507023	507242	KD2-H4K8ac_peak_4725	10.42398	RNH1_ENSG00000023191
11	518437	518738	KD2-H4K8ac_peak_4726	5.71749	
11	518990	519363	KD2-H4K8ac_peak_4727	13.09607	
11	535803	536316	KD2-H4K8ac_peak_4728	14.30077	HRAS_ENSG00000174775
11	554290	555052	KD2-H4K8ac_peak_4729	10.5847	
11	555297	555545	KD2-H4K8ac_peak_4730	6.65673	
11	560302	560659	KD2-H4K8ac_peak_4731	15.08926	C11orf35_ENSG00000185522;RASSF7_ENSG00000099849
11	568747	569315	KD2-H4K8ac_peak_4732	20.2003	MIR210HG_ENSG00000247095;MIR210_ENSG00000199038
11	576203	576689	KD2-H4K8ac_peak_4733	7.49599	PHRF1_ENSG00000070047
11	616183	616377	KD2-H4K8ac_peak_4734	7.51054	IRF7_ENSG00000185507
11	694619	694918	KD2-H4K8ac_peak_4735	11.33751	EPS8L2_ENSG00000177106;TMEM80_ENSG00000177042
11	695379	695593	KD2-H4K8ac_peak_4736	14.71663	EPS8L2_ENSG00000177106;TMEM80_ENSG00000177042

11	695871	696341	KD2-H4K8ac_peak_4737	7.18615	TMEM80_ENSG00000177042
11	710860	711065	KD2-H4K8ac_peak_4738	10.08707	
11	726175	726403	KD2-H4K8ac_peak_4739	7.76329	AP006621.9_ENSG00000269915
11	727669	728109	KD2-H4K8ac_peak_4740	9.16531	AP006621.9_ENSG00000269915
11	728428	728631	KD2-H4K8ac_peak_4741	9.11031	
11	746877	747236	KD2-H4K8ac_peak_4742	9.57101	TALDO1_ENSG00000177156
11	747495	748056	KD2-H4K8ac_peak_4743	9.57101	TALDO1_ENSG00000177156
11	777270	777911	KD2-H4K8ac_peak_4744	8.58725	PDDC1_ENSG00000177225;AP006621.5_ENSG00000255284
11	796626	796857	KD2-H4K8ac_peak_4745	7.167	
11	797519	798181	KD2-H4K8ac_peak_4746	13.81781	SLC25A22_ENSG00000177542
11	798401	798683	KD2-H4K8ac_peak_4747	8.30974	SLC25A22_ENSG00000177542
11	804535	805065	KD2-H4K8ac_peak_4748	11.00004	
11	805451	805807	KD2-H4K8ac_peak_4749	14.80472	
11	808900	809436	KD2-H4K8ac_peak_4750	11.05467	PIDD_ENSG00000177595;RPLP2_ENSG00000177600
11	810143	810432	KD2-H4K8ac_peak_4751	6.58902	PIDD_ENSG00000177595;RPLP2_ENSG00000177600
11	818937	819404	KD2-H4K8ac_peak_4752	16.3889	PNPLA2_ENSG00000177666
11	819617	819825	KD2-H4K8ac_peak_4753	9.1707	PNPLA2_ENSG00000177666
11	842375	842764	KD2-H4K8ac_peak_4754	6.84261	POLR2L_ENSG00000177700;TSPAN4_ENSG00000214063
11	849399	849630	KD2-H4K8ac_peak_4755	6.9556	
11	854533	855087	KD2-H4K8ac_peak_4756	6.25869	
11	855966	856713	KD2-H4K8ac_peak_4757	11.36939	
11	891036	891346	KD2-H4K8ac_peak_4758	5.88845	
11	910985	911653	KD2-H4K8ac_peak_4759	12.46522	
11	918952	919505	KD2-H4K8ac_peak_4760	12.25733	
11	924342	925595	KD2-H4K8ac_peak_4761	7.76104	AP2A2_ENSG00000183020
11	1330443	1330785	KD2-H4K8ac_peak_4762	11.02863	TOLLIP_ENSG00000078902;TOLLIP-AS1_ENSG00000255153
11	1331144	1331587	KD2-H4K8ac_peak_4763	10.35678	TOLLIP_ENSG00000078902;TOLLIP-AS1_ENSG00000255153
11	1356873	1357068	KD2-H4K8ac_peak_4764	6.30229	
11	1358220	1358422	KD2-H4K8ac_peak_4765	8.17902	
11	1403462	1405012	KD2-H4K8ac_peak_4766	16.63953	
11	1410520	1410730	KD2-H4K8ac_peak_4767	7.28628	BRSK2_ENSG00000174672
11	1479550	1480037	KD2-H4K8ac_peak_4768	8.94469	
11	1524612	1525648	KD2-H4K8ac_peak_4769	14.00658	
11	1567984	1568624	KD2-H4K8ac_peak_4770	10.96455	
11	1592759	1593085	KD2-H4K8ac_peak_4771	8.53092	DUSP8_ENSG00000184545;KRTAP5-AS1_ENSG00000233930
11	1593728	1594124	KD2-H4K8ac_peak_4772	13.1706	DUSP8_ENSG00000184545
11	1649807	1650132	KD2-H4K8ac_peak_4773	6.66359	KRTAP5-5_ENSG00000185940
11	1659450	1659968	KD2-H4K8ac_peak_4774	8.74596	
11	1660569	1660961	KD2-H4K8ac_peak_4775	13.73635	
11	1661635	1662108	KD2-H4K8ac_peak_4776	9.11031	
11	1674241	1674690	KD2-H4K8ac_peak_4777	15.039	
11	1680837	1681215	KD2-H4K8ac_peak_4778	6.79289	
11	1714713	1715595	KD2-H4K8ac_peak_4779	26.58409	
11	1765441	1765802	KD2-H4K8ac_peak_4780	8.26945	
11	1766143	1766381	KD2-H4K8ac_peak_4781	5.75757	
11	1770018	1770286	KD2-H4K8ac_peak_4782	6.9556	

11	1784708	1785141	KD2-H4K8ac_peak_4783	12.93209	CTSD_ENSG00000117984;AC068580.5_ENSG00000229512;AC068580.1_ENSG00000265587
11	1785400	1785857	KD2-H4K8ac_peak_4784	10.77651	CTSD_ENSG00000117984;AC068580.5_ENSG00000229512;AC068580.1_ENSG00000265587
11	1847772	1848495	KD2-H4K8ac_peak_4785	8.23274	SYT8_ENSG00000149043
11	1848770	1849059	KD2-H4K8ac_peak_4786	8.4716	SYT8_ENSG00000149043
11	1853308	1853896	KD2-H4K8ac_peak_4787	5.84991	
11	1855397	1855932	KD2-H4K8ac_peak_4788	9.03107	
11	1858541	1859512	KD2-H4K8ac_peak_4789	11.05467	TNNI2_ENSG00000130598
11	1884622	1885122	KD2-H4K8ac_peak_4790	7.13615	AC051649.12_ENSG00000249086
11	1885805	1886033	KD2-H4K8ac_peak_4791	8.53092	AC051649.12_ENSG00000249086
11	1886267	1886464	KD2-H4K8ac_peak_4792	5.20998	AC051649.12_ENSG00000249086
11	1895960	1896372	KD2-H4K8ac_peak_4793	14.39799	
11	1896753	1896969	KD2-H4K8ac_peak_4794	6.66359	
11	1897395	1898073	KD2-H4K8ac_peak_4795	10.80014	
11	1898357	1899543	KD2-H4K8ac_peak_4796	15.40891	
11	1903182	1904059	KD2-H4K8ac_peak_4797	10.43145	
11	1945882	1946249	KD2-H4K8ac_peak_4798	4.01115	
11	1960260	1960523	KD2-H4K8ac_peak_4799	7.69287	
11	1967642	1968197	KD2-H4K8ac_peak_4800	14.80472	MRPL23_ENSG00000214026
11	1968596	1970114	KD2-H4K8ac_peak_4801	29.13005	MRPL23_ENSG00000214026
11	1974179	1974580	KD2-H4K8ac_peak_4802	9.40886	
11	1974784	1975077	KD2-H4K8ac_peak_4803	5.61067	
11	1975339	1975592	KD2-H4K8ac_peak_4804	10.73134	
11	1975898	1976141	KD2-H4K8ac_peak_4805	4.91674	
11	1977211	1978235	KD2-H4K8ac_peak_4806	15.05364	
11	1978593	1978798	KD2-H4K8ac_peak_4807	4.06758	
11	1981354	1981844	KD2-H4K8ac_peak_4808	6.48071	
11	1982120	1982420	KD2-H4K8ac_peak_4809	6.52841	
11	1983771	1984181	KD2-H4K8ac_peak_4810	8.14802	
11	1986805	1987006	KD2-H4K8ac_peak_4811	7.146	
11	1988050	1988344	KD2-H4K8ac_peak_4812	3.90565	
11	1988620	1988959	KD2-H4K8ac_peak_4813	10.21366	
11	1989462	1991357	KD2-H4K8ac_peak_4814	15.66774	
11	1991815	1992904	KD2-H4K8ac_peak_4815	14.40719	
11	1997994	1998801	KD2-H4K8ac_peak_4816	9.64735	
11	1999031	2000108	KD2-H4K8ac_peak_4817	24.60055	
11	2000625	2004935	KD2-H4K8ac_peak_4818	17.46525	
11	2005131	2005579	KD2-H4K8ac_peak_4819	5.50645	
11	2006055	2006261	KD2-H4K8ac_peak_4820	7.74292	
11	2009309	2012679	KD2-H4K8ac_peak_4821	17.83665	MRPL23-AS1_ENSG00000226416;AC051649.6_ENSG00000232987
11	2013962	2015301	KD2-H4K8ac_peak_4822	10.65724	
11	2015716	2016029	KD2-H4K8ac_peak_4823	14.80079	
11	2017244	2017548	KD2-H4K8ac_peak_4824	5.13893	
11	2018401	2018952	KD2-H4K8ac_peak_4825	7.82935	
11	2177531	2177756	KD2-H4K8ac_peak_4826	10.65724	
11	2189986	2191025	KD2-H4K8ac_peak_4827	10.0692	
11	2191299	2191826	KD2-H4K8ac_peak_4828	8.17902	

11	2195842	2196039	KD2-H4K8ac_peak_4829	6.40683	
11	2207272	2207650	KD2-H4K8ac_peak_4830	6.40297	
11	2211271	2212273	KD2-H4K8ac_peak_4831	8.4716	
11	2212502	2212920	KD2-H4K8ac_peak_4832	3.86308	
11	2213383	2214523	KD2-H4K8ac_peak_4833	21.11201	
11	2214781	2214978	KD2-H4K8ac_peak_4834	11.27376	
11	2215734	2215980	KD2-H4K8ac_peak_4835	5.30799	
11	2326476	2326932	KD2-H4K8ac_peak_4836	7.27716	
11	2335742	2336195	KD2-H4K8ac_peak_4837	10.95611	
11	2336521	2337308	KD2-H4K8ac_peak_4838	8.30974	
11	2420605	2421511	KD2-H4K8ac_peak_4839	29.96261	TSSC4_ENSG00000184281
11	2422047	2422241	KD2-H4K8ac_peak_4840	8.85813	TSSC4_ENSG00000184281
11	2429911	2430150	KD2-H4K8ac_peak_4841	5.95537	
11	2481950	2482269	KD2-H4K8ac_peak_4842	6.30626	
11	2482525	2482926	KD2-H4K8ac_peak_4843	6.89708	
11	2483187	2483425	KD2-H4K8ac_peak_4844	5.61067	
11	2603479	2603836	KD2-H4K8ac_peak_4845	6.67081	
11	2604202	2604442	KD2-H4K8ac_peak_4846	7.47462	
11	2695314	2695521	KD2-H4K8ac_peak_4847	9.59814	
11	2721521	2722019	KD2-H4K8ac_peak_4848	11.05467	KCNQ1OT1_ENSG00000269821
11	2724199	2724672	KD2-H4K8ac_peak_4849	6.66692	
11	2725043	2725323	KD2-H4K8ac_peak_4850	5.45396	
11	2729548	2730104	KD2-H4K8ac_peak_4851	15.22237	
11	2730698	2730911	KD2-H4K8ac_peak_4852	5.77265	
11	2733858	2734069	KD2-H4K8ac_peak_4853	7.47462	
11	2734692	2735961	KD2-H4K8ac_peak_4854	15.01306	
11	2739102	2739634	KD2-H4K8ac_peak_4855	8.08344	
11	2750291	2750508	KD2-H4K8ac_peak_4856	6.0605	
11	2772361	2772654	KD2-H4K8ac_peak_4857	3.68875	
11	2793041	2793365	KD2-H4K8ac_peak_4858	6.15357	COX6CP18_ENSG00000236041
11	2797459	2798256	KD2-H4K8ac_peak_4859	14.80472	
11	2798718	2802226	KD2-H4K8ac_peak_4860	22.59108	
11	2828580	2828802	KD2-H4K8ac_peak_4861	6.66692	
11	2905190	2905861	KD2-H4K8ac_peak_4862	6.0073	
11	2907479	2907930	KD2-H4K8ac_peak_4863	8.83582	CDKN1C_ENSG00000129757
11	2922884	2923139	KD2-H4K8ac_peak_4864	12.36441	
11	2923581	2923779	KD2-H4K8ac_peak_4865	6.66359	
11	3013004	3013207	KD2-H4K8ac_peak_4866	8.36514	NAP1L4_ENSG00000205531
11	3013856	3014277	KD2-H4K8ac_peak_4867	8.86099	NAP1L4_ENSG00000205531
11	3022364	3022908	KD2-H4K8ac_peak_4868	5.92591	
11	3077934	3078600	KD2-H4K8ac_peak_4869	16.40694	CARS_ENSG00000110619
11	3078801	3079339	KD2-H4K8ac_peak_4870	19.3524	CARS_ENSG00000110619
11	3175255	3175449	KD2-H4K8ac_peak_4871	4.8485	
11	3181243	3181554	KD2-H4K8ac_peak_4872	5.88845	
11	3253261	3253759	KD2-H4K8ac_peak_4873	7.76329	MRGPRE_ENSG00000184350
11	3399852	3400342	KD2-H4K8ac_peak_4874	8.58725	ZNF195_ENSG00000005801

11	3443243	3443706	KD2-H4K8ac_peak_4875	12.84642	FAM86GP_ENSG00000166492
11	3464099	3464617	KD2-H4K8ac_peak_4876	7.12754	
11	3818490	3818704	KD2-H4K8ac_peak_4877	5.72327	NUP98_ENSG00000110713;PGAP2_ENSG00000148985
11	3819294	3819770	KD2-H4K8ac_peak_4878	6.84261	NUP98_ENSG00000110713;PGAP2_ENSG00000148985
11	3828980	3829327	KD2-H4K8ac_peak_4879	5.20908	
11	3829860	3830221	KD2-H4K8ac_peak_4880	8.51832	
11	3862413	3862849	KD2-H4K8ac_peak_4881	14.00658	RHOG_ENSG00000177105
11	3876518	3876717	KD2-H4K8ac_peak_4882	6.66692	AC090587.5_ENSG00000228661;STIM1_ENSG00000167323;AC090587.4_ENSG00000229368;MIR4687_ENSG00000263421
11	3876914	3877115	KD2-H4K8ac_peak_4883	8.6308	AC090587.5_ENSG00000228661;MIR4687_ENSG00000263421
11	4115307	4115824	KD2-H4K8ac_peak_4884	8.34752	RRM1_ENSG00000167325
11	4208385	4208829	KD2-H4K8ac_peak_4885	14.15075	RP11-23F23.2_ENSG00000254480
11	4414459	4414901	KD2-H4K8ac_peak_4886	9.95119	TRIM21_ENSG00000132109
11	4629061	4629262	KD2-H4K8ac_peak_4887	9.34989	TRIM68_ENSG00000167333
11	6439930	6440921	KD2-H4K8ac_peak_4888	9.95759	APBB1_ENSG00000166313
11	6502842	6503175	KD2-H4K8ac_peak_4889	5.80191	ARFIP2_ENSG00000132254;TIMM10B_ENSG00000132286;TIMM10B_ENSG00000265264
11	6607735	6608733	KD2-H4K8ac_peak_4890	15.32867	
11	6625038	6625394	KD2-H4K8ac_peak_4891	11.86082	RRP8_ENSG00000132275;RP11-732A19.8_ENSG00000254400;ILK_ENSG00000166333
11	6632902	6633382	KD2-H4K8ac_peak_4892	7.90806	TAF10_ENSG00000166337
11	6633627	6634059	KD2-H4K8ac_peak_4893	9.63487	TAF10_ENSG00000166337
11	6667390	6667785	KD2-H4K8ac_peak_4894	7.18916	
11	6668218	6668698	KD2-H4K8ac_peak_4895	8.10216	
11	6669057	6669249	KD2-H4K8ac_peak_4896	4.77079	
11	6676089	6676953	KD2-H4K8ac_peak_4897	9.73431	DCHS1_ENSG00000166341
11	6677227	6677511	KD2-H4K8ac_peak_4898	9.1707	DCHS1_ENSG00000166341
11	6704098	6704568	KD2-H4K8ac_peak_4899	13.57066	MRPL17_ENSG00000158042
11	6705361	6705728	KD2-H4K8ac_peak_4900	5.20908	MRPL17_ENSG00000158042
11	7041108	7041354	KD2-H4K8ac_peak_4901	7.13615	ZNF214_ENSG00000149050;NLRP14_ENSG00000158077
11	7041760	7042159	KD2-H4K8ac_peak_4902	9.95119	ZNF214_ENSG00000149050;NLRP14_ENSG00000158077
11	7174145	7174359	KD2-H4K8ac_peak_4903	12.42547	
11	7597001	7597373	KD2-H4K8ac_peak_4904	8.30974	
11	7597686	7599188	KD2-H4K8ac_peak_4905	8.85813	
11	8040174	8040871	KD2-H4K8ac_peak_4906	10.46264	TUB_ENSG00000166402
11	8054386	8054820	KD2-H4K8ac_peak_4907	10.43145	
11	8078050	8078681	KD2-H4K8ac_peak_4908	7.47462	
11	8080622	8081035	KD2-H4K8ac_peak_4909	5.57044	RP11-236J17.5_ENSG00000248332
11	8087565	8089273	KD2-H4K8ac_peak_4910	6.40297	
11	8089725	8090550	KD2-H4K8ac_peak_4911	15.61313	
11	8091340	8091655	KD2-H4K8ac_peak_4912	6.40297	
11	8102173	8102583	KD2-H4K8ac_peak_4913	14.12454	
11	8102866	8103488	KD2-H4K8ac_peak_4914	8.02571	
11	8103784	8103990	KD2-H4K8ac_peak_4915	8.23796	
11	8104503	8104738	KD2-H4K8ac_peak_4916	7.76104	
11	8107046	8107272	KD2-H4K8ac_peak_4917	6.24114	
11	8190464	8190710	KD2-H4K8ac_peak_4918	9.41619	RIC3_ENSG00000166405;RP11-379P15.1_ENSG00000246820
11	8227204	8227803	KD2-H4K8ac_peak_4919	3.90565	
11	8228579	8229284	KD2-H4K8ac_peak_4920	12.84642	

11	8249739	8250804	KD2-H4K8ac_peak_4921	8.82366	
11	8251409	8253702	KD2-H4K8ac_peak_4922	17.60835	
11	8253961	8255290	KD2-H4K8ac_peak_4923	21.01814	
11	8255987	8256730	KD2-H4K8ac_peak_4924	12.14337	
11	8258008	8258208	KD2-H4K8ac_peak_4925	6.13909	
11	8260830	8261444	KD2-H4K8ac_peak_4926	9.1707	
11	8263513	8264353	KD2-H4K8ac_peak_4927	6.98244	
11	8264596	8265446	KD2-H4K8ac_peak_4928	16.67374	
11	8266124	8266500	KD2-H4K8ac_peak_4929	7.37678	
11	8283811	8285075	KD2-H4K8ac_peak_4930	16.57619	
11	8289405	8289600	KD2-H4K8ac_peak_4931	6.62457	LMO1_ENSG00000166407
11	8289846	8290287	KD2-H4K8ac_peak_4932	8.88082	LMO1_ENSG00000166407
11	8304008	8304581	KD2-H4K8ac_peak_4933	4.211	
11	8305321	8305820	KD2-H4K8ac_peak_4934	25.8903	
11	8311184	8311399	KD2-H4K8ac_peak_4935	6.99815	
11	8350601	8351158	KD2-H4K8ac_peak_4936	9.95119	
11	8357513	8357855	KD2-H4K8ac_peak_4937	8.79106	
11	8358278	8358580	KD2-H4K8ac_peak_4938	6.99815	
11	8365049	8365253	KD2-H4K8ac_peak_4939	4.37815	
11	8376296	8377046	KD2-H4K8ac_peak_4940	18.29344	
11	8615642	8616083	KD2-H4K8ac_peak_4941	10.20046	STK33_ENSG00000130413
11	8692946	8693231	KD2-H4K8ac_peak_4942	4.42765	TRIM66_ENSG00000166436
11	8703883	8704139	KD2-H4K8ac_peak_4943	11.28831	RPL27A_ENSG00000166441
11	8704384	8704929	KD2-H4K8ac_peak_4944	12.10889	RPL27A_ENSG00000166441;SNORA3_ENSG00000200983
11	8854232	8854507	KD2-H4K8ac_peak_4945	10.0746	
11	8892235	8892710	KD2-H4K8ac_peak_4946	8.85813	
11	8932836	8933159	KD2-H4K8ac_peak_4947	9.20193	ST5_ENSG00000166444;AKIP1_ENSG00000166452
11	8985662	8986668	KD2-H4K8ac_peak_4948	11.28831	TMEM9B_ENSG00000175348;TMEM9B-AS1_ENSG00000254860
11	9088066	9088318	KD2-H4K8ac_peak_4949	5.08628	
11	9112593	9112847	KD2-H4K8ac_peak_4950	5.88562	MIR5691_ENSG00000264984
11	9286296	9286824	KD2-H4K8ac_peak_4951	6.83936	DENND5A_ENSG00000184014
11	9287228	9287451	KD2-H4K8ac_peak_4952	4.44527	DENND5A_ENSG00000184014
11	9335818	9336082	KD2-H4K8ac_peak_4953	7.07117	TMEM41B_ENSG00000166471
11	9336546	9337136	KD2-H4K8ac_peak_4954	13.81781	TMEM41B_ENSG00000166471
11	9385047	9385256	KD2-H4K8ac_peak_4955	6.36319	
11	9385528	9386298	KD2-H4K8ac_peak_4956	13.3695	
11	9405521	9405852	KD2-H4K8ac_peak_4957	10.29344	IPO7_ENSG00000205339
11	9406281	9406829	KD2-H4K8ac_peak_4958	19.55721	IPO7_ENSG00000205339
11	9481770	9482127	KD2-H4K8ac_peak_4959	7.73264	AC132192.1_ENSG00000268403;ZNF143_ENSG00000166478
11	9482706	9483187	KD2-H4K8ac_peak_4960	23.33626	AC132192.1_ENSG00000268403;ZNF143_ENSG00000166478
11	9573558	9573827	KD2-H4K8ac_peak_4961	13.09607	
11	9595449	9595864	KD2-H4K8ac_peak_4962	6.99194	WEE1_ENSG00000166483
11	9685005	9685511	KD2-H4K8ac_peak_4963	9.11031	SWAP70_ENSG00000133789
11	9779448	9780450	KD2-H4K8ac_peak_4964	17.43561	SBF2-AS1_ENSG00000246273;RP11-540A21.2_ENSG00000245522
11	9780662	9780990	KD2-H4K8ac_peak_4965	13.75159	SBF2-AS1_ENSG00000246273;RP11-540A21.2_ENSG00000245522
11	10315207	10316490	KD2-H4K8ac_peak_4966	16.64426	SBF2_ENSG00000133812

11	10342138	10342340	KD2-H4K8ac_peak_4967	4.42765	
11	10429265	10429809	KD2-H4K8ac_peak_4968	6.78668	
11	10476744	10478097	KD2-H4K8ac_peak_4969	9.36516	
11	10562319	10562686	KD2-H4K8ac_peak_4970	9.07715	RNF141_ENSG00000110315;MRVI1-AS1_ENSG00000177112
11	10562932	10563125	KD2-H4K8ac_peak_4971	7.92775	RNF141_ENSG00000110315;MRVI1-AS1_ENSG00000177112
11	10715269	10715582	KD2-H4K8ac_peak_4972	9.65086	MRVI1_ENSG00000072952
11	10750691	10750910	KD2-H4K8ac_peak_4973	12.09025	
11	10757057	10757304	KD2-H4K8ac_peak_4974	7.18916	
11	10763086	10763330	KD2-H4K8ac_peak_4975	7.13615	
11	10764025	10764291	KD2-H4K8ac_peak_4976	4.42765	
11	10772002	10772247	KD2-H4K8ac_peak_4977	7.11925	CTR9_ENSG00000198730
11	10772476	10773227	KD2-H4K8ac_peak_4978	8.16906	CTR9_ENSG00000198730
11	10790578	10790800	KD2-H4K8ac_peak_4979	6.16888	
11	10800420	10800647	KD2-H4K8ac_peak_4980	6.24747	
11	10806129	10806590	KD2-H4K8ac_peak_4981	7.36933	
11	10829609	10830100	KD2-H4K8ac_peak_4982	15.25814	RP11-685M7.3_ENSG00000246308;EIF4G2_ENSG00000110321
11	10878996	10879525	KD2-H4K8ac_peak_4983	9.18237	ZBED5_ENSG00000236287;ZBED5-AS1_ENSG00000247271
11	10879986	10880592	KD2-H4K8ac_peak_4984	10.30284	ZBED5_ENSG00000236287;ZBED5-AS1_ENSG00000247271
11	10952284	10952519	KD2-H4K8ac_peak_4985	7.98621	
11	10952850	10953513	KD2-H4K8ac_peak_4986	7.74292	
11	11643869	11644072	KD2-H4K8ac_peak_4987	6.99815	GALNT18_ENSG00000110328
11	11862948	11864112	KD2-H4K8ac_peak_4988	15.58442	USP47_ENSG00000170242
11	11985923	11986122	KD2-H4K8ac_peak_4989	6.25869	
11	11989505	11990240	KD2-H4K8ac_peak_4990	12.00597	
11	11990598	11991640	KD2-H4K8ac_peak_4991	11.24702	
11	11998109	11998462	KD2-H4K8ac_peak_4992	3.90565	
11	12000297	12000709	KD2-H4K8ac_peak_4993	12.25498	
11	12001563	12002260	KD2-H4K8ac_peak_4994	10.87093	
11	12002623	12002873	KD2-H4K8ac_peak_4995	6.99815	
11	12003544	12003956	KD2-H4K8ac_peak_4996	4.68541	
11	12030407	12030860	KD2-H4K8ac_peak_4997	10.86378	DKK3_ENSG00000050165
11	12310539	12310783	KD2-H4K8ac_peak_4998	7.27716	
11	12341242	12341493	KD2-H4K8ac_peak_4999	15.34166	
11	12352556	12352784	KD2-H4K8ac_peak_5000	8.1233	
11	12378156	12378387	KD2-H4K8ac_peak_5001	5.04237	
11	12399150	12399868	KD2-H4K8ac_peak_5002	13.4748	PARVA_ENSG00000197702
11	12455744	12456362	KD2-H4K8ac_peak_5003	7.1181	
11	12695388	12695663	KD2-H4K8ac_peak_5004	13.70753	TEAD1_ENSG00000187079
11	12696602	12696951	KD2-H4K8ac_peak_5005	10.17698	TEAD1_ENSG00000187079
11	12824038	12824352	KD2-H4K8ac_peak_5006	5.14125	
11	12835680	12836176	KD2-H4K8ac_peak_5007	7.76329	
11	12838455	12838700	KD2-H4K8ac_peak_5008	6.66692	
11	12866630	12866846	KD2-H4K8ac_peak_5009	6.99815	
11	12903487	12903779	KD2-H4K8ac_peak_5010	7.26089	
11	13351060	13351383	KD2-H4K8ac_peak_5011	5.54647	
11	13353022	13353362	KD2-H4K8ac_peak_5012	5.45396	

11	13484357	13484693	KD2-H4K8ac_peak_5013	8.10216	BTBD10_ENSG00000148925
11	13484973	13485631	KD2-H4K8ac_peak_5014	7.59643	BTBD10_ENSG00000148925
11	13689401	13689800	KD2-H4K8ac_peak_5015	15.22643	FAR1_ENSG00000197601
11	13690321	13690731	KD2-H4K8ac_peak_5016	5.66705	FAR1_ENSG00000197601;FAR1-IT1_ENSG00000254791
11	14223658	14223953	KD2-H4K8ac_peak_5017	4.42765	
11	14380062	14380645	KD2-H4K8ac_peak_5018	11.05407	
11	14402700	14403192	KD2-H4K8ac_peak_5019	4.91674	
11	14520984	14521299	KD2-H4K8ac_peak_5020	4.42765	COPB1_ENSG00000129083
11	14541635	14541876	KD2-H4K8ac_peak_5021	15.59774	PSMA1_ENSG00000256206
11	14664901	14665111	KD2-H4K8ac_peak_5022	8.62078	PSMA1_ENSG00000129084;PDE3B_ENSG00000152270
11	14665479	14665904	KD2-H4K8ac_peak_5023	7.28827	PSMA1_ENSG00000129084;PDE3B_ENSG00000152270
11	14913368	14913738	KD2-H4K8ac_peak_5024	14.63458	CYP2R1_ENSG00000186104
11	15637391	15637589	KD2-H4K8ac_peak_5025	7.05051	
11	15642573	15642944	KD2-H4K8ac_peak_5026	7.76329	RP11-531H8.2_ENSG00000254789
11	15644438	15644789	KD2-H4K8ac_peak_5027	5.557	RP11-531H8.2_ENSG00000254789
11	15671909	15672302	KD2-H4K8ac_peak_5028	6.30229	
11	15771316	15771514	KD2-H4K8ac_peak_5029	6.16888	
11	15866727	15867055	KD2-H4K8ac_peak_5030	4.77079	
11	16628548	16628751	KD2-H4K8ac_peak_5031	5.14125	
11	16758739	16758983	KD2-H4K8ac_peak_5032	5.25071	
11	17035270	17035809	KD2-H4K8ac_peak_5033	7.20567	PLEKHA7_ENSG00000166689;OR7E14P_ENSG00000184669
11	17036089	17036386	KD2-H4K8ac_peak_5034	5.14125	PLEKHA7_ENSG00000166689;OR7E14P_ENSG00000184669
11	17098826	17099055	KD2-H4K8ac_peak_5035	9.06369	RPS13_ENSG00000110700
11	17100151	17100388	KD2-H4K8ac_peak_5036	4.67342	RPS13_ENSG00000110700
11	17229201	17229457	KD2-H4K8ac_peak_5037	13.13965	PIK3C2A_ENSG00000011405;NUCB2_ENSG00000070081
11	17229693	17230159	KD2-H4K8ac_peak_5038	8.55271	PIK3C2A_ENSG00000011405;NUCB2_ENSG00000070081
11	17297684	17298075	KD2-H4K8ac_peak_5039	8.36432	
11	17298333	17298676	KD2-H4K8ac_peak_5040	9.6247	
11	17373762	17374044	KD2-H4K8ac_peak_5041	5.77029	NCR3LG1_ENSG00000188211
11	17411529	17411812	KD2-H4K8ac_peak_5042	6.66692	KCNJ11_ENSG00000187486
11	17436969	17437228	KD2-H4K8ac_peak_5043	7.59643	
11	17497703	17498013	KD2-H4K8ac_peak_5044	6.76298	ABCC8_ENSG00000006071
11	18034958	18035222	KD2-H4K8ac_peak_5045	6.60094	SERGEF_ENSG00000129158
11	18127254	18127560	KD2-H4K8ac_peak_5046	13.95627	SAAL1_ENSG00000166788;HIGD1AP5_ENSG00000255254
11	18404865	18406378	KD2-H4K8ac_peak_5047	9.36516	
11	18415104	18416711	KD2-H4K8ac_peak_5048	9.402	LDHA_ENSG00000134333
11	18548006	18548376	KD2-H4K8ac_peak_5049	10.77651	TSG101_ENSG00000074319
11	18609901	18610474	KD2-H4K8ac_peak_5050	10.87093	UEVLD_ENSG00000151116
11	18655428	18655622	KD2-H4K8ac_peak_5051	6.0073	SPTY2D1_ENSG00000179119
11	18656218	18656567	KD2-H4K8ac_peak_5052	6.56211	SPTY2D1_ENSG00000179119
11	18727400	18728617	KD2-H4K8ac_peak_5053	11.60333	RP11-1081L13.4_ENSG00000254966
11	18743349	18743707	KD2-H4K8ac_peak_5054	6.90231	
11	19137886	19138157	KD2-H4K8ac_peak_5055	7.59643	ZDHHC13_ENSG00000177054
11	19138393	19139031	KD2-H4K8ac_peak_5056	20.46575	ZDHHC13_ENSG00000177054
11	19262840	19263158	KD2-H4K8ac_peak_5057	12.57143	E2F8_ENSG00000129173
11	19464017	19464217	KD2-H4K8ac_peak_5058	6.0073	

11	19581760	19581964	KD2-H4K8ac_peak_5059	5.32933	
11	19586155	19586362	KD2-H4K8ac_peak_5060	7.73264	
11	19595281	19595476	KD2-H4K8ac_peak_5061	5.88845	
11	19598446	19599162	KD2-H4K8ac_peak_5062	8.21084	
11	19617703	19618044	KD2-H4K8ac_peak_5063	11.28831	
11	19619362	19619910	KD2-H4K8ac_peak_5064	26.15943	
11	19733621	19734141	KD2-H4K8ac_peak_5065	9.1707	RP11-359E10.1_ENSG00000270607
11	19734880	19735307	KD2-H4K8ac_peak_5066	6.25869	RP11-359E10.1_ENSG00000270607
11	19735798	19736009	KD2-H4K8ac_peak_5067	4.42765	
11	19738585	19738862	KD2-H4K8ac_peak_5068	5.30799	
11	19744336	19744742	KD2-H4K8ac_peak_5069	5.75757	
11	19745999	19746195	KD2-H4K8ac_peak_5070	5.9696	
11	19746754	19747014	KD2-H4K8ac_peak_5071	6.11333	
11	19760172	19761356	KD2-H4K8ac_peak_5072	10.87093	
11	19813761	19814015	KD2-H4K8ac_peak_5073	10.87093	
11	19948811	19949143	KD2-H4K8ac_peak_5074	4.86824	
11	19953324	19953664	KD2-H4K8ac_peak_5075	5.3778	
11	20285706	20285936	KD2-H4K8ac_peak_5076	6.16888	
11	20408766	20408970	KD2-H4K8ac_peak_5077	11.24702	PRMT3_ENSG00000185238
11	20409294	20409845	KD2-H4K8ac_peak_5078	11.04256	PRMT3_ENSG00000185238
11	20412850	20413072	KD2-H4K8ac_peak_5079	8.94469	
11	20441440	20441716	KD2-H4K8ac_peak_5080	7.27716	
11	20474391	20474731	KD2-H4K8ac_peak_5081	5.14125	
11	20478432	20478699	KD2-H4K8ac_peak_5082	5.73839	
11	20482574	20482799	KD2-H4K8ac_peak_5083	5.25071	
11	20533914	20534108	KD2-H4K8ac_peak_5084	7.47462	
11	20566403	20566613	KD2-H4K8ac_peak_5085	5.10276	
11	20616495	20616718	KD2-H4K8ac_peak_5086	6.0073	
11	20617668	20618008	KD2-H4K8ac_peak_5087	6.85193	
11	20631062	20631594	KD2-H4K8ac_peak_5088	9.1707	
11	20691585	20691819	KD2-H4K8ac_peak_5089	7.67504	NELL1_ENSG00000165973
11	22213954	22214761	KD2-H4K8ac_peak_5090	10.05606	ANO5_ENSG00000171714
11	22646377	22647368	KD2-H4K8ac_peak_5091	12.88108	FANCF_ENSG00000183161;AC103801.2_ENSG00000229387;GAS2_ENSG00000148935
11	22687828	22688055	KD2-H4K8ac_peak_5092	7.27716	
11	22688256	22688539	KD2-H4K8ac_peak_5093	13.39763	
11	24518345	24518605	KD2-H4K8ac_peak_5094	5.88845	LUZP2_ENSG00000187398
11	26848961	26849160	KD2-H4K8ac_peak_5095	4.37708	
11	26927138	26927331	KD2-H4K8ac_peak_5096	5.25071	
11	26974231	26975085	KD2-H4K8ac_peak_5097	13.68542	
11	27034354	27034657	KD2-H4K8ac_peak_5098	4.37708	
11	27528055	27528286	KD2-H4K8ac_peak_5099	22.28045	LIN7C_ENSG00000148943;BDNF-AS_ENSG00000245573
11	27528489	27528822	KD2-H4K8ac_peak_5100	7.13615	LIN7C_ENSG00000148943;BDNF-AS_ENSG00000245573
11	27740514	27740899	KD2-H4K8ac_peak_5101	10.17486	
11	28131865	28132114	KD2-H4K8ac_peak_5102	12.57143	
11	30343774	30344369	KD2-H4K8ac_peak_5103	7.12754	ARL14EP_ENSG00000152219
11	30344736	30345022	KD2-H4K8ac_peak_5104	13.21293	ARL14EP_ENSG00000152219

11	30448021	30448241	KD2-H4K8ac_peak_5105	7.08474	RP4-710M3.2_ENSG00000255480
11	30606745	30607163	KD2-H4K8ac_peak_5106	8.30974	
11	30607520	30608091	KD2-H4K8ac_peak_5107	12.00273	MPPED2_ENSG00000066382
11	30882450	30883082	KD2-H4K8ac_peak_5108	8.1233	
11	30891668	30891867	KD2-H4K8ac_peak_5109	6.68439	
11	32112075	32112290	KD2-H4K8ac_peak_5110	4.0528	
11	32112542	32113808	KD2-H4K8ac_peak_5111	6.25869	
11	32604638	32605133	KD2-H4K8ac_peak_5112	12.88108	EIF3M_ENSG00000149100
11	32605818	32606045	KD2-H4K8ac_peak_5113	6.0073	EIF3M_ENSG00000149100
11	32913677	32914499	KD2-H4K8ac_peak_5114	6.0605	QSER1_ENSG00000060749
11	32914801	32915418	KD2-H4K8ac_peak_5115	10.05606	QSER1_ENSG00000060749
11	33036970	33037303	KD2-H4K8ac_peak_5116	11.66477	DEPDC7_ENSG00000121690
11	33037619	33037816	KD2-H4K8ac_peak_5117	7.40314	DEPDC7_ENSG00000121690
11	33060214	33061894	KD2-H4K8ac_peak_5118	14.68449	TCP11L1_ENSG00000176148
11	33182615	33182957	KD2-H4K8ac_peak_5119	17.43705	CSTF3_ENSG00000176102;CSTF3-AS1_ENSG00000247151
11	33277562	33277755	KD2-H4K8ac_peak_5120	6.54442	HIPK3_ENSG00000110422
11	33278390	33278674	KD2-H4K8ac_peak_5121	8.4939	HIPK3_ENSG00000110422
11	33278986	33279685	KD2-H4K8ac_peak_5122	17.19888	HIPK3_ENSG00000110422
11	33397231	33398352	KD2-H4K8ac_peak_5123	6.25869	
11	33713435	33713647	KD2-H4K8ac_peak_5124	7.01964	
11	33757616	33757923	KD2-H4K8ac_peak_5125	5.14125	CD59_ENSG00000085063
11	33758176	33758510	KD2-H4K8ac_peak_5126	3.75035	CD59_ENSG00000085063
11	33796376	33796593	KD2-H4K8ac_peak_5127	5.59859	FBXO3_ENSG00000110429;RP11-646J21.5_ENSG00000254508
11	33800099	33800322	KD2-H4K8ac_peak_5128	9.47671	
11	33850448	33850884	KD2-H4K8ac_peak_5129	7.40111	
11	34073029	34073476	KD2-H4K8ac_peak_5130	8.70727	CAPRIN1_ENSG00000135387
11	34074441	34075083	KD2-H4K8ac_peak_5131	10.96455	
11	34127248	34127553	KD2-H4K8ac_peak_5132	6.24114	NAT10_ENSG00000135372
11	34180301	34180545	KD2-H4K8ac_peak_5133	5.80374	
11	34181553	34181890	KD2-H4K8ac_peak_5134	13.73574	
11	34182402	34182665	KD2-H4K8ac_peak_5135	11.89502	
11	34206144	34206393	KD2-H4K8ac_peak_5136	4.9025	
11	34336783	34337203	KD2-H4K8ac_peak_5137	6.90231	
11	34447694	34447986	KD2-H4K8ac_peak_5138	10.72656	
11	34459893	34460283	KD2-H4K8ac_peak_5139	14.00658	CAT_ENSG00000121691
11	34937279	34937943	KD2-H4K8ac_peak_5140	14.8083	APIP_ENSG00000149089;PDHX_ENSG00000110435
11	34938224	34938664	KD2-H4K8ac_peak_5141	17.14886	APIP_ENSG00000149089;PDHX_ENSG00000110435
11	35160350	35161029	KD2-H4K8ac_peak_5142	9.16531	RP4-607I7.1_ENSG00000255521;CD44_ENSG00000026508
11	35179163	35179369	KD2-H4K8ac_peak_5143	4.42765	
11	35441315	35441561	KD2-H4K8ac_peak_5144	6.25869	SLC1A2_ENSG00000110436;RP4-683L5.1_ENSG00000255542
11	35639572	35639812	KD2-H4K8ac_peak_5145	8.52559	FJX1_ENSG00000179431
11	35640006	35640960	KD2-H4K8ac_peak_5146	10.29736	FJX1_ENSG00000179431
11	35684358	35685027	KD2-H4K8ac_peak_5147	21.50391	TRIM44_ENSG00000166326
11	35965849	35966240	KD2-H4K8ac_peak_5148	7.70067	LDLRAD3_ENSG00000179241
11	36040984	36041180	KD2-H4K8ac_peak_5149	7.13615	
11	36185416	36185848	KD2-H4K8ac_peak_5150	4.211	

11	36311061	36311394	KD2-H4K8ac_peak_5151	4.77079	COMMD9_ENSG00000110442
11	36531386	36531743	KD2-H4K8ac_peak_5152	8.34752	TRAF6_ENSG00000175104;RAG1_ENSG00000166349
11	36554601	36554801	KD2-H4K8ac_peak_5153	7.27716	
11	36586894	36587168	KD2-H4K8ac_peak_5154	7.8711	
11	36590722	36590963	KD2-H4K8ac_peak_5155	7.26029	
11	36616256	36616481	KD2-H4K8ac_peak_5156	5.9696	C11orf74_ENSG00000166352
11	36638214	36638455	KD2-H4K8ac_peak_5157	8.1233	
11	36640448	36640686	KD2-H4K8ac_peak_5158	9.15329	
11	36648713	36649029	KD2-H4K8ac_peak_5159	7.12754	
11	36649692	36649918	KD2-H4K8ac_peak_5160	6.16888	
11	36652464	36652908	KD2-H4K8ac_peak_5161	9.59147	
11	36814791	36815032	KD2-H4K8ac_peak_5162	7.27716	
11	42983632	42983918	KD2-H4K8ac_peak_5163	4.37708	
11	43333636	43333876	KD2-H4K8ac_peak_5164	6.25869	API5_ENSG00000166181
11	43380521	43380842	KD2-H4K8ac_peak_5165	11.50346	RP11-484D2.2_ENSG00000254907;TTC17_ENSG00000052841
11	43569068	43569478	KD2-H4K8ac_peak_5166	6.66692	
11	43569718	43569997	KD2-H4K8ac_peak_5167	3.75035	
11	43665884	43666077	KD2-H4K8ac_peak_5168	6.40683	
11	43685614	43686028	KD2-H4K8ac_peak_5169	6.75638	
11	43901879	43902856	KD2-H4K8ac_peak_5170	17.38513	RP11-613D13.5_ENSG00000246250;ALKBH3_ENSG00000166199
11	43923496	43923730	KD2-H4K8ac_peak_5171	4.86824	
11	43925254	43925448	KD2-H4K8ac_peak_5172	4.11746	
11	43926539	43926989	KD2-H4K8ac_peak_5173	7.45501	
11	43928001	43928304	KD2-H4K8ac_peak_5174	11.95211	
11	43928641	43929846	KD2-H4K8ac_peak_5175	7.27716	
11	43933442	43934006	KD2-H4K8ac_peak_5176	9.15069	
11	43941545	43941761	KD2-H4K8ac_peak_5177	5.57044	ALKBH3-AS1_ENSG00000244926;RP11-613D13.4_ENSG00000254409
11	43942589	43942858	KD2-H4K8ac_peak_5178	5.40356	ALKBH3-AS1_ENSG00000244926;RP11-613D13.4_ENSG00000254409
11	43943181	43943990	KD2-H4K8ac_peak_5179	7.47462	ALKBH3-AS1_ENSG00000244926;RP11-613D13.4_ENSG00000254409
11	43944369	43944880	KD2-H4K8ac_peak_5180	6.25869	
11	43945792	43947451	KD2-H4K8ac_peak_5181	15.05364	C11orf96_ENSG00000187479
11	43948266	43948630	KD2-H4K8ac_peak_5182	19.01066	
11	43949090	43949409	KD2-H4K8ac_peak_5183	5.47227	
11	43950103	43950475	KD2-H4K8ac_peak_5184	9.90914	
11	43954928	43955498	KD2-H4K8ac_peak_5185	10.65724	
11	43956337	43956745	KD2-H4K8ac_peak_5186	7.67504	
11	43962607	43963217	KD2-H4K8ac_peak_5187	18.47871	
11	43963678	43964167	KD2-H4K8ac_peak_5188	15.10495	
11	43964374	43965072	KD2-H4K8ac_peak_5189	15.13261	
11	43965526	43966327	KD2-H4K8ac_peak_5190	7.07117	
11	43966589	43967208	KD2-H4K8ac_peak_5191	6.83936	
11	43969229	43969526	KD2-H4K8ac_peak_5192	7.43041	RP11-613D13.8_ENSG00000244953
11	43970175	43970632	KD2-H4K8ac_peak_5193	9.73431	
11	43983587	43983876	KD2-H4K8ac_peak_5194	6.31143	
11	43984566	43985034	KD2-H4K8ac_peak_5195	4.211	
11	44008815	44009458	KD2-H4K8ac_peak_5196	11.50346	

11	44009807	44010098	KD2-H4K8ac_peak_5197	7.67504	
11	44011061	44011637	KD2-H4K8ac_peak_5198	10.96455	
11	44026171	44027124	KD2-H4K8ac_peak_5199	11.95211	
11	44027326	44027724	KD2-H4K8ac_peak_5200	6.91644	
11	44088004	44088263	KD2-H4K8ac_peak_5201	11.05407	ACCS_ENSG00000110455
11	44307442	44307653	KD2-H4K8ac_peak_5202	4.37815	
11	44559907	44560153	KD2-H4K8ac_peak_5203	14.8083	
11	44586245	44586712	KD2-H4K8ac_peak_5204	5.88845	CD82_ENSG00000085117
11	44614156	44614365	KD2-H4K8ac_peak_5205	6.56926	
11	44614775	44615233	KD2-H4K8ac_peak_5206	4.86324	
11	44617634	44617859	KD2-H4K8ac_peak_5207	4.211	
11	44624411	44624850	KD2-H4K8ac_peak_5208	5.25667	
11	44638701	44638916	KD2-H4K8ac_peak_5209	7.48822	
11	44649848	44650090	KD2-H4K8ac_peak_5210	6.83936	RPL34P22_ENSG00000244267
11	44656310	44657046	KD2-H4K8ac_peak_5211	6.2781	
11	44715590	44716135	KD2-H4K8ac_peak_5212	8.10216	
11	44724737	44724949	KD2-H4K8ac_peak_5213	5.08628	
11	44733752	44734069	KD2-H4K8ac_peak_5214	7.28628	
11	44747997	44748335	KD2-H4K8ac_peak_5215	13.73635	TSPAN18_ENSG00000157570
11	44748838	44749323	KD2-H4K8ac_peak_5216	6.40297	TSPAN18_ENSG00000157570
11	44764682	44765188	KD2-H4K8ac_peak_5217	9.08816	
11	44765656	44766034	KD2-H4K8ac_peak_5218	5.58725	
11	44774773	44775124	KD2-H4K8ac_peak_5219	11.05407	
11	44775680	44776570	KD2-H4K8ac_peak_5220	10.72656	
11	44776925	44777137	KD2-H4K8ac_peak_5221	7.51054	
11	44777337	44777727	KD2-H4K8ac_peak_5222	9.63487	
11	44782699	44783162	KD2-H4K8ac_peak_5223	9.1707	
11	44783477	44783797	KD2-H4K8ac_peak_5224	6.66692	
11	44797806	44798190	KD2-H4K8ac_peak_5225	5.73016	
11	44798456	44798984	KD2-H4K8ac_peak_5226	4.54006	
11	44802025	44802462	KD2-H4K8ac_peak_5227	7.95934	
11	44805172	44805401	KD2-H4K8ac_peak_5228	7.70289	
11	44805946	44806218	KD2-H4K8ac_peak_5229	9.16531	
11	44861326	44861639	KD2-H4K8ac_peak_5230	8.37231	
11	44915364	44915870	KD2-H4K8ac_peak_5231	6.05809	
11	44971918	44972407	KD2-H4K8ac_peak_5232	6.67538	TP53I11_ENSG00000175274
11	45047716	45048014	KD2-H4K8ac_peak_5233	8.26945	
11	45167692	45168251	KD2-H4K8ac_peak_5234	16.57619	
11	45168881	45169305	KD2-H4K8ac_peak_5235	13.12288	
11	45179765	45180455	KD2-H4K8ac_peak_5236	6.85193	
11	45182774	45183085	KD2-H4K8ac_peak_5237	6.16747	
11	45201609	45202932	KD2-H4K8ac_peak_5238	15.58442	
11	45307681	45308085	KD2-H4K8ac_peak_5239	3.75035	SYT13_ENSG00000019505
11	45376375	45376716	KD2-H4K8ac_peak_5240	10.21366	RP11-430H10.1_ENSG00000254427
11	45432417	45433279	KD2-H4K8ac_peak_5241	15.22643	
11	45582022	45582638	KD2-H4K8ac_peak_5242	16.8435	

11	45793192	45793390	KD2-H4K8ac_peak_5243	6.56926	CTD-2210P24.4_ENSG00000205106
11	45826208	45826525	KD2-H4K8ac_peak_5244	6.30607	SLC35C1_ENSG00000181830
11	45921410	45921604	KD2-H4K8ac_peak_5245	10.44656	
11	45939061	45939528	KD2-H4K8ac_peak_5246	8.01894	PEX16_ENSG00000121680
11	45939906	45940212	KD2-H4K8ac_peak_5247	8.23274	PEX16_ENSG00000121680
11	45943803	45945100	KD2-H4K8ac_peak_5248	12.99417	GYLTL1B_ENSG00000165905
11	46073149	46073350	KD2-H4K8ac_peak_5249	8.1233	
11	46141411	46141637	KD2-H4K8ac_peak_5250	10.21502	
11	46143493	46143721	KD2-H4K8ac_peak_5251	12.99417	PHF21A_ENSG00000135365
11	46158235	46158518	KD2-H4K8ac_peak_5252	6.16888	
11	46259720	46259945	KD2-H4K8ac_peak_5253	6.65673	CTD-2589M5.5_ENSG00000254639
11	46264618	46264893	KD2-H4K8ac_peak_5254	8.28234	
11	46265157	46265447	KD2-H4K8ac_peak_5255	14.65304	
11	46337631	46337892	KD2-H4K8ac_peak_5256	6.56926	
11	46384253	46384692	KD2-H4K8ac_peak_5257	5.90414	
11	46388670	46388872	KD2-H4K8ac_peak_5258	4.37815	
11	46391332	46391579	KD2-H4K8ac_peak_5259	4.87217	
11	46401472	46401781	KD2-H4K8ac_peak_5260	8.85813	MDK_ENSG00000110492
11	46402198	46403071	KD2-H4K8ac_peak_5261	23.67389	MDK_ENSG00000110492
11	46403326	46404033	KD2-H4K8ac_peak_5262	19.23713	
11	46615207	46615436	KD2-H4K8ac_peak_5263	8.30974	AMBRA1_ENSG00000110497
11	46639135	46639763	KD2-H4K8ac_peak_5264	14.8083	HARBI1_ENSG00000180423;ATG13_ENSG00000175224
11	46722349	46723101	KD2-H4K8ac_peak_5265	12.79505	ARHGAP1_ENSG00000175220;ZNF408_ENSG00000175213
11	46739846	46740253	KD2-H4K8ac_peak_5266	4.77079	F2_ENSG00000180210
11	46867160	46867696	KD2-H4K8ac_peak_5267	7.45501	CKAP5_ENSG00000175216;LRP4-AS1_ENSG00000247675
11	46957536	46957813	KD2-H4K8ac_peak_5268	8.1233	C11orf49_ENSG00000149179
11	47119903	47120209	KD2-H4K8ac_peak_5269	4.01115	
11	47197892	47198272	KD2-H4K8ac_peak_5270	9.36665	ARFGAP2_ENSG00000149182
11	47206370	47206702	KD2-H4K8ac_peak_5271	4.42765	
11	47207233	47208458	KD2-H4K8ac_peak_5272	10.77651	PACSIN3_ENSG00000165912
11	47208813	47209137	KD2-H4K8ac_peak_5273	9.36516	PACSIN3_ENSG00000165912
11	47236601	47237269	KD2-H4K8ac_peak_5274	15.59774	DDB2_ENSG00000134574
11	47270037	47271306	KD2-H4K8ac_peak_5275	7.76329	ACP2_ENSG00000134575;NR1H3_ENSG00000025434
11	47290265	47291052	KD2-H4K8ac_peak_5276	5.54647	MADD_ENSG00000110514
11	47421345	47422205	KD2-H4K8ac_peak_5277	11.92978	MIR4487_ENSG00000264583
11	47428864	47429892	KD2-H4K8ac_peak_5278	7.76104	SLC39A13_ENSG00000165915;RP11-750H9.5_ENSG00000255197
11	47430189	47430654	KD2-H4K8ac_peak_5279	13.5195	RP11-750H9.5_ENSG00000255197
11	47447260	47447974	KD2-H4K8ac_peak_5280	11.09936	PSMC3_ENSG00000165916
11	47574075	47574472	KD2-H4K8ac_peak_5281	5.72657	
11	47574934	47575136	KD2-H4K8ac_peak_5282	9.36516	
11	47586606	47587710	KD2-H4K8ac_peak_5283	20.47312	CELF1_ENSG00000149187;NDUFS3_ENSG00000213619;PTPMT1_ENSG00000110536
11	47600665	47601093	KD2-H4K8ac_peak_5284	7.07117	KBTBD4_ENSG00000123444
11	47615585	47615816	KD2-H4K8ac_peak_5285	4.86324	C1QTNF4_ENSG00000172247
11	47616044	47616255	KD2-H4K8ac_peak_5286	7.13615	C1QTNF4_ENSG00000172247
11	47663591	47663996	KD2-H4K8ac_peak_5287	7.47462	MTCH2_ENSG00000109919
11	47736329	47736916	KD2-H4K8ac_peak_5288	6.40297	AGBL2_ENSG00000165923

11	47788294	47788589	KD2-H4K8ac_peak_5289	7.28628	FNBP4_ENSG00000109920
11	47789311	47790021	KD2-H4K8ac_peak_5290	11.92978	FNBP4_ENSG00000109920
11	47790430	47790680	KD2-H4K8ac_peak_5291	4.67342	
11	47848741	47848935	KD2-H4K8ac_peak_5292	9.15329	
11	47869642	47870562	KD2-H4K8ac_peak_5293	12.93209	NUP160_ENSG00000030066
11	47936082	47936342	KD2-H4K8ac_peak_5294	4.86324	
11	47939175	47939536	KD2-H4K8ac_peak_5295	7.146	
11	47940137	47940445	KD2-H4K8ac_peak_5296	6.39197	
11	48001523	48001801	KD2-H4K8ac_peak_5297	9.98013	PTPRJ_ENSG00000149177
11	48131281	48131537	KD2-H4K8ac_peak_5298	10.96455	
11	48131853	48132090	KD2-H4K8ac_peak_5299	8.17902	
11	50257051	50257300	KD2-H4K8ac_peak_5300	7.12754	RP11-347H15.4_ENSG00000254518
11	55640533	55640735	KD2-H4K8ac_peak_5301	6.10699	
11	56865814	56866173	KD2-H4K8ac_peak_5302	8.97719	
11	56923799	56924110	KD2-H4K8ac_peak_5303	6.40683	
11	57005065	57005287	KD2-H4K8ac_peak_5304	5.57044	APLNR_ENSG00000134817
11	57071714	57072032	KD2-H4K8ac_peak_5305	9.72244	
11	57091558	57091805	KD2-H4K8ac_peak_5306	6.98292	TNKS1BP1_ENSG00000149115
11	57102966	57103246	KD2-H4K8ac_peak_5307	10.96455	SSRP1_ENSG00000149136
11	57103471	57104101	KD2-H4K8ac_peak_5308	10.05606	SSRP1_ENSG00000149136
11	57112924	57113255	KD2-H4K8ac_peak_5309	12.25733	
11	57117107	57117376	KD2-H4K8ac_peak_5310	9.20193	
11	57228000	57228417	KD2-H4K8ac_peak_5311	12.93209	RTN4RL2_ENSG00000186907
11	57243642	57244796	KD2-H4K8ac_peak_5312	16.8435	RP11-624G17.3_ENSG00000255301
11	57249611	57250568	KD2-H4K8ac_peak_5313	14.69247	
11	57266925	57267149	KD2-H4K8ac_peak_5314	6.83936	
11	57281518	57282368	KD2-H4K8ac_peak_5315	7.95563	SLC43A1_ENSG00000149150
11	57282904	57283459	KD2-H4K8ac_peak_5316	7.64093	SLC43A1_ENSG00000149150
11	57297624	57298106	KD2-H4K8ac_peak_5317	7.36799	TIMM10_ENSG00000134809
11	57335295	57335734	KD2-H4K8ac_peak_5318	6.65673	UBE2L6_ENSG00000156587
11	57365586	57365944	KD2-H4K8ac_peak_5319	9.34989	SERPING1_ENSG00000149131
11	57404949	57405646	KD2-H4K8ac_peak_5320	8.86099	AP000662.4_ENSG00000254602
11	57405879	57406186	KD2-H4K8ac_peak_5321	7.36799	AP000662.4_ENSG00000254602
11	57435499	57436117	KD2-H4K8ac_peak_5322	8.85813	ZDHHC5_ENSG00000156599
11	57479418	57479613	KD2-H4K8ac_peak_5323	8.26945	MED19_ENSG00000156603;TMX2_ENSG00000213593;TMX2-CTNND1_ENSG00000254462
11	57480108	57480425	KD2-H4K8ac_peak_5324	12.06346	MED19_ENSG00000156603;TMX2_ENSG00000213593;TMX2-CTNND1_ENSG00000254462
11	57508385	57508675	KD2-H4K8ac_peak_5325	9.1707	RP11-691N7.6_ENSG00000254732;C11orf31_ENSG00000211450
11	57509053	57509456	KD2-H4K8ac_peak_5326	7.51054	RP11-691N7.6_ENSG00000254732;C11orf31_ENSG00000211450
11	57528461	57528811	KD2-H4K8ac_peak_5327	9.23578	
11	58346108	58346330	KD2-H4K8ac_peak_5328	8.55271	LPXN_ENSG00000110031;ZFP91_ENSG00000186660;ZFP91-CNTF_ENSG00000255073
11	58346688	58347430	KD2-H4K8ac_peak_5329	19.93588	LPXN_ENSG00000110031;ZFP91_ENSG00000186660;ZFP91-CNTF_ENSG00000255073
11	58672591	58673106	KD2-H4K8ac_peak_5330	9.11818	AP001652.1_ENSG00000224130
11	58873773	58874001	KD2-H4K8ac_peak_5331	5.88845	FAM111B_ENSG00000189057
11	58910170	58910561	KD2-H4K8ac_peak_5332	7.27716	AP001258.4_ENSG00000245571;AP001258.5_ENSG00000255381;FAM111A_ENSG00000166801
11	59304391	59304640	KD2-H4K8ac_peak_5333	12.42547	
11	59316565	59316811	KD2-H4K8ac_peak_5334	11.15509	

11	59323285	59323564	KD2-H4K8ac_peak_5335	11.05407	
11	59327973	59328348	KD2-H4K8ac_peak_5336	9.51688	
11	59333292	59333693	KD2-H4K8ac_peak_5337	6.2781	AP000442.4_ENSG00000255008
11	59522959	59523193	KD2-H4K8ac_peak_5338	9.95119	AP000640.10_ENSG00000254477
11	59528727	59528996	KD2-H4K8ac_peak_5339	13.57066	
11	59529825	59530181	KD2-H4K8ac_peak_5340	9.16531	
11	59577503	59579061	KD2-H4K8ac_peak_5341	16.32216	MRPL16_ENSG00000166902
11	60608387	60608591	KD2-H4K8ac_peak_5342	4.66652	CCDC86_ENSG00000110104
11	60608989	60609373	KD2-H4K8ac_peak_5343	8.23274	CCDC86_ENSG00000110104
11	60673687	60674009	KD2-H4K8ac_peak_5344	10.21366	PRPF19_ENSG00000110107;RP11-881M11.2_ENSG00000257052
11	60680775	60681205	KD2-H4K8ac_peak_5345	8.23274	RP11-881M11.1_ENSG00000256944;TMEM109_ENSG00000110108
11	60681661	60682000	KD2-H4K8ac_peak_5346	7.76329	RP11-881M11.1_ENSG00000256944;TMEM109_ENSG00000110108
11	60691451	60691757	KD2-H4K8ac_peak_5347	5.57044	TMEM132A_ENSG00000006118
11	60691984	60692684	KD2-H4K8ac_peak_5348	12.36441	RP11-881M11.4_ENSG00000256196;TMEM132A_ENSG00000006118
11	60749009	60749384	KD2-H4K8ac_peak_5349	5.77265	
11	60774891	60775603	KD2-H4K8ac_peak_5350	8.55271	
11	60888906	60889141	KD2-H4K8ac_peak_5351	13.08184	
11	60893261	60893686	KD2-H4K8ac_peak_5352	6.25014	
11	60927847	60928187	KD2-H4K8ac_peak_5353	4.20044	VPS37C_ENSG00000167987
11	60928875	60929566	KD2-H4K8ac_peak_5354	22.21705	VPS37C_ENSG00000167987
11	60930070	60930379	KD2-H4K8ac_peak_5355	5.57044	VPS37C_ENSG00000167987
11	61061446	61061719	KD2-H4K8ac_peak_5356	4.70206	
11	61062017	61063195	KD2-H4K8ac_peak_5357	11.28765	VWCE_ENSG00000167992
11	61100779	61101448	KD2-H4K8ac_peak_5358	12.00597	DAK_ENSG00000149476
11	61104148	61104451	KD2-H4K8ac_peak_5359	10.44656	
11	61113208	61113444	KD2-H4K8ac_peak_5360	10.87093	
11	61128824	61129732	KD2-H4K8ac_peak_5361	12.82393	CYB561A3_ENSG00000162144;TMEM138_ENSG00000149483
11	61159352	61160425	KD2-H4K8ac_peak_5362	12.06346	TMEM216_ENSG00000187049
11	61196994	61197439	KD2-H4K8ac_peak_5363	15.01306	CPSF7_ENSG00000149532;RP11-286N22.8_ENSG00000256591;SDHAF2_ENSG00000167985
11	61197663	61197916	KD2-H4K8ac_peak_5364	6.25869	CPSF7_ENSG00000149532;RP11-286N22.8_ENSG00000256591;SDHAF2_ENSG00000167985
11	61297028	61297220	KD2-H4K8ac_peak_5365	5.20908	
11	61298931	61299184	KD2-H4K8ac_peak_5366	4.46868	
11	61309615	61310469	KD2-H4K8ac_peak_5367	10.72313	RP11-794G24.1_ENSG00000256443
11	61322404	61323001	KD2-H4K8ac_peak_5368	8.26565	
11	61447351	61447698	KD2-H4K8ac_peak_5369	12.08081	
11	61485396	61485967	KD2-H4K8ac_peak_5370	10.54073	
11	61486209	61486626	KD2-H4K8ac_peak_5371	15.58963	
11	61514801	61515028	KD2-H4K8ac_peak_5372	5.14125	
11	61520113	61520526	KD2-H4K8ac_peak_5373	7.47462	MYRF_ENSG00000124920
11	61530135	61530725	KD2-H4K8ac_peak_5374	7.95934	
11	61532744	61533067	KD2-H4K8ac_peak_5375	3.75035	
11	61539747	61540210	KD2-H4K8ac_peak_5376	9.05772	
11	61559542	61560923	KD2-H4K8ac_peak_5377	15.61313	TMEM258_ENSG00000134825;MIR611_ENSG00000207601;FEN1_ENSG00000168496;FADS2_ENSG00000134824
11	61582305	61582603	KD2-H4K8ac_peak_5378	11.81678	MIR1908_ENSG00000222326
11	61582966	61584300	KD2-H4K8ac_peak_5379	18.54875	MIR1908_ENSG00000222326
11	61584712	61585046	KD2-H4K8ac_peak_5380	13.56049	

11	61594116	61595295	KD2-H4K8ac_peak_5381	16.09057	
11	61595575	61596792	KD2-H4K8ac_peak_5382	19.33018	FADS1_ENSG00000149485
11	61638397	61639006	KD2-H4K8ac_peak_5383	4.67342	
11	61658376	61659788	KD2-H4K8ac_peak_5384	20.88898	FADS3_ENSG00000221968
11	61684623	61685464	KD2-H4K8ac_peak_5385	8.85813	
11	61690457	61691090	KD2-H4K8ac_peak_5386	3.83717	
11	61714970	61715171	KD2-H4K8ac_peak_5387	6.25869	
11	61717321	61718003	KD2-H4K8ac_peak_5388	15.18544	BEST1_ENSG00000167995
11	61734842	61735053	KD2-H4K8ac_peak_5389	12.79505	FTH1_ENSG00000167996;AP003733.1_ENSG00000269089
11	61735340	61736114	KD2-H4K8ac_peak_5390	8.53092	FTH1_ENSG00000167996;AP003733.1_ENSG00000269089
11	61738946	61739304	KD2-H4K8ac_peak_5391	5.88845	
11	61880275	61880469	KD2-H4K8ac_peak_5392	4.54006	
11	61891552	61891880	KD2-H4K8ac_peak_5393	5.9696	INCENP_ENSG00000149503
11	62104980	62105447	KD2-H4K8ac_peak_5394	9.41619	RP11-703H8.7_ENSG00000255118
11	62149249	62149518	KD2-H4K8ac_peak_5395	5.57044	
11	62162857	62163102	KD2-H4K8ac_peak_5396	5.88845	
11	62165563	62165927	KD2-H4K8ac_peak_5397	8.17902	
11	62167344	62167692	KD2-H4K8ac_peak_5398	4.56666	
11	62168433	62169280	KD2-H4K8ac_peak_5399	8.30974	
11	62340965	62341357	KD2-H4K8ac_peak_5400	9.89126	
11	62341746	62342105	KD2-H4K8ac_peak_5401	8.79106	EEF1G_ENSG00000254772
11	62359461	62359727	KD2-H4K8ac_peak_5402	7.69287	MIR3654_ENSG00000255508;TUT1_ENSG00000149016
11	62368665	62369269	KD2-H4K8ac_peak_5403	9.78882	MTA2_ENSG00000149480
11	62379717	62380044	KD2-H4K8ac_peak_5404	6.97919	EML3_ENSG00000149499;ROM1_ENSG00000149489
11	62413680	62414034	KD2-H4K8ac_peak_5405	9.26408	GANAB_ENSG00000089597
11	62420984	62421188	KD2-H4K8ac_peak_5406	14.15075	INTS5_ENSG00000185085
11	62432189	62432640	KD2-H4K8ac_peak_5407	7.96322	RP11-831H9.11_ENSG00000255432;METTL12_ENSG00000214756;SNORA57_ENSG00000206597
11	62432934	62433211	KD2-H4K8ac_peak_5408	6.2781	RP11-831H9.11_ENSG00000255432;METTL12_ENSG00000214756;SNORA57_ENSG00000206597
11	62438973	62439710	KD2-H4K8ac_peak_5409	9.20193	C11orf48_ENSG00000162194
11	62446264	62447084	KD2-H4K8ac_peak_5410	11.66477	UBXN1_ENSG00000162191
11	62447983	62448606	KD2-H4K8ac_peak_5411	5.30799	
11	62477216	62477664	KD2-H4K8ac_peak_5412	17.27647	BSCL2_ENSG00000168000
11	62494234	62494899	KD2-H4K8ac_peak_5413	17.27647	HNRNPUL2-BSCL2_ENSG00000234857;HNRNPUL2_ENSG00000214753;TTC9C_ENSG00000162222
11	62495638	62495850	KD2-H4K8ac_peak_5414	15.9754	HNRNPUL2-BSCL2_ENSG00000234857;HNRNPUL2_ENSG00000214753;TTC9C_ENSG00000162222
11	62520902	62521190	KD2-H4K8ac_peak_5415	4.77079	ZBTB3_ENSG00000185670
11	62528573	62528769	KD2-H4K8ac_peak_5416	5.75452	POLR2G_ENSG00000168002
11	62538143	62538489	KD2-H4K8ac_peak_5417	7.36799	RP11-727F15.11_ENSG00000267811;TAF6L_ENSG00000162227
11	62538887	62539148	KD2-H4K8ac_peak_5418	8.58725	RP11-727F15.11_ENSG00000267811;TAF6L_ENSG00000162227
11	62554372	62554709	KD2-H4K8ac_peak_5419	8.58069	RP11-727F15.12_ENSG00000269176;TMEM179B_ENSG00000185475
11	62558707	62559376	KD2-H4K8ac_peak_5420	9.95119	TMEM223_ENSG00000168569
11	62598607	62598995	KD2-H4K8ac_peak_5421	5.54647	STX5_ENSG00000162236;RP11-727F15.9_ENSG00000256690
11	62606647	62607003	KD2-H4K8ac_peak_5422	7.92775	
11	62608294	62608509	KD2-H4K8ac_peak_5423	7.79898	WDR74_ENSG00000133316;RNU2-2P_ENSG00000222328
11	62609603	62610147	KD2-H4K8ac_peak_5424	11.2938	WDR74_ENSG00000133316;RNU2-2P_ENSG00000222328
11	62623688	62623956	KD2-H4K8ac_peak_5425	8.28234	SNHG1_ENSG00000255717;SLC3A2_ENSG00000168003
11	62624465	62624962	KD2-H4K8ac_peak_5426	6.66692	SLC3A2_ENSG00000168003

11	62648575	62648830	KD2-H4K8ac_peak_5427	5.75757	
11	62677100	62677332	KD2-H4K8ac_peak_5428	5.82799	AP000438.2_ENSG00000257002
11	62677712	62678501	KD2-H4K8ac_peak_5429	7.70289	AP000438.2_ENSG00000257002
11	62679920	62680333	KD2-H4K8ac_peak_5430	8.17902	
11	62688498	62688709	KD2-H4K8ac_peak_5431	4.86324	CHRM1_ENSG00000168539
11	62688911	62689118	KD2-H4K8ac_peak_5432	5.30799	CHRM1_ENSG00000168539
11	62784475	62785100	KD2-H4K8ac_peak_5433	7.47462	
11	63438296	63439056	KD2-H4K8ac_peak_5434	15.05364	ATL3_ENSG00000184743
11	63439352	63439610	KD2-H4K8ac_peak_5435	6.84261	ATL3_ENSG00000184743
11	63449039	63449314	KD2-H4K8ac_peak_5436	8.55271	RTN3_ENSG00000133318
11	63535274	63536075	KD2-H4K8ac_peak_5437	9.59814	C11orf95_ENSG00000188070;RP11-466C23.4_ENSG00000255651
11	63536373	63537402	KD2-H4K8ac_peak_5438	18.75146	C11orf95_ENSG00000188070;RP11-466C23.4_ENSG00000255651
11	63551881	63552080	KD2-H4K8ac_peak_5439	6.1228	
11	63581085	63581451	KD2-H4K8ac_peak_5440	7.51054	C11orf84_ENSG00000168005
11	63606626	63606845	KD2-H4K8ac_peak_5441	7.98621	MARK2_ENSG00000072518
11	63683377	63684447	KD2-H4K8ac_peak_5442	14.8083	RCOR2_ENSG00000167771
11	63684791	63685416	KD2-H4K8ac_peak_5443	19.42408	RCOR2_ENSG00000167771
11	63685765	63687636	KD2-H4K8ac_peak_5444	13.80019	
11	63687941	63688273	KD2-H4K8ac_peak_5445	6.53538	
11	63742140	63742375	KD2-H4K8ac_peak_5446	5.75757	COX8A_ENSG00000176340;AP000721.4_ENSG00000256100
11	63753082	63754491	KD2-H4K8ac_peak_5447	8.10216	OTUB1_ENSG00000167770
11	63775078	63775541	KD2-H4K8ac_peak_5448	4.86324	
11	63812155	63812419	KD2-H4K8ac_peak_5449	6.62541	
11	63812838	63813323	KD2-H4K8ac_peak_5450	8.0372	
11	63813617	63813958	KD2-H4K8ac_peak_5451	7.95563	
11	63820105	63821374	KD2-H4K8ac_peak_5452	10.94055	
11	63822496	63823767	KD2-H4K8ac_peak_5453	14.4394	
11	63823992	63824757	KD2-H4K8ac_peak_5454	9.67696	
11	63824978	63825290	KD2-H4K8ac_peak_5455	4.86324	
11	63825668	63825979	KD2-H4K8ac_peak_5456	11.7608	
11	63826473	63828467	KD2-H4K8ac_peak_5457	18.31034	
11	63828808	63829081	KD2-H4K8ac_peak_5458	16.57619	
11	63829571	63829771	KD2-H4K8ac_peak_5459	7.35057	
11	63830927	63831150	KD2-H4K8ac_peak_5460	4.52804	
11	63831485	63832647	KD2-H4K8ac_peak_5461	6.67538	
11	63834935	63835346	KD2-H4K8ac_peak_5462	10.77651	
11	63835648	63835883	KD2-H4K8ac_peak_5463	5.18937	
11	63836111	63836307	KD2-H4K8ac_peak_5464	6.24992	
11	63841465	63841837	KD2-H4K8ac_peak_5465	6.20524	
11	63844568	63844808	KD2-H4K8ac_peak_5466	7.42964	
11	63845106	63845792	KD2-H4K8ac_peak_5467	14.17488	
11	63846113	63846787	KD2-H4K8ac_peak_5468	9.90914	
11	63850091	63850499	KD2-H4K8ac_peak_5469	6.40297	RP11-21A7A.4_ENSG00000256481
11	63851516	63852771	KD2-H4K8ac_peak_5470	19.06706	
11	63853115	63853350	KD2-H4K8ac_peak_5471	10.0665	
11	63853563	63854079	KD2-H4K8ac_peak_5472	9.9355	

11	63854631	63855073	KD2-H4K8ac_peak_5473	5.25269	
11	63855679	63855957	KD2-H4K8ac_peak_5474	8.55581	
11	63856358	63856734	KD2-H4K8ac_peak_5475	9.1707	
11	63858217	63858453	KD2-H4K8ac_peak_5476	6.65673	
11	63863255	63864799	KD2-H4K8ac_peak_5477	9.36516	
11	63869064	63869486	KD2-H4K8ac_peak_5478	5.30799	
11	63869744	63870158	KD2-H4K8ac_peak_5479	6.29509	FLRT1_ENSG00000126500
11	63871124	63871383	KD2-H4K8ac_peak_5480	9.80315	FLRT1_ENSG00000126500
11	63900343	63900854	KD2-H4K8ac_peak_5481	9.74423	
11	63911594	63911826	KD2-H4K8ac_peak_5482	7.1733	
11	63912413	63913524	KD2-H4K8ac_peak_5483	7.07117	
11	63915536	63915747	KD2-H4K8ac_peak_5484	7.45629	
11	63917025	63917285	KD2-H4K8ac_peak_5485	6.27245	
11	63932744	63934188	KD2-H4K8ac_peak_5486	17.70889	MACROD1_ENSG00000133315
11	63952539	63953082	KD2-H4K8ac_peak_5487	6.65673	STIP1_ENSG00000168439
11	63953906	63954243	KD2-H4K8ac_peak_5488	4.71229	
11	63993306	63993564	KD2-H4K8ac_peak_5489	6.84261	TRPT1_ENSG00000149743;NUDT22_ENSG00000149761
11	63993815	63994083	KD2-H4K8ac_peak_5490	12.93007	TRPT1_ENSG00000149743;NUDT22_ENSG00000149761
11	64001532	64001778	KD2-H4K8ac_peak_5491	7.27716	RP11-783K16.14_ENSG00000256116;VEGFB_ENSG00000173511
11	64002235	64002836	KD2-H4K8ac_peak_5492	15.93117	RP11-783K16.14_ENSG00000256116;VEGFB_ENSG00000173511
11	64007886	64008737	KD2-H4K8ac_peak_5493	16.54162	FKBP2_ENSG00000173486
11	64009202	64009867	KD2-H4K8ac_peak_5494	10.43145	FKBP2_ENSG00000173486
11	64013817	64014276	KD2-H4K8ac_peak_5495	15.08752	PPP1R14B_ENSG00000173457;RP11-783K16.5_ENSG00000256940
11	64015096	64015387	KD2-H4K8ac_peak_5496	11.55418	PPP1R14B_ENSG00000173457
11	64015861	64016210	KD2-H4K8ac_peak_5497	5.75452	RP11-783K16.13_ENSG00000257086
11	64017865	64019468	KD2-H4K8ac_peak_5498	11.22681	RP11-783K16.13_ENSG00000257086;PLCB3_ENSG00000149782
11	64035749	64037362	KD2-H4K8ac_peak_5499	11.05467	GPR137_ENSG00000173264
11	64037702	64038296	KD2-H4K8ac_peak_5500	9.63487	GPR137_ENSG00000173264
11	64038967	64039562	KD2-H4K8ac_peak_5501	13.95627	
11	64051618	64052131	KD2-H4K8ac_peak_5502	14.22723	BAD_ENSG00000002330
11	64052452	64052862	KD2-H4K8ac_peak_5503	11.24702	BAD_ENSG00000002330
11	64053463	64053760	KD2-H4K8ac_peak_5504	10.74343	
11	64072031	64072440	KD2-H4K8ac_peak_5505	9.70019	ESRRA_ENSG00000173153
11	64073720	64073973	KD2-H4K8ac_peak_5506	4.22101	ESRRA_ENSG00000173153
11	64084392	64084913	KD2-H4K8ac_peak_5507	12.99417	TRMT112_ENSG00000173113;PRDX5_ENSG00000126432
11	64085162	64085368	KD2-H4K8ac_peak_5508	4.54006	TRMT112_ENSG00000173113;PRDX5_ENSG00000126432
11	64085703	64086248	KD2-H4K8ac_peak_5509	10.5847	TRMT112_ENSG00000173113;PRDX5_ENSG00000126432
11	64122769	64123057	KD2-H4K8ac_peak_5510	9.36516	
11	64125992	64127042	KD2-H4K8ac_peak_5511	11.44756	RPS6KA4_ENSG00000162302
11	64140705	64141056	KD2-H4K8ac_peak_5512	5.14125	
11	64215860	64216090	KD2-H4K8ac_peak_5513	6.39197	AP003774.4_ENSG00000181908
11	64216600	64216898	KD2-H4K8ac_peak_5514	7.74292	AP003774.4_ENSG00000181908
11	64258335	64258556	KD2-H4K8ac_peak_5515	5.57044	
11	64333967	64335554	KD2-H4K8ac_peak_5516	12.84642	
11	64336205	64336448	KD2-H4K8ac_peak_5517	3.72574	
11	64388526	64388859	KD2-H4K8ac_peak_5518	7.51945	

11	64404514	64404719	KD2-H4K8ac_peak_5519	4.42765	
11	64405250	64405804	KD2-H4K8ac_peak_5520	12.08081	
11	64455585	64455898	KD2-H4K8ac_peak_5521	9.51625	
11	64462590	64462834	KD2-H4K8ac_peak_5522	6.25869	
11	64490778	64491786	KD2-H4K8ac_peak_5523	20.02161	NRXN2_ENSG00000110076
11	64497600	64497851	KD2-H4K8ac_peak_5524	5.20908	
11	64511819	64512242	KD2-H4K8ac_peak_5525	6.85193	RASGRP2_ENSG00000068831
11	64512499	64512807	KD2-H4K8ac_peak_5526	6.66359	RASGRP2_ENSG00000068831
11	64513027	64513519	KD2-H4K8ac_peak_5527	8.88082	RASGRP2_ENSG00000068831
11	64545346	64546057	KD2-H4K8ac_peak_5528	16.64426	SF1_ENSG00000168066;AP001462.6_ENSG00000269038
11	64546943	64547217	KD2-H4K8ac_peak_5529	7.76329	SF1_ENSG00000168066;AP001462.6_ENSG00000269038
11	64571347	64571584	KD2-H4K8ac_peak_5530	4.87807	MAP4K2_ENSG00000168067
11	64578394	64578597	KD2-H4K8ac_peak_5531	5.82799	MEN1_ENSG00000133895
11	64611932	64612543	KD2-H4K8ac_peak_5532	11.09936	CDC42BPG_ENSG00000171219
11	64624173	64624453	KD2-H4K8ac_peak_5533	5.08628	
11	64646377	64646867	KD2-H4K8ac_peak_5534	6.78242	
11	64655261	64655462	KD2-H4K8ac_peak_5535	11.22681	EHD1_ENSG00000110047
11	64655689	64655887	KD2-H4K8ac_peak_5536	4.86324	EHD1_ENSG00000110047
11	64684229	64684585	KD2-H4K8ac_peak_5537	6.25869	ATG2A_ENSG00000110046;PPP2R5B_ENSG00000068971
11	64690699	64691531	KD2-H4K8ac_peak_5538	6.40297	
11	64691772	64692052	KD2-H4K8ac_peak_5539	14.68449	
11	64692280	64692632	KD2-H4K8ac_peak_5540	5.81222	
11	64781195	64782446	KD2-H4K8ac_peak_5541	20.5798	ARL2_ENSG00000213465;RP11-399J13.3_ENSG00000273003
11	64851035	64851462	KD2-H4K8ac_peak_5542	7.36799	CDCA5_ENSG00000146670;ZFPL1_ENSG00000162300
11	64852037	64852287	KD2-H4K8ac_peak_5543	6.99815	CDCA5_ENSG00000146670;ZFPL1_ENSG00000162300
11	64863078	64863946	KD2-H4K8ac_peak_5544	17.55491	
11	64879428	64880768	KD2-H4K8ac_peak_5545	16.49944	AP003068.9_ENSG00000254501;TM7SF2_ENSG00000149809
11	64883964	64885056	KD2-H4K8ac_peak_5546	15.81711	ZNHIT2_ENSG00000174276;AP003068.12_ENSG00000255173
11	64901463	64902477	KD2-H4K8ac_peak_5547	5.75757	SYVN1_ENSG00000162298
11	64902732	64902941	KD2-H4K8ac_peak_5548	4.211	SYVN1_ENSG00000162298
11	64903353	64903641	KD2-H4K8ac_peak_5549	5.54647	
11	64917836	64918891	KD2-H4K8ac_peak_5550	11.92978	
11	64948264	64949088	KD2-H4K8ac_peak_5551	11.79585	AP003068.23_ENSG00000254614;CAPN1_ENSG00000014216
11	64949421	64949827	KD2-H4K8ac_peak_5552	12.08081	AP003068.23_ENSG00000254614
11	64992526	64993788	KD2-H4K8ac_peak_5553	10.30284	
11	65057175	65057597	KD2-H4K8ac_peak_5554	7.58939	
11	65100713	65101144	KD2-H4K8ac_peak_5555	15.61313	DPF2_ENSG00000133884
11	65101471	65101706	KD2-H4K8ac_peak_5556	9.18237	DPF2_ENSG00000133884
11	65121575	65121986	KD2-H4K8ac_peak_5557	7.40314	TIGD3_ENSG00000173825
11	65122316	65122510	KD2-H4K8ac_peak_5558	6.45083	TIGD3_ENSG00000173825
11	65144804	65145037	KD2-H4K8ac_peak_5559	5.65802	
11	65149200	65150062	KD2-H4K8ac_peak_5560	3.82395	
11	65150374	65150895	KD2-H4K8ac_peak_5561	10.53377	SLC25A45_ENSG00000162241
11	65153557	65153984	KD2-H4K8ac_peak_5562	7.76104	FRMD8_ENSG00000126391
11	65183460	65183940	KD2-H4K8ac_peak_5563	6.9556	
11	65185998	65187159	KD2-H4K8ac_peak_5564	16.34834	

11	65189425	65189706	KD2-H4K8ac_peak_5565	8.52559	NEAT1_ENSG00000245532
11	65220546	65220756	KD2-H4K8ac_peak_5566	8.44476	
11	65222248	65222637	KD2-H4K8ac_peak_5567	10.17486	AP000769.1_ENSG00000173727
11	65244432	65244916	KD2-H4K8ac_peak_5568	10.43145	
11	65245193	65245456	KD2-H4K8ac_peak_5569	8.86099	
11	65253612	65253807	KD2-H4K8ac_peak_5570	5.82799	
11	65254517	65254780	KD2-H4K8ac_peak_5571	8.17557	
11	65255529	65255867	KD2-H4K8ac_peak_5572	5.01002	
11	65256350	65256978	KD2-H4K8ac_peak_5573	9.59905	
11	65264446	65264647	KD2-H4K8ac_peak_5574	8.79106	MALAT1_ENSG00000251562
11	65265279	65266365	KD2-H4K8ac_peak_5575	17.8868	MALAT1_ENSG00000251562;AP000769.7_ENSG00000270117
11	65324911	65325157	KD2-H4K8ac_peak_5576	12.99417	
11	65325385	65325794	KD2-H4K8ac_peak_5577	13.58431	LTBP3_ENSG00000168056
11	65326063	65326897	KD2-H4K8ac_peak_5578	10.77651	LTBP3_ENSG00000168056
11	65327431	65328124	KD2-H4K8ac_peak_5579	9.89126	
11	65328603	65328857	KD2-H4K8ac_peak_5580	7.45501	
11	65337246	65337777	KD2-H4K8ac_peak_5581	7.36799	SSSCA1-AS1_ENSG00000260233;SSSCA1_ENSG00000173465
11	65338105	65338350	KD2-H4K8ac_peak_5582	7.07117	SSSCA1-AS1_ENSG00000260233;SSSCA1_ENSG00000173465
11	65343541	65344042	KD2-H4K8ac_peak_5583	9.07715	EHBP1L1_ENSG00000173442
11	65381203	65382048	KD2-H4K8ac_peak_5584	11.03331	MAP3K11_ENSG00000173327
11	65383309	65383555	KD2-H4K8ac_peak_5585	14.78033	MAP3K11_ENSG00000173327;PCNXL3_ENSG00000197136
11	65405700	65406137	KD2-H4K8ac_peak_5586	6.97919	SIPA1_ENSG00000213445
11	65414407	65415176	KD2-H4K8ac_peak_5587	8.04448	
11	65420165	65420396	KD2-H4K8ac_peak_5588	9.95119	
11	65420668	65421522	KD2-H4K8ac_peak_5589	14.4458	
11	65430833	65431170	KD2-H4K8ac_peak_5590	9.36516	RELA_ENSG00000173039
11	65478096	65478360	KD2-H4K8ac_peak_5591	4.82979	
11	65479499	65479916	KD2-H4K8ac_peak_5592	8.4716	KAT5_ENSG00000172977
11	65487923	65488241	KD2-H4K8ac_peak_5593	9.34195	RNASEH2C_ENSG00000172922
11	65488638	65488888	KD2-H4K8ac_peak_5594	7.26029	RNASEH2C_ENSG00000172922
11	65548534	65549283	KD2-H4K8ac_peak_5595	29.70907	AP5B1_ENSG00000254470
11	65585308	65585660	KD2-H4K8ac_peak_5596	9.1707	
11	65600531	65601336	KD2-H4K8ac_peak_5597	9.95119	SNX32_ENSG00000172803
11	65624982	65625644	KD2-H4K8ac_peak_5598	15.57998	MUS81_ENSG00000172732
11	65627186	65627683	KD2-H4K8ac_peak_5599	5.01002	
11	65628041	65628438	KD2-H4K8ac_peak_5600	12.13241	
11	65639449	65640406	KD2-H4K8ac_peak_5601	15.81711	EFEMP2_ENSG00000172638
11	65640705	65640910	KD2-H4K8ac_peak_5602	4.06758	EFEMP2_ENSG00000172638
11	65641311	65641668	KD2-H4K8ac_peak_5603	4.67342	EFEMP2_ENSG00000172638
11	65656144	65656418	KD2-H4K8ac_peak_5604	20.17563	FIBP_ENSG00000172500
11	65656920	65657150	KD2-H4K8ac_peak_5605	7.40111	FIBP_ENSG00000172500;CCDC85B_ENSG00000175602
11	65660984	65661753	KD2-H4K8ac_peak_5606	14.93459	
11	65667509	65667875	KD2-H4K8ac_peak_5607	12.46946	FOSL1_ENSG00000175592
11	65679641	65679848	KD2-H4K8ac_peak_5608	4.86324	
11	65685942	65686459	KD2-H4K8ac_peak_5609	18.7894	C11orf68_ENSG00000175573;DRAP1_ENSG00000175550
11	65720842	65721218	KD2-H4K8ac_peak_5610	6.99815	

11	65728167	65728937	KD2-H4K8ac_peak_5611	11.92978	SART1_ENSG00000175467
11	65729220	65729513	KD2-H4K8ac_peak_5612	9.65086	SART1_ENSG00000175467
11	65789822	65790518	KD2-H4K8ac_peak_5613	7.76329	
11	65816231	65816590	KD2-H4K8ac_peak_5614	9.1994	GAL3ST3_ENSG00000175229
11	65818665	65819527	KD2-H4K8ac_peak_5615	12.13241	SF3B2_ENSG00000087365
11	65819902	65820102	KD2-H4K8ac_peak_5616	7.0754	snoU13_ENSG00000238752
11	65837795	65838344	KD2-H4K8ac_peak_5617	12.84642	RP11-1167A19.2_ENSG00000255038;PACS1_ENSG00000175115
11	65885861	65886098	KD2-H4K8ac_peak_5618	10.1591	
11	65886401	65886655	KD2-H4K8ac_peak_5619	4.42765	
11	65887279	65887482	KD2-H4K8ac_peak_5620	9.11031	
11	66024657	66025050	KD2-H4K8ac_peak_5621	6.40297	KLC2_ENSG00000174996
11	66035313	66035661	KD2-H4K8ac_peak_5622	6.55628	RP11-867G23.2_ENSG00000254762;RAB1B_ENSG00000174903
11	66036081	66036701	KD2-H4K8ac_peak_5623	12.93253	RP11-867G23.2_ENSG00000254762;RAB1B_ENSG00000174903
11	66045092	66046237	KD2-H4K8ac_peak_5624	11.89502	RP11-867G23.3_ENSG00000245156;RP11-867G23.4_ENSG00000254452;CNIH2_ENSG00000174871
11	66056183	66056553	KD2-H4K8ac_peak_5625	14.00658	YIF1A_ENSG00000174851
11	66058833	66059184	KD2-H4K8ac_peak_5626	5.88845	TMEM151A_ENSG00000179292
11	66059465	66060003	KD2-H4K8ac_peak_5627	9.1707	TMEM151A_ENSG00000179292
11	66079132	66079598	KD2-H4K8ac_peak_5628	5.88845	RP11-867G23.13_ENSG00000254458
11	66080665	66082543	KD2-H4K8ac_peak_5629	14.08365	RP11-867G23.13_ENSG00000254458
11	66084193	66086290	KD2-H4K8ac_peak_5630	17.83665	CD248_ENSG00000174807
11	66093761	66094122	KD2-H4K8ac_peak_5631	6.25869	
11	66096094	66096413	KD2-H4K8ac_peak_5632	14.67919	
11	66103922	66104184	KD2-H4K8ac_peak_5633	6.25869	RIN1_ENSG00000174791
11	66112105	66112483	KD2-H4K8ac_peak_5634	4.54006	BRMS1_ENSG00000174744
11	66114500	66114971	KD2-H4K8ac_peak_5635	4.04573	B3GNT1_ENSG00000174684;RP11-867G23.8_ENSG00000255468
11	66115868	66116070	KD2-H4K8ac_peak_5636	8.09303	B3GNT1_ENSG00000174684;RP11-867G23.8_ENSG00000255468
11	66138471	66139583	KD2-H4K8ac_peak_5637	13.81781	SLC29A2_ENSG00000174669
11	66205829	66206282	KD2-H4K8ac_peak_5638	13.80019	
11	66233889	66234133	KD2-H4K8ac_peak_5639	5.9696	MRPL11_ENSG00000174547;PELI3_ENSG00000174516
11	66247082	66247598	KD2-H4K8ac_peak_5640	5.4779	CTD-3074O7.5_ENSG00000255517;DPP3_ENSG00000254986
11	66248021	66248282	KD2-H4K8ac_peak_5641	8.55271	CTD-3074O7.5_ENSG00000255517;DPP3_ENSG00000254986
11	66277665	66278005	KD2-H4K8ac_peak_5642	9.11031	BBS1_ENSG00000174483
11	66278272	66278566	KD2-H4K8ac_peak_5643	7.59643	BBS1_ENSG00000174483
11	66313212	66313437	KD2-H4K8ac_peak_5644	6.66359	ZDHHC24_ENSG00000174165;ACTN3_ENSG00000248746
11	66335792	66336391	KD2-H4K8ac_peak_5645	13.81781	CTSF_ENSG00000174080
11	66360692	66360898	KD2-H4K8ac_peak_5646	5.54647	CCDC87_ENSG00000182791;CCS_ENSG00000173992
11	66383008	66383795	KD2-H4K8ac_peak_5647	6.99815	RNU4-39P_ENSG00000199325;RBM14_ENSG00000239306;RBM4_ENSG00000173933;RBM14-RBM4_ENSG00000248643
11	66444873	66445195	KD2-H4K8ac_peak_5648	6.40297	RBM4B_ENSG00000173914
11	66494726	66495606	KD2-H4K8ac_peak_5649	9.95119	
11	66495905	66496364	KD2-H4K8ac_peak_5650	19.72117	SPTBN2_ENSG00000173898
11	66511861	66512124	KD2-H4K8ac_peak_5651	8.79106	C11orf80_ENSG00000173715
11	66512352	66512684	KD2-H4K8ac_peak_5652	10.19949	C11orf80_ENSG00000173715
11	66610008	66611469	KD2-H4K8ac_peak_5653	12.82393	RCE1_ENSG00000173653
11	66623051	66623781	KD2-H4K8ac_peak_5654	8.10216	LRFN4_ENSG00000173621
11	66624070	66624990	KD2-H4K8ac_peak_5655	9.65086	LRFN4_ENSG00000173621
11	66741882	66742173	KD2-H4K8ac_peak_5656	6.31143	C11orf86_ENSG00000173237

11	66742722	66742919	KD2-H4K8ac_peak_5657	6.4095	C11orf86_ENSG00000173237
11	66838952	66839234	KD2-H4K8ac_peak_5658	5.54647	
11	66883018	66883249	KD2-H4K8ac_peak_5659	4.56666	
11	66885748	66886202	KD2-H4K8ac_peak_5660	9.36516	KDM2A_ENSG00000173120
11	66886767	66887172	KD2-H4K8ac_peak_5661	13.73635	KDM2A_ENSG00000173120
11	67028643	67029951	KD2-H4K8ac_peak_5662	15.16343	
11	67030377	67030868	KD2-H4K8ac_peak_5663	10.05606	
11	67033438	67033693	KD2-H4K8ac_peak_5664	5.57044	ADRBK1_ENSG00000173020
11	67034227	67034658	KD2-H4K8ac_peak_5665	9.16531	ADRBK1_ENSG00000173020
11	67055043	67055759	KD2-H4K8ac_peak_5666	8.53092	ANKRD13D_ENSG00000172932
11	67056047	67056377	KD2-H4K8ac_peak_5667	10.66719	ANKRD13D_ENSG00000172932
11	67070587	67072064	KD2-H4K8ac_peak_5668	16.29614	SSH3_ENSG00000172830
11	67084108	67085232	KD2-H4K8ac_peak_5669	13.36684	
11	67120524	67121000	KD2-H4K8ac_peak_5670	10.19949	
11	67124014	67124333	KD2-H4K8ac_peak_5671	8.30974	POLD4_ENSG00000175482
11	67139787	67140026	KD2-H4K8ac_peak_5672	4.52514	
11	67140659	67140913	KD2-H4K8ac_peak_5673	5.30799	AP003419.11_ENSG00000256514;CLCF1_ENSG00000175505
11	67168716	67169257	KD2-H4K8ac_peak_5674	13.73635	
11	67169609	67170113	KD2-H4K8ac_peak_5675	7.76329	
11	67183164	67183390	KD2-H4K8ac_peak_5676	8.17902	CARNS1_ENSG00000172508
11	67188254	67188592	KD2-H4K8ac_peak_5677	6.67092	PPP1CA_ENSG00000172531
11	67195391	67195795	KD2-H4K8ac_peak_5678	16.57619	RPS6KB2_ENSG00000175634
11	67195993	67196195	KD2-H4K8ac_peak_5679	8.83582	RPS6KB2_ENSG00000175634
11	67231513	67232692	KD2-H4K8ac_peak_5680	15.08751	
11	67236305	67236600	KD2-H4K8ac_peak_5681	8.55271	TMEM134_ENSG00000172663
11	67236884	67237141	KD2-H4K8ac_peak_5682	12.84642	TMEM134_ENSG00000172663
11	67250640	67251275	KD2-H4K8ac_peak_5683	3.86308	AIP_ENSG00000110711
11	67272624	67272844	KD2-H4K8ac_peak_5684	6.65673	PITPNM1_ENSG00000110697
11	67274998	67275588	KD2-H4K8ac_peak_5685	14.66631	CDK2AP2_ENSG00000167797
11	67275928	67276445	KD2-H4K8ac_peak_5686	15.81711	CDK2AP2_ENSG00000167797
11	67350429	67350720	KD2-H4K8ac_peak_5687	12.78794	GSTP1_ENSG00000084207
11	67351090	67352051	KD2-H4K8ac_peak_5688	18.05501	GSTP1_ENSG00000084207
11	67373606	67374178	KD2-H4K8ac_peak_5689	9.15329	C11orf72_ENSG00000184224;RP11-655M14.12_ENSG00000255119;NDUFV1_ENSG00000167792
11	67374496	67375151	KD2-H4K8ac_peak_5690	7.47462	C11orf72_ENSG00000184224;NDUFV1_ENSG00000167792
11	67396533	67397238	KD2-H4K8ac_peak_5691	8.4716	NUDT8_ENSG00000167799
11	67398037	67398267	KD2-H4K8ac_peak_5692	5.75757	NUDT8_ENSG00000167799
11	67573619	67573958	KD2-H4K8ac_peak_5693	8.17902	FAM86C2P_ENSG00000160172
11	67593366	67593676	KD2-H4K8ac_peak_5694	9.34989	
11	67770978	67772359	KD2-H4K8ac_peak_5695	16.61426	UNC93B1_ENSG00000110057
11	67777478	67777954	KD2-H4K8ac_peak_5696	9.08816	
11	67797105	67797368	KD2-H4K8ac_peak_5697	9.06369	NDUFS8_ENSG00000110717;RP5-901A4.1_ENSG00000255306
11	67798170	67798503	KD2-H4K8ac_peak_5698	10.35678	NDUFS8_ENSG00000110717;RP5-901A4.1_ENSG00000255306
11	67807638	67808712	KD2-H4K8ac_peak_5699	12.00597	
11	67889132	67889826	KD2-H4K8ac_peak_5700	17.75427	CHKA_ENSG00000110721;CTD-2655K5.1_ENSG00000255236
11	67980902	67982144	KD2-H4K8ac_peak_5701	29.97849	SUV420H1_ENSG00000110066
11	67982418	67982613	KD2-H4K8ac_peak_5702	6.31143	

11	68025171	68025460	KD2-H4K8ac_peak_5703	5.85291	
11	68039027	68039311	KD2-H4K8ac_peak_5704	6.90231	C11orf24_ENSG00000171067
11	68039674	68039984	KD2-H4K8ac_peak_5705	6.40874	C11orf24_ENSG00000171067
11	68064974	68065357	KD2-H4K8ac_peak_5706	7.76329	
11	68079250	68080645	KD2-H4K8ac_peak_5707	14.93224	LRP5_ENSG00000162337
11	68080884	68081085	KD2-H4K8ac_peak_5708	4.06758	LRP5_ENSG00000162337
11	68081902	68082258	KD2-H4K8ac_peak_5709	4.77079	
11	68093810	68094013	KD2-H4K8ac_peak_5710	4.38478	
11	68095585	68096071	KD2-H4K8ac_peak_5711	6.58902	
11	68137801	68138201	KD2-H4K8ac_peak_5712	4.56666	
11	68138653	68138865	KD2-H4K8ac_peak_5713	6.60096	
11	68140386	68141043	KD2-H4K8ac_peak_5714	17.55491	
11	68141305	68141833	KD2-H4K8ac_peak_5715	15.31552	
11	68142318	68142868	KD2-H4K8ac_peak_5716	9.96798	
11	68207763	68208303	KD2-H4K8ac_peak_5717	9.89126	
11	68208733	68208962	KD2-H4K8ac_peak_5718	8.4716	
11	68424784	68425080	KD2-H4K8ac_peak_5719	7.47462	
11	68425600	68425983	KD2-H4K8ac_peak_5720	8.93255	
11	68426248	68426448	KD2-H4K8ac_peak_5721	8.36514	
11	68427013	68428073	KD2-H4K8ac_peak_5722	11.90231	
11	68428601	68429526	KD2-H4K8ac_peak_5723	8.36432	
11	68430886	68431314	KD2-H4K8ac_peak_5724	6.05809	
11	68445420	68445655	KD2-H4K8ac_peak_5725	5.88845	
11	68451581	68452388	KD2-H4K8ac_peak_5726	18.64825	GAL_ENSG00000069482
11	68518673	68518983	KD2-H4K8ac_peak_5727	16.8435	MTL5_ENSG00000132749
11	68608189	68609088	KD2-H4K8ac_peak_5728	12.57143	
11	68622197	68623330	KD2-H4K8ac_peak_5729	15.69572	
11	68657719	68658219	KD2-H4K8ac_peak_5730	4.06758	
11	68671446	68672135	KD2-H4K8ac_peak_5731	6.84261	MRPL21_ENSG00000197345;IGHMBP2_ENSG00000132740
11	68761813	68762102	KD2-H4K8ac_peak_5732	4.97693	
11	68768982	68769189	KD2-H4K8ac_peak_5733	5.97274	RP11-554A11.4_ENSG00000261625
11	68781168	68782705	KD2-H4K8ac_peak_5734	14.48084	MRGPRF_ENSG00000172935
11	68816411	68816999	KD2-H4K8ac_peak_5735	22.21705	TPCN2_ENSG00000162341
11	68872811	68873335	KD2-H4K8ac_peak_5736	11.05467	
11	68873870	68874425	KD2-H4K8ac_peak_5737	5.38581	
11	68883655	68883971	KD2-H4K8ac_peak_5738	4.01115	
11	68884468	68884660	KD2-H4K8ac_peak_5739	5.43522	
11	68891402	68891623	KD2-H4K8ac_peak_5740	4.86324	
11	68922631	68922832	KD2-H4K8ac_peak_5741	6.36375	RP11-554A11.9_ENSG00000259799
11	68923179	68924839	KD2-H4K8ac_peak_5742	20.46575	RP11-554A11.9_ENSG00000259799
11	68925037	68926211	KD2-H4K8ac_peak_5743	12.40345	
11	68946733	68947003	KD2-H4K8ac_peak_5744	9.11031	
11	68950585	68951384	KD2-H4K8ac_peak_5745	11.94983	
11	68953189	68953744	KD2-H4K8ac_peak_5746	16.61426	
11	68954677	68954911	KD2-H4K8ac_peak_5747	12.57297	
11	68955185	68955546	KD2-H4K8ac_peak_5748	9.08816	

11	68960016	68960895	KD2-H4K8ac_peak_5749	4.54006	
11	68967830	68968287	KD2-H4K8ac_peak_5750	5.01002	
11	68979711	68980040	KD2-H4K8ac_peak_5751	4.9025	
11	68985454	68986008	KD2-H4K8ac_peak_5752	4.86324	
11	69064857	69065424	KD2-H4K8ac_peak_5753	10.42398	
11	69189050	69189479	KD2-H4K8ac_peak_5754	9.47671	
11	69195390	69196014	KD2-H4K8ac_peak_5755	8.79106	
11	69257445	69258041	KD2-H4K8ac_peak_5756	9.95119	
11	69258343	69259144	KD2-H4K8ac_peak_5757	18.41623	AP000439.2_ENSG00000255606
11	69263373	69263606	KD2-H4K8ac_peak_5758	4.04573	
11	69264495	69264741	KD2-H4K8ac_peak_5759	8.53092	
11	69265020	69265286	KD2-H4K8ac_peak_5760	8.78496	
11	69449011	69449856	KD2-H4K8ac_peak_5761	14.81583	
11	69452667	69453692	KD2-H4K8ac_peak_5762	13.9234	
11	69455078	69455657	KD2-H4K8ac_peak_5763	8.02296	CCND1_ENSG00000110092
11	69456880	69457238	KD2-H4K8ac_peak_5764	15.17328	
11	69457477	69458146	KD2-H4K8ac_peak_5765	6.16103	
11	69459238	69459430	KD2-H4K8ac_peak_5766	5.9696	
11	69489637	69490027	KD2-H4K8ac_peak_5767	14.43704	ORAOV1_ENSG00000149716
11	69514075	69514278	KD2-H4K8ac_peak_5768	6.82976	
11	69518538	69519227	KD2-H4K8ac_peak_5769	10.74343	FGF19_ENSG00000162344
11	69707253	69708186	KD2-H4K8ac_peak_5770	11.92978	
11	69708510	69708724	KD2-H4K8ac_peak_5771	8.58069	
11	70048320	70049189	KD2-H4K8ac_peak_5772	11.70505	FADD_ENSG00000168040
11	70049495	70049842	KD2-H4K8ac_peak_5773	10.30284	FADD_ENSG00000168040
11	70116211	70116539	KD2-H4K8ac_peak_5774	5.13615	PPFIA1_ENSG00000131626
11	70243868	70244228	KD2-H4K8ac_peak_5775	4.68059	AP000487.5_ENSG00000246889;CTTN_ENSG00000085733
11	70244651	70244993	KD2-H4K8ac_peak_5776	12.23	AP000487.5_ENSG00000246889;CTTN_ENSG00000085733
11	70457191	70457552	KD2-H4K8ac_peak_5777	4.54838	
11	70508036	70508745	KD2-H4K8ac_peak_5778	8.83582	
11	70601480	70601769	KD2-H4K8ac_peak_5779	5.88845	
11	71158791	71159442	KD2-H4K8ac_peak_5780	9.31907	RP11-660L16.2_ENSG00000254682
11	71159723	71160253	KD2-H4K8ac_peak_5781	7.76329	RP11-660L16.2_ENSG00000254682
11	71199293	71199593	KD2-H4K8ac_peak_5782	11.81257	
11	71211441	71211868	KD2-H4K8ac_peak_5783	7.27716	
11	71476255	71476583	KD2-H4K8ac_peak_5784	16.11042	
11	71493298	71493589	KD2-H4K8ac_peak_5785	4.77079	
11	71497511	71497760	KD2-H4K8ac_peak_5786	12.3187	FAM86C1_ENSG00000158483
11	71524452	71524860	KD2-H4K8ac_peak_5787	17.55491	
11	71640152	71640361	KD2-H4K8ac_peak_5788	7.47462	RP11-849H4.2_ENSG00000254469;RNF121_ENSG00000137522
11	71749720	71749992	KD2-H4K8ac_peak_5789	5.51783	
11	71750397	71751353	KD2-H4K8ac_peak_5790	5.82799	
11	71752716	71752999	KD2-H4K8ac_peak_5791	7.92775	
11	71791993	71792554	KD2-H4K8ac_peak_5792	12.74171	NUMA1_ENSG00000137497;LRTOMT_ENSG00000184154
11	71813727	71814345	KD2-H4K8ac_peak_5793	18.94636	LAMTOR1_ENSG00000149357;snoU13_ENSG00000238768
11	71814587	71815261	KD2-H4K8ac_peak_5794	13.09607	LAMTOR1_ENSG00000149357;snoU13_ENSG00000238768

11	71823992	71824672	KD2-H4K8ac_peak_5795	9.11031	ANAPC15_ENSG00000110200
11	71934652	71935474	KD2-H4K8ac_peak_5796	20.02161	INPPL1_ENSG00000165458
11	71935792	71936273	KD2-H4K8ac_peak_5797	7.47462	
11	71948668	71949163	KD2-H4K8ac_peak_5798	7.146	
11	71950793	71951380	KD2-H4K8ac_peak_5799	16.57619	
11	71951777	71952174	KD2-H4K8ac_peak_5800	9.57101	
11	71953576	71953786	KD2-H4K8ac_peak_5801	6.35295	
11	71954033	71954407	KD2-H4K8ac_peak_5802	7.40111	
11	71954648	71954853	KD2-H4K8ac_peak_5803	6.56211	
11	71955317	71956540	KD2-H4K8ac_peak_5804	15.53798	PHOX2A_ENSG00000165462
11	72017118	72017657	KD2-H4K8ac_peak_5805	7.76329	
11	72018608	72019556	KD2-H4K8ac_peak_5806	11.60333	
11	72019972	72022245	KD2-H4K8ac_peak_5807	13.09607	
11	72022515	72022957	KD2-H4K8ac_peak_5808	7.47462	
11	72029766	72030032	KD2-H4K8ac_peak_5809	10.65724	
11	72031424	72032013	KD2-H4K8ac_peak_5810	9.34989	
11	72033395	72033655	KD2-H4K8ac_peak_5811	6.0073	
11	72035661	72036097	KD2-H4K8ac_peak_5812	7.47462	
11	72037937	72038493	KD2-H4K8ac_peak_5813	4.67342	
11	72039364	72039649	KD2-H4K8ac_peak_5814	3.7063	
11	72045255	72045926	KD2-H4K8ac_peak_5815	10.43145	
11	72046250	72046576	KD2-H4K8ac_peak_5816	6.58902	
11	72052011	72052225	KD2-H4K8ac_peak_5817	7.79741	
11	72060476	72060813	KD2-H4K8ac_peak_5818	16.09057	
11	72061185	72061486	KD2-H4K8ac_peak_5819	9.51625	RP11-45F15.1_ENSG00000256739
11	72064550	72064798	KD2-H4K8ac_peak_5820	6.81873	RP11-45F15.2_ENSG00000255672
11	72067270	72068299	KD2-H4K8ac_peak_5821	10.72656	
11	72068854	72069148	KD2-H4K8ac_peak_5822	13.31016	
11	72069375	72069781	KD2-H4K8ac_peak_5823	9.47671	
11	72071014	72071293	KD2-H4K8ac_peak_5824	5.54647	
11	72071523	72071835	KD2-H4K8ac_peak_5825	10.1591	
11	72079832	72080748	KD2-H4K8ac_peak_5826	11.30637	
11	72081652	72082418	KD2-H4K8ac_peak_5827	13.80319	
11	72083949	72084254	KD2-H4K8ac_peak_5828	9.96798	
11	72122049	72122452	KD2-H4K8ac_peak_5829	7.146	RP11-7N14.1_ENSG00000256403
11	72123209	72123768	KD2-H4K8ac_peak_5830	11.28831	
11	72145012	72145571	KD2-H4K8ac_peak_5831	12.84642	CLPB_ENSG00000162129
11	72146043	72146240	KD2-H4K8ac_peak_5832	11.05407	CLPB_ENSG00000162129
11	72182608	72183073	KD2-H4K8ac_peak_5833	9.8758	
11	72336729	72337480	KD2-H4K8ac_peak_5834	14.08365	
11	72339816	72340423	KD2-H4K8ac_peak_5835	8.58069	
11	72342625	72342836	KD2-H4K8ac_peak_5836	5.63186	
11	72343076	72343474	KD2-H4K8ac_peak_5837	8.87852	
11	72423564	72423780	KD2-H4K8ac_peak_5838	6.33462	
11	72432592	72433040	KD2-H4K8ac_peak_5839	12.65397	
11	72433460	72433932	KD2-H4K8ac_peak_5840	6.24114	

11	72463048	72463370	KD2-H4K8ac_peak_5841	5.9696	
11	72493039	72493603	KD2-H4K8ac_peak_5842	5.07021	MIR4692_ENSG00000265064
11	72503955	72504219	KD2-H4K8ac_peak_5843	4.211	ARAP1_ENSG00000186635;STARD10_ENSG00000214530
11	72505294	72505501	KD2-H4K8ac_peak_5844	5.94347	ARAP1_ENSG00000186635;STARD10_ENSG00000214530
11	72525064	72525318	KD2-H4K8ac_peak_5845	6.24114	ATG16L2_ENSG00000168010
11	72532683	72533098	KD2-H4K8ac_peak_5846	15.57998	
11	72533473	72534159	KD2-H4K8ac_peak_5847	9.6247	
11	72740761	72740986	KD2-H4K8ac_peak_5848	6.56926	
11	72852859	72853272	KD2-H4K8ac_peak_5849	5.59859	FCHSD2_ENSG00000137478
11	72854009	72854587	KD2-H4K8ac_peak_5850	8.56219	FCHSD2_ENSG00000137478
11	72929319	72929521	KD2-H4K8ac_peak_5851	8.30974	P2RY2_ENSG00000175591
11	72953444	72954377	KD2-H4K8ac_peak_5852	8.85813	
11	72983321	72984007	KD2-H4K8ac_peak_5853	11.8457	
11	72998650	72999016	KD2-H4K8ac_peak_5854	5.88845	
11	73000356	73000608	KD2-H4K8ac_peak_5855	6.20982	
11	73018082	73018389	KD2-H4K8ac_peak_5856	10.54933	ARHGEF17_ENSG00000110237
11	73019007	73020290	KD2-H4K8ac_peak_5857	7.07117	RP11-800A3.7_ENSG00000257038;ARHGEF17_ENSG00000110237
11	73024207	73024420	KD2-H4K8ac_peak_5858	8.66368	
11	73032155	73032637	KD2-H4K8ac_peak_5859	6.48242	
11	73033337	73033748	KD2-H4K8ac_peak_5860	7.76329	
11	73034400	73034956	KD2-H4K8ac_peak_5861	10.46874	
11	73042134	73042424	KD2-H4K8ac_peak_5862	5.88845	
11	73044631	73044922	KD2-H4K8ac_peak_5863	4.85384	
11	73045754	73046397	KD2-H4K8ac_peak_5864	5.30799	
11	73086782	73087163	KD2-H4K8ac_peak_5865	5.9696	RELT_ENSG00000054967
11	73310362	73310735	KD2-H4K8ac_peak_5866	8.4084	
11	73358693	73358962	KD2-H4K8ac_peak_5867	5.61067	
11	73471427	73472097	KD2-H4K8ac_peak_5868	17.16047	RAB6A_ENSG00000175582;RP11-707G14.1_ENSG00000256034
11	73472620	73473035	KD2-H4K8ac_peak_5869	7.67504	RAB6A_ENSG00000175582
11	73490279	73490501	KD2-H4K8ac_peak_5870	7.27716	
11	73491144	73491613	KD2-H4K8ac_peak_5871	10.21502	
11	73578523	73578727	KD2-H4K8ac_peak_5872	7.18916	
11	73587455	73587848	KD2-H4K8ac_peak_5873	9.63487	COA4_ENSG00000181924;PAAF1_ENSG00000175575
11	73693210	73693474	KD2-H4K8ac_peak_5874	8.23274	UCP2_ENSG00000175567
11	73694557	73695079	KD2-H4K8ac_peak_5875	10.43145	UCP2_ENSG00000175567
11	73881519	73881897	KD2-H4K8ac_peak_5876	11.1952	C2CD3_ENSG00000168014;PPME1_ENSG00000214517
11	74109683	74110426	KD2-H4K8ac_peak_5877	10.19949	PGM2L1_ENSG00000165434;MIR548AL_ENSG00000264402
11	74114692	74114910	KD2-H4K8ac_peak_5878	6.94255	
11	74204414	74205018	KD2-H4K8ac_peak_5879	9.57101	LIPT2_ENSG00000175536;AP001372.2_ENSG00000254837;POLD3_ENSG00000077514
11	74383633	74384040	KD2-H4K8ac_peak_5880	6.83936	
11	74384546	74384780	KD2-H4K8ac_peak_5881	6.66692	
11	74394611	74394804	KD2-H4K8ac_peak_5882	5.61067	
11	74459216	74459541	KD2-H4K8ac_peak_5883	5.54647	RNF169_ENSG00000166439
11	74460018	74460358	KD2-H4K8ac_peak_5884	11.92978	RNF169_ENSG00000166439
11	74659893	74660088	KD2-H4K8ac_peak_5885	10.43145	XRRA1_ENSG00000166435;SPCS2_ENSG00000118363
11	74660375	74660578	KD2-H4K8ac_peak_5886	6.25869	XRRA1_ENSG00000166435;SPCS2_ENSG00000118363

11	74699266	74699460	KD2-H4K8ac_peak_5887	8.17902	NEU3_ENSG00000162139
11	74953024	74953235	KD2-H4K8ac_peak_5888	4.35724	CTD-2562J17.4_ENSG00000255136
11	75012638	75013269	KD2-H4K8ac_peak_5889	8.59579	
11	75013601	75013989	KD2-H4K8ac_peak_5890	8.1718	
11	75014355	75014904	KD2-H4K8ac_peak_5891	5.54647	
11	75022822	75023472	KD2-H4K8ac_peak_5892	10.87093	
11	75050124	75050349	KD2-H4K8ac_peak_5893	8.23796	
11	75050853	75052044	KD2-H4K8ac_peak_5894	11.50346	
11	75062349	75063293	KD2-H4K8ac_peak_5895	11.83301	ARRB1_ENSG00000137486
11	75095432	75095678	KD2-H4K8ac_peak_5896	8.17902	
11	75110054	75110282	KD2-H4K8ac_peak_5897	6.92624	RPS3_ENSG00000149273
11	75110618	75110856	KD2-H4K8ac_peak_5898	12.82393	RPS3_ENSG00000149273;SNORD15A_ENSG00000206941
11	75138762	75138981	KD2-H4K8ac_peak_5899	7.31433	
11	75140727	75141809	KD2-H4K8ac_peak_5900	12.82393	KLHL35_ENSG00000149243
11	75181953	75182174	KD2-H4K8ac_peak_5901	6.56211	
11	75236445	75236876	KD2-H4K8ac_peak_5902	7.36799	GDPD5_ENSG00000158555
11	75251255	75251618	KD2-H4K8ac_peak_5903	4.67342	
11	75258865	75259201	KD2-H4K8ac_peak_5904	7.27716	
11	75264839	75265330	KD2-H4K8ac_peak_5905	6.99815	
11	75265856	75266669	KD2-H4K8ac_peak_5906	13.3695	
11	75268535	75268772	KD2-H4K8ac_peak_5907	6.25869	
11	75272648	75274701	KD2-H4K8ac_peak_5908	16.31471	SERPINH1_ENSG00000149257
11	75293372	75293655	KD2-H4K8ac_peak_5909	6.25869	CTD-2530H12.4_ENSG00000255326
11	75293904	75295530	KD2-H4K8ac_peak_5910	29.65657	CTD-2530H12.4_ENSG00000255326
11	75339010	75339651	KD2-H4K8ac_peak_5911	14.84786	
11	75378445	75379030	KD2-H4K8ac_peak_5912	8.55271	
11	75379429	75379648	KD2-H4K8ac_peak_5913	6.83936	MAP6_ENSG00000171533
11	75479213	75480209	KD2-H4K8ac_peak_5914	10.46874	CTD-2530H12.1_ENSG00000247867
11	75526403	75526629	KD2-H4K8ac_peak_5915	4.54006	RP11-535A19.2_ENSG00000255507;UVRAG_ENSG00000198382
11	75880732	75880925	KD2-H4K8ac_peak_5916	6.50927	
11	75891795	75892149	KD2-H4K8ac_peak_5917	4.77079	
11	75896920	75897306	KD2-H4K8ac_peak_5918	5.14125	
11	75945676	75946092	KD2-H4K8ac_peak_5919	9.1707	
11	76091619	76092108	KD2-H4K8ac_peak_5920	6.40297	PRKRIR_ENSG00000137492;RP11-111M22.2_ENSG00000179240
11	76155281	76155527	KD2-H4K8ac_peak_5921	9.8758	RP11-111M22.3_ENSG00000255135;C11orf30_ENSG00000158636
11	76156151	76156362	KD2-H4K8ac_peak_5922	7.36799	RP11-111M22.3_ENSG00000255135;C11orf30_ENSG00000158636
11	76314773	76315227	KD2-H4K8ac_peak_5923	7.76329	
11	76315425	76316042	KD2-H4K8ac_peak_5924	8.59579	
11	76325185	76325444	KD2-H4K8ac_peak_5925	4.66652	
11	76327024	76327328	KD2-H4K8ac_peak_5926	10.08707	
11	76382008	76382318	KD2-H4K8ac_peak_5927	8.85813	LRRC32_ENSG00000137507
11	76432682	76432945	KD2-H4K8ac_peak_5928	10.05606	
11	76493333	76493543	KD2-H4K8ac_peak_5929	12.84642	TSKU_ENSG00000182704;RP11-21L23.3_ENSG00000255100
11	76494367	76494861	KD2-H4K8ac_peak_5930	11.24702	RP11-21L23.3_ENSG00000255100
11	76509931	76510235	KD2-H4K8ac_peak_5931	4.06758	
11	76750646	76750910	KD2-H4K8ac_peak_5932	7.96322	

11	76776945	76777268	KD2-H4K8ac_peak_5933	6.05809	CAPN5_ENSG00000149260
11	76782659	76783163	KD2-H4K8ac_peak_5934	7.64093	
11	76784628	76785881	KD2-H4K8ac_peak_5935	6.0605	
11	76792647	76793533	KD2-H4K8ac_peak_5936	6.67092	
11	76838014	76838755	KD2-H4K8ac_peak_5937	10.46264	MYO7A_ENSG00000137474
11	76839329	76839800	KD2-H4K8ac_peak_5938	7.99241	MYO7A_ENSG00000137474
11	76851303	76851518	KD2-H4K8ac_peak_5939	5.14125	
11	77184107	77184741	KD2-H4K8ac_peak_5940	19.72117	PAK1_ENSG00000149269;DKFZP434E1119_ENSG00000268635
11	77184937	77185584	KD2-H4K8ac_peak_5941	9.65086	PAK1_ENSG00000149269;DKFZP434E1119_ENSG00000268635
11	77299825	77300035	KD2-H4K8ac_peak_5942	7.13615	AQP11_ENSG00000178301
11	77300294	77301321	KD2-H4K8ac_peak_5943	13.44374	AQP11_ENSG00000178301
11	77348423	77349070	KD2-H4K8ac_peak_5944	16.09057	CLNS1A_ENSG00000074201
11	77349587	77349990	KD2-H4K8ac_peak_5945	6.30229	CLNS1A_ENSG00000074201
11	77530882	77531708	KD2-H4K8ac_peak_5946	12.93209	RSF1_ENSG00000048649;AAMDC_ENSG00000087884
11	77574192	77574459	KD2-H4K8ac_peak_5947	5.55317	
11	77742358	77742761	KD2-H4K8ac_peak_5948	7.47462	
11	77750421	77750995	KD2-H4K8ac_peak_5949	6.25869	
11	77751593	77751904	KD2-H4K8ac_peak_5950	4.49837	
11	77790532	77791144	KD2-H4K8ac_peak_5951	15.57998	NDUFC2-KCTD14_ENSG00000259112;NDUFC2_ENSG00000151366
11	77850079	77850571	KD2-H4K8ac_peak_5952	6.99815	ALG8_ENSG00000159063;KCTD21-AS1_ENSG00000246174
11	77872591	77872984	KD2-H4K8ac_peak_5953	6.66692	
11	77873315	77873980	KD2-H4K8ac_peak_5954	10.21502	
11	77879823	77880110	KD2-H4K8ac_peak_5955	5.9696	
11	77899421	77899638	KD2-H4K8ac_peak_5956	8.88082	KCTD21_ENSG00000188997;USP35_ENSG00000118369
11	78031086	78031456	KD2-H4K8ac_peak_5957	4.86324	
11	78064112	78064424	KD2-H4K8ac_peak_5958	6.0073	
11	78129117	78129482	KD2-H4K8ac_peak_5959	6.20982	GAB2_ENSG00000033327
11	78130316	78130786	KD2-H4K8ac_peak_5960	5.20998	GAB2_ENSG00000033327
11	78131095	78131459	KD2-H4K8ac_peak_5961	17.83665	
11	78132448	78132919	KD2-H4K8ac_peak_5962	13.02323	
11	78137068	78137737	KD2-H4K8ac_peak_5963	7.40633	
11	78440910	78441454	KD2-H4K8ac_peak_5964	4.42765	
11	78445655	78445907	KD2-H4K8ac_peak_5965	5.57044	
11	78447017	78447281	KD2-H4K8ac_peak_5966	8.30974	
11	78450730	78451272	KD2-H4K8ac_peak_5967	17.42905	
11	78452277	78453058	KD2-H4K8ac_peak_5968	11.60333	
11	78453744	78454053	KD2-H4K8ac_peak_5969	9.16531	
11	78454264	78454479	KD2-H4K8ac_peak_5970	6.50882	
11	78458015	78458220	KD2-H4K8ac_peak_5971	4.0809	
11	78458419	78458624	KD2-H4K8ac_peak_5972	5.34636	
11	78461610	78461835	KD2-H4K8ac_peak_5973	6.25869	
11	78483468	78483726	KD2-H4K8ac_peak_5974	7.12754	
11	78496187	78496380	KD2-H4K8ac_peak_5975	5.57044	
11	78521408	78521633	KD2-H4K8ac_peak_5976	5.88562	
11	78541325	78541619	KD2-H4K8ac_peak_5977	4.98318	
11	78563523	78564110	KD2-H4K8ac_peak_5978	6.65673	

11	78565034	78565422	KD2-H4K8ac_peak_5979	7.27716	
11	78567683	78568418	KD2-H4K8ac_peak_5980	5.32137	
11	78569189	78569574	KD2-H4K8ac_peak_5981	4.66652	
11	78572498	78573020	KD2-H4K8ac_peak_5982	4.77079	
11	78577659	78577892	KD2-H4K8ac_peak_5983	8.1233	
11	78642763	78643012	KD2-H4K8ac_peak_5984	8.02571	
11	78661152	78661485	KD2-H4K8ac_peak_5985	7.29632	
11	78661859	78662149	KD2-H4K8ac_peak_5986	6.51023	
11	78692939	78693578	KD2-H4K8ac_peak_5987	10.46264	
11	78693933	78694142	KD2-H4K8ac_peak_5988	10.19949	
11	78694540	78694778	KD2-H4K8ac_peak_5989	9.16605	
11	78699700	78700016	KD2-H4K8ac_peak_5990	12.60538	
11	78728150	78728468	KD2-H4K8ac_peak_5991	6.98517	
11	78728854	78729140	KD2-H4K8ac_peak_5992	7.47462	
11	78774049	78774330	KD2-H4K8ac_peak_5993	5.75757	
11	78776803	78777130	KD2-H4K8ac_peak_5994	5.13893	
11	78777743	78778421	KD2-H4K8ac_peak_5995	9.1707	
11	78779300	78779539	KD2-H4K8ac_peak_5996	4.42765	
11	78783239	78783528	KD2-H4K8ac_peak_5997	10.81728	
11	78785569	78785951	KD2-H4K8ac_peak_5998	5.88562	
11	78788212	78788424	KD2-H4K8ac_peak_5999	10.08707	
11	78788991	78789289	KD2-H4K8ac_peak_6000	5.54647	
11	78790118	78790662	KD2-H4K8ac_peak_6001	12.99417	
11	78790969	78791702	KD2-H4K8ac_peak_6002	9.36516	
11	78798978	78799250	KD2-H4K8ac_peak_6003	5.20998	
11	78801800	78801999	KD2-H4K8ac_peak_6004	4.75875	
11	78816449	78816684	KD2-H4K8ac_peak_6005	6.40683	
11	78821281	78821479	KD2-H4K8ac_peak_6006	7.27716	
11	78831765	78832186	KD2-H4K8ac_peak_6007	6.66692	
11	78832495	78832804	KD2-H4K8ac_peak_6008	8.01894	
11	79149520	79149838	KD2-H4K8ac_peak_6009	7.76329	
11	79151428	79151975	KD2-H4K8ac_peak_6010	5.01002	TENM4_ENSG00000149256
11	82444091	82444291	KD2-H4K8ac_peak_6011	8.5092	FAM181B_ENSG00000182103
11	82444503	82445228	KD2-H4K8ac_peak_6012	9.1707	FAM181B_ENSG00000182103
11	82501681	82501939	KD2-H4K8ac_peak_6013	4.9025	RP11-718B12.2_ENSG00000255382
11	82503730	82503928	KD2-H4K8ac_peak_6014	7.27716	
11	82719160	82719367	KD2-H4K8ac_peak_6015	9.59147	
11	82783178	82783427	KD2-H4K8ac_peak_6016	7.49599	RAB30_ENSG00000137502;RAB30-AS1_ENSG00000246067
11	82866671	82867020	KD2-H4K8ac_peak_6017	3.75035	
11	82903903	82904208	KD2-H4K8ac_peak_6018	5.25071	RP11-727A23.5_ENSG00000247137;ANKRD42_ENSG00000137494
11	82904405	82904606	KD2-H4K8ac_peak_6019	12.42761	RP11-727A23.5_ENSG00000247137;ANKRD42_ENSG00000137494
11	82996773	82996979	KD2-H4K8ac_peak_6020	9.41619	CCDC90B_ENSG00000137500;RP11-727A23.10_ENSG00000255234
11	84032908	84033161	KD2-H4K8ac_peak_6021	4.37708	
11	85566233	85566770	KD2-H4K8ac_peak_6022	12.57297	AP000974.1_ENSG00000215504;CCDC83_ENSG00000150676
11	85779339	85779543	KD2-H4K8ac_peak_6023	6.09229	
11	85780233	85780848	KD2-H4K8ac_peak_6024	10.29344	PICALM_ENSG00000073921

11	85955617	85956025	KD2-H4K8ac_peak_6025	9.36516	EED_ENSG00000074266
11	86013339	86013774	KD2-H4K8ac_peak_6026	12.30509	C11orf73_ENSG00000149196
11	86382801	86383106	KD2-H4K8ac_peak_6027	7.76329	ME3_ENSG00000151376
11	86383359	86383756	KD2-H4K8ac_peak_6028	8.37231	ME3_ENSG00000151376
11	86510793	86511880	KD2-H4K8ac_peak_6029	17.17257	
11	86666045	86666304	KD2-H4K8ac_peak_6030	3.90565	FZD4_ENSG00000174804;RP11-736K20.6_ENSG00000246523
11	86748259	86748794	KD2-H4K8ac_peak_6031	7.76329	TMEM135_ENSG00000166575
11	86749005	86749457	KD2-H4K8ac_peak_6032	13.09607	TMEM135_ENSG00000166575
11	88070548	88070861	KD2-H4K8ac_peak_6033	4.211	CTSC_ENSG00000109861
11	89224362	89225162	KD2-H4K8ac_peak_6034	13.09607	
11	89867563	89867936	KD2-H4K8ac_peak_6035	6.66692	
11	91665266	91665505	KD2-H4K8ac_peak_6036	6.9809	
11	91959670	91960205	KD2-H4K8ac_peak_6037	5.54647	
11	92504156	92504390	KD2-H4K8ac_peak_6038	5.14125	
11	92562229	92562430	KD2-H4K8ac_peak_6039	8.74596	
11	92584867	92585177	KD2-H4K8ac_peak_6040	7.01964	
11	92712841	92713205	KD2-H4K8ac_peak_6041	5.57044	
11	92930752	92931005	KD2-H4K8ac_peak_6042	14.43794	SLC36A4_ENSG00000180773
11	93063622	93064131	KD2-H4K8ac_peak_6043	7.11824	CCDC67_ENSG00000165325
11	93394314	93394630	KD2-H4K8ac_peak_6044	6.76298	KIAA1731_ENSG00000166004
11	93394877	93395078	KD2-H4K8ac_peak_6045	12.66412	KIAA1731_ENSG00000166004
11	93474100	93474472	KD2-H4K8ac_peak_6046	9.89126	C11orf54_ENSG00000182919
11	93517545	93518056	KD2-H4K8ac_peak_6047	14.8083	TAF1D_ENSG00000166012;MED17_ENSG00000042429
11	93582854	93583766	KD2-H4K8ac_peak_6048	11.92978	VSTM5_ENSG00000214376
11	93861541	93861810	KD2-H4K8ac_peak_6049	12.88108	PANX1_ENSG00000110218
11	93862083	93863170	KD2-H4K8ac_peak_6050	20.58734	PANX1_ENSG00000110218
11	93875196	93875420	KD2-H4K8ac_peak_6051	3.75035	
11	94276874	94277766	KD2-H4K8ac_peak_6052	11.24702	PIWIL4_ENSG00000134627;FUT4_ENSG00000196371
11	94472711	94472941	KD2-H4K8ac_peak_6053	3.75035	RP11-867G2.8_ENSG00000255929
11	94473349	94474011	KD2-H4K8ac_peak_6054	9.1707	RP11-867G2.8_ENSG00000255929
11	94474224	94474432	KD2-H4K8ac_peak_6055	4.211	RP11-867G2.8_ENSG00000255929
11	94500617	94501156	KD2-H4K8ac_peak_6056	7.35686	
11	94501690	94502236	KD2-H4K8ac_peak_6057	10.96455	
11	94657955	94658344	KD2-H4K8ac_peak_6058	6.15357	RP11-856F16.2_ENSG00000256469
11	94667514	94667798	KD2-H4K8ac_peak_6059	7.40111	
11	94668020	94668447	KD2-H4K8ac_peak_6060	5.73839	
11	94800113	94801251	KD2-H4K8ac_peak_6061	11.89502	SRSF8_ENSG00000180771;SRSF8_ENSG00000271885
11	94822601	94822889	KD2-H4K8ac_peak_6062	6.65673	ENDOD1_ENSG00000149218
11	94965289	94965687	KD2-H4K8ac_peak_6063	6.25869	SESN3_ENSG00000149212
11	94986471	94986940	KD2-H4K8ac_peak_6064	6.16888	
11	95522322	95522722	KD2-H4K8ac_peak_6065	11.92978	FAM76B_ENSG00000077458;CEP57_ENSG00000166037
11	95523637	95524048	KD2-H4K8ac_peak_6066	15.22643	FAM76B_ENSG00000077458;CEP57_ENSG00000166037
11	95656725	95657661	KD2-H4K8ac_peak_6067	11.92978	MTMR2_ENSG00000087053
11	95976639	95977237	KD2-H4K8ac_peak_6068	14.74047	
11	96179990	96180381	KD2-H4K8ac_peak_6069	4.42765	
11	96552732	96553018	KD2-H4K8ac_peak_6070	5.25071	

11	97617061	97617256	KD2-H4K8ac_peak_6071	5.71749	
11	101785830	1.02E+08	KD2-H4K8ac_peak_6072	9.15329	ANGPTL5_ENSG00000187151;KIAA1377_ENSG00000110318
11	102188071	1.02E+08	KD2-H4K8ac_peak_6073	5.88845	BIRC3_ENSG00000023445
11	102217518	1.02E+08	KD2-H4K8ac_peak_6074	7.36799	BIRC2_ENSG00000110330
11	102218065	1.02E+08	KD2-H4K8ac_peak_6075	6.66692	BIRC2_ENSG00000110330
11	102322981	1.02E+08	KD2-H4K8ac_peak_6076	14.8083	RP11-315O6.1_ENSG00000255337
11	102962437	1.03E+08	KD2-H4K8ac_peak_6077	7.40111	DCUN1D5_ENSG00000137692
11	102963071	1.03E+08	KD2-H4K8ac_peak_6078	8.1718	DCUN1D5_ENSG00000137692
11	103149388	1.03E+08	KD2-H4K8ac_peak_6079	4.01115	
11	103401744	1.03E+08	KD2-H4K8ac_peak_6080	7.12754	
11	103406849	1.03E+08	KD2-H4K8ac_peak_6081	6.36375	
11	103408264	1.03E+08	KD2-H4K8ac_peak_6082	7.12754	
11	104034636	1.04E+08	KD2-H4K8ac_peak_6083	6.66692	PDGFD_ENSG00000170962
11	104035503	1.04E+08	KD2-H4K8ac_peak_6084	5.57044	PDGFD_ENSG00000170962
11	105481047	1.05E+08	KD2-H4K8ac_peak_6085	9.73431	GRIA4_ENSG00000152578
11	105947471	1.06E+08	KD2-H4K8ac_peak_6086	4.211	KBTBD3_ENSG00000182359
11	107328218	1.07E+08	KD2-H4K8ac_peak_6087	10.86378	CWF19L2_ENSG00000152404
11	107436196	1.07E+08	KD2-H4K8ac_peak_6088	6.25869	ALKBH8_ENSG00000137760
11	107798472	1.08E+08	KD2-H4K8ac_peak_6089	5.54647	SLC35F2_ENSG00000110660;RAB39A_ENSG00000179331
11	107826894	1.08E+08	KD2-H4K8ac_peak_6090	4.44748	
11	107828435	1.08E+08	KD2-H4K8ac_peak_6091	5.54647	
11	107859220	1.08E+08	KD2-H4K8ac_peak_6092	7.42204	
11	107879729	1.08E+08	KD2-H4K8ac_peak_6093	16.49944	CUL5_ENSG00000166266
11	107992355	1.08E+08	KD2-H4K8ac_peak_6094	10.87093	RP11-144G7.2_ENSG00000255467;ACAT1_ENSG00000075239
11	108093756	1.08E+08	KD2-H4K8ac_peak_6095	9.15329	NPAT_ENSG00000149308;ATM_ENSG00000149311
11	108336888	1.08E+08	KD2-H4K8ac_peak_6096	7.27716	
11	108337714	1.08E+08	KD2-H4K8ac_peak_6097	12.88108	C11orf65_ENSG00000166323
11	108368777	1.08E+08	KD2-H4K8ac_peak_6098	8.53092	KDELC2_ENSG00000178202
11	108535014	1.09E+08	KD2-H4K8ac_peak_6099	5.14125	DDX10_ENSG00000178105
11	108536024	1.09E+08	KD2-H4K8ac_peak_6100	8.1233	DDX10_ENSG00000178105
11	108837999	1.09E+08	KD2-H4K8ac_peak_6101	8.97719	
11	108856596	1.09E+08	KD2-H4K8ac_peak_6102	8.30974	
11	109377217	1.09E+08	KD2-H4K8ac_peak_6103	4.77079	
11	109963690	1.1E+08	KD2-H4K8ac_peak_6104	15.58442	ZC3H12C_ENSG00000149289
11	110079915	1.1E+08	KD2-H4K8ac_peak_6105	5.14125	
11	110166855	1.1E+08	KD2-H4K8ac_peak_6106	7.76104	RDX_ENSG00000137710
11	110268826	1.1E+08	KD2-H4K8ac_peak_6107	9.1707	
11	110300435	1.1E+08	KD2-H4K8ac_peak_6108	6.84261	FDX1_ENSG00000137714
11	111170181	1.11E+08	KD2-H4K8ac_peak_6109	8.1233	COLCA2_ENSG00000214290
11	111307378	1.11E+08	KD2-H4K8ac_peak_6110	7.40111	
11	111383271	1.11E+08	KD2-H4K8ac_peak_6111	8.02571	BTG4_ENSG00000137707;MIR34B_ENSG00000207811;MIR34C_ENSG00000207562
11	111473257	1.11E+08	KD2-H4K8ac_peak_6112	3.83717	SIK2_ENSG00000170145
11	111504443	1.12E+08	KD2-H4K8ac_peak_6113	7.51935	
11	111601834	1.12E+08	KD2-H4K8ac_peak_6114	5.57044	
11	111604712	1.12E+08	KD2-H4K8ac_peak_6115	6.56926	
11	111605182	1.12E+08	KD2-H4K8ac_peak_6116	7.47462	

11	111665108	1.12E+08	KD2-H4K8ac_peak_6117	6.24114	
11	111668578	1.12E+08	KD2-H4K8ac_peak_6118	4.77079	
11	111741627	1.12E+08	KD2-H4K8ac_peak_6119	10.19949	ALG9_ENSG00000086848
11	111749430	1.12E+08	KD2-H4K8ac_peak_6120	4.42765	ALG9_ENSG00000258529;C11orf1_ENSG00000137720
11	111749966	1.12E+08	KD2-H4K8ac_peak_6121	15.53798	ALG9_ENSG00000258529;C11orf1_ENSG00000137720
11	111797197	1.12E+08	KD2-H4K8ac_peak_6122	13.09607	DIXDC1_ENSG00000150764
11	111895518	1.12E+08	KD2-H4K8ac_peak_6123	10.73134	DLAT_ENSG00000150768
11	111944148	1.12E+08	KD2-H4K8ac_peak_6124	9.15329	PIH1D2_ENSG00000150773;C11orf57_ENSG00000150776
11	111945077	1.12E+08	KD2-H4K8ac_peak_6125	8.85813	PIH1D2_ENSG00000150773;C11orf57_ENSG00000150776
11	111957074	1.12E+08	KD2-H4K8ac_peak_6126	12.23	TIMM8B_ENSG00000150779;SDHD_ENSG00000204370;SDHD_ENSG00000255292
11	111957735	1.12E+08	KD2-H4K8ac_peak_6127	4.54006	TIMM8B_ENSG00000150779;SDHD_ENSG00000204370;SDHD_ENSG00000255292
11	112096340	1.12E+08	KD2-H4K8ac_peak_6128	20.96279	PTS_ENSG00000150787
11	112135418	1.12E+08	KD2-H4K8ac_peak_6129	5.71749	
11	112647765	1.13E+08	KD2-H4K8ac_peak_6130	12.42761	
11	112648878	1.13E+08	KD2-H4K8ac_peak_6131	7.79898	
11	112655567	1.13E+08	KD2-H4K8ac_peak_6132	6.25869	
11	112704920	1.13E+08	KD2-H4K8ac_peak_6133	8.85813	
11	112705639	1.13E+08	KD2-H4K8ac_peak_6134	13.34404	
11	112706721	1.13E+08	KD2-H4K8ac_peak_6135	10.46264	
11	112708100	1.13E+08	KD2-H4K8ac_peak_6136	8.4939	
11	112752510	1.13E+08	KD2-H4K8ac_peak_6137	5.88845	
11	112832551	1.13E+08	KD2-H4K8ac_peak_6138	5.54647	NCAM1_ENSG00000149294
11	112833452	1.13E+08	KD2-H4K8ac_peak_6139	7.70289	RP11-629G13.1_ENSG00000247416
11	112834225	1.13E+08	KD2-H4K8ac_peak_6140	7.47462	RP11-629G13.1_ENSG00000247416
11	113009476	1.13E+08	KD2-H4K8ac_peak_6141	3.76318	
11	113071150	1.13E+08	KD2-H4K8ac_peak_6142	5.71749	
11	113072482	1.13E+08	KD2-H4K8ac_peak_6143	9.82729	
11	113073895	1.13E+08	KD2-H4K8ac_peak_6144	7.44366	
11	113144096	1.13E+08	KD2-H4K8ac_peak_6145	5.14125	NCAM1-AS1_ENSG00000227487
11	113185463	1.13E+08	KD2-H4K8ac_peak_6146	5.71749	RP11-839D17.3_ENSG00000255129;TTC12_ENSG00000149292
11	113308357	1.13E+08	KD2-H4K8ac_peak_6147	7.32463	
11	113313707	1.13E+08	KD2-H4K8ac_peak_6148	6.25014	
11	113316662	1.13E+08	KD2-H4K8ac_peak_6149	5.61069	
11	113420482	1.13E+08	KD2-H4K8ac_peak_6150	9.1707	
11	113424859	1.13E+08	KD2-H4K8ac_peak_6151	6.56926	
11	113447048	1.13E+08	KD2-H4K8ac_peak_6152	4.42765	
11	113448015	1.13E+08	KD2-H4K8ac_peak_6153	9.36516	
11	113448934	1.13E+08	KD2-H4K8ac_peak_6154	10.0692	
11	113450102	1.13E+08	KD2-H4K8ac_peak_6155	5.88845	
11	113459969	1.13E+08	KD2-H4K8ac_peak_6156	7.79558	
11	113460611	1.13E+08	KD2-H4K8ac_peak_6157	5.73839	
11	113462566	1.13E+08	KD2-H4K8ac_peak_6158	7.0983	
11	113644033	1.14E+08	KD2-H4K8ac_peak_6159	12.3187	ZW10_ENSG00000086827
11	113745948	1.14E+08	KD2-H4K8ac_peak_6160	8.5092	USP28_ENSG00000048028
11	113845859	1.14E+08	KD2-H4K8ac_peak_6161	4.77079	HTR3A_ENSG00000166736
11	114099165	1.14E+08	KD2-H4K8ac_peak_6162	8.1233	

11	114151650	1.14E+08	KD2-H4K8ac_peak_6163	12.82393	
11	114270720	1.14E+08	KD2-H4K8ac_peak_6164	6.84261	C11orf71_ENSG00000180425;RBM7_ENSG00000076053;RP11-212D19.4_ENSG00000255663
11	114271396	1.14E+08	KD2-H4K8ac_peak_6165	10.63441	C11orf71_ENSG00000180425;RBM7_ENSG00000076053;RP11-212D19.4_ENSG00000255663
11	114645841	1.15E+08	KD2-H4K8ac_peak_6166	6.40683	
11	114954387	1.15E+08	KD2-H4K8ac_peak_6167	4.06758	
11	114999149	1.15E+08	KD2-H4K8ac_peak_6168	4.77079	ACA59_ENSG00000252870
11	115071277	1.15E+08	KD2-H4K8ac_peak_6169	7.22457	
11	115374033	1.15E+08	KD2-H4K8ac_peak_6170	6.45083	
11	115447685	1.15E+08	KD2-H4K8ac_peak_6171	8.09303	RP11-136I14.4_ENSG00000255903
11	115450604	1.15E+08	KD2-H4K8ac_peak_6172	9.31907	
11	115451379	1.15E+08	KD2-H4K8ac_peak_6173	5.405	
11	115452546	1.15E+08	KD2-H4K8ac_peak_6174	7.96322	RP11-136I14.5_ENSG00000255689
11	115453352	1.15E+08	KD2-H4K8ac_peak_6175	7.27716	RP11-136I14.5_ENSG00000255689
11	115476674	1.15E+08	KD2-H4K8ac_peak_6176	5.14125	
11	115478445	1.15E+08	KD2-H4K8ac_peak_6177	5.9696	
11	115513600	1.16E+08	KD2-H4K8ac_peak_6178	4.59912	
11	115516942	1.16E+08	KD2-H4K8ac_peak_6179	8.26945	
11	115529795	1.16E+08	KD2-H4K8ac_peak_6180	9.36516	AP000797.3_ENSG00000256717
11	115613290	1.16E+08	KD2-H4K8ac_peak_6181	6.16747	
11	115614469	1.16E+08	KD2-H4K8ac_peak_6182	8.30974	
11	115615868	1.16E+08	KD2-H4K8ac_peak_6183	9.07715	
11	115616777	1.16E+08	KD2-H4K8ac_peak_6184	11.89502	
11	115619491	1.16E+08	KD2-H4K8ac_peak_6185	10.47747	
11	115630346	1.16E+08	KD2-H4K8ac_peak_6186	6.99815	LINC00900_ENSG00000246100
11	115655805	1.16E+08	KD2-H4K8ac_peak_6187	8.21084	
11	115830098	1.16E+08	KD2-H4K8ac_peak_6188	4.01115	
11	115846232	1.16E+08	KD2-H4K8ac_peak_6189	4.67342	
11	115931349	1.16E+08	KD2-H4K8ac_peak_6190	7.10552	
11	115932328	1.16E+08	KD2-H4K8ac_peak_6191	9.95119	
11	115935491	1.16E+08	KD2-H4K8ac_peak_6192	7.36799	
11	115944657	1.16E+08	KD2-H4K8ac_peak_6193	5.29555	
11	115948597	1.16E+08	KD2-H4K8ac_peak_6194	7.62364	
11	116044763	1.16E+08	KD2-H4K8ac_peak_6195	5.21139	
11	116048282	1.16E+08	KD2-H4K8ac_peak_6196	6.09229	
11	116051116	1.16E+08	KD2-H4K8ac_peak_6197	7.47462	
11	116051725	1.16E+08	KD2-H4K8ac_peak_6198	12.57143	
11	116063694	1.16E+08	KD2-H4K8ac_peak_6199	7.20567	
11	116064139	1.16E+08	KD2-H4K8ac_peak_6200	6.50927	
11	116069745	1.16E+08	KD2-H4K8ac_peak_6201	7.45501	
11	116078453	1.16E+08	KD2-H4K8ac_peak_6202	10.65724	
11	116095326	1.16E+08	KD2-H4K8ac_peak_6203	7.71478	
11	116095876	1.16E+08	KD2-H4K8ac_peak_6204	5.73839	
11	116147567	1.16E+08	KD2-H4K8ac_peak_6205	5.01002	
11	116182209	1.16E+08	KD2-H4K8ac_peak_6206	7.08474	
11	116183054	1.16E+08	KD2-H4K8ac_peak_6207	10.0692	
11	116183521	1.16E+08	KD2-H4K8ac_peak_6208	8.17902	

11	116226299	1.16E+08	KD2-H4K8ac_peak_6209	4.42765	
11	116243060	1.16E+08	KD2-H4K8ac_peak_6210	8.16133	
11	116253231	1.16E+08	KD2-H4K8ac_peak_6211	5.25269	
11	116255603	1.16E+08	KD2-H4K8ac_peak_6212	13.73635	
11	116256499	1.16E+08	KD2-H4K8ac_peak_6213	8.53092	
11	116284536	1.16E+08	KD2-H4K8ac_peak_6214	6.99815	
11	116287993	1.16E+08	KD2-H4K8ac_peak_6215	8.02571	
11	116295468	1.16E+08	KD2-H4K8ac_peak_6216	6.04107	
11	116299276	1.16E+08	KD2-H4K8ac_peak_6217	7.73264	
11	116333017	1.16E+08	KD2-H4K8ac_peak_6218	4.60576	
11	116369583	1.16E+08	KD2-H4K8ac_peak_6219	7.76104	
11	116370767	1.16E+08	KD2-H4K8ac_peak_6220	16.54484	AP001891.1_ENSG00000236437
11	116373393	1.16E+08	KD2-H4K8ac_peak_6221	8.79106	
11	116374218	1.16E+08	KD2-H4K8ac_peak_6222	7.20567	
11	116375502	1.16E+08	KD2-H4K8ac_peak_6223	13.95627	
11	116377136	1.16E+08	KD2-H4K8ac_peak_6224	15.61313	
11	116411450	1.16E+08	KD2-H4K8ac_peak_6225	6.00243	
11	116427827	1.16E+08	KD2-H4K8ac_peak_6226	7.95563	
11	116437882	1.16E+08	KD2-H4K8ac_peak_6227	3.72305	
11	116439712	1.16E+08	KD2-H4K8ac_peak_6228	7.79898	
11	116450162	1.16E+08	KD2-H4K8ac_peak_6229	12.99417	
11	116511706	1.17E+08	KD2-H4K8ac_peak_6230	7.36799	
11	116561919	1.17E+08	KD2-H4K8ac_peak_6231	6.69844	
11	116573403	1.17E+08	KD2-H4K8ac_peak_6232	4.86324	
11	116573896	1.17E+08	KD2-H4K8ac_peak_6233	12.00273	
11	116602060	1.17E+08	KD2-H4K8ac_peak_6234	6.40683	
11	116643275	1.17E+08	KD2-H4K8ac_peak_6235	12.82393	BUD13_ENSG00000137656;AP006216.10_ENSG00000226645
11	116644008	1.17E+08	KD2-H4K8ac_peak_6236	11.49574	BUD13_ENSG00000137656;AP006216.10_ENSG00000226645
11	116658501	1.17E+08	KD2-H4K8ac_peak_6237	7.44324	ZNF259_ENSG00000109917
11	116699563	1.17E+08	KD2-H4K8ac_peak_6238	4.06758	APOC3_ENSG00000110245
11	116700116	1.17E+08	KD2-H4K8ac_peak_6239	11.79585	APOC3_ENSG00000110245
11	116968825	1.17E+08	KD2-H4K8ac_peak_6240	9.36516	SIK3_ENSG00000160584;AP000936.4_ENSG00000224077
11	117014519	1.17E+08	KD2-H4K8ac_peak_6241	7.76329	RP11-109L13.5_ENSG00000254678;PAFAH1B2_ENSG00000168092
11	117103659	1.17E+08	KD2-H4K8ac_peak_6242	10.43145	PCSK7_ENSG00000160613;RNF214_ENSG00000167257
11	117186251	1.17E+08	KD2-H4K8ac_peak_6243	11.14658	BACE1_ENSG00000186318;CEP164_ENSG00000110274
11	117198625	1.17E+08	KD2-H4K8ac_peak_6244	14.56955	AP000892.4_ENSG00000250699
11	117249439	1.17E+08	KD2-H4K8ac_peak_6245	4.24345	
11	117313766	1.17E+08	KD2-H4K8ac_peak_6246	18.54875	
11	117315960	1.17E+08	KD2-H4K8ac_peak_6247	6.66359	
11	117358756	1.17E+08	KD2-H4K8ac_peak_6248	12.84642	
11	117360244	1.17E+08	KD2-H4K8ac_peak_6249	13.50024	
11	117361111	1.17E+08	KD2-H4K8ac_peak_6250	8.73935	
11	117368603	1.17E+08	KD2-H4K8ac_peak_6251	6.98517	
11	117372136	1.17E+08	KD2-H4K8ac_peak_6252	5.30799	
11	117381673	1.17E+08	KD2-H4K8ac_peak_6253	5.95537	
11	117382623	1.17E+08	KD2-H4K8ac_peak_6254	8.53092	

11	117383756	1.17E+08	KD2-H4K8ac_peak_6255	5.54647	
11	117395633	1.17E+08	KD2-H4K8ac_peak_6256	8.30974	
11	117550156	1.18E+08	KD2-H4K8ac_peak_6257	7.01658	
11	117588730	1.18E+08	KD2-H4K8ac_peak_6258	5.20908	
11	117679792	1.18E+08	KD2-H4K8ac_peak_6259	10.05606	
11	117687624	1.18E+08	KD2-H4K8ac_peak_6260	10.65724	DSCAML1_ENSG00000177103
11	117740912	1.18E+08	KD2-H4K8ac_peak_6261	4.54006	
11	117746255	1.18E+08	KD2-H4K8ac_peak_6262	12.08081	FXYD6-FXYD2_ENSG00000255245;FXYD6_ENSG00000137726
11	117856794	1.18E+08	KD2-H4K8ac_peak_6263	8.85813	IL10RA_ENSG00000110324
11	118016521	1.18E+08	KD2-H4K8ac_peak_6264	16.73922	
11	118122504	1.18E+08	KD2-H4K8ac_peak_6265	14.74047	MPZL3_ENSG00000160588
11	118229658	1.18E+08	KD2-H4K8ac_peak_6266	18.94636	UBE4A_ENSG00000110344
11	118271237	1.18E+08	KD2-H4K8ac_peak_6267	11.89502	RP11-770J1.5_ENSG00000254873;ATP5L_ENSG00000167283
11	118305462	1.18E+08	KD2-H4K8ac_peak_6268	11.24702	RP11-770J1.4_ENSG00000255384
11	118306381	1.18E+08	KD2-H4K8ac_peak_6269	14.84212	RP11-770J1.4_ENSG00000255384;KMT2A_ENSG00000118058
11	118307240	1.18E+08	KD2-H4K8ac_peak_6270	4.06758	KMT2A_ENSG00000118058
11	118401271	1.18E+08	KD2-H4K8ac_peak_6271	12.08081	RP11-770J1.3_ENSG00000255435;TMEM25_ENSG00000149582
11	118442520	1.18E+08	KD2-H4K8ac_peak_6272	15.96836	IFT46_ENSG00000118096;ARCN1_ENSG00000095139
11	118443152	1.18E+08	KD2-H4K8ac_peak_6273	13.95627	IFT46_ENSG00000118096;ARCN1_ENSG00000095139
11	118478415	1.18E+08	KD2-H4K8ac_peak_6274	6.36375	
11	118479271	1.18E+08	KD2-H4K8ac_peak_6275	5.54647	
11	118491503	1.18E+08	KD2-H4K8ac_peak_6276	8.2413	
11	118492120	1.18E+08	KD2-H4K8ac_peak_6277	9.58924	
11	118494190	1.18E+08	KD2-H4K8ac_peak_6278	5.54647	
11	118559870	1.19E+08	KD2-H4K8ac_peak_6279	7.36799	AP002954.6_ENSG00000255239
11	118561166	1.19E+08	KD2-H4K8ac_peak_6280	8.88082	AP002954.6_ENSG00000255239
11	118575899	1.19E+08	KD2-H4K8ac_peak_6281	3.75035	AP002954.4_ENSG00000255422
11	118661073	1.19E+08	KD2-H4K8ac_peak_6282	18.54875	DDX6_ENSG00000110367
11	118778035	1.19E+08	KD2-H4K8ac_peak_6283	8.55271	
11	118780270	1.19E+08	KD2-H4K8ac_peak_6284	11.92978	MIR4492_ENSG00000264211
11	118787057	1.19E+08	KD2-H4K8ac_peak_6285	6.56211	
11	118789940	1.19E+08	KD2-H4K8ac_peak_6286	13.31016	
11	118795850	1.19E+08	KD2-H4K8ac_peak_6287	9.20193	BCL9L_ENSG00000186174;UPK2_ENSG00000110375
11	118800269	1.19E+08	KD2-H4K8ac_peak_6288	4.86324	
11	118868181	1.19E+08	KD2-H4K8ac_peak_6289	6.8836	RP11-110I1.12_ENSG00000255121;CCDC84_ENSG00000186166
11	118868929	1.19E+08	KD2-H4K8ac_peak_6290	11.28831	RP11-110I1.12_ENSG00000255121;CCDC84_ENSG00000186166
11	118888626	1.19E+08	KD2-H4K8ac_peak_6291	15.90329	RPS25_ENSG00000118181;TRAPPC4_ENSG00000196655;MIR3656_ENSG00000266398
11	118889564	1.19E+08	KD2-H4K8ac_peak_6292	11.02863	RPS25_ENSG00000118181;TRAPPC4_ENSG00000196655;MIR3656_ENSG00000266398
11	118900969	1.19E+08	KD2-H4K8ac_peak_6293	7.83489	SLC37A4_ENSG00000137700
11	118927387	1.19E+08	KD2-H4K8ac_peak_6294	8.30974	HYOU1_ENSG00000149428
11	118928152	1.19E+08	KD2-H4K8ac_peak_6295	13.56049	HYOU1_ENSG00000149428
11	118955320	1.19E+08	KD2-H4K8ac_peak_6296	12.83435	HMBS_ENSG00000256269
11	118956423	1.19E+08	KD2-H4K8ac_peak_6297	6.30626	HMBS_ENSG00000256269
11	118965082	1.19E+08	KD2-H4K8ac_peak_6298	14.98671	H2AFX_ENSG00000188486
11	118972175	1.19E+08	KD2-H4K8ac_peak_6299	10.46264	C2CD2L_ENSG00000172375
11	118973010	1.19E+08	KD2-H4K8ac_peak_6300	4.98072	C2CD2L_ENSG00000172375

11	118977643	1.19E+08	KD2-H4K8ac_peak_6301	6.65673	
11	119015217	1.19E+08	KD2-H4K8ac_peak_6302	5.88845	
11	119015894	1.19E+08	KD2-H4K8ac_peak_6303	5.71749	
11	119019603	1.19E+08	KD2-H4K8ac_peak_6304	14.48922	ABCG4_ENSG00000172350
11	119039462	1.19E+08	KD2-H4K8ac_peak_6305	20.96279	
11	119075871	1.19E+08	KD2-H4K8ac_peak_6306	7.47462	CBL_ENSG00000110395
11	119076718	1.19E+08	KD2-H4K8ac_peak_6307	9.1707	CBL_ENSG00000110395
11	119187324	1.19E+08	KD2-H4K8ac_peak_6308	8.23274	
11	119208590	1.19E+08	KD2-H4K8ac_peak_6309	5.72327	RP11-334E6.10_ENSG00000245385
11	119210613	1.19E+08	KD2-H4K8ac_peak_6310	12.93209	
11	119234814	1.19E+08	KD2-H4K8ac_peak_6311	13.31016	
11	119239015	1.19E+08	KD2-H4K8ac_peak_6312	5.29555	
11	119239470	1.19E+08	KD2-H4K8ac_peak_6313	11.05467	
11	119246305	1.19E+08	KD2-H4K8ac_peak_6314	8.3261	
11	119293483	1.19E+08	KD2-H4K8ac_peak_6315	5.9696	
11	119294065	1.19E+08	KD2-H4K8ac_peak_6316	9.96798	
11	119352377	1.19E+08	KD2-H4K8ac_peak_6317	5.57044	
11	119438367	1.19E+08	KD2-H4K8ac_peak_6318	8.23274	
11	119439417	1.19E+08	KD2-H4K8ac_peak_6319	7.146	
11	119455091	1.19E+08	KD2-H4K8ac_peak_6320	8.16906	
11	119514309	1.2E+08	KD2-H4K8ac_peak_6321	6.4095	
11	119541937	1.2E+08	KD2-H4K8ac_peak_6322	13.5195	
11	119555216	1.2E+08	KD2-H4K8ac_peak_6323	6.20982	
11	119564345	1.2E+08	KD2-H4K8ac_peak_6324	6.25014	
11	119570678	1.2E+08	KD2-H4K8ac_peak_6325	6.77511	
11	119572237	1.2E+08	KD2-H4K8ac_peak_6326	6.04107	
11	119573544	1.2E+08	KD2-H4K8ac_peak_6327	7.07117	
11	119579557	1.2E+08	KD2-H4K8ac_peak_6328	16.57619	CTD-2523D13.1_ENSG00000255247
11	119583472	1.2E+08	KD2-H4K8ac_peak_6329	4.93687	
11	119600363	1.2E+08	KD2-H4K8ac_peak_6330	9.34989	PVRL1_ENSG00000110400;CTD-2523D13.2_ENSG00000254854
11	119613256	1.2E+08	KD2-H4K8ac_peak_6331	14.83804	
11	119634951	1.2E+08	KD2-H4K8ac_peak_6332	4.42765	
11	119650527	1.2E+08	KD2-H4K8ac_peak_6333	6.40297	
11	119660089	1.2E+08	KD2-H4K8ac_peak_6334	5.51783	
11	119702744	1.2E+08	KD2-H4K8ac_peak_6335	7.70289	
11	119734717	1.2E+08	KD2-H4K8ac_peak_6336	6.67092	
11	119745384	1.2E+08	KD2-H4K8ac_peak_6337	9.1707	
11	119759874	1.2E+08	KD2-H4K8ac_peak_6338	7.1181	
11	119761330	1.2E+08	KD2-H4K8ac_peak_6339	5.75757	
11	119762863	1.2E+08	KD2-H4K8ac_peak_6340	4.67342	
11	119764038	1.2E+08	KD2-H4K8ac_peak_6341	9.402	
11	119777945	1.2E+08	KD2-H4K8ac_peak_6342	8.42997	
11	119779286	1.2E+08	KD2-H4K8ac_peak_6343	5.88845	
11	119806557	1.2E+08	KD2-H4K8ac_peak_6344	6.2159	
11	119872170	1.2E+08	KD2-H4K8ac_peak_6345	7.27716	
11	119896132	1.2E+08	KD2-H4K8ac_peak_6346	4.42765	

11	119933142	1.2E+08	KD2-H4K8ac_peak_6347	5.04237	
11	119938542	1.2E+08	KD2-H4K8ac_peak_6348	9.89126	
11	120039488	1.2E+08	KD2-H4K8ac_peak_6349	10.94736	AP000679.2_ENSG00000176984
11	120055560	1.2E+08	KD2-H4K8ac_peak_6350	11.92978	TRIM29_ENSG00000137699
11	120056468	1.2E+08	KD2-H4K8ac_peak_6351	8.58725	TRIM29_ENSG00000137699
11	120058850	1.2E+08	KD2-H4K8ac_peak_6352	7.09076	
11	120063767	1.2E+08	KD2-H4K8ac_peak_6353	7.47462	
11	120080965	1.2E+08	KD2-H4K8ac_peak_6354	11.89502	OAF_ENSG00000184232
11	120081544	1.2E+08	KD2-H4K8ac_peak_6355	4.54006	OAF_ENSG00000184232
11	120100043	1.2E+08	KD2-H4K8ac_peak_6356	6.81873	
11	120195614	1.2E+08	KD2-H4K8ac_peak_6357	10.35678	TMEM136_ENSG00000181264
11	120206422	1.2E+08	KD2-H4K8ac_peak_6358	14.98671	ARHGEF12_ENSG00000196914
11	120207168	1.2E+08	KD2-H4K8ac_peak_6359	4.37809	ARHGEF12_ENSG00000196914
11	120387519	1.2E+08	KD2-H4K8ac_peak_6360	8.17902	
11	120388106	1.2E+08	KD2-H4K8ac_peak_6361	4.42765	
11	120398071	1.2E+08	KD2-H4K8ac_peak_6362	9.65086	
11	120398634	1.2E+08	KD2-H4K8ac_peak_6363	4.38478	
11	120409458	1.2E+08	KD2-H4K8ac_peak_6364	16.93803	
11	120411616	1.2E+08	KD2-H4K8ac_peak_6365	4.44527	
11	120425211	1.2E+08	KD2-H4K8ac_peak_6366	3.75035	
11	120426103	1.2E+08	KD2-H4K8ac_peak_6367	9.65086	
11	120429751	1.2E+08	KD2-H4K8ac_peak_6368	8.09472	
11	120754584	1.21E+08	KD2-H4K8ac_peak_6369	8.02571	
11	120856570	1.21E+08	KD2-H4K8ac_peak_6370	8.37043	
11	120894869	1.21E+08	KD2-H4K8ac_peak_6371	6.84261	TBCEL_ENSG00000154114
11	121297753	1.21E+08	KD2-H4K8ac_peak_6372	6.66692	RP11-142I2.1_ENSG00000271022
11	121317584	1.21E+08	KD2-H4K8ac_peak_6373	4.62507	
11	121322430	1.21E+08	KD2-H4K8ac_peak_6374	11.86082	RP11-730K11.1_ENSG00000246790;SORL1_ENSG00000137642
11	121336961	1.21E+08	KD2-H4K8ac_peak_6375	6.67092	
11	121527195	1.22E+08	KD2-H4K8ac_peak_6376	9.90914	
11	121541878	1.22E+08	KD2-H4K8ac_peak_6377	7.47462	
11	121543262	1.22E+08	KD2-H4K8ac_peak_6378	5.73839	
11	121593002	1.22E+08	KD2-H4K8ac_peak_6379	8.55271	
11	122029539	1.22E+08	KD2-H4K8ac_peak_6380	7.12754	
11	122030046	1.22E+08	KD2-H4K8ac_peak_6381	6.16888	
11	122073893	1.22E+08	KD2-H4K8ac_peak_6382	10.0746	
11	122236715	1.22E+08	KD2-H4K8ac_peak_6383	3.75035	
11	122311225	1.22E+08	KD2-H4K8ac_peak_6384	9.1707	
11	122452194	1.22E+08	KD2-H4K8ac_peak_6385	5.43522	
11	122753372	1.23E+08	KD2-H4K8ac_peak_6386	9.36516	C11orf63_ENSG00000109944
11	122855193	1.23E+08	KD2-H4K8ac_peak_6387	9.96798	
11	122933407	1.23E+08	KD2-H4K8ac_peak_6388	20.5798	HSPA8_ENSG00000109971
11	123005640	1.23E+08	KD2-H4K8ac_peak_6389	5.15777	
11	123007392	1.23E+08	KD2-H4K8ac_peak_6390	12.06346	RP11-762B21.5_ENSG00000255342
11	123008030	1.23E+08	KD2-H4K8ac_peak_6391	5.88845	RP11-762B21.5_ENSG00000255342
11	123008687	1.23E+08	KD2-H4K8ac_peak_6392	5.14125	

11	123009400	1.23E+08	KD2-H4K8ac_peak_6393	9.19305	
11	123010219	1.23E+08	KD2-H4K8ac_peak_6394	9.36516	
11	123016326	1.23E+08	KD2-H4K8ac_peak_6395	5.13615	
11	123065265	1.23E+08	KD2-H4K8ac_peak_6396	15.58442	CLMP_ENSG00000166250
11	123066514	1.23E+08	KD2-H4K8ac_peak_6397	11.05407	CLMP_ENSG00000166250
11	123072453	1.23E+08	KD2-H4K8ac_peak_6398	8.17902	
11	123074146	1.23E+08	KD2-H4K8ac_peak_6399	10.58496	
11	123221835	1.23E+08	KD2-H4K8ac_peak_6400	9.1707	
11	123229179	1.23E+08	KD2-H4K8ac_peak_6401	10.05606	
11	123348735	1.23E+08	KD2-H4K8ac_peak_6402	9.89126	
11	123380250	1.23E+08	KD2-H4K8ac_peak_6403	6.65673	
11	123380768	1.23E+08	KD2-H4K8ac_peak_6404	5.81222	
11	123382192	1.23E+08	KD2-H4K8ac_peak_6405	6.25869	
11	123395712	1.23E+08	KD2-H4K8ac_peak_6406	8.55271	GRAMD1B_ENSG00000023171
11	123431716	1.23E+08	KD2-H4K8ac_peak_6407	5.54647	
11	123525487	1.24E+08	KD2-H4K8ac_peak_6408	9.63487	SCN3B_ENSG00000166257
11	123611844	1.24E+08	KD2-H4K8ac_peak_6409	11.92978	ZNF202_ENSG00000166261
11	124492302	1.24E+08	KD2-H4K8ac_peak_6410	5.20998	TBRG1_ENSG00000154144
11	124544125	1.25E+08	KD2-H4K8ac_peak_6411	6.66692	SPA17_ENSG00000064199
11	124587953	1.25E+08	KD2-H4K8ac_peak_6412	12.79505	
11	124588791	1.25E+08	KD2-H4K8ac_peak_6413	10.08707	
11	124609380	1.25E+08	KD2-H4K8ac_peak_6414	15.53798	NRGN_ENSG00000154146
11	124615898	1.25E+08	KD2-H4K8ac_peak_6415	5.90098	RP11-677M14.2_ENSG00000255045
11	124632417	1.25E+08	KD2-H4K8ac_peak_6416	4.06758	ESAM_ENSG00000149564
11	124670087	1.25E+08	KD2-H4K8ac_peak_6417	9.71513	MSANTD2_ENSG00000120458;RP11-677M14.7_ENSG00000245498
11	124713125	1.25E+08	KD2-H4K8ac_peak_6418	11.05467	AP000866.1_ENSG00000215942
11	124733121	1.25E+08	KD2-H4K8ac_peak_6419	6.40683	
11	124745902	1.25E+08	KD2-H4K8ac_peak_6420	16.33377	
11	124747003	1.25E+08	KD2-H4K8ac_peak_6421	13.44374	
11	124790941	1.25E+08	KD2-H4K8ac_peak_6422	18.54875	
11	124824084	1.25E+08	KD2-H4K8ac_peak_6423	11.75669	CCDC15_ENSG00000149548
11	124980934	1.25E+08	KD2-H4K8ac_peak_6424	6.83936	TMEM218_ENSG00000150433
11	124981920	1.25E+08	KD2-H4K8ac_peak_6425	14.29602	TMEM218_ENSG00000150433;KRT18P59_ENSG00000187686
11	125035260	1.25E+08	KD2-H4K8ac_peak_6426	4.8888	RP11-687M24.5_ENSG00000254880;PKNOX2_ENSG00000165495
11	125132774	1.25E+08	KD2-H4K8ac_peak_6427	10.87093	
11	125274724	1.25E+08	KD2-H4K8ac_peak_6428	5.71749	
11	125303769	1.25E+08	KD2-H4K8ac_peak_6429	7.38933	
11	125321218	1.25E+08	KD2-H4K8ac_peak_6430	6.72021	
11	125364920	1.25E+08	KD2-H4K8ac_peak_6431	6.05809	FEZ1_ENSG00000149557;AP000708.1_ENSG00000255537
11	125365876	1.25E+08	KD2-H4K8ac_peak_6432	4.86324	FEZ1_ENSG00000149557;AP000708.1_ENSG00000255537
11	125438709	1.25E+08	KD2-H4K8ac_peak_6433	8.45238	EI24_ENSG00000149547
11	125439415	1.25E+08	KD2-H4K8ac_peak_6434	20.88898	EI24_ENSG00000149547
11	125461162	1.25E+08	KD2-H4K8ac_peak_6435	6.2781	STT3A_ENSG00000134910
11	125461651	1.25E+08	KD2-H4K8ac_peak_6436	4.42765	STT3A-AS1_ENSG00000254671;STT3A_ENSG00000134910
11	125462882	1.25E+08	KD2-H4K8ac_peak_6437	6.05809	STT3A-AS1_ENSG00000254671
11	125495151	1.25E+08	KD2-H4K8ac_peak_6438	18.5703	CHEK1_ENSG00000149554

11	125757027	1.26E+08	KD2-H4K8ac_peak_6439	33.81557	
11	125820122	1.26E+08	KD2-H4K8ac_peak_6440	11.04256	RP11-680F20.6_ENSG00000254967
11	125821707	1.26E+08	KD2-H4K8ac_peak_6441	7.27281	
11	125823278	1.26E+08	KD2-H4K8ac_peak_6442	10.46874	
11	125932311	1.26E+08	KD2-H4K8ac_peak_6443	7.40314	CDON_ENSG00000064309
11	125934087	1.26E+08	KD2-H4K8ac_peak_6444	7.83489	CDON_ENSG00000064309
11	125949479	1.26E+08	KD2-H4K8ac_peak_6445	10.90543	
11	125950317	1.26E+08	KD2-H4K8ac_peak_6446	8.10216	
11	125950866	1.26E+08	KD2-H4K8ac_peak_6447	8.26945	
11	125951302	1.26E+08	KD2-H4K8ac_peak_6448	6.25869	
11	125953728	1.26E+08	KD2-H4K8ac_peak_6449	8.19585	
11	125954179	1.26E+08	KD2-H4K8ac_peak_6450	5.54647	
11	125980476	1.26E+08	KD2-H4K8ac_peak_6451	9.36516	
11	125981733	1.26E+08	KD2-H4K8ac_peak_6452	12.82393	
11	125982728	1.26E+08	KD2-H4K8ac_peak_6453	13.86993	
11	125983921	1.26E+08	KD2-H4K8ac_peak_6454	22.4399	
11	125985331	1.26E+08	KD2-H4K8ac_peak_6455	15.69572	
11	126011184	1.26E+08	KD2-H4K8ac_peak_6456	16.07006	
11	126081024	1.26E+08	KD2-H4K8ac_peak_6457	5.1707	RPUSD4_ENSG00000165526;FAM118B_ENSG00000197798
11	126152321	1.26E+08	KD2-H4K8ac_peak_6458	13.73635	snoU13_ENSG00000238855;TIRAP_ENSG00000150455
11	126173489	1.26E+08	KD2-H4K8ac_peak_6459	9.95119	RP11-712L6.5_ENSG00000255062;DCPS_ENSG00000110063
11	126174738	1.26E+08	KD2-H4K8ac_peak_6460	3.97079	RP11-712L6.5_ENSG00000255062
11	126224784	1.26E+08	KD2-H4K8ac_peak_6461	15.74255	ST3GAL4_ENSG00000110080
11	126256141	1.26E+08	KD2-H4K8ac_peak_6462	8.88549	
11	126263022	1.26E+08	KD2-H4K8ac_peak_6463	4.9025	
11	126350394	1.26E+08	KD2-H4K8ac_peak_6464	14.99434	
11	126382738	1.26E+08	KD2-H4K8ac_peak_6465	8.02571	
11	126383352	1.26E+08	KD2-H4K8ac_peak_6466	3.90565	
11	126388558	1.26E+08	KD2-H4K8ac_peak_6467	9.16531	
11	126391070	1.26E+08	KD2-H4K8ac_peak_6468	7.26089	
11	126413613	1.26E+08	KD2-H4K8ac_peak_6469	9.76863	KIRREL3-AS1_ENSG00000257271
11	126455282	1.26E+08	KD2-H4K8ac_peak_6470	8.5781	
11	126859994	1.27E+08	KD2-H4K8ac_peak_6471	5.72327	
11	126873103	1.27E+08	KD2-H4K8ac_peak_6472	5.88845	KIRREL3_ENSG00000149571;KIRREL3-AS3_ENSG00000218109;AP002833.1_ENSG00000256527
11	126951728	1.27E+08	KD2-H4K8ac_peak_6473	5.19569	
11	127063579	1.27E+08	KD2-H4K8ac_peak_6474	12.36249	
11	127065384	1.27E+08	KD2-H4K8ac_peak_6475	6.44277	
11	127321592	1.27E+08	KD2-H4K8ac_peak_6476	7.79558	
11	127330926	1.27E+08	KD2-H4K8ac_peak_6477	9.15329	
11	128049346	1.28E+08	KD2-H4K8ac_peak_6478	8.1718	RP11-702B10.2_ENSG00000273415
11	128104517	1.28E+08	KD2-H4K8ac_peak_6479	10.1591	
11	128105006	1.28E+08	KD2-H4K8ac_peak_6480	6.40683	
11	128106805	1.28E+08	KD2-H4K8ac_peak_6481	17.84477	
11	128116645	1.28E+08	KD2-H4K8ac_peak_6482	15.22643	
11	128160254	1.28E+08	KD2-H4K8ac_peak_6483	9.11031	
11	128261409	1.28E+08	KD2-H4K8ac_peak_6484	6.8836	

11	128262391	1.28E+08	KD2-H4K8ac_peak_6485	6.31143	
11	128290292	1.28E+08	KD2-H4K8ac_peak_6486	6.99815	
11	128291765	1.28E+08	KD2-H4K8ac_peak_6487	8.26945	
11	128292980	1.28E+08	KD2-H4K8ac_peak_6488	5.98124	
11	128294424	1.28E+08	KD2-H4K8ac_peak_6489	5.45396	
11	128325502	1.28E+08	KD2-H4K8ac_peak_6490	7.70289	
11	128376572	1.28E+08	KD2-H4K8ac_peak_6491	6.00226	
11	128391588	1.28E+08	KD2-H4K8ac_peak_6492	12.78794	
11	128392529	1.28E+08	KD2-H4K8ac_peak_6493	12.79505	
11	128480972	1.28E+08	KD2-H4K8ac_peak_6494	7.72801	
11	128646776	1.29E+08	KD2-H4K8ac_peak_6495	8.17902	
11	128765610	1.29E+08	KD2-H4K8ac_peak_6496	5.34946	
11	128774944	1.29E+08	KD2-H4K8ac_peak_6497	11.8457	C11orf45_ENSG00000174370
11	129149662	1.29E+08	KD2-H4K8ac_peak_6498	10.65724	ARHGAP32_ENSG00000134909
11	129685809	1.3E+08	KD2-H4K8ac_peak_6499	15.61313	TMEM45B_ENSG00000151715
11	129765009	1.3E+08	KD2-H4K8ac_peak_6500	8.02571	NFRKB_ENSG00000170322
11	129838738	1.3E+08	KD2-H4K8ac_peak_6501	8.79557	
11	129871507	1.3E+08	KD2-H4K8ac_peak_6502	13.87818	PRDM10_ENSG00000170325;LINC00167_ENSG00000233220
11	129938512	1.3E+08	KD2-H4K8ac_peak_6503	10.96455	APLP2_ENSG00000084234
11	130083181	1.3E+08	KD2-H4K8ac_peak_6504	9.11031	
11	130185089	1.3E+08	KD2-H4K8ac_peak_6505	8.55271	ZBTB44_ENSG00000196323;RP11-121M22.1_ENSG00000175773
11	130785760	1.31E+08	KD2-H4K8ac_peak_6506	16.49944	SNX19_ENSG00000120451
11	130786614	1.31E+08	KD2-H4K8ac_peak_6507	5.57044	SNX19_ENSG00000120451
11	132640559	1.33E+08	KD2-H4K8ac_peak_6508	10.21502	
11	132662355	1.33E+08	KD2-H4K8ac_peak_6509	10.63441	
11	133096954	1.33E+08	KD2-H4K8ac_peak_6510	5.54647	
11	133798418	1.34E+08	KD2-H4K8ac_peak_6511	5.14125	
11	133799205	1.34E+08	KD2-H4K8ac_peak_6512	5.30799	
11	133804683	1.34E+08	KD2-H4K8ac_peak_6513	6.58902	
11	133805195	1.34E+08	KD2-H4K8ac_peak_6514	26.99824	
11	133807226	1.34E+08	KD2-H4K8ac_peak_6515	3.82509	
11	133815785	1.34E+08	KD2-H4K8ac_peak_6516	13.9152	
11	133816989	1.34E+08	KD2-H4K8ac_peak_6517	9.65086	
11	133827259	1.34E+08	KD2-H4K8ac_peak_6518	11.92978	IGSF9B_ENSG00000080854;AP000911.1_ENSG00000264674
11	133836120	1.34E+08	KD2-H4K8ac_peak_6519	4.67342	
11	133902596	1.34E+08	KD2-H4K8ac_peak_6520	5.13615	RP11-713P17.3_ENSG00000204241
11	133903574	1.34E+08	KD2-H4K8ac_peak_6521	10.99466	
11	133904221	1.34E+08	KD2-H4K8ac_peak_6522	11.72064	
11	133907015	1.34E+08	KD2-H4K8ac_peak_6523	4.28151	RP11-713P17.4_ENSG00000254648
11	133910388	1.34E+08	KD2-H4K8ac_peak_6524	5.66317	
11	133990902	1.34E+08	KD2-H4K8ac_peak_6525	6.76298	
11	134093549	1.34E+08	KD2-H4K8ac_peak_6526	6.40683	VPS26B_ENSG00000151502
11	134094146	1.34E+08	KD2-H4K8ac_peak_6527	9.16531	NCAPD3_ENSG00000151503;VPS26B_ENSG00000151502
11	134094621	1.34E+08	KD2-H4K8ac_peak_6528	13.70753	NCAPD3_ENSG00000151503;VPS26B_ENSG00000151502
11	134095337	1.34E+08	KD2-H4K8ac_peak_6529	5.88845	NCAPD3_ENSG00000151503;VPS26B_ENSG00000151502
11	134123523	1.34E+08	KD2-H4K8ac_peak_6530	4.98072	THYN1_ENSG00000151500;ACAD8_ENSG00000151498

11	134202012	1.34E+08	KD2-H4K8ac_peak_6531	11.43863	
11	134281636	1.34E+08	KD2-H4K8ac_peak_6532	8.30974	B3GAT1_ENSG00000109956
11	134874150	1.35E+08	KD2-H4K8ac_peak_6533	8.41122	
11	134874820	1.35E+08	KD2-H4K8ac_peak_6534	9.73163	
11	134878545	1.35E+08	KD2-H4K8ac_peak_6535	7.12754	
12	73753	74083	KD2-H4K8ac_peak_6536	25.98527	AC215219.1_ENSG00000238823;ABC7-42389800N19.1_ENSG00000226210
12	79861	80118	KD2-H4K8ac_peak_6537	5.13615	
12	187115	187571	KD2-H4K8ac_peak_6538	12.41575	
12	262149	262350	KD2-H4K8ac_peak_6539	5.51783	
12	275223	275475	KD2-H4K8ac_peak_6540	7.47462	RP11-598F7.5_ENSG00000256694
12	281570	282177	KD2-H4K8ac_peak_6541	6.65673	
12	283613	283987	KD2-H4K8ac_peak_6542	9.20193	
12	284184	284464	KD2-H4K8ac_peak_6543	7.95563	
12	284854	285502	KD2-H4K8ac_peak_6544	7.70067	
12	286513	287001	KD2-H4K8ac_peak_6545	8.4716	
12	288131	288368	KD2-H4K8ac_peak_6546	4.95872	
12	297721	298364	KD2-H4K8ac_peak_6547	17.19888	
12	312463	312904	KD2-H4K8ac_peak_6548	7.76329	RP11-283I3.2_ENSG00000256577
12	313208	313646	KD2-H4K8ac_peak_6549	4.54006	RP11-283I3.2_ENSG00000256577
12	329314	329526	KD2-H4K8ac_peak_6550	5.82799	
12	510165	510428	KD2-H4K8ac_peak_6551	8.37231	
12	678616	678980	KD2-H4K8ac_peak_6552	9.36516	
12	679220	680004	KD2-H4K8ac_peak_6553	15.47237	
12	684524	684725	KD2-H4K8ac_peak_6554	8.1718	
12	685015	685885	KD2-H4K8ac_peak_6555	21.26856	
12	699640	699900	KD2-H4K8ac_peak_6556	7.44366	RNU7-103P_ENSG00000238370;RP5-1154L15.1_ENSG00000255825
12	700243	700684	KD2-H4K8ac_peak_6557	7.40111	RP5-1154L15.1_ENSG00000255825
12	701566	701865	KD2-H4K8ac_peak_6558	8.59579	
12	719275	719642	KD2-H4K8ac_peak_6559	5.14125	
12	752166	752527	KD2-H4K8ac_peak_6560	11.09837	
12	1057802	1058004	KD2-H4K8ac_peak_6561	7.15405	
12	1058374	1058807	KD2-H4K8ac_peak_6562	19.03298	
12	1059276	1059569	KD2-H4K8ac_peak_6563	6.65673	
12	1099712	1099966	KD2-H4K8ac_peak_6564	11.04256	RAD52_ENSG00000002016;RP11-359B12.2_ENSG00000250132;ERC1_ENSG00000082805
12	1164146	1164509	KD2-H4K8ac_peak_6565	6.16888	
12	1393829	1394102	KD2-H4K8ac_peak_6566	6.16888	
12	1639074	1639481	KD2-H4K8ac_peak_6567	7.36799	WNT5B_ENSG00000111186
12	1703088	1703509	KD2-H4K8ac_peak_6568	6.99815	FBXL14_ENSG00000171823
12	1703789	1704258	KD2-H4K8ac_peak_6569	10.46264	FBXL14_ENSG00000171823
12	1738555	1738865	KD2-H4K8ac_peak_6570	16.61426	
12	1739137	1739892	KD2-H4K8ac_peak_6571	11.05467	
12	1770686	1770914	KD2-H4K8ac_peak_6572	8.10216	
12	1771127	1771484	KD2-H4K8ac_peak_6573	7.70289	
12	1799720	1799928	KD2-H4K8ac_peak_6574	7.95563	
12	1800324	1800632	KD2-H4K8ac_peak_6575	7.1181	
12	1905499	1906103	KD2-H4K8ac_peak_6576	13.73635	

12	1913926	1914569	KD2-H4K8ac_peak_6577	18.41034	
12	1914775	1915078	KD2-H4K8ac_peak_6578	4.37815	
12	1935887	1936176	KD2-H4K8ac_peak_6579	5.86783	
12	1944955	1945714	KD2-H4K8ac_peak_6580	6.40297	
12	1946216	1946943	KD2-H4K8ac_peak_6581	4.44527	
12	1947946	1948168	KD2-H4K8ac_peak_6582	5.66705	
12	2113143	2113573	KD2-H4K8ac_peak_6583	11.04256	DCP1B_ENSG00000151065;RP5-1096D14.6_ENSG00000203593
12	2123753	2124129	KD2-H4K8ac_peak_6584	5.45396	
12	2144351	2144609	KD2-H4K8ac_peak_6585	4.91674	
12	2162190	2162741	KD2-H4K8ac_peak_6586	7.27716	
12	2348779	2348997	KD2-H4K8ac_peak_6587	6.25014	
12	2392752	2393385	KD2-H4K8ac_peak_6588	7.9076	
12	2393578	2393983	KD2-H4K8ac_peak_6589	6.2781	
12	2692409	2692769	KD2-H4K8ac_peak_6590	5.61067	
12	2903316	2903707	KD2-H4K8ac_peak_6591	5.9696	CBX3P4_ENSG00000256030;FKBP4_ENSG00000004478
12	2904271	2904988	KD2-H4K8ac_peak_6592	5.9696	CBX3P4_ENSG00000256030;FKBP4_ENSG00000004478
12	2921855	2922049	KD2-H4K8ac_peak_6593	10.43145	RP4-816N1.6_ENSG00000258325;ITFG2_ENSG00000111203
12	2985799	2986101	KD2-H4K8ac_peak_6594	4.44527	FOXM1_ENSG00000111206;RHNO1_ENSG00000171792;TULP3_ENSG00000078246
12	2986474	2986826	KD2-H4K8ac_peak_6595	11.34353	FOXM1_ENSG00000111206;TULP3_ENSG00000078246
12	2999188	2999804	KD2-H4K8ac_peak_6596	11.50346	
12	3067904	3068868	KD2-H4K8ac_peak_6597	5.54647	TEAD4_ENSG00000197905
12	3112811	3113160	KD2-H4K8ac_peak_6598	8.09472	
12	3138293	3138606	KD2-H4K8ac_peak_6599	3.85282	
12	3185945	3186361	KD2-H4K8ac_peak_6600	7.76104	TSPAN9_ENSG00000011105
12	3212822	3213279	KD2-H4K8ac_peak_6601	10.46264	
12	3213625	3213860	KD2-H4K8ac_peak_6602	6.16747	
12	3214422	3214882	KD2-H4K8ac_peak_6603	20.02161	
12	3216553	3216928	KD2-H4K8ac_peak_6604	9.11031	
12	3313535	3313885	KD2-H4K8ac_peak_6605	9.33795	
12	3325786	3325985	KD2-H4K8ac_peak_6606	4.8888	
12	3327528	3327781	KD2-H4K8ac_peak_6607	9.11031	
12	3328479	3328715	KD2-H4K8ac_peak_6608	8.4084	
12	3333484	3333739	KD2-H4K8ac_peak_6609	8.94469	
12	3390197	3391054	KD2-H4K8ac_peak_6610	15.039	
12	3474862	3475111	KD2-H4K8ac_peak_6611	6.36375	
12	3713450	3714176	KD2-H4K8ac_peak_6612	5.30799	
12	3861842	3862598	KD2-H4K8ac_peak_6613	26.59781	
12	3862861	3863323	KD2-H4K8ac_peak_6614	4.42765	
12	3981672	3982149	KD2-H4K8ac_peak_6615	8.02571	PARP11_ENSG00000111224;RP11-664D1.1_ENSG00000256862
12	4429758	4429966	KD2-H4K8ac_peak_6616	8.1233	C12orf5_ENSG00000078237
12	4430452	4430694	KD2-H4K8ac_peak_6617	7.36799	C12orf5_ENSG00000078237
12	4524597	4524796	KD2-H4K8ac_peak_6618	4.44923	
12	4526679	4527059	KD2-H4K8ac_peak_6619	7.47462	
12	4550500	4550907	KD2-H4K8ac_peak_6620	4.42765	
12	4714136	4714372	KD2-H4K8ac_peak_6621	10.63441	RP11-500M8.7_ENSG00000272921
12	4757703	4758103	KD2-H4K8ac_peak_6622	6.25869	AKAP3_ENSG00000111254;NDUFA9_ENSG00000139180

12	4758423	4758719	KD2-H4K8ac_peak_6623	4.211	AKAP3_ENSG00000111254;NDUFA9_ENSG00000139180
12	6194157	6194407	KD2-H4K8ac_peak_6624	5.88845	
12	6223808	6224017	KD2-H4K8ac_peak_6625	9.35804	
12	6267550	6267771	KD2-H4K8ac_peak_6626	7.45501	
12	6290825	6291431	KD2-H4K8ac_peak_6627	7.47462	
12	6291625	6292023	KD2-H4K8ac_peak_6628	12.06346	
12	6305040	6305446	KD2-H4K8ac_peak_6629	8.62078	
12	6309050	6309296	KD2-H4K8ac_peak_6630	9.63487	CD9_ENSG00000010278
12	6356501	6356871	KD2-H4K8ac_peak_6631	12.27142	
12	6387226	6387999	KD2-H4K8ac_peak_6632	25.71759	
12	6388226	6388466	KD2-H4K8ac_peak_6633	6.13909	
12	6426814	6427040	KD2-H4K8ac_peak_6634	5.10276	
12	6450915	6451143	KD2-H4K8ac_peak_6635	5.29352	TNFRSF1A_ENSG00000067182
12	6560164	6560575	KD2-H4K8ac_peak_6636	11.86082	CD27-AS1_ENSG00000215039;TAPBPL_ENSG00000139192
12	6560774	6561458	KD2-H4K8ac_peak_6637	6.99815	CD27-AS1_ENSG00000215039;TAPBPL_ENSG00000139192
12	6580036	6580823	KD2-H4K8ac_peak_6638	9.402	VAMP1_ENSG00000139190
12	6602022	6602319	KD2-H4K8ac_peak_6639	9.1707	MRPL51_ENSG00000111639;NCAPD2_ENSG00000010292
12	6602660	6602883	KD2-H4K8ac_peak_6640	9.6247	MRPL51_ENSG00000111639;NCAPD2_ENSG00000010292
12	6642543	6643437	KD2-H4K8ac_peak_6641	31.33361	RP5-940J5.3_ENSG00000255966;GAPDH_ENSG00000111640
12	6643782	6644164	KD2-H4K8ac_peak_6642	7.95563	GAPDH_ENSG00000111640
12	6644683	6644924	KD2-H4K8ac_peak_6643	5.75452	
12	6677690	6678163	KD2-H4K8ac_peak_6644	11.24702	NOP2_ENSG00000111641
12	6716307	6716525	KD2-H4K8ac_peak_6645	16.32216	CHD4_ENSG00000111642
12	6717696	6718363	KD2-H4K8ac_peak_6646	5.30799	
12	6721615	6722087	KD2-H4K8ac_peak_6647	8.83582	
12	6722453	6722794	KD2-H4K8ac_peak_6648	43.30787	
12	6723197	6723830	KD2-H4K8ac_peak_6649	14.68449	
12	6741815	6742084	KD2-H4K8ac_peak_6650	5.84991	
12	6771616	6771906	KD2-H4K8ac_peak_6651	8.17902	ING4_ENSG00000111653;RP4-761J14.8_ENSG00000219410
12	6772726	6773443	KD2-H4K8ac_peak_6652	5.88845	ING4_ENSG00000111653;RP4-761J14.8_ENSG00000219410
12	6797698	6798558	KD2-H4K8ac_peak_6653	34.98954	ZNF384_ENSG00000126746
12	6808938	6810401	KD2-H4K8ac_peak_6654	20.15307	PIANP_ENSG00000139200
12	6833661	6833861	KD2-H4K8ac_peak_6655	4.86324	COPS7A_ENSG00000111652
12	6862384	6862643	KD2-H4K8ac_peak_6656	9.63487	
12	6872678	6873495	KD2-H4K8ac_peak_6657	10.7186	
12	6874752	6875211	KD2-H4K8ac_peak_6658	12.17278	PTMS_ENSG00000159335
12	6876265	6876654	KD2-H4K8ac_peak_6659	5.07021	MLF2_ENSG00000089693
12	6937651	6938173	KD2-H4K8ac_peak_6660	5.59859	LEPREL2_ENSG00000110811
12	6938523	6938775	KD2-H4K8ac_peak_6661	4.06758	LEPREL2_ENSG00000110811
12	6976005	6976491	KD2-H4K8ac_peak_6662	7.27716	TPI1_ENSG00000111669
12	6976747	6977194	KD2-H4K8ac_peak_6663	16.63953	TPI1_ENSG00000111669
12	6982209	6982452	KD2-H4K8ac_peak_6664	6.56211	RPL13P5_ENSG00000240370;LRRC23_ENSG00000010626
12	6982745	6982953	KD2-H4K8ac_peak_6665	6.66692	RPL13P5_ENSG00000240370;LRRC23_ENSG00000010626
12	7000584	7000907	KD2-H4K8ac_peak_6666	14.27217	
12	7022936	7024017	KD2-H4K8ac_peak_6667	18.5703	ENO2_ENSG00000111674
12	7024408	7024732	KD2-H4K8ac_peak_6668	5.45396	

12	7033768	7034213	KD2-H4K8ac_peak_6669	4.67342	ATN1_ENSG00000111676
12	7034729	7035289	KD2-H4K8ac_peak_6670	7.47462	
12	7036567	7037107	KD2-H4K8ac_peak_6671	14.15075	
12	7046068	7047191	KD2-H4K8ac_peak_6672	23.38267	
12	7052339	7052661	KD2-H4K8ac_peak_6673	14.48084	C12orf57_ENSG00000111678;RNU7-1_ENSG00000238923
12	7053206	7053823	KD2-H4K8ac_peak_6674	13.80019	U47924.31_ENSG00000272173;RNU7-1_ENSG00000238923
12	7070959	7071823	KD2-H4K8ac_peak_6675	9.36516	EMG1_ENSG00000126749;U47924.27_ENSG00000257084
12	7074021	7074220	KD2-H4K8ac_peak_6676	5.90414	MIR141_ENSG00000207708;U47924.29_ENSG00000271969
12	7079225	7079785	KD2-H4K8ac_peak_6677	15.13261	PHB2_ENSG00000215021
12	7125921	7126133	KD2-H4K8ac_peak_6678	8.10216	LPCAT3_ENSG00000111684
12	7244973	7245169	KD2-H4K8ac_peak_6679	8.74596	C1R_ENSG00000159403
12	7341826	7342128	KD2-H4K8ac_peak_6680	11.92978	RP11-273B20.3_ENSG00000255572;PEX5_ENSG00000139197
12	7342351	7342544	KD2-H4K8ac_peak_6681	7.95563	RP11-273B20.3_ENSG00000255572
12	7521914	7522272	KD2-H4K8ac_peak_6682	6.16888	
12	8179518	8180031	KD2-H4K8ac_peak_6683	8.23274	
12	8185675	8186038	KD2-H4K8ac_peak_6684	4.67342	FOXJ2_ENSG00000065970
12	8332899	8333252	KD2-H4K8ac_peak_6685	11.24702	FAM66C_ENSG00000226711
12	8396401	8396695	KD2-H4K8ac_peak_6686	3.82395	FAM86FP_ENSG00000164845
12	8849851	8851101	KD2-H4K8ac_peak_6687	10.96455	
12	9065643	9065921	KD2-H4K8ac_peak_6688	5.57044	PHC1_ENSG00000111752
12	9103645	9103924	KD2-H4K8ac_peak_6689	4.7849	
12	9800176	9801277	KD2-H4K8ac_peak_6690	7.76104	RP11-705C15.4_ENSG00000256442
12	9892196	9892445	KD2-H4K8ac_peak_6691	5.20908	
12	10516888	10517451	KD2-H4K8ac_peak_6692	6.16888	RP11-277P12.20_ENSG00000245648
12	10766844	10767099	KD2-H4K8ac_peak_6693	7.12754	MAGOHB_ENSG00000111196
12	11323768	11324156	KD2-H4K8ac_peak_6694	24.3087	PRR4_ENSG00000111215;TAS2R14_ENSG00000212127;RP11-785H5.2_ENSG00000256712;RP11-785H5.1_ENSG00000256537
12	11801566	11802628	KD2-H4K8ac_peak_6695	14.66631	ETV6_ENSG00000139083
12	11802821	11803067	KD2-H4K8ac_peak_6696	14.05403	ETV6_ENSG00000139083
12	11829269	11829611	KD2-H4K8ac_peak_6697	5.25071	
12	12161983	12162262	KD2-H4K8ac_peak_6698	6.40683	
12	12185938	12186161	KD2-H4K8ac_peak_6699	6.25869	
12	12235109	12235332	KD2-H4K8ac_peak_6700	11.30637	
12	12419710	12420510	KD2-H4K8ac_peak_6701	25.35008	LRP6_ENSG00000070018
12	12502714	12503469	KD2-H4K8ac_peak_6702	12.93209	MANSC1_ENSG00000111261
12	12510173	12510467	KD2-H4K8ac_peak_6703	5.30799	LOH12CR2_ENSG00000205791;LOH12CR1_ENSG00000165714
12	12664173	12664727	KD2-H4K8ac_peak_6704	4.42765	
12	12686453	12686662	KD2-H4K8ac_peak_6705	4.19473	
12	12714574	12714987	KD2-H4K8ac_peak_6706	16.57619	DUSP16_ENSG00000111266
12	12715709	12716038	KD2-H4K8ac_peak_6707	5.13615	DUSP16_ENSG00000111266
12	12849348	12849540	KD2-H4K8ac_peak_6708	7.45501	GPR19_ENSG00000183150
12	12869283	12870074	KD2-H4K8ac_peak_6709	28.21903	
12	12870330	12870726	KD2-H4K8ac_peak_6710	18.31034	
12	12876635	12878234	KD2-H4K8ac_peak_6711	13.80019	APOLD1_ENSG00000178878;RP11-180M15.4_ENSG00000256658
12	12939567	12939813	KD2-H4K8ac_peak_6712	5.57044	
12	12940034	12940576	KD2-H4K8ac_peak_6713	19.88671	
12	12944620	12944824	KD2-H4K8ac_peak_6714	6.72334	

12	13197106	13197728	KD2-H4K8ac_peak_6715	8.82366	KIAA1467_ENSG00000084444
12	13253417	13254795	KD2-H4K8ac_peak_6716	26.74774	
12	13408991	13409327	KD2-H4K8ac_peak_6717	4.01115	
12	14393299	14393628	KD2-H4K8ac_peak_6718	10.96455	
12	14411106	14411670	KD2-H4K8ac_peak_6719	6.36375	
12	14538021	14538364	KD2-H4K8ac_peak_6720	10.21502	
12	14720415	14720730	KD2-H4K8ac_peak_6721	6.76298	PLBD1_ENSG00000121316;RP11-695J4.2_ENSG00000256751
12	14956054	14956349	KD2-H4K8ac_peak_6722	5.30799	WBP11_ENSG00000084463;C12orf60_ENSG00000182993
12	15475573	15476033	KD2-H4K8ac_peak_6723	9.11891	PTPRO_ENSG00000151490
12	15942077	15942279	KD2-H4K8ac_peak_6724	5.75757	
12	15942633	15943168	KD2-H4K8ac_peak_6725	12.23	
12	16034752	16035182	KD2-H4K8ac_peak_6726	14.8083	EPS8_ENSG00000151491;STRAP_ENSG00000023734
12	16035530	16035772	KD2-H4K8ac_peak_6727	6.76298	EPS8_ENSG00000151491;STRAP_ENSG00000023734
12	16064295	16064516	KD2-H4K8ac_peak_6728	9.63487	DERA_ENSG00000023697
12	16757885	16758087	KD2-H4K8ac_peak_6729	8.17902	
12	19282622	19283387	KD2-H4K8ac_peak_6730	18.62998	PLEKHA5_ENSG00000052126
12	19563031	19563228	KD2-H4K8ac_peak_6731	6.75638	
12	19563534	19563846	KD2-H4K8ac_peak_6732	4.92515	
12	19592619	19593059	KD2-H4K8ac_peak_6733	12.84642	
12	19726192	19727325	KD2-H4K8ac_peak_6734	7.47462	
12	20057165	20057500	KD2-H4K8ac_peak_6735	5.75452	
12	20097593	20097870	KD2-H4K8ac_peak_6736	4.52514	
12	20125380	20125666	KD2-H4K8ac_peak_6737	6.2781	AC087237.1_ENSG00000221679
12	20421942	20422245	KD2-H4K8ac_peak_6738	6.40683	
12	20521471	20521881	KD2-H4K8ac_peak_6739	7.27716	PDE3A_ENSG00000172572
12	20522214	20523154	KD2-H4K8ac_peak_6740	22.62254	RP11-284H19.1_ENSG00000256879;PDE3A_ENSG00000172572
12	20741622	20742115	KD2-H4K8ac_peak_6741	6.40683	
12	20782784	20782984	KD2-H4K8ac_peak_6742	3.75035	
12	21590531	21590745	KD2-H4K8ac_peak_6743	6.25869	PYROXD1_ENSG00000121350
12	21590951	21591379	KD2-H4K8ac_peak_6744	6.65673	PYROXD1_ENSG00000121350
12	21653868	21654291	KD2-H4K8ac_peak_6745	7.12754	RECQL_ENSG00000004700;GOLT1B_ENSG00000111711
12	21654787	21654986	KD2-H4K8ac_peak_6746	9.34989	RECQL_ENSG00000004700;GOLT1B_ENSG00000111711
12	21810349	21810711	KD2-H4K8ac_peak_6747	7.07117	
12	22199194	22199587	KD2-H4K8ac_peak_6748	34.26109	CMAS_ENSG00000111726
12	22697630	22697859	KD2-H4K8ac_peak_6749	5.53033	C2CD5_ENSG00000111731
12	22777337	22777647	KD2-H4K8ac_peak_6750	4.20044	RP11-268P4.4_ENSG00000257023;ETNK1_ENSG00000139163
12	22778206	22778439	KD2-H4K8ac_peak_6751	6.20982	RP11-268P4.4_ENSG00000257023;ETNK1_ENSG00000139163
12	23250557	23250892	KD2-H4K8ac_peak_6752	4.42765	
12	24714905	24716318	KD2-H4K8ac_peak_6753	9.63487	RP11-444D3.1_ENSG00000255864
12	25101554	25102956	KD2-H4K8ac_peak_6754	16.8435	BCAT1_ENSG00000060982;RP11-662I13.2_ENSG00000255921
12	25403317	25403794	KD2-H4K8ac_peak_6755	8.55271	KRAS_ENSG00000133703
12	25517288	25517511	KD2-H4K8ac_peak_6756	9.11818	
12	25538733	25538985	KD2-H4K8ac_peak_6757	6.40297	
12	25805459	25806281	KD2-H4K8ac_peak_6758	8.23062	
12	25807395	25807804	KD2-H4K8ac_peak_6759	5.88845	
12	25808617	25808931	KD2-H4K8ac_peak_6760	3.75035	

12	25927367	25927620	KD2-H4K8ac_peak_6761	5.75452	
12	26110972	26112375	KD2-H4K8ac_peak_6762	15.22643	RASSF8-AS1_ENSG00000246695;RASSF8_ENSG00000123094
12	26139304	26139498	KD2-H4K8ac_peak_6763	4.37708	
12	26170465	26170696	KD2-H4K8ac_peak_6764	4.86824	
12	26266650	26267206	KD2-H4K8ac_peak_6765	6.31143	
12	26348702	26349171	KD2-H4K8ac_peak_6766	8.53092	
12	26379473	26379747	KD2-H4K8ac_peak_6767	5.4779	
12	26986799	26987093	KD2-H4K8ac_peak_6768	5.14125	ITPR2_ENSG00000123104
12	27007072	27007310	KD2-H4K8ac_peak_6769	6.40683	
12	27023082	27023276	KD2-H4K8ac_peak_6770	6.66692	
12	27023582	27023793	KD2-H4K8ac_peak_6771	4.52326	
12	27024269	27024468	KD2-H4K8ac_peak_6772	6.40683	
12	27091447	27091798	KD2-H4K8ac_peak_6773	10.21366	ASUN_ENSG00000064102;FGFR1OP2_ENSG00000111790
12	27167544	27167889	KD2-H4K8ac_peak_6774	8.00457	TM7SF3_ENSG00000064115
12	27396689	27397039	KD2-H4K8ac_peak_6775	6.20982	STK38L_ENSG00000211455
12	27397266	27397650	KD2-H4K8ac_peak_6776	10.05606	STK38L_ENSG00000211455
12	27485386	27485659	KD2-H4K8ac_peak_6777	5.01002	ARNTL2_ENSG00000029153
12	27485860	27486318	KD2-H4K8ac_peak_6778	8.83582	ARNTL2_ENSG00000029153
12	27676426	27676654	KD2-H4K8ac_peak_6779	5.22731	PPFIBP1_ENSG00000110841
12	27863791	27864333	KD2-H4K8ac_peak_6780	18.12133	RP11-1060J15.4_ENSG00000256377;MRPS35_ENSG00000061794
12	27932361	27933636	KD2-H4K8ac_peak_6781	11.04256	KLHL42_ENSG00000087448;RP11-860B13.1_ENSG00000256747
12	27970900	27971296	KD2-H4K8ac_peak_6782	5.14125	
12	28342965	28343629	KD2-H4K8ac_peak_6783	15.66774	RP11-967K21.1_ENSG00000247934
12	29301489	29302684	KD2-H4K8ac_peak_6784	19.91242	RP11-946L16.1_ENSG00000257258;FAR2_ENSG00000064763
12	29533669	29534049	KD2-H4K8ac_peak_6785	11.92978	ERGIC2_ENSG00000087502
12	29878844	29879196	KD2-H4K8ac_peak_6786	8.1233	
12	29936522	29937281	KD2-H4K8ac_peak_6787	8.86099	TMTC1_ENSG00000133687
12	30848232	30848713	KD2-H4K8ac_peak_6788	12.36441	IPO8_ENSG00000133704
12	30849463	30849682	KD2-H4K8ac_peak_6789	7.40314	IPO8_ENSG00000133704
12	30907314	30907555	KD2-H4K8ac_peak_6790	9.20193	CAPRIN2_ENSG00000110888;RP11-77I22.2_ENSG00000246331
12	30908113	30908644	KD2-H4K8ac_peak_6791	8.55271	CAPRIN2_ENSG00000110888;RP11-77I22.2_ENSG00000246331
12	30949103	30950017	KD2-H4K8ac_peak_6792	12.82393	
12	31078963	31080338	KD2-H4K8ac_peak_6793	15.58442	TSPAN11_ENSG00000110900
12	31388032	31388650	KD2-H4K8ac_peak_6794	5.77029	
12	31477232	31478659	KD2-H4K8ac_peak_6795	7.07117	AC024940.1_ENSG00000177340
12	31742769	31743968	KD2-H4K8ac_peak_6796	22.66567	DENND5B_ENSG00000170456;DENND5B-AS1_ENSG00000255867
12	31873917	31874366	KD2-H4K8ac_peak_6797	6.31143	
12	31875556	31875947	KD2-H4K8ac_peak_6798	6.0073	
12	32112411	32112736	KD2-H4K8ac_peak_6799	5.75452	KIAA1551_ENSG00000174718
12	32146937	32147343	KD2-H4K8ac_peak_6800	5.19569	
12	32246651	32246892	KD2-H4K8ac_peak_6801	4.01115	
12	32259161	32260502	KD2-H4K8ac_peak_6802	22.36554	RP11-843B15.2_ENSG00000257530;BICD1_ENSG00000151746
12	32334049	32334350	KD2-H4K8ac_peak_6803	6.9809	
12	32543786	32544188	KD2-H4K8ac_peak_6804	10.05606	
12	32653749	32654179	KD2-H4K8ac_peak_6805	5.57044	
12	32831417	32831965	KD2-H4K8ac_peak_6806	9.43685	DNM1L_ENSG00000087470

12	32908281	32908796	KD2-H4K8ac_peak_6807	19.33018	YARS2_ENSG00000139131
12	33030966	33031370	KD2-H4K8ac_peak_6808	6.30229	
12	33049507	33049710	KD2-H4K8ac_peak_6809	7.47462	PKP2_ENSG00000057294
12	33049949	33050259	KD2-H4K8ac_peak_6810	16.32216	PKP2_ENSG00000057294
12	34175448	34175938	KD2-H4K8ac_peak_6811	4.77079	ALG10_ENSG00000139133
12	34496245	34496878	KD2-H4K8ac_peak_6812	12.17278	
12	34498179	34498837	KD2-H4K8ac_peak_6813	5.30799	
12	34499142	34499660	KD2-H4K8ac_peak_6814	5.57044	
12	38710674	38711010	KD2-H4K8ac_peak_6815	11.1952	ALG10B_ENSG00000175548
12	39300159	39300549	KD2-H4K8ac_peak_6816	12.80176	CPNE8_ENSG00000139117;RP11-396F22.1_ENSG00000257718
12	39300746	39300953	KD2-H4K8ac_peak_6817	5.14125	CPNE8_ENSG00000139117;RP11-396F22.1_ENSG00000257718
12	39436384	39436616	KD2-H4K8ac_peak_6818	7.12754	
12	39441914	39442141	KD2-H4K8ac_peak_6819	6.00243	
12	39783695	39784074	KD2-H4K8ac_peak_6820	9.07071	
12	39785545	39785878	KD2-H4K8ac_peak_6821	4.9025	
12	39786110	39786562	KD2-H4K8ac_peak_6822	5.88845	
12	39786844	39787091	KD2-H4K8ac_peak_6823	6.64891	
12	39789304	39789609	KD2-H4K8ac_peak_6824	8.79557	
12	39836020	39836736	KD2-H4K8ac_peak_6825	7.36799	KIF21A_ENSG00000139116;AC121334.1_ENSG00000252974
12	39837212	39837623	KD2-H4K8ac_peak_6826	12.82393	KIF21A_ENSG00000139116;AC121334.1_ENSG00000252974
12	40618188	40619147	KD2-H4K8ac_peak_6827	14.48922	AC079630.4_ENSG00000225342
12	42326019	42326300	KD2-H4K8ac_peak_6828	6.65673	RP11-630C16.2_ENSG00000257239
12	42326626	42326928	KD2-H4K8ac_peak_6829	9.15329	RP11-630C16.2_ENSG00000257239
12	42538068	42538453	KD2-H4K8ac_peak_6830	9.1707	GXYLT1_ENSG00000151233
12	42631709	42632008	KD2-H4K8ac_peak_6831	10.72656	PPHLN1_ENSG00000134283;YAF2_ENSG00000015153
12	42632291	42632560	KD2-H4K8ac_peak_6832	10.87093	PPHLN1_ENSG00000134283;YAF2_ENSG00000015153
12	42876914	42877502	KD2-H4K8ac_peak_6833	8.30974	
12	43876194	43876552	KD2-H4K8ac_peak_6834	6.16888	
12	44152058	44152337	KD2-H4K8ac_peak_6835	8.30974	PUS7L_ENSG00000129317;IRAK4_ENSG00000198001
12	44152949	44153154	KD2-H4K8ac_peak_6836	5.29555	PUS7L_ENSG00000129317;IRAK4_ENSG00000198001
12	44199729	44200062	KD2-H4K8ac_peak_6837	15.81711	TWF1_ENSG00000151239
12	44200345	44200640	KD2-H4K8ac_peak_6838	6.00226	TWF1_ENSG00000151239
12	45269853	45270054	KD2-H4K8ac_peak_6839	7.12754	
12	45609496	45609715	KD2-H4K8ac_peak_6840	7.40314	PLEKHA8P1_ENSG00000134297;ANO6_ENSG00000177119
12	45610160	45610897	KD2-H4K8ac_peak_6841	17.16047	PLEKHA8P1_ENSG00000134297;ANO6_ENSG00000177119
12	46120788	46120987	KD2-H4K8ac_peak_6842	6.90231	LINC00938_ENSG00000273015
12	46123085	46123371	KD2-H4K8ac_peak_6843	21.52958	ARID2_ENSG00000189079
12	46123625	46124506	KD2-H4K8ac_peak_6844	11.15509	ARID2_ENSG00000189079
12	46383916	46384321	KD2-H4K8ac_peak_6845	10.43145	
12	46384805	46385007	KD2-H4K8ac_peak_6846	6.65673	SCAF11_ENSG00000139218
12	46385525	46385766	KD2-H4K8ac_peak_6847	8.59579	SCAF11_ENSG00000139218
12	46661064	46663863	KD2-H4K8ac_peak_6848	12.30509	SLC38A1_ENSG00000111371
12	46765508	46766231	KD2-H4K8ac_peak_6849	8.30974	SLC38A2_ENSG00000134294;RP11-474P2.2_ENSG00000258096
12	46776286	46777072	KD2-H4K8ac_peak_6850	12.82393	RP11-96H19.1_ENSG00000257261
12	46777504	46777844	KD2-H4K8ac_peak_6851	10.21366	RP11-96H19.1_ENSG00000257261
12	48099463	48099695	KD2-H4K8ac_peak_6852	5.14125	RPAP3_ENSG00000005175;RP1-197B17.3_ENSG00000257433

12	48131218	48131661	KD2-H4K8ac_peak_6853	3.75035	
12	48134782	48135302	KD2-H4K8ac_peak_6854	12.93253	
12	48146845	48147130	KD2-H4K8ac_peak_6855	6.66692	SLC48A1_ENSG00000211584
12	48153000	48153474	KD2-H4K8ac_peak_6856	8.10216	
12	48167140	48167342	KD2-H4K8ac_peak_6857	5.14125	
12	48196101	48196320	KD2-H4K8ac_peak_6858	5.6755	
12	48201698	48202430	KD2-H4K8ac_peak_6859	5.54647	
12	48212897	48213530	KD2-H4K8ac_peak_6860	13.95627	
12	48213831	48214355	KD2-H4K8ac_peak_6861	8.16906	
12	48222866	48223194	KD2-H4K8ac_peak_6862	5.88845	
12	48384507	48386138	KD2-H4K8ac_peak_6863	10.96455	
12	48386332	48386553	KD2-H4K8ac_peak_6864	6.45018	
12	48499352	48500509	KD2-H4K8ac_peak_6865	13.81781	SENP1_ENSG00000079387;PFKM_ENSG00000152556
12	48576670	48576884	KD2-H4K8ac_peak_6866	4.54006	C12orf68_ENSG00000177875
12	48577418	48577668	KD2-H4K8ac_peak_6867	5.54647	C12orf68_ENSG00000177875
12	48592316	48592846	KD2-H4K8ac_peak_6868	4.33139	DKFZP779L1853_ENSG00000269514
12	48690515	48690834	KD2-H4K8ac_peak_6869	5.30799	
12	48744128	48745251	KD2-H4K8ac_peak_6870	15.039	ZNF641_ENSG00000167528;RP11-370I10.6_ENSG00000257735
12	49111005	49111306	KD2-H4K8ac_peak_6871	13.09607	CCNT1_ENSG00000129315
12	49182542	49184174	KD2-H4K8ac_peak_6872	21.75906	ADCY6_ENSG00000174233;RP11-579D7.4_ENSG00000257660
12	49208662	49209659	KD2-H4K8ac_peak_6873	15.61791	
12	49211502	49213096	KD2-H4K8ac_peak_6874	14.27217	
12	49245522	49245922	KD2-H4K8ac_peak_6875	10.43145	DDX23_ENSG00000174243
12	49259284	49259528	KD2-H4K8ac_peak_6876	7.40314	RND1_ENSG00000172602
12	49276531	49277898	KD2-H4K8ac_peak_6877	7.47462	
12	49350993	49351227	KD2-H4K8ac_peak_6878	18.31034	RP11-302B13.5_ENSG00000272822;ARF3_ENSG00000134287
12	49391771	49392499	KD2-H4K8ac_peak_6879	8.83582	DDN_ENSG00000181418;RP11-386G11.5_ENSG00000257913
12	49411730	49412510	KD2-H4K8ac_peak_6880	15.52826	PRKAG1_ENSG00000181929
12	49413283	49413517	KD2-H4K8ac_peak_6881	8.30974	PRKAG1_ENSG00000181929
12	49453464	49455234	KD2-H4K8ac_peak_6882	14.39182	KMT2D_ENSG00000167548
12	49481957	49482473	KD2-H4K8ac_peak_6883	10.86378	
12	49505281	49505569	KD2-H4K8ac_peak_6884	4.65111	LMBR1L_ENSG00000139636
12	49524236	49524543	KD2-H4K8ac_peak_6885	8.86099	TUBA1B_ENSG00000123416
12	49524762	49525151	KD2-H4K8ac_peak_6886	16.71655	TUBA1B_ENSG00000123416
12	49582343	49582643	KD2-H4K8ac_peak_6887	9.34989	TUBA1A_ENSG00000167552;TUBA1C_ENSG00000167553
12	49608378	49608659	KD2-H4K8ac_peak_6888	8.16133	
12	49617168	49617372	KD2-H4K8ac_peak_6889	4.59928	
12	49617713	49618015	KD2-H4K8ac_peak_6890	9.72244	
12	49626437	49627090	KD2-H4K8ac_peak_6891	7.40111	
12	49627995	49628769	KD2-H4K8ac_peak_6892	7.67504	
12	49686222	49687684	KD2-H4K8ac_peak_6893	16.87121	PRPH_ENSG00000135406
12	49688130	49688531	KD2-H4K8ac_peak_6894	6.0073	
12	49715566	49716030	KD2-H4K8ac_peak_6895	7.94441	TROAP_ENSG00000135451
12	49716259	49716736	KD2-H4K8ac_peak_6896	8.59579	TROAP_ENSG00000135451
12	49717102	49717447	KD2-H4K8ac_peak_6897	8.28234	RP11-161H23.9_ENSG00000258334;TROAP_ENSG00000135451
12	49730251	49730907	KD2-H4K8ac_peak_6898	7.36799	C1QL4_ENSG00000186897

12	49741617	49742244	KD2-H4K8ac_peak_6899	16.15695	DNAJC22_ENSG00000178401
12	49759812	49760141	KD2-H4K8ac_peak_6900	10.29736	SPATS2_ENSG00000123352
12	49761600	49761813	KD2-H4K8ac_peak_6901	4.47369	
12	49932557	49932858	KD2-H4K8ac_peak_6902	4.52514	KCNH3_ENSG00000135519
12	49961202	49962283	KD2-H4K8ac_peak_6903	10.30284	MCRS1_ENSG00000187778;PRPF40B_ENSG00000110844
12	50066874	50067455	KD2-H4K8ac_peak_6904	5.30799	
12	50067826	50068218	KD2-H4K8ac_peak_6905	5.07901	
12	50100271	50100579	KD2-H4K8ac_peak_6906	5.08628	TMBIM6_ENSG00000139644
12	50100831	50101210	KD2-H4K8ac_peak_6907	10.53377	FMNL3_ENSG00000161791;TMBIM6_ENSG00000139644
12	50134437	50134678	KD2-H4K8ac_peak_6908	4.35346	
12	50135614	50135845	KD2-H4K8ac_peak_6909	8.82366	
12	50170340	50170792	KD2-H4K8ac_peak_6910	9.15329	
12	50236298	50236817	KD2-H4K8ac_peak_6911	11.24702	BCDIN3D_ENSG00000186666
12	50237113	50237432	KD2-H4K8ac_peak_6912	5.88845	BCDIN3D_ENSG00000186666
12	50261640	50262527	KD2-H4K8ac_peak_6913	10.7186	
12	50263419	50264045	KD2-H4K8ac_peak_6914	9.36665	
12	50276420	50276624	KD2-H4K8ac_peak_6915	4.98072	
12	50298153	50298360	KD2-H4K8ac_peak_6916	5.32397	FAIM2_ENSG00000135472
12	50339236	50339452	KD2-H4K8ac_peak_6917	9.11031	
12	50361005	50361348	KD2-H4K8ac_peak_6918	14.39182	AQP6_ENSG00000086159
12	50361595	50361889	KD2-H4K8ac_peak_6919	7.40314	AQP6_ENSG00000086159
12	50369072	50369311	KD2-H4K8ac_peak_6920	4.35724	
12	50374029	50374231	KD2-H4K8ac_peak_6921	5.59859	
12	50377293	50377589	KD2-H4K8ac_peak_6922	6.04107	
12	50380443	50380690	KD2-H4K8ac_peak_6923	5.95537	
12	50418975	50419234	KD2-H4K8ac_peak_6924	8.25022	
12	50419631	50420180	KD2-H4K8ac_peak_6925	4.42765	
12	50435276	50435491	KD2-H4K8ac_peak_6926	3.75035	
12	50444970	50445299	KD2-H4K8ac_peak_6927	4.211	
12	50451149	50451346	KD2-H4K8ac_peak_6928	9.18237	ASIC1_ENSG00000110881
12	50451993	50452210	KD2-H4K8ac_peak_6929	4.64388	ASIC1_ENSG00000110881
12	50479118	50479382	KD2-H4K8ac_peak_6930	20.96279	SMARCD1_ENSG00000066117
12	50503966	50504366	KD2-H4K8ac_peak_6931	5.57044	
12	50504578	50505454	KD2-H4K8ac_peak_6932	12.93253	COX14_ENSG00000178449;RP4-605O3.4_ENSG00000272368
12	50560678	50560929	KD2-H4K8ac_peak_6933	7.07117	CERS5_ENSG00000139624
12	50676962	50677962	KD2-H4K8ac_peak_6934	9.51625	LIMA1_ENSG00000050405;AC008147.2_ENSG00000203433
12	50793265	50793848	KD2-H4K8ac_peak_6935	7.47462	
12	50794821	50795239	KD2-H4K8ac_peak_6936	7.76329	
12	50898837	50899085	KD2-H4K8ac_peak_6937	6.2781	DIP2B_ENSG00000066084
12	51157917	51158720	KD2-H4K8ac_peak_6938	21.26856	ATF1_ENSG00000123268
12	51419627	51420107	KD2-H4K8ac_peak_6939	7.28628	
12	51420432	51420932	KD2-H4K8ac_peak_6940	7.69287	
12	51441688	51441900	KD2-H4K8ac_peak_6941	9.31907	LETMD1_ENSG00000050426
12	51442132	51442578	KD2-H4K8ac_peak_6942	7.70289	LETMD1_ENSG00000050426
12	51566631	51566895	KD2-H4K8ac_peak_6943	19.54082	TFCP2_ENSG00000135457
12	51567128	51567557	KD2-H4K8ac_peak_6944	8.85813	TFCP2_ENSG00000135457

12	51611964	51612800	KD2-H4K8ac_peak_6945	14.57259	POU6F1_ENSG00000184271
12	51632008	51632246	KD2-H4K8ac_peak_6946	7.32463	DAZAP2_ENSG00000183283
12	51632646	51632948	KD2-H4K8ac_peak_6947	14.99434	DAZAP2_ENSG00000183283
12	51663821	51664156	KD2-H4K8ac_peak_6948	5.54647	SMAGP_ENSG00000170545
12	51664371	51664656	KD2-H4K8ac_peak_6949	7.1181	SMAGP_ENSG00000170545
12	51765155	51766104	KD2-H4K8ac_peak_6950	8.30974	
12	51781460	51781926	KD2-H4K8ac_peak_6951	5.14125	
12	51784887	51785844	KD2-H4K8ac_peak_6952	12.08081	GALNT6_ENSG00000139629;SLC4A8_ENSG00000050438
12	51800435	51800629	KD2-H4K8ac_peak_6953	5.14125	
12	51801382	51801600	KD2-H4K8ac_peak_6954	10.63725	
12	51818176	51818404	KD2-H4K8ac_peak_6955	7.47462	RP11-607P23.1_ENSG00000271065
12	51818690	51819053	KD2-H4K8ac_peak_6956	18.94636	RP11-607P23.1_ENSG00000271065
12	51941062	51941254	KD2-H4K8ac_peak_6957	5.22731	
12	51984186	51984742	KD2-H4K8ac_peak_6958	5.88845	SCN8A_ENSG00000196876
12	52207737	52208897	KD2-H4K8ac_peak_6959	14.66631	
12	52214877	52215427	KD2-H4K8ac_peak_6960	15.69572	RP11-923I11.7_ENSG00000261308
12	52239141	52239449	KD2-H4K8ac_peak_6961	5.14125	
12	52240918	52241135	KD2-H4K8ac_peak_6962	5.88845	RP11-923I11.5_ENSG00000259887
12	52242027	52242450	KD2-H4K8ac_peak_6963	11.80283	RP11-923I11.5_ENSG00000259887
12	52243037	52243289	KD2-H4K8ac_peak_6964	8.09303	
12	52262524	52263445	KD2-H4K8ac_peak_6965	13.70753	
12	52304580	52304781	KD2-H4K8ac_peak_6966	4.67342	
12	52306028	52307872	KD2-H4K8ac_peak_6967	6.20982	
12	52309002	52309275	KD2-H4K8ac_peak_6968	7.76569	
12	52311411	52311979	KD2-H4K8ac_peak_6969	12.57143	
12	52312860	52313072	KD2-H4K8ac_peak_6970	6.40297	
12	52314926	52315128	KD2-H4K8ac_peak_6971	6.27719	
12	52332479	52332909	KD2-H4K8ac_peak_6972	9.11031	
12	52334168	52334648	KD2-H4K8ac_peak_6973	6.25869	
12	52345466	52345981	KD2-H4K8ac_peak_6974	13.73635	ACVR1B_ENSG00000135503
12	52387159	52387384	KD2-H4K8ac_peak_6975	4.77079	
12	52400821	52401072	KD2-H4K8ac_peak_6976	5.82799	GRASP_ENSG00000161835
12	52403912	52404580	KD2-H4K8ac_peak_6977	11.05407	
12	52414426	52414619	KD2-H4K8ac_peak_6978	5.88845	
12	52416529	52417242	KD2-H4K8ac_peak_6979	6.8836	NR4A1_ENSG00000123358
12	52419115	52419501	KD2-H4K8ac_peak_6980	8.4814	
12	52419733	52419977	KD2-H4K8ac_peak_6981	7.65815	
12	52430489	52430808	KD2-H4K8ac_peak_6982	4.67342	
12	52431124	52431486	KD2-H4K8ac_peak_6983	9.63487	
12	52444282	52444952	KD2-H4K8ac_peak_6984	9.96941	
12	52462902	52463299	KD2-H4K8ac_peak_6985	9.31907	C12orf44_ENSG00000123395
12	52463898	52464118	KD2-H4K8ac_peak_6986	10.0692	C12orf44_ENSG00000123395
12	52545918	52546129	KD2-H4K8ac_peak_6987	4.37709	
12	52568279	52568474	KD2-H4K8ac_peak_6988	6.30626	
12	52568753	52569153	KD2-H4K8ac_peak_6989	5.29555	
12	52569347	52569842	KD2-H4K8ac_peak_6990	8.95692	

12	52576198	52576445	KD2-H4K8ac_peak_6991	7.67504	
12	52577105	52577458	KD2-H4K8ac_peak_6992	4.21775	
12	52577723	52578275	KD2-H4K8ac_peak_6993	11.03352	
12	52579953	52580467	KD2-H4K8ac_peak_6994	6.65673	
12	52592506	52593697	KD2-H4K8ac_peak_6995	13.44374	
12	52593915	52594153	KD2-H4K8ac_peak_6996	7.69287	
12	52594487	52594690	KD2-H4K8ac_peak_6997	5.02474	
12	52596041	52596445	KD2-H4K8ac_peak_6998	6.65673	
12	52596670	52597005	KD2-H4K8ac_peak_6999	8.55581	
12	52597246	52597639	KD2-H4K8ac_peak_7000	5.14125	
12	52600852	52601249	KD2-H4K8ac_peak_7001	6.66692	
12	52637950	52638384	KD2-H4K8ac_peak_7002	9.1994	
12	53399259	53399849	KD2-H4K8ac_peak_7003	11.43863	EIF4B_ENSG00000063046
12	53400336	53400692	KD2-H4K8ac_peak_7004	11.60333	EIF4B_ENSG00000063046
12	53440547	53440885	KD2-H4K8ac_peak_7005	13.73635	TENC1_ENSG00000111077
12	53441270	53441837	KD2-H4K8ac_peak_7006	13.81781	TENC1_ENSG00000111077
12	53443032	53443499	KD2-H4K8ac_peak_7007	8.79106	
12	53443790	53444229	KD2-H4K8ac_peak_7008	3.97079	
12	53447778	53448194	KD2-H4K8ac_peak_7009	5.54647	RP11-983P16.4_ENSG00000257337
12	53472659	53472990	KD2-H4K8ac_peak_7010	14.48084	SPRYD3_ENSG00000167778
12	53473350	53474484	KD2-H4K8ac_peak_7011	10.19949	SPRYD3_ENSG00000167778
12	53490888	53492132	KD2-H4K8ac_peak_7012	11.03331	IGFBP6_ENSG00000167779
12	53492428	53492670	KD2-H4K8ac_peak_7013	5.557	
12	53573618	53574188	KD2-H4K8ac_peak_7014	14.05403	ZNF740_ENSG00000139651;CSAD_ENSG00000139631
12	53614483	53614735	KD2-H4K8ac_peak_7015	4.06758	
12	53615086	53615967	KD2-H4K8ac_peak_7016	10.7186	
12	53616369	53617148	KD2-H4K8ac_peak_7017	10.96455	
12	53625135	53625996	KD2-H4K8ac_peak_7018	15.25274	RARG_ENSG00000172819
12	53626263	53626768	KD2-H4K8ac_peak_7019	6.2781	RARG_ENSG00000172819
12	53627029	53628083	KD2-H4K8ac_peak_7020	12.84642	RARG_ENSG00000172819
12	53645146	53645675	KD2-H4K8ac_peak_7021	7.36799	MFSD5_ENSG00000182544
12	53645925	53646480	KD2-H4K8ac_peak_7022	10.43145	MFSD5_ENSG00000182544
12	53661567	53661892	KD2-H4K8ac_peak_7023	5.08628	ESPL1_ENSG00000135476
12	53693059	53693905	KD2-H4K8ac_peak_7024	8.26945	RP11-680A11.5_ENSG00000257605;C12orf10_ENSG00000139637
12	53715634	53715964	KD2-H4K8ac_peak_7025	6.30229	
12	53717974	53718248	KD2-H4K8ac_peak_7026	6.83936	AAAS_ENSG00000094914
12	53718517	53720119	KD2-H4K8ac_peak_7027	12.93209	AAAS_ENSG00000094914
12	53732525	53732851	KD2-H4K8ac_peak_7028	8.02571	
12	53738447	53738913	KD2-H4K8ac_peak_7029	7.28827	SP7_ENSG00000170374
12	53739550	53739959	KD2-H4K8ac_peak_7030	7.70289	SP7_ENSG00000170374
12	53772996	53773210	KD2-H4K8ac_peak_7031	8.10216	SP1_ENSG00000185591
12	53774084	53774335	KD2-H4K8ac_peak_7032	8.33649	SP1_ENSG00000185591
12	53784156	53784446	KD2-H4K8ac_peak_7033	14.00658	
12	53785224	53785450	KD2-H4K8ac_peak_7034	9.02573	
12	53808527	53808755	KD2-H4K8ac_peak_7035	6.94255	
12	53809154	53809751	KD2-H4K8ac_peak_7036	8.17902	

12	53810602	53810814	KD2-H4K8ac_peak_7037	5.75452	
12	53811819	53812164	KD2-H4K8ac_peak_7038	7.70289	
12	53834195	53834610	KD2-H4K8ac_peak_7039	6.24747	PRR13_ENSG00000205352;RP11-793H13.8_ENSG00000257379;PCBP2_ENSG00000197111
12	53846146	53846410	KD2-H4K8ac_peak_7040	12.82393	
12	53892842	53893074	KD2-H4K8ac_peak_7041	11.96169	MAP3K12_ENSG00000139625
12	53894809	53895992	KD2-H4K8ac_peak_7042	15.61791	MAP3K12_ENSG00000139625;RP11-793H13.11_ENSG00000270175;TARBP2_ENSG00000139546
12	54069152	54069826	KD2-H4K8ac_peak_7043	8.30974	
12	54070154	54070807	KD2-H4K8ac_peak_7044	19.38373	ATP5G2_ENSG00000135390
12	54121592	54121951	KD2-H4K8ac_peak_7045	6.66692	CALCOCO1_ENSG00000012822
12	54379104	54379316	KD2-H4K8ac_peak_7046	5.51783	RP11-834C11.12_ENSG00000273049;HOXC-AS3_ENSG00000251151;HOXC10_ENSG00000180818
12	54393488	54393702	KD2-H4K8ac_peak_7047	6.25869	HOXC-AS1_ENSG00000250451
12	54394056	54394600	KD2-H4K8ac_peak_7048	11.8457	HOXC-AS1_ENSG00000250451
12	54399989	54400198	KD2-H4K8ac_peak_7049	9.23578	
12	54409405	54409781	KD2-H4K8ac_peak_7050	3.75035	HOXC4_ENSG00000198353
12	54411384	54411677	KD2-H4K8ac_peak_7051	8.86391	HOXC4_ENSG00000198353;RP11-834C11.14_ENSG00000273046
12	54412009	54412732	KD2-H4K8ac_peak_7052	14.99434	AC012531.25_ENSG00000260597
12	54413020	54413508	KD2-H4K8ac_peak_7053	16.69872	AC012531.25_ENSG00000260597
12	54414918	54415328	KD2-H4K8ac_peak_7054	6.0073	
12	54417913	54418152	KD2-H4K8ac_peak_7055	4.70206	
12	54422853	54423069	KD2-H4K8ac_peak_7056	7.76329	
12	54426955	54427517	KD2-H4K8ac_peak_7057	9.36516	HOXC5_ENSG00000172789;MIR615_ENSG00000207571
12	54427900	54428580	KD2-H4K8ac_peak_7058	7.90806	MIR615_ENSG00000207571
12	54446348	54447484	KD2-H4K8ac_peak_7059	16.15695	HOXC4_ENSG00000273266
12	54447797	54448057	KD2-H4K8ac_peak_7060	8.55271	HOXC4_ENSG00000273266
12	54474392	54474598	KD2-H4K8ac_peak_7061	7.18916	
12	54476058	54476306	KD2-H4K8ac_peak_7062	5.88845	RP11-834C11.7_ENSG00000250654;RP11-834C11.6_ENSG00000249388
12	54483418	54483731	KD2-H4K8ac_peak_7063	5.14125	
12	54483994	54484395	KD2-H4K8ac_peak_7064	11.05407	
12	54519906	54520491	KD2-H4K8ac_peak_7065	7.07117	RP11-834C11.5_ENSG00000250432;RP11-834C11.4_ENSG00000250742
12	54521149	54521521	KD2-H4K8ac_peak_7066	11.92978	
12	54529181	54529534	KD2-H4K8ac_peak_7067	9.36665	
12	54566273	54567955	KD2-H4K8ac_peak_7068	5.38581	
12	54582417	54582667	KD2-H4K8ac_peak_7069	8.37231	SMUG1_ENSG00000123415
12	54583031	54583512	KD2-H4K8ac_peak_7070	10.59312	SMUG1_ENSG00000123415
12	54588561	54588902	KD2-H4K8ac_peak_7071	4.211	
12	54589500	54589700	KD2-H4K8ac_peak_7072	7.31433	
12	54589904	54590148	KD2-H4K8ac_peak_7073	6.56211	
12	54594985	54595183	KD2-H4K8ac_peak_7074	7.99241	
12	54652949	54653374	KD2-H4K8ac_peak_7075	11.38361	
12	54654022	54654355	KD2-H4K8ac_peak_7076	10.29344	
12	54717819	54718358	KD2-H4K8ac_peak_7077	4.54006	
12	54745412	54746924	KD2-H4K8ac_peak_7078	12.08081	RP11-753H16.3_ENSG00000258137;RP11-753H16.5_ENSG00000258086
12	54747452	54747752	KD2-H4K8ac_peak_7079	8.17902	RP11-753H16.3_ENSG00000258137;RP11-753H16.5_ENSG00000258086
12	54750910	54751163	KD2-H4K8ac_peak_7080	3.75035	
12	54752653	54752850	KD2-H4K8ac_peak_7081	8.85813	
12	54753451	54753654	KD2-H4K8ac_peak_7082	4.67342	

12	54784920	54785274	KD2-H4K8ac_peak_7083	5.52329	ZNF385A_ENSG00000161642
12	56040190	56040497	KD2-H4K8ac_peak_7084	14.15075	OR10AE3P_ENSG00000272837
12	56109361	56110420	KD2-H4K8ac_peak_7085	11.92978	ITGA7_ENSG00000135424;RP11-644F5.10_ENSG00000258311;BLOC1S1_ENSG00000135441
12	56122069	56123416	KD2-H4K8ac_peak_7086	12.84642	CD63_ENSG00000135404;RP11-644F5.11_ENSG00000258056
12	56123643	56123926	KD2-H4K8ac_peak_7087	10.87093	CD63_ENSG00000135404;RP11-644F5.11_ENSG00000258056
12	56131703	56132212	KD2-H4K8ac_peak_7088	13.2669	
12	56211063	56211438	KD2-H4K8ac_peak_7089	5.20998	SARNP_ENSG00000205323;ORMDL2_ENSG00000123353
12	56222801	56223062	KD2-H4K8ac_peak_7090	10.05606	TMEM198B_ENSG00000182796
12	56223667	56224117	KD2-H4K8ac_peak_7091	10.96455	DNAJC14_ENSG00000135392;TMEM198B_ENSG00000182796
12	56241364	56241648	KD2-H4K8ac_peak_7092	8.26945	
12	56242271	56242566	KD2-H4K8ac_peak_7093	11.05407	
12	56320463	56322209	KD2-H4K8ac_peak_7094	17.8868	DGKA_ENSG00000065357
12	56325221	56325541	KD2-H4K8ac_peak_7095	5.75757	WIBG_ENSG00000170473
12	56334349	56334601	KD2-H4K8ac_peak_7096	6.40683	
12	56367904	56368185	KD2-H4K8ac_peak_7097	10.29842	PMEL_ENSG00000185664;RAB5B_ENSG00000111540
12	56401291	56401849	KD2-H4K8ac_peak_7098	6.65673	IKZF4_ENSG00000123411
12	56414392	56414840	KD2-H4K8ac_peak_7099	20.88898	
12	56415144	56416138	KD2-H4K8ac_peak_7100	18.09077	
12	56435946	56436181	KD2-H4K8ac_peak_7101	12.08081	RP11-603J24.4_ENSG00000257449;RPS26_ENSG00000197728
12	56472998	56473567	KD2-H4K8ac_peak_7102	12.84642	ERBB3_ENSG00000065361
12	56473767	56474261	KD2-H4K8ac_peak_7103	15.59774	ERBB3_ENSG00000065361
12	56498402	56498751	KD2-H4K8ac_peak_7104	6.67538	PA2G4_ENSG00000170515
12	56509270	56509875	KD2-H4K8ac_peak_7105	10.96455	RPL41_ENSG00000229117
12	56512123	56512418	KD2-H4K8ac_peak_7106	10.53377	ZC3H10_ENSG00000135482;ESYT1_ENSG00000139641
12	56522139	56522332	KD2-H4K8ac_peak_7107	6.90231	
12	56545633	56546240	KD2-H4K8ac_peak_7108	5.75757	MYL6B_ENSG00000196465
12	56551232	56551836	KD2-H4K8ac_peak_7109	8.20667	RP11-603J24.14_ENSG00000257809;MYL6_ENSG00000092841
12	56583840	56584038	KD2-H4K8ac_peak_7110	9.36516	RP11-977G19.5_ENSG00000258199;SMARCC2_ENSG00000139613
12	56587178	56587377	KD2-H4K8ac_peak_7111	3.99369	
12	56615369	56615577	KD2-H4K8ac_peak_7112	4.86324	RNF41_ENSG00000181852;NABP2_ENSG00000139579
12	56615881	56616425	KD2-H4K8ac_peak_7113	9.96798	RNF41_ENSG00000181852;NABP2_ENSG00000139579
12	56617546	56617791	KD2-H4K8ac_peak_7114	7.98621	
12	56618246	56618744	KD2-H4K8ac_peak_7115	6.97919	
12	56652276	56652477	KD2-H4K8ac_peak_7116	12.70959	ANKRD52_ENSG00000139645
12	56660232	56660852	KD2-H4K8ac_peak_7117	8.55271	COQ10A_ENSG00000135469
12	56661085	56661537	KD2-H4K8ac_peak_7118	6.2781	COQ10A_ENSG00000135469
12	56693605	56694084	KD2-H4K8ac_peak_7119	8.55271	CS_ENSG00000062485;RP11-977G19.11_ENSG00000257303
12	56710007	56710633	KD2-H4K8ac_peak_7120	5.75757	RP11-977G19.10_ENSG00000144785;CNPY2_ENSG00000257727
12	56726699	56727140	KD2-H4K8ac_peak_7121	9.26408	PAN2_ENSG00000135473
12	56728120	56728518	KD2-H4K8ac_peak_7122	11.92978	PAN2_ENSG00000135473
12	56729138	56729330	KD2-H4K8ac_peak_7123	4.35346	
12	56753443	56753842	KD2-H4K8ac_peak_7124	7.146	STAT2_ENSG00000170581
12	56841901	56842131	KD2-H4K8ac_peak_7125	8.53092	
12	56842717	56843228	KD2-H4K8ac_peak_7126	8.55271	TIMELESS_ENSG00000111602
12	56843876	56844143	KD2-H4K8ac_peak_7127	4.77079	TIMELESS_ENSG00000111602
12	56862470	56862808	KD2-H4K8ac_peak_7128	4.67342	MIP_ENSG00000135517;SPRYD4_ENSG00000176422

12	56881804	56882127	KD2-H4K8ac_peak_7129	8.2413	
12	56882361	56882607	KD2-H4K8ac_peak_7130	7.47462	
12	57023753	57023996	KD2-H4K8ac_peak_7131	6.66359	
12	57028054	57028565	KD2-H4K8ac_peak_7132	4.19765	
12	57029788	57030047	KD2-H4K8ac_peak_7133	11.47498	BAZ2A_ENSG00000076108
12	57081611	57081913	KD2-H4K8ac_peak_7134	12.93209	PTGES3_ENSG00000110958
12	57082397	57082873	KD2-H4K8ac_peak_7135	13.73635	PTGES3_ENSG00000110958
12	57118386	57119039	KD2-H4K8ac_peak_7136	12.93253	
12	57146493	57146704	KD2-H4K8ac_peak_7137	6.31143	PRIM1_ENSG00000198056;HSD17B6_ENSG00000025423
12	57399742	57400284	KD2-H4K8ac_peak_7138	15.039	ZBTB39_ENSG00000166860
12	57420960	57421274	KD2-H4K8ac_peak_7139	5.54647	
12	57479786	57480176	KD2-H4K8ac_peak_7140	8.30094	
12	57480762	57481033	KD2-H4K8ac_peak_7141	9.63487	TMEM194A_ENSG00000166881
12	57481252	57481633	KD2-H4K8ac_peak_7142	19.72117	TMEM194A_ENSG00000166881
12	57481995	57482345	KD2-H4K8ac_peak_7143	4.86324	TMEM194A_ENSG00000166881;NAB2_ENSG00000166886
12	57505406	57505771	KD2-H4K8ac_peak_7144	5.57044	
12	57506083	57506318	KD2-H4K8ac_peak_7145	6.98292	
12	57521412	57522105	KD2-H4K8ac_peak_7146	10.96455	LRP1_ENSG00000123384
12	57529683	57529931	KD2-H4K8ac_peak_7147	4.86324	
12	57545834	57546482	KD2-H4K8ac_peak_7148	7.79741	
12	57546680	57547302	KD2-H4K8ac_peak_7149	8.85813	
12	57547806	57548700	KD2-H4K8ac_peak_7150	8.56219	
12	57552073	57552282	KD2-H4K8ac_peak_7151	7.13615	
12	57631764	57632023	KD2-H4K8ac_peak_7152	7.76329	
12	57632251	57632922	KD2-H4K8ac_peak_7153	9.46843	
12	57633151	57634320	KD2-H4K8ac_peak_7154	25.87783	NDUFA4L2_ENSG00000185633
12	57824219	57824621	KD2-H4K8ac_peak_7155	8.85813	R3HDM2_ENSG00000179912;RP11-756H6.1_ENSG00000258001
12	57824900	57825437	KD2-H4K8ac_peak_7156	10.0692	R3HDM2_ENSG00000179912;RP11-756H6.1_ENSG00000258001
12	57848446	57849038	KD2-H4K8ac_peak_7157	12.74183	
12	57849272	57849685	KD2-H4K8ac_peak_7158	5.01002	
12	57881020	57881616	KD2-H4K8ac_peak_7159	17.37958	ARHGAP9_ENSG00000123329
12	57881840	57882369	KD2-H4K8ac_peak_7160	18.5703	ARHGAP9_ENSG00000123329
12	57914752	57915640	KD2-H4K8ac_peak_7161	13.73635	DDIT3_ENSG00000175197;MBD6_ENSG00000166987
12	57940344	57940962	KD2-H4K8ac_peak_7162	16.61426	DCTN2_ENSG00000175203
12	57943184	57943521	KD2-H4K8ac_peak_7163	4.04573	KIF5A_ENSG00000155980
12	57946500	57946803	KD2-H4K8ac_peak_7164	7.01658	
12	57984478	57984839	KD2-H4K8ac_peak_7165	5.01002	PIP4K2C_ENSG00000166908
12	57998204	57999025	KD2-H4K8ac_peak_7166	14.66631	DTX3_ENSG00000178498
12	58004230	58005737	KD2-H4K8ac_peak_7167	20.46575	ARHGEF25_ENSG00000240771
12	58026084	58026290	KD2-H4K8ac_peak_7168	5.30799	B4GALNT1_ENSG00000135454
12	58026615	58026957	KD2-H4K8ac_peak_7169	15.22643	B4GALNT1_ENSG00000135454
12	58087273	58087479	KD2-H4K8ac_peak_7170	5.20908	OS9_ENSG00000135506
12	58119637	58119893	KD2-H4K8ac_peak_7171	7.36799	RP11-571M6.8_ENSG00000257499;AGAP2-AS1_ENSG00000255737
12	58120142	58121248	KD2-H4K8ac_peak_7172	13.31016	RP11-571M6.8_ENSG00000257499;AGAP2-AS1_ENSG00000255737
12	58131794	58132061	KD2-H4K8ac_peak_7173	5.57044	TSPAN31_ENSG00000135452
12	58137909	58138557	KD2-H4K8ac_peak_7174	9.11031	

12	58138810	58139060	KD2-H4K8ac_peak_7175	18.89109	
12	58145363	58146080	KD2-H4K8ac_peak_7176	11.00372	
12	58148174	58148379	KD2-H4K8ac_peak_7177	5.14125	MARCH9_ENSG00000139266
12	58148895	58149095	KD2-H4K8ac_peak_7178	12.46264	CDK4_ENSG00000135446;MARCH9_ENSG00000139266
12	58166064	58167028	KD2-H4K8ac_peak_7179	17.19888	METTL1_ENSG00000037897;METTL21B_ENSG00000123427;RP11-571M6.15_ENSG00000257921
12	58234071	58234355	KD2-H4K8ac_peak_7180	7.84888	
12	58238896	58239460	KD2-H4K8ac_peak_7181	8.55271	
12	58240028	58240713	KD2-H4K8ac_peak_7182	12.00597	CTDSP2_ENSG00000175215
12	58281728	58281928	KD2-H4K8ac_peak_7183	4.77079	
12	58282425	58282634	KD2-H4K8ac_peak_7184	7.47462	
12	58283488	58283856	KD2-H4K8ac_peak_7185	6.24114	
12	58287071	58287858	KD2-H4K8ac_peak_7186	9.63487	
12	58288235	58288562	KD2-H4K8ac_peak_7187	9.36516	
12	58290059	58291033	KD2-H4K8ac_peak_7188	17.09549	RP11-620J15.2_ENSG00000245651
12	58298813	58299055	KD2-H4K8ac_peak_7189	3.75035	
12	62653510	62653979	KD2-H4K8ac_peak_7190	11.89502	USP15_ENSG00000135655
12	62860683	62861035	KD2-H4K8ac_peak_7191	5.59859	MON2_ENSG00000061987
12	62996052	62997743	KD2-H4K8ac_peak_7192	14.98936	C12orf61_ENSG00000221949;RP11-631N16.2_ENSG00000257354;MIRLET7I_ENSG00000199179
12	63025715	63026673	KD2-H4K8ac_peak_7193	16.64426	
12	63104865	63105139	KD2-H4K8ac_peak_7194	5.88845	
12	63108805	63109222	KD2-H4K8ac_peak_7195	6.00226	
12	64061848	64062924	KD2-H4K8ac_peak_7196	11.3394	DPY19L2_ENSG00000177990
12	64173672	64174243	KD2-H4K8ac_peak_7197	11.05407	TMEM5_ENSG00000118600
12	64237234	64237460	KD2-H4K8ac_peak_7198	12.74171	SRGAP1_ENSG00000196935
12	64237969	64238519	KD2-H4K8ac_peak_7199	11.05407	SRGAP1_ENSG00000196935
12	64331608	64331864	KD2-H4K8ac_peak_7200	6.05809	
12	64333799	64334120	KD2-H4K8ac_peak_7201	6.8836	
12	64334343	64334545	KD2-H4K8ac_peak_7202	6.00243	
12	64338545	64338771	KD2-H4K8ac_peak_7203	11.30637	
12	64347206	64347505	KD2-H4K8ac_peak_7204	6.16888	
12	64616174	64616574	KD2-H4K8ac_peak_7205	5.4779	C12orf66_ENSG00000174206;RPS11P6_ENSG00000243024
12	64797550	64797970	KD2-H4K8ac_peak_7206	5.75452	XPOT_ENSG00000184575
12	64798335	64798834	KD2-H4K8ac_peak_7207	15.57998	XPOT_ENSG00000184575
12	64800215	64800453	KD2-H4K8ac_peak_7208	4.42765	
12	64845524	64845738	KD2-H4K8ac_peak_7209	10.05606	TBK1_ENSG00000183735
12	64845931	64846307	KD2-H4K8ac_peak_7210	11.28831	TBK1_ENSG00000183735
12	64936535	64936761	KD2-H4K8ac_peak_7211	9.11031	
12	64977328	64977734	KD2-H4K8ac_peak_7212	7.40111	
12	64978900	64979111	KD2-H4K8ac_peak_7213	4.3015	
12	65004279	65004994	KD2-H4K8ac_peak_7214	11.90517	RASSF3_ENSG00000153179
12	65063355	65063555	KD2-H4K8ac_peak_7215	7.167	
12	65065992	65066445	KD2-H4K8ac_peak_7216	15.81711	
12	65076150	65076402	KD2-H4K8ac_peak_7217	8.5781	
12	65076808	65077005	KD2-H4K8ac_peak_7218	5.88845	
12	65153944	65154197	KD2-H4K8ac_peak_7219	7.33266	GNS_ENSG00000135677;RP11-629N8.3_ENSG00000215159;snoU13_ENSG00000238592
12	65562832	65563212	KD2-H4K8ac_peak_7220	10.43145	LEMD3_ENSG00000174106

12	65563501	65564047	KD2-H4K8ac_peak_7221	15.58442	LEMD3_ENSG00000174106
12	65672287	65672710	KD2-H4K8ac_peak_7222	7.40314	MSRB3_ENSG00000174099
12	66563216	66564191	KD2-H4K8ac_peak_7223	19.41412	TMBIM4_ENSG00000228144;TMBIM4_ENSG00000155957
12	66629201	66629405	KD2-H4K8ac_peak_7224	5.25071	RBMS1P1_ENSG00000225422;AC078889.1_ENSG00000266539
12	67663187	67664130	KD2-H4K8ac_peak_7225	6.66359	CAND1_ENSG00000111530
12	68042581	68043866	KD2-H4K8ac_peak_7226	15.22643	DYRK2_ENSG00000127334
12	68120431	68121390	KD2-H4K8ac_peak_7227	8.1233	
12	68122495	68122804	KD2-H4K8ac_peak_7228	6.64891	RP11-43N5.1_ENSG00000255970
12	68123129	68123391	KD2-H4K8ac_peak_7229	6.16888	RP11-43N5.1_ENSG00000255970
12	68639247	68640152	KD2-H4K8ac_peak_7230	10.63441	
12	68725760	68726089	KD2-H4K8ac_peak_7231	15.69572	MDM1_ENSG00000111554
12	68800093	68800292	KD2-H4K8ac_peak_7232	5.57044	
12	69004228	69004427	KD2-H4K8ac_peak_7233	11.05407	RAP1B_ENSG00000127314
12	69080790	69081377	KD2-H4K8ac_peak_7234	15.9336	RP11-637A17.2_ENSG00000247363;NUP107_ENSG00000111581
12	69139355	69139717	KD2-H4K8ac_peak_7235	6.25869	SLC35E3_ENSG00000175782
12	69201275	69201728	KD2-H4K8ac_peak_7236	5.9696	MDM2_ENSG00000135679
12	69202034	69202287	KD2-H4K8ac_peak_7237	12.14909	MDM2_ENSG00000135679
12	69633432	69634256	KD2-H4K8ac_peak_7238	15.69572	CPSF6_ENSG00000111605
12	69724889	69725275	KD2-H4K8ac_peak_7239	7.12754	
12	69753547	69753782	KD2-H4K8ac_peak_7240	11.24702	YEATS4_ENSG00000127337
12	69864204	69864412	KD2-H4K8ac_peak_7241	16.15695	FRS2_ENSG00000166225
12	69977955	69978359	KD2-H4K8ac_peak_7242	5.53095	MIR3913-1_ENSG00000264405;CCT2_ENSG00000166226
12	69979266	69979534	KD2-H4K8ac_peak_7243	11.24702	MIR3913-1_ENSG00000264405;CCT2_ENSG00000166226
12	70132719	70132962	KD2-H4K8ac_peak_7244	7.36799	RP11-588G21.2_ENSG00000247131;RAB3IP_ENSG00000127328
12	70272891	70273208	KD2-H4K8ac_peak_7245	3.75035	
12	70636813	70637861	KD2-H4K8ac_peak_7246	6.84261	RP11-611E13.2_ENSG00000257815;CNOT2_ENSG00000111596
12	71113435	71113644	KD2-H4K8ac_peak_7247	6.00226	
12	71951945	71952385	KD2-H4K8ac_peak_7248	5.1313	
12	71957049	71957435	KD2-H4K8ac_peak_7249	12.3187	
12	72057229	72057578	KD2-H4K8ac_peak_7250	12.46522	THAP2_ENSG00000173451
12	72058174	72058437	KD2-H4K8ac_peak_7251	7.76329	
12	72079219	72079466	KD2-H4K8ac_peak_7252	9.16531	TMEM19_ENSG00000139291
12	72079877	72080089	KD2-H4K8ac_peak_7253	7.76329	TMEM19_ENSG00000139291
12	72147812	72148053	KD2-H4K8ac_peak_7254	3.80759	RAB21_ENSG00000080371
12	72148702	72149249	KD2-H4K8ac_peak_7255	5.01002	RAB21_ENSG00000080371
12	72232802	72233438	KD2-H4K8ac_peak_7256	12.93209	TBC1D15_ENSG00000121749
12	72666685	72666922	KD2-H4K8ac_peak_7257	6.40683	
12	74931665	74931879	KD2-H4K8ac_peak_7258	6.04107	ATXN7L3B_ENSG00000253719;RP11-56G10.2_ENSG00000257386
12	75785032	75785260	KD2-H4K8ac_peak_7259	10.05606	CAPS2_ENSG00000180881
12	75904995	75905338	KD2-H4K8ac_peak_7260	13.57066	KRR1_ENSG00000111615
12	75905968	75906288	KD2-H4K8ac_peak_7261	4.77079	KRR1_ENSG00000111615
12	75986475	75987027	KD2-H4K8ac_peak_7262	7.47462	
12	76101101	76101319	KD2-H4K8ac_peak_7263	8.1718	
12	76116266	76116474	KD2-H4K8ac_peak_7264	5.57044	
12	76204999	76205546	KD2-H4K8ac_peak_7265	8.41122	
12	76235269	76235600	KD2-H4K8ac_peak_7266	5.57044	

12	76247789	76248001	KD2-H4K8ac_peak_7267	4.211	
12	76353871	76354083	KD2-H4K8ac_peak_7268	4.59928	
12	76395518	76395969	KD2-H4K8ac_peak_7269	8.25022	
12	76408665	76408910	KD2-H4K8ac_peak_7270	10.86378	
12	76423910	76424149	KD2-H4K8ac_peak_7271	5.61067	RP11-290L1.3_ENSG00000257453
12	76425646	76426142	KD2-H4K8ac_peak_7272	11.32417	RP11-290L1.2_ENSG00000257839
12	76477955	76478370	KD2-H4K8ac_peak_7273	12.82393	NAP1L1_ENSG00000187109
12	76742344	76743095	KD2-H4K8ac_peak_7274	8.26945	BBS10_ENSG00000179941
12	76953045	76953261	KD2-H4K8ac_peak_7275	5.9696	OSBPL8_ENSG00000091039
12	76954802	76954994	KD2-H4K8ac_peak_7276	5.29352	
12	77158062	77158506	KD2-H4K8ac_peak_7277	15.61313	ZDHHC17_ENSG00000186908
12	77272983	77273518	KD2-H4K8ac_peak_7278	17.42214	CSRP2_ENSG00000175183
12	77336783	77336979	KD2-H4K8ac_peak_7279	6.40683	
12	77345066	77345403	KD2-H4K8ac_peak_7280	9.82729	
12	77458987	77459255	KD2-H4K8ac_peak_7281	8.23274	E2F7_ENSG00000165891
12	78490458	78490725	KD2-H4K8ac_peak_7282	4.9025	
12	79257271	79258168	KD2-H4K8ac_peak_7283	14.45402	SYT1_ENSG00000067715
12	80328369	80328564	KD2-H4K8ac_peak_7284	6.84261	PPP1R12A_ENSG00000058272;RP11-84G21.1_ENSG00000257557
12	80329070	80329423	KD2-H4K8ac_peak_7285	15.13261	PPP1R12A_ENSG00000058272;RP11-84G21.1_ENSG00000257557
12	82751661	82752560	KD2-H4K8ac_peak_7286	15.53798	CCDC59_ENSG00000133773;METTL25_ENSG00000127720
12	83102451	83102672	KD2-H4K8ac_peak_7287	11.30637	
12	83210796	83211064	KD2-H4K8ac_peak_7288	9.72244	
12	84902132	84902403	KD2-H4K8ac_peak_7289	5.25071	
12	85711802	85712063	KD2-H4K8ac_peak_7290	10.21502	RP11-408B11.2_ENSG00000258815
12	88429386	88429703	KD2-H4K8ac_peak_7291	7.27716	
12	88535453	88535784	KD2-H4K8ac_peak_7292	7.70289	CEP290_ENSG00000198707;TMTC3_ENSG00000139324
12	88536205	88536732	KD2-H4K8ac_peak_7293	6.94255	CEP290_ENSG00000198707;TMTC3_ENSG00000139324
12	88889281	88889619	KD2-H4K8ac_peak_7294	5.73839	
12	88960384	88960592	KD2-H4K8ac_peak_7295	4.77079	
12	88973874	88974082	KD2-H4K8ac_peak_7296	6.30229	KITLG_ENSG00000049130
12	88974321	88974579	KD2-H4K8ac_peak_7297	12.57865	KITLG_ENSG00000049130;RNU1-117P_ENSG00000252850
12	89410715	89411022	KD2-H4K8ac_peak_7298	12.06346	
12	89493059	89493413	KD2-H4K8ac_peak_7299	4.77079	
12	89495363	89495619	KD2-H4K8ac_peak_7300	4.211	
12	89511359	89511721	KD2-H4K8ac_peak_7301	4.37708	
12	89604228	89604430	KD2-H4K8ac_peak_7302	7.12754	
12	89670752	89670994	KD2-H4K8ac_peak_7303	5.58333	
12	89671860	89672493	KD2-H4K8ac_peak_7304	5.75452	
12	89673364	89673675	KD2-H4K8ac_peak_7305	7.48133	
12	89739507	89739864	KD2-H4K8ac_peak_7306	6.66692	
12	89745168	89745401	KD2-H4K8ac_peak_7307	9.36665	
12	89745595	89745871	KD2-H4K8ac_peak_7308	4.37809	
12	89746545	89748707	KD2-H4K8ac_peak_7309	12.93209	DUSP6_ENSG00000139318
12	89919338	89920002	KD2-H4K8ac_peak_7310	11.24702	POC1B_ENSG00000139323;GALNT4_ENSG00000257594;POC1B-GALNT4_ENSG00000259075;RP11-734K2.4_ENSG00000270344
12	89920216	89920594	KD2-H4K8ac_peak_7311	4.44748	POC1B_ENSG00000139323;GALNT4_ENSG00000257594;POC1B-GALNT4_ENSG00000259075;RP11-734K2.4_ENSG00000270344
12	91950328	91951137	KD2-H4K8ac_peak_7312	9.16531	

12	92540142	92540346	KD2-H4K8ac_peak_7313	6.60096	BTG1_ENSG00000133639;RP11-796E2.4_ENSG00000245904
12	92618590	92618810	KD2-H4K8ac_peak_7314	8.17902	
12	92690803	92691018	KD2-H4K8ac_peak_7315	4.59928	
12	92758191	92758421	KD2-H4K8ac_peak_7316	11.05407	
12	92805699	92805898	KD2-H4K8ac_peak_7317	5.73839	
12	93494030	93494372	KD2-H4K8ac_peak_7318	3.75035	
12	93578377	93578642	KD2-H4K8ac_peak_7319	7.12754	
12	93693580	93693808	KD2-H4K8ac_peak_7320	9.06369	
12	93770320	93770924	KD2-H4K8ac_peak_7321	9.68448	RP11-486A14.2_ENSG00000257252;NUDT4_ENSG00000173598
12	93771985	93772198	KD2-H4K8ac_peak_7322	6.0605	RP11-486A14.2_ENSG00000257252;NUDT4_ENSG00000173598
12	93834962	93835676	KD2-H4K8ac_peak_7323	16.15695	UBE2N_ENSG00000177889
12	93836001	93836342	KD2-H4K8ac_peak_7324	6.40297	UBE2N_ENSG00000177889
12	93860769	93861004	KD2-H4K8ac_peak_7325	8.02571	MRPL42_ENSG00000198015
12	93861321	93861713	KD2-H4K8ac_peak_7326	13.81781	MRPL42_ENSG00000198015
12	93964226	93964497	KD2-H4K8ac_peak_7327	8.4716	SOCS2_ENSG00000120833
12	93964914	93965479	KD2-H4K8ac_peak_7328	12.08081	SOCS2-AS1_ENSG00000246985
12	93965709	93966029	KD2-H4K8ac_peak_7329	11.05407	SOCS2-AS1_ENSG00000246985
12	93984211	93984418	KD2-H4K8ac_peak_7330	5.14125	
12	93985245	93985452	KD2-H4K8ac_peak_7331	5.54647	
12	94070214	94070878	KD2-H4K8ac_peak_7332	10.25088	CRADD_ENSG00000169372
12	94071286	94071697	KD2-H4K8ac_peak_7333	9.36665	CRADD_ENSG00000169372
12	94072808	94073053	KD2-H4K8ac_peak_7334	6.78668	
12	94076888	94077081	KD2-H4K8ac_peak_7335	5.75452	
12	94135432	94135706	KD2-H4K8ac_peak_7336	5.3778	
12	94179171	94179560	KD2-H4K8ac_peak_7337	7.12754	
12	94288259	94288591	KD2-H4K8ac_peak_7338	8.83582	
12	94495484	94496039	KD2-H4K8ac_peak_7339	24.1491	
12	94496405	94496609	KD2-H4K8ac_peak_7340	5.54647	
12	94558859	94559051	KD2-H4K8ac_peak_7341	4.66652	
12	94656363	94656782	KD2-H4K8ac_peak_7342	5.13615	
12	94853753	94854350	KD2-H4K8ac_peak_7343	20.08519	CCDC41_ENSG00000173588
12	94927032	94927267	KD2-H4K8ac_peak_7344	11.89502	
12	95267115	95267372	KD2-H4K8ac_peak_7345	7.76329	
12	95267588	95267968	KD2-H4K8ac_peak_7346	12.81138	
12	95610800	95611109	KD2-H4K8ac_peak_7347	5.84991	FGD6_ENSG00000180263;VEZT_ENSG00000028203
12	95611643	95612042	KD2-H4K8ac_peak_7348	7.0754	FGD6_ENSG00000180263;VEZT_ENSG00000028203
12	95868038	95868302	KD2-H4K8ac_peak_7349	9.20193	METAP2_ENSG00000111142
12	95941967	95942469	KD2-H4K8ac_peak_7350	10.96455	
12	96252817	96253053	KD2-H4K8ac_peak_7351	6.8713	RP11-536G4.2_ENSG00000258343;SNRPF_ENSG00000139343
12	96336348	96336977	KD2-H4K8ac_peak_7352	28.04957	CCDC38_ENSG00000165972;AMDHD1_ENSG00000139344
12	96337182	96337713	KD2-H4K8ac_peak_7353	5.14125	CCDC38_ENSG00000165972;AMDHD1_ENSG00000139344
12	96360496	96360896	KD2-H4K8ac_peak_7354	4.42765	
12	96428958	96429218	KD2-H4K8ac_peak_7355	5.01002	
12	96514850	96515151	KD2-H4K8ac_peak_7356	4.0528	
12	96546103	96546297	KD2-H4K8ac_peak_7357	13.81781	
12	96546829	96547132	KD2-H4K8ac_peak_7358	5.30799	

12	96587756	96588452	KD2-H4K8ac_peak_7359	14.68449	ELK3_ENSG00000111145
12	96588758	96589261	KD2-H4K8ac_peak_7360	4.86324	ELK3_ENSG00000111145
12	96634297	96634576	KD2-H4K8ac_peak_7361	10.0692	
12	96793258	96794168	KD2-H4K8ac_peak_7362	6.60096	CDK17_ENSG00000059758
12	96795333	96795658	KD2-H4K8ac_peak_7363	7.55771	CDK17_ENSG00000059758
12	97300659	97301244	KD2-H4K8ac_peak_7364	14.43704	NEDD1_ENSG00000139350
12	98885021	98885221	KD2-H4K8ac_peak_7365	7.12754	
12	98897068	98897952	KD2-H4K8ac_peak_7366	8.55271	RP11-181C3.1_ENSG00000245017
12	98908833	98909126	KD2-H4K8ac_peak_7367	10.19949	TMPO_ENSG00000120802
12	98909469	98910242	KD2-H4K8ac_peak_7368	9.57101	TMPO-AS1_ENSG00000257167;TMPO_ENSG00000120802
12	98986916	98987134	KD2-H4K8ac_peak_7369	6.2781	SLC25A3_ENSG00000075415
12	98987528	98988086	KD2-H4K8ac_peak_7370	8.4716	SLC25A3_ENSG00000075415
12	99037873	99038731	KD2-H4K8ac_peak_7371	12.26331	IKBIP_ENSG00000166130;APAF1_ENSG00000120868
12	99039083	99039508	KD2-H4K8ac_peak_7372	14.45402	IKBIP_ENSG00000166130;APAF1_ENSG00000120868
12	99139283	99139607	KD2-H4K8ac_peak_7373	8.5781	
12	100536997	1.01E+08	KD2-H4K8ac_peak_7374	9.59147	UHRF1BP1L_ENSG00000111647;RP11-175P13.2_ENSG00000257696
12	100557163	1.01E+08	KD2-H4K8ac_peak_7375	6.37652	
12	100592931	1.01E+08	KD2-H4K8ac_peak_7376	12.06346	RP11-175P13.3_ENSG00000257489;ACTR6_ENSG00000075089
12	100661183	1.01E+08	KD2-H4K8ac_peak_7377	9.89126	DEPDC4_ENSG00000166153;SCYL2_ENSG00000136021
12	101801886	1.02E+08	KD2-H4K8ac_peak_7378	10.96455	ARL1_ENSG00000120805;RP11-321F8.4_ENSG00000257543
12	102091820	1.02E+08	KD2-H4K8ac_peak_7379	7.20567	
12	102224178	1.02E+08	KD2-H4K8ac_peak_7380	10.0692	GNPTAB_ENSG00000111670
12	102270928	1.02E+08	KD2-H4K8ac_peak_7381	22.36554	DRAM1_ENSG00000136048
12	102455382	1.02E+08	KD2-H4K8ac_peak_7382	8.02571	CCDC53_ENSG00000120860
12	103889487	1.04E+08	KD2-H4K8ac_peak_7383	6.83936	C12orf42_ENSG00000179088
12	103969519	1.04E+08	KD2-H4K8ac_peak_7384	6.99815	
12	104323019	1.04E+08	KD2-H4K8ac_peak_7385	9.1707	RP11-642P15.1_ENSG00000214198;HSP90B1_ENSG00000166598;MIR3652_ENSG00000265072
12	104324226	1.04E+08	KD2-H4K8ac_peak_7386	6.5996	RP11-642P15.1_ENSG00000214198;HSP90B1_ENSG00000166598;MIR3652_ENSG00000265072
12	104350477	1.04E+08	KD2-H4K8ac_peak_7387	7.27716	
12	104351179	1.04E+08	KD2-H4K8ac_peak_7388	5.88845	
12	104359681	1.04E+08	KD2-H4K8ac_peak_7389	11.92978	C12orf73_ENSG00000204954;TDG_ENSG00000139372
12	104443670	1.04E+08	KD2-H4K8ac_peak_7390	8.53581	
12	104531230	1.05E+08	KD2-H4K8ac_peak_7391	26.15943	NFYB_ENSG00000120837
12	104532217	1.05E+08	KD2-H4K8ac_peak_7392	15.81711	NFYB_ENSG00000120837
12	104680802	1.05E+08	KD2-H4K8ac_peak_7393	6.30229	
12	105038251	1.05E+08	KD2-H4K8ac_peak_7394	5.73839	
12	105062601	1.05E+08	KD2-H4K8ac_peak_7395	13.2669	
12	105118331	1.05E+08	KD2-H4K8ac_peak_7396	8.87298	
12	105351910	1.05E+08	KD2-H4K8ac_peak_7397	14.00658	SLC41A2_ENSG00000136052
12	105352835	1.05E+08	KD2-H4K8ac_peak_7398	8.02571	SLC41A2_ENSG00000136052
12	105500946	1.06E+08	KD2-H4K8ac_peak_7399	8.74596	RP11-61E11.2_ENSG00000257999;KIAA1033_ENSG00000136051
12	105501606	1.06E+08	KD2-H4K8ac_peak_7400	6.25869	RP11-61E11.2_ENSG00000257999;KIAA1033_ENSG00000136051
12	105629523	1.06E+08	KD2-H4K8ac_peak_7401	15.58442	APPL2_ENSG00000136044;C12orf75_ENSG00000235162
12	105630289	1.06E+08	KD2-H4K8ac_peak_7402	7.70289	APPL2_ENSG00000136044
12	105724508	1.06E+08	KD2-H4K8ac_peak_7403	12.99417	
12	106532284	1.07E+08	KD2-H4K8ac_peak_7404	22.07309	NUAK1_ENSG00000074590

12	106640994	1.07E+08	KD2-H4K8ac_peak_7405	9.20193	RP11-651L5.2_ENSG00000258355
12	106641458	1.07E+08	KD2-H4K8ac_peak_7406	21.6161	RP11-651L5.2_ENSG00000258355
12	106751575	1.07E+08	KD2-H4K8ac_peak_7407	14.8083	POLR3B_ENSG00000013503
12	107167156	1.07E+08	KD2-H4K8ac_peak_7408	7.27716	RIC8B_ENSG00000111785
12	107168118	1.07E+08	KD2-H4K8ac_peak_7409	8.85813	RP11-144F15.1_ENSG00000257545;RIC8B_ENSG00000111785
12	107523980	1.08E+08	KD2-H4K8ac_peak_7410	12.00273	
12	107525854	1.08E+08	KD2-H4K8ac_peak_7411	9.11031	
12	107533187	1.08E+08	KD2-H4K8ac_peak_7412	9.63925	
12	107764997	1.08E+08	KD2-H4K8ac_peak_7413	7.26029	
12	107766731	1.08E+08	KD2-H4K8ac_peak_7414	7.11925	
12	107767471	1.08E+08	KD2-H4K8ac_peak_7415	6.40297	SNORD74_ENSG00000200897
12	107796157	1.08E+08	KD2-H4K8ac_peak_7416	6.4095	
12	107800125	1.08E+08	KD2-H4K8ac_peak_7417	7.26029	
12	107900956	1.08E+08	KD2-H4K8ac_peak_7418	4.49837	
12	108001756	1.08E+08	KD2-H4K8ac_peak_7419	7.95563	
12	108039115	1.08E+08	KD2-H4K8ac_peak_7420	11.79585	
12	108040481	1.08E+08	KD2-H4K8ac_peak_7421	5.81222	
12	108048127	1.08E+08	KD2-H4K8ac_peak_7422	8.83749	
12	108048713	1.08E+08	KD2-H4K8ac_peak_7423	5.14125	
12	108049535	1.08E+08	KD2-H4K8ac_peak_7424	6.56211	
12	108079610	1.08E+08	KD2-H4K8ac_peak_7425	9.65086	PWP1_ENSG00000136045
12	108154441	1.08E+08	KD2-H4K8ac_peak_7426	11.92978	PRDM4_ENSG00000110851
12	108155259	1.08E+08	KD2-H4K8ac_peak_7427	6.76298	PRDM4_ENSG00000110851
12	108297313	1.08E+08	KD2-H4K8ac_peak_7428	7.47462	RP11-554D14.2_ENSG00000258072
12	108485640	1.08E+08	KD2-H4K8ac_peak_7429	6.40683	
12	108692386	1.09E+08	KD2-H4K8ac_peak_7430	4.211	
12	108699500	1.09E+08	KD2-H4K8ac_peak_7431	6.56211	
12	108704454	1.09E+08	KD2-H4K8ac_peak_7432	5.75757	
12	108705395	1.09E+08	KD2-H4K8ac_peak_7433	5.9696	
12	108706104	1.09E+08	KD2-H4K8ac_peak_7434	5.61067	
12	108764493	1.09E+08	KD2-H4K8ac_peak_7435	5.54647	
12	108774924	1.09E+08	KD2-H4K8ac_peak_7436	5.10276	
12	108779269	1.09E+08	KD2-H4K8ac_peak_7437	6.14257	
12	108787562	1.09E+08	KD2-H4K8ac_peak_7438	7.79898	
12	108788217	1.09E+08	KD2-H4K8ac_peak_7439	5.54647	
12	108793494	1.09E+08	KD2-H4K8ac_peak_7440	5.15777	
12	108908538	1.09E+08	KD2-H4K8ac_peak_7441	9.06369	FICD_ENSG00000198855
12	108954528	1.09E+08	KD2-H4K8ac_peak_7442	12.06346	SART3_ENSG00000075856
12	108960154	1.09E+08	KD2-H4K8ac_peak_7443	4.87807	
12	108983024	1.09E+08	KD2-H4K8ac_peak_7444	5.54647	
12	108990697	1.09E+08	KD2-H4K8ac_peak_7445	7.20567	TMEM119_ENSG00000183160
12	108992051	1.09E+08	KD2-H4K8ac_peak_7446	5.94405	TMEM119_ENSG00000183160
12	109003495	1.09E+08	KD2-H4K8ac_peak_7447	4.06758	
12	109126043	1.09E+08	KD2-H4K8ac_peak_7448	14.8083	CORO1C_ENSG00000110880
12	109167617	1.09E+08	KD2-H4K8ac_peak_7449	7.167	
12	109220468	1.09E+08	KD2-H4K8ac_peak_7450	11.89502	

12	109233573	1.09E+08	KD2-H4K8ac_peak_7451	5.5051	
12	109250815	1.09E+08	KD2-H4K8ac_peak_7452	10.08707	SSH1_ENSG00000084112
12	109251530	1.09E+08	KD2-H4K8ac_peak_7453	19.3524	SSH1_ENSG00000084112;DAO_ENSG00000110887
12	109489974	1.09E+08	KD2-H4K8ac_peak_7454	21.01976	USP30-AS1_ENSG00000256262
12	109530370	1.1E+08	KD2-H4K8ac_peak_7455	7.01658	ALKBH2_ENSG00000189046
12	109548464	1.1E+08	KD2-H4K8ac_peak_7456	19.1048	RP11-968O1.5_ENSG00000256139
12	109553525	1.1E+08	KD2-H4K8ac_peak_7457	7.63551	ACACB_ENSG00000076555
12	109728463	1.1E+08	KD2-H4K8ac_peak_7458	15.59774	
12	109729718	1.1E+08	KD2-H4K8ac_peak_7459	12.08081	
12	109742565	1.1E+08	KD2-H4K8ac_peak_7460	5.71749	
12	109746810	1.1E+08	KD2-H4K8ac_peak_7461	6.5996	FOXN4_ENSG00000139445
12	109747428	1.1E+08	KD2-H4K8ac_peak_7462	10.73134	FOXN4_ENSG00000139445
12	109915062	1.1E+08	KD2-H4K8ac_peak_7463	11.92978	KCTD10_ENSG00000110906;UBE3B_ENSG00000151148
12	109960025	1.1E+08	KD2-H4K8ac_peak_7464	6.84261	
12	109961648	1.1E+08	KD2-H4K8ac_peak_7465	5.30799	
12	109967988	1.1E+08	KD2-H4K8ac_peak_7466	5.82859	
12	109968576	1.1E+08	KD2-H4K8ac_peak_7467	8.33304	
12	109972215	1.1E+08	KD2-H4K8ac_peak_7468	6.72624	
12	109978842	1.1E+08	KD2-H4K8ac_peak_7469	7.79741	
12	109982874	1.1E+08	KD2-H4K8ac_peak_7470	8.74596	
12	110010916	1.1E+08	KD2-H4K8ac_peak_7471	16.15695	MMAB_ENSG00000139428;MVK_ENSG00000110921
12	110028954	1.1E+08	KD2-H4K8ac_peak_7472	5.47943	
12	110035886	1.1E+08	KD2-H4K8ac_peak_7473	7.38799	
12	110043161	1.1E+08	KD2-H4K8ac_peak_7474	7.98621	
12	110043765	1.1E+08	KD2-H4K8ac_peak_7475	9.63487	
12	110049556	1.1E+08	KD2-H4K8ac_peak_7476	9.1707	
12	110059603	1.1E+08	KD2-H4K8ac_peak_7477	7.76329	
12	110060940	1.1E+08	KD2-H4K8ac_peak_7478	5.54647	
12	110080693	1.1E+08	KD2-H4K8ac_peak_7479	4.01115	
12	110082044	1.1E+08	KD2-H4K8ac_peak_7480	5.20908	
12	110083385	1.1E+08	KD2-H4K8ac_peak_7481	9.80695	
12	110086269	1.1E+08	KD2-H4K8ac_peak_7482	5.82799	
12	110092611	1.1E+08	KD2-H4K8ac_peak_7483	9.1707	
12	110093734	1.1E+08	KD2-H4K8ac_peak_7484	7.146	
12	110133792	1.1E+08	KD2-H4K8ac_peak_7485	5.57044	
12	110138531	1.1E+08	KD2-H4K8ac_peak_7486	13.07128	
12	110157829	1.1E+08	KD2-H4K8ac_peak_7487	9.18237	
12	110169910	1.1E+08	KD2-H4K8ac_peak_7488	7.1181	
12	110175812	1.1E+08	KD2-H4K8ac_peak_7489	7.67504	
12	110177828	1.1E+08	KD2-H4K8ac_peak_7490	5.56396	
12	110181004	1.1E+08	KD2-H4K8ac_peak_7491	3.75035	
12	110181740	1.1E+08	KD2-H4K8ac_peak_7492	5.45396	
12	110190667	1.1E+08	KD2-H4K8ac_peak_7493	6.66692	
12	110192083	1.1E+08	KD2-H4K8ac_peak_7494	4.8402	
12	110196778	1.1E+08	KD2-H4K8ac_peak_7495	11.92978	
12	110197770	1.1E+08	KD2-H4K8ac_peak_7496	7.40111	

12	110257124	1.1E+08	KD2-H4K8ac_peak_7497	5.71749	
12	110270846	1.1E+08	KD2-H4K8ac_peak_7498	10.86378	TRPV4_ENSG00000111199;MIR4497_ENSG00000263510
12	110337383	1.1E+08	KD2-H4K8ac_peak_7499	10.21366	TCHP_ENSG00000139437
12	110433716	1.1E+08	KD2-H4K8ac_peak_7500	15.22643	GIT2_ENSG00000139436
12	110434320	1.1E+08	KD2-H4K8ac_peak_7501	11.59946	GIT2_ENSG00000139436
12	110436508	1.1E+08	KD2-H4K8ac_peak_7502	9.73431	ANKRD13A_ENSG00000076513
12	110436943	1.1E+08	KD2-H4K8ac_peak_7503	16.57619	ANKRD13A_ENSG00000076513
12	110485883	1.1E+08	KD2-H4K8ac_peak_7504	7.36799	
12	110486704	1.1E+08	KD2-H4K8ac_peak_7505	10.05606	
12	110562259	1.11E+08	KD2-H4K8ac_peak_7506	9.36516	IFT81_ENSG00000122970
12	110707617	1.11E+08	KD2-H4K8ac_peak_7507	5.1313	
12	110718398	1.11E+08	KD2-H4K8ac_peak_7508	10.77651	ATP2A2_ENSG00000174437
12	110841103	1.11E+08	KD2-H4K8ac_peak_7509	6.36375	ANAPC7_ENSG00000196510
12	110887745	1.11E+08	KD2-H4K8ac_peak_7510	7.28628	ARPC3_ENSG00000111229
12	110906424	1.11E+08	KD2-H4K8ac_peak_7511	21.2844	GPN3_ENSG00000111231;FAM216A_ENSG00000204856
12	111020730	1.11E+08	KD2-H4K8ac_peak_7512	10.29344	PPTC7_ENSG00000196850
12	111051072	1.11E+08	KD2-H4K8ac_peak_7513	8.52559	TCTN1_ENSG00000204852
12	111116882	1.11E+08	KD2-H4K8ac_peak_7514	5.58333	
12	111126786	1.11E+08	KD2-H4K8ac_peak_7515	6.31143	
12	111131758	1.11E+08	KD2-H4K8ac_peak_7516	4.211	
12	111132292	1.11E+08	KD2-H4K8ac_peak_7517	4.98072	
12	111137196	1.11E+08	KD2-H4K8ac_peak_7518	6.35843	
12	111137660	1.11E+08	KD2-H4K8ac_peak_7519	8.02571	
12	111180460	1.11E+08	KD2-H4K8ac_peak_7520	7.47462	PPP1CC_ENSG00000186298
12	111298734	1.11E+08	KD2-H4K8ac_peak_7521	6.16888	
12	111394999	1.11E+08	KD2-H4K8ac_peak_7522	6.16888	RP1-46F2.3_ENSG00000258240
12	111395839	1.11E+08	KD2-H4K8ac_peak_7523	6.31143	RP1-46F2.3_ENSG00000258240
12	111470697	1.11E+08	KD2-H4K8ac_peak_7524	5.9696	CUX2_ENSG00000111249
12	111472006	1.11E+08	KD2-H4K8ac_peak_7525	13.02811	CUX2_ENSG00000111249
12	111494450	1.11E+08	KD2-H4K8ac_peak_7526	6.1228	
12	111616834	1.12E+08	KD2-H4K8ac_peak_7527	14.04094	
12	111618379	1.12E+08	KD2-H4K8ac_peak_7528	7.74292	
12	111623517	1.12E+08	KD2-H4K8ac_peak_7529	9.1707	
12	111623999	1.12E+08	KD2-H4K8ac_peak_7530	10.21066	
12	111646807	1.12E+08	KD2-H4K8ac_peak_7531	4.77079	
12	111649784	1.12E+08	KD2-H4K8ac_peak_7532	7.22457	
12	111662467	1.12E+08	KD2-H4K8ac_peak_7533	8.26945	
12	111685813	1.12E+08	KD2-H4K8ac_peak_7534	5.14125	
12	111806417	1.12E+08	KD2-H4K8ac_peak_7535	5.75757	FAM109A_ENSG00000198324;RP3-473L9.4_ENSG00000257595
12	111842883	1.12E+08	KD2-H4K8ac_peak_7536	3.90565	SH2B3_ENSG00000111252
12	111844245	1.12E+08	KD2-H4K8ac_peak_7537	4.211	SH2B3_ENSG00000111252
12	111847821	1.12E+08	KD2-H4K8ac_peak_7538	4.211	
12	111867845	1.12E+08	KD2-H4K8ac_peak_7539	8.86391	
12	112035789	1.12E+08	KD2-H4K8ac_peak_7540	6.56926	
12	112036716	1.12E+08	KD2-H4K8ac_peak_7541	12.57865	ATXN2_ENSG00000204842;RP11-686G8.2_ENSG00000258099
12	112123308	1.12E+08	KD2-H4K8ac_peak_7542	9.61895	BRAP_ENSG00000089234;ACAD10_ENSG00000111271

12	112123950	1.12E+08	KD2-H4K8ac_peak_7543	6.99815	BRAP_ENSG00000089234;ACAD10_ENSG00000111271
12	112204387	1.12E+08	KD2-H4K8ac_peak_7544	5.94405	ALDH2_ENSG00000111275
12	112204799	1.12E+08	KD2-H4K8ac_peak_7545	5.1707	ALDH2_ENSG00000111275
12	112279254	1.12E+08	KD2-H4K8ac_peak_7546	5.14125	AC003029.1_ENSG00000248594;MAPKAPK5_ENSG00000089022
12	112279830	1.12E+08	KD2-H4K8ac_peak_7547	9.20193	MAPKAPK5-AS1_ENSG00000234608;AC003029.1_ENSG00000248594;MAPKAPK5_ENSG00000089022
12	112450092	1.12E+08	KD2-H4K8ac_peak_7548	10.19949	TMEM116_ENSG00000198270;ERP29_ENSG00000089248
12	112451321	1.12E+08	KD2-H4K8ac_peak_7549	7.90806	TMEM116_ENSG00000198270;ERP29_ENSG00000089248
12	112546080	1.13E+08	KD2-H4K8ac_peak_7550	12.84642	NAA25_ENSG00000111300
12	112554043	1.13E+08	KD2-H4K8ac_peak_7551	5.30799	
12	112554656	1.13E+08	KD2-H4K8ac_peak_7552	6.0073	
12	112785035	1.13E+08	KD2-H4K8ac_peak_7553	5.64449	
12	112820359	1.13E+08	KD2-H4K8ac_peak_7554	7.49599	HECTD4_ENSG00000173064
12	112825422	1.13E+08	KD2-H4K8ac_peak_7555	9.15329	
12	112846561	1.13E+08	KD2-H4K8ac_peak_7556	13.3695	
12	112856744	1.13E+08	KD2-H4K8ac_peak_7557	13.12288	RPL6_ENSG00000089009;PTPN11_ENSG00000179295
12	113242037	1.13E+08	KD2-H4K8ac_peak_7558	5.14125	
12	113258402	1.13E+08	KD2-H4K8ac_peak_7559	11.5673	
12	113260183	1.13E+08	KD2-H4K8ac_peak_7560	12.06346	
12	113273642	1.13E+08	KD2-H4K8ac_peak_7561	4.75764	
12	113280377	1.13E+08	KD2-H4K8ac_peak_7562	6.4095	
12	113328363	1.13E+08	KD2-H4K8ac_peak_7563	11.24422	
12	113330086	1.13E+08	KD2-H4K8ac_peak_7564	3.8301	
12	113495012	1.13E+08	KD2-H4K8ac_peak_7565	8.5092	DTX1_ENSG00000135144
12	113622967	1.14E+08	KD2-H4K8ac_peak_7566	6.0605	DDX54_ENSG00000123064;RITA1_ENSG00000139405
12	113682354	1.14E+08	KD2-H4K8ac_peak_7567	18.88045	
12	113683967	1.14E+08	KD2-H4K8ac_peak_7568	9.95119	
12	113698065	1.14E+08	KD2-H4K8ac_peak_7569	10.12553	
12	113737276	1.14E+08	KD2-H4K8ac_peak_7570	7.70289	
12	113772645	1.14E+08	KD2-H4K8ac_peak_7571	10.43145	
12	113795866	1.14E+08	KD2-H4K8ac_peak_7572	9.63487	PLBD2_ENSG00000151176
12	113796442	1.14E+08	KD2-H4K8ac_peak_7573	7.69287	SLC8B1_ENSG00000089060;PLBD2_ENSG00000151176
12	114075460	1.14E+08	KD2-H4K8ac_peak_7574	6.40683	
12	114203706	1.14E+08	KD2-H4K8ac_peak_7575	7.74292	
12	114278528	1.14E+08	KD2-H4K8ac_peak_7576	5.14125	
12	115122460	1.15E+08	KD2-H4K8ac_peak_7577	5.54647	TBX3_ENSG00000135111
12	115123070	1.15E+08	KD2-H4K8ac_peak_7578	7.01964	
12	115180176	1.15E+08	KD2-H4K8ac_peak_7579	3.7063	
12	115307283	1.15E+08	KD2-H4K8ac_peak_7580	5.15777	
12	115450254	1.15E+08	KD2-H4K8ac_peak_7581	12.26331	
12	115452072	1.15E+08	KD2-H4K8ac_peak_7582	6.16888	
12	116715695	1.17E+08	KD2-H4K8ac_peak_7583	12.57297	MED13L_ENSG00000123066
12	116796470	1.17E+08	KD2-H4K8ac_peak_7584	9.47671	
12	116803989	1.17E+08	KD2-H4K8ac_peak_7585	10.65724	
12	116809882	1.17E+08	KD2-H4K8ac_peak_7586	8.66888	
12	116810383	1.17E+08	KD2-H4K8ac_peak_7587	9.06703	
12	116811536	1.17E+08	KD2-H4K8ac_peak_7588	5.57044	

12	117150928	1.17E+08	KD2-H4K8ac_peak_7589	6.56926	
12	117175589	1.17E+08	KD2-H4K8ac_peak_7590	11.70505	C12orf49_ENSG00000111412;RNFT2_ENSG00000135119
12	117256819	1.17E+08	KD2-H4K8ac_peak_7591	7.36799	
12	117259092	1.17E+08	KD2-H4K8ac_peak_7592	7.12754	
12	117299608	1.17E+08	KD2-H4K8ac_peak_7593	6.14257	
12	117319358	1.17E+08	KD2-H4K8ac_peak_7594	12.99417	HRK_ENSG00000135116
12	117348527	1.17E+08	KD2-H4K8ac_peak_7595	6.25869	FBXW8_ENSG00000174989
12	117370237	1.17E+08	KD2-H4K8ac_peak_7596	6.66692	
12	117383209	1.17E+08	KD2-H4K8ac_peak_7597	7.67496	
12	117396627	1.17E+08	KD2-H4K8ac_peak_7598	7.47462	
12	117397306	1.17E+08	KD2-H4K8ac_peak_7599	6.00226	
12	117398467	1.17E+08	KD2-H4K8ac_peak_7600	5.58324	
12	117399933	1.17E+08	KD2-H4K8ac_peak_7601	5.405	
12	117400642	1.17E+08	KD2-H4K8ac_peak_7602	7.69287	
12	117402305	1.17E+08	KD2-H4K8ac_peak_7603	8.02571	
12	117405099	1.17E+08	KD2-H4K8ac_peak_7604	4.57371	
12	117407089	1.17E+08	KD2-H4K8ac_peak_7605	8.4939	
12	117408016	1.17E+08	KD2-H4K8ac_peak_7606	8.26945	
12	117417658	1.17E+08	KD2-H4K8ac_peak_7607	8.17902	
12	117440161	1.17E+08	KD2-H4K8ac_peak_7608	5.32137	
12	117465639	1.17E+08	KD2-H4K8ac_peak_7609	6.69204	
12	117483372	1.17E+08	KD2-H4K8ac_peak_7610	13.73635	
12	117536745	1.18E+08	KD2-H4K8ac_peak_7611	19.59051	TESC_ENSG00000088992;RP11-103B5.2_ENSG00000258285
12	117556666	1.18E+08	KD2-H4K8ac_peak_7612	7.40111	
12	117627778	1.18E+08	KD2-H4K8ac_peak_7613	8.5092	FBXO21_ENSG00000135108
12	117876845	1.18E+08	KD2-H4K8ac_peak_7614	10.20046	
12	118406839	1.18E+08	KD2-H4K8ac_peak_7615	5.54647	KSR2_ENSG00000171435
12	118498374	1.18E+08	KD2-H4K8ac_peak_7616	7.67504	
12	118541095	1.19E+08	KD2-H4K8ac_peak_7617	14.80472	
12	118542022	1.19E+08	KD2-H4K8ac_peak_7618	5.88845	
12	118573971	1.19E+08	KD2-H4K8ac_peak_7619	7.96322	VSIG10_ENSG00000176834;PEBP1_ENSG00000089220
12	118619769	1.19E+08	KD2-H4K8ac_peak_7620	5.25071	
12	118627617	1.19E+08	KD2-H4K8ac_peak_7621	4.54006	
12	118769703	1.19E+08	KD2-H4K8ac_peak_7622	6.16888	
12	118813955	1.19E+08	KD2-H4K8ac_peak_7623	13.88933	RP11-131L12.2_ENSG00000270482;SUDS3_ENSG00000111707
12	119177727	1.19E+08	KD2-H4K8ac_peak_7624	6.66692	
12	119199847	1.19E+08	KD2-H4K8ac_peak_7625	6.40683	RP11-3L23.2_ENSG00000256750
12	119212012	1.19E+08	KD2-H4K8ac_peak_7626	7.22457	RP11-357K6.1_ENSG00000255618
12	119443989	1.19E+08	KD2-H4K8ac_peak_7627	4.01115	
12	119613955	1.2E+08	KD2-H4K8ac_peak_7628	6.94954	RP11-64B16.3_ENSG00000256884
12	119616730	1.2E+08	KD2-H4K8ac_peak_7629	4.54037	HSPB8_ENSG00000152137
12	119726938	1.2E+08	KD2-H4K8ac_peak_7630	9.11031	
12	120105101	1.2E+08	KD2-H4K8ac_peak_7631	9.89126	RP11-768F21.1_ENSG00000248636;PRKAB1_ENSG00000111725
12	120105770	1.2E+08	KD2-H4K8ac_peak_7632	12.08081	RP11-768F21.1_ENSG00000248636;PRKAB1_ENSG00000111725
12	120314716	1.2E+08	KD2-H4K8ac_peak_7633	13.56967	CIT_ENSG00000122966
12	120315305	1.2E+08	KD2-H4K8ac_peak_7634	11.05467	CIT_ENSG00000122966

12	120531295	1.21E+08	KD2-H4K8ac_peak_7635	9.34989	
12	120553871	1.21E+08	KD2-H4K8ac_peak_7636	5.51783	RAB35_ENSG00000111737
12	120554820	1.21E+08	KD2-H4K8ac_peak_7637	5.54647	RAB35_ENSG00000111737
12	120632201	1.21E+08	KD2-H4K8ac_peak_7638	14.63726	GCN1L1_ENSG00000089154
12	120662557	1.21E+08	KD2-H4K8ac_peak_7639	11.50346	
12	120672994	1.21E+08	KD2-H4K8ac_peak_7640	5.20908	
12	120730031	1.21E+08	KD2-H4K8ac_peak_7641	5.13615	RNU4-2_ENSG00000202538;RNU4-1_ENSG00000200795
12	120731130	1.21E+08	KD2-H4K8ac_peak_7642	7.48133	RNU4-1_ENSG00000200795
12	120754976	1.21E+08	KD2-H4K8ac_peak_7643	7.45501	
12	120799035	1.21E+08	KD2-H4K8ac_peak_7644	17.88027	
12	120801164	1.21E+08	KD2-H4K8ac_peak_7645	12.60538	
12	120805797	1.21E+08	KD2-H4K8ac_peak_7646	16.15695	MSI1_ENSG00000135097;RPS27P25_ENSG00000239881
12	120807185	1.21E+08	KD2-H4K8ac_peak_7647	10.19949	MSI1_ENSG00000135097;RPS27P25_ENSG00000239881
12	120884313	1.21E+08	KD2-H4K8ac_peak_7648	12.00273	TRIAP1_ENSG00000170855;GATC_ENSG00000257218
12	120906767	1.21E+08	KD2-H4K8ac_peak_7649	9.90914	SRSF9_ENSG00000111786;DYNLL1_ENSG00000088986
12	120907191	1.21E+08	KD2-H4K8ac_peak_7650	9.63487	SRSF9_ENSG00000111786;DYNLL1_ENSG00000088986
12	120907739	1.21E+08	KD2-H4K8ac_peak_7651	6.67092	SRSF9_ENSG00000111786;DYNLL1_ENSG00000088986
12	121022407	1.21E+08	KD2-H4K8ac_peak_7652	14.84212	
12	121086744	1.21E+08	KD2-H4K8ac_peak_7653	12.42761	
12	121107270	1.21E+08	KD2-H4K8ac_peak_7654	5.63633	
12	121124330	1.21E+08	KD2-H4K8ac_peak_7655	16.8435	MLEC_ENSG00000110917
12	121146434	1.21E+08	KD2-H4K8ac_peak_7656	7.59643	
12	121147492	1.21E+08	KD2-H4K8ac_peak_7657	5.9696	UNC119B_ENSG00000175970
12	121163670	1.21E+08	KD2-H4K8ac_peak_7658	12.82393	ACADS_ENSG00000122971
12	121341696	1.21E+08	KD2-H4K8ac_peak_7659	16.73922	SPPL3_ENSG00000157837
12	121453711	1.21E+08	KD2-H4K8ac_peak_7660	11.50346	C12orf43_ENSG00000157895
12	121570822	1.22E+08	KD2-H4K8ac_peak_7661	9.36516	P2RX7_ENSG00000089041
12	121647031	1.22E+08	KD2-H4K8ac_peak_7662	11.2938	P2RX4_ENSG00000135124
12	121647688	1.22E+08	KD2-H4K8ac_peak_7663	17.83665	P2RX4_ENSG00000135124
12	121734761	1.22E+08	KD2-H4K8ac_peak_7664	9.63487	CAMKK2_ENSG00000110931
12	121789794	1.22E+08	KD2-H4K8ac_peak_7665	7.96322	
12	121829209	1.22E+08	KD2-H4K8ac_peak_7666	4.211	RP11-711D18.2_ENSG00000258435
12	121837396	1.22E+08	KD2-H4K8ac_peak_7667	7.90806	ANAPC5_ENSG00000089053;RNF34_ENSG00000170633
12	121838004	1.22E+08	KD2-H4K8ac_peak_7668	12.11734	ANAPC5_ENSG00000089053;RNF34_ENSG00000170633
12	121904720	1.22E+08	KD2-H4K8ac_peak_7669	6.56211	
12	121905524	1.22E+08	KD2-H4K8ac_peak_7670	9.78882	
12	121975773	1.22E+08	KD2-H4K8ac_peak_7671	4.67342	
12	122018667	1.22E+08	KD2-H4K8ac_peak_7672	8.17902	KDM2B_ENSG00000089094;RP13-941N14.1_ENSG00000256742
12	122020404	1.22E+08	KD2-H4K8ac_peak_7673	10.68173	
12	122027826	1.22E+08	KD2-H4K8ac_peak_7674	15.7388	
12	122063991	1.22E+08	KD2-H4K8ac_peak_7675	8.58725	ORAI1_ENSG00000182500
12	122064986	1.22E+08	KD2-H4K8ac_peak_7676	4.67342	ORAI1_ENSG00000182500
12	122096766	1.22E+08	KD2-H4K8ac_peak_7677	7.96194	
12	122101854	1.22E+08	KD2-H4K8ac_peak_7678	4.42765	
12	122110221	1.22E+08	KD2-H4K8ac_peak_7679	18.47652	MORN3_ENSG00000139714
12	122119714	1.22E+08	KD2-H4K8ac_peak_7680	8.26945	

12	122125195	1.22E+08	KD2-H4K8ac_peak_7681	15.99047	
12	122204931	1.22E+08	KD2-H4K8ac_peak_7682	4.26235	
12	122214268	1.22E+08	KD2-H4K8ac_peak_7683	7.52168	
12	122231385	1.22E+08	KD2-H4K8ac_peak_7684	10.43145	
12	122234378	1.22E+08	KD2-H4K8ac_peak_7685	6.05809	RP11-347I19.8_ENSG00000272849
12	122235403	1.22E+08	KD2-H4K8ac_peak_7686	7.96322	RP11-347I19.8_ENSG00000272849
12	122236934	1.22E+08	KD2-H4K8ac_peak_7687	5.405	
12	122237724	1.22E+08	KD2-H4K8ac_peak_7688	30.35433	
12	122241318	1.22E+08	KD2-H4K8ac_peak_7689	15.61313	SETD1B_ENSG00000139718;RHOF_ENSG00000139725;AC084018.1_ENSG00000212694
12	122277043	1.22E+08	KD2-H4K8ac_peak_7690	15.039	
12	122311105	1.22E+08	KD2-H4K8ac_peak_7691	6.40297	
12	122459328	1.22E+08	KD2-H4K8ac_peak_7692	10.0692	
12	122460269	1.22E+08	KD2-H4K8ac_peak_7693	7.52168	
12	122461018	1.22E+08	KD2-H4K8ac_peak_7694	4.37815	
12	122501625	1.23E+08	KD2-H4K8ac_peak_7695	9.16531	AC156455.1_ENSG00000256546
12	122502299	1.23E+08	KD2-H4K8ac_peak_7696	6.99815	
12	122516055	1.23E+08	KD2-H4K8ac_peak_7697	10.96455	MLXIP_ENSG00000175727
12	122516773	1.23E+08	KD2-H4K8ac_peak_7698	9.96798	MLXIP_ENSG00000175727
12	122667621	1.23E+08	KD2-H4K8ac_peak_7699	16.64426	
12	122685959	1.23E+08	KD2-H4K8ac_peak_7700	8.52559	
12	122687762	1.23E+08	KD2-H4K8ac_peak_7701	8.10216	B3GNT4_ENSG00000176383
12	122688366	1.23E+08	KD2-H4K8ac_peak_7702	13.80019	B3GNT4_ENSG00000176383
12	122710018	1.23E+08	KD2-H4K8ac_peak_7703	5.92591	
12	122712130	1.23E+08	KD2-H4K8ac_peak_7704	10.21366	DIABLO_ENSG00000184047
12	122713258	1.23E+08	KD2-H4K8ac_peak_7705	8.26945	
12	122714365	1.23E+08	KD2-H4K8ac_peak_7706	8.42997	
12	122715295	1.23E+08	KD2-H4K8ac_peak_7707	7.54952	
12	122750603	1.23E+08	KD2-H4K8ac_peak_7708	10.66719	RP11-512M8.5_ENSG00000256861;VPS33A_ENSG00000139719
12	122840267	1.23E+08	KD2-H4K8ac_peak_7709	8.00457	
12	122906529	1.23E+08	KD2-H4K8ac_peak_7710	18.37277	CLIP1_ENSG00000130779
12	122907453	1.23E+08	KD2-H4K8ac_peak_7711	11.86082	CLIP1_ENSG00000130779
12	123011872	1.23E+08	KD2-H4K8ac_peak_7712	10.65724	RSRC2_ENSG00000111011;KNTC1_ENSG00000184445
12	123237440	1.23E+08	KD2-H4K8ac_peak_7713	8.4716	DENR_ENSG00000139726
12	123259054	1.23E+08	KD2-H4K8ac_peak_7714	9.89126	CCDC62_ENSG00000130783
12	123318598	1.23E+08	KD2-H4K8ac_peak_7715	7.47462	HIP1R_ENSG00000130787
12	123338111	1.23E+08	KD2-H4K8ac_peak_7716	7.47462	
12	123346892	1.23E+08	KD2-H4K8ac_peak_7717	6.15357	
12	123458704	1.23E+08	KD2-H4K8ac_peak_7718	6.51023	OGFOD2_ENSG00000111325;RP11-197N18.2_ENSG00000256028
12	123459325	1.23E+08	KD2-H4K8ac_peak_7719	20.88898	OGFOD2_ENSG00000111325;RP11-197N18.2_ENSG00000256028
12	123464246	1.23E+08	KD2-H4K8ac_peak_7720	16.8435	ABCB9_ENSG00000150967;ARL6IP4_ENSG00000182196
12	123634834	1.24E+08	KD2-H4K8ac_peak_7721	7.81657	PITPNM2_ENSG00000090975
12	123717434	1.24E+08	KD2-H4K8ac_peak_7722	13.48341	C12orf65_ENSG00000130921
12	123752504	1.24E+08	KD2-H4K8ac_peak_7723	4.37809	
12	123752934	1.24E+08	KD2-H4K8ac_peak_7724	10.05606	
12	123753889	1.24E+08	KD2-H4K8ac_peak_7725	6.66359	
12	123754398	1.24E+08	KD2-H4K8ac_peak_7726	17.42905	CDK2AP1_ENSG00000111328

12	123756400	1.24E+08	KD2-H4K8ac_peak_7727	16.12525	CDK2AP1_ENSG00000111328
12	123848969	1.24E+08	KD2-H4K8ac_peak_7728	8.62078	SBNO1_ENSG00000139697;hsa-mir-8072_ENSG00000256092
12	123867887	1.24E+08	KD2-H4K8ac_peak_7729	15.52424	SETD8_ENSG00000183955
12	123874095	1.24E+08	KD2-H4K8ac_peak_7730	4.77079	
12	123920619	1.24E+08	KD2-H4K8ac_peak_7731	6.99815	RILPL2_ENSG00000150977
12	123942677	1.24E+08	KD2-H4K8ac_peak_7732	16.04168	SNRNP35_ENSG00000184209
12	123943259	1.24E+08	KD2-H4K8ac_peak_7733	8.30974	
12	123953975	1.24E+08	KD2-H4K8ac_peak_7734	4.77079	
12	124017613	1.24E+08	KD2-H4K8ac_peak_7735	5.88845	RILPL1_ENSG00000188026
12	124018331	1.24E+08	KD2-H4K8ac_peak_7736	18.5703	RILPL1_ENSG00000188026
12	124055326	1.24E+08	KD2-H4K8ac_peak_7737	8.97719	
12	124068392	1.24E+08	KD2-H4K8ac_peak_7738	5.08628	TMED2_ENSG00000086598
12	124086045	1.24E+08	KD2-H4K8ac_peak_7739	19.35989	DDX55_ENSG00000111364
12	124117966	1.24E+08	KD2-H4K8ac_peak_7740	4.98072	EIF2B1_ENSG00000111361;GTF2H3_ENSG00000111358
12	124196191	1.24E+08	KD2-H4K8ac_peak_7741	4.06758	ATP6V0A2_ENSG00000185344
12	124196958	1.24E+08	KD2-H4K8ac_peak_7742	12.00194	ATP6V0A2_ENSG00000185344
12	124229153	1.24E+08	KD2-H4K8ac_peak_7743	5.07241	
12	124456618	1.24E+08	KD2-H4K8ac_peak_7744	15.25814	CCDC92_ENSG00000119242;RP11-214K3.18_ENSG00000270095;ZNF664_ENSG00000179195;FAM101A_ENSG00000178882
12	124457787	1.24E+08	KD2-H4K8ac_peak_7745	9.96798	CCDC92_ENSG00000119242;FAM101A_ENSG00000178882
12	124505670	1.25E+08	KD2-H4K8ac_peak_7746	5.77029	
12	124534506	1.25E+08	KD2-H4K8ac_peak_7747	4.77079	
12	124773902	1.25E+08	KD2-H4K8ac_peak_7748	6.65673	
12	124779426	1.25E+08	KD2-H4K8ac_peak_7749	6.25869	
12	124854270	1.25E+08	KD2-H4K8ac_peak_7750	7.36799	
12	124872747	1.25E+08	KD2-H4K8ac_peak_7751	9.58924	
12	124873530	1.25E+08	KD2-H4K8ac_peak_7752	11.60333	
12	124877063	1.25E+08	KD2-H4K8ac_peak_7753	7.28827	
12	124881053	1.25E+08	KD2-H4K8ac_peak_7754	8.79106	
12	124895934	1.25E+08	KD2-H4K8ac_peak_7755	7.07117	
12	124897073	1.25E+08	KD2-H4K8ac_peak_7756	6.67474	
12	124906641	1.25E+08	KD2-H4K8ac_peak_7757	7.76104	
12	124911563	1.25E+08	KD2-H4K8ac_peak_7758	12.66465	
12	124913452	1.25E+08	KD2-H4K8ac_peak_7759	11.50346	
12	124947889	1.25E+08	KD2-H4K8ac_peak_7760	7.27716	
12	124989748	1.25E+08	KD2-H4K8ac_peak_7761	13.50024	
12	124990355	1.25E+08	KD2-H4K8ac_peak_7762	13.61173	
12	124991377	1.25E+08	KD2-H4K8ac_peak_7763	7.28628	
12	124991921	1.25E+08	KD2-H4K8ac_peak_7764	17.08129	
12	124992886	1.25E+08	KD2-H4K8ac_peak_7765	5.88457	
12	124994613	1.25E+08	KD2-H4K8ac_peak_7766	11.28765	
12	124997543	1.25E+08	KD2-H4K8ac_peak_7767	5.54647	RP11-83B20.1_ENSG00000214650
12	125002162	1.25E+08	KD2-H4K8ac_peak_7768	6.66359	
12	125002837	1.25E+08	KD2-H4K8ac_peak_7769	12.53053	
12	125009067	1.25E+08	KD2-H4K8ac_peak_7770	4.95193	
12	125016123	1.25E+08	KD2-H4K8ac_peak_7771	6.56211	
12	125017135	1.25E+08	KD2-H4K8ac_peak_7772	6.66692	

12	125027900	1.25E+08	KD2-H4K8ac_peak_7773	10.5847	
12	125029583	1.25E+08	KD2-H4K8ac_peak_7774	6.60096	
12	125051308	1.25E+08	KD2-H4K8ac_peak_7775	12.60538	NCOR2_ENSG00000196498
12	125052380	1.25E+08	KD2-H4K8ac_peak_7776	4.67342	NCOR2_ENSG00000196498
12	125053363	1.25E+08	KD2-H4K8ac_peak_7777	10.46874	
12	125087605	1.25E+08	KD2-H4K8ac_peak_7778	6.0605	
12	125089322	1.25E+08	KD2-H4K8ac_peak_7779	10.19723	
12	125102266	1.25E+08	KD2-H4K8ac_peak_7780	9.80315	
12	125117122	1.25E+08	KD2-H4K8ac_peak_7781	5.51783	
12	125136534	1.25E+08	KD2-H4K8ac_peak_7782	7.35057	
12	125139194	1.25E+08	KD2-H4K8ac_peak_7783	9.57101	
12	125140036	1.25E+08	KD2-H4K8ac_peak_7784	12.79505	
12	125145392	1.25E+08	KD2-H4K8ac_peak_7785	8.59301	
12	125145843	1.25E+08	KD2-H4K8ac_peak_7786	7.95563	
12	125160180	1.25E+08	KD2-H4K8ac_peak_7787	4.37815	
12	125166139	1.25E+08	KD2-H4K8ac_peak_7788	5.9696	
12	125169258	1.25E+08	KD2-H4K8ac_peak_7789	7.28577	
12	125198717	1.25E+08	KD2-H4K8ac_peak_7790	5.88845	
12	125199787	1.25E+08	KD2-H4K8ac_peak_7791	11.90517	
12	125204567	1.25E+08	KD2-H4K8ac_peak_7792	7.96322	
12	125226732	1.25E+08	KD2-H4K8ac_peak_7793	10.50156	
12	125230270	1.25E+08	KD2-H4K8ac_peak_7794	15.23486	
12	125239706	1.25E+08	KD2-H4K8ac_peak_7795	5.37864	
12	125271684	1.25E+08	KD2-H4K8ac_peak_7796	4.98072	
12	125318487	1.25E+08	KD2-H4K8ac_peak_7797	5.9696	
12	125326194	1.25E+08	KD2-H4K8ac_peak_7798	5.75757	
12	125348629	1.25E+08	KD2-H4K8ac_peak_7799	18.37277	
12	125383220	1.25E+08	KD2-H4K8ac_peak_7800	6.10699	
12	125398821	1.25E+08	KD2-H4K8ac_peak_7801	11.70505	MIR5188_ENSG00000265345
12	125425877	1.25E+08	KD2-H4K8ac_peak_7802	3.91903	
12	125473368	1.25E+08	KD2-H4K8ac_peak_7803	9.89126	DHX37_ENSG00000150990
12	125478261	1.25E+08	KD2-H4K8ac_peak_7804	7.67504	BRI3BP_ENSG00000184992
12	125534717	1.26E+08	KD2-H4K8ac_peak_7805	9.89126	
12	125549670	1.26E+08	KD2-H4K8ac_peak_7806	6.65673	AACS_ENSG00000081760
12	127630910	1.28E+08	KD2-H4K8ac_peak_7807	5.9696	RP11-575F12.2_ENSG00000256001
12	129307796	1.29E+08	KD2-H4K8ac_peak_7808	8.26945	SLC15A4_ENSG00000139370
12	129308722	1.29E+08	KD2-H4K8ac_peak_7809	8.53092	SLC15A4_ENSG00000139370
12	130554833	1.31E+08	KD2-H4K8ac_peak_7810	6.40683	RP11-474D1.2_ENSG00000256906
12	130822390	1.31E+08	KD2-H4K8ac_peak_7811	14.81929	PIWIL1_ENSG00000125207
12	130874917	1.31E+08	KD2-H4K8ac_peak_7812	16.8435	
12	130910296	1.31E+08	KD2-H4K8ac_peak_7813	3.91903	
12	130947740	1.31E+08	KD2-H4K8ac_peak_7814	6.03202	
12	131041658	1.31E+08	KD2-H4K8ac_peak_7815	4.52514	
12	131056255	1.31E+08	KD2-H4K8ac_peak_7816	11.89502	
12	131061099	1.31E+08	KD2-H4K8ac_peak_7817	10.96455	
12	131070212	1.31E+08	KD2-H4K8ac_peak_7818	7.79898	

12	131097849	1.31E+08	KD2-H4K8ac_peak_7819	6.80488	
12	131102858	1.31E+08	KD2-H4K8ac_peak_7820	6.25869	
12	131104612	1.31E+08	KD2-H4K8ac_peak_7821	16.822	
12	131106027	1.31E+08	KD2-H4K8ac_peak_7822	11.92978	
12	131107055	1.31E+08	KD2-H4K8ac_peak_7823	7.42204	
12	131117958	1.31E+08	KD2-H4K8ac_peak_7824	3.90565	
12	131118764	1.31E+08	KD2-H4K8ac_peak_7825	9.23578	
12	131170500	1.31E+08	KD2-H4K8ac_peak_7826	7.95563	
12	131198906	1.31E+08	KD2-H4K8ac_peak_7827	7.76329	
12	131200309	1.31E+08	KD2-H4K8ac_peak_7828	8.30974	RIMBP2_ENSG00000060709;RP11-662M24.2_ENSG00000256725
12	131201149	1.31E+08	KD2-H4K8ac_peak_7829	12.74183	RIMBP2_ENSG00000060709;RP11-662M24.2_ENSG00000256725
12	131214160	1.31E+08	KD2-H4K8ac_peak_7830	9.59147	
12	131232047	1.31E+08	KD2-H4K8ac_peak_7831	7.22936	
12	131238898	1.31E+08	KD2-H4K8ac_peak_7832	5.71749	
12	131356000	1.31E+08	KD2-H4K8ac_peak_7833	9.41619	RAN_ENSG00000132341
12	131356825	1.31E+08	KD2-H4K8ac_peak_7834	19.35989	RAN_ENSG00000132341
12	131436245	1.31E+08	KD2-H4K8ac_peak_7835	5.13615	
12	131438496	1.31E+08	KD2-H4K8ac_peak_7836	7.42204	GPR133_ENSG00000111452
12	131589490	1.32E+08	KD2-H4K8ac_peak_7837	6.25869	
12	131853049	1.32E+08	KD2-H4K8ac_peak_7838	6.99815	
12	132108951	1.32E+08	KD2-H4K8ac_peak_7839	5.45396	
12	132126406	1.32E+08	KD2-H4K8ac_peak_7840	6.36319	
12	132194881	1.32E+08	KD2-H4K8ac_peak_7841	11.09936	SFSWAP_ENSG00000061936
12	132280054	1.32E+08	KD2-H4K8ac_peak_7842	4.01115	
12	132345251	1.32E+08	KD2-H4K8ac_peak_7843	18.94636	
12	132348062	1.32E+08	KD2-H4K8ac_peak_7844	9.1707	
12	132348628	1.32E+08	KD2-H4K8ac_peak_7845	6.66692	
12	132352781	1.32E+08	KD2-H4K8ac_peak_7846	4.211	
12	132397419	1.32E+08	KD2-H4K8ac_peak_7847	12.57143	
12	132413095	1.32E+08	KD2-H4K8ac_peak_7848	8.4716	RP11-417L19.4_ENSG00000255992;PUS1_ENSG00000177192
12	132413840	1.32E+08	KD2-H4K8ac_peak_7849	7.76329	RP11-417L19.4_ENSG00000255992;PUS1_ENSG00000177192
12	132434531	1.32E+08	KD2-H4K8ac_peak_7850	12.46522	EP400_ENSG00000183495
12	132568887	1.33E+08	KD2-H4K8ac_peak_7851	10.19949	EP400NL_ENSG00000185684
12	132628319	1.33E+08	KD2-H4K8ac_peak_7852	6.65673	DDX51_ENSG00000185163;NOC4L_ENSG00000184967
12	133066135	1.33E+08	KD2-H4K8ac_peak_7853	4.06758	FBRSL1_ENSG00000112787
12	133136736	1.33E+08	KD2-H4K8ac_peak_7854	11.05467	
12	133176641	1.33E+08	KD2-H4K8ac_peak_7855	9.85259	
12	133177343	1.33E+08	KD2-H4K8ac_peak_7856	4.86324	
12	133180590	1.33E+08	KD2-H4K8ac_peak_7857	6.66692	
12	133263378	1.33E+08	KD2-H4K8ac_peak_7858	15.22643	POLE_ENSG00000177084;PXMP2_ENSG00000176894;RP13-672B3.2_ENSG00000256632
12	133286706	1.33E+08	KD2-H4K8ac_peak_7859	13.72842	PGAM5_ENSG00000247077
12	133338049	1.33E+08	KD2-H4K8ac_peak_7860	11.05467	ANKLE2_ENSG00000176915
12	133338794	1.33E+08	KD2-H4K8ac_peak_7861	11.03331	ANKLE2_ENSG00000176915
12	133404768	1.33E+08	KD2-H4K8ac_peak_7862	6.64891	GOLGA3_ENSG00000090615
12	133405416	1.33E+08	KD2-H4K8ac_peak_7863	18.91876	GOLGA3_ENSG00000090615
12	133463820	1.33E+08	KD2-H4K8ac_peak_7864	10.65724	RP11-46H11.12_ENSG00000236617

12	133464431	1.33E+08	KD2-H4K8ac_peak_7865	22.04674	RP11-46H11.12_ENSG00000236617
12	133485404	1.33E+08	KD2-H4K8ac_peak_7866	4.86324	
12	133706481	1.34E+08	KD2-H4K8ac_peak_7867	13.27022	ZNF891_ENSG00000214029;ZNF10_ENSG00000256223;ZNF268_ENSG00000090612;CTD-2140B24.4_ENSG00000256825
12	133757838	1.34E+08	KD2-H4K8ac_peak_7868	18.54875	
13	20138833	20139203	KD2-H4K8ac_peak_7869	6.16888	CASC4P1_ENSG00000238132
13	20175435	20176647	KD2-H4K8ac_peak_7870	12.93209	
13	20356315	20356894	KD2-H4K8ac_peak_7871	10.30284	PSPC1_ENSG00000121390
13	20438105	20439033	KD2-H4K8ac_peak_7872	17.55491	ZMYM5_ENSG00000132950
13	20531587	20532500	KD2-H4K8ac_peak_7873	20.18584	ZMYM2_ENSG00000121741
13	20533481	20534176	KD2-H4K8ac_peak_7874	9.15069	ZMYM2_ENSG00000121741
13	20692566	20692922	KD2-H4K8ac_peak_7875	6.40297	
13	20701714	20703048	KD2-H4K8ac_peak_7876	9.57101	
13	21099768	21099999	KD2-H4K8ac_peak_7877	7.47462	CRYL1_ENSG00000165475
13	21277205	21277487	KD2-H4K8ac_peak_7878	4.77079	IL17D_ENSG00000172458;AL161772.1_ENSG00000265710
13	21347465	21347896	KD2-H4K8ac_peak_7879	6.20982	N6AMT2_ENSG00000150456
13	21348258	21348592	KD2-H4K8ac_peak_7880	9.96798	N6AMT2_ENSG00000150456
13	21476523	21477388	KD2-H4K8ac_peak_7881	15.59774	XPO4_ENSG00000132953
13	21714755	21715011	KD2-H4K8ac_peak_7882	5.9696	SNORD27_ENSG00000252128;SAP18_ENSG00000150459
13	21750413	21750633	KD2-H4K8ac_peak_7883	7.36799	SKA3_ENSG00000165480;MRP63_ENSG00000173141
13	21750911	21751279	KD2-H4K8ac_peak_7884	4.54006	SKA3_ENSG00000165480;MRP63_ENSG00000173141
13	22032946	22033368	KD2-H4K8ac_peak_7885	8.30974	ZDHHC20_ENSG00000180776
13	22033774	22034034	KD2-H4K8ac_peak_7886	6.0073	ZDHHC20_ENSG00000180776
13	22177746	22178233	KD2-H4K8ac_peak_7887	12.08081	MICU2_ENSG00000165487
13	24040559	24041038	KD2-H4K8ac_peak_7888	8.5092	LINC00327_ENSG00000232977
13	24041263	24041466	KD2-H4K8ac_peak_7889	8.5781	LINC00327_ENSG00000232977
13	24730375	24730568	KD2-H4K8ac_peak_7890	8.88549	
13	25086546	25087265	KD2-H4K8ac_peak_7891	13.36684	PARP4_ENSG00000102699
13	25115807	25116077	KD2-H4K8ac_peak_7892	6.0605	
13	25496617	25496954	KD2-H4K8ac_peak_7893	10.0692	CENPJ_ENSG00000151849
13	25497232	25498262	KD2-H4K8ac_peak_7894	13.36684	CENPJ_ENSG00000151849
13	25861261	25861959	KD2-H4K8ac_peak_7895	13.09607	MTMR6_ENSG00000139505
13	25874968	25875468	KD2-H4K8ac_peak_7896	12.82393	RP11-271M24.2_ENSG00000260509;NUPL1_ENSG00000139496
13	26795540	26795783	KD2-H4K8ac_peak_7897	6.2781	
13	26796139	26796640	KD2-H4K8ac_peak_7898	21.12734	RNF6_ENSG00000127870
13	26827957	26828675	KD2-H4K8ac_peak_7899	18.54875	CDK8_ENSG00000132964
13	27131265	27131530	KD2-H4K8ac_peak_7900	7.48133	WASF3_ENSG00000132970
13	27132144	27132382	KD2-H4K8ac_peak_7901	7.36799	WASF3_ENSG00000132970
13	27745221	27745501	KD2-H4K8ac_peak_7902	11.05407	USP12_ENSG00000152484;USP12-AS2_ENSG00000230641
13	27746318	27746640	KD2-H4K8ac_peak_7903	12.42547	USP12_ENSG00000152484;USP12-AS2_ENSG00000230641
13	27825603	27826207	KD2-H4K8ac_peak_7904	11.05467	RPL21_ENSG00000122026
13	27845129	27845491	KD2-H4K8ac_peak_7905	5.82799	RASL11A_ENSG00000122035
13	27998213	27998492	KD2-H4K8ac_peak_7906	6.83936	GTF3A_ENSG00000122034
13	28024044	28025319	KD2-H4K8ac_peak_7907	21.75906	MTIF3_ENSG00000122033
13	28194951	28195317	KD2-H4K8ac_peak_7908	5.14125	LNX2_ENSG00000139517;POLR1D_ENSG00000186184
13	28196094	28196294	KD2-H4K8ac_peak_7909	8.16906	
13	28712703	28713271	KD2-H4K8ac_peak_7910	5.3157	PAN3-AS1_ENSG00000261485;PAN3_ENSG00000152520

13	29232700	29233157	KD2-H4K8ac_peak_7911	8.26945	POMP_ENSG00000132963
13	29233351	29233687	KD2-H4K8ac_peak_7912	6.99815	POMP_ENSG00000132963
13	29293418	29293860	KD2-H4K8ac_peak_7913	5.57044	SLC46A3_ENSG00000139508
13	30168948	30169748	KD2-H4K8ac_peak_7914	19.35989	SLC7A1_ENSG00000139514
13	30316891	30317132	KD2-H4K8ac_peak_7915	6.40683	
13	30424294	30424741	KD2-H4K8ac_peak_7916	12.82393	UBL3_ENSG00000122042
13	30766886	30767228	KD2-H4K8ac_peak_7917	6.78668	
13	30881305	30882097	KD2-H4K8ac_peak_7918	22.59108	KATNAL1_ENSG00000102781
13	30937946	30938287	KD2-H4K8ac_peak_7919	4.211	
13	30969254	30969560	KD2-H4K8ac_peak_7920	8.55271	
13	31019767	31020265	KD2-H4K8ac_peak_7921	6.8836	
13	31038340	31038729	KD2-H4K8ac_peak_7922	6.40683	
13	31039642	31039971	KD2-H4K8ac_peak_7923	8.30974	
13	31191306	31191608	KD2-H4K8ac_peak_7924	8.53092	HMGB1_ENSG00000189403;USPL1_ENSG00000132952
13	31248340	31248736	KD2-H4K8ac_peak_7925	5.88845	
13	31437450	31437656	KD2-H4K8ac_peak_7926	6.56926	
13	31445282	31445710	KD2-H4K8ac_peak_7927	5.01262	
13	31489822	31490033	KD2-H4K8ac_peak_7928	4.42765	
13	31735178	31735775	KD2-H4K8ac_peak_7929	6.99815	HSPH1_ENSG00000120694
13	31774075	31774383	KD2-H4K8ac_peak_7930	19.03298	B3GALTL_ENSG00000187676
13	31783586	31783818	KD2-H4K8ac_peak_7931	11.2938	
13	32244675	32244918	KD2-H4K8ac_peak_7932	7.01964	
13	32438842	32439260	KD2-H4K8ac_peak_7933	9.59147	
13	32884036	32884244	KD2-H4K8ac_peak_7934	7.12754	
13	32889257	32889498	KD2-H4K8ac_peak_7935	13.50024	ZAR1L_ENSG00000189167;BRCA2_ENSG00000139618
13	32889762	32889991	KD2-H4K8ac_peak_7936	14.8083	ZAR1L_ENSG00000189167;BRCA2_ENSG00000139618
13	33001462	33002169	KD2-H4K8ac_peak_7937	8.85813	N4BP2L1_ENSG00000139597
13	33002396	33002712	KD2-H4K8ac_peak_7938	7.40314	N4BP2L1_ENSG00000139597
13	33113286	33113731	KD2-H4K8ac_peak_7939	6.94255	N4BP2L2_ENSG00000244754
13	33160760	33160964	KD2-H4K8ac_peak_7940	6.66692	PDS5B_ENSG00000083642
13	33514725	33514929	KD2-H4K8ac_peak_7941	4.86324	
13	33590051	33590829	KD2-H4K8ac_peak_7942	8.5781	KL_ENSG00000133116
13	33764375	33764585	KD2-H4K8ac_peak_7943	6.00226	
13	33778102	33778383	KD2-H4K8ac_peak_7944	4.211	
13	33780653	33780881	KD2-H4K8ac_peak_7945	7.22457	
13	34111823	34112102	KD2-H4K8ac_peak_7946	8.1233	
13	34116223	34116448	KD2-H4K8ac_peak_7947	5.10276	
13	34458883	34459290	KD2-H4K8ac_peak_7948	10.05606	
13	34492140	34492369	KD2-H4K8ac_peak_7949	3.91903	
13	35324411	35324669	KD2-H4K8ac_peak_7950	6.16888	
13	36044703	36045532	KD2-H4K8ac_peak_7951	15.66774	
13	36049332	36050272	KD2-H4K8ac_peak_7952	9.11031	MAB21L1_ENSG00000180660
13	36052474	36053150	KD2-H4K8ac_peak_7953	17.91241	
13	36247250	36247653	KD2-H4K8ac_peak_7954	8.17902	
13	36289068	36289309	KD2-H4K8ac_peak_7955	4.20044	
13	36290902	36291137	KD2-H4K8ac_peak_7956	5.08628	

13	36294713	36295128	KD2-H4K8ac_peak_7957	7.89389	
13	36295657	36295897	KD2-H4K8ac_peak_7958	7.36799	
13	36358899	36359097	KD2-H4K8ac_peak_7959	6.66692	
13	36359449	36359683	KD2-H4K8ac_peak_7960	9.89126	
13	36360608	36360812	KD2-H4K8ac_peak_7961	6.16888	
13	36364017	36364260	KD2-H4K8ac_peak_7962	7.27716	
13	36383750	36384376	KD2-H4K8ac_peak_7963	7.08474	
13	36562254	36562579	KD2-H4K8ac_peak_7964	7.75499	
13	36606104	36606338	KD2-H4K8ac_peak_7965	6.83936	
13	36871625	36872289	KD2-H4K8ac_peak_7966	5.13615	SOHLH2_ENSG00000120669;CCDC169-SOHLH2_ENSG00000250709;CCDC169_ENSG00000242715
13	36920369	36920917	KD2-H4K8ac_peak_7967	18.54875	SPG20OS_ENSG00000120664
13	37006292	37006496	KD2-H4K8ac_peak_7968	5.61067	CCNA1_ENSG00000133101
13	37248262	37248556	KD2-H4K8ac_peak_7969	5.45396	SERTM1_ENSG00000180440
13	37393449	37394107	KD2-H4K8ac_peak_7970	8.55271	RFXAP_ENSG00000133111
13	37494046	37495041	KD2-H4K8ac_peak_7971	12.78794	SMAD9_ENSG00000120693
13	37573083	37573416	KD2-H4K8ac_peak_7972	12.36441	ALG5_ENSG00000120697;EXOSC8_ENSG00000120699
13	37573979	37574728	KD2-H4K8ac_peak_7973	8.41122	ALG5_ENSG00000120697
13	37575066	37575581	KD2-H4K8ac_peak_7974	5.14125	ALG5_ENSG00000120697
13	37633535	37633753	KD2-H4K8ac_peak_7975	10.0692	SUPT20H_ENSG00000102710
13	39489909	39490146	KD2-H4K8ac_peak_7976	6.66692	
13	39612576	39612938	KD2-H4K8ac_peak_7977	6.36375	PROSER1_ENSG00000120685;NHLRC3_ENSG00000188811
13	40177030	40177238	KD2-H4K8ac_peak_7978	7.76329	LHFP_ENSG00000183722
13	40229904	40230199	KD2-H4K8ac_peak_7979	10.05606	COG6_ENSG00000133103
13	40467380	40467579	KD2-H4K8ac_peak_7980	5.45396	
13	40764246	40764510	KD2-H4K8ac_peak_7981	3.75035	
13	40957324	40957632	KD2-H4K8ac_peak_7982	16.73922	
13	41238417	41239298	KD2-H4K8ac_peak_7983	7.27716	
13	41345522	41345942	KD2-H4K8ac_peak_7984	9.15329	MRPS31_ENSG00000102738
13	41363296	41363524	KD2-H4K8ac_peak_7985	8.28234	SLC25A15_ENSG00000102743
13	41363865	41364545	KD2-H4K8ac_peak_7986	13.87818	SLC25A15_ENSG00000102743
13	41483966	41484331	KD2-H4K8ac_peak_7987	4.70206	
13	41489899	41490418	KD2-H4K8ac_peak_7988	5.16439	
13	41495981	41496370	KD2-H4K8ac_peak_7989	6.65673	TPTE2P5_ENSG00000168852;SUGT1P3_ENSG00000239827
13	41706410	41706695	KD2-H4K8ac_peak_7990	8.17902	KBTBD6_ENSG00000165572
13	41768109	41768648	KD2-H4K8ac_peak_7991	17.70889	KBTBD7_ENSG00000120696
13	41885449	41885941	KD2-H4K8ac_peak_7992	12.93209	NAA16_ENSG00000172766
13	42534789	42535761	KD2-H4K8ac_peak_7993	24.9142	VWA8_ENSG00000102763
13	42845441	42846024	KD2-H4K8ac_peak_7994	12.93209	AKAP11_ENSG00000023516
13	42846339	42846736	KD2-H4K8ac_peak_7995	4.86324	AKAP11_ENSG00000023516
13	43597349	43598170	KD2-H4K8ac_peak_7996	9.36516	DNAJC15_ENSG00000120675
13	44361297	44361867	KD2-H4K8ac_peak_7997	11.03331	ENOX1_ENSG00000120658;RP11-168P13.1_ENSG00000270522
13	44453151	44453826	KD2-H4K8ac_peak_7998	19.91242	CCDC122_ENSG00000151773;LACC1_ENSG00000179630
13	44454096	44454328	KD2-H4K8ac_peak_7999	8.17902	CCDC122_ENSG00000151773;LACC1_ENSG00000179630
13	44715398	44716046	KD2-H4K8ac_peak_8000	8.58725	SMIM2-AS1_ENSG00000227258
13	44834055	44834424	KD2-H4K8ac_peak_8001	10.68173	
13	44846156	44846423	KD2-H4K8ac_peak_8002	6.40683	

13	44848164	44848503	KD2-H4K8ac_peak_8003	6.99815	
13	44880870	44881077	KD2-H4K8ac_peak_8004	6.99815	
13	44905707	44905904	KD2-H4K8ac_peak_8005	10.46264	
13	44946718	44948558	KD2-H4K8ac_peak_8006	24.48793	SERP2_ENSG00000151778
13	44971545	44971767	KD2-H4K8ac_peak_8007	12.84642	
13	44977620	44978096	KD2-H4K8ac_peak_8008	4.67342	
13	44978347	44979538	KD2-H4K8ac_peak_8009	8.5092	LINC01071_ENSG00000237361
13	45009655	45009870	KD2-H4K8ac_peak_8010	7.27716	
13	45150846	45151295	KD2-H4K8ac_peak_8011	19.43192	TSC22D1_ENSG00000102804
13	45151707	45152266	KD2-H4K8ac_peak_8012	9.63487	TSC22D1_ENSG00000102804
13	45307833	45308119	KD2-H4K8ac_peak_8013	6.56926	
13	45338545	45338748	KD2-H4K8ac_peak_8014	7.72801	
13	45468128	45468386	KD2-H4K8ac_peak_8015	8.94469	
13	45493240	45493438	KD2-H4K8ac_peak_8016	9.15069	
13	45563091	45564069	KD2-H4K8ac_peak_8017	15.69572	NUFIP1_ENSG00000083635;GPALPP1_ENSG00000133114
13	45884983	45885239	KD2-H4K8ac_peak_8018	6.81873	
13	45914855	45915222	KD2-H4K8ac_peak_8019	13.80019	TPT1_ENSG00000133112;RP11-290D2.6_ENSG00000273149;TPT1-AS1_ENSG00000170919
13	45915707	45916011	KD2-H4K8ac_peak_8020	12.42761	TPT1_ENSG00000133112;TPT1-AS1_ENSG00000170919
13	46038330	46038703	KD2-H4K8ac_peak_8021	13.09607	COG3_ENSG00000136152
13	46039153	46039455	KD2-H4K8ac_peak_8022	8.36432	COG3_ENSG00000136152
13	46397776	46398088	KD2-H4K8ac_peak_8023	6.24747	
13	46626475	46626848	KD2-H4K8ac_peak_8024	18.5703	ZC3H13_ENSG00000123200;CPB2-AS1_ENSG00000235903
13	46786171	46786491	KD2-H4K8ac_peak_8025	5.9696	LCP1_ENSG00000136167;LRRC63_ENSG00000173988
13	47126259	47126602	KD2-H4K8ac_peak_8026	5.20908	LRCH1_ENSG00000136141
13	47126917	47127160	KD2-H4K8ac_peak_8027	13.80019	LRCH1_ENSG00000136141
13	47204661	47204871	KD2-H4K8ac_peak_8028	6.16888	
13	47211491	47211799	KD2-H4K8ac_peak_8029	9.15329	
13	47370352	47371274	KD2-H4K8ac_peak_8030	13.73635	ESD_ENSG00000139684
13	47372021	47372231	KD2-H4K8ac_peak_8031	8.00457	ESD_ENSG00000139684
13	48574934	48575400	KD2-H4K8ac_peak_8032	19.9503	SUCLA2-AS1_ENSG00000227848
13	48575810	48576466	KD2-H4K8ac_peak_8033	6.00226	SUCLA2-AS1_ENSG00000227848
13	48610996	48611301	KD2-H4K8ac_peak_8034	4.75764	SUCLA2_ENSG00000136143;NUDT15_ENSG00000136159
13	48611862	48612062	KD2-H4K8ac_peak_8035	8.55271	SUCLA2_ENSG00000136143;NUDT15_ENSG00000136159
13	48612274	48612555	KD2-H4K8ac_peak_8036	5.88845	SUCLA2_ENSG00000136143;NUDT15_ENSG00000136159
13	48668672	48669167	KD2-H4K8ac_peak_8037	8.30974	MED4_ENSG00000136146
13	48669570	48669804	KD2-H4K8ac_peak_8038	11.30637	MED4_ENSG00000136146
13	48807456	48807767	KD2-H4K8ac_peak_8039	7.76104	ITM2B_ENSG00000136156
13	48877370	48877742	KD2-H4K8ac_peak_8040	6.20982	LINC00441_ENSG00000231473;RB1_ENSG00000139687
13	48877961	48878368	KD2-H4K8ac_peak_8041	12.74171	LINC00441_ENSG00000231473;RB1_ENSG00000139687
13	49105991	49106758	KD2-H4K8ac_peak_8042	7.40111	RCBTB2_ENSG00000136161
13	49106966	49107791	KD2-H4K8ac_peak_8043	15.81711	RCBTB2_ENSG00000136161
13	49116630	49116827	KD2-H4K8ac_peak_8044	5.43522	
13	49549248	49550627	KD2-H4K8ac_peak_8045	7.76329	FNDC3A_ENSG00000102531
13	49684118	49684325	KD2-H4K8ac_peak_8046	7.12754	
13	49766475	49766739	KD2-H4K8ac_peak_8047	4.3289	
13	49822215	49822452	KD2-H4K8ac_peak_8048	6.76298	CDADC1_ENSG00000102543

13	50017866	50018062	KD2-H4K8ac_peak_8049	9.20193	CAB39L_ENSG00000102547;SETDB2_ENSG00000136169
13	50159261	50160498	KD2-H4K8ac_peak_8050	17.8868	RCBTB1_ENSG00000136144
13	50199742	50200032	KD2-H4K8ac_peak_8051	6.40683	
13	50265816	50266148	KD2-H4K8ac_peak_8052	9.59147	EBPL_ENSG00000123179
13	50366090	50366630	KD2-H4K8ac_peak_8053	5.59859	KPNA3_ENSG00000102753
13	50366881	50367084	KD2-H4K8ac_peak_8054	11.04256	KPNA3_ENSG00000102753
13	50510990	50511226	KD2-H4K8ac_peak_8055	8.17902	SPRYD7_ENSG00000123178
13	50570114	50570879	KD2-H4K8ac_peak_8056	12.00273	TRIM13_ENSG00000204977;MIR3613_ENSG00000264864
13	50571201	50571588	KD2-H4K8ac_peak_8057	12.96672	MIR3613_ENSG00000264864
13	50655602	50655930	KD2-H4K8ac_peak_8058	5.54647	DLEU1_ENSG00000176124
13	50698046	50698534	KD2-H4K8ac_peak_8059	14.00658	
13	50699068	50699652	KD2-H4K8ac_peak_8060	11.24702	DLEU2_ENSG00000231607
13	50699875	50700415	KD2-H4K8ac_peak_8061	20.76543	DLEU2_ENSG00000231607
13	50774605	50774946	KD2-H4K8ac_peak_8062	5.30799	
13	50775596	50775798	KD2-H4K8ac_peak_8063	5.57044	
13	51103628	51103957	KD2-H4K8ac_peak_8064	7.1181	
13	51168729	51169015	KD2-H4K8ac_peak_8065	6.31143	
13	51264429	51264797	KD2-H4K8ac_peak_8066	4.42765	
13	51375381	51375590	KD2-H4K8ac_peak_8067	7.79558	
13	51417073	51417394	KD2-H4K8ac_peak_8068	4.42765	DLEU7_ENSG00000186047
13	51483674	51484760	KD2-H4K8ac_peak_8069	17.55491	RNASEH2B-AS1_ENSG00000233672;RNASEH2B_ENSG00000136104
13	51795988	51796619	KD2-H4K8ac_peak_8070	15.81711	FAM124A_ENSG00000150510
13	51821082	51821486	KD2-H4K8ac_peak_8071	7.40314	
13	51872819	51873135	KD2-H4K8ac_peak_8072	12.42547	
13	52026171	52027110	KD2-H4K8ac_peak_8073	12.81138	INTS6-AS1_ENSG00000236778
13	52027557	52027987	KD2-H4K8ac_peak_8074	9.11031	INTS6_ENSG00000102786;RPS4XP16_ENSG00000224892
13	52157527	52158009	KD2-H4K8ac_peak_8075	12.14909	WDFY2_ENSG00000139668
13	52193288	52193484	KD2-H4K8ac_peak_8076	4.04247	
13	52194403	52194872	KD2-H4K8ac_peak_8077	12.25733	
13	52195092	52195339	KD2-H4K8ac_peak_8078	4.57371	
13	52198865	52199145	KD2-H4K8ac_peak_8079	5.25667	
13	52202196	52202682	KD2-H4K8ac_peak_8080	25.6154	
13	52206458	52206663	KD2-H4K8ac_peak_8081	4.67737	
13	52356398	52356604	KD2-H4K8ac_peak_8082	4.86324	
13	52361376	52361640	KD2-H4K8ac_peak_8083	8.30114	
13	52377986	52378568	KD2-H4K8ac_peak_8084	22.0091	DHRS12_ENSG00000102796;RP11-327P2.5_ENSG00000231856
13	52733269	52733757	KD2-H4K8ac_peak_8085	16.57619	NEK3_ENSG00000136098
13	52768310	52769254	KD2-H4K8ac_peak_8086	11.43353	MRPS31P5_ENSG00000243406
13	52980047	52980726	KD2-H4K8ac_peak_8087	10.05606	THSD1_ENSG00000136114
13	53024244	53024726	KD2-H4K8ac_peak_8088	23.72814	VPS36_ENSG00000136100
13	53174008	53174445	KD2-H4K8ac_peak_8089	7.59643	
13	53191033	53191315	KD2-H4K8ac_peak_8090	9.1707	HNRNPA1L2_ENSG00000139675;MRPS31P4_ENSG00000250299
13	53226988	53227356	KD2-H4K8ac_peak_8091	8.65175	SUGT1_ENSG00000165416
13	53643064	53643297	KD2-H4K8ac_peak_8092	8.62078	
13	53775879	53776156	KD2-H4K8ac_peak_8093	6.66692	
13	59853210	59853413	KD2-H4K8ac_peak_8094	6.00226	

13	60738284	60738599	KD2-H4K8ac_peak_8095	8.55271	DIAPH3_ENSG00000139734
13	60970679	60972005	KD2-H4K8ac_peak_8096	14.55656	TDRD3_ENSG00000083544
13	67231169	67231466	KD2-H4K8ac_peak_8097	8.1233	
13	67240345	67240583	KD2-H4K8ac_peak_8098	4.77079	
13	67804844	67805055	KD2-H4K8ac_peak_8099	7.18615	PCDH9_ENSG00000184226
13	72439194	72440923	KD2-H4K8ac_peak_8100	9.01262	DACH1_ENSG00000165659
13	73098119	73098328	KD2-H4K8ac_peak_8101	5.25071	
13	73109053	73109357	KD2-H4K8ac_peak_8102	3.99369	
13	73271095	73271341	KD2-H4K8ac_peak_8103	5.04237	
13	73301279	73301725	KD2-H4K8ac_peak_8104	9.34989	MZT1_ENSG00000204899;BORA_ENSG00000136122
13	74339628	74339919	KD2-H4K8ac_peak_8105	4.86324	
13	74708739	74709621	KD2-H4K8ac_peak_8106	30.77376	KLF12_ENSG00000118922
13	74709836	74710657	KD2-H4K8ac_peak_8107	8.59301	
13	74862611	74863110	KD2-H4K8ac_peak_8108	5.75452	RNY1P5_ENSG00000206617
13	75129538	75129736	KD2-H4K8ac_peak_8109	5.29555	
13	75794268	75794513	KD2-H4K8ac_peak_8110	5.43522	
13	75875461	75875707	KD2-H4K8ac_peak_8111	6.16888	
13	76032304	76032639	KD2-H4K8ac_peak_8112	5.20908	
13	76055785	76056058	KD2-H4K8ac_peak_8113	4.68806	TBC1D4_ENSG00000136111
13	76111564	76111881	KD2-H4K8ac_peak_8114	5.88845	
13	76112297	76112545	KD2-H4K8ac_peak_8115	4.67342	
13	76123972	76124302	KD2-H4K8ac_peak_8116	7.40111	COMMD6_ENSG00000188243;UCHL3_ENSG00000118939;RP11-29G8.3_ENSG00000261553
13	76209665	76210234	KD2-H4K8ac_peak_8117	12.06346	RP11-173B14.5_ENSG00000261105
13	76210473	76210964	KD2-H4K8ac_peak_8118	4.44527	RP11-173B14.5_ENSG00000261105
13	76387548	76387858	KD2-H4K8ac_peak_8119	6.16888	
13	77459510	77460253	KD2-H4K8ac_peak_8120	6.40683	KCTD12_ENSG00000178695;AC000403.1_ENSG00000264908
13	77565613	77565907	KD2-H4K8ac_peak_8121	6.85193	CLN5_ENSG00000102805
13	77600947	77601247	KD2-H4K8ac_peak_8122	14.66631	FBXL3_ENSG00000005812
13	77903519	77904114	KD2-H4K8ac_peak_8123	10.08707	
13	78271904	78272121	KD2-H4K8ac_peak_8124	5.30799	SLAIN1_ENSG00000139737;MIR3665_ENSG00000266325
13	78899267	78899511	KD2-H4K8ac_peak_8125	9.15329	
13	79232790	79233172	KD2-H4K8ac_peak_8126	13.09607	RNF219_ENSG00000152193
13	79233888	79234682	KD2-H4K8ac_peak_8127	9.15329	RNF219_ENSG00000152193
13	79979798	79980967	KD2-H4K8ac_peak_8128	10.29842	RBM26_ENSG00000139746;RBM26-AS1_ENSG00000227354
13	80055083	80055854	KD2-H4K8ac_peak_8129	20.78681	NDFIP2-AS1_ENSG00000232132;NDFIP2_ENSG00000102471
13	80232730	80233060	KD2-H4K8ac_peak_8130	4.06758	
13	80255049	80255405	KD2-H4K8ac_peak_8131	8.88549	
13	80372440	80372920	KD2-H4K8ac_peak_8132	7.47462	
13	80377156	80377517	KD2-H4K8ac_peak_8133	7.36933	
13	80448851	80449063	KD2-H4K8ac_peak_8134	5.16439	
13	80509879	80510210	KD2-H4K8ac_peak_8135	9.15069	
13	80510881	80511273	KD2-H4K8ac_peak_8136	9.11031	
13	80914068	80915813	KD2-H4K8ac_peak_8137	17.75427	SPRY2_ENSG00000136158
13	80916639	80917123	KD2-H4K8ac_peak_8138	13.9234	
13	80917356	80917554	KD2-H4K8ac_peak_8139	4.76709	
13	82739706	82740145	KD2-H4K8ac_peak_8140	14.74047	

13	85840243	85841372	KD2-H4K8ac_peak_8141	8.17902	
13	91999164	91999639	KD2-H4K8ac_peak_8142	5.54647	MIR17HG_ENSG00000215417
13	91999965	92001221	KD2-H4K8ac_peak_8143	33.14727	MIR17HG_ENSG00000215417
13	93015675	93015990	KD2-H4K8ac_peak_8144	9.15329	
13	93879200	93880973	KD2-H4K8ac_peak_8145	10.43145	GPC6_ENSG00000183098
13	94534449	94534661	KD2-H4K8ac_peak_8146	7.12754	
13	95107442	95107635	KD2-H4K8ac_peak_8147	4.42765	
13	95200709	95201658	KD2-H4K8ac_peak_8148	13.81781	
13	95202079	95202711	KD2-H4K8ac_peak_8149	11.70505	
13	95248003	95248444	KD2-H4K8ac_peak_8150	14.98936	TGDS_ENSG00000088451
13	95248838	95249085	KD2-H4K8ac_peak_8151	11.43353	TGDS_ENSG00000088451
13	95253586	95253783	KD2-H4K8ac_peak_8152	8.83582	GPR180_ENSG00000152749
13	95254191	95254594	KD2-H4K8ac_peak_8153	7.76329	GPR180_ENSG00000152749
13	95655380	95655653	KD2-H4K8ac_peak_8154	5.16439	
13	95953208	95953633	KD2-H4K8ac_peak_8155	5.30799	ABCC4_ENSG00000125257
13	96296430	96296780	KD2-H4K8ac_peak_8156	10.05606	DZIP1_ENSG00000134874
13	96297090	96297317	KD2-H4K8ac_peak_8157	7.29632	DZIP1_ENSG00000134874
13	96705244	96705561	KD2-H4K8ac_peak_8158	8.85813	UGGT2_ENSG00000102595
13	96705960	96706163	KD2-H4K8ac_peak_8159	7.51935	UGGT2_ENSG00000102595
13	97887189	97887479	KD2-H4K8ac_peak_8160	5.57044	
13	98085869	98086080	KD2-H4K8ac_peak_8161	10.87093	RAP2A_ENSG00000125249
13	98627650	98628683	KD2-H4K8ac_peak_8162	16.8435	
13	98628935	98629427	KD2-H4K8ac_peak_8163	10.19949	
13	98794942	98795220	KD2-H4K8ac_peak_8164	5.9696	FARP1_ENSG00000152767
13	98795703	98796390	KD2-H4K8ac_peak_8165	8.55271	FARP1_ENSG00000152767
13	98824480	98824723	KD2-H4K8ac_peak_8166	5.32933	
13	99131911	99132189	KD2-H4K8ac_peak_8167	5.7068	
13	99229506	99230189	KD2-H4K8ac_peak_8168	13.80019	STK24_ENSG00000102572;STK24-AS1_ENSG00000224418
13	99274880	99275168	KD2-H4K8ac_peak_8169	7.40111	
13	99404801	99405035	KD2-H4K8ac_peak_8170	6.9809	SLC15A1_ENSG00000088386
13	99473388	99473656	KD2-H4K8ac_peak_8171	11.05467	
13	99738516	99739851	KD2-H4K8ac_peak_8172	10.43145	DOCK9_ENSG00000088387;DOCK9-AS2_ENSG00000260992
13	99740121	99740361	KD2-H4K8ac_peak_8173	5.9696	DOCK9-AS2_ENSG00000260992
13	99852351	99852887	KD2-H4K8ac_peak_8174	25.6154	UBAC2-AS1_ENSG00000228889;UBAC2_ENSG00000134882
13	99853112	99853647	KD2-H4K8ac_peak_8175	14.66631	UBAC2-AS1_ENSG00000228889;UBAC2_ENSG00000134882
13	99964041	99964280	KD2-H4K8ac_peak_8176	6.99815	
13	99964893	99965399	KD2-H4K8ac_peak_8177	5.88845	
13	100150092	1E+08	KD2-H4K8ac_peak_8178	10.72656	
13	100151139	1E+08	KD2-H4K8ac_peak_8179	7.27716	
13	100153812	1E+08	KD2-H4K8ac_peak_8180	5.30799	LINC00449_ENSG00000203441;TM9SF2_ENSG00000125304
13	100258996	1E+08	KD2-H4K8ac_peak_8181	18.5703	CLYBL_ENSG00000125246
13	100547680	1.01E+08	KD2-H4K8ac_peak_8182	4.37708	
13	100740525	1.01E+08	KD2-H4K8ac_peak_8183	5.57044	PCCA_ENSG00000175198
13	101199744	1.01E+08	KD2-H4K8ac_peak_8184	6.0073	
13	101240662	1.01E+08	KD2-H4K8ac_peak_8185	14.56955	GGACT_ENSG00000134864
13	101326749	1.01E+08	KD2-H4K8ac_peak_8186	12.13241	TMTC4_ENSG00000125247

13	102068997	1.02E+08	KD2-H4K8ac_peak_8187	7.12754	NALCN_ENSG00000102452
13	102970420	1.03E+08	KD2-H4K8ac_peak_8188	4.86324	
13	103046417	1.03E+08	KD2-H4K8ac_peak_8189	16.09057	FGF14-IT1_ENSG00000243319;FGF14-AS2_ENSG00000272143
13	103052542	1.03E+08	KD2-H4K8ac_peak_8190	7.76104	FGF14_ENSG00000102466
13	103244527	1.03E+08	KD2-H4K8ac_peak_8191	10.0692	
13	103248718	1.03E+08	KD2-H4K8ac_peak_8192	16.57619	TPP2_ENSG00000134900
13	103249522	1.03E+08	KD2-H4K8ac_peak_8193	5.1628	TPP2_ENSG00000134900
13	103426301	1.03E+08	KD2-H4K8ac_peak_8194	10.31695	TEX30_ENSG00000151287
13	103451556	1.03E+08	KD2-H4K8ac_peak_8195	6.66359	KDELC1_ENSG00000134901;BIVM_ENSG00000134897
13	103497305	1.03E+08	KD2-H4K8ac_peak_8196	9.73431	ERCC5_ENSG00000134899
13	104618151	1.05E+08	KD2-H4K8ac_peak_8197	10.0692	
13	104657916	1.05E+08	KD2-H4K8ac_peak_8198	7.12754	
13	104659716	1.05E+08	KD2-H4K8ac_peak_8199	7.27716	
13	105135747	1.05E+08	KD2-H4K8ac_peak_8200	7.27716	
13	105925295	1.06E+08	KD2-H4K8ac_peak_8201	14.45402	
13	106589402	1.07E+08	KD2-H4K8ac_peak_8202	4.37708	
13	107186658	1.07E+08	KD2-H4K8ac_peak_8203	9.72244	EFNB2_ENSG00000125266
13	107187546	1.07E+08	KD2-H4K8ac_peak_8204	6.56211	EFNB2_ENSG00000125266
13	107219842	1.07E+08	KD2-H4K8ac_peak_8205	11.89502	
13	107570034	1.08E+08	KD2-H4K8ac_peak_8206	22.21127	
13	107572090	1.08E+08	KD2-H4K8ac_peak_8207	6.40683	
13	107823401	1.08E+08	KD2-H4K8ac_peak_8208	7.12754	
13	108029401	1.08E+08	KD2-H4K8ac_peak_8209	6.25869	
13	108518560	1.09E+08	KD2-H4K8ac_peak_8210	4.77079	FAM155A_ENSG00000204442
13	108519251	1.09E+08	KD2-H4K8ac_peak_8211	9.36516	FAM155A_ENSG00000204442
13	108520116	1.09E+08	KD2-H4K8ac_peak_8212	4.76709	
13	108869918	1.09E+08	KD2-H4K8ac_peak_8213	6.55628	LIG4_ENSG00000174405;ABHD13_ENSG00000139826
13	109792873	1.1E+08	KD2-H4K8ac_peak_8214	8.09113	
13	109798870	1.1E+08	KD2-H4K8ac_peak_8215	8.09113	
13	109986761	1.1E+08	KD2-H4K8ac_peak_8216	8.2413	
13	109994368	1.1E+08	KD2-H4K8ac_peak_8217	11.30637	
13	109996957	1.1E+08	KD2-H4K8ac_peak_8218	13.81781	
13	109998245	1.1E+08	KD2-H4K8ac_peak_8219	22.21127	
13	110008321	1.1E+08	KD2-H4K8ac_peak_8220	5.45396	
13	110013500	1.1E+08	KD2-H4K8ac_peak_8221	6.40683	
13	110015798	1.1E+08	KD2-H4K8ac_peak_8222	5.57044	
13	110021616	1.1E+08	KD2-H4K8ac_peak_8223	6.65673	
13	110294993	1.1E+08	KD2-H4K8ac_peak_8224	5.16439	
13	110318800	1.1E+08	KD2-H4K8ac_peak_8225	9.8758	
13	110321344	1.1E+08	KD2-H4K8ac_peak_8226	7.76104	
13	110322302	1.1E+08	KD2-H4K8ac_peak_8227	12.84642	
13	110352151	1.1E+08	KD2-H4K8ac_peak_8228	6.36375	
13	110396319	1.1E+08	KD2-H4K8ac_peak_8229	3.97079	
13	110433953	1.1E+08	KD2-H4K8ac_peak_8230	18.7894	
13	110436671	1.1E+08	KD2-H4K8ac_peak_8231	11.35142	
13	110439498	1.1E+08	KD2-H4K8ac_peak_8232	5.54647	IRS2_ENSG00000185950

13	110521708	1.11E+08	KD2-H4K8ac_peak_8233	3.75035	
13	110791065	1.11E+08	KD2-H4K8ac_peak_8234	6.25869	
13	110918261	1.11E+08	KD2-H4K8ac_peak_8235	10.0692	
13	110959019	1.11E+08	KD2-H4K8ac_peak_8236	18.51132	COL4A1_ENSG00000187498;COL4A2_ENSG00000134871
13	111024029	1.11E+08	KD2-H4K8ac_peak_8237	9.08816	
13	111118747	1.11E+08	KD2-H4K8ac_peak_8238	9.76452	
13	111214279	1.11E+08	KD2-H4K8ac_peak_8239	7.27716	RAB20_ENSG00000139832
13	111267147	1.11E+08	KD2-H4K8ac_peak_8240	11.04256	CARKD_ENSG00000213995
13	111267977	1.11E+08	KD2-H4K8ac_peak_8241	15.53798	CARKD_ENSG00000213995
13	111280545	1.11E+08	KD2-H4K8ac_peak_8242	6.66692	
13	111358074	1.11E+08	KD2-H4K8ac_peak_8243	10.0692	
13	111358609	1.11E+08	KD2-H4K8ac_peak_8244	10.19949	
13	111364187	1.11E+08	KD2-H4K8ac_peak_8245	7.86151	ING1_ENSG00000153487
13	111364895	1.11E+08	KD2-H4K8ac_peak_8246	31.533	CARS2_ENSG00000134905;ING1_ENSG00000153487
13	111365623	1.11E+08	KD2-H4K8ac_peak_8247	10.96455	CARS2_ENSG00000134905;ING1_ENSG00000153487
13	111566540	1.12E+08	KD2-H4K8ac_peak_8248	11.09936	ANKRD10_ENSG00000088448
13	111568068	1.12E+08	KD2-H4K8ac_peak_8249	8.30974	ANKRD10_ENSG00000088448
13	111767585	1.12E+08	KD2-H4K8ac_peak_8250	4.67342	ARHGEF7-AS2_ENSG00000235875;ARHGEF7_ENSG00000102606
13	111768306	1.12E+08	KD2-H4K8ac_peak_8251	8.17902	ARHGEF7-AS2_ENSG00000235875
13	111805533	1.12E+08	KD2-H4K8ac_peak_8252	12.82393	
13	111806082	1.12E+08	KD2-H4K8ac_peak_8253	10.46264	
13	113184736	1.13E+08	KD2-H4K8ac_peak_8254	8.37043	
13	113187380	1.13E+08	KD2-H4K8ac_peak_8255	20.50833	
13	113242020	1.13E+08	KD2-H4K8ac_peak_8256	17.55491	TUBGCP3_ENSG00000126216
13	113343817	1.13E+08	KD2-H4K8ac_peak_8257	10.98959	ATP11A_ENSG00000068650
13	113344465	1.13E+08	KD2-H4K8ac_peak_8258	5.13615	ATP11A_ENSG00000068650
13	113547859	1.14E+08	KD2-H4K8ac_peak_8259	8.62963	MCF2L_ENSG00000126217
13	113548270	1.14E+08	KD2-H4K8ac_peak_8260	6.66692	MCF2L_ENSG00000126217
13	113623177	1.14E+08	KD2-H4K8ac_peak_8261	5.30799	MCF2L-AS1_ENSG00000235280
13	113656032	1.14E+08	KD2-H4K8ac_peak_8262	18.25771	
13	113776186	1.14E+08	KD2-H4K8ac_peak_8263	10.05606	F10_ENSG00000126218
13	113777148	1.14E+08	KD2-H4K8ac_peak_8264	15.5861	F10_ENSG00000126218
13	113807667	1.14E+08	KD2-H4K8ac_peak_8265	9.8758	
13	113862592	1.14E+08	KD2-H4K8ac_peak_8266	17.70889	PCID2_ENSG00000126226;CUL4A_ENSG00000139842
13	113864012	1.14E+08	KD2-H4K8ac_peak_8267	15.04693	PCID2_ENSG00000126226
13	113868881	1.14E+08	KD2-H4K8ac_peak_8268	5.21139	
13	113950573	1.14E+08	KD2-H4K8ac_peak_8269	6.25869	LAMP1_ENSG00000185896
13	113950969	1.14E+08	KD2-H4K8ac_peak_8270	22.06226	LAMP1_ENSG00000185896
13	113951633	1.14E+08	KD2-H4K8ac_peak_8271	9.20193	LAMP1_ENSG00000185896
13	114017761	1.14E+08	KD2-H4K8ac_peak_8272	9.36516	GRTP1_ENSG00000139835
13	114018800	1.14E+08	KD2-H4K8ac_peak_8273	8.33649	GRTP1_ENSG00000139835
13	114048547	1.14E+08	KD2-H4K8ac_peak_8274	7.76329	
13	114061930	1.14E+08	KD2-H4K8ac_peak_8275	17.28876	
13	114075924	1.14E+08	KD2-H4K8ac_peak_8276	4.42765	
13	114076831	1.14E+08	KD2-H4K8ac_peak_8277	6.40683	
13	114145376	1.14E+08	KD2-H4K8ac_peak_8278	9.20193	DCUN1D2_ENSG00000150401;TMCO3_ENSG00000150403

13	114198285	1.14E+08	KD2-H4K8ac_peak_8279	18.94636	
13	114199979	1.14E+08	KD2-H4K8ac_peak_8280	9.11031	
13	114239176	1.14E+08	KD2-H4K8ac_peak_8281	9.06369	TFDP1_ENSG00000198176
13	114273826	1.14E+08	KD2-H4K8ac_peak_8282	9.16531	
13	114312302	1.14E+08	KD2-H4K8ac_peak_8283	7.70289	ATP4B_ENSG00000186009
13	114429578	1.14E+08	KD2-H4K8ac_peak_8284	8.55271	
13	114445256	1.14E+08	KD2-H4K8ac_peak_8285	7.22457	
13	114461770	1.14E+08	KD2-H4K8ac_peak_8286	12.08081	TMEM255B_ENSG00000184497
13	114504509	1.15E+08	KD2-H4K8ac_peak_8287	10.7186	
13	114523618	1.15E+08	KD2-H4K8ac_peak_8288	9.96941	
13	114524863	1.15E+08	KD2-H4K8ac_peak_8289	7.69287	
13	114540456	1.15E+08	KD2-H4K8ac_peak_8290	6.52179	
13	114564461	1.15E+08	KD2-H4K8ac_peak_8291	9.6261	
13	114566101	1.15E+08	KD2-H4K8ac_peak_8292	8.85813	GAS6_ENSG00000183087;GAS6-AS2_ENSG00000272695
13	114566676	1.15E+08	KD2-H4K8ac_peak_8293	14.00658	GAS6_ENSG00000183087;GAS6-AS2_ENSG00000272695
13	114638041	1.15E+08	KD2-H4K8ac_peak_8294	5.43522	
13	114829033	1.15E+08	KD2-H4K8ac_peak_8295	8.7325	
13	114829602	1.15E+08	KD2-H4K8ac_peak_8296	14.27217	
13	114830712	1.15E+08	KD2-H4K8ac_peak_8297	16.822	
13	114844323	1.15E+08	KD2-H4K8ac_peak_8298	7.1181	
13	114863310	1.15E+08	KD2-H4K8ac_peak_8299	9.1707	
13	114864128	1.15E+08	KD2-H4K8ac_peak_8300	4.01115	
13	114864821	1.15E+08	KD2-H4K8ac_peak_8301	11.6141	
13	114875154	1.15E+08	KD2-H4K8ac_peak_8302	7.89389	RASA3-IT1_ENSG00000232487
13	114898172	1.15E+08	KD2-H4K8ac_peak_8303	8.79106	RASA3_ENSG00000185989
13	114999929	1.15E+08	KD2-H4K8ac_peak_8304	14.8083	CDC16_ENSG00000130177
13	115046490	1.15E+08	KD2-H4K8ac_peak_8305	7.73264	UPF3A_ENSG00000169062
13	115079548	1.15E+08	KD2-H4K8ac_peak_8306	6.45083	CHAMP1_ENSG00000198824
13	115080024	1.15E+08	KD2-H4K8ac_peak_8307	9.63487	CHAMP1_ENSG00000198824
14	20801069	20801364	KD2-H4K8ac_peak_8308	6.40683	CCNB1IP1_ENSG00000100814
14	20810723	20811026	KD2-H4K8ac_peak_8309	3.75035	RPPH1_ENSG00000259001;PARP2_ENSG00000129484
14	20922751	20922956	KD2-H4K8ac_peak_8310	5.4779	OSGEP_ENSG00000092094;APEX1_ENSG00000100823
14	20929135	20929562	KD2-H4K8ac_peak_8311	11.30637	TMEM55B_ENSG00000165782
14	20930197	20930396	KD2-H4K8ac_peak_8312	7.40111	TMEM55B_ENSG00000165782
14	20937686	20938034	KD2-H4K8ac_peak_8313	4.56666	PNP_ENSG00000198805
14	21150045	21150885	KD2-H4K8ac_peak_8314	18.5703	
14	21151087	21151309	KD2-H4K8ac_peak_8315	12.93209	RNASE4_ENSG00000258818
14	21458198	21458515	KD2-H4K8ac_peak_8316	6.40297	METTL17_ENSG00000165792
14	21492714	21493157	KD2-H4K8ac_peak_8317	13.9234	TPPP2_ENSG00000179636;AL161668.5_ENSG00000258604
14	21493613	21493838	KD2-H4K8ac_peak_8318	4.67342	
14	21536913	21538191	KD2-H4K8ac_peak_8319	11.79585	NDRG2_ENSG00000165795;ARHGEF40_ENSG00000165801
14	21538594	21538940	KD2-H4K8ac_peak_8320	4.211	NDRG2_ENSG00000165795;ARHGEF40_ENSG00000165801
14	21539265	21539712	KD2-H4K8ac_peak_8321	9.65086	NDRG2_ENSG00000165795;ARHGEF40_ENSG00000165801
14	21565665	21567333	KD2-H4K8ac_peak_8322	10.86378	TMEM253_ENSG00000232070
14	21571307	21571660	KD2-H4K8ac_peak_8323	9.51625	
14	21575265	21575489	KD2-H4K8ac_peak_8324	7.167	RP11-998D10.4_ENSG00000178107

14	21659337	21659547	KD2-H4K8ac_peak_8325	4.37708	
14	21660133	21660575	KD2-H4K8ac_peak_8326	7.36799	
14	21738099	21738960	KD2-H4K8ac_peak_8327	11.89502	HNRNPC_ENSG00000092199
14	21769307	21769615	KD2-H4K8ac_peak_8328	4.06758	
14	21851586	21851955	KD2-H4K8ac_peak_8329	10.96455	SUPT16H_ENSG00000092201;RP11-524O1.4_ENSG00000260830
14	21852693	21852901	KD2-H4K8ac_peak_8330	6.25869	SUPT16H_ENSG00000092201;RP11-524O1.4_ENSG00000260830
14	21945434	21945638	KD2-H4K8ac_peak_8331	5.82799	RAB2B_ENSG00000129472;TOX4_ENSG00000092203
14	21979067	21979286	KD2-H4K8ac_peak_8332	8.10216	METTL3_ENSG00000165819
14	21979861	21980133	KD2-H4K8ac_peak_8333	12.00273	METTL3_ENSG00000165819
14	21993521	21993759	KD2-H4K8ac_peak_8334	4.77079	
14	23026084	23026454	KD2-H4K8ac_peak_8335	5.9696	AE000662.93_ENSG00000259054;AE000662.92_ENSG00000259003
14	23235936	23236595	KD2-H4K8ac_peak_8336	9.36516	CTD-2555K7.2_ENSG00000258458;OXA1L_ENSG00000155463
14	23291559	23291756	KD2-H4K8ac_peak_8337	5.57044	AL135998.1_ENSG00000215306
14	23298191	23298911	KD2-H4K8ac_peak_8338	13.81781	MRPL52_ENSG00000172590;SLC7A7_ENSG00000155465
14	23299136	23299552	KD2-H4K8ac_peak_8339	9.4551	MRPL52_ENSG00000172590;SLC7A7_ENSG00000155465
14	23317972	23318622	KD2-H4K8ac_peak_8340	7.67504	
14	23322077	23322318	KD2-H4K8ac_peak_8341	5.57044	Y_RNA_ENSG00000212335
14	23341119	23341594	KD2-H4K8ac_peak_8342	8.23274	LRP10_ENSG00000197324
14	23355822	23356499	KD2-H4K8ac_peak_8343	16.53429	
14	23357272	23357512	KD2-H4K8ac_peak_8344	5.54647	
14	23387864	23388286	KD2-H4K8ac_peak_8345	7.07117	RBM23_ENSG00000100461;PRMT5-AS1_ENSG00000237054
14	23399851	23400233	KD2-H4K8ac_peak_8346	9.96798	
14	23426125	23426794	KD2-H4K8ac_peak_8347	8.85813	HAUS4_ENSG00000092036;MIR4707_ENSG00000265037
14	23451409	23452114	KD2-H4K8ac_peak_8348	6.84261	RP11-298I3.5_ENSG00000259132;AJUBA_ENSG00000129474;RP11-298I3.4_ENSG00000258457
14	23475992	23476233	KD2-H4K8ac_peak_8349	4.84794	
14	23479401	23479666	KD2-H4K8ac_peak_8350	6.22239	C14orf93_ENSG00000100802
14	23503723	23504418	KD2-H4K8ac_peak_8351	8.4716	PSMB5_ENSG00000100804;AL132780.1_ENSG00000207765
14	23505064	23505348	KD2-H4K8ac_peak_8352	6.31143	PSMB5_ENSG00000100804
14	23526994	23527197	KD2-H4K8ac_peak_8353	4.67342	CDH24_ENSG00000139880
14	23563910	23564303	KD2-H4K8ac_peak_8354	25.32841	ACIN1_ENSG00000100813;C14orf119_ENSG00000179933
14	23564756	23565006	KD2-H4K8ac_peak_8355	6.30229	ACIN1_ENSG00000100813;C14orf119_ENSG00000179933
14	23755655	23756032	KD2-H4K8ac_peak_8356	4.86324	
14	23764032	23764266	KD2-H4K8ac_peak_8357	7.79898	RP11-124D2.7_ENSG00000270433
14	23770568	23770778	KD2-H4K8ac_peak_8358	9.95119	
14	23776096	23776560	KD2-H4K8ac_peak_8359	5.01002	BCL2L2-PABPN1_ENSG00000258643
14	23789724	23790157	KD2-H4K8ac_peak_8360	12.84642	PABPN1_ENSG00000100836
14	23790718	23791326	KD2-H4K8ac_peak_8361	8.4716	AL049829.1_ENSG00000268702;PABPN1_ENSG00000100836
14	23820912	23821165	KD2-H4K8ac_peak_8362	4.86324	SLC22A17_ENSG00000092096
14	23821776	23821999	KD2-H4K8ac_peak_8363	6.66692	SLC22A17_ENSG00000092096
14	23822529	23822763	KD2-H4K8ac_peak_8364	7.47462	SLC22A17_ENSG00000092096
14	23833285	23835517	KD2-H4K8ac_peak_8365	11.58876	EFS_ENSG00000100842
14	23835791	23836334	KD2-H4K8ac_peak_8366	9.20193	EFS_ENSG00000100842
14	23938096	23938447	KD2-H4K8ac_peak_8367	5.14125	NGDN_ENSG00000129460
14	23981358	23981689	KD2-H4K8ac_peak_8368	17.43705	RP11-66N24.4_ENSG00000157306
14	24008139	24008335	KD2-H4K8ac_peak_8369	4.37708	
14	24020863	24021439	KD2-H4K8ac_peak_8370	9.11031	

14	24024983	24025962	KD2-H4K8ac_peak_8371	12.93209	ZFHX2_ENSG00000136367;THTPA_ENSG00000259431
14	24031586	24031822	KD2-H4K8ac_peak_8372	5.11586	
14	24036783	24037171	KD2-H4K8ac_peak_8373	13.70753	AP1G2_ENSG00000213983
14	24058586	24059171	KD2-H4K8ac_peak_8374	12.26331	
14	24422638	24423382	KD2-H4K8ac_peak_8375	10.7186	DHRS4_ENSG00000157326
14	24438705	24438924	KD2-H4K8ac_peak_8376	9.15329	DHRS4L2_ENSG00000187630
14	24475810	24476757	KD2-H4K8ac_peak_8377	19.43192	
14	24505328	24506040	KD2-H4K8ac_peak_8378	9.63487	RP11-468E2.9_ENSG00000225766
14	24520700	24522040	KD2-H4K8ac_peak_8379	5.85291	LRRC16B_ENSG00000186648
14	24550168	24551140	KD2-H4K8ac_peak_8380	12.57865	
14	24562622	24563108	KD2-H4K8ac_peak_8381	11.92978	PCK2_ENSG00000100889
14	24563567	24564054	KD2-H4K8ac_peak_8382	7.146	PCK2_ENSG00000100889
14	24582706	24583153	KD2-H4K8ac_peak_8383	6.65673	DCAF11_ENSG00000100897
14	24604807	24605131	KD2-H4K8ac_peak_8384	10.0692	PSME1_ENSG00000092010
14	24605416	24605789	KD2-H4K8ac_peak_8385	10.16972	PSME1_ENSG00000092010
14	24616168	24616452	KD2-H4K8ac_peak_8386	20.29027	PSME2_ENSG00000100911;RNF31_ENSG00000092098
14	24630664	24630974	KD2-H4K8ac_peak_8387	7.70289	IRF9_ENSG00000213928
14	24640583	24640912	KD2-H4K8ac_peak_8388	3.97079	REC8_ENSG00000100918
14	24657691	24657891	KD2-H4K8ac_peak_8389	11.09936	IPO4_ENSG00000196497
14	24664111	24665344	KD2-H4K8ac_peak_8390	11.50346	
14	24682142	24682597	KD2-H4K8ac_peak_8391	6.30229	TM9SF1_ENSG00000100926;TM9SF1_ENSG00000254692;CHMP4A_ENSG00000254505
14	24684864	24685103	KD2-H4K8ac_peak_8392	6.76298	AL136419.6_ENSG00000260669;MDP1_ENSG00000213920
14	24701149	24701444	KD2-H4K8ac_peak_8393	13.13965	NEDD8-MDP1_ENSG00000255526;NEDD8_ENSG00000129559;GMPR2_ENSG00000100938
14	24701809	24702677	KD2-H4K8ac_peak_8394	14.8083	NEDD8-MDP1_ENSG00000255526;NEDD8_ENSG00000129559;GMPR2_ENSG00000100938
14	24712354	24712921	KD2-H4K8ac_peak_8395	6.25869	TINF2_ENSG00000092330
14	24739975	24740714	KD2-H4K8ac_peak_8396	22.77389	RABGGTA_ENSG00000100949
14	24740931	24741277	KD2-H4K8ac_peak_8397	5.59859	RABGGTA_ENSG00000100949
14	24768601	24769257	KD2-H4K8ac_peak_8398	8.53092	DHRS1_ENSG00000157379;NOP9_ENSG00000196943
14	24801636	24801953	KD2-H4K8ac_peak_8399	5.13615	
14	24835796	24836419	KD2-H4K8ac_peak_8400	5.14125	NFATC4_ENSG00000100968
14	24836702	24837098	KD2-H4K8ac_peak_8401	26.85324	
14	24837884	24838867	KD2-H4K8ac_peak_8402	17.45005	
14	24868193	24868649	KD2-H4K8ac_peak_8403	7.36799	NYNRIN_ENSG00000205978
14	24869111	24869421	KD2-H4K8ac_peak_8404	4.42765	
14	24898089	24898519	KD2-H4K8ac_peak_8405	4.33139	KHNYN_ENSG00000100441
14	24898891	24899692	KD2-H4K8ac_peak_8406	10.29842	CBLN3_ENSG00000139899;KHNYN_ENSG00000100441
14	24911647	24911888	KD2-H4K8ac_peak_8407	7.38799	SDR39U1_ENSG00000100445
14	24912246	24912698	KD2-H4K8ac_peak_8408	8.37231	SDR39U1_ENSG00000100445
14	27104190	27104485	KD2-H4K8ac_peak_8409	6.56926	
14	31027836	31028743	KD2-H4K8ac_peak_8410	9.1707	G2E3_ENSG00000092140
14	31090574	31090842	KD2-H4K8ac_peak_8411	5.71749	SCFD1_ENSG00000092108
14	31343051	31344491	KD2-H4K8ac_peak_8412	15.58442	COCH_ENSG00000100473
14	31494698	31495565	KD2-H4K8ac_peak_8413	23.52296	STRN3_ENSG00000196792;AP4S1_ENSG00000100478
14	31676093	31676937	KD2-H4K8ac_peak_8414	15.039	HECTD1_ENSG00000092148
14	31677168	31677698	KD2-H4K8ac_peak_8415	9.1707	HECTD1_ENSG00000092148
14	31677970	31678241	KD2-H4K8ac_peak_8416	9.11031	HECTD1_ENSG00000092148

14	31926035	31926572	KD2-H4K8ac_peak_8417	14.8083	RP11-176H8.1_ENSG00000203546;DTD2_ENSG00000129480
14	32030627	32030893	KD2-H4K8ac_peak_8418	16.73922	CTD-2213F21.3_ENSG00000257155
14	32420064	32420314	KD2-H4K8ac_peak_8419	5.72327	
14	32420811	32421067	KD2-H4K8ac_peak_8420	3.90565	
14	32546571	32546861	KD2-H4K8ac_peak_8421	5.9696	ARHGAP5-AS1_ENSG00000258655
14	32724330	32724706	KD2-H4K8ac_peak_8422	5.88845	
14	32897515	32897728	KD2-H4K8ac_peak_8423	4.86324	
14	34407374	34407740	KD2-H4K8ac_peak_8424	6.25869	
14	34429034	34429266	KD2-H4K8ac_peak_8425	12.06346	
14	34529390	34529969	KD2-H4K8ac_peak_8426	12.82393	
14	34930926	34931390	KD2-H4K8ac_peak_8427	6.66692	SPTSSA_ENSG00000165389
14	35009078	35009582	KD2-H4K8ac_peak_8428	18.25771	EAPP_ENSG00000129518
14	35098891	35099681	KD2-H4K8ac_peak_8429	14.98671	SNX6_ENSG00000129515
14	35183501	35184071	KD2-H4K8ac_peak_8430	9.95119	CFL2_ENSG00000165410
14	35191831	35192168	KD2-H4K8ac_peak_8431	6.51023	
14	35342903	35344424	KD2-H4K8ac_peak_8432	17.38513	BAZ1A_ENSG00000198604;RP11-73E17.2_ENSG00000258738
14	35515625	35516055	KD2-H4K8ac_peak_8433	7.13615	
14	35591253	35592094	KD2-H4K8ac_peak_8434	11.90231	PPP2R3C_ENSG00000092020;KIAA0391_ENSG00000100890;KIAA0391_ENSG00000258790
14	35760333	35760968	KD2-H4K8ac_peak_8435	8.1718	AL121594.1_ENSG00000265530
14	35761624	35762018	KD2-H4K8ac_peak_8436	21.09577	AL121594.1_ENSG00000265530
14	35802747	35802981	KD2-H4K8ac_peak_8437	10.08707	
14	35811079	35811642	KD2-H4K8ac_peak_8438	7.36799	
14	35845848	35846049	KD2-H4K8ac_peak_8439	8.1233	
14	35854093	35854307	KD2-H4K8ac_peak_8440	6.1846	
14	35873395	35874466	KD2-H4K8ac_peak_8441	16.3889	NFKBIA_ENSG00000100906
14	36002573	36003122	KD2-H4K8ac_peak_8442	8.55271	INSM2_ENSG00000168348
14	36003438	36005075	KD2-H4K8ac_peak_8443	13.81781	INSM2_ENSG00000168348
14	36012920	36013119	KD2-H4K8ac_peak_8444	7.12754	
14	36030082	36030354	KD2-H4K8ac_peak_8445	9.15329	
14	36040773	36041035	KD2-H4K8ac_peak_8446	5.15777	
14	36789572	36789815	KD2-H4K8ac_peak_8447	10.05606	MBIP_ENSG00000151332
14	39643639	39644143	KD2-H4K8ac_peak_8448	10.21502	RP11-407N17.4_ENSG00000259083;PNN_ENSG00000100941
14	39736301	39736587	KD2-H4K8ac_peak_8449	11.05407	RP11-407N17.5_ENSG00000258940
14	39901421	39901640	KD2-H4K8ac_peak_8450	8.1718	FBXO33_ENSG00000165355
14	39901942	39902152	KD2-H4K8ac_peak_8451	7.01964	FBXO33_ENSG00000165355
14	45381349	45381703	KD2-H4K8ac_peak_8452	11.30637	RP11-857B24.5_ENSG00000258949
14	45603203	45603590	KD2-H4K8ac_peak_8453	5.25071	FKBP3_ENSG00000100442
14	50052823	50053070	KD2-H4K8ac_peak_8454	10.05606	RN7SL1_ENSG00000258486
14	50065142	50065905	KD2-H4K8ac_peak_8455	12.27142	RPS29_ENSG00000213741;AL139099.1_ENSG00000253459;LRR1_ENSG00000165501;RHOQP1_ENSG00000258568
14	50066305	50066656	KD2-H4K8ac_peak_8456	3.87414	RPS29_ENSG00000213741;AL139099.1_ENSG00000253459;LRR1_ENSG00000165501;RHOQP1_ENSG00000258568
14	50100317	50101039	KD2-H4K8ac_peak_8457	11.24702	DNAAF2_ENSG00000165506
14	50101245	50101479	KD2-H4K8ac_peak_8458	7.36799	DNAAF2_ENSG00000165506
14	50154689	50155361	KD2-H4K8ac_peak_8459	22.62254	POLE2_ENSG00000100479
14	50159539	50160128	KD2-H4K8ac_peak_8460	18.5703	KLHDC1_ENSG00000197776
14	50233497	50233855	KD2-H4K8ac_peak_8461	7.47462	KLHDC2_ENSG00000165516
14	50234108	50234540	KD2-H4K8ac_peak_8462	11.70879	KLHDC2_ENSG00000165516

14	50234904	50235560	KD2-H4K8ac_peak_8463	13.36684	KLHDC2_ENSG00000165516
14	50319070	50319597	KD2-H4K8ac_peak_8464	13.56049	NEMF_ENSG00000165525
14	50319848	50320480	KD2-H4K8ac_peak_8465	8.59579	NEMF_ENSG00000165525;RN7SL3_ENSG00000266037
14	50359356	50359552	KD2-H4K8ac_peak_8466	8.85813	ARF6_ENSG00000165527
14	50359876	50360238	KD2-H4K8ac_peak_8467	11.83301	ARF6_ENSG00000165527
14	50469854	50470476	KD2-H4K8ac_peak_8468	14.48084	
14	50527638	50528138	KD2-H4K8ac_peak_8469	12.25733	
14	50528912	50529561	KD2-H4K8ac_peak_8470	10.19949	
14	50697937	50698493	KD2-H4K8ac_peak_8471	9.65086	SOS2_ENSG00000100485
14	50698728	50699095	KD2-H4K8ac_peak_8472	4.86324	SOS2_ENSG00000100485
14	50778649	50778867	KD2-H4K8ac_peak_8473	9.63487	L2HGDH_ENSG00000087299;ATP5S_ENSG00000125375
14	50779459	50779728	KD2-H4K8ac_peak_8474	6.25869	L2HGDH_ENSG00000087299;ATP5S_ENSG00000125375
14	50863253	50864047	KD2-H4K8ac_peak_8475	10.43145	RP11-247L20.3_ENSG00000258857
14	50999022	50999312	KD2-H4K8ac_peak_8476	11.60333	ATL1_ENSG00000198513
14	50999529	50999747	KD2-H4K8ac_peak_8477	5.88845	ATL1_ENSG00000198513
14	51026992	51027904	KD2-H4K8ac_peak_8478	13.9234	MAP4K5_ENSG00000012983
14	51134750	51134966	KD2-H4K8ac_peak_8479	8.4716	SAV1_ENSG00000151748
14	51297531	51298367	KD2-H4K8ac_peak_8480	19.9503	NIN_ENSG00000100503
14	51326846	51327136	KD2-H4K8ac_peak_8481	17.43705	
14	51338773	51339153	KD2-H4K8ac_peak_8482	6.66692	ABHD12B_ENSG00000131969
14	51422063	51422416	KD2-H4K8ac_peak_8483	8.1233	
14	51467305	51467546	KD2-H4K8ac_peak_8484	5.25071	
14	51560198	51560421	KD2-H4K8ac_peak_8485	5.73839	
14	51561994	51562393	KD2-H4K8ac_peak_8486	18.41184	TRIM9_ENSG00000100505
14	51562619	51562834	KD2-H4K8ac_peak_8487	5.14125	TRIM9_ENSG00000100505
14	51695137	51695356	KD2-H4K8ac_peak_8488	7.22457	
14	51707033	51707407	KD2-H4K8ac_peak_8489	6.25869	TMX1_ENSG00000139921
14	51861101	51861785	KD2-H4K8ac_peak_8490	7.40111	
14	51863864	51864067	KD2-H4K8ac_peak_8491	6.0073	RP11-255G12.3_ENSG00000258808
14	52117958	52118366	KD2-H4K8ac_peak_8492	16.07006	
14	52326774	52327248	KD2-H4K8ac_peak_8493	10.99365	
14	52455243	52456059	KD2-H4K8ac_peak_8494	10.02417	C14orf166_ENSG00000087302
14	52456324	52456601	KD2-H4K8ac_peak_8495	15.61313	C14orf166_ENSG00000087302
14	52536045	52536263	KD2-H4K8ac_peak_8496	7.50083	NID2_ENSG00000087303
14	53161743	53162492	KD2-H4K8ac_peak_8497	11.79585	ERO1L_ENSG00000197930
14	53163242	53163453	KD2-H4K8ac_peak_8498	4.04247	ERO1L_ENSG00000197930
14	53163766	53163983	KD2-H4K8ac_peak_8499	6.16888	
14	53172800	53173067	KD2-H4K8ac_peak_8500	15.9336	PSMC6_ENSG00000100519
14	53173998	53174262	KD2-H4K8ac_peak_8501	7.96322	PSMC6_ENSG00000100519
14	53257754	53258125	KD2-H4K8ac_peak_8502	5.13615	GNPNAT1_ENSG00000100522
14	53297199	53297631	KD2-H4K8ac_peak_8503	6.54442	
14	53299398	53299609	KD2-H4K8ac_peak_8504	6.61376	
14	53302334	53303016	KD2-H4K8ac_peak_8505	13.13969	
14	53320668	53320900	KD2-H4K8ac_peak_8506	6.64891	
14	53417487	53417693	KD2-H4K8ac_peak_8507	8.28234	
14	53418151	53418903	KD2-H4K8ac_peak_8508	8.85813	FERMT2_ENSG00000073712

14	53445579	53445813	KD2-H4K8ac_peak_8509	9.27888	
14	53454593	53454848	KD2-H4K8ac_peak_8510	4.86324	
14	53461747	53462017	KD2-H4K8ac_peak_8511	4.37708	
14	53618938	53619299	KD2-H4K8ac_peak_8512	5.14125	DDHD1_ENSG00000100523;AL356020.1_ENSG00000266552;RP11-547D23.1_ENSG00000258731
14	53619624	53619904	KD2-H4K8ac_peak_8513	9.402	DDHD1_ENSG00000100523;AL356020.1_ENSG00000266552;RP11-547D23.1_ENSG00000258731
14	53620123	53620505	KD2-H4K8ac_peak_8514	10.65724	DDHD1_ENSG00000100523;AL356020.1_ENSG00000266552;RP11-547D23.1_ENSG00000258731
14	54655232	54655691	KD2-H4K8ac_peak_8515	8.00457	
14	54689952	54690450	KD2-H4K8ac_peak_8516	8.45238	
14	54809956	54810296	KD2-H4K8ac_peak_8517	11.03331	
14	54811195	54811683	KD2-H4K8ac_peak_8518	8.79106	
14	54814230	54814691	KD2-H4K8ac_peak_8519	6.8836	
14	54863052	54863402	KD2-H4K8ac_peak_8520	14.45402	CDKN3_ENSG00000100526
14	54863918	54864141	KD2-H4K8ac_peak_8521	6.65673	CDKN3_ENSG00000100526
14	54907617	54907975	KD2-H4K8ac_peak_8522	8.34752	CNIH1_ENSG00000100528
14	54955045	54955666	KD2-H4K8ac_peak_8523	9.1707	GMFB_ENSG00000197045
14	54956368	54956575	KD2-H4K8ac_peak_8524	4.06758	GMFB_ENSG00000197045
14	54976696	54977019	KD2-H4K8ac_peak_8525	9.36516	CGRRF1_ENSG00000100532
14	55031973	55032495	KD2-H4K8ac_peak_8526	9.34989	
14	55032864	55033677	KD2-H4K8ac_peak_8527	12.06346	SAMD4A_ENSG00000020577
14	55033969	55034299	KD2-H4K8ac_peak_8528	6.98292	SAMD4A_ENSG00000020577
14	55227020	55227215	KD2-H4K8ac_peak_8529	5.88845	
14	55251622	55251921	KD2-H4K8ac_peak_8530	4.9025	
14	55252138	55252571	KD2-H4K8ac_peak_8531	4.211	
14	55274303	55275098	KD2-H4K8ac_peak_8532	13.73635	
14	55275611	55276372	KD2-H4K8ac_peak_8533	6.25869	
14	55283820	55284241	KD2-H4K8ac_peak_8534	11.05407	
14	55284576	55285320	KD2-H4K8ac_peak_8535	10.29842	
14	55286033	55286932	KD2-H4K8ac_peak_8536	20.08519	
14	55287322	55287635	KD2-H4K8ac_peak_8537	6.56926	
14	55303414	55303740	KD2-H4K8ac_peak_8538	6.91644	
14	55344475	55344725	KD2-H4K8ac_peak_8539	4.8888	MIR4308_ENSG00000265432
14	55346421	55346625	KD2-H4K8ac_peak_8540	7.26029	
14	55349796	55349999	KD2-H4K8ac_peak_8541	6.33462	
14	55350373	55350599	KD2-H4K8ac_peak_8542	8.17902	
14	55352378	55352626	KD2-H4K8ac_peak_8543	5.53033	FDPSP3_ENSG00000258872
14	55355569	55355988	KD2-H4K8ac_peak_8544	6.66692	
14	55389211	55389507	KD2-H4K8ac_peak_8545	10.21502	
14	55390015	55390410	KD2-H4K8ac_peak_8546	8.1233	
14	55494042	55494309	KD2-H4K8ac_peak_8547	7.76329	WDHD1_ENSG00000198554;SOCS4_ENSG00000180008
14	55517640	55518153	KD2-H4K8ac_peak_8548	11.86082	MAPK1IP1L_ENSG00000168175
14	55518497	55518724	KD2-H4K8ac_peak_8549	6.65673	MAPK1IP1L_ENSG00000168175
14	55540971	55541615	KD2-H4K8ac_peak_8550	8.00457	
14	55558531	55559165	KD2-H4K8ac_peak_8551	12.09025	
14	55559714	55560019	KD2-H4K8ac_peak_8552	8.74596	
14	55568472	55568766	KD2-H4K8ac_peak_8553	5.57044	
14	55595656	55595887	KD2-H4K8ac_peak_8554	6.99815	

14	55596262	55596576	KD2-H4K8ac_peak_8555	10.19949	
14	55738181	55738784	KD2-H4K8ac_peak_8556	8.55271	RP11-665C16.6_ENSG00000258413;FBXO34_ENSG00000178974
14	55877969	55879038	KD2-H4K8ac_peak_8557	14.56955	ATG14_ENSG00000126775
14	55921663	55922033	KD2-H4K8ac_peak_8558	7.40633	
14	56046575	56046799	KD2-H4K8ac_peak_8559	12.80176	KTN1-AS1_ENSG00000186615
14	56047385	56047582	KD2-H4K8ac_peak_8560	10.96455	KTN1-AS1_ENSG00000186615
14	57046308	57046509	KD2-H4K8ac_peak_8561	8.55271	
14	57734902	57735617	KD2-H4K8ac_peak_8562	14.45402	EXOC5_ENSG00000070367;AP5M1_ENSG00000053770
14	57735924	57736235	KD2-H4K8ac_peak_8563	4.86324	EXOC5_ENSG00000070367;AP5M1_ENSG00000053770
14	57810349	57810716	KD2-H4K8ac_peak_8564	6.16888	
14	57856592	57857137	KD2-H4K8ac_peak_8565	7.76329	NAA30_ENSG00000139977
14	57857336	57857780	KD2-H4K8ac_peak_8566	6.60096	NAA30_ENSG00000139977
14	58618383	58618803	KD2-H4K8ac_peak_8567	6.40683	
14	58666376	58667207	KD2-H4K8ac_peak_8568	10.96455	ACTR10_ENSG00000131966
14	58711776	58712005	KD2-H4K8ac_peak_8569	6.25869	PSMA3_ENSG00000100567
14	58766069	58766264	KD2-H4K8ac_peak_8570	7.76329	ARID4A_ENSG00000032219
14	58862248	58863237	KD2-H4K8ac_peak_8571	28.94181	RP11-517O13.3_ENSG00000258658;TOMM20L_ENSG00000196860
14	58893481	58893872	KD2-H4K8ac_peak_8572	18.1686	TIMM9_ENSG00000100575;KIAA0586_ENSG00000100578
14	59104859	59105133	KD2-H4K8ac_peak_8573	3.90565	
14	59931211	59932042	KD2-H4K8ac_peak_8574	4.67342	GPR135_ENSG00000181619
14	59950641	59950957	KD2-H4K8ac_peak_8575	8.79106	L3HYPDH_ENSG00000126790;JKAMP_ENSG00000050130
14	59951283	59951507	KD2-H4K8ac_peak_8576	13.59098	L3HYPDH_ENSG00000126790;JKAMP_ENSG00000050130
14	60097158	60097616	KD2-H4K8ac_peak_8577	4.67342	
14	60337206	60338022	KD2-H4K8ac_peak_8578	8.10216	RTN1_ENSG00000139970
14	60386243	60386903	KD2-H4K8ac_peak_8579	4.67342	RP11-62H20.1_ENSG00000261120;LRRC9_ENSG00000131951
14	60558248	60558468	KD2-H4K8ac_peak_8580	10.46264	RP11-16B13.1_ENSG00000258553;PCNXL4_ENSG00000126773
14	60558738	60559223	KD2-H4K8ac_peak_8581	4.44527	RP11-16B13.1_ENSG00000258553;PCNXL4_ENSG00000126773
14	60631734	60632220	KD2-H4K8ac_peak_8582	9.89383	
14	60715453	60715745	KD2-H4K8ac_peak_8583	20.31556	CTD-2184C24.2_ENSG00000254718
14	60716156	60716398	KD2-H4K8ac_peak_8584	5.30799	CTD-2184C24.2_ENSG00000254718
14	60778569	60778775	KD2-H4K8ac_peak_8585	8.02571	
14	60779889	60780146	KD2-H4K8ac_peak_8586	8.00457	
14	60822271	60822529	KD2-H4K8ac_peak_8587	6.16888	
14	60824447	60824693	KD2-H4K8ac_peak_8588	6.16888	
14	60825220	60825632	KD2-H4K8ac_peak_8589	7.12754	
14	60981064	60981647	KD2-H4K8ac_peak_8590	6.25869	C14orf39_ENSG00000179008;RP11-1042B17.3_ENSG00000258670
14	60981897	60982201	KD2-H4K8ac_peak_8591	11.60333	C14orf39_ENSG00000179008;RP11-1042B17.3_ENSG00000258670
14	61092413	61092637	KD2-H4K8ac_peak_8592	6.25869	
14	61104094	61104389	KD2-H4K8ac_peak_8593	4.06758	
14	61123133	61124616	KD2-H4K8ac_peak_8594	12.84642	SIX1_ENSG00000126778
14	61190302	61190842	KD2-H4K8ac_peak_8595	6.99815	SIX4_ENSG00000100625
14	61447524	61447722	KD2-H4K8ac_peak_8596	9.16531	TRMT5_ENSG00000126814;SLC38A6_ENSG00000139974
14	61718900	61719327	KD2-H4K8ac_peak_8597	6.03693	
14	61736562	61736804	KD2-H4K8ac_peak_8598	7.79558	
14	61747104	61747880	KD2-H4K8ac_peak_8599	9.34195	TMEM30B_ENSG00000182107
14	61787854	61788722	KD2-H4K8ac_peak_8600	9.63487	RP11-902B17.1_ENSG00000247287

14	61934056	61934467	KD2-H4K8ac_peak_8601	7.62364	
14	61935723	61936302	KD2-H4K8ac_peak_8602	6.39197	
14	61936498	61938641	KD2-H4K8ac_peak_8603	6.40297	
14	61939260	61940553	KD2-H4K8ac_peak_8604	8.30974	
14	61942734	61943175	KD2-H4K8ac_peak_8605	9.50015	
14	61944561	61944772	KD2-H4K8ac_peak_8606	6.99815	
14	61946507	61946921	KD2-H4K8ac_peak_8607	4.70388	
14	61950327	61950684	KD2-H4K8ac_peak_8608	6.40683	
14	62043000	62043348	KD2-H4K8ac_peak_8609	4.86324	
14	62043655	62044120	KD2-H4K8ac_peak_8610	8.19585	
14	62050783	62051015	KD2-H4K8ac_peak_8611	6.27931	
14	62052403	62052927	KD2-H4K8ac_peak_8612	5.82799	
14	62053634	62053866	KD2-H4K8ac_peak_8613	7.52168	
14	62074589	62075062	KD2-H4K8ac_peak_8614	5.59859	
14	62077225	62077421	KD2-H4K8ac_peak_8615	6.40683	
14	62162302	62163088	KD2-H4K8ac_peak_8616	13.35993	HIF1A-AS1_ENSG00000258777;HIF1A_ENSG00000100644
14	62278812	62279086	KD2-H4K8ac_peak_8617	7.47462	CTD-2277K2.1_ENSG00000258882
14	62279507	62280022	KD2-H4K8ac_peak_8618	12.82393	CTD-2277K2.1_ENSG00000258882
14	63989435	63990033	KD2-H4K8ac_peak_8619	10.96455	
14	63993645	63993871	KD2-H4K8ac_peak_8620	4.01115	
14	64009644	64010021	KD2-H4K8ac_peak_8621	10.96455	PPP2R5E_ENSG00000154001;CTD-2302E22.4_ENSG00000261242
14	64010358	64010688	KD2-H4K8ac_peak_8622	10.0692	PPP2R5E_ENSG00000154001;CTD-2302E22.4_ENSG00000261242
14	64108092	64108533	KD2-H4K8ac_peak_8623	9.89126	WDR89_ENSG00000140006
14	64194495	64194718	KD2-H4K8ac_peak_8624	5.9696	SGPP1_ENSG00000126821
14	64319805	64320553	KD2-H4K8ac_peak_8625	11.05407	SYNE2_ENSG00000054654
14	64760715	64761135	KD2-H4K8ac_peak_8626	11.05407	
14	64804703	64805256	KD2-H4K8ac_peak_8627	6.65673	ESR2_ENSG00000140009;RP11-544I20.2_ENSG00000214770
14	64805779	64806085	KD2-H4K8ac_peak_8628	11.92978	ESR2_ENSG00000140009;RP11-544I20.2_ENSG00000214770
14	64806417	64806754	KD2-H4K8ac_peak_8629	9.16531	
14	64854066	64854606	KD2-H4K8ac_peak_8630	10.20046	MTHFD1_ENSG00000100714
14	64932181	64932487	KD2-H4K8ac_peak_8631	10.96455	AKAP5_ENSG00000179841
14	64967465	64967708	KD2-H4K8ac_peak_8632	8.17902	
14	64970003	64970330	KD2-H4K8ac_peak_8633	9.20193	ZBTB1_ENSG00000126804
14	64970710	64971940	KD2-H4K8ac_peak_8634	17.70837	ZBTB25_ENSG00000089775;ZBTB1_ENSG00000126804
14	65006078	65006665	KD2-H4K8ac_peak_8635	9.95119	RP11-973N13.4_ENSG00000259116
14	65007452	65007798	KD2-H4K8ac_peak_8636	10.87093	RP11-973N13.4_ENSG00000259116
14	65016632	65016890	KD2-H4K8ac_peak_8637	9.36516	PPP1R36_ENSG00000165807
14	65103062	65103419	KD2-H4K8ac_peak_8638	7.36799	
14	65152666	65152881	KD2-H4K8ac_peak_8639	5.53095	
14	65170791	65171684	KD2-H4K8ac_peak_8640	11.24702	PLEKHG3_ENSG00000126822
14	65224929	65225247	KD2-H4K8ac_peak_8641	10.69383	
14	65229772	65230176	KD2-H4K8ac_peak_8642	11.05467	
14	65230598	65231033	KD2-H4K8ac_peak_8643	8.30974	
14	65231281	65231637	KD2-H4K8ac_peak_8644	12.00273	
14	65290467	65290685	KD2-H4K8ac_peak_8645	3.97079	
14	65346407	65346943	KD2-H4K8ac_peak_8646	4.86324	SPTB_ENSG00000070182

14	65380787	65381553	KD2-H4K8ac_peak_8647	19.94588	CHURC1_ENSG00000258289;FNTB_ENSG00000257365;CHURC1-FNTB_ENSG00000125954
14	65438411	65438982	KD2-H4K8ac_peak_8648	7.07117	RAB15_ENSG00000139998
14	65439189	65439745	KD2-H4K8ac_peak_8649	12.08081	RAB15_ENSG00000139998
14	65454073	65454307	KD2-H4K8ac_peak_8650	5.13615	
14	65569604	65570170	KD2-H4K8ac_peak_8651	8.1233	MAX_ENSG00000125952
14	65665119	65665382	KD2-H4K8ac_peak_8652	5.25071	
14	65682135	65682478	KD2-H4K8ac_peak_8653	3.87414	
14	65696325	65696713	KD2-H4K8ac_peak_8654	11.05467	
14	65878653	65879198	KD2-H4K8ac_peak_8655	8.10216	
14	65937108	65937353	KD2-H4K8ac_peak_8656	4.42765	MIR625_ENSG00000207781
14	66257229	66257440	KD2-H4K8ac_peak_8657	6.25014	
14	66700663	66701078	KD2-H4K8ac_peak_8658	8.79557	
14	66974240	66975163	KD2-H4K8ac_peak_8659	16.58076	RP11-72M17.1_ENSG00000258561;GPHN_ENSG00000171723
14	67826133	67826448	KD2-H4K8ac_peak_8660	5.88845	ATP6V1D_ENSG00000100554;EIF2S1_ENSG00000134001
14	67878479	67879214	KD2-H4K8ac_peak_8661	9.1707	PLEK2_ENSG00000100558
14	67999536	68000469	KD2-H4K8ac_peak_8662	17.8868	TMEM229B_ENSG00000198133;PLEKHH1_ENSG00000054690
14	68067148	68067669	KD2-H4K8ac_peak_8663	7.59794	PIGH_ENSG00000100564
14	68141082	68142204	KD2-H4K8ac_peak_8664	12.30509	VTI1B_ENSG00000100568
14	68162038	68162401	KD2-H4K8ac_peak_8665	13.80019	RDH11_ENSG00000072042
14	68162781	68163091	KD2-H4K8ac_peak_8666	5.57044	RDH11_ENSG00000072042
14	68283069	68283269	KD2-H4K8ac_peak_8667	10.43145	ZFYVE26_ENSG00000072121
14	68283880	68284466	KD2-H4K8ac_peak_8668	7.51935	ZFYVE26_ENSG00000072121
14	68285806	68286136	KD2-H4K8ac_peak_8669	13.62729	RAD51B_ENSG00000182185
14	68286605	68286858	KD2-H4K8ac_peak_8670	4.86324	RAD51B_ENSG00000182185
14	69005991	69006230	KD2-H4K8ac_peak_8671	6.16888	
14	69052480	69053173	KD2-H4K8ac_peak_8672	7.0754	
14	69101207	69101441	KD2-H4K8ac_peak_8673	10.39154	
14	69121301	69121911	KD2-H4K8ac_peak_8674	19.41056	
14	69122190	69122849	KD2-H4K8ac_peak_8675	8.26945	
14	69155444	69155648	KD2-H4K8ac_peak_8676	5.43522	
14	69156026	69156479	KD2-H4K8ac_peak_8677	8.17902	
14	69157344	69157605	KD2-H4K8ac_peak_8678	9.19305	
14	69253943	69254239	KD2-H4K8ac_peak_8679	6.16888	
14	69262102	69263188	KD2-H4K8ac_peak_8680	9.01262	ZFP36L1_ENSG00000185650
14	69273375	69274361	KD2-H4K8ac_peak_8681	13.56967	
14	69282673	69283566	KD2-H4K8ac_peak_8682	13.80019	
14	69291203	69291746	KD2-H4K8ac_peak_8683	15.52826	
14	69291990	69292259	KD2-H4K8ac_peak_8684	6.91644	
14	69316218	69316487	KD2-H4K8ac_peak_8685	9.15329	
14	69404335	69404676	KD2-H4K8ac_peak_8686	12.51591	
14	69405753	69406268	KD2-H4K8ac_peak_8687	4.42765	
14	69418705	69419106	KD2-H4K8ac_peak_8688	5.73016	
14	69443874	69444241	KD2-H4K8ac_peak_8689	4.49003	
14	69446264	69446486	KD2-H4K8ac_peak_8690	14.38358	ACTN1_ENSG00000072110;ACTN1-AS1_ENSG00000259062
14	69480889	69481232	KD2-H4K8ac_peak_8691	6.0073	
14	69505599	69505842	KD2-H4K8ac_peak_8692	8.5092	

14	69618502	69619084	KD2-H4K8ac_peak_8693	9.95119	DCAF5_ENSG00000139990
14	69620026	69620508	KD2-H4K8ac_peak_8694	11.50346	DCAF5_ENSG00000139990
14	69658226	69658840	KD2-H4K8ac_peak_8695	11.24702	EXD2_ENSG00000081177
14	69726236	69726749	KD2-H4K8ac_peak_8696	8.79106	RP11-363J20.2_ENSG00000258520;GALNT16_ENSG00000100626
14	69727429	69727632	KD2-H4K8ac_peak_8697	4.39858	RP11-363J20.2_ENSG00000258520
14	69754338	69754747	KD2-H4K8ac_peak_8698	8.26945	
14	69759024	69759235	KD2-H4K8ac_peak_8699	5.57044	
14	69780033	69780260	KD2-H4K8ac_peak_8700	7.12754	
14	69864448	69864944	KD2-H4K8ac_peak_8701	9.20193	ERH_ENSG00000100632;SLC39A9_ENSG00000029364
14	69865708	69865955	KD2-H4K8ac_peak_8702	5.57044	ERH_ENSG00000100632;SLC39A9_ENSG00000029364
14	69967033	69967686	KD2-H4K8ac_peak_8703	8.94469	
14	69972451	69972670	KD2-H4K8ac_peak_8704	10.65724	
14	70026201	70026512	KD2-H4K8ac_peak_8705	6.56926	
14	70078501	70078795	KD2-H4K8ac_peak_8706	6.66692	KIAA0247_ENSG00000100647
14	70159841	70160053	KD2-H4K8ac_peak_8707	5.88845	
14	70178595	70178886	KD2-H4K8ac_peak_8708	4.86324	
14	70193456	70194155	KD2-H4K8ac_peak_8709	12.84642	SRSF5_ENSG00000100650
14	70233899	70234369	KD2-H4K8ac_peak_8710	16.53429	
14	70561923	70562409	KD2-H4K8ac_peak_8711	7.95563	
14	70562680	70562890	KD2-H4K8ac_peak_8712	6.56211	
14	70653379	70653865	KD2-H4K8ac_peak_8713	6.66692	RP11-486O13.2_ENSG00000258422
14	70721251	70721562	KD2-H4K8ac_peak_8714	7.76329	
14	70826055	70827070	KD2-H4K8ac_peak_8715	13.92307	COX16_ENSG00000133983;RNU2-51P_ENSG00000222640
14	71066929	71067192	KD2-H4K8ac_peak_8716	6.16888	MED6_ENSG00000133997
14	71067697	71068322	KD2-H4K8ac_peak_8717	9.15329	MED6_ENSG00000133997
14	71108110	71109120	KD2-H4K8ac_peak_8718	14.15075	CTD-2540L5.5_ENSG00000259115;CTD-2540L5.6_ENSG00000245466;TTC9_ENSG00000133985
14	71288497	71288891	KD2-H4K8ac_peak_8719	8.30974	
14	71374304	71374939	KD2-H4K8ac_peak_8720	8.10216	PCNX_ENSG00000100731
14	71699214	71699406	KD2-H4K8ac_peak_8721	7.01964	
14	71703662	71704004	KD2-H4K8ac_peak_8722	4.77079	
14	71787491	71788442	KD2-H4K8ac_peak_8723	6.87217	RP1-261D10.2_ENSG00000259146;SIPA1L1_ENSG00000197555
14	71807548	71807984	KD2-H4K8ac_peak_8724	6.1228	
14	72052858	72053246	KD2-H4K8ac_peak_8725	7.11925	
14	72062732	72063037	KD2-H4K8ac_peak_8726	9.15329	
14	72095671	72095995	KD2-H4K8ac_peak_8727	6.25869	
14	73146463	73146683	KD2-H4K8ac_peak_8728	5.3778	
14	73203985	73204234	KD2-H4K8ac_peak_8729	7.27716	
14	73205003	73205344	KD2-H4K8ac_peak_8730	11.05407	
14	73206791	73207018	KD2-H4K8ac_peak_8731	7.40111	
14	73208341	73209625	KD2-H4K8ac_peak_8732	13.73635	
14	73602717	73603048	KD2-H4K8ac_peak_8733	12.23	PSEN1_ENSG00000080815
14	73603358	73603562	KD2-H4K8ac_peak_8734	4.84206	PSEN1_ENSG00000080815
14	73928985	73929460	KD2-H4K8ac_peak_8735	5.14125	NUMB_ENSG00000133961;RP1-240K6.3_ENSG00000251393
14	73957087	73957467	KD2-H4K8ac_peak_8736	12.00597	AC005280.1_ENSG00000170468;C14orf169_ENSG00000255242
14	73957737	73958255	KD2-H4K8ac_peak_8737	13.70605	AC005280.1_ENSG00000170468;C14orf169_ENSG00000255242
14	74035703	74036877	KD2-H4K8ac_peak_8738	23.28866	

14	74058446	74059071	KD2-H4K8ac_peak_8739	10.72313	ACOT4_ENSG00000177465
14	74079280	74079636	KD2-H4K8ac_peak_8740	8.10216	
14	74110096	74110811	KD2-H4K8ac_peak_8741	4.54006	DNAL1_ENSG00000119661
14	74180556	74180989	KD2-H4K8ac_peak_8742	9.1707	PNMA1_ENSG00000176903
14	74185591	74185819	KD2-H4K8ac_peak_8743	7.22457	
14	74253573	74254500	KD2-H4K8ac_peak_8744	12.44769	RP5-1021I20.1_ENSG00000259065
14	74318744	74319195	KD2-H4K8ac_peak_8745	8.94469	PTGR2_ENSG00000140043;RP5-1021I20.4_ENSG00000258653
14	74319526	74319731	KD2-H4K8ac_peak_8746	3.97079	PTGR2_ENSG00000140043;RP5-1021I20.4_ENSG00000258653
14	74352545	74352811	KD2-H4K8ac_peak_8747	11.05407	ZNF410_ENSG00000119725
14	74353294	74354084	KD2-H4K8ac_peak_8748	11.05467	ZNF410_ENSG00000119725
14	74417059	74417283	KD2-H4K8ac_peak_8749	6.40297	FAM161B_ENSG00000156050;COQ6_ENSG00000119723
14	74485391	74485904	KD2-H4K8ac_peak_8750	17.50963	ENTPD5_ENSG00000187097;CCDC176_ENSG00000119636
14	74723863	74724176	KD2-H4K8ac_peak_8751	5.13615	
14	74725094	74725501	KD2-H4K8ac_peak_8752	4.66652	
14	74725707	74725943	KD2-H4K8ac_peak_8753	6.40683	
14	74769382	74769654	KD2-H4K8ac_peak_8754	15.01306	ABCD4_ENSG00000119688;VRTN_ENSG00000133980
14	74769923	74770410	KD2-H4K8ac_peak_8755	9.1707	ABCD4_ENSG00000119688;VRTN_ENSG00000133980
14	74869091	74869332	KD2-H4K8ac_peak_8756	6.8836	
14	74959752	74959960	KD2-H4K8ac_peak_8757	10.0692	NPC2_ENSG00000119655;ISCA2_ENSG00000165898
14	74968148	74968406	KD2-H4K8ac_peak_8758	9.11031	
14	75003085	75003278	KD2-H4K8ac_peak_8759	7.36933	
14	75036688	75037046	KD2-H4K8ac_peak_8760	7.1181	
14	75044507	75044919	KD2-H4K8ac_peak_8761	6.66692	
14	75061915	75062372	KD2-H4K8ac_peak_8762	6.13909	
14	75078452	75079306	KD2-H4K8ac_peak_8763	8.16906	LTBP2_ENSG00000119681
14	75103901	75104213	KD2-H4K8ac_peak_8764	4.86324	
14	75179535	75179767	KD2-H4K8ac_peak_8765	10.96455	AREL1_ENSG00000119682;AC007956.1_ENSG00000214670;SNORA7_ENSG00000222604;FCF1_ENSG00000119616
14	75229282	75229513	KD2-H4K8ac_peak_8766	13.39973	YLPM1_ENSG00000119596
14	75230106	75230435	KD2-H4K8ac_peak_8767	13.81781	YLPM1_ENSG00000119596
14	75348247	75348908	KD2-H4K8ac_peak_8768	17.38513	DLST_ENSG00000119689
14	75389420	75389915	KD2-H4K8ac_peak_8769	13.62729	RPS6KL1_ENSG00000198208
14	75421928	75422665	KD2-H4K8ac_peak_8770	7.36799	PGF_ENSG00000119630
14	75439833	75440360	KD2-H4K8ac_peak_8771	8.23274	
14	75444277	75444489	KD2-H4K8ac_peak_8772	7.92775	
14	75445382	75445619	KD2-H4K8ac_peak_8773	9.3793	
14	75446451	75446832	KD2-H4K8ac_peak_8774	8.5092	
14	75469860	75470151	KD2-H4K8ac_peak_8775	11.86082	EIF2B2_ENSG00000119718
14	75517322	75518122	KD2-H4K8ac_peak_8776	12.08081	MLH3_ENSG00000119684
14	75535075	75535320	KD2-H4K8ac_peak_8777	7.22936	ACYP1_ENSG00000119640
14	75535905	75536211	KD2-H4K8ac_peak_8778	7.28628	ACYP1_ENSG00000119640
14	75536431	75537014	KD2-H4K8ac_peak_8779	8.79106	ACYP1_ENSG00000119640
14	75546634	75546832	KD2-H4K8ac_peak_8780	5.57044	
14	75593307	75594144	KD2-H4K8ac_peak_8781	13.70753	NEK9_ENSG00000119638;RP11-950C14.7_ENSG00000259138
14	75642635	75643469	KD2-H4K8ac_peak_8782	13.81781	TMED10_ENSG00000170348
14	75703904	75704105	KD2-H4K8ac_peak_8783	6.94255	
14	75743689	75744333	KD2-H4K8ac_peak_8784	7.0983	

14	75745225	75745465	KD2-H4K8ac_peak_8785	12.80176	FOS_ENSG00000170345
14	75894275	75895378	KD2-H4K8ac_peak_8786	21.29027	RP11-293M10.6_ENSG00000259319;JDP2_ENSG00000140044
14	75900853	75901124	KD2-H4K8ac_peak_8787	5.20908	
14	75901770	75902480	KD2-H4K8ac_peak_8788	10.53377	
14	76102891	76103407	KD2-H4K8ac_peak_8789	4.06758	
14	76445195	76445456	KD2-H4K8ac_peak_8790	8.2413	
14	76449230	76449469	KD2-H4K8ac_peak_8791	7.96322	TGFB3_ENSG00000119699
14	76617730	76618022	KD2-H4K8ac_peak_8792	5.45396	GPATCH2L_ENSG00000089916
14	77176935	77177266	KD2-H4K8ac_peak_8793	5.00214	
14	77183817	77184023	KD2-H4K8ac_peak_8794	5.14125	
14	77185905	77186506	KD2-H4K8ac_peak_8795	11.05407	
14	77186928	77187213	KD2-H4K8ac_peak_8796	5.54647	
14	77227402	77229025	KD2-H4K8ac_peak_8797	14.39182	RP11-99E15.2_ENSG00000258569;VASH1_ENSG00000071246
14	77278765	77279152	KD2-H4K8ac_peak_8798	16.71291	
14	77371247	77371473	KD2-H4K8ac_peak_8799	6.99815	
14	77379719	77380363	KD2-H4K8ac_peak_8800	16.29309	
14	77383290	77383493	KD2-H4K8ac_peak_8801	7.51945	
14	77383742	77384674	KD2-H4K8ac_peak_8802	15.27939	
14	77385374	77386332	KD2-H4K8ac_peak_8803	21.01976	
14	77386575	77386994	KD2-H4K8ac_peak_8804	13.73635	
14	77387191	77387468	KD2-H4K8ac_peak_8805	12.2118	
14	77387701	77387947	KD2-H4K8ac_peak_8806	5.95537	
14	77389194	77389648	KD2-H4K8ac_peak_8807	5.13893	
14	77390307	77391083	KD2-H4K8ac_peak_8808	9.16531	RP11-488C13.4_ENSG00000259058
14	77398802	77399198	KD2-H4K8ac_peak_8809	7.40314	
14	77401937	77402158	KD2-H4K8ac_peak_8810	6.66692	
14	77402663	77402995	KD2-H4K8ac_peak_8811	5.57044	
14	77403199	77403519	KD2-H4K8ac_peak_8812	4.76709	
14	77403848	77405170	KD2-H4K8ac_peak_8813	10.17486	
14	77406001	77406275	KD2-H4K8ac_peak_8814	6.2781	
14	77406536	77406898	KD2-H4K8ac_peak_8815	7.10552	
14	77408478	77408875	KD2-H4K8ac_peak_8816	4.42765	
14	77412733	77413591	KD2-H4K8ac_peak_8817	8.86099	
14	77414211	77415092	KD2-H4K8ac_peak_8818	10.19949	
14	77417349	77417828	KD2-H4K8ac_peak_8819	9.24289	
14	77419169	77419485	KD2-H4K8ac_peak_8820	5.9696	
14	77419787	77420442	KD2-H4K8ac_peak_8821	15.01306	
14	77421093	77421974	KD2-H4K8ac_peak_8822	11.8457	
14	77422200	77422786	KD2-H4K8ac_peak_8823	9.63487	
14	77423170	77423421	KD2-H4K8ac_peak_8824	14.66123	
14	77424069	77424349	KD2-H4K8ac_peak_8825	9.20193	
14	77424663	77425634	KD2-H4K8ac_peak_8826	5.30799	RP11-7F17.7_ENSG00000258602
14	77426732	77427167	KD2-H4K8ac_peak_8827	8.30974	RP11-7F17.7_ENSG00000258602
14	77427999	77428345	KD2-H4K8ac_peak_8828	6.0073	
14	77458950	77459366	KD2-H4K8ac_peak_8829	6.16747	
14	77493189	77493553	KD2-H4K8ac_peak_8830	5.66705	

14	77493978	77495025	KD2-H4K8ac_peak_8831	11.41236	IRF2BPL_ENSG00000119669
14	77498981	77500406	KD2-H4K8ac_peak_8832	21.11201	
14	77502265	77502486	KD2-H4K8ac_peak_8833	6.36319	
14	77503773	77504684	KD2-H4K8ac_peak_8834	9.76863	
14	77506095	77506343	KD2-H4K8ac_peak_8835	6.60096	
14	77507090	77507563	KD2-H4K8ac_peak_8836	13.13965	RP11-7F17.5_ENSG00000246548
14	77512605	77512889	KD2-H4K8ac_peak_8837	8.30974	
14	77516507	77516774	KD2-H4K8ac_peak_8838	7.29632	
14	77517482	77517874	KD2-H4K8ac_peak_8839	9.95119	
14	77518359	77518609	KD2-H4K8ac_peak_8840	6.99815	
14	77518834	77519463	KD2-H4K8ac_peak_8841	10.96455	
14	77590985	77591545	KD2-H4K8ac_peak_8842	4.64018	
14	77624077	77624722	KD2-H4K8ac_peak_8843	10.29842	
14	77624992	77625518	KD2-H4K8ac_peak_8844	18.54875	
14	77647410	77647756	KD2-H4K8ac_peak_8845	8.10216	
14	77648711	77648924	KD2-H4K8ac_peak_8846	5.9696	
14	77786866	77787162	KD2-H4K8ac_peak_8847	11.89502	POMT2_ENSG00000009830;GSTZ1_ENSG00000100577
14	77787394	77787592	KD2-H4K8ac_peak_8848	11.05407	POMT2_ENSG00000009830;GSTZ1_ENSG00000100577
14	77843133	77843390	KD2-H4K8ac_peak_8849	13.81781	TMED8_ENSG00000100580;SAMD15_ENSG00000100583
14	77924483	77924946	KD2-H4K8ac_peak_8850	9.16531	VIPAS39_ENSG00000151445;AHSA1_ENSG00000100591
14	78082696	78082930	KD2-H4K8ac_peak_8851	5.92591	SPTLC2_ENSG00000100596
14	78121594	78121866	KD2-H4K8ac_peak_8852	4.42765	
14	78173763	78174220	KD2-H4K8ac_peak_8853	5.01002	ALKBH1_ENSG00000100601;SLIRP_ENSG00000119705
14	78174510	78174791	KD2-H4K8ac_peak_8854	8.52559	ALKBH1_ENSG00000100601;SLIRP_ENSG00000119705
14	78227057	78227439	KD2-H4K8ac_peak_8855	17.8868	SNW1_ENSG00000100603;C14orf178_ENSG00000197734;AC008372.1_ENSG00000268208
14	78227775	78228275	KD2-H4K8ac_peak_8856	4.37815	SNW1_ENSG00000100603;C14orf178_ENSG00000197734;AC008372.1_ENSG00000268208
14	78265732	78266078	KD2-H4K8ac_peak_8857	8.30974	ADCK1_ENSG00000063761
14	78266275	78266882	KD2-H4K8ac_peak_8858	14.4458	ADCK1_ENSG00000063761
14	78345733	78346039	KD2-H4K8ac_peak_8859	7.04492	
14	78347599	78347813	KD2-H4K8ac_peak_8860	8.30974	
14	78349990	78350726	KD2-H4K8ac_peak_8861	14.66631	
14	78379760	78380016	KD2-H4K8ac_peak_8862	5.86783	
14	78388460	78388671	KD2-H4K8ac_peak_8863	5.61067	
14	78390775	78391117	KD2-H4K8ac_peak_8864	7.27716	
14	78432316	78432651	KD2-H4K8ac_peak_8865	9.15069	
14	78618791	78619176	KD2-H4K8ac_peak_8866	4.01115	
14	79745230	79745496	KD2-H4K8ac_peak_8867	4.42765	
14	81408354	81408586	KD2-H4K8ac_peak_8868	5.57044	
14	81421776	81421974	KD2-H4K8ac_peak_8869	9.15069	TSHR_ENSG00000165409
14	81636018	81637013	KD2-H4K8ac_peak_8870	11.79585	RP11-114N19.3_ENSG00000258999
14	81687435	81687638	KD2-H4K8ac_peak_8871	5.71749	GTF2A1_ENSG00000165417
14	81687851	81688255	KD2-H4K8ac_peak_8872	7.27716	GTF2A1_ENSG00000165417
14	82001022	82001291	KD2-H4K8ac_peak_8873	6.75638	SEL1L_ENSG00000071537
14	86400892	86401153	KD2-H4K8ac_peak_8874	4.37708	CTD-2341M24.1_ENSG00000258733
14	88459082	88459467	KD2-H4K8ac_peak_8875	8.28234	GALC_ENSG00000054983
14	88459694	88460153	KD2-H4K8ac_peak_8876	13.44374	GALC_ENSG00000054983

14	88790186	88790471	KD2-H4K8ac_peak_8877	11.89502	
14	88851671	88852295	KD2-H4K8ac_peak_8878	9.15069	SPATA7_ENSG00000042317
14	89020807	89021577	KD2-H4K8ac_peak_8879	8.26945	PTPN21_ENSG00000070778
14	89029513	89029799	KD2-H4K8ac_peak_8880	9.11891	ZC3H14_ENSG00000100722
14	89258534	89259185	KD2-H4K8ac_peak_8881	11.02863	EML5_ENSG00000165521
14	89259403	89259849	KD2-H4K8ac_peak_8882	8.85813	EML5_ENSG00000165521
14	89281447	89281694	KD2-H4K8ac_peak_8883	6.16888	
14	89290835	89291481	KD2-H4K8ac_peak_8884	8.1718	TTC8_ENSG00000165533
14	89292427	89292836	KD2-H4K8ac_peak_8885	6.40683	
14	89358231	89358425	KD2-H4K8ac_peak_8886	9.15329	
14	89365960	89367126	KD2-H4K8ac_peak_8887	8.90387	
14	89368086	89368722	KD2-H4K8ac_peak_8888	9.63487	
14	89368982	89369247	KD2-H4K8ac_peak_8889	4.42765	
14	89371328	89371714	KD2-H4K8ac_peak_8890	8.85813	
14	89450826	89451140	KD2-H4K8ac_peak_8891	5.1628	
14	89451721	89451957	KD2-H4K8ac_peak_8892	9.36516	
14	89453296	89453849	KD2-H4K8ac_peak_8893	9.402	
14	89463833	89464112	KD2-H4K8ac_peak_8894	11.81678	
14	89465575	89465857	KD2-H4K8ac_peak_8895	5.88845	
14	89506990	89507316	KD2-H4K8ac_peak_8896	10.53377	
14	89507824	89508177	KD2-H4K8ac_peak_8897	6.4095	
14	89678368	89678984	KD2-H4K8ac_peak_8898	6.66692	
14	89883692	89884385	KD2-H4K8ac_peak_8899	16.23438	FOXN3-AS1_ENSG00000258920
14	90085580	90085905	KD2-H4K8ac_peak_8900	9.63925	FOXN3_ENSG00000053254
14	90091412	90092496	KD2-H4K8ac_peak_8901	10.06887	
14	90127100	90127409	KD2-H4K8ac_peak_8902	10.08707	
14	90167703	90167947	KD2-H4K8ac_peak_8903	10.19949	
14	90168143	90168366	KD2-H4K8ac_peak_8904	7.76104	
14	90422352	90422860	KD2-H4K8ac_peak_8905	7.69294	
14	90625221	90625454	KD2-H4K8ac_peak_8906	3.72574	
14	90722955	90723349	KD2-H4K8ac_peak_8907	17.55491	PSMC1_ENSG00000100764
14	90796984	90798280	KD2-H4K8ac_peak_8908	10.96455	NRDE2_ENSG00000119720
14	90848605	90848897	KD2-H4K8ac_peak_8909	5.45396	
14	90849142	90849351	KD2-H4K8ac_peak_8910	5.02474	
14	90849843	90850706	KD2-H4K8ac_peak_8911	19.42408	
14	90862824	90863157	KD2-H4K8ac_peak_8912	9.20193	CALM1_ENSG00000198668
14	90917364	90918110	KD2-H4K8ac_peak_8913	13.73635	
14	90918788	90919105	KD2-H4K8ac_peak_8914	5.9696	
14	90919306	90919513	KD2-H4K8ac_peak_8915	5.75757	
14	90919976	90920731	KD2-H4K8ac_peak_8916	25.95849	RP11-1078H9.1_ENSG00000258678;LINC00642_ENSG00000233208
14	90920992	90921578	KD2-H4K8ac_peak_8917	10.44656	RP11-1078H9.1_ENSG00000258678;LINC00642_ENSG00000233208
14	90978375	90978775	KD2-H4K8ac_peak_8918	4.98072	
14	90988915	90989341	KD2-H4K8ac_peak_8919	12.23	
14	91082219	91082687	KD2-H4K8ac_peak_8920	8.17902	
14	91164201	91164424	KD2-H4K8ac_peak_8921	8.17902	RP11-661G16.2_ENSG00000258716
14	91165157	91165472	KD2-H4K8ac_peak_8922	7.27716	

14	91282172	91282611	KD2-H4K8ac_peak_8923	9.36516	TTC7B_ENSG00000165914
14	91283134	91283447	KD2-H4K8ac_peak_8924	5.54647	TTC7B_ENSG00000165914
14	91294941	91295430	KD2-H4K8ac_peak_8925	8.04448	CTD-3035D6.2_ENSG00000258884
14	91580049	91580409	KD2-H4K8ac_peak_8926	5.30799	
14	91580667	91580865	KD2-H4K8ac_peak_8927	6.05809	
14	91581087	91581312	KD2-H4K8ac_peak_8928	5.88845	
14	91636381	91636933	KD2-H4K8ac_peak_8929	11.9209	
14	91642981	91643212	KD2-H4K8ac_peak_8930	5.88845	
14	91716006	91716470	KD2-H4K8ac_peak_8931	8.23274	
14	91719300	91720692	KD2-H4K8ac_peak_8932	17.8868	GPR68_ENSG00000119714
14	91730513	91731291	KD2-H4K8ac_peak_8933	6.25869	
14	91736753	91736964	KD2-H4K8ac_peak_8934	4.98318	
14	91746127	91746416	KD2-H4K8ac_peak_8935	3.93917	
14	91749628	91750165	KD2-H4K8ac_peak_8936	5.4779	
14	91751596	91752482	KD2-H4K8ac_peak_8937	9.77606	
14	91790186	91790766	KD2-H4K8ac_peak_8938	8.58725	
14	91842940	91843144	KD2-H4K8ac_peak_8939	6.69844	
14	91845542	91846050	KD2-H4K8ac_peak_8940	7.82935	
14	91865212	91865409	KD2-H4K8ac_peak_8941	5.14125	
14	91884443	91884796	KD2-H4K8ac_peak_8942	3.82059	CCDC88C_ENSG00000015133;RP11-895M11.3_ENSG00000258798;RP11-895M11.2_ENSG00000258446
14	91976025	91977274	KD2-H4K8ac_peak_8943	10.66719	SMEK1_ENSG00000100796
14	92505827	92506309	KD2-H4K8ac_peak_8944	15.22643	TRIP11_ENSG00000100815
14	92572645	92572900	KD2-H4K8ac_peak_8945	11.05407	ATXN3_ENSG00000066427
14	92573268	92573694	KD2-H4K8ac_peak_8946	15.9336	ATXN3_ENSG00000066427
14	92587674	92588910	KD2-H4K8ac_peak_8947	24.85162	NDUFB1_ENSG00000183648;CPSF2_ENSG00000165934
14	93214493	93214865	KD2-H4K8ac_peak_8948	6.56211	LGMN_ENSG00000100600
14	93260797	93261216	KD2-H4K8ac_peak_8949	14.48922	GOLGA5_ENSG00000066455
14	93341430	93341712	KD2-H4K8ac_peak_8950	5.15777	
14	93372728	93372945	KD2-H4K8ac_peak_8951	6.00226	RP11-862G15.2_ENSG00000258499
14	93373608	93373840	KD2-H4K8ac_peak_8952	4.77079	RP11-862G15.2_ENSG00000258499
14	93383120	93383647	KD2-H4K8ac_peak_8953	11.09936	
14	93384202	93384624	KD2-H4K8ac_peak_8954	9.63487	
14	93389586	93389974	KD2-H4K8ac_peak_8955	6.97919	CHGA_ENSG00000100604
14	93411843	93412137	KD2-H4K8ac_peak_8956	7.40314	
14	93424833	93425098	KD2-H4K8ac_peak_8957	7.76569	
14	93426205	93427199	KD2-H4K8ac_peak_8958	9.16531	
14	93427406	93427630	KD2-H4K8ac_peak_8959	5.82799	
14	93429810	93430099	KD2-H4K8ac_peak_8960	7.64093	
14	93430878	93431310	KD2-H4K8ac_peak_8961	5.9696	
14	93452826	93453019	KD2-H4K8ac_peak_8962	7.40111	
14	93453504	93454096	KD2-H4K8ac_peak_8963	6.60096	
14	93457653	93457889	KD2-H4K8ac_peak_8964	7.372	
14	93464606	93464844	KD2-H4K8ac_peak_8965	6.60096	
14	93472452	93473015	KD2-H4K8ac_peak_8966	7.82935	
14	93474113	93474630	KD2-H4K8ac_peak_8967	8.10216	
14	93474999	93475922	KD2-H4K8ac_peak_8968	16.61426	

14	93476245	93477309	KD2-H4K8ac_peak_8969	14.67919	
14	93478383	93479565	KD2-H4K8ac_peak_8970	8.10216	
14	93489347	93489559	KD2-H4K8ac_peak_8971	6.83936	
14	93490177	93490932	KD2-H4K8ac_peak_8972	11.86082	
14	93512227	93512425	KD2-H4K8ac_peak_8973	6.25869	
14	93557937	93558304	KD2-H4K8ac_peak_8974	8.52881	
14	93581093	93581289	KD2-H4K8ac_peak_8975	7.10552	
14	93581886	93582978	KD2-H4K8ac_peak_8976	14.68449	ITPK1_ENSG00000100605
14	93651429	93651743	KD2-H4K8ac_peak_8977	9.36516	MOAP1_ENSG00000165943;TMEM251_ENSG00000153485;RP11-371E8.4_ENSG00000259066
14	93672280	93672643	KD2-H4K8ac_peak_8978	6.57893	C14orf142_ENSG00000170270;UBR7_ENSG00000012963
14	93696989	93697774	KD2-H4K8ac_peak_8979	17.8868	
14	93798728	93799359	KD2-H4K8ac_peak_8980	13.81781	BTBD7_ENSG00000011114;UNC79_ENSG00000133958
14	93896649	93896976	KD2-H4K8ac_peak_8981	4.42765	
14	94211917	94213065	KD2-H4K8ac_peak_8982	5.75757	
14	94213265	94213599	KD2-H4K8ac_peak_8983	11.36939	
14	94214803	94215013	KD2-H4K8ac_peak_8984	7.11824	
14	94215379	94215641	KD2-H4K8ac_peak_8985	5.94405	
14	94254465	94255275	KD2-H4K8ac_peak_8986	6.20982	PRIMA1_ENSG00000175785
14	94255576	94256016	KD2-H4K8ac_peak_8987	8.79106	PRIMA1_ENSG00000175785
14	94359237	94359929	KD2-H4K8ac_peak_8988	6.99815	
14	94372631	94373109	KD2-H4K8ac_peak_8989	8.1718	
14	94407850	94408053	KD2-H4K8ac_peak_8990	7.26029	
14	94492163	94492432	KD2-H4K8ac_peak_8991	4.76709	OTUB2_ENSG00000089723
14	94492828	94493287	KD2-H4K8ac_peak_8992	9.63487	OTUB2_ENSG00000089723
14	95235905	95236458	KD2-H4K8ac_peak_8993	14.15075	GSC_ENSG00000133937
14	95403349	95403872	KD2-H4K8ac_peak_8994	14.8083	
14	95469497	95469759	KD2-H4K8ac_peak_8995	4.66652	
14	95473045	95473317	KD2-H4K8ac_peak_8996	6.8836	
14	95474213	95474406	KD2-H4K8ac_peak_8997	6.78668	
14	95475701	95475902	KD2-H4K8ac_peak_8998	3.75035	
14	95537441	95537667	KD2-H4K8ac_peak_8999	6.64891	
14	95562391	95562583	KD2-H4K8ac_peak_9000	9.15329	
14	95622911	95623788	KD2-H4K8ac_peak_9001	7.76329	DICER1_ENSG00000100697;DICER1-AS1_ENSG00000235706
14	95624039	95624704	KD2-H4K8ac_peak_9002	10.19949	DICER1_ENSG00000100697;DICER1-AS1_ENSG00000235706
14	95786505	95786986	KD2-H4K8ac_peak_9003	13.09607	CLMN_ENSG00000165959
14	95875229	95875576	KD2-H4K8ac_peak_9004	10.96455	LINC00341_ENSG00000229645
14	95947889	95948367	KD2-H4K8ac_peak_9005	6.81873	
14	95963048	95963458	KD2-H4K8ac_peak_9006	4.52326	
14	95982329	95982609	KD2-H4K8ac_peak_9007	6.83936	RP11-1070N10.3_ENSG00000258572
14	95983009	95983562	KD2-H4K8ac_peak_9008	5.32713	RP11-1070N10.3_ENSG00000258572
14	95986466	95986980	KD2-H4K8ac_peak_9009	5.15777	
14	96505800	96506300	KD2-H4K8ac_peak_9010	11.92978	C14orf132_ENSG00000227051
14	96555883	96556614	KD2-H4K8ac_peak_9011	4.86324	
14	96557861	96558300	KD2-H4K8ac_peak_9012	7.98621	
14	96561759	96562006	KD2-H4K8ac_peak_9013	6.99815	
14	96563545	96563787	KD2-H4K8ac_peak_9014	4.77079	

14	96583810	96584135	KD2-H4K8ac_peak_9015	7.47462	
14	96760759	96761009	KD2-H4K8ac_peak_9016	4.37708	
14	96829235	96829608	KD2-H4K8ac_peak_9017	6.25869	ATG2B_ENSG00000066739;GSKIP_ENSG00000100744
14	96857515	96857847	KD2-H4K8ac_peak_9018	10.29842	AK7_ENSG00000140057
14	96968068	96969358	KD2-H4K8ac_peak_9019	13.73635	RP11-872J21.3_ENSG00000260806;PAPOLA_ENSG00000090060
14	97059390	97059677	KD2-H4K8ac_peak_9020	6.65673	RP11-433J8.1_ENSG00000258702
14	97081122	97081318	KD2-H4K8ac_peak_9021	5.14125	
14	97091020	97091216	KD2-H4K8ac_peak_9022	7.27716	
14	97228872	97229120	KD2-H4K8ac_peak_9023	8.1233	
14	97263750	97264186	KD2-H4K8ac_peak_9024	12.25733	VRK1_ENSG00000100749
14	99946685	99947029	KD2-H4K8ac_peak_9025	8.5092	SETD3_ENSG00000183576;CCNK_ENSG00000090061
14	99947795	99948100	KD2-H4K8ac_peak_9026	12.42547	SETD3_ENSG00000183576;CCNK_ENSG00000090061
14	100004763	1E+08	KD2-H4K8ac_peak_9027	6.1298	
14	100007838	1E+08	KD2-H4K8ac_peak_9028	5.4497	
14	100026766	1E+08	KD2-H4K8ac_peak_9029	7.70067	
14	100027991	1E+08	KD2-H4K8ac_peak_9030	6.16103	
14	100031321	1E+08	KD2-H4K8ac_peak_9031	12.75059	
14	100038249	1E+08	KD2-H4K8ac_peak_9032	4.54006	
14	100039190	1E+08	KD2-H4K8ac_peak_9033	10.19949	
14	100057485	1E+08	KD2-H4K8ac_peak_9034	7.69287	
14	100071014	1E+08	KD2-H4K8ac_peak_9035	16.25886	CCDC85C_ENSG00000205476;RP11-543C4.1_ENSG00000247970
14	100111275	1E+08	KD2-H4K8ac_peak_9036	7.36799	HHIPL1_ENSG00000182218
14	100196594	1E+08	KD2-H4K8ac_peak_9037	8.16906	
14	100239507	1E+08	KD2-H4K8ac_peak_9038	6.65673	
14	100240103	1E+08	KD2-H4K8ac_peak_9039	11.79585	
14	100259091	1E+08	KD2-H4K8ac_peak_9040	17.8868	
14	100259882	1E+08	KD2-H4K8ac_peak_9041	9.59147	
14	100276388	1E+08	KD2-H4K8ac_peak_9042	5.82799	
14	100437291	1E+08	KD2-H4K8ac_peak_9043	8.26945	EVL_ENSG00000196405
14	100438297	1E+08	KD2-H4K8ac_peak_9044	10.87093	EVL_ENSG00000196405
14	100609609	1.01E+08	KD2-H4K8ac_peak_9045	5.53033	
14	100658604	1.01E+08	KD2-H4K8ac_peak_9046	27.41885	
14	100659539	1.01E+08	KD2-H4K8ac_peak_9047	24.39841	
14	100680108	1.01E+08	KD2-H4K8ac_peak_9048	23.70459	
14	100705888	1.01E+08	KD2-H4K8ac_peak_9049	6.25869	RP11-638I2.4_ENSG00000258982
14	100751862	1.01E+08	KD2-H4K8ac_peak_9050	21.2844	
14	100772392	1.01E+08	KD2-H4K8ac_peak_9051	7.76104	SLC25A29_ENSG00000197119
14	100773068	1.01E+08	KD2-H4K8ac_peak_9052	8.86099	SLC25A29_ENSG00000197119;MIR345_ENSG00000198984
14	100841910	1.01E+08	KD2-H4K8ac_peak_9053	10.05606	WARS_ENSG00000140105;WDR25_ENSG00000176473
14	100850248	1.01E+08	KD2-H4K8ac_peak_9054	13.70605	
14	100852031	1.01E+08	KD2-H4K8ac_peak_9055	7.86151	
14	100852894	1.01E+08	KD2-H4K8ac_peak_9056	10.30284	
14	100853809	1.01E+08	KD2-H4K8ac_peak_9057	9.40857	
14	100855836	1.01E+08	KD2-H4K8ac_peak_9058	11.81678	
14	100857006	1.01E+08	KD2-H4K8ac_peak_9059	8.76725	
14	100857575	1.01E+08	KD2-H4K8ac_peak_9060	9.16531	

14	100860026	1.01E+08	KD2-H4K8ac_peak_9061	7.07117	
14	100864022	1.01E+08	KD2-H4K8ac_peak_9062	8.49556	
14	100867525	1.01E+08	KD2-H4K8ac_peak_9063	17.42905	
14	100871680	1.01E+08	KD2-H4K8ac_peak_9064	5.88845	RP11-362L22.1_ENSG00000258620
14	100872348	1.01E+08	KD2-H4K8ac_peak_9065	10.19949	RP11-362L22.1_ENSG00000258620
14	100873832	1.01E+08	KD2-H4K8ac_peak_9066	6.25869	RP11-362L22.1_ENSG00000258620
14	100876438	1.01E+08	KD2-H4K8ac_peak_9067	10.08874	
14	100879154	1.01E+08	KD2-H4K8ac_peak_9068	10.0692	
14	100882960	1.01E+08	KD2-H4K8ac_peak_9069	13.44374	
14	100884100	1.01E+08	KD2-H4K8ac_peak_9070	11.89502	
14	100886075	1.01E+08	KD2-H4K8ac_peak_9071	10.47747	
14	100887121	1.01E+08	KD2-H4K8ac_peak_9072	5.88845	
14	100888743	1.01E+08	KD2-H4K8ac_peak_9073	11.82518	
14	100889441	1.01E+08	KD2-H4K8ac_peak_9074	8.90799	
14	100890159	1.01E+08	KD2-H4K8ac_peak_9075	13.92465	
14	100890843	1.01E+08	KD2-H4K8ac_peak_9076	5.94405	
14	100894106	1.01E+08	KD2-H4K8ac_peak_9077	11.08187	
14	100894612	1.01E+08	KD2-H4K8ac_peak_9078	3.83717	
14	100895465	1.01E+08	KD2-H4K8ac_peak_9079	13.05834	
14	100897020	1.01E+08	KD2-H4K8ac_peak_9080	5.9696	
14	100902902	1.01E+08	KD2-H4K8ac_peak_9081	10.0665	
14	100905167	1.01E+08	KD2-H4K8ac_peak_9082	19.98162	
14	100906497	1.01E+08	KD2-H4K8ac_peak_9083	12.23	
14	100907612	1.01E+08	KD2-H4K8ac_peak_9084	28.03233	
14	100909323	1.01E+08	KD2-H4K8ac_peak_9085	10.19949	
14	100910201	1.01E+08	KD2-H4K8ac_peak_9086	11.04256	
14	100911859	1.01E+08	KD2-H4K8ac_peak_9087	8.14467	
14	100913536	1.01E+08	KD2-H4K8ac_peak_9088	7.54952	
14	100915469	1.01E+08	KD2-H4K8ac_peak_9089	4.86324	
14	100998486	1.01E+08	KD2-H4K8ac_peak_9090	4.42765	
14	101000985	1.01E+08	KD2-H4K8ac_peak_9091	5.81222	
14	101001808	1.01E+08	KD2-H4K8ac_peak_9092	6.99815	
14	101004103	1.01E+08	KD2-H4K8ac_peak_9093	5.50645	CTD-2062F14.3_ENSG00000259031
14	101007843	1.01E+08	KD2-H4K8ac_peak_9094	9.65086	
14	101008622	1.01E+08	KD2-H4K8ac_peak_9095	5.82799	
14	101033165	1.01E+08	KD2-H4K8ac_peak_9096	5.31717	
14	101033856	1.01E+08	KD2-H4K8ac_peak_9097	17.27647	
14	101036318	1.01E+08	KD2-H4K8ac_peak_9098	4.44527	
14	101043914	1.01E+08	KD2-H4K8ac_peak_9099	9.51688	
14	101051962	1.01E+08	KD2-H4K8ac_peak_9100	8.85813	BEGAIN_ENSG00000183092
14	101054040	1.01E+08	KD2-H4K8ac_peak_9101	7.49599	BEGAIN_ENSG00000183092
14	101055097	1.01E+08	KD2-H4K8ac_peak_9102	5.13615	CTD-2644I21.1_ENSG00000258576
14	101055995	1.01E+08	KD2-H4K8ac_peak_9103	4.42765	CTD-2644I21.1_ENSG00000258576
14	101077366	1.01E+08	KD2-H4K8ac_peak_9104	4.76709	
14	101079569	1.01E+08	KD2-H4K8ac_peak_9105	8.86391	
14	101102832	1.01E+08	KD2-H4K8ac_peak_9106	4.30319	

14	101110841	1.01E+08	KD2-H4K8ac_peak_9107	6.05995	
14	101120116	1.01E+08	KD2-H4K8ac_peak_9108	3.90565	
14	101121186	1.01E+08	KD2-H4K8ac_peak_9109	9.34195	
14	101123318	1.01E+08	KD2-H4K8ac_peak_9110	7.37678	LINC00523_ENSG00000196273
14	101124580	1.01E+08	KD2-H4K8ac_peak_9111	7.76329	LINC00523_ENSG00000196273
14	101126390	1.01E+08	KD2-H4K8ac_peak_9112	5.42727	
14	101129223	1.01E+08	KD2-H4K8ac_peak_9113	6.65673	
14	101130669	1.01E+08	KD2-H4K8ac_peak_9114	6.65673	
14	101135011	1.01E+08	KD2-H4K8ac_peak_9115	7.76329	
14	101136526	1.01E+08	KD2-H4K8ac_peak_9116	9.58942	
14	101138727	1.01E+08	KD2-H4K8ac_peak_9117	18.70664	
14	101140744	1.01E+08	KD2-H4K8ac_peak_9118	12.57297	
14	101142279	1.01E+08	KD2-H4K8ac_peak_9119	16.58205	
14	101143793	1.01E+08	KD2-H4K8ac_peak_9120	20.30085	RP11-566J3.2_ENSG00000258717
14	101146056	1.01E+08	KD2-H4K8ac_peak_9121	5.75757	RP11-566J3.2_ENSG00000258717
14	101146835	1.01E+08	KD2-H4K8ac_peak_9122	7.70067	RP11-566J3.2_ENSG00000258717
14	101147493	1.01E+08	KD2-H4K8ac_peak_9123	5.9696	
14	101148383	1.01E+08	KD2-H4K8ac_peak_9124	5.00593	
14	101150913	1.01E+08	KD2-H4K8ac_peak_9125	3.75035	
14	101153179	1.01E+08	KD2-H4K8ac_peak_9126	15.09202	
14	101155085	1.01E+08	KD2-H4K8ac_peak_9127	6.04107	
14	101156877	1.01E+08	KD2-H4K8ac_peak_9128	12.00273	
14	101158954	1.01E+08	KD2-H4K8ac_peak_9129	9.36516	
14	101160667	1.01E+08	KD2-H4K8ac_peak_9130	5.56396	
14	101163423	1.01E+08	KD2-H4K8ac_peak_9131	6.65673	
14	101164402	1.01E+08	KD2-H4K8ac_peak_9132	5.9696	
14	101164937	1.01E+08	KD2-H4K8ac_peak_9133	7.93362	
14	101172466	1.01E+08	KD2-H4K8ac_peak_9134	6.99815	
14	101175007	1.01E+08	KD2-H4K8ac_peak_9135	15.58442	
14	101177328	1.01E+08	KD2-H4K8ac_peak_9136	10.87093	
14	101189148	1.01E+08	KD2-H4K8ac_peak_9137	8.52559	
14	101192645	1.01E+08	KD2-H4K8ac_peak_9138	7.96322	DLK1_ENSG00000185559
14	101193289	1.01E+08	KD2-H4K8ac_peak_9139	5.9696	
14	101194260	1.01E+08	KD2-H4K8ac_peak_9140	5.14125	
14	101195083	1.01E+08	KD2-H4K8ac_peak_9141	6.25869	
14	101196785	1.01E+08	KD2-H4K8ac_peak_9142	5.14125	
14	101234462	1.01E+08	KD2-H4K8ac_peak_9143	6.65673	
14	101235370	1.01E+08	KD2-H4K8ac_peak_9144	9.36516	
14	101236607	1.01E+08	KD2-H4K8ac_peak_9145	18.7894	
14	101237907	1.01E+08	KD2-H4K8ac_peak_9146	16.49944	
14	101241831	1.01E+08	KD2-H4K8ac_peak_9147	22.32344	
14	101244471	1.01E+08	KD2-H4K8ac_peak_9148	24.23593	MEG3_ENSG00000214548
14	101249371	1.01E+08	KD2-H4K8ac_peak_9149	29.27429	
14	101250754	1.01E+08	KD2-H4K8ac_peak_9150	17.8868	
14	101253522	1.01E+08	KD2-H4K8ac_peak_9151	3.97079	
14	101254921	1.01E+08	KD2-H4K8ac_peak_9152	8.52881	

14	101289994	1.01E+08	KD2-H4K8ac_peak_9153	5.54647	
14	101306065	1.01E+08	KD2-H4K8ac_peak_9154	6.05809	
14	101306766	1.01E+08	KD2-H4K8ac_peak_9155	11.77304	
14	101308160	1.01E+08	KD2-H4K8ac_peak_9156	14.69247	
14	101311302	1.01E+08	KD2-H4K8ac_peak_9157	7.13615	
14	101363053	1.01E+08	KD2-H4K8ac_peak_9158	11.03331	
14	101364909	1.01E+08	KD2-H4K8ac_peak_9159	16.57619	
14	101366155	1.01E+08	KD2-H4K8ac_peak_9160	6.99815	
14	101366704	1.01E+08	KD2-H4K8ac_peak_9161	11.04256	AL117190.1_ENSG00000221077
14	101371078	1.01E+08	KD2-H4K8ac_peak_9162	12.46522	
14	101371602	1.01E+08	KD2-H4K8ac_peak_9163	7.76329	
14	101472536	1.01E+08	KD2-H4K8ac_peak_9164	5.14125	
14	101474296	1.01E+08	KD2-H4K8ac_peak_9165	11.90231	
14	101475950	1.01E+08	KD2-H4K8ac_peak_9166	10.29842	
14	101538975	1.02E+08	KD2-H4K8ac_peak_9167	14.60723	
14	101539993	1.02E+08	KD2-H4K8ac_peak_9168	10.53377	
14	101577870	1.02E+08	KD2-H4K8ac_peak_9169	8.23274	
14	101592336	1.02E+08	KD2-H4K8ac_peak_9170	8.05311	
14	101593062	1.02E+08	KD2-H4K8ac_peak_9171	12.84642	
14	101597200	1.02E+08	KD2-H4K8ac_peak_9172	9.9302	
14	101597870	1.02E+08	KD2-H4K8ac_peak_9173	13.07128	
14	101601205	1.02E+08	KD2-H4K8ac_peak_9174	8.37231	
14	101601921	1.02E+08	KD2-H4K8ac_peak_9175	9.1707	
14	101603425	1.02E+08	KD2-H4K8ac_peak_9176	6.90458	
14	101616401	1.02E+08	KD2-H4K8ac_peak_9177	3.75035	
14	101642397	1.02E+08	KD2-H4K8ac_peak_9178	4.66652	
14	101690095	1.02E+08	KD2-H4K8ac_peak_9179	4.211	
14	101691566	1.02E+08	KD2-H4K8ac_peak_9180	7.30812	
14	101696761	1.02E+08	KD2-H4K8ac_peak_9181	13.95627	
14	101699325	1.02E+08	KD2-H4K8ac_peak_9182	9.1707	
14	101701351	1.02E+08	KD2-H4K8ac_peak_9183	6.35843	
14	101925251	1.02E+08	KD2-H4K8ac_peak_9184	5.9696	
14	101992195	1.02E+08	KD2-H4K8ac_peak_9185	7.32463	
14	102050840	1.02E+08	KD2-H4K8ac_peak_9186	8.85813	
14	102055876	1.02E+08	KD2-H4K8ac_peak_9187	11.24702	
14	102088790	1.02E+08	KD2-H4K8ac_peak_9188	4.67342	
14	102093989	1.02E+08	KD2-H4K8ac_peak_9189	7.36799	
14	102095008	1.02E+08	KD2-H4K8ac_peak_9190	12.08081	
14	102095643	1.02E+08	KD2-H4K8ac_peak_9191	13.81781	
14	102173217	1.02E+08	KD2-H4K8ac_peak_9192	11.24422	
14	102174162	1.02E+08	KD2-H4K8ac_peak_9193	6.30229	
14	102241874	1.02E+08	KD2-H4K8ac_peak_9194	5.54647	
14	102247424	1.02E+08	KD2-H4K8ac_peak_9195	12.74514	
14	102278585	1.02E+08	KD2-H4K8ac_peak_9196	4.54838	
14	102305245	1.02E+08	KD2-H4K8ac_peak_9197	10.05606	CTD-2017C7.1_ENSG00000256705
14	102307698	1.02E+08	KD2-H4K8ac_peak_9198	20.02161	

14	102325435	1.02E+08	KD2-H4K8ac_peak_9199	7.27716	
14	102343443	1.02E+08	KD2-H4K8ac_peak_9200	8.59579	
14	102413245	1.02E+08	KD2-H4K8ac_peak_9201	7.51054	RP11-1017G21.5_ENSG00000271780
14	102414704	1.02E+08	KD2-H4K8ac_peak_9202	13.50024	RP11-1017G21.5_ENSG00000271780
14	102428252	1.02E+08	KD2-H4K8ac_peak_9203	7.01494	
14	102429013	1.02E+08	KD2-H4K8ac_peak_9204	6.30626	
14	102431167	1.02E+08	KD2-H4K8ac_peak_9205	9.65086	DYNC1H1_ENSG00000197102
14	102552935	1.03E+08	KD2-H4K8ac_peak_9206	6.40297	
14	102553666	1.03E+08	KD2-H4K8ac_peak_9207	17.8868	
14	102606289	1.03E+08	KD2-H4K8ac_peak_9208	7.51054	HSP90AA1_ENSG00000080824;WDR20_ENSG00000140153
14	102638875	1.03E+08	KD2-H4K8ac_peak_9209	6.16888	
14	102652956	1.03E+08	KD2-H4K8ac_peak_9210	7.47462	
14	102653871	1.03E+08	KD2-H4K8ac_peak_9211	7.27716	
14	102656304	1.03E+08	KD2-H4K8ac_peak_9212	5.51783	
14	102729378	1.03E+08	KD2-H4K8ac_peak_9213	13.81781	
14	102780763	1.03E+08	KD2-H4K8ac_peak_9214	8.33304	
14	102782897	1.03E+08	KD2-H4K8ac_peak_9215	5.13893	ZNF839_ENSG00000022976
14	102783636	1.03E+08	KD2-H4K8ac_peak_9216	9.08816	ZNF839_ENSG00000022976
14	102785773	1.03E+08	KD2-H4K8ac_peak_9217	12.93253	
14	102829384	1.03E+08	KD2-H4K8ac_peak_9218	10.29842	CINP_ENSG00000100865;TECPR2_ENSG00000196663
14	102976408	1.03E+08	KD2-H4K8ac_peak_9219	9.1707	ANKRD9_ENSG00000156381
14	102982986	1.03E+08	KD2-H4K8ac_peak_9220	18.59584	
14	102990398	1.03E+08	KD2-H4K8ac_peak_9221	18.94636	
14	102991308	1.03E+08	KD2-H4K8ac_peak_9222	4.86324	
14	103005106	1.03E+08	KD2-H4K8ac_peak_9223	4.56666	MIR4309_ENSG00000266015
14	103006576	1.03E+08	KD2-H4K8ac_peak_9224	7.07117	MIR4309_ENSG00000266015
14	103012036	1.03E+08	KD2-H4K8ac_peak_9225	5.88845	CTD-2555C10.3_ENSG00000259230
14	103043884	1.03E+08	KD2-H4K8ac_peak_9226	5.57044	
14	103046402	1.03E+08	KD2-H4K8ac_peak_9227	8.94469	
14	103057966	1.03E+08	KD2-H4K8ac_peak_9228	12.82393	RCOR1_ENSG00000089902
14	103150110	1.03E+08	KD2-H4K8ac_peak_9229	5.57044	
14	103315121	1.03E+08	KD2-H4K8ac_peak_9230	5.88845	
14	103315911	1.03E+08	KD2-H4K8ac_peak_9231	5.7068	
14	103396294	1.03E+08	KD2-H4K8ac_peak_9232	6.30229	
14	103523301	1.04E+08	KD2-H4K8ac_peak_9233	6.44277	CDC42BPB_ENSG00000198752
14	103541739	1.04E+08	KD2-H4K8ac_peak_9234	11.79585	
14	103542342	1.04E+08	KD2-H4K8ac_peak_9235	8.79106	
14	103557409	1.04E+08	KD2-H4K8ac_peak_9236	20.88898	
14	103578774	1.04E+08	KD2-H4K8ac_peak_9237	5.82799	
14	103587609	1.04E+08	KD2-H4K8ac_peak_9238	8.86099	LINC00677_ENSG00000259717;TNFAIP2_ENSG00000185215
14	103590330	1.04E+08	KD2-H4K8ac_peak_9239	4.8888	LINC00677_ENSG00000259717;TNFAIP2_ENSG00000185215
14	103593090	1.04E+08	KD2-H4K8ac_peak_9240	8.85813	
14	103593615	1.04E+08	KD2-H4K8ac_peak_9241	7.43893	
14	103598689	1.04E+08	KD2-H4K8ac_peak_9242	9.09777	
14	103617912	1.04E+08	KD2-H4K8ac_peak_9243	6.94954	
14	103673761	1.04E+08	KD2-H4K8ac_peak_9244	9.95119	RP11-736N17.9_ENSG00000270705

14	103701648	1.04E+08	KD2-H4K8ac_peak_9245	4.73064	
14	103726190	1.04E+08	KD2-H4K8ac_peak_9246	6.99815	
14	103744417	1.04E+08	KD2-H4K8ac_peak_9247	4.01115	RAP2CP1_ENSG00000270938
14	103745068	1.04E+08	KD2-H4K8ac_peak_9248	11.29366	RAP2CP1_ENSG00000270938
14	103799649	1.04E+08	KD2-H4K8ac_peak_9249	23.33626	RP11-45P15.4_ENSG00000259775;EIF5_ENSG00000100664
14	103800613	1.04E+08	KD2-H4K8ac_peak_9250	8.58725	EIF5_ENSG00000100664
14	103840901	1.04E+08	KD2-H4K8ac_peak_9251	6.60096	RP11-600F24.1_ENSG00000243904
14	103850823	1.04E+08	KD2-H4K8ac_peak_9252	16.822	MARK3_ENSG00000075413
14	103851781	1.04E+08	KD2-H4K8ac_peak_9253	6.0605	MARK3_ENSG00000075413
14	103985218	1.04E+08	KD2-H4K8ac_peak_9254	4.40553	
14	103987684	1.04E+08	KD2-H4K8ac_peak_9255	4.67342	
14	103988643	1.04E+08	KD2-H4K8ac_peak_9256	5.20908	CKB_ENSG00000166165
14	103989334	1.04E+08	KD2-H4K8ac_peak_9257	13.73635	CKB_ENSG00000166165
14	103994415	1.04E+08	KD2-H4K8ac_peak_9258	6.40297	RP11-600F24.7_ENSG00000260285;TRMT61A_ENSG00000166166
14	103995595	1.04E+08	KD2-H4K8ac_peak_9259	8.2413	RP11-600F24.7_ENSG00000260285;TRMT61A_ENSG00000166166
14	104028499	1.04E+08	KD2-H4K8ac_peak_9260	16.64426	BAG5_ENSG00000166170;KLC1_ENSG00000126214;RP11-73M18.2_ENSG00000256500;APOPT1_ENSG00000256053
14	104094469	1.04E+08	KD2-H4K8ac_peak_9261	16.57619	
14	104096102	1.04E+08	KD2-H4K8ac_peak_9262	10.95611	
14	104163062	1.04E+08	KD2-H4K8ac_peak_9263	4.77079	RP11-73M18.8_ENSG00000269958
14	104181360	1.04E+08	KD2-H4K8ac_peak_9264	8.04448	XRCC3_ENSG00000126215;ZFYVE21_ENSG00000100711
14	104182401	1.04E+08	KD2-H4K8ac_peak_9265	8.58069	XRCC3_ENSG00000126215;ZFYVE21_ENSG00000100711
14	104314156	1.04E+08	KD2-H4K8ac_peak_9266	6.66359	PPP1R13B_ENSG00000088808;LINC00637_ENSG00000258735
14	104321338	1.04E+08	KD2-H4K8ac_peak_9267	7.49599	
14	104336352	1.04E+08	KD2-H4K8ac_peak_9268	7.27716	
14	104346146	1.04E+08	KD2-H4K8ac_peak_9269	6.36375	CTD-2134A5.4_ENSG00000258534
14	104347100	1.04E+08	KD2-H4K8ac_peak_9270	11.24702	CTD-2134A5.4_ENSG00000258534
14	104348047	1.04E+08	KD2-H4K8ac_peak_9271	11.09936	
14	104349044	1.04E+08	KD2-H4K8ac_peak_9272	11.05467	
14	104389528	1.04E+08	KD2-H4K8ac_peak_9273	7.10552	
14	104427284	1.04E+08	KD2-H4K8ac_peak_9274	6.40683	
14	104560407	1.05E+08	KD2-H4K8ac_peak_9275	8.86099	
14	104601300	1.05E+08	KD2-H4K8ac_peak_9276	6.24114	
14	104601955	1.05E+08	KD2-H4K8ac_peak_9277	6.98244	
14	104602778	1.05E+08	KD2-H4K8ac_peak_9278	4.30033	CTD-2223O18.1_ENSG00000258748
14	104603601	1.05E+08	KD2-H4K8ac_peak_9279	4.49087	CTD-2223O18.1_ENSG00000258748
14	104604121	1.05E+08	KD2-H4K8ac_peak_9280	9.63487	CTD-2223O18.1_ENSG00000258748;KIF26A_ENSG00000066735
14	104604928	1.05E+08	KD2-H4K8ac_peak_9281	5.38581	CTD-2223O18.1_ENSG00000258748;KIF26A_ENSG00000066735
14	104613328	1.05E+08	KD2-H4K8ac_peak_9282	7.02364	
14	104615056	1.05E+08	KD2-H4K8ac_peak_9283	19.1417	
14	104616904	1.05E+08	KD2-H4K8ac_peak_9284	7.92775	
14	104623961	1.05E+08	KD2-H4K8ac_peak_9285	10.01546	
14	104625608	1.05E+08	KD2-H4K8ac_peak_9286	5.64429	
14	104626044	1.05E+08	KD2-H4K8ac_peak_9287	4.68059	
14	104626613	1.05E+08	KD2-H4K8ac_peak_9288	5.56864	
14	104649493	1.05E+08	KD2-H4K8ac_peak_9289	4.00051	
14	104650304	1.05E+08	KD2-H4K8ac_peak_9290	7.47462	

14	104651216	1.05E+08	KD2-H4K8ac_peak_9291	5.88845	
14	104661940	1.05E+08	KD2-H4K8ac_peak_9292	4.75875	
14	104667041	1.05E+08	KD2-H4K8ac_peak_9293	13.9152	
14	104672019	1.05E+08	KD2-H4K8ac_peak_9294	7.76329	
14	104673231	1.05E+08	KD2-H4K8ac_peak_9295	11.82518	
14	104682333	1.05E+08	KD2-H4K8ac_peak_9296	4.53319	
14	104686085	1.05E+08	KD2-H4K8ac_peak_9297	6.10575	
14	104687124	1.05E+08	KD2-H4K8ac_peak_9298	15.61313	
14	104688771	1.05E+08	KD2-H4K8ac_peak_9299	24.17855	RP11-260M19.2_ENSG00000258913
14	104689964	1.05E+08	KD2-H4K8ac_peak_9300	23.04547	RP11-260M19.2_ENSG00000258913
14	104690733	1.05E+08	KD2-H4K8ac_peak_9301	30.09832	RP11-260M19.2_ENSG00000258913
14	104692023	1.05E+08	KD2-H4K8ac_peak_9302	4.67342	
14	104741752	1.05E+08	KD2-H4K8ac_peak_9303	8.44476	
14	104746196	1.05E+08	KD2-H4K8ac_peak_9304	8.55271	
14	104747092	1.05E+08	KD2-H4K8ac_peak_9305	5.9696	
14	104757136	1.05E+08	KD2-H4K8ac_peak_9306	14.68449	
14	104758660	1.05E+08	KD2-H4K8ac_peak_9307	24.56058	
14	104760582	1.05E+08	KD2-H4K8ac_peak_9308	22.32344	
14	104763242	1.05E+08	KD2-H4K8ac_peak_9309	20.30085	
14	104768037	1.05E+08	KD2-H4K8ac_peak_9310	8.3771	
14	104769375	1.05E+08	KD2-H4K8ac_peak_9311	5.14125	
14	104821556	1.05E+08	KD2-H4K8ac_peak_9312	9.1707	
14	104822308	1.05E+08	KD2-H4K8ac_peak_9313	5.54647	
14	104834365	1.05E+08	KD2-H4K8ac_peak_9314	7.47462	
14	104835184	1.05E+08	KD2-H4K8ac_peak_9315	5.9696	
14	104836449	1.05E+08	KD2-H4K8ac_peak_9316	8.52881	
14	104849745	1.05E+08	KD2-H4K8ac_peak_9317	13.73635	
14	104887689	1.05E+08	KD2-H4K8ac_peak_9318	8.56219	
14	104888301	1.05E+08	KD2-H4K8ac_peak_9319	6.83936	
14	104890505	1.05E+08	KD2-H4K8ac_peak_9320	7.38799	
14	104891752	1.05E+08	KD2-H4K8ac_peak_9321	4.211	
14	104893512	1.05E+08	KD2-H4K8ac_peak_9322	7.40314	
14	104902228	1.05E+08	KD2-H4K8ac_peak_9323	15.76765	
14	104927293	1.05E+08	KD2-H4K8ac_peak_9324	13.81781	
14	104928060	1.05E+08	KD2-H4K8ac_peak_9325	22.07309	
14	104976420	1.05E+08	KD2-H4K8ac_peak_9326	4.54037	
14	104977083	1.05E+08	KD2-H4K8ac_peak_9327	11.86082	
14	105020925	1.05E+08	KD2-H4K8ac_peak_9328	5.51783	
14	105028544	1.05E+08	KD2-H4K8ac_peak_9329	6.40425	
14	105029561	1.05E+08	KD2-H4K8ac_peak_9330	22.59108	
14	105032500	1.05E+08	KD2-H4K8ac_peak_9331	6.48242	
14	105033907	1.05E+08	KD2-H4K8ac_peak_9332	6.30229	
14	105046770	1.05E+08	KD2-H4K8ac_peak_9333	9.91109	C14orf180_ENSG00000184601
14	105047649	1.05E+08	KD2-H4K8ac_peak_9334	14.07846	
14	105050588	1.05E+08	KD2-H4K8ac_peak_9335	5.9696	
14	105053493	1.05E+08	KD2-H4K8ac_peak_9336	14.96478	

14	105055409	1.05E+08	KD2-H4K8ac_peak_9337	6.76298	RP11-614O9.1_ENSG00000259037
14	105065985	1.05E+08	KD2-H4K8ac_peak_9338	9.36516	
14	105067099	1.05E+08	KD2-H4K8ac_peak_9339	6.7498	
14	105067777	1.05E+08	KD2-H4K8ac_peak_9340	5.96443	
14	105070663	1.05E+08	KD2-H4K8ac_peak_9341	7.9265	TMEM179_ENSG00000258986
14	105071416	1.05E+08	KD2-H4K8ac_peak_9342	8.30094	TMEM179_ENSG00000258986
14	105106118	1.05E+08	KD2-H4K8ac_peak_9343	6.0073	
14	105106612	1.05E+08	KD2-H4K8ac_peak_9344	15.22643	
14	105116455	1.05E+08	KD2-H4K8ac_peak_9345	10.19949	
14	105155348	1.05E+08	KD2-H4K8ac_peak_9346	10.46264	INF2_ENSG00000203485;RP11-982M15.5_ENSG00000258858
14	105190446	1.05E+08	KD2-H4K8ac_peak_9347	12.47162	ADSSL1_ENSG00000185100
14	105191012	1.05E+08	KD2-H4K8ac_peak_9348	8.56219	ADSSL1_ENSG00000185100
14	105218531	1.05E+08	KD2-H4K8ac_peak_9349	9.26408	SIVA1_ENSG00000184990
14	105261143	1.05E+08	KD2-H4K8ac_peak_9350	10.59312	AKT1_ENSG00000142208
14	105262192	1.05E+08	KD2-H4K8ac_peak_9351	14.81929	AKT1_ENSG00000142208
14	105265945	1.05E+08	KD2-H4K8ac_peak_9352	20.2903	ZBTB42_ENSG00000179627
14	105282022	1.05E+08	KD2-H4K8ac_peak_9353	9.1707	
14	105282514	1.05E+08	KD2-H4K8ac_peak_9354	20.97251	
14	105292123	1.05E+08	KD2-H4K8ac_peak_9355	10.94736	
14	105293806	1.05E+08	KD2-H4K8ac_peak_9356	6.97919	
14	105331072	1.05E+08	KD2-H4K8ac_peak_9357	8.55271	CEP170B_ENSG00000099814
14	105332001	1.05E+08	KD2-H4K8ac_peak_9358	7.13615	CEP170B_ENSG00000099814
14	105348961	1.05E+08	KD2-H4K8ac_peak_9359	5.05318	
14	105399365	1.05E+08	KD2-H4K8ac_peak_9360	14.17488	
14	105445006	1.05E+08	KD2-H4K8ac_peak_9361	6.56211	AHNAK2_ENSG00000185567
14	105451201	1.05E+08	KD2-H4K8ac_peak_9362	7.51945	C14orf79_ENSG00000140104
14	105486873	1.05E+08	KD2-H4K8ac_peak_9363	9.08816	CDCA4_ENSG00000170779
14	105496620	1.05E+08	KD2-H4K8ac_peak_9364	8.17902	
14	105525042	1.06E+08	KD2-H4K8ac_peak_9365	7.22457	
14	105527636	1.06E+08	KD2-H4K8ac_peak_9366	6.48713	
14	105559180	1.06E+08	KD2-H4K8ac_peak_9367	17.38513	RP11-44N21.1_ENSG00000257556
14	105647147	1.06E+08	KD2-H4K8ac_peak_9368	10.30284	RP11-44N21.4_ENSG00000257622;NUDT14_ENSG00000183828
14	105714219	1.06E+08	KD2-H4K8ac_peak_9369	4.86324	BTBD6_ENSG00000184887
14	105759365	1.06E+08	KD2-H4K8ac_peak_9370	8.36432	
14	105767085	1.06E+08	KD2-H4K8ac_peak_9371	10.53377	PACS2_ENSG00000179364
14	105768011	1.06E+08	KD2-H4K8ac_peak_9372	6.66692	
14	105779629	1.06E+08	KD2-H4K8ac_peak_9373	12.82393	
14	105781261	1.06E+08	KD2-H4K8ac_peak_9374	7.47462	BRF1_ENSG00000185024
14	105790938	1.06E+08	KD2-H4K8ac_peak_9375	5.29352	
14	105864002	1.06E+08	KD2-H4K8ac_peak_9376	21.09577	TEX22_ENSG00000226174
14	105877521	1.06E+08	KD2-H4K8ac_peak_9377	11.89502	
14	105881106	1.06E+08	KD2-H4K8ac_peak_9378	12.00273	
14	105885442	1.06E+08	KD2-H4K8ac_peak_9379	33.81557	RP11-521B24.3_ENSG00000251602;MTA1_ENSG00000182979
14	105886763	1.06E+08	KD2-H4K8ac_peak_9380	8.55271	RP11-521B24.3_ENSG00000251602;MTA1_ENSG00000182979
14	105887952	1.06E+08	KD2-H4K8ac_peak_9381	7.40314	
14	105937363	1.06E+08	KD2-H4K8ac_peak_9382	5.13615	RP11-521B24.5_ENSG00000257270

14	105938182	1.06E+08	KD2-H4K8ac_peak_9383	6.43014	CRIP2_ENSG00000182809
14	105938874	1.06E+08	KD2-H4K8ac_peak_9384	6.4562	CRIP2_ENSG00000182809
14	105940006	1.06E+08	KD2-H4K8ac_peak_9385	12.74183	CRIP2_ENSG00000182809
14	105941213	1.06E+08	KD2-H4K8ac_peak_9386	9.20193	
14	105943069	1.06E+08	KD2-H4K8ac_peak_9387	15.61313	
14	105947444	1.06E+08	KD2-H4K8ac_peak_9388	8.16906	
14	105948751	1.06E+08	KD2-H4K8ac_peak_9389	3.90565	
14	105951796	1.06E+08	KD2-H4K8ac_peak_9390	7.44324	CRIP1_ENSG00000213145;CRIP1_ENSG00000257341
14	105956231	1.06E+08	KD2-H4K8ac_peak_9391	13.13969	C14orf80_ENSG00000185347
14	105992373	1.06E+08	KD2-H4K8ac_peak_9392	5.88845	TMEM121_ENSG00000184986
14	105993166	1.06E+08	KD2-H4K8ac_peak_9393	9.83789	TMEM121_ENSG00000184986
14	106004175	1.06E+08	KD2-H4K8ac_peak_9394	4.86324	ATP5G1P1_ENSG00000230157
14	106025703	1.06E+08	KD2-H4K8ac_peak_9395	6.40297	
14	106146197	1.06E+08	KD2-H4K8ac_peak_9396	9.15329	
14	106854923	1.07E+08	KD2-H4K8ac_peak_9397	11.15509	
14	106936170	1.07E+08	KD2-H4K8ac_peak_9398	11.04694	
14	106938104	1.07E+08	KD2-H4K8ac_peak_9399	20.02161	LINC00221_ENSG00000187156
15	22544590	22545178	KD2-H4K8ac_peak_9400	5.57044	RP11-603B24.2_ENSG00000259098
15	22832889	22833142	KD2-H4K8ac_peak_9401	10.65724	TUBGCP5_ENSG00000153575
15	22833537	22833985	KD2-H4K8ac_peak_9402	21.60954	TUBGCP5_ENSG00000153575
15	22892166	22892544	KD2-H4K8ac_peak_9403	8.4716	CYFIP1_ENSG00000068793
15	23034120	23034323	KD2-H4K8ac_peak_9404	9.96798	NIPA2_ENSG00000140157
15	23034699	23035233	KD2-H4K8ac_peak_9405	10.21366	NIPA2_ENSG00000140157
15	23085933	23086156	KD2-H4K8ac_peak_9406	7.36799	
15	23086689	23087002	KD2-H4K8ac_peak_9407	5.14125	
15	23114997	23115201	KD2-H4K8ac_peak_9408	9.08816	RP11-566K19.6_ENSG00000259344
15	23810864	23811177	KD2-H4K8ac_peak_9409	10.21502	MKRN3_ENSG00000179455
15	25683677	25684091	KD2-H4K8ac_peak_9410	17.27647	UBE3A_ENSG00000114062
15	25684422	25684769	KD2-H4K8ac_peak_9411	11.05407	UBE3A_ENSG00000114062
15	26042757	26043022	KD2-H4K8ac_peak_9412	6.66692	
15	26095508	26095700	KD2-H4K8ac_peak_9413	7.12754	
15	26178889	26179105	KD2-H4K8ac_peak_9414	6.07178	
15	26915235	26915446	KD2-H4K8ac_peak_9415	7.12754	
15	27018435	27018852	KD2-H4K8ac_peak_9416	5.30799	
15	27136804	27137023	KD2-H4K8ac_peak_9417	7.47462	
15	27332019	27332250	KD2-H4K8ac_peak_9418	5.9696	
15	27818927	27819194	KD2-H4K8ac_peak_9419	5.88845	
15	28235251	28235657	KD2-H4K8ac_peak_9420	6.05809	
15	28266578	28266888	KD2-H4K8ac_peak_9421	5.57044	
15	28297153	28297347	KD2-H4K8ac_peak_9422	7.47462	
15	28351583	28351836	KD2-H4K8ac_peak_9423	6.65673	
15	28699802	28700256	KD2-H4K8ac_peak_9424	15.28459	
15	28834079	28834437	KD2-H4K8ac_peak_9425	26.96144	HERC2P9_ENSG00000206149
15	28982725	28983205	KD2-H4K8ac_peak_9426	5.54647	GOLGA8M_ENSG00000188626;RP11-578F21.6_ENSG00000261480;WHAMMP2_ENSG00000248334
15	29077487	29077828	KD2-H4K8ac_peak_9427	10.46264	
15	29130600	29131527	KD2-H4K8ac_peak_9428	14.66631	APBA2_ENSG00000034053

15	29131861	29132213	KD2-H4K8ac_peak_9429	10.19949	
15	29157532	29157893	KD2-H4K8ac_peak_9430	5.88845	
15	29246886	29247263	KD2-H4K8ac_peak_9431	5.82869	
15	29414105	29414308	KD2-H4K8ac_peak_9432	4.211	
15	29519665	29519889	KD2-H4K8ac_peak_9433	7.07117	
15	29561669	29561964	KD2-H4K8ac_peak_9434	12.08081	NDNL2_ENSG00000185115
15	29638373	29638684	KD2-H4K8ac_peak_9435	4.42765	
15	29743753	29744016	KD2-H4K8ac_peak_9436	6.9809	
15	29980502	29980754	KD2-H4K8ac_peak_9437	5.40356	
15	30038280	30038475	KD2-H4K8ac_peak_9438	8.1233	
15	30260554	30261447	KD2-H4K8ac_peak_9439	15.5861	TJP1_ENSG00000104067
15	30488302	30488667	KD2-H4K8ac_peak_9440	12.81138	AC026150.5_ENSG00000225930
15	30659356	30659743	KD2-H4K8ac_peak_9441	9.1707	
15	31196157	31196354	KD2-H4K8ac_peak_9442	12.08081	FAN1_ENSG00000198690
15	31283995	31284506	KD2-H4K8ac_peak_9443	11.89502	MTMR10_ENSG00000166912
15	31505588	31505833	KD2-H4K8ac_peak_9444	8.17902	
15	31507862	31508393	KD2-H4K8ac_peak_9445	29.10149	RP11-16E12.1_ENSG00000259448
15	31529291	31529919	KD2-H4K8ac_peak_9446	10.8368	
15	31556005	31556295	KD2-H4K8ac_peak_9447	5.02474	
15	31572282	31572623	KD2-H4K8ac_peak_9448	5.53033	
15	31592572	31592930	KD2-H4K8ac_peak_9449	8.52559	
15	31593318	31593614	KD2-H4K8ac_peak_9450	14.275	
15	31594166	31594388	KD2-H4K8ac_peak_9451	7.42204	
15	31598197	31598397	KD2-H4K8ac_peak_9452	5.75757	
15	31618067	31618603	KD2-H4K8ac_peak_9453	11.92978	KLF13_ENSG00000169926
15	31619077	31620827	KD2-H4K8ac_peak_9454	11.89502	KLF13_ENSG00000169926
15	31637478	31637680	KD2-H4K8ac_peak_9455	5.61377	
15	31645128	31645348	KD2-H4K8ac_peak_9456	6.65673	
15	31664162	31664511	KD2-H4K8ac_peak_9457	6.75424	
15	31683689	31684406	KD2-H4K8ac_peak_9458	8.26565	
15	31690465	31691534	KD2-H4K8ac_peak_9459	14.66631	
15	31691785	31692038	KD2-H4K8ac_peak_9460	5.92591	
15	31727400	31727801	KD2-H4K8ac_peak_9461	7.27716	
15	31732820	31734079	KD2-H4K8ac_peak_9462	19.3524	
15	31792253	31793024	KD2-H4K8ac_peak_9463	9.1707	
15	32163131	32163622	KD2-H4K8ac_peak_9464	10.33529	OTUD7A_ENSG00000169918
15	32180002	32180194	KD2-H4K8ac_peak_9465	4.211	
15	32266781	32266975	KD2-H4K8ac_peak_9466	6.40683	
15	32267335	32267765	KD2-H4K8ac_peak_9467	11.89502	
15	32322001	32322523	KD2-H4K8ac_peak_9468	8.55271	CHRNA7_ENSG00000175344
15	32322750	32322988	KD2-H4K8ac_peak_9469	8.16906	CHRNA7_ENSG00000175344
15	32383885	32384104	KD2-H4K8ac_peak_9470	5.14125	
15	32385493	32385764	KD2-H4K8ac_peak_9471	7.27716	
15	32392264	32392456	KD2-H4K8ac_peak_9472	5.66317	
15	33312081	33312287	KD2-H4K8ac_peak_9473	4.37708	
15	33359725	33360761	KD2-H4K8ac_peak_9474	10.8368	

15	33418265	33418611	KD2-H4K8ac_peak_9475	4.77079	
15	33440629	33440868	KD2-H4K8ac_peak_9476	7.05877	
15	33451154	33451384	KD2-H4K8ac_peak_9477	7.45501	
15	33451997	33452580	KD2-H4K8ac_peak_9478	8.17902	
15	33453501	33454103	KD2-H4K8ac_peak_9479	13.34404	
15	33931100	33931309	KD2-H4K8ac_peak_9480	5.73839	
15	34330423	34331483	KD2-H4K8ac_peak_9481	22.21705	AVEN_ENSG00000169857
15	34501826	34502180	KD2-H4K8ac_peak_9482	9.20193	KATNBL1_ENSG00000134152
15	34515753	34516929	KD2-H4K8ac_peak_9483	10.7855	EMC4_ENSG00000128463
15	34517232	34517546	KD2-H4K8ac_peak_9484	19.9503	EMC4_ENSG00000128463
15	34634900	34635176	KD2-H4K8ac_peak_9485	7.12754	NOP10_ENSG00000182117;NUTM1_ENSG00000184507
15	34635654	34635900	KD2-H4K8ac_peak_9486	13.92307	NOP10_ENSG00000182117;NUTM1_ENSG00000184507
15	34658986	34659203	KD2-H4K8ac_peak_9487	9.34989	LPCAT4_ENSG00000176454
15	34659544	34660182	KD2-H4K8ac_peak_9488	13.81781	LPCAT4_ENSG00000176454
15	34729107	34729487	KD2-H4K8ac_peak_9489	8.17902	
15	34787129	34787394	KD2-H4K8ac_peak_9490	4.56666	RP11-1H8.3_ENSG00000261559
15	34806170	34806625	KD2-H4K8ac_peak_9491	4.211	
15	34806866	34807308	KD2-H4K8ac_peak_9492	6.25869	
15	34874796	34875009	KD2-H4K8ac_peak_9493	10.77651	
15	34875246	34876612	KD2-H4K8ac_peak_9494	5.26288	
15	35280133	35280447	KD2-H4K8ac_peak_9495	8.4716	ZNF770_ENSG00000198146
15	35280650	35280850	KD2-H4K8ac_peak_9496	7.36799	ZNF770_ENSG00000198146
15	35837948	35838917	KD2-H4K8ac_peak_9497	13.3695	DPH6_ENSG00000134146;DPH6-AS1_ENSG00000248079
15	36163512	36163709	KD2-H4K8ac_peak_9498	8.1233	
15	36871875	36872429	KD2-H4K8ac_peak_9499	9.36516	C15orf41_ENSG00000186073
15	37388002	37389020	KD2-H4K8ac_peak_9500	9.51625	
15	37389334	37390415	KD2-H4K8ac_peak_9501	6.99815	
15	37390928	37391284	KD2-H4K8ac_peak_9502	4.86324	RP11-128A17.1_ENSG00000259460
15	37393090	37394170	KD2-H4K8ac_peak_9503	11.92978	MEIS2_ENSG00000134138
15	37394504	37394801	KD2-H4K8ac_peak_9504	5.9696	
15	37396639	37396966	KD2-H4K8ac_peak_9505	6.00226	
15	37398844	37399108	KD2-H4K8ac_peak_9506	6.8836	
15	37402598	37403068	KD2-H4K8ac_peak_9507	6.66359	
15	37511375	37511608	KD2-H4K8ac_peak_9508	11.89502	
15	37514736	37515091	KD2-H4K8ac_peak_9509	8.04763	
15	38086324	38086553	KD2-H4K8ac_peak_9510	7.12754	
15	38113199	38114013	KD2-H4K8ac_peak_9511	9.02371	
15	38116098	38116327	KD2-H4K8ac_peak_9512	7.27716	
15	38172076	38172488	KD2-H4K8ac_peak_9513	5.10276	
15	38364921	38365449	KD2-H4K8ac_peak_9514	11.00004	RP11-1008C21.2_ENSG00000236914
15	38544303	38545258	KD2-H4K8ac_peak_9515	11.92978	SPRED1_ENSG00000166068
15	38746450	38746823	KD2-H4K8ac_peak_9516	12.99417	FAM98B_ENSG00000171262
15	38856750	38857414	KD2-H4K8ac_peak_9517	8.02571	RASGRP1_ENSG00000172575
15	39169989	39170439	KD2-H4K8ac_peak_9518	5.57044	
15	39172438	39172853	KD2-H4K8ac_peak_9519	5.14125	
15	39188268	39188551	KD2-H4K8ac_peak_9520	11.92209	

15	39522875	39523208	KD2-H4K8ac_peak_9521	9.11031	
15	39655549	39655756	KD2-H4K8ac_peak_9522	5.25071	
15	39872606	39873083	KD2-H4K8ac_peak_9523	11.03331	THBS1_ENSG00000137801
15	40074848	40075302	KD2-H4K8ac_peak_9524	6.16888	FSIP1_ENSG00000150667;RP11-37C7.3_ENSG00000261136
15	40211864	40213547	KD2-H4K8ac_peak_9525	12.82393	GPR176_ENSG00000166073;RP11-325N19.3_ENSG00000246863
15	40225678	40225980	KD2-H4K8ac_peak_9526	8.37043	EIF2AK4_ENSG00000128829
15	40226402	40226686	KD2-H4K8ac_peak_9527	4.70206	EIF2AK4_ENSG00000128829
15	40331076	40331282	KD2-H4K8ac_peak_9528	6.98244	SRP14_ENSG00000140319;SRP14-AS1_ENSG00000248508
15	40360444	40360672	KD2-H4K8ac_peak_9529	5.10505	
15	40361559	40362170	KD2-H4K8ac_peak_9530	10.44656	
15	40373982	40374479	KD2-H4K8ac_peak_9531	6.83936	
15	40384854	40385513	KD2-H4K8ac_peak_9532	11.19034	
15	40394672	40395546	KD2-H4K8ac_peak_9533	8.29	
15	40395798	40396004	KD2-H4K8ac_peak_9534	4.06758	
15	40401425	40401622	KD2-H4K8ac_peak_9535	6.55628	BMF_ENSG00000104081
15	40452573	40453022	KD2-H4K8ac_peak_9536	10.08707	BUB1B_ENSG00000156970
15	40453386	40453684	KD2-H4K8ac_peak_9537	4.211	BUB1B_ENSG00000156970
15	40530814	40531769	KD2-H4K8ac_peak_9538	9.11031	
15	40574165	40574385	KD2-H4K8ac_peak_9539	5.54647	ANKRD63_ENSG00000230778
15	40574689	40575192	KD2-H4K8ac_peak_9540	6.65673	ANKRD63_ENSG00000230778
15	40583218	40584042	KD2-H4K8ac_peak_9541	15.81711	
15	40594028	40594472	KD2-H4K8ac_peak_9542	7.07117	
15	40594733	40595027	KD2-H4K8ac_peak_9543	8.02571	
15	40616333	40616588	KD2-H4K8ac_peak_9544	4.67342	LINC00984_ENSG00000259330
15	40631926	40633481	KD2-H4K8ac_peak_9545	12.13241	C15orf52_ENSG00000188549
15	40649938	40650296	KD2-H4K8ac_peak_9546	10.74057	DISP2_ENSG00000140323
15	40660539	40660907	KD2-H4K8ac_peak_9547	6.1846	RP11-64K12.4_ENSG00000259368
15	40674734	40675006	KD2-H4K8ac_peak_9548	10.87093	KNSTRN_ENSG00000128944
15	40675205	40675652	KD2-H4K8ac_peak_9549	9.51625	KNSTRN_ENSG00000128944
15	40727933	40729442	KD2-H4K8ac_peak_9550	17.93813	
15	40733491	40733707	KD2-H4K8ac_peak_9551	4.86324	
15	40762597	40763012	KD2-H4K8ac_peak_9552	8.10216	CHST14_ENSG00000169105
15	40763387	40763939	KD2-H4K8ac_peak_9553	11.80283	CHST14_ENSG00000169105
15	40856850	40857086	KD2-H4K8ac_peak_9554	6.65673	C15orf57_ENSG00000128891
15	40886211	40886909	KD2-H4K8ac_peak_9555	14.81583	CASC5_ENSG00000137812
15	40987009	40987231	KD2-H4K8ac_peak_9556	15.69572	RAD51-AS1_ENSG00000245849;RAD51_ENSG00000051180
15	40987950	40988165	KD2-H4K8ac_peak_9557	3.87414	RAD51-AS1_ENSG00000245849;RAD51_ENSG00000051180
15	41047171	41047369	KD2-H4K8ac_peak_9558	8.17902	RMDN3_ENSG00000137824
15	41047642	41047942	KD2-H4K8ac_peak_9559	5.57044	RMDN3_ENSG00000137824
15	41049793	41050039	KD2-H4K8ac_peak_9560	8.2413	
15	41061012	41061967	KD2-H4K8ac_peak_9561	12.17278	C15orf62_ENSG00000188277
15	41098356	41098621	KD2-H4K8ac_peak_9562	4.47369	ZFYVE19_ENSG00000166140
15	41099198	41099609	KD2-H4K8ac_peak_9563	15.82743	DNAJC17_ENSG00000104129;ZFYVE19_ENSG00000166140
15	41135976	41136397	KD2-H4K8ac_peak_9564	6.2781	RP11-532F12.5_ENSG00000261183;SPINT1_ENSG00000166145
15	41149648	41150260	KD2-H4K8ac_peak_9565	7.47462	
15	41166059	41166516	KD2-H4K8ac_peak_9566	8.16906	RHOV_ENSG00000104140;AC025166.1_ENSG00000264256

15	41185885	41186267	KD2-H4K8ac_peak_9567	7.146	VPS18_ENSG00000104142
15	41198906	41199967	KD2-H4K8ac_peak_9568	11.80283	RP11-540O11.1_ENSG00000251161
15	41203581	41203856	KD2-H4K8ac_peak_9569	5.29555	
15	41233428	41234645	KD2-H4K8ac_peak_9570	16.8435	
15	41244551	41245413	KD2-H4K8ac_peak_9571	17.09549	CHAC1_ENSG00000128965
15	41408269	41408673	KD2-H4K8ac_peak_9572	10.45683	INO80_ENSG00000128908
15	41409077	41409389	KD2-H4K8ac_peak_9573	5.20998	INO80_ENSG00000128908
15	41522502	41523164	KD2-H4K8ac_peak_9574	5.405	EXD1_ENSG00000178997;CHP1_ENSG00000187446
15	41523478	41523701	KD2-H4K8ac_peak_9575	8.16906	EXD1_ENSG00000178997;CHP1_ENSG00000187446
15	41575530	41575885	KD2-H4K8ac_peak_9576	10.43145	OIP5-AS1_ENSG00000247556
15	41576259	41576519	KD2-H4K8ac_peak_9577	7.07117	OIP5-AS1_ENSG00000247556
15	41624488	41624751	KD2-H4K8ac_peak_9578	14.56955	OIP5_ENSG00000104147;NUSAP1_ENSG00000137804
15	41625116	41625360	KD2-H4K8ac_peak_9579	5.88845	OIP5_ENSG00000104147;NUSAP1_ENSG00000137804
15	41694977	41695270	KD2-H4K8ac_peak_9580	9.51625	NDUFAF1_ENSG00000137806
15	41709491	41709786	KD2-H4K8ac_peak_9581	8.10216	
15	41785492	41785877	KD2-H4K8ac_peak_9582	8.85813	ITPKA_ENSG00000137825
15	41836141	41836408	KD2-H4K8ac_peak_9583	9.20193	RPAP1_ENSG00000103932
15	41836772	41837066	KD2-H4K8ac_peak_9584	6.66692	RPAP1_ENSG00000103932
15	41851400	41851618	KD2-H4K8ac_peak_9585	9.16531	
15	41856637	41857137	KD2-H4K8ac_peak_9586	7.48822	
15	41913045	41914082	KD2-H4K8ac_peak_9587	5.01002	MGA_ENSG00000174197
15	41952135	41953339	KD2-H4K8ac_peak_9588	15.06766	
15	42066792	42067082	KD2-H4K8ac_peak_9589	11.50346	MAPKBP1_ENSG00000137802
15	42120317	42120578	KD2-H4K8ac_peak_9590	11.05467	RP11-23P13.4_ENSG00000250379;PLA2G4B_ENSG00000243708;JMJD7_ENSG00000243789;JMJD7-PLA2G4B_ENSG00000168970
15	42186357	42187560	KD2-H4K8ac_peak_9591	9.63487	SPTBN5_ENSG00000137877
15	42376874	42377197	KD2-H4K8ac_peak_9592	6.0073	
15	42500012	42500362	KD2-H4K8ac_peak_9593	7.95563	VPS39_ENSG00000166887;RP11-546B15.1_ENSG00000261002
15	42565872	42567096	KD2-H4K8ac_peak_9594	7.36799	TMEM87A_ENSG00000103978;GANC_ENSG00000214013
15	42782940	42783271	KD2-H4K8ac_peak_9595	12.70959	ZNF106_ENSG00000103994;SNAP23_ENSG00000092531
15	42787996	42788231	KD2-H4K8ac_peak_9596	8.02571	
15	42841104	42841395	KD2-H4K8ac_peak_9597	6.28103	LRRC57_ENSG00000180979;HAUS2_ENSG00000137814
15	42866841	42867203	KD2-H4K8ac_peak_9598	8.59579	STARD9_ENSG00000159433
15	43028884	43029203	KD2-H4K8ac_peak_9599	6.65673	CDAN1_ENSG00000140326
15	43029450	43029648	KD2-H4K8ac_peak_9600	11.05467	CDAN1_ENSG00000140326
15	43213467	43213776	KD2-H4K8ac_peak_9601	7.27716	TTBK2_ENSG00000128881
15	43397819	43398180	KD2-H4K8ac_peak_9602	8.59579	UBR1_ENSG00000159459
15	43425014	43426380	KD2-H4K8ac_peak_9603	10.19949	
15	43476940	43477225	KD2-H4K8ac_peak_9604	4.19473	RP11-473C18.3_ENSG00000261687;CCNDBP1_ENSG00000166946
15	43621904	43622386	KD2-H4K8ac_peak_9605	11.04256	LCMT2_ENSG00000168806;ADAL_ENSG00000168803
15	43662564	43663107	KD2-H4K8ac_peak_9606	13.34404	ZSCAN29_ENSG00000140265
15	43663371	43663581	KD2-H4K8ac_peak_9607	5.85291	ZSCAN29_ENSG00000140265
15	43800336	43800672	KD2-H4K8ac_peak_9608	4.42765	
15	43801491	43801945	KD2-H4K8ac_peak_9609	10.05606	TP53BP1_ENSG00000067369
15	43802568	43802810	KD2-H4K8ac_peak_9610	8.23274	TP53BP1_ENSG00000067369;MAP1A_ENSG00000166963
15	44037952	44038299	KD2-H4K8ac_peak_9611	6.60096	PDIA3_ENSG00000167004;CATSPER2P1_ENSG00000205771
15	44083757	44084189	KD2-H4K8ac_peak_9612	8.26945	

15	44084764	44084991	KD2-H4K8ac_peak_9613	8.4716	MIR1282_ENSG00000221792
15	44092085	44092525	KD2-H4K8ac_peak_9614	13.07235	RP11-296A16.1_ENSG00000262560;SERINC4_ENSG00000184716
15	44117455	44117752	KD2-H4K8ac_peak_9615	9.02573	MFAP1_ENSG00000140259
15	44118773	44119375	KD2-H4K8ac_peak_9616	9.16531	WDR76_ENSG00000092470
15	44195896	44196390	KD2-H4K8ac_peak_9617	8.17902	
15	44486725	44487114	KD2-H4K8ac_peak_9618	10.86378	FRMD5_ENSG00000171877
15	44487703	44488161	KD2-H4K8ac_peak_9619	8.10216	FRMD5_ENSG00000171877
15	44580951	44581261	KD2-H4K8ac_peak_9620	11.24702	CASC4_ENSG00000166734
15	44829370	44829933	KD2-H4K8ac_peak_9621	19.47291	EIF3J-AS1_ENSG00000179523;EIF3J_ENSG00000104131
15	44955171	44955727	KD2-H4K8ac_peak_9622	14.43794	SPG11_ENSG00000104133
15	44956065	44956640	KD2-H4K8ac_peak_9623	9.34195	SPG11_ENSG00000104133
15	45003295	45003502	KD2-H4K8ac_peak_9624	8.02571	PATL2_ENSG00000229474;B2M_ENSG00000166710
15	45004095	45004300	KD2-H4K8ac_peak_9625	8.17902	PATL2_ENSG00000229474;B2M_ENSG00000166710
15	45020546	45021014	KD2-H4K8ac_peak_9626	11.89502	TRIM69_ENSG00000185880
15	45021280	45021670	KD2-H4K8ac_peak_9627	17.43705	TRIM69_ENSG00000185880
15	45076386	45076919	KD2-H4K8ac_peak_9628	13.2739	
15	45114532	45114981	KD2-H4K8ac_peak_9629	5.88845	
15	45271650	45271947	KD2-H4K8ac_peak_9630	4.37708	
15	45275408	45275796	KD2-H4K8ac_peak_9631	7.12754	
15	45277401	45277607	KD2-H4K8ac_peak_9632	5.57044	
15	45314940	45315181	KD2-H4K8ac_peak_9633	13.35993	SORD_ENSG00000140263
15	45315429	45315718	KD2-H4K8ac_peak_9634	7.92775	SORD_ENSG00000140263
15	45369932	45370409	KD2-H4K8ac_peak_9635	7.70289	
15	45410013	45410308	KD2-H4K8ac_peak_9636	12.84642	
15	45458748	45459141	KD2-H4K8ac_peak_9637	17.8737	CTD-2651B20.1_ENSG00000259539
15	45459408	45460231	KD2-H4K8ac_peak_9638	8.80432	CTD-2651B20.1_ENSG00000259539
15	45460632	45461726	KD2-H4K8ac_peak_9639	10.74343	CTD-2651B20.1_ENSG00000259539;RP11-519G16.2_ENSG00000259519
15	45479295	45479516	KD2-H4K8ac_peak_9640	5.61067	
15	45492797	45493284	KD2-H4K8ac_peak_9641	13.57115	SHF_ENSG00000138606;CTD-2651B20.6_ENSG00000260035
15	45669961	45671015	KD2-H4K8ac_peak_9642	15.13261	
15	45694106	45694385	KD2-H4K8ac_peak_9643	6.85193	GATM_ENSG00000171766;SPATA5L1_ENSG00000171763
15	45694608	45695412	KD2-H4K8ac_peak_9644	11.28831	GATM_ENSG00000171766;SPATA5L1_ENSG00000171763
15	45814680	45815575	KD2-H4K8ac_peak_9645	15.59774	SLC30A4_ENSG00000104154
15	45878908	45879961	KD2-H4K8ac_peak_9646	4.84794	BLOC1S6_ENSG00000104164;RP11-96O20.4_ENSG00000260170
15	45926869	45927482	KD2-H4K8ac_peak_9647	8.83582	
15	45997508	45997721	KD2-H4K8ac_peak_9648	5.22731	RP11-718O11.1_ENSG00000259200
15	46373085	46373891	KD2-H4K8ac_peak_9649	12.42547	
15	46569995	46570458	KD2-H4K8ac_peak_9650	8.1233	
15	47476563	47477301	KD2-H4K8ac_peak_9651	17.17257	SEMA6D_ENSG00000137872
15	47477754	47477952	KD2-H4K8ac_peak_9652	7.13615	
15	47697283	47697533	KD2-H4K8ac_peak_9653	7.75499	
15	47703889	47704188	KD2-H4K8ac_peak_9654	7.27716	
15	48392818	48393092	KD2-H4K8ac_peak_9655	7.01964	
15	48470033	48470507	KD2-H4K8ac_peak_9656	14.80472	MYEF2_ENSG00000104177
15	48470710	48470920	KD2-H4K8ac_peak_9657	9.72244	MYEF2_ENSG00000104177
15	48623391	48624037	KD2-H4K8ac_peak_9658	13.73635	RP11-154J22.1_ENSG00000259488;DUT_ENSG00000128951

15	48829522	48829818	KD2-H4K8ac_peak_9659	5.14125	
15	48937595	48937824	KD2-H4K8ac_peak_9660	8.2413	FBN1_ENSG00000166147;RP11-227D13.1_ENSG00000259705
15	48938311	48938654	KD2-H4K8ac_peak_9661	16.64426	FBN1_ENSG00000166147;RP11-227D13.1_ENSG00000259705
15	49102841	49103150	KD2-H4K8ac_peak_9662	9.63487	CEP152_ENSG00000103995;RP11-485O10.3_ENSG00000259700
15	49170383	49170768	KD2-H4K8ac_peak_9663	11.1952	EID1_ENSG00000255302;AC012379.1_ENSG00000235883
15	49255419	49255916	KD2-H4K8ac_peak_9664	6.04107	SHC4_ENSG00000185634
15	49268258	49268908	KD2-H4K8ac_peak_9665	14.63458	
15	49338192	49338520	KD2-H4K8ac_peak_9666	5.59859	SECISBP2L_ENSG00000138593
15	49447543	49447745	KD2-H4K8ac_peak_9667	8.16906	NDUFAF4P1_ENSG00000259467;COPS2_ENSG00000166200;GALK2_ENSG00000156958
15	49462210	49462717	KD2-H4K8ac_peak_9668	8.17902	MIR4716_ENSG00000264210
15	49913301	49913514	KD2-H4K8ac_peak_9669	11.30637	FAM227B_ENSG00000166262;DTWD1_ENSG00000104047
15	50474251	50474531	KD2-H4K8ac_peak_9670	5.08628	ATP8B4_ENSG00000104043;SLC27A2_ENSG00000140284
15	50646431	50647989	KD2-H4K8ac_peak_9671	22.21705	GABPB1_ENSG00000104064;GABPB1-AS1_ENSG00000244879
15	50715735	50716073	KD2-H4K8ac_peak_9672	17.25154	USP8_ENSG00000138592
15	50716630	50716897	KD2-H4K8ac_peak_9673	11.43353	USP8_ENSG00000138592
15	50978575	50978920	KD2-H4K8ac_peak_9674	6.40297	TRPM7_ENSG00000092439
15	50979127	50979483	KD2-H4K8ac_peak_9675	11.22681	TRPM7_ENSG00000092439
15	51057487	51058402	KD2-H4K8ac_peak_9676	16.73922	SPPL2A_ENSG00000138600
15	51200078	51201341	KD2-H4K8ac_peak_9677	14.8083	AP4E1_ENSG00000081014
15	51914536	51915533	KD2-H4K8ac_peak_9678	10.65724	DMXL2_ENSG00000104093
15	51973313	51973692	KD2-H4K8ac_peak_9679	5.88845	SCG3_ENSG00000104112
15	52029592	52030313	KD2-H4K8ac_peak_9680	9.63487	
15	52043359	52043633	KD2-H4K8ac_peak_9681	6.99194	LYSMD2_ENSG00000140280;TMOD2_ENSG00000128872
15	52121253	52122465	KD2-H4K8ac_peak_9682	15.81711	TMOD3_ENSG00000138594
15	52263617	52263932	KD2-H4K8ac_peak_9683	6.0605	LEO1_ENSG00000166477
15	52264329	52264577	KD2-H4K8ac_peak_9684	6.66692	LEO1_ENSG00000166477
15	52310865	52311646	KD2-H4K8ac_peak_9685	16.54484	CTD-2650P22.1_ENSG00000259438
15	52319121	52319380	KD2-H4K8ac_peak_9686	4.40093	
15	52471643	52471849	KD2-H4K8ac_peak_9687	8.52559	RP11-430B1.2_ENSG00000259577
15	52472244	52472607	KD2-H4K8ac_peak_9688	8.4716	RP11-430B1.2_ENSG00000259577
15	52587416	52588008	KD2-H4K8ac_peak_9689	16.61426	MYO5C_ENSG00000128833
15	52820696	52821565	KD2-H4K8ac_peak_9690	11.24702	MYO5A_ENSG00000197535
15	52860793	52861841	KD2-H4K8ac_peak_9691	9.36516	ARPP19_ENSG00000128989
15	52970676	52971474	KD2-H4K8ac_peak_9692	4.06758	
15	53082689	53082941	KD2-H4K8ac_peak_9693	10.94736	ONECUT1_ENSG00000169856
15	54124215	54124700	KD2-H4K8ac_peak_9694	5.43522	
15	55488526	55488965	KD2-H4K8ac_peak_9695	4.54006	RSL24D1_ENSG00000137876
15	55581758	55582590	KD2-H4K8ac_peak_9696	11.60333	
15	55610712	55611748	KD2-H4K8ac_peak_9697	17.21106	RAB27A_ENSG00000069974;RP11-139H15.1_ENSG00000225973;PIGB_ENSG00000069943
15	55700845	55701355	KD2-H4K8ac_peak_9698	4.42765	CCPG1_ENSG00000260916;C15orf65_ENSG00000261652
15	55790924	55791576	KD2-H4K8ac_peak_9699	4.35724	DYX1C1-CCPG1_ENSG00000261771
15	55879746	55881348	KD2-H4K8ac_peak_9700	16.07006	PYGO1_ENSG00000171016
15	55960047	55960249	KD2-H4K8ac_peak_9701	6.40683	
15	55962703	55962950	KD2-H4K8ac_peak_9702	7.12754	
15	56034766	56035722	KD2-H4K8ac_peak_9703	16.49944	PRTG_ENSG00000166450
15	56076902	56077342	KD2-H4K8ac_peak_9704	10.1591	

15	56078729	56078961	KD2-H4K8ac_peak_9705	10.34425	
15	56091485	56091859	KD2-H4K8ac_peak_9706	7.44366	
15	56111293	56111500	KD2-H4K8ac_peak_9707	6.31143	
15	56285350	56285722	KD2-H4K8ac_peak_9708	6.66692	NEDD4_ENSG00000069869
15	56535606	56536572	KD2-H4K8ac_peak_9709	17.566	RFX7_ENSG00000181827;TEX9_ENSG00000151575
15	57025153	57026214	KD2-H4K8ac_peak_9710	20.02161	
15	57041679	57041900	KD2-H4K8ac_peak_9711	5.25071	
15	57179108	57179330	KD2-H4K8ac_peak_9712	8.55271	
15	57179570	57180282	KD2-H4K8ac_peak_9713	8.23274	
15	57210302	57210663	KD2-H4K8ac_peak_9714	9.20193	ZNF280D_ENSG00000137871;TCF12_ENSG00000140262
15	57210889	57211431	KD2-H4K8ac_peak_9715	10.77651	ZNF280D_ENSG00000137871;TCF12_ENSG00000140262
15	57211661	57212312	KD2-H4K8ac_peak_9716	5.88845	ZNF280D_ENSG00000137871;TCF12_ENSG00000140262
15	57599084	57599282	KD2-H4K8ac_peak_9717	7.76329	
15	57668348	57668904	KD2-H4K8ac_peak_9718	11.92978	CGNL1_ENSG00000128849
15	57680065	57680384	KD2-H4K8ac_peak_9719	5.01002	
15	57750827	57751032	KD2-H4K8ac_peak_9720	8.94415	
15	57788987	57789436	KD2-H4K8ac_peak_9721	9.72244	
15	57811933	57812128	KD2-H4K8ac_peak_9722	5.61377	
15	57884207	57884525	KD2-H4K8ac_peak_9723	16.09057	GCOM1_ENSG00000137878;MYZAP_ENSG00000263155;POLR2M_ENSG00000255529
15	57918285	57918482	KD2-H4K8ac_peak_9724	4.14296	
15	57988506	57988752	KD2-H4K8ac_peak_9725	7.13615	
15	57998140	57998381	KD2-H4K8ac_peak_9726	6.56211	
15	57998922	57999401	KD2-H4K8ac_peak_9727	17.38513	
15	58357692	58357952	KD2-H4K8ac_peak_9728	5.61067	CTD-2330J20.2_ENSG00000259285
15	58624159	58624353	KD2-H4K8ac_peak_9729	4.42765	
15	58624572	58625260	KD2-H4K8ac_peak_9730	8.5781	
15	58856957	58857296	KD2-H4K8ac_peak_9731	4.9025	
15	59063557	59064119	KD2-H4K8ac_peak_9732	7.0754	RP11-30K9.6_ENSG00000245975;FAM63B_ENSG00000128923
15	59225273	59225726	KD2-H4K8ac_peak_9733	12.30509	SLTM_ENSG00000137776
15	59226096	59226586	KD2-H4K8ac_peak_9734	6.36375	SLTM_ENSG00000137776
15	59397388	59397700	KD2-H4K8ac_peak_9735	10.68431	CCNB2_ENSG00000157456
15	59463337	59463555	KD2-H4K8ac_peak_9736	6.65673	MIR2116_ENSG00000253030
15	59663995	59665116	KD2-H4K8ac_peak_9737	10.2048	MYO1E_ENSG00000157483;FAM81A_ENSG00000157470
15	59981130	59981403	KD2-H4K8ac_peak_9738	5.94405	RP11-361D15.2_ENSG00000259238;BNIP2_ENSG00000140299
15	59994134	59994395	KD2-H4K8ac_peak_9739	7.12754	
15	60158246	60158493	KD2-H4K8ac_peak_9740	7.27716	
15	60159570	60159816	KD2-H4K8ac_peak_9741	5.25071	
15	60651683	60651945	KD2-H4K8ac_peak_9742	8.41122	
15	60666140	60666351	KD2-H4K8ac_peak_9743	8.70735	
15	60666949	60667187	KD2-H4K8ac_peak_9744	10.19949	
15	60770962	60771322	KD2-H4K8ac_peak_9745	13.50024	NARG2_ENSG00000128915;RP11-219B17.1_ENSG00000245534
15	60884267	60884553	KD2-H4K8ac_peak_9746	5.20998	
15	60884882	60885171	KD2-H4K8ac_peak_9747	7.07117	
15	61126859	61127073	KD2-H4K8ac_peak_9748	5.57044	
15	61494320	61494545	KD2-H4K8ac_peak_9749	6.75488	
15	61496183	61496424	KD2-H4K8ac_peak_9750	7.11925	

15	61521693	61522077	KD2-H4K8ac_peak_9751	9.11031	RORA_ENSG00000069667
15	62206617	62206934	KD2-H4K8ac_peak_9752	4.9025	RN7SL613P_ENSG00000264376
15	62207422	62207623	KD2-H4K8ac_peak_9753	5.88845	RN7SL613P_ENSG00000264376
15	62360410	62361011	KD2-H4K8ac_peak_9754	17.8868	
15	62403279	62403779	KD2-H4K8ac_peak_9755	7.76329	
15	62681641	62681907	KD2-H4K8ac_peak_9756	6.03693	TLN2_ENSG00000171914
15	62682219	62682936	KD2-H4K8ac_peak_9757	13.12288	TLN2_ENSG00000171914
15	62917309	62917750	KD2-H4K8ac_peak_9758	9.11031	
15	62917969	62918209	KD2-H4K8ac_peak_9759	4.01115	
15	63074636	63074875	KD2-H4K8ac_peak_9760	9.31907	
15	63221276	63221468	KD2-H4K8ac_peak_9761	6.00226	
15	63227540	63227858	KD2-H4K8ac_peak_9762	5.29555	
15	63340747	63341104	KD2-H4K8ac_peak_9763	7.0754	RP11-244F12.3_ENSG00000259498
15	63413483	63413848	KD2-H4K8ac_peak_9764	3.87414	LACTB_ENSG00000103642
15	63449828	63450308	KD2-H4K8ac_peak_9765	11.66477	RPS27L_ENSG00000185088
15	63481132	63481553	KD2-H4K8ac_peak_9766	16.32216	RAB8B_ENSG00000166128
15	63569236	63569496	KD2-H4K8ac_peak_9767	6.0073	
15	63569829	63570177	KD2-H4K8ac_peak_9768	12.06346	
15	63728965	63729635	KD2-H4K8ac_peak_9769	7.76104	
15	63796070	63796377	KD2-H4K8ac_peak_9770	5.4779	USP3_ENSG00000140455
15	63796857	63797670	KD2-H4K8ac_peak_9771	17.75427	USP3_ENSG00000140455
15	63892731	63893004	KD2-H4K8ac_peak_9772	3.86308	USP3-AS1_ENSG00000259248
15	63893276	63893692	KD2-H4K8ac_peak_9773	7.76104	USP3-AS1_ENSG00000259248
15	64126297	64126800	KD2-H4K8ac_peak_9774	14.15383	HERC1_ENSG00000103657
15	64237756	64238454	KD2-H4K8ac_peak_9775	7.76329	
15	64386409	64386964	KD2-H4K8ac_peak_9776	10.99365	FAM96A_ENSG00000166797;SNX1_ENSG00000028528
15	64388324	64388575	KD2-H4K8ac_peak_9777	9.96798	
15	64439432	64439979	KD2-H4K8ac_peak_9778	12.61258	
15	64647810	64648315	KD2-H4K8ac_peak_9779	7.67504	CSNK1G1_ENSG00000169118
15	65067064	65067556	KD2-H4K8ac_peak_9780	10.78136	RBPMS2_ENSG00000166831
15	65068047	65068311	KD2-H4K8ac_peak_9781	10.70006	RBPMS2_ENSG00000166831
15	65104651	65104939	KD2-H4K8ac_peak_9782	6.30229	
15	65105768	65106005	KD2-H4K8ac_peak_9783	8.30974	
15	65118493	65118714	KD2-H4K8ac_peak_9784	4.37709	PIF1_ENSG00000140451
15	65132985	65133833	KD2-H4K8ac_peak_9785	16.09057	AC069368.3_ENSG00000249240;PLEKHO2_ENSG00000241839
15	65149302	65149510	KD2-H4K8ac_peak_9786	10.29344	
15	65203657	65203852	KD2-H4K8ac_peak_9787	5.9696	ANKDD1A_ENSG00000166839
15	65281622	65282175	KD2-H4K8ac_peak_9788	20.96279	SPG21_ENSG00000090487
15	65284172	65284369	KD2-H4K8ac_peak_9789	6.16888	
15	65321391	65321892	KD2-H4K8ac_peak_9790	6.66692	MTFMT_ENSG00000103707
15	65359990	65360586	KD2-H4K8ac_peak_9791	8.86391	
15	65425353	65426086	KD2-H4K8ac_peak_9792	10.96455	PDCD7_ENSG00000090470
15	65426409	65426914	KD2-H4K8ac_peak_9793	6.98292	PDCD7_ENSG00000090470
15	65477369	65478142	KD2-H4K8ac_peak_9794	13.81781	CLPX_ENSG00000166855
15	65578496	65578880	KD2-H4K8ac_peak_9795	8.55271	SNORA24_ENSG00000206903
15	65595951	65596481	KD2-H4K8ac_peak_9796	6.0073	RNU5B-1_ENSG00000200156

15	65626579	65627136	KD2-H4K8ac_peak_9797	8.53092	
15	65628870	65629798	KD2-H4K8ac_peak_9798	10.05606	
15	65638844	65639185	KD2-H4K8ac_peak_9799	9.05418	
15	65660865	65661169	KD2-H4K8ac_peak_9800	4.86324	
15	65662096	65662314	KD2-H4K8ac_peak_9801	4.12757	
15	65669630	65670236	KD2-H4K8ac_peak_9802	7.76329	IGDCC3_ENSG00000174498
15	65677389	65678027	KD2-H4K8ac_peak_9803	11.52628	
15	65714165	65714360	KD2-H4K8ac_peak_9804	6.25869	
15	65809702	65810008	KD2-H4K8ac_peak_9805	11.80283	DPP8_ENSG00000074603
15	65810286	65810637	KD2-H4K8ac_peak_9806	5.20908	DPP8_ENSG00000074603
15	65822943	65823424	KD2-H4K8ac_peak_9807	15.82362	PTPLAD1_ENSG00000074696
15	65871782	65872010	KD2-H4K8ac_peak_9808	8.93112	
15	65873507	65873724	KD2-H4K8ac_peak_9809	12.20507	
15	65902523	65902721	KD2-H4K8ac_peak_9810	7.79558	VWA9_ENSG00000138614;SLC24A1_ENSG00000074621
15	65902927	65903443	KD2-H4K8ac_peak_9811	18.5703	VWA9_ENSG00000138614;SLC24A1_ENSG00000074621
15	66125233	66125494	KD2-H4K8ac_peak_9812	9.1707	
15	66161883	66162232	KD2-H4K8ac_peak_9813	8.28234	
15	66246851	66247185	KD2-H4K8ac_peak_9814	6.14257	
15	66430006	66430585	KD2-H4K8ac_peak_9815	5.20908	
15	66431005	66431472	KD2-H4K8ac_peak_9816	5.28072	
15	66470936	66471154	KD2-H4K8ac_peak_9817	5.57044	
15	66504647	66505009	KD2-H4K8ac_peak_9818	7.70289	
15	66523550	66523781	KD2-H4K8ac_peak_9819	6.40683	
15	66585694	66586675	KD2-H4K8ac_peak_9820	10.5953	RP11-653J6.1_ENSG00000261318;DIS3L_ENSG00000166938
15	66788960	66789251	KD2-H4K8ac_peak_9821	4.66652	SNAPC5_ENSG00000174446;MIR4512_ENSG00000266589
15	66796891	66797717	KD2-H4K8ac_peak_9822	9.36516	ZWILCH_ENSG00000174442
15	66922959	66923592	KD2-H4K8ac_peak_9823	8.4716	
15	66924024	66924301	KD2-H4K8ac_peak_9824	7.76329	
15	66924641	66924843	KD2-H4K8ac_peak_9825	7.69287	
15	66925286	66926058	KD2-H4K8ac_peak_9826	8.55271	
15	66939709	66940058	KD2-H4K8ac_peak_9827	8.59579	
15	66971590	66972210	KD2-H4K8ac_peak_9828	12.10284	
15	66992920	66993368	KD2-H4K8ac_peak_9829	14.70804	
15	67000076	67000284	KD2-H4K8ac_peak_9830	5.88845	
15	67065185	67065401	KD2-H4K8ac_peak_9831	6.89708	
15	67066352	67066676	KD2-H4K8ac_peak_9832	8.34752	
15	67113481	67113686	KD2-H4K8ac_peak_9833	4.91674	
15	67134659	67135418	KD2-H4K8ac_peak_9834	12.06346	
15	67142944	67143270	KD2-H4K8ac_peak_9835	4.42765	
15	67228633	67228963	KD2-H4K8ac_peak_9836	8.30974	
15	67318179	67318392	KD2-H4K8ac_peak_9837	8.17902	
15	67357190	67357644	KD2-H4K8ac_peak_9838	9.63487	
15	67358205	67358520	KD2-H4K8ac_peak_9839	7.47462	
15	67358913	67359138	KD2-H4K8ac_peak_9840	6.25869	
15	67413595	67413829	KD2-H4K8ac_peak_9841	5.14125	
15	67481848	67482068	KD2-H4K8ac_peak_9842	9.31907	

15	67813051	67813284	KD2-H4K8ac_peak_9843	7.96322	IQCH-AS1_ENSG00000259673;C15orf61_ENSG00000189227
15	67813621	67814132	KD2-H4K8ac_peak_9844	7.76329	IQCH-AS1_ENSG00000259673;C15orf61_ENSG00000189227
15	67835067	67835761	KD2-H4K8ac_peak_9845	15.22643	RP11-502I4.3_ENSG00000270964;MAP2K5_ENSG00000137764
15	68125983	68126220	KD2-H4K8ac_peak_9846	7.36799	RP11-34F13.3_ENSG00000259410
15	68155958	68156257	KD2-H4K8ac_peak_9847	8.10216	
15	68176740	68177426	KD2-H4K8ac_peak_9848	8.94469	
15	68260053	68260617	KD2-H4K8ac_peak_9849	9.96798	
15	68260862	68261090	KD2-H4K8ac_peak_9850	4.01115	
15	68491792	68492041	KD2-H4K8ac_peak_9851	5.32137	
15	68502731	68503148	KD2-H4K8ac_peak_9852	4.98318	
15	68521594	68521993	KD2-H4K8ac_peak_9853	13.56049	RP11-315D16.2_ENSG00000260007;AC107871.1_ENSG00000264779
15	68569406	68570643	KD2-H4K8ac_peak_9854	18.31034	RP11-315D16.4_ENSG00000260657;FEM1B_ENSG00000169018
15	68598798	68598993	KD2-H4K8ac_peak_9855	6.85193	
15	68599346	68599570	KD2-H4K8ac_peak_9856	5.29555	
15	68891054	68891450	KD2-H4K8ac_peak_9857	5.9696	
15	68993018	68993429	KD2-H4K8ac_peak_9858	5.75452	
15	69109617	69109848	KD2-H4K8ac_peak_9859	6.37652	SPESP1_ENSG00000258484
15	69110079	69112835	KD2-H4K8ac_peak_9860	20.88898	ANP32A_ENSG00000140350;SPESP1_ENSG00000258484
15	69323767	69324032	KD2-H4K8ac_peak_9861	8.30974	
15	69366457	69367429	KD2-H4K8ac_peak_9862	8.30974	
15	69373733	69374088	KD2-H4K8ac_peak_9863	8.17902	RP11-809H16.5_ENSG00000259222
15	69453031	69453262	KD2-H4K8ac_peak_9864	10.53377	AC026512.1_ENSG00000266374
15	69590905	69591124	KD2-H4K8ac_peak_9865	5.9696	RP11-352D13.6_ENSG00000261634;PAQR5_ENSG00000137819
15	69754682	69755004	KD2-H4K8ac_peak_9866	7.76104	RP11-253M7.4_ENSG00000259215;RP11-279F6.1_ENSG00000245750
15	69853307	69853709	KD2-H4K8ac_peak_9867	5.55317	
15	70168405	70168700	KD2-H4K8ac_peak_9868	6.99815	
15	70169188	70169417	KD2-H4K8ac_peak_9869	9.11031	
15	70173706	70173921	KD2-H4K8ac_peak_9870	4.77079	
15	70354197	70354391	KD2-H4K8ac_peak_9871	6.13909	
15	70388732	70389153	KD2-H4K8ac_peak_9872	3.86308	
15	70389381	70389754	KD2-H4K8ac_peak_9873	9.36516	TLE3_ENSG00000140332
15	70390979	70391848	KD2-H4K8ac_peak_9874	13.80019	TLE3_ENSG00000140332
15	70392651	70393064	KD2-H4K8ac_peak_9875	7.67504	
15	70444513	70444908	KD2-H4K8ac_peak_9876	4.42765	
15	70446200	70446499	KD2-H4K8ac_peak_9877	14.98671	
15	70446808	70447250	KD2-H4K8ac_peak_9878	12.69604	
15	70457649	70457853	KD2-H4K8ac_peak_9879	5.9696	
15	70462669	70462909	KD2-H4K8ac_peak_9880	8.52559	
15	70478026	70479193	KD2-H4K8ac_peak_9881	17.43705	
15	70488043	70489008	KD2-H4K8ac_peak_9882	17.19888	
15	70491235	70491677	KD2-H4K8ac_peak_9883	6.8108	AC011648.1_ENSG00000259252
15	70540621	70541058	KD2-H4K8ac_peak_9884	6.67092	
15	70543363	70544113	KD2-H4K8ac_peak_9885	6.60096	
15	70544682	70545097	KD2-H4K8ac_peak_9886	6.66692	
15	70556050	70556273	KD2-H4K8ac_peak_9887	12.86466	
15	70565433	70565670	KD2-H4K8ac_peak_9888	9.5326	

15	70593241	70593485	KD2-H4K8ac_peak_9889	4.37708	
15	70615577	70615930	KD2-H4K8ac_peak_9890	7.22457	
15	70616867	70617153	KD2-H4K8ac_peak_9891	6.68439	
15	70629308	70629632	KD2-H4K8ac_peak_9892	6.31143	
15	70632744	70633349	KD2-H4K8ac_peak_9893	9.402	
15	70634022	70634601	KD2-H4K8ac_peak_9894	8.90871	
15	70649898	70650180	KD2-H4K8ac_peak_9895	6.40683	
15	70650423	70650680	KD2-H4K8ac_peak_9896	6.56926	
15	70666757	70666984	KD2-H4K8ac_peak_9897	4.98072	
15	70667355	70668070	KD2-H4K8ac_peak_9898	6.30229	
15	70668417	70668766	KD2-H4K8ac_peak_9899	4.44923	
15	70669755	70669990	KD2-H4K8ac_peak_9900	12.84642	
15	70670740	70671160	KD2-H4K8ac_peak_9901	6.99815	
15	70677420	70677679	KD2-H4K8ac_peak_9902	6.81873	
15	70677939	70678486	KD2-H4K8ac_peak_9903	9.63487	
15	70679569	70680589	KD2-H4K8ac_peak_9904	9.1707	
15	70726472	70726719	KD2-H4K8ac_peak_9905	4.10597	
15	70770885	70771148	KD2-H4K8ac_peak_9906	6.83936	
15	70818849	70819884	KD2-H4K8ac_peak_9907	8.59579	
15	70850007	70850220	KD2-H4K8ac_peak_9908	5.57044	
15	70851634	70852038	KD2-H4K8ac_peak_9909	5.6755	
15	70898059	70898581	KD2-H4K8ac_peak_9910	7.76329	
15	71146231	71146460	KD2-H4K8ac_peak_9911	11.24702	LARP6_ENSG00000166173;LRRC49_ENSG00000137821
15	71146662	71146897	KD2-H4K8ac_peak_9912	13.70605	LARP6_ENSG00000166173
15	71184375	71184714	KD2-H4K8ac_peak_9913	10.87093	THAP10_ENSG00000129028
15	71184920	71185374	KD2-H4K8ac_peak_9914	6.65673	THAP10_ENSG00000129028
15	72448076	72448479	KD2-H4K8ac_peak_9915	4.86324	RP11-2I17.4_ENSG00000260173
15	72489547	72489757	KD2-H4K8ac_peak_9916	4.66652	GRAMD2_ENSG00000175318
15	72522416	72522704	KD2-H4K8ac_peak_9917	13.9234	
15	72523873	72524160	KD2-H4K8ac_peak_9918	8.10216	PKM_ENSG00000067225
15	72564528	72564874	KD2-H4K8ac_peak_9919	14.69247	PARP6_ENSG00000137817
15	72667871	72668266	KD2-H4K8ac_peak_9920	11.92978	RP11-106M3.2_ENSG00000260729;HEXA_ENSG00000213614;HEXA-AS1_ENSG00000260339
15	72668521	72669178	KD2-H4K8ac_peak_9921	6.40297	RP11-106M3.2_ENSG00000260729;HEXA_ENSG00000213614;HEXA-AS1_ENSG00000260339
15	72766107	72766500	KD2-H4K8ac_peak_9922	13.73635	ARIH1_ENSG00000166233
15	72766713	72767120	KD2-H4K8ac_peak_9923	9.96798	RP11-1007O24.3_ENSG00000261423;ARIH1_ENSG00000166233
15	72930206	72930452	KD2-H4K8ac_peak_9924	7.12754	RP11-1006G14.2_ENSG00000259783;RP11-1006G14.3_ENSG00000259909
15	72963080	72963362	KD2-H4K8ac_peak_9925	11.05407	
15	72978211	72978498	KD2-H4K8ac_peak_9926	13.34404	HIGD2B_ENSG00000175202;BBS4_ENSG00000140463
15	73075682	73076020	KD2-H4K8ac_peak_9927	6.66359	ADPGK-AS1_ENSG00000260898
15	73076338	73076593	KD2-H4K8ac_peak_9928	7.67504	
15	73077364	73077556	KD2-H4K8ac_peak_9929	6.56926	ADPGK_ENSG00000159322
15	73088856	73089871	KD2-H4K8ac_peak_9930	10.05606	
15	73343782	73344322	KD2-H4K8ac_peak_9931	7.76104	NEO1_ENSG00000067141
15	73344947	73345372	KD2-H4K8ac_peak_9932	7.22457	NEO1_ENSG00000067141
15	73926049	73926669	KD2-H4K8ac_peak_9933	5.08628	NPTN_ENSG00000156642
15	73976116	73977187	KD2-H4K8ac_peak_9934	26.59781	CD276_ENSG00000103855

15	74044704	74044958	KD2-H4K8ac_peak_9935	6.56211	C15orf59_ENSG00000205363
15	74067428	74067625	KD2-H4K8ac_peak_9936	6.47962	
15	74231532	74231883	KD2-H4K8ac_peak_9937	5.54647	
15	74236380	74237079	KD2-H4K8ac_peak_9938	12.82393	
15	74238196	74238529	KD2-H4K8ac_peak_9939	4.211	
15	74242847	74243142	KD2-H4K8ac_peak_9940	8.28234	
15	74243350	74243593	KD2-H4K8ac_peak_9941	9.95119	
15	74243926	74245239	KD2-H4K8ac_peak_9942	13.80019	
15	74265145	74265947	KD2-H4K8ac_peak_9943	11.24702	
15	74275098	74275544	KD2-H4K8ac_peak_9944	7.70289	
15	74284845	74285165	KD2-H4K8ac_peak_9945	9.1707	
15	74286621	74286917	KD2-H4K8ac_peak_9946	7.22936	STOML1_ENSG00000067221;PML_ENSG00000140464
15	74287489	74287698	KD2-H4K8ac_peak_9947	6.53915	STOML1_ENSG00000067221;PML_ENSG00000140464
15	74392370	74392566	KD2-H4K8ac_peak_9948	8.55271	ISLR2_ENSG00000167178
15	74420515	74422280	KD2-H4K8ac_peak_9949	16.57619	RP11-247C2.2_ENSG00000248540
15	74422751	74423298	KD2-H4K8ac_peak_9950	9.20502	
15	74428446	74428718	KD2-H4K8ac_peak_9951	7.04698	
15	74429075	74429496	KD2-H4K8ac_peak_9952	16.43913	
15	74431253	74431475	KD2-H4K8ac_peak_9953	5.82799	
15	74466213	74466690	KD2-H4K8ac_peak_9954	3.90565	ISLR_ENSG00000129009
15	74466904	74468463	KD2-H4K8ac_peak_9955	9.62006	ISLR_ENSG00000129009
15	74468677	74469089	KD2-H4K8ac_peak_9956	8.73935	
15	74472138	74472380	KD2-H4K8ac_peak_9957	5.00593	RP11-665J16.1_ENSG00000261543
15	74492080	74492444	KD2-H4K8ac_peak_9958	4.42643	
15	74493332	74494086	KD2-H4K8ac_peak_9959	11.23023	RP11-60L3.1_ENSG00000259264
15	74494301	74495360	KD2-H4K8ac_peak_9960	13.95627	RP11-60L3.1_ENSG00000259264
15	74495630	74495916	KD2-H4K8ac_peak_9961	9.91109	RP11-60L3.1_ENSG00000259264
15	74496162	74497103	KD2-H4K8ac_peak_9962	10.33736	
15	74514059	74515051	KD2-H4K8ac_peak_9963	7.49599	
15	74515309	74515868	KD2-H4K8ac_peak_9964	10.19949	
15	74516237	74516918	KD2-H4K8ac_peak_9965	21.50391	
15	74518125	74519460	KD2-H4K8ac_peak_9966	10.97565	
15	74519721	74520033	KD2-H4K8ac_peak_9967	6.25869	
15	74520284	74520978	KD2-H4K8ac_peak_9968	7.76329	
15	74521453	74521925	KD2-H4K8ac_peak_9969	7.76104	
15	74526200	74526814	KD2-H4K8ac_peak_9970	7.76329	
15	74531497	74532080	KD2-H4K8ac_peak_9971	5.75757	
15	74532964	74533967	KD2-H4K8ac_peak_9972	6.25869	
15	74534506	74534985	KD2-H4K8ac_peak_9973	6.20982	
15	74535227	74535438	KD2-H4K8ac_peak_9974	7.36799	
15	74537543	74537766	KD2-H4K8ac_peak_9975	14.4458	
15	74543396	74543934	KD2-H4K8ac_peak_9976	8.30974	
15	74551424	74551807	KD2-H4K8ac_peak_9977	6.99815	
15	74568727	74569261	KD2-H4K8ac_peak_9978	5.66317	
15	74575503	74575776	KD2-H4K8ac_peak_9979	9.95119	
15	74581661	74582060	KD2-H4K8ac_peak_9980	5.30799	

15	74582549	74583183	KD2-H4K8ac_peak_9981	8.58619	
15	74584189	74585267	KD2-H4K8ac_peak_9982	13.81781	
15	74585867	74586385	KD2-H4K8ac_peak_9983	16.3889	
15	74586814	74587306	KD2-H4K8ac_peak_9984	6.99815	
15	74589195	74590426	KD2-H4K8ac_peak_9985	8.30974	
15	74591423	74591622	KD2-H4K8ac_peak_9986	9.03107	
15	74593350	74593854	KD2-H4K8ac_peak_9987	11.80002	
15	74594463	74595195	KD2-H4K8ac_peak_9988	8.88082	RP11-60L3.2_ENSG00000261384
15	74595492	74596213	KD2-H4K8ac_peak_9989	11.55163	RP11-60L3.2_ENSG00000261384
15	74597059	74597316	KD2-H4K8ac_peak_9990	8.82006	
15	74599263	74599504	KD2-H4K8ac_peak_9991	4.211	
15	74600805	74601242	KD2-H4K8ac_peak_9992	5.30799	
15	74604427	74604723	KD2-H4K8ac_peak_9993	6.0605	
15	74605157	74605608	KD2-H4K8ac_peak_9994	6.82618	
15	74610615	74611104	KD2-H4K8ac_peak_9995	9.62006	
15	74613241	74613507	KD2-H4K8ac_peak_9996	10.19949	
15	74615007	74615374	KD2-H4K8ac_peak_9997	19.7067	
15	74667062	74667276	KD2-H4K8ac_peak_9998	4.56666	
15	74725822	74726131	KD2-H4K8ac_peak_9999	5.92591	SEMA7A_ENSG00000138623
15	74726479	74726703	KD2-H4K8ac_peak_10000	4.211	SEMA7A_ENSG00000138623
15	74753189	74753401	KD2-H4K8ac_peak_10001	5.57044	UBL7_ENSG00000138629;UBL7-AS1_ENSG00000247240
15	74753674	74754012	KD2-H4K8ac_peak_10002	7.27716	UBL7_ENSG00000138629;UBL7-AS1_ENSG00000247240
15	74832896	74833210	KD2-H4K8ac_peak_10003	5.54647	ARID3B_ENSG00000179361
15	74833590	74833832	KD2-H4K8ac_peak_10004	12.93209	ARID3B_ENSG00000179361
15	74834105	74834325	KD2-H4K8ac_peak_10005	9.402	ARID3B_ENSG00000179361
15	74890931	74891371	KD2-H4K8ac_peak_10006	10.43145	CLK3_ENSG00000179335
15	74906642	74907020	KD2-H4K8ac_peak_10007	9.08816	CTD-3154N5.1_ENSG00000260919
15	74907317	74907936	KD2-H4K8ac_peak_10008	18.39704	CTD-3154N5.1_ENSG00000260919
15	74908286	74908719	KD2-H4K8ac_peak_10009	7.90806	CTD-3154N5.1_ENSG00000260919
15	74988006	74988917	KD2-H4K8ac_peak_10010	13.35993	EDC3_ENSG00000179151
15	75018388	75018813	KD2-H4K8ac_peak_10011	7.146	CYP1A1_ENSG00000140465
15	75074504	75075099	KD2-H4K8ac_peak_10012	11.89502	CSK_ENSG00000103653
15	75135114	75135449	KD2-H4K8ac_peak_10013	13.3695	ULK3_ENSG00000140474
15	75135690	75135979	KD2-H4K8ac_peak_10014	11.00372	ULK3_ENSG00000140474
15	75165445	75165668	KD2-H4K8ac_peak_10015	9.35804	SCAMP2_ENSG00000140497
15	75165898	75166237	KD2-H4K8ac_peak_10016	8.55552	SCAMP2_ENSG00000140497
15	75166498	75166697	KD2-H4K8ac_peak_10017	6.40683	SCAMP2_ENSG00000140497
15	75181414	75182961	KD2-H4K8ac_peak_10018	12.84642	MPI_ENSG00000178802
15	75199434	75199663	KD2-H4K8ac_peak_10019	7.36799	FAM219B_ENSG00000178761
15	75230728	75231181	KD2-H4K8ac_peak_10020	18.95762	COX5A_ENSG00000178741
15	75248153	75248535	KD2-H4K8ac_peak_10021	8.28234	
15	75248741	75250063	KD2-H4K8ac_peak_10022	19.35989	RPP25_ENSG00000178718;SCAMP5_ENSG00000198794
15	75287924	75288552	KD2-H4K8ac_peak_10023	11.58876	
15	75315980	75316294	KD2-H4K8ac_peak_10024	11.89502	RP11-151H2.1_ENSG00000260483;PPCDC_ENSG00000138621
15	75339062	75340843	KD2-H4K8ac_peak_10025	16.3486	
15	75471568	75471976	KD2-H4K8ac_peak_10026	6.66692	

15	75472754	75473224	KD2-H4K8ac_peak_10027	11.68645	
15	75487985	75488373	KD2-H4K8ac_peak_10028	12.30509	C15orf39_ENSG00000167173
15	75570865	75571058	KD2-H4K8ac_peak_10029	6.99815	
15	75628423	75628873	KD2-H4K8ac_peak_10030	11.50346	COMMD4_ENSG00000140365
15	75639660	75639898	KD2-H4K8ac_peak_10031	6.40297	NEIL1_ENSG00000140398
15	75640200	75640690	KD2-H4K8ac_peak_10032	7.43893	NEIL1_ENSG00000140398
15	75660494	75660917	KD2-H4K8ac_peak_10033	14.68449	MAN2C1_ENSG00000140400;RP11-817O13.8_ENSG00000260274
15	75746001	75746293	KD2-H4K8ac_peak_10034	10.44656	
15	75746696	75747205	KD2-H4K8ac_peak_10035	9.43437	SIN3A_ENSG00000169375
15	75870667	75871225	KD2-H4K8ac_peak_10036	6.0605	PTPN9_ENSG00000169410
15	75917064	75918029	KD2-H4K8ac_peak_10037	23.27293	SNUPN_ENSG00000169371;CTD-2026K11.3_ENSG00000260235
15	75918278	75918764	KD2-H4K8ac_peak_10038	7.76104	SNUPN_ENSG00000169371
15	75931993	75932486	KD2-H4K8ac_peak_10039	9.96798	
15	75940250	75940891	KD2-H4K8ac_peak_10040	5.9696	IMP3_ENSG00000177971;SNX33_ENSG00000173548
15	75976569	75976774	KD2-H4K8ac_peak_10041	7.52168	
15	75986098	75986689	KD2-H4K8ac_peak_10042	7.76104	
15	75987340	75987776	KD2-H4K8ac_peak_10043	5.72327	
15	75991934	75992487	KD2-H4K8ac_peak_10044	11.89502	
15	75993255	75994017	KD2-H4K8ac_peak_10045	9.96798	
15	75998417	75998770	KD2-H4K8ac_peak_10046	5.61067	
15	76001610	76001988	KD2-H4K8ac_peak_10047	7.47462	
15	76002502	76003073	KD2-H4K8ac_peak_10048	8.30974	
15	76030643	76030880	KD2-H4K8ac_peak_10049	8.10216	DNM1P35_ENSG00000246877;RP11-24M17.4_ENSG00000260288;AC019294.1_ENSG00000256530
15	76031190	76031692	KD2-H4K8ac_peak_10050	12.14909	DNM1P35_ENSG00000246877;AC019294.1_ENSG00000256530
15	76050635	76051437	KD2-H4K8ac_peak_10051	9.63487	
15	76078748	76078993	KD2-H4K8ac_peak_10052	6.65673	
15	76135074	76135440	KD2-H4K8ac_peak_10053	14.8083	UBE2Q2_ENSG00000140367
15	76135993	76136582	KD2-H4K8ac_peak_10054	11.04256	UBE2Q2_ENSG00000140367
15	76195729	76196623	KD2-H4K8ac_peak_10055	20.30085	FBXO22_ENSG00000167196
15	76461155	76461523	KD2-H4K8ac_peak_10056	7.27716	
15	76603402	76603647	KD2-H4K8ac_peak_10057	6.55628	ETFA_ENSG00000140374
15	76628349	76628769	KD2-H4K8ac_peak_10058	15.09202	ISL2_ENSG00000159556
15	77197311	77197673	KD2-H4K8ac_peak_10059	9.95342	SCAPER_ENSG00000140386
15	77224186	77224602	KD2-H4K8ac_peak_10060	5.14125	RCN2_ENSG00000117906
15	77286901	77287891	KD2-H4K8ac_peak_10061	8.10216	
15	77328756	77329036	KD2-H4K8ac_peak_10062	4.26087	
15	77362758	77363079	KD2-H4K8ac_peak_10063	7.70289	
15	77713270	77713712	KD2-H4K8ac_peak_10064	6.40297	PEAK1_ENSG00000173517;HMG20A_ENSG00000140382
15	77788920	77789160	KD2-H4K8ac_peak_10065	4.49837	
15	77789403	77789939	KD2-H4K8ac_peak_10066	5.13615	
15	77790802	77791028	KD2-H4K8ac_peak_10067	6.56926	
15	77821870	77822227	KD2-H4K8ac_peak_10068	6.37852	
15	77835127	77836254	KD2-H4K8ac_peak_10069	13.02811	
15	77861349	77861874	KD2-H4K8ac_peak_10070	8.4627	RP11-307C19.2_ENSG00000259420
15	77869758	77870003	KD2-H4K8ac_peak_10071	4.86324	
15	77895684	77896217	KD2-H4K8ac_peak_10072	5.88845	

15	77896424	77896854	KD2-H4K8ac_peak_10073	16.89856	
15	77897088	77898151	KD2-H4K8ac_peak_10074	11.03331	
15	77898896	77899841	KD2-H4K8ac_peak_10075	12.14909	
15	77900169	77900403	KD2-H4K8ac_peak_10076	11.02863	
15	77900617	77900992	KD2-H4K8ac_peak_10077	4.42765	
15	77925446	77925717	KD2-H4K8ac_peak_10078	10.44656	
15	78031294	78031505	KD2-H4K8ac_peak_10079	6.69204	
15	78175820	78176165	KD2-H4K8ac_peak_10080	4.77079	
15	78179483	78179783	KD2-H4K8ac_peak_10081	3.75035	
15	78181569	78181876	KD2-H4K8ac_peak_10082	4.211	
15	78267292	78268712	KD2-H4K8ac_peak_10083	8.53092	ADAMTS7P3_ENSG00000261143
15	78281150	78281661	KD2-H4K8ac_peak_10084	7.51945	
15	78283137	78283778	KD2-H4K8ac_peak_10085	8.10216	
15	78284553	78284837	KD2-H4K8ac_peak_10086	8.79106	RP11-114H24.6_ENSG00000259792
15	78285893	78286920	KD2-H4K8ac_peak_10087	15.69572	RP11-114H24.6_ENSG00000259792
15	78289296	78289640	KD2-H4K8ac_peak_10088	7.13615	
15	78289920	78290907	KD2-H4K8ac_peak_10089	8.59579	
15	78360963	78361179	KD2-H4K8ac_peak_10090	4.01115	
15	78369497	78370358	KD2-H4K8ac_peak_10091	12.84642	TBC1D2B_ENSG00000167202;SH2D7_ENSG00000183476
15	78424025	78424411	KD2-H4K8ac_peak_10092	7.38799	CIB2_ENSG00000136425;IDH3A_ENSG00000166411
15	78592577	78593159	KD2-H4K8ac_peak_10093	6.66692	WDR61_ENSG00000140395
15	78601219	78601467	KD2-H4K8ac_peak_10094	9.34989	
15	78607966	78608261	KD2-H4K8ac_peak_10095	7.20567	
15	78608595	78609434	KD2-H4K8ac_peak_10096	9.89383	
15	78614248	78615463	KD2-H4K8ac_peak_10097	15.64162	
15	78623784	78624010	KD2-H4K8ac_peak_10098	9.11818	
15	78684685	78684993	KD2-H4K8ac_peak_10099	5.45396	
15	78729858	78730201	KD2-H4K8ac_peak_10100	9.96798	IREB2_ENSG00000136381
15	78730646	78731034	KD2-H4K8ac_peak_10101	11.82518	IREB2_ENSG00000136381
15	78831883	78832279	KD2-H4K8ac_peak_10102	12.88108	PSMA4_ENSG00000041357
15	78832487	78833147	KD2-H4K8ac_peak_10103	10.21366	PSMA4_ENSG00000041357
15	78909871	78910074	KD2-H4K8ac_peak_10104	6.03353	
15	78910418	78910745	KD2-H4K8ac_peak_10105	9.73431	
15	78911706	78913280	KD2-H4K8ac_peak_10106	14.27217	CHRNA3_ENSG00000080644
15	78913543	78913922	KD2-H4K8ac_peak_10107	8.30974	CHRNA3_ENSG00000080644
15	78917467	78917882	KD2-H4K8ac_peak_10108	12.00273	RP11-335K5.2_ENSG00000259555
15	78918256	78918617	KD2-H4K8ac_peak_10109	7.86151	RP11-335K5.2_ENSG00000259555
15	78930487	78930750	KD2-H4K8ac_peak_10110	10.21366	
15	78931718	78932217	KD2-H4K8ac_peak_10111	8.7325	
15	78932460	78933092	KD2-H4K8ac_peak_10112	11.92978	
15	78933382	78934347	KD2-H4K8ac_peak_10113	23.33626	
15	78953083	78953299	KD2-H4K8ac_peak_10114	4.77079	RP11-160C18.2_ENSG00000261303;RPL18P11_ENSG00000213307
15	78960324	78961147	KD2-H4K8ac_peak_10115	5.30799	
15	78962010	78962287	KD2-H4K8ac_peak_10116	7.82935	
15	78963266	78963548	KD2-H4K8ac_peak_10117	9.1707	
15	78964820	78965411	KD2-H4K8ac_peak_10118	24.30324	

15	78965680	78966659	KD2-H4K8ac_peak_10119	18.37277	
15	78967243	78967997	KD2-H4K8ac_peak_10120	10.513	
15	78968246	78968992	KD2-H4K8ac_peak_10121	10.19949	
15	78971414	78972213	KD2-H4K8ac_peak_10122	13.58614	
15	78972941	78973876	KD2-H4K8ac_peak_10123	21.76228	
15	78974663	78975147	KD2-H4K8ac_peak_10124	5.30799	
15	78975352	78976025	KD2-H4K8ac_peak_10125	8.85813	
15	78976266	78976534	KD2-H4K8ac_peak_10126	4.35991	
15	78977681	78979076	KD2-H4K8ac_peak_10127	16.54162	
15	78983415	78984285	KD2-H4K8ac_peak_10128	8.00457	
15	78986156	78986404	KD2-H4K8ac_peak_10129	5.7068	
15	78986978	78987218	KD2-H4K8ac_peak_10130	4.75764	
15	79001387	79001736	KD2-H4K8ac_peak_10131	6.8836	
15	79002014	79002538	KD2-H4K8ac_peak_10132	6.65673	
15	79029788	79030004	KD2-H4K8ac_peak_10133	5.15777	
15	79043683	79045388	KD2-H4K8ac_peak_10134	12.84642	RP11-160C18.4_ENSG00000238166
15	79046000	79047627	KD2-H4K8ac_peak_10135	12.36441	
15	79049233	79050148	KD2-H4K8ac_peak_10136	13.70753	
15	79050448	79051380	KD2-H4K8ac_peak_10137	8.93761	
15	79051634	79052559	KD2-H4K8ac_peak_10138	19.16083	
15	79052961	79053172	KD2-H4K8ac_peak_10139	8.28234	
15	79053422	79053991	KD2-H4K8ac_peak_10140	6.40297	
15	79055892	79056490	KD2-H4K8ac_peak_10141	6.65673	
15	79102423	79102855	KD2-H4K8ac_peak_10142	3.90565	ADAMTS7_ENSG00000136378;MORF4L1_ENSG00000185787
15	79104013	79104676	KD2-H4K8ac_peak_10143	16.61426	ADAMTS7_ENSG00000136378
15	79164672	79164935	KD2-H4K8ac_peak_10144	9.36516	
15	79165371	79165695	KD2-H4K8ac_peak_10145	14.8083	
15	79222953	79223273	KD2-H4K8ac_peak_10146	9.16531	
15	79237021	79237357	KD2-H4K8ac_peak_10147	8.28234	
15	79288708	79288937	KD2-H4K8ac_peak_10148	8.23274	
15	79603065	79603271	KD2-H4K8ac_peak_10149	7.13615	TMED3_ENSG00000166557
15	79603504	79604088	KD2-H4K8ac_peak_10150	15.61313	TMED3_ENSG00000166557
15	80146446	80146796	KD2-H4K8ac_peak_10151	5.30799	
15	80188807	80189017	KD2-H4K8ac_peak_10152	6.86699	MTHFS_ENSG00000136371
15	80189285	80189610	KD2-H4K8ac_peak_10153	5.75452	MTHFS_ENSG00000136371
15	80215191	80215936	KD2-H4K8ac_peak_10154	12.93209	ST20-MTHFS_ENSG00000259332;ST20_ENSG00000180953;C15orf37_ENSG00000259642;C15ORF37_ENSG00000257028
15	80216422	80216832	KD2-H4K8ac_peak_10155	5.71749	ST20-MTHFS_ENSG00000259332;ST20_ENSG00000180953;C15ORF37_ENSG00000257028
15	80352020	80352627	KD2-H4K8ac_peak_10156	11.79585	ZFAND6_ENSG00000086666
15	80695529	80697303	KD2-H4K8ac_peak_10157	13.80019	RP11-210M15.2_ENSG00000259495;ARNT2_ENSG00000172379
15	80986930	80987364	KD2-H4K8ac_peak_10158	16.8435	RP11-28H5.2_ENSG00000271983
15	80987672	80988458	KD2-H4K8ac_peak_10159	11.60333	
15	81292841	81294009	KD2-H4K8ac_peak_10160	17.30994	MESDC1_ENSG00000140406
15	81452611	81452863	KD2-H4K8ac_peak_10161	4.67342	IL16_ENSG00000172349
15	82042065	82042494	KD2-H4K8ac_peak_10162	8.55271	
15	82042803	82043099	KD2-H4K8ac_peak_10163	4.211	
15	82043670	82043872	KD2-H4K8ac_peak_10164	6.90458	

15	82045885	82046164	KD2-H4K8ac_peak_10165	9.11031	
15	82201853	82202091	KD2-H4K8ac_peak_10166	5.84991	
15	82206808	82207065	KD2-H4K8ac_peak_10167	4.06758	
15	82337220	82337645	KD2-H4K8ac_peak_10168	5.13893	MEX3B_ENSG00000183496;AC026956.1_ENSG00000222521
15	82337917	82338355	KD2-H4K8ac_peak_10169	7.6718	MEX3B_ENSG00000183496
15	82338766	82339314	KD2-H4K8ac_peak_10170	24.1491	MEX3B_ENSG00000183496
15	82339766	82340387	KD2-H4K8ac_peak_10171	9.63487	
15	82405090	82405330	KD2-H4K8ac_peak_10172	9.81419	
15	82420751	82420952	KD2-H4K8ac_peak_10173	5.54647	
15	82554773	82554990	KD2-H4K8ac_peak_10174	9.20193	EFTUD1_ENSG00000140598;FAM154B_ENSG00000188659
15	83240193	83240400	KD2-H4K8ac_peak_10175	5.405	
15	83315878	83316905	KD2-H4K8ac_peak_10176	13.51987	CPEB1_ENSG00000214575;RP11-752G15.3_ENSG00000259462
15	83378838	83379399	KD2-H4K8ac_peak_10177	4.67342	AP3B2_ENSG00000103723
15	83418621	83419146	KD2-H4K8ac_peak_10178	8.17902	RP11-752G15.6_ENSG00000250988
15	83477553	83477812	KD2-H4K8ac_peak_10179	5.25071	WHAMM_ENSG00000156232
15	83478415	83478838	KD2-H4K8ac_peak_10180	5.72327	WHAMM_ENSG00000156232
15	83519458	83519749	KD2-H4K8ac_peak_10181	9.16531	
15	83620978	83621599	KD2-H4K8ac_peak_10182	5.13615	
15	83654374	83655466	KD2-H4K8ac_peak_10183	8.85813	HOMER2_ENSG00000103942;FAM103A1_ENSG00000169612
15	83679900	83680317	KD2-H4K8ac_peak_10184	8.58069	C15orf40_ENSG00000169609
15	83680554	83681160	KD2-H4K8ac_peak_10185	9.65086	C15orf40_ENSG00000169609;RP11-382A20.7_ENSG00000260608
15	83735568	83736657	KD2-H4K8ac_peak_10186	15.25814	BTBD1_ENSG00000064726;MIR4515_ENSG00000263643
15	83776213	83776440	KD2-H4K8ac_peak_10187	4.66652	TM6SF1_ENSG00000136404
15	83876001	83877248	KD2-H4K8ac_peak_10188	19.33018	HDGFRP3_ENSG00000166503
15	84748649	84749203	KD2-H4K8ac_peak_10189	10.96455	RP11-342A23.1_ENSG00000259609;EFTUD1P1_ENSG00000259404
15	84833325	84833619	KD2-H4K8ac_peak_10190	8.02571	
15	84864638	84865055	KD2-H4K8ac_peak_10191	7.59794	
15	84976278	84976550	KD2-H4K8ac_peak_10192	20.91724	UBE2Q2P11_ENSG00000259538;RP11-182J1.13_ENSG00000259774
15	85043464	85043686	KD2-H4K8ac_peak_10193	11.30637	UBE2Q2P12_ENSG00000197627;RP11-182J1.10_ENSG00000259551
15	85113060	85113980	KD2-H4K8ac_peak_10194	8.55271	LINC00933_ENSG00000259728;UBE2Q2P1_ENSG00000189136
15	85144308	85144733	KD2-H4K8ac_peak_10195	10.43145	ZSCAN2_ENSG00000176371
15	85174012	85174391	KD2-H4K8ac_peak_10196	6.83936	SCAND2P_ENSG00000176700
15	85197182	85197396	KD2-H4K8ac_peak_10197	12.08081	WDR73_ENSG00000177082
15	85197690	85197937	KD2-H4K8ac_peak_10198	9.09777	WDR73_ENSG00000177082
15	85201957	85202193	KD2-H4K8ac_peak_10199	10.19949	NMB_ENSG00000197696
15	85258729	85258936	KD2-H4K8ac_peak_10200	5.08628	
15	85259478	85259696	KD2-H4K8ac_peak_10201	11.05467	SEC11A_ENSG00000140612
15	85259898	85260502	KD2-H4K8ac_peak_10202	8.37043	SEC11A_ENSG00000140612
15	85291020	85292433	KD2-H4K8ac_peak_10203	16.29309	ZNF592_ENSG00000166716
15	85470181	85470446	KD2-H4K8ac_peak_10204	5.57044	
15	85498797	85499021	KD2-H4K8ac_peak_10205	9.16531	
15	85523681	85524408	KD2-H4K8ac_peak_10206	5.9696	PDE8A_ENSG00000073417
15	85524704	85525216	KD2-H4K8ac_peak_10207	12.00597	
15	85588599	85588820	KD2-H4K8ac_peak_10208	4.37709	
15	85698763	85698992	KD2-H4K8ac_peak_10209	9.15329	
15	85924257	85924617	KD2-H4K8ac_peak_10210	13.34404	AKAP13_ENSG00000170776

15	86158531	86158743	KD2-H4K8ac_peak_10211	4.01115	
15	86163285	86163505	KD2-H4K8ac_peak_10212	5.25071	
15	86323271	86323700	KD2-H4K8ac_peak_10213	12.52363	
15	86329434	86329832	KD2-H4K8ac_peak_10214	8.30974	
15	86374423	86374629	KD2-H4K8ac_peak_10215	5.61067	
15	87114639	87114854	KD2-H4K8ac_peak_10216	5.71749	
15	87115633	87115972	KD2-H4K8ac_peak_10217	9.91109	
15	88266587	88267247	KD2-H4K8ac_peak_10218	5.15777	
15	89089358	89090108	KD2-H4K8ac_peak_10219	25.71759	DET1_ENSG00000140543
15	89090437	89090805	KD2-H4K8ac_peak_10220	7.12754	DET1_ENSG00000140543
15	89114891	89115128	KD2-H4K8ac_peak_10221	10.19949	
15	89115612	89115869	KD2-H4K8ac_peak_10222	6.31143	
15	89164630	89164992	KD2-H4K8ac_peak_10223	12.42761	AEN_ENSG00000181026
15	89182124	89182583	KD2-H4K8ac_peak_10224	7.27716	
15	89440992	89441229	KD2-H4K8ac_peak_10225	8.98093	
15	89560575	89561193	KD2-H4K8ac_peak_10226	10.86318	
15	89628908	89629266	KD2-H4K8ac_peak_10227	4.67342	
15	89630728	89631224	KD2-H4K8ac_peak_10228	12.00273	RP11-326A19.3_ENSG00000261407;ABHD2_ENSG00000140526
15	89904942	89905162	KD2-H4K8ac_peak_10229	6.25014	LINC00925_ENSG00000255571
15	89905786	89906026	KD2-H4K8ac_peak_10230	9.34989	LINC00925_ENSG00000255571
15	89910390	89911494	KD2-H4K8ac_peak_10231	7.07117	MIR9-3_ENSG00000207819
15	89927716	89928000	KD2-H4K8ac_peak_10232	5.18553	
15	89932492	89932959	KD2-H4K8ac_peak_10233	11.92978	
15	89942559	89943929	KD2-H4K8ac_peak_10234	14.68449	
15	89992556	89992814	KD2-H4K8ac_peak_10235	3.86308	
15	90118855	90119265	KD2-H4K8ac_peak_10236	8.84009	TICRR_ENSG00000140534
15	90198378	90199050	KD2-H4K8ac_peak_10237	7.40314	KIF7_ENSG00000166813
15	90208696	90209637	KD2-H4K8ac_peak_10238	10.72313	
15	90233816	90234340	KD2-H4K8ac_peak_10239	12.99417	PEX11A_ENSG00000166821;WDR93_ENSG00000140527
15	90293476	90294903	KD2-H4K8ac_peak_10240	23.68358	MESP1_ENSG00000166823;MRPL15P1_ENSG00000259441
15	90319027	90320775	KD2-H4K8ac_peak_10241	15.96909	
15	90357681	90358375	KD2-H4K8ac_peak_10242	6.66359	ANPEP_ENSG00000166825
15	90358676	90358896	KD2-H4K8ac_peak_10243	4.35724	ANPEP_ENSG00000166825
15	90436986	90438085	KD2-H4K8ac_peak_10244	11.05467	AP3S2_ENSG00000157823
15	90456021	90457016	KD2-H4K8ac_peak_10245	13.56049	C15orf38-AP3S2_ENSG00000250021;C15orf38_ENSG00000242498
15	90506644	90506850	KD2-H4K8ac_peak_10246	4.77079	
15	90543456	90544840	KD2-H4K8ac_peak_10247	20.17674	ZNF710_ENSG00000140548
15	90545221	90545492	KD2-H4K8ac_peak_10248	8.41122	ZNF710_ENSG00000140548
15	90645144	90645645	KD2-H4K8ac_peak_10249	5.38581	IDH2_ENSG00000182054;CTD-2315E11.1_ENSG00000259685
15	90701360	90702179	KD2-H4K8ac_peak_10250	12.93209	
15	90717247	90717457	KD2-H4K8ac_peak_10251	8.59579	
15	90718268	90718770	KD2-H4K8ac_peak_10252	16.11042	
15	90719021	90719278	KD2-H4K8ac_peak_10253	7.5554	
15	90724949	90725331	KD2-H4K8ac_peak_10254	5.05318	
15	90734876	90735746	KD2-H4K8ac_peak_10255	11.1952	
15	90736263	90736516	KD2-H4K8ac_peak_10256	9.68068	

15	90744165	90744894	KD2-H4K8ac_peak_10257	7.36799	
15	90777467	90777665	KD2-H4K8ac_peak_10258	11.33854	CIB1_ENSG00000185043;GDPGP1_ENSG00000183208
15	90808721	90809134	KD2-H4K8ac_peak_10259	10.78621	NGRN_ENSG00000182768
15	90863521	90863722	KD2-H4K8ac_peak_10260	7.36799	
15	90894777	90895289	KD2-H4K8ac_peak_10261	13.02811	ZNF774_ENSG00000196391
15	90895567	90895764	KD2-H4K8ac_peak_10262	7.07117	ZNF774_ENSG00000196391
15	90930922	90931168	KD2-H4K8ac_peak_10263	11.05467	IQGAP1_ENSG00000140575
15	91065236	91065449	KD2-H4K8ac_peak_10264	5.88845	
15	91072087	91073408	KD2-H4K8ac_peak_10265	13.3695	CRTC3_ENSG00000140577
15	91208319	91209414	KD2-H4K8ac_peak_10266	24.93342	
15	91259988	91260227	KD2-H4K8ac_peak_10267	6.0073	RP11-387D10.2_ENSG00000259736;BLM_ENSG00000197299
15	91384309	91384518	KD2-H4K8ac_peak_10268	5.40356	
15	91414270	91414658	KD2-H4K8ac_peak_10269	11.03331	
15	91438221	91439588	KD2-H4K8ac_peak_10270	8.4716	
15	91445613	91445849	KD2-H4K8ac_peak_10271	10.05606	MAN2A2_ENSG00000196547
15	91446157	91446715	KD2-H4K8ac_peak_10272	6.0605	MAN2A2_ENSG00000196547
15	91473469	91473788	KD2-H4K8ac_peak_10273	10.05606	UNC45A_ENSG00000140553
15	91475226	91475752	KD2-H4K8ac_peak_10274	12.57865	HDDC3_ENSG00000184508
15	91498193	91498821	KD2-H4K8ac_peak_10275	8.53092	AC068831.6_ENSG00000258384;RCCD1_ENSG00000166965
15	91499389	91499754	KD2-H4K8ac_peak_10276	5.88845	AC068831.6_ENSG00000258384
15	91500001	91500644	KD2-H4K8ac_peak_10277	11.76021	
15	91537514	91537725	KD2-H4K8ac_peak_10278	8.65175	
15	91565424	91565747	KD2-H4K8ac_peak_10279	6.99815	VPS33B_ENSG00000184056;AC068831.10_ENSG00000214432
15	91566591	91566840	KD2-H4K8ac_peak_10280	6.25869	VPS33B_ENSG00000184056;AC068831.10_ENSG00000214432
15	92396580	92397430	KD2-H4K8ac_peak_10281	9.73431	SLCO3A1_ENSG00000176463
15	92493454	92493683	KD2-H4K8ac_peak_10282	4.01115	
15	92936695	92936911	KD2-H4K8ac_peak_10283	6.2781	ST8SIA2_ENSG00000140557
15	92937367	92937677	KD2-H4K8ac_peak_10284	6.36375	ST8SIA2_ENSG00000140557
15	92937963	92938565	KD2-H4K8ac_peak_10285	9.95119	ST8SIA2_ENSG00000140557
15	93042458	93043084	KD2-H4K8ac_peak_10286	7.70289	
15	93064805	93065042	KD2-H4K8ac_peak_10287	5.25071	
15	93177900	93178166	KD2-H4K8ac_peak_10288	5.61377	
15	93198823	93199235	KD2-H4K8ac_peak_10289	6.66692	
15	93257505	93258109	KD2-H4K8ac_peak_10290	9.1707	
15	93277031	93277353	KD2-H4K8ac_peak_10291	8.55271	RP11-386M24.4_ENSG00000258741
15	93363270	93363514	KD2-H4K8ac_peak_10292	7.22457	
15	93364212	93364497	KD2-H4K8ac_peak_10293	6.85193	
15	93366128	93366499	KD2-H4K8ac_peak_10294	4.42765	
15	93375660	93375994	KD2-H4K8ac_peak_10295	6.25869	
15	93377109	93377420	KD2-H4K8ac_peak_10296	6.16888	
15	93413444	93413706	KD2-H4K8ac_peak_10297	8.17902	
15	93425307	93425509	KD2-H4K8ac_peak_10298	4.77079	AC013394.2_ENSG00000272888
15	93426093	93426666	KD2-H4K8ac_peak_10299	15.59774	AC013394.2_ENSG00000272888;CHD2_ENSG00000173575
15	93460446	93460656	KD2-H4K8ac_peak_10300	7.12754	
15	93615803	93616173	KD2-H4K8ac_peak_10301	8.79106	
15	94404437	94404676	KD2-H4K8ac_peak_10302	6.66692	

15	94774834	94775034	KD2-H4K8ac_peak_10303	5.51783	MCTP2_ENSG00000140563
15	96883969	96884290	KD2-H4K8ac_peak_10304	10.05606	
15	97121938	97122206	KD2-H4K8ac_peak_10305	4.37708	
15	99190698	99191266	KD2-H4K8ac_peak_10306	8.4716	RP11-35O15.1_ENSG00000259424;IGF1R_ENSG00000140443
15	99191809	99192011	KD2-H4K8ac_peak_10307	6.85103	IGF1R_ENSG00000140443
15	99192292	99192706	KD2-H4K8ac_peak_10308	12.00273	IGF1R_ENSG00000140443
15	99192933	99193288	KD2-H4K8ac_peak_10309	9.20193	IGF1R_ENSG00000140443
15	99193505	99193980	KD2-H4K8ac_peak_10310	5.13615	
15	99250798	99250999	KD2-H4K8ac_peak_10311	5.6755	
15	99270825	99271112	KD2-H4K8ac_peak_10312	8.53092	
15	99278109	99278750	KD2-H4K8ac_peak_10313	8.30974	
15	99279683	99280248	KD2-H4K8ac_peak_10314	11.59946	
15	99280451	99280659	KD2-H4K8ac_peak_10315	5.73839	
15	99281006	99281217	KD2-H4K8ac_peak_10316	9.1707	
15	99287811	99288176	KD2-H4K8ac_peak_10317	6.31143	
15	99341516	99341825	KD2-H4K8ac_peak_10318	4.11746	
15	99557880	99559141	KD2-H4K8ac_peak_10319	7.36799	
15	99602400	99603068	KD2-H4K8ac_peak_10320	29.60293	
15	99619644	99619986	KD2-H4K8ac_peak_10321	7.13615	
15	99640566	99640828	KD2-H4K8ac_peak_10322	3.97079	
15	99645529	99645723	KD2-H4K8ac_peak_10323	4.7158	RP11-654A16.3_ENSG00000259475
15	99663566	99663900	KD2-H4K8ac_peak_10324	12.99417	
15	99664309	99664651	KD2-H4K8ac_peak_10325	7.98621	
15	99665096	99665801	KD2-H4K8ac_peak_10326	8.94469	
15	99791743	99792058	KD2-H4K8ac_peak_10327	12.06346	TTC23_ENSG00000103852;LRRC28_ENSG00000168904
15	99942815	99943084	KD2-H4K8ac_peak_10328	5.22731	
15	99943342	99943539	KD2-H4K8ac_peak_10329	8.1233	
15	100026500	1E+08	KD2-H4K8ac_peak_10330	10.30284	
15	100105290	1E+08	KD2-H4K8ac_peak_10331	13.70753	
15	100273320	1E+08	KD2-H4K8ac_peak_10332	14.98671	LYSMD4_ENSG00000183060
15	100273898	1E+08	KD2-H4K8ac_peak_10333	9.36516	LYSMD4_ENSG00000183060
15	100347410	1E+08	KD2-H4K8ac_peak_10334	5.57044	DNM1P46_ENSG00000182397;CTD-2054N24.2_ENSG00000259363
15	100537536	1.01E+08	KD2-H4K8ac_peak_10335	7.47462	
15	100735200	1.01E+08	KD2-H4K8ac_peak_10336	4.44527	
15	100742663	1.01E+08	KD2-H4K8ac_peak_10337	8.01894	
15	100874783	1.01E+08	KD2-H4K8ac_peak_10338	7.5554	
15	100875327	1.01E+08	KD2-H4K8ac_peak_10339	5.9696	
15	100890077	1.01E+08	KD2-H4K8ac_peak_10340	10.29842	SPATA41_ENSG00000189419
15	100913591	1.01E+08	KD2-H4K8ac_peak_10341	3.75035	RP11-168G16.2_ENSG00000259430
15	101142120	1.01E+08	KD2-H4K8ac_peak_10342	13.56049	ASB7_ENSG00000183475
15	101142817	1.01E+08	KD2-H4K8ac_peak_10343	13.81781	LINS_ENSG00000140471;ASB7_ENSG00000183475
15	101198701	1.01E+08	KD2-H4K8ac_peak_10344	5.15777	
15	101203117	1.01E+08	KD2-H4K8ac_peak_10345	8.45238	
15	101208117	1.01E+08	KD2-H4K8ac_peak_10346	12.30969	
15	101209634	1.01E+08	KD2-H4K8ac_peak_10347	8.30974	
15	101210853	1.01E+08	KD2-H4K8ac_peak_10348	4.06758	

15	101303481	1.01E+08	KD2-H4K8ac_peak_10349	8.30974	
15	101305375	1.01E+08	KD2-H4K8ac_peak_10350	6.8713	
15	101306009	1.01E+08	KD2-H4K8ac_peak_10351	18.24798	
15	101307682	1.01E+08	KD2-H4K8ac_peak_10352	9.33795	
15	101308527	1.01E+08	KD2-H4K8ac_peak_10353	8.56219	
15	101309732	1.01E+08	KD2-H4K8ac_peak_10354	11.89502	
15	101310607	1.01E+08	KD2-H4K8ac_peak_10355	5.66317	
15	101311274	1.01E+08	KD2-H4K8ac_peak_10356	14.56955	
15	101312465	1.01E+08	KD2-H4K8ac_peak_10357	8.23062	
15	101314164	1.01E+08	KD2-H4K8ac_peak_10358	12.84642	
15	101320721	1.01E+08	KD2-H4K8ac_peak_10359	8.16906	
15	101322108	1.01E+08	KD2-H4K8ac_peak_10360	12.00597	
15	101322653	1.01E+08	KD2-H4K8ac_peak_10361	6.99815	
15	101370477	1.01E+08	KD2-H4K8ac_peak_10362	11.05407	
15	101371944	1.01E+08	KD2-H4K8ac_peak_10363	12.25733	
15	101418601	1.01E+08	KD2-H4K8ac_peak_10364	28.21903	ALDH1A3_ENSG00000184254
15	101420629	1.01E+08	KD2-H4K8ac_peak_10365	10.0692	
15	101437406	1.01E+08	KD2-H4K8ac_peak_10366	6.2781	
15	101438305	1.01E+08	KD2-H4K8ac_peak_10367	5.51783	
15	101440963	1.01E+08	KD2-H4K8ac_peak_10368	7.98621	
15	101458551	1.01E+08	KD2-H4K8ac_peak_10369	8.4716	RP11-66B24.4_ENSG00000259583;LRRK1_ENSG00000154237
15	101459207	1.01E+08	KD2-H4K8ac_peak_10370	9.24683	RP11-66B24.4_ENSG00000259583;LRRK1_ENSG00000154237
15	101459733	1.01E+08	KD2-H4K8ac_peak_10371	10.43145	RP11-66B24.4_ENSG00000259583;LRRK1_ENSG00000154237
15	101484445	1.01E+08	KD2-H4K8ac_peak_10372	4.01115	
15	101653618	1.02E+08	KD2-H4K8ac_peak_10373	5.21139	
15	101656139	1.02E+08	KD2-H4K8ac_peak_10374	7.40111	RP11-424I19.1_ENSG00000259365
15	101661035	1.02E+08	KD2-H4K8ac_peak_10375	5.4779	
15	101662488	1.02E+08	KD2-H4K8ac_peak_10376	7.40314	
15	101666007	1.02E+08	KD2-H4K8ac_peak_10377	18.21424	
15	101666739	1.02E+08	KD2-H4K8ac_peak_10378	14.8083	
15	101668050	1.02E+08	KD2-H4K8ac_peak_10379	9.1707	
15	101669367	1.02E+08	KD2-H4K8ac_peak_10380	6.40683	
15	101673728	1.02E+08	KD2-H4K8ac_peak_10381	5.71749	
15	101677591	1.02E+08	KD2-H4K8ac_peak_10382	4.42765	
15	101678293	1.02E+08	KD2-H4K8ac_peak_10383	13.09607	
15	101683221	1.02E+08	KD2-H4K8ac_peak_10384	5.14125	
15	101688525	1.02E+08	KD2-H4K8ac_peak_10385	29.18655	
15	101689893	1.02E+08	KD2-H4K8ac_peak_10386	9.96941	
15	101703405	1.02E+08	KD2-H4K8ac_peak_10387	6.56211	
15	101791296	1.02E+08	KD2-H4K8ac_peak_10388	10.87093	CHSY1_ENSG00000131873
15	101791937	1.02E+08	KD2-H4K8ac_peak_10389	4.80593	CHSY1_ENSG00000131873
15	101792372	1.02E+08	KD2-H4K8ac_peak_10390	10.96455	CHSY1_ENSG00000131873
15	101835753	1.02E+08	KD2-H4K8ac_peak_10391	7.40111	SNRPA1_ENSG00000131876;RP11-299G20.2_ENSG00000259172
15	101918837	1.02E+08	KD2-H4K8ac_peak_10392	5.1313	
15	101989884	1.02E+08	KD2-H4K8ac_peak_10393	3.7063	
15	101990338	1.02E+08	KD2-H4K8ac_peak_10394	6.84261	

15	101990761	1.02E+08	KD2-H4K8ac_peak_10395	9.95119	
15	101991460	1.02E+08	KD2-H4K8ac_peak_10396	4.91516	
15	101993413	1.02E+08	KD2-H4K8ac_peak_10397	7.73418	
15	102009705	1.02E+08	KD2-H4K8ac_peak_10398	8.17902	
15	102028949	1.02E+08	KD2-H4K8ac_peak_10399	4.86824	
15	102029978	1.02E+08	KD2-H4K8ac_peak_10400	8.17902	
15	102042030	1.02E+08	KD2-H4K8ac_peak_10401	10.58496	
15	102152999	1.02E+08	KD2-H4K8ac_peak_10402	6.66692	
15	102154046	1.02E+08	KD2-H4K8ac_peak_10403	7.07117	
15	102159199	1.02E+08	KD2-H4K8ac_peak_10404	8.94469	
15	102192234	1.02E+08	KD2-H4K8ac_peak_10405	5.30799	TM2D3_ENSG00000184277
15	102192734	1.02E+08	KD2-H4K8ac_peak_10406	10.01546	TM2D3_ENSG00000184277
15	102285471	1.02E+08	KD2-H4K8ac_peak_10407	8.1233	RP11-89K11.1_ENSG00000259658
15	102286542	1.02E+08	KD2-H4K8ac_peak_10408	5.57044	RP11-89K11.1_ENSG00000259658
16	103357	103557	KD2-H4K8ac_peak_10409	9.96798	POLR3K_ENSG00000161980;SNRNP25_ENSG00000161981
16	103930	104176	KD2-H4K8ac_peak_10410	13.12288	POLR3K_ENSG00000161980;SNRNP25_ENSG00000161981
16	122100	122364	KD2-H4K8ac_peak_10411	8.59301	
16	127219	128149	KD2-H4K8ac_peak_10412	30.13194	RHBDF1_ENSG00000007384;MPG_ENSG00000103152
16	188070	188324	KD2-H4K8ac_peak_10413	11.05407	NPRL3_ENSG00000103148
16	188828	189242	KD2-H4K8ac_peak_10414	8.74596	NPRL3_ENSG00000103148
16	278836	279763	KD2-H4K8ac_peak_10415	22.65017	LUC7L_ENSG00000007392
16	283624	283909	KD2-H4K8ac_peak_10416	6.36319	ITFG3_ENSG00000167930
16	284282	284683	KD2-H4K8ac_peak_10417	13.6756	ITFG3_ENSG00000167930
16	284926	285208	KD2-H4K8ac_peak_10418	7.76329	ITFG3_ENSG00000167930
16	324986	325568	KD2-H4K8ac_peak_10419	9.51688	RGS11_ENSG00000076344
16	325883	326554	KD2-H4K8ac_peak_10420	9.31907	RGS11_ENSG00000076344
16	329964	330393	KD2-H4K8ac_peak_10421	9.57101	
16	330714	331444	KD2-H4K8ac_peak_10422	18.80827	
16	401902	402656	KD2-H4K8ac_peak_10423	6.51023	AXIN1_ENSG00000103126
16	420893	421264	KD2-H4K8ac_peak_10424	5.54647	MRPL28_ENSG00000086504
16	431507	431774	KD2-H4K8ac_peak_10425	8.85813	Z97634.3_ENSG00000236829
16	432155	432602	KD2-H4K8ac_peak_10426	16.23676	Z97634.3_ENSG00000236829
16	446656	446997	KD2-H4K8ac_peak_10427	12.42761	NME4_ENSG00000103202
16	447388	447597	KD2-H4K8ac_peak_10428	5.4779	NME4_ENSG00000103202
16	451983	452523	KD2-H4K8ac_peak_10429	8.30974	DECR2_ENSG00000242612
16	474774	475451	KD2-H4K8ac_peak_10430	9.89126	RAB11FIP3_ENSG00000090565
16	475695	476125	KD2-H4K8ac_peak_10431	10.53596	RAB11FIP3_ENSG00000090565
16	576664	577863	KD2-H4K8ac_peak_10432	10.19949	CAPN15_ENSG00000103326
16	578175	578688	KD2-H4K8ac_peak_10433	7.47462	CAPN15_ENSG00000103326
16	615033	616065	KD2-H4K8ac_peak_10434	11.8457	PIGQ_ENSG00000007541;NHLRC4_ENSG00000257108
16	616395	616912	KD2-H4K8ac_peak_10435	8.74082	PIGQ_ENSG00000007541;NHLRC4_ENSG00000257108
16	617414	617615	KD2-H4K8ac_peak_10436	4.70564	PIGQ_ENSG00000007541;NHLRC4_ENSG00000257108
16	619307	619861	KD2-H4K8ac_peak_10437	8.86099	
16	638707	639771	KD2-H4K8ac_peak_10438	12.14909	RAB40C_ENSG00000197562
16	679216	679820	KD2-H4K8ac_peak_10439	5.14125	WFIKKN1_ENSG00000127578
16	681232	681599	KD2-H4K8ac_peak_10440	6.92624	

16	685962	686218	KD2-H4K8ac_peak_10441	7.47462	C16orf13_ENSG00000130731
16	686460	687032	KD2-H4K8ac_peak_10442	13.67958	C16orf13_ENSG00000130731
16	687400	687973	KD2-H4K8ac_peak_10443	7.36799	
16	698391	698641	KD2-H4K8ac_peak_10444	6.16747	AL022341.3_ENSG00000228201;WDR90_ENSG00000161996
16	698841	699224	KD2-H4K8ac_peak_10445	9.34989	AL022341.3_ENSG00000228201;WDR90_ENSG00000161996
16	699525	699881	KD2-H4K8ac_peak_10446	5.30799	AL022341.3_ENSG00000228201;WDR90_ENSG00000161996
16	709808	710630	KD2-H4K8ac_peak_10447	9.57101	
16	711245	711643	KD2-H4K8ac_peak_10448	5.70596	
16	717394	717651	KD2-H4K8ac_peak_10449	8.55271	RHOT2_ENSG00000140983
16	718154	718687	KD2-H4K8ac_peak_10450	10.33736	RHOT2_ENSG00000140983
16	725514	725862	KD2-H4K8ac_peak_10451	7.36799	RHBDL1_ENSG00000103269
16	726145	726383	KD2-H4K8ac_peak_10452	5.00593	RHBDL1_ENSG00000103269
16	730104	730583	KD2-H4K8ac_peak_10453	12.82393	LA16c-313D11.9_ENSG00000260394;STUB1_ENSG00000103266
16	734026	734257	KD2-H4K8ac_peak_10454	9.18237	JMJD8_ENSG00000161999
16	739894	741044	KD2-H4K8ac_peak_10455	7.07117	WDR24_ENSG00000127580;LA16c-313D11.12_ENSG00000261659
16	759990	760338	KD2-H4K8ac_peak_10456	8.80397	LA16c-380A1.1_ENSG00000259840
16	760637	760914	KD2-H4K8ac_peak_10457	5.56396	LA16c-380A1.1_ENSG00000259840
16	766205	766465	KD2-H4K8ac_peak_10458	4.06758	
16	770640	771374	KD2-H4K8ac_peak_10459	17.83665	FAM173A_ENSG00000103254
16	771676	771907	KD2-H4K8ac_peak_10460	5.84991	
16	776535	776895	KD2-H4K8ac_peak_10461	11.24702	CCDC78_ENSG00000162004;HAGHL_ENSG00000103253
16	777776	778106	KD2-H4K8ac_peak_10462	7.40314	CCDC78_ENSG00000162004;HAGHL_ENSG00000103253
16	778529	778851	KD2-H4K8ac_peak_10463	14.38358	
16	779088	779302	KD2-H4K8ac_peak_10464	5.11586	
16	781417	781669	KD2-H4K8ac_peak_10465	5.57044	
16	790575	790974	KD2-H4K8ac_peak_10466	8.04448	NARFL_ENSG00000103245
16	791185	791752	KD2-H4K8ac_peak_10467	11.24702	NARFL_ENSG00000103245
16	838036	838319	KD2-H4K8ac_peak_10468	12.99417	RPUSD1_ENSG00000007376;CHTF18_ENSG00000127586
16	838734	839060	KD2-H4K8ac_peak_10469	7.36799	RPUSD1_ENSG00000007376;CHTF18_ENSG00000127586
16	857152	857781	KD2-H4K8ac_peak_10470	27.2501	
16	857981	858272	KD2-H4K8ac_peak_10471	11.33854	
16	936190	936455	KD2-H4K8ac_peak_10472	4.211	
16	1020347	1020880	KD2-H4K8ac_peak_10473	26.3019	
16	1358654	1358882	KD2-H4K8ac_peak_10474	10.96455	
16	1359186	1359466	KD2-H4K8ac_peak_10475	5.85291	
16	1359683	1360080	KD2-H4K8ac_peak_10476	8.28234	
16	1401305	1402320	KD2-H4K8ac_peak_10477	11.89502	TSR3_ENSG00000007520;GNPTG_ENSG00000090581
16	1429358	1429592	KD2-H4K8ac_peak_10478	10.73134	AL031721.1_ENSG00000265529
16	1429886	1430132	KD2-H4K8ac_peak_10479	7.47462	AL031721.1_ENSG00000265529
16	1464015	1464547	KD2-H4K8ac_peak_10480	12.63341	UNKL_ENSG00000059145
16	1470337	1470684	KD2-H4K8ac_peak_10481	10.24981	
16	1471023	1471672	KD2-H4K8ac_peak_10482	8.30974	
16	1475645	1475870	KD2-H4K8ac_peak_10483	3.87922	
16	1480179	1481456	KD2-H4K8ac_peak_10484	12.57143	C16orf91_ENSG00000174109
16	1524512	1526007	KD2-H4K8ac_peak_10485	30.32576	CLCN7_ENSG00000103249
16	1580457	1581105	KD2-H4K8ac_peak_10486	7.36799	

16	1581472	1581820	KD2-H4K8ac_peak_10487	7.11824	
16	1660066	1660672	KD2-H4K8ac_peak_10488	11.3394	
16	1660934	1661284	KD2-H4K8ac_peak_10489	7.76329	IFT140_ENSG00000187535
16	1664710	1665135	KD2-H4K8ac_peak_10490	14.8083	
16	1727278	1728017	KD2-H4K8ac_peak_10491	6.15357	HN1L_ENSG00000206053
16	1728444	1728720	KD2-H4K8ac_peak_10492	13.36684	HN1L_ENSG00000206053
16	1820736	1821685	KD2-H4K8ac_peak_10493	15.04266	NME3_ENSG00000103024
16	1822532	1823850	KD2-H4K8ac_peak_10494	21.12734	NME3_ENSG00000103024;MRPS34_ENSG00000074071;EME2_ENSG00000197774
16	1831974	1832360	KD2-H4K8ac_peak_10495	8.59579	NUBP2_ENSG00000095906
16	1833030	1833276	KD2-H4K8ac_peak_10496	5.01002	NUBP2_ENSG00000095906
16	1876231	1876457	KD2-H4K8ac_peak_10497	7.47462	HAGH_ENSG00000063854;FAHD1_ENSG00000180185
16	1876978	1877692	KD2-H4K8ac_peak_10498	13.80019	HAGH_ENSG00000063854;FAHD1_ENSG00000180185
16	1922423	1922729	KD2-H4K8ac_peak_10499	5.45396	
16	1954070	1954469	KD2-H4K8ac_peak_10500	9.63487	
16	1958462	1958751	KD2-H4K8ac_peak_10501	6.2781	
16	1959410	1959859	KD2-H4K8ac_peak_10502	6.25869	
16	1960117	1960605	KD2-H4K8ac_peak_10503	4.92711	
16	1979372	1979744	KD2-H4K8ac_peak_10504	5.9696	
16	1979954	1980246	KD2-H4K8ac_peak_10505	9.95119	
16	1982081	1982919	KD2-H4K8ac_peak_10506	8.55271	
16	1992824	1993084	KD2-H4K8ac_peak_10507	5.75757	MSRB1_ENSG00000198736
16	1993532	1994236	KD2-H4K8ac_peak_10508	9.20388	MSRB1_ENSG00000198736
16	2008657	2009196	KD2-H4K8ac_peak_10509	9.63487	NDUFB10_ENSG00000140990
16	2009600	2009941	KD2-H4K8ac_peak_10510	11.24702	NDUFB10_ENSG00000140990
16	2014418	2014772	KD2-H4K8ac_peak_10511	8.85813	AC005363.9_ENSG00000255513;RPS2_ENSG00000140988;SNHG9_ENSG00000255198
16	2015096	2015453	KD2-H4K8ac_peak_10512	10.08707	RPS2_ENSG00000140988;SNHG9_ENSG00000255198
16	2021407	2021852	KD2-H4K8ac_peak_10513	6.67092	TBL3_ENSG00000183751
16	2022110	2022507	KD2-H4K8ac_peak_10514	11.24702	TBL3_ENSG00000183751
16	2033469	2033925	KD2-H4K8ac_peak_10515	6.04107	NOXO1_ENSG00000196408;GFER_ENSG00000127554
16	2034264	2034854	KD2-H4K8ac_peak_10516	10.87093	NOXO1_ENSG00000196408;GFER_ENSG00000127554
16	2039665	2040926	KD2-H4K8ac_peak_10517	8.28234	AC005606.14_ENSG00000261790;SYNGR3_ENSG00000127561
16	2059269	2059597	KD2-H4K8ac_peak_10518	18.11402	ZNF598_ENSG00000167962;NPW_ENSG00000183971
16	2059916	2060825	KD2-H4K8ac_peak_10519	13.87397	ZNF598_ENSG00000167962;NPW_ENSG00000183971
16	2073475	2073831	KD2-H4K8ac_peak_10520	9.63487	
16	2083866	2084291	KD2-H4K8ac_peak_10521	8.66368	
16	2086170	2086611	KD2-H4K8ac_peak_10522	7.07117	
16	2087912	2088283	KD2-H4K8ac_peak_10523	7.47462	
16	2088555	2089477	KD2-H4K8ac_peak_10524	10.86378	
16	2089881	2090490	KD2-H4K8ac_peak_10525	9.08816	
16	2097573	2097825	KD2-H4K8ac_peak_10526	13.73635	NTHL1_ENSG00000065057;TSC2_ENSG00000103197
16	2098020	2098621	KD2-H4K8ac_peak_10527	9.51625	NTHL1_ENSG00000065057;TSC2_ENSG00000103197
16	2171842	2172042	KD2-H4K8ac_peak_10528	7.34308	
16	2172336	2173505	KD2-H4K8ac_peak_10529	15.40852	
16	2173732	2175023	KD2-H4K8ac_peak_10530	33.7704	
16	2175361	2176384	KD2-H4K8ac_peak_10531	15.20126	
16	2178358	2179009	KD2-H4K8ac_peak_10532	9.94337	

16	2198260	2198476	KD2-H4K8ac_peak_10533	7.74292	
16	2199685	2199961	KD2-H4K8ac_peak_10534	4.86324	
16	2205840	2206211	KD2-H4K8ac_peak_10535	4.60576	RP11-304L19.5_ENSG00000260260;SNORD60_ENSG00000206630;TRAF7_ENSG00000131653
16	2245532	2245781	KD2-H4K8ac_peak_10536	6.33462	CASKIN1_ENSG00000167971
16	2246263	2246496	KD2-H4K8ac_peak_10537	4.211	CASKIN1_ENSG00000167971
16	2254892	2255647	KD2-H4K8ac_peak_10538	11.24702	MLST8_ENSG00000167965;AC009065.3_ENSG00000252958
16	2265230	2265449	KD2-H4K8ac_peak_10539	8.30974	PGP_ENSG00000184207
16	2265753	2266179	KD2-H4K8ac_peak_10540	14.48084	PGP_ENSG00000184207
16	2273676	2274279	KD2-H4K8ac_peak_10541	17.94825	E4F1_ENSG00000167967
16	2301350	2301542	KD2-H4K8ac_peak_10542	10.77651	ECI1_ENSG00000167969
16	2317546	2318648	KD2-H4K8ac_peak_10543	13.59098	RNPS1_ENSG00000205937;AC009065.2_ENSG00000207715;MIR940_ENSG00000260778
16	2389894	2390516	KD2-H4K8ac_peak_10544	4.84794	ABCA3_ENSG00000167972;ABCA17P_ENSG00000238098
16	2391145	2391357	KD2-H4K8ac_peak_10545	10.65724	ABCA3_ENSG00000167972
16	2417854	2418054	KD2-H4K8ac_peak_10546	7.27716	
16	2478351	2479093	KD2-H4K8ac_peak_10547	11.92978	CCNF_ENSG00000162063
16	2479482	2479702	KD2-H4K8ac_peak_10548	9.81419	CCNF_ENSG00000162063
16	2510514	2510712	KD2-H4K8ac_peak_10549	5.29555	RP11-715J22.4_ENSG00000260874;C16orf59_ENSG00000162062
16	2517147	2519332	KD2-H4K8ac_peak_10550	19.33018	RP11-715J22.2_ENSG00000259895
16	2519579	2519964	KD2-H4K8ac_peak_10551	6.16883	
16	2520206	2520413	KD2-H4K8ac_peak_10552	5.64429	
16	2521333	2522362	KD2-H4K8ac_peak_10553	10.43145	NTN3_ENSG00000162068
16	2523779	2524388	KD2-H4K8ac_peak_10554	8.86099	TBC1D24_ENSG00000162065
16	2532290	2532496	KD2-H4K8ac_peak_10555	6.85193	
16	2533157	2533695	KD2-H4K8ac_peak_10556	5.20998	
16	2562801	2563080	KD2-H4K8ac_peak_10557	6.56211	ATP6V0C_ENSG00000185883;ATP6C_ENSG00000259784
16	2563311	2563859	KD2-H4K8ac_peak_10558	12.08081	ATP6V0C_ENSG00000185883;ATP6C_ENSG00000259784
16	2564182	2564404	KD2-H4K8ac_peak_10559	5.51783	ATP6V0C_ENSG00000185883;ATP6C_ENSG00000259784
16	2581554	2581919	KD2-H4K8ac_peak_10560	14.12454	CEMP1_ENSG00000205923;MIR3178_ENSG00000266232
16	2582725	2583115	KD2-H4K8ac_peak_10561	10.70006	MIR3178_ENSG00000266232
16	2653445	2654017	KD2-H4K8ac_peak_10562	4.86324	AC141586.5_ENSG00000215154
16	2723136	2723380	KD2-H4K8ac_peak_10563	11.97761	ERVK13-1_ENSG00000260565
16	2731542	2732318	KD2-H4K8ac_peak_10564	10.0692	KCTD5_ENSG00000167977
16	2732592	2733016	KD2-H4K8ac_peak_10565	12.00273	KCTD5_ENSG00000167977
16	2769415	2770072	KD2-H4K8ac_peak_10566	8.23274	PRSS27_ENSG00000172382
16	2770396	2771302	KD2-H4K8ac_peak_10567	9.63487	PRSS27_ENSG00000172382
16	2771607	2771939	KD2-H4K8ac_peak_10568	11.05407	
16	2801785	2802487	KD2-H4K8ac_peak_10569	20.90514	SRRM2-AS1_ENSG00000205913;SRRM2_ENSG00000167978
16	2802730	2802938	KD2-H4K8ac_peak_10570	11.89502	SRRM2-AS1_ENSG00000205913;SRRM2_ENSG00000167978
16	2803170	2803409	KD2-H4K8ac_peak_10571	7.19807	SRRM2-AS1_ENSG00000205913;SRRM2_ENSG00000167978
16	2826787	2827158	KD2-H4K8ac_peak_10572	16.07006	TCEB2_ENSG00000103363
16	2827369	2828236	KD2-H4K8ac_peak_10573	18.94636	TCEB2_ENSG00000103363
16	2848486	2848684	KD2-H4K8ac_peak_10574	7.01658	PRSS41_ENSG00000215148
16	2902307	2902721	KD2-H4K8ac_peak_10575	14.22723	
16	2917881	2918759	KD2-H4K8ac_peak_10576	13.80019	LA16c-325D7.2_ENSG00000263280
16	2933310	2933521	KD2-H4K8ac_peak_10577	15.13261	FLYWCH2_ENSG00000162076
16	2953871	2954191	KD2-H4K8ac_peak_10578	5.54647	

16	2954659	2955330	KD2-H4K8ac_peak_10579	8.5092	
16	2962130	2962379	KD2-H4K8ac_peak_10580	14.48084	FLYWCH1_ENSG00000059122
16	3013652	3014375	KD2-H4K8ac_peak_10581	12.82393	KREMEN2_ENSG00000131650
16	3018650	3019021	KD2-H4K8ac_peak_10582	9.59905	PAQR4_ENSG00000162073
16	3029949	3030450	KD2-H4K8ac_peak_10583	15.40367	PKMYT1_ENSG00000127564
16	3030655	3031585	KD2-H4K8ac_peak_10584	20.30085	PKMYT1_ENSG00000127564
16	3033556	3033765	KD2-H4K8ac_peak_10585	15.43566	
16	3053449	3053811	KD2-H4K8ac_peak_10586	8.82366	
16	3073721	3074253	KD2-H4K8ac_peak_10587	12.47554	HCFC1R1_ENSG00000103145;THOC6_ENSG00000131652
16	3109053	3110144	KD2-H4K8ac_peak_10588	33.81493	RP11-473M20.7_ENSG00000261971
16	3155463	3156538	KD2-H4K8ac_peak_10589	18.54875	RP11-473M20.11_ENSG00000263011
16	3161981	3163107	KD2-H4K8ac_peak_10590	16.49944	ZNF205_ENSG00000122386
16	3173892	3174160	KD2-H4K8ac_peak_10591	6.90231	
16	3175078	3175384	KD2-H4K8ac_peak_10592	4.75875	
16	3185163	3185430	KD2-H4K8ac_peak_10593	12.74171	RP11-473M20.14_ENSG00000263072
16	3199957	3200364	KD2-H4K8ac_peak_10594	6.65673	
16	3239252	3239551	KD2-H4K8ac_peak_10595	6.20982	
16	3314078	3314431	KD2-H4K8ac_peak_10596	8.85813	ZNF263_ENSG00000006194
16	3331846	3332086	KD2-H4K8ac_peak_10597	4.77079	
16	3332591	3333241	KD2-H4K8ac_peak_10598	9.63487	
16	3333633	3334015	KD2-H4K8ac_peak_10599	5.9696	
16	3354840	3356153	KD2-H4K8ac_peak_10600	9.63487	TIGD7_ENSG00000140993;ZNF75A_ENSG00000162086
16	3481656	3482102	KD2-H4K8ac_peak_10601	4.86824	
16	3493810	3494097	KD2-H4K8ac_peak_10602	7.27716	ZNF597_ENSG00000167981;NAA60_ENSG00000122390
16	3507577	3508599	KD2-H4K8ac_peak_10603	28.28058	LA16c-306E5.3_ENSG00000263212
16	3661266	3661507	KD2-H4K8ac_peak_10604	9.71513	SLX4_ENSG00000188827;DNASE1_ENSG00000213918
16	3661826	3662122	KD2-H4K8ac_peak_10605	11.92978	SLX4_ENSG00000188827;DNASE1_ENSG00000213918
16	3767061	3767914	KD2-H4K8ac_peak_10606	16.25886	TRAP1_ENSG00000126602
16	3930210	3930437	KD2-H4K8ac_peak_10607	8.30974	CREBBP_ENSG00000005339
16	3930911	3931548	KD2-H4K8ac_peak_10608	18.54875	CREBBP_ENSG00000005339
16	4097880	4098083	KD2-H4K8ac_peak_10609	7.05051	
16	4098554	4098931	KD2-H4K8ac_peak_10610	3.75035	
16	4165848	4166342	KD2-H4K8ac_peak_10611	5.88562	ADCY9_ENSG00000162104
16	4166702	4167173	KD2-H4K8ac_peak_10612	15.58442	ADCY9_ENSG00000162104
16	4233415	4233755	KD2-H4K8ac_peak_10613	13.12288	RP11-95P2.3_ENSG00000263105
16	4234253	4234622	KD2-H4K8ac_peak_10614	6.20982	RP11-95P2.3_ENSG00000263105
16	4250161	4250372	KD2-H4K8ac_peak_10615	7.27716	
16	4313966	4314282	KD2-H4K8ac_peak_10616	12.23	
16	4321327	4321866	KD2-H4K8ac_peak_10617	12.93209	
16	4322117	4322634	KD2-H4K8ac_peak_10618	10.96455	TFAP4_ENSG00000090447
16	4323544	4323874	KD2-H4K8ac_peak_10619	10.17341	TFAP4_ENSG00000090447
16	4356606	4357535	KD2-H4K8ac_peak_10620	8.79106	
16	4357827	4358425	KD2-H4K8ac_peak_10621	13.9234	
16	4364517	4365923	KD2-H4K8ac_peak_10622	10.05606	
16	4366241	4366458	KD2-H4K8ac_peak_10623	4.54006	
16	4377319	4377720	KD2-H4K8ac_peak_10624	11.89502	AC005356.1_ENSG00000262686

16	4378030	4378851	KD2-H4K8ac_peak_10625	13.81781	AC005356.1_ENSG00000262686
16	4421871	4422210	KD2-H4K8ac_peak_10626	7.93362	VASN_ENSG00000168140
16	4433784	4434913	KD2-H4K8ac_peak_10627	10.19949	
16	4441474	4441950	KD2-H4K8ac_peak_10628	9.09777	
16	4442179	4444103	KD2-H4K8ac_peak_10629	22.21705	
16	4444992	4445303	KD2-H4K8ac_peak_10630	5.80191	
16	4466110	4466524	KD2-H4K8ac_peak_10631	11.33751	
16	4474992	4475752	KD2-H4K8ac_peak_10632	6.20982	CORO7_ENSG00000262246;DNAJA3_ENSG00000103423
16	4476175	4476380	KD2-H4K8ac_peak_10633	5.82799	CORO7_ENSG00000262246;DNAJA3_ENSG00000103423
16	4524025	4524471	KD2-H4K8ac_peak_10634	7.07117	HMOX2_ENSG00000103415
16	4524972	4525269	KD2-H4K8ac_peak_10635	6.97919	HMOX2_ENSG00000103415
16	4526511	4526770	KD2-H4K8ac_peak_10636	6.36375	
16	4587537	4588387	KD2-H4K8ac_peak_10637	10.44656	CDIP1_ENSG00000089486
16	4588976	4589353	KD2-H4K8ac_peak_10638	6.66692	CDIP1_ENSG00000089486
16	4664671	4666154	KD2-H4K8ac_peak_10639	14.4458	UBALD1_ENSG00000153443;MGRN1_ENSG00000102858
16	4666610	4666984	KD2-H4K8ac_peak_10640	7.96322	MGRN1_ENSG00000102858
16	4674357	4674597	KD2-H4K8ac_peak_10641	5.13615	
16	4675035	4675455	KD2-H4K8ac_peak_10642	10.53377	
16	4732842	4733070	KD2-H4K8ac_peak_10643	6.66692	
16	4783834	4784042	KD2-H4K8ac_peak_10644	9.06369	ANKS3_ENSG00000168096;C16orf71_ENSG00000166246
16	4817427	4818048	KD2-H4K8ac_peak_10645	6.2781	ZNF500_ENSG00000103199
16	4821120	4821525	KD2-H4K8ac_peak_10646	5.15777	
16	4822136	4822770	KD2-H4K8ac_peak_10647	7.27716	
16	4852317	4852942	KD2-H4K8ac_peak_10648	8.55271	ROGDI_ENSG00000067836
16	4897002	4897233	KD2-H4K8ac_peak_10649	7.38799	UBN1_ENSG00000118900
16	4993291	4993709	KD2-H4K8ac_peak_10650	6.30229	
16	5083186	5083908	KD2-H4K8ac_peak_10651	10.53377	NAGPA_ENSG00000103174;ALG1_ENSG00000033011
16	5121882	5122165	KD2-H4K8ac_peak_10652	12.93253	
16	5147359	5147687	KD2-H4K8ac_peak_10653	13.56049	FAM86A_ENSG00000118894
16	5148608	5148897	KD2-H4K8ac_peak_10654	8.10216	FAM86A_ENSG00000118894;RP11-10K17.6_ENSG00000267070
16	5552179	5552483	KD2-H4K8ac_peak_10655	8.52559	
16	5553025	5553753	KD2-H4K8ac_peak_10656	7.70289	
16	5554814	5555379	KD2-H4K8ac_peak_10657	4.67342	
16	5912874	5913118	KD2-H4K8ac_peak_10658	10.46264	
16	8714864	8715136	KD2-H4K8ac_peak_10659	6.14257	METTL22_ENSG00000067365
16	8715614	8715816	KD2-H4K8ac_peak_10660	8.10216	METTL22_ENSG00000067365
16	8767940	8768217	KD2-H4K8ac_peak_10661	6.65673	ABAT_ENSG00000183044
16	8806882	8807087	KD2-H4K8ac_peak_10662	8.6308	
16	8891214	8891423	KD2-H4K8ac_peak_10663	10.17698	TMEM186_ENSG00000184857
16	8891732	8892183	KD2-H4K8ac_peak_10664	7.47462	TMEM186_ENSG00000184857
16	8918942	8919199	KD2-H4K8ac_peak_10665	9.27888	
16	8962542	8962806	KD2-H4K8ac_peak_10666	5.59859	RP11-77H9.6_ENSG00000261481;CARHSP1_ENSG00000153048
16	8963470	8963775	KD2-H4K8ac_peak_10667	9.36516	RP11-77H9.6_ENSG00000261481;CARHSP1_ENSG00000153048
16	8974021	8974216	KD2-H4K8ac_peak_10668	8.59579	
16	9058010	9058204	KD2-H4K8ac_peak_10669	7.95563	USP7_ENSG00000187555
16	9107026	9107223	KD2-H4K8ac_peak_10670	5.14125	

16	9146646	9147079	KD2-H4K8ac_peak_10671	10.05606	
16	9147472	9147674	KD2-H4K8ac_peak_10672	5.57044	
16	9183832	9184621	KD2-H4K8ac_peak_10673	16.57619	C16orf72_ENSG00000182831
16	9185628	9186603	KD2-H4K8ac_peak_10674	12.93209	C16orf72_ENSG00000182831
16	9504989	9505197	KD2-H4K8ac_peak_10675	6.03693	
16	9505420	9505707	KD2-H4K8ac_peak_10676	6.90458	
16	10480057	10480265	KD2-H4K8ac_peak_10677	10.0692	
16	10671327	10671667	KD2-H4K8ac_peak_10678	9.02573	RP11-27M24.2_ENSG00000260310
16	10672247	10672961	KD2-H4K8ac_peak_10679	7.22457	RP11-27M24.2_ENSG00000260310
16	10673686	10674122	KD2-H4K8ac_peak_10680	5.30799	EMP2_ENSG00000213853
16	10674762	10674963	KD2-H4K8ac_peak_10681	4.54006	EMP2_ENSG00000213853
16	10707928	10708125	KD2-H4K8ac_peak_10682	6.06203	
16	10710502	10711174	KD2-H4K8ac_peak_10683	16.61426	
16	10711387	10711856	KD2-H4K8ac_peak_10684	5.14125	
16	10713153	10713505	KD2-H4K8ac_peak_10685	10.43145	
16	10835230	10835479	KD2-H4K8ac_peak_10686	8.17902	
16	10837130	10837431	KD2-H4K8ac_peak_10687	6.06203	NUBP1_ENSG00000103274
16	10837668	10837923	KD2-H4K8ac_peak_10688	15.47237	NUBP1_ENSG00000103274
16	10912234	10912958	KD2-H4K8ac_peak_10689	19.93588	TVP23A_ENSG00000166676
16	10921810	10922168	KD2-H4K8ac_peak_10690	4.211	
16	10922905	10923200	KD2-H4K8ac_peak_10691	4.84794	
16	11012327	11012559	KD2-H4K8ac_peak_10692	5.54647	
16	11035974	11036253	KD2-H4K8ac_peak_10693	9.20193	DEXI_ENSG00000182108
16	11038501	11038788	KD2-H4K8ac_peak_10694	6.0605	CLEC16A_ENSG00000038532
16	11223848	11224256	KD2-H4K8ac_peak_10695	4.211	
16	11349763	11349978	KD2-H4K8ac_peak_10696	9.96798	SOCS1_ENSG00000185338
16	11350206	11350543	KD2-H4K8ac_peak_10697	11.79585	SOCS1_ENSG00000185338
16	11391632	11391957	KD2-H4K8ac_peak_10698	11.92978	
16	11397553	11398258	KD2-H4K8ac_peak_10699	13.39763	
16	11490367	11490777	KD2-H4K8ac_peak_10700	8.10216	
16	11588440	11589012	KD2-H4K8ac_peak_10701	11.05407	
16	11589297	11589540	KD2-H4K8ac_peak_10702	10.0746	
16	11590095	11590658	KD2-H4K8ac_peak_10703	8.1331	
16	11590876	11591704	KD2-H4K8ac_peak_10704	17.64174	
16	11592071	11592311	KD2-H4K8ac_peak_10705	9.11031	
16	11680152	11681293	KD2-H4K8ac_peak_10706	19.42408	
16	11761855	11762073	KD2-H4K8ac_peak_10707	7.76104	SNN_ENSG00000184602
16	11837035	11837264	KD2-H4K8ac_peak_10708	8.23274	TXNDC11_ENSG00000153066
16	11890701	11891068	KD2-H4K8ac_peak_10709	8.85813	ZC3H7A_ENSG00000122299
16	11891452	11891996	KD2-H4K8ac_peak_10710	6.25869	ZC3H7A_ENSG00000122299
16	12010086	12010604	KD2-H4K8ac_peak_10711	12.25733	GSPT1_ENSG00000103342;AC007216.1_ENSG00000266163
16	12069927	12070463	KD2-H4K8ac_peak_10712	7.90806	RP11-166B2.1_ENSG00000234719;SNX29_ENSG00000048471
16	12072066	12072287	KD2-H4K8ac_peak_10713	4.7849	RP11-166B2.7_ENSG00000260488
16	12849842	12850088	KD2-H4K8ac_peak_10714	4.01115	
16	12897255	12897736	KD2-H4K8ac_peak_10715	10.46264	CPPED1_ENSG00000103381
16	14014083	14014459	KD2-H4K8ac_peak_10716	7.76104	ERCC4_ENSG00000175595

16	14096741	14097103	KD2-H4K8ac_peak_10717	7.70289	
16	14164436	14165430	KD2-H4K8ac_peak_10718	13.73635	MKL2_ENSG00000186260
16	14396255	14396507	KD2-H4K8ac_peak_10719	6.0073	RP11-65J21.3_ENSG00000262454
16	14723687	14724060	KD2-H4K8ac_peak_10720	11.92978	
16	14724384	14724842	KD2-H4K8ac_peak_10721	7.70289	
16	14726010	14726420	KD2-H4K8ac_peak_10722	6.30229	PARN_ENSG00000140694;BFAR_ENSG00000103429
16	14726856	14727219	KD2-H4K8ac_peak_10723	10.46874	PARN_ENSG00000140694;BFAR_ENSG00000103429
16	14918610	14918851	KD2-H4K8ac_peak_10724	8.1233	ABCC6P2_ENSG00000255277
16	15069022	15069405	KD2-H4K8ac_peak_10725	5.92195	PDXDC1_ENSG00000179889
16	15149130	15149871	KD2-H4K8ac_peak_10726	17.8868	NTAN1_ENSG00000157045
16	15187708	15188092	KD2-H4K8ac_peak_10727	13.09607	RRN3_ENSG00000085721;RP11-72I8.1_ENSG00000260735
16	15188300	15188856	KD2-H4K8ac_peak_10728	10.86378	RRN3_ENSG00000085721;RP11-72I8.1_ENSG00000260735
16	15235412	15236512	KD2-H4K8ac_peak_10729	8.83582	
16	15236731	15238135	KD2-H4K8ac_peak_10730	12.93209	
16	15238432	15239541	KD2-H4K8ac_peak_10731	16.25886	
16	15239846	15240288	KD2-H4K8ac_peak_10732	4.54006	
16	15241074	15241835	KD2-H4K8ac_peak_10733	7.27716	
16	15248928	15249273	KD2-H4K8ac_peak_10734	16.15695	PKD1P6_ENSG00000250251;hsa-mir-3180-4_ENSG00000264115;MIR3180-4_ENSG00000257391
16	15489587	15490603	KD2-H4K8ac_peak_10735	13.34404	RP11-1021N1.1_ENSG00000261130;MPV17L_ENSG00000156968
16	15527436	15528156	KD2-H4K8ac_peak_10736	12.68903	C16orf45_ENSG00000166780
16	15736569	15736890	KD2-H4K8ac_peak_10737	11.05467	KIAA0430_ENSG00000166783;NDE1_ENSG00000072864;MIR484_ENSG00000272213
16	15743279	15743882	KD2-H4K8ac_peak_10738	7.36933	
16	15744223	15744481	KD2-H4K8ac_peak_10739	7.92775	
16	15982767	15983567	KD2-H4K8ac_peak_10740	8.23274	FOPNL_ENSG00000133393
16	16036682	16037137	KD2-H4K8ac_peak_10741	5.88845	
16	16043015	16043324	KD2-H4K8ac_peak_10742	9.02573	ABCC1_ENSG00000103222
16	16043646	16044126	KD2-H4K8ac_peak_10743	13.17969	ABCC1_ENSG00000103222
16	16084490	16084859	KD2-H4K8ac_peak_10744	5.5051	
16	16243675	16243986	KD2-H4K8ac_peak_10745	3.90565	
16	16244237	16244584	KD2-H4K8ac_peak_10746	4.52514	
16	16268919	16269127	KD2-H4K8ac_peak_10747	4.98072	
16	16317317	16317562	KD2-H4K8ac_peak_10748	18.94636	ABCC6_ENSG00000091262;RP11-517A5.7_ENSG00000262332
16	17151912	17152119	KD2-H4K8ac_peak_10749	3.65557	
16	17468780	17469082	KD2-H4K8ac_peak_10750	4.12757	
16	17499012	17499705	KD2-H4K8ac_peak_10751	9.32235	
16	17564336	17564704	KD2-H4K8ac_peak_10752	4.84206	XYLT1_ENSG00000103489
16	17564956	17565165	KD2-H4K8ac_peak_10753	9.36516	XYLT1_ENSG00000103489
16	17684077	17685636	KD2-H4K8ac_peak_10754	15.5861	
16	17686395	17687021	KD2-H4K8ac_peak_10755	4.42765	
16	17701327	17701894	KD2-H4K8ac_peak_10756	6.40683	
16	17813147	17813344	KD2-H4K8ac_peak_10757	7.51935	
16	17839234	17839812	KD2-H4K8ac_peak_10758	5.43522	
16	17840438	17840643	KD2-H4K8ac_peak_10759	6.60096	
16	17841609	17841830	KD2-H4K8ac_peak_10760	8.70735	
16	17842198	17842629	KD2-H4K8ac_peak_10761	9.8758	
16	17868081	17868398	KD2-H4K8ac_peak_10762	9.11031	

16	17870735	17871147	KD2-H4K8ac_peak_10763	7.27716	
16	18065956	18066699	KD2-H4K8ac_peak_10764	8.37231	
16	18801153	18801619	KD2-H4K8ac_peak_10765	15.13261	RPS15A_ENSG00000134419
16	18801869	18802345	KD2-H4K8ac_peak_10766	13.44374	RPS15A_ENSG00000134419
16	18812479	18812782	KD2-H4K8ac_peak_10767	8.23274	RP11-1035H13.3_ENSG00000260342;ARL6IP1_ENSG00000170540
16	18813241	18813807	KD2-H4K8ac_peak_10768	5.88845	RP11-1035H13.3_ENSG00000260342;ARL6IP1_ENSG00000170540;RP11-1035H13.2_ENSG00000260017
16	18937209	18937647	KD2-H4K8ac_peak_10769	5.59859	SMG1_ENSG00000157106;CTD-2288F12.1_ENSG00000260352
16	18938345	18938958	KD2-H4K8ac_peak_10770	9.15329	SMG1_ENSG00000157106;CTD-2288F12.1_ENSG00000260352
16	18981029	18981225	KD2-H4K8ac_peak_10771	4.01115	
16	18995260	18995913	KD2-H4K8ac_peak_10772	13.88933	TMC7_ENSG00000170537
16	19079358	19079898	KD2-H4K8ac_peak_10773	12.06346	RP11-626G11.5_ENSG00000261465;COQ7_ENSG00000167186
16	19097512	19097926	KD2-H4K8ac_peak_10774	12.17278	RP11-626G11.4_ENSG00000260430
16	19124809	19125914	KD2-H4K8ac_peak_10775	15.27939	ITPRIPL2_ENSG00000205730;CTD-2349B8.1_ENSG00000261427
16	19179609	19180421	KD2-H4K8ac_peak_10776	12.36441	SYT17_ENSG00000103528
16	19243932	19244146	KD2-H4K8ac_peak_10777	5.20908	
16	19359053	19359592	KD2-H4K8ac_peak_10778	15.39543	
16	19364307	19364510	KD2-H4K8ac_peak_10779	4.67737	
16	19566024	19566398	KD2-H4K8ac_peak_10780	5.29555	C16orf62_ENSG00000103544
16	19567087	19567540	KD2-H4K8ac_peak_10781	18.80827	C16orf62_ENSG00000103544
16	19729072	19729486	KD2-H4K8ac_peak_10782	14.66631	KNOP1_ENSG00000103550
16	19729834	19730334	KD2-H4K8ac_peak_10783	5.54647	KNOP1_ENSG00000103550
16	20817255	20817695	KD2-H4K8ac_peak_10784	10.43145	AC004381.6_ENSG00000005189
16	20818011	20818614	KD2-H4K8ac_peak_10785	3.97079	AC004381.6_ENSG00000005189
16	20911971	20912573	KD2-H4K8ac_peak_10786	16.71655	ERI2_ENSG00000196678;DCUN1D3_ENSG00000188215;LYRM1_ENSG00000102897
16	21169985	21170255	KD2-H4K8ac_peak_10787	7.27716	DNAH3_ENSG00000158486;TMEM159_ENSG00000011638
16	21275538	21275806	KD2-H4K8ac_peak_10788	6.66692	
16	21289334	21289823	KD2-H4K8ac_peak_10789	8.74596	
16	21294995	21295259	KD2-H4K8ac_peak_10790	9.20193	
16	21513249	21513491	KD2-H4K8ac_peak_10791	7.28628	
16	21522870	21523274	KD2-H4K8ac_peak_10792	7.28628	
16	21531089	21531738	KD2-H4K8ac_peak_10793	9.67696	CTD-2547E10.2_ENSG00000180747;SLC7A5P2_ENSG00000258186
16	21531944	21533234	KD2-H4K8ac_peak_10794	35.76486	CTD-2547E10.2_ENSG00000180747;SLC7A5P2_ENSG00000258186
16	21566606	21566800	KD2-H4K8ac_peak_10795	7.81948	
16	21568327	21568557	KD2-H4K8ac_peak_10796	7.25164	
16	21610401	21610691	KD2-H4K8ac_peak_10797	23.33626	
16	21610915	21611431	KD2-H4K8ac_peak_10798	11.05467	
16	21675184	21675593	KD2-H4K8ac_peak_10799	4.86324	
16	21678589	21678901	KD2-H4K8ac_peak_10800	6.40683	
16	21831355	21831700	KD2-H4K8ac_peak_10801	7.76329	RRN3P1_ENSG00000248124
16	21964752	21965118	KD2-H4K8ac_peak_10802	10.65724	UQCRC2_ENSG00000140740
16	22012550	22012745	KD2-H4K8ac_peak_10803	4.06758	PDZD9_ENSG00000155714
16	22018878	22019326	KD2-H4K8ac_peak_10804	15.81711	C16orf52_ENSG00000185716
16	22019679	22020032	KD2-H4K8ac_peak_10805	10.96455	C16orf52_ENSG00000185716
16	22207921	22208122	KD2-H4K8ac_peak_10806	5.15777	
16	22216515	22216781	KD2-H4K8ac_peak_10807	6.56926	EEF2K_ENSG00000103319
16	22217671	22217928	KD2-H4K8ac_peak_10808	15.41687	EEF2K_ENSG00000103319

16	22308764	22309231	KD2-H4K8ac_peak_10809	15.61313	POLR3E_ENSG00000058600
16	22385163	22385831	KD2-H4K8ac_peak_10810	12.06346	RP11-21M24.2_ENSG00000260790
16	22448472	22448919	KD2-H4K8ac_peak_10811	8.10216	CDR2_ENSG00000140743;RRN3P3_ENSG00000257122;SMG1P1_ENSG00000237296
16	22774952	22775205	KD2-H4K8ac_peak_10812	9.11031	
16	22967112	22967530	KD2-H4K8ac_peak_10813	6.83936	
16	22979962	22980186	KD2-H4K8ac_peak_10814	4.77079	
16	23038160	23038458	KD2-H4K8ac_peak_10815	12.84642	
16	23159783	23160050	KD2-H4K8ac_peak_10816	4.86324	USP31_ENSG00000103404
16	23160406	23160603	KD2-H4K8ac_peak_10817	6.24114	USP31_ENSG00000103404
16	23463978	23465042	KD2-H4K8ac_peak_10818	17.43705	COG7_ENSG00000168434;CTD-2270L9.4_ENSG00000260136
16	23521359	23521686	KD2-H4K8ac_peak_10819	9.06369	
16	23568414	23568665	KD2-H4K8ac_peak_10820	12.74171	EARS2_ENSG00000103356;UBFD1_ENSG00000103353
16	23569118	23569426	KD2-H4K8ac_peak_10821	9.41619	EARS2_ENSG00000103356;UBFD1_ENSG00000103353
16	23607240	23607589	KD2-H4K8ac_peak_10822	10.73134	NDUFAB1_ENSG00000004779
16	23652218	23652574	KD2-H4K8ac_peak_10823	6.30229	PALB2_ENSG00000083093;DCTN5_ENSG00000166847
16	23652800	23653116	KD2-H4K8ac_peak_10824	7.96322	PALB2_ENSG00000083093;DCTN5_ENSG00000166847
16	23690335	23690795	KD2-H4K8ac_peak_10825	5.405	
16	24550951	24551253	KD2-H4K8ac_peak_10826	10.09333	
16	24676293	24676520	KD2-H4K8ac_peak_10827	7.01964	
16	24682166	24682564	KD2-H4K8ac_peak_10828	8.30974	AC012317.1_ENSG00000224310
16	24697212	24697711	KD2-H4K8ac_peak_10829	6.0073	
16	24697941	24698216	KD2-H4K8ac_peak_10830	8.25022	
16	24739414	24739616	KD2-H4K8ac_peak_10831	6.40683	
16	24740175	24740481	KD2-H4K8ac_peak_10832	11.15509	TNRC6A_ENSG00000090905
16	24740706	24741430	KD2-H4K8ac_peak_10833	12.93209	TNRC6A_ENSG00000090905
16	24864954	24865305	KD2-H4K8ac_peak_10834	5.51783	
16	25026037	25026622	KD2-H4K8ac_peak_10835	7.83489	ARHGAP17_ENSG00000140750;RP11-266L9.1_ENSG00000260714
16	25043123	25043370	KD2-H4K8ac_peak_10836	11.86082	RP11-266L9.4_ENSG00000262587
16	25077498	25078073	KD2-H4K8ac_peak_10837	5.14125	RP11-266L9.5_ENSG00000262155
16	25078306	25078534	KD2-H4K8ac_peak_10838	9.1707	RP11-266L9.5_ENSG00000262155
16	25117942	25118963	KD2-H4K8ac_peak_10839	9.63487	
16	25236965	25237218	KD2-H4K8ac_peak_10840	11.20531	
16	25268928	25270113	KD2-H4K8ac_peak_10841	17.70837	ZKSCAN2_ENSG00000155592
16	27215345	27215749	KD2-H4K8ac_peak_10842	18.33962	KDM8_ENSG00000155666
16	27246967	27247495	KD2-H4K8ac_peak_10843	6.50927	
16	27279504	27279942	KD2-H4K8ac_peak_10844	5.9696	NSMCE1_ENSG00000169189;CTD-3203P2.2_ENSG00000245888
16	27280238	27280987	KD2-H4K8ac_peak_10845	11.43863	NSMCE1_ENSG00000169189;CTD-3203P2.2_ENSG00000245888
16	27560746	27561185	KD2-H4K8ac_peak_10846	7.70289	GTF3C1_ENSG00000077235;KIAA0556_ENSG00000047578
16	27734379	27734639	KD2-H4K8ac_peak_10847	4.9025	Y_RNA_ENSG00000201371
16	27794326	27794600	KD2-H4K8ac_peak_10848	8.30974	
16	27795653	27796919	KD2-H4K8ac_peak_10849	10.19949	
16	27937494	27938002	KD2-H4K8ac_peak_10850	6.40683	
16	27939604	27939852	KD2-H4K8ac_peak_10851	5.15777	
16	27956756	27957041	KD2-H4K8ac_peak_10852	4.06758	
16	28222725	28223285	KD2-H4K8ac_peak_10853	15.10495	XPO6_ENSG00000169180
16	28223648	28223896	KD2-H4K8ac_peak_10854	9.51688	XPO6_ENSG00000169180

16	28289276	28289593	KD2-H4K8ac_peak_10855	8.85813	
16	28289967	28290256	KD2-H4K8ac_peak_10856	6.66692	
16	28302241	28302479	KD2-H4K8ac_peak_10857	6.60096	RP11-57A19.2_ENSG00000246465
16	28302834	28303455	KD2-H4K8ac_peak_10858	8.93255	RP11-57A19.2_ENSG00000246465;SBK1_ENSG00000188322
16	28303933	28304226	KD2-H4K8ac_peak_10859	9.76863	RP11-57A19.2_ENSG00000246465;SBK1_ENSG00000188322
16	28304457	28304739	KD2-H4K8ac_peak_10860	7.11824	SBK1_ENSG00000188322
16	28502741	28503669	KD2-H4K8ac_peak_10861	12.69604	CLN3_ENSG00000261832
16	28504002	28504515	KD2-H4K8ac_peak_10862	8.52559	CLN3_ENSG00000261832
16	28507794	28507988	KD2-H4K8ac_peak_10863	6.0073	CLN3_ENSG00000188603
16	28564650	28564939	KD2-H4K8ac_peak_10864	8.10216	CCDC101_ENSG00000176476
16	28565241	28565507	KD2-H4K8ac_peak_10865	18.41184	CCDC101_ENSG00000176476
16	28565840	28566083	KD2-H4K8ac_peak_10866	5.88845	CCDC101_ENSG00000176476
16	28833524	28833832	KD2-H4K8ac_peak_10867	6.85193	RP11-1348G14.5_ENSG00000260796;ATXN2L_ENSG00000168488
16	28834567	28835188	KD2-H4K8ac_peak_10868	18.37277	ATXN2L_ENSG00000168488
16	28857121	28857609	KD2-H4K8ac_peak_10869	11.8457	TUFM_ENSG00000178952;SH2B1_ENSG00000178188
16	28873689	28873916	KD2-H4K8ac_peak_10870	9.68068	RP11-22P6.2_ENSG00000261766
16	28874115	28874570	KD2-H4K8ac_peak_10871	9.36516	RP11-22P6.2_ENSG00000261766
16	28875311	28875597	KD2-H4K8ac_peak_10872	7.76104	RP11-22P6.2_ENSG00000261766
16	28890621	28891112	KD2-H4K8ac_peak_10873	8.10216	ATP2A1_ENSG00000196296;RP11-22P6.3_ENSG00000260442
16	28891375	28892147	KD2-H4K8ac_peak_10874	20.8421	RP11-22P6.3_ENSG00000260442;SNORA43_ENSG00000252461
16	28913184	28913411	KD2-H4K8ac_peak_10875	4.211	
16	28936391	28937045	KD2-H4K8ac_peak_10876	10.7186	
16	28941945	28942210	KD2-H4K8ac_peak_10877	7.05877	
16	28956557	28956871	KD2-H4K8ac_peak_10878	9.89383	
16	28961405	28961903	KD2-H4K8ac_peak_10879	9.08816	NFATC2IP_ENSG00000176953
16	28962338	28962724	KD2-H4K8ac_peak_10880	16.49944	NFATC2IP_ENSG00000176953
16	28986292	28986677	KD2-H4K8ac_peak_10881	8.4716	SPNS1_ENSG00000169682;RP11-264B17.3_ENSG00000261067
16	29040699	29040916	KD2-H4K8ac_peak_10882	5.43522	
16	29127549	29127746	KD2-H4K8ac_peak_10883	4.77079	RP11-426C22.5_ENSG00000260517
16	29140695	29140972	KD2-H4K8ac_peak_10884	3.97991	
16	29143918	29144119	KD2-H4K8ac_peak_10885	5.30799	
16	29169157	29170163	KD2-H4K8ac_peak_10886	8.82366	
16	29180659	29181608	KD2-H4K8ac_peak_10887	15.25814	
16	29186806	29187758	KD2-H4K8ac_peak_10888	13.9152	
16	29188145	29189573	KD2-H4K8ac_peak_10889	22.21705	
16	29189808	29190299	KD2-H4K8ac_peak_10890	4.51154	
16	29190533	29192188	KD2-H4K8ac_peak_10891	15.25814	
16	29193844	29194956	KD2-H4K8ac_peak_10892	4.44527	
16	29195760	29196087	KD2-H4K8ac_peak_10893	4.86324	
16	29197614	29198019	KD2-H4K8ac_peak_10894	5.88845	
16	29210718	29211035	KD2-H4K8ac_peak_10895	4.8888	
16	29212176	29212586	KD2-H4K8ac_peak_10896	6.99815	
16	29212809	29213018	KD2-H4K8ac_peak_10897	8.55271	
16	29214159	29214513	KD2-H4K8ac_peak_10898	5.59859	
16	29231890	29232188	KD2-H4K8ac_peak_10899	7.27716	RP11-426C22.4_ENSG00000259807
16	29233385	29233775	KD2-H4K8ac_peak_10900	6.99815	

16	29239779	29240152	KD2-H4K8ac_peak_10901	4.42765	
16	29266477	29267069	KD2-H4K8ac_peak_10902	9.36516	
16	29268854	29269078	KD2-H4K8ac_peak_10903	5.30799	
16	29270333	29272527	KD2-H4K8ac_peak_10904	18.54875	
16	29272828	29273042	KD2-H4K8ac_peak_10905	8.85813	
16	29273278	29273869	KD2-H4K8ac_peak_10906	20.32753	
16	29275470	29276661	KD2-H4K8ac_peak_10907	14.69247	RP11-426C22.6_ENSG00000260953
16	29303170	29303474	KD2-H4K8ac_peak_10908	6.99815	
16	29311688	29312135	KD2-H4K8ac_peak_10909	6.13909	
16	29325513	29325972	KD2-H4K8ac_peak_10910	6.72021	
16	29348792	29348991	KD2-H4K8ac_peak_10911	5.88845	
16	29614669	29614887	KD2-H4K8ac_peak_10912	6.40683	
16	29618416	29618639	KD2-H4K8ac_peak_10913	6.0073	
16	29624749	29624943	KD2-H4K8ac_peak_10914	7.146	SLC7A5P1_ENSG00000260727
16	29625242	29625547	KD2-H4K8ac_peak_10915	8.55271	SLC7A5P1_ENSG00000260727
16	29687758	29688120	KD2-H4K8ac_peak_10916	6.86812	
16	29690661	29690910	KD2-H4K8ac_peak_10917	7.51054	
16	29691418	29691722	KD2-H4K8ac_peak_10918	7.95563	
16	29740410	29740710	KD2-H4K8ac_peak_10919	7.98152	
16	29800974	29801773	KD2-H4K8ac_peak_10920	15.52826	KIF22_ENSG00000079616
16	29802078	29802611	KD2-H4K8ac_peak_10921	14.15075	KIF22_ENSG00000079616
16	29816119	29816711	KD2-H4K8ac_peak_10922	13.87818	MAZ_ENSG00000103495
16	29818005	29819153	KD2-H4K8ac_peak_10923	18.54875	MAZ_ENSG00000103495;AC009133.15_ENSG00000259952
16	29822943	29823321	KD2-H4K8ac_peak_10924	5.59859	AC009133.14_ENSG00000238045;PRRT2_ENSG00000167371
16	29823661	29824327	KD2-H4K8ac_peak_10925	8.30974	AC009133.14_ENSG00000238045;PRRT2_ENSG00000167371
16	29826621	29826902	KD2-H4K8ac_peak_10926	7.76329	PAGR1_ENSG00000185928;PAGR1_ENSG00000263136
16	29827468	29828270	KD2-H4K8ac_peak_10927	12.32775	PAGR1_ENSG00000185928;PAGR1_ENSG00000263136
16	29874155	29874433	KD2-H4K8ac_peak_10928	7.74292	CDIPT_ENSG00000103502;CDIPT-AS1_ENSG00000214725
16	29875010	29875647	KD2-H4K8ac_peak_10929	15.13261	CDIPT_ENSG00000103502;CDIPT-AS1_ENSG00000214725
16	29912417	29912650	KD2-H4K8ac_peak_10930	4.9025	ASPHD1_ENSG00000174939
16	29937689	29937932	KD2-H4K8ac_peak_10931	10.05606	KCTD13_ENSG00000174943;CTD-2574D22.2_ENSG00000247735
16	29938139	29938342	KD2-H4K8ac_peak_10932	4.52326	KCTD13_ENSG00000174943;CTD-2574D22.2_ENSG00000247735
16	29972705	29973184	KD2-H4K8ac_peak_10933	8.53092	
16	29985263	29985587	KD2-H4K8ac_peak_10934	9.10868	TAOK2_ENSG00000149930
16	30006517	30007334	KD2-H4K8ac_peak_10935	12.30509	HIRIP3_ENSG00000149929;INO80E_ENSG00000169592
16	30007717	30008331	KD2-H4K8ac_peak_10936	7.36799	HIRIP3_ENSG00000149929
16	30022545	30022788	KD2-H4K8ac_peak_10937	6.40297	
16	30032462	30032965	KD2-H4K8ac_peak_10938	14.15075	
16	30042416	30042875	KD2-H4K8ac_peak_10939	7.07117	
16	30043088	30043456	KD2-H4K8ac_peak_10940	7.0754	
16	30063937	30064305	KD2-H4K8ac_peak_10941	9.84972	FAM57B_ENSG00000149926;ALDOA_ENSG00000149925
16	30064563	30064987	KD2-H4K8ac_peak_10942	3.75035	FAM57B_ENSG00000149926;ALDOA_ENSG00000149925
16	30075023	30075270	KD2-H4K8ac_peak_10943	5.75757	
16	30075661	30076854	KD2-H4K8ac_peak_10944	13.27022	
16	30086520	30086991	KD2-H4K8ac_peak_10945	7.76329	PPP4C_ENSG00000149923
16	30102411	30102641	KD2-H4K8ac_peak_10946	3.97079	TBX6_ENSG00000149922

16	30103461	30104278	KD2-H4K8ac_peak_10947	20.10577	TBX6_ENSG00000149922
16	30107806	30108258	KD2-H4K8ac_peak_10948	18.59584	YPEL3_ENSG00000090238;RP11-455F5.3_ENSG00000250616
16	30133766	30134410	KD2-H4K8ac_peak_10949	16.57619	MAPK3_ENSG00000102882
16	30134705	30135328	KD2-H4K8ac_peak_10950	11.7296	MAPK3_ENSG00000102882
16	30149413	30149891	KD2-H4K8ac_peak_10951	5.57044	
16	30194203	30194788	KD2-H4K8ac_peak_10952	10.19949	CORO1A_ENSG00000102879
16	30195534	30195738	KD2-H4K8ac_peak_10953	9.36516	RP11-455F5.5_ENSG00000261416
16	30366168	30366501	KD2-H4K8ac_peak_10954	5.13615	CD2BP2_ENSG00000169217;RP11-347C12.10_ENSG00000260219
16	30366841	30367429	KD2-H4K8ac_peak_10955	11.90231	CD2BP2_ENSG00000169217;RP11-347C12.10_ENSG00000260219
16	30381408	30381708	KD2-H4K8ac_peak_10956	8.10216	TBC1D10B_ENSG00000169221;MYLPF_ENSG00000180209
16	30382714	30383033	KD2-H4K8ac_peak_10957	8.04448	MYLPF_ENSG00000180209
16	30388551	30389509	KD2-H4K8ac_peak_10958	10.80014	ZNF48_ENSG00000180035
16	30404747	30406225	KD2-H4K8ac_peak_10959	15.61313	
16	30406851	30407321	KD2-H4K8ac_peak_10960	6.67538	SEPT1_ENSG00000180096
16	30411188	30411384	KD2-H4K8ac_peak_10961	5.34636	
16	30411819	30412307	KD2-H4K8ac_peak_10962	6.75585	
16	30418284	30418676	KD2-H4K8ac_peak_10963	12.88108	ZNF771_ENSG00000179965
16	30419043	30419249	KD2-H4K8ac_peak_10964	9.73431	ZNF771_ENSG00000179965
16	30428834	30429120	KD2-H4K8ac_peak_10965	10.73134	
16	30440788	30441328	KD2-H4K8ac_peak_10966	21.09577	DCTPP1_ENSG00000179958
16	30441793	30442283	KD2-H4K8ac_peak_10967	10.19949	DCTPP1_ENSG00000179958
16	30456868	30457158	KD2-H4K8ac_peak_10968	16.57619	SEPHS2_ENSG00000179918
16	30457712	30457925	KD2-H4K8ac_peak_10969	5.21139	SEPHS2_ENSG00000179918
16	30537487	30537902	KD2-H4K8ac_peak_10970	6.25869	ZNF768_ENSG00000169957
16	30538207	30538672	KD2-H4K8ac_peak_10971	25.95849	ZNF768_ENSG00000169957
16	30545254	30546189	KD2-H4K8ac_peak_10972	24.30324	ZNF747_ENSG00000169955;ZNF747_ENSG00000261459;AC002310.12_ENSG00000235560
16	30546398	30546609	KD2-H4K8ac_peak_10973	4.37809	ZNF747_ENSG00000169955;ZNF747_ENSG00000261459;AC002310.12_ENSG00000235560
16	30569182	30569684	KD2-H4K8ac_peak_10974	16.67374	AC002310.13_ENSG00000260869;ZNF764_ENSG00000169951
16	30569994	30570618	KD2-H4K8ac_peak_10975	13.73635	AC002310.13_ENSG00000260869;ZNF764_ENSG00000169951
16	30582166	30583150	KD2-H4K8ac_peak_10976	16.86242	ZNF688_ENSG00000229809;AC002310.7_ENSG00000239791
16	30583804	30584031	KD2-H4K8ac_peak_10977	6.0073	ZNF688_ENSG00000229809;AC002310.7_ENSG00000239791
16	30596452	30597005	KD2-H4K8ac_peak_10978	27.97933	ZNF785_ENSG00000197162;RP11-146F11.5_ENSG00000260167
16	30597336	30597806	KD2-H4K8ac_peak_10979	8.94469	ZNF785_ENSG00000197162;RP11-146F11.5_ENSG00000260167
16	30621063	30621652	KD2-H4K8ac_peak_10980	14.17488	
16	30639553	30640069	KD2-H4K8ac_peak_10981	6.31143	
16	30644955	30645325	KD2-H4K8ac_peak_10982	5.54647	
16	30645888	30646379	KD2-H4K8ac_peak_10983	11.89502	
16	30661420	30661655	KD2-H4K8ac_peak_10984	7.76104	PRR14_ENSG00000156858
16	30662418	30662805	KD2-H4K8ac_peak_10985	6.52179	PRR14_ENSG00000156858
16	30670365	30670756	KD2-H4K8ac_peak_10986	8.65175	FBRS_ENSG00000156860
16	30670978	30671298	KD2-H4K8ac_peak_10987	9.95119	
16	30709141	30709343	KD2-H4K8ac_peak_10988	9.07715	SRCAP_ENSG00000080603
16	30709575	30710353	KD2-H4K8ac_peak_10989	12.65012	RP11-146F11.1_ENSG00000261840;SRCAP_ENSG00000080603
16	30759072	30759371	KD2-H4K8ac_peak_10990	6.40297	PHKG2_ENSG00000156873
16	30772981	30773260	KD2-H4K8ac_peak_10991	8.17902	C16orf93_ENSG00000196118;RNF40_ENSG00000103549
16	30773674	30774042	KD2-H4K8ac_peak_10992	8.55271	C16orf93_ENSG00000196118;RNF40_ENSG00000103549

16	30786394	30786683	KD2-H4K8ac_peak_10993	6.99815	
16	30786900	30787634	KD2-H4K8ac_peak_10994	10.43145	
16	30797843	30799181	KD2-H4K8ac_peak_10995	21.6161	ZNF629_ENSG00000102870
16	30817005	30817413	KD2-H4K8ac_peak_10996	10.29842	
16	30824776	30825664	KD2-H4K8ac_peak_10997	13.36684	
16	30885914	30886585	KD2-H4K8ac_peak_10998	7.11824	MIR4519_ENSG00000260083;MIR4519_ENSG00000265991
16	30886812	30887688	KD2-H4K8ac_peak_10999	6.66692	MIR4519_ENSG00000260083;MIR4519_ENSG00000265991
16	30904878	30905325	KD2-H4K8ac_peak_11000	9.98013	BCL7C_ENSG00000099385;MIR762_ENSG00000211591
16	30907580	30907817	KD2-H4K8ac_peak_11001	7.36799	CTF1_ENSG00000150281
16	30908017	30908288	KD2-H4K8ac_peak_11002	4.06758	CTF1_ENSG00000150281
16	30913311	30913508	KD2-H4K8ac_peak_11003	9.16605	
16	30913744	30914274	KD2-H4K8ac_peak_11004	5.14125	
16	30915351	30915876	KD2-H4K8ac_peak_11005	7.36799	
16	30932294	30932583	KD2-H4K8ac_peak_11006	5.77265	
16	30933260	30933719	KD2-H4K8ac_peak_11007	5.1628	FBXL19-AS1_ENSG00000260852;FBXL19_ENSG00000099364
16	30934223	30934434	KD2-H4K8ac_peak_11008	4.68059	FBXL19-AS1_ENSG00000260852;FBXL19_ENSG00000099364
16	30934875	30935803	KD2-H4K8ac_peak_11009	19.87796	FBXL19-AS1_ENSG00000260852;FBXL19_ENSG00000099364
16	30938930	30939274	KD2-H4K8ac_peak_11010	5.88845	
16	30960458	30960769	KD2-H4K8ac_peak_11011	9.65086	AC135048.13_ENSG00000261487;ORAI3_ENSG00000175938
16	30965335	30965636	KD2-H4K8ac_peak_11012	5.02516	
16	30968323	30969469	KD2-H4K8ac_peak_11013	15.61313	SETD1A_ENSG00000099381
16	31008003	31008197	KD2-H4K8ac_peak_11014	6.16888	
16	31022291	31022514	KD2-H4K8ac_peak_11015	5.54647	STX1B_ENSG00000099365
16	31044091	31044670	KD2-H4K8ac_peak_11016	19.1048	STX4_ENSG00000103496
16	31084841	31085068	KD2-H4K8ac_peak_11017	11.00004	ZNF668_ENSG00000167394;ZNF646_ENSG00000167395
16	31085766	31085967	KD2-H4K8ac_peak_11018	12.08081	ZNF668_ENSG00000167394;ZNF646_ENSG00000167395
16	31105747	31105988	KD2-H4K8ac_peak_11019	8.95692	RP11-196G11.1_ENSG00000255439
16	31106483	31106712	KD2-H4K8ac_peak_11020	7.69287	RP11-196G11.1_ENSG00000255439;VKORC1_ENSG00000167397
16	31116618	31116854	KD2-H4K8ac_peak_11021	8.87298	BCKDK_ENSG00000103507
16	31117205	31117557	KD2-H4K8ac_peak_11022	5.38581	BCKDK_ENSG00000103507
16	31119818	31120305	KD2-H4K8ac_peak_11023	5.4779	AC135050.1_ENSG00000252809
16	31153937	31154531	KD2-H4K8ac_peak_11024	20.0792	
16	31190794	31191084	KD2-H4K8ac_peak_11025	10.05606	FUS_ENSG00000089280
16	31439432	31439697	KD2-H4K8ac_peak_11026	5.88845	COX6A2_ENSG00000156885
16	31453341	31454355	KD2-H4K8ac_peak_11027	10.19949	ZNF843_ENSG00000176723
16	31470235	31471064	KD2-H4K8ac_peak_11028	15.9754	RP11-452L6.5_ENSG00000260267;ARMC5_ENSG00000140691
16	31471652	31472020	KD2-H4K8ac_peak_11029	4.66652	RP11-452L6.5_ENSG00000260267
16	31482818	31484304	KD2-H4K8ac_peak_11030	20.78681	TGFB1I1_ENSG00000140682
16	31499178	31499403	KD2-H4K8ac_peak_11031	6.97919	AC026471.6_ENSG00000260740
16	31519314	31520032	KD2-H4K8ac_peak_11032	16.04168	C16orf58_ENSG00000140688;RP11-452L6.7_ENSG00000260625
16	31711867	31712285	KD2-H4K8ac_peak_11033	13.44374	CTD-2358C21.3_ENSG00000260568;CLUHP3_ENSG00000131797
16	31724651	31724981	KD2-H4K8ac_peak_11034	12.80176	ZNF720_ENSG00000197302
16	32200172	32200475	KD2-H4K8ac_peak_11035	5.25071	HERC2P4_ENSG00000230267;RP11-17M15.1_ENSG00000260847;RP11-56L13.4_ENSG00000260649
16	32265562	32266020	KD2-H4K8ac_peak_11036	9.20193	RP11-56L13.7_ENSG00000260575
16	33318917	33319172	KD2-H4K8ac_peak_11037	5.88845	
16	33509221	33509647	KD2-H4K8ac_peak_11038	14.99434	

16	34441380	34441794	KD2-H4K8ac_peak_11039	6.40297	RP11-244B22.5_ENSG00000261274;RP11-488I20.8_ENSG00000260958
16	34489969	34490526	KD2-H4K8ac_peak_11040	9.72244	RARRES2P6_ENSG00000261010
16	34586706	34587256	KD2-H4K8ac_peak_11041	11.89502	RP11-488I20.4_ENSG00000260480
16	34740076	34740362	KD2-H4K8ac_peak_11042	7.81657	RP11-80F22.15_ENSG00000260857
16	34741008	34741405	KD2-H4K8ac_peak_11043	8.34752	RP11-80F22.15_ENSG00000260857;RP11-80F22.2_ENSG00000269622
16	46654855	46655150	KD2-H4K8ac_peak_11044	3.97079	SHCBP1_ENSG00000171241
16	46663593	46663818	KD2-H4K8ac_peak_11045	7.47462	
16	46723263	46723984	KD2-H4K8ac_peak_11046	11.24702	VPS35_ENSG00000069329;ORC6_ENSG00000091651
16	46864462	46865044	KD2-H4K8ac_peak_11047	10.46264	C16orf87_ENSG00000155330
16	46917602	46917818	KD2-H4K8ac_peak_11048	10.30284	GPT2_ENSG00000166123
16	46918316	46919062	KD2-H4K8ac_peak_11049	8.94469	GPT2_ENSG00000166123
16	47006889	47007469	KD2-H4K8ac_peak_11050	9.1707	DNAJA2_ENSG00000069345;RP11-169E6.1_ENSG00000261173
16	47007719	47008012	KD2-H4K8ac_peak_11051	9.95119	DNAJA2_ENSG00000069345;RP11-169E6.1_ENSG00000261173
16	47071350	47071782	KD2-H4K8ac_peak_11052	10.7186	
16	47072200	47072425	KD2-H4K8ac_peak_11053	8.09472	
16	47176770	47178329	KD2-H4K8ac_peak_11054	15.22643	NETO2_ENSG00000171208;RP11-329J18.2_ENSG00000260281
16	47494730	47495393	KD2-H4K8ac_peak_11055	11.89502	PHKB_ENSG00000102893
16	47746571	47746810	KD2-H4K8ac_peak_11056	7.70289	
16	47747648	47747943	KD2-H4K8ac_peak_11057	7.47462	
16	48277654	48277857	KD2-H4K8ac_peak_11058	7.27716	LONP2_ENSG00000102910
16	48278248	48278583	KD2-H4K8ac_peak_11059	6.36375	LONP2_ENSG00000102910
16	48418319	48419289	KD2-H4K8ac_peak_11060	20.5798	
16	48419512	48419937	KD2-H4K8ac_peak_11061	8.16906	
16	48565240	48565757	KD2-H4K8ac_peak_11062	16.61426	
16	48644309	48644650	KD2-H4K8ac_peak_11063	7.67504	
16	48656765	48657153	KD2-H4K8ac_peak_11064	5.08628	RP11-42I10.1_ENSG00000260086
16	48657425	48657852	KD2-H4K8ac_peak_11065	7.76329	RP11-42I10.1_ENSG00000260086
16	49316032	49316443	KD2-H4K8ac_peak_11066	14.15075	CBLN1_ENSG00000102924
16	49526114	49526608	KD2-H4K8ac_peak_11067	11.86082	
16	49557377	49557790	KD2-H4K8ac_peak_11068	6.69844	
16	49558004	49558596	KD2-H4K8ac_peak_11069	14.14575	
16	49558814	49559604	KD2-H4K8ac_peak_11070	13.30514	
16	49560889	49561184	KD2-H4K8ac_peak_11071	4.211	
16	49627510	49627786	KD2-H4K8ac_peak_11072	3.75035	
16	49628645	49629193	KD2-H4K8ac_peak_11073	10.41994	
16	49630757	49631193	KD2-H4K8ac_peak_11074	11.24702	
16	49633681	49634163	KD2-H4K8ac_peak_11075	7.96322	
16	49635607	49635978	KD2-H4K8ac_peak_11076	5.16215	
16	49654653	49655679	KD2-H4K8ac_peak_11077	17.8868	
16	49673029	49673993	KD2-H4K8ac_peak_11078	7.47462	
16	49674882	49675339	KD2-H4K8ac_peak_11079	6.65673	
16	49686260	49686562	KD2-H4K8ac_peak_11080	7.76329	
16	49697414	49698435	KD2-H4K8ac_peak_11081	17.27647	
16	49698733	49699272	KD2-H4K8ac_peak_11082	9.95342	
16	49699491	49699683	KD2-H4K8ac_peak_11083	4.86324	
16	49730313	49731016	KD2-H4K8ac_peak_11084	12.84642	

16	49731806	49732162	KD2-H4K8ac_peak_11085	6.20982	
16	49732661	49733456	KD2-H4K8ac_peak_11086	12.78794	
16	49733676	49733962	KD2-H4K8ac_peak_11087	11.92978	
16	49734223	49734694	KD2-H4K8ac_peak_11088	6.65673	
16	49776574	49776782	KD2-H4K8ac_peak_11089	5.82799	
16	49778046	49778410	KD2-H4K8ac_peak_11090	4.86324	
16	49786053	49786274	KD2-H4K8ac_peak_11091	12.00597	
16	49788739	49789252	KD2-H4K8ac_peak_11092	7.76104	
16	49810380	49810742	KD2-H4K8ac_peak_11093	4.77079	
16	49843717	49844049	KD2-H4K8ac_peak_11094	7.76329	
16	49851117	49851565	KD2-H4K8ac_peak_11095	6.56211	AC027348.1_ENSG00000265708
16	49855413	49855960	KD2-H4K8ac_peak_11096	7.36799	
16	49856245	49856490	KD2-H4K8ac_peak_11097	7.58939	
16	49867786	49867981	KD2-H4K8ac_peak_11098	5.57044	
16	49868440	49869817	KD2-H4K8ac_peak_11099	11.89502	
16	49880412	49880614	KD2-H4K8ac_peak_11100	5.88845	
16	49888546	49889409	KD2-H4K8ac_peak_11101	15.81711	
16	49889681	49889920	KD2-H4K8ac_peak_11102	9.1707	
16	49890557	49891065	KD2-H4K8ac_peak_11103	5.405	ZNF423_ENSG00000102935
16	49891766	49892334	KD2-H4K8ac_peak_11104	8.3261	ZNF423_ENSG00000102935
16	49892565	49893193	KD2-H4K8ac_peak_11105	7.44324	ZNF423_ENSG00000102935
16	49900238	49900670	KD2-H4K8ac_peak_11106	11.24702	
16	49900953	49901456	KD2-H4K8ac_peak_11107	11.92978	
16	49902139	49902348	KD2-H4K8ac_peak_11108	4.12757	
16	49910323	49910572	KD2-H4K8ac_peak_11109	10.2048	
16	49917097	49917390	KD2-H4K8ac_peak_11110	5.15777	
16	49955101	49955811	KD2-H4K8ac_peak_11111	8.30974	
16	50059228	50059533	KD2-H4K8ac_peak_11112	7.07117	CNEP1R1_ENSG00000205423
16	50099937	50100188	KD2-H4K8ac_peak_11113	15.82362	RP11-429P3.3_ENSG00000259843;HEATR3_ENSG00000155393
16	50186799	50187007	KD2-H4K8ac_peak_11114	6.99815	PAPD5_ENSG00000121274
16	50278638	50278919	KD2-H4K8ac_peak_11115	9.72244	
16	50279226	50279770	KD2-H4K8ac_peak_11116	10.90946	ADCY7_ENSG00000121281
16	50280057	50280695	KD2-H4K8ac_peak_11117	8.30974	ADCY7_ENSG00000121281
16	50281039	50281251	KD2-H4K8ac_peak_11118	6.2159	ADCY7_ENSG00000121281
16	50299607	50300086	KD2-H4K8ac_peak_11119	4.86324	
16	50300444	50300825	KD2-H4K8ac_peak_11120	13.36684	
16	50331787	50332107	KD2-H4K8ac_peak_11121	4.44527	
16	50402368	50402885	KD2-H4K8ac_peak_11122	9.36516	BRD7_ENSG00000166164;RP11-21B23.1_ENSG00000261393
16	50424171	50424855	KD2-H4K8ac_peak_11123	8.85813	
16	50425164	50425546	KD2-H4K8ac_peak_11124	14.00658	
16	50502281	50502494	KD2-H4K8ac_peak_11125	9.95759	
16	50727270	50727664	KD2-H4K8ac_peak_11126	8.55271	NOD2_ENSG00000167207
16	50776189	50776393	KD2-H4K8ac_peak_11127	4.44527	RP11-327F22.1_ENSG00000260929;CYLD_ENSG00000083799;hsa-mir-3181_ENSG00000264947
16	53088334	53088759	KD2-H4K8ac_peak_11128	10.43145	CHD9_ENSG00000177200
16	53088985	53089296	KD2-H4K8ac_peak_11129	7.07117	CHD9_ENSG00000177200
16	53114210	53114490	KD2-H4K8ac_peak_11130	10.53377	

16	53118593	53119470	KD2-H4K8ac_peak_11131	8.85813	
16	53119892	53120521	KD2-H4K8ac_peak_11132	8.30974	
16	53124373	53124706	KD2-H4K8ac_peak_11133	9.11031	
16	53125002	53125446	KD2-H4K8ac_peak_11134	6.54442	
16	53126705	53127001	KD2-H4K8ac_peak_11135	5.77029	
16	53127953	53128279	KD2-H4K8ac_peak_11136	7.79558	
16	53131766	53132807	KD2-H4K8ac_peak_11137	6.25869	
16	53134448	53134721	KD2-H4K8ac_peak_11138	8.09113	
16	53163898	53164346	KD2-H4K8ac_peak_11139	7.51054	
16	53467849	53468185	KD2-H4K8ac_peak_11140	6.25869	RBL2_ENSG00000103479
16	53536581	53537030	KD2-H4K8ac_peak_11141	6.40683	
16	53537582	53538473	KD2-H4K8ac_peak_11142	11.95211	AKTIP_ENSG00000166971
16	53544163	53544444	KD2-H4K8ac_peak_11143	8.17902	
16	54096896	54097088	KD2-H4K8ac_peak_11144	5.14125	
16	54210334	54210564	KD2-H4K8ac_peak_11145	5.14125	
16	54227567	54227816	KD2-H4K8ac_peak_11146	4.24345	
16	54228065	54228735	KD2-H4K8ac_peak_11147	11.89502	
16	54311331	54311574	KD2-H4K8ac_peak_11148	10.0692	
16	54311884	54312546	KD2-H4K8ac_peak_11149	7.12754	
16	54319751	54320407	KD2-H4K8ac_peak_11150	12.74171	IRX3_ENSG00000177508
16	54320960	54321396	KD2-H4K8ac_peak_11151	20.30085	IRX3_ENSG00000177508
16	54962375	54963030	KD2-H4K8ac_peak_11152	20.58734	CRNDE_ENSG00000245694
16	54964020	54964389	KD2-H4K8ac_peak_11153	9.11031	CRNDE_ENSG00000245694;IRX5_ENSG00000176842
16	54964991	54965481	KD2-H4K8ac_peak_11154	11.2938	IRX5_ENSG00000176842
16	54966090	54966460	KD2-H4K8ac_peak_11155	5.82869	
16	55297100	55297525	KD2-H4K8ac_peak_11156	12.14738	
16	55357559	55358407	KD2-H4K8ac_peak_11157	14.00658	IRX6_ENSG00000159387
16	55407147	55408004	KD2-H4K8ac_peak_11158	9.1707	
16	55413615	55413895	KD2-H4K8ac_peak_11159	4.9025	
16	55513178	55513726	KD2-H4K8ac_peak_11160	9.36516	
16	55513981	55514240	KD2-H4K8ac_peak_11161	4.86324	
16	55676721	55677276	KD2-H4K8ac_peak_11162	8.51832	
16	55684152	55684590	KD2-H4K8ac_peak_11163	9.4551	
16	55684974	55685171	KD2-H4K8ac_peak_11164	8.59579	
16	55686519	55686920	KD2-H4K8ac_peak_11165	12.56039	
16	55689091	55689634	KD2-H4K8ac_peak_11166	4.8888	SLC6A2_ENSG00000103546
16	55689983	55690925	KD2-H4K8ac_peak_11167	12.08081	SLC6A2_ENSG00000103546
16	55978434	55978756	KD2-H4K8ac_peak_11168	6.16888	
16	56135527	56135850	KD2-H4K8ac_peak_11169	9.23578	
16	56291718	56291954	KD2-H4K8ac_peak_11170	5.45396	
16	56292423	56293163	KD2-H4K8ac_peak_11171	12.82393	
16	56351876	56352101	KD2-H4K8ac_peak_11172	6.05809	
16	56458953	56459232	KD2-H4K8ac_peak_11173	9.36516	AMFR_ENSG00000159461
16	56484840	56485201	KD2-H4K8ac_peak_11174	9.1707	NUDT21_ENSG00000167005;OGFOD1_ENSG00000087263
16	56485679	56485884	KD2-H4K8ac_peak_11175	3.75035	NUDT21_ENSG00000167005;OGFOD1_ENSG00000087263
16	56553586	56553862	KD2-H4K8ac_peak_11176	10.08707	BBS2_ENSG00000125124

16	56642014	56642356	KD2-H4K8ac_peak_11177	16.49944	MT2A_ENSG00000125148
16	56644045	56644237	KD2-H4K8ac_peak_11178	5.53095	
16	56691616	56691825	KD2-H4K8ac_peak_11179	5.57044	MT1F_ENSG00000198417
16	56715769	56716027	KD2-H4K8ac_peak_11180	12.08081	MT1X_ENSG00000187193;RP11-343H19.2_ENSG00000259827
16	56716399	56716716	KD2-H4K8ac_peak_11181	7.76329	MT1X_ENSG00000187193;RP11-343H19.2_ENSG00000259827
16	56743188	56743628	KD2-H4K8ac_peak_11182	6.00226	
16	56764105	56764486	KD2-H4K8ac_peak_11183	6.65673	RP11-343H19.1_ENSG00000261302;NUP93_ENSG00000102900
16	56905242	56905457	KD2-H4K8ac_peak_11184	4.41495	
16	56908030	56908746	KD2-H4K8ac_peak_11185	8.10216	
16	56910053	56910279	KD2-H4K8ac_peak_11186	7.40314	
16	56964703	56965044	KD2-H4K8ac_peak_11187	4.91674	HERPUD1_ENSG00000051108
16	56965332	56965776	KD2-H4K8ac_peak_11188	25.84576	HERPUD1_ENSG00000051108
16	56966134	56966684	KD2-H4K8ac_peak_11189	7.74292	HERPUD1_ENSG00000051108
16	57023169	57023369	KD2-H4K8ac_peak_11190	6.97919	NLRC5_ENSG00000140853
16	57093387	57093586	KD2-H4K8ac_peak_11191	5.53095	RP11-322D14.2_ENSG00000260145
16	57095079	57095885	KD2-H4K8ac_peak_11192	20.97251	
16	57125501	57125700	KD2-H4K8ac_peak_11193	6.04107	RP11-407G23.1_ENSG00000260148;CPNE2_ENSG00000140848
16	57125981	57126970	KD2-H4K8ac_peak_11194	8.4716	RP11-407G23.1_ENSG00000260148;CPNE2_ENSG00000140848
16	57149581	57149815	KD2-H4K8ac_peak_11195	7.98152	
16	57181132	57181347	KD2-H4K8ac_peak_11196	4.42765	
16	57219471	57219706	KD2-H4K8ac_peak_11197	7.76329	FAM192A_ENSG00000172775;RSPRY1_ENSG00000159579
16	57220277	57220698	KD2-H4K8ac_peak_11198	11.89502	FAM192A_ENSG00000172775;RSPRY1_ENSG00000159579
16	57279217	57279472	KD2-H4K8ac_peak_11199	15.58442	ARL2BP_ENSG00000102931
16	57296847	57297248	KD2-H4K8ac_peak_11200	5.54647	
16	57307250	57307476	KD2-H4K8ac_peak_11201	6.37852	
16	57318037	57318703	KD2-H4K8ac_peak_11202	16.822	PLLP_ENSG00000102934
16	57405262	57406641	KD2-H4K8ac_peak_11203	12.14909	CX3CL1_ENSG00000006210
16	57450160	57451007	KD2-H4K8ac_peak_11204	17.43561	
16	57453290	57453744	KD2-H4K8ac_peak_11205	6.7498	
16	57480652	57481142	KD2-H4K8ac_peak_11206	8.85813	CIAPIN1_ENSG00000005194;COQ9_ENSG00000088682
16	57481446	57481818	KD2-H4K8ac_peak_11207	12.93209	CIAPIN1_ENSG00000005194;COQ9_ENSG00000088682
16	57507175	57507471	KD2-H4K8ac_peak_11208	3.90565	
16	57513009	57513513	KD2-H4K8ac_peak_11209	8.58725	
16	57513994	57515279	KD2-H4K8ac_peak_11210	13.34709	
16	57517703	57517990	KD2-H4K8ac_peak_11211	9.36982	
16	57518317	57518522	KD2-H4K8ac_peak_11212	4.72599	
16	57537425	57537967	KD2-H4K8ac_peak_11213	7.372	
16	57539220	57539437	KD2-H4K8ac_peak_11214	5.40498	
16	57541165	57541570	KD2-H4K8ac_peak_11215	9.20193	
16	57541825	57542778	KD2-H4K8ac_peak_11216	7.28628	
16	57543216	57543763	KD2-H4K8ac_peak_11217	11.02863	
16	57544389	57545023	KD2-H4K8ac_peak_11218	8.23274	
16	57548685	57549184	KD2-H4K8ac_peak_11219	9.06432	
16	57567733	57567947	KD2-H4K8ac_peak_11220	6.08655	
16	57568994	57569231	KD2-H4K8ac_peak_11221	8.26945	
16	57570213	57571116	KD2-H4K8ac_peak_11222	14.95275	CCDC102A_ENSG00000135736

16	57728725	57729063	KD2-H4K8ac_peak_11223	9.11031	CCDC135_ENSG00000159625
16	57730039	57730444	KD2-H4K8ac_peak_11224	6.99815	
16	57768396	57769126	KD2-H4K8ac_peak_11225	15.81711	KATNB1_ENSG00000140854
16	57836033	57836286	KD2-H4K8ac_peak_11226	10.53377	
16	57836588	57837471	KD2-H4K8ac_peak_11227	12.88108	
16	58018396	58018862	KD2-H4K8ac_peak_11228	8.55271	
16	58033880	58034542	KD2-H4K8ac_peak_11229	7.76329	ZNF319_ENSG00000166188;USB1_ENSG00000103005
16	58034744	58035023	KD2-H4K8ac_peak_11230	6.90231	ZNF319_ENSG00000166188
16	58058838	58059547	KD2-H4K8ac_peak_11231	9.2116	MMP15_ENSG00000102996
16	58067125	58067468	KD2-H4K8ac_peak_11232	7.48958	
16	58069140	58069359	KD2-H4K8ac_peak_11233	5.07021	
16	58163827	58164118	KD2-H4K8ac_peak_11234	10.87093	C16orf80_ENSG00000070761;RP11-459F6.3_ENSG00000260927
16	58231395	58231589	KD2-H4K8ac_peak_11235	7.67504	CSNK2A2_ENSG00000070770;RP11-459F6.1_ENSG00000260867
16	58232237	58232555	KD2-H4K8ac_peak_11236	11.05467	CSNK2A2_ENSG00000070770
16	58270032	58270434	KD2-H4K8ac_peak_11237	12.42761	
16	58283908	58284108	KD2-H4K8ac_peak_11238	6.13909	
16	58426136	58426972	KD2-H4K8ac_peak_11239	10.43145	
16	58497012	58497682	KD2-H4K8ac_peak_11240	6.55628	NDRG4_ENSG00000103034
16	58497908	58498116	KD2-H4K8ac_peak_11241	7.07117	
16	58527351	58527981	KD2-H4K8ac_peak_11242	5.85704	
16	58533154	58533703	KD2-H4K8ac_peak_11243	6.99815	
16	58535472	58535690	KD2-H4K8ac_peak_11244	5.66705	
16	58548925	58549220	KD2-H4K8ac_peak_11245	9.73431	SETD6_ENSG00000103037
16	58549468	58549964	KD2-H4K8ac_peak_11246	9.95119	SETD6_ENSG00000103037
16	58663167	58663714	KD2-H4K8ac_peak_11247	9.95119	CNOT1_ENSG00000125107
16	58664121	58664870	KD2-H4K8ac_peak_11248	8.55271	CNOT1_ENSG00000125107
16	58718117	58718567	KD2-H4K8ac_peak_11249	10.19949	SLC38A7_ENSG00000103042
16	58718773	58719067	KD2-H4K8ac_peak_11250	9.72244	SLC38A7_ENSG00000103042
16	58767646	58768058	KD2-H4K8ac_peak_11251	7.76104	GOT2_ENSG00000125166;RP11-410D17.2_ENSG00000245768
16	58768398	58768892	KD2-H4K8ac_peak_11252	9.3973	GOT2_ENSG00000125166;RP11-410D17.2_ENSG00000245768
16	64649656	64649851	KD2-H4K8ac_peak_11253	4.01115	
16	65156145	65157017	KD2-H4K8ac_peak_11254	12.42761	
16	65157265	65157946	KD2-H4K8ac_peak_11255	11.89502	
16	65690404	65690776	KD2-H4K8ac_peak_11256	5.15777	
16	65696761	65697023	KD2-H4K8ac_peak_11257	9.76452	
16	65789600	65789817	KD2-H4K8ac_peak_11258	5.47943	
16	65910836	65911035	KD2-H4K8ac_peak_11259	4.37708	
16	66223068	66223606	KD2-H4K8ac_peak_11260	4.77079	
16	66304212	66304728	KD2-H4K8ac_peak_11261	15.74662	
16	66442375	66442860	KD2-H4K8ac_peak_11262	5.88845	LINC00920_ENSG00000246898
16	66550821	66551525	KD2-H4K8ac_peak_11263	8.00457	RP11-403P17.5_ENSG00000260851
16	66583181	66584219	KD2-H4K8ac_peak_11264	20.88898	Y_RNA_ENSG00000261519
16	66585904	66586382	KD2-H4K8ac_peak_11265	14.80472	TK2_ENSG00000166548;CKLF_ENSG00000217555;CKLF-CMTM1_ENSG00000254788
16	66586576	66586955	KD2-H4K8ac_peak_11266	9.36516	TK2_ENSG00000166548;CKLF_ENSG00000217555;CKLF-CMTM1_ENSG00000254788
16	66624916	66625117	KD2-H4K8ac_peak_11267	5.64449	
16	66637585	66637945	KD2-H4K8ac_peak_11268	5.14125	CMTM3_ENSG00000140931

16	66638139	66638388	KD2-H4K8ac_peak_11269	7.76329	CMTM3_ENSG00000140931
16	66638640	66639565	KD2-H4K8ac_peak_11270	7.36799	CMTM3_ENSG00000140931
16	66729997	66730392	KD2-H4K8ac_peak_11271	6.25869	CMTM4_ENSG00000183723
16	66785079	66785919	KD2-H4K8ac_peak_11272	5.9696	DYNC1LI2_ENSG00000135720;RP11-61A14.4_ENSG00000246777
16	66863672	66863949	KD2-H4K8ac_peak_11273	4.77079	
16	66864404	66864783	KD2-H4K8ac_peak_11274	12.30509	
16	66906814	66907084	KD2-H4K8ac_peak_11275	7.36799	NAE1_ENSG00000159593
16	66913533	66914262	KD2-H4K8ac_peak_11276	16.49944	
16	66914588	66914980	KD2-H4K8ac_peak_11277	3.86308	
16	66933345	66933632	KD2-H4K8ac_peak_11278	7.36799	
16	66934021	66934472	KD2-H4K8ac_peak_11279	7.76329	
16	66967845	66968193	KD2-H4K8ac_peak_11280	7.76329	FAM96B_ENSG00000166595;CES2_ENSG00000172831
16	66995172	66995586	KD2-H4K8ac_peak_11281	13.9234	CES3_ENSG00000172828
16	67034422	67034826	KD2-H4K8ac_peak_11282	15.53798	
16	67047087	67048374	KD2-H4K8ac_peak_11283	10.7186	
16	67052232	67052434	KD2-H4K8ac_peak_11284	5.54647	RN7SL543P_ENSG00000265918
16	67062397	67062844	KD2-H4K8ac_peak_11285	9.1707	CBFB_ENSG00000067955
16	67063113	67063615	KD2-H4K8ac_peak_11286	11.92978	CBFB_ENSG00000067955
16	67109775	67109984	KD2-H4K8ac_peak_11287	4.01115	
16	67140692	67141023	KD2-H4K8ac_peak_11288	8.23274	
16	67143163	67143579	KD2-H4K8ac_peak_11289	7.76104	C16orf70_ENSG00000125149
16	67183959	67185108	KD2-H4K8ac_peak_11290	9.06369	B3GNT9_ENSG00000237172
16	67185383	67185668	KD2-H4K8ac_peak_11291	6.60096	B3GNT9_ENSG00000237172
16	67193306	67193668	KD2-H4K8ac_peak_11292	6.25225	TRADD_ENSG00000102871;FBXL8_ENSG00000135722
16	67195078	67195323	KD2-H4K8ac_peak_11293	5.9696	TRADD_ENSG00000102871
16	67196696	67197153	KD2-H4K8ac_peak_11294	15.96909	HSF4_ENSG00000102878;RP11-5A19.5_ENSG00000265690
16	67197718	67198401	KD2-H4K8ac_peak_11295	11.04256	HSF4_ENSG00000102878;RP11-5A19.5_ENSG00000265690
16	67198694	67198991	KD2-H4K8ac_peak_11296	5.30799	
16	67203575	67204291	KD2-H4K8ac_peak_11297	8.55581	NOL3_ENSG00000140939
16	67208355	67209226	KD2-H4K8ac_peak_11298	12.08081	
16	67217642	67217891	KD2-H4K8ac_peak_11299	21.52156	KIAA0895L_ENSG00000196123
16	67226225	67226432	KD2-H4K8ac_peak_11300	6.0605	E2F4_ENSG00000205250
16	67261077	67261363	KD2-H4K8ac_peak_11301	5.30799	LRRC29_ENSG00000125122;AC040160.1_ENSG00000237102;TMEM208_ENSG00000168701
16	67279250	67280644	KD2-H4K8ac_peak_11302	8.55271	FHOD1_ENSG00000135723
16	67282090	67283350	KD2-H4K8ac_peak_11303	17.70889	FHOD1_ENSG00000135723
16	67312792	67313901	KD2-H4K8ac_peak_11304	12.57297	
16	67360231	67360641	KD2-H4K8ac_peak_11305	11.50346	KCTD19_ENSG00000168676;LRRC36_ENSG00000159708
16	67361001	67361264	KD2-H4K8ac_peak_11306	9.73163	KCTD19_ENSG00000168676;LRRC36_ENSG00000159708
16	67429942	67430271	KD2-H4K8ac_peak_11307	11.43863	
16	67430539	67430863	KD2-H4K8ac_peak_11308	8.73351	
16	67450003	67451241	KD2-H4K8ac_peak_11309	14.80472	ZDHHC1_ENSG00000159714
16	67515350	67516443	KD2-H4K8ac_peak_11310	7.95563	ATP6V0D1_ENSG00000159720;RP11-297D21.4_ENSG00000270049
16	67562371	67563583	KD2-H4K8ac_peak_11311	15.9754	
16	67564329	67564845	KD2-H4K8ac_peak_11312	13.31016	
16	67572424	67572651	KD2-H4K8ac_peak_11313	5.51783	
16	67596465	67597395	KD2-H4K8ac_peak_11314	11.24702	CTD-2012K14.6_ENSG00000261386;CTD-2012K14.7_ENSG00000259804;CTCF_ENSG00000102974

16	67678809	67679528	KD2-H4K8ac_peak_11315	12.30509	RLTPR_ENSG00000159753
16	67694385	67694638	KD2-H4K8ac_peak_11316	10.05606	ACD_ENSG00000102977;PARD6A_ENSG00000102981
16	67700072	67700498	KD2-H4K8ac_peak_11317	6.65673	ENKD1_ENSG00000124074;C16orf86_ENSG00000159761
16	67700867	67701490	KD2-H4K8ac_peak_11318	6.24114	ENKD1_ENSG00000124074;C16orf86_ENSG00000159761
16	67840049	67840400	KD2-H4K8ac_peak_11319	8.4716	RANBP10_ENSG00000141084;TSNAXIP1_ENSG00000102904
16	67867893	67868096	KD2-H4K8ac_peak_11320	7.93362	
16	67875954	67876780	KD2-H4K8ac_peak_11321	12.70959	THAP11_ENSG00000168286
16	67880114	67880551	KD2-H4K8ac_peak_11322	11.89502	NUTF2_ENSG00000102898
16	67907022	67907648	KD2-H4K8ac_peak_11323	14.68449	EDC4_ENSG00000038358
16	67927224	67927784	KD2-H4K8ac_peak_11324	12.30509	PSKH1_ENSG00000159792
16	67969828	67970053	KD2-H4K8ac_peak_11325	11.92978	CTC-479C5.12_ENSG00000261884;PSMB10_ENSG00000205220
16	67970391	67970733	KD2-H4K8ac_peak_11326	6.76298	CTC-479C5.12_ENSG00000261884;PSMB10_ENSG00000205220
16	68002070	68002442	KD2-H4K8ac_peak_11327	6.30229	
16	68002725	68003097	KD2-H4K8ac_peak_11328	7.36799	SLC12A4_ENSG00000124067
16	68026905	68027684	KD2-H4K8ac_peak_11329	18.37277	
16	68056728	68057297	KD2-H4K8ac_peak_11330	11.90231	DDX28_ENSG00000182810
16	68117911	68119042	KD2-H4K8ac_peak_11331	29.13005	NFATC3_ENSG00000072736
16	68119544	68119965	KD2-H4K8ac_peak_11332	5.57044	NFATC3_ENSG00000072736
16	68232582	68232823	KD2-H4K8ac_peak_11333	4.49837	RP11-96D1.9_ENSG00000262514
16	68271150	68271351	KD2-H4K8ac_peak_11334	8.16906	ESRP2_ENSG00000103067;RP11-96D1.6_ENSG00000261469
16	68271821	68272077	KD2-H4K8ac_peak_11335	5.38581	ESRP2_ENSG00000103067;RP11-96D1.6_ENSG00000261469
16	68297758	68298661	KD2-H4K8ac_peak_11336	9.63487	SLC7A6_ENSG00000103064
16	68344237	68344748	KD2-H4K8ac_peak_11337	6.52179	SLC7A6OS_ENSG00000103061;PRMT7_ENSG00000132600
16	68440675	68440899	KD2-H4K8ac_peak_11338	8.23274	
16	68554404	68554901	KD2-H4K8ac_peak_11339	5.54647	
16	68563242	68563436	KD2-H4K8ac_peak_11340	4.3015	ZFP90_ENSG00000184939
16	68563685	68564481	KD2-H4K8ac_peak_11341	11.92978	ZFP90_ENSG00000184939
16	68573228	68574281	KD2-H4K8ac_peak_11342	12.74171	
16	68806565	68806779	KD2-H4K8ac_peak_11343	5.71749	
16	68877437	68877892	KD2-H4K8ac_peak_11344	10.96455	TANGO6_ENSG00000103047
16	69123454	69123976	KD2-H4K8ac_peak_11345	5.71749	
16	69165714	69166051	KD2-H4K8ac_peak_11346	9.57101	CHTF8_ENSG00000168802;CIRH1A_ENSG00000141076
16	69166274	69166765	KD2-H4K8ac_peak_11347	12.70959	CHTF8_ENSG00000168802
16	69221129	69221573	KD2-H4K8ac_peak_11348	8.55271	SNTB2_ENSG00000168807
16	69345370	69345722	KD2-H4K8ac_peak_11349	7.76104	VPS4A_ENSG00000132612
16	69363930	69364389	KD2-H4K8ac_peak_11350	6.40297	PDF_ENSG00000258429
16	69364729	69364961	KD2-H4K8ac_peak_11351	10.96455	PDF_ENSG00000258429
16	69372720	69373412	KD2-H4K8ac_peak_11352	4.44527	COG8_ENSG00000213380;NIP7_ENSG00000132603
16	69373727	69374357	KD2-H4K8ac_peak_11353	7.76104	COG8_ENSG00000213380;NIP7_ENSG00000132603
16	69419284	69420424	KD2-H4K8ac_peak_11354	15.82743	
16	69430259	69430482	KD2-H4K8ac_peak_11355	6.40683	
16	69458696	69458896	KD2-H4K8ac_peak_11356	4.8485	CYB5B_ENSG00000103018
16	69529573	69529912	KD2-H4K8ac_peak_11357	7.07117	
16	69530242	69530541	KD2-H4K8ac_peak_11358	8.42997	
16	69599389	69599644	KD2-H4K8ac_peak_11359	18.24798	NFAT5_ENSG00000102908;MIR1538_ENSG00000223109
16	69599944	69600171	KD2-H4K8ac_peak_11360	8.52559	NFAT5_ENSG00000102908;MIR1538_ENSG00000223109

16	69760020	69760213	KD2-H4K8ac_peak_11361	5.9696	NQO1_ENSG00000181019;CTD-2033A16.3_ENSG00000262136
16	69760679	69760883	KD2-H4K8ac_peak_11362	7.92775	NQO1_ENSG00000181019;CTD-2033A16.3_ENSG00000262136
16	69761169	69761433	KD2-H4K8ac_peak_11363	8.83582	NQO1_ENSG00000181019;CTD-2033A16.3_ENSG00000262136
16	69788419	69788753	KD2-H4K8ac_peak_11364	11.30637	NOB1_ENSG00000141101
16	69795892	69796231	KD2-H4K8ac_peak_11365	6.2159	WWP2_ENSG00000198373
16	69796447	69796895	KD2-H4K8ac_peak_11366	12.06346	WWP2_ENSG00000198373
16	69855186	69855473	KD2-H4K8ac_peak_11367	5.75757	
16	69864399	69864945	KD2-H4K8ac_peak_11368	11.05407	
16	69865844	69866037	KD2-H4K8ac_peak_11369	6.66692	
16	69866633	69867025	KD2-H4K8ac_peak_11370	7.27716	
16	69867901	69868245	KD2-H4K8ac_peak_11371	6.65673	
16	69923846	69924947	KD2-H4K8ac_peak_11372	14.15075	
16	70147855	70148052	KD2-H4K8ac_peak_11373	6.66692	PDPR_ENSG00000090857
16	70285441	70285779	KD2-H4K8ac_peak_11374	8.83582	EXOSC6_ENSG00000223496
16	70322551	70322914	KD2-H4K8ac_peak_11375	5.55317	AARS_ENSG00000090861;DDX19B_ENSG00000157349
16	70323825	70324556	KD2-H4K8ac_peak_11376	8.30974	AARS_ENSG00000090861;DDX19B_ENSG00000157349
16	70333249	70333609	KD2-H4K8ac_peak_11377	4.86324	RP11-529K1.3_ENSG00000260537
16	70380249	70380548	KD2-H4K8ac_peak_11378	8.10216	RP11-529K1.2_ENSG00000261777;DDX19A_ENSG00000168872
16	70414293	70415144	KD2-H4K8ac_peak_11379	15.61313	RP11-529K1.4_ENSG00000260111
16	70415601	70415827	KD2-H4K8ac_peak_11380	6.84261	
16	70461865	70462132	KD2-H4K8ac_peak_11381	9.11031	
16	70463061	70463381	KD2-H4K8ac_peak_11382	8.17902	
16	70463709	70464031	KD2-H4K8ac_peak_11383	4.86324	
16	70473353	70473697	KD2-H4K8ac_peak_11384	9.6247	ST3GAL2_ENSG00000157350
16	70488323	70488955	KD2-H4K8ac_peak_11385	10.66719	FUK_ENSG00000157353
16	70557792	70558174	KD2-H4K8ac_peak_11386	9.1707	COG4_ENSG00000103051;SF3B3_ENSG00000189091
16	70720038	70720564	KD2-H4K8ac_peak_11387	11.3394	MTSS1L_ENSG00000132613
16	70725557	70725809	KD2-H4K8ac_peak_11388	7.95563	
16	70729637	70730094	KD2-H4K8ac_peak_11389	9.13143	
16	70736891	70737834	KD2-H4K8ac_peak_11390	6.53538	
16	70747314	70747566	KD2-H4K8ac_peak_11391	10.01478	
16	70748753	70748998	KD2-H4K8ac_peak_11392	8.55271	
16	70750178	70750856	KD2-H4K8ac_peak_11393	6.92624	
16	70770983	70771221	KD2-H4K8ac_peak_11394	5.4779	
16	70771795	70772455	KD2-H4K8ac_peak_11395	8.21852	
16	70834497	70834969	KD2-H4K8ac_peak_11396	10.87093	VAC14_ENSG00000103043
16	71264690	71265107	KD2-H4K8ac_peak_11397	7.12754	HYDIN_ENSG00000157423
16	71322899	71323238	KD2-H4K8ac_peak_11398	10.05606	CMTR2_ENSG00000180917
16	71323553	71323818	KD2-H4K8ac_peak_11399	7.22457	CMTR2_ENSG00000180917
16	71495640	71496411	KD2-H4K8ac_peak_11400	11.55163	ZNF23_ENSG00000167377;RP11-510M2.2_ENSG00000247324
16	71518043	71518800	KD2-H4K8ac_peak_11401	20.88898	
16	71598974	71599386	KD2-H4K8ac_peak_11402	6.04107	RP11-432I5.1_ENSG00000260886;ZNF19_ENSG00000157429
16	71757947	71758156	KD2-H4K8ac_peak_11403	9.1707	PHLPP2_ENSG00000040199;RP11-432I5.8_ENSG00000261513
16	71842379	71842787	KD2-H4K8ac_peak_11404	8.30974	AP1G1_ENSG00000166747
16	71843195	71843541	KD2-H4K8ac_peak_11405	10.58496	AP1G1_ENSG00000166747
16	71879272	71879690	KD2-H4K8ac_peak_11406	6.66359	ATXN1L_ENSG00000224470;IST1_ENSG00000182149

16	71917504	71918006	KD2-H4K8ac_peak_11407	16.86242	
16	71918969	71919292	KD2-H4K8ac_peak_11408	5.13893	
16	71928903	71929243	KD2-H4K8ac_peak_11409	10.21366	ZNF821_ENSG00000102984
16	71929455	71929888	KD2-H4K8ac_peak_11410	15.13261	ZNF821_ENSG00000102984
16	72041899	72042173	KD2-H4K8ac_peak_11411	8.1718	DHODH_ENSG00000102967
16	72127208	72127458	KD2-H4K8ac_peak_11412	14.84212	TXNL4B_ENSG00000140830;DHX38_ENSG00000140829
16	72127934	72128186	KD2-H4K8ac_peak_11413	5.13615	TXNL4B_ENSG00000140830;DHX38_ENSG00000140829
16	72158566	72158930	KD2-H4K8ac_peak_11414	10.53596	
16	72229604	72229802	KD2-H4K8ac_peak_11415	6.00226	
16	72231306	72231983	KD2-H4K8ac_peak_11416	9.95119	
16	72232332	72232662	KD2-H4K8ac_peak_11417	10.72656	
16	72233008	72233491	KD2-H4K8ac_peak_11418	6.84261	
16	72233855	72234519	KD2-H4K8ac_peak_11419	10.0692	
16	72698309	72698768	KD2-H4K8ac_peak_11420	5.88845	AC004158.2_ENSG00000261008;RP5-991G20.1_ENSG00000259768
16	72867054	72867287	KD2-H4K8ac_peak_11421	4.81242	
16	73081605	73081955	KD2-H4K8ac_peak_11422	11.03331	
16	73082257	73082559	KD2-H4K8ac_peak_11423	6.66692	
16	73087229	73087649	KD2-H4K8ac_peak_11424	10.29344	
16	73091421	73091977	KD2-H4K8ac_peak_11425	16.79606	
16	73092183	73093033	KD2-H4K8ac_peak_11426	9.36516	ZFHX3_ENSG00000140836
16	73096897	73097199	KD2-H4K8ac_peak_11427	7.76329	
16	73105441	73105901	KD2-H4K8ac_peak_11428	6.65673	
16	73204331	73204551	KD2-H4K8ac_peak_11429	5.4779	
16	73205619	73206393	KD2-H4K8ac_peak_11430	10.53377	
16	73207430	73207716	KD2-H4K8ac_peak_11431	7.62364	
16	73218267	73218569	KD2-H4K8ac_peak_11432	7.47462	
16	73337965	73338295	KD2-H4K8ac_peak_11433	6.25869	
16	74330751	74331484	KD2-H4K8ac_peak_11434	12.93209	AC009120.4_ENSG00000261404;PSMD7_ENSG00000103035
16	74401585	74402013	KD2-H4K8ac_peak_11435	4.44527	RP11-252A24.2_ENSG00000214331;RP11-252A24.3_ENSG00000261079
16	74430435	74430690	KD2-H4K8ac_peak_11436	7.76329	
16	74640444	74640963	KD2-H4K8ac_peak_11437	14.00658	
16	74700323	74700716	KD2-H4K8ac_peak_11438	12.42761	RFWD3_ENSG00000168411;RP11-144N1.1_ENSG00000262904
16	74776036	74776230	KD2-H4K8ac_peak_11439	4.57371	
16	74808269	74808639	KD2-H4K8ac_peak_11440	8.74596	FA2H_ENSG00000103089
16	75019361	75019796	KD2-H4K8ac_peak_11441	6.66692	
16	75032997	75033343	KD2-H4K8ac_peak_11442	16.8435	WDR59_ENSG00000103091;ZNRF1_ENSG00000186187
16	75100533	75100735	KD2-H4K8ac_peak_11443	8.00457	
16	75103241	75103480	KD2-H4K8ac_peak_11444	10.73467	
16	75182482	75182827	KD2-H4K8ac_peak_11445	7.11824	ZFP1_ENSG00000184517
16	75232106	75232440	KD2-H4K8ac_peak_11446	4.01115	
16	75232817	75233315	KD2-H4K8ac_peak_11447	6.07178	
16	75273614	75274016	KD2-H4K8ac_peak_11448	4.68541	
16	75297869	75298160	KD2-H4K8ac_peak_11449	6.53538	
16	75299316	75299669	KD2-H4K8ac_peak_11450	7.146	
16	75300036	75300694	KD2-H4K8ac_peak_11451	19.93398	
16	75331306	75331599	KD2-H4K8ac_peak_11452	7.94441	

16	75455071	75455292	KD2-H4K8ac_peak_11453	9.07071	
16	75466968	75467371	KD2-H4K8ac_peak_11454	17.17257	CFDP1_ENSG00000153774
16	75467738	75468048	KD2-H4K8ac_peak_11455	8.00457	CFDP1_ENSG00000153774
16	75478009	75478239	KD2-H4K8ac_peak_11456	4.77079	
16	75497908	75498496	KD2-H4K8ac_peak_11457	8.88082	RP11-77K12.1_ENSG00000261717;TMEM170A_ENSG00000166822
16	75550656	75551140	KD2-H4K8ac_peak_11458	17.46525	RP11-77K12.5_ENSG00000262583
16	75590496	75590804	KD2-H4K8ac_peak_11459	5.14125	TMEM231_ENSG00000205084
16	75655889	75656115	KD2-H4K8ac_peak_11460	5.40638	
16	75656702	75657060	KD2-H4K8ac_peak_11461	6.13909	ADAT1_ENSG00000065457
16	75681292	75681515	KD2-H4K8ac_peak_11462	7.76329	TERF2IP_ENSG00000166848
16	75682312	75682962	KD2-H4K8ac_peak_11463	8.82366	KARS_ENSG00000065427;TERF2IP_ENSG00000166848
16	77224152	77224551	KD2-H4K8ac_peak_11464	19.91242	MON1B_ENSG00000103111
16	77245788	77246064	KD2-H4K8ac_peak_11465	6.54442	
16	77246319	77246800	KD2-H4K8ac_peak_11466	7.13615	
16	77787593	77787827	KD2-H4K8ac_peak_11467	6.16888	
16	77797963	77798203	KD2-H4K8ac_peak_11468	4.14296	
16	78132484	78132831	KD2-H4K8ac_peak_11469	8.28994	WWOX_ENSG00000186153
16	78133590	78133965	KD2-H4K8ac_peak_11470	10.05606	WWOX_ENSG00000186153
16	78602489	78602690	KD2-H4K8ac_peak_11471	6.07178	
16	79804074	79804381	KD2-H4K8ac_peak_11472	9.59147	RP11-345M22.2_ENSG00000261390
16	79872380	79872580	KD2-H4K8ac_peak_11473	8.00457	
16	80574978	80575333	KD2-H4K8ac_peak_11474	6.0073	DYNLRB2_ENSG00000168589
16	80934185	80934703	KD2-H4K8ac_peak_11475	5.55317	
16	81040153	81040756	KD2-H4K8ac_peak_11476	9.63487	CENPN_ENSG00000166451
16	81069506	81070018	KD2-H4K8ac_peak_11477	10.72656	RP11-303E16.3_ENSG00000260213;ATMIN_ENSG00000166454
16	81110968	81111324	KD2-H4K8ac_peak_11478	7.76104	C16orf46_ENSG00000166455;RP11-303E16.7_ENSG00000245059
16	81130390	81130722	KD2-H4K8ac_peak_11479	4.03256	GCSH_ENSG00000140905
16	81348661	81349358	KD2-H4K8ac_peak_11480	10.87093	GAN_ENSG00000261609
16	81437522	81437883	KD2-H4K8ac_peak_11481	9.65086	
16	81465454	81465996	KD2-H4K8ac_peak_11482	5.46431	
16	81477713	81478029	KD2-H4K8ac_peak_11483	9.1707	CMIP_ENSG00000153815
16	81478569	81478864	KD2-H4K8ac_peak_11484	10.82243	CMIP_ENSG00000153815
16	81520984	81521233	KD2-H4K8ac_peak_11485	6.0073	
16	81547891	81548862	KD2-H4K8ac_peak_11486	8.93255	
16	81549086	81550074	KD2-H4K8ac_peak_11487	14.68449	
16	81552717	81553169	KD2-H4K8ac_peak_11488	6.36375	
16	81572928	81573307	KD2-H4K8ac_peak_11489	6.2781	
16	81643465	81644367	KD2-H4K8ac_peak_11490	7.67496	
16	81658691	81658923	KD2-H4K8ac_peak_11491	5.57044	
16	81702037	81702339	KD2-H4K8ac_peak_11492	4.86324	
16	81763102	81763332	KD2-H4K8ac_peak_11493	8.09472	
16	81763526	81764017	KD2-H4K8ac_peak_11494	12.88108	
16	81764214	81764540	KD2-H4K8ac_peak_11495	18.7894	
16	81765436	81765636	KD2-H4K8ac_peak_11496	6.72021	
16	81779300	81779947	KD2-H4K8ac_peak_11497	13.95627	
16	82203403	82203750	KD2-H4K8ac_peak_11498	14.48084	MPHOSPH6_ENSG00000135698;CTD-2588J6.2_ENSG00000261029

16	82203982	82204340	KD2-H4K8ac_peak_11499	8.10216	MPHOSPH6_ENSG00000135698;CTD-2588J6.2_ENSG00000261029
16	83840990	83841402	KD2-H4K8ac_peak_11500	7.1181	RP11-483P21.2_ENSG00000260228;HSBP1_ENSG00000230989
16	83841648	83842008	KD2-H4K8ac_peak_11501	11.50346	RP11-483P21.2_ENSG00000260228;HSBP1_ENSG00000230989
16	83932145	83932375	KD2-H4K8ac_peak_11502	10.96455	MLYCD_ENSG00000103150
16	83932818	83933391	KD2-H4K8ac_peak_11503	10.19949	MLYCD_ENSG00000103150
16	83986190	83987048	KD2-H4K8ac_peak_11504	6.65673	
16	84002305	84002773	KD2-H4K8ac_peak_11505	7.13615	NECAB2_ENSG00000103154
16	84150740	84151115	KD2-H4K8ac_peak_11506	6.16883	MBTPS1_ENSG00000140943;RP11-505K9.1_ENSG00000260018
16	84178464	84178713	KD2-H4K8ac_peak_11507	11.54737	HSDL1_ENSG00000103160;DNAAF1_ENSG00000154099
16	84220230	84220629	KD2-H4K8ac_peak_11508	15.22643	TAF1C_ENSG00000103168
16	84267768	84268221	KD2-H4K8ac_peak_11509	8.30974	
16	84268470	84268702	KD2-H4K8ac_peak_11510	9.06703	
16	84538529	84538808	KD2-H4K8ac_peak_11511	5.01002	
16	84681435	84681864	KD2-H4K8ac_peak_11512	7.47462	KLHL36_ENSG00000135686
16	84682174	84682554	KD2-H4K8ac_peak_11513	4.86324	KLHL36_ENSG00000135686
16	84733148	84734183	KD2-H4K8ac_peak_11514	17.69527	USP10_ENSG00000103194
16	84860156	84860369	KD2-H4K8ac_peak_11515	8.10216	
16	85044568	85045023	KD2-H4K8ac_peak_11516	8.85813	ZDHHC7_ENSG00000153786
16	85045282	85045692	KD2-H4K8ac_peak_11517	8.79106	ZDHHC7_ENSG00000153786
16	85067543	85067765	KD2-H4K8ac_peak_11518	4.37708	
16	85145956	85146488	KD2-H4K8ac_peak_11519	4.86324	FAM92B_ENSG00000153789
16	85160259	85160627	KD2-H4K8ac_peak_11520	6.99815	
16	85169921	85170120	KD2-H4K8ac_peak_11521	5.45396	
16	85170728	85171562	KD2-H4K8ac_peak_11522	12.82393	
16	85183435	85184144	KD2-H4K8ac_peak_11523	6.66692	
16	85184863	85185267	KD2-H4K8ac_peak_11524	7.76329	
16	85185569	85186260	KD2-H4K8ac_peak_11525	15.82362	
16	85191274	85192432	KD2-H4K8ac_peak_11526	10.73134	
16	85202273	85202529	KD2-H4K8ac_peak_11527	6.84261	
16	85203219	85203553	KD2-H4K8ac_peak_11528	8.23274	
16	85207587	85208485	KD2-H4K8ac_peak_11529	9.59905	
16	85230741	85230977	KD2-H4K8ac_peak_11530	15.57998	
16	85235786	85236430	KD2-H4K8ac_peak_11531	10.7186	
16	85236787	85237342	KD2-H4K8ac_peak_11532	13.73635	
16	85239142	85240227	KD2-H4K8ac_peak_11533	12.60538	
16	85240630	85242157	KD2-H4K8ac_peak_11534	19.55721	
16	85253211	85253793	KD2-H4K8ac_peak_11535	9.57101	
16	85254336	85255706	KD2-H4K8ac_peak_11536	20.8421	
16	85262794	85263022	KD2-H4K8ac_peak_11537	6.30229	
16	85268501	85270047	KD2-H4K8ac_peak_11538	21.16019	
16	85271989	85272218	KD2-H4K8ac_peak_11539	4.67342	
16	85280382	85281031	KD2-H4K8ac_peak_11540	7.167	
16	85283784	85284023	KD2-H4K8ac_peak_11541	6.30229	
16	85292465	85292718	KD2-H4K8ac_peak_11542	4.8888	
16	85303010	85303461	KD2-H4K8ac_peak_11543	6.80057	
16	85304468	85304924	KD2-H4K8ac_peak_11544	7.22167	

16	85317734	85318143	KD2-H4K8ac_peak_11545	6.8108	
16	85319479	85319846	KD2-H4K8ac_peak_11546	8.36514	
16	85320984	85321446	KD2-H4K8ac_peak_11547	4.52514	
16	85335293	85335694	KD2-H4K8ac_peak_11548	4.54006	
16	85348261	85348515	KD2-H4K8ac_peak_11549	5.28072	
16	85382094	85382525	KD2-H4K8ac_peak_11550	6.55628	
16	85382996	85384131	KD2-H4K8ac_peak_11551	15.05364	
16	85388100	85388697	KD2-H4K8ac_peak_11552	10.37168	
16	85389407	85390051	KD2-H4K8ac_peak_11553	6.20982	
16	85390404	85391278	KD2-H4K8ac_peak_11554	7.47462	RP11-680G10.1_ENSG00000261567
16	85392331	85393070	KD2-H4K8ac_peak_11555	8.58069	
16	85395018	85395248	KD2-H4K8ac_peak_11556	9.87106	
16	85399494	85400091	KD2-H4K8ac_peak_11557	9.57101	
16	85400578	85400851	KD2-H4K8ac_peak_11558	8.79106	
16	85409977	85410378	KD2-H4K8ac_peak_11559	11.48808	
16	85411229	85412917	KD2-H4K8ac_peak_11560	11.28831	
16	85415972	85416214	KD2-H4K8ac_peak_11561	6.8713	
16	85428110	85428535	KD2-H4K8ac_peak_11562	5.02474	
16	85429562	85430788	KD2-H4K8ac_peak_11563	10.29842	
16	85478388	85479049	KD2-H4K8ac_peak_11564	10.73134	
16	85479398	85479605	KD2-H4K8ac_peak_11565	8.28234	
16	85480003	85480895	KD2-H4K8ac_peak_11566	14.64974	
16	85481801	85483266	KD2-H4K8ac_peak_11567	7.11824	
16	85496459	85496779	KD2-H4K8ac_peak_11568	8.85813	
16	85506654	85506874	KD2-H4K8ac_peak_11569	7.28827	
16	85507823	85508087	KD2-H4K8ac_peak_11570	5.08628	
16	85508708	85509063	KD2-H4K8ac_peak_11571	6.92624	
16	85509565	85510014	KD2-H4K8ac_peak_11572	9.66321	
16	85549166	85549414	KD2-H4K8ac_peak_11573	4.85088	
16	85563185	85563668	KD2-H4K8ac_peak_11574	6.99815	
16	85586972	85587706	KD2-H4K8ac_peak_11575	12.57143	
16	85588155	85588972	KD2-H4K8ac_peak_11576	13.81781	
16	85590075	85590275	KD2-H4K8ac_peak_11577	9.36516	
16	85595304	85595591	KD2-H4K8ac_peak_11578	4.06758	
16	85607983	85608563	KD2-H4K8ac_peak_11579	10.96455	
16	85615852	85616170	KD2-H4K8ac_peak_11580	4.86324	
16	85619910	85620538	KD2-H4K8ac_peak_11581	7.93362	
16	85626933	85627155	KD2-H4K8ac_peak_11582	14.57937	
16	85627467	85627660	KD2-H4K8ac_peak_11583	6.67081	
16	85639978	85640394	KD2-H4K8ac_peak_11584	15.40891	
16	85640757	85641481	KD2-H4K8ac_peak_11585	9.63487	
16	85644540	85644739	KD2-H4K8ac_peak_11586	6.62541	GSE1_ENSG00000131149
16	85644969	85645788	KD2-H4K8ac_peak_11587	17.41726	GSE1_ENSG00000131149
16	85646450	85647271	KD2-H4K8ac_peak_11588	15.61313	
16	85648027	85648738	KD2-H4K8ac_peak_11589	14.95369	
16	85649989	85650315	KD2-H4K8ac_peak_11590	3.97079	

16	85659117	85659724	KD2-H4K8ac_peak_11591	6.04107	
16	85683663	85684295	KD2-H4K8ac_peak_11592	7.44324	
16	85685025	85686630	KD2-H4K8ac_peak_11593	10.66719	
16	85687106	85687462	KD2-H4K8ac_peak_11594	5.13615	
16	85832634	85832854	KD2-H4K8ac_peak_11595	7.20567	EMC8_ENSG00000131148;COX4I1_ENSG00000131143
16	85833344	85833889	KD2-H4K8ac_peak_11596	8.4716	EMC8_ENSG00000131148
16	86588400	86588728	KD2-H4K8ac_peak_11597	11.24702	MTHFSD_ENSG00000103248
16	86588984	86589782	KD2-H4K8ac_peak_11598	15.81711	MTHFSD_ENSG00000103248
16	87350667	87350870	KD2-H4K8ac_peak_11599	11.04256	C16orf95_ENSG00000260456;RP11-178L8.7_ENSG00000270006
16	87387096	87387299	KD2-H4K8ac_peak_11600	5.15777	
16	87397798	87398428	KD2-H4K8ac_peak_11601	5.88845	
16	87417955	87418641	KD2-H4K8ac_peak_11602	8.55271	MAP1LC3B_ENSG00000140941
16	87421640	87421918	KD2-H4K8ac_peak_11603	8.30974	
16	87425067	87425653	KD2-H4K8ac_peak_11604	10.7186	FBXO31_ENSG00000103264
16	87491799	87492036	KD2-H4K8ac_peak_11605	5.405	
16	87492350	87492771	KD2-H4K8ac_peak_11606	6.60096	
16	87498467	87498774	KD2-H4K8ac_peak_11607	12.42761	
16	87499072	87499344	KD2-H4K8ac_peak_11608	5.14125	
16	87500003	87500534	KD2-H4K8ac_peak_11609	14.23999	
16	87503244	87504105	KD2-H4K8ac_peak_11610	9.1707	
16	87511371	87511571	KD2-H4K8ac_peak_11611	7.1181	
16	87526979	87527494	KD2-H4K8ac_peak_11612	10.19949	RP11-482M8.1_ENSG00000260750
16	87551906	87552657	KD2-H4K8ac_peak_11613	7.47462	
16	87584324	87584695	KD2-H4K8ac_peak_11614	6.40683	
16	87590347	87590635	KD2-H4K8ac_peak_11615	11.43863	
16	87604310	87604679	KD2-H4K8ac_peak_11616	9.36665	
16	87615978	87616426	KD2-H4K8ac_peak_11617	5.14125	
16	87622627	87622967	KD2-H4K8ac_peak_11618	6.66692	
16	87755726	87756672	KD2-H4K8ac_peak_11619	11.92978	
16	87799367	87800729	KD2-H4K8ac_peak_11620	13.13969	KLHDC4_ENSG00000104731
16	87812163	87813195	KD2-H4K8ac_peak_11621	21.50391	RP4-536B24.4_ENSG00000260498
16	87813558	87813792	KD2-H4K8ac_peak_11622	5.14125	RP4-536B24.4_ENSG00000260498
16	87839596	87841562	KD2-H4K8ac_peak_11623	16.67374	RP4-536B24.3_ENSG00000260177
16	87885863	87886140	KD2-H4K8ac_peak_11624	8.30974	
16	87886932	87887248	KD2-H4K8ac_peak_11625	13.31016	
16	87888533	87888746	KD2-H4K8ac_peak_11626	4.8888	
16	87888991	87889329	KD2-H4K8ac_peak_11627	5.76151	
16	87904971	87905201	KD2-H4K8ac_peak_11628	7.31433	
16	87905600	87905905	KD2-H4K8ac_peak_11629	10.05606	
16	87952934	87953228	KD2-H4K8ac_peak_11630	5.14125	
16	87958372	87958584	KD2-H4K8ac_peak_11631	3.75035	
16	87958899	87959175	KD2-H4K8ac_peak_11632	5.45396	
16	87978731	87980141	KD2-H4K8ac_peak_11633	6.66692	CTD-3057O21.1_ENSG00000260443
16	87985150	87985715	KD2-H4K8ac_peak_11634	15.58442	
16	88075568	88075923	KD2-H4K8ac_peak_11635	7.76104	
16	88076384	88076906	KD2-H4K8ac_peak_11636	8.98477	

16	88096098	88097242	KD2-H4K8ac_peak_11637	11.92978	
16	88110175	88110539	KD2-H4K8ac_peak_11638	13.30586	
16	88111424	88112184	KD2-H4K8ac_peak_11639	8.86099	
16	88125096	88125908	KD2-H4K8ac_peak_11640	11.24702	
16	88126257	88126854	KD2-H4K8ac_peak_11641	11.89502	
16	88133909	88134516	KD2-H4K8ac_peak_11642	10.77651	RP11-863P13.4_ENSG00000205037
16	88153597	88154617	KD2-H4K8ac_peak_11643	9.16531	
16	88166586	88166959	KD2-H4K8ac_peak_11644	3.97079	
16	88167574	88168034	KD2-H4K8ac_peak_11645	5.14125	
16	88168629	88168874	KD2-H4K8ac_peak_11646	10.61986	
16	88169068	88169273	KD2-H4K8ac_peak_11647	11.24702	
16	88170378	88170577	KD2-H4K8ac_peak_11648	6.25869	
16	88175280	88175875	KD2-H4K8ac_peak_11649	4.54006	
16	88176209	88177181	KD2-H4K8ac_peak_11650	10.17341	
16	88177492	88177896	KD2-H4K8ac_peak_11651	21.2844	
16	88179060	88179763	KD2-H4K8ac_peak_11652	10.15155	
16	88180057	88180390	KD2-H4K8ac_peak_11653	6.67538	
16	88183008	88183326	KD2-H4K8ac_peak_11654	9.90914	
16	88227867	88228099	KD2-H4K8ac_peak_11655	5.14125	RP11-863P13.2_ENSG00000261816;LA16c-444G7.2_ENSG00000260420
16	88228455	88228657	KD2-H4K8ac_peak_11656	12.42761	LA16c-444G7.2_ENSG00000260420
16	88261821	88262045	KD2-H4K8ac_peak_11657	5.57044	
16	88265286	88265962	KD2-H4K8ac_peak_11658	7.47462	
16	88266228	88267005	KD2-H4K8ac_peak_11659	6.20982	
16	88268010	88268356	KD2-H4K8ac_peak_11660	11.05467	LA16c-444G7.1_ENSG00000261273
16	88268624	88269091	KD2-H4K8ac_peak_11661	11.05467	LA16c-444G7.1_ENSG00000261273
16	88271389	88271608	KD2-H4K8ac_peak_11662	10.70006	
16	88271806	88272218	KD2-H4K8ac_peak_11663	12.93253	
16	88273775	88274157	KD2-H4K8ac_peak_11664	6.98292	
16	88295932	88296270	KD2-H4K8ac_peak_11665	6.05995	
16	88296725	88297277	KD2-H4K8ac_peak_11666	8.26945	
16	88307911	88308607	KD2-H4K8ac_peak_11667	5.01002	
16	88308878	88309518	KD2-H4K8ac_peak_11668	15.53798	
16	88309720	88310080	KD2-H4K8ac_peak_11669	7.13615	
16	88449425	88449642	KD2-H4K8ac_peak_11670	5.30799	
16	88471777	88472940	KD2-H4K8ac_peak_11671	12.84642	
16	88503564	88503851	KD2-H4K8ac_peak_11672	12.70959	
16	88515550	88515956	KD2-H4K8ac_peak_11673	10.12356	
16	88522073	88522284	KD2-H4K8ac_peak_11674	6.20982	
16	88527701	88527959	KD2-H4K8ac_peak_11675	7.07512	
16	88529403	88530142	KD2-H4K8ac_peak_11676	7.86151	
16	88531794	88532374	KD2-H4K8ac_peak_11677	11.09936	
16	88564241	88564433	KD2-H4K8ac_peak_11678	5.57044	
16	88571113	88571421	KD2-H4K8ac_peak_11679	7.57979	
16	88579117	88579606	KD2-H4K8ac_peak_11680	14.56955	
16	88625059	88625286	KD2-H4K8ac_peak_11681	5.88845	
16	88636922	88637216	KD2-H4K8ac_peak_11682	8.55271	ZC3H18_ENSG00000158545

16	88716753	88717482	KD2-H4K8ac_peak_11683	10.4923	CYBA_ENSG00000051523
16	88729057	88729379	KD2-H4K8ac_peak_11684	7.47462	MVD_ENSG00000167508;SNAI3-AS1_ENSG00000260630
16	88794588	88794869	KD2-H4K8ac_peak_11685	6.84261	
16	88796497	88796716	KD2-H4K8ac_peak_11686	6.25869	RP5-1142A6.2_ENSG00000224888
16	88797337	88797611	KD2-H4K8ac_peak_11687	9.07715	RP5-1142A6.2_ENSG00000224888
16	88797886	88798202	KD2-H4K8ac_peak_11688	9.96798	RP5-1142A6.2_ENSG00000224888
16	88869514	88869933	KD2-H4K8ac_peak_11689	17.55491	CDT1_ENSG00000167513
16	88870247	88870475	KD2-H4K8ac_peak_11690	8.85813	CDT1_ENSG00000167513
16	88877987	88878298	KD2-H4K8ac_peak_11691	12.96152	APRT_ENSG00000198931
16	88878498	88879153	KD2-H4K8ac_peak_11692	18.37277	APRT_ENSG00000198931
16	88922770	88922967	KD2-H4K8ac_peak_11693	9.51625	GALNS_ENSG00000141012;TRAPPC2L_ENSG00000167515
16	88923575	88924009	KD2-H4K8ac_peak_11694	19.93588	GALNS_ENSG00000141012;TRAPPC2L_ENSG00000167515
16	89137633	89139494	KD2-H4K8ac_peak_11695	9.95119	
16	89159597	89160701	KD2-H4K8ac_peak_11696	12.46522	
16	89216792	89217264	KD2-H4K8ac_peak_11697	12.40345	
16	89218010	89218335	KD2-H4K8ac_peak_11698	6.24114	
16	89233583	89233912	KD2-H4K8ac_peak_11699	14.43704	RP11-46C24.3_ENSG00000205015
16	89283779	89284529	KD2-H4K8ac_peak_11700	20.02161	RP11-46C24.7_ENSG00000259877;ZNF778_ENSG00000170100
16	89306716	89307416	KD2-H4K8ac_peak_11701	11.07495	
16	89467987	89468234	KD2-H4K8ac_peak_11702	11.52086	
16	89568541	89568734	KD2-H4K8ac_peak_11703	4.77079	
16	89573922	89575225	KD2-H4K8ac_peak_11704	18.94636	
16	89625788	89626030	KD2-H4K8ac_peak_11705	6.30229	
16	89626365	89626912	KD2-H4K8ac_peak_11706	9.63487	RPL13_ENSG00000167526;SNORD68_ENSG00000200084
16	89627134	89627743	KD2-H4K8ac_peak_11707	14.88905	RPL13_ENSG00000167526;SNORD68_ENSG00000200084
16	89668699	89669074	KD2-H4K8ac_peak_11708	4.42765	
16	89675122	89675410	KD2-H4K8ac_peak_11709	5.29352	
16	89681948	89682280	KD2-H4K8ac_peak_11710	5.14125	
16	89682800	89683209	KD2-H4K8ac_peak_11711	4.77079	
16	89723635	89724065	KD2-H4K8ac_peak_11712	9.41619	CHMP1A_ENSG00000131165;SPATA33_ENSG00000167523
16	89753175	89753511	KD2-H4K8ac_peak_11713	5.38581	RP11-368I7.4_ENSG00000260259
16	89772208	89772830	KD2-H4K8ac_peak_11714	8.98093	
16	89786601	89787753	KD2-H4K8ac_peak_11715	23.17589	VPS9D1_ENSG00000075399;ZNF276_ENSG00000158805
16	89882562	89883688	KD2-H4K8ac_peak_11716	18.37277	SPIRE2_ENSG00000204991;FANCA_ENSG00000187741
16	89894378	89894669	KD2-H4K8ac_peak_11717	20.46575	
16	89894947	89895154	KD2-H4K8ac_peak_11718	8.2413	
16	89939397	89939888	KD2-H4K8ac_peak_11719	13.31016	TCF25_ENSG00000141002
16	89976271	89976816	KD2-H4K8ac_peak_11720	6.25869	
16	89984262	89985141	KD2-H4K8ac_peak_11721	11.28765	AC092143.1_ENSG00000256390;TUBB3_ENSG00000198211
16	89988775	89989560	KD2-H4K8ac_peak_11722	10.40376	RP11-566K11.4_ENSG00000259006;TUBB3_ENSG00000258947
16	89990309	89990573	KD2-H4K8ac_peak_11723	3.97079	
16	90014096	90015026	KD2-H4K8ac_peak_11724	10.19949	DEF8_ENSG00000140995
16	90038279	90039707	KD2-H4K8ac_peak_11725	19.7067	CENPBD1_ENSG00000177946;AFG3L1P_ENSG00000223959
16	90085160	90085809	KD2-H4K8ac_peak_11726	11.05155	DBNDD1_ENSG00000003249;GAS8_ENSG00000141013
16	90086048	90086509	KD2-H4K8ac_peak_11727	9.49967	DBNDD1_ENSG00000003249;GAS8_ENSG00000141013
16	90087451	90087694	KD2-H4K8ac_peak_11728	6.30229	DBNDD1_ENSG00000003249

16	90089087	90089524	KD2-H4K8ac_peak_11729	8.5092	
16	90113026	90114138	KD2-H4K8ac_peak_11730	15.61313	URAHP_ENSG00000222019
16	90148702	90149462	KD2-H4K8ac_peak_11731	8.30974	
17	29283	31196	KD2-H4K8ac_peak_11732	16.8435	DOC2B_ENSG00000272636
17	31503	31728	KD2-H4K8ac_peak_11733	5.51783	DOC2B_ENSG00000272636
17	147943	148189	KD2-H4K8ac_peak_11734	4.24345	
17	150448	150761	KD2-H4K8ac_peak_11735	15.22643	
17	160035	160377	KD2-H4K8ac_peak_11736	4.42765	
17	183863	184146	KD2-H4K8ac_peak_11737	7.40111	
17	202285	202562	KD2-H4K8ac_peak_11738	7.146	RP11-1260E13.2_ENSG00000262294
17	235244	235570	KD2-H4K8ac_peak_11739	14.68449	RPH3AL_ENSG00000181031
17	235783	236240	KD2-H4K8ac_peak_11740	4.06758	RPH3AL_ENSG00000181031
17	259224	259730	KD2-H4K8ac_peak_11741	12.42761	C17orf97_ENSG00000187624
17	500377	500850	KD2-H4K8ac_peak_11742	9.11031	
17	503454	504402	KD2-H4K8ac_peak_11743	9.31907	
17	505827	506050	KD2-H4K8ac_peak_11744	10.96455	
17	511972	512164	KD2-H4K8ac_peak_11745	9.11031	
17	618263	618467	KD2-H4K8ac_peak_11746	5.53033	
17	634761	635515	KD2-H4K8ac_peak_11747	11.24702	FAM57A_ENSG00000167695
17	635745	636707	KD2-H4K8ac_peak_11748	16.32216	FAM57A_ENSG00000167695
17	655048	655370	KD2-H4K8ac_peak_11749	12.57297	DBIL5P_ENSG00000231784
17	655676	656309	KD2-H4K8ac_peak_11750	9.26408	GEMIN4_ENSG00000179409;DBIL5P_ENSG00000231784
17	685194	685395	KD2-H4K8ac_peak_11751	5.75452	RNMTL1_ENSG00000171861
17	685663	685958	KD2-H4K8ac_peak_11752	5.30799	RNMTL1_ENSG00000171861
17	883316	883697	KD2-H4K8ac_peak_11753	6.25869	NXN_ENSG00000167693
17	899506	899871	KD2-H4K8ac_peak_11754	10.63725	TIMM22_ENSG00000177370
17	933737	934648	KD2-H4K8ac_peak_11755	4.211	
17	974480	974858	KD2-H4K8ac_peak_11756	5.53033	
17	980980	981233	KD2-H4K8ac_peak_11757	6.67092	
17	981458	982070	KD2-H4K8ac_peak_11758	10.29842	
17	982530	982860	KD2-H4K8ac_peak_11759	7.34308	
17	987326	987568	KD2-H4K8ac_peak_11760	5.88845	
17	991248	991532	KD2-H4K8ac_peak_11761	4.211	
17	1011543	1011747	KD2-H4K8ac_peak_11762	5.58725	
17	1012143	1012976	KD2-H4K8ac_peak_11763	15.59774	
17	1014712	1014968	KD2-H4K8ac_peak_11764	4.8888	
17	1057281	1057878	KD2-H4K8ac_peak_11765	11.43863	
17	1083540	1083974	KD2-H4K8ac_peak_11766	6.52179	
17	1090314	1090590	KD2-H4K8ac_peak_11767	7.76329	
17	1106896	1107105	KD2-H4K8ac_peak_11768	6.13909	
17	1154980	1155257	KD2-H4K8ac_peak_11769	7.15405	
17	1155836	1156184	KD2-H4K8ac_peak_11770	5.54647	
17	1171144	1172223	KD2-H4K8ac_peak_11771	20.2003	
17	1302774	1303289	KD2-H4K8ac_peak_11772	17.05794	YWHAE_ENSG00000108953
17	1303734	1304162	KD2-H4K8ac_peak_11773	6.66692	YWHAE_ENSG00000108953
17	1358936	1359496	KD2-H4K8ac_peak_11774	7.47462	

17	1359799	1360185	KD2-H4K8ac_peak_11775	6.40297	
17	1388907	1389770	KD2-H4K8ac_peak_11776	8.16906	
17	1390962	1391258	KD2-H4K8ac_peak_11777	4.44527	
17	1394254	1395576	KD2-H4K8ac_peak_11778	4.91674	MYO1C_ENSG00000197879
17	1419600	1419836	KD2-H4K8ac_peak_11779	4.67342	INPP5K_ENSG00000132376;PITPNA-AS1_ENSG00000236618
17	1420290	1420726	KD2-H4K8ac_peak_11780	5.88845	INPP5K_ENSG00000132376;PITPNA-AS1_ENSG00000236618
17	1477158	1477562	KD2-H4K8ac_peak_11781	5.74496	
17	1532350	1533228	KD2-H4K8ac_peak_11782	12.30509	SLC43A2_ENSG00000167703
17	1551812	1552197	KD2-H4K8ac_peak_11783	5.14125	
17	1587751	1588120	KD2-H4K8ac_peak_11784	15.61313	PRPF8_ENSG00000174231
17	1588431	1588756	KD2-H4K8ac_peak_11785	6.07178	PRPF8_ENSG00000174231
17	1613311	1613518	KD2-H4K8ac_peak_11786	10.44656	TLCD2_ENSG00000185561
17	1619949	1620395	KD2-H4K8ac_peak_11787	27.36011	MIR22HG_ENSG00000186594;WDR81_ENSG00000167716
17	1620610	1621410	KD2-H4K8ac_peak_11788	14.8314	MIR22HG_ENSG00000186594;WDR81_ENSG00000167716
17	1627491	1627766	KD2-H4K8ac_peak_11789	6.87784	
17	1628079	1628391	KD2-H4K8ac_peak_11790	9.57101	
17	1658713	1659147	KD2-H4K8ac_peak_11791	7.28827	
17	1665313	1666427	KD2-H4K8ac_peak_11792	12.69325	SERPINF1_ENSG00000132386
17	1666938	1667648	KD2-H4K8ac_peak_11793	9.26408	
17	1670133	1670384	KD2-H4K8ac_peak_11794	6.25869	
17	1802129	1802537	KD2-H4K8ac_peak_11795	9.11031	
17	1846152	1846391	KD2-H4K8ac_peak_11796	6.36375	
17	1864500	1864694	KD2-H4K8ac_peak_11797	4.42765	
17	1899174	1899601	KD2-H4K8ac_peak_11798	9.1707	CTD-2545H1.2_ENSG00000262445
17	1944757	1945138	KD2-H4K8ac_peak_11799	9.56945	OVCA2_ENSG00000262664
17	1945355	1946089	KD2-H4K8ac_peak_11800	8.16906	OVCA2_ENSG00000262664;RP11-667K14.4_ENSG00000262533
17	1953388	1954199	KD2-H4K8ac_peak_11801	11.05467	MIR132_ENSG00000267200;MIR212_ENSG00000267195
17	1966202	1966399	KD2-H4K8ac_peak_11802	6.66692	
17	1971372	1971661	KD2-H4K8ac_peak_11803	9.68068	
17	1971885	1972406	KD2-H4K8ac_peak_11804	6.65673	
17	1978157	1979489	KD2-H4K8ac_peak_11805	9.36516	
17	1980102	1980748	KD2-H4K8ac_peak_11806	11.00372	
17	1985047	1985398	KD2-H4K8ac_peak_11807	8.10216	
17	1985861	1986073	KD2-H4K8ac_peak_11808	7.146	
17	1986690	1986919	KD2-H4K8ac_peak_11809	10.78136	
17	1987478	1988356	KD2-H4K8ac_peak_11810	7.74292	
17	2119169	2119590	KD2-H4K8ac_peak_11811	9.92477	AC130689.5_ENSG00000236457
17	2206668	2207118	KD2-H4K8ac_peak_11812	8.55271	SMG6_ENSG00000070366;SRR_ENSG00000167720
17	2216543	2216810	KD2-H4K8ac_peak_11813	3.63462	
17	2238917	2239668	KD2-H4K8ac_peak_11814	4.33139	
17	2240443	2241237	KD2-H4K8ac_peak_11815	7.36799	TSR1_ENSG00000167721;SGSM2_ENSG00000141258
17	2283078	2283508	KD2-H4K8ac_peak_11816	5.34636	RP1-59D14.5_ENSG00000263345
17	2295559	2295761	KD2-H4K8ac_peak_11817	6.13909	
17	2296678	2297027	KD2-H4K8ac_peak_11818	7.92775	
17	2297663	2297876	KD2-H4K8ac_peak_11819	5.63428	
17	2302431	2302780	KD2-H4K8ac_peak_11820	18.70363	

17	2304570	2304857	KD2-H4K8ac_peak_11821	11.39234	MNT_ENSG00000070444
17	2305126	2305319	KD2-H4K8ac_peak_11822	7.13615	MNT_ENSG00000070444
17	2414626	2415090	KD2-H4K8ac_peak_11823	6.40683	METTL16_ENSG00000127804
17	2496930	2497664	KD2-H4K8ac_peak_11824	12.17278	PAFAH1B1_ENSG00000007168
17	2589121	2589322	KD2-H4K8ac_peak_11825	4.77079	RP11-74E22.5_ENSG00000272770
17	2614453	2614942	KD2-H4K8ac_peak_11826	9.72244	RP11-74E22.3_ENSG00000262050
17	2615160	2616050	KD2-H4K8ac_peak_11827	15.22643	CLUH_ENSG00000132361;RP11-74E22.3_ENSG00000262050
17	2679496	2680008	KD2-H4K8ac_peak_11828	11.43863	RAP1GAP2_ENSG00000132359
17	2680675	2680876	KD2-H4K8ac_peak_11829	4.77079	RAP1GAP2_ENSG00000132359
17	2750920	2751148	KD2-H4K8ac_peak_11830	7.22457	
17	2755886	2756897	KD2-H4K8ac_peak_11831	9.20193	
17	2804430	2804753	KD2-H4K8ac_peak_11832	7.372	
17	2812969	2813202	KD2-H4K8ac_peak_11833	6.60096	
17	2814604	2814879	KD2-H4K8ac_peak_11834	7.73418	
17	2815218	2815774	KD2-H4K8ac_peak_11835	11.27037	
17	2828712	2829710	KD2-H4K8ac_peak_11836	10.19949	
17	2832063	2832772	KD2-H4K8ac_peak_11837	14.275	
17	2843579	2843825	KD2-H4K8ac_peak_11838	8.10216	
17	2855673	2855868	KD2-H4K8ac_peak_11839	7.67496	
17	2862579	2862825	KD2-H4K8ac_peak_11840	6.66692	
17	2863099	2863325	KD2-H4K8ac_peak_11841	8.5092	
17	2863646	2864024	KD2-H4K8ac_peak_11842	9.67696	
17	2864447	2864644	KD2-H4K8ac_peak_11843	4.86324	
17	2866895	2867311	KD2-H4K8ac_peak_11844	7.76329	
17	2868855	2869332	KD2-H4K8ac_peak_11845	18.5703	CTD-3060P21.1_ENSG00000262884
17	2874071	2874273	KD2-H4K8ac_peak_11846	8.30974	
17	2906269	2907495	KD2-H4K8ac_peak_11847	8.30974	
17	2907838	2908761	KD2-H4K8ac_peak_11848	11.04256	
17	2951719	2952136	KD2-H4K8ac_peak_11849	11.05467	
17	2952476	2952842	KD2-H4K8ac_peak_11850	19.43192	
17	3438785	3439173	KD2-H4K8ac_peak_11851	5.43522	
17	3539705	3540474	KD2-H4K8ac_peak_11852	17.8868	SHPK_ENSG00000262304;SHPK_ENSG00000197417;CTNS_ENSG00000040531
17	3571023	3571802	KD2-H4K8ac_peak_11853	10.7186	TAX1BP3_ENSG00000213977;EMC6_ENSG00000127774
17	3572190	3572439	KD2-H4K8ac_peak_11854	8.85813	TAX1BP3_ENSG00000213977;EMC6_ENSG00000127774
17	3598079	3598864	KD2-H4K8ac_peak_11855	13.9234	P2RX5-TAX1BP3_ENSG00000257950;P2RX5_ENSG00000083454
17	3626785	3627115	KD2-H4K8ac_peak_11856	18.7894	GSG2_ENSG00000177602
17	3748818	3749453	KD2-H4K8ac_peak_11857	8.55271	C17orf85_ENSG00000074356
17	3781897	3782418	KD2-H4K8ac_peak_11858	5.88845	
17	3782903	3783447	KD2-H4K8ac_peak_11859	8.02571	
17	3796540	3797107	KD2-H4K8ac_peak_11860	11.09936	
17	3830432	3830836	KD2-H4K8ac_peak_11861	5.32137	
17	3848272	3848641	KD2-H4K8ac_peak_11862	16.49944	
17	3879821	3880185	KD2-H4K8ac_peak_11863	4.3015	RP11-459C13.1_ENSG00000263312
17	4047053	4047638	KD2-H4K8ac_peak_11864	16.822	ZZEF1_ENSG00000074755;CYB5D2_ENSG00000167740
17	4064471	4064676	KD2-H4K8ac_peak_11865	5.45396	
17	4166913	4167614	KD2-H4K8ac_peak_11866	14.8083	ANKFY1_ENSG00000185722

17	4268798	4269597	KD2-H4K8ac_peak_11867	11.89502	UBE2G1_ENSG00000132388
17	4270247	4270496	KD2-H4K8ac_peak_11868	4.44748	UBE2G1_ENSG00000132388
17	4378428	4379439	KD2-H4K8ac_peak_11869	8.4084	
17	4436775	4437027	KD2-H4K8ac_peak_11870	9.33795	
17	4437253	4438363	KD2-H4K8ac_peak_11871	12.08081	
17	4458815	4459444	KD2-H4K8ac_peak_11872	13.59098	MYBBP1A_ENSG00000132382
17	4491721	4492186	KD2-H4K8ac_peak_11873	5.54647	
17	4606989	4607364	KD2-H4K8ac_peak_11874	5.54647	PELP1_ENSG00000141456;RP11-314A20.2_ENSG00000244184
17	4612281	4612765	KD2-H4K8ac_peak_11875	10.77651	
17	4614032	4614376	KD2-H4K8ac_peak_11876	11.86082	ARRB2_ENSG00000141480
17	4634190	4634479	KD2-H4K8ac_peak_11877	4.06758	MED11_ENSG00000161920
17	4693096	4693675	KD2-H4K8ac_peak_11878	13.80019	
17	4698845	4699225	KD2-H4K8ac_peak_11879	12.86276	PSMB6_ENSG00000142507
17	4709966	4710295	KD2-H4K8ac_peak_11880	16.8435	RP11-81A22.5_ENSG00000262165;PLD2_ENSG00000129219
17	4711045	4711259	KD2-H4K8ac_peak_11881	7.07117	RP11-81A22.5_ENSG00000262165;PLD2_ENSG00000129219
17	4736721	4737164	KD2-H4K8ac_peak_11882	15.58442	MINK1_ENSG00000141503
17	4811713	4811947	KD2-H4K8ac_peak_11883	4.86324	
17	4812643	4813130	KD2-H4K8ac_peak_11884	7.13615	
17	4837553	4837777	KD2-H4K8ac_peak_11885	6.66692	
17	4842951	4843184	KD2-H4K8ac_peak_11886	8.4716	SLC25A11_ENSG00000108528;RNF167_ENSG00000108523
17	4843507	4843823	KD2-H4K8ac_peak_11887	15.25814	SLC25A11_ENSG00000108528;RNF167_ENSG00000108523
17	4850897	4851720	KD2-H4K8ac_peak_11888	13.81781	PFN1_ENSG00000108518;ENO3_ENSG00000108515
17	4852447	4854439	KD2-H4K8ac_peak_11889	17.43561	PFN1_ENSG00000108518
17	4869865	4870354	KD2-H4K8ac_peak_11890	15.76765	SPAG7_ENSG00000091640;RP5-1050D4.3_ENSG00000262429
17	4870885	4871106	KD2-H4K8ac_peak_11891	8.2413	SPAG7_ENSG00000091640;RP5-1050D4.3_ENSG00000262429
17	4871512	4872534	KD2-H4K8ac_peak_11892	8.16906	SPAG7_ENSG00000091640;RP5-1050D4.3_ENSG00000262429
17	4890117	4890828	KD2-H4K8ac_peak_11893	9.34195	CAMTA2_ENSG00000108509;RP5-1050D4.4_ENSG00000262678;AC004771.1_ENSG00000203562;RP5-1050D4.5_ENSG00000262227
17	4891115	4891435	KD2-H4K8ac_peak_11894	5.9696	CAMTA2_ENSG00000108509;AC004771.1_ENSG00000203562;RP5-1050D4.5_ENSG00000262227
17	4900360	4901137	KD2-H4K8ac_peak_11895	15.82362	INCA1_ENSG00000196388;KIF1C_ENSG00000129250
17	4934561	4935412	KD2-H4K8ac_peak_11896	13.81781	
17	4938644	4938846	KD2-H4K8ac_peak_11897	4.37709	
17	4980741	4982203	KD2-H4K8ac_peak_11898	16.8435	RP11-46I8.3_ENSG00000262693;ZFP3_ENSG00000180787
17	5014719	5015051	KD2-H4K8ac_peak_11899	9.048	AC012146.7_ENSG00000234327
17	5015641	5016184	KD2-H4K8ac_peak_11900	4.04573	AC012146.7_ENSG00000234327
17	5094796	5095122	KD2-H4K8ac_peak_11901	15.20126	ZNF594_ENSG00000180626;RP11-333E1.1_ENSG00000261879
17	5095378	5095796	KD2-H4K8ac_peak_11902	7.146	ZNF594_ENSG00000180626;RP11-333E1.1_ENSG00000261879
17	5175740	5175977	KD2-H4K8ac_peak_11903	5.73839	
17	5323321	5323802	KD2-H4K8ac_peak_11904	14.275	NUP88_ENSG00000108559;RPAIN_ENSG00000129197
17	5372536	5372865	KD2-H4K8ac_peak_11905	9.95119	DHX33_ENSG00000005100;CTC-524C5.5_ENSG00000262099
17	5389877	5390149	KD2-H4K8ac_peak_11906	14.68449	DERL2_ENSG00000072849;MIS12_ENSG00000167842
17	6346501	6346978	KD2-H4K8ac_peak_11907	11.49574	FAM64A_ENSG00000129195
17	6358063	6358727	KD2-H4K8ac_peak_11908	10.05606	
17	6543122	6544241	KD2-H4K8ac_peak_11909	15.61313	KIAA0753_ENSG00000198920;TXNDC17_ENSG00000129235
17	6555109	6555893	KD2-H4K8ac_peak_11910	33.59052	MED31_ENSG00000108590;C17orf100_ENSG00000212734;AC004706.1_ENSG00000256806
17	6915393	6916422	KD2-H4K8ac_peak_11911	5.75757	AC027763.2_ENSG00000215067;RNASEK_ENSG00000219200;RNASEK-C17orf49_ENSG00000161939
17	6917423	6917920	KD2-H4K8ac_peak_11912	13.56049	AC040977.1_ENSG00000269871;C17orf49_ENSG00000258315

17	6921604	6922046	KD2-H4K8ac_peak_11913	11.43353	RP11-589P10.7_ENSG00000267047;MIR497HG_ENSG00000267532
17	6922652	6923813	KD2-H4K8ac_peak_11914	20.01779	RP11-589P10.7_ENSG00000267047;MIR497HG_ENSG00000267532
17	6925198	6925707	KD2-H4K8ac_peak_11915	6.25869	BCL6B_ENSG00000161940
17	6938619	6939015	KD2-H4K8ac_peak_11916	5.88845	SLC16A13_ENSG00000174327
17	6939343	6939881	KD2-H4K8ac_peak_11917	11.66477	SLC16A13_ENSG00000174327
17	6945513	6945793	KD2-H4K8ac_peak_11918	4.40093	
17	6946785	6947557	KD2-H4K8ac_peak_11919	6.5996	SLC16A11_ENSG00000174326
17	7080386	7080870	KD2-H4K8ac_peak_11920	6.90231	
17	7081070	7081920	KD2-H4K8ac_peak_11921	8.52559	ASGR1_ENSG00000141505
17	7107651	7107888	KD2-H4K8ac_peak_11922	11.07495	
17	7121039	7121769	KD2-H4K8ac_peak_11923	22.94649	ACADVL_ENSG00000072778
17	7122714	7123004	KD2-H4K8ac_peak_11924	5.54647	DLG4_ENSG00000132535
17	7123310	7123997	KD2-H4K8ac_peak_11925	14.22723	DLG4_ENSG00000132535
17	7141264	7141508	KD2-H4K8ac_peak_11926	20.58734	
17	7142071	7142794	KD2-H4K8ac_peak_11927	22.76112	PHF23_ENSG00000040633
17	7145386	7145632	KD2-H4K8ac_peak_11928	7.76329	GABARAP_ENSG00000170296
17	7154490	7155289	KD2-H4K8ac_peak_11929	22.39508	CTDNEP1_ENSG00000175826;ELP5_ENSG00000170291
17	7155523	7156098	KD2-H4K8ac_peak_11930	9.95119	CTDNEP1_ENSG00000175826;ELP5_ENSG00000170291
17	7164322	7164942	KD2-H4K8ac_peak_11931	12.70959	RP1-4G17.5_ENSG00000262302
17	7197630	7197924	KD2-H4K8ac_peak_11932	8.55271	YBX2_ENSG00000006047
17	7198212	7198565	KD2-H4K8ac_peak_11933	19.559	YBX2_ENSG00000006047
17	7199732	7200198	KD2-H4K8ac_peak_11934	4.67342	
17	7209559	7210109	KD2-H4K8ac_peak_11935	8.59579	EIF5A_ENSG00000132507
17	7210988	7211843	KD2-H4K8ac_peak_11936	17.70889	EIF5A_ENSG00000132507
17	7218150	7219582	KD2-H4K8ac_peak_11937	14.68449	GPS2_ENSG00000132522
17	7233515	7234272	KD2-H4K8ac_peak_11938	22.21705	NEURL4_ENSG00000215041;AC026954.6_ENSG00000224647
17	7254393	7255778	KD2-H4K8ac_peak_11939	9.67696	KCTD11_ENSG00000213859
17	7284102	7284529	KD2-H4K8ac_peak_11940	6.66692	TNK1_ENSG00000174292
17	7286950	7288225	KD2-H4K8ac_peak_11941	15.01306	
17	7297988	7298304	KD2-H4K8ac_peak_11942	6.84261	
17	7307984	7309316	KD2-H4K8ac_peak_11943	8.94469	TMEM256-PLSCR3_ENSG00000187838;C17orf61-PLSCR3_ENSG00000262481;TMEM256_ENSG00000205544;NLGN2_ENSG000001699
17	7339182	7339547	KD2-H4K8ac_peak_11944	7.40314	TMEM102_ENSG00000181284
17	7342875	7344358	KD2-H4K8ac_peak_11945	11.98173	RP11-104H15.10_ENSG00000272884
17	7358299	7358699	KD2-H4K8ac_peak_11946	7.96322	
17	7358942	7359243	KD2-H4K8ac_peak_11947	7.47462	
17	7382500	7382836	KD2-H4K8ac_peak_11948	18.80827	
17	7383074	7383914	KD2-H4K8ac_peak_11949	9.36516	SLC35G6_ENSG00000259224
17	7386483	7386717	KD2-H4K8ac_peak_11950	6.67538	ZBTB4_ENSG00000174282;POLR2A_ENSG00000181222
17	7451633	7452809	KD2-H4K8ac_peak_11951	21.50391	TNFSF12_ENSG00000239697;TNFSF12-TNFSF13_ENSG00000248871
17	7460361	7460908	KD2-H4K8ac_peak_11952	6.56211	TNFSF13_ENSG00000161955
17	7461291	7461574	KD2-H4K8ac_peak_11953	5.9696	TNFSF13_ENSG00000161955
17	7464711	7465095	KD2-H4K8ac_peak_11954	8.4716	SENP3_ENSG00000161956
17	7465335	7465713	KD2-H4K8ac_peak_11955	12.37938	SENP3_ENSG00000161956;SENP3-EIF4A1_ENSG00000265500
17	7476250	7477038	KD2-H4K8ac_peak_11956	7.70067	EIF4A1_ENSG00000161960;SNORA67_ENSG00000264772;SNORA48_ENSG00000209582
17	7486240	7486985	KD2-H4K8ac_peak_11957	26.15791	AC113189.5_ENSG00000233223;MPDU1_ENSG00000129255
17	7487714	7488003	KD2-H4K8ac_peak_11958	5.14125	AC113189.5_ENSG00000233223;MPDU1_ENSG00000129255

17	7518674	7518924	KD2-H4K8ac_peak_11959	16.87121	FXR2_ENSG00000129245
17	7553701	7553894	KD2-H4K8ac_peak_11960	10.08707	
17	7554683	7555275	KD2-H4K8ac_peak_11961	9.96798	
17	7589794	7590769	KD2-H4K8ac_peak_11962	8.55271	TP53_ENSG00000141510;RP11-199F11.2_ENSG00000262251;WRAP53_ENSG00000141499
17	7591766	7592127	KD2-H4K8ac_peak_11963	7.49599	TP53_ENSG00000141510
17	7608557	7608867	KD2-H4K8ac_peak_11964	8.88082	EFNB3_ENSG00000108947
17	7619158	7620464	KD2-H4K8ac_peak_11965	5.54647	DNAH2_ENSG00000183914
17	7620687	7620913	KD2-H4K8ac_peak_11966	5.88845	DNAH2_ENSG00000183914
17	7746486	7746712	KD2-H4K8ac_peak_11967	8.42997	
17	7760018	7760254	KD2-H4K8ac_peak_11968	5.54647	CYB5D1_ENSG00000182224
17	7786116	7786537	KD2-H4K8ac_peak_11969	5.54647	
17	7788218	7790144	KD2-H4K8ac_peak_11970	21.11201	LSMD1_ENSG00000183011;CHD3_ENSG00000170004
17	7791193	7792176	KD2-H4K8ac_peak_11971	15.58442	
17	7819158	7819933	KD2-H4K8ac_peak_11972	18.37277	AC025335.1_ENSG00000179859
17	7832602	7833059	KD2-H4K8ac_peak_11973	8.37231	KCNAB3_ENSG00000170049;RP11-1099M24.7_ENSG00000262730
17	7835594	7835923	KD2-H4K8ac_peak_11974	12.00273	TRAPPC1_ENSG00000170043;CNTROB_ENSG00000170037
17	7891803	7892135	KD2-H4K8ac_peak_11975	6.36319	
17	7893255	7893504	KD2-H4K8ac_peak_11976	7.92775	
17	7919274	7920058	KD2-H4K8ac_peak_11977	6.25869	
17	7982944	7983334	KD2-H4K8ac_peak_11978	5.08628	AC129492.6_ENSG00000214999
17	8021061	8021301	KD2-H4K8ac_peak_11979	8.55271	
17	8054103	8054456	KD2-H4K8ac_peak_11980	6.16747	
17	8066596	8067865	KD2-H4K8ac_peak_11981	15.59774	RP11-599B13.6_ENSG00000263620;VAMP2_ENSG00000220205
17	8079867	8080448	KD2-H4K8ac_peak_11982	6.66692	TMEM107_ENSG00000179029;RP11-599B13.7_ENSG00000266824
17	8093903	8094110	KD2-H4K8ac_peak_11983	5.20908	C17orf59_ENSG00000196544
17	8095852	8096108	KD2-H4K8ac_peak_11984	9.12849	
17	8113389	8113848	KD2-H4K8ac_peak_11985	9.1707	AURKB_ENSG00000178999
17	8123461	8123712	KD2-H4K8ac_peak_11986	3.75035	
17	8125036	8125329	KD2-H4K8ac_peak_11987	8.85813	
17	8126334	8127574	KD2-H4K8ac_peak_11988	8.82366	LINC00324_ENSG00000178977
17	8150985	8151270	KD2-H4K8ac_peak_11989	11.05407	CTC1_ENSG00000178971;PFAS_ENSG00000178921
17	8151910	8152330	KD2-H4K8ac_peak_11990	7.69287	CTC1_ENSG00000178971;PFAS_ENSG00000178921
17	8191325	8191519	KD2-H4K8ac_peak_11991	6.13909	RANGRF_ENSG00000108961
17	8198240	8198604	KD2-H4K8ac_peak_11992	7.96322	SLC25A35_ENSG00000125434
17	8198885	8199509	KD2-H4K8ac_peak_11993	14.66631	SLC25A35_ENSG00000125434
17	8207849	8208043	KD2-H4K8ac_peak_11994	4.65111	
17	8230138	8230414	KD2-H4K8ac_peak_11995	6.99815	
17	8250406	8250933	KD2-H4K8ac_peak_11996	8.17902	
17	8279432	8279930	KD2-H4K8ac_peak_11997	5.9696	KRBA2_ENSG00000184619
17	8286095	8286352	KD2-H4K8ac_peak_11998	6.56926	RP11-849F2.7_ENSG00000263809;RPL26_ENSG00000161970
17	8286677	8287689	KD2-H4K8ac_peak_11999	14.15075	RP11-849F2.7_ENSG00000263809;RPL26_ENSG00000161970
17	8338723	8338941	KD2-H4K8ac_peak_12000	9.96798	
17	8339328	8339623	KD2-H4K8ac_peak_12001	5.4919	
17	8467732	8467939	KD2-H4K8ac_peak_12002	6.82976	
17	8532751	8533337	KD2-H4K8ac_peak_12003	12.84642	MYH10_ENSG00000133026
17	8534454	8534911	KD2-H4K8ac_peak_12004	8.10216	MYH10_ENSG00000133026

17	8583744	8583950	KD2-H4K8ac_peak_12005	5.54647	
17	8585107	8585703	KD2-H4K8ac_peak_12006	11.10075	
17	8601544	8601998	KD2-H4K8ac_peak_12007	7.01494	
17	8649647	8649855	KD2-H4K8ac_peak_12008	7.27716	
17	9144559	9144782	KD2-H4K8ac_peak_12009	3.75035	
17	9478814	9479070	KD2-H4K8ac_peak_12010	9.1707	STX8_ENSG00000170310;WDR16_ENSG00000166596
17	10018730	10019077	KD2-H4K8ac_peak_12011	12.81138	
17	10030979	10031481	KD2-H4K8ac_peak_12012	7.40111	
17	10600448	10600777	KD2-H4K8ac_peak_12013	6.66692	SCO1_ENSG00000133028;ADPRM_ENSG00000170222
17	10632777	10633448	KD2-H4K8ac_peak_12014	12.08081	TMEM220_ENSG00000187824;CTC-297N7.5_ENSG00000263400
17	10652806	10653259	KD2-H4K8ac_peak_12015	6.31143	
17	10654672	10654955	KD2-H4K8ac_peak_12016	5.52329	
17	10748030	10748388	KD2-H4K8ac_peak_12017	5.25071	
17	11900356	11900750	KD2-H4K8ac_peak_12018	13.92307	ZNF18_ENSG00000154957
17	11900966	11901573	KD2-H4K8ac_peak_12019	16.94317	ZNF18_ENSG00000154957
17	11924324	11924633	KD2-H4K8ac_peak_12020	14.66631	RPL21P122_ENSG00000241185;MAP2K4_ENSG00000065559
17	12692803	12693035	KD2-H4K8ac_peak_12021	9.1707	RP11-1090M7.1_ENSG00000265489;ARHGAP44_ENSG00000006740
17	12920871	12921325	KD2-H4K8ac_peak_12022	9.89126	ELAC2_ENSG00000006744
17	13972267	13972673	KD2-H4K8ac_peak_12023	7.95563	COX10-AS1_ENSG00000236088;COX10_ENSG00000006695
17	13972929	13973127	KD2-H4K8ac_peak_12024	6.40683	COX10-AS1_ENSG00000236088;COX10_ENSG00000006695
17	15163866	15164566	KD2-H4K8ac_peak_12025	11.03331	RP11-849N15.1_ENSG00000265445
17	15197441	15197726	KD2-H4K8ac_peak_12026	9.50015	
17	15244589	15244888	KD2-H4K8ac_peak_12027	5.25071	TEKT3_ENSG00000125409
17	15301316	15301601	KD2-H4K8ac_peak_12028	8.23274	
17	15302457	15303397	KD2-H4K8ac_peak_12029	8.55271	
17	15405758	15405975	KD2-H4K8ac_peak_12030	6.8836	CDRT4_ENSG00000239704
17	15443156	15443418	KD2-H4K8ac_peak_12031	6.25869	
17	15466535	15466772	KD2-H4K8ac_peak_12032	8.17902	TVP23C-CDRT4_ENSG00000259024;TVP23C_ENSG00000175106
17	15554252	15554963	KD2-H4K8ac_peak_12033	8.55271	RP11-385D13.1_ENSG00000251537;RP11-640I15.1_ENSG00000266261
17	15586932	15587952	KD2-H4K8ac_peak_12034	17.27647	TRIM16_ENSG00000221926
17	15672835	15673209	KD2-H4K8ac_peak_12035	4.44748	
17	15689561	15690019	KD2-H4K8ac_peak_12036	9.36516	MEIS3P1_ENSG00000179277
17	15847729	15848949	KD2-H4K8ac_peak_12037	12.99417	ADORA2B_ENSG00000170425
17	16118426	16119886	KD2-H4K8ac_peak_12038	14.275	
17	16120252	16120957	KD2-H4K8ac_peak_12039	10.43145	NCOR1_ENSG00000141027
17	16256237	16256570	KD2-H4K8ac_peak_12040	8.30974	CENPV_ENSG00000166582
17	16283458	16283881	KD2-H4K8ac_peak_12041	8.79106	UBB_ENSG00000170315
17	16290356	16290911	KD2-H4K8ac_peak_12042	7.44324	
17	16310172	16310653	KD2-H4K8ac_peak_12043	9.36516	
17	16311343	16311668	KD2-H4K8ac_peak_12044	8.85813	
17	16312121	16312326	KD2-H4K8ac_peak_12045	8.1718	
17	16322110	16322568	KD2-H4K8ac_peak_12046	5.57044	
17	16331637	16331841	KD2-H4K8ac_peak_12047	6.65673	AC093484.4_ENSG00000239203
17	16341240	16341463	KD2-H4K8ac_peak_12048	7.99241	FAM211A-AS1_ENSG00000175061
17	16341688	16341894	KD2-H4K8ac_peak_12049	5.4919	FAM211A-AS1_ENSG00000175061
17	16342430	16342726	KD2-H4K8ac_peak_12050	7.59794	FAM211A-AS1_ENSG00000175061

17	16354003	16354195	KD2-H4K8ac_peak_12051	4.35346	
17	16389511	16389859	KD2-H4K8ac_peak_12052	8.56219	
17	16390901	16391127	KD2-H4K8ac_peak_12053	5.60968	
17	16395130	16395392	KD2-H4K8ac_peak_12054	8.17902	FAM211A_ENSG00000181350
17	16395656	16395958	KD2-H4K8ac_peak_12055	9.31907	FAM211A_ENSG00000181350
17	16424175	16424399	KD2-H4K8ac_peak_12056	7.47462	
17	16439423	16440120	KD2-H4K8ac_peak_12057	10.46264	
17	16472039	16472480	KD2-H4K8ac_peak_12058	13.77586	ZNF287_ENSG00000141040
17	16487876	16488169	KD2-H4K8ac_peak_12059	3.97079	
17	16556622	16557087	KD2-H4K8ac_peak_12060	20.42458	ZNF624_ENSG00000197566;RP11-92B11.4_ENSG00000264765
17	16883673	16883894	KD2-H4K8ac_peak_12061	8.1233	AC104024.1_ENSG00000230709
17	16905363	16905682	KD2-H4K8ac_peak_12062	10.46264	
17	16912230	16912460	KD2-H4K8ac_peak_12063	5.88845	
17	16928592	16929017	KD2-H4K8ac_peak_12064	6.76298	
17	16931386	16932908	KD2-H4K8ac_peak_12065	10.43145	
17	16934256	16934685	KD2-H4K8ac_peak_12066	7.76329	
17	16935218	16935889	KD2-H4K8ac_peak_12067	6.0073	
17	16940002	16940322	KD2-H4K8ac_peak_12068	11.22681	
17	16979406	16979631	KD2-H4K8ac_peak_12069	3.99369	
17	17109129	17109472	KD2-H4K8ac_peak_12070	7.76104	PLD6_ENSG00000179598
17	17140236	17140733	KD2-H4K8ac_peak_12071	21.6161	RP11-45M22.4_ENSG00000264187;FLCN_ENSG00000154803;RP11-45M22.5_ENSG00000266498
17	17184214	17184525	KD2-H4K8ac_peak_12072	17.8868	COPS3_ENSG00000141030
17	17206713	17207349	KD2-H4K8ac_peak_12073	15.81711	NT5M_ENSG00000205309
17	17254693	17255820	KD2-H4K8ac_peak_12074	15.039	
17	17289049	17289405	KD2-H4K8ac_peak_12075	8.6308	
17	17306887	17307716	KD2-H4K8ac_peak_12076	7.76329	
17	17379456	17380068	KD2-H4K8ac_peak_12077	14.74047	MED9_ENSG00000141026
17	17380377	17380794	KD2-H4K8ac_peak_12078	8.55271	MED9_ENSG00000141026
17	17399362	17400683	KD2-H4K8ac_peak_12079	19.559	RASD1_ENSG00000108551
17	17418089	17418775	KD2-H4K8ac_peak_12080	5.13615	
17	17470720	17470922	KD2-H4K8ac_peak_12081	8.02571	
17	17494239	17494957	KD2-H4K8ac_peak_12082	11.05467	PEMT_ENSG00000133027;RP11-524F11.2_ENSG00000264666
17	17495265	17495886	KD2-H4K8ac_peak_12083	11.92978	PEMT_ENSG00000133027;RP11-524F11.2_ENSG00000264666
17	17566522	17566985	KD2-H4K8ac_peak_12084	6.2781	
17	17584774	17586263	KD2-H4K8ac_peak_12085	11.79585	RAI1_ENSG00000108557
17	17588587	17588812	KD2-H4K8ac_peak_12086	6.66692	
17	17589371	17589809	KD2-H4K8ac_peak_12087	8.17902	
17	17596451	17597012	KD2-H4K8ac_peak_12088	11.89502	
17	17597286	17598106	KD2-H4K8ac_peak_12089	14.68449	
17	17641281	17641473	KD2-H4K8ac_peak_12090	5.23279	
17	17641907	17642212	KD2-H4K8ac_peak_12091	6.66692	
17	17643081	17643582	KD2-H4K8ac_peak_12092	6.67092	
17	17647494	17647703	KD2-H4K8ac_peak_12093	3.75035	
17	17649152	17649568	KD2-H4K8ac_peak_12094	6.20282	
17	17652145	17652344	KD2-H4K8ac_peak_12095	6.84261	
17	17652706	17653188	KD2-H4K8ac_peak_12096	6.30607	

17	17655932	17656283	KD2-H4K8ac_peak_12097	8.70496	
17	17673040	17673313	KD2-H4K8ac_peak_12098	7.13615	RAI1-AS1_ENSG00000237328
17	17685519	17685798	KD2-H4K8ac_peak_12099	8.86099	
17	17686022	17686244	KD2-H4K8ac_peak_12100	14.06758	
17	17686850	17687086	KD2-H4K8ac_peak_12101	13.15684	
17	17687489	17687708	KD2-H4K8ac_peak_12102	7.74434	
17	17691143	17691430	KD2-H4K8ac_peak_12103	4.67342	
17	17693103	17693681	KD2-H4K8ac_peak_12104	20.79845	
17	17694096	17695361	KD2-H4K8ac_peak_12105	17.8868	
17	17725838	17726123	KD2-H4K8ac_peak_12106	8.79106	
17	17727369	17727790	KD2-H4K8ac_peak_12107	11.47352	
17	17735041	17735921	KD2-H4K8ac_peak_12108	8.93761	
17	17739000	17739407	KD2-H4K8ac_peak_12109	10.30284	SREBF1_ENSG00000072310
17	17739753	17741185	KD2-H4K8ac_peak_12110	20.76319	SREBF1_ENSG00000072310
17	17742973	17743407	KD2-H4K8ac_peak_12111	5.32397	
17	17743663	17744244	KD2-H4K8ac_peak_12112	7.76104	
17	17749364	17749973	KD2-H4K8ac_peak_12113	10.19949	
17	17776823	17777034	KD2-H4K8ac_peak_12114	5.57044	
17	17780698	17780943	KD2-H4K8ac_peak_12115	4.8485	
17	17781230	17781857	KD2-H4K8ac_peak_12116	5.66705	
17	17804362	17804942	KD2-H4K8ac_peak_12117	8.23274	
17	17815419	17815699	KD2-H4K8ac_peak_12118	13.09607	
17	17825184	17825680	KD2-H4K8ac_peak_12119	4.77079	
17	17911799	17912111	KD2-H4K8ac_peak_12120	7.27716	
17	17929331	17929530	KD2-H4K8ac_peak_12121	6.45018	
17	17990592	17991062	KD2-H4K8ac_peak_12122	10.87093	DRG2_ENSG00000108591
17	17991321	17991795	KD2-H4K8ac_peak_12123	9.36516	DRG2_ENSG00000108591
17	18085615	18086551	KD2-H4K8ac_peak_12124	13.87818	ALKBH5_ENSG00000091542
17	18127900	18128610	KD2-H4K8ac_peak_12125	12.88108	
17	18162216	18162543	KD2-H4K8ac_peak_12126	4.8485	FLII_ENSG00000177731
17	18164132	18164391	KD2-H4K8ac_peak_12127	4.06758	MIEF2_ENSG00000177427
17	18217653	18218236	KD2-H4K8ac_peak_12128	7.36799	TOP3A_ENSG00000177302;SMCR8_ENSG00000176994
17	18218436	18219178	KD2-H4K8ac_peak_12129	17.42905	TOP3A_ENSG00000177302;SMCR8_ENSG00000176994
17	18280624	18281013	KD2-H4K8ac_peak_12130	6.56926	EVPLL_ENSG00000214860
17	18314849	18315208	KD2-H4K8ac_peak_12131	9.1707	RP1-37N7.3_ENSG00000220161
17	18585166	18585547	KD2-H4K8ac_peak_12132	12.36441	ZNF286B_ENSG00000249459
17	18601424	18601843	KD2-H4K8ac_peak_12133	7.67504	TRIM16L_ENSG00000108448
17	18684620	18684866	KD2-H4K8ac_peak_12134	7.76104	TVP23B_ENSG00000171928
17	18761501	18762084	KD2-H4K8ac_peak_12135	18.54875	
17	19044757	19045002	KD2-H4K8ac_peak_12136	17.14886	
17	19140751	19141409	KD2-H4K8ac_peak_12137	8.86099	
17	19165153	19165469	KD2-H4K8ac_peak_12138	6.0073	
17	19265498	19265982	KD2-H4K8ac_peak_12139	12.08081	
17	19268673	19268917	KD2-H4K8ac_peak_12140	4.86324	
17	19280750	19281482	KD2-H4K8ac_peak_12141	10.43145	B9D1_ENSG00000108641;MAPK7_ENSG00000166484
17	19287073	19287996	KD2-H4K8ac_peak_12142	11.09936	

17	19288386	19288767	KD2-H4K8ac_peak_12143	10.43145	
17	19289144	19289470	KD2-H4K8ac_peak_12144	4.06758	
17	19289934	19290218	KD2-H4K8ac_peak_12145	9.36516	MFAP4_ENSG00000166482
17	19291635	19292192	KD2-H4K8ac_peak_12146	9.63487	
17	19294777	19295031	KD2-H4K8ac_peak_12147	11.11133	
17	19304761	19305158	KD2-H4K8ac_peak_12148	5.30799	
17	19313771	19314734	KD2-H4K8ac_peak_12149	9.1707	RNF112_ENSG00000128482
17	19314950	19315504	KD2-H4K8ac_peak_12150	10.96455	RNF112_ENSG00000128482;CTB-187M2.2_ENSG00000265126
17	19350605	19351133	KD2-H4K8ac_peak_12151	11.05467	RPS2P46_ENSG00000189343
17	19410538	19410767	KD2-H4K8ac_peak_12152	7.01658	
17	19436769	19437013	KD2-H4K8ac_peak_12153	4.7849	
17	19437210	19437700	KD2-H4K8ac_peak_12154	4.86324	
17	19482697	19483771	KD2-H4K8ac_peak_12155	10.19949	AC025627.7_ENSG00000228983
17	19551082	19551884	KD2-H4K8ac_peak_12156	12.93209	ALDH3A2_ENSG00000072210
17	19647486	19648105	KD2-H4K8ac_peak_12157	18.54875	
17	19648415	19648968	KD2-H4K8ac_peak_12158	8.16906	
17	19665766	19665967	KD2-H4K8ac_peak_12159	9.72244	
17	19770088	19771763	KD2-H4K8ac_peak_12160	13.80019	ULK2_ENSG00000083290
17	19880535	19881650	KD2-H4K8ac_peak_12161	16.54484	AKAP10_ENSG00000108599
17	19896337	19896715	KD2-H4K8ac_peak_12162	10.43145	
17	19911969	19913278	KD2-H4K8ac_peak_12163	17.19888	RP11-209D14.2_ENSG00000261033;SPECC1_ENSG00000128487
17	19998444	19999023	KD2-H4K8ac_peak_12164	6.40683	
17	20203243	20203676	KD2-H4K8ac_peak_12165	9.16531	
17	20203876	20204077	KD2-H4K8ac_peak_12166	6.66692	
17	20217292	20217625	KD2-H4K8ac_peak_12167	5.64449	
17	20284920	20285588	KD2-H4K8ac_peak_12168	5.73839	
17	20486802	20487182	KD2-H4K8ac_peak_12169	5.73839	
17	20492186	20492620	KD2-H4K8ac_peak_12170	9.72244	MEIS3P2_ENSG00000188013
17	20755776	20756035	KD2-H4K8ac_peak_12171	8.23274	
17	20811099	20811343	KD2-H4K8ac_peak_12172	6.66692	
17	20811623	20811879	KD2-H4K8ac_peak_12173	6.66692	
17	20945888	20946294	KD2-H4K8ac_peak_12174	20.18584	USP22_ENSG00000124422
17	20946787	20947067	KD2-H4K8ac_peak_12175	19.11513	USP22_ENSG00000124422
17	20979011	20979241	KD2-H4K8ac_peak_12176	8.00457	AC087393.1_ENSG00000236819
17	21029775	21030119	KD2-H4K8ac_peak_12177	5.57044	
17	21030474	21030686	KD2-H4K8ac_peak_12178	3.97079	
17	21098581	21098832	KD2-H4K8ac_peak_12179	8.1331	
17	21116991	21117415	KD2-H4K8ac_peak_12180	17.17257	TMEM11_ENSG00000178307;AC087294.2_ENSG00000235530
17	21117715	21118388	KD2-H4K8ac_peak_12181	6.99815	TMEM11_ENSG00000178307;AC087294.2_ENSG00000235530
17	21156918	21157534	KD2-H4K8ac_peak_12182	7.9076	C17orf103_ENSG00000154035
17	21178845	21179084	KD2-H4K8ac_peak_12183	8.16906	
17	21220092	21220831	KD2-H4K8ac_peak_12184	10.44656	
17	21279753	21280042	KD2-H4K8ac_peak_12185	5.557	KCNJ12_ENSG00000184185
17	21414621	21414937	KD2-H4K8ac_peak_12186	6.66692	
17	21454549	21454859	KD2-H4K8ac_peak_12187	9.51625	RP11-822E23.3_ENSG00000266673
17	21792428	21792834	KD2-H4K8ac_peak_12188	12.25733	RP11-1109M24.16_ENSG00000263609;RP11-1109M24.5_ENSG00000264956

17	21825341	21825723	KD2-H4K8ac_peak_12189	4.86324	FAM27L_ENSG00000178130;RP11-1109M24.14_ENSG00000264811
17	25620400	25621399	KD2-H4K8ac_peak_12190	10.87093	MIR4522_ENSG00000263583;WSB1_ENSG00000109046
17	25659496	25659919	KD2-H4K8ac_peak_12191	9.34195	RP11-173M1.4_ENSG00000266313
17	25660190	25660393	KD2-H4K8ac_peak_12192	10.29842	RP11-173M1.4_ENSG00000266313
17	25680271	25680868	KD2-H4K8ac_peak_12193	25.10776	RP11-173M1.5_ENSG00000265683
17	25681085	25681403	KD2-H4K8ac_peak_12194	8.4716	
17	25707060	25707330	KD2-H4K8ac_peak_12195	8.10216	
17	25707605	25708071	KD2-H4K8ac_peak_12196	5.75757	
17	25708429	25708701	KD2-H4K8ac_peak_12197	5.63633	
17	25782784	25783697	KD2-H4K8ac_peak_12198	18.53838	KSR1_ENSG00000141068
17	25886722	25886958	KD2-H4K8ac_peak_12199	4.86324	
17	25887359	25887584	KD2-H4K8ac_peak_12200	8.1718	
17	25898362	25898567	KD2-H4K8ac_peak_12201	4.211	
17	25904325	25905090	KD2-H4K8ac_peak_12202	12.30509	
17	25912788	25913054	KD2-H4K8ac_peak_12203	4.8888	
17	25981463	25981742	KD2-H4K8ac_peak_12204	8.5781	
17	26120373	26121019	KD2-H4K8ac_peak_12205	15.39543	
17	26219907	26220270	KD2-H4K8ac_peak_12206	13.73635	RP1-66C13.4_ENSG00000266202
17	26554126	26554899	KD2-H4K8ac_peak_12207	16.49944	PYY2_ENSG00000237575
17	26662837	26663065	KD2-H4K8ac_peak_12208	9.20193	IFT20_ENSG00000109083;TNFAIP1_ENSG00000109079
17	26684016	26684489	KD2-H4K8ac_peak_12209	9.06369	POLDIP2_ENSG00000004142;TMEM199_ENSG00000244045;CTB-96E2.3_ENSG00000258924
17	26684724	26685073	KD2-H4K8ac_peak_12210	9.95119	POLDIP2_ENSG00000004142;TMEM199_ENSG00000244045;CTB-96E2.3_ENSG00000258924
17	26698759	26699511	KD2-H4K8ac_peak_12211	14.8083	VTN_ENSG00000109072
17	26732382	26732685	KD2-H4K8ac_peak_12212	4.0528	CTD-2350C19.2_ENSG00000265254
17	26897866	26898445	KD2-H4K8ac_peak_12213	6.30229	PIGS_ENSG00000087111
17	26971601	26972116	KD2-H4K8ac_peak_12214	12.08081	KIAA0100_ENSG00000007202
17	26972375	26972735	KD2-H4K8ac_peak_12215	8.85813	KIAA0100_ENSG00000007202
17	26988826	26989618	KD2-H4K8ac_peak_12216	8.10216	SDF2_ENSG00000132581;SUPT6H_ENSG00000109111
17	27038054	27038298	KD2-H4K8ac_peak_12217	13.73635	PROCA1_ENSG00000167525
17	27038618	27039032	KD2-H4K8ac_peak_12218	8.4716	PROCA1_ENSG00000167525
17	27046180	27046533	KD2-H4K8ac_peak_12219	6.78668	RAB34_ENSG00000109113;RPL23A_ENSG00000198242
17	27047012	27047266	KD2-H4K8ac_peak_12220	12.88108	RPL23A_ENSG00000198242;SNORD42B_ENSG00000238423
17	27052807	27053171	KD2-H4K8ac_peak_12221	6.66692	NEK8_ENSG00000160602
17	27053444	27053679	KD2-H4K8ac_peak_12222	6.66692	NEK8_ENSG00000160602;AC010761.14_ENSG00000267729
17	27054440	27055596	KD2-H4K8ac_peak_12223	6.56211	TLCD1_ENSG00000160606;AC010761.14_ENSG00000267729
17	27071154	27071674	KD2-H4K8ac_peak_12224	8.26945	TRAF4_ENSG00000076604
17	27139170	27139393	KD2-H4K8ac_peak_12225	13.70753	
17	27169325	27170077	KD2-H4K8ac_peak_12226	7.47462	
17	27181120	27182364	KD2-H4K8ac_peak_12227	14.98936	FAM222B_ENSG00000173065;ERAL1_ENSG00000132591
17	27224093	27224997	KD2-H4K8ac_peak_12228	15.20126	FLOT2_ENSG00000132589;RP11-20B24.4_ENSG00000266642
17	27229380	27229906	KD2-H4K8ac_peak_12229	10.19949	DHRS13_ENSG00000167536
17	27275898	27276101	KD2-H4K8ac_peak_12230	6.83936	
17	27276420	27277043	KD2-H4K8ac_peak_12231	4.30033	PIPOX_ENSG00000179761
17	27277539	27278297	KD2-H4K8ac_peak_12232	9.06432	PIPOX_ENSG00000179761;PHF12_ENSG00000109118
17	27279008	27279292	KD2-H4K8ac_peak_12233	5.9696	PHF12_ENSG00000109118
17	27370044	27370263	KD2-H4K8ac_peak_12234	4.8485	

17	27375907	27376437	KD2-H4K8ac_peak_12235	7.13615	
17	27429706	27430855	KD2-H4K8ac_peak_12236	11.9948	
17	27503627	27503874	KD2-H4K8ac_peak_12237	11.86082	
17	27506593	27507157	KD2-H4K8ac_peak_12238	7.47462	MYO18A_ENSG00000196535
17	27507541	27507899	KD2-H4K8ac_peak_12239	8.30974	MYO18A_ENSG00000196535
17	27619869	27620548	KD2-H4K8ac_peak_12240	9.96798	NUFIP2_ENSG00000108256
17	27621378	27621757	KD2-H4K8ac_peak_12241	4.86324	NUFIP2_ENSG00000108256
17	27717025	27718044	KD2-H4K8ac_peak_12242	22.21705	MIR4523_ENSG00000264808;TAOK1_ENSG00000160551;MIR4523_ENSG00000263719
17	27893832	27894025	KD2-H4K8ac_peak_12243	6.47962	ABHD15_ENSG00000168792;TP53I13_ENSG00000167543
17	27895798	27896203	KD2-H4K8ac_peak_12244	9.73431	RP11-68I3.4_ENSG00000264290
17	27916806	27917046	KD2-H4K8ac_peak_12245	8.55271	ANKRD13B_ENSG00000198720
17	27917319	27917659	KD2-H4K8ac_peak_12246	5.71781	ANKRD13B_ENSG00000198720
17	27917891	27918703	KD2-H4K8ac_peak_12247	8.53092	RP11-68I3.7_ENSG00000264647
17	27918926	27919169	KD2-H4K8ac_peak_12248	5.72327	RP11-68I3.7_ENSG00000264647
17	27919435	27920360	KD2-H4K8ac_peak_12249	12.47554	GIT1_ENSG00000108262;RP11-68I3.7_ENSG00000264647
17	27920628	27921162	KD2-H4K8ac_peak_12250	7.76329	GIT1_ENSG00000108262
17	27923821	27924027	KD2-H4K8ac_peak_12251	4.37815	
17	27940563	27940771	KD2-H4K8ac_peak_12252	3.72574	
17	27950089	27950349	KD2-H4K8ac_peak_12253	11.37483	CORO6_ENSG00000167549;RP11-68I3.10_ENSG00000264007
17	27971194	27971745	KD2-H4K8ac_peak_12254	9.1707	
17	28033915	28034201	KD2-H4K8ac_peak_12255	6.36319	
17	28038681	28039008	KD2-H4K8ac_peak_12256	6.22239	
17	28048366	28048623	KD2-H4K8ac_peak_12257	4.01115	
17	28048942	28049252	KD2-H4K8ac_peak_12258	9.84972	
17	28051245	28051450	KD2-H4K8ac_peak_12259	8.59579	
17	28053119	28053562	KD2-H4K8ac_peak_12260	5.54647	
17	28072292	28072517	KD2-H4K8ac_peak_12261	5.86783	
17	28088017	28088684	KD2-H4K8ac_peak_12262	8.55271	RP11-82O19.1_ENSG00000263657
17	28106947	28107148	KD2-H4K8ac_peak_12263	7.12754	
17	28431830	28432428	KD2-H4K8ac_peak_12264	6.16888	
17	28443097	28443683	KD2-H4K8ac_peak_12265	10.0692	RP11-1148O4.2_ENSG00000265394;MIR3184_ENSG00000266919;MIR423_ENSG00000199071;NSRP1_ENSG00000126653
17	28444044	28444240	KD2-H4K8ac_peak_12266	7.76329	RP11-1148O4.2_ENSG00000265394;MIR3184_ENSG00000266919;MIR423_ENSG00000199071
17	28618559	28619472	KD2-H4K8ac_peak_12267	11.24702	BLMH_ENSG00000108578
17	28705379	28705687	KD2-H4K8ac_peak_12268	4.86324	CPD_ENSG00000108582
17	28705983	28706300	KD2-H4K8ac_peak_12269	11.43353	CPD_ENSG00000108582
17	28803917	28804759	KD2-H4K8ac_peak_12270	14.8083	GOSR1_ENSG00000108587
17	28927477	28928393	KD2-H4K8ac_peak_12271	11.00004	SMURF2P1_ENSG00000248121
17	28994103	28994553	KD2-H4K8ac_peak_12272	8.53092	
17	28994780	28995104	KD2-H4K8ac_peak_12273	22.21705	
17	28996210	28996464	KD2-H4K8ac_peak_12274	8.78496	
17	29035096	29035443	KD2-H4K8ac_peak_12275	6.67538	SUZ12P_ENSG00000264538
17	29036092	29036491	KD2-H4K8ac_peak_12276	8.33649	SUZ12P_ENSG00000264538
17	29036692	29036956	KD2-H4K8ac_peak_12277	7.76329	SUZ12P_ENSG00000264538
17	29151967	29152976	KD2-H4K8ac_peak_12278	8.26945	CRLF3_ENSG00000176390
17	29232846	29233826	KD2-H4K8ac_peak_12279	11.80283	TEFM_ENSG00000172171;ADAP2_ENSG00000184060
17	29421460	29421816	KD2-H4K8ac_peak_12280	7.67504	RP11-848P1.5_ENSG00000264107;MIR4733_ENSG00000265444;NF1_ENSG00000196712

17	29717817	29718539	KD2-H4K8ac_peak_12281	8.10216	RAB11FIP4_ENSG00000131242
17	29719202	29719514	KD2-H4K8ac_peak_12282	8.30974	RAB11FIP4_ENSG00000131242
17	29752722	29753151	KD2-H4K8ac_peak_12283	10.96455	
17	29876343	29876924	KD2-H4K8ac_peak_12284	11.02863	
17	29885458	29885740	KD2-H4K8ac_peak_12285	5.77265	
17	29886323	29886652	KD2-H4K8ac_peak_12286	11.05467	MIR193A_ENSG00000207614
17	29908441	29908735	KD2-H4K8ac_peak_12287	7.07117	
17	30185916	30186226	KD2-H4K8ac_peak_12288	15.61313	COPRS_ENSG00000172301
17	30229099	30229429	KD2-H4K8ac_peak_12289	12.06346	UTP6_ENSG00000108651
17	30263278	30263670	KD2-H4K8ac_peak_12290	9.36516	SUZ12_ENSG00000178691
17	30264094	30264466	KD2-H4K8ac_peak_12291	10.05606	SUZ12_ENSG00000178691
17	30334002	30334408	KD2-H4K8ac_peak_12292	12.84642	RP11-640N20.1_ENSG00000264300;LRRC37B_ENSG00000185158
17	30396035	30396723	KD2-H4K8ac_peak_12293	6.1846	
17	30410515	30411222	KD2-H4K8ac_peak_12294	21.10246	RP11-640N20.6_ENSG00000264164
17	30432844	30433038	KD2-H4K8ac_peak_12295	6.15357	
17	30438399	30438659	KD2-H4K8ac_peak_12296	9.1707	CTD-2095E4.1_ENSG00000265574
17	30468969	30469360	KD2-H4K8ac_peak_12297	13.09607	AC090616.2_ENSG00000214708;RHOT1_ENSG00000126858
17	30592372	30593048	KD2-H4K8ac_peak_12298	3.90565	RHBDL3_ENSG00000141314
17	30602138	30602400	KD2-H4K8ac_peak_12299	4.44527	
17	30607876	30608623	KD2-H4K8ac_peak_12300	6.20982	RP11-443G13.2_ENSG00000263674
17	30668513	30669231	KD2-H4K8ac_peak_12301	15.10495	
17	30676284	30676496	KD2-H4K8ac_peak_12302	5.61067	C17orf75_ENSG00000108666;MIR632_ENSG00000207928;ZNF207_ENSG00000010244
17	30677323	30677595	KD2-H4K8ac_peak_12303	6.99815	C17orf75_ENSG00000108666;MIR632_ENSG00000207928;ZNF207_ENSG00000010244
17	30771565	30772055	KD2-H4K8ac_peak_12304	8.04448	PSMD11_ENSG00000108671
17	30813140	30813374	KD2-H4K8ac_peak_12305	8.4716	CDK5R1_ENSG00000176749
17	30814174	30814578	KD2-H4K8ac_peak_12306	9.34195	CDK5R1_ENSG00000176749
17	30845916	30846742	KD2-H4K8ac_peak_12307	15.47237	RP11-466A19.6_ENSG00000263717;RP11-466A19.3_ENSG00000266599
17	31149441	31149934	KD2-H4K8ac_peak_12308	14.43704	
17	31203777	31204189	KD2-H4K8ac_peak_12309	15.69572	MYO1D_ENSG00000176658;AC084809.2_ENSG00000226377
17	31254363	31255508	KD2-H4K8ac_peak_12310	16.54484	TMEM98_ENSG00000006042
17	32940747	32941119	KD2-H4K8ac_peak_12311	5.82799	
17	32943553	32943910	KD2-H4K8ac_peak_12312	12.3187	
17	32947202	32947522	KD2-H4K8ac_peak_12313	5.6755	
17	33107647	33107845	KD2-H4K8ac_peak_12314	4.67737	
17	33108136	33108596	KD2-H4K8ac_peak_12315	5.43522	
17	33287760	33289125	KD2-H4K8ac_peak_12316	15.81711	ZNF830_ENSG00000198783
17	33307581	33308142	KD2-H4K8ac_peak_12317	8.80432	LIG3_ENSG00000005156;CCT6B_ENSG00000132141
17	33410950	33411250	KD2-H4K8ac_peak_12318	6.99815	
17	33415905	33416222	KD2-H4K8ac_peak_12319	6.20982	RFFL_ENSG00000092871
17	33416516	33417055	KD2-H4K8ac_peak_12320	6.65673	RFFL_ENSG00000092871
17	33446072	33446821	KD2-H4K8ac_peak_12321	14.15075	
17	33468770	33469290	KD2-H4K8ac_peak_12322	14.00658	NLE1_ENSG00000073536
17	33469548	33469939	KD2-H4K8ac_peak_12323	8.37231	NLE1_ENSG00000073536
17	33894119	33894516	KD2-H4K8ac_peak_12324	16.94317	RP11-1094M14.1_ENSG00000242660;RP11-1094M14.11_ENSG00000267321
17	33895163	33895399	KD2-H4K8ac_peak_12325	10.17698	RP11-1094M14.11_ENSG00000267321
17	33905127	33905581	KD2-H4K8ac_peak_12326	15.06766	PEX12_ENSG00000108733;AP2B1_ENSG00000006125

17	33905775	33906044	KD2-H4K8ac_peak_12327	11.03331	PEX12_ENSG00000108733;AP2B1_ENSG00000006125
17	33913845	33914073	KD2-H4K8ac_peak_12328	9.96798	
17	34091429	34091697	KD2-H4K8ac_peak_12329	6.0605	
17	34101306	34102137	KD2-H4K8ac_peak_12330	9.89383	
17	34128066	34128582	KD2-H4K8ac_peak_12331	7.76329	
17	34128866	34129545	KD2-H4K8ac_peak_12332	14.63262	
17	34135507	34136183	KD2-H4K8ac_peak_12333	4.35346	AC015849.12_ENSG00000267578;TAF15_ENSG00000172660
17	34136534	34136997	KD2-H4K8ac_peak_12334	10.53377	AC015849.12_ENSG00000267578;TAF15_ENSG00000172660
17	34838500	34838705	KD2-H4K8ac_peak_12335	5.30799	
17	34839062	34839565	KD2-H4K8ac_peak_12336	13.35993	
17	34841711	34842245	KD2-H4K8ac_peak_12337	9.73431	ZNHIT3_ENSG00000108278
17	34890472	34890672	KD2-H4K8ac_peak_12338	6.40297	PIGW_ENSG00000184886
17	34900376	34900619	KD2-H4K8ac_peak_12339	8.85813	GGNBP2_ENSG00000005955
17	34947783	34948162	KD2-H4K8ac_peak_12340	9.16531	DHRS11_ENSG00000108272
17	34948400	34948625	KD2-H4K8ac_peak_12341	9.1707	DHRS11_ENSG00000108272
17	34957061	34957852	KD2-H4K8ac_peak_12342	8.28234	MRM1_ENSG00000129282
17	34958083	34958550	KD2-H4K8ac_peak_12343	10.17341	MRM1_ENSG00000129282
17	34982637	34982877	KD2-H4K8ac_peak_12344	6.24747	
17	34998183	34998408	KD2-H4K8ac_peak_12345	6.76298	
17	35036590	35037336	KD2-H4K8ac_peak_12346	8.34752	
17	35076902	35077166	KD2-H4K8ac_peak_12347	6.99815	
17	35078746	35079058	KD2-H4K8ac_peak_12348	9.20193	CTD-3194G12.2_ENSG00000267785
17	35083573	35084303	KD2-H4K8ac_peak_12349	15.81711	
17	35085138	35085843	KD2-H4K8ac_peak_12350	20.47312	
17	35086104	35086339	KD2-H4K8ac_peak_12351	8.59579	
17	35086658	35087030	KD2-H4K8ac_peak_12352	7.1181	
17	35087913	35088185	KD2-H4K8ac_peak_12353	5.57044	
17	35096011	35096256	KD2-H4K8ac_peak_12354	3.75035	
17	35096486	35096687	KD2-H4K8ac_peak_12355	4.01115	
17	35165177	35166067	KD2-H4K8ac_peak_12356	14.4458	
17	35305745	35306022	KD2-H4K8ac_peak_12357	13.31016	AATF_ENSG00000108270
17	35306306	35306784	KD2-H4K8ac_peak_12358	5.13615	AATF_ENSG00000108270
17	35716237	35716666	KD2-H4K8ac_peak_12359	7.44324	
17	35766430	35766885	KD2-H4K8ac_peak_12360	16.32216	ACACA_ENSG00000132142;TADA2A_ENSG00000108264
17	35849454	35849785	KD2-H4K8ac_peak_12361	8.4716	DUSP14_ENSG00000161326
17	35969178	35969390	KD2-H4K8ac_peak_12362	8.02571	SYNRG_ENSG00000006114;RP11-697E22.1_ENSG00000267542
17	36003215	36003414	KD2-H4K8ac_peak_12363	6.66692	DDX52_ENSG00000141141;RP11-697E22.2_ENSG00000267668
17	36401535	36401772	KD2-H4K8ac_peak_12364	5.59859	
17	36413578	36414396	KD2-H4K8ac_peak_12365	11.03331	
17	36442620	36442934	KD2-H4K8ac_peak_12366	9.11031	
17	36452293	36452666	KD2-H4K8ac_peak_12367	7.40314	MRPL45_ENSG00000174100
17	36507300	36507618	KD2-H4K8ac_peak_12368	6.65673	SOCS7_ENSG00000174111
17	36507952	36508301	KD2-H4K8ac_peak_12369	5.9696	SOCS7_ENSG00000174111
17	36571192	36572311	KD2-H4K8ac_peak_12370	13.80019	
17	36572532	36572843	KD2-H4K8ac_peak_12371	5.82869	
17	36574864	36575361	KD2-H4K8ac_peak_12372	10.96455	

17	36575972	36576780	KD2-H4K8ac_peak_12373	6.69204	
17	36577302	36577530	KD2-H4K8ac_peak_12374	8.02571	
17	36584263	36584495	KD2-H4K8ac_peak_12375	8.37231	ARHGAP23_ENSG00000225485
17	36609929	36610191	KD2-H4K8ac_peak_12376	5.37864	
17	36620866	36621491	KD2-H4K8ac_peak_12377	10.08707	
17	36665980	36666794	KD2-H4K8ac_peak_12378	11.50346	
17	36741498	36741812	KD2-H4K8ac_peak_12379	5.30494	
17	36761213	36761436	KD2-H4K8ac_peak_12380	4.42498	SRCIN1_ENSG00000017373
17	36761884	36763078	KD2-H4K8ac_peak_12381	11.24702	SRCIN1_ENSG00000017373
17	36830017	36830228	KD2-H4K8ac_peak_12382	5.49478	C17orf96_ENSG00000179294
17	36830648	36831217	KD2-H4K8ac_peak_12383	7.28827	C17orf96_ENSG00000179294
17	36849854	36850105	KD2-H4K8ac_peak_12384	11.66477	
17	36850460	36850701	KD2-H4K8ac_peak_12385	14.48084	
17	36857865	36859247	KD2-H4K8ac_peak_12386	17.17257	MIR4734_ENSG00000265930;CTB-58E17.1_ENSG00000261005
17	36859923	36861280	KD2-H4K8ac_peak_12387	15.13261	MLLT6_ENSG00000108292
17	36861519	36862353	KD2-H4K8ac_peak_12388	12.00273	CTB-58E17.3_ENSG00000266040;MLLT6_ENSG00000108292
17	36881137	36881329	KD2-H4K8ac_peak_12389	5.84991	
17	36881664	36882134	KD2-H4K8ac_peak_12390	7.146	
17	36886043	36886365	KD2-H4K8ac_peak_12391	12.84642	CISD3_ENSG00000230055
17	36886634	36887018	KD2-H4K8ac_peak_12392	10.08707	CISD3_ENSG00000230055
17	36898394	36898772	KD2-H4K8ac_peak_12393	5.74496	
17	36899750	36900080	KD2-H4K8ac_peak_12394	8.59579	
17	36903626	36904431	KD2-H4K8ac_peak_12395	16.23676	
17	36904949	36905988	KD2-H4K8ac_peak_12396	12.2118	PCGF2_ENSG00000056661;CTB-58E17.5_ENSG00000227450
17	36906197	36906550	KD2-H4K8ac_peak_12397	12.12738	PCGF2_ENSG00000056661;CTB-58E17.5_ENSG00000227450
17	36906751	36906948	KD2-H4K8ac_peak_12398	5.20998	PCGF2_ENSG00000056661
17	36955331	36955672	KD2-H4K8ac_peak_12399	6.0605	PIP4K2B_ENSG00000141720
17	36955944	36956179	KD2-H4K8ac_peak_12400	12.08081	PIP4K2B_ENSG00000141720
17	36981355	36982604	KD2-H4K8ac_peak_12401	8.4716	CWC25_ENSG00000108296;MIR4727_ENSG00000263791
17	37009702	37009928	KD2-H4K8ac_peak_12402	8.79106	RPL23_ENSG00000125691;SNORA21_ENSG00000199293
17	37010530	37010820	KD2-H4K8ac_peak_12403	9.1707	RPL23_ENSG00000125691;RPS20P35_ENSG00000244086
17	37011073	37011296	KD2-H4K8ac_peak_12404	6.66692	RPL23_ENSG00000125691;RPS20P35_ENSG00000244086
17	37025688	37026153	KD2-H4K8ac_peak_12405	27.50934	LASP1_ENSG00000002834
17	37026987	37027960	KD2-H4K8ac_peak_12406	8.52559	LASP1_ENSG00000002834
17	37213358	37213789	KD2-H4K8ac_peak_12407	7.28628	CTD-2206N4.4_ENSG00000263818
17	37321034	37321804	KD2-H4K8ac_peak_12408	5.9696	
17	37353570	37353882	KD2-H4K8ac_peak_12409	14.275	CACNB1_ENSG00000067191
17	37354247	37354712	KD2-H4K8ac_peak_12410	9.908	CACNB1_ENSG00000067191
17	37355557	37355990	KD2-H4K8ac_peak_12411	8.86787	RPL19_ENSG00000108298
17	37356707	37356908	KD2-H4K8ac_peak_12412	8.30974	RPL19_ENSG00000108298
17	37365948	37366491	KD2-H4K8ac_peak_12413	6.36375	
17	37381813	37382174	KD2-H4K8ac_peak_12414	7.18916	STAC2_ENSG00000141750
17	37382524	37382801	KD2-H4K8ac_peak_12415	5.10505	STAC2_ENSG00000141750
17	37386977	37387692	KD2-H4K8ac_peak_12416	8.10216	
17	37387976	37388353	KD2-H4K8ac_peak_12417	6.65673	
17	37392716	37393154	KD2-H4K8ac_peak_12418	8.94469	

17	37607248	37607460	KD2-H4K8ac_peak_12419	5.9696	MED1_ENSG00000125686
17	37607831	37608145	KD2-H4K8ac_peak_12420	16.03995	MED1_ENSG00000125686
17	37617078	37617391	KD2-H4K8ac_peak_12421	6.76298	CDK12_ENSG00000167258
17	37729903	37730447	KD2-H4K8ac_peak_12422	5.88845	
17	37773620	37775333	KD2-H4K8ac_peak_12423	18.5703	AC087491.2_ENSG00000214546
17	37792750	37793774	KD2-H4K8ac_peak_12424	10.29842	STARD3_ENSG00000131748
17	37817149	37817356	KD2-H4K8ac_peak_12425	7.98621	
17	37823646	37823990	KD2-H4K8ac_peak_12426	7.79741	PNMT_ENSG00000141744
17	37824220	37825086	KD2-H4K8ac_peak_12427	15.58442	PNMT_ENSG00000141744
17	37844683	37845082	KD2-H4K8ac_peak_12428	7.76329	ERBB2_ENSG00000141736
17	37886482	37886981	KD2-H4K8ac_peak_12429	13.83097	MIEN1_ENSG00000141741
17	37909841	37911370	KD2-H4K8ac_peak_12430	18.37277	
17	38084189	38084548	KD2-H4K8ac_peak_12431	9.34195	ORMDL3_ENSG00000172057;RP11-387H17.4_ENSG00000264968
17	38108819	38109068	KD2-H4K8ac_peak_12432	8.55271	
17	38109684	38110113	KD2-H4K8ac_peak_12433	11.05467	
17	38115494	38115955	KD2-H4K8ac_peak_12434	19.6416	
17	38136342	38136635	KD2-H4K8ac_peak_12435	8.17902	PSMD3_ENSG00000108344
17	38182971	38183371	KD2-H4K8ac_peak_12436	6.63018	SNORD124_ENSG00000238793
17	38210058	38210473	KD2-H4K8ac_peak_12437	4.211	
17	38210785	38211151	KD2-H4K8ac_peak_12438	11.05467	
17	38219207	38219646	KD2-H4K8ac_peak_12439	4.06758	
17	38224612	38224988	KD2-H4K8ac_peak_12440	6.55628	
17	38225528	38226135	KD2-H4K8ac_peak_12441	10.65724	
17	38228021	38228488	KD2-H4K8ac_peak_12442	15.14522	
17	38269704	38270169	KD2-H4K8ac_peak_12443	9.43685	
17	38270386	38270668	KD2-H4K8ac_peak_12444	6.69204	
17	38277692	38278078	KD2-H4K8ac_peak_12445	4.211	MSL1_ENSG00000188895
17	38278546	38279565	KD2-H4K8ac_peak_12446	13.88933	MSL1_ENSG00000188895
17	38295832	38296079	KD2-H4K8ac_peak_12447	3.90565	CASC3_ENSG00000108349
17	38296790	38297380	KD2-H4K8ac_peak_12448	21.11201	CASC3_ENSG00000108349
17	38333799	38334074	KD2-H4K8ac_peak_12449	5.88845	RAPGEFL1_ENSG00000108352
17	38347485	38347691	KD2-H4K8ac_peak_12450	8.30974	
17	38348109	38348452	KD2-H4K8ac_peak_12451	9.5456	
17	38462802	38463259	KD2-H4K8ac_peak_12452	7.993	
17	38463682	38463876	KD2-H4K8ac_peak_12453	6.01098	
17	38464161	38464388	KD2-H4K8ac_peak_12454	8.26129	
17	38465550	38466494	KD2-H4K8ac_peak_12455	10.7186	RARA_ENSG00000131759
17	38468362	38468744	KD2-H4K8ac_peak_12456	7.98621	
17	38472278	38472480	KD2-H4K8ac_peak_12457	4.86324	
17	38473188	38473617	KD2-H4K8ac_peak_12458	5.38581	
17	38478754	38478950	KD2-H4K8ac_peak_12459	4.40553	
17	38486695	38486950	KD2-H4K8ac_peak_12460	5.33892	
17	38496980	38497466	KD2-H4K8ac_peak_12461	4.211	
17	38497765	38498173	KD2-H4K8ac_peak_12462	5.08628	
17	38498708	38499076	KD2-H4K8ac_peak_12463	4.56666	CTD-2267D19.2_ENSG00000265666
17	38500348	38500554	KD2-H4K8ac_peak_12464	5.08628	CTD-2267D19.2_ENSG00000265666

17	38500773	38501133	KD2-H4K8ac_peak_12465	5.59859	
17	38501621	38502235	KD2-H4K8ac_peak_12466	5.75757	
17	38503721	38503931	KD2-H4K8ac_peak_12467	6.52841	
17	38505856	38506359	KD2-H4K8ac_peak_12468	7.95563	
17	38506644	38507161	KD2-H4K8ac_peak_12469	8.55271	
17	38507598	38508604	KD2-H4K8ac_peak_12470	20.5798	
17	38510409	38510647	KD2-H4K8ac_peak_12471	8.30974	
17	38510909	38511278	KD2-H4K8ac_peak_12472	4.86324	
17	38512472	38512772	KD2-H4K8ac_peak_12473	7.76329	
17	38519296	38519699	KD2-H4K8ac_peak_12474	7.95563	GJD3_ENSG00000183153
17	38574535	38574974	KD2-H4K8ac_peak_12475	6.99194	TOP2A_ENSG00000131747
17	38576194	38576536	KD2-H4K8ac_peak_12476	5.30494	
17	38589796	38590178	KD2-H4K8ac_peak_12477	11.92978	
17	38599027	38600277	KD2-H4K8ac_peak_12478	16.49944	IGFBP4_ENSG00000141753
17	38616218	38616668	KD2-H4K8ac_peak_12479	12.65397	
17	38617421	38617753	KD2-H4K8ac_peak_12480	15.61313	
17	38618396	38619163	KD2-H4K8ac_peak_12481	13.12288	
17	38619717	38619988	KD2-H4K8ac_peak_12482	6.99815	
17	38627442	38627694	KD2-H4K8ac_peak_12483	4.56666	
17	38632097	38632335	KD2-H4K8ac_peak_12484	8.79106	
17	38646245	38647287	KD2-H4K8ac_peak_12485	14.83804	
17	38647767	38648119	KD2-H4K8ac_peak_12486	4.42765	
17	38692645	38693180	KD2-H4K8ac_peak_12487	4.06758	
17	38694634	38695153	KD2-H4K8ac_peak_12488	10.88013	
17	38696116	38696916	KD2-H4K8ac_peak_12489	10.34742	
17	38697741	38698940	KD2-H4K8ac_peak_12490	19.93398	
17	38699261	38701082	KD2-H4K8ac_peak_12491	18.94636	
17	38701910	38702428	KD2-H4K8ac_peak_12492	10.34742	
17	38703591	38704113	KD2-H4K8ac_peak_12493	6.40297	
17	38704844	38705543	KD2-H4K8ac_peak_12494	15.61313	
17	38705916	38706380	KD2-H4K8ac_peak_12495	6.66692	
17	38712879	38713263	KD2-H4K8ac_peak_12496	6.16883	
17	38713560	38714339	KD2-H4K8ac_peak_12497	14.88905	
17	38715024	38715958	KD2-H4K8ac_peak_12498	11.94415	
17	38716874	38717083	KD2-H4K8ac_peak_12499	6.04107	
17	38803691	38804891	KD2-H4K8ac_peak_12500	16.822	SMARCE1_ENSG00000073584
17	38974897	38975230	KD2-H4K8ac_peak_12501	7.12754	TMEM99_ENSG00000167920
17	38975597	38975936	KD2-H4K8ac_peak_12502	5.25071	TMEM99_ENSG00000167920
17	39677458	39677871	KD2-H4K8ac_peak_12503	6.93012	KRT15_ENSG00000171346
17	39679873	39680462	KD2-H4K8ac_peak_12504	8.79106	
17	39683489	39683937	KD2-H4K8ac_peak_12505	7.47462	KRT19_ENSG00000171345
17	39685604	39686498	KD2-H4K8ac_peak_12506	15.25274	
17	39686907	39687558	KD2-H4K8ac_peak_12507	14.48084	
17	39696458	39696974	KD2-H4K8ac_peak_12508	9.9363	
17	39700688	39701291	KD2-H4K8ac_peak_12509	12.65964	
17	39704224	39704491	KD2-H4K8ac_peak_12510	6.66692	

17	39704843	39705581	KD2-H4K8ac_peak_12511	11.05467	
17	39790964	39791249	KD2-H4K8ac_peak_12512	7.29632	
17	39822965	39823352	KD2-H4K8ac_peak_12513	8.23274	
17	39845270	39845493	KD2-H4K8ac_peak_12514	8.58725	EIF1_ENSG00000173812
17	39894495	39895229	KD2-H4K8ac_peak_12515	14.23999	
17	39942602	39942876	KD2-H4K8ac_peak_12516	5.00278	JUP_ENSG00000173801
17	39968065	39969785	KD2-H4K8ac_peak_12517	12.57143	LEPREL4_ENSG00000141696;FKBP10_ENSG00000141756
17	39992060	39992386	KD2-H4K8ac_peak_12518	11.92978	KLHL10_ENSG00000161594;NT5C3B_ENSG00000141698
17	40021304	40021573	KD2-H4K8ac_peak_12519	8.85813	KLHL11_ENSG00000178502
17	40051604	40051889	KD2-H4K8ac_peak_12520	8.78496	
17	40062689	40063064	KD2-H4K8ac_peak_12521	9.16531	
17	40074483	40074931	KD2-H4K8ac_peak_12522	9.57101	
17	40075597	40076133	KD2-H4K8ac_peak_12523	20.42362	
17	40086398	40086670	KD2-H4K8ac_peak_12524	8.4716	ACLY_ENSG00000131473;TTC25_ENSG00000204815
17	40117958	40118349	KD2-H4K8ac_peak_12525	7.40111	CNP_ENSG00000173786
17	40118826	40119382	KD2-H4K8ac_peak_12526	6.0605	CNP_ENSG00000173786
17	40169051	40170269	KD2-H4K8ac_peak_12527	20.71987	
17	40202023	40202817	KD2-H4K8ac_peak_12528	16.07006	
17	40219879	40220211	KD2-H4K8ac_peak_12529	6.5996	
17	40250081	40250415	KD2-H4K8ac_peak_12530	6.66692	
17	40253917	40254181	KD2-H4K8ac_peak_12531	4.67342	
17	40273006	40273231	KD2-H4K8ac_peak_12532	8.79106	KAT2A_ENSG00000108773
17	40306740	40306976	KD2-H4K8ac_peak_12533	12.79505	CTD-2132N18.3_ENSG00000267261;RAB5C_ENSG00000108774;RP11-358B23.1_ENSG00000267658
17	40307311	40307707	KD2-H4K8ac_peak_12534	10.53377	CTD-2132N18.3_ENSG00000267261;RAB5C_ENSG00000108774;RP11-358B23.1_ENSG00000267658
17	40313244	40313694	KD2-H4K8ac_peak_12535	10.0692	
17	40336318	40336599	KD2-H4K8ac_peak_12536	6.25869	HCRT_ENSG00000161610
17	40346641	40346975	KD2-H4K8ac_peak_12537	10.43145	GHDC_ENSG00000167925
17	40427853	40429295	KD2-H4K8ac_peak_12538	16.95399	STAT5B_ENSG00000173757
17	40452361	40452570	KD2-H4K8ac_peak_12539	7.67496	
17	40540920	40541125	KD2-H4K8ac_peak_12540	8.55271	STAT3_ENSG00000168610
17	40610490	40610743	KD2-H4K8ac_peak_12541	8.30974	ATP6V0A1_ENSG00000033627
17	40610998	40611252	KD2-H4K8ac_peak_12542	12.25733	ATP6V0A1_ENSG00000033627
17	40684139	40684415	KD2-H4K8ac_peak_12543	18.5703	
17	40687843	40688122	KD2-H4K8ac_peak_12544	4.0528	RP11-400F19.8_ENSG00000266929
17	40688329	40688766	KD2-H4K8ac_peak_12545	8.10216	RP11-400F19.8_ENSG00000266929
17	40706133	40707127	KD2-H4K8ac_peak_12546	11.02863	RP11-400F19.6_ENSG00000266962
17	40713506	40713928	KD2-H4K8ac_peak_12547	8.10216	COASY_ENSG00000068120
17	40714331	40714638	KD2-H4K8ac_peak_12548	9.34195	COASY_ENSG00000068120
17	40718537	40718844	KD2-H4K8ac_peak_12549	8.4716	MLX_ENSG00000108788
17	40719227	40719534	KD2-H4K8ac_peak_12550	12.23	MLX_ENSG00000108788
17	40729467	40729686	KD2-H4K8ac_peak_12551	8.16906	PSMC3IP_ENSG00000131470
17	40761764	40761965	KD2-H4K8ac_peak_12552	8.26945	TUBG1_ENSG00000131462;FAM134C_ENSG00000141699
17	40785323	40785629	KD2-H4K8ac_peak_12553	6.31143	
17	40810894	40811146	KD2-H4K8ac_peak_12554	4.56666	TUBG2_ENSG00000037042
17	40811384	40811840	KD2-H4K8ac_peak_12555	8.10216	TUBG2_ENSG00000037042
17	40829487	40829911	KD2-H4K8ac_peak_12556	11.6867	PLEKHH3_ENSG00000068137

17	40830700	40832588	KD2-H4K8ac_peak_12557	25.8903	CTD-3193K9.4_ENSG00000267042
17	40834792	40835238	KD2-H4K8ac_peak_12558	8.3479	CCR10_ENSG00000184451;CNTNAP1_ENSG00000108797
17	40896440	40896658	KD2-H4K8ac_peak_12559	5.4779	EZH1_ENSG00000108799
17	40896870	40897369	KD2-H4K8ac_peak_12560	5.75757	EZH1_ENSG00000108799
17	40912639	40913899	KD2-H4K8ac_peak_12561	16.54484	RAMP2-AS1_ENSG00000197291
17	40915185	40915567	KD2-H4K8ac_peak_12562	8.30974	
17	40924576	40925021	KD2-H4K8ac_peak_12563	7.98621	VPS25_ENSG00000131475
17	40925533	40925910	KD2-H4K8ac_peak_12564	11.43863	VPS25_ENSG00000131475
17	40932163	40933157	KD2-H4K8ac_peak_12565	8.83749	WNK4_ENSG00000126562
17	40950430	40950663	KD2-H4K8ac_peak_12566	12.08081	COA3_ENSG00000183978;CNTD1_ENSG00000176563
17	40951010	40951231	KD2-H4K8ac_peak_12567	9.41619	COA3_ENSG00000183978;CNTD1_ENSG00000176563
17	40975824	40976875	KD2-H4K8ac_peak_12568	9.63487	PSME3_ENSG00000131467
17	40984220	40984532	KD2-H4K8ac_peak_12569	6.66692	BECN1_ENSG00000126581
17	40984803	40985838	KD2-H4K8ac_peak_12570	11.05467	BECN1_ENSG00000126581
17	41116042	41116452	KD2-H4K8ac_peak_12571	8.00457	AARSD1_ENSG00000266967
17	41116676	41116962	KD2-H4K8ac_peak_12572	6.00226	AARSD1_ENSG00000266967
17	41117279	41117639	KD2-H4K8ac_peak_12573	5.57044	AARSD1_ENSG00000266967
17	41131376	41131569	KD2-H4K8ac_peak_12574	4.01115	PTGES3L-AARSD1_ENSG00000108825;PTGES3L_ENSG00000267060
17	41150522	41150978	KD2-H4K8ac_peak_12575	6.56211	Y_RNA_ENSG00000200127;RPL27_ENSG00000131469
17	41173788	41174225	KD2-H4K8ac_peak_12576	5.87879	
17	41176853	41177050	KD2-H4K8ac_peak_12577	6.25869	VAT1_ENSG00000108828;RND2_ENSG00000108830
17	41322511	41323001	KD2-H4K8ac_peak_12578	7.27716	RP11-242D8.1_ENSG00000267002;RP11-242D8.3_ENSG00000267340;NBR1_ENSG00000188554
17	41392217	41393243	KD2-H4K8ac_peak_12579	12.93209	
17	41400880	41401117	KD2-H4K8ac_peak_12580	11.29	
17	41439348	41439948	KD2-H4K8ac_peak_12581	17.76996	
17	41445948	41447082	KD2-H4K8ac_peak_12582	11.92978	
17	41464827	41465182	KD2-H4K8ac_peak_12583	7.69294	
17	41471629	41471970	KD2-H4K8ac_peak_12584	5.95537	
17	41475897	41476132	KD2-H4K8ac_peak_12585	7.74292	ARL4D_ENSG00000175906
17	41496558	41497052	KD2-H4K8ac_peak_12586	7.47462	
17	41560513	41561011	KD2-H4K8ac_peak_12587	9.19305	DHX8_ENSG00000067596
17	41622110	41622463	KD2-H4K8ac_peak_12588	8.41122	RP11-392O1.4_ENSG00000267747
17	41622672	41623705	KD2-H4K8ac_peak_12589	17.63891	RP11-392O1.4_ENSG00000267747
17	41669576	41670090	KD2-H4K8ac_peak_12590	5.32397	
17	41723106	41723853	KD2-H4K8ac_peak_12591	9.15069	
17	41754902	41755341	KD2-H4K8ac_peak_12592	13.56049	
17	41791217	41791604	KD2-H4K8ac_peak_12593	5.30799	
17	41797632	41798278	KD2-H4K8ac_peak_12594	23.72814	RP11-209M4.1_ENSG00000267253
17	41855784	41856265	KD2-H4K8ac_peak_12595	16.8435	DUSP3_ENSG00000108861
17	41909823	41911127	KD2-H4K8ac_peak_12596	11.05467	MPP3_ENSG00000161647
17	41978168	41978574	KD2-H4K8ac_peak_12597	4.44527	
17	41985609	41985863	KD2-H4K8ac_peak_12598	5.51783	
17	42015183	42015485	KD2-H4K8ac_peak_12599	8.10216	RP11-527L4.2_ENSG00000261514
17	42016047	42016286	KD2-H4K8ac_peak_12600	6.66692	RP11-527L4.2_ENSG00000261514
17	42051500	42051953	KD2-H4K8ac_peak_12601	4.86324	
17	42055017	42055248	KD2-H4K8ac_peak_12602	8.05667	

17	42061086	42061713	KD2-H4K8ac_peak_12603	13.13965	
17	42091651	42092301	KD2-H4K8ac_peak_12604	17.55491	
17	42100958	42101562	KD2-H4K8ac_peak_12605	13.70753	TMEM101_ENSG00000091947
17	42143701	42143945	KD2-H4K8ac_peak_12606	13.80019	
17	42144419	42144625	KD2-H4K8ac_peak_12607	11.79585	LSM12_ENSG00000161654
17	42148360	42148605	KD2-H4K8ac_peak_12608	7.36799	G6PC3_ENSG00000141349
17	42173158	42173583	KD2-H4K8ac_peak_12609	9.36516	
17	42188274	42188534	KD2-H4K8ac_peak_12610	9.34989	
17	42200641	42200926	KD2-H4K8ac_peak_12611	7.40314	HDAC5_ENSG00000108840
17	42218602	42219059	KD2-H4K8ac_peak_12612	9.1707	C17orf53_ENSG00000125319
17	42219378	42219607	KD2-H4K8ac_peak_12613	10.0692	C17orf53_ENSG00000125319
17	42264375	42264862	KD2-H4K8ac_peak_12614	9.1707	ASB16-AS1_ENSG00000267080;TMUB2_ENSG00000168591
17	42276343	42277352	KD2-H4K8ac_peak_12615	10.96455	ATXN7L3_ENSG00000087152;CTB-175E5.7_ENSG00000267394
17	42277856	42278145	KD2-H4K8ac_peak_12616	7.76104	ATXN7L3_ENSG00000087152
17	42295533	42295780	KD2-H4K8ac_peak_12617	7.36799	
17	42296117	42296958	KD2-H4K8ac_peak_12618	6.50449	
17	42297263	42298221	KD2-H4K8ac_peak_12619	10.43145	UBTF_ENSG00000108312;RP5-882C2.2_ENSG00000260793
17	42298437	42298888	KD2-H4K8ac_peak_12620	6.66692	UBTF_ENSG00000108312;RP5-882C2.2_ENSG00000260793
17	42324182	42324403	KD2-H4K8ac_peak_12621	7.27716	
17	42324644	42325137	KD2-H4K8ac_peak_12622	11.97761	
17	42386231	42386471	KD2-H4K8ac_peak_12623	6.30229	RUNDC3A_ENSG00000108309
17	42421499	42422268	KD2-H4K8ac_peak_12624	9.51625	GRN_ENSG00000030582
17	42462338	42462743	KD2-H4K8ac_peak_12625	10.98959	
17	42462947	42463284	KD2-H4K8ac_peak_12626	22.76112	
17	42580343	42581751	KD2-H4K8ac_peak_12627	9.36516	GPATCH8_ENSG00000186566
17	42610955	42611537	KD2-H4K8ac_peak_12628	11.70505	
17	42633845	42634385	KD2-H4K8ac_peak_12629	6.83936	FZD2_ENSG00000180340
17	42645583	42645888	KD2-H4K8ac_peak_12630	8.21852	
17	42676912	42677270	KD2-H4K8ac_peak_12631	5.32137	
17	42684843	42687080	KD2-H4K8ac_peak_12632	18.54875	
17	42733817	42734025	KD2-H4K8ac_peak_12633	6.25869	C17orf104_ENSG00000180336
17	42734311	42734574	KD2-H4K8ac_peak_12634	6.66692	C17orf104_ENSG00000180336
17	42766701	42767011	KD2-H4K8ac_peak_12635	7.07117	CCDC43_ENSG00000180329
17	42767319	42767541	KD2-H4K8ac_peak_12636	5.34636	CCDC43_ENSG00000180329
17	42784996	42785801	KD2-H4K8ac_peak_12637	5.82799	DBF4B_ENSG00000161692
17	42786231	42786534	KD2-H4K8ac_peak_12638	5.13615	DBF4B_ENSG00000161692
17	42835910	42836410	KD2-H4K8ac_peak_12639	14.8083	ADAM11_ENSG00000073670
17	42836636	42837363	KD2-H4K8ac_peak_12640	6.99815	ADAM11_ENSG00000073670
17	42856712	42857196	KD2-H4K8ac_peak_12641	14.68449	
17	42907442	42908119	KD2-H4K8ac_peak_12642	11.92978	GJC1_ENSG00000182963
17	42976756	42977046	KD2-H4K8ac_peak_12643	9.048	EFTUD2_ENSG00000108883;CCDC103_ENSG00000167131;FAM187A_ENSG00000214447
17	42998531	42998889	KD2-H4K8ac_peak_12644	6.83936	
17	43000729	43001349	KD2-H4K8ac_peak_12645	7.07512	
17	43034288	43034530	KD2-H4K8ac_peak_12646	5.14125	
17	43045139	43045509	KD2-H4K8ac_peak_12647	7.69287	C1QL1_ENSG00000131094
17	43047572	43047975	KD2-H4K8ac_peak_12648	16.15695	

17	43095688	43095908	KD2-H4K8ac_peak_12649	8.30974	
17	43096105	43097276	KD2-H4K8ac_peak_12650	14.4458	
17	43099169	43099411	KD2-H4K8ac_peak_12651	8.16906	
17	43099642	43099966	KD2-H4K8ac_peak_12652	9.08816	
17	43107614	43107978	KD2-H4K8ac_peak_12653	6.47962	
17	43108210	43108794	KD2-H4K8ac_peak_12654	8.30974	
17	43109573	43110418	KD2-H4K8ac_peak_12655	7.76104	
17	43128164	43128850	KD2-H4K8ac_peak_12656	10.05606	NMT1_ENSG00000136448
17	43129471	43129785	KD2-H4K8ac_peak_12657	14.98671	NMT1_ENSG00000136448
17	43130095	43130351	KD2-H4K8ac_peak_12658	7.26089	
17	43138108	43138368	KD2-H4K8ac_peak_12659	12.46522	DCAKD_ENSG00000172992
17	43205811	43206026	KD2-H4K8ac_peak_12660	4.42765	
17	43213051	43213758	KD2-H4K8ac_peak_12661	8.4716	
17	43224338	43225413	KD2-H4K8ac_peak_12662	14.70748	HEXIM1_ENSG00000186834
17	43238453	43238887	KD2-H4K8ac_peak_12663	13.95627	AC002117.1_ENSG00000224505;HEXIM2_ENSG00000168517
17	43239366	43240210	KD2-H4K8ac_peak_12664	7.47462	AC002117.1_ENSG00000224505
17	43248605	43249494	KD2-H4K8ac_peak_12665	9.89383	RP13-890H12.2_ENSG00000267288
17	43250194	43250723	KD2-H4K8ac_peak_12666	12.08081	
17	43275049	43275683	KD2-H4K8ac_peak_12667	12.08081	
17	43298763	43299696	KD2-H4K8ac_peak_12668	6.40297	CTD-2020K17.1_ENSG00000267121;FMNL1_ENSG00000184922
17	43318863	43319216	KD2-H4K8ac_peak_12669	5.01002	CTD-2020K17.3_ENSG00000233175
17	43324055	43324409	KD2-H4K8ac_peak_12670	5.20908	MAP3K14-AS1_ENSG00000267278
17	43365544	43366288	KD2-H4K8ac_peak_12671	7.49599	
17	43367834	43368052	KD2-H4K8ac_peak_12672	4.44527	
17	43370168	43370433	KD2-H4K8ac_peak_12673	4.56666	
17	43370895	43371414	KD2-H4K8ac_peak_12674	8.5092	
17	43376984	43377212	KD2-H4K8ac_peak_12675	3.75035	
17	43419061	43419638	KD2-H4K8ac_peak_12676	7.36799	
17	43449082	43449466	KD2-H4K8ac_peak_12677	9.63487	CTB-39G8.2_ENSG00000267446
17	43472315	43472854	KD2-H4K8ac_peak_12678	7.45501	
17	43502840	43503213	KD2-H4K8ac_peak_12679	8.55271	
17	43508961	43509258	KD2-H4K8ac_peak_12680	9.44423	
17	43514083	43514795	KD2-H4K8ac_peak_12681	23.33626	
17	43567688	43568014	KD2-H4K8ac_peak_12682	6.40297	PLEKHM1_ENSG00000225190
17	43568503	43568895	KD2-H4K8ac_peak_12683	5.92591	PLEKHM1_ENSG00000225190
17	43661698	43663316	KD2-H4K8ac_peak_12684	26.39451	DND1P1_ENSG00000264070
17	43697756	43698247	KD2-H4K8ac_peak_12685	7.36799	CRHR1-IT1_ENSG00000204650
17	43816946	43817272	KD2-H4K8ac_peak_12686	5.13893	
17	43903903	43904197	KD2-H4K8ac_peak_12687	5.48272	
17	43926160	43926979	KD2-H4K8ac_peak_12688	5.38581	
17	43970584	43971093	KD2-H4K8ac_peak_12689	8.49564	MAPT_ENSG00000186868
17	43971288	43971705	KD2-H4K8ac_peak_12690	28.09984	MAPT_ENSG00000186868
17	43971944	43972378	KD2-H4K8ac_peak_12691	17.75427	MAPT-AS1_ENSG00000264589;MAPT_ENSG00000186868
17	44269922	44270734	KD2-H4K8ac_peak_12692	17.90608	KANSL1-AS1_ENSG00000214401
17	44270981	44271387	KD2-H4K8ac_peak_12693	9.67696	KANSL1-AS1_ENSG00000214401
17	44302273	44302683	KD2-H4K8ac_peak_12694	11.50346	KANSL1_ENSG00000120071

17	44336830	44337022	KD2-H4K8ac_peak_12695	4.77079	RP11-259G18.3_ENSG00000262539
17	44343764	44344141	KD2-H4K8ac_peak_12696	11.89502	RP11-259G18.1_ENSG00000261575
17	44344414	44344850	KD2-H4K8ac_peak_12697	12.84642	RP11-259G18.1_ENSG00000261575
17	44847700	44849088	KD2-H4K8ac_peak_12698	23.04547	
17	44849318	44849911	KD2-H4K8ac_peak_12699	19.25648	
17	44886688	44887189	KD2-H4K8ac_peak_12700	14.27217	
17	44913164	44913366	KD2-H4K8ac_peak_12701	5.38581	
17	44999717	45000059	KD2-H4K8ac_peak_12702	4.57782	RP11-63A1.1_ENSG00000261886;GOSR2_ENSG00000108433;RP11-156P1.2_ENSG00000262633
17	45000291	45000887	KD2-H4K8ac_peak_12703	11.22667	RP11-63A1.1_ENSG00000261886;GOSR2_ENSG00000108433;RP11-156P1.2_ENSG00000262633
17	45144511	45144854	KD2-H4K8ac_peak_12704	16.54484	
17	45177178	45177543	KD2-H4K8ac_peak_12705	9.57101	RP11-156P1.3_ENSG00000262879
17	45178275	45178516	KD2-H4K8ac_peak_12706	7.45629	RP11-156P1.3_ENSG00000262879
17	45220231	45220441	KD2-H4K8ac_peak_12707	4.06758	
17	45331393	45331893	KD2-H4K8ac_peak_12708	8.26945	ITGB3_ENSG00000259207;ITGB3_ENSG00000259753
17	45353431	45353800	KD2-H4K8ac_peak_12709	5.59859	
17	45400758	45401585	KD2-H4K8ac_peak_12710	9.26408	RP11-290H9.4_ENSG00000263293;EFCAB13_ENSG00000178852
17	45607946	45608300	KD2-H4K8ac_peak_12711	9.63487	
17	45608796	45609353	KD2-H4K8ac_peak_12712	16.58076	
17	45726141	45726694	KD2-H4K8ac_peak_12713	13.20052	RP11-580I16.2_ENSG00000263766;KPNB1_ENSG00000108424
17	45727625	45727897	KD2-H4K8ac_peak_12714	19.30951	RP11-580I16.2_ENSG00000263766;KPNB1_ENSG00000108424
17	45728197	45728638	KD2-H4K8ac_peak_12715	6.57664	
17	45765166	45765377	KD2-H4K8ac_peak_12716	5.405	
17	45771641	45772063	KD2-H4K8ac_peak_12717	11.0821	TBKBP1_ENSG00000198933
17	45772330	45772615	KD2-H4K8ac_peak_12718	8.85813	TBKBP1_ENSG00000198933
17	45804327	45804555	KD2-H4K8ac_peak_12719	6.40297	
17	45866976	45867491	KD2-H4K8ac_peak_12720	6.91449	
17	45898271	45899016	KD2-H4K8ac_peak_12721	6.66359	OSBPL7_ENSG00000006025
17	45899592	45900177	KD2-H4K8ac_peak_12722	10.44656	OSBPL7_ENSG00000006025
17	45908623	45908853	KD2-H4K8ac_peak_12723	9.57101	LRRC46_ENSG00000141294;MRPL10_ENSG00000159111
17	45917982	45919318	KD2-H4K8ac_peak_12724	16.29636	SCRN2_ENSG00000141295
17	45972602	45973105	KD2-H4K8ac_peak_12725	7.44247	RP11-6N17.4_ENSG00000264920;SP2_ENSG00000167182
17	45973615	45974322	KD2-H4K8ac_peak_12726	13.23341	RP11-6N17.4_ENSG00000264920;SP2_ENSG00000167182
17	46018354	46018714	KD2-H4K8ac_peak_12727	11.61757	AC003665.1_ENSG00000234494;PNPO_ENSG00000108439
17	46018971	46019846	KD2-H4K8ac_peak_12728	15.57998	AC003665.1_ENSG00000234494;PNPO_ENSG00000108439
17	46026951	46027524	KD2-H4K8ac_peak_12729	7.76329	
17	46047560	46047933	KD2-H4K8ac_peak_12730	6.45083	
17	46048504	46049302	KD2-H4K8ac_peak_12731	14.99434	
17	46059286	46059671	KD2-H4K8ac_peak_12732	11.82518	
17	46060093	46060884	KD2-H4K8ac_peak_12733	9.63487	
17	46064363	46064892	KD2-H4K8ac_peak_12734	14.45144	
17	46065216	46065648	KD2-H4K8ac_peak_12735	12.76142	
17	46068250	46069125	KD2-H4K8ac_peak_12736	25.00847	
17	46070687	46070904	KD2-H4K8ac_peak_12737	9.20193	
17	46072111	46072486	KD2-H4K8ac_peak_12738	8.58725	
17	46072767	46073047	KD2-H4K8ac_peak_12739	8.4716	RP11-6N17.10_ENSG00000264701
17	46073361	46073637	KD2-H4K8ac_peak_12740	11.3474	RP11-6N17.10_ENSG00000264701

17	46075996	46076211	KD2-H4K8ac_peak_12741	6.74189	
17	46077042	46077266	KD2-H4K8ac_peak_12742	8.30671	
17	46079039	46080185	KD2-H4K8ac_peak_12743	11.44756	
17	46080498	46081034	KD2-H4K8ac_peak_12744	12.82393	
17	46081371	46082901	KD2-H4K8ac_peak_12745	13.80019	
17	46088675	46089444	KD2-H4K8ac_peak_12746	29.06047	
17	46089942	46090913	KD2-H4K8ac_peak_12747	27.57364	
17	46091189	46092320	KD2-H4K8ac_peak_12748	15.51374	
17	46092531	46092999	KD2-H4K8ac_peak_12749	15.66774	
17	46095292	46095667	KD2-H4K8ac_peak_12750	9.357	
17	46100799	46101532	KD2-H4K8ac_peak_12751	5.38581	
17	46104133	46104366	KD2-H4K8ac_peak_12752	9.18237	
17	46114466	46114950	KD2-H4K8ac_peak_12753	10.24981	COPZ2_ENSG00000005243;MIR152_ENSG00000207947
17	46115378	46115610	KD2-H4K8ac_peak_12754	6.99815	COPZ2_ENSG00000005243;MIR152_ENSG00000207947
17	46125178	46125403	KD2-H4K8ac_peak_12755	9.08072	RP5-890E16.2_ENSG00000263412;NFE2L1_ENSG00000082641
17	46125733	46126315	KD2-H4K8ac_peak_12756	18.37277	RP5-890E16.2_ENSG00000263412;NFE2L1_ENSG00000082641
17	46178980	46179224	KD2-H4K8ac_peak_12757	9.8795	CBX1_ENSG00000108468
17	46184903	46185300	KD2-H4K8ac_peak_12758	11.44756	
17	46968502	46968696	KD2-H4K8ac_peak_12759	7.47462	
17	46969259	46969657	KD2-H4K8ac_peak_12760	15.039	ATP5G1_ENSG00000159199
17	46985883	46986192	KD2-H4K8ac_peak_12761	6.52179	SUMO2P17_ENSG00000248278;UBE2Z_ENSG00000159202
17	47021491	47022991	KD2-H4K8ac_peak_12762	30.35433	SNF8_ENSG00000159210
17	47269425	47270197	KD2-H4K8ac_peak_12763	13.73635	
17	47438766	47439541	KD2-H4K8ac_peak_12764	14.63726	ZNF652_ENSG00000198740;RP11-1079K10.3_ENSG00000248714
17	47439804	47440249	KD2-H4K8ac_peak_12765	7.49599	ZNF652_ENSG00000198740
17	47493163	47493360	KD2-H4K8ac_peak_12766	6.4562	PHB_ENSG00000167085;Y_RNA_ENSG00000207127
17	47513144	47513364	KD2-H4K8ac_peak_12767	5.58725	
17	47532639	47533084	KD2-H4K8ac_peak_12768	8.84009	
17	47547754	47548871	KD2-H4K8ac_peak_12769	7.07117	
17	47572247	47572494	KD2-H4K8ac_peak_12770	15.44609	NGFR_ENSG00000064300
17	47572860	47573094	KD2-H4K8ac_peak_12771	5.81222	NGFR_ENSG00000064300
17	47573528	47573755	KD2-H4K8ac_peak_12772	4.22101	NGFR_ENSG00000064300
17	47586135	47586516	KD2-H4K8ac_peak_12773	7.74292	
17	47591857	47592246	KD2-H4K8ac_peak_12774	11.32417	
17	47592601	47594373	KD2-H4K8ac_peak_12775	14.80472	
17	47596936	47597165	KD2-H4K8ac_peak_12776	9.56945	
17	47597482	47597752	KD2-H4K8ac_peak_12777	5.72657	
17	47601551	47601792	KD2-H4K8ac_peak_12778	7.76329	
17	47633318	47633777	KD2-H4K8ac_peak_12779	11.80283	
17	47646973	47647234	KD2-H4K8ac_peak_12780	8.10216	
17	47754568	47754871	KD2-H4K8ac_peak_12781	6.66692	SPOP_ENSG00000121067
17	47784769	47785539	KD2-H4K8ac_peak_12782	13.77586	SLC35B1_ENSG00000121073;RP11-613C6.2_ENSG00000250751
17	47865225	47865587	KD2-H4K8ac_peak_12783	5.66503	FAM117A_ENSG00000121104;KAT7_ENSG00000136504
17	47866071	47866306	KD2-H4K8ac_peak_12784	15.75541	FAM117A_ENSG00000121104;KAT7_ENSG00000136504
17	47928898	47929095	KD2-H4K8ac_peak_12785	3.90565	
17	48045694	48046438	KD2-H4K8ac_peak_12786	12.23	DLX4_ENSG00000108813

17	48075856	48076388	KD2-H4K8ac_peak_12787	11.03331	
17	48133115	48133801	KD2-H4K8ac_peak_12788	12.14909	RP11-1094H24.4_ENSG00000246640;ITGA3_ENSG00000005884
17	48172828	48173199	KD2-H4K8ac_peak_12789	5.4779	PDK2_ENSG00000005882
17	48179203	48179431	KD2-H4K8ac_peak_12790	4.42765	RP5-875H18.4_ENSG00000250282
17	48189255	48189823	KD2-H4K8ac_peak_12791	12.88702	
17	48205943	48207895	KD2-H4K8ac_peak_12792	18.37277	SAMD14_ENSG00000167100
17	48227520	48227878	KD2-H4K8ac_peak_12793	6.62457	PPP1R9B_ENSG00000108819
17	48228629	48229135	KD2-H4K8ac_peak_12794	15.27939	PPP1R9B_ENSG00000108819
17	48229577	48230321	KD2-H4K8ac_peak_12795	11.55418	
17	48237927	48238807	KD2-H4K8ac_peak_12796	8.65175	RP11-893F2.13_ENSG00000253730
17	48239143	48239633	KD2-H4K8ac_peak_12797	16.49944	RP11-893F2.13_ENSG00000253730
17	48250671	48251237	KD2-H4K8ac_peak_12798	9.53451	
17	48254422	48254675	KD2-H4K8ac_peak_12799	6.84261	
17	48255156	48256380	KD2-H4K8ac_peak_12800	20.18381	
17	48278055	48278596	KD2-H4K8ac_peak_12801	9.57101	COL1A1_ENSG00000108821
17	48284320	48284989	KD2-H4K8ac_peak_12802	10.46874	
17	48285600	48285843	KD2-H4K8ac_peak_12803	4.75537	RP11-893F2.5_ENSG00000249406
17	48286156	48286524	KD2-H4K8ac_peak_12804	5.4919	RP11-893F2.5_ENSG00000249406
17	48287182	48288569	KD2-H4K8ac_peak_12805	20.49868	RP11-893F2.5_ENSG00000249406
17	48298257	48298456	KD2-H4K8ac_peak_12806	4.86324	
17	48350144	48350511	KD2-H4K8ac_peak_12807	4.84206	
17	48350752	48350971	KD2-H4K8ac_peak_12808	8.43115	
17	48423084	48423291	KD2-H4K8ac_peak_12809	12.50666	XYLT2_ENSG00000015532
17	48423519	48424304	KD2-H4K8ac_peak_12810	11.7608	XYLT2_ENSG00000015532
17	48449775	48450989	KD2-H4K8ac_peak_12811	12.57865	MRPL27_ENSG00000108826;EME1_ENSG00000154920
17	48474168	48474755	KD2-H4K8ac_peak_12812	9.63487	LRRC59_ENSG00000108829;RP1-117B12.4_ENSG00000253102
17	48474986	48475502	KD2-H4K8ac_peak_12813	8.63879	LRRC59_ENSG00000108829
17	48502957	48503283	KD2-H4K8ac_peak_12814	9.98324	ACSF2_ENSG00000167107
17	48503570	48504482	KD2-H4K8ac_peak_12815	12.70959	ACSF2_ENSG00000167107
17	48546242	48546452	KD2-H4K8ac_peak_12816	3.87368	CHAD_ENSG00000136457
17	48555282	48556519	KD2-H4K8ac_peak_12817	15.2334	RSAD1_ENSG00000136444
17	48585361	48586354	KD2-H4K8ac_peak_12818	13.65567	RP11-94C24.6_ENSG00000249451;MYCBPAP_ENSG00000136449
17	48619078	48619439	KD2-H4K8ac_peak_12819	10.51355	SPATA20_ENSG00000006282
17	48624526	48625027	KD2-H4K8ac_peak_12820	20.7096	
17	48637089	48637294	KD2-H4K8ac_peak_12821	8.25469	
17	48642029	48643362	KD2-H4K8ac_peak_12822	17.39368	RP11-94C24.11_ENSG00000250976
17	48670384	48670593	KD2-H4K8ac_peak_12823	3.69421	
17	48678909	48679875	KD2-H4K8ac_peak_12824	16.81258	
17	48681355	48681915	KD2-H4K8ac_peak_12825	11.44756	
17	48682159	48682570	KD2-H4K8ac_peak_12826	7.92775	
17	48686098	48686368	KD2-H4K8ac_peak_12827	5.54647	
17	48687214	48687457	KD2-H4K8ac_peak_12828	5.65317	
17	48697871	48698534	KD2-H4K8ac_peak_12829	8.37358	
17	48705468	48705856	KD2-H4K8ac_peak_12830	10.43145	
17	48712106	48712486	KD2-H4K8ac_peak_12831	10.23258	ABCC3_ENSG00000108846
17	48735491	48735963	KD2-H4K8ac_peak_12832	4.211	

17	48737467	48738743	KD2-H4K8ac_peak_12833	11.54384	
17	48739024	48739323	KD2-H4K8ac_peak_12834	4.86966	
17	48766962	48767185	KD2-H4K8ac_peak_12835	8.16906	
17	48767767	48768426	KD2-H4K8ac_peak_12836	14.48922	
17	48784466	48785198	KD2-H4K8ac_peak_12837	11.50346	ANKRD40_ENSG00000154945
17	48785981	48786182	KD2-H4K8ac_peak_12838	4.67342	ANKRD40_ENSG00000154945
17	48786405	48786897	KD2-H4K8ac_peak_12839	16.25886	
17	48796233	48796749	KD2-H4K8ac_peak_12840	6.87217	LUC7L3_ENSG00000108848
17	48797003	48797397	KD2-H4K8ac_peak_12841	16.86242	LUC7L3_ENSG00000108848
17	48910078	48912232	KD2-H4K8ac_peak_12842	9.95759	WFIKKN2_ENSG00000173714
17	48944176	48944666	KD2-H4K8ac_peak_12843	5.78173	TOB1_ENSG00000141232;TOB1-AS1_ENSG00000229980
17	48981593	48983194	KD2-H4K8ac_peak_12844	12.85233	
17	48992451	48992731	KD2-H4K8ac_peak_12845	13.5461	
17	48995235	48997711	KD2-H4K8ac_peak_12846	35.03449	
17	49000707	49001293	KD2-H4K8ac_peak_12847	5.75757	
17	49006974	49007542	KD2-H4K8ac_peak_12848	7.07117	
17	49008312	49008580	KD2-H4K8ac_peak_12849	6.31838	
17	49008773	49009219	KD2-H4K8ac_peak_12850	25.83468	
17	49009649	49010171	KD2-H4K8ac_peak_12851	8.53092	
17	49012426	49013080	KD2-H4K8ac_peak_12852	5.98692	
17	49019857	49020077	KD2-H4K8ac_peak_12853	7.36799	
17	49021389	49021939	KD2-H4K8ac_peak_12854	21.46903	RP11-700H6.1_ENSG00000247011;RP11-700H6.2_ENSG00000251665
17	49025111	49025767	KD2-H4K8ac_peak_12855	11.04256	
17	49026609	49027230	KD2-H4K8ac_peak_12856	13.12288	
17	49027711	49027959	KD2-H4K8ac_peak_12857	7.81657	
17	49030938	49032936	KD2-H4K8ac_peak_12858	25.84288	
17	49126754	49127104	KD2-H4K8ac_peak_12859	4.42765	
17	49198519	49199218	KD2-H4K8ac_peak_12860	8.59579	SPAG9_ENSG00000008294
17	49229590	49229918	KD2-H4K8ac_peak_12861	7.07117	NME1_ENSG00000239672
17	49230269	49230547	KD2-H4K8ac_peak_12862	6.65673	NME1_ENSG00000239672;NME1-NME2_ENSG00000243678;NME2_ENSG00000011052
17	49231023	49231365	KD2-H4K8ac_peak_12863	6.99815	NME1_ENSG00000239672;NME1-NME2_ENSG00000243678;NME2_ENSG00000011052
17	49242724	49243532	KD2-H4K8ac_peak_12864	11.82518	
17	49243817	49244337	KD2-H4K8ac_peak_12865	6.01128	
17	49337065	49337392	KD2-H4K8ac_peak_12866	12.00597	MBTD1_ENSG00000011258;UTP18_ENSG00000011260
17	49337977	49338317	KD2-H4K8ac_peak_12867	13.65567	MBTD1_ENSG00000011258;UTP18_ENSG00000011260
17	49432493	49432989	KD2-H4K8ac_peak_12868	12.13241	
17	49577334	49577570	KD2-H4K8ac_peak_12869	9.36516	
17	49585305	49585601	KD2-H4K8ac_peak_12870	5.54647	
17	50558715	50558938	KD2-H4K8ac_peak_12871	6.16888	
17	52937427	52937763	KD2-H4K8ac_peak_12872	10.05606	
17	52977332	52978746	KD2-H4K8ac_peak_12873	11.24702	TOM1L1_ENSG00000141198
17	52981503	52981695	KD2-H4K8ac_peak_12874	7.146	
17	53045750	53045988	KD2-H4K8ac_peak_12875	7.92775	COX11_ENSG00000166260;STXBP4_ENSG00000166263
17	53046200	53046643	KD2-H4K8ac_peak_12876	16.71655	COX11_ENSG00000166260;STXBP4_ENSG00000166263
17	53341147	53341459	KD2-H4K8ac_peak_12877	3.87368	HLF_ENSG00000108924
17	53498147	53498489	KD2-H4K8ac_peak_12878	6.48713	MMD_ENSG00000108960

17	53498805	53499224	KD2-H4K8ac_peak_12879	11.80283	MMD_ENSG00000108960
17	53507561	53508286	KD2-H4K8ac_peak_12880	11.05467	
17	53510586	53510790	KD2-H4K8ac_peak_12881	6.08655	
17	53517352	53517558	KD2-H4K8ac_peak_12882	8.55271	
17	53518286	53518688	KD2-H4K8ac_peak_12883	12.84642	
17	53644867	53645371	KD2-H4K8ac_peak_12884	6.05809	
17	53753040	53753340	KD2-H4K8ac_peak_12885	8.30974	
17	53766670	53767170	KD2-H4K8ac_peak_12886	5.20908	
17	53776373	53776625	KD2-H4K8ac_peak_12887	5.92591	
17	53784343	53784854	KD2-H4K8ac_peak_12888	6.99815	
17	53828567	53828806	KD2-H4K8ac_peak_12889	3.82059	PCTP_ENSG00000141179
17	54910936	54911229	KD2-H4K8ac_peak_12890	12.88702	DGKE_ENSG00000153933
17	54991187	54991965	KD2-H4K8ac_peak_12891	24.82509	TRIM25_ENSG00000121060
17	55037461	55037882	KD2-H4K8ac_peak_12892	15.01306	COIL_ENSG00000121058
17	55038126	55038355	KD2-H4K8ac_peak_12893	12.08081	COIL_ENSG00000121058
17	55056522	55056730	KD2-H4K8ac_peak_12894	9.908	
17	55087628	55087821	KD2-H4K8ac_peak_12895	5.88845	
17	55162642	55162977	KD2-H4K8ac_peak_12896	17.19699	RP11-166P13.3_ENSG00000263004;AKAP1_ENSG00000121057
17	55163182	55163584	KD2-H4K8ac_peak_12897	5.75757	RP11-166P13.3_ENSG00000263004;AKAP1_ENSG00000121057
17	55174917	55175233	KD2-H4K8ac_peak_12898	3.97079	
17	55210910	55211252	KD2-H4K8ac_peak_12899	10.19949	
17	55212925	55213488	KD2-H4K8ac_peak_12900	7.51054	
17	55213831	55214053	KD2-H4K8ac_peak_12901	7.20567	
17	55332781	55333769	KD2-H4K8ac_peak_12902	14.69247	MSI2_ENSG00000153944
17	55363776	55364000	KD2-H4K8ac_peak_12903	4.67342	
17	55370601	55370826	KD2-H4K8ac_peak_12904	6.34074	
17	55443400	55443917	KD2-H4K8ac_peak_12905	6.90231	
17	55447606	55447958	KD2-H4K8ac_peak_12906	8.53092	
17	55456270	55456530	KD2-H4K8ac_peak_12907	7.76329	
17	55457713	55457932	KD2-H4K8ac_peak_12908	7.07154	
17	55458526	55458896	KD2-H4K8ac_peak_12909	6.50449	
17	55497915	55498107	KD2-H4K8ac_peak_12910	8.4716	
17	55498777	55499144	KD2-H4K8ac_peak_12911	10.83984	
17	55515372	55515675	KD2-H4K8ac_peak_12912	21.12734	
17	55516082	55516461	KD2-H4K8ac_peak_12913	6.16883	
17	55517491	55517721	KD2-H4K8ac_peak_12914	10.58745	
17	55518182	55518806	KD2-H4K8ac_peak_12915	8.79891	
17	55520485	55520893	KD2-H4K8ac_peak_12916	4.84794	
17	55543499	55543809	KD2-H4K8ac_peak_12917	4.86324	
17	55582547	55582769	KD2-H4K8ac_peak_12918	9.28804	
17	55583737	55584357	KD2-H4K8ac_peak_12919	12.93105	
17	55586753	55586998	KD2-H4K8ac_peak_12920	7.7192	
17	55594715	55595427	KD2-H4K8ac_peak_12921	15.82362	
17	55601388	55601590	KD2-H4K8ac_peak_12922	7.76329	RP11-118E18.2_ENSG00000266100
17	55604818	55605049	KD2-H4K8ac_peak_12923	6.99815	
17	55609298	55609719	KD2-H4K8ac_peak_12924	8.58069	

17	55609983	55610775	KD2-H4K8ac_peak_12925	9.34989
17	55610973	55611292	KD2-H4K8ac_peak_12926	15.05364
17	55611743	55613225	KD2-H4K8ac_peak_12927	14.73375
17	55613694	55614784	KD2-H4K8ac_peak_12928	21.83552
17	55615168	55616009	KD2-H4K8ac_peak_12929	27.69612
17	55616701	55616920	KD2-H4K8ac_peak_12930	12.79505
17	55617140	55617508	KD2-H4K8ac_peak_12931	14.69247
17	55617796	55618443	KD2-H4K8ac_peak_12932	12.69561
17	55618753	55620532	KD2-H4K8ac_peak_12933	12.82393
17	55621369	55621820	KD2-H4K8ac_peak_12934	8.30974
17	55622027	55622955	KD2-H4K8ac_peak_12935	12.70308
17	55623511	55624792	KD2-H4K8ac_peak_12936	23.13674
17	55626420	55626646	KD2-H4K8ac_peak_12937	6.99825
17	55627048	55627416	KD2-H4K8ac_peak_12938	7.43893
17	55628514	55629670	KD2-H4K8ac_peak_12939	20.76696
17	55630454	55630738	KD2-H4K8ac_peak_12940	11.05467
17	55630994	55631265	KD2-H4K8ac_peak_12941	8.81774
17	55632179	55633043	KD2-H4K8ac_peak_12942	9.95119
17	55635723	55636245	KD2-H4K8ac_peak_12943	9.34195
17	55637014	55637367	KD2-H4K8ac_peak_12944	14.57327
17	55637639	55639273	KD2-H4K8ac_peak_12945	13.77586
17	55639597	55640353	KD2-H4K8ac_peak_12946	14.48776
17	55640763	55641416	KD2-H4K8ac_peak_12947	13.42798
17	55641798	55642326	KD2-H4K8ac_peak_12948	9.53451
17	55642682	55643478	KD2-H4K8ac_peak_12949	13.3695
17	55643805	55644494	KD2-H4K8ac_peak_12950	18.96365
17	55645041	55646324	KD2-H4K8ac_peak_12951	11.05467
17	55647661	55647960	KD2-H4K8ac_peak_12952	8.60771
17	55648337	55648902	KD2-H4K8ac_peak_12953	8.5564
17	55649161	55649551	KD2-H4K8ac_peak_12954	10.43145
17	55649887	55650493	KD2-H4K8ac_peak_12955	8.63354
17	55651886	55652108	KD2-H4K8ac_peak_12956	7.76104
17	55652645	55653161	KD2-H4K8ac_peak_12957	5.85291
17	55656651	55657089	KD2-H4K8ac_peak_12958	8.86099
17	55661031	55661250	KD2-H4K8ac_peak_12959	7.90806
17	55661761	55662491	KD2-H4K8ac_peak_12960	5.94405
17	55662899	55663219	KD2-H4K8ac_peak_12961	5.72657
17	55663994	55664658	KD2-H4K8ac_peak_12962	10.39103
17	55665389	55665829	KD2-H4K8ac_peak_12963	10.01546
17	55671518	55671807	KD2-H4K8ac_peak_12964	6.76298
17	55673317	55674178	KD2-H4K8ac_peak_12965	3.83717
17	55674993	55675261	KD2-H4K8ac_peak_12966	4.54006
17	55675988	55677317	KD2-H4K8ac_peak_12967	15.66774
17	55678353	55678887	KD2-H4K8ac_peak_12968	6.7209
17	55680066	55680750	KD2-H4K8ac_peak_12969	9.18237
17	55681308	55682245	KD2-H4K8ac_peak_12970	14.99434

17	55682499	55682947	KD2-H4K8ac_peak_12971	7.73264	
17	55683149	55683913	KD2-H4K8ac_peak_12972	13.35993	
17	55684767	55685270	KD2-H4K8ac_peak_12973	5.96768	RP11-118E18.4_ENSG00000263499
17	55685524	55685923	KD2-H4K8ac_peak_12974	4.33139	RP11-118E18.4_ENSG00000263499
17	55691322	55691804	KD2-H4K8ac_peak_12975	7.13615	
17	55696614	55696911	KD2-H4K8ac_peak_12976	4.37809	
17	55697992	55698499	KD2-H4K8ac_peak_12977	13.58614	
17	55699441	55700337	KD2-H4K8ac_peak_12978	10.83984	
17	55700686	55700986	KD2-H4K8ac_peak_12979	9.94905	
17	55701805	55704067	KD2-H4K8ac_peak_12980	17.42905	
17	55713902	55714286	KD2-H4K8ac_peak_12981	5.13615	
17	55727086	55727383	KD2-H4K8ac_peak_12982	4.86324	
17	55738954	55739438	KD2-H4K8ac_peak_12983	9.29151	
17	55740714	55742106	KD2-H4K8ac_peak_12984	12.40421	
17	55742433	55742726	KD2-H4K8ac_peak_12985	6.93515	
17	55743077	55743927	KD2-H4K8ac_peak_12986	13.39388	
17	55744510	55745654	KD2-H4K8ac_peak_12987	14.17488	
17	55746541	55746810	KD2-H4K8ac_peak_12988	9.26408	
17	55747068	55747803	KD2-H4K8ac_peak_12989	9.95119	
17	55748400	55748889	KD2-H4K8ac_peak_12990	10.17341	
17	55749885	55750080	KD2-H4K8ac_peak_12991	5.48272	
17	55750632	55750824	KD2-H4K8ac_peak_12992	6.16883	
17	55752424	55752675	KD2-H4K8ac_peak_12993	8.10216	
17	55777046	55777291	KD2-H4K8ac_peak_12994	8.28234	
17	55785539	55786163	KD2-H4K8ac_peak_12995	11.28765	
17	55817589	55817995	KD2-H4K8ac_peak_12996	5.75757	
17	55818287	55818595	KD2-H4K8ac_peak_12997	5.26288	
17	55818933	55819205	KD2-H4K8ac_peak_12998	5.06602	
17	55825482	55825845	KD2-H4K8ac_peak_12999	9.34195	
17	55826227	55826676	KD2-H4K8ac_peak_13000	5.34636	
17	55827522	55828619	KD2-H4K8ac_peak_13001	24.23147	
17	55828834	55829547	KD2-H4K8ac_peak_13002	13.354	
17	55829986	55830411	KD2-H4K8ac_peak_13003	11.9364	
17	55835046	55835904	KD2-H4K8ac_peak_13004	5.3157	
17	55836393	55836650	KD2-H4K8ac_peak_13005	10.7186	
17	55839035	55841312	KD2-H4K8ac_peak_13006	12.93209	
17	55857635	55858107	KD2-H4K8ac_peak_13007	5.01002	
17	55858731	55860640	KD2-H4K8ac_peak_13008	15.82743	
17	55860900	55861105	KD2-H4K8ac_peak_13009	12.30509	
17	55861625	55861885	KD2-H4K8ac_peak_13010	3.82395	
17	55886883	55887085	KD2-H4K8ac_peak_13011	5.13615	
17	55887360	55887662	KD2-H4K8ac_peak_13012	6.04107	
17	55888034	55888630	KD2-H4K8ac_peak_13013	21.6161	
17	55889005	55889524	KD2-H4K8ac_peak_13014	19.55721	
17	55909049	55909332	KD2-H4K8ac_peak_13015	7.70067	
17	55909952	55910793	KD2-H4K8ac_peak_13016	11.92978	

17	55923572	55923774	KD2-H4K8ac_peak_13017	6.22746	
17	55927690	55928037	KD2-H4K8ac_peak_13018	5.38581	MRPS23_ENSG00000181610
17	55945286	55945501	KD2-H4K8ac_peak_13019	7.20116	
17	55981987	55982312	KD2-H4K8ac_peak_13020	10.33975	
17	56032060	56032624	KD2-H4K8ac_peak_13021	25.00847	CUEDC1_ENSG00000180891
17	56032880	56033178	KD2-H4K8ac_peak_13022	7.73264	CUEDC1_ENSG00000180891
17	56065019	56066454	KD2-H4K8ac_peak_13023	23.72814	VEZF1_ENSG00000136451
17	56083964	56084979	KD2-H4K8ac_peak_13024	45.30175	SRSF1_ENSG00000136450
17	56085227	56085481	KD2-H4K8ac_peak_13025	9.95342	SRSF1_ENSG00000136450
17	56159973	56160548	KD2-H4K8ac_peak_13026	20.40378	RP11-159D12.10_ENSG00000266290;DYNLL2_ENSG00000264364
17	56296195	56296564	KD2-H4K8ac_peak_13027	8.86099	MKS1_ENSG00000011143;LPO_ENSG00000167419
17	56296811	56297042	KD2-H4K8ac_peak_13028	12.13241	MKS1_ENSG00000011143;LPO_ENSG00000167419
17	56326697	56327104	KD2-H4K8ac_peak_13029	5.13615	
17	56401200	56402482	KD2-H4K8ac_peak_13030	22.92963	BZRAP1-AS1_ENSG00000265148
17	56405963	56406171	KD2-H4K8ac_peak_13031	13.12288	BZRAP1_ENSG00000005379
17	56416303	56417063	KD2-H4K8ac_peak_13032	7.09076	
17	56429111	56430110	KD2-H4K8ac_peak_13033	16.54484	SUPT4H1_ENSG00000213246
17	56591485	56591915	KD2-H4K8ac_peak_13034	18.43734	
17	56594788	56595174	KD2-H4K8ac_peak_13035	12.88702	MTMR4_ENSG00000108389
17	56595864	56596068	KD2-H4K8ac_peak_13036	7.36799	MTMR4_ENSG00000108389
17	56596334	56596810	KD2-H4K8ac_peak_13037	6.84261	RP11-112H10.4_ENSG00000264672
17	56597136	56597668	KD2-H4K8ac_peak_13038	14.06758	RP11-112H10.4_ENSG00000264672
17	56608761	56609624	KD2-H4K8ac_peak_13039	10.44656	
17	56708489	56708786	KD2-H4K8ac_peak_13040	5.75757	U3_ENSG00000212195
17	56709605	56710099	KD2-H4K8ac_peak_13041	11.92978	U3_ENSG00000212195
17	56737077	56737289	KD2-H4K8ac_peak_13042	7.67504	RNU1-60P_ENSG00000202077
17	56769183	56770327	KD2-H4K8ac_peak_13043	12.37731	TEX14_ENSG00000121101;RAD51C_ENSG00000108384
17	56833284	56833787	KD2-H4K8ac_peak_13044	8.85813	PPM1E_ENSG00000175175
17	56863400	56863674	KD2-H4K8ac_peak_13045	13.9234	
17	57183604	57184077	KD2-H4K8ac_peak_13046	15.27939	TRIM37_ENSG00000108395;AC099850.1_ENSG00000224738
17	57184397	57184846	KD2-H4K8ac_peak_13047	13.27022	TRIM37_ENSG00000108395;AC099850.1_ENSG00000224738
17	57231231	57231454	KD2-H4K8ac_peak_13048	7.77027	
17	57231705	57232180	KD2-H4K8ac_peak_13049	6.20982	SKA2_ENSG00000182628;PRR11_ENSG00000068489
17	57242890	57243108	KD2-H4K8ac_peak_13050	6.90231	
17	57286731	57287212	KD2-H4K8ac_peak_13051	14.00658	SMG8_ENSG00000167447
17	57287409	57287869	KD2-H4K8ac_peak_13052	13.92465	SMG8_ENSG00000167447
17	57298027	57298694	KD2-H4K8ac_peak_13053	7.28628	GDPD1_ENSG00000153982
17	57443047	57443363	KD2-H4K8ac_peak_13054	4.75875	MIR4729_ENSG00000263857
17	57448693	57449069	KD2-H4K8ac_peak_13055	8.79106	
17	57464209	57464541	KD2-H4K8ac_peak_13056	9.07715	
17	57465240	57465568	KD2-H4K8ac_peak_13057	12.17278	
17	57501177	57501663	KD2-H4K8ac_peak_13058	5.30799	
17	57502632	57503511	KD2-H4K8ac_peak_13059	8.85813	
17	57577023	57577309	KD2-H4K8ac_peak_13060	8.10216	
17	57577512	57578042	KD2-H4K8ac_peak_13061	17.19888	
17	57590824	57591018	KD2-H4K8ac_peak_13062	7.62364	

17	57591993	57592423	KD2-H4K8ac_peak_13063	6.98292	
17	57623845	57624234	KD2-H4K8ac_peak_13064	11.35142	
17	57625037	57625616	KD2-H4K8ac_peak_13065	7.36799	
17	57642546	57642864	KD2-H4K8ac_peak_13066	18.51132	DHX40_ENSG00000108406
17	57696107	57697518	KD2-H4K8ac_peak_13067	18.95603	CLTC_ENSG00000141367
17	57784118	57784733	KD2-H4K8ac_peak_13068	13.42721	PTRH2_ENSG00000141378;VMP1_ENSG00000062716
17	57970526	57970906	KD2-H4K8ac_peak_13069	19.92174	TUBD1_ENSG00000108423;RPS6KB1_ENSG00000108443
17	58042370	58042836	KD2-H4K8ac_peak_13070	6.97919	RNFT1_ENSG00000189050;RP11-178C3.2_ENSG00000267302
17	58114785	58115156	KD2-H4K8ac_peak_13071	6.80998	
17	58155737	58156248	KD2-H4K8ac_peak_13072	17.27647	HEATR6_ENSG00000068097;CTD-2319I12.2_ENSG00000267416
17	58156470	58156827	KD2-H4K8ac_peak_13073	5.92591	HEATR6_ENSG00000068097;CTD-2319I12.2_ENSG00000267416
17	58157051	58157423	KD2-H4K8ac_peak_13074	4.08993	HEATR6_ENSG00000068097;CTD-2319I12.2_ENSG00000267416
17	58213180	58213926	KD2-H4K8ac_peak_13075	17.09929	CTD-2319I12.4_ENSG00000267248
17	58219262	58219460	KD2-H4K8ac_peak_13076	6.77511	
17	58220478	58220853	KD2-H4K8ac_peak_13077	9.63487	
17	58227204	58227494	KD2-H4K8ac_peak_13078	5.75757	CA4_ENSG00000167434
17	58469028	58469338	KD2-H4K8ac_peak_13079	11.83301	C17orf64_ENSG00000141371
17	58469586	58470785	KD2-H4K8ac_peak_13080	7.49599	C17orf64_ENSG00000141371
17	58498755	58499445	KD2-H4K8ac_peak_13081	11.00372	USP32_ENSG00000170832
17	58547618	58548000	KD2-H4K8ac_peak_13082	5.54647	
17	58602939	58603559	KD2-H4K8ac_peak_13083	11.7608	APPBP2_ENSG00000062725;RP11-15E18.1_ENSG00000259349
17	58676766	58677356	KD2-H4K8ac_peak_13084	12.00597	PPM1D_ENSG00000170836
17	58677684	58678176	KD2-H4K8ac_peak_13085	12.40345	PPM1D_ENSG00000170836
17	58908355	58908825	KD2-H4K8ac_peak_13086	7.76329	
17	59096452	59096661	KD2-H4K8ac_peak_13087	7.40111	
17	59142532	59142904	KD2-H4K8ac_peak_13088	4.75764	
17	59213341	59213540	KD2-H4K8ac_peak_13089	6.60096	RP11-136H19.1_ENSG00000267667
17	59218812	59219156	KD2-H4K8ac_peak_13090	8.23796	
17	59220829	59221507	KD2-H4K8ac_peak_13091	14.38358	
17	59223370	59223970	KD2-H4K8ac_peak_13092	7.76104	
17	59224371	59224618	KD2-H4K8ac_peak_13093	4.44527	
17	59225783	59225989	KD2-H4K8ac_peak_13094	6.25869	
17	59235337	59235762	KD2-H4K8ac_peak_13095	4.71229	
17	59238079	59238377	KD2-H4K8ac_peak_13096	6.66692	
17	59239569	59239929	KD2-H4K8ac_peak_13097	7.70289	
17	59240455	59240732	KD2-H4K8ac_peak_13098	10.61986	
17	59273424	59273871	KD2-H4K8ac_peak_13099	4.54006	
17	59281184	59281437	KD2-H4K8ac_peak_13100	5.51783	
17	59392421	59392644	KD2-H4K8ac_peak_13101	7.49599	
17	59392848	59393074	KD2-H4K8ac_peak_13102	8.82366	
17	59402397	59402947	KD2-H4K8ac_peak_13103	6.36375	
17	59403480	59403743	KD2-H4K8ac_peak_13104	4.04573	
17	59457332	59457698	KD2-H4K8ac_peak_13105	4.54006	
17	59458701	59459755	KD2-H4K8ac_peak_13106	10.4745	RP11-332H18.3_ENSG00000266934
17	59460146	59460995	KD2-H4K8ac_peak_13107	16.79057	RP11-332H18.3_ENSG00000266934
17	59461280	59462387	KD2-H4K8ac_peak_13108	17.69527	

17	59462639	59463140	KD2-H4K8ac_peak_13109	16.67374	
17	59463597	59464065	KD2-H4K8ac_peak_13110	8.58725	
17	59464350	59464772	KD2-H4K8ac_peak_13111	7.64096	
17	59466727	59467199	KD2-H4K8ac_peak_13112	5.64429	
17	59474141	59475030	KD2-H4K8ac_peak_13113	13.3695	
17	59475308	59475695	KD2-H4K8ac_peak_13114	9.20193	
17	59476280	59476908	KD2-H4K8ac_peak_13115	8.16823	RP11-332H18.5_ENSG00000267131;TBX2_ENSG00000121068
17	59478074	59478531	KD2-H4K8ac_peak_13116	6.52179	RP11-332H18.5_ENSG00000267131;TBX2_ENSG00000121068
17	59480513	59480711	KD2-H4K8ac_peak_13117	4.30804	
17	59488600	59488904	KD2-H4K8ac_peak_13118	4.30033	RP11-332H18.4_ENSG00000267280;C17orf82_ENSG00000187013
17	59489223	59489733	KD2-H4K8ac_peak_13119	21.04966	RP11-332H18.4_ENSG00000267280;C17orf82_ENSG00000187013
17	59489991	59490665	KD2-H4K8ac_peak_13120	18.15393	C17orf82_ENSG00000187013
17	59493095	59494238	KD2-H4K8ac_peak_13121	29.1097	
17	59494602	59494816	KD2-H4K8ac_peak_13122	5.3157	
17	59539360	59539574	KD2-H4K8ac_peak_13123	4.52514	
17	59544803	59545873	KD2-H4K8ac_peak_13124	5.01002	
17	59562803	59564178	KD2-H4K8ac_peak_13125	15.93337	
17	59564486	59564944	KD2-H4K8ac_peak_13126	5.49478	
17	59565311	59565882	KD2-H4K8ac_peak_13127	13.65567	
17	59566845	59567328	KD2-H4K8ac_peak_13128	6.99815	
17	59573793	59574346	KD2-H4K8ac_peak_13129	9.94337	
17	59574554	59574860	KD2-H4K8ac_peak_13130	8.79106	
17	59634939	59635166	KD2-H4K8ac_peak_13131	6.99815	
17	59652537	59652849	KD2-H4K8ac_peak_13132	5.405	
17	59661182	59661519	KD2-H4K8ac_peak_13133	6.99815	
17	59834603	59834907	KD2-H4K8ac_peak_13134	4.98072	
17	59835146	59835349	KD2-H4K8ac_peak_13135	7.48822	
17	59864962	59865248	KD2-H4K8ac_peak_13136	6.27931	
17	59940495	59940821	KD2-H4K8ac_peak_13137	6.20982	BRIP1_ENSG00000136492
17	60004891	60005259	KD2-H4K8ac_peak_13138	15.75541	INTS2_ENSG00000108506
17	60005519	60005950	KD2-H4K8ac_peak_13139	7.76329	INTS2_ENSG00000108506
17	60142893	60143255	KD2-H4K8ac_peak_13140	12.57865	MED13_ENSG00000108510
17	60197668	60197866	KD2-H4K8ac_peak_13141	4.44923	
17	60215750	60216069	KD2-H4K8ac_peak_13142	9.26402	
17	60500816	60501568	KD2-H4K8ac_peak_13143	11.3923	METTL2A_ENSG00000087995
17	60547382	60547874	KD2-H4K8ac_peak_13144	7.20567	
17	60555228	60555934	KD2-H4K8ac_peak_13145	17.55491	
17	60556177	60557053	KD2-H4K8ac_peak_13146	23.87808	
17	60704263	60705883	KD2-H4K8ac_peak_13147	12.93862	MRC2_ENSG00000011028
17	60706309	60706909	KD2-H4K8ac_peak_13148	5.65324	
17	60721431	60722290	KD2-H4K8ac_peak_13149	11.24702	
17	60729438	60731680	KD2-H4K8ac_peak_13150	26.61491	
17	60771727	60771933	KD2-H4K8ac_peak_13151	6.17833	
17	60780855	60781163	KD2-H4K8ac_peak_13152	8.63033	
17	60781367	60782268	KD2-H4K8ac_peak_13153	18.73301	
17	60783008	60783352	KD2-H4K8ac_peak_13154	5.30799	

17	60827438	60828392	KD2-H4K8ac_peak_13155	11.00004	
17	60856245	60856657	KD2-H4K8ac_peak_13156	4.06758	
17	60856965	60857264	KD2-H4K8ac_peak_13157	4.42765	
17	60906072	60906324	KD2-H4K8ac_peak_13158	8.36514	
17	60946705	60947041	KD2-H4K8ac_peak_13159	6.99815	
17	60947960	60948192	KD2-H4K8ac_peak_13160	6.25869	
17	60970307	60970577	KD2-H4K8ac_peak_13161	6.05809	
17	61000305	61000757	KD2-H4K8ac_peak_13162	7.93362	
17	61001618	61002049	KD2-H4K8ac_peak_13163	5.14125	
17	61004133	61004373	KD2-H4K8ac_peak_13164	4.36736	
17	61042513	61043435	KD2-H4K8ac_peak_13165	9.26408	RP11-180P8.1_ENSG00000263931
17	61504965	61505157	KD2-H4K8ac_peak_13166	5.1685	
17	61505391	61505625	KD2-H4K8ac_peak_13167	8.49556	
17	61506736	61506958	KD2-H4K8ac_peak_13168	4.54006	
17	61508759	61509298	KD2-H4K8ac_peak_13169	9.05131	RP11-269G24.3_ENSG00000263644;RP11-269G24.4_ENSG00000265282
17	61509753	61510599	KD2-H4K8ac_peak_13170	15.62033	RP11-269G24.4_ENSG00000265282
17	61510925	61512866	KD2-H4K8ac_peak_13171	23.98831	
17	61515946	61516699	KD2-H4K8ac_peak_13172	9.44322	
17	61517377	61520045	KD2-H4K8ac_peak_13173	18.53991	
17	61520468	61520894	KD2-H4K8ac_peak_13174	15.48103	
17	61521094	61522729	KD2-H4K8ac_peak_13175	17.61538	
17	61522961	61525612	KD2-H4K8ac_peak_13176	35.30524	CYB561_ENSG00000008283
17	61528112	61528315	KD2-H4K8ac_peak_13177	6.84261	
17	61533021	61534082	KD2-H4K8ac_peak_13178	10.43145	RP11-269G24.6_ENSG00000265971
17	61552519	61552961	KD2-H4K8ac_peak_13179	5.9696	
17	61553762	61554539	KD2-H4K8ac_peak_13180	22.68518	ACE_ENSG00000159640
17	61554879	61555427	KD2-H4K8ac_peak_13181	17.32967	ACE_ENSG00000159640
17	61627004	61627454	KD2-H4K8ac_peak_13182	12.53675	DCAF7_ENSG00000136485
17	61627950	61628495	KD2-H4K8ac_peak_13183	11.7608	DCAF7_ENSG00000136485
17	61677729	61678673	KD2-H4K8ac_peak_13184	29.49632	TACO1_ENSG00000136463
17	61698889	61699281	KD2-H4K8ac_peak_13185	11.05155	MAP3K3_ENSG00000198909
17	61752840	61753154	KD2-H4K8ac_peak_13186	3.86308	
17	61776447	61777418	KD2-H4K8ac_peak_13187	8.36432	
17	61777646	61778482	KD2-H4K8ac_peak_13188	24.97616	LIMD2_ENSG00000136490
17	61797686	61797959	KD2-H4K8ac_peak_13189	6.30626	
17	61818879	61819109	KD2-H4K8ac_peak_13190	8.18084	STRADA_ENSG00000266173
17	61819561	61820165	KD2-H4K8ac_peak_13191	10.53377	STRADA_ENSG00000266173
17	61851261	61851505	KD2-H4K8ac_peak_13192	14.80472	DDX42_ENSG00000198231
17	61851706	61852060	KD2-H4K8ac_peak_13193	16.49944	DDX42_ENSG00000198231
17	61903910	61904264	KD2-H4K8ac_peak_13194	11.50346	PSMC5_ENSG00000087191
17	61904567	61905293	KD2-H4K8ac_peak_13195	20.18381	PSMC5_ENSG00000087191
17	61917017	61917402	KD2-H4K8ac_peak_13196	13.35993	RN7SL805P_ENSG00000264756
17	61918400	61918688	KD2-H4K8ac_peak_13197	6.66359	
17	61919166	61920146	KD2-H4K8ac_peak_13198	21.46903	SMARCD2_ENSG00000108604
17	61920668	61920942	KD2-H4K8ac_peak_13199	10.19949	SMARCD2_ENSG00000108604
17	62075114	62075884	KD2-H4K8ac_peak_13200	7.76104	C17orf72_ENSG00000224383

17	62076259	62076588	KD2-H4K8ac_peak_13201	4.50891	RP11-214C8.2_ENSG00000264954;C17orf72_ENSG00000224383
17	62082763	62083530	KD2-H4K8ac_peak_13202	8.6475	
17	62083989	62084299	KD2-H4K8ac_peak_13203	8.5092	
17	62206919	62207452	KD2-H4K8ac_peak_13204	9.36516	ERN1_ENSG00000178607
17	62207761	62208095	KD2-H4K8ac_peak_13205	7.76329	ERN1_ENSG00000178607
17	62223439	62223858	KD2-H4K8ac_peak_13206	5.75757	SNORA76_ENSG00000266402;SNORD104_ENSG00000199753
17	62267820	62268075	KD2-H4K8ac_peak_13207	6.67092	
17	62317693	62317985	KD2-H4K8ac_peak_13208	5.54647	
17	62340815	62341296	KD2-H4K8ac_peak_13209	10.7186	TEX2_ENSG00000136478
17	62492707	62493154	KD2-H4K8ac_peak_13210	8.4716	POLG2_ENSG00000256525
17	62501675	62501902	KD2-H4K8ac_peak_13211	16.54484	CEP95_ENSG00000258890
17	62502828	62503374	KD2-H4K8ac_peak_13212	14.63726	CEP95_ENSG00000258890;DDX5_ENSG00000108654
17	62657675	62658400	KD2-H4K8ac_peak_13213	21.12734	SMURF2_ENSG00000108854
17	62658595	62658912	KD2-H4K8ac_peak_13214	8.3261	SMURF2_ENSG00000108854
17	62703441	62703868	KD2-H4K8ac_peak_13215	4.37809	
17	62772204	62772946	KD2-H4K8ac_peak_13216	9.57101	
17	62832746	62833232	KD2-H4K8ac_peak_13217	8.58725	PLEKHM1P_ENSG00000214176
17	62833446	62834432	KD2-H4K8ac_peak_13218	11.90231	PLEKHM1P_ENSG00000214176
17	62834737	62834996	KD2-H4K8ac_peak_13219	6.75424	
17	62900325	62900541	KD2-H4K8ac_peak_13220	7.47462	
17	62914914	62916516	KD2-H4K8ac_peak_13221	19.80635	LRRC37A3_ENSG00000176809
17	62961927	62962398	KD2-H4K8ac_peak_13222	17.70889	
17	62971025	62971575	KD2-H4K8ac_peak_13223	16.14659	AMZ2P1_ENSG00000214174
17	62971871	62972674	KD2-H4K8ac_peak_13224	8.5251	AMZ2P1_ENSG00000214174
17	62981860	62982058	KD2-H4K8ac_peak_13225	8.80225	
17	63052011	63052283	KD2-H4K8ac_peak_13226	7.59183	GNA13_ENSG00000120063
17	63052488	63053632	KD2-H4K8ac_peak_13227	20.5911	GNA13_ENSG00000120063
17	63096172	63096687	KD2-H4K8ac_peak_13228	5.38581	RP11-160O5.1_ENSG00000263470
17	63097007	63097331	KD2-H4K8ac_peak_13229	7.76104	RP11-160O5.1_ENSG00000263470
17	63118891	63119448	KD2-H4K8ac_peak_13230	6.25869	
17	63119660	63120091	KD2-H4K8ac_peak_13231	7.40314	
17	63169688	63170320	KD2-H4K8ac_peak_13232	7.36799	
17	63175912	63176359	KD2-H4K8ac_peak_13233	6.84261	
17	63224379	63224573	KD2-H4K8ac_peak_13234	6.36375	
17	63527490	63527885	KD2-H4K8ac_peak_13235	8.21852	
17	63556691	63557057	KD2-H4K8ac_peak_13236	14.74638	AXIN2_ENSG00000168646
17	64187727	64188586	KD2-H4K8ac_peak_13237	13.13969	CEP112_ENSG00000154240
17	64297984	64298343	KD2-H4K8ac_peak_13238	11.52628	PRKCA_ENSG00000154229
17	64321677	64322168	KD2-H4K8ac_peak_13239	4.67342	
17	64346387	64346630	KD2-H4K8ac_peak_13240	5.71781	
17	64361390	64361595	KD2-H4K8ac_peak_13241	6.93012	
17	64363365	64363599	KD2-H4K8ac_peak_13242	8.26129	
17	64369987	64371093	KD2-H4K8ac_peak_13243	21.12734	
17	64379845	64380055	KD2-H4K8ac_peak_13244	8.51832	
17	64393865	64394117	KD2-H4K8ac_peak_13245	14.60723	
17	64411875	64412101	KD2-H4K8ac_peak_13246	4.211	RP11-4F22.2_ENSG00000264630

17	64429603	64430173	KD2-H4K8ac_peak_13247	10.77651	
17	64659025	64659532	KD2-H4K8ac_peak_13248	11.05467	
17	64724375	64724629	KD2-H4K8ac_peak_13249	4.70206	
17	64760245	64760538	KD2-H4K8ac_peak_13250	4.98072	
17	64761063	64761398	KD2-H4K8ac_peak_13251	6.66692	
17	64771414	64771875	KD2-H4K8ac_peak_13252	6.65673	
17	64830927	64831567	KD2-H4K8ac_peak_13253	17.42905	CACNG5_ENSG00000075429
17	64901042	64901567	KD2-H4K8ac_peak_13254	8.55271	
17	64902594	64902910	KD2-H4K8ac_peak_13255	10.6665	
17	64948960	64949720	KD2-H4K8ac_peak_13256	8.86099	
17	64950058	64950382	KD2-H4K8ac_peak_13257	5.54647	
17	64960048	64961577	KD2-H4K8ac_peak_13258	18.73205	CACNG4_ENSG00000075461
17	64962134	64962797	KD2-H4K8ac_peak_13259	8.85813	
17	64963351	64963756	KD2-H4K8ac_peak_13260	9.26287	
17	64963979	64964444	KD2-H4K8ac_peak_13261	5.85291	
17	64965171	64965602	KD2-H4K8ac_peak_13262	12.30509	
17	64967175	64967888	KD2-H4K8ac_peak_13263	12.48406	
17	64968194	64969233	KD2-H4K8ac_peak_13264	10.31523	
17	64969440	64969962	KD2-H4K8ac_peak_13265	6.5969	
17	64970359	64970574	KD2-H4K8ac_peak_13266	5.98692	
17	64971815	64972443	KD2-H4K8ac_peak_13267	11.20059	
17	64972885	64973146	KD2-H4K8ac_peak_13268	7.00136	
17	64973597	64974027	KD2-H4K8ac_peak_13269	8.85813	
17	64974292	64975452	KD2-H4K8ac_peak_13270	15.50901	
17	64982096	64982863	KD2-H4K8ac_peak_13271	10.35678	
17	64983650	64984124	KD2-H4K8ac_peak_13272	7.50083	
17	64984344	64985067	KD2-H4K8ac_peak_13273	12.65012	
17	64987090	64987606	KD2-H4K8ac_peak_13274	5.6644	
17	65013241	65013505	KD2-H4K8ac_peak_13275	4.44527	
17	65013747	65016495	KD2-H4K8ac_peak_13276	21.04966	
17	65017082	65017946	KD2-H4K8ac_peak_13277	8.80432	RP11-349A8.3_ENSG00000264491
17	65018312	65018837	KD2-H4K8ac_peak_13278	6.48713	RP11-349A8.3_ENSG00000264491
17	65023230	65023564	KD2-H4K8ac_peak_13279	6.11999	
17	65115303	65115578	KD2-H4K8ac_peak_13280	5.60968	
17	65118322	65118738	KD2-H4K8ac_peak_13281	7.76104	
17	65120523	65120720	KD2-H4K8ac_peak_13282	4.01115	
17	65121876	65122344	KD2-H4K8ac_peak_13283	10.87093	
17	65122714	65122929	KD2-H4K8ac_peak_13284	6.90231	
17	65123764	65124153	KD2-H4K8ac_peak_13285	8.55271	
17	65241092	65242553	KD2-H4K8ac_peak_13286	9.01262	HELZ_ENSG00000198265;RP11-401F2.3_ENSG00000266473
17	65242853	65243169	KD2-H4K8ac_peak_13287	5.3608	HELZ_ENSG00000198265
17	65317812	65318374	KD2-H4K8ac_peak_13288	10.16028	
17	65362291	65362692	KD2-H4K8ac_peak_13289	14.43794	PSMD12_ENSG00000197170
17	65362926	65363548	KD2-H4K8ac_peak_13290	10.0997	PSMD12_ENSG00000197170
17	65373063	65374931	KD2-H4K8ac_peak_13291	15.84211	PITPNC1_ENSG00000154217
17	65439230	65439542	KD2-H4K8ac_peak_13292	4.84206	

17	65440746	65441033	KD2-H4K8ac_peak_13293	5.01002	
17	65525049	65525242	KD2-H4K8ac_peak_13294	10.29842	
17	65600497	65601452	KD2-H4K8ac_peak_13295	12.17278	
17	65603174	65604078	KD2-H4K8ac_peak_13296	7.9042	
17	65604488	65604721	KD2-H4K8ac_peak_13297	5.18937	
17	65712922	65713186	KD2-H4K8ac_peak_13298	6.69844	NOL11_ENSG00000130935
17	65713396	65713677	KD2-H4K8ac_peak_13299	9.96798	NOL11_ENSG00000130935
17	65714099	65714440	KD2-H4K8ac_peak_13300	9.20193	NOL11_ENSG00000130935
17	65775254	65775580	KD2-H4K8ac_peak_13301	17.51891	
17	65821219	65821421	KD2-H4K8ac_peak_13302	8.58725	BPTF_ENSG00000171634
17	65821847	65822498	KD2-H4K8ac_peak_13303	13.59098	BPTF_ENSG00000171634
17	65988887	65989674	KD2-H4K8ac_peak_13304	15.61313	C17orf58_ENSG00000186665
17	65989969	65990495	KD2-H4K8ac_peak_13305	14.99434	C17orf58_ENSG00000186665
17	66016241	66016701	KD2-H4K8ac_peak_13306	17.42905	
17	66031899	66032291	KD2-H4K8ac_peak_13307	9.67696	KPNA2_ENSG00000182481
17	66097271	66097629	KD2-H4K8ac_peak_13308	8.88428	AC145343.2_ENSG00000265055;LINC00674_ENSG00000237854
17	66097861	66098224	KD2-H4K8ac_peak_13309	10.09705	AC145343.2_ENSG00000265055;LINC00674_ENSG00000237854
17	66201283	66202387	KD2-H4K8ac_peak_13310	27.10442	
17	66243579	66243953	KD2-H4K8ac_peak_13311	12.48406	RP11-147L13.2_ENSG00000265100;AMZ2_ENSG00000196704
17	66244235	66244564	KD2-H4K8ac_peak_13312	19.24845	RP11-147L13.2_ENSG00000265100;AMZ2_ENSG00000196704
17	66287640	66288366	KD2-H4K8ac_peak_13313	7.07117	SLC16A6_ENSG00000108932
17	66507567	66508366	KD2-H4K8ac_peak_13314	9.048	PRKAR1A_ENSG00000108946
17	66508578	66509220	KD2-H4K8ac_peak_13315	10.84707	PRKAR1A_ENSG00000108946
17	67322803	67323237	KD2-H4K8ac_peak_13316	8.23274	ABCA5_ENSG00000154265
17	67323444	67323837	KD2-H4K8ac_peak_13317	17.53289	ABCA5_ENSG00000154265
17	67410250	67411048	KD2-H4K8ac_peak_13318	15.40891	MAP2K6_ENSG00000108984
17	67447717	67447975	KD2-H4K8ac_peak_13319	6.05809	
17	68165082	68165335	KD2-H4K8ac_peak_13320	8.58725	KCNJ2-AS1_ENSG00000267365;KCNJ2_ENSG00000123700
17	68266651	68266914	KD2-H4K8ac_peak_13321	6.24747	
17	69159836	69160102	KD2-H4K8ac_peak_13322	6.9809	
17	69161146	69161339	KD2-H4K8ac_peak_13323	7.44366	
17	69177010	69177209	KD2-H4K8ac_peak_13324	5.57044	
17	69282860	69283105	KD2-H4K8ac_peak_13325	8.17902	
17	69434987	69435247	KD2-H4K8ac_peak_13326	7.75499	
17	69867438	69867789	KD2-H4K8ac_peak_13327	5.20908	
17	69972405	69972614	KD2-H4K8ac_peak_13328	6.39197	
17	70028611	70028902	KD2-H4K8ac_peak_13329	5.20998	
17	70116245	70116439	KD2-H4K8ac_peak_13330	6.69354	SOX9_ENSG00000125398
17	70123037	70123682	KD2-H4K8ac_peak_13331	7.10552	
17	70216315	70216718	KD2-H4K8ac_peak_13332	8.02571	
17	70345453	70345746	KD2-H4K8ac_peak_13333	7.76329	
17	70346102	70346351	KD2-H4K8ac_peak_13334	6.40297	
17	70359745	70359946	KD2-H4K8ac_peak_13335	6.36375	
17	70373272	70373678	KD2-H4K8ac_peak_13336	13.12288	
17	70374201	70374436	KD2-H4K8ac_peak_13337	8.86099	
17	70374832	70375240	KD2-H4K8ac_peak_13338	8.85813	

17	70375550	70375850	KD2-H4K8ac_peak_13339	4.37809	
17	70377075	70377363	KD2-H4K8ac_peak_13340	5.30799	
17	70378237	70378465	KD2-H4K8ac_peak_13341	6.30607	
17	70403008	70403265	KD2-H4K8ac_peak_13342	6.13909	
17	70419169	70419670	KD2-H4K8ac_peak_13343	9.34989	
17	70564175	70564471	KD2-H4K8ac_peak_13344	4.68059	
17	70588641	70588870	KD2-H4K8ac_peak_13345	4.54006	
17	70589125	70589423	KD2-H4K8ac_peak_13346	8.28234	
17	70774229	70774481	KD2-H4K8ac_peak_13347	6.53538	
17	71007430	71007646	KD2-H4K8ac_peak_13348	4.86324	
17	71045327	71045530	KD2-H4K8ac_peak_13349	7.40111	
17	71088679	71089375	KD2-H4K8ac_peak_13350	11.90231	SLC39A11_ENSG00000133195
17	71114436	71114946	KD2-H4K8ac_peak_13351	8.26129	
17	71115985	71116204	KD2-H4K8ac_peak_13352	7.12213	
17	71151218	71151412	KD2-H4K8ac_peak_13353	6.62541	
17	71151714	71151971	KD2-H4K8ac_peak_13354	13.73635	
17	71152212	71152478	KD2-H4K8ac_peak_13355	10.29842	
17	71187960	71188211	KD2-H4K8ac_peak_13356	8.56219	COG1_ENSG00000166685
17	71188541	71188828	KD2-H4K8ac_peak_13357	14.43794	COG1_ENSG00000166685
17	71189226	71189675	KD2-H4K8ac_peak_13358	32.7515	COG1_ENSG00000166685
17	71228017	71228372	KD2-H4K8ac_peak_13359	8.85813	C17orf80_ENSG00000141219
17	71228726	71229020	KD2-H4K8ac_peak_13360	13.13969	C17orf80_ENSG00000141219
17	71252084	71252310	KD2-H4K8ac_peak_13361	4.44527	
17	71281406	71281883	KD2-H4K8ac_peak_13362	7.70543	
17	71305831	71306698	KD2-H4K8ac_peak_13363	8.84009	
17	71306972	71307294	KD2-H4K8ac_peak_13364	12.40345	
17	71307778	71307979	KD2-H4K8ac_peak_13365	9.77335	CDC42EP4_ENSG00000179604
17	71308361	71308677	KD2-H4K8ac_peak_13366	13.3695	CDC42EP4_ENSG00000179604
17	71331403	71331611	KD2-H4K8ac_peak_13367	5.88845	
17	71332773	71332995	KD2-H4K8ac_peak_13368	5.30799	
17	71333329	71333526	KD2-H4K8ac_peak_13369	10.46874	
17	71337554	71338199	KD2-H4K8ac_peak_13370	15.29724	
17	71338414	71339362	KD2-H4K8ac_peak_13371	32.20234	
17	71339714	71340948	KD2-H4K8ac_peak_13372	12.13241	
17	71343676	71343923	KD2-H4K8ac_peak_13373	4.71835	
17	71344967	71345776	KD2-H4K8ac_peak_13374	12.15474	
17	71352209	71353237	KD2-H4K8ac_peak_13375	11.44756	
17	71353821	71354495	KD2-H4K8ac_peak_13376	25.51536	
17	71354691	71355119	KD2-H4K8ac_peak_13377	7.28628	
17	71355533	71356219	KD2-H4K8ac_peak_13378	9.56945	
17	71357323	71357852	KD2-H4K8ac_peak_13379	5.96384	
17	71361812	71362005	KD2-H4K8ac_peak_13380	5.22256	
17	71362503	71362899	KD2-H4K8ac_peak_13381	6.20038	
17	71365662	71366081	KD2-H4K8ac_peak_13382	10.59979	
17	71477069	71477318	KD2-H4K8ac_peak_13383	5.54647	
17	71485625	71485866	KD2-H4K8ac_peak_13384	4.06758	

17	71487921	71488952	KD2-H4K8ac_peak_13385	11.17159	
17	71489827	71490068	KD2-H4K8ac_peak_13386	7.57681	
17	71529208	71529664	KD2-H4K8ac_peak_13387	8.4716	
17	71596073	71596299	KD2-H4K8ac_peak_13388	3.85826	
17	71609835	71610193	KD2-H4K8ac_peak_13389	6.40297	
17	71715313	71715809	KD2-H4K8ac_peak_13390	8.95692	
17	71766098	71766719	KD2-H4K8ac_peak_13391	7.69287	
17	71781714	71781953	KD2-H4K8ac_peak_13392	6.1298	AC125421.1_ENSG00000234721
17	71823773	71823991	KD2-H4K8ac_peak_13393	6.67092	LINC00469_ENSG00000177338
17	71948762	71949341	KD2-H4K8ac_peak_13394	8.10216	
17	72143014	72143252	KD2-H4K8ac_peak_13395	12.25498	
17	72143626	72143848	KD2-H4K8ac_peak_13396	5.1628	
17	72148775	72149071	KD2-H4K8ac_peak_13397	5.13615	
17	72149644	72150023	KD2-H4K8ac_peak_13398	5.75757	RP11-101O21.1_ENSG00000266357
17	72198815	72199051	KD2-H4K8ac_peak_13399	7.07117	RPL38_ENSG00000172809
17	72199876	72200143	KD2-H4K8ac_peak_13400	9.24683	RPL38_ENSG00000172809
17	72209728	72210110	KD2-H4K8ac_peak_13401	5.49478	CTD-2514K5.2_ENSG00000246731;TTYH2_ENSG00000141540
17	72239078	72239862	KD2-H4K8ac_peak_13402	8.4716	
17	72240120	72240584	KD2-H4K8ac_peak_13403	10.53377	
17	72321893	72322238	KD2-H4K8ac_peak_13404	5.66705	KIF19_ENSG00000196169
17	72357403	72357912	KD2-H4K8ac_peak_13405	7.79741	BTBD17_ENSG00000204347
17	72364693	72365101	KD2-H4K8ac_peak_13406	11.35142	
17	72375846	72376136	KD2-H4K8ac_peak_13407	8.4716	
17	72449739	72450082	KD2-H4K8ac_peak_13408	11.82518	
17	72450474	72451051	KD2-H4K8ac_peak_13409	6.14438	
17	72453362	72453769	KD2-H4K8ac_peak_13410	9.95119	
17	72628322	72628902	KD2-H4K8ac_peak_13411	9.34989	
17	72741096	72741347	KD2-H4K8ac_peak_13412	7.49599	
17	72744086	72744531	KD2-H4K8ac_peak_13413	12.70959	MIR3615_ENSG00000266036;MIR3615_ENSG00000264624;SLC9A3R1_ENSG00000109062
17	72745461	72745780	KD2-H4K8ac_peak_13414	4.54006	MIR3615_ENSG00000266036;MIR3615_ENSG00000264624;SLC9A3R1_ENSG00000109062
17	72753892	72754571	KD2-H4K8ac_peak_13415	5.78173	
17	72754879	72756881	KD2-H4K8ac_peak_13416	16.54484	
17	72757219	72757822	KD2-H4K8ac_peak_13417	5.94405	
17	72772068	72772399	KD2-H4K8ac_peak_13418	13.02811	NAT9_ENSG00000109065;TMEM104_ENSG00000109066
17	72838538	72838837	KD2-H4K8ac_peak_13419	15.17328	
17	72868834	72869085	KD2-H4K8ac_peak_13420	13.21979	FDXR_ENSG00000161513
17	72869652	72870126	KD2-H4K8ac_peak_13421	13.31016	FDXR_ENSG00000161513
17	72870400	72870633	KD2-H4K8ac_peak_13422	4.70206	
17	72967807	72968231	KD2-H4K8ac_peak_13423	11.3923	
17	72968480	72968771	KD2-H4K8ac_peak_13424	13.75159	HID1_ENSG00000167861
17	72977906	72978358	KD2-H4K8ac_peak_13425	17.10757	
17	72978590	72979041	KD2-H4K8ac_peak_13426	11.7608	
17	72983803	72984326	KD2-H4K8ac_peak_13427	8.86099	CDR2L_ENSG00000109089
17	72986192	72986936	KD2-H4K8ac_peak_13428	4.33139	
17	73006409	73006634	KD2-H4K8ac_peak_13429	5.01002	
17	73006959	73007154	KD2-H4K8ac_peak_13430	9.89383	

17	73008157	73009360	KD2-H4K8ac_peak_13431	21.14153	ICT1_ENSG00000167862
17	73030674	73031189	KD2-H4K8ac_peak_13432	16.49944	
17	73031462	73032740	KD2-H4K8ac_peak_13433	15.57998	
17	73042679	73042977	KD2-H4K8ac_peak_13434	5.94405	ATP5H_ENSG00000167863
17	73043425	73043879	KD2-H4K8ac_peak_13435	21.60954	ATP5H_ENSG00000167863
17	73087087	73087279	KD2-H4K8ac_peak_13436	6.40297	
17	73091100	73091319	KD2-H4K8ac_peak_13437	5.54647	
17	73105397	73106521	KD2-H4K8ac_peak_13438	15.40241	ARMC7_ENSG00000125449
17	73127208	73128550	KD2-H4K8ac_peak_13439	20.26332	NT5C_ENSG00000125458
17	73149214	73149633	KD2-H4K8ac_peak_13440	9.96798	
17	73149900	73150279	KD2-H4K8ac_peak_13441	8.36432	
17	73178453	73179048	KD2-H4K8ac_peak_13442	34.12383	SUMO2_ENSG00000188612
17	73179361	73179833	KD2-H4K8ac_peak_13443	11.83301	SUMO2_ENSG00000188612
17	73200820	73201354	KD2-H4K8ac_peak_13444	10.43145	NUP85_ENSG00000125450
17	73201605	73202168	KD2-H4K8ac_peak_13445	8.48413	NUP85_ENSG00000125450
17	73257253	73258519	KD2-H4K8ac_peak_13446	14.71125	GGA3_ENSG00000125447;MRPS7_ENSG00000125445
17	73266101	73266418	KD2-H4K8ac_peak_13447	5.94405	MIF4GD_ENSG00000125457
17	73266750	73268843	KD2-H4K8ac_peak_13448	26.38304	MIF4GD_ENSG00000125457;RP11-649A18.12_ENSG00000263843
17	73269241	73269703	KD2-H4K8ac_peak_13449	11.3923	
17	73285804	73286129	KD2-H4K8ac_peak_13450	8.58069	SLC25A19_ENSG00000125454
17	73389467	73389774	KD2-H4K8ac_peak_13451	9.76863	RP11-16C1.1_ENSG00000265342
17	73452157	73452439	KD2-H4K8ac_peak_13452	11.50346	
17	73452775	73453504	KD2-H4K8ac_peak_13453	25.68735	
17	73511112	73511576	KD2-H4K8ac_peak_13454	10.80014	CASKIN2_ENSG00000177303;TSEN54_ENSG00000182173
17	73521416	73521651	KD2-H4K8ac_peak_13455	7.74292	
17	73536261	73536495	KD2-H4K8ac_peak_13456	4.27645	
17	73553279	73554543	KD2-H4K8ac_peak_13457	11.50346	
17	73584545	73585286	KD2-H4K8ac_peak_13458	31.95181	MYO15B_ENSG00000266714
17	73585571	73586223	KD2-H4K8ac_peak_13459	22.0091	
17	73622348	73622740	KD2-H4K8ac_peak_13460	5.30799	
17	73662899	73663216	KD2-H4K8ac_peak_13461	9.36665	RECQL5_ENSG00000108469;SAP30BP_ENSG00000161526
17	73663442	73663867	KD2-H4K8ac_peak_13462	21.01814	RECQL5_ENSG00000108469;SAP30BP_ENSG00000161526
17	73690116	73690376	KD2-H4K8ac_peak_13463	5.53033	
17	73717160	73717402	KD2-H4K8ac_peak_13464	7.27281	ITGB4_ENSG00000132470
17	73745720	73746083	KD2-H4K8ac_peak_13465	14.17488	
17	73749255	73750516	KD2-H4K8ac_peak_13466	15.74985	
17	73760447	73761246	KD2-H4K8ac_peak_13467	11.44887	GALK1_ENSG00000108479
17	73761513	73761763	KD2-H4K8ac_peak_13468	8.55271	GALK1_ENSG00000108479
17	73775295	73775797	KD2-H4K8ac_peak_13469	13.65567	
17	73779232	73779461	KD2-H4K8ac_peak_13470	6.74189	
17	73780262	73780623	KD2-H4K8ac_peak_13471	11.61757	MIR4738_ENSG00000263565;UNK_ENSG00000132478
17	73780845	73781931	KD2-H4K8ac_peak_13472	23.17425	H3F3B_ENSG00000132475;MIR4738_ENSG00000263565;UNK_ENSG00000132478
17	73822895	73825195	KD2-H4K8ac_peak_13473	14.30822	
17	73840496	73840715	KD2-H4K8ac_peak_13474	4.37809	UNC13D_ENSG00000092929
17	73851896	73852355	KD2-H4K8ac_peak_13475	4.91674	WBP2_ENSG00000132471
17	73865708	73866298	KD2-H4K8ac_peak_13476	5.9696	

17	73892351	73893498	KD2-H4K8ac_peak_13477	9.08072	TRIM65_ENSG00000141569;RP11-552F3.10_ENSG00000267342
17	73900427	73900789	KD2-H4K8ac_peak_13478	18.80827	
17	73901038	73901652	KD2-H4K8ac_peak_13479	17.84477	
17	73936836	73937094	KD2-H4K8ac_peak_13480	15.76765	FBF1_ENSG00000188878
17	73974876	73975133	KD2-H4K8ac_peak_13481	7.73264	ACOX1_ENSG00000161533;TEN1_ENSG00000257949;TEN1-CDK3_ENSG00000261408
17	73975355	73976454	KD2-H4K8ac_peak_13482	20.18381	ACOX1_ENSG00000161533;TEN1_ENSG00000257949;TEN1-CDK3_ENSG00000261408
17	73996448	73997064	KD2-H4K8ac_peak_13483	13.81781	CDK3_ENSG00000250506
17	74025919	74026320	KD2-H4K8ac_peak_13484	4.85908	
17	74068769	74069236	KD2-H4K8ac_peak_13485	25.34026	SRP68_ENSG00000167881;ZACN_ENSG00000186919
17	74076068	74076448	KD2-H4K8ac_peak_13486	5.54647	
17	74099935	74100982	KD2-H4K8ac_peak_13487	21.23512	
17	74117465	74118245	KD2-H4K8ac_peak_13488	10.05606	EXOC7_ENSG00000182473
17	74235838	74236116	KD2-H4K8ac_peak_13489	5.65324	RNF157_ENSG00000141576
17	74236931	74237404	KD2-H4K8ac_peak_13490	9.71513	RNF157_ENSG00000141576
17	74256282	74256481	KD2-H4K8ac_peak_13491	6.30229	
17	74349100	74350262	KD2-H4K8ac_peak_13492	18.79324	
17	74350574	74351228	KD2-H4K8ac_peak_13493	8.26129	
17	74364118	74364647	KD2-H4K8ac_peak_13494	4.54006	
17	74370279	74370617	KD2-H4K8ac_peak_13495	5.02584	
17	74380044	74380627	KD2-H4K8ac_peak_13496	6.8713	PRPSAP1_ENSG00000161542
17	74382091	74382394	KD2-H4K8ac_peak_13497	9.41619	
17	74448730	74449269	KD2-H4K8ac_peak_13498	30.32576	UBE2O_ENSG00000175931;AANAT_ENSG00000129673
17	74493621	74494234	KD2-H4K8ac_peak_13499	9.26402	
17	74497206	74497431	KD2-H4K8ac_peak_13500	7.92775	RHBDF2_ENSG00000129667
17	74497685	74498228	KD2-H4K8ac_peak_13501	14.03283	RHBDF2_ENSG00000129667
17	74502259	74503253	KD2-H4K8ac_peak_13502	11.00372	
17	74524467	74524667	KD2-H4K8ac_peak_13503	5.26288	PRCD_ENSG00000214140
17	74525222	74525497	KD2-H4K8ac_peak_13504	9.98869	
17	74525871	74527994	KD2-H4K8ac_peak_13505	9.26408	
17	74532466	74532685	KD2-H4K8ac_peak_13506	4.30033	
17	74533204	74533645	KD2-H4K8ac_peak_13507	7.44247	
17	74533945	74534912	KD2-H4K8ac_peak_13508	28.67307	
17	74536435	74536917	KD2-H4K8ac_peak_13509	17.90608	
17	74537313	74537707	KD2-H4K8ac_peak_13510	8.63879	
17	74538640	74539923	KD2-H4K8ac_peak_13511	21.12524	
17	74540382	74541682	KD2-H4K8ac_peak_13512	30.55453	
17	74541883	74542665	KD2-H4K8ac_peak_13513	8.58725	
17	74543075	74543414	KD2-H4K8ac_peak_13514	4.28151	
17	74549833	74550122	KD2-H4K8ac_peak_13515	6.27245	
17	74550556	74550813	KD2-H4K8ac_peak_13516	3.97079	
17	74553127	74553647	KD2-H4K8ac_peak_13517	9.56945	RP11-666A8.8_ENSG00000267546;SNHG16_ENSG00000163597
17	74667041	74667700	KD2-H4K8ac_peak_13518	16.21671	RP11-318A15.2_ENSG00000261335
17	74682369	74682600	KD2-H4K8ac_peak_13519	5.19103	
17	74683095	74683309	KD2-H4K8ac_peak_13520	4.33139	
17	74694097	74694363	KD2-H4K8ac_peak_13521	9.81188	
17	74696768	74697207	KD2-H4K8ac_peak_13522	13.95627	

17	74697596	74697915	KD2-H4K8ac_peak_13523	7.81657	
17	74705599	74706070	KD2-H4K8ac_peak_13524	13.21979	
17	74706356	74706653	KD2-H4K8ac_peak_13525	5.9696	MXRA7_ENSG00000182534
17	74722220	74722452	KD2-H4K8ac_peak_13526	7.92775	JMJD6_ENSG00000070495;METTL23_ENSG00000181038
17	74722967	74723452	KD2-H4K8ac_peak_13527	12.85233	JMJD6_ENSG00000070495;METTL23_ENSG00000181038
17	74732631	74733371	KD2-H4K8ac_peak_13528	17.09929	SRSF2_ENSG00000161547;MFSD11_ENSG00000092931;MIR636_ENSG00000207556
17	74733731	74733948	KD2-H4K8ac_peak_13529	13.18326	SRSF2_ENSG00000161547
17	74769796	74770139	KD2-H4K8ac_peak_13530	6.05809	
17	74787557	74787919	KD2-H4K8ac_peak_13531	13.58614	
17	74816766	74817051	KD2-H4K8ac_peak_13532	6.76298	
17	74830574	74830807	KD2-H4K8ac_peak_13533	7.49217	
17	74836034	74836456	KD2-H4K8ac_peak_13534	16.74555	
17	74836691	74837116	KD2-H4K8ac_peak_13535	6.84261	
17	74837684	74838133	KD2-H4K8ac_peak_13536	7.79068	
17	74838465	74838777	KD2-H4K8ac_peak_13537	5.01002	
17	74839576	74841383	KD2-H4K8ac_peak_13538	16.12602	
17	74863958	74865245	KD2-H4K8ac_peak_13539	8.58725	MGAT5B_ENSG00000167889
17	74964861	74965514	KD2-H4K8ac_peak_13540	11.44887	RP11-87G24.3_ENSG00000267568
17	74966130	74966339	KD2-H4K8ac_peak_13541	12.93007	RP11-87G24.3_ENSG00000267568
17	75052188	75052427	KD2-H4K8ac_peak_13542	8.4716	
17	75082795	75083004	KD2-H4K8ac_peak_13543	12.79505	LINC00338_ENSG00000234912;SEC14L1_ENSG00000129657;AC015815.3_ENSG00000203316
17	75083202	75083484	KD2-H4K8ac_peak_13544	4.88306	LINC00338_ENSG00000234912;SEC14L1_ENSG00000129657;AC015815.3_ENSG00000203316
17	75084112	75085208	KD2-H4K8ac_peak_13545	26.49677	AC015815.3_ENSG00000203316
17	75136240	75136669	KD2-H4K8ac_peak_13546	11.03172	
17	75242852	75243602	KD2-H4K8ac_peak_13547	14.18821	
17	75251308	75251747	KD2-H4K8ac_peak_13548	7.69287	
17	75261564	75261785	KD2-H4K8ac_peak_13549	6.13909	RP11-285E9.5_ENSG00000265121
17	75277016	75277272	KD2-H4K8ac_peak_13550	10.29842	RP11-285E9.6_ENSG00000263718;SEPT9_ENSG00000184640
17	75277572	75277969	KD2-H4K8ac_peak_13551	9.95119	RP11-285E9.6_ENSG00000263718;SEPT9_ENSG00000184640
17	75281787	75282450	KD2-H4K8ac_peak_13552	5.59859	
17	75282700	75283219	KD2-H4K8ac_peak_13553	8.02118	
17	75283545	75284630	KD2-H4K8ac_peak_13554	14.80472	
17	75301186	75301536	KD2-H4K8ac_peak_13555	4.39858	
17	75302371	75302675	KD2-H4K8ac_peak_13556	3.97079	
17	75303914	75305544	KD2-H4K8ac_peak_13557	27.81685	
17	75305926	75306617	KD2-H4K8ac_peak_13558	5.75757	
17	75306827	75307057	KD2-H4K8ac_peak_13559	6.6792	
17	75310692	75311014	KD2-H4K8ac_peak_13560	11.52765	
17	75311779	75312767	KD2-H4K8ac_peak_13561	13.95627	
17	75318662	75320086	KD2-H4K8ac_peak_13562	9.44322	
17	75327753	75328315	KD2-H4K8ac_peak_13563	21.60954	
17	75346982	75347189	KD2-H4K8ac_peak_13564	6.99194	
17	75347483	75348895	KD2-H4K8ac_peak_13565	8.63033	
17	75350521	75351034	KD2-H4K8ac_peak_13566	7.76329	
17	75351550	75351783	KD2-H4K8ac_peak_13567	6.40297	
17	75352845	75353119	KD2-H4K8ac_peak_13568	11.3394	

17	75358634	75359349	KD2-H4K8ac_peak_13569	10.7186	
17	75359586	75359955	KD2-H4K8ac_peak_13570	10.35678	
17	75360371	75361305	KD2-H4K8ac_peak_13571	19.93398	
17	75361713	75362251	KD2-H4K8ac_peak_13572	6.84261	
17	75362560	75362833	KD2-H4K8ac_peak_13573	7.0754	
17	75363177	75363382	KD2-H4K8ac_peak_13574	4.54006	
17	75368704	75369022	KD2-H4K8ac_peak_13575	8.18476	
17	75369467	75370412	KD2-H4K8ac_peak_13576	8.86099	
17	75371716	75372062	KD2-H4K8ac_peak_13577	8.52921	
17	75372397	75373088	KD2-H4K8ac_peak_13578	8.86099	RP11-936I5.1_ENSG00000266998
17	75373899	75374379	KD2-H4K8ac_peak_13579	10.54778	RP11-936I5.1_ENSG00000266998
17	75383001	75383321	KD2-H4K8ac_peak_13580	5.86865	
17	75384110	75384398	KD2-H4K8ac_peak_13581	7.49599	
17	75385060	75386275	KD2-H4K8ac_peak_13582	24.83142	
17	75415512	75415817	KD2-H4K8ac_peak_13583	4.00527	
17	75416626	75416834	KD2-H4K8ac_peak_13584	6.0605	
17	75417502	75417848	KD2-H4K8ac_peak_13585	9.16767	
17	75419621	75419985	KD2-H4K8ac_peak_13586	6.44479	
17	75420562	75421038	KD2-H4K8ac_peak_13587	5.94405	
17	75421339	75421737	KD2-H4K8ac_peak_13588	15.82743	
17	75422106	75422679	KD2-H4K8ac_peak_13589	8.01338	
17	75425907	75426158	KD2-H4K8ac_peak_13590	5.59859	
17	75426581	75427012	KD2-H4K8ac_peak_13591	6.84261	
17	75428695	75429930	KD2-H4K8ac_peak_13592	27.01413	
17	75430128	75430713	KD2-H4K8ac_peak_13593	8.58725	
17	75431925	75432299	KD2-H4K8ac_peak_13594	4.79789	
17	75432612	75432938	KD2-H4K8ac_peak_13595	9.01262	
17	75433559	75434051	KD2-H4K8ac_peak_13596	5.69399	
17	75436790	75437543	KD2-H4K8ac_peak_13597	3.90565	Y_RNA_ENSG00000200651
17	75447186	75447717	KD2-H4K8ac_peak_13598	9.1511	
17	75471539	75471930	KD2-H4K8ac_peak_13599	5.81222	
17	75475376	75476588	KD2-H4K8ac_peak_13600	10.76532	
17	75524212	75525463	KD2-H4K8ac_peak_13601	21.2844	
17	75568282	75568542	KD2-H4K8ac_peak_13602	5.92591	
17	75686396	75686692	KD2-H4K8ac_peak_13603	9.29151	
17	75686959	75687325	KD2-H4K8ac_peak_13604	8.65175	
17	75687661	75687996	KD2-H4K8ac_peak_13605	6.65673	
17	75693006	75693247	KD2-H4K8ac_peak_13606	5.36437	
17	75695624	75696084	KD2-H4K8ac_peak_13607	5.84991	
17	75696630	75696837	KD2-H4K8ac_peak_13608	7.76104	
17	75720115	75720406	KD2-H4K8ac_peak_13609	4.42765	
17	75722895	75723138	KD2-H4K8ac_peak_13610	7.1181	
17	75723427	75723703	KD2-H4K8ac_peak_13611	14.43206	RP11-316M20.1_ENSG00000267790
17	75723971	75724171	KD2-H4K8ac_peak_13612	6.16747	RP11-316M20.1_ENSG00000267790
17	75724497	75724733	KD2-H4K8ac_peak_13613	5.95537	RP11-316M20.1_ENSG00000267790
17	75730254	75730641	KD2-H4K8ac_peak_13614	8.88428	

17	75731093	75731834	KD2-H4K8ac_peak_13615	20.18584	
17	75732244	75733046	KD2-H4K8ac_peak_13616	6.40297	
17	75768865	75769292	KD2-H4K8ac_peak_13617	7.47462	
17	75776910	75777146	KD2-H4K8ac_peak_13618	7.92873	
17	75799514	75800038	KD2-H4K8ac_peak_13619	6.0605	
17	75801145	75801348	KD2-H4K8ac_peak_13620	6.41015	
17	75859917	75860557	KD2-H4K8ac_peak_13621	9.95342	
17	75861612	75862050	KD2-H4K8ac_peak_13622	5.59859	
17	75868548	75868780	KD2-H4K8ac_peak_13623	4.211	
17	75870053	75870907	KD2-H4K8ac_peak_13624	6.40297	
17	75876108	75876714	KD2-H4K8ac_peak_13625	6.98244	
17	75877097	75877817	KD2-H4K8ac_peak_13626	28.64145	FLJ45079_ENSG00000204283
17	75878572	75879014	KD2-H4K8ac_peak_13627	5.75757	FLJ45079_ENSG00000204283
17	75879298	75879667	KD2-H4K8ac_peak_13628	6.20982	FLJ45079_ENSG00000204283
17	75880030	75880663	KD2-H4K8ac_peak_13629	8.86099	
17	75911686	75911899	KD2-H4K8ac_peak_13630	7.70289	
17	75919500	75919769	KD2-H4K8ac_peak_13631	3.75035	
17	75923840	75924306	KD2-H4K8ac_peak_13632	6.66692	
17	75926121	75926671	KD2-H4K8ac_peak_13633	8.49564	
17	75927197	75927833	KD2-H4K8ac_peak_13634	10.43145	
17	75954110	75954376	KD2-H4K8ac_peak_13635	7.73264	
17	75954797	75955141	KD2-H4K8ac_peak_13636	14.48922	
17	75955415	75955621	KD2-H4K8ac_peak_13637	8.04448	
17	76035766	76035980	KD2-H4K8ac_peak_13638	7.44324	
17	76036922	76037707	KD2-H4K8ac_peak_13639	12.46522	
17	76113412	76114324	KD2-H4K8ac_peak_13640	13.21045	
17	76121895	76122213	KD2-H4K8ac_peak_13641	5.13615	
17	76124521	76124822	KD2-H4K8ac_peak_13642	9.24683	
17	76140093	76140494	KD2-H4K8ac_peak_13643	6.28228	
17	76141259	76141548	KD2-H4K8ac_peak_13644	5.30799	C17orf99_ENSG00000187997
17	76164078	76164524	KD2-H4K8ac_peak_13645	5.19622	SYNGR2_ENSG00000108639
17	76193928	76194135	KD2-H4K8ac_peak_13646	5.48272	
17	76207466	76207704	KD2-H4K8ac_peak_13647	6.22746	RP11-219G17.6_ENSG00000267624
17	76209565	76210907	KD2-H4K8ac_peak_13648	20.53952	BIRC5_ENSG00000089685
17	76219614	76220132	KD2-H4K8ac_peak_13649	10.09361	AC087645.1_ENSG00000268310
17	76221226	76221874	KD2-H4K8ac_peak_13650	7.36799	AC087645.1_ENSG00000268310
17	76227257	76227964	KD2-H4K8ac_peak_13651	11.92978	TMEM235_ENSG00000204278
17	76228170	76228835	KD2-H4K8ac_peak_13652	7.96136	TMEM235_ENSG00000204278
17	76230415	76230917	KD2-H4K8ac_peak_13653	10.77651	
17	76232360	76232746	KD2-H4K8ac_peak_13654	9.63487	
17	76233167	76234521	KD2-H4K8ac_peak_13655	13.02811	
17	76249951	76250629	KD2-H4K8ac_peak_13656	26.03179	THA1P_ENSG00000267676
17	76253104	76253351	KD2-H4K8ac_peak_13657	4.06758	
17	76253682	76254786	KD2-H4K8ac_peak_13658	22.7745	
17	76255118	76255423	KD2-H4K8ac_peak_13659	5.64497	
17	76259269	76259806	KD2-H4K8ac_peak_13660	6.65673	

17	76265716	76266082	KD2-H4K8ac_peak_13661	8.01338	
17	76309832	76310599	KD2-H4K8ac_peak_13662	11.92978	
17	76318886	76319222	KD2-H4K8ac_peak_13663	4.67342	
17	76320525	76321148	KD2-H4K8ac_peak_13664	7.36799	
17	76337556	76338584	KD2-H4K8ac_peak_13665	13.95627	
17	76347526	76347984	KD2-H4K8ac_peak_13666	9.08816	
17	76348626	76348898	KD2-H4K8ac_peak_13667	9.6247	
17	76355394	76356198	KD2-H4K8ac_peak_13668	8.58725	SOCS3_ENSG00000184557;RP11-806H10.4_ENSG00000266970
17	76356583	76356926	KD2-H4K8ac_peak_13669	17.43368	SOCS3_ENSG00000184557;RP11-806H10.4_ENSG00000266970
17	76365662	76366079	KD2-H4K8ac_peak_13670	7.44324	
17	76373986	76375145	KD2-H4K8ac_peak_13671	10.09705	PGS1_ENSG00000087157
17	76495123	76495573	KD2-H4K8ac_peak_13672	6.7498	DNAH17-AS1_ENSG00000268470
17	76584762	76585062	KD2-H4K8ac_peak_13673	10.16972	
17	76587366	76587579	KD2-H4K8ac_peak_13674	5.88845	
17	76588488	76589089	KD2-H4K8ac_peak_13675	5.75757	
17	76703648	76704319	KD2-H4K8ac_peak_13676	9.05772	
17	76711941	76712339	KD2-H4K8ac_peak_13677	7.95563	
17	76713190	76713467	KD2-H4K8ac_peak_13678	12.70959	
17	76713694	76714026	KD2-H4K8ac_peak_13679	8.85813	
17	76778675	76779185	KD2-H4K8ac_peak_13680	14.30077	CYTH1_ENSG00000108669
17	76837179	76837573	KD2-H4K8ac_peak_13681	24.77129	USP36_ENSG00000055483
17	76871791	76871989	KD2-H4K8ac_peak_13682	6.25869	
17	76878246	76878481	KD2-H4K8ac_peak_13683	10.31695	
17	76878844	76880316	KD2-H4K8ac_peak_13684	22.28045	
17	76880612	76881448	KD2-H4K8ac_peak_13685	8.84009	
17	76887670	76888089	KD2-H4K8ac_peak_13686	5.30799	
17	76910696	76910979	KD2-H4K8ac_peak_13687	5.56864	
17	76912550	76912963	KD2-H4K8ac_peak_13688	5.3157	
17	76921595	76921992	KD2-H4K8ac_peak_13689	20.26332	TIMP2_ENSG00000035862
17	76922230	76922583	KD2-H4K8ac_peak_13690	9.84999	TIMP2_ENSG00000035862
17	76928927	76930400	KD2-H4K8ac_peak_13691	13.65567	
17	76930727	76932224	KD2-H4K8ac_peak_13692	18.14508	
17	76932525	76932955	KD2-H4K8ac_peak_13693	8.01475	
17	76975415	76976004	KD2-H4K8ac_peak_13694	13.62458	LGALS3BP_ENSG00000108679
17	76976293	76977337	KD2-H4K8ac_peak_13695	15.94391	LGALS3BP_ENSG00000108679
17	77005546	77006635	KD2-H4K8ac_peak_13696	20.5798	CANT1_ENSG00000171302
17	77020184	77020842	KD2-H4K8ac_peak_13697	6.40874	
17	77023292	77024111	KD2-H4K8ac_peak_13698	12.13241	C1QTNF1-AS1_ENSG00000265096
17	77024657	77024922	KD2-H4K8ac_peak_13699	5.40065	C1QTNF1-AS1_ENSG00000265096
17	77042376	77043155	KD2-H4K8ac_peak_13700	9.03583	
17	77043426	77043706	KD2-H4K8ac_peak_13701	4.86324	
17	77070365	77070600	KD2-H4K8ac_peak_13702	6.30607	ENGASE_ENSG00000167280
17	77071061	77071264	KD2-H4K8ac_peak_13703	5.26288	ENGASE_ENSG00000167280
17	77111227	77111562	KD2-H4K8ac_peak_13704	3.86308	
17	77207746	77208187	KD2-H4K8ac_peak_13705	6.16883	
17	77210597	77211780	KD2-H4K8ac_peak_13706	15.93507	

17	77669810	77670168	KD2-H4K8ac_peak_13707	8.10216	
17	77682174	77682504	KD2-H4K8ac_peak_13708	6.05809	
17	77701524	77703414	KD2-H4K8ac_peak_13709	22.92963	
17	77715479	77716394	KD2-H4K8ac_peak_13710	11.05467	
17	77750975	77751722	KD2-H4K8ac_peak_13711	17.42905	CBX2_ENSG00000173894
17	77752035	77752709	KD2-H4K8ac_peak_13712	4.76255	CBX2_ENSG00000173894
17	77764790	77765195	KD2-H4K8ac_peak_13713	6.84261	
17	77765406	77765722	KD2-H4K8ac_peak_13714	9.63487	
17	77771633	77772320	KD2-H4K8ac_peak_13715	24.02151	
17	77783302	77783957	KD2-H4K8ac_peak_13716	17.42905	AC100791.2_ENSG00000238331
17	77784261	77784466	KD2-H4K8ac_peak_13717	14.06194	AC100791.2_ENSG00000238331
17	77784892	77786036	KD2-H4K8ac_peak_13718	23.7234	AC100791.2_ENSG00000238331
17	77786231	77788148	KD2-H4K8ac_peak_13719	46.70645	
17	77805288	77806199	KD2-H4K8ac_peak_13720	21.12734	
17	77813492	77813920	KD2-H4K8ac_peak_13721	15.40367	CBX4_ENSG00000141582
17	77817131	77818363	KD2-H4K8ac_peak_13722	19.25648	
17	77818852	77819096	KD2-H4K8ac_peak_13723	5.85291	
17	77903944	77904567	KD2-H4K8ac_peak_13724	15.82743	
17	77965778	77966124	KD2-H4K8ac_peak_13725	15.86619	CTD-2529O21.1_ENSG00000262172
17	78009276	78009613	KD2-H4K8ac_peak_13726	15.67427	TBC1D16_ENSG00000167291;CCDC40_ENSG00000141519
17	78009834	78011033	KD2-H4K8ac_peak_13727	15.81243	TBC1D16_ENSG00000167291;CCDC40_ENSG00000141519
17	78056182	78056910	KD2-H4K8ac_peak_13728	8.86099	
17	78070468	78071215	KD2-H4K8ac_peak_13729	8.80432	
17	78073919	78074274	KD2-H4K8ac_peak_13730	5.98282	
17	78074903	78076382	KD2-H4K8ac_peak_13731	24.77129	GAA_ENSG00000171298
17	78079484	78079679	KD2-H4K8ac_peak_13732	5.92591	
17	78099844	78100085	KD2-H4K8ac_peak_13733	6.92624	
17	78120446	78120810	KD2-H4K8ac_peak_13734	11.28831	EIF4A3_ENSG00000141543
17	78121232	78121657	KD2-H4K8ac_peak_13735	8.58725	EIF4A3_ENSG00000141543
17	78193625	78194563	KD2-H4K8ac_peak_13736	14.83804	SGSH_ENSG00000181523;SLC26A11_ENSG00000181045
17	78233631	78234276	KD2-H4K8ac_peak_13737	17.42905	RNF213_ENSG00000173821
17	78234680	78235767	KD2-H4K8ac_peak_13738	15.82743	RNF213_ENSG00000173821
17	78363995	78364416	KD2-H4K8ac_peak_13739	6.25869	
17	78388675	78389518	KD2-H4K8ac_peak_13740	14.73375	CTD-2047H16.4_ENSG00000263069;ENDOV_ENSG00000173818
17	78427759	78428535	KD2-H4K8ac_peak_13741	28.53052	CTD-2526A2.2_ENSG00000260369
17	78428756	78429330	KD2-H4K8ac_peak_13742	14.85549	CTD-2526A2.2_ENSG00000260369
17	78505732	78506200	KD2-H4K8ac_peak_13743	7.95563	
17	78517964	78518185	KD2-H4K8ac_peak_13744	12.23	RPTOR_ENSG00000141564
17	78518733	78519458	KD2-H4K8ac_peak_13745	11.3923	RPTOR_ENSG00000141564
17	78667501	78668029	KD2-H4K8ac_peak_13746	8.21523	
17	78670366	78670594	KD2-H4K8ac_peak_13747	3.97079	
17	78807488	78807711	KD2-H4K8ac_peak_13748	8.16906	
17	78808335	78808591	KD2-H4K8ac_peak_13749	6.67538	
17	78832202	78832428	KD2-H4K8ac_peak_13750	5.1707	
17	78832758	78833177	KD2-H4K8ac_peak_13751	17.09929	
17	78833408	78834479	KD2-H4K8ac_peak_13752	14.43794	

17	78835667	78836097	KD2-H4K8ac_peak_13753	6.48713	
17	78932874	78933346	KD2-H4K8ac_peak_13754	6.0605	
17	78965302	78966106	KD2-H4K8ac_peak_13755	18.22876	CHMP6_ENSG00000176108
17	79008086	79008485	KD2-H4K8ac_peak_13756	9.95119	BAIAP2-AS1_ENSG00000226137;BAIAP2_ENSG00000175866
17	79009335	79009720	KD2-H4K8ac_peak_13757	8.88082	BAIAP2-AS1_ENSG00000226137;BAIAP2_ENSG00000175866
17	79031036	79031246	KD2-H4K8ac_peak_13758	4.33139	
17	79041549	79041929	KD2-H4K8ac_peak_13759	7.92775	
17	79048979	79049223	KD2-H4K8ac_peak_13760	7.36799	
17	79049503	79049775	KD2-H4K8ac_peak_13761	7.92775	
17	79050610	79051046	KD2-H4K8ac_peak_13762	6.40297	
17	79056854	79057081	KD2-H4K8ac_peak_13763	6.97919	
17	79071187	79071554	KD2-H4K8ac_peak_13764	7.36799	
17	79088844	79089362	KD2-H4K8ac_peak_13765	5.75757	
17	79116725	79117133	KD2-H4K8ac_peak_13766	10.7186	
17	79138935	79139912	KD2-H4K8ac_peak_13767	12.14337	AATK_ENSG00000181409;AATK-AS1_ENSG00000225180
17	79140384	79140872	KD2-H4K8ac_peak_13768	10.77651	AATK_ENSG00000181409
17	79196206	79196743	KD2-H4K8ac_peak_13769	16.21436	AZI1_ENSG00000141577
17	79197148	79197517	KD2-H4K8ac_peak_13770	20.30085	AZI1_ENSG00000141577
17	79212471	79212779	KD2-H4K8ac_peak_13771	8.18084	ENTHD2_ENSG00000167302;C17orf89_ENSG00000224877
17	79213187	79213993	KD2-H4K8ac_peak_13772	19.28826	ENTHD2_ENSG00000167302;C17orf89_ENSG00000224877
17	79218199	79218439	KD2-H4K8ac_peak_13773	5.9696	
17	79226784	79227037	KD2-H4K8ac_peak_13774	6.40297	
17	79268483	79269008	KD2-H4K8ac_peak_13775	11.04256	SLC38A10_ENSG00000157637
17	79269234	79269685	KD2-H4K8ac_peak_13776	15.50901	SLC38A10_ENSG00000157637
17	79276889	79277242	KD2-H4K8ac_peak_13777	6.20982	
17	79282679	79284956	KD2-H4K8ac_peak_13778	18.40451	LINC00482_ENSG00000185168
17	79285196	79286082	KD2-H4K8ac_peak_13779	10.49196	
17	79287375	79289407	KD2-H4K8ac_peak_13780	15.82743	
17	79290288	79290752	KD2-H4K8ac_peak_13781	9.36908	
17	79291843	79292674	KD2-H4K8ac_peak_13782	13.80019	
17	79295733	79296280	KD2-H4K8ac_peak_13783	5.29282	
17	79296833	79297356	KD2-H4K8ac_peak_13784	21.09086	
17	79297663	79298312	KD2-H4K8ac_peak_13785	7.44247	
17	79301898	79304220	KD2-H4K8ac_peak_13786	11.03172	TMEM105_ENSG00000185332
17	79304789	79305439	KD2-H4K8ac_peak_13787	11.44979	TMEM105_ENSG00000185332
17	79305693	79306102	KD2-H4K8ac_peak_13788	11.01051	
17	79310897	79311136	KD2-H4K8ac_peak_13789	9.95119	
17	79311458	79312011	KD2-H4K8ac_peak_13790	8.86099	
17	79312246	79312461	KD2-H4K8ac_peak_13791	9.15555	
17	79313068	79313808	KD2-H4K8ac_peak_13792	16.74606	
17	79314188	79314723	KD2-H4K8ac_peak_13793	7.92775	
17	79315003	79315700	KD2-H4K8ac_peak_13794	9.87655	
17	79316597	79317603	KD2-H4K8ac_peak_13795	9.01262	
17	79317906	79319801	KD2-H4K8ac_peak_13796	23.04547	
17	79320289	79320599	KD2-H4K8ac_peak_13797	10.72313	
17	79367001	79367704	KD2-H4K8ac_peak_13798	8.03676	

17	79392820	79393487	KD2-H4K8ac_peak_13799	14.34343	
17	79393710	79394355	KD2-H4K8ac_peak_13800	9.95759	
17	79396608	79396918	KD2-H4K8ac_peak_13801	6.99815	
17	79403214	79403485	KD2-H4K8ac_peak_13802	7.4599	
17	79404461	79405162	KD2-H4K8ac_peak_13803	18.31034	
17	79424076	79424367	KD2-H4K8ac_peak_13804	5.68393	
17	79438253	79439397	KD2-H4K8ac_peak_13805	12.57865	
17	79439631	79440051	KD2-H4K8ac_peak_13806	6.30607	
17	79448165	79448400	KD2-H4K8ac_peak_13807	7.11824	
17	79448755	79449420	KD2-H4K8ac_peak_13808	6.84261	
17	79451038	79452286	KD2-H4K8ac_peak_13809	14.73375	
17	79454505	79455664	KD2-H4K8ac_peak_13810	17.42905	
17	79461789	79462106	KD2-H4K8ac_peak_13811	8.79106	
17	79479361	79479788	KD2-H4K8ac_peak_13812	13.674	RP13-766D20.1_ENSG00000229947
17	79479986	79480609	KD2-H4K8ac_peak_13813	23.87808	RP13-766D20.1_ENSG00000229947;RP13-766D20.2_ENSG00000229848
17	79481366	79481945	KD2-H4K8ac_peak_13814	14.3323	RP13-766D20.2_ENSG00000229848
17	79482182	79482393	KD2-H4K8ac_peak_13815	12.30509	
17	79482633	79482986	KD2-H4K8ac_peak_13816	7.49599	
17	79518781	79520017	KD2-H4K8ac_peak_13817	22.28045	C17orf70_ENSG00000185504
17	79603693	79604064	KD2-H4K8ac_peak_13818	12.13241	TSPAN10_ENSG00000182612
17	79604580	79604804	KD2-H4K8ac_peak_13819	4.42765	TSPAN10_ENSG00000182612
17	79633241	79633568	KD2-H4K8ac_peak_13820	11.83301	OXLD1_ENSG00000204237;CCDC137_ENSG00000185298
17	79633814	79634351	KD2-H4K8ac_peak_13821	14.73375	OXLD1_ENSG00000204237;CCDC137_ENSG00000185298
17	79650341	79650730	KD2-H4K8ac_peak_13822	6.70962	ARL16_ENSG00000214087;HGS_ENSG00000185359
17	79651145	79651469	KD2-H4K8ac_peak_13823	14.10488	ARL16_ENSG00000214087;HGS_ENSG00000185359
17	79669982	79671239	KD2-H4K8ac_peak_13824	31.75202	RP13-1032I1.7_ENSG00000262049;MRPL12_ENSG00000262814;SLC25A10_ENSG00000262660;SLC25A10_ENSG00000183048
17	79677533	79677826	KD2-H4K8ac_peak_13825	5.9696	
17	79678112	79678475	KD2-H4K8ac_peak_13826	5.75757	
17	79678864	79679168	KD2-H4K8ac_peak_13827	11.05155	
17	79772913	79773494	KD2-H4K8ac_peak_13828	5.9696	
17	79790472	79790963	KD2-H4K8ac_peak_13829	15.81243	FAM195B_ENSG00000225663
17	79791273	79791929	KD2-H4K8ac_peak_13830	14.43794	FAM195B_ENSG00000225663;PPP1R27_ENSG00000182676
17	79818026	79818349	KD2-H4K8ac_peak_13831	9.47033	P4HB_ENSG00000185624
17	79819059	79819624	KD2-H4K8ac_peak_13832	14.39182	P4HB_ENSG00000185624
17	79824719	79825394	KD2-H4K8ac_peak_13833	9.19219	RP11-498C9.3_ENSG00000262413
17	79825813	79826539	KD2-H4K8ac_peak_13834	8.86099	RP11-498C9.3_ENSG00000262413
17	79828104	79828964	KD2-H4K8ac_peak_13835	14.17488	ARHGDIA_ENSG00000141522
17	79829333	79830328	KD2-H4K8ac_peak_13836	33.44672	ARHGDIA_ENSG00000141522
17	79848310	79849422	KD2-H4K8ac_peak_13837	11.28765	ALYREF_ENSG00000183684;ANAPC11_ENSG00000141552
17	79849686	79850303	KD2-H4K8ac_peak_13838	5.4779	ALYREF_ENSG00000183684
17	79859684	79860635	KD2-H4K8ac_peak_13839	10.29842	
17	79876149	79876561	KD2-H4K8ac_peak_13840	17.53289	
17	79884618	79885039	KD2-H4K8ac_peak_13841	9.11891	MAFG_ENSG00000197063;MAFG-AS1_ENSG00000265688
17	79885844	79886748	KD2-H4K8ac_peak_13842	11.86355	MAFG_ENSG00000197063;MAFG-AS1_ENSG00000265688
17	79894573	79895089	KD2-H4K8ac_peak_13843	16.58186	
17	79895327	79896239	KD2-H4K8ac_peak_13844	21.79955	

17	79910604	79910798	KD2-H4K8ac_peak_13845	5.30799	
17	79917200	79917480	KD2-H4K8ac_peak_13846	14.99434	
17	79924474	79925966	KD2-H4K8ac_peak_13847	17.90608	RP11-498C9.17_ENSG00000264735
17	79926162	79926503	KD2-H4K8ac_peak_13848	3.83717	
17	79926702	79926917	KD2-H4K8ac_peak_13849	7.07117	
17	79934590	79934809	KD2-H4K8ac_peak_13850	4.93968	ASPSCR1_ENSG00000169696
17	79935472	79936172	KD2-H4K8ac_peak_13851	11.10856	ASPSCR1_ENSG00000169696
17	79949129	79951081	KD2-H4K8ac_peak_13852	14.85549	
17	79970522	79970770	KD2-H4K8ac_peak_13853	5.98692	
17	79971202	79971697	KD2-H4K8ac_peak_13854	4.28151	
17	79981293	79981597	KD2-H4K8ac_peak_13855	8.84009	STRA13_ENSG00000169689;LRRC45_ENSG00000169683
17	79988711	79989347	KD2-H4K8ac_peak_13856	8.63033	RAC3_ENSG00000169750
17	79995028	79995625	KD2-H4K8ac_peak_13857	19.04925	DCXR_ENSG00000169738;RP13-650J16.1_ENSG00000264569
17	80008811	80009509	KD2-H4K8ac_peak_13858	22.04674	RFNG_ENSG00000169733;GPS1_ENSG00000169727
17	80009846	80010203	KD2-H4K8ac_peak_13859	12.08081	RFNG_ENSG00000169733
17	80023270	80024650	KD2-H4K8ac_peak_13860	23.83902	DUS1L_ENSG00000169718
17	80055377	80056023	KD2-H4K8ac_peak_13861	4.72493	FASN_ENSG00000169710
17	80056386	80057842	KD2-H4K8ac_peak_13862	21.12734	FASN_ENSG00000169710
17	80062950	80063201	KD2-H4K8ac_peak_13863	9.26408	
17	80063547	80064991	KD2-H4K8ac_peak_13864	14.69327	
17	80163968	80164223	KD2-H4K8ac_peak_13865	9.01262	
17	80170367	80171610	KD2-H4K8ac_peak_13866	16.50872	CCDC57_ENSG00000176155;RP13-516M14.2_ENSG00000264548
17	80186150	80186813	KD2-H4K8ac_peak_13867	6.99815	SLC16A3_ENSG00000141526
17	80187032	80187302	KD2-H4K8ac_peak_13868	12.32775	SLC16A3_ENSG00000141526
17	80199089	80199602	KD2-H4K8ac_peak_13869	7.73264	
17	80231007	80231961	KD2-H4K8ac_peak_13870	13.94213	CSNK1D_ENSG00000141551
17	80240574	80240767	KD2-H4K8ac_peak_13871	6.30607	
17	80241542	80241747	KD2-H4K8ac_peak_13872	7.39141	
17	80250149	80250617	KD2-H4K8ac_peak_13873	12.53675	RP13-516M14.4_ENSG00000265692;RP13-516M14.1_ENSG00000260563
17	80251499	80251877	KD2-H4K8ac_peak_13874	8.85813	RP13-516M14.4_ENSG00000265692;RP13-516M14.1_ENSG00000260563
17	80255294	80256697	KD2-H4K8ac_peak_13875	32.47076	
17	80259252	80259498	KD2-H4K8ac_peak_13876	8.50556	
17	80290286	80290571	KD2-H4K8ac_peak_13877	6.18657	
17	80316819	80317286	KD2-H4K8ac_peak_13878	8.55271	TEX19_ENSG00000182459
17	80376234	80376816	KD2-H4K8ac_peak_13879	20.18584	OGFOD3_ENSG00000181396;HEXDC_ENSG00000169660
17	80408228	80408629	KD2-H4K8ac_peak_13880	7.76104	C17orf62_ENSG00000178927
17	80416091	80416379	KD2-H4K8ac_peak_13881	29.64378	RP13-20L14.6_ENSG00000265458;NARF_ENSG00000141562
17	80451990	80452312	KD2-H4K8ac_peak_13882	9.63487	
17	80454699	80455089	KD2-H4K8ac_peak_13883	11.27376	
17	80455451	80455675	KD2-H4K8ac_peak_13884	12.4684	
17	80476647	80477389	KD2-H4K8ac_peak_13885	32.65156	FOXK2_ENSG00000141568
17	80550398	80550683	KD2-H4K8ac_peak_13886	3.62612	
17	80550944	80551841	KD2-H4K8ac_peak_13887	17.53289	
17	80569078	80569292	KD2-H4K8ac_peak_13888	6.67092	
17	80605823	80606272	KD2-H4K8ac_peak_13889	10.35678	WDR45B_ENSG00000141580
17	80606819	80607089	KD2-H4K8ac_peak_13890	11.26685	WDR45B_ENSG00000141580

17	80622543	80622874	KD2-H4K8ac_peak_13891	6.08185	
17	80655497	80655759	KD2-H4K8ac_peak_13892	7.40314	RAB40B_ENSG00000141542
17	80667899	80668191	KD2-H4K8ac_peak_13893	3.87922	
17	80692484	80693875	KD2-H4K8ac_peak_13894	19.28826	FN3K_ENSG00000167363;RP11-388C12.5_ENSG00000263321
17	80709073	80709375	KD2-H4K8ac_peak_13895	8.4716	TBCD_ENSG00000141556
17	80709689	80710435	KD2-H4K8ac_peak_13896	9.36516	TBCD_ENSG00000141556
17	80751447	80752198	KD2-H4K8ac_peak_13897	9.63487	
17	80787867	80788828	KD2-H4K8ac_peak_13898	20.30085	
17	80824384	80824778	KD2-H4K8ac_peak_13899	5.3157	
17	80824982	80825238	KD2-H4K8ac_peak_13900	9.36665	
17	80838428	80838658	KD2-H4K8ac_peak_13901	5.1707	
17	80839308	80839587	KD2-H4K8ac_peak_13902	8.79106	
17	81009151	81009503	KD2-H4K8ac_peak_13903	10.66719	B3GNTL1_ENSG00000175711
17	81009896	81010397	KD2-H4K8ac_peak_13904	15.19625	B3GNTL1_ENSG00000175711
17	81027955	81028163	KD2-H4K8ac_peak_13905	5.78354	
17	81036859	81037307	KD2-H4K8ac_peak_13906	8.58725	METRNL_ENSG00000176845
18	158107	158358	KD2-H4K8ac_peak_13907	10.21502	USP14_ENSG00000101557
18	158561	159048	KD2-H4K8ac_peak_13908	13.09607	USP14_ENSG00000101557
18	268147	268846	KD2-H4K8ac_peak_13909	10.19949	THOC1_ENSG00000079134;RP11-705O1.8_ENSG00000263884
18	657214	657505	KD2-H4K8ac_peak_13910	8.82427	C18orf56_ENSG00000176912;RP11-806L2.5_ENSG00000263727;TYMS_ENSG00000176890
18	657806	658232	KD2-H4K8ac_peak_13911	5.01002	C18orf56_ENSG00000176912;RP11-806L2.5_ENSG00000263727;TYMS_ENSG00000176890
18	658668	658934	KD2-H4K8ac_peak_13912	6.8836	C18orf56_ENSG00000176912
18	812592	812950	KD2-H4K8ac_peak_13913	7.27716	YES1_ENSG00000176105;RP11-672L10.6_ENSG00000273355
18	2570593	2570886	KD2-H4K8ac_peak_13914	7.12754	METTL4_ENSG00000101574;NDC80_ENSG00000080986
18	2571084	2571381	KD2-H4K8ac_peak_13915	8.1233	METTL4_ENSG00000101574;NDC80_ENSG00000080986
18	2655291	2655523	KD2-H4K8ac_peak_13916	7.49599	CBX3P2_ENSG00000266405;SMCHD1_ENSG00000101596
18	2655967	2656184	KD2-H4K8ac_peak_13917	8.10216	CBX3P2_ENSG00000266405;SMCHD1_ENSG00000101596
18	2905845	2906870	KD2-H4K8ac_peak_13918	11.24702	
18	3012535	3013119	KD2-H4K8ac_peak_13919	10.19949	LPIN2_ENSG00000101577
18	3013864	3014355	KD2-H4K8ac_peak_13920	13.4559	LPIN2_ENSG00000101577
18	3049714	3049908	KD2-H4K8ac_peak_13921	5.25071	
18	3246878	3247267	KD2-H4K8ac_peak_13922	7.47462	RP13-270P17.3_ENSG00000272688;MYL12A_ENSG00000101608
18	3247855	3248157	KD2-H4K8ac_peak_13923	7.07117	RP13-270P17.3_ENSG00000272688;MYL12A_ENSG00000101608
18	3261666	3262278	KD2-H4K8ac_peak_13924	15.9336	RP13-270P17.1_ENSG00000264235;MYL12B_ENSG00000118680
18	3297187	3297482	KD2-H4K8ac_peak_13925	16.57619	
18	3450250	3450917	KD2-H4K8ac_peak_13926	7.44324	
18	3499027	3499397	KD2-H4K8ac_peak_13927	9.95119	
18	3530257	3530483	KD2-H4K8ac_peak_13928	7.59643	
18	3594662	3595121	KD2-H4K8ac_peak_13929	6.25869	DLGAP1-AS1_ENSG00000177337
18	3595945	3596156	KD2-H4K8ac_peak_13930	6.08655	
18	3621761	3622261	KD2-H4K8ac_peak_13931	5.25071	
18	3624017	3624310	KD2-H4K8ac_peak_13932	4.24345	
18	3625176	3625400	KD2-H4K8ac_peak_13933	8.09113	
18	5237113	5238546	KD2-H4K8ac_peak_13934	6.66359	RP11-835E18.5_ENSG00000265091;LINC00526_ENSG00000264575
18	5294742	5295873	KD2-H4K8ac_peak_13935	13.57066	
18	5296404	5296611	KD2-H4K8ac_peak_13936	7.40111	ZBTB14_ENSG00000198081

18	5890266	5890625	KD2-H4K8ac_peak_13937	9.15069	
18	5895622	5896312	KD2-H4K8ac_peak_13938	6.57893	TMEM200C_ENSG00000206432
18	6414230	6415466	KD2-H4K8ac_peak_13939	13.80019	L3MBTL4_ENSG00000154655
18	6728992	6729226	KD2-H4K8ac_peak_13940	6.57893	RP11-91I8.3_ENSG00000266441;ARHGAP28_ENSG00000088756
18	6729645	6730025	KD2-H4K8ac_peak_13941	6.66692	RP11-91I8.3_ENSG00000266441;ARHGAP28_ENSG00000088756
18	6781252	6781777	KD2-H4K8ac_peak_13942	8.1718	
18	6970591	6970813	KD2-H4K8ac_peak_13943	8.59579	
18	7118049	7118250	KD2-H4K8ac_peak_13944	7.76329	LAMA1_ENSG00000101680
18	7383081	7383303	KD2-H4K8ac_peak_13945	5.73839	
18	7567786	7568142	KD2-H4K8ac_peak_13946	5.72327	
18	8139327	8139527	KD2-H4K8ac_peak_13947	4.35659	
18	8253124	8253352	KD2-H4K8ac_peak_13948	4.70206	
18	8255703	8255972	KD2-H4K8ac_peak_13949	4.77079	
18	8260398	8261356	KD2-H4K8ac_peak_13950	7.27716	
18	8367406	8367767	KD2-H4K8ac_peak_13951	4.67342	RP11-789C17.3_ENSG00000266149
18	8397025	8397279	KD2-H4K8ac_peak_13952	9.1707	
18	8608759	8609026	KD2-H4K8ac_peak_13953	5.9696	RAB12_ENSG00000206418
18	8614956	8615208	KD2-H4K8ac_peak_13954	5.82799	
18	8656821	8657302	KD2-H4K8ac_peak_13955	8.00457	
18	8657607	8657801	KD2-H4K8ac_peak_13956	6.05809	
18	8661615	8661833	KD2-H4K8ac_peak_13957	9.98013	
18	8704553	8704891	KD2-H4K8ac_peak_13958	8.30974	SOGA2_ENSG00000168502
18	8705642	8705928	KD2-H4K8ac_peak_13959	12.3187	SOGA2_ENSG00000168502
18	8765467	8765706	KD2-H4K8ac_peak_13960	6.76298	
18	8766149	8766349	KD2-H4K8ac_peak_13961	4.37815	
18	8766645	8767341	KD2-H4K8ac_peak_13962	11.75669	
18	8792770	8793007	KD2-H4K8ac_peak_13963	5.9696	
18	8794283	8794558	KD2-H4K8ac_peak_13964	5.88845	
18	9017603	9017885	KD2-H4K8ac_peak_13965	6.2781	
18	9102749	9102974	KD2-H4K8ac_peak_13966	14.68449	NDUFV2_ENSG00000178127;RP11-21J18.1_ENSG00000265257
18	9334770	9335219	KD2-H4K8ac_peak_13967	14.43704	RP11-888D10.3_ENSG00000264964;TWSG1_ENSG00000128791
18	9474147	9474495	KD2-H4K8ac_peak_13968	8.74596	RP11-61L19.2_ENSG00000273335;RALBP1_ENSG00000017797
18	9474785	9475143	KD2-H4K8ac_peak_13969	7.62396	RP11-61L19.2_ENSG00000273335;RALBP1_ENSG00000017797
18	9475926	9476187	KD2-H4K8ac_peak_13970	5.54647	RALBP1_ENSG00000017797
18	9613693	9614541	KD2-H4K8ac_peak_13971	21.10246	PPP4R1_ENSG00000154845;RP11-692N5.1_ENSG00000263627
18	9614756	9615336	KD2-H4K8ac_peak_13972	22.36554	PPP4R1_ENSG00000154845;RP11-692N5.1_ENSG00000263627
18	9707915	9708280	KD2-H4K8ac_peak_13973	5.1707	RAB31_ENSG00000168461
18	9803656	9804265	KD2-H4K8ac_peak_13974	10.19949	
18	9914065	9914787	KD2-H4K8ac_peak_13975	20.08519	VAPA_ENSG00000101558
18	10168315	10168507	KD2-H4K8ac_peak_13976	7.45501	
18	10246231	10246674	KD2-H4K8ac_peak_13977	7.70289	
18	10454635	10455226	KD2-H4K8ac_peak_13978	6.85193	APCDD1_ENSG00000154856
18	10526194	10526419	KD2-H4K8ac_peak_13979	4.211	NAPG_ENSG00000134265
18	11908522	11909188	KD2-H4K8ac_peak_13980	8.30974	MPPE1_ENSG00000154889;RP11-820I16.4_ENSG00000273141
18	12059294	12059489	KD2-H4K8ac_peak_13981	5.57044	
18	12076312	12076801	KD2-H4K8ac_peak_13982	9.63487	RP11-815J4.6_ENSG00000256616

18	12271324	12271694	KD2-H4K8ac_peak_13983	10.46264	
18	12307816	12308077	KD2-H4K8ac_peak_13984	12.08081	TUBB6_ENSG00000176014
18	12308433	12308787	KD2-H4K8ac_peak_13985	4.91674	TUBB6_ENSG00000176014
18	12376721	12377153	KD2-H4K8ac_peak_13986	6.65673	AFG3L2_ENSG00000141385
18	12407965	12408574	KD2-H4K8ac_peak_13987	11.86082	SLMO1_ENSG00000141391
18	12419935	12420487	KD2-H4K8ac_peak_13988	13.70605	
18	12420854	12421281	KD2-H4K8ac_peak_13989	16.8435	
18	12658157	12658705	KD2-H4K8ac_peak_13990	6.99815	SPIRE1_ENSG00000134278;AP005482.1_ENSG00000215527;PSMG2_ENSG00000128789
18	12702380	12703367	KD2-H4K8ac_peak_13991	7.49599	CEP76_ENSG00000101624
18	12883263	12884191	KD2-H4K8ac_peak_13992	17.43705	
18	12884437	12884640	KD2-H4K8ac_peak_13993	6.40683	
18	12948012	12948869	KD2-H4K8ac_peak_13994	12.06346	SEH1L_ENSG00000085415
18	12991416	12991637	KD2-H4K8ac_peak_13995	7.76104	RP11-773H22.4_ENSG00000266969;CEP192_ENSG00000101639
18	13136789	13137414	KD2-H4K8ac_peak_13996	10.43145	
18	13217039	13217840	KD2-H4K8ac_peak_13997	7.146	RP11-794M8.1_ENSG00000267239;LDLRAD4_ENSG00000168675
18	13218053	13218588	KD2-H4K8ac_peak_13998	7.96322	LDLRAD4_ENSG00000168675
18	13223432	13223642	KD2-H4K8ac_peak_13999	8.5092	
18	13223889	13224248	KD2-H4K8ac_peak_14000	6.99815	
18	13225387	13225608	KD2-H4K8ac_peak_14001	8.82427	
18	13265755	13265989	KD2-H4K8ac_peak_14002	5.14125	
18	13500303	13500608	KD2-H4K8ac_peak_14003	6.47635	RP11-53B2.5_ENSG00000267366
18	13503371	13503658	KD2-H4K8ac_peak_14004	4.77079	
18	13570285	13570504	KD2-H4K8ac_peak_14005	4.42765	
18	13583732	13583993	KD2-H4K8ac_peak_14006	7.27716	
18	13584341	13584583	KD2-H4K8ac_peak_14007	6.54442	
18	13726167	13726409	KD2-H4K8ac_peak_14008	3.75035	FAM210A_ENSG00000177150;RNMT_ENSG00000101654
18	13726841	13727318	KD2-H4K8ac_peak_14009	7.47462	FAM210A_ENSG00000177150;RNMT_ENSG00000101654
18	14132159	14132391	KD2-H4K8ac_peak_14010	10.86378	ZNF519_ENSG00000175322
18	14132863	14133087	KD2-H4K8ac_peak_14011	8.74596	ZNF519_ENSG00000175322
18	18690977	18691622	KD2-H4K8ac_peak_14012	8.55271	ROCK1_ENSG00000067900
18	19179832	19180634	KD2-H4K8ac_peak_14013	8.16906	ESCO1_ENSG00000141446
18	19181168	19181613	KD2-H4K8ac_peak_14014	11.89502	ESCO1_ENSG00000141446
18	19191853	19192111	KD2-H4K8ac_peak_14015	13.88933	SNRPD1_ENSG00000167088
18	19192322	19192686	KD2-H4K8ac_peak_14016	10.96455	SNRPD1_ENSG00000167088
18	19320829	19321319	KD2-H4K8ac_peak_14017	13.95627	
18	20513419	20513667	KD2-H4K8ac_peak_14018	15.40891	RP11-739L10.1_ENSG00000265943;MIR4741_ENSG00000264817
18	20714981	20715258	KD2-H4K8ac_peak_14019	5.66705	CABLES1_ENSG00000134508
18	20715569	20716512	KD2-H4K8ac_peak_14020	5.1628	AC105247.1_ENSG00000222999
18	20723406	20724101	KD2-H4K8ac_peak_14021	5.9696	
18	20736017	20736365	KD2-H4K8ac_peak_14022	13.44374	
18	20757524	20757743	KD2-H4K8ac_peak_14023	5.57044	
18	20839635	20840385	KD2-H4K8ac_peak_14024	17.19888	RP11-17J14.2_ENSG00000266495
18	21032761	21032969	KD2-H4K8ac_peak_14025	7.69294	RIOK3_ENSG00000101782
18	21083540	21083775	KD2-H4K8ac_peak_14026	4.06758	C18orf8_ENSG00000141452
18	21166576	21166783	KD2-H4K8ac_peak_14027	11.43863	NPC1_ENSG00000141458
18	21269521	21270042	KD2-H4K8ac_peak_14028	4.44527	LAMA3_ENSG00000053747

18	21352157	21352365	KD2-H4K8ac_peak_14029	7.79898	
18	21594254	21595356	KD2-H4K8ac_peak_14030	11.09936	RP11-403A21.2_ENSG00000264745
18	21684110	21684430	KD2-H4K8ac_peak_14031	9.98013	
18	21718967	21719336	KD2-H4K8ac_peak_14032	5.54647	CABYR_ENSG00000154040
18	21968393	21968670	KD2-H4K8ac_peak_14033	9.63776	
18	21977387	21977724	KD2-H4K8ac_peak_14034	13.12288	OSBPL1A_ENSG00000141447
18	21997223	21997435	KD2-H4K8ac_peak_14035	5.88845	
18	22005978	22007193	KD2-H4K8ac_peak_14036	19.43192	IMPACT_ENSG00000154059
18	22929561	22929847	KD2-H4K8ac_peak_14037	4.84794	
18	22930228	22930491	KD2-H4K8ac_peak_14038	6.8836	
18	22930772	22931162	KD2-H4K8ac_peak_14039	7.12754	ZNF521_ENSG00000198795
18	22931656	22932038	KD2-H4K8ac_peak_14040	7.47462	ZNF521_ENSG00000198795
18	23184983	23185178	KD2-H4K8ac_peak_14041	9.89126	
18	23670308	23670785	KD2-H4K8ac_peak_14042	11.02863	SS18_ENSG00000141380
18	24127972	24128494	KD2-H4K8ac_peak_14043	9.89126	
18	24129075	24129382	KD2-H4K8ac_peak_14044	9.07715	
18	24129886	24130086	KD2-H4K8ac_peak_14045	7.146	
18	24229445	24229780	KD2-H4K8ac_peak_14046	8.5092	
18	24235546	24237321	KD2-H4K8ac_peak_14047	15.61313	KCTD1_ENSG00000134504;AQP4-AS1_ENSG00000260372
18	24265439	24265705	KD2-H4K8ac_peak_14048	6.2781	
18	24443273	24443678	KD2-H4K8ac_peak_14049	6.40297	
18	25755748	25756151	KD2-H4K8ac_peak_14050	9.1707	
18	25757330	25758552	KD2-H4K8ac_peak_14051	14.98671	CDH2_ENSG00000170558
18	29264783	29266155	KD2-H4K8ac_peak_14052	9.1707	B4GALT6_ENSG00000118276;RP11-549B18.1_ENSG00000259985
18	29303716	29304695	KD2-H4K8ac_peak_14053	11.30637	LRRC37A7P_ENSG00000265158
18	29522496	29523031	KD2-H4K8ac_peak_14054	9.20193	RP11-326K13.4_ENSG00000263823
18	29598586	29599516	KD2-H4K8ac_peak_14055	12.00273	RNF125_ENSG00000101695;RP11-53I6.2_ENSG00000263917
18	29671581	29672259	KD2-H4K8ac_peak_14056	8.55271	RP11-53I6.3_ENSG00000265008;RNF138_ENSG00000134758
18	30051048	30051244	KD2-H4K8ac_peak_14057	10.17341	GAREM_ENSG00000141441
18	30349917	30350305	KD2-H4K8ac_peak_14058	5.54647	AC012123.1_ENSG00000228835
18	30350739	30351114	KD2-H4K8ac_peak_14059	6.55628	AC012123.1_ENSG00000228835
18	31020444	31020650	KD2-H4K8ac_peak_14060	9.59147	CCDC178_ENSG00000166960
18	31157867	31158212	KD2-H4K8ac_peak_14061	10.19949	RP11-258B16.1_ENSG00000267642;ASXL3_ENSG00000141431
18	31158607	31158941	KD2-H4K8ac_peak_14062	22.07309	RP11-258B16.1_ENSG00000267642;ASXL3_ENSG00000141431
18	31739342	31739915	KD2-H4K8ac_peak_14063	6.65673	
18	31802404	31802998	KD2-H4K8ac_peak_14064	9.96798	RP11-379L18.1_ENSG00000267746
18	31803226	31803605	KD2-H4K8ac_peak_14065	5.72327	RP11-379L18.1_ENSG00000267746
18	32828322	32828613	KD2-H4K8ac_peak_14066	4.16871	
18	32847059	32847279	KD2-H4K8ac_peak_14067	8.1233	
18	32870306	32870588	KD2-H4K8ac_peak_14068	7.76329	ZSCAN30_ENSG00000186814;RP11-158H5.7_ENSG00000268573;ZNF271_ENSG00000257267
18	32924072	32924366	KD2-H4K8ac_peak_14069	22.0091	
18	32956705	32957095	KD2-H4K8ac_peak_14070	10.7186	ZNF396_ENSG00000186496
18	33161088	33161420	KD2-H4K8ac_peak_14071	11.89502	GALNT1_ENSG00000141429
18	33161908	33162252	KD2-H4K8ac_peak_14072	7.12754	GALNT1_ENSG00000141429
18	33214277	33214485	KD2-H4K8ac_peak_14073	8.1233	
18	33530127	33530415	KD2-H4K8ac_peak_14074	5.59859	

18	33597015	33597220	KD2-H4K8ac_peak_14075	8.1718	
18	33647728	33647955	KD2-H4K8ac_peak_14076	5.71749	RPRD1A_ENSG00000141425
18	33680290	33680482	KD2-H4K8ac_peak_14077	7.12754	
18	33708958	33709248	KD2-H4K8ac_peak_14078	14.68449	SLC39A6_ENSG00000141424;ELP2_ENSG00000134759
18	33709521	33709765	KD2-H4K8ac_peak_14079	7.92775	SLC39A6_ENSG00000141424;ELP2_ENSG00000134759
18	33767207	33767823	KD2-H4K8ac_peak_14080	8.53092	RP11-49I11.1_ENSG00000260552;MOCOS_ENSG00000075643
18	33876821	33877160	KD2-H4K8ac_peak_14081	6.40297	FHOD3_ENSG00000134775
18	33877839	33878083	KD2-H4K8ac_peak_14082	5.9696	FHOD3_ENSG00000134775
18	33961960	33962260	KD2-H4K8ac_peak_14083	7.47462	
18	34273192	34273506	KD2-H4K8ac_peak_14084	12.42547	
18	34408590	34408837	KD2-H4K8ac_peak_14085	4.86324	TPGS2_ENSG00000134779;KIAA1328_ENSG00000150477
18	34409196	34409481	KD2-H4K8ac_peak_14086	4.86324	TPGS2_ENSG00000134779;KIAA1328_ENSG00000150477
18	34914344	34914818	KD2-H4K8ac_peak_14087	4.06758	
18	34973201	34973610	KD2-H4K8ac_peak_14088	7.36799	
18	34974291	34974853	KD2-H4K8ac_peak_14089	8.21852	
18	35009793	35010065	KD2-H4K8ac_peak_14090	6.98292	
18	35025154	35025847	KD2-H4K8ac_peak_14091	7.47462	
18	35029817	35030369	KD2-H4K8ac_peak_14092	7.76329	
18	35030872	35031180	KD2-H4K8ac_peak_14093	10.86378	
18	35031592	35031963	KD2-H4K8ac_peak_14094	5.42867	
18	35044502	35044703	KD2-H4K8ac_peak_14095	5.20998	
18	35099479	35100207	KD2-H4K8ac_peak_14096	6.9556	
18	35115566	35115780	KD2-H4K8ac_peak_14097	7.89389	
18	35128554	35128752	KD2-H4K8ac_peak_14098	4.75875	
18	35146785	35147252	KD2-H4K8ac_peak_14099	5.75452	CELF4_ENSG00000101489
18	37421178	37421820	KD2-H4K8ac_peak_14100	6.99815	
18	38951460	38951727	KD2-H4K8ac_peak_14101	9.02371	
18	39254885	39255103	KD2-H4K8ac_peak_14102	6.40683	
18	39255350	39255574	KD2-H4K8ac_peak_14103	6.40683	
18	39772635	39772854	KD2-H4K8ac_peak_14104	7.12754	
18	39879242	39879574	KD2-H4K8ac_peak_14105	6.00226	
18	42259120	42259774	KD2-H4K8ac_peak_14106	15.52424	RP11-456K23.1_ENSG00000267414;SETBP1_ENSG00000152217
18	42260076	42260666	KD2-H4K8ac_peak_14107	11.50346	RP11-456K23.1_ENSG00000267414;SETBP1_ENSG00000152217
18	42260970	42261265	KD2-H4K8ac_peak_14108	5.57044	SETBP1_ENSG00000152217
18	42295822	42296064	KD2-H4K8ac_peak_14109	4.37708	
18	42322266	42322571	KD2-H4K8ac_peak_14110	7.8711	
18	42323249	42323622	KD2-H4K8ac_peak_14111	12.74171	
18	42324375	42325226	KD2-H4K8ac_peak_14112	13.81781	
18	42451196	42451541	KD2-H4K8ac_peak_14113	8.1233	
18	43168430	43168725	KD2-H4K8ac_peak_14114	5.34946	
18	43417505	43417763	KD2-H4K8ac_peak_14115	4.01115	
18	43546933	43547241	KD2-H4K8ac_peak_14116	6.20982	EPG5_ENSG00000152223
18	43547471	43548018	KD2-H4K8ac_peak_14117	6.67092	EPG5_ENSG00000152223
18	43607870	43608441	KD2-H4K8ac_peak_14118	12.93209	RP11-8H2.1_ENSG00000267293
18	43651840	43652051	KD2-H4K8ac_peak_14119	4.44527	PSTPIP2_ENSG00000152229
18	43683669	43683871	KD2-H4K8ac_peak_14120	6.90231	ATP5A1_ENSG00000152234;HAUS1_ENSG00000152240

18	43753274	43753857	KD2-H4K8ac_peak_14121	15.9754	C18orf25_ENSG00000152242
18	43914293	43914582	KD2-H4K8ac_peak_14122	18.31034	
18	43915706	43916099	KD2-H4K8ac_peak_14123	4.77079	
18	43918848	43919081	KD2-H4K8ac_peak_14124	5.14125	
18	43999327	43999786	KD2-H4K8ac_peak_14125	6.30229	
18	44004233	44004724	KD2-H4K8ac_peak_14126	7.96194	
18	44013457	44013768	KD2-H4K8ac_peak_14127	5.7068	
18	44054779	44055152	KD2-H4K8ac_peak_14128	3.87414	
18	44203493	44203716	KD2-H4K8ac_peak_14129	6.2781	
18	44496974	44497376	KD2-H4K8ac_peak_14130	8.85813	KATNAL2_ENSG00000167216
18	44497592	44497892	KD2-H4K8ac_peak_14131	11.92978	KATNAL2_ENSG00000167216
18	44498300	44498521	KD2-H4K8ac_peak_14132	5.25071	KATNAL2_ENSG00000167216
18	44676983	44677691	KD2-H4K8ac_peak_14133	15.7388	HDHD2_ENSG00000167220
18	45321621	45322210	KD2-H4K8ac_peak_14134	6.68439	
18	45325890	45326262	KD2-H4K8ac_peak_14135	5.26591	
18	45456516	45456844	KD2-H4K8ac_peak_14136	8.55271	SMAD2_ENSG00000175387
18	45457178	45457443	KD2-H4K8ac_peak_14137	6.97919	SMAD2_ENSG00000175387
18	45457742	45458003	KD2-H4K8ac_peak_14138	15.61313	SMAD2_ENSG00000175387
18	45533278	45533527	KD2-H4K8ac_peak_14139	5.97274	
18	45534002	45535149	KD2-H4K8ac_peak_14140	20.88898	
18	45548826	45549113	KD2-H4K8ac_peak_14141	12.84642	
18	45557693	45557887	KD2-H4K8ac_peak_14142	5.29555	
18	45560642	45561069	KD2-H4K8ac_peak_14143	10.19949	
18	45561524	45562010	KD2-H4K8ac_peak_14144	7.69287	
18	45562216	45562976	KD2-H4K8ac_peak_14145	11.92978	
18	45563245	45563794	KD2-H4K8ac_peak_14146	6.56211	
18	45586704	45586925	KD2-H4K8ac_peak_14147	7.27716	
18	45592147	45592637	KD2-H4K8ac_peak_14148	9.08816	
18	45593120	45593437	KD2-H4K8ac_peak_14149	8.10216	
18	45598770	45599499	KD2-H4K8ac_peak_14150	14.84212	
18	45599857	45600288	KD2-H4K8ac_peak_14151	5.85291	
18	45600587	45600806	KD2-H4K8ac_peak_14152	5.21035	
18	45611481	45611727	KD2-H4K8ac_peak_14153	6.24747	
18	45662331	45662728	KD2-H4K8ac_peak_14154	6.65673	
18	45663081	45663426	KD2-H4K8ac_peak_14155	6.66692	
18	45687742	45688030	KD2-H4K8ac_peak_14156	15.53798	
18	45688275	45688750	KD2-H4K8ac_peak_14157	6.66692	
18	45754727	45755003	KD2-H4K8ac_peak_14158	9.3973	
18	45756619	45756826	KD2-H4K8ac_peak_14159	8.30974	
18	45820316	45820684	KD2-H4K8ac_peak_14160	8.02571	
18	45846530	45846723	KD2-H4K8ac_peak_14161	8.21084	
18	45868788	45869149	KD2-H4K8ac_peak_14162	5.557	
18	46065028	46065304	KD2-H4K8ac_peak_14163	22.50339	CTIF_ENSG00000134030
18	46066282	46066567	KD2-H4K8ac_peak_14164	4.57782	CTIF_ENSG00000134030
18	46102287	46102538	KD2-H4K8ac_peak_14165	6.25869	
18	46119378	46119570	KD2-H4K8ac_peak_14166	4.73064	

18	46268348	46268938	KD2-H4K8ac_peak_14167	6.90231	
18	46277562	46278214	KD2-H4K8ac_peak_14168	4.91516	
18	46306182	46308108	KD2-H4K8ac_peak_14169	12.00273	
18	46308356	46308727	KD2-H4K8ac_peak_14170	10.43145	
18	46314069	46314447	KD2-H4K8ac_peak_14171	4.56666	
18	46355076	46355583	KD2-H4K8ac_peak_14172	6.40297	
18	46359460	46359683	KD2-H4K8ac_peak_14173	5.14125	
18	46367537	46367747	KD2-H4K8ac_peak_14174	6.66692	
18	46377418	46378119	KD2-H4K8ac_peak_14175	12.68903	
18	46379570	46379840	KD2-H4K8ac_peak_14176	5.45396	
18	46384994	46385323	KD2-H4K8ac_peak_14177	6.99194	
18	46385739	46388104	KD2-H4K8ac_peak_14178	8.85813	
18	46393277	46393789	KD2-H4K8ac_peak_14179	8.59579	
18	46402540	46402813	KD2-H4K8ac_peak_14180	5.88845	
18	46455018	46455678	KD2-H4K8ac_peak_14181	9.43437	
18	46456690	46457234	KD2-H4K8ac_peak_14182	10.96455	
18	46458110	46458831	KD2-H4K8ac_peak_14183	6.25869	
18	46459854	46460380	KD2-H4K8ac_peak_14184	8.83582	
18	46466968	46467256	KD2-H4K8ac_peak_14185	6.40683	
18	46477889	46478337	KD2-H4K8ac_peak_14186	8.23274	SMAD7_ENSG00000101665
18	46478650	46479586	KD2-H4K8ac_peak_14187	19.39602	
18	46501712	46502126	KD2-H4K8ac_peak_14188	6.20982	
18	46502422	46503111	KD2-H4K8ac_peak_14189	14.48084	
18	46523222	46523507	KD2-H4K8ac_peak_14190	9.1707	
18	46524807	46525284	KD2-H4K8ac_peak_14191	6.66692	
18	46537478	46538015	KD2-H4K8ac_peak_14192	13.09607	
18	46578914	46579462	KD2-H4K8ac_peak_14193	14.68449	
18	46580039	46580508	KD2-H4K8ac_peak_14194	8.30974	
18	46587623	46588165	KD2-H4K8ac_peak_14195	6.25869	
18	46598795	46599183	KD2-H4K8ac_peak_14196	7.12754	
18	46986551	46987032	KD2-H4K8ac_peak_14197	12.17278	DYM_ENSG00000141627
18	47013354	47013562	KD2-H4K8ac_peak_14198	7.07117	C18orf32_ENSG00000177576;RP11-110H1.4_ENSG00000263916;MIR1539_ENSG00000265496
18	47339688	47340610	KD2-H4K8ac_peak_14199	12.42761	ACAA2_ENSG00000167315;SCARNA17_ENSG00000267322;SCARNA17_ENSG00000251992;SCARNA18_ENSG00000252139
18	47807511	47807758	KD2-H4K8ac_peak_14200	6.0605	MBD1_ENSG00000141644
18	47808289	47808618	KD2-H4K8ac_peak_14201	13.95627	MBD1_ENSG00000141644
18	47814505	47814712	KD2-H4K8ac_peak_14202	4.211	CXXC1_ENSG00000154832
18	47824081	47825144	KD2-H4K8ac_peak_14203	12.08081	
18	48086116	48086316	KD2-H4K8ac_peak_14204	6.13909	MAPK4_ENSG00000141639
18	48086587	48086906	KD2-H4K8ac_peak_14205	4.06758	MAPK4_ENSG00000141639
18	48404933	48406020	KD2-H4K8ac_peak_14206	16.61426	ME2_ENSG00000082212
18	48493563	48493902	KD2-H4K8ac_peak_14207	6.00226	ELAC1_ENSG00000141642;RP11-729L2.2_ENSG00000267699;SMAD4_ENSG00000141646
18	48494192	48494926	KD2-H4K8ac_peak_14208	20.0792	ELAC1_ENSG00000141642;RP11-729L2.2_ENSG00000267699;SMAD4_ENSG00000141646
18	48556621	48557107	KD2-H4K8ac_peak_14209	21.75906	
18	48722438	48723894	KD2-H4K8ac_peak_14210	13.87818	
18	48724495	48725776	KD2-H4K8ac_peak_14211	12.42761	
18	49180937	49181479	KD2-H4K8ac_peak_14212	6.16888	

18	49193400	49193965	KD2-H4K8ac_peak_14213	4.211	
18	51795451	51795733	KD2-H4K8ac_peak_14214	8.26945	POLI_ENSG00000101751
18	51795979	51796197	KD2-H4K8ac_peak_14215	10.05606	POLI_ENSG00000101751
18	51796476	51796707	KD2-H4K8ac_peak_14216	5.61067	POLI_ENSG00000101751
18	51883589	51884148	KD2-H4K8ac_peak_14217	12.70959	STARD6_ENSG00000174448;C18orf54_ENSG00000166845
18	51884540	51884856	KD2-H4K8ac_peak_14218	9.31907	STARD6_ENSG00000174448;C18orf54_ENSG00000166845
18	52968462	52968796	KD2-H4K8ac_peak_14219	4.77079	
18	52987978	52988229	KD2-H4K8ac_peak_14220	8.1233	
18	52988738	52989028	KD2-H4K8ac_peak_14221	5.88845	
18	52989780	52990023	KD2-H4K8ac_peak_14222	12.06346	
18	53066031	53066258	KD2-H4K8ac_peak_14223	5.10276	
18	53073905	53074213	KD2-H4K8ac_peak_14224	9.97423	
18	53090994	53091303	KD2-H4K8ac_peak_14225	5.57044	
18	53255194	53255388	KD2-H4K8ac_peak_14226	8.17902	
18	53257208	53257579	KD2-H4K8ac_peak_14227	8.55271	
18	53446406	53446680	KD2-H4K8ac_peak_14228	7.22457	
18	53446926	53447232	KD2-H4K8ac_peak_14229	7.146	
18	53530125	53530320	KD2-H4K8ac_peak_14230	4.35659	
18	54305356	54305681	KD2-H4K8ac_peak_14231	5.59859	
18	54306806	54307001	KD2-H4K8ac_peak_14232	5.57044	
18	54729961	54730179	KD2-H4K8ac_peak_14233	7.12754	
18	55019718	55020712	KD2-H4K8ac_peak_14234	9.36621	
18	55021074	55021718	KD2-H4K8ac_peak_14235	7.76329	
18	55101922	55102395	KD2-H4K8ac_peak_14236	6.40683	ONECUT2_ENSG00000119547;AC090340.1_ENSG00000266636
18	55105382	55105845	KD2-H4K8ac_peak_14237	10.46264	
18	55253553	55253784	KD2-H4K8ac_peak_14238	8.88082	FECH_ENSG00000066926
18	55288628	55289002	KD2-H4K8ac_peak_14239	7.59643	NARS_ENSG00000134440
18	55296861	55297456	KD2-H4K8ac_peak_14240	19.9503	RP11-35G9.3_ENSG00000267040
18	55297671	55297870	KD2-H4K8ac_peak_14241	5.73839	RP11-35G9.3_ENSG00000267040
18	55583488	55583849	KD2-H4K8ac_peak_14242	9.51625	
18	55914806	55915023	KD2-H4K8ac_peak_14243	6.16888	
18	56338782	56339062	KD2-H4K8ac_peak_14244	12.06346	RP11-126O1.5_ENSG00000267226;MALT1_ENSG00000172175
18	56530141	56531373	KD2-H4K8ac_peak_14245	20.341	ZNF532_ENSG00000074657
18	57145304	57145522	KD2-H4K8ac_peak_14246	17.42214	
18	57169279	57169481	KD2-H4K8ac_peak_14247	6.8836	
18	57217748	57218093	KD2-H4K8ac_peak_14248	4.24345	
18	57221253	57221485	KD2-H4K8ac_peak_14249	8.04763	
18	57236054	57236430	KD2-H4K8ac_peak_14250	5.14125	
18	57237996	57238206	KD2-H4K8ac_peak_14251	6.56211	
18	57283831	57284225	KD2-H4K8ac_peak_14252	5.57044	
18	57504425	57504723	KD2-H4K8ac_peak_14253	9.15329	
18	57851444	57851692	KD2-H4K8ac_peak_14254	4.37708	
18	57864828	57865124	KD2-H4K8ac_peak_14255	6.16888	
18	59139317	59139545	KD2-H4K8ac_peak_14256	5.88845	
18	59515852	59516198	KD2-H4K8ac_peak_14257	4.47369	
18	59523574	59524124	KD2-H4K8ac_peak_14258	5.13615	

18	59560624	59560899	KD2-H4K8ac_peak_14259	4.37809	RNF152_ENSG00000176641
18	59561237	59562160	KD2-H4K8ac_peak_14260	16.07006	RNF152_ENSG00000176641
18	59854712	59855039	KD2-H4K8ac_peak_14261	5.92591	PIGN_ENSG00000197563;KIAA1468_ENSG00000134444
18	60189619	60189967	KD2-H4K8ac_peak_14262	7.40111	ZCCHC2_ENSG00000141664
18	60190968	60191607	KD2-H4K8ac_peak_14263	7.27716	ZCCHC2_ENSG00000141664
18	60193758	60194018	KD2-H4K8ac_peak_14264	19.88671	
18	60331378	60331650	KD2-H4K8ac_peak_14265	6.16888	
18	60381914	60382124	KD2-H4K8ac_peak_14266	5.29555	PHLPP1_ENSG00000081913
18	60756384	60756706	KD2-H4K8ac_peak_14267	7.72801	
18	60758272	60758550	KD2-H4K8ac_peak_14268	12.60983	
18	60809870	60810729	KD2-H4K8ac_peak_14269	6.64891	
18	60986670	60988539	KD2-H4K8ac_peak_14270	8.85813	BCL2_ENSG00000171791
18	61035149	61035560	KD2-H4K8ac_peak_14271	11.54737	KDSR_ENSG00000119537;RP11-635N19.1_ENSG00000267390
18	61090020	61090328	KD2-H4K8ac_peak_14272	15.81711	VPS4B_ENSG00000119541
18	61603750	61604156	KD2-H4K8ac_peak_14273	9.07715	
18	61616602	61617008	KD2-H4K8ac_peak_14274	6.64891	HMSD_ENSG00000221887
18	61637116	61637666	KD2-H4K8ac_peak_14275	16.49944	SERPINB8_ENSG00000166401
18	61638067	61638384	KD2-H4K8ac_peak_14276	16.25886	SERPINB8_ENSG00000166401
18	63144320	63144523	KD2-H4K8ac_peak_14277	6.16888	
18	63417841	63418278	KD2-H4K8ac_peak_14278	7.27716	CDH7_ENSG00000081138
18	65176030	65176714	KD2-H4K8ac_peak_14279	8.1233	
18	66381876	66382268	KD2-H4K8ac_peak_14280	14.00658	TMX3_ENSG00000166479;CCDC102B_ENSG00000150636
18	67067780	67068529	KD2-H4K8ac_peak_14281	7.49599	DOK6_ENSG00000206052
18	67872686	67873122	KD2-H4K8ac_peak_14282	10.46264	RTTN_ENSG00000176225
18	67873584	67873898	KD2-H4K8ac_peak_14283	4.59928	RTTN_ENSG00000176225
18	67955681	67957045	KD2-H4K8ac_peak_14284	24.051	SOCS6_ENSG00000170677
18	67963634	67963851	KD2-H4K8ac_peak_14285	5.6755	
18	68083153	68083971	KD2-H4K8ac_peak_14286	10.94087	
18	68135844	68136160	KD2-H4K8ac_peak_14287	12.38154	
18	68138331	68138653	KD2-H4K8ac_peak_14288	5.54647	
18	68266591	68266905	KD2-H4K8ac_peak_14289	10.21502	
18	68650197	68650440	KD2-H4K8ac_peak_14290	4.76709	
18	68695005	68695819	KD2-H4K8ac_peak_14291	4.91674	RP11-529J17.1_ENSG00000265639
18	68696149	68696901	KD2-H4K8ac_peak_14292	12.88108	RP11-529J17.1_ENSG00000265639
18	69566930	69567353	KD2-H4K8ac_peak_14293	9.36516	
18	70209386	70209695	KD2-H4K8ac_peak_14294	5.92591	
18	70210445	70210656	KD2-H4K8ac_peak_14295	6.94255	
18	70210889	70211172	KD2-H4K8ac_peak_14296	12.38154	
18	71814826	71815519	KD2-H4K8ac_peak_14297	12.88108	FBXO15_ENSG00000141665;TIMM21_ENSG00000075336
18	71922575	71923013	KD2-H4K8ac_peak_14298	5.88845	
18	71924776	71925098	KD2-H4K8ac_peak_14299	4.42765	
18	71936862	71937405	KD2-H4K8ac_peak_14300	9.63776	
18	71946224	71946751	KD2-H4K8ac_peak_14301	7.45501	
18	72162880	72163224	KD2-H4K8ac_peak_14302	8.28994	CNDP2_ENSG00000133313
18	72163839	72164099	KD2-H4K8ac_peak_14303	10.96455	CNDP2_ENSG00000133313
18	72264465	72264732	KD2-H4K8ac_peak_14304	6.25869	ZNF407_ENSG00000215421

18	72264966	72265189	KD2-H4K8ac_peak_14305	4.20044	LINC00909_ENSG00000264247;ZNF407_ENSG00000215421
18	72265390	72266047	KD2-H4K8ac_peak_14306	17.16047	LINC00909_ENSG00000264247;ZNF407_ENSG00000215421
18	72515100	72515338	KD2-H4K8ac_peak_14307	6.31143	
18	72837284	72838203	KD2-H4K8ac_peak_14308	21.11201	
18	72920780	72921054	KD2-H4K8ac_peak_14309	12.00597	ZADH2_ENSG00000180011
18	72921268	72921857	KD2-H4K8ac_peak_14310	12.2054	ZADH2_ENSG00000180011;TSHZ1_ENSG00000179981
18	72923742	72924076	KD2-H4K8ac_peak_14311	24.94195	
18	72956479	72956894	KD2-H4K8ac_peak_14312	15.64848	
18	72957433	72958080	KD2-H4K8ac_peak_14313	6.2159	
18	72959093	72959319	KD2-H4K8ac_peak_14314	6.31143	
18	72961023	72961332	KD2-H4K8ac_peak_14315	10.87093	
18	73627872	73628603	KD2-H4K8ac_peak_14316	14.74047	
18	74162985	74163496	KD2-H4K8ac_peak_14317	7.59643	
18	74207403	74207820	KD2-H4K8ac_peak_14318	6.66692	ZNF516_ENSG00000101493;RP11-17M16.1_ENSG00000220032
18	74499418	74499740	KD2-H4K8ac_peak_14319	5.43522	
18	74513820	74514134	KD2-H4K8ac_peak_14320	8.23274	
18	74533781	74534655	KD2-H4K8ac_peak_14321	15.03918	RP11-162A12.2_ENSG00000264278;ZNF236_ENSG00000130856
18	74534937	74535377	KD2-H4K8ac_peak_14322	17.8868	RP11-162A12.2_ENSG00000264278;ZNF236_ENSG00000130856
18	74535670	74535973	KD2-H4K8ac_peak_14323	4.06758	
18	76828573	76829141	KD2-H4K8ac_peak_14324	17.8868	ATP9B_ENSG00000166377
18	76829569	76829799	KD2-H4K8ac_peak_14325	5.54647	ATP9B_ENSG00000166377
18	76981370	76981617	KD2-H4K8ac_peak_14326	5.10276	
18	77010899	77011317	KD2-H4K8ac_peak_14327	7.12754	
18	77438735	77439242	KD2-H4K8ac_peak_14328	12.99417	RP11-567M16.3_ENSG00000178412;CTDP1_ENSG00000060069
18	77711815	77712510	KD2-H4K8ac_peak_14329	10.19949	PQLC1_ENSG00000122490
18	77724152	77724759	KD2-H4K8ac_peak_14330	11.05407	HSBP1L1_ENSG00000226742
18	77748088	77748503	KD2-H4K8ac_peak_14331	16.07006	
18	77748711	77749336	KD2-H4K8ac_peak_14332	11.2938	
18	77793348	77793712	KD2-H4K8ac_peak_14333	6.65673	TXNL4A_ENSG00000141759;RP11-795F19.5_ENSG00000267127;RBFA_ENSG00000101546
18	77794495	77794768	KD2-H4K8ac_peak_14334	6.66692	TXNL4A_ENSG00000141759;RP11-795F19.5_ENSG00000267127;RBFA_ENSG00000101546
18	77866014	77866349	KD2-H4K8ac_peak_14335	10.0746	ADNP2_ENSG00000101544
18	77866920	77867379	KD2-H4K8ac_peak_14336	21.96198	ADNP2_ENSG00000101544
18	78004049	78004277	KD2-H4K8ac_peak_14337	8.09303	
18	78005099	78005295	KD2-H4K8ac_peak_14338	6.65673	PARD6G_ENSG00000178184
19	290513	291272	KD2-H4K8ac_peak_14339	10.7186	PPAP2C_ENSG00000141934
19	291728	292209	KD2-H4K8ac_peak_14340	12.99417	PPAP2C_ENSG00000141934
19	344152	345449	KD2-H4K8ac_peak_14341	11.89502	MIER2_ENSG00000105556
19	402229	402876	KD2-H4K8ac_peak_14342	14.15075	
19	408715	409464	KD2-H4K8ac_peak_14343	10.17341	C2CD4C_ENSG00000183186
19	448192	448430	KD2-H4K8ac_peak_14344	8.30974	
19	460039	461248	KD2-H4K8ac_peak_14345	9.96798	SHC2_ENSG00000129946
19	480072	480311	KD2-H4K8ac_peak_14346	11.66127	
19	483452	483727	KD2-H4K8ac_peak_14347	5.88845	
19	488100	488898	KD2-H4K8ac_peak_14348	7.36799	MADCAM1_ENSG00000099866
19	506398	507045	KD2-H4K8ac_peak_14349	12.08081	AC005775.2_ENSG00000266933;TPGS1_ENSG00000141933
19	507567	507768	KD2-H4K8ac_peak_14350	6.76298	AC005775.2_ENSG00000266933;TPGS1_ENSG00000141933

19	524105	524667	KD2-H4K8ac_peak_14351	12.84642	
19	530339	530707	KD2-H4K8ac_peak_14352	7.14562	
19	531221	531538	KD2-H4K8ac_peak_14353	10.46874	CDC34_ENSG00000099804
19	531826	532103	KD2-H4K8ac_peak_14354	4.67342	CDC34_ENSG00000099804
19	572656	572994	KD2-H4K8ac_peak_14355	14.99078	AC009005.2_ENSG00000267751
19	588348	588837	KD2-H4K8ac_peak_14356	5.57044	
19	589132	589726	KD2-H4K8ac_peak_14357	9.72244	HCN2_ENSG00000099822
19	609154	609805	KD2-H4K8ac_peak_14358	7.96322	
19	610882	611596	KD2-H4K8ac_peak_14359	15.25274	
19	633112	633457	KD2-H4K8ac_peak_14360	5.53033	POLRMT_ENSG00000099821
19	634126	634385	KD2-H4K8ac_peak_14361	7.38933	POLRMT_ENSG00000099821
19	662815	663074	KD2-H4K8ac_peak_14362	5.66705	RNF126_ENSG00000070423;AC004156.3_ENSG00000267666
19	663491	663952	KD2-H4K8ac_peak_14363	11.05467	RNF126_ENSG00000070423;AC004156.3_ENSG00000267666
19	679361	680290	KD2-H4K8ac_peak_14364	11.9948	
19	694948	695294	KD2-H4K8ac_peak_14365	6.07178	PRSS57_ENSG00000185198
19	708191	708742	KD2-H4K8ac_peak_14366	10.76768	PALM_ENSG00000099864
19	709081	709299	KD2-H4K8ac_peak_14367	9.07715	PALM_ENSG00000099864
19	709636	710497	KD2-H4K8ac_peak_14368	10.21366	PALM_ENSG00000099864
19	719539	719832	KD2-H4K8ac_peak_14369	9.11031	
19	720878	721249	KD2-H4K8ac_peak_14370	4.67342	
19	778812	779120	KD2-H4K8ac_peak_14371	3.75035	
19	787583	787893	KD2-H4K8ac_peak_14372	5.82799	AC006273.7_ENSG00000266927
19	788712	790035	KD2-H4K8ac_peak_14373	16.5604	
19	792115	792471	KD2-H4K8ac_peak_14374	6.55245	
19	796434	796958	KD2-H4K8ac_peak_14375	18.33962	PTBP1_ENSG00000011304
19	797450	797717	KD2-H4K8ac_peak_14376	22.12955	PTBP1_ENSG00000011304
19	821306	822895	KD2-H4K8ac_peak_14377	16.64426	LPPR3_ENSG00000129951
19	892382	892657	KD2-H4K8ac_peak_14378	4.86324	MED16_ENSG00000175221;RNU6-9_ENSG00000207507
19	892955	893175	KD2-H4K8ac_peak_14379	17.76093	MED16_ENSG00000175221;RNU6-9_ENSG00000207507
19	893691	894301	KD2-H4K8ac_peak_14380	5.65802	MED16_ENSG00000175221;RNU6-9_ENSG00000207507
19	913284	913793	KD2-H4K8ac_peak_14381	8.86099	R3HDM4_ENSG00000198858
19	916950	917384	KD2-H4K8ac_peak_14382	6.85193	KISS1R_ENSG00000116014
19	924803	925911	KD2-H4K8ac_peak_14383	16.61426	ARID3A_ENSG00000116017
19	926287	926811	KD2-H4K8ac_peak_14384	7.69287	ARID3A_ENSG00000116017
19	927081	927469	KD2-H4K8ac_peak_14385	7.57979	
19	932570	933274	KD2-H4K8ac_peak_14386	10.30284	
19	933509	933784	KD2-H4K8ac_peak_14387	20.70767	
19	934515	935330	KD2-H4K8ac_peak_14388	6.82618	
19	940396	940833	KD2-H4K8ac_peak_14389	10.7186	
19	941227	941450	KD2-H4K8ac_peak_14390	8.10216	
19	945360	945758	KD2-H4K8ac_peak_14391	6.85193	
19	956520	956844	KD2-H4K8ac_peak_14392	5.73016	
19	983730	984858	KD2-H4K8ac_peak_14393	17.75427	WDR18_ENSG00000065268
19	1020605	1021067	KD2-H4K8ac_peak_14394	9.08816	TMEM259_ENSG00000182087;RNU6-2_ENSG00000207357
19	1026235	1026470	KD2-H4K8ac_peak_14395	8.79106	CNN2_ENSG00000064666
19	1026813	1027214	KD2-H4K8ac_peak_14396	7.51054	CNN2_ENSG00000064666

19	1027800	1028481	KD2-H4K8ac_peak_14397	15.10495	
19	1028812	1029074	KD2-H4K8ac_peak_14398	11.05407	
19	1074491	1074742	KD2-H4K8ac_peak_14399	9.20193	
19	1095617	1096260	KD2-H4K8ac_peak_14400	8.28234	POLR2E_ENSG00000099817
19	1104164	1104362	KD2-H4K8ac_peak_14401	8.16133	GPX4_ENSG00000167468
19	1104564	1104889	KD2-H4K8ac_peak_14402	9.73431	GPX4_ENSG00000167468
19	1170197	1170519	KD2-H4K8ac_peak_14403	6.0073	
19	1173149	1173351	KD2-H4K8ac_peak_14404	4.84794	SBNO2_ENSG00000064932
19	1174452	1174812	KD2-H4K8ac_peak_14405	10.72313	SBNO2_ENSG00000064932
19	1180271	1180568	KD2-H4K8ac_peak_14406	7.5554	
19	1194158	1194503	KD2-H4K8ac_peak_14407	11.2938	
19	1205243	1205487	KD2-H4K8ac_peak_14408	14.06758	
19	1237047	1237893	KD2-H4K8ac_peak_14409	13.70753	C19orf26_ENSG00000099625;AC004221.2_ENSG00000267778
19	1238163	1238587	KD2-H4K8ac_peak_14410	6.50882	C19orf26_ENSG00000099625;AC004221.2_ENSG00000267778
19	1239751	1240276	KD2-H4K8ac_peak_14411	11.92978	
19	1241817	1242110	KD2-H4K8ac_peak_14412	13.80019	ATP5D_ENSG00000099624
19	1248095	1248404	KD2-H4K8ac_peak_14413	4.06758	MIDN_ENSG00000167470
19	1248670	1248878	KD2-H4K8ac_peak_14414	6.97919	MIDN_ENSG00000167470
19	1249099	1249593	KD2-H4K8ac_peak_14415	26.43409	MIDN_ENSG00000167470
19	1249999	1251227	KD2-H4K8ac_peak_14416	9.36516	
19	1251503	1252187	KD2-H4K8ac_peak_14417	6.40297	
19	1252561	1252930	KD2-H4K8ac_peak_14418	10.7679	
19	1260317	1260827	KD2-H4K8ac_peak_14419	11.80283	CIRBP_ENSG00000099622
19	1261177	1261405	KD2-H4K8ac_peak_14420	8.67843	
19	1266764	1267050	KD2-H4K8ac_peak_14421	6.04107	
19	1269317	1269683	KD2-H4K8ac_peak_14422	26.99824	CIRBP-AS1_ENSG00000267493
19	1275117	1275357	KD2-H4K8ac_peak_14423	10.19949	C19orf24_ENSG00000228300
19	1275551	1275800	KD2-H4K8ac_peak_14424	18.16578	C19orf24_ENSG00000228300
19	1285312	1286468	KD2-H4K8ac_peak_14425	11.50346	MUM1_ENSG00000160953;EFNA2_ENSG00000099617
19	1315708	1316542	KD2-H4K8ac_peak_14426	8.10216	
19	1324993	1325285	KD2-H4K8ac_peak_14427	5.66705	
19	1325484	1325921	KD2-H4K8ac_peak_14428	9.402	
19	1331686	1332041	KD2-H4K8ac_peak_14429	4.86324	
19	1350225	1350570	KD2-H4K8ac_peak_14430	5.13893	
19	1354081	1354755	KD2-H4K8ac_peak_14431	24.23147	
19	1355053	1355361	KD2-H4K8ac_peak_14432	8.55271	
19	1383927	1384334	KD2-H4K8ac_peak_14433	12.93209	NDUFS7_ENSG00000115286
19	1400761	1401973	KD2-H4K8ac_peak_14434	15.50259	GAMT_ENSG00000130005
19	1407666	1408280	KD2-H4K8ac_peak_14435	12.10889	DAZAP1_ENSG00000071626
19	1444919	1445203	KD2-H4K8ac_peak_14436	10.0089	
19	1445407	1445859	KD2-H4K8ac_peak_14437	9.95119	APC2_ENSG00000115266
19	1446297	1446781	KD2-H4K8ac_peak_14438	3.97079	APC2_ENSG00000115266
19	1449254	1449866	KD2-H4K8ac_peak_14439	10.19723	
19	1450240	1450544	KD2-H4K8ac_peak_14440	6.35843	
19	1478555	1478893	KD2-H4K8ac_peak_14441	5.54647	C19orf25_ENSG00000119559
19	1479399	1479997	KD2-H4K8ac_peak_14442	6.66359	C19orf25_ENSG00000119559

19	1480205	1480455	KD2-H4K8ac_peak_14443	7.42204	C19orf25_ENSG00000119559
19	1489688	1490256	KD2-H4K8ac_peak_14444	5.64429	PCSK4_ENSG00000115257;REEP6_ENSG00000115255
19	1491227	1491531	KD2-H4K8ac_peak_14445	7.98621	PCSK4_ENSG00000115257;REEP6_ENSG00000115255
19	1507252	1507720	KD2-H4K8ac_peak_14446	10.05606	CTB-25B13.9_ENSG00000267092
19	1507976	1508171	KD2-H4K8ac_peak_14447	7.27716	CTB-25B13.9_ENSG00000267092
19	1566814	1567678	KD2-H4K8ac_peak_14448	12.96152	MEX3D_ENSG00000181588
19	1591366	1592289	KD2-H4K8ac_peak_14449	12.30509	MBD3_ENSG00000071655
19	1592484	1592764	KD2-H4K8ac_peak_14450	18.82114	MBD3_ENSG00000071655
19	1593118	1593532	KD2-H4K8ac_peak_14451	7.4599	MBD3_ENSG00000071655
19	1604958	1605394	KD2-H4K8ac_peak_14452	10.46264	UQCR11_ENSG00000267059;UQCR11_ENSG00000127540
19	1605733	1606614	KD2-H4K8ac_peak_14453	9.1707	UQCR11_ENSG00000267059;UQCR11_ENSG00000127540
19	1651254	1652356	KD2-H4K8ac_peak_14454	14.15075	TCF3_ENSG00000071564
19	1652674	1652917	KD2-H4K8ac_peak_14455	26.96144	TCF3_ENSG00000071564
19	1653212	1653421	KD2-H4K8ac_peak_14456	14.22723	TCF3_ENSG00000071564
19	1672198	1672466	KD2-H4K8ac_peak_14457	10.96455	
19	1725322	1725665	KD2-H4K8ac_peak_14458	8.86099	
19	1725907	1726219	KD2-H4K8ac_peak_14459	9.96798	
19	1795999	1796366	KD2-H4K8ac_peak_14460	12.84642	
19	1811862	1812223	KD2-H4K8ac_peak_14461	11.05407	ATP8B3_ENSG00000130270
19	1847904	1848734	KD2-H4K8ac_peak_14462	12.08081	REXO1_ENSG00000079313
19	1854980	1855260	KD2-H4K8ac_peak_14463	6.99815	
19	1855650	1855856	KD2-H4K8ac_peak_14464	5.01002	
19	1873328	1874096	KD2-H4K8ac_peak_14465	5.13615	CTB-31O20.9_ENSG00000267232
19	1876491	1876926	KD2-H4K8ac_peak_14466	18.29344	CTB-31O20.2_ENSG00000261526
19	1885780	1885997	KD2-H4K8ac_peak_14467	12.28999	ABHD17A_ENSG00000129968
19	1904423	1905140	KD2-H4K8ac_peak_14468	15.81711	SCAMP4_ENSG00000227500;ADAT3_ENSG00000213638
19	1909923	1910141	KD2-H4K8ac_peak_14469	4.211	
19	1929930	1930261	KD2-H4K8ac_peak_14470	8.17902	
19	1940190	1940686	KD2-H4K8ac_peak_14471	11.60101	CSNK1G2_ENSG00000133275
19	1940909	1941442	KD2-H4K8ac_peak_14472	13.9234	CSNK1G2_ENSG00000133275
19	2015275	2015496	KD2-H4K8ac_peak_14473	12.84642	
19	2016220	2016956	KD2-H4K8ac_peak_14474	6.66692	
19	2032399	2032962	KD2-H4K8ac_peak_14475	6.25869	
19	2041563	2041971	KD2-H4K8ac_peak_14476	9.048	
19	2042498	2043095	KD2-H4K8ac_peak_14477	9.18237	
19	2050461	2050748	KD2-H4K8ac_peak_14478	7.47462	MKNK2_ENSG00000099875
19	2051459	2052569	KD2-H4K8ac_peak_14479	11.92978	MKNK2_ENSG00000099875
19	2056396	2056629	KD2-H4K8ac_peak_14480	7.86151	
19	2058144	2058379	KD2-H4K8ac_peak_14481	5.13893	
19	2059491	2060257	KD2-H4K8ac_peak_14482	4.37809	
19	2060483	2061474	KD2-H4K8ac_peak_14483	24.93342	
19	2061753	2062251	KD2-H4K8ac_peak_14484	14.4458	
19	2064895	2065186	KD2-H4K8ac_peak_14485	8.08344	
19	2096076	2096570	KD2-H4K8ac_peak_14486	8.01338	MOB3A_ENSG00000172081;IZUMO4_ENSG00000099840
19	2150822	2151522	KD2-H4K8ac_peak_14487	7.74292	
19	2163159	2163442	KD2-H4K8ac_peak_14488	7.67504	DOT1L_ENSG00000104885

19	2180876	2181431	KD2-H4K8ac_peak_14489	6.30229	
19	2221723	2221946	KD2-H4K8ac_peak_14490	3.82509	
19	2235527	2236222	KD2-H4K8ac_peak_14491	15.69395	SF3A2_ENSG00000104897
19	2236864	2237200	KD2-H4K8ac_peak_14492	11.90231	PLEKHJ1_ENSG00000104886;SF3A2_ENSG00000104897
19	2243083	2243340	KD2-H4K8ac_peak_14493	5.13615	
19	2244569	2244776	KD2-H4K8ac_peak_14494	5.61067	
19	2255457	2256352	KD2-H4K8ac_peak_14495	5.9696	
19	2256840	2257157	KD2-H4K8ac_peak_14496	5.31717	
19	2266441	2266686	KD2-H4K8ac_peak_14497	6.72624	
19	2269034	2269228	KD2-H4K8ac_peak_14498	10.19949	JSRP1_ENSG00000167476;OAZ1_ENSG00000104904
19	2282232	2282573	KD2-H4K8ac_peak_14499	5.54647	C19orf35_ENSG00000188305
19	2294213	2294426	KD2-H4K8ac_peak_14500	10.05606	
19	2307830	2308027	KD2-H4K8ac_peak_14501	8.79106	LINGO3_ENSG00000220008
19	2308262	2308628	KD2-H4K8ac_peak_14502	8.30974	LINGO3_ENSG00000220008
19	2316176	2316520	KD2-H4K8ac_peak_14503	6.60096	
19	2328716	2329256	KD2-H4K8ac_peak_14504	9.96798	LSM7_ENSG00000130332;SPPL2B_ENSG00000005206
19	2427206	2427877	KD2-H4K8ac_peak_14505	23.78494	TIMM13_ENSG00000099800
19	2428289	2428626	KD2-H4K8ac_peak_14506	9.1707	TIMM13_ENSG00000099800
19	2456415	2456890	KD2-H4K8ac_peak_14507	15.13261	LMNB2_ENSG00000176619
19	2461562	2463450	KD2-H4K8ac_peak_14508	20.47312	AC005624.2_ENSG00000267201
19	2542899	2544390	KD2-H4K8ac_peak_14509	8.55271	
19	2579006	2579209	KD2-H4K8ac_peak_14510	5.30799	
19	2599370	2599569	KD2-H4K8ac_peak_14511	8.41122	
19	2601975	2602227	KD2-H4K8ac_peak_14512	6.85193	
19	2611054	2611265	KD2-H4K8ac_peak_14513	9.55262	CTC-265F19.2_ENSG00000267412
19	2721108	2722093	KD2-H4K8ac_peak_14514	11.33854	DIRAS1_ENSG00000176490
19	2749038	2749360	KD2-H4K8ac_peak_14515	4.211	
19	2782947	2783248	KD2-H4K8ac_peak_14516	12.14337	SGTA_ENSG00000104969
19	2785571	2785852	KD2-H4K8ac_peak_14517	11.19034	THOP1_ENSG00000172009
19	2819909	2820231	KD2-H4K8ac_peak_14518	12.82393	ZNF554_ENSG00000172006
19	2840557	2841170	KD2-H4K8ac_peak_14519	10.43145	ZNF555_ENSG00000186300
19	2841543	2841931	KD2-H4K8ac_peak_14520	5.4779	ZNF555_ENSG00000186300
19	2867344	2867543	KD2-H4K8ac_peak_14521	9.02371	ZNF556_ENSG00000172000
19	2899949	2900148	KD2-H4K8ac_peak_14522	4.67342	ZNF57_ENSG00000171970
19	2945273	2945793	KD2-H4K8ac_peak_14523	8.85813	ZNF77_ENSG00000175691
19	2977572	2977856	KD2-H4K8ac_peak_14524	10.08707	TLE6_ENSG00000104953
19	2982580	2982915	KD2-H4K8ac_peak_14525	10.21366	
19	2983238	2983700	KD2-H4K8ac_peak_14526	10.17486	
19	2986867	2987102	KD2-H4K8ac_peak_14527	8.5092	
19	3035191	3035487	KD2-H4K8ac_peak_14528	6.05809	
19	3061105	3061797	KD2-H4K8ac_peak_14529	7.76104	
19	3062601	3063081	KD2-H4K8ac_peak_14530	8.86099	AES_ENSG00000104964
19	3067665	3068003	KD2-H4K8ac_peak_14531	14.48084	
19	3093474	3094093	KD2-H4K8ac_peak_14532	13.80019	GNA11_ENSG00000088256
19	3109502	3109719	KD2-H4K8ac_peak_14533	6.06203	
19	3184410	3185073	KD2-H4K8ac_peak_14534	11.05467	NCLN_ENSG00000125912

19	3186011	3186237	KD2-H4K8ac_peak_14535	5.30799	NCLN_ENSG00000125912
19	3224711	3224990	KD2-H4K8ac_peak_14536	10.68173	CELF5_ENSG00000161082
19	3275701	3275945	KD2-H4K8ac_peak_14537	5.38581	
19	3336738	3337421	KD2-H4K8ac_peak_14538	10.17486	
19	3344012	3344224	KD2-H4K8ac_peak_14539	9.63487	
19	3346166	3346532	KD2-H4K8ac_peak_14540	4.44748	
19	3358602	3359406	KD2-H4K8ac_peak_14541	9.1707	NFIC_ENSG00000141905
19	3359649	3360177	KD2-H4K8ac_peak_14542	15.20126	NFIC_ENSG00000141905
19	3360757	3361352	KD2-H4K8ac_peak_14543	7.07117	
19	3365584	3366276	KD2-H4K8ac_peak_14544	11.70879	
19	3366606	3367975	KD2-H4K8ac_peak_14545	19.25648	
19	3375694	3376247	KD2-H4K8ac_peak_14546	8.02571	
19	3386549	3386932	KD2-H4K8ac_peak_14547	14.16977	
19	3388116	3388378	KD2-H4K8ac_peak_14548	7.11824	
19	3388627	3388865	KD2-H4K8ac_peak_14549	8.96519	
19	3412813	3413019	KD2-H4K8ac_peak_14550	3.75035	
19	3423234	3423922	KD2-H4K8ac_peak_14551	9.16531	
19	3427914	3428728	KD2-H4K8ac_peak_14552	7.79898	
19	3434338	3434835	KD2-H4K8ac_peak_14553	6.76298	
19	3435421	3435767	KD2-H4K8ac_peak_14554	7.76329	
19	3441129	3442553	KD2-H4K8ac_peak_14555	15.58442	
19	3442843	3443097	KD2-H4K8ac_peak_14556	7.96322	
19	3458881	3459129	KD2-H4K8ac_peak_14557	9.70057	
19	3459371	3459635	KD2-H4K8ac_peak_14558	10.65724	
19	3500214	3500868	KD2-H4K8ac_peak_14559	11.04256	DOHH_ENSG00000129932;RN7SL866P_ENSG00000264159
19	3501305	3501528	KD2-H4K8ac_peak_14560	6.40683	DOHH_ENSG00000129932;RN7SL866P_ENSG00000264159
19	3505453	3506014	KD2-H4K8ac_peak_14561	14.93459	FZR1_ENSG00000105325
19	3506359	3507055	KD2-H4K8ac_peak_14562	12.2118	FZR1_ENSG00000105325
19	3557775	3558064	KD2-H4K8ac_peak_14563	13.70753	AC005786.5_ENSG00000267231
19	3572078	3572393	KD2-H4K8ac_peak_14564	7.36799	HMG20B_ENSG00000064961
19	3585780	3585995	KD2-H4K8ac_peak_14565	6.25869	GIPC3_ENSG00000179855
19	3606206	3606475	KD2-H4K8ac_peak_14566	3.97079	TBXA2R_ENSG00000006638;CACTIN-AS1_ENSG00000226800
19	3606689	3607825	KD2-H4K8ac_peak_14567	13.73635	TBXA2R_ENSG00000006638;CACTIN-AS1_ENSG00000226800
19	3626561	3627037	KD2-H4K8ac_peak_14568	10.17341	CACTIN_ENSG00000105298
19	3665698	3666168	KD2-H4K8ac_peak_14569	6.72624	
19	3700176	3700409	KD2-H4K8ac_peak_14570	9.43685	PIP5K1C_ENSG00000186111
19	3761539	3762008	KD2-H4K8ac_peak_14571	5.54647	APBA3_ENSG00000011132;MRPL54_ENSG00000183617
19	3762665	3762908	KD2-H4K8ac_peak_14572	13.9234	APBA3_ENSG00000011132;MRPL54_ENSG00000183617
19	3826041	3826237	KD2-H4K8ac_peak_14573	6.25014	
19	3970695	3971034	KD2-H4K8ac_peak_14574	12.74171	DAPK3_ENSG00000167657
19	3971386	3971670	KD2-H4K8ac_peak_14575	10.21366	DAPK3_ENSG00000167657
19	3984950	3985222	KD2-H4K8ac_peak_14576	5.88845	EEF2_ENSG00000167658
19	3986570	3986978	KD2-H4K8ac_peak_14577	4.42765	
19	3990487	3990873	KD2-H4K8ac_peak_14578	5.30799	
19	3991142	3991570	KD2-H4K8ac_peak_14579	7.0983	
19	4007758	4008123	KD2-H4K8ac_peak_14580	16.61426	PIAS4_ENSG00000105229

19	4014433	4014933	KD2-H4K8ac_peak_14581	6.94249	
19	4015452	4015880	KD2-H4K8ac_peak_14582	6.60094	
19	4066265	4066816	KD2-H4K8ac_peak_14583	16.8435	ZBTB7A_ENSG00000178951
19	4067146	4067812	KD2-H4K8ac_peak_14584	10.43145	ZBTB7A_ENSG00000178951
19	4123717	4124076	KD2-H4K8ac_peak_14585	10.33529	MAP2K2_ENSG00000126934
19	4124316	4124508	KD2-H4K8ac_peak_14586	7.47462	MAP2K2_ENSG00000126934
19	4139834	4140305	KD2-H4K8ac_peak_14587	5.43522	
19	4140598	4140878	KD2-H4K8ac_peak_14588	4.01115	
19	4181953	4182493	KD2-H4K8ac_peak_14589	8.16906	SIRT6_ENSG00000077463;ANKRD24_ENSG00000089847
19	4224188	4224628	KD2-H4K8ac_peak_14590	7.01658	
19	4246368	4246780	KD2-H4K8ac_peak_14591	14.56955	CCDC94_ENSG00000105248
19	4276660	4277316	KD2-H4K8ac_peak_14592	9.73431	
19	4277689	4278459	KD2-H4K8ac_peak_14593	12.36441	SHD_ENSG00000105251
19	4278779	4280445	KD2-H4K8ac_peak_14594	21.6161	SHD_ENSG00000105251
19	4291243	4291670	KD2-H4K8ac_peak_14595	8.49564	
19	4301357	4301555	KD2-H4K8ac_peak_14596	5.58324	TMIGD2_ENSG00000167664
19	4302213	4302978	KD2-H4K8ac_peak_14597	5.54647	TMIGD2_ENSG00000167664
19	4304280	4304495	KD2-H4K8ac_peak_14598	7.27716	FSD1_ENSG00000105255
19	4327051	4327260	KD2-H4K8ac_peak_14599	8.83582	
19	4328725	4329052	KD2-H4K8ac_peak_14600	11.89502	
19	4342176	4342677	KD2-H4K8ac_peak_14601	29.1888	STAP2_ENSG00000178078;MPND_ENSG00000008382
19	4343096	4343407	KD2-H4K8ac_peak_14602	14.98936	STAP2_ENSG00000178078;MPND_ENSG00000008382
19	4352809	4353185	KD2-H4K8ac_peak_14603	10.0692	AC007292.4_ENSG00000269807
19	4373197	4373528	KD2-H4K8ac_peak_14604	6.25869	
19	4374297	4375145	KD2-H4K8ac_peak_14605	7.92775	
19	4402685	4403137	KD2-H4K8ac_peak_14606	17.27647	CHAF1A_ENSG00000167670
19	4458258	4458527	KD2-H4K8ac_peak_14607	6.40297	UBXN6_ENSG00000167671
19	4474907	4475191	KD2-H4K8ac_peak_14608	11.05467	
19	4556117	4556340	KD2-H4K8ac_peak_14609	5.14125	
19	4557028	4557254	KD2-H4K8ac_peak_14610	10.21366	
19	4584243	4584845	KD2-H4K8ac_peak_14611	9.57101	
19	4635985	4636670	KD2-H4K8ac_peak_14612	21.60954	
19	4638435	4638731	KD2-H4K8ac_peak_14613	9.96798	TNFAIP8L1_ENSG00000185361
19	4669799	4670309	KD2-H4K8ac_peak_14614	11.1952	C19orf10_ENSG00000074842
19	4723349	4723745	KD2-H4K8ac_peak_14615	11.51672	DPP9_ENSG00000142002
19	4738349	4738712	KD2-H4K8ac_peak_14616	7.70289	
19	4742258	4742563	KD2-H4K8ac_peak_14617	5.14125	
19	4742787	4743834	KD2-H4K8ac_peak_14618	7.76104	
19	4791918	4792612	KD2-H4K8ac_peak_14619	8.28234	AC005523.3_ENSG00000268536;FEM1A_ENSG00000141965
19	4803671	4803872	KD2-H4K8ac_peak_14620	6.45018	
19	4809458	4809681	KD2-H4K8ac_peak_14621	7.08474	
19	4815955	4816707	KD2-H4K8ac_peak_14622	22.04674	
19	4830839	4831191	KD2-H4K8ac_peak_14623	8.5781	TICAM1_ENSG00000127666
19	4831510	4832226	KD2-H4K8ac_peak_14624	19.55721	TICAM1_ENSG00000127666
19	4908988	4909329	KD2-H4K8ac_peak_14625	5.51783	
19	4910119	4910557	KD2-H4K8ac_peak_14626	11.89502	

19	4968351	4968544	KD2-H4K8ac_peak_14627	5.14125	KDM4B_ENSG00000127663
19	4969163	4969469	KD2-H4K8ac_peak_14628	9.59814	KDM4B_ENSG00000127663
19	5037458	5037910	KD2-H4K8ac_peak_14629	4.54006	
19	5090798	5091067	KD2-H4K8ac_peak_14630	7.11824	
19	5092395	5092734	KD2-H4K8ac_peak_14631	4.85908	
19	5093295	5093497	KD2-H4K8ac_peak_14632	5.29555	
19	5093780	5094094	KD2-H4K8ac_peak_14633	4.82973	
19	5107333	5107730	KD2-H4K8ac_peak_14634	5.49497	
19	5292936	5293210	KD2-H4K8ac_peak_14635	14.66631	
19	5293456	5293814	KD2-H4K8ac_peak_14636	15.58442	
19	5308033	5309211	KD2-H4K8ac_peak_14637	11.14658	
19	5340323	5340733	KD2-H4K8ac_peak_14638	10.43145	PTPRS_ENSG00000105426
19	5436083	5436666	KD2-H4K8ac_peak_14639	13.57066	
19	5436925	5437276	KD2-H4K8ac_peak_14640	20.88898	
19	5480177	5480533	KD2-H4K8ac_peak_14641	6.25869	
19	5553645	5554657	KD2-H4K8ac_peak_14642	11.05407	
19	5578108	5578371	KD2-H4K8ac_peak_14643	7.76329	
19	5622410	5622770	KD2-H4K8ac_peak_14644	12.70959	SAFB_ENSG00000160633
19	5623193	5623577	KD2-H4K8ac_peak_14645	6.16883	SAFB2_ENSG00000130254;SAFB_ENSG00000160633
19	5679991	5680382	KD2-H4K8ac_peak_14646	8.3261	C19orf70_ENSG00000174917;HSD11B1L_ENSG00000167733
19	5690379	5690904	KD2-H4K8ac_peak_14647	6.40297	
19	5719638	5720144	KD2-H4K8ac_peak_14648	14.69247	LONP1_ENSG00000196365;CATSPERD_ENSG00000174898
19	5720781	5721526	KD2-H4K8ac_peak_14649	10.17341	LONP1_ENSG00000196365;CATSPERD_ENSG00000174898
19	5773038	5773841	KD2-H4K8ac_peak_14650	10.96455	
19	5790832	5791172	KD2-H4K8ac_peak_14651	16.04168	DUS3L_ENSG00000141994
19	5791476	5791768	KD2-H4K8ac_peak_14652	10.29344	DUS3L_ENSG00000141994
19	5798150	5798352	KD2-H4K8ac_peak_14653	6.25869	
19	5799519	5800139	KD2-H4K8ac_peak_14654	3.90565	
19	5801933	5802195	KD2-H4K8ac_peak_14655	5.1313	
19	5803691	5804031	KD2-H4K8ac_peak_14656	8.30974	
19	5804513	5804888	KD2-H4K8ac_peak_14657	8.66888	
19	5805745	5805937	KD2-H4K8ac_peak_14658	9.73163	
19	5827136	5827660	KD2-H4K8ac_peak_14659	6.05809	
19	5827903	5828123	KD2-H4K8ac_peak_14660	7.76104	
19	5828371	5828713	KD2-H4K8ac_peak_14661	9.07715	
19	5828952	5830013	KD2-H4K8ac_peak_14662	10.96455	
19	5893061	5894488	KD2-H4K8ac_peak_14663	9.36516	
19	5894805	5895320	KD2-H4K8ac_peak_14664	9.95342	
19	5903458	5903746	KD2-H4K8ac_peak_14665	15.76765	FUT5_ENSG00000130383;AC024592.12_ENSG00000267740;NDUFA11_ENSG00000174886
19	5927725	5927933	KD2-H4K8ac_peak_14666	7.07117	
19	5933068	5934309	KD2-H4K8ac_peak_14667	6.84261	
19	5935824	5936564	KD2-H4K8ac_peak_14668	10.43601	
19	5977781	5978065	KD2-H4K8ac_peak_14669	14.4458	RANBP3_ENSG00000031823;CTC-232P5.1_ENSG00000266983
19	5978387	5978668	KD2-H4K8ac_peak_14670	17.83665	RANBP3_ENSG00000031823;CTC-232P5.1_ENSG00000266983
19	5994274	5995460	KD2-H4K8ac_peak_14671	13.13969	
19	6200101	6200382	KD2-H4K8ac_peak_14672	6.56211	RFX2_ENSG00000087903

19	6279447	6279655	KD2-H4K8ac_peak_14673	11.05467	MLLT1_ENSG00000130382
19	6361551	6362044	KD2-H4K8ac_peak_14674	20.5798	CTB-180A7.3_ENSG00000269802;CLPP_ENSG00000125656
19	6372861	6373134	KD2-H4K8ac_peak_14675	13.44374	ALKBH7_ENSG00000125652
19	6373935	6374166	KD2-H4K8ac_peak_14676	5.18447	
19	6393616	6394298	KD2-H4K8ac_peak_14677	12.57865	GTF2F1_ENSG00000125651;CTB-180A7.8_ENSG00000214347
19	6424247	6424772	KD2-H4K8ac_peak_14678	5.58324	KHSRP_ENSG00000088247
19	6425575	6426051	KD2-H4K8ac_peak_14679	14.71663	KHSRP_ENSG00000088247
19	6459463	6459772	KD2-H4K8ac_peak_14680	11.60333	
19	6477159	6477372	KD2-H4K8ac_peak_14681	7.33266	
19	6502215	6503004	KD2-H4K8ac_peak_14682	12.74171	TUBB4A_ENSG00000104833
19	6504349	6504745	KD2-H4K8ac_peak_14683	8.23062	
19	6737366	6737575	KD2-H4K8ac_peak_14684	14.68449	GPR108_ENSG00000125734;TRIP10_ENSG00000125733
19	6738132	6739401	KD2-H4K8ac_peak_14685	9.1707	GPR108_ENSG00000125734;TRIP10_ENSG00000125733
19	7068888	7069086	KD2-H4K8ac_peak_14686	4.86324	ZNF557_ENSG00000130544
19	7069450	7069997	KD2-H4K8ac_peak_14687	12.82393	ZNF557_ENSG00000130544
19	7098980	7099207	KD2-H4K8ac_peak_14688	9.57101	
19	7099651	7099849	KD2-H4K8ac_peak_14689	5.3778	
19	7198344	7198734	KD2-H4K8ac_peak_14690	10.05606	
19	7444769	7445468	KD2-H4K8ac_peak_14691	7.40111	CTD-2207O23.3_ENSG00000268861
19	7459529	7459761	KD2-H4K8ac_peak_14692	9.47671	CTB-133G6.2_ENSG00000267852;ARHGEF18_ENSG00000104880
19	7460075	7460325	KD2-H4K8ac_peak_14693	13.35993	CTB-133G6.2_ENSG00000267852;ARHGEF18_ENSG00000104880
19	7547137	7547373	KD2-H4K8ac_peak_14694	7.18615	
19	7547568	7547863	KD2-H4K8ac_peak_14695	3.90565	
19	7548331	7548965	KD2-H4K8ac_peak_14696	8.58069	
19	7554050	7554469	KD2-H4K8ac_peak_14697	9.11031	
19	7558864	7559847	KD2-H4K8ac_peak_14698	8.85813	
19	7565620	7565973	KD2-H4K8ac_peak_14699	12.23	
19	7566203	7566598	KD2-H4K8ac_peak_14700	5.75757	
19	7571327	7571562	KD2-H4K8ac_peak_14701	7.47462	CTD-2207O23.12_ENSG00000267952
19	7571797	7572039	KD2-H4K8ac_peak_14702	5.98124	CTD-2207O23.12_ENSG00000267952
19	7573177	7573397	KD2-H4K8ac_peak_14703	6.24114	
19	7579506	7579800	KD2-H4K8ac_peak_14704	9.11031	ZNF358_ENSG00000198816
19	7580046	7580951	KD2-H4K8ac_peak_14705	18.5703	ZNF358_ENSG00000198816
19	7581202	7581669	KD2-H4K8ac_peak_14706	14.69317	
19	7598276	7598802	KD2-H4K8ac_peak_14707	8.85813	CTD-2207O23.10_ENSG00000268614;PNPLA6_ENSG00000032444
19	7600670	7601074	KD2-H4K8ac_peak_14708	14.99434	
19	7615701	7615940	KD2-H4K8ac_peak_14709	9.12849	
19	7616513	7616985	KD2-H4K8ac_peak_14710	6.60096	
19	7660318	7660678	KD2-H4K8ac_peak_14711	5.14125	CAMSAP3_ENSG00000076826
19	7661118	7661560	KD2-H4K8ac_peak_14712	9.11031	CAMSAP3_ENSG00000076826
19	7681203	7681400	KD2-H4K8ac_peak_14713	5.14125	
19	7684055	7684298	KD2-H4K8ac_peak_14714	9.20193	
19	7684546	7685145	KD2-H4K8ac_peak_14715	7.07117	
19	7693987	7694388	KD2-H4K8ac_peak_14716	8.85813	XAB2_ENSG00000076924;PET100_ENSG00000229833;CTD-3214H19.4_ENSG00000268400
19	7694706	7695133	KD2-H4K8ac_peak_14717	9.73431	XAB2_ENSG00000076924;PET100_ENSG00000229833;CTD-3214H19.4_ENSG00000268400
19	7745232	7746412	KD2-H4K8ac_peak_14718	12.88108	TRAPPC5_ENSG00000181029

19	7894036	7894377	KD2-H4K8ac_peak_14719	12.05219	EVI5L_ENSG00000142459
19	7895151	7895360	KD2-H4K8ac_peak_14720	6.8836	EVI5L_ENSG00000142459
19	7938952	7939366	KD2-H4K8ac_peak_14721	7.76104	CTD-3193O13.9_ENSG00000183248
19	7939720	7940199	KD2-H4K8ac_peak_14722	7.45501	CTD-3193O13.9_ENSG00000183248
19	7968057	7968353	KD2-H4K8ac_peak_14723	8.10216	AC010336.1_ENSG00000214248;RN7SL115P_ENSG00000264166;MAP2K7_ENSG00000076984
19	7981327	7981818	KD2-H4K8ac_peak_14724	8.55271	TGFBR3L_ENSG00000260001
19	7984569	7985033	KD2-H4K8ac_peak_14725	12.36441	CTD-3193O13.1_ENSG00000260500;SNAPC2_ENSG00000104976
19	7990423	7990809	KD2-H4K8ac_peak_14726	12.08081	CTXN1_ENSG00000178531;CTD-3193O13.8_ENSG00000269139
19	8007766	8008543	KD2-H4K8ac_peak_14727	16.11042	TIMM44_ENSG00000104980
19	8070790	8071020	KD2-H4K8ac_peak_14728	5.59859	ELAVL1_ENSG00000066044
19	8115085	8115394	KD2-H4K8ac_peak_14729	10.05606	
19	8273278	8273853	KD2-H4K8ac_peak_14730	14.8083	
19	8274168	8274898	KD2-H4K8ac_peak_14731	9.34989	
19	8305716	8305984	KD2-H4K8ac_peak_14732	3.75035	
19	8334063	8334373	KD2-H4K8ac_peak_14733	10.21502	
19	8372945	8373203	KD2-H4K8ac_peak_14734	5.38581	CD320_ENSG00000167775
19	8385544	8386208	KD2-H4K8ac_peak_14735	12.08081	NDUFA7_ENSG00000167774;NDUFA7_ENSG00000267855;RPS28_ENSG00000233927
19	8407775	8407968	KD2-H4K8ac_peak_14736	5.9696	KANK3_ENSG00000186994
19	8454475	8454735	KD2-H4K8ac_peak_14737	9.1707	RAB11B-AS1_ENSG00000269386;MIR4999_ENSG00000265390;RAB11B_ENSG00000185236
19	8455271	8455612	KD2-H4K8ac_peak_14738	28.09984	RAB11B-AS1_ENSG00000269386;RAB11B_ENSG00000185236
19	8478252	8478481	KD2-H4K8ac_peak_14739	8.30974	MARCH2_ENSG00000099785
19	8509020	8509302	KD2-H4K8ac_peak_14740	8.74596	HNRNPM_ENSG00000099783
19	8509997	8510629	KD2-H4K8ac_peak_14741	8.52559	HNRNPM_ENSG00000099783
19	8570574	8571634	KD2-H4K8ac_peak_14742	14.45402	
19	8577911	8579688	KD2-H4K8ac_peak_14743	18.31034	ZNF414_ENSG00000133250
19	8591024	8591489	KD2-H4K8ac_peak_14744	7.47462	
19	8674225	8674678	KD2-H4K8ac_peak_14745	10.7186	ADAMTS10_ENSG00000142303
19	8675444	8675801	KD2-H4K8ac_peak_14746	6.66359	ADAMTS10_ENSG00000142303
19	8942605	8942868	KD2-H4K8ac_peak_14747	8.16906	ZNF558_ENSG00000167785;MBD3L1_ENSG00000170948;CTD-2529P6.4_ENSG00000269400
19	9247769	9248021	KD2-H4K8ac_peak_14748	8.00457	
19	9251089	9251367	KD2-H4K8ac_peak_14749	15.9336	ZNF317_ENSG00000130803
19	9394112	9394386	KD2-H4K8ac_peak_14750	5.54647	
19	9420193	9420779	KD2-H4K8ac_peak_14751	6.65673	ZNF699_ENSG00000196110
19	9421320	9421517	KD2-H4K8ac_peak_14752	5.07901	ZNF699_ENSG00000196110
19	9434440	9434725	KD2-H4K8ac_peak_14753	7.76329	ZNF559_ENSG00000188321;ZNF559-ZNF177_ENSG00000270011;ZNF177_ENSG00000188629
19	9434972	9435282	KD2-H4K8ac_peak_14754	24.85162	ZNF559_ENSG00000188321;ZNF559-ZNF177_ENSG00000270011;ZNF177_ENSG00000188629
19	9517447	9517760	KD2-H4K8ac_peak_14755	5.57044	
19	9517954	9518146	KD2-H4K8ac_peak_14756	6.31143	
19	9546351	9546915	KD2-H4K8ac_peak_14757	9.15069	ZNF266_ENSG00000174652
19	9648900	9649533	KD2-H4K8ac_peak_14758	9.11031	ZNF426_ENSG00000130818
19	9694759	9695494	KD2-H4K8ac_peak_14759	10.35678	ZNF121_ENSG00000197961
19	9731676	9732107	KD2-H4K8ac_peak_14760	8.16906	ZNF561_ENSG00000171469;C19orf82_ENSG00000267106
19	9785489	9786605	KD2-H4K8ac_peak_14761	8.16906	ZNF562_ENSG00000171466
19	9878855	9879241	KD2-H4K8ac_peak_14762	9.43685	
19	9896572	9896934	KD2-H4K8ac_peak_14763	12.84642	
19	9929103	9929342	KD2-H4K8ac_peak_14764	6.99815	

19	9930397	9930845	KD2-H4K8ac_peak_14765	7.27716	SNORA70_ENSG00000200237;RPL10P15_ENSG00000267119;AC008752.1_ENSG00000216102
19	9938058	9938410	KD2-H4K8ac_peak_14766	10.19949	FBXL12_ENSG00000127452;UBL5_ENSG00000198258
19	9938656	9938943	KD2-H4K8ac_peak_14767	13.70753	FBXL12_ENSG00000127452;UBL5_ENSG00000198258
19	10024094	10024395	KD2-H4K8ac_peak_14768	5.88845	
19	10024855	10025243	KD2-H4K8ac_peak_14769	7.35686	
19	10046797	10047087	KD2-H4K8ac_peak_14770	13.73635	OLFM2_ENSG00000105088
19	10047338	10047632	KD2-H4K8ac_peak_14771	9.08816	OLFM2_ENSG00000105088
19	10056184	10057014	KD2-H4K8ac_peak_14772	11.86082	
19	10196306	10197389	KD2-H4K8ac_peak_14773	16.33377	C19orf66_ENSG00000130813
19	10207062	10207561	KD2-H4K8ac_peak_14774	25.46877	
19	10216521	10216763	KD2-H4K8ac_peak_14775	16.09057	PPAN-P2RY11_ENSG00000243207;PPAN_ENSG00000130810
19	10217076	10217719	KD2-H4K8ac_peak_14776	19.91242	PPAN-P2RY11_ENSG00000243207;PPAN_ENSG00000130810;SNORD105_ENSG00000209645
19	10229923	10230508	KD2-H4K8ac_peak_14777	15.82362	EIF3G_ENSG00000130811
19	10230733	10230954	KD2-H4K8ac_peak_14778	8.85813	EIF3G_ENSG00000130811
19	10249027	10249563	KD2-H4K8ac_peak_14779	7.76104	
19	10305394	10305613	KD2-H4K8ac_peak_14780	11.89502	
19	10305912	10307009	KD2-H4K8ac_peak_14781	13.01932	
19	10337030	10337533	KD2-H4K8ac_peak_14782	7.27716	
19	10341087	10341942	KD2-H4K8ac_peak_14783	12.14909	DNMT1_ENSG00000130816;S1PR2_ENSG00000267534;MIR4322_ENSG00000264266
19	10346951	10347418	KD2-H4K8ac_peak_14784	8.30974	
19	10361950	10362220	KD2-H4K8ac_peak_14785	8.30974	MRPL4_ENSG00000105364
19	10426246	10426519	KD2-H4K8ac_peak_14786	4.91674	FDX1L_ENSG00000267673;CTD-2369P2.10_ENSG00000167807
19	10443942	10444158	KD2-H4K8ac_peak_14787	7.98621	RAVER1_ENSG00000161847
19	10490549	10491226	KD2-H4K8ac_peak_14788	19.33018	TYK2_ENSG00000105397
19	10491545	10491823	KD2-H4K8ac_peak_14789	9.31907	TYK2_ENSG00000105397
19	10515052	10516087	KD2-H4K8ac_peak_14790	8.85813	MIR1181_ENSG00000221566
19	10527051	10527273	KD2-H4K8ac_peak_14791	4.37809	PDE4A_ENSG00000065989
19	10589827	10590258	KD2-H4K8ac_peak_14792	10.43145	
19	10612393	10614266	KD2-H4K8ac_peak_14793	19.3691	KEAP1_ENSG00000079999
19	10654076	10654410	KD2-H4K8ac_peak_14794	6.16747	ATG4D_ENSG00000130734
19	10654708	10655024	KD2-H4K8ac_peak_14795	5.75757	ATG4D_ENSG00000130734
19	10676180	10676596	KD2-H4K8ac_peak_14796	12.57143	KRI1_ENSG00000129347
19	10679974	10680343	KD2-H4K8ac_peak_14797	5.9696	CDKN2D_ENSG00000129355
19	10682423	10683060	KD2-H4K8ac_peak_14798	8.26945	
19	10713244	10713882	KD2-H4K8ac_peak_14799	7.36799	SLC44A2_ENSG00000129353
19	10736272	10736689	KD2-H4K8ac_peak_14800	10.17341	
19	10755039	10755314	KD2-H4K8ac_peak_14801	3.72574	
19	10763972	10764448	KD2-H4K8ac_peak_14802	9.95342	ILF3-AS1_ENSG00000267100;ILF3_ENSG00000129351
19	10765028	10765359	KD2-H4K8ac_peak_14803	11.24702	ILF3-AS1_ENSG00000267100;ILF3_ENSG00000129351
19	10765580	10766042	KD2-H4K8ac_peak_14804	5.9696	ILF3_ENSG00000129351
19	10811607	10811874	KD2-H4K8ac_peak_14805	9.31907	QTRT1_ENSG00000213339
19	10812187	10812531	KD2-H4K8ac_peak_14806	6.20982	QTRT1_ENSG00000213339
19	10828840	10829174	KD2-H4K8ac_peak_14807	6.65673	DNM2_ENSG00000079805;MIR638_ENSG00000207972
19	10927285	10927709	KD2-H4K8ac_peak_14808	6.82618	MIR199A1_ENSG00000207752
19	10928051	10928392	KD2-H4K8ac_peak_14809	10.05606	MIR199A1_ENSG00000207752
19	10946638	10946836	KD2-H4K8ac_peak_14810	9.61895	TMED1_ENSG00000099203;C19orf38_ENSG00000214212

19	10947128	10947384	KD2-H4K8ac_peak_14811	10.17341	TMED1_ENSG00000099203;C19orf38_ENSG00000214212
19	10974825	10975084	KD2-H4K8ac_peak_14812	7.40111	
19	10980855	10981395	KD2-H4K8ac_peak_14813	5.75757	CARM1_ENSG00000142453
19	10982426	10982794	KD2-H4K8ac_peak_14814	15.81711	CARM1_ENSG00000142453
19	10990102	10990329	KD2-H4K8ac_peak_14815	10.17486	
19	10998954	10999148	KD2-H4K8ac_peak_14816	7.47462	
19	11038918	11039312	KD2-H4K8ac_peak_14817	11.51672	YIPF2_ENSG00000130733;C19orf52_ENSG00000142444
19	11039631	11040017	KD2-H4K8ac_peak_14818	5.01002	YIPF2_ENSG00000130733;C19orf52_ENSG00000142444
19	11047598	11047856	KD2-H4K8ac_peak_14819	8.1718	
19	11070619	11070849	KD2-H4K8ac_peak_14820	6.34383	SMARCA4_ENSG00000127616
19	11071083	11071336	KD2-H4K8ac_peak_14821	14.15075	SMARCA4_ENSG00000127616
19	11071768	11072408	KD2-H4K8ac_peak_14822	13.81781	SMARCA4_ENSG00000127616
19	11196123	11196585	KD2-H4K8ac_peak_14823	5.51783	
19	11247887	11248154	KD2-H4K8ac_peak_14824	5.54647	
19	11249446	11249711	KD2-H4K8ac_peak_14825	9.94337	
19	11249917	11250183	KD2-H4K8ac_peak_14826	6.25869	
19	11252759	11253040	KD2-H4K8ac_peak_14827	4.06758	
19	11253446	11253927	KD2-H4K8ac_peak_14828	13.95627	
19	11266068	11266425	KD2-H4K8ac_peak_14829	17.70889	SPC24_ENSG00000161888
19	11305589	11305957	KD2-H4K8ac_peak_14830	6.60096	
19	11307987	11308518	KD2-H4K8ac_peak_14831	9.36516	KANK2_ENSG00000197256
19	11373288	11373548	KD2-H4K8ac_peak_14832	6.25869	DOCK6_ENSG00000130158
19	11403875	11404220	KD2-H4K8ac_peak_14833	6.1846	
19	11434698	11435001	KD2-H4K8ac_peak_14834	8.30974	CTC-510F12.4_ENSG00000267174
19	11435408	11435940	KD2-H4K8ac_peak_14835	7.27716	CTC-510F12.4_ENSG00000267174
19	11456047	11456359	KD2-H4K8ac_peak_14836	7.51935	RAB3D_ENSG00000105514;TMEM205_ENSG00000105518;CCDC159_ENSG00000183401
19	11457383	11457886	KD2-H4K8ac_peak_14837	9.72244	RAB3D_ENSG00000105514;TMEM205_ENSG00000105518
19	11465524	11465910	KD2-H4K8ac_peak_14838	12.06346	DKFZP761J1410_ENSG00000105520
19	11484593	11484900	KD2-H4K8ac_peak_14839	8.16133	CTD-2342J14.6_ENSG00000267277;SWSAP1_ENSG00000173928
19	11493834	11494448	KD2-H4K8ac_peak_14840	6.40297	EPOR_ENSG00000187266
19	11494676	11494943	KD2-H4K8ac_peak_14841	11.86082	EPOR_ENSG00000187266
19	11529074	11529545	KD2-H4K8ac_peak_14842	7.36799	
19	11530168	11530492	KD2-H4K8ac_peak_14843	6.13909	
19	11545689	11545888	KD2-H4K8ac_peak_14844	5.14125	CCDC151_ENSG00000198003;PRKCSH_ENSG00000130175
19	11546502	11546965	KD2-H4K8ac_peak_14845	4.06758	CCDC151_ENSG00000198003;PRKCSH_ENSG00000130175
19	11562464	11562768	KD2-H4K8ac_peak_14846	4.86324	
19	11589869	11590271	KD2-H4K8ac_peak_14847	10.61986	
19	11590775	11591045	KD2-H4K8ac_peak_14848	10.53377	ELAVL3_ENSG00000196361
19	11591508	11591737	KD2-H4K8ac_peak_14849	6.83936	ELAVL3_ENSG00000196361
19	11593162	11593476	KD2-H4K8ac_peak_14850	11.27376	
19	11616209	11616601	KD2-H4K8ac_peak_14851	16.3889	CTC-398G3.6_ENSG00000267477;ZNF653_ENSG00000161914
19	11616851	11617203	KD2-H4K8ac_peak_14852	12.82393	CTC-398G3.6_ENSG00000267477;ZNF653_ENSG00000161914
19	11640215	11640544	KD2-H4K8ac_peak_14853	8.66368	ECSIT_ENSG00000130159
19	11646338	11646940	KD2-H4K8ac_peak_14854	8.30974	
19	11651305	11651864	KD2-H4K8ac_peak_14855	5.23853	
19	11670360	11670590	KD2-H4K8ac_peak_14856	7.44366	ELOF1_ENSG00000130165;ZNF627_ENSG00000198551

19	11698718	11699022	KD2-H4K8ac_peak_14857	4.42765	
19	11708312	11708843	KD2-H4K8ac_peak_14858	5.19622	
19	11805412	11805619	KD2-H4K8ac_peak_14859	4.03256	
19	11850036	11850279	KD2-H4K8ac_peak_14860	7.28827	ZNF823_ENSG00000197933
19	11877296	11877609	KD2-H4K8ac_peak_14861	8.26945	ZNF441_ENSG00000197044
19	11908881	11909133	KD2-H4K8ac_peak_14862	10.87093	CTC-499B15.7_ENSG00000267646;ZNF491_ENSG00000177599
19	11909469	11909698	KD2-H4K8ac_peak_14863	6.84261	CTC-499B15.7_ENSG00000267646;ZNF491_ENSG00000177599
19	11925170	11925767	KD2-H4K8ac_peak_14864	12.84642	ZNF440_ENSG00000171295
19	11959034	11959349	KD2-H4K8ac_peak_14865	8.93112	ZNF439_ENSG00000171291
19	11959676	11960098	KD2-H4K8ac_peak_14866	9.11031	ZNF439_ENSG00000171291
19	12034918	12035170	KD2-H4K8ac_peak_14867	4.66652	ZNF700_ENSG00000196757;ZNF763_ENSG00000197054;ZNF763_ENSG00000267179
19	12035966	12036419	KD2-H4K8ac_peak_14868	12.84642	ZNF700_ENSG00000196757;ZNF763_ENSG00000197054;ZNF763_ENSG00000267179
19	12074463	12074674	KD2-H4K8ac_peak_14869	5.45396	
19	12075960	12076319	KD2-H4K8ac_peak_14870	8.55271	
19	12250755	12251080	KD2-H4K8ac_peak_14871	4.06758	ZNF20_ENSG00000132010
19	12267051	12267401	KD2-H4K8ac_peak_14872	7.49599	ZNF625-ZNF20_ENSG00000213297;ZNF625_ENSG00000257591
19	12267780	12268069	KD2-H4K8ac_peak_14873	6.36375	ZNF625-ZNF20_ENSG00000213297;ZNF625_ENSG00000257591
19	12273946	12274550	KD2-H4K8ac_peak_14874	24.00936	ZNF136_ENSG00000196646
19	12406183	12406617	KD2-H4K8ac_peak_14875	8.33649	ZNF44_ENSG00000197857
19	12444675	12445109	KD2-H4K8ac_peak_14876	17.76996	ZNF563_ENSG00000188868
19	12551560	12551781	KD2-H4K8ac_peak_14877	8.65175	CTD-3105H18.16_ENSG00000268870;ZNF443_ENSG00000180855
19	12552181	12552435	KD2-H4K8ac_peak_14878	6.0073	CTD-3105H18.16_ENSG00000268870;ZNF443_ENSG00000180855
19	12606309	12606826	KD2-H4K8ac_peak_14879	22.07309	
19	12661773	12662262	KD2-H4K8ac_peak_14880	13.95627	ZNF709_ENSG00000196826;ZNF564_ENSG00000249709
19	12721258	12721459	KD2-H4K8ac_peak_14881	8.17902	ZNF791_ENSG00000173875
19	12721808	12722302	KD2-H4K8ac_peak_14882	25.23999	ZNF791_ENSG00000173875
19	12750621	12750917	KD2-H4K8ac_peak_14883	4.77079	ZNF490_ENSG00000188033
19	12751156	12751371	KD2-H4K8ac_peak_14884	9.08816	ZNF490_ENSG00000188033
19	12779930	12780182	KD2-H4K8ac_peak_14885	8.85813	CTD-2192J16.24_ENSG00000269590
19	12792439	12792644	KD2-H4K8ac_peak_14886	5.59859	DHPS_ENSG00000095059
19	12792855	12793125	KD2-H4K8ac_peak_14887	15.52826	DHPS_ENSG00000095059
19	12832112	12832422	KD2-H4K8ac_peak_14888	6.36375	
19	12833044	12833353	KD2-H4K8ac_peak_14889	16.95032	
19	12834973	12835282	KD2-H4K8ac_peak_14890	15.13261	TNPO2_ENSG00000105576
19	12845229	12845454	KD2-H4K8ac_peak_14891	16.25886	C19orf43_ENSG00000123144
19	12845786	12846226	KD2-H4K8ac_peak_14892	10.43145	C19orf43_ENSG00000123144
19	12847416	12847615	KD2-H4K8ac_peak_14893	7.31433	ASNA1_ENSG00000198356
19	12848362	12848683	KD2-H4K8ac_peak_14894	9.57101	ASNA1_ENSG00000198356
19	12867919	12868465	KD2-H4K8ac_peak_14895	10.19949	
19	12868676	12869007	KD2-H4K8ac_peak_14896	12.93209	
19	12886023	12886313	KD2-H4K8ac_peak_14897	9.96798	
19	12888550	12890493	KD2-H4K8ac_peak_14898	14.8083	
19	12893108	12893426	KD2-H4K8ac_peak_14899	5.75757	
19	12893830	12894082	KD2-H4K8ac_peak_14900	16.49944	
19	12894439	12895281	KD2-H4K8ac_peak_14901	8.55271	CTD-2659N19.9_ENSG00000267212
19	12896484	12896731	KD2-H4K8ac_peak_14902	5.53033	CTD-2659N19.9_ENSG00000267212

19	12897167	12897602	KD2-H4K8ac_peak_14903	12.67903	MIR5684_ENSG00000263800
19	12898041	12898688	KD2-H4K8ac_peak_14904	4.8485	MIR5684_ENSG00000263800
19	12899284	12901216	KD2-H4K8ac_peak_14905	17.43561	
19	12912231	12913432	KD2-H4K8ac_peak_14906	10.43145	PRDX2_ENSG00000167815
19	12916819	12917199	KD2-H4K8ac_peak_14907	11.86082	RNASEH2A_ENSG00000104889
19	12917488	12917963	KD2-H4K8ac_peak_14908	5.54647	RNASEH2A_ENSG00000104889
19	12944872	12945294	KD2-H4K8ac_peak_14909	5.9696	RTBDN_ENSG00000132026;MAST1_ENSG00000105613
19	12946140	12946460	KD2-H4K8ac_peak_14910	5.73016	RTBDN_ENSG00000132026
19	12949358	12949576	KD2-H4K8ac_peak_14911	7.47462	
19	12958136	12958407	KD2-H4K8ac_peak_14912	5.18447	
19	12991520	12992086	KD2-H4K8ac_peak_14913	6.24114	DNASE2_ENSG00000105612;CTD-2265O21.7_ENSG00000267735
19	12995811	12996355	KD2-H4K8ac_peak_14914	10.0692	
19	13029624	13030028	KD2-H4K8ac_peak_14915	7.79898	SYCE2_ENSG00000161860
19	13044208	13044482	KD2-H4K8ac_peak_14916	9.95119	FARSA_ENSG00000179115
19	13044705	13044980	KD2-H4K8ac_peak_14917	12.21163	FARSA_ENSG00000179115
19	13048438	13048988	KD2-H4K8ac_peak_14918	9.63487	CALR_ENSG00000179218
19	13056798	13057162	KD2-H4K8ac_peak_14919	8.86099	RAD23A_ENSG00000179262
19	13064293	13064589	KD2-H4K8ac_peak_14920	8.52921	
19	13064858	13065148	KD2-H4K8ac_peak_14921	9.1707	
19	13067569	13068635	KD2-H4K8ac_peak_14922	15.81711	GADD45GIP1_ENSG00000179271
19	13076038	13076623	KD2-H4K8ac_peak_14923	14.4458	DAND5_ENSG00000179284
19	13079008	13079246	KD2-H4K8ac_peak_14924	6.56211	
19	13080364	13080946	KD2-H4K8ac_peak_14925	10.09333	
19	13085304	13086257	KD2-H4K8ac_peak_14926	17.09929	
19	13106675	13106956	KD2-H4K8ac_peak_14927	4.97693	NFIX_ENSG00000008441
19	13129239	13129624	KD2-H4K8ac_peak_14928	12.0857	
19	13132984	13133521	KD2-H4K8ac_peak_14929	11.05467	
19	13164404	13165773	KD2-H4K8ac_peak_14930	12.23	
19	13166117	13166882	KD2-H4K8ac_peak_14931	19.87796	
19	13167080	13167337	KD2-H4K8ac_peak_14932	11.11797	
19	13168164	13168367	KD2-H4K8ac_peak_14933	6.66692	
19	13170781	13171715	KD2-H4K8ac_peak_14934	8.4716	
19	13172055	13172250	KD2-H4K8ac_peak_14935	3.75035	
19	13174777	13175215	KD2-H4K8ac_peak_14936	4.86324	
19	13180352	13180556	KD2-H4K8ac_peak_14937	7.12213	
19	13189105	13189720	KD2-H4K8ac_peak_14938	9.5326	
19	13190374	13190926	KD2-H4K8ac_peak_14939	10.30284	
19	13200168	13200781	KD2-H4K8ac_peak_14940	10.0997	
19	13201239	13202117	KD2-H4K8ac_peak_14941	13.81781	
19	13202417	13202848	KD2-H4K8ac_peak_14942	10.30284	
19	13203110	13204042	KD2-H4K8ac_peak_14943	20.2003	
19	13204305	13204704	KD2-H4K8ac_peak_14944	6.20982	
19	13204924	13206567	KD2-H4K8ac_peak_14945	15.38498	
19	13206763	13206990	KD2-H4K8ac_peak_14946	6.25869	
19	13212994	13214490	KD2-H4K8ac_peak_14947	15.59774	LYL1_ENSG00000104903
19	13215176	13215441	KD2-H4K8ac_peak_14948	6.20982	

19	13227147	13227532	KD2-H4K8ac_peak_14949	12.93209	TRMT1_ENSG00000104907
19	13229240	13229511	KD2-H4K8ac_peak_14950	5.88845	TRMT1_ENSG00000104907;NACC1_ENSG00000160877
19	13261380	13261721	KD2-H4K8ac_peak_14951	15.20126	STX10_ENSG00000104915;IER2_ENSG00000160888
19	13262914	13263159	KD2-H4K8ac_peak_14952	7.30812	
19	13273538	13273754	KD2-H4K8ac_peak_14953	10.19949	
19	13274116	13274812	KD2-H4K8ac_peak_14954	15.58442	
19	13275104	13275892	KD2-H4K8ac_peak_14955	9.63487	
19	13278299	13278914	KD2-H4K8ac_peak_14956	10.21366	
19	13283018	13283556	KD2-H4K8ac_peak_14957	13.44374	
19	13292582	13293269	KD2-H4K8ac_peak_14958	10.25088	
19	13842652	13842930	KD2-H4K8ac_peak_14959	11.05467	CCDC130_ENSG00000104957
19	13858164	13858444	KD2-H4K8ac_peak_14960	7.146	
19	13884595	13884814	KD2-H4K8ac_peak_14961	5.28072	CTB-5E10.3_ENSG00000267633;C19orf53_ENSG00000104979
19	13885333	13885675	KD2-H4K8ac_peak_14962	18.54875	CTB-5E10.3_ENSG00000267633;C19orf53_ENSG00000104979
19	13906258	13906751	KD2-H4K8ac_peak_14963	19.55721	ZSWIM4_ENSG00000132003;CTD-3252C9.2_ENSG00000267582
19	13949560	13950476	KD2-H4K8ac_peak_14964	15.95195	
19	13951141	13951567	KD2-H4K8ac_peak_14965	10.46874	
19	13953353	13953617	KD2-H4K8ac_peak_14966	8.55581	
19	13953876	13954357	KD2-H4K8ac_peak_14967	18.62675	
19	13954625	13955167	KD2-H4K8ac_peak_14968	5.30799	
19	13957443	13958939	KD2-H4K8ac_peak_14969	13.70753	
19	13961030	13961285	KD2-H4K8ac_peak_14970	10.19949	
19	13961990	13962465	KD2-H4K8ac_peak_14971	7.26089	
19	13972187	13972932	KD2-H4K8ac_peak_14972	26.59781	NANOS3_ENSG00000187556
19	13975609	13976028	KD2-H4K8ac_peak_14973	17.06312	
19	13976390	13976931	KD2-H4K8ac_peak_14974	11.05467	
19	13991220	13991755	KD2-H4K8ac_peak_14975	11.79585	
19	14016298	14016855	KD2-H4K8ac_peak_14976	12.57143	C19orf57_ENSG00000132016;CC2D1A_ENSG00000132024
19	14017075	14017583	KD2-H4K8ac_peak_14977	13.50024	C19orf57_ENSG00000132016;CC2D1A_ENSG00000132024
19	14048526	14049334	KD2-H4K8ac_peak_14978	21.00776	
19	14063312	14063516	KD2-H4K8ac_peak_14979	15.58456	PODNL1_ENSG00000132000;DCAF15_ENSG00000132017
19	14063731	14064046	KD2-H4K8ac_peak_14980	12.22449	PODNL1_ENSG00000132000;DCAF15_ENSG00000132017
19	14096794	14098083	KD2-H4K8ac_peak_14981	16.71655	
19	14104257	14104496	KD2-H4K8ac_peak_14982	9.1707	
19	14116810	14117021	KD2-H4K8ac_peak_14983	7.47462	RFX1_ENSG00000132005
19	14117587	14118553	KD2-H4K8ac_peak_14984	17.27647	RFX1_ENSG00000132005
19	14142572	14143012	KD2-H4K8ac_peak_14985	9.95119	CTB-55O6.4_ENSG00000267670;IL27RA_ENSG00000104998
19	14168164	14168378	KD2-H4K8ac_peak_14986	10.05606	
19	14182488	14183016	KD2-H4K8ac_peak_14987	12.82393	hsa-mir-1199_ENSG00000141854
19	14191808	14192026	KD2-H4K8ac_peak_14988	6.0605	
19	14202023	14202369	KD2-H4K8ac_peak_14989	8.30974	SAMD1_ENSG00000141858
19	14229022	14229614	KD2-H4K8ac_peak_14990	10.04868	PRKACA_ENSG00000072062;CTB-55O6.10_ENSG00000267783
19	14248064	14248412	KD2-H4K8ac_peak_14991	8.2413	ASF1B_ENSG00000105011;CTB-55O6.12_ENSG00000267169
19	14261203	14261537	KD2-H4K8ac_peak_14992	5.57044	
19	14317802	14318831	KD2-H4K8ac_peak_14993	19.8737	LPHN1_ENSG00000072071
19	14320041	14320894	KD2-H4K8ac_peak_14994	18.94636	

19	14358664	14359257	KD2-H4K8ac_peak_14995	13.73635	
19	14359562	14360393	KD2-H4K8ac_peak_14996	37.97899	
19	14404007	14404351	KD2-H4K8ac_peak_14997	6.56926	CTD-2189E23.1_ENSG00000267723
19	14477444	14477763	KD2-H4K8ac_peak_14998	4.01115	
19	14490050	14490348	KD2-H4K8ac_peak_14999	5.98764	CD97_ENSG00000123146
19	14492240	14492869	KD2-H4K8ac_peak_15000	10.77651	CD97_ENSG00000123146
19	14494950	14495216	KD2-H4K8ac_peak_15001	6.40683	
19	14529699	14530129	KD2-H4K8ac_peak_15002	5.92591	DDX39A_ENSG00000123136
19	14530475	14531075	KD2-H4K8ac_peak_15003	12.84642	DDX39A_ENSG00000123136
19	14543789	14544654	KD2-H4K8ac_peak_15004	16.8435	PKN1_ENSG00000123143
19	14545206	14545604	KD2-H4K8ac_peak_15005	7.70067	
19	14583437	14583749	KD2-H4K8ac_peak_15006	5.30799	
19	14585846	14586116	KD2-H4K8ac_peak_15007	11.90231	PTGER1_ENSG00000160951
19	14607338	14608084	KD2-H4K8ac_peak_15008	11.31076	GIPC1_ENSG00000123159
19	14628833	14629164	KD2-H4K8ac_peak_15009	11.24702	TECR_ENSG00000099797
19	14629607	14629877	KD2-H4K8ac_peak_15010	9.36516	
19	14682438	14682849	KD2-H4K8ac_peak_15011	13.80019	NDUFB7_ENSG00000099795
19	14800662	14800884	KD2-H4K8ac_peak_15012	9.1707	EMR3_ENSG00000131355;ZNF333_ENSG00000160961
19	15217572	15218427	KD2-H4K8ac_peak_15013	8.1233	SYDE1_ENSG00000105137
19	15218624	15219082	KD2-H4K8ac_peak_15014	9.11031	SYDE1_ENSG00000105137
19	15235696	15236461	KD2-H4K8ac_peak_15015	8.10216	AC003956.1_ENSG00000268564;ILVBL_ENSG00000105135
19	15237072	15237278	KD2-H4K8ac_peak_15016	7.22936	ILVBL_ENSG00000105135
19	15306487	15306681	KD2-H4K8ac_peak_15017	9.94337	
19	15310628	15312145	KD2-H4K8ac_peak_15018	7.76329	NOTCH3_ENSG00000074181
19	15333566	15334040	KD2-H4K8ac_peak_15019	10.05606	AC004257.1_ENSG00000269635
19	15334670	15334976	KD2-H4K8ac_peak_15020	10.72313	
19	15442827	15443217	KD2-H4K8ac_peak_15021	11.89502	BRD4_ENSG00000141867
19	15443468	15443766	KD2-H4K8ac_peak_15022	10.29842	BRD4_ENSG00000141867
19	15490117	15490395	KD2-H4K8ac_peak_15023	9.97578	AKAP8_ENSG00000105127;AC005785.2_ENSG00000268189
19	15490930	15491546	KD2-H4K8ac_peak_15024	11.89502	AKAP8_ENSG00000105127
19	15529403	15529766	KD2-H4K8ac_peak_15025	7.76329	AKAP8L_ENSG00000011243
19	15543094	15543290	KD2-H4K8ac_peak_15026	7.76329	
19	15544234	15544648	KD2-H4K8ac_peak_15027	6.83936	
19	15559962	15560707	KD2-H4K8ac_peak_15028	14.69247	WIZ_ENSG00000011451;MIR1470_ENSG00000269782
19	15562571	15562903	KD2-H4K8ac_peak_15029	5.88845	
19	15947478	15947811	KD2-H4K8ac_peak_15030	5.9696	AC004510.3_ENSG00000267308
19	16186925	16187919	KD2-H4K8ac_peak_15031	9.90914	
19	16188116	16189382	KD2-H4K8ac_peak_15032	8.79106	
19	16221386	16222076	KD2-H4K8ac_peak_15033	20.31556	RAB8A_ENSG00000167461
19	16222864	16223182	KD2-H4K8ac_peak_15034	12.17278	RAB8A_ENSG00000167461
19	16295586	16295917	KD2-H4K8ac_peak_15035	16.07006	FAM32A_ENSG00000105058
19	16296254	16296671	KD2-H4K8ac_peak_15036	9.36516	FAM32A_ENSG00000105058
19	16308771	16309308	KD2-H4K8ac_peak_15037	13.80019	AP1M1_ENSG00000072958
19	16567245	16567951	KD2-H4K8ac_peak_15038	6.40683	
19	16568322	16569130	KD2-H4K8ac_peak_15039	16.8435	
19	16583136	16583447	KD2-H4K8ac_peak_15040	6.76298	EPS15L1_ENSG00000127527

19	16606624	16606899	KD2-H4K8ac_peak_15041	16.3889	CALR3_ENSG00000269058;C19orf44_ENSG00000105072
19	16652860	16653242	KD2-H4K8ac_peak_15042	13.70605	CHERP_ENSG00000085872
19	16653474	16653700	KD2-H4K8ac_peak_15043	8.86099	CHERP_ENSG00000085872
19	16682776	16683174	KD2-H4K8ac_peak_15044	9.96798	SLC35E1_ENSG00000127526
19	16699374	16699661	KD2-H4K8ac_peak_15045	6.99815	CTD-3222D19.5_ENSG00000269578
19	16738647	16738965	KD2-H4K8ac_peak_15046	7.27716	CTD-3222D19.2_ENSG00000141979;MED26_ENSG00000105085
19	16739405	16740128	KD2-H4K8ac_peak_15047	15.06766	CTD-3222D19.2_ENSG00000141979;MED26_ENSG00000105085
19	16771288	16771838	KD2-H4K8ac_peak_15048	8.58725	CTC-429P9.4_ENSG00000268790;SMIM7_ENSG00000214046;TMEM38A_ENSG00000072954
19	16809490	16809792	KD2-H4K8ac_peak_15049	8.70735	
19	16813266	16813491	KD2-H4K8ac_peak_15050	7.36799	
19	16935937	16936277	KD2-H4K8ac_peak_15051	10.29344	
19	16939742	16940916	KD2-H4K8ac_peak_15052	10.0692	SIN3B_ENSG00000127511
19	17007107	17007394	KD2-H4K8ac_peak_15053	6.66692	
19	17185963	17186223	KD2-H4K8ac_peak_15054	11.34353	HAUS8_ENSG00000131351;MYO9B_ENSG00000099331
19	17186691	17186934	KD2-H4K8ac_peak_15055	6.34383	HAUS8_ENSG00000131351;MYO9B_ENSG00000099331
19	17236635	17237142	KD2-H4K8ac_peak_15056	5.4779	
19	17246926	17247927	KD2-H4K8ac_peak_15057	10.05606	
19	17325289	17326452	KD2-H4K8ac_peak_15058	23.17589	USE1_ENSG00000053501
19	17336547	17336844	KD2-H4K8ac_peak_15059	4.211	OCEL1_ENSG00000099330
19	17337467	17337737	KD2-H4K8ac_peak_15060	7.40314	OCEL1_ENSG00000099330
19	17355736	17356660	KD2-H4K8ac_peak_15061	8.28234	NR2F6_ENSG00000160113
19	17357039	17357908	KD2-H4K8ac_peak_15062	13.31016	NR2F6_ENSG00000160113
19	17392247	17392626	KD2-H4K8ac_peak_15063	9.48511	USHBP1_ENSG00000130307;ANKLE1_ENSG00000160117
19	17413925	17414142	KD2-H4K8ac_peak_15064	5.75757	
19	17414472	17414665	KD2-H4K8ac_peak_15065	10.69383	
19	17415609	17415948	KD2-H4K8ac_peak_15066	6.62541	
19	17416663	17417194	KD2-H4K8ac_peak_15067	15.61313	
19	17445109	17445545	KD2-H4K8ac_peak_15068	18.33962	ANO8_ENSG00000074855;GTPBP3_ENSG00000130299
19	17457173	17457582	KD2-H4K8ac_peak_15069	5.88845	
19	17459234	17459898	KD2-H4K8ac_peak_15070	7.38933	
19	17506233	17506475	KD2-H4K8ac_peak_15071	7.79898	
19	17514542	17514921	KD2-H4K8ac_peak_15072	6.94954	
19	17530000	17530247	KD2-H4K8ac_peak_15073	5.9696	CTD-2521M24.4_ENSG00000254503;CTD-2521M24.8_ENSG00000269053
19	17530941	17531564	KD2-H4K8ac_peak_15074	11.92978	CTD-2521M24.8_ENSG00000269053
19	17558863	17559698	KD2-H4K8ac_peak_15075	5.30799	TMEM221_ENSG00000188051
19	17580318	17580522	KD2-H4K8ac_peak_15076	6.66692	SLC27A1_ENSG00000130304
19	17621864	17622125	KD2-H4K8ac_peak_15077	9.63925	CTD-3131K8.2_ENSG00000269439
19	17622452	17622987	KD2-H4K8ac_peak_15078	20.18381	CTD-3131K8.2_ENSG00000269439
19	17666552	17667187	KD2-H4K8ac_peak_15079	11.05407	COLGALT1_ENSG00000130309
19	17716192	17716636	KD2-H4K8ac_peak_15080	11.09936	
19	17716931	17717174	KD2-H4K8ac_peak_15081	21.2844	
19	17798753	17798968	KD2-H4K8ac_peak_15082	8.52559	UNC13A_ENSG00000130477
19	17799299	17799524	KD2-H4K8ac_peak_15083	6.00226	UNC13A_ENSG00000130477
19	17829967	17830594	KD2-H4K8ac_peak_15084	8.86099	MAP1S_ENSG00000130479
19	17858618	17859092	KD2-H4K8ac_peak_15085	8.94469	FCHO1_ENSG00000130475
19	17905628	17906065	KD2-H4K8ac_peak_15086	5.00278	B3GNT3_ENSG00000179913

19	17940761	17940977	KD2-H4K8ac_peak_15087	5.30799	
19	17941187	17941466	KD2-H4K8ac_peak_15088	5.57044	
19	17942033	17942385	KD2-H4K8ac_peak_15089	7.47462	
19	17952149	17952585	KD2-H4K8ac_peak_15090	6.24114	
19	17953217	17953690	KD2-H4K8ac_peak_15091	9.08816	
19	17958325	17958999	KD2-H4K8ac_peak_15092	11.68645	JAK3_ENSG00000105639
19	17969953	17970309	KD2-H4K8ac_peak_15093	7.1181	RPL18A_ENSG00000105640
19	17970762	17971361	KD2-H4K8ac_peak_15094	12.93209	RPL18A_ENSG00000105640
19	17985335	17985557	KD2-H4K8ac_peak_15095	5.88845	
19	18042873	18044260	KD2-H4K8ac_peak_15096	18.5703	CCDC124_ENSG00000007080
19	18061784	18062278	KD2-H4K8ac_peak_15097	6.85193	KCNN1_ENSG00000105642
19	18062671	18063714	KD2-H4K8ac_peak_15098	14.93224	KCNN1_ENSG00000105642
19	18111871	18112667	KD2-H4K8ac_peak_15099	8.30974	ARRDC2_ENSG00000105643
19	18119077	18119409	KD2-H4K8ac_peak_15100	11.2938	
19	18130998	18131615	KD2-H4K8ac_peak_15101	9.15069	
19	18131829	18132121	KD2-H4K8ac_peak_15102	5.64449	
19	18132441	18132689	KD2-H4K8ac_peak_15103	11.14658	CTB-52I2.4_ENSG00000268032
19	18133268	18133807	KD2-H4K8ac_peak_15104	5.01002	CTB-52I2.4_ENSG00000268032
19	18220586	18221044	KD2-H4K8ac_peak_15105	7.69287	
19	18228836	18229036	KD2-H4K8ac_peak_15106	6.90231	
19	18262651	18263148	KD2-H4K8ac_peak_15107	11.89502	AC007192.6_ENSG00000269145;PIK3R2_ENSG00000105647;PIK3R2_ENSG00000268173
19	18264058	18264364	KD2-H4K8ac_peak_15108	4.44527	PIK3R2_ENSG00000105647;PIK3R2_ENSG00000268173
19	18267460	18267812	KD2-H4K8ac_peak_15109	9.07715	
19	18268249	18268492	KD2-H4K8ac_peak_15110	11.50346	
19	18271853	18272239	KD2-H4K8ac_peak_15111	11.92978	
19	18284225	18284471	KD2-H4K8ac_peak_15112	11.09936	IFI30_ENSG00000216490
19	18315626	18316317	KD2-H4K8ac_peak_15113	12.08557	RAB3A_ENSG00000105649;AC068499.10_ENSG00000268650
19	18334747	18335293	KD2-H4K8ac_peak_15114	18.95762	
19	18335626	18336346	KD2-H4K8ac_peak_15115	11.92978	
19	18336631	18337186	KD2-H4K8ac_peak_15116	7.69294	
19	18343894	18344105	KD2-H4K8ac_peak_15117	4.54006	
19	18391907	18392188	KD2-H4K8ac_peak_15118	4.84794	JUND_ENSG00000130522;MIR3188_ENSG00000267959
19	18392818	18393124	KD2-H4K8ac_peak_15119	9.36665	JUND_ENSG00000130522;MIR3188_ENSG00000267959
19	18393350	18393845	KD2-H4K8ac_peak_15120	4.44527	JUND_ENSG00000130522;MIR3188_ENSG00000267959
19	18402200	18403606	KD2-H4K8ac_peak_15121	13.87818	
19	18404119	18404717	KD2-H4K8ac_peak_15122	15.81711	
19	18414151	18414450	KD2-H4K8ac_peak_15123	6.67474	
19	18414938	18415162	KD2-H4K8ac_peak_15124	5.54647	
19	18415402	18416472	KD2-H4K8ac_peak_15125	17.46467	
19	18433294	18433842	KD2-H4K8ac_peak_15126	15.01306	LSM4_ENSG00000130520
19	18434299	18434533	KD2-H4K8ac_peak_15127	6.83936	LSM4_ENSG00000130520
19	18439961	18440217	KD2-H4K8ac_peak_15128	7.94441	
19	18450681	18450906	KD2-H4K8ac_peak_15129	6.40297	PGPEP1_ENSG00000130517
19	18474406	18474641	KD2-H4K8ac_peak_15130	6.95649	
19	18475019	18475592	KD2-H4K8ac_peak_15131	17.8868	
19	18476174	18477061	KD2-H4K8ac_peak_15132	10.87093	

19	18479876	18480764	KD2-H4K8ac_peak_15133	11.05467	
19	18484224	18484825	KD2-H4K8ac_peak_15134	8.79106	GDF15_ENSG00000130513
19	18485024	18485371	KD2-H4K8ac_peak_15135	4.79789	GDF15_ENSG00000130513
19	18496863	18497059	KD2-H4K8ac_peak_15136	5.9696	MIR3189_ENSG00000264175
19	18499145	18499594	KD2-H4K8ac_peak_15137	5.72327	
19	18526479	18526730	KD2-H4K8ac_peak_15138	4.211	
19	18526983	18527716	KD2-H4K8ac_peak_15139	6.0605	
19	18527982	18528549	KD2-H4K8ac_peak_15140	17.10757	
19	18528761	18529212	KD2-H4K8ac_peak_15141	11.92978	SSBP4_ENSG00000130511
19	18529503	18530022	KD2-H4K8ac_peak_15142	7.49599	SSBP4_ENSG00000130511
19	18530259	18530767	KD2-H4K8ac_peak_15143	7.73264	SSBP4_ENSG00000130511
19	18539690	18540068	KD2-H4K8ac_peak_15144	5.54647	
19	18542795	18543010	KD2-H4K8ac_peak_15145	7.40111	
19	18543572	18544379	KD2-H4K8ac_peak_15146	8.85813	
19	18544595	18545197	KD2-H4K8ac_peak_15147	8.26945	
19	18547494	18547722	KD2-H4K8ac_peak_15148	4.49003	
19	18548492	18549747	KD2-H4K8ac_peak_15149	11.03331	ISYNA1_ENSG00000105655
19	18654631	18655254	KD2-H4K8ac_peak_15150	17.86168	FKBP8_ENSG00000105701
19	18668710	18668983	KD2-H4K8ac_peak_15151	17.75427	KXD1_ENSG00000105700
19	18699579	18699923	KD2-H4K8ac_peak_15152	13.73635	C19orf60_ENSG00000006015
19	18705371	18705987	KD2-H4K8ac_peak_15153	6.65673	
19	18723263	18724124	KD2-H4K8ac_peak_15154	9.95119	
19	18748131	18748329	KD2-H4K8ac_peak_15155	5.51783	KLHL26_ENSG00000167487
19	18788135	18788345	KD2-H4K8ac_peak_15156	5.91443	
19	18793994	18794258	KD2-H4K8ac_peak_15157	11.43863	CRTC1_ENSG00000105662
19	18854490	18854884	KD2-H4K8ac_peak_15158	6.24114	
19	18868115	18868453	KD2-H4K8ac_peak_15159	7.47462	
19	18872977	18873324	KD2-H4K8ac_peak_15160	6.65673	
19	18874566	18875486	KD2-H4K8ac_peak_15161	6.66692	
19	18906459	18906752	KD2-H4K8ac_peak_15162	5.54647	
19	18942145	18942527	KD2-H4K8ac_peak_15163	9.96798	UPF1_ENSG00000005007
19	18942828	18943106	KD2-H4K8ac_peak_15164	11.92978	UPF1_ENSG00000005007
19	18959775	18959995	KD2-H4K8ac_peak_15165	7.20567	
19	19029826	19030151	KD2-H4K8ac_peak_15166	11.22681	COPE_ENSG00000105669;DDX49_ENSG00000105671
19	19050589	19051432	KD2-H4K8ac_peak_15167	5.405	HOMER3_ENSG00000051128;AC005932.1_ENSG00000269019
19	19143907	19144831	KD2-H4K8ac_peak_15168	12.84642	SUGP2_ENSG00000064607;ARMC6_ENSG00000105676
19	19248821	19249225	KD2-H4K8ac_peak_15169	12.84642	TMEM161A_ENSG00000064545
19	19256312	19256966	KD2-H4K8ac_peak_15170	11.24702	
19	19303031	19303322	KD2-H4K8ac_peak_15171	18.95762	MEF2BNB-MEF2B_ENSG00000064489;MEF2B_ENSG00000213999;MEF2BNB_ENSG00000254901;RFXANK_ENSG00000064490
19	19313825	19314084	KD2-H4K8ac_peak_15172	7.27716	NR2C2AP_ENSG00000184162
19	19431666	19432021	KD2-H4K8ac_peak_15173	18.86697	SUGP1_ENSG00000105705;MAU2_ENSG00000129933
19	19495990	19496217	KD2-H4K8ac_peak_15174	5.94405	GATAD2A_ENSG00000167491
19	19497248	19497523	KD2-H4K8ac_peak_15175	4.8485	GATAD2A_ENSG00000167491
19	19516756	19517097	KD2-H4K8ac_peak_15176	14.74047	
19	19626187	19626799	KD2-H4K8ac_peak_15177	14.4458	TSSK6_ENSG00000178093;NDUFA13_ENSG00000186010;YJEFN3_ENSG00000250067;CTC-260F20.3_ENSG00000258674
19	19649220	19649430	KD2-H4K8ac_peak_15178	5.20908	CILP2_ENSG00000160161

19	19651499	19652049	KD2-H4K8ac_peak_15179	6.25869	
19	19657504	19657754	KD2-H4K8ac_peak_15180	7.47462	
19	19738592	19739863	KD2-H4K8ac_peak_15181	12.82393	LPAR2_ENSG00000064547
19	19754074	19754693	KD2-H4K8ac_peak_15182	6.66692	GMIP_ENSG00000089639
19	19774223	19774480	KD2-H4K8ac_peak_15183	14.27217	ATP13A1_ENSG00000105726
19	19774687	19775470	KD2-H4K8ac_peak_15184	12.99417	ATP13A1_ENSG00000105726
19	19779639	19780219	KD2-H4K8ac_peak_15185	9.95119	ZNF101_ENSG00000181896
19	19843349	19843745	KD2-H4K8ac_peak_15186	5.1707	ZNF14_ENSG00000105708
19	19844564	19844761	KD2-H4K8ac_peak_15187	7.76329	ZNF14_ENSG00000105708
19	19887591	19887909	KD2-H4K8ac_peak_15188	4.86324	LINC00663_ENSG00000266904;CTC-559E9.6_ENSG00000267419
19	19932103	19932522	KD2-H4K8ac_peak_15189	11.22681	CTC-559E9.4_ENSG00000267581;ZNF506_ENSG00000081665
19	19976761	19977145	KD2-H4K8ac_peak_15190	8.26945	ZNF253_ENSG00000256771
19	19977402	19977596	KD2-H4K8ac_peak_15191	9.11031	ZNF253_ENSG00000256771
19	20011833	20012253	KD2-H4K8ac_peak_15192	10.65724	ZNF93_ENSG00000184635;AC007204.2_ENSG00000267268
19	20162465	20162845	KD2-H4K8ac_peak_15193	9.1707	
19	20163357	20164115	KD2-H4K8ac_peak_15194	8.17902	
19	20348896	20349166	KD2-H4K8ac_peak_15195	11.09936	
19	20607261	20607695	KD2-H4K8ac_peak_15196	9.8758	ZNF826P_ENSG00000231205
19	20747979	20748514	KD2-H4K8ac_peak_15197	3.97079	ZNF737_ENSG00000237440
19	20844014	20844289	KD2-H4K8ac_peak_15198	11.05467	CTC-513N18.7_ENSG00000269110;ZNF626_ENSG00000188171
19	21106556	21106887	KD2-H4K8ac_peak_15199	6.24747	ZNF85_ENSG00000105750
19	21119811	21120066	KD2-H4K8ac_peak_15200	8.1233	
19	21182777	21183167	KD2-H4K8ac_peak_15201	12.40345	
19	21203041	21203256	KD2-H4K8ac_peak_15202	9.36516	ZNF430_ENSG00000118620
19	21265081	21265424	KD2-H4K8ac_peak_15203	8.1233	ZNF714_ENSG00000160352
19	21324897	21325288	KD2-H4K8ac_peak_15204	9.15329	ZNF431_ENSG00000196705
19	21511794	21512034	KD2-H4K8ac_peak_15205	7.36799	ZNF708_ENSG00000182141
19	21512645	21512845	KD2-H4K8ac_peak_15206	3.72574	ZNF708_ENSG00000182141
19	21541901	21542235	KD2-H4K8ac_peak_15207	9.08816	ZNF738_ENSG00000172687
19	21579985	21580320	KD2-H4K8ac_peak_15208	4.86324	ZNF493_ENSG00000196268
19	21688521	21688812	KD2-H4K8ac_peak_15209	7.67504	
19	21949887	21950321	KD2-H4K8ac_peak_15210	13.81781	ZNF100_ENSG00000197020
19	22018375	22018938	KD2-H4K8ac_peak_15211	12.99417	
19	22034582	22034831	KD2-H4K8ac_peak_15212	7.76329	ZNF43_ENSG00000198521
19	22816597	22816832	KD2-H4K8ac_peak_15213	10.0692	ZNF492_ENSG00000229676
19	23253436	23253717	KD2-H4K8ac_peak_15214	11.81257	CTD-2291D10.3_ENSG00000269543
19	23257511	23257813	KD2-H4K8ac_peak_15215	7.22457	CTD-2291D10.4_ENSG00000267886;ZNF730_ENSG00000183850
19	23258052	23258683	KD2-H4K8ac_peak_15216	10.0692	CTD-2291D10.4_ENSG00000267886;ZNF730_ENSG00000183850
19	23432941	23433141	KD2-H4K8ac_peak_15217	8.10216	ZNF724P_ENSG00000196081
19	23456563	23456886	KD2-H4K8ac_peak_15218	6.84261	RP11-15H20.7_ENSG00000269107;RP11-15H20.6_ENSG00000213971
19	23598399	23598686	KD2-H4K8ac_peak_15219	10.63441	CTB-175P5.4_ENSG00000269416
19	23599147	23599400	KD2-H4K8ac_peak_15220	11.86708	CTB-175P5.4_ENSG00000269416
19	23869295	23869914	KD2-H4K8ac_peak_15221	12.81138	ZNF675_ENSG00000197372
19	23941056	23941569	KD2-H4K8ac_peak_15222	6.25869	ZNF681_ENSG00000196172
19	24097138	24097470	KD2-H4K8ac_peak_15223	8.1233	ZNF726_ENSG00000213967
19	24097780	24098108	KD2-H4K8ac_peak_15224	4.211	ZNF726_ENSG00000213967

19	24215558	24215827	KD2-H4K8ac_peak_15225	6.61376	CTD-2017D11.1_ENSG00000268362;ZNF254_ENSG00000213096
19	24216353	24216672	KD2-H4K8ac_peak_15226	6.40297	CTD-2017D11.1_ENSG00000268362;ZNF254_ENSG00000213096
19	24270087	24270481	KD2-H4K8ac_peak_15227	9.55735	
19	28284048	28284253	KD2-H4K8ac_peak_15228	7.47462	LINC00662_ENSG00000261824;CTC-459F4.3_ENSG00000267575;LLNLF-65H9.1_ENSG00000272635
19	28284470	28285561	KD2-H4K8ac_peak_15229	11.2938	LINC00662_ENSG00000261824;CTC-459F4.3_ENSG00000267575;LLNLF-65H9.1_ENSG00000272635
19	30096422	30096925	KD2-H4K8ac_peak_15230	6.29608	
19	30155942	30156156	KD2-H4K8ac_peak_15231	12.82393	PLEKHF1_ENSG00000166289
19	30205837	30206685	KD2-H4K8ac_peak_15232	11.24702	C19orf12_ENSG00000131943
19	30266453	30266693	KD2-H4K8ac_peak_15233	5.15777	
19	30301910	30302567	KD2-H4K8ac_peak_15234	16.8435	CCNE1_ENSG00000105173
19	30363945	30364802	KD2-H4K8ac_peak_15235	18.64825	
19	30370283	30370533	KD2-H4K8ac_peak_15236	5.14125	
19	30433553	30433846	KD2-H4K8ac_peak_15237	8.1233	
19	30562194	30562584	KD2-H4K8ac_peak_15238	6.0073	
19	30601425	30601713	KD2-H4K8ac_peak_15239	4.77079	
19	30602519	30602864	KD2-H4K8ac_peak_15240	15.66774	
19	30622979	30623872	KD2-H4K8ac_peak_15241	7.83489	
19	30625444	30625776	KD2-H4K8ac_peak_15242	6.2781	
19	30626017	30626844	KD2-H4K8ac_peak_15243	10.29344	
19	30627981	30628527	KD2-H4K8ac_peak_15244	8.75125	
19	30640765	30640963	KD2-H4K8ac_peak_15245	4.66652	
19	30641179	30641411	KD2-H4K8ac_peak_15246	4.98072	
19	30642493	30642717	KD2-H4K8ac_peak_15247	5.14125	
19	30643540	30644299	KD2-H4K8ac_peak_15248	19.25648	
19	30644710	30644934	KD2-H4K8ac_peak_15249	7.47462	
19	30646421	30647155	KD2-H4K8ac_peak_15250	9.89383	
19	30648109	30648419	KD2-H4K8ac_peak_15251	6.85193	
19	30664836	30665205	KD2-H4K8ac_peak_15252	7.79898	
19	30716945	30717317	KD2-H4K8ac_peak_15253	13.6756	
19	30865704	30866447	KD2-H4K8ac_peak_15254	7.47462	
19	31170502	31170709	KD2-H4K8ac_peak_15255	6.1228	
19	31176645	31177112	KD2-H4K8ac_peak_15256	4.97693	
19	31178048	31178339	KD2-H4K8ac_peak_15257	7.27716	
19	31183494	31184232	KD2-H4K8ac_peak_15258	11.2938	
19	31210280	31211459	KD2-H4K8ac_peak_15259	15.5861	
19	31229982	31230240	KD2-H4K8ac_peak_15260	5.08628	
19	31231237	31231538	KD2-H4K8ac_peak_15261	7.27716	
19	31233510	31233774	KD2-H4K8ac_peak_15262	10.05606	
19	31234168	31235035	KD2-H4K8ac_peak_15263	11.05467	
19	31400040	31400363	KD2-H4K8ac_peak_15264	6.99815	
19	31754253	31754513	KD2-H4K8ac_peak_15265	5.14125	
19	31829711	31830773	KD2-H4K8ac_peak_15266	6.99815	
19	31841166	31841661	KD2-H4K8ac_peak_15267	11.89502	TSHZ3_ENSG00000121297
19	31872164	31872432	KD2-H4K8ac_peak_15268	4.35346	
19	32596692	32597034	KD2-H4K8ac_peak_15269	7.22457	AC011518.1_ENSG00000267204
19	32715145	32715470	KD2-H4K8ac_peak_15270	4.42765	

19	32836203	32837316	KD2-H4K8ac_peak_15271	22.46823	ZNF507_ENSG00000168813
19	32895515	32896230	KD2-H4K8ac_peak_15272	11.55418	AC007773.2_ENSG00000267213;AC007773.3_ENSG00000269093;DPY19L3_ENSG00000178904
19	32896752	32896971	KD2-H4K8ac_peak_15273	8.16906	AC007773.2_ENSG00000267213;AC007773.3_ENSG00000269093;DPY19L3_ENSG00000178904
19	33072202	33072521	KD2-H4K8ac_peak_15274	14.80472	PDCD5_ENSG00000105185
19	33165585	33166054	KD2-H4K8ac_peak_15275	6.76298	RGS9BP_ENSG00000186326
19	33182835	33183135	KD2-H4K8ac_peak_15276	5.01002	CTD-2538C1.2_ENSG00000267475;NUDT19_ENSG00000213965
19	33183365	33183866	KD2-H4K8ac_peak_15277	12.96672	CTD-2538C1.2_ENSG00000267475;NUDT19_ENSG00000213965
19	33555939	33556239	KD2-H4K8ac_peak_15278	13.9234	RHPN2_ENSG00000131941
19	33571521	33572280	KD2-H4K8ac_peak_15279	10.43145	GPATCH1_ENSG00000076650
19	33667398	33667747	KD2-H4K8ac_peak_15280	9.20193	AC008738.2_ENSG00000264355;LRP3_ENSG00000130881
19	33684708	33685020	KD2-H4K8ac_peak_15281	5.47943	
19	33685909	33686186	KD2-H4K8ac_peak_15282	6.13909	
19	33717507	33717951	KD2-H4K8ac_peak_15283	6.27931	SLC7A10_ENSG00000130876
19	33751767	33752026	KD2-H4K8ac_peak_15284	8.94469	
19	33764491	33764696	KD2-H4K8ac_peak_15285	5.88845	
19	33793134	33793388	KD2-H4K8ac_peak_15286	5.92591	CEBPA_ENSG00000245848;AC008738.1_ENSG00000230259;CTD-2540B15.7_ENSG00000267727;CEBPA-AS1_ENSG00000267296;CEB[illegible]
19	33793738	33793974	KD2-H4K8ac_peak_15287	4.33139	CEBPA_ENSG00000245848;AC008738.1_ENSG00000230259;CEBPA-AS1_ENSG00000267296;CEBPA-AS1_ENSG00000178863
19	33864028	33864417	KD2-H4K8ac_peak_15288	15.52826	CEBPG_ENSG00000153879
19	33864684	33865050	KD2-H4K8ac_peak_15289	13.73635	CEBPG_ENSG00000153879
19	33925561	33928252	KD2-H4K8ac_peak_15290	13.80019	
19	33928680	33929679	KD2-H4K8ac_peak_15291	10.05606	
19	33994033	33994866	KD2-H4K8ac_peak_15292	13.70753	
19	33995251	33996539	KD2-H4K8ac_peak_15293	17.34875	
19	33998020	33998404	KD2-H4K8ac_peak_15294	5.30799	
19	33998745	33999208	KD2-H4K8ac_peak_15295	8.18084	
19	34012210	34012484	KD2-H4K8ac_peak_15296	12.04009	PEPD_ENSG00000124299
19	34012903	34013382	KD2-H4K8ac_peak_15297	5.54647	PEPD_ENSG00000124299
19	34175921	34176113	KD2-H4K8ac_peak_15298	5.63428	
19	34177410	34177703	KD2-H4K8ac_peak_15299	5.405	
19	34178022	34178245	KD2-H4K8ac_peak_15300	8.10216	
19	34178762	34179399	KD2-H4K8ac_peak_15301	9.61895	
19	34179910	34180861	KD2-H4K8ac_peak_15302	7.1181	
19	34215459	34215913	KD2-H4K8ac_peak_15303	8.10216	
19	34218766	34218992	KD2-H4K8ac_peak_15304	6.25869	
19	34285673	34286009	KD2-H4K8ac_peak_15305	5.54647	KCTD15_ENSG00000153885
19	34286306	34286902	KD2-H4K8ac_peak_15306	11.92978	KCTD15_ENSG00000153885
19	34287229	34287569	KD2-H4K8ac_peak_15307	12.30509	KCTD15_ENSG00000153885
19	34287789	34288471	KD2-H4K8ac_peak_15308	12.82202	KCTD15_ENSG00000153885
19	34353338	34353863	KD2-H4K8ac_peak_15309	8.37231	
19	34354231	34354569	KD2-H4K8ac_peak_15310	4.42765	
19	34359656	34360289	KD2-H4K8ac_peak_15311	7.07117	
19	34370810	34371300	KD2-H4K8ac_peak_15312	15.25814	
19	34396573	34397124	KD2-H4K8ac_peak_15313	25.36636	
19	34397382	34397693	KD2-H4K8ac_peak_15314	7.67504	
19	34624729	34624921	KD2-H4K8ac_peak_15315	8.00457	
19	34625501	34625955	KD2-H4K8ac_peak_15316	17.25154	

19	34662713	34663239	KD2-H4K8ac_peak_15317	12.08081	LSM14A_ENSG00000257103
19	34663436	34663981	KD2-H4K8ac_peak_15318	24.52509	LSM14A_ENSG00000257103
19	34745657	34746156	KD2-H4K8ac_peak_15319	11.92978	KIAA0355_ENSG00000166398
19	34797883	34798204	KD2-H4K8ac_peak_15320	5.86783	
19	34850409	34851019	KD2-H4K8ac_peak_15321	10.77651	AC010504.2_ENSG00000267219;GPI_ENSG00000105220
19	34855557	34856504	KD2-H4K8ac_peak_15322	13.73635	
19	34894927	34895616	KD2-H4K8ac_peak_15323	14.66631	PDCD2L_ENSG00000126249
19	34918587	34919163	KD2-H4K8ac_peak_15324	6.97919	CTD-2588C8.8_ENSG00000267024;UBA2_ENSG00000126261
19	35137045	35137299	KD2-H4K8ac_peak_15325	7.76329	SCGB2B3P_ENSG00000269811
19	35167715	35168398	KD2-H4K8ac_peak_15326	5.75757	AC020910.2_ENSG00000256383;ZNF302_ENSG00000089335
19	35168665	35169097	KD2-H4K8ac_peak_15327	9.57101	AC020910.2_ENSG00000256383;ZNF302_ENSG00000089335
19	35223832	35224639	KD2-H4K8ac_peak_15328	6.97919	ZNF181_ENSG00000197841
19	35225068	35225872	KD2-H4K8ac_peak_15329	23.68363	ZNF181_ENSG00000197841
19	35263724	35264038	KD2-H4K8ac_peak_15330	9.89126	ZNF599_ENSG00000153896
19	35454524	35455204	KD2-H4K8ac_peak_15331	5.9696	ZNF792_ENSG00000180884
19	35490533	35491029	KD2-H4K8ac_peak_15332	9.11891	CTD-2527I21.7_ENSG00000269303
19	35491319	35491894	KD2-H4K8ac_peak_15333	17.70889	CTD-2527I21.7_ENSG00000269303
19	35524869	35525166	KD2-H4K8ac_peak_15334	5.88845	
19	35584936	35585218	KD2-H4K8ac_peak_15335	9.72244	
19	35615134	35616468	KD2-H4K8ac_peak_15336	18.80827	
19	35626113	35626352	KD2-H4K8ac_peak_15337	5.54647	
19	35758093	35758978	KD2-H4K8ac_peak_15338	10.96455	USF2_ENSG00000105698
19	35759486	35759758	KD2-H4K8ac_peak_15339	15.22643	USF2_ENSG00000105698
19	35760159	35760475	KD2-H4K8ac_peak_15340	11.05467	USF2_ENSG00000105698
19	36036187	36036846	KD2-H4K8ac_peak_15341	11.92978	AD000090.2_ENSG00000236144;TMEM147_ENSG00000105677
19	36103069	36103458	KD2-H4K8ac_peak_15342	11.3394	AC002115.9_ENSG00000267626;HAUS5_ENSG00000249115
19	36119261	36119673	KD2-H4K8ac_peak_15343	9.08816	RBM42_ENSG00000126254
19	36120043	36120261	KD2-H4K8ac_peak_15344	8.10216	RBM42_ENSG00000126254
19	36134009	36134655	KD2-H4K8ac_peak_15345	17.42905	
19	36134870	36135287	KD2-H4K8ac_peak_15346	7.76329	
19	36138534	36138833	KD2-H4K8ac_peak_15347	8.33649	COX6B1_ENSG00000126267
19	36192834	36193268	KD2-H4K8ac_peak_15348	14.68449	
19	36193581	36194095	KD2-H4K8ac_peak_15349	5.88845	
19	36206813	36207732	KD2-H4K8ac_peak_15350	5.9696	
19	36209062	36209429	KD2-H4K8ac_peak_15351	5.01002	KMT2B_ENSG00000105663;KMT2B_ENSG00000272333
19	36231140	36231393	KD2-H4K8ac_peak_15352	5.82799	
19	36231807	36232210	KD2-H4K8ac_peak_15353	5.13615	
19	36235978	36236251	KD2-H4K8ac_peak_15354	13.73635	AD000671.6_ENSG00000267120;U2AF1L4_ENSG00000161265;PSENEN_ENSG00000205155;AC002398.9_ENSG00000188223
19	36236645	36237055	KD2-H4K8ac_peak_15355	6.60096	AD000671.6_ENSG00000267120;U2AF1L4_ENSG00000161265;PSENEN_ENSG00000205155;AC002398.9_ENSG00000188223
19	36247718	36248652	KD2-H4K8ac_peak_15356	16.822	HSPB6_ENSG00000004776;C19orf55_ENSG00000167595
19	36249070	36249436	KD2-H4K8ac_peak_15357	13.72842	HSPB6_ENSG00000004776;C19orf55_ENSG00000167595
19	36266466	36266923	KD2-H4K8ac_peak_15358	8.59579	
19	36358768	36359323	KD2-H4K8ac_peak_15359	9.36516	NPHS1_ENSG00000161270;APLP1_ENSG00000105290
19	36359980	36360227	KD2-H4K8ac_peak_15360	5.30799	NPHS1_ENSG00000161270
19	36389604	36389984	KD2-H4K8ac_peak_15361	6.40683	
19	36391774	36392210	KD2-H4K8ac_peak_15362	7.26089	NFKBID_ENSG00000167604

19	36394407	36394685	KD2-H4K8ac_peak_15363	6.66692	
19	36426139	36426334	KD2-H4K8ac_peak_15364	5.20908	LRFN3_ENSG00000126243
19	36426600	36427126	KD2-H4K8ac_peak_15365	6.56211	LRFN3_ENSG00000126243
19	36427580	36427772	KD2-H4K8ac_peak_15366	9.51688	
19	36478577	36478826	KD2-H4K8ac_peak_15367	5.57044	
19	36484731	36485033	KD2-H4K8ac_peak_15368	5.14125	
19	36485718	36485917	KD2-H4K8ac_peak_15369	8.83582	SDHAF1_ENSG00000205138
19	36486162	36486481	KD2-H4K8ac_peak_15370	5.30799	SDHAF1_ENSG00000205138
19	36499365	36500892	KD2-H4K8ac_peak_15371	14.8083	SYNE4_ENSG00000181392
19	36504636	36504873	KD2-H4K8ac_peak_15372	5.75452	AC002116.8_ENSG00000248101;ALKBH6_ENSG00000239382;AC002116.7_ENSG00000267698
19	36522913	36523681	KD2-H4K8ac_peak_15373	7.94441	CLIP3_ENSG00000105270
19	36523932	36524162	KD2-H4K8ac_peak_15374	10.86378	CLIP3_ENSG00000105270
19	36605209	36605599	KD2-H4K8ac_peak_15375	6.65673	POLR2I_ENSG00000105258;TBCB_ENSG00000105254
19	36606435	36606843	KD2-H4K8ac_peak_15376	4.54006	POLR2I_ENSG00000105258
19	36630310	36630813	KD2-H4K8ac_peak_15377	10.05081	CAPNS1_ENSG00000126247
19	36748219	36748959	KD2-H4K8ac_peak_15378	9.96798	
19	36822881	36823309	KD2-H4K8ac_peak_15379	11.05407	LINC00665_ENSG00000232677
19	36870252	36870724	KD2-H4K8ac_peak_15380	11.05467	ZFP14_ENSG00000142065
19	36908978	36909926	KD2-H4K8ac_peak_15381	14.80472	ZFP82_ENSG00000181007
19	36979869	36980781	KD2-H4K8ac_peak_15382	16.71655	ZNF566_ENSG00000186017;CTD-2630F21.1_ENSG00000267309
19	37064006	37064238	KD2-H4K8ac_peak_15383	10.0692	AC092295.7_ENSG00000233527
19	37064516	37064749	KD2-H4K8ac_peak_15384	11.89502	AC092295.7_ENSG00000233527
19	37095904	37096595	KD2-H4K8ac_peak_15385	9.08816	ZNF529_ENSG00000186020;ZNF382_ENSG00000161298
19	37178564	37178780	KD2-H4K8ac_peak_15386	9.1707	AC074138.3_ENSG00000225975;ZNF567_ENSG00000189042
19	37263489	37263951	KD2-H4K8ac_peak_15387	8.55271	ZNF850_ENSG00000267041;CTD-2162K18.4_ENSG00000267260
19	37287786	37288046	KD2-H4K8ac_peak_15388	5.93446	CTD-2162K18.5_ENSG00000267254
19	37288471	37288800	KD2-H4K8ac_peak_15389	11.05467	CTD-2162K18.5_ENSG00000267254
19	37329016	37329870	KD2-H4K8ac_peak_15390	11.05467	
19	37406693	37407180	KD2-H4K8ac_peak_15391	14.68449	ZNF829_ENSG00000185869;ZNF568_ENSG00000198453
19	37463370	37464314	KD2-H4K8ac_peak_15392	16.71291	
19	37569374	37569577	KD2-H4K8ac_peak_15393	10.53377	CTD-2293H3.2_ENSG00000267345
19	37701889	37702083	KD2-H4K8ac_peak_15394	10.29344	CTC-454I21.3_ENSG00000267360
19	37742854	37743068	KD2-H4K8ac_peak_15395	12.84642	AC012309.5_ENSG00000226686
19	37794908	37795169	KD2-H4K8ac_peak_15396	4.01115	
19	37808882	37809098	KD2-H4K8ac_peak_15397	8.30974	
19	37825708	37825955	KD2-H4K8ac_peak_15398	8.82366	
19	37861388	37861746	KD2-H4K8ac_peak_15399	5.75452	ZNF527_ENSG00000189164
19	37957890	37958302	KD2-H4K8ac_peak_15400	6.65673	ZNF570_ENSG00000171827
19	37959267	37960778	KD2-H4K8ac_peak_15401	19.85702	ZNF569_ENSG00000196437;ZNF570_ENSG00000171827
19	37996801	37997162	KD2-H4K8ac_peak_15402	7.47462	CTD-3064H18.1_ENSG00000266916;ZNF793_ENSG00000188227
19	37997359	37997888	KD2-H4K8ac_peak_15403	5.53033	CTD-3064H18.1_ENSG00000266916;ZNF793_ENSG00000188227
19	38041802	38042606	KD2-H4K8ac_peak_15404	9.63487	ZNF540_ENSG00000171817
19	38145627	38146621	KD2-H4K8ac_peak_15405	13.9234	
19	38146820	38147170	KD2-H4K8ac_peak_15406	8.85813	
19	38182868	38183116	KD2-H4K8ac_peak_15407	7.96322	ZFP30_ENSG00000120784;ZNF781_ENSG00000196381
19	38210122	38210549	KD2-H4K8ac_peak_15408	18.24717	CTD-2528L19.4_ENSG00000267552;ZNF607_ENSG00000198182

19	38269855	38270060	KD2-H4K8ac_peak_15409	7.47462	CTD-2554C21.1_ENSG00000267422
19	38396907	38397155	KD2-H4K8ac_peak_15410	8.82366	WDR87_ENSG00000171804;SIPA1L3_ENSG00000105738
19	38397477	38397702	KD2-H4K8ac_peak_15411	13.50024	WDR87_ENSG00000171804;SIPA1L3_ENSG00000105738
19	38397936	38398200	KD2-H4K8ac_peak_15412	10.0692	WDR87_ENSG00000171804;SIPA1L3_ENSG00000105738
19	38493645	38494010	KD2-H4K8ac_peak_15413	8.55271	
19	38494687	38495148	KD2-H4K8ac_peak_15414	10.63441	
19	38495895	38496481	KD2-H4K8ac_peak_15415	8.17902	
19	38644160	38644402	KD2-H4K8ac_peak_15416	4.49003	
19	38724687	38725054	KD2-H4K8ac_peak_15417	13.12288	
19	38741932	38742318	KD2-H4K8ac_peak_15418	5.54647	
19	38742529	38742727	KD2-H4K8ac_peak_15419	10.12356	
19	38746409	38747107	KD2-H4K8ac_peak_15420	7.40111	PPP1R14A_ENSG00000167641
19	38755040	38755264	KD2-H4K8ac_peak_15421	6.48713	
19	38761292	38761576	KD2-H4K8ac_peak_15422	3.75035	
19	38779117	38779640	KD2-H4K8ac_peak_15423	5.54647	CTB-102L5.4_ENSG00000267748
19	38810039	38811095	KD2-H4K8ac_peak_15424	7.76104	KCNK6_ENSG00000099337
19	38812115	38812406	KD2-H4K8ac_peak_15425	4.211	
19	38864360	38865060	KD2-H4K8ac_peak_15426	6.60096	PSMD8_ENSG00000099341
19	38865463	38865956	KD2-H4K8ac_peak_15427	9.72244	PSMD8_ENSG00000099341
19	38877330	38877548	KD2-H4K8ac_peak_15428	6.83936	
19	38878116	38878580	KD2-H4K8ac_peak_15429	7.76329	GGN_ENSG00000179168;SPRED3_ENSG00000188766
19	38879367	38880026	KD2-H4K8ac_peak_15430	9.96798	GGN_ENSG00000179168;SPRED3_ENSG00000188766
19	38893872	38894860	KD2-H4K8ac_peak_15431	10.43145	FAM98C_ENSG00000130244
19	38998258	38998494	KD2-H4K8ac_peak_15432	9.76452	
19	39005614	39006422	KD2-H4K8ac_peak_15433	13.92307	
19	39034260	39034591	KD2-H4K8ac_peak_15434	9.90914	
19	39055915	39056357	KD2-H4K8ac_peak_15435	7.40314	
19	39108516	39109538	KD2-H4K8ac_peak_15436	11.24702	EIF3K_ENSG00000178982;MAP4K1_ENSG00000104814
19	39137525	39137808	KD2-H4K8ac_peak_15437	7.1181	ACTN4_ENSG00000130402
19	39183054	39183264	KD2-H4K8ac_peak_15438	9.08816	CTB-186G2.4_ENSG00000267375
19	39226894	39227319	KD2-H4K8ac_peak_15439	4.42765	
19	39227782	39228291	KD2-H4K8ac_peak_15440	5.7068	CTD-2540F13.2_ENSG00000267892
19	39321860	39322400	KD2-H4K8ac_peak_15441	8.30974	ECH1_ENSG00000104823
19	39340261	39340629	KD2-H4K8ac_peak_15442	22.66567	
19	39341425	39342337	KD2-H4K8ac_peak_15443	6.99815	HNRNPL_ENSG00000104824
19	39342871	39343733	KD2-H4K8ac_peak_15444	13.81781	HNRNPL_ENSG00000104824
19	39353989	39354282	KD2-H4K8ac_peak_15445	4.42765	
19	39359961	39360168	KD2-H4K8ac_peak_15446	5.54647	CTC-360G5.6_ENSG00000269050
19	39368728	39368956	KD2-H4K8ac_peak_15447	8.79106	RINL_ENSG00000187994
19	39408898	39409416	KD2-H4K8ac_peak_15448	6.00226	
19	39430331	39430532	KD2-H4K8ac_peak_15449	5.57044	
19	39465770	39466653	KD2-H4K8ac_peak_15450	6.40297	FBXO17_ENSG00000269190
19	39466995	39467312	KD2-H4K8ac_peak_15451	9.08816	FBXO17_ENSG00000269190
19	39522385	39522877	KD2-H4K8ac_peak_15452	10.19949	FBXO27_ENSG00000161243
19	39523098	39523466	KD2-H4K8ac_peak_15453	4.8888	FBXO27_ENSG00000161243
19	39616501	39616766	KD2-H4K8ac_peak_15454	6.99815	PAK4_ENSG00000130669

19	39630020	39630227	KD2-H4K8ac_peak_15455	3.75035	
19	39635767	39636200	KD2-H4K8ac_peak_15456	7.08474	
19	39658113	39658340	KD2-H4K8ac_peak_15457	5.61067	
19	39662978	39663366	KD2-H4K8ac_peak_15458	6.57664	
19	39687604	39687871	KD2-H4K8ac_peak_15459	8.23062	NCCRP1_ENSG00000188505
19	39811055	39811514	KD2-H4K8ac_peak_15460	7.47462	CTC-246B18.8_ENSG00000268262
19	39832174	39832522	KD2-H4K8ac_peak_15461	5.54647	GMFG_ENSG00000130755;CTC-246B18.10_ENSG00000269246;SAMD4B_ENSG00000179134
19	39881360	39881723	KD2-H4K8ac_peak_15462	17.38513	PAF1_ENSG00000006712;MED29_ENSG00000063322
19	39892616	39893788	KD2-H4K8ac_peak_15463	11.79585	
19	39893995	39894305	KD2-H4K8ac_peak_15464	12.74183	
19	39900062	39900957	KD2-H4K8ac_peak_15465	22.21705	MIR4530_ENSG00000266559
19	39903278	39904017	KD2-H4K8ac_peak_15466	7.59794	PLEKHG2_ENSG00000090924
19	39926225	39926586	KD2-H4K8ac_peak_15467	7.76329	RPS16_ENSG00000105193;SUPT5H_ENSG00000196235
19	39926900	39927191	KD2-H4K8ac_peak_15468	6.30229	RPS16_ENSG00000105193;SUPT5H_ENSG00000196235
19	39936294	39936663	KD2-H4K8ac_peak_15469	5.34636	
19	39970775	39971678	KD2-H4K8ac_peak_15470	12.3187	TIMM50_ENSG00000105197
19	39988442	39988901	KD2-H4K8ac_peak_15471	4.44527	DLL3_ENSG00000090932
19	39989693	39990054	KD2-H4K8ac_peak_15472	9.1707	DLL3_ENSG00000090932
19	39997546	39998635	KD2-H4K8ac_peak_15473	8.4716	
19	40006077	40006593	KD2-H4K8ac_peak_15474	4.91674	SELV_ENSG00000186838
19	40030132	40030756	KD2-H4K8ac_peak_15475	11.24702	EID2_ENSG00000176396
19	40314802	40315043	KD2-H4K8ac_peak_15476	4.76709	
19	40315534	40315868	KD2-H4K8ac_peak_15477	11.05407	
19	40323913	40324141	KD2-H4K8ac_peak_15478	5.01002	DYRK1B_ENSG00000105204
19	40324488	40324754	KD2-H4K8ac_peak_15479	14.68449	DYRK1B_ENSG00000105204
19	40365999	40366725	KD2-H4K8ac_peak_15480	9.96798	
19	40476381	40476775	KD2-H4K8ac_peak_15481	15.59774	PSMC4_ENSG00000013275
19	40477177	40477429	KD2-H4K8ac_peak_15482	5.30799	PSMC4_ENSG00000013275
19	40487005	40487213	KD2-H4K8ac_peak_15483	4.77079	
19	40502460	40502809	KD2-H4K8ac_peak_15484	5.14125	
19	40503120	40503336	KD2-H4K8ac_peak_15485	7.47462	
19	40561644	40562071	KD2-H4K8ac_peak_15486	12.23	ZNF780B_ENSG00000128000
19	40596430	40597142	KD2-H4K8ac_peak_15487	11.24702	AC005614.5_ENSG00000269749;ZNF780A_ENSG00000197782;AC005614.3_ENSG00000269296
19	40696510	40697562	KD2-H4K8ac_peak_15488	8.1235	MAP3K10_ENSG00000130758
19	40698026	40698352	KD2-H4K8ac_peak_15489	6.8713	MAP3K10_ENSG00000130758
19	40790483	40790696	KD2-H4K8ac_peak_15490	5.54647	AKT2_ENSG00000105221
19	40791647	40792216	KD2-H4K8ac_peak_15491	11.89502	AKT2_ENSG00000105221
19	40853251	40853457	KD2-H4K8ac_peak_15492	8.84031	C19orf47_ENSG00000160392;PLD3_ENSG00000105223
19	40909751	40910204	KD2-H4K8ac_peak_15493	12.26331	
19	40926821	40927126	KD2-H4K8ac_peak_15494	9.95119	
19	40927390	40927660	KD2-H4K8ac_peak_15495	9.65086	
19	40931464	40931710	KD2-H4K8ac_peak_15496	11.79585	SERTAD1_ENSG00000197019
19	40938838	40940423	KD2-H4K8ac_peak_15497	10.19949	
19	40950919	40951306	KD2-H4K8ac_peak_15498	5.53033	SERTAD3_ENSG00000167565
19	40973229	40973451	KD2-H4K8ac_peak_15499	9.33782	
19	41035454	41036805	KD2-H4K8ac_peak_15500	14.8083	

19	41047253	41047492	KD2-H4K8ac_peak_15501	8.30974	
19	41054885	41055145	KD2-H4K8ac_peak_15502	6.25869	
19	41076011	41076354	KD2-H4K8ac_peak_15503	5.20998	
19	41081864	41082376	KD2-H4K8ac_peak_15504	14.84212	SHKBP1_ENSG00000160410
19	41082801	41083299	KD2-H4K8ac_peak_15505	8.80225	SHKBP1_ENSG00000160410
19	41107308	41107665	KD2-H4K8ac_peak_15506	8.30974	
19	41109716	41109936	KD2-H4K8ac_peak_15507	6.20982	
19	41115610	41115848	KD2-H4K8ac_peak_15508	11.00372	
19	41116243	41117755	KD2-H4K8ac_peak_15509	5.54647	
19	41169429	41169882	KD2-H4K8ac_peak_15510	8.30974	
19	41196845	41197141	KD2-H4K8ac_peak_15511	4.91674	NUMBL_ENSG00000105245
19	41222128	41222607	KD2-H4K8ac_peak_15512	4.06758	ITPKC_ENSG00000086544
19	41255123	41256339	KD2-H4K8ac_peak_15513	11.60333	SNRPA_ENSG00000077312
19	41256757	41256996	KD2-H4K8ac_peak_15514	17.8868	C19orf54_ENSG00000188493;SNRPA_ENSG00000077312
19	41282958	41283262	KD2-H4K8ac_peak_15515	7.01494	RAB4B_ENSG00000167578
19	41303624	41304052	KD2-H4K8ac_peak_15516	4.211	
19	41316657	41316911	KD2-H4K8ac_peak_15517	4.42765	CYP2T2P_ENSG00000233622
19	41317159	41317733	KD2-H4K8ac_peak_15518	16.32216	CYP2T2P_ENSG00000233622
19	41767929	41768305	KD2-H4K8ac_peak_15519	9.02573	HNRNPUL1_ENSG00000105323
19	41769115	41769479	KD2-H4K8ac_peak_15520	8.30974	HNRNPUL1_ENSG00000105323
19	41770300	41771071	KD2-H4K8ac_peak_15521	14.99434	
19	41815865	41816299	KD2-H4K8ac_peak_15522	8.53092	CCDC97_ENSG00000142039
19	41829313	41829918	KD2-H4K8ac_peak_15523	7.36799	
19	41831566	41832359	KD2-H4K8ac_peak_15524	12.97552	
19	41837002	41837308	KD2-H4K8ac_peak_15525	8.28994	
19	41869022	41869555	KD2-H4K8ac_peak_15526	14.93224	B9D2_ENSG00000123810
19	41870327	41870699	KD2-H4K8ac_peak_15527	9.07715	B9D2_ENSG00000123810
19	41882332	41882646	KD2-H4K8ac_peak_15528	12.46264	
19	41903773	41904198	KD2-H4K8ac_peak_15529	5.88845	EXOSC5_ENSG00000077348
19	41933059	41933286	KD2-H4K8ac_peak_15530	8.98477	
19	41933646	41934496	KD2-H4K8ac_peak_15531	10.19949	B3GNT8_ENSG00000177191
19	41944929	41945808	KD2-H4K8ac_peak_15532	11.05407	ATP5SL_ENSG00000105341
19	41946041	41946370	KD2-H4K8ac_peak_15533	8.5781	ATP5SL_ENSG00000105341
19	42364360	42364863	KD2-H4K8ac_peak_15534	14.80472	RPS19_ENSG00000105372
19	42386792	42387778	KD2-H4K8ac_peak_15535	26.59744	ARHGEF1_ENSG00000076928
19	42443897	42445084	KD2-H4K8ac_peak_15536	14.15075	
19	42455833	42456171	KD2-H4K8ac_peak_15537	5.88845	
19	42463095	42463437	KD2-H4K8ac_peak_15538	10.53377	RABAC1_ENSG00000105404
19	42497940	42498194	KD2-H4K8ac_peak_15539	7.27716	
19	42500530	42500977	KD2-H4K8ac_peak_15540	16.93985	ATP1A3_ENSG00000105409
19	42501197	42501639	KD2-H4K8ac_peak_15541	13.80019	ATP1A3_ENSG00000105409
19	42502544	42503369	KD2-H4K8ac_peak_15542	8.17902	ATP1A3_ENSG00000105409
19	42572981	42573186	KD2-H4K8ac_peak_15543	4.66652	GRIK5_ENSG00000105737;ZNF574_ENSG00000105732
19	42573902	42574905	KD2-H4K8ac_peak_15544	9.87284	GRIK5_ENSG00000105737
19	42579185	42579927	KD2-H4K8ac_peak_15545	7.92775	
19	42580404	42580608	KD2-H4K8ac_peak_15546	5.13615	

19	42595404	42595608	KD2-H4K8ac_peak_15547	7.12754	
19	42633456	42633926	KD2-H4K8ac_peak_15548	10.96455	
19	42654058	42654425	KD2-H4K8ac_peak_15549	3.97079	
19	42654978	42655317	KD2-H4K8ac_peak_15550	8.30974	
19	42657877	42658126	KD2-H4K8ac_peak_15551	9.26823	
19	42658936	42659322	KD2-H4K8ac_peak_15552	7.1181	
19	42721359	42722327	KD2-H4K8ac_peak_15553	15.09202	
19	42746353	42746753	KD2-H4K8ac_peak_15554	10.513	AC006486.1_ENSG00000204957;GSK3A_ENSG00000105723
19	42747042	42747314	KD2-H4K8ac_peak_15555	11.88036	AC006486.1_ENSG00000204957;GSK3A_ENSG00000105723
19	42758063	42758898	KD2-H4K8ac_peak_15556	9.03107	AC006486.9_ENSG00000268643;ERF_ENSG00000105722
19	42759809	42760270	KD2-H4K8ac_peak_15557	10.30284	AC006486.9_ENSG00000268643;ERF_ENSG00000105722
19	42771860	42772249	KD2-H4K8ac_peak_15558	10.65724	CIC_ENSG00000079432
19	42782074	42782356	KD2-H4K8ac_peak_15559	6.22746	
19	42784749	42785021	KD2-H4K8ac_peak_15560	9.95119	
19	42785333	42785771	KD2-H4K8ac_peak_15561	10.7186	
19	42786100	42786667	KD2-H4K8ac_peak_15562	6.0605	
19	42806068	42806721	KD2-H4K8ac_peak_15563	3.90565	PAFAH1B3_ENSG00000079462;PRR19_ENSG00000188368
19	42807343	42807751	KD2-H4K8ac_peak_15564	8.30094	PAFAH1B3_ENSG00000079462
19	42817601	42818170	KD2-H4K8ac_peak_15565	17.70889	TMEM145_ENSG00000167619
19	42900693	42900956	KD2-H4K8ac_peak_15566	10.44656	LIPE-AS1_ENSG00000213904
19	42927287	42928252	KD2-H4K8ac_peak_15567	13.65567	CTB-50E14.4_ENSG00000268605
19	43979222	43979422	KD2-H4K8ac_peak_15568	8.30974	
19	44008031	44008256	KD2-H4K8ac_peak_15569	9.36516	PHLDB3_ENSG00000176531
19	44008585	44008985	KD2-H4K8ac_peak_15570	6.40297	PHLDB3_ENSG00000176531
19	44036293	44037244	KD2-H4K8ac_peak_15571	13.81781	
19	44080879	44081317	KD2-H4K8ac_peak_15572	5.14125	PINLYP_ENSG00000234465
19	44084899	44085474	KD2-H4K8ac_peak_15573	4.86324	XRCC1_ENSG00000073050
19	44100612	44100823	KD2-H4K8ac_peak_15574	6.20982	IRGQ_ENSG00000167378;ZNF576_ENSG00000124444;SRRM5_ENSG00000226763
19	44123500	44124277	KD2-H4K8ac_peak_15575	10.7186	ZNF428_ENSG00000131116
19	44143563	44143803	KD2-H4K8ac_peak_15576	10.0692	CADM4_ENSG00000105767
19	44144211	44144628	KD2-H4K8ac_peak_15577	4.37809	CADM4_ENSG00000105767
19	44174171	44174471	KD2-H4K8ac_peak_15578	12.84642	PLAUR_ENSG00000011422
19	44237024	44237230	KD2-H4K8ac_peak_15579	6.44277	
19	44259326	44259691	KD2-H4K8ac_peak_15580	10.17486	SMG9_ENSG00000105771
19	44259915	44260170	KD2-H4K8ac_peak_15581	14.07373	SMG9_ENSG00000105771
19	44278318	44279155	KD2-H4K8ac_peak_15582	8.28234	
19	44281769	44282453	KD2-H4K8ac_peak_15583	10.78136	
19	44330921	44331339	KD2-H4K8ac_peak_15584	15.22643	LYPD5_ENSG00000159871;ZNF283_ENSG00000167637
19	44406079	44406413	KD2-H4K8ac_peak_15585	12.57143	ZNF404_ENSG00000176222;RP11-15A1.3_ENSG00000267058;RP11-15A1.2_ENSG00000267191
19	44439048	44439278	KD2-H4K8ac_peak_15586	6.25869	ZNF45_ENSG00000124459
19	44439544	44439892	KD2-H4K8ac_peak_15587	8.30974	ZNF45_ENSG00000124459
19	44455473	44455695	KD2-H4K8ac_peak_15588	4.211	ZNF221_ENSG00000159905
19	44529011	44529339	KD2-H4K8ac_peak_15589	10.05606	AC067968.3_ENSG00000267144;ZNF222_ENSG00000159885;ZNF223_ENSG00000267022
19	44529581	44529804	KD2-H4K8ac_peak_15590	9.89126	AC067968.3_ENSG00000267144;ZNF222_ENSG00000159885;ZNF223_ENSG00000267022
19	44555293	44555850	KD2-H4K8ac_peak_15591	8.5092	ZNF223_ENSG00000178386
19	44576422	44576813	KD2-H4K8ac_peak_15592	7.13615	ZNF284_ENSG00000186026

19	44617647	44618142	KD2-H4K8ac_peak_15593	9.16531	AC084219.4_ENSG00000186019
19	44645748	44645943	KD2-H4K8ac_peak_15594	5.13615	ZNF234_ENSG00000263002
19	44669264	44669585	KD2-H4K8ac_peak_15595	18.78112	ZNF226_ENSG00000167380
19	44711314	44712005	KD2-H4K8ac_peak_15596	9.36516	ZNF227_ENSG00000131115
19	44716083	44716336	KD2-H4K8ac_peak_15597	8.00457	
19	44860224	44860767	KD2-H4K8ac_peak_15598	10.05606	
19	44906293	44906664	KD2-H4K8ac_peak_15599	5.57044	CTC-512J12.6_ENSG00000267173;ZNF285_ENSG00000267508
19	44952249	44952633	KD2-H4K8ac_peak_15600	17.14886	CTC-512J12.4_ENSG00000267188;ZNF229_ENSG00000167383
19	45004318	45005367	KD2-H4K8ac_peak_15601	26.77453	ZNF180_ENSG00000167384
19	45146425	45147401	KD2-H4K8ac_peak_15602	14.8083	PVR_ENSG00000073008
19	45349591	45349850	KD2-H4K8ac_peak_15603	5.13615	PVRL2_ENSG00000130202
19	45394557	45394969	KD2-H4K8ac_peak_15604	7.92775	CTB-129P6.4_ENSG00000267282;TOMM40_ENSG00000130204
19	45408721	45409245	KD2-H4K8ac_peak_15605	6.25869	APOE_ENSG00000130203
19	45414408	45414632	KD2-H4K8ac_peak_15606	5.71749	
19	45417698	45418361	KD2-H4K8ac_peak_15607	8.4716	APOC1_ENSG00000130208
19	45429797	45430808	KD2-H4K8ac_peak_15608	10.43145	APOC1P1_ENSG00000214855
19	45454665	45454891	KD2-H4K8ac_peak_15609	7.76335	
19	45457982	45458443	KD2-H4K8ac_peak_15610	7.76329	CTB-129P6.11_ENSG00000267114;CLPTM1_ENSG00000104853
19	45458755	45459052	KD2-H4K8ac_peak_15611	7.07117	CLPTM1_ENSG00000104853
19	45541552	45542162	KD2-H4K8ac_peak_15612	14.8083	CLASRP_ENSG00000104859
19	45542366	45542677	KD2-H4K8ac_peak_15613	11.60333	CLASRP_ENSG00000104859
19	45581914	45582326	KD2-H4K8ac_peak_15614	9.48012	GEMIN7_ENSG00000142252;MARK4_ENSG00000007047
19	45582585	45583034	KD2-H4K8ac_peak_15615	6.65673	GEMIN7_ENSG00000142252;MARK4_ENSG00000007047
19	45594242	45594663	KD2-H4K8ac_peak_15616	11.89502	CTB-179K24.3_ENSG00000267348;PPP1R37_ENSG00000104866
19	45595553	45596004	KD2-H4K8ac_peak_15617	7.40111	PPP1R37_ENSG00000104866
19	45639785	45640277	KD2-H4K8ac_peak_15618	11.84007	AC005757.6_ENSG00000267044
19	45664974	45665383	KD2-H4K8ac_peak_15619	6.40683	
19	45682499	45682831	KD2-H4K8ac_peak_15620	6.66692	BLOC1S3_ENSG00000189114;AC005779.2_ENSG00000267545
19	45751654	45752213	KD2-H4K8ac_peak_15621	7.40111	
19	45753783	45754398	KD2-H4K8ac_peak_15622	5.01002	
19	45779426	45779744	KD2-H4K8ac_peak_15623	3.75035	
19	45814929	45815226	KD2-H4K8ac_peak_15624	6.0073	
19	45826662	45827409	KD2-H4K8ac_peak_15625	12.06346	CKM_ENSG00000104879
19	45874244	45874590	KD2-H4K8ac_peak_15626	7.36799	ERCC2_ENSG00000104884
19	45895125	45895365	KD2-H4K8ac_peak_15627	3.87368	
19	45905993	45906232	KD2-H4K8ac_peak_15628	5.21139	
19	45908052	45908673	KD2-H4K8ac_peak_15629	10.08707	PPP1R13L_ENSG00000104881;CD3EAP_ENSG00000117877
19	45926303	45926655	KD2-H4K8ac_peak_15630	5.30799	
19	45927436	45927840	KD2-H4K8ac_peak_15631	13.87818	
19	45931739	45932331	KD2-H4K8ac_peak_15632	10.43145	
19	45942898	45943399	KD2-H4K8ac_peak_15633	10.7186	
19	45943628	45943841	KD2-H4K8ac_peak_15634	6.25869	
19	45946934	45947204	KD2-H4K8ac_peak_15635	8.85813	
19	45947743	45947957	KD2-H4K8ac_peak_15636	7.47462	
19	45953575	45953862	KD2-H4K8ac_peak_15637	6.25869	
19	45959184	45959664	KD2-H4K8ac_peak_15638	13.73635	

19	45970512	45970885	KD2-H4K8ac_peak_15639	12.08081	FOSB_ENSG00000125740
19	45988827	45989135	KD2-H4K8ac_peak_15640	22.21127	
19	45999888	46000184	KD2-H4K8ac_peak_15641	17.07338	RTN2_ENSG00000125744
19	46000490	46000757	KD2-H4K8ac_peak_15642	8.98477	RTN2_ENSG00000125744
19	46009134	46009401	KD2-H4K8ac_peak_15643	4.42765	VASP_ENSG00000125753
19	46010304	46010554	KD2-H4K8ac_peak_15644	5.53033	VASP_ENSG00000125753
19	46010770	46011066	KD2-H4K8ac_peak_15645	15.25274	VASP_ENSG00000125753
19	46012860	46013075	KD2-H4K8ac_peak_15646	6.19483	
19	46086950	46087484	KD2-H4K8ac_peak_15647	6.85193	
19	46087812	46088053	KD2-H4K8ac_peak_15648	19.43192	
19	46088414	46088627	KD2-H4K8ac_peak_15649	5.45396	
19	46110191	46110547	KD2-H4K8ac_peak_15650	6.20982	
19	46110911	46111198	KD2-H4K8ac_peak_15651	8.58069	
19	46118980	46119211	KD2-H4K8ac_peak_15652	7.76329	
19	46119572	46119903	KD2-H4K8ac_peak_15653	18.25771	
19	46136139	46136584	KD2-H4K8ac_peak_15654	12.86276	
19	46148380	46148692	KD2-H4K8ac_peak_15655	15.69839	EML2_ENSG00000125746
19	46149085	46149279	KD2-H4K8ac_peak_15656	5.45396	EML2_ENSG00000125746
19	46174456	46174665	KD2-H4K8ac_peak_15657	9.1707	
19	46180949	46181553	KD2-H4K8ac_peak_15658	11.68645	
19	46184525	46185166	KD2-H4K8ac_peak_15659	6.99815	
19	46234471	46235250	KD2-H4K8ac_peak_15660	14.15075	FBXO46_ENSG00000177051
19	46271400	46271810	KD2-H4K8ac_peak_15661	5.30799	SIX5_ENSG00000177045;AC074212.6_ENSG00000267395
19	46273071	46273343	KD2-H4K8ac_peak_15662	9.93893	SIX5_ENSG00000177045
19	46295526	46295973	KD2-H4K8ac_peak_15663	14.68449	DMWD_ENSG00000185800
19	46318943	46320248	KD2-H4K8ac_peak_15664	12.84642	RSPH6A_ENSG00000104941
19	46366678	46367911	KD2-H4K8ac_peak_15665	10.43145	SYMPK_ENSG00000125755;FOXA3_ENSG00000170608
19	46388929	46389853	KD2-H4K8ac_peak_15666	10.96455	IRF2BP1_ENSG00000170604
19	46390216	46390746	KD2-H4K8ac_peak_15667	10.19949	IRF2BP1_ENSG00000170604
19	46406185	46406633	KD2-H4K8ac_peak_15668	13.95627	MYPOP_ENSG00000176182
19	46431287	46431527	KD2-H4K8ac_peak_15669	7.12754	
19	46456246	46456465	KD2-H4K8ac_peak_15670	4.9025	
19	46497259	46497578	KD2-H4K8ac_peak_15671	5.57044	CCDC61_ENSG00000104983
19	46502679	46503012	KD2-H4K8ac_peak_15672	5.75757	
19	46800493	46800804	KD2-H4K8ac_peak_15673	4.86324	HIF3A_ENSG00000124440
19	46801719	46801988	KD2-H4K8ac_peak_15674	11.02863	
19	46804379	46804651	KD2-H4K8ac_peak_15675	6.55628	
19	46849765	46850069	KD2-H4K8ac_peak_15676	7.67504	PPP5C_ENSG00000011485
19	46850410	46850680	KD2-H4K8ac_peak_15677	9.26408	PPP5C_ENSG00000011485
19	46919385	46919774	KD2-H4K8ac_peak_15678	11.5673	
19	46990850	46991221	KD2-H4K8ac_peak_15679	7.05051	
19	46998820	46999379	KD2-H4K8ac_peak_15680	9.6589	PNMAL2_ENSG00000204851
19	47049640	47049946	KD2-H4K8ac_peak_15681	10.30284	
19	47103492	47104161	KD2-H4K8ac_peak_15682	9.95342	PPP5D1_ENSG00000230510;AC011551.3_ENSG00000268423;CALM3_ENSG00000160014
19	47104644	47105055	KD2-H4K8ac_peak_15683	11.05467	PPP5D1_ENSG00000230510;AC011551.3_ENSG00000268423;CALM3_ENSG00000160014
19	47117292	47117491	KD2-H4K8ac_peak_15684	9.51625	

19	47124380	47125166	KD2-H4K8ac_peak_15685	13.3695	
19	47125379	47125659	KD2-H4K8ac_peak_15686	10.19949	
19	47126877	47127621	KD2-H4K8ac_peak_15687	7.67504	PTGIR_ENSG00000160013
19	47128525	47128856	KD2-H4K8ac_peak_15688	7.36799	PTGIR_ENSG00000160013
19	47129051	47131320	KD2-H4K8ac_peak_15689	8.85813	PTGIR_ENSG00000160013
19	47132298	47132953	KD2-H4K8ac_peak_15690	8.55271	
19	47133260	47133567	KD2-H4K8ac_peak_15691	5.75757	
19	47133769	47135699	KD2-H4K8ac_peak_15692	12.57297	
19	47136397	47136926	KD2-H4K8ac_peak_15693	7.48958	
19	47137825	47138533	KD2-H4K8ac_peak_15694	12.44942	GNG8_ENSG00000167414
19	47142092	47142337	KD2-H4K8ac_peak_15695	8.10216	
19	47142634	47143446	KD2-H4K8ac_peak_15696	8.88082	
19	47143689	47143949	KD2-H4K8ac_peak_15697	8.69797	
19	47217200	47217570	KD2-H4K8ac_peak_15698	8.4716	
19	47217811	47218213	KD2-H4K8ac_peak_15699	6.99815	
19	47219269	47219480	KD2-H4K8ac_peak_15700	4.67342	PRKD2_ENSG00000105287
19	47220510	47220890	KD2-H4K8ac_peak_15701	14.19272	PRKD2_ENSG00000105287
19	47249346	47249670	KD2-H4K8ac_peak_15702	16.57619	STRN4_ENSG00000090372;FKRP_ENSG00000181027
19	47249897	47250220	KD2-H4K8ac_peak_15703	8.10216	STRN4_ENSG00000090372;FKRP_ENSG00000181027
19	47289683	47290029	KD2-H4K8ac_peak_15704	9.1707	
19	47290798	47291028	KD2-H4K8ac_peak_15705	6.90231	SLC1A5_ENSG00000105281
19	47315945	47316801	KD2-H4K8ac_peak_15706	10.96455	
19	47332955	47333325	KD2-H4K8ac_peak_15707	10.0692	
19	47336873	47337393	KD2-H4K8ac_peak_15708	5.14125	
19	47337769	47338131	KD2-H4K8ac_peak_15709	7.13615	
19	47353799	47354062	KD2-H4K8ac_peak_15710	8.55271	AP2S1_ENSG00000042753
19	47359875	47360117	KD2-H4K8ac_peak_15711	6.66692	
19	47363726	47364086	KD2-H4K8ac_peak_15712	18.24798	
19	47364369	47364587	KD2-H4K8ac_peak_15713	11.20531	
19	47461223	47461502	KD2-H4K8ac_peak_15714	8.85813	
19	47507208	47507543	KD2-H4K8ac_peak_15715	4.42765	
19	47516499	47517018	KD2-H4K8ac_peak_15716	9.36516	
19	47538517	47539025	KD2-H4K8ac_peak_15717	5.88845	
19	47539259	47539789	KD2-H4K8ac_peak_15718	5.54647	
19	47552377	47552695	KD2-H4K8ac_peak_15719	10.0692	TMEM160_ENSG00000130748
19	47614159	47614575	KD2-H4K8ac_peak_15720	6.84261	
19	47614795	47616216	KD2-H4K8ac_peak_15721	26.43146	ZC3H4_ENSG00000130749;SAE1_ENSG00000142230
19	47616623	47616873	KD2-H4K8ac_peak_15722	8.43115	ZC3H4_ENSG00000130749;SAE1_ENSG00000142230
19	47714363	47714645	KD2-H4K8ac_peak_15723	6.24747	
19	47729827	47730404	KD2-H4K8ac_peak_15724	17.43705	MIR3191_ENSG00000265134
19	47730764	47731798	KD2-H4K8ac_peak_15725	14.8083	MIR3191_ENSG00000265134
19	47734586	47734884	KD2-H4K8ac_peak_15726	6.65673	
19	47743043	47743464	KD2-H4K8ac_peak_15727	8.69797	
19	47743666	47744440	KD2-H4K8ac_peak_15728	9.1707	
19	47747314	47747511	KD2-H4K8ac_peak_15729	9.16531	
19	47747777	47748079	KD2-H4K8ac_peak_15730	11.60333	

19	47758234	47758507	KD2-H4K8ac_peak_15731	6.66692	CCDC9_ENSG00000105321
19	47777666	47777867	KD2-H4K8ac_peak_15732	11.48808	PRR24_ENSG00000257704
19	47852097	47852355	KD2-H4K8ac_peak_15733	6.84261	DHX34_ENSG00000134815
19	47922363	47922623	KD2-H4K8ac_peak_15734	8.4716	MEIS3_ENSG00000105419
19	47922979	47923499	KD2-H4K8ac_peak_15735	9.89126	MEIS3_ENSG00000105419
19	47924389	47924868	KD2-H4K8ac_peak_15736	7.76104	
19	47925188	47925758	KD2-H4K8ac_peak_15737	5.57044	
19	47974846	47975043	KD2-H4K8ac_peak_15738	8.55271	SLC8A2_ENSG00000118160
19	47987960	47988171	KD2-H4K8ac_peak_15739	6.66692	KPTN_ENSG00000118162;NAPA-AS1_ENSG00000268061
19	47996230	47996631	KD2-H4K8ac_peak_15740	5.54647	
19	47997015	47997836	KD2-H4K8ac_peak_15741	7.40314	
19	47998347	47998559	KD2-H4K8ac_peak_15742	9.73431	
19	47998764	47999071	KD2-H4K8ac_peak_15743	6.17833	
19	48018899	48019428	KD2-H4K8ac_peak_15744	9.77606	NAPA_ENSG00000105402
19	48103891	48104511	KD2-H4K8ac_peak_15745	12.88108	
19	48111463	48111835	KD2-H4K8ac_peak_15746	10.65724	CTD-2571L23.8_ENSG00000268746
19	48221615	48223595	KD2-H4K8ac_peak_15747	23.64558	
19	48226131	48226781	KD2-H4K8ac_peak_15748	8.17902	
19	48233186	48233384	KD2-H4K8ac_peak_15749	11.30637	
19	48233614	48234058	KD2-H4K8ac_peak_15750	9.15329	
19	48246242	48247015	KD2-H4K8ac_peak_15751	13.31016	
19	48248065	48248570	KD2-H4K8ac_peak_15752	7.74292	
19	48280946	48281159	KD2-H4K8ac_peak_15753	5.51783	SEPW1_ENSG00000178980
19	48281427	48282150	KD2-H4K8ac_peak_15754	10.43145	SEPW1_ENSG00000178980
19	48613296	48614170	KD2-H4K8ac_peak_15755	14.00658	PLA2G4C_ENSG00000105499
19	48673294	48673852	KD2-H4K8ac_peak_15756	8.58725	LIG1_ENSG00000105486;C19orf68_ENSG00000185453
19	48674121	48674408	KD2-H4K8ac_peak_15757	7.59794	LIG1_ENSG00000105486;C19orf68_ENSG00000185453
19	48758421	48758617	KD2-H4K8ac_peak_15758	6.99815	CARD8_ENSG00000105483;CTC-241F20.3_ENSG00000268001
19	48758961	48759180	KD2-H4K8ac_peak_15759	10.19949	CARD8_ENSG00000105483;CTC-241F20.3_ENSG00000268001
19	48774132	48774438	KD2-H4K8ac_peak_15760	7.33266	CTC-241F20.4_ENSG00000268186
19	48775121	48775449	KD2-H4K8ac_peak_15761	10.17486	CTC-241F20.4_ENSG00000268186
19	48794300	48794560	KD2-H4K8ac_peak_15762	8.17902	
19	48824741	48825091	KD2-H4K8ac_peak_15763	12.37731	CCDC114_ENSG00000105479;EMP3_ENSG00000142227
19	48825424	48825772	KD2-H4K8ac_peak_15764	7.40314	CCDC114_ENSG00000105479;EMP3_ENSG00000142227
19	48833880	48834129	KD2-H4K8ac_peak_15765	8.55271	
19	48836367	48836845	KD2-H4K8ac_peak_15766	6.66692	
19	48837197	48837552	KD2-H4K8ac_peak_15767	8.1718	
19	48866633	48867132	KD2-H4K8ac_peak_15768	7.76104	TMEM143_ENSG00000161558;SYNGR4_ENSG00000105467
19	48867671	48868316	KD2-H4K8ac_peak_15769	11.75669	TMEM143_ENSG00000161558;SYNGR4_ENSG00000105467
19	48874157	48874430	KD2-H4K8ac_peak_15770	3.97079	
19	48876270	48876630	KD2-H4K8ac_peak_15771	6.40297	
19	48893876	48894251	KD2-H4K8ac_peak_15772	8.5092	KDELR1_ENSG00000105438
19	48896640	48897535	KD2-H4K8ac_peak_15773	9.36516	GRIN2D_ENSG00000105464
19	48897916	48898156	KD2-H4K8ac_peak_15774	5.54647	GRIN2D_ENSG00000105464
19	48899856	48900150	KD2-H4K8ac_peak_15775	4.42765	
19	48901370	48902017	KD2-H4K8ac_peak_15776	13.2669	

19	48903321	48903724	KD2-H4K8ac_peak_15777	5.42867	
19	48922421	48923036	KD2-H4K8ac_peak_15778	5.14125	
19	48947676	48948278	KD2-H4K8ac_peak_15779	7.47462	GRWD1_ENSG00000105447
19	48949122	48949475	KD2-H4K8ac_peak_15780	5.9696	GRWD1_ENSG00000105447
19	48971703	48972072	KD2-H4K8ac_peak_15781	8.94469	CTC-273B12.5_ENSG00000268530;CYTH2_ENSG00000105443
19	48972645	48972941	KD2-H4K8ac_peak_15782	11.05155	CTC-273B12.5_ENSG00000268530;CYTH2_ENSG00000105443
19	48973322	48973520	KD2-H4K8ac_peak_15783	5.20998	CTC-273B12.5_ENSG00000268530
19	48996889	48997188	KD2-H4K8ac_peak_15784	6.20982	
19	49016762	49017749	KD2-H4K8ac_peak_15785	22.28018	LMTK3_ENSG00000142235;CTC-273B12.10_ENSG00000269814
19	49092480	49092762	KD2-H4K8ac_peak_15786	7.27716	
19	49111784	49112402	KD2-H4K8ac_peak_15787	18.5703	
19	49112665	49112982	KD2-H4K8ac_peak_15788	7.40111	
19	49121893	49122297	KD2-H4K8ac_peak_15789	5.75757	RPL18_ENSG00000063177;SPHK2_ENSG00000063176
19	49122784	49123116	KD2-H4K8ac_peak_15790	9.03107	RPL18_ENSG00000063177;SPHK2_ENSG00000063176
19	49126967	49127250	KD2-H4K8ac_peak_15791	6.0073	AC022154.7_ENSG00000268093
19	49138114	49138450	KD2-H4K8ac_peak_15792	9.34989	
19	49141700	49142396	KD2-H4K8ac_peak_15793	9.44423	SEC1P_ENSG00000232871
19	49148607	49149294	KD2-H4K8ac_peak_15794	10.46874	CA11_ENSG00000063180
19	49149539	49149936	KD2-H4K8ac_peak_15795	25.36636	CA11_ENSG00000063180
19	49157369	49158385	KD2-H4K8ac_peak_15796	7.07117	
19	49164624	49164872	KD2-H4K8ac_peak_15797	7.40314	
19	49178075	49178762	KD2-H4K8ac_peak_15798	8.4716	
19	49218725	49219141	KD2-H4K8ac_peak_15799	5.02584	
19	49220187	49220480	KD2-H4K8ac_peak_15800	4.86324	
19	49222902	49223858	KD2-H4K8ac_peak_15801	8.23796	MAMSTR_ENSG00000176909
19	49224169	49224379	KD2-H4K8ac_peak_15802	10.87093	
19	49227867	49228094	KD2-H4K8ac_peak_15803	5.54647	
19	49242326	49242883	KD2-H4K8ac_peak_15804	13.80019	
19	49250030	49250801	KD2-H4K8ac_peak_15805	13.56049	IZUMO1_ENSG00000182264
19	49255733	49256766	KD2-H4K8ac_peak_15806	11.05467	
19	49285257	49285494	KD2-H4K8ac_peak_15807	6.94255	
19	49297856	49298744	KD2-H4K8ac_peak_15808	13.09607	
19	49299120	49299328	KD2-H4K8ac_peak_15809	9.1707	
19	49313637	49313969	KD2-H4K8ac_peak_15810	6.66692	BCAT2_ENSG00000105552
19	49314178	49314459	KD2-H4K8ac_peak_15811	4.211	BCAT2_ENSG00000105552
19	49338711	49339058	KD2-H4K8ac_peak_15812	7.49599	HSD17B14_ENSG00000087076
19	49339599	49339796	KD2-H4K8ac_peak_15813	6.0073	HSD17B14_ENSG00000087076
19	49340601	49340969	KD2-H4K8ac_peak_15814	15.99047	HSD17B14_ENSG00000087076
19	49371269	49371519	KD2-H4K8ac_peak_15815	6.86812	PLEKHA4_ENSG00000105559
19	49371867	49372185	KD2-H4K8ac_peak_15816	5.75757	PLEKHA4_ENSG00000105559
19	49374958	49375434	KD2-H4K8ac_peak_15817	13.53715	PPP1R15A_ENSG00000087074
19	49375698	49376075	KD2-H4K8ac_peak_15818	13.70753	PPP1R15A_ENSG00000087074
19	49399064	49399537	KD2-H4K8ac_peak_15819	15.57998	
19	49458285	49458667	KD2-H4K8ac_peak_15820	8.16906	BAX_ENSG00000087088
19	49466694	49467249	KD2-H4K8ac_peak_15821	6.72021	CTD-2639E6.9_ENSG00000267898
19	49521190	49521615	KD2-H4K8ac_peak_15822	10.21366	LHB_ENSG00000104826

19	49522958	49523243	KD2-H4K8ac_peak_15823	4.211	CTB-60B18.10_ENSG00000268655
19	49560310	49560505	KD2-H4K8ac_peak_15824	4.42765	
19	49560731	49561124	KD2-H4K8ac_peak_15825	5.88845	CGB7_ENSG00000196337
19	49587902	49588292	KD2-H4K8ac_peak_15826	8.53581	SNRNP70_ENSG00000104852
19	49588720	49589303	KD2-H4K8ac_peak_15827	12.84642	SNRNP70_ENSG00000104852
19	49617290	49617828	KD2-H4K8ac_peak_15828	10.86378	LIN7B_ENSG00000104863
19	49622970	49623383	KD2-H4K8ac_peak_15829	10.90543	C19orf73_ENSG00000221916;PPFIA3_ENSG00000177380
19	49630797	49631263	KD2-H4K8ac_peak_15830	8.86099	
19	49645304	49645525	KD2-H4K8ac_peak_15831	6.15357	
19	49653989	49654239	KD2-H4K8ac_peak_15832	10.96455	
19	49660621	49660918	KD2-H4K8ac_peak_15833	9.06703	TRPM4_ENSG00000130529
19	49864461	49864768	KD2-H4K8ac_peak_15834	17.83205	TEAD2_ENSG00000074219;DKKL1_ENSG00000104901
19	49865116	49865628	KD2-H4K8ac_peak_15835	15.66774	TEAD2_ENSG00000074219;DKKL1_ENSG00000104901
19	49865844	49866080	KD2-H4K8ac_peak_15836	9.11031	TEAD2_ENSG00000074219;DKKL1_ENSG00000104901
19	49954543	49954942	KD2-H4K8ac_peak_15837	9.1707	
19	49956910	49957251	KD2-H4K8ac_peak_15838	8.10216	PIH1D1_ENSG00000104872;ALDH16A1_ENSG00000161618
19	49977112	49977630	KD2-H4K8ac_peak_15839	9.90914	FLT3LG_ENSG00000090554;CTD-3148I10.15_ENSG00000273189
19	49979405	49979636	KD2-H4K8ac_peak_15840	7.19807	
19	49982349	49983079	KD2-H4K8ac_peak_15841	9.11031	
19	49989861	49990546	KD2-H4K8ac_peak_15842	15.039	RPL13A_ENSG00000142541
19	49990881	49991334	KD2-H4K8ac_peak_15843	9.34989	RPL13A_ENSG00000142541
19	49998572	49999212	KD2-H4K8ac_peak_15844	13.80019	RPS11_ENSG00000142534
19	49999723	49999923	KD2-H4K8ac_peak_15845	12.57143	RPS11_ENSG00000142534
19	50015430	50016017	KD2-H4K8ac_peak_15846	11.05407	
19	50016446	50016866	KD2-H4K8ac_peak_15847	5.30799	
19	50030671	50030914	KD2-H4K8ac_peak_15848	7.76329	RCN3_ENSG00000142552
19	50084006	50084846	KD2-H4K8ac_peak_15849	11.24422	PRRG2_ENSG00000126460
19	50088087	50088419	KD2-H4K8ac_peak_15850	8.37231	
19	50094464	50094695	KD2-H4K8ac_peak_15851	12.85889	NOSIP_ENSG00000142546;PRR12_ENSG00000126464
19	50095052	50095250	KD2-H4K8ac_peak_15852	5.80249	PRR12_ENSG00000126464
19	50143042	50143317	KD2-H4K8ac_peak_15853	8.66888	RRAS_ENSG00000126458
19	50143919	50145044	KD2-H4K8ac_peak_15854	22.12789	RRAS_ENSG00000126458;SCAF1_ENSG00000126461
19	50145502	50146143	KD2-H4K8ac_peak_15855	12.82393	SCAF1_ENSG00000126461
19	50167949	50168149	KD2-H4K8ac_peak_15856	5.30799	IRF3_ENSG00000126456;BCL2L12_ENSG00000126453
19	50169387	50169665	KD2-H4K8ac_peak_15857	4.19765	IRF3_ENSG00000126456;BCL2L12_ENSG00000126453
19	50178623	50178950	KD2-H4K8ac_peak_15858	5.405	PRMT1_ENSG00000126457
19	50180588	50181260	KD2-H4K8ac_peak_15859	14.275	
19	50194471	50195119	KD2-H4K8ac_peak_15860	6.40297	CTB-33G10.6_ENSG00000268677;CPT1C_ENSG00000169169
19	50195365	50195631	KD2-H4K8ac_peak_15861	13.56049	
19	50216100	50216374	KD2-H4K8ac_peak_15862	7.47462	
19	50216638	50216920	KD2-H4K8ac_peak_15863	5.69023	
19	50268970	50269260	KD2-H4K8ac_peak_15864	9.9363	AP2A1_ENSG00000196961
19	50305955	50306380	KD2-H4K8ac_peak_15865	14.43704	
19	50315706	50316780	KD2-H4K8ac_peak_15866	18.94636	
19	50317693	50317892	KD2-H4K8ac_peak_15867	7.70289	
19	50321355	50321869	KD2-H4K8ac_peak_15868	8.30974	FUZ_ENSG00000010361;MED25_ENSG00000104973

19	50353386	50353601	KD2-H4K8ac_peak_15869	5.04761	PTOV1_ENSG00000104960
19	50370326	50370782	KD2-H4K8ac_peak_15870	7.49599	PNKP_ENSG00000039650
19	50371466	50371794	KD2-H4K8ac_peak_15871	6.98302	PNKP_ENSG00000039650
19	50372389	50372600	KD2-H4K8ac_peak_15872	6.99815	
19	50373181	50373553	KD2-H4K8ac_peak_15873	19.33018	
19	50379465	50379902	KD2-H4K8ac_peak_15874	22.07161	TBC1D17_ENSG00000104946
19	50380103	50380609	KD2-H4K8ac_peak_15875	24.29798	TBC1D17_ENSG00000104946
19	50394350	50394721	KD2-H4K8ac_peak_15876	6.40683	
19	50432131	50433251	KD2-H4K8ac_peak_15877	23.8572	IL4I1_ENSG00000104951;NUP62_ENSG00000213024;ATF5_ENSG00000169136
19	50528204	50528588	KD2-H4K8ac_peak_15878	14.8083	VRK3_ENSG00000105053;ZNF473_ENSG00000142528
19	50815844	50816056	KD2-H4K8ac_peak_15879	7.40633	
19	50818574	50818775	KD2-H4K8ac_peak_15880	8.30974	
19	50828695	50828897	KD2-H4K8ac_peak_15881	10.08874	
19	50836409	50837068	KD2-H4K8ac_peak_15882	5.54647	KCNC3_ENSG00000131398
19	50837265	50837545	KD2-H4K8ac_peak_15883	10.87093	KCNC3_ENSG00000131398
19	50861025	50861595	KD2-H4K8ac_peak_15884	7.13615	
19	50872047	50872519	KD2-H4K8ac_peak_15885	7.47462	CTB-191K22.6_ENSG00000269392
19	50872865	50873316	KD2-H4K8ac_peak_15886	7.47462	CTB-191K22.6_ENSG00000269392
19	50878873	50879246	KD2-H4K8ac_peak_15887	5.30799	
19	50879772	50879998	KD2-H4K8ac_peak_15888	15.25814	
19	50886639	50886965	KD2-H4K8ac_peak_15889	4.42765	POLD1_ENSG00000062822
19	50935529	50935835	KD2-H4K8ac_peak_15890	9.43685	MYBPC2_ENSG00000086967
19	50964239	50964523	KD2-H4K8ac_peak_15891	7.95563	
19	50964924	50965154	KD2-H4K8ac_peak_15892	7.45501	
19	50979022	50979629	KD2-H4K8ac_peak_15893	9.63487	FAM71E1_ENSG00000142530;EMC10_ENSG00000161671
19	50979829	50980085	KD2-H4K8ac_peak_15894	10.65724	FAM71E1_ENSG00000142530;EMC10_ENSG00000161671
19	51003527	51003883	KD2-H4K8ac_peak_15895	5.57044	
19	51013637	51013860	KD2-H4K8ac_peak_15896	5.34636	JOSD2_ENSG00000161677
19	51014106	51014359	KD2-H4K8ac_peak_15897	7.49599	JOSD2_ENSG00000161677
19	51015785	51016189	KD2-H4K8ac_peak_15898	4.62142	
19	51016706	51017180	KD2-H4K8ac_peak_15899	10.50156	ASPDH_ENSG00000204653
19	51017376	51018461	KD2-H4K8ac_peak_15900	12.84642	ASPDH_ENSG00000204653
19	51071300	51071528	KD2-H4K8ac_peak_15901	9.36516	LRRC4B_ENSG00000131409
19	51109843	51110056	KD2-H4K8ac_peak_15902	6.16888	
19	51110797	51111051	KD2-H4K8ac_peak_15903	7.12754	
19	51142241	51142446	KD2-H4K8ac_peak_15904	8.5092	
19	51143173	51143567	KD2-H4K8ac_peak_15905	13.9234	
19	51153144	51153498	KD2-H4K8ac_peak_15906	8.00457	C19orf81_ENSG00000235034
19	51198872	51199254	KD2-H4K8ac_peak_15907	11.59946	
19	51222860	51223063	KD2-H4K8ac_peak_15908	9.59147	SHANK1_ENSG00000161681
19	51227596	51228056	KD2-H4K8ac_peak_15909	8.66888	
19	51228462	51228743	KD2-H4K8ac_peak_15910	10.19949	
19	51307583	51308157	KD2-H4K8ac_peak_15911	9.36516	C19orf48_ENSG00000167747
19	51308354	51308586	KD2-H4K8ac_peak_15912	11.05407	C19orf48_ENSG00000167747
19	51308816	51309021	KD2-H4K8ac_peak_15913	7.12754	C19orf48_ENSG00000167747
19	51612195	51612566	KD2-H4K8ac_peak_15914	10.87093	CTU1_ENSG00000142544

19	51815102	51815471	KD2-H4K8ac_peak_15915	11.05407	
19	51843460	51843752	KD2-H4K8ac_peak_15916	6.40297	CTD-2616J11.16_ENSG00000267905
19	51871212	51872278	KD2-H4K8ac_peak_15917	5.85291	CTD-2616J11.11_ENSG00000269403;CLDND2_ENSG00000160318
19	51896977	51897309	KD2-H4K8ac_peak_15918	4.37809	CTD-2616J11.14_ENSG00000268889
19	51897841	51898068	KD2-H4K8ac_peak_15919	7.27716	CTD-2616J11.14_ENSG00000268889
19	52097150	52097426	KD2-H4K8ac_peak_15920	4.24345	AC018755.1_ENSG00000167765;AC018755.16_ENSG00000269388
19	52097871	52098457	KD2-H4K8ac_peak_15921	11.22681	AC018755.1_ENSG00000167765;AC018755.16_ENSG00000269388
19	52191548	52191848	KD2-H4K8ac_peak_15922	7.70289	
19	52192664	52193159	KD2-H4K8ac_peak_15923	9.1707	
19	52193457	52194026	KD2-H4K8ac_peak_15924	6.40297	
19	52207585	52207838	KD2-H4K8ac_peak_15925	12.00597	
19	52407817	52408198	KD2-H4K8ac_peak_15926	5.71749	ZNF649_ENSG00000198093
19	52429752	52430261	KD2-H4K8ac_peak_15927	10.0692	ZNF613_ENSG00000176024
19	52511557	52512218	KD2-H4K8ac_peak_15928	8.74596	ZNF615_ENSG00000197619
19	52531177	52532141	KD2-H4K8ac_peak_15929	14.15075	
19	52642754	52643166	KD2-H4K8ac_peak_15930	10.96455	ZNF616_ENSG00000204611
19	52674292	52674832	KD2-H4K8ac_peak_15931	5.88845	CTC-471J1.8_ENSG00000267827;ZNF836_ENSG00000196267;CTD-2525I3.2_ENSG00000267927
19	52692521	52692867	KD2-H4K8ac_peak_15932	6.07178	PPP2R1A_ENSG00000105568
19	52693359	52693936	KD2-H4K8ac_peak_15933	10.05606	PPP2R1A_ENSG00000105568
19	52694218	52694412	KD2-H4K8ac_peak_15934	6.16888	PPP2R1A_ENSG00000105568
19	52772209	52772457	KD2-H4K8ac_peak_15935	5.10276	ZNF766_ENSG00000196214
19	52772968	52773328	KD2-H4K8ac_peak_15936	16.8435	ZNF766_ENSG00000196214
19	52800503	52800945	KD2-H4K8ac_peak_15937	16.32216	CTD-2525I3.5_ENSG00000269102;ZNF480_ENSG00000198464
19	52839698	52840428	KD2-H4K8ac_peak_15938	9.51625	ZNF610_ENSG00000167554
19	53029973	53030658	KD2-H4K8ac_peak_15939	5.73839	ZNF808_ENSG00000198482
19	53031007	53031639	KD2-H4K8ac_peak_15940	9.1707	ZNF808_ENSG00000198482
19	53072962	53073273	KD2-H4K8ac_peak_15941	6.66692	
19	53104792	53105096	KD2-H4K8ac_peak_15942	6.0073	
19	53140900	53141565	KD2-H4K8ac_peak_15943	14.74047	
19	53193080	53193315	KD2-H4K8ac_peak_15944	6.85193	ZNF83_ENSG00000167766
19	53360994	53361556	KD2-H4K8ac_peak_15945	5.88845	ZNF28_ENSG00000198538;ZNF468_ENSG00000204604
19	53465833	53466034	KD2-H4K8ac_peak_15946	6.25869	ZNF816_ENSG00000180257;ZNF321P_ENSG00000221874
19	53466572	53466790	KD2-H4K8ac_peak_15947	4.8888	ZNF816_ENSG00000180257;ZNF321P_ENSG00000221874
19	53606084	53606389	KD2-H4K8ac_peak_15948	10.8368	ZNF160_ENSG00000170949
19	53636253	53636447	KD2-H4K8ac_peak_15949	5.14125	ZNF415_ENSG00000170954
19	53696054	53696588	KD2-H4K8ac_peak_15950	7.12754	ZNF665_ENSG00000197497
19	53700427	53700803	KD2-H4K8ac_peak_15951	3.97079	CTD-2245F17.3_ENSG00000269051
19	53868448	53868725	KD2-H4K8ac_peak_15952	7.146	ZNF525_ENSG00000203326
19	53897815	53898030	KD2-H4K8ac_peak_15953	8.58725	
19	53898640	53898992	KD2-H4K8ac_peak_15954	8.75125	
19	54023461	54023875	KD2-H4K8ac_peak_15955	8.59579	ZNF331_ENSG00000130844
19	54041289	54041605	KD2-H4K8ac_peak_15956	4.0528	
19	54369159	54369546	KD2-H4K8ac_peak_15957	16.54162	MYADM_ENSG00000179820
19	54369781	54370533	KD2-H4K8ac_peak_15958	6.25869	MYADM_ENSG00000179820
19	54372215	54372479	KD2-H4K8ac_peak_15959	5.13615	AC008753.6_ENSG00000228323
19	54382955	54383240	KD2-H4K8ac_peak_15960	7.83489	PRKCG_ENSG00000126583

19	54385562	54385770	KD2-H4K8ac_peak_15961	5.9696	
19	54392829	54393264	KD2-H4K8ac_peak_15962	7.47462	
19	54409946	54410140	KD2-H4K8ac_peak_15963	10.1591	
19	54411533	54411742	KD2-H4K8ac_peak_15964	4.211	CACNG7_ENSG00000105605
19	54411973	54412289	KD2-H4K8ac_peak_15965	4.77079	CACNG7_ENSG00000105605
19	54412719	54413196	KD2-H4K8ac_peak_15966	10.2048	CACNG7_ENSG00000105605
19	54449026	54449224	KD2-H4K8ac_peak_15967	7.27716	
19	54461075	54461460	KD2-H4K8ac_peak_15968	6.00226	
19	54461868	54462205	KD2-H4K8ac_peak_15969	11.20997	
19	54464990	54465610	KD2-H4K8ac_peak_15970	7.27716	CACNG8_ENSG00000142408
19	54605371	54605929	KD2-H4K8ac_peak_15971	17.70889	OSCAR_ENSG00000170909;NDUFA3_ENSG00000170906
19	54606204	54606632	KD2-H4K8ac_peak_15972	10.19949	OSCAR_ENSG00000170909;NDUFA3_ENSG00000170906
19	54618012	54618433	KD2-H4K8ac_peak_15973	6.0073	TFPT_ENSG00000105619;PRPF31_ENSG00000105618
19	54618810	54619003	KD2-H4K8ac_peak_15974	5.82799	TFPT_ENSG00000105619;PRPF31_ENSG00000105618
19	54641496	54642150	KD2-H4K8ac_peak_15975	17.43705	CNOT3_ENSG00000088038
19	54694140	54694444	KD2-H4K8ac_peak_15976	11.50346	MBOAT7_ENSG00000125505;TSEN34_ENSG00000170892
19	54694766	54695339	KD2-H4K8ac_peak_15977	9.402	TSEN34_ENSG00000170892
19	54703748	54703962	KD2-H4K8ac_peak_15978	7.13615	AC012314.20_ENSG00000223660;RPS9_ENSG00000170889
19	54704190	54704535	KD2-H4K8ac_peak_15979	9.08816	AC012314.20_ENSG00000223660;RPS9_ENSG00000170889
19	54959455	54959979	KD2-H4K8ac_peak_15980	6.40297	LENG8-AS1_ENSG00000226696;LENG8_ENSG00000167615
19	54960456	54960837	KD2-H4K8ac_peak_15981	5.30799	LENG8-AS1_ENSG00000226696;LENG8_ENSG00000167615
19	54976118	54976591	KD2-H4K8ac_peak_15982	11.9948	
19	55591204	55592069	KD2-H4K8ac_peak_15983	12.30509	
19	55593666	55593887	KD2-H4K8ac_peak_15984	5.34636	
19	55629252	55629632	KD2-H4K8ac_peak_15985	13.73635	PPP1R12C_ENSG00000125503
19	55630043	55630262	KD2-H4K8ac_peak_15986	3.97079	
19	55658013	55658592	KD2-H4K8ac_peak_15987	14.98671	
19	55691236	55691432	KD2-H4K8ac_peak_15988	4.42498	SYT5_ENSG00000129990
19	55718355	55718566	KD2-H4K8ac_peak_15989	5.13615	
19	55728038	55728609	KD2-H4K8ac_peak_15990	9.36516	CTD-2587H24.10_ENSG00000267649
19	55770017	55770320	KD2-H4K8ac_peak_15991	8.85813	PPP6R1_ENSG00000105063
19	55771199	55771590	KD2-H4K8ac_peak_15992	4.67342	PPP6R1_ENSG00000105063
19	55790652	55790881	KD2-H4K8ac_peak_15993	7.8711	HSPBP1_ENSG00000133265
19	55791906	55792109	KD2-H4K8ac_peak_15994	11.05467	HSPBP1_ENSG00000133265
19	55794736	55795091	KD2-H4K8ac_peak_15995	5.88845	
19	55795501	55795996	KD2-H4K8ac_peak_15996	16.8435	
19	55812760	55813902	KD2-H4K8ac_peak_15997	10.1591	
19	55831390	55831696	KD2-H4K8ac_peak_15998	5.9696	
19	55850161	55850527	KD2-H4K8ac_peak_15999	7.74292	SUV420H2_ENSG00000133247;AC020922.1_ENSG00000267531
19	55851573	55852043	KD2-H4K8ac_peak_16000	5.76151	SUV420H2_ENSG00000133247;AC020922.1_ENSG00000267531
19	55885466	55885689	KD2-H4K8ac_peak_16001	7.22457	
19	55894751	55894990	KD2-H4K8ac_peak_16002	10.7186	TMEM238_ENSG00000233493
19	55895277	55895737	KD2-H4K8ac_peak_16003	11.00004	TMEM238_ENSG00000233493;RPL28_ENSG00000108107
19	55896214	55896603	KD2-H4K8ac_peak_16004	6.20982	TMEM238_ENSG00000233493;RPL28_ENSG00000108107
19	55897660	55897860	KD2-H4K8ac_peak_16005	5.32397	RPL28_ENSG00000108107
19	55918725	55919434	KD2-H4K8ac_peak_16006	12.93253	UBE2S_ENSG00000108106

19	55919849	55920429	KD2-H4K8ac_peak_16007	10.29842	UBE2S_ENSG00000108106
19	55951660	55952240	KD2-H4K8ac_peak_16008	18.54875	
19	55963870	55964217	KD2-H4K8ac_peak_16009	6.25869	
19	55972626	55972915	KD2-H4K8ac_peak_16010	11.76021	ISOC2_ENSG00000063241
19	55986952	55988164	KD2-H4K8ac_peak_16011	22.25695	CTD-2537I9.16_ENSG00000269859;ZNF628_ENSG00000197483
19	55995963	55996720	KD2-H4K8ac_peak_16012	12.78054	NAT14_ENSG00000090971
19	55997894	55998399	KD2-H4K8ac_peak_16013	7.30812	
19	56028588	56028888	KD2-H4K8ac_peak_16014	11.89502	
19	56091611	56092177	KD2-H4K8ac_peak_16015	11.92978	ZNF579_ENSG00000218891
19	56092381	56092991	KD2-H4K8ac_peak_16016	10.59312	ZNF579_ENSG00000218891
19	56096920	56097164	KD2-H4K8ac_peak_16017	10.19949	
19	56097430	56097762	KD2-H4K8ac_peak_16018	13.70753	
19	56099221	56099462	KD2-H4K8ac_peak_16019	5.57044	
19	56110623	56110886	KD2-H4K8ac_peak_16020	21.11201	ZNF524_ENSG00000171443
19	56111117	56111589	KD2-H4K8ac_peak_16021	11.24702	ZNF524_ENSG00000171443
19	56111995	56112377	KD2-H4K8ac_peak_16022	8.33649	FIZ1_ENSG00000179943;ZNF524_ENSG00000171443
19	56116034	56116393	KD2-H4K8ac_peak_16023	10.96455	ZNF865_ENSG00000261221
19	56117066	56117417	KD2-H4K8ac_peak_16024	9.63487	ZNF865_ENSG00000261221
19	56145704	56146002	KD2-H4K8ac_peak_16025	6.40297	ZNF580_ENSG00000213015;ZNF581_ENSG00000171425
19	56146428	56146756	KD2-H4K8ac_peak_16026	12.51591	ZNF580_ENSG00000213015;ZNF581_ENSG00000171425
19	56151683	56152035	KD2-H4K8ac_peak_16027	6.52179	CCDC106_ENSG00000173581
19	56152368	56152941	KD2-H4K8ac_peak_16028	14.95369	CCDC106_ENSG00000173581
19	56155035	56155412	KD2-H4K8ac_peak_16029	7.74292	
19	56158965	56160042	KD2-H4K8ac_peak_16030	9.36665	
19	56164803	56165021	KD2-H4K8ac_peak_16031	7.45629	U2AF2_ENSG00000063244
19	56165336	56165613	KD2-H4K8ac_peak_16032	5.30799	U2AF2_ENSG00000063244
19	56165940	56166172	KD2-H4K8ac_peak_16033	9.20193	U2AF2_ENSG00000063244
19	56166384	56166737	KD2-H4K8ac_peak_16034	20.03864	U2AF2_ENSG00000063244
19	56631761	56632264	KD2-H4K8ac_peak_16035	11.20531	ZNF787_ENSG00000142409
19	56825620	56825814	KD2-H4K8ac_peak_16036	7.98152	AC006116.17_ENSG00000267549
19	56826020	56826235	KD2-H4K8ac_peak_16037	11.89502	AC006116.17_ENSG00000267549
19	56826709	56827129	KD2-H4K8ac_peak_16038	11.90231	AC006116.17_ENSG00000267549;Y_RNA_ENSG00000200646
19	56879164	56880124	KD2-H4K8ac_peak_16039	10.53377	ZSCAN5A_ENSG00000131848;AC006116.21_ENSG00000267606;ZNF542_ENSG00000240225
19	56904636	56904905	KD2-H4K8ac_peak_16040	10.0692	ZNF582_ENSG00000018869;ZNF582-AS1_ENSG00000267454
19	56905140	56905375	KD2-H4K8ac_peak_16041	9.1707	ZNF582_ENSG00000018869;ZNF582-AS1_ENSG00000267454
19	56915058	56915940	KD2-H4K8ac_peak_16042	37.48141	
19	56988974	56989391	KD2-H4K8ac_peak_16043	5.75757	ZNF667_ENSG00000198046;ZNF667-AS1_ENSG00000166770
19	57019218	57019455	KD2-H4K8ac_peak_16044	13.73635	ZNF471_ENSG00000196263
19	57050418	57050617	KD2-H4K8ac_peak_16045	7.79898	ZFP28_ENSG00000196867;AC005498.3_ENSG00000267421
19	57078431	57079543	KD2-H4K8ac_peak_16046	12.57297	AC007228.11_ENSG00000269696;ZNF470_ENSG00000197016
19	57106679	57107060	KD2-H4K8ac_peak_16047	22.21705	ZNF71_ENSG00000197951
19	57182549	57183095	KD2-H4K8ac_peak_16048	7.76329	ZNF835_ENSG00000127903;AC007228.5_ENSG00000268352
19	57702470	57702735	KD2-H4K8ac_peak_16049	10.17341	ZNF264_ENSG00000083844
19	57751142	57751753	KD2-H4K8ac_peak_16050	10.05606	ZNF805_ENSG00000204524
19	57752045	57752661	KD2-H4K8ac_peak_16051	7.36799	ZNF805_ENSG00000204524
19	57791328	57791614	KD2-H4K8ac_peak_16052	13.27022	CTC-444N24.6_ENSG00000267871;ZNF460_ENSG00000197714

19	57791932	57792215	KD2-H4K8ac_peak_16053	7.11824	CTC-444N24.6_ENSG00000267871;ZNF460_ENSG00000197714
19	57831953	57832381	KD2-H4K8ac_peak_16054	10.7186	ZNF543_ENSG00000178229
19	57861187	57861425	KD2-H4K8ac_peak_16055	6.94255	
19	57862113	57862423	KD2-H4K8ac_peak_16056	10.8368	CTC-444N24.13_ENSG00000268678;ZNF304_ENSG00000131845
19	57862664	57863102	KD2-H4K8ac_peak_16057	8.30974	CTC-444N24.13_ENSG00000268678;ZNF304_ENSG00000131845
19	57946206	57946404	KD2-H4K8ac_peak_16058	4.42765	ZNF749_ENSG00000186230
19	57946832	57947381	KD2-H4K8ac_peak_16059	5.30799	ZNF749_ENSG00000186230
19	57988559	57988825	KD2-H4K8ac_peak_16060	10.73467	AC004076.9_ENSG00000268163;ZNF772_ENSG00000197128;AC003005.2_ENSG00000268266
19	57989134	57989344	KD2-H4K8ac_peak_16061	7.01964	AC004076.9_ENSG00000268163;ZNF772_ENSG00000197128;AC003005.2_ENSG00000268266
19	57998764	57999516	KD2-H4K8ac_peak_16062	16.8435	ZNF419_ENSG00000105136;AC003005.4_ENSG00000268107
19	58038761	58039162	KD2-H4K8ac_peak_16063	8.85813	ZNF549_ENSG00000121406
19	58070805	58071148	KD2-H4K8ac_peak_16064	7.36799	ZNF550_ENSG00000251369
19	58089834	58090184	KD2-H4K8ac_peak_16065	6.60096	ZNF416_ENSG00000083817;ZIK1_ENSG00000171649
19	58094959	58095300	KD2-H4K8ac_peak_16066	5.92591	
19	58125702	58126105	KD2-H4K8ac_peak_16067	7.76329	ZNF134_ENSG00000213762
19	58175655	58176208	KD2-H4K8ac_peak_16068	8.02571	AC003682.17_ENSG00000269097
19	58192725	58193060	KD2-H4K8ac_peak_16069	4.77079	ZNF551_ENSG00000204519;AC003006.7_ENSG00000269026
19	58193444	58193851	KD2-H4K8ac_peak_16070	6.45083	ZNF551_ENSG00000204519;AC003006.7_ENSG00000269026
19	58258263	58258729	KD2-H4K8ac_peak_16071	7.47462	ZNF776_ENSG00000152443
19	58315030	58315381	KD2-H4K8ac_peak_16072	21.10246	
19	58325875	58326163	KD2-H4K8ac_peak_16073	6.2781	ZNF552_ENSG00000178935
19	58331148	58331436	KD2-H4K8ac_peak_16074	12.57297	CTD-2583A14.10_ENSG00000268750;ZNF587B_ENSG00000269343
19	58361226	58361859	KD2-H4K8ac_peak_16075	10.05606	ZNF587_ENSG00000198466
19	58427590	58427898	KD2-H4K8ac_peak_16076	22.12789	CTD-2583A14.9_ENSG00000269476;ZNF417_ENSG00000173480
19	58458681	58458909	KD2-H4K8ac_peak_16077	7.51054	ZNF256_ENSG00000152454
19	58513550	58514799	KD2-H4K8ac_peak_16078	22.04674	ZNF606_ENSG00000166704;CTD-2368P22.1_ENSG00000176593
19	58545550	58545758	KD2-H4K8ac_peak_16079	5.51783	ZSCAN1_ENSG00000152467
19	58609221	58609578	KD2-H4K8ac_peak_16080	26.59781	
19	58661779	58662068	KD2-H4K8ac_peak_16081	4.37809	
19	58666178	58666425	KD2-H4K8ac_peak_16082	12.00597	ZNF329_ENSG00000181894
19	58666662	58667135	KD2-H4K8ac_peak_16083	10.96455	ZNF329_ENSG00000181894
19	58693970	58694196	KD2-H4K8ac_peak_16084	9.07071	ZNF274_ENSG00000171606
19	58694449	58695336	KD2-H4K8ac_peak_16085	11.05467	ZNF274_ENSG00000171606
19	58789831	58790032	KD2-H4K8ac_peak_16086	7.07117	CTD-3138B18.5_ENSG00000268516;ZNF8_ENSG00000083842;ZNF8_ENSG00000273439;AC010642.1_ENSG00000267216
19	58790400	58790675	KD2-H4K8ac_peak_16087	6.80488	CTD-3138B18.5_ENSG00000268516;ZNF8_ENSG00000083842;ZNF8_ENSG00000273439;AC010642.1_ENSG00000267216
19	58790956	58791180	KD2-H4K8ac_peak_16088	8.55271	CTD-3138B18.5_ENSG00000268516;ZNF8_ENSG00000083842;ZNF8_ENSG00000273439;AC010642.1_ENSG00000267216
19	58816773	58817193	KD2-H4K8ac_peak_16089	7.81657	ERVK3-1_ENSG00000142396
19	58838441	58839028	KD2-H4K8ac_peak_16090	7.36799	ZSCAN22_ENSG00000182318
19	58872720	58873704	KD2-H4K8ac_peak_16091	12.79505	CTD-2619J13.8_ENSG00000268230;ZNF497_ENSG00000174586;CTD-2619J13.3_ENSG00000269054
19	58891535	58892400	KD2-H4K8ac_peak_16092	12.92667	ZNF837_ENSG00000152475
19	58897331	58898389	KD2-H4K8ac_peak_16093	13.27022	RPS5_ENSG00000083845;MIR4754_ENSG00000266640
19	58912130	58912480	KD2-H4K8ac_peak_16094	7.28827	CTD-2619J13.13_ENSG00000268307;ZNF584_ENSG00000171574
19	58920137	58920686	KD2-H4K8ac_peak_16095	10.19949	CTD-2619J13.14_ENSG00000232098
19	58950946	58951490	KD2-H4K8ac_peak_16096	12.30509	ZNF132_ENSG00000131849;CTD-2619J13.19_ENSG00000269473
19	58951807	58952316	KD2-H4K8ac_peak_16097	17.8868	ZNF132_ENSG00000131849;CTD-2619J13.19_ENSG00000269473
19	58962998	58963416	KD2-H4K8ac_peak_16098	6.36375	ZNF324B_ENSG00000249471

19	58978808	58979025	KD2-H4K8ac_peak_16099	5.63633	ZNF324_ENSG00000083812
19	58987579	58988341	KD2-H4K8ac_peak_16100	14.69247	CTD-2619J13.23_ENSG00000269106
19	59010996	59011296	KD2-H4K8ac_peak_16101	7.96322	
19	59025032	59025308	KD2-H4K8ac_peak_16102	7.07117	
19	59030368	59031144	KD2-H4K8ac_peak_16103	13.70753	
19	59031511	59031852	KD2-H4K8ac_peak_16104	7.36799	
19	59032113	59032312	KD2-H4K8ac_peak_16105	6.68439	
19	59066072	59066383	KD2-H4K8ac_peak_16106	20.64015	CHMP2A_ENSG00000130724
19	59066707	59067021	KD2-H4K8ac_peak_16107	6.55628	CHMP2A_ENSG00000130724
19	59070578	59070928	KD2-H4K8ac_peak_16108	11.90231	UBE2M_ENSG00000130725;AC016629.8_ENSG00000267858
19	59071121	59071799	KD2-H4K8ac_peak_16109	12.30509	UBE2M_ENSG00000130725;AC016629.8_ENSG00000267858
19	59073688	59074726	KD2-H4K8ac_peak_16110	10.78621	
19	59083825	59084067	KD2-H4K8ac_peak_16111	9.1707	MZF1_ENSG00000099326;CENPBD1P1_ENSG00000213753
19	59084277	59084740	KD2-H4K8ac_peak_16112	11.24702	MZF1_ENSG00000099326;CENPBD1P1_ENSG00000213753
19	59087117	59087416	KD2-H4K8ac_peak_16113	8.58069	
2	263988	264551	KD2-H4K8ac_peak_16114	18.43081	ACP1_ENSG00000143727
2	264986	265481	KD2-H4K8ac_peak_16115	12.57297	SH3YL1_ENSG00000035115;ACP1_ENSG00000143727
2	385251	385454	KD2-H4K8ac_peak_16116	6.66692	
2	609701	609939	KD2-H4K8ac_peak_16117	5.20908	
2	636630	637127	KD2-H4K8ac_peak_16118	9.63487	
2	677151	677922	KD2-H4K8ac_peak_16119	27.43474	TMEM18_ENSG00000151353;AC092159.2_ENSG00000233296
2	690016	690252	KD2-H4K8ac_peak_16120	5.75757	
2	764300	764518	KD2-H4K8ac_peak_16121	3.75035	
2	844421	844890	KD2-H4K8ac_peak_16122	14.68449	
2	945749	946064	KD2-H4K8ac_peak_16123	12.99417	AC116614.1_ENSG00000235688;SNTG2_ENSG00000172554
2	946571	946924	KD2-H4K8ac_peak_16124	8.2413	AC116614.1_ENSG00000235688;SNTG2_ENSG00000172554
2	1231223	1231469	KD2-H4K8ac_peak_16125	6.72624	
2	1251165	1251410	KD2-H4K8ac_peak_16126	6.40683	
2	1286794	1287531	KD2-H4K8ac_peak_16127	4.86324	
2	1370326	1370706	KD2-H4K8ac_peak_16128	8.4716	
2	1514177	1514369	KD2-H4K8ac_peak_16129	4.52514	
2	1544821	1545252	KD2-H4K8ac_peak_16130	4.06758	
2	1545628	1545991	KD2-H4K8ac_peak_16131	4.37809	
2	1546562	1546767	KD2-H4K8ac_peak_16132	5.81222	
2	1554276	1554860	KD2-H4K8ac_peak_16133	5.54647	
2	1595711	1595948	KD2-H4K8ac_peak_16134	4.04573	
2	1598166	1598846	KD2-H4K8ac_peak_16135	7.38799	
2	1609324	1609560	KD2-H4K8ac_peak_16136	4.13134	
2	1711696	1712479	KD2-H4K8ac_peak_16137	6.84261	
2	1718036	1718481	KD2-H4K8ac_peak_16138	8.1235	
2	1719217	1719613	KD2-H4K8ac_peak_16139	8.55271	
2	1719835	1720098	KD2-H4K8ac_peak_16140	6.65673	
2	1734025	1734557	KD2-H4K8ac_peak_16141	6.40297	
2	1735183	1735594	KD2-H4K8ac_peak_16142	8.90799	
2	1747674	1748110	KD2-H4K8ac_peak_16143	14.48922	PXDN_ENSG00000130508
2	1748356	1748923	KD2-H4K8ac_peak_16144	5.6845	PXDN_ENSG00000130508

2	1820518	1820718	KD2-H4K8ac_peak_16145	4.86324	
2	1870928	1871124	KD2-H4K8ac_peak_16146	5.82799	
2	1972565	1972770	KD2-H4K8ac_peak_16147	8.1235	
2	2110656	2111030	KD2-H4K8ac_peak_16148	6.30607	
2	2111651	2112060	KD2-H4K8ac_peak_16149	7.20567	
2	2112956	2113180	KD2-H4K8ac_peak_16150	7.07117	
2	2114537	2115165	KD2-H4K8ac_peak_16151	8.85813	
2	2116635	2117348	KD2-H4K8ac_peak_16152	5.4779	
2	2156950	2157315	KD2-H4K8ac_peak_16153	4.67342	
2	2192911	2193202	KD2-H4K8ac_peak_16154	7.81657	
2	2195558	2196018	KD2-H4K8ac_peak_16155	5.72327	
2	2196794	2197066	KD2-H4K8ac_peak_16156	5.54647	
2	2256882	2257074	KD2-H4K8ac_peak_16157	6.2781	
2	2275321	2275570	KD2-H4K8ac_peak_16158	4.06758	
2	2326042	2326408	KD2-H4K8ac_peak_16159	3.75035	
2	2327872	2328577	KD2-H4K8ac_peak_16160	6.90231	
2	2329955	2330161	KD2-H4K8ac_peak_16161	6.66692	
2	2335812	2336092	KD2-H4K8ac_peak_16162	4.211	MYT1L_ENSG00000186487
2	2336357	2336620	KD2-H4K8ac_peak_16163	5.57044	
2	2337111	2337385	KD2-H4K8ac_peak_16164	13.62729	
2	2467933	2468389	KD2-H4K8ac_peak_16165	6.25869	
2	2527374	2527647	KD2-H4K8ac_peak_16166	4.53319	
2	2585491	2585746	KD2-H4K8ac_peak_16167	7.0754	
2	2616997	2617238	KD2-H4K8ac_peak_16168	10.19949	
2	2617620	2618491	KD2-H4K8ac_peak_16169	11.05467	
2	2737812	2738033	KD2-H4K8ac_peak_16170	5.82799	
2	2751737	2751984	KD2-H4K8ac_peak_16171	6.50882	
2	2791084	2791315	KD2-H4K8ac_peak_16172	6.67092	
2	2849557	2850912	KD2-H4K8ac_peak_16173	11.28831	
2	2851237	2851484	KD2-H4K8ac_peak_16174	8.87149	
2	2851997	2852490	KD2-H4K8ac_peak_16175	13.10449	
2	2852788	2855022	KD2-H4K8ac_peak_16176	22.66567	
2	2855662	2856060	KD2-H4K8ac_peak_16177	9.11876	
2	2870983	2871180	KD2-H4K8ac_peak_16178	4.211	
2	2922794	2923507	KD2-H4K8ac_peak_16179	10.87093	
2	2923943	2924144	KD2-H4K8ac_peak_16180	4.44527	
2	2937162	2937731	KD2-H4K8ac_peak_16181	12.30509	
2	2937975	2938338	KD2-H4K8ac_peak_16182	5.30799	
2	2979713	2980011	KD2-H4K8ac_peak_16183	6.99815	
2	3045060	3045399	KD2-H4K8ac_peak_16184	9.55894	
2	3045917	3046357	KD2-H4K8ac_peak_16185	5.78173	
2	3046672	3047002	KD2-H4K8ac_peak_16186	7.38799	
2	3080809	3081884	KD2-H4K8ac_peak_16187	15.69572	
2	3101627	3101957	KD2-H4K8ac_peak_16188	6.93012	
2	3103006	3103850	KD2-H4K8ac_peak_16189	7.146	
2	3104316	3105286	KD2-H4K8ac_peak_16190	21.09086	

2	3105716	3107228	KD2-H4K8ac_peak_16191	26.9893	
2	3108083	3108581	KD2-H4K8ac_peak_16192	20.17267	
2	3109497	3110288	KD2-H4K8ac_peak_16193	9.91109	
2	3111943	3112270	KD2-H4K8ac_peak_16194	9.20193	
2	3115561	3115814	KD2-H4K8ac_peak_16195	8.30974	
2	3118293	3118508	KD2-H4K8ac_peak_16196	5.14125	
2	3121135	3121944	KD2-H4K8ac_peak_16197	8.02571	
2	3129243	3130587	KD2-H4K8ac_peak_16198	22.03039	AC019118.2_ENSG00000234423
2	3130809	3131079	KD2-H4K8ac_peak_16199	9.26408	
2	3131545	3132063	KD2-H4K8ac_peak_16200	21.46903	
2	3132596	3133510	KD2-H4K8ac_peak_16201	7.73264	
2	3133873	3135697	KD2-H4K8ac_peak_16202	14.63458	
2	3204729	3204941	KD2-H4K8ac_peak_16203	9.62006	
2	3285740	3286253	KD2-H4K8ac_peak_16204	3.97079	
2	3286973	3287374	KD2-H4K8ac_peak_16205	6.25869	
2	3305714	3305934	KD2-H4K8ac_peak_16206	7.54159	TSSC1-IT1_ENSG00000224885
2	3306265	3306494	KD2-H4K8ac_peak_16207	7.07117	
2	3306722	3307361	KD2-H4K8ac_peak_16208	17.09549	
2	3316836	3317830	KD2-H4K8ac_peak_16209	9.95759	
2	3318442	3318769	KD2-H4K8ac_peak_16210	9.8391	
2	3381236	3381452	KD2-H4K8ac_peak_16211	9.20193	TSSC1_ENSG00000032389
2	3381987	3382371	KD2-H4K8ac_peak_16212	8.88082	TSSC1_ENSG00000032389
2	3451827	3452690	KD2-H4K8ac_peak_16213	15.61381	
2	3463438	3463699	KD2-H4K8ac_peak_16214	6.45063	
2	3497070	3497597	KD2-H4K8ac_peak_16215	5.3608	
2	3522197	3522590	KD2-H4K8ac_peak_16216	7.25989	ADI1_ENSG00000182551;AC142528.1_ENSG00000235078
2	3523037	3523861	KD2-H4K8ac_peak_16217	24.70186	ADI1_ENSG00000182551;AC142528.1_ENSG00000235078
2	3605517	3605920	KD2-H4K8ac_peak_16218	13.02811	RNASEH1_ENSG00000171865;RNASEH1-AS1_ENSG00000234171
2	3606166	3606829	KD2-H4K8ac_peak_16219	11.05467	RNASEH1_ENSG00000171865;RNASEH1-AS1_ENSG00000234171
2	3622228	3622721	KD2-H4K8ac_peak_16220	18.40451	RPS7_ENSG00000171863
2	3622925	3623465	KD2-H4K8ac_peak_16221	11.66477	RPS7_ENSG00000171863
2	3632687	3633387	KD2-H4K8ac_peak_16222	10.72313	
2	3633604	3633962	KD2-H4K8ac_peak_16223	4.85908	
2	3642064	3643293	KD2-H4K8ac_peak_16224	10.09705	COLEC11_ENSG00000118004
2	3650096	3650431	KD2-H4K8ac_peak_16225	9.48511	
2	3650688	3651821	KD2-H4K8ac_peak_16226	12.93209	AC010907.2_ENSG00000237370
2	3652202	3652399	KD2-H4K8ac_peak_16227	7.11824	AC010907.2_ENSG00000237370
2	3653326	3654627	KD2-H4K8ac_peak_16228	20.64015	
2	3655192	3655690	KD2-H4K8ac_peak_16229	11.24702	
2	3656176	3656637	KD2-H4K8ac_peak_16230	3.83717	
2	3657150	3657584	KD2-H4K8ac_peak_16231	6.65673	
2	3681144	3681387	KD2-H4K8ac_peak_16232	5.20998	
2	3697817	3698517	KD2-H4K8ac_peak_16233	10.20046	
2	3751441	3751919	KD2-H4K8ac_peak_16234	14.48084	AC010907.5_ENSG00000224661;DCDC2C_ENSG00000214866
2	5619471	5619741	KD2-H4K8ac_peak_16235	8.4716	
2	5660626	5660822	KD2-H4K8ac_peak_16236	6.24114	

2	5661747	5662193	KD2-H4K8ac_peak_16237	6.83936	
2	5663523	5663716	KD2-H4K8ac_peak_16238	4.54838	
2	5679302	5680109	KD2-H4K8ac_peak_16239	10.59312	
2	5806363	5806785	KD2-H4K8ac_peak_16240	4.86324	
2	5813435	5814215	KD2-H4K8ac_peak_16241	17.75427	
2	5830941	5831388	KD2-H4K8ac_peak_16242	9.95119	AC107057.2_ENSG00000224128;AC108025.2_ENSG00000230090
2	5831594	5832309	KD2-H4K8ac_peak_16243	14.68449	AC107057.2_ENSG00000224128;AC108025.2_ENSG00000230090;SOX11_ENSG00000176887
2	5833051	5833372	KD2-H4K8ac_peak_16244	7.50083	SOX11_ENSG00000176887
2	5836062	5837282	KD2-H4K8ac_peak_16245	9.1707	AC010729.1_ENSG00000242540
2	5979299	5979986	KD2-H4K8ac_peak_16246	6.25869	
2	5981326	5981548	KD2-H4K8ac_peak_16247	6.0605	
2	5982723	5982927	KD2-H4K8ac_peak_16248	3.75035	
2	5990869	5991229	KD2-H4K8ac_peak_16249	5.34946	
2	6052715	6052953	KD2-H4K8ac_peak_16250	9.36516	
2	6067056	6067495	KD2-H4K8ac_peak_16251	6.65673	
2	6072946	6073365	KD2-H4K8ac_peak_16252	8.4814	LINC01105_ENSG00000232044
2	6078624	6078835	KD2-H4K8ac_peak_16253	4.71229	
2	6120639	6121140	KD2-H4K8ac_peak_16254	8.87852	
2	6121454	6122131	KD2-H4K8ac_peak_16255	11.05155	
2	6209637	6209833	KD2-H4K8ac_peak_16256	7.54952	
2	6212859	6213178	KD2-H4K8ac_peak_16257	8.4716	
2	6213781	6214080	KD2-H4K8ac_peak_16258	5.88845	
2	6214378	6214812	KD2-H4K8ac_peak_16259	13.35153	
2	6217246	6217475	KD2-H4K8ac_peak_16260	5.88845	
2	6220711	6221036	KD2-H4K8ac_peak_16261	11.28831	
2	6223024	6223423	KD2-H4K8ac_peak_16262	8.34752	
2	6251643	6251878	KD2-H4K8ac_peak_16263	7.15405	
2	6271548	6271840	KD2-H4K8ac_peak_16264	11.75669	
2	6324914	6325133	KD2-H4K8ac_peak_16265	3.75035	
2	6392714	6392912	KD2-H4K8ac_peak_16266	6.82976	
2	6429500	6430049	KD2-H4K8ac_peak_16267	9.26287	
2	6430795	6431170	KD2-H4K8ac_peak_16268	9.57101	
2	6450796	6451302	KD2-H4K8ac_peak_16269	15.61313	
2	6451547	6451808	KD2-H4K8ac_peak_16270	4.86324	
2	6651047	6651278	KD2-H4K8ac_peak_16271	8.80225	
2	6662097	6662390	KD2-H4K8ac_peak_16272	5.16215	
2	6663608	6663864	KD2-H4K8ac_peak_16273	8.30974	
2	6665586	6666123	KD2-H4K8ac_peak_16274	12.70959	
2	6675052	6675253	KD2-H4K8ac_peak_16275	5.64449	
2	6910200	6910644	KD2-H4K8ac_peak_16276	8.85813	LINC00487_ENSG00000205837
2	6912237	6912855	KD2-H4K8ac_peak_16277	9.96798	
2	6968205	6968420	KD2-H4K8ac_peak_16278	7.44366	
2	7005166	7006182	KD2-H4K8ac_peak_16279	13.97093	CMPK2_ENSG00000134326;RSAD2_ENSG00000134321
2	7057077	7057376	KD2-H4K8ac_peak_16280	9.36516	RNF144A_ENSG00000151692
2	7057646	7058620	KD2-H4K8ac_peak_16281	11.24702	RNF144A-AS1_ENSG00000228203;RNF144A_ENSG00000151692
2	7133503	7133799	KD2-H4K8ac_peak_16282	5.30799	

2	7134760	7135257	KD2-H4K8ac_peak_16283	7.76329	
2	7147603	7149053	KD2-H4K8ac_peak_16284	8.35267	
2	7149265	7149532	KD2-H4K8ac_peak_16285	5.32397	
2	7155166	7155593	KD2-H4K8ac_peak_16286	5.32397	
2	7157104	7157318	KD2-H4K8ac_peak_16287	8.01338	
2	7158150	7158589	KD2-H4K8ac_peak_16288	13.73635	
2	7160208	7160429	KD2-H4K8ac_peak_16289	4.211	
2	7237810	7238184	KD2-H4K8ac_peak_16290	11.58876	
2	8333980	8334243	KD2-H4K8ac_peak_16291	6.2781	
2	8369665	8369921	KD2-H4K8ac_peak_16292	7.52168	
2	8422249	8422588	KD2-H4K8ac_peak_16293	4.67342	
2	8442631	8442947	KD2-H4K8ac_peak_16294	8.85813	
2	8675870	8676302	KD2-H4K8ac_peak_16295	4.37809	
2	8676591	8676829	KD2-H4K8ac_peak_16296	8.10216	
2	8677049	8677364	KD2-H4K8ac_peak_16297	9.91109	
2	8679775	8680027	KD2-H4K8ac_peak_16298	11.38464	
2	8680357	8680707	KD2-H4K8ac_peak_16299	7.92775	
2	8683718	8684240	KD2-H4K8ac_peak_16300	9.78882	
2	8685597	8685835	KD2-H4K8ac_peak_16301	4.211	
2	8816920	8817814	KD2-H4K8ac_peak_16302	8.55271	
2	8818340	8818715	KD2-H4K8ac_peak_16303	7.36799	ID2_ENSG00000115738
2	8819198	8819788	KD2-H4K8ac_peak_16304	8.63033	ID2_ENSG00000115738
2	8825112	8825868	KD2-H4K8ac_peak_16305	10.43145	
2	8833666	8834066	KD2-H4K8ac_peak_16306	8.85813	
2	8977306	8977670	KD2-H4K8ac_peak_16307	6.66692	KIDINS220_ENSG00000134313
2	9143548	9143848	KD2-H4K8ac_peak_16308	10.45683	MBOAT2_ENSG00000143797
2	9144249	9144744	KD2-H4K8ac_peak_16309	13.95627	MBOAT2_ENSG00000143797
2	9201438	9201636	KD2-H4K8ac_peak_16310	6.25869	
2	9324075	9324473	KD2-H4K8ac_peak_16311	9.36665	
2	9346360	9346726	KD2-H4K8ac_peak_16312	28.09984	ASAP2_ENSG00000151693
2	9346920	9347997	KD2-H4K8ac_peak_16313	16.8435	ASAP2_ENSG00000151693
2	9358423	9358620	KD2-H4K8ac_peak_16314	4.62142	
2	9370527	9370776	KD2-H4K8ac_peak_16315	5.98692	
2	9371753	9372064	KD2-H4K8ac_peak_16316	4.44527	
2	9374392	9374633	KD2-H4K8ac_peak_16317	9.41741	
2	9374836	9375147	KD2-H4K8ac_peak_16318	5.81222	
2	9375621	9376317	KD2-H4K8ac_peak_16319	8.0287	
2	9377652	9377901	KD2-H4K8ac_peak_16320	5.81222	
2	9386834	9387303	KD2-H4K8ac_peak_16321	13.13965	
2	9387750	9387986	KD2-H4K8ac_peak_16322	8.10216	
2	9389194	9389518	KD2-H4K8ac_peak_16323	7.51682	
2	9391203	9391515	KD2-H4K8ac_peak_16324	8.64831	
2	9476696	9476958	KD2-H4K8ac_peak_16325	5.38504	
2	9526621	9526869	KD2-H4K8ac_peak_16326	4.90805	
2	9563141	9563610	KD2-H4K8ac_peak_16327	22.93827	ITGB1BP1_ENSG00000119185;CPSF3_ENSG00000119203
2	9563892	9564318	KD2-H4K8ac_peak_16328	7.73264	ITGB1BP1_ENSG00000119185;CPSF3_ENSG00000119203

2	9614132	9615246	KD2-H4K8ac_peak_16329	20.18381	IAH1_ENSG00000134330
2	9695433	9695826	KD2-H4K8ac_peak_16330	12.37938	ADAM17_ENSG00000151694;RP11-214N9.1_ENSG00000271855
2	9696113	9696690	KD2-H4K8ac_peak_16331	21.52156	ADAM17_ENSG00000151694;RP11-214N9.1_ENSG00000271855
2	9770401	9771092	KD2-H4K8ac_peak_16332	12.08081	YWHAQ_ENSG00000134308
2	9771500	9771787	KD2-H4K8ac_peak_16333	12.70959	YWHAQ_ENSG00000134308
2	9830466	9830700	KD2-H4K8ac_peak_16334	7.57979	
2	9893892	9894378	KD2-H4K8ac_peak_16335	5.14125	
2	9940114	9940478	KD2-H4K8ac_peak_16336	7.74292	
2	9983155	9983840	KD2-H4K8ac_peak_16337	14.30822	TAF1B_ENSG00000115750
2	10092098	10092485	KD2-H4K8ac_peak_16338	10.66719	
2	10146593	10146894	KD2-H4K8ac_peak_16339	7.62396	AC010969.1_ENSG00000188525
2	10149876	10150200	KD2-H4K8ac_peak_16340	4.73897	
2	10183769	10184210	KD2-H4K8ac_peak_16341	12.93209	KLF11_ENSG00000172059
2	10197691	10197990	KD2-H4K8ac_peak_16342	4.86574	
2	10219753	10220197	KD2-H4K8ac_peak_16343	6.60096	CYS1_ENSG00000205795
2	10259864	10260359	KD2-H4K8ac_peak_16344	9.67696	RP11-254F7.4_ENSG00000272524
2	10260631	10260853	KD2-H4K8ac_peak_16345	9.78882	RP11-254F7.4_ENSG00000272524
2	10262125	10262643	KD2-H4K8ac_peak_16346	6.40874	RRM2_ENSG00000171848
2	10310656	10310911	KD2-H4K8ac_peak_16347	7.07117	
2	10394574	10395269	KD2-H4K8ac_peak_16348	8.59579	
2	10425444	10427484	KD2-H4K8ac_peak_16349	27.99951	
2	10442263	10442796	KD2-H4K8ac_peak_16350	10.70697	HPCAL1_ENSG00000115756
2	10471347	10471568	KD2-H4K8ac_peak_16351	4.37815	
2	10482773	10482981	KD2-H4K8ac_peak_16352	3.86308	
2	10483716	10484142	KD2-H4K8ac_peak_16353	5.13615	
2	10487861	10488111	KD2-H4K8ac_peak_16354	4.56666	
2	10501117	10501387	KD2-H4K8ac_peak_16355	6.65673	
2	10502574	10502948	KD2-H4K8ac_peak_16356	8.55271	
2	10529397	10529604	KD2-H4K8ac_peak_16357	4.98639	
2	10530774	10531463	KD2-H4K8ac_peak_16358	10.39103	
2	10531942	10532423	KD2-H4K8ac_peak_16359	7.36799	
2	10532790	10533146	KD2-H4K8ac_peak_16360	9.26408	
2	10533609	10535113	KD2-H4K8ac_peak_16361	9.87773	
2	10544564	10545473	KD2-H4K8ac_peak_16362	8.04448	
2	10587842	10588567	KD2-H4K8ac_peak_16363	16.67374	ODC1_ENSG00000115758;SNORA80B_ENSG00000206633;RP11-320M2.1_ENSG00000257135
2	10588818	10589244	KD2-H4K8ac_peak_16364	26.9893	ODC1_ENSG00000115758;RP11-320M2.1_ENSG00000257135
2	10688719	10689030	KD2-H4K8ac_peak_16365	17.8737	
2	10689432	10689642	KD2-H4K8ac_peak_16366	3.86308	
2	10689928	10690214	KD2-H4K8ac_peak_16367	4.0809	
2	10829865	10830987	KD2-H4K8ac_peak_16368	8.25648	NOL10_ENSG00000115761;RN7SL832P_ENSG00000243819
2	10952057	10952500	KD2-H4K8ac_peak_16369	4.33139	
2	10955644	10955838	KD2-H4K8ac_peak_16370	6.76298	RNU7-176P_ENSG00000238962
2	11008635	11009186	KD2-H4K8ac_peak_16371	9.6247	
2	11052165	11052498	KD2-H4K8ac_peak_16372	5.9696	KCNF1_ENSG00000162975
2	11075463	11075655	KD2-H4K8ac_peak_16373	4.35724	
2	11270360	11270582	KD2-H4K8ac_peak_16374	9.61895	

2	11272344	11272948	KD2-H4K8ac_peak_16375	7.36799	AC062028.1_ENSG00000145063;C2orf50_ENSG00000150873
2	11273192	11273744	KD2-H4K8ac_peak_16376	10.21366	AC062028.1_ENSG00000145063;C2orf50_ENSG00000150873
2	11294842	11295178	KD2-H4K8ac_peak_16377	9.36516	PQLC3_ENSG00000162976
2	11295587	11296028	KD2-H4K8ac_peak_16378	13.12288	PQLC3_ENSG00000162976
2	11484178	11484691	KD2-H4K8ac_peak_16379	13.3695	
2	11484970	11485670	KD2-H4K8ac_peak_16380	26.92276	
2	11527424	11527800	KD2-H4K8ac_peak_16381	7.43041	
2	11605560	11606201	KD2-H4K8ac_peak_16382	6.52179	E2F6_ENSG00000169016
2	11606517	11606815	KD2-H4K8ac_peak_16383	6.20982	E2F6_ENSG00000169016
2	11622265	11622791	KD2-H4K8ac_peak_16384	13.92465	
2	11623005	11623740	KD2-H4K8ac_peak_16385	11.3923	
2	11780636	11780832	KD2-H4K8ac_peak_16386	4.8888	
2	11822091	11822289	KD2-H4K8ac_peak_16387	11.50346	AC106875.1_ENSG00000228496
2	11836724	11837905	KD2-H4K8ac_peak_16388	11.80283	
2	11886157	11886627	KD2-H4K8ac_peak_16389	7.49599	AC012456.4_ENSG00000230790
2	11886893	11887357	KD2-H4K8ac_peak_16390	9.95119	
2	11969792	11970284	KD2-H4K8ac_peak_16391	6.18657	
2	12070626	12071154	KD2-H4K8ac_peak_16392	8.30974	
2	12222782	12223012	KD2-H4K8ac_peak_16393	6.40297	
2	12856691	12856891	KD2-H4K8ac_peak_16394	9.65086	TRIB2_ENSG00000071575
2	12857118	12857675	KD2-H4K8ac_peak_16395	8.86099	TRIB2_ENSG00000071575
2	13087723	13088080	KD2-H4K8ac_peak_16396	14.15075	
2	13088769	13089640	KD2-H4K8ac_peak_16397	12.06346	
2	14453978	14454175	KD2-H4K8ac_peak_16398	7.38933	
2	14772273	14772683	KD2-H4K8ac_peak_16399	12.08081	FAM84A_ENSG00000162981
2	14774165	14774529	KD2-H4K8ac_peak_16400	5.38581	AC011897.1_ENSG00000265629
2	14774879	14775659	KD2-H4K8ac_peak_16401	16.33377	AC011897.1_ENSG00000265629
2	15701596	15702123	KD2-H4K8ac_peak_16402	8.55271	NBAS_ENSG00000151779
2	15731190	15732420	KD2-H4K8ac_peak_16403	16.25682	DDX1_ENSG00000079785
2	15965910	15966218	KD2-H4K8ac_peak_16404	5.30799	
2	15982028	15982292	KD2-H4K8ac_peak_16405	10.31695	
2	15982668	15983008	KD2-H4K8ac_peak_16406	10.92268	
2	15983390	15984446	KD2-H4K8ac_peak_16407	23.24123	
2	15986321	15986536	KD2-H4K8ac_peak_16408	8.2413	
2	16031575	16031842	KD2-H4K8ac_peak_16409	10.84242	
2	16080211	16080419	KD2-H4K8ac_peak_16410	7.18615	MYCN_ENSG00000134323
2	16080907	16081425	KD2-H4K8ac_peak_16411	4.28151	MYCNOS_ENSG00000233718;MYCN_ENSG00000134323
2	16124317	16125304	KD2-H4K8ac_peak_16412	13.10449	
2	16210432	16210708	KD2-H4K8ac_peak_16413	5.88845	
2	16216869	16217088	KD2-H4K8ac_peak_16414	7.04492	
2	16234775	16235287	KD2-H4K8ac_peak_16415	9.63487	
2	16378531	16379338	KD2-H4K8ac_peak_16416	16.33377	
2	16380345	16380830	KD2-H4K8ac_peak_16417	10.46874	SNORA40_ENSG00000251704
2	16408040	16408246	KD2-H4K8ac_peak_16418	7.76329	
2	16513141	16513385	KD2-H4K8ac_peak_16419	5.88845	
2	16516261	16516481	KD2-H4K8ac_peak_16420	6.24114	

2	16517040	16517295	KD2-H4K8ac_peak_16421	8.83582	
2	16575449	16575724	KD2-H4K8ac_peak_16422	8.4716	
2	16689308	16689607	KD2-H4K8ac_peak_16423	11.76169	
2	17180538	17180743	KD2-H4K8ac_peak_16424	5.14125	
2	17181813	17182061	KD2-H4K8ac_peak_16425	11.07495	
2	17190506	17190831	KD2-H4K8ac_peak_16426	6.72334	
2	17192100	17192362	KD2-H4K8ac_peak_16427	9.59814	
2	17199175	17199412	KD2-H4K8ac_peak_16428	4.3015	
2	17212361	17212609	KD2-H4K8ac_peak_16429	5.88845	
2	17264452	17264702	KD2-H4K8ac_peak_16430	10.05606	
2	17266631	17266865	KD2-H4K8ac_peak_16431	6.92624	
2	17267844	17268355	KD2-H4K8ac_peak_16432	10.05606	
2	17490581	17490892	KD2-H4K8ac_peak_16433	4.06758	
2	17719420	17719660	KD2-H4K8ac_peak_16434	4.89359	VSNL1_ENSG00000163032
2	17720381	17720701	KD2-H4K8ac_peak_16435	9.95119	VSNL1_ENSG00000163032
2	17721422	17721701	KD2-H4K8ac_peak_16436	17.67328	
2	17751986	17752193	KD2-H4K8ac_peak_16437	4.89656	
2	17934689	17935889	KD2-H4K8ac_peak_16438	21.11201	GEN1_ENSG00000178295
2	18741376	18741822	KD2-H4K8ac_peak_16439	10.39576	RDH14_ENSG00000240857
2	18742467	18742735	KD2-H4K8ac_peak_16440	5.98124	RDH14_ENSG00000240857
2	18784969	18785275	KD2-H4K8ac_peak_16441	4.20044	
2	19547401	19547708	KD2-H4K8ac_peak_16442	12.13241	MIR4757_ENSG00000266738;AC092594.1_ENSG00000236204
2	20101009	20102224	KD2-H4K8ac_peak_16443	14.17488	TTC32_ENSG00000183891;RP11-79O8.1_ENSG00000271991
2	20103071	20103269	KD2-H4K8ac_peak_16444	7.07512	
2	20123719	20124318	KD2-H4K8ac_peak_16445	10.05606	
2	20190035	20190294	KD2-H4K8ac_peak_16446	14.39182	WDR35_ENSG00000118965;AC079145.4_ENSG00000227210
2	20210934	20211176	KD2-H4K8ac_peak_16447	7.47462	
2	20211747	20212915	KD2-H4K8ac_peak_16448	21.09086	MATN3_ENSG00000132031
2	20251640	20252675	KD2-H4K8ac_peak_16449	20.10577	LAPTM4A_ENSG00000068697;RP11-644K8.1_ENSG00000223734
2	20266086	20266535	KD2-H4K8ac_peak_16450	5.59859	
2	20270248	20270947	KD2-H4K8ac_peak_16451	11.9364	
2	20271241	20271467	KD2-H4K8ac_peak_16452	7.28628	
2	20272470	20273165	KD2-H4K8ac_peak_16453	9.95342	
2	20274020	20275027	KD2-H4K8ac_peak_16454	7.11824	
2	20335509	20335713	KD2-H4K8ac_peak_16455	5.72327	
2	20349118	20349733	KD2-H4K8ac_peak_16456	10.19723	
2	20351085	20351277	KD2-H4K8ac_peak_16457	5.53095	
2	20363977	20364231	KD2-H4K8ac_peak_16458	3.90565	
2	20366981	20367622	KD2-H4K8ac_peak_16459	8.07129	
2	20368355	20369057	KD2-H4K8ac_peak_16460	13.95627	
2	20422333	20422954	KD2-H4K8ac_peak_16461	4.84794	
2	20423254	20424661	KD2-H4K8ac_peak_16462	14.66259	SDC1_ENSG00000115884
2	20425085	20425573	KD2-H4K8ac_peak_16463	8.21852	SDC1_ENSG00000115884
2	20550120	20550519	KD2-H4K8ac_peak_16464	24.3087	
2	20550871	20551497	KD2-H4K8ac_peak_16465	14.07412	PUM2_ENSG00000055917
2	20589902	20590310	KD2-H4K8ac_peak_16466	7.74292	

2	20604000	20604246	KD2-H4K8ac_peak_16467	8.30974	
2	20619111	20619337	KD2-H4K8ac_peak_16468	5.33892	
2	20624427	20624838	KD2-H4K8ac_peak_16469	6.30229	
2	20646056	20646602	KD2-H4K8ac_peak_16470	9.20193	RHOB_ENSG00000143878
2	20646875	20647316	KD2-H4K8ac_peak_16471	17.27897	RHOB_ENSG00000143878
2	20666121	20666419	KD2-H4K8ac_peak_16472	5.29555	
2	20691607	20691864	KD2-H4K8ac_peak_16473	8.36432	
2	20706437	20706649	KD2-H4K8ac_peak_16474	4.38478	
2	20798789	20799160	KD2-H4K8ac_peak_16475	9.36516	
2	20817743	20818002	KD2-H4K8ac_peak_16476	4.35724	
2	20834259	20834585	KD2-H4K8ac_peak_16477	6.92611	
2	20834960	20835558	KD2-H4K8ac_peak_16478	8.55271	
2	20835861	20836121	KD2-H4K8ac_peak_16479	8.59224	
2	20850566	20850846	KD2-H4K8ac_peak_16480	11.7608	HS1BP3_ENSG00000118960
2	20851062	20851679	KD2-H4K8ac_peak_16481	14.07846	HS1BP3_ENSG00000118960
2	21021842	21022788	KD2-H4K8ac_peak_16482	15.2334	C2orf43_ENSG00000118961
2	21023582	21023783	KD2-H4K8ac_peak_16483	5.30494	C2orf43_ENSG00000118961
2	21213007	21213253	KD2-H4K8ac_peak_16484	6.97919	
2	21214117	21214326	KD2-H4K8ac_peak_16485	5.66705	
2	21506179	21506409	KD2-H4K8ac_peak_16486	4.67342	
2	21856423	21856662	KD2-H4K8ac_peak_16487	10.0692	
2	22258459	22258690	KD2-H4K8ac_peak_16488	4.77079	
2	22552159	22552545	KD2-H4K8ac_peak_16489	4.211	
2	23573321	23575277	KD2-H4K8ac_peak_16490	24.75714	
2	23607529	23607887	KD2-H4K8ac_peak_16491	11.79585	KLHL29_ENSG00000119771
2	23608534	23608726	KD2-H4K8ac_peak_16492	6.62457	KLHL29_ENSG00000119771
2	23755573	23755847	KD2-H4K8ac_peak_16493	5.9696	
2	23768028	23768782	KD2-H4K8ac_peak_16494	11.00372	
2	23769783	23770002	KD2-H4K8ac_peak_16495	6.24114	
2	23771820	23772094	KD2-H4K8ac_peak_16496	9.18237	
2	23772810	23773002	KD2-H4K8ac_peak_16497	4.21775	
2	23787271	23787576	KD2-H4K8ac_peak_16498	5.80191	
2	23841841	23842251	KD2-H4K8ac_peak_16499	11.05467	
2	23859320	23860869	KD2-H4K8ac_peak_16500	17.42905	
2	23891518	23892613	KD2-H4K8ac_peak_16501	12.88702	
2	23893015	23893458	KD2-H4K8ac_peak_16502	8.85813	
2	23922131	23922681	KD2-H4K8ac_peak_16503	7.76329	
2	24109872	24110085	KD2-H4K8ac_peak_16504	4.37815	
2	24149246	24149891	KD2-H4K8ac_peak_16505	13.22091	ATAD2B_ENSG00000119778;UBXN2A_ENSG00000173960
2	24150191	24150393	KD2-H4K8ac_peak_16506	16.57619	ATAD2B_ENSG00000119778;UBXN2A_ENSG00000173960
2	24162322	24163734	KD2-H4K8ac_peak_16507	23.72814	
2	24270542	24271339	KD2-H4K8ac_peak_16508	12.36441	
2	24271879	24272201	KD2-H4K8ac_peak_16509	5.01002	C2orf44_ENSG00000163026;FKBP1B_ENSG00000119782
2	24272617	24273056	KD2-H4K8ac_peak_16510	9.26408	C2orf44_ENSG00000163026;FKBP1B_ENSG00000119782
2	24299756	24300157	KD2-H4K8ac_peak_16511	8.4716	SF3B14_ENSG00000115128;FAM228B_ENSG00000219626
2	24306411	24306668	KD2-H4K8ac_peak_16512	15.70138	

2	24306935	24308236	KD2-H4K8ac_peak_16513	14.27217	TP53I3_ENSG00000115129
2	24345601	24347041	KD2-H4K8ac_peak_16514	16.29636	PFN4_ENSG00000176732;RP11-507M3.1_ENSG00000266118
2	24582787	24584484	KD2-H4K8ac_peak_16515	18.73085	ITSN2_ENSG00000198399
2	24713132	24714337	KD2-H4K8ac_peak_16516	9.96798	NCOA1_ENSG00000084676
2	25015378	25016836	KD2-H4K8ac_peak_16517	16.67374	PTRHD1_ENSG00000184924;CENPO_ENSG00000138092
2	25142398	25142989	KD2-H4K8ac_peak_16518	8.21852	ADCY3_ENSG00000138031
2	25151504	25151776	KD2-H4K8ac_peak_16519	5.14125	
2	25193939	25195643	KD2-H4K8ac_peak_16520	25.75599	DNAJC27_ENSG00000115137;DNAJC27-AS1_ENSG00000224165
2	25207508	25207701	KD2-H4K8ac_peak_16521	6.30626	
2	25237636	25237843	KD2-H4K8ac_peak_16522	5.88845	
2	25265356	25265781	KD2-H4K8ac_peak_16523	3.82059	EFR3B_ENSG00000084710
2	25424598	25425027	KD2-H4K8ac_peak_16524	5.51783	
2	25474166	25475122	KD2-H4K8ac_peak_16525	32.38585	
2	25475442	25476115	KD2-H4K8ac_peak_16526	10.66719	
2	25480693	25480891	KD2-H4K8ac_peak_16527	5.48743	
2	25508318	25508624	KD2-H4K8ac_peak_16528	5.66705	
2	25512487	25512854	KD2-H4K8ac_peak_16529	5.75757	
2	25513732	25514072	KD2-H4K8ac_peak_16530	8.36432	
2	25514278	25515247	KD2-H4K8ac_peak_16531	16.29636	
2	25516635	25517059	KD2-H4K8ac_peak_16532	10.43145	
2	25517265	25518419	KD2-H4K8ac_peak_16533	14.73375	
2	25533286	25533897	KD2-H4K8ac_peak_16534	17.66411	
2	25534660	25535378	KD2-H4K8ac_peak_16535	12.82393	
2	25536961	25538504	KD2-H4K8ac_peak_16536	20.30085	
2	25558206	25558475	KD2-H4K8ac_peak_16537	9.46843	
2	25563071	25564785	KD2-H4K8ac_peak_16538	17.43561	DNMT3A_ENSG00000119772
2	25565070	25565836	KD2-H4K8ac_peak_16539	19.25648	DNMT3A_ENSG00000119772
2	25584547	25584807	KD2-H4K8ac_peak_16540	7.30812	
2	25599425	25600594	KD2-H4K8ac_peak_16541	13.73635	AC012074.2_ENSG00000235072
2	25600791	25601301	KD2-H4K8ac_peak_16542	5.76151	
2	25739584	25739787	KD2-H4K8ac_peak_16543	9.66321	
2	25895639	25896391	KD2-H4K8ac_peak_16544	19.55721	DTNB_ENSG00000138101
2	25896634	25897065	KD2-H4K8ac_peak_16545	12.13241	DTNB_ENSG00000138101
2	25970944	25971147	KD2-H4K8ac_peak_16546	3.87414	
2	26100817	26101112	KD2-H4K8ac_peak_16547	9.19869	ASXL2_ENSG00000143970
2	26174447	26174666	KD2-H4K8ac_peak_16548	10.53377	
2	26204696	26205341	KD2-H4K8ac_peak_16549	9.67696	KIF3C_ENSG00000084731
2	26205658	26206234	KD2-H4K8ac_peak_16550	13.22091	KIF3C_ENSG00000084731
2	26256144	26256633	KD2-H4K8ac_peak_16551	13.70605	RAB10_ENSG00000084733
2	26257001	26257551	KD2-H4K8ac_peak_16552	12.66412	RAB10_ENSG00000084733
2	26396043	26396458	KD2-H4K8ac_peak_16553	14.68449	GAREML_ENSG00000157833
2	26401537	26402160	KD2-H4K8ac_peak_16554	12.08081	
2	26467251	26468087	KD2-H4K8ac_peak_16555	12.57865	HADHA_ENSG00000084754
2	26553195	26553535	KD2-H4K8ac_peak_16556	9.16531	
2	26568253	26568613	KD2-H4K8ac_peak_16557	13.30514	
2	26619282	26619615	KD2-H4K8ac_peak_16558	5.23279	

2	26624872	26625414	KD2-H4K8ac_peak_16559	4.79789	DRC1_ENSG00000157856
2	26700349	26700927	KD2-H4K8ac_peak_16560	6.30229	
2	26784947	26785766	KD2-H4K8ac_peak_16561	14.68449	C2orf70_ENSG00000173557
2	26875671	26876380	KD2-H4K8ac_peak_16562	14.80472	
2	26943338	26943729	KD2-H4K8ac_peak_16563	6.94249	
2	26973035	26973639	KD2-H4K8ac_peak_16564	7.7031	
2	26976072	26976266	KD2-H4K8ac_peak_16565	7.19631	
2	26986343	26987609	KD2-H4K8ac_peak_16566	18.33962	SLC35F6_ENSG00000213699;CENPA_ENSG00000115163
2	27008992	27009376	KD2-H4K8ac_peak_16567	15.19625	
2	27069429	27069796	KD2-H4K8ac_peak_16568	10.72313	DPYSL5_ENSG00000157851
2	27070387	27070776	KD2-H4K8ac_peak_16569	7.57336	DPYSL5_ENSG00000157851
2	27071005	27071836	KD2-H4K8ac_peak_16570	12.48406	DPYSL5_ENSG00000157851
2	27072255	27073035	KD2-H4K8ac_peak_16571	11.24702	
2	27134628	27135017	KD2-H4K8ac_peak_16572	3.97079	
2	27194079	27194331	KD2-H4K8ac_peak_16573	8.3771	MAPRE3_ENSG00000084764
2	27255373	27255629	KD2-H4K8ac_peak_16574	7.44324	TMEM214_ENSG00000119777
2	27272349	27273110	KD2-H4K8ac_peak_16575	13.21979	AGBL5-AS1_ENSG00000231636
2	27274178	27275289	KD2-H4K8ac_peak_16576	20.5832	
2	27294127	27294503	KD2-H4K8ac_peak_16577	10.78621	OST4_ENSG00000228474
2	27294720	27296034	KD2-H4K8ac_peak_16578	19.72564	OST4_ENSG00000228474
2	27298344	27298592	KD2-H4K8ac_peak_16579	7.92775	
2	27300056	27301007	KD2-H4K8ac_peak_16580	10.7186	EMILIN1_ENSG00000138080
2	27301528	27302206	KD2-H4K8ac_peak_16581	10.35678	EMILIN1_ENSG00000138080
2	27308437	27309396	KD2-H4K8ac_peak_16582	13.95627	KHK_ENSG00000138030
2	27318681	27319256	KD2-H4K8ac_peak_16583	14.63726	
2	27341305	27342564	KD2-H4K8ac_peak_16584	19.92174	CGREF1_ENSG00000138028
2	27346074	27347223	KD2-H4K8ac_peak_16585	13.22091	ABHD1_ENSG00000143994
2	27357773	27358381	KD2-H4K8ac_peak_16586	19.54082	PREB_ENSG00000138073
2	27371619	27371955	KD2-H4K8ac_peak_16587	5.54647	TCF23_ENSG00000163792
2	27434936	27435662	KD2-H4K8ac_peak_16588	9.20193	SLC5A6_ENSG00000138074;ATRAID_ENSG00000138085
2	27439807	27440095	KD2-H4K8ac_peak_16589	4.7158	CAD_ENSG00000084774
2	27440389	27440772	KD2-H4K8ac_peak_16590	8.80432	CAD_ENSG00000084774
2	27477810	27478208	KD2-H4K8ac_peak_16591	7.76104	
2	27485541	27485833	KD2-H4K8ac_peak_16592	5.30799	
2	27487304	27488031	KD2-H4K8ac_peak_16593	14.43794	
2	27526106	27526299	KD2-H4K8ac_peak_16594	9.1511	
2	27545203	27546320	KD2-H4K8ac_peak_16595	12.93209	
2	27578962	27580662	KD2-H4K8ac_peak_16596	20.02161	GTF3C2_ENSG00000115207;AC074117.10_ENSG00000234072
2	27592809	27593109	KD2-H4K8ac_peak_16597	6.40874	EIF2B4_ENSG00000115211;SNX17_ENSG00000115234
2	27593512	27594017	KD2-H4K8ac_peak_16598	14.99434	EIF2B4_ENSG00000115211;SNX17_ENSG00000115234
2	27600896	27601093	KD2-H4K8ac_peak_16599	5.8127	
2	27601519	27601833	KD2-H4K8ac_peak_16600	5.30799	
2	27602843	27603602	KD2-H4K8ac_peak_16601	14.30822	ZNF513_ENSG00000163795
2	27603843	27604419	KD2-H4K8ac_peak_16602	10.54778	ZNF513_ENSG00000163795
2	27631941	27632385	KD2-H4K8ac_peak_16603	7.54635	PPM1G_ENSG00000115241
2	27632664	27632921	KD2-H4K8ac_peak_16604	14.80472	PPM1G_ENSG00000115241

2	27633243	27633610	KD2-H4K8ac_peak_16605	7.07117	PPM1G_ENSG00000115241
2	27651089	27651360	KD2-H4K8ac_peak_16606	12.70959	NRBP1_ENSG00000115216
2	27651562	27651865	KD2-H4K8ac_peak_16607	15.05445	NRBP1_ENSG00000115216
2	27717369	27718485	KD2-H4K8ac_peak_16608	11.50346	FNDC4_ENSG00000115226
2	27804952	27805684	KD2-H4K8ac_peak_16609	7.92775	AC074091.1_ENSG00000221531;ZNF512_ENSG00000243943;RP11-158I13.2_ENSG00000259080
2	27851347	27851961	KD2-H4K8ac_peak_16610	11.50346	GPN1_ENSG00000198522;CCDC121_ENSG00000176714
2	27886209	27887360	KD2-H4K8ac_peak_16611	16.49944	SUPT7L_ENSG00000119760;SLC4A1AP_ENSG00000163798
2	27937776	27938651	KD2-H4K8ac_peak_16612	16.67374	AC074091.13_ENSG00000205334
2	27960686	27960909	KD2-H4K8ac_peak_16613	5.4779	
2	27961113	27961807	KD2-H4K8ac_peak_16614	18.97568	
2	27975358	27975578	KD2-H4K8ac_peak_16615	5.91443	
2	27994632	27994874	KD2-H4K8ac_peak_16616	10.78621	MRPL33_ENSG00000243147
2	28112861	28113088	KD2-H4K8ac_peak_16617	7.74292	BRE_ENSG00000158019;RBKS_ENSG00000171174
2	28113688	28114421	KD2-H4K8ac_peak_16618	15.82362	BRE_ENSG00000158019;RBKS_ENSG00000171174
2	28548329	28549214	KD2-H4K8ac_peak_16619	8.90799	
2	28557173	28557693	KD2-H4K8ac_peak_16620	9.34989	
2	28569764	28570190	KD2-H4K8ac_peak_16621	8.16212	
2	28577999	28578405	KD2-H4K8ac_peak_16622	8.16906	
2	28578650	28580158	KD2-H4K8ac_peak_16623	20.76319	
2	28589201	28589965	KD2-H4K8ac_peak_16624	5.59859	
2	28612930	28614052	KD2-H4K8ac_peak_16625	19.54082	
2	28614926	28615564	KD2-H4K8ac_peak_16626	11.24702	FOSL2_ENSG00000075426
2	28616260	28616453	KD2-H4K8ac_peak_16627	4.20044	FOSL2_ENSG00000075426
2	28789305	28789691	KD2-H4K8ac_peak_16628	14.63458	
2	28886338	28886732	KD2-H4K8ac_peak_16629	6.30607	AC074011.2_ENSG00000230730
2	28887510	28887776	KD2-H4K8ac_peak_16630	10.7186	AC074011.2_ENSG00000230730
2	28973582	28974544	KD2-H4K8ac_peak_16631	15.82362	PPP1CB_ENSG00000213639
2	28974924	28975395	KD2-H4K8ac_peak_16632	14.99434	PPP1CB_ENSG00000213639
2	29033725	29034152	KD2-H4K8ac_peak_16633	11.05155	RP11-713D19.1_ENSG00000273233
2	29092757	29093031	KD2-H4K8ac_peak_16634	7.70633	TRMT61B_ENSG00000171103
2	29093528	29094197	KD2-H4K8ac_peak_16635	5.4779	TRMT61B_ENSG00000171103
2	29116914	29117227	KD2-H4K8ac_peak_16636	12.82393	WDR43_ENSG00000163811
2	29117613	29118105	KD2-H4K8ac_peak_16637	19.07436	WDR43_ENSG00000163811
2	29177809	29178387	KD2-H4K8ac_peak_16638	7.87496	
2	29181823	29182136	KD2-H4K8ac_peak_16639	8.10216	
2	29191309	29191508	KD2-H4K8ac_peak_16640	4.42765	
2	29245559	29246050	KD2-H4K8ac_peak_16641	4.56666	
2	29265071	29265342	KD2-H4K8ac_peak_16642	8.10216	
2	29265600	29266427	KD2-H4K8ac_peak_16643	8.6202	
2	29275928	29276438	KD2-H4K8ac_peak_16644	15.58442	
2	29287736	29288716	KD2-H4K8ac_peak_16645	6.84261	
2	29291575	29291978	KD2-H4K8ac_peak_16646	5.13615	
2	29293369	29293721	KD2-H4K8ac_peak_16647	5.9696	
2	29299175	29299440	KD2-H4K8ac_peak_16648	5.54647	
2	29337728	29338953	KD2-H4K8ac_peak_16649	33.10165	
2	29340262	29340474	KD2-H4K8ac_peak_16650	7.24717	

2	29418390	29418589	KD2-H4K8ac_peak_16651	7.45501
2	29420162	29420457	KD2-H4K8ac_peak_16652	7.79558
2	29447382	29448484	KD2-H4K8ac_peak_16653	4.84794
2	29471278	29471536	KD2-H4K8ac_peak_16654	6.16
2	29471778	29473348	KD2-H4K8ac_peak_16655	20.64015
2	29474018	29474922	KD2-H4K8ac_peak_16656	8.85813
2	29483353	29483680	KD2-H4K8ac_peak_16657	7.76104
2	29484000	29485400	KD2-H4K8ac_peak_16658	14.95275
2	29486165	29488356	KD2-H4K8ac_peak_16659	13.70605
2	29488587	29488887	KD2-H4K8ac_peak_16660	9.00811
2	29489510	29490506	KD2-H4K8ac_peak_16661	18.53991
2	29490990	29491845	KD2-H4K8ac_peak_16662	7.0728
2	29492064	29492939	KD2-H4K8ac_peak_16663	12.40345
2	29493227	29494115	KD2-H4K8ac_peak_16664	10.05953
2	29494439	29494968	KD2-H4K8ac_peak_16665	13.73635
2	29495215	29495418	KD2-H4K8ac_peak_16666	4.9532
2	29495852	29496905	KD2-H4K8ac_peak_16667	21.12734
2	29497301	29497855	KD2-H4K8ac_peak_16668	9.63487
2	29498094	29498617	KD2-H4K8ac_peak_16669	12.30509
2	29498896	29499456	KD2-H4K8ac_peak_16670	6.65673
2	29504056	29504415	KD2-H4K8ac_peak_16671	9.41741
2	29622360	29622665	KD2-H4K8ac_peak_16672	6.84261
2	29623584	29623776	KD2-H4K8ac_peak_16673	4.64799
2	29666265	29666501	KD2-H4K8ac_peak_16674	4.67342
2	29697724	29697935	KD2-H4K8ac_peak_16675	4.98072
2	29703789	29704079	KD2-H4K8ac_peak_16676	10.19949
2	29720189	29720389	KD2-H4K8ac_peak_16677	5.30799
2	29738751	29739602	KD2-H4K8ac_peak_16678	16.71655
2	29739905	29740191	KD2-H4K8ac_peak_16679	11.33751
2	29740675	29740873	KD2-H4K8ac_peak_16680	6.84261
2	29741320	29741610	KD2-H4K8ac_peak_16681	8.79106
2	29742032	29742292	KD2-H4K8ac_peak_16682	5.5478
2	29742555	29742821	KD2-H4K8ac_peak_16683	6.99825
2	29750298	29750842	KD2-H4K8ac_peak_16684	10.19949
2	29751245	29752843	KD2-H4K8ac_peak_16685	8.16906
2	29783459	29783662	KD2-H4K8ac_peak_16686	9.30297
2	29784592	29785974	KD2-H4K8ac_peak_16687	10.96455
2	29793380	29793639	KD2-H4K8ac_peak_16688	7.90197
2	29797516	29797972	KD2-H4K8ac_peak_16689	7.81472
2	29798582	29798880	KD2-H4K8ac_peak_16690	6.22071
2	29801387	29802280	KD2-H4K8ac_peak_16691	14.15383
2	29802595	29802839	KD2-H4K8ac_peak_16692	5.73924
2	29803732	29804472	KD2-H4K8ac_peak_16693	11.67741
2	29805957	29806165	KD2-H4K8ac_peak_16694	7.41896
2	29811082	29811321	KD2-H4K8ac_peak_16695	7.20116
2	29835419	29835695	KD2-H4K8ac_peak_16696	9.20808

2	29836979	29837271	KD2-H4K8ac_peak_16697	7.30812	
2	29838812	29839170	KD2-H4K8ac_peak_16698	11.83301	
2	29840351	29840572	KD2-H4K8ac_peak_16699	4.2447	
2	30039141	30039442	KD2-H4K8ac_peak_16700	5.21035	
2	30057667	30057955	KD2-H4K8ac_peak_16701	4.42765	
2	30248131	30248353	KD2-H4K8ac_peak_16702	6.15163	
2	30249211	30249857	KD2-H4K8ac_peak_16703	15.42656	
2	30251715	30251910	KD2-H4K8ac_peak_16704	6.42941	
2	30307868	30308100	KD2-H4K8ac_peak_16705	8.17902	
2	30338123	30338390	KD2-H4K8ac_peak_16706	9.07715	
2	30453482	30453763	KD2-H4K8ac_peak_16707	4.74647	LBH_ENSG00000213626
2	30524289	30524800	KD2-H4K8ac_peak_16708	7.47462	
2	30645783	30645992	KD2-H4K8ac_peak_16709	6.25869	
2	30669520	30670670	KD2-H4K8ac_peak_16710	11.97691	LCLAT1_ENSG00000172954
2	30736906	30737168	KD2-H4K8ac_peak_16711	4.44748	
2	30912294	30912701	KD2-H4K8ac_peak_16712	5.9696	
2	30960229	30960495	KD2-H4K8ac_peak_16713	7.61026	
2	30980036	30980323	KD2-H4K8ac_peak_16714	6.04107	
2	30981265	30981555	KD2-H4K8ac_peak_16715	7.42964	
2	31094594	31094954	KD2-H4K8ac_peak_16716	9.36516	
2	31138392	31138655	KD2-H4K8ac_peak_16717	4.01115	
2	31216669	31217072	KD2-H4K8ac_peak_16718	9.08816	
2	31345041	31345233	KD2-H4K8ac_peak_16719	4.01115	
2	31353077	31353320	KD2-H4K8ac_peak_16720	5.14125	
2	31360217	31361797	KD2-H4K8ac_peak_16721	12.93209	
2	31457301	31457808	KD2-H4K8ac_peak_16722	8.28234	CAPN14_ENSG00000214711;EHD3_ENSG00000013016
2	32234747	32235081	KD2-H4K8ac_peak_16723	4.86324	
2	32235445	32235645	KD2-H4K8ac_peak_16724	12.90757	MEMO1_ENSG00000162959
2	32235903	32236475	KD2-H4K8ac_peak_16725	21.60954	MEMO1_ENSG00000162959
2	32264991	32265188	KD2-H4K8ac_peak_16726	9.6247	DPY30_ENSG00000162961
2	32288789	32289191	KD2-H4K8ac_peak_16727	10.74343	SPAST_ENSG00000021574
2	32390277	32390619	KD2-H4K8ac_peak_16728	9.26408	RP11-563N4.1_ENSG00000272716;SLC30A6_ENSG00000152683
2	32501737	32502026	KD2-H4K8ac_peak_16729	5.9696	YIPF4_ENSG00000119820
2	32502220	32502664	KD2-H4K8ac_peak_16730	11.89502	YIPF4_ENSG00000119820
2	32503009	32503358	KD2-H4K8ac_peak_16731	10.7186	YIPF4_ENSG00000119820
2	32581489	32581947	KD2-H4K8ac_peak_16732	18.33962	BIRC6_ENSG00000115760
2	32582352	32582647	KD2-H4K8ac_peak_16733	10.43145	BIRC6_ENSG00000115760
2	32853257	32853586	KD2-H4K8ac_peak_16734	6.40297	TTC27_ENSG00000018699
2	33063947	33064262	KD2-H4K8ac_peak_16735	4.56666	
2	33824630	33824950	KD2-H4K8ac_peak_16736	9.06369	FAM98A_ENSG00000119812
2	36824911	36825266	KD2-H4K8ac_peak_16737	30.35433	
2	37193402	37193633	KD2-H4K8ac_peak_16738	13.59098	STRN_ENSG00000115808
2	37311720	37311927	KD2-H4K8ac_peak_16739	7.47462	HEATR5B_ENSG00000008869;GPATCH11_ENSG00000152133
2	37383891	37384792	KD2-H4K8ac_peak_16740	11.7608	EIF2AK2_ENSG00000055332
2	37422991	37423411	KD2-H4K8ac_peak_16741	8.85813	SULT6B1_ENSG00000138068;CEBPZ-AS1_ENSG00000218739
2	37423638	37424131	KD2-H4K8ac_peak_16742	8.4716	SULT6B1_ENSG00000138068;CEBPZ-AS1_ENSG00000218739

2	37457888	37458160	KD2-H4K8ac_peak_16743	6.72624	CEBPZ_ENSG00000115816;NDUFAF7_ENSG00000003509
2	37458395	37459145	KD2-H4K8ac_peak_16744	12.13241	CEBPZ_ENSG00000115816;NDUFAF7_ENSG00000003509
2	37551307	37551883	KD2-H4K8ac_peak_16745	10.86378	PRKD3_ENSG00000115825;AC007391.2_ENSG00000232028
2	37552104	37552599	KD2-H4K8ac_peak_16746	8.4716	PRKD3_ENSG00000115825;AC007391.2_ENSG00000232028
2	37781752	37782266	KD2-H4K8ac_peak_16747	4.76709	
2	37787523	37788325	KD2-H4K8ac_peak_16748	6.08655	
2	37868024	37868382	KD2-H4K8ac_peak_16749	5.54647	
2	37894186	37894410	KD2-H4K8ac_peak_16750	5.12715	
2	37898455	37900103	KD2-H4K8ac_peak_16751	16.54484	
2	38082418	38083164	KD2-H4K8ac_peak_16752	7.57336	
2	38083696	38084302	KD2-H4K8ac_peak_16753	9.57101	
2	38151944	38153265	KD2-H4K8ac_peak_16754	17.38513	
2	38153828	38154034	KD2-H4K8ac_peak_16755	3.75035	
2	38157658	38157851	KD2-H4K8ac_peak_16756	5.54647	
2	38302678	38304274	KD2-H4K8ac_peak_16757	23.72665	CYP1B1-AS1_ENSG00000232973
2	38332777	38333086	KD2-H4K8ac_peak_16758	5.59859	
2	38333559	38333907	KD2-H4K8ac_peak_16759	6.35843	
2	38334841	38335047	KD2-H4K8ac_peak_16760	5.30799	
2	38336352	38336579	KD2-H4K8ac_peak_16761	6.27719	CYP1B1_ENSG00000138061
2	38343173	38343431	KD2-H4K8ac_peak_16762	7.94441	
2	38346288	38346950	KD2-H4K8ac_peak_16763	9.98013	
2	38441110	38441437	KD2-H4K8ac_peak_16764	5.92967	
2	38446285	38446526	KD2-H4K8ac_peak_16765	8.30974	
2	38603823	38604394	KD2-H4K8ac_peak_16766	15.61313	ATL2_ENSG00000119787
2	38762781	38763698	KD2-H4K8ac_peak_16767	24.67911	RP11-541E12.1_ENSG00000271443
2	38829160	38830032	KD2-H4K8ac_peak_16768	26.37453	AC011247.3_ENSG00000235586;HNRNPLL_ENSG00000143889
2	38892792	38892997	KD2-H4K8ac_peak_16769	6.24114	GALM_ENSG00000143891
2	38977677	38978448	KD2-H4K8ac_peak_16770	13.3695	SRSF7_ENSG00000115875;GEMIN6_ENSG00000152147
2	38981961	38982159	KD2-H4K8ac_peak_16771	5.14125	
2	39004732	39005147	KD2-H4K8ac_peak_16772	12.57865	
2	39005549	39005851	KD2-H4K8ac_peak_16773	5.92591	
2	39102717	39103002	KD2-H4K8ac_peak_16774	10.43145	DHX57_ENSG00000163214;MORN2_ENSG00000188010
2	39162819	39163033	KD2-H4K8ac_peak_16775	7.27716	
2	39288560	39288759	KD2-H4K8ac_peak_16776	6.40683	
2	39347689	39348572	KD2-H4K8ac_peak_16777	15.58442	
2	39349136	39349377	KD2-H4K8ac_peak_16778	6.13909	
2	39350900	39351431	KD2-H4K8ac_peak_16779	11.92978	SOS1_ENSG00000115904
2	39351942	39352289	KD2-H4K8ac_peak_16780	4.35724	SOS1_ENSG00000115904
2	39470730	39472117	KD2-H4K8ac_peak_16781	12.66138	
2	39664068	39664354	KD2-H4K8ac_peak_16782	19.33018	MAP4K3_ENSG00000011566;AC007246.3_ENSG00000231312
2	39664637	39665334	KD2-H4K8ac_peak_16783	6.40297	MAP4K3_ENSG00000011566;AC007246.3_ENSG00000231312
2	39893079	39893615	KD2-H4K8ac_peak_16784	13.12288	TMEM178A_ENSG00000152154
2	40005974	40006358	KD2-H4K8ac_peak_16785	27.01413	THUMPD2_ENSG00000138050
2	40006559	40007038	KD2-H4K8ac_peak_16786	8.58069	THUMPD2_ENSG00000138050
2	40558785	40559152	KD2-H4K8ac_peak_16787	4.86324	
2	40658496	40659006	KD2-H4K8ac_peak_16788	9.65086	

2	40679100	40679556	KD2-H4K8ac_peak_16789	13.50024	
2	41737228	41737476	KD2-H4K8ac_peak_16790	3.96965	
2	42068216	42068673	KD2-H4K8ac_peak_16791	17.53289	
2	42105240	42105473	KD2-H4K8ac_peak_16792	6.30229	
2	42106752	42107014	KD2-H4K8ac_peak_16793	5.01002	
2	42180731	42180954	KD2-H4K8ac_peak_16794	5.9696	C2orf91_ENSG00000205086
2	42181228	42181480	KD2-H4K8ac_peak_16795	4.67342	C2orf91_ENSG00000205086
2	42182001	42182994	KD2-H4K8ac_peak_16796	20.26332	C2orf91_ENSG00000205086
2	42190348	42190604	KD2-H4K8ac_peak_16797	5.3608	
2	42191373	42191577	KD2-H4K8ac_peak_16798	5.405	
2	42193821	42194044	KD2-H4K8ac_peak_16799	6.55628	
2	42273514	42273733	KD2-H4K8ac_peak_16800	10.35678	
2	42273959	42274935	KD2-H4K8ac_peak_16801	17.43561	PKDCC_ENSG00000162878
2	42275256	42275529	KD2-H4K8ac_peak_16802	4.54006	PKDCC_ENSG00000162878
2	42343827	42344021	KD2-H4K8ac_peak_16803	3.89905	
2	42345139	42345650	KD2-H4K8ac_peak_16804	10.86378	
2	42353023	42353286	KD2-H4K8ac_peak_16805	6.40297	
2	42355332	42355603	KD2-H4K8ac_peak_16806	6.92611	
2	42356523	42356892	KD2-H4K8ac_peak_16807	5.405	
2	42359257	42359695	KD2-H4K8ac_peak_16808	8.86099	
2	42360466	42361505	KD2-H4K8ac_peak_16809	19.77698	
2	42363560	42363774	KD2-H4K8ac_peak_16810	6.25869	
2	42364119	42364695	KD2-H4K8ac_peak_16811	8.18929	
2	42365449	42365820	KD2-H4K8ac_peak_16812	4.86324	
2	42367840	42369137	KD2-H4K8ac_peak_16813	12.36441	
2	42369992	42370364	KD2-H4K8ac_peak_16814	12.53053	
2	42371072	42371426	KD2-H4K8ac_peak_16815	6.36375	
2	42374562	42374836	KD2-H4K8ac_peak_16816	7.1181	
2	42395979	42396295	KD2-H4K8ac_peak_16817	5.78173	EML4_ENSG00000143924
2	42396566	42396832	KD2-H4K8ac_peak_16818	8.3261	AC083949.1_ENSG00000224875;EML4_ENSG00000143924
2	42400685	42400898	KD2-H4K8ac_peak_16819	4.70206	
2	42587904	42588616	KD2-H4K8ac_peak_16820	10.19949	
2	42588875	42589072	KD2-H4K8ac_peak_16821	9.90914	
2	42595966	42596326	KD2-H4K8ac_peak_16822	6.0073	
2	42596553	42596800	KD2-H4K8ac_peak_16823	8.26945	
2	42598830	42599349	KD2-H4K8ac_peak_16824	11.50346	
2	42603540	42603748	KD2-H4K8ac_peak_16825	9.95119	
2	42720495	42722009	KD2-H4K8ac_peak_16826	11.32417	KCNG3_ENSG00000171126;MTA3_ENSG00000057935
2	42737121	42737643	KD2-H4K8ac_peak_16827	8.6475	
2	42795197	42795544	KD2-H4K8ac_peak_16828	8.63033	
2	42795831	42796674	KD2-H4K8ac_peak_16829	23.36419	
2	42831114	42831346	KD2-H4K8ac_peak_16830	5.28072	
2	42917987	42918214	KD2-H4K8ac_peak_16831	5.57044	
2	43012746	43012953	KD2-H4K8ac_peak_16832	5.72327	
2	43037008	43037564	KD2-H4K8ac_peak_16833	26.00609	
2	43037875	43039460	KD2-H4K8ac_peak_16834	34.25782	

2	43055039	43055498	KD2-H4K8ac_peak_16835	5.4779	AC098824.6_ENSG00000232202
2	43082724	43082959	KD2-H4K8ac_peak_16836	4.211	
2	43084217	43084469	KD2-H4K8ac_peak_16837	8.10216	
2	43085435	43085714	KD2-H4K8ac_peak_16838	11.48808	
2	43132649	43134616	KD2-H4K8ac_peak_16839	12.70959	
2	43152082	43152320	KD2-H4K8ac_peak_16840	7.07117	
2	43153013	43153271	KD2-H4K8ac_peak_16841	8.86099	
2	43154082	43154491	KD2-H4K8ac_peak_16842	6.40808	
2	43154697	43155086	KD2-H4K8ac_peak_16843	8.10216	
2	43156956	43157225	KD2-H4K8ac_peak_16844	4.44527	
2	43194582	43195234	KD2-H4K8ac_peak_16845	4.73897	
2	43202051	43202778	KD2-H4K8ac_peak_16846	13.27022	
2	43227764	43228086	KD2-H4K8ac_peak_16847	4.37815	
2	43251404	43251735	KD2-H4K8ac_peak_16848	10.84242	
2	43268396	43268694	KD2-H4K8ac_peak_16849	8.73935	
2	43269278	43269494	KD2-H4K8ac_peak_16850	5.75757	
2	43273428	43273726	KD2-H4K8ac_peak_16851	4.44527	
2	43294798	43295550	KD2-H4K8ac_peak_16852	7.92775	
2	43307762	43308211	KD2-H4K8ac_peak_16853	10.19949	
2	43309077	43309707	KD2-H4K8ac_peak_16854	18.37277	
2	43309934	43310797	KD2-H4K8ac_peak_16855	17.30994	
2	43312436	43312702	KD2-H4K8ac_peak_16856	4.28801	
2	43367471	43367663	KD2-H4K8ac_peak_16857	4.67342	
2	43382998	43383277	KD2-H4K8ac_peak_16858	6.60096	
2	43395184	43395416	KD2-H4K8ac_peak_16859	5.48272	
2	43395627	43396000	KD2-H4K8ac_peak_16860	5.01002	
2	43437259	43437526	KD2-H4K8ac_peak_16861	4.54006	
2	43444800	43445307	KD2-H4K8ac_peak_16862	8.86099	
2	43445533	43446003	KD2-H4K8ac_peak_16863	6.84261	
2	43446819	43447516	KD2-H4K8ac_peak_16864	12.53675	
2	43447938	43448346	KD2-H4K8ac_peak_16865	6.20038	
2	43452642	43453753	KD2-H4K8ac_peak_16866	12.10046	ZFP36L2_ENSG00000152518
2	43454006	43454907	KD2-H4K8ac_peak_16867	20.10577	ZFP36L2_ENSG00000152518
2	43476459	43476724	KD2-H4K8ac_peak_16868	4.06758	
2	43478712	43479355	KD2-H4K8ac_peak_16869	6.66359	
2	43494358	43494670	KD2-H4K8ac_peak_16870	5.15342	
2	43495608	43496705	KD2-H4K8ac_peak_16871	11.24702	
2	43503897	43504428	KD2-H4K8ac_peak_16872	4.08993	
2	43505221	43505604	KD2-H4K8ac_peak_16873	4.3437	
2	43540086	43540423	KD2-H4K8ac_peak_16874	4.57371	
2	43540676	43541094	KD2-H4K8ac_peak_16875	10.05606	
2	43546218	43546426	KD2-H4K8ac_peak_16876	6.99815	
2	43555758	43555956	KD2-H4K8ac_peak_16877	6.25869	
2	43572824	43573276	KD2-H4K8ac_peak_16878	6.25225	
2	43573474	43573992	KD2-H4K8ac_peak_16879	12.69561	
2	43576085	43576499	KD2-H4K8ac_peak_16880	10.53377	

2	43620576	43620795	KD2-H4K8ac_peak_16881	6.25869	
2	43748401	43748788	KD2-H4K8ac_peak_16882	6.25869	
2	43822606	43823126	KD2-H4K8ac_peak_16883	6.84261	THADA_ENSG00000115970
2	43823373	43823719	KD2-H4K8ac_peak_16884	16.95399	THADA_ENSG00000115970
2	43864536	43864801	KD2-H4K8ac_peak_16885	5.38581	PLEKHH2_ENSG00000152527
2	44001246	44001502	KD2-H4K8ac_peak_16886	9.95119	DYNC2LI1_ENSG00000138036
2	44059144	44059430	KD2-H4K8ac_peak_16887	5.88845	
2	44222712	44223091	KD2-H4K8ac_peak_16888	11.04256	LRPPRC_ENSG00000138095
2	44223621	44224379	KD2-H4K8ac_peak_16889	14.275	LRPPRC_ENSG00000138095
2	44271575	44271810	KD2-H4K8ac_peak_16890	16.57619	
2	44314262	44314472	KD2-H4K8ac_peak_16891	10.43145	
2	44314769	44315138	KD2-H4K8ac_peak_16892	7.36799	
2	44394546	44394912	KD2-H4K8ac_peak_16893	11.28831	PPM1B_ENSG00000138032
2	44588500	44588924	KD2-H4K8ac_peak_16894	7.76329	PREPL_ENSG00000138078;CAMKMT_ENSG00000143919
2	44589131	44589683	KD2-H4K8ac_peak_16895	8.55271	PREPL_ENSG00000138078;CAMKMT_ENSG00000143919
2	44906540	44906841	KD2-H4K8ac_peak_16896	4.91516	
2	44907880	44908073	KD2-H4K8ac_peak_16897	6.62457	
2	44932311	44932673	KD2-H4K8ac_peak_16898	7.40314	
2	44933082	44933805	KD2-H4K8ac_peak_16899	9.36516	
2	44935817	44936088	KD2-H4K8ac_peak_16900	6.99815	
2	44941850	44942197	KD2-H4K8ac_peak_16901	3.86308	
2	44995616	44995924	KD2-H4K8ac_peak_16902	8.4716	
2	44999280	44999525	KD2-H4K8ac_peak_16903	14.56272	
2	45160746	45160946	KD2-H4K8ac_peak_16904	6.27245	
2	45161358	45163523	KD2-H4K8ac_peak_16905	15.58442	
2	45164527	45164821	KD2-H4K8ac_peak_16906	7.18615	
2	45165480	45166575	KD2-H4K8ac_peak_16907	8.58725	RP11-89K21.1_ENSG00000259439
2	45167832	45168526	KD2-H4K8ac_peak_16908	17.42905	SIX3-AS1_ENSG00000236502;SIX3_ENSG00000138083
2	45169230	45170576	KD2-H4K8ac_peak_16909	13.35993	SIX3-AS1_ENSG00000236502;SIX3_ENSG00000138083
2	45170970	45171314	KD2-H4K8ac_peak_16910	12.08081	
2	45178965	45179371	KD2-H4K8ac_peak_16911	5.63142	
2	45237293	45237684	KD2-H4K8ac_peak_16912	5.78173	SIX2_ENSG00000170577
2	45240835	45241568	KD2-H4K8ac_peak_16913	17.67328	AC093702.1_ENSG00000231156
2	45396288	45397439	KD2-H4K8ac_peak_16914	20.18584	AC009236.2_ENSG00000231054
2	45417487	45418102	KD2-H4K8ac_peak_16915	8.4716	
2	45451793	45452114	KD2-H4K8ac_peak_16916	5.88845	
2	45483979	45484185	KD2-H4K8ac_peak_16917	5.01002	
2	45484470	45484710	KD2-H4K8ac_peak_16918	9.30599	
2	45794473	45794713	KD2-H4K8ac_peak_16919	8.82427	
2	45837937	45838278	KD2-H4K8ac_peak_16920	7.28827	
2	45838701	45839162	KD2-H4K8ac_peak_16921	5.72327	SRBD1_ENSG00000068784
2	45870489	45870948	KD2-H4K8ac_peak_16922	10.35678	
2	45877758	45878607	KD2-H4K8ac_peak_16923	7.07117	PRKCE_ENSG00000171132
2	45878883	45879200	KD2-H4K8ac_peak_16924	9.03583	PRKCE_ENSG00000171132
2	45879424	45879624	KD2-H4K8ac_peak_16925	5.01002	PRKCE_ENSG00000171132
2	45976061	45976433	KD2-H4K8ac_peak_16926	5.07021	

2	45998237	45998669	KD2-H4K8ac_peak_16927	6.2781	
2	46010440	46011033	KD2-H4K8ac_peak_16928	7.54952	
2	46013815	46014532	KD2-H4K8ac_peak_16929	7.47462	
2	46039639	46039852	KD2-H4K8ac_peak_16930	4.42765	
2	46110347	46111005	KD2-H4K8ac_peak_16931	11.90231	
2	46463017	46463604	KD2-H4K8ac_peak_16932	17.55491	
2	46465092	46465302	KD2-H4K8ac_peak_16933	8.55271	
2	46523939	46524331	KD2-H4K8ac_peak_16934	7.11824	
2	46534571	46534780	KD2-H4K8ac_peak_16935	5.20908	
2	46541475	46541681	KD2-H4K8ac_peak_16936	6.36375	
2	46542347	46542626	KD2-H4K8ac_peak_16937	6.13909	
2	46546726	46547012	KD2-H4K8ac_peak_16938	6.85193	
2	46554903	46555371	KD2-H4K8ac_peak_16939	3.97079	
2	46556657	46558134	KD2-H4K8ac_peak_16940	10.40376	
2	46558825	46559362	KD2-H4K8ac_peak_16941	12.36528	
2	46559712	46560111	KD2-H4K8ac_peak_16942	7.92775	
2	46561202	46561742	KD2-H4K8ac_peak_16943	11.24702	
2	46562927	46563867	KD2-H4K8ac_peak_16944	5.59859	
2	46564393	46564643	KD2-H4K8ac_peak_16945	4.54006	
2	46565495	46566031	KD2-H4K8ac_peak_16946	11.50346	
2	46566783	46567107	KD2-H4K8ac_peak_16947	10.43145	
2	46567738	46568201	KD2-H4K8ac_peak_16948	10.54778	
2	46569033	46569336	KD2-H4K8ac_peak_16949	11.03268	
2	46604248	46604515	KD2-H4K8ac_peak_16950	4.44527	
2	46604861	46605247	KD2-H4K8ac_peak_16951	7.29931	
2	46687353	46687558	KD2-H4K8ac_peak_16952	7.07117	
2	46727188	46727401	KD2-H4K8ac_peak_16953	7.22919	RP11-417F21.2_ENSG00000253515
2	46768958	46769709	KD2-H4K8ac_peak_16954	12.13241	ATP6V1E2_ENSG00000250565;RHOQ_ENSG00000119729
2	46770737	46771021	KD2-H4K8ac_peak_16955	4.67342	
2	46843666	46844144	KD2-H4K8ac_peak_16956	14.17488	PIGF_ENSG00000151665;CRIPT_ENSG00000119878
2	46925019	46925858	KD2-H4K8ac_peak_16957	8.16906	SOCS5_ENSG00000171150
2	46926436	46926821	KD2-H4K8ac_peak_16958	21.12734	SOCS5_ENSG00000171150
2	47073831	47074354	KD2-H4K8ac_peak_16959	16.49944	
2	47074696	47075297	KD2-H4K8ac_peak_16960	5.72657	
2	47075618	47076040	KD2-H4K8ac_peak_16961	4.84206	
2	47076989	47077375	KD2-H4K8ac_peak_16962	9.34195	
2	47101257	47101539	KD2-H4K8ac_peak_16963	4.54006	
2	47142353	47142887	KD2-H4K8ac_peak_16964	18.33962	TTC7A_ENSG00000068724
2	47143088	47143824	KD2-H4K8ac_peak_16965	9.95119	TTC7A_ENSG00000068724
2	47167826	47168225	KD2-H4K8ac_peak_16966	16.33377	MCFD2_ENSG00000180398
2	47168509	47168931	KD2-H4K8ac_peak_16967	6.8713	MCFD2_ENSG00000180398
2	47210378	47210869	KD2-H4K8ac_peak_16968	7.76674	
2	47213665	47214173	KD2-H4K8ac_peak_16969	15.58442	
2	47214405	47214647	KD2-H4K8ac_peak_16970	5.4779	
2	47215046	47215263	KD2-H4K8ac_peak_16971	10.7186	
2	47215849	47216068	KD2-H4K8ac_peak_16972	8.5333	

2	47216534	47216752	KD2-H4K8ac_peak_16973	8.85813	
2	47223802	47224037	KD2-H4K8ac_peak_16974	4.37809	
2	47225185	47225400	KD2-H4K8ac_peak_16975	3.63069	
2	47227455	47229815	KD2-H4K8ac_peak_16976	10.35678	
2	47230187	47231422	KD2-H4K8ac_peak_16977	20.67053	
2	47235085	47236706	KD2-H4K8ac_peak_16978	13.65567	
2	47254210	47254522	KD2-H4K8ac_peak_16979	5.88127	
2	47254764	47254959	KD2-H4K8ac_peak_16980	7.90806	
2	47258786	47259784	KD2-H4K8ac_peak_16981	11.28765	
2	47260371	47262013	KD2-H4K8ac_peak_16982	20.78173	
2	47267482	47268179	KD2-H4K8ac_peak_16983	15.2334	AC093732.1_ENSG00000233845
2	47268467	47268825	KD2-H4K8ac_peak_16984	8.78814	AC093732.1_ENSG00000233845
2	47269962	47270408	KD2-H4K8ac_peak_16985	12.13241	
2	47270649	47271308	KD2-H4K8ac_peak_16986	11.55163	
2	47276847	47277296	KD2-H4K8ac_peak_16987	4.78037	
2	47282146	47284378	KD2-H4K8ac_peak_16988	22.03039	
2	47284599	47286070	KD2-H4K8ac_peak_16989	16.22559	
2	47286432	47287052	KD2-H4K8ac_peak_16990	5.01002	
2	47287972	47288746	KD2-H4K8ac_peak_16991	6.66359	
2	47288973	47289388	KD2-H4K8ac_peak_16992	14.99434	
2	47290181	47290451	KD2-H4K8ac_peak_16993	8.388	
2	47290754	47291864	KD2-H4K8ac_peak_16994	30.11673	
2	47295109	47295444	KD2-H4K8ac_peak_16995	6.59919	AC073283.7_ENSG00000225187
2	47296213	47296923	KD2-H4K8ac_peak_16996	4.91674	
2	47297281	47297803	KD2-H4K8ac_peak_16997	8.94905	
2	47298265	47298603	KD2-H4K8ac_peak_16998	6.67538	
2	47298938	47299885	KD2-H4K8ac_peak_16999	9.11891	
2	47300161	47300705	KD2-H4K8ac_peak_17000	6.40297	
2	47302857	47303054	KD2-H4K8ac_peak_17001	4.69702	
2	47306817	47307264	KD2-H4K8ac_peak_17002	13.12288	
2	47309991	47310326	KD2-H4K8ac_peak_17003	9.54616	
2	47310551	47311750	KD2-H4K8ac_peak_17004	17.67328	
2	47312972	47313665	KD2-H4K8ac_peak_17005	27.25041	
2	47314218	47315690	KD2-H4K8ac_peak_17006	26.40805	
2	47315999	47317375	KD2-H4K8ac_peak_17007	24.80313	
2	47317641	47317878	KD2-H4K8ac_peak_17008	13.73635	
2	47322470	47322662	KD2-H4K8ac_peak_17009	6.67092	
2	47402965	47403536	KD2-H4K8ac_peak_17010	13.95627	RP11-761B3.1_ENSG00000273269;CALM2_ENSG00000143933
2	47403748	47404666	KD2-H4K8ac_peak_17011	8.58725	RP11-761B3.1_ENSG00000273269;CALM2_ENSG00000143933
2	47419701	47420064	KD2-H4K8ac_peak_17012	6.65673	
2	47432123	47432599	KD2-H4K8ac_peak_17013	8.28234	
2	47463944	47464688	KD2-H4K8ac_peak_17014	10.40889	
2	47465910	47466157	KD2-H4K8ac_peak_17015	8.11222	
2	47466644	47467569	KD2-H4K8ac_peak_17016	12.10889	
2	47470454	47471106	KD2-H4K8ac_peak_17017	11.50346	
2	47471311	47471674	KD2-H4K8ac_peak_17018	10.53377	

2	47491430	47492440	KD2-H4K8ac_peak_17019	12.30509	
2	47492638	47492938	KD2-H4K8ac_peak_17020	7.36799	
2	47501770	47501976	KD2-H4K8ac_peak_17021	10.24494	
2	47510539	47510945	KD2-H4K8ac_peak_17022	9.91109	
2	47529590	47530063	KD2-H4K8ac_peak_17023	8.33304	
2	47537221	47537577	KD2-H4K8ac_peak_17024	11.00004	
2	47538321	47538877	KD2-H4K8ac_peak_17025	11.03268	
2	47546537	47547012	KD2-H4K8ac_peak_17026	6.99815	
2	47571527	47571811	KD2-H4K8ac_peak_17027	7.76329	AC073283.4_ENSG00000234690;EPCAM_ENSG00000119888
2	47572791	47573220	KD2-H4K8ac_peak_17028	4.13134	AC073283.4_ENSG00000234690;EPCAM_ENSG00000119888
2	47596156	47596959	KD2-H4K8ac_peak_17029	12.96152	
2	47629337	47629546	KD2-H4K8ac_peak_17030	6.90231	MSH2_ENSG00000095002
2	47629817	47630017	KD2-H4K8ac_peak_17031	7.25989	MSH2_ENSG00000095002
2	47630344	47630634	KD2-H4K8ac_peak_17032	13.52195	MSH2_ENSG00000095002
2	47747678	47748882	KD2-H4K8ac_peak_17033	16.14659	
2	47994956	47995166	KD2-H4K8ac_peak_17034	7.49599	
2	48009324	48010115	KD2-H4K8ac_peak_17035	22.12955	RNU6-688P_ENSG00000251872
2	48010378	48010617	KD2-H4K8ac_peak_17036	5.94405	
2	48132154	48132799	KD2-H4K8ac_peak_17037	10.19949	FBXO11_ENSG00000138081;AC079807.2_ENSG00000233230
2	48133029	48133608	KD2-H4K8ac_peak_17038	12.42761	FBXO11_ENSG00000138081;AC079807.2_ENSG00000233230
2	48333188	48333454	KD2-H4K8ac_peak_17039	8.33649	
2	48338667	48339796	KD2-H4K8ac_peak_17040	17.43561	
2	48541968	48542590	KD2-H4K8ac_peak_17041	9.01262	FOXN2_ENSG00000170802
2	48647374	48647798	KD2-H4K8ac_peak_17042	5.38581	
2	48667358	48667671	KD2-H4K8ac_peak_17043	8.10216	RP11-191L17.1_ENSG00000272663;PPP1R21_ENSG00000162869
2	48757395	48757920	KD2-H4K8ac_peak_17044	4.211	STON1_ENSG00000243244;STON1-GTF2A1L_ENSG00000068781
2	51259436	51259844	KD2-H4K8ac_peak_17045	4.42765	NRXN1_ENSG00000179915;AC007682.1_ENSG00000231918
2	53994203	53994415	KD2-H4K8ac_peak_17046	5.57044	CHAC2_ENSG00000143942
2	53994958	53995206	KD2-H4K8ac_peak_17047	7.22457	CHAC2_ENSG00000143942
2	54013706	54013932	KD2-H4K8ac_peak_17048	4.67342	ERLEC1_ENSG00000068912
2	54197741	54198762	KD2-H4K8ac_peak_17049	14.15075	PSME4_ENSG00000068878;ACYP2_ENSG00000170634
2	54342509	54342967	KD2-H4K8ac_peak_17050	19.51835	
2	54557333	54557856	KD2-H4K8ac_peak_17051	7.76329	C2orf73_ENSG00000177994
2	54558063	54558589	KD2-H4K8ac_peak_17052	6.66692	C2orf73_ENSG00000177994
2	54682798	54683234	KD2-H4K8ac_peak_17053	12.06346	SPTBN1_ENSG00000115306
2	54684423	54684948	KD2-H4K8ac_peak_17054	10.08874	
2	55276326	55276853	KD2-H4K8ac_peak_17055	7.73264	
2	55277049	55277539	KD2-H4K8ac_peak_17056	8.53092	
2	55338713	55339231	KD2-H4K8ac_peak_17057	8.04763	RTN4_ENSG00000115310
2	55459982	55460281	KD2-H4K8ac_peak_17058	5.29555	CLHC1_ENSG00000162994;RPS27A_ENSG00000143947
2	55495805	55496825	KD2-H4K8ac_peak_17059	9.59147	MTIF2_ENSG00000085760
2	55646377	55646817	KD2-H4K8ac_peak_17060	9.11031	CCDC88A_ENSG00000115355
2	55746116	55746459	KD2-H4K8ac_peak_17061	5.54647	CCDC104_ENSG00000163001
2	55746870	55747090	KD2-H4K8ac_peak_17062	4.54006	CCDC104_ENSG00000163001
2	55844472	55844756	KD2-H4K8ac_peak_17063	16.822	RP11-554J4.1_ENSG00000272606
2	55845195	55845407	KD2-H4K8ac_peak_17064	5.86783	SMEK2_ENSG00000138041;RP11-554J4.1_ENSG00000272606

2	57948310	57948505	KD2-H4K8ac_peak_17065	4.42765	
2	57987253	57987447	KD2-H4K8ac_peak_17066	10.53377	
2	58468084	58468420	KD2-H4K8ac_peak_17067	9.20193	FANCL_ENSG00000115392;RP11-334G22.1_ENSG00000273063
2	58654766	58655092	KD2-H4K8ac_peak_17068	6.31143	LINC01122_ENSG00000233723
2	58655866	58656302	KD2-H4K8ac_peak_17069	9.11031	LINC01122_ENSG00000233723
2	60776597	60776834	KD2-H4K8ac_peak_17070	6.94954	
2	60781177	60781460	KD2-H4K8ac_peak_17071	5.32933	BCL11A_ENSG00000119866
2	60808734	60809032	KD2-H4K8ac_peak_17072	8.86099	
2	61108208	61108506	KD2-H4K8ac_peak_17073	12.17278	AC010733.4_ENSG00000228414;REL_ENSG00000162924
2	61243977	61244758	KD2-H4K8ac_peak_17074	9.63487	PUS10_ENSG00000162927;PEX13_ENSG00000162928
2	61293054	61293671	KD2-H4K8ac_peak_17075	17.42214	KIAA1841_ENSG00000162929
2	61371615	61372011	KD2-H4K8ac_peak_17076	9.11031	AC016747.3_ENSG00000212978;C2orf74_ENSG00000237651
2	61372631	61372944	KD2-H4K8ac_peak_17077	5.14125	AC016747.3_ENSG00000212978;C2orf74_ENSG00000237651
2	61403739	61405299	KD2-H4K8ac_peak_17078	14.68449	AHSA2_ENSG00000173209
2	61407633	61407919	KD2-H4K8ac_peak_17079	7.70289	
2	61697409	61697839	KD2-H4K8ac_peak_17080	14.42504	USP34_ENSG00000115464;RP11-355B11.2_ENSG00000270820
2	61698559	61698851	KD2-H4K8ac_peak_17081	4.42765	USP34_ENSG00000115464;RP11-355B11.2_ENSG00000270820
2	61764975	61765322	KD2-H4K8ac_peak_17082	6.8713	XPO1_ENSG00000082898
2	61766036	61766300	KD2-H4K8ac_peak_17083	5.30799	XPO1_ENSG00000082898
2	61921574	61921817	KD2-H4K8ac_peak_17084	7.49599	
2	62080922	62081145	KD2-H4K8ac_peak_17085	15.52826	FAM161A_ENSG00000170264
2	62081847	62082685	KD2-H4K8ac_peak_17086	5.88845	FAM161A_ENSG00000170264
2	62115535	62115984	KD2-H4K8ac_peak_17087	8.55271	CCT4_ENSG00000115484;COMMD1_ENSG00000173163
2	62403614	62404019	KD2-H4K8ac_peak_17088	4.211	
2	62404237	62404500	KD2-H4K8ac_peak_17089	4.211	
2	62422462	62423089	KD2-H4K8ac_peak_17090	13.09607	B3GNT2_ENSG00000170340
2	62423283	62423834	KD2-H4K8ac_peak_17091	12.00273	B3GNT2_ENSG00000170340
2	62520789	62521534	KD2-H4K8ac_peak_17092	17.16047	
2	62561545	62561770	KD2-H4K8ac_peak_17093	7.44366	
2	62568704	62568900	KD2-H4K8ac_peak_17094	6.40683	
2	62638928	62639149	KD2-H4K8ac_peak_17095	11.30637	
2	62683865	62684973	KD2-H4K8ac_peak_17096	16.11042	
2	62732493	62733612	KD2-H4K8ac_peak_17097	24.94195	
2	63277347	63277556	KD2-H4K8ac_peak_17098	6.03693	OTX1_ENSG00000115507
2	63278360	63278634	KD2-H4K8ac_peak_17099	6.31143	
2	63816136	63816566	KD2-H4K8ac_peak_17100	10.05606	MDH1_ENSG00000014641
2	64068193	64068778	KD2-H4K8ac_peak_17101	12.84642	UGP2_ENSG00000169764
2	64246049	64246982	KD2-H4K8ac_peak_17102	11.50346	VPS54_ENSG00000143952
2	64280328	64280702	KD2-H4K8ac_peak_17103	9.1707	
2	64370818	64371442	KD2-H4K8ac_peak_17104	13.9234	PELI1_ENSG00000197329;AC074289.1_ENSG00000225889
2	64454944	64455296	KD2-H4K8ac_peak_17105	4.01115	
2	64455597	64455826	KD2-H4K8ac_peak_17106	5.54647	
2	64631340	64631583	KD2-H4K8ac_peak_17107	4.24756	
2	64681328	64682278	KD2-H4K8ac_peak_17108	15.52826	AC008074.3_ENSG00000223935;LGALSL_ENSG00000119862
2	64751582	64751889	KD2-H4K8ac_peak_17109	12.00597	RP11-568N6.1_ENSG00000260101;AFTPH_ENSG00000119844
2	64852736	64852971	KD2-H4K8ac_peak_17110	5.10505	

2	64854775	64854997	KD2-H4K8ac_peak_17111	5.21139	
2	64880544	64880943	KD2-H4K8ac_peak_17112	12.22449	
2	64881485	64881844	KD2-H4K8ac_peak_17113	6.84261	
2	64978645	64979010	KD2-H4K8ac_peak_17114	10.05606	SERTAD2_ENSG00000179833
2	64995047	64995635	KD2-H4K8ac_peak_17115	20.5798	
2	65084885	65085350	KD2-H4K8ac_peak_17116	4.211	
2	65085636	65085946	KD2-H4K8ac_peak_17117	5.75452	
2	65091311	65091664	KD2-H4K8ac_peak_17118	6.44277	AC007880.1_ENSG00000234572
2	65092289	65093087	KD2-H4K8ac_peak_17119	13.34404	
2	65100873	65101072	KD2-H4K8ac_peak_17120	8.30974	
2	65101315	65101522	KD2-H4K8ac_peak_17121	4.7849	
2	65186814	65187066	KD2-H4K8ac_peak_17122	8.17902	AC007386.3_ENSG00000227394
2	65215552	65216351	KD2-H4K8ac_peak_17123	19.55721	SLC1A4_ENSG00000115902
2	65216574	65217073	KD2-H4K8ac_peak_17124	11.92978	SLC1A4_ENSG00000115902
2	65356732	65357167	KD2-H4K8ac_peak_17125	10.1591	RAB1A_ENSG00000138069
2	65357878	65358320	KD2-H4K8ac_peak_17126	5.30799	RAB1A_ENSG00000138069
2	65454414	65454613	KD2-H4K8ac_peak_17127	5.54647	ACTR2_ENSG00000138071
2	65454989	65455283	KD2-H4K8ac_peak_17128	13.70605	ACTR2_ENSG00000138071
2	65579023	65579372	KD2-H4K8ac_peak_17129	5.25071	
2	65581352	65581562	KD2-H4K8ac_peak_17130	7.12754	
2	65594051	65594289	KD2-H4K8ac_peak_17131	4.04247	
2	65658314	65658525	KD2-H4K8ac_peak_17132	6.30229	
2	65658749	65659049	KD2-H4K8ac_peak_17133	4.91674	SPRED2_ENSG00000198369
2	65662780	65663488	KD2-H4K8ac_peak_17134	11.55418	AC074391.1_ENSG00000204929
2	65693158	65693649	KD2-H4K8ac_peak_17135	8.30974	
2	66072171	66072398	KD2-H4K8ac_peak_17136	4.04247	
2	66296853	66297491	KD2-H4K8ac_peak_17137	12.06346	
2	66652789	66653721	KD2-H4K8ac_peak_17138	15.19698	
2	66661138	66662092	KD2-H4K8ac_peak_17139	18.94636	MEIS1-AS3_ENSG00000226819;MEIS1_ENSG00000143995
2	66664754	66665783	KD2-H4K8ac_peak_17140	10.81728	
2	66666361	66667060	KD2-H4K8ac_peak_17141	9.63487	MEIS1-AS2_ENSG00000230749
2	66802766	66803425	KD2-H4K8ac_peak_17142	6.0073	
2	66804703	66804960	KD2-H4K8ac_peak_17143	4.59928	
2	67190287	67190707	KD2-H4K8ac_peak_17144	12.80176	
2	67215296	67215841	KD2-H4K8ac_peak_17145	8.04763	
2	67232830	67233023	KD2-H4K8ac_peak_17146	5.59859	
2	67234905	67235100	KD2-H4K8ac_peak_17147	7.22457	
2	67440662	67440876	KD2-H4K8ac_peak_17148	10.0692	
2	67545262	67545686	KD2-H4K8ac_peak_17149	6.94954	
2	67546424	67546682	KD2-H4K8ac_peak_17150	7.47462	
2	67551907	67552390	KD2-H4K8ac_peak_17151	5.57044	AC023115.4_ENSG00000236605
2	67623689	67623909	KD2-H4K8ac_peak_17152	8.17902	ETAA1_ENSG00000143971
2	67624674	67625131	KD2-H4K8ac_peak_17153	6.84261	ETAA1_ENSG00000143971
2	67877042	67877373	KD2-H4K8ac_peak_17154	5.88845	
2	67893286	67893851	KD2-H4K8ac_peak_17155	8.04763	
2	67913369	67913573	KD2-H4K8ac_peak_17156	5.88845	

2	67928541	67928890	KD2-H4K8ac_peak_17157	8.1233	
2	67945279	67945481	KD2-H4K8ac_peak_17158	6.16888	
2	67981808	67982019	KD2-H4K8ac_peak_17159	4.42765	
2	67982424	67982672	KD2-H4K8ac_peak_17160	7.01658	
2	67983098	67984215	KD2-H4K8ac_peak_17161	4.86324	
2	68069530	68069740	KD2-H4K8ac_peak_17162	6.56211	
2	68072908	68073330	KD2-H4K8ac_peak_17163	7.0983	
2	68271593	68271883	KD2-H4K8ac_peak_17164	8.30974	
2	68274613	68274836	KD2-H4K8ac_peak_17165	4.211	
2	68289800	68290421	KD2-H4K8ac_peak_17166	11.30637	
2	68384324	68384893	KD2-H4K8ac_peak_17167	8.30974	WDR92_ENSG00000243667;PNO1_ENSG00000115946
2	68479142	68479569	KD2-H4K8ac_peak_17168	5.88845	RP11-474G23.3_ENSG00000273064
2	68546150	68546466	KD2-H4K8ac_peak_17169	11.24702	CNRIP1_ENSG00000119865
2	68546664	68547021	KD2-H4K8ac_peak_17170	11.80283	CNRIP1_ENSG00000119865
2	68694791	68695041	KD2-H4K8ac_peak_17171	9.34195	FBXO48_ENSG00000204923;APLF_ENSG00000169621
2	68870164	68870892	KD2-H4K8ac_peak_17172	8.74596	PROKR1_ENSG00000169618
2	69027007	69027434	KD2-H4K8ac_peak_17173	9.08816	
2	69170895	69171569	KD2-H4K8ac_peak_17174	18.94636	
2	69240485	69240782	KD2-H4K8ac_peak_17175	11.05467	ANTXR1_ENSG00000169604
2	69532945	69533268	KD2-H4K8ac_peak_17176	5.43522	
2	69533625	69534631	KD2-H4K8ac_peak_17177	9.36516	
2	69614062	69614289	KD2-H4K8ac_peak_17178	5.75757	GFPT1_ENSG00000198380
2	69664195	69664443	KD2-H4K8ac_peak_17179	6.98244	NFU1_ENSG00000169599
2	69664818	69665444	KD2-H4K8ac_peak_17180	6.90231	NFU1_ENSG00000169599
2	69871468	69871817	KD2-H4K8ac_peak_17181	10.0692	ANXA4_ENSG00000196975
2	69968668	69969510	KD2-H4K8ac_peak_17182	10.19949	
2	70056192	70057129	KD2-H4K8ac_peak_17183	21.69575	GMCL1_ENSG00000087338
2	70120439	70120839	KD2-H4K8ac_peak_17184	5.30799	SNRNP27_ENSG00000124380
2	70140812	70141049	KD2-H4K8ac_peak_17185	3.75035	snoU13_ENSG00000238465
2	70141320	70141629	KD2-H4K8ac_peak_17186	11.05467	
2	70298251	70298744	KD2-H4K8ac_peak_17187	5.6755	
2	70312364	70312557	KD2-H4K8ac_peak_17188	4.86324	
2	70313129	70314503	KD2-H4K8ac_peak_17189	12.57865	PCBP1_ENSG00000169564
2	70335915	70336434	KD2-H4K8ac_peak_17190	9.06703	
2	70350703	70353161	KD2-H4K8ac_peak_17191	20.88898	AC016700.5_ENSG00000231327
2	70355341	70355534	KD2-H4K8ac_peak_17192	6.44277	
2	70368683	70369688	KD2-H4K8ac_peak_17193	31.88004	
2	70370428	70371127	KD2-H4K8ac_peak_17194	8.30974	
2	70417641	70418136	KD2-H4K8ac_peak_17195	14.8083	
2	70418360	70418714	KD2-H4K8ac_peak_17196	5.20908	
2	70475259	70475716	KD2-H4K8ac_peak_17197	22.00006	C2orf42_ENSG00000115998;TIA1_ENSG00000116001
2	70484887	70485110	KD2-H4K8ac_peak_17198	9.96798	PCYOX1_ENSG00000116005
2	70521065	70521589	KD2-H4K8ac_peak_17199	20.78681	SNRPG_ENSG00000143977
2	70528658	70529147	KD2-H4K8ac_peak_17200	13.57407	FAM136A_ENSG00000035141;AC022201.5_ENSG00000233849
2	70530806	70531031	KD2-H4K8ac_peak_17201	10.34425	
2	70704864	70705163	KD2-H4K8ac_peak_17202	7.12754	

2	70781002	70781202	KD2-H4K8ac_peak_17203	6.25869	TGFA_ENSG00000163235
2	70947962	70948160	KD2-H4K8ac_peak_17204	6.00226	
2	70994290	70995304	KD2-H4K8ac_peak_17205	6.65673	ADD2_ENSG00000075340
2	71127092	71127525	KD2-H4K8ac_peak_17206	7.40314	VAX2_ENSG00000116035
2	71166037	71166240	KD2-H4K8ac_peak_17207	4.01796	
2	71203800	71204092	KD2-H4K8ac_peak_17208	6.40683	
2	71227845	71228171	KD2-H4K8ac_peak_17209	5.59859	
2	71228527	71228945	KD2-H4K8ac_peak_17210	5.9696	
2	71270603	71270892	KD2-H4K8ac_peak_17211	14.45402	
2	71294835	71295280	KD2-H4K8ac_peak_17212	6.65673	RP11-467P9.1_ENSG00000272735
2	71295821	71296013	KD2-H4K8ac_peak_17213	7.44324	RP11-467P9.1_ENSG00000272735
2	71357737	71358098	KD2-H4K8ac_peak_17214	11.05407	MCEE_ENSG00000124370;MPHOSPH10_ENSG00000124383
2	71453881	71454112	KD2-H4K8ac_peak_17215	7.69287	PAIP2B_ENSG00000124374
2	71503771	71504115	KD2-H4K8ac_peak_17216	5.88845	ZNF638_ENSG00000075292
2	71558943	71559449	KD2-H4K8ac_peak_17217	12.93209	
2	73114168	73115005	KD2-H4K8ac_peak_17218	7.76104	SPR_ENSG00000116096
2	73298021	73298274	KD2-H4K8ac_peak_17219	7.50083	
2	73299130	73299388	KD2-H4K8ac_peak_17220	6.25869	
2	73402736	73403161	KD2-H4K8ac_peak_17221	7.40314	
2	73404130	73404385	KD2-H4K8ac_peak_17222	6.2781	
2	73441116	73441689	KD2-H4K8ac_peak_17223	28.02409	SMYD5_ENSG00000135632
2	73461479	73461965	KD2-H4K8ac_peak_17224	14.66631	CCT7_ENSG00000135624
2	73511167	73511383	KD2-H4K8ac_peak_17225	4.91674	FBXO41_ENSG00000163013
2	73511729	73512358	KD2-H4K8ac_peak_17226	8.79106	FBXO41_ENSG00000163013
2	73964735	73965430	KD2-H4K8ac_peak_17227	8.55271	TPRKB_ENSG00000144034
2	74056164	74056653	KD2-H4K8ac_peak_17228	16.61426	STAMBP_ENSG00000124356
2	74154058	74154288	KD2-H4K8ac_peak_17229	9.36516	DGUOK_ENSG00000114956
2	74211162	74212286	KD2-H4K8ac_peak_17230	20.02161	AC073046.25_ENSG00000235499
2	74212603	74213218	KD2-H4K8ac_peak_17231	14.07373	AC073046.25_ENSG00000235499;RP11-711M9.1_ENSG00000255989
2	74347288	74347761	KD2-H4K8ac_peak_17232	25.10776	FNBP1P1_ENSG00000257800
2	74375219	74375686	KD2-H4K8ac_peak_17233	5.13615	BOLA3_ENSG00000163170;BOLA3-AS1_ENSG00000225439
2	74406433	74406905	KD2-H4K8ac_peak_17234	7.27716	MOB1A_ENSG00000114978
2	74425811	74426291	KD2-H4K8ac_peak_17235	22.59108	MTHFD2_ENSG00000065911
2	74618614	74618900	KD2-H4K8ac_peak_17236	7.76329	DCTN1_ENSG00000204843
2	74619543	74619830	KD2-H4K8ac_peak_17237	5.08628	DCTN1_ENSG00000204843
2	74648457	74648707	KD2-H4K8ac_peak_17238	14.06758	C2orf81_ENSG00000159239;WDR54_ENSG00000005448
2	74649024	74649757	KD2-H4K8ac_peak_17239	11.89502	C2orf81_ENSG00000159239;WDR54_ENSG00000005448
2	74667179	74668543	KD2-H4K8ac_peak_17240	14.80472	
2	74684126	74684334	KD2-H4K8ac_peak_17241	8.55271	
2	74684592	74685045	KD2-H4K8ac_peak_17242	18.88045	WBP1_ENSG00000239779
2	74685652	74686256	KD2-H4K8ac_peak_17243	11.43863	WBP1_ENSG00000239779
2	74692014	74692450	KD2-H4K8ac_peak_17244	7.28628	MOGS_ENSG00000115275
2	74692675	74692993	KD2-H4K8ac_peak_17245	6.69204	MOGS_ENSG00000115275
2	74699247	74700678	KD2-H4K8ac_peak_17246	9.20193	MRPL53_ENSG00000204822
2	74709633	74709959	KD2-H4K8ac_peak_17247	7.18615	CCDC142_ENSG00000135637;TTC31_ENSG00000115282
2	74726073	74726273	KD2-H4K8ac_peak_17248	4.06758	AC005041.17_ENSG00000258821

2	74734985	74735345	KD2-H4K8ac_peak_17249	30.61401	PCGF1_ENSG00000115289
2	74735574	74735897	KD2-H4K8ac_peak_17250	8.55552	PCGF1_ENSG00000115289
2	74739522	74742535	KD2-H4K8ac_peak_17251	15.82362	TLX2_ENSG00000115297
2	74756369	74756767	KD2-H4K8ac_peak_17252	10.29842	AUP1_ENSG00000115307;HTRA2_ENSG00000115317
2	74776233	74776936	KD2-H4K8ac_peak_17253	10.76532	DOK1_ENSG00000115325
2	74780261	74781087	KD2-H4K8ac_peak_17254	9.402	
2	74781301	74782046	KD2-H4K8ac_peak_17255	12.17278	LOXL3_ENSG00000115318
2	74782285	74782546	KD2-H4K8ac_peak_17256	4.86324	LOXL3_ENSG00000115318
2	74874234	74874577	KD2-H4K8ac_peak_17257	5.25071	M1AP_ENSG00000159374
2	74880745	74881050	KD2-H4K8ac_peak_17258	8.53581	SEMA4F_ENSG00000135622
2	74881407	74881993	KD2-H4K8ac_peak_17259	13.73635	SEMA4F_ENSG00000135622
2	75005687	75006263	KD2-H4K8ac_peak_17260	6.40297	
2	75044017	75044241	KD2-H4K8ac_peak_17261	9.11031	
2	75061920	75062224	KD2-H4K8ac_peak_17262	9.65086	RP11-259N19.1_ENSG00000272711;HK2_ENSG00000159399
2	75088087	75088349	KD2-H4K8ac_peak_17263	6.37652	
2	75185392	75186218	KD2-H4K8ac_peak_17264	13.80019	POLE4_ENSG00000115350
2	75720157	75720447	KD2-H4K8ac_peak_17265	5.88845	
2	75835967	75836337	KD2-H4K8ac_peak_17266	9.89126	
2	75873653	75874218	KD2-H4K8ac_peak_17267	14.17488	MRPL19_ENSG00000115364
2	75937731	75938335	KD2-H4K8ac_peak_17268	7.30812	GCFC2_ENSG00000005436
2	76015456	76015749	KD2-H4K8ac_peak_17269	7.96194	
2	76026875	76027076	KD2-H4K8ac_peak_17270	6.40683	
2	76300374	76300825	KD2-H4K8ac_peak_17271	5.57044	
2	76303028	76303246	KD2-H4K8ac_peak_17272	6.16888	
2	76497105	76497437	KD2-H4K8ac_peak_17273	6.16888	
2	76537459	76537891	KD2-H4K8ac_peak_17274	6.16888	
2	76829628	76829824	KD2-H4K8ac_peak_17275	7.12754	
2	76911571	76912315	KD2-H4K8ac_peak_17276	6.40683	
2	76918126	76918500	KD2-H4K8ac_peak_17277	5.13615	
2	79006380	79006613	KD2-H4K8ac_peak_17278	6.07178	
2	80276924	80277128	KD2-H4K8ac_peak_17279	4.01115	
2	80593412	80593720	KD2-H4K8ac_peak_17280	7.12754	
2	81708454	81708788	KD2-H4K8ac_peak_17281	5.71749	
2	85132309	85132586	KD2-H4K8ac_peak_17282	9.63487	TMSB10_ENSG00000034510
2	85132823	85133520	KD2-H4K8ac_peak_17283	15.58442	TRABD2A_ENSG00000186854;TMSB10_ENSG00000034510
2	85153589	85154083	KD2-H4K8ac_peak_17284	4.8888	
2	85197182	85197988	KD2-H4K8ac_peak_17285	8.30974	KCMF1_ENSG00000176407
2	85198434	85198778	KD2-H4K8ac_peak_17286	9.1707	KCMF1_ENSG00000176407
2	85199028	85199374	KD2-H4K8ac_peak_17287	5.75452	KCMF1_ENSG00000176407
2	85359829	85360032	KD2-H4K8ac_peak_17288	6.0073	TCF7L1_ENSG00000152284
2	85443030	85444215	KD2-H4K8ac_peak_17289	11.05407	
2	85472543	85472736	KD2-H4K8ac_peak_17290	5.75452	
2	85481499	85483614	KD2-H4K8ac_peak_17291	16.57619	
2	85483834	85485043	KD2-H4K8ac_peak_17292	19.12154	
2	85494128	85494346	KD2-H4K8ac_peak_17293	6.4095	
2	85555208	85555628	KD2-H4K8ac_peak_17294	6.99815	TGOLN2_ENSG00000152291

2	85581957	85582158	KD2-H4K8ac_peak_17295	4.06758	RETSAT_ENSG00000042445;ELMOD3_ENSG00000115459
2	85644798	85646661	KD2-H4K8ac_peak_17296	24.23147	CAPG_ENSG00000042493;SH2D6_ENSG00000152292
2	85648076	85648376	KD2-H4K8ac_peak_17297	7.59794	
2	85657599	85657856	KD2-H4K8ac_peak_17298	4.42765	
2	85660051	85662071	KD2-H4K8ac_peak_17299	14.83804	Y_RNA_ENSG00000207207
2	85662292	85663101	KD2-H4K8ac_peak_17300	7.69287	Y_RNA_ENSG00000207207
2	85663316	85665072	KD2-H4K8ac_peak_17301	17.75427	
2	85665480	85666083	KD2-H4K8ac_peak_17302	16.54484	
2	85672640	85673785	KD2-H4K8ac_peak_17303	13.65567	
2	85673999	85674196	KD2-H4K8ac_peak_17304	6.67538	
2	85674551	85674925	KD2-H4K8ac_peak_17305	5.14125	
2	85750395	85751285	KD2-H4K8ac_peak_17306	8.45238	
2	85751612	85752869	KD2-H4K8ac_peak_17307	16.73922	
2	85764799	85765789	KD2-H4K8ac_peak_17308	15.2334	MAT2A_ENSG00000168906
2	85766363	85767015	KD2-H4K8ac_peak_17309	13.73635	MAT2A_ENSG00000168906
2	85788304	85788523	KD2-H4K8ac_peak_17310	4.77079	GGCX_ENSG00000115486;VAMP8_ENSG00000118640
2	85788809	85789108	KD2-H4K8ac_peak_17311	6.85193	GGCX_ENSG00000115486;VAMP8_ENSG00000118640
2	85811366	85811871	KD2-H4K8ac_peak_17312	6.0605	VAMP5_ENSG00000168899
2	85822293	85822593	KD2-H4K8ac_peak_17313	9.36665	RNF181_ENSG00000168894
2	85822934	85823208	KD2-H4K8ac_peak_17314	10.20046	RNF181_ENSG00000168894
2	85829405	85829734	KD2-H4K8ac_peak_17315	7.27716	TMEM150A_ENSG00000168890;USP39_ENSG00000168883
2	85838884	85839159	KD2-H4K8ac_peak_17316	10.17486	C2orf68_ENSG00000168887
2	85839576	85839962	KD2-H4K8ac_peak_17317	11.79585	C2orf68_ENSG00000168887
2	85842642	85843089	KD2-H4K8ac_peak_17318	8.7325	
2	85968499	85969259	KD2-H4K8ac_peak_17319	6.20982	
2	85981490	85981822	KD2-H4K8ac_peak_17320	4.82973	
2	85985895	85986132	KD2-H4K8ac_peak_17321	4.56666	
2	85996760	85997375	KD2-H4K8ac_peak_17322	7.20116	
2	85997788	85998166	KD2-H4K8ac_peak_17323	4.66531	
2	85998501	85999069	KD2-H4K8ac_peak_17324	4.54006	
2	85999944	86000798	KD2-H4K8ac_peak_17325	10.46874	
2	86022059	86022334	KD2-H4K8ac_peak_17326	5.88845	
2	86022775	86023024	KD2-H4K8ac_peak_17327	5.08628	
2	86041135	86041367	KD2-H4K8ac_peak_17328	6.66692	AC105053.3_ENSG00000229498
2	86054369	86054614	KD2-H4K8ac_peak_17329	11.75669	
2	86054957	86055183	KD2-H4K8ac_peak_17330	6.2159	
2	86115695	86116126	KD2-H4K8ac_peak_17331	4.67342	ST3GAL5_ENSG00000115525;AC105053.4_ENSG00000232504
2	86116490	86116792	KD2-H4K8ac_peak_17332	7.13615	ST3GAL5_ENSG00000115525;AC105053.4_ENSG00000232504
2	86262811	86263093	KD2-H4K8ac_peak_17333	5.77905	
2	86263689	86264586	KD2-H4K8ac_peak_17334	7.81657	
2	86333388	86333628	KD2-H4K8ac_peak_17335	10.0692	POLR1A_ENSG00000068654;PTCD3_ENSG00000132300
2	86422267	86422524	KD2-H4K8ac_peak_17336	13.80019	IMMT_ENSG00000132305;RP11-301O19.1_ENSG00000273080
2	86422955	86423423	KD2-H4K8ac_peak_17337	7.76104	IMMT_ENSG00000132305;RP11-301O19.1_ENSG00000273080
2	86426169	86426390	KD2-H4K8ac_peak_17338	9.89126	MRPL35_ENSG00000132313
2	86476193	86476462	KD2-H4K8ac_peak_17339	5.08628	
2	86564161	86564807	KD2-H4K8ac_peak_17340	13.2739	REEP1_ENSG00000068615

2	86595362	86595576	KD2-H4K8ac_peak_17341	8.1233	
2	86789824	86790036	KD2-H4K8ac_peak_17342	9.6247	AC015971.2_ENSG00000228363
2	86790843	86791298	KD2-H4K8ac_peak_17343	9.08816	
2	86850270	86850630	KD2-H4K8ac_peak_17344	6.84261	RNF103_ENSG00000239305
2	86851317	86851715	KD2-H4K8ac_peak_17345	5.45396	RNF103_ENSG00000239305
2	87033619	87033933	KD2-H4K8ac_peak_17346	9.15329	
2	87034270	87034650	KD2-H4K8ac_peak_17347	5.75452	CD8A_ENSG00000153563
2	87303188	87303778	KD2-H4K8ac_peak_17348	8.37231	AC125232.1_ENSG00000231259
2	87304058	87304282	KD2-H4K8ac_peak_17349	14.15075	AC125232.1_ENSG00000231259
2	87568260	87568534	KD2-H4K8ac_peak_17350	7.146	
2	87568930	87569600	KD2-H4K8ac_peak_17351	17.51891	
2	87800559	87800805	KD2-H4K8ac_peak_17352	10.21502	
2	87877298	87877828	KD2-H4K8ac_peak_17353	10.21502	
2	87892950	87893278	KD2-H4K8ac_peak_17354	9.11031	
2	87893537	87893785	KD2-H4K8ac_peak_17355	10.21502	
2	87894083	87894493	KD2-H4K8ac_peak_17356	16.57619	
2	87894697	87894920	KD2-H4K8ac_peak_17357	9.63487	
2	87903487	87903714	KD2-H4K8ac_peak_17358	11.30637	
2	87934067	87934768	KD2-H4K8ac_peak_17359	11.30637	
2	87937115	87937344	KD2-H4K8ac_peak_17360	5.88845	
2	88316118	88316880	KD2-H4K8ac_peak_17361	10.21502	RNU2-63P_ENSG00000222724
2	88354654	88355254	KD2-H4K8ac_peak_17362	6.66359	KRCC1_ENSG00000172086
2	88499350	88499666	KD2-H4K8ac_peak_17363	7.59643	
2	88509724	88509961	KD2-H4K8ac_peak_17364	5.90098	
2	88802612	88803139	KD2-H4K8ac_peak_17365	15.58442	
2	88849340	88849734	KD2-H4K8ac_peak_17366	5.75452	
2	88927452	88927730	KD2-H4K8ac_peak_17367	7.07117	EIF2AK3_ENSG00000172071;AC062029.1_ENSG00000234028
2	88991306	88991598	KD2-H4K8ac_peak_17368	13.9234	RPIA_ENSG00000153574
2	91761774	91762325	KD2-H4K8ac_peak_17369	17.84074	
2	91762866	91763588	KD2-H4K8ac_peak_17370	12.49766	
2	91845547	91846087	KD2-H4K8ac_peak_17371	14.89775	
2	91846677	91846905	KD2-H4K8ac_peak_17372	6.20982	
2	91847146	91848474	KD2-H4K8ac_peak_17373	19.00088	AC027612.6_ENSG00000143429
2	95742709	95742939	KD2-H4K8ac_peak_17374	7.1181	
2	95787097	95788043	KD2-H4K8ac_peak_17375	12.82393	
2	95824483	95825174	KD2-H4K8ac_peak_17376	10.65724	
2	95825584	95826098	KD2-H4K8ac_peak_17377	10.0692	
2	95830863	95831350	KD2-H4K8ac_peak_17378	6.40297	ZNF514_ENSG00000144026;ZNF2_ENSG00000163067
2	95872521	95873687	KD2-H4K8ac_peak_17379	16.8435	AC092835.2_ENSG00000233757
2	96067786	96068296	KD2-H4K8ac_peak_17380	8.28234	FAHD2A_ENSG00000115042
2	96068520	96068982	KD2-H4K8ac_peak_17381	8.2413	FAHD2A_ENSG00000115042
2	96192224	96192922	KD2-H4K8ac_peak_17382	11.02863	RP11-440D17.3_ENSG00000272913
2	96658324	96658569	KD2-H4K8ac_peak_17383	12.42547	ANKRD36C_ENSG00000174501
2	96675609	96676182	KD2-H4K8ac_peak_17384	9.63487	FAHD2CP_ENSG00000231584
2	96755731	96756060	KD2-H4K8ac_peak_17385	10.53377	
2	96756658	96757260	KD2-H4K8ac_peak_17386	4.66652	

2	96811847	96813157	KD2-H4K8ac_peak_17387	17.70889	DUSP2_ENSG00000158050;AC012307.2_ENSG00000228873
2	96813357	96813906	KD2-H4K8ac_peak_17388	13.13965	AC012307.2_ENSG00000228873
2	96814249	96814501	KD2-H4K8ac_peak_17389	5.00796	
2	96874214	96874534	KD2-H4K8ac_peak_17390	12.66412	STARD7_ENSG00000084090;STARD7-AS1_ENSG00000204685
2	96915632	96915845	KD2-H4K8ac_peak_17391	7.74292	
2	96931367	96931675	KD2-H4K8ac_peak_17392	10.65724	TMEM127_ENSG00000135956;CIAO1_ENSG00000144021
2	96932056	96932294	KD2-H4K8ac_peak_17393	8.10216	TMEM127_ENSG00000135956;CIAO1_ENSG00000144021
2	96970831	96971179	KD2-H4K8ac_peak_17394	9.90914	SNRNP200_ENSG00000144028
2	96971378	96972020	KD2-H4K8ac_peak_17395	8.86099	SNRNP200_ENSG00000144028
2	96972511	96972712	KD2-H4K8ac_peak_17396	6.66692	
2	96986630	96987213	KD2-H4K8ac_peak_17397	8.10216	AC021188.4_ENSG00000230747
2	96987438	96987794	KD2-H4K8ac_peak_17398	6.25869	AC021188.4_ENSG00000230747
2	96990574	96990972	KD2-H4K8ac_peak_17399	7.98621	ITPRIPL1_ENSG00000198885
2	97000992	97001413	KD2-H4K8ac_peak_17400	7.76329	NCAPH_ENSG00000121152
2	97197612	97197849	KD2-H4K8ac_peak_17401	9.65086	RP11-363D14.1_ENSG00000235480
2	97303559	97303790	KD2-H4K8ac_peak_17402	10.05606	
2	97308228	97308677	KD2-H4K8ac_peak_17403	5.94347	KANSL3_ENSG00000114982;FER1L5_ENSG00000249715
2	97351246	97351744	KD2-H4K8ac_peak_17404	8.23274	
2	97406382	97406782	KD2-H4K8ac_peak_17405	8.5781	LMAN2L_ENSG00000114988
2	97426036	97426370	KD2-H4K8ac_peak_17406	9.06369	CNNM4_ENSG00000158158
2	97466324	97466589	KD2-H4K8ac_peak_17407	6.31143	
2	97481501	97481737	KD2-H4K8ac_peak_17408	5.75757	CNNM3_ENSG00000168763
2	97482029	97482276	KD2-H4K8ac_peak_17409	14.8083	CNNM3_ENSG00000168763
2	97523209	97523682	KD2-H4K8ac_peak_17410	12.40345	ANKRD23_ENSG00000163126;ANKRD39_ENSG00000213337
2	97524312	97524607	KD2-H4K8ac_peak_17411	5.75452	ANKRD23_ENSG00000163126;ANKRD39_ENSG00000213337
2	97534111	97535742	KD2-H4K8ac_peak_17412	18.23876	SEMA4C_ENSG00000168758
2	97535936	97536353	KD2-H4K8ac_peak_17413	9.34195	SEMA4C_ENSG00000168758
2	97572226	97573835	KD2-H4K8ac_peak_17414	9.36516	
2	97734142	97734390	KD2-H4K8ac_peak_17415	5.25071	
2	97751676	97751881	KD2-H4K8ac_peak_17416	7.12754	
2	97760753	97761365	KD2-H4K8ac_peak_17417	14.74047	FAHD2B_ENSG00000144199
2	97778933	97779137	KD2-H4K8ac_peak_17418	9.90914	ANKRD36_ENSG00000135976
2	98206979	98207300	KD2-H4K8ac_peak_17419	8.10216	ANKRD36B_ENSG00000196912
2	98280012	98280306	KD2-H4K8ac_peak_17420	11.89502	ACTR1B_ENSG00000115073;LINC01125_ENSG00000228486;RNU4-8P_ENSG00000201806
2	98280734	98281192	KD2-H4K8ac_peak_17421	11.1952	ACTR1B_ENSG00000115073;LINC01125_ENSG00000228486;RNU4-8P_ENSG00000201806
2	98326600	98326907	KD2-H4K8ac_peak_17422	5.1313	
2	98329480	98329756	KD2-H4K8ac_peak_17423	5.14125	ZAP70_ENSG00000115085
2	98329949	98330235	KD2-H4K8ac_peak_17424	8.02571	ZAP70_ENSG00000115085
2	98611884	98612636	KD2-H4K8ac_peak_17425	8.55271	TMEM131_ENSG00000075568
2	98613122	98613377	KD2-H4K8ac_peak_17426	4.84206	TMEM131_ENSG00000075568
2	99060739	99061012	KD2-H4K8ac_peak_17427	5.57044	INPP4A_ENSG00000040933
2	99081980	99082188	KD2-H4K8ac_peak_17428	6.75424	
2	99224450	99225355	KD2-H4K8ac_peak_17429	10.43145	COA5_ENSG00000183513;UNC50_ENSG00000115446
2	99347794	99348279	KD2-H4K8ac_peak_17430	10.0692	MGAT4A_ENSG00000071073
2	99422170	99422565	KD2-H4K8ac_peak_17431	5.57044	
2	99438660	99439816	KD2-H4K8ac_peak_17432	10.19949	

2	99528838	99529079	KD2-H4K8ac_peak_17433	9.40857	
2	99552939	99553138	KD2-H4K8ac_peak_17434	6.05809	KIAA1211L_ENSG00000196872
2	99757787	99758508	KD2-H4K8ac_peak_17435	11.43353	C2orf15_ENSG00000241962;C2ORF15_ENSG00000273045
2	99771101	99771324	KD2-H4K8ac_peak_17436	6.85193	TSGA10_ENSG00000135951;LIPT1_ENSG00000144182;MRPL30_ENSG00000273155
2	99771612	99772071	KD2-H4K8ac_peak_17437	4.86324	TSGA10_ENSG00000135951;LIPT1_ENSG00000144182;MRPL30_ENSG00000273155
2	99954037	99954368	KD2-H4K8ac_peak_17438	7.0754	EIF5B_ENSG00000158417
2	100105766	1E+08	KD2-H4K8ac_peak_17439	5.54647	REV1_ENSG00000135945
2	100252001	1E+08	KD2-H4K8ac_peak_17440	12.08081	
2	100261488	1E+08	KD2-H4K8ac_peak_17441	9.41046	
2	100326487	1E+08	KD2-H4K8ac_peak_17442	4.59928	
2	100485256	1E+08	KD2-H4K8ac_peak_17443	7.27716	
2	100496805	1E+08	KD2-H4K8ac_peak_17444	6.03693	
2	100507003	1.01E+08	KD2-H4K8ac_peak_17445	6.94954	
2	100602234	1.01E+08	KD2-H4K8ac_peak_17446	3.80759	
2	100721384	1.01E+08	KD2-H4K8ac_peak_17447	18.41184	AC092667.2_ENSG00000230393
2	100754152	1.01E+08	KD2-H4K8ac_peak_17448	5.9696	
2	100814218	1.01E+08	KD2-H4K8ac_peak_17449	5.75757	
2	100815011	1.01E+08	KD2-H4K8ac_peak_17450	7.51054	
2	100859046	1.01E+08	KD2-H4K8ac_peak_17451	4.86324	
2	101033694	1.01E+08	KD2-H4K8ac_peak_17452	9.20193	CHST10_ENSG00000115526
2	101178484	1.01E+08	KD2-H4K8ac_peak_17453	10.21502	PDCL3_ENSG00000115539
2	101179463	1.01E+08	KD2-H4K8ac_peak_17454	16.32216	PDCL3_ENSG00000115539
2	101610997	1.02E+08	KD2-H4K8ac_peak_17455	4.57371	
2	101618258	1.02E+08	KD2-H4K8ac_peak_17456	24.051	AC016738.4_ENSG00000223947;RPL31_ENSG00000071082
2	101618819	1.02E+08	KD2-H4K8ac_peak_17457	7.76329	AC016738.4_ENSG00000223947;RPL31_ENSG00000071082
2	101726807	1.02E+08	KD2-H4K8ac_peak_17458	4.73064	
2	101729037	1.02E+08	KD2-H4K8ac_peak_17459	7.40111	
2	101731918	1.02E+08	KD2-H4K8ac_peak_17460	5.54647	
2	101768139	1.02E+08	KD2-H4K8ac_peak_17461	7.47462	RP11-299H21.1_ENSG00000272902
2	101868797	1.02E+08	KD2-H4K8ac_peak_17462	7.12754	TBC1D8_ENSG00000204634;CNOT11_ENSG00000158435
2	101869383	1.02E+08	KD2-H4K8ac_peak_17463	16.8435	TBC1D8_ENSG00000204634;CNOT11_ENSG00000158435
2	101924958	1.02E+08	KD2-H4K8ac_peak_17464	10.86378	MIR5696_ENSG00000264857;RNF149_ENSG00000163162
2	102090742	1.02E+08	KD2-H4K8ac_peak_17465	12.80176	RFX8_ENSG00000196460
2	102112186	1.02E+08	KD2-H4K8ac_peak_17466	6.00226	
2	102313309	1.02E+08	KD2-H4K8ac_peak_17467	15.16343	MAP4K4_ENSG00000071054
2	102314317	1.02E+08	KD2-H4K8ac_peak_17468	14.15075	
2	102420889	1.02E+08	KD2-H4K8ac_peak_17469	9.07071	
2	102422950	1.02E+08	KD2-H4K8ac_peak_17470	10.63441	
2	103353026	1.03E+08	KD2-H4K8ac_peak_17471	5.53033	MFSD9_ENSG00000135953;TMEM182_ENSG00000170417
2	104994972	1.05E+08	KD2-H4K8ac_peak_17472	9.59147	
2	105305822	1.05E+08	KD2-H4K8ac_peak_17473	7.12754	
2	105459055	1.05E+08	KD2-H4K8ac_peak_17474	7.94441	
2	105460145	1.05E+08	KD2-H4K8ac_peak_17475	9.6247	
2	105460999	1.05E+08	KD2-H4K8ac_peak_17476	12.3187	
2	105471428	1.05E+08	KD2-H4K8ac_peak_17477	10.1591	POU3F3_ENSG00000198914
2	105654644	1.06E+08	KD2-H4K8ac_peak_17478	6.36375	AC010884.1_ENSG00000224509;MRPS9_ENSG00000135972

2	105759846	1.06E+08	KD2-H4K8ac_peak_17479	5.14125	AC104655.3_ENSG00000226508
2	105760506	1.06E+08	KD2-H4K8ac_peak_17480	16.09057	AC104655.3_ENSG00000226508
2	105946218	1.06E+08	KD2-H4K8ac_peak_17481	21.11201	TGFBRAP1_ENSG00000135966
2	106014859	1.06E+08	KD2-H4K8ac_peak_17482	14.69247	
2	106054783	1.06E+08	KD2-H4K8ac_peak_17483	9.96798	FHL2_ENSG00000115641
2	106360901	1.06E+08	KD2-H4K8ac_peak_17484	14.45402	NCK2_ENSG00000071051
2	106681783	1.07E+08	KD2-H4K8ac_peak_17485	10.21502	
2	106747027	1.07E+08	KD2-H4K8ac_peak_17486	5.57044	
2	106809918	1.07E+08	KD2-H4K8ac_peak_17487	20.91724	UXS1_ENSG00000115652
2	106811013	1.07E+08	KD2-H4K8ac_peak_17488	6.66692	UXS1_ENSG00000115652
2	107191976	1.07E+08	KD2-H4K8ac_peak_17489	6.25869	
2	107195387	1.07E+08	KD2-H4K8ac_peak_17490	4.35346	
2	107196653	1.07E+08	KD2-H4K8ac_peak_17491	11.86082	
2	107198582	1.07E+08	KD2-H4K8ac_peak_17492	6.68439	
2	107502636	1.08E+08	KD2-H4K8ac_peak_17493	8.55271	ST6GAL2_ENSG00000144057
2	107503288	1.08E+08	KD2-H4K8ac_peak_17494	9.65086	ST6GAL2_ENSG00000144057
2	108993763	1.09E+08	KD2-H4K8ac_peak_17495	6.05809	SULT1C4_ENSG00000198075
2	108994447	1.09E+08	KD2-H4K8ac_peak_17496	5.75757	SULT1C4_ENSG00000198075
2	109065031	1.09E+08	KD2-H4K8ac_peak_17497	16.822	GCC2_ENSG00000135968
2	109150390	1.09E+08	KD2-H4K8ac_peak_17498	5.38581	AC012487.2_ENSG00000214184;LIMS1_ENSG00000169756
2	109150897	1.09E+08	KD2-H4K8ac_peak_17499	9.96798	AC012487.2_ENSG00000214184;LIMS1_ENSG00000169756
2	109334824	1.09E+08	KD2-H4K8ac_peak_17500	8.94469	RANBP2_ENSG00000153201
2	109336043	1.09E+08	KD2-H4K8ac_peak_17501	10.53377	RANBP2_ENSG00000153201
2	109403294	1.09E+08	KD2-H4K8ac_peak_17502	7.01964	CCDC138_ENSG00000163006
2	109787306	1.1E+08	KD2-H4K8ac_peak_17503	10.25088	
2	109788353	1.1E+08	KD2-H4K8ac_peak_17504	7.76104	
2	109790326	1.1E+08	KD2-H4K8ac_peak_17505	7.11925	
2	109875315	1.1E+08	KD2-H4K8ac_peak_17506	6.34383	
2	110062959	1.1E+08	KD2-H4K8ac_peak_17507	5.75757	
2	110064368	1.1E+08	KD2-H4K8ac_peak_17508	6.44277	
2	110064792	1.1E+08	KD2-H4K8ac_peak_17509	7.44366	
2	110066539	1.1E+08	KD2-H4K8ac_peak_17510	5.58333	
2	110088633	1.1E+08	KD2-H4K8ac_peak_17511	6.40683	
2	110090240	1.1E+08	KD2-H4K8ac_peak_17512	7.40111	
2	110371995	1.1E+08	KD2-H4K8ac_peak_17513	6.67092	SEPT10_ENSG00000186522;SOWAHC_ENSG00000198142
2	110450036	1.1E+08	KD2-H4K8ac_peak_17514	8.17902	
2	110858230	1.11E+08	KD2-H4K8ac_peak_17515	20.88898	
2	110859385	1.11E+08	KD2-H4K8ac_peak_17516	16.8435	
2	110873225	1.11E+08	KD2-H4K8ac_peak_17517	4.42765	MALL_ENSG00000144063
2	110962928	1.11E+08	KD2-H4K8ac_peak_17518	6.16888	NPHP1_ENSG00000144061
2	110969758	1.11E+08	KD2-H4K8ac_peak_17519	11.86082	
2	111435867	1.11E+08	KD2-H4K8ac_peak_17520	3.97079	BUB1_ENSG00000169679
2	111527561	1.12E+08	KD2-H4K8ac_peak_17521	10.05606	
2	111627189	1.12E+08	KD2-H4K8ac_peak_17522	7.79898	
2	111660113	1.12E+08	KD2-H4K8ac_peak_17523	4.67342	
2	111877649	1.12E+08	KD2-H4K8ac_peak_17524	14.275	BCL2L11_ENSG00000153094

2	112022191	1.12E+08	KD2-H4K8ac_peak_17525	5.88845	AC068491.2_ENSG00000229118
2	112069834	1.12E+08	KD2-H4K8ac_peak_17526	8.1233	
2	112070638	1.12E+08	KD2-H4K8ac_peak_17527	7.27716	
2	112073272	1.12E+08	KD2-H4K8ac_peak_17528	7.12754	
2	112112971	1.12E+08	KD2-H4K8ac_peak_17529	19.6416	
2	112123760	1.12E+08	KD2-H4K8ac_peak_17530	10.72656	
2	112221216	1.12E+08	KD2-H4K8ac_peak_17531	6.16888	
2	112271121	1.12E+08	KD2-H4K8ac_peak_17532	5.57044	
2	112280658	1.12E+08	KD2-H4K8ac_peak_17533	8.30974	
2	112283796	1.12E+08	KD2-H4K8ac_peak_17534	6.79289	
2	112293171	1.12E+08	KD2-H4K8ac_peak_17535	5.25071	
2	112641408	1.13E+08	KD2-H4K8ac_peak_17536	6.99194	ANAPC1_ENSG00000153107
2	112655606	1.13E+08	KD2-H4K8ac_peak_17537	9.36516	MERTK_ENSG00000153208
2	112676540	1.13E+08	KD2-H4K8ac_peak_17538	5.10505	
2	112732009	1.13E+08	KD2-H4K8ac_peak_17539	4.01115	
2	113011968	1.13E+08	KD2-H4K8ac_peak_17540	5.88845	ZC3H8_ENSG00000144161
2	113012830	1.13E+08	KD2-H4K8ac_peak_17541	9.11031	ZC3H8_ENSG00000144161
2	113017473	1.13E+08	KD2-H4K8ac_peak_17542	9.80695	
2	113033219	1.13E+08	KD2-H4K8ac_peak_17543	15.69572	ZC3H6_ENSG00000188177
2	113238982	1.13E+08	KD2-H4K8ac_peak_17544	16.40694	TTL_ENSG00000114999
2	113299491	1.13E+08	KD2-H4K8ac_peak_17545	12.36441	POLR1B_ENSG00000125630
2	113341511	1.13E+08	KD2-H4K8ac_peak_17546	10.7186	CHCHD5_ENSG00000125611
2	113342642	1.13E+08	KD2-H4K8ac_peak_17547	9.34989	CHCHD5_ENSG00000125611
2	113357250	1.13E+08	KD2-H4K8ac_peak_17548	6.57893	
2	113383911	1.13E+08	KD2-H4K8ac_peak_17549	16.61426	
2	113403563	1.13E+08	KD2-H4K8ac_peak_17550	6.40297	AC079922.3_ENSG00000237753;SLC20A1_ENSG00000144136
2	113484022	1.13E+08	KD2-H4K8ac_peak_17551	10.19949	
2	113956978	1.14E+08	KD2-H4K8ac_peak_17552	5.51783	
2	114082908	1.14E+08	KD2-H4K8ac_peak_17553	5.25071	
2	114341192	1.14E+08	KD2-H4K8ac_peak_17554	42.89502	MIR1302-3_ENSG00000221055;WASH2P_ENSG00000146556
2	114341942	1.14E+08	KD2-H4K8ac_peak_17555	13.57066	WASH2P_ENSG00000146556
2	114646682	1.15E+08	KD2-H4K8ac_peak_17556	11.30637	RP11-141B14.1_ENSG00000270019;ACTR3_ENSG00000115091
2	114647707	1.15E+08	KD2-H4K8ac_peak_17557	8.17902	RP11-141B14.1_ENSG00000270019;ACTR3_ENSG00000115091
2	117511901	1.18E+08	KD2-H4K8ac_peak_17558	8.30974	
2	118449543	1.18E+08	KD2-H4K8ac_peak_17559	4.86324	
2	118470459	1.18E+08	KD2-H4K8ac_peak_17560	8.74596	
2	118472370	1.18E+08	KD2-H4K8ac_peak_17561	6.4095	
2	118476297	1.18E+08	KD2-H4K8ac_peak_17562	5.75452	
2	118477866	1.18E+08	KD2-H4K8ac_peak_17563	13.09607	
2	118529426	1.19E+08	KD2-H4K8ac_peak_17564	4.01115	
2	118530171	1.19E+08	KD2-H4K8ac_peak_17565	10.0746	
2	118760298	1.19E+08	KD2-H4K8ac_peak_17566	10.21502	
2	118771268	1.19E+08	KD2-H4K8ac_peak_17567	8.59579	CCDC93_ENSG00000125633
2	118771866	1.19E+08	KD2-H4K8ac_peak_17568	10.21502	CCDC93_ENSG00000125633
2	118900286	1.19E+08	KD2-H4K8ac_peak_17569	8.1233	
2	118943267	1.19E+08	KD2-H4K8ac_peak_17570	10.96455	AC093901.1_ENSG00000226856

2	118944122	1.19E+08	KD2-H4K8ac_peak_17571	5.75757	AC093901.1_ENSG00000226856
2	119427668	1.19E+08	KD2-H4K8ac_peak_17572	5.57044	
2	119981061	1.2E+08	KD2-H4K8ac_peak_17573	6.65673	STEAP3_ENSG00000115107
2	119998225	1.2E+08	KD2-H4K8ac_peak_17574	8.26945	
2	120124027	1.2E+08	KD2-H4K8ac_peak_17575	5.00278	C2orf76_ENSG00000186132;DBI_ENSG00000155368
2	120188953	1.2E+08	KD2-H4K8ac_peak_17576	10.05606	
2	120234209	1.2E+08	KD2-H4K8ac_peak_17577	5.3778	AC013275.2_ENSG00000231013
2	120242400	1.2E+08	KD2-H4K8ac_peak_17578	6.98302	
2	120302093	1.2E+08	KD2-H4K8ac_peak_17579	8.30974	PCDP1_ENSG00000163075
2	120436804	1.2E+08	KD2-H4K8ac_peak_17580	9.11031	TMEM177_ENSG00000144120
2	120517568	1.21E+08	KD2-H4K8ac_peak_17581	7.27716	PTPN4_ENSG00000088179
2	120980421	1.21E+08	KD2-H4K8ac_peak_17582	15.58442	TMEM185B_ENSG00000226479
2	121009842	1.21E+08	KD2-H4K8ac_peak_17583	5.88845	
2	121010555	1.21E+08	KD2-H4K8ac_peak_17584	9.36516	
2	121199411	1.21E+08	KD2-H4K8ac_peak_17585	4.211	
2	121200078	1.21E+08	KD2-H4K8ac_peak_17586	6.8836	
2	121334205	1.21E+08	KD2-H4K8ac_peak_17587	8.10216	
2	121492739	1.21E+08	KD2-H4K8ac_peak_17588	9.72244	
2	121499194	1.21E+08	KD2-H4K8ac_peak_17589	17.42905	
2	121578107	1.22E+08	KD2-H4K8ac_peak_17590	6.36375	
2	121662832	1.22E+08	KD2-H4K8ac_peak_17591	10.05081	
2	121673584	1.22E+08	KD2-H4K8ac_peak_17592	6.4562	
2	122003532	1.22E+08	KD2-H4K8ac_peak_17593	4.59912	
2	122287771	1.22E+08	KD2-H4K8ac_peak_17594	6.60096	RP11-204L24.2_ENSG00000265451;RNU4ATAC_ENSG00000264229
2	122364597	1.22E+08	KD2-H4K8ac_peak_17595	7.76329	
2	122406542	1.22E+08	KD2-H4K8ac_peak_17596	7.76104	CLASP1_ENSG00000074054;NIFK-AS1_ENSG00000236859
2	122407393	1.22E+08	KD2-H4K8ac_peak_17597	6.66692	CLASP1_ENSG00000074054;NIFK-AS1_ENSG00000236859
2	122494115	1.22E+08	KD2-H4K8ac_peak_17598	9.63487	NIFK_ENSG00000155438;TSN_ENSG00000211460
2	122495024	1.22E+08	KD2-H4K8ac_peak_17599	13.81781	NIFK_ENSG00000155438;TSN_ENSG00000211460
2	122513271	1.23E+08	KD2-H4K8ac_peak_17600	10.96455	
2	122529174	1.23E+08	KD2-H4K8ac_peak_17601	8.37231	
2	122530086	1.23E+08	KD2-H4K8ac_peak_17602	7.79898	
2	122532114	1.23E+08	KD2-H4K8ac_peak_17603	5.61067	
2	125065065	1.25E+08	KD2-H4K8ac_peak_17604	7.47462	
2	125072646	1.25E+08	KD2-H4K8ac_peak_17605	7.22457	
2	125074933	1.25E+08	KD2-H4K8ac_peak_17606	8.74596	
2	125076424	1.25E+08	KD2-H4K8ac_peak_17607	8.37043	
2	125083639	1.25E+08	KD2-H4K8ac_peak_17608	5.15777	
2	125161482	1.25E+08	KD2-H4K8ac_peak_17609	6.16888	
2	127534483	1.28E+08	KD2-H4K8ac_peak_17610	4.42498	
2	127615180	1.28E+08	KD2-H4K8ac_peak_17611	6.00226	
2	127615986	1.28E+08	KD2-H4K8ac_peak_17612	7.18916	
2	127704191	1.28E+08	KD2-H4K8ac_peak_17613	5.57044	
2	127729392	1.28E+08	KD2-H4K8ac_peak_17614	3.90565	
2	127733913	1.28E+08	KD2-H4K8ac_peak_17615	5.9696	
2	127782680	1.28E+08	KD2-H4K8ac_peak_17616	11.28831	RP11-521O16.1_ENSG00000260634;RP11-521O16.2_ENSG00000260163

2	127807446	1.28E+08	KD2-H4K8ac_peak_17617	10.01478	
2	127844766	1.28E+08	KD2-H4K8ac_peak_17618	10.19949	
2	127863245	1.28E+08	KD2-H4K8ac_peak_17619	5.26288	BIN1_ENSG00000136717
2	127864810	1.28E+08	KD2-H4K8ac_peak_17620	4.14655	BIN1_ENSG00000136717
2	127976254	1.28E+08	KD2-H4K8ac_peak_17621	5.92591	
2	127976723	1.28E+08	KD2-H4K8ac_peak_17622	8.85813	CYP27C1_ENSG00000186684
2	127977511	1.28E+08	KD2-H4K8ac_peak_17623	16.8435	CYP27C1_ENSG00000186684
2	128051348	1.28E+08	KD2-H4K8ac_peak_17624	14.56955	ERCC3_ENSG00000163161
2	128052011	1.28E+08	KD2-H4K8ac_peak_17625	9.47671	ERCC3_ENSG00000163161
2	128145077	1.28E+08	KD2-H4K8ac_peak_17626	26.96144	MAP3K2_ENSG00000169967
2	128145760	1.28E+08	KD2-H4K8ac_peak_17627	14.15075	MAP3K2_ENSG00000169967;AC068282.3_ENSG00000236682
2	128165723	1.28E+08	KD2-H4K8ac_peak_17628	15.22643	
2	128283654	1.28E+08	KD2-H4K8ac_peak_17629	11.02863	IWS1_ENSG00000163166
2	128334370	1.28E+08	KD2-H4K8ac_peak_17630	4.42765	
2	128391901	1.28E+08	KD2-H4K8ac_peak_17631	4.44923	
2	128406936	1.28E+08	KD2-H4K8ac_peak_17632	9.08816	
2	128409007	1.28E+08	KD2-H4K8ac_peak_17633	11.92978	
2	128410308	1.28E+08	KD2-H4K8ac_peak_17634	5.59859	
2	128432293	1.28E+08	KD2-H4K8ac_peak_17635	6.25869	
2	128453132	1.28E+08	KD2-H4K8ac_peak_17636	6.25869	
2	128458101	1.28E+08	KD2-H4K8ac_peak_17637	8.59301	SFT2D3_ENSG00000173349
2	128459113	1.28E+08	KD2-H4K8ac_peak_17638	9.95342	SFT2D3_ENSG00000173349
2	128568469	1.29E+08	KD2-H4K8ac_peak_17639	14.27217	WDR33_ENSG00000136709
2	128568922	1.29E+08	KD2-H4K8ac_peak_17640	15.59774	WDR33_ENSG00000136709
2	128615329	1.29E+08	KD2-H4K8ac_peak_17641	12.25733	POLR2D_ENSG00000144231
2	128643257	1.29E+08	KD2-H4K8ac_peak_17642	6.31143	AMMECR1L_ENSG00000144233;RP11-395A13.2_ENSG00000272667
2	128643963	1.29E+08	KD2-H4K8ac_peak_17643	5.9696	AMMECR1L_ENSG00000144233;RP11-395A13.2_ENSG00000272667
2	128785398	1.29E+08	KD2-H4K8ac_peak_17644	5.01002	SAP130_ENSG00000136715
2	128786072	1.29E+08	KD2-H4K8ac_peak_17645	5.75452	SAP130_ENSG00000136715
2	128990858	1.29E+08	KD2-H4K8ac_peak_17646	14.12659	
2	129040008	1.29E+08	KD2-H4K8ac_peak_17647	8.66368	
2	129076765	1.29E+08	KD2-H4K8ac_peak_17648	9.89383	HS6ST1_ENSG00000136720
2	129079641	1.29E+08	KD2-H4K8ac_peak_17649	20.18381	
2	129105277	1.29E+08	KD2-H4K8ac_peak_17650	6.99815	
2	129136227	1.29E+08	KD2-H4K8ac_peak_17651	6.25869	
2	129137399	1.29E+08	KD2-H4K8ac_peak_17652	5.34636	
2	129147985	1.29E+08	KD2-H4K8ac_peak_17653	4.06722	
2	129164844	1.29E+08	KD2-H4K8ac_peak_17654	3.97079	
2	129170804	1.29E+08	KD2-H4K8ac_peak_17655	5.72327	
2	129179029	1.29E+08	KD2-H4K8ac_peak_17656	6.60096	
2	129180763	1.29E+08	KD2-H4K8ac_peak_17657	11.1952	
2	129182805	1.29E+08	KD2-H4K8ac_peak_17658	13.88933	
2	129198546	1.29E+08	KD2-H4K8ac_peak_17659	11.56469	
2	129206342	1.29E+08	KD2-H4K8ac_peak_17660	5.9696	
2	129206780	1.29E+08	KD2-H4K8ac_peak_17661	13.73635	
2	129208680	1.29E+08	KD2-H4K8ac_peak_17662	13.22195	

2	129215392	1.29E+08	KD2-H4K8ac_peak_17663	6.34383	
2	129252552	1.29E+08	KD2-H4K8ac_peak_17664	6.36375	
2	129381396	1.29E+08	KD2-H4K8ac_peak_17665	10.21502	
2	129382232	1.29E+08	KD2-H4K8ac_peak_17666	9.15329	
2	129494558	1.29E+08	KD2-H4K8ac_peak_17667	6.40297	
2	130566211	1.31E+08	KD2-H4K8ac_peak_17668	8.53581	
2	130635603	1.31E+08	KD2-H4K8ac_peak_17669	15.00387	AC079776.1_ENSG00000229536
2	130687172	1.31E+08	KD2-H4K8ac_peak_17670	5.00796	
2	130713350	1.31E+08	KD2-H4K8ac_peak_17671	7.44324	
2	130938780	1.31E+08	KD2-H4K8ac_peak_17672	7.07117	MZT2B_ENSG00000152082
2	130939540	1.31E+08	KD2-H4K8ac_peak_17673	10.43145	MZT2B_ENSG00000152082;SMPD4_ENSG00000136699;AC018804.1_ENSG00000265280
2	130940418	1.31E+08	KD2-H4K8ac_peak_17674	10.87093	SMPD4_ENSG00000136699;AC018804.1_ENSG00000265280
2	130941575	1.31E+08	KD2-H4K8ac_peak_17675	6.99815	AC018804.1_ENSG00000265280
2	131099177	1.31E+08	KD2-H4K8ac_peak_17676	9.1707	CCDC115_ENSG00000136710;IMP4_ENSG00000136718
2	131113658	1.31E+08	KD2-H4K8ac_peak_17677	12.93209	PTPN18_ENSG00000072135
2	131128805	1.31E+08	KD2-H4K8ac_peak_17678	5.4779	
2	131130304	1.31E+08	KD2-H4K8ac_peak_17679	6.84261	
2	131149017	1.31E+08	KD2-H4K8ac_peak_17680	12.93253	
2	131484157	1.31E+08	KD2-H4K8ac_peak_17681	11.05467	
2	131485475	1.31E+08	KD2-H4K8ac_peak_17682	9.51625	GPR148_ENSG00000173302
2	131502479	1.32E+08	KD2-H4K8ac_peak_17683	9.1707	
2	131513938	1.32E+08	KD2-H4K8ac_peak_17684	5.30799	AMER3_ENSG00000178171;AC140481.8_ENSG00000229797
2	131576925	1.32E+08	KD2-H4K8ac_peak_17685	6.2781	
2	131792488	1.32E+08	KD2-H4K8ac_peak_17686	10.19949	
2	131850523	1.32E+08	KD2-H4K8ac_peak_17687	12.23	FAM168B_ENSG00000152102
2	131861553	1.32E+08	KD2-H4K8ac_peak_17688	8.41122	PLEKHB2_ENSG00000115762
2	131862089	1.32E+08	KD2-H4K8ac_peak_17689	5.61067	PLEKHB2_ENSG00000115762
2	131863082	1.32E+08	KD2-H4K8ac_peak_17690	6.24114	PLEKHB2_ENSG00000115762
2	132182934	1.32E+08	KD2-H4K8ac_peak_17691	5.13341	GNAQP1_ENSG00000214077
2	132249579	1.32E+08	KD2-H4K8ac_peak_17692	22.21705	MZT2A_ENSG00000173272;MIR4784_ENSG00000265575;AC093838.4_ENSG00000152117
2	132250527	1.32E+08	KD2-H4K8ac_peak_17693	8.85813	MZT2A_ENSG00000173272;AC093838.4_ENSG00000152117
2	132285137	1.32E+08	KD2-H4K8ac_peak_17694	7.40111	CCDC74A_ENSG00000163040
2	132290299	1.32E+08	KD2-H4K8ac_peak_17695	5.4779	
2	132291485	1.32E+08	KD2-H4K8ac_peak_17696	8.7325	
2	132439461	1.32E+08	KD2-H4K8ac_peak_17697	14.06758	
2	132559166	1.33E+08	KD2-H4K8ac_peak_17698	8.5092	C2orf27B_ENSG00000186825
2	133104143	1.33E+08	KD2-H4K8ac_peak_17699	6.36375	RP11-725P16.2_ENSG00000272769
2	133105087	1.33E+08	KD2-H4K8ac_peak_17700	22.92795	RP11-725P16.2_ENSG00000272769
2	133110362	1.33E+08	KD2-H4K8ac_peak_17701	20.61349	FAM201B_ENSG00000230992
2	134023055	1.34E+08	KD2-H4K8ac_peak_17702	13.73635	AC010890.1_ENSG00000226953
2	134357234	1.34E+08	KD2-H4K8ac_peak_17703	9.15329	
2	134469903	1.34E+08	KD2-H4K8ac_peak_17704	14.45402	
2	134534592	1.35E+08	KD2-H4K8ac_peak_17705	5.25071	
2	134877952	1.35E+08	KD2-H4K8ac_peak_17706	11.09936	MGAT5_ENSG00000152127
2	134878857	1.35E+08	KD2-H4K8ac_peak_17707	5.54647	
2	134935362	1.35E+08	KD2-H4K8ac_peak_17708	10.21502	

2	135092596	1.35E+08	KD2-H4K8ac_peak_17709	4.12757	
2	135168576	1.35E+08	KD2-H4K8ac_peak_17710	6.40683	
2	135169404	1.35E+08	KD2-H4K8ac_peak_17711	9.5456	
2	135257046	1.35E+08	KD2-H4K8ac_peak_17712	6.90458	
2	135279705	1.35E+08	KD2-H4K8ac_peak_17713	4.06758	
2	135281158	1.35E+08	KD2-H4K8ac_peak_17714	5.7068	
2	135332302	1.35E+08	KD2-H4K8ac_peak_17715	7.12754	
2	135675485	1.36E+08	KD2-H4K8ac_peak_17716	5.23853	CCNT2-AS1_ENSG00000224043;CCNT2_ENSG00000082258
2	135676503	1.36E+08	KD2-H4K8ac_peak_17717	4.37809	CCNT2-AS1_ENSG00000224043;CCNT2_ENSG00000082258
2	135810038	1.36E+08	KD2-H4K8ac_peak_17718	11.05407	RAB3GAP1_ENSG00000115839
2	136499516	1.37E+08	KD2-H4K8ac_peak_17719	7.36799	UBXN4_ENSG00000144224
2	136633514	1.37E+08	KD2-H4K8ac_peak_17720	7.12754	MCM6_ENSG00000076003
2	136742772	1.37E+08	KD2-H4K8ac_peak_17721	19.51835	DARS_ENSG00000115866;AC093391.2_ENSG00000231890
2	136743341	1.37E+08	KD2-H4K8ac_peak_17722	8.55271	DARS_ENSG00000115866;AC093391.2_ENSG00000231890
2	136824978	1.37E+08	KD2-H4K8ac_peak_17723	3.74443	
2	139258707	1.39E+08	KD2-H4K8ac_peak_17724	9.34195	AC097721.2_ENSG00000228043;SPOPL_ENSG00000144228
2	142888819	1.43E+08	KD2-H4K8ac_peak_17725	4.86324	LRP1B_ENSG00000168702
2	144459103	1.44E+08	KD2-H4K8ac_peak_17726	4.77079	
2	144694416	1.45E+08	KD2-H4K8ac_peak_17727	9.63487	AC016910.1_ENSG00000232377
2	145089346	1.45E+08	KD2-H4K8ac_peak_17728	13.57066	GTDC1_ENSG00000121964
2	145218998	1.45E+08	KD2-H4K8ac_peak_17729	7.48133	
2	145273415	1.45E+08	KD2-H4K8ac_peak_17730	15.22643	
2	145274200	1.45E+08	KD2-H4K8ac_peak_17731	9.63487	ZEB2-AS1_ENSG00000238057
2	145280471	1.45E+08	KD2-H4K8ac_peak_17732	8.30974	ZEB2_ENSG00000169554
2	148601454	1.49E+08	KD2-H4K8ac_peak_17733	27.41885	ACVR2A_ENSG00000121989
2	149401885	1.49E+08	KD2-H4K8ac_peak_17734	15.66774	EPC2_ENSG00000135999
2	149423649	1.49E+08	KD2-H4K8ac_peak_17735	4.49837	
2	149632029	1.5E+08	KD2-H4K8ac_peak_17736	4.86324	KIF5C_ENSG00000168280
2	149632844	1.5E+08	KD2-H4K8ac_peak_17737	13.92307	KIF5C_ENSG00000168280
2	149633830	1.5E+08	KD2-H4K8ac_peak_17738	5.54647	
2	149640803	1.5E+08	KD2-H4K8ac_peak_17739	6.40683	
2	149894996	1.5E+08	KD2-H4K8ac_peak_17740	8.30974	LYPD6B_ENSG00000150556
2	150187123	1.5E+08	KD2-H4K8ac_peak_17741	8.88082	LYPD6_ENSG00000187123
2	150443951	1.5E+08	KD2-H4K8ac_peak_17742	6.55628	MMADHC_ENSG00000168288;AC144449.1_ENSG00000231969
2	151184197	1.51E+08	KD2-H4K8ac_peak_17743	8.17902	
2	151342747	1.51E+08	KD2-H4K8ac_peak_17744	8.5092	
2	151669780	1.52E+08	KD2-H4K8ac_peak_17745	8.55271	
2	151733180	1.52E+08	KD2-H4K8ac_peak_17746	6.40683	
2	151741370	1.52E+08	KD2-H4K8ac_peak_17747	8.5781	
2	151743412	1.52E+08	KD2-H4K8ac_peak_17748	3.75035	
2	151770849	1.52E+08	KD2-H4K8ac_peak_17749	4.37708	
2	151838690	1.52E+08	KD2-H4K8ac_peak_17750	7.15405	AC023469.2_ENSG00000228064
2	151853013	1.52E+08	KD2-H4K8ac_peak_17751	16.86926	
2	152094945	1.52E+08	KD2-H4K8ac_peak_17752	5.73839	
2	152117579	1.52E+08	KD2-H4K8ac_peak_17753	14.43794	RBM43_ENSG00000184898
2	152266509	1.52E+08	KD2-H4K8ac_peak_17754	8.59579	RIF1_ENSG00000080345

2	152684528	1.53E+08	KD2-H4K8ac_peak_17755	7.76329	ARL5A_ENSG00000162980
2	152955431	1.53E+08	KD2-H4K8ac_peak_17756	10.0692	CACNB4_ENSG00000182389;AC079790.2_ENSG00000225214
2	153031780	1.53E+08	KD2-H4K8ac_peak_17757	6.65673	STAM2_ENSG00000115145
2	153191766	1.53E+08	KD2-H4K8ac_peak_17758	10.05606	FMNL2_ENSG00000157827
2	153573417	1.54E+08	KD2-H4K8ac_peak_17759	10.08707	PRPF40A_ENSG00000196504;ARL6IP6_ENSG00000177917
2	153574204	1.54E+08	KD2-H4K8ac_peak_17760	9.048	PRPF40A_ENSG00000196504;ARL6IP6_ENSG00000177917
2	154078407	1.54E+08	KD2-H4K8ac_peak_17761	6.66359	
2	154727948	1.55E+08	KD2-H4K8ac_peak_17762	8.10216	GALNT13_ENSG00000144278
2	154728501	1.55E+08	KD2-H4K8ac_peak_17763	10.53596	GALNT13_ENSG00000144278
2	154985809	1.55E+08	KD2-H4K8ac_peak_17764	5.25071	
2	154986370	1.55E+08	KD2-H4K8ac_peak_17765	6.8836	
2	155163466	1.55E+08	KD2-H4K8ac_peak_17766	6.16888	
2	155553851	1.56E+08	KD2-H4K8ac_peak_17767	6.85193	AC061961.2_ENSG00000235949;KCNJ3_ENSG00000162989
2	157292086	1.57E+08	KD2-H4K8ac_peak_17768	15.40367	GPD2_ENSG00000115159
2	158485438	1.58E+08	KD2-H4K8ac_peak_17769	9.11031	ACVR1C_ENSG00000123612
2	158694192	1.59E+08	KD2-H4K8ac_peak_17770	4.77079	
2	158733135	1.59E+08	KD2-H4K8ac_peak_17771	12.42761	ACVR1_ENSG00000115170;UPP2_ENSG00000007001;RP11-546J1.1_ENSG00000270557
2	158858799	1.59E+08	KD2-H4K8ac_peak_17772	10.0692	
2	159823690	1.6E+08	KD2-H4K8ac_peak_17773	18.80403	TANC1_ENSG00000115183
2	159911487	1.6E+08	KD2-H4K8ac_peak_17774	4.75764	
2	160142791	1.6E+08	KD2-H4K8ac_peak_17775	6.24114	WDSUB1_ENSG00000196151
2	160471914	1.6E+08	KD2-H4K8ac_peak_17776	13.81781	BAZ2B_ENSG00000123636;AC009506.1_ENSG00000224152
2	160568630	1.61E+08	KD2-H4K8ac_peak_17777	7.36799	AC009961.3_ENSG00000226266;MARCH7_ENSG00000136536
2	160569067	1.61E+08	KD2-H4K8ac_peak_17778	13.35993	AC009961.3_ENSG00000226266;MARCH7_ENSG00000136536
2	160654323	1.61E+08	KD2-H4K8ac_peak_17779	13.9234	CD302_ENSG00000241399
2	161264583	1.61E+08	KD2-H4K8ac_peak_17780	7.05051	MIR4785_ENSG00000263948
2	161349003	1.61E+08	KD2-H4K8ac_peak_17781	11.70505	RBMS1_ENSG00000153250
2	161349757	1.61E+08	KD2-H4K8ac_peak_17782	10.96455	RBMS1_ENSG00000153250
2	161777652	1.62E+08	KD2-H4K8ac_peak_17783	7.12754	
2	162016128	1.62E+08	KD2-H4K8ac_peak_17784	5.14125	AC009313.1_ENSG00000224467
2	162016967	1.62E+08	KD2-H4K8ac_peak_17785	10.96455	AC009313.1_ENSG00000224467
2	162094211	1.62E+08	KD2-H4K8ac_peak_17786	4.42765	
2	162095124	1.62E+08	KD2-H4K8ac_peak_17787	18.37277	
2	162164984	1.62E+08	KD2-H4K8ac_peak_17788	13.70605	PSMD14_ENSG00000115233
2	164592737	1.65E+08	KD2-H4K8ac_peak_17789	13.3695	FIGN_ENSG00000182263
2	165477640	1.65E+08	KD2-H4K8ac_peak_17790	5.30799	GRB14_ENSG00000115290
2	165687536	1.66E+08	KD2-H4K8ac_peak_17791	9.15329	
2	165697473	1.66E+08	KD2-H4K8ac_peak_17792	5.98692	AC019181.2_ENSG00000233255
2	165698318	1.66E+08	KD2-H4K8ac_peak_17793	6.20982	
2	165747602	1.66E+08	KD2-H4K8ac_peak_17794	6.64891	
2	165926744	1.66E+08	KD2-H4K8ac_peak_17795	10.21502	
2	167037893	1.67E+08	KD2-H4K8ac_peak_17796	7.59643	RN7SKP152_ENSG00000222376
2	167232024	1.67E+08	KD2-H4K8ac_peak_17797	5.54647	SCN9A_ENSG00000169432
2	167232881	1.67E+08	KD2-H4K8ac_peak_17798	5.71749	SCN9A_ENSG00000169432
2	167275922	1.67E+08	KD2-H4K8ac_peak_17799	6.16888	
2	167350620	1.67E+08	KD2-H4K8ac_peak_17800	6.99815	SCN7A_ENSG00000136546

2	167532942	1.68E+08	KD2-H4K8ac_peak_17801	7.01964	
2	168149035	1.68E+08	KD2-H4K8ac_peak_17802	14.99434	AC074363.1_ENSG00000228222
2	168964625	1.69E+08	KD2-H4K8ac_peak_17803	8.1233	
2	169103174	1.69E+08	KD2-H4K8ac_peak_17804	8.59579	
2	169103656	1.69E+08	KD2-H4K8ac_peak_17805	16.32216	STK39_ENSG00000198648
2	169203444	1.69E+08	KD2-H4K8ac_peak_17806	5.54647	
2	169294507	1.69E+08	KD2-H4K8ac_peak_17807	10.77174	
2	169295573	1.69E+08	KD2-H4K8ac_peak_17808	11.44456	
2	169313331	1.69E+08	KD2-H4K8ac_peak_17809	8.85813	CERS6_ENSG00000172292
2	169747171	1.7E+08	KD2-H4K8ac_peak_17810	5.15777	
2	169769587	1.7E+08	KD2-H4K8ac_peak_17811	9.02371	SPC25_ENSG00000152253
2	169770096	1.7E+08	KD2-H4K8ac_peak_17812	8.1233	SPC25_ENSG00000152253
2	170440911	1.7E+08	KD2-H4K8ac_peak_17813	8.26945	PPIG_ENSG00000138398
2	170551084	1.71E+08	KD2-H4K8ac_peak_17814	9.89383	CCDC173_ENSG00000154479;PHOSPHO2_ENSG00000144362;KLHL23_ENSG00000213160
2	170570262	1.71E+08	KD2-H4K8ac_peak_17815	7.27716	
2	170572112	1.71E+08	KD2-H4K8ac_peak_17816	5.88845	
2	170572996	1.71E+08	KD2-H4K8ac_peak_17817	13.81781	
2	170574090	1.71E+08	KD2-H4K8ac_peak_17818	10.0692	
2	170590303	1.71E+08	KD2-H4K8ac_peak_17819	13.44374	
2	170654692	1.71E+08	KD2-H4K8ac_peak_17820	7.71478	
2	170681084	1.71E+08	KD2-H4K8ac_peak_17821	10.29344	METTL5_ENSG00000138382
2	170681761	1.71E+08	KD2-H4K8ac_peak_17822	4.86324	METTL5_ENSG00000138382
2	170684053	1.71E+08	KD2-H4K8ac_peak_17823	8.4716	UBR3_ENSG00000144357
2	171627630	1.72E+08	KD2-H4K8ac_peak_17824	7.76329	AC007405.4_ENSG00000234350;AC007405.6_ENSG00000239467
2	171729058	1.72E+08	KD2-H4K8ac_peak_17825	4.77079	
2	171784750	1.72E+08	KD2-H4K8ac_peak_17826	9.1707	GORASP2_ENSG00000115806
2	171785777	1.72E+08	KD2-H4K8ac_peak_17827	11.02863	GORASP2_ENSG00000115806
2	171829860	1.72E+08	KD2-H4K8ac_peak_17828	22.25695	
2	172016463	1.72E+08	KD2-H4K8ac_peak_17829	8.23274	
2	172017626	1.72E+08	KD2-H4K8ac_peak_17830	7.12754	
2	172290857	1.72E+08	KD2-H4K8ac_peak_17831	9.41619	METTL8_ENSG00000123600;DCAF17_ENSG00000115827
2	172378995	1.72E+08	KD2-H4K8ac_peak_17832	8.55271	CYBRD1_ENSG00000071967
2	172543999	1.73E+08	KD2-H4K8ac_peak_17833	8.94469	DYNC1I2_ENSG00000077380
2	172544457	1.73E+08	KD2-H4K8ac_peak_17834	7.36799	DYNC1I2_ENSG00000077380
2	172864542	1.73E+08	KD2-H4K8ac_peak_17835	5.9696	SLC25A12_ENSG00000115840;METAP1D_ENSG00000172878
2	173099450	1.73E+08	KD2-H4K8ac_peak_17836	6.07178	
2	173099888	1.73E+08	KD2-H4K8ac_peak_17837	13.57066	
2	173291942	1.73E+08	KD2-H4K8ac_peak_17838	6.13909	ITGA6_ENSG00000091409
2	173292362	1.73E+08	KD2-H4K8ac_peak_17839	4.33139	ITGA6_ENSG00000091409;AC078883.4_ENSG00000226963
2	173420273	1.73E+08	KD2-H4K8ac_peak_17840	8.5092	AC093818.1_ENSG00000225205;PDK1_ENSG00000152256
2	173420892	1.73E+08	KD2-H4K8ac_peak_17841	8.52559	AC093818.1_ENSG00000225205;PDK1_ENSG00000152256
2	173591173	1.74E+08	KD2-H4K8ac_peak_17842	5.01262	
2	173600295	1.74E+08	KD2-H4K8ac_peak_17843	7.92775	RAPGEF4-AS1_ENSG00000228016;RAPGEF4_ENSG00000091428
2	173940284	1.74E+08	KD2-H4K8ac_peak_17844	15.13261	MLTK_ENSG00000091436
2	174219200	1.74E+08	KD2-H4K8ac_peak_17845	9.20193	AC092573.2_ENSG00000236391;CDCA7_ENSG00000144354
2	174313634	1.74E+08	KD2-H4K8ac_peak_17846	9.36516	

2	174828739	1.75E+08	KD2-H4K8ac_peak_17847	10.72656	
2	174829687	1.75E+08	KD2-H4K8ac_peak_17848	11.02863	SP3_ENSG00000172845
2	174830298	1.75E+08	KD2-H4K8ac_peak_17849	6.40297	SP3_ENSG00000172845
2	174855799	1.75E+08	KD2-H4K8ac_peak_17850	6.85193	
2	174888571	1.75E+08	KD2-H4K8ac_peak_17851	5.38581	
2	174889223	1.75E+08	KD2-H4K8ac_peak_17852	4.37815	RP11-394I13.1_ENSG00000260868
2	174904391	1.75E+08	KD2-H4K8ac_peak_17853	8.55271	
2	175112889	1.75E+08	KD2-H4K8ac_peak_17854	12.57143	OLA1_ENSG00000138430
2	175113531	1.75E+08	KD2-H4K8ac_peak_17855	14.66631	OLA1_ENSG00000138430
2	175399035	1.75E+08	KD2-H4K8ac_peak_17856	6.40683	
2	175432574	1.75E+08	KD2-H4K8ac_peak_17857	7.91974	
2	175450554	1.75E+08	KD2-H4K8ac_peak_17858	5.16439	
2	175528656	1.76E+08	KD2-H4K8ac_peak_17859	7.27716	
2	175546854	1.76E+08	KD2-H4K8ac_peak_17860	16.49944	WIPF1_ENSG00000115935
2	175637094	1.76E+08	KD2-H4K8ac_peak_17861	10.72656	
2	175651836	1.76E+08	KD2-H4K8ac_peak_17862	7.59643	AC018890.4_ENSG00000237104
2	175653479	1.76E+08	KD2-H4K8ac_peak_17863	9.15329	AC018890.4_ENSG00000237104
2	175869478	1.76E+08	KD2-H4K8ac_peak_17864	17.51891	CHN1_ENSG00000128656
2	176032181	1.76E+08	KD2-H4K8ac_peak_17865	10.53377	ATF2_ENSG00000115966;MIR933_ENSG00000215973;AC096649.2_ENSG00000229750
2	176045666	1.76E+08	KD2-H4K8ac_peak_17866	9.61895	
2	176046756	1.76E+08	KD2-H4K8ac_peak_17867	7.20567	
2	176749416	1.77E+08	KD2-H4K8ac_peak_17868	3.75035	
2	176753809	1.77E+08	KD2-H4K8ac_peak_17869	4.42765	
2	176866462	1.77E+08	KD2-H4K8ac_peak_17870	14.8083	KIAA1715_ENSG00000144320
2	176867197	1.77E+08	KD2-H4K8ac_peak_17871	13.81781	KIAA1715_ENSG00000144320
2	176957329	1.77E+08	KD2-H4K8ac_peak_17872	8.23062	HOXD13_ENSG00000128714
2	176969460	1.77E+08	KD2-H4K8ac_peak_17873	6.8836	HOXD11_ENSG00000128713
2	176986672	1.77E+08	KD2-H4K8ac_peak_17874	16.12525	HOXD9_ENSG00000128709
2	176994050	1.77E+08	KD2-H4K8ac_peak_17875	23.70459	HOXD8_ENSG00000175879
2	176994710	1.77E+08	KD2-H4K8ac_peak_17876	9.34989	HOXD8_ENSG00000175879
2	177000544	1.77E+08	KD2-H4K8ac_peak_17877	8.85813	HOXD3_ENSG00000128652
2	177001139	1.77E+08	KD2-H4K8ac_peak_17878	13.95627	HOXD-AS2_ENSG00000237380;HOXD3_ENSG00000128652
2	177021198	1.77E+08	KD2-H4K8ac_peak_17879	6.66692	
2	177021944	1.77E+08	KD2-H4K8ac_peak_17880	7.49599	
2	177022976	1.77E+08	KD2-H4K8ac_peak_17881	5.01002	
2	177024130	1.77E+08	KD2-H4K8ac_peak_17882	6.36375	
2	177052999	1.77E+08	KD2-H4K8ac_peak_17883	8.4716	HOXD-AS1_ENSG00000224189;HOXD1_ENSG00000128645
2	177053461	1.77E+08	KD2-H4K8ac_peak_17884	11.55418	HOXD-AS1_ENSG00000224189;HOXD1_ENSG00000128645
2	177134204	1.77E+08	KD2-H4K8ac_peak_17885	7.92775	MTX2_ENSG00000128654
2	177329619	1.77E+08	KD2-H4K8ac_peak_17886	4.77079	
2	177419105	1.77E+08	KD2-H4K8ac_peak_17887	8.30974	
2	177440213	1.77E+08	KD2-H4K8ac_peak_17888	14.55656	
2	177453483	1.77E+08	KD2-H4K8ac_peak_17889	5.25071	
2	177502101	1.78E+08	KD2-H4K8ac_peak_17890	19.9503	LINC01116_ENSG00000163364;LINC01117_ENSG00000224577
2	177903413	1.78E+08	KD2-H4K8ac_peak_17891	6.75638	
2	178029183	1.78E+08	KD2-H4K8ac_peak_17892	6.16888	AC079305.11_ENSG00000236501

2	178076886	1.78E+08	KD2-H4K8ac_peak_17893	6.65673	AC079305.8_ENSG00000229337;HNRNPA3_ENSG00000170144;MIR4444-2_ENSG00000263721
2	178077543	1.78E+08	KD2-H4K8ac_peak_17894	8.85813	AC079305.8_ENSG00000229337;HNRNPA3_ENSG00000170144;MIR4444-2_ENSG00000263721
2	178128425	1.78E+08	KD2-H4K8ac_peak_17895	17.17257	AC079305.10_ENSG00000222043
2	178129667	1.78E+08	KD2-H4K8ac_peak_17896	11.05407	AC079305.10_ENSG00000222043
2	178256981	1.78E+08	KD2-H4K8ac_peak_17897	7.49599	NFE2L2_ENSG00000116044;AC074286.1_ENSG00000213963;AGPS_ENSG00000018510
2	178387353	1.78E+08	KD2-H4K8ac_peak_17898	7.12754	
2	178393749	1.78E+08	KD2-H4K8ac_peak_17899	5.14125	
2	178412599	1.78E+08	KD2-H4K8ac_peak_17900	7.40633	
2	178416720	1.78E+08	KD2-H4K8ac_peak_17901	12.57865	TTC30B_ENSG00000196659
2	178483105	1.78E+08	KD2-H4K8ac_peak_17902	5.38581	TTC30A_ENSG00000197557
2	178483523	1.78E+08	KD2-H4K8ac_peak_17903	10.87093	TTC30A_ENSG00000197557
2	178623337	1.79E+08	KD2-H4K8ac_peak_17904	6.65673	
2	178872498	1.79E+08	KD2-H4K8ac_peak_17905	6.40683	
2	178937564	1.79E+08	KD2-H4K8ac_peak_17906	15.13261	
2	178976649	1.79E+08	KD2-H4K8ac_peak_17907	4.9025	RBM45_ENSG00000155636
2	178977236	1.79E+08	KD2-H4K8ac_peak_17908	5.13615	RBM45_ENSG00000155636
2	179058588	1.79E+08	KD2-H4K8ac_peak_17909	8.74596	OSBPL6_ENSG00000079156
2	179277951	1.79E+08	KD2-H4K8ac_peak_17910	11.24702	AC009948.5_ENSG00000223960
2	179315601	1.79E+08	KD2-H4K8ac_peak_17911	12.13241	PRKRA_ENSG00000180228;DFNB59_ENSG00000204311
2	179316307	1.79E+08	KD2-H4K8ac_peak_17912	14.275	PRKRA_ENSG00000180228;DFNB59_ENSG00000204311
2	179345263	1.79E+08	KD2-H4K8ac_peak_17913	8.55271	PLEKHA3_ENSG00000116095
2	180129157	1.8E+08	KD2-H4K8ac_peak_17914	8.30974	SESTD1_ENSG00000187231
2	180725878	1.81E+08	KD2-H4K8ac_peak_17915	5.51783	ZNF385B_ENSG00000144331;MIR1258_ENSG00000221240
2	180871535	1.81E+08	KD2-H4K8ac_peak_17916	11.24702	CWC22_ENSG00000163510
2	181844760	1.82E+08	KD2-H4K8ac_peak_17917	17.25154	AC104076.3_ENSG00000236153
2	181845769	1.82E+08	KD2-H4K8ac_peak_17918	13.34404	AC104076.3_ENSG00000236153
2	182321290	1.82E+08	KD2-H4K8ac_peak_17919	8.10216	ITGA4_ENSG00000115232
2	182322128	1.82E+08	KD2-H4K8ac_peak_17920	12.99417	ITGA4_ENSG00000115232
2	182521334	1.83E+08	KD2-H4K8ac_peak_17921	9.31907	
2	182545565	1.83E+08	KD2-H4K8ac_peak_17922	5.54647	CERKL_ENSG00000188452;NEUROD1_ENSG00000162992
2	182548256	1.83E+08	KD2-H4K8ac_peak_17923	11.89502	AC013733.3_ENSG00000234595
2	183580571	1.84E+08	KD2-H4K8ac_peak_17924	10.43145	DNAJC10_ENSG00000077232
2	183731202	1.84E+08	KD2-H4K8ac_peak_17925	10.86378	FRZB_ENSG00000162998
2	183902299	1.84E+08	KD2-H4K8ac_peak_17926	11.79585	NCKAP1_ENSG00000061676
2	183903623	1.84E+08	KD2-H4K8ac_peak_17927	4.42765	NCKAP1_ENSG00000061676
2	183989193	1.84E+08	KD2-H4K8ac_peak_17928	8.55271	
2	185462497	1.85E+08	KD2-H4K8ac_peak_17929	4.37815	ZNF804A_ENSG00000170396
2	185463346	1.85E+08	KD2-H4K8ac_peak_17930	6.20982	ZNF804A_ENSG00000170396
2	187350450	1.87E+08	KD2-H4K8ac_peak_17931	10.63441	ZC3H15_ENSG00000065548
2	187351077	1.87E+08	KD2-H4K8ac_peak_17932	6.99815	ZC3H15_ENSG00000065548
2	187454378	1.87E+08	KD2-H4K8ac_peak_17933	14.00658	ITGAV_ENSG00000138448
2	187558501	1.88E+08	KD2-H4K8ac_peak_17934	8.30974	FAM171B_ENSG00000144369
2	187713638	1.88E+08	KD2-H4K8ac_peak_17935	6.99815	ZSWIM2_ENSG00000163012
2	188859188	1.89E+08	KD2-H4K8ac_peak_17936	4.06758	
2	189156323	1.89E+08	KD2-H4K8ac_peak_17937	11.92978	GULP1_ENSG00000144366
2	189483415	1.89E+08	KD2-H4K8ac_peak_17938	7.12754	

2	190119261	1.9E+08	KD2-H4K8ac_peak_17939	7.12754	
2	190306070	1.9E+08	KD2-H4K8ac_peak_17940	9.97423	WDR75_ENSG00000115368
2	190444960	1.9E+08	KD2-H4K8ac_peak_17941	10.43145	
2	190467589	1.9E+08	KD2-H4K8ac_peak_17942	9.15329	
2	190539001	1.91E+08	KD2-H4K8ac_peak_17943	14.15075	ANKAR_ENSG00000151687
2	190627040	1.91E+08	KD2-H4K8ac_peak_17944	8.17902	OSGEPL1_ENSG00000128694;OSGEPL1-AS1_ENSG00000253559
2	190648492	1.91E+08	KD2-H4K8ac_peak_17945	8.55271	ORMDL1_ENSG00000128699;PMS1_ENSG00000064933
2	191044959	1.91E+08	KD2-H4K8ac_peak_17946	9.89126	
2	191208001	1.91E+08	KD2-H4K8ac_peak_17947	6.56211	HIBCH_ENSG00000198130;INPP1_ENSG00000151689
2	191208433	1.91E+08	KD2-H4K8ac_peak_17948	10.53377	HIBCH_ENSG00000198130;INPP1_ENSG00000151689
2	191272733	1.91E+08	KD2-H4K8ac_peak_17949	12.84642	MFSD6_ENSG00000151690
2	191399086	1.91E+08	KD2-H4K8ac_peak_17950	16.57619	TMEM194B_ENSG00000189362;AC093388.3_ENSG00000233654
2	191492657	1.91E+08	KD2-H4K8ac_peak_17951	13.92307	
2	191513023	1.92E+08	KD2-H4K8ac_peak_17952	10.96455	
2	191513414	1.92E+08	KD2-H4K8ac_peak_17953	6.40683	
2	191513870	1.92E+08	KD2-H4K8ac_peak_17954	7.47462	
2	191885275	1.92E+08	KD2-H4K8ac_peak_17955	13.95627	STAT1_ENSG00000115415;AC067945.4_ENSG00000231858
2	192110222	1.92E+08	KD2-H4K8ac_peak_17956	9.6247	AC092614.2_ENSG00000227542;MYO1B_ENSG00000128641
2	192344514	1.92E+08	KD2-H4K8ac_peak_17957	5.53033	
2	192365695	1.92E+08	KD2-H4K8ac_peak_17958	13.56049	
2	193059853	1.93E+08	KD2-H4K8ac_peak_17959	9.73431	TMEFF2_ENSG00000144339
2	196313076	1.96E+08	KD2-H4K8ac_peak_17960	7.12754	AC064834.1_ENSG00000224099
2	196315160	1.96E+08	KD2-H4K8ac_peak_17961	4.01115	
2	196316344	1.96E+08	KD2-H4K8ac_peak_17962	5.16439	
2	196520700	1.97E+08	KD2-H4K8ac_peak_17963	4.211	
2	196522003	1.97E+08	KD2-H4K8ac_peak_17964	12.74171	
2	197457163	1.97E+08	KD2-H4K8ac_peak_17965	11.66477	HECW2_ENSG00000138411
2	197466478	1.97E+08	KD2-H4K8ac_peak_17966	12.42547	
2	197504141	1.98E+08	KD2-H4K8ac_peak_17967	9.59147	CCDC150_ENSG00000144395
2	197664002	1.98E+08	KD2-H4K8ac_peak_17968	4.86324	GTF3C3_ENSG00000119041
2	197664776	1.98E+08	KD2-H4K8ac_peak_17969	4.0528	GTF3C3_ENSG00000119041
2	197791607	1.98E+08	KD2-H4K8ac_peak_17970	4.54006	PGAP1_ENSG00000197121
2	197853500	1.98E+08	KD2-H4K8ac_peak_17971	6.72021	
2	197861363	1.98E+08	KD2-H4K8ac_peak_17972	8.70735	
2	197915692	1.98E+08	KD2-H4K8ac_peak_17973	5.9696	
2	197917587	1.98E+08	KD2-H4K8ac_peak_17974	9.59814	
2	197922360	1.98E+08	KD2-H4K8ac_peak_17975	7.15405	
2	197955381	1.98E+08	KD2-H4K8ac_peak_17976	6.40683	
2	197956017	1.98E+08	KD2-H4K8ac_peak_17977	10.86378	
2	197960163	1.98E+08	KD2-H4K8ac_peak_17978	3.75035	
2	197973159	1.98E+08	KD2-H4K8ac_peak_17979	7.01964	
2	198008468	1.98E+08	KD2-H4K8ac_peak_17980	7.12754	
2	198010290	1.98E+08	KD2-H4K8ac_peak_17981	8.1233	
2	198028953	1.98E+08	KD2-H4K8ac_peak_17982	3.75035	
2	198043641	1.98E+08	KD2-H4K8ac_peak_17983	6.40683	
2	198045001	1.98E+08	KD2-H4K8ac_peak_17984	5.46431	

2	198049606	1.98E+08	KD2-H4K8ac_peak_17985	6.2781	
2	198111517	1.98E+08	KD2-H4K8ac_peak_17986	10.05606	
2	198112343	1.98E+08	KD2-H4K8ac_peak_17987	10.95611	
2	198144190	1.98E+08	KD2-H4K8ac_peak_17988	6.66692	
2	198174534	1.98E+08	KD2-H4K8ac_peak_17989	9.28039	ANKRD44_ENSG00000065413;AC010746.3_ENSG00000225979
2	198224764	1.98E+08	KD2-H4K8ac_peak_17990	8.10216	
2	198242500	1.98E+08	KD2-H4K8ac_peak_17991	6.99815	
2	198243674	1.98E+08	KD2-H4K8ac_peak_17992	5.30799	
2	198299385	1.98E+08	KD2-H4K8ac_peak_17993	6.40683	SF3B1_ENSG00000115524
2	198300831	1.98E+08	KD2-H4K8ac_peak_17994	5.73839	
2	198364279	1.98E+08	KD2-H4K8ac_peak_17995	9.34989	HSPE1_ENSG00000115541;HSPE1-MOB4_ENSG00000270757
2	198364881	1.98E+08	KD2-H4K8ac_peak_17996	9.36516	HSPE1_ENSG00000115541;HSPE1-MOB4_ENSG00000270757
2	198380330	1.98E+08	KD2-H4K8ac_peak_17997	14.98936	HSPD1_ENSG00000144381;MOB4_ENSG00000115540
2	198380865	1.98E+08	KD2-H4K8ac_peak_17998	11.79585	HSPD1_ENSG00000144381;MOB4_ENSG00000115540
2	198569648	1.99E+08	KD2-H4K8ac_peak_17999	8.37231	MARS2_ENSG00000247626
2	200323778	2E+08	KD2-H4K8ac_peak_18000	9.11031	
2	200787508	2.01E+08	KD2-H4K8ac_peak_18001	6.16888	
2	200819053	2.01E+08	KD2-H4K8ac_peak_18002	7.12754	C2orf47_ENSG00000162972
2	200819914	2.01E+08	KD2-H4K8ac_peak_18003	18.41184	TYW5_ENSG00000162971;C2orf47_ENSG00000162972
2	201171454	2.01E+08	KD2-H4K8ac_peak_18004	7.07117	SPATS2L_ENSG00000196141
2	201374185	2.01E+08	KD2-H4K8ac_peak_18005	13.73635	SGOL2_ENSG00000163535
2	201374961	2.01E+08	KD2-H4K8ac_peak_18006	7.76329	SGOL2_ENSG00000163535
2	201676309	2.02E+08	KD2-H4K8ac_peak_18007	12.57143	AC007163.6_ENSG00000230408;BZW1_ENSG00000082153
2	201728794	2.02E+08	KD2-H4K8ac_peak_18008	22.66567	CLK1_ENSG00000013441;Y_RNA_ENSG00000252759
2	201729674	2.02E+08	KD2-H4K8ac_peak_18009	14.27981	CLK1_ENSG00000013441
2	201754141	2.02E+08	KD2-H4K8ac_peak_18010	13.80019	PPIL3_ENSG00000240344;NIF3L1_ENSG00000196290
2	201754690	2.02E+08	KD2-H4K8ac_peak_18011	6.40683	PPIL3_ENSG00000240344;NIF3L1_ENSG00000196290
2	201827964	2.02E+08	KD2-H4K8ac_peak_18012	13.13969	ORC2_ENSG00000115942;AC005037.3_ENSG00000183308
2	201980485	2.02E+08	KD2-H4K8ac_peak_18013	13.31016	CFLAR_ENSG00000003402
2	202316519	2.02E+08	KD2-H4K8ac_peak_18014	13.09607	TRAK2_ENSG00000115993
2	202330358	2.02E+08	KD2-H4K8ac_peak_18015	7.59643	
2	202507320	2.03E+08	KD2-H4K8ac_peak_18016	19.35989	TMEM237_ENSG00000155755
2	202517809	2.03E+08	KD2-H4K8ac_peak_18017	4.42765	
2	202645411	2.03E+08	KD2-H4K8ac_peak_18018	7.0754	ALS2_ENSG00000003393
2	202679744	2.03E+08	KD2-H4K8ac_peak_18019	8.1233	
2	202832127	2.03E+08	KD2-H4K8ac_peak_18020	6.99815	
2	202849676	2.03E+08	KD2-H4K8ac_peak_18021	7.47462	
2	202850829	2.03E+08	KD2-H4K8ac_peak_18022	4.37709	
2	202855436	2.03E+08	KD2-H4K8ac_peak_18023	4.7849	
2	202860831	2.03E+08	KD2-H4K8ac_peak_18024	5.9696	
2	202862547	2.03E+08	KD2-H4K8ac_peak_18025	18.5703	
2	202879959	2.03E+08	KD2-H4K8ac_peak_18026	5.14125	
2	202881398	2.03E+08	KD2-H4K8ac_peak_18027	6.94954	
2	202885075	2.03E+08	KD2-H4K8ac_peak_18028	7.70289	
2	202885850	2.03E+08	KD2-H4K8ac_peak_18029	8.05667	
2	202897396	2.03E+08	KD2-H4K8ac_peak_18030	4.44527	RP11-107N15.1_ENSG00000273209

2	202898608	2.03E+08	KD2-H4K8ac_peak_18031	12.69604	RP11-107N15.1_ENSG00000273209;FZD7_ENSG00000155760
2	202904079	2.03E+08	KD2-H4K8ac_peak_18032	5.80249	
2	203005795	2.03E+08	KD2-H4K8ac_peak_18033	10.21502	
2	203102651	2.03E+08	KD2-H4K8ac_peak_18034	11.05407	SUMO1_ENSG00000116030;AC079354.2_ENSG00000267889
2	203129761	2.03E+08	KD2-H4K8ac_peak_18035	8.86391	NOP58_ENSG00000055044
2	203130511	2.03E+08	KD2-H4K8ac_peak_18036	9.63925	NOP58_ENSG00000055044
2	203239816	2.03E+08	KD2-H4K8ac_peak_18037	9.6589	RP11-686O6.2_ENSG00000273456;BMPR2_ENSG00000204217
2	203241175	2.03E+08	KD2-H4K8ac_peak_18038	4.26235	RP11-686O6.2_ENSG00000273456;BMPR2_ENSG00000204217
2	203453713	2.03E+08	KD2-H4K8ac_peak_18039	4.37809	
2	203499092	2.03E+08	KD2-H4K8ac_peak_18040	4.86324	FAM117B_ENSG00000138439
2	203499829	2.04E+08	KD2-H4K8ac_peak_18041	10.19949	FAM117B_ENSG00000138439
2	203736268	2.04E+08	KD2-H4K8ac_peak_18042	10.17341	ICA1L_ENSG00000163596
2	203776657	2.04E+08	KD2-H4K8ac_peak_18043	7.48133	CARF_ENSG00000138380
2	204103724	2.04E+08	KD2-H4K8ac_peak_18044	14.8083	CYP20A1_ENSG00000119004
2	204192344	2.04E+08	KD2-H4K8ac_peak_18045	12.42761	ABI2_ENSG00000138443
2	204399507	2.04E+08	KD2-H4K8ac_peak_18046	6.84261	RAPH1_ENSG00000173166
2	204400259	2.04E+08	KD2-H4K8ac_peak_18047	4.44527	RAPH1_ENSG00000173166
2	205318671	2.05E+08	KD2-H4K8ac_peak_18048	5.9696	
2	205409716	2.05E+08	KD2-H4K8ac_peak_18049	7.44324	PARD3B_ENSG00000116117
2	205410298	2.05E+08	KD2-H4K8ac_peak_18050	5.30799	PARD3B_ENSG00000116117
2	205889661	2.06E+08	KD2-H4K8ac_peak_18051	5.15777	
2	206055177	2.06E+08	KD2-H4K8ac_peak_18052	4.37708	
2	206477471	2.06E+08	KD2-H4K8ac_peak_18053	7.27716	
2	206595560	2.07E+08	KD2-H4K8ac_peak_18054	9.51625	
2	206598266	2.07E+08	KD2-H4K8ac_peak_18055	9.11031	
2	206610521	2.07E+08	KD2-H4K8ac_peak_18056	4.44527	
2	206611116	2.07E+08	KD2-H4K8ac_peak_18057	7.1181	
2	206949696	2.07E+08	KD2-H4K8ac_peak_18058	20.46575	INO80D_ENSG00000114933;AC007383.3_ENSG00000227946
2	207023716	2.07E+08	KD2-H4K8ac_peak_18059	10.65724	NDUFS1_ENSG00000023228;EEF1B2_ENSG00000114942
2	207024605	2.07E+08	KD2-H4K8ac_peak_18060	8.10216	NDUFS1_ENSG00000023228;EEF1B2_ENSG00000114942
2	207082547	2.07E+08	KD2-H4K8ac_peak_18061	4.73064	GPR1_ENSG00000183671
2	207083299	2.07E+08	KD2-H4K8ac_peak_18062	6.51023	GPR1_ENSG00000183671
2	207084342	2.07E+08	KD2-H4K8ac_peak_18063	7.27716	
2	207139657	2.07E+08	KD2-H4K8ac_peak_18064	8.59579	ZDBF2_ENSG00000204186
2	207308325	2.07E+08	KD2-H4K8ac_peak_18065	8.30974	ADAM23_ENSG00000114948
2	207629793	2.08E+08	KD2-H4K8ac_peak_18066	11.09936	MDH1B_ENSG00000138400;FASTKD2_ENSG00000118246
2	207646584	2.08E+08	KD2-H4K8ac_peak_18067	6.07178	
2	207847172	2.08E+08	KD2-H4K8ac_peak_18068	6.40683	
2	207847992	2.08E+08	KD2-H4K8ac_peak_18069	5.43522	
2	207931024	2.08E+08	KD2-H4K8ac_peak_18070	6.54442	
2	207950113	2.08E+08	KD2-H4K8ac_peak_18071	6.66692	
2	207950991	2.08E+08	KD2-H4K8ac_peak_18072	8.17902	
2	207953043	2.08E+08	KD2-H4K8ac_peak_18073	7.13615	
2	208024474	2.08E+08	KD2-H4K8ac_peak_18074	9.31907	
2	208025555	2.08E+08	KD2-H4K8ac_peak_18075	5.08628	
2	208026348	2.08E+08	KD2-H4K8ac_peak_18076	6.56926	

2	208028960	2.08E+08	KD2-H4K8ac_peak_18077	6.40297	
2	208031166	2.08E+08	KD2-H4K8ac_peak_18078	11.80283	KLF7_ENSG00000118263
2	208116055	2.08E+08	KD2-H4K8ac_peak_18079	4.211	
2	208116754	2.08E+08	KD2-H4K8ac_peak_18080	11.03331	
2	208120528	2.08E+08	KD2-H4K8ac_peak_18081	9.63487	
2	208122313	2.08E+08	KD2-H4K8ac_peak_18082	4.86324	
2	208175135	2.08E+08	KD2-H4K8ac_peak_18083	6.15357	
2	208184264	2.08E+08	KD2-H4K8ac_peak_18084	6.07178	
2	208295524	2.08E+08	KD2-H4K8ac_peak_18085	7.12754	
2	208301191	2.08E+08	KD2-H4K8ac_peak_18086	9.8758	
2	208302865	2.08E+08	KD2-H4K8ac_peak_18087	6.66692	
2	208305480	2.08E+08	KD2-H4K8ac_peak_18088	3.96965	
2	208310091	2.08E+08	KD2-H4K8ac_peak_18089	7.12754	
2	208330460	2.08E+08	KD2-H4K8ac_peak_18090	5.61377	
2	208331114	2.08E+08	KD2-H4K8ac_peak_18091	7.40633	
2	208333438	2.08E+08	KD2-H4K8ac_peak_18092	5.01002	
2	208334397	2.08E+08	KD2-H4K8ac_peak_18093	7.47462	
2	208335746	2.08E+08	KD2-H4K8ac_peak_18094	7.36933	
2	208336991	2.08E+08	KD2-H4K8ac_peak_18095	6.54442	
2	208339078	2.08E+08	KD2-H4K8ac_peak_18096	12.20507	
2	208393835	2.08E+08	KD2-H4K8ac_peak_18097	16.09057	AC007879.5_ENSG00000223725;CREB1_ENSG00000118260
2	208489453	2.08E+08	KD2-H4K8ac_peak_18098	16.8435	METTL21A_ENSG00000144401
2	208490760	2.08E+08	KD2-H4K8ac_peak_18099	10.7186	METTL21A_ENSG00000144401
2	208661755	2.09E+08	KD2-H4K8ac_peak_18100	5.93446	
2	208890010	2.09E+08	KD2-H4K8ac_peak_18101	8.94469	PLEKHM3_ENSG00000178385
2	209129717	2.09E+08	KD2-H4K8ac_peak_18102	10.0692	IDH1_ENSG00000138413;PIKFYVE_ENSG00000115020
2	209131053	2.09E+08	KD2-H4K8ac_peak_18103	10.43145	IDH1_ENSG00000138413;PIKFYVE_ENSG00000115020
2	209224981	2.09E+08	KD2-H4K8ac_peak_18104	10.30284	AC012362.3_ENSG00000213081;PTH2R_ENSG00000144407
2	209794696	2.1E+08	KD2-H4K8ac_peak_18105	7.12754	
2	210288152	2.1E+08	KD2-H4K8ac_peak_18106	24.94195	MAP2_ENSG00000078018
2	210867410	2.11E+08	KD2-H4K8ac_peak_18107	5.88845	RPE_ENSG00000197713
2	211089582	2.11E+08	KD2-H4K8ac_peak_18108	7.69287	ACADL_ENSG00000115361
2	214016089	2.14E+08	KD2-H4K8ac_peak_18109	7.01964	IKZF2_ENSG00000030419
2	214016676	2.14E+08	KD2-H4K8ac_peak_18110	7.47462	IKZF2_ENSG00000030419;RP11-105N14.1_ENSG00000270659
2	214149225	2.14E+08	KD2-H4K8ac_peak_18111	4.57782	AC079610.2_ENSG00000196096;SPAG16_ENSG00000144451
2	215548674	2.16E+08	KD2-H4K8ac_peak_18112	10.21502	AC107218.3_ENSG00000197585
2	216175862	2.16E+08	KD2-H4K8ac_peak_18113	7.47462	ATIC_ENSG00000138363
2	216176392	2.16E+08	KD2-H4K8ac_peak_18114	17.70837	ATIC_ENSG00000138363
2	216301055	2.16E+08	KD2-H4K8ac_peak_18115	8.17902	FN1_ENSG00000115414;AC012462.1_ENSG00000230695
2	216946238	2.17E+08	KD2-H4K8ac_peak_18116	10.46874	TMEM169_ENSG00000163449
2	216972516	2.17E+08	KD2-H4K8ac_peak_18117	4.42765	XRCC5_ENSG00000079246
2	216973126	2.17E+08	KD2-H4K8ac_peak_18118	12.09025	XRCC5_ENSG00000079246
2	216974182	2.17E+08	KD2-H4K8ac_peak_18119	10.0746	
2	216979871	2.17E+08	KD2-H4K8ac_peak_18120	7.67504	
2	217080835	2.17E+08	KD2-H4K8ac_peak_18121	5.88845	RP11-566E18.3_ENSG00000260804
2	217171775	2.17E+08	KD2-H4K8ac_peak_18122	6.16888	

2	217277236	2.17E+08	KD2-H4K8ac_peak_18123	10.84707	AC098820.2_ENSG00000235042;SMARCAL1_ENSG00000138375
2	217362860	2.17E+08	KD2-H4K8ac_peak_18124	6.30229	AC098820.3_ENSG00000232485;RPL37A_ENSG00000197756
2	217363661	2.17E+08	KD2-H4K8ac_peak_18125	8.58725	AC098820.3_ENSG00000232485;RPL37A_ENSG00000197756
2	217460410	2.17E+08	KD2-H4K8ac_peak_18126	5.88845	
2	217483282	2.17E+08	KD2-H4K8ac_peak_18127	10.86378	
2	217485022	2.17E+08	KD2-H4K8ac_peak_18128	13.77612	
2	217486610	2.17E+08	KD2-H4K8ac_peak_18129	8.52559	
2	217487274	2.17E+08	KD2-H4K8ac_peak_18130	8.30974	
2	217497642	2.17E+08	KD2-H4K8ac_peak_18131	8.28234	IGFBP2_ENSG00000115457
2	217499821	2.18E+08	KD2-H4K8ac_peak_18132	5.88845	
2	217500593	2.18E+08	KD2-H4K8ac_peak_18133	7.99241	
2	217564324	2.18E+08	KD2-H4K8ac_peak_18134	7.40314	
2	217599644	2.18E+08	KD2-H4K8ac_peak_18135	5.45396	
2	217656483	2.18E+08	KD2-H4K8ac_peak_18136	12.60538	
2	217659029	2.18E+08	KD2-H4K8ac_peak_18137	4.87807	RNA5SP120_ENSG00000222832
2	218112944	2.18E+08	KD2-H4K8ac_peak_18138	4.42765	
2	218155284	2.18E+08	KD2-H4K8ac_peak_18139	7.27716	
2	218375765	2.18E+08	KD2-H4K8ac_peak_18140	8.30974	
2	218458582	2.18E+08	KD2-H4K8ac_peak_18141	5.16439	
2	218486629	2.18E+08	KD2-H4K8ac_peak_18142	5.88845	
2	218501563	2.19E+08	KD2-H4K8ac_peak_18143	5.13615	
2	218594354	2.19E+08	KD2-H4K8ac_peak_18144	6.66692	
2	218787400	2.19E+08	KD2-H4K8ac_peak_18145	5.63633	
2	218797892	2.19E+08	KD2-H4K8ac_peak_18146	16.71655	
2	218807192	2.19E+08	KD2-H4K8ac_peak_18147	3.97079	
2	218867441	2.19E+08	KD2-H4K8ac_peak_18148	9.63487	TNS1_ENSG00000079308
2	218874334	2.19E+08	KD2-H4K8ac_peak_18149	21.42203	
2	218875983	2.19E+08	KD2-H4K8ac_peak_18150	13.50024	
2	218880967	2.19E+08	KD2-H4K8ac_peak_18151	13.03787	
2	218883165	2.19E+08	KD2-H4K8ac_peak_18152	8.80225	
2	218899154	2.19E+08	KD2-H4K8ac_peak_18153	5.88845	RUFY4_ENSG00000188282
2	218933810	2.19E+08	KD2-H4K8ac_peak_18154	7.38933	
2	219082097	2.19E+08	KD2-H4K8ac_peak_18155	14.98671	ARPC2_ENSG00000163466
2	219124226	2.19E+08	KD2-H4K8ac_peak_18156	6.13909	GPBAR1_ENSG00000179921
2	219232196	2.19E+08	KD2-H4K8ac_peak_18157	7.47462	AC021016.6_ENSG00000225062
2	219259587	2.19E+08	KD2-H4K8ac_peak_18158	4.20044	
2	219260319	2.19E+08	KD2-H4K8ac_peak_18159	5.1707	
2	219263112	2.19E+08	KD2-H4K8ac_peak_18160	11.50346	CTDSP1_ENSG00000144579;RP11-378A13.2_ENSG00000273361
2	219264788	2.19E+08	KD2-H4K8ac_peak_18161	14.70748	RP11-378A13.2_ENSG00000273361
2	219271421	2.19E+08	KD2-H4K8ac_peak_18162	9.63487	
2	219272469	2.19E+08	KD2-H4K8ac_peak_18163	7.146	
2	219432712	2.19E+08	KD2-H4K8ac_peak_18164	6.25869	USP37_ENSG00000135913;RQCD1_ENSG00000144580
2	219433348	2.19E+08	KD2-H4K8ac_peak_18165	11.1952	USP37_ENSG00000135913;RQCD1_ENSG00000144580
2	219469389	2.19E+08	KD2-H4K8ac_peak_18166	9.63925	
2	219472447	2.19E+08	KD2-H4K8ac_peak_18167	7.36799	PLCD4_ENSG00000115556
2	219524024	2.2E+08	KD2-H4K8ac_peak_18168	9.95119	ZNF142_ENSG00000115568;BCS1L_ENSG00000074582

2	219524490	2.2E+08	KD2-H4K8ac_peak_18169	8.4716	ZNF142_ENSG00000115568
2	219536909	2.2E+08	KD2-H4K8ac_peak_18170	8.55271	RNF25_ENSG00000163481;STK36_ENSG00000163482
2	219574794	2.2E+08	KD2-H4K8ac_peak_18171	6.66359	TTLL4_ENSG00000135912
2	219575670	2.2E+08	KD2-H4K8ac_peak_18172	7.76329	TTLL4_ENSG00000135912
2	219720175	2.2E+08	KD2-H4K8ac_peak_18173	15.59774	
2	219724651	2.2E+08	KD2-H4K8ac_peak_18174	9.40857	WNT6_ENSG00000115596
2	219745292	2.2E+08	KD2-H4K8ac_peak_18175	9.96798	WNT10A_ENSG00000135925
2	219757670	2.2E+08	KD2-H4K8ac_peak_18176	11.50346	
2	219761374	2.2E+08	KD2-H4K8ac_peak_18177	8.49556	
2	219773488	2.2E+08	KD2-H4K8ac_peak_18178	12.42761	
2	219823645	2.2E+08	KD2-H4K8ac_peak_18179	10.21366	CDK5R2_ENSG00000171450
2	219824435	2.2E+08	KD2-H4K8ac_peak_18180	10.30284	CDK5R2_ENSG00000171450
2	219827424	2.2E+08	KD2-H4K8ac_peak_18181	6.16883	AC097468.7_ENSG00000235024
2	219828274	2.2E+08	KD2-H4K8ac_peak_18182	7.40314	
2	219848622	2.2E+08	KD2-H4K8ac_peak_18183	9.95119	FEV_ENSG00000163497
2	219861108	2.2E+08	KD2-H4K8ac_peak_18184	9.14807	
2	219862066	2.2E+08	KD2-H4K8ac_peak_18185	7.28827	
2	219863074	2.2E+08	KD2-H4K8ac_peak_18186	10.74508	
2	219864126	2.2E+08	KD2-H4K8ac_peak_18187	6.0605	
2	219865156	2.2E+08	KD2-H4K8ac_peak_18188	9.95119	MIR375_ENSG00000198973
2	219866932	2.2E+08	KD2-H4K8ac_peak_18189	13.50024	MIR375_ENSG00000198973;AC097468.4_ENSG00000224090
2	219882476	2.2E+08	KD2-H4K8ac_peak_18190	9.20388	
2	220025049	2.2E+08	KD2-H4K8ac_peak_18191	11.60333	NHEJ1_ENSG00000187736
2	220040993	2.2E+08	KD2-H4K8ac_peak_18192	18.5703	CNPPD1_ENSG00000115649;FAM134A_ENSG00000144567
2	220043088	2.2E+08	KD2-H4K8ac_peak_18193	11.89502	CNPPD1_ENSG00000115649
2	220083089	2.2E+08	KD2-H4K8ac_peak_18194	9.01262	ABCB6_ENSG00000115657
2	220083919	2.2E+08	KD2-H4K8ac_peak_18195	8.83582	ABCB6_ENSG00000115657
2	220094632	2.2E+08	KD2-H4K8ac_peak_18196	5.20998	ATG9A_ENSG00000198925;ANKZF1_ENSG00000163516
2	220110263	2.2E+08	KD2-H4K8ac_peak_18197	6.84261	STK16_ENSG00000115661
2	220144150	2.2E+08	KD2-H4K8ac_peak_18198	9.90914	DNAJB2_ENSG00000135924
2	220159167	2.2E+08	KD2-H4K8ac_peak_18199	13.87818	MIR153-1_ENSG00000207647
2	220253315	2.2E+08	KD2-H4K8ac_peak_18200	8.30974	AC053503.4_ENSG00000229525
2	220306377	2.2E+08	KD2-H4K8ac_peak_18201	7.07117	
2	220310551	2.2E+08	KD2-H4K8ac_peak_18202	6.9556	
2	220317270	2.2E+08	KD2-H4K8ac_peak_18203	9.89126	
2	220318754	2.2E+08	KD2-H4K8ac_peak_18204	10.30284	
2	220324433	2.2E+08	KD2-H4K8ac_peak_18205	11.79585	
2	220336950	2.2E+08	KD2-H4K8ac_peak_18206	7.5554	
2	220341492	2.2E+08	KD2-H4K8ac_peak_18207	12.06346	
2	220362453	2.2E+08	KD2-H4K8ac_peak_18208	14.00658	RP11-316O14.1_ENSG00000268603;GMPPA_ENSG00000144591
2	220366783	2.2E+08	KD2-H4K8ac_peak_18209	7.47462	
2	220369209	2.2E+08	KD2-H4K8ac_peak_18210	7.96322	
2	220370098	2.2E+08	KD2-H4K8ac_peak_18211	12.57865	
2	220373208	2.2E+08	KD2-H4K8ac_peak_18212	11.11797	
2	220373743	2.2E+08	KD2-H4K8ac_peak_18213	13.81781	
2	220374272	2.2E+08	KD2-H4K8ac_peak_18214	9.34195	

2	220375121	2.2E+08	KD2-H4K8ac_peak_18215	6.84261	
2	220375772	2.2E+08	KD2-H4K8ac_peak_18216	5.54647	
2	220377302	2.2E+08	KD2-H4K8ac_peak_18217	9.34989	ASIC4_ENSG00000072182
2	220378610	2.2E+08	KD2-H4K8ac_peak_18218	8.30114	ASIC4_ENSG00000072182
2	220380444	2.2E+08	KD2-H4K8ac_peak_18219	5.92591	AC053503.11_ENSG00000227432
2	220385081	2.2E+08	KD2-H4K8ac_peak_18220	7.36799	
2	220385860	2.2E+08	KD2-H4K8ac_peak_18221	16.822	
2	220387340	2.2E+08	KD2-H4K8ac_peak_18222	6.58902	
2	220390973	2.2E+08	KD2-H4K8ac_peak_18223	6.16883	
2	220394648	2.2E+08	KD2-H4K8ac_peak_18224	7.07117	
2	220408800	2.2E+08	KD2-H4K8ac_peak_18225	10.05606	CHPF_ENSG00000123989;TMEM198_ENSG00000188760
2	220435536	2.2E+08	KD2-H4K8ac_peak_18226	11.92978	OBSL1_ENSG00000124006
2	220437087	2.2E+08	KD2-H4K8ac_peak_18227	12.80176	OBSL1_ENSG00000124006
2	220461021	2.2E+08	KD2-H4K8ac_peak_18228	6.99815	
2	220461852	2.2E+08	KD2-H4K8ac_peak_18229	7.76329	STK11IP_ENSG00000144589
2	220491851	2.2E+08	KD2-H4K8ac_peak_18230	6.0605	AC009955.8_ENSG00000228973;SLC4A3_ENSG00000114923
2	220492433	2.2E+08	KD2-H4K8ac_peak_18231	9.95119	SLC4A3_ENSG00000114923
2	223185224	2.23E+08	KD2-H4K8ac_peak_18232	6.00243	
2	223289153	2.23E+08	KD2-H4K8ac_peak_18233	5.14125	SGPP2_ENSG00000163082
2	223535959	2.24E+08	KD2-H4K8ac_peak_18234	9.95119	MOGAT1_ENSG00000124003
2	223859940	2.24E+08	KD2-H4K8ac_peak_18235	7.27716	
2	224477872	2.24E+08	KD2-H4K8ac_peak_18236	10.05606	
2	224702063	2.25E+08	KD2-H4K8ac_peak_18237	15.32867	AP1S3_ENSG00000152056
2	224809159	2.25E+08	KD2-H4K8ac_peak_18238	4.06758	WDFY1_ENSG00000085449
2	224809634	2.25E+08	KD2-H4K8ac_peak_18239	10.29842	WDFY1_ENSG00000085449
2	224872790	2.25E+08	KD2-H4K8ac_peak_18240	5.64449	
2	224874059	2.25E+08	KD2-H4K8ac_peak_18241	8.37231	
2	224903720	2.25E+08	KD2-H4K8ac_peak_18242	7.96322	SERPINE2_ENSG00000135919
2	224904176	2.25E+08	KD2-H4K8ac_peak_18243	9.63487	SERPINE2_ENSG00000135919
2	225306926	2.25E+08	KD2-H4K8ac_peak_18244	7.12754	
2	225449612	2.25E+08	KD2-H4K8ac_peak_18245	4.42765	CUL3_ENSG00000036257
2	225847073	2.26E+08	KD2-H4K8ac_peak_18246	8.97719	
2	225847807	2.26E+08	KD2-H4K8ac_peak_18247	3.75035	
2	225848988	2.26E+08	KD2-H4K8ac_peak_18248	10.0746	
2	225906744	2.26E+08	KD2-H4K8ac_peak_18249	4.52514	DOCK10_ENSG00000135905
2	226263277	2.26E+08	KD2-H4K8ac_peak_18250	6.16888	AC067961.1_ENSG00000232784
2	226307600	2.26E+08	KD2-H4K8ac_peak_18251	4.01115	
2	227655887	2.28E+08	KD2-H4K8ac_peak_18252	8.82366	
2	227666328	2.28E+08	KD2-H4K8ac_peak_18253	5.80056	
2	227700107	2.28E+08	KD2-H4K8ac_peak_18254	20.88898	RHBDD1_ENSG00000144468
2	227700853	2.28E+08	KD2-H4K8ac_peak_18255	9.63487	RHBDD1_ENSG00000144468
2	228189382	2.28E+08	KD2-H4K8ac_peak_18256	3.87368	AC097662.2_ENSG00000236432;MFF_ENSG00000168958
2	228190185	2.28E+08	KD2-H4K8ac_peak_18257	6.52179	AC097662.2_ENSG00000236432;MFF_ENSG00000168958
2	228336917	2.28E+08	KD2-H4K8ac_peak_18258	7.12754	MIR5703_ENSG00000266382;AGFG1_ENSG00000173744
2	230786272	2.31E+08	KD2-H4K8ac_peak_18259	11.86082	FBXO36_ENSG00000153832
2	230787332	2.31E+08	KD2-H4K8ac_peak_18260	9.51625	TRIP12_ENSG00000153827;FBXO36_ENSG00000153832

2	230932476	2.31E+08	KD2-H4K8ac_peak_18261	10.19949	SLC16A14_ENSG00000163053
2	231084693	2.31E+08	KD2-H4K8ac_peak_18262	4.0528	
2	231577595	2.32E+08	KD2-H4K8ac_peak_18263	18.41184	CAB39_ENSG00000135932
2	231712793	2.32E+08	KD2-H4K8ac_peak_18264	15.22643	
2	231728990	2.32E+08	KD2-H4K8ac_peak_18265	5.30799	ITM2C_ENSG00000135916
2	231729810	2.32E+08	KD2-H4K8ac_peak_18266	10.08707	ITM2C_ENSG00000135916
2	231730540	2.32E+08	KD2-H4K8ac_peak_18267	6.85193	
2	231733135	2.32E+08	KD2-H4K8ac_peak_18268	25.8903	
2	231734706	2.32E+08	KD2-H4K8ac_peak_18269	8.85813	
2	231851266	2.32E+08	KD2-H4K8ac_peak_18270	5.75452	
2	231852338	2.32E+08	KD2-H4K8ac_peak_18271	10.02417	
2	231902249	2.32E+08	KD2-H4K8ac_peak_18272	14.69247	C2orf72_ENSG00000204128
2	231902842	2.32E+08	KD2-H4K8ac_peak_18273	7.76104	C2orf72_ENSG00000204128
2	231921754	2.32E+08	KD2-H4K8ac_peak_18274	7.81657	PSMD1_ENSG00000173692
2	232050956	2.32E+08	KD2-H4K8ac_peak_18275	5.57044	
2	232056965	2.32E+08	KD2-H4K8ac_peak_18276	6.0073	
2	232062442	2.32E+08	KD2-H4K8ac_peak_18277	8.74596	ARMC9_ENSG00000135931
2	232134736	2.32E+08	KD2-H4K8ac_peak_18278	5.30799	
2	232220535	2.32E+08	KD2-H4K8ac_peak_18279	11.70879	
2	232222110	2.32E+08	KD2-H4K8ac_peak_18280	5.82869	
2	232229524	2.32E+08	KD2-H4K8ac_peak_18281	8.58725	
2	232230426	2.32E+08	KD2-H4K8ac_peak_18282	7.32463	
2	232235738	2.32E+08	KD2-H4K8ac_peak_18283	5.43522	
2	232253049	2.32E+08	KD2-H4K8ac_peak_18284	6.99815	AC017104.6_ENSG00000224376
2	232260439	2.32E+08	KD2-H4K8ac_peak_18285	5.405	B3GNT7_ENSG00000156966
2	232264948	2.32E+08	KD2-H4K8ac_peak_18286	12.60538	
2	232276783	2.32E+08	KD2-H4K8ac_peak_18287	7.36799	
2	232328726	2.32E+08	KD2-H4K8ac_peak_18288	7.47462	
2	232329545	2.32E+08	KD2-H4K8ac_peak_18289	9.08816	
2	232347425	2.32E+08	KD2-H4K8ac_peak_18290	9.26408	NCL_ENSG00000115053
2	232379309	2.32E+08	KD2-H4K8ac_peak_18291	10.77651	LINC00471_ENSG00000181798
2	232394742	2.32E+08	KD2-H4K8ac_peak_18292	7.36799	NMUR1_ENSG00000171596
2	232395424	2.32E+08	KD2-H4K8ac_peak_18293	12.93253	NMUR1_ENSG00000171596
2	232459809	2.32E+08	KD2-H4K8ac_peak_18294	4.67342	
2	232468614	2.32E+08	KD2-H4K8ac_peak_18295	13.22091	
2	232469665	2.32E+08	KD2-H4K8ac_peak_18296	11.50346	
2	232477076	2.32E+08	KD2-H4K8ac_peak_18297	20.8599	
2	232478020	2.32E+08	KD2-H4K8ac_peak_18298	17.70837	
2	232520144	2.33E+08	KD2-H4K8ac_peak_18299	8.10216	
2	232544962	2.33E+08	KD2-H4K8ac_peak_18300	16.57619	
2	232545982	2.33E+08	KD2-H4K8ac_peak_18301	13.22091	
2	232547669	2.33E+08	KD2-H4K8ac_peak_18302	15.04266	
2	232552281	2.33E+08	KD2-H4K8ac_peak_18303	5.92591	
2	232570873	2.33E+08	KD2-H4K8ac_peak_18304	17.84477	MGC4771_ENSG00000269363;PTMA_ENSG00000187514
2	232572034	2.33E+08	KD2-H4K8ac_peak_18305	16.67374	MGC4771_ENSG00000269363;PTMA_ENSG00000187514
2	232573371	2.33E+08	KD2-H4K8ac_peak_18306	12.93209	

2	232574835	2.33E+08	KD2-H4K8ac_peak_18307	6.25869	
2	232645542	2.33E+08	KD2-H4K8ac_peak_18308	7.13615	COPS7B_ENSG00000144524
2	232650668	2.33E+08	KD2-H4K8ac_peak_18309	18.31034	PDE6D_ENSG00000156973
2	232825363	2.33E+08	KD2-H4K8ac_peak_18310	5.57044	DIS3L2_ENSG00000144535
2	232825782	2.33E+08	KD2-H4K8ac_peak_18311	13.12288	DIS3L2_ENSG00000144535
2	233072100	2.33E+08	KD2-H4K8ac_peak_18312	5.25071	
2	233308801	2.33E+08	KD2-H4K8ac_peak_18313	9.16531	
2	233367189	2.33E+08	KD2-H4K8ac_peak_18314	17.8868	
2	233370323	2.33E+08	KD2-H4K8ac_peak_18315	4.86324	
2	233388765	2.33E+08	KD2-H4K8ac_peak_18316	9.16531	
2	233397565	2.33E+08	KD2-H4K8ac_peak_18317	6.75488	
2	233414898	2.33E+08	KD2-H4K8ac_peak_18318	14.68449	TIGD1_ENSG00000221944;EIF4E2_ENSG00000135930;MIR5001_ENSG00000266620
2	233415444	2.33E+08	KD2-H4K8ac_peak_18319	15.66774	TIGD1_ENSG00000221944;EIF4E2_ENSG00000135930;MIR5001_ENSG00000266620
2	233470298	2.33E+08	KD2-H4K8ac_peak_18320	16.80494	EFHD1_ENSG00000115468
2	233497876	2.33E+08	KD2-H4K8ac_peak_18321	8.55271	
2	233498433	2.33E+08	KD2-H4K8ac_peak_18322	7.69287	
2	233561160	2.34E+08	KD2-H4K8ac_peak_18323	5.61377	GIGYF2_ENSG00000204120
2	233561581	2.34E+08	KD2-H4K8ac_peak_18324	5.14125	GIGYF2_ENSG00000204120
2	233562077	2.34E+08	KD2-H4K8ac_peak_18325	15.61313	GIGYF2_ENSG00000204120
2	233741009	2.34E+08	KD2-H4K8ac_peak_18326	8.30974	
2	233926529	2.34E+08	KD2-H4K8ac_peak_18327	10.02431	
2	234160379	2.34E+08	KD2-H4K8ac_peak_18328	8.55271	
2	234262207	2.34E+08	KD2-H4K8ac_peak_18329	12.81138	RP11-400N9.1_ENSG00000259793;DGKD_ENSG00000077044;AC019221.4_ENSG00000243637
2	234474890	2.34E+08	KD2-H4K8ac_peak_18330	12.74171	USP40_ENSG00000085982
2	234608645	2.35E+08	KD2-H4K8ac_peak_18331	6.16888	
2	234762612	2.35E+08	KD2-H4K8ac_peak_18332	7.28827	HJURP_ENSG00000123485
2	234906423	2.35E+08	KD2-H4K8ac_peak_18333	4.9025	
2	235241988	2.35E+08	KD2-H4K8ac_peak_18334	10.0692	
2	235242955	2.35E+08	KD2-H4K8ac_peak_18335	8.74596	
2	235244962	2.35E+08	KD2-H4K8ac_peak_18336	10.29344	
2	235404065	2.35E+08	KD2-H4K8ac_peak_18337	5.14125	
2	235405899	2.35E+08	KD2-H4K8ac_peak_18338	22.38694	ARL4C_ENSG00000188042
2	235842090	2.36E+08	KD2-H4K8ac_peak_18339	6.16888	
2	235860130	2.36E+08	KD2-H4K8ac_peak_18340	10.98959	SH3BP4_ENSG00000130147
2	235908879	2.36E+08	KD2-H4K8ac_peak_18341	7.70289	
2	235920322	2.36E+08	KD2-H4K8ac_peak_18342	7.13615	
2	235958521	2.36E+08	KD2-H4K8ac_peak_18343	10.96455	
2	235960096	2.36E+08	KD2-H4K8ac_peak_18344	13.63179	
2	236133429	2.36E+08	KD2-H4K8ac_peak_18345	5.15777	
2	236174288	2.36E+08	KD2-H4K8ac_peak_18346	4.77079	
2	236401607	2.36E+08	KD2-H4K8ac_peak_18347	13.09607	AGAP1_ENSG00000157985
2	236402777	2.36E+08	KD2-H4K8ac_peak_18348	11.14658	AGAP1_ENSG00000157985
2	236403421	2.36E+08	KD2-H4K8ac_peak_18349	4.77079	AGAP1_ENSG00000157985
2	236413558	2.36E+08	KD2-H4K8ac_peak_18350	6.36319	AGAP1-IT1_ENSG00000235529
2	236567633	2.37E+08	KD2-H4K8ac_peak_18351	6.2781	
2	236578423	2.37E+08	KD2-H4K8ac_peak_18352	7.69294	

2	236579257	2.37E+08	KD2-H4K8ac_peak_18353	3.91903	
2	237993937	2.38E+08	KD2-H4K8ac_peak_18354	11.03331	AC105760.2_ENSG00000227252;COPS8_ENSG00000198612
2	238206321	2.38E+08	KD2-H4K8ac_peak_18355	5.57044	
2	238207208	2.38E+08	KD2-H4K8ac_peak_18356	5.14125	
2	238341773	2.38E+08	KD2-H4K8ac_peak_18357	13.01932	
2	238360861	2.38E+08	KD2-H4K8ac_peak_18358	11.03212	
2	238382988	2.38E+08	KD2-H4K8ac_peak_18359	16.57619	
2	238391740	2.38E+08	KD2-H4K8ac_peak_18360	4.211	
2	238535631	2.39E+08	KD2-H4K8ac_peak_18361	23.43329	LRRFIP1_ENSG00000124831
2	238536289	2.39E+08	KD2-H4K8ac_peak_18362	9.63487	LRRFIP1_ENSG00000124831
2	238600103	2.39E+08	KD2-H4K8ac_peak_18363	8.23274	
2	238600849	2.39E+08	KD2-H4K8ac_peak_18364	8.59579	
2	238610411	2.39E+08	KD2-H4K8ac_peak_18365	6.24114	
2	238611363	2.39E+08	KD2-H4K8ac_peak_18366	6.56211	
2	238612219	2.39E+08	KD2-H4K8ac_peak_18367	5.34636	
2	238767391	2.39E+08	KD2-H4K8ac_peak_18368	16.49944	RAMP1_ENSG00000132329
2	238771354	2.39E+08	KD2-H4K8ac_peak_18369	6.67092	
2	238773125	2.39E+08	KD2-H4K8ac_peak_18370	7.92775	
2	238774172	2.39E+08	KD2-H4K8ac_peak_18371	10.01478	
2	238774773	2.39E+08	KD2-H4K8ac_peak_18372	6.30229	
2	238778871	2.39E+08	KD2-H4K8ac_peak_18373	7.95563	SNORD39_ENSG00000263723
2	238780837	2.39E+08	KD2-H4K8ac_peak_18374	5.54647	
2	238783719	2.39E+08	KD2-H4K8ac_peak_18375	5.59859	
2	238784837	2.39E+08	KD2-H4K8ac_peak_18376	14.4458	
2	238786237	2.39E+08	KD2-H4K8ac_peak_18377	7.44324	
2	238791046	2.39E+08	KD2-H4K8ac_peak_18378	14.68449	
2	238793160	2.39E+08	KD2-H4K8ac_peak_18379	27.60769	
2	238795844	2.39E+08	KD2-H4K8ac_peak_18380	5.49478	
2	238796545	2.39E+08	KD2-H4K8ac_peak_18381	6.25869	
2	238802989	2.39E+08	KD2-H4K8ac_peak_18382	14.38934	
2	238804169	2.39E+08	KD2-H4K8ac_peak_18383	10.21366	
2	238827016	2.39E+08	KD2-H4K8ac_peak_18384	6.86812	
2	238830722	2.39E+08	KD2-H4K8ac_peak_18385	5.30799	
2	238831709	2.39E+08	KD2-H4K8ac_peak_18386	9.1707	
2	238832970	2.39E+08	KD2-H4K8ac_peak_18387	10.86378	
2	238875071	2.39E+08	KD2-H4K8ac_peak_18388	10.53377	UBE2F_ENSG00000184182;UBE2F-SCLY_ENSG00000258984
2	238875700	2.39E+08	KD2-H4K8ac_peak_18389	8.30974	UBE2F_ENSG00000184182;UBE2F-SCLY_ENSG00000258984
2	238968902	2.39E+08	KD2-H4K8ac_peak_18390	9.20193	SCLY_ENSG00000132330
2	239001935	2.39E+08	KD2-H4K8ac_peak_18391	4.52039	
2	239009152	2.39E+08	KD2-H4K8ac_peak_18392	5.47943	ESPNL_ENSG00000144488
2	239047323	2.39E+08	KD2-H4K8ac_peak_18393	5.51783	KLHL30_ENSG00000168427
2	239047942	2.39E+08	KD2-H4K8ac_peak_18394	6.14031	KLHL30_ENSG00000168427
2	239067621	2.39E+08	KD2-H4K8ac_peak_18395	8.85813	FAM132B_ENSG00000178752
2	239111725	2.39E+08	KD2-H4K8ac_peak_18396	13.80019	ILKAP_ENSG00000132323
2	239143152	2.39E+08	KD2-H4K8ac_peak_18397	12.10889	
2	239147661	2.39E+08	KD2-H4K8ac_peak_18398	6.60096	

2	239148919	2.39E+08	KD2-H4K8ac_peak_18399	12.08557	HES6_ENSG00000144485
2	239149744	2.39E+08	KD2-H4K8ac_peak_18400	4.86324	HES6_ENSG00000144485
2	239152070	2.39E+08	KD2-H4K8ac_peak_18401	4.67342	
2	239229371	2.39E+08	KD2-H4K8ac_peak_18402	7.96322	TRAF3IP1_ENSG00000204104
2	239335142	2.39E+08	KD2-H4K8ac_peak_18403	15.2334	ASB1_ENSG00000065802;AC016999.2_ENSG00000229915
2	239431595	2.39E+08	KD2-H4K8ac_peak_18404	7.40111	
2	239432073	2.39E+08	KD2-H4K8ac_peak_18405	7.36799	
2	239756166	2.4E+08	KD2-H4K8ac_peak_18406	9.36621	TWIST2_ENSG00000233608
2	239957240	2.4E+08	KD2-H4K8ac_peak_18407	11.05467	
2	239983554	2.4E+08	KD2-H4K8ac_peak_18408	3.75035	
2	240000412	2.4E+08	KD2-H4K8ac_peak_18409	4.42765	
2	240003770	2.4E+08	KD2-H4K8ac_peak_18410	11.86082	
2	240004486	2.4E+08	KD2-H4K8ac_peak_18411	5.57044	
2	240014764	2.4E+08	KD2-H4K8ac_peak_18412	8.53092	
2	240015802	2.4E+08	KD2-H4K8ac_peak_18413	6.35843	
2	240018463	2.4E+08	KD2-H4K8ac_peak_18414	10.61986	
2	240019023	2.4E+08	KD2-H4K8ac_peak_18415	5.88845	
2	240134274	2.4E+08	KD2-H4K8ac_peak_18416	3.75035	
2	240161047	2.4E+08	KD2-H4K8ac_peak_18417	4.80593	
2	240162628	2.4E+08	KD2-H4K8ac_peak_18418	8.10216	
2	240174023	2.4E+08	KD2-H4K8ac_peak_18419	5.14125	
2	240184364	2.4E+08	KD2-H4K8ac_peak_18420	4.42765	
2	240187072	2.4E+08	KD2-H4K8ac_peak_18421	3.75035	
2	240196295	2.4E+08	KD2-H4K8ac_peak_18422	5.51783	
2	240196827	2.4E+08	KD2-H4K8ac_peak_18423	10.29842	
2	240197804	2.4E+08	KD2-H4K8ac_peak_18424	25.8903	
2	240198443	2.4E+08	KD2-H4K8ac_peak_18425	17.19888	
2	240231567	2.4E+08	KD2-H4K8ac_peak_18426	4.8888	
2	240251007	2.4E+08	KD2-H4K8ac_peak_18427	5.54647	
2	240270683	2.4E+08	KD2-H4K8ac_peak_18428	6.25869	
2	240323099	2.4E+08	KD2-H4K8ac_peak_18429	8.58725	HDAC4_ENSG00000068024;AC062017.1_ENSG00000222020
2	240508890	2.41E+08	KD2-H4K8ac_peak_18430	4.12757	
2	240597895	2.41E+08	KD2-H4K8ac_peak_18431	5.54647	
2	240639029	2.41E+08	KD2-H4K8ac_peak_18432	4.67342	
2	240648270	2.41E+08	KD2-H4K8ac_peak_18433	5.75452	
2	240964835	2.41E+08	KD2-H4K8ac_peak_18434	20.88898	NDUFA10_ENSG00000130414
2	241075807	2.41E+08	KD2-H4K8ac_peak_18435	9.95119	MYEOV2_ENSG00000172428
2	241083465	2.41E+08	KD2-H4K8ac_peak_18436	6.40683	OTOS_ENSG00000178602
2	241190273	2.41E+08	KD2-H4K8ac_peak_18437	5.54647	
2	241198412	2.41E+08	KD2-H4K8ac_peak_18438	6.0073	
2	241247482	2.41E+08	KD2-H4K8ac_peak_18439	6.47635	
2	241289224	2.41E+08	KD2-H4K8ac_peak_18440	5.08628	
2	241290940	2.41E+08	KD2-H4K8ac_peak_18441	5.75757	
2	241292311	2.41E+08	KD2-H4K8ac_peak_18442	10.96455	
2	241374270	2.41E+08	KD2-H4K8ac_peak_18443	11.82518	GPC1_ENSG00000063660
2	241391372	2.41E+08	KD2-H4K8ac_peak_18444	15.19879	AC110619.1_ENSG00000255735

2	241499857	2.42E+08	KD2-H4K8ac_peak_18445	9.48511	DUSP28_ENSG00000188542
2	241505631	2.42E+08	KD2-H4K8ac_peak_18446	12.74171	RNPEPL1_ENSG00000142327
2	241506119	2.42E+08	KD2-H4K8ac_peak_18447	5.14125	RNPEPL1_ENSG00000142327
2	241507302	2.42E+08	KD2-H4K8ac_peak_18448	10.50587	ANKMY1_ENSG00000144504
2	241507981	2.42E+08	KD2-H4K8ac_peak_18449	12.96672	ANKMY1_ENSG00000144504
2	241525799	2.42E+08	KD2-H4K8ac_peak_18450	13.83097	CAPN10-AS1_ENSG00000260942;CAPN10_ENSG00000142330
2	241562219	2.42E+08	KD2-H4K8ac_peak_18451	7.19807	
2	241760169	2.42E+08	KD2-H4K8ac_peak_18452	17.16047	KIF1A_ENSG00000130294
2	241771130	2.42E+08	KD2-H4K8ac_peak_18453	6.13909	
2	241842273	2.42E+08	KD2-H4K8ac_peak_18454	5.20908	
2	241853336	2.42E+08	KD2-H4K8ac_peak_18455	5.53033	
2	241856256	2.42E+08	KD2-H4K8ac_peak_18456	5.88845	
2	241897186	2.42E+08	KD2-H4K8ac_peak_18457	5.30799	
2	241899949	2.42E+08	KD2-H4K8ac_peak_18458	8.59579	
2	241902034	2.42E+08	KD2-H4K8ac_peak_18459	6.65673	
2	241904896	2.42E+08	KD2-H4K8ac_peak_18460	5.57044	
2	241937577	2.42E+08	KD2-H4K8ac_peak_18461	8.17557	SNED1_ENSG00000162804
2	241949050	2.42E+08	KD2-H4K8ac_peak_18462	14.05403	AC093585.6_ENSG00000229996
2	242041505	2.42E+08	KD2-H4K8ac_peak_18463	9.95119	MTERFD2_ENSG00000122085
2	242088599	2.42E+08	KD2-H4K8ac_peak_18464	7.40111	PASK_ENSG00000115687;PPP1R7_ENSG00000115685
2	242089980	2.42E+08	KD2-H4K8ac_peak_18465	13.95627	PASK_ENSG00000115687;PPP1R7_ENSG00000115685
2	242156971	2.42E+08	KD2-H4K8ac_peak_18466	4.44527	
2	242162959	2.42E+08	KD2-H4K8ac_peak_18467	5.88845	
2	242211563	2.42E+08	KD2-H4K8ac_peak_18468	4.42765	
2	242212364	2.42E+08	KD2-H4K8ac_peak_18469	5.88845	
2	242244592	2.42E+08	KD2-H4K8ac_peak_18470	6.67081	
2	242254182	2.42E+08	KD2-H4K8ac_peak_18471	6.8713	SEPT2_ENSG00000168385
2	242254800	2.42E+08	KD2-H4K8ac_peak_18472	10.43145	SEPT2_ENSG00000168385
2	242255386	2.42E+08	KD2-H4K8ac_peak_18473	8.55271	HDLBP_ENSG00000115677;SEPT2_ENSG00000168385
2	242294638	2.42E+08	KD2-H4K8ac_peak_18474	10.05606	FARP2_ENSG00000006607
2	242295814	2.42E+08	KD2-H4K8ac_peak_18475	7.47462	FARP2_ENSG00000006607
2	242447718	2.42E+08	KD2-H4K8ac_peak_18476	10.96455	
2	242448310	2.42E+08	KD2-H4K8ac_peak_18477	13.35993	STK25_ENSG00000115694
2	242480391	2.42E+08	KD2-H4K8ac_peak_18478	7.79898	
2	242498216	2.42E+08	KD2-H4K8ac_peak_18479	7.76335	BOK-AS1_ENSG00000234235;BOK_ENSG00000176720
2	242576184	2.43E+08	KD2-H4K8ac_peak_18480	13.62318	THAP4_ENSG00000176946;ATG4B_ENSG00000168397
2	242625759	2.43E+08	KD2-H4K8ac_peak_18481	9.11891	DTYMK_ENSG00000168393
2	242626519	2.43E+08	KD2-H4K8ac_peak_18482	15.69572	DTYMK_ENSG00000168393
2	242640449	2.43E+08	KD2-H4K8ac_peak_18483	9.73431	ING5_ENSG00000168395
2	242640954	2.43E+08	KD2-H4K8ac_peak_18484	7.28827	ING5_ENSG00000168395
2	242668047	2.43E+08	KD2-H4K8ac_peak_18485	7.01658	
2	242673450	2.43E+08	KD2-H4K8ac_peak_18486	5.4779	D2HGDH_ENSG00000180902
2	242674121	2.43E+08	KD2-H4K8ac_peak_18487	8.4716	D2HGDH_ENSG00000180902;AC114730.8_ENSG00000215692
2	242703878	2.43E+08	KD2-H4K8ac_peak_18488	10.0692	
2	242707239	2.43E+08	KD2-H4K8ac_peak_18489	5.1313	
2	242714241	2.43E+08	KD2-H4K8ac_peak_18490	12.06346	

2	242737136	2.43E+08	KD2-H4K8ac_peak_18491	5.64449	
2	242741242	2.43E+08	KD2-H4K8ac_peak_18492	5.14125	AC114730.5_ENSG00000215023
2	242742145	2.43E+08	KD2-H4K8ac_peak_18493	6.99815	AC114730.5_ENSG00000215023
2	242743045	2.43E+08	KD2-H4K8ac_peak_18494	20.18584	
2	242749275	2.43E+08	KD2-H4K8ac_peak_18495	6.16888	NEU4_ENSG00000204099
2	242749712	2.43E+08	KD2-H4K8ac_peak_18496	9.95119	NEU4_ENSG00000204099
2	242750560	2.43E+08	KD2-H4K8ac_peak_18497	12.36441	AC114730.3_ENSG00000224272;NEU4_ENSG00000204099
2	242785800	2.43E+08	KD2-H4K8ac_peak_18498	11.30637	AC114730.2_ENSG00000235151
2	242786268	2.43E+08	KD2-H4K8ac_peak_18499	16.32216	AC114730.2_ENSG00000235151
2	242796248	2.43E+08	KD2-H4K8ac_peak_18500	8.55271	
2	242800640	2.43E+08	KD2-H4K8ac_peak_18501	7.07117	PDCD1_ENSG00000188389
2	242810333	2.43E+08	KD2-H4K8ac_peak_18502	4.66652	
2	242810841	2.43E+08	KD2-H4K8ac_peak_18503	4.70388	CXXC11_ENSG00000188011
2	242822648	2.43E+08	KD2-H4K8ac_peak_18504	22.18958	AC131097.3_ENSG00000233806
2	243011195	2.43E+08	KD2-H4K8ac_peak_18505	9.11031	
20	247324	247977	KD2-H4K8ac_peak_18506	4.54006	RP5-1103G7.10_ENSG00000272874
20	270719	270961	KD2-H4K8ac_peak_18507	10.21502	C20orf96_ENSG00000196476
20	278281	278925	KD2-H4K8ac_peak_18508	9.07715	ZCCHC3_ENSG00000177764;AL034548.1_ENSG00000247315
20	290439	291139	KD2-H4K8ac_peak_18509	11.89502	
20	306316	306865	KD2-H4K8ac_peak_18510	10.96455	SOX12_ENSG00000177732
20	307114	307591	KD2-H4K8ac_peak_18511	6.25869	SOX12_ENSG00000177732
20	327244	328298	KD2-H4K8ac_peak_18512	17.19888	RP5-1103G7.4_ENSG00000225377;NRSN2_ENSG00000125841
20	328595	328904	KD2-H4K8ac_peak_18513	16.64426	RP5-1103G7.4_ENSG00000225377
20	340849	341189	KD2-H4K8ac_peak_18514	4.77079	
20	343084	343442	KD2-H4K8ac_peak_18515	4.42765	
20	344123	344566	KD2-H4K8ac_peak_18516	6.00226	
20	361453	362566	KD2-H4K8ac_peak_18517	12.00597	TRIB3_ENSG00000101255
20	388708	389619	KD2-H4K8ac_peak_18518	18.80403	RBCK1_ENSG00000125826
20	524047	524631	KD2-H4K8ac_peak_18519	15.61313	CSNK2A1_ENSG00000101266
20	524877	525207	KD2-H4K8ac_peak_18520	10.33529	CSNK2A1_ENSG00000101266
20	633418	634004	KD2-H4K8ac_peak_18521	13.2669	SRXN1_ENSG00000271303
20	655787	656325	KD2-H4K8ac_peak_18522	8.59579	RP5-850E9.3_ENSG00000270299;SCRT2_ENSG00000215397
20	656864	657097	KD2-H4K8ac_peak_18523	7.8711	RP5-850E9.3_ENSG00000270299;SCRT2_ENSG00000215397
20	657454	657704	KD2-H4K8ac_peak_18524	7.13615	SCRT2_ENSG00000215397
20	680202	680522	KD2-H4K8ac_peak_18525	5.61067	
20	680868	681062	KD2-H4K8ac_peak_18526	5.30799	
20	690005	690330	KD2-H4K8ac_peak_18527	5.32137	
20	690646	690894	KD2-H4K8ac_peak_18528	8.30974	
20	708652	708996	KD2-H4K8ac_peak_18529	3.90565	
20	814324	814555	KD2-H4K8ac_peak_18530	14.8083	FAM110A_ENSG00000125898
20	825419	826087	KD2-H4K8ac_peak_18531	12.30509	
20	982961	983246	KD2-H4K8ac_peak_18532	9.11031	RSPO4_ENSG00000101282
20	1099257	1099862	KD2-H4K8ac_peak_18533	19.9503	
20	1165571	1166004	KD2-H4K8ac_peak_18534	5.82799	TMEM74B_ENSG00000125895
20	1206017	1206256	KD2-H4K8ac_peak_18535	7.27716	RAD21L1_ENSG00000244588
20	1206860	1207225	KD2-H4K8ac_peak_18536	12.32214	RAD21L1_ENSG00000244588

20	1246552	1246777	KD2-H4K8ac_peak_18537	5.57044	SNPH_ENSG00000101298
20	1256005	1256261	KD2-H4K8ac_peak_18538	4.42765	
20	1294032	1294243	KD2-H4K8ac_peak_18539	6.66692	
20	1306111	1306500	KD2-H4K8ac_peak_18540	14.56955	SDCBP2-AS1_ENSG00000234684
20	1374244	1374444	KD2-H4K8ac_peak_18541	11.2938	FKBP1A_ENSG00000088832
20	1447093	1447460	KD2-H4K8ac_peak_18542	11.89502	
20	1447952	1448155	KD2-H4K8ac_peak_18543	9.02573	
20	1757614	1757837	KD2-H4K8ac_peak_18544	8.83582	
20	1875077	1875704	KD2-H4K8ac_peak_18545	7.47462	SIRPA_ENSG00000198053
20	2082489	2084037	KD2-H4K8ac_peak_18546	9.6247	STK35_ENSG00000125834
20	2187588	2188133	KD2-H4K8ac_peak_18547	8.86099	RP11-128M1.1_ENSG00000226644
20	2329187	2329454	KD2-H4K8ac_peak_18548	6.07178	
20	2450764	2451406	KD2-H4K8ac_peak_18549	12.30509	SNRPB_ENSG00000125835
20	2489034	2489671	KD2-H4K8ac_peak_18550	8.55271	RP4-734P14.4_ENSG00000256566
20	2489903	2490289	KD2-H4K8ac_peak_18551	7.76104	RP4-734P14.4_ENSG00000256566
20	2505200	2505874	KD2-H4K8ac_peak_18552	17.8868	ZNF343_ENSG00000088876
20	2515101	2515737	KD2-H4K8ac_peak_18553	9.36516	
20	2632523	2633046	KD2-H4K8ac_peak_18554	16.32216	NOP56_ENSG00000101361;MIR1292_ENSG00000221062
20	2633470	2633970	KD2-H4K8ac_peak_18555	10.1591	NOP56_ENSG00000101361;MIR1292_ENSG00000221062;SNORD110_ENSG00000221116
20	2644339	2644769	KD2-H4K8ac_peak_18556	5.13615	IDH3B_ENSG00000101365;RP4-686C3.7_ENSG00000228293
20	2645004	2645354	KD2-H4K8ac_peak_18557	6.27931	IDH3B_ENSG00000101365;RP4-686C3.7_ENSG00000228293
20	2672518	2673262	KD2-H4K8ac_peak_18558	14.15075	EBF4_ENSG00000088881
20	2673539	2673826	KD2-H4K8ac_peak_18559	5.88562	EBF4_ENSG00000088881
20	2780017	2780444	KD2-H4K8ac_peak_18560	6.25869	CPXM1_ENSG00000088882
20	2780752	2781204	KD2-H4K8ac_peak_18561	10.7186	CPXM1_ENSG00000088882
20	2781791	2782369	KD2-H4K8ac_peak_18562	10.05606	CPXM1_ENSG00000088882
20	2820368	2821280	KD2-H4K8ac_peak_18563	25.6154	PCED1A_ENSG00000132635;VPS16_ENSG00000215305
20	2852933	2854618	KD2-H4K8ac_peak_18564	13.09607	
20	3026154	3026538	KD2-H4K8ac_peak_18565	7.11824	MRPS26_ENSG00000125901
20	3026753	3027439	KD2-H4K8ac_peak_18566	7.07117	MRPS26_ENSG00000125901
20	3140187	3140490	KD2-H4K8ac_peak_18567	11.03331	UBOX5_ENSG00000185019;FASTKD5_ENSG00000215251
20	3140709	3140930	KD2-H4K8ac_peak_18568	6.25869	UBOX5_ENSG00000185019;FASTKD5_ENSG00000215251
20	3183899	3184196	KD2-H4K8ac_peak_18569	4.211	
20	3184415	3185247	KD2-H4K8ac_peak_18570	14.84212	DDRGK1_ENSG00000198171
20	3190137	3190808	KD2-H4K8ac_peak_18571	6.40297	ITPA_ENSG00000125877
20	3219962	3220226	KD2-H4K8ac_peak_18572	7.43893	SLC4A11_ENSG00000088836
20	3321268	3321519	KD2-H4K8ac_peak_18573	9.1707	
20	3387887	3388237	KD2-H4K8ac_peak_18574	8.17902	C20orf194_ENSG00000088854
20	3451048	3451483	KD2-H4K8ac_peak_18575	5.59859	ATRN_ENSG00000088812
20	3660099	3660434	KD2-H4K8ac_peak_18576	5.557	
20	3729893	3730460	KD2-H4K8ac_peak_18577	7.51054	
20	3748709	3749317	KD2-H4K8ac_peak_18578	13.87818	C20orf27_ENSG00000101220
20	3758203	3759395	KD2-H4K8ac_peak_18579	12.08081	
20	3766541	3766860	KD2-H4K8ac_peak_18580	9.89126	CENPB_ENSG00000125817;CDC25B_ENSG00000101224
20	3776121	3776782	KD2-H4K8ac_peak_18581	7.92775	
20	3777114	3777712	KD2-H4K8ac_peak_18582	11.86082	

20	3788199	3788976	KD2-H4K8ac_peak_18583	8.85813	
20	3791328	3791587	KD2-H4K8ac_peak_18584	5.57044	
20	3792689	3793396	KD2-H4K8ac_peak_18585	11.39383	
20	3799439	3799697	KD2-H4K8ac_peak_18586	7.48133	
20	3800905	3801378	KD2-H4K8ac_peak_18587	15.57998	AP5S1_ENSG00000125843
20	3826858	3827295	KD2-H4K8ac_peak_18588	9.34195	MAVS_ENSG00000088888
20	3827578	3827917	KD2-H4K8ac_peak_18589	6.20982	MAVS_ENSG00000088888
20	3868752	3869741	KD2-H4K8ac_peak_18590	20.97251	RP11-119B16.2_ENSG00000229539;PANK2_ENSG00000125779
20	3870118	3870378	KD2-H4K8ac_peak_18591	9.01262	RP11-119B16.2_ENSG00000229539;PANK2_ENSG00000125779
20	4066127	4066473	KD2-H4K8ac_peak_18592	14.8083	
20	4128991	4129309	KD2-H4K8ac_peak_18593	9.11031	
20	4129818	4130232	KD2-H4K8ac_peak_18594	4.67342	
20	4148138	4148337	KD2-H4K8ac_peak_18595	5.75452	
20	4152614	4153245	KD2-H4K8ac_peak_18596	8.85813	
20	4667300	4667502	KD2-H4K8ac_peak_18597	6.90231	PRNP_ENSG00000171867
20	4741435	4741999	KD2-H4K8ac_peak_18598	18.5703	
20	4803880	4804175	KD2-H4K8ac_peak_18599	10.05606	RASSF2_ENSG00000101265
20	4804529	4804856	KD2-H4K8ac_peak_18600	7.28628	RASSF2_ENSG00000101265
20	5093037	5094142	KD2-H4K8ac_peak_18601	10.05606	TMEM230_ENSG00000089063;Y_RNA_ENSG00000252367
20	5100248	5100534	KD2-H4K8ac_peak_18602	5.30799	
20	5107665	5108048	KD2-H4K8ac_peak_18603	4.86324	PCNA_ENSG00000132646;CDS2_ENSG00000101290
20	5277440	5277643	KD2-H4K8ac_peak_18604	4.01115	
20	5285720	5285945	KD2-H4K8ac_peak_18605	7.40111	
20	5372960	5373571	KD2-H4K8ac_peak_18606	5.88845	
20	5391882	5392758	KD2-H4K8ac_peak_18607	8.25022	
20	5429511	5429759	KD2-H4K8ac_peak_18608	5.15777	
20	5484801	5485258	KD2-H4K8ac_peak_18609	5.57044	LINC00654_ENSG00000205181
20	5591082	5591551	KD2-H4K8ac_peak_18610	20.5798	GPCPD1_ENSG00000125772
20	5591856	5592140	KD2-H4K8ac_peak_18611	6.90231	GPCPD1_ENSG00000125772
20	5730298	5731001	KD2-H4K8ac_peak_18612	10.43145	C20orf196_ENSG00000171984
20	5731202	5732078	KD2-H4K8ac_peak_18613	8.17902	C20orf196_ENSG00000171984
20	5787579	5787778	KD2-H4K8ac_peak_18614	7.44366	
20	5810845	5811223	KD2-H4K8ac_peak_18615	4.59928	
20	5891709	5891944	KD2-H4K8ac_peak_18616	9.11031	CHGB_ENSG00000089199
20	5892193	5892552	KD2-H4K8ac_peak_18617	18.41184	CHGB_ENSG00000089199
20	5930898	5931097	KD2-H4K8ac_peak_18618	12.06346	TRMT6_ENSG00000089195;MCM8_ENSG00000125885
20	5986839	5987205	KD2-H4K8ac_peak_18619	7.36799	CRLS1_ENSG00000088766
20	7999925	8000338	KD2-H4K8ac_peak_18620	20.02161	TMX4_ENSG00000125827;RP5-971N18.3_ENSG00000229766
20	8001679	8001872	KD2-H4K8ac_peak_18621	4.01115	
20	8112887	8113376	KD2-H4K8ac_peak_18622	13.36684	PLCB1_ENSG00000182621
20	8412480	8412729	KD2-H4K8ac_peak_18623	11.86708	
20	8625077	8625272	KD2-H4K8ac_peak_18624	4.11746	
20	9047965	9048329	KD2-H4K8ac_peak_18625	12.06346	AL121898.1_ENSG00000252819
20	9048893	9049362	KD2-H4K8ac_peak_18626	15.13261	PLCB4_ENSG00000101333
20	9145633	9145851	KD2-H4K8ac_peak_18627	5.25071	
20	9495118	9495327	KD2-H4K8ac_peak_18628	7.01964	RP5-1119D9.4_ENSG00000225988;LAMP5_ENSG00000125869

20	9496275	9497015	KD2-H4K8ac_peak_18629	6.65673	RP5-1119D9.4_ENSG00000225988
20	9497241	9497536	KD2-H4K8ac_peak_18630	5.88845	
20	10015772	10016052	KD2-H4K8ac_peak_18631	5.9696	
20	10198382	10198648	KD2-H4K8ac_peak_18632	5.30799	SNAP25_ENSG00000132639
20	10258369	10258607	KD2-H4K8ac_peak_18633	7.12754	
20	10288337	10288640	KD2-H4K8ac_peak_18634	4.54838	
20	10291110	10291512	KD2-H4K8ac_peak_18635	4.59928	
20	10414309	10414863	KD2-H4K8ac_peak_18636	11.66477	MKKS_ENSG00000125863
20	10415954	10416176	KD2-H4K8ac_peak_18637	9.08816	SLX4IP_ENSG00000149346
20	10531270	10531483	KD2-H4K8ac_peak_18638	4.01115	
20	10617254	10617462	KD2-H4K8ac_peak_18639	6.56926	
20	10623017	10623218	KD2-H4K8ac_peak_18640	4.47369	
20	10624566	10624808	KD2-H4K8ac_peak_18641	10.19949	
20	10632050	10632527	KD2-H4K8ac_peak_18642	5.05318	
20	10633124	10633381	KD2-H4K8ac_peak_18643	4.98072	
20	10652282	10652589	KD2-H4K8ac_peak_18644	7.27716	RP11-103J8.1_ENSG00000270792
20	10655196	10655737	KD2-H4K8ac_peak_18645	8.94469	JAG1_ENSG00000101384
20	11871611	11872268	KD2-H4K8ac_peak_18646	10.43145	BTBD3_ENSG00000132640
20	13619163	13619513	KD2-H4K8ac_peak_18647	4.86324	TASP1_ENSG00000089123
20	13619754	13620039	KD2-H4K8ac_peak_18648	8.37231	TASP1_ENSG00000089123
20	13765676	13766160	KD2-H4K8ac_peak_18649	14.43704	ESF1_ENSG00000089048;NDUFAF5_ENSG00000101247
20	13975508	13975860	KD2-H4K8ac_peak_18650	5.13615	MACROD2_ENSG00000172264
20	14207993	14208216	KD2-H4K8ac_peak_18651	4.37708	
20	16330972	16331289	KD2-H4K8ac_peak_18652	10.0692	
20	16553803	16554002	KD2-H4K8ac_peak_18653	9.36516	KIF16B_ENSG00000089177
20	16628078	16628325	KD2-H4K8ac_peak_18654	8.09303	
20	16633324	16633727	KD2-H4K8ac_peak_18655	5.14125	
20	16635092	16635437	KD2-H4K8ac_peak_18656	6.40683	
20	16709982	16710503	KD2-H4K8ac_peak_18657	16.54162	SNRPB2_ENSG00000125870
20	16710709	16711286	KD2-H4K8ac_peak_18658	15.01306	SNRPB2_ENSG00000125870;RP4-705D16.3_ENSG00000228809
20	17485019	17485232	KD2-H4K8ac_peak_18659	8.85813	
20	17485867	17486238	KD2-H4K8ac_peak_18660	5.57044	
20	17511849	17512128	KD2-H4K8ac_peak_18661	5.405	
20	17550347	17550608	KD2-H4K8ac_peak_18662	6.66692	BFSP1_ENSG00000125864;DSTN_ENSG00000125868
20	17550842	17551239	KD2-H4K8ac_peak_18663	8.2413	BFSP1_ENSG00000125864;DSTN_ENSG00000125868
20	17617142	17617346	KD2-H4K8ac_peak_18664	6.25869	
20	17626727	17626926	KD2-H4K8ac_peak_18665	5.3778	
20	17663224	17663453	KD2-H4K8ac_peak_18666	15.19698	RRBP1_ENSG00000125844
20	17787304	17787836	KD2-H4K8ac_peak_18667	7.22457	
20	17790413	17790611	KD2-H4K8ac_peak_18668	7.12754	
20	17854366	17854585	KD2-H4K8ac_peak_18669	10.53377	
20	17948627	17950055	KD2-H4K8ac_peak_18670	12.08081	SNX5_ENSG00000089006;MGME1_ENSG00000125871
20	17988348	17988747	KD2-H4K8ac_peak_18671	5.54647	
20	18117778	18119134	KD2-H4K8ac_peak_18672	28.93328	PET117_ENSG00000232838;CSRP2BP_ENSG00000149474
20	18268154	18268732	KD2-H4K8ac_peak_18673	5.72327	ZNF133_ENSG00000125846
20	18394090	18394444	KD2-H4K8ac_peak_18674	4.86824	

20	18548100	18548306	KD2-H4K8ac_peak_18675	11.05407	LINC00493_ENSG00000232388
20	18568756	18569074	KD2-H4K8ac_peak_18676	10.19949	DTD1_ENSG00000125821
20	18774773	18774974	KD2-H4K8ac_peak_18677	9.96798	LINC00652_ENSG00000179935;RP5-1068E13.7_ENSG00000273148
20	19738008	19738660	KD2-H4K8ac_peak_18678	7.76329	AL121761.2_ENSG00000268628;RP1-122P22.2_ENSG00000233895
20	19956009	19956442	KD2-H4K8ac_peak_18679	5.54647	
20	19997317	19997713	KD2-H4K8ac_peak_18680	8.02571	NAA20_ENSG00000173418
20	19998203	19998566	KD2-H4K8ac_peak_18681	7.74292	NAA20_ENSG00000173418
20	20032649	20033037	KD2-H4K8ac_peak_18682	14.15075	C20orf26_ENSG00000089101
20	20175997	20176252	KD2-H4K8ac_peak_18683	7.96322	
20	20184590	20184841	KD2-H4K8ac_peak_18684	6.72021	
20	20213988	20214195	KD2-H4K8ac_peak_18685	8.59579	
20	21081382	21083160	KD2-H4K8ac_peak_18686	18.53838	
20	21086036	21087160	KD2-H4K8ac_peak_18687	12.88108	LINC00237_ENSG00000225127
20	21272337	21272934	KD2-H4K8ac_peak_18688	13.73635	
20	21284000	21284344	KD2-H4K8ac_peak_18689	14.68449	XRN2_ENSG00000088930
20	23330810	23331160	KD2-H4K8ac_peak_18690	25.35008	RP3-322G13.7_ENSG00000234832;NXT1_ENSG00000132661
20	23331536	23331728	KD2-H4K8ac_peak_18691	7.49599	RP3-322G13.7_ENSG00000234832;NXT1_ENSG00000132661
20	23338365	23338716	KD2-H4K8ac_peak_18692	8.1331	RP3-322G13.5_ENSG00000232645
20	23342127	23342430	KD2-H4K8ac_peak_18693	7.76329	GZF1_ENSG00000125812
20	23401342	23402020	KD2-H4K8ac_peak_18694	9.72244	NAPB_ENSG00000125814
20	23402292	23402734	KD2-H4K8ac_peak_18695	7.40111	NAPB_ENSG00000125814
20	23618158	23618561	KD2-H4K8ac_peak_18696	16.51005	CST3_ENSG00000101439
20	24973044	24973300	KD2-H4K8ac_peak_18697	12.93209	APMAP_ENSG00000101474
20	24973560	24973909	KD2-H4K8ac_peak_18698	8.52559	APMAP_ENSG00000101474
20	24975705	24975913	KD2-H4K8ac_peak_18699	6.03693	
20	25176447	25176713	KD2-H4K8ac_peak_18700	6.76298	ENTPD6_ENSG00000197586
20	25228909	25229362	KD2-H4K8ac_peak_18701	15.64127	PYGB_ENSG00000100994
20	25290280	25290762	KD2-H4K8ac_peak_18702	7.40633	
20	25370459	25370981	KD2-H4K8ac_peak_18703	13.44374	ABHD12_ENSG00000100997
20	25512709	25512908	KD2-H4K8ac_peak_18704	4.98072	
20	25564797	25565071	KD2-H4K8ac_peak_18705	6.75638	
20	25565383	25566135	KD2-H4K8ac_peak_18706	18.94636	NINL_ENSG00000101004
20	25566346	25566898	KD2-H4K8ac_peak_18707	8.26945	NINL_ENSG00000101004
20	25604852	25605146	KD2-H4K8ac_peak_18708	7.07117	NANP_ENSG00000170191;ZNF337-AS1_ENSG00000213742
20	25605594	25605815	KD2-H4K8ac_peak_18709	9.06703	NANP_ENSG00000170191;ZNF337-AS1_ENSG00000213742
20	25677017	25677404	KD2-H4K8ac_peak_18710	8.85813	ZNF337_ENSG00000130684
20	25834883	25835143	KD2-H4K8ac_peak_18711	11.43353	
20	25846338	25847181	KD2-H4K8ac_peak_18712	9.89126	
20	25848491	25848755	KD2-H4K8ac_peak_18713	4.42765	FAM182B_ENSG00000175170
20	25967585	25967821	KD2-H4K8ac_peak_18714	5.88845	
20	25990137	25990579	KD2-H4K8ac_peak_18715	11.30637	RP4-760C5.3_ENSG00000231081
20	29518170	29518765	KD2-H4K8ac_peak_18716	13.50024	
20	29518969	29519418	KD2-H4K8ac_peak_18717	6.25869	
20	29611238	29611713	KD2-H4K8ac_peak_18718	11.05467	FRG1B_ENSG00000149531
20	29612032	29612468	KD2-H4K8ac_peak_18719	12.93209	FRG1B_ENSG00000149531
20	30102326	30102598	KD2-H4K8ac_peak_18720	20.67053	HM13_ENSG00000101294

20	30157687	30158044	KD2-H4K8ac_peak_18721	7.76329	
20	30158332	30158552	KD2-H4K8ac_peak_18722	5.54647	
20	30160145	30160685	KD2-H4K8ac_peak_18723	11.92978	HM13-AS1_ENSG00000230613
20	30161297	30161611	KD2-H4K8ac_peak_18724	5.30799	HM13-AS1_ENSG00000230613
20	30175355	30175992	KD2-H4K8ac_peak_18725	10.86378	
20	30182904	30183115	KD2-H4K8ac_peak_18726	6.53915	
20	30191515	30192020	KD2-H4K8ac_peak_18727	10.54874	
20	30196081	30196412	KD2-H4K8ac_peak_18728	11.43863	
20	30200461	30200662	KD2-H4K8ac_peak_18729	10.2519	
20	30200943	30201278	KD2-H4K8ac_peak_18730	4.66652	
20	30225384	30225855	KD2-H4K8ac_peak_18731	10.17341	COX4I2_ENSG00000131055
20	30226422	30227046	KD2-H4K8ac_peak_18732	9.34989	COX4I2_ENSG00000131055
20	30310847	30311749	KD2-H4K8ac_peak_18733	18.37277	BCL2L1_ENSG00000171552
20	30423831	30424199	KD2-H4K8ac_peak_18734	9.09777	
20	30424669	30424878	KD2-H4K8ac_peak_18735	4.0528	
20	30430213	30431094	KD2-H4K8ac_peak_18736	7.13615	
20	30440256	30440853	KD2-H4K8ac_peak_18737	7.48133	
20	30449627	30449951	KD2-H4K8ac_peak_18738	12.37731	
20	30450295	30450567	KD2-H4K8ac_peak_18739	4.7158	
20	30458035	30458950	KD2-H4K8ac_peak_18740	12.14909	DUSP15_ENSG00000149599;TTLL9_ENSG00000131044
20	30468141	30468429	KD2-H4K8ac_peak_18741	3.75035	
20	30498706	30499319	KD2-H4K8ac_peak_18742	6.50927	
20	30539392	30539656	KD2-H4K8ac_peak_18743	5.75757	PDRG1_ENSG00000088356
20	30555943	30556193	KD2-H4K8ac_peak_18744	7.69287	XKR7_ENSG00000260903
20	30595863	30596064	KD2-H4K8ac_peak_18745	5.88845	RNA5SP481_ENSG00000252623
20	30606172	30607207	KD2-H4K8ac_peak_18746	6.20982	
20	30679634	30680207	KD2-H4K8ac_peak_18747	9.82729	
20	30697008	30698347	KD2-H4K8ac_peak_18748	10.43145	TM9SF4_ENSG00000101337
20	30777658	30778193	KD2-H4K8ac_peak_18749	16.54162	TSPY26P_ENSG00000235217
20	30795013	30795507	KD2-H4K8ac_peak_18750	23.33626	POFUT1_ENSG00000101346
20	30795731	30796129	KD2-H4K8ac_peak_18751	10.96455	POFUT1_ENSG00000101346
20	30864845	30865836	KD2-H4K8ac_peak_18752	11.80283	KIF3B_ENSG00000101350;AL121897.1_ENSG00000264308
20	30946210	30946431	KD2-H4K8ac_peak_18753	4.67342	ASXL1_ENSG00000171456
20	30946649	30947061	KD2-H4K8ac_peak_18754	5.59859	ASXL1_ENSG00000171456
20	31033552	31033746	KD2-H4K8ac_peak_18755	6.25869	
20	31034110	31034375	KD2-H4K8ac_peak_18756	7.07117	
20	31036308	31036763	KD2-H4K8ac_peak_18757	8.82006	RP5-1184F4.5_ENSG00000236772
20	31037397	31037625	KD2-H4K8ac_peak_18758	6.35843	RP5-1184F4.5_ENSG00000236772
20	31039130	31039343	KD2-H4K8ac_peak_18759	5.13615	
20	31043916	31044862	KD2-H4K8ac_peak_18760	8.4716	
20	31071065	31071264	KD2-H4K8ac_peak_18761	10.0692	
20	31071677	31072742	KD2-H4K8ac_peak_18762	36.03116	
20	31084486	31084804	KD2-H4K8ac_peak_18763	9.36516	
20	31085251	31085795	KD2-H4K8ac_peak_18764	5.4779	
20	31086389	31086923	KD2-H4K8ac_peak_18765	9.63925	
20	31094585	31094858	KD2-H4K8ac_peak_18766	8.05311	

20	31096930	31097123	KD2-H4K8ac_peak_18767	4.211	
20	31104529	31105119	KD2-H4K8ac_peak_18768	22.12955	
20	31105398	31106797	KD2-H4K8ac_peak_18769	16.54162	
20	31109209	31109539	KD2-H4K8ac_peak_18770	6.29509	
20	31110591	31111048	KD2-H4K8ac_peak_18771	6.67092	
20	31111339	31111577	KD2-H4K8ac_peak_18772	5.49497	
20	31114998	31116589	KD2-H4K8ac_peak_18773	13.81781	
20	31123904	31124198	KD2-H4K8ac_peak_18774	11.79585	
20	31169216	31169599	KD2-H4K8ac_peak_18775	7.40111	RP11-410N8.1_ENSG00000228156
20	31170548	31171227	KD2-H4K8ac_peak_18776	10.46264	RP11-410N8.1_ENSG00000228156
20	31171527	31171827	KD2-H4K8ac_peak_18777	12.2973	
20	31195701	31196114	KD2-H4K8ac_peak_18778	9.05418	
20	31214690	31215992	KD2-H4K8ac_peak_18779	12.79505	
20	31226459	31226733	KD2-H4K8ac_peak_18780	4.06758	
20	31252390	31252790	KD2-H4K8ac_peak_18781	5.15777	
20	31260608	31260815	KD2-H4K8ac_peak_18782	7.79898	C20orf203_ENSG00000198547
20	31268904	31269489	KD2-H4K8ac_peak_18783	4.86324	
20	31273784	31274004	KD2-H4K8ac_peak_18784	5.51783	
20	31330475	31330951	KD2-H4K8ac_peak_18785	5.9696	COMMD7_ENSG00000149600
20	31349542	31349877	KD2-H4K8ac_peak_18786	11.64359	DNMT3B_ENSG00000088305
20	31406983	31407357	KD2-H4K8ac_peak_18787	10.86378	MAPRE1_ENSG00000101367
20	31407780	31408244	KD2-H4K8ac_peak_18788	10.29344	MAPRE1_ENSG00000101367
20	31864473	31864736	KD2-H4K8ac_peak_18789	6.82976	
20	31989091	31989313	KD2-H4K8ac_peak_18790	9.73431	CDK5RAP1_ENSG00000101391
20	31989744	31990162	KD2-H4K8ac_peak_18791	9.59814	CDK5RAP1_ENSG00000101391
20	32077198	32077558	KD2-H4K8ac_peak_18792	7.47462	CBFA2T2_ENSG00000078699
20	32077975	32078264	KD2-H4K8ac_peak_18793	8.79106	CBFA2T2_ENSG00000078699
20	32086188	32086808	KD2-H4K8ac_peak_18794	11.60333	
20	32152338	32152579	KD2-H4K8ac_peak_18795	5.45396	
20	32156751	32157033	KD2-H4K8ac_peak_18796	4.59928	
20	32157767	32158088	KD2-H4K8ac_peak_18797	9.11031	
20	32162207	32162602	KD2-H4K8ac_peak_18798	10.0692	
20	32163498	32163944	KD2-H4K8ac_peak_18799	10.05606	
20	32168346	32168581	KD2-H4K8ac_peak_18800	6.91644	
20	32251180	32251439	KD2-H4K8ac_peak_18801	8.52921	
20	32251664	32252220	KD2-H4K8ac_peak_18802	8.10216	
20	32254264	32254457	KD2-H4K8ac_peak_18803	6.45083	ACTL10_ENSG00000182584
20	32261349	32261637	KD2-H4K8ac_peak_18804	6.48242	NECAB3_ENSG00000125967;RP1-63M2.5_ENSG00000271803
20	32261941	32262146	KD2-H4K8ac_peak_18805	4.91674	NECAB3_ENSG00000125967;RP1-63M2.5_ENSG00000271803
20	32274567	32274827	KD2-H4K8ac_peak_18806	3.95782	E2F1_ENSG00000101412
20	32281636	32282252	KD2-H4K8ac_peak_18807	18.72657	
20	32283759	32284227	KD2-H4K8ac_peak_18808	5.14125	
20	32284735	32285100	KD2-H4K8ac_peak_18809	6.56926	
20	32291124	32291371	KD2-H4K8ac_peak_18810	3.87414	
20	32307769	32308024	KD2-H4K8ac_peak_18811	11.09936	PXMP4_ENSG00000101417
20	32308346	32309039	KD2-H4K8ac_peak_18812	14.74047	PXMP4_ENSG00000101417

20	32319202	32319704	KD2-H4K8ac_peak_18813	5.75757	RP4-553F4.2_ENSG00000229188;ZNF341_ENSG00000131061
20	32319927	32320603	KD2-H4K8ac_peak_18814	17.17257	RP4-553F4.2_ENSG00000229188;ZNF341_ENSG00000131061
20	32376159	32376664	KD2-H4K8ac_peak_18815	7.54952	
20	32377131	32377385	KD2-H4K8ac_peak_18816	10.21366	
20	32399199	32399640	KD2-H4K8ac_peak_18817	5.53033	RP4-553F4.6_ENSG00000230753;CHMP4B_ENSG00000101421
20	32426962	32427180	KD2-H4K8ac_peak_18818	7.13615	
20	32430157	32430520	KD2-H4K8ac_peak_18819	5.14125	
20	32433610	32433806	KD2-H4K8ac_peak_18820	4.44748	
20	32447701	32447905	KD2-H4K8ac_peak_18821	4.19341	
20	32450652	32451230	KD2-H4K8ac_peak_18822	7.74292	
20	32451820	32452220	KD2-H4K8ac_peak_18823	10.30284	
20	32452735	32454259	KD2-H4K8ac_peak_18824	12.78794	
20	32516093	32516294	KD2-H4K8ac_peak_18825	3.75035	
20	32581502	32582257	KD2-H4K8ac_peak_18826	9.89383	RP5-1125A11.1_ENSG00000228265;RALY_ENSG00000125970
20	32699399	32699971	KD2-H4K8ac_peak_18827	11.04256	EIF2S2_ENSG00000125977
20	32700423	32700831	KD2-H4K8ac_peak_18828	7.52168	EIF2S2_ENSG00000125977
20	32890480	32890729	KD2-H4K8ac_peak_18829	6.0605	
20	32951208	32951658	KD2-H4K8ac_peak_18830	7.36799	ITCH_ENSG00000078747
20	33103415	33104527	KD2-H4K8ac_peak_18831	12.99417	DYNLRB1_ENSG00000125971
20	33145883	33146332	KD2-H4K8ac_peak_18832	12.08081	
20	33146563	33146877	KD2-H4K8ac_peak_18833	9.95119	
20	33264218	33264446	KD2-H4K8ac_peak_18834	5.54647	PIGU_ENSG00000101464
20	33291507	33292947	KD2-H4K8ac_peak_18835	14.12454	TP53INP2_ENSG00000078804
20	33297058	33297505	KD2-H4K8ac_peak_18836	4.37809	
20	33412574	33413865	KD2-H4K8ac_peak_18837	12.08081	NCOA6_ENSG00000198646
20	33543139	33544481	KD2-H4K8ac_peak_18838	13.73635	GSS_ENSG00000100983
20	33568386	33568727	KD2-H4K8ac_peak_18839	8.6308	
20	33680164	33680576	KD2-H4K8ac_peak_18840	6.60096	TRPC4AP_ENSG00000100991
20	33734425	33735056	KD2-H4K8ac_peak_18841	7.76104	
20	33754141	33754584	KD2-H4K8ac_peak_18842	6.66692	
20	33754780	33755362	KD2-H4K8ac_peak_18843	9.15329	
20	33864948	33865504	KD2-H4K8ac_peak_18844	7.44324	EDEM2_ENSG00000088298;MMP24-AS1_ENSG00000126005
20	33872082	33872370	KD2-H4K8ac_peak_18845	9.95119	EIF6_ENSG00000242372;FAM83C-AS1_ENSG00000235214
20	33872691	33873489	KD2-H4K8ac_peak_18846	7.49599	EIF6_ENSG00000242372;FAM83C-AS1_ENSG00000235214
20	33883845	33884211	KD2-H4K8ac_peak_18847	4.68932	
20	33999267	33999732	KD2-H4K8ac_peak_18848	8.58069	UQCC1_ENSG00000101019
20	34000011	34000352	KD2-H4K8ac_peak_18849	13.50024	UQCC1_ENSG00000101019
20	34026932	34027240	KD2-H4K8ac_peak_18850	6.64891	
20	34041930	34042425	KD2-H4K8ac_peak_18851	6.56211	GDF5_ENSG00000125965;MIR1289-1_ENSG00000221763;CEP250_ENSG00000126001
20	34043015	34043308	KD2-H4K8ac_peak_18852	5.75757	GDF5_ENSG00000125965;CEP250_ENSG00000126001
20	34129877	34130422	KD2-H4K8ac_peak_18853	14.68449	ERGIC3_ENSG00000125991
20	34189031	34189448	KD2-H4K8ac_peak_18854	5.57044	
20	34203772	34204011	KD2-H4K8ac_peak_18855	4.30033	SPAG4_ENSG00000061656
20	34252346	34252811	KD2-H4K8ac_peak_18856	20.0792	CPNE1_ENSG00000214078;RBM12_ENSG00000244462
20	34253665	34253963	KD2-H4K8ac_peak_18857	4.42765	CPNE1_ENSG00000214078;RBM12_ENSG00000244462
20	34286859	34287233	KD2-H4K8ac_peak_18858	10.65724	NFS1_ENSG00000244005;ROMO1_ENSG00000125995

20	34329676	34330107	KD2-H4K8ac_peak_18859	8.36432	RBM39_ENSG00000131051
20	34359518	34359748	KD2-H4K8ac_peak_18860	11.05467	PHF20_ENSG00000025293
20	34541860	34542175	KD2-H4K8ac_peak_18861	10.46874	
20	34579603	34579914	KD2-H4K8ac_peak_18862	7.91974	
20	34638389	34638833	KD2-H4K8ac_peak_18863	10.20046	LINC00657_ENSG00000260032
20	34639077	34639629	KD2-H4K8ac_peak_18864	16.09057	LINC00657_ENSG00000260032
20	34652287	34652615	KD2-H4K8ac_peak_18865	18.29344	
20	34680118	34680428	KD2-H4K8ac_peak_18866	11.05407	EPB41L1_ENSG00000088367
20	34681192	34681581	KD2-H4K8ac_peak_18867	6.40683	
20	34742039	34742327	KD2-H4K8ac_peak_18868	8.85813	
20	34742705	34742928	KD2-H4K8ac_peak_18869	5.9696	RP11-234K24.3_ENSG00000232406
20	34823950	34824183	KD2-H4K8ac_peak_18870	4.30033	AAR2_ENSG00000131043
20	34824453	34824717	KD2-H4K8ac_peak_18871	6.66692	AAR2_ENSG00000131043
20	34861937	34862414	KD2-H4K8ac_peak_18872	3.83717	
20	34863622	34863871	KD2-H4K8ac_peak_18873	6.40297	
20	34894274	34894935	KD2-H4K8ac_peak_18874	6.25869	DLGAP4_ENSG00000080845
20	35064368	35064674	KD2-H4K8ac_peak_18875	5.82799	
20	35088962	35089509	KD2-H4K8ac_peak_18876	19.55721	
20	35090261	35090951	KD2-H4K8ac_peak_18877	13.2669	
20	35200971	35201772	KD2-H4K8ac_peak_18878	13.49029	RP5-977B1.7_ENSG00000232907;TGIF2_ENSG00000118707
20	35202059	35202779	KD2-H4K8ac_peak_18879	6.40297	RP5-977B1.7_ENSG00000232907;TGIF2_ENSG00000118707;TGIF2-C20orf24_ENSG00000259399
20	35203005	35203304	KD2-H4K8ac_peak_18880	10.96455	TGIF2-C20orf24_ENSG00000259399
20	35233575	35233884	KD2-H4K8ac_peak_18881	8.55271	RP5-977B1.11_ENSG00000259716;C20orf24_ENSG00000101084
20	35234189	35234606	KD2-H4K8ac_peak_18882	13.73635	C20orf24_ENSG00000101084
20	35401724	35402161	KD2-H4K8ac_peak_18883	10.65724	DSN1_ENSG00000149636
20	35402398	35402648	KD2-H4K8ac_peak_18884	9.36516	DSN1_ENSG00000149636
20	35402856	35403175	KD2-H4K8ac_peak_18885	10.65724	DSN1_ENSG00000149636
20	35463598	35464062	KD2-H4K8ac_peak_18886	12.84642	
20	35491415	35491948	KD2-H4K8ac_peak_18887	11.02863	SOGA1_ENSG00000149639
20	35492258	35492908	KD2-H4K8ac_peak_18888	9.65086	SOGA1_ENSG00000149639
20	35515185	35515609	KD2-H4K8ac_peak_18889	10.87093	
20	35580481	35580947	KD2-H4K8ac_peak_18890	7.45501	SAMHD1_ENSG00000101347
20	35723606	35723895	KD2-H4K8ac_peak_18891	7.40111	RBL1_ENSG00000080839;RBL1_ENSG00000269846
20	35724124	35725203	KD2-H4K8ac_peak_18892	12.82393	RBL1_ENSG00000080839;RBL1_ENSG00000269846
20	35806467	35806689	KD2-H4K8ac_peak_18893	6.30229	RPN2_ENSG00000118705
20	35806984	35807246	KD2-H4K8ac_peak_18894	9.36516	MROH8_ENSG00000101353;RPN2_ENSG00000118705
20	35807515	35808096	KD2-H4K8ac_peak_18895	13.73635	MROH8_ENSG00000101353;RPN2_ENSG00000118705
20	35831346	35831547	KD2-H4K8ac_peak_18896	5.15777	
20	35849158	35849436	KD2-H4K8ac_peak_18897	5.77029	
20	35849659	35849885	KD2-H4K8ac_peak_18898	10.0692	
20	35860290	35860814	KD2-H4K8ac_peak_18899	5.9696	
20	35863122	35863467	KD2-H4K8ac_peak_18900	13.80019	
20	35863864	35864332	KD2-H4K8ac_peak_18901	15.48665	
20	35865819	35867112	KD2-H4K8ac_peak_18902	12.99417	
20	35867962	35868402	KD2-H4K8ac_peak_18903	5.14125	
20	35869026	35869231	KD2-H4K8ac_peak_18904	7.27716	

20	35870370	35870646	KD2-H4K8ac_peak_18905	7.9076	
20	35871841	35872478	KD2-H4K8ac_peak_18906	14.49045	
20	35872902	35873337	KD2-H4K8ac_peak_18907	9.55735	
20	35879920	35880231	KD2-H4K8ac_peak_18908	4.42765	
20	35887783	35888122	KD2-H4K8ac_peak_18909	10.08707	
20	35896522	35896855	KD2-H4K8ac_peak_18910	7.76329	
20	35905041	35905797	KD2-H4K8ac_peak_18911	8.17557	
20	35917555	35917912	KD2-H4K8ac_peak_18912	13.83528	MANBAL_ENSG00000101363
20	35918127	35918445	KD2-H4K8ac_peak_18913	11.04256	MANBAL_ENSG00000101363
20	35931339	35931622	KD2-H4K8ac_peak_18914	5.02756	
20	35940251	35940473	KD2-H4K8ac_peak_18915	7.75971	
20	35942026	35942955	KD2-H4K8ac_peak_18916	12.79505	
20	35943760	35945472	KD2-H4K8ac_peak_18917	15.40367	
20	35946516	35947688	KD2-H4K8ac_peak_18918	12.84642	
20	35954325	35955139	KD2-H4K8ac_peak_18919	12.5605	
20	35956872	35957626	KD2-H4K8ac_peak_18920	5.9696	
20	35958735	35959012	KD2-H4K8ac_peak_18921	6.24114	
20	35959509	35960557	KD2-H4K8ac_peak_18922	14.8083	
20	35960841	35962594	KD2-H4K8ac_peak_18923	24.80313	
20	35964191	35964882	KD2-H4K8ac_peak_18924	11.24702	
20	35965251	35965522	KD2-H4K8ac_peak_18925	11.89502	
20	35965859	35966064	KD2-H4K8ac_peak_18926	6.0073	
20	35968883	35969353	KD2-H4K8ac_peak_18927	11.9948	
20	35969914	35970234	KD2-H4K8ac_peak_18928	5.32933	
20	35972966	35973208	KD2-H4K8ac_peak_18929	4.95872	SRC_ENSG00000197122
20	35973567	35974077	KD2-H4K8ac_peak_18930	10.87093	SRC_ENSG00000197122
20	35974287	35974677	KD2-H4K8ac_peak_18931	8.4716	
20	35974940	35975342	KD2-H4K8ac_peak_18932	13.12288	
20	36007613	36007868	KD2-H4K8ac_peak_18933	7.167	
20	36013288	36013729	KD2-H4K8ac_peak_18934	5.54647	
20	36014206	36014713	KD2-H4K8ac_peak_18935	5.57044	
20	36015160	36015589	KD2-H4K8ac_peak_18936	7.27716	
20	36020778	36021125	KD2-H4K8ac_peak_18937	5.30799	
20	36021450	36021779	KD2-H4K8ac_peak_18938	4.68806	
20	36022252	36022584	KD2-H4K8ac_peak_18939	6.28103	
20	36023368	36024597	KD2-H4K8ac_peak_18940	21.38135	
20	36024937	36027253	KD2-H4K8ac_peak_18941	14.74638	
20	36034650	36035069	KD2-H4K8ac_peak_18942	15.05364	
20	36035768	36036854	KD2-H4K8ac_peak_18943	7.36799	
20	36038033	36038298	KD2-H4K8ac_peak_18944	15.039	
20	36051333	36051575	KD2-H4K8ac_peak_18945	4.42765	
20	36145973	36146242	KD2-H4K8ac_peak_18946	5.18447	
20	36147364	36147655	KD2-H4K8ac_peak_18947	4.23819	
20	36156685	36156926	KD2-H4K8ac_peak_18948	6.67092	BLCAP_ENSG00000166619
20	36162474	36162899	KD2-H4K8ac_peak_18949	8.79106	
20	36192567	36192895	KD2-H4K8ac_peak_18950	7.40111	

20	36226476	36226723	KD2-H4K8ac_peak_18951	4.84794	
20	36340192	36340505	KD2-H4K8ac_peak_18952	5.14125	
20	36531496	36531897	KD2-H4K8ac_peak_18953	12.84642	VSTM2L_ENSG00000132821
20	36539877	36540202	KD2-H4K8ac_peak_18954	6.60096	
20	36540754	36541103	KD2-H4K8ac_peak_18955	7.74292	
20	36543547	36543816	KD2-H4K8ac_peak_18956	8.2413	
20	36544420	36544982	KD2-H4K8ac_peak_18957	6.25869	
20	36616539	36616783	KD2-H4K8ac_peak_18958	5.5051	
20	36661307	36661627	KD2-H4K8ac_peak_18959	10.46264	TTI1_ENSG00000101407;RPRD1B_ENSG00000101413
20	36661922	36662364	KD2-H4K8ac_peak_18960	12.96152	TTI1_ENSG00000101407;RPRD1B_ENSG00000101413
20	36695630	36695932	KD2-H4K8ac_peak_18961	8.30094	
20	36723694	36723893	KD2-H4K8ac_peak_18962	7.70289	
20	36737852	36738158	KD2-H4K8ac_peak_18963	15.13261	
20	36739355	36739582	KD2-H4K8ac_peak_18964	4.54006	
20	36739869	36740119	KD2-H4K8ac_peak_18965	7.70289	
20	36751560	36751789	KD2-H4K8ac_peak_18966	9.16531	
20	36755305	36755574	KD2-H4K8ac_peak_18967	4.211	
20	36767948	36768290	KD2-H4K8ac_peak_18968	10.74057	
20	36768739	36768946	KD2-H4K8ac_peak_18969	6.42097	
20	36779878	36780441	KD2-H4K8ac_peak_18970	10.21366	
20	36781215	36781914	KD2-H4K8ac_peak_18971	13.51732	
20	36793228	36793771	KD2-H4K8ac_peak_18972	9.31907	
20	36888808	36889048	KD2-H4K8ac_peak_18973	9.63487	KIAA1755_ENSG00000149633;BPI_ENSG00000101425
20	37063573	37063943	KD2-H4K8ac_peak_18974	10.66719	SNHG17_ENSG00000196756;SNORA71D_ENSG00000200354
20	37074859	37075066	KD2-H4K8ac_peak_18975	4.70206	SNHG11_ENSG00000174365
20	37075349	37075857	KD2-H4K8ac_peak_18976	13.12288	SNHG11_ENSG00000174365
20	37101522	37101798	KD2-H4K8ac_peak_18977	13.95627	RALGAPB_ENSG00000170471
20	37302508	37302988	KD2-H4K8ac_peak_18978	6.65673	
20	37303277	37303778	KD2-H4K8ac_peak_18979	10.86378	
20	37360080	37360299	KD2-H4K8ac_peak_18980	10.96455	
20	37377150	37377357	KD2-H4K8ac_peak_18981	10.43145	ACTR5_ENSG00000101442
20	37434920	37435113	KD2-H4K8ac_peak_18982	3.87414	PPP1R16B_ENSG00000101445
20	37435727	37436038	KD2-H4K8ac_peak_18983	7.27716	
20	37485001	37485216	KD2-H4K8ac_peak_18984	6.9809	
20	37490669	37490891	KD2-H4K8ac_peak_18985	7.47462	
20	37493173	37494158	KD2-H4K8ac_peak_18986	7.51054	
20	37494384	37495349	KD2-H4K8ac_peak_18987	17.8868	
20	37495789	37496129	KD2-H4K8ac_peak_18988	10.58016	
20	37499375	37500072	KD2-H4K8ac_peak_18989	6.25869	
20	37501846	37503444	KD2-H4K8ac_peak_18990	13.56049	RN7SL116P_ENSG00000240474
20	37504326	37504847	KD2-H4K8ac_peak_18991	10.38558	
20	37505825	37506222	KD2-H4K8ac_peak_18992	9.58924	
20	37507572	37507853	KD2-H4K8ac_peak_18993	6.25869	
20	37509730	37510275	KD2-H4K8ac_peak_18994	13.43171	
20	37511555	37511886	KD2-H4K8ac_peak_18995	12.93209	
20	37512371	37512867	KD2-H4K8ac_peak_18996	7.93362	

20	37513878	37514117	KD2-H4K8ac_peak_18997	4.89359	
20	37518255	37518635	KD2-H4K8ac_peak_18998	8.26945	
20	37522896	37523152	KD2-H4K8ac_peak_18999	7.07512	
20	37590321	37590680	KD2-H4K8ac_peak_19000	4.0528	RP4-616B8.4_ENSG00000261431;DHX35_ENSG00000101452
20	37591013	37591434	KD2-H4K8ac_peak_19001	21.11201	RP4-616B8.4_ENSG00000261431;DHX35_ENSG00000101452
20	37678676	37679456	KD2-H4K8ac_peak_19002	8.52559	
20	38589669	38589946	KD2-H4K8ac_peak_19003	4.89656	
20	38907489	38907700	KD2-H4K8ac_peak_19004	5.57044	
20	38915504	38915985	KD2-H4K8ac_peak_19005	9.15329	
20	39317258	39317740	KD2-H4K8ac_peak_19006	12.06346	MAFB_ENSG00000204103
20	39318109	39318755	KD2-H4K8ac_peak_19007	11.89502	MAFB_ENSG00000204103
20	39525463	39525726	KD2-H4K8ac_peak_19008	6.72021	
20	39531897	39532198	KD2-H4K8ac_peak_19009	6.14257	
20	39597732	39598055	KD2-H4K8ac_peak_19010	5.25071	
20	39658254	39658454	KD2-H4K8ac_peak_19011	6.24114	TOP1_ENSG00000198900
20	39765140	39765432	KD2-H4K8ac_peak_19012	5.08628	PLCG1_ENSG00000124181
20	39765645	39765988	KD2-H4K8ac_peak_19013	22.66567	RP1-1J6.2_ENSG00000226648;PLCG1_ENSG00000124181
20	39766190	39766886	KD2-H4K8ac_peak_19014	12.93209	RP1-1J6.2_ENSG00000226648;PLCG1_ENSG00000124181
20	39945515	39946201	KD2-H4K8ac_peak_19015	11.92978	ZHX3_ENSG00000174306
20	39946562	39946807	KD2-H4K8ac_peak_19016	9.36516	ZHX3_ENSG00000174306
20	40015733	40015931	KD2-H4K8ac_peak_19017	9.11031	
20	40016165	40016430	KD2-H4K8ac_peak_19018	4.211	
20	40246659	40246918	KD2-H4K8ac_peak_19019	12.42761	CHD6_ENSG00000124177
20	40247468	40247774	KD2-H4K8ac_peak_19020	13.73635	CHD6_ENSG00000124177
20	40321059	40322106	KD2-H4K8ac_peak_19021	7.76329	
20	42018851	42019085	KD2-H4K8ac_peak_19022	6.07178	
20	42073960	42074165	KD2-H4K8ac_peak_19023	7.45501	
20	42074851	42075217	KD2-H4K8ac_peak_19024	14.48084	
20	42075518	42075947	KD2-H4K8ac_peak_19025	7.40111	
20	42077250	42077457	KD2-H4K8ac_peak_19026	4.42765	
20	42086582	42087338	KD2-H4K8ac_peak_19027	23.72814	SRSF6_ENSG00000124193
20	42135895	42136668	KD2-H4K8ac_peak_19028	13.88933	L3MBTL1_ENSG00000185513
20	42218821	42219041	KD2-H4K8ac_peak_19029	6.65673	IFT52_ENSG00000101052
20	42219632	42219936	KD2-H4K8ac_peak_19030	12.00273	IFT52_ENSG00000101052
20	42285714	42286109	KD2-H4K8ac_peak_19031	6.40874	
20	42286339	42286598	KD2-H4K8ac_peak_19032	7.07117	
20	42295803	42296167	KD2-H4K8ac_peak_19033	6.56211	MYBL2_ENSG00000101057
20	42375405	42375653	KD2-H4K8ac_peak_19034	6.2781	
20	42542969	42543348	KD2-H4K8ac_peak_19035	14.66631	TOX2_ENSG00000124191
20	42544692	42545118	KD2-H4K8ac_peak_19036	7.74292	
20	42554006	42554341	KD2-H4K8ac_peak_19037	6.44277	
20	42587839	42588079	KD2-H4K8ac_peak_19038	5.4497	
20	42591029	42591314	KD2-H4K8ac_peak_19039	12.77963	
20	42637613	42637907	KD2-H4K8ac_peak_19040	5.57044	
20	42638304	42638604	KD2-H4K8ac_peak_19041	6.40297	
20	42639168	42640003	KD2-H4K8ac_peak_19042	14.8083	

20	42640817	42641200	KD2-H4K8ac_peak_19043	7.47462	
20	42642896	42643390	KD2-H4K8ac_peak_19044	9.16531	
20	42644538	42646718	KD2-H4K8ac_peak_19045	14.69247	
20	42647160	42647558	KD2-H4K8ac_peak_19046	9.05418	
20	42647924	42648363	KD2-H4K8ac_peak_19047	12.74183	
20	42649165	42649460	KD2-H4K8ac_peak_19048	7.76329	
20	42649713	42650055	KD2-H4K8ac_peak_19049	5.14125	
20	42651188	42651503	KD2-H4K8ac_peak_19050	12.3187	
20	42655334	42655604	KD2-H4K8ac_peak_19051	9.1707	
20	42656004	42656260	KD2-H4K8ac_peak_19052	5.54647	
20	42661089	42661452	KD2-H4K8ac_peak_19053	12.16946	
20	42667803	42668096	KD2-H4K8ac_peak_19054	9.36516	
20	42703647	42703924	KD2-H4K8ac_peak_19055	11.02863	
20	42704306	42704751	KD2-H4K8ac_peak_19056	9.1707	
20	42710158	42710525	KD2-H4K8ac_peak_19057	9.40857	
20	42839033	42839332	KD2-H4K8ac_peak_19058	4.54006	OSER1_ENSG00000132823;OSER1-AS1_ENSG00000223891
20	42875902	42876261	KD2-H4K8ac_peak_19059	11.89502	GDAP1L1_ENSG00000124194
20	42924679	42924930	KD2-H4K8ac_peak_19060	4.65111	
20	42939436	42939796	KD2-H4K8ac_peak_19061	10.7186	FITM2_ENSG00000197296
20	42940063	42940415	KD2-H4K8ac_peak_19062	5.1707	FITM2_ENSG00000197296
20	42964705	42965061	KD2-H4K8ac_peak_19063	4.42765	R3HDML_ENSG00000101074
20	43031185	43031460	KD2-H4K8ac_peak_19064	7.76329	
20	43104023	43105274	KD2-H4K8ac_peak_19065	13.73635	TTPAL_ENSG00000124120
20	43159954	43160384	KD2-H4K8ac_peak_19066	10.73134	PKIG_ENSG00000168734
20	43270627	43271202	KD2-H4K8ac_peak_19067	5.88845	
20	43271493	43271719	KD2-H4K8ac_peak_19068	9.402	
20	43280022	43280771	KD2-H4K8ac_peak_19069	15.25814	ADA_ENSG00000196839
20	43367638	43368352	KD2-H4K8ac_peak_19070	8.55271	
20	43374310	43375014	KD2-H4K8ac_peak_19071	11.79585	RP11-445H22.4_ENSG00000244558;KCNK15_ENSG00000124249
20	43388668	43388886	KD2-H4K8ac_peak_19072	7.76329	
20	43389914	43390311	KD2-H4K8ac_peak_19073	3.97079	
20	43390768	43391055	KD2-H4K8ac_peak_19074	8.16906	
20	43392018	43392245	KD2-H4K8ac_peak_19075	6.11333	
20	43438194	43438459	KD2-H4K8ac_peak_19076	4.54006	RIMS4_ENSG00000101098
20	43438666	43438947	KD2-H4K8ac_peak_19077	6.30229	RIMS4_ENSG00000101098
20	43439252	43439671	KD2-H4K8ac_peak_19078	7.49599	RIMS4_ENSG00000101098
20	43496058	43496314	KD2-H4K8ac_peak_19079	9.15329	
20	43514462	43514682	KD2-H4K8ac_peak_19080	10.66719	YWHAB_ENSG00000166913
20	43538774	43539132	KD2-H4K8ac_peak_19081	13.34404	PABPC1L_ENSG00000101104
20	43539425	43539650	KD2-H4K8ac_peak_19082	7.12754	PABPC1L_ENSG00000101104
20	43588730	43588991	KD2-H4K8ac_peak_19083	11.03331	TOMM34_ENSG00000025772
20	43594564	43595000	KD2-H4K8ac_peak_19084	7.1181	STK4-AS1_ENSG00000227477;STK4_ENSG00000101109
20	43595199	43595508	KD2-H4K8ac_peak_19085	8.16906	STK4-AS1_ENSG00000227477;STK4_ENSG00000101109
20	43921855	43922499	KD2-H4K8ac_peak_19086	9.68068	
20	43970940	43971140	KD2-H4K8ac_peak_19087	8.10216	
20	43976602	43976983	KD2-H4K8ac_peak_19088	13.1684	SDC4_ENSG00000124145

20	43991081	43991392	KD2-H4K8ac_peak_19089	12.0857	SYS1_ENSG00000204070;SYS1-DBNDD2_ENSG00000254806
20	44034640	44036017	KD2-H4K8ac_peak_19090	8.86099	TP53TG5_ENSG00000124251;DBNDD2_ENSG00000244274
20	44044137	44044599	KD2-H4K8ac_peak_19091	6.31143	PIGT_ENSG00000124155
20	44044805	44045196	KD2-H4K8ac_peak_19092	9.73431	PIGT_ENSG00000124155
20	44099262	44099717	KD2-H4K8ac_peak_19093	4.06758	WFDC2_ENSG00000101443
20	44400048	44400683	KD2-H4K8ac_peak_19094	15.5861	
20	44420022	44420445	KD2-H4K8ac_peak_19095	7.47462	WFDC3_ENSG00000124116;DNTTIP1_ENSG00000101457
20	44441326	44441817	KD2-H4K8ac_peak_19096	6.52179	UBE2C_ENSG00000175063
20	44462067	44462318	KD2-H4K8ac_peak_19097	5.75757	TNNC2_ENSG00000101470;SNX21_ENSG00000124104
20	44462785	44463239	KD2-H4K8ac_peak_19098	7.95563	TNNC2_ENSG00000101470;SNX21_ENSG00000124104
20	44485738	44485952	KD2-H4K8ac_peak_19099	8.55271	ACOT8_ENSG00000101473;ZSWIM3_ENSG00000132801
20	44486313	44486830	KD2-H4K8ac_peak_19100	13.9234	ACOT8_ENSG00000101473;ZSWIM3_ENSG00000132801
20	44508970	44509557	KD2-H4K8ac_peak_19101	6.36319	ZSWIM1_ENSG00000168612
20	44518925	44520190	KD2-H4K8ac_peak_19102	20.96279	NEURL2_ENSG00000124257;CTSA_ENSG00000064601
20	44540859	44541434	KD2-H4K8ac_peak_19103	8.09472	PLTP_ENSG00000100979
20	44563088	44563713	KD2-H4K8ac_peak_19104	16.57619	PCIF1_ENSG00000100982
20	44599848	44601050	KD2-H4K8ac_peak_19105	19.43192	ZNF335_ENSG00000198026
20	44601537	44601749	KD2-H4K8ac_peak_19106	6.24747	ZNF335_ENSG00000198026
20	44650288	44650528	KD2-H4K8ac_peak_19107	14.56955	RP11-465L10.10_ENSG00000204044;SLC12A5_ENSG00000124140
20	44718074	44718575	KD2-H4K8ac_peak_19108	17.42905	NCOA5_ENSG00000124160
20	44767645	44768399	KD2-H4K8ac_peak_19109	10.96455	
20	44768679	44769054	KD2-H4K8ac_peak_19110	6.66359	
20	44777691	44777912	KD2-H4K8ac_peak_19111	4.33002	
20	44960206	44960556	KD2-H4K8ac_peak_19112	5.20908	
20	44960992	44961192	KD2-H4K8ac_peak_19113	5.13615	
20	44962803	44963005	KD2-H4K8ac_peak_19114	3.75035	
20	44992622	44993067	KD2-H4K8ac_peak_19115	30.37291	SLC35C2_ENSG00000080189
20	45141835	45142159	KD2-H4K8ac_peak_19116	13.12288	ZNF334_ENSG00000198185
20	45190390	45190586	KD2-H4K8ac_peak_19117	5.57044	
20	45190808	45191215	KD2-H4K8ac_peak_19118	9.63487	
20	45191483	45191690	KD2-H4K8ac_peak_19119	10.96455	
20	45200741	45201012	KD2-H4K8ac_peak_19120	6.25869	
20	45337934	45338848	KD2-H4K8ac_peak_19121	18.54875	SLC2A10_ENSG00000197496
20	45479055	45479320	KD2-H4K8ac_peak_19122	8.1233	
20	45592266	45592486	KD2-H4K8ac_peak_19123	7.40314	
20	45599893	45600098	KD2-H4K8ac_peak_19124	6.00243	
20	45660490	45660687	KD2-H4K8ac_peak_19125	3.75035	
20	45664560	45664843	KD2-H4K8ac_peak_19126	9.1707	
20	45870828	45871089	KD2-H4K8ac_peak_19127	3.69804	
20	45891052	45891270	KD2-H4K8ac_peak_19128	4.44923	
20	45898562	45898821	KD2-H4K8ac_peak_19129	5.25071	
20	45981339	45981614	KD2-H4K8ac_peak_19130	6.40297	RP4-569M23.2_ENSG00000231119
20	45985659	45986849	KD2-H4K8ac_peak_19131	29.92854	ZMYND8_ENSG00000101040
20	45987497	45987922	KD2-H4K8ac_peak_19132	8.85813	
20	45988860	45990437	KD2-H4K8ac_peak_19133	11.05467	
20	45991203	45991469	KD2-H4K8ac_peak_19134	5.82859	

20	46096959	46097153	KD2-H4K8ac_peak_19135	6.2781	
20	46121470	46121680	KD2-H4K8ac_peak_19136	4.75764	RNU6-563P_ENSG00000201742
20	46130768	46131026	KD2-H4K8ac_peak_19137	10.30284	NCOA3_ENSG00000124151
20	46204938	46205133	KD2-H4K8ac_peak_19138	5.55317	
20	46336223	46336487	KD2-H4K8ac_peak_19139	9.96941	
20	46339206	46339479	KD2-H4K8ac_peak_19140	7.35686	
20	46429100	46429818	KD2-H4K8ac_peak_19141	4.211	
20	46586050	46586718	KD2-H4K8ac_peak_19142	14.12659	RP11-347D21.2_ENSG00000228503
20	46601366	46601607	KD2-H4K8ac_peak_19143	4.42765	
20	46653951	46654225	KD2-H4K8ac_peak_19144	7.47462	
20	46915774	46916066	KD2-H4K8ac_peak_19145	6.56926	
20	46996261	46996662	KD2-H4K8ac_peak_19146	6.40683	
20	47096406	47096795	KD2-H4K8ac_peak_19147	6.72021	
20	47236719	47237089	KD2-H4K8ac_peak_19148	9.20193	
20	47298684	47299172	KD2-H4K8ac_peak_19149	7.61026	
20	47367899	47368097	KD2-H4K8ac_peak_19150	6.40683	
20	47444801	47445228	KD2-H4K8ac_peak_19151	14.63458	PREX1_ENSG00000124126
20	47463234	47463667	KD2-H4K8ac_peak_19152	5.01002	
20	47537470	47537930	KD2-H4K8ac_peak_19153	5.30799	ARFGEF2_ENSG00000124198
20	47538337	47539197	KD2-H4K8ac_peak_19154	9.89126	ARFGEF2_ENSG00000124198
20	47662948	47663214	KD2-H4K8ac_peak_19155	12.15474	RP1-155G6.4_ENSG00000227431;CSE1L_ENSG00000124207
20	47804407	47804630	KD2-H4K8ac_peak_19156	6.00226	STAU1_ENSG00000124214
20	47835070	47835358	KD2-H4K8ac_peak_19157	5.57044	DDX27_ENSG00000124228
20	47894700	47894950	KD2-H4K8ac_peak_19158	9.63487	ZNFX1_ENSG00000124201;ZFAS1_ENSG00000177410;SNORD12C_ENSG00000209042
20	47962264	47962480	KD2-H4K8ac_peak_19159	4.86824	
20	48004321	48004888	KD2-H4K8ac_peak_19160	5.30799	
20	48066621	48066864	KD2-H4K8ac_peak_19161	7.59643	
20	48184075	48184564	KD2-H4K8ac_peak_19162	15.25814	PTGIS_ENSG00000124212
20	48330060	48330340	KD2-H4K8ac_peak_19163	19.39805	B4GALT5_ENSG00000158470
20	48364191	48364394	KD2-H4K8ac_peak_19164	6.03693	
20	48364650	48364848	KD2-H4K8ac_peak_19165	5.88845	
20	48429075	48429644	KD2-H4K8ac_peak_19166	11.70879	SLC9A8_ENSG00000197818
20	48531645	48532792	KD2-H4K8ac_peak_19167	16.822	SPATA2_ENSG00000158480
20	48551878	48552605	KD2-H4K8ac_peak_19168	12.08081	RNF114_ENSG00000124226
20	48595763	48596342	KD2-H4K8ac_peak_19169	8.55271	
20	48605790	48607195	KD2-H4K8ac_peak_19170	9.90914	
20	48630441	48631375	KD2-H4K8ac_peak_19171	8.55271	
20	48631663	48632532	KD2-H4K8ac_peak_19172	12.57143	
20	48632827	48633171	KD2-H4K8ac_peak_19173	8.4716	
20	48636155	48636363	KD2-H4K8ac_peak_19174	6.05809	
20	48645858	48647055	KD2-H4K8ac_peak_19175	12.42761	
20	48657079	48657949	KD2-H4K8ac_peak_19176	6.65673	TRERNA1_ENSG00000231265
20	48663462	48663748	KD2-H4K8ac_peak_19177	8.10216	
20	48669255	48669705	KD2-H4K8ac_peak_19178	4.06758	
20	48669942	48670727	KD2-H4K8ac_peak_19179	4.54006	
20	48672294	48672516	KD2-H4K8ac_peak_19180	10.05606	

20	48673871	48674075	KD2-H4K8ac_peak_19181	3.97079	
20	48687333	48687547	KD2-H4K8ac_peak_19182	3.65228	
20	48692326	48692555	KD2-H4K8ac_peak_19183	8.21084	
20	48729200	48730270	KD2-H4K8ac_peak_19184	9.95119	
20	48732847	48733386	KD2-H4K8ac_peak_19185	11.35142	UBE2V1_ENSG00000244687
20	48769766	48770133	KD2-H4K8ac_peak_19186	4.06758	TMEM189-UBE2V1_ENSG00000124208;TMEM189_ENSG00000240849
20	48770495	48770728	KD2-H4K8ac_peak_19187	6.84261	TMEM189-UBE2V1_ENSG00000124208;TMEM189_ENSG00000240849
20	48770950	48771166	KD2-H4K8ac_peak_19188	4.86324	TMEM189-UBE2V1_ENSG00000124208;TMEM189_ENSG00000240849
20	48782803	48783127	KD2-H4K8ac_peak_19189	6.94954	RP11-112L6.3_ENSG00000237595;RP11-112L6.2_ENSG00000234698
20	48806565	48806903	KD2-H4K8ac_peak_19190	7.76329	CEBPB_ENSG00000172216
20	48807674	48808732	KD2-H4K8ac_peak_19191	8.16906	CEBPB_ENSG00000172216
20	48809545	48809781	KD2-H4K8ac_peak_19192	11.5673	
20	48890182	48890388	KD2-H4K8ac_peak_19193	6.30229	
20	48912887	48913089	KD2-H4K8ac_peak_19194	5.88845	
20	48920213	48921270	KD2-H4K8ac_peak_19195	9.06369	
20	48921609	48921841	KD2-H4K8ac_peak_19196	5.92591	
20	48922905	48923160	KD2-H4K8ac_peak_19197	6.60096	
20	48925443	48925825	KD2-H4K8ac_peak_19198	13.70753	
20	48995301	48995822	KD2-H4K8ac_peak_19199	5.14125	
20	49031611	49032321	KD2-H4K8ac_peak_19200	15.61313	
20	49032586	49032856	KD2-H4K8ac_peak_19201	6.90231	
20	49115605	49115954	KD2-H4K8ac_peak_19202	8.02571	
20	49126283	49126577	KD2-H4K8ac_peak_19203	6.84261	PTPN1_ENSG00000196396
20	49244804	49245631	KD2-H4K8ac_peak_19204	12.60538	
20	49252264	49252461	KD2-H4K8ac_peak_19205	8.98477	
20	49271244	49271442	KD2-H4K8ac_peak_19206	5.51783	
20	49272387	49272696	KD2-H4K8ac_peak_19207	5.37864	
20	49307580	49308167	KD2-H4K8ac_peak_19208	7.76329	FAM65C_ENSG00000042062
20	49332288	49332504	KD2-H4K8ac_peak_19209	4.04247	
20	49341166	49341649	KD2-H4K8ac_peak_19210	11.05407	
20	49347189	49347623	KD2-H4K8ac_peak_19211	15.13261	PARD6B_ENSG00000124171
20	49348026	49348400	KD2-H4K8ac_peak_19212	10.46874	PARD6B_ENSG00000124171
20	49379857	49380184	KD2-H4K8ac_peak_19213	11.03331	
20	49407051	49407870	KD2-H4K8ac_peak_19214	14.19272	
20	49411848	49412098	KD2-H4K8ac_peak_19215	9.70057	BCAS4_ENSG00000124243
20	49546704	49547735	KD2-H4K8ac_peak_19216	11.80497	ADNP_ENSG00000101126;RP5-914P20.5_ENSG00000259456
20	49575537	49575856	KD2-H4K8ac_peak_19217	7.36799	DPM1_ENSG00000000419;MOCS3_ENSG00000124217
20	49639263	49640367	KD2-H4K8ac_peak_19218	15.82362	KCNG1_ENSG00000026559
20	49711647	49711852	KD2-H4K8ac_peak_19219	7.87794	
20	49737177	49737651	KD2-H4K8ac_peak_19220	5.8536	
20	49742518	49742739	KD2-H4K8ac_peak_19221	8.02571	
20	49749052	49749275	KD2-H4K8ac_peak_19222	5.54647	RPSAP1_ENSG00000228820
20	49755487	49755911	KD2-H4K8ac_peak_19223	5.88845	
20	49772843	49773047	KD2-H4K8ac_peak_19224	10.46264	
20	49774688	49774959	KD2-H4K8ac_peak_19225	5.32933	
20	49854483	49854729	KD2-H4K8ac_peak_19226	5.57044	

20	50029092	50029390	KD2-H4K8ac_peak_19227	11.05407	
20	50061405	50061859	KD2-H4K8ac_peak_19228	7.70289	
20	50073788	50074033	KD2-H4K8ac_peak_19229	8.37043	
20	50079300	50079603	KD2-H4K8ac_peak_19230	8.59579	
20	50416962	50417245	KD2-H4K8ac_peak_19231	6.1228	
20	50417565	50418611	KD2-H4K8ac_peak_19232	20.47312	SALL4_ENSG00000101115
20	50602088	50602484	KD2-H4K8ac_peak_19233	10.21502	
20	50807688	50808961	KD2-H4K8ac_peak_19234	13.22091	
20	51098749	51098964	KD2-H4K8ac_peak_19235	5.71749	
20	51512808	51513000	KD2-H4K8ac_peak_19236	4.3015	
20	51576121	51576334	KD2-H4K8ac_peak_19237	4.56666	
20	51589965	51590287	KD2-H4K8ac_peak_19238	5.30799	
20	51787059	51787589	KD2-H4K8ac_peak_19239	7.27716	
20	52110544	52110843	KD2-H4K8ac_peak_19240	6.2159	
20	52209484	52210187	KD2-H4K8ac_peak_19241	10.86378	
20	52210579	52210775	KD2-H4K8ac_peak_19242	10.63441	
20	52238255	52238483	KD2-H4K8ac_peak_19243	5.57044	
20	52239923	52240466	KD2-H4K8ac_peak_19244	6.99815	
20	52354329	52354561	KD2-H4K8ac_peak_19245	7.12754	
20	52530812	52531691	KD2-H4K8ac_peak_19246	6.16888	
20	52532078	52532358	KD2-H4K8ac_peak_19247	8.30974	
20	52533149	52533377	KD2-H4K8ac_peak_19248	4.98072	
20	52558703	52559056	KD2-H4K8ac_peak_19249	4.86324	
20	52630534	52630920	KD2-H4K8ac_peak_19250	5.25071	
20	52824325	52825151	KD2-H4K8ac_peak_19251	9.20193	PFDN4_ENSG00000101132
20	53298506	53298827	KD2-H4K8ac_peak_19252	6.2781	
20	53459086	53459338	KD2-H4K8ac_peak_19253	8.10216	
20	53653102	53653315	KD2-H4K8ac_peak_19254	8.02571	
20	53711148	53711380	KD2-H4K8ac_peak_19255	8.45238	
20	54052983	54053220	KD2-H4K8ac_peak_19256	7.12754	
20	54053848	54054095	KD2-H4K8ac_peak_19257	11.30637	
20	54056014	54056225	KD2-H4K8ac_peak_19258	7.79898	
20	54092918	54093126	KD2-H4K8ac_peak_19259	7.27716	
20	54096528	54097298	KD2-H4K8ac_peak_19260	4.44527	
20	54365361	54365567	KD2-H4K8ac_peak_19261	8.1233	
20	54763148	54763409	KD2-H4K8ac_peak_19262	6.31143	
20	54935261	54935474	KD2-H4K8ac_peak_19263	7.47462	
20	54967045	54967237	KD2-H4K8ac_peak_19264	8.55271	AURKA_ENSG00000087586;CSTF1_ENSG00000101138
20	54967775	54968076	KD2-H4K8ac_peak_19265	4.211	AURKA_ENSG00000087586;CSTF1_ENSG00000101138
20	55033393	55033932	KD2-H4K8ac_peak_19266	6.94255	RPL39P_ENSG00000228601
20	55776778	55777161	KD2-H4K8ac_peak_19267	7.27716	
20	55796220	55796426	KD2-H4K8ac_peak_19268	9.1994	
20	55816369	55816825	KD2-H4K8ac_peak_19269	10.0692	
20	55817021	55818770	KD2-H4K8ac_peak_19270	15.22643	
20	55819076	55819729	KD2-H4K8ac_peak_19271	11.22681	
20	55822073	55822277	KD2-H4K8ac_peak_19272	6.72334	

20	55824265	55824512	KD2-H4K8ac_peak_19273	6.40683	
20	55826035	55826295	KD2-H4K8ac_peak_19274	3.82395	
20	55826500	55826847	KD2-H4K8ac_peak_19275	9.06369	
20	55835462	55836150	KD2-H4K8ac_peak_19276	5.58324	
20	55840273	55841280	KD2-H4K8ac_peak_19277	9.16531	BMP7_ENSG00000101144;RP4-813D12.3_ENSG00000226308
20	55841918	55842266	KD2-H4K8ac_peak_19278	6.66692	BMP7_ENSG00000101144;RP4-813D12.3_ENSG00000226308
20	55848208	55848456	KD2-H4K8ac_peak_19279	6.76298	
20	55848690	55849428	KD2-H4K8ac_peak_19280	6.72334	
20	55926396	55926809	KD2-H4K8ac_peak_19281	9.36516	RAE1_ENSG00000101146
20	55965620	55966252	KD2-H4K8ac_peak_19282	9.06369	RBM38_ENSG00000132819
20	55966579	55966878	KD2-H4K8ac_peak_19283	7.47462	RBM38_ENSG00000132819
20	56015720	56016784	KD2-H4K8ac_peak_19284	7.36799	
20	56025814	56026390	KD2-H4K8ac_peak_19285	7.40314	
20	56027493	56027705	KD2-H4K8ac_peak_19286	5.43522	
20	56046438	56046713	KD2-H4K8ac_peak_19287	5.14125	
20	56171809	56172120	KD2-H4K8ac_peak_19288	11.02863	
20	56203655	56204035	KD2-H4K8ac_peak_19289	11.43863	
20	56241652	56242557	KD2-H4K8ac_peak_19290	5.01002	
20	56242958	56243300	KD2-H4K8ac_peak_19291	6.60096	
20	56243698	56244058	KD2-H4K8ac_peak_19292	4.211	
20	56245286	56245634	KD2-H4K8ac_peak_19293	4.06758	
20	56247568	56247998	KD2-H4K8ac_peak_19294	7.20567	
20	56257186	56257652	KD2-H4K8ac_peak_19295	8.33649	
20	56259428	56259766	KD2-H4K8ac_peak_19296	11.92978	
20	56260756	56261760	KD2-H4K8ac_peak_19297	7.47462	
20	56284347	56284557	KD2-H4K8ac_peak_19298	7.11925	
20	56321819	56322364	KD2-H4K8ac_peak_19299	9.1707	
20	56323188	56323394	KD2-H4K8ac_peak_19300	5.75757	
20	56324067	56324607	KD2-H4K8ac_peak_19301	12.93253	
20	56383293	56383510	KD2-H4K8ac_peak_19302	5.75452	
20	56412639	56412859	KD2-H4K8ac_peak_19303	5.61067	
20	56413149	56413802	KD2-H4K8ac_peak_19304	16.57619	
20	56414355	56415373	KD2-H4K8ac_peak_19305	11.66477	
20	56441770	56441962	KD2-H4K8ac_peak_19306	6.8836	
20	56457516	56457989	KD2-H4K8ac_peak_19307	14.06758	
20	56504938	56505501	KD2-H4K8ac_peak_19308	5.88845	
20	56569007	56569292	KD2-H4K8ac_peak_19309	11.86082	
20	56574417	56574685	KD2-H4K8ac_peak_19310	4.06758	
20	56576234	56576428	KD2-H4K8ac_peak_19311	4.86324	
20	56576801	56577045	KD2-H4K8ac_peak_19312	8.26945	
20	56613183	56613685	KD2-H4K8ac_peak_19313	15.81711	
20	56614493	56615474	KD2-H4K8ac_peak_19314	9.95119	
20	56618123	56618462	KD2-H4K8ac_peak_19315	10.99466	
20	56619848	56620451	KD2-H4K8ac_peak_19316	10.70006	
20	56621316	56621786	KD2-H4K8ac_peak_19317	18.82114	
20	56622477	56623190	KD2-H4K8ac_peak_19318	13.81781	

20	56662219	56662413	KD2-H4K8ac_peak_19319	4.42765	
20	56706075	56706375	KD2-H4K8ac_peak_19320	4.44923	
20	56749478	56750059	KD2-H4K8ac_peak_19321	7.07117	
20	56750607	56750857	KD2-H4K8ac_peak_19322	11.09936	
20	56803134	56803637	KD2-H4K8ac_peak_19323	9.08816	ANKRD60_ENSG00000124227
20	56883745	56884517	KD2-H4K8ac_peak_19324	16.32216	PPP4R1L_ENSG00000124224;RAB22A_ENSG00000124209
20	56884796	56885237	KD2-H4K8ac_peak_19325	15.81711	PPP4R1L_ENSG00000124224;RAB22A_ENSG00000124209
20	56964406	56964798	KD2-H4K8ac_peak_19326	8.10216	VAPB_ENSG00000124164
20	57032940	57033152	KD2-H4K8ac_peak_19327	7.40314	
20	57046205	57046459	KD2-H4K8ac_peak_19328	6.84261	
20	57046977	57047328	KD2-H4K8ac_peak_19329	5.1707	
20	57186482	57187026	KD2-H4K8ac_peak_19330	7.27716	
20	57210250	57210606	KD2-H4K8ac_peak_19331	5.57044	MGC4294_ENSG00000268941
20	57225036	57225515	KD2-H4K8ac_peak_19332	4.67342	STX16_ENSG00000124222;STX16-NPEPL1_ENSG00000254995
20	57225944	57226669	KD2-H4K8ac_peak_19333	15.82362	STX16_ENSG00000124222;STX16-NPEPL1_ENSG00000254995
20	57452298	57452967	KD2-H4K8ac_peak_19334	13.92307	RP1-309F20.4_ENSG00000270951
20	57454987	57455317	KD2-H4K8ac_peak_19335	6.05809	
20	57464024	57464243	KD2-H4K8ac_peak_19336	6.56211	RP1-309F20.3_ENSG00000225806
20	57465578	57465876	KD2-H4K8ac_peak_19337	6.52179	
20	57466202	57466442	KD2-H4K8ac_peak_19338	4.56666	
20	57466793	57467316	KD2-H4K8ac_peak_19339	5.14125	
20	57473764	57473978	KD2-H4K8ac_peak_19340	6.4095	
20	57555523	57556145	KD2-H4K8ac_peak_19341	7.76329	NELFCD_ENSG00000101158
20	57556387	57556585	KD2-H4K8ac_peak_19342	10.46874	NELFCD_ENSG00000101158
20	57607081	57607307	KD2-H4K8ac_peak_19343	6.40874	ATP5E_ENSG00000124172
20	57617242	57617781	KD2-H4K8ac_peak_19344	5.08628	SLMO2_ENSG00000101166
20	57618126	57618556	KD2-H4K8ac_peak_19345	5.9696	SLMO2_ENSG00000101166
20	57638493	57638717	KD2-H4K8ac_peak_19346	7.12754	
20	57797021	57797487	KD2-H4K8ac_peak_19347	8.53092	
20	57893542	57893736	KD2-H4K8ac_peak_19348	7.48958	
20	57896910	57897221	KD2-H4K8ac_peak_19349	4.70206	
20	57931283	57931706	KD2-H4K8ac_peak_19350	6.2781	
20	57965908	57966229	KD2-H4K8ac_peak_19351	7.95563	
20	57974616	57974901	KD2-H4K8ac_peak_19352	3.85282	
20	57978365	57978901	KD2-H4K8ac_peak_19353	13.36684	
20	57987664	57987887	KD2-H4K8ac_peak_19354	4.67342	
20	57999110	57999328	KD2-H4K8ac_peak_19355	5.14125	
20	58001584	58001788	KD2-H4K8ac_peak_19356	5.14125	
20	58146828	58147385	KD2-H4K8ac_peak_19357	13.73635	
20	58152220	58152763	KD2-H4K8ac_peak_19358	6.83936	PHACTR3_ENSG00000087495
20	58178946	58179447	KD2-H4K8ac_peak_19359	12.84642	
20	58179744	58180810	KD2-H4K8ac_peak_19360	6.65673	
20	58508376	58508741	KD2-H4K8ac_peak_19361	13.36684	SYCP2_ENSG00000196074;FAM217B_ENSG00000196227
20	58508970	58509179	KD2-H4K8ac_peak_19362	8.4716	SYCP2_ENSG00000196074;FAM217B_ENSG00000196227
20	58514593	58514791	KD2-H4K8ac_peak_19363	6.2159	PPP1R3D_ENSG00000132825
20	58516101	58516339	KD2-H4K8ac_peak_19364	5.9696	PPP1R3D_ENSG00000132825

20	60640665	60640858	KD2-H4K8ac_peak_19365	12.04009	TAF4_ENSG00000130699;hsa-mir-3195_ENSG00000265306
20	60641527	60641756	KD2-H4K8ac_peak_19366	6.66692	TAF4_ENSG00000130699
20	60697628	60697906	KD2-H4K8ac_peak_19367	4.42765	LSM14B_ENSG00000149657
20	60717769	60718789	KD2-H4K8ac_peak_19368	20.6423	PSMA7_ENSG00000101182;SS18L1_ENSG00000184402
20	60719103	60719364	KD2-H4K8ac_peak_19369	10.86318	PSMA7_ENSG00000101182;SS18L1_ENSG00000184402
20	60751536	60752265	KD2-H4K8ac_peak_19370	12.06346	
20	60752675	60752938	KD2-H4K8ac_peak_19371	7.47462	
20	60757574	60758738	KD2-H4K8ac_peak_19372	14.8083	MTG2_ENSG00000101181
20	60785088	60785376	KD2-H4K8ac_peak_19373	5.02474	
20	60785618	60786079	KD2-H4K8ac_peak_19374	7.47462	
20	60877570	60877777	KD2-H4K8ac_peak_19375	8.79106	ADRM1_ENSG00000130706
20	60878119	60878689	KD2-H4K8ac_peak_19376	7.74292	ADRM1_ENSG00000130706
20	60927087	60927616	KD2-H4K8ac_peak_19377	9.49967	RP11-157P1.5_ENSG00000228812
20	60928205	60928885	KD2-H4K8ac_peak_19378	12.00597	RP11-157P1.5_ENSG00000228812
20	60929530	60930465	KD2-H4K8ac_peak_19379	10.73134	
20	60930718	60931085	KD2-H4K8ac_peak_19380	9.57101	
20	60931619	60932489	KD2-H4K8ac_peak_19381	6.20982	
20	60941805	60943935	KD2-H4K8ac_peak_19382	18.86697	LAMA5_ENSG00000130702
20	60945614	60946065	KD2-H4K8ac_peak_19383	8.16906	
20	60947572	60947980	KD2-H4K8ac_peak_19384	5.94136	
20	60961430	60961846	KD2-H4K8ac_peak_19385	8.85813	RPS21_ENSG00000171858
20	60962286	60962866	KD2-H4K8ac_peak_19386	11.09936	RPS21_ENSG00000171858
20	60981880	60982151	KD2-H4K8ac_peak_19387	10.08707	CABLES2_ENSG00000149679
20	60982510	60983015	KD2-H4K8ac_peak_19388	19.55721	CABLES2_ENSG00000149679
20	60983286	60983538	KD2-H4K8ac_peak_19389	5.54647	CABLES2_ENSG00000149679
20	60985526	60985929	KD2-H4K8ac_peak_19390	5.75757	
20	61071037	61071420	KD2-H4K8ac_peak_19391	7.29632	
20	61265392	61265666	KD2-H4K8ac_peak_19392	6.2781	RP13-30A9.2_ENSG00000229882
20	61270175	61270438	KD2-H4K8ac_peak_19393	8.1718	
20	61273248	61273653	KD2-H4K8ac_peak_19394	7.73264	SLCO4A1_ENSG00000101187
20	61273849	61274779	KD2-H4K8ac_peak_19395	8.59301	RP11-93B14.6_ENSG00000167046;SLCO4A1_ENSG00000101187
20	61278308	61278545	KD2-H4K8ac_peak_19396	9.96798	
20	61279182	61279900	KD2-H4K8ac_peak_19397	7.40314	
20	61280437	61280710	KD2-H4K8ac_peak_19398	5.9696	
20	61281163	61281409	KD2-H4K8ac_peak_19399	7.44324	
20	61281729	61282348	KD2-H4K8ac_peak_19400	16.71655	
20	61283103	61284364	KD2-H4K8ac_peak_19401	11.24702	
20	61306031	61306660	KD2-H4K8ac_peak_19402	17.63891	
20	61307039	61307297	KD2-H4K8ac_peak_19403	5.88845	
20	61343030	61343241	KD2-H4K8ac_peak_19404	7.07117	
20	61407953	61408235	KD2-H4K8ac_peak_19405	7.96322	
20	61408667	61408947	KD2-H4K8ac_peak_19406	8.17902	
20	61415766	61416095	KD2-H4K8ac_peak_19407	4.77079	
20	61416954	61417167	KD2-H4K8ac_peak_19408	4.42765	
20	61417449	61417715	KD2-H4K8ac_peak_19409	4.67342	
20	61426870	61427332	KD2-H4K8ac_peak_19410	16.89856	MRGBP_ENSG00000101189

20	61427876	61428107	KD2-H4K8ac_peak_19411	13.21541	MRGBP_ENSG00000101189
20	61435535	61435912	KD2-H4K8ac_peak_19412	14.8083	OGFR_ENSG00000060491
20	61448733	61451757	KD2-H4K8ac_peak_19413	13.95627	
20	61454876	61455105	KD2-H4K8ac_peak_19414	6.45083	
20	61456017	61456338	KD2-H4K8ac_peak_19415	8.55271	
20	61456649	61457093	KD2-H4K8ac_peak_19416	5.9696	
20	61507153	61507870	KD2-H4K8ac_peak_19417	13.44374	
20	61508386	61508769	KD2-H4K8ac_peak_19418	5.88845	
20	61557254	61557834	KD2-H4K8ac_peak_19419	9.95119	
20	61568863	61569210	KD2-H4K8ac_peak_19420	11.80283	DIDO1_ENSG00000101191;GID8_ENSG00000101193
20	61569488	61569968	KD2-H4K8ac_peak_19421	27.41885	DIDO1_ENSG00000101191;GID8_ENSG00000101193
20	61846802	61847241	KD2-H4K8ac_peak_19422	4.67342	YTHDF1_ENSG00000149658
20	61847829	61848099	KD2-H4K8ac_peak_19423	9.95119	YTHDF1_ENSG00000149658
20	61904216	61904710	KD2-H4K8ac_peak_19424	7.47462	NKAIN4_ENSG00000101198;ARFGAP1_ENSG00000101199
20	61940696	61940957	KD2-H4K8ac_peak_19425	4.86324	
20	62047552	62048064	KD2-H4K8ac_peak_19426	12.84642	
20	62048355	62048613	KD2-H4K8ac_peak_19427	8.4716	
20	62084481	62085320	KD2-H4K8ac_peak_19428	12.82393	
20	62085564	62086527	KD2-H4K8ac_peak_19429	21.14153	
20	62086735	62087212	KD2-H4K8ac_peak_19430	15.12992	
20	62087448	62088227	KD2-H4K8ac_peak_19431	17.51891	
20	62088584	62089651	KD2-H4K8ac_peak_19432	20.30085	
20	62089888	62090307	KD2-H4K8ac_peak_19433	6.12046	
20	62093640	62094139	KD2-H4K8ac_peak_19434	5.8057	
20	62094561	62095189	KD2-H4K8ac_peak_19435	10.20041	
20	62095647	62095872	KD2-H4K8ac_peak_19436	7.28827	
20	62102511	62102846	KD2-H4K8ac_peak_19437	6.30607	
20	62103342	62103950	KD2-H4K8ac_peak_19438	3.97079	KCNQ2_ENSG00000075043
20	62104481	62104799	KD2-H4K8ac_peak_19439	7.76329	KCNQ2_ENSG00000075043
20	62113388	62113808	KD2-H4K8ac_peak_19440	8.23274	
20	62114181	62114427	KD2-H4K8ac_peak_19441	4.7158	
20	62115156	62115514	KD2-H4K8ac_peak_19442	6.66692	
20	62128022	62128280	KD2-H4K8ac_peak_19443	5.75452	
20	62129615	62130962	KD2-H4K8ac_peak_19444	17.8868	EEF1A2_ENSG00000101210
20	62133653	62134261	KD2-H4K8ac_peak_19445	11.24702	RP4-697K14.3_ENSG00000230226
20	62150936	62151271	KD2-H4K8ac_peak_19446	6.65673	PPDPF_ENSG00000125534
20	62151617	62151933	KD2-H4K8ac_peak_19447	11.8457	PPDPF_ENSG00000125534
20	62167702	62168402	KD2-H4K8ac_peak_19448	10.7679	PTK6_ENSG00000101213
20	62169050	62169446	KD2-H4K8ac_peak_19449	9.91109	PTK6_ENSG00000101213
20	62184018	62184519	KD2-H4K8ac_peak_19450	5.59859	C20orf195_ENSG00000125531
20	62185177	62185487	KD2-H4K8ac_peak_19451	6.99815	C20orf195_ENSG00000125531
20	62205671	62206202	KD2-H4K8ac_peak_19452	9.63487	HELZ2_ENSG00000130589
20	62257644	62258782	KD2-H4K8ac_peak_19453	20.78681	GMEB2_ENSG00000101216;CTD-3184A7.4_ENSG00000232442
20	62283981	62284709	KD2-H4K8ac_peak_19454	5.01002	STMN3_ENSG00000197457
20	62285383	62285923	KD2-H4K8ac_peak_19455	6.8713	STMN3_ENSG00000197457
20	62289041	62290153	KD2-H4K8ac_peak_19456	11.50346	RTEL1_ENSG00000258366;RTEL1-TNFRSF6B_ENSG00000026036

20	62338623	62340013	KD2-H4K8ac_peak_19457	13.70753	ARFRP1_ENSG00000101246;ZGPAT_ENSG00000197114;RP4-583P15.15_ENSG00000273154
20	62406416	62406860	KD2-H4K8ac_peak_19458	4.91674	
20	62417843	62418145	KD2-H4K8ac_peak_19459	6.56926	
20	62462829	62463032	KD2-H4K8ac_peak_19460	7.47462	ZBTB46_ENSG00000130584
20	62465761	62466302	KD2-H4K8ac_peak_19461	9.08816	
20	62466554	62466822	KD2-H4K8ac_peak_19462	9.20193	
20	62485638	62487101	KD2-H4K8ac_peak_19463	10.30284	
20	62495904	62496279	KD2-H4K8ac_peak_19464	5.349	TPD52L2_ENSG00000101150
20	62496760	62497033	KD2-H4K8ac_peak_19465	7.49599	TPD52L2_ENSG00000101150
20	62587213	62587703	KD2-H4K8ac_peak_19466	16.57619	UCKL1_ENSG00000198276
20	62588377	62588613	KD2-H4K8ac_peak_19467	5.59859	UCKL1_ENSG00000198276
20	62600571	62601189	KD2-H4K8ac_peak_19468	11.05467	
20	62601428	62601785	KD2-H4K8ac_peak_19469	14.87555	
20	62610273	62612906	KD2-H4K8ac_peak_19470	14.8083	SAMD10_ENSG00000130590;PRPF6_ENSG00000101161
20	62670114	62670847	KD2-H4K8ac_peak_19471	14.12659	
20	62673303	62674027	KD2-H4K8ac_peak_19472	16.8435	
20	62685523	62685824	KD2-H4K8ac_peak_19473	11.24702	
20	62693853	62694330	KD2-H4K8ac_peak_19474	28.26254	
20	62709929	62710144	KD2-H4K8ac_peak_19475	5.82859	
20	62710535	62711158	KD2-H4K8ac_peak_19476	14.23999	RGS19_ENSG00000171700;OPRL1_ENSG00000125510
20	62715133	62715618	KD2-H4K8ac_peak_19477	8.10216	C20orf201_ENSG00000171695
20	62733705	62733922	KD2-H4K8ac_peak_19478	7.74292	
20	62778136	62778589	KD2-H4K8ac_peak_19479	10.05606	
20	62785919	62786412	KD2-H4K8ac_peak_19480	8.86099	
20	62786986	62788012	KD2-H4K8ac_peak_19481	5.30799	
20	62886532	62886920	KD2-H4K8ac_peak_19482	9.01262	PCMTD2_ENSG00000203880
20	62887154	62887392	KD2-H4K8ac_peak_19483	6.48713	PCMTD2_ENSG00000203880
21	10596378	10596795	KD2-H4K8ac_peak_19484	3.82395	
21	10597131	10598417	KD2-H4K8ac_peak_19485	9.44322	
21	10602023	10602672	KD2-H4K8ac_peak_19486	9.56945	
21	15435699	15435931	KD2-H4K8ac_peak_19487	4.42765	ANKRD20A18P_ENSG00000249493
21	17443194	17443601	KD2-H4K8ac_peak_19488	5.57044	LINC00478_ENSG00000215386
21	17791053	17791524	KD2-H4K8ac_peak_19489	6.16888	AP001172.3_ENSG00000270139
21	17960543	17960736	KD2-H4K8ac_peak_19490	5.20908	
21	18827961	18828173	KD2-H4K8ac_peak_19491	6.82976	
21	18885047	18885884	KD2-H4K8ac_peak_19492	12.08081	CXADR_ENSG00000154639
21	18984314	18984541	KD2-H4K8ac_peak_19493	4.8888	BTG3_ENSG00000154640
21	18984918	18985122	KD2-H4K8ac_peak_19494	6.25869	BTG3_ENSG00000154640
21	18985369	18985873	KD2-H4K8ac_peak_19495	26.621	BTG3_ENSG00000154640
21	19191135	19191999	KD2-H4K8ac_peak_19496	16.8435	C21orf91_ENSG00000154642
21	22370076	22370503	KD2-H4K8ac_peak_19497	6.66359	NCAM2_ENSG00000154654
21	24116178	24116406	KD2-H4K8ac_peak_19498	7.27716	
21	26979442	26979813	KD2-H4K8ac_peak_19499	16.54484	MRPL39_ENSG00000154719
21	27011146	27012292	KD2-H4K8ac_peak_19500	10.65724	JAM2_ENSG00000154721
21	27106916	27107233	KD2-H4K8ac_peak_19501	10.05606	ATP5J_ENSG00000154723;GABPA_ENSG00000154727
21	27107453	27108382	KD2-H4K8ac_peak_19502	24.85162	ATP5J_ENSG00000154723;GABPA_ENSG00000154727

21	27162636	27163133	KD2-H4K8ac_peak_19503	7.59643	
21	27541664	27542171	KD2-H4K8ac_peak_19504	7.38799	
21	27542404	27543360	KD2-H4K8ac_peak_19505	6.55628	APP_ENSG00000142192;AP000230.1_ENSG00000273492
21	27926186	27926400	KD2-H4K8ac_peak_19506	9.15329	
21	27959658	27959926	KD2-H4K8ac_peak_19507	5.07241	
21	28218003	28219155	KD2-H4K8ac_peak_19508	6.40297	ADAMTS1_ENSG00000154734
21	28338280	28338650	KD2-H4K8ac_peak_19509	4.06758	ADAMTS5_ENSG00000154736
21	28339707	28340248	KD2-H4K8ac_peak_19510	15.22643	ADAMTS5_ENSG00000154736
21	29630664	29630932	KD2-H4K8ac_peak_19511	5.57044	
21	29642352	29642581	KD2-H4K8ac_peak_19512	8.1233	
21	29706763	29706966	KD2-H4K8ac_peak_19513	6.16888	
21	29707598	29707856	KD2-H4K8ac_peak_19514	5.14125	
21	29731526	29731755	KD2-H4K8ac_peak_19515	6.00226	
21	29754209	29754461	KD2-H4K8ac_peak_19516	5.14125	
21	30257462	30257676	KD2-H4K8ac_peak_19517	14.8083	N6AMT1_ENSG00000156239
21	30375195	30375413	KD2-H4K8ac_peak_19518	8.55271	
21	30391292	30391549	KD2-H4K8ac_peak_19519	6.99815	RWDD2B_ENSG00000156253
21	30391846	30392176	KD2-H4K8ac_peak_19520	10.96455	RWDD2B_ENSG00000156253
21	30396302	30396656	KD2-H4K8ac_peak_19521	7.45501	RP1-100J12.1_ENSG00000273254;USP16_ENSG00000156256
21	30397002	30397215	KD2-H4K8ac_peak_19522	14.80472	RP1-100J12.1_ENSG00000273254;USP16_ENSG00000156256
21	30445510	30445931	KD2-H4K8ac_peak_19523	12.88108	CCT8_ENSG00000156261
21	30565744	30566243	KD2-H4K8ac_peak_19524	6.40683	LINC00189_ENSG00000215533;BACH1_ENSG00000156273
21	30670600	30671865	KD2-H4K8ac_peak_19525	11.05467	
21	32518312	32518552	KD2-H4K8ac_peak_19526	5.6755	
21	32536500	32536704	KD2-H4K8ac_peak_19527	6.40683	
21	32544156	32544468	KD2-H4K8ac_peak_19528	9.11031	
21	32546433	32546641	KD2-H4K8ac_peak_19529	5.10505	
21	32546978	32547594	KD2-H4K8ac_peak_19530	10.54933	
21	32932082	32932603	KD2-H4K8ac_peak_19531	14.43704	TIAM1_ENSG00000156299;AP000251.3_ENSG00000237594
21	33032039	33032264	KD2-H4K8ac_peak_19532	8.30974	AP000253.1_ENSG00000234509;SOD1_ENSG00000142168
21	33103803	33104201	KD2-H4K8ac_peak_19533	8.17902	SCAF4_ENSG00000156304
21	33104636	33105072	KD2-H4K8ac_peak_19534	9.01262	SCAF4_ENSG00000156304
21	33244924	33246453	KD2-H4K8ac_peak_19535	15.57998	HUNK_ENSG00000142149
21	33319112	33319381	KD2-H4K8ac_peak_19536	7.47462	
21	33507059	33507310	KD2-H4K8ac_peak_19537	6.00243	
21	33507989	33508264	KD2-H4K8ac_peak_19538	7.63551	
21	33651963	33652188	KD2-H4K8ac_peak_19539	4.01115	MIS18A_ENSG00000159055
21	33765034	33765258	KD2-H4K8ac_peak_19540	11.05467	URB1_ENSG00000142207;C21orf119_ENSG00000256073
21	33765495	33766041	KD2-H4K8ac_peak_19541	9.89126	URB1_ENSG00000142207;C21orf119_ENSG00000256073
21	33783787	33784224	KD2-H4K8ac_peak_19542	11.09936	EVA1C_ENSG00000166979
21	33784422	33784639	KD2-H4K8ac_peak_19543	5.13615	EVA1C_ENSG00000166979
21	33941959	33942157	KD2-H4K8ac_peak_19544	5.53033	
21	33984475	33985830	KD2-H4K8ac_peak_19545	21.52958	AP000275.65_ENSG00000265590;C21orf59_ENSG00000159079
21	33986168	33986491	KD2-H4K8ac_peak_19546	6.42097	C21orf59_ENSG00000159079
21	34099765	34100251	KD2-H4K8ac_peak_19547	6.99815	SYNJ1_ENSG00000159082;PAXBP1-AS1_ENSG00000238197
21	34100458	34100739	KD2-H4K8ac_peak_19548	19.72117	SYNJ1_ENSG00000159082;PAXBP1-AS1_ENSG00000238197

21	34143319	34143998	KD2-H4K8ac_peak_19549	10.0692	PAXBP1_ENSG00000159086;C21orf49_ENSG00000205930
21	34602288	34602623	KD2-H4K8ac_peak_19550	9.36516	IFNAR2_ENSG00000159110
21	34638723	34638948	KD2-H4K8ac_peak_19551	11.05467	IL10RB-AS1_ENSG00000223799;IL10RB_ENSG00000243646
21	34696703	34697060	KD2-H4K8ac_peak_19552	9.63487	IFNAR1_ENSG00000142166
21	34697382	34697712	KD2-H4K8ac_peak_19553	5.01002	IFNAR1_ENSG00000142166
21	34775430	34776725	KD2-H4K8ac_peak_19554	10.43145	IFNGR2_ENSG00000159128
21	34851876	34852109	KD2-H4K8ac_peak_19555	5.54647	TMEM50B_ENSG00000142188
21	34852665	34852861	KD2-H4K8ac_peak_19556	6.66692	TMEM50B_ENSG00000142188
21	34863649	34864463	KD2-H4K8ac_peak_19557	15.22643	DNAJC28_ENSG00000177692
21	34961287	34961540	KD2-H4K8ac_peak_19558	6.72334	DONSON_ENSG00000159147
21	35014875	35015504	KD2-H4K8ac_peak_19559	10.05606	CRYZL1_ENSG00000205758;ITSN1_ENSG00000205726
21	35287678	35287971	KD2-H4K8ac_peak_19560	10.65724	ATP5O_ENSG00000241837;LINC00649_ENSG00000237945
21	35288421	35288684	KD2-H4K8ac_peak_19561	5.4919	ATP5O_ENSG00000241837;LINC00649_ENSG00000237945
21	35401904	35402339	KD2-H4K8ac_peak_19562	6.56926	
21	35444872	35445586	KD2-H4K8ac_peak_19563	40.814	MRPS6_ENSG00000243927;SLC5A3_ENSG00000272962;SLC5A3_ENSG00000198743;AP000320.7_ENSG00000272657
21	35576756	35577055	KD2-H4K8ac_peak_19564	7.12754	AP000318.2_ENSG00000214955
21	35577343	35577591	KD2-H4K8ac_peak_19565	10.21502	AP000318.2_ENSG00000214955
21	35747056	35747623	KD2-H4K8ac_peak_19566	5.94405	AP000320.6_ENSG00000225555;SMIM11_ENSG00000205670
21	35986998	35987520	KD2-H4K8ac_peak_19567	14.15075	RCAN1_ENSG00000159200
21	37432110	37433181	KD2-H4K8ac_peak_19568	7.76329	AP000688.11_ENSG00000236677
21	37442311	37442685	KD2-H4K8ac_peak_19569	13.21979	CBR1_ENSG00000159228
21	37485387	37485817	KD2-H4K8ac_peak_19570	4.86324	
21	37501985	37502216	KD2-H4K8ac_peak_19571	6.40683	MEMO1P1_ENSG00000226054
21	37502469	37502994	KD2-H4K8ac_peak_19572	8.1331	MEMO1P1_ENSG00000226054
21	37507317	37508019	KD2-H4K8ac_peak_19573	6.99815	CBR3_ENSG00000159231
21	37529269	37529584	KD2-H4K8ac_peak_19574	5.82799	CBR3-AS1_ENSG00000236830;DOPEY2_ENSG00000142197
21	37632679	37632906	KD2-H4K8ac_peak_19575	5.16439	
21	37691558	37692323	KD2-H4K8ac_peak_19576	11.03331	AP000692.10_ENSG00000273199;MORC3_ENSG00000159256
21	37692616	37693166	KD2-H4K8ac_peak_19577	10.96455	AP000692.10_ENSG00000273199;MORC3_ENSG00000159256
21	37694662	37695059	KD2-H4K8ac_peak_19578	7.05877	
21	37757724	37758183	KD2-H4K8ac_peak_19579	10.88859	CHAF1B_ENSG00000159259
21	37833344	37833619	KD2-H4K8ac_peak_19580	4.19473	
21	38120294	38120646	KD2-H4K8ac_peak_19581	4.44527	
21	38338231	38338934	KD2-H4K8ac_peak_19582	17.8868	AP000704.5_ENSG00000224790
21	38339225	38339713	KD2-H4K8ac_peak_19583	14.45402	AP000704.5_ENSG00000224790
21	38361725	38362941	KD2-H4K8ac_peak_19584	13.70753	HLCS_ENSG00000159267
21	38381978	38382269	KD2-H4K8ac_peak_19585	10.05606	
21	38382613	38382812	KD2-H4K8ac_peak_19586	5.557	
21	38416290	38416536	KD2-H4K8ac_peak_19587	7.0983	
21	38444825	38445309	KD2-H4K8ac_peak_19588	11.04256	PIGP_ENSG00000185808;TTC3_ENSG00000182670
21	38445626	38446082	KD2-H4K8ac_peak_19589	7.18615	PIGP_ENSG00000185808;TTC3_ENSG00000182670
21	38592709	38593501	KD2-H4K8ac_peak_19590	5.26288	AP001432.14_ENSG00000242553
21	38593788	38594097	KD2-H4K8ac_peak_19591	5.20998	AP001432.14_ENSG00000242553
21	38640099	38640555	KD2-H4K8ac_peak_19592	9.57101	DSCR3_ENSG00000157538;AP001412.1_ENSG00000272948
21	38738154	38738571	KD2-H4K8ac_peak_19593	18.54875	AP001437.1_ENSG00000273210;DYRK1A_ENSG00000157540
21	38739159	38740527	KD2-H4K8ac_peak_19594	13.80019	AP001437.1_ENSG00000273210

21	38935631	38936148	KD2-H4K8ac_peak_19595	15.20126	
21	38936427	38936639	KD2-H4K8ac_peak_19596	7.51054	
21	38936934	38937239	KD2-H4K8ac_peak_19597	7.67504	
21	40177943	40178243	KD2-H4K8ac_peak_19598	11.92978	ETS2_ENSG00000157557
21	40684993	40686384	KD2-H4K8ac_peak_19599	18.91876	BRWD1-IT2_ENSG00000255568
21	40720298	40720973	KD2-H4K8ac_peak_19600	28.21903	HMGN1_ENSG00000205581
21	40721548	40721845	KD2-H4K8ac_peak_19601	9.18237	HMGN1_ENSG00000205581
21	40758211	40758481	KD2-H4K8ac_peak_19602	9.72244	
21	40817327	40817574	KD2-H4K8ac_peak_19603	8.30974	LCA5L_ENSG00000157578;SH3BGR_ENSG00000185437
21	40817835	40818051	KD2-H4K8ac_peak_19604	8.16133	LCA5L_ENSG00000157578;SH3BGR_ENSG00000185437
21	40984685	40985030	KD2-H4K8ac_peak_19605	6.25869	C21orf88_ENSG00000184809
21	41032536	41032801	KD2-H4K8ac_peak_19606	5.26591	
21	41045065	41045368	KD2-H4K8ac_peak_19607	7.27716	
21	41047366	41047796	KD2-H4K8ac_peak_19608	9.1707	
21	41048579	41048986	KD2-H4K8ac_peak_19609	11.92209	
21	41549859	41550054	KD2-H4K8ac_peak_19610	5.15777	
21	42213522	42213840	KD2-H4K8ac_peak_19611	6.07178	
21	43298717	43298941	KD2-H4K8ac_peak_19612	9.19305	PRDM15_ENSG00000141956;AP001619.2_ENSG00000227698
21	43299403	43300051	KD2-H4K8ac_peak_19613	24.48793	PRDM15_ENSG00000141956
21	43373348	43374616	KD2-H4K8ac_peak_19614	8.83582	C2CD2_ENSG00000157617
21	43430293	43431237	KD2-H4K8ac_peak_19615	13.80019	ZBTB21_ENSG00000173276;ZNF295-AS1_ENSG00000237232
21	43576913	43577149	KD2-H4K8ac_peak_19616	5.45396	
21	43640598	43640815	KD2-H4K8ac_peak_19617	4.86324	RNA5SP492_ENSG00000223262
21	43654611	43655493	KD2-H4K8ac_peak_19618	5.9696	
21	43916729	43917053	KD2-H4K8ac_peak_19619	11.86082	RSPH1_ENSG00000160188;SLC37A1_ENSG00000160190;AP001625.4_ENSG00000239930
21	43933599	43934346	KD2-H4K8ac_peak_19620	9.15069	
21	43991077	43991472	KD2-H4K8ac_peak_19621	4.42765	
21	44012811	44013485	KD2-H4K8ac_peak_19622	5.30799	
21	44034917	44035150	KD2-H4K8ac_peak_19623	6.66692	AP001626.1_ENSG00000225431
21	44072909	44073550	KD2-H4K8ac_peak_19624	14.69247	PDE9A_ENSG00000160191
21	44073854	44074508	KD2-H4K8ac_peak_19625	5.38581	PDE9A_ENSG00000160191
21	44201446	44201988	KD2-H4K8ac_peak_19626	9.15329	AP001628.7_ENSG00000233754
21	44202205	44202729	KD2-H4K8ac_peak_19627	12.42547	AP001628.7_ENSG00000233754
21	44298924	44299532	KD2-H4K8ac_peak_19628	17.43705	WDR4_ENSG00000160193;NDUFV3_ENSG00000160194
21	44299797	44300234	KD2-H4K8ac_peak_19629	12.26331	WDR4_ENSG00000160193;NDUFV3_ENSG00000160194
21	44312917	44313190	KD2-H4K8ac_peak_19630	8.55271	
21	44313481	44313832	KD2-H4K8ac_peak_19631	7.146	
21	44393525	44394277	KD2-H4K8ac_peak_19632	21.2844	PKNOX1_ENSG00000160199
21	44394787	44395056	KD2-H4K8ac_peak_19633	13.80019	PKNOX1_ENSG00000160199
21	44395307	44395530	KD2-H4K8ac_peak_19634	11.03331	PKNOX1_ENSG00000160199
21	44494755	44495154	KD2-H4K8ac_peak_19635	5.98692	
21	44495431	44495908	KD2-H4K8ac_peak_19636	11.28831	
21	44496446	44496854	KD2-H4K8ac_peak_19637	7.36799	CBS_ENSG00000160200
21	44527222	44527644	KD2-H4K8ac_peak_19638	11.3394	U2AF1_ENSG00000160201
21	44527875	44528120	KD2-H4K8ac_peak_19639	11.09936	U2AF1_ENSG00000160201
21	44537213	44537439	KD2-H4K8ac_peak_19640	7.27716	

21	44598420	44599138	KD2-H4K8ac_peak_19641	6.60096	
21	44777937	44778129	KD2-H4K8ac_peak_19642	4.54838	
21	44779863	44780104	KD2-H4K8ac_peak_19643	5.69023	
21	44780333	44780559	KD2-H4K8ac_peak_19644	7.99241	
21	44781563	44782062	KD2-H4K8ac_peak_19645	6.65673	AP001046.5_ENSG00000237989
21	44782865	44783078	KD2-H4K8ac_peak_19646	8.36514	AP001046.5_ENSG00000237989;AP001046.6_ENSG00000225637
21	44783370	44783665	KD2-H4K8ac_peak_19647	6.66692	AP001046.6_ENSG00000225637
21	44787069	44787523	KD2-H4K8ac_peak_19648	9.76863	
21	44803575	44803826	KD2-H4K8ac_peak_19649	8.53581	
21	44847439	44847692	KD2-H4K8ac_peak_19650	8.16906	SIK1_ENSG00000142178
21	44870757	44871027	KD2-H4K8ac_peak_19651	10.7186	
21	44871543	44871798	KD2-H4K8ac_peak_19652	7.36799	
21	44906114	44906494	KD2-H4K8ac_peak_19653	4.98072	
21	44936431	44936720	KD2-H4K8ac_peak_19654	4.54006	
21	44938237	44938458	KD2-H4K8ac_peak_19655	8.94469	
21	45025976	45026379	KD2-H4K8ac_peak_19656	7.70289	
21	45078563	45079200	KD2-H4K8ac_peak_19657	7.28628	HSF2BP_ENSG00000160207;RRP1B_ENSG00000160208
21	45138174	45138722	KD2-H4K8ac_peak_19658	14.48084	PDXK_ENSG00000160209
21	45139051	45139248	KD2-H4K8ac_peak_19659	6.65673	PDXK_ENSG00000160209
21	45147932	45148924	KD2-H4K8ac_peak_19660	13.50024	
21	45195999	45196708	KD2-H4K8ac_peak_19661	10.43145	CSTB_ENSG00000160213
21	45208818	45209067	KD2-H4K8ac_peak_19662	5.9696	RRP1_ENSG00000160214
21	45209508	45209756	KD2-H4K8ac_peak_19663	8.95692	RRP1_ENSG00000160214
21	45284189	45284681	KD2-H4K8ac_peak_19664	20.15307	AGPAT3_ENSG00000160216
21	45284980	45285884	KD2-H4K8ac_peak_19665	17.06312	AGPAT3_ENSG00000160216
21	45431735	45432032	KD2-H4K8ac_peak_19666	12.23	TRAPPC10_ENSG00000160218
21	45432271	45432483	KD2-H4K8ac_peak_19667	7.36799	TRAPPC10_ENSG00000160218
21	45553040	45553319	KD2-H4K8ac_peak_19668	15.81711	C21orf33_ENSG00000160221
21	45553578	45553911	KD2-H4K8ac_peak_19669	10.43145	C21orf33_ENSG00000160221
21	45719470	45720871	KD2-H4K8ac_peak_19670	15.52424	PFKL_ENSG00000141959
21	45759395	45759866	KD2-H4K8ac_peak_19671	10.53377	C21orf2_ENSG00000160226;AP001062.9_ENSG00000232969
21	45857519	45858709	KD2-H4K8ac_peak_19672	14.8083	snoZ6_ENSG00000266692;snoZ6_ENSG00000264452
21	45870250	45870493	KD2-H4K8ac_peak_19673	7.13615	
21	45906260	45906467	KD2-H4K8ac_peak_19674	7.87794	AP001065.15_ENSG00000228709
21	45936777	45936984	KD2-H4K8ac_peak_19675	7.69287	C21orf90_ENSG00000182912
21	46185924	46186151	KD2-H4K8ac_peak_19676	6.9556	
21	46237900	46238514	KD2-H4K8ac_peak_19677	18.37277	SUMO3_ENSG00000184900
21	46265876	46266085	KD2-H4K8ac_peak_19678	9.68448	
21	46293011	46293572	KD2-H4K8ac_peak_19679	10.0692	PTTG1IP_ENSG00000183255
21	46294011	46294830	KD2-H4K8ac_peak_19680	7.76329	PTTG1IP_ENSG00000183255
21	46359357	46360340	KD2-H4K8ac_peak_19681	17.16047	C21orf67_ENSG00000183250;FAM207A_ENSG00000160256
21	46386705	46387295	KD2-H4K8ac_peak_19682	11.50346	
21	46400314	46400549	KD2-H4K8ac_peak_19683	6.16883	
21	46400813	46401133	KD2-H4K8ac_peak_19684	5.70596	
21	46404996	46405636	KD2-H4K8ac_peak_19685	10.7186	
21	46406628	46407389	KD2-H4K8ac_peak_19686	7.69287	

21	46408936	46409155	KD2-H4K8ac_peak_19687	5.54647	
21	46409983	46410353	KD2-H4K8ac_peak_19688	8.79106	
21	46438245	46439397	KD2-H4K8ac_peak_19689	8.10216	
21	46494050	46494347	KD2-H4K8ac_peak_19690	14.8083	SSR4P1_ENSG00000235374;ADARB1_ENSG00000197381
21	46494779	46495226	KD2-H4K8ac_peak_19691	8.59579	SSR4P1_ENSG00000235374
21	46513134	46513408	KD2-H4K8ac_peak_19692	6.05809	
21	46573350	46573836	KD2-H4K8ac_peak_19693	8.41122	
21	46575170	46575378	KD2-H4K8ac_peak_19694	7.22457	
21	46578183	46578781	KD2-H4K8ac_peak_19695	12.85889	
21	46623064	46623292	KD2-H4K8ac_peak_19696	10.29344	
21	46668806	46669028	KD2-H4K8ac_peak_19697	5.57044	
21	46708002	46708626	KD2-H4K8ac_peak_19698	9.72244	POFUT2_ENSG00000186866;BX322557.10_ENSG00000215447
21	46712530	46712801	KD2-H4K8ac_peak_19699	5.82799	LINC00205_ENSG00000223768
21	46769745	46770071	KD2-H4K8ac_peak_19700	3.91903	
21	46772643	46773006	KD2-H4K8ac_peak_19701	9.34989	
21	46779317	46779916	KD2-H4K8ac_peak_19702	12.06346	
21	46780280	46780692	KD2-H4K8ac_peak_19703	4.73543	
21	46781012	46781896	KD2-H4K8ac_peak_19704	9.73431	
21	46824387	46824599	KD2-H4K8ac_peak_19705	6.66692	COL18A1_ENSG00000182871
21	46825397	46825597	KD2-H4K8ac_peak_19706	5.94347	COL18A1_ENSG00000182871
21	46825917	46826238	KD2-H4K8ac_peak_19707	7.07117	COL18A1_ENSG00000182871
21	46851523	46851818	KD2-H4K8ac_peak_19708	7.95934	
21	46885891	46886369	KD2-H4K8ac_peak_19709	4.35346	
21	46887147	46887454	KD2-H4K8ac_peak_19710	7.40111	
21	46897763	46898126	KD2-H4K8ac_peak_19711	12.30509	
21	46961411	46961929	KD2-H4K8ac_peak_19712	9.1707	
21	46962785	46963390	KD2-H4K8ac_peak_19713	14.09907	SLC19A1_ENSG00000173638
21	46974407	46974920	KD2-H4K8ac_peak_19714	8.21084	
21	46975580	46976044	KD2-H4K8ac_peak_19715	14.15075	
21	46991856	46992434	KD2-H4K8ac_peak_19716	10.16666	
21	47035807	47036647	KD2-H4K8ac_peak_19717	9.1994	
21	47058855	47059320	KD2-H4K8ac_peak_19718	7.67504	
21	47059731	47060127	KD2-H4K8ac_peak_19719	9.20193	
21	47060325	47061152	KD2-H4K8ac_peak_19720	9.16531	
21	47061372	47061675	KD2-H4K8ac_peak_19721	3.90565	
21	47062070	47062708	KD2-H4K8ac_peak_19722	11.16154	PCBP3_ENSG00000183570
21	47123362	47124490	KD2-H4K8ac_peak_19723	12.82393	
21	47124910	47125555	KD2-H4K8ac_peak_19724	11.05467	
21	47126027	47126617	KD2-H4K8ac_peak_19725	6.36375	
21	47128724	47128926	KD2-H4K8ac_peak_19726	5.58324	
21	47145409	47145686	KD2-H4K8ac_peak_19727	9.16531	
21	47146429	47146712	KD2-H4K8ac_peak_19728	5.08628	
21	47167360	47167668	KD2-H4K8ac_peak_19729	8.85813	
21	47186333	47186650	KD2-H4K8ac_peak_19730	5.14125	
21	47240029	47240263	KD2-H4K8ac_peak_19731	5.75757	
21	47281066	47281340	KD2-H4K8ac_peak_19732	8.98477	

21	47307336	47307596	KD2-H4K8ac_peak_19733	6.92624	
21	47314371	47314572	KD2-H4K8ac_peak_19734	7.27716	
21	47390233	47391685	KD2-H4K8ac_peak_19735	23.59427	
21	47392124	47394796	KD2-H4K8ac_peak_19736	17.55491	
21	47498701	47499356	KD2-H4K8ac_peak_19737	8.99803	
21	47499905	47500414	KD2-H4K8ac_peak_19738	13.47439	
21	47517302	47518853	KD2-H4K8ac_peak_19739	12.14909	AP001471.1_ENSG00000227438;COL6A2_ENSG00000142173
21	47548602	47549154	KD2-H4K8ac_peak_19740	5.34636	
21	47573545	47573909	KD2-H4K8ac_peak_19741	5.13615	
21	47604709	47604993	KD2-H4K8ac_peak_19742	6.40683	SPATC1L_ENSG00000160284;AP001468.58_ENSG00000228404
21	47614354	47614936	KD2-H4K8ac_peak_19743	9.34195	AP001468.1_ENSG00000235878
21	47649151	47649702	KD2-H4K8ac_peak_19744	11.05467	LSS_ENSG00000160285;MCM3AP-AS1_ENSG00000215424
21	47705947	47706404	KD2-H4K8ac_peak_19745	6.98244	MCM3AP_ENSG00000160294;YBEY_ENSG00000182362
21	47714922	47715511	KD2-H4K8ac_peak_19746	10.05606	
21	47743143	47743718	KD2-H4K8ac_peak_19747	8.55271	PCNT_ENSG00000160299;C21orf58_ENSG00000160298
21	47744042	47744389	KD2-H4K8ac_peak_19748	31.533	PCNT_ENSG00000160299;C21orf58_ENSG00000160298
21	47744665	47745252	KD2-H4K8ac_peak_19749	14.48084	PCNT_ENSG00000160299;C21orf58_ENSG00000160298
21	47878133	47878577	KD2-H4K8ac_peak_19750	11.50346	DIP2A_ENSG00000160305
21	47878895	47879135	KD2-H4K8ac_peak_19751	10.21366	DIP2A_ENSG00000160305
21	48054298	48054731	KD2-H4K8ac_peak_19752	6.85193	PRMT2_ENSG00000160310
21	48055122	48055869	KD2-H4K8ac_peak_19753	19.43192	PRMT2_ENSG00000160310
21	48087674	48087933	KD2-H4K8ac_peak_19754	7.07117	
22	17601760	17602070	KD2-H4K8ac_peak_19755	11.39383	CECR6_ENSG00000183307;AC006946.15_ENSG00000235478
22	17602545	17602901	KD2-H4K8ac_peak_19756	11.24702	CECR6_ENSG00000183307;AC006946.15_ENSG00000235478
22	17639675	17640055	KD2-H4K8ac_peak_19757	8.30974	CECR5-AS1_ENSG00000185837
22	17640290	17640686	KD2-H4K8ac_peak_19758	10.21366	CECR5-AS1_ENSG00000185837
22	17652176	17653559	KD2-H4K8ac_peak_19759	10.7186	
22	17679875	17680281	KD2-H4K8ac_peak_19760	8.28994	
22	17739079	17739470	KD2-H4K8ac_peak_19761	4.80593	
22	17745003	17745329	KD2-H4K8ac_peak_19762	7.47462	
22	18009046	18009276	KD2-H4K8ac_peak_19763	7.76104	
22	18061220	18061433	KD2-H4K8ac_peak_19764	5.88845	
22	18111120	18111475	KD2-H4K8ac_peak_19765	11.89502	ATP6V1E1_ENSG00000131100;BCL2L13_ENSG00000099968
22	18111773	18112081	KD2-H4K8ac_peak_19766	7.01964	ATP6V1E1_ENSG00000131100;BCL2L13_ENSG00000099968
22	18121056	18121882	KD2-H4K8ac_peak_19767	9.36516	
22	18256875	18257924	KD2-H4K8ac_peak_19768	12.66412	BID_ENSG00000015475
22	18483979	18484216	KD2-H4K8ac_peak_19769	11.2938	
22	18484816	18485339	KD2-H4K8ac_peak_19770	6.65673	
22	18560987	18561257	KD2-H4K8ac_peak_19771	5.59859	XXbac-B476C20.9_ENSG00000225335;PEX26_ENSG00000215193
22	18592938	18593697	KD2-H4K8ac_peak_19772	5.72327	TUBA8_ENSG00000183785
22	18632280	18632554	KD2-H4K8ac_peak_19773	4.77079	USP18_ENSG00000184979
22	18893293	18893641	KD2-H4K8ac_peak_19774	10.29842	DGCR6_ENSG00000183628
22	18893909	18894135	KD2-H4K8ac_peak_19775	5.75757	DGCR6_ENSG00000183628
22	18904215	18904440	KD2-H4K8ac_peak_19776	5.14125	
22	18910232	18910435	KD2-H4K8ac_peak_19777	4.54006	
22	18923528	18923755	KD2-H4K8ac_peak_19778	8.02571	PRODH_ENSG00000100033

22	18955472	18956000	KD2-H4K8ac_peak_19779	5.9696	
22	18957212	18958956	KD2-H4K8ac_peak_19780	14.8083	DGCR5_ENSG00000237517
22	19109653	19109899	KD2-H4K8ac_peak_19781	15.22643	DGCR2_ENSG00000070413;AC004471.9_ENSG00000223461
22	19110322	19110720	KD2-H4K8ac_peak_19782	6.99815	DGCR2_ENSG00000070413
22	19158537	19159665	KD2-H4K8ac_peak_19783	27.59	AC004463.6_ENSG00000260924
22	19165949	19166230	KD2-H4K8ac_peak_19784	11.3394	SLC25A1_ENSG00000100075
22	19278638	19279205	KD2-H4K8ac_peak_19785	9.8758	CLTCL1_ENSG00000070371
22	19279478	19279688	KD2-H4K8ac_peak_19786	12.26331	CLTCL1_ENSG00000070371
22	19418849	19419097	KD2-H4K8ac_peak_19787	7.96322	MRPL40_ENSG00000185608
22	19419319	19419826	KD2-H4K8ac_peak_19788	15.69572	MRPL40_ENSG00000185608
22	19434744	19435180	KD2-H4K8ac_peak_19789	18.94636	HIRA_ENSG00000100084;C22orf39_ENSG00000242259;AC000068.5_ENSG00000185065
22	19435479	19435783	KD2-H4K8ac_peak_19790	7.76329	HIRA_ENSG00000100084;C22orf39_ENSG00000242259;AC000068.5_ENSG00000185065
22	19683829	19684048	KD2-H4K8ac_peak_19791	9.61895	
22	19701018	19701244	KD2-H4K8ac_peak_19792	6.25869	SEPT5_ENSG00000184702
22	19701471	19701885	KD2-H4K8ac_peak_19793	14.66631	SEPT5_ENSG00000184702
22	19702091	19702306	KD2-H4K8ac_peak_19794	6.76298	SEPT5_ENSG00000184702
22	19705418	19705617	KD2-H4K8ac_peak_19795	8.4716	
22	19705997	19706250	KD2-H4K8ac_peak_19796	6.04107	
22	19732853	19733436	KD2-H4K8ac_peak_19797	5.08628	
22	19799559	19800193	KD2-H4K8ac_peak_19798	7.146	
22	19832053	19832379	KD2-H4K8ac_peak_19799	10.54933	
22	19842660	19843282	KD2-H4K8ac_peak_19800	14.59156	GNB1L_ENSG00000185838;C22orf29_ENSG00000215012
22	19878833	19879162	KD2-H4K8ac_peak_19801	9.08816	
22	19879465	19879682	KD2-H4K8ac_peak_19802	11.00004	
22	19880000	19880703	KD2-H4K8ac_peak_19803	10.77651	
22	19883939	19884513	KD2-H4K8ac_peak_19804	6.66692	
22	19928904	19929998	KD2-H4K8ac_peak_19805	18.94636	TXNRD2_ENSG00000184470;COMT_ENSG00000093010
22	19930433	19930648	KD2-H4K8ac_peak_19806	5.14125	
22	19974327	19974643	KD2-H4K8ac_peak_19807	5.9696	
22	19984514	19984840	KD2-H4K8ac_peak_19808	8.55271	
22	19985747	19986666	KD2-H4K8ac_peak_19809	9.402	
22	20004030	20004227	KD2-H4K8ac_peak_19810	8.10216	ARVCF_ENSG00000099889;TANGO2_ENSG00000183597
22	20008107	20008378	KD2-H4K8ac_peak_19811	12.93209	
22	20066667	20067413	KD2-H4K8ac_peak_19812	5.9696	DGCR8_ENSG00000128191
22	20067815	20068259	KD2-H4K8ac_peak_19813	12.79505	DGCR8_ENSG00000128191
22	20103961	20104205	KD2-H4K8ac_peak_19814	11.43353	TRMT2A_ENSG00000099899;RANBP1_ENSG00000099901
22	20105195	20105552	KD2-H4K8ac_peak_19815	15.58442	TRMT2A_ENSG00000099899
22	20118215	20118433	KD2-H4K8ac_peak_19816	5.34636	
22	20118830	20119183	KD2-H4K8ac_peak_19817	12.44942	
22	20142126	20142909	KD2-H4K8ac_peak_19818	13.44374	
22	20143293	20143516	KD2-H4K8ac_peak_19819	6.60096	
22	20143734	20143946	KD2-H4K8ac_peak_19820	5.54647	
22	20144809	20145464	KD2-H4K8ac_peak_19821	4.67342	
22	20194315	20194643	KD2-H4K8ac_peak_19822	4.80593	LINC00896_ENSG00000236499
22	20198043	20198754	KD2-H4K8ac_peak_19823	8.05667	
22	20199178	20199933	KD2-H4K8ac_peak_19824	10.63725	

22	20255293	20255762	KD2-H4K8ac_peak_19825	8.10216	
22	20256062	20256772	KD2-H4K8ac_peak_19826	10.78136	
22	20307748	20308189	KD2-H4K8ac_peak_19827	9.57101	DGCR6L_ENSG00000128185;XXbac-B444P24.14_ENSG00000273139
22	20747347	20748121	KD2-H4K8ac_peak_19828	13.80019	ZNF74_ENSG00000185252
22	20748508	20748830	KD2-H4K8ac_peak_19829	11.05467	ZNF74_ENSG00000185252
22	20772748	20772963	KD2-H4K8ac_peak_19830	6.65673	
22	20776263	20777651	KD2-H4K8ac_peak_19831	12.82393	
22	20791843	20792045	KD2-H4K8ac_peak_19832	5.30799	SCARF2_ENSG00000244486
22	20792292	20792722	KD2-H4K8ac_peak_19833	13.80019	SCARF2_ENSG00000244486
22	20810866	20811407	KD2-H4K8ac_peak_19834	4.86324	
22	20849393	20849962	KD2-H4K8ac_peak_19835	4.91674	KLHL22_ENSG00000099910;MED15_ENSG00000099917
22	20860915	20861699	KD2-H4K8ac_peak_19836	7.76329	
22	20885777	20885970	KD2-H4K8ac_peak_19837	4.57782	
22	20886963	20887177	KD2-H4K8ac_peak_19838	10.7186	
22	20887450	20887699	KD2-H4K8ac_peak_19839	6.05809	
22	20888609	20889091	KD2-H4K8ac_peak_19840	7.09076	
22	20890337	20890551	KD2-H4K8ac_peak_19841	10.84242	
22	20891440	20891906	KD2-H4K8ac_peak_19842	8.56219	
22	21056521	21056909	KD2-H4K8ac_peak_19843	5.835	
22	21270473	21271378	KD2-H4K8ac_peak_19844	12.06346	CRKL_ENSG00000099942
22	21271790	21271993	KD2-H4K8ac_peak_19845	5.59859	CRKL_ENSG00000099942
22	21319622	21319830	KD2-H4K8ac_peak_19846	4.67342	AIFM3_ENSG00000183773
22	21336296	21337137	KD2-H4K8ac_peak_19847	7.90806	XXbac-B135H6.18_ENSG00000272829
22	21355596	21355959	KD2-H4K8ac_peak_19848	7.92775	THAP7_ENSG00000184436;THAP7-AS1_ENSG00000230513
22	21356165	21356893	KD2-H4K8ac_peak_19849	18.25771	THAP7_ENSG00000184436;THAP7-AS1_ENSG00000230513
22	21357250	21357558	KD2-H4K8ac_peak_19850	9.36516	THAP7_ENSG00000184436
22	21368200	21368425	KD2-H4K8ac_peak_19851	7.49599	TUBA3FP_ENSG00000161149
22	21368792	21369004	KD2-H4K8ac_peak_19852	6.36375	TUBA3FP_ENSG00000161149
22	21369284	21370463	KD2-H4K8ac_peak_19853	16.09057	TUBA3FP_ENSG00000161149
22	21400252	21400623	KD2-H4K8ac_peak_19854	9.1707	P2RX6P_ENSG00000206145;AC002472.13_ENSG00000187905
22	21457315	21457539	KD2-H4K8ac_peak_19855	15.22643	BCRP2_ENSG00000169668
22	21770730	21770934	KD2-H4K8ac_peak_19856	5.88845	HIC2_ENSG00000169635
22	21921470	21921711	KD2-H4K8ac_peak_19857	6.83936	
22	21922053	21922617	KD2-H4K8ac_peak_19858	17.43561	
22	21983305	21983615	KD2-H4K8ac_peak_19859	10.08707	YDJC_ENSG00000161179
22	21983820	21984240	KD2-H4K8ac_peak_19860	15.58442	YDJC_ENSG00000161179
22	21984532	21984794	KD2-H4K8ac_peak_19861	12.00273	YDJC_ENSG00000161179
22	21996629	21996830	KD2-H4K8ac_peak_19862	7.1181	SDF2L1_ENSG00000128228
22	22005799	22006236	KD2-H4K8ac_peak_19863	10.46874	PPIL2_ENSG00000100023
22	22006545	22007303	KD2-H4K8ac_peak_19864	7.0754	PPIL2_ENSG00000100023;MIR301B_ENSG00000212102;MIR130B_ENSG00000207751
22	22011475	22012746	KD2-H4K8ac_peak_19865	13.26049	
22	22019556	22020846	KD2-H4K8ac_peak_19866	20.42362	
22	22089757	22090014	KD2-H4K8ac_peak_19867	11.09936	YPEL1_ENSG00000100027
22	22090521	22090827	KD2-H4K8ac_peak_19868	5.01002	YPEL1_ENSG00000100027
22	22222147	22222370	KD2-H4K8ac_peak_19869	10.96455	MAPK1_ENSG00000100030
22	22306617	22307156	KD2-H4K8ac_peak_19870	8.59579	PPM1F_ENSG00000100034

22	22652561	22652873	KD2-H4K8ac_peak_19871	11.92978	
22	22862697	22863103	KD2-H4K8ac_peak_19872	6.8713	ZNF280B_ENSG00000198477
22	22899657	22899999	KD2-H4K8ac_peak_19873	13.74404	
22	22901374	22901603	KD2-H4K8ac_peak_19874	10.05606	PRAME_ENSG00000185686;LL22NC03-63E9.3_ENSG00000220891
22	22901891	22902193	KD2-H4K8ac_peak_19875	7.98621	PRAME_ENSG00000185686;LL22NC03-63E9.3_ENSG00000220891
22	22993886	22994233	KD2-H4K8ac_peak_19876	5.15777	
22	23411856	23412257	KD2-H4K8ac_peak_19877	8.30974	GNAZ_ENSG00000128266
22	23412664	23413339	KD2-H4K8ac_peak_19878	7.99241	GNAZ_ENSG00000128266
22	23453518	23453879	KD2-H4K8ac_peak_19879	5.71749	
22	23455563	23455857	KD2-H4K8ac_peak_19880	6.30229	
22	23483829	23484111	KD2-H4K8ac_peak_19881	5.30799	
22	23522160	23522472	KD2-H4K8ac_peak_19882	7.47462	BCR_ENSG00000186716;BCRP8_ENSG00000236794
22	23744510	23744814	KD2-H4K8ac_peak_19883	8.28234	ZDHHC8P1_ENSG00000133519
22	23745051	23745596	KD2-H4K8ac_peak_19884	17.51891	ZDHHC8P1_ENSG00000133519
22	23778262	23778696	KD2-H4K8ac_peak_19885	12.17278	
22	23778935	23779362	KD2-H4K8ac_peak_19886	5.59859	
22	23779584	23779868	KD2-H4K8ac_peak_19887	4.22101	
22	23799094	23799968	KD2-H4K8ac_peak_19888	7.76329	
22	23803042	23803261	KD2-H4K8ac_peak_19889	7.22936	
22	23806703	23806995	KD2-H4K8ac_peak_19890	6.94255	
22	23843053	23843258	KD2-H4K8ac_peak_19891	4.98072	
22	23856824	23857084	KD2-H4K8ac_peak_19892	11.51672	
22	23860881	23861344	KD2-H4K8ac_peak_19893	9.6247	
22	23862787	23865898	KD2-H4K8ac_peak_19894	30.32576	
22	23875120	23875480	KD2-H4K8ac_peak_19895	5.20908	
22	23876604	23876808	KD2-H4K8ac_peak_19896	6.56211	
22	23907847	23908838	KD2-H4K8ac_peak_19897	9.28039	AP000345.2_ENSG00000224277
22	24058891	24060103	KD2-H4K8ac_peak_19898	11.04256	GUSBP11_ENSG00000228315;KB-1572G7.2_ENSG00000273000;AP000347.2_ENSG00000272578
22	24093465	24094522	KD2-H4K8ac_peak_19899	12.93209	ZNF70_ENSG00000187792
22	24109905	24110814	KD2-H4K8ac_peak_19900	9.34989	CHCHD10_ENSG00000250479;MMP11_ENSG00000099953
22	24113357	24113928	KD2-H4K8ac_peak_19901	6.72624	
22	24114589	24115413	KD2-H4K8ac_peak_19902	13.70605	
22	24128522	24129008	KD2-H4K8ac_peak_19903	17.42905	SMARCB1_ENSG00000099956
22	24129201	24129522	KD2-H4K8ac_peak_19904	32.77198	SMARCB1_ENSG00000099956
22	24168100	24168302	KD2-H4K8ac_peak_19905	6.90231	
22	24187307	24187994	KD2-H4K8ac_peak_19906	7.36799	
22	24188307	24188762	KD2-H4K8ac_peak_19907	13.34898	
22	24189075	24189409	KD2-H4K8ac_peak_19908	6.04107	
22	24191144	24191796	KD2-H4K8ac_peak_19909	16.14659	
22	24198888	24199167	KD2-H4K8ac_peak_19910	10.63441	KB-1125A3.11_ENSG00000272973;SLC2A11_ENSG00000133460
22	24199383	24199653	KD2-H4K8ac_peak_19911	5.4779	KB-1125A3.11_ENSG00000272973;SLC2A11_ENSG00000133460
22	24200135	24200476	KD2-H4K8ac_peak_19912	9.63487	KB-1125A3.11_ENSG00000272973
22	24236027	24237602	KD2-H4K8ac_peak_19913	12.69604	MIF_ENSG00000240972
22	24255613	24256135	KD2-H4K8ac_peak_19914	14.74047	
22	24359582	24360027	KD2-H4K8ac_peak_19915	9.36516	
22	24360376	24360580	KD2-H4K8ac_peak_19916	3.87414	

22	24407862	24408085	KD2-H4K8ac_peak_19917	8.16906	CABIN1_ENSG00000099991
22	24638444	24638811	KD2-H4K8ac_peak_19918	7.40111	
22	24639380	24640813	KD2-H4K8ac_peak_19919	13.60392	GGT5_ENSG00000099998
22	24641221	24641931	KD2-H4K8ac_peak_19920	9.20193	GGT5_ENSG00000099998
22	24643084	24643912	KD2-H4K8ac_peak_19921	7.49599	
22	24655188	24655490	KD2-H4K8ac_peak_19922	9.11031	
22	24660926	24661170	KD2-H4K8ac_peak_19923	6.09229	
22	24666868	24667383	KD2-H4K8ac_peak_19924	12.93209	AP000354.2_ENSG00000215464;SPECC1L_ENSG00000100014;SPECC1L-ADORA2A_ENSG00000258555
22	24720650	24720928	KD2-H4K8ac_peak_19925	5.14125	
22	24755493	24755730	KD2-H4K8ac_peak_19926	7.12754	
22	24775871	24776118	KD2-H4K8ac_peak_19927	9.11031	
22	24789073	24789428	KD2-H4K8ac_peak_19928	3.90565	
22	24797893	24798532	KD2-H4K8ac_peak_19929	12.2973	
22	24799543	24800840	KD2-H4K8ac_peak_19930	14.67919	
22	24802280	24802530	KD2-H4K8ac_peak_19931	4.06758	
22	24803097	24803516	KD2-H4K8ac_peak_19932	6.99815	
22	24803887	24804135	KD2-H4K8ac_peak_19933	9.09777	
22	24811737	24812035	KD2-H4K8ac_peak_19934	7.20567	
22	24812490	24812941	KD2-H4K8ac_peak_19935	6.55628	ADORA2A_ENSG00000128271
22	24816432	24816624	KD2-H4K8ac_peak_19936	5.72327	
22	24819625	24819828	KD2-H4K8ac_peak_19937	9.90319	
22	24820059	24820656	KD2-H4K8ac_peak_19938	12.70308	
22	24828872	24829334	KD2-H4K8ac_peak_19939	4.56666	
22	24840342	24841850	KD2-H4K8ac_peak_19940	7.76104	
22	24845527	24846463	KD2-H4K8ac_peak_19941	11.64359	
22	24919146	24919903	KD2-H4K8ac_peak_19942	7.95563	
22	24920265	24920507	KD2-H4K8ac_peak_19943	7.49599	
22	24950586	24951078	KD2-H4K8ac_peak_19944	5.75452	SNRPD3_ENSG00000100028;GUCD1_ENSG00000138867
22	24951428	24952212	KD2-H4K8ac_peak_19945	17.94825	SNRPD3_ENSG00000100028;GUCD1_ENSG00000138867
22	24984548	24984921	KD2-H4K8ac_peak_19946	4.68059	
22	24988519	24989410	KD2-H4K8ac_peak_19947	16.49944	FAM211B_ENSG00000178026
22	25018854	25019257	KD2-H4K8ac_peak_19948	6.31143	
22	25029224	25029424	KD2-H4K8ac_peak_19949	8.85813	BCRP3_ENSG00000215481
22	25201868	25202641	KD2-H4K8ac_peak_19950	8.4716	SGSM1_ENSG00000167037
22	25289367	25289573	KD2-H4K8ac_peak_19951	6.47962	
22	25289989	25290266	KD2-H4K8ac_peak_19952	5.75757	
22	25346211	25346418	KD2-H4K8ac_peak_19953	9.40857	
22	25348247	25348502	KD2-H4K8ac_peak_19954	5.29555	KIAA1671_ENSG00000197077
22	25363489	25363869	KD2-H4K8ac_peak_19955	5.91443	
22	25366175	25366455	KD2-H4K8ac_peak_19956	10.43145	
22	25372582	25372832	KD2-H4K8ac_peak_19957	5.02474	
22	25373327	25373666	KD2-H4K8ac_peak_19958	7.40111	
22	25457629	25457834	KD2-H4K8ac_peak_19959	4.42765	
22	25464257	25464589	KD2-H4K8ac_peak_19960	6.97919	
22	25616374	25616579	KD2-H4K8ac_peak_19961	6.8836	CRYBB2_ENSG00000244752
22	25631712	25631906	KD2-H4K8ac_peak_19962	10.63441	

22	25816431	25816655	KD2-H4K8ac_peak_19963	8.1233	
22	25816919	25817199	KD2-H4K8ac_peak_19964	13.57066	
22	25871329	25871523	KD2-H4K8ac_peak_19965	4.01115	CTA-390C10.10_ENSG00000272977
22	25871960	25872564	KD2-H4K8ac_peak_19966	9.15329	CTA-390C10.10_ENSG00000272977
22	25877067	25877271	KD2-H4K8ac_peak_19967	10.21502	
22	25878865	25879136	KD2-H4K8ac_peak_19968	4.77079	
22	25888571	25889160	KD2-H4K8ac_peak_19969	8.1233	
22	25960183	25960744	KD2-H4K8ac_peak_19970	13.02811	CTA-407F11.8_ENSG00000234884;ADRBK2_ENSG00000100077
22	25961052	25961345	KD2-H4K8ac_peak_19971	15.61313	CTA-407F11.8_ENSG00000234884;ADRBK2_ENSG00000100077
22	26091971	26092244	KD2-H4K8ac_peak_19972	8.55271	
22	26093495	26093816	KD2-H4K8ac_peak_19973	5.75757	
22	26143914	26144440	KD2-H4K8ac_peak_19974	6.66692	
22	26165063	26165479	KD2-H4K8ac_peak_19975	6.16888	
22	26171301	26171565	KD2-H4K8ac_peak_19976	10.96455	
22	26175718	26176508	KD2-H4K8ac_peak_19977	13.80019	
22	26177650	26177869	KD2-H4K8ac_peak_19978	5.88845	
22	26178233	26178518	KD2-H4K8ac_peak_19979	7.27716	
22	26339209	26339434	KD2-H4K8ac_peak_19980	7.44366	
22	26564361	26564680	KD2-H4K8ac_peak_19981	5.46431	SEZ6L_ENSG00000100095
22	26824829	26825042	KD2-H4K8ac_peak_19982	6.40297	ASPHD2_ENSG00000128203
22	26825401	26825606	KD2-H4K8ac_peak_19983	5.20998	ASPHD2_ENSG00000128203
22	26879331	26879785	KD2-H4K8ac_peak_19984	20.02161	HPS4_ENSG00000100099;SRRD_ENSG00000100104
22	26908594	26909064	KD2-H4K8ac_peak_19985	7.27716	TFIP11_ENSG00000100109;CTA-445C9.14_ENSG00000261188
22	27013133	27013521	KD2-H4K8ac_peak_19986	6.27931	CRYBB1_ENSG00000100122
22	27013864	27014408	KD2-H4K8ac_peak_19987	8.01894	CRYBB1_ENSG00000100122
22	27041023	27041674	KD2-H4K8ac_peak_19988	9.89126	ISCA2P1_ENSG00000226912;MIAT_ENSG00000225783
22	27044066	27044693	KD2-H4K8ac_peak_19989	11.49574	
22	27053037	27053230	KD2-H4K8ac_peak_19990	10.19949	
22	27053551	27053761	KD2-H4K8ac_peak_19991	5.9696	
22	27054455	27055224	KD2-H4K8ac_peak_19992	13.56049	
22	27065648	27065910	KD2-H4K8ac_peak_19993	9.11031	
22	27067912	27068896	KD2-H4K8ac_peak_19994	13.73635	CTA-373H7.7_ENSG00000206028;CTA-211A9.5_ENSG00000244625
22	27069128	27069524	KD2-H4K8ac_peak_19995	7.76329	CTA-373H7.7_ENSG00000206028;CTA-211A9.5_ENSG00000244625
22	27152780	27153150	KD2-H4K8ac_peak_19996	9.89126	
22	28111315	28111670	KD2-H4K8ac_peak_19997	4.77079	RP1-213J1P__B.1_ENSG00000227838;RP11-375H17.1_ENSG00000226169
22	28197222	28198489	KD2-H4K8ac_peak_19998	14.8083	MN1_ENSG00000169184
22	28314989	28315181	KD2-H4K8ac_peak_19999	5.61067	PITPNB_ENSG00000180957;TTC28-AS1_ENSG00000235954
22	28315573	28315881	KD2-H4K8ac_peak_20000	7.98621	PITPNB_ENSG00000180957;TTC28-AS1_ENSG00000235954;MIR3199-2_ENSG00000264073
22	28411464	28411821	KD2-H4K8ac_peak_20001	7.146	
22	28413065	28413446	KD2-H4K8ac_peak_20002	7.48309	
22	28413765	28414112	KD2-H4K8ac_peak_20003	8.30974	
22	28416897	28417109	KD2-H4K8ac_peak_20004	4.211	
22	28430220	28430739	KD2-H4K8ac_peak_20005	12.93253	
22	29026084	29026372	KD2-H4K8ac_peak_20006	4.01115	
22	29076014	29076540	KD2-H4K8ac_peak_20007	12.79505	TTC28_ENSG00000100154
22	29138097	29138406	KD2-H4K8ac_peak_20008	12.84642	CHEK2_ENSG00000183765;HSCB_ENSG00000100209

22	29168044	29168421	KD2-H4K8ac_peak_20009	7.76329	CCDC117_ENSG00000159873
22	29168762	29168962	KD2-H4K8ac_peak_20010	9.51625	CCDC117_ENSG00000159873
22	29196159	29196446	KD2-H4K8ac_peak_20011	5.9696	XBP1_ENSG00000100219;CTA-292E10.6_ENSG00000226471
22	29280076	29280296	KD2-H4K8ac_peak_20012	6.60096	ZNRF3_ENSG00000183579
22	29319854	29320165	KD2-H4K8ac_peak_20013	5.25071	
22	29426776	29427019	KD2-H4K8ac_peak_20014	5.14125	ZNRF3-AS1_ENSG00000177993
22	29427580	29427790	KD2-H4K8ac_peak_20015	5.54647	ZNRF3-AS1_ENSG00000177993
22	29469061	29469842	KD2-H4K8ac_peak_20016	8.10216	KREMEN1_ENSG00000183762
22	29601518	29601809	KD2-H4K8ac_peak_20017	10.73134	EMID1_ENSG00000186998
22	29663625	29664828	KD2-H4K8ac_peak_20018	23.72814	RHBDD3_ENSG00000100263;EWSR1_ENSG00000182944
22	29702019	29703430	KD2-H4K8ac_peak_20019	13.73635	GAS2L1_ENSG00000185340
22	29714590	29714848	KD2-H4K8ac_peak_20020	11.05056	RASL10A_ENSG00000100276
22	29784017	29784542	KD2-H4K8ac_peak_20021	17.76996	
22	29876246	29876981	KD2-H4K8ac_peak_20022	10.66719	NEFH_ENSG00000100285
22	29877588	29877872	KD2-H4K8ac_peak_20023	4.42765	
22	29949223	29949646	KD2-H4K8ac_peak_20024	12.65056	
22	29950079	29950710	KD2-H4K8ac_peak_20025	7.167	THOC5_ENSG00000100296
22	29976024	29977086	KD2-H4K8ac_peak_20026	11.22681	NIPSNAP1_ENSG00000184117
22	29977560	29977902	KD2-H4K8ac_peak_20027	5.01002	NIPSNAP1_ENSG00000184117
22	29998691	29998884	KD2-H4K8ac_peak_20028	6.56211	NF2_ENSG00000186575
22	29999727	30000029	KD2-H4K8ac_peak_20029	11.55418	NF2_ENSG00000186575
22	30101217	30101796	KD2-H4K8ac_peak_20030	6.72464	
22	30106418	30106714	KD2-H4K8ac_peak_20031	4.93968	
22	30107667	30107882	KD2-H4K8ac_peak_20032	4.52514	
22	30162595	30162838	KD2-H4K8ac_peak_20033	8.30974	ZMAT5_ENSG00000100319;UQCR10_ENSG00000184076
22	30163393	30163604	KD2-H4K8ac_peak_20034	11.05056	ZMAT5_ENSG00000100319;UQCR10_ENSG00000184076
22	30279225	30279508	KD2-H4K8ac_peak_20035	9.71513	MTMR3_ENSG00000100330
22	30604872	30605108	KD2-H4K8ac_peak_20036	5.54647	
22	30641889	30642651	KD2-H4K8ac_peak_20037	16.18205	LIF_ENSG00000128342;RP1-102K2.8_ENSG00000268812
22	30646835	30647233	KD2-H4K8ac_peak_20038	4.41495	
22	30648598	30649102	KD2-H4K8ac_peak_20039	6.66359	
22	30649676	30650139	KD2-H4K8ac_peak_20040	5.54647	
22	30650386	30650982	KD2-H4K8ac_peak_20041	9.01743	
22	30651912	30652567	KD2-H4K8ac_peak_20042	5.37864	
22	30686935	30687372	KD2-H4K8ac_peak_20043	7.47462	
22	30752615	30752862	KD2-H4K8ac_peak_20044	17.27647	SF3A1_ENSG00000099995;CCDC157_ENSG00000187860
22	30782560	30783353	KD2-H4K8ac_peak_20045	9.63487	
22	30783586	30784086	KD2-H4K8ac_peak_20046	9.34195	
22	30793027	30793479	KD2-H4K8ac_peak_20047	6.30607	SEC14L2_ENSG00000100003
22	30819024	30819847	KD2-H4K8ac_peak_20048	9.63487	
22	30820285	30821190	KD2-H4K8ac_peak_20049	13.46824	MTFP1_ENSG00000242114
22	30821447	30822019	KD2-H4K8ac_peak_20050	15.25814	MTFP1_ENSG00000242114
22	30831082	30831301	KD2-H4K8ac_peak_20051	12.42547	RP4-539M6.22_ENSG00000273428;AC004832.1_ENSG00000203606
22	30831664	30832312	KD2-H4K8ac_peak_20052	11.30637	RP4-539M6.22_ENSG00000273428;AC004832.1_ENSG00000203606
22	30840947	30841233	KD2-H4K8ac_peak_20053	9.73431	
22	30987476	30988091	KD2-H4K8ac_peak_20054	9.10868	

22	31001325	31001695	KD2-H4K8ac_peak_20055	7.1181	
22	31003269	31003470	KD2-H4K8ac_peak_20056	5.05318	PES1_ENSG00000100029;TCN2_ENSG00000185339
22	31030684	31031428	KD2-H4K8ac_peak_20057	13.73635	SLC35E4_ENSG00000100036
22	31064099	31064479	KD2-H4K8ac_peak_20058	7.38799	DUSP18_ENSG00000167065
22	31070966	31071382	KD2-H4K8ac_peak_20059	8.17902	
22	31072117	31072328	KD2-H4K8ac_peak_20060	5.75452	
22	31090132	31090520	KD2-H4K8ac_peak_20061	7.27716	OSBP2_ENSG00000184792
22	31090937	31091228	KD2-H4K8ac_peak_20062	8.58725	
22	31271984	31272643	KD2-H4K8ac_peak_20063	5.88845	
22	31275485	31275812	KD2-H4K8ac_peak_20064	6.99815	
22	31364488	31365919	KD2-H4K8ac_peak_20065	23.29435	MORC2_ENSG00000133422;TUG1_ENSG00000253352
22	31455799	31456029	KD2-H4K8ac_peak_20066	4.77079	RN7SL633P_ENSG00000240186
22	31480718	31481680	KD2-H4K8ac_peak_20067	10.05606	
22	31502933	31503494	KD2-H4K8ac_peak_20068	5.9696	
22	31518654	31518846	KD2-H4K8ac_peak_20069	7.36799	INPP5J_ENSG00000185133
22	31521372	31521750	KD2-H4K8ac_peak_20070	11.43353	
22	31539169	31539498	KD2-H4K8ac_peak_20071	4.47369	
22	31555626	31556045	KD2-H4K8ac_peak_20072	11.43353	MIR3928_ENSG00000264141;RNF185_ENSG00000138942
22	31625461	31625942	KD2-H4K8ac_peak_20073	5.57044	Y_RNA_ENSG00000202019
22	31739980	31740174	KD2-H4K8ac_peak_20074	7.51005	
22	31742112	31742510	KD2-H4K8ac_peak_20075	7.67504	PATZ1_ENSG00000100105;AC005003.1_ENSG00000213888
22	31742735	31743790	KD2-H4K8ac_peak_20076	16.67374	PATZ1_ENSG00000100105;AC005003.1_ENSG00000213888
22	31745812	31746152	KD2-H4K8ac_peak_20077	3.75035	
22	31795087	31795312	KD2-H4K8ac_peak_20078	7.76329	DRG1_ENSG00000185721
22	31795735	31796030	KD2-H4K8ac_peak_20079	7.67504	DRG1_ENSG00000185721
22	31885224	31885552	KD2-H4K8ac_peak_20080	9.402	SFI1_ENSG00000198089
22	31885916	31886325	KD2-H4K8ac_peak_20081	17.94825	
22	31892102	31892357	KD2-H4K8ac_peak_20082	8.82366	EIF4ENIF1_ENSG00000184708
22	31892585	31892887	KD2-H4K8ac_peak_20083	4.44527	EIF4ENIF1_ENSG00000184708
22	32025862	32026292	KD2-H4K8ac_peak_20084	10.54073	
22	32061258	32061582	KD2-H4K8ac_peak_20085	6.27931	
22	32145312	32145504	KD2-H4K8ac_peak_20086	5.20998	PRR14L_ENSG00000183530
22	32145745	32146064	KD2-H4K8ac_peak_20087	9.20193	PRR14L_ENSG00000183530
22	32148354	32148566	KD2-H4K8ac_peak_20088	8.66368	
22	32149393	32149773	KD2-H4K8ac_peak_20089	14.56955	DEPDC5_ENSG00000100150
22	32150030	32150297	KD2-H4K8ac_peak_20090	12.14909	DEPDC5_ENSG00000100150
22	32324846	32325185	KD2-H4K8ac_peak_20091	4.06758	
22	32325609	32325941	KD2-H4K8ac_peak_20092	9.26823	
22	32339431	32340267	KD2-H4K8ac_peak_20093	15.52424	YWHAH_ENSG00000128245
22	32340505	32341395	KD2-H4K8ac_peak_20094	22.07309	YWHAH_ENSG00000128245;C22orf24_ENSG00000128254
22	32601310	32601518	KD2-H4K8ac_peak_20095	4.52326	RFPL2_ENSG00000128253;RP1-90G24.10_ENSG00000242082
22	32869779	32870020	KD2-H4K8ac_peak_20096	7.27716	FBXO7_ENSG00000100225
22	32870788	32871145	KD2-H4K8ac_peak_20097	11.55163	FBXO7_ENSG00000100225
22	32928310	32928635	KD2-H4K8ac_peak_20098	7.146	
22	33140828	33141049	KD2-H4K8ac_peak_20099	4.17128	
22	33276034	33276541	KD2-H4K8ac_peak_20100	4.84794	

22	33277495	33278352	KD2-H4K8ac_peak_20101	24.47492	
22	33279500	33280104	KD2-H4K8ac_peak_20102	4.86324	
22	33281836	33282039	KD2-H4K8ac_peak_20103	5.1313	
22	33282908	33283147	KD2-H4K8ac_peak_20104	7.01658	
22	33299496	33299697	KD2-H4K8ac_peak_20105	5.14125	
22	33300328	33301502	KD2-H4K8ac_peak_20106	7.9265	
22	33301872	33302124	KD2-H4K8ac_peak_20107	5.9696	
22	33307351	33307778	KD2-H4K8ac_peak_20108	5.14125	
22	33313572	33314049	KD2-H4K8ac_peak_20109	6.66359	
22	33314854	33315336	KD2-H4K8ac_peak_20110	10.43145	
22	33346096	33347360	KD2-H4K8ac_peak_20111	8.16906	
22	33347587	33347818	KD2-H4K8ac_peak_20112	9.34989	
22	33348097	33348692	KD2-H4K8ac_peak_20113	7.47462	
22	33352834	33353141	KD2-H4K8ac_peak_20114	6.2781	
22	33353600	33354375	KD2-H4K8ac_peak_20115	9.11031	
22	33359848	33360135	KD2-H4K8ac_peak_20116	8.62078	
22	33383249	33383590	KD2-H4K8ac_peak_20117	4.42765	
22	33387845	33388238	KD2-H4K8ac_peak_20118	9.51625	
22	33396550	33396751	KD2-H4K8ac_peak_20119	8.59579	
22	33454582	33454882	KD2-H4K8ac_peak_20120	12.85889	SYN3_ENSG00000185666
22	34317580	34317966	KD2-H4K8ac_peak_20121	7.47462	LARGE_ENSG00000133424;LL22NC03-32F9.1_ENSG00000273082
22	35552834	35553124	KD2-H4K8ac_peak_20122	8.1233	
22	35653526	35654039	KD2-H4K8ac_peak_20123	12.00597	HMGXB4_ENSG00000100281
22	35746609	35746810	KD2-H4K8ac_peak_20124	9.56945	
22	35777090	35777575	KD2-H4K8ac_peak_20125	13.31016	HMOX1_ENSG00000100292
22	35795187	35795714	KD2-H4K8ac_peak_20126	8.53092	MCM5_ENSG00000100297
22	35848426	35849114	KD2-H4K8ac_peak_20127	6.99815	
22	35863718	35864313	KD2-H4K8ac_peak_20128	13.09607	
22	35885096	35885530	KD2-H4K8ac_peak_20129	10.05606	
22	35919374	35919771	KD2-H4K8ac_peak_20130	10.53596	
22	35920263	35920553	KD2-H4K8ac_peak_20131	5.6755	
22	35922011	35922585	KD2-H4K8ac_peak_20132	8.09472	RP4-569D19.8_ENSG00000273044
22	35936100	35936431	KD2-H4K8ac_peak_20133	9.66321	RASD2_ENSG00000100302
22	35982792	35983101	KD2-H4K8ac_peak_20134	6.9556	
22	35988625	35988947	KD2-H4K8ac_peak_20135	9.242	
22	36001752	36002119	KD2-H4K8ac_peak_20136	14.27217	
22	36002469	36002831	KD2-H4K8ac_peak_20137	11.05467	
22	36003247	36003893	KD2-H4K8ac_peak_20138	5.88845	
22	36034981	36035256	KD2-H4K8ac_peak_20139	5.45396	MB_ENSG00000198125
22	36312351	36312806	KD2-H4K8ac_peak_20140	7.47462	
22	36366402	36366869	KD2-H4K8ac_peak_20141	7.13615	
22	36370404	36371040	KD2-H4K8ac_peak_20142	6.30229	
22	36404599	36404801	KD2-H4K8ac_peak_20143	4.57371	
22	36413276	36413502	KD2-H4K8ac_peak_20144	7.47462	
22	36414517	36414795	KD2-H4K8ac_peak_20145	4.01115	
22	36424042	36424938	KD2-H4K8ac_peak_20146	12.2054	RBFOX2_ENSG00000100320

22	36430843	36431119	KD2-H4K8ac_peak_20147	8.37231	
22	36433246	36433817	KD2-H4K8ac_peak_20148	8.85813	
22	36461518	36461793	KD2-H4K8ac_peak_20149	4.06758	
22	36462156	36463036	KD2-H4K8ac_peak_20150	6.66359	
22	36506220	36506690	KD2-H4K8ac_peak_20151	8.94469	
22	36597404	36598443	KD2-H4K8ac_peak_20152	6.40297	
22	36634919	36635228	KD2-H4K8ac_peak_20153	3.90565	APOL2_ENSG00000128335
22	36651087	36651542	KD2-H4K8ac_peak_20154	10.46264	
22	36783596	36783887	KD2-H4K8ac_peak_20155	4.67342	MYH9_ENSG00000100345;RP4-633O19__A.1_ENSG00000223695
22	36828592	36828800	KD2-H4K8ac_peak_20156	4.66652	
22	36850856	36851902	KD2-H4K8ac_peak_20157	13.70605	RP5-1119A7.14_ENSG00000228719
22	36877055	36878075	KD2-H4K8ac_peak_20158	15.81711	TXN2_ENSG00000100348
22	36878405	36878624	KD2-H4K8ac_peak_20159	8.55271	TXN2_ENSG00000100348
22	36902286	36902840	KD2-H4K8ac_peak_20160	10.05081	FOXRED2_ENSG00000100350
22	36903505	36903717	KD2-H4K8ac_peak_20161	4.8888	FOXRED2_ENSG00000100350
22	36925873	36926513	KD2-H4K8ac_peak_20162	7.36799	EIF3D_ENSG00000100353
22	36966110	36966321	KD2-H4K8ac_peak_20163	5.88845	
22	37055901	37056719	KD2-H4K8ac_peak_20164	6.40683	
22	37139947	37140433	KD2-H4K8ac_peak_20165	6.0073	
22	37172757	37173057	KD2-H4K8ac_peak_20166	10.19949	IFT27_ENSG00000100360
22	37373448	37374011	KD2-H4K8ac_peak_20167	7.47462	
22	37376630	37377165	KD2-H4K8ac_peak_20168	5.73839	
22	37415296	37415743	KD2-H4K8ac_peak_20169	22.21705	TST_ENSG00000128311;MPST_ENSG00000128309
22	37415961	37416329	KD2-H4K8ac_peak_20170	11.92978	TST_ENSG00000128311;MPST_ENSG00000128309
22	37430094	37430340	KD2-H4K8ac_peak_20171	6.40683	
22	37447819	37448091	KD2-H4K8ac_peak_20172	11.66477	KCTD17_ENSG00000100379
22	37562876	37563138	KD2-H4K8ac_peak_20173	7.0754	RP1-151B14.6_ENSG00000235237
22	37583048	37583408	KD2-H4K8ac_peak_20174	8.09303	
22	37583716	37584214	KD2-H4K8ac_peak_20175	10.43145	
22	37594932	37595401	KD2-H4K8ac_peak_20176	15.69572	C1QTNF6_ENSG00000133466
22	37595741	37596125	KD2-H4K8ac_peak_20177	14.39182	C1QTNF6_ENSG00000133466
22	37607946	37608145	KD2-H4K8ac_peak_20178	7.27716	SSTR3_ENSG00000183473
22	37608642	37608894	KD2-H4K8ac_peak_20179	7.47462	SSTR3_ENSG00000183473
22	37625092	37625390	KD2-H4K8ac_peak_20180	4.95193	
22	37663300	37663953	KD2-H4K8ac_peak_20181	16.11042	
22	37740878	37742382	KD2-H4K8ac_peak_20182	8.10216	
22	37767374	37768063	KD2-H4K8ac_peak_20183	5.57044	RP1-63G5.8_ENSG00000272694
22	37816403	37816704	KD2-H4K8ac_peak_20184	8.30974	
22	37860076	37860316	KD2-H4K8ac_peak_20185	6.8836	
22	37879767	37880191	KD2-H4K8ac_peak_20186	9.51688	
22	37882145	37882745	KD2-H4K8ac_peak_20187	5.88845	MFNG_ENSG00000100060
22	37883060	37883307	KD2-H4K8ac_peak_20188	5.88845	MFNG_ENSG00000100060
22	37904447	37905790	KD2-H4K8ac_peak_20189	14.22723	
22	37914816	37915338	KD2-H4K8ac_peak_20190	9.34195	CARD10_ENSG00000100065
22	37955987	37956315	KD2-H4K8ac_peak_20191	9.36516	CDC42EP1_ENSG00000128283
22	37957040	37957806	KD2-H4K8ac_peak_20192	10.05606	CDC42EP1_ENSG00000128283

22	38004982	38005411	KD2-H4K8ac_peak_20193	10.84242	GGA1_ENSG00000100083
22	38029180	38029566	KD2-H4K8ac_peak_20194	11.28765	
22	38031497	38031785	KD2-H4K8ac_peak_20195	4.90805	SH3BP1_ENSG00000100092
22	38035119	38035746	KD2-H4K8ac_peak_20196	5.4779	
22	38070502	38071389	KD2-H4K8ac_peak_20197	8.10216	LGALS1_ENSG00000100097
22	38071713	38072080	KD2-H4K8ac_peak_20198	5.98764	LGALS1_ENSG00000100097
22	38072444	38072967	KD2-H4K8ac_peak_20199	5.13615	LGALS1_ENSG00000100097
22	38081771	38083110	KD2-H4K8ac_peak_20200	14.275	
22	38093071	38093590	KD2-H4K8ac_peak_20201	8.85813	TRIOBP_ENSG00000100106
22	38137087	38137303	KD2-H4K8ac_peak_20202	6.30607	
22	38141747	38142028	KD2-H4K8ac_peak_20203	18.54875	
22	38142347	38143042	KD2-H4K8ac_peak_20204	11.80283	
22	38147634	38148262	KD2-H4K8ac_peak_20205	7.146	
22	38148670	38149047	KD2-H4K8ac_peak_20206	9.1707	
22	38198086	38198837	KD2-H4K8ac_peak_20207	19.88442	
22	38200016	38201129	KD2-H4K8ac_peak_20208	23.08992	H1F0_ENSG00000189060
22	38214474	38214930	KD2-H4K8ac_peak_20209	9.23578	
22	38240031	38240322	KD2-H4K8ac_peak_20210	8.16906	MIR658_ENSG00000207945
22	38244421	38245212	KD2-H4K8ac_peak_20211	9.51625	ANKRD54_ENSG00000100124;MIR659_ENSG00000207696;EIF3L_ENSG00000100129
22	38245465	38245724	KD2-H4K8ac_peak_20212	10.19949	ANKRD54_ENSG00000100124;EIF3L_ENSG00000100129
22	38301299	38303020	KD2-H4K8ac_peak_20213	12.93209	MICALL1_ENSG00000100139
22	38453194	38453596	KD2-H4K8ac_peak_20214	9.15329	PICK1_ENSG00000100151
22	38476878	38477470	KD2-H4K8ac_peak_20215	8.1233	
22	38669088	38669317	KD2-H4K8ac_peak_20216	8.1233	TMEM184B_ENSG00000198792
22	38712886	38713300	KD2-H4K8ac_peak_20217	10.21502	
22	38713558	38714078	KD2-H4K8ac_peak_20218	4.42765	
22	38851765	38852113	KD2-H4K8ac_peak_20219	7.12754	KCNJ4_ENSG00000168135
22	38857327	38857584	KD2-H4K8ac_peak_20220	9.02371	
22	38901510	38902152	KD2-H4K8ac_peak_20221	8.1718	
22	38902720	38903543	KD2-H4K8ac_peak_20222	8.59579	DDX17_ENSG00000100201
22	38965792	38965987	KD2-H4K8ac_peak_20223	7.12754	DMC1_ENSG00000100206
22	38966484	38966741	KD2-H4K8ac_peak_20224	9.15329	DMC1_ENSG00000100206
22	39052425	39052917	KD2-H4K8ac_peak_20225	13.57066	FAM227A_ENSG00000184949;CBY1_ENSG00000100211
22	39078108	39078443	KD2-H4K8ac_peak_20226	7.12754	RP3-508I15.9_ENSG00000228274;TOMM22_ENSG00000100216
22	39102004	39102206	KD2-H4K8ac_peak_20227	8.1233	GTPBP1_ENSG00000100226
22	39151550	39151820	KD2-H4K8ac_peak_20228	7.27716	
22	39152215	39152462	KD2-H4K8ac_peak_20229	6.66692	
22	39158853	39159091	KD2-H4K8ac_peak_20230	5.07241	
22	39165090	39165374	KD2-H4K8ac_peak_20231	4.77079	
22	39190559	39190778	KD2-H4K8ac_peak_20232	10.21502	SUN2_ENSG00000100242;DNAL4_ENSG00000100246
22	39268854	39269171	KD2-H4K8ac_peak_20233	12.06346	CBX6_ENSG00000183741
22	39269891	39270088	KD2-H4K8ac_peak_20234	7.12754	
22	39409758	39409989	KD2-H4K8ac_peak_20235	7.27716	APOBEC3C_ENSG00000244509;APOBEC3D_ENSG00000243811
22	39416565	39416791	KD2-H4K8ac_peak_20236	7.27716	
22	39417334	39417649	KD2-H4K8ac_peak_20237	4.42765	
22	39436830	39437034	KD2-H4K8ac_peak_20238	11.30637	APOBEC3F_ENSG00000128394;APOBEC3G_ENSG00000239713

22	39493281	39493610	KD2-H4K8ac_peak_20239	8.1233	APOBEC3H_ENSG00000100298
22	39541367	39541621	KD2-H4K8ac_peak_20240	8.17902	
22	39715956	39716383	KD2-H4K8ac_peak_20241	10.21502	RPL3_ENSG00000100316;SNORD43_ENSG00000263764
22	39744954	39745637	KD2-H4K8ac_peak_20242	5.45396	SYNGR1_ENSG00000100321
22	39795276	39795479	KD2-H4K8ac_peak_20243	6.16888	TAB1_ENSG00000100324
22	39795809	39796120	KD2-H4K8ac_peak_20244	17.14886	TAB1_ENSG00000100324
22	39833708	39833948	KD2-H4K8ac_peak_20245	11.30637	
22	39852827	39853099	KD2-H4K8ac_peak_20246	10.08707	MGAT3_ENSG00000128268
22	39853531	39854333	KD2-H4K8ac_peak_20247	5.88845	MGAT3_ENSG00000128268
22	39886478	39886689	KD2-H4K8ac_peak_20248	8.1233	
22	39897844	39898116	KD2-H4K8ac_peak_20249	9.59147	
22	39915525	39915997	KD2-H4K8ac_peak_20250	4.42765	ATF4_ENSG00000128272
22	39916633	39916927	KD2-H4K8ac_peak_20251	5.30799	ATF4_ENSG00000128272
22	39928379	39928688	KD2-H4K8ac_peak_20252	11.00372	RPS19BP1_ENSG00000187051
22	41032288	41032645	KD2-H4K8ac_peak_20253	7.13615	MKL1_ENSG00000196588
22	41033111	41033368	KD2-H4K8ac_peak_20254	7.59643	MKL1_ENSG00000196588
22	41047911	41048114	KD2-H4K8ac_peak_20255	8.1233	
22	41048318	41048528	KD2-H4K8ac_peak_20256	6.16888	
22	41252323	41252540	KD2-H4K8ac_peak_20257	4.3015	ST13_ENSG00000100380;XPNPEP3_ENSG00000196236
22	41346661	41346967	KD2-H4K8ac_peak_20258	5.57044	RBX1_ENSG00000100387
22	41417976	41418817	KD2-H4K8ac_peak_20259	7.47462	
22	41487852	41488599	KD2-H4K8ac_peak_20260	9.11031	EP300_ENSG00000100393;MIR1281_ENSG00000221160
22	41601412	41601615	KD2-H4K8ac_peak_20261	5.57044	L3MBTL2_ENSG00000100395
22	41798030	41798683	KD2-H4K8ac_peak_20262	8.1233	
22	41809178	41809963	KD2-H4K8ac_peak_20263	11.30637	
22	41843312	41844296	KD2-H4K8ac_peak_20264	10.30284	TOB2_ENSG00000183864
22	41956573	41956782	KD2-H4K8ac_peak_20265	4.37708	CSDC2_ENSG00000172346
22	42017345	42017671	KD2-H4K8ac_peak_20266	12.42547	DESI1_ENSG00000100418;XRCC6_ENSG00000196419
22	42062930	42063197	KD2-H4K8ac_peak_20267	4.42765	
22	42195922	42196247	KD2-H4K8ac_peak_20268	7.27716	CCDC134_ENSG00000100147
22	42228309	42228608	KD2-H4K8ac_peak_20269	8.1718	SREBF2_ENSG00000198911
22	42229467	42229828	KD2-H4K8ac_peak_20270	8.30974	RP5-821D11.7_ENSG00000184068;SREBF2_ENSG00000198911
22	42469859	42470580	KD2-H4K8ac_peak_20271	9.11031	FAM109B_ENSG00000177096
22	42475076	42475475	KD2-H4K8ac_peak_20272	12.42547	SMDT1_ENSG00000183172
22	42739294	42739523	KD2-H4K8ac_peak_20273	8.1233	TCF20_ENSG00000100207
22	42743504	42743808	KD2-H4K8ac_peak_20274	6.16888	
22	42761496	42761932	KD2-H4K8ac_peak_20275	5.14125	
22	42808871	42809070	KD2-H4K8ac_peak_20276	6.16888	
22	42839832	42840081	KD2-H4K8ac_peak_20277	5.25071	
22	42977683	42977954	KD2-H4K8ac_peak_20278	7.12754	RRP7B_ENSG00000182841
22	43539639	43539892	KD2-H4K8ac_peak_20279	6.16888	MCAT_ENSG00000100294
22	43547042	43547407	KD2-H4K8ac_peak_20280	6.66692	TSPO_ENSG00000100300
22	43547637	43547842	KD2-H4K8ac_peak_20281	6.07178	TSPO_ENSG00000100300
22	43660638	43660834	KD2-H4K8ac_peak_20282	4.01115	
22	43685789	43686239	KD2-H4K8ac_peak_20283	9.10868	
22	44208353	44208689	KD2-H4K8ac_peak_20284	4.42765	EFCAB6_ENSG00000186976;RP3-388M5.9_ENSG00000261251

22	44319758	44320376	KD2-H4K8ac_peak_20285	4.86324	PNPLA3_ENSG00000100344
22	44350914	44351534	KD2-H4K8ac_peak_20286	13.80019	SAMM50_ENSG00000100347
22	44419912	44420737	KD2-H4K8ac_peak_20287	17.70837	
22	44444519	44445363	KD2-H4K8ac_peak_20288	7.36799	
22	44791033	44791418	KD2-H4K8ac_peak_20289	8.55271	
22	44893448	44894096	KD2-H4K8ac_peak_20290	35.93287	LDOC1L_ENSG00000188636
22	44894489	44894761	KD2-H4K8ac_peak_20291	9.77606	LDOC1L_ENSG00000188636
22	45036086	45036472	KD2-H4K8ac_peak_20292	7.70289	
22	45054349	45054624	KD2-H4K8ac_peak_20293	4.70206	
22	45064069	45064439	KD2-H4K8ac_peak_20294	7.76329	PRR5_ENSG00000186654
22	45072442	45073403	KD2-H4K8ac_peak_20295	12.06346	
22	45081563	45081804	KD2-H4K8ac_peak_20296	7.13615	
22	45097555	45097825	KD2-H4K8ac_peak_20297	8.02571	PRR5-ARHGAP8_ENSG00000248405;ARHGAP8_ENSG00000241484
22	45124970	45125502	KD2-H4K8ac_peak_20298	8.1235	
22	45125778	45126145	KD2-H4K8ac_peak_20299	12.93007	
22	45148136	45148364	KD2-H4K8ac_peak_20300	11.80497	
22	45154111	45154310	KD2-H4K8ac_peak_20301	5.61377	
22	45403759	45404142	KD2-H4K8ac_peak_20302	7.8711	
22	45405305	45405635	KD2-H4K8ac_peak_20303	7.36799	PHF21B_ENSG00000056487
22	45559323	45559699	KD2-H4K8ac_peak_20304	11.95211	CTA-217C2.1_ENSG00000226328;NUP50_ENSG00000093000
22	45559947	45560212	KD2-H4K8ac_peak_20305	8.17902	CTA-217C2.1_ENSG00000226328;NUP50_ENSG00000093000
22	45608077	45608527	KD2-H4K8ac_peak_20306	10.21366	
22	45637009	45637523	KD2-H4K8ac_peak_20307	15.09202	KIAA0930_ENSG00000100364
22	45641945	45642147	KD2-H4K8ac_peak_20308	5.14125	
22	45659449	45659698	KD2-H4K8ac_peak_20309	10.96455	CTA-268H5.14_ENSG00000273287
22	45660027	45660236	KD2-H4K8ac_peak_20310	8.26945	CTA-268H5.14_ENSG00000273287
22	45663760	45664009	KD2-H4K8ac_peak_20311	7.27716	
22	45665413	45665635	KD2-H4K8ac_peak_20312	4.76709	
22	45704915	45705331	KD2-H4K8ac_peak_20313	7.98621	FAM118A_ENSG00000100376
22	45897964	45898646	KD2-H4K8ac_peak_20314	12.82393	FBLN1_ENSG00000077942
22	45899831	45900336	KD2-H4K8ac_peak_20315	7.372	
22	45900589	45901058	KD2-H4K8ac_peak_20316	9.65086	
22	46067211	46068410	KD2-H4K8ac_peak_20317	15.40891	ATXN10_ENSG00000130638
22	46282177	46282418	KD2-H4K8ac_peak_20318	10.54933	
22	46390940	46391220	KD2-H4K8ac_peak_20319	12.93253	
22	46392309	46392656	KD2-H4K8ac_peak_20320	8.5092	
22	46393016	46393494	KD2-H4K8ac_peak_20321	11.15405	
22	46393709	46393953	KD2-H4K8ac_peak_20322	6.10575	
22	46402561	46404438	KD2-H4K8ac_peak_20323	10.01546	
22	46409487	46410305	KD2-H4K8ac_peak_20324	15.20126	CITF22-92A6.1_ENSG00000273145
22	46436521	46436750	KD2-H4K8ac_peak_20325	7.76329	
22	46439696	46441306	KD2-H4K8ac_peak_20326	9.11031	LINC00899_ENSG00000231711
22	46448890	46450090	KD2-H4K8ac_peak_20327	20.30085	C22orf26_ENSG00000182257;FLJ27365_ENSG00000197182;RP6-109B7.5_ENSG00000273289;RP6-109B7.3_ENSG00000241990
22	46450293	46451331	KD2-H4K8ac_peak_20328	11.05467	C22orf26_ENSG00000182257;FLJ27365_ENSG00000197182;RP6-109B7.3_ENSG00000241990
22	46451593	46452221	KD2-H4K8ac_peak_20329	14.70748	
22	46454713	46455024	KD2-H4K8ac_peak_20330	10.43145	RP6-109B7.2_ENSG00000231010

22	46460141	46460490	KD2-H4K8ac_peak_20331	12.25733	
22	46462544	46463107	KD2-H4K8ac_peak_20332	9.63925	
22	46465151	46465347	KD2-H4K8ac_peak_20333	8.83582	RP6-109B7.4_ENSG00000235159
22	46465985	46466885	KD2-H4K8ac_peak_20334	22.25695	RP6-109B7.4_ENSG00000235159
22	46467244	46468535	KD2-H4K8ac_peak_20335	10.86378	
22	46474900	46475112	KD2-H4K8ac_peak_20336	4.44527	
22	46475503	46475700	KD2-H4K8ac_peak_20337	5.81222	
22	46477383	46477672	KD2-H4K8ac_peak_20338	11.00085	
22	46479987	46480444	KD2-H4K8ac_peak_20339	5.92591	
22	46480665	46480915	KD2-H4K8ac_peak_20340	8.4716	
22	46481207	46482441	KD2-H4K8ac_peak_20341	8.4716	
22	46483373	46483688	KD2-H4K8ac_peak_20342	4.52039	
22	46518653	46518885	KD2-H4K8ac_peak_20343	6.97919	
22	46545850	46546106	KD2-H4K8ac_peak_20344	8.85813	PPARA_ENSG00000186951
22	46546795	46547198	KD2-H4K8ac_peak_20345	9.36516	PPARA_ENSG00000186951
22	46629169	46629449	KD2-H4K8ac_peak_20346	7.10552	
22	46641431	46641784	KD2-H4K8ac_peak_20347	4.33139	
22	46645731	46646844	KD2-H4K8ac_peak_20348	14.43794	CDPF1_ENSG00000205643
22	46663385	46663722	KD2-H4K8ac_peak_20349	6.25869	TTC38_ENSG00000075234
22	46663990	46664286	KD2-H4K8ac_peak_20350	8.82366	TTC38_ENSG00000075234
22	46692188	46692526	KD2-H4K8ac_peak_20351	14.70748	GTSE1_ENSG00000075218
22	46692858	46693328	KD2-H4K8ac_peak_20352	8.30974	GTSE1_ENSG00000075218
22	46731183	46732038	KD2-H4K8ac_peak_20353	16.822	
22	46956853	46957153	KD2-H4K8ac_peak_20354	11.75669	
22	46957629	46958035	KD2-H4K8ac_peak_20355	8.90387	
22	46971729	46973662	KD2-H4K8ac_peak_20356	22.21705	GRAMD4_ENSG00000075240
22	46984060	46984521	KD2-H4K8ac_peak_20357	4.42765	
22	46991537	46991888	KD2-H4K8ac_peak_20358	8.53092	
22	47008414	47008637	KD2-H4K8ac_peak_20359	5.08628	
22	47015369	47015703	KD2-H4K8ac_peak_20360	5.20998	
22	47016019	47016845	KD2-H4K8ac_peak_20361	14.69247	
22	47017589	47017834	KD2-H4K8ac_peak_20362	5.98124	
22	47133515	47134100	KD2-H4K8ac_peak_20363	13.73635	CERK_ENSG00000100422
22	47134351	47134904	KD2-H4K8ac_peak_20364	8.55271	CERK_ENSG00000100422
22	47158572	47159108	KD2-H4K8ac_peak_20365	13.95627	CTA-29F11.1_ENSG00000260708;TBC1D22A_ENSG00000054611
22	48027538	48027873	KD2-H4K8ac_peak_20366	7.47462	LINC00898_ENSG00000205634;RP11-191L9.4_ENSG00000224271
22	49031101	49031296	KD2-H4K8ac_peak_20367	6.16888	
22	49035823	49036137	KD2-H4K8ac_peak_20368	8.1233	
22	49406909	49407856	KD2-H4K8ac_peak_20369	8.94415	
22	49408138	49408427	KD2-H4K8ac_peak_20370	11.70879	
22	49410377	49411677	KD2-H4K8ac_peak_20371	7.27716	
22	49423207	49423440	KD2-H4K8ac_peak_20372	9.36516	
22	49463731	49463961	KD2-H4K8ac_peak_20373	10.34425	
22	49473035	49473647	KD2-H4K8ac_peak_20374	6.56211	
22	49545709	49546486	KD2-H4K8ac_peak_20375	8.30974	
22	49546793	49547476	KD2-H4K8ac_peak_20376	7.47462	

22	49547964	49549033	KD2-H4K8ac_peak_20377	15.06766	
22	50220633	50220997	KD2-H4K8ac_peak_20378	7.146	BRD1_ENSG00000100425
22	50221598	50221956	KD2-H4K8ac_peak_20379	19.11513	BRD1_ENSG00000100425
22	50243205	50243401	KD2-H4K8ac_peak_20380	4.42765	
22	50246763	50247123	KD2-H4K8ac_peak_20381	10.92268	ZBED4_ENSG00000100426
22	50312335	50312579	KD2-H4K8ac_peak_20382	18.62998	ALG12_ENSG00000182858;CRELD2_ENSG00000184164
22	50328681	50328989	KD2-H4K8ac_peak_20383	13.03787	
22	50339661	50339926	KD2-H4K8ac_peak_20384	7.47462	
22	50340827	50341038	KD2-H4K8ac_peak_20385	4.73064	
22	50353592	50353932	KD2-H4K8ac_peak_20386	17.75813	PIM3_ENSG00000198355
22	50354142	50354776	KD2-H4K8ac_peak_20387	15.49029	PIM3_ENSG00000198355
22	50552952	50553473	KD2-H4K8ac_peak_20388	10.63441	
22	50553991	50554425	KD2-H4K8ac_peak_20389	8.66387	
22	50584329	50584629	KD2-H4K8ac_peak_20390	5.08628	
22	50608195	50608426	KD2-H4K8ac_peak_20391	13.5195	PANX2_ENSG00000073150
22	50623686	50624089	KD2-H4K8ac_peak_20392	9.65086	TRABD_ENSG00000170638
22	50638496	50638736	KD2-H4K8ac_peak_20393	5.51783	RP3-402G11.26_ENSG00000273253;SELO_ENSG00000073169
22	50638955	50639220	KD2-H4K8ac_peak_20394	16.88883	RP3-402G11.26_ENSG00000273253;SELO_ENSG00000073169
22	50639461	50640205	KD2-H4K8ac_peak_20395	10.86378	RP3-402G11.26_ENSG00000273253;SELO_ENSG00000073169
22	50689250	50690234	KD2-H4K8ac_peak_20396	15.40891	HDAC10_ENSG00000100429
22	50698923	50700329	KD2-H4K8ac_peak_20397	10.05606	MAPK12_ENSG00000188130
22	50708170	50708441	KD2-H4K8ac_peak_20398	8.55271	MAPK11_ENSG00000185386
22	50708918	50710147	KD2-H4K8ac_peak_20399	10.66719	MAPK11_ENSG00000185386
22	50710535	50710772	KD2-H4K8ac_peak_20400	13.09607	
22	50746427	50746773	KD2-H4K8ac_peak_20401	8.4716	PLXNB2_ENSG00000196576
22	50765625	50766492	KD2-H4K8ac_peak_20402	18.39704	DENND6B_ENSG00000205593
22	50781188	50781427	KD2-H4K8ac_peak_20403	7.96322	PPP6R2_ENSG00000100239
22	50781788	50782049	KD2-H4K8ac_peak_20404	19.72117	PPP6R2_ENSG00000100239
22	50913696	50914093	KD2-H4K8ac_peak_20405	9.41619	SBF1_ENSG00000100241
22	50919707	50920825	KD2-H4K8ac_peak_20406	9.78882	ADM2_ENSG00000128165
22	50945771	50946009	KD2-H4K8ac_peak_20407	7.47462	LMF2_ENSG00000100258;NCAPH2_ENSG00000025770
22	50946671	50946894	KD2-H4K8ac_peak_20408	19.1048	LMF2_ENSG00000100258;NCAPH2_ENSG00000025770
22	50963501	50963935	KD2-H4K8ac_peak_20409	6.84261	SCO2_ENSG00000130489
22	50964237	50965357	KD2-H4K8ac_peak_20410	12.82393	SCO2_ENSG00000130489
22	50969792	50970006	KD2-H4K8ac_peak_20411	6.99194	
22	50970313	50971123	KD2-H4K8ac_peak_20412	9.95119	ODF3B_ENSG00000177989
22	51020625	51021210	KD2-H4K8ac_peak_20413	9.26408	CHKB-CPT1B_ENSG00000254413;CHKB-AS1_ENSG00000205559
22	51066090	51066495	KD2-H4K8ac_peak_20414	5.9696	ARSA_ENSG00000100299
22	51194892	51195099	KD2-H4K8ac_peak_20415	7.01964	RPL23AP82_ENSG00000184319
22	51195368	51195623	KD2-H4K8ac_peak_20416	8.59579	RPL23AP82_ENSG00000184319
3	238925	240173	KD2-H4K8ac_peak_20417	10.0746	CHL1-AS2_ENSG00000224318;CHL1_ENSG00000134121
3	334362	334634	KD2-H4K8ac_peak_20418	5.15777	
3	2342367	2342564	KD2-H4K8ac_peak_20419	6.40683	
3	2458196	2458639	KD2-H4K8ac_peak_20420	9.63487	
3	2492444	2492651	KD2-H4K8ac_peak_20421	7.12754	
3	2552591	2553374	KD2-H4K8ac_peak_20422	12.26331	

3	3168275	3168470	KD2-H4K8ac_peak_20423	10.19949	IL5RA_ENSG00000091181;TRNT1_ENSG00000072756
3	3168959	3169162	KD2-H4K8ac_peak_20424	6.13909	IL5RA_ENSG00000091181;TRNT1_ENSG00000072756
3	3840376	3840758	KD2-H4K8ac_peak_20425	20.76534	LRRN1_ENSG00000175928
3	3841147	3841593	KD2-H4K8ac_peak_20426	6.40683	LRRN1_ENSG00000175928
3	3842572	3842772	KD2-H4K8ac_peak_20427	4.42765	
3	4345030	4345716	KD2-H4K8ac_peak_20428	10.87093	SETMAR_ENSG00000170364
3	4345976	4346286	KD2-H4K8ac_peak_20429	7.27716	SETMAR_ENSG00000170364
3	4508508	4509456	KD2-H4K8ac_peak_20430	9.11031	SUMF1_ENSG00000144455
3	4534557	4534761	KD2-H4K8ac_peak_20431	7.95563	ITPR1-AS1_ENSG00000231249;ITPR1_ENSG00000150995
3	4544280	4544562	KD2-H4K8ac_peak_20432	4.86324	
3	4547027	4547254	KD2-H4K8ac_peak_20433	8.83582	
3	4549415	4549657	KD2-H4K8ac_peak_20434	6.31143	
3	4550295	4550598	KD2-H4K8ac_peak_20435	5.29352	
3	4551464	4551749	KD2-H4K8ac_peak_20436	6.00226	
3	4552578	4553365	KD2-H4K8ac_peak_20437	5.88845	
3	4553609	4554139	KD2-H4K8ac_peak_20438	13.03344	
3	4555374	4555821	KD2-H4K8ac_peak_20439	15.5861	
3	4564570	4564940	KD2-H4K8ac_peak_20440	5.25667	
3	4572785	4573218	KD2-H4K8ac_peak_20441	7.27716	
3	4574933	4575426	KD2-H4K8ac_peak_20442	6.83936	
3	4757224	4757473	KD2-H4K8ac_peak_20443	5.15777	
3	4910315	4910854	KD2-H4K8ac_peak_20444	11.58876	
3	5067911	5068126	KD2-H4K8ac_peak_20445	10.53377	
3	5069040	5069654	KD2-H4K8ac_peak_20446	8.1233	
3	5163981	5164366	KD2-H4K8ac_peak_20447	15.81711	ARL8B_ENSG00000134108
3	5229506	5229786	KD2-H4K8ac_peak_20448	6.52179	AC026202.3_ENSG00000233912;AC026202.1_ENSG00000268509;EDEM1_ENSG00000134109
3	5292817	5293062	KD2-H4K8ac_peak_20449	6.16888	MIR4790_ENSG00000265180
3	6902318	6902577	KD2-H4K8ac_peak_20450	9.1707	
3	6908438	6908734	KD2-H4K8ac_peak_20451	4.76709	
3	8570956	8571210	KD2-H4K8ac_peak_20452	4.59928	
3	8571603	8572227	KD2-H4K8ac_peak_20453	6.0073	
3	8596102	8596792	KD2-H4K8ac_peak_20454	15.99047	
3	8597044	8597264	KD2-H4K8ac_peak_20455	8.94469	
3	8598347	8598624	KD2-H4K8ac_peak_20456	7.167	
3	8665052	8665371	KD2-H4K8ac_peak_20457	7.07117	
3	8690504	8691927	KD2-H4K8ac_peak_20458	17.51891	
3	8693400	8694317	KD2-H4K8ac_peak_20459	8.53092	
3	8694596	8694807	KD2-H4K8ac_peak_20460	6.8108	
3	8699388	8700332	KD2-H4K8ac_peak_20461	22.9953	
3	8701015	8701386	KD2-H4K8ac_peak_20462	14.70804	
3	8703788	8704164	KD2-H4K8ac_peak_20463	6.66692	
3	8706132	8706329	KD2-H4K8ac_peak_20464	5.57044	
3	8717401	8717624	KD2-H4K8ac_peak_20465	4.44923	
3	8810831	8811121	KD2-H4K8ac_peak_20466	7.96322	OXTR_ENSG00000180914
3	8826720	8826952	KD2-H4K8ac_peak_20467	8.94469	
3	8827364	8827642	KD2-H4K8ac_peak_20468	4.86824	

3	8834506	8834864	KD2-H4K8ac_peak_20469	9.6247	
3	9143822	9144264	KD2-H4K8ac_peak_20470	4.211	
3	9244022	9244547	KD2-H4K8ac_peak_20471	12.42761	
3	9259448	9259697	KD2-H4K8ac_peak_20472	7.70289	SRGAP3-AS3_ENSG00000227929
3	9404408	9404970	KD2-H4K8ac_peak_20473	10.19949	SRGAP3_ENSG00000196220;RP11-380O24.1_ENSG00000254485;THUMPD3_ENSG00000134077
3	9438004	9438432	KD2-H4K8ac_peak_20474	5.51783	SETD5_ENSG00000168137
3	9439640	9439938	KD2-H4K8ac_peak_20475	6.65673	SETD5-AS1_ENSG00000206573;SETD5_ENSG00000168137
3	9595988	9596231	KD2-H4K8ac_peak_20476	4.8888	LHFPL4_ENSG00000156959
3	9772427	9772827	KD2-H4K8ac_peak_20477	5.54647	BRPF1_ENSG00000156983
3	9773220	9773821	KD2-H4K8ac_peak_20478	12.36441	BRPF1_ENSG00000156983
3	9791169	9791390	KD2-H4K8ac_peak_20479	5.13615	OGG1_ENSG00000114026
3	9807423	9807655	KD2-H4K8ac_peak_20480	5.90098	
3	9811238	9811583	KD2-H4K8ac_peak_20481	9.63487	CAMK1_ENSG00000134072
3	9811868	9812267	KD2-H4K8ac_peak_20482	11.79585	CAMK1_ENSG00000134072
3	9833801	9834565	KD2-H4K8ac_peak_20483	23.67389	TADA3_ENSG00000171148;ARPC4_ENSG00000241553;ARPC4-TTLL3_ENSG00000250151
3	9851361	9852705	KD2-H4K8ac_peak_20484	16.40694	
3	9885136	9885569	KD2-H4K8ac_peak_20485	4.54006	RPUSD3_ENSG00000156990
3	9906921	9907120	KD2-H4K8ac_peak_20486	4.01115	
3	9913167	9913377	KD2-H4K8ac_peak_20487	4.70206	
3	9931673	9932034	KD2-H4K8ac_peak_20488	7.8711	JAGN1_ENSG00000171135
3	9932330	9932869	KD2-H4K8ac_peak_20489	11.80283	JAGN1_ENSG00000171135
3	9955458	9955822	KD2-H4K8ac_peak_20490	7.14562	
3	9956890	9957869	KD2-H4K8ac_peak_20491	16.8471	IL17RC_ENSG00000163702
3	9958925	9959233	KD2-H4K8ac_peak_20492	5.9696	IL17RC_ENSG00000163702
3	9974154	9975248	KD2-H4K8ac_peak_20493	11.89502	CRELD1_ENSG00000163703
3	9987675	9988054	KD2-H4K8ac_peak_20494	5.54647	
3	10028117	10028473	KD2-H4K8ac_peak_20495	5.9696	EMC3-AS1_ENSG00000180385
3	10052434	10052743	KD2-H4K8ac_peak_20496	8.23274	EMC3_ENSG00000125037;AC022007.5_ENSG00000206567
3	10067628	10068676	KD2-H4K8ac_peak_20497	6.40683	CIDECP_ENSG00000186162;FANCD2_ENSG00000144554
3	10156430	10156833	KD2-H4K8ac_peak_20498	6.99815	BRK1_ENSG00000254999
3	10157509	10157716	KD2-H4K8ac_peak_20499	6.99815	BRK1_ENSG00000254999
3	10182940	10183923	KD2-H4K8ac_peak_20500	16.8435	VHL_ENSG00000134086;snoU13_ENSG00000238345
3	10206649	10206928	KD2-H4K8ac_peak_20501	6.06203	IRAK2_ENSG00000134070
3	10276193	10276507	KD2-H4K8ac_peak_20502	8.17902	
3	10362514	10362786	KD2-H4K8ac_peak_20503	11.05407	SEC13_ENSG00000157020
3	10396421	10396700	KD2-H4K8ac_peak_20504	6.56211	
3	10546521	10546870	KD2-H4K8ac_peak_20505	7.146	
3	10857498	10858499	KD2-H4K8ac_peak_20506	9.95119	SLC6A11_ENSG00000132164
3	10968301	10969698	KD2-H4K8ac_peak_20507	11.66477	
3	10969976	10970263	KD2-H4K8ac_peak_20508	6.00226	
3	10970611	10971027	KD2-H4K8ac_peak_20509	8.09472	
3	11033403	11033850	KD2-H4K8ac_peak_20510	9.1707	SLC6A1_ENSG00000157103
3	11138529	11138841	KD2-H4K8ac_peak_20511	6.12046	
3	11237796	11238070	KD2-H4K8ac_peak_20512	8.00457	
3	11314095	11314442	KD2-H4K8ac_peak_20513	28.28058	ATG7_ENSG00000197548
3	11489703	11489916	KD2-H4K8ac_peak_20514	4.01115	

3	11680662	11680876	KD2-H4K8ac_peak_20515	5.29555	
3	11684118	11684481	KD2-H4K8ac_peak_20516	13.70753	
3	11684690	11684891	KD2-H4K8ac_peak_20517	9.1707	
3	11760519	11760995	KD2-H4K8ac_peak_20518	9.1707	
3	11812343	11812717	KD2-H4K8ac_peak_20519	4.0528	
3	11888041	11888314	KD2-H4K8ac_peak_20520	4.42765	TAMM41_ENSG00000144559
3	11996591	11996962	KD2-H4K8ac_peak_20521	6.16888	
3	12222018	12222252	KD2-H4K8ac_peak_20522	4.7849	
3	12521165	12521395	KD2-H4K8ac_peak_20523	6.60096	
3	12521599	12521937	KD2-H4K8ac_peak_20524	9.57101	
3	12525308	12525692	KD2-H4K8ac_peak_20525	12.99417	TSEN2_ENSG00000154743
3	12526085	12526348	KD2-H4K8ac_peak_20526	3.86308	TSEN2_ENSG00000154743
3	12595463	12595873	KD2-H4K8ac_peak_20527	11.05407	
3	12597181	12597514	KD2-H4K8ac_peak_20528	8.28994	MKRN2_ENSG00000075975
3	12597838	12598392	KD2-H4K8ac_peak_20529	12.00597	MKRN2_ENSG00000075975
3	12705073	12705587	KD2-H4K8ac_peak_20530	15.76765	RAF1_ENSG00000132155
3	12706413	12706684	KD2-H4K8ac_peak_20531	4.42765	RAF1_ENSG00000132155
3	12837676	12838582	KD2-H4K8ac_peak_20532	16.822	CAND2_ENSG00000144712
3	12858632	12859119	KD2-H4K8ac_peak_20533	10.29344	
3	12882491	12882998	KD2-H4K8ac_peak_20534	12.84642	RPL32_ENSG00000144713;SNORA7A_ENSG00000207496
3	12917402	12917680	KD2-H4K8ac_peak_20535	14.69247	
3	12964721	12964938	KD2-H4K8ac_peak_20536	4.66652	
3	12996648	12996856	KD2-H4K8ac_peak_20537	5.85704	
3	13009517	13009822	KD2-H4K8ac_peak_20538	6.14031	
3	13063499	13063883	KD2-H4K8ac_peak_20539	11.76021	
3	13064283	13064759	KD2-H4K8ac_peak_20540	7.36799	
3	13064966	13065329	KD2-H4K8ac_peak_20541	4.211	
3	13082024	13082326	KD2-H4K8ac_peak_20542	6.25869	
3	13083058	13083868	KD2-H4K8ac_peak_20543	12.74171	
3	13284992	13285264	KD2-H4K8ac_peak_20544	9.08816	
3	13302091	13302345	KD2-H4K8ac_peak_20545	5.94347	
3	13338311	13338552	KD2-H4K8ac_peak_20546	4.211	
3	13461138	13461668	KD2-H4K8ac_peak_20547	13.77586	NUP210_ENSG00000132182
3	13461881	13462487	KD2-H4K8ac_peak_20548	17.94825	NUP210_ENSG00000132182
3	13493506	13493938	KD2-H4K8ac_peak_20549	9.06369	
3	13494152	13494355	KD2-H4K8ac_peak_20550	6.31143	
3	13521009	13521540	KD2-H4K8ac_peak_20551	13.35993	HDAC11-AS1_ENSG00000244502;HDAC11_ENSG00000163517
3	13521965	13522185	KD2-H4K8ac_peak_20552	5.92591	HDAC11-AS1_ENSG00000244502;HDAC11_ENSG00000163517
3	13526363	13526577	KD2-H4K8ac_peak_20553	6.99815	
3	13554562	13554852	KD2-H4K8ac_peak_20554	6.65673	
3	13560798	13561045	KD2-H4K8ac_peak_20555	5.32933	
3	13590234	13590487	KD2-H4K8ac_peak_20556	12.00273	
3	13590713	13591004	KD2-H4K8ac_peak_20557	14.38358	
3	13606144	13606540	KD2-H4K8ac_peak_20558	4.86324	
3	13610294	13610977	KD2-H4K8ac_peak_20559	5.54647	
3	13615478	13615939	KD2-H4K8ac_peak_20560	11.92978	

3	13616166	13617224	KD2-H4K8ac_peak_20561	11.89502	
3	13624906	13625430	KD2-H4K8ac_peak_20562	5.52329	
3	13696547	13696831	KD2-H4K8ac_peak_20563	5.9696	
3	13824319	13824585	KD2-H4K8ac_peak_20564	9.59147	
3	13936212	13936894	KD2-H4K8ac_peak_20565	16.05022	
3	14165162	14166180	KD2-H4K8ac_peak_20566	18.29344	CHCHD4_ENSG00000163528;TMEM43_ENSG00000170876
3	14219691	14220008	KD2-H4K8ac_peak_20567	8.4716	XPC_ENSG00000154767;LSM3_ENSG00000170860
3	14257226	14257664	KD2-H4K8ac_peak_20568	4.59928	
3	14382753	14383102	KD2-H4K8ac_peak_20569	4.211	
3	14443534	14443855	KD2-H4K8ac_peak_20570	14.06758	SLC6A6_ENSG00000131389
3	14480525	14480788	KD2-H4K8ac_peak_20571	8.16133	
3	14481748	14482003	KD2-H4K8ac_peak_20572	6.16747	
3	14576314	14576508	KD2-H4K8ac_peak_20573	5.71749	
3	14581414	14581904	KD2-H4K8ac_peak_20574	10.08707	
3	14582390	14582902	KD2-H4K8ac_peak_20575	4.67342	GRIP2_ENSG00000144596
3	14644041	14644466	KD2-H4K8ac_peak_20576	7.76329	
3	14692438	14692776	KD2-H4K8ac_peak_20577	8.04763	CCDC174_ENSG00000154781
3	14988716	14988952	KD2-H4K8ac_peak_20578	7.76329	FGD5-AS1_ENSG00000225733;NR2C2_ENSG00000177463
3	15064410	15064753	KD2-H4K8ac_peak_20579	5.49367	
3	15106401	15106747	KD2-H4K8ac_peak_20580	4.44527	MRPS25_ENSG00000131368
3	15140385	15140628	KD2-H4K8ac_peak_20581	16.1002	ZFYVE20_ENSG00000131381
3	15140847	15141047	KD2-H4K8ac_peak_20582	9.16531	ZFYVE20_ENSG00000131381
3	15247782	15248120	KD2-H4K8ac_peak_20583	6.65673	COL6A4P1_ENSG00000230524;CAPN7_ENSG00000131375
3	15373685	15373907	KD2-H4K8ac_peak_20584	5.80191	
3	15374196	15374431	KD2-H4K8ac_peak_20585	8.53092	
3	15403382	15403590	KD2-H4K8ac_peak_20586	4.66652	
3	15468614	15469408	KD2-H4K8ac_peak_20587	13.80019	EAF1_ENSG00000144597
3	15502950	15503341	KD2-H4K8ac_peak_20588	3.75035	
3	15642608	15643001	KD2-H4K8ac_peak_20589	8.55271	HACL1_ENSG00000131373;BTD_ENSG00000169814
3	15901597	15902334	KD2-H4K8ac_peak_20590	14.45402	ANKRD28_ENSG00000206560
3	16508952	16509530	KD2-H4K8ac_peak_20591	12.05219	
3	16510922	16511340	KD2-H4K8ac_peak_20592	8.85813	
3	16551223	16551706	KD2-H4K8ac_peak_20593	8.4627	
3	16553545	16553775	KD2-H4K8ac_peak_20594	5.30799	
3	16554044	16555010	KD2-H4K8ac_peak_20595	9.01262	RFTN1_ENSG00000131378
3	16555255	16555589	KD2-H4K8ac_peak_20596	8.16906	RFTN1_ENSG00000131378
3	16557757	16558096	KD2-H4K8ac_peak_20597	8.55271	
3	16559518	16560025	KD2-H4K8ac_peak_20598	6.33462	
3	16561415	16561755	KD2-H4K8ac_peak_20599	7.27716	
3	16592485	16592783	KD2-H4K8ac_peak_20600	11.89502	
3	16925877	16926320	KD2-H4K8ac_peak_20601	20.8593	
3	17783463	17784036	KD2-H4K8ac_peak_20602	16.822	
3	18155092	18155755	KD2-H4K8ac_peak_20603	7.27716	
3	18167289	18167629	KD2-H4K8ac_peak_20604	5.57044	
3	18168659	18169060	KD2-H4K8ac_peak_20605	12.06346	
3	18169922	18170295	KD2-H4K8ac_peak_20606	5.73839	

3	18180486	18180980	KD2-H4K8ac_peak_20607	7.01964	
3	18465485	18465679	KD2-H4K8ac_peak_20608	6.83936	
3	18467149	18467663	KD2-H4K8ac_peak_20609	11.05407	
3	19188718	19188980	KD2-H4K8ac_peak_20610	10.53377	KCNH8_ENSG00000183960
3	19189190	19190115	KD2-H4K8ac_peak_20611	8.55271	KCNH8_ENSG00000183960
3	19275963	19276361	KD2-H4K8ac_peak_20612	10.05606	
3	19987578	19988298	KD2-H4K8ac_peak_20613	4.42765	EFHB_ENSG00000163576;RAB5A_ENSG00000144566
3	19988748	19989204	KD2-H4K8ac_peak_20614	6.76298	EFHB_ENSG00000163576;RAB5A_ENSG00000144566
3	20065268	20065462	KD2-H4K8ac_peak_20615	4.3015	
3	20081283	20081660	KD2-H4K8ac_peak_20616	5.29555	KAT2B_ENSG00000114166
3	20589501	20589705	KD2-H4K8ac_peak_20617	5.16439	
3	22492935	22493162	KD2-H4K8ac_peak_20618	8.17902	
3	23244703	23245010	KD2-H4K8ac_peak_20619	10.96455	UBE2E2-AS1_ENSG00000233153;UBE2E2_ENSG00000182247
3	23804816	23805339	KD2-H4K8ac_peak_20620	4.211	
3	23847604	23848707	KD2-H4K8ac_peak_20621	12.84642	UBE2E1-AS1_ENSG00000223791;UBE2E1_ENSG00000170142
3	23851955	23852592	KD2-H4K8ac_peak_20622	11.90231	
3	23958694	23959078	KD2-H4K8ac_peak_20623	4.91674	RPL15_ENSG00000174748
3	23986339	23986539	KD2-H4K8ac_peak_20624	11.89502	NR1D2_ENSG00000174738
3	24034656	24034882	KD2-H4K8ac_peak_20625	4.42765	
3	24536382	24536677	KD2-H4K8ac_peak_20626	6.66692	THRB_ENSG00000151090;THRB-AS1_ENSG00000228791
3	24562780	24563823	KD2-H4K8ac_peak_20627	8.10216	MIR4792_ENSG00000265028
3	25469236	25469560	KD2-H4K8ac_peak_20628	9.11031	
3	25469791	25469989	KD2-H4K8ac_peak_20629	12.06346	
3	25494295	25494502	KD2-H4K8ac_peak_20630	5.40638	
3	25495097	25495391	KD2-H4K8ac_peak_20631	6.09229	
3	25496820	25497233	KD2-H4K8ac_peak_20632	6.44277	
3	25510297	25510673	KD2-H4K8ac_peak_20633	4.77079	
3	25515053	25515265	KD2-H4K8ac_peak_20634	7.12754	
3	25705676	25706327	KD2-H4K8ac_peak_20635	14.98671	TOP2B_ENSG00000077097;MIR4442_ENSG00000264219
3	25706708	25706933	KD2-H4K8ac_peak_20636	4.9025	TOP2B_ENSG00000077097;MIR4442_ENSG00000264219
3	25825165	25825414	KD2-H4K8ac_peak_20637	5.88845	OXSM_ENSG00000151093
3	25831851	25832062	KD2-H4K8ac_peak_20638	6.0073	
3	27167091	27167489	KD2-H4K8ac_peak_20639	9.15329	
3	27525075	27525830	KD2-H4K8ac_peak_20640	8.26945	SLC4A7_ENSG00000033867
3	27526220	27526489	KD2-H4K8ac_peak_20641	12.42547	SLC4A7_ENSG00000033867
3	27601829	27602069	KD2-H4K8ac_peak_20642	8.00457	
3	27673990	27674879	KD2-H4K8ac_peak_20643	15.9336	AC098614.2_ENSG00000213846
3	27770281	27770514	KD2-H4K8ac_peak_20644	4.42765	
3	28007426	28007877	KD2-H4K8ac_peak_20645	7.12754	
3	28283158	28283482	KD2-H4K8ac_peak_20646	21.26856	CMC1_ENSG00000187118
3	28390122	28390535	KD2-H4K8ac_peak_20647	6.40874	AZI2_ENSG00000163512;ZCWPW2_ENSG00000206559
3	28616763	28617210	KD2-H4K8ac_peak_20648	11.43863	LINC00693_ENSG00000228214
3	28617425	28617636	KD2-H4K8ac_peak_20649	5.4779	
3	28686535	28686762	KD2-H4K8ac_peak_20650	6.16888	
3	28886475	28886714	KD2-H4K8ac_peak_20651	4.86324	
3	28958445	28958654	KD2-H4K8ac_peak_20652	7.27716	

3	29322135	29322602	KD2-H4K8ac_peak_20653	7.27716	RBMS3_ENSG00000144642
3	29327151	29327500	KD2-H4K8ac_peak_20654	5.54647	
3	29425665	29425883	KD2-H4K8ac_peak_20655	8.1233	
3	29495060	29495353	KD2-H4K8ac_peak_20656	6.65673	
3	29566398	29566596	KD2-H4K8ac_peak_20657	6.07178	
3	29683266	29683789	KD2-H4K8ac_peak_20658	13.57066	RBMS3-AS2_ENSG00000203506
3	29691846	29692038	KD2-H4K8ac_peak_20659	9.15329	
3	30647982	30648629	KD2-H4K8ac_peak_20660	9.90914	TGFBR2_ENSG00000163513
3	30723077	30723636	KD2-H4K8ac_peak_20661	4.95402	
3	31574164	31574369	KD2-H4K8ac_peak_20662	8.1235	STT3B_ENSG00000163527
3	31789448	31789679	KD2-H4K8ac_peak_20663	7.75499	
3	32147529	32147862	KD2-H4K8ac_peak_20664	10.17341	GPD1L_ENSG00000152642
3	32148281	32148539	KD2-H4K8ac_peak_20665	4.86324	
3	32280474	32280716	KD2-H4K8ac_peak_20666	6.97919	RP11-384L8.1_ENSG00000261572;CMTM8_ENSG00000170293
3	32433503	32433880	KD2-H4K8ac_peak_20667	6.65673	CMTM7_ENSG00000153551
3	32442736	32443404	KD2-H4K8ac_peak_20668	24.89958	
3	32443706	32443977	KD2-H4K8ac_peak_20669	9.44423	
3	32543796	32544306	KD2-H4K8ac_peak_20670	4.86324	CMTM6_ENSG00000091317
3	32544704	32545052	KD2-H4K8ac_peak_20671	4.42765	CMTM6_ENSG00000091317
3	32611578	32612219	KD2-H4K8ac_peak_20672	11.60333	DYNC1LI1_ENSG00000144635
3	32726785	32727094	KD2-H4K8ac_peak_20673	10.87093	CNOT10_ENSG00000182973
3	32822098	32822961	KD2-H4K8ac_peak_20674	6.40683	
3	32851515	32851766	KD2-H4K8ac_peak_20675	5.22731	
3	32858164	32858862	KD2-H4K8ac_peak_20676	10.96455	TRIM71_ENSG00000206557
3	32944502	32944708	KD2-H4K8ac_peak_20677	6.0073	
3	33111870	33112235	KD2-H4K8ac_peak_20678	3.75035	
3	33138254	33138571	KD2-H4K8ac_peak_20679	12.06346	TMPPE_ENSG00000188167
3	33138912	33139268	KD2-H4K8ac_peak_20680	9.15069	TMPPE_ENSG00000188167
3	33139574	33139804	KD2-H4K8ac_peak_20681	8.17902	
3	33155500	33155984	KD2-H4K8ac_peak_20682	7.93948	CRTAP_ENSG00000170275
3	33318246	33318472	KD2-H4K8ac_peak_20683	5.43522	FBXL2_ENSG00000153558
3	33482381	33482784	KD2-H4K8ac_peak_20684	11.04256	UBP1_ENSG00000153560
3	33759318	33759649	KD2-H4K8ac_peak_20685	7.49599	CLASP2_ENSG00000163539
3	33760200	33760421	KD2-H4K8ac_peak_20686	9.11031	CLASP2_ENSG00000163539
3	35831057	35831573	KD2-H4K8ac_peak_20687	8.1233	
3	36422015	36422263	KD2-H4K8ac_peak_20688	7.62396	STAC_ENSG00000144681
3	36552908	36553595	KD2-H4K8ac_peak_20689	6.40683	
3	36554533	36555103	KD2-H4K8ac_peak_20690	8.1233	
3	36562847	36563054	KD2-H4K8ac_peak_20691	9.97423	
3	36563311	36563530	KD2-H4K8ac_peak_20692	4.16871	
3	36563932	36564428	KD2-H4K8ac_peak_20693	11.04694	
3	36565427	36566085	KD2-H4K8ac_peak_20694	5.14125	
3	36738661	36739035	KD2-H4K8ac_peak_20695	9.31907	
3	36756045	36756247	KD2-H4K8ac_peak_20696	4.49837	
3	36805460	36805669	KD2-H4K8ac_peak_20697	6.13909	
3	36805898	36806109	KD2-H4K8ac_peak_20698	7.62396	

3	36966294	36966557	KD2-H4K8ac_peak_20699	5.54647	
3	37034181	37034544	KD2-H4K8ac_peak_20700	9.11891	EPM2AIP1_ENSG00000178567;MLH1_ENSG00000076242
3	37217465	37218279	KD2-H4K8ac_peak_20701	20.02161	
3	37283443	37283680	KD2-H4K8ac_peak_20702	6.64891	GOLGA4_ENSG00000144674
3	37284113	37284694	KD2-H4K8ac_peak_20703	16.3889	RP11-259K5.2_ENSG00000270194;AC097359.1_ENSG00000176354;GOLGA4_ENSG00000144674
3	37493655	37494294	KD2-H4K8ac_peak_20704	8.58069	ITGA9_ENSG00000144668
3	37518706	37519209	KD2-H4K8ac_peak_20705	4.42765	
3	37520887	37521101	KD2-H4K8ac_peak_20706	6.8108	
3	37532555	37532862	KD2-H4K8ac_peak_20707	7.70289	
3	37533635	37534073	KD2-H4K8ac_peak_20708	14.48084	
3	37535345	37535876	KD2-H4K8ac_peak_20709	11.05407	
3	37562197	37562555	KD2-H4K8ac_peak_20710	7.70289	
3	37563865	37564292	KD2-H4K8ac_peak_20711	4.86324	
3	37579284	37579538	KD2-H4K8ac_peak_20712	4.70206	
3	37580050	37580276	KD2-H4K8ac_peak_20713	6.40297	
3	37587006	37587361	KD2-H4K8ac_peak_20714	7.76329	
3	37741548	37741764	KD2-H4K8ac_peak_20715	6.09229	
3	37742737	37743147	KD2-H4K8ac_peak_20716	7.47462	
3	37814088	37814437	KD2-H4K8ac_peak_20717	4.211	
3	37901482	37901891	KD2-H4K8ac_peak_20718	12.14909	
3	37903463	37904008	KD2-H4K8ac_peak_20719	6.66692	AC093415.2_ENSG00000235257;CTDSPL_ENSG00000144677
3	37964397	37964794	KD2-H4K8ac_peak_20720	6.98292	
3	37977312	37977638	KD2-H4K8ac_peak_20721	5.45396	
3	37988225	37988855	KD2-H4K8ac_peak_20722	8.17902	
3	38035344	38036081	KD2-H4K8ac_peak_20723	9.67696	
3	38065564	38065783	KD2-H4K8ac_peak_20724	4.77079	
3	38070220	38070429	KD2-H4K8ac_peak_20725	6.45018	PLCD1_ENSG00000187091
3	38071382	38071592	KD2-H4K8ac_peak_20726	8.21852	PLCD1_ENSG00000187091
3	38178937	38179171	KD2-H4K8ac_peak_20727	7.70289	ACAA1_ENSG00000060971;MYD88_ENSG00000172936
3	38179763	38181272	KD2-H4K8ac_peak_20728	14.69247	MYD88_ENSG00000172936
3	38355436	38355735	KD2-H4K8ac_peak_20729	5.71749	
3	38495605	38496881	KD2-H4K8ac_peak_20730	9.95119	ACVR2B-AS1_ENSG00000229589;ACVR2B_ENSG00000114739
3	38537906	38538236	KD2-H4K8ac_peak_20731	4.91674	EXOG_ENSG00000157036
3	38583926	38584126	KD2-H4K8ac_peak_20732	4.66652	
3	38617069	38617298	KD2-H4K8ac_peak_20733	5.20908	
3	38620886	38621698	KD2-H4K8ac_peak_20734	7.36799	
3	38622162	38622418	KD2-H4K8ac_peak_20735	12.06346	
3	38624934	38625402	KD2-H4K8ac_peak_20736	6.66692	
3	38690306	38691163	KD2-H4K8ac_peak_20737	9.51625	SCN5A_ENSG00000183873
3	38764662	38765092	KD2-H4K8ac_peak_20738	7.1181	
3	39092919	39093299	KD2-H4K8ac_peak_20739	8.04448	WDR48_ENSG00000114742
3	39093571	39093853	KD2-H4K8ac_peak_20740	9.63487	WDR48_ENSG00000114742
3	39148692	39149055	KD2-H4K8ac_peak_20741	13.81781	GORASP1_ENSG00000114745;TTC21A_ENSG00000168026
3	39189225	39189418	KD2-H4K8ac_peak_20742	8.26945	
3	39194690	39195426	KD2-H4K8ac_peak_20743	10.05606	CSRNP1_ENSG00000144655
3	39219570	39219776	KD2-H4K8ac_peak_20744	7.76104	

3	39222464	39222699	KD2-H4K8ac_peak_20745	9.89126	
3	39424367	39425320	KD2-H4K8ac_peak_20746	6.99815	SLC25A38_ENSG00000144659
3	39543775	39544351	KD2-H4K8ac_peak_20747	15.81711	
3	39794644	39794904	KD2-H4K8ac_peak_20748	6.10699	
3	39851142	39851514	KD2-H4K8ac_peak_20749	15.13261	MYRIP_ENSG00000170011
3	39948368	39948647	KD2-H4K8ac_peak_20750	4.77079	
3	39950357	39950677	KD2-H4K8ac_peak_20751	8.17902	
3	40085418	40085760	KD2-H4K8ac_peak_20752	11.30637	
3	40350396	40350999	KD2-H4K8ac_peak_20753	11.80283	EIF1B_ENSG00000114784
3	40351379	40351878	KD2-H4K8ac_peak_20754	7.76104	EIF1B_ENSG00000114784
3	40498943	40499167	KD2-H4K8ac_peak_20755	3.82395	RPL14_ENSG00000188846
3	40517641	40517899	KD2-H4K8ac_peak_20756	6.16888	ZNF619_ENSG00000177873
3	40518350	40518869	KD2-H4K8ac_peak_20757	16.32216	ZNF619_ENSG00000177873
3	40547019	40547613	KD2-H4K8ac_peak_20758	17.42905	ZNF620_ENSG00000177842
3	40565974	40566254	KD2-H4K8ac_peak_20759	11.05467	ZNF621_ENSG00000172888
3	40566476	40566891	KD2-H4K8ac_peak_20760	7.76104	ZNF621_ENSG00000172888
3	40657108	40657660	KD2-H4K8ac_peak_20761	7.40314	
3	40762276	40762476	KD2-H4K8ac_peak_20762	4.92515	
3	41038448	41038968	KD2-H4K8ac_peak_20763	6.40683	
3	41240188	41240851	KD2-H4K8ac_peak_20764	17.75427	
3	42004143	42004411	KD2-H4K8ac_peak_20765	10.08707	ULK4_ENSG00000168038
3	42054512	42055031	KD2-H4K8ac_peak_20766	13.73635	TRAK1_ENSG00000182606
3	42055226	42055651	KD2-H4K8ac_peak_20767	8.4716	TRAK1_ENSG00000182606
3	42065429	42065650	KD2-H4K8ac_peak_20768	4.42765	
3	42084588	42084949	KD2-H4K8ac_peak_20769	5.9696	
3	42623082	42623363	KD2-H4K8ac_peak_20770	5.9696	SS18L2_ENSG00000008324
3	42623710	42624078	KD2-H4K8ac_peak_20771	11.43353	SS18L2_ENSG00000008324
3	42631105	42631933	KD2-H4K8ac_peak_20772	6.83936	
3	42642225	42642576	KD2-H4K8ac_peak_20773	5.54647	SEC22C_ENSG00000093183;NKTR_ENSG00000114857
3	42694260	42694473	KD2-H4K8ac_peak_20774	3.76273	ZBTB47_ENSG00000114853
3	42695401	42695693	KD2-H4K8ac_peak_20775	11.79585	ZBTB47_ENSG00000114853;RP4-613B23.1_ENSG00000230084
3	42695965	42696850	KD2-H4K8ac_peak_20776	13.73635	ZBTB47_ENSG00000114853;RP4-613B23.1_ENSG00000230084
3	42845611	42845826	KD2-H4K8ac_peak_20777	6.24114	HIGD1A_ENSG00000181061;ACKR2_ENSG00000144648
3	42922173	42922954	KD2-H4K8ac_peak_20778	10.66719	
3	42947135	42947778	KD2-H4K8ac_peak_20779	7.146	ZNF662_ENSG00000182983
3	43060706	43060913	KD2-H4K8ac_peak_20780	6.90458	
3	43147200	43147494	KD2-H4K8ac_peak_20781	13.09607	POMGNT2_ENSG00000144647
3	43327679	43328307	KD2-H4K8ac_peak_20782	13.57066	SNRK_ENSG00000163788
3	43663045	43663439	KD2-H4K8ac_peak_20783	6.65673	
3	43664060	43664633	KD2-H4K8ac_peak_20784	5.88845	
3	43731665	43732225	KD2-H4K8ac_peak_20785	8.30974	ANO10_ENSG00000160746;ABHD5_ENSG00000011198
3	43732526	43732974	KD2-H4K8ac_peak_20786	7.76329	ANO10_ENSG00000160746;ABHD5_ENSG00000011198
3	43811220	43811988	KD2-H4K8ac_peak_20787	4.37809	
3	43812642	43813456	KD2-H4K8ac_peak_20788	4.91674	
3	43814281	43814602	KD2-H4K8ac_peak_20789	6.60096	
3	43814964	43815259	KD2-H4K8ac_peak_20790	9.63487	

3	43827076	43827681	KD2-H4K8ac_peak_20791	7.07512	
3	43828281	43828581	KD2-H4K8ac_peak_20792	5.405	
3	43924873	43925370	KD2-H4K8ac_peak_20793	4.44527	
3	43994332	43994602	KD2-H4K8ac_peak_20794	7.40111	
3	44036740	44037526	KD2-H4K8ac_peak_20795	5.64429	RP4-555D20.3_ENSG00000271192
3	44039970	44040602	KD2-H4K8ac_peak_20796	10.43145	RP4-555D20.4_ENSG00000272121
3	44063296	44063795	KD2-H4K8ac_peak_20797	7.01964	
3	44379350	44380142	KD2-H4K8ac_peak_20798	12.08081	RP11-424N24.2_ENSG00000271937;TCAIM_ENSG00000179152
3	44480723	44481163	KD2-H4K8ac_peak_20799	5.88845	
3	44518849	44519901	KD2-H4K8ac_peak_20800	22.36554	ZNF445_ENSG00000185219
3	44552331	44552951	KD2-H4K8ac_peak_20801	12.06346	ZNF852_ENSG00000178917
3	44596095	44597087	KD2-H4K8ac_peak_20802	11.50346	ZKSCAN7_ENSG00000196345
3	44626429	44626766	KD2-H4K8ac_peak_20803	9.95119	ZNF197_ENSG00000186448
3	44666162	44666378	KD2-H4K8ac_peak_20804	10.1591	ZNF197-AS1_ENSG00000233509
3	44666607	44666857	KD2-H4K8ac_peak_20805	15.61313	ZNF197-AS1_ENSG00000233509
3	44689952	44690676	KD2-H4K8ac_peak_20806	16.72355	ZNF35_ENSG00000169981
3	44726786	44727081	KD2-H4K8ac_peak_20807	6.65673	RP11-944L7.4_ENSG00000236869
3	44753722	44753957	KD2-H4K8ac_peak_20808	6.16888	ZNF502_ENSG00000196653
3	44754189	44754600	KD2-H4K8ac_peak_20809	10.96455	ZNF502_ENSG00000196653
3	44770562	44771486	KD2-H4K8ac_peak_20810	12.42761	ZNF501_ENSG00000186446
3	44902877	44903948	KD2-H4K8ac_peak_20811	13.73635	TMEM42_ENSG00000169964;MIR564_ENSG00000207783
3	45037495	45037699	KD2-H4K8ac_peak_20812	4.67342	
3	45208522	45208846	KD2-H4K8ac_peak_20813	8.5781	
3	45379960	45380377	KD2-H4K8ac_peak_20814	5.835	
3	45422987	45423478	KD2-H4K8ac_peak_20815	10.46264	
3	45429528	45429821	KD2-H4K8ac_peak_20816	8.23274	LARS2_ENSG00000011376
3	45430143	45430337	KD2-H4K8ac_peak_20817	6.65673	LARS2_ENSG00000011376
3	45624567	45625028	KD2-H4K8ac_peak_20818	9.59147	
3	45634957	45636034	KD2-H4K8ac_peak_20819	11.92978	
3	45730918	45731202	KD2-H4K8ac_peak_20820	7.76329	LIMD1-AS1_ENSG00000230530;SACM1L_ENSG00000211456
3	45837777	45838107	KD2-H4K8ac_peak_20821	8.30974	SLC6A20_ENSG00000163817
3	45849663	45849902	KD2-H4K8ac_peak_20822	6.94255	
3	45883311	45883528	KD2-H4K8ac_peak_20823	7.59643	
3	45883798	45884313	KD2-H4K8ac_peak_20824	12.80176	
3	46036751	46037255	KD2-H4K8ac_peak_20825	7.45501	FYCO1_ENSG00000163820
3	46530459	46530688	KD2-H4K8ac_peak_20826	8.23274	
3	46607496	46607741	KD2-H4K8ac_peak_20827	8.4716	
3	46650899	46651110	KD2-H4K8ac_peak_20828	4.01115	
3	46705091	46705362	KD2-H4K8ac_peak_20829	9.89126	
3	46735295	46735585	KD2-H4K8ac_peak_20830	17.55491	ALS2CL_ENSG00000178038
3	46736960	46737240	KD2-H4K8ac_peak_20831	8.52881	
3	46741661	46742073	KD2-H4K8ac_peak_20832	12.40345	TMIE_ENSG00000181585
3	46742668	46743477	KD2-H4K8ac_peak_20833	11.92978	TMIE_ENSG00000181585
3	46886085	46886291	KD2-H4K8ac_peak_20834	5.14125	
3	46886906	46887370	KD2-H4K8ac_peak_20835	15.05364	
3	46887676	46888511	KD2-H4K8ac_peak_20836	18.86697	

3	46951405	46951627	KD2-H4K8ac_peak_20837	5.4779	
3	47018544	47018785	KD2-H4K8ac_peak_20838	9.63925	
3	47020370	47020992	KD2-H4K8ac_peak_20839	10.29344	NBEAL2_ENSG00000160796
3	47021214	47021409	KD2-H4K8ac_peak_20840	8.30974	NBEAL2_ENSG00000160796
3	47050168	47050493	KD2-H4K8ac_peak_20841	4.76709	
3	47051187	47051717	KD2-H4K8ac_peak_20842	15.40891	
3	47204872	47205214	KD2-H4K8ac_peak_20843	6.0605	SETD2_ENSG00000181555;KIF9-AS1_ENSG00000227398
3	47324130	47324828	KD2-H4K8ac_peak_20844	6.20982	KIF9_ENSG00000088727;KLHL18_ENSG00000114648
3	47462749	47463323	KD2-H4K8ac_peak_20845	6.99815	
3	47516975	47517288	KD2-H4K8ac_peak_20846	5.61067	
3	47517570	47517959	KD2-H4K8ac_peak_20847	18.80827	SCAP_ENSG00000114650
3	47518172	47518443	KD2-H4K8ac_peak_20848	6.25869	SCAP_ENSG00000114650
3	47554497	47554778	KD2-H4K8ac_peak_20849	6.20982	ELP6_ENSG00000163832
3	47555020	47555820	KD2-H4K8ac_peak_20850	5.59859	ELP6_ENSG00000163832
3	47823067	47823289	KD2-H4K8ac_peak_20851	9.41619	SMARCC1_ENSG00000173473
3	47823661	47824009	KD2-H4K8ac_peak_20852	11.80283	SMARCC1_ENSG00000173473
3	47830149	47830657	KD2-H4K8ac_peak_20853	9.402	
3	47844149	47845290	KD2-H4K8ac_peak_20854	22.76112	DHX30_ENSG00000132153
3	47866490	47867067	KD2-H4K8ac_peak_20855	18.39704	
3	48130573	48130793	KD2-H4K8ac_peak_20856	11.50346	MAP4_ENSG00000047849
3	48192363	48192745	KD2-H4K8ac_peak_20857	9.15329	
3	48193032	48193598	KD2-H4K8ac_peak_20858	6.07178	
3	48228920	48229428	KD2-H4K8ac_peak_20859	9.31907	CDC25A_ENSG00000164045
3	48230176	48230689	KD2-H4K8ac_peak_20860	19.43192	CDC25A_ENSG00000164045
3	48236334	48236531	KD2-H4K8ac_peak_20861	4.01115	
3	48274542	48274866	KD2-H4K8ac_peak_20862	6.30229	
3	48281807	48282054	KD2-H4K8ac_peak_20863	5.53095	ZNF589_ENSG00000164048
3	48342371	48343409	KD2-H4K8ac_peak_20864	13.3695	NME6_ENSG00000172113
3	48392850	48393161	KD2-H4K8ac_peak_20865	4.75764	
3	48411285	48411661	KD2-H4K8ac_peak_20866	6.0073	
3	48469569	48470095	KD2-H4K8ac_peak_20867	11.15071	
3	48470343	48471809	KD2-H4K8ac_peak_20868	15.40891	PLXNB1_ENSG00000164050
3	48481059	48481459	KD2-H4K8ac_peak_20869	10.05606	CCDC51_ENSG00000164051;TMA7_ENSG00000232112
3	48481733	48482213	KD2-H4K8ac_peak_20870	7.96322	CCDC51_ENSG00000164051;TMA7_ENSG00000232112
3	48487770	48488395	KD2-H4K8ac_peak_20871	11.05467	RP11-24C3.2_ENSG00000244380;ATRIP_ENSG00000164053
3	48507217	48507842	KD2-H4K8ac_peak_20872	10.29842	TREX1_ENSG00000213689
3	48540912	48542396	KD2-H4K8ac_peak_20873	13.73635	SHISA5_ENSG00000164054
3	48586007	48586490	KD2-H4K8ac_peak_20874	9.1707	
3	48598314	48598999	KD2-H4K8ac_peak_20875	6.99815	PFKFB4_ENSG00000114268
3	48632456	48632732	KD2-H4K8ac_peak_20876	6.83936	COL7A1_ENSG00000114270
3	48646646	48647785	KD2-H4K8ac_peak_20877	13.59098	UQCRC1_ENSG00000010256
3	48672173	48672855	KD2-H4K8ac_peak_20878	9.34989	SLC26A6_ENSG00000225697
3	48673321	48673570	KD2-H4K8ac_peak_20879	5.405	SLC26A6_ENSG00000225697
3	48700612	48701014	KD2-H4K8ac_peak_20880	11.82518	CELSR3_ENSG00000008300;RP11-148G20.1_ENSG00000228350
3	48722929	48723261	KD2-H4K8ac_peak_20881	6.99815	NCKIPSD_ENSG00000213672
3	48754223	48754544	KD2-H4K8ac_peak_20882	11.2938	

3	48755041	48755360	KD2-H4K8ac_peak_20883	9.73431	
3	48777261	48777720	KD2-H4K8ac_peak_20884	9.34195	IP6K2_ENSG00000068745
3	48936614	48936822	KD2-H4K8ac_peak_20885	4.42765	SLC25A20_ENSG00000178537
3	48955970	48956473	KD2-H4K8ac_peak_20886	6.66692	ARIH2OS_ENSG00000221883;ARIH2_ENSG00000177479
3	49035399	49036130	KD2-H4K8ac_peak_20887	7.27716	
3	49043641	49044563	KD2-H4K8ac_peak_20888	9.36516	WDR6_ENSG00000178252
3	49044866	49045239	KD2-H4K8ac_peak_20889	14.12659	WDR6_ENSG00000178252
3	49055663	49056236	KD2-H4K8ac_peak_20890	18.94636	
3	49058236	49058775	KD2-H4K8ac_peak_20891	11.8696	DALRD3_ENSG00000178149;MIR425_ENSG00000199032;NDUFAF3_ENSG00000178057;MIR191_ENSG00000207605
3	49059142	49060099	KD2-H4K8ac_peak_20892	23.70459	DALRD3_ENSG00000178149
3	49131081	49131424	KD2-H4K8ac_peak_20893	6.67538	QRICH1_ENSG00000198218
3	49141807	49142152	KD2-H4K8ac_peak_20894	18.37277	QARS_ENSG00000172053
3	49157700	49158948	KD2-H4K8ac_peak_20895	14.68449	USP19_ENSG00000172046
3	49170732	49171116	KD2-H4K8ac_peak_20896	9.81419	LAMB2_ENSG00000172037
3	49203075	49204456	KD2-H4K8ac_peak_20897	10.7186	CCDC71_ENSG00000177352
3	49208362	49208598	KD2-H4K8ac_peak_20898	6.45083	KLHDC8B_ENSG00000185909
3	49209127	49209363	KD2-H4K8ac_peak_20899	6.60096	KLHDC8B_ENSG00000185909
3	49210187	49210571	KD2-H4K8ac_peak_20900	8.16906	
3	49211637	49211843	KD2-H4K8ac_peak_20901	6.99194	
3	49377036	49377410	KD2-H4K8ac_peak_20902	12.08081	USP4_ENSG00000114316
3	49377700	49378644	KD2-H4K8ac_peak_20903	13.73635	USP4_ENSG00000114316
3	49395351	49395756	KD2-H4K8ac_peak_20904	12.70959	GPX1_ENSG00000233276
3	49395981	49396603	KD2-H4K8ac_peak_20905	10.73134	GPX1_ENSG00000233276
3	49448866	49449338	KD2-H4K8ac_peak_20906	11.89502	TCTA_ENSG00000145022
3	49449781	49450051	KD2-H4K8ac_peak_20907	22.78932	RHOA_ENSG00000067560;TCTA_ENSG00000145022
3	49459349	49459655	KD2-H4K8ac_peak_20908	7.69287	AMT_ENSG00000145020;NICN1-AS1_ENSG00000235261
3	49466536	49467039	KD2-H4K8ac_peak_20909	10.96455	NICN1_ENSG00000145029
3	49467468	49467811	KD2-H4K8ac_peak_20910	7.76329	NICN1_ENSG00000145029
3	49506899	49507394	KD2-H4K8ac_peak_20911	7.76329	DAG1_ENSG00000173402
3	49507889	49508279	KD2-H4K8ac_peak_20912	8.30974	
3	49576808	49577210	KD2-H4K8ac_peak_20913	9.16531	
3	49577567	49578111	KD2-H4K8ac_peak_20914	11.79585	
3	49591429	49591725	KD2-H4K8ac_peak_20915	7.07117	BSN-AS2_ENSG00000226913;BSN_ENSG00000164061
3	49647322	49647682	KD2-H4K8ac_peak_20916	6.40683	
3	49710777	49711156	KD2-H4K8ac_peak_20917	15.36189	APEH_ENSG00000164062
3	49724424	49724859	KD2-H4K8ac_peak_20918	5.54647	
3	49726184	49727251	KD2-H4K8ac_peak_20919	12.17278	MST1_ENSG00000173531;RNF123_ENSG00000164068
3	49760985	49761324	KD2-H4K8ac_peak_20920	4.44527	AMIGO3_ENSG00000176020;GMPPB_ENSG00000173540
3	49761701	49762239	KD2-H4K8ac_peak_20921	10.86378	AMIGO3_ENSG00000176020;GMPPB_ENSG00000173540
3	49823661	49823940	KD2-H4K8ac_peak_20922	15.25814	IP6K1_ENSG00000176095
3	49824250	49824575	KD2-H4K8ac_peak_20923	19.91242	IP6K1_ENSG00000176095
3	49840728	49840993	KD2-H4K8ac_peak_20924	11.92978	FAM212A_ENSG00000185614
3	49843105	49843605	KD2-H4K8ac_peak_20925	17.55491	MIR5193_ENSG00000263506
3	49844046	49844714	KD2-H4K8ac_peak_20926	10.7186	MIR5193_ENSG00000263506
3	49845074	49845418	KD2-H4K8ac_peak_20927	5.50645	
3	49845653	49845890	KD2-H4K8ac_peak_20928	6.20524	

3	49894114	49894479	KD2-H4K8ac_peak_20929	18.29344	TRAIP_ENSG00000183763
3	49906969	49907542	KD2-H4K8ac_peak_20930	9.70019	CAMKV_ENSG00000164076
3	49910528	49910971	KD2-H4K8ac_peak_20931	5.88845	
3	49967459	49968170	KD2-H4K8ac_peak_20932	20.24043	MON1A_ENSG00000164077
3	49976946	49977266	KD2-H4K8ac_peak_20933	14.8083	RBM6_ENSG00000004534
3	50125622	50126028	KD2-H4K8ac_peak_20934	7.146	RBM5_ENSG00000003756
3	50126464	50126895	KD2-H4K8ac_peak_20935	8.65175	RBM5_ENSG00000003756
3	50163055	50163496	KD2-H4K8ac_peak_20936	10.05606	
3	50175336	50175644	KD2-H4K8ac_peak_20937	5.14125	
3	50176027	50176454	KD2-H4K8ac_peak_20938	6.66692	
3	50187429	50187689	KD2-H4K8ac_peak_20939	6.25869	
3	50188068	50188338	KD2-H4K8ac_peak_20940	4.55978	
3	50188627	50189564	KD2-H4K8ac_peak_20941	6.52179	
3	50191873	50192191	KD2-H4K8ac_peak_20942	7.69287	SEMA3F_ENSG00000001617
3	50242422	50242940	KD2-H4K8ac_peak_20943	7.76329	SLC38A3_ENSG00000188338
3	50263827	50264411	KD2-H4K8ac_peak_20944	7.49599	GNAI2_ENSG00000114353
3	50265131	50265855	KD2-H4K8ac_peak_20945	14.68449	
3	50275834	50276165	KD2-H4K8ac_peak_20946	5.88845	U73169.1_ENSG00000213600
3	50282973	50283730	KD2-H4K8ac_peak_20947	15.40891	
3	50283981	50284184	KD2-H4K8ac_peak_20948	3.90565	
3	50297165	50297449	KD2-H4K8ac_peak_20949	9.73431	U73166.2_ENSG00000230454
3	50297750	50298791	KD2-H4K8ac_peak_20950	23.17425	U73166.2_ENSG00000230454
3	50329407	50329821	KD2-H4K8ac_peak_20951	14.03725	IFRD2_ENSG00000214706
3	50330267	50330680	KD2-H4K8ac_peak_20952	6.40297	IFRD2_ENSG00000214706
3	50336355	50336600	KD2-H4K8ac_peak_20953	6.66359	HYAL3_ENSG00000186792;NAT6_ENSG00000243477
3	50340856	50341207	KD2-H4K8ac_peak_20954	5.91842	
3	50358363	50359159	KD2-H4K8ac_peak_20955	9.95119	
3	50359443	50359660	KD2-H4K8ac_peak_20956	8.10216	HYAL2_ENSG00000068001
3	50374376	50374795	KD2-H4K8ac_peak_20957	12.93209	
3	50375185	50375682	KD2-H4K8ac_peak_20958	8.55271	
3	50375987	50376439	KD2-H4K8ac_peak_20959	6.60096	
3	50396552	50396886	KD2-H4K8ac_peak_20960	8.4716	TMEM115_ENSG00000126062
3	50405761	50405979	KD2-H4K8ac_peak_20961	7.40111	
3	50412182	50412652	KD2-H4K8ac_peak_20962	7.76329	
3	50413671	50413890	KD2-H4K8ac_peak_20963	5.54647	
3	50414961	50415325	KD2-H4K8ac_peak_20964	7.40314	
3	50415662	50416306	KD2-H4K8ac_peak_20965	4.54006	
3	50416716	50417487	KD2-H4K8ac_peak_20966	9.43857	
3	50417714	50418832	KD2-H4K8ac_peak_20967	26.8781	
3	50419164	50419888	KD2-H4K8ac_peak_20968	14.17923	
3	50420192	50420987	KD2-H4K8ac_peak_20969	9.26287	
3	50422442	50423283	KD2-H4K8ac_peak_20970	13.21979	
3	50423631	50424194	KD2-H4K8ac_peak_20971	8.2413	
3	50424450	50425464	KD2-H4K8ac_peak_20972	16.95399	
3	50425697	50428267	KD2-H4K8ac_peak_20973	17.42905	
3	50428603	50430305	KD2-H4K8ac_peak_20974	17.43561	

3	50431958	50432267	KD2-H4K8ac_peak_20975	5.06602	
3	50432800	50433013	KD2-H4K8ac_peak_20976	5.06602	
3	50462599	50463055	KD2-H4K8ac_peak_20977	9.06369	
3	50463373	50465767	KD2-H4K8ac_peak_20978	13.77586	
3	50466020	50466688	KD2-H4K8ac_peak_20979	17.97634	
3	50467073	50468357	KD2-H4K8ac_peak_20980	21.07675	
3	50468834	50469251	KD2-H4K8ac_peak_20981	12.82393	
3	50469447	50471057	KD2-H4K8ac_peak_20982	7.49599	
3	50471259	50472776	KD2-H4K8ac_peak_20983	10.19949	
3	50473168	50474287	KD2-H4K8ac_peak_20984	8.34752	
3	50474505	50476250	KD2-H4K8ac_peak_20985	10.46843	
3	50477101	50477339	KD2-H4K8ac_peak_20986	4.67342	
3	50540593	50540799	KD2-H4K8ac_peak_20987	5.14125	CACNA2D2_ENSG00000007402
3	50541243	50541731	KD2-H4K8ac_peak_20988	8.30974	CACNA2D2_ENSG00000007402
3	50550553	50550857	KD2-H4K8ac_peak_20989	5.61067	
3	50552671	50553223	KD2-H4K8ac_peak_20990	11.05467	
3	50553717	50554937	KD2-H4K8ac_peak_20991	13.73635	
3	50562737	50563186	KD2-H4K8ac_peak_20992	7.95934	
3	50569874	50570140	KD2-H4K8ac_peak_20993	5.01002	
3	50604704	50605181	KD2-H4K8ac_peak_20994	12.70959	
3	50605816	50606041	KD2-H4K8ac_peak_20995	8.56219	HEMK1_ENSG00000114735
3	50606327	50607497	KD2-H4K8ac_peak_20996	12.82393	C3orf18_ENSG00000088543;HEMK1_ENSG00000114735
3	50638267	50638536	KD2-H4K8ac_peak_20997	5.34636	
3	50638840	50639403	KD2-H4K8ac_peak_20998	5.4779	
3	50639615	50639830	KD2-H4K8ac_peak_20999	8.99803	
3	50648996	50649397	KD2-H4K8ac_peak_21000	5.75757	CISH_ENSG00000114737;MAPKAPK3_ENSG00000114738
3	50668953	50669442	KD2-H4K8ac_peak_21001	5.57044	
3	51421608	51422410	KD2-H4K8ac_peak_21002	18.54875	MANF_ENSG00000145050
3	51422785	51423192	KD2-H4K8ac_peak_21003	4.06758	MANF_ENSG00000145050
3	51428765	51429267	KD2-H4K8ac_peak_21004	9.20193	RBM15B_ENSG00000179837
3	51534641	51535040	KD2-H4K8ac_peak_21005	5.54647	VPRBP_ENSG00000145041
3	51571910	51573331	KD2-H4K8ac_peak_21006	11.05407	
3	51704794	51705031	KD2-H4K8ac_peak_21007	6.66692	
3	51705289	51705855	KD2-H4K8ac_peak_21008	17.27647	
3	51846466	51846691	KD2-H4K8ac_peak_21009	5.15777	
3	51976398	51976875	KD2-H4K8ac_peak_21010	12.30509	RRP9_ENSG00000114767;PARP3_ENSG00000041880
3	51990286	51991048	KD2-H4K8ac_peak_21011	7.47462	GPR62_ENSG00000180929
3	52001012	52002307	KD2-H4K8ac_peak_21012	28.09167	PCBP4_ENSG00000090097
3	52002601	52002866	KD2-H4K8ac_peak_21013	9.05418	PCBP4_ENSG00000090097
3	52003606	52003862	KD2-H4K8ac_peak_21014	3.76327	
3	52007736	52007960	KD2-H4K8ac_peak_21015	6.65673	RP11-155D18.14_ENSG00000272762;RP11-155D18.12_ENSG00000254782
3	52008222	52009386	KD2-H4K8ac_peak_21016	15.61313	RP11-155D18.14_ENSG00000272762;RP11-155D18.12_ENSG00000254782;ACY1_ENSG00000243989;ABHD14A-ACY1_ENSG0000011[illegible]
3	52017091	52018140	KD2-H4K8ac_peak_21017	16.822	ABHD14B_ENSG00000114779
3	52029505	52029863	KD2-H4K8ac_peak_21018	10.05606	RPL29_ENSG00000162244
3	52039001	52040855	KD2-H4K8ac_peak_21019	10.43145	
3	52050183	52050390	KD2-H4K8ac_peak_21020	5.98692	

3	52053229	52053640	KD2-H4K8ac_peak_21021	5.82799	
3	52055164	52056510	KD2-H4K8ac_peak_21022	17.55491	
3	52057772	52058043	KD2-H4K8ac_peak_21023	10.08707	
3	52059950	52060775	KD2-H4K8ac_peak_21024	8.26945	
3	52062239	52064969	KD2-H4K8ac_peak_21025	22.21705	
3	52065730	52066369	KD2-H4K8ac_peak_21026	14.84786	
3	52066639	52067769	KD2-H4K8ac_peak_21027	18.86697	
3	52070103	52070330	KD2-H4K8ac_peak_21028	5.30799	
3	52071567	52071867	KD2-H4K8ac_peak_21029	9.02745	
3	52075560	52075949	KD2-H4K8ac_peak_21030	4.211	
3	52079335	52079719	KD2-H4K8ac_peak_21031	6.53538	
3	52090820	52091156	KD2-H4K8ac_peak_21032	5.02474	DUSP7_ENSG00000164086
3	52091677	52091957	KD2-H4K8ac_peak_21033	6.75585	
3	52096731	52097684	KD2-H4K8ac_peak_21034	8.23274	LINC00696_ENSG00000256097
3	52100165	52100557	KD2-H4K8ac_peak_21035	5.13615	
3	52101271	52102114	KD2-H4K8ac_peak_21036	14.1139	
3	52102381	52103265	KD2-H4K8ac_peak_21037	13.12288	
3	52103609	52104100	KD2-H4K8ac_peak_21038	5.54647	
3	52104327	52104580	KD2-H4K8ac_peak_21039	7.95563	
3	52122229	52122731	KD2-H4K8ac_peak_21040	7.47462	
3	52142874	52143169	KD2-H4K8ac_peak_21041	7.76329	
3	52231466	52232488	KD2-H4K8ac_peak_21042	16.09057	ALAS1_ENSG00000023330
3	52273329	52274597	KD2-H4K8ac_peak_21043	9.402	TLR9_ENSG00000239732;TWF2_ENSG00000247596;RP5-1157M23.2_ENSG00000243224
3	52279483	52280315	KD2-H4K8ac_peak_21044	4.91674	PPM1M_ENSG00000164088
3	52312078	52312505	KD2-H4K8ac_peak_21045	5.9696	
3	52313268	52313553	KD2-H4K8ac_peak_21046	12.82393	
3	52321130	52321543	KD2-H4K8ac_peak_21047	7.67504	WDR82_ENSG00000164091
3	52321831	52322728	KD2-H4K8ac_peak_21048	10.96455	WDR82_ENSG00000164091
3	52331765	52332880	KD2-H4K8ac_peak_21049	8.10216	
3	52333189	52334615	KD2-H4K8ac_peak_21050	21.75906	
3	52340926	52341499	KD2-H4K8ac_peak_21051	11.92978	
3	52342145	52342411	KD2-H4K8ac_peak_21052	5.9696	
3	52345000	52345330	KD2-H4K8ac_peak_21053	14.72123	
3	52345841	52346222	KD2-H4K8ac_peak_21054	12.65012	
3	52443632	52443949	KD2-H4K8ac_peak_21055	11.92978	BAP1_ENSG00000163930;PHF7_ENSG00000010318
3	52444619	52444899	KD2-H4K8ac_peak_21056	9.35804	BAP1_ENSG00000163930;PHF7_ENSG00000010318
3	52480931	52481125	KD2-H4K8ac_peak_21057	4.8888	
3	52489078	52489485	KD2-H4K8ac_peak_21058	13.30586	TNNC1_ENSG00000114854;NISCH_ENSG00000010322
3	52490282	52490475	KD2-H4K8ac_peak_21059	4.78225	
3	52558701	52558925	KD2-H4K8ac_peak_21060	7.40314	
3	52564847	52565300	KD2-H4K8ac_peak_21061	7.36799	
3	52566874	52567637	KD2-H4K8ac_peak_21062	10.15155	SMIM4_ENSG00000168273
3	52568037	52568663	KD2-H4K8ac_peak_21063	13.48341	NT5DC2_ENSG00000168268;SMIM4_ENSG00000168273
3	52719702	52719936	KD2-H4K8ac_peak_21064	12.57143	PBRM1_ENSG00000163939
3	52720244	52720517	KD2-H4K8ac_peak_21065	5.88845	PBRM1_ENSG00000163939
3	52739407	52740448	KD2-H4K8ac_peak_21066	11.24702	SPCS1_ENSG00000114902

3	52805271	52805875	KD2-H4K8ac_peak_21067	4.52039	NEK4_ENSG00000114904
3	52809276	52809537	KD2-H4K8ac_peak_21068	4.42643	
3	53019789	53020063	KD2-H4K8ac_peak_21069	14.74047	
3	53079680	53080360	KD2-H4K8ac_peak_21070	14.8083	SFMBT1_ENSG00000163935
3	53107294	53107906	KD2-H4K8ac_peak_21071	9.34989	
3	53164145	53164417	KD2-H4K8ac_peak_21072	15.58442	RFT1_ENSG00000163933
3	53164655	53165146	KD2-H4K8ac_peak_21073	8.23274	RFT1_ENSG00000163933
3	53194565	53194832	KD2-H4K8ac_peak_21074	5.14125	
3	53195265	53195582	KD2-H4K8ac_peak_21075	8.94469	
3	53270473	53270843	KD2-H4K8ac_peak_21076	4.67342	
3	53303639	53303972	KD2-H4K8ac_peak_21077	6.66692	
3	53304230	53304936	KD2-H4K8ac_peak_21078	15.00387	
3	53381155	53381519	KD2-H4K8ac_peak_21079	14.80472	DCP1A_ENSG00000162290
3	53381806	53382220	KD2-H4K8ac_peak_21080	11.28831	DCP1A_ENSG00000162290
3	53527955	53528564	KD2-H4K8ac_peak_21081	9.63487	CACNA1D_ENSG00000157388
3	53568979	53569183	KD2-H4K8ac_peak_21082	3.75035	
3	53569483	53570041	KD2-H4K8ac_peak_21083	4.70388	
3	53705023	53705428	KD2-H4K8ac_peak_21084	8.94469	
3	53707876	53708152	KD2-H4K8ac_peak_21085	5.86783	
3	53807354	53807614	KD2-H4K8ac_peak_21086	15.06766	
3	53807920	53808444	KD2-H4K8ac_peak_21087	10.0692	
3	53829719	53830019	KD2-H4K8ac_peak_21088	7.40314	
3	53879779	53880884	KD2-H4K8ac_peak_21089	10.96455	CHDH_ENSG00000016391;IL17RB_ENSG00000056736
3	53915935	53916131	KD2-H4K8ac_peak_21090	7.57336	ACTR8_ENSG00000113812;AC012467.1_ENSG00000222888
3	53925537	53925736	KD2-H4K8ac_peak_21091	6.30607	SELK_ENSG00000113811
3	54081054	54081656	KD2-H4K8ac_peak_21092	6.30626	
3	54180553	54180786	KD2-H4K8ac_peak_21093	11.1952	
3	54965196	54965434	KD2-H4K8ac_peak_21094	9.11818	
3	55522632	55522862	KD2-H4K8ac_peak_21095	6.36375	WNT5A-AS1_ENSG00000244586
3	56320007	56320292	KD2-H4K8ac_peak_21096	6.16888	
3	56501775	56502438	KD2-H4K8ac_peak_21097	7.76329	ERC2_ENSG00000187672
3	56591380	56591616	KD2-H4K8ac_peak_21098	7.40314	CCDC66_ENSG00000180376
3	56716404	56717648	KD2-H4K8ac_peak_21099	29.48993	FAM208A_ENSG00000163946
3	57010794	57011290	KD2-H4K8ac_peak_21100	4.77079	
3	57011490	57011781	KD2-H4K8ac_peak_21101	9.06703	
3	57019629	57019844	KD2-H4K8ac_peak_21102	3.72574	
3	57020459	57020710	KD2-H4K8ac_peak_21103	5.14125	
3	57155215	57155957	KD2-H4K8ac_peak_21104	8.02571	
3	57157151	57157493	KD2-H4K8ac_peak_21105	10.46264	
3	57159038	57159709	KD2-H4K8ac_peak_21106	5.88845	
3	57160511	57161116	KD2-H4K8ac_peak_21107	6.11333	
3	57163233	57163784	KD2-H4K8ac_peak_21108	7.72801	
3	57164017	57164709	KD2-H4K8ac_peak_21109	6.04107	
3	57165358	57166344	KD2-H4K8ac_peak_21110	10.19949	
3	57168573	57169102	KD2-H4K8ac_peak_21111	10.0089	RP11-157F20.1_ENSG00000229863
3	57172636	57172832	KD2-H4K8ac_peak_21112	7.5554	

3	57177144	57178166	KD2-H4K8ac_peak_21113	13.62729	
3	57178512	57179233	KD2-H4K8ac_peak_21114	7.27716	
3	57182777	57183240	KD2-H4K8ac_peak_21115	6.44277	
3	57184546	57184799	KD2-H4K8ac_peak_21116	5.45396	
3	57185736	57186520	KD2-H4K8ac_peak_21117	13.1258	
3	57187903	57188183	KD2-H4K8ac_peak_21118	8.70727	
3	57191203	57191500	KD2-H4K8ac_peak_21119	11.43863	
3	57193466	57194249	KD2-H4K8ac_peak_21120	7.146	
3	57194510	57194729	KD2-H4K8ac_peak_21121	7.146	
3	57196047	57196404	KD2-H4K8ac_peak_21122	6.22239	
3	57198849	57199370	KD2-H4K8ac_peak_21123	12.16946	
3	57203868	57204631	KD2-H4K8ac_peak_21124	9.51625	IL17RD_ENSG00000144730
3	57251720	57251920	KD2-H4K8ac_peak_21125	7.12754	
3	57261896	57262287	KD2-H4K8ac_peak_21126	9.96798	APPL1_ENSG00000157500
3	57540591	57540989	KD2-H4K8ac_peak_21127	6.11333	
3	57541549	57541761	KD2-H4K8ac_peak_21128	8.16906	PDE12_ENSG00000174840
3	57542043	57542376	KD2-H4K8ac_peak_21129	7.90806	PDE12_ENSG00000174840
3	57582827	57583048	KD2-H4K8ac_peak_21130	7.98621	ARF4_ENSG00000168374;RP11-755B10.4_ENSG00000272146
3	57678488	57678769	KD2-H4K8ac_peak_21131	13.81781	DENND6A_ENSG00000174839;RP11-755B10.3_ENSG00000241933
3	57679194	57679398	KD2-H4K8ac_peak_21132	4.42765	DENND6A_ENSG00000174839;RP11-755B10.3_ENSG00000241933
3	57937050	57937378	KD2-H4K8ac_peak_21133	7.47462	
3	57993638	57994445	KD2-H4K8ac_peak_21134	23.8815	FLNB_ENSG00000136068
3	58000588	58000791	KD2-H4K8ac_peak_21135	4.54006	
3	58163656	58164032	KD2-H4K8ac_peak_21136	8.85813	
3	58291502	58291769	KD2-H4K8ac_peak_21137	14.56955	RPP14_ENSG00000163684
3	58292028	58292220	KD2-H4K8ac_peak_21138	10.08707	RPP14_ENSG00000163684
3	58317960	58318563	KD2-H4K8ac_peak_21139	12.82393	PXK_ENSG00000168297
3	58419271	58419503	KD2-H4K8ac_peak_21140	11.22681	PDHB_ENSG00000168291
3	58419734	58419984	KD2-H4K8ac_peak_21141	10.43145	PDHB_ENSG00000168291
3	58477019	58477230	KD2-H4K8ac_peak_21142	7.76329	RP11-359I18.5_ENSG00000272360;KCTD6_ENSG00000168301
3	58477895	58478289	KD2-H4K8ac_peak_21143	5.75757	RP11-359I18.5_ENSG00000272360;KCTD6_ENSG00000168301
3	58570870	58571516	KD2-H4K8ac_peak_21144	5.54647	
3	59035305	59035727	KD2-H4K8ac_peak_21145	20.47312	C3orf67_ENSG00000163689
3	59235838	59236079	KD2-H4K8ac_peak_21146	5.73839	
3	59593010	59593214	KD2-H4K8ac_peak_21147	5.80249	
3	59686976	59687275	KD2-H4K8ac_peak_21148	4.77079	
3	59693097	59693294	KD2-H4K8ac_peak_21149	5.13341	
3	59695519	59696118	KD2-H4K8ac_peak_21150	10.12553	
3	59719248	59719915	KD2-H4K8ac_peak_21151	11.86082	
3	59758982	59759567	KD2-H4K8ac_peak_21152	7.27716	
3	59761113	59761320	KD2-H4K8ac_peak_21153	5.29352	
3	59803570	59803784	KD2-H4K8ac_peak_21154	5.80249	
3	61236625	61237089	KD2-H4K8ac_peak_21155	17.8868	FHIT_ENSG00000189283
3	61237526	61237817	KD2-H4K8ac_peak_21156	7.71478	FHIT_ENSG00000189283
3	61318768	61319101	KD2-H4K8ac_peak_21157	10.72656	
3	61546319	61546560	KD2-H4K8ac_peak_21158	7.22936	PTPRG_ENSG00000144724

3	61546847	61547064	KD2-H4K8ac_peak_21159	6.66692	PTPRG_ENSG00000144724
3	61579903	61580280	KD2-H4K8ac_peak_21160	5.14125	
3	62304747	62305054	KD2-H4K8ac_peak_21161	4.211	C3orf14_ENSG00000114405
3	62554864	62555103	KD2-H4K8ac_peak_21162	6.40683	
3	62572198	62572433	KD2-H4K8ac_peak_21163	6.31143	
3	63001086	63001417	KD2-H4K8ac_peak_21164	4.59928	
3	63072199	63072746	KD2-H4K8ac_peak_21165	6.16888	
3	63328780	63329369	KD2-H4K8ac_peak_21166	8.5781	
3	63330402	63330609	KD2-H4K8ac_peak_21167	6.40683	
3	63848964	63849491	KD2-H4K8ac_peak_21168	17.55491	THOC7_ENSG00000163634;ATXN7_ENSG00000163635
3	63849757	63850367	KD2-H4K8ac_peak_21169	15.53798	THOC7_ENSG00000163634;ATXN7_ENSG00000163635
3	64008932	64009128	KD2-H4K8ac_peak_21170	10.29842	PSMD6_ENSG00000163636
3	64136204	64136497	KD2-H4K8ac_peak_21171	6.27931	
3	64210804	64211121	KD2-H4K8ac_peak_21172	5.71749	
3	64430860	64431388	KD2-H4K8ac_peak_21173	5.45396	PRICKLE2_ENSG00000163637;RP11-14D22.2_ENSG00000243993;RP11-14D22.1_ENSG00000244564
3	64628317	64628665	KD2-H4K8ac_peak_21174	7.47462	
3	64658699	64659153	KD2-H4K8ac_peak_21175	10.06887	
3	64661075	64661425	KD2-H4K8ac_peak_21176	4.42765	
3	64663026	64663225	KD2-H4K8ac_peak_21177	5.29555	
3	64671773	64672020	KD2-H4K8ac_peak_21178	6.90231	
3	64673891	64674451	KD2-H4K8ac_peak_21179	8.86099	ADAMTS9_ENSG00000163638
3	64843283	64843692	KD2-H4K8ac_peak_21180	7.75499	
3	64844706	64845044	KD2-H4K8ac_peak_21181	10.0746	
3	65156335	65156694	KD2-H4K8ac_peak_21182	7.12754	
3	65194955	65195172	KD2-H4K8ac_peak_21183	6.2781	
3	65196774	65197118	KD2-H4K8ac_peak_21184	5.14125	
3	65216426	65217015	KD2-H4K8ac_peak_21185	5.54647	
3	65218158	65218422	KD2-H4K8ac_peak_21186	5.88845	AC104331.1_ENSG00000264871
3	65545416	65545673	KD2-H4K8ac_peak_21187	6.1846	
3	65582335	65582832	KD2-H4K8ac_peak_21188	10.29344	
3	65583306	65584126	KD2-H4K8ac_peak_21189	14.66631	
3	66024157	66024413	KD2-H4K8ac_peak_21190	7.51054	MAGI1_ENSG00000151276
3	66024902	66025634	KD2-H4K8ac_peak_21191	14.84212	MAGI1_ENSG00000151276
3	66063407	66063700	KD2-H4K8ac_peak_21192	5.51783	
3	66448698	66449124	KD2-H4K8ac_peak_21193	6.63018	
3	66550912	66551113	KD2-H4K8ac_peak_21194	4.68059	LRIG1_ENSG00000144749
3	67704499	67705669	KD2-H4K8ac_peak_21195	12.82393	SUCLG2_ENSG00000172340;RP11-81N13.1_ENSG00000241316
3	69062081	69063190	KD2-H4K8ac_peak_21196	18.7894	EOGT_ENSG00000163378;CTD-2013N24.2_ENSG00000244513
3	69101852	69102269	KD2-H4K8ac_peak_21197	5.20908	TMF1_ENSG00000144747
3	69128980	69129284	KD2-H4K8ac_peak_21198	13.81781	UBA3_ENSG00000144744
3	69133470	69133899	KD2-H4K8ac_peak_21199	10.21502	ARL6IP5_ENSG00000144746
3	69134235	69134553	KD2-H4K8ac_peak_21200	11.05407	ARL6IP5_ENSG00000144746
3	69503587	69503835	KD2-H4K8ac_peak_21201	6.56926	
3	69788649	69788903	KD2-H4K8ac_peak_21202	6.56211	MITF_ENSG00000187098
3	71032103	71032328	KD2-H4K8ac_peak_21203	6.40683	
3	71102549	71102829	KD2-H4K8ac_peak_21204	5.30799	

3	71113582	71114039	KD2-H4K8ac_peak_21205	10.21502	
3	71114249	71114791	KD2-H4K8ac_peak_21206	12.42547	
3	71150101	71150602	KD2-H4K8ac_peak_21207	6.40683	
3	71215483	71215685	KD2-H4K8ac_peak_21208	5.25071	
3	71296400	71296786	KD2-H4K8ac_peak_21209	8.62078	
3	71592610	71592868	KD2-H4K8ac_peak_21210	6.56211	
3	71630564	71631013	KD2-H4K8ac_peak_21211	6.66692	
3	71631543	71633104	KD2-H4K8ac_peak_21212	9.73431	FOXP1_ENSG00000114861;RP11-154H23.3_ENSG00000270562
3	71774291	71774484	KD2-H4K8ac_peak_21213	4.19765	
3	71774717	71775004	KD2-H4K8ac_peak_21214	9.95119	
3	71802560	71803812	KD2-H4K8ac_peak_21215	16.822	EIF4E3_ENSG00000163412;GPR27_ENSG00000170837
3	71834367	71834619	KD2-H4K8ac_peak_21216	9.36516	PROK2_ENSG00000163421
3	72005573	72005825	KD2-H4K8ac_peak_21217	5.45396	
3	72201009	72201793	KD2-H4K8ac_peak_21218	5.88845	LINC00870_ENSG00000243083
3	72226999	72227196	KD2-H4K8ac_peak_21219	5.6755	RP11-433A10.3_ENSG00000272774
3	72304515	72304746	KD2-H4K8ac_peak_21220	5.88845	
3	72337666	72337911	KD2-H4K8ac_peak_21221	5.20908	
3	72338114	72338818	KD2-H4K8ac_peak_21222	8.26945	
3	72345573	72346030	KD2-H4K8ac_peak_21223	7.36799	
3	72353049	72353449	KD2-H4K8ac_peak_21224	6.99815	
3	72360311	72360530	KD2-H4K8ac_peak_21225	5.08628	
3	72385964	72386273	KD2-H4K8ac_peak_21226	9.89383	
3	72496518	72496953	KD2-H4K8ac_peak_21227	14.12659	RYBP_ENSG00000163602
3	72619669	72619863	KD2-H4K8ac_peak_21228	7.47462	
3	72818625	72818856	KD2-H4K8ac_peak_21229	8.59579	
3	72897304	72897547	KD2-H4K8ac_peak_21230	6.65673	
3	72937277	72937739	KD2-H4K8ac_peak_21231	6.66692	GXYLT2_ENSG00000172986
3	73045121	73045673	KD2-H4K8ac_peak_21232	3.86308	PPP4R2_ENSG00000163605
3	73046026	73046506	KD2-H4K8ac_peak_21233	10.7186	PPP4R2_ENSG00000163605
3	73242029	73242241	KD2-H4K8ac_peak_21234	9.11031	
3	73400065	73400319	KD2-H4K8ac_peak_21235	8.74596	
3	73481299	73481696	KD2-H4K8ac_peak_21236	5.54647	
3	73519667	73519890	KD2-H4K8ac_peak_21237	7.01964	
3	73520135	73521128	KD2-H4K8ac_peak_21238	19.6416	
3	73522192	73522394	KD2-H4K8ac_peak_21239	4.37708	
3	73673528	73673952	KD2-H4K8ac_peak_21240	8.12706	PDZRN3_ENSG00000121440
3	74382051	74382243	KD2-H4K8ac_peak_21241	5.22731	
3	74663700	74664135	KD2-H4K8ac_peak_21242	7.27716	
3	75485036	75485229	KD2-H4K8ac_peak_21243	7.40314	FAM86DP_ENSG00000244026;RP11-803B1.8_ENSG00000272690
3	75834013	75834691	KD2-H4K8ac_peak_21244	26.9893	ZNF717_ENSG00000227124
3	75835103	75835336	KD2-H4K8ac_peak_21245	4.95402	ZNF717_ENSG00000227124
3	75955936	75956184	KD2-H4K8ac_peak_21246	8.10216	ROBO2_ENSG00000185008
3	77088703	77089110	KD2-H4K8ac_peak_21247	6.60096	
3	81810200	81811143	KD2-H4K8ac_peak_21248	14.275	GBE1_ENSG00000114480
3	85553715	85554127	KD2-H4K8ac_peak_21249	15.5861	
3	87275926	87276321	KD2-H4K8ac_peak_21250	7.40111	MIR4795_ENSG00000264119;CHMP2B_ENSG00000083937

3	87276581	87276864	KD2-H4K8ac_peak_21251	8.4716	CHMP2B_ENSG00000083937
3	88107574	88108061	KD2-H4K8ac_peak_21252	4.54006	RP11-159G9.5_ENSG00000229729
3	88108440	88108772	KD2-H4K8ac_peak_21253	13.39763	RP11-159G9.5_ENSG00000229729
3	88198344	88198961	KD2-H4K8ac_peak_21254	9.36516	CGGBP1_ENSG00000163320;C3orf38_ENSG00000179021
3	88199170	88199563	KD2-H4K8ac_peak_21255	15.22643	CGGBP1_ENSG00000163320;C3orf38_ENSG00000179021
3	93531330	93531547	KD2-H4K8ac_peak_21256	5.01262	
3	93692561	93693136	KD2-H4K8ac_peak_21257	6.99815	PROS1_ENSG00000184500
3	93693358	93693550	KD2-H4K8ac_peak_21258	5.77029	PROS1_ENSG00000184500
3	93781906	93782108	KD2-H4K8ac_peak_21259	13.92465	DHFRL1_ENSG00000178700;NSUN3_ENSG00000178694
3	93782559	93782965	KD2-H4K8ac_peak_21260	4.37815	DHFRL1_ENSG00000178700;NSUN3_ENSG00000178694
3	94131291	94131488	KD2-H4K8ac_peak_21261	7.12754	
3	94150810	94151064	KD2-H4K8ac_peak_21262	7.12754	
3	96147463	96147714	KD2-H4K8ac_peak_21263	7.12754	
3	96532307	96532690	KD2-H4K8ac_peak_21264	6.60096	EPHA6_ENSG00000080224
3	97483109	97483831	KD2-H4K8ac_peak_21265	8.55271	ARL6_ENSG00000113966
3	97540925	97541516	KD2-H4K8ac_peak_21266	9.6589	CRYBG3_ENSG00000233280
3	97691604	97691976	KD2-H4K8ac_peak_21267	5.58333	MINA_ENSG00000170854
3	98312697	98313101	KD2-H4K8ac_peak_21268	8.98477	CPOX_ENSG00000080819
3	98451628	98452192	KD2-H4K8ac_peak_21269	11.04256	ST3GAL6-AS1_ENSG00000239445;ST3GAL6_ENSG00000064225
3	98482604	98483057	KD2-H4K8ac_peak_21270	8.97719	
3	98685816	98686087	KD2-H4K8ac_peak_21271	9.02371	
3	99594858	99595230	KD2-H4K8ac_peak_21272	5.13615	
3	99978958	99979257	KD2-H4K8ac_peak_21273	13.68542	TBC1D23_ENSG00000036054
3	99979876	99980084	KD2-H4K8ac_peak_21274	6.0605	TBC1D23_ENSG00000036054
3	100053687	1E+08	KD2-H4K8ac_peak_21275	9.95119	NIT2_ENSG00000114021
3	100119351	1E+08	KD2-H4K8ac_peak_21276	10.65724	TOMM70A_ENSG00000154174;LNP1_ENSG00000206535
3	100210816	1E+08	KD2-H4K8ac_peak_21277	8.23274	TMEM45A_ENSG00000181458
3	100428393	1E+08	KD2-H4K8ac_peak_21278	8.59579	TFG_ENSG00000114354
3	100428803	1E+08	KD2-H4K8ac_peak_21279	11.15509	TFG_ENSG00000114354
3	101231386	1.01E+08	KD2-H4K8ac_peak_21280	14.74047	SENP7_ENSG00000138468
3	101280315	1.01E+08	KD2-H4K8ac_peak_21281	14.43704	TRMT10C_ENSG00000174173
3	101293222	1.01E+08	KD2-H4K8ac_peak_21282	17.16047	PCNP_ENSG00000081154
3	101395391	1.01E+08	KD2-H4K8ac_peak_21283	9.242	ZBTB11_ENSG00000066422;ZBTB11-AS1_ENSG00000256628
3	101405258	1.01E+08	KD2-H4K8ac_peak_21284	10.17341	RPL24_ENSG00000114391
3	101443959	1.01E+08	KD2-H4K8ac_peak_21285	5.14125	
3	101497714	1.01E+08	KD2-H4K8ac_peak_21286	9.34195	NXPE3_ENSG00000144815
3	101567733	1.02E+08	KD2-H4K8ac_peak_21287	11.43353	
3	101568563	1.02E+08	KD2-H4K8ac_peak_21288	6.36375	
3	102440166	1.02E+08	KD2-H4K8ac_peak_21289	14.15075	
3	102929642	1.03E+08	KD2-H4K8ac_peak_21290	7.01964	
3	103623193	1.04E+08	KD2-H4K8ac_peak_21291	10.05606	
3	104628063	1.05E+08	KD2-H4K8ac_peak_21292	6.40683	
3	104675866	1.05E+08	KD2-H4K8ac_peak_21293	5.45396	
3	104700564	1.05E+08	KD2-H4K8ac_peak_21294	5.57044	
3	104711514	1.05E+08	KD2-H4K8ac_peak_21295	7.12754	
3	105074188	1.05E+08	KD2-H4K8ac_peak_21296	5.14125	

3	105085058	1.05E+08	KD2-H4K8ac_peak_21297	5.835	ALCAM_ENSG00000170017
3	105587261	1.06E+08	KD2-H4K8ac_peak_21298	7.49599	CBLB_ENSG00000114423
3	105588046	1.06E+08	KD2-H4K8ac_peak_21299	7.76329	CBLB_ENSG00000114423
3	106117766	1.06E+08	KD2-H4K8ac_peak_21300	5.57044	
3	106545155	1.07E+08	KD2-H4K8ac_peak_21301	6.2159	
3	107149936	1.07E+08	KD2-H4K8ac_peak_21302	14.80472	RP11-115H18.1_ENSG00000273125
3	107241207	1.07E+08	KD2-H4K8ac_peak_21303	4.81242	BBX_ENSG00000114439
3	107241907	1.07E+08	KD2-H4K8ac_peak_21304	7.76329	BBX_ENSG00000114439
3	107646176	1.08E+08	KD2-H4K8ac_peak_21305	6.2781	
3	107706843	1.08E+08	KD2-H4K8ac_peak_21306	12.85889	
3	107810032	1.08E+08	KD2-H4K8ac_peak_21307	12.82393	CD47_ENSG00000196776
3	108228709	1.08E+08	KD2-H4K8ac_peak_21308	8.25022	
3	111578168	1.12E+08	KD2-H4K8ac_peak_21309	5.13615	
3	111717651	1.12E+08	KD2-H4K8ac_peak_21310	7.95563	
3	111718355	1.12E+08	KD2-H4K8ac_peak_21311	4.67342	
3	111804734	1.12E+08	KD2-H4K8ac_peak_21312	9.08816	C3orf52_ENSG00000114529
3	112022262	1.12E+08	KD2-H4K8ac_peak_21313	6.64891	RP11-90K6.1_ENSG00000239482
3	112022745	1.12E+08	KD2-H4K8ac_peak_21314	7.27716	
3	112052004	1.12E+08	KD2-H4K8ac_peak_21315	9.11031	CD200_ENSG00000091972
3	112052998	1.12E+08	KD2-H4K8ac_peak_21316	6.66692	
3	112280504	1.12E+08	KD2-H4K8ac_peak_21317	13.73635	ATG3_ENSG00000144848;SLC35A5_ENSG00000138459
3	112280945	1.12E+08	KD2-H4K8ac_peak_21318	7.70289	ATG3_ENSG00000144848;SLC35A5_ENSG00000138459
3	112353870	1.12E+08	KD2-H4K8ac_peak_21319	6.56926	
3	112361977	1.12E+08	KD2-H4K8ac_peak_21320	7.63551	
3	112709480	1.13E+08	KD2-H4K8ac_peak_21321	7.76329	RP11-484K9.4_ENSG00000272844;GTPBP8_ENSG00000163607
3	112738705	1.13E+08	KD2-H4K8ac_peak_21322	6.56211	C3orf17_ENSG00000163608;RP11-572M11.4_ENSG00000240057
3	112867453	1.13E+08	KD2-H4K8ac_peak_21323	8.30974	
3	112870699	1.13E+08	KD2-H4K8ac_peak_21324	6.66692	
3	112883916	1.13E+08	KD2-H4K8ac_peak_21325	13.09607	
3	112885085	1.13E+08	KD2-H4K8ac_peak_21326	4.44748	
3	112888034	1.13E+08	KD2-H4K8ac_peak_21327	6.56211	
3	112888699	1.13E+08	KD2-H4K8ac_peak_21328	13.34404	
3	112911891	1.13E+08	KD2-H4K8ac_peak_21329	6.16888	
3	112929747	1.13E+08	KD2-H4K8ac_peak_21330	9.59147	BOC_ENSG00000144857
3	112930486	1.13E+08	KD2-H4K8ac_peak_21331	5.75757	BOC_ENSG00000144857
3	112940614	1.13E+08	KD2-H4K8ac_peak_21332	9.1707	
3	112960232	1.13E+08	KD2-H4K8ac_peak_21333	6.30229	
3	112961171	1.13E+08	KD2-H4K8ac_peak_21334	10.48739	
3	112967687	1.13E+08	KD2-H4K8ac_peak_21335	5.51783	
3	112994700	1.13E+08	KD2-H4K8ac_peak_21336	10.0692	
3	113251691	1.13E+08	KD2-H4K8ac_peak_21337	7.76104	SIDT1_ENSG00000072858
3	113464259	1.13E+08	KD2-H4K8ac_peak_21338	11.22681	NAA50_ENSG00000121579;ATP6V1A_ENSG00000114573
3	113666761	1.14E+08	KD2-H4K8ac_peak_21339	11.05467	RP11-255E6.6_ENSG00000273394;ZDHHC23_ENSG00000184307
3	113774946	1.14E+08	KD2-H4K8ac_peak_21340	11.05407	KIAA1407_ENSG00000163617
3	113871465	1.14E+08	KD2-H4K8ac_peak_21341	9.16531	
3	114119435	1.14E+08	KD2-H4K8ac_peak_21342	9.15329	

3	114129489	1.14E+08	KD2-H4K8ac_peak_21343	6.16888	
3	114173218	1.14E+08	KD2-H4K8ac_peak_21344	3.75035	RP11-197K3.1_ENSG00000242290
3	114343460	1.14E+08	KD2-H4K8ac_peak_21345	4.01115	
3	114865408	1.15E+08	KD2-H4K8ac_peak_21346	12.42547	ZBTB20_ENSG00000181722
3	114866765	1.15E+08	KD2-H4K8ac_peak_21347	7.12754	ZBTB20_ENSG00000181722
3	115113612	1.15E+08	KD2-H4K8ac_peak_21348	4.01115	
3	115238449	1.15E+08	KD2-H4K8ac_peak_21349	5.57044	
3	115377390	1.15E+08	KD2-H4K8ac_peak_21350	18.31034	RP11-326J18.1_ENSG00000241596
3	115427483	1.15E+08	KD2-H4K8ac_peak_21351	8.30974	
3	115430389	1.15E+08	KD2-H4K8ac_peak_21352	9.11818	
3	115503065	1.16E+08	KD2-H4K8ac_peak_21353	6.84261	
3	115837090	1.16E+08	KD2-H4K8ac_peak_21354	7.12754	
3	115851024	1.16E+08	KD2-H4K8ac_peak_21355	7.12754	
3	115855600	1.16E+08	KD2-H4K8ac_peak_21356	4.37708	
3	116649238	1.17E+08	KD2-H4K8ac_peak_21357	14.29602	
3	116739985	1.17E+08	KD2-H4K8ac_peak_21358	6.40683	
3	117250099	1.17E+08	KD2-H4K8ac_peak_21359	8.1233	
3	117640125	1.18E+08	KD2-H4K8ac_peak_21360	6.82976	
3	117641104	1.18E+08	KD2-H4K8ac_peak_21361	6.16888	
3	117648287	1.18E+08	KD2-H4K8ac_peak_21362	7.0983	
3	117715820	1.18E+08	KD2-H4K8ac_peak_21363	11.60333	LSAMP_ENSG00000185565;RP11-384F7.2_ENSG00000239268
3	118220013	1.18E+08	KD2-H4K8ac_peak_21364	5.88845	
3	118959453	1.19E+08	KD2-H4K8ac_peak_21365	5.88845	B4GALT4_ENSG00000121578
3	118995100	1.19E+08	KD2-H4K8ac_peak_21366	4.77079	
3	119011535	1.19E+08	KD2-H4K8ac_peak_21367	6.99815	
3	119012866	1.19E+08	KD2-H4K8ac_peak_21368	14.275	ARHGAP31_ENSG00000031081
3	119041348	1.19E+08	KD2-H4K8ac_peak_21369	12.74171	ARHGAP31-AS1_ENSG00000241155
3	119050679	1.19E+08	KD2-H4K8ac_peak_21370	8.83582	
3	119051103	1.19E+08	KD2-H4K8ac_peak_21371	11.92978	
3	119052924	1.19E+08	KD2-H4K8ac_peak_21372	13.09607	
3	119078390	1.19E+08	KD2-H4K8ac_peak_21373	7.13615	
3	119080429	1.19E+08	KD2-H4K8ac_peak_21374	6.2781	
3	119083431	1.19E+08	KD2-H4K8ac_peak_21375	4.86324	
3	119085586	1.19E+08	KD2-H4K8ac_peak_21376	6.40683	
3	119182157	1.19E+08	KD2-H4K8ac_peak_21377	10.05606	
3	119182684	1.19E+08	KD2-H4K8ac_peak_21378	8.00457	
3	119187540	1.19E+08	KD2-H4K8ac_peak_21379	9.63487	TMEM39A_ENSG00000176142
3	119270711	1.19E+08	KD2-H4K8ac_peak_21380	5.15777	
3	119298303	1.19E+08	KD2-H4K8ac_peak_21381	10.96455	RP11-190C22.9_ENSG00000272967;ADPRH_ENSG00000144843
3	119349556	1.19E+08	KD2-H4K8ac_peak_21382	3.80759	
3	119351829	1.19E+08	KD2-H4K8ac_peak_21383	9.27888	
3	119356057	1.19E+08	KD2-H4K8ac_peak_21384	8.86391	
3	119357277	1.19E+08	KD2-H4K8ac_peak_21385	8.17902	
3	119359451	1.19E+08	KD2-H4K8ac_peak_21386	20.09573	
3	119378783	1.19E+08	KD2-H4K8ac_peak_21387	15.22643	
3	119422170	1.19E+08	KD2-H4K8ac_peak_21388	9.96798	MAATS1_ENSG00000183833

3	119812790	1.2E+08	KD2-H4K8ac_peak_21389	14.8083	GSK3B_ENSG00000082701;RP11-18H7.1_ENSG00000242622
3	119814151	1.2E+08	KD2-H4K8ac_peak_21390	24.8283	GSK3B_ENSG00000082701;RP11-18H7.1_ENSG00000242622
3	120003865	1.2E+08	KD2-H4K8ac_peak_21391	13.70605	GPR156_ENSG00000175697
3	120067705	1.2E+08	KD2-H4K8ac_peak_21392	6.16883	LRRC58_ENSG00000163428;RP11-174O3.3_ENSG00000240661
3	120069605	1.2E+08	KD2-H4K8ac_peak_21393	9.02573	
3	120169736	1.2E+08	KD2-H4K8ac_peak_21394	10.43145	FSTL1_ENSG00000163430
3	120278135	1.2E+08	KD2-H4K8ac_peak_21395	4.67342	
3	120461105	1.2E+08	KD2-H4K8ac_peak_21396	17.16047	RABL3_ENSG00000144840;GTF2E1_ENSG00000153767
3	121468086	1.21E+08	KD2-H4K8ac_peak_21397	8.55271	GOLGB1_ENSG00000173230
3	122101854	1.22E+08	KD2-H4K8ac_peak_21398	13.92307	CCDC58_ENSG00000160124;FAM162A_ENSG00000114023
3	122135147	1.22E+08	KD2-H4K8ac_peak_21399	7.20567	WDR5B_ENSG00000196981;RP11-299J3.8_ENSG00000272758;RP11-299J3.6_ENSG00000242531
3	122135606	1.22E+08	KD2-H4K8ac_peak_21400	4.86324	WDR5B_ENSG00000196981;RP11-299J3.8_ENSG00000272758;RP11-299J3.6_ENSG00000242531
3	122282485	1.22E+08	KD2-H4K8ac_peak_21401	17.43705	PARP9_ENSG00000138496;DTX3L_ENSG00000163840
3	122399637	1.22E+08	KD2-H4K8ac_peak_21402	10.46264	PARP14_ENSG00000173193
3	122512358	1.23E+08	KD2-H4K8ac_peak_21403	6.99815	HSPBAP1_ENSG00000169087
3	122513956	1.23E+08	KD2-H4K8ac_peak_21404	12.93209	DIRC2_ENSG00000138463
3	122647643	1.23E+08	KD2-H4K8ac_peak_21405	15.14522	
3	122649084	1.23E+08	KD2-H4K8ac_peak_21406	5.9696	
3	122654652	1.23E+08	KD2-H4K8ac_peak_21407	9.70057	
3	122656087	1.23E+08	KD2-H4K8ac_peak_21408	9.44423	
3	122659977	1.23E+08	KD2-H4K8ac_peak_21409	12.65397	
3	122691966	1.23E+08	KD2-H4K8ac_peak_21410	7.47462	
3	122693984	1.23E+08	KD2-H4K8ac_peak_21411	7.76329	
3	122695014	1.23E+08	KD2-H4K8ac_peak_21412	4.57699	
3	122695545	1.23E+08	KD2-H4K8ac_peak_21413	6.66692	
3	122697399	1.23E+08	KD2-H4K8ac_peak_21414	4.06758	
3	122711882	1.23E+08	KD2-H4K8ac_peak_21415	4.54006	
3	122715633	1.23E+08	KD2-H4K8ac_peak_21416	6.83936	
3	122745720	1.23E+08	KD2-H4K8ac_peak_21417	6.24114	
3	122746817	1.23E+08	KD2-H4K8ac_peak_21418	10.19949	SEMA5B_ENSG00000082684
3	122785943	1.23E+08	KD2-H4K8ac_peak_21419	12.70959	PDIA5_ENSG00000065485
3	122800924	1.23E+08	KD2-H4K8ac_peak_21420	13.34404	
3	122803077	1.23E+08	KD2-H4K8ac_peak_21421	3.75035	
3	122803888	1.23E+08	KD2-H4K8ac_peak_21422	10.0692	
3	122872662	1.23E+08	KD2-H4K8ac_peak_21423	7.89389	
3	122920248	1.23E+08	KD2-H4K8ac_peak_21424	6.65673	SEC22A_ENSG00000121542
3	122920849	1.23E+08	KD2-H4K8ac_peak_21425	13.3695	SEC22A_ENSG00000121542
3	123303575	1.23E+08	KD2-H4K8ac_peak_21426	12.57143	PTPLB_ENSG00000206527;MYLK-AS1_ENSG00000239523
3	123304153	1.23E+08	KD2-H4K8ac_peak_21427	5.72327	PTPLB_ENSG00000206527;MYLK-AS1_ENSG00000239523
3	123492481	1.23E+08	KD2-H4K8ac_peak_21428	5.25071	
3	123602644	1.24E+08	KD2-H4K8ac_peak_21429	4.42765	MYLK_ENSG00000065534
3	123751418	1.24E+08	KD2-H4K8ac_peak_21430	16.49944	
3	123752199	1.24E+08	KD2-H4K8ac_peak_21431	29.22161	
3	123779510	1.24E+08	KD2-H4K8ac_peak_21432	9.31907	
3	123876617	1.24E+08	KD2-H4K8ac_peak_21433	9.15329	
3	123894045	1.24E+08	KD2-H4K8ac_peak_21434	7.0754	

3	124001690	1.24E+08	KD2-H4K8ac_peak_21435	10.81728	
3	124015387	1.24E+08	KD2-H4K8ac_peak_21436	6.66692	
3	124017419	1.24E+08	KD2-H4K8ac_peak_21437	9.51625	
3	124035897	1.24E+08	KD2-H4K8ac_peak_21438	7.22457	
3	124039265	1.24E+08	KD2-H4K8ac_peak_21439	5.57044	
3	124085604	1.24E+08	KD2-H4K8ac_peak_21440	5.19569	
3	124449498	1.24E+08	KD2-H4K8ac_peak_21441	5.88845	UMPS_ENSG00000114491
3	124490251	1.24E+08	KD2-H4K8ac_peak_21442	6.22746	
3	124491839	1.24E+08	KD2-H4K8ac_peak_21443	5.88845	
3	124492395	1.24E+08	KD2-H4K8ac_peak_21444	5.30799	
3	124553744	1.25E+08	KD2-H4K8ac_peak_21445	7.28827	
3	124605321	1.25E+08	KD2-H4K8ac_peak_21446	4.86324	
3	124606343	1.25E+08	KD2-H4K8ac_peak_21447	7.44324	
3	124762523	1.25E+08	KD2-H4K8ac_peak_21448	3.97079	
3	124774454	1.25E+08	KD2-H4K8ac_peak_21449	9.402	HEG1_ENSG00000173706
3	124839458	1.25E+08	KD2-H4K8ac_peak_21450	6.65673	
3	124860151	1.25E+08	KD2-H4K8ac_peak_21451	17.70837	
3	125093364	1.25E+08	KD2-H4K8ac_peak_21452	6.5996	ZNF148_ENSG00000163848
3	125093944	1.25E+08	KD2-H4K8ac_peak_21453	17.43705	ZNF148_ENSG00000163848
3	125238825	1.25E+08	KD2-H4K8ac_peak_21454	13.34404	SNX4_ENSG00000114520
3	125315335	1.25E+08	KD2-H4K8ac_peak_21455	7.05051	
3	125477556	1.25E+08	KD2-H4K8ac_peak_21456	8.30974	
3	125634345	1.26E+08	KD2-H4K8ac_peak_21457	6.25869	FAM86JP_ENSG00000171084
3	125709369	1.26E+08	KD2-H4K8ac_peak_21458	8.17902	
3	125802344	1.26E+08	KD2-H4K8ac_peak_21459	8.55271	RP11-124N2.1_ENSG00000250012
3	125819782	1.26E+08	KD2-H4K8ac_peak_21460	4.59928	SLC41A3_ENSG00000114544
3	125889965	1.26E+08	KD2-H4K8ac_peak_21461	7.12754	
3	125900064	1.26E+08	KD2-H4K8ac_peak_21462	4.42765	
3	125931540	1.26E+08	KD2-H4K8ac_peak_21463	14.8083	
3	125979945	1.26E+08	KD2-H4K8ac_peak_21464	6.61376	
3	125986133	1.26E+08	KD2-H4K8ac_peak_21465	5.88845	RP11-71E19.1_ENSG00000250934
3	125986556	1.26E+08	KD2-H4K8ac_peak_21466	4.12757	RP11-71E19.1_ENSG00000250934
3	126006272	1.26E+08	KD2-H4K8ac_peak_21467	5.88845	
3	126074274	1.26E+08	KD2-H4K8ac_peak_21468	9.34989	
3	126075103	1.26E+08	KD2-H4K8ac_peak_21469	5.85704	KLF15_ENSG00000163884
3	126107866	1.26E+08	KD2-H4K8ac_peak_21470	11.43353	
3	126194474	1.26E+08	KD2-H4K8ac_peak_21471	24.48793	ZXDC_ENSG00000070476
3	126195228	1.26E+08	KD2-H4K8ac_peak_21472	5.53033	ZXDC_ENSG00000070476
3	126200066	1.26E+08	KD2-H4K8ac_peak_21473	6.25869	
3	126248004	1.26E+08	KD2-H4K8ac_peak_21474	7.82935	
3	126373293	1.26E+08	KD2-H4K8ac_peak_21475	12.84642	TXNRD3_ENSG00000197763
3	126397613	1.26E+08	KD2-H4K8ac_peak_21476	9.15069	
3	126398336	1.26E+08	KD2-H4K8ac_peak_21477	7.76329	
3	126402549	1.26E+08	KD2-H4K8ac_peak_21478	9.27888	
3	126423193	1.26E+08	KD2-H4K8ac_peak_21479	4.36736	CHCHD6_ENSG00000159685
3	126645910	1.27E+08	KD2-H4K8ac_peak_21480	7.27716	

3	126656252	1.27E+08	KD2-H4K8ac_peak_21481	5.20908	
3	126678401	1.27E+08	KD2-H4K8ac_peak_21482	5.13615	
3	126690642	1.27E+08	KD2-H4K8ac_peak_21483	5.57044	
3	126701241	1.27E+08	KD2-H4K8ac_peak_21484	8.16906	
3	126702242	1.27E+08	KD2-H4K8ac_peak_21485	4.76709	
3	126719983	1.27E+08	KD2-H4K8ac_peak_21486	12.44942	
3	126721278	1.27E+08	KD2-H4K8ac_peak_21487	8.85813	
3	126726946	1.27E+08	KD2-H4K8ac_peak_21488	5.47227	
3	126729324	1.27E+08	KD2-H4K8ac_peak_21489	9.56573	
3	126730558	1.27E+08	KD2-H4K8ac_peak_21490	8.7723	
3	126733598	1.27E+08	KD2-H4K8ac_peak_21491	5.90414	
3	126792003	1.27E+08	KD2-H4K8ac_peak_21492	5.9696	
3	126807566	1.27E+08	KD2-H4K8ac_peak_21493	6.72624	
3	126852911	1.27E+08	KD2-H4K8ac_peak_21494	11.68645	
3	126854552	1.27E+08	KD2-H4K8ac_peak_21495	9.18237	
3	126889902	1.27E+08	KD2-H4K8ac_peak_21496	11.90231	
3	126892333	1.27E+08	KD2-H4K8ac_peak_21497	13.83097	
3	127023465	1.27E+08	KD2-H4K8ac_peak_21498	7.59643	
3	127089718	1.27E+08	KD2-H4K8ac_peak_21499	7.70289	
3	127145075	1.27E+08	KD2-H4K8ac_peak_21500	5.64449	
3	127174024	1.27E+08	KD2-H4K8ac_peak_21501	5.54647	
3	127174715	1.27E+08	KD2-H4K8ac_peak_21502	9.6247	
3	127265988	1.27E+08	KD2-H4K8ac_peak_21503	7.146	
3	127271089	1.27E+08	KD2-H4K8ac_peak_21504	6.09229	
3	127294211	1.27E+08	KD2-H4K8ac_peak_21505	5.54647	
3	127309781	1.27E+08	KD2-H4K8ac_peak_21506	15.81711	
3	127316666	1.27E+08	KD2-H4K8ac_peak_21507	13.70753	TPRA1_ENSG00000163870;MCM2_ENSG00000073111
3	127348065	1.27E+08	KD2-H4K8ac_peak_21508	14.45402	PODXL2_ENSG00000114631
3	127390755	1.27E+08	KD2-H4K8ac_peak_21509	15.59774	ABTB1_ENSG00000114626
3	127391977	1.27E+08	KD2-H4K8ac_peak_21510	6.99815	ABTB1_ENSG00000114626
3	127406924	1.27E+08	KD2-H4K8ac_peak_21511	9.51625	
3	127408063	1.27E+08	KD2-H4K8ac_peak_21512	12.16946	
3	127414817	1.27E+08	KD2-H4K8ac_peak_21513	7.95563	
3	127415440	1.27E+08	KD2-H4K8ac_peak_21514	13.27022	
3	127417665	1.27E+08	KD2-H4K8ac_peak_21515	6.40297	
3	127425723	1.27E+08	KD2-H4K8ac_peak_21516	7.27716	
3	127436288	1.27E+08	KD2-H4K8ac_peak_21517	6.33462	
3	127462595	1.27E+08	KD2-H4K8ac_peak_21518	4.9025	
3	127500572	1.28E+08	KD2-H4K8ac_peak_21519	12.13163	
3	127503297	1.28E+08	KD2-H4K8ac_peak_21520	20.0792	
3	127505601	1.28E+08	KD2-H4K8ac_peak_21521	9.63487	
3	127506214	1.28E+08	KD2-H4K8ac_peak_21522	9.1707	
3	127515952	1.28E+08	KD2-H4K8ac_peak_21523	4.01115	
3	127542272	1.28E+08	KD2-H4K8ac_peak_21524	7.96322	
3	127725106	1.28E+08	KD2-H4K8ac_peak_21525	8.17902	
3	127740845	1.28E+08	KD2-H4K8ac_peak_21526	4.37708	

3	127771359	1.28E+08	KD2-H4K8ac_peak_21527	4.44527	SEC61A1_ENSG00000058262
3	127842337	1.28E+08	KD2-H4K8ac_peak_21528	5.9696	
3	127842997	1.28E+08	KD2-H4K8ac_peak_21529	14.29602	
3	127872380	1.28E+08	KD2-H4K8ac_peak_21530	10.0692	RUVBL1_ENSG00000175792;EEFSEC_ENSG00000132394
3	127886290	1.28E+08	KD2-H4K8ac_peak_21531	6.60096	
3	127888145	1.28E+08	KD2-H4K8ac_peak_21532	4.64388	
3	127953132	1.28E+08	KD2-H4K8ac_peak_21533	3.93917	
3	127955556	1.28E+08	KD2-H4K8ac_peak_21534	7.61026	
3	127957809	1.28E+08	KD2-H4K8ac_peak_21535	5.9696	
3	127983030	1.28E+08	KD2-H4K8ac_peak_21536	8.02571	
3	127994801	1.28E+08	KD2-H4K8ac_peak_21537	5.96443	
3	127995649	1.28E+08	KD2-H4K8ac_peak_21538	7.11824	
3	127996407	1.28E+08	KD2-H4K8ac_peak_21539	16.822	
3	127998116	1.28E+08	KD2-H4K8ac_peak_21540	16.3889	
3	127999214	1.28E+08	KD2-H4K8ac_peak_21541	8.73351	
3	128000755	1.28E+08	KD2-H4K8ac_peak_21542	10.19949	
3	128001756	1.28E+08	KD2-H4K8ac_peak_21543	11.89502	
3	128005943	1.28E+08	KD2-H4K8ac_peak_21544	5.71749	
3	128035901	1.28E+08	KD2-H4K8ac_peak_21545	8.09231	
3	128036455	1.28E+08	KD2-H4K8ac_peak_21546	9.61895	
3	128073885	1.28E+08	KD2-H4K8ac_peak_21547	4.06758	
3	128113492	1.28E+08	KD2-H4K8ac_peak_21548	10.77651	
3	128116045	1.28E+08	KD2-H4K8ac_peak_21549	4.85062	
3	128117379	1.28E+08	KD2-H4K8ac_peak_21550	12.58841	
3	128118496	1.28E+08	KD2-H4K8ac_peak_21551	6.99815	
3	128124788	1.28E+08	KD2-H4K8ac_peak_21552	7.3948	
3	128125899	1.28E+08	KD2-H4K8ac_peak_21553	8.80432	
3	128128399	1.28E+08	KD2-H4K8ac_peak_21554	24.91915	
3	128130808	1.28E+08	KD2-H4K8ac_peak_21555	23.04547	
3	128131769	1.28E+08	KD2-H4K8ac_peak_21556	13.22555	
3	128133876	1.28E+08	KD2-H4K8ac_peak_21557	5.4779	
3	128134455	1.28E+08	KD2-H4K8ac_peak_21558	6.8713	
3	128135029	1.28E+08	KD2-H4K8ac_peak_21559	6.66359	
3	128139703	1.28E+08	KD2-H4K8ac_peak_21560	8.23274	
3	128144116	1.28E+08	KD2-H4K8ac_peak_21561	4.93687	
3	128144920	1.28E+08	KD2-H4K8ac_peak_21562	13.81781	
3	128146410	1.28E+08	KD2-H4K8ac_peak_21563	4.86324	
3	128151200	1.28E+08	KD2-H4K8ac_peak_21564	7.6718	
3	128154043	1.28E+08	KD2-H4K8ac_peak_21565	8.55271	
3	128173276	1.28E+08	KD2-H4K8ac_peak_21566	10.21366	
3	128174742	1.28E+08	KD2-H4K8ac_peak_21567	29.36444	
3	128176364	1.28E+08	KD2-H4K8ac_peak_21568	21.07675	
3	128178305	1.28E+08	KD2-H4K8ac_peak_21569	10.73134	
3	128186081	1.28E+08	KD2-H4K8ac_peak_21570	12.57865	DNAJB8_ENSG00000179407
3	128187769	1.28E+08	KD2-H4K8ac_peak_21571	15.76765	
3	128189358	1.28E+08	KD2-H4K8ac_peak_21572	12.57297	

3	128194076	1.28E+08	KD2-H4K8ac_peak_21573	7.99241	
3	128200966	1.28E+08	KD2-H4K8ac_peak_21574	6.27719	
3	128201674	1.28E+08	KD2-H4K8ac_peak_21575	7.76329	
3	128202376	1.28E+08	KD2-H4K8ac_peak_21576	4.37809	
3	128205883	1.28E+08	KD2-H4K8ac_peak_21577	6.24992	
3	128206519	1.28E+08	KD2-H4K8ac_peak_21578	12.14719	
3	128207326	1.28E+08	KD2-H4K8ac_peak_21579	6.75585	RP11-475N22.4_ENSG00000244300
3	128207783	1.28E+08	KD2-H4K8ac_peak_21580	14.06758	RP11-475N22.4_ENSG00000244300
3	128209827	1.28E+08	KD2-H4K8ac_peak_21581	6.60096	
3	128210376	1.28E+08	KD2-H4K8ac_peak_21582	13.018	GATA2_ENSG00000179348
3	128212197	1.28E+08	KD2-H4K8ac_peak_21583	9.1707	GATA2_ENSG00000179348
3	128214585	1.28E+08	KD2-H4K8ac_peak_21584	11.03331	
3	128215191	1.28E+08	KD2-H4K8ac_peak_21585	13.73635	
3	128236554	1.28E+08	KD2-H4K8ac_peak_21586	6.1228	
3	128268144	1.28E+08	KD2-H4K8ac_peak_21587	8.10216	
3	128303280	1.28E+08	KD2-H4K8ac_peak_21588	5.9696	
3	128326745	1.28E+08	KD2-H4K8ac_peak_21589	8.30974	
3	128336040	1.28E+08	KD2-H4K8ac_peak_21590	15.58442	
3	128336745	1.28E+08	KD2-H4K8ac_peak_21591	12.70983	
3	128369154	1.28E+08	KD2-H4K8ac_peak_21592	13.13969	
3	128369988	1.28E+08	KD2-H4K8ac_peak_21593	10.96455	
3	128370496	1.28E+08	KD2-H4K8ac_peak_21594	4.26087	
3	128398832	1.28E+08	KD2-H4K8ac_peak_21595	10.59312	RPN1_ENSG00000163902
3	128399744	1.28E+08	KD2-H4K8ac_peak_21596	6.97919	RPN1_ENSG00000163902
3	128400335	1.28E+08	KD2-H4K8ac_peak_21597	4.98072	RPN1_ENSG00000163902
3	128420254	1.28E+08	KD2-H4K8ac_peak_21598	8.10216	
3	128421190	1.28E+08	KD2-H4K8ac_peak_21599	6.1846	
3	128444476	1.28E+08	KD2-H4K8ac_peak_21600	9.34989	RAB7A_ENSG00000075785
3	128445104	1.28E+08	KD2-H4K8ac_peak_21601	10.19949	RAB7A_ENSG00000075785
3	128564581	1.29E+08	KD2-H4K8ac_peak_21602	7.07117	
3	128583168	1.29E+08	KD2-H4K8ac_peak_21603	9.16531	
3	128598512	1.29E+08	KD2-H4K8ac_peak_21604	9.07715	ACAD9_ENSG00000177646
3	128712431	1.29E+08	KD2-H4K8ac_peak_21605	10.0692	
3	128780790	1.29E+08	KD2-H4K8ac_peak_21606	5.57044	
3	128840884	1.29E+08	KD2-H4K8ac_peak_21607	8.70727	RAB43_ENSG00000172780;RP11-434H6.6_ENSG00000273174
3	128880053	1.29E+08	KD2-H4K8ac_peak_21608	6.66692	ISY1-RAB43_ENSG00000261796;ISY1_ENSG00000240682
3	128902002	1.29E+08	KD2-H4K8ac_peak_21609	7.07117	CNBP_ENSG00000169714
3	128902958	1.29E+08	KD2-H4K8ac_peak_21610	8.85813	CNBP_ENSG00000169714
3	128997130	1.29E+08	KD2-H4K8ac_peak_21611	8.26945	HMCES_ENSG00000183624
3	129033566	1.29E+08	KD2-H4K8ac_peak_21612	6.40683	H1FX-AS1_ENSG00000206417
3	129033971	1.29E+08	KD2-H4K8ac_peak_21613	13.12288	H1FX_ENSG00000184897;H1FX-AS1_ENSG00000206417
3	129035375	1.29E+08	KD2-H4K8ac_peak_21614	16.87121	H1FX_ENSG00000184897
3	129085134	1.29E+08	KD2-H4K8ac_peak_21615	7.1181	
3	129104654	1.29E+08	KD2-H4K8ac_peak_21616	4.42765	
3	129158556	1.29E+08	KD2-H4K8ac_peak_21617	8.4716	MBD4_ENSG00000129071;IFT122_ENSG00000163913
3	129159197	1.29E+08	KD2-H4K8ac_peak_21618	7.52168	MBD4_ENSG00000129071;IFT122_ENSG00000163913

3	129276773	1.29E+08	KD2-H4K8ac_peak_21619	7.67504	
3	129277457	1.29E+08	KD2-H4K8ac_peak_21620	7.47462	
3	129279120	1.29E+08	KD2-H4K8ac_peak_21621	9.61895	
3	129279864	1.29E+08	KD2-H4K8ac_peak_21622	10.96455	
3	129280652	1.29E+08	KD2-H4K8ac_peak_21623	11.09936	
3	129284237	1.29E+08	KD2-H4K8ac_peak_21624	5.88845	
3	129285617	1.29E+08	KD2-H4K8ac_peak_21625	8.44476	
3	129315243	1.29E+08	KD2-H4K8ac_peak_21626	8.83582	
3	129316673	1.29E+08	KD2-H4K8ac_peak_21627	4.211	
3	129325212	1.29E+08	KD2-H4K8ac_peak_21628	6.11333	PLXND1_ENSG00000004399
3	129341114	1.29E+08	KD2-H4K8ac_peak_21629	6.64891	
3	129409523	1.29E+08	KD2-H4K8ac_peak_21630	5.73839	
3	129611815	1.3E+08	KD2-H4K8ac_peak_21631	10.0692	TMCC1_ENSG00000172765;TMCC1-AS1_ENSG00000271270
3	129612731	1.3E+08	KD2-H4K8ac_peak_21632	5.57044	TMCC1_ENSG00000172765;TMCC1-AS1_ENSG00000271270
3	129831114	1.3E+08	KD2-H4K8ac_peak_21633	7.76329	FAM86HP_ENSG00000253540;RP11-77P16.4_ENSG00000249846
3	130466514	1.3E+08	KD2-H4K8ac_peak_21634	4.77079	PIK3R4_ENSG00000196455
3	130745298	1.31E+08	KD2-H4K8ac_peak_21635	11.24702	ASTE1_ENSG00000034533;NEK11_ENSG00000114670
3	131099945	1.31E+08	KD2-H4K8ac_peak_21636	10.96455	RP11-933H2.4_ENSG00000250608;NUDT16_ENSG00000198585
3	131100680	1.31E+08	KD2-H4K8ac_peak_21637	7.76329	RP11-933H2.4_ENSG00000250608;NUDT16_ENSG00000198585
3	131221468	1.31E+08	KD2-H4K8ac_peak_21638	10.77651	MRPL3_ENSG00000114686
3	131222216	1.31E+08	KD2-H4K8ac_peak_21639	6.66692	MRPL3_ENSG00000114686
3	132122514	1.32E+08	KD2-H4K8ac_peak_21640	8.02571	
3	132124575	1.32E+08	KD2-H4K8ac_peak_21641	10.25088	
3	132135573	1.32E+08	KD2-H4K8ac_peak_21642	6.85193	DNAJC13_ENSG00000138246
3	132136407	1.32E+08	KD2-H4K8ac_peak_21643	16.53429	DNAJC13_ENSG00000138246
3	132378400	1.32E+08	KD2-H4K8ac_peak_21644	5.54647	ACAD11_ENSG00000240303
3	132379219	1.32E+08	KD2-H4K8ac_peak_21645	9.61895	ACAD11_ENSG00000240303
3	132756703	1.33E+08	KD2-H4K8ac_peak_21646	11.43353	RP11-469L4.1_ENSG00000249725;TMEM108_ENSG00000144868
3	132757294	1.33E+08	KD2-H4K8ac_peak_21647	6.65673	TMEM108_ENSG00000144868
3	132759846	1.33E+08	KD2-H4K8ac_peak_21648	7.13615	
3	132842715	1.33E+08	KD2-H4K8ac_peak_21649	10.88859	
3	132965335	1.33E+08	KD2-H4K8ac_peak_21650	10.0692	
3	132983581	1.33E+08	KD2-H4K8ac_peak_21651	4.77079	
3	132985277	1.33E+08	KD2-H4K8ac_peak_21652	6.4095	
3	132986850	1.33E+08	KD2-H4K8ac_peak_21653	14.69317	
3	132988668	1.33E+08	KD2-H4K8ac_peak_21654	7.08474	
3	133005295	1.33E+08	KD2-H4K8ac_peak_21655	5.58333	
3	133380409	1.33E+08	KD2-H4K8ac_peak_21656	12.99417	TOPBP1_ENSG00000163781;TFP1_ENSG00000242337
3	133380999	1.33E+08	KD2-H4K8ac_peak_21657	7.75499	TOPBP1_ENSG00000163781;TFP1_ENSG00000242337
3	133614295	1.34E+08	KD2-H4K8ac_peak_21658	12.84642	RAB6B_ENSG00000154917
3	133646012	1.34E+08	KD2-H4K8ac_peak_21659	12.88108	
3	133675059	1.34E+08	KD2-H4K8ac_peak_21660	13.44374	
3	133969213	1.34E+08	KD2-H4K8ac_peak_21661	4.211	RYK_ENSG00000163785
3	133970853	1.34E+08	KD2-H4K8ac_peak_21662	4.211	
3	134082361	1.34E+08	KD2-H4K8ac_peak_21663	10.86378	
3	134093065	1.34E+08	KD2-H4K8ac_peak_21664	7.36799	AMOTL2_ENSG00000114019

3	134125145	1.34E+08	KD2-H4K8ac_peak_21665	11.00372	
3	134125787	1.34E+08	KD2-H4K8ac_peak_21666	6.31838	
3	134203883	1.34E+08	KD2-H4K8ac_peak_21667	7.47462	CEP63_ENSG00000182923
3	134204589	1.34E+08	KD2-H4K8ac_peak_21668	9.16531	ANAPC13_ENSG00000129055;CEP63_ENSG00000182923
3	134205016	1.34E+08	KD2-H4K8ac_peak_21669	9.63487	ANAPC13_ENSG00000129055;CEP63_ENSG00000182923
3	134336618	1.34E+08	KD2-H4K8ac_peak_21670	11.2938	
3	134337863	1.34E+08	KD2-H4K8ac_peak_21671	7.9076	
3	134957072	1.35E+08	KD2-H4K8ac_peak_21672	7.79558	
3	135914282	1.36E+08	KD2-H4K8ac_peak_21673	17.16047	MSL2_ENSG00000174579
3	135915503	1.36E+08	KD2-H4K8ac_peak_21674	13.65567	MSL2_ENSG00000174579
3	135968757	1.36E+08	KD2-H4K8ac_peak_21675	13.09607	PCCB_ENSG00000114054
3	136470293	1.36E+08	KD2-H4K8ac_peak_21676	16.57619	STAG1_ENSG00000118007;RP11-102M11.2_ENSG00000261758
3	136471603	1.36E+08	KD2-H4K8ac_peak_21677	17.8868	STAG1_ENSG00000118007;RP11-102M11.2_ENSG00000261758
3	136538031	1.37E+08	KD2-H4K8ac_peak_21678	6.13909	SLC35G2_ENSG00000168917
3	136538926	1.37E+08	KD2-H4K8ac_peak_21679	6.25869	
3	137307164	1.37E+08	KD2-H4K8ac_peak_21680	7.12754	
3	137308029	1.37E+08	KD2-H4K8ac_peak_21681	4.42765	
3	137833976	1.38E+08	KD2-H4K8ac_peak_21682	8.36432	DZIP1L_ENSG00000158163
3	137894091	1.38E+08	KD2-H4K8ac_peak_21683	10.29344	DBR1_ENSG00000138231
3	137906354	1.38E+08	KD2-H4K8ac_peak_21684	9.89383	ARMC8_ENSG00000114098
3	138048148	1.38E+08	KD2-H4K8ac_peak_21685	9.16531	NME9_ENSG00000181322
3	138066993	1.38E+08	KD2-H4K8ac_peak_21686	15.22643	MRAS_ENSG00000158186
3	138327329	1.38E+08	KD2-H4K8ac_peak_21687	14.68449	FAIM_ENSG00000158234
3	138553426	1.39E+08	KD2-H4K8ac_peak_21688	10.17486	PIK3CB_ENSG00000051382
3	138553927	1.39E+08	KD2-H4K8ac_peak_21689	10.21366	PIK3CB_ENSG00000051382
3	139062110	1.39E+08	KD2-H4K8ac_peak_21690	12.91654	
3	139108812	1.39E+08	KD2-H4K8ac_peak_21691	5.34636	COPB2_ENSG00000184432;RP11-319G6.1_ENSG00000248932
3	139109307	1.39E+08	KD2-H4K8ac_peak_21692	7.01964	COPB2_ENSG00000184432;RP11-319G6.1_ENSG00000248932
3	139253542	1.39E+08	KD2-H4K8ac_peak_21693	9.89383	
3	139256528	1.39E+08	KD2-H4K8ac_peak_21694	6.83165	RBP1_ENSG00000114115
3	139258066	1.39E+08	KD2-H4K8ac_peak_21695	6.38689	RBP1_ENSG00000114115
3	139258763	1.39E+08	KD2-H4K8ac_peak_21696	13.80019	RBP1_ENSG00000114115
3	139260014	1.39E+08	KD2-H4K8ac_peak_21697	5.1707	
3	139654426	1.4E+08	KD2-H4K8ac_peak_21698	3.75035	CLSTN2_ENSG00000158258
3	140660156	1.41E+08	KD2-H4K8ac_peak_21699	7.45501	SLC25A36_ENSG00000114120
3	140660732	1.41E+08	KD2-H4K8ac_peak_21700	14.98936	SLC25A36_ENSG00000114120
3	140661199	1.41E+08	KD2-H4K8ac_peak_21701	6.40683	SLC25A36_ENSG00000114120
3	140769769	1.41E+08	KD2-H4K8ac_peak_21702	7.51054	SPSB4_ENSG00000175093
3	140771615	1.41E+08	KD2-H4K8ac_peak_21703	5.18553	
3	140802122	1.41E+08	KD2-H4K8ac_peak_21704	8.74596	
3	140949865	1.41E+08	KD2-H4K8ac_peak_21705	21.26856	
3	141029928	1.41E+08	KD2-H4K8ac_peak_21706	6.66692	
3	141112292	1.41E+08	KD2-H4K8ac_peak_21707	9.68448	
3	141120835	1.41E+08	KD2-H4K8ac_peak_21708	12.99417	
3	141205300	1.41E+08	KD2-H4K8ac_peak_21709	7.59643	RASA2_ENSG00000155903
3	141206005	1.41E+08	KD2-H4K8ac_peak_21710	9.36516	RASA2_ENSG00000155903

3	141378524	1.41E+08	KD2-H4K8ac_peak_21711	8.23274	
3	141457207	1.41E+08	KD2-H4K8ac_peak_21712	5.9696	RNF7_ENSG00000114125
3	141594729	1.42E+08	KD2-H4K8ac_peak_21713	6.66692	ATP1B3_ENSG00000069849
3	141595956	1.42E+08	KD2-H4K8ac_peak_21714	5.51783	ATP1B3_ENSG00000069849
3	141868612	1.42E+08	KD2-H4K8ac_peak_21715	11.05467	TFDP2_ENSG00000114126
3	141943802	1.42E+08	KD2-H4K8ac_peak_21716	11.92978	GK5_ENSG00000175066
3	142166482	1.42E+08	KD2-H4K8ac_peak_21717	5.9696	XRN1_ENSG00000114127
3	142166940	1.42E+08	KD2-H4K8ac_peak_21718	8.37231	XRN1_ENSG00000114127
3	142297147	1.42E+08	KD2-H4K8ac_peak_21719	12.99417	ATR_ENSG00000175054
3	142442962	1.42E+08	KD2-H4K8ac_peak_21720	12.82393	TRPC1_ENSG00000144935
3	142607415	1.43E+08	KD2-H4K8ac_peak_21721	7.18615	PCOLCE2_ENSG00000163710
3	142607979	1.43E+08	KD2-H4K8ac_peak_21722	6.05809	PCOLCE2_ENSG00000163710
3	142682036	1.43E+08	KD2-H4K8ac_peak_21723	5.30799	PAQR9_ENSG00000188582;RP11-372E1.6_ENSG00000241570;U2SURP_ENSG00000163714
3	142720480	1.43E+08	KD2-H4K8ac_peak_21724	14.15075	RP11-91G21.1_ENSG00000268129
3	145051096	1.45E+08	KD2-H4K8ac_peak_21725	5.30799	
3	147110236	1.47E+08	KD2-H4K8ac_peak_21726	7.95563	ZIC1_ENSG00000152977
3	147111108	1.47E+08	KD2-H4K8ac_peak_21727	7.51054	ZIC1_ENSG00000152977
3	147138991	1.47E+08	KD2-H4K8ac_peak_21728	9.41046	
3	148709537	1.49E+08	KD2-H4K8ac_peak_21729	11.08187	GYG1_ENSG00000163754
3	148804536	1.49E+08	KD2-H4K8ac_peak_21730	8.02571	HLTF_ENSG00000071794;HLTF-AS1_ENSG00000239718
3	149265136	1.49E+08	KD2-H4K8ac_peak_21731	5.57044	
3	149266086	1.49E+08	KD2-H4K8ac_peak_21732	4.42765	
3	149272535	1.49E+08	KD2-H4K8ac_peak_21733	7.13615	
3	149375209	1.49E+08	KD2-H4K8ac_peak_21734	5.77029	WWTR1-AS1_ENSG00000241313
3	149375900	1.49E+08	KD2-H4K8ac_peak_21735	8.94469	
3	149470011	1.49E+08	KD2-H4K8ac_peak_21736	9.65086	COMMD2_ENSG00000114744
3	149530424	1.5E+08	KD2-H4K8ac_peak_21737	16.33377	RNF13_ENSG00000082996
3	149687440	1.5E+08	KD2-H4K8ac_peak_21738	5.16439	
3	149688009	1.5E+08	KD2-H4K8ac_peak_21739	23.68363	AC117395.1_ENSG00000268175
3	150126215	1.5E+08	KD2-H4K8ac_peak_21740	10.19949	TSC22D2_ENSG00000196428
3	150263787	1.5E+08	KD2-H4K8ac_peak_21741	12.66412	EIF2A_ENSG00000144895
3	150320801	1.5E+08	KD2-H4K8ac_peak_21742	8.52559	SERP1_ENSG00000120742;SELT_ENSG00000198843
3	150421383	1.5E+08	KD2-H4K8ac_peak_21743	4.42765	FAM194A_ENSG00000163645;RP11-103G8.2_ENSG00000240137
3	150479732	1.5E+08	KD2-H4K8ac_peak_21744	6.94954	SIAH2-AS1_ENSG00000244265
3	150481392	1.5E+08	KD2-H4K8ac_peak_21745	13.36684	SIAH2_ENSG00000181788
3	150803578	1.51E+08	KD2-H4K8ac_peak_21746	7.44324	MED12L_ENSG00000144893
3	150934239	1.51E+08	KD2-H4K8ac_peak_21747	6.16888	
3	151870458	1.52E+08	KD2-H4K8ac_peak_21748	8.5781	RP11-246A10.1_ENSG00000239941
3	151986318	1.52E+08	KD2-H4K8ac_peak_21749	11.05407	MBNL1-AS1_ENSG00000229619
3	152880105	1.53E+08	KD2-H4K8ac_peak_21750	6.97919	RP11-529G21.2_ENSG00000244268;RAP2B_ENSG00000181467
3	152880981	1.53E+08	KD2-H4K8ac_peak_21751	4.67342	RAP2B_ENSG00000181467
3	154041889	1.54E+08	KD2-H4K8ac_peak_21752	15.7388	DHX36_ENSG00000174953
3	154797500	1.55E+08	KD2-H4K8ac_peak_21753	10.21502	
3	155462098	1.55E+08	KD2-H4K8ac_peak_21754	10.46264	PLCH1_ENSG00000114805;AC104472.1_ENSG00000214919
3	155523625	1.56E+08	KD2-H4K8ac_peak_21755	8.55271	C3orf33_ENSG00000174928
3	155588586	1.56E+08	KD2-H4K8ac_peak_21756	11.05467	GMPS_ENSG00000163655

3	156101141	1.56E+08	KD2-H4K8ac_peak_21757	5.43522	
3	156272599	1.56E+08	KD2-H4K8ac_peak_21758	6.65673	SSR3_ENSG00000114850
3	156273200	1.56E+08	KD2-H4K8ac_peak_21759	8.1718	SSR3_ENSG00000114850
3	156533812	1.57E+08	KD2-H4K8ac_peak_21760	7.49599	LINC00886_ENSG00000240875
3	156543758	1.57E+08	KD2-H4K8ac_peak_21761	9.34195	LEKR1_ENSG00000197980
3	156806494	1.57E+08	KD2-H4K8ac_peak_21762	4.54006	LINC00881_ENSG00000241135
3	156877328	1.57E+08	KD2-H4K8ac_peak_21763	14.56955	CCNL1_ENSG00000163660
3	156878307	1.57E+08	KD2-H4K8ac_peak_21764	6.13177	CCNL1_ENSG00000163660
3	156892080	1.57E+08	KD2-H4K8ac_peak_21765	6.40297	RNA5SP146_ENSG00000222675;RP11-550I24.2_ENSG00000243176
3	156893533	1.57E+08	KD2-H4K8ac_peak_21766	4.44923	RP11-550I24.2_ENSG00000243176
3	157074116	1.57E+08	KD2-H4K8ac_peak_21767	4.37708	
3	157155370	1.57E+08	KD2-H4K8ac_peak_21768	18.5703	PTX3_ENSG00000163661
3	157155947	1.57E+08	KD2-H4K8ac_peak_21769	6.66692	
3	157251048	1.57E+08	KD2-H4K8ac_peak_21770	5.57044	VEPH1_ENSG00000197415
3	157260562	1.57E+08	KD2-H4K8ac_peak_21771	6.20982	C3orf55_ENSG00000174899
3	157336956	1.57E+08	KD2-H4K8ac_peak_21772	6.31143	
3	157588586	1.58E+08	KD2-H4K8ac_peak_21773	6.16888	
3	157590425	1.58E+08	KD2-H4K8ac_peak_21774	12.42547	
3	157723338	1.58E+08	KD2-H4K8ac_peak_21775	7.12754	
3	157822964	1.58E+08	KD2-H4K8ac_peak_21776	5.9696	SHOX2_ENSG00000168779;RSRC1_ENSG00000174891
3	157824911	1.58E+08	KD2-H4K8ac_peak_21777	5.71749	SHOX2_ENSG00000168779
3	157827867	1.58E+08	KD2-H4K8ac_peak_21778	11.89502	
3	158086077	1.58E+08	KD2-H4K8ac_peak_21779	6.40683	
3	158201780	1.58E+08	KD2-H4K8ac_peak_21780	6.16888	
3	158205293	1.58E+08	KD2-H4K8ac_peak_21781	6.25869	
3	158449472	1.58E+08	KD2-H4K8ac_peak_21782	8.85813	RARRES1_ENSG00000118849;MFSD1_ENSG00000118855;RP11-379F4.4_ENSG00000240207
3	158518992	1.59E+08	KD2-H4K8ac_peak_21783	7.07117	RP11-379F4.9_ENSG00000272247
3	159476362	1.59E+08	KD2-H4K8ac_peak_21784	4.75764	
3	159481014	1.59E+08	KD2-H4K8ac_peak_21785	6.99815	
3	159481884	1.59E+08	KD2-H4K8ac_peak_21786	9.63487	
3	159557806	1.6E+08	KD2-H4K8ac_peak_21787	5.01002	SCHIP1_ENSG00000151967
3	159569491	1.6E+08	KD2-H4K8ac_peak_21788	4.9025	
3	159570734	1.6E+08	KD2-H4K8ac_peak_21789	11.75669	
3	159574035	1.6E+08	KD2-H4K8ac_peak_21790	9.36516	
3	159574900	1.6E+08	KD2-H4K8ac_peak_21791	5.05318	
3	159578410	1.6E+08	KD2-H4K8ac_peak_21792	5.14125	
3	159597502	1.6E+08	KD2-H4K8ac_peak_21793	3.75035	
3	159706555	1.6E+08	KD2-H4K8ac_peak_21794	6.67538	IL12A_ENSG00000168811
3	159935578	1.6E+08	KD2-H4K8ac_peak_21795	7.12754	
3	159943987	1.6E+08	KD2-H4K8ac_peak_21796	5.14125	IL12A-AS1_ENSG00000244040;C3orf80_ENSG00000180044;RP11-431I8.1_ENSG00000244078
3	160117780	1.6E+08	KD2-H4K8ac_peak_21797	9.65086	IFT80_ENSG00000068885;SMC4_ENSG00000113810
3	160283605	1.6E+08	KD2-H4K8ac_peak_21798	12.84642	KPNA4_ENSG00000186432;KRT8P12_ENSG00000229320
3	160472589	1.6E+08	KD2-H4K8ac_peak_21799	3.75035	RP11-16N11.2_ENSG00000260572;PPM1L_ENSG00000163590
3	160603398	1.61E+08	KD2-H4K8ac_peak_21800	8.1233	
3	160939157	1.61E+08	KD2-H4K8ac_peak_21801	7.47462	
3	161089776	1.61E+08	KD2-H4K8ac_peak_21802	6.04107	SPTSSB_ENSG00000196542

3	161090318	1.61E+08	KD2-H4K8ac_peak_21803	4.91674	SPTSSB_ENSG00000196542
3	167452228	1.67E+08	KD2-H4K8ac_peak_21804	10.05606	PDCD10_ENSG00000114209;SERPINI1_ENSG00000163536
3	167453077	1.67E+08	KD2-H4K8ac_peak_21805	5.75757	PDCD10_ENSG00000114209;SERPINI1_ENSG00000163536
3	167812865	1.68E+08	KD2-H4K8ac_peak_21806	7.76329	GOLIM4_ENSG00000173905
3	167967519	1.68E+08	KD2-H4K8ac_peak_21807	7.07117	EGFEM1P_ENSG00000206120
3	169380028	1.69E+08	KD2-H4K8ac_peak_21808	7.76329	
3	169482039	1.69E+08	KD2-H4K8ac_peak_21809	10.96455	TERC_ENSG00000270141
3	169487142	1.69E+08	KD2-H4K8ac_peak_21810	13.39763	ACTRT3_ENSG00000184378;RP11-816J6.3_ENSG00000269889
3	169490662	1.69E+08	KD2-H4K8ac_peak_21811	11.05467	MYNN_ENSG00000085274
3	169491262	1.69E+08	KD2-H4K8ac_peak_21812	7.07117	MYNN_ENSG00000085274
3	169529977	1.7E+08	KD2-H4K8ac_peak_21813	17.27897	LRRC34_ENSG00000171757
3	169684805	1.7E+08	KD2-H4K8ac_peak_21814	9.36516	RP11-379K17.4_ENSG00000239219;SEC62_ENSG00000008952
3	169755708	1.7E+08	KD2-H4K8ac_peak_21815	9.11031	GPR160_ENSG00000173890
3	169898973	1.7E+08	KD2-H4K8ac_peak_21816	10.21366	PHC3_ENSG00000173889
3	169939373	1.7E+08	KD2-H4K8ac_peak_21817	7.59643	PRKCI_ENSG00000163558
3	169939977	1.7E+08	KD2-H4K8ac_peak_21818	10.72656	PRKCI_ENSG00000163558
3	170074022	1.7E+08	KD2-H4K8ac_peak_21819	10.29736	SKIL_ENSG00000136603
3	170075516	1.7E+08	KD2-H4K8ac_peak_21820	9.63487	SKIL_ENSG00000136603
3	170143460	1.7E+08	KD2-H4K8ac_peak_21821	5.57044	
3	170166512	1.7E+08	KD2-H4K8ac_peak_21822	5.25071	
3	170304041	1.7E+08	KD2-H4K8ac_peak_21823	8.74596	SLC7A14_ENSG00000013293
3	170368937	1.7E+08	KD2-H4K8ac_peak_21824	5.45396	
3	170588209	1.71E+08	KD2-H4K8ac_peak_21825	4.8888	RPL22L1_ENSG00000163584
3	171178738	1.71E+08	KD2-H4K8ac_peak_21826	8.97719	TNIK_ENSG00000154310
3	171528426	1.72E+08	KD2-H4K8ac_peak_21827	4.42765	PLD1_ENSG00000075651;PP13439_ENSG00000183657
3	171757536	1.72E+08	KD2-H4K8ac_peak_21828	4.54006	FNDC3B_ENSG00000075420
3	171758482	1.72E+08	KD2-H4K8ac_peak_21829	12.00273	
3	172428586	1.72E+08	KD2-H4K8ac_peak_21830	5.88845	NCEH1_ENSG00000144959
3	172468578	1.72E+08	KD2-H4K8ac_peak_21831	7.76329	ECT2_ENSG00000114346
3	173115525	1.73E+08	KD2-H4K8ac_peak_21832	11.92978	
3	173302843	1.73E+08	KD2-H4K8ac_peak_21833	5.14125	
3	176914213	1.77E+08	KD2-H4K8ac_peak_21834	5.14125	TBL1XR1_ENSG00000177565
3	176914886	1.77E+08	KD2-H4K8ac_peak_21835	9.41046	TBL1XR1_ENSG00000177565
3	176915551	1.77E+08	KD2-H4K8ac_peak_21836	20.42458	TBL1XR1_ENSG00000177565
3	177074823	1.77E+08	KD2-H4K8ac_peak_21837	7.70289	
3	177076664	1.77E+08	KD2-H4K8ac_peak_21838	24.85162	
3	177079568	1.77E+08	KD2-H4K8ac_peak_21839	9.97423	
3	177234145	1.77E+08	KD2-H4K8ac_peak_21840	5.14125	
3	177244054	1.77E+08	KD2-H4K8ac_peak_21841	10.17698	
3	177261909	1.77E+08	KD2-H4K8ac_peak_21842	5.14125	
3	177264221	1.77E+08	KD2-H4K8ac_peak_21843	10.0692	
3	177265307	1.77E+08	KD2-H4K8ac_peak_21844	6.40683	
3	177278701	1.77E+08	KD2-H4K8ac_peak_21845	8.1233	
3	177281467	1.77E+08	KD2-H4K8ac_peak_21846	4.86824	
3	177308863	1.77E+08	KD2-H4K8ac_peak_21847	6.66692	
3	177314388	1.77E+08	KD2-H4K8ac_peak_21848	7.71478	

3	177315075	1.77E+08	KD2-H4K8ac_peak_21849	8.1718	
3	177430534	1.77E+08	KD2-H4K8ac_peak_21850	5.45396	
3	177469780	1.77E+08	KD2-H4K8ac_peak_21851	7.01964	
3	177472275	1.77E+08	KD2-H4K8ac_peak_21852	6.40297	
3	177541535	1.78E+08	KD2-H4K8ac_peak_21853	7.13615	
3	177543273	1.78E+08	KD2-H4K8ac_peak_21854	7.70289	
3	177593872	1.78E+08	KD2-H4K8ac_peak_21855	4.42765	
3	177652025	1.78E+08	KD2-H4K8ac_peak_21856	6.16888	RP11-2L8.1_ENSG00000225790
3	177796655	1.78E+08	KD2-H4K8ac_peak_21857	6.31143	
3	177801233	1.78E+08	KD2-H4K8ac_peak_21858	8.45238	
3	177802422	1.78E+08	KD2-H4K8ac_peak_21859	5.64449	
3	177803160	1.78E+08	KD2-H4K8ac_peak_21860	8.17902	
3	177899571	1.78E+08	KD2-H4K8ac_peak_21861	11.75669	
3	178006798	1.78E+08	KD2-H4K8ac_peak_21862	9.11818	
3	178008813	1.78E+08	KD2-H4K8ac_peak_21863	4.01115	
3	178011466	1.78E+08	KD2-H4K8ac_peak_21864	8.94469	
3	178038510	1.78E+08	KD2-H4K8ac_peak_21865	6.16888	
3	178789097	1.79E+08	KD2-H4K8ac_peak_21866	8.55271	ZMAT3_ENSG00000172667
3	178850197	1.79E+08	KD2-H4K8ac_peak_21867	6.16888	
3	178866172	1.79E+08	KD2-H4K8ac_peak_21868	11.89502	RP11-360P21.2_ENSG00000229102;PIK3CA_ENSG00000121879
3	178978901	1.79E+08	KD2-H4K8ac_peak_21869	12.13241	LRRFIP1P1_ENSG00000240429
3	179040293	1.79E+08	KD2-H4K8ac_peak_21870	8.12964	ZNF639_ENSG00000121864
3	179040809	1.79E+08	KD2-H4K8ac_peak_21871	10.4923	ZNF639_ENSG00000121864
3	179064696	1.79E+08	KD2-H4K8ac_peak_21872	6.60096	MFN1_ENSG00000171109
3	179065538	1.79E+08	KD2-H4K8ac_peak_21873	10.02417	MFN1_ENSG00000171109
3	179168681	1.79E+08	KD2-H4K8ac_peak_21874	16.09057	GNB4_ENSG00000114450
3	179169549	1.79E+08	KD2-H4K8ac_peak_21875	5.14125	GNB4_ENSG00000114450;RP11-145M9.2_ENSG00000239255
3	179322643	1.79E+08	KD2-H4K8ac_peak_21876	12.84642	MRPL47_ENSG00000136522;NDUFB5_ENSG00000136521
3	179370469	1.79E+08	KD2-H4K8ac_peak_21877	9.1707	USP13_ENSG00000058056
3	179371014	1.79E+08	KD2-H4K8ac_peak_21878	5.1707	USP13_ENSG00000058056
3	179660001	1.8E+08	KD2-H4K8ac_peak_21879	10.96455	
3	180320048	1.8E+08	KD2-H4K8ac_peak_21880	10.53377	RP11-496B10.3_ENSG00000239774;TTC14_ENSG00000163728
3	180397363	1.8E+08	KD2-H4K8ac_peak_21881	15.9336	CCDC39-AS1_ENSG00000243187
3	180588010	1.81E+08	KD2-H4K8ac_peak_21882	9.15329	CCDC39_ENSG00000145075
3	180630074	1.81E+08	KD2-H4K8ac_peak_21883	16.86926	
3	180707181	1.81E+08	KD2-H4K8ac_peak_21884	9.36516	DNAJC19_ENSG00000205981;SOX2-OT_ENSG00000242808
3	181429049	1.81E+08	KD2-H4K8ac_peak_21885	6.14257	SOX2_ENSG00000181449
3	181598661	1.82E+08	KD2-H4K8ac_peak_21886	6.25869	
3	182082327	1.82E+08	KD2-H4K8ac_peak_21887	4.76709	RP11-338L18.1_ENSG00000242012
3	182400528	1.82E+08	KD2-H4K8ac_peak_21888	4.42765	
3	182443587	1.82E+08	KD2-H4K8ac_peak_21889	14.16941	
3	182444337	1.82E+08	KD2-H4K8ac_peak_21890	6.9809	
3	182510807	1.83E+08	KD2-H4K8ac_peak_21891	13.57066	RP11-225N10.1_ENSG00000240063;ATP11B_ENSG00000058063
3	182511383	1.83E+08	KD2-H4K8ac_peak_21892	12.06346	RP11-225N10.1_ENSG00000240063;ATP11B_ENSG00000058063
3	182638411	1.83E+08	KD2-H4K8ac_peak_21893	9.15329	
3	182697623	1.83E+08	KD2-H4K8ac_peak_21894	9.57101	

3	182880403	1.83E+08	KD2-H4K8ac_peak_21895	5.54647	LAMP3_ENSG00000078081
3	182971802	1.83E+08	KD2-H4K8ac_peak_21896	18.54875	B3GNT5_ENSG00000176597
3	183104612	1.83E+08	KD2-H4K8ac_peak_21897	6.54442	
3	183165475	1.83E+08	KD2-H4K8ac_peak_21898	6.05809	LINC00888_ENSG00000240024
3	183195664	1.83E+08	KD2-H4K8ac_peak_21899	7.40111	
3	183196467	1.83E+08	KD2-H4K8ac_peak_21900	6.0073	
3	183353975	1.83E+08	KD2-H4K8ac_peak_21901	4.86324	KLHL24_ENSG00000114796
3	183415037	1.83E+08	KD2-H4K8ac_peak_21902	6.83936	YEATS2_ENSG00000163872
3	183415627	1.83E+08	KD2-H4K8ac_peak_21903	14.68449	YEATS2_ENSG00000163872
3	183602277	1.84E+08	KD2-H4K8ac_peak_21904	11.89502	PARL_ENSG00000175193;RP11-315J22.5_ENSG00000234371
3	183602878	1.84E+08	KD2-H4K8ac_peak_21905	8.59579	PARL_ENSG00000175193;RP11-315J22.5_ENSG00000234371
3	183735384	1.84E+08	KD2-H4K8ac_peak_21906	13.56049	ABCC5_ENSG00000114770
3	183735868	1.84E+08	KD2-H4K8ac_peak_21907	16.73922	ABCC5_ENSG00000114770
3	183852796	1.84E+08	KD2-H4K8ac_peak_21908	8.58069	RP11-778D9.12_ENSG00000272721;EIF2B5_ENSG00000145191
3	183872463	1.84E+08	KD2-H4K8ac_peak_21909	10.0692	DVL3_ENSG00000161202
3	183873823	1.84E+08	KD2-H4K8ac_peak_21910	5.15777	DVL3_ENSG00000161202
3	183874290	1.84E+08	KD2-H4K8ac_peak_21911	5.88845	
3	183892698	1.84E+08	KD2-H4K8ac_peak_21912	11.92978	AP2M1_ENSG00000161203
3	183903222	1.84E+08	KD2-H4K8ac_peak_21913	7.40314	ABCF3_ENSG00000161204
3	183928408	1.84E+08	KD2-H4K8ac_peak_21914	5.25071	
3	183929951	1.84E+08	KD2-H4K8ac_peak_21915	9.15329	
3	183966070	1.84E+08	KD2-H4K8ac_peak_21916	8.83582	ALG3_ENSG00000214160
3	183967599	1.84E+08	KD2-H4K8ac_peak_21917	6.44277	ALG3_ENSG00000214160;ECE2_ENSG00000145194
3	184016010	1.84E+08	KD2-H4K8ac_peak_21918	10.96455	PSMD2_ENSG00000175166
3	184032523	1.84E+08	KD2-H4K8ac_peak_21919	11.43863	EIF4G1_ENSG00000114867
3	184054128	1.84E+08	KD2-H4K8ac_peak_21920	10.87093	FAM131A_ENSG00000175182
3	184079075	1.84E+08	KD2-H4K8ac_peak_21921	6.25869	CLCN2_ENSG00000114859;POLR2H_ENSG00000163882
3	184079586	1.84E+08	KD2-H4K8ac_peak_21922	10.43145	CLCN2_ENSG00000114859;POLR2H_ENSG00000163882
3	184080277	1.84E+08	KD2-H4K8ac_peak_21923	23.78494	CLCN2_ENSG00000114859;POLR2H_ENSG00000163882
3	184163010	1.84E+08	KD2-H4K8ac_peak_21924	4.01115	
3	184243117	1.84E+08	KD2-H4K8ac_peak_21925	12.74171	
3	184279637	1.84E+08	KD2-H4K8ac_peak_21926	7.20116	EPHB3_ENSG00000182580
3	184286156	1.84E+08	KD2-H4K8ac_peak_21927	14.69247	
3	184286967	1.84E+08	KD2-H4K8ac_peak_21928	8.10216	
3	184291996	1.84E+08	KD2-H4K8ac_peak_21929	7.84589	
3	184292624	1.84E+08	KD2-H4K8ac_peak_21930	17.86695	
3	184309974	1.84E+08	KD2-H4K8ac_peak_21931	11.79585	
3	184310957	1.84E+08	KD2-H4K8ac_peak_21932	14.16977	
3	184315417	1.84E+08	KD2-H4K8ac_peak_21933	18.19257	
3	184316636	1.84E+08	KD2-H4K8ac_peak_21934	5.9696	
3	184320714	1.84E+08	KD2-H4K8ac_peak_21935	6.40683	
3	184352853	1.84E+08	KD2-H4K8ac_peak_21936	9.15069	
3	184353589	1.84E+08	KD2-H4K8ac_peak_21937	18.94636	
3	184360790	1.84E+08	KD2-H4K8ac_peak_21938	6.25869	
3	184376414	1.84E+08	KD2-H4K8ac_peak_21939	9.36516	
3	184377758	1.84E+08	KD2-H4K8ac_peak_21940	6.20982	

3	184395298	1.84E+08	KD2-H4K8ac_peak_21941	7.76335	
3	184396827	1.84E+08	KD2-H4K8ac_peak_21942	13.70753	
3	184402847	1.84E+08	KD2-H4K8ac_peak_21943	6.40683	
3	184409764	1.84E+08	KD2-H4K8ac_peak_21944	7.27716	
3	184410668	1.84E+08	KD2-H4K8ac_peak_21945	5.88845	
3	184429100	1.84E+08	KD2-H4K8ac_peak_21946	16.61426	MAGEF1_ENSG00000177383
3	184430111	1.84E+08	KD2-H4K8ac_peak_21947	9.36621	MAGEF1_ENSG00000177383
3	184434553	1.84E+08	KD2-H4K8ac_peak_21948	5.57044	
3	184443193	1.84E+08	KD2-H4K8ac_peak_21949	6.64891	
3	184460474	1.84E+08	KD2-H4K8ac_peak_21950	9.28039	RP11-329B9.3_ENSG00000273403;RP11-329B9.5_ENSG00000272922
3	184490731	1.84E+08	KD2-H4K8ac_peak_21951	7.83489	RP11-329B9.1_ENSG00000229433
3	184491207	1.84E+08	KD2-H4K8ac_peak_21952	6.25869	RP11-329B9.1_ENSG00000229433
3	184512974	1.85E+08	KD2-H4K8ac_peak_21953	12.84642	
3	184788355	1.85E+08	KD2-H4K8ac_peak_21954	7.45501	
3	184870337	1.85E+08	KD2-H4K8ac_peak_21955	10.05606	C3orf70_ENSG00000187068
3	184893486	1.85E+08	KD2-H4K8ac_peak_21956	7.1181	
3	185000318	1.85E+08	KD2-H4K8ac_peak_21957	5.57044	EHHADH_ENSG00000113790;MAP3K13_ENSG00000073803
3	185119785	1.85E+08	KD2-H4K8ac_peak_21958	10.53596	
3	185216258	1.85E+08	KD2-H4K8ac_peak_21959	9.63487	TMEM41A_ENSG00000163900
3	185277063	1.85E+08	KD2-H4K8ac_peak_21960	11.92209	
3	185655359	1.86E+08	KD2-H4K8ac_peak_21961	15.69572	TRA2B_ENSG00000136527
3	185661202	1.86E+08	KD2-H4K8ac_peak_21962	9.34989	
3	185815275	1.86E+08	KD2-H4K8ac_peak_21963	6.05809	
3	185826135	1.86E+08	KD2-H4K8ac_peak_21964	6.85193	
3	185828099	1.86E+08	KD2-H4K8ac_peak_21965	8.55271	ETV5_ENSG00000244405
3	185927680	1.86E+08	KD2-H4K8ac_peak_21966	11.89502	
3	185977649	1.86E+08	KD2-H4K8ac_peak_21967	4.3289	
3	186501100	1.87E+08	KD2-H4K8ac_peak_21968	7.76329	RP11-573D15.9_ENSG00000263826;EIF4A2_ENSG00000156976;SNORD2_ENSG00000238942
3	186524398	1.87E+08	KD2-H4K8ac_peak_21969	8.4716	RFC4_ENSG00000163918;RP11-573D15.3_ENSG00000231724
3	186648594	1.87E+08	KD2-H4K8ac_peak_21970	9.02371	ST6GAL1_ENSG00000073849
3	186856662	1.87E+08	KD2-H4K8ac_peak_21971	8.30974	
3	191046119	1.91E+08	KD2-H4K8ac_peak_21972	24.85162	UTS2B_ENSG00000188958;CCDC50_ENSG00000152492
3	192958716	1.93E+08	KD2-H4K8ac_peak_21973	7.40314	HRASLS_ENSG00000127252
3	193310187	1.93E+08	KD2-H4K8ac_peak_21974	7.15405	ATP13A4_ENSG00000127249;RP11-175P19.2_ENSG00000236297;OPA1_ENSG00000198836
3	193534684	1.94E+08	KD2-H4K8ac_peak_21975	4.49837	
3	193721364	1.94E+08	KD2-H4K8ac_peak_21976	10.87093	RP11-699L21.1_ENSG00000214146
3	193788032	1.94E+08	KD2-H4K8ac_peak_21977	22.93827	RP11-407B7.1_ENSG00000230102
3	193789319	1.94E+08	KD2-H4K8ac_peak_21978	7.40633	RP11-407B7.1_ENSG00000230102
3	193801208	1.94E+08	KD2-H4K8ac_peak_21979	7.76335	
3	193810892	1.94E+08	KD2-H4K8ac_peak_21980	9.41046	
3	193813264	1.94E+08	KD2-H4K8ac_peak_21981	8.4939	
3	193819701	1.94E+08	KD2-H4K8ac_peak_21982	5.07241	
3	193852157	1.94E+08	KD2-H4K8ac_peak_21983	14.66631	
3	193855572	1.94E+08	KD2-H4K8ac_peak_21984	6.45083	
3	193858544	1.94E+08	KD2-H4K8ac_peak_21985	11.04256	
3	193859506	1.94E+08	KD2-H4K8ac_peak_21986	14.98671	

3	193967622	1.94E+08	KD2-H4K8ac_peak_21987	4.06758	RP11-513G11.4_ENSG00000225742
3	193973221	1.94E+08	KD2-H4K8ac_peak_21988	5.42867	
3	193988390	1.94E+08	KD2-H4K8ac_peak_21989	12.46522	
3	193989288	1.94E+08	KD2-H4K8ac_peak_21990	18.62675	
3	193991136	1.94E+08	KD2-H4K8ac_peak_21991	10.19949	
3	193994485	1.94E+08	KD2-H4K8ac_peak_21992	3.97079	RP11-513G11.2_ENSG00000228271
3	193995154	1.94E+08	KD2-H4K8ac_peak_21993	8.10216	RP11-513G11.2_ENSG00000228271
3	194018029	1.94E+08	KD2-H4K8ac_peak_21994	5.30799	
3	194030508	1.94E+08	KD2-H4K8ac_peak_21995	4.24345	LINC00887_ENSG00000214145
3	194033267	1.94E+08	KD2-H4K8ac_peak_21996	4.03256	
3	194102275	1.94E+08	KD2-H4K8ac_peak_21997	5.57044	
3	194207850	1.94E+08	KD2-H4K8ac_peak_21998	14.00658	LINC00884_ENSG00000233058
3	194353417	1.94E+08	KD2-H4K8ac_peak_21999	11.05467	TMEM44_ENSG00000145014;AC046143.3_ENSG00000229334
3	194354419	1.94E+08	KD2-H4K8ac_peak_22000	7.47462	TMEM44_ENSG00000145014;AC046143.3_ENSG00000229334
3	194393419	1.94E+08	KD2-H4K8ac_peak_22001	4.77079	LSG1_ENSG00000041802
3	194395994	1.94E+08	KD2-H4K8ac_peak_22002	8.30974	
3	194407696	1.94E+08	KD2-H4K8ac_peak_22003	5.91443	
3	194413186	1.94E+08	KD2-H4K8ac_peak_22004	8.1718	
3	194489208	1.94E+08	KD2-H4K8ac_peak_22005	11.89502	AC090505.4_ENSG00000230401
3	194573747	1.95E+08	KD2-H4K8ac_peak_22006	6.0073	
3	194592360	1.95E+08	KD2-H4K8ac_peak_22007	10.8368	
3	194601609	1.95E+08	KD2-H4K8ac_peak_22008	7.47462	
3	194651577	1.95E+08	KD2-H4K8ac_peak_22009	4.59928	
3	194673692	1.95E+08	KD2-H4K8ac_peak_22010	6.2781	
3	194733455	1.95E+08	KD2-H4K8ac_peak_22011	4.01115	
3	194734278	1.95E+08	KD2-H4K8ac_peak_22012	8.4939	
3	194796568	1.95E+08	KD2-H4K8ac_peak_22013	6.2781	
3	194873691	1.95E+08	KD2-H4K8ac_peak_22014	8.26945	
3	194875602	1.95E+08	KD2-H4K8ac_peak_22015	4.0528	
3	194882551	1.95E+08	KD2-H4K8ac_peak_22016	6.25869	
3	194899396	1.95E+08	KD2-H4K8ac_peak_22017	6.34383	
3	194900708	1.95E+08	KD2-H4K8ac_peak_22018	5.53033	
3	194930187	1.95E+08	KD2-H4K8ac_peak_22019	7.38799	
3	194930756	1.95E+08	KD2-H4K8ac_peak_22020	10.01478	
3	194967388	1.95E+08	KD2-H4K8ac_peak_22021	6.2159	
3	194974727	1.95E+08	KD2-H4K8ac_peak_22022	14.99078	
3	194991182	1.95E+08	KD2-H4K8ac_peak_22023	14.56955	XXYLT1_ENSG00000173950
3	195163209	1.95E+08	KD2-H4K8ac_peak_22024	8.5092	ACAP2_ENSG00000114331
3	195269710	1.95E+08	KD2-H4K8ac_peak_22025	10.19949	PPP1R2_ENSG00000184203;AC091633.3_ENSG00000223711
3	195384363	1.95E+08	KD2-H4K8ac_peak_22026	16.8435	AC069513.4_ENSG00000229178;LINC00969_ENSG00000242086;SDHAP2_ENSG00000215837
3	195474474	1.95E+08	KD2-H4K8ac_peak_22027	6.97919	
3	195618531	1.96E+08	KD2-H4K8ac_peak_22028	4.70206	
3	195619095	1.96E+08	KD2-H4K8ac_peak_22029	6.90231	
3	195619847	1.96E+08	KD2-H4K8ac_peak_22030	5.01002	
3	195620678	1.96E+08	KD2-H4K8ac_peak_22031	6.18308	
3	195622576	1.96E+08	KD2-H4K8ac_peak_22032	7.0762	

3	195623947	1.96E+08	KD2-H4K8ac_peak_22033	9.90319	
3	195636762	1.96E+08	KD2-H4K8ac_peak_22034	7.76329	
3	195641681	1.96E+08	KD2-H4K8ac_peak_22035	6.83936	
3	195682172	1.96E+08	KD2-H4K8ac_peak_22036	9.15329	
3	195717421	1.96E+08	KD2-H4K8ac_peak_22037	17.43368	SDHAP1_ENSG00000185485
3	195808534	1.96E+08	KD2-H4K8ac_peak_22038	10.77651	TFRC_ENSG00000072274
3	195809676	1.96E+08	KD2-H4K8ac_peak_22039	7.40111	TFRC_ENSG00000072274
3	195913561	1.96E+08	KD2-H4K8ac_peak_22040	9.34989	
3	195946527	1.96E+08	KD2-H4K8ac_peak_22041	5.75452	
3	196014835	1.96E+08	KD2-H4K8ac_peak_22042	19.72117	PCYT1A_ENSG00000161217
3	196044416	1.96E+08	KD2-H4K8ac_peak_22043	9.1707	RP11-447L10.1_ENSG00000272741;TCTEX1D2_ENSG00000213123;TM4SF19-AS1_ENSG00000235897
3	196045277	1.96E+08	KD2-H4K8ac_peak_22044	8.10216	RP11-447L10.1_ENSG00000272741;TCTEX1D2_ENSG00000213123;TM4SF19-AS1_ENSG00000235897
3	196045796	1.96E+08	KD2-H4K8ac_peak_22045	6.56211	RP11-447L10.1_ENSG00000272741;TCTEX1D2_ENSG00000213123;TM4SF19-AS1_ENSG00000235897
3	196047167	1.96E+08	KD2-H4K8ac_peak_22046	6.94255	
3	196158507	1.96E+08	KD2-H4K8ac_peak_22047	5.01002	UBXN7_ENSG00000163960;UBXN7-AS1_ENSG00000225822
3	196230893	1.96E+08	KD2-H4K8ac_peak_22048	15.9336	RNF168_ENSG00000163961
3	196255452	1.96E+08	KD2-H4K8ac_peak_22049	16.32216	AC092933.4_ENSG00000213358
3	196295028	1.96E+08	KD2-H4K8ac_peak_22050	8.02571	WDR53_ENSG00000185798;FBXO45_ENSG00000174013
3	196295620	1.96E+08	KD2-H4K8ac_peak_22051	5.9696	WDR53_ENSG00000185798;FBXO45_ENSG00000174013
3	196358648	1.96E+08	KD2-H4K8ac_peak_22052	16.23438	LINC01063_ENSG00000232065
3	196366201	1.96E+08	KD2-H4K8ac_peak_22053	9.65086	NRROS_ENSG00000174004;PIGX_ENSG00000163964
3	196439256	1.96E+08	KD2-H4K8ac_peak_22054	10.53377	CEP19_ENSG00000174007
3	196466976	1.96E+08	KD2-H4K8ac_peak_22055	9.63487	PAK2_ENSG00000180370
3	196668863	1.97E+08	KD2-H4K8ac_peak_22056	15.01306	NCBP2_ENSG00000114503;NCBP2-AS2_ENSG00000270170
3	196669583	1.97E+08	KD2-H4K8ac_peak_22057	8.10216	NCBP2_ENSG00000114503;NCBP2-AS2_ENSG00000270170
3	196695418	1.97E+08	KD2-H4K8ac_peak_22058	9.63487	PIGZ_ENSG00000119227
3	196729282	1.97E+08	KD2-H4K8ac_peak_22059	15.81711	
3	196755697	1.97E+08	KD2-H4K8ac_peak_22060	15.58442	MFI2_ENSG00000163975
3	196757287	1.97E+08	KD2-H4K8ac_peak_22061	7.95934	MFI2_ENSG00000163975
3	197024409	1.97E+08	KD2-H4K8ac_peak_22062	9.11031	DLG1-AS1_ENSG00000227375
3	197025200	1.97E+08	KD2-H4K8ac_peak_22063	9.36516	DLG1_ENSG00000075711;DLG1-AS1_ENSG00000227375
3	197025753	1.97E+08	KD2-H4K8ac_peak_22064	5.54647	DLG1_ENSG00000075711;DLG1-AS1_ENSG00000227375
3	197062740	1.97E+08	KD2-H4K8ac_peak_22065	6.36375	
3	197152962	1.97E+08	KD2-H4K8ac_peak_22066	4.87807	
3	197153596	1.97E+08	KD2-H4K8ac_peak_22067	7.146	
3	197185150	1.97E+08	KD2-H4K8ac_peak_22068	9.15329	AC128709.3_ENSG00000235126;AC128709.2_ENSG00000237167
3	197191496	1.97E+08	KD2-H4K8ac_peak_22069	3.75035	
3	197192185	1.97E+08	KD2-H4K8ac_peak_22070	5.77265	
3	197193509	1.97E+08	KD2-H4K8ac_peak_22071	8.86099	
3	197194320	1.97E+08	KD2-H4K8ac_peak_22072	10.46264	
3	197211652	1.97E+08	KD2-H4K8ac_peak_22073	5.60968	
3	197214025	1.97E+08	KD2-H4K8ac_peak_22074	7.64093	
3	197215244	1.97E+08	KD2-H4K8ac_peak_22075	7.96322	
3	197216042	1.97E+08	KD2-H4K8ac_peak_22076	9.1707	
3	197216838	1.97E+08	KD2-H4K8ac_peak_22077	4.36417	
3	197219353	1.97E+08	KD2-H4K8ac_peak_22078	4.56666	

3	197220019	1.97E+08	KD2-H4K8ac_peak_22079	6.66692	
3	197229066	1.97E+08	KD2-H4K8ac_peak_22080	5.64429	
3	197230067	1.97E+08	KD2-H4K8ac_peak_22081	5.78354	
3	197231351	1.97E+08	KD2-H4K8ac_peak_22082	6.65673	
3	197232370	1.97E+08	KD2-H4K8ac_peak_22083	5.13615	
3	197232851	1.97E+08	KD2-H4K8ac_peak_22084	4.67342	RP13-616I3.1_ENSG00000272989;AC128709.1_ENSG00000250443
3	197233505	1.97E+08	KD2-H4K8ac_peak_22085	5.02584	RP13-616I3.1_ENSG00000272989;AC128709.1_ENSG00000250443
3	197236270	1.97E+08	KD2-H4K8ac_peak_22086	13.3695	
3	197246062	1.97E+08	KD2-H4K8ac_peak_22087	8.79106	
3	197247950	1.97E+08	KD2-H4K8ac_peak_22088	6.56211	
3	197282077	1.97E+08	KD2-H4K8ac_peak_22089	10.66719	
3	197354907	1.97E+08	KD2-H4K8ac_peak_22090	12.25733	AC024560.3_ENSG00000214135
3	197382616	1.97E+08	KD2-H4K8ac_peak_22091	12.42547	
3	197391730	1.97E+08	KD2-H4K8ac_peak_22092	8.1718	AC024560.2_ENSG00000236833
3	197476197	1.97E+08	KD2-H4K8ac_peak_22093	10.29344	KIAA0226_ENSG00000145016
3	197476869	1.97E+08	KD2-H4K8ac_peak_22094	14.12659	KIAA0226_ENSG00000145016
3	197518212	1.98E+08	KD2-H4K8ac_peak_22095	13.87818	LRCH3_ENSG00000186001
3	197687162	1.98E+08	KD2-H4K8ac_peak_22096	9.08816	IQCG_ENSG00000114473;LMLN_ENSG00000185621
4	52256	52457	KD2-H4K8ac_peak_22097	4.8136	ZNF595_ENSG00000197701
4	52772	52995	KD2-H4K8ac_peak_22098	16.1315	ZNF595_ENSG00000197701
4	53261	53806	KD2-H4K8ac_peak_22099	16.31433	ZNF595_ENSG00000197701
4	330009	331211	KD2-H4K8ac_peak_22100	13.81781	RP11-478C6.1_ENSG00000251535;ZNF141_ENSG00000131127
4	467295	467904	KD2-H4K8ac_peak_22101	25.36636	ABCA11P_ENSG00000251595
4	468397	468799	KD2-H4K8ac_peak_22102	6.79289	ABCA11P_ENSG00000251595
4	493048	493702	KD2-H4K8ac_peak_22103	16.09057	ZNF721_ENSG00000182903;PIGG_ENSG00000174227
4	503187	503389	KD2-H4K8ac_peak_22104	8.1233	
4	657081	658914	KD2-H4K8ac_peak_22105	17.55491	
4	667328	667716	KD2-H4K8ac_peak_22106	15.34166	ATP5I_ENSG00000169020;MYL5_ENSG00000215375
4	668357	668980	KD2-H4K8ac_peak_22107	12.08081	ATP5I_ENSG00000169020;MYL5_ENSG00000215375
4	674623	675201	KD2-H4K8ac_peak_22108	12.2118	
4	677520	677857	KD2-H4K8ac_peak_22109	8.23274	
4	678055	678691	KD2-H4K8ac_peak_22110	13.80019	
4	699658	699997	KD2-H4K8ac_peak_22111	13.12288	PCGF3_ENSG00000185619
4	775332	775594	KD2-H4K8ac_peak_22112	17.16047	RP11-440L14.1_ENSG00000249592
4	776410	777152	KD2-H4K8ac_peak_22113	14.62911	RP11-440L14.1_ENSG00000249592
4	925733	926051	KD2-H4K8ac_peak_22114	9.6589	GAK_ENSG00000178950;TMEM175_ENSG00000127419
4	967526	967984	KD2-H4K8ac_peak_22115	11.50346	
4	980334	980579	KD2-H4K8ac_peak_22116	7.47462	DGKQ_ENSG00000145214;IDUA_ENSG00000127415
4	980888	981245	KD2-H4K8ac_peak_22117	6.65673	DGKQ_ENSG00000145214;IDUA_ENSG00000127415
4	1187817	1188740	KD2-H4K8ac_peak_22118	14.8083	
4	1241761	1242467	KD2-H4K8ac_peak_22119	12.82393	CTBP1-AS2_ENSG00000196810
4	1243030	1243909	KD2-H4K8ac_peak_22120	10.66719	CTBP1_ENSG00000159692;CTBP1-AS2_ENSG00000196810
4	1282510	1282906	KD2-H4K8ac_peak_22121	6.64891	MAEA_ENSG00000090316
4	1283122	1283423	KD2-H4K8ac_peak_22122	5.75757	MAEA_ENSG00000090316
4	1283726	1284034	KD2-H4K8ac_peak_22123	10.96455	MAEA_ENSG00000090316
4	1340101	1340646	KD2-H4K8ac_peak_22124	7.36799	UVSSA_ENSG00000163945

4	1341094	1341688	KD2-H4K8ac_peak_22125	7.49599	UVSSA_ENSG00000163945
4	1647167	1647500	KD2-H4K8ac_peak_22126	12.40345	
4	1685518	1685926	KD2-H4K8ac_peak_22127	8.10216	FAM53A_ENSG00000174137;Y_RNA_ENSG00000207009
4	1686339	1686920	KD2-H4K8ac_peak_22128	16.8435	FAM53A_ENSG00000174137
4	1714622	1714953	KD2-H4K8ac_peak_22129	9.1707	SLBP_ENSG00000163950;RP11-572O17.1_ENSG00000270195
4	1722045	1722951	KD2-H4K8ac_peak_22130	11.79585	TMEM129_ENSG00000168936;TACC3_ENSG00000013810
4	1756010	1756292	KD2-H4K8ac_peak_22131	7.13615	
4	1756694	1756928	KD2-H4K8ac_peak_22132	8.17902	
4	1768474	1769601	KD2-H4K8ac_peak_22133	12.84642	
4	1857378	1857801	KD2-H4K8ac_peak_22134	9.96798	LETM1_ENSG00000168924
4	1858240	1859179	KD2-H4K8ac_peak_22135	8.55271	LETM1_ENSG00000168924
4	1871838	1872677	KD2-H4K8ac_peak_22136	20.18381	RN7SL671P_ENSG00000240455;WHSC1_ENSG00000109685
4	1873228	1873856	KD2-H4K8ac_peak_22137	13.80019	WHSC1_ENSG00000109685
4	1985480	1985733	KD2-H4K8ac_peak_22138	4.56666	
4	2010407	2011358	KD2-H4K8ac_peak_22139	10.19949	
4	2043162	2043532	KD2-H4K8ac_peak_22140	9.91109	NELFA_ENSG00000185049;C4orf48_ENSG00000243449
4	2044012	2044415	KD2-H4K8ac_peak_22141	6.90231	NELFA_ENSG00000185049;C4orf48_ENSG00000243449
4	2060014	2060685	KD2-H4K8ac_peak_22142	12.86276	NAT8L_ENSG00000185818
4	2262970	2263195	KD2-H4K8ac_peak_22143	7.47462	MXD4_ENSG00000123933
4	2264363	2264693	KD2-H4K8ac_peak_22144	9.01262	MXD4_ENSG00000123933
4	2293789	2294265	KD2-H4K8ac_peak_22145	6.99815	
4	2414664	2415542	KD2-H4K8ac_peak_22146	7.47462	
4	2420034	2420253	KD2-H4K8ac_peak_22147	10.46874	ZFYVE28_ENSG00000159733;RP11-503N18.1_ENSG00000206113
4	2463867	2464494	KD2-H4K8ac_peak_22148	7.47462	RNF4_ENSG00000063978
4	2470128	2470620	KD2-H4K8ac_peak_22149	17.19888	
4	2470900	2471470	KD2-H4K8ac_peak_22150	12.46522	
4	2536841	2537897	KD2-H4K8ac_peak_22151	11.05467	
4	2538617	2539164	KD2-H4K8ac_peak_22152	5.66705	
4	2600538	2600825	KD2-H4K8ac_peak_22153	5.88845	
4	2757590	2758033	KD2-H4K8ac_peak_22154	11.05467	TNIP2_ENSG00000168884
4	2758418	2758922	KD2-H4K8ac_peak_22155	5.88845	TNIP2_ENSG00000168884
4	2770583	2770937	KD2-H4K8ac_peak_22156	8.30974	
4	2789647	2789929	KD2-H4K8ac_peak_22157	6.62541	
4	2790403	2790642	KD2-H4K8ac_peak_22158	9.02745	
4	2796360	2796666	KD2-H4K8ac_peak_22159	9.06369	
4	2800289	2800976	KD2-H4K8ac_peak_22160	9.08816	
4	2813782	2814224	KD2-H4K8ac_peak_22161	5.14125	
4	2819464	2820593	KD2-H4K8ac_peak_22162	22.7107	
4	2844682	2845374	KD2-H4K8ac_peak_22163	13.36684	ADD1_ENSG00000087274
4	2935363	2936060	KD2-H4K8ac_peak_22164	11.51672	MFSD10_ENSG00000109736;NOP14-AS1_ENSG00000249673
4	2936841	2937272	KD2-H4K8ac_peak_22165	7.96322	MFSD10_ENSG00000109736;NOP14-AS1_ENSG00000249673
4	2964681	2965080	KD2-H4K8ac_peak_22166	11.60333	NOP14_ENSG00000087269;GRK4_ENSG00000125388
4	2965424	2965790	KD2-H4K8ac_peak_22167	4.35724	NOP14_ENSG00000087269;GRK4_ENSG00000125388
4	3018699	3018964	KD2-H4K8ac_peak_22168	4.77079	
4	3075166	3075648	KD2-H4K8ac_peak_22169	12.30509	HTT-AS_ENSG00000251075;HTT_ENSG00000197386
4	3194765	3195021	KD2-H4K8ac_peak_22170	9.15329	

4	3196189	3196446	KD2-H4K8ac_peak_22171	7.12754	
4	3465362	3465607	KD2-H4K8ac_peak_22172	7.22457	DOK7_ENSG00000175920
4	3494557	3495440	KD2-H4K8ac_peak_22173	6.65673	
4	3534356	3534814	KD2-H4K8ac_peak_22174	11.86082	LRPAP1_ENSG00000163956
4	3587031	3587253	KD2-H4K8ac_peak_22175	5.9696	
4	3587781	3588043	KD2-H4K8ac_peak_22176	8.06145	
4	3588472	3588692	KD2-H4K8ac_peak_22177	6.76298	
4	3615720	3616639	KD2-H4K8ac_peak_22178	9.34195	
4	3659817	3660774	KD2-H4K8ac_peak_22179	8.52559	
4	3708758	3709547	KD2-H4K8ac_peak_22180	5.75757	
4	3709802	3710254	KD2-H4K8ac_peak_22181	8.4716	
4	3732743	3732978	KD2-H4K8ac_peak_22182	6.37652	
4	3735053	3735287	KD2-H4K8ac_peak_22183	5.23853	
4	3747969	3748168	KD2-H4K8ac_peak_22184	9.1707	
4	3748632	3748987	KD2-H4K8ac_peak_22185	4.85384	
4	3767170	3767573	KD2-H4K8ac_peak_22186	4.8402	ADRA2C_ENSG00000184160
4	3782614	3782880	KD2-H4K8ac_peak_22187	4.44527	
4	3787636	3787945	KD2-H4K8ac_peak_22188	7.18615	
4	3829263	3830190	KD2-H4K8ac_peak_22189	14.8083	
4	3957955	3958277	KD2-H4K8ac_peak_22190	12.06346	AC226119.5_ENSG00000253917;FAM86EP_ENSG00000251669
4	4249409	4249885	KD2-H4K8ac_peak_22191	11.05407	TMEM128_ENSG00000132406
4	4292046	4292238	KD2-H4K8ac_peak_22192	9.91109	LYAR_ENSG00000145220;ZBTB49_ENSG00000168826
4	4388296	4388527	KD2-H4K8ac_peak_22193	8.04448	
4	4388977	4389176	KD2-H4K8ac_peak_22194	6.67092	
4	4543374	4543703	KD2-H4K8ac_peak_22195	17.16047	STX18_ENSG00000168818;STX18-AS1_ENSG00000247708
4	4543899	4544187	KD2-H4K8ac_peak_22196	10.05606	STX18_ENSG00000168818;STX18-AS1_ENSG00000247708
4	5710029	5710539	KD2-H4K8ac_peak_22197	6.15357	EVC2_ENSG00000173040
4	5712420	5712707	KD2-H4K8ac_peak_22198	8.94469	EVC_ENSG00000072840
4	5712933	5713570	KD2-H4K8ac_peak_22199	8.55271	EVC_ENSG00000072840
4	5889461	5890235	KD2-H4K8ac_peak_22200	11.04256	
4	5890492	5890763	KD2-H4K8ac_peak_22201	5.88845	
4	5894194	5894398	KD2-H4K8ac_peak_22202	6.72624	CRMP1_ENSG00000072832
4	5957995	5958473	KD2-H4K8ac_peak_22203	5.14125	
4	6163668	6164206	KD2-H4K8ac_peak_22204	8.30974	
4	6173395	6173665	KD2-H4K8ac_peak_22205	6.4095	
4	6177346	6177582	KD2-H4K8ac_peak_22206	7.05051	
4	6179611	6179879	KD2-H4K8ac_peak_22207	7.27716	
4	6200864	6201225	KD2-H4K8ac_peak_22208	7.13615	
4	6201900	6202169	KD2-H4K8ac_peak_22209	6.99815	JAKMIP1_ENSG00000152969;RP11-586D19.1_ENSG00000249896
4	6202440	6202699	KD2-H4K8ac_peak_22210	20.42362	JAKMIP1_ENSG00000152969;RP11-586D19.1_ENSG00000249896
4	6223894	6224158	KD2-H4K8ac_peak_22211	4.67342	
4	6271633	6271837	KD2-H4K8ac_peak_22212	8.6308	WFS1_ENSG00000109501
4	6310650	6311167	KD2-H4K8ac_peak_22213	9.20193	
4	6418594	6418853	KD2-H4K8ac_peak_22214	8.74596	
4	6577036	6577388	KD2-H4K8ac_peak_22215	6.65673	MAN2B2_ENSG00000013288
4	6641461	6642152	KD2-H4K8ac_peak_22216	4.44527	MRFAP1_ENSG00000179010

4	6674663	6674887	KD2-H4K8ac_peak_22217	7.07117	RP11-539L10.3_ENSG00000251580;AC093323.3_ENSG00000170846
4	6711180	6711511	KD2-H4K8ac_peak_22218	8.55271	MRFAP1L1_ENSG00000178988
4	6711851	6712088	KD2-H4K8ac_peak_22219	8.5781	MRFAP1L1_ENSG00000178988
4	6717905	6718462	KD2-H4K8ac_peak_22220	9.20193	BLOC1S4_ENSG00000186222
4	6751399	6751717	KD2-H4K8ac_peak_22221	4.0528	
4	6763219	6763688	KD2-H4K8ac_peak_22222	11.92978	
4	6773072	6773461	KD2-H4K8ac_peak_22223	4.77079	
4	6783891	6784257	KD2-H4K8ac_peak_22224	11.05467	KIAA0232_ENSG00000170871
4	6784490	6785261	KD2-H4K8ac_peak_22225	13.9234	
4	6911209	6911894	KD2-H4K8ac_peak_22226	12.08081	TBC1D14_ENSG00000132405
4	7045322	7045638	KD2-H4K8ac_peak_22227	9.70057	CCDC96_ENSG00000173013
4	7069465	7069673	KD2-H4K8ac_peak_22228	5.405	GRPEL1_ENSG00000109519
4	7075140	7075351	KD2-H4K8ac_peak_22229	6.56926	
4	7103504	7103835	KD2-H4K8ac_peak_22230	13.92465	
4	7141439	7141638	KD2-H4K8ac_peak_22231	4.40093	
4	7207958	7208247	KD2-H4K8ac_peak_22232	5.91443	
4	7208701	7209416	KD2-H4K8ac_peak_22233	18.68247	
4	7209740	7209981	KD2-H4K8ac_peak_22234	7.40314	
4	7227305	7229265	KD2-H4K8ac_peak_22235	14.43704	
4	7253854	7254115	KD2-H4K8ac_peak_22236	5.557	
4	7399399	7399794	KD2-H4K8ac_peak_22237	4.77079	
4	7818342	7818582	KD2-H4K8ac_peak_22238	10.58016	
4	7820709	7820909	KD2-H4K8ac_peak_22239	8.69797	
4	7821843	7822037	KD2-H4K8ac_peak_22240	7.99241	
4	7882297	7882509	KD2-H4K8ac_peak_22241	5.57044	
4	7887883	7888132	KD2-H4K8ac_peak_22242	5.15777	
4	7894416	7894624	KD2-H4K8ac_peak_22243	10.08707	
4	7894954	7895196	KD2-H4K8ac_peak_22244	8.76725	
4	7896230	7896458	KD2-H4K8ac_peak_22245	5.75757	
4	7899277	7899487	KD2-H4K8ac_peak_22246	5.11586	
4	7899872	7900134	KD2-H4K8ac_peak_22247	6.75424	
4	7904444	7905352	KD2-H4K8ac_peak_22248	8.10216	
4	7906505	7906834	KD2-H4K8ac_peak_22249	4.42765	
4	7907483	7907833	KD2-H4K8ac_peak_22250	14.66631	
4	7912263	7912682	KD2-H4K8ac_peak_22251	11.47476	
4	7940993	7941412	KD2-H4K8ac_peak_22252	8.04448	AFAP1_ENSG00000196526;AC097381.1_ENSG00000228919
4	7951272	7951720	KD2-H4K8ac_peak_22253	11.86708	
4	7989436	7989673	KD2-H4K8ac_peak_22254	7.27716	
4	8012025	8012325	KD2-H4K8ac_peak_22255	9.36516	
4	8052006	8052987	KD2-H4K8ac_peak_22256	12.82393	
4	8129753	8130009	KD2-H4K8ac_peak_22257	5.51783	
4	8160882	8161094	KD2-H4K8ac_peak_22258	9.08816	ABLIM2_ENSG00000163995
4	8190977	8191292	KD2-H4K8ac_peak_22259	4.44527	
4	8193178	8193509	KD2-H4K8ac_peak_22260	5.97274	
4	8194308	8194514	KD2-H4K8ac_peak_22261	8.82427	
4	8200151	8200772	KD2-H4K8ac_peak_22262	8.85813	

4	8201090	8201764	KD2-H4K8ac_peak_22263	7.70067	
4	8207502	8207731	KD2-H4K8ac_peak_22264	4.67342	
4	8224691	8224892	KD2-H4K8ac_peak_22265	6.83936	
4	8230153	8230850	KD2-H4K8ac_peak_22266	5.32397	
4	8244278	8244668	KD2-H4K8ac_peak_22267	10.05606	
4	8261592	8261833	KD2-H4K8ac_peak_22268	6.39197	
4	8263602	8263798	KD2-H4K8ac_peak_22269	6.22239	
4	8268709	8269026	KD2-H4K8ac_peak_22270	5.57044	
4	8274528	8275191	KD2-H4K8ac_peak_22271	7.79741	
4	8275502	8275707	KD2-H4K8ac_peak_22272	5.14125	
4	8275966	8276431	KD2-H4K8ac_peak_22273	11.79585	
4	8276892	8277511	KD2-H4K8ac_peak_22274	5.75757	
4	8287456	8287663	KD2-H4K8ac_peak_22275	6.66692	
4	8317837	8318327	KD2-H4K8ac_peak_22276	4.98072	
4	8342465	8342809	KD2-H4K8ac_peak_22277	5.14125	
4	8598762	8599013	KD2-H4K8ac_peak_22278	4.36417	
4	8706479	8707555	KD2-H4K8ac_peak_22279	7.47462	
4	8873961	8874335	KD2-H4K8ac_peak_22280	4.42765	HMX1_ENSG00000215612
4	8894574	8894845	KD2-H4K8ac_peak_22281	10.63441	
4	8910189	8910447	KD2-H4K8ac_peak_22282	4.73064	
4	8912363	8913457	KD2-H4K8ac_peak_22283	7.29632	
4	9153843	9154260	KD2-H4K8ac_peak_22284	10.0692	FAM86KP_ENSG00000163612
4	9155155	9155392	KD2-H4K8ac_peak_22285	6.76298	FAM86KP_ENSG00000163612
4	9382663	9383070	KD2-H4K8ac_peak_22286	10.21502	
4	9533996	9534203	KD2-H4K8ac_peak_22287	7.59643	
4	10020607	10020804	KD2-H4K8ac_peak_22288	7.47462	
4	10118112	10118425	KD2-H4K8ac_peak_22289	8.37231	WDR1_ENSG00000071127;RNA5SP155_ENSG00000223086
4	12610990	12611446	KD2-H4K8ac_peak_22290	10.05606	
4	13143969	13144242	KD2-H4K8ac_peak_22291	9.23578	
4	13485399	13485814	KD2-H4K8ac_peak_22292	8.00457	RAB28_ENSG00000157869
4	13486401	13486598	KD2-H4K8ac_peak_22293	10.1591	RAB28_ENSG00000157869
4	13628956	13629294	KD2-H4K8ac_peak_22294	6.45083	BOD1L1_ENSG00000038219;MIR5091_ENSG00000266240
4	13655928	13656254	KD2-H4K8ac_peak_22295	5.75452	RP11-341G5.1_ENSG00000250634
4	15003147	15003569	KD2-H4K8ac_peak_22296	4.77079	CPEB2-AS1_ENSG00000247624;CPEB2_ENSG00000137449
4	15004164	15004729	KD2-H4K8ac_peak_22297	9.51625	CPEB2-AS1_ENSG00000247624;CPEB2_ENSG00000137449
4	15005028	15005671	KD2-H4K8ac_peak_22298	6.60096	CPEB2_ENSG00000137449
4	15005911	15006686	KD2-H4K8ac_peak_22299	6.25869	
4	15022559	15022766	KD2-H4K8ac_peak_22300	9.15329	
4	15134791	15135113	KD2-H4K8ac_peak_22301	11.30637	
4	15149061	15150069	KD2-H4K8ac_peak_22302	10.1591	
4	15150285	15150816	KD2-H4K8ac_peak_22303	7.51935	
4	15481144	15481510	KD2-H4K8ac_peak_22304	15.39543	
4	15656545	15656874	KD2-H4K8ac_peak_22305	10.05606	
4	15657163	15657530	KD2-H4K8ac_peak_22306	13.95627	
4	15683467	15683831	KD2-H4K8ac_peak_22307	16.32216	FBXL5_ENSG00000118564;FAM200B_ENSG00000237765
4	15704349	15704989	KD2-H4K8ac_peak_22308	10.96455	BST1_ENSG00000109743

4	16084327	16084865	KD2-H4K8ac_peak_22309	5.9696	
4	16227713	16227980	KD2-H4K8ac_peak_22310	7.27716	TAPT1-AS1_ENSG00000263327
4	16228281	16228821	KD2-H4K8ac_peak_22311	16.63953	TAPT1_ENSG00000169762;TAPT1-AS1_ENSG00000263327
4	17128474	17128748	KD2-H4K8ac_peak_22312	6.16888	
4	17491565	17492168	KD2-H4K8ac_peak_22313	5.14125	
4	17513273	17513654	KD2-H4K8ac_peak_22314	7.07117	QDPR_ENSG00000151552
4	17614866	17615396	KD2-H4K8ac_peak_22315	6.82976	AC006160.5_ENSG00000249502;MED28_ENSG00000118579
4	17616387	17616785	KD2-H4K8ac_peak_22316	12.88108	AC006160.5_ENSG00000249502;MED28_ENSG00000118579
4	17669506	17669703	KD2-H4K8ac_peak_22317	6.16888	
4	17672775	17673149	KD2-H4K8ac_peak_22318	11.30637	
4	17811994	17812200	KD2-H4K8ac_peak_22319	6.25869	DCAF16_ENSG00000163257;NCAPG_ENSG00000109805
4	18022930	18023262	KD2-H4K8ac_peak_22320	8.1718	LCORL_ENSG00000178177
4	18320850	18321212	KD2-H4K8ac_peak_22321	11.30637	
4	20701215	20701667	KD2-H4K8ac_peak_22322	5.25071	
4	20702111	20702333	KD2-H4K8ac_peak_22323	7.45501	
4	22517107	22517623	KD2-H4K8ac_peak_22324	14.87555	GPR125_ENSG00000152990
4	22517831	22518216	KD2-H4K8ac_peak_22325	11.75669	GPR125_ENSG00000152990
4	24585611	24586054	KD2-H4K8ac_peak_22326	17.19888	DHX15_ENSG00000109606
4	24586536	24586858	KD2-H4K8ac_peak_22327	13.39763	DHX15_ENSG00000109606
4	25161271	25161488	KD2-H4K8ac_peak_22328	6.76298	SEPSECS_ENSG00000109618;PI4K2B_ENSG00000038210
4	25161737	25162011	KD2-H4K8ac_peak_22329	9.95119	SEPSECS_ENSG00000109618;PI4K2B_ENSG00000038210
4	25235677	25236088	KD2-H4K8ac_peak_22330	12.08081	
4	25377973	25378545	KD2-H4K8ac_peak_22331	11.05407	ANAPC4_ENSG00000053900
4	25378872	25379093	KD2-H4K8ac_peak_22332	7.36799	ANAPC4_ENSG00000053900
4	25914993	25915230	KD2-H4K8ac_peak_22333	9.96798	
4	26321484	26321817	KD2-H4K8ac_peak_22334	9.15069	
4	26805197	26805407	KD2-H4K8ac_peak_22335	4.06758	
4	26858846	26859140	KD2-H4K8ac_peak_22336	8.5092	STIM2_ENSG00000109689
4	26859467	26859677	KD2-H4K8ac_peak_22337	6.97919	STIM2_ENSG00000109689
4	26862598	26862860	KD2-H4K8ac_peak_22338	6.25869	RP11-293A21.1_ENSG00000240005
4	30836949	30837261	KD2-H4K8ac_peak_22339	7.12754	
4	37675380	37675636	KD2-H4K8ac_peak_22340	6.66692	
4	37688965	37689331	KD2-H4K8ac_peak_22341	5.77029	RELL1_ENSG00000181826
4	37828344	37828709	KD2-H4K8ac_peak_22342	12.00597	PGM2_ENSG00000169299
4	37892568	37892800	KD2-H4K8ac_peak_22343	5.54647	TBC1D1_ENSG00000065882
4	37979196	37979422	KD2-H4K8ac_peak_22344	8.86099	
4	38321740	38322147	KD2-H4K8ac_peak_22345	8.1233	
4	38384863	38385095	KD2-H4K8ac_peak_22346	4.211	
4	38664661	38665346	KD2-H4K8ac_peak_22347	7.76104	KLF3_ENSG00000109787
4	38665937	38666484	KD2-H4K8ac_peak_22348	9.50015	RP11-617D20.1_ENSG00000231160;AC021860.1_ENSG00000196355;KLF3_ENSG00000109787
4	38869030	38869963	KD2-H4K8ac_peak_22349	7.07117	FAM114A1_ENSG00000197712;MIR574_ENSG00000207944
4	39038911	39039109	KD2-H4K8ac_peak_22350	5.07901	
4	39046245	39047007	KD2-H4K8ac_peak_22351	11.89502	KLHL5_ENSG00000109790
4	39367590	39368584	KD2-H4K8ac_peak_22352	7.40314	RFC1_ENSG00000035928
4	39460740	39461119	KD2-H4K8ac_peak_22353	11.05407	RPL9_ENSG00000163682;LIAS_ENSG00000121897
4	39528601	39528963	KD2-H4K8ac_peak_22354	8.28234	UGDH_ENSG00000109814;UGDH-AS1_ENSG00000249348

4	39529362	39530032	KD2-H4K8ac_peak_22355	17.27897	UGDH_ENSG00000109814;UGDH-AS1_ENSG00000249348
4	39641158	39641392	KD2-H4K8ac_peak_22356	6.00226	SMIM14_ENSG00000163683;RP11-539G18.2_ENSG00000255458
4	39699362	39699594	KD2-H4K8ac_peak_22357	8.17902	UBE2K_ENSG00000078140
4	39699804	39700344	KD2-H4K8ac_peak_22358	11.79585	UBE2K_ENSG00000078140
4	39978642	39979490	KD2-H4K8ac_peak_22359	7.07117	PDS5A_ENSG00000121892
4	39979819	39980073	KD2-H4K8ac_peak_22360	7.44324	PDS5A_ENSG00000121892
4	40055991	40056206	KD2-H4K8ac_peak_22361	4.12757	
4	40057471	40058188	KD2-H4K8ac_peak_22362	12.36441	RP11-333E13.4_ENSG00000205794;N4BP2_ENSG00000078177
4	40058395	40059196	KD2-H4K8ac_peak_22363	12.93253	RP11-333E13.4_ENSG00000205794;N4BP2_ENSG00000078177
4	40266723	40267422	KD2-H4K8ac_peak_22364	5.40638	RP11-395I6.2_ENSG00000250338
4	40318889	40319107	KD2-H4K8ac_peak_22365	5.54647	AC195454.1_ENSG00000249241
4	40631592	40631865	KD2-H4K8ac_peak_22366	7.83656	
4	40751709	40752425	KD2-H4K8ac_peak_22367	6.40297	NSUN7_ENSG00000179299
4	40884242	40884443	KD2-H4K8ac_peak_22368	7.18916	
4	41218546	41219042	KD2-H4K8ac_peak_22369	20.42362	APBB2_ENSG00000163697
4	41259059	41259517	KD2-H4K8ac_peak_22370	13.13965	UCHL1-AS1_ENSG00000251173;UCHL1_ENSG00000154277
4	41361159	41361782	KD2-H4K8ac_peak_22371	17.19888	LIMCH1_ENSG00000064042
4	41361985	41362605	KD2-H4K8ac_peak_22372	6.66359	LIMCH1_ENSG00000064042
4	41362894	41363582	KD2-H4K8ac_peak_22373	11.89502	
4	41386225	41386878	KD2-H4K8ac_peak_22374	7.29632	
4	41468116	41468333	KD2-H4K8ac_peak_22375	7.05051	
4	41592175	41592454	KD2-H4K8ac_peak_22376	7.70289	
4	41640622	41640871	KD2-H4K8ac_peak_22377	6.94255	
4	41689054	41689483	KD2-H4K8ac_peak_22378	7.12754	
4	41733654	41733940	KD2-H4K8ac_peak_22379	4.77079	
4	41747395	41748096	KD2-H4K8ac_peak_22380	11.03331	
4	41748324	41749626	KD2-H4K8ac_peak_22381	11.89502	RP11-227F19.1_ENSG00000250467
4	41750334	41750631	KD2-H4K8ac_peak_22382	7.38933	PHOX2B_ENSG00000109132;RP11-227F19.1_ENSG00000250467
4	41751749	41752021	KD2-H4K8ac_peak_22383	10.96455	PHOX2B_ENSG00000109132;RP11-227F19.2_ENSG00000249122
4	41752792	41753065	KD2-H4K8ac_peak_22384	6.56211	RP11-227F19.2_ENSG00000249122
4	41753262	41754161	KD2-H4K8ac_peak_22385	20.57085	RP11-227F19.2_ENSG00000249122
4	41786056	41786434	KD2-H4K8ac_peak_22386	5.25071	
4	41787076	41787375	KD2-H4K8ac_peak_22387	14.74047	
4	41790055	41790247	KD2-H4K8ac_peak_22388	6.40683	
4	41823160	41823488	KD2-H4K8ac_peak_22389	4.76709	
4	41836733	41836967	KD2-H4K8ac_peak_22390	9.34989	
4	41848393	41848662	KD2-H4K8ac_peak_22391	8.17902	
4	41857706	41857960	KD2-H4K8ac_peak_22392	6.99815	
4	41864486	41864817	KD2-H4K8ac_peak_22393	8.1718	
4	41865297	41865711	KD2-H4K8ac_peak_22394	6.0073	
4	41868323	41869537	KD2-H4K8ac_peak_22395	10.05606	
4	41875394	41875841	KD2-H4K8ac_peak_22396	11.90231	
4	41876527	41876756	KD2-H4K8ac_peak_22397	12.00597	
4	41877531	41877779	KD2-H4K8ac_peak_22398	4.53319	
4	41879250	41880003	KD2-H4K8ac_peak_22399	15.50259	
4	41880297	41881444	KD2-H4K8ac_peak_22400	9.26408	

4	41881654	41884524	KD2-H4K8ac_peak_22401	23.67389	LINC00682_ENSG00000245870
4	41885195	41885399	KD2-H4K8ac_peak_22402	4.82973	LINC00682_ENSG00000245870
4	41888532	41888767	KD2-H4K8ac_peak_22403	3.83999	
4	41937269	41937792	KD2-H4K8ac_peak_22404	6.20982	TMEM33_ENSG00000109133
4	42152662	42152902	KD2-H4K8ac_peak_22405	5.30799	
4	42153320	42153621	KD2-H4K8ac_peak_22406	9.08816	
4	42154002	42154913	KD2-H4K8ac_peak_22407	9.65086	BEND4_ENSG00000188848
4	42307621	42307960	KD2-H4K8ac_peak_22408	4.06758	
4	42309823	42310350	KD2-H4K8ac_peak_22409	14.52995	
4	42310872	42311444	KD2-H4K8ac_peak_22410	9.73431	
4	42312268	42312545	KD2-H4K8ac_peak_22411	6.99815	
4	42312928	42313183	KD2-H4K8ac_peak_22412	8.58069	
4	42317588	42317780	KD2-H4K8ac_peak_22413	4.77079	
4	42485461	42486046	KD2-H4K8ac_peak_22414	9.50015	
4	42658698	42659044	KD2-H4K8ac_peak_22415	9.51625	ATP8A1_ENSG00000124406;RP11-109E24.2_ENSG00000260519
4	42659399	42659780	KD2-H4K8ac_peak_22416	10.43145	ATP8A1_ENSG00000124406;RP11-109E24.2_ENSG00000260519
4	42660162	42660456	KD2-H4K8ac_peak_22417	8.66387	RP11-109E24.2_ENSG00000260519
4	42677418	42677743	KD2-H4K8ac_peak_22418	5.57044	
4	44680471	44680664	KD2-H4K8ac_peak_22419	6.66692	YIPF7_ENSG00000177752;GUF1_ENSG00000151806
4	44728293	44728530	KD2-H4K8ac_peak_22420	11.2938	GNPDA2_ENSG00000163281
4	46996147	46996345	KD2-H4K8ac_peak_22421	5.57044	GABRA4_ENSG00000109158;GABRB1_ENSG00000163288
4	47465229	47465700	KD2-H4K8ac_peak_22422	19.6416	COMMD8_ENSG00000169019
4	47915824	47916423	KD2-H4K8ac_peak_22423	4.91674	NFXL1_ENSG00000170448;NIPAL1_ENSG00000163293
4	48272047	48272362	KD2-H4K8ac_peak_22424	6.25869	TEC_ENSG00000135605
4	48343659	48344181	KD2-H4K8ac_peak_22425	10.77651	SLAIN2_ENSG00000109171
4	48484415	48484639	KD2-H4K8ac_peak_22426	5.57044	SLC10A4_ENSG00000145248
4	48485537	48486433	KD2-H4K8ac_peak_22427	13.87818	SLC10A4_ENSG00000145248
4	48781328	48782203	KD2-H4K8ac_peak_22428	9.36516	FRYL_ENSG00000075539
4	48782748	48783092	KD2-H4K8ac_peak_22429	6.40683	FRYL_ENSG00000075539
4	48832681	48832888	KD2-H4K8ac_peak_22430	11.70505	
4	48833166	48833552	KD2-H4K8ac_peak_22431	12.13241	
4	48908019	48909602	KD2-H4K8ac_peak_22432	10.29842	OCIAD2_ENSG00000145247
4	52861893	52862299	KD2-H4K8ac_peak_22433	8.1233	
4	52903916	52904164	KD2-H4K8ac_peak_22434	10.21502	SGCB_ENSG00000163069
4	52904408	52904950	KD2-H4K8ac_peak_22435	6.40297	SGCB_ENSG00000163069
4	53524881	53525312	KD2-H4K8ac_peak_22436	10.96455	USP46_ENSG00000109189;USP46-AS1_ENSG00000248866
4	53525593	53526355	KD2-H4K8ac_peak_22437	9.1707	USP46_ENSG00000109189;USP46-AS1_ENSG00000248866
4	53578727	53579199	KD2-H4K8ac_peak_22438	8.34752	DANCR_ENSG00000226950;MIR4449_ENSG00000264585;SNORA26_ENSG00000212588
4	53588123	53588473	KD2-H4K8ac_peak_22439	12.00273	
4	53589283	53589646	KD2-H4K8ac_peak_22440	7.12754	
4	53617200	53617506	KD2-H4K8ac_peak_22441	5.45396	ERVMER34-1_ENSG00000226887;AC104066.1_ENSG00000266656
4	53617734	53618142	KD2-H4K8ac_peak_22442	5.54647	ERVMER34-1_ENSG00000226887;AC104066.1_ENSG00000266656
4	53706818	53707226	KD2-H4K8ac_peak_22443	6.47635	
4	53707737	53707951	KD2-H4K8ac_peak_22444	4.86324	
4	53708229	53708432	KD2-H4K8ac_peak_22445	5.45396	
4	53727911	53728297	KD2-H4K8ac_peak_22446	12.84642	RASL11B_ENSG00000128045

4	53730667	53731067	KD2-H4K8ac_peak_22447	7.13615	
4	53731585	53732207	KD2-H4K8ac_peak_22448	8.69797	
4	53733810	53734881	KD2-H4K8ac_peak_22449	9.242	
4	53736128	53736926	KD2-H4K8ac_peak_22450	6.66692	
4	53858417	53858838	KD2-H4K8ac_peak_22451	6.14031	
4	53861422	53862081	KD2-H4K8ac_peak_22452	11.11809	
4	53863062	53863816	KD2-H4K8ac_peak_22453	10.08707	
4	53915551	53915765	KD2-H4K8ac_peak_22454	4.95193	
4	53916129	53916332	KD2-H4K8ac_peak_22455	5.71749	
4	54231579	54231818	KD2-H4K8ac_peak_22456	6.16888	SCFD2_ENSG00000184178
4	54243894	54244431	KD2-H4K8ac_peak_22457	9.8758	FIP1L1_ENSG00000145216
4	54567447	54568151	KD2-H4K8ac_peak_22458	8.94469	LNX1_ENSG00000072201
4	54568593	54568790	KD2-H4K8ac_peak_22459	4.86324	
4	54569180	54569627	KD2-H4K8ac_peak_22460	8.6308	
4	54615545	54615802	KD2-H4K8ac_peak_22461	4.01115	
4	54772140	54772564	KD2-H4K8ac_peak_22462	4.211	
4	54930206	54930693	KD2-H4K8ac_peak_22463	4.67342	CHIC2_ENSG00000109220
4	54957940	54958599	KD2-H4K8ac_peak_22464	9.1707	
4	55062845	55063114	KD2-H4K8ac_peak_22465	6.16888	
4	55063483	55063927	KD2-H4K8ac_peak_22466	5.45396	
4	55093581	55093825	KD2-H4K8ac_peak_22467	10.46264	
4	55094231	55094560	KD2-H4K8ac_peak_22468	4.42765	PDGFRA_ENSG00000134853
4	55099718	55099981	KD2-H4K8ac_peak_22469	6.76298	
4	55149275	55149595	KD2-H4K8ac_peak_22470	5.73839	
4	55953566	55953879	KD2-H4K8ac_peak_22471	7.47462	
4	56262307	56262729	KD2-H4K8ac_peak_22472	11.75669	SRD5A3-AS1_ENSG00000249700;TMEM165_ENSG00000134851
4	56411914	56412634	KD2-H4K8ac_peak_22473	12.42761	CLOCK_ENSG00000134852;RP11-528I4.2_ENSG00000272969
4	56412987	56413501	KD2-H4K8ac_peak_22474	6.40297	CLOCK_ENSG00000134852;RP11-528I4.2_ENSG00000272969
4	56502076	56502334	KD2-H4K8ac_peak_22475	9.65086	NMU_ENSG00000109255
4	56502800	56503113	KD2-H4K8ac_peak_22476	6.99815	NMU_ENSG00000109255
4	56719934	56720311	KD2-H4K8ac_peak_22477	10.29344	EXOC1_ENSG00000090989
4	56815076	56815351	KD2-H4K8ac_peak_22478	13.80019	CEP135_ENSG00000174799
4	56914807	56916139	KD2-H4K8ac_peak_22479	23.55663	
4	57014454	57014717	KD2-H4K8ac_peak_22480	7.27716	
4	57015063	57015473	KD2-H4K8ac_peak_22481	5.49367	
4	57102037	57102234	KD2-H4K8ac_peak_22482	5.93446	
4	57108697	57109032	KD2-H4K8ac_peak_22483	12.36633	
4	57162700	57162898	KD2-H4K8ac_peak_22484	5.51783	
4	57253394	57253885	KD2-H4K8ac_peak_22485	11.75669	AASDH_ENSG00000157426;RP11-646I6.5_ENSG00000269921
4	57411044	57411420	KD2-H4K8ac_peak_22486	10.0746	
4	57737505	57738343	KD2-H4K8ac_peak_22487	10.0692	
4	57774839	57775299	KD2-H4K8ac_peak_22488	6.99815	REST_ENSG00000084093
4	57843238	57843609	KD2-H4K8ac_peak_22489	5.32933	POLR2B_ENSG00000047315
4	57844163	57844658	KD2-H4K8ac_peak_22490	9.37314	NOA1_ENSG00000084092;POLR2B_ENSG00000047315
4	57922862	57923059	KD2-H4K8ac_peak_22491	5.77029	
4	58023358	58023662	KD2-H4K8ac_peak_22492	4.77079	

4	58030003	58030591	KD2-H4K8ac_peak_22493	7.67504	
4	60721817	60722095	KD2-H4K8ac_peak_22494	5.25071	
4	66864203	66864927	KD2-H4K8ac_peak_22495	14.74047	RP11-25H12.1_ENSG00000249413
4	69215976	69216432	KD2-H4K8ac_peak_22496	5.14125	YTHDC1_ENSG00000083896
4	71553107	71553845	KD2-H4K8ac_peak_22497	16.49944	UTP3_ENSG00000132467
4	71554311	71554711	KD2-H4K8ac_peak_22498	4.86324	UTP3_ENSG00000132467
4	71570106	71571539	KD2-H4K8ac_peak_22499	7.76104	RP11-46J23.1_ENSG00000272986;RUFY3_ENSG00000018189
4	71705173	71705593	KD2-H4K8ac_peak_22500	20.8593	GRSF1_ENSG00000132463
4	71767571	71767894	KD2-H4K8ac_peak_22501	10.05606	MOB1B_ENSG00000173542
4	71768184	71768607	KD2-H4K8ac_peak_22502	11.1952	MOB1B_ENSG00000173542
4	71858852	71859120	KD2-H4K8ac_peak_22503	4.86324	DCK_ENSG00000156136
4	71859374	71859861	KD2-H4K8ac_peak_22504	9.20193	
4	72898096	72898440	KD2-H4K8ac_peak_22505	5.25071	NPFFR2_ENSG00000056291
4	73177788	73178057	KD2-H4K8ac_peak_22506	8.17902	
4	73284838	73285334	KD2-H4K8ac_peak_22507	8.17902	
4	73292412	73292608	KD2-H4K8ac_peak_22508	8.88549	
4	73434742	73435493	KD2-H4K8ac_peak_22509	11.05407	ADAMTS3_ENSG00000156140
4	73934954	73935433	KD2-H4K8ac_peak_22510	14.63458	COX18_ENSG00000163626
4	74123855	74124468	KD2-H4K8ac_peak_22511	8.02571	ANKRD17_ENSG00000132466;RP11-692D12.1_ENSG00000250220
4	74124683	74125246	KD2-H4K8ac_peak_22512	9.63487	ANKRD17_ENSG00000132466;RP11-692D12.1_ENSG00000250220
4	76439005	76439441	KD2-H4K8ac_peak_22513	10.73467	RCHY1_ENSG00000163743;THAP6_ENSG00000174796
4	76439703	76439940	KD2-H4K8ac_peak_22514	14.66631	RCHY1_ENSG00000163743;THAP6_ENSG00000174796
4	76555379	76555583	KD2-H4K8ac_peak_22515	6.25869	CDKL2_ENSG00000138769
4	76597959	76598528	KD2-H4K8ac_peak_22516	13.80019	
4	76598847	76599359	KD2-H4K8ac_peak_22517	5.88845	
4	76650067	76650531	KD2-H4K8ac_peak_22518	10.96455	G3BP2_ENSG00000138757;USO1_ENSG00000138768
4	76911647	76911877	KD2-H4K8ac_peak_22519	6.66692	SDAD1_ENSG00000198301
4	76912331	76912581	KD2-H4K8ac_peak_22520	10.7186	SDAD1_ENSG00000198301
4	77118352	77118709	KD2-H4K8ac_peak_22521	6.56926	
4	77120849	77121256	KD2-H4K8ac_peak_22522	6.31143	
4	77134497	77135768	KD2-H4K8ac_peak_22523	18.70522	SCARB2_ENSG00000138760;FAM47E_ENSG00000189157
4	77155165	77155863	KD2-H4K8ac_peak_22524	9.72244	
4	77172602	77173498	KD2-H4K8ac_peak_22525	12.84642	FAM47E-STBD1_ENSG00000272414;FAM47E-STBD1_ENSG00000118804
4	77227295	77227633	KD2-H4K8ac_peak_22526	6.52179	
4	77228183	77228471	KD2-H4K8ac_peak_22527	7.73264	
4	77342078	77342716	KD2-H4K8ac_peak_22528	11.92978	CCDC158_ENSG00000163749
4	77501782	77502080	KD2-H4K8ac_peak_22529	6.8836	
4	77504189	77504693	KD2-H4K8ac_peak_22530	12.06346	
4	77610274	77611127	KD2-H4K8ac_peak_22531	13.73635	
4	77870966	77871205	KD2-H4K8ac_peak_22532	12.82393	SEPT11_ENSG00000138758
4	77871579	77872028	KD2-H4K8ac_peak_22533	7.76329	SEPT11_ENSG00000138758
4	77996691	77996951	KD2-H4K8ac_peak_22534	14.74047	CCNI_ENSG00000118816
4	77997185	77997473	KD2-H4K8ac_peak_22535	19.85702	CCNI_ENSG00000118816
4	78078072	78078300	KD2-H4K8ac_peak_22536	12.36441	CCNG2_ENSG00000138764
4	78078594	78078955	KD2-H4K8ac_peak_22537	6.84261	CCNG2_ENSG00000138764
4	78740271	78741226	KD2-H4K8ac_peak_22538	10.72656	CNOT6L_ENSG00000138767

4	78741606	78741841	KD2-H4K8ac_peak_22539	6.13909	CNOT6L_ENSG00000138767
4	78976834	78978007	KD2-H4K8ac_peak_22540	13.70753	FRAS1_ENSG00000138759
4	78980102	78980477	KD2-H4K8ac_peak_22541	4.86324	
4	79126171	79126657	KD2-H4K8ac_peak_22542	7.12754	
4	79472768	79473329	KD2-H4K8ac_peak_22543	17.17257	ANXA3_ENSG00000138772
4	79696624	79697059	KD2-H4K8ac_peak_22544	6.84261	RP11-109G23.3_ENSG00000260278;BMP2K_ENSG00000138756
4	79697264	79697915	KD2-H4K8ac_peak_22545	9.11031	RP11-109G23.3_ENSG00000260278;BMP2K_ENSG00000138756
4	79860038	79860521	KD2-H4K8ac_peak_22546	14.15075	PAQR3_ENSG00000163291
4	79971852	79972133	KD2-H4K8ac_peak_22547	9.15329	
4	80993138	80994406	KD2-H4K8ac_peak_22548	11.8457	
4	81117898	81118090	KD2-H4K8ac_peak_22549	5.54647	
4	81187740	81188239	KD2-H4K8ac_peak_22550	8.10216	FGF5_ENSG00000138675
4	83294591	83295108	KD2-H4K8ac_peak_22551	9.36516	HNRNPD_ENSG00000138668;RP11-127B20.3_ENSG00000272677
4	83295511	83295794	KD2-H4K8ac_peak_22552	14.8083	HNRNPD_ENSG00000138668;RP11-127B20.3_ENSG00000272677
4	83350307	83350540	KD2-H4K8ac_peak_22553	12.3187	HNRNPDL_ENSG00000152795
4	83350892	83351350	KD2-H4K8ac_peak_22554	10.19949	HNRNPDL_ENSG00000152795;ENOPH1_ENSG00000145293
4	83482851	83483436	KD2-H4K8ac_peak_22555	9.96798	TMEM150C_ENSG00000249242
4	83651819	83652038	KD2-H4K8ac_peak_22556	6.31143	
4	83812440	83812762	KD2-H4K8ac_peak_22557	12.91654	
4	83934653	83934904	KD2-H4K8ac_peak_22558	10.08874	LIN54_ENSG00000189308
4	83955525	83955834	KD2-H4K8ac_peak_22559	8.1233	COPS4_ENSG00000138663
4	84205681	84205957	KD2-H4K8ac_peak_22560	19.3524	COQ2_ENSG00000173085
4	84376648	84376951	KD2-H4K8ac_peak_22561	10.19949	HELQ_ENSG00000163312;MRPS18C_ENSG00000163319
4	84405820	84406144	KD2-H4K8ac_peak_22562	6.65673	
4	84406505	84406709	KD2-H4K8ac_peak_22563	6.2781	
4	85886964	85888065	KD2-H4K8ac_peak_22564	19.6416	WDFY3_ENSG00000163625;WDFY3-AS2_ENSG00000180769
4	86396367	86396635	KD2-H4K8ac_peak_22565	5.14125	ARHGAP24_ENSG00000138639
4	86941685	86941880	KD2-H4K8ac_peak_22566	4.211	
4	87813334	87813672	KD2-H4K8ac_peak_22567	6.08439	
4	87856320	87856573	KD2-H4K8ac_peak_22568	5.9696	C4orf36_ENSG00000163633;AFF1_ENSG00000172493
4	87927656	87928260	KD2-H4K8ac_peak_22569	8.10216	
4	87989981	87990261	KD2-H4K8ac_peak_22570	4.47369	
4	88133513	88133763	KD2-H4K8ac_peak_22571	5.16439	
4	88141115	88141600	KD2-H4K8ac_peak_22572	5.92591	
4	88311132	88311602	KD2-H4K8ac_peak_22573	10.72656	HSD17B11_ENSG00000198189
4	88312580	88312986	KD2-H4K8ac_peak_22574	6.8836	HSD17B11_ENSG00000198189
4	88343288	88343528	KD2-H4K8ac_peak_22575	5.94405	NUDT9_ENSG00000170502
4	88441002	88441349	KD2-H4K8ac_peak_22576	8.1233	
4	88928897	88929348	KD2-H4K8ac_peak_22577	5.84991	PKD2_ENSG00000118762
4	89204904	89205195	KD2-H4K8ac_peak_22578	10.29344	PPM1K_ENSG00000163644;RP11-10L7.1_ENSG00000246375
4	89300228	89300637	KD2-H4K8ac_peak_22579	8.4716	HERC6_ENSG00000138642
4	89378410	89378943	KD2-H4K8ac_peak_22580	5.30799	HERC5_ENSG00000138646
4	89444306	89445249	KD2-H4K8ac_peak_22581	16.15695	PYURF_ENSG00000145337
4	89619216	89619430	KD2-H4K8ac_peak_22582	4.86324	NAP1L5_ENSG00000177432
4	89744330	89744629	KD2-H4K8ac_peak_22583	8.1233	
4	90228671	90229522	KD2-H4K8ac_peak_22584	10.43145	GPRIN3_ENSG00000185477

4	90757994	90758866	KD2-H4K8ac_peak_22585	13.57066	SNCA_ENSG00000145335;RP11-67M1.1_ENSG00000247775
4	91049040	91049592	KD2-H4K8ac_peak_22586	11.04256	CCSER1_ENSG00000184305
4	93227069	93227359	KD2-H4K8ac_peak_22587	6.07178	
4	95127795	95128019	KD2-H4K8ac_peak_22588	4.42765	RP11-363G15.2_ENSG00000246541;SMARCAD1_ENSG00000163104
4	95128783	95129817	KD2-H4K8ac_peak_22589	13.57066	RP11-363G15.2_ENSG00000246541;SMARCAD1_ENSG00000163104
4	95569581	95570420	KD2-H4K8ac_peak_22590	9.51625	
4	95572201	95572503	KD2-H4K8ac_peak_22591	5.57044	
4	95573294	95573580	KD2-H4K8ac_peak_22592	9.15329	
4	95575439	95576020	KD2-H4K8ac_peak_22593	6.40683	
4	95601177	95601560	KD2-H4K8ac_peak_22594	7.83489	
4	95678542	95680540	KD2-H4K8ac_peak_22595	32.24865	RP11-168E14.1_ENSG00000249599;BMPR1B_ENSG00000138696
4	95681644	95681892	KD2-H4K8ac_peak_22596	7.59643	
4	95736254	95736494	KD2-H4K8ac_peak_22597	5.01262	
4	95746301	95746533	KD2-H4K8ac_peak_22598	9.11818	
4	95972562	95972759	KD2-H4K8ac_peak_22599	9.15329	
4	95980186	95980422	KD2-H4K8ac_peak_22600	10.0746	
4	95983011	95983225	KD2-H4K8ac_peak_22601	8.17902	
4	96115071	96115301	KD2-H4K8ac_peak_22602	6.90458	
4	96435844	96436093	KD2-H4K8ac_peak_22603	4.42765	
4	96441772	96442213	KD2-H4K8ac_peak_22604	12.42547	
4	96442482	96442899	KD2-H4K8ac_peak_22605	6.16888	
4	96444218	96444491	KD2-H4K8ac_peak_22606	10.0692	
4	96456654	96456852	KD2-H4K8ac_peak_22607	4.42765	
4	96457327	96457543	KD2-H4K8ac_peak_22608	10.21502	
4	96468383	96469518	KD2-H4K8ac_peak_22609	16.11042	UNC5C_ENSG00000182168;RP11-710C12.1_ENSG00000271474
4	96469726	96470014	KD2-H4K8ac_peak_22610	12.08081	UNC5C_ENSG00000182168;RP11-710C12.1_ENSG00000271474
4	96470466	96471063	KD2-H4K8ac_peak_22611	12.08081	UNC5C_ENSG00000182168;RP11-710C12.1_ENSG00000271474
4	99063889	99064456	KD2-H4K8ac_peak_22612	4.67342	STPG2_ENSG00000163116
4	99181379	99182256	KD2-H4K8ac_peak_22613	9.95119	RP11-323J4.1_ENSG00000214559;RAP1GDS1_ENSG00000138698
4	99182654	99183109	KD2-H4K8ac_peak_22614	9.59147	RP11-323J4.1_ENSG00000214559;RAP1GDS1_ENSG00000138698
4	99580069	99580276	KD2-H4K8ac_peak_22615	7.36799	TSPAN5_ENSG00000168785;RP11-1299A16.3_ENSG00000260641
4	99849622	99850217	KD2-H4K8ac_peak_22616	10.43145	RP11-571L19.7_ENSG00000263923;AC019131.1_ENSG00000238449
4	99850579	99851215	KD2-H4K8ac_peak_22617	4.7849	EIF4E_ENSG00000151247;RP11-571L19.7_ENSG00000263923;AC019131.1_ENSG00000238449
4	99916907	99917423	KD2-H4K8ac_peak_22618	9.24683	METAP1_ENSG00000164024
4	100010198	1E+08	KD2-H4K8ac_peak_22619	9.15329	ADH5_ENSG00000197894;RP11-696N14.1_ENSG00000246090
4	100484009	1E+08	KD2-H4K8ac_peak_22620	10.53377	TRMT10A_ENSG00000145331;MTTP_ENSG00000138823
4	100484942	1E+08	KD2-H4K8ac_peak_22621	18.91876	TRMT10A_ENSG00000145331;MTTP_ENSG00000138823
4	100765034	1.01E+08	KD2-H4K8ac_peak_22622	6.16888	
4	100772115	1.01E+08	KD2-H4K8ac_peak_22623	8.1233	
4	100815197	1.01E+08	KD2-H4K8ac_peak_22624	8.85813	LAMTOR3_ENSG00000109270
4	100815911	1.01E+08	KD2-H4K8ac_peak_22625	7.40111	LAMTOR3_ENSG00000109270
4	100867392	1.01E+08	KD2-H4K8ac_peak_22626	6.99815	DNAJB14_ENSG00000164031
4	100870422	1.01E+08	KD2-H4K8ac_peak_22627	14.15075	H2AFZ_ENSG00000164032;RP11-15B17.1_ENSG00000245322
4	102963606	1.03E+08	KD2-H4K8ac_peak_22628	5.16439	
4	103089884	1.03E+08	KD2-H4K8ac_peak_22629	3.75035	
4	103265714	1.03E+08	KD2-H4K8ac_peak_22630	6.99815	

4	103352177	1.03E+08	KD2-H4K8ac_peak_22631	13.57066	SLC39A8_ENSG00000138821
4	103422108	1.03E+08	KD2-H4K8ac_peak_22632	15.40891	AF213884.2_ENSG00000260651;NFKB1_ENSG00000109320
4	103748171	1.04E+08	KD2-H4K8ac_peak_22633	19.87796	RP11-10L12.4_ENSG00000246560
4	103997475	1.04E+08	KD2-H4K8ac_peak_22634	13.50024	
4	104119727	1.04E+08	KD2-H4K8ac_peak_22635	6.16888	CENPE_ENSG00000138778
4	105411940	1.05E+08	KD2-H4K8ac_peak_22636	5.25071	AC093628.1_ENSG00000272082;AC004053.1_ENSG00000245384
4	105412687	1.05E+08	KD2-H4K8ac_peak_22637	26.19661	AC093628.1_ENSG00000272082;AC004053.1_ENSG00000245384
4	105415943	1.05E+08	KD2-H4K8ac_peak_22638	4.42765	CXXC4_ENSG00000168772
4	106067518	1.06E+08	KD2-H4K8ac_peak_22639	13.34404	TET2_ENSG00000168769
4	106394661	1.06E+08	KD2-H4K8ac_peak_22640	7.07117	PPA2_ENSG00000138777
4	106629525	1.07E+08	KD2-H4K8ac_peak_22641	12.06346	GSTCD_ENSG00000138780
4	106630029	1.07E+08	KD2-H4K8ac_peak_22642	4.42765	GSTCD_ENSG00000138780
4	107236771	1.07E+08	KD2-H4K8ac_peak_22643	7.76329	AIMP1_ENSG00000164022
4	107237648	1.07E+08	KD2-H4K8ac_peak_22644	4.77079	AIMP1_ENSG00000164022
4	107446556	1.07E+08	KD2-H4K8ac_peak_22645	11.89502	
4	107956486	1.08E+08	KD2-H4K8ac_peak_22646	6.40683	
4	108132712	1.08E+08	KD2-H4K8ac_peak_22647	7.27716	
4	108134160	1.08E+08	KD2-H4K8ac_peak_22648	8.1233	
4	108179408	1.08E+08	KD2-H4K8ac_peak_22649	5.25071	RP11-713M6.2_ENSG00000251081
4	108299479	1.08E+08	KD2-H4K8ac_peak_22650	3.75035	
4	108641121	1.09E+08	KD2-H4K8ac_peak_22651	8.30974	PAPSS1_ENSG00000138801
4	108642755	1.09E+08	KD2-H4K8ac_peak_22652	6.64891	
4	108852067	1.09E+08	KD2-H4K8ac_peak_22653	5.30799	CYP2U1_ENSG00000155016
4	108852777	1.09E+08	KD2-H4K8ac_peak_22654	3.86308	CYP2U1_ENSG00000155016
4	108911084	1.09E+08	KD2-H4K8ac_peak_22655	11.15509	HADH_ENSG00000138796
4	109093671	1.09E+08	KD2-H4K8ac_peak_22656	13.70753	
4	109541853	1.1E+08	KD2-H4K8ac_peak_22657	6.65673	RPL34-AS1_ENSG00000234492;RPL34_ENSG00000109475
4	110354417	1.1E+08	KD2-H4K8ac_peak_22658	12.17278	SEC24B-AS1_ENSG00000247950;SEC24B_ENSG00000138802
4	110480809	1.1E+08	KD2-H4K8ac_peak_22659	9.20193	CCDC109B_ENSG00000005059
4	110623577	1.11E+08	KD2-H4K8ac_peak_22660	8.98477	CASP6_ENSG00000138794
4	110624379	1.11E+08	KD2-H4K8ac_peak_22661	6.84261	CASP6_ENSG00000138794
4	110650719	1.11E+08	KD2-H4K8ac_peak_22662	11.70505	PLA2G12A_ENSG00000123739
4	110735550	1.11E+08	KD2-H4K8ac_peak_22663	10.21502	RP11-602N24.3_ENSG00000272795;GAR1_ENSG00000109534
4	110737109	1.11E+08	KD2-H4K8ac_peak_22664	5.43522	RP11-602N24.3_ENSG00000272795;GAR1_ENSG00000109534
4	111114523	1.11E+08	KD2-H4K8ac_peak_22665	6.57893	
4	111117154	1.11E+08	KD2-H4K8ac_peak_22666	8.85813	
4	111119822	1.11E+08	KD2-H4K8ac_peak_22667	4.67342	ELOVL6_ENSG00000170522
4	111120796	1.11E+08	KD2-H4K8ac_peak_22668	6.0073	ELOVL6_ENSG00000170522
4	113152190	1.13E+08	KD2-H4K8ac_peak_22669	7.95563	RP11-73K9.2_ENSG00000260526;AP1AR_ENSG00000138660
4	113153035	1.13E+08	KD2-H4K8ac_peak_22670	7.40314	RP11-73K9.2_ENSG00000260526;AP1AR_ENSG00000138660
4	113206845	1.13E+08	KD2-H4K8ac_peak_22671	6.0605	TIFA_ENSG00000145365;ALPK1_ENSG00000073331
4	113247014	1.13E+08	KD2-H4K8ac_peak_22672	5.88845	
4	113269501	1.13E+08	KD2-H4K8ac_peak_22673	4.77079	
4	113298926	1.13E+08	KD2-H4K8ac_peak_22674	6.56926	
4	113430961	1.13E+08	KD2-H4K8ac_peak_22675	11.50346	
4	113435665	1.13E+08	KD2-H4K8ac_peak_22676	5.30799	NEUROG2_ENSG00000178403;RP11-402J6.1_ENSG00000249509

4	113436774	1.13E+08	KD2-H4K8ac_peak_22677	5.75757	NEUROG2_ENSG00000178403;RP11-402J6.1_ENSG00000249509
4	113438195	1.13E+08	KD2-H4K8ac_peak_22678	4.67342	NEUROG2_ENSG00000178403
4	113441871	1.13E+08	KD2-H4K8ac_peak_22679	10.96455	
4	113443033	1.13E+08	KD2-H4K8ac_peak_22680	5.15777	
4	113451543	1.13E+08	KD2-H4K8ac_peak_22681	6.66692	RP11-402J6.2_ENSG00000248439
4	113454820	1.13E+08	KD2-H4K8ac_peak_22682	4.42765	
4	113478606	1.13E+08	KD2-H4K8ac_peak_22683	6.16888	
4	113483144	1.13E+08	KD2-H4K8ac_peak_22684	8.30974	
4	113626735	1.14E+08	KD2-H4K8ac_peak_22685	11.30637	RP11-148B6.2_ENSG00000250046
4	113627294	1.14E+08	KD2-H4K8ac_peak_22686	10.21502	RP11-148B6.2_ENSG00000250046
4	113629164	1.14E+08	KD2-H4K8ac_peak_22687	8.17902	
4	113817043	1.14E+08	KD2-H4K8ac_peak_22688	3.75035	
4	113817770	1.14E+08	KD2-H4K8ac_peak_22689	9.59147	
4	113833024	1.14E+08	KD2-H4K8ac_peak_22690	7.22457	
4	113866671	1.14E+08	KD2-H4K8ac_peak_22691	9.11031	
4	113893890	1.14E+08	KD2-H4K8ac_peak_22692	6.16888	
4	113897392	1.14E+08	KD2-H4K8ac_peak_22693	6.94255	
4	114006307	1.14E+08	KD2-H4K8ac_peak_22694	4.8402	
4	114683194	1.15E+08	KD2-H4K8ac_peak_22695	13.81781	CAMK2D_ENSG00000145349
4	114900524	1.15E+08	KD2-H4K8ac_peak_22696	4.8888	ARSJ_ENSG00000180801
4	118954436	1.19E+08	KD2-H4K8ac_peak_22697	11.24702	NDST3_ENSG00000164100
4	119186484	1.19E+08	KD2-H4K8ac_peak_22698	6.05809	
4	119199411	1.19E+08	KD2-H4K8ac_peak_22699	7.27716	SNHG8_ENSG00000269893
4	119199977	1.19E+08	KD2-H4K8ac_peak_22700	8.09113	SNHG8_ENSG00000269893
4	119231913	1.19E+08	KD2-H4K8ac_peak_22701	6.07178	
4	119249984	1.19E+08	KD2-H4K8ac_peak_22702	5.75757	
4	119258562	1.19E+08	KD2-H4K8ac_peak_22703	9.02371	
4	119268797	1.19E+08	KD2-H4K8ac_peak_22704	7.05877	
4	119273268	1.19E+08	KD2-H4K8ac_peak_22705	7.49599	PRSS12_ENSG00000164099
4	119274093	1.19E+08	KD2-H4K8ac_peak_22706	5.13615	PRSS12_ENSG00000164099
4	119274642	1.19E+08	KD2-H4K8ac_peak_22707	8.37231	PRSS12_ENSG00000164099
4	119319128	1.19E+08	KD2-H4K8ac_peak_22708	6.79289	
4	119321579	1.19E+08	KD2-H4K8ac_peak_22709	6.16888	NDUFS5P5_ENSG00000230671
4	119512951	1.2E+08	KD2-H4K8ac_peak_22710	9.59147	RP11-384K6.6_ENSG00000260404
4	119592763	1.2E+08	KD2-H4K8ac_peak_22711	9.11031	
4	119593252	1.2E+08	KD2-H4K8ac_peak_22712	4.77079	
4	119604729	1.2E+08	KD2-H4K8ac_peak_22713	7.22457	
4	119606071	1.2E+08	KD2-H4K8ac_peak_22714	6.8836	METTL14_ENSG00000145388
4	119690891	1.2E+08	KD2-H4K8ac_peak_22715	7.12754	
4	119693759	1.2E+08	KD2-H4K8ac_peak_22716	7.27716	
4	119727625	1.2E+08	KD2-H4K8ac_peak_22717	6.27931	
4	119756955	1.2E+08	KD2-H4K8ac_peak_22718	7.07117	
4	119786136	1.2E+08	KD2-H4K8ac_peak_22719	19.6416	
4	119802681	1.2E+08	KD2-H4K8ac_peak_22720	6.31143	
4	119804904	1.2E+08	KD2-H4K8ac_peak_22721	6.16888	
4	119831304	1.2E+08	KD2-H4K8ac_peak_22722	6.16888	

4	120133926	1.2E+08	KD2-H4K8ac_peak_22723	6.20982	RP11-455G16.1_ENSG00000178636;USP53_ENSG00000145390
4	120222178	1.2E+08	KD2-H4K8ac_peak_22724	6.65673	
4	120549850	1.21E+08	KD2-H4K8ac_peak_22725	6.0073	PDE5A_ENSG00000138735
4	120817369	1.21E+08	KD2-H4K8ac_peak_22726	7.12754	
4	120970943	1.21E+08	KD2-H4K8ac_peak_22727	7.59643	
4	121843389	1.22E+08	KD2-H4K8ac_peak_22728	15.58442	PRDM5_ENSG00000138738
4	121992619	1.22E+08	KD2-H4K8ac_peak_22729	3.97079	NDNF_ENSG00000173376;RP11-647P12.1_ENSG00000250344
4	121993983	1.22E+08	KD2-H4K8ac_peak_22730	8.17902	NDNF_ENSG00000173376
4	122744306	1.23E+08	KD2-H4K8ac_peak_22731	6.8836	CCNA2_ENSG00000145386
4	122791164	1.23E+08	KD2-H4K8ac_peak_22732	9.402	BBS7_ENSG00000138686;RP11-63B13.1_ENSG00000261501
4	123747862	1.24E+08	KD2-H4K8ac_peak_22733	9.36516	FGF2_ENSG00000138685;AC021205.1_ENSG00000253069
4	124666184	1.25E+08	KD2-H4K8ac_peak_22734	5.25071	RPL21P50_ENSG00000228489;AC096732.2_ENSG00000226655
4	125633084	1.26E+08	KD2-H4K8ac_peak_22735	13.9234	ANKRD50_ENSG00000151458
4	127490365	1.27E+08	KD2-H4K8ac_peak_22736	10.21502	
4	128543890	1.29E+08	KD2-H4K8ac_peak_22737	16.23438	INTU_ENSG00000164066
4	128554153	1.29E+08	KD2-H4K8ac_peak_22738	5.57044	
4	128702897	1.29E+08	KD2-H4K8ac_peak_22739	13.3695	HSPA4L_ENSG00000164070
4	128885774	1.29E+08	KD2-H4K8ac_peak_22740	5.1707	C4orf29_ENSG00000164074
4	128886584	1.29E+08	KD2-H4K8ac_peak_22741	11.2938	MFSD8_ENSG00000164073;C4orf29_ENSG00000164074
4	128981755	1.29E+08	KD2-H4K8ac_peak_22742	28.92332	LARP1B_ENSG00000138709
4	128982524	1.29E+08	KD2-H4K8ac_peak_22743	5.9696	LARP1B_ENSG00000138709
4	129209248	1.29E+08	KD2-H4K8ac_peak_22744	8.26945	PGRMC2_ENSG00000164040
4	129308551	1.29E+08	KD2-H4K8ac_peak_22745	5.45396	
4	129474321	1.29E+08	KD2-H4K8ac_peak_22746	18.5703	RP11-130C6.1_ENSG00000273077
4	129522215	1.3E+08	KD2-H4K8ac_peak_22747	12.06346	RP11-184M15.2_ENSG00000248802
4	129730953	1.3E+08	KD2-H4K8ac_peak_22748	7.70289	JADE1_ENSG00000077684
4	129732506	1.3E+08	KD2-H4K8ac_peak_22749	12.14909	
4	129733446	1.3E+08	KD2-H4K8ac_peak_22750	8.10216	
4	130065931	1.3E+08	KD2-H4K8ac_peak_22751	9.02371	
4	133265266	1.33E+08	KD2-H4K8ac_peak_22752	5.57044	
4	133266292	1.33E+08	KD2-H4K8ac_peak_22753	7.12754	
4	135122609	1.35E+08	KD2-H4K8ac_peak_22754	5.25071	PABPC4L_ENSG00000254535
4	137316437	1.37E+08	KD2-H4K8ac_peak_22755	7.01964	RP11-775H9.2_ENSG00000251567
4	138310082	1.38E+08	KD2-H4K8ac_peak_22756	7.12754	
4	138529218	1.39E+08	KD2-H4K8ac_peak_22757	8.1233	
4	139812943	1.4E+08	KD2-H4K8ac_peak_22758	6.16888	
4	139832385	1.4E+08	KD2-H4K8ac_peak_22759	7.95563	
4	139836414	1.4E+08	KD2-H4K8ac_peak_22760	11.42902	
4	140004588	1.4E+08	KD2-H4K8ac_peak_22761	9.16531	
4	140099764	1.4E+08	KD2-H4K8ac_peak_22762	7.45501	
4	140223161	1.4E+08	KD2-H4K8ac_peak_22763	8.79106	NDUFC1_ENSG00000109390;NAA15_ENSG00000164134
4	140374445	1.4E+08	KD2-H4K8ac_peak_22764	28.29986	RP11-83A24.2_ENSG00000273247;RAB33B_ENSG00000172007
4	140476643	1.4E+08	KD2-H4K8ac_peak_22765	13.70605	RP11-342I1.2_ENSG00000272717
4	140654650	1.41E+08	KD2-H4K8ac_peak_22766	5.23279	
4	140656643	1.41E+08	KD2-H4K8ac_peak_22767	18.94636	
4	140659166	1.41E+08	KD2-H4K8ac_peak_22768	4.89359	

4	140661322	1.41E+08	KD2-H4K8ac_peak_22769	6.65673	
4	140661911	1.41E+08	KD2-H4K8ac_peak_22770	14.66631	
4	140697621	1.41E+08	KD2-H4K8ac_peak_22771	5.88845	
4	140699580	1.41E+08	KD2-H4K8ac_peak_22772	4.42765	
4	140701848	1.41E+08	KD2-H4K8ac_peak_22773	8.98093	
4	140703647	1.41E+08	KD2-H4K8ac_peak_22774	8.16133	
4	140704519	1.41E+08	KD2-H4K8ac_peak_22775	5.88845	
4	140707852	1.41E+08	KD2-H4K8ac_peak_22776	9.1707	
4	140735469	1.41E+08	KD2-H4K8ac_peak_22777	7.79898	
4	140737238	1.41E+08	KD2-H4K8ac_peak_22778	8.79557	
4	140746287	1.41E+08	KD2-H4K8ac_peak_22779	6.44277	
4	140754277	1.41E+08	KD2-H4K8ac_peak_22780	3.97079	
4	140760464	1.41E+08	KD2-H4K8ac_peak_22781	4.66652	
4	140766164	1.41E+08	KD2-H4K8ac_peak_22782	6.31143	
4	140784672	1.41E+08	KD2-H4K8ac_peak_22783	4.77079	
4	140786967	1.41E+08	KD2-H4K8ac_peak_22784	12.00597	
4	140788945	1.41E+08	KD2-H4K8ac_peak_22785	7.22457	
4	140797704	1.41E+08	KD2-H4K8ac_peak_22786	4.42765	
4	140800928	1.41E+08	KD2-H4K8ac_peak_22787	5.57044	
4	140809282	1.41E+08	KD2-H4K8ac_peak_22788	6.47635	
4	140810164	1.41E+08	KD2-H4K8ac_peak_22789	4.211	
4	140811398	1.41E+08	KD2-H4K8ac_peak_22790	8.55271	
4	140841718	1.41E+08	KD2-H4K8ac_peak_22791	6.9809	
4	140842909	1.41E+08	KD2-H4K8ac_peak_22792	5.835	
4	140846114	1.41E+08	KD2-H4K8ac_peak_22793	6.16888	
4	140850250	1.41E+08	KD2-H4K8ac_peak_22794	6.03693	
4	140854057	1.41E+08	KD2-H4K8ac_peak_22795	8.66387	
4	140857677	1.41E+08	KD2-H4K8ac_peak_22796	6.40683	
4	140862969	1.41E+08	KD2-H4K8ac_peak_22797	9.11031	
4	140864423	1.41E+08	KD2-H4K8ac_peak_22798	8.1233	
4	140876938	1.41E+08	KD2-H4K8ac_peak_22799	7.45501	
4	140925461	1.41E+08	KD2-H4K8ac_peak_22800	9.11031	
4	140926161	1.41E+08	KD2-H4K8ac_peak_22801	5.6755	
4	140974468	1.41E+08	KD2-H4K8ac_peak_22802	6.47635	
4	141016676	1.41E+08	KD2-H4K8ac_peak_22803	5.88845	
4	141056168	1.41E+08	KD2-H4K8ac_peak_22804	4.77079	
4	141056979	1.41E+08	KD2-H4K8ac_peak_22805	7.27716	
4	141071889	1.41E+08	KD2-H4K8ac_peak_22806	23.43329	
4	141073153	1.41E+08	KD2-H4K8ac_peak_22807	3.86308	
4	141073880	1.41E+08	KD2-H4K8ac_peak_22808	6.40297	
4	141075436	1.41E+08	KD2-H4K8ac_peak_22809	13.09607	MAML3_ENSG00000196782
4	141157724	1.41E+08	KD2-H4K8ac_peak_22810	7.27716	
4	141159093	1.41E+08	KD2-H4K8ac_peak_22811	13.80019	
4	141160683	1.41E+08	KD2-H4K8ac_peak_22812	6.56926	
4	141164090	1.41E+08	KD2-H4K8ac_peak_22813	6.66692	
4	141173150	1.41E+08	KD2-H4K8ac_peak_22814	18.40451	

4	141294018	1.41E+08	KD2-H4K8ac_peak_22815	7.01964	RP11-425I13.3_ENSG00000196951
4	141294833	1.41E+08	KD2-H4K8ac_peak_22816	10.65724	RP11-425I13.3_ENSG00000196951
4	141348096	1.41E+08	KD2-H4K8ac_peak_22817	9.73431	CLGN_ENSG00000153132
4	141444827	1.41E+08	KD2-H4K8ac_peak_22818	7.27716	ELMOD2_ENSG00000179387
4	141445355	1.41E+08	KD2-H4K8ac_peak_22819	12.57143	ELMOD2_ENSG00000179387
4	141489786	1.41E+08	KD2-H4K8ac_peak_22820	5.54647	UCP1_ENSG00000109424
4	141530787	1.42E+08	KD2-H4K8ac_peak_22821	11.30637	
4	141609453	1.42E+08	KD2-H4K8ac_peak_22822	7.47462	
4	141677271	1.42E+08	KD2-H4K8ac_peak_22823	8.3261	TBC1D9_ENSG00000109436;RP11-102N12.3_ENSG00000273472
4	141823935	1.42E+08	KD2-H4K8ac_peak_22824	4.42765	
4	141846722	1.42E+08	KD2-H4K8ac_peak_22825	7.70289	
4	141855246	1.42E+08	KD2-H4K8ac_peak_22826	10.68173	
4	142053408	1.42E+08	KD2-H4K8ac_peak_22827	5.13615	
4	142054165	1.42E+08	KD2-H4K8ac_peak_22828	7.76329	
4	142141475	1.42E+08	KD2-H4K8ac_peak_22829	4.06758	ZNF330_ENSG00000109445
4	142557464	1.43E+08	KD2-H4K8ac_peak_22830	7.47462	IL15_ENSG00000164136
4	143043303	1.43E+08	KD2-H4K8ac_peak_22831	5.57044	
4	144106118	1.44E+08	KD2-H4K8ac_peak_22832	9.402	RP11-284M14.1_ENSG00000250326;USP38_ENSG00000170185
4	144256725	1.44E+08	KD2-H4K8ac_peak_22833	14.275	GAB1_ENSG00000109458
4	144257393	1.44E+08	KD2-H4K8ac_peak_22834	8.53092	GAB1_ENSG00000109458
4	144434605	1.44E+08	KD2-H4K8ac_peak_22835	10.29344	SMARCA5_ENSG00000153147;SMARCA5-AS1_ENSG00000245112
4	144444299	1.44E+08	KD2-H4K8ac_peak_22836	6.16888	
4	144480555	1.44E+08	KD2-H4K8ac_peak_22837	13.80019	GUSBP5_ENSG00000236296
4	146019507	1.46E+08	KD2-H4K8ac_peak_22838	7.27716	ANAPC10_ENSG00000164162;ABCE1_ENSG00000164163
4	146101042	1.46E+08	KD2-H4K8ac_peak_22839	14.84212	OTUD4_ENSG00000164164
4	146402971	1.46E+08	KD2-H4K8ac_peak_22840	16.09057	SMAD1_ENSG00000170365
4	146539945	1.47E+08	KD2-H4K8ac_peak_22841	11.86082	MMAA_ENSG00000151611
4	146540665	1.47E+08	KD2-H4K8ac_peak_22842	4.42765	
4	146653950	1.47E+08	KD2-H4K8ac_peak_22843	22.21705	
4	146857237	1.47E+08	KD2-H4K8ac_peak_22844	14.3374	
4	146858391	1.47E+08	KD2-H4K8ac_peak_22845	10.43145	
4	147157705	1.47E+08	KD2-H4K8ac_peak_22846	5.43522	
4	147158929	1.47E+08	KD2-H4K8ac_peak_22847	4.42765	
4	147162755	1.47E+08	KD2-H4K8ac_peak_22848	6.9809	RP11-6L6.7_ENSG00000251010
4	147163437	1.47E+08	KD2-H4K8ac_peak_22849	10.19949	RP11-6L6.7_ENSG00000251010
4	147443675	1.47E+08	KD2-H4K8ac_peak_22850	10.19949	SLC10A7_ENSG00000120519
4	147569082	1.48E+08	KD2-H4K8ac_peak_22851	7.12754	
4	148000917	1.48E+08	KD2-H4K8ac_peak_22852	4.49837	
4	148538677	1.49E+08	KD2-H4K8ac_peak_22853	9.06369	RP11-425A23.1_ENSG00000251298;TMEM184C_ENSG00000164168
4	148643919	1.49E+08	KD2-H4K8ac_peak_22854	5.13341	
4	148652866	1.49E+08	KD2-H4K8ac_peak_22855	7.18615	ARHGAP10_ENSG00000071205
4	148653510	1.49E+08	KD2-H4K8ac_peak_22856	9.41619	ARHGAP10_ENSG00000071205
4	148783099	1.49E+08	KD2-H4K8ac_peak_22857	6.66692	
4	149206516	1.49E+08	KD2-H4K8ac_peak_22858	8.84031	
4	149363874	1.49E+08	KD2-H4K8ac_peak_22859	5.75757	
4	150998325	1.51E+08	KD2-H4K8ac_peak_22860	6.31143	DCLK2_ENSG00000170390

4	150999489	1.51E+08	KD2-H4K8ac_peak_22861	10.21366	DCLK2_ENSG00000170390
4	151000291	1.51E+08	KD2-H4K8ac_peak_22862	4.06758	DCLK2_ENSG00000170390
4	151936598	1.52E+08	KD2-H4K8ac_peak_22863	11.80283	LRBA_ENSG00000198589
4	152019254	1.52E+08	KD2-H4K8ac_peak_22864	5.6755	
4	152019852	1.52E+08	KD2-H4K8ac_peak_22865	9.89126	RPS3A_ENSG00000145425
4	152020861	1.52E+08	KD2-H4K8ac_peak_22866	3.90565	RPS3A_ENSG00000145425
4	152246293	1.52E+08	KD2-H4K8ac_peak_22867	10.77651	SH3D19_ENSG00000109686
4	152329698	1.52E+08	KD2-H4K8ac_peak_22868	9.15069	RP11-610P16.1_ENSG00000251611;FAM160A1_ENSG00000164142
4	152330581	1.52E+08	KD2-H4K8ac_peak_22869	6.0073	RP11-610P16.1_ENSG00000251611;FAM160A1_ENSG00000164142
4	152463158	1.52E+08	KD2-H4K8ac_peak_22870	10.39154	
4	152596143	1.53E+08	KD2-H4K8ac_peak_22871	5.57044	RP11-164P12.3_ENSG00000251455
4	152681470	1.53E+08	KD2-H4K8ac_peak_22872	10.63441	PET112_ENSG00000059691
4	152682400	1.53E+08	KD2-H4K8ac_peak_22873	6.79289	PET112_ENSG00000059691
4	153168881	1.53E+08	KD2-H4K8ac_peak_22874	7.22457	
4	153457617	1.53E+08	KD2-H4K8ac_peak_22875	6.83936	FBXW7_ENSG00000109670;MIR4453_ENSG00000268471
4	153600910	1.54E+08	KD2-H4K8ac_peak_22876	5.54647	TMEM154_ENSG00000170006
4	153700429	1.54E+08	KD2-H4K8ac_peak_22877	13.70753	TIGD4_ENSG00000169989;ARFIP1_ENSG00000164144
4	153701209	1.54E+08	KD2-H4K8ac_peak_22878	10.29344	TIGD4_ENSG00000169989;ARFIP1_ENSG00000164144
4	154004064	1.54E+08	KD2-H4K8ac_peak_22879	8.84031	
4	154048839	1.54E+08	KD2-H4K8ac_peak_22880	7.51935	
4	154049603	1.54E+08	KD2-H4K8ac_peak_22881	10.72656	
4	154060301	1.54E+08	KD2-H4K8ac_peak_22882	3.75035	
4	154063074	1.54E+08	KD2-H4K8ac_peak_22883	8.30974	
4	154063686	1.54E+08	KD2-H4K8ac_peak_22884	11.00314	
4	154064365	1.54E+08	KD2-H4K8ac_peak_22885	12.84642	
4	154067439	1.54E+08	KD2-H4K8ac_peak_22886	3.75035	
4	154072609	1.54E+08	KD2-H4K8ac_peak_22887	6.86699	TRIM2_ENSG00000109654
4	154073207	1.54E+08	KD2-H4K8ac_peak_22888	9.63776	TRIM2_ENSG00000109654
4	154073691	1.54E+08	KD2-H4K8ac_peak_22889	19.33282	TRIM2_ENSG00000109654
4	154077594	1.54E+08	KD2-H4K8ac_peak_22890	6.44277	
4	154143395	1.54E+08	KD2-H4K8ac_peak_22891	7.27716	
4	154144012	1.54E+08	KD2-H4K8ac_peak_22892	11.92978	
4	154170664	1.54E+08	KD2-H4K8ac_peak_22893	7.76329	
4	154182340	1.54E+08	KD2-H4K8ac_peak_22894	7.11925	
4	154240614	1.54E+08	KD2-H4K8ac_peak_22895	4.77079	
4	154386738	1.54E+08	KD2-H4K8ac_peak_22896	6.99815	KIAA0922_ENSG00000121210
4	154605721	1.55E+08	KD2-H4K8ac_peak_22897	6.64891	
4	154680532	1.55E+08	KD2-H4K8ac_peak_22898	17.8868	RNF175_ENSG00000145428
4	156298323	1.56E+08	KD2-H4K8ac_peak_22899	7.12754	MAP9_ENSG00000164114
4	156513222	1.57E+08	KD2-H4K8ac_peak_22900	6.64891	
4	156587913	1.57E+08	KD2-H4K8ac_peak_22901	7.92775	GUCY1A3_ENSG00000164116
4	156680238	1.57E+08	KD2-H4K8ac_peak_22902	12.00273	GUCY1B3_ENSG00000061918
4	156680985	1.57E+08	KD2-H4K8ac_peak_22903	3.90565	GUCY1B3_ENSG00000061918
4	156874497	1.57E+08	KD2-H4K8ac_peak_22904	12.57297	CTSO_ENSG00000256043
4	157211304	1.57E+08	KD2-H4K8ac_peak_22905	6.16888	
4	157997504	1.58E+08	KD2-H4K8ac_peak_22906	5.92591	

4	159220044	1.59E+08	KD2-H4K8ac_peak_22907	4.35346	
4	159644161	1.6E+08	KD2-H4K8ac_peak_22908	4.54006	PPID_ENSG00000171497
4	159644669	1.6E+08	KD2-H4K8ac_peak_22909	17.14886	PPID_ENSG00000171497
4	159689137	1.6E+08	KD2-H4K8ac_peak_22910	10.21502	FNIP2_ENSG00000052795
4	159689604	1.6E+08	KD2-H4K8ac_peak_22911	15.61313	FNIP2_ENSG00000052795
4	159745666	1.6E+08	KD2-H4K8ac_peak_22912	4.211	
4	159791551	1.6E+08	KD2-H4K8ac_peak_22913	6.40297	
4	160023637	1.6E+08	KD2-H4K8ac_peak_22914	8.2413	
4	160024320	1.6E+08	KD2-H4K8ac_peak_22915	5.01002	RAPGEF2_ENSG00000109756
4	160423155	1.6E+08	KD2-H4K8ac_peak_22916	5.40638	
4	160427300	1.6E+08	KD2-H4K8ac_peak_22917	5.57044	U3_ENSG00000251979
4	164087619	1.64E+08	KD2-H4K8ac_peak_22918	8.79106	NAF1_ENSG00000145414
4	164088198	1.64E+08	KD2-H4K8ac_peak_22919	10.05606	NAF1_ENSG00000145414
4	164414798	1.64E+08	KD2-H4K8ac_peak_22920	4.37708	TMA16_ENSG00000198498
4	164415323	1.64E+08	KD2-H4K8ac_peak_22921	14.8083	TMA16_ENSG00000198498
4	165877961	1.66E+08	KD2-H4K8ac_peak_22922	6.40683	FAM218A_ENSG00000250486
4	166128204	1.66E+08	KD2-H4K8ac_peak_22923	11.05407	TMEM192_ENSG00000170088;KLHL2_ENSG00000109466
4	166248865	1.66E+08	KD2-H4K8ac_peak_22924	15.039	MSMO1_ENSG00000052802
4	169239566	1.69E+08	KD2-H4K8ac_peak_22925	12.84642	DDX60_ENSG00000137628
4	169401253	1.69E+08	KD2-H4K8ac_peak_22926	17.43705	
4	169555307	1.7E+08	KD2-H4K8ac_peak_22927	4.7849	
4	169563592	1.7E+08	KD2-H4K8ac_peak_22928	8.59579	
4	169569268	1.7E+08	KD2-H4K8ac_peak_22929	5.40638	RP11-610J23.1_ENSG00000249623
4	169579517	1.7E+08	KD2-H4K8ac_peak_22930	5.64449	
4	169752990	1.7E+08	KD2-H4K8ac_peak_22931	12.00273	RP11-635L1.3_ENSG00000249609
4	169930557	1.7E+08	KD2-H4K8ac_peak_22932	6.90231	CBR4_ENSG00000145439;RP11-483A20.3_ENSG00000251445
4	169936470	1.7E+08	KD2-H4K8ac_peak_22933	7.55771	
4	169959916	1.7E+08	KD2-H4K8ac_peak_22934	5.01262	
4	170006571	1.7E+08	KD2-H4K8ac_peak_22935	6.51023	
4	170191810	1.7E+08	KD2-H4K8ac_peak_22936	16.57619	SH3RF1_ENSG00000154447
4	170533405	1.71E+08	KD2-H4K8ac_peak_22937	5.9696	NEK1_ENSG00000137601;CLCN3_ENSG00000109572
4	170713176	1.71E+08	KD2-H4K8ac_peak_22938	9.73431	PTGES3P3_ENSG00000229204
4	170728475	1.71E+08	KD2-H4K8ac_peak_22939	5.57044	
4	170947214	1.71E+08	KD2-H4K8ac_peak_22940	11.77304	
4	170947729	1.71E+08	KD2-H4K8ac_peak_22941	10.96455	
4	171010953	1.71E+08	KD2-H4K8ac_peak_22942	9.402	
4	174089986	1.74E+08	KD2-H4K8ac_peak_22943	16.73922	RP11-10K16.1_ENSG00000245213;GALNT7_ENSG00000109586
4	174255042	1.74E+08	KD2-H4K8ac_peak_22944	16.32216	HMGB2_ENSG00000164104
4	174255978	1.74E+08	KD2-H4K8ac_peak_22945	8.85813	HMGB2_ENSG00000164104
4	174411922	1.74E+08	KD2-H4K8ac_peak_22946	8.17902	
4	174427875	1.74E+08	KD2-H4K8ac_peak_22947	10.17341	
4	174429631	1.74E+08	KD2-H4K8ac_peak_22948	8.95692	
4	174430413	1.74E+08	KD2-H4K8ac_peak_22949	9.91109	
4	174431531	1.74E+08	KD2-H4K8ac_peak_22950	4.52804	
4	174432133	1.74E+08	KD2-H4K8ac_peak_22951	7.24717	
4	174437751	1.74E+08	KD2-H4K8ac_peak_22952	7.36799	

4	174439042	1.74E+08	KD2-H4K8ac_peak_22953	9.1707	
4	174439644	1.74E+08	KD2-H4K8ac_peak_22954	11.52628	
4	174442801	1.74E+08	KD2-H4K8ac_peak_22955	6.65673	
4	174443771	1.74E+08	KD2-H4K8ac_peak_22956	5.61067	
4	174444585	1.74E+08	KD2-H4K8ac_peak_22957	6.67092	
4	174446459	1.74E+08	KD2-H4K8ac_peak_22958	6.9039	
4	174450710	1.74E+08	KD2-H4K8ac_peak_22959	5.13615	HAND2_ENSG00000164107
4	174451760	1.74E+08	KD2-H4K8ac_peak_22960	6.76298	HAND2_ENSG00000164107
4	174452395	1.74E+08	KD2-H4K8ac_peak_22961	16.95032	
4	174453987	1.74E+08	KD2-H4K8ac_peak_22962	7.09076	
4	174458826	1.74E+08	KD2-H4K8ac_peak_22963	14.66631	
4	174857082	1.75E+08	KD2-H4K8ac_peak_22964	4.59928	
4	174910018	1.75E+08	KD2-H4K8ac_peak_22965	8.17902	
4	174925959	1.75E+08	KD2-H4K8ac_peak_22966	6.03693	
4	174929234	1.75E+08	KD2-H4K8ac_peak_22967	5.835	
4	174931835	1.75E+08	KD2-H4K8ac_peak_22968	10.21502	
4	174986391	1.75E+08	KD2-H4K8ac_peak_22969	4.62142	
4	175055514	1.75E+08	KD2-H4K8ac_peak_22970	7.12754	
4	175073580	1.75E+08	KD2-H4K8ac_peak_22971	4.04247	
4	175138163	1.75E+08	KD2-H4K8ac_peak_22972	11.1952	
4	175204928	1.75E+08	KD2-H4K8ac_peak_22973	4.44527	FBXO8_ENSG00000164117;CEP44_ENSG00000164118
4	176986594	1.77E+08	KD2-H4K8ac_peak_22974	11.15509	WDR17_ENSG00000150627
4	176987162	1.77E+08	KD2-H4K8ac_peak_22975	4.42765	WDR17_ENSG00000150627
4	177241318	1.77E+08	KD2-H4K8ac_peak_22976	4.52514	RP11-87F15.2_ENSG00000248980;SPCS3_ENSG00000129128
4	178363336	1.78E+08	KD2-H4K8ac_peak_22977	8.16906	AGA_ENSG00000038002
4	178364127	1.78E+08	KD2-H4K8ac_peak_22978	9.15069	AGA_ENSG00000038002
4	181881468	1.82E+08	KD2-H4K8ac_peak_22979	6.40683	
4	182887605	1.83E+08	KD2-H4K8ac_peak_22980	6.40683	
4	183012499	1.83E+08	KD2-H4K8ac_peak_22981	10.17698	
4	183065405	1.83E+08	KD2-H4K8ac_peak_22982	19.3524	AC108142.1_ENSG00000177822;TENM3_ENSG00000218336;RP11-402C9.1_ENSG00000248266
4	183369249	1.83E+08	KD2-H4K8ac_peak_22983	10.43145	
4	183727484	1.84E+08	KD2-H4K8ac_peak_22984	7.07117	
4	183799849	1.84E+08	KD2-H4K8ac_peak_22985	4.06758	
4	183801354	1.84E+08	KD2-H4K8ac_peak_22986	5.45396	
4	183802047	1.84E+08	KD2-H4K8ac_peak_22987	6.2781	RP11-188P17.2_ENSG00000272646
4	183837630	1.84E+08	KD2-H4K8ac_peak_22988	16.57619	DCTD_ENSG00000129187
4	183838673	1.84E+08	KD2-H4K8ac_peak_22989	13.44374	DCTD_ENSG00000129187
4	184019395	1.84E+08	KD2-H4K8ac_peak_22990	11.92978	WWC2-AS2_ENSG00000251359;WWC2_ENSG00000151718
4	184062873	1.84E+08	KD2-H4K8ac_peak_22991	4.57371	
4	184319035	1.84E+08	KD2-H4K8ac_peak_22992	26.15943	
4	184365036	1.84E+08	KD2-H4K8ac_peak_22993	13.81781	CDKN2AIP_ENSG00000168564
4	184366018	1.84E+08	KD2-H4K8ac_peak_22994	6.67092	CDKN2AIP_ENSG00000168564
4	184426845	1.84E+08	KD2-H4K8ac_peak_22995	13.88933	ING2_ENSG00000168556
4	184579814	1.85E+08	KD2-H4K8ac_peak_22996	15.59774	RWDD4_ENSG00000182552;TRAPPC11_ENSG00000168538
4	184643374	1.85E+08	KD2-H4K8ac_peak_22997	11.89502	
4	184719186	1.85E+08	KD2-H4K8ac_peak_22998	6.30229	

4	184797741	1.85E+08	KD2-H4K8ac_peak_22999	6.90231	
4	184827827	1.85E+08	KD2-H4K8ac_peak_23000	11.86082	
4	184986168	1.85E+08	KD2-H4K8ac_peak_23001	6.1846	
4	184987185	1.85E+08	KD2-H4K8ac_peak_23002	6.31143	
4	184988746	1.85E+08	KD2-H4K8ac_peak_23003	4.67342	
4	185015737	1.85E+08	KD2-H4K8ac_peak_23004	5.08628	
4	185018355	1.85E+08	KD2-H4K8ac_peak_23005	5.87879	
4	185184911	1.85E+08	KD2-H4K8ac_peak_23006	9.97423	
4	185264922	1.85E+08	KD2-H4K8ac_peak_23007	4.06758	
4	185373329	1.85E+08	KD2-H4K8ac_peak_23008	3.96965	
4	185393892	1.85E+08	KD2-H4K8ac_peak_23009	15.20126	IRF2_ENSG00000168310;RP11-326I11.5_ENSG00000270426
4	185395990	1.85E+08	KD2-H4K8ac_peak_23010	9.73431	IRF2_ENSG00000168310;RP11-326I11.3_ENSG00000271646
4	185458437	1.85E+08	KD2-H4K8ac_peak_23011	7.47462	RP11-326I11.4_ENSG00000271538
4	185570000	1.86E+08	KD2-H4K8ac_peak_23012	5.30799	CASP3_ENSG00000164305;PRIMPOL_ENSG00000164306
4	185570849	1.86E+08	KD2-H4K8ac_peak_23013	10.0692	CASP3_ENSG00000164305;PRIMPOL_ENSG00000164306
4	185669576	1.86E+08	KD2-H4K8ac_peak_23014	4.57371	
4	185705733	1.86E+08	KD2-H4K8ac_peak_23015	5.14125	
4	185707070	1.86E+08	KD2-H4K8ac_peak_23016	6.65673	
4	185746487	1.86E+08	KD2-H4K8ac_peak_23017	5.61067	
4	185747398	1.86E+08	KD2-H4K8ac_peak_23018	6.98244	ACSL1_ENSG00000151726
4	185775223	1.86E+08	KD2-H4K8ac_peak_23019	5.15777	
4	186048211	1.86E+08	KD2-H4K8ac_peak_23020	9.63487	
4	186063880	1.86E+08	KD2-H4K8ac_peak_23021	7.96322	SLC25A4_ENSG00000151729
4	186070752	1.86E+08	KD2-H4K8ac_peak_23022	6.4095	
4	186124613	1.86E+08	KD2-H4K8ac_peak_23023	8.85813	SNX25_ENSG00000109762
4	186130883	1.86E+08	KD2-H4K8ac_peak_23024	6.25869	KIAA1430_ENSG00000164323
4	186266768	1.86E+08	KD2-H4K8ac_peak_23025	5.3778	
4	186317170	1.86E+08	KD2-H4K8ac_peak_23026	8.85813	LRP2BP_ENSG00000109771;ANKRD37_ENSG00000186352
4	186347233	1.86E+08	KD2-H4K8ac_peak_23027	5.75757	UFSP2_ENSG00000109775;C4orf47_ENSG00000205129
4	186392732	1.86E+08	KD2-H4K8ac_peak_23028	4.54006	CCDC110_ENSG00000168491;RP11-279O9.4_ENSG00000249679
4	186456227	1.86E+08	KD2-H4K8ac_peak_23029	12.57828	PDLIM3_ENSG00000154553
4	186494796	1.86E+08	KD2-H4K8ac_peak_23030	10.05606	
4	186559869	1.87E+08	KD2-H4K8ac_peak_23031	8.17902	
4	186732138	1.87E+08	KD2-H4K8ac_peak_23032	10.0746	
4	186768825	1.87E+08	KD2-H4K8ac_peak_23033	6.25869	
4	186831563	1.87E+08	KD2-H4K8ac_peak_23034	7.96194	
4	186877287	1.87E+08	KD2-H4K8ac_peak_23035	5.9696	SORBS2_ENSG00000154556
4	186942674	1.87E+08	KD2-H4K8ac_peak_23036	13.56967	
4	186951251	1.87E+08	KD2-H4K8ac_peak_23037	11.24702	
4	187025976	1.87E+08	KD2-H4K8ac_peak_23038	10.53377	FAM149A_ENSG00000109794
4	187066209	1.87E+08	KD2-H4K8ac_peak_23039	7.76329	
4	187111846	1.87E+08	KD2-H4K8ac_peak_23040	16.57619	AC110771.1_ENSG00000269302;CYP4V2_ENSG00000145476
4	187476576	1.87E+08	KD2-H4K8ac_peak_23041	4.42765	RP11-215A19.2_ENSG00000272297;MTNR1A_ENSG00000168412
4	187644494	1.88E+08	KD2-H4K8ac_peak_23042	10.21502	
4	187647594	1.88E+08	KD2-H4K8ac_peak_23043	5.88845	FAT1_ENSG00000083857
4	190224447	1.9E+08	KD2-H4K8ac_peak_23044	6.40683	

4	190504346	1.91E+08	KD2-H4K8ac_peak_23045	7.55771	
4	190508935	1.91E+08	KD2-H4K8ac_peak_23046	7.01964	
4	190520523	1.91E+08	KD2-H4K8ac_peak_23047	9.11818	
4	190521245	1.91E+08	KD2-H4K8ac_peak_23048	16.57619	
4	190522359	1.91E+08	KD2-H4K8ac_peak_23049	5.10276	
4	190522953	1.91E+08	KD2-H4K8ac_peak_23050	9.16531	
4	190524368	1.91E+08	KD2-H4K8ac_peak_23051	7.12754	
4	190825891	1.91E+08	KD2-H4K8ac_peak_23052	6.64891	
4	190861416	1.91E+08	KD2-H4K8ac_peak_23053	20.02161	AF146191.4_ENSG00000245685;FRG1_ENSG00000109536
4	190862128	1.91E+08	KD2-H4K8ac_peak_23054	7.76329	AF146191.4_ENSG00000245685;FRG1_ENSG00000109536
5	53773	54206	KD2-H4K8ac_peak_23055	7.12754	
5	90630	92054	KD2-H4K8ac_peak_23056	24.47492	CTD-2231H16.1_ENSG00000249430
5	218471	219023	KD2-H4K8ac_peak_23057	15.81711	CCDC127_ENSG00000164366;SDHA_ENSG00000073578
5	271240	271470	KD2-H4K8ac_peak_23058	9.34195	CTD-2083E4.6_ENSG00000248925;PDCD6_ENSG00000249915
5	316160	316357	KD2-H4K8ac_peak_23059	4.42765	
5	344199	344579	KD2-H4K8ac_peak_23060	18.5703	
5	373375	374646	KD2-H4K8ac_peak_23061	16.32216	
5	442224	442609	KD2-H4K8ac_peak_23062	6.04107	C5orf55_ENSG00000221990;EXOC3_ENSG00000180104
5	442861	443158	KD2-H4K8ac_peak_23063	5.75757	C5orf55_ENSG00000221990;EXOC3_ENSG00000180104
5	443471	443820	KD2-H4K8ac_peak_23064	7.167	C5orf55_ENSG00000221990;EXOC3_ENSG00000180104
5	472504	473120	KD2-H4K8ac_peak_23065	17.19888	CTD-2228K2.5_ENSG00000188242;CTD-2228K2.7_ENSG00000225138
5	473418	473659	KD2-H4K8ac_peak_23066	6.65673	CTD-2228K2.5_ENSG00000188242;CTD-2228K2.7_ENSG00000225138
5	612018	612260	KD2-H4K8ac_peak_23067	13.31016	RP11-310P5.1_ENSG00000249650;CEP72_ENSG00000112877
5	612626	612938	KD2-H4K8ac_peak_23068	10.96455	RP11-310P5.1_ENSG00000249650;CEP72_ENSG00000112877
5	693636	693968	KD2-H4K8ac_peak_23069	15.9336	TPPP_ENSG00000171368
5	785008	785591	KD2-H4K8ac_peak_23070	15.66774	
5	850378	850590	KD2-H4K8ac_peak_23071	6.99815	ZDHHC11_ENSG00000188818
5	892430	892896	KD2-H4K8ac_peak_23072	11.92978	BRD9_ENSG00000028310;TRIP13_ENSG00000071539
5	893323	893532	KD2-H4K8ac_peak_23073	9.402	BRD9_ENSG00000028310;TRIP13_ENSG00000071539
5	947006	947319	KD2-H4K8ac_peak_23074	8.02571	RP11-661C8.2_ENSG00000248949
5	947518	947711	KD2-H4K8ac_peak_23075	6.66692	
5	1295592	1295794	KD2-H4K8ac_peak_23076	5.88845	TERT_ENSG00000164362
5	1344586	1344782	KD2-H4K8ac_peak_23077	6.05809	CLPTM1L_ENSG00000049656
5	1345316	1345615	KD2-H4K8ac_peak_23078	11.09936	CLPTM1L_ENSG00000049656
5	1386295	1386538	KD2-H4K8ac_peak_23079	9.36665	
5	1467758	1468153	KD2-H4K8ac_peak_23080	4.92515	
5	1523727	1525065	KD2-H4K8ac_peak_23081	28.94181	LPCAT1_ENSG00000153395
5	1594772	1595225	KD2-H4K8ac_peak_23082	19.55721	SDHAP3_ENSG00000185986;CTD-2012J19.3_ENSG00000271119
5	1633553	1634928	KD2-H4K8ac_peak_23083	12.36441	RP11-43F13.1_ENSG00000188002
5	1799467	1799740	KD2-H4K8ac_peak_23084	4.67342	
5	1800359	1800565	KD2-H4K8ac_peak_23085	4.37815	MRPL36_ENSG00000171421;NDUFS6_ENSG00000145494
5	1801594	1801848	KD2-H4K8ac_peak_23086	12.93209	MRPL36_ENSG00000171421;NDUFS6_ENSG00000145494
5	5026282	5026711	KD2-H4K8ac_peak_23087	7.76329	
5	5270966	5271415	KD2-H4K8ac_peak_23088	6.2781	
5	5272721	5272915	KD2-H4K8ac_peak_23089	7.12754	
5	5273212	5273493	KD2-H4K8ac_peak_23090	6.16888	

5	5299201	5299514	KD2-H4K8ac_peak_23091	4.66652	
5	5318139	5318465	KD2-H4K8ac_peak_23092	8.17902	
5	5422534	5423428	KD2-H4K8ac_peak_23093	11.22681	
5	5512150	5512388	KD2-H4K8ac_peak_23094	8.17902	
5	5712310	5713513	KD2-H4K8ac_peak_23095	9.63487	
5	6312385	6312646	KD2-H4K8ac_peak_23096	6.31143	
5	6360473	6360751	KD2-H4K8ac_peak_23097	9.23578	
5	6378843	6379742	KD2-H4K8ac_peak_23098	15.5861	MED10_ENSG00000133398
5	6632926	6633338	KD2-H4K8ac_peak_23099	5.64429	NSUN2_ENSG00000037474;SRD5A1_ENSG00000145545
5	6633844	6634267	KD2-H4K8ac_peak_23100	3.90092	NSUN2_ENSG00000037474;SRD5A1_ENSG00000145545
5	6704815	6705036	KD2-H4K8ac_peak_23101	6.66692	
5	6712248	6712972	KD2-H4K8ac_peak_23102	8.85813	
5	6766215	6766542	KD2-H4K8ac_peak_23103	9.06369	RP11-332J15.3_ENSG00000251365
5	6772812	6773536	KD2-H4K8ac_peak_23104	11.05407	
5	7851015	7851545	KD2-H4K8ac_peak_23105	11.05407	C5orf49_ENSG00000215217;MTRR_ENSG00000124275
5	7868753	7869039	KD2-H4K8ac_peak_23106	4.86324	FASTKD3_ENSG00000124279
5	7869388	7869764	KD2-H4K8ac_peak_23107	6.97919	FASTKD3_ENSG00000124279
5	8457894	8458209	KD2-H4K8ac_peak_23108	8.4716	RP11-480D4.1_ENSG00000245729;RP11-480D4.2_ENSG00000249159
5	9954095	9954293	KD2-H4K8ac_peak_23109	6.8836	
5	10249655	10249926	KD2-H4K8ac_peak_23110	18.41507	FAM173B_ENSG00000150756;CCT5_ENSG00000150753
5	10250571	10250990	KD2-H4K8ac_peak_23111	13.12288	FAM173B_ENSG00000150756;CCT5_ENSG00000150753
5	10307597	10308001	KD2-H4K8ac_peak_23112	9.20193	CMBL_ENSG00000164237
5	10352981	10353660	KD2-H4K8ac_peak_23113	14.39547	CTD-2256P15.2_ENSG00000259802;MARCH6_ENSG00000145495
5	10353949	10354282	KD2-H4K8ac_peak_23114	11.50346	CTD-2256P15.2_ENSG00000259802;MARCH6_ENSG00000145495
5	10527558	10528192	KD2-H4K8ac_peak_23115	7.40111	
5	10601755	10602466	KD2-H4K8ac_peak_23116	8.59579	
5	10654955	10655315	KD2-H4K8ac_peak_23117	10.02417	RP11-54F2.1_ENSG00000251196;RP11-215G15.5_ENSG00000272016
5	10662684	10662921	KD2-H4K8ac_peak_23118	6.00226	
5	10760794	10761182	KD2-H4K8ac_peak_23119	9.11031	DAP_ENSG00000112977;CTD-2154B17.4_ENSG00000272324
5	10761636	10761899	KD2-H4K8ac_peak_23120	8.52559	DAP_ENSG00000112977;CTD-2154B17.4_ENSG00000272324
5	12574090	12574577	KD2-H4K8ac_peak_23121	8.94469	RP11-308B16.2_ENSG00000248783;CT49_ENSG00000248131
5	14011236	14011465	KD2-H4K8ac_peak_23122	7.13615	
5	14142795	14143157	KD2-H4K8ac_peak_23123	11.09936	TRIO_ENSG00000038382
5	14250307	14250659	KD2-H4K8ac_peak_23124	13.81781	
5	14410228	14410421	KD2-H4K8ac_peak_23125	7.47462	
5	14416929	14417231	KD2-H4K8ac_peak_23126	15.81711	
5	14417931	14419123	KD2-H4K8ac_peak_23127	16.63953	
5	14441155	14441749	KD2-H4K8ac_peak_23128	6.36375	
5	14448394	14448789	KD2-H4K8ac_peak_23129	6.2781	
5	14581444	14581728	KD2-H4K8ac_peak_23130	6.66692	FAM105A_ENSG00000145569
5	14581959	14582620	KD2-H4K8ac_peak_23131	5.45396	FAM105A_ENSG00000145569
5	14594583	14595019	KD2-H4K8ac_peak_23132	12.2054	
5	14664304	14665181	KD2-H4K8ac_peak_23133	12.06346	CTD-2165H16.4_ENSG00000261360;FAM105B_ENSG00000154124
5	14871440	14871881	KD2-H4K8ac_peak_23134	9.36516	ANKH_ENSG00000154122;CTB-40H15.4_ENSG00000272057
5	14872201	14872670	KD2-H4K8ac_peak_23135	19.6416	ANKH_ENSG00000154122;CTB-40H15.4_ENSG00000272057
5	15500293	15500645	KD2-H4K8ac_peak_23136	9.08816	FBXL7_ENSG00000183580

5	15960645	15961206	KD2-H4K8ac_peak_23137	5.88845	
5	16420211	16420458	KD2-H4K8ac_peak_23138	5.07241	
5	16465153	16465352	KD2-H4K8ac_peak_23139	9.50015	ZNF622_ENSG00000173545
5	16465575	16466387	KD2-H4K8ac_peak_23140	17.91241	ZNF622_ENSG00000173545
5	16568805	16569014	KD2-H4K8ac_peak_23141	7.12754	
5	16616725	16617644	KD2-H4K8ac_peak_23142	13.50024	FAM134B_ENSG00000154153;RP11-260E18.1_ENSG00000246214;CTC-461F20.1_ENSG00000250415
5	16936157	16936405	KD2-H4K8ac_peak_23143	8.52559	MYO10_ENSG00000145555
5	16936602	16936967	KD2-H4K8ac_peak_23144	7.59643	MYO10_ENSG00000145555
5	17217769	17218040	KD2-H4K8ac_peak_23145	8.17902	AC091878.1_ENSG00000215196
5	17218505	17218774	KD2-H4K8ac_peak_23146	5.71749	
5	17219399	17219814	KD2-H4K8ac_peak_23147	6.07178	
5	17380223	17380519	KD2-H4K8ac_peak_23148	4.77079	
5	21481733	21482366	KD2-H4K8ac_peak_23149	11.00597	
5	21482659	21482916	KD2-H4K8ac_peak_23150	14.38886	
5	31682949	31683149	KD2-H4K8ac_peak_23151	6.24747	
5	31700927	31701685	KD2-H4K8ac_peak_23152	10.29344	
5	31702210	31703295	KD2-H4K8ac_peak_23153	18.5461	
5	31758448	31758881	KD2-H4K8ac_peak_23154	6.05809	
5	32173855	32174306	KD2-H4K8ac_peak_23155	7.49599	GOLPH3_ENSG00000113384;CTD-2186M15.3_ENSG00000272086
5	32174537	32174969	KD2-H4K8ac_peak_23156	24.23593	GOLPH3_ENSG00000113384;CTD-2186M15.3_ENSG00000272086
5	32190544	32190772	KD2-H4K8ac_peak_23157	6.79289	
5	32312479	32313000	KD2-H4K8ac_peak_23158	16.8435	MTMR12_ENSG00000150712
5	32444216	32445447	KD2-H4K8ac_peak_23159	9.36516	ZFR_ENSG00000056097
5	32585224	32585423	KD2-H4K8ac_peak_23160	6.2781	
5	32585771	32586087	KD2-H4K8ac_peak_23161	11.92978	
5	33441144	33441376	KD2-H4K8ac_peak_23162	10.87093	TARS_ENSG00000113407;CTD-2203K17.1_ENSG00000249572
5	34007738	34008139	KD2-H4K8ac_peak_23163	10.96455	AMACR_ENSG00000242110
5	34008413	34008606	KD2-H4K8ac_peak_23164	6.40683	AMACR_ENSG00000242110
5	34191461	34193732	KD2-H4K8ac_peak_23165	49.08989	
5	34244986	34245291	KD2-H4K8ac_peak_23166	18.38518	RP11-1023L17.1_ENSG00000215158
5	34656515	34657220	KD2-H4K8ac_peak_23167	7.12754	RAI14_ENSG00000039560;CTD-2024P10.1_ENSG00000250234
5	34838814	34839205	KD2-H4K8ac_peak_23168	8.52559	CTD-2517O10.6_ENSG00000272323;TTC23L_ENSG00000205838
5	34839549	34839745	KD2-H4K8ac_peak_23169	8.30974	CTD-2517O10.6_ENSG00000272323;TTC23L_ENSG00000205838
5	34915869	34916205	KD2-H4K8ac_peak_23170	18.29344	BRIX1_ENSG00000113460
5	34930031	34930374	KD2-H4K8ac_peak_23171	9.92477	DNAJC21_ENSG00000168724
5	35617481	35617795	KD2-H4K8ac_peak_23172	5.54647	SPEF2_ENSG00000152582
5	35618370	35618569	KD2-H4K8ac_peak_23173	6.40683	SPEF2_ENSG00000152582
5	36079644	36079867	KD2-H4K8ac_peak_23174	6.16888	
5	36151588	36151925	KD2-H4K8ac_peak_23175	5.51783	LMBRD2_ENSG00000164187;SKP2_ENSG00000145604
5	36157533	36157740	KD2-H4K8ac_peak_23176	5.57044	
5	36159062	36159569	KD2-H4K8ac_peak_23177	5.15777	
5	36241396	36242170	KD2-H4K8ac_peak_23178	13.13969	NADK2_ENSG00000152620
5	36242842	36243168	KD2-H4K8ac_peak_23179	13.68542	NADK2_ENSG00000152620
5	36690197	36690572	KD2-H4K8ac_peak_23180	9.89126	
5	36876188	36876740	KD2-H4K8ac_peak_23181	10.29842	NIPBL_ENSG00000164190
5	37249099	37249371	KD2-H4K8ac_peak_23182	10.72656	C5orf42_ENSG00000197603

5	37249602	37250206	KD2-H4K8ac_peak_23183	9.20193	C5orf42_ENSG00000197603
5	37379466	37379695	KD2-H4K8ac_peak_23184	6.25869	WDR70_ENSG00000082068
5	38258382	38259405	KD2-H4K8ac_peak_23185	5.88845	EGFLAM_ENSG00000164318
5	38317866	38318088	KD2-H4K8ac_peak_23186	8.79557	
5	38556219	38556564	KD2-H4K8ac_peak_23187	7.59643	LIFR-AS1_ENSG00000244968
5	39073672	39074434	KD2-H4K8ac_peak_23188	10.08707	RICTOR_ENSG00000164327
5	39074682	39075218	KD2-H4K8ac_peak_23189	6.99815	RICTOR_ENSG00000164327
5	40755526	40755868	KD2-H4K8ac_peak_23190	12.93209	TTC33_ENSG00000113638
5	40798573	40798847	KD2-H4K8ac_peak_23191	6.65673	PRKAA1_ENSG00000132356
5	40834949	40835256	KD2-H4K8ac_peak_23192	7.67504	RPL37_ENSG00000145592
5	40835560	40835913	KD2-H4K8ac_peak_23193	12.17278	RPL37_ENSG00000145592
5	41371631	41372007	KD2-H4K8ac_peak_23194	4.70388	
5	41509732	41510226	KD2-H4K8ac_peak_23195	7.22457	PLCXD3_ENSG00000182836
5	41786236	41786429	KD2-H4K8ac_peak_23196	6.40683	
5	41791922	41792218	KD2-H4K8ac_peak_23197	11.70505	
5	41792752	41792998	KD2-H4K8ac_peak_23198	5.43522	
5	41795269	41795467	KD2-H4K8ac_peak_23199	5.57044	
5	41870190	41871069	KD2-H4K8ac_peak_23200	16.25886	OXCT1_ENSG00000083720;OXCT1-AS1_ENSG00000248668
5	41904563	41904818	KD2-H4K8ac_peak_23201	5.54647	C5orf51_ENSG00000205765
5	41924964	41925938	KD2-H4K8ac_peak_23202	24.58052	FBXO4_ENSG00000151876
5	42757476	42757729	KD2-H4K8ac_peak_23203	8.1233	CCDC152_ENSG00000198865
5	43007288	43008672	KD2-H4K8ac_peak_23204	7.07117	CTD-2201E18.5_ENSG00000271788
5	43009614	43010015	KD2-H4K8ac_peak_23205	6.40683	
5	43066047	43066967	KD2-H4K8ac_peak_23206	11.70505	CTD-2201E18.3_ENSG00000177738;ZNF131_ENSG00000172262
5	43067196	43067774	KD2-H4K8ac_peak_23207	14.66631	CTD-2201E18.3_ENSG00000177738
5	43120710	43121511	KD2-H4K8ac_peak_23208	6.65673	
5	43121814	43122097	KD2-H4K8ac_peak_23209	5.57044	
5	43311187	43311396	KD2-H4K8ac_peak_23210	5.80249	
5	43483500	43483919	KD2-H4K8ac_peak_23211	8.17902	C5orf28_ENSG00000151881;RP11-159F24.3_ENSG00000249492
5	43484212	43484515	KD2-H4K8ac_peak_23212	15.06766	C5orf28_ENSG00000151881;RP11-159F24.3_ENSG00000249492
5	43521993	43522607	KD2-H4K8ac_peak_23213	11.30637	
5	43556078	43557139	KD2-H4K8ac_peak_23214	23.22963	PAIP1_ENSG00000172239
5	43557398	43558015	KD2-H4K8ac_peak_23215	8.23274	PAIP1_ENSG00000172239
5	43602746	43603574	KD2-H4K8ac_peak_23216	11.05056	NNT-AS1_ENSG00000248092;NNT_ENSG00000112992
5	44809101	44809746	KD2-H4K8ac_peak_23217	9.11031	RP11-53O19.1_ENSG00000251141;MRPS30_ENSG00000112996
5	49736865	49737468	KD2-H4K8ac_peak_23218	5.13615	
5	49962273	49962566	KD2-H4K8ac_peak_23219	6.40683	PARP8_ENSG00000151883
5	49963017	49963875	KD2-H4K8ac_peak_23220	13.73635	
5	50259042	50259567	KD2-H4K8ac_peak_23221	6.99815	
5	50259834	50260166	KD2-H4K8ac_peak_23222	11.00314	
5	50262121	50262980	KD2-H4K8ac_peak_23223	9.11031	CTD-2089N3.1_ENSG00000250360
5	50264334	50264856	KD2-H4K8ac_peak_23224	7.01494	CTD-2089N3.3_ENSG00000248918
5	50265077	50265902	KD2-H4K8ac_peak_23225	18.94636	CTD-2089N3.2_ENSG00000251573;CTD-2089N3.3_ENSG00000248918
5	50480981	50481198	KD2-H4K8ac_peak_23226	5.16439	
5	50575725	50575951	KD2-H4K8ac_peak_23227	5.25071	
5	50673180	50673586	KD2-H4K8ac_peak_23228	6.16888	

5	50674135	50674449	KD2-H4K8ac_peak_23229	4.67342	
5	50678399	50679040	KD2-H4K8ac_peak_23230	11.28831	CTD-2314G24.2_ENSG00000259663;ISL1_ENSG00000016082
5	50679622	50679856	KD2-H4K8ac_peak_23231	6.06203	CTD-2314G24.2_ENSG00000259663;ISL1_ENSG00000016082
5	50683187	50683507	KD2-H4K8ac_peak_23232	6.65673	
5	50684610	50685049	KD2-H4K8ac_peak_23233	7.18916	
5	50685417	50685730	KD2-H4K8ac_peak_23234	13.44374	
5	50686193	50686395	KD2-H4K8ac_peak_23235	12.17278	
5	50694659	50694894	KD2-H4K8ac_peak_23236	8.30974	
5	50764233	50764677	KD2-H4K8ac_peak_23237	7.01964	
5	51282176	51282467	KD2-H4K8ac_peak_23238	9.02371	
5	51283526	51283943	KD2-H4K8ac_peak_23239	7.47462	
5	51284370	51284929	KD2-H4K8ac_peak_23240	9.59147	
5	51285540	51286171	KD2-H4K8ac_peak_23241	7.12754	
5	51287798	51288000	KD2-H4K8ac_peak_23242	5.86783	
5	51288308	51288596	KD2-H4K8ac_peak_23243	13.57066	
5	51291008	51291333	KD2-H4K8ac_peak_23244	5.43522	
5	51292091	51292322	KD2-H4K8ac_peak_23245	7.45501	
5	51294136	51294411	KD2-H4K8ac_peak_23246	5.57044	
5	51296081	51296422	KD2-H4K8ac_peak_23247	9.15329	
5	51654280	51654534	KD2-H4K8ac_peak_23248	9.15329	
5	51859859	51860309	KD2-H4K8ac_peak_23249	4.42765	
5	52096177	52096685	KD2-H4K8ac_peak_23250	7.27716	
5	52285229	52285579	KD2-H4K8ac_peak_23251	13.92307	CTD-2175A23.1_ENSG00000249899;ITGA2_ENSG00000164171
5	52405799	52406186	KD2-H4K8ac_peak_23252	5.01002	MOCS2_ENSG00000164172;CTD-2366F13.1_ENSG00000247796
5	53606028	53606247	KD2-H4K8ac_peak_23253	4.42765	ARL15_ENSG00000185305
5	53606611	53606928	KD2-H4K8ac_peak_23254	6.25869	ARL15_ENSG00000185305
5	53813650	53814248	KD2-H4K8ac_peak_23255	10.19949	SNX18_ENSG00000178996
5	54603837	54604073	KD2-H4K8ac_peak_23256	9.65086	DHX29_ENSG00000067248;SKIV2L2_ENSG00000039123
5	54830278	54830818	KD2-H4K8ac_peak_23257	13.77586	PPAP2A_ENSG00000067113
5	55007846	55008118	KD2-H4K8ac_peak_23258	9.65086	
5	55117662	55117855	KD2-H4K8ac_peak_23259	4.33139	
5	55290477	55290737	KD2-H4K8ac_peak_23260	9.95119	IL6ST_ENSG00000134352;CTD-2031P19.3_ENSG00000227908
5	55310534	55310816	KD2-H4K8ac_peak_23261	4.01115	
5	55567101	55567406	KD2-H4K8ac_peak_23262	5.54647	
5	55569297	55569565	KD2-H4K8ac_peak_23263	8.17902	
5	55776615	55778135	KD2-H4K8ac_peak_23264	8.4716	CTC-236F12.4_ENSG00000248727
5	55790244	55790490	KD2-H4K8ac_peak_23265	8.17902	
5	56012330	56012565	KD2-H4K8ac_peak_23266	4.35346	
5	56058842	56059565	KD2-H4K8ac_peak_23267	9.82729	
5	56060411	56060671	KD2-H4K8ac_peak_23268	4.42765	
5	56110327	56110893	KD2-H4K8ac_peak_23269	4.86324	MAP3K1_ENSG00000095015
5	56111375	56112935	KD2-H4K8ac_peak_23270	16.8435	MAP3K1_ENSG00000095015
5	56131998	56132194	KD2-H4K8ac_peak_23271	4.01115	
5	56205015	56205586	KD2-H4K8ac_peak_23272	17.55491	AC008937.3_ENSG00000225230;SETD9_ENSG00000155542
5	56205937	56206332	KD2-H4K8ac_peak_23273	7.47462	AC008937.3_ENSG00000225230;SETD9_ENSG00000155542
5	56247387	56247898	KD2-H4K8ac_peak_23274	20.53952	

5	56248147	56248581	KD2-H4K8ac_peak_23275	9.63487	
5	56407856	56408153	KD2-H4K8ac_peak_23276	8.1233	
5	56469015	56469312	KD2-H4K8ac_peak_23277	9.72244	GPBP1_ENSG00000062194
5	56469734	56470522	KD2-H4K8ac_peak_23278	7.36799	GPBP1_ENSG00000062194
5	57373013	57373208	KD2-H4K8ac_peak_23279	5.25071	
5	57756067	57756326	KD2-H4K8ac_peak_23280	6.60096	PLK2_ENSG00000145632
5	57877827	57878316	KD2-H4K8ac_peak_23281	6.25869	RAB3C_ENSG00000152932
5	57962216	57962502	KD2-H4K8ac_peak_23282	5.25071	
5	58187294	58187717	KD2-H4K8ac_peak_23283	6.27931	
5	58422620	58423237	KD2-H4K8ac_peak_23284	7.27716	
5	58505131	58505342	KD2-H4K8ac_peak_23285	5.73839	
5	58894436	58894718	KD2-H4K8ac_peak_23286	6.66692	
5	58955726	58955918	KD2-H4K8ac_peak_23287	9.15329	
5	58957207	58957716	KD2-H4K8ac_peak_23288	6.14257	
5	59995383	59995907	KD2-H4K8ac_peak_23289	10.21366	DEPDC1B_ENSG00000035499
5	59996104	59996349	KD2-H4K8ac_peak_23290	11.24702	DEPDC1B_ENSG00000035499
5	60325491	60325740	KD2-H4K8ac_peak_23291	5.73839	
5	60457820	60458021	KD2-H4K8ac_peak_23292	4.42765	SMIM15_ENSG00000188725;CTC-436P18.1_ENSG00000251279
5	60458437	60458908	KD2-H4K8ac_peak_23293	17.17257	SMIM15_ENSG00000188725;CTC-436P18.1_ENSG00000251279
5	60570956	60571179	KD2-H4K8ac_peak_23294	8.66387	
5	60626683	60626952	KD2-H4K8ac_peak_23295	13.13969	
5	60627196	60627462	KD2-H4K8ac_peak_23296	4.06758	ZSWIM6_ENSG00000130449
5	60629085	60629362	KD2-H4K8ac_peak_23297	5.14125	ZSWIM6_ENSG00000130449
5	61601238	61601798	KD2-H4K8ac_peak_23298	13.9234	KIF2A_ENSG00000068796
5	61602055	61602681	KD2-H4K8ac_peak_23299	6.99815	KIF2A_ENSG00000068796
5	61700092	61700380	KD2-H4K8ac_peak_23300	5.61067	DIMT1_ENSG00000086189;IPO11_ENSG00000086200
5	61708673	61708980	KD2-H4K8ac_peak_23301	4.85908	
5	64064658	64065139	KD2-H4K8ac_peak_23302	4.8888	SREK1IP1_ENSG00000153006;CWC27_ENSG00000153015
5	64330751	64331955	KD2-H4K8ac_peak_23303	9.89126	
5	64357491	64357730	KD2-H4K8ac_peak_23304	8.17902	
5	64398571	64399312	KD2-H4K8ac_peak_23305	7.76329	
5	64778858	64779124	KD2-H4K8ac_peak_23306	7.79558	
5	64919917	64920392	KD2-H4K8ac_peak_23307	10.72656	TRAPPC13_ENSG00000113597;CTC-534A2.2_ENSG00000253251
5	65017587	65017860	KD2-H4K8ac_peak_23308	8.66387	NLN_ENSG00000123213
5	65220780	65221366	KD2-H4K8ac_peak_23309	12.08081	CTD-2033C11.1_ENSG00000269961;ERBB2IP_ENSG00000112851
5	65221665	65222135	KD2-H4K8ac_peak_23310	17.67328	CTD-2033C11.1_ENSG00000269961;ERBB2IP_ENSG00000112851
5	65440126	65440361	KD2-H4K8ac_peak_23311	8.58725	AC025442.3_ENSG00000253744
5	66299645	66299945	KD2-H4K8ac_peak_23312	6.40297	MAST4-AS1_ENSG00000229666
5	66300675	66301150	KD2-H4K8ac_peak_23313	9.15329	MAST4-AS1_ENSG00000229666
5	66497807	66498001	KD2-H4K8ac_peak_23314	9.23578	
5	66498743	66499034	KD2-H4K8ac_peak_23315	3.96965	
5	66499253	66499709	KD2-H4K8ac_peak_23316	9.41046	
5	66500667	66500987	KD2-H4K8ac_peak_23317	4.98072	
5	66511407	66512024	KD2-H4K8ac_peak_23318	4.06758	
5	66563207	66564040	KD2-H4K8ac_peak_23319	12.08081	CTD-2187J20.1_ENSG00000251206
5	66564820	66565654	KD2-H4K8ac_peak_23320	4.44527	CTD-2187J20.1_ENSG00000251206

5	67294656	67294884	KD2-H4K8ac_peak_23321	4.77079	
5	67316274	67316542	KD2-H4K8ac_peak_23322	6.64891	
5	67510944	67511393	KD2-H4K8ac_peak_23323	9.11031	PIK3R1_ENSG00000145675
5	67533813	67534017	KD2-H4K8ac_peak_23324	6.16888	
5	67545877	67546186	KD2-H4K8ac_peak_23325	12.06346	
5	67552560	67553191	KD2-H4K8ac_peak_23326	6.50927	
5	67555314	67555507	KD2-H4K8ac_peak_23327	5.88562	
5	67583862	67584230	KD2-H4K8ac_peak_23328	5.71749	
5	67584846	67585100	KD2-H4K8ac_peak_23329	6.40683	
5	67702566	67702849	KD2-H4K8ac_peak_23330	7.12754	
5	67724219	67724468	KD2-H4K8ac_peak_23331	4.37708	
5	67829313	67829567	KD2-H4K8ac_peak_23332	10.21502	CTC-537E7.2_ENSG00000248359
5	67830274	67830628	KD2-H4K8ac_peak_23333	7.07117	CTC-537E7.2_ENSG00000248359
5	67834437	67834675	KD2-H4K8ac_peak_23334	6.00226	
5	67857146	67857426	KD2-H4K8ac_peak_23335	9.15329	
5	67952943	67953234	KD2-H4K8ac_peak_23336	5.43522	
5	68170798	68171010	KD2-H4K8ac_peak_23337	6.66692	
5	68253274	68253514	KD2-H4K8ac_peak_23338	4.77079	
5	68291200	68291494	KD2-H4K8ac_peak_23339	4.42765	
5	68327356	68327639	KD2-H4K8ac_peak_23340	7.27716	
5	68331175	68331429	KD2-H4K8ac_peak_23341	6.00243	
5	68332505	68332737	KD2-H4K8ac_peak_23342	10.72656	
5	68389210	68389535	KD2-H4K8ac_peak_23343	5.88845	SLC30A5_ENSG00000145740
5	68389881	68390420	KD2-H4K8ac_peak_23344	14.39547	SLC30A5_ENSG00000145740
5	68485427	68485626	KD2-H4K8ac_peak_23345	11.24702	CENPH_ENSG00000153044
5	68513690	68513961	KD2-H4K8ac_peak_23346	12.23	MRPS36_ENSG00000134056
5	68628302	68629398	KD2-H4K8ac_peak_23347	14.56955	CCDC125_ENSG00000183323;CHCHD2P2_ENSG00000215006
5	68665192	68665891	KD2-H4K8ac_peak_23348	8.93954	TAF9_ENSG00000085231;RAD17_ENSG00000152942
5	68788187	68788480	KD2-H4K8ac_peak_23349	5.51783	OCLN_ENSG00000197822
5	68789512	68789804	KD2-H4K8ac_peak_23350	3.97079	
5	68855547	68855816	KD2-H4K8ac_peak_23351	6.2781	GTF2H2C_ENSG00000183474
5	69344948	69345205	KD2-H4K8ac_peak_23352	7.12754	SMN2_ENSG00000205571
5	70751557	70751929	KD2-H4K8ac_peak_23353	7.76329	BDP1_ENSG00000145734
5	70881738	70882069	KD2-H4K8ac_peak_23354	5.88845	
5	70882673	70882955	KD2-H4K8ac_peak_23355	6.55628	MCCC2_ENSG00000131844
5	70883205	70883430	KD2-H4K8ac_peak_23356	11.02863	MCCC2_ENSG00000131844
5	70989832	70990197	KD2-H4K8ac_peak_23357	5.73839	
5	71361715	71361935	KD2-H4K8ac_peak_23358	6.25869	
5	71402784	71403079	KD2-H4K8ac_peak_23359	8.10216	MAP1B_ENSG00000131711
5	71403477	71404969	KD2-H4K8ac_peak_23360	16.64426	MAP1B_ENSG00000131711
5	71461851	71462194	KD2-H4K8ac_peak_23361	6.2781	
5	71603840	71604088	KD2-H4K8ac_peak_23362	7.07117	
5	71616301	71616918	KD2-H4K8ac_peak_23363	7.36799	MRPS27_ENSG00000113048;PTCD2_ENSG00000049883
5	71747866	71748205	KD2-H4K8ac_peak_23364	4.77079	
5	71855344	71855543	KD2-H4K8ac_peak_23365	4.49837	
5	71985524	71985729	KD2-H4K8ac_peak_23366	5.19569	

5	71988377	71988723	KD2-H4K8ac_peak_23367	5.16439	
5	72112571	72113143	KD2-H4K8ac_peak_23368	12.30509	CTD-2631K10.1_ENSG00000249085;TNPO1_ENSG00000083312
5	72251093	72251628	KD2-H4K8ac_peak_23369	10.05606	CTD-2376I4.1_ENSG00000271926;CTD-2376I4.2_ENSG00000272081;FCHO2_ENSG00000157107
5	72511962	72512325	KD2-H4K8ac_peak_23370	4.86324	
5	72715548	72715755	KD2-H4K8ac_peak_23371	8.5781	
5	72793910	72794735	KD2-H4K8ac_peak_23372	22.5974	RP11-79P5.9_ENSG00000272525;BTF3_ENSG00000145741
5	72860865	72861193	KD2-H4K8ac_peak_23373	4.86324	ANKRA2_ENSG00000164331;UTP15_ENSG00000164338
5	73480385	73480813	KD2-H4K8ac_peak_23374	6.40683	
5	73729094	73729296	KD2-H4K8ac_peak_23375	6.14257	
5	73936174	73937101	KD2-H4K8ac_peak_23376	7.81657	ENC1_ENSG00000171617;HEXB_ENSG00000049860
5	73980627	73980890	KD2-H4K8ac_peak_23377	5.66705	
5	73981174	73981493	KD2-H4K8ac_peak_23378	5.9696	
5	74061852	74062120	KD2-H4K8ac_peak_23379	7.01964	NSA2_ENSG00000164346
5	74062551	74062881	KD2-H4K8ac_peak_23380	10.29344	GFM2_ENSG00000164347;NSA2_ENSG00000164346
5	74063119	74063568	KD2-H4K8ac_peak_23381	10.72656	GFM2_ENSG00000164347;NSA2_ENSG00000164346
5	74161635	74162968	KD2-H4K8ac_peak_23382	14.68449	FAM169A_ENSG00000198780;CTD-2377O17.1_ENSG00000271714
5	74348368	74348660	KD2-H4K8ac_peak_23383	6.25869	RP11-229C3.2_ENSG00000250889
5	74531444	74531816	KD2-H4K8ac_peak_23384	5.15777	ANKRD31_ENSG00000145700
5	74532311	74532515	KD2-H4K8ac_peak_23385	8.30974	ANKRD31_ENSG00000145700
5	74629524	74630001	KD2-H4K8ac_peak_23386	6.90458	
5	74632300	74632598	KD2-H4K8ac_peak_23387	7.22457	CTD-2235C13.2_ENSG00000247372;HMGCR_ENSG00000113161
5	74952514	74952791	KD2-H4K8ac_peak_23388	5.45396	
5	74959462	74960335	KD2-H4K8ac_peak_23389	7.27716	
5	74962732	74963383	KD2-H4K8ac_peak_23390	9.11031	
5	75013015	75013219	KD2-H4K8ac_peak_23391	8.10216	POC5_ENSG00000152359
5	75013545	75014055	KD2-H4K8ac_peak_23392	11.2938	POC5_ENSG00000152359
5	75327000	75327356	KD2-H4K8ac_peak_23393	9.11031	
5	75377722	75377952	KD2-H4K8ac_peak_23394	5.57044	
5	75379282	75380322	KD2-H4K8ac_peak_23395	10.87093	CTC-235G5.3_ENSG00000248127;SV2C_ENSG00000122012
5	75380583	75380998	KD2-H4K8ac_peak_23396	10.05606	CTC-235G5.3_ENSG00000248127
5	75551832	75552111	KD2-H4K8ac_peak_23397	9.11818	
5	75668226	75668498	KD2-H4K8ac_peak_23398	8.17902	
5	75698817	75699459	KD2-H4K8ac_peak_23399	10.08707	IQGAP2_ENSG00000145703
5	76011106	76011379	KD2-H4K8ac_peak_23400	6.42097	CTD-2384B11.2_ENSG00000225407;F2R_ENSG00000181104
5	76011966	76012298	KD2-H4K8ac_peak_23401	9.51625	CTD-2384B11.2_ENSG00000225407;F2R_ENSG00000181104
5	76114860	76115272	KD2-H4K8ac_peak_23402	6.35843	F2RL1_ENSG00000164251
5	76373004	76373565	KD2-H4K8ac_peak_23403	9.36516	
5	76382747	76382961	KD2-H4K8ac_peak_23404	8.16906	ZBED3_ENSG00000132846;ZBED3-AS1_ENSG00000250802
5	76383749	76383987	KD2-H4K8ac_peak_23405	3.69804	ZBED3_ENSG00000132846
5	76475884	76476322	KD2-H4K8ac_peak_23406	5.9696	
5	76651400	76651693	KD2-H4K8ac_peak_23407	4.66652	
5	76787838	76788046	KD2-H4K8ac_peak_23408	8.85813	
5	76788450	76788747	KD2-H4K8ac_peak_23409	8.79557	
5	77071700	77071944	KD2-H4K8ac_peak_23410	4.39858	
5	77147171	77147989	KD2-H4K8ac_peak_23411	23.33626	
5	77148556	77148797	KD2-H4K8ac_peak_23412	9.89126	

5	77268457	77268777	KD2-H4K8ac_peak_23413	6.85193	
5	77590809	77591727	KD2-H4K8ac_peak_23414	8.30974	AP3B1_ENSG00000132842
5	77655426	77656184	KD2-H4K8ac_peak_23415	6.25869	CTD-2037K23.2_ENSG00000245556;SCAMP1_ENSG00000085365
5	77944199	77945178	KD2-H4K8ac_peak_23416	11.05467	
5	77967158	77967368	KD2-H4K8ac_peak_23417	7.87794	
5	78099730	78099927	KD2-H4K8ac_peak_23418	7.96322	
5	78101234	78101438	KD2-H4K8ac_peak_23419	6.2159	
5	78102540	78102856	KD2-H4K8ac_peak_23420	4.01115	
5	78126296	78126509	KD2-H4K8ac_peak_23421	4.01115	
5	78202913	78203666	KD2-H4K8ac_peak_23422	6.06203	
5	78208987	78209249	KD2-H4K8ac_peak_23423	9.9302	
5	78280170	78281794	KD2-H4K8ac_peak_23424	18.62998	ARSB_ENSG00000113273
5	78365963	78366448	KD2-H4K8ac_peak_23425	7.29632	BHMT2_ENSG00000132840
5	78531467	78531733	KD2-H4K8ac_peak_23426	5.01002	DMGDH_ENSG00000132837;JMY_ENSG00000152409
5	78809440	78810346	KD2-H4K8ac_peak_23427	21.09577	HOMER1_ENSG00000152413
5	78907712	78908083	KD2-H4K8ac_peak_23428	15.69572	PAPD4_ENSG00000164329
5	78908351	78908876	KD2-H4K8ac_peak_23429	6.65673	PAPD4_ENSG00000164329
5	79152438	79152630	KD2-H4K8ac_peak_23430	5.25071	
5	79286478	79287022	KD2-H4K8ac_peak_23431	11.24702	MTX3_ENSG00000177034;THBS4_ENSG00000113296
5	79330842	79331569	KD2-H4K8ac_peak_23432	11.05467	
5	79405854	79406236	KD2-H4K8ac_peak_23433	10.0692	
5	79476955	79477276	KD2-H4K8ac_peak_23434	7.79898	
5	79552088	79552468	KD2-H4K8ac_peak_23435	8.17902	SERINC5_ENSG00000164300
5	79702883	79703230	KD2-H4K8ac_peak_23436	8.84031	ZFYVE16_ENSG00000039319
5	79703435	79703679	KD2-H4K8ac_peak_23437	9.11031	ZFYVE16_ENSG00000039319
5	79703892	79704219	KD2-H4K8ac_peak_23438	9.89126	ZFYVE16_ENSG00000039319
5	79783145	79783550	KD2-H4K8ac_peak_23439	4.20044	CTD-2015H6.3_ENSG00000249042;FAM151B_ENSG00000152380
5	79784040	79784239	KD2-H4K8ac_peak_23440	5.02474	CTD-2015H6.3_ENSG00000249042;FAM151B_ENSG00000152380
5	79950555	79950777	KD2-H4K8ac_peak_23441	7.27716	DHFR_ENSG00000228716;MSH3_ENSG00000113318
5	80256139	80256723	KD2-H4K8ac_peak_23442	7.20567	CTC-459I6.1_ENSG00000251450;RASGRF2_ENSG00000113319
5	80436317	80436553	KD2-H4K8ac_peak_23443	3.75035	
5	80480529	80481524	KD2-H4K8ac_peak_23444	10.05606	
5	80597488	80597711	KD2-H4K8ac_peak_23445	11.02863	CKMT2-AS1_ENSG00000247572;ZCCHC9_ENSG00000131732
5	80874814	80875169	KD2-H4K8ac_peak_23446	6.16888	
5	81047370	81048062	KD2-H4K8ac_peak_23447	25.6154	SSBP2_ENSG00000145687
5	81145617	81145903	KD2-H4K8ac_peak_23448	3.75035	
5	81146542	81147022	KD2-H4K8ac_peak_23449	9.15069	CTD-2249K22.1_ENSG00000249483
5	81147244	81147596	KD2-H4K8ac_peak_23450	5.01002	CTD-2249K22.1_ENSG00000249483
5	81267874	81268423	KD2-H4K8ac_peak_23451	16.54484	ATG10_ENSG00000152348
5	81573714	81574008	KD2-H4K8ac_peak_23452	13.56049	RPS23_ENSG00000186468
5	81574717	81574949	KD2-H4K8ac_peak_23453	10.86378	RPS23_ENSG00000186468;ATP6AP1L_ENSG00000205464
5	81683539	81683828	KD2-H4K8ac_peak_23454	14.98671	
5	81684153	81684432	KD2-H4K8ac_peak_23455	8.1718	
5	81692278	81692477	KD2-H4K8ac_peak_23456	4.77079	
5	81832972	81833167	KD2-H4K8ac_peak_23457	8.53581	
5	81833917	81834510	KD2-H4K8ac_peak_23458	6.05809	

5	81834938	81835267	KD2-H4K8ac_peak_23459	4.211	
5	82331075	82331322	KD2-H4K8ac_peak_23460	8.94469	
5	82344658	82344854	KD2-H4K8ac_peak_23461	5.25071	
5	82372816	82373131	KD2-H4K8ac_peak_23462	7.27716	TMEM167A_ENSG00000174695;XRCC4_ENSG00000152422
5	82373494	82373745	KD2-H4K8ac_peak_23463	7.36799	TMEM167A_ENSG00000174695;XRCC4_ENSG00000152422
5	82768246	82769080	KD2-H4K8ac_peak_23464	9.36516	VCAN_ENSG00000038427
5	83214751	83215110	KD2-H4K8ac_peak_23465	5.25071	
5	84959059	84959394	KD2-H4K8ac_peak_23466	4.86324	CTC-480C2.1_ENSG00000250874
5	85865338	85865673	KD2-H4K8ac_peak_23467	7.1181	
5	85913059	85913410	KD2-H4K8ac_peak_23468	7.12754	COX7C_ENSG00000127184
5	85913801	85914651	KD2-H4K8ac_peak_23469	17.43705	COX7C_ENSG00000127184
5	86563253	86563502	KD2-H4K8ac_peak_23470	15.22643	RASA1_ENSG00000145715
5	86808123	86808414	KD2-H4K8ac_peak_23471	10.21502	
5	87564121	87564568	KD2-H4K8ac_peak_23472	17.76996	TMEM161B_ENSG00000164180;TMEM161B-AS1_ENSG00000247828
5	87800114	87800314	KD2-H4K8ac_peak_23473	8.1233	
5	87956248	87956629	KD2-H4K8ac_peak_23474	5.54647	
5	87970154	87970681	KD2-H4K8ac_peak_23475	14.06758	
5	88185204	88185600	KD2-H4K8ac_peak_23476	5.53033	
5	89704768	89705509	KD2-H4K8ac_peak_23477	11.05407	CETN3_ENSG00000153140;AC093510.1_ENSG00000255647
5	89769911	89770223	KD2-H4K8ac_peak_23478	8.34752	MBLAC2_ENSG00000176055
5	89825091	89825364	KD2-H4K8ac_peak_23479	12.00597	LYSMD3_ENSG00000176018;GPR98_ENSG00000164199
5	90575186	90575402	KD2-H4K8ac_peak_23480	9.11031	
5	90609085	90609284	KD2-H4K8ac_peak_23481	3.61633	LUCAT1_ENSG00000248323;RP11-213H15.4_ENSG00000271762
5	90610593	90611047	KD2-H4K8ac_peak_23482	7.74292	LUCAT1_ENSG00000248323;RP11-213H15.4_ENSG00000271762
5	90647793	90648190	KD2-H4K8ac_peak_23483	7.67504	
5	90676509	90676721	KD2-H4K8ac_peak_23484	5.30799	
5	92757303	92757692	KD2-H4K8ac_peak_23485	4.86324	
5	92914584	92914828	KD2-H4K8ac_peak_23486	5.32397	
5	92915837	92916181	KD2-H4K8ac_peak_23487	7.38799	
5	92919700	92920205	KD2-H4K8ac_peak_23488	11.89502	NR2F1_ENSG00000175745
5	92920442	92921109	KD2-H4K8ac_peak_23489	9.98013	NR2F1-AS1_ENSG00000237187
5	92923469	92924113	KD2-H4K8ac_peak_23490	7.76329	
5	92932681	92932873	KD2-H4K8ac_peak_23491	4.7849	
5	92934362	92934677	KD2-H4K8ac_peak_23492	9.15069	
5	92937793	92938467	KD2-H4K8ac_peak_23493	10.21502	
5	92939625	92940141	KD2-H4K8ac_peak_23494	10.21502	
5	92956599	92956835	KD2-H4K8ac_peak_23495	8.4716	MIR2277_ENSG00000251725
5	93804900	93805189	KD2-H4K8ac_peak_23496	17.14886	
5	93808240	93808436	KD2-H4K8ac_peak_23497	10.08707	
5	94110531	94110746	KD2-H4K8ac_peak_23498	6.16888	
5	94114084	94114315	KD2-H4K8ac_peak_23499	5.57044	
5	94890912	94891132	KD2-H4K8ac_peak_23500	9.65086	TTC37_ENSG00000198677;ARSK_ENSG00000164291
5	94955612	94957374	KD2-H4K8ac_peak_23501	14.8083	GPR150_ENSG00000178015
5	95066683	95066941	KD2-H4K8ac_peak_23502	6.30229	
5	95067206	95067707	KD2-H4K8ac_peak_23503	7.44324	CTD-2154I11.2_ENSG00000250240
5	95296316	95296511	KD2-H4K8ac_peak_23504	3.75035	

5	95297123	95297338	KD2-H4K8ac_peak_23505	6.25869	ELL2_ENSG00000118985;CTD-2337A12.1_ENSG00000251314
5	96038588	96038807	KD2-H4K8ac_peak_23506	7.83656	
5	96142814	96143821	KD2-H4K8ac_peak_23507	13.95627	ERAP1_ENSG00000164307
5	96144191	96144415	KD2-H4K8ac_peak_23508	6.65673	ERAP1_ENSG00000164307
5	96270610	96271276	KD2-H4K8ac_peak_23509	6.40297	LNPEP_ENSG00000113441;CTD-2260A17.2_ENSG00000247121
5	96518932	96519285	KD2-H4K8ac_peak_23510	4.9025	RP11-155G15.2_ENSG00000251513;RIOK2_ENSG00000058729
5	96519752	96520186	KD2-H4K8ac_peak_23511	17.91241	RP11-155G15.2_ENSG00000251513;RIOK2_ENSG00000058729
5	98104513	98104770	KD2-H4K8ac_peak_23512	6.45083	RGMB_ENSG00000174136
5	98105141	98105344	KD2-H4K8ac_peak_23513	5.66705	RGMB_ENSG00000174136
5	98263966	98264549	KD2-H4K8ac_peak_23514	11.29366	CTD-2007H13.3_ENSG00000248489
5	98264966	98265281	KD2-H4K8ac_peak_23515	5.84991	CTD-2007H13.3_ENSG00000248489
5	101385745	1.01E+08	KD2-H4K8ac_peak_23516	4.01115	
5	101631405	1.02E+08	KD2-H4K8ac_peak_23517	6.66359	SLCO4C1_ENSG00000173930
5	101632096	1.02E+08	KD2-H4K8ac_peak_23518	5.9696	SLCO4C1_ENSG00000173930
5	102089808	1.02E+08	KD2-H4K8ac_peak_23519	10.21366	PAM_ENSG00000145730
5	102090560	1.02E+08	KD2-H4K8ac_peak_23520	14.275	PAM_ENSG00000145730
5	102097840	1.02E+08	KD2-H4K8ac_peak_23521	9.15329	
5	102594597	1.03E+08	KD2-H4K8ac_peak_23522	13.20052	C5orf30_ENSG00000181751
5	102780251	1.03E+08	KD2-H4K8ac_peak_23523	4.77079	
5	102898229	1.03E+08	KD2-H4K8ac_peak_23524	11.05407	NUDT12_ENSG00000112874
5	103576831	1.04E+08	KD2-H4K8ac_peak_23525	5.57044	
5	106802226	1.07E+08	KD2-H4K8ac_peak_23526	4.89656	
5	106850581	1.07E+08	KD2-H4K8ac_peak_23527	7.146	
5	106851115	1.07E+08	KD2-H4K8ac_peak_23528	10.05606	
5	107004909	1.07E+08	KD2-H4K8ac_peak_23529	5.60968	
5	107005810	1.07E+08	KD2-H4K8ac_peak_23530	17.8868	EFNA5_ENSG00000184349
5	107009207	1.07E+08	KD2-H4K8ac_peak_23531	5.9696	
5	107419415	1.07E+08	KD2-H4K8ac_peak_23532	5.25071	
5	107716406	1.08E+08	KD2-H4K8ac_peak_23533	9.63487	FBXL17_ENSG00000145743
5	108063675	1.08E+08	KD2-H4K8ac_peak_23534	8.36432	LINC01023_ENSG00000272523
5	108083944	1.08E+08	KD2-H4K8ac_peak_23535	13.87818	FER_ENSG00000151422
5	108084691	1.08E+08	KD2-H4K8ac_peak_23536	10.43145	
5	109025673	1.09E+08	KD2-H4K8ac_peak_23537	5.405	MAN2A1_ENSG00000112893
5	110427328	1.1E+08	KD2-H4K8ac_peak_23538	8.37231	CTC-551A13.2_ENSG00000253613;WDR36_ENSG00000134987
5	110559325	1.11E+08	KD2-H4K8ac_peak_23539	15.039	CAMK4_ENSG00000152495
5	110560183	1.11E+08	KD2-H4K8ac_peak_23540	5.00796	CAMK4_ENSG00000152495
5	110571578	1.11E+08	KD2-H4K8ac_peak_23541	4.77079	
5	110720329	1.11E+08	KD2-H4K8ac_peak_23542	7.12754	
5	110721201	1.11E+08	KD2-H4K8ac_peak_23543	12.42547	
5	110722433	1.11E+08	KD2-H4K8ac_peak_23544	15.61313	
5	110789076	1.11E+08	KD2-H4K8ac_peak_23545	9.11031	
5	110868388	1.11E+08	KD2-H4K8ac_peak_23546	6.16888	
5	110886462	1.11E+08	KD2-H4K8ac_peak_23547	3.75035	
5	111017373	1.11E+08	KD2-H4K8ac_peak_23548	11.05467	
5	111093194	1.11E+08	KD2-H4K8ac_peak_23549	19.33282	
5	111094304	1.11E+08	KD2-H4K8ac_peak_23550	8.17902	

5	111398976	1.11E+08	KD2-H4K8ac_peak_23551	5.64449	
5	111678370	1.12E+08	KD2-H4K8ac_peak_23552	9.02371	
5	111755795	1.12E+08	KD2-H4K8ac_peak_23553	6.35843	EPB41L4A_ENSG00000129595;EPB41L4A-AS2_ENSG00000268358
5	112042788	1.12E+08	KD2-H4K8ac_peak_23554	7.70289	APC_ENSG00000134982
5	112043324	1.12E+08	KD2-H4K8ac_peak_23555	15.81711	APC_ENSG00000134982
5	112196384	1.12E+08	KD2-H4K8ac_peak_23556	5.88845	CTC-487M23.8_ENSG00000272869;SRP19_ENSG00000153037
5	112197003	1.12E+08	KD2-H4K8ac_peak_23557	15.53798	CTC-487M23.8_ENSG00000272869;SRP19_ENSG00000153037
5	112256971	1.12E+08	KD2-H4K8ac_peak_23558	16.09057	REEP5_ENSG00000129625
5	112394029	1.12E+08	KD2-H4K8ac_peak_23559	10.30284	
5	112395266	1.12E+08	KD2-H4K8ac_peak_23560	15.13261	
5	112452770	1.12E+08	KD2-H4K8ac_peak_23561	8.41122	
5	112454537	1.12E+08	KD2-H4K8ac_peak_23562	5.75757	
5	112538774	1.13E+08	KD2-H4K8ac_peak_23563	15.10495	
5	112823710	1.13E+08	KD2-H4K8ac_peak_23564	8.10216	MCC_ENSG00000171444
5	112849106	1.13E+08	KD2-H4K8ac_peak_23565	18.38518	YTHDC2_ENSG00000047188
5	114504834	1.15E+08	KD2-H4K8ac_peak_23566	8.98477	
5	114515085	1.15E+08	KD2-H4K8ac_peak_23567	14.84212	TRIM36_ENSG00000152503
5	114516108	1.15E+08	KD2-H4K8ac_peak_23568	12.82393	TRIM36_ENSG00000152503
5	114598209	1.15E+08	KD2-H4K8ac_peak_23569	6.25869	PGGT1B_ENSG00000164219;CTC-428G20.6_ENSG00000271797
5	114599207	1.15E+08	KD2-H4K8ac_peak_23570	7.12754	PGGT1B_ENSG00000164219
5	114880022	1.15E+08	KD2-H4K8ac_peak_23571	14.15075	FEM1C_ENSG00000145780
5	114880755	1.15E+08	KD2-H4K8ac_peak_23572	6.40683	FEM1C_ENSG00000145780
5	114961275	1.15E+08	KD2-H4K8ac_peak_23573	12.82393	TICAM2_ENSG00000243414;TMED7-TICAM2_ENSG00000251201
5	115177065	1.15E+08	KD2-H4K8ac_peak_23574	8.10216	ATG12_ENSG00000145782;AP3S1_ENSG00000177879
5	115297459	1.15E+08	KD2-H4K8ac_peak_23575	4.211	AQPEP_ENSG00000172901
5	115420444	1.15E+08	KD2-H4K8ac_peak_23576	6.65673	CTD-2287O16.5_ENSG00000271918;COMMD10_ENSG00000145781
5	115912077	1.16E+08	KD2-H4K8ac_peak_23577	8.16906	
5	116074134	1.16E+08	KD2-H4K8ac_peak_23578	9.06703	
5	116640284	1.17E+08	KD2-H4K8ac_peak_23579	5.25071	
5	118323982	1.18E+08	KD2-H4K8ac_peak_23580	17.43705	DTWD2_ENSG00000169570
5	118406841	1.18E+08	KD2-H4K8ac_peak_23581	8.55271	CTB-161M19.4_ENSG00000249494
5	118747466	1.19E+08	KD2-H4K8ac_peak_23582	8.1233	
5	118788231	1.19E+08	KD2-H4K8ac_peak_23583	3.75035	HSD17B4_ENSG00000133835
5	119799657	1.2E+08	KD2-H4K8ac_peak_23584	8.59579	PRR16_ENSG00000184838
5	121296614	1.21E+08	KD2-H4K8ac_peak_23585	6.40683	SRFBP1_ENSG00000151304
5	121413127	1.21E+08	KD2-H4K8ac_peak_23586	4.56666	LOX_ENSG00000113083
5	122110818	1.22E+08	KD2-H4K8ac_peak_23587	9.08816	AC093267.1_ENSG00000212114;SNX2_ENSG00000205302
5	122181002	1.22E+08	KD2-H4K8ac_peak_23588	18.62998	
5	122506471	1.23E+08	KD2-H4K8ac_peak_23589	7.12754	
5	122758624	1.23E+08	KD2-H4K8ac_peak_23590	8.34752	CEP120_ENSG00000168944
5	122847952	1.23E+08	KD2-H4K8ac_peak_23591	17.14886	CSNK1G3_ENSG00000151292
5	123121362	1.23E+08	KD2-H4K8ac_peak_23592	5.43522	
5	123149792	1.23E+08	KD2-H4K8ac_peak_23593	3.91903	
5	123211328	1.23E+08	KD2-H4K8ac_peak_23594	5.14125	
5	123392612	1.23E+08	KD2-H4K8ac_peak_23595	10.48739	
5	123461195	1.23E+08	KD2-H4K8ac_peak_23596	6.40683	

5	123462763	1.23E+08	KD2-H4K8ac_peak_23597	9.15329	
5	123463935	1.23E+08	KD2-H4K8ac_peak_23598	7.83656	
5	123730425	1.24E+08	KD2-H4K8ac_peak_23599	8.26945	CTC-369A16.2_ENSG00000251421;CTC-369A16.3_ENSG00000251662
5	123731130	1.24E+08	KD2-H4K8ac_peak_23600	7.59643	CTC-369A16.2_ENSG00000251421;CTC-369A16.3_ENSG00000251662
5	123916544	1.24E+08	KD2-H4K8ac_peak_23601	10.29344	
5	123934712	1.24E+08	KD2-H4K8ac_peak_23602	6.40683	
5	123973815	1.24E+08	KD2-H4K8ac_peak_23603	6.40683	
5	123995711	1.24E+08	KD2-H4K8ac_peak_23604	5.63633	
5	124041649	1.24E+08	KD2-H4K8ac_peak_23605	4.77079	
5	124042741	1.24E+08	KD2-H4K8ac_peak_23606	13.57066	RP11-43D2.2_ENSG00000249112
5	124079321	1.24E+08	KD2-H4K8ac_peak_23607	6.39197	
5	124079810	1.24E+08	KD2-H4K8ac_peak_23608	14.68449	
5	124084836	1.24E+08	KD2-H4K8ac_peak_23609	7.13615	ZNF608_ENSG00000168916
5	124085318	1.24E+08	KD2-H4K8ac_peak_23610	5.61377	ZNF608_ENSG00000168916
5	124341904	1.24E+08	KD2-H4K8ac_peak_23611	4.77079	
5	124682292	1.25E+08	KD2-H4K8ac_peak_23612	5.88845	
5	124747770	1.25E+08	KD2-H4K8ac_peak_23613	6.56211	
5	124819487	1.25E+08	KD2-H4K8ac_peak_23614	4.86824	
5	124912422	1.25E+08	KD2-H4K8ac_peak_23615	4.01115	
5	125398583	1.25E+08	KD2-H4K8ac_peak_23616	7.22936	
5	125758841	1.26E+08	KD2-H4K8ac_peak_23617	10.05606	
5	125936146	1.26E+08	KD2-H4K8ac_peak_23618	11.79585	PHAX_ENSG00000164902
5	125936659	1.26E+08	KD2-H4K8ac_peak_23619	19.87796	PHAX_ENSG00000164902
5	126111462	1.26E+08	KD2-H4K8ac_peak_23620	6.25869	RP11-434D11.4_ENSG00000251072;LMNB1_ENSG00000113368
5	126112277	1.26E+08	KD2-H4K8ac_peak_23621	7.79558	RP11-434D11.4_ENSG00000251072;LMNB1_ENSG00000113368
5	126112890	1.26E+08	KD2-H4K8ac_peak_23622	11.24702	RP11-434D11.4_ENSG00000251072;LMNB1_ENSG00000113368
5	126253051	1.26E+08	KD2-H4K8ac_peak_23623	7.12754	
5	126366544	1.26E+08	KD2-H4K8ac_peak_23624	5.1628	MARCH3_ENSG00000173926
5	126409065	1.26E+08	KD2-H4K8ac_peak_23625	7.1181	C5orf63_ENSG00000164241
5	126565056	1.27E+08	KD2-H4K8ac_peak_23626	5.14125	
5	126852854	1.27E+08	KD2-H4K8ac_peak_23627	10.21502	PRRC1_ENSG00000164244
5	127418478	1.27E+08	KD2-H4K8ac_peak_23628	18.54875	SLC12A2_ENSG00000064651;CTC-228N24.3_ENSG00000245937
5	127419115	1.27E+08	KD2-H4K8ac_peak_23629	7.95563	SLC12A2_ENSG00000064651;CTC-228N24.3_ENSG00000245937
5	128300647	1.28E+08	KD2-H4K8ac_peak_23630	7.36799	
5	128399454	1.28E+08	KD2-H4K8ac_peak_23631	7.27716	
5	128429945	1.28E+08	KD2-H4K8ac_peak_23632	10.08707	ISOC1_ENSG00000066583
5	128430558	1.28E+08	KD2-H4K8ac_peak_23633	8.85813	ISOC1_ENSG00000066583
5	128795344	1.29E+08	KD2-H4K8ac_peak_23634	6.25869	ADAMTS19-AS1_ENSG00000249421;ADAMTS19_ENSG00000145808
5	128796744	1.29E+08	KD2-H4K8ac_peak_23635	10.05606	ADAMTS19-AS1_ENSG00000249421;ADAMTS19_ENSG00000145808
5	130500307	1.31E+08	KD2-H4K8ac_peak_23636	12.3187	
5	130501102	1.31E+08	KD2-H4K8ac_peak_23637	4.67342	
5	130599798	1.31E+08	KD2-H4K8ac_peak_23638	9.36516	
5	130971156	1.31E+08	KD2-H4K8ac_peak_23639	11.75669	RAPGEF6_ENSG00000158987
5	131132135	1.31E+08	KD2-H4K8ac_peak_23640	10.46264	CTC-432M15.3_ENSG00000273217;FNIP1_ENSG00000217128
5	131132993	1.31E+08	KD2-H4K8ac_peak_23641	6.83936	CTC-432M15.3_ENSG00000273217;FNIP1_ENSG00000217128
5	131347099	1.31E+08	KD2-H4K8ac_peak_23642	7.76104	ACSL6_ENSG00000164398;AC034228.2_ENSG00000231585

5	131563331	1.32E+08	KD2-H4K8ac_peak_23643	17.43705	
5	131592859	1.32E+08	KD2-H4K8ac_peak_23644	13.9234	PDLIM4_ENSG00000131435
5	131593536	1.32E+08	KD2-H4K8ac_peak_23645	8.69797	PDLIM4_ENSG00000131435
5	131595088	1.32E+08	KD2-H4K8ac_peak_23646	13.73635	
5	131605234	1.32E+08	KD2-H4K8ac_peak_23647	5.13615	
5	131628819	1.32E+08	KD2-H4K8ac_peak_23648	11.50346	SLC22A4_ENSG00000197208
5	131705232	1.32E+08	KD2-H4K8ac_peak_23649	9.20193	AC034220.3_ENSG00000233006;SLC22A5_ENSG00000197375
5	131746054	1.32E+08	KD2-H4K8ac_peak_23650	25.44388	C5orf56_ENSG00000197536
5	131755798	1.32E+08	KD2-H4K8ac_peak_23651	14.36023	
5	131825727	1.32E+08	KD2-H4K8ac_peak_23652	10.19949	IRF1_ENSG00000125347
5	131826758	1.32E+08	KD2-H4K8ac_peak_23653	5.00278	IRF1_ENSG00000125347
5	131830877	1.32E+08	KD2-H4K8ac_peak_23654	11.7296	
5	131862406	1.32E+08	KD2-H4K8ac_peak_23655	5.57044	
5	131887158	1.32E+08	KD2-H4K8ac_peak_23656	7.12754	
5	131892284	1.32E+08	KD2-H4K8ac_peak_23657	9.63487	IL5_ENSG00000113525;RAD50_ENSG00000113522
5	131892695	1.32E+08	KD2-H4K8ac_peak_23658	9.20193	IL5_ENSG00000113525;RAD50_ENSG00000113522
5	131991772	1.32E+08	KD2-H4K8ac_peak_23659	8.10216	IL13_ENSG00000169194
5	132072807	1.32E+08	KD2-H4K8ac_peak_23660	5.9696	KIF3A_ENSG00000131437
5	132073441	1.32E+08	KD2-H4K8ac_peak_23661	4.20044	KIF3A_ENSG00000131437
5	132082826	1.32E+08	KD2-H4K8ac_peak_23662	11.09936	CCNI2_ENSG00000205089
5	132111936	1.32E+08	KD2-H4K8ac_peak_23663	6.24747	
5	132112410	1.32E+08	KD2-H4K8ac_peak_23664	6.40297	
5	132112964	1.32E+08	KD2-H4K8ac_peak_23665	13.73635	
5	132148918	1.32E+08	KD2-H4K8ac_peak_23666	8.37231	SOWAHA_ENSG00000198944
5	132165860	1.32E+08	KD2-H4K8ac_peak_23667	20.76319	SHROOM1_ENSG00000164403
5	132166452	1.32E+08	KD2-H4K8ac_peak_23668	8.10216	SHROOM1_ENSG00000164403
5	132201584	1.32E+08	KD2-H4K8ac_peak_23669	15.25814	GDF9_ENSG00000164404;UQCRQ_ENSG00000164405
5	132298609	1.32E+08	KD2-H4K8ac_peak_23670	9.89126	AFF4_ENSG00000072364;CTC-350I8.1_ENSG00000272023
5	132299448	1.32E+08	KD2-H4K8ac_peak_23671	11.05467	AFF4_ENSG00000072364;CTC-350I8.1_ENSG00000272023
5	132361813	1.32E+08	KD2-H4K8ac_peak_23672	14.8083	ZCCHC10_ENSG00000155329
5	132386631	1.32E+08	KD2-H4K8ac_peak_23673	12.82393	HSPA4_ENSG00000170606
5	132387766	1.32E+08	KD2-H4K8ac_peak_23674	8.16906	HSPA4_ENSG00000170606
5	132579843	1.33E+08	KD2-H4K8ac_peak_23675	6.40683	CTB-49A3.5_ENSG00000249478
5	133257594	1.33E+08	KD2-H4K8ac_peak_23676	7.40111	
5	133262314	1.33E+08	KD2-H4K8ac_peak_23677	9.72244	
5	133303646	1.33E+08	KD2-H4K8ac_peak_23678	10.73134	C5orf15_ENSG00000113583
5	133339802	1.33E+08	KD2-H4K8ac_peak_23679	9.16531	VDAC1_ENSG00000213585;CTB-113I20.2_ENSG00000271737
5	133340589	1.33E+08	KD2-H4K8ac_peak_23680	14.69247	VDAC1_ENSG00000213585;CTB-113I20.2_ENSG00000271737
5	133439872	1.33E+08	KD2-H4K8ac_peak_23681	7.96322	
5	133440901	1.33E+08	KD2-H4K8ac_peak_23682	7.47462	
5	133442315	1.33E+08	KD2-H4K8ac_peak_23683	16.61426	
5	133443593	1.33E+08	KD2-H4K8ac_peak_23684	12.00597	
5	133460419	1.33E+08	KD2-H4K8ac_peak_23685	8.58069	
5	133512683	1.34E+08	KD2-H4K8ac_peak_23686	12.57297	SKP1_ENSG00000113558
5	133561336	1.34E+08	KD2-H4K8ac_peak_23687	18.37277	CTD-2410N18.5_ENSG00000272772;PPP2CA_ENSG00000113575;MIR3661_ENSG00000266751;CTD-2410N18.3_ENSG00000270177
5	133562152	1.34E+08	KD2-H4K8ac_peak_23688	11.05467	CTD-2410N18.5_ENSG00000272772;PPP2CA_ENSG00000113575;MIR3661_ENSG00000266751;CTD-2410N18.3_ENSG00000270177

5	133702568	1.34E+08	KD2-H4K8ac_peak_23689	5.75757	
5	133703119	1.34E+08	KD2-H4K8ac_peak_23690	8.17902	
5	133707290	1.34E+08	KD2-H4K8ac_peak_23691	5.59859	CDKL3_ENSG00000006837;CTD-2410N18.4_ENSG00000273345;UBE2B_ENSG00000119048
5	133747163	1.34E+08	KD2-H4K8ac_peak_23692	9.20193	CDKN2AIPNL_ENSG00000237190
5	133747755	1.34E+08	KD2-H4K8ac_peak_23693	8.4716	CDKN2AIPNL_ENSG00000237190
5	133801250	1.34E+08	KD2-H4K8ac_peak_23694	13.08184	
5	133842158	1.34E+08	KD2-H4K8ac_peak_23695	5.30494	AC005355.2_ENSG00000251169
5	133859753	1.34E+08	KD2-H4K8ac_peak_23696	23.2707	JADE2_ENSG00000043143
5	133861404	1.34E+08	KD2-H4K8ac_peak_23697	11.03331	
5	133862318	1.34E+08	KD2-H4K8ac_peak_23698	6.24114	
5	133888845	1.34E+08	KD2-H4K8ac_peak_23699	5.01002	
5	133890494	1.34E+08	KD2-H4K8ac_peak_23700	10.66719	
5	133904697	1.34E+08	KD2-H4K8ac_peak_23701	7.1181	
5	133922661	1.34E+08	KD2-H4K8ac_peak_23702	7.76329	
5	133968752	1.34E+08	KD2-H4K8ac_peak_23703	4.06758	
5	133984572	1.34E+08	KD2-H4K8ac_peak_23704	6.99194	SAR1B_ENSG00000152700;SEC24A_ENSG00000113615
5	134072891	1.34E+08	KD2-H4K8ac_peak_23705	7.76329	CAMLG_ENSG00000164615
5	134074372	1.34E+08	KD2-H4K8ac_peak_23706	7.07117	CAMLG_ENSG00000164615
5	134093423	1.34E+08	KD2-H4K8ac_peak_23707	11.60333	DDX46_ENSG00000145833
5	134094582	1.34E+08	KD2-H4K8ac_peak_23708	5.88845	DDX46_ENSG00000145833
5	134181701	1.34E+08	KD2-H4K8ac_peak_23709	14.275	C5orf24_ENSG00000181904
5	134209476	1.34E+08	KD2-H4K8ac_peak_23710	13.95627	TXNDC15_ENSG00000113621
5	134240100	1.34E+08	KD2-H4K8ac_peak_23711	16.57619	PCBD2_ENSG00000132570
5	134526801	1.35E+08	KD2-H4K8ac_peak_23712	15.61313	
5	134734414	1.35E+08	KD2-H4K8ac_peak_23713	22.12789	H2AFY_ENSG00000113648;CTC-203F4.2_ENSG00000270021
5	135336802	1.35E+08	KD2-H4K8ac_peak_23714	6.40683	
5	135467795	1.35E+08	KD2-H4K8ac_peak_23715	10.96455	SMAD5_ENSG00000113658
5	135468612	1.35E+08	KD2-H4K8ac_peak_23716	11.03331	SMAD5_ENSG00000113658
5	136515362	1.37E+08	KD2-H4K8ac_peak_23717	5.52329	
5	136709062	1.37E+08	KD2-H4K8ac_peak_23718	10.11379	
5	136711370	1.37E+08	KD2-H4K8ac_peak_23719	7.18916	
5	136834284	1.37E+08	KD2-H4K8ac_peak_23720	10.1591	
5	136834740	1.37E+08	KD2-H4K8ac_peak_23721	11.05407	
5	136900247	1.37E+08	KD2-H4K8ac_peak_23722	7.79741	
5	136901828	1.37E+08	KD2-H4K8ac_peak_23723	11.09936	
5	136954336	1.37E+08	KD2-H4K8ac_peak_23724	4.37709	
5	137089565	1.37E+08	KD2-H4K8ac_peak_23725	6.24114	HNRNPA0_ENSG00000177733
5	137090174	1.37E+08	KD2-H4K8ac_peak_23726	28.03233	HNRNPA0_ENSG00000177733
5	137224321	1.37E+08	KD2-H4K8ac_peak_23727	8.04448	RP11-381K20.2_ENSG00000250159;PKD2L2_ENSG00000078795
5	137368405	1.37E+08	KD2-H4K8ac_peak_23728	15.81711	RP11-325L7.1_ENSG00000246323
5	137371983	1.37E+08	KD2-H4K8ac_peak_23729	6.83936	
5	137475120	1.37E+08	KD2-H4K8ac_peak_23730	7.59643	NME5_ENSG00000112981
5	137549520	1.38E+08	KD2-H4K8ac_peak_23731	4.24345	CDC23_ENSG00000094880
5	137577472	1.38E+08	KD2-H4K8ac_peak_23732	14.68449	
5	137609779	1.38E+08	KD2-H4K8ac_peak_23733	10.05606	GFRA3_ENSG00000146013
5	137673477	1.38E+08	KD2-H4K8ac_peak_23734	11.04256	CDC25C_ENSG00000158402

5	137774486	1.38E+08	KD2-H4K8ac_peak_23735	7.69287	REEP2_ENSG00000132563
5	137775856	1.38E+08	KD2-H4K8ac_peak_23736	6.66692	
5	137784785	1.38E+08	KD2-H4K8ac_peak_23737	10.96455	
5	137785909	1.38E+08	KD2-H4K8ac_peak_23738	13.82493	
5	137799479	1.38E+08	KD2-H4K8ac_peak_23739	12.30509	
5	137826978	1.38E+08	KD2-H4K8ac_peak_23740	19.33018	
5	137829708	1.38E+08	KD2-H4K8ac_peak_23741	3.97079	
5	137878310	1.38E+08	KD2-H4K8ac_peak_23742	9.63487	ETF1_ENSG00000120705
5	137910751	1.38E+08	KD2-H4K8ac_peak_23743	13.59098	HSPA9_ENSG00000113013
5	137911598	1.38E+08	KD2-H4K8ac_peak_23744	13.80019	HSPA9_ENSG00000113013
5	137946295	1.38E+08	KD2-H4K8ac_peak_23745	18.85772	CTNNA1_ENSG00000044115
5	138061071	1.38E+08	KD2-H4K8ac_peak_23746	7.05051	
5	138088542	1.38E+08	KD2-H4K8ac_peak_23747	26.59781	AC034243.1_ENSG00000253404
5	138264280	1.38E+08	KD2-H4K8ac_peak_23748	11.6141	
5	138281273	1.38E+08	KD2-H4K8ac_peak_23749	10.05606	
5	138283593	1.38E+08	KD2-H4K8ac_peak_23750	6.34074	
5	138284234	1.38E+08	KD2-H4K8ac_peak_23751	6.31785	
5	138284920	1.38E+08	KD2-H4K8ac_peak_23752	13.00656	
5	138286074	1.38E+08	KD2-H4K8ac_peak_23753	9.95119	
5	138288357	1.38E+08	KD2-H4K8ac_peak_23754	14.12659	
5	138289610	1.38E+08	KD2-H4K8ac_peak_23755	6.0605	
5	138291270	1.38E+08	KD2-H4K8ac_peak_23756	6.25869	
5	138292151	1.38E+08	KD2-H4K8ac_peak_23757	6.36319	
5	138515024	1.39E+08	KD2-H4K8ac_peak_23758	6.2781	
5	138533602	1.39E+08	KD2-H4K8ac_peak_23759	7.36799	
5	138597982	1.39E+08	KD2-H4K8ac_peak_23760	7.45501	
5	138629482	1.39E+08	KD2-H4K8ac_peak_23761	18.54875	SIL1_ENSG00000120725
5	138677588	1.39E+08	KD2-H4K8ac_peak_23762	21.2844	PAIP2_ENSG00000120727
5	138739964	1.39E+08	KD2-H4K8ac_peak_23763	10.17341	SPATA24_ENSG00000170469
5	138774911	1.39E+08	KD2-H4K8ac_peak_23764	6.66359	
5	138775593	1.39E+08	KD2-H4K8ac_peak_23765	4.86324	
5	138897267	1.39E+08	KD2-H4K8ac_peak_23766	13.80019	
5	138939989	1.39E+08	KD2-H4K8ac_peak_23767	6.40297	
5	138940995	1.39E+08	KD2-H4K8ac_peak_23768	8.88082	
5	139014867	1.39E+08	KD2-H4K8ac_peak_23769	5.92591	
5	139017213	1.39E+08	KD2-H4K8ac_peak_23770	13.13969	
5	139027957	1.39E+08	KD2-H4K8ac_peak_23771	10.19949	CTD-3224K15.2_ENSG00000250635
5	139029213	1.39E+08	KD2-H4K8ac_peak_23772	6.90231	CTD-3224K15.2_ENSG00000250635
5	139038950	1.39E+08	KD2-H4K8ac_peak_23773	8.85813	
5	139040657	1.39E+08	KD2-H4K8ac_peak_23774	5.75757	
5	139041520	1.39E+08	KD2-H4K8ac_peak_23775	6.40297	
5	139046818	1.39E+08	KD2-H4K8ac_peak_23776	4.0528	
5	139047893	1.39E+08	KD2-H4K8ac_peak_23777	12.48986	
5	139049223	1.39E+08	KD2-H4K8ac_peak_23778	8.28234	
5	139050908	1.39E+08	KD2-H4K8ac_peak_23779	8.23274	
5	139056594	1.39E+08	KD2-H4K8ac_peak_23780	7.20116	

5	139057964	1.39E+08	KD2-H4K8ac_peak_23781	6.36375	
5	139066590	1.39E+08	KD2-H4K8ac_peak_23782	4.72599	
5	139076155	1.39E+08	KD2-H4K8ac_peak_23783	22.21705	
5	139087176	1.39E+08	KD2-H4K8ac_peak_23784	3.7063	
5	139087932	1.39E+08	KD2-H4K8ac_peak_23785	8.4716	
5	139089126	1.39E+08	KD2-H4K8ac_peak_23786	19.28826	
5	139126076	1.39E+08	KD2-H4K8ac_peak_23787	12.08081	CTB-35F21.2_ENSG00000249131
5	139126973	1.39E+08	KD2-H4K8ac_peak_23788	13.59098	
5	139138246	1.39E+08	KD2-H4K8ac_peak_23789	9.6247	
5	139145278	1.39E+08	KD2-H4K8ac_peak_23790	6.83936	
5	139227537	1.39E+08	KD2-H4K8ac_peak_23791	15.69839	CTB-35F21.4_ENSG00000250692
5	139228018	1.39E+08	KD2-H4K8ac_peak_23792	15.47237	CTB-35F21.4_ENSG00000250692
5	139260328	1.39E+08	KD2-H4K8ac_peak_23793	6.8836	
5	139273128	1.39E+08	KD2-H4K8ac_peak_23794	6.90231	
5	139421414	1.39E+08	KD2-H4K8ac_peak_23795	8.17902	
5	139422076	1.39E+08	KD2-H4K8ac_peak_23796	3.75035	NRG2_ENSG00000158458
5	139423082	1.39E+08	KD2-H4K8ac_peak_23797	10.1591	NRG2_ENSG00000158458
5	139486712	1.39E+08	KD2-H4K8ac_peak_23798	11.90231	LINC01024_ENSG00000245146;PURA_ENSG00000185129
5	139493109	1.39E+08	KD2-H4K8ac_peak_23799	9.36516	
5	139554121	1.4E+08	KD2-H4K8ac_peak_23800	17.00119	CYSTM1_ENSG00000120306
5	139682373	1.4E+08	KD2-H4K8ac_peak_23801	11.66477	PFDN1_ENSG00000113068
5	139683060	1.4E+08	KD2-H4K8ac_peak_23802	8.30974	PFDN1_ENSG00000113068
5	139742947	1.4E+08	KD2-H4K8ac_peak_23803	6.76298	
5	139752963	1.4E+08	KD2-H4K8ac_peak_23804	7.79898	
5	139780287	1.4E+08	KD2-H4K8ac_peak_23805	10.96455	CTC-329D1.2_ENSG00000249637;ANKHD1_ENSG00000131503;ANKHD1-EIF4EBP3_ENSG00000254996
5	139781491	1.4E+08	KD2-H4K8ac_peak_23806	11.2938	CTC-329D1.2_ENSG00000249637;ANKHD1_ENSG00000131503;ANKHD1-EIF4EBP3_ENSG00000254996
5	139927399	1.4E+08	KD2-H4K8ac_peak_23807	5.92967	EIF4EBP3_ENSG00000243056
5	139937171	1.4E+08	KD2-H4K8ac_peak_23808	17.16047	SRA1_ENSG00000213523
5	140019150	1.4E+08	KD2-H4K8ac_peak_23809	7.28827	TMCO6_ENSG00000113119
5	140026915	1.4E+08	KD2-H4K8ac_peak_23810	6.83936	NDUFA2_ENSG00000131495;IK_ENSG00000113141;MIR3655_ENSG00000264052
5	140027454	1.4E+08	KD2-H4K8ac_peak_23811	7.26029	NDUFA2_ENSG00000131495;IK_ENSG00000113141;MIR3655_ENSG00000264052
5	140043790	1.4E+08	KD2-H4K8ac_peak_23812	5.30799	WDR55_ENSG00000120314
5	140186727	1.4E+08	KD2-H4K8ac_peak_23813	7.22457	PCDHA4_ENSG00000204967
5	140346151	1.4E+08	KD2-H4K8ac_peak_23814	8.04448	PCDHAC2_ENSG00000243232
5	140380995	1.4E+08	KD2-H4K8ac_peak_23815	9.89283	
5	140382251	1.4E+08	KD2-H4K8ac_peak_23816	6.00226	
5	140700057	1.41E+08	KD2-H4K8ac_peak_23817	8.55271	TAF7_ENSG00000178913;AC005618.1_ENSG00000255729
5	140762333	1.41E+08	KD2-H4K8ac_peak_23818	8.04763	PCDHGA7_ENSG00000253537
5	140887631	1.41E+08	KD2-H4K8ac_peak_23819	5.13615	
5	140888602	1.41E+08	KD2-H4K8ac_peak_23820	9.402	
5	140892171	1.41E+08	KD2-H4K8ac_peak_23821	6.20982	
5	140893407	1.41E+08	KD2-H4K8ac_peak_23822	8.85813	
5	140898186	1.41E+08	KD2-H4K8ac_peak_23823	6.63018	
5	140903660	1.41E+08	KD2-H4K8ac_peak_23824	8.1718	
5	140904576	1.41E+08	KD2-H4K8ac_peak_23825	4.211	
5	140905181	1.41E+08	KD2-H4K8ac_peak_23826	9.1707	

5	140914507	1.41E+08	KD2-H4K8ac_peak_23827	9.98013	
5	140936923	1.41E+08	KD2-H4K8ac_peak_23828	9.4551	CTD-2024I7.13_ENSG00000246422
5	140939055	1.41E+08	KD2-H4K8ac_peak_23829	5.34946	
5	140939859	1.41E+08	KD2-H4K8ac_peak_23830	4.42765	
5	141030511	1.41E+08	KD2-H4K8ac_peak_23831	7.13615	FCHSD1_ENSG00000197948
5	141060796	1.41E+08	KD2-H4K8ac_peak_23832	8.86099	ARAP3_ENSG00000120318
5	141190990	1.41E+08	KD2-H4K8ac_peak_23833	11.2938	
5	141201762	1.41E+08	KD2-H4K8ac_peak_23834	5.57044	
5	141217670	1.41E+08	KD2-H4K8ac_peak_23835	4.3437	
5	141221062	1.41E+08	KD2-H4K8ac_peak_23836	8.98093	
5	141228551	1.41E+08	KD2-H4K8ac_peak_23837	20.5798	
5	141230378	1.41E+08	KD2-H4K8ac_peak_23838	16.75834	
5	141262601	1.41E+08	KD2-H4K8ac_peak_23839	7.70289	
5	141293896	1.41E+08	KD2-H4K8ac_peak_23840	6.40297	
5	141302505	1.41E+08	KD2-H4K8ac_peak_23841	16.49944	KIAA0141_ENSG00000081791
5	141339867	1.41E+08	KD2-H4K8ac_peak_23842	8.74596	
5	141392175	1.41E+08	KD2-H4K8ac_peak_23843	12.08081	GNPDA1_ENSG00000113552
5	141443656	1.41E+08	KD2-H4K8ac_peak_23844	12.99417	
5	141487883	1.41E+08	KD2-H4K8ac_peak_23845	12.93209	NDFIP1_ENSG00000131507
5	141546086	1.42E+08	KD2-H4K8ac_peak_23846	4.52326	
5	141547443	1.42E+08	KD2-H4K8ac_peak_23847	7.36933	
5	141548587	1.42E+08	KD2-H4K8ac_peak_23848	7.27716	
5	141559138	1.42E+08	KD2-H4K8ac_peak_23849	19.49525	
5	141560621	1.42E+08	KD2-H4K8ac_peak_23850	12.3187	
5	141561740	1.42E+08	KD2-H4K8ac_peak_23851	7.72801	
5	141562992	1.42E+08	KD2-H4K8ac_peak_23852	4.86324	
5	141564200	1.42E+08	KD2-H4K8ac_peak_23853	4.06758	
5	141565124	1.42E+08	KD2-H4K8ac_peak_23854	11.05407	
5	141566407	1.42E+08	KD2-H4K8ac_peak_23855	10.72656	
5	141640516	1.42E+08	KD2-H4K8ac_peak_23856	6.91644	
5	141667325	1.42E+08	KD2-H4K8ac_peak_23857	4.54006	
5	141669974	1.42E+08	KD2-H4K8ac_peak_23858	5.92967	
5	141703804	1.42E+08	KD2-H4K8ac_peak_23859	6.40683	AC005592.2_ENSG00000231185
5	141704859	1.42E+08	KD2-H4K8ac_peak_23860	13.1684	SPRY4_ENSG00000187678;AC005592.2_ENSG00000231185
5	141705468	1.42E+08	KD2-H4K8ac_peak_23861	24.1491	SPRY4_ENSG00000187678;AC005592.2_ENSG00000231185
5	141735611	1.42E+08	KD2-H4K8ac_peak_23862	18.94636	
5	141874541	1.42E+08	KD2-H4K8ac_peak_23863	9.8758	
5	142064050	1.42E+08	KD2-H4K8ac_peak_23864	4.70388	
5	142064603	1.42E+08	KD2-H4K8ac_peak_23865	4.44748	
5	142065445	1.42E+08	KD2-H4K8ac_peak_23866	13.80319	
5	142066237	1.42E+08	KD2-H4K8ac_peak_23867	10.1591	
5	142068159	1.42E+08	KD2-H4K8ac_peak_23868	8.45238	
5	142069050	1.42E+08	KD2-H4K8ac_peak_23869	10.58496	
5	142077057	1.42E+08	KD2-H4K8ac_peak_23870	6.00226	FGF1_ENSG00000113578
5	142078416	1.42E+08	KD2-H4K8ac_peak_23871	5.20908	FGF1_ENSG00000113578
5	142092882	1.42E+08	KD2-H4K8ac_peak_23872	9.47671	

5	142189029	1.42E+08	KD2-H4K8ac_peak_23873	9.11031	
5	142782941	1.43E+08	KD2-H4K8ac_peak_23874	4.24345	
5	142784481	1.43E+08	KD2-H4K8ac_peak_23875	10.43145	
5	142813238	1.43E+08	KD2-H4K8ac_peak_23876	6.68439	
5	142814753	1.43E+08	KD2-H4K8ac_peak_23877	5.3778	NR3C1_ENSG00000113580
5	143265575	1.43E+08	KD2-H4K8ac_peak_23878	7.47462	
5	143397807	1.43E+08	KD2-H4K8ac_peak_23879	9.11818	
5	143407219	1.43E+08	KD2-H4K8ac_peak_23880	9.11031	
5	143411832	1.43E+08	KD2-H4K8ac_peak_23881	5.54647	
5	145214318	1.45E+08	KD2-H4K8ac_peak_23882	14.10697	PRELID2_ENSG00000186314
5	145561911	1.46E+08	KD2-H4K8ac_peak_23883	7.92775	LARS_ENSG00000133706
5	145582689	1.46E+08	KD2-H4K8ac_peak_23884	8.59579	RBM27_ENSG00000091009
5	145583214	1.46E+08	KD2-H4K8ac_peak_23885	6.99815	RBM27_ENSG00000091009
5	145826959	1.46E+08	KD2-H4K8ac_peak_23886	19.33282	TCERG1_ENSG00000113649
5	145945561	1.46E+08	KD2-H4K8ac_peak_23887	5.71749	
5	146614332	1.47E+08	KD2-H4K8ac_peak_23888	5.14125	CTC-255N20.1_ENSG00000250343;STK32A_ENSG00000169302
5	146833678	1.47E+08	KD2-H4K8ac_peak_23889	10.65724	
5	146859525	1.47E+08	KD2-H4K8ac_peak_23890	6.57893	
5	146888653	1.47E+08	KD2-H4K8ac_peak_23891	8.85813	DPYSL3_ENSG00000113657
5	147699571	1.48E+08	KD2-H4K8ac_peak_23892	11.75669	SPINK9_ENSG00000204909
5	147763563	1.48E+08	KD2-H4K8ac_peak_23893	16.32216	RP11-373N22.3_ENSG00000247199;FBXO38_ENSG00000145868
5	148033423	1.48E+08	KD2-H4K8ac_peak_23894	6.99815	
5	148205736	1.48E+08	KD2-H4K8ac_peak_23895	11.43353	ADRB2_ENSG00000169252
5	148331757	1.48E+08	KD2-H4K8ac_peak_23896	5.57044	
5	148651464	1.49E+08	KD2-H4K8ac_peak_23897	5.30799	AFAP1L1_ENSG00000157510
5	148697151	1.49E+08	KD2-H4K8ac_peak_23898	5.54647	
5	148699901	1.49E+08	KD2-H4K8ac_peak_23899	11.89502	
5	148723970	1.49E+08	KD2-H4K8ac_peak_23900	6.05809	GRPEL2_ENSG00000164284
5	148725118	1.49E+08	KD2-H4K8ac_peak_23901	8.85813	GRPEL2_ENSG00000164284
5	148761017	1.49E+08	KD2-H4K8ac_peak_23902	7.76329	
5	148765093	1.49E+08	KD2-H4K8ac_peak_23903	9.31907	
5	148785776	1.49E+08	KD2-H4K8ac_peak_23904	11.80497	MIR143HG_ENSG00000249669;AC131025.8_ENSG00000253864
5	148786308	1.49E+08	KD2-H4K8ac_peak_23905	7.83489	MIR143HG_ENSG00000249669;AC131025.8_ENSG00000253864
5	148786890	1.49E+08	KD2-H4K8ac_peak_23906	8.86391	MIR143HG_ENSG00000249669;AC131025.8_ENSG00000253864
5	148792301	1.49E+08	KD2-H4K8ac_peak_23907	5.82869	
5	148799682	1.49E+08	KD2-H4K8ac_peak_23908	9.34195	
5	148800326	1.49E+08	KD2-H4K8ac_peak_23909	13.70605	
5	148802615	1.49E+08	KD2-H4K8ac_peak_23910	13.81781	
5	148930643	1.49E+08	KD2-H4K8ac_peak_23911	10.17341	CSNK1A1_ENSG00000113712;ARHGEF37_ENSG00000183111
5	148960571	1.49E+08	KD2-H4K8ac_peak_23912	16.57619	
5	149026293	1.49E+08	KD2-H4K8ac_peak_23913	10.21502	
5	149109951	1.49E+08	KD2-H4K8ac_peak_23914	6.25869	PPARGC1B_ENSG00000155846
5	149185759	1.49E+08	KD2-H4K8ac_peak_23915	7.146	
5	149339780	1.49E+08	KD2-H4K8ac_peak_23916	12.23	SLC26A2_ENSG00000155850
5	149340370	1.49E+08	KD2-H4K8ac_peak_23917	9.73431	SLC26A2_ENSG00000155850
5	149379703	1.49E+08	KD2-H4K8ac_peak_23918	6.84261	TIGD6_ENSG00000164296;HMGXB3_ENSG00000113716

5	149380308	1.49E+08	KD2-H4K8ac_peak_23919	9.61895	TIGD6_ENSG00000164296;HMGXB3_ENSG00000113716
5	149492093	1.49E+08	KD2-H4K8ac_peak_23920	4.62142	CSF1R_ENSG00000182578
5	149517831	1.5E+08	KD2-H4K8ac_peak_23921	3.97079	
5	149518583	1.5E+08	KD2-H4K8ac_peak_23922	5.92591	
5	149519806	1.5E+08	KD2-H4K8ac_peak_23923	5.63186	
5	149523157	1.5E+08	KD2-H4K8ac_peak_23924	5.71781	
5	149526676	1.5E+08	KD2-H4K8ac_peak_23925	5.14125	
5	149533372	1.5E+08	KD2-H4K8ac_peak_23926	8.2413	
5	149535087	1.5E+08	KD2-H4K8ac_peak_23927	8.4716	PDGFRB_ENSG00000113721
5	149535663	1.5E+08	KD2-H4K8ac_peak_23928	6.16103	PDGFRB_ENSG00000113721
5	149536877	1.5E+08	KD2-H4K8ac_peak_23929	12.79505	
5	149538182	1.5E+08	KD2-H4K8ac_peak_23930	7.95563	
5	149539405	1.5E+08	KD2-H4K8ac_peak_23931	8.85813	
5	149660231	1.5E+08	KD2-H4K8ac_peak_23932	4.01115	
5	149696982	1.5E+08	KD2-H4K8ac_peak_23933	6.85193	
5	149737308	1.5E+08	KD2-H4K8ac_peak_23934	9.20193	TCOF1_ENSG00000070814
5	149789688	1.5E+08	KD2-H4K8ac_peak_23935	10.72656	
5	149828937	1.5E+08	KD2-H4K8ac_peak_23936	5.13615	RPS14_ENSG00000164587
5	149837718	1.5E+08	KD2-H4K8ac_peak_23937	5.42867	
5	149864857	1.5E+08	KD2-H4K8ac_peak_23938	19.55721	CTC-367J11.1_ENSG00000254333;NDST1_ENSG00000070614
5	149883416	1.5E+08	KD2-H4K8ac_peak_23939	6.15399	
5	149891603	1.5E+08	KD2-H4K8ac_peak_23940	4.36736	
5	149950670	1.5E+08	KD2-H4K8ac_peak_23941	7.27716	
5	149953913	1.5E+08	KD2-H4K8ac_peak_23942	20.47312	
5	149962007	1.5E+08	KD2-H4K8ac_peak_23943	9.36516	
5	149963195	1.5E+08	KD2-H4K8ac_peak_23944	4.06758	
5	149964196	1.5E+08	KD2-H4K8ac_peak_23945	7.47462	
5	149969737	1.5E+08	KD2-H4K8ac_peak_23946	10.17486	
5	149970494	1.5E+08	KD2-H4K8ac_peak_23947	9.65086	
5	149972124	1.5E+08	KD2-H4K8ac_peak_23948	4.89359	
5	149973728	1.5E+08	KD2-H4K8ac_peak_23949	5.14125	
5	149975449	1.5E+08	KD2-H4K8ac_peak_23950	10.23436	
5	149977566	1.5E+08	KD2-H4K8ac_peak_23951	7.59183	
5	149978397	1.5E+08	KD2-H4K8ac_peak_23952	5.10665	
5	149979701	1.5E+08	KD2-H4K8ac_peak_23953	13.87818	SYNPO_ENSG00000171992
5	149980636	1.5E+08	KD2-H4K8ac_peak_23954	13.92465	SYNPO_ENSG00000171992
5	149994914	1.5E+08	KD2-H4K8ac_peak_23955	6.76298	CTB-140J7.2_ENSG00000253852
5	149997950	1.5E+08	KD2-H4K8ac_peak_23956	9.20193	
5	150004502	1.5E+08	KD2-H4K8ac_peak_23957	6.99815	
5	150024530	1.5E+08	KD2-H4K8ac_peak_23958	7.22919	
5	150035828	1.5E+08	KD2-H4K8ac_peak_23959	26.05481	
5	150051052	1.5E+08	KD2-H4K8ac_peak_23960	19.33018	CTC-345K18.2_ENSG00000250309
5	150080187	1.5E+08	KD2-H4K8ac_peak_23961	26.77453	RBM22_ENSG00000086589
5	150137729	1.5E+08	KD2-H4K8ac_peak_23962	5.57044	DCTN4_ENSG00000132912
5	150138365	1.5E+08	KD2-H4K8ac_peak_23963	9.34195	DCTN4_ENSG00000132912
5	150158108	1.5E+08	KD2-H4K8ac_peak_23964	5.30799	SMIM3_ENSG00000256235;AC010441.1_ENSG00000269636

5	150460325	1.5E+08	KD2-H4K8ac_peak_23965	15.57998	
5	150536842	1.51E+08	KD2-H4K8ac_peak_23966	7.0754	ANXA6_ENSG00000197043;CTB-17P3.4_ENSG00000254298
5	150827195	1.51E+08	KD2-H4K8ac_peak_23967	13.21979	
5	151066352	1.51E+08	KD2-H4K8ac_peak_23968	6.83936	SPARC_ENSG00000113140
5	151138387	1.51E+08	KD2-H4K8ac_peak_23969	4.37815	
5	151150356	1.51E+08	KD2-H4K8ac_peak_23970	15.16343	G3BP1_ENSG00000145907
5	151151934	1.51E+08	KD2-H4K8ac_peak_23971	8.02571	ATOX1_ENSG00000177556
5	153418222	1.53E+08	KD2-H4K8ac_peak_23972	11.70505	FAM114A2_ENSG00000055147;MFAP3_ENSG00000037749
5	153569444	1.54E+08	KD2-H4K8ac_peak_23973	11.92978	GALNT10_ENSG00000164574
5	153596973	1.54E+08	KD2-H4K8ac_peak_23974	6.9809	
5	153825680	1.54E+08	KD2-H4K8ac_peak_23975	6.60096	SAP30L-AS1_ENSG00000245275;SAP30L_ENSG00000164576
5	153847658	1.54E+08	KD2-H4K8ac_peak_23976	7.1181	
5	153849760	1.54E+08	KD2-H4K8ac_peak_23977	4.42498	
5	153853248	1.54E+08	KD2-H4K8ac_peak_23978	11.55418	
5	153854057	1.54E+08	KD2-H4K8ac_peak_23979	7.73418	
5	153855103	1.54E+08	KD2-H4K8ac_peak_23980	7.40111	
5	153856930	1.54E+08	KD2-H4K8ac_peak_23981	7.92775	HAND1_ENSG00000113196
5	153858228	1.54E+08	KD2-H4K8ac_peak_23982	4.36736	HAND1_ENSG00000113196
5	153862405	1.54E+08	KD2-H4K8ac_peak_23983	4.44527	
5	153863468	1.54E+08	KD2-H4K8ac_peak_23984	11.24702	
5	153864058	1.54E+08	KD2-H4K8ac_peak_23985	9.20193	
5	153865158	1.54E+08	KD2-H4K8ac_peak_23986	7.47462	CTB-158E9.1_ENSG00000254293
5	153874685	1.54E+08	KD2-H4K8ac_peak_23987	6.40683	
5	153884717	1.54E+08	KD2-H4K8ac_peak_23988	14.12659	
5	153885462	1.54E+08	KD2-H4K8ac_peak_23989	12.57297	
5	153890348	1.54E+08	KD2-H4K8ac_peak_23990	6.0073	
5	153894349	1.54E+08	KD2-H4K8ac_peak_23991	6.56211	
5	153895384	1.54E+08	KD2-H4K8ac_peak_23992	6.8836	
5	153895840	1.54E+08	KD2-H4K8ac_peak_23993	9.11031	
5	153897119	1.54E+08	KD2-H4K8ac_peak_23994	14.8083	
5	153898548	1.54E+08	KD2-H4K8ac_peak_23995	15.22643	
5	153900704	1.54E+08	KD2-H4K8ac_peak_23996	7.95934	
5	153901303	1.54E+08	KD2-H4K8ac_peak_23997	10.96455	
5	153902479	1.54E+08	KD2-H4K8ac_peak_23998	14.98671	
5	153903587	1.54E+08	KD2-H4K8ac_peak_23999	14.72959	
5	153904328	1.54E+08	KD2-H4K8ac_peak_24000	5.61067	
5	153905876	1.54E+08	KD2-H4K8ac_peak_24001	7.64093	
5	153921138	1.54E+08	KD2-H4K8ac_peak_24002	8.00457	
5	153948577	1.54E+08	KD2-H4K8ac_peak_24003	7.11925	
5	153967211	1.54E+08	KD2-H4K8ac_peak_24004	8.17902	
5	153975438	1.54E+08	KD2-H4K8ac_peak_24005	6.56926	MIR3141_ENSG00000264760
5	153988812	1.54E+08	KD2-H4K8ac_peak_24006	5.08628	
5	153990123	1.54E+08	KD2-H4K8ac_peak_24007	9.11891	
5	154061723	1.54E+08	KD2-H4K8ac_peak_24008	5.30799	
5	154062587	1.54E+08	KD2-H4K8ac_peak_24009	13.88933	
5	154134192	1.54E+08	KD2-H4K8ac_peak_24010	10.29842	

5	154134938	1.54E+08	KD2-H4K8ac_peak_24011	13.36684	
5	154194005	1.54E+08	KD2-H4K8ac_peak_24012	5.29352	
5	154214200	1.54E+08	KD2-H4K8ac_peak_24013	4.8888	
5	154236275	1.54E+08	KD2-H4K8ac_peak_24014	11.55418	CNOT8_ENSG00000155508
5	154317224	1.54E+08	KD2-H4K8ac_peak_24015	4.37815	GEMIN5_ENSG00000082516
5	154317891	1.54E+08	KD2-H4K8ac_peak_24016	6.85193	GEMIN5_ENSG00000082516
5	154320432	1.54E+08	KD2-H4K8ac_peak_24017	6.65673	MRPL22_ENSG00000082515
5	155754029	1.56E+08	KD2-H4K8ac_peak_24018	4.77079	
5	156569430	1.57E+08	KD2-H4K8ac_peak_24019	12.84642	HAVCR2_ENSG00000135077;ITK_ENSG00000113263
5	156570363	1.57E+08	KD2-H4K8ac_peak_24020	8.25022	HAVCR2_ENSG00000135077;ITK_ENSG00000113263
5	156692704	1.57E+08	KD2-H4K8ac_peak_24021	5.84991	CTC-248O19.1_ENSG00000253653;CYFIP2_ENSG00000055163
5	156693148	1.57E+08	KD2-H4K8ac_peak_24022	8.16906	CTC-248O19.1_ENSG00000253653;CYFIP2_ENSG00000055163
5	156811345	1.57E+08	KD2-H4K8ac_peak_24023	10.05606	CTB-47B11.3_ENSG00000248544
5	156814784	1.57E+08	KD2-H4K8ac_peak_24024	10.58016	
5	156870506	1.57E+08	KD2-H4K8ac_peak_24025	5.14125	
5	156871418	1.57E+08	KD2-H4K8ac_peak_24026	5.29555	
5	156876618	1.57E+08	KD2-H4K8ac_peak_24027	3.75035	
5	156886964	1.57E+08	KD2-H4K8ac_peak_24028	12.30509	CTB-109A12.1_ENSG00000251405;NIPAL4_ENSG00000172548
5	157002345	1.57E+08	KD2-H4K8ac_peak_24029	11.05467	ADAM19_ENSG00000135074
5	157098626	1.57E+08	KD2-H4K8ac_peak_24030	5.71749	SOX30_ENSG00000039600;C5orf52_ENSG00000187658
5	157145788	1.57E+08	KD2-H4K8ac_peak_24031	6.66692	
5	157285544	1.57E+08	KD2-H4K8ac_peak_24032	17.14886	CLINT1_ENSG00000113282
5	157376092	1.57E+08	KD2-H4K8ac_peak_24033	6.06203	
5	157388079	1.57E+08	KD2-H4K8ac_peak_24034	5.57044	
5	157910469	1.58E+08	KD2-H4K8ac_peak_24035	6.40683	
5	158448342	1.58E+08	KD2-H4K8ac_peak_24036	8.26945	
5	158522139	1.59E+08	KD2-H4K8ac_peak_24037	10.19949	
5	158527837	1.59E+08	KD2-H4K8ac_peak_24038	4.03256	RP11-175K6.1_ENSG00000245812
5	158635975	1.59E+08	KD2-H4K8ac_peak_24039	7.47462	RNF145_ENSG00000145860;CTB-11I22.1_ENSG00000253256
5	158636675	1.59E+08	KD2-H4K8ac_peak_24040	6.66692	RNF145_ENSG00000145860;CTB-11I22.1_ENSG00000253256
5	158689643	1.59E+08	KD2-H4K8ac_peak_24041	11.89502	UBLCP1_ENSG00000164332
5	158758538	1.59E+08	KD2-H4K8ac_peak_24042	17.17257	IL12B_ENSG00000113302
5	159435528	1.59E+08	KD2-H4K8ac_peak_24043	11.1952	Y_RNA_ENSG00000199398;TTC1_ENSG00000113312
5	159545954	1.6E+08	KD2-H4K8ac_peak_24044	16.8435	PWWP2A_ENSG00000170234
5	159591386	1.6E+08	KD2-H4K8ac_peak_24045	14.63458	
5	159626420	1.6E+08	KD2-H4K8ac_peak_24046	15.57998	
5	159714040	1.6E+08	KD2-H4K8ac_peak_24047	5.72327	
5	159738708	1.6E+08	KD2-H4K8ac_peak_24048	14.66631	
5	159743119	1.6E+08	KD2-H4K8ac_peak_24049	7.59643	
5	159771938	1.6E+08	KD2-H4K8ac_peak_24050	15.22643	CCNJL_ENSG00000135083
5	159797375	1.6E+08	KD2-H4K8ac_peak_24051	4.42765	C1QTNF2_ENSG00000145861
5	159826784	1.6E+08	KD2-H4K8ac_peak_24052	13.73635	C5orf54_ENSG00000221886
5	162886702	1.63E+08	KD2-H4K8ac_peak_24053	9.36516	NUDCD2_ENSG00000170584;HMMR_ENSG00000072571
5	162887720	1.63E+08	KD2-H4K8ac_peak_24054	7.48133	NUDCD2_ENSG00000170584;HMMR_ENSG00000072571
5	162930895	1.63E+08	KD2-H4K8ac_peak_24055	8.88549	MAT2B_ENSG00000038274
5	162931317	1.63E+08	KD2-H4K8ac_peak_24056	5.54647	

5	163087143	1.63E+08	KD2-H4K8ac_peak_24057	11.70505	
5	164008366	1.64E+08	KD2-H4K8ac_peak_24058	5.14125	
5	167589251	1.68E+08	KD2-H4K8ac_peak_24059	11.03331	
5	167589900	1.68E+08	KD2-H4K8ac_peak_24060	8.23274	
5	167605011	1.68E+08	KD2-H4K8ac_peak_24061	5.92591	
5	167718336	1.68E+08	KD2-H4K8ac_peak_24062	5.75757	WWC1_ENSG00000113645
5	167912929	1.68E+08	KD2-H4K8ac_peak_24063	10.1591	RARS_ENSG00000113643
5	167913542	1.68E+08	KD2-H4K8ac_peak_24064	7.1181	RARS_ENSG00000113643
5	167956035	1.68E+08	KD2-H4K8ac_peak_24065	16.09057	FBLL1_ENSG00000188573
5	167956823	1.68E+08	KD2-H4K8ac_peak_24066	6.20982	FBLL1_ENSG00000188573
5	168006068	1.68E+08	KD2-H4K8ac_peak_24067	8.16906	PANK3_ENSG00000120137
5	168080624	1.68E+08	KD2-H4K8ac_peak_24068	10.0692	CTC-558O2.2_ENSG00000254192
5	168084576	1.68E+08	KD2-H4K8ac_peak_24069	4.211	
5	168182981	1.68E+08	KD2-H4K8ac_peak_24070	12.74183	
5	168184084	1.68E+08	KD2-H4K8ac_peak_24071	7.73418	
5	168185538	1.68E+08	KD2-H4K8ac_peak_24072	4.86324	
5	168186395	1.68E+08	KD2-H4K8ac_peak_24073	13.72842	
5	168189465	1.68E+08	KD2-H4K8ac_peak_24074	6.99815	
5	168190620	1.68E+08	KD2-H4K8ac_peak_24075	4.44923	
5	168193378	1.68E+08	KD2-H4K8ac_peak_24076	12.36633	
5	168194251	1.68E+08	KD2-H4K8ac_peak_24077	17.59645	MIR218-2_ENSG00000207739
5	168198014	1.68E+08	KD2-H4K8ac_peak_24078	10.43145	
5	168199928	1.68E+08	KD2-H4K8ac_peak_24079	8.55271	
5	168200580	1.68E+08	KD2-H4K8ac_peak_24080	7.56441	
5	168201873	1.68E+08	KD2-H4K8ac_peak_24081	5.9696	
5	168206351	1.68E+08	KD2-H4K8ac_peak_24082	4.211	
5	168224129	1.68E+08	KD2-H4K8ac_peak_24083	8.30974	
5	168241012	1.68E+08	KD2-H4K8ac_peak_24084	4.44527	
5	168271118	1.68E+08	KD2-H4K8ac_peak_24085	21.36133	
5	168275229	1.68E+08	KD2-H4K8ac_peak_24086	8.52559	
5	168278848	1.68E+08	KD2-H4K8ac_peak_24087	5.88845	
5	168303099	1.68E+08	KD2-H4K8ac_peak_24088	5.71749	
5	168307185	1.68E+08	KD2-H4K8ac_peak_24089	6.25869	
5	168308832	1.68E+08	KD2-H4K8ac_peak_24090	5.45396	
5	168309934	1.68E+08	KD2-H4K8ac_peak_24091	11.09936	
5	168311870	1.68E+08	KD2-H4K8ac_peak_24092	14.48084	
5	168312778	1.68E+08	KD2-H4K8ac_peak_24093	7.96322	
5	168314027	1.68E+08	KD2-H4K8ac_peak_24094	8.44476	
5	168314591	1.68E+08	KD2-H4K8ac_peak_24095	5.88845	
5	168316860	1.68E+08	KD2-H4K8ac_peak_24096	8.09472	
5	168322014	1.68E+08	KD2-H4K8ac_peak_24097	8.30974	
5	168323771	1.68E+08	KD2-H4K8ac_peak_24098	7.76329	
5	168324218	1.68E+08	KD2-H4K8ac_peak_24099	7.42204	
5	168324733	1.68E+08	KD2-H4K8ac_peak_24100	14.59844	
5	168326213	1.68E+08	KD2-H4K8ac_peak_24101	8.55271	
5	168346042	1.68E+08	KD2-H4K8ac_peak_24102	6.40683	

5	168363110	1.68E+08	KD2-H4K8ac_peak_24103	8.28994	
5	168369693	1.68E+08	KD2-H4K8ac_peak_24104	6.1846	
5	168371243	1.68E+08	KD2-H4K8ac_peak_24105	5.71749	
5	168372321	1.68E+08	KD2-H4K8ac_peak_24106	11.00314	
5	168374456	1.68E+08	KD2-H4K8ac_peak_24107	12.06346	
5	169010843	1.69E+08	KD2-H4K8ac_peak_24108	11.89502	SPDL1_ENSG00000040275
5	169372989	1.69E+08	KD2-H4K8ac_peak_24109	5.26591	
5	169853625	1.7E+08	KD2-H4K8ac_peak_24110	9.16531	
5	170025199	1.7E+08	KD2-H4K8ac_peak_24111	6.40683	
5	170033739	1.7E+08	KD2-H4K8ac_peak_24112	8.80225	
5	170178964	1.7E+08	KD2-H4K8ac_peak_24113	8.10216	
5	170179602	1.7E+08	KD2-H4K8ac_peak_24114	6.40297	
5	170209600	1.7E+08	KD2-H4K8ac_peak_24115	5.88845	
5	170289074	1.7E+08	KD2-H4K8ac_peak_24116	7.76329	RANBP17_ENSG00000204764
5	170702975	1.71E+08	KD2-H4K8ac_peak_24117	5.25071	
5	170735384	1.71E+08	KD2-H4K8ac_peak_24118	7.76329	TLX3_ENSG00000164438
5	170762593	1.71E+08	KD2-H4K8ac_peak_24119	5.13341	
5	170813383	1.71E+08	KD2-H4K8ac_peak_24120	9.63925	MIR3912_ENSG00000264249;NPM1_ENSG00000181163
5	170814045	1.71E+08	KD2-H4K8ac_peak_24121	9.242	MIR3912_ENSG00000264249;NPM1_ENSG00000181163
5	170864445	1.71E+08	KD2-H4K8ac_peak_24122	3.75035	
5	171071473	1.71E+08	KD2-H4K8ac_peak_24123	6.40683	
5	171432877	1.71E+08	KD2-H4K8ac_peak_24124	9.63487	FBXW11_ENSG00000072803
5	171434086	1.71E+08	KD2-H4K8ac_peak_24125	9.20193	FBXW11_ENSG00000072803
5	171531001	1.72E+08	KD2-H4K8ac_peak_24126	5.58333	
5	171533450	1.72E+08	KD2-H4K8ac_peak_24127	8.94415	
5	171538333	1.72E+08	KD2-H4K8ac_peak_24128	6.99815	
5	171541951	1.72E+08	KD2-H4K8ac_peak_24129	4.211	
5	171571433	1.72E+08	KD2-H4K8ac_peak_24130	4.86324	
5	171614498	1.72E+08	KD2-H4K8ac_peak_24131	8.59579	STK10_ENSG00000072786
5	171615449	1.72E+08	KD2-H4K8ac_peak_24132	8.55271	STK10_ENSG00000072786
5	171672381	1.72E+08	KD2-H4K8ac_peak_24133	9.15329	
5	171710859	1.72E+08	KD2-H4K8ac_peak_24134	14.06758	UBTD2_ENSG00000168246
5	171711335	1.72E+08	KD2-H4K8ac_peak_24135	8.36432	UBTD2_ENSG00000168246
5	171880338	1.72E+08	KD2-H4K8ac_peak_24136	7.76329	SH3PXD2B_ENSG00000174705
5	171881751	1.72E+08	KD2-H4K8ac_peak_24137	10.01478	SH3PXD2B_ENSG00000174705
5	171977325	1.72E+08	KD2-H4K8ac_peak_24138	7.70289	
5	171978045	1.72E+08	KD2-H4K8ac_peak_24139	6.99815	
5	172067763	1.72E+08	KD2-H4K8ac_peak_24140	8.10216	NEURL1B_ENSG00000214357
5	172068324	1.72E+08	KD2-H4K8ac_peak_24141	7.38799	NEURL1B_ENSG00000214357
5	172086658	1.72E+08	KD2-H4K8ac_peak_24142	12.88108	
5	172092135	1.72E+08	KD2-H4K8ac_peak_24143	11.00314	
5	172133796	1.72E+08	KD2-H4K8ac_peak_24144	6.16888	
5	172134406	1.72E+08	KD2-H4K8ac_peak_24145	12.06346	
5	172192046	1.72E+08	KD2-H4K8ac_peak_24146	10.0692	
5	172197557	1.72E+08	KD2-H4K8ac_peak_24147	10.30284	DUSP1_ENSG00000120129
5	172198513	1.72E+08	KD2-H4K8ac_peak_24148	9.08816	DUSP1_ENSG00000120129

5	172199196	1.72E+08	KD2-H4K8ac_peak_24149	18.31034	DUSP1_ENSG00000120129
5	172385594	1.72E+08	KD2-H4K8ac_peak_24150	11.43353	CTC-308K20.1_ENSG00000204758;RPL26L1_ENSG00000037241
5	172410264	1.72E+08	KD2-H4K8ac_peak_24151	6.45083	ATP6V0E1_ENSG00000113732
5	172571272	1.73E+08	KD2-H4K8ac_peak_24152	7.76329	BNIP1_ENSG00000113734;CTC-209H22.3_ENSG00000253172
5	172710744	1.73E+08	KD2-H4K8ac_peak_24153	5.54647	
5	172719060	1.73E+08	KD2-H4K8ac_peak_24154	6.92624	RNA5SP200_ENSG00000252169
5	172750360	1.73E+08	KD2-H4K8ac_peak_24155	7.67504	
5	172754228	1.73E+08	KD2-H4K8ac_peak_24156	8.17557	
5	172755733	1.73E+08	KD2-H4K8ac_peak_24157	4.04573	STC2_ENSG00000113739
5	172756175	1.73E+08	KD2-H4K8ac_peak_24158	13.09607	STC2_ENSG00000113739
5	173043182	1.73E+08	KD2-H4K8ac_peak_24159	16.8435	BOD1_ENSG00000145919
5	173043900	1.73E+08	KD2-H4K8ac_peak_24160	7.40633	BOD1_ENSG00000145919
5	173060845	1.73E+08	KD2-H4K8ac_peak_24161	3.80759	
5	173071135	1.73E+08	KD2-H4K8ac_peak_24162	11.05407	
5	173124218	1.73E+08	KD2-H4K8ac_peak_24163	5.40356	
5	173125423	1.73E+08	KD2-H4K8ac_peak_24164	5.08628	
5	173126018	1.73E+08	KD2-H4K8ac_peak_24165	7.96322	
5	173127331	1.73E+08	KD2-H4K8ac_peak_24166	8.59579	
5	173146639	1.73E+08	KD2-H4K8ac_peak_24167	14.66631	
5	173147353	1.73E+08	KD2-H4K8ac_peak_24168	8.30974	
5	173148579	1.73E+08	KD2-H4K8ac_peak_24169	9.08816	
5	173149942	1.73E+08	KD2-H4K8ac_peak_24170	4.211	
5	173180202	1.73E+08	KD2-H4K8ac_peak_24171	6.13909	
5	173184716	1.73E+08	KD2-H4K8ac_peak_24172	11.94983	
5	173209237	1.73E+08	KD2-H4K8ac_peak_24173	8.1233	
5	173314569	1.73E+08	KD2-H4K8ac_peak_24174	11.3394	CPEB4_ENSG00000113742
5	173400435	1.73E+08	KD2-H4K8ac_peak_24175	8.55271	C5orf47_ENSG00000185056
5	173510023	1.74E+08	KD2-H4K8ac_peak_24176	8.37231	
5	173510583	1.74E+08	KD2-H4K8ac_peak_24177	6.25869	
5	173511045	1.74E+08	KD2-H4K8ac_peak_24178	12.80176	
5	173547366	1.74E+08	KD2-H4K8ac_peak_24179	4.67342	
5	173594638	1.74E+08	KD2-H4K8ac_peak_24180	5.25071	
5	173618148	1.74E+08	KD2-H4K8ac_peak_24181	7.1181	
5	173622154	1.74E+08	KD2-H4K8ac_peak_24182	6.66692	
5	173648783	1.74E+08	KD2-H4K8ac_peak_24183	8.17902	
5	173659460	1.74E+08	KD2-H4K8ac_peak_24184	6.0073	
5	173661574	1.74E+08	KD2-H4K8ac_peak_24185	5.14125	
5	173663832	1.74E+08	KD2-H4K8ac_peak_24186	6.56211	
5	173738477	1.74E+08	KD2-H4K8ac_peak_24187	7.74292	
5	173738904	1.74E+08	KD2-H4K8ac_peak_24188	15.40367	
5	173750898	1.74E+08	KD2-H4K8ac_peak_24189	6.40683	
5	173756102	1.74E+08	KD2-H4K8ac_peak_24190	3.83717	CTC-430J12.2_ENSG00000249722
5	173763966	1.74E+08	KD2-H4K8ac_peak_24191	6.85193	RP11-267A15.1_ENSG00000249306
5	173811515	1.74E+08	KD2-H4K8ac_peak_24192	5.88845	
5	173991518	1.74E+08	KD2-H4K8ac_peak_24193	4.42765	
5	174035718	1.74E+08	KD2-H4K8ac_peak_24194	7.13615	

5	174150862	1.74E+08	KD2-H4K8ac_peak_24195	6.14257	MSX2_ENSG00000120149
5	174178028	1.74E+08	KD2-H4K8ac_peak_24196	10.7186	CTD-2532K18.1_ENSG00000251670;MIR4634_ENSG00000266890
5	174905597	1.75E+08	KD2-H4K8ac_peak_24197	12.57143	
5	175223649	1.75E+08	KD2-H4K8ac_peak_24198	10.73134	CPLX2_ENSG00000145920
5	175288433	1.75E+08	KD2-H4K8ac_peak_24199	4.54006	
5	175335613	1.75E+08	KD2-H4K8ac_peak_24200	3.75035	
5	175336082	1.75E+08	KD2-H4K8ac_peak_24201	9.15329	
5	175395018	1.75E+08	KD2-H4K8ac_peak_24202	11.7608	
5	175561429	1.76E+08	KD2-H4K8ac_peak_24203	11.05467	
5	175662424	1.76E+08	KD2-H4K8ac_peak_24204	6.40683	
5	175788311	1.76E+08	KD2-H4K8ac_peak_24205	8.10216	KIAA1191_ENSG00000122203
5	175792120	1.76E+08	KD2-H4K8ac_peak_24206	4.86324	ARL10_ENSG00000175414
5	175792531	1.76E+08	KD2-H4K8ac_peak_24207	14.98671	ARL10_ENSG00000175414
5	175815207	1.76E+08	KD2-H4K8ac_peak_24208	10.95625	NOP16_ENSG00000048162;HIGD2A_ENSG00000146066
5	175843733	1.76E+08	KD2-H4K8ac_peak_24209	6.05809	CLTB_ENSG00000175416
5	175874296	1.76E+08	KD2-H4K8ac_peak_24210	6.65673	FAF2_ENSG00000113194
5	175875430	1.76E+08	KD2-H4K8ac_peak_24211	7.76329	FAF2_ENSG00000113194
5	175962642	1.76E+08	KD2-H4K8ac_peak_24212	20.02161	RNF44_ENSG00000146083
5	175969741	1.76E+08	KD2-H4K8ac_peak_24213	10.37746	CDHR2_ENSG00000074276
5	175970890	1.76E+08	KD2-H4K8ac_peak_24214	7.45501	
5	176037314	1.76E+08	KD2-H4K8ac_peak_24215	24.02151	GPRIN1_ENSG00000169258
5	176037835	1.76E+08	KD2-H4K8ac_peak_24216	7.86151	GPRIN1_ENSG00000169258
5	176073809	1.76E+08	KD2-H4K8ac_peak_24217	14.66631	TSPAN17_ENSG00000048140
5	176074498	1.76E+08	KD2-H4K8ac_peak_24218	9.77606	TSPAN17_ENSG00000048140
5	176165939	1.76E+08	KD2-H4K8ac_peak_24219	6.14031	RP11-375B1.2_ENSG00000251446
5	176166443	1.76E+08	KD2-H4K8ac_peak_24220	16.40085	RP11-375B1.2_ENSG00000251446
5	176220229	1.76E+08	KD2-H4K8ac_peak_24221	6.55628	
5	176244335	1.76E+08	KD2-H4K8ac_peak_24222	13.13969	
5	176262168	1.76E+08	KD2-H4K8ac_peak_24223	3.75035	
5	176264300	1.76E+08	KD2-H4K8ac_peak_24224	4.211	
5	176267578	1.76E+08	KD2-H4K8ac_peak_24225	8.42997	
5	176271221	1.76E+08	KD2-H4K8ac_peak_24226	5.54647	
5	176271904	1.76E+08	KD2-H4K8ac_peak_24227	9.36516	
5	176289912	1.76E+08	KD2-H4K8ac_peak_24228	5.51783	
5	176293099	1.76E+08	KD2-H4K8ac_peak_24229	4.62548	
5	176310859	1.76E+08	KD2-H4K8ac_peak_24230	8.2413	
5	176433348	1.76E+08	KD2-H4K8ac_peak_24231	8.16906	
5	176433992	1.76E+08	KD2-H4K8ac_peak_24232	4.44748	
5	176449790	1.76E+08	KD2-H4K8ac_peak_24233	7.49599	UIMC1_ENSG00000087206;ZNF346_ENSG00000113761
5	176514174	1.77E+08	KD2-H4K8ac_peak_24234	3.75035	FGFR4_ENSG00000160867
5	176541196	1.77E+08	KD2-H4K8ac_peak_24235	10.2048	
5	176560124	1.77E+08	KD2-H4K8ac_peak_24236	25.36636	NSD1_ENSG00000165671
5	176730234	1.77E+08	KD2-H4K8ac_peak_24237	11.3394	RAB24_ENSG00000169228;PRELID1_ENSG00000169230
5	176778286	1.77E+08	KD2-H4K8ac_peak_24238	15.58442	LMAN2_ENSG00000169223
5	176779104	1.77E+08	KD2-H4K8ac_peak_24239	9.20193	LMAN2_ENSG00000169223
5	176789901	1.77E+08	KD2-H4K8ac_peak_24240	9.1707	

5	176793051	1.77E+08	KD2-H4K8ac_peak_24241	18.25771	
5	176794371	1.77E+08	KD2-H4K8ac_peak_24242	5.59859	
5	176797822	1.77E+08	KD2-H4K8ac_peak_24243	6.22239	
5	176817857	1.77E+08	KD2-H4K8ac_peak_24244	12.82393	
5	176829245	1.77E+08	KD2-H4K8ac_peak_24245	13.50024	GRK6_ENSG00000198055
5	176830142	1.77E+08	KD2-H4K8ac_peak_24246	5.38581	GRK6_ENSG00000198055
5	176831647	1.77E+08	KD2-H4K8ac_peak_24247	8.5251	
5	176852613	1.77E+08	KD2-H4K8ac_peak_24248	8.55271	
5	176853876	1.77E+08	KD2-H4K8ac_peak_24249	7.70289	
5	176873180	1.77E+08	KD2-H4K8ac_peak_24250	11.05467	PRR7-AS1_ENSG00000246334;PRR7_ENSG00000131188
5	176880963	1.77E+08	KD2-H4K8ac_peak_24251	8.58725	
5	176881956	1.77E+08	KD2-H4K8ac_peak_24252	8.23274	
5	176882443	1.77E+08	KD2-H4K8ac_peak_24253	4.79789	
5	176899777	1.77E+08	KD2-H4K8ac_peak_24254	17.83665	DBN1_ENSG00000113758
5	176901070	1.77E+08	KD2-H4K8ac_peak_24255	6.41015	DBN1_ENSG00000113758
5	176906918	1.77E+08	KD2-H4K8ac_peak_24256	11.89502	
5	176924132	1.77E+08	KD2-H4K8ac_peak_24257	7.07117	PDLIM7_ENSG00000196923
5	176927602	1.77E+08	KD2-H4K8ac_peak_24258	5.51783	
5	176928293	1.77E+08	KD2-H4K8ac_peak_24259	7.13615	
5	176943639	1.77E+08	KD2-H4K8ac_peak_24260	11.05467	DDX41_ENSG00000183258
5	176944257	1.77E+08	KD2-H4K8ac_peak_24261	5.9696	DDX41_ENSG00000183258
5	176981204	1.77E+08	KD2-H4K8ac_peak_24262	7.98621	FAM193B_ENSG00000146067;RP11-1277A3.3_ENSG00000272459
5	176981755	1.77E+08	KD2-H4K8ac_peak_24263	6.40683	FAM193B_ENSG00000146067;RP11-1277A3.3_ENSG00000272459
5	177019272	1.77E+08	KD2-H4K8ac_peak_24264	12.17278	TMED9_ENSG00000184840
5	177026425	1.77E+08	KD2-H4K8ac_peak_24265	16.14659	B4GALT7_ENSG00000027847
5	177540308	1.78E+08	KD2-H4K8ac_peak_24266	9.16531	N4BP3_ENSG00000145911
5	177548324	1.78E+08	KD2-H4K8ac_peak_24267	5.51783	
5	177556749	1.78E+08	KD2-H4K8ac_peak_24268	4.66652	RMND5B_ENSG00000145916
5	177557244	1.78E+08	KD2-H4K8ac_peak_24269	8.16906	RMND5B_ENSG00000145916
5	177591055	1.78E+08	KD2-H4K8ac_peak_24270	14.48084	
5	177614035	1.78E+08	KD2-H4K8ac_peak_24271	8.30974	GMCL1P1_ENSG00000244234
5	177630925	1.78E+08	KD2-H4K8ac_peak_24272	5.30799	HNRNPAB_ENSG00000197451
5	177631596	1.78E+08	KD2-H4K8ac_peak_24273	9.61895	HNRNPAB_ENSG00000197451
5	177658899	1.78E+08	KD2-H4K8ac_peak_24274	25.71759	PHYKPL_ENSG00000175309
5	177758437	1.78E+08	KD2-H4K8ac_peak_24275	6.30229	
5	177763978	1.78E+08	KD2-H4K8ac_peak_24276	9.20193	
5	177767512	1.78E+08	KD2-H4K8ac_peak_24277	4.77079	
5	177775404	1.78E+08	KD2-H4K8ac_peak_24278	6.65673	
5	177783645	1.78E+08	KD2-H4K8ac_peak_24279	9.59147	
5	177799037	1.78E+08	KD2-H4K8ac_peak_24280	5.40356	
5	177809244	1.78E+08	KD2-H4K8ac_peak_24281	11.07495	
5	177824071	1.78E+08	KD2-H4K8ac_peak_24282	5.14125	
5	177825878	1.78E+08	KD2-H4K8ac_peak_24283	11.05407	
5	177841161	1.78E+08	KD2-H4K8ac_peak_24284	5.49367	
5	177855858	1.78E+08	KD2-H4K8ac_peak_24285	9.89383	
5	177861013	1.78E+08	KD2-H4K8ac_peak_24286	9.95119	

5	177862273	1.78E+08	KD2-H4K8ac_peak_24287	12.52363	
5	177877133	1.78E+08	KD2-H4K8ac_peak_24288	9.73431	
5	177896524	1.78E+08	KD2-H4K8ac_peak_24289	6.40683	
5	178016638	1.78E+08	KD2-H4K8ac_peak_24290	7.45501	COL23A1_ENSG00000050767
5	178053722	1.78E+08	KD2-H4K8ac_peak_24291	11.00004	
5	178054294	1.78E+08	KD2-H4K8ac_peak_24292	15.82362	
5	178157884	1.78E+08	KD2-H4K8ac_peak_24293	6.57893	ZNF354A_ENSG00000169131
5	178287043	1.78E+08	KD2-H4K8ac_peak_24294	7.95563	ZNF354B_ENSG00000178338
5	178322314	1.78E+08	KD2-H4K8ac_peak_24295	17.42905	ZFP2_ENSG00000198939
5	178367498	1.78E+08	KD2-H4K8ac_peak_24296	10.94087	ZNF454_ENSG00000178187
5	178450227	1.78E+08	KD2-H4K8ac_peak_24297	19.33282	ZNF879_ENSG00000234284
5	178487419	1.78E+08	KD2-H4K8ac_peak_24298	10.96455	ZNF354C_ENSG00000177932
5	178593889	1.79E+08	KD2-H4K8ac_peak_24299	6.65673	
5	178688675	1.79E+08	KD2-H4K8ac_peak_24300	4.67342	
5	178694308	1.79E+08	KD2-H4K8ac_peak_24301	6.18308	
5	178696772	1.79E+08	KD2-H4K8ac_peak_24302	6.90231	
5	178697216	1.79E+08	KD2-H4K8ac_peak_24303	8.55271	
5	178712431	1.79E+08	KD2-H4K8ac_peak_24304	9.96798	
5	178712911	1.79E+08	KD2-H4K8ac_peak_24305	7.70289	
5	178713334	1.79E+08	KD2-H4K8ac_peak_24306	5.40356	
5	178714080	1.79E+08	KD2-H4K8ac_peak_24307	9.70057	
5	178714817	1.79E+08	KD2-H4K8ac_peak_24308	9.36516	
5	178721405	1.79E+08	KD2-H4K8ac_peak_24309	5.88845	
5	178728841	1.79E+08	KD2-H4K8ac_peak_24310	6.40683	
5	178735765	1.79E+08	KD2-H4K8ac_peak_24311	6.25869	
5	178736879	1.79E+08	KD2-H4K8ac_peak_24312	8.85813	
5	178740891	1.79E+08	KD2-H4K8ac_peak_24313	29.10149	
5	178742171	1.79E+08	KD2-H4K8ac_peak_24314	8.16133	
5	178748339	1.79E+08	KD2-H4K8ac_peak_24315	5.88845	
5	178749660	1.79E+08	KD2-H4K8ac_peak_24316	10.19949	
5	178750317	1.79E+08	KD2-H4K8ac_peak_24317	6.99815	
5	178757598	1.79E+08	KD2-H4K8ac_peak_24318	7.59643	
5	178772622	1.79E+08	KD2-H4K8ac_peak_24319	6.98302	ADAMTS2_ENSG00000087116
5	178862829	1.79E+08	KD2-H4K8ac_peak_24320	5.88845	
5	178864553	1.79E+08	KD2-H4K8ac_peak_24321	7.62396	
5	178865397	1.79E+08	KD2-H4K8ac_peak_24322	14.27217	
5	178977215	1.79E+08	KD2-H4K8ac_peak_24323	18.54875	RUFY1_ENSG00000176783
5	178986230	1.79E+08	KD2-H4K8ac_peak_24324	11.03331	
5	179051041	1.79E+08	KD2-H4K8ac_peak_24325	12.14909	
5	179105103	1.79E+08	KD2-H4K8ac_peak_24326	8.53092	CANX_ENSG00000127022;HMGB3P22_ENSG00000225051
5	179105806	1.79E+08	KD2-H4K8ac_peak_24327	12.74171	CANX_ENSG00000127022;HMGB3P22_ENSG00000225051
5	179126051	1.79E+08	KD2-H4K8ac_peak_24328	5.13615	
5	179159896	1.79E+08	KD2-H4K8ac_peak_24329	8.58069	MAML1_ENSG00000161021
5	179222162	1.79E+08	KD2-H4K8ac_peak_24330	6.99815	
5	179223434	1.79E+08	KD2-H4K8ac_peak_24331	8.30974	
5	179234075	1.79E+08	KD2-H4K8ac_peak_24332	16.15695	MGAT4B_ENSG00000161013;SQSTM1_ENSG00000161011

5	179245714	1.79E+08	KD2-H4K8ac_peak_24333	9.20193	
5	179246474	1.79E+08	KD2-H4K8ac_peak_24334	11.80283	
5	179247403	1.79E+08	KD2-H4K8ac_peak_24335	14.63726	
5	179247995	1.79E+08	KD2-H4K8ac_peak_24336	4.19765	
5	179286057	1.79E+08	KD2-H4K8ac_peak_24337	13.81781	CTC-241N9.1_ENSG00000245317
5	179334217	1.79E+08	KD2-H4K8ac_peak_24338	6.65673	TBC1D9B_ENSG00000197226
5	179335456	1.79E+08	KD2-H4K8ac_peak_24339	6.85193	TBC1D9B_ENSG00000197226
5	179498157	1.79E+08	KD2-H4K8ac_peak_24340	7.47462	RNF130_ENSG00000113269
5	179498865	1.79E+08	KD2-H4K8ac_peak_24341	16.71655	RNF130_ENSG00000113269
5	179517121	1.8E+08	KD2-H4K8ac_peak_24342	13.36684	
5	179518056	1.8E+08	KD2-H4K8ac_peak_24343	7.22457	
5	179604281	1.8E+08	KD2-H4K8ac_peak_24344	10.77651	
5	179635592	1.8E+08	KD2-H4K8ac_peak_24345	7.63551	RASGEF1C_ENSG00000146090
5	179636404	1.8E+08	KD2-H4K8ac_peak_24346	10.17698	RASGEF1C_ENSG00000146090
5	179716958	1.8E+08	KD2-H4K8ac_peak_24347	10.17698	
5	179718374	1.8E+08	KD2-H4K8ac_peak_24348	11.81257	MAPK9_ENSG00000050748
5	179719225	1.8E+08	KD2-H4K8ac_peak_24349	7.47462	MAPK9_ENSG00000050748;CTB-129O4.1_ENSG00000248367
5	179741003	1.8E+08	KD2-H4K8ac_peak_24350	6.47635	
5	179808082	1.8E+08	KD2-H4K8ac_peak_24351	6.55628	
5	179808915	1.8E+08	KD2-H4K8ac_peak_24352	6.40297	
5	179921484	1.8E+08	KD2-H4K8ac_peak_24353	8.1235	CNOT6_ENSG00000113300
5	180229997	1.8E+08	KD2-H4K8ac_peak_24354	14.43704	
5	180236910	1.8E+08	KD2-H4K8ac_peak_24355	16.8435	
5	180258018	1.8E+08	KD2-H4K8ac_peak_24356	11.80283	LINC00847_ENSG00000245060
5	180287461	1.8E+08	KD2-H4K8ac_peak_24357	15.81711	ZFP62_ENSG00000196670
5	180586864	1.81E+08	KD2-H4K8ac_peak_24358	9.26408	
5	180588277	1.81E+08	KD2-H4K8ac_peak_24359	5.13615	
5	180611368	1.81E+08	KD2-H4K8ac_peak_24360	4.8888	
5	180617853	1.81E+08	KD2-H4K8ac_peak_24361	6.17833	CTC-338M12.2_ENSG00000248473;CTC-338M12.5_ENSG00000250222
5	180618910	1.81E+08	KD2-H4K8ac_peak_24362	11.79585	CTC-338M12.2_ENSG00000248473;CTC-338M12.5_ENSG00000250222
5	180634064	1.81E+08	KD2-H4K8ac_peak_24363	5.43522	CTC-338M12.1_ENSG00000248514
5	180649055	1.81E+08	KD2-H4K8ac_peak_24364	8.17557	TRIM41_ENSG00000146063;MIR4638_ENSG00000264732
5	180650701	1.81E+08	KD2-H4K8ac_peak_24365	11.03331	
5	180670638	1.81E+08	KD2-H4K8ac_peak_24366	11.58876	SNORD95_ENSG00000264549
5	180672655	1.81E+08	KD2-H4K8ac_peak_24367	9.63487	CTC-338M12.4_ENSG00000233937
5	180673548	1.81E+08	KD2-H4K8ac_peak_24368	11.24702	CTC-338M12.4_ENSG00000233937
5	180688258	1.81E+08	KD2-H4K8ac_peak_24369	11.16154	TRIM52_ENSG00000183718;TRIM52-AS1_ENSG00000248275
5	180689348	1.81E+08	KD2-H4K8ac_peak_24370	5.14125	
6	291603	292338	KD2-H4K8ac_peak_24371	23.13674	DUSP22_ENSG00000112679
6	692885	693671	KD2-H4K8ac_peak_24372	14.12659	EXOC2_ENSG00000112685
6	1604439	1604631	KD2-H4K8ac_peak_24373	5.75452	
6	1609269	1609955	KD2-H4K8ac_peak_24374	13.49029	FOXC1_ENSG00000054598
6	1611560	1612132	KD2-H4K8ac_peak_24375	4.86324	FOXC1_ENSG00000054598
6	1766841	1767237	KD2-H4K8ac_peak_24376	6.16888	
6	1801602	1801879	KD2-H4K8ac_peak_24377	4.42765	
6	1802201	1802399	KD2-H4K8ac_peak_24378	6.8836	

6	1804471	1804782	KD2-H4K8ac_peak_24379	10.21502	
6	1821408	1821600	KD2-H4K8ac_peak_24380	5.57044	
6	1855484	1855816	KD2-H4K8ac_peak_24381	4.59928	
6	1903730	1904091	KD2-H4K8ac_peak_24382	6.83936	
6	1904747	1904969	KD2-H4K8ac_peak_24383	9.08816	
6	2634380	2634789	KD2-H4K8ac_peak_24384	10.19949	C6orf195_ENSG00000164385
6	2764988	2765252	KD2-H4K8ac_peak_24385	11.86082	WRNIP1_ENSG00000124535
6	2765877	2766126	KD2-H4K8ac_peak_24386	7.90806	WRNIP1_ENSG00000124535
6	2766333	2766699	KD2-H4K8ac_peak_24387	6.65673	WRNIP1_ENSG00000124535
6	2841987	2842224	KD2-H4K8ac_peak_24388	9.10868	SERPINB1_ENSG00000021355
6	2875600	2876368	KD2-H4K8ac_peak_24389	13.57066	
6	2876571	2876906	KD2-H4K8ac_peak_24390	6.16888	
6	2971149	2971594	KD2-H4K8ac_peak_24391	16.71655	SERPINB6_ENSG00000124570
6	2971818	2972329	KD2-H4K8ac_peak_24392	8.10216	SERPINB6_ENSG00000124570
6	2988412	2988669	KD2-H4K8ac_peak_24393	12.42761	LINC01011_ENSG00000244041;NQO2_ENSG00000124588
6	2989000	2989647	KD2-H4K8ac_peak_24394	12.79505	LINC01011_ENSG00000244041;NQO2_ENSG00000124588
6	2991245	2991451	KD2-H4K8ac_peak_24395	3.75035	
6	3000149	3000694	KD2-H4K8ac_peak_24396	11.05407	RP1-90J20.8_ENSG00000224846
6	3053762	3054389	KD2-H4K8ac_peak_24397	9.1707	
6	3064162	3064888	KD2-H4K8ac_peak_24398	6.72334	RIPK1_ENSG00000137275
6	3068772	3069529	KD2-H4K8ac_peak_24399	10.76532	RP1-40E16.12_ENSG00000272277
6	3157545	3158279	KD2-H4K8ac_peak_24400	19.47291	TUBB2A_ENSG00000137267
6	3162884	3163330	KD2-H4K8ac_peak_24401	18.37277	
6	3195193	3195700	KD2-H4K8ac_peak_24402	7.76329	RP1-40E16.9_ENSG00000230269
6	3209530	3209973	KD2-H4K8ac_peak_24403	10.0089	
6	3228139	3228746	KD2-H4K8ac_peak_24404	20.64015	
6	3229108	3230086	KD2-H4K8ac_peak_24405	6.67092	
6	3231388	3232265	KD2-H4K8ac_peak_24406	14.8083	TUBB2B_ENSG00000137285;PSMG4_ENSG00000180822
6	3259267	3259675	KD2-H4K8ac_peak_24407	7.76329	
6	4018438	4018738	KD2-H4K8ac_peak_24408	4.91674	RP3-406P24.4_ENSG00000272248
6	4021607	4021968	KD2-H4K8ac_peak_24409	10.21366	RP3-406P24.3_ENSG00000230648;PRPF4B_ENSG00000112739
6	4135380	4135635	KD2-H4K8ac_peak_24410	6.66692	ECI2_ENSG00000198721;RP3-400B16.4_ENSG00000254821;RP3-400B16.1_ENSG00000234817
6	4136229	4136580	KD2-H4K8ac_peak_24411	6.25869	ECI2_ENSG00000198721;RP3-400B16.4_ENSG00000254821;RP3-400B16.1_ENSG00000234817
6	4775370	4775564	KD2-H4K8ac_peak_24412	5.30799	RP3-430A16.1_ENSG00000236336
6	4974315	4974567	KD2-H4K8ac_peak_24413	5.82799	
6	5004441	5004789	KD2-H4K8ac_peak_24414	8.1233	RPP40_ENSG00000124787
6	5026486	5027053	KD2-H4K8ac_peak_24415	4.211	
6	5261526	5262165	KD2-H4K8ac_peak_24416	9.65086	LYRM4_ENSG00000214113;FARS2_ENSG00000145982
6	5604212	5604423	KD2-H4K8ac_peak_24417	9.15329	
6	5663964	5664388	KD2-H4K8ac_peak_24418	12.80176	
6	5744219	5744438	KD2-H4K8ac_peak_24419	8.16133	
6	5752793	5753132	KD2-H4K8ac_peak_24420	6.66692	
6	5938013	5938582	KD2-H4K8ac_peak_24421	8.1718	
6	7052073	7052475	KD2-H4K8ac_peak_24422	5.57044	
6	7107489	7107701	KD2-H4K8ac_peak_24423	4.8402	RREB1_ENSG00000124782
6	7140991	7141245	KD2-H4K8ac_peak_24424	8.5781	

6	7314006	7314206	KD2-H4K8ac_peak_24425	5.30799	
6	7389651	7390436	KD2-H4K8ac_peak_24426	9.89126	CAGE1_ENSG00000164304;RIOK1_ENSG00000124784
6	7468766	7469050	KD2-H4K8ac_peak_24427	9.11031	
6	7910120	7910412	KD2-H4K8ac_peak_24428	10.41994	
6	7911209	7911604	KD2-H4K8ac_peak_24429	7.81657	
6	8064133	8064570	KD2-H4K8ac_peak_24430	12.82393	BLOC1S5-TXNDC5_ENSG00000259040;BLOC1S5_ENSG00000188428
6	8102152	8103133	KD2-H4K8ac_peak_24431	9.11031	EEF1E1-BLOC1S5_ENSG00000265818;EEF1E1_ENSG00000124802
6	8237532	8237790	KD2-H4K8ac_peak_24432	5.29555	
6	8435479	8436360	KD2-H4K8ac_peak_24433	14.55656	SLC35B3_ENSG00000124786
6	10404861	10405196	KD2-H4K8ac_peak_24434	17.94825	
6	10510937	10511133	KD2-H4K8ac_peak_24435	6.16888	
6	10659945	10660702	KD2-H4K8ac_peak_24436	7.36799	
6	10720387	10721200	KD2-H4K8ac_peak_24437	8.28234	
6	10723437	10723707	KD2-H4K8ac_peak_24438	6.66099	TMEM14C_ENSG00000111843
6	10747387	10747748	KD2-H4K8ac_peak_24439	4.98072	RP11-421M1.8_ENSG00000272097;TMEM14B_ENSG00000137210;SYCP2L_ENSG00000153157;RP11-637O19.3_ENSG00000272162
6	11043920	11044505	KD2-H4K8ac_peak_24440	8.17902	ELOVL2_ENSG00000197977;ELOVL2-AS1_ENSG00000230314
6	11094086	11094727	KD2-H4K8ac_peak_24441	21.6161	SMIM13_ENSG00000224531
6	11537981	11538898	KD2-H4K8ac_peak_24442	12.36441	TMEM170B_ENSG00000205269
6	12008520	12008805	KD2-H4K8ac_peak_24443	5.835	RP11-456H18.2_ENSG00000229896;HIVEP1_ENSG00000095951
6	12011902	12012429	KD2-H4K8ac_peak_24444	10.29344	
6	12012622	12013113	KD2-H4K8ac_peak_24445	5.14125	
6	13328916	13329304	KD2-H4K8ac_peak_24446	14.8083	TBC1D7_ENSG00000145979
6	13527226	13527692	KD2-H4K8ac_peak_24447	4.211	
6	13558679	13558981	KD2-H4K8ac_peak_24448	4.211	
6	13574463	13575514	KD2-H4K8ac_peak_24449	13.9234	SIRT5_ENSG00000124523
6	13614963	13615219	KD2-H4K8ac_peak_24450	6.25869	RP1-223E5.4_ENSG00000261071;NOL7_ENSG00000225921
6	13711620	13711997	KD2-H4K8ac_peak_24451	10.95611	RANBP9_ENSG00000010017
6	13712207	13712597	KD2-H4K8ac_peak_24452	6.25869	RANBP9_ENSG00000010017
6	13814073	13814753	KD2-H4K8ac_peak_24453	17.55491	MCUR1_ENSG00000050393
6	13860221	13860756	KD2-H4K8ac_peak_24454	4.42765	
6	13873771	13874555	KD2-H4K8ac_peak_24455	9.63487	
6	13924226	13924424	KD2-H4K8ac_peak_24456	5.43522	RNF182_ENSG00000180537
6	13924636	13925038	KD2-H4K8ac_peak_24457	7.12754	RNF182_ENSG00000180537
6	14117414	14118365	KD2-H4K8ac_peak_24458	19.77886	CD83_ENSG00000112149
6	14210960	14211273	KD2-H4K8ac_peak_24459	11.90231	
6	14211964	14212213	KD2-H4K8ac_peak_24460	6.99815	
6	14515887	14516124	KD2-H4K8ac_peak_24461	5.04237	
6	14519533	14519811	KD2-H4K8ac_peak_24462	5.93446	
6	14621598	14621831	KD2-H4K8ac_peak_24463	5.6755	
6	14694196	14694428	KD2-H4K8ac_peak_24464	4.3015	
6	15245072	15245433	KD2-H4K8ac_peak_24465	3.86308	RP11-560J1.2_ENSG00000271888
6	15245690	15246174	KD2-H4K8ac_peak_24466	17.8868	RP11-560J1.2_ENSG00000271888;JARID2_ENSG00000008083
6	15338044	15338250	KD2-H4K8ac_peak_24467	4.98318	
6	15342435	15342636	KD2-H4K8ac_peak_24468	10.21502	
6	15662483	15662851	KD2-H4K8ac_peak_24469	5.405	DTNBP1_ENSG00000047579
6	15663569	15663763	KD2-H4K8ac_peak_24470	4.9025	DTNBP1_ENSG00000047579

6	15880700	15880977	KD2-H4K8ac_peak_24471	6.27931	
6	15883648	15883957	KD2-H4K8ac_peak_24472	4.04247	
6	16129376	16129641	KD2-H4K8ac_peak_24473	8.23274	MYLIP_ENSG00000007944
6	16160059	16160267	KD2-H4K8ac_peak_24474	7.51935	RP1-13D10.2_ENSG00000218073
6	16238596	16239040	KD2-H4K8ac_peak_24475	5.57044	GMPR_ENSG00000137198
6	16239705	16239936	KD2-H4K8ac_peak_24476	10.25088	GMPR_ENSG00000137198
6	16337893	16338250	KD2-H4K8ac_peak_24477	8.05667	
6	16345665	16345930	KD2-H4K8ac_peak_24478	4.66652	
6	16346334	16346640	KD2-H4K8ac_peak_24479	6.90231	
6	16760659	16761086	KD2-H4K8ac_peak_24480	17.70837	ATXN1_ENSG00000124788;RP1-151F17.1_ENSG00000229931
6	17280747	17280990	KD2-H4K8ac_peak_24481	6.16888	RBM24_ENSG00000112183
6	17282104	17282300	KD2-H4K8ac_peak_24482	6.25869	RBM24_ENSG00000112183
6	17393470	17394301	KD2-H4K8ac_peak_24483	7.27716	CAP2_ENSG00000112186
6	17580887	17581267	KD2-H4K8ac_peak_24484	6.84261	SUMO2P13_ENSG00000218359
6	17599873	17600344	KD2-H4K8ac_peak_24485	4.42765	FAM8A1_ENSG00000137414
6	17600670	17601020	KD2-H4K8ac_peak_24486	10.63441	FAM8A1_ENSG00000137414
6	17706394	17706920	KD2-H4K8ac_peak_24487	5.13615	NUP153_ENSG00000124789;RP11-500C11.3_ENSG00000272269
6	17708248	17708509	KD2-H4K8ac_peak_24488	8.37043	
6	17987504	17987807	KD2-H4K8ac_peak_24489	8.26945	KIF13A_ENSG00000137177
6	17988016	17988379	KD2-H4K8ac_peak_24490	12.25733	KIF13A_ENSG00000137177
6	18155032	18155225	KD2-H4K8ac_peak_24491	8.05667	TPMT_ENSG00000137364;KDM1B_ENSG00000165097
6	18155758	18155977	KD2-H4K8ac_peak_24492	5.4779	TPMT_ENSG00000137364;KDM1B_ENSG00000165097
6	18264014	18264664	KD2-H4K8ac_peak_24493	8.55552	DEK_ENSG00000124795
6	18264918	18265529	KD2-H4K8ac_peak_24494	4.211	DEK_ENSG00000124795
6	19601022	19601235	KD2-H4K8ac_peak_24495	5.07241	
6	20212297	20212640	KD2-H4K8ac_peak_24496	15.52826	MBOAT1_ENSG00000172197;RP11-239H6.2_ENSG00000227803
6	20402308	20403140	KD2-H4K8ac_peak_24497	19.91242	E2F3_ENSG00000112242
6	20403930	20404292	KD2-H4K8ac_peak_24498	21.26856	
6	20534047	20534321	KD2-H4K8ac_peak_24499	5.96828	CDKAL1_ENSG00000145996
6	20673852	20674181	KD2-H4K8ac_peak_24500	4.01115	
6	20679340	20679563	KD2-H4K8ac_peak_24501	6.16888	
6	20691241	20691508	KD2-H4K8ac_peak_24502	6.16888	
6	21004935	21005141	KD2-H4K8ac_peak_24503	6.66692	
6	21198968	21199255	KD2-H4K8ac_peak_24504	4.37815	
6	21200887	21201106	KD2-H4K8ac_peak_24505	9.55735	
6	21241090	21241434	KD2-H4K8ac_peak_24506	5.57044	
6	21244290	21244580	KD2-H4K8ac_peak_24507	8.30974	
6	21381958	21382607	KD2-H4K8ac_peak_24508	8.17902	
6	21383206	21383425	KD2-H4K8ac_peak_24509	6.66692	
6	21384679	21384954	KD2-H4K8ac_peak_24510	9.98013	
6	21394726	21394929	KD2-H4K8ac_peak_24511	4.77079	
6	21500747	21501498	KD2-H4K8ac_peak_24512	7.33266	
6	21587104	21588955	KD2-H4K8ac_peak_24513	14.27217	
6	21589235	21589524	KD2-H4K8ac_peak_24514	6.09229	
6	21595130	21595322	KD2-H4K8ac_peak_24515	7.47462	
6	21595582	21596135	KD2-H4K8ac_peak_24516	5.75757	

6	21665884	21666492	KD2-H4K8ac_peak_24517	9.02573	CASC15_ENSG00000272168
6	21666691	21666955	KD2-H4K8ac_peak_24518	8.10216	
6	21667326	21667808	KD2-H4K8ac_peak_24519	4.42765	
6	21949643	21949982	KD2-H4K8ac_peak_24520	6.9809	
6	22000050	22000524	KD2-H4K8ac_peak_24521	7.27716	
6	22146967	22147597	KD2-H4K8ac_peak_24522	8.30974	CASC14_ENSG00000260455
6	22148218	22148425	KD2-H4K8ac_peak_24523	7.47462	CASC14_ENSG00000260455
6	22327153	22327420	KD2-H4K8ac_peak_24524	12.06346	
6	22363861	22364278	KD2-H4K8ac_peak_24525	4.42765	
6	22928106	22928338	KD2-H4K8ac_peak_24526	13.39763	
6	23004575	23004870	KD2-H4K8ac_peak_24527	8.85813	
6	23179802	23180184	KD2-H4K8ac_peak_24528	6.07178	
6	23180611	23180844	KD2-H4K8ac_peak_24529	6.16888	
6	23182972	23183197	KD2-H4K8ac_peak_24530	7.12754	
6	23281754	23282323	KD2-H4K8ac_peak_24531	8.87298	
6	23347703	23348257	KD2-H4K8ac_peak_24532	11.89502	
6	23412396	23412655	KD2-H4K8ac_peak_24533	9.15329	
6	23443317	23443746	KD2-H4K8ac_peak_24534	8.17902	
6	23444838	23445183	KD2-H4K8ac_peak_24535	7.12754	
6	23446113	23446342	KD2-H4K8ac_peak_24536	6.16888	
6	23446992	23447242	KD2-H4K8ac_peak_24537	7.12754	
6	23447532	23447834	KD2-H4K8ac_peak_24538	6.16888	
6	23523284	23523481	KD2-H4K8ac_peak_24539	5.13615	
6	23657436	23658132	KD2-H4K8ac_peak_24540	8.28994	
6	24191160	24191399	KD2-H4K8ac_peak_24541	6.16888	
6	24402613	24402960	KD2-H4K8ac_peak_24542	4.42765	MRS2_ENSG00000124532
6	24403236	24403534	KD2-H4K8ac_peak_24543	14.00658	MRS2_ENSG00000124532
6	24494919	24496046	KD2-H4K8ac_peak_24544	9.89383	GPLD1_ENSG00000112293;ALDH5A1_ENSG00000112294
6	24631161	24631555	KD2-H4K8ac_peak_24545	6.14257	
6	24646576	24646935	KD2-H4K8ac_peak_24546	11.30637	KIAA0319_ENSG00000137261
6	24666781	24667018	KD2-H4K8ac_peak_24547	6.40297	TDP2_ENSG00000111802;ACOT13_ENSG00000112304
6	24720545	24721005	KD2-H4K8ac_peak_24548	10.0692	C6orf62_ENSG00000112308
6	24721757	24722214	KD2-H4K8ac_peak_24549	9.1707	C6orf62_ENSG00000112308
6	24774722	24775418	KD2-H4K8ac_peak_24550	7.76104	GMNN_ENSG00000112312
6	25121977	25122383	KD2-H4K8ac_peak_24551	7.12754	
6	25279298	25279794	KD2-H4K8ac_peak_24552	5.59859	LRRC16A_ENSG00000079691
6	26020746	26021116	KD2-H4K8ac_peak_24553	10.08707	HIST1H3A_ENSG00000198366;HIST1H4A_ENSG00000196176
6	26028945	26029165	KD2-H4K8ac_peak_24554	7.70289	
6	26043302	26043598	KD2-H4K8ac_peak_24555	6.40683	HIST1H2BB_ENSG00000196226;U91328.2_ENSG00000234816
6	26044793	26045143	KD2-H4K8ac_peak_24556	11.2938	HIST1H2BB_ENSG00000196226;U91328.2_ENSG00000234816;HIST1H3C_ENSG00000196532
6	26057377	26057580	KD2-H4K8ac_peak_24557	10.1591	HIST1H1C_ENSG00000187837
6	26123600	26124125	KD2-H4K8ac_peak_24558	10.29344	HIST1H2BC_ENSG00000180596;HIST1H2AC_ENSG00000180573
6	26172537	26173015	KD2-H4K8ac_peak_24559	5.01002	
6	26184114	26184388	KD2-H4K8ac_peak_24560	6.40683	HIST1H2BE_ENSG00000197697
6	26188444	26189055	KD2-H4K8ac_peak_24561	5.71749	HIST1H4D_ENSG00000188987
6	26215690	26215926	KD2-H4K8ac_peak_24562	5.07241	HIST1H2BG_ENSG00000187990

6	26218048	26218704	KD2-H4K8ac_peak_24563	8.45238	HIST1H2AE_ENSG00000168274
6	26234575	26234811	KD2-H4K8ac_peak_24564	6.90231	HIST1H1D_ENSG00000124575
6	26249970	26250634	KD2-H4K8ac_peak_24565	5.9696	HIST1H3F_ENSG00000256316
6	26272289	26272755	KD2-H4K8ac_peak_24566	16.8184	HIST1H3G_ENSG00000256018;HIST1H2APS4_ENSG00000218690;HIST1H2BI_ENSG00000168242
6	26364166	26364553	KD2-H4K8ac_peak_24567	4.211	BTN3A2_ENSG00000186470
6	26383607	26384163	KD2-H4K8ac_peak_24568	12.57828	BTN2A2_ENSG00000124508
6	26421109	26421415	KD2-H4K8ac_peak_24569	14.43704	BTN2A3P_ENSG00000124549
6	26458239	26458605	KD2-H4K8ac_peak_24570	11.05407	BTN2A1_ENSG00000112763
6	26473907	26474316	KD2-H4K8ac_peak_24571	6.64891	
6	26501706	26502063	KD2-H4K8ac_peak_24572	8.1233	BTN1A1_ENSG00000124557
6	26520648	26520914	KD2-H4K8ac_peak_24573	7.96194	RNU6-502P_ENSG00000199289
6	26537007	26537204	KD2-H4K8ac_peak_24574	6.53915	
6	26538361	26538918	KD2-H4K8ac_peak_24575	12.76041	HMGN4_ENSG00000182952
6	26550382	26551154	KD2-H4K8ac_peak_24576	6.56211	
6	26569364	26569670	KD2-H4K8ac_peak_24577	5.25071	
6	26572516	26572912	KD2-H4K8ac_peak_24578	6.8836	
6	26596198	26596449	KD2-H4K8ac_peak_24579	10.21502	ABT1_ENSG00000146109
6	26597185	26597501	KD2-H4K8ac_peak_24580	10.19949	ABT1_ENSG00000146109
6	26924458	26924708	KD2-H4K8ac_peak_24581	29.60293	GUSBP2_ENSG00000241549;LINC00240_ENSG00000224843
6	26987507	26988042	KD2-H4K8ac_peak_24582	16.09057	TRNAI2_ENSG00000238621
6	27094480	27094775	KD2-H4K8ac_peak_24583	7.22457	
6	27100066	27100357	KD2-H4K8ac_peak_24584	11.15509	HIST1H2BJ_ENSG00000124635;HIST1H2AG_ENSG00000196787
6	27113260	27113685	KD2-H4K8ac_peak_24585	7.12754	HIST1H2BK_ENSG00000197903
6	27114156	27114377	KD2-H4K8ac_peak_24586	7.70289	HIST1H2BK_ENSG00000197903;HIST1H2AH_ENSG00000184825
6	27145264	27145856	KD2-H4K8ac_peak_24587	10.21502	
6	27146193	27146501	KD2-H4K8ac_peak_24588	7.12754	
6	27342648	27342862	KD2-H4K8ac_peak_24589	8.5092	ZNF391_ENSG00000124613
6	27356201	27357279	KD2-H4K8ac_peak_24590	8.58725	
6	27440406	27440696	KD2-H4K8ac_peak_24591	10.19949	ZNF184_ENSG00000096654
6	27655608	27655822	KD2-H4K8ac_peak_24592	8.5781	
6	27661425	27661642	KD2-H4K8ac_peak_24593	5.71749	
6	27706057	27706271	KD2-H4K8ac_peak_24594	7.12754	RP1-97D16.1_ENSG00000216915
6	27740493	27740689	KD2-H4K8ac_peak_24595	10.21502	
6	27775309	27775579	KD2-H4K8ac_peak_24596	6.25869	HIST1H4PS1_ENSG00000217862;HIST1H2BL_ENSG00000185130;HIST1H2AI_ENSG00000196747
6	27783492	27784014	KD2-H4K8ac_peak_24597	4.67342	HIST1H2AJ_ENSG00000182611;HIST1H2BM_ENSG00000196374
6	27794589	27794809	KD2-H4K8ac_peak_24598	6.60096	
6	27805379	27805984	KD2-H4K8ac_peak_24599	10.19949	HIST1H2AK_ENSG00000184348;HIST1H2BN_ENSG00000233822
6	27833232	27833459	KD2-H4K8ac_peak_24600	5.14125	HIST1H2AL_ENSG00000198374
6	27838007	27838204	KD2-H4K8ac_peak_24601	5.60968	
6	27840754	27841112	KD2-H4K8ac_peak_24602	8.02571	HIST1H3I_ENSG00000182572;HIST1H4L_ENSG00000198558
6	27842900	27843157	KD2-H4K8ac_peak_24603	6.31143	
6	27859798	27860153	KD2-H4K8ac_peak_24604	5.51783	HIST1H3J_ENSG00000197153;HIST1H2AM_ENSG00000233224
6	27870448	27870641	KD2-H4K8ac_peak_24605	6.40683	
6	28048012	28048225	KD2-H4K8ac_peak_24606	4.35346	RP1-313I6.12_ENSG00000272009;ZNF165_ENSG00000197279
6	28048768	28048969	KD2-H4K8ac_peak_24607	10.0746	RP1-313I6.12_ENSG00000272009;ZNF165_ENSG00000197279
6	28058836	28059224	KD2-H4K8ac_peak_24608	3.75035	ZSCAN12P1_ENSG00000219891

6	28072323	28072833	KD2-H4K8ac_peak_24609	8.55271	
6	28092366	28092687	KD2-H4K8ac_peak_24610	11.70505	ZSCAN16_ENSG00000196812
6	28104702	28105162	KD2-H4K8ac_peak_24611	21.60865	ZSCAN16-AS1_ENSG00000269293;RP1-265C24.8_ENSG00000261839
6	28109385	28109989	KD2-H4K8ac_peak_24612	6.04107	ZKSCAN8_ENSG00000198315
6	28129606	28129806	KD2-H4K8ac_peak_24613	8.86099	ZNF192P1_ENSG00000226314
6	28185993	28186659	KD2-H4K8ac_peak_24614	10.05606	TOB2P1_ENSG00000176933
6	28191939	28192251	KD2-H4K8ac_peak_24615	9.15329	ZSCAN9_ENSG00000137185
6	28193182	28193434	KD2-H4K8ac_peak_24616	8.2413	ZSCAN9_ENSG00000137185
6	28219411	28220018	KD2-H4K8ac_peak_24617	12.42547	
6	28220323	28220866	KD2-H4K8ac_peak_24618	12.25733	
6	28249076	28249712	KD2-H4K8ac_peak_24619	11.70505	PGBD1_ENSG00000137338
6	28302793	28303132	KD2-H4K8ac_peak_24620	8.1233	
6	28303486	28304180	KD2-H4K8ac_peak_24621	14.15075	
6	28317729	28317959	KD2-H4K8ac_peak_24622	5.54647	ZKSCAN3_ENSG00000189298
6	28410963	28411208	KD2-H4K8ac_peak_24623	15.28177	ZSCAN23_ENSG00000187987
6	28510042	28510808	KD2-H4K8ac_peak_24624	9.11031	
6	28554789	28555112	KD2-H4K8ac_peak_24625	8.5092	RP5-1186N24.3_ENSG00000246350
6	28565790	28566099	KD2-H4K8ac_peak_24626	4.9025	
6	28574753	28575124	KD2-H4K8ac_peak_24627	4.42765	
6	28589044	28589393	KD2-H4K8ac_peak_24628	8.1233	
6	28834142	28835053	KD2-H4K8ac_peak_24629	6.65673	
6	28890869	28891694	KD2-H4K8ac_peak_24630	16.8435	TRIM27_ENSG00000204713
6	28891915	28892301	KD2-H4K8ac_peak_24631	8.23062	TRIM27_ENSG00000204713
6	28948618	28948885	KD2-H4K8ac_peak_24632	9.15329	
6	28953733	28953966	KD2-H4K8ac_peak_24633	6.40683	HCG15_ENSG00000227214;HCG16_ENSG00000244349
6	29595354	29595772	KD2-H4K8ac_peak_24634	19.72117	
6	29601092	29601348	KD2-H4K8ac_peak_24635	10.96455	GABBR1_ENSG00000204681
6	29617565	29617802	KD2-H4K8ac_peak_24636	10.21502	
6	29909945	29910137	KD2-H4K8ac_peak_24637	12.42547	HLA-A_ENSG00000206503;HCG4P5_ENSG00000227766
6	29933037	29933262	KD2-H4K8ac_peak_24638	6.76298	
6	29944255	29944698	KD2-H4K8ac_peak_24639	10.08707	
6	30028530	30028898	KD2-H4K8ac_peak_24640	4.42765	ZNRD1-AS1_ENSG00000204623
6	30029152	30029504	KD2-H4K8ac_peak_24641	6.05809	ZNRD1-AS1_ENSG00000204623
6	30181463	30182282	KD2-H4K8ac_peak_24642	12.00597	TRIM26_ENSG00000234127
6	30226838	30228211	KD2-H4K8ac_peak_24643	16.11042	HLA-L_ENSG00000243753
6	30293944	30295400	KD2-H4K8ac_peak_24644	28.31632	HCG17_ENSG00000270604;HCG18_ENSG00000231074;TRIM39_ENSG00000204599
6	30313074	30313368	KD2-H4K8ac_peak_24645	7.98621	RPP21_ENSG00000241370
6	30457336	30457786	KD2-H4K8ac_peak_24646	6.25869	HLA-E_ENSG00000204592
6	30483825	30484142	KD2-H4K8ac_peak_24647	4.14296	XXbac-BPG249D20.9_ENSG00000235781
6	30524489	30525449	KD2-H4K8ac_peak_24648	12.13241	GNL1_ENSG00000204590;PRR3_ENSG00000204576
6	30583788	30584101	KD2-H4K8ac_peak_24649	8.23062	AL662800.1_ENSG00000222894
6	30584526	30584756	KD2-H4K8ac_peak_24650	9.51625	AL662800.1_ENSG00000222894;MRPS18B_ENSG00000204568
6	30585468	30585717	KD2-H4K8ac_peak_24651	4.77079	PPP1R10_ENSG00000204569;MRPS18B_ENSG00000204568
6	30594709	30594971	KD2-H4K8ac_peak_24652	9.36516	ATAT1_ENSG00000137343
6	30614953	30615520	KD2-H4K8ac_peak_24653	18.22876	C6orf136_ENSG00000204564;AL662800.2_ENSG00000266183
6	30650067	30650291	KD2-H4K8ac_peak_24654	4.95872	

6	30654703	30654969	KD2-H4K8ac_peak_24655	15.52826	PPP1R18_ENSG00000146112
6	30655254	30655751	KD2-H4K8ac_peak_24656	11.92978	PPP1R18_ENSG00000146112
6	30658357	30658633	KD2-H4K8ac_peak_24657	4.89656	NRM_ENSG00000137404
6	30684750	30684990	KD2-H4K8ac_peak_24658	11.92978	MDC1_ENSG00000137337
6	30688620	30689004	KD2-H4K8ac_peak_24659	12.5605	TUBB_ENSG00000196230
6	30709893	30710936	KD2-H4K8ac_peak_24660	12.84642	FLOT1_ENSG00000137312;XXbac-BPG252P9.10_ENSG00000272273
6	30737364	30737633	KD2-H4K8ac_peak_24661	8.00457	
6	30746919	30747227	KD2-H4K8ac_peak_24662	6.60096	
6	30747537	30747745	KD2-H4K8ac_peak_24663	4.42765	
6	30748134	30748480	KD2-H4K8ac_peak_24664	6.63018	
6	30748794	30749625	KD2-H4K8ac_peak_24665	16.58076	
6	30845302	30846087	KD2-H4K8ac_peak_24666	11.05407	
6	30851310	30851927	KD2-H4K8ac_peak_24667	8.16906	
6	30875288	30875672	KD2-H4K8ac_peak_24668	6.24114	RN7SL175P_ENSG00000264731;GTF2H4_ENSG00000213780;VARS2_ENSG00000137411
6	30882539	30882838	KD2-H4K8ac_peak_24669	10.45683	
6	31164755	31165387	KD2-H4K8ac_peak_24670	10.0692	XXbac-BPG299F13.17_ENSG00000272501;HCG27_ENSG00000206344
6	31239137	31239856	KD2-H4K8ac_peak_24671	10.29344	HLA-C_ENSG00000204525
6	31509190	31509652	KD2-H4K8ac_peak_24672	11.24422	DDX39B_ENSG00000198563;SNORD84_ENSG00000265236;DDX39B-AS1_ENSG00000234006
6	31515537	31516156	KD2-H4K8ac_peak_24673	7.76104	ATP6V1G2_ENSG00000213760;NFKBIL1_ENSG00000204498
6	31548377	31549536	KD2-H4K8ac_peak_24674	9.20193	LTB_ENSG00000227507
6	31587282	31587524	KD2-H4K8ac_peak_24675	6.99815	PRRC2A_ENSG00000204469
6	31587889	31588099	KD2-H4K8ac_peak_24676	14.00658	PRRC2A_ENSG00000204469
6	31588575	31589447	KD2-H4K8ac_peak_24677	13.13969	PRRC2A_ENSG00000204469
6	31620928	31621135	KD2-H4K8ac_peak_24678	5.71749	BAG6_ENSG00000204463;APOM_ENSG00000204444
6	31621550	31621772	KD2-H4K8ac_peak_24679	5.73839	
6	31628004	31628465	KD2-H4K8ac_peak_24680	8.2413	C6orf47_ENSG00000204439
6	31632940	31633595	KD2-H4K8ac_peak_24681	12.84642	GPANK1_ENSG00000204438;CSNK2B_ENSG00000204435;CSNK2B-LY6G5B-1181_ENSG00000263020
6	31633890	31634255	KD2-H4K8ac_peak_24682	9.36516	GPANK1_ENSG00000204438;CSNK2B_ENSG00000204435;CSNK2B-LY6G5B-1181_ENSG00000263020
6	31648180	31648574	KD2-H4K8ac_peak_24683	7.27716	
6	31648944	31649311	KD2-H4K8ac_peak_24684	9.20193	
6	31671540	31672032	KD2-H4K8ac_peak_24685	10.44656	ABHD16A_ENSG00000204427
6	31703987	31704579	KD2-H4K8ac_peak_24686	9.19305	
6	31707096	31707321	KD2-H4K8ac_peak_24687	7.22457	CLIC1_ENSG00000213719;MSH5_ENSG00000204410;MSH5-SAPCD1_ENSG00000255152
6	31707876	31708763	KD2-H4K8ac_peak_24688	5.9696	CLIC1_ENSG00000213719;MSH5_ENSG00000204410;MSH5-SAPCD1_ENSG00000255152
6	31744878	31745082	KD2-H4K8ac_peak_24689	9.51625	VWA7_ENSG00000204396
6	31762624	31763738	KD2-H4K8ac_peak_24690	11.24702	VARS_ENSG00000204394
6	31763937	31764287	KD2-H4K8ac_peak_24691	9.26408	VARS_ENSG00000204394
6	31774012	31774328	KD2-H4K8ac_peak_24692	5.26591	LSM2_ENSG00000204392
6	31781637	31782004	KD2-H4K8ac_peak_24693	6.85193	
6	31789233	31789587	KD2-H4K8ac_peak_24694	8.1233	
6	31801761	31802254	KD2-H4K8ac_peak_24695	9.1707	C6orf48_ENSG00000204387;SNORD48_ENSG00000201823
6	31869324	31869884	KD2-H4K8ac_peak_24696	13.9438	ZBTB12_ENSG00000204366
6	31870217	31870890	KD2-H4K8ac_peak_24697	8.90387	ZBTB12_ENSG00000204366
6	31926200	31926669	KD2-H4K8ac_peak_24698	10.09333	NELFE_ENSG00000204356;SKIV2L_ENSG00000204351
6	31938677	31940888	KD2-H4K8ac_peak_24699	17.16047	DXO_ENSG00000204348;STK19_ENSG00000204344
6	32016160	32016457	KD2-H4K8ac_peak_24700	4.42765	

6	32095695	32095905	KD2-H4K8ac_peak_24701	5.54647	ATF6B_ENSG00000213676
6	32096599	32096941	KD2-H4K8ac_peak_24702	5.14125	ATF6B_ENSG00000213676
6	32098573	32098863	KD2-H4K8ac_peak_24703	10.0692	FKBPL_ENSG00000204315
6	32121511	32121718	KD2-H4K8ac_peak_24704	10.05606	PRRT1_ENSG00000204314;PPT2_ENSG00000221988;PPT2-EGFL8_ENSG00000258388
6	32121996	32122258	KD2-H4K8ac_peak_24705	11.05407	PRRT1_ENSG00000204314;PPT2_ENSG00000221988;PPT2-EGFL8_ENSG00000258388
6	32143468	32143778	KD2-H4K8ac_peak_24706	13.09607	
6	32144970	32145443	KD2-H4K8ac_peak_24707	11.05056	AGPAT1_ENSG00000204310;RNF5_ENSG00000204308
6	32158117	32158501	KD2-H4K8ac_peak_24708	8.55271	PBX2_ENSG00000204304
6	32162988	32163273	KD2-H4K8ac_peak_24709	4.06758	GPSM3_ENSG00000213654
6	32163868	32164261	KD2-H4K8ac_peak_24710	25.94184	GPSM3_ENSG00000213654
6	32164617	32165417	KD2-H4K8ac_peak_24711	7.28628	
6	32806316	32806549	KD2-H4K8ac_peak_24712	15.53082	TAP2_ENSG00000250264;TAP2_ENSG00000204267
6	32861777	32862717	KD2-H4K8ac_peak_24713	13.70753	
6	32935841	32936240	KD2-H4K8ac_peak_24714	16.01525	HLA-DMA_ENSG00000204257;BRD2_ENSG00000204256
6	32937135	32939075	KD2-H4K8ac_peak_24715	7.11824	HLA-DMA_ENSG00000204257;BRD2_ENSG00000204256;BRD2-IT1_ENSG00000223837
6	33129449	33129863	KD2-H4K8ac_peak_24716	12.57297	
6	33167925	33168434	KD2-H4K8ac_peak_24717	10.05606	RXRB_ENSG00000204231;RNY4P10_ENSG00000202441;SLC39A7_ENSG00000112473
6	33172769	33172982	KD2-H4K8ac_peak_24718	10.0692	HSD17B8_ENSG00000204228
6	33175205	33176451	KD2-H4K8ac_peak_24719	7.28628	MIR219-1_ENSG00000199036;RING1_ENSG00000204227
6	33181435	33181631	KD2-H4K8ac_peak_24720	9.63487	
6	33216469	33216922	KD2-H4K8ac_peak_24721	5.14125	HCG25_ENSG00000232940
6	33238883	33239583	KD2-H4K8ac_peak_24722	15.25814	VPS52_ENSG00000223501;RPS18_ENSG00000231500
6	33239939	33240265	KD2-H4K8ac_peak_24723	10.05606	VPS52_ENSG00000223501;RPS18_ENSG00000231500
6	33244967	33245532	KD2-H4K8ac_peak_24724	7.36799	B3GALT4_ENSG00000235863
6	33257602	33257862	KD2-H4K8ac_peak_24725	7.89389	WDR46_ENSG00000227057;PFDN6_ENSG00000204220
6	33266579	33267129	KD2-H4K8ac_peak_24726	12.93209	
6	33290090	33290286	KD2-H4K8ac_peak_24727	6.69204	
6	33291117	33291878	KD2-H4K8ac_peak_24728	4.86324	
6	33359709	33359920	KD2-H4K8ac_peak_24729	8.2413	KIFC1_ENSG00000237649
6	33377664	33377898	KD2-H4K8ac_peak_24730	4.86324	PHF1_ENSG00000112511
6	33378526	33379220	KD2-H4K8ac_peak_24731	10.11379	PHF1_ENSG00000112511
6	33385369	33385563	KD2-H4K8ac_peak_24732	6.30607	CUTA_ENSG00000112514
6	33386484	33386989	KD2-H4K8ac_peak_24733	10.11379	CUTA_ENSG00000112514;SYNGAP1_ENSG00000197283
6	33392937	33393354	KD2-H4K8ac_peak_24734	10.1591	
6	33454092	33454366	KD2-H4K8ac_peak_24735	6.40683	
6	33539907	33540197	KD2-H4K8ac_peak_24736	7.70289	
6	33547612	33547900	KD2-H4K8ac_peak_24737	8.16906	BAK1_ENSG00000030110
6	33553124	33553850	KD2-H4K8ac_peak_24738	6.84261	
6	33574743	33575104	KD2-H4K8ac_peak_24739	6.25869	
6	33575777	33576382	KD2-H4K8ac_peak_24740	9.77606	
6	33577793	33578517	KD2-H4K8ac_peak_24741	10.46264	
6	33578772	33579440	KD2-H4K8ac_peak_24742	9.36516	
6	33588493	33588962	KD2-H4K8ac_peak_24743	18.29344	ITPR3_ENSG00000096433
6	33623405	33623667	KD2-H4K8ac_peak_24744	9.68068	
6	33624866	33625115	KD2-H4K8ac_peak_24745	5.43522	
6	33678551	33678840	KD2-H4K8ac_peak_24746	5.51783	UQCC2_ENSG00000137288

6	33729551	33729948	KD2-H4K8ac_peak_24747	5.14125	
6	33756139	33756379	KD2-H4K8ac_peak_24748	6.99194	LEMD2_ENSG00000161904
6	33756589	33756855	KD2-H4K8ac_peak_24749	10.29842	LEMD2_ENSG00000161904
6	33757351	33757607	KD2-H4K8ac_peak_24750	3.90565	LEMD2_ENSG00000161904
6	33783451	33783757	KD2-H4K8ac_peak_24751	13.88933	
6	33811221	33811461	KD2-H4K8ac_peak_24752	7.76335	
6	34122477	34122836	KD2-H4K8ac_peak_24753	7.47462	GRM4_ENSG00000124493
6	34164270	34164766	KD2-H4K8ac_peak_24754	8.16906	
6	34191325	34191672	KD2-H4K8ac_peak_24755	11.60333	
6	34192087	34192429	KD2-H4K8ac_peak_24756	9.63487	
6	34204192	34204514	KD2-H4K8ac_peak_24757	10.30284	HMGA1_ENSG00000137309
6	34205388	34206049	KD2-H4K8ac_peak_24758	13.95627	HMGA1_ENSG00000137309
6	34216496	34216717	KD2-H4K8ac_peak_24759	8.1235	C6orf1_ENSG00000186577
6	34359635	34360306	KD2-H4K8ac_peak_24760	15.82743	NUDT3_ENSG00000272325
6	34393398	34393790	KD2-H4K8ac_peak_24761	5.92591	RPS10-NUDT3_ENSG00000270800;RPS10_ENSG00000124614
6	34394283	34394674	KD2-H4K8ac_peak_24762	12.91654	RPS10-NUDT3_ENSG00000270800;RPS10_ENSG00000124614
6	34433472	34433672	KD2-H4K8ac_peak_24763	10.05606	PACSIN1_ENSG00000124507
6	34433910	34434190	KD2-H4K8ac_peak_24764	4.66652	PACSIN1_ENSG00000124507
6	34482706	34482976	KD2-H4K8ac_peak_24765	5.88845	
6	34493063	34494593	KD2-H4K8ac_peak_24766	10.7186	
6	34664816	34665073	KD2-H4K8ac_peak_24767	6.66692	C6orf106_ENSG00000196821;RP11-140K17.3_ENSG00000272288
6	34677738	34678261	KD2-H4K8ac_peak_24768	4.62507	
6	34724016	34724828	KD2-H4K8ac_peak_24769	14.8083	SNRPC_ENSG00000124562
6	34725316	34725531	KD2-H4K8ac_peak_24770	12.06346	SNRPC_ENSG00000124562
6	34759476	34760654	KD2-H4K8ac_peak_24771	13.74404	UHRF1BP1_ENSG00000065060
6	34807406	34807735	KD2-H4K8ac_peak_24772	4.211	
6	34856788	34857446	KD2-H4K8ac_peak_24773	14.98936	TAF11_ENSG00000064995;ANKS1A_ENSG00000064999
6	35226789	35227029	KD2-H4K8ac_peak_24774	4.54037	ZNF76_ENSG00000065029
6	35227583	35227785	KD2-H4K8ac_peak_24775	16.40478	ZNF76_ENSG00000065029
6	35286013	35286220	KD2-H4K8ac_peak_24776	6.67092	
6	35286441	35286699	KD2-H4K8ac_peak_24777	3.75035	
6	35420307	35420713	KD2-H4K8ac_peak_24778	6.40683	FANCE_ENSG00000112039
6	35435638	35436026	KD2-H4K8ac_peak_24779	6.25869	RPL10A_ENSG00000198755
6	35436227	35436731	KD2-H4K8ac_peak_24780	9.63487	RPL10A_ENSG00000198755
6	35464009	35464401	KD2-H4K8ac_peak_24781	5.30799	TEAD3_ENSG00000007866
6	35464959	35465587	KD2-H4K8ac_peak_24782	13.21979	TEAD3_ENSG00000007866
6	35499733	35500254	KD2-H4K8ac_peak_24783	6.40683	
6	35501460	35502020	KD2-H4K8ac_peak_24784	6.94954	
6	35503578	35503784	KD2-H4K8ac_peak_24785	5.88845	
6	35656291	35656640	KD2-H4K8ac_peak_24786	9.34195	
6	35687076	35688584	KD2-H4K8ac_peak_24787	11.92978	
6	35695123	35696947	KD2-H4K8ac_peak_24788	15.58442	FKBP5_ENSG00000096060
6	35699463	35700045	KD2-H4K8ac_peak_24789	8.4716	
6	35704819	35705373	KD2-H4K8ac_peak_24790	15.53798	RP3-510O8.4_ENSG00000232909;ARMC12_ENSG00000157343
6	35705587	35705942	KD2-H4K8ac_peak_24791	6.66692	RP3-510O8.4_ENSG00000232909;ARMC12_ENSG00000157343
6	35744247	35744915	KD2-H4K8ac_peak_24792	18.29344	CLPSL2_ENSG00000196748

6	35772925	35773295	KD2-H4K8ac_peak_24793	4.86324	LHFPL5_ENSG00000197753
6	35888128	35888378	KD2-H4K8ac_peak_24794	6.99815	SRPK1_ENSG00000096063
6	35889021	35889267	KD2-H4K8ac_peak_24795	10.08707	SRPK1_ENSG00000096063
6	35995639	35995996	KD2-H4K8ac_peak_24796	10.19949	MAPK14_ENSG00000112062
6	36098779	36099464	KD2-H4K8ac_peak_24797	8.16906	
6	36099865	36100733	KD2-H4K8ac_peak_24798	6.40297	
6	36164210	36164756	KD2-H4K8ac_peak_24799	12.82393	RP1-179N16.6_ENSG00000246982;BRPF3_ENSG00000096070
6	36213444	36213888	KD2-H4K8ac_peak_24800	7.1181	
6	36309037	36309270	KD2-H4K8ac_peak_24801	7.40633	
6	36328193	36328603	KD2-H4K8ac_peak_24802	7.96322	
6	36409911	36410148	KD2-H4K8ac_peak_24803	6.51023	PXT1_ENSG00000179165;KCTD20_ENSG00000112078
6	36410909	36411250	KD2-H4K8ac_peak_24804	5.54647	PXT1_ENSG00000179165;KCTD20_ENSG00000112078
6	36514905	36515141	KD2-H4K8ac_peak_24805	7.44324	STK38_ENSG00000112079
6	36515604	36516029	KD2-H4K8ac_peak_24806	4.49837	STK38_ENSG00000112079
6	36602100	36602472	KD2-H4K8ac_peak_24807	5.80056	
6	36605255	36605538	KD2-H4K8ac_peak_24808	5.05318	
6	36617421	36617947	KD2-H4K8ac_peak_24809	9.11031	
6	36634761	36635278	KD2-H4K8ac_peak_24810	6.25869	
6	36644075	36644419	KD2-H4K8ac_peak_24811	5.9696	CDKN1A_ENSG00000124762
6	36761637	36761869	KD2-H4K8ac_peak_24812	4.24345	
6	36842195	36842778	KD2-H4K8ac_peak_24813	15.13261	PPIL1_ENSG00000137168
6	36843181	36843550	KD2-H4K8ac_peak_24814	3.75035	PPIL1_ENSG00000137168
6	36852988	36853490	KD2-H4K8ac_peak_24815	5.57044	
6	36953620	36954517	KD2-H4K8ac_peak_24816	12.30509	MTCH1_ENSG00000137409
6	36965550	36965789	KD2-H4K8ac_peak_24817	4.81242	
6	37045036	37046064	KD2-H4K8ac_peak_24818	11.92209	
6	37069932	37070181	KD2-H4K8ac_peak_24819	9.1707	
6	37071068	37071385	KD2-H4K8ac_peak_24820	4.42765	
6	37077524	37077953	KD2-H4K8ac_peak_24821	4.95402	
6	37099071	37099339	KD2-H4K8ac_peak_24822	5.9696	
6	37189560	37190487	KD2-H4K8ac_peak_24823	11.2938	
6	37225636	37226261	KD2-H4K8ac_peak_24824	9.34989	TMEM217_ENSG00000172738;TBC1D22B_ENSG00000065491
6	37321291	37321608	KD2-H4K8ac_peak_24825	5.25071	RNF8_ENSG00000112130
6	37321836	37322386	KD2-H4K8ac_peak_24826	10.96455	RNF8_ENSG00000112130
6	37400243	37401382	KD2-H4K8ac_peak_24827	13.95627	CMTR1_ENSG00000137200
6	37458151	37458805	KD2-H4K8ac_peak_24828	7.96322	
6	37467327	37467523	KD2-H4K8ac_peak_24829	5.9696	CCDC167_ENSG00000198937
6	37483411	37483742	KD2-H4K8ac_peak_24830	6.65673	
6	37484048	37485085	KD2-H4K8ac_peak_24831	6.66359	
6	37504125	37504517	KD2-H4K8ac_peak_24832	6.99815	
6	37533079	37534151	KD2-H4K8ac_peak_24833	8.83582	
6	37543597	37544018	KD2-H4K8ac_peak_24834	7.83489	
6	37592730	37593573	KD2-H4K8ac_peak_24835	9.67696	
6	37767996	37768428	KD2-H4K8ac_peak_24836	10.43925	
6	37786113	37786732	KD2-H4K8ac_peak_24837	3.90565	RP3-441A12.1_ENSG00000225945;ZFAND3_ENSG00000156639
6	37786960	37787578	KD2-H4K8ac_peak_24838	18.94636	RP3-441A12.1_ENSG00000225945;ZFAND3_ENSG00000156639

6	37787993	37788195	KD2-H4K8ac_peak_24839	8.30974	RP3-441A12.1_ENSG00000225945;ZFAND3_ENSG00000156639
6	38023076	38023381	KD2-H4K8ac_peak_24840	7.05877	
6	38137885	38138095	KD2-H4K8ac_peak_24841	7.27716	
6	38235192	38235617	KD2-H4K8ac_peak_24842	4.86824	
6	38325105	38325308	KD2-H4K8ac_peak_24843	6.82976	
6	38326855	38327131	KD2-H4K8ac_peak_24844	4.86324	AL031905.1_ENSG00000238716
6	38607023	38608040	KD2-H4K8ac_peak_24845	7.73264	BTBD9_ENSG00000183826
6	38670400	38670875	KD2-H4K8ac_peak_24846	19.09174	
6	38682906	38683346	KD2-H4K8ac_peak_24847	4.67342	RP3-503A6.2_ENSG00000236355;DNAH8_ENSG00000124721
6	38943123	38943366	KD2-H4K8ac_peak_24848	6.64891	
6	39016069	39016892	KD2-H4K8ac_peak_24849	18.94636	
6	39026667	39027609	KD2-H4K8ac_peak_24850	7.27716	
6	39082604	39082813	KD2-H4K8ac_peak_24851	7.36799	SAYSD1_ENSG00000112167
6	39196916	39197558	KD2-H4K8ac_peak_24852	11.70505	KCNK5_ENSG00000164626
6	39399472	39399790	KD2-H4K8ac_peak_24853	9.89126	
6	39692865	39693102	KD2-H4K8ac_peak_24854	9.16531	KIF6_ENSG00000164627
6	39693342	39693834	KD2-H4K8ac_peak_24855	14.66631	KIF6_ENSG00000164627
6	39798419	39798646	KD2-H4K8ac_peak_24856	4.65111	
6	39901865	39902435	KD2-H4K8ac_peak_24857	10.43145	MOCS1_ENSG00000124615
6	40317694	40317889	KD2-H4K8ac_peak_24858	3.75035	
6	40374231	40374904	KD2-H4K8ac_peak_24859	9.1707	RNU6-250P_ENSG00000252767
6	40377530	40377978	KD2-H4K8ac_peak_24860	10.05606	
6	40378954	40379648	KD2-H4K8ac_peak_24861	22.25031	
6	40392408	40393087	KD2-H4K8ac_peak_24862	18.31034	
6	40404268	40404534	KD2-H4K8ac_peak_24863	9.72244	
6	40405721	40406477	KD2-H4K8ac_peak_24864	5.9696	
6	40407166	40407579	KD2-H4K8ac_peak_24865	6.66692	
6	40410192	40410623	KD2-H4K8ac_peak_24866	11.03331	
6	40411682	40411908	KD2-H4K8ac_peak_24867	10.17486	
6	40445244	40445492	KD2-H4K8ac_peak_24868	4.54838	
6	40452114	40452365	KD2-H4K8ac_peak_24869	4.42765	
6	40458740	40458966	KD2-H4K8ac_peak_24870	6.35295	
6	40467809	40468009	KD2-H4K8ac_peak_24871	6.75488	
6	40478439	40478676	KD2-H4K8ac_peak_24872	4.77079	
6	40535874	40536114	KD2-H4K8ac_peak_24873	4.01115	
6	40554166	40554409	KD2-H4K8ac_peak_24874	6.20982	LRFN2_ENSG00000156564
6	40598305	40599028	KD2-H4K8ac_peak_24875	9.1707	
6	40639348	40639640	KD2-H4K8ac_peak_24876	7.27716	
6	40674119	40674324	KD2-H4K8ac_peak_24877	7.27716	
6	40686898	40687380	KD2-H4K8ac_peak_24878	13.35153	
6	40687746	40687969	KD2-H4K8ac_peak_24879	6.40297	
6	40688512	40688916	KD2-H4K8ac_peak_24880	11.92978	
6	40689524	40690037	KD2-H4K8ac_peak_24881	13.6476	
6	40722444	40722884	KD2-H4K8ac_peak_24882	6.65673	
6	40764933	40765241	KD2-H4K8ac_peak_24883	5.60968	
6	40837021	40837379	KD2-H4K8ac_peak_24884	7.96322	

6	40848714	40848970	KD2-H4K8ac_peak_24885	8.88549	
6	40854642	40854914	KD2-H4K8ac_peak_24886	4.57371	
6	40856427	40856684	KD2-H4K8ac_peak_24887	6.00226	
6	40858115	40858472	KD2-H4K8ac_peak_24888	10.05606	
6	40995474	40996507	KD2-H4K8ac_peak_24889	12.82393	
6	41039672	41040137	KD2-H4K8ac_peak_24890	12.08081	NFYA_ENSG00000001167
6	41040944	41041402	KD2-H4K8ac_peak_24891	12.93209	NFYA_ENSG00000001167
6	41395739	41395958	KD2-H4K8ac_peak_24892	9.08816	
6	41455949	41456189	KD2-H4K8ac_peak_24893	5.30799	
6	41456643	41457140	KD2-H4K8ac_peak_24894	4.42765	
6	41471525	41472440	KD2-H4K8ac_peak_24895	13.81781	
6	41487703	41487927	KD2-H4K8ac_peak_24896	13.57066	
6	41513383	41513762	KD2-H4K8ac_peak_24897	14.80472	FOXP4_ENSG00000137166
6	41570633	41570878	KD2-H4K8ac_peak_24898	4.211	
6	41605032	41606024	KD2-H4K8ac_peak_24899	14.72959	MDFI_ENSG00000112559
6	41606416	41606688	KD2-H4K8ac_peak_24900	10.7186	
6	41642909	41643159	KD2-H4K8ac_peak_24901	6.30229	
6	41702726	41703242	KD2-H4K8ac_peak_24902	6.33462	TFEB_ENSG00000112561
6	41703581	41703927	KD2-H4K8ac_peak_24903	6.2781	TFEB_ENSG00000112561
6	41748811	41749224	KD2-H4K8ac_peak_24904	4.67342	PRICKLE4_ENSG00000124593
6	41753888	41754258	KD2-H4K8ac_peak_24905	14.98936	FRS3_ENSG00000137218
6	41754494	41755266	KD2-H4K8ac_peak_24906	8.16906	FRS3_ENSG00000137218;TOMM6_ENSG00000214736
6	41755460	41755961	KD2-H4K8ac_peak_24907	15.52826	TOMM6_ENSG00000214736
6	41862472	41862679	KD2-H4K8ac_peak_24908	8.30974	USP49_ENSG00000164663
6	41888462	41888779	KD2-H4K8ac_peak_24909	5.02474	MED20_ENSG00000124641;BYSL_ENSG00000112578
6	41889013	41889870	KD2-H4K8ac_peak_24910	14.98936	MED20_ENSG00000124641;BYSL_ENSG00000112578
6	41909843	41910270	KD2-H4K8ac_peak_24911	5.9696	
6	42015819	42016969	KD2-H4K8ac_peak_24912	12.74171	
6	42017789	42018037	KD2-H4K8ac_peak_24913	8.28994	CCND3_ENSG00000112576;TAF8_ENSG00000137413
6	42018279	42018501	KD2-H4K8ac_peak_24914	12.81138	CCND3_ENSG00000112576;TAF8_ENSG00000137413
6	42086139	42086441	KD2-H4K8ac_peak_24915	6.66692	
6	42104471	42104798	KD2-H4K8ac_peak_24916	12.42761	
6	42104999	42105384	KD2-H4K8ac_peak_24917	13.63179	
6	42108998	42110178	KD2-H4K8ac_peak_24918	10.29344	C6orf132_ENSG00000188112
6	42125941	42126230	KD2-H4K8ac_peak_24919	5.13615	
6	42134489	42134806	KD2-H4K8ac_peak_24920	5.10505	
6	42145612	42146482	KD2-H4K8ac_peak_24921	11.79585	
6	42185177	42185500	KD2-H4K8ac_peak_24922	5.54647	MRPS10_ENSG00000048544
6	42419492	42419726	KD2-H4K8ac_peak_24923	5.57044	TRERF1_ENSG00000124496
6	42420104	42420439	KD2-H4K8ac_peak_24924	12.08081	TRERF1_ENSG00000124496
6	42421090	42421551	KD2-H4K8ac_peak_24925	4.211	
6	42500215	42500461	KD2-H4K8ac_peak_24926	10.53596	
6	42515558	42516068	KD2-H4K8ac_peak_24927	9.1707	
6	42531810	42532406	KD2-H4K8ac_peak_24928	12.06346	UBR2_ENSG00000024048
6	42713251	42713666	KD2-H4K8ac_peak_24929	8.55271	TBCC_ENSG00000124659
6	42714176	42714506	KD2-H4K8ac_peak_24930	8.01894	TBCC_ENSG00000124659

6	42715240	42715447	KD2-H4K8ac_peak_24931	6.40683	TBCC_ENSG00000124659
6	42750356	42751766	KD2-H4K8ac_peak_24932	37.36644	
6	42846553	42847201	KD2-H4K8ac_peak_24933	8.02571	RPL7L1_ENSG00000146223
6	42847506	42848167	KD2-H4K8ac_peak_24934	6.85193	RPL7L1_ENSG00000146223
6	42858289	42858522	KD2-H4K8ac_peak_24935	13.48341	C6orf226_ENSG00000221821
6	42859023	42859480	KD2-H4K8ac_peak_24936	5.9696	C6orf226_ENSG00000221821
6	42896060	42897561	KD2-H4K8ac_peak_24937	12.82393	CNPY3_ENSG00000137161
6	42928650	42928847	KD2-H4K8ac_peak_24938	6.66692	GNMT_ENSG00000124713
6	42946517	42947464	KD2-H4K8ac_peak_24939	11.05467	PEX6_ENSG00000124587
6	42951664	42951995	KD2-H4K8ac_peak_24940	7.95563	PPP2R5D_ENSG00000112640
6	42988603	42989233	KD2-H4K8ac_peak_24941	9.20193	RRP36_ENSG00000124541
6	42989691	42990154	KD2-H4K8ac_peak_24942	7.65815	RRP36_ENSG00000124541
6	43020981	43021594	KD2-H4K8ac_peak_24943	13.80019	CUL7_ENSG00000044090
6	43026914	43027134	KD2-H4K8ac_peak_24944	7.76329	MRPL2_ENSG00000112651
6	43027462	43027824	KD2-H4K8ac_peak_24945	5.30799	MRPL2_ENSG00000112651
6	43043485	43043852	KD2-H4K8ac_peak_24946	9.08816	PTK7_ENSG00000112655
6	43044624	43044983	KD2-H4K8ac_peak_24947	8.59579	PTK7_ENSG00000112655
6	43082076	43082773	KD2-H4K8ac_peak_24948	8.10216	
6	43083155	43083607	KD2-H4K8ac_peak_24949	10.77174	
6	43087372	43087610	KD2-H4K8ac_peak_24950	4.40093	
6	43089743	43090185	KD2-H4K8ac_peak_24951	7.69287	
6	43090620	43091208	KD2-H4K8ac_peak_24952	4.86324	
6	43091451	43092890	KD2-H4K8ac_peak_24953	13.73635	
6	43093582	43093870	KD2-H4K8ac_peak_24954	6.61611	
6	43110716	43111003	KD2-H4K8ac_peak_24955	6.63018	
6	43149645	43150446	KD2-H4K8ac_peak_24956	6.25869	CUL9_ENSG00000112659
6	43196507	43197608	KD2-H4K8ac_peak_24957	23.83732	DNPH1_ENSG00000112667
6	43210962	43211751	KD2-H4K8ac_peak_24958	23.38883	TTBK1_ENSG00000146216
6	43214673	43214990	KD2-H4K8ac_peak_24959	11.60333	
6	43215293	43215589	KD2-H4K8ac_peak_24960	8.55271	
6	43336516	43337368	KD2-H4K8ac_peak_24961	8.10216	ZNF318_ENSG00000171467
6	43337592	43338063	KD2-H4K8ac_peak_24962	10.05606	ZNF318_ENSG00000171467
6	43394520	43395910	KD2-H4K8ac_peak_24963	15.039	ABCC10_ENSG00000124574
6	43444631	43444980	KD2-H4K8ac_peak_24964	6.82976	TJAP1_ENSG00000137221
6	43478402	43478679	KD2-H4K8ac_peak_24965	7.70289	LRRC73_ENSG00000204052;POLR1C_ENSG00000171453
6	43484258	43484621	KD2-H4K8ac_peak_24966	13.9234	YIPF3_ENSG00000137207
6	43543344	43543707	KD2-H4K8ac_peak_24967	8.85813	XPO5_ENSG00000124571;POLH_ENSG00000170734
6	43596323	43596874	KD2-H4K8ac_peak_24968	18.5703	GTPBP2_ENSG00000172432;MAD2L1BP_ENSG00000124688
6	43597309	43597614	KD2-H4K8ac_peak_24969	14.66631	GTPBP2_ENSG00000172432;MAD2L1BP_ENSG00000124688
6	43598062	43598394	KD2-H4K8ac_peak_24970	11.11809	MAD2L1BP_ENSG00000124688
6	43603128	43603478	KD2-H4K8ac_peak_24971	12.79505	
6	43655179	43655962	KD2-H4K8ac_peak_24972	10.05606	MRPS18A_ENSG00000096080
6	43682233	43682494	KD2-H4K8ac_peak_24973	4.86324	
6	43683620	43684273	KD2-H4K8ac_peak_24974	5.54647	
6	43736682	43736918	KD2-H4K8ac_peak_24975	4.58615	
6	43739240	43740219	KD2-H4K8ac_peak_24976	9.63487	RP1-261G23.7_ENSG00000272114

6	43742861	43743290	KD2-H4K8ac_peak_24977	10.0665	
6	43774963	43775434	KD2-H4K8ac_peak_24978	4.86324	
6	43822588	43822956	KD2-H4K8ac_peak_24979	5.80374	
6	43824135	43824568	KD2-H4K8ac_peak_24980	10.58016	
6	43858569	43858764	KD2-H4K8ac_peak_24981	4.77079	
6	43868435	43868770	KD2-H4K8ac_peak_24982	6.99815	
6	43869121	43869386	KD2-H4K8ac_peak_24983	7.47462	
6	43871081	43871363	KD2-H4K8ac_peak_24984	5.88845	
6	43879013	43879248	KD2-H4K8ac_peak_24985	5.88845	
6	43895006	43895297	KD2-H4K8ac_peak_24986	6.35843	
6	43905192	43905916	KD2-H4K8ac_peak_24987	8.5092	
6	43906363	43906731	KD2-H4K8ac_peak_24988	9.1707	
6	43907055	43907456	KD2-H4K8ac_peak_24989	9.16531	
6	43908926	43909520	KD2-H4K8ac_peak_24990	7.76329	
6	43909882	43910120	KD2-H4K8ac_peak_24991	5.08628	
6	43910417	43910722	KD2-H4K8ac_peak_24992	7.95563	
6	43910945	43911290	KD2-H4K8ac_peak_24993	9.36516	
6	43918801	43919022	KD2-H4K8ac_peak_24994	6.27931	
6	43919480	43919750	KD2-H4K8ac_peak_24995	6.25869	
6	43936093	43936331	KD2-H4K8ac_peak_24996	6.13909	
6	43957223	43959333	KD2-H4K8ac_peak_24997	11.02863	
6	43970357	43970834	KD2-H4K8ac_peak_24998	6.43014	
6	43992466	43992839	KD2-H4K8ac_peak_24999	6.83936	
6	44011291	44012214	KD2-H4K8ac_peak_25000	7.76104	
6	44018321	44019792	KD2-H4K8ac_peak_25001	10.96455	
6	44020144	44021096	KD2-H4K8ac_peak_25002	7.146	
6	44021970	44022431	KD2-H4K8ac_peak_25003	5.59859	
6	44022626	44022877	KD2-H4K8ac_peak_25004	8.1235	
6	44024424	44025179	KD2-H4K8ac_peak_25005	13.27022	
6	44040451	44040944	KD2-H4K8ac_peak_25006	7.07117	RP5-1120P11.3_ENSG00000231881
6	44041420	44041860	KD2-H4K8ac_peak_25007	5.3157	RP5-1120P11.1_ENSG00000237686;RP5-1120P11.3_ENSG00000231881
6	44044638	44044981	KD2-H4K8ac_peak_25008	12.74171	
6	44050692	44050932	KD2-H4K8ac_peak_25009	8.14467	
6	44095431	44095653	KD2-H4K8ac_peak_25010	9.06369	MRPL14_ENSG00000180992;TMEM63B_ENSG00000137216
6	44186929	44187822	KD2-H4K8ac_peak_25011	12.79505	SLC29A1_ENSG00000112759
6	44204960	44205229	KD2-H4K8ac_peak_25012	5.14125	
6	44205598	44205811	KD2-H4K8ac_peak_25013	15.40891	
6	44213367	44214111	KD2-H4K8ac_peak_25014	10.05606	HSP90AB1_ENSG00000096384
6	44214344	44214587	KD2-H4K8ac_peak_25015	7.36799	HSP90AB1_ENSG00000096384
6	44215038	44215381	KD2-H4K8ac_peak_25016	4.52514	HSP90AB1_ENSG00000096384
6	44224890	44225102	KD2-H4K8ac_peak_25017	8.55271	SLC35B2_ENSG00000157593
6	44233102	44233823	KD2-H4K8ac_peak_25018	6.25869	NFKBIE_ENSG00000146232
6	44237866	44238089	KD2-H4K8ac_peak_25019	13.11967	TMEM151B_ENSG00000178233
6	44238422	44239038	KD2-H4K8ac_peak_25020	8.02571	TMEM151B_ENSG00000178233
6	44280836	44281064	KD2-H4K8ac_peak_25021	8.85813	AARS2_ENSG00000124608
6	44310198	44310875	KD2-H4K8ac_peak_25022	18.94636	SPATS1_ENSG00000249481

6	44355401	44355644	KD2-H4K8ac_peak_25023	8.55271	CDC5L_ENSG00000096401
6	44683205	44683501	KD2-H4K8ac_peak_25024	8.80225	
6	45345281	45345587	KD2-H4K8ac_peak_25025	11.09936	SUPT3H_ENSG00000196284
6	45345975	45346450	KD2-H4K8ac_peak_25026	4.211	SUPT3H_ENSG00000196284
6	47205603	47205844	KD2-H4K8ac_peak_25027	7.51935	
6	47209700	47209961	KD2-H4K8ac_peak_25028	8.17902	
6	47277160	47277440	KD2-H4K8ac_peak_25029	6.8836	TNFRSF21_ENSG00000146072
6	47445038	47445332	KD2-H4K8ac_peak_25030	6.83936	RP11-385F7.1_ENSG00000270761;CD2AP_ENSG00000198087
6	48036118	48036345	KD2-H4K8ac_peak_25031	6.16888	PTCHD4_ENSG00000244694
6	49430497	49430914	KD2-H4K8ac_peak_25032	14.74047	MUT_ENSG00000146085;CENPQ_ENSG00000031691
6	50692339	50692577	KD2-H4K8ac_peak_25033	5.57044	
6	50787268	50788074	KD2-H4K8ac_peak_25034	16.57619	TFAP2B_ENSG00000008196
6	50788710	50788963	KD2-H4K8ac_peak_25035	6.25869	
6	50789850	50790432	KD2-H4K8ac_peak_25036	10.08874	
6	50790634	50791621	KD2-H4K8ac_peak_25037	12.93209	
6	50793483	50794121	KD2-H4K8ac_peak_25038	10.72656	
6	50816985	50817315	KD2-H4K8ac_peak_25039	4.211	
6	50817719	50818786	KD2-H4K8ac_peak_25040	13.74404	
6	50857322	50857563	KD2-H4K8ac_peak_25041	4.37708	
6	50858416	50858663	KD2-H4K8ac_peak_25042	5.25071	
6	50903031	50903322	KD2-H4K8ac_peak_25043	6.25869	
6	50904909	50905123	KD2-H4K8ac_peak_25044	8.17902	
6	51124846	51125049	KD2-H4K8ac_peak_25045	7.01964	
6	51125796	51126292	KD2-H4K8ac_peak_25046	6.16888	
6	51128980	51129244	KD2-H4K8ac_peak_25047	9.15329	
6	51130091	51130582	KD2-H4K8ac_peak_25048	7.27716	
6	51418534	51418751	KD2-H4K8ac_peak_25049	10.53377	
6	51445210	51445436	KD2-H4K8ac_peak_25050	7.12754	
6	51474444	51474652	KD2-H4K8ac_peak_25051	5.25071	
6	51540783	51541019	KD2-H4K8ac_peak_25052	16.32216	
6	51544499	51544767	KD2-H4K8ac_peak_25053	7.27716	
6	51682271	51682753	KD2-H4K8ac_peak_25054	7.87794	
6	51708061	51708418	KD2-H4K8ac_peak_25055	7.91974	
6	51814419	51814679	KD2-H4K8ac_peak_25056	4.01115	
6	51857402	51857674	KD2-H4K8ac_peak_25057	6.25869	
6	51873832	51874136	KD2-H4K8ac_peak_25058	12.3187	
6	51880372	51880928	KD2-H4K8ac_peak_25059	6.40683	
6	51881153	51881364	KD2-H4K8ac_peak_25060	9.15329	
6	51882142	51882442	KD2-H4K8ac_peak_25061	5.9696	
6	51883436	51883663	KD2-H4K8ac_peak_25062	7.12754	
6	51885100	51885465	KD2-H4K8ac_peak_25063	6.16888	
6	52149085	52149473	KD2-H4K8ac_peak_25064	6.65673	MCM3_ENSG00000112118
6	52226961	52227721	KD2-H4K8ac_peak_25065	7.11824	PAQR8_ENSG00000170915
6	52284612	52284838	KD2-H4K8ac_peak_25066	8.79106	EFHC1_ENSG00000096093
6	52377190	52377507	KD2-H4K8ac_peak_25067	6.75638	
6	52382937	52383190	KD2-H4K8ac_peak_25068	8.04448	

6	52411911	52412238	KD2-H4K8ac_peak_25069	8.1718	
6	52416862	52417132	KD2-H4K8ac_peak_25070	4.211	
6	52436223	52436512	KD2-H4K8ac_peak_25071	4.01115	
6	52441663	52442473	KD2-H4K8ac_peak_25072	18.54875	TRAM2_ENSG00000065308;TRAM2-AS1_ENSG00000225791
6	52461575	52461796	KD2-H4K8ac_peak_25073	6.94954	
6	52529289	52529481	KD2-H4K8ac_peak_25074	6.66692	RP1-152L7.5_ENSG00000216775
6	52535357	52536328	KD2-H4K8ac_peak_25075	14.15075	TMEM14A_ENSG00000096092
6	52859544	52859861	KD2-H4K8ac_peak_25076	7.40314	GSTA4_ENSG00000170899;RN7SK_ENSG00000202198
6	52926191	52926606	KD2-H4K8ac_peak_25077	13.80019	ICK_ENSG00000112144
6	52930371	52930630	KD2-H4K8ac_peak_25078	8.85813	
6	53213049	53213688	KD2-H4K8ac_peak_25079	4.54006	ELOVL5_ENSG00000012660
6	53223653	53224183	KD2-H4K8ac_peak_25080	18.86697	
6	53409233	53409749	KD2-H4K8ac_peak_25081	5.30799	
6	53413050	53413549	KD2-H4K8ac_peak_25082	12.05219	
6	53469146	53469349	KD2-H4K8ac_peak_25083	8.1233	
6	53516357	53517411	KD2-H4K8ac_peak_25084	6.85193	
6	53658857	53659049	KD2-H4K8ac_peak_25085	6.64891	RP13-476E20.1_ENSG00000228614;LRRC1_ENSG00000137269
6	53659267	53659548	KD2-H4K8ac_peak_25086	13.56049	RP13-476E20.1_ENSG00000228614;LRRC1_ENSG00000137269
6	53659862	53660099	KD2-H4K8ac_peak_25087	7.13615	RP13-476E20.1_ENSG00000228614;LRRC1_ENSG00000137269
6	56707921	56708410	KD2-H4K8ac_peak_25088	10.43145	RP11-472M19.2_ENSG00000231441
6	56708717	56709042	KD2-H4K8ac_peak_25089	11.92209	RP11-472M19.2_ENSG00000231441
6	56716074	56716307	KD2-H4K8ac_peak_25090	9.1707	
6	56819076	56820169	KD2-H4K8ac_peak_25091	14.56955	DST_ENSG00000151914;BEND6_ENSG00000151917
6	57037176	57037797	KD2-H4K8ac_peak_25092	11.92978	BAG2_ENSG00000112208
6	57086741	57086968	KD2-H4K8ac_peak_25093	8.4716	RAB23_ENSG00000112210
6	58287421	58287649	KD2-H4K8ac_peak_25094	12.06346	LINC00680_ENSG00000215190
6	58287966	58288599	KD2-H4K8ac_peak_25095	9.15329	LINC00680_ENSG00000215190
6	64282627	64283293	KD2-H4K8ac_peak_25096	5.01002	RP5-1148A21.3_ENSG00000266680
6	64346293	64346569	KD2-H4K8ac_peak_25097	8.23274	PHF3_ENSG00000118482
6	70311266	70311556	KD2-H4K8ac_peak_25098	5.13341	
6	70506416	70507583	KD2-H4K8ac_peak_25099	10.58496	LMBRD1_ENSG00000168216
6	70577304	70577621	KD2-H4K8ac_peak_25100	8.26945	COL19A1_ENSG00000082293
6	71122511	71122746	KD2-H4K8ac_peak_25101	9.08816	RP11-462G2.2_ENSG00000224349;FAM135A_ENSG00000082269
6	71123299	71123800	KD2-H4K8ac_peak_25102	6.97919	RP11-462G2.2_ENSG00000224349;FAM135A_ENSG00000082269
6	71275965	71276307	KD2-H4K8ac_peak_25103	7.22457	C6orf57_ENSG00000154079
6	71276692	71277046	KD2-H4K8ac_peak_25104	8.85813	C6orf57_ENSG00000154079
6	71376709	71377253	KD2-H4K8ac_peak_25105	5.71749	SMAP1_ENSG00000112305
6	71997795	71998398	KD2-H4K8ac_peak_25106	14.68449	OGFRL1_ENSG00000119900
6	71998593	71999076	KD2-H4K8ac_peak_25107	13.81781	OGFRL1_ENSG00000119900
6	72596895	72597097	KD2-H4K8ac_peak_25108	6.99815	RIMS1_ENSG00000079841
6	72926307	72926619	KD2-H4K8ac_peak_25109	8.00457	
6	73331422	73331740	KD2-H4K8ac_peak_25110	7.1181	KCNQ5_ENSG00000185760
6	73331992	73332463	KD2-H4K8ac_peak_25111	14.69065	KCNQ5_ENSG00000185760
6	73332831	73333062	KD2-H4K8ac_peak_25112	10.21502	
6	73972932	73973445	KD2-H4K8ac_peak_25113	16.32216	AC019205.1_ENSG00000263378;RP11-257K9.8_ENSG00000243501;RP11-398K22.12_ENSG00000229852
6	74008795	74009729	KD2-H4K8ac_peak_25114	8.4716	

6	74161207	74162252	KD2-H4K8ac_peak_25115	12.80176	MB21D1_ENSG00000164430
6	74170917	74171140	KD2-H4K8ac_peak_25116	8.30974	MTO1_ENSG00000135297
6	74171502	74171873	KD2-H4K8ac_peak_25117	15.7388	MTO1_ENSG00000135297
6	74230160	74230714	KD2-H4K8ac_peak_25118	8.85813	
6	74231152	74231552	KD2-H4K8ac_peak_25119	24.19623	
6	74289062	74289371	KD2-H4K8ac_peak_25120	9.15329	
6	74363302	74363537	KD2-H4K8ac_peak_25121	11.1952	SLC17A5_ENSG00000119899
6	76058617	76059594	KD2-H4K8ac_peak_25122	11.70505	
6	76311271	76312505	KD2-H4K8ac_peak_25123	7.28628	SENP6_ENSG00000112701
6	76458321	76458731	KD2-H4K8ac_peak_25124	5.01002	MYO6_ENSG00000196586
6	79576824	79577088	KD2-H4K8ac_peak_25125	8.17902	IRAK1BP1_ENSG00000146243
6	79577295	79577609	KD2-H4K8ac_peak_25126	11.2938	IRAK1BP1_ENSG00000146243
6	79786965	79787265	KD2-H4K8ac_peak_25127	7.27716	PHIP_ENSG00000146247
6	79787745	79788844	KD2-H4K8ac_peak_25128	10.96455	PHIP_ENSG00000146247
6	80487883	80488496	KD2-H4K8ac_peak_25129	9.50015	
6	80518717	80518934	KD2-H4K8ac_peak_25130	4.49837	
6	80520823	80521039	KD2-H4K8ac_peak_25131	7.36799	
6	80521563	80522224	KD2-H4K8ac_peak_25132	7.40633	
6	80579480	80579887	KD2-H4K8ac_peak_25133	11.30637	
6	80657462	80657681	KD2-H4K8ac_peak_25134	13.81781	ELOVL4_ENSG00000118402
6	80713905	80714138	KD2-H4K8ac_peak_25135	9.96798	TTK_ENSG00000112742
6	80816461	80816757	KD2-H4K8ac_peak_25136	10.96455	BCKDHB_ENSG00000083123
6	80921080	80921508	KD2-H4K8ac_peak_25137	4.86324	
6	82463284	82463590	KD2-H4K8ac_peak_25138	5.45396	FAM46A_ENSG00000112773
6	82750180	82750390	KD2-H4K8ac_peak_25139	7.27716	
6	82957150	82958007	KD2-H4K8ac_peak_25140	7.07117	IBTK_ENSG00000005700
6	83775356	83775924	KD2-H4K8ac_peak_25141	5.9696	UBE3D_ENSG00000118420
6	83902611	83902866	KD2-H4K8ac_peak_25142	8.1233	PGM3_ENSG00000013375;RWDD2A_ENSG00000013392
6	84140415	84141388	KD2-H4K8ac_peak_25143	20.02161	ME1_ENSG00000065833
6	84322265	84322541	KD2-H4K8ac_peak_25144	9.15329	
6	84325885	84326345	KD2-H4K8ac_peak_25145	4.77079	
6	84417938	84418360	KD2-H4K8ac_peak_25146	4.44527	
6	84563047	84563767	KD2-H4K8ac_peak_25147	10.96455	RIPPLY2_ENSG00000203877
6	84729682	84729941	KD2-H4K8ac_peak_25148	4.86824	
6	84737111	84737335	KD2-H4K8ac_peak_25149	6.8836	
6	84743256	84744064	KD2-H4K8ac_peak_25150	20.02161	MRAP2_ENSG00000135324
6	84937423	84937880	KD2-H4K8ac_peak_25151	8.74596	KIAA1009_ENSG00000135315
6	86302865	86303450	KD2-H4K8ac_peak_25152	5.01002	SNX14_ENSG00000135317
6	86303792	86304216	KD2-H4K8ac_peak_25153	10.96455	SNX14_ENSG00000135317
6	86351954	86352486	KD2-H4K8ac_peak_25154	11.79585	
6	86352766	86353061	KD2-H4K8ac_peak_25155	16.48166	SYNCRIP_ENSG00000135316
6	86353430	86353939	KD2-H4K8ac_peak_25156	6.40683	SYNCRIP_ENSG00000135316
6	86387953	86388270	KD2-H4K8ac_peak_25157	5.49367	SNHG5_ENSG00000203875
6	86388828	86389099	KD2-H4K8ac_peak_25158	9.08816	SNHG5_ENSG00000203875
6	87246636	87246833	KD2-H4K8ac_peak_25159	5.14125	
6	87305207	87305523	KD2-H4K8ac_peak_25160	6.16888	

6	87647039	87647686	KD2-H4K8ac_peak_25161	8.10216	HTR1E_ENSG00000168830
6	87861530	87862347	KD2-H4K8ac_peak_25162	12.08081	ZNF292_ENSG00000188994
6	87864641	87864879	KD2-H4K8ac_peak_25163	5.15777	RP11-393I2.4_ENSG00000272008
6	87865294	87866018	KD2-H4K8ac_peak_25164	30.13194	RP11-393I2.4_ENSG00000272008
6	88031459	88031915	KD2-H4K8ac_peak_25165	7.47462	SMIM8_ENSG00000111850;RP1-102H19.8_ENSG00000226524
6	88032370	88032659	KD2-H4K8ac_peak_25166	9.11031	SMIM8_ENSG00000111850;RP1-102H19.8_ENSG00000226524
6	88182449	88182920	KD2-H4K8ac_peak_25167	13.73635	
6	88411045	88411907	KD2-H4K8ac_peak_25168	12.17278	AKIRIN2_ENSG00000135334
6	88875723	88876915	KD2-H4K8ac_peak_25169	10.05606	CNR1_ENSG00000118432
6	89672906	89673232	KD2-H4K8ac_peak_25170	6.45083	RNGTT_ENSG00000111880
6	89790796	89791122	KD2-H4K8ac_peak_25171	7.40314	RP11-63L7.5_ENSG00000271931;PNRC1_ENSG00000146278
6	89827104	89827663	KD2-H4K8ac_peak_25172	11.79585	SRSF12_ENSG00000154548
6	89827914	89828331	KD2-H4K8ac_peak_25173	6.30229	SRSF12_ENSG00000154548
6	89855575	89856388	KD2-H4K8ac_peak_25174	8.58725	PM20D2_ENSG00000146281
6	90062170	90063302	KD2-H4K8ac_peak_25175	15.58442	UBE2J1_ENSG00000198833
6	90121897	90122672	KD2-H4K8ac_peak_25176	11.03331	RRAGD_ENSG00000025039
6	90122912	90123149	KD2-H4K8ac_peak_25177	5.20908	RRAGD_ENSG00000025039
6	90142466	90142692	KD2-H4K8ac_peak_25178	5.75452	ANKRD6_ENSG00000135299
6	90142935	90143291	KD2-H4K8ac_peak_25179	4.84794	ANKRD6_ENSG00000135299
6	90240291	90240501	KD2-H4K8ac_peak_25180	5.52329	
6	90245675	90246324	KD2-H4K8ac_peak_25181	7.27716	
6	90348240	90348434	KD2-H4K8ac_peak_25182	7.76104	LYRM2_ENSG00000083099
6	90529070	90529454	KD2-H4K8ac_peak_25183	5.42867	MDN1_ENSG00000112159
6	90529676	90530004	KD2-H4K8ac_peak_25184	4.67342	MDN1_ENSG00000112159
6	90538366	90539075	KD2-H4K8ac_peak_25185	6.40297	CASP8AP2_ENSG00000118412
6	90539634	90539898	KD2-H4K8ac_peak_25186	11.05467	CASP8AP2_ENSG00000118412
6	90555825	90556057	KD2-H4K8ac_peak_25187	6.07178	
6	90689740	90690253	KD2-H4K8ac_peak_25188	9.1707	
6	90695356	90695899	KD2-H4K8ac_peak_25189	19.31583	
6	90696941	90697151	KD2-H4K8ac_peak_25190	5.43522	
6	90734732	90734935	KD2-H4K8ac_peak_25191	8.79106	
6	90856866	90857260	KD2-H4K8ac_peak_25192	7.27716	
6	91006881	91007334	KD2-H4K8ac_peak_25193	14.56955	BACH2_ENSG00000112182
[truncated: 391,461 more chars]
