# Supplementary material for: SIRT2 functions as a histone delactylase and inhibits the proliferation and migration of neuroblastoma cells
Source: Cell Discov. 2022 Jun 7;8:54. doi: 10.1038/s41421-022-00398-y (PMC9174446; doi:10.1038/s41421-022-00398-y)
Supplement: Supplementary file 5 — Supplementary Table S5 [file 41421_2022_398_MOESM5_ESM.pdf]

Table S5. ChIP-seq peaks and annotated genes by anti-H4K8la antibody in SIRT2 KD SH-SY5Y cells.

chr	start	end	peakid	-LOG(pvalue	gene
1	714361	714601	KD2-H4K8la_peak_1	9.0413	RP11-206L10.2_ENSG00000228327;RP11-206L10.9_ENSG00000237491
1	877517	878123	KD2-H4K8la_peak_2	9.07903	
1	879156	879425	KD2-H4K8la_peak_3	8.66402	
1	896086	896475	KD2-H4K8la_peak_4	7.10162	KLHL17_ENSG00000187961
1	941579	941786	KD2-H4K8la_peak_5	6.38565	
1	1016386	1016644	KD2-H4K8la_peak_6	8.15906	
1	1080163	1080478	KD2-H4K8la_peak_7	6.86458	
1	1093099	1093385	KD2-H4K8la_peak_8	6.86458	
1	1167795	1168113	KD2-H4K8la_peak_9	6.68593	SDF4_ENSG00000078808;B3GALT6_ENSG00000176022
1	1209460	1209938	KD2-H4K8la_peak_10	8.56099	UBE2J2_ENSG00000160087;RP5-902P8.10_ENSG00000230415
1	1240711	1241271	KD2-H4K8la_peak_11	8.11415	
1	1259674	1259933	KD2-H4K8la_peak_12	7.12014	CPSF3L_ENSG00000127054
1	1295507	1295739	KD2-H4K8la_peak_13	8.0333	
1	1310302	1310505	KD2-H4K8la_peak_14	8.3574	AURKAIP1_ENSG00000175756
1	1311090	1311775	KD2-H4K8la_peak_15	12.07084	AURKAIP1_ENSG00000175756
1	1334321	1334624	KD2-H4K8la_peak_16	9.76928	CCNL2_ENSG00000221978;RP4-758J18.2_ENSG00000224870
1	1355719	1355946	KD2-H4K8la_peak_17	9.61894	RP4-758J18.7_ENSG00000225905
1	1363001	1363271	KD2-H4K8la_peak_18	6.98292	
1	1365895	1366255	KD2-H4K8la_peak_19	5.00908	
1	1397530	1397769	KD2-H4K8la_peak_20	8.84474	
1	1398042	1398358	KD2-H4K8la_peak_21	5.84082	
1	1510516	1510894	KD2-H4K8la_peak_22	10.5885	SSU72_ENSG00000160075;AL645728.1_ENSG00000215014
1	1550887	1551114	KD2-H4K8la_peak_23	14.7348	RP11-345P4.9_ENSG00000272106;MIB2_ENSG00000197530
1	1821986	1822414	KD2-H4K8la_peak_24	11.52764	GNB1_ENSG00000078369;RP1-140A9.1_ENSG00000231050
1	1875000	1875488	KD2-H4K8la_peak_25	6.3399	
1	1976079	1976378	KD2-H4K8la_peak_26	13.37883	
1	2126372	2126660	KD2-H4K8la_peak_27	5.02488	
1	2137048	2137286	KD2-H4K8la_peak_28	6.1189	
1	2143700	2143955	KD2-H4K8la_peak_29	9.18682	C1orf86_ENSG00000162585;RP11-181G12.5_ENSG00000243558;RP11-181G12.4_ENSG00000234396
1	2144200	2144759	KD2-H4K8la_peak_30	9.37576	C1orf86_ENSG00000162585;RP11-181G12.5_ENSG00000243558;RP11-181G12.4_ENSG00000234396;AL590822.1_ENSG00000203301
1	2245943	2246267	KD2-H4K8la_peak_31	13.27512	
1	2246839	2247316	KD2-H4K8la_peak_32	9.05604	
1	2405681	2406352	KD2-H4K8la_peak_33	12.32641	
1	2406638	2407052	KD2-H4K8la_peak_34	7.92775	
1	2413729	2414239	KD2-H4K8la_peak_35	8.16787	
1	2475848	2476274	KD2-H4K8la_peak_36	9.61894	
1	2574092	2574590	KD2-H4K8la_peak_37	15.75739	
1	3086723	3087073	KD2-H4K8la_peak_38	8.76082	
1	3433097	3433467	KD2-H4K8la_peak_39	5.89088	
1	3447560	3447758	KD2-H4K8la_peak_40	5.3107	
1	3566200	3566494	KD2-H4K8la_peak_41	9.27415	
1	3568220	3568880	KD2-H4K8la_peak_42	12.83096	WRAP73_ENSG00000116213;TP73_ENSG00000078900
1	3712630	3712838	KD2-H4K8la_peak_43	7.92775	LRRC47_ENSG00000130764
1	6260289	6260644	KD2-H4K8la_peak_44	9.27415	

1	6550773	6551055	KD2-H4K8la_peak_45	6.47589	
1	6761410	6761785	KD2-H4K8la_peak_46	7.41831	DNAJC11_ENSG00000007923
1	6762314	6762532	KD2-H4K8la_peak_47	6.04152	DNAJC11_ENSG00000007923
1	7401241	7401441	KD2-H4K8la_peak_48	8.72694	
1	7537757	7538101	KD2-H4K8la_peak_49	8.37064	
1	7594527	7594818	KD2-H4K8la_peak_50	7.49467	
1	7616557	7616828	KD2-H4K8la_peak_51	7.92775	
1	7618519	7618768	KD2-H4K8la_peak_52	6.20477	
1	7646615	7647003	KD2-H4K8la_peak_53	10.43707	
1	7727953	7728183	KD2-H4K8la_peak_54	9.99681	
1	7729021	7729479	KD2-H4K8la_peak_55	5.79108	
1	7740494	7740724	KD2-H4K8la_peak_56	6.04899	
1	7740965	7741421	KD2-H4K8la_peak_57	10.59328	
1	8086622	8087392	KD2-H4K8la_peak_58	14.15376	ERRFI1_ENSG00000116285;RP11-431K24.1_ENSG00000238290
1	8230277	8230523	KD2-H4K8la_peak_59	7.56395	
1	8378177	8378728	KD2-H4K8la_peak_60	13.26459	SLC45A1_ENSG00000162426
1	9488324	9488764	KD2-H4K8la_peak_61	6.96161	
1	9554963	9555356	KD2-H4K8la_peak_62	8.12466	
1	9663619	9663873	KD2-H4K8la_peak_63	4.68264	
1	9884400	9884867	KD2-H4K8la_peak_64	6.13507	CLSTN1_ENSG00000171603
1	1.1E+07	1.1E+07	KD2-H4K8la_peak_65	6.38565	
1	1.1E+07	1.1E+07	KD2-H4K8la_peak_66	8.76116	
1	1.1E+07	1.1E+07	KD2-H4K8la_peak_67	5.58883	
1	1.1E+07	1.1E+07	KD2-H4K8la_peak_68	9.20163	
1	1.1E+07	1.1E+07	KD2-H4K8la_peak_69	7.97806	
1	1.1E+07	1.1E+07	KD2-H4K8la_peak_70	11.87032	
1	1.1E+07	1.1E+07	KD2-H4K8la_peak_71	12.13455	
1	1.1E+07	1.1E+07	KD2-H4K8la_peak_72	5.67596	
1	1.1E+07	1.1E+07	KD2-H4K8la_peak_73	9.27415	
1	1.1E+07	1.1E+07	KD2-H4K8la_peak_74	9.61894	
1	1.1E+07	1.1E+07	KD2-H4K8la_peak_75	7.49467	EXOSC10_ENSG00000171824;RP4-635E18.6_ENSG00000230337
1	1.1E+07	1.1E+07	KD2-H4K8la_peak_76	9.38376	EXOSC10_ENSG00000171824;RP4-635E18.6_ENSG00000230337
1	1.1E+07	1.1E+07	KD2-H4K8la_peak_77	12.1221	UBIAD1_ENSG00000120942
1	1.1E+07	1.1E+07	KD2-H4K8la_peak_78	7.22716	
1	1.2E+07	1.2E+07	KD2-H4K8la_peak_79	6.13507	FBXO6_ENSG00000116663
1	1.2E+07	1.2E+07	KD2-H4K8la_peak_80	6.13507	
1	1.2E+07	1.2E+07	KD2-H4K8la_peak_81	9.66714	
1	1.2E+07	1.2E+07	KD2-H4K8la_peak_82	16.21274	
1	1.4E+07	1.4E+07	KD2-H4K8la_peak_83	10.20357	PRDM2_ENSG00000116731
1	1.5E+07	1.5E+07	KD2-H4K8la_peak_84	7.61975	
1	1.6E+07	1.6E+07	KD2-H4K8la_peak_85	6.64804	
1	1.6E+07	1.6E+07	KD2-H4K8la_peak_86	10.04111	
1	1.6E+07	1.6E+07	KD2-H4K8la_peak_87	12.97817	RP4-680D5.9_ENSG00000271742;PLEKHM2_ENSG00000116786;AL121992.1_ENSG00000264048
1	1.6E+07	1.6E+07	KD2-H4K8la_peak_88	7.41831	
1	1.6E+07	1.6E+07	KD2-H4K8la_peak_89	5.66328	CLCNKA_ENSG00000186510
1	1.6E+07	1.6E+07	KD2-H4K8la_peak_90	6.2946	

1	1.7E+07	1.7E+07	KD2-H4K8la_peak_91	12.52637	
1	1.7E+07	1.7E+07	KD2-H4K8la_peak_92	10.04111	FBXO42_ENSG00000037637;SZRD1_ENSG00000055070
1	1.7E+07	1.7E+07	KD2-H4K8la_peak_93	6.13507	
1	1.7E+07	1.7E+07	KD2-H4K8la_peak_94	5.29444	NECAP2_ENSG00000157191
1	1.7E+07	1.7E+07	KD2-H4K8la_peak_95	18.85804	NBPF1_ENSG00000219481
1	1.7E+07	1.7E+07	KD2-H4K8la_peak_96	8.83002	CROCCP2_ENSG00000215908;MST1P2_ENSG00000186301
1	1.7E+07	1.7E+07	KD2-H4K8la_peak_97	13.63087	CROCCP2_ENSG00000215908;MST1P2_ENSG00000186301
1	1.7E+07	1.7E+07	KD2-H4K8la_peak_98	7.43997	
1	1.7E+07	1.7E+07	KD2-H4K8la_peak_99	10.47118	RNU1-2_ENSG00000207005
1	1.7E+07	1.7E+07	KD2-H4K8la_peak_100	16.1269	
1	1.7E+07	1.7E+07	KD2-H4K8la_peak_101	5.84385	
1	1.7E+07	1.7E+07	KD2-H4K8la_peak_102	10.20357	
1	1.8E+07	1.8E+07	KD2-H4K8la_peak_103	10.7833	
1	1.8E+07	1.8E+07	KD2-H4K8la_peak_104	8.37064	ARHGEF10L_ENSG00000074964
1	1.8E+07	1.8E+07	KD2-H4K8la_peak_105	5.64331	
1	1.8E+07	1.8E+07	KD2-H4K8la_peak_106	8.52847	
1	1.8E+07	1.8E+07	KD2-H4K8la_peak_107	5.43278	
1	2E+07	2E+07	KD2-H4K8la_peak_108	6.3399	EMC1_ENSG00000127463;MRTO4_ENSG00000053372
1	2E+07	2E+07	KD2-H4K8la_peak_109	6.83927	AKR7A2_ENSG00000053371;PQLC2_ENSG00000040487
1	2E+07	2E+07	KD2-H4K8la_peak_110	11.36807	
1	2E+07	2E+07	KD2-H4K8la_peak_111	6.3399	HTR6_ENSG00000158748
1	2E+07	2E+07	KD2-H4K8la_peak_112	9.35473	TMCO4_ENSG00000162542
1	2E+07	2E+07	KD2-H4K8la_peak_113	7.13237	OTUD3_ENSG00000169914
1	2.1E+07	2.1E+07	KD2-H4K8la_peak_114	10.20357	
1	2.1E+07	2.1E+07	KD2-H4K8la_peak_115	9.90319	
1	2.2E+07	2.2E+07	KD2-H4K8la_peak_116	8.76082	
1	2.2E+07	2.2E+07	KD2-H4K8la_peak_117	8.7731	
1	2.2E+07	2.2E+07	KD2-H4K8la_peak_118	5.05146	
1	2.2E+07	2.2E+07	KD2-H4K8la_peak_119	6.96161	
1	2.2E+07	2.2E+07	KD2-H4K8la_peak_120	7.41831	ALPL_ENSG00000162551
1	2.2E+07	2.2E+07	KD2-H4K8la_peak_121	4.98702	
1	2.2E+07	2.2E+07	KD2-H4K8la_peak_122	9.0413	RP1-224A6.3_ENSG00000228397;LINC00339_ENSG00000218510
1	2.2E+07	2.2E+07	KD2-H4K8la_peak_123	5.43278	RP1-224A6.3_ENSG00000228397;LINC00339_ENSG00000218510
1	2.3E+07	2.3E+07	KD2-H4K8la_peak_124	8.27395	
1	2.3E+07	2.3E+07	KD2-H4K8la_peak_125	4.86896	
1	2.3E+07	2.3E+07	KD2-H4K8la_peak_126	6.10429	
1	2.3E+07	2.3E+07	KD2-H4K8la_peak_127	5.42108	
1	2.3E+07	2.3E+07	KD2-H4K8la_peak_128	7.49467	ZBTB40_ENSG00000184677
1	2.3E+07	2.3E+07	KD2-H4K8la_peak_129	7.13237	EPHB2_ENSG00000133216
1	2.4E+07	2.4E+07	KD2-H4K8la_peak_130	7.12014	E2F2_ENSG00000007968
1	2.4E+07	2.4E+07	KD2-H4K8la_peak_131	4.30663	
1	2.4E+07	2.4E+07	KD2-H4K8la_peak_132	12.32641	
1	2.4E+07	2.4E+07	KD2-H4K8la_peak_133	10.91646	
1	2.4E+07	2.4E+07	KD2-H4K8la_peak_134	7.13237	
1	2.4E+07	2.4E+07	KD2-H4K8la_peak_135	6.3399	TCEB3_ENSG00000011007
1	2.4E+07	2.4E+07	KD2-H4K8la_peak_136	6.96161	RP5-886K2.3_ENSG00000236810;PITHD1_ENSG00000057757

1	2.4E+07	2.4E+07	KD2-H4K8la_peak_137	8.6948	
1	2.5E+07	2.5E+07	KD2-H4K8la_peak_138	6.13507	RCAN3_ENSG00000117602
1	2.5E+07	2.5E+07	KD2-H4K8la_peak_139	10.28981	
1	2.6E+07	2.6E+07	KD2-H4K8la_peak_140	5.58883	SYF2_ENSG00000117614
1	2.6E+07	2.6E+07	KD2-H4K8la_peak_141	7.58011	
1	2.6E+07	2.6E+07	KD2-H4K8la_peak_142	8.74872	
1	2.6E+07	2.6E+07	KD2-H4K8la_peak_143	15.92725	
1	2.6E+07	2.6E+07	KD2-H4K8la_peak_144	10.23822	PAFAH2_ENSG00000158006
1	2.6E+07	2.6E+07	KD2-H4K8la_peak_145	4.86896	
1	2.7E+07	2.7E+07	KD2-H4K8la_peak_146	6.68593	
1	2.7E+07	2.7E+07	KD2-H4K8la_peak_147	6.89612	PIGV_ENSG00000060642
1	2.7E+07	2.7E+07	KD2-H4K8la_peak_148	4.66279	PIGV_ENSG00000060642
1	2.7E+07	2.7E+07	KD2-H4K8la_peak_149	5.27672	ZDHHC18_ENSG00000204160
1	2.7E+07	2.7E+07	KD2-H4K8la_peak_150	5.67034	GPATCH3_ENSG00000198746;NUDC_ENSG00000090273
1	2.7E+07	2.7E+07	KD2-H4K8la_peak_151	8.24531	
1	2.7E+07	2.7E+07	KD2-H4K8la_peak_152	6.5142	
1	2.8E+07	2.8E+07	KD2-H4K8la_peak_153	9.32091	RP11-40H20.4_ENSG00000224311;WDTC1_ENSG00000142784
1	2.8E+07	2.8E+07	KD2-H4K8la_peak_154	6.13528	
1	2.8E+07	2.8E+07	KD2-H4K8la_peak_155	8.50453	
1	2.8E+07	2.8E+07	KD2-H4K8la_peak_156	11.50202	WASF2_ENSG00000158195
1	2.8E+07	2.8E+07	KD2-H4K8la_peak_157	11.06859	
1	2.8E+07	2.8E+07	KD2-H4K8la_peak_158	12.39776	
1	2.8E+07	2.8E+07	KD2-H4K8la_peak_159	7.81267	
1	2.8E+07	2.8E+07	KD2-H4K8la_peak_160	11.6827	RP11-288L9.1_ENSG00000233975
1	2.8E+07	2.8E+07	KD2-H4K8la_peak_161	7.49467	FAM76A_ENSG00000009780
1	2.8E+07	2.8E+07	KD2-H4K8la_peak_162	5.28047	PPP1R8_ENSG00000117751
1	2.9E+07	2.9E+07	KD2-H4K8la_peak_163	13.95774	
1	2.9E+07	2.9E+07	KD2-H4K8la_peak_164	7.13185	
1	2.9E+07	2.9E+07	KD2-H4K8la_peak_165	7.49467	MED18_ENSG00000130772
1	2.9E+07	2.9E+07	KD2-H4K8la_peak_166	9.5329	PHACTR4_ENSG00000204138
1	2.9E+07	2.9E+07	KD2-H4K8la_peak_167	6.13528	
1	2.9E+07	2.9E+07	KD2-H4K8la_peak_168	8.487	TRNAU1AP_ENSG00000180098
1	2.9E+07	2.9E+07	KD2-H4K8la_peak_169	8.07102	RNU11_ENSG00000270103
1	2.9E+07	2.9E+07	KD2-H4K8la_peak_170	7.8597	GMEB1_ENSG00000162419
1	2.9E+07	2.9E+07	KD2-H4K8la_peak_171	15.92725	GMEB1_ENSG00000162419
1	2.9E+07	2.9E+07	KD2-H4K8la_peak_172	7.92775	YTHDF2_ENSG00000198492
1	2.9E+07	2.9E+07	KD2-H4K8la_peak_173	6.72794	
1	2.9E+07	2.9E+07	KD2-H4K8la_peak_174	6.64804	EPB41_ENSG00000159023
1	3E+07	3E+07	KD2-H4K8la_peak_175	9.90319	SRSF4_ENSG00000116350
1	3E+07	3E+07	KD2-H4K8la_peak_176	4.7338	SRSF4_ENSG00000116350
1	3E+07	3E+07	KD2-H4K8la_peak_177	7.12014	
1	3E+07	3E+07	KD2-H4K8la_peak_178	6.86458	
1	3E+07	3E+07	KD2-H4K8la_peak_179	4.86896	
1	3E+07	3E+07	KD2-H4K8la_peak_180	17.48572	
1	3E+07	3E+07	KD2-H4K8la_peak_181	6.64804	
1	3E+07	3E+07	KD2-H4K8la_peak_182	11.20123	

1	3E+07	3E+07	KD2-H4K8la_peak_183	5.83285	
1	3E+07	3E+07	KD2-H4K8la_peak_184	12.03112	
1	3E+07	3E+07	KD2-H4K8la_peak_185	4.94337	
1	3.1E+07	3.1E+07	KD2-H4K8la_peak_186	13.6415	
1	3.1E+07	3.1E+07	KD2-H4K8la_peak_187	6.3399	
1	3.1E+07	3.1E+07	KD2-H4K8la_peak_188	6.13507	SDC3_ENSG00000162512
1	3.2E+07	3.2E+07	KD2-H4K8la_peak_189	9.61894	
1	3.2E+07	3.2E+07	KD2-H4K8la_peak_190	11.87091	
1	3.2E+07	3.2E+07	KD2-H4K8la_peak_191	9.0516	
1	3.2E+07	3.2E+07	KD2-H4K8la_peak_192	10.39665	HCRTR1_ENSG00000121764
1	3.2E+07	3.2E+07	KD2-H4K8la_peak_193	6.36118	
1	3.2E+07	3.2E+07	KD2-H4K8la_peak_194	6.25288	
1	3.2E+07	3.2E+07	KD2-H4K8la_peak_195	7.13237	
1	3.2E+07	3.2E+07	KD2-H4K8la_peak_196	7.49467	SPOCD1_ENSG00000134668
1	3.2E+07	3.2E+07	KD2-H4K8la_peak_197	6.3399	
1	3.3E+07	3.3E+07	KD2-H4K8la_peak_198	12.13455	RP4-622L5.7_ENSG00000224066;IQCC_ENSG00000160051
1	3.3E+07	3.3E+07	KD2-H4K8la_peak_199	5.55184	MARCKSL1_ENSG00000175130
1	3.3E+07	3.3E+07	KD2-H4K8la_peak_200	13.27013	KIAA1522_ENSG00000162522
1	3.3E+07	3.3E+07	KD2-H4K8la_peak_201	9.7321	YARS_ENSG00000134684
1	3.3E+07	3.3E+07	KD2-H4K8la_peak_202	4.30663	
1	3.3E+07	3.3E+07	KD2-H4K8la_peak_203	5.27164	TMEM54_ENSG00000121900
1	3.3E+07	3.3E+07	KD2-H4K8la_peak_204	5.58883	FKSG48_ENSG00000267885
1	3.4E+07	3.4E+07	KD2-H4K8la_peak_205	8.13727	
1	3.6E+07	3.6E+07	KD2-H4K8la_peak_206	8.26997	SFPQ_ENSG00000116560
1	3.6E+07	3.6E+07	KD2-H4K8la_peak_207	7.12014	
1	3.6E+07	3.6E+07	KD2-H4K8la_peak_208	15.92725	
1	3.6E+07	3.6E+07	KD2-H4K8la_peak_209	7.12014	C1orf216_ENSG00000142686
1	3.6E+07	3.6E+07	KD2-H4K8la_peak_210	7.13237	
1	3.6E+07	3.6E+07	KD2-H4K8la_peak_211	4.66279	AGO4_ENSG00000134698
1	3.7E+07	3.7E+07	KD2-H4K8la_peak_212	5.58883	
1	3.7E+07	3.7E+07	KD2-H4K8la_peak_213	7.3993	COL8A2_ENSG00000171812
1	3.7E+07	3.7E+07	KD2-H4K8la_peak_214	5.5977	
1	3.7E+07	3.7E+07	KD2-H4K8la_peak_215	15.80488	
1	3.8E+07	3.8E+07	KD2-H4K8la_peak_216	7.13237	
1	3.8E+07	3.8E+07	KD2-H4K8la_peak_217	6.2278	FHL3_ENSG00000183386
1	4E+07	4E+07	KD2-H4K8la_peak_218	6.95378	
1	4.1E+07	4.1E+07	KD2-H4K8la_peak_219	5.63532	
1	4.1E+07	4.1E+07	KD2-H4K8la_peak_220	11.15747	RP1-39G22.7_ENSG00000259943;ZMPSTE24_ENSG00000084073
1	4.1E+07	4.1E+07	KD2-H4K8la_peak_221	18.96048	CTPS1_ENSG00000171793
1	4.1E+07	4.1E+07	KD2-H4K8la_peak_222	7.77341	CTPS1_ENSG00000171793
1	4.2E+07	4.2E+07	KD2-H4K8la_peak_223	8.39919	SCMH1_ENSG00000010803;RP11-399E6.1_ENSG00000235358
1	4.2E+07	4.2E+07	KD2-H4K8la_peak_224	12.23212	
1	4.2E+07	4.2E+07	KD2-H4K8la_peak_225	7.38202	
1	4.2E+07	4.2E+07	KD2-H4K8la_peak_226	5.99671	
1	4.2E+07	4.2E+07	KD2-H4K8la_peak_227	5.4856	
1	4.2E+07	4.2E+07	KD2-H4K8la_peak_228	12.25633	

1	4.2E+07	4.2E+07	KD2-H4K8la_peak_229	6.18994	
1	4.3E+07	4.3E+07	KD2-H4K8la_peak_230	10.04111	ZMYND12_ENSG00000066185;PPCS_ENSG00000127125
1	4.3E+07	4.3E+07	KD2-H4K8la_peak_231	9.27415	RP11-342M1.3_ENSG00000228192;ZNF691_ENSG00000164011
1	4.3E+07	4.3E+07	KD2-H4K8la_peak_232	10.73719	
1	4.3E+07	4.3E+07	KD2-H4K8la_peak_233	8.07102	
1	4.4E+07	4.4E+07	KD2-H4K8la_peak_234	6.25288	
1	4.4E+07	4.4E+07	KD2-H4K8la_peak_235	14.07018	
1	4.4E+07	4.4E+07	KD2-H4K8la_peak_236	4.36246	MED8_ENSG00000159479;SZT2_ENSG00000198198
1	4.4E+07	4.4E+07	KD2-H4K8la_peak_237	6.79079	HYI_ENSG00000178922;HYI-AS1_ENSG00000229348
1	4.4E+07	4.4E+07	KD2-H4K8la_peak_238	9.10568	
1	4.4E+07	4.4E+07	KD2-H4K8la_peak_239	7.17272	SLC6A9_ENSG00000196517
1	4.5E+07	4.5E+07	KD2-H4K8la_peak_240	7.90495	
1	4.5E+07	4.5E+07	KD2-H4K8la_peak_241	12.32641	
1	4.5E+07	4.5E+07	KD2-H4K8la_peak_242	6.61963	
1	4.5E+07	4.5E+07	KD2-H4K8la_peak_243	8.43222	PTCH2_ENSG00000117425;RP5-882O7.1_ENSG00000226499
1	4.6E+07	4.6E+07	KD2-H4K8la_peak_244	7.51014	IPP_ENSG00000197429
1	4.6E+07	4.6E+07	KD2-H4K8la_peak_245	6.60114	IPP_ENSG00000197429
1	4.7E+07	4.7E+07	KD2-H4K8la_peak_246	5.33721	
1	4.7E+07	4.7E+07	KD2-H4K8la_peak_247	16.56946	
1	4.7E+07	4.7E+07	KD2-H4K8la_peak_248	10.25348	
1	4.7E+07	4.7E+07	KD2-H4K8la_peak_249	14.5688	LRRC41_ENSG00000132128;UQCRH_ENSG00000173660
1	4.7E+07	4.7E+07	KD2-H4K8la_peak_250	6.64643	NSUN4_ENSG00000117481
1	4.7E+07	4.7E+07	KD2-H4K8la_peak_251	7.3993	NSUN4_ENSG00000117481
1	4.7E+07	4.7E+07	KD2-H4K8la_peak_252	8.79494	
1	4.7E+07	4.7E+07	KD2-H4K8la_peak_253	6.64804	
1	4.7E+07	4.7E+07	KD2-H4K8la_peak_254	15.42498	EFCAB14_ENSG00000159658
1	4.8E+07	4.8E+07	KD2-H4K8la_peak_255	6.61963	CMPK1_ENSG00000162368
1	5.1E+07	5.1E+07	KD2-H4K8la_peak_256	11.35192	
1	5.1E+07	5.1E+07	KD2-H4K8la_peak_257	11.06859	
1	5.2E+07	5.2E+07	KD2-H4K8la_peak_258	5.05146	RP11-275F13.1_ENSG00000261664
1	5.2E+07	5.2E+07	KD2-H4K8la_peak_259	10.07718	NRD1_ENSG00000078618
1	5.3E+07	5.3E+07	KD2-H4K8la_peak_260	6.86458	TXNDC12_ENSG00000117862;BTF3L4_ENSG00000134717
1	5.3E+07	5.3E+07	KD2-H4K8la_peak_261	8.76116	RP11-25O10.2_ENSG00000272371;ZCCHC11_ENSG00000134744
1	5.3E+07	5.3E+07	KD2-H4K8la_peak_262	9.27415	FAM159A_ENSG00000182183
1	5.4E+07	5.4E+07	KD2-H4K8la_peak_263	8.86558	MAGOH_ENSG00000162385;RP5-1024G6.5_ENSG00000226754
1	5.4E+07	5.4E+07	KD2-H4K8la_peak_264	8.16787	LRP8_ENSG00000157193;RP4-784A16.5_ENSG00000225675
1	5.4E+07	5.4E+07	KD2-H4K8la_peak_265	6.61495	RP11-117D22.2_ENSG00000230138
1	5.4E+07	5.4E+07	KD2-H4K8la_peak_266	5.83285	
1	5.4E+07	5.4E+07	KD2-H4K8la_peak_267	5.83285	
1	5.5E+07	5.5E+07	KD2-H4K8la_peak_268	5.67596	
1	5.5E+07	5.5E+07	KD2-H4K8la_peak_269	11.36807	
1	5.5E+07	5.5E+07	KD2-H4K8la_peak_270	6.4136	ACOT11_ENSG00000162390
1	5.5E+07	5.5E+07	KD2-H4K8la_peak_271	8.1293	ACOT11_ENSG00000162390
1	5.5E+07	5.5E+07	KD2-H4K8la_peak_272	7.06427	
1	5.6E+07	5.6E+07	KD2-H4K8la_peak_273	6.57426	USP24_ENSG00000162402
1	5.6E+07	5.6E+07	KD2-H4K8la_peak_274	5.29444	USP24_ENSG00000162402

1	5.6E+07	5.6E+07	KD2-H4K8la_peak_275	9.49266	
1	5.7E+07	5.7E+07	KD2-H4K8la_peak_276	6.3399	
1	5.7E+07	5.7E+07	KD2-H4K8la_peak_277	5.43278	PPAP2B_ENSG00000162407;PRKAA2_ENSG00000162409
1	5.9E+07	5.9E+07	KD2-H4K8la_peak_278	12.67978	
1	6E+07	6E+07	KD2-H4K8la_peak_279	8.39813	FGGY_ENSG00000172456
1	6E+07	6E+07	KD2-H4K8la_peak_280	4.6555	HOOK1_ENSG00000134709
1	6.1E+07	6.1E+07	KD2-H4K8la_peak_281	6.2278	
1	6.1E+07	6.1E+07	KD2-H4K8la_peak_282	6.70626	
1	6.2E+07	6.2E+07	KD2-H4K8la_peak_283	13.2369	
1	6.2E+07	6.2E+07	KD2-H4K8la_peak_284	8.73956	RP11-430G17.3_ENSG00000271200;INADL_ENSG00000132849
1	6.3E+07	6.3E+07	KD2-H4K8la_peak_285	7.86224	USP1_ENSG00000162607
1	6.3E+07	6.3E+07	KD2-H4K8la_peak_286	6.70626	ATG4C_ENSG00000125703
1	6.4E+07	6.4E+07	KD2-H4K8la_peak_287	7.81267	ALG6_ENSG00000088035
1	6.5E+07	6.5E+07	KD2-H4K8la_peak_288	7.26721	
1	6.6E+07	6.6E+07	KD2-H4K8la_peak_289	13.18631	
1	6.8E+07	6.8E+07	KD2-H4K8la_peak_290	5.91129	
1	6.8E+07	6.8E+07	KD2-H4K8la_peak_291	8.07102	
1	6.8E+07	6.8E+07	KD2-H4K8la_peak_292	6.2278	GADD45A_ENSG00000116717
1	6.8E+07	6.8E+07	KD2-H4K8la_peak_293	8.13727	
1	7.1E+07	7.1E+07	KD2-H4K8la_peak_294	6.3399	ANKRD13C_ENSG00000118454;HHLA3_ENSG00000197568
1	7.3E+07	7.3E+07	KD2-H4K8la_peak_295	5.72892	
1	7.5E+07	7.5E+07	KD2-H4K8la_peak_296	4.86896	LRRIQ3_ENSG00000162620;FPGT_ENSG00000254685;FPGT-TNNI3K_ENSG00000259030;TNNI3K_ENSG00000116783
1	7.6E+07	7.6E+07	KD2-H4K8la_peak_297	7.56395	
1	7.8E+07	7.8E+07	KD2-H4K8la_peak_298	10.04111	PIGK_ENSG00000142892
1	8.4E+07	8.4E+07	KD2-H4K8la_peak_299	5.72892	TTLL7_ENSG00000137941
1	8.5E+07	8.5E+07	KD2-H4K8la_peak_300	6.57319	SAMD13_ENSG00000203943
1	8.7E+07	8.7E+07	KD2-H4K8la_peak_301	5.72892	
1	8.7E+07	8.7E+07	KD2-H4K8la_peak_302	6.86458	RP4-612B15.3_ENSG00000261737
1	9E+07	9E+07	KD2-H4K8la_peak_303	11.77509	LRRC8B_ENSG00000197147
1	9E+07	9E+07	KD2-H4K8la_peak_304	5.05146	
1	9.1E+07	9.1E+07	KD2-H4K8la_peak_305	9.6708	ZNF644_ENSG00000122482
1	9.3E+07	9.3E+07	KD2-H4K8la_peak_306	4.57647	
1	9.4E+07	9.4E+07	KD2-H4K8la_peak_307	7.72421	FNBP1L_ENSG00000137942
1	9.4E+07	9.4E+07	KD2-H4K8la_peak_308	11.81691	
1	9.4E+07	9.4E+07	KD2-H4K8la_peak_309	10.00175	
1	9.4E+07	9.4E+07	KD2-H4K8la_peak_310	6.96161	BCAR3_ENSG00000137936;MIR760_ENSG00000211575;RP4-561L24.3_ENSG00000260464
1	9.4E+07	9.4E+07	KD2-H4K8la_peak_311	6.60114	DNTTIP2_ENSG00000067334
1	9.5E+07	9.5E+07	KD2-H4K8la_peak_312	7.49467	
1	9.5E+07	9.5E+07	KD2-H4K8la_peak_313	9.90319	
1	9.5E+07	9.5E+07	KD2-H4K8la_peak_314	10.20357	LINC01057_ENSG00000224081;SLC44A3_ENSG00000143036
1	9.6E+07	9.6E+07	KD2-H4K8la_peak_315	6.22999	RP11-14O19.2_ENSG00000237954
1	9.7E+07	9.7E+07	KD2-H4K8la_peak_316	8.37064	PTBP2_ENSG00000117569
1	9.9E+07	9.9E+07	KD2-H4K8la_peak_317	8.24908	
1	9.9E+07	9.9E+07	KD2-H4K8la_peak_318	4.94337	LPPR5_ENSG00000117598;RP5-896L10.1_ENSG00000232825
1	9.9E+07	9.9E+07	KD2-H4K8la_peak_319	5.72892	
1	1E+08	1E+08	KD2-H4K8la_peak_320	4.35346	RP5-884G6.2_ENSG00000228084;SLC35A3_ENSG00000117620

1	1E+08	1E+08	KD2-H4K8la_peak_321	8.7731	RP11-305E17.6_ENSG00000224616;RTCA_ENSG00000137996
1	1E+08	1E+08	KD2-H4K8la_peak_322	6.36118	EXTL2_ENSG00000162694;RP4-549L20.3_ENSG00000273204;SLC30A7_ENSG00000162695
1	1.1E+08	1.1E+08	KD2-H4K8la_peak_323	10.98662	
1	1.1E+08	1.1E+08	KD2-H4K8la_peak_324	11.24937	PRMT6_ENSG00000198890
1	1.1E+08	1.1E+08	KD2-H4K8la_peak_325	5.98303	VAV3_ENSG00000134215;VAV3-AS1_ENSG00000230489
1	1.1E+08	1.1E+08	KD2-H4K8la_peak_326	5.36015	STXBP3_ENSG00000116266
1	1.1E+08	1.1E+08	KD2-H4K8la_peak_327	9.0413	TAF13_ENSG00000197780
1	1.1E+08	1.1E+08	KD2-H4K8la_peak_328	8.72694	
1	1.1E+08	1.1E+08	KD2-H4K8la_peak_329	5.16795	CSF1_ENSG00000184371
1	1.1E+08	1.1E+08	KD2-H4K8la_peak_330	8.07044	AHCYL1_ENSG00000168710
1	1.1E+08	1.1E+08	KD2-H4K8la_peak_331	7.49467	RP4-773N10.4_ENSG00000258634
1	1.1E+08	1.1E+08	KD2-H4K8la_peak_332	8.16787	
1	1.1E+08	1.1E+08	KD2-H4K8la_peak_333	7.49467	
1	1.1E+08	1.1E+08	KD2-H4K8la_peak_334	14.52454	CAPZA1_ENSG00000116489
1	1.1E+08	1.1E+08	KD2-H4K8la_peak_335	6.04152	
1	1.1E+08	1.1E+08	KD2-H4K8la_peak_336	4.45136	RP11-31F15.2_ENSG00000238198;LRIG2_ENSG00000198799
1	1.1E+08	1.1E+08	KD2-H4K8la_peak_337	6.04312	
1	1.2E+08	1.2E+08	KD2-H4K8la_peak_338	14.40178	BCAS2_ENSG00000116752
1	1.2E+08	1.2E+08	KD2-H4K8la_peak_339	12.06531	CSDE1_ENSG00000009307
1	1.2E+08	1.2E+08	KD2-H4K8la_peak_340	9.6708	NGF_ENSG00000134259
1	1.2E+08	1.2E+08	KD2-H4K8la_peak_341	6.36118	ATP1A1OS_ENSG00000203865
1	1.2E+08	1.2E+08	KD2-H4K8la_peak_342	6.86458	CD58_ENSG00000116815
1	1.2E+08	1.2E+08	KD2-H4K8la_peak_343	9.27415	
1	1.2E+08	1.2E+08	KD2-H4K8la_peak_344	10.39665	
1	1.4E+08	1.4E+08	KD2-H4K8la_peak_345	11.15747	RP11-435B5.4_ENSG00000185044
1	1.4E+08	1.4E+08	KD2-H4K8la_peak_346	8.37064	
1	1.5E+08	1.5E+08	KD2-H4K8la_peak_347	9.5503	RP11-458D21.1_ENSG00000233396;RNVU1-6_ENSG00000201558
1	1.5E+08	1.5E+08	KD2-H4K8la_peak_348	6.75222	TXNIP_ENSG00000117289
1	1.5E+08	1.5E+08	KD2-H4K8la_peak_349	6.3399	GNRHR2_ENSG00000211451;PEX11B_ENSG00000131779
1	1.5E+08	1.5E+08	KD2-H4K8la_peak_350	6.13507	NUDT17_ENSG00000186364
1	1.5E+08	1.5E+08	KD2-H4K8la_peak_351	5.28047	PRKAB2_ENSG00000131791;RP11-337C18.8_ENSG00000237188;RP11-337C18.10_ENSG00000273071
1	1.5E+08	1.5E+08	KD2-H4K8la_peak_352	12.11591	FMO5_ENSG00000131781;CHD1L_ENSG00000131778
1	1.5E+08	1.5E+08	KD2-H4K8la_peak_353	7.92775	BCL9_ENSG00000116128
1	1.5E+08	1.5E+08	KD2-H4K8la_peak_354	6.61495	ACP6_ENSG00000162836
1	1.5E+08	1.5E+08	KD2-H4K8la_peak_355	7.06923	RP11-14N7.2_ENSG00000232527
1	1.5E+08	1.5E+08	KD2-H4K8la_peak_356	6.56022	
1	1.5E+08	1.5E+08	KD2-H4K8la_peak_357	10.44868	
1	1.5E+08	1.5E+08	KD2-H4K8la_peak_358	8.3497	PLEKHO1_ENSG00000023902
1	1.5E+08	1.5E+08	KD2-H4K8la_peak_359	8.84265	
1	1.5E+08	1.5E+08	KD2-H4K8la_peak_360	8.00569	ANP32E_ENSG00000143401
1	1.5E+08	1.5E+08	KD2-H4K8la_peak_361	9.41649	
1	1.5E+08	1.5E+08	KD2-H4K8la_peak_362	11.24289	PRPF3_ENSG00000117360
1	1.5E+08	1.5E+08	KD2-H4K8la_peak_363	7.12014	RPRD2_ENSG00000163125
1	1.5E+08	1.5E+08	KD2-H4K8la_peak_364	8.39919	TARS2_ENSG00000143374
1	1.5E+08	1.5E+08	KD2-H4K8la_peak_365	10.47452	SETDB1_ENSG00000143379
1	1.5E+08	1.5E+08	KD2-H4K8la_peak_366	9.12246	CERS2_ENSG00000143418

1	1.5E+08	1.5E+08	KD2-H4K8la_peak_367	13.31601	CDC42SE1_ENSG00000197622;GABPB2_ENSG00000143458
1	1.5E+08	1.5E+08	KD2-H4K8la_peak_368	9.05604	
1	1.5E+08	1.5E+08	KD2-H4K8la_peak_369	9.6708	PSMB4_ENSG00000159377
1	1.5E+08	1.5E+08	KD2-H4K8la_peak_370	6.64804	
1	1.5E+08	1.5E+08	KD2-H4K8la_peak_371	5.33301	MRPL9_ENSG00000143436;OAZ3_ENSG00000143450
1	1.5E+08	1.5E+08	KD2-H4K8la_peak_372	5.99671	S100A6_ENSG00000197956
1	1.5E+08	1.5E+08	KD2-H4K8la_peak_373	8.39919	
1	1.5E+08	1.5E+08	KD2-H4K8la_peak_374	6.63954	S100A2_ENSG00000196754
1	1.5E+08	1.5E+08	KD2-H4K8la_peak_375	13.00097	S100A13_ENSG00000189171;RP1-178F15.5_ENSG00000271853;RP1-178F15.4_ENSG00000272030;CHTOP_ENSG00000160679
1	1.5E+08	1.5E+08	KD2-H4K8la_peak_376	6.73603	
1	1.5E+08	1.5E+08	KD2-H4K8la_peak_377	6.6875	Y_RNA_ENSG00000199565
1	1.5E+08	1.5E+08	KD2-H4K8la_peak_378	14.44261	INTS3_ENSG00000143624
1	1.5E+08	1.5E+08	KD2-H4K8la_peak_379	8.88448	CRTC2_ENSG00000160741
1	1.5E+08	1.5E+08	KD2-H4K8la_peak_380	11.28887	
1	1.5E+08	1.5E+08	KD2-H4K8la_peak_381	5.20532	C1orf43_ENSG00000143612
1	1.5E+08	1.5E+08	KD2-H4K8la_peak_382	9.23326	ATP8B2_ENSG00000143515
1	1.5E+08	1.5E+08	KD2-H4K8la_peak_383	10.31182	UBE2Q1_ENSG00000160714
1	1.5E+08	1.5E+08	KD2-H4K8la_peak_384	6.34086	PMVK_ENSG00000163344;RP11-307C12.13_ENSG00000270361
1	1.5E+08	1.5E+08	KD2-H4K8la_peak_385	5.79108	RP11-307C12.12_ENSG00000271380
1	1.5E+08	1.5E+08	KD2-H4K8la_peak_386	6.69137	
1	1.5E+08	1.5E+08	KD2-H4K8la_peak_387	10.84044	
1	1.6E+08	1.6E+08	KD2-H4K8la_peak_388	5.54909	
1	1.6E+08	1.6E+08	KD2-H4K8la_peak_389	10.92814	
1	1.6E+08	1.6E+08	KD2-H4K8la_peak_390	11.57314	EFNA1_ENSG00000169242
1	1.6E+08	1.6E+08	KD2-H4K8la_peak_391	8.48488	
1	1.6E+08	1.6E+08	KD2-H4K8la_peak_392	13.84838	KRTCAP2_ENSG00000163463;TRIM46_ENSG00000163462
1	1.6E+08	1.6E+08	KD2-H4K8la_peak_393	12.50352	KRTCAP2_ENSG00000163463;TRIM46_ENSG00000163462
1	1.6E+08	1.6E+08	KD2-H4K8la_peak_394	9.7321	MUC1_ENSG00000185499
1	1.6E+08	1.6E+08	KD2-H4K8la_peak_395	5.58883	MUC1_ENSG00000185499;MIR92B_ENSG00000271748
1	1.6E+08	1.6E+08	KD2-H4K8la_peak_396	10.02595	THBS3_ENSG00000169231;MTX1_ENSG00000173171
1	1.6E+08	1.6E+08	KD2-H4K8la_peak_397	7.79412	THBS3_ENSG00000169231;MTX1_ENSG00000173171
1	1.6E+08	1.6E+08	KD2-H4K8la_peak_398	8.47421	SCAMP3_ENSG00000116521
1	1.6E+08	1.6E+08	KD2-H4K8la_peak_399	6.29651	FDPS_ENSG00000160752
1	1.6E+08	1.6E+08	KD2-H4K8la_peak_400	5.43278	
1	1.6E+08	1.6E+08	KD2-H4K8la_peak_401	8.27596	RXFP4_ENSG00000173080
1	1.6E+08	1.6E+08	KD2-H4K8la_peak_402	5.99671	RXFP4_ENSG00000173080
1	1.6E+08	1.6E+08	KD2-H4K8la_peak_403	8.39919	SSR2_ENSG00000163479
1	1.6E+08	1.6E+08	KD2-H4K8la_peak_404	14.15376	UBQLN4_ENSG00000160803;LAMTOR2_ENSG00000116586
1	1.6E+08	1.6E+08	KD2-H4K8la_peak_405	8.66164	LAMTOR2_ENSG00000116586
1	1.6E+08	1.6E+08	KD2-H4K8la_peak_406	9.77334	MEX3A_ENSG00000254726;LMNA_ENSG00000160789
1	1.6E+08	1.6E+08	KD2-H4K8la_peak_407	8.48488	MEX3A_ENSG00000254726;LMNA_ENSG00000160789
1	1.6E+08	1.6E+08	KD2-H4K8la_peak_408	6.56022	MEX3A_ENSG00000254726;LMNA_ENSG00000160789
1	1.6E+08	1.6E+08	KD2-H4K8la_peak_409	7.35976	
1	1.6E+08	1.6E+08	KD2-H4K8la_peak_410	6.69137	PAQR6_ENSG00000160781
1	1.6E+08	1.6E+08	KD2-H4K8la_peak_411	4.6555	TSACC_ENSG00000163467
1	1.6E+08	1.6E+08	KD2-H4K8la_peak_412	8.95597	

1	1.6E+08	1.6E+08	KD2-H4K8la_peak_413	6.02599	MIR9-1_ENSG00000207933
1	1.6E+08	1.6E+08	KD2-H4K8la_peak_414	6.78512	
1	1.6E+08	1.6E+08	KD2-H4K8la_peak_415	5.33301	
1	1.6E+08	1.6E+08	KD2-H4K8la_peak_416	7.77341	RP11-98G7.1_ENSG00000236947
1	1.6E+08	1.6E+08	KD2-H4K8la_peak_417	14.89556	RP11-98G7.1_ENSG00000236947
1	1.6E+08	1.6E+08	KD2-H4K8la_peak_418	11.68081	RP11-66D17.3_ENSG00000237588
1	1.6E+08	1.6E+08	KD2-H4K8la_peak_419	9.40293	CRABP2_ENSG00000143320
1	1.6E+08	1.6E+08	KD2-H4K8la_peak_420	6.4136	CRABP2_ENSG00000143320
1	1.6E+08	1.6E+08	KD2-H4K8la_peak_421	13.60057	
1	1.6E+08	1.6E+08	KD2-H4K8la_peak_422	7.49897	MRPL24_ENSG00000143314
1	1.6E+08	1.6E+08	KD2-H4K8la_peak_423	7.61975	
1	1.6E+08	1.6E+08	KD2-H4K8la_peak_424	11.92959	PRCC_ENSG00000143294
1	1.6E+08	1.6E+08	KD2-H4K8la_peak_425	9.77334	HDGF_ENSG00000143321
1	1.6E+08	1.6E+08	KD2-H4K8la_peak_426	8.21187	INSRR_ENSG00000027644
1	1.6E+08	1.6E+08	KD2-H4K8la_peak_427	5.29561	
1	1.6E+08	1.6E+08	KD2-H4K8la_peak_428	8.84474	ARHGEF11_ENSG00000132694
1	1.6E+08	1.6E+08	KD2-H4K8la_peak_429	7.13237	
1	1.6E+08	1.6E+08	KD2-H4K8la_peak_430	9.98668	
1	1.6E+08	1.6E+08	KD2-H4K8la_peak_431	13.13367	RP11-574F21.2_ENSG00000228606
1	1.6E+08	1.6E+08	KD2-H4K8la_peak_432	5.28047	
1	1.6E+08	1.6E+08	KD2-H4K8la_peak_433	14.44261	DCAF8_ENSG00000132716;DCAF8_ENSG00000258465
1	1.6E+08	1.6E+08	KD2-H4K8la_peak_434	4.75945	COPA_ENSG00000122218;NCSTN_ENSG00000162736
1	1.6E+08	1.6E+08	KD2-H4K8la_peak_435	6.40658	KLHDC9_ENSG00000162755
1	1.6E+08	1.6E+08	KD2-H4K8la_peak_436	6.09539	UFC1_ENSG00000143222
1	1.6E+08	1.6E+08	KD2-H4K8la_peak_437	5.90392	
1	1.6E+08	1.6E+08	KD2-H4K8la_peak_438	8.48577	RP11-297K8.2_ENSG00000224985;USP21_ENSG00000143258
1	1.6E+08	1.6E+08	KD2-H4K8la_peak_439	6.86458	B4GALT3_ENSG00000158850
1	1.6E+08	1.6E+08	KD2-H4K8la_peak_440	7.16303	NDUFS2_ENSG00000158864;ADAMTS4_ENSG00000158859
1	1.6E+08	1.6E+08	KD2-H4K8la_peak_441	8.0333	
1	1.6E+08	1.6E+08	KD2-H4K8la_peak_442	5.94474	
1	1.6E+08	1.6E+08	KD2-H4K8la_peak_443	5.6499	
1	1.6E+08	1.6E+08	KD2-H4K8la_peak_444	7.9076	SDHC_ENSG00000143252
1	1.6E+08	1.6E+08	KD2-H4K8la_peak_445	7.61975	SDHC_ENSG00000143252
1	1.6E+08	1.6E+08	KD2-H4K8la_peak_446	10.04113	
1	1.6E+08	1.6E+08	KD2-H4K8la_peak_447	12.32641	
1	1.6E+08	1.6E+08	KD2-H4K8la_peak_448	15.63685	
1	1.6E+08	1.6E+08	KD2-H4K8la_peak_449	8.3497	
1	1.6E+08	1.6E+08	KD2-H4K8la_peak_450	9.61894	UHMK1_ENSG00000152332
1	1.6E+08	1.6E+08	KD2-H4K8la_peak_451	8.89364	
1	1.6E+08	1.6E+08	KD2-H4K8la_peak_452	7.3993	
1	1.6E+08	1.6E+08	KD2-H4K8la_peak_453	10.70284	
1	1.6E+08	1.6E+08	KD2-H4K8la_peak_454	7.68525	
1	1.6E+08	1.6E+08	KD2-H4K8la_peak_455	11.15747	
1	1.6E+08	1.6E+08	KD2-H4K8la_peak_456	9.27415	
1	1.7E+08	1.7E+08	KD2-H4K8la_peak_457	11.40266	TMCO1_ENSG00000143183;UCK2_ENSG00000143179
1	1.7E+08	1.7E+08	KD2-H4K8la_peak_458	5.18385	

1	1.7E+08	1.7E+08	KD2-H4K8la_peak_459	5.78071	
1	1.7E+08	1.7E+08	KD2-H4K8la_peak_460	8.37064	KLHL20_ENSG00000076321
1	1.7E+08	1.7E+08	KD2-H4K8la_peak_461	6.13507	RC3H1_ENSG00000135870;RP11-160H22.3_ENSG00000224977
1	1.7E+08	1.7E+08	KD2-H4K8la_peak_462	6.98743	CACYBP_ENSG00000116161
1	1.7E+08	1.7E+08	KD2-H4K8la_peak_463	5.33301	
1	1.7E+08	1.7E+08	KD2-H4K8la_peak_464	8.06229	MRPS14_ENSG00000120333
1	1.8E+08	1.8E+08	KD2-H4K8la_peak_465	18.26015	C1orf220_ENSG00000213057;C1ORF220_ENSG00000184909
1	1.8E+08	1.8E+08	KD2-H4K8la_peak_466	8.76116	
1	1.8E+08	1.8E+08	KD2-H4K8la_peak_467	5.45079	
1	1.8E+08	1.8E+08	KD2-H4K8la_peak_468	6.4136	TDRD5_ENSG00000162782
1	1.8E+08	1.8E+08	KD2-H4K8la_peak_469	11.42932	FAM163A_ENSG00000143340
1	1.8E+08	1.8E+08	KD2-H4K8la_peak_470	10.04397	
1	1.8E+08	1.8E+08	KD2-H4K8la_peak_471	10.49982	
1	1.8E+08	1.8E+08	KD2-H4K8la_peak_472	6.57176	
1	1.8E+08	1.8E+08	KD2-H4K8la_peak_473	9.06396	
1	1.8E+08	1.8E+08	KD2-H4K8la_peak_474	12.55003	
1	1.8E+08	1.8E+08	KD2-H4K8la_peak_475	12.44878	
1	1.8E+08	1.8E+08	KD2-H4K8la_peak_476	12.05405	
1	1.8E+08	1.8E+08	KD2-H4K8la_peak_477	5.33301	
1	1.8E+08	1.8E+08	KD2-H4K8la_peak_478	9.67249	
1	1.8E+08	1.8E+08	KD2-H4K8la_peak_479	7.12014	
1	1.8E+08	1.8E+08	KD2-H4K8la_peak_480	11.7362	
1	1.8E+08	1.8E+08	KD2-H4K8la_peak_481	15.08271	
1	1.8E+08	1.8E+08	KD2-H4K8la_peak_482	12.12477	RP11-317P15.4_ENSG00000261504
1	1.8E+08	1.8E+08	KD2-H4K8la_peak_483	4.30663	
1	1.8E+08	1.8E+08	KD2-H4K8la_peak_484	5.28047	DHX9_ENSG00000135829
1	1.8E+08	1.8E+08	KD2-H4K8la_peak_485	10.47118	LAMC1_ENSG00000135862
1	1.8E+08	1.8E+08	KD2-H4K8la_peak_486	14.23294	SMG7-AS1_ENSG00000232860;SMG7_ENSG00000116698
1	1.8E+08	1.8E+08	KD2-H4K8la_peak_487	8.13557	SMG7-AS1_ENSG00000232860;SMG7_ENSG00000116698
1	1.8E+08	1.8E+08	KD2-H4K8la_peak_488	5.33301	COLGALT2_ENSG00000198756
1	1.9E+08	1.9E+08	KD2-H4K8la_peak_489	5.84082	RNF2_ENSG00000121481
1	1.9E+08	1.9E+08	KD2-H4K8la_peak_490	18.22154	TRMT1L_ENSG00000121486;SWT1_ENSG00000116668
1	1.9E+08	1.9E+08	KD2-H4K8la_peak_491	6.86458	IVNS1ABP_ENSG00000116679
1	1.9E+08	1.9E+08	KD2-H4K8la_peak_492	5.90392	IVNS1ABP_ENSG00000116679;GS1-279B7.2_ENSG00000273004
1	1.9E+08	1.9E+08	KD2-H4K8la_peak_493	9.66714	CDC73_ENSG00000134371
1	2E+08	2E+08	KD2-H4K8la_peak_494	4.30663	
1	2E+08	2E+08	KD2-H4K8la_peak_495	6.2278	AL450244.1_ENSG00000263805
1	2E+08	2E+08	KD2-H4K8la_peak_496	6.59654	ZNF281_ENSG00000162702
1	2E+08	2E+08	KD2-H4K8la_peak_497	7.61975	DDX59_ENSG00000118197;RP11-92G12.3_ENSG00000260088
1	2E+08	2E+08	KD2-H4K8la_peak_498	6.68593	CAMSAP2_ENSG00000118200
1	2E+08	2E+08	KD2-H4K8la_peak_499	9.87983	
1	2E+08	2E+08	KD2-H4K8la_peak_500	8.73956	
1	2E+08	2E+08	KD2-H4K8la_peak_501	5.58883	
1	2E+08	2E+08	KD2-H4K8la_peak_502	8.84474	
1	2E+08	2E+08	KD2-H4K8la_peak_503	11.40266	SHISA4_ENSG00000198892
1	2E+08	2E+08	KD2-H4K8la_peak_504	7.69026	SHISA4_ENSG00000198892

1	2E+08	2E+08	KD2-H4K8la_peak_505	13.47548	TIMM17A_ENSG00000134375
1	2E+08	2E+08	KD2-H4K8la_peak_506	12.72886	RNPEP_ENSG00000176393
1	2E+08	2E+08	KD2-H4K8la_peak_507	8.0333	ARL8A_ENSG00000143862
1	2E+08	2E+08	KD2-H4K8la_peak_508	6.40658	ARL8A_ENSG00000143862
1	2E+08	2E+08	KD2-H4K8la_peak_509	7.90495	
1	2E+08	2E+08	KD2-H4K8la_peak_510	7.36635	
1	2E+08	2E+08	KD2-H4K8la_peak_511	5.58883	
1	2E+08	2E+08	KD2-H4K8la_peak_512	8.48488	
1	2E+08	2E+08	KD2-H4K8la_peak_513	8.16916	RP11-480I12.10_ENSG00000260021;PCAT6_ENSG00000228288
1	2E+08	2E+08	KD2-H4K8la_peak_514	12.19151	KLHL12_ENSG00000117153
1	2E+08	2E+08	KD2-H4K8la_peak_515	14.44261	ADIPOR1_ENSG00000159346
1	2E+08	2E+08	KD2-H4K8la_peak_516	9.61894	CYB5R1_ENSG00000159348
1	2E+08	2E+08	KD2-H4K8la_peak_517	5.28047	
1	2E+08	2E+08	KD2-H4K8la_peak_518	5.27164	
1	2E+08	2E+08	KD2-H4K8la_peak_519	8.89364	
1	2E+08	2E+08	KD2-H4K8la_peak_520	8.00843	
1	2E+08	2E+08	KD2-H4K8la_peak_521	6.93796	
1	2E+08	2E+08	KD2-H4K8la_peak_522	9.01952	SNRPE_ENSG00000182004
1	2E+08	2E+08	KD2-H4K8la_peak_523	8.31696	
1	2E+08	2E+08	KD2-H4K8la_peak_524	8.76116	
1	2E+08	2E+08	KD2-H4K8la_peak_525	6.20477	ETNK2_ENSG00000143845
1	2E+08	2E+08	KD2-H4K8la_peak_526	7.3993	
1	2E+08	2E+08	KD2-H4K8la_peak_527	6.3399	
1	2E+08	2E+08	KD2-H4K8la_peak_528	8.50453	
1	2E+08	2E+08	KD2-H4K8la_peak_529	7.49458	PIK3C2B_ENSG00000133056
1	2E+08	2E+08	KD2-H4K8la_peak_530	11.7362	MDM4_ENSG00000198625
1	2.1E+08	2.1E+08	KD2-H4K8la_peak_531	6.56022	
1	2.1E+08	2.1E+08	KD2-H4K8la_peak_532	14.96976	AL583832.1_ENSG00000251696
1	2.1E+08	2.1E+08	KD2-H4K8la_peak_533	13.18253	
1	2.1E+08	2.1E+08	KD2-H4K8la_peak_534	5.43354	
1	2.1E+08	2.1E+08	KD2-H4K8la_peak_535	7.65547	
1	2.1E+08	2.1E+08	KD2-H4K8la_peak_536	14.25411	ELK4_ENSG00000158711
1	2.1E+08	2.1E+08	KD2-H4K8la_peak_537	7.30722	SLC45A3_ENSG00000158715
1	2.1E+08	2.1E+08	KD2-H4K8la_peak_538	8.4237	
1	2.1E+08	2.1E+08	KD2-H4K8la_peak_539	10.31182	C1orf132_ENSG00000203709
1	2.1E+08	2.1E+08	KD2-H4K8la_peak_540	12.93869	
1	2.1E+08	2.1E+08	KD2-H4K8la_peak_541	6.35646	
1	2.1E+08	2.1E+08	KD2-H4K8la_peak_542	4.98386	
1	2.1E+08	2.1E+08	KD2-H4K8la_peak_543	5.09794	
1	2.1E+08	2.1E+08	KD2-H4K8la_peak_544	7.12014	
1	2.1E+08	2.1E+08	KD2-H4K8la_peak_545	8.03267	C1orf74_ENSG00000162757
1	2.1E+08	2.1E+08	KD2-H4K8la_peak_546	7.12014	SYT14_ENSG00000143469
1	2.1E+08	2.1E+08	KD2-H4K8la_peak_547	12.97817	SERTAD4-AS1_ENSG00000203706;SERTAD4_ENSG00000082497
1	2.1E+08	2.1E+08	KD2-H4K8la_peak_548	13.05299	
1	2.1E+08	2.1E+08	KD2-H4K8la_peak_549	4.75945	
1	2.1E+08	2.1E+08	KD2-H4K8la_peak_550	8.84265	RP11-359E8.3_ENSG00000223649

1	2.1E+08	2.1E+08	KD2-H4K8la_peak_551	6.68593	
1	2.1E+08	2.1E+08	KD2-H4K8la_peak_552	14.15376	RP11-354K1.2_ENSG00000228792
1	2.1E+08	2.1E+08	KD2-H4K8la_peak_553	5.84385	LPGAT1_ENSG00000123684;RP11-552D8.1_ENSG00000229258
1	2.1E+08	2.1E+08	KD2-H4K8la_peak_554	7.79412	TMEM206_ENSG00000065600
1	2.1E+08	2.1E+08	KD2-H4K8la_peak_555	5.33301	NENF_ENSG00000117691
1	2.1E+08	2.1E+08	KD2-H4K8la_peak_556	10.66741	RP11-61J19.4_ENSG00000260805
1	2.1E+08	2.1E+08	KD2-H4K8la_peak_557	11.36807	
1	2.1E+08	2.1E+08	KD2-H4K8la_peak_558	10.27876	NSL1_ENSG00000117697;TATDN3_ENSG00000203705
1	2.1E+08	2.1E+08	KD2-H4K8la_peak_559	6.77116	FLVCR1-AS1_ENSG00000198468;FLVCR1_ENSG00000162769
1	2.1E+08	2.1E+08	KD2-H4K8la_peak_560	6.2278	
1	2.1E+08	2.1E+08	KD2-H4K8la_peak_561	4.6555	RPS6KC1_ENSG00000136643
1	2.1E+08	2.1E+08	KD2-H4K8la_peak_562	6.93796	RPS6KC1_ENSG00000136643
1	2.1E+08	2.1E+08	KD2-H4K8la_peak_563	11.02635	
1	2.1E+08	2.1E+08	KD2-H4K8la_peak_564	13.04837	PROX1_ENSG00000117707
1	2.1E+08	2.1E+08	KD2-H4K8la_peak_565	6.64804	
1	2.1E+08	2.1E+08	KD2-H4K8la_peak_566	13.04837	SMYD2_ENSG00000143499
1	2.1E+08	2.1E+08	KD2-H4K8la_peak_567	8.7731	PTPN14_ENSG00000152104
1	2.2E+08	2.2E+08	KD2-H4K8la_peak_568	8.07102	
1	2.2E+08	2.2E+08	KD2-H4K8la_peak_569	9.92519	GPATCH2_ENSG00000092978;SPATA17_ENSG00000162814
1	2.2E+08	2.2E+08	KD2-H4K8la_peak_570	13.38201	RRP15_ENSG00000067533
1	2.2E+08	2.2E+08	KD2-H4K8la_peak_571	9.72904	
1	2.2E+08	2.2E+08	KD2-H4K8la_peak_572	6.64804	RP11-135J2.4_ENSG00000228063;LYPLAL1_ENSG00000143353
1	2.2E+08	2.2E+08	KD2-H4K8la_peak_573	5.22418	EPRS_ENSG00000136628
1	2.2E+08	2.2E+08	KD2-H4K8la_peak_574	6.20793	RAB3GAP2_ENSG00000118873
1	2.2E+08	2.2E+08	KD2-H4K8la_peak_575	9.99681	MARK1_ENSG00000116141
1	2.2E+08	2.2E+08	KD2-H4K8la_peak_576	7.84781	MARK1_ENSG00000116141
1	2.2E+08	2.2E+08	KD2-H4K8la_peak_577	7.34033	
1	2.2E+08	2.2E+08	KD2-H4K8la_peak_578	8.77936	
1	2.2E+08	2.2E+08	KD2-H4K8la_peak_579	7.12014	TAF1A_ENSG00000143498;RP11-378J18.3_ENSG00000225265
1	2.2E+08	2.2E+08	KD2-H4K8la_peak_580	11.81269	RP11-452F19.3_ENSG00000228106;DISP1_ENSG00000154309
1	2.2E+08	2.2E+08	KD2-H4K8la_peak_581	5.47284	
1	2.2E+08	2.2E+08	KD2-H4K8la_peak_582	6.27787	TLR5_ENSG00000187554
1	2.2E+08	2.2E+08	KD2-H4K8la_peak_583	4.94771	
1	2.2E+08	2.2E+08	KD2-H4K8la_peak_584	10.92814	FBXO28_ENSG00000143756
1	2.2E+08	2.2E+08	KD2-H4K8la_peak_585	4.68264	
1	2.3E+08	2.3E+08	KD2-H4K8la_peak_586	9.61894	ENAH_ENSG00000154380
1	2.3E+08	2.3E+08	KD2-H4K8la_peak_587	12.14568	ENAH_ENSG00000154380
1	2.3E+08	2.3E+08	KD2-H4K8la_peak_588	4.68264	TMEM63A_ENSG00000196187
1	2.3E+08	2.3E+08	KD2-H4K8la_peak_589	4.7932	RP4-559A3.7_ENSG00000255835;PYCR2_ENSG00000143811
1	2.3E+08	2.3E+08	KD2-H4K8la_peak_590	5.99671	RP4-559A3.7_ENSG00000255835;PYCR2_ENSG00000143811
1	2.3E+08	2.3E+08	KD2-H4K8la_peak_591	10.66741	SDE2_ENSG00000143751
1	2.3E+08	2.3E+08	KD2-H4K8la_peak_592	7.3993	RP11-396C23.4_ENSG00000272562;H3F3A_ENSG00000163041
1	2.3E+08	2.3E+08	KD2-H4K8la_peak_593	9.41838	
1	2.3E+08	2.3E+08	KD2-H4K8la_peak_594	17.08706	
1	2.3E+08	2.3E+08	KD2-H4K8la_peak_595	11.84538	ACBD3_ENSG00000182827
1	2.3E+08	2.3E+08	KD2-H4K8la_peak_596	7.0396	ACBD3_ENSG00000182827

1	2.3E+08	2.3E+08	KD2-H4K8la_peak_597	10.66741	LIN9_ENSG00000183814
1	2.3E+08	2.3E+08	KD2-H4K8la_peak_598	9.20163	LIN9_ENSG00000183814
1	2.3E+08	2.3E+08	KD2-H4K8la_peak_599	6.53067	
1	2.3E+08	2.3E+08	KD2-H4K8la_peak_600	5.43278	
1	2.3E+08	2.3E+08	KD2-H4K8la_peak_601	13.34201	
1	2.3E+08	2.3E+08	KD2-H4K8la_peak_602	12.12151	ZNF678_ENSG00000181450
1	2.3E+08	2.3E+08	KD2-H4K8la_peak_603	7.12014	SNAP47_ENSG00000143740
1	2.3E+08	2.3E+08	KD2-H4K8la_peak_604	5.23773	
1	2.3E+08	2.3E+08	KD2-H4K8la_peak_605	12.97817	
1	2.3E+08	2.3E+08	KD2-H4K8la_peak_606	7.68525	ARF1_ENSG00000143761
1	2.3E+08	2.3E+08	KD2-H4K8la_peak_607	9.58134	MRPL55_ENSG00000162910
1	2.3E+08	2.3E+08	KD2-H4K8la_peak_608	7.85888	GUK1_ENSG00000143774
1	2.3E+08	2.3E+08	KD2-H4K8la_peak_609	4.32503	GUK1_ENSG00000143774
1	2.3E+08	2.3E+08	KD2-H4K8la_peak_610	5.67596	
1	2.3E+08	2.3E+08	KD2-H4K8la_peak_611	8.1293	
1	2.3E+08	2.3E+08	KD2-H4K8la_peak_612	4.6555	
1	2.3E+08	2.3E+08	KD2-H4K8la_peak_613	8.89364	
1	2.3E+08	2.3E+08	KD2-H4K8la_peak_614	5.22418	RP5-956O18.2_ENSG00000227006
1	2.3E+08	2.3E+08	KD2-H4K8la_peak_615	8.00843	RP5-956O18.3_ENSG00000224407
1	2.3E+08	2.3E+08	KD2-H4K8la_peak_616	7.3993	COG2_ENSG00000135775
1	2.3E+08	2.3E+08	KD2-H4K8la_peak_617	7.49467	RP11-295G20.2_ENSG00000233461;TSNAX-DISC1_ENSG00000270106;TSNAX_ENSG00000116918
1	2.3E+08	2.3E+08	KD2-H4K8la_peak_618	6.35646	MAP10_ENSG00000212916
1	2.3E+08	2.3E+08	KD2-H4K8la_peak_619	9.67249	
1	2.3E+08	2.3E+08	KD2-H4K8la_peak_620	8.24908	SLC35F3_ENSG00000183780
1	2.3E+08	2.3E+08	KD2-H4K8la_peak_621	7.67081	
1	2.3E+08	2.3E+08	KD2-H4K8la_peak_622	5.33301	RP5-827C21.4_ENSG00000231663;COA6_ENSG00000168275
1	2.3E+08	2.3E+08	KD2-H4K8la_peak_623	4.6555	TARBP1_ENSG00000059588
1	2.3E+08	2.3E+08	KD2-H4K8la_peak_624	5.43278	
1	2.3E+08	2.3E+08	KD2-H4K8la_peak_625	7.30722	
1	2.3E+08	2.3E+08	KD2-H4K8la_peak_626	9.52146	
1	2.3E+08	2.3E+08	KD2-H4K8la_peak_627	10.20357	
1	2.3E+08	2.3E+08	KD2-H4K8la_peak_628	10.20357	
1	2.3E+08	2.3E+08	KD2-H4K8la_peak_629	10.50811	
1	2.3E+08	2.3E+08	KD2-H4K8la_peak_630	5.27164	
1	2.4E+08	2.4E+08	KD2-H4K8la_peak_631	8.13557	TOMM20_ENSG00000173726
1	2.4E+08	2.4E+08	KD2-H4K8la_peak_632	8.89364	
1	2.4E+08	2.4E+08	KD2-H4K8la_peak_633	6.13507	
1	2.4E+08	2.4E+08	KD2-H4K8la_peak_634	5.99671	
1	2.4E+08	2.4E+08	KD2-H4K8la_peak_635	9.37943	
1	2.4E+08	2.4E+08	KD2-H4K8la_peak_636	9.61894	LGALS8-AS1_ENSG00000223776
1	2.4E+08	2.4E+08	KD2-H4K8la_peak_637	12.77989	
1	2.4E+08	2.4E+08	KD2-H4K8la_peak_638	7.51014	
1	2.4E+08	2.4E+08	KD2-H4K8la_peak_639	7.41438	
1	2.4E+08	2.4E+08	KD2-H4K8la_peak_640	7.13528	
1	2.4E+08	2.4E+08	KD2-H4K8la_peak_641	8.37064	
1	2.4E+08	2.4E+08	KD2-H4K8la_peak_642	7.11805	

1	2.4E+08	2.4E+08	KD2-H4K8la_peak_643	7.61975	
1	2.4E+08	2.4E+08	KD2-H4K8la_peak_644	7.61975	
1	2.4E+08	2.4E+08	KD2-H4K8la_peak_645	7.06586	
1	2.4E+08	2.4E+08	KD2-H4K8la_peak_646	9.40293	RP11-527D7.1_ENSG00000225554
1	2.4E+08	2.4E+08	KD2-H4K8la_peak_647	7.12014	
1	2.4E+08	2.4E+08	KD2-H4K8la_peak_648	6.13507	
1	2.4E+08	2.4E+08	KD2-H4K8la_peak_649	5.33301	
1	2.4E+08	2.4E+08	KD2-H4K8la_peak_650	11.44218	
1	2.4E+08	2.4E+08	KD2-H4K8la_peak_651	14.07018	RP11-278H7.1_ENSG00000226828
1	2.4E+08	2.4E+08	KD2-H4K8la_peak_652	9.19451	
1	2.4E+08	2.4E+08	KD2-H4K8la_peak_653	5.83285	
1	2.5E+08	2.5E+08	KD2-H4K8la_peak_654	10.16316	HNRNPU_ENSG00000153187;RP11-11N7.4_ENSG00000273175
1	2.5E+08	2.5E+08	KD2-H4K8la_peak_655	6.82292	
1	2.5E+08	2.5E+08	KD2-H4K8la_peak_656	10.02595	RP11-156E8.1_ENSG00000272195;EFCAB2_ENSG00000203666
1	2.5E+08	2.5E+08	KD2-H4K8la_peak_657	5.24027	KIF26B_ENSG00000162849
1	2.5E+08	2.5E+08	KD2-H4K8la_peak_658	8.66164	
1	2.5E+08	2.5E+08	KD2-H4K8la_peak_659	13.03265	
1	2.5E+08	2.5E+08	KD2-H4K8la_peak_660	5.27164	RP11-522M21.2_ENSG00000238224
1	2.5E+08	2.5E+08	KD2-H4K8la_peak_661	4.86896	
1	2.5E+08	2.5E+08	KD2-H4K8la_peak_662	7.49467	
1	2.5E+08	2.5E+08	KD2-H4K8la_peak_663	10.27876	SCCPDH_ENSG00000143653
1	2.5E+08	2.5E+08	KD2-H4K8la_peak_664	10.64143	ZNF669_ENSG00000188295
1	2.5E+08	2.5E+08	KD2-H4K8la_peak_665	5.79108	
1	2.5E+08	2.5E+08	KD2-H4K8la_peak_666	8.75982	C1orf229_ENSG00000221953
1	2.5E+08	2.5E+08	KD2-H4K8la_peak_667	9.9149	ZNF496_ENSG00000162714
1	2.5E+08	2.5E+08	KD2-H4K8la_peak_668	7.16303	ZNF496_ENSG00000162714
1	2.5E+08	2.5E+08	KD2-H4K8la_peak_669	12.85404	
10	180670	181194	KD2-H4K8la_peak_670	5.7631	ZMYND11_ENSG00000015171
10	1033896	1034183	KD2-H4K8la_peak_671	5.58883	AL359878.1_ENSG00000205740;GTPBP4_ENSG00000107937
10	1102266	1102496	KD2-H4K8la_peak_672	6.36118	
10	1314839	1315445	KD2-H4K8la_peak_673	7.49467	
10	1315658	1316063	KD2-H4K8la_peak_674	14.40178	
10	1316953	1317311	KD2-H4K8la_peak_675	12.21541	
10	1332412	1332771	KD2-H4K8la_peak_676	5.52913	
10	1334694	1335064	KD2-H4K8la_peak_677	13.95047	
10	2267472	2267847	KD2-H4K8la_peak_678	13.3197	
10	4868505	4868774	KD2-H4K8la_peak_679	9.11321	
10	5932364	5932619	KD2-H4K8la_peak_680	7.56395	ANKRD16_ENSG00000134461;FBXO18_ENSG00000134452
10	6622534	6622738	KD2-H4K8la_peak_681	5.05146	PRKCQ_ENSG00000065675;PRKCQ-AS1_ENSG00000237943
10	7310506	7310729	KD2-H4K8la_peak_682	8.60796	
10	7860551	7860750	KD2-H4K8la_peak_683	5.58883	TAF3_ENSG00000165632
10	8091800	8092061	KD2-H4K8la_peak_684	6.04152	
10	8092783	8093014	KD2-H4K8la_peak_685	11.15747	RP11-379F12.4_ENSG00000232638
10	8093225	8093887	KD2-H4K8la_peak_686	5.58883	RP11-379F12.4_ENSG00000232638
10	8100497	8100957	KD2-H4K8la_peak_687	7.89224	
10	8138473	8138701	KD2-H4K8la_peak_688	5.52913	

10	8286954	8287160	KD2-H4K8la_peak_689	7.23116	
10	8683484	8683835	KD2-H4K8la_peak_690	5.51108	
10	9280179	9280457	KD2-H4K8la_peak_691	10.99007	
10	1.2E+07	1.2E+07	KD2-H4K8la_peak_692	10.04111	
10	1.3E+07	1.3E+07	KD2-H4K8la_peak_693	8.04432	RP11-730A19.7_ENSG00000237540
10	1.3E+07	1.3E+07	KD2-H4K8la_peak_694	13.91009	
10	1.3E+07	1.3E+07	KD2-H4K8la_peak_695	5.24865	
10	1.4E+07	1.4E+07	KD2-H4K8la_peak_696	8.43222	BEND7_ENSG00000165626;RP11-214D15.2_ENSG00000227175
10	1.5E+07	1.5E+07	KD2-H4K8la_peak_697	9.49266	RP11-398C13.6_ENSG00000272853;SUV39H2_ENSG00000152455
10	1.8E+07	1.8E+07	KD2-H4K8la_peak_698	6.2278	
10	1.9E+07	1.9E+07	KD2-H4K8la_peak_699	6.26214	
10	2E+07	2E+07	KD2-H4K8la_peak_700	9.86881	
10	2.2E+07	2.2E+07	KD2-H4K8la_peak_701	9.9701	SKIDA1_ENSG00000180592
10	2.2E+07	2.2E+07	KD2-H4K8la_peak_702	9.05604	
10	2.3E+07	2.3E+07	KD2-H4K8la_peak_703	13.91009	BMI1_ENSG00000168283
10	2.3E+07	2.3E+07	KD2-H4K8la_peak_704	4.69635	
10	2.3E+07	2.3E+07	KD2-H4K8la_peak_705	7.68279	RP11-573G6.10_ENSG00000272366
10	2.3E+07	2.3E+07	KD2-H4K8la_peak_706	12.92798	PIP4K2A_ENSG00000150867
10	2.3E+07	2.3E+07	KD2-H4K8la_peak_707	11.06859	MSRB2_ENSG00000148450
10	2.4E+07	2.4E+07	KD2-H4K8la_peak_708	8.31696	OTUD1_ENSG00000165312
10	2.5E+07	2.5E+07	KD2-H4K8la_peak_709	6.13507	PRTFDC1_ENSG00000099256;RP11-165A20.3_ENSG00000273107
10	2.8E+07	2.8E+07	KD2-H4K8la_peak_710	4.53273	
10	2.9E+07	2.9E+07	KD2-H4K8la_peak_711	6.26214	
10	2.9E+07	2.9E+07	KD2-H4K8la_peak_712	5.79108	WAC-AS1_ENSG00000254635;WAC_ENSG00000095787
10	3.1E+07	3.1E+07	KD2-H4K8la_peak_713	12.1221	
10	3.1E+07	3.1E+07	KD2-H4K8la_peak_714	9.86881	ZNF438_ENSG00000183621
10	3.2E+07	3.2E+07	KD2-H4K8la_peak_715	9.08387	
10	3.3E+07	3.3E+07	KD2-H4K8la_peak_716	15.62171	RP11-135A24.4_ENSG00000233825;AL391839.1_ENSG00000222309;RP11-135A24.2_ENSG00000229327
10	3.3E+07	3.3E+07	KD2-H4K8la_peak_717	9.86881	CCDC7_ENSG00000216937
10	3.3E+07	3.3E+07	KD2-H4K8la_peak_718	8.43222	CCDC7_ENSG00000216937
10	3.3E+07	3.3E+07	KD2-H4K8la_peak_719	11.40266	RP11-462L8.1_ENSG00000229656
10	3.5E+07	3.5E+07	KD2-H4K8la_peak_720	6.70626	
10	3.5E+07	3.5E+07	KD2-H4K8la_peak_721	11.36807	PARD3_ENSG00000148498;PARD3-AS1_ENSG00000226386
10	3.9E+07	3.9E+07	KD2-H4K8la_peak_722	10.04111	SEPT7P9_ENSG00000120555
10	3.9E+07	3.9E+07	KD2-H4K8la_peak_723	11.97807	SEPT7P9_ENSG00000120555
10	4.3E+07	4.3E+07	KD2-H4K8la_peak_724	7.79412	RP11-124O11.1_ENSG00000234944
10	4.3E+07	4.3E+07	KD2-H4K8la_peak_725	7.49467	
10	4.3E+07	4.3E+07	KD2-H4K8la_peak_726	5.05146	
10	4.3E+07	4.3E+07	KD2-H4K8la_peak_727	5.6499	
10	4.3E+07	4.3E+07	KD2-H4K8la_peak_728	9.0413	
10	4.3E+07	4.3E+07	KD2-H4K8la_peak_729	10.20357	
10	4.3E+07	4.3E+07	KD2-H4K8la_peak_730	5.22418	
10	4.3E+07	4.3E+07	KD2-H4K8la_peak_731	5.33301	
10	4.3E+07	4.3E+07	KD2-H4K8la_peak_732	9.35473	RP11-124O11.2_ENSG00000229630
10	4.3E+07	4.3E+07	KD2-H4K8la_peak_733	9.40293	
10	4.4E+07	4.4E+07	KD2-H4K8la_peak_734	7.22716	

10	4.4E+07	4.4E+07	KD2-H4K8la_peak_735	6.8348	
10	4.4E+07	4.4E+07	KD2-H4K8la_peak_736	7.64387	
10	4.4E+07	4.4E+07	KD2-H4K8la_peak_737	5.35972	
10	4.4E+07	4.4E+07	KD2-H4K8la_peak_738	6.92459	RET_ENSG00000165731
10	4.4E+07	4.4E+07	KD2-H4K8la_peak_739	8.944	RP11-351D16.3_ENSG00000273008;CSGALNACT2_ENSG00000169826
10	4.4E+07	4.4E+07	KD2-H4K8la_peak_740	19.77487	
10	4.4E+07	4.4E+07	KD2-H4K8la_peak_741	8.50453	HNRNPF_ENSG00000169813
10	4.4E+07	4.4E+07	KD2-H4K8la_peak_742	7.13237	RP11-517P14.2_ENSG00000230555
10	4.4E+07	4.4E+07	KD2-H4K8la_peak_743	5.90392	RP11-517P14.2_ENSG00000230555
10	4.4E+07	4.4E+07	KD2-H4K8la_peak_744	6.13507	ZNF239_ENSG00000196793
10	4.4E+07	4.4E+07	KD2-H4K8la_peak_745	4.86896	CAP1P2_ENSG00000232004;ZNF485_ENSG00000198298
10	4.4E+07	4.4E+07	KD2-H4K8la_peak_746	6.76424	ZNF32_ENSG00000169740
10	4.4E+07	4.4E+07	KD2-H4K8la_peak_747	6.0136	ZNF32_ENSG00000169740
10	4.6E+07	4.6E+07	KD2-H4K8la_peak_748	7.13237	
10	4.6E+07	4.6E+07	KD2-H4K8la_peak_749	6.28277	ZFAND4_ENSG00000172671
10	4.6E+07	4.6E+07	KD2-H4K8la_peak_750	19.28322	FAM21FP_ENSG00000237840;RP11-671E7.1_ENSG00000228702;FAM21C_ENSG00000172661
10	4.7E+07	4.7E+07	KD2-H4K8la_peak_751	7.61975	
10	4.7E+07	4.7E+07	KD2-H4K8la_peak_752	8.11213	
10	4.7E+07	4.7E+07	KD2-H4K8la_peak_753	8.81931	GPRIN2_ENSG00000204175
10	4.7E+07	4.7E+07	KD2-H4K8la_peak_754	10.91646	
10	4.7E+07	4.7E+07	KD2-H4K8la_peak_755	11.04415	
10	4.7E+07	4.7E+07	KD2-H4K8la_peak_756	7.58308	
10	5E+07	5E+07	KD2-H4K8la_peak_757	4.36246	
10	5E+07	5E+07	KD2-H4K8la_peak_758	6.2278	
10	5.1E+07	5.1E+07	KD2-H4K8la_peak_759	6.13507	C10orf71-AS1_ENSG00000236208;C10orf71_ENSG00000177354
10	5.1E+07	5.1E+07	KD2-H4K8la_peak_760	5.58883	
10	5.1E+07	5.1E+07	KD2-H4K8la_peak_761	5.33301	
10	5.1E+07	5.1E+07	KD2-H4K8la_peak_762	5.84082	OGDHL_ENSG00000197444
10	5.1E+07	5.2E+07	KD2-H4K8la_peak_763	6.3399	
10	5.2E+07	5.2E+07	KD2-H4K8la_peak_764	8.77936	RP11-324H6.5_ENSG00000235618;FAM21A_ENSG00000099290
10	5.2E+07	5.2E+07	KD2-H4K8la_peak_765	9.19986	ASAH2B_ENSG00000204147
10	6E+07	6E+07	KD2-H4K8la_peak_766	10.04111	UBE2D1_ENSG00000072401
10	6.2E+07	6.2E+07	KD2-H4K8la_peak_767	10.98662	ANK3_ENSG00000151150;RP11-131N11.4_ENSG00000254271
10	6.6E+07	6.6E+07	KD2-H4K8la_peak_768	8.35736	
10	7E+07	7E+07	KD2-H4K8la_peak_769	18.59289	SLC25A16_ENSG00000122912
10	7E+07	7E+07	KD2-H4K8la_peak_770	7.91061	CCAR1_ENSG00000060339
10	7.1E+07	7.1E+07	KD2-H4K8la_peak_771	8.63473	DDX50_ENSG00000107625
10	7.1E+07	7.1E+07	KD2-H4K8la_peak_772	9.49266	DDX21_ENSG00000165732
10	7.1E+07	7.1E+07	KD2-H4K8la_peak_773	8.37064	KIAA1279_ENSG00000198954
10	7.1E+07	7.1E+07	KD2-H4K8la_peak_774	8.26997	
10	7.1E+07	7.1E+07	KD2-H4K8la_peak_775	13.20018	
10	7.1E+07	7.1E+07	KD2-H4K8la_peak_776	6.75892	
10	7.1E+07	7.1E+07	KD2-H4K8la_peak_777	8.07102	
10	7.2E+07	7.2E+07	KD2-H4K8la_peak_778	8.76116	
10	7.2E+07	7.2E+07	KD2-H4K8la_peak_779	7.90495	H2AFY2_ENSG00000099284
10	7.2E+07	7.2E+07	KD2-H4K8la_peak_780	12.36938	AIFM2_ENSG00000042286

10	7.2E+07	7.2E+07	KD2-H4K8la_peak_781	8.6948	SAR1A_ENSG00000079332
10	7.2E+07	7.2E+07	KD2-H4K8la_peak_782	7.56826	
10	7.2E+07	7.2E+07	KD2-H4K8la_peak_783	8.76116	
10	7.2E+07	7.2E+07	KD2-H4K8la_peak_784	6.0136	PALD1_ENSG00000107719
10	7.2E+07	7.2E+07	KD2-H4K8la_peak_785	6.36118	
10	7.3E+07	7.3E+07	KD2-H4K8la_peak_786	11.60966	
10	7.3E+07	7.3E+07	KD2-H4K8la_peak_787	22.21576	
10	7.3E+07	7.3E+07	KD2-H4K8la_peak_788	13.27013	
10	7.3E+07	7.3E+07	KD2-H4K8la_peak_789	6.3399	
10	7.3E+07	7.3E+07	KD2-H4K8la_peak_790	5.27164	
10	7.3E+07	7.3E+07	KD2-H4K8la_peak_791	4.66473	
10	7.3E+07	7.3E+07	KD2-H4K8la_peak_792	6.01825	
10	7.3E+07	7.3E+07	KD2-H4K8la_peak_793	7.61975	
10	7.3E+07	7.3E+07	KD2-H4K8la_peak_794	8.06101	
10	7.3E+07	7.3E+07	KD2-H4K8la_peak_795	6.40658	
10	7.3E+07	7.3E+07	KD2-H4K8la_peak_796	10.39965	
10	7.3E+07	7.3E+07	KD2-H4K8la_peak_797	6.40658	
10	7.3E+07	7.3E+07	KD2-H4K8la_peak_798	6.68593	
10	7.3E+07	7.3E+07	KD2-H4K8la_peak_799	6.66345	
10	7.3E+07	7.3E+07	KD2-H4K8la_peak_800	5.80342	
10	7.3E+07	7.3E+07	KD2-H4K8la_peak_801	8.50453	
10	7.3E+07	7.3E+07	KD2-H4K8la_peak_802	6.52199	
10	7.3E+07	7.3E+07	KD2-H4K8la_peak_803	11.15747	
10	7.3E+07	7.3E+07	KD2-H4K8la_peak_804	9.61894	
10	7.3E+07	7.3E+07	KD2-H4K8la_peak_805	6.56022	SLC29A3_ENSG00000198246
10	7.3E+07	7.3E+07	KD2-H4K8la_peak_806	9.37943	
10	7.3E+07	7.3E+07	KD2-H4K8la_peak_807	4.66473	
10	7.3E+07	7.3E+07	KD2-H4K8la_peak_808	8.38878	
10	7.3E+07	7.3E+07	KD2-H4K8la_peak_809	6.19992	C10orf105_ENSG00000214688
10	7.4E+07	7.4E+07	KD2-H4K8la_peak_810	4.86896	PSAP_ENSG00000197746
10	7.4E+07	7.4E+07	KD2-H4K8la_peak_811	5.88616	
10	7.4E+07	7.4E+07	KD2-H4K8la_peak_812	7.49467	
10	7.4E+07	7.4E+07	KD2-H4K8la_peak_813	5.67596	
10	7.4E+07	7.4E+07	KD2-H4K8la_peak_814	5.30989	
10	7.4E+07	7.4E+07	KD2-H4K8la_peak_815	7.56395	
10	7.4E+07	7.4E+07	KD2-H4K8la_peak_816	8.16787	
10	7.4E+07	7.4E+07	KD2-H4K8la_peak_817	6.76703	
10	7.4E+07	7.4E+07	KD2-H4K8la_peak_818	12.30425	
10	7.4E+07	7.4E+07	KD2-H4K8la_peak_819	8.1293	
10	7.4E+07	7.4E+07	KD2-H4K8la_peak_820	6.68593	
10	7.4E+07	7.4E+07	KD2-H4K8la_peak_821	14.42249	
10	7.4E+07	7.4E+07	KD2-H4K8la_peak_822	6.2278	DNAJB12_ENSG00000148719
10	7.5E+07	7.5E+07	KD2-H4K8la_peak_823	5.468	
10	7.6E+07	7.6E+07	KD2-H4K8la_peak_824	5.7631	ZSWIM8_ENSG00000214655
10	7.7E+07	7.7E+07	KD2-H4K8la_peak_825	10.49982	KAT6B_ENSG00000156650
10	7.7E+07	7.7E+07	KD2-H4K8la_peak_826	10.74378	KAT6B_ENSG00000156650

10	7.7E+07	7.7E+07	KD2-H4K8la_peak_827	6.03964	
10	7.7E+07	7.7E+07	KD2-H4K8la_peak_828	8.76116	
10	7.7E+07	7.7E+07	KD2-H4K8la_peak_829	6.70626	RP11-399K21.10_ENSG00000236842
10	8E+07	8E+07	KD2-H4K8la_peak_830	7.12014	
10	8E+07	8E+07	KD2-H4K8la_peak_831	6.13507	
10	8E+07	8E+07	KD2-H4K8la_peak_832	6.68593	RP11-90J7.2_ENSG00000229543
10	8E+07	8E+07	KD2-H4K8la_peak_833	4.90979	
10	8E+07	8E+07	KD2-H4K8la_peak_834	4.75945	
10	8E+07	8E+07	KD2-H4K8la_peak_835	8.07102	
10	8E+07	8E+07	KD2-H4K8la_peak_836	6.64804	
10	8E+07	8E+07	KD2-H4K8la_peak_837	11.90654	
10	8E+07	8E+07	KD2-H4K8la_peak_838	9.49266	
10	8E+07	8E+07	KD2-H4K8la_peak_839	6.68593	
10	8.1E+07	8.1E+07	KD2-H4K8la_peak_840	10.47452	
10	8.1E+07	8.1E+07	KD2-H4K8la_peak_841	5.05146	
10	8.1E+07	8.1E+07	KD2-H4K8la_peak_842	4.66473	
10	8.1E+07	8.1E+07	KD2-H4K8la_peak_843	4.30663	
10	8.1E+07	8.1E+07	KD2-H4K8la_peak_844	7.49467	
10	8.1E+07	8.1E+07	KD2-H4K8la_peak_845	11.72187	
10	8.1E+07	8.1E+07	KD2-H4K8la_peak_846	7.22716	
10	8.1E+07	8.1E+07	KD2-H4K8la_peak_847	5.6499	
10	8.1E+07	8.1E+07	KD2-H4K8la_peak_848	10.84057	
10	8.1E+07	8.1E+07	KD2-H4K8la_peak_849	5.83285	
10	8.1E+07	8.1E+07	KD2-H4K8la_peak_850	6.3399	
10	8.1E+07	8.1E+07	KD2-H4K8la_peak_851	5.24027	
10	8.1E+07	8.1E+07	KD2-H4K8la_peak_852	6.9399	
10	8.1E+07	8.1E+07	KD2-H4K8la_peak_853	8.52304	
10	8.1E+07	8.1E+07	KD2-H4K8la_peak_854	8.48488	
10	8.1E+07	8.1E+07	KD2-H4K8la_peak_855	8.76116	
10	8.1E+07	8.1E+07	KD2-H4K8la_peak_856	6.45303	
10	8.1E+07	8.1E+07	KD2-H4K8la_peak_857	7.06427	
10	8.1E+07	8.1E+07	KD2-H4K8la_peak_858	7.06427	
10	8.1E+07	8.1E+07	KD2-H4K8la_peak_859	12.62032	
10	8.1E+07	8.1E+07	KD2-H4K8la_peak_860	7.12014	
10	8.1E+07	8.1E+07	KD2-H4K8la_peak_861	6.13507	PPIF_ENSG00000108179
10	8.1E+07	8.1E+07	KD2-H4K8la_peak_862	5.05146	
10	8.2E+07	8.2E+07	KD2-H4K8la_peak_863	11.40266	MBL1P_ENSG00000242600
10	8.2E+07	8.2E+07	KD2-H4K8la_peak_864	6.3399	FAM213A_ENSG00000122378
10	8.8E+07	8.8E+07	KD2-H4K8la_peak_865	6.54311	
10	8.8E+07	8.8E+07	KD2-H4K8la_peak_866	5.7631	
10	8.9E+07	8.9E+07	KD2-H4K8la_peak_867	5.58883	
10	9.3E+07	9.3E+07	KD2-H4K8la_peak_868	16.25311	HECTD2_ENSG00000165338
10	9.4E+07	9.4E+07	KD2-H4K8la_peak_869	5.7631	
10	9.4E+07	9.4E+07	KD2-H4K8la_peak_870	13.20018	CPEB3_ENSG00000107864;MARCH5_ENSG00000198060
10	9.5E+07	9.5E+07	KD2-H4K8la_peak_871	9.11321	
10	9.6E+07	9.6E+07	KD2-H4K8la_peak_872	7.12014	SLC35G1_ENSG00000176273

10	9.6E+07	9.6E+07	KD2-H4K8la_peak_873	5.67034	TBC1D12_ENSG00000108239
10	9.7E+07	9.7E+07	KD2-H4K8la_peak_874	11.70864	PAWRP1_ENSG00000225533
10	9.8E+07	9.8E+07	KD2-H4K8la_peak_875	12.14099	CCNJ_ENSG00000107443
10	9.8E+07	9.8E+07	KD2-H4K8la_peak_876	4.75945	TM9SF3_ENSG00000077147
10	9.8E+07	9.8E+07	KD2-H4K8la_peak_877	7.61975	PIK3AP1_ENSG00000155629
10	9.9E+07	9.9E+07	KD2-H4K8la_peak_878	6.69599	
10	9.9E+07	9.9E+07	KD2-H4K8la_peak_879	7.12014	AL355490.1_ENSG00000224474;PGAM1_ENSG00000171314
10	9.9E+07	9.9E+07	KD2-H4K8la_peak_880	6.85533	MORN4_ENSG00000171160
10	9.9E+07	9.9E+07	KD2-H4K8la_peak_881	13.79336	
10	9.9E+07	9.9E+07	KD2-H4K8la_peak_882	11.45561	MARVELD1_ENSG00000155254
10	9.9E+07	9.9E+07	KD2-H4K8la_peak_883	8.76082	ZFYVE27_ENSG00000155256
10	1E+08	1E+08	KD2-H4K8la_peak_884	7.4233	
10	1E+08	1E+08	KD2-H4K8la_peak_885	10.31941	
10	1E+08	1E+08	KD2-H4K8la_peak_886	9.27415	R3HCC1L_ENSG00000166024
10	1E+08	1E+08	KD2-H4K8la_peak_887	5.58883	LOXL4_ENSG00000138131
10	1E+08	1E+08	KD2-H4K8la_peak_888	5.60379	
10	1E+08	1E+08	KD2-H4K8la_peak_889	6.63409	CWF19L1_ENSG00000095485
10	1E+08	1E+08	KD2-H4K8la_peak_890	6.3399	SEC31B_ENSG00000075826;NDUFB8_ENSG00000255339;NDUFB8_ENSG00000166136;HIF1AN_ENSG00000166135
10	1E+08	1E+08	KD2-H4K8la_peak_891	6.64804	
10	1E+08	1E+08	KD2-H4K8la_peak_892	8.07044	
10	1E+08	1E+08	KD2-H4K8la_peak_893	6.26214	
10	1E+08	1E+08	KD2-H4K8la_peak_894	13.84838	
10	1E+08	1E+08	KD2-H4K8la_peak_895	6.71169	
10	1E+08	1E+08	KD2-H4K8la_peak_896	9.03222	
10	1E+08	1E+08	KD2-H4K8la_peak_897	6.47589	BTRC_ENSG00000166167
10	1E+08	1E+08	KD2-H4K8la_peak_898	8.37064	DPCD_ENSG00000166171
10	1E+08	1E+08	KD2-H4K8la_peak_899	10.92814	FBXW4_ENSG00000107829
10	1E+08	1E+08	KD2-H4K8la_peak_900	5.43278	C10orf76_ENSG00000120029
10	1E+08	1E+08	KD2-H4K8la_peak_901	6.03112	HPS6_ENSG00000166189
10	1E+08	1E+08	KD2-H4K8la_peak_902	6.5142	NOLC1_ENSG00000166197
10	1E+08	1E+08	KD2-H4K8la_peak_903	7.81267	GBF1_ENSG00000107862
10	1E+08	1E+08	KD2-H4K8la_peak_904	10.31182	CUEDC2_ENSG00000107874
10	1E+08	1E+08	KD2-H4K8la_peak_905	14.40178	TMEM180_ENSG00000138111
10	1E+08	1E+08	KD2-H4K8la_peak_906	6.39807	
10	1E+08	1E+08	KD2-H4K8la_peak_907	4.75945	
10	1E+08	1E+08	KD2-H4K8la_peak_908	9.27415	RP11-47A8.5_ENSG00000272933
10	1E+08	1E+08	KD2-H4K8la_peak_909	12.07084	RP11-47A8.5_ENSG00000272933;TRIM8_ENSG00000171206
10	1E+08	1E+08	KD2-H4K8la_peak_910	5.33301	AS3MT_ENSG00000214435
10	1.1E+08	1.1E+08	KD2-H4K8la_peak_911	6.3399	TAF5_ENSG00000148835
10	1.1E+08	1.1E+08	KD2-H4K8la_peak_912	12.23212	
10	1.1E+08	1.1E+08	KD2-H4K8la_peak_913	6.64804	
10	1.1E+08	1.1E+08	KD2-H4K8la_peak_914	8.40055	SH3PXD2A_ENSG00000107957
10	1.1E+08	1.1E+08	KD2-H4K8la_peak_915	10.98662	SORCS1_ENSG00000108018
10	1.1E+08	1.1E+08	KD2-H4K8la_peak_916	10.36292	
10	1.1E+08	1.1E+08	KD2-H4K8la_peak_917	6.57319	SMNDC1_ENSG00000119953
10	1.1E+08	1.1E+08	KD2-H4K8la_peak_918	9.96333	

10	1.1E+08	1.1E+08	KD2-H4K8la_peak_919	9.41442	GPAM_ENSG00000119927
10	1.2E+08	1.2E+08	KD2-H4K8la_peak_920	7.12014	DCLRE1A_ENSG00000198924;NHLRC2_ENSG00000196865
10	1.2E+08	1.2E+08	KD2-H4K8la_peak_921	6.70626	
10	1.2E+08	1.2E+08	KD2-H4K8la_peak_922	4.86896	HSPA12A_ENSG00000165868
10	1.2E+08	1.2E+08	KD2-H4K8la_peak_923	8.37064	
10	1.2E+08	1.2E+08	KD2-H4K8la_peak_924	7.61975	CACUL1_ENSG00000151893
10	1.2E+08	1.2E+08	KD2-H4K8la_peak_925	6.28277	
10	1.2E+08	1.2E+08	KD2-H4K8la_peak_926	5.83285	EIF3A_ENSG00000107581
10	1.2E+08	1.2E+08	KD2-H4K8la_peak_927	6.3399	SFXN4_ENSG00000183605
10	1.2E+08	1.2E+08	KD2-H4K8la_peak_928	9.0516	RGS10_ENSG00000148908
10	1.2E+08	1.2E+08	KD2-H4K8la_peak_929	6.3399	TIAL1_ENSG00000151923
10	1.2E+08	1.2E+08	KD2-H4K8la_peak_930	15.48964	
10	1.2E+08	1.2E+08	KD2-H4K8la_peak_931	6.25288	
10	1.2E+08	1.2E+08	KD2-H4K8la_peak_932	6.4077	FGFR2_ENSG00000066468
10	1.2E+08	1.2E+08	KD2-H4K8la_peak_933	8.77936	
10	1.2E+08	1.2E+08	KD2-H4K8la_peak_934	5.89088	
10	1.2E+08	1.2E+08	KD2-H4K8la_peak_935	6.2278	
10	1.2E+08	1.2E+08	KD2-H4K8la_peak_936	7.94013	PLEKHA1_ENSG00000107679
10	1.2E+08	1.2E+08	KD2-H4K8la_peak_937	10.20357	C10orf88_ENSG00000119965;PSTK_ENSG00000179988
10	1.2E+08	1.2E+08	KD2-H4K8la_peak_938	4.86896	BUB3_ENSG00000154473
10	1.3E+08	1.3E+08	KD2-H4K8la_peak_939	6.0136	
10	1.3E+08	1.3E+08	KD2-H4K8la_peak_940	8.37064	
10	1.3E+08	1.3E+08	KD2-H4K8la_peak_941	5.9366	
10	1.3E+08	1.3E+08	KD2-H4K8la_peak_942	8.8932	
10	1.3E+08	1.3E+08	KD2-H4K8la_peak_943	11.40266	FAM175B_ENSG00000165660
10	1.3E+08	1.3E+08	KD2-H4K8la_peak_944	8.7731	
10	1.3E+08	1.3E+08	KD2-H4K8la_peak_945	9.27415	
10	1.3E+08	1.3E+08	KD2-H4K8la_peak_946	4.86896	RP11-383C5.4_ENSG00000224023;RP11-383C5.5_ENSG00000234134;EDRF1_ENSG00000107938
10	1.3E+08	1.3E+08	KD2-H4K8la_peak_947	12.58104	UROS_ENSG00000188690;BCCIP_ENSG00000107949
10	1.3E+08	1.3E+08	KD2-H4K8la_peak_948	7.30722	DHX32_ENSG00000089876;FANK1_ENSG00000203780
10	1.3E+08	1.3E+08	KD2-H4K8la_peak_949	10.04111	ADAM12_ENSG00000148848
10	1.3E+08	1.3E+08	KD2-H4K8la_peak_950	5.16597	
10	1.3E+08	1.3E+08	KD2-H4K8la_peak_951	8.48085	
10	1.3E+08	1.3E+08	KD2-H4K8la_peak_952	8.39919	MGMT_ENSG00000170430
10	1.3E+08	1.3E+08	KD2-H4K8la_peak_953	5.7631	
10	1.3E+08	1.3E+08	KD2-H4K8la_peak_954	9.05604	
10	1.3E+08	1.3E+08	KD2-H4K8la_peak_955	10.98662	BNIP3_ENSG00000176171
10	1.3E+08	1.3E+08	KD2-H4K8la_peak_956	6.92098	
10	1.3E+08	1.3E+08	KD2-H4K8la_peak_957	9.53224	
10	1.3E+08	1.3E+08	KD2-H4K8la_peak_958	6.2278	
10	1.3E+08	1.3E+08	KD2-H4K8la_peak_959	8.86391	PWWP2B_ENSG00000171813
10	1.3E+08	1.3E+08	KD2-H4K8la_peak_960	7.01227	PWWP2B_ENSG00000171813
10	1.3E+08	1.3E+08	KD2-H4K8la_peak_961	7.61975	RP11-432J24.5_ENSG00000226900
10	1.3E+08	1.3E+08	KD2-H4K8la_peak_962	7.92775	
10	1.4E+08	1.4E+08	KD2-H4K8la_peak_963	10.30333	
10	1.4E+08	1.4E+08	KD2-H4K8la_peak_964	7.22716	PAOX_ENSG00000148832

10	1.4E+08	1.4E+08	KD2-H4K8la_peak_965	5.83285	MTG1_ENSG00000148824
10	1.4E+08	1.4E+08	KD2-H4K8la_peak_966	4.66473	MTG1_ENSG00000148824
11	268419	268655	KD2-H4K8la_peak_967	8.65116	
11	288903	289100	KD2-H4K8la_peak_968	4.86896	ATHL1_ENSG00000142102
11	355672	356049	KD2-H4K8la_peak_969	25.0981	
11	406567	406961	KD2-H4K8la_peak_970	7.72421	
11	450548	450751	KD2-H4K8la_peak_971	6.77116	
11	535818	536038	KD2-H4K8la_peak_972	10.52448	
11	554656	555020	KD2-H4K8la_peak_973	7.78021	
11	560249	560495	KD2-H4K8la_peak_974	5.31813	C11orf35_ENSG00000185522;RASSF7_ENSG00000099849
11	568956	569283	KD2-H4K8la_peak_975	8.89364	MIR210HG_ENSG00000247095;MIR210_ENSG00000199038
11	726305	726516	KD2-H4K8la_peak_976	8.0374	AP006621.9_ENSG00000269915
11	727803	728117	KD2-H4K8la_peak_977	10.23822	AP006621.9_ENSG00000269915
11	797651	798001	KD2-H4K8la_peak_978	7.12014	SLC25A22_ENSG00000177542
11	911061	911395	KD2-H4K8la_peak_979	8.39919	
11	1331256	1331479	KD2-H4K8la_peak_980	10.66741	TOLLIP_ENSG00000078902;TOLLIP-AS1_ENSG00000255153
11	1358214	1358450	KD2-H4K8la_peak_981	6.2278	
11	1403783	1404396	KD2-H4K8la_peak_982	10.52788	
11	1410506	1410727	KD2-H4K8la_peak_983	7.79412	BRSK2_ENSG00000174672
11	1568211	1568609	KD2-H4K8la_peak_984	9.05604	
11	1593819	1594183	KD2-H4K8la_peak_985	4.69635	DUSP8_ENSG00000184545
11	1659542	1659920	KD2-H4K8la_peak_986	10.11868	
11	1674222	1674749	KD2-H4K8la_peak_987	6.36118	
11	1714856	1715257	KD2-H4K8la_peak_988	9.19986	
11	1769990	1770206	KD2-H4K8la_peak_989	7.62867	
11	1784801	1785138	KD2-H4K8la_peak_990	8.39919	CTSD_ENSG00000117984;AC068580.5_ENSG00000229512;AC068580.1_ENSG00000265587
11	1898422	1898712	KD2-H4K8la_peak_991	10.01526	
11	1899089	1899551	KD2-H4K8la_peak_992	10.77072	
11	1968617	1969567	KD2-H4K8la_peak_993	19.53802	MRPL23_ENSG00000214026
11	1970615	1970888	KD2-H4K8la_peak_994	8.37064	
11	1977377	1977688	KD2-H4K8la_peak_995	6.75222	
11	1977958	1978162	KD2-H4K8la_peak_996	6.68593	
11	1989344	1990326	KD2-H4K8la_peak_997	8.53077	
11	1990792	1991163	KD2-H4K8la_peak_998	14.44261	
11	1991814	1992592	KD2-H4K8la_peak_999	8.15033	
11	1998999	1999385	KD2-H4K8la_peak_1000	9.95824	
11	2000771	2001134	KD2-H4K8la_peak_1001	9.77334	
11	2001339	2001915	KD2-H4K8la_peak_1002	7.7007	
11	2003075	2003559	KD2-H4K8la_peak_1003	9.52146	
11	2003915	2004135	KD2-H4K8la_peak_1004	9.20163	
11	2004477	2005612	KD2-H4K8la_peak_1005	12.21336	
11	2009378	2009767	KD2-H4K8la_peak_1006	9.9701	
11	2010017	2010512	KD2-H4K8la_peak_1007	6.40658	MRPL23-AS1_ENSG00000226416
11	2011377	2012069	KD2-H4K8la_peak_1008	7.92775	MRPL23-AS1_ENSG00000226416;AC051649.6_ENSG00000232987
11	2012269	2012604	KD2-H4K8la_peak_1009	6.83387	AC051649.6_ENSG00000232987
11	2015602	2016047	KD2-H4K8la_peak_1010	11.75972	

11	2190637	2190916	KD2-H4K8la_peak_1011	8.93385	
11	2211468	2211707	KD2-H4K8la_peak_1012	4.69635	
11	2213603	2214077	KD2-H4K8la_peak_1013	14.30428	
11	2216531	2216787	KD2-H4K8la_peak_1014	6.77116	
11	2336681	2337195	KD2-H4K8la_peak_1015	8.37064	
11	2420637	2421060	KD2-H4K8la_peak_1016	7.49467	TSSC4_ENSG00000184281
11	2421287	2421488	KD2-H4K8la_peak_1017	10.20357	TSSC4_ENSG00000184281
11	2724990	2725446	KD2-H4K8la_peak_1018	4.38315	
11	2772209	2772446	KD2-H4K8la_peak_1019	4.86896	
11	2799399	2799781	KD2-H4K8la_peak_1020	12.97817	
11	2800294	2800571	KD2-H4K8la_peak_1021	5.8369	
11	2801298	2801701	KD2-H4K8la_peak_1022	11.07667	
11	2801926	2802140	KD2-H4K8la_peak_1023	8.44557	
11	2905476	2905850	KD2-H4K8la_peak_1024	5.05146	
11	3078110	3078368	KD2-H4K8la_peak_1025	7.90495	CARS_ENSG00000110619
11	3078966	3079233	KD2-H4K8la_peak_1026	10.00612	CARS_ENSG00000110619
11	3829984	3830189	KD2-H4K8la_peak_1027	7.13237	
11	4208505	4208756	KD2-H4K8la_peak_1028	8.07102	RP11-23F23.2_ENSG00000254480
11	4414587	4414803	KD2-H4K8la_peak_1029	5.99671	TRIM21_ENSG00000132109
11	6495488	6495809	KD2-H4K8la_peak_1030	5.43466	TRIM3_ENSG00000110171
11	6608336	6608549	KD2-H4K8la_peak_1031	5.95337	
11	6704266	6704468	KD2-H4K8la_peak_1032	8.6948	MRPL17_ENSG00000158042
11	8040824	8041133	KD2-H4K8la_peak_1033	7.12014	TUB_ENSG00000166402
11	8254394	8255069	KD2-H4K8la_peak_1034	13.85872	
11	8259123	8259353	KD2-H4K8la_peak_1035	9.02706	
11	8263605	8263953	KD2-H4K8la_peak_1036	6.86458	
11	8305437	8305705	KD2-H4K8la_peak_1037	8.76116	
11	8350875	8351162	KD2-H4K8la_peak_1038	7.30722	
11	8351678	8351890	KD2-H4K8la_peak_1039	6.3399	
11	8376475	8376787	KD2-H4K8la_peak_1040	4.30663	
11	8703816	8704018	KD2-H4K8la_peak_1041	6.20793	RPL27A_ENSG00000166441
11	8704458	8704761	KD2-H4K8la_peak_1042	6.9399	RPL27A_ENSG00000166441
11	8892253	8892485	KD2-H4K8la_peak_1043	7.12014	
11	9482962	9483167	KD2-H4K8la_peak_1044	8.76082	AC132192.1_ENSG00000268403
11	9685169	9685376	KD2-H4K8la_peak_1045	6.79079	SWAP70_ENSG00000133789
11	9779403	9779670	KD2-H4K8la_peak_1046	9.27415	SBF2-AS1_ENSG00000246273
11	1.1E+07	1.1E+07	KD2-H4K8la_peak_1047	7.65547	RP11-685M7.3_ENSG00000246308;EIF4G2_ENSG00000110321
11	1.2E+07	1.2E+07	KD2-H4K8la_peak_1048	7.90495	USP47_ENSG00000170242
11	1.2E+07	1.2E+07	KD2-H4K8la_peak_1049	12.76394	
11	1.2E+07	1.2E+07	KD2-H4K8la_peak_1050	10.58561	
11	1.3E+07	1.3E+07	KD2-H4K8la_peak_1051	7.30722	TEAD1_ENSG00000187079
11	1.3E+07	1.3E+07	KD2-H4K8la_peak_1052	4.53273	
11	1.6E+07	1.6E+07	KD2-H4K8la_peak_1053	9.22403	
11	1.7E+07	1.7E+07	KD2-H4K8la_peak_1054	6.70626	
11	1.7E+07	1.7E+07	KD2-H4K8la_peak_1055	5.05146	PLEKHA7_ENSG00000166689;OR7E14P_ENSG00000184669
11	1.8E+07	1.8E+07	KD2-H4K8la_peak_1056	7.22716	SAAL1_ENSG00000166788;HIGD1AP5_ENSG00000255254

11	1.8E+07	1.8E+07	KD2-H4K8la_peak_1057	11.06859	
11	1.9E+07	1.9E+07	KD2-H4K8la_peak_1058	10.07718	RP11-1081L13.4_ENSG00000254966
11	2E+07	2E+07	KD2-H4K8la_peak_1059	13.23927	
11	2E+07	2E+07	KD2-H4K8la_peak_1060	14.45289	
11	2E+07	2E+07	KD2-H4K8la_peak_1061	7.92775	
11	2.1E+07	2.1E+07	KD2-H4K8la_peak_1062	5.60379	
11	2.1E+07	2.1E+07	KD2-H4K8la_peak_1063	9.27415	
11	2.7E+07	2.7E+07	KD2-H4K8la_peak_1064	8.07102	
11	2.7E+07	2.7E+07	KD2-H4K8la_peak_1065	5.22084	
11	2.8E+07	2.8E+07	KD2-H4K8la_peak_1066	8.37064	LIN7C_ENSG00000148943;BDNF-AS_ENSG00000245573
11	3E+07	3E+07	KD2-H4K8la_peak_1067	11.81487	ARL14EP_ENSG00000152219
11	3.1E+07	3.1E+07	KD2-H4K8la_peak_1068	6.36118	MPPED2_ENSG00000066382
11	3.2E+07	3.2E+07	KD2-H4K8la_peak_1069	8.37064	
11	3.3E+07	3.3E+07	KD2-H4K8la_peak_1070	7.49467	EIF3M_ENSG00000149100
11	3.4E+07	3.4E+07	KD2-H4K8la_peak_1071	12.63129	CAPRIN1_ENSG00000135387
11	3.4E+07	3.4E+07	KD2-H4K8la_peak_1072	9.8025	
11	3.5E+07	3.5E+07	KD2-H4K8la_peak_1073	7.22716	RP4-607I7.1_ENSG00000255521;CD44_ENSG00000026508
11	3.6E+07	3.6E+07	KD2-H4K8la_peak_1074	15.19386	TRIM44_ENSG00000166326
11	4.3E+07	4.3E+07	KD2-H4K8la_peak_1075	6.57319	
11	4.4E+07	4.4E+07	KD2-H4K8la_peak_1076	11.06859	
11	4.4E+07	4.4E+07	KD2-H4K8la_peak_1077	5.14476	ALKBH3-AS1_ENSG00000244926;RP11-613D13.4_ENSG00000254409
11	4.4E+07	4.4E+07	KD2-H4K8la_peak_1078	8.48577	C11orf96_ENSG00000187479
11	4.4E+07	4.4E+07	KD2-H4K8la_peak_1079	7.92775	C11orf96_ENSG00000187479
11	4.4E+07	4.4E+07	KD2-H4K8la_peak_1080	8.8621	
11	4.4E+07	4.4E+07	KD2-H4K8la_peak_1081	11.15747	
11	4.4E+07	4.4E+07	KD2-H4K8la_peak_1082	6.61963	
11	4.4E+07	4.4E+07	KD2-H4K8la_peak_1083	15.09309	
11	4.4E+07	4.4E+07	KD2-H4K8la_peak_1084	5.78071	
11	4.4E+07	4.4E+07	KD2-H4K8la_peak_1085	5.30989	
11	4.4E+07	4.4E+07	KD2-H4K8la_peak_1086	4.72651	
11	4.5E+07	4.5E+07	KD2-H4K8la_peak_1087	6.48366	
11	4.5E+07	4.5E+07	KD2-H4K8la_peak_1088	9.70817	
11	4.5E+07	4.5E+07	KD2-H4K8la_peak_1089	10.36292	RP11-45A12.1_ENSG00000255079
11	4.5E+07	4.5E+07	KD2-H4K8la_peak_1090	6.13507	TSPAN18_ENSG00000157570
11	4.5E+07	4.5E+07	KD2-H4K8la_peak_1091	10.04111	
11	4.5E+07	4.5E+07	KD2-H4K8la_peak_1092	10.2573	
11	4.5E+07	4.5E+07	KD2-H4K8la_peak_1093	5.05146	
11	4.5E+07	4.5E+07	KD2-H4K8la_peak_1094	7.22716	
11	4.5E+07	4.5E+07	KD2-H4K8la_peak_1095	17.22831	
11	4.6E+07	4.6E+07	KD2-H4K8la_peak_1096	7.49467	
11	4.6E+07	4.6E+07	KD2-H4K8la_peak_1097	6.83387	GYLTL1B_ENSG00000165905
11	4.6E+07	4.6E+07	KD2-H4K8la_peak_1098	4.30663	CREB3L1_ENSG00000157613
11	4.6E+07	4.6E+07	KD2-H4K8la_peak_1099	7.3993	MDK_ENSG00000110492
11	4.6E+07	4.6E+07	KD2-H4K8la_peak_1100	9.37943	MDK_ENSG00000110492
11	4.6E+07	4.6E+07	KD2-H4K8la_peak_1101	11.40266	
11	4.7E+07	4.7E+07	KD2-H4K8la_peak_1102	9.38376	CKAP5_ENSG00000175216;LRP4-AS1_ENSG00000247675

11	4.7E+07	4.7E+07	KD2-H4K8la_peak_1103	7.72421	C11orf49_ENSG00000149179
11	4.7E+07	4.7E+07	KD2-H4K8la_peak_1104	7.83588	PACSIN3_ENSG00000165912
11	4.7E+07	4.7E+07	KD2-H4K8la_peak_1105	6.3399	PSMC3_ENSG00000165916
11	4.8E+07	4.8E+07	KD2-H4K8la_peak_1106	11.36807	CELF1_ENSG00000149187;NDUFS3_ENSG00000213619;PTPMT1_ENSG00000110536
11	4.8E+07	4.8E+07	KD2-H4K8la_peak_1107	4.69635	PTPRJ_ENSG00000149177
11	5.7E+07	5.7E+07	KD2-H4K8la_peak_1108	9.77649	
11	5.7E+07	5.7E+07	KD2-H4K8la_peak_1109	8.07102	RP11-624G17.3_ENSG00000255301
11	5.7E+07	5.7E+07	KD2-H4K8la_peak_1110	10.11868	
11	5.7E+07	5.7E+07	KD2-H4K8la_peak_1111	5.39166	TIMM10_ENSG00000134809
11	5.7E+07	5.7E+07	KD2-H4K8la_peak_1112	8.76116	AP000662.4_ENSG00000254602
11	5.7E+07	5.7E+07	KD2-H4K8la_peak_1113	6.13507	AP000662.4_ENSG00000254602
11	5.7E+07	5.7E+07	KD2-H4K8la_peak_1114	5.36015	MED19_ENSG00000156603;TMX2_ENSG00000213593;TMX2-CTNND1_ENSG00000254462
11	5.8E+07	5.8E+07	KD2-H4K8la_peak_1115	9.27765	ZFP91_ENSG00000186660;ZFP91-CNTF_ENSG00000255073
11	5.9E+07	5.9E+07	KD2-H4K8la_peak_1116	7.72421	
11	5.9E+07	5.9E+07	KD2-H4K8la_peak_1117	4.57867	
11	6E+07	6E+07	KD2-H4K8la_peak_1118	13.20018	MRPL16_ENSG00000166902
11	6.1E+07	6.1E+07	KD2-H4K8la_peak_1119	7.12014	CCDC86_ENSG00000110104
11	6.1E+07	6.1E+07	KD2-H4K8la_peak_1120	9.6708	RP11-881M11.1_ENSG00000256944;TMEM109_ENSG00000110108
11	6.1E+07	6.1E+07	KD2-H4K8la_peak_1121	5.56351	TMEM132A_ENSG00000006118
11	6.1E+07	6.1E+07	KD2-H4K8la_peak_1122	5.99671	VWCE_ENSG00000167992
11	6.1E+07	6.1E+07	KD2-H4K8la_peak_1123	5.66328	
11	6.1E+07	6.1E+07	KD2-H4K8la_peak_1124	9.49266	TMEM216_ENSG00000187049
11	6.1E+07	6.1E+07	KD2-H4K8la_peak_1125	7.3993	CPSF7_ENSG00000149532;RP11-286N22.8_ENSG00000256591;SDHAF2_ENSG00000167985
11	6.1E+07	6.1E+07	KD2-H4K8la_peak_1126	5.47284	
11	6.2E+07	6.2E+07	KD2-H4K8la_peak_1127	8.43222	MYRF_ENSG00000124920
11	6.2E+07	6.2E+07	KD2-H4K8la_peak_1128	9.14903	TMEM258_ENSG00000134825;MIR611_ENSG00000207601;FEN1_ENSG00000168496;FADS2_ENSG00000134824
11	6.2E+07	6.2E+07	KD2-H4K8la_peak_1129	9.78176	FADS1_ENSG00000149485
11	6.2E+07	6.2E+07	KD2-H4K8la_peak_1130	5.05146	FADS3_ENSG00000221968
11	6.2E+07	6.2E+07	KD2-H4K8la_peak_1131	7.13185	FADS3_ENSG00000221968
11	6.2E+07	6.2E+07	KD2-H4K8la_peak_1132	6.11881	BEST1_ENSG00000167995
11	6.2E+07	6.2E+07	KD2-H4K8la_peak_1133	9.61894	INCENP_ENSG00000149503
11	6.2E+07	6.2E+07	KD2-H4K8la_peak_1134	8.79494	
11	6.2E+07	6.2E+07	KD2-H4K8la_peak_1135	5.83285	MIR3654_ENSG00000255508;TUT1_ENSG00000149016
11	6.2E+07	6.2E+07	KD2-H4K8la_peak_1136	5.76341	MTA2_ENSG00000149480
11	6.2E+07	6.2E+07	KD2-H4K8la_peak_1137	6.64804	MTA2_ENSG00000149480
11	6.2E+07	6.2E+07	KD2-H4K8la_peak_1138	13.47548	BSCL2_ENSG00000168000
11	6.3E+07	6.3E+07	KD2-H4K8la_peak_1139	5.83285	
11	6.4E+07	6.4E+07	KD2-H4K8la_peak_1140	17.45444	C11orf95_ENSG00000188070;RP11-466C23.4_ENSG00000255651
11	6.4E+07	6.4E+07	KD2-H4K8la_peak_1141	12.32641	RCOR2_ENSG00000167771
11	6.4E+07	6.4E+07	KD2-H4K8la_peak_1142	6.68593	
11	6.4E+07	6.4E+07	KD2-H4K8la_peak_1143	6.25288	
11	6.4E+07	6.4E+07	KD2-H4K8la_peak_1144	5.43278	
11	6.4E+07	6.4E+07	KD2-H4K8la_peak_1145	7.61975	OTUB1_ENSG00000167770
11	6.4E+07	6.4E+07	KD2-H4K8la_peak_1146	7.30722	
11	6.4E+07	6.4E+07	KD2-H4K8la_peak_1147	7.98235	
11	6.4E+07	6.4E+07	KD2-H4K8la_peak_1148	8.71024	

11	6.4E+07	6.4E+07	KD2-H4K8la_peak_1149	6.37643	
11	6.4E+07	6.4E+07	KD2-H4K8la_peak_1150	6.86458	
11	6.4E+07	6.4E+07	KD2-H4K8la_peak_1151	8.66164	
11	6.4E+07	6.4E+07	KD2-H4K8la_peak_1152	13.04289	
11	6.4E+07	6.4E+07	KD2-H4K8la_peak_1153	9.02706	
11	6.4E+07	6.4E+07	KD2-H4K8la_peak_1154	4.55308	
11	6.4E+07	6.4E+07	KD2-H4K8la_peak_1155	6.83387	
11	6.4E+07	6.4E+07	KD2-H4K8la_peak_1156	7.81267	MACROD1_ENSG00000133315
11	6.4E+07	6.4E+07	KD2-H4K8la_peak_1157	8.07044	MACROD1_ENSG00000133315
11	6.4E+07	6.4E+07	KD2-H4K8la_peak_1158	6.31081	RP11-783K16.14_ENSG00000256116;VEGFB_ENSG00000173511
11	6.4E+07	6.4E+07	KD2-H4K8la_peak_1159	7.61975	FKBP2_ENSG00000173486
11	6.4E+07	6.4E+07	KD2-H4K8la_peak_1160	10.00612	PPP1R14B_ENSG00000173457;RP11-783K16.5_ENSG00000256940
11	6.4E+07	6.4E+07	KD2-H4K8la_peak_1161	7.90495	GPR137_ENSG00000173264
11	6.4E+07	6.4E+07	KD2-H4K8la_peak_1162	5.98041	GPR137_ENSG00000173264
11	6.4E+07	6.4E+07	KD2-H4K8la_peak_1163	10.20357	BAD_ENSG00000002330
11	6.4E+07	6.4E+07	KD2-H4K8la_peak_1164	5.12859	RPS6KA4_ENSG00000162302
11	6.4E+07	6.4E+07	KD2-H4K8la_peak_1165	7.49467	
11	6.4E+07	6.4E+07	KD2-H4K8la_peak_1166	8.12466	
11	6.4E+07	6.4E+07	KD2-H4K8la_peak_1167	19.53802	NRXN2_ENSG00000110076
11	6.5E+07	6.5E+07	KD2-H4K8la_peak_1168	8.13557	RASGRP2_ENSG00000068831
11	6.5E+07	6.5E+07	KD2-H4K8la_peak_1169	15.75739	SF1_ENSG00000168066;AP001462.6_ENSG00000269038
11	6.5E+07	6.5E+07	KD2-H4K8la_peak_1170	11.15747	ARL2_ENSG00000213465;RP11-399J13.3_ENSG00000273003
11	6.5E+07	6.5E+07	KD2-H4K8la_peak_1171	6.86458	CDCA5_ENSG00000146670;ZFPL1_ENSG00000162300
11	6.5E+07	6.5E+07	KD2-H4K8la_peak_1172	6.64804	
11	6.5E+07	6.5E+07	KD2-H4K8la_peak_1173	5.99671	
11	6.5E+07	6.5E+07	KD2-H4K8la_peak_1174	6.68593	ZNHIT2_ENSG00000174276;AP003068.12_ENSG00000255173
11	6.5E+07	6.5E+07	KD2-H4K8la_peak_1175	13.3186	AP003068.23_ENSG00000254614;CAPN1_ENSG00000014216
11	6.5E+07	6.5E+07	KD2-H4K8la_peak_1176	7.13237	
11	6.5E+07	6.5E+07	KD2-H4K8la_peak_1177	5.43278	
11	6.5E+07	6.5E+07	KD2-H4K8la_peak_1178	4.30663	NEAT1_ENSG00000245532
11	6.5E+07	6.5E+07	KD2-H4K8la_peak_1179	8.944	
11	6.5E+07	6.5E+07	KD2-H4K8la_peak_1180	9.61894	LTBP3_ENSG00000168056
11	6.5E+07	6.5E+07	KD2-H4K8la_peak_1181	5.33301	EHBP1L1_ENSG00000173442
11	6.5E+07	6.5E+07	KD2-H4K8la_peak_1182	6.81115	MAP3K11_ENSG00000173327;PCNXL3_ENSG00000197136
11	6.6E+07	6.6E+07	KD2-H4K8la_peak_1183	11.98921	AP5B1_ENSG00000254470
11	6.6E+07	6.6E+07	KD2-H4K8la_peak_1184	5.26241	EFEMP2_ENSG00000172638
11	6.6E+07	6.6E+07	KD2-H4K8la_peak_1185	10.70453	
11	6.6E+07	6.6E+07	KD2-H4K8la_peak_1186	6.28686	GAL3ST3_ENSG00000175229
11	6.6E+07	6.6E+07	KD2-H4K8la_peak_1187	8.12466	SF3B2_ENSG00000087365
11	6.6E+07	6.6E+07	KD2-H4K8la_peak_1188	20.66643	RP11-867G23.13_ENSG00000254458
11	6.6E+07	6.6E+07	KD2-H4K8la_peak_1189	6.03112	CD248_ENSG00000174807
11	6.6E+07	6.6E+07	KD2-H4K8la_peak_1190	5.83285	CD248_ENSG00000174807
11	6.6E+07	6.6E+07	KD2-H4K8la_peak_1191	8.76116	BRMS1_ENSG00000174744
11	6.6E+07	6.6E+07	KD2-H4K8la_peak_1192	9.05604	SLC29A2_ENSG00000174669
11	6.6E+07	6.6E+07	KD2-H4K8la_peak_1193	10.49982	SPTBN2_ENSG00000173898
11	6.7E+07	6.7E+07	KD2-H4K8la_peak_1194	7.78021	LRFN4_ENSG00000173621

11	6.7E+07	6.7E+07	KD2-H4K8la_peak_1195	8.76116	KDM2A_ENSG00000173120
11	6.7E+07	6.7E+07	KD2-H4K8la_peak_1196	8.71041	
11	6.7E+07	6.7E+07	KD2-H4K8la_peak_1197	7.12014	
11	6.7E+07	6.7E+07	KD2-H4K8la_peak_1198	8.59373	SSH3_ENSG00000172830
11	6.7E+07	6.7E+07	KD2-H4K8la_peak_1199	8.59735	
11	6.7E+07	6.7E+07	KD2-H4K8la_peak_1200	6.88801	
11	6.7E+07	6.7E+07	KD2-H4K8la_peak_1201	6.31081	CARNS1_ENSG00000172508
11	6.7E+07	6.7E+07	KD2-H4K8la_peak_1202	7.49467	
11	6.7E+07	6.7E+07	KD2-H4K8la_peak_1203	5.28047	AIP_ENSG00000110711
11	6.7E+07	6.7E+07	KD2-H4K8la_peak_1204	10.20357	CDK2AP2_ENSG00000167797
11	6.7E+07	6.7E+07	KD2-H4K8la_peak_1205	11.65011	GSTP1_ENSG00000084207
11	6.7E+07	6.7E+07	KD2-H4K8la_peak_1206	9.86881	C11orf72_ENSG00000184224;RP11-655M14.12_ENSG00000255119;NDUFV1_ENSG00000167792
11	6.8E+07	6.8E+07	KD2-H4K8la_peak_1207	9.38376	UNC93B1_ENSG00000110057
11	6.8E+07	6.8E+07	KD2-H4K8la_peak_1208	9.61894	
11	6.8E+07	6.8E+07	KD2-H4K8la_peak_1209	16.68107	SUV420H1_ENSG00000110066
11	6.8E+07	6.8E+07	KD2-H4K8la_peak_1210	10.58561	LRP5_ENSG00000162337
11	6.8E+07	6.8E+07	KD2-H4K8la_peak_1211	7.92775	
11	6.8E+07	6.8E+07	KD2-H4K8la_peak_1212	6.77116	
11	6.8E+07	6.8E+07	KD2-H4K8la_peak_1213	9.27415	
11	6.8E+07	6.8E+07	KD2-H4K8la_peak_1214	10.47118	
11	6.8E+07	6.8E+07	KD2-H4K8la_peak_1215	12.43263	GAL_ENSG00000069482
11	6.9E+07	6.9E+07	KD2-H4K8la_peak_1216	7.12014	
11	6.9E+07	6.9E+07	KD2-H4K8la_peak_1217	7.61975	TPCN2_ENSG00000162341
11	6.9E+07	6.9E+07	KD2-H4K8la_peak_1218	4.90979	RP11-554A11.9_ENSG00000259799
11	6.9E+07	6.9E+07	KD2-H4K8la_peak_1219	23.64423	RP11-554A11.9_ENSG00000259799
11	6.9E+07	6.9E+07	KD2-H4K8la_peak_1220	6.86458	
11	6.9E+07	6.9E+07	KD2-H4K8la_peak_1221	11.85985	
11	6.9E+07	6.9E+07	KD2-H4K8la_peak_1222	5.99671	
11	6.9E+07	6.9E+07	KD2-H4K8la_peak_1223	11.28887	
11	6.9E+07	6.9E+07	KD2-H4K8la_peak_1224	6.0136	
11	6.9E+07	6.9E+07	KD2-H4K8la_peak_1225	7.13237	
11	6.9E+07	6.9E+07	KD2-H4K8la_peak_1226	13.29476	
11	7E+07	7E+07	KD2-H4K8la_peak_1227	8.16787	FADD_ENSG00000168040
11	7.1E+07	7.1E+07	KD2-H4K8la_peak_1228	6.32736	
11	7.1E+07	7.1E+07	KD2-H4K8la_peak_1229	10.97039	FAM86C1_ENSG00000158483
11	7.2E+07	7.2E+07	KD2-H4K8la_peak_1230	8.76116	NUMA1_ENSG00000137497;LRTOMT_ENSG00000184154
11	7.2E+07	7.2E+07	KD2-H4K8la_peak_1231	6.89612	LAMTOR1_ENSG00000149357;snoU13_ENSG00000238768
11	7.2E+07	7.2E+07	KD2-H4K8la_peak_1232	8.487	ANAPC15_ENSG00000110200
11	7.2E+07	7.2E+07	KD2-H4K8la_peak_1233	8.59561	
11	7.2E+07	7.2E+07	KD2-H4K8la_peak_1234	7.86398	
11	7.2E+07	7.2E+07	KD2-H4K8la_peak_1235	7.61975	PHOX2A_ENSG00000165462
11	7.2E+07	7.2E+07	KD2-H4K8la_peak_1236	7.51014	
11	7.2E+07	7.2E+07	KD2-H4K8la_peak_1237	9.0413	
11	7.2E+07	7.2E+07	KD2-H4K8la_peak_1238	5.67034	
11	7.2E+07	7.2E+07	KD2-H4K8la_peak_1239	9.54333	
11	7.3E+07	7.3E+07	KD2-H4K8la_peak_1240	8.3574	

11	7.3E+07	7.3E+07	KD2-H4K8la_peak_1241	6.43694	
11	7.3E+07	7.3E+07	KD2-H4K8la_peak_1242	15.53091	RAB6A_ENSG00000175582;RP11-707G14.1_ENSG00000256034
11	7.3E+07	7.3E+07	KD2-H4K8la_peak_1243	13.73665	RAB6A_ENSG00000175582
11	7.4E+07	7.4E+07	KD2-H4K8la_peak_1244	13.27013	LIPT2_ENSG00000175536;AP001372.2_ENSG00000254837;POLD3_ENSG00000077514
11	7.5E+07	7.5E+07	KD2-H4K8la_peak_1245	10.28981	
11	7.5E+07	7.5E+07	KD2-H4K8la_peak_1246	5.89088	
11	7.5E+07	7.5E+07	KD2-H4K8la_peak_1247	6.75222	ARRB1_ENSG00000137486
11	7.5E+07	7.5E+07	KD2-H4K8la_peak_1248	8.65988	RPS3_ENSG00000149273
11	7.5E+07	7.5E+07	KD2-H4K8la_peak_1249	9.61894	KLHL35_ENSG00000149243
11	7.5E+07	7.5E+07	KD2-H4K8la_peak_1250	14.15376	CTD-2530H12.4_ENSG00000255326
11	7.5E+07	7.5E+07	KD2-H4K8la_peak_1251	6.61963	
11	7.6E+07	7.6E+07	KD2-H4K8la_peak_1252	8.16787	
11	7.7E+07	7.7E+07	KD2-H4K8la_peak_1253	5.83285	
11	7.7E+07	7.7E+07	KD2-H4K8la_peak_1254	7.60756	PAK1_ENSG00000149269;DKFZP434E1119_ENSG00000268635
11	7.8E+07	7.8E+07	KD2-H4K8la_peak_1255	7.92775	ALG8_ENSG00000159063;KCTD21-AS1_ENSG00000246174
11	7.8E+07	7.8E+07	KD2-H4K8la_peak_1256	7.14575	
11	7.9E+07	7.9E+07	KD2-H4K8la_peak_1257	8.73956	
11	8.6E+07	8.6E+07	KD2-H4K8la_peak_1258	7.41831	AP000974.1_ENSG00000215504;CCDC83_ENSG00000150676
11	8.6E+07	8.6E+07	KD2-H4K8la_peak_1259	5.05146	ME3_ENSG00000151376
11	9E+07	9E+07	KD2-H4K8la_peak_1260	7.83588	CHORDC1_ENSG00000110172
11	9.3E+07	9.3E+07	KD2-H4K8la_peak_1261	6.98136	CCDC67_ENSG00000165325
11	9.3E+07	9.3E+07	KD2-H4K8la_peak_1262	7.77341	KIAA1731_ENSG00000166004
11	9.4E+07	9.4E+07	KD2-H4K8la_peak_1263	8.44557	TAF1D_ENSG00000166012;MED17_ENSG00000042429
11	9.4E+07	9.4E+07	KD2-H4K8la_peak_1264	10.47118	VSTM5_ENSG00000214376
11	9.4E+07	9.4E+07	KD2-H4K8la_peak_1265	8.76082	PANX1_ENSG00000110218
11	9.5E+07	9.5E+07	KD2-H4K8la_peak_1266	11.40266	
11	9.5E+07	9.5E+07	KD2-H4K8la_peak_1267	10.20357	SRSF8_ENSG00000180771;SRSF8_ENSG00000271885
11	9.6E+07	9.6E+07	KD2-H4K8la_peak_1268	10.99007	
11	9.8E+07	9.8E+07	KD2-H4K8la_peak_1269	5.67034	
11	9.8E+07	9.8E+07	KD2-H4K8la_peak_1270	8.77936	
11	1E+08	1E+08	KD2-H4K8la_peak_1271	5.36015	RP11-315O6.1_ENSG00000255337
11	1.1E+08	1.1E+08	KD2-H4K8la_peak_1272	8.35736	MSANTD4_ENSG00000170903
11	1.1E+08	1.1E+08	KD2-H4K8la_peak_1273	9.66714	SLC35F2_ENSG00000110660;RAB39A_ENSG00000179331
11	1.1E+08	1.1E+08	KD2-H4K8la_peak_1274	5.05146	KDELC2_ENSG00000178202
11	1.1E+08	1.1E+08	KD2-H4K8la_peak_1275	10.73719	KDELC2_ENSG00000178202
11	1.1E+08	1.1E+08	KD2-H4K8la_peak_1276	5.36015	DDX10_ENSG00000178105
11	1.1E+08	1.1E+08	KD2-H4K8la_peak_1277	7.72421	
11	1.1E+08	1.1E+08	KD2-H4K8la_peak_1278	10.83873	COLCA2_ENSG00000214290
11	1.1E+08	1.1E+08	KD2-H4K8la_peak_1279	7.23932	PTS_ENSG00000150787
11	1.1E+08	1.1E+08	KD2-H4K8la_peak_1280	10.49169	
11	1.1E+08	1.1E+08	KD2-H4K8la_peak_1281	8.96921	
11	1.1E+08	1.1E+08	KD2-H4K8la_peak_1282	6.67305	
11	1.2E+08	1.2E+08	KD2-H4K8la_peak_1283	5.83285	RP11-136I14.5_ENSG00000255689
11	1.2E+08	1.2E+08	KD2-H4K8la_peak_1284	8.76116	AP000797.3_ENSG00000256717
11	1.2E+08	1.2E+08	KD2-H4K8la_peak_1285	7.12014	
11	1.2E+08	1.2E+08	KD2-H4K8la_peak_1286	6.3399	

11	1.2E+08	1.2E+08	KD2-H4K8la_peak_1287	8.12466	LINC00900_ENSG00000246100
11	1.2E+08	1.2E+08	KD2-H4K8la_peak_1288	4.86896	
11	1.2E+08	1.2E+08	KD2-H4K8la_peak_1289	5.05146	
11	1.2E+08	1.2E+08	KD2-H4K8la_peak_1290	7.59874	
11	1.2E+08	1.2E+08	KD2-H4K8la_peak_1291	4.6555	AP001891.1_ENSG00000236437
11	1.2E+08	1.2E+08	KD2-H4K8la_peak_1292	5.43278	
11	1.2E+08	1.2E+08	KD2-H4K8la_peak_1293	8.80046	APOA5_ENSG00000110243
11	1.2E+08	1.2E+08	KD2-H4K8la_peak_1294	10.97039	SIK3_ENSG00000160584;AP000936.4_ENSG00000224077
11	1.2E+08	1.2E+08	KD2-H4K8la_peak_1295	6.2278	BACE1_ENSG00000186318
11	1.2E+08	1.2E+08	KD2-H4K8la_peak_1296	6.96161	
11	1.2E+08	1.2E+08	KD2-H4K8la_peak_1297	9.9701	
11	1.2E+08	1.2E+08	KD2-H4K8la_peak_1298	8.76116	
11	1.2E+08	1.2E+08	KD2-H4K8la_peak_1299	4.85784	
11	1.2E+08	1.2E+08	KD2-H4K8la_peak_1300	9.05604	DSCAML1_ENSG00000177103
11	1.2E+08	1.2E+08	KD2-H4K8la_peak_1301	7.72421	MPZL3_ENSG00000160588
11	1.2E+08	1.2E+08	KD2-H4K8la_peak_1302	4.30663	UBE4A_ENSG00000110344
11	1.2E+08	1.2E+08	KD2-H4K8la_peak_1303	8.37064	RP11-770J1.5_ENSG00000254873;ATP5L_ENSG00000167283
11	1.2E+08	1.2E+08	KD2-H4K8la_peak_1304	14.15376	DDX6_ENSG00000110367
11	1.2E+08	1.2E+08	KD2-H4K8la_peak_1305	9.38376	DDX6_ENSG00000110367
11	1.2E+08	1.2E+08	KD2-H4K8la_peak_1306	12.1221	SLC37A4_ENSG00000137700
11	1.2E+08	1.2E+08	KD2-H4K8la_peak_1307	7.51014	H2AFX_ENSG00000188486
11	1.2E+08	1.2E+08	KD2-H4K8la_peak_1308	6.2278	
11	1.2E+08	1.2E+08	KD2-H4K8la_peak_1309	9.35473	
11	1.2E+08	1.2E+08	KD2-H4K8la_peak_1310	9.27415	
11	1.2E+08	1.2E+08	KD2-H4K8la_peak_1311	4.30663	
11	1.2E+08	1.2E+08	KD2-H4K8la_peak_1312	5.05146	
11	1.2E+08	1.2E+08	KD2-H4K8la_peak_1313	6.25495	
11	1.2E+08	1.2E+08	KD2-H4K8la_peak_1314	5.33301	
11	1.2E+08	1.2E+08	KD2-H4K8la_peak_1315	6.96161	
11	1.2E+08	1.2E+08	KD2-H4K8la_peak_1316	8.43222	
11	1.2E+08	1.2E+08	KD2-H4K8la_peak_1317	6.46253	
11	1.2E+08	1.2E+08	KD2-H4K8la_peak_1318	5.21483	AP000679.2_ENSG00000176984
11	1.2E+08	1.2E+08	KD2-H4K8la_peak_1319	13.29266	
11	1.2E+08	1.2E+08	KD2-H4K8la_peak_1320	8.8263	
11	1.2E+08	1.2E+08	KD2-H4K8la_peak_1321	8.7731	
11	1.2E+08	1.2E+08	KD2-H4K8la_peak_1322	6.16656	
11	1.2E+08	1.2E+08	KD2-H4K8la_peak_1323	6.13507	TBCEL_ENSG00000154114
11	1.2E+08	1.2E+08	KD2-H4K8la_peak_1324	9.27765	
11	1.2E+08	1.2E+08	KD2-H4K8la_peak_1325	7.61975	CLMP_ENSG00000166250
11	1.2E+08	1.2E+08	KD2-H4K8la_peak_1326	16.45749	CLMP_ENSG00000166250
11	1.2E+08	1.2E+08	KD2-H4K8la_peak_1327	6.39807	
11	1.2E+08	1.2E+08	KD2-H4K8la_peak_1328	10.02595	
11	1.2E+08	1.2E+08	KD2-H4K8la_peak_1329	7.51302	TMEM218_ENSG00000150433;KRT18P59_ENSG00000187686
11	1.3E+08	1.3E+08	KD2-H4K8la_peak_1330	8.15906	EI24_ENSG00000149547
11	1.3E+08	1.3E+08	KD2-H4K8la_peak_1331	12.83096	EI24_ENSG00000149547
11	1.3E+08	1.3E+08	KD2-H4K8la_peak_1332	8.23729	CHEK1_ENSG00000149554

11	1.3E+08	1.3E+08	KD2-H4K8la_peak_1333	6.36286	
11	1.3E+08	1.3E+08	KD2-H4K8la_peak_1334	6.2278	CDON_ENSG00000064309
11	1.3E+08	1.3E+08	KD2-H4K8la_peak_1335	8.37064	
11	1.3E+08	1.3E+08	KD2-H4K8la_peak_1336	6.28686	
11	1.3E+08	1.3E+08	KD2-H4K8la_peak_1337	5.41777	
11	1.3E+08	1.3E+08	KD2-H4K8la_peak_1338	10.04111	
11	1.3E+08	1.3E+08	KD2-H4K8la_peak_1339	5.36015	
11	1.3E+08	1.3E+08	KD2-H4K8la_peak_1340	10.73332	ST3GAL4_ENSG00000110080
11	1.3E+08	1.3E+08	KD2-H4K8la_peak_1341	7.72519	
11	1.3E+08	1.3E+08	KD2-H4K8la_peak_1342	6.03964	
11	1.3E+08	1.3E+08	KD2-H4K8la_peak_1343	9.94613	
11	1.3E+08	1.3E+08	KD2-H4K8la_peak_1344	6.70626	RP11-702B10.2_ENSG00000273415
11	1.3E+08	1.3E+08	KD2-H4K8la_peak_1345	11.7362	
11	1.3E+08	1.3E+08	KD2-H4K8la_peak_1346	7.13237	
11	1.3E+08	1.3E+08	KD2-H4K8la_peak_1347	10.27876	PRDM10_ENSG00000170325;LINC00167_ENSG00000233220
11	1.3E+08	1.3E+08	KD2-H4K8la_peak_1348	8.37064	APLP2_ENSG00000084234
11	1.3E+08	1.3E+08	KD2-H4K8la_peak_1349	6.70626	SNX19_ENSG00000120451
11	1.3E+08	1.3E+08	KD2-H4K8la_peak_1350	6.97699	
11	1.3E+08	1.3E+08	KD2-H4K8la_peak_1351	11.52725	
11	1.3E+08	1.3E+08	KD2-H4K8la_peak_1352	7.90495	
11	1.3E+08	1.3E+08	KD2-H4K8la_peak_1353	5.83285	IGSF9B_ENSG00000080854;AP000911.1_ENSG00000264674
12	73831	74121	KD2-H4K8la_peak_1354	5.79784	AC215219.1_ENSG00000238823;ABC7-42389800N19.1_ENSG00000226210
12	284268	284527	KD2-H4K8la_peak_1355	7.06427	
12	286751	287008	KD2-H4K8la_peak_1356	6.68593	
12	679781	680150	KD2-H4K8la_peak_1357	9.00481	
12	701577	701815	KD2-H4K8la_peak_1358	6.89612	
12	1058483	1058770	KD2-H4K8la_peak_1359	9.90319	
12	1739302	1739506	KD2-H4K8la_peak_1360	4.75945	
12	1752445	1752754	KD2-H4K8la_peak_1361	12.92115	
12	1905635	1905837	KD2-H4K8la_peak_1362	6.3399	
12	1913961	1914399	KD2-H4K8la_peak_1363	8.26997	
12	1935735	1935969	KD2-H4K8la_peak_1364	7.26721	
12	3862306	3862614	KD2-H4K8la_peak_1365	9.11321	
12	3981865	3982198	KD2-H4K8la_peak_1366	5.16795	PARP11_ENSG00000111224;RP11-664D1.1_ENSG00000256862
12	6387421	6387831	KD2-H4K8la_peak_1367	10.04111	
12	6602114	6602322	KD2-H4K8la_peak_1368	4.30663	MRPL51_ENSG00000111639;NCAPD2_ENSG00000010292
12	6642780	6642985	KD2-H4K8la_peak_1369	10.49982	RP5-940J5.3_ENSG00000255966;GAPDH_ENSG00000111640
12	6716265	6716483	KD2-H4K8la_peak_1370	7.13237	CHD4_ENSG00000111642
12	6717852	6718188	KD2-H4K8la_peak_1371	6.88801	
12	6721854	6722053	KD2-H4K8la_peak_1372	11.60966	
12	6722469	6722778	KD2-H4K8la_peak_1373	18.46931	
12	6723445	6723851	KD2-H4K8la_peak_1374	6.86458	
12	6797750	6798001	KD2-H4K8la_peak_1375	5.7631	ZNF384_ENSG00000126746
12	6798255	6798512	KD2-H4K8la_peak_1376	19.30767	ZNF384_ENSG00000126746
12	6809517	6809754	KD2-H4K8la_peak_1377	10.11868	PIANP_ENSG00000139200
12	6976769	6977105	KD2-H4K8la_peak_1378	13.91009	TPI1_ENSG00000111669

12	7023066	7023970	KD2-H4K8la_peak_1379	12.1221	ENO2_ENSG00000111674
12	7046363	7047122	KD2-H4K8la_peak_1380	8.93385	
12	7053361	7053644	KD2-H4K8la_peak_1381	7.12014	U47924.31_ENSG00000272173;RNU7-1_ENSG00000238923
12	7079345	7079799	KD2-H4K8la_peak_1382	13.47548	PHB2_ENSG00000215021
12	7261050	7261425	KD2-H4K8la_peak_1383	4.86896	C1RL_ENSG00000139178;C1RL-AS1_ENSG00000205885
12	1.3E+07	1.3E+07	KD2-H4K8la_peak_1384	9.0413	
12	1.3E+07	1.3E+07	KD2-H4K8la_peak_1385	11.60966	
12	1.3E+07	1.3E+07	KD2-H4K8la_peak_1386	8.77936	
12	1.6E+07	1.6E+07	KD2-H4K8la_peak_1387	8.59561	
12	2E+07	2E+07	KD2-H4K8la_peak_1388	5.58883	
12	2E+07	2E+07	KD2-H4K8la_peak_1389	6.22999	
12	2.1E+07	2.1E+07	KD2-H4K8la_peak_1390	5.58883	RP11-284H19.1_ENSG00000256879;PDE3A_ENSG00000172572
12	2.2E+07	2.2E+07	KD2-H4K8la_peak_1391	14.15376	CMAS_ENSG00000111726
12	2.3E+07	2.3E+07	KD2-H4K8la_peak_1392	6.26214	
12	2.5E+07	2.5E+07	KD2-H4K8la_peak_1393	6.2278	RP11-444D3.1_ENSG00000255864
12	2.6E+07	2.6E+07	KD2-H4K8la_peak_1394	8.77936	RASSF8_ENSG00000123094
12	2.8E+07	2.8E+07	KD2-H4K8la_peak_1395	10.03312	RP11-1060J15.4_ENSG00000256377;MRPS35_ENSG00000061794
12	2.9E+07	2.9E+07	KD2-H4K8la_peak_1396	11.06859	RP11-946L16.1_ENSG00000257258;FAR2_ENSG00000064763
12	3.1E+07	3.1E+07	KD2-H4K8la_peak_1397	4.30663	
12	3.1E+07	3.1E+07	KD2-H4K8la_peak_1398	8.39919	TSPAN11_ENSG00000110900
12	3.2E+07	3.2E+07	KD2-H4K8la_peak_1399	4.79631	
12	3.2E+07	3.2E+07	KD2-H4K8la_peak_1400	21.33961	RP11-843B15.2_ENSG00000257530;BICD1_ENSG00000151746
12	3.3E+07	3.3E+07	KD2-H4K8la_peak_1401	5.05146	
12	3.4E+07	3.4E+07	KD2-H4K8la_peak_1402	6.57319	
12	3.9E+07	3.9E+07	KD2-H4K8la_peak_1403	8.86558	ALG10B_ENSG00000175548
12	4.2E+07	4.2E+07	KD2-H4K8la_peak_1404	7.81267	RP11-630C16.2_ENSG00000257239
12	4.4E+07	4.4E+07	KD2-H4K8la_peak_1405	4.53273	TWF1_ENSG00000151239
12	4.6E+07	4.6E+07	KD2-H4K8la_peak_1406	6.36118	PLEKHA8P1_ENSG00000134297;ANO6_ENSG00000177119
12	4.6E+07	4.6E+07	KD2-H4K8la_peak_1407	6.64804	ARID2_ENSG00000189079
12	4.7E+07	4.7E+07	KD2-H4K8la_peak_1408	6.48366	SLC38A2_ENSG00000134294;RP11-474P2.2_ENSG00000258096
12	4.8E+07	4.8E+07	KD2-H4K8la_peak_1409	6.86458	
12	4.8E+07	4.8E+07	KD2-H4K8la_peak_1410	4.44972	
12	4.8E+07	4.8E+07	KD2-H4K8la_peak_1411	8.37064	
12	4.9E+07	4.9E+07	KD2-H4K8la_peak_1412	6.57319	SENP1_ENSG00000079387
12	4.9E+07	4.9E+07	KD2-H4K8la_peak_1413	9.90319	CCNT1_ENSG00000129315
12	4.9E+07	4.9E+07	KD2-H4K8la_peak_1414	8.72694	ADCY6_ENSG00000174233;RP11-579D7.4_ENSG00000257660
12	4.9E+07	4.9E+07	KD2-H4K8la_peak_1415	8.944	
12	4.9E+07	4.9E+07	KD2-H4K8la_peak_1416	8.59735	DDN_ENSG00000181418;RP11-386G11.5_ENSG00000257913
12	4.9E+07	4.9E+07	KD2-H4K8la_peak_1417	10.97039	PRKAG1_ENSG00000181929
12	5E+07	5E+07	KD2-H4K8la_peak_1418	13.47548	TUBA1B_ENSG00000123416
12	5E+07	5E+07	KD2-H4K8la_peak_1419	5.84082	TUBA1A_ENSG00000167552;TUBA1C_ENSG00000167553
12	5E+07	5E+07	KD2-H4K8la_peak_1420	11.06333	PRPH_ENSG00000135406
12	5E+07	5E+07	KD2-H4K8la_peak_1421	6.60352	TROAP_ENSG00000135451
12	5E+07	5E+07	KD2-H4K8la_peak_1422	5.05146	MCRS1_ENSG00000187778;PRPF40B_ENSG00000110844
12	5E+07	5E+07	KD2-H4K8la_peak_1423	8.12466	
12	5E+07	5E+07	KD2-H4K8la_peak_1424	6.42514	

12	5E+07	5E+07	KD2-H4K8la_peak_1425	6.64804	
12	5E+07	5E+07	KD2-H4K8la_peak_1426	13.95533	
12	5.1E+07	5.1E+07	KD2-H4K8la_peak_1427	7.12014	ATF1_ENSG00000123268
12	5.2E+07	5.2E+07	KD2-H4K8la_peak_1428	6.77116	POU6F1_ENSG00000184271
12	5.2E+07	5.2E+07	KD2-H4K8la_peak_1429	4.30663	
12	5.2E+07	5.2E+07	KD2-H4K8la_peak_1430	5.58883	SMAGP_ENSG00000170545
12	5.2E+07	5.2E+07	KD2-H4K8la_peak_1431	6.04152	GALNT6_ENSG00000139629;SLC4A8_ENSG00000050438
12	5.2E+07	5.2E+07	KD2-H4K8la_peak_1432	15.92725	RP11-923I11.7_ENSG00000261308
12	5.2E+07	5.2E+07	KD2-H4K8la_peak_1433	11.40266	
12	5.2E+07	5.2E+07	KD2-H4K8la_peak_1434	11.06859	
12	5.2E+07	5.2E+07	KD2-H4K8la_peak_1435	9.73848	ACVR1B_ENSG00000135503
12	5.2E+07	5.2E+07	KD2-H4K8la_peak_1436	8.8293	
12	5.3E+07	5.3E+07	KD2-H4K8la_peak_1437	6.12473	
12	5.3E+07	5.3E+07	KD2-H4K8la_peak_1438	7.92067	
12	5.3E+07	5.3E+07	KD2-H4K8la_peak_1439	5.05146	EIF4B_ENSG00000063046
12	5.3E+07	5.3E+07	KD2-H4K8la_peak_1440	5.43278	TENC1_ENSG00000111077
12	5.3E+07	5.3E+07	KD2-H4K8la_peak_1441	9.05604	
12	5.3E+07	5.3E+07	KD2-H4K8la_peak_1442	8.12047	
12	5.4E+07	5.4E+07	KD2-H4K8la_peak_1443	9.88732	RARG_ENSG00000172819
12	5.4E+07	5.4E+07	KD2-H4K8la_peak_1444	7.81267	RARG_ENSG00000172819
12	5.4E+07	5.4E+07	KD2-H4K8la_peak_1445	5.98775	
12	5.4E+07	5.4E+07	KD2-H4K8la_peak_1446	5.78738	AAAS_ENSG00000094914
12	5.4E+07	5.4E+07	KD2-H4K8la_peak_1447	12.43981	AAAS_ENSG00000094914
12	5.4E+07	5.4E+07	KD2-H4K8la_peak_1448	6.42514	
12	5.4E+07	5.4E+07	KD2-H4K8la_peak_1449	4.53273	
12	5.4E+07	5.4E+07	KD2-H4K8la_peak_1450	14.76753	ATP5G2_ENSG00000135390
12	5.4E+07	5.4E+07	KD2-H4K8la_peak_1451	5.90392	
12	5.4E+07	5.4E+07	KD2-H4K8la_peak_1452	4.69635	AC012531.25_ENSG00000260597
12	5.4E+07	5.4E+07	KD2-H4K8la_peak_1453	6.2278	AC012531.25_ENSG00000260597
12	5.4E+07	5.4E+07	KD2-H4K8la_peak_1454	4.53273	
12	5.4E+07	5.4E+07	KD2-H4K8la_peak_1455	5.50657	HOXC5_ENSG00000172789;MIR615_ENSG00000207571
12	5.4E+07	5.4E+07	KD2-H4K8la_peak_1456	5.6499	HOXC4_ENSG00000273266
12	5.4E+07	5.4E+07	KD2-H4K8la_peak_1457	7.13237	
12	5.6E+07	5.6E+07	KD2-H4K8la_peak_1458	8.44557	DNAJC14_ENSG00000135392;TMEM198B_ENSG00000182796
12	5.6E+07	5.6E+07	KD2-H4K8la_peak_1459	7.81267	
12	5.6E+07	5.6E+07	KD2-H4K8la_peak_1460	8.07102	
12	5.7E+07	5.7E+07	KD2-H4K8la_peak_1461	4.90979	RNF41_ENSG00000181852;NABP2_ENSG00000139579
12	5.7E+07	5.7E+07	KD2-H4K8la_peak_1462	5.5899	RNF41_ENSG00000181852;NABP2_ENSG00000139579
12	5.7E+07	5.7E+07	KD2-H4K8la_peak_1463	4.7496	PAN2_ENSG00000135473
12	5.7E+07	5.7E+07	KD2-H4K8la_peak_1464	10.27876	
12	5.7E+07	5.7E+07	KD2-H4K8la_peak_1465	9.27765	PRIM1_ENSG00000198056;HSD17B6_ENSG00000025423
12	5.7E+07	5.7E+07	KD2-H4K8la_peak_1466	8.12466	TMEM194A_ENSG00000166881;NAB2_ENSG00000166886
12	5.8E+07	5.8E+07	KD2-H4K8la_peak_1467	5.43278	
12	5.8E+07	5.8E+07	KD2-H4K8la_peak_1468	10.46101	
12	5.8E+07	5.8E+07	KD2-H4K8la_peak_1469	8.62669	
12	5.8E+07	5.8E+07	KD2-H4K8la_peak_1470	7.49467	KIF5A_ENSG00000155980

12	5.8E+07	5.8E+07	KD2-H4K8la_peak_1471	9.6708	DTX3_ENSG00000178498
12	5.8E+07	5.8E+07	KD2-H4K8la_peak_1472	17.60548	ARHGEF25_ENSG00000240771
12	5.8E+07	5.8E+07	KD2-H4K8la_peak_1473	4.75945	
12	5.8E+07	5.8E+07	KD2-H4K8la_peak_1474	4.57647	
12	5.8E+07	5.8E+07	KD2-H4K8la_peak_1475	4.86896	
12	5.8E+07	5.8E+07	KD2-H4K8la_peak_1476	12.12477	RP11-620J15.2_ENSG00000245651
12	6.4E+07	6.4E+07	KD2-H4K8la_peak_1477	9.61894	DPY19L2_ENSG00000177990
12	6.4E+07	6.4E+07	KD2-H4K8la_peak_1478	4.53273	
12	6.4E+07	6.4E+07	KD2-H4K8la_peak_1479	9.73848	
12	6.8E+07	6.8E+07	KD2-H4K8la_peak_1480	6.11881	CAND1_ENSG00000111530
12	6.9E+07	6.9E+07	KD2-H4K8la_peak_1481	5.83285	
12	6.9E+07	6.9E+07	KD2-H4K8la_peak_1482	7.13528	MDM2_ENSG00000135679
12	7.1E+07	7.1E+07	KD2-H4K8la_peak_1483	9.05604	RP11-611E13.2_ENSG00000257815;CNOT2_ENSG00000111596
12	7.2E+07	7.2E+07	KD2-H4K8la_peak_1484	5.7631	
12	7.7E+07	7.7E+07	KD2-H4K8la_peak_1485	10.28981	BBS10_ENSG00000179941
12	8.6E+07	8.6E+07	KD2-H4K8la_peak_1486	8.77936	RP11-408B11.2_ENSG00000258815
12	8.9E+07	8.9E+07	KD2-H4K8la_peak_1487	6.13528	
12	9E+07	9E+07	KD2-H4K8la_peak_1488	4.36246	DUSP6_ENSG00000139318
12	9.2E+07	9.2E+07	KD2-H4K8la_peak_1489	5.36015	
12	9.3E+07	9.3E+07	KD2-H4K8la_peak_1490	6.64804	
12	9.4E+07	9.4E+07	KD2-H4K8la_peak_1491	6.3399	UBE2N_ENSG00000177889
12	9.4E+07	9.4E+07	KD2-H4K8la_peak_1492	8.37064	SOCS2-AS1_ENSG00000246985
12	9.4E+07	9.4E+07	KD2-H4K8la_peak_1493	6.88801	CRADD_ENSG00000169372
12	9.4E+07	9.4E+07	KD2-H4K8la_peak_1494	11.06859	
12	9.5E+07	9.5E+07	KD2-H4K8la_peak_1495	6.89612	
12	9.5E+07	9.5E+07	KD2-H4K8la_peak_1496	6.57319	CCDC41_ENSG00000173588
12	9.6E+07	9.6E+07	KD2-H4K8la_peak_1497	7.22716	
12	9.6E+07	9.6E+07	KD2-H4K8la_peak_1498	13.04837	CCDC38_ENSG00000165972;AMDHD1_ENSG00000139344
12	9.7E+07	9.7E+07	KD2-H4K8la_peak_1499	7.13237	NEDD1_ENSG00000139350
12	1E+08	1E+08	KD2-H4K8la_peak_1500	8.86558	DRAM1_ENSG00000136048
12	1E+08	1E+08	KD2-H4K8la_peak_1501	5.83285	RP11-642P15.1_ENSG00000214198;HSP90B1_ENSG00000166598;MIR3652_ENSG00000265072
12	1E+08	1E+08	KD2-H4K8la_peak_1502	19.04072	NFYB_ENSG00000120837
12	1.1E+08	1.1E+08	KD2-H4K8la_peak_1503	8.12466	SLC41A2_ENSG00000136052
12	1.1E+08	1.1E+08	KD2-H4K8la_peak_1504	12.31762	NUAK1_ENSG00000074590
12	1.1E+08	1.1E+08	KD2-H4K8la_peak_1505	5.33301	SNORD74_ENSG00000200897
12	1.1E+08	1.1E+08	KD2-H4K8la_peak_1506	6.61963	
12	1.1E+08	1.1E+08	KD2-H4K8la_peak_1507	5.83285	
12	1.1E+08	1.1E+08	KD2-H4K8la_peak_1508	8.07102	
12	1.1E+08	1.1E+08	KD2-H4K8la_peak_1509	10.00612	SSH1_ENSG00000084112;DAO_ENSG00000110887
12	1.1E+08	1.1E+08	KD2-H4K8la_peak_1510	6.13528	
12	1.1E+08	1.1E+08	KD2-H4K8la_peak_1511	10.45647	
12	1.1E+08	1.1E+08	KD2-H4K8la_peak_1512	10.00175	FOXN4_ENSG00000139445
12	1.1E+08	1.1E+08	KD2-H4K8la_peak_1513	5.05146	
12	1.1E+08	1.1E+08	KD2-H4K8la_peak_1514	7.12497	
12	1.1E+08	1.1E+08	KD2-H4K8la_peak_1515	4.66473	
12	1.1E+08	1.1E+08	KD2-H4K8la_peak_1516	6.2278	

12	1.1E+08	1.1E+08	KD2-H4K8la_peak_1517	8.43222	
12	1.1E+08	1.1E+08	KD2-H4K8la_peak_1518	7.49467	
12	1.1E+08	1.1E+08	KD2-H4K8la_peak_1519	9.73848	TRPV4_ENSG00000111199;MIR4497_ENSG00000263510
12	1.1E+08	1.1E+08	KD2-H4K8la_peak_1520	5.36015	GIT2_ENSG00000139436
12	1.1E+08	1.1E+08	KD2-H4K8la_peak_1521	7.16004	GIT2_ENSG00000139436
12	1.1E+08	1.1E+08	KD2-H4K8la_peak_1522	6.89612	C12orf76_ENSG00000174456
12	1.1E+08	1.1E+08	KD2-H4K8la_peak_1523	5.58883	
12	1.1E+08	1.1E+08	KD2-H4K8la_peak_1524	10.11868	
12	1.1E+08	1.1E+08	KD2-H4K8la_peak_1525	13.85872	
12	1.1E+08	1.1E+08	KD2-H4K8la_peak_1526	8.7731	
12	1.1E+08	1.1E+08	KD2-H4K8la_peak_1527	5.58883	
12	1.1E+08	1.1E+08	KD2-H4K8la_peak_1528	4.94337	
12	1.1E+08	1.1E+08	KD2-H4K8la_peak_1529	7.68279	
12	1.1E+08	1.1E+08	KD2-H4K8la_peak_1530	11.77509	MAPKAPK5-AS1_ENSG00000234608;AC003029.1_ENSG00000248594;MAPKAPK5_ENSG00000089022
12	1.1E+08	1.1E+08	KD2-H4K8la_peak_1531	6.57319	NAA25_ENSG00000111300
12	1.1E+08	1.1E+08	KD2-H4K8la_peak_1532	6.2278	NAA25_ENSG00000111300
12	1.1E+08	1.1E+08	KD2-H4K8la_peak_1533	6.2278	
12	1.1E+08	1.1E+08	KD2-H4K8la_peak_1534	5.43278	
12	1.1E+08	1.1E+08	KD2-H4K8la_peak_1535	7.96951	
12	1.1E+08	1.1E+08	KD2-H4K8la_peak_1536	8.07102	
12	1.2E+08	1.2E+08	KD2-H4K8la_peak_1537	4.80784	
12	1.2E+08	1.2E+08	KD2-H4K8la_peak_1538	7.60756	
12	1.2E+08	1.2E+08	KD2-H4K8la_peak_1539	5.72892	
12	1.2E+08	1.2E+08	KD2-H4K8la_peak_1540	7.04184	
12	1.2E+08	1.2E+08	KD2-H4K8la_peak_1541	9.49266	TESC_ENSG00000088992;RP11-103B5.2_ENSG00000258285
12	1.2E+08	1.2E+08	KD2-H4K8la_peak_1542	6.86458	
12	1.2E+08	1.2E+08	KD2-H4K8la_peak_1543	6.28277	VSIG10_ENSG00000176834;PEBP1_ENSG00000089220
12	1.2E+08	1.2E+08	KD2-H4K8la_peak_1544	4.30663	RP11-131L12.2_ENSG00000270482;SUDS3_ENSG00000111707
12	1.2E+08	1.2E+08	KD2-H4K8la_peak_1545	8.39919	RP11-768F21.1_ENSG00000248636;PRKAB1_ENSG00000111725
12	1.2E+08	1.2E+08	KD2-H4K8la_peak_1546	7.19298	
12	1.2E+08	1.2E+08	KD2-H4K8la_peak_1547	10.00612	GCN1L1_ENSG00000089154
12	1.2E+08	1.2E+08	KD2-H4K8la_peak_1548	7.72421	
12	1.2E+08	1.2E+08	KD2-H4K8la_peak_1549	9.55813	
12	1.2E+08	1.2E+08	KD2-H4K8la_peak_1550	5.89088	MSI1_ENSG00000135097;RPS27P25_ENSG00000239881
12	1.2E+08	1.2E+08	KD2-H4K8la_peak_1551	7.3993	MSI1_ENSG00000135097;RPS27P25_ENSG00000239881
12	1.2E+08	1.2E+08	KD2-H4K8la_peak_1552	9.35473	TRIAP1_ENSG00000170855;GATC_ENSG00000257218
12	1.2E+08	1.2E+08	KD2-H4K8la_peak_1553	7.61975	SRSF9_ENSG00000111786;DYNLL1_ENSG00000088986
12	1.2E+08	1.2E+08	KD2-H4K8la_peak_1554	11.5908	COQ5_ENSG00000110871;RNF10_ENSG00000022840
12	1.2E+08	1.2E+08	KD2-H4K8la_peak_1555	7.13237	P2RX4_ENSG00000135124
12	1.2E+08	1.2E+08	KD2-H4K8la_peak_1556	6.51326	
12	1.2E+08	1.2E+08	KD2-H4K8la_peak_1557	8.89875	MORN3_ENSG00000139714
12	1.2E+08	1.2E+08	KD2-H4K8la_peak_1558	12.97817	
12	1.2E+08	1.2E+08	KD2-H4K8la_peak_1559	7.90495	SETD1B_ENSG00000139718;RHOF_ENSG00000139725;AC084018.1_ENSG00000212694
12	1.2E+08	1.2E+08	KD2-H4K8la_peak_1560	7.06427	SETD1B_ENSG00000139718;RHOF_ENSG00000139725;AC084018.1_ENSG00000212694
12	1.2E+08	1.2E+08	KD2-H4K8la_peak_1561	12.1221	
12	1.2E+08	1.2E+08	KD2-H4K8la_peak_1562	10.20357	OGFOD2_ENSG00000111325;RP11-197N18.2_ENSG00000256028

12	1.2E+08	1.2E+08	KD2-H4K8la_peak_1563	7.30722	ARL6IP4_ENSG00000182196
12	1.2E+08	1.2E+08	KD2-H4K8la_peak_1564	6.68593	
12	1.2E+08	1.2E+08	KD2-H4K8la_peak_1565	6.47589	
12	1.2E+08	1.2E+08	KD2-H4K8la_peak_1566	8.39919	CDK2AP1_ENSG00000111328
12	1.2E+08	1.2E+08	KD2-H4K8la_peak_1567	9.27415	
12	1.2E+08	1.2E+08	KD2-H4K8la_peak_1568	7.81267	RILPL1_ENSG00000188026
12	1.2E+08	1.2E+08	KD2-H4K8la_peak_1569	9.35473	DDX55_ENSG00000111364
12	1.2E+08	1.2E+08	KD2-H4K8la_peak_1570	7.30722	CCDC92_ENSG00000119242;FAM101A_ENSG00000178882
12	1.2E+08	1.2E+08	KD2-H4K8la_peak_1571	5.33721	
12	1.2E+08	1.2E+08	KD2-H4K8la_peak_1572	6.97066	
12	1.2E+08	1.2E+08	KD2-H4K8la_peak_1573	12.55003	
12	1.3E+08	1.3E+08	KD2-H4K8la_peak_1574	15.82244	NCOR2_ENSG00000196498
12	1.3E+08	1.3E+08	KD2-H4K8la_peak_1575	7.07162	
12	1.3E+08	1.3E+08	KD2-H4K8la_peak_1576	7.92775	
12	1.3E+08	1.3E+08	KD2-H4K8la_peak_1577	11.17433	
12	1.3E+08	1.3E+08	KD2-H4K8la_peak_1578	7.49467	
12	1.3E+08	1.3E+08	KD2-H4K8la_peak_1579	6.11881	SLC15A4_ENSG00000139370
12	1.3E+08	1.3E+08	KD2-H4K8la_peak_1580	10.03312	
12	1.3E+08	1.3E+08	KD2-H4K8la_peak_1581	6.3399	
12	1.3E+08	1.3E+08	KD2-H4K8la_peak_1582	9.70343	
12	1.3E+08	1.3E+08	KD2-H4K8la_peak_1583	9.9701	
12	1.3E+08	1.3E+08	KD2-H4K8la_peak_1584	9.81837	
12	1.3E+08	1.3E+08	KD2-H4K8la_peak_1585	5.27164	RAN_ENSG00000132341
12	1.3E+08	1.3E+08	KD2-H4K8la_peak_1586	7.12014	RP13-507P19.1_ENSG00000256484
12	1.3E+08	1.3E+08	KD2-H4K8la_peak_1587	13.2369	SFSWAP_ENSG00000061936
12	1.3E+08	1.3E+08	KD2-H4K8la_peak_1588	10.8046	
12	1.3E+08	1.3E+08	KD2-H4K8la_peak_1589	6.57426	
12	1.3E+08	1.3E+08	KD2-H4K8la_peak_1590	10.20357	
12	1.3E+08	1.3E+08	KD2-H4K8la_peak_1591	6.2278	POLE_ENSG00000177084;PXMP2_ENSG00000176894;RP13-672B3.2_ENSG00000256632
12	1.3E+08	1.3E+08	KD2-H4K8la_peak_1592	6.42514	PGAM5_ENSG00000247077
12	1.3E+08	1.3E+08	KD2-H4K8la_peak_1593	8.24908	RP11-46H11.12_ENSG00000236617
13	2.1E+07	2.1E+07	KD2-H4K8la_peak_1594	10.49982	ZMYM2_ENSG00000121741
13	2.1E+07	2.1E+07	KD2-H4K8la_peak_1595	7.61975	XPO4_ENSG00000132953
13	2.2E+07	2.2E+07	KD2-H4K8la_peak_1596	6.70626	MIPEPP3_ENSG00000233325
13	2.2E+07	2.2E+07	KD2-H4K8la_peak_1597	8.4393	ZDHHC20_ENSG00000180776
13	2.6E+07	2.6E+07	KD2-H4K8la_peak_1598	8.13557	RP11-271M24.2_ENSG00000260509;NUPL1_ENSG00000139496
13	2.8E+07	2.8E+07	KD2-H4K8la_peak_1599	15.01131	MTIF3_ENSG00000122033
13	3E+07	3E+07	KD2-H4K8la_peak_1600	8.44557	SLC7A1_ENSG00000139514
13	3.1E+07	3.1E+07	KD2-H4K8la_peak_1601	7.13237	KATNAL1_ENSG00000102781
13	3.1E+07	3.1E+07	KD2-H4K8la_peak_1602	5.48079	
13	3.1E+07	3.1E+07	KD2-H4K8la_peak_1603	9.0413	
13	3.2E+07	3.2E+07	KD2-H4K8la_peak_1604	7.23116	
13	3.3E+07	3.3E+07	KD2-H4K8la_peak_1605	5.60379	
13	3.4E+07	3.4E+07	KD2-H4K8la_peak_1606	9.0413	KL_ENSG00000133116
13	3.6E+07	3.6E+07	KD2-H4K8la_peak_1607	13.91009	
13	3.7E+07	3.7E+07	KD2-H4K8la_peak_1608	5.58883	SPG20OS_ENSG00000120664

13	3.7E+07	3.7E+07	KD2-H4K8la_peak_1609	5.14476	SMAD9_ENSG00000120693
13	4.1E+07	4.1E+07	KD2-H4K8la_peak_1610	6.70626	MRPS31_ENSG00000102738
13	4.2E+07	4.2E+07	KD2-H4K8la_peak_1611	9.0516	KBTBD7_ENSG00000120696
13	4.3E+07	4.3E+07	KD2-H4K8la_peak_1612	7.23116	VWA8_ENSG00000102763
13	4.4E+07	4.4E+07	KD2-H4K8la_peak_1613	6.70626	
13	4.4E+07	4.4E+07	KD2-H4K8la_peak_1614	7.12014	CCDC122_ENSG00000151773;LACC1_ENSG00000179630
13	4.5E+07	4.5E+07	KD2-H4K8la_peak_1615	10.20357	SERP2_ENSG00000151778
13	4.5E+07	4.5E+07	KD2-H4K8la_peak_1616	5.66717	
13	4.9E+07	4.9E+07	KD2-H4K8la_peak_1617	6.61963	SUCLA2-AS1_ENSG00000227848
13	4.9E+07	4.9E+07	KD2-H4K8la_peak_1618	7.72421	MED4_ENSG00000136146
13	4.9E+07	4.9E+07	KD2-H4K8la_peak_1619	5.43278	RCBTB2_ENSG00000136161
13	5E+07	5E+07	KD2-H4K8la_peak_1620	5.67596	FNDC3A_ENSG00000102531
13	5.1E+07	5.1E+07	KD2-H4K8la_peak_1621	6.88507	DLEU2_ENSG00000231607
13	5.1E+07	5.1E+07	KD2-H4K8la_peak_1622	6.40658	RNASEH2B-AS1_ENSG00000233672;RNASEH2B_ENSG00000136104
13	5.2E+07	5.2E+07	KD2-H4K8la_peak_1623	6.64804	INTS6-AS1_ENSG00000236778
13	5.2E+07	5.2E+07	KD2-H4K8la_peak_1624	4.45136	
13	5.2E+07	5.2E+07	KD2-H4K8la_peak_1625	7.81267	
13	5.2E+07	5.2E+07	KD2-H4K8la_peak_1626	12.11081	
13	5.2E+07	5.2E+07	KD2-H4K8la_peak_1627	7.34033	
13	5.3E+07	5.3E+07	KD2-H4K8la_peak_1628	4.75945	MRPS31P5_ENSG00000243406
13	5.3E+07	5.3E+07	KD2-H4K8la_peak_1629	13.13367	VPS36_ENSG00000136100
13	7.2E+07	7.2E+07	KD2-H4K8la_peak_1630	6.57426	DACH1_ENSG00000165659
13	7.5E+07	7.5E+07	KD2-H4K8la_peak_1631	8.37064	KLF12_ENSG00000118922
13	7.6E+07	7.6E+07	KD2-H4K8la_peak_1632	9.20163	RP11-173B14.5_ENSG00000261105
13	7.7E+07	7.7E+07	KD2-H4K8la_peak_1633	6.28277	KCTD12_ENSG00000178695;AC000403.1_ENSG00000264908
13	7.8E+07	7.8E+07	KD2-H4K8la_peak_1634	10.98662	CLN5_ENSG00000102805
13	7.8E+07	7.8E+07	KD2-H4K8la_peak_1635	8.43222	
13	7.9E+07	7.9E+07	KD2-H4K8la_peak_1636	7.23116	RNF219_ENSG00000152193
13	8.1E+07	8.1E+07	KD2-H4K8la_peak_1637	5.36015	
13	8.1E+07	8.1E+07	KD2-H4K8la_peak_1638	4.52633	
13	8.1E+07	8.1E+07	KD2-H4K8la_peak_1639	6.64804	SPRY2_ENSG00000136158
13	8.1E+07	8.1E+07	KD2-H4K8la_peak_1640	11.15747	SPRY2_ENSG00000136158
13	8.1E+07	8.1E+07	KD2-H4K8la_peak_1641	5.16795	
13	8.6E+07	8.6E+07	KD2-H4K8la_peak_1642	10.34978	
13	9.2E+07	9.2E+07	KD2-H4K8la_peak_1643	12.14099	MIR17HG_ENSG00000215417
13	9.5E+07	9.5E+07	KD2-H4K8la_peak_1644	9.27765	
13	9.5E+07	9.5E+07	KD2-H4K8la_peak_1645	6.3399	TGDS_ENSG00000088451
13	9.9E+07	9.9E+07	KD2-H4K8la_peak_1646	4.72651	STK24_ENSG00000102572;STK24-AS1_ENSG00000224418
13	9.9E+07	9.9E+07	KD2-H4K8la_peak_1647	4.86896	
13	1E+08	1E+08	KD2-H4K8la_peak_1648	8.73956	UBAC2-AS1_ENSG00000228889;UBAC2_ENSG00000134882
13	1E+08	1E+08	KD2-H4K8la_peak_1649	7.61975	UBAC2-AS1_ENSG00000228889;UBAC2_ENSG00000134882
13	1E+08	1E+08	KD2-H4K8la_peak_1650	8.13557	GGACT_ENSG00000134864
13	1E+08	1E+08	KD2-H4K8la_peak_1651	10.62798	FGF14-IT1_ENSG00000243319;FGF14-AS2_ENSG00000272143
13	1E+08	1E+08	KD2-H4K8la_peak_1652	9.86881	ERCC5_ENSG00000134899
13	1.1E+08	1.1E+08	KD2-H4K8la_peak_1653	5.72892	
13	1.1E+08	1.1E+08	KD2-H4K8la_peak_1654	12.14099	

13	1.1E+08	1.1E+08	KD2-H4K8la_peak_1655	7.65547	
13	1.1E+08	1.1E+08	KD2-H4K8la_peak_1656	5.83285	
13	1.1E+08	1.1E+08	KD2-H4K8la_peak_1657	6.70626	
13	1.1E+08	1.1E+08	KD2-H4K8la_peak_1658	8.07102	
13	1.1E+08	1.1E+08	KD2-H4K8la_peak_1659	8.73956	
13	1.1E+08	1.1E+08	KD2-H4K8la_peak_1660	6.64804	
13	1.1E+08	1.1E+08	KD2-H4K8la_peak_1661	8.6199	
13	1.1E+08	1.1E+08	KD2-H4K8la_peak_1662	6.3399	IRS2_ENSG00000185950
13	1.1E+08	1.1E+08	KD2-H4K8la_peak_1663	8.77936	
13	1.1E+08	1.1E+08	KD2-H4K8la_peak_1664	10.03265	COL4A1_ENSG00000187498;COL4A2_ENSG00000134871
13	1.1E+08	1.1E+08	KD2-H4K8la_peak_1665	8.66164	CARKD_ENSG00000213995
13	1.1E+08	1.1E+08	KD2-H4K8la_peak_1666	20.66643	CARS2_ENSG00000134905;ING1_ENSG00000153487
13	1.1E+08	1.1E+08	KD2-H4K8la_peak_1667	5.43278	ANKRD10_ENSG00000088448
13	1.1E+08	1.1E+08	KD2-H4K8la_peak_1668	8.07102	
13	1.1E+08	1.1E+08	KD2-H4K8la_peak_1669	6.61495	TUBGCP3_ENSG00000126216
13	1.1E+08	1.1E+08	KD2-H4K8la_peak_1670	5.28047	TUBGCP3_ENSG00000126216
13	1.1E+08	1.1E+08	KD2-H4K8la_peak_1671	9.27415	MCF2L_ENSG00000126217
13	1.1E+08	1.1E+08	KD2-H4K8la_peak_1672	4.3268	
13	1.1E+08	1.1E+08	KD2-H4K8la_peak_1673	16.7435	
13	1.1E+08	1.1E+08	KD2-H4K8la_peak_1674	7.4908	
13	1.1E+08	1.1E+08	KD2-H4K8la_peak_1675	10.28981	PCID2_ENSG00000126226;CUL4A_ENSG00000139842
13	1.1E+08	1.1E+08	KD2-H4K8la_peak_1676	12.85404	LAMP1_ENSG00000185896
13	1.1E+08	1.1E+08	KD2-H4K8la_peak_1677	8.37064	GRTP1_ENSG00000139835
13	1.1E+08	1.1E+08	KD2-H4K8la_peak_1678	6.64804	
13	1.1E+08	1.1E+08	KD2-H4K8la_peak_1679	5.36015	
13	1.1E+08	1.1E+08	KD2-H4K8la_peak_1680	9.05604	
13	1.1E+08	1.1E+08	KD2-H4K8la_peak_1681	6.76424	
13	1.1E+08	1.1E+08	KD2-H4K8la_peak_1682	7.33892	
13	1.1E+08	1.1E+08	KD2-H4K8la_peak_1683	4.78385	
14	2E+07	2E+07	KD2-H4K8la_peak_1684	4.44972	
14	2.1E+07	2.1E+07	KD2-H4K8la_peak_1685	4.79631	TMEM55B_ENSG00000165782
14	2.1E+07	2.1E+07	KD2-H4K8la_peak_1686	7.13237	
14	2.1E+07	2.1E+07	KD2-H4K8la_peak_1687	10.04111	TPPP2_ENSG00000179636;AL161668.5_ENSG00000258604
14	2.2E+07	2.2E+07	KD2-H4K8la_peak_1688	4.30663	ARHGEF40_ENSG00000165801
14	2.2E+07	2.2E+07	KD2-H4K8la_peak_1689	7.72421	
14	2.2E+07	2.2E+07	KD2-H4K8la_peak_1690	8.07102	HNRNPC_ENSG00000092199
14	2.3E+07	2.3E+07	KD2-H4K8la_peak_1691	5.30505	MRPL52_ENSG00000172590;SLC7A7_ENSG00000155465
14	2.3E+07	2.3E+07	KD2-H4K8la_peak_1692	7.11805	
14	2.3E+07	2.3E+07	KD2-H4K8la_peak_1693	7.92775	HAUS4_ENSG00000092036;MIR4707_ENSG00000265037
14	2.4E+07	2.4E+07	KD2-H4K8la_peak_1694	7.61975	PABPN1_ENSG00000100836
14	2.4E+07	2.4E+07	KD2-H4K8la_peak_1695	6.03112	EFS_ENSG00000100842
14	2.4E+07	2.4E+07	KD2-H4K8la_peak_1696	5.67034	RP11-66N24.4_ENSG00000157306
14	2.4E+07	2.4E+07	KD2-H4K8la_peak_1697	11.97807	
14	2.4E+07	2.4E+07	KD2-H4K8la_peak_1698	13.3197	DHRS4L2_ENSG00000187630
14	2.4E+07	2.4E+07	KD2-H4K8la_peak_1699	8.43222	
14	2.5E+07	2.5E+07	KD2-H4K8la_peak_1700	8.16787	

14	2.5E+07	2.5E+07	KD2-H4K8la_peak_1701	10.62798	TM9SF1_ENSG00000100926;TM9SF1_ENSG00000254692;CHMP4A_ENSG00000254505
14	2.5E+07	2.5E+07	KD2-H4K8la_peak_1702	14.64566	
14	2.5E+07	2.5E+07	KD2-H4K8la_peak_1703	7.59044	
14	3.1E+07	3.1E+07	KD2-H4K8la_peak_1704	6.86458	COCH_ENSG00000100473
14	3.1E+07	3.1E+07	KD2-H4K8la_peak_1705	10.99007	STRN3_ENSG00000196792;AP4S1_ENSG00000100478
14	3.2E+07	3.2E+07	KD2-H4K8la_peak_1706	8.12466	HECTD1_ENSG00000092148
14	3.5E+07	3.5E+07	KD2-H4K8la_peak_1707	8.07102	
14	3.5E+07	3.5E+07	KD2-H4K8la_peak_1708	7.49467	EAPP_ENSG00000129518
14	3.6E+07	3.6E+07	KD2-H4K8la_peak_1709	8.37064	INSM2_ENSG00000168348
14	5E+07	5E+07	KD2-H4K8la_peak_1710	6.19992	RPS29_ENSG00000213741;AL139099.1_ENSG00000253459;LRR1_ENSG00000165501
14	5E+07	5E+07	KD2-H4K8la_peak_1711	6.64804	KLHDC1_ENSG00000197776
14	5E+07	5E+07	KD2-H4K8la_peak_1712	11.15182	KLHDC2_ENSG00000165516
14	5E+07	5E+07	KD2-H4K8la_peak_1713	4.75945	
14	5.1E+07	5.1E+07	KD2-H4K8la_peak_1714	7.13237	
14	5.1E+07	5.1E+07	KD2-H4K8la_peak_1715	5.83285	RP11-247L20.3_ENSG00000258857
14	5.1E+07	5.1E+07	KD2-H4K8la_peak_1716	9.38376	MAP4K5_ENSG00000012983
14	5.1E+07	5.1E+07	KD2-H4K8la_peak_1717	10.00612	NIN_ENSG00000100503
14	5.2E+07	5.2E+07	KD2-H4K8la_peak_1718	4.30663	C14orf166_ENSG00000087302
14	5.3E+07	5.3E+07	KD2-H4K8la_peak_1719	7.72421	
14	5.3E+07	5.3E+07	KD2-H4K8la_peak_1720	8.12466	PSMC6_ENSG00000100519
14	5.5E+07	5.5E+07	KD2-H4K8la_peak_1721	6.3399	GMFB_ENSG00000197045
14	5.5E+07	5.5E+07	KD2-H4K8la_peak_1722	5.16597	
14	5.5E+07	5.5E+07	KD2-H4K8la_peak_1723	11.24937	
14	5.5E+07	5.5E+07	KD2-H4K8la_peak_1724	12.25633	
14	5.5E+07	5.5E+07	KD2-H4K8la_peak_1725	6.13507	MIR4308_ENSG00000265432
14	5.6E+07	5.6E+07	KD2-H4K8la_peak_1726	5.84082	
14	5.6E+07	5.6E+07	KD2-H4K8la_peak_1727	5.05146	ATG14_ENSG00000126775
14	5.8E+07	5.8E+07	KD2-H4K8la_peak_1728	8.07102	EXOC5_ENSG00000070367;AP5M1_ENSG00000053770
14	5.9E+07	5.9E+07	KD2-H4K8la_peak_1729	9.9701	RP11-517O13.3_ENSG00000258658;TOMM20L_ENSG00000196860
14	6.1E+07	6.1E+07	KD2-H4K8la_peak_1730	7.72421	
14	6.1E+07	6.1E+07	KD2-H4K8la_peak_1731	9.72904	SIX1_ENSG00000126778
14	6.2E+07	6.2E+07	KD2-H4K8la_peak_1732	4.86896	
14	6.2E+07	6.2E+07	KD2-H4K8la_peak_1733	5.43278	
14	6.2E+07	6.2E+07	KD2-H4K8la_peak_1734	8.07102	
14	6.2E+07	6.2E+07	KD2-H4K8la_peak_1735	7.22716	
14	6.2E+07	6.2E+07	KD2-H4K8la_peak_1736	7.92775	
14	6.2E+07	6.2E+07	KD2-H4K8la_peak_1737	6.3399	CTD-2277K2.1_ENSG00000258882
14	6.4E+07	6.4E+07	KD2-H4K8la_peak_1738	12.62032	
14	6.4E+07	6.4E+07	KD2-H4K8la_peak_1739	7.19298	PPP2R5E_ENSG00000154001;CTD-2302E22.4_ENSG00000261242
14	6.4E+07	6.4E+07	KD2-H4K8la_peak_1740	5.83285	SYNE2_ENSG00000054654
14	6.5E+07	6.5E+07	KD2-H4K8la_peak_1741	5.99671	ZBTB1_ENSG00000126804
14	6.5E+07	6.5E+07	KD2-H4K8la_peak_1742	12.32641	ZBTB25_ENSG00000089775;ZBTB1_ENSG00000126804
14	6.5E+07	6.5E+07	KD2-H4K8la_peak_1743	7.49467	
14	6.5E+07	6.5E+07	KD2-H4K8la_peak_1744	8.13727	SPTB_ENSG00000070182
14	6.5E+07	6.5E+07	KD2-H4K8la_peak_1745	5.8369	RAB15_ENSG00000139998
14	6.8E+07	6.8E+07	KD2-H4K8la_peak_1746	5.05146	ATP6V1D_ENSG00000100554;EIF2S1_ENSG00000134001

14	6.8E+07	6.8E+07	KD2-H4K8la_peak_1747	9.78176	PIGH_ENSG00000100564
14	6.8E+07	6.8E+07	KD2-H4K8la_peak_1748	7.05576	RAD51B_ENSG00000182185
14	6.9E+07	6.9E+07	KD2-H4K8la_peak_1749	5.49688	ZFP36L1_ENSG00000185650
14	6.9E+07	6.9E+07	KD2-H4K8la_peak_1750	12.1221	
14	6.9E+07	6.9E+07	KD2-H4K8la_peak_1751	6.86458	
14	7E+07	7E+07	KD2-H4K8la_peak_1752	7.49467	SRSF5_ENSG00000100650
14	7.1E+07	7.1E+07	KD2-H4K8la_peak_1753	8.23729	COX16_ENSG00000133983;RNU2-51P_ENSG00000222640
14	7.1E+07	7.1E+07	KD2-H4K8la_peak_1754	6.3399	CTD-2540L5.5_ENSG00000259115;CTD-2540L5.6_ENSG00000245466;TTC9_ENSG00000133985
14	7.2E+07	7.2E+07	KD2-H4K8la_peak_1755	8.77936	
14	7.4E+07	7.4E+07	KD2-H4K8la_peak_1756	8.07102	RBM25_ENSG00000119707
14	7.4E+07	7.4E+07	KD2-H4K8la_peak_1757	7.13237	AC005280.1_ENSG00000170468;C14orf169_ENSG00000255242
14	7.4E+07	7.4E+07	KD2-H4K8la_peak_1758	7.13528	ACOT4_ENSG00000177465
14	7.4E+07	7.4E+07	KD2-H4K8la_peak_1759	11.77509	PTGR2_ENSG00000140043;RP5-1021I20.4_ENSG00000258653
14	7.4E+07	7.4E+07	KD2-H4K8la_peak_1760	5.36015	ZNF410_ENSG00000119725
14	7.4E+07	7.4E+07	KD2-H4K8la_peak_1761	9.65288	ENTPD5_ENSG00000187097;CCDC176_ENSG00000119636
14	7.5E+07	7.5E+07	KD2-H4K8la_peak_1762	8.37064	ABCD4_ENSG00000119688;VRTN_ENSG00000133980
14	7.5E+07	7.5E+07	KD2-H4K8la_peak_1763	11.5908	AREL1_ENSG00000119682;AC007956.1_ENSG00000214670;SNORA7_ENSG00000222604;FCF1_ENSG00000119616
14	7.5E+07	7.5E+07	KD2-H4K8la_peak_1764	10.28981	DLST_ENSG00000119689
14	7.5E+07	7.5E+07	KD2-H4K8la_peak_1765	7.20846	
14	7.6E+07	7.6E+07	KD2-H4K8la_peak_1766	8.59561	ACYP1_ENSG00000119640
14	7.6E+07	7.6E+07	KD2-H4K8la_peak_1767	9.27765	NEK9_ENSG00000119638;RP11-950C14.7_ENSG00000259138
14	7.6E+07	7.6E+07	KD2-H4K8la_peak_1768	6.86458	NEK9_ENSG00000119638;RP11-950C14.7_ENSG00000259138
14	7.7E+07	7.7E+07	KD2-H4K8la_peak_1769	5.83285	
14	7.7E+07	7.7E+07	KD2-H4K8la_peak_1770	6.64804	RP11-99E15.2_ENSG00000258569;VASH1_ENSG00000071246
14	7.7E+07	7.7E+07	KD2-H4K8la_peak_1771	4.30663	VASH1_ENSG00000071246
14	7.7E+07	7.7E+07	KD2-H4K8la_peak_1772	8.79494	
14	7.7E+07	7.7E+07	KD2-H4K8la_peak_1773	6.20793	
14	7.7E+07	7.7E+07	KD2-H4K8la_peak_1774	7.12014	
14	7.7E+07	7.7E+07	KD2-H4K8la_peak_1775	7.81267	
14	7.7E+07	7.7E+07	KD2-H4K8la_peak_1776	5.43278	
14	7.7E+07	7.7E+07	KD2-H4K8la_peak_1777	5.84082	
14	7.7E+07	7.7E+07	KD2-H4K8la_peak_1778	7.12014	RP11-7F17.7_ENSG00000258602
14	7.7E+07	7.7E+07	KD2-H4K8la_peak_1779	10.20357	IRF2BPL_ENSG00000119669
14	7.7E+07	7.7E+07	KD2-H4K8la_peak_1780	10.11868	
14	7.8E+07	7.8E+07	KD2-H4K8la_peak_1781	7.11895	
14	7.8E+07	7.8E+07	KD2-H4K8la_peak_1782	9.0516	
14	7.8E+07	7.8E+07	KD2-H4K8la_peak_1783	7.83588	
14	7.8E+07	7.8E+07	KD2-H4K8la_peak_1784	16.13355	POMT2_ENSG00000009830;GSTZ1_ENSG00000100577
14	7.8E+07	7.8E+07	KD2-H4K8la_peak_1785	8.12466	TMED8_ENSG00000100580;SAMD15_ENSG00000100583
14	7.8E+07	7.8E+07	KD2-H4K8la_peak_1786	7.49467	SNW1_ENSG00000100603;C14orf178_ENSG00000197734;AC008372.1_ENSG00000268208
14	7.8E+07	7.8E+07	KD2-H4K8la_peak_1787	6.3399	
14	8.8E+07	8.8E+07	KD2-H4K8la_peak_1788	9.0516	GALC_ENSG00000054983
14	8.9E+07	8.9E+07	KD2-H4K8la_peak_1789	11.06859	SPATA7_ENSG00000042317
14	8.9E+07	8.9E+07	KD2-H4K8la_peak_1790	5.43278	
14	8.9E+07	8.9E+07	KD2-H4K8la_peak_1791	5.58883	
14	9E+07	9E+07	KD2-H4K8la_peak_1792	4.86896	FOXN3-AS1_ENSG00000258920

14	9.1E+07	9.1E+07	KD2-H4K8la_peak_1793	8.76116	
14	9.1E+07	9.1E+07	KD2-H4K8la_peak_1794	9.78176	
14	9.1E+07	9.1E+07	KD2-H4K8la_peak_1795	14.23294	RP11-1078H9.1_ENSG00000258678;LINC00642_ENSG00000233208
14	9.1E+07	9.1E+07	KD2-H4K8la_peak_1796	5.99671	
14	9.1E+07	9.1E+07	KD2-H4K8la_peak_1797	8.76116	TTC7B_ENSG00000165914
14	9.2E+07	9.2E+07	KD2-H4K8la_peak_1798	17.34333	GPR68_ENSG00000119714
14	9.2E+07	9.2E+07	KD2-H4K8la_peak_1799	8.37064	
14	9.2E+07	9.2E+07	KD2-H4K8la_peak_1800	5.99671	SMEK1_ENSG00000100796
14	9.3E+07	9.3E+07	KD2-H4K8la_peak_1801	9.86881	NDUFB1_ENSG00000183648;CPSF2_ENSG00000165934
14	9.3E+07	9.3E+07	KD2-H4K8la_peak_1802	8.39919	
14	9.3E+07	9.3E+07	KD2-H4K8la_peak_1803	6.3399	
14	9.3E+07	9.3E+07	KD2-H4K8la_peak_1804	7.06427	
14	9.4E+07	9.4E+07	KD2-H4K8la_peak_1805	4.69635	ITPK1_ENSG00000100605
14	9.4E+07	9.4E+07	KD2-H4K8la_peak_1806	8.37064	
14	9.4E+07	9.4E+07	KD2-H4K8la_peak_1807	5.63442	
14	9.4E+07	9.4E+07	KD2-H4K8la_peak_1808	7.90495	
14	9.4E+07	9.4E+07	KD2-H4K8la_peak_1809	6.25288	PRIMA1_ENSG00000175785
14	9.4E+07	9.4E+07	KD2-H4K8la_peak_1810	6.20477	OTUB2_ENSG00000089723
14	9.5E+07	9.5E+07	KD2-H4K8la_peak_1811	8.34387	GSC_ENSG00000133937
14	9.5E+07	9.5E+07	KD2-H4K8la_peak_1812	11.15747	
14	9.6E+07	9.6E+07	KD2-H4K8la_peak_1813	7.8597	SNHG10_ENSG00000247092
14	1E+08	1E+08	KD2-H4K8la_peak_1814	9.37943	
14	1E+08	1E+08	KD2-H4K8la_peak_1815	6.3399	CCDC85C_ENSG00000205476;RP11-543C4.1_ENSG00000247970
14	1E+08	1E+08	KD2-H4K8la_peak_1816	9.37943	
14	1E+08	1E+08	KD2-H4K8la_peak_1817	11.06859	
14	1E+08	1E+08	KD2-H4K8la_peak_1818	4.72651	
14	1E+08	1E+08	KD2-H4K8la_peak_1819	14.33193	
14	1E+08	1E+08	KD2-H4K8la_peak_1820	12.06531	
14	1E+08	1E+08	KD2-H4K8la_peak_1821	8.39919	
14	1E+08	1E+08	KD2-H4K8la_peak_1822	8.13557	
14	1E+08	1E+08	KD2-H4K8la_peak_1823	11.06859	
14	1E+08	1E+08	KD2-H4K8la_peak_1824	6.03112	
14	1E+08	1E+08	KD2-H4K8la_peak_1825	9.5503	
14	1E+08	1E+08	KD2-H4K8la_peak_1826	8.30479	
14	1E+08	1E+08	KD2-H4K8la_peak_1827	6.86458	
14	1E+08	1E+08	KD2-H4K8la_peak_1828	9.37943	
14	1E+08	1E+08	KD2-H4K8la_peak_1829	9.02706	
14	1E+08	1E+08	KD2-H4K8la_peak_1830	8.59042	
14	1E+08	1E+08	KD2-H4K8la_peak_1831	6.25288	
14	1E+08	1E+08	KD2-H4K8la_peak_1832	6.61576	
14	1E+08	1E+08	KD2-H4K8la_peak_1833	13.04837	
14	1E+08	1E+08	KD2-H4K8la_peak_1834	7.86505	
14	1E+08	1E+08	KD2-H4K8la_peak_1835	12.11591	
14	1E+08	1E+08	KD2-H4K8la_peak_1836	10.4679	
14	1E+08	1E+08	KD2-H4K8la_peak_1837	7.01227	
14	1E+08	1E+08	KD2-H4K8la_peak_1838	9.6656	

14	1E+08	1E+08	KD2-H4K8la_peak_1839	6.2381	
14	1E+08	1E+08	KD2-H4K8la_peak_1840	7.92775	
14	1E+08	1E+08	KD2-H4K8la_peak_1841	7.19244	
14	1E+08	1E+08	KD2-H4K8la_peak_1842	6.47589	
14	1E+08	1E+08	KD2-H4K8la_peak_1843	6.11881	
14	1E+08	1E+08	KD2-H4K8la_peak_1844	5.67596	
14	1E+08	1E+08	KD2-H4K8la_peak_1845	6.61495	
14	1E+08	1E+08	KD2-H4K8la_peak_1846	12.14568	
14	1E+08	1E+08	KD2-H4K8la_peak_1847	19.16587	
14	1E+08	1E+08	KD2-H4K8la_peak_1848	7.92775	
14	1E+08	1E+08	KD2-H4K8la_peak_1849	8.66164	
14	1E+08	1E+08	KD2-H4K8la_peak_1850	13.37883	
14	1E+08	1E+08	KD2-H4K8la_peak_1851	13.47548	MEG3_ENSG00000214548
14	1E+08	1E+08	KD2-H4K8la_peak_1852	9.61894	MEG3_ENSG00000214548
14	1E+08	1E+08	KD2-H4K8la_peak_1853	11.4939	
14	1E+08	1E+08	KD2-H4K8la_peak_1854	6.85986	
14	1E+08	1E+08	KD2-H4K8la_peak_1855	26.07412	
14	1E+08	1E+08	KD2-H4K8la_peak_1856	7.92775	
14	1E+08	1E+08	KD2-H4K8la_peak_1857	5.83285	
14	1E+08	1E+08	KD2-H4K8la_peak_1858	4.86896	
14	1E+08	1E+08	KD2-H4K8la_peak_1859	6.13507	
14	1E+08	1E+08	KD2-H4K8la_peak_1860	6.47266	
14	1E+08	1E+08	KD2-H4K8la_peak_1861	7.12014	AL117190.1_ENSG00000221077
14	1E+08	1E+08	KD2-H4K8la_peak_1862	7.55141	AL132709.1_ENSG00000230805
14	1E+08	1E+08	KD2-H4K8la_peak_1863	5.36015	
14	1E+08	1E+08	KD2-H4K8la_peak_1864	5.98041	
14	1E+08	1E+08	KD2-H4K8la_peak_1865	7.51302	
14	1E+08	1E+08	KD2-H4K8la_peak_1866	12.1221	
14	1E+08	1E+08	KD2-H4K8la_peak_1867	5.83285	
14	1E+08	1E+08	KD2-H4K8la_peak_1868	7.65547	RP11-1017G21.5_ENSG00000271780
14	1E+08	1E+08	KD2-H4K8la_peak_1869	5.05146	
14	1E+08	1E+08	KD2-H4K8la_peak_1870	4.79631	
14	1E+08	1E+08	KD2-H4K8la_peak_1871	6.83387	
14	1E+08	1E+08	KD2-H4K8la_peak_1872	15.16808	
14	1E+08	1E+08	KD2-H4K8la_peak_1873	5.90392	MIR4309_ENSG00000266015
14	1E+08	1E+08	KD2-H4K8la_peak_1874	7.49467	CTD-2555C10.3_ENSG00000259230
14	1E+08	1E+08	KD2-H4K8la_peak_1875	11.15182	
14	1E+08	1E+08	KD2-H4K8la_peak_1876	11.7362	RCOR1_ENSG00000089902
14	1E+08	1E+08	KD2-H4K8la_peak_1877	12.1221	
14	1E+08	1E+08	KD2-H4K8la_peak_1878	4.86896	
14	1E+08	1E+08	KD2-H4K8la_peak_1879	7.30347	LINC00677_ENSG00000259717;TNFAIP2_ENSG00000185215
14	1E+08	1E+08	KD2-H4K8la_peak_1880	11.45154	RAP2CP1_ENSG00000270938
14	1E+08	1E+08	KD2-H4K8la_peak_1881	7.92775	RP11-45P15.4_ENSG00000259775;EIF5_ENSG00000100664
14	1E+08	1E+08	KD2-H4K8la_peak_1882	7.51014	RP11-600F24.7_ENSG00000260285;TRMT61A_ENSG00000166166
14	1E+08	1E+08	KD2-H4K8la_peak_1883	8.12466	BAG5_ENSG00000166170;KLC1_ENSG00000126214;RP11-73M18.2_ENSG00000256500;APOPT1_ENSG00000256053
14	1E+08	1E+08	KD2-H4K8la_peak_1884	13.13367	

14	1E+08	1E+08	KD2-H4K8la_peak_1885	7.92775	
14	1E+08	1E+08	KD2-H4K8la_peak_1886	6.61495	
14	1E+08	1E+08	KD2-H4K8la_peak_1887	5.90392	
14	1E+08	1E+08	KD2-H4K8la_peak_1888	5.43278	
14	1E+08	1E+08	KD2-H4K8la_peak_1889	5.58883	
14	1E+08	1E+08	KD2-H4K8la_peak_1890	14.90676	RP11-260M19.2_ENSG00000258913
14	1E+08	1E+08	KD2-H4K8la_peak_1891	8.39919	RP11-260M19.2_ENSG00000258913
14	1E+08	1E+08	KD2-H4K8la_peak_1892	8.48488	
14	1E+08	1E+08	KD2-H4K8la_peak_1893	6.3399	
14	1E+08	1E+08	KD2-H4K8la_peak_1894	8.83119	
14	1E+08	1E+08	KD2-H4K8la_peak_1895	8.37064	
14	1E+08	1E+08	KD2-H4K8la_peak_1896	7.12972	
14	1E+08	1E+08	KD2-H4K8la_peak_1897	11.7362	
14	1E+08	1E+08	KD2-H4K8la_peak_1898	6.54523	
14	1E+08	1E+08	KD2-H4K8la_peak_1899	5.71505	
14	1E+08	1E+08	KD2-H4K8la_peak_1900	10.27876	
14	1E+08	1E+08	KD2-H4K8la_peak_1901	11.45154	
14	1E+08	1E+08	KD2-H4K8la_peak_1902	9.61894	
14	1E+08	1E+08	KD2-H4K8la_peak_1903	7.49467	
14	1.1E+08	1.1E+08	KD2-H4K8la_peak_1904	9.98668	
14	1.1E+08	1.1E+08	KD2-H4K8la_peak_1905	10.87112	
14	1.1E+08	1.1E+08	KD2-H4K8la_peak_1906	5.58883	INF2_ENSG00000203485
14	1.1E+08	1.1E+08	KD2-H4K8la_peak_1907	4.6555	SIVA1_ENSG00000184990
14	1.1E+08	1.1E+08	KD2-H4K8la_peak_1908	9.41442	AKT1_ENSG00000142208
14	1.1E+08	1.1E+08	KD2-H4K8la_peak_1909	7.25503	ZBTB42_ENSG00000179627
14	1.1E+08	1.1E+08	KD2-H4K8la_peak_1910	11.28887	
14	1.1E+08	1.1E+08	KD2-H4K8la_peak_1911	11.15747	C14orf79_ENSG00000140104
14	1.1E+08	1.1E+08	KD2-H4K8la_peak_1912	8.24908	CDCA4_ENSG00000170779
14	1.1E+08	1.1E+08	KD2-H4K8la_peak_1913	9.10568	RP11-44N21.1_ENSG00000257556
14	1.1E+08	1.1E+08	KD2-H4K8la_peak_1914	5.67596	BTBD6_ENSG00000184887
14	1.1E+08	1.1E+08	KD2-H4K8la_peak_1915	12.69796	
14	1.1E+08	1.1E+08	KD2-H4K8la_peak_1916	6.86458	TEX22_ENSG00000226174
14	1.1E+08	1.1E+08	KD2-H4K8la_peak_1917	9.38376	TEX22_ENSG00000226174
14	1.1E+08	1.1E+08	KD2-H4K8la_peak_1918	9.9701	RP11-521B24.3_ENSG00000251602;MTA1_ENSG00000182979
14	1.1E+08	1.1E+08	KD2-H4K8la_peak_1919	6.13507	RP11-521B24.3_ENSG00000251602;MTA1_ENSG00000182979
14	1.1E+08	1.1E+08	KD2-H4K8la_peak_1920	6.95378	
14	1.1E+08	1.1E+08	KD2-H4K8la_peak_1921	8.59561	
15	2.3E+07	2.3E+07	KD2-H4K8la_peak_1922	9.0516	TUBGCP5_ENSG00000153575
15	2.3E+07	2.3E+07	KD2-H4K8la_peak_1923	5.48079	
15	2.3E+07	2.3E+07	KD2-H4K8la_peak_1924	4.86896	
15	2.4E+07	2.4E+07	KD2-H4K8la_peak_1925	7.13237	MKRN3_ENSG00000179455
15	2.6E+07	2.6E+07	KD2-H4K8la_peak_1926	9.44217	UBE3A_ENSG00000114062
15	2.7E+07	2.7E+07	KD2-H4K8la_peak_1927	5.48079	
15	2.9E+07	2.9E+07	KD2-H4K8la_peak_1928	10.16316	APBA2_ENSG00000034053
15	3E+07	3E+07	KD2-H4K8la_peak_1929	6.89612	TJP1_ENSG00000104067
15	3E+07	3E+07	KD2-H4K8la_peak_1930	5.05146	AC026150.5_ENSG00000225930

15	3.2E+07	3.2E+07	KD2-H4K8la_peak_1931	4.35346	
15	3.2E+07	3.2E+07	KD2-H4K8la_peak_1932	8.12466	KLF13_ENSG00000169926
15	3.2E+07	3.2E+07	KD2-H4K8la_peak_1933	5.52913	KLF13_ENSG00000169926
15	3.2E+07	3.2E+07	KD2-H4K8la_peak_1934	7.12014	
15	3.2E+07	3.2E+07	KD2-H4K8la_peak_1935	9.20163	
15	3.2E+07	3.2E+07	KD2-H4K8la_peak_1936	6.2278	
15	3.3E+07	3.3E+07	KD2-H4K8la_peak_1937	9.90319	
15	3.4E+07	3.4E+07	KD2-H4K8la_peak_1938	5.72892	
15	3.5E+07	3.5E+07	KD2-H4K8la_peak_1939	9.0413	EMC4_ENSG00000128463
15	3.5E+07	3.5E+07	KD2-H4K8la_peak_1940	6.89612	EMC4_ENSG00000128463
15	3.7E+07	3.7E+07	KD2-H4K8la_peak_1941	6.89612	
15	3.8E+07	3.8E+07	KD2-H4K8la_peak_1942	5.49688	RP11-1008C21.2_ENSG00000236914
15	4E+07	4E+07	KD2-H4K8la_peak_1943	5.67596	THBS1_ENSG00000137801
15	4E+07	4E+07	KD2-H4K8la_peak_1944	9.20163	GPR176_ENSG00000166073;RP11-325N19.3_ENSG00000246863
15	4.1E+07	4.1E+07	KD2-H4K8la_peak_1945	8.72694	DISP2_ENSG00000140323
15	4.1E+07	4.1E+07	KD2-H4K8la_peak_1946	9.34957	
15	4.1E+07	4.1E+07	KD2-H4K8la_peak_1947	10.20357	CHST14_ENSG00000169105
15	4.1E+07	4.1E+07	KD2-H4K8la_peak_1948	6.47589	RPUSD2_ENSG00000166133
15	4.1E+07	4.1E+07	KD2-H4K8la_peak_1949	9.9701	RAD51-AS1_ENSG00000245849;RAD51_ENSG00000051180
15	4.1E+07	4.1E+07	KD2-H4K8la_peak_1950	7.13237	RP11-532F12.5_ENSG00000261183;SPINT1_ENSG00000166145
15	4.1E+07	4.1E+07	KD2-H4K8la_peak_1951	7.49467	
15	4.1E+07	4.1E+07	KD2-H4K8la_peak_1952	8.16787	
15	4.1E+07	4.1E+07	KD2-H4K8la_peak_1953	5.80814	CHAC1_ENSG00000128965
15	4.2E+07	4.2E+07	KD2-H4K8la_peak_1954	4.94337	EXD1_ENSG00000178997;CHP1_ENSG00000187446
15	4.2E+07	4.2E+07	KD2-H4K8la_peak_1955	6.86458	EXD1_ENSG00000178997;CHP1_ENSG00000187446
15	4.2E+07	4.2E+07	KD2-H4K8la_peak_1956	7.83588	
15	4.2E+07	4.2E+07	KD2-H4K8la_peak_1957	8.65116	
15	4.2E+07	4.2E+07	KD2-H4K8la_peak_1958	5.99671	SPTBN5_ENSG00000137877
15	4.3E+07	4.3E+07	KD2-H4K8la_peak_1959	7.92775	VPS39_ENSG00000166887;RP11-546B15.1_ENSG00000261002
15	4.4E+07	4.4E+07	KD2-H4K8la_peak_1960	7.33815	
15	4.4E+07	4.4E+07	KD2-H4K8la_peak_1961	7.92775	PDIA3_ENSG00000167004
15	4.4E+07	4.4E+07	KD2-H4K8la_peak_1962	9.98668	PDIA3_ENSG00000167004;CATSPER2P1_ENSG00000205771
15	4.4E+07	4.4E+07	KD2-H4K8la_peak_1963	9.69375	RP11-296A16.1_ENSG00000262560;SERINC4_ENSG00000184716
15	4.4E+07	4.4E+07	KD2-H4K8la_peak_1964	8.12466	FRMD5_ENSG00000171877
15	4.5E+07	4.5E+07	KD2-H4K8la_peak_1965	5.36015	EIF3J-AS1_ENSG00000179523;EIF3J_ENSG00000104131
15	4.5E+07	4.5E+07	KD2-H4K8la_peak_1966	5.16795	
15	4.5E+07	4.5E+07	KD2-H4K8la_peak_1967	9.14592	CTD-2651B20.1_ENSG00000259539
15	4.5E+07	4.5E+07	KD2-H4K8la_peak_1968	5.79108	CTD-2651B20.1_ENSG00000259539
15	4.6E+07	4.6E+07	KD2-H4K8la_peak_1969	5.89088	
15	4.6E+07	4.6E+07	KD2-H4K8la_peak_1970	8.77936	
15	4.7E+07	4.7E+07	KD2-H4K8la_peak_1971	11.36807	SEMA6D_ENSG00000137872
15	4.9E+07	4.9E+07	KD2-H4K8la_peak_1972	13.38201	
15	5.1E+07	5.1E+07	KD2-H4K8la_peak_1973	12.13455	GABPB1_ENSG00000104064;GABPB1-AS1_ENSG00000244879
15	5.1E+07	5.1E+07	KD2-H4K8la_peak_1974	4.6555	GABPB1_ENSG00000104064;GABPB1-AS1_ENSG00000244879
15	5.1E+07	5.1E+07	KD2-H4K8la_peak_1975	12.211	USP8_ENSG00000138592
15	5.1E+07	5.1E+07	KD2-H4K8la_peak_1976	7.51302	USP8_ENSG00000138592

15	5.1E+07	5.1E+07	KD2-H4K8la_peak_1977	9.27415	AP4E1_ENSG00000081014
15	5.2E+07	5.2E+07	KD2-H4K8la_peak_1978	7.13237	LEO1_ENSG00000166477
15	5.3E+07	5.3E+07	KD2-H4K8la_peak_1979	12.32641	MYO5C_ENSG00000128833
15	5.6E+07	5.6E+07	KD2-H4K8la_peak_1980	7.72421	
15	5.7E+07	5.7E+07	KD2-H4K8la_peak_1981	6.89612	ZNF280D_ENSG00000137871;TCF12_ENSG00000140262
15	5.8E+07	5.8E+07	KD2-H4K8la_peak_1982	9.0413	
15	5.9E+07	5.9E+07	KD2-H4K8la_peak_1983	10.04111	CCNB2_ENSG00000157456
15	6.2E+07	6.2E+07	KD2-H4K8la_peak_1984	4.30663	RORA_ENSG00000069667
15	6.2E+07	6.2E+07	KD2-H4K8la_peak_1985	11.15747	VPS13C_ENSG00000129003;RP11-643M14.1_ENSG00000259251
15	6.2E+07	6.2E+07	KD2-H4K8la_peak_1986	5.58883	
15	6.3E+07	6.3E+07	KD2-H4K8la_peak_1987	9.27415	TLN2_ENSG00000171914
15	6.3E+07	6.3E+07	KD2-H4K8la_peak_1988	5.72892	
15	6.3E+07	6.3E+07	KD2-H4K8la_peak_1989	9.10568	RPS27L_ENSG00000185088
15	6.4E+07	6.4E+07	KD2-H4K8la_peak_1990	7.13237	
15	6.4E+07	6.4E+07	KD2-H4K8la_peak_1991	4.66473	USP3-AS1_ENSG00000259248
15	6.4E+07	6.4E+07	KD2-H4K8la_peak_1992	10.00612	
15	6.5E+07	6.5E+07	KD2-H4K8la_peak_1993	6.83387	MTFMT_ENSG00000103707
15	6.6E+07	6.6E+07	KD2-H4K8la_peak_1994	7.51302	
15	6.6E+07	6.6E+07	KD2-H4K8la_peak_1995	4.53273	
15	6.6E+07	6.6E+07	KD2-H4K8la_peak_1996	9.10568	PTPLAD1_ENSG00000074696
15	6.6E+07	6.6E+07	KD2-H4K8la_peak_1997	7.60756	
15	6.7E+07	6.7E+07	KD2-H4K8la_peak_1998	6.86458	
15	6.7E+07	6.7E+07	KD2-H4K8la_peak_1999	6.88801	
15	6.7E+07	6.7E+07	KD2-H4K8la_peak_2000	13.20018	
15	6.7E+07	6.7E+07	KD2-H4K8la_peak_2001	9.27415	
15	6.8E+07	6.8E+07	KD2-H4K8la_peak_2002	7.12014	RP11-502I4.3_ENSG00000270964;MAP2K5_ENSG00000137764
15	6.8E+07	6.8E+07	KD2-H4K8la_peak_2003	5.40949	
15	6.9E+07	6.9E+07	KD2-H4K8la_peak_2004	5.43278	SPESP1_ENSG00000258484
15	6.9E+07	6.9E+07	KD2-H4K8la_peak_2005	5.47284	SPESP1_ENSG00000258484
15	7E+07	7E+07	KD2-H4K8la_peak_2006	11.40266	TLE3_ENSG00000140332
15	7E+07	7E+07	KD2-H4K8la_peak_2007	5.1913	
15	7E+07	7E+07	KD2-H4K8la_peak_2008	9.04068	
15	7E+07	7E+07	KD2-H4K8la_peak_2009	5.28646	
15	7E+07	7E+07	KD2-H4K8la_peak_2010	11.24937	
15	7E+07	7E+07	KD2-H4K8la_peak_2011	5.69126	AC011648.1_ENSG00000259252
15	7.1E+07	7.1E+07	KD2-H4K8la_peak_2012	7.13237	
15	7.1E+07	7.1E+07	KD2-H4K8la_peak_2013	6.66491	
15	7.1E+07	7.1E+07	KD2-H4K8la_peak_2014	7.19298	CT62_ENSG00000225362
15	7.3E+07	7.3E+07	KD2-H4K8la_peak_2015	7.56826	
15	7.3E+07	7.3E+07	KD2-H4K8la_peak_2016	6.64804	
15	7.4E+07	7.4E+07	KD2-H4K8la_peak_2017	8.44557	CD276_ENSG00000103855
15	7.4E+07	7.4E+07	KD2-H4K8la_peak_2018	8.39919	
15	7.4E+07	7.4E+07	KD2-H4K8la_peak_2019	12.32641	RP11-247C2.2_ENSG00000248540
15	7.4E+07	7.4E+07	KD2-H4K8la_peak_2020	6.20793	
15	7.5E+07	7.5E+07	KD2-H4K8la_peak_2021	17.60548	
15	7.5E+07	7.5E+07	KD2-H4K8la_peak_2022	5.73914	

15	7.5E+07	7.5E+07	KD2-H4K8la_peak_2023	8.88448	
15	7.5E+07	7.5E+07	KD2-H4K8la_peak_2024	9.9701	
15	7.5E+07	7.5E+07	KD2-H4K8la_peak_2025	13.68324	
15	7.5E+07	7.5E+07	KD2-H4K8la_peak_2026	6.77116	RP11-60L3.2_ENSG00000261384
15	7.5E+07	7.5E+07	KD2-H4K8la_peak_2027	11.21444	
15	7.5E+07	7.5E+07	KD2-H4K8la_peak_2028	4.30663	SEMA7A_ENSG00000138623
15	7.5E+07	7.5E+07	KD2-H4K8la_peak_2029	6.20477	CTD-3154N5.1_ENSG00000260919
15	7.5E+07	7.5E+07	KD2-H4K8la_peak_2030	11.15747	CSK_ENSG00000103653
15	7.5E+07	7.5E+07	KD2-H4K8la_peak_2031	7.3993	ULK3_ENSG00000140474
15	7.5E+07	7.5E+07	KD2-H4K8la_peak_2032	4.75945	COX5A_ENSG00000178741
15	7.5E+07	7.5E+07	KD2-H4K8la_peak_2033	10.28981	COX5A_ENSG00000178741
15	7.5E+07	7.5E+07	KD2-H4K8la_peak_2034	9.9701	RP11-151H2.1_ENSG00000260483;PPCDC_ENSG00000138621
15	7.5E+07	7.5E+07	KD2-H4K8la_peak_2035	6.78512	
15	7.6E+07	7.6E+07	KD2-H4K8la_peak_2036	8.06229	NEIL1_ENSG00000140398
15	7.6E+07	7.6E+07	KD2-H4K8la_peak_2037	12.12477	MAN2C1_ENSG00000140400;RP11-817O13.8_ENSG00000260274
15	7.6E+07	7.6E+07	KD2-H4K8la_peak_2038	7.55141	SNUPN_ENSG00000169371
15	7.6E+07	7.6E+07	KD2-H4K8la_peak_2039	5.6499	
15	7.6E+07	7.6E+07	KD2-H4K8la_peak_2040	5.43278	
15	7.6E+07	7.6E+07	KD2-H4K8la_peak_2041	8.07102	
15	7.7E+07	7.7E+07	KD2-H4K8la_peak_2042	5.83285	RCN2_ENSG00000117906
15	7.7E+07	7.7E+07	KD2-H4K8la_peak_2043	10.74378	
15	7.8E+07	7.8E+07	KD2-H4K8la_peak_2044	10.8046	
15	7.8E+07	7.8E+07	KD2-H4K8la_peak_2045	6.61963	
15	7.8E+07	7.8E+07	KD2-H4K8la_peak_2046	14.71613	ADAMTS7P3_ENSG00000261143
15	7.8E+07	7.8E+07	KD2-H4K8la_peak_2047	8.76082	RP11-114H24.6_ENSG00000259792
15	7.8E+07	7.8E+07	KD2-H4K8la_peak_2048	8.07102	
15	7.9E+07	7.9E+07	KD2-H4K8la_peak_2049	7.61975	
15	7.9E+07	7.9E+07	KD2-H4K8la_peak_2050	13.01988	
15	7.9E+07	7.9E+07	KD2-H4K8la_peak_2051	4.30663	
15	7.9E+07	7.9E+07	KD2-H4K8la_peak_2052	13.11108	CHRNA3_ENSG00000080644
15	7.9E+07	7.9E+07	KD2-H4K8la_peak_2053	6.3399	
15	7.9E+07	7.9E+07	KD2-H4K8la_peak_2054	5.58883	
15	7.9E+07	7.9E+07	KD2-H4K8la_peak_2055	5.64417	
15	7.9E+07	7.9E+07	KD2-H4K8la_peak_2056	9.05604	
15	7.9E+07	7.9E+07	KD2-H4K8la_peak_2057	9.61894	
15	7.9E+07	7.9E+07	KD2-H4K8la_peak_2058	9.05604	
15	7.9E+07	7.9E+07	KD2-H4K8la_peak_2059	10.02595	
15	7.9E+07	7.9E+07	KD2-H4K8la_peak_2060	5.27164	
15	7.9E+07	7.9E+07	KD2-H4K8la_peak_2061	4.94337	
15	7.9E+07	7.9E+07	KD2-H4K8la_peak_2062	7.51014	RP11-160C18.4_ENSG00000238166
15	7.9E+07	7.9E+07	KD2-H4K8la_peak_2063	13.27013	
15	7.9E+07	7.9E+07	KD2-H4K8la_peak_2064	9.05604	
15	7.9E+07	7.9E+07	KD2-H4K8la_peak_2065	8.76116	
15	7.9E+07	7.9E+07	KD2-H4K8la_peak_2066	9.44217	
15	7.9E+07	7.9E+07	KD2-H4K8la_peak_2067	7.82875	
15	7.9E+07	7.9E+07	KD2-H4K8la_peak_2068	12.35598	ADAMTS7_ENSG00000136378

15	7.9E+07	7.9E+07	KD2-H4K8la_peak_2069	8.43222	
15	8.1E+07	8.1E+07	KD2-H4K8la_peak_2070	6.13507	RP11-210M15.2_ENSG00000259495;ARNT2_ENSG00000172379
15	8.1E+07	8.1E+07	KD2-H4K8la_peak_2071	8.487	RP11-210M15.2_ENSG00000259495;ARNT2_ENSG00000172379
15	8.1E+07	8.1E+07	KD2-H4K8la_peak_2072	7.12014	
15	8.1E+07	8.1E+07	KD2-H4K8la_peak_2073	6.96161	
15	8.1E+07	8.1E+07	KD2-H4K8la_peak_2074	8.76116	MESDC1_ENSG00000140406
15	8.2E+07	8.2E+07	KD2-H4K8la_peak_2075	10.98662	MEX3B_ENSG00000183496
15	8.3E+07	8.3E+07	KD2-H4K8la_peak_2076	5.72892	WHAMM_ENSG00000156232
15	8.4E+07	8.4E+07	KD2-H4K8la_peak_2077	7.86224	C15orf40_ENSG00000169609
15	8.4E+07	8.4E+07	KD2-H4K8la_peak_2078	6.3399	BTBD1_ENSG00000064726;MIR4515_ENSG00000263643
15	8.5E+07	8.5E+07	KD2-H4K8la_peak_2079	14.52454	UBE2Q2P11_ENSG00000259538;RP11-182J1.13_ENSG00000259774
15	8.5E+07	8.5E+07	KD2-H4K8la_peak_2080	5.58883	LINC00933_ENSG00000259728;UBE2Q2P1_ENSG00000189136
15	8.5E+07	8.5E+07	KD2-H4K8la_peak_2081	6.19813	SEC11A_ENSG00000140612
15	8.9E+07	8.9E+07	KD2-H4K8la_peak_2082	4.53763	AEN_ENSG00000181026
15	8.9E+07	8.9E+07	KD2-H4K8la_peak_2083	6.26214	
15	9E+07	9E+07	KD2-H4K8la_peak_2084	6.63409	
15	9E+07	9E+07	KD2-H4K8la_peak_2085	10.00612	RP11-326A19.3_ENSG00000261407;ABHD2_ENSG00000140526
15	9E+07	9E+07	KD2-H4K8la_peak_2086	9.9701	MIR9-3_ENSG00000207819
15	9E+07	9E+07	KD2-H4K8la_peak_2087	5.58883	
15	9E+07	9E+07	KD2-H4K8la_peak_2088	6.13507	
15	9E+07	9E+07	KD2-H4K8la_peak_2089	7.61975	MESP1_ENSG00000166823
15	9E+07	9E+07	KD2-H4K8la_peak_2090	10.71653	
15	9E+07	9E+07	KD2-H4K8la_peak_2091	9.10568	ANPEP_ENSG00000166825
15	9E+07	9E+07	KD2-H4K8la_peak_2092	8.43222	C15orf38-AP3S2_ENSG00000250021;C15orf38_ENSG00000242498
15	9.1E+07	9.1E+07	KD2-H4K8la_peak_2093	11.65627	ZNF710_ENSG00000140548
15	9.1E+07	9.1E+07	KD2-H4K8la_peak_2094	10.23822	
15	9.1E+07	9.1E+07	KD2-H4K8la_peak_2095	7.92775	
15	9.2E+07	9.2E+07	KD2-H4K8la_peak_2096	6.93796	
15	9.3E+07	9.3E+07	KD2-H4K8la_peak_2097	4.35346	
15	9.3E+07	9.3E+07	KD2-H4K8la_peak_2098	5.05146	
15	9.9E+07	9.9E+07	KD2-H4K8la_peak_2099	7.41484	
15	9.9E+07	9.9E+07	KD2-H4K8la_peak_2100	5.05146	
15	1E+08	1E+08	KD2-H4K8la_peak_2101	6.64804	
15	1E+08	1E+08	KD2-H4K8la_peak_2102	11.06859	
15	1E+08	1E+08	KD2-H4K8la_peak_2103	13.01988	
15	1E+08	1E+08	KD2-H4K8la_peak_2104	8.73887	
15	1E+08	1E+08	KD2-H4K8la_peak_2105	6.96161	ASB7_ENSG00000183475
15	1E+08	1E+08	KD2-H4K8la_peak_2106	9.0261	
15	1E+08	1E+08	KD2-H4K8la_peak_2107	5.16795	
15	1E+08	1E+08	KD2-H4K8la_peak_2108	13.23192	
15	1E+08	1E+08	KD2-H4K8la_peak_2109	14.47885	
15	1E+08	1E+08	KD2-H4K8la_peak_2110	7.81267	
15	1E+08	1E+08	KD2-H4K8la_peak_2111	4.75945	
15	1E+08	1E+08	KD2-H4K8la_peak_2112	15.42498	
15	1E+08	1E+08	KD2-H4K8la_peak_2113	8.07102	
15	1E+08	1E+08	KD2-H4K8la_peak_2114	6.88801	

15	1E+08	1E+08	KD2-H4K8la_peak_2115	10.04111	
15	1E+08	1E+08	KD2-H4K8la_peak_2116	8.76116	
15	1E+08	1E+08	KD2-H4K8la_peak_2117	13.81576	
15	1E+08	1E+08	KD2-H4K8la_peak_2118	7.41438	
15	1E+08	1E+08	KD2-H4K8la_peak_2119	13.2369	
15	1E+08	1E+08	KD2-H4K8la_peak_2120	7.19298	
15	1E+08	1E+08	KD2-H4K8la_peak_2121	15.01131	
15	1E+08	1E+08	KD2-H4K8la_peak_2122	7.3993	
15	1E+08	1E+08	KD2-H4K8la_peak_2123	17.00685	
15	1E+08	1E+08	KD2-H4K8la_peak_2124	6.19813	
15	1E+08	1E+08	KD2-H4K8la_peak_2125	13.3197	RP11-89K11.1_ENSG00000259658
16	127151	128062	KD2-H4K8la_peak_2126	11.15747	RHBDF1_ENSG00000007384;MPG_ENSG00000103152
16	188116	188315	KD2-H4K8la_peak_2127	8.37064	NPRL3_ENSG00000103148
16	284166	284573	KD2-H4K8la_peak_2128	7.31804	ITFG3_ENSG00000167930
16	325027	325600	KD2-H4K8la_peak_2129	7.41438	RGS11_ENSG00000076344
16	325996	326218	KD2-H4K8la_peak_2130	6.28277	RGS11_ENSG00000076344
16	330101	330299	KD2-H4K8la_peak_2131	7.3993	
16	330729	330960	KD2-H4K8la_peak_2132	11.52725	
16	432186	432451	KD2-H4K8la_peak_2133	9.88732	Z97634.3_ENSG00000236829
16	446849	447048	KD2-H4K8la_peak_2134	6.96161	NME4_ENSG00000103202
16	475931	476316	KD2-H4K8la_peak_2135	7.19544	RAB11FIP3_ENSG00000090565
16	616474	616838	KD2-H4K8la_peak_2136	5.98041	PIGQ_ENSG00000007541;NHLRC4_ENSG00000257108
16	698911	699151	KD2-H4K8la_peak_2137	6.25288	AL022341.3_ENSG00000228201;WDR90_ENSG00000161996
16	709805	710050	KD2-H4K8la_peak_2138	5.69126	
16	718223	718615	KD2-H4K8la_peak_2139	12.23212	RHOT2_ENSG00000140983
16	739652	740291	KD2-H4K8la_peak_2140	9.00481	WDR24_ENSG00000127580;LA16c-313D11.12_ENSG00000261659
16	740534	740938	KD2-H4K8la_peak_2141	8.39919	WDR24_ENSG00000127580
16	770657	770861	KD2-H4K8la_peak_2142	8.50453	FAM173A_ENSG00000103254
16	771718	771919	KD2-H4K8la_peak_2143	4.60174	
16	791195	791908	KD2-H4K8la_peak_2144	7.61975	NARFL_ENSG00000103245
16	857211	857683	KD2-H4K8la_peak_2145	12.1221	
16	1020585	1020817	KD2-H4K8la_peak_2146	6.36118	
16	1401439	1401714	KD2-H4K8la_peak_2147	7.49467	TSR3_ENSG00000007520;GNPTG_ENSG00000090581
16	1470341	1470604	KD2-H4K8la_peak_2148	7.48323	
16	1471559	1471855	KD2-H4K8la_peak_2149	6.25495	
16	1480571	1481024	KD2-H4K8la_peak_2150	5.83285	
16	1524648	1524971	KD2-H4K8la_peak_2151	6.89612	CLCN7_ENSG00000103249
16	1664695	1665048	KD2-H4K8la_peak_2152	8.76116	
16	1823294	1823858	KD2-H4K8la_peak_2153	10.12382	MRPS34_ENSG00000074071;EME2_ENSG00000197774
16	1877312	1877567	KD2-H4K8la_peak_2154	7.92775	HAGH_ENSG00000063854;FAHD1_ENSG00000180185
16	1922710	1922926	KD2-H4K8la_peak_2155	8.86558	
16	1959474	1959938	KD2-H4K8la_peak_2156	5.43278	
16	1979996	1980219	KD2-H4K8la_peak_2157	8.13557	
16	1982446	1982810	KD2-H4K8la_peak_2158	10.98662	
16	2009782	2010016	KD2-H4K8la_peak_2159	5.8369	NDUFB10_ENSG00000140990
16	2088545	2089009	KD2-H4K8la_peak_2160	21.13867	

16	2089883	2090571	KD2-H4K8la_peak_2161	8.44557	
16	2172720	2172920	KD2-H4K8la_peak_2162	7.72519	
16	2173391	2175042	KD2-H4K8la_peak_2163	12.85404	
16	2175477	2175905	KD2-H4K8la_peak_2164	7.12014	
16	2198839	2199045	KD2-H4K8la_peak_2165	4.66473	
16	2265912	2266226	KD2-H4K8la_peak_2166	4.94337	
16	2272870	2273280	KD2-H4K8la_peak_2167	9.98668	E4F1_ENSG00000167967
16	2301854	2302064	KD2-H4K8la_peak_2168	11.20341	ECI1_ENSG00000167969
16	2391172	2391369	KD2-H4K8la_peak_2169	8.12466	ABCA3_ENSG00000167972
16	2478591	2478955	KD2-H4K8la_peak_2170	9.55813	CCNF_ENSG00000162063
16	2479532	2479791	KD2-H4K8la_peak_2171	9.6656	CCNF_ENSG00000162063
16	2517168	2517555	KD2-H4K8la_peak_2172	5.93538	RP11-715J22.2_ENSG00000259895
16	2517878	2518281	KD2-H4K8la_peak_2173	10.80993	RP11-715J22.2_ENSG00000259895
16	2518881	2519088	KD2-H4K8la_peak_2174	11.81269	RP11-715J22.2_ENSG00000259895
16	2519633	2519831	KD2-H4K8la_peak_2175	6.9399	
16	2653511	2654025	KD2-H4K8la_peak_2176	5.58883	AC141586.5_ENSG00000215154
16	2732639	2732944	KD2-H4K8la_peak_2177	8.12466	KCTD5_ENSG00000167977
16	2770636	2771057	KD2-H4K8la_peak_2178	6.86458	PRSS27_ENSG00000172382
16	2775000	2775210	KD2-H4K8la_peak_2179	6.01294	
16	2803177	2803430	KD2-H4K8la_peak_2180	7.87573	SRRM2-AS1_ENSG00000205913;SRRM2_ENSG00000167978
16	2826800	2827085	KD2-H4K8la_peak_2181	9.73848	TCEB2_ENSG00000103363
16	2827517	2827914	KD2-H4K8la_peak_2182	8.7731	TCEB2_ENSG00000103363
16	2902437	2902665	KD2-H4K8la_peak_2183	8.0046	
16	2917889	2918111	KD2-H4K8la_peak_2184	10.27876	LA16c-325D7.2_ENSG00000263280
16	3018652	3018953	KD2-H4K8la_peak_2185	8.37064	PAQR4_ENSG00000162073
16	3030824	3031179	KD2-H4K8la_peak_2186	7.12014	PKMYT1_ENSG00000127564
16	3109651	3110121	KD2-H4K8la_peak_2187	7.13185	RP11-473M20.7_ENSG00000261971
16	3151195	3151393	KD2-H4K8la_peak_2188	8.487	
16	3156156	3156425	KD2-H4K8la_peak_2189	14.23294	RP11-473M20.11_ENSG00000263011
16	3162648	3162987	KD2-H4K8la_peak_2190	6.13507	ZNF205_ENSG00000122386
16	3215711	3216004	KD2-H4K8la_peak_2191	6.04152	
16	3355160	3355386	KD2-H4K8la_peak_2192	11.52725	TIGD7_ENSG00000140993;ZNF75A_ENSG00000162086
16	3355651	3356036	KD2-H4K8la_peak_2193	14.67686	TIGD7_ENSG00000140993;ZNF75A_ENSG00000162086
16	3931025	3931608	KD2-H4K8la_peak_2194	9.20163	CREBBP_ENSG00000005339
16	4233436	4233794	KD2-H4K8la_peak_2195	6.68593	RP11-95P2.3_ENSG00000263105
16	4322200	4322732	KD2-H4K8la_peak_2196	6.13507	TFAP4_ENSG00000090447
16	4365259	4365708	KD2-H4K8la_peak_2197	9.27415	
16	4378173	4378750	KD2-H4K8la_peak_2198	5.58883	AC005356.1_ENSG00000262686
16	4441681	4441950	KD2-H4K8la_peak_2199	10.91315	
16	4442195	4442658	KD2-H4K8la_peak_2200	10.20357	
16	4665119	4665510	KD2-H4K8la_peak_2201	9.67249	UBALD1_ENSG00000153443;MGRN1_ENSG00000102858
16	4819039	4819245	KD2-H4K8la_peak_2202	8.76116	
16	5553211	5553542	KD2-H4K8la_peak_2203	5.83285	
16	8715604	8715812	KD2-H4K8la_peak_2204	6.86458	METTL22_ENSG00000067365
16	9184015	9184544	KD2-H4K8la_peak_2205	9.67249	C16orf72_ENSG00000182831
16	1.1E+07	1.1E+07	KD2-H4K8la_peak_2206	6.64804	

16	1.1E+07	1.1E+07	KD2-H4K8la_peak_2207	13.73665	TVP23A_ENSG00000166676
16	1.1E+07	1.1E+07	KD2-H4K8la_peak_2208	5.89088	
16	1.1E+07	1.1E+07	KD2-H4K8la_peak_2209	5.67596	SOCS1_ENSG00000185338
16	1.1E+07	1.1E+07	KD2-H4K8la_peak_2210	8.65116	
16	1.2E+07	1.2E+07	KD2-H4K8la_peak_2211	8.65116	
16	1.2E+07	1.2E+07	KD2-H4K8la_peak_2212	5.33301	
16	1.2E+07	1.2E+07	KD2-H4K8la_peak_2213	11.30277	
16	1.5E+07	1.5E+07	KD2-H4K8la_peak_2214	5.58883	
16	1.5E+07	1.5E+07	KD2-H4K8la_peak_2215	8.37064	NTAN1_ENSG00000157045
16	1.5E+07	1.5E+07	KD2-H4K8la_peak_2216	11.52725	
16	1.5E+07	1.5E+07	KD2-H4K8la_peak_2217	8.50453	
16	1.5E+07	1.5E+07	KD2-H4K8la_peak_2218	9.66714	
16	1.5E+07	1.5E+07	KD2-H4K8la_peak_2219	7.49467	RP11-1021N1.1_ENSG00000261130;MPV17L_ENSG00000156968
16	1.6E+07	1.6E+07	KD2-H4K8la_peak_2220	7.16004	C16orf45_ENSG00000166780
16	1.7E+07	1.7E+07	KD2-H4K8la_peak_2221	5.83285	
16	1.8E+07	1.8E+07	KD2-H4K8la_peak_2222	5.72892	
16	1.9E+07	1.9E+07	KD2-H4K8la_peak_2223	8.487	TMC7_ENSG00000170537
16	1.9E+07	1.9E+07	KD2-H4K8la_peak_2224	6.39383	
16	2E+07	2E+07	KD2-H4K8la_peak_2225	7.30722	C16orf62_ENSG00000103544
16	2.1E+07	2.1E+07	KD2-H4K8la_peak_2226	10.12162	ERI2_ENSG00000196678;DCUN1D3_ENSG00000188215;LYRM1_ENSG00000102897
16	2.2E+07	2.2E+07	KD2-H4K8la_peak_2227	6.03683	CTD-2547E10.2_ENSG00000180747;SLC7A5P2_ENSG00000258186
16	2.2E+07	2.2E+07	KD2-H4K8la_peak_2228	4.51055	EEF2K_ENSG00000103319
16	2.2E+07	2.2E+07	KD2-H4K8la_peak_2229	6.36118	POLR3E_ENSG00000058600
16	2.3E+07	2.3E+07	KD2-H4K8la_peak_2230	6.3399	
16	2.4E+07	2.4E+07	KD2-H4K8la_peak_2231	5.67596	
16	2.5E+07	2.5E+07	KD2-H4K8la_peak_2232	6.60352	ARHGAP17_ENSG00000140750;RP11-266L9.1_ENSG00000260714
16	2.5E+07	2.5E+07	KD2-H4K8la_peak_2233	8.37064	RP11-266L9.5_ENSG00000262155
16	2.7E+07	2.7E+07	KD2-H4K8la_peak_2234	10.19807	
16	2.7E+07	2.7E+07	KD2-H4K8la_peak_2235	16.8445	NSMCE1_ENSG00000169189;CTD-3203P2.2_ENSG00000245888
16	2.8E+07	2.8E+07	KD2-H4K8la_peak_2236	9.37943	
16	2.9E+07	2.9E+07	KD2-H4K8la_peak_2237	22.53907	ATXN2L_ENSG00000168488
16	2.9E+07	2.9E+07	KD2-H4K8la_peak_2238	7.30722	RP11-22P6.2_ENSG00000261766
16	2.9E+07	2.9E+07	KD2-H4K8la_peak_2239	8.37064	RP11-22P6.3_ENSG00000260442
16	2.9E+07	2.9E+07	KD2-H4K8la_peak_2240	7.55141	
16	2.9E+07	2.9E+07	KD2-H4K8la_peak_2241	6.47589	NFATC2IP_ENSG00000176953
16	2.9E+07	2.9E+07	KD2-H4K8la_peak_2242	6.61495	
16	2.9E+07	2.9E+07	KD2-H4K8la_peak_2243	10.04113	
16	2.9E+07	2.9E+07	KD2-H4K8la_peak_2244	8.37064	
16	2.9E+07	2.9E+07	KD2-H4K8la_peak_2245	9.61894	
16	2.9E+07	2.9E+07	KD2-H4K8la_peak_2246	6.2278	
16	2.9E+07	2.9E+07	KD2-H4K8la_peak_2247	8.76116	
16	2.9E+07	2.9E+07	KD2-H4K8la_peak_2248	8.95597	
16	2.9E+07	2.9E+07	KD2-H4K8la_peak_2249	6.13507	
16	2.9E+07	2.9E+07	KD2-H4K8la_peak_2250	5.83285	
16	2.9E+07	2.9E+07	KD2-H4K8la_peak_2251	10.70453	
16	3E+07	3E+07	KD2-H4K8la_peak_2252	9.20163	MAZ_ENSG00000103495;AC009133.15_ENSG00000259952

16	3E+07	3E+07	KD2-H4K8la_peak_2253	9.35473	CDIPT_ENSG00000103502;CDIPT-AS1_ENSG00000214725
16	3E+07	3E+07	KD2-H4K8la_peak_2254	9.0516	KCTD13_ENSG00000174943;CTD-2574D22.2_ENSG00000247735
16	3E+07	3E+07	KD2-H4K8la_peak_2255	5.33301	
16	3E+07	3E+07	KD2-H4K8la_peak_2256	9.64487	YPEL3_ENSG00000090238;RP11-455F5.3_ENSG00000250616
16	3E+07	3E+07	KD2-H4K8la_peak_2257	6.86759	MAPK3_ENSG00000102882
16	3E+07	3E+07	KD2-H4K8la_peak_2258	4.37527	
16	3E+07	3E+07	KD2-H4K8la_peak_2259	10.74378	CORO1A_ENSG00000102879
16	3E+07	3E+07	KD2-H4K8la_peak_2260	4.66473	CD2BP2_ENSG00000169217;RP11-347C12.10_ENSG00000260219
16	3.1E+07	3.1E+07	KD2-H4K8la_peak_2261	6.40658	ZNF768_ENSG00000169957
16	3.1E+07	3.1E+07	KD2-H4K8la_peak_2262	11.99163	ZNF747_ENSG00000169955;ZNF747_ENSG00000261459;AC002310.12_ENSG00000235560
16	3.1E+07	3.1E+07	KD2-H4K8la_peak_2263	6.86458	AC002310.13_ENSG00000260869;ZNF764_ENSG00000169951
16	3.1E+07	3.1E+07	KD2-H4K8la_peak_2264	10.02595	AC002310.13_ENSG00000260869;ZNF764_ENSG00000169951
16	3.1E+07	3.1E+07	KD2-H4K8la_peak_2265	6.47266	ZNF688_ENSG00000229809;AC002310.7_ENSG00000239791
16	3.1E+07	3.1E+07	KD2-H4K8la_peak_2266	9.27415	ZNF785_ENSG00000197162;RP11-146F11.5_ENSG00000260167
16	3.1E+07	3.1E+07	KD2-H4K8la_peak_2267	5.05146	
16	3.1E+07	3.1E+07	KD2-H4K8la_peak_2268	7.49467	
16	3.1E+07	3.1E+07	KD2-H4K8la_peak_2269	12.17279	RP11-146F11.1_ENSG00000261840;SRCAP_ENSG00000080603
16	3.1E+07	3.1E+07	KD2-H4K8la_peak_2270	7.49467	
16	3.1E+07	3.1E+07	KD2-H4K8la_peak_2271	10.00612	ZNF629_ENSG00000102870
16	3.1E+07	3.1E+07	KD2-H4K8la_peak_2272	7.7007	
16	3.1E+07	3.1E+07	KD2-H4K8la_peak_2273	8.76082	
16	3.1E+07	3.1E+07	KD2-H4K8la_peak_2274	9.73495	BCL7C_ENSG00000099385;MIR762_ENSG00000211591
16	3.1E+07	3.1E+07	KD2-H4K8la_peak_2275	8.16787	AC106782.20_ENSG00000262721;CTF1_ENSG00000150281
16	3.1E+07	3.1E+07	KD2-H4K8la_peak_2276	7.13185	
16	3.1E+07	3.1E+07	KD2-H4K8la_peak_2277	7.06427	STX1B_ENSG00000099365
16	3.1E+07	3.1E+07	KD2-H4K8la_peak_2278	12.36938	STX4_ENSG00000103496
16	3.1E+07	3.1E+07	KD2-H4K8la_peak_2279	9.6656	BCKDK_ENSG00000103507
16	3.1E+07	3.1E+07	KD2-H4K8la_peak_2280	15.58875	
16	3.1E+07	3.1E+07	KD2-H4K8la_peak_2281	7.49467	ZNF843_ENSG00000176723
16	3.1E+07	3.1E+07	KD2-H4K8la_peak_2282	5.83285	
16	3.2E+07	3.2E+07	KD2-H4K8la_peak_2283	9.90319	ZNF720_ENSG00000197302
16	3.2E+07	3.2E+07	KD2-H4K8la_peak_2284	6.56022	RP11-56L13.7_ENSG00000260575
16	3.4E+07	3.4E+07	KD2-H4K8la_peak_2285	7.82372	RARRES2P6_ENSG00000261010
16	3.5E+07	3.5E+07	KD2-H4K8la_peak_2286	5.58883	RP11-488I20.4_ENSG00000260480
16	3.5E+07	3.5E+07	KD2-H4K8la_peak_2287	4.86896	RP11-488I20.2_ENSG00000260291
16	4.7E+07	4.7E+07	KD2-H4K8la_peak_2288	7.13237	NETO2_ENSG00000171208;RP11-329J18.2_ENSG00000260281
16	4.8E+07	4.8E+07	KD2-H4K8la_peak_2289	10.49982	
16	4.9E+07	4.9E+07	KD2-H4K8la_peak_2290	7.51014	
16	5E+07	5E+07	KD2-H4K8la_peak_2291	11.21258	
16	5E+07	5E+07	KD2-H4K8la_peak_2292	6.64804	
16	5E+07	5E+07	KD2-H4K8la_peak_2293	5.83285	
16	5E+07	5E+07	KD2-H4K8la_peak_2294	7.94927	
16	5E+07	5E+07	KD2-H4K8la_peak_2295	13.11108	
16	5E+07	5E+07	KD2-H4K8la_peak_2296	10.04113	
16	5E+07	5E+07	KD2-H4K8la_peak_2297	8.39919	
16	5E+07	5E+07	KD2-H4K8la_peak_2298	10.20357	

16	5E+07	5E+07	KD2-H4K8la_peak_2299	4.30663	
16	5E+07	5E+07	KD2-H4K8la_peak_2300	7.13237	ADCY7_ENSG00000121281
16	5E+07	5E+07	KD2-H4K8la_peak_2301	7.13237	ADCY7_ENSG00000121281
16	5E+07	5E+07	KD2-H4K8la_peak_2302	12.97817	
16	5.3E+07	5.3E+07	KD2-H4K8la_peak_2303	7.13237	
16	5.3E+07	5.3E+07	KD2-H4K8la_peak_2304	6.2278	
16	5.3E+07	5.3E+07	KD2-H4K8la_peak_2305	5.95337	
16	5.5E+07	5.5E+07	KD2-H4K8la_peak_2306	4.53273	
16	5.6E+07	5.6E+07	KD2-H4K8la_peak_2307	5.30505	
16	5.6E+07	5.6E+07	KD2-H4K8la_peak_2308	9.29932	
16	5.6E+07	5.6E+07	KD2-H4K8la_peak_2309	7.3993	RP11-461O7.1_ENSG00000246379;GNAO1_ENSG00000087258
16	5.7E+07	5.7E+07	KD2-H4K8la_peak_2310	8.43222	BBS2_ENSG00000125124
16	5.7E+07	5.7E+07	KD2-H4K8la_peak_2311	8.13557	HERPUD1_ENSG00000051108
16	5.7E+07	5.7E+07	KD2-H4K8la_peak_2312	13.11108	
16	5.7E+07	5.7E+07	KD2-H4K8la_peak_2313	9.14988	RP11-407G23.1_ENSG00000260148;CPNE2_ENSG00000140848
16	5.7E+07	5.7E+07	KD2-H4K8la_peak_2314	5.05146	
16	5.8E+07	5.8E+07	KD2-H4K8la_peak_2315	5.58883	
16	5.8E+07	5.8E+07	KD2-H4K8la_peak_2316	7.61975	
16	5.8E+07	5.8E+07	KD2-H4K8la_peak_2317	10.0408	
16	5.8E+07	5.8E+07	KD2-H4K8la_peak_2318	8.06229	CCDC102A_ENSG00000135736
16	5.8E+07	5.8E+07	KD2-H4K8la_peak_2319	9.96333	CCDC135_ENSG00000159625
16	5.8E+07	5.8E+07	KD2-H4K8la_peak_2320	7.06427	
16	6.7E+07	6.7E+07	KD2-H4K8la_peak_2321	15.91733	Y_RNA_ENSG00000261519
16	6.7E+07	6.7E+07	KD2-H4K8la_peak_2322	9.20163	TK2_ENSG00000166548;CKLF_ENSG00000217555;CKLF-CMTM1_ENSG00000254788
16	6.7E+07	6.7E+07	KD2-H4K8la_peak_2323	6.3399	DYNC1LI2_ENSG00000135720;RP11-61A14.4_ENSG00000246777
16	6.7E+07	6.7E+07	KD2-H4K8la_peak_2324	7.19298	CES3_ENSG00000172828
16	6.7E+07	6.7E+07	KD2-H4K8la_peak_2325	6.64804	
16	6.7E+07	6.7E+07	KD2-H4K8la_peak_2326	5.23773	
16	6.7E+07	6.7E+07	KD2-H4K8la_peak_2327	11.06859	CBFB_ENSG00000067955
16	6.7E+07	6.7E+07	KD2-H4K8la_peak_2328	8.43222	B3GNT9_ENSG00000237172
16	6.7E+07	6.7E+07	KD2-H4K8la_peak_2329	7.56395	
16	6.7E+07	6.7E+07	KD2-H4K8la_peak_2330	13.73095	
16	6.8E+07	6.8E+07	KD2-H4K8la_peak_2331	6.83387	ATP6V0D1_ENSG00000159720;RP11-297D21.4_ENSG00000270049
16	6.8E+07	6.8E+07	KD2-H4K8la_peak_2332	7.61975	CTD-2012K14.6_ENSG00000261386;CTD-2012K14.7_ENSG00000259804;CTCF_ENSG00000102974
16	6.8E+07	6.8E+07	KD2-H4K8la_peak_2333	11.40266	NUTF2_ENSG00000102898
16	6.8E+07	6.8E+07	KD2-H4K8la_peak_2334	9.5329	EDC4_ENSG00000038358
16	6.8E+07	6.8E+07	KD2-H4K8la_peak_2335	7.79412	SLC12A4_ENSG00000124067
16	6.8E+07	6.8E+07	KD2-H4K8la_peak_2336	11.20341	DDX28_ENSG00000182810
16	6.8E+07	6.8E+07	KD2-H4K8la_peak_2337	11.15747	NFATC3_ENSG00000072736
16	6.8E+07	6.8E+07	KD2-H4K8la_peak_2338	6.13507	SLC7A6_ENSG00000103064
16	6.9E+07	6.9E+07	KD2-H4K8la_peak_2339	7.12014	ZFP90_ENSG00000184939
16	6.9E+07	6.9E+07	KD2-H4K8la_peak_2340	11.06859	
16	6.9E+07	6.9E+07	KD2-H4K8la_peak_2341	10.49982	PDF_ENSG00000258429
16	7E+07	7E+07	KD2-H4K8la_peak_2342	5.70367	
16	7E+07	7E+07	KD2-H4K8la_peak_2343	6.36044	
16	7E+07	7E+07	KD2-H4K8la_peak_2344	6.3399	AARS_ENSG00000090861;DDX19B_ENSG00000157349

16	7E+07	7E+07	KD2-H4K8la_peak_2345	6.61495	
16	7.1E+07	7.1E+07	KD2-H4K8la_peak_2346	6.70626	HYDIN_ENSG00000157423
16	7.1E+07	7.1E+07	KD2-H4K8la_peak_2347	4.75945	RP11-510M2.2_ENSG00000247324
16	7.2E+07	7.2E+07	KD2-H4K8la_peak_2348	9.0413	
16	7.2E+07	7.2E+07	KD2-H4K8la_peak_2349	4.86896	AP1G1_ENSG00000166747
16	7.2E+07	7.2E+07	KD2-H4K8la_peak_2350	6.2278	AP1G1_ENSG00000166747
16	7.2E+07	7.2E+07	KD2-H4K8la_peak_2351	4.6555	ATXN1L_ENSG00000224470;IST1_ENSG00000182149
16	7.2E+07	7.2E+07	KD2-H4K8la_peak_2352	9.35473	ZNF821_ENSG00000102984
16	7.2E+07	7.2E+07	KD2-H4K8la_peak_2353	7.83588	
16	7.3E+07	7.3E+07	KD2-H4K8la_peak_2354	5.36015	
16	7.4E+07	7.4E+07	KD2-H4K8la_peak_2355	10.34978	
16	7.5E+07	7.5E+07	KD2-H4K8la_peak_2356	4.86896	
16	7.5E+07	7.5E+07	KD2-H4K8la_peak_2357	11.20341	
16	7.5E+07	7.5E+07	KD2-H4K8la_peak_2358	10.02595	
16	7.5E+07	7.5E+07	KD2-H4K8la_peak_2359	5.52913	CFDP1_ENSG00000153774
16	7.7E+07	7.7E+07	KD2-H4K8la_peak_2360	7.60756	
16	7.7E+07	7.7E+07	KD2-H4K8la_peak_2361	6.3399	MON1B_ENSG00000103111
16	7.7E+07	7.7E+07	KD2-H4K8la_peak_2362	5.22084	
16	7.7E+07	7.7E+07	KD2-H4K8la_peak_2363	8.07102	
16	8.2E+07	8.2E+07	KD2-H4K8la_peak_2364	11.60966	
16	8.2E+07	8.2E+07	KD2-H4K8la_peak_2365	7.61975	
16	8.2E+07	8.2E+07	KD2-H4K8la_peak_2366	7.12497	
16	8.4E+07	8.4E+07	KD2-H4K8la_peak_2367	6.71169	RP11-483P21.2_ENSG00000260228;HSBP1_ENSG00000230989
16	8.5E+07	8.5E+07	KD2-H4K8la_peak_2368	6.86458	USP10_ENSG00000103194
16	8.5E+07	8.5E+07	KD2-H4K8la_peak_2369	5.83285	ZDHHC7_ENSG00000153786
16	8.5E+07	8.5E+07	KD2-H4K8la_peak_2370	5.43278	
16	8.5E+07	8.5E+07	KD2-H4K8la_peak_2371	5.58883	
16	8.5E+07	8.5E+07	KD2-H4K8la_peak_2372	9.70817	
16	8.5E+07	8.5E+07	KD2-H4K8la_peak_2373	9.18773	
16	8.5E+07	8.5E+07	KD2-H4K8la_peak_2374	7.12014	
16	8.5E+07	8.5E+07	KD2-H4K8la_peak_2375	8.37064	
16	8.5E+07	8.5E+07	KD2-H4K8la_peak_2376	6.86458	
16	8.5E+07	8.5E+07	KD2-H4K8la_peak_2377	5.27164	
16	8.5E+07	8.5E+07	KD2-H4K8la_peak_2378	12.03112	
16	8.5E+07	8.5E+07	KD2-H4K8la_peak_2379	4.75945	
16	8.5E+07	8.5E+07	KD2-H4K8la_peak_2380	6.00056	
16	8.5E+07	8.5E+07	KD2-H4K8la_peak_2381	7.92775	
16	8.5E+07	8.5E+07	KD2-H4K8la_peak_2382	4.96993	
16	8.6E+07	8.6E+07	KD2-H4K8la_peak_2383	6.64804	
16	8.6E+07	8.6E+07	KD2-H4K8la_peak_2384	5.57469	
16	8.6E+07	8.6E+07	KD2-H4K8la_peak_2385	8.37064	
16	8.6E+07	8.6E+07	KD2-H4K8la_peak_2386	7.12014	
16	8.6E+07	8.6E+07	KD2-H4K8la_peak_2387	12.32641	
16	8.7E+07	8.7E+07	KD2-H4K8la_peak_2388	6.64804	MTHFSD_ENSG00000103248
16	8.7E+07	8.7E+07	KD2-H4K8la_peak_2389	14.23294	FBXO31_ENSG00000103264
16	8.8E+07	8.8E+07	KD2-H4K8la_peak_2390	10.49982	RP11-482M8.1_ENSG00000260750

16	8.8E+07	8.8E+07	KD2-H4K8la_peak_2391	6.3399	
16	8.8E+07	8.8E+07	KD2-H4K8la_peak_2392	10.70061	KLHDC4_ENSG00000104731
16	8.8E+07	8.8E+07	KD2-H4K8la_peak_2393	11.20123	RP4-536B24.4_ENSG00000260498
16	8.8E+07	8.8E+07	KD2-H4K8la_peak_2394	6.0136	
16	8.8E+07	8.8E+07	KD2-H4K8la_peak_2395	7.61975	
16	8.8E+07	8.8E+07	KD2-H4K8la_peak_2396	9.06396	
16	8.8E+07	8.8E+07	KD2-H4K8la_peak_2397	7.53947	
16	8.8E+07	8.8E+07	KD2-H4K8la_peak_2398	13.13367	
16	8.8E+07	8.8E+07	KD2-H4K8la_peak_2399	9.73848	
16	8.8E+07	8.8E+07	KD2-H4K8la_peak_2400	7.61975	LA16c-444G7.1_ENSG00000261273
16	8.8E+07	8.8E+07	KD2-H4K8la_peak_2401	7.45205	LA16c-444G7.1_ENSG00000261273
16	8.8E+07	8.8E+07	KD2-H4K8la_peak_2402	5.27164	
16	8.9E+07	8.9E+07	KD2-H4K8la_peak_2403	7.19244	
16	8.9E+07	8.9E+07	KD2-H4K8la_peak_2404	11.21258	
16	8.9E+07	8.9E+07	KD2-H4K8la_peak_2405	6.96161	
16	8.9E+07	8.9E+07	KD2-H4K8la_peak_2406	8.12466	ZC3H18_ENSG00000158545
16	8.9E+07	8.9E+07	KD2-H4K8la_peak_2407	10.04111	GALNS_ENSG00000141012;TRAPPC2L_ENSG00000167515
16	8.9E+07	8.9E+07	KD2-H4K8la_peak_2408	18.73383	
16	8.9E+07	8.9E+07	KD2-H4K8la_peak_2409	9.18773	
16	8.9E+07	8.9E+07	KD2-H4K8la_peak_2410	14.30066	
16	9E+07	9E+07	KD2-H4K8la_peak_2411	8.39919	SPIRE2_ENSG00000204991;FANCA_ENSG00000187741
16	9E+07	9E+07	KD2-H4K8la_peak_2412	4.86896	
16	9E+07	9E+07	KD2-H4K8la_peak_2413	11.15747	CENPBD1_ENSG00000177946;AFG3L1P_ENSG00000223959
16	9E+07	9E+07	KD2-H4K8la_peak_2414	8.66164	DBNDD1_ENSG00000003249;GAS8_ENSG00000141013
16	9E+07	9E+07	KD2-H4K8la_peak_2415	6.86458	URAHP_ENSG00000222019
17	30330	30791	KD2-H4K8la_peak_2416	6.48828	DOC2B_ENSG00000272636
17	259395	259675	KD2-H4K8la_peak_2417	10.36292	C17orf97_ENSG00000187624
17	503548	503843	KD2-H4K8la_peak_2418	5.83285	
17	635148	635428	KD2-H4K8la_peak_2419	4.69635	FAM57A_ENSG00000167695
17	635846	636270	KD2-H4K8la_peak_2420	7.09021	FAM57A_ENSG00000167695
17	636480	636677	KD2-H4K8la_peak_2421	6.22999	FAM57A_ENSG00000167695
17	685187	685440	KD2-H4K8la_peak_2422	6.96161	RNMTL1_ENSG00000171861
17	1057493	1057710	KD2-H4K8la_peak_2423	9.10568	
17	1154970	1155212	KD2-H4K8la_peak_2424	5.92398	
17	1302873	1303284	KD2-H4K8la_peak_2425	13.86277	YWHAE_ENSG00000108953
17	1359060	1359412	KD2-H4K8la_peak_2426	7.9397	
17	1478377	1478730	KD2-H4K8la_peak_2427	5.52913	
17	1551858	1552117	KD2-H4K8la_peak_2428	9.27415	
17	1587841	1588102	KD2-H4K8la_peak_2429	7.12014	PRPF8_ENSG00000174231
17	1620035	1620334	KD2-H4K8la_peak_2430	10.51221	MIR22HG_ENSG00000186594;WDR81_ENSG00000167716
17	1627499	1627731	KD2-H4K8la_peak_2431	8.47421	
17	1665429	1666342	KD2-H4K8la_peak_2432	10.12872	SERPINF1_ENSG00000132386
17	1846226	1846440	KD2-H4K8la_peak_2433	7.65547	
17	1978804	1979282	KD2-H4K8la_peak_2434	7.61975	
17	2296771	2297015	KD2-H4K8la_peak_2435	5.24027	
17	2614101	2614579	KD2-H4K8la_peak_2436	4.53763	

17	2615235	2615913	KD2-H4K8la_peak_2437	10.20357	CLUH_ENSG00000132361;RP11-74E22.3_ENSG00000262050
17	2828921	2829249	KD2-H4K8la_peak_2438	10.20357	
17	2906755	2907260	KD2-H4K8la_peak_2439	7.49467	
17	3598078	3598404	KD2-H4K8la_peak_2440	5.05146	
17	3599524	3599723	KD2-H4K8la_peak_2441	6.28277	P2RX5-TAX1BP3_ENSG00000257950;P2RX5_ENSG00000083454
17	3749047	3749367	KD2-H4K8la_peak_2442	6.40658	C17orf85_ENSG00000074356
17	3796630	3797131	KD2-H4K8la_peak_2443	8.24908	
17	3848372	3848682	KD2-H4K8la_peak_2444	13.5227	
17	4045852	4046151	KD2-H4K8la_peak_2445	5.90392	ZZEF1_ENSG00000074755;CYB5D2_ENSG00000167740
17	4269073	4269274	KD2-H4K8la_peak_2446	5.83285	UBE2G1_ENSG00000132388
17	4378645	4378956	KD2-H4K8la_peak_2447	7.19298	
17	4437368	4438119	KD2-H4K8la_peak_2448	15.19386	
17	4458973	4459478	KD2-H4K8la_peak_2449	6.57426	MYBBP1A_ENSG00000132382
17	4607056	4607263	KD2-H4K8la_peak_2450	5.58883	PELP1_ENSG00000141456;RP11-314A20.2_ENSG00000244184
17	4812653	4813109	KD2-H4K8la_peak_2451	8.37064	
17	4981356	4981573	KD2-H4K8la_peak_2452	7.12014	RP11-46I8.3_ENSG00000262693;ZFP3_ENSG00000180787
17	5390402	5390675	KD2-H4K8la_peak_2453	8.43222	DERL2_ENSG00000072849;MIS12_ENSG00000167842
17	6346459	6346716	KD2-H4K8la_peak_2454	11.20575	
17	6358565	6358771	KD2-H4K8la_peak_2455	6.13507	
17	6555175	6555651	KD2-H4K8la_peak_2456	6.2278	MED31_ENSG00000108590;C17orf100_ENSG00000212734;AC004706.1_ENSG00000256806
17	6922476	6922913	KD2-H4K8la_peak_2457	6.95378	RP11-589P10.7_ENSG00000267047;MIR497HG_ENSG00000267532
17	6923630	6923832	KD2-H4K8la_peak_2458	7.12014	RP11-589P10.7_ENSG00000267047;MIR497HG_ENSG00000267532
17	6939357	6939779	KD2-H4K8la_peak_2459	5.84082	SLC16A13_ENSG00000174327
17	7142100	7142665	KD2-H4K8la_peak_2460	8.43222	PHF23_ENSG00000040633
17	7154568	7155285	KD2-H4K8la_peak_2461	13.6415	CTDNEP1_ENSG00000175826;ELP5_ENSG00000170291
17	7155986	7156205	KD2-H4K8la_peak_2462	4.75945	CTDNEP1_ENSG00000175826
17	7209530	7209993	KD2-H4K8la_peak_2463	8.43222	EIF5A_ENSG00000132507
17	7211566	7211810	KD2-H4K8la_peak_2464	6.61963	
17	7218227	7218475	KD2-H4K8la_peak_2465	9.20163	GPS2_ENSG00000132522
17	7233548	7234333	KD2-H4K8la_peak_2466	8.76116	NEURL4_ENSG00000215041;AC026954.6_ENSG00000224647
17	7284527	7284735	KD2-H4K8la_peak_2467	4.53273	TNK1_ENSG00000174292
17	7287648	7287977	KD2-H4K8la_peak_2468	6.86458	
17	7308283	7308533	KD2-H4K8la_peak_2469	6.88801	TMEM256-PLSCR3_ENSG00000187838;C17orf61-PLSCR3_ENSG00000262481;TMEM256_ENSG00000205544;NLGN2_ENSG00000169992
17	7359926	7360154	KD2-H4K8la_peak_2470	8.12466	
17	7382519	7382906	KD2-H4K8la_peak_2471	7.30722	
17	7383106	7383444	KD2-H4K8la_peak_2472	5.58883	
17	7452457	7452754	KD2-H4K8la_peak_2473	9.27415	TNFSF12_ENSG00000239697;TNFSF12-TNFSF13_ENSG00000248871
17	7476261	7476780	KD2-H4K8la_peak_2474	9.9149	EIF4A1_ENSG00000161960;SNORA67_ENSG00000264772
17	7486341	7486915	KD2-H4K8la_peak_2475	10.28981	AC113189.5_ENSG00000233223;MPDU1_ENSG00000129255
17	7788294	7788524	KD2-H4K8la_peak_2476	9.27415	LSMD1_ENSG00000183011;CHD3_ENSG00000170004
17	7819486	7819982	KD2-H4K8la_peak_2477	5.43278	AC025335.1_ENSG00000179859
17	7982947	7983228	KD2-H4K8la_peak_2478	6.47589	AC129492.6_ENSG00000214999
17	8066675	8067075	KD2-H4K8la_peak_2479	9.35473	RP11-599B13.6_ENSG00000263620;VAMP2_ENSG00000220205
17	8095901	8096164	KD2-H4K8la_peak_2480	8.8293	
17	8103828	8104230	KD2-H4K8la_peak_2481	5.78071	
17	8113457	8113681	KD2-H4K8la_peak_2482	6.89612	AURKB_ENSG00000178999

17	8250466	8250812	KD2-H4K8la_peak_2483	6.2278	
17	1.2E+07	1.2E+07	KD2-H4K8la_peak_2484	10.04111	ZNF18_ENSG00000154957
17	1.2E+07	1.2E+07	KD2-H4K8la_peak_2485	7.30722	RPL21P122_ENSG00000241185;MAP2K4_ENSG00000065559
17	1.5E+07	1.5E+07	KD2-H4K8la_peak_2486	6.3399	
17	1.6E+07	1.6E+07	KD2-H4K8la_peak_2487	6.64804	TRIM16_ENSG00000221926
17	1.6E+07	1.6E+07	KD2-H4K8la_peak_2488	9.86881	MEIS3P1_ENSG00000179277
17	1.6E+07	1.6E+07	KD2-H4K8la_peak_2489	8.37064	MEIS3P1_ENSG00000179277
17	1.6E+07	1.6E+07	KD2-H4K8la_peak_2490	11.77509	ADORA2B_ENSG00000170425
17	1.6E+07	1.6E+07	KD2-H4K8la_peak_2491	6.25288	ADORA2B_ENSG00000170425
17	1.6E+07	1.6E+07	KD2-H4K8la_peak_2492	8.16787	
17	1.6E+07	1.6E+07	KD2-H4K8la_peak_2493	9.70817	UBB_ENSG00000170315
17	1.6E+07	1.6E+07	KD2-H4K8la_peak_2494	7.96951	
17	1.7E+07	1.7E+07	KD2-H4K8la_peak_2495	9.05604	ZNF624_ENSG00000197566;RP11-92B11.4_ENSG00000264765
17	1.7E+07	1.7E+07	KD2-H4K8la_peak_2496	7.92775	
17	1.7E+07	1.7E+07	KD2-H4K8la_peak_2497	10.90862	
17	1.7E+07	1.7E+07	KD2-H4K8la_peak_2498	5.36015	
17	1.7E+07	1.7E+07	KD2-H4K8la_peak_2499	15.75402	MED9_ENSG00000141026
17	1.8E+07	1.8E+07	KD2-H4K8la_peak_2500	7.30722	RAI1_ENSG00000108557
17	1.8E+07	1.8E+07	KD2-H4K8la_peak_2501	13.89769	RAI1_ENSG00000108557
17	1.8E+07	1.8E+07	KD2-H4K8la_peak_2502	10.46101	
17	1.8E+07	1.8E+07	KD2-H4K8la_peak_2503	6.85638	
17	1.8E+07	1.8E+07	KD2-H4K8la_peak_2504	7.3993	
17	1.8E+07	1.8E+07	KD2-H4K8la_peak_2505	12.32641	
17	1.8E+07	1.8E+07	KD2-H4K8la_peak_2506	10.61716	
17	1.8E+07	1.8E+07	KD2-H4K8la_peak_2507	6.61576	SREBF1_ENSG00000072310
17	1.8E+07	1.8E+07	KD2-H4K8la_peak_2508	6.3399	DRG2_ENSG00000108591
17	1.8E+07	1.8E+07	KD2-H4K8la_peak_2509	8.76116	DRG2_ENSG00000108591
17	1.8E+07	1.8E+07	KD2-H4K8la_peak_2510	5.98775	
17	1.8E+07	1.8E+07	KD2-H4K8la_peak_2511	6.86458	TOP3A_ENSG00000177302;SMCR8_ENSG00000176994
17	1.8E+07	1.8E+07	KD2-H4K8la_peak_2512	5.83285	RP1-37N7.3_ENSG00000220161
17	1.9E+07	1.9E+07	KD2-H4K8la_peak_2513	10.28981	TRIM16L_ENSG00000108448
17	1.9E+07	1.9E+07	KD2-H4K8la_peak_2514	5.7631	RNF112_ENSG00000128482
17	1.9E+07	1.9E+07	KD2-H4K8la_peak_2515	7.92775	AC025627.7_ENSG00000228983
17	2E+07	2E+07	KD2-H4K8la_peak_2516	6.71169	ALDH3A2_ENSG00000072210
17	2E+07	2E+07	KD2-H4K8la_peak_2517	12.13455	
17	2E+07	2E+07	KD2-H4K8la_peak_2518	7.61975	ULK2_ENSG00000083290
17	2E+07	2E+07	KD2-H4K8la_peak_2519	7.19244	ULK2_ENSG00000083290
17	2E+07	2E+07	KD2-H4K8la_peak_2520	5.33512	
17	2E+07	2E+07	KD2-H4K8la_peak_2521	8.39919	AKAP10_ENSG00000108599
17	2E+07	2E+07	KD2-H4K8la_peak_2522	9.55729	AKAP10_ENSG00000108599
17	2.1E+07	2.1E+07	KD2-H4K8la_peak_2523	9.86881	
17	2.1E+07	2.1E+07	KD2-H4K8la_peak_2524	11.28887	USP22_ENSG00000124422
17	2.1E+07	2.1E+07	KD2-H4K8la_peak_2525	5.83285	TMEM11_ENSG00000178307;AC087294.2_ENSG00000235530
17	2.6E+07	2.6E+07	KD2-H4K8la_peak_2526	8.76116	RP11-173M1.5_ENSG00000265683
17	2.7E+07	2.7E+07	KD2-H4K8la_peak_2527	4.66473	
17	2.7E+07	2.7E+07	KD2-H4K8la_peak_2528	7.48323	IFT20_ENSG00000109083;TNFAIP1_ENSG00000109079

17	2.7E+07	2.7E+07	KD2-H4K8la_peak_2529	5.05146	VTN_ENSG00000109072
17	2.7E+07	2.7E+07	KD2-H4K8la_peak_2530	12.39776	FAM222B_ENSG00000173065;ERAL1_ENSG00000132591
17	2.7E+07	2.7E+07	KD2-H4K8la_peak_2531	9.45339	FAM222B_ENSG00000173065;ERAL1_ENSG00000132591
17	2.7E+07	2.7E+07	KD2-H4K8la_peak_2532	4.81796	FLOT2_ENSG00000132589;RP11-20B24.4_ENSG00000266642
17	2.7E+07	2.7E+07	KD2-H4K8la_peak_2533	5.86121	
17	2.7E+07	2.7E+07	KD2-H4K8la_peak_2534	5.67034	
17	2.7E+07	2.7E+07	KD2-H4K8la_peak_2535	8.8293	
17	2.8E+07	2.8E+07	KD2-H4K8la_peak_2536	5.05146	MYO18A_ENSG00000196535
17	2.8E+07	2.8E+07	KD2-H4K8la_peak_2537	11.06859	MIR4523_ENSG00000264808;TAOK1_ENSG00000160551;MIR4523_ENSG00000263719
17	2.8E+07	2.8E+07	KD2-H4K8la_peak_2538	5.21483	
17	2.9E+07	2.9E+07	KD2-H4K8la_peak_2539	9.20163	BLMH_ENSG00000108578
17	2.9E+07	2.9E+07	KD2-H4K8la_peak_2540	11.06859	
17	3E+07	3E+07	KD2-H4K8la_peak_2541	6.86458	RAB11FIP4_ENSG00000131242
17	3E+07	3E+07	KD2-H4K8la_peak_2542	7.49467	
17	3E+07	3E+07	KD2-H4K8la_peak_2543	12.32641	MIR193A_ENSG00000207614
17	3E+07	3E+07	KD2-H4K8la_peak_2544	23.77242	RP11-640N20.6_ENSG00000264164
17	3.1E+07	3.1E+07	KD2-H4K8la_peak_2545	5.58883	PSMD11_ENSG00000108671
17	3.1E+07	3.1E+07	KD2-H4K8la_peak_2546	8.07044	RP11-466A19.6_ENSG00000263717;RP11-466A19.3_ENSG00000266599
17	3.1E+07	3.1E+07	KD2-H4K8la_peak_2547	7.72421	
17	3.3E+07	3.3E+07	KD2-H4K8la_peak_2548	5.36015	ZNF830_ENSG00000198783
17	3.3E+07	3.3E+07	KD2-H4K8la_peak_2549	5.43278	LIG3_ENSG00000005156;CCT6B_ENSG00000132141
17	3.4E+07	3.4E+07	KD2-H4K8la_peak_2550	9.72904	RP11-1094M14.1_ENSG00000242660;RP11-1094M14.11_ENSG00000267321
17	3.4E+07	3.4E+07	KD2-H4K8la_peak_2551	10.04111	
17	3.5E+07	3.5E+07	KD2-H4K8la_peak_2552	4.75945	PIGW_ENSG00000184886
17	3.5E+07	3.5E+07	KD2-H4K8la_peak_2553	7.12014	
17	3.5E+07	3.5E+07	KD2-H4K8la_peak_2554	7.3993	
17	3.5E+07	3.5E+07	KD2-H4K8la_peak_2555	6.64804	
17	3.6E+07	3.6E+07	KD2-H4K8la_peak_2556	8.07044	
17	3.7E+07	3.7E+07	KD2-H4K8la_peak_2557	7.83588	
17	3.7E+07	3.7E+07	KD2-H4K8la_peak_2558	10.31941	
17	3.7E+07	3.7E+07	KD2-H4K8la_peak_2559	5.56351	SRCIN1_ENSG00000017373
17	3.7E+07	3.7E+07	KD2-H4K8la_peak_2560	6.3399	SRCIN1_ENSG00000017373
17	3.7E+07	3.7E+07	KD2-H4K8la_peak_2561	7.51014	
17	3.7E+07	3.7E+07	KD2-H4K8la_peak_2562	6.71169	MLLT6_ENSG00000108292
17	3.7E+07	3.7E+07	KD2-H4K8la_peak_2563	8.37064	
17	3.7E+07	3.7E+07	KD2-H4K8la_peak_2564	10.21747	PCGF2_ENSG00000056661;CTB-58E17.5_ENSG00000227450
17	3.7E+07	3.7E+07	KD2-H4K8la_peak_2565	6.64804	RPL23_ENSG00000125691;RPS20P35_ENSG00000244086
17	3.7E+07	3.7E+07	KD2-H4K8la_peak_2566	4.53273	
17	3.8E+07	3.8E+07	KD2-H4K8la_peak_2567	6.2624	MED1_ENSG00000125686
17	3.8E+07	3.8E+07	KD2-H4K8la_peak_2568	9.0413	
17	3.8E+07	3.8E+07	KD2-H4K8la_peak_2569	10.20357	AC087491.2_ENSG00000214546
17	3.8E+07	3.8E+07	KD2-H4K8la_peak_2570	7.3993	PNMT_ENSG00000141744
17	3.8E+07	3.8E+07	KD2-H4K8la_peak_2571	11.40266	
17	3.8E+07	3.8E+07	KD2-H4K8la_peak_2572	9.37576	
17	3.8E+07	3.8E+07	KD2-H4K8la_peak_2573	8.76116	ORMDL3_ENSG00000172057;RP11-387H17.4_ENSG00000264968
17	3.8E+07	3.8E+07	KD2-H4K8la_peak_2574	7.22716	

17	3.8E+07	3.8E+07	KD2-H4K8la_peak_2575	9.11321	
17	3.8E+07	3.8E+07	KD2-H4K8la_peak_2576	6.11881	
17	3.8E+07	3.8E+07	KD2-H4K8la_peak_2577	7.01227	
17	3.8E+07	3.8E+07	KD2-H4K8la_peak_2578	7.01227	
17	3.9E+07	3.9E+07	KD2-H4K8la_peak_2579	8.26997	
17	3.9E+07	3.9E+07	KD2-H4K8la_peak_2580	6.3399	IGFBP4_ENSG00000141753
17	3.9E+07	3.9E+07	KD2-H4K8la_peak_2581	12.65402	
17	3.9E+07	3.9E+07	KD2-H4K8la_peak_2582	6.3399	
17	3.9E+07	3.9E+07	KD2-H4K8la_peak_2583	6.17271	
17	3.9E+07	3.9E+07	KD2-H4K8la_peak_2584	11.7374	
17	3.9E+07	3.9E+07	KD2-H4K8la_peak_2585	7.61975	
17	3.9E+07	3.9E+07	KD2-H4K8la_peak_2586	10.21747	
17	3.9E+07	3.9E+07	KD2-H4K8la_peak_2587	7.19244	
17	3.9E+07	3.9E+07	KD2-H4K8la_peak_2588	6.56022	
17	3.9E+07	3.9E+07	KD2-H4K8la_peak_2589	12.92898	
17	4E+07	4E+07	KD2-H4K8la_peak_2590	6.16656	
17	4E+07	4E+07	KD2-H4K8la_peak_2591	10.90862	
17	4E+07	4E+07	KD2-H4K8la_peak_2592	7.49467	
17	4E+07	4E+07	KD2-H4K8la_peak_2593	5.58883	
17	4E+07	4E+07	KD2-H4K8la_peak_2594	5.7631	STAT5B_ENSG00000173757
17	4E+07	4E+07	KD2-H4K8la_peak_2595	8.84474	STAT5B_ENSG00000173757
17	4.1E+07	4.1E+07	KD2-H4K8la_peak_2596	5.67596	RP11-400F19.6_ENSG00000266962
17	4.1E+07	4.1E+07	KD2-H4K8la_peak_2597	6.40658	COASY_ENSG00000068120
17	4.1E+07	4.1E+07	KD2-H4K8la_peak_2598	8.59561	COASY_ENSG00000068120
17	4.1E+07	4.1E+07	KD2-H4K8la_peak_2599	6.3399	
17	4.1E+07	4.1E+07	KD2-H4K8la_peak_2600	13.27013	CTD-3193K9.4_ENSG00000267042
17	4.1E+07	4.1E+07	KD2-H4K8la_peak_2601	6.86458	RAMP2-AS1_ENSG00000197291
17	4.1E+07	4.1E+07	KD2-H4K8la_peak_2602	9.73848	Y_RNA_ENSG00000200127;RPL27_ENSG00000131469
17	4.1E+07	4.1E+07	KD2-H4K8la_peak_2603	9.38376	
17	4.1E+07	4.1E+07	KD2-H4K8la_peak_2604	7.47988	
17	4.2E+07	4.2E+07	KD2-H4K8la_peak_2605	9.27415	DHX8_ENSG00000067596
17	4.2E+07	4.2E+07	KD2-H4K8la_peak_2606	8.37064	RP11-392O1.4_ENSG00000267747
17	4.2E+07	4.2E+07	KD2-H4K8la_peak_2607	10.49169	
17	4.2E+07	4.2E+07	KD2-H4K8la_peak_2608	8.73956	
17	4.2E+07	4.2E+07	KD2-H4K8la_peak_2609	12.13455	RP11-209M4.1_ENSG00000267253
17	4.2E+07	4.2E+07	KD2-H4K8la_peak_2610	9.27415	DUSP3_ENSG00000108861
17	4.2E+07	4.2E+07	KD2-H4K8la_peak_2611	13.13367	RP11-527L4.2_ENSG00000261514
17	4.2E+07	4.2E+07	KD2-H4K8la_peak_2612	6.66491	
17	4.2E+07	4.2E+07	KD2-H4K8la_peak_2613	10.27876	
17	4.2E+07	4.2E+07	KD2-H4K8la_peak_2614	11.7362	
17	4.2E+07	4.2E+07	KD2-H4K8la_peak_2615	8.39919	
17	4.2E+07	4.2E+07	KD2-H4K8la_peak_2616	7.49467	C17orf53_ENSG00000125319
17	4.2E+07	4.2E+07	KD2-H4K8la_peak_2617	6.64804	ASB16-AS1_ENSG00000267080;TMUB2_ENSG00000168591
17	4.2E+07	4.2E+07	KD2-H4K8la_peak_2618	5.9366	ATXN7L3_ENSG00000087152;CTB-175E5.7_ENSG00000267394
17	4.2E+07	4.2E+07	KD2-H4K8la_peak_2619	8.76116	ATXN7L3_ENSG00000087152
17	4.2E+07	4.2E+07	KD2-H4K8la_peak_2620	8.43222	

17	4.2E+07	4.2E+07	KD2-H4K8la_peak_2621	5.7631	GRN_ENSG00000030582
17	4.2E+07	4.2E+07	KD2-H4K8la_peak_2622	7.56395	
17	4.3E+07	4.3E+07	KD2-H4K8la_peak_2623	6.2278	GPATCH8_ENSG00000186566
17	4.3E+07	4.3E+07	KD2-H4K8la_peak_2624	7.52934	GPATCH8_ENSG00000186566
17	4.3E+07	4.3E+07	KD2-H4K8la_peak_2625	8.76116	
17	4.3E+07	4.3E+07	KD2-H4K8la_peak_2626	9.98668	
17	4.3E+07	4.3E+07	KD2-H4K8la_peak_2627	7.30722	ADAM11_ENSG00000073670
17	4.3E+07	4.3E+07	KD2-H4K8la_peak_2628	5.05146	ADAM11_ENSG00000073670
17	4.3E+07	4.3E+07	KD2-H4K8la_peak_2629	5.43278	GJC1_ENSG00000182963
17	4.3E+07	4.3E+07	KD2-H4K8la_peak_2630	14.26267	
17	4.3E+07	4.3E+07	KD2-H4K8la_peak_2631	8.13557	
17	4.3E+07	4.3E+07	KD2-H4K8la_peak_2632	7.51014	
17	4.3E+07	4.3E+07	KD2-H4K8la_peak_2633	4.59516	HEXIM1_ENSG00000186834
17	4.3E+07	4.3E+07	KD2-H4K8la_peak_2634	7.94013	
17	4.3E+07	4.3E+07	KD2-H4K8la_peak_2635	7.01227	
17	4.4E+07	4.4E+07	KD2-H4K8la_peak_2636	6.3399	
17	4.4E+07	4.4E+07	KD2-H4K8la_peak_2637	10.48625	DND1P1_ENSG00000264070
17	4.4E+07	4.4E+07	KD2-H4K8la_peak_2638	6.4136	DND1P1_ENSG00000264070
17	4.4E+07	4.4E+07	KD2-H4K8la_peak_2639	16.21274	MAPT_ENSG00000186868
17	4.4E+07	4.4E+07	KD2-H4K8la_peak_2640	7.49467	RP11-259G18.1_ENSG00000261575
17	4.5E+07	4.5E+07	KD2-H4K8la_peak_2641	17.3629	
17	4.5E+07	4.5E+07	KD2-H4K8la_peak_2642	12.62032	
17	4.5E+07	4.5E+07	KD2-H4K8la_peak_2643	10.9753	RP11-156P1.3_ENSG00000262879
17	4.5E+07	4.5E+07	KD2-H4K8la_peak_2644	11.24937	RP11-290H9.4_ENSG00000263293;EFCAB13_ENSG00000178852
17	4.6E+07	4.6E+07	KD2-H4K8la_peak_2645	5.43278	
17	4.6E+07	4.6E+07	KD2-H4K8la_peak_2646	7.59044	LRRC46_ENSG00000141294;MRPL10_ENSG00000159111
17	4.6E+07	4.6E+07	KD2-H4K8la_peak_2647	8.24671	SCRN2_ENSG00000141295
17	4.6E+07	4.6E+07	KD2-H4K8la_peak_2648	5.90392	RP11-6N17.4_ENSG00000264920;SP2_ENSG00000167182
17	4.6E+07	4.6E+07	KD2-H4K8la_peak_2649	11.20341	AC003665.1_ENSG00000234494;PNPO_ENSG00000108439
17	4.6E+07	4.6E+07	KD2-H4K8la_peak_2650	8.1293	
17	4.6E+07	4.6E+07	KD2-H4K8la_peak_2651	7.75076	
17	4.6E+07	4.6E+07	KD2-H4K8la_peak_2652	14.75465	
17	4.6E+07	4.6E+07	KD2-H4K8la_peak_2653	17.48572	
17	4.6E+07	4.6E+07	KD2-H4K8la_peak_2654	5.22418	
17	4.6E+07	4.6E+07	KD2-H4K8la_peak_2655	6.56022	
17	4.6E+07	4.6E+07	KD2-H4K8la_peak_2656	13.5227	
17	4.6E+07	4.6E+07	KD2-H4K8la_peak_2657	9.06396	COPZ2_ENSG00000005243;MIR152_ENSG00000207947
17	4.6E+07	4.6E+07	KD2-H4K8la_peak_2658	4.67224	RP5-890E16.2_ENSG00000263412;NFE2L1_ENSG00000082641
17	4.6E+07	4.6E+07	KD2-H4K8la_peak_2659	6.86458	RP5-890E16.2_ENSG00000263412;NFE2L1_ENSG00000082641
17	4.7E+07	4.7E+07	KD2-H4K8la_peak_2660	9.0516	ATP5G1_ENSG00000159199
17	4.7E+07	4.7E+07	KD2-H4K8la_peak_2661	11.40266	SNF8_ENSG00000159210
17	4.7E+07	4.7E+07	KD2-H4K8la_peak_2662	9.67249	ZNF652_ENSG00000198740;RP11-1079K10.3_ENSG00000248714
17	4.8E+07	4.8E+07	KD2-H4K8la_peak_2663	6.20477	
17	4.8E+07	4.8E+07	KD2-H4K8la_peak_2664	5.23773	RP11-613C6.2_ENSG00000250751
17	4.8E+07	4.8E+07	KD2-H4K8la_peak_2665	8.7731	DLX4_ENSG00000108813
17	4.8E+07	4.8E+07	KD2-H4K8la_peak_2666	6.68593	

17	4.8E+07	4.8E+07	KD2-H4K8la_peak_2667	8.26997	
17	4.8E+07	4.8E+07	KD2-H4K8la_peak_2668	5.23773	RP11-893F2.13_ENSG00000253730
17	4.8E+07	4.8E+07	KD2-H4K8la_peak_2669	9.31176	
17	4.8E+07	4.8E+07	KD2-H4K8la_peak_2670	9.78176	RP11-893F2.5_ENSG00000249406
17	4.8E+07	4.8E+07	KD2-H4K8la_peak_2671	14.5688	RP11-893F2.5_ENSG00000249406
17	4.9E+07	4.9E+07	KD2-H4K8la_peak_2672	9.5329	ACSF2_ENSG00000167107
17	4.9E+07	4.9E+07	KD2-H4K8la_peak_2673	7.23932	RSAD1_ENSG00000136444
17	4.9E+07	4.9E+07	KD2-H4K8la_peak_2674	11.05666	RP11-94C24.6_ENSG00000249451;MYCBPAP_ENSG00000136449
17	4.9E+07	4.9E+07	KD2-H4K8la_peak_2675	7.33892	RP11-94C24.6_ENSG00000249451;MYCBPAP_ENSG00000136449
17	4.9E+07	4.9E+07	KD2-H4K8la_peak_2676	13.89767	
17	4.9E+07	4.9E+07	KD2-H4K8la_peak_2677	6.68593	ANKRD40_ENSG00000154945
17	4.9E+07	4.9E+07	KD2-H4K8la_peak_2678	10.90862	
17	4.9E+07	4.9E+07	KD2-H4K8la_peak_2679	13.27915	
17	4.9E+07	4.9E+07	KD2-H4K8la_peak_2680	9.03222	
17	4.9E+07	4.9E+07	KD2-H4K8la_peak_2681	7.79412	
17	4.9E+07	4.9E+07	KD2-H4K8la_peak_2682	14.22324	RP11-700H6.1_ENSG00000247011;RP11-700H6.2_ENSG00000251665
17	4.9E+07	4.9E+07	KD2-H4K8la_peak_2683	11.20341	
17	4.9E+07	4.9E+07	KD2-H4K8la_peak_2684	10.49982	SPAG9_ENSG00000008294
17	4.9E+07	4.9E+07	KD2-H4K8la_peak_2685	9.78176	
17	4.9E+07	4.9E+07	KD2-H4K8la_peak_2686	13.62642	MBTD1_ENSG00000011258;UTP18_ENSG00000011260
17	5.4E+07	5.4E+07	KD2-H4K8la_peak_2687	9.9701	
17	5.5E+07	5.5E+07	KD2-H4K8la_peak_2688	9.66714	TRIM25_ENSG00000121060
17	5.5E+07	5.5E+07	KD2-H4K8la_peak_2689	5.84082	
17	5.5E+07	5.5E+07	KD2-H4K8la_peak_2690	8.39919	MSI2_ENSG00000153944
17	5.5E+07	5.5E+07	KD2-H4K8la_peak_2691	5.09794	
17	5.5E+07	5.5E+07	KD2-H4K8la_peak_2692	6.74498	
17	5.6E+07	5.6E+07	KD2-H4K8la_peak_2693	13.37883	
17	5.6E+07	5.6E+07	KD2-H4K8la_peak_2694	10.61716	
17	5.6E+07	5.6E+07	KD2-H4K8la_peak_2695	7.91061	
17	5.6E+07	5.6E+07	KD2-H4K8la_peak_2696	7.63122	
17	5.6E+07	5.6E+07	KD2-H4K8la_peak_2697	7.3993	
17	5.6E+07	5.6E+07	KD2-H4K8la_peak_2698	11.81691	
17	5.6E+07	5.6E+07	KD2-H4K8la_peak_2699	12.70652	
17	5.6E+07	5.6E+07	KD2-H4K8la_peak_2700	8.76116	
17	5.6E+07	5.6E+07	KD2-H4K8la_peak_2701	5.67596	
17	5.6E+07	5.6E+07	KD2-H4K8la_peak_2702	10.20357	
17	5.6E+07	5.6E+07	KD2-H4K8la_peak_2703	7.72519	
17	5.6E+07	5.6E+07	KD2-H4K8la_peak_2704	12.74311	
17	5.6E+07	5.6E+07	KD2-H4K8la_peak_2705	8.59561	
17	5.6E+07	5.6E+07	KD2-H4K8la_peak_2706	6.3399	
17	5.6E+07	5.6E+07	KD2-H4K8la_peak_2707	9.92993	
17	5.6E+07	5.6E+07	KD2-H4K8la_peak_2708	7.3993	
17	5.6E+07	5.6E+07	KD2-H4K8la_peak_2709	8.48488	
17	5.6E+07	5.6E+07	KD2-H4K8la_peak_2710	7.77341	
17	5.6E+07	5.6E+07	KD2-H4K8la_peak_2711	9.61894	
17	5.6E+07	5.6E+07	KD2-H4K8la_peak_2712	5.43264	

17	5.6E+07	5.6E+07	KD2-H4K8la_peak_2713	6.61296	
17	5.6E+07	5.6E+07	KD2-H4K8la_peak_2714	6.09309	
17	5.6E+07	5.6E+07	KD2-H4K8la_peak_2715	14.7348	
17	5.6E+07	5.6E+07	KD2-H4K8la_peak_2716	11.7362	
17	5.6E+07	5.6E+07	KD2-H4K8la_peak_2717	10.0147	
17	5.6E+07	5.6E+07	KD2-H4K8la_peak_2718	6.47266	
17	5.6E+07	5.6E+07	KD2-H4K8la_peak_2719	9.61894	
17	5.6E+07	5.6E+07	KD2-H4K8la_peak_2720	12.98337	
17	5.6E+07	5.6E+07	KD2-H4K8la_peak_2721	10.22817	
17	5.6E+07	5.6E+07	KD2-H4K8la_peak_2722	8.1293	
17	5.6E+07	5.6E+07	KD2-H4K8la_peak_2723	9.20163	
17	5.6E+07	5.6E+07	KD2-H4K8la_peak_2724	8.40421	
17	5.6E+07	5.6E+07	KD2-H4K8la_peak_2725	7.94013	
17	5.6E+07	5.6E+07	KD2-H4K8la_peak_2726	13.27013	
17	5.6E+07	5.6E+07	KD2-H4K8la_peak_2727	8.15033	
17	5.6E+07	5.6E+07	KD2-H4K8la_peak_2728	9.87752	
17	5.6E+07	5.6E+07	KD2-H4K8la_peak_2729	6.13507	
17	5.6E+07	5.6E+07	KD2-H4K8la_peak_2730	5.70367	
17	5.6E+07	5.6E+07	KD2-H4K8la_peak_2731	7.68525	
17	5.6E+07	5.6E+07	KD2-H4K8la_peak_2732	8.76116	
17	5.6E+07	5.6E+07	KD2-H4K8la_peak_2733	5.33711	
17	5.6E+07	5.6E+07	KD2-H4K8la_peak_2734	7.12308	CUEDC1_ENSG00000180891
17	5.6E+07	5.6E+07	KD2-H4K8la_peak_2735	8.95597	SRSF1_ENSG00000136450
17	5.6E+07	5.6E+07	KD2-H4K8la_peak_2736	6.4136	RP11-159D12.10_ENSG00000266290;DYNLL2_ENSG00000264364
17	5.6E+07	5.6E+07	KD2-H4K8la_peak_2737	6.57176	MKS1_ENSG00000011143;LPO_ENSG00000167419
17	5.6E+07	5.6E+07	KD2-H4K8la_peak_2738	11.42932	BZRAP1-AS1_ENSG00000265148
17	5.6E+07	5.6E+07	KD2-H4K8la_peak_2739	5.24676	
17	5.6E+07	5.6E+07	KD2-H4K8la_peak_2740	6.98743	SUPT4H1_ENSG00000213246
17	5.7E+07	5.7E+07	KD2-H4K8la_peak_2741	14.7348	
17	5.7E+07	5.7E+07	KD2-H4K8la_peak_2742	10.47118	TEX14_ENSG00000121101;RAD51C_ENSG00000108384
17	5.7E+07	5.7E+07	KD2-H4K8la_peak_2743	11.57314	PPM1E_ENSG00000175175
17	5.7E+07	5.7E+07	KD2-H4K8la_peak_2744	8.39919	SMG8_ENSG00000167447
17	5.7E+07	5.7E+07	KD2-H4K8la_peak_2745	5.58883	
17	5.7E+07	5.7E+07	KD2-H4K8la_peak_2746	7.56395	
17	5.8E+07	5.8E+07	KD2-H4K8la_peak_2747	10.11868	
17	5.8E+07	5.8E+07	KD2-H4K8la_peak_2748	11.25108	
17	5.8E+07	5.8E+07	KD2-H4K8la_peak_2749	8.76116	
17	5.8E+07	5.8E+07	KD2-H4K8la_peak_2750	6.38565	CLTC_ENSG00000141367
17	5.8E+07	5.8E+07	KD2-H4K8la_peak_2751	5.5326	PTRH2_ENSG00000141378;VMP1_ENSG00000062716
17	5.8E+07	5.8E+07	KD2-H4K8la_peak_2752	11.16073	TUBD1_ENSG00000108423;RPS6KB1_ENSG00000108443
17	5.8E+07	5.8E+07	KD2-H4K8la_peak_2753	5.36015	
17	5.8E+07	5.8E+07	KD2-H4K8la_peak_2754	6.97699	C17orf64_ENSG00000141371
17	5.8E+07	5.8E+07	KD2-H4K8la_peak_2755	8.13557	USP32_ENSG00000170832
17	5.9E+07	5.9E+07	KD2-H4K8la_peak_2756	7.19244	PPM1D_ENSG00000170836
17	5.9E+07	5.9E+07	KD2-H4K8la_peak_2757	12.3825	RP11-136H19.1_ENSG00000267667
17	5.9E+07	5.9E+07	KD2-H4K8la_peak_2758	9.34957	

17	5.9E+07	5.9E+07	KD2-H4K8la_peak_2759	8.39919	
17	5.9E+07	5.9E+07	KD2-H4K8la_peak_2760	5.83285	
17	5.9E+07	5.9E+07	KD2-H4K8la_peak_2761	9.45281	
17	5.9E+07	5.9E+07	KD2-H4K8la_peak_2762	6.59357	
17	5.9E+07	5.9E+07	KD2-H4K8la_peak_2763	8.39919	RP11-332H18.3_ENSG00000266934
17	5.9E+07	5.9E+07	KD2-H4K8la_peak_2764	11.63682	RP11-332H18.3_ENSG00000266934
17	5.9E+07	5.9E+07	KD2-H4K8la_peak_2765	8.24671	
17	5.9E+07	5.9E+07	KD2-H4K8la_peak_2766	9.34828	
17	5.9E+07	5.9E+07	KD2-H4K8la_peak_2767	5.84385	
17	5.9E+07	5.9E+07	KD2-H4K8la_peak_2768	9.19451	
17	5.9E+07	5.9E+07	KD2-H4K8la_peak_2769	8.03267	RP11-332H18.4_ENSG00000267280;C17orf82_ENSG00000187013
17	5.9E+07	5.9E+07	KD2-H4K8la_peak_2770	7.84882	C17orf82_ENSG00000187013
17	5.9E+07	5.9E+07	KD2-H4K8la_peak_2771	9.44217	
17	6E+07	6E+07	KD2-H4K8la_peak_2772	8.84265	
17	6E+07	6E+07	KD2-H4K8la_peak_2773	4.6555	
17	6E+07	6E+07	KD2-H4K8la_peak_2774	8.0374	
17	6E+07	6E+07	KD2-H4K8la_peak_2775	6.13507	
17	6E+07	6E+07	KD2-H4K8la_peak_2776	10.97465	INTS2_ENSG00000108506
17	6E+07	6E+07	KD2-H4K8la_peak_2777	11.87032	MED13_ENSG00000108510
17	6E+07	6E+07	KD2-H4K8la_peak_2778	5.64331	
17	6.1E+07	6.1E+07	KD2-H4K8la_peak_2779	8.13557	
17	6.1E+07	6.1E+07	KD2-H4K8la_peak_2780	18.43157	
17	6.1E+07	6.1E+07	KD2-H4K8la_peak_2781	9.87983	MRC2_ENSG00000011028
17	6.1E+07	6.1E+07	KD2-H4K8la_peak_2782	6.13507	
17	6.1E+07	6.1E+07	KD2-H4K8la_peak_2783	13.27013	
17	6.1E+07	6.1E+07	KD2-H4K8la_peak_2784	9.19451	
17	6.1E+07	6.1E+07	KD2-H4K8la_peak_2785	9.5329	
17	6.2E+07	6.2E+07	KD2-H4K8la_peak_2786	5.83285	
17	6.2E+07	6.2E+07	KD2-H4K8la_peak_2787	7.21572	RP11-269G24.4_ENSG00000265282
17	6.2E+07	6.2E+07	KD2-H4K8la_peak_2788	8.50781	
17	6.2E+07	6.2E+07	KD2-H4K8la_peak_2789	14.89648	
17	6.2E+07	6.2E+07	KD2-H4K8la_peak_2790	11.70246	
17	6.2E+07	6.2E+07	KD2-H4K8la_peak_2791	6.56022	CYB561_ENSG00000008283
17	6.2E+07	6.2E+07	KD2-H4K8la_peak_2792	19.14893	CYB561_ENSG00000008283
17	6.2E+07	6.2E+07	KD2-H4K8la_peak_2793	5.22616	TACO1_ENSG00000136463
17	6.2E+07	6.2E+07	KD2-H4K8la_peak_2794	20.04954	LIMD2_ENSG00000136490
17	6.2E+07	6.2E+07	KD2-H4K8la_peak_2795	6.57426	
17	6.2E+07	6.2E+07	KD2-H4K8la_peak_2796	5.91374	C17orf72_ENSG00000224383
17	6.3E+07	6.3E+07	KD2-H4K8la_peak_2797	4.44613	
17	6.3E+07	6.3E+07	KD2-H4K8la_peak_2798	5.90392	
17	6.3E+07	6.3E+07	KD2-H4K8la_peak_2799	12.83706	LRRC37A3_ENSG00000176809
17	6.3E+07	6.3E+07	KD2-H4K8la_peak_2800	9.18773	AMZ2P1_ENSG00000214174
17	6.3E+07	6.3E+07	KD2-H4K8la_peak_2801	5.80814	AMZ2P1_ENSG00000214174
17	6.3E+07	6.3E+07	KD2-H4K8la_peak_2802	7.94013	GNA13_ENSG00000120063
17	6.3E+07	6.3E+07	KD2-H4K8la_peak_2803	8.44557	
17	6.4E+07	6.4E+07	KD2-H4K8la_peak_2804	6.20793	AXIN2_ENSG00000168646

17	6.4E+07	6.4E+07	KD2-H4K8la_peak_2805	7.77341	
17	6.5E+07	6.5E+07	KD2-H4K8la_peak_2806	6.3399	
17	6.5E+07	6.5E+07	KD2-H4K8la_peak_2807	10.10182	CACNG4_ENSG00000075461
17	6.5E+07	6.5E+07	KD2-H4K8la_peak_2808	12.77347	
17	6.5E+07	6.5E+07	KD2-H4K8la_peak_2809	4.6555	
17	6.5E+07	6.5E+07	KD2-H4K8la_peak_2810	5.0802	
17	6.5E+07	6.5E+07	KD2-H4K8la_peak_2811	6.00056	
17	6.5E+07	6.5E+07	KD2-H4K8la_peak_2812	7.86224	
17	6.5E+07	6.5E+07	KD2-H4K8la_peak_2813	6.59654	
17	6.5E+07	6.5E+07	KD2-H4K8la_peak_2814	12.55003	
17	6.5E+07	6.5E+07	KD2-H4K8la_peak_2815	13.76639	
17	6.5E+07	6.5E+07	KD2-H4K8la_peak_2816	5.24027	RP11-349A8.3_ENSG00000264491
17	6.5E+07	6.5E+07	KD2-H4K8la_peak_2817	5.22616	HELZ_ENSG00000198265;RP11-401F2.3_ENSG00000266473
17	6.5E+07	6.5E+07	KD2-H4K8la_peak_2818	5.468	HELZ_ENSG00000198265
17	6.5E+07	6.5E+07	KD2-H4K8la_peak_2819	5.23773	PSMD12_ENSG00000197170
17	6.5E+07	6.5E+07	KD2-H4K8la_peak_2820	8.4237	PITPNC1_ENSG00000154217
17	6.6E+07	6.6E+07	KD2-H4K8la_peak_2821	8.00701	
17	6.6E+07	6.6E+07	KD2-H4K8la_peak_2822	10.59328	
17	6.6E+07	6.6E+07	KD2-H4K8la_peak_2823	6.03112	NOL11_ENSG00000130935
17	6.6E+07	6.6E+07	KD2-H4K8la_peak_2824	11.24289	BPTF_ENSG00000171634
17	6.6E+07	6.6E+07	KD2-H4K8la_peak_2825	4.6555	C17orf58_ENSG00000186665
17	6.6E+07	6.6E+07	KD2-H4K8la_peak_2826	5.58883	
17	6.6E+07	6.6E+07	KD2-H4K8la_peak_2827	5.43278	
17	6.7E+07	6.7E+07	KD2-H4K8la_peak_2828	5.67596	ABCA5_ENSG00000154265
17	6.7E+07	6.7E+07	KD2-H4K8la_peak_2829	8.13557	ABCA5_ENSG00000154265
17	6.7E+07	6.7E+07	KD2-H4K8la_peak_2830	7.23932	MAP2K6_ENSG00000108984
17	6.9E+07	6.9E+07	KD2-H4K8la_peak_2831	5.33364	RP11-238F2.1_ENSG00000271239
17	7.1E+07	7.1E+07	KD2-H4K8la_peak_2832	5.29444	
17	7.1E+07	7.1E+07	KD2-H4K8la_peak_2833	8.00569	SLC39A11_ENSG00000133195
17	7.1E+07	7.1E+07	KD2-H4K8la_peak_2834	6.86458	COG1_ENSG00000166685
17	7.1E+07	7.1E+07	KD2-H4K8la_peak_2835	8.69391	
17	7.1E+07	7.1E+07	KD2-H4K8la_peak_2836	15.52202	
17	7.1E+07	7.1E+07	KD2-H4K8la_peak_2837	7.60503	
17	7.1E+07	7.1E+07	KD2-H4K8la_peak_2838	12.50352	
17	7.2E+07	7.2E+07	KD2-H4K8la_peak_2839	5.57299	
17	7.2E+07	7.2E+07	KD2-H4K8la_peak_2840	9.87983	
17	7.2E+07	7.2E+07	KD2-H4K8la_peak_2841	5.27164	
17	7.3E+07	7.3E+07	KD2-H4K8la_peak_2842	9.5503	MIR3615_ENSG00000266036;MIR3615_ENSG00000264624;SLC9A3R1_ENSG00000109062
17	7.3E+07	7.3E+07	KD2-H4K8la_peak_2843	7.36752	CDR2L_ENSG00000109089
17	7.3E+07	7.3E+07	KD2-H4K8la_peak_2844	7.79412	ICT1_ENSG00000167862
17	7.3E+07	7.3E+07	KD2-H4K8la_peak_2845	5.33301	
17	7.3E+07	7.3E+07	KD2-H4K8la_peak_2846	11.87032	
17	7.3E+07	7.3E+07	KD2-H4K8la_peak_2847	6.59654	ATP5H_ENSG00000167863
17	7.3E+07	7.3E+07	KD2-H4K8la_peak_2848	9.48529	ARMC7_ENSG00000125449
17	7.3E+07	7.3E+07	KD2-H4K8la_peak_2849	6.03112	NT5C_ENSG00000125458
17	7.3E+07	7.3E+07	KD2-H4K8la_peak_2850	13.27013	SUMO2_ENSG00000188612

17	7.3E+07	7.3E+07	KD2-H4K8la_peak_2851	15.77733	SUMO2_ENSG00000188612
17	7.3E+07	7.3E+07	KD2-H4K8la_peak_2852	7.86224	GGA3_ENSG00000125447;MRPS7_ENSG00000125445
17	7.3E+07	7.3E+07	KD2-H4K8la_peak_2853	8.89364	MIF4GD_ENSG00000125457;RP11-649A18.12_ENSG00000263843
17	7.3E+07	7.3E+07	KD2-H4K8la_peak_2854	7.30347	MIF4GD_ENSG00000125457;RP11-649A18.12_ENSG00000263843
17	7.3E+07	7.3E+07	KD2-H4K8la_peak_2855	8.3497	
17	7.4E+07	7.4E+07	KD2-H4K8la_peak_2856	10.4679	CASKIN2_ENSG00000177303;TSEN54_ENSG00000182173
17	7.4E+07	7.4E+07	KD2-H4K8la_peak_2857	4.69635	
17	7.4E+07	7.4E+07	KD2-H4K8la_peak_2858	6.83387	
17	7.4E+07	7.4E+07	KD2-H4K8la_peak_2859	23.25607	MYO15B_ENSG00000266714
17	7.4E+07	7.4E+07	KD2-H4K8la_peak_2860	12.83706	
17	7.4E+07	7.4E+07	KD2-H4K8la_peak_2861	5.66318	
17	7.4E+07	7.4E+07	KD2-H4K8la_peak_2862	11.7374	
17	7.4E+07	7.4E+07	KD2-H4K8la_peak_2863	8.3497	WBP2_ENSG00000132471
17	7.4E+07	7.4E+07	KD2-H4K8la_peak_2864	8.48488	TRIM65_ENSG00000141569;RP11-552F3.10_ENSG00000267342
17	7.4E+07	7.4E+07	KD2-H4K8la_peak_2865	11.24289	
17	7.4E+07	7.4E+07	KD2-H4K8la_peak_2866	12.13455	CDK3_ENSG00000250506
17	7.4E+07	7.4E+07	KD2-H4K8la_peak_2867	21.32197	SRP68_ENSG00000167881;ZACN_ENSG00000186919
17	7.4E+07	7.4E+07	KD2-H4K8la_peak_2868	11.7362	
17	7.4E+07	7.4E+07	KD2-H4K8la_peak_2869	4.68264	RNF157_ENSG00000141576
17	7.4E+07	7.4E+07	KD2-H4K8la_peak_2870	5.99975	PRPSAP1_ENSG00000161542
17	7.4E+07	7.4E+07	KD2-H4K8la_peak_2871	6.13507	
17	7.5E+07	7.5E+07	KD2-H4K8la_peak_2872	6.47266	
17	7.5E+07	7.5E+07	KD2-H4K8la_peak_2873	10.94155	
17	7.5E+07	7.5E+07	KD2-H4K8la_peak_2874	11.92959	
17	7.5E+07	7.5E+07	KD2-H4K8la_peak_2875	11.55303	
17	7.5E+07	7.5E+07	KD2-H4K8la_peak_2876	11.62424	
17	7.5E+07	7.5E+07	KD2-H4K8la_peak_2877	16.34466	
17	7.5E+07	7.5E+07	KD2-H4K8la_peak_2878	15.79607	
17	7.5E+07	7.5E+07	KD2-H4K8la_peak_2879	7.0396	
17	7.5E+07	7.5E+07	KD2-H4K8la_peak_2880	11.90654	SRSF2_ENSG00000161547;MFSD11_ENSG00000092931;MIR636_ENSG00000207556
17	7.5E+07	7.5E+07	KD2-H4K8la_peak_2881	4.96683	
17	7.5E+07	7.5E+07	KD2-H4K8la_peak_2882	7.80361	
17	7.5E+07	7.5E+07	KD2-H4K8la_peak_2883	11.64946	AC015815.3_ENSG00000203316
17	7.5E+07	7.5E+07	KD2-H4K8la_peak_2884	14.26347	
17	7.5E+07	7.5E+07	KD2-H4K8la_peak_2885	9.20163	RP11-285E9.6_ENSG00000263718;SEPT9_ENSG00000184640
17	7.5E+07	7.5E+07	KD2-H4K8la_peak_2886	6.39807	
17	7.5E+07	7.5E+07	KD2-H4K8la_peak_2887	12.77347	
17	7.5E+07	7.5E+07	KD2-H4K8la_peak_2888	11.28887	
17	7.5E+07	7.5E+07	KD2-H4K8la_peak_2889	7.55141	
17	7.5E+07	7.5E+07	KD2-H4K8la_peak_2890	9.18773	
17	7.5E+07	7.5E+07	KD2-H4K8la_peak_2891	6.72313	
17	7.5E+07	7.5E+07	KD2-H4K8la_peak_2892	7.9957	RP11-936I5.1_ENSG00000266998
17	7.5E+07	7.5E+07	KD2-H4K8la_peak_2893	10.34952	RP11-936I5.1_ENSG00000266998
17	7.5E+07	7.5E+07	KD2-H4K8la_peak_2894	10.87112	
17	7.5E+07	7.5E+07	KD2-H4K8la_peak_2895	14.61956	
17	7.5E+07	7.5E+07	KD2-H4K8la_peak_2896	11.18137	

17	7.5E+07	7.5E+07	KD2-H4K8la_peak_2897	7.36235	
17	7.5E+07	7.5E+07	KD2-H4K8la_peak_2898	7.60503	
17	7.6E+07	7.6E+07	KD2-H4K8la_peak_2899	4.86896	
17	7.6E+07	7.6E+07	KD2-H4K8la_peak_2900	13.51092	
17	7.6E+07	7.6E+07	KD2-H4K8la_peak_2901	5.43278	
17	7.6E+07	7.6E+07	KD2-H4K8la_peak_2902	6.13507	
17	7.6E+07	7.6E+07	KD2-H4K8la_peak_2903	10.20834	
17	7.6E+07	7.6E+07	KD2-H4K8la_peak_2904	21.14996	FLJ45079_ENSG00000204283
17	7.6E+07	7.6E+07	KD2-H4K8la_peak_2905	8.3068	
17	7.6E+07	7.6E+07	KD2-H4K8la_peak_2906	9.67249	
17	7.6E+07	7.6E+07	KD2-H4K8la_peak_2907	5.24027	
17	7.6E+07	7.6E+07	KD2-H4K8la_peak_2908	6.44746	
17	7.6E+07	7.6E+07	KD2-H4K8la_peak_2909	5.26403	SYNGR2_ENSG00000108639
17	7.6E+07	7.6E+07	KD2-H4K8la_peak_2910	9.44217	BIRC5_ENSG00000089685
17	7.6E+07	7.6E+07	KD2-H4K8la_peak_2911	6.53039	TMEM235_ENSG00000204278
17	7.6E+07	7.6E+07	KD2-H4K8la_peak_2912	6.68593	
17	7.6E+07	7.6E+07	KD2-H4K8la_peak_2913	9.20163	
17	7.6E+07	7.6E+07	KD2-H4K8la_peak_2914	7.78365	THA1P_ENSG00000267676
17	7.6E+07	7.6E+07	KD2-H4K8la_peak_2915	10.50851	
17	7.6E+07	7.6E+07	KD2-H4K8la_peak_2916	8.59561	
17	7.6E+07	7.6E+07	KD2-H4K8la_peak_2917	16.12381	
17	7.6E+07	7.6E+07	KD2-H4K8la_peak_2918	5.40012	PGS1_ENSG00000087157
17	7.7E+07	7.7E+07	KD2-H4K8la_peak_2919	6.19992	
17	7.7E+07	7.7E+07	KD2-H4K8la_peak_2920	5.58883	CYTH1_ENSG00000108669
17	7.7E+07	7.7E+07	KD2-H4K8la_peak_2921	5.80814	USP36_ENSG00000055483
17	7.7E+07	7.7E+07	KD2-H4K8la_peak_2922	5.81654	
17	7.7E+07	7.7E+07	KD2-H4K8la_peak_2923	6.75222	
17	7.7E+07	7.7E+07	KD2-H4K8la_peak_2924	13.00097	TIMP2_ENSG00000035862
17	7.7E+07	7.7E+07	KD2-H4K8la_peak_2925	7.0396	
17	7.7E+07	7.7E+07	KD2-H4K8la_peak_2926	14.19183	LGALS3BP_ENSG00000108679
17	7.7E+07	7.7E+07	KD2-H4K8la_peak_2927	11.15747	CANT1_ENSG00000171302
17	7.7E+07	7.7E+07	KD2-H4K8la_peak_2928	6.3399	
17	7.8E+07	7.8E+07	KD2-H4K8la_peak_2929	18.43157	
17	7.8E+07	7.8E+07	KD2-H4K8la_peak_2930	10.66741	
17	7.8E+07	7.8E+07	KD2-H4K8la_peak_2931	12.85504	CBX2_ENSG00000173894
17	7.8E+07	7.8E+07	KD2-H4K8la_peak_2932	6.59654	CBX2_ENSG00000173894
17	7.8E+07	7.8E+07	KD2-H4K8la_peak_2933	6.86458	
17	7.8E+07	7.8E+07	KD2-H4K8la_peak_2934	16.55399	
17	7.8E+07	7.8E+07	KD2-H4K8la_peak_2935	15.95674	
17	7.8E+07	7.8E+07	KD2-H4K8la_peak_2936	4.69635	
17	7.8E+07	7.8E+07	KD2-H4K8la_peak_2937	7.94013	
17	7.8E+07	7.8E+07	KD2-H4K8la_peak_2938	6.06216	CTD-2529O21.1_ENSG00000262172
17	7.8E+07	7.8E+07	KD2-H4K8la_peak_2939	7.94013	
17	7.8E+07	7.8E+07	KD2-H4K8la_peak_2940	9.77334	GAA_ENSG00000171298
17	7.8E+07	7.8E+07	KD2-H4K8la_peak_2941	7.3993	RNF213_ENSG00000173821
17	7.8E+07	7.8E+07	KD2-H4K8la_peak_2942	4.68264	RNF213_ENSG00000173821

17	7.8E+07	7.8E+07	KD2-H4K8la_peak_2943	17.56185	CTD-2526A2.2_ENSG00000260369
17	7.8E+07	7.8E+07	KD2-H4K8la_peak_2944	9.14592	CTD-2526A2.2_ENSG00000260369
17	7.9E+07	7.9E+07	KD2-H4K8la_peak_2945	9.29385	RPTOR_ENSG00000141564
17	7.9E+07	7.9E+07	KD2-H4K8la_peak_2946	9.92226	RPTOR_ENSG00000141564
17	7.9E+07	7.9E+07	KD2-H4K8la_peak_2947	5.23773	
17	7.9E+07	7.9E+07	KD2-H4K8la_peak_2948	11.31083	
17	7.9E+07	7.9E+07	KD2-H4K8la_peak_2949	6.98743	
17	7.9E+07	7.9E+07	KD2-H4K8la_peak_2950	12.44878	CHMP6_ENSG00000176108
17	7.9E+07	7.9E+07	KD2-H4K8la_peak_2951	6.85108	
17	7.9E+07	7.9E+07	KD2-H4K8la_peak_2952	8.22514	AATK_ENSG00000181409;AATK-AS1_ENSG00000225180
17	7.9E+07	7.9E+07	KD2-H4K8la_peak_2953	9.10568	AATK_ENSG00000181409
17	7.9E+07	7.9E+07	KD2-H4K8la_peak_2954	17.30105	AZI1_ENSG00000141577
17	7.9E+07	7.9E+07	KD2-H4K8la_peak_2955	9.92226	ENTHD2_ENSG00000167302;C17orf89_ENSG00000224877
17	7.9E+07	7.9E+07	KD2-H4K8la_peak_2956	9.9149	SLC38A10_ENSG00000157637
17	7.9E+07	7.9E+07	KD2-H4K8la_peak_2957	15.96001	LINC00482_ENSG00000185168
17	7.9E+07	7.9E+07	KD2-H4K8la_peak_2958	6.47266	
17	7.9E+07	7.9E+07	KD2-H4K8la_peak_2959	8.39919	
17	7.9E+07	7.9E+07	KD2-H4K8la_peak_2960	4.75945	
17	7.9E+07	7.9E+07	KD2-H4K8la_peak_2961	11.74575	
17	7.9E+07	7.9E+07	KD2-H4K8la_peak_2962	7.56738	TMEM105_ENSG00000185332
17	7.9E+07	7.9E+07	KD2-H4K8la_peak_2963	8.62939	
17	7.9E+07	7.9E+07	KD2-H4K8la_peak_2964	7.3993	
17	7.9E+07	7.9E+07	KD2-H4K8la_peak_2965	10.48445	
17	7.9E+07	7.9E+07	KD2-H4K8la_peak_2966	5.73529	
17	7.9E+07	7.9E+07	KD2-H4K8la_peak_2967	5.90392	
17	7.9E+07	7.9E+07	KD2-H4K8la_peak_2968	9.48547	
17	7.9E+07	7.9E+07	KD2-H4K8la_peak_2969	8.3574	
17	7.9E+07	7.9E+07	KD2-H4K8la_peak_2970	4.68264	
17	7.9E+07	7.9E+07	KD2-H4K8la_peak_2971	8.50453	
17	7.9E+07	7.9E+07	KD2-H4K8la_peak_2972	6.13507	
17	7.9E+07	7.9E+07	KD2-H4K8la_peak_2973	4.95845	
17	7.9E+07	7.9E+07	KD2-H4K8la_peak_2974	9.67249	
17	7.9E+07	7.9E+07	KD2-H4K8la_peak_2975	5.86422	
17	7.9E+07	7.9E+07	KD2-H4K8la_peak_2976	7.79412	
17	7.9E+07	7.9E+07	KD2-H4K8la_peak_2977	14.22324	
17	7.9E+07	7.9E+07	KD2-H4K8la_peak_2978	7.12308	RP13-766D20.1_ENSG00000229947;RP13-766D20.2_ENSG00000229848
17	8E+07	8E+07	KD2-H4K8la_peak_2979	9.87983	
17	8E+07	8E+07	KD2-H4K8la_peak_2980	14.55919	OXLD1_ENSG00000204237;CCDC137_ENSG00000185298
17	8E+07	8E+07	KD2-H4K8la_peak_2981	7.94013	RP13-1032I1.7_ENSG00000262049;MRPL12_ENSG00000262814;SLC25A10_ENSG00000262660;SLC25A10_ENSG00000183048
17	8E+07	8E+07	KD2-H4K8la_peak_2982	6.03112	
17	8E+07	8E+07	KD2-H4K8la_peak_2983	9.19451	FAM195B_ENSG00000225663
17	8E+07	8E+07	KD2-H4K8la_peak_2984	5.90392	P4HB_ENSG00000185624
17	8E+07	8E+07	KD2-H4K8la_peak_2985	6.95908	RP11-498C9.3_ENSG00000262413
17	8E+07	8E+07	KD2-H4K8la_peak_2986	5.48061	ARHGDIA_ENSG00000141522
17	8E+07	8E+07	KD2-H4K8la_peak_2987	24.81934	ARHGDIA_ENSG00000141522
17	8E+07	8E+07	KD2-H4K8la_peak_2988	7.48323	ALYREF_ENSG00000183684;ANAPC11_ENSG00000141552

17	8E+07	8E+07	KD2-H4K8la_peak_2989	9.03222	
17	8E+07	8E+07	KD2-H4K8la_peak_2990	7.84781	
17	8E+07	8E+07	KD2-H4K8la_peak_2991	10.27305	RP11-498C9.17_ENSG00000264735
17	8E+07	8E+07	KD2-H4K8la_peak_2992	9.86736	
17	8E+07	8E+07	KD2-H4K8la_peak_2993	13.27013	
17	8E+07	8E+07	KD2-H4K8la_peak_2994	7.61975	RFNG_ENSG00000169733;GPS1_ENSG00000169727
17	8E+07	8E+07	KD2-H4K8la_peak_2995	15.63685	DUS1L_ENSG00000169718
17	8E+07	8E+07	KD2-H4K8la_peak_2996	15.47627	FASN_ENSG00000169710
17	8E+07	8E+07	KD2-H4K8la_peak_2997	6.2413	
17	8E+07	8E+07	KD2-H4K8la_peak_2998	7.12308	
17	8E+07	8E+07	KD2-H4K8la_peak_2999	8.29695	CCDC57_ENSG00000176155;RP13-516M14.2_ENSG00000264548
17	8E+07	8E+07	KD2-H4K8la_peak_3000	9.67249	CSNK1D_ENSG00000141551
17	8E+07	8E+07	KD2-H4K8la_peak_3001	10.47118	
17	8E+07	8E+07	KD2-H4K8la_peak_3002	7.49897	
17	8.1E+07	8.1E+07	KD2-H4K8la_peak_3003	8.48488	WDR45B_ENSG00000141580
17	8.1E+07	8.1E+07	KD2-H4K8la_peak_3004	8.39919	RP11-388C12.1_ENSG00000263063;FN3KRP_ENSG00000141560
17	8.1E+07	8.1E+07	KD2-H4K8la_peak_3005	6.56022	FN3K_ENSG00000167363;RP11-388C12.5_ENSG00000263321
17	8.1E+07	8.1E+07	KD2-H4K8la_peak_3006	4.86896	B3GNTL1_ENSG00000175711
17	8.1E+07	8.1E+07	KD2-H4K8la_peak_3007	12.24258	B3GNTL1_ENSG00000175711
18	812508	812758	KD2-H4K8la_peak_3008	9.11321	YES1_ENSG00000176105;RP11-672L10.6_ENSG00000273355
18	2906845	2907131	KD2-H4K8la_peak_3009	8.06229	
18	3246750	3246966	KD2-H4K8la_peak_3010	5.36015	RP13-270P17.3_ENSG00000272688;MYL12A_ENSG00000101608
18	3594600	3594835	KD2-H4K8la_peak_3011	4.86896	DLGAP1-AS1_ENSG00000177337
18	3621985	3622189	KD2-H4K8la_peak_3012	7.72421	
18	5238304	5238598	KD2-H4K8la_peak_3013	7.83588	RP11-835E18.5_ENSG00000265091;LINC00526_ENSG00000264575
18	5895751	5896046	KD2-H4K8la_peak_3014	8.19799	TMEM200C_ENSG00000206432
18	8704715	8704990	KD2-H4K8la_peak_3015	8.24908	SOGA2_ENSG00000168502
18	8751628	8751866	KD2-H4K8la_peak_3016	4.53273	
18	9803893	9804172	KD2-H4K8la_peak_3017	8.76116	
18	1E+07	1E+07	KD2-H4K8la_peak_3018	6.11881	APCDD1_ENSG00000154856
18	1.3E+07	1.3E+07	KD2-H4K8la_peak_3019	4.66473	CEP76_ENSG00000101624
18	1.3E+07	1.3E+07	KD2-H4K8la_peak_3020	8.07102	
18	1.3E+07	1.3E+07	KD2-H4K8la_peak_3021	8.65116	
18	1.3E+07	1.3E+07	KD2-H4K8la_peak_3022	7.55141	
18	1.3E+07	1.3E+07	KD2-H4K8la_peak_3023	7.56395	LDLRAD4_ENSG00000168675
18	1.4E+07	1.4E+07	KD2-H4K8la_peak_3024	8.07102	RP11-53B2.5_ENSG00000267366
18	1.4E+07	1.4E+07	KD2-H4K8la_peak_3025	8.37064	FAM210A_ENSG00000177150;RNMT_ENSG00000101654
18	1.4E+07	1.4E+07	KD2-H4K8la_peak_3026	9.0413	ZNF519_ENSG00000175322
18	2.1E+07	2.1E+07	KD2-H4K8la_peak_3027	6.71169	RP11-17J14.2_ENSG00000266495
18	2.1E+07	2.1E+07	KD2-H4K8la_peak_3028	6.3399	TMEM241_ENSG00000134490
18	2.1E+07	2.1E+07	KD2-H4K8la_peak_3029	5.83285	
18	2.4E+07	2.4E+07	KD2-H4K8la_peak_3030	11.40266	KCTD1_ENSG00000134504;AQP4-AS1_ENSG00000260372
18	2.6E+07	2.6E+07	KD2-H4K8la_peak_3031	4.30663	CDH2_ENSG00000170558
18	2.9E+07	2.9E+07	KD2-H4K8la_peak_3032	8.37064	B4GALT6_ENSG00000118276;RP11-549B18.1_ENSG00000259985
18	3E+07	3E+07	KD2-H4K8la_peak_3033	5.69935	RP11-53I6.2_ENSG00000263917
18	3.4E+07	3.4E+07	KD2-H4K8la_peak_3034	11.28887	SLC39A6_ENSG00000141424;ELP2_ENSG00000134759

18	3.4E+07	3.4E+07	KD2-H4K8la_peak_3035	8.43222	FHOD3_ENSG00000134775
18	4.2E+07	4.2E+07	KD2-H4K8la_peak_3036	8.8263	RP11-456K23.1_ENSG00000267414;SETBP1_ENSG00000152217
18	4.2E+07	4.2E+07	KD2-H4K8la_peak_3037	6.66491	
18	4.6E+07	4.6E+07	KD2-H4K8la_peak_3038	15.04077	
18	4.6E+07	4.6E+07	KD2-H4K8la_peak_3039	5.30989	
18	4.6E+07	4.6E+07	KD2-H4K8la_peak_3040	8.37064	
18	4.6E+07	4.6E+07	KD2-H4K8la_peak_3041	6.88801	
18	4.6E+07	4.6E+07	KD2-H4K8la_peak_3042	6.86458	
18	4.6E+07	4.6E+07	KD2-H4K8la_peak_3043	7.51014	
18	4.6E+07	4.6E+07	KD2-H4K8la_peak_3044	5.05146	
18	4.7E+07	4.7E+07	KD2-H4K8la_peak_3045	7.61975	
18	4.7E+07	4.7E+07	KD2-H4K8la_peak_3046	8.12466	
18	4.7E+07	4.7E+07	KD2-H4K8la_peak_3047	5.52913	ACAA2_ENSG00000167315;SCARNA17_ENSG00000267322;SCARNA17_ENSG00000251992;SCARNA18_ENSG00000252139
18	4.8E+07	4.8E+07	KD2-H4K8la_peak_3048	7.92775	ELAC1_ENSG00000141642;RP11-729L2.2_ENSG00000267699;SMAD4_ENSG00000141646
18	4.9E+07	4.9E+07	KD2-H4K8la_peak_3049	11.77509	
18	4.9E+07	4.9E+07	KD2-H4K8la_peak_3050	7.60756	
18	5.3E+07	5.3E+07	KD2-H4K8la_peak_3051	7.12014	
18	5.3E+07	5.3E+07	KD2-H4K8la_peak_3052	5.33301	
18	5.5E+07	5.5E+07	KD2-H4K8la_peak_3053	5.72892	
18	5.5E+07	5.5E+07	KD2-H4K8la_peak_3054	5.83285	RP11-35G9.3_ENSG00000267040
18	5.6E+07	5.6E+07	KD2-H4K8la_peak_3055	13.20018	RP11-126O1.5_ENSG00000267226;MALT1_ENSG00000172175
18	5.7E+07	5.7E+07	KD2-H4K8la_peak_3056	12.74483	
18	6.1E+07	6.1E+07	KD2-H4K8la_peak_3057	10.36292	VPS4B_ENSG00000119541
18	6.8E+07	6.8E+07	KD2-H4K8la_peak_3058	7.12014	SOCS6_ENSG00000170677
18	6.8E+07	6.8E+07	KD2-H4K8la_peak_3059	12.1221	SOCS6_ENSG00000170677
18	6.8E+07	6.8E+07	KD2-H4K8la_peak_3060	4.60523	
18	7.2E+07	7.2E+07	KD2-H4K8la_peak_3061	8.487	FBXO15_ENSG00000141665;TIMM21_ENSG00000075336
18	7.2E+07	7.2E+07	KD2-H4K8la_peak_3062	8.30878	
18	7.2E+07	7.2E+07	KD2-H4K8la_peak_3063	6.11881	LINC00909_ENSG00000264247;ZNF407_ENSG00000215421
18	7.3E+07	7.3E+07	KD2-H4K8la_peak_3064	9.38376	
18	7.4E+07	7.4E+07	KD2-H4K8la_peak_3065	10.99007	
18	7.5E+07	7.5E+07	KD2-H4K8la_peak_3066	7.12014	RNU6-346P_ENSG00000252097
18	7.5E+07	7.5E+07	KD2-H4K8la_peak_3067	6.64804	RP11-162A12.2_ENSG00000264278;ZNF236_ENSG00000130856
18	7.5E+07	7.5E+07	KD2-H4K8la_peak_3068	8.76116	RP11-162A12.2_ENSG00000264278;ZNF236_ENSG00000130856
18	7.7E+07	7.7E+07	KD2-H4K8la_peak_3069	8.43222	ATP9B_ENSG00000166377
18	7.7E+07	7.7E+07	KD2-H4K8la_peak_3070	6.13507	ATP9B_ENSG00000166377
18	7.8E+07	7.8E+07	KD2-H4K8la_peak_3071	6.3399	PQLC1_ENSG00000122490
18	7.8E+07	7.8E+07	KD2-H4K8la_peak_3072	6.89612	HSBP1L1_ENSG00000226742
19	290924	291181	KD2-H4K8la_peak_3073	5.83285	PPAP2C_ENSG00000141934
19	291728	291975	KD2-H4K8la_peak_3074	8.37064	PPAP2C_ENSG00000141934
19	344986	345183	KD2-H4K8la_peak_3075	8.37064	MIER2_ENSG00000105556
19	402367	402601	KD2-H4K8la_peak_3076	6.2278	
19	448129	448355	KD2-H4K8la_peak_3077	9.73848	
19	524232	524723	KD2-H4K8la_peak_3078	11.60966	
19	662809	663055	KD2-H4K8la_peak_3079	6.20477	RNF126_ENSG00000070423;AC004156.3_ENSG00000267666
19	679522	679827	KD2-H4K8la_peak_3080	6.95378	

19	680092	680299	KD2-H4K8la_peak_3081	10.49982	
19	719647	719979	KD2-H4K8la_peak_3082	8.07102	
19	720765	721113	KD2-H4K8la_peak_3083	4.75945	
19	789157	789646	KD2-H4K8la_peak_3084	10.02595	
19	796163	796871	KD2-H4K8la_peak_3085	14.22324	PTBP1_ENSG00000011304
19	821539	822728	KD2-H4K8la_peak_3086	8.79494	LPPR3_ENSG00000129951
19	924895	925396	KD2-H4K8la_peak_3087	10.36292	ARID3A_ENSG00000116017
19	932840	933127	KD2-H4K8la_peak_3088	8.37064	
19	1074394	1074659	KD2-H4K8la_peak_3089	7.23932	
19	1180528	1180806	KD2-H4K8la_peak_3090	6.39623	
19	1205236	1205452	KD2-H4K8la_peak_3091	8.00569	
19	1239706	1240049	KD2-H4K8la_peak_3092	12.32641	
19	1261742	1261952	KD2-H4K8la_peak_3093	5.86882	
19	1269358	1269589	KD2-H4K8la_peak_3094	14.71706	CIRBP-AS1_ENSG00000267493
19	1275530	1275815	KD2-H4K8la_peak_3095	22.92734	C19orf24_ENSG00000228300
19	1285385	1285604	KD2-H4K8la_peak_3096	5.84385	MUM1_ENSG00000160953;EFNA2_ENSG00000099617
19	1354513	1354741	KD2-H4K8la_peak_3097	8.39919	
19	1444766	1445087	KD2-H4K8la_peak_3098	5.83285	
19	1446298	1446506	KD2-H4K8la_peak_3099	7.61975	APC2_ENSG00000115266
19	1479590	1479994	KD2-H4K8la_peak_3100	9.00481	C19orf25_ENSG00000119559
19	1567349	1567588	KD2-H4K8la_peak_3101	7.30722	MEX3D_ENSG00000181588
19	1605018	1605304	KD2-H4K8la_peak_3102	7.12014	UQCR11_ENSG00000267059;UQCR11_ENSG00000127540
19	1725377	1725586	KD2-H4K8la_peak_3103	6.86458	
19	1811962	1812186	KD2-H4K8la_peak_3104	7.10162	ATP8B3_ENSG00000130270
19	1847961	1848662	KD2-H4K8la_peak_3105	12.07697	REXO1_ENSG00000079313
19	1876486	1876788	KD2-H4K8la_peak_3106	9.0516	CTB-31O20.2_ENSG00000261526
19	2016319	2016543	KD2-H4K8la_peak_3107	5.83285	
19	2042740	2043039	KD2-H4K8la_peak_3108	5.63442	
19	2051495	2051884	KD2-H4K8la_peak_3109	13.27013	MKNK2_ENSG00000099875
19	2052152	2052368	KD2-H4K8la_peak_3110	5.05146	MKNK2_ENSG00000099875
19	2056310	2056550	KD2-H4K8la_peak_3111	9.52279	
19	2060526	2061220	KD2-H4K8la_peak_3112	8.89364	
19	2061866	2062495	KD2-H4K8la_peak_3113	12.85404	
19	2235944	2236195	KD2-H4K8la_peak_3114	7.83588	SF3A2_ENSG00000104897
19	2328768	2329023	KD2-H4K8la_peak_3115	9.37943	LSM7_ENSG00000130332;SPPL2B_ENSG00000005206
19	2427231	2427486	KD2-H4K8la_peak_3116	10.90862	TIMM13_ENSG00000099800
19	2456525	2456829	KD2-H4K8la_peak_3117	8.16787	LMNB2_ENSG00000176619
19	2461807	2462037	KD2-H4K8la_peak_3118	5.52913	AC005624.2_ENSG00000267201
19	2462830	2463059	KD2-H4K8la_peak_3119	8.33785	AC005624.2_ENSG00000267201
19	2543147	2543424	KD2-H4K8la_peak_3120	11.06859	
19	2820054	2820300	KD2-H4K8la_peak_3121	6.3399	ZNF554_ENSG00000172006
19	2866709	2866933	KD2-H4K8la_peak_3122	5.36015	ZNF556_ENSG00000172000
19	2867338	2867606	KD2-H4K8la_peak_3123	7.13237	ZNF556_ENSG00000172000
19	2983437	2983643	KD2-H4K8la_peak_3124	4.75924	
19	3062593	3062857	KD2-H4K8la_peak_3125	8.59561	AES_ENSG00000104964
19	3358915	3359262	KD2-H4K8la_peak_3126	9.27415	NFIC_ENSG00000141905

19	3367641	3367911	KD2-H4K8la_peak_3127	9.11321	
19	3434293	3434736	KD2-H4K8la_peak_3128	6.97699	
19	3441377	3441705	KD2-H4K8la_peak_3129	6.13507	
19	3442822	3443025	KD2-H4K8la_peak_3130	9.60611	
19	3501321	3501534	KD2-H4K8la_peak_3131	7.49467	DOHH_ENSG00000129932;RN7SL866P_ENSG00000264159
19	3505623	3505897	KD2-H4K8la_peak_3132	7.75048	FZR1_ENSG00000105325
19	3506474	3506752	KD2-H4K8la_peak_3133	5.57469	FZR1_ENSG00000105325
19	3607097	3607298	KD2-H4K8la_peak_3134	7.30722	TBXA2R_ENSG00000006638;CACTIN-AS1_ENSG00000226800
19	3985053	3985424	KD2-H4K8la_peak_3135	6.36118	EEF2_ENSG00000167658
19	4007828	4008070	KD2-H4K8la_peak_3136	7.22716	PIAS4_ENSG00000105229
19	4066399	4066751	KD2-H4K8la_peak_3137	13.63083	ZBTB7A_ENSG00000178951
19	4140577	4141080	KD2-H4K8la_peak_3138	6.26214	
19	4182051	4182489	KD2-H4K8la_peak_3139	4.66473	SIRT6_ENSG00000077463;ANKRD24_ENSG00000089847
19	4246230	4246781	KD2-H4K8la_peak_3140	10.74378	CCDC94_ENSG00000105248
19	4277684	4278435	KD2-H4K8la_peak_3141	8.24908	SHD_ENSG00000105251
19	4279397	4280426	KD2-H4K8la_peak_3142	7.61975	SHD_ENSG00000105251
19	4342177	4342648	KD2-H4K8la_peak_3143	8.73956	STAP2_ENSG00000178078;MPND_ENSG00000008382
19	4402718	4403130	KD2-H4K8la_peak_3144	11.87032	CHAF1A_ENSG00000167670
19	4670713	4671093	KD2-H4K8la_peak_3145	7.92775	C19orf10_ENSG00000074842
19	4723500	4723773	KD2-H4K8la_peak_3146	6.86458	DPP9_ENSG00000142002
19	4815409	4815614	KD2-H4K8la_peak_3147	7.49467	
19	4816067	4816858	KD2-H4K8la_peak_3148	15.10098	
19	4908993	4909295	KD2-H4K8la_peak_3149	9.0516	
19	5107813	5108136	KD2-H4K8la_peak_3150	6.16608	
19	5340378	5340630	KD2-H4K8la_peak_3151	7.61975	PTPRS_ENSG00000105426
19	5437065	5437273	KD2-H4K8la_peak_3152	7.13237	
19	5772864	5773411	KD2-H4K8la_peak_3153	7.30722	
19	5827326	5827559	KD2-H4K8la_peak_3154	6.96161	
19	5994563	5994987	KD2-H4K8la_peak_3155	5.43278	
19	6361626	6361883	KD2-H4K8la_peak_3156	7.12014	CTB-180A7.3_ENSG00000269802;CLPP_ENSG00000125656
19	6424419	6424621	KD2-H4K8la_peak_3157	7.01227	KHSRP_ENSG00000088247
19	6504542	6504768	KD2-H4K8la_peak_3158	7.1623	
19	6739207	6739447	KD2-H4K8la_peak_3159	10.90862	
19	7433187	7433448	KD2-H4K8la_peak_3160	7.09021	
19	7558831	7559226	KD2-H4K8la_peak_3161	13.20018	C19orf45_ENSG00000198723
19	7580169	7580449	KD2-H4K8la_peak_3162	8.86558	ZNF358_ENSG00000198816
19	7581201	7581556	KD2-H4K8la_peak_3163	13.40081	
19	7598367	7598579	KD2-H4K8la_peak_3164	11.36807	CTD-2207O23.10_ENSG00000268614;PNPLA6_ENSG00000032444
19	7600790	7601136	KD2-H4K8la_peak_3165	8.39919	
19	7615686	7615907	KD2-H4K8la_peak_3166	7.97203	
19	7684570	7684997	KD2-H4K8la_peak_3167	5.99671	
19	7745344	7745579	KD2-H4K8la_peak_3168	5.83285	TRAPPC5_ENSG00000181029
19	7745816	7746073	KD2-H4K8la_peak_3169	5.52913	TRAPPC5_ENSG00000181029
19	8008278	8008475	KD2-H4K8la_peak_3170	6.89612	TIMM44_ENSG00000104980
19	8273505	8273764	KD2-H4K8la_peak_3171	7.49467	
19	8385892	8386090	KD2-H4K8la_peak_3172	6.68593	NDUFA7_ENSG00000167774;NDUFA7_ENSG00000267855;RPS28_ENSG00000233927

19	8455348	8455553	KD2-H4K8la_peak_3173	8.39919	RAB11B-AS1_ENSG00000269386;RAB11B_ENSG00000185236
19	8570647	8571045	KD2-H4K8la_peak_3174	7.8597	
19	8577992	8578569	KD2-H4K8la_peak_3175	9.98668	ZNF414_ENSG00000133250
19	9517340	9517715	KD2-H4K8la_peak_3176	8.65116	
19	9546393	9546854	KD2-H4K8la_peak_3177	8.86558	ZNF266_ENSG00000174652
19	9648850	9649538	KD2-H4K8la_peak_3178	11.20575	ZNF426_ENSG00000130818
19	9649855	9650060	KD2-H4K8la_peak_3179	9.38376	ZNF426_ENSG00000130818
19	9786296	9786531	KD2-H4K8la_peak_3180	11.5908	ZNF562_ENSG00000171466
19	1E+07	1E+07	KD2-H4K8la_peak_3181	6.07357	
19	1E+07	1E+07	KD2-H4K8la_peak_3182	8.94515	C19orf66_ENSG00000130813
19	1E+07	1E+07	KD2-H4K8la_peak_3183	8.12466	PPAN-P2RY11_ENSG00000243207;PPAN_ENSG00000130810
19	1E+07	1E+07	KD2-H4K8la_peak_3184	10.02595	TYK2_ENSG00000105397
19	1.1E+07	1.1E+07	KD2-H4K8la_peak_3185	7.65547	
19	1.1E+07	1.1E+07	KD2-H4K8la_peak_3186	7.23932	
19	1.1E+07	1.1E+07	KD2-H4K8la_peak_3187	6.68593	
19	1.1E+07	1.1E+07	KD2-H4K8la_peak_3188	9.20163	KEAP1_ENSG00000079999
19	1.1E+07	1.1E+07	KD2-H4K8la_peak_3189	5.05146	CARM1_ENSG00000142453
19	1.1E+07	1.1E+07	KD2-H4K8la_peak_3190	8.78735	SMARCA4_ENSG00000127616
19	1.1E+07	1.1E+07	KD2-H4K8la_peak_3191	4.86896	
19	1.1E+07	1.1E+07	KD2-H4K8la_peak_3192	5.51108	CTC-510F12.2_ENSG00000267082
19	1.1E+07	1.1E+07	KD2-H4K8la_peak_3193	5.22084	DKFZP761J1410_ENSG00000105520
19	1.2E+07	1.2E+07	KD2-H4K8la_peak_3194	7.13237	
19	1.2E+07	1.2E+07	KD2-H4K8la_peak_3195	14.30153	ZNF700_ENSG00000196757;ZNF763_ENSG00000197054;ZNF763_ENSG00000267179
19	1.2E+07	1.2E+07	KD2-H4K8la_peak_3196	5.33301	ZNF563_ENSG00000188868
19	1.3E+07	1.3E+07	KD2-H4K8la_peak_3197	9.27415	
19	1.3E+07	1.3E+07	KD2-H4K8la_peak_3198	9.15019	ZNF490_ENSG00000188033
19	1.3E+07	1.3E+07	KD2-H4K8la_peak_3199	7.54643	DHPS_ENSG00000095059
19	1.3E+07	1.3E+07	KD2-H4K8la_peak_3200	5.468	ASNA1_ENSG00000198356
19	1.3E+07	1.3E+07	KD2-H4K8la_peak_3201	9.61894	
19	1.3E+07	1.3E+07	KD2-H4K8la_peak_3202	6.61576	
19	1.3E+07	1.3E+07	KD2-H4K8la_peak_3203	9.73848	RNASEH2A_ENSG00000104889
19	1.3E+07	1.3E+07	KD2-H4K8la_peak_3204	5.33301	MAST1_ENSG00000105613
19	1.3E+07	1.3E+07	KD2-H4K8la_peak_3205	8.07102	SYCE2_ENSG00000161860
19	1.3E+07	1.3E+07	KD2-H4K8la_peak_3206	7.59044	FARSA_ENSG00000179115
19	1.3E+07	1.3E+07	KD2-H4K8la_peak_3207	10.20357	GADD45GIP1_ENSG00000179271
19	1.3E+07	1.3E+07	KD2-H4K8la_peak_3208	9.87983	
19	1.3E+07	1.3E+07	KD2-H4K8la_peak_3209	11.52725	
19	1.3E+07	1.3E+07	KD2-H4K8la_peak_3210	5.33301	
19	1.3E+07	1.3E+07	KD2-H4K8la_peak_3211	4.44412	
19	1.3E+07	1.3E+07	KD2-H4K8la_peak_3212	9.5329	
19	1.3E+07	1.3E+07	KD2-H4K8la_peak_3213	6.98743	
19	1.3E+07	1.3E+07	KD2-H4K8la_peak_3214	8.43222	LYL1_ENSG00000104903
19	1.3E+07	1.3E+07	KD2-H4K8la_peak_3215	9.19986	
19	1.3E+07	1.3E+07	KD2-H4K8la_peak_3216	7.61975	
19	1.3E+07	1.3E+07	KD2-H4K8la_peak_3217	5.36015	
19	1.4E+07	1.4E+07	KD2-H4K8la_peak_3218	11.15182	ZSWIM4_ENSG00000132003

19	1.4E+07	1.4E+07	KD2-H4K8la_peak_3219	6.57426	
19	1.4E+07	1.4E+07	KD2-H4K8la_peak_3220	9.63836	
19	1.4E+07	1.4E+07	KD2-H4K8la_peak_3221	7.22716	
19	1.4E+07	1.4E+07	KD2-H4K8la_peak_3222	11.15747	
19	1.4E+07	1.4E+07	KD2-H4K8la_peak_3223	4.30663	
19	1.4E+07	1.4E+07	KD2-H4K8la_peak_3224	11.77509	NANOS3_ENSG00000187556
19	1.4E+07	1.4E+07	KD2-H4K8la_peak_3225	13.29476	
19	1.4E+07	1.4E+07	KD2-H4K8la_peak_3226	6.3399	
19	1.4E+07	1.4E+07	KD2-H4K8la_peak_3227	7.51302	
19	1.4E+07	1.4E+07	KD2-H4K8la_peak_3228	6.25288	RFX1_ENSG00000132005
19	1.4E+07	1.4E+07	KD2-H4K8la_peak_3229	9.0516	SAMD1_ENSG00000141858
19	1.4E+07	1.4E+07	KD2-H4K8la_peak_3230	7.01227	PRKACA_ENSG00000072062;CTB-55O6.10_ENSG00000267783
19	1.4E+07	1.4E+07	KD2-H4K8la_peak_3231	10.62798	
19	1.4E+07	1.4E+07	KD2-H4K8la_peak_3232	10.49982	
19	1.4E+07	1.4E+07	KD2-H4K8la_peak_3233	10.20357	
19	1.4E+07	1.4E+07	KD2-H4K8la_peak_3234	10.00612	
19	1.5E+07	1.5E+07	KD2-H4K8la_peak_3235	13.13367	DDX39A_ENSG00000123136
19	1.5E+07	1.5E+07	KD2-H4K8la_peak_3236	8.44557	PKN1_ENSG00000123143
19	1.5E+07	1.5E+07	KD2-H4K8la_peak_3237	5.90392	
19	1.5E+07	1.5E+07	KD2-H4K8la_peak_3238	9.73848	DNAJB1_ENSG00000132002;MIR639_ENSG00000207707
19	1.5E+07	1.5E+07	KD2-H4K8la_peak_3239	6.64804	BRD4_ENSG00000141867
19	1.6E+07	1.6E+07	KD2-H4K8la_peak_3240	7.92775	WIZ_ENSG00000011451;MIR1470_ENSG00000269782
19	1.6E+07	1.6E+07	KD2-H4K8la_peak_3241	7.61975	
19	1.6E+07	1.6E+07	KD2-H4K8la_peak_3242	4.30663	
19	1.6E+07	1.6E+07	KD2-H4K8la_peak_3243	10.00612	RAB8A_ENSG00000167461
19	1.6E+07	1.6E+07	KD2-H4K8la_peak_3244	7.30722	RAB8A_ENSG00000167461
19	1.6E+07	1.6E+07	KD2-H4K8la_peak_3245	12.13455	FAM32A_ENSG00000105058
19	1.7E+07	1.7E+07	KD2-H4K8la_peak_3246	5.90392	CTC-429P9.4_ENSG00000268790;SMIM7_ENSG00000214046;TMEM38A_ENSG00000072954
19	1.7E+07	1.7E+07	KD2-H4K8la_peak_3247	4.73646	
19	1.7E+07	1.7E+07	KD2-H4K8la_peak_3248	7.51014	USE1_ENSG00000053501
19	1.7E+07	1.7E+07	KD2-H4K8la_peak_3249	4.86896	OCEL1_ENSG00000099330
19	1.7E+07	1.7E+07	KD2-H4K8la_peak_3250	8.22219	NR2F6_ENSG00000160113
19	1.7E+07	1.7E+07	KD2-H4K8la_peak_3251	13.13367	
19	1.7E+07	1.7E+07	KD2-H4K8la_peak_3252	7.38202	
19	1.8E+07	1.8E+07	KD2-H4K8la_peak_3253	9.27415	
19	1.8E+07	1.8E+07	KD2-H4K8la_peak_3254	4.75945	CTD-2521M24.8_ENSG00000269053
19	1.8E+07	1.8E+07	KD2-H4K8la_peak_3255	5.83285	UNC13A_ENSG00000130477
19	1.8E+07	1.8E+07	KD2-H4K8la_peak_3256	6.72794	JAK3_ENSG00000105639
19	1.8E+07	1.8E+07	KD2-H4K8la_peak_3257	6.86458	RPL18A_ENSG00000105640
19	1.8E+07	1.8E+07	KD2-H4K8la_peak_3258	6.89612	KCNN1_ENSG00000105642
19	1.8E+07	1.8E+07	KD2-H4K8la_peak_3259	7.51302	
19	1.8E+07	1.8E+07	KD2-H4K8la_peak_3260	11.77509	
19	1.8E+07	1.8E+07	KD2-H4K8la_peak_3261	8.89364	
19	1.8E+07	1.8E+07	KD2-H4K8la_peak_3262	8.50453	
19	1.8E+07	1.8E+07	KD2-H4K8la_peak_3263	8.89364	
19	1.8E+07	1.8E+07	KD2-H4K8la_peak_3264	6.13507	JUND_ENSG00000130522;MIR3188_ENSG00000267959

19	1.8E+07	1.8E+07	KD2-H4K8la_peak_3265	10.49982	
19	1.8E+07	1.8E+07	KD2-H4K8la_peak_3266	12.49088	
19	1.8E+07	1.8E+07	KD2-H4K8la_peak_3267	6.86458	
19	1.8E+07	1.8E+07	KD2-H4K8la_peak_3268	6.61963	
19	1.9E+07	1.9E+07	KD2-H4K8la_peak_3269	5.66328	SSBP4_ENSG00000130511
19	1.9E+07	1.9E+07	KD2-H4K8la_peak_3270	9.61894	
19	1.9E+07	1.9E+07	KD2-H4K8la_peak_3271	6.64804	
19	1.9E+07	1.9E+07	KD2-H4K8la_peak_3272	7.41484	FKBP8_ENSG00000105701
19	1.9E+07	1.9E+07	KD2-H4K8la_peak_3273	10.89081	C19orf60_ENSG00000006015
19	1.9E+07	1.9E+07	KD2-H4K8la_peak_3274	9.61894	CRTC1_ENSG00000105662
19	1.9E+07	1.9E+07	KD2-H4K8la_peak_3275	10.00612	
19	1.9E+07	1.9E+07	KD2-H4K8la_peak_3276	7.61975	
19	1.9E+07	1.9E+07	KD2-H4K8la_peak_3277	6.96161	SUGP2_ENSG00000064607;ARMC6_ENSG00000105676
19	1.9E+07	1.9E+07	KD2-H4K8la_peak_3278	8.76116	SUGP1_ENSG00000105705;MAU2_ENSG00000129933
19	2E+07	2E+07	KD2-H4K8la_peak_3279	4.69635	TSSK6_ENSG00000178093;NDUFA13_ENSG00000186010;YJEFN3_ENSG00000250067;CTC-260F20.3_ENSG00000258674
19	2E+07	2E+07	KD2-H4K8la_peak_3280	9.05604	GMIP_ENSG00000089639
19	2E+07	2E+07	KD2-H4K8la_peak_3281	6.88801	
19	2.1E+07	2.1E+07	KD2-H4K8la_peak_3282	11.70864	ZNF826P_ENSG00000231205
19	2.3E+07	2.3E+07	KD2-H4K8la_peak_3283	6.2278	CTD-2291D10.4_ENSG00000267886;ZNF730_ENSG00000183850
19	3E+07	3E+07	KD2-H4K8la_peak_3284	7.33892	
19	3.1E+07	3.1E+07	KD2-H4K8la_peak_3285	4.53273	
19	3.1E+07	3.1E+07	KD2-H4K8la_peak_3286	7.81267	
19	3.1E+07	3.1E+07	KD2-H4K8la_peak_3287	6.86458	
19	3.1E+07	3.1E+07	KD2-H4K8la_peak_3288	7.34033	
19	3.1E+07	3.1E+07	KD2-H4K8la_peak_3289	7.8597	
19	3.1E+07	3.1E+07	KD2-H4K8la_peak_3290	4.30663	
19	3.1E+07	3.1E+07	KD2-H4K8la_peak_3291	5.83285	
19	3.2E+07	3.2E+07	KD2-H4K8la_peak_3292	5.6499	
19	3.3E+07	3.3E+07	KD2-H4K8la_peak_3293	7.19298	ZNF507_ENSG00000168813
19	3.3E+07	3.3E+07	KD2-H4K8la_peak_3294	8.07102	CTD-2538C1.2_ENSG00000267475;NUDT19_ENSG00000213965
19	3.3E+07	3.3E+07	KD2-H4K8la_peak_3295	6.2278	CTD-2085J24.5_ENSG00000267245
19	3.4E+07	3.4E+07	KD2-H4K8la_peak_3296	9.6708	
19	3.4E+07	3.4E+07	KD2-H4K8la_peak_3297	9.11321	CEBPG_ENSG00000153879
19	3.4E+07	3.4E+07	KD2-H4K8la_peak_3298	8.8293	
19	3.4E+07	3.4E+07	KD2-H4K8la_peak_3299	7.61975	
19	3.5E+07	3.5E+07	KD2-H4K8la_peak_3300	9.0413	
19	3.5E+07	3.5E+07	KD2-H4K8la_peak_3301	6.60114	
19	3.5E+07	3.5E+07	KD2-H4K8la_peak_3302	11.69149	LSM14A_ENSG00000257103
19	3.5E+07	3.5E+07	KD2-H4K8la_peak_3303	5.67034	
19	3.5E+07	3.5E+07	KD2-H4K8la_peak_3304	6.2278	CTD-2588C8.8_ENSG00000267024;UBA2_ENSG00000126261
19	3.5E+07	3.5E+07	KD2-H4K8la_peak_3305	5.36015	ZNF181_ENSG00000197841
19	3.5E+07	3.5E+07	KD2-H4K8la_peak_3306	9.11321	ZNF181_ENSG00000197841
19	3.5E+07	3.5E+07	KD2-H4K8la_peak_3307	6.57319	ZNF599_ENSG00000153896
19	3.5E+07	3.5E+07	KD2-H4K8la_peak_3308	4.38865	CTD-2527I21.7_ENSG00000269303
19	3.6E+07	3.6E+07	KD2-H4K8la_peak_3309	7.90495	
19	3.6E+07	3.6E+07	KD2-H4K8la_peak_3310	8.37064	

19	3.6E+07	3.6E+07	KD2-H4K8la_peak_3311	12.166	USF2_ENSG00000105698
19	3.6E+07	3.6E+07	KD2-H4K8la_peak_3312	7.65547	RBM42_ENSG00000126254
19	3.6E+07	3.6E+07	KD2-H4K8la_peak_3313	5.05146	
19	3.6E+07	3.6E+07	KD2-H4K8la_peak_3314	4.69635	
19	3.6E+07	3.6E+07	KD2-H4K8la_peak_3315	8.76116	AD000671.6_ENSG00000267120;U2AF1L4_ENSG00000161265;PSENEN_ENSG00000205155;AC002398.9_ENSG00000188223
19	3.6E+07	3.6E+07	KD2-H4K8la_peak_3316	6.83387	HSPB6_ENSG00000004776;C19orf55_ENSG00000167595
19	3.6E+07	3.6E+07	KD2-H4K8la_peak_3317	4.75945	LRFN3_ENSG00000126243
19	3.6E+07	3.7E+07	KD2-H4K8la_peak_3318	9.0516	SYNE4_ENSG00000181392
19	3.7E+07	3.7E+07	KD2-H4K8la_peak_3319	10.49982	
19	3.7E+07	3.7E+07	KD2-H4K8la_peak_3320	8.37064	ZFP14_ENSG00000142065
19	3.7E+07	3.7E+07	KD2-H4K8la_peak_3321	5.16795	
19	3.7E+07	3.7E+07	KD2-H4K8la_peak_3322	6.25288	
19	3.8E+07	3.8E+07	KD2-H4K8la_peak_3323	7.19298	CTC-454I21.3_ENSG00000267360
19	3.8E+07	3.8E+07	KD2-H4K8la_peak_3324	7.56826	ZNF569_ENSG00000196437;ZNF570_ENSG00000171827
19	3.8E+07	3.8E+07	KD2-H4K8la_peak_3325	8.65116	ZNF569_ENSG00000196437
19	3.8E+07	3.8E+07	KD2-H4K8la_peak_3326	6.0136	
19	3.8E+07	3.8E+07	KD2-H4K8la_peak_3327	7.33892	WDR87_ENSG00000171804;SIPA1L3_ENSG00000105738
19	3.8E+07	3.8E+07	KD2-H4K8la_peak_3328	6.28277	
19	3.9E+07	3.9E+07	KD2-H4K8la_peak_3329	17.44668	
19	3.9E+07	3.9E+07	KD2-H4K8la_peak_3330	4.86896	PPP1R14A_ENSG00000167641
19	3.9E+07	3.9E+07	KD2-H4K8la_peak_3331	7.61975	KCNK6_ENSG00000099337
19	3.9E+07	3.9E+07	KD2-H4K8la_peak_3332	5.05146	SPRED3_ENSG00000188766
19	3.9E+07	3.9E+07	KD2-H4K8la_peak_3333	5.33364	
19	3.9E+07	3.9E+07	KD2-H4K8la_peak_3334	7.77341	EIF3K_ENSG00000178982;MAP4K1_ENSG00000104814
19	3.9E+07	3.9E+07	KD2-H4K8la_peak_3335	5.67596	HNRNPL_ENSG00000104824
19	3.9E+07	3.9E+07	KD2-H4K8la_peak_3336	6.57319	CCER2_ENSG00000262484
19	3.9E+07	3.9E+07	KD2-H4K8la_peak_3337	4.30663	
19	4E+07	4E+07	KD2-H4K8la_peak_3338	4.72651	PAK4_ENSG00000130669
19	4E+07	4E+07	KD2-H4K8la_peak_3339	6.3399	CTC-246B18.8_ENSG00000268262
19	4E+07	4E+07	KD2-H4K8la_peak_3340	5.58883	
19	4E+07	4E+07	KD2-H4K8la_peak_3341	6.3399	DLL3_ENSG00000090932
19	4E+07	4E+07	KD2-H4K8la_peak_3342	5.83285	
19	4.1E+07	4.1E+07	KD2-H4K8la_peak_3343	7.12014	
19	4.1E+07	4.1E+07	KD2-H4K8la_peak_3344	6.70058	
19	4.1E+07	4.1E+07	KD2-H4K8la_peak_3345	7.65547	SHKBP1_ENSG00000160410
19	4.1E+07	4.1E+07	KD2-H4K8la_peak_3346	5.09889	SHKBP1_ENSG00000160410
19	4.1E+07	4.1E+07	KD2-H4K8la_peak_3347	12.13455	
19	4.1E+07	4.1E+07	KD2-H4K8la_peak_3348	7.30491	
19	4.2E+07	4.2E+07	KD2-H4K8la_peak_3349	17.98201	
19	4.2E+07	4.2E+07	KD2-H4K8la_peak_3350	15.19386	ARHGEF1_ENSG00000076928
19	4.2E+07	4.2E+07	KD2-H4K8la_peak_3351	12.36938	
19	4.2E+07	4.2E+07	KD2-H4K8la_peak_3352	8.39919	
19	4.2E+07	4.2E+07	KD2-H4K8la_peak_3353	5.468	RABAC1_ENSG00000105404
19	4.2E+07	4.2E+07	KD2-H4K8la_peak_3354	7.51014	
19	4.3E+07	4.3E+07	KD2-H4K8la_peak_3355	7.13237	ATP1A3_ENSG00000105409
19	4.3E+07	4.3E+07	KD2-H4K8la_peak_3356	5.3107	ATP1A3_ENSG00000105409

19	4.3E+07	4.3E+07	KD2-H4K8la_peak_3357	9.27415	GRIK5_ENSG00000105737
19	4.3E+07	4.3E+07	KD2-H4K8la_peak_3358	6.86759	
19	4.3E+07	4.3E+07	KD2-H4K8la_peak_3359	8.0374	PAFAH1B3_ENSG00000079462
19	4.3E+07	4.3E+07	KD2-H4K8la_peak_3360	10.28981	MEGF8_ENSG00000105429
19	4.4E+07	4.4E+07	KD2-H4K8la_peak_3361	5.89088	ZNF404_ENSG00000176222;RP11-15A1.3_ENSG00000267058;RP11-15A1.2_ENSG00000267191
19	4.5E+07	4.5E+07	KD2-H4K8la_peak_3362	4.86896	ZNF223_ENSG00000178386
19	4.5E+07	4.5E+07	KD2-H4K8la_peak_3363	7.72421	CTC-512J12.4_ENSG00000267188;ZNF229_ENSG00000167383
19	4.5E+07	4.5E+07	KD2-H4K8la_peak_3364	6.64804	APOC1P1_ENSG00000214855
19	4.6E+07	4.6E+07	KD2-H4K8la_peak_3365	6.0136	RELB_ENSG00000104856
19	4.6E+07	4.6E+07	KD2-H4K8la_peak_3366	9.0413	CLASRP_ENSG00000104859
19	4.6E+07	4.6E+07	KD2-H4K8la_peak_3367	9.38376	PPP1R37_ENSG00000104866
19	4.6E+07	4.6E+07	KD2-H4K8la_peak_3368	10.16645	RTN2_ENSG00000125744
19	4.6E+07	4.6E+07	KD2-H4K8la_peak_3369	8.39919	
19	4.6E+07	4.6E+07	KD2-H4K8la_peak_3370	11.52197	DMWD_ENSG00000185800
19	4.6E+07	4.6E+07	KD2-H4K8la_peak_3371	7.64828	SYMPK_ENSG00000125755;FOXA3_ENSG00000170608
19	4.7E+07	4.7E+07	KD2-H4K8la_peak_3372	7.49467	PTGIR_ENSG00000160013
19	4.7E+07	4.7E+07	KD2-H4K8la_peak_3373	5.47284	
19	4.7E+07	4.7E+07	KD2-H4K8la_peak_3374	8.83119	
19	4.7E+07	4.7E+07	KD2-H4K8la_peak_3375	9.63836	GNG8_ENSG00000167414
19	4.7E+07	4.7E+07	KD2-H4K8la_peak_3376	6.64804	
19	4.7E+07	4.7E+07	KD2-H4K8la_peak_3377	13.27013	
19	4.8E+07	4.8E+07	KD2-H4K8la_peak_3378	5.58883	
19	4.8E+07	4.8E+07	KD2-H4K8la_peak_3379	8.07102	TMEM160_ENSG00000130748
19	4.8E+07	4.8E+07	KD2-H4K8la_peak_3380	14.23294	
19	4.8E+07	4.8E+07	KD2-H4K8la_peak_3381	14.25411	ZC3H4_ENSG00000130749;SAE1_ENSG00000142230
19	4.8E+07	4.8E+07	KD2-H4K8la_peak_3382	15.69409	MIR3191_ENSG00000265134
19	4.8E+07	4.8E+07	KD2-H4K8la_peak_3383	13.45069	MIR3191_ENSG00000265134
19	4.8E+07	4.8E+07	KD2-H4K8la_peak_3384	6.3399	MEIS3_ENSG00000105419
19	4.8E+07	4.8E+07	KD2-H4K8la_peak_3385	8.76116	
19	4.8E+07	4.8E+07	KD2-H4K8la_peak_3386	8.43222	NAPA_ENSG00000105402
19	4.8E+07	4.8E+07	KD2-H4K8la_peak_3387	4.30663	
19	4.8E+07	4.8E+07	KD2-H4K8la_peak_3388	6.77116	
19	4.9E+07	4.9E+07	KD2-H4K8la_peak_3389	6.96161	PLA2G4C_ENSG00000105499
19	4.9E+07	4.9E+07	KD2-H4K8la_peak_3390	5.36015	PLA2G4C_ENSG00000105499
19	4.9E+07	4.9E+07	KD2-H4K8la_peak_3391	9.27415	
19	4.9E+07	4.9E+07	KD2-H4K8la_peak_3392	7.13237	
19	4.9E+07	4.9E+07	KD2-H4K8la_peak_3393	8.27395	TMEM143_ENSG00000161558;SYNGR4_ENSG00000105467
19	4.9E+07	4.9E+07	KD2-H4K8la_peak_3394	14.35104	
19	4.9E+07	4.9E+07	KD2-H4K8la_peak_3395	11.25405	LMTK3_ENSG00000142235;CTC-273B12.10_ENSG00000269814
19	4.9E+07	4.9E+07	KD2-H4K8la_peak_3396	7.13237	
19	4.9E+07	4.9E+07	KD2-H4K8la_peak_3397	9.29932	
19	4.9E+07	4.9E+07	KD2-H4K8la_peak_3398	8.98765	RPL18_ENSG00000063177;SPHK2_ENSG00000063176
19	4.9E+07	4.9E+07	KD2-H4K8la_peak_3399	9.61894	CA11_ENSG00000063180
19	4.9E+07	4.9E+07	KD2-H4K8la_peak_3400	6.25288	
19	4.9E+07	4.9E+07	KD2-H4K8la_peak_3401	9.0516	
19	4.9E+07	4.9E+07	KD2-H4K8la_peak_3402	6.96161	

19	4.9E+07	4.9E+07	KD2-H4K8la_peak_3403	10.07718	
19	4.9E+07	4.9E+07	KD2-H4K8la_peak_3404	10.02595	
19	5E+07	5E+07	KD2-H4K8la_peak_3405	6.46158	
19	5E+07	5E+07	KD2-H4K8la_peak_3406	9.8606	TRPM4_ENSG00000130529
19	5E+07	5E+07	KD2-H4K8la_peak_3407	10.15823	TEAD2_ENSG00000074219;DKKL1_ENSG00000104901
19	5E+07	5E+07	KD2-H4K8la_peak_3408	10.49982	TEAD2_ENSG00000074219;DKKL1_ENSG00000104901
19	5E+07	5E+07	KD2-H4K8la_peak_3409	6.39623	TEAD2_ENSG00000074219;DKKL1_ENSG00000104901
19	5E+07	5E+07	KD2-H4K8la_peak_3410	10.49982	PIH1D1_ENSG00000104872;ALDH16A1_ENSG00000161618
19	5E+07	5E+07	KD2-H4K8la_peak_3411	4.53273	
19	5E+07	5E+07	KD2-H4K8la_peak_3412	17.16422	RPL13A_ENSG00000142541
19	5E+07	5E+07	KD2-H4K8la_peak_3413	8.89364	RPS11_ENSG00000142534
19	5E+07	5E+07	KD2-H4K8la_peak_3414	6.19992	
19	5E+07	5E+07	KD2-H4K8la_peak_3415	9.94613	PRRG2_ENSG00000126460
19	5E+07	5E+07	KD2-H4K8la_peak_3416	4.53273	
19	5E+07	5E+07	KD2-H4K8la_peak_3417	6.96161	RRAS_ENSG00000126458;SCAF1_ENSG00000126461
19	5E+07	5E+07	KD2-H4K8la_peak_3418	5.43278	
19	5E+07	5E+07	KD2-H4K8la_peak_3419	8.76116	IL4I1_ENSG00000104951;NUP62_ENSG00000213024;ATF5_ENSG00000169136
19	5E+07	5E+07	KD2-H4K8la_peak_3420	10.90354	IL4I1_ENSG00000104951;NUP62_ENSG00000213024;ATF5_ENSG00000169136
19	5.1E+07	5.1E+07	KD2-H4K8la_peak_3421	13.27013	VRK3_ENSG00000105053;ZNF473_ENSG00000142528
19	5.1E+07	5.1E+07	KD2-H4K8la_peak_3422	7.13237	
19	5.1E+07	5.1E+07	KD2-H4K8la_peak_3423	7.49467	CTB-191K22.6_ENSG00000269392
19	5.1E+07	5.1E+07	KD2-H4K8la_peak_3424	13.6415	
19	5.1E+07	5.1E+07	KD2-H4K8la_peak_3425	11.15747	FAM71E1_ENSG00000142530;EMC10_ENSG00000161671
19	5.1E+07	5.1E+07	KD2-H4K8la_peak_3426	4.86896	ASPDH_ENSG00000204653
19	5.1E+07	5.1E+07	KD2-H4K8la_peak_3427	7.68916	ASPDH_ENSG00000204653
19	5.1E+07	5.1E+07	KD2-H4K8la_peak_3428	10.20357	
19	5.1E+07	5.1E+07	KD2-H4K8la_peak_3429	8.13727	
19	5.2E+07	5.2E+07	KD2-H4K8la_peak_3430	8.43222	CTU1_ENSG00000142544
19	5.2E+07	5.2E+07	KD2-H4K8la_peak_3431	15.62171	
19	5.2E+07	5.2E+07	KD2-H4K8la_peak_3432	7.72421	ZNF175_ENSG00000105497
19	5.2E+07	5.2E+07	KD2-H4K8la_peak_3433	5.83285	AC018755.1_ENSG00000167765;AC018755.16_ENSG00000269388
19	5.2E+07	5.2E+07	KD2-H4K8la_peak_3434	5.33301	
19	5.2E+07	5.2E+07	KD2-H4K8la_peak_3435	6.57319	ZNF350_ENSG00000256683
19	5.3E+07	5.3E+07	KD2-H4K8la_peak_3436	10.99007	ZNF808_ENSG00000198482
19	5.3E+07	5.3E+07	KD2-H4K8la_peak_3437	7.3993	ZNF83_ENSG00000167766
19	5.4E+07	5.4E+07	KD2-H4K8la_peak_3438	5.58883	ZNF415_ENSG00000170954
19	5.4E+07	5.4E+07	KD2-H4K8la_peak_3439	10.99007	ZNF665_ENSG00000197497
19	5.4E+07	5.4E+07	KD2-H4K8la_peak_3440	8.07102	ZNF813_ENSG00000198346
19	5.4E+07	5.4E+07	KD2-H4K8la_peak_3441	8.12466	ZNF331_ENSG00000130844
19	5.4E+07	5.4E+07	KD2-H4K8la_peak_3442	7.13237	
19	5.4E+07	5.4E+07	KD2-H4K8la_peak_3443	7.13237	CACNG7_ENSG00000105605
19	5.4E+07	5.4E+07	KD2-H4K8la_peak_3444	8.00675	CACNG7_ENSG00000105605
19	5.5E+07	5.5E+07	KD2-H4K8la_peak_3445	7.81267	OSCAR_ENSG00000170909;NDUFA3_ENSG00000170906
19	5.5E+07	5.5E+07	KD2-H4K8la_peak_3446	6.68593	OSCAR_ENSG00000170909;NDUFA3_ENSG00000170906
19	5.5E+07	5.5E+07	KD2-H4K8la_peak_3447	15.68123	CNOT3_ENSG00000088038
19	5.5E+07	5.5E+07	KD2-H4K8la_peak_3448	6.09229	

19	5.6E+07	5.6E+07	KD2-H4K8la_peak_3449	6.3399	
19	5.6E+07	5.6E+07	KD2-H4K8la_peak_3450	5.49688	PPP6R1_ENSG00000105063
19	5.6E+07	5.6E+07	KD2-H4K8la_peak_3451	16.25311	
19	5.6E+07	5.6E+07	KD2-H4K8la_peak_3452	8.86391	IL11_ENSG00000095752
19	5.6E+07	5.6E+07	KD2-H4K8la_peak_3453	5.84385	TMEM238_ENSG00000233493;RPL28_ENSG00000108107
19	5.6E+07	5.6E+07	KD2-H4K8la_peak_3454	10.76886	ISOC2_ENSG00000063241
19	5.6E+07	5.6E+07	KD2-H4K8la_peak_3455	9.27415	CTD-2537I9.16_ENSG00000269859;ZNF628_ENSG00000197483
19	5.6E+07	5.6E+07	KD2-H4K8la_peak_3456	7.35742	NAT14_ENSG00000090971
19	5.6E+07	5.6E+07	KD2-H4K8la_peak_3457	6.68593	
19	5.6E+07	5.6E+07	KD2-H4K8la_peak_3458	7.13237	
19	5.6E+07	5.6E+07	KD2-H4K8la_peak_3459	9.61894	ZNF579_ENSG00000218891
19	5.6E+07	5.6E+07	KD2-H4K8la_peak_3460	9.0413	ZNF579_ENSG00000218891
19	5.6E+07	5.6E+07	KD2-H4K8la_peak_3461	10.90862	
19	5.6E+07	5.6E+07	KD2-H4K8la_peak_3462	6.25495	FIZ1_ENSG00000179943;ZNF524_ENSG00000171443
19	5.6E+07	5.6E+07	KD2-H4K8la_peak_3463	8.43222	ZNF865_ENSG00000261221
19	5.6E+07	5.6E+07	KD2-H4K8la_peak_3464	10.00612	ZNF580_ENSG00000213015;ZNF581_ENSG00000171425
19	5.6E+07	5.6E+07	KD2-H4K8la_peak_3465	7.77341	CCDC106_ENSG00000173581
19	5.6E+07	5.6E+07	KD2-H4K8la_peak_3466	6.3399	U2AF2_ENSG00000063244
19	5.7E+07	5.7E+07	KD2-H4K8la_peak_3467	4.53273	
19	5.8E+07	5.8E+07	KD2-H4K8la_peak_3468	6.46158	ZNF304_ENSG00000131845
19	5.8E+07	5.8E+07	KD2-H4K8la_peak_3469	8.76082	CTC-444N24.13_ENSG00000268678;ZNF304_ENSG00000131845
19	5.8E+07	5.8E+07	KD2-H4K8la_peak_3470	7.23116	ZNF749_ENSG00000186230
19	5.8E+07	5.8E+07	KD2-H4K8la_peak_3471	9.36457	AC004076.9_ENSG00000268163;ZNF772_ENSG00000197128;AC003005.2_ENSG00000268266
19	5.8E+07	5.8E+07	KD2-H4K8la_peak_3472	11.06859	ZNF419_ENSG00000105136;AC003005.4_ENSG00000268107
19	5.8E+07	5.8E+07	KD2-H4K8la_peak_3473	5.05146	
19	5.8E+07	5.8E+07	KD2-H4K8la_peak_3474	8.76082	AC003682.17_ENSG00000269097
19	5.8E+07	5.8E+07	KD2-H4K8la_peak_3475	8.944	ZNF551_ENSG00000204519;AC003006.7_ENSG00000269026
19	5.8E+07	5.8E+07	KD2-H4K8la_peak_3476	6.57319	ZNF776_ENSG00000152443
19	5.8E+07	5.8E+07	KD2-H4K8la_peak_3477	5.83285	ZNF587_ENSG00000198466
19	5.9E+07	5.9E+07	KD2-H4K8la_peak_3478	9.6708	ZNF606_ENSG00000166704;CTD-2368P22.1_ENSG00000176593
19	5.9E+07	5.9E+07	KD2-H4K8la_peak_3479	5.90392	ZNF606_ENSG00000166704;CTD-2368P22.1_ENSG00000176593
19	5.9E+07	5.9E+07	KD2-H4K8la_peak_3480	10.97039	ZNF274_ENSG00000171606
19	5.9E+07	5.9E+07	KD2-H4K8la_peak_3481	12.43263	RPS5_ENSG00000083845;MIR4754_ENSG00000266640
19	5.9E+07	5.9E+07	KD2-H4K8la_peak_3482	6.68593	CTD-2619J13.13_ENSG00000268307;ZNF584_ENSG00000171574
19	5.9E+07	5.9E+07	KD2-H4K8la_peak_3483	8.76116	ZNF132_ENSG00000131849;CTD-2619J13.19_ENSG00000269473
19	5.9E+07	5.9E+07	KD2-H4K8la_peak_3484	7.97203	
19	5.9E+07	5.9E+07	KD2-H4K8la_peak_3485	8.944	CHMP2A_ENSG00000130724
19	5.9E+07	5.9E+07	KD2-H4K8la_peak_3486	7.23932	
2	264312	264527	KD2-H4K8la_peak_3487	8.3497	ACP1_ENSG00000143727
2	265098	265310	KD2-H4K8la_peak_3488	5.43278	ACP1_ENSG00000143727
2	844524	844781	KD2-H4K8la_peak_3489	10.16316	
2	945764	946061	KD2-H4K8la_peak_3490	7.51014	AC116614.1_ENSG00000235688;SNTG2_ENSG00000172554
2	1286929	1287440	KD2-H4K8la_peak_3491	4.30663	
2	1598396	1598602	KD2-H4K8la_peak_3492	6.57176	
2	1718020	1718282	KD2-H4K8la_peak_3493	7.3993	
2	2194592	2194811	KD2-H4K8la_peak_3494	5.66328	

2	2617956	2618225	KD2-H4K8la_peak_3495	7.56395	
2	2661123	2661341	KD2-H4K8la_peak_3496	6.28686	AC018685.1_ENSG00000234929
2	2850510	2850749	KD2-H4K8la_peak_3497	8.22268	
2	2852760	2853124	KD2-H4K8la_peak_3498	9.40293	
2	2853717	2854034	KD2-H4K8la_peak_3499	5.25645	
2	2922951	2923227	KD2-H4K8la_peak_3500	9.05604	
2	3104289	3104521	KD2-H4K8la_peak_3501	7.0396	
2	3105816	3106700	KD2-H4K8la_peak_3502	25.17273	
2	3129601	3130428	KD2-H4K8la_peak_3503	9.92226	AC019118.2_ENSG00000234423
2	3134684	3134998	KD2-H4K8la_peak_3504	12.14696	
2	3306957	3307352	KD2-H4K8la_peak_3505	18.06211	
2	3381958	3382518	KD2-H4K8la_peak_3506	7.12972	TSSC1_ENSG00000032389;TRAPPC12_ENSG00000171853
2	3452427	3452632	KD2-H4K8la_peak_3507	6.72304	
2	3522205	3522579	KD2-H4K8la_peak_3508	8.4237	ADI1_ENSG00000182551;AC142528.1_ENSG00000235078
2	3523072	3523306	KD2-H4K8la_peak_3509	11.74575	ADI1_ENSG00000182551;AC142528.1_ENSG00000235078
2	3622252	3622674	KD2-H4K8la_peak_3510	10.94155	RPS7_ENSG00000171863
2	3633036	3633259	KD2-H4K8la_peak_3511	10.12382	
2	3642498	3642735	KD2-H4K8la_peak_3512	7.68525	COLEC11_ENSG00000118004
2	3642953	3643163	KD2-H4K8la_peak_3513	6.41605	COLEC11_ENSG00000118004
2	3651029	3651388	KD2-H4K8la_peak_3514	7.23932	AC010907.2_ENSG00000237370
2	5125277	5125501	KD2-H4K8la_peak_3515	6.2278	
2	5813452	5813748	KD2-H4K8la_peak_3516	7.65547	
2	5837031	5837272	KD2-H4K8la_peak_3517	7.12014	AC010729.1_ENSG00000242540
2	6220725	6221008	KD2-H4K8la_peak_3518	10.47118	
2	6271471	6271730	KD2-H4K8la_peak_3519	9.0413	
2	6429477	6429854	KD2-H4K8la_peak_3520	6.75222	
2	6877465	6877684	KD2-H4K8la_peak_3521	6.26214	
2	7057148	7057354	KD2-H4K8la_peak_3522	6.38565	RNF144A_ENSG00000151692
2	8677127	8677351	KD2-H4K8la_peak_3523	7.49897	
2	8679879	8680107	KD2-H4K8la_peak_3524	9.06396	
2	9143504	9143748	KD2-H4K8la_peak_3525	8.06229	MBOAT2_ENSG00000143797
2	9144266	9144743	KD2-H4K8la_peak_3526	12.97817	MBOAT2_ENSG00000143797
2	9345971	9346199	KD2-H4K8la_peak_3527	7.61975	ASAP2_ENSG00000151693
2	9347169	9347559	KD2-H4K8la_peak_3528	4.75945	ASAP2_ENSG00000151693
2	9376140	9376346	KD2-H4K8la_peak_3529	8.58767	
2	9614153	9615245	KD2-H4K8la_peak_3530	11.7374	IAH1_ENSG00000134330
2	9983057	9983363	KD2-H4K8la_peak_3531	8.89364	TAF1B_ENSG00000115750
2	1E+07	1E+07	KD2-H4K8la_peak_3532	8.61463	
2	1E+07	1E+07	KD2-H4K8la_peak_3533	18.4179	
2	1E+07	1E+07	KD2-H4K8la_peak_3534	11.81269	
2	1E+07	1E+07	KD2-H4K8la_peak_3535	11.50359	HPCAL1_ENSG00000115756
2	1.1E+07	1.1E+07	KD2-H4K8la_peak_3536	4.69635	
2	1.1E+07	1.1E+07	KD2-H4K8la_peak_3537	7.61975	
2	1.1E+07	1.1E+07	KD2-H4K8la_peak_3538	4.63265	
2	1.1E+07	1.1E+07	KD2-H4K8la_peak_3539	17.22831	ODC1_ENSG00000115758;RP11-320M2.1_ENSG00000257135
2	1.1E+07	1.1E+07	KD2-H4K8la_peak_3540	6.43451	

2	1.1E+07	1.1E+07	KD2-H4K8la_peak_3541	6.13507	NOL10_ENSG00000115761;RN7SL832P_ENSG00000243819
2	1.1E+07	1.1E+07	KD2-H4K8la_peak_3542	10.22817	
2	1.1E+07	1.1E+07	KD2-H4K8la_peak_3543	9.20163	
2	1.2E+07	1.2E+07	KD2-H4K8la_peak_3544	12.12824	
2	1.2E+07	1.2E+07	KD2-H4K8la_peak_3545	7.86224	
2	1.3E+07	1.3E+07	KD2-H4K8la_peak_3546	9.0413	
2	1.5E+07	1.5E+07	KD2-H4K8la_peak_3547	5.47767	AC011897.1_ENSG00000265629
2	1.6E+07	1.6E+07	KD2-H4K8la_peak_3548	9.29385	
2	1.6E+07	1.6E+07	KD2-H4K8la_peak_3549	9.20163	
2	1.6E+07	1.6E+07	KD2-H4K8la_peak_3550	6.2381	
2	1.6E+07	1.6E+07	KD2-H4K8la_peak_3551	12.11591	
2	1.6E+07	1.6E+07	KD2-H4K8la_peak_3552	7.3993	
2	1.7E+07	1.7E+07	KD2-H4K8la_peak_3553	4.6668	
2	1.8E+07	1.8E+07	KD2-H4K8la_peak_3554	6.19992	
2	1.8E+07	1.8E+07	KD2-H4K8la_peak_3555	7.92775	GEN1_ENSG00000178295
2	1.9E+07	1.9E+07	KD2-H4K8la_peak_3556	8.80749	RDH14_ENSG00000240857
2	2E+07	2E+07	KD2-H4K8la_peak_3557	12.24258	TTC32_ENSG00000183891;RP11-79O8.1_ENSG00000271991
2	2E+07	2E+07	KD2-H4K8la_peak_3558	9.92226	MATN3_ENSG00000132031
2	2E+07	2E+07	KD2-H4K8la_peak_3559	6.47266	
2	2E+07	2E+07	KD2-H4K8la_peak_3560	11.18137	
2	2.1E+07	2.1E+07	KD2-H4K8la_peak_3561	11.46073	PUM2_ENSG00000055917
2	2.1E+07	2.1E+07	KD2-H4K8la_peak_3562	7.49467	
2	2.1E+07	2.1E+07	KD2-H4K8la_peak_3563	9.67249	RHOB_ENSG00000143878
2	2.1E+07	2.1E+07	KD2-H4K8la_peak_3564	5.0364	HS1BP3_ENSG00000118960
2	2.1E+07	2.1E+07	KD2-H4K8la_peak_3565	5.05146	
2	2.4E+07	2.4E+07	KD2-H4K8la_peak_3566	10.49982	
2	2.4E+07	2.4E+07	KD2-H4K8la_peak_3567	10.00129	KLHL29_ENSG00000119771
2	2.4E+07	2.4E+07	KD2-H4K8la_peak_3568	12.62032	
2	2.4E+07	2.4E+07	KD2-H4K8la_peak_3569	9.60842	
2	2.4E+07	2.4E+07	KD2-H4K8la_peak_3570	7.92775	
2	2.4E+07	2.4E+07	KD2-H4K8la_peak_3571	5.99671	
2	2.4E+07	2.4E+07	KD2-H4K8la_peak_3572	5.83285	
2	2.4E+07	2.4E+07	KD2-H4K8la_peak_3573	5.58883	
2	2.4E+07	2.4E+07	KD2-H4K8la_peak_3574	5.33721	C2orf44_ENSG00000163026;FKBP1B_ENSG00000119782
2	2.4E+07	2.4E+07	KD2-H4K8la_peak_3575	12.97817	PFN4_ENSG00000176732
2	2.4E+07	2.4E+07	KD2-H4K8la_peak_3576	8.0538	PFN4_ENSG00000176732;RP11-507M3.1_ENSG00000266118
2	2.5E+07	2.5E+07	KD2-H4K8la_peak_3577	9.46185	ITSN2_ENSG00000198399
2	2.5E+07	2.5E+07	KD2-H4K8la_peak_3578	11.73315	NCOA1_ENSG00000084676
2	2.5E+07	2.5E+07	KD2-H4K8la_peak_3579	14.10219	
2	2.6E+07	2.6E+07	KD2-H4K8la_peak_3580	12.12037	
2	2.6E+07	2.6E+07	KD2-H4K8la_peak_3581	6.76424	
2	2.6E+07	2.6E+07	KD2-H4K8la_peak_3582	6.13507	
2	2.6E+07	2.6E+07	KD2-H4K8la_peak_3583	9.67249	
2	2.6E+07	2.6E+07	KD2-H4K8la_peak_3584	9.67249	DNMT3A_ENSG00000119772
2	2.6E+07	2.6E+07	KD2-H4K8la_peak_3585	9.92226	DTNB_ENSG00000138101
2	2.6E+07	2.6E+07	KD2-H4K8la_peak_3586	7.12308	KIF3C_ENSG00000084731

2	2.7E+07	2.7E+07	KD2-H4K8la_peak_3587	5.3312	
2	2.7E+07	2.7E+07	KD2-H4K8la_peak_3588	6.47266	C2orf70_ENSG00000173557
2	2.7E+07	2.7E+07	KD2-H4K8la_peak_3589	5.41777	
2	2.7E+07	2.7E+07	KD2-H4K8la_peak_3590	10.02595	
2	2.7E+07	2.7E+07	KD2-H4K8la_peak_3591	13.38201	SLC35F6_ENSG00000213699;CENPA_ENSG00000115163
2	2.7E+07	2.7E+07	KD2-H4K8la_peak_3592	8.89364	SLC35F6_ENSG00000213699;CENPA_ENSG00000115163
2	2.7E+07	2.7E+07	KD2-H4K8la_peak_3593	8.66164	
2	2.7E+07	2.7E+07	KD2-H4K8la_peak_3594	8.48577	
2	2.7E+07	2.7E+07	KD2-H4K8la_peak_3595	5.29444	TMEM214_ENSG00000119777
2	2.7E+07	2.7E+07	KD2-H4K8la_peak_3596	9.70817	AGBL5-AS1_ENSG00000231636
2	2.7E+07	2.7E+07	KD2-H4K8la_peak_3597	12.59787	
2	2.7E+07	2.7E+07	KD2-H4K8la_peak_3598	9.9149	OST4_ENSG00000228474
2	2.7E+07	2.7E+07	KD2-H4K8la_peak_3599	6.9399	EMILIN1_ENSG00000138080
2	2.7E+07	2.7E+07	KD2-H4K8la_peak_3600	6.68593	KHK_ENSG00000138030
2	2.7E+07	2.7E+07	KD2-H4K8la_peak_3601	6.75222	
2	2.7E+07	2.7E+07	KD2-H4K8la_peak_3602	9.14592	CGREF1_ENSG00000138028
2	2.7E+07	2.7E+07	KD2-H4K8la_peak_3603	7.68525	ABHD1_ENSG00000143994
2	2.7E+07	2.7E+07	KD2-H4K8la_peak_3604	13.17297	PREB_ENSG00000138073
2	2.7E+07	2.7E+07	KD2-H4K8la_peak_3605	10.94155	
2	2.8E+07	2.8E+07	KD2-H4K8la_peak_3606	8.59042	
2	2.8E+07	2.8E+07	KD2-H4K8la_peak_3607	9.27415	GTF3C2_ENSG00000115207;AC074117.10_ENSG00000234072
2	2.8E+07	2.8E+07	KD2-H4K8la_peak_3608	9.61894	GTF3C2_ENSG00000115207;AC074117.10_ENSG00000234072
2	2.8E+07	2.8E+07	KD2-H4K8la_peak_3609	10.87112	FNDC4_ENSG00000115226
2	2.8E+07	2.8E+07	KD2-H4K8la_peak_3610	6.27787	AC074091.1_ENSG00000221531;ZNF512_ENSG00000243943;RP11-158I13.2_ENSG00000259080
2	2.8E+07	2.8E+07	KD2-H4K8la_peak_3611	8.06229	SUPT7L_ENSG00000119760;SLC4A1AP_ENSG00000163798
2	2.8E+07	2.8E+07	KD2-H4K8la_peak_3612	9.10568	AC074091.13_ENSG00000205334
2	2.8E+07	2.8E+07	KD2-H4K8la_peak_3613	6.68593	AC074091.13_ENSG00000205334
2	2.8E+07	2.8E+07	KD2-H4K8la_peak_3614	5.70367	
2	2.8E+07	2.8E+07	KD2-H4K8la_peak_3615	6.25288	RBKS_ENSG00000171174
2	2.9E+07	2.9E+07	KD2-H4K8la_peak_3616	10.87112	
2	2.9E+07	2.9E+07	KD2-H4K8la_peak_3617	6.68593	
2	2.9E+07	2.9E+07	KD2-H4K8la_peak_3618	7.3993	PPP1CB_ENSG00000213639
2	2.9E+07	2.9E+07	KD2-H4K8la_peak_3619	10.87112	
2	2.9E+07	2.9E+07	KD2-H4K8la_peak_3620	16.33239	
2	2.9E+07	2.9E+07	KD2-H4K8la_peak_3621	11.45154	
2	2.9E+07	2.9E+07	KD2-H4K8la_peak_3622	6.68593	
2	2.9E+07	2.9E+07	KD2-H4K8la_peak_3623	11.00891	
2	2.9E+07	2.9E+07	KD2-H4K8la_peak_3624	8.16787	
2	2.9E+07	2.9E+07	KD2-H4K8la_peak_3625	5.79108	
2	2.9E+07	2.9E+07	KD2-H4K8la_peak_3626	10.45168	
2	2.9E+07	2.9E+07	KD2-H4K8la_peak_3627	7.23932	
2	2.9E+07	2.9E+07	KD2-H4K8la_peak_3628	9.46422	
2	2.9E+07	2.9E+07	KD2-H4K8la_peak_3629	7.53542	
2	2.9E+07	2.9E+07	KD2-H4K8la_peak_3630	7.28149	
2	2.9E+07	2.9E+07	KD2-H4K8la_peak_3631	14.7348	
2	2.9E+07	2.9E+07	KD2-H4K8la_peak_3632	4.89452	

2	3E+07	3E+07	KD2-H4K8la_peak_3633	11.24937	
2	3E+07	3E+07	KD2-H4K8la_peak_3634	7.7786	
2	3E+07	3E+07	KD2-H4K8la_peak_3635	8.61032	
2	3E+07	3E+07	KD2-H4K8la_peak_3636	7.12014	
2	3E+07	3E+07	KD2-H4K8la_peak_3637	7.10506	
2	3E+07	3E+07	KD2-H4K8la_peak_3638	8.39919	
2	3E+07	3E+07	KD2-H4K8la_peak_3639	8.75981	
2	3E+07	3E+07	KD2-H4K8la_peak_3640	5.40012	
2	3.1E+07	3.1E+07	KD2-H4K8la_peak_3641	5.23773	CAPN14_ENSG00000214711;EHD3_ENSG00000013016
2	3.2E+07	3.2E+07	KD2-H4K8la_peak_3642	6.13507	RP11-563N4.1_ENSG00000272716;SLC30A6_ENSG00000152683
2	3.3E+07	3.3E+07	KD2-H4K8la_peak_3643	4.6555	BIRC6_ENSG00000115760
2	3.3E+07	3.3E+07	KD2-H4K8la_peak_3644	10.87112	BIRC6_ENSG00000115760
2	3.7E+07	3.7E+07	KD2-H4K8la_peak_3645	6.38565	STRN_ENSG00000115808
2	3.7E+07	3.7E+07	KD2-H4K8la_peak_3646	11.40266	SULT6B1_ENSG00000138068;CEBPZ-AS1_ENSG00000218739
2	3.8E+07	3.8E+07	KD2-H4K8la_peak_3647	6.61963	PRKD3_ENSG00000115825;AC007391.2_ENSG00000232028
2	3.8E+07	3.8E+07	KD2-H4K8la_peak_3648	7.0396	
2	3.8E+07	3.8E+07	KD2-H4K8la_peak_3649	10.37746	
2	3.8E+07	3.8E+07	KD2-H4K8la_peak_3650	5.50657	
2	3.8E+07	3.8E+07	KD2-H4K8la_peak_3651	5.84082	
2	3.8E+07	3.8E+07	KD2-H4K8la_peak_3652	8.39919	
2	3.8E+07	3.8E+07	KD2-H4K8la_peak_3653	8.76116	CYP1B1-AS1_ENSG00000232973
2	3.9E+07	3.9E+07	KD2-H4K8la_peak_3654	15.19386	ATL2_ENSG00000119787
2	3.9E+07	3.9E+07	KD2-H4K8la_peak_3655	20.05453	RP11-541E12.1_ENSG00000271443
2	3.9E+07	3.9E+07	KD2-H4K8la_peak_3656	14.11447	AC011247.3_ENSG00000235586;HNRNPLL_ENSG00000143889
2	3.9E+07	3.9E+07	KD2-H4K8la_peak_3657	5.84385	
2	3.9E+07	3.9E+07	KD2-H4K8la_peak_3658	6.3399	SOS1_ENSG00000115904
2	4E+07	4E+07	KD2-H4K8la_peak_3659	7.12014	TMEM178A_ENSG00000152154
2	4.2E+07	4.2E+07	KD2-H4K8la_peak_3660	5.83285	
2	4.2E+07	4.2E+07	KD2-H4K8la_peak_3661	8.16916	EML4_ENSG00000143924
2	4.3E+07	4.3E+07	KD2-H4K8la_peak_3662	13.00097	
2	4.3E+07	4.3E+07	KD2-H4K8la_peak_3663	6.4136	KCNG3_ENSG00000171126;MTA3_ENSG00000057935
2	4.3E+07	4.3E+07	KD2-H4K8la_peak_3664	12.35598	
2	4.3E+07	4.3E+07	KD2-H4K8la_peak_3665	9.78176	
2	4.3E+07	4.3E+07	KD2-H4K8la_peak_3666	5.43278	
2	4.3E+07	4.3E+07	KD2-H4K8la_peak_3667	8.13557	
2	4.3E+07	4.3E+07	KD2-H4K8la_peak_3668	6.56022	
2	4.3E+07	4.3E+07	KD2-H4K8la_peak_3669	8.13557	
2	4.4E+07	4.4E+07	KD2-H4K8la_peak_3670	7.49467	
2	4.4E+07	4.4E+07	KD2-H4K8la_peak_3671	8.37064	
2	4.4E+07	4.4E+07	KD2-H4K8la_peak_3672	5.28047	
2	4.4E+07	4.4E+07	KD2-H4K8la_peak_3673	4.86896	
2	4.4E+07	4.4E+07	KD2-H4K8la_peak_3674	16.12381	LRPPRC_ENSG00000138095
2	4.4E+07	4.4E+07	KD2-H4K8la_peak_3675	9.27415	
2	4.5E+07	4.5E+07	KD2-H4K8la_peak_3676	5.24321	
2	4.5E+07	4.5E+07	KD2-H4K8la_peak_3677	7.61975	
2	4.5E+07	4.5E+07	KD2-H4K8la_peak_3678	5.27164	RP11-89K21.1_ENSG00000259439

2	4.5E+07	4.5E+07	KD2-H4K8la_peak_3679	8.96792	RP11-89K21.1_ENSG00000259439
2	4.5E+07	4.5E+07	KD2-H4K8la_peak_3680	4.69635	SIX3-AS1_ENSG00000236502;SIX3_ENSG00000138083
2	4.5E+07	4.5E+07	KD2-H4K8la_peak_3681	6.57176	SIX3-AS1_ENSG00000236502;SIX3_ENSG00000138083
2	4.5E+07	4.5E+07	KD2-H4K8la_peak_3682	5.27164	
2	4.5E+07	4.5E+07	KD2-H4K8la_peak_3683	10.02595	AC093702.1_ENSG00000231156
2	4.7E+07	4.7E+07	KD2-H4K8la_peak_3684	5.55274	
2	4.7E+07	4.7E+07	KD2-H4K8la_peak_3685	10.34952	
2	4.7E+07	4.7E+07	KD2-H4K8la_peak_3686	5.7062	
2	4.7E+07	4.7E+07	KD2-H4K8la_peak_3687	10.47118	PIGF_ENSG00000151665;CRIPT_ENSG00000119878
2	4.7E+07	4.7E+07	KD2-H4K8la_peak_3688	4.80784	
2	4.7E+07	4.7E+07	KD2-H4K8la_peak_3689	9.19986	SOCS5_ENSG00000171150
2	4.7E+07	4.7E+07	KD2-H4K8la_peak_3690	5.99671	
2	4.7E+07	4.7E+07	KD2-H4K8la_peak_3691	10.39508	
2	4.7E+07	4.7E+07	KD2-H4K8la_peak_3692	8.0333	
2	4.7E+07	4.7E+07	KD2-H4K8la_peak_3693	8.06229	
2	4.7E+07	4.7E+07	KD2-H4K8la_peak_3694	14.26347	
2	4.7E+07	4.7E+07	KD2-H4K8la_peak_3695	18.83567	
2	4.7E+07	4.7E+07	KD2-H4K8la_peak_3696	10.47118	
2	4.7E+07	4.7E+07	KD2-H4K8la_peak_3697	7.3993	
2	4.7E+07	4.7E+07	KD2-H4K8la_peak_3698	15.7313	
2	4.7E+07	4.7E+07	KD2-H4K8la_peak_3699	10.71653	
2	4.7E+07	4.7E+07	KD2-H4K8la_peak_3700	5.43278	
2	4.7E+07	4.7E+07	KD2-H4K8la_peak_3701	8.66164	
2	4.7E+07	4.7E+07	KD2-H4K8la_peak_3702	8.66164	
2	4.7E+07	4.7E+07	KD2-H4K8la_peak_3703	9.20163	
2	4.7E+07	4.7E+07	KD2-H4K8la_peak_3704	10.49982	
2	4.7E+07	4.7E+07	KD2-H4K8la_peak_3705	14.23294	
2	4.7E+07	4.7E+07	KD2-H4K8la_peak_3706	9.5329	RP11-761B3.1_ENSG00000273269;CALM2_ENSG00000143933
2	4.7E+07	4.7E+07	KD2-H4K8la_peak_3707	7.49897	
2	4.7E+07	4.7E+07	KD2-H4K8la_peak_3708	11.00891	
2	4.7E+07	4.7E+07	KD2-H4K8la_peak_3709	8.13557	
2	4.8E+07	4.8E+07	KD2-H4K8la_peak_3710	6.86458	
2	4.8E+07	4.8E+07	KD2-H4K8la_peak_3711	7.23932	
2	4.8E+07	4.8E+07	KD2-H4K8la_peak_3712	8.77961	MSH2_ENSG00000095002
2	4.8E+07	4.8E+07	KD2-H4K8la_peak_3713	9.44217	
2	4.8E+07	4.8E+07	KD2-H4K8la_peak_3714	6.71169	RNU6-688P_ENSG00000251872
2	4.8E+07	4.8E+07	KD2-H4K8la_peak_3715	12.62032	
2	5.5E+07	5.5E+07	KD2-H4K8la_peak_3716	6.64804	
2	6.1E+07	6.1E+07	KD2-H4K8la_peak_3717	6.28277	AC010733.4_ENSG00000228414;REL_ENSG00000162924
2	6.1E+07	6.1E+07	KD2-H4K8la_peak_3718	7.83588	AHSA2_ENSG00000173209
2	6.4E+07	6.4E+07	KD2-H4K8la_peak_3719	7.30722	VPS54_ENSG00000143952
2	6.5E+07	6.5E+07	KD2-H4K8la_peak_3720	6.64804	AC008074.3_ENSG00000223935;LGALSL_ENSG00000119862
2	6.5E+07	6.5E+07	KD2-H4K8la_peak_3721	6.89612	
2	6.5E+07	6.5E+07	KD2-H4K8la_peak_3722	6.3399	
2	6.5E+07	6.5E+07	KD2-H4K8la_peak_3723	10.87898	
2	6.5E+07	6.5E+07	KD2-H4K8la_peak_3724	11.40266	SLC1A4_ENSG00000115902

2	6.6E+07	6.6E+07	KD2-H4K8la_peak_3725	7.60756	
2	6.7E+07	6.7E+07	KD2-H4K8la_peak_3726	6.0136	MEIS1-AS3_ENSG00000226819;MEIS1_ENSG00000143995
2	6.8E+07	6.8E+07	KD2-H4K8la_peak_3727	8.60796	
2	6.8E+07	6.8E+07	KD2-H4K8la_peak_3728	10.16645	RP11-474G23.3_ENSG00000273064
2	6.9E+07	6.9E+07	KD2-H4K8la_peak_3729	8.76116	
2	7E+07	7E+07	KD2-H4K8la_peak_3730	8.76116	NFU1_ENSG00000169599
2	7E+07	7E+07	KD2-H4K8la_peak_3731	6.83387	
2	7E+07	7E+07	KD2-H4K8la_peak_3732	10.20357	AC016700.5_ENSG00000231327
2	7E+07	7E+07	KD2-H4K8la_peak_3733	14.61815	AC016700.5_ENSG00000231327
2	7E+07	7E+07	KD2-H4K8la_peak_3734	23.67682	
2	7E+07	7E+07	KD2-H4K8la_peak_3735	9.14903	
2	7E+07	7E+07	KD2-H4K8la_peak_3736	12.06531	C2orf42_ENSG00000115998;TIA1_ENSG00000116001
2	7.1E+07	7.1E+07	KD2-H4K8la_peak_3737	13.79336	SNRPG_ENSG00000143977
2	7.1E+07	7.1E+07	KD2-H4K8la_peak_3738	6.72733	
2	7.3E+07	7.3E+07	KD2-H4K8la_peak_3739	13.1338	SMYD5_ENSG00000135632
2	7.3E+07	7.3E+07	KD2-H4K8la_peak_3740	6.64804	CCT7_ENSG00000135624
2	7.4E+07	7.4E+07	KD2-H4K8la_peak_3741	9.27415	FBXO41_ENSG00000163013
2	7.4E+07	7.4E+07	KD2-H4K8la_peak_3742	5.52913	STAMBP_ENSG00000124356
2	7.4E+07	7.4E+07	KD2-H4K8la_peak_3743	8.8293	AC073046.25_ENSG00000235499
2	7.4E+07	7.4E+07	KD2-H4K8la_peak_3744	9.84972	AC073046.25_ENSG00000235499;RP11-711M9.1_ENSG00000255989
2	7.4E+07	7.4E+07	KD2-H4K8la_peak_3745	7.81267	BOLA3_ENSG00000163170;BOLA3-AS1_ENSG00000225439
2	7.4E+07	7.4E+07	KD2-H4K8la_peak_3746	12.14568	MTHFD2_ENSG00000065911
2	7.5E+07	7.5E+07	KD2-H4K8la_peak_3747	6.96161	DCTN1_ENSG00000204843
2	7.5E+07	7.5E+07	KD2-H4K8la_peak_3748	5.99671	
2	7.5E+07	7.5E+07	KD2-H4K8la_peak_3749	6.51326	MRPL53_ENSG00000204822
2	7.5E+07	7.5E+07	KD2-H4K8la_peak_3750	9.6708	PCGF1_ENSG00000115289
2	7.5E+07	7.5E+07	KD2-H4K8la_peak_3751	7.49467	TLX2_ENSG00000115297
2	7.5E+07	7.5E+07	KD2-H4K8la_peak_3752	5.90392	DOK1_ENSG00000115325
2	7.5E+07	7.5E+07	KD2-H4K8la_peak_3753	7.49467	RP11-259N19.1_ENSG00000272711;HK2_ENSG00000159399
2	7.6E+07	7.6E+07	KD2-H4K8la_peak_3754	6.56573	
2	7.7E+07	7.7E+07	KD2-H4K8la_peak_3755	8.65116	
2	7.9E+07	7.9E+07	KD2-H4K8la_peak_3756	4.79631	
2	8.5E+07	8.5E+07	KD2-H4K8la_peak_3757	5.40949	
2	8.5E+07	8.5E+07	KD2-H4K8la_peak_3758	6.64804	KCMF1_ENSG00000176407
2	8.5E+07	8.5E+07	KD2-H4K8la_peak_3759	7.8597	KCMF1_ENSG00000176407
2	8.5E+07	8.5E+07	KD2-H4K8la_peak_3760	6.71169	
2	8.5E+07	8.5E+07	KD2-H4K8la_peak_3761	6.75548	
2	8.6E+07	8.6E+07	KD2-H4K8la_peak_3762	8.59561	CAPG_ENSG00000042493;SH2D6_ENSG00000152292
2	8.6E+07	8.6E+07	KD2-H4K8la_peak_3763	10.37746	
2	8.6E+07	8.6E+07	KD2-H4K8la_peak_3764	4.67224	
2	8.6E+07	8.6E+07	KD2-H4K8la_peak_3765	5.11209	
2	8.6E+07	8.6E+07	KD2-H4K8la_peak_3766	12.67978	
2	8.6E+07	8.6E+07	KD2-H4K8la_peak_3767	5.90392	VAMP5_ENSG00000168899
2	8.6E+07	8.6E+07	KD2-H4K8la_peak_3768	4.75945	C2orf68_ENSG00000168887
2	8.6E+07	8.6E+07	KD2-H4K8la_peak_3769	6.70058	
2	8.6E+07	8.6E+07	KD2-H4K8la_peak_3770	5.45079	

2	8.6E+07	8.6E+07	KD2-H4K8la_peak_3771	5.83285	MRPL35_ENSG00000132313
2	8.8E+07	8.8E+07	KD2-H4K8la_peak_3772	6.01825	
2	8.8E+07	8.8E+07	KD2-H4K8la_peak_3773	7.72421	
2	9.2E+07	9.2E+07	KD2-H4K8la_peak_3774	5.14539	
2	9.2E+07	9.2E+07	KD2-H4K8la_peak_3775	6.64532	
2	9.2E+07	9.2E+07	KD2-H4K8la_peak_3776	8.74373	
2	9.6E+07	9.6E+07	KD2-H4K8la_peak_3777	5.30989	
2	9.7E+07	9.7E+07	KD2-H4K8la_peak_3778	10.99007	ANKRD36C_ENSG00000174501
2	9.7E+07	9.7E+07	KD2-H4K8la_peak_3779	5.84082	FAHD2CP_ENSG00000231584
2	9.7E+07	9.7E+07	KD2-H4K8la_peak_3780	11.44733	DUSP2_ENSG00000158050;AC012307.2_ENSG00000228873
2	9.7E+07	9.7E+07	KD2-H4K8la_peak_3781	6.64804	NCAPH_ENSG00000121152
2	9.7E+07	9.7E+07	KD2-H4K8la_peak_3782	5.99671	
2	9.8E+07	9.8E+07	KD2-H4K8la_peak_3783	6.83927	ANKRD23_ENSG00000163126;ANKRD39_ENSG00000213337
2	9.8E+07	9.8E+07	KD2-H4K8la_peak_3784	8.76116	
2	9.8E+07	9.8E+07	KD2-H4K8la_peak_3785	7.65547	SEMA4C_ENSG00000168758
2	9.8E+07	9.8E+07	KD2-H4K8la_peak_3786	9.72904	FAHD2B_ENSG00000144199
2	9.9E+07	9.9E+07	KD2-H4K8la_peak_3787	7.8597	TMEM131_ENSG00000075568
2	9.9E+07	9.9E+07	KD2-H4K8la_peak_3788	9.27415	
2	1E+08	1E+08	KD2-H4K8la_peak_3789	6.56656	
2	1E+08	1E+08	KD2-H4K8la_peak_3790	5.67034	AC092667.2_ENSG00000230393
2	1E+08	1E+08	KD2-H4K8la_peak_3791	14.33193	AC016738.4_ENSG00000223947;RPL31_ENSG00000071082
2	1E+08	1E+08	KD2-H4K8la_peak_3792	5.58883	TBC1D8_ENSG00000204634;CNOT11_ENSG00000158435
2	1E+08	1E+08	KD2-H4K8la_peak_3793	12.13455	RNF149_ENSG00000163162
2	1E+08	1E+08	KD2-H4K8la_peak_3794	7.64828	MAP4K4_ENSG00000071054
2	1E+08	1E+08	KD2-H4K8la_peak_3795	6.61963	MAP4K4_ENSG00000071054
2	1.1E+08	1.1E+08	KD2-H4K8la_peak_3796	6.0136	
2	1.1E+08	1.1E+08	KD2-H4K8la_peak_3797	5.28047	
2	1.1E+08	1.1E+08	KD2-H4K8la_peak_3798	10.44395	
2	1.1E+08	1.1E+08	KD2-H4K8la_peak_3799	6.64804	
2	1.1E+08	1.1E+08	KD2-H4K8la_peak_3800	7.13237	NPHP1_ENSG00000144061
2	1.1E+08	1.1E+08	KD2-H4K8la_peak_3801	6.61963	
2	1.1E+08	1.1E+08	KD2-H4K8la_peak_3802	5.05146	
2	1.1E+08	1.1E+08	KD2-H4K8la_peak_3803	6.2278	
2	1.1E+08	1.1E+08	KD2-H4K8la_peak_3804	12.13455	ZC3H6_ENSG00000188177
2	1.1E+08	1.1E+08	KD2-H4K8la_peak_3805	5.83285	CHCHD5_ENSG00000125611
2	1.1E+08	1.1E+08	KD2-H4K8la_peak_3806	7.83588	
2	1.1E+08	1.1E+08	KD2-H4K8la_peak_3807	27.75889	MIR1302-3_ENSG00000221055;WASH2P_ENSG00000146556
2	1.1E+08	1.1E+08	KD2-H4K8la_peak_3808	5.36015	
2	1.2E+08	1.2E+08	KD2-H4K8la_peak_3809	7.49467	
2	1.2E+08	1.2E+08	KD2-H4K8la_peak_3810	8.07102	TMEM177_ENSG00000144120
2	1.2E+08	1.2E+08	KD2-H4K8la_peak_3811	7.3993	TMEM185B_ENSG00000226479
2	1.2E+08	1.2E+08	KD2-H4K8la_peak_3812	8.00569	TMEM185B_ENSG00000226479
2	1.2E+08	1.2E+08	KD2-H4K8la_peak_3813	8.13557	
2	1.2E+08	1.2E+08	KD2-H4K8la_peak_3814	8.98765	
2	1.2E+08	1.2E+08	KD2-H4K8la_peak_3815	8.37064	NIFK_ENSG00000155438;TSN_ENSG00000211460
2	1.3E+08	1.3E+08	KD2-H4K8la_peak_3816	4.3268	

2	1.3E+08	1.3E+08	KD2-H4K8la_peak_3817	8.944	RP11-521O16.1_ENSG00000260634;RP11-521O16.2_ENSG00000260163
2	1.3E+08	1.3E+08	KD2-H4K8la_peak_3818	5.83285	CYP27C1_ENSG00000186684
2	1.3E+08	1.3E+08	KD2-H4K8la_peak_3819	6.11881	ERCC3_ENSG00000163161
2	1.3E+08	1.3E+08	KD2-H4K8la_peak_3820	13.20018	MAP3K2_ENSG00000169967
2	1.3E+08	1.3E+08	KD2-H4K8la_peak_3821	7.13237	
2	1.3E+08	1.3E+08	KD2-H4K8la_peak_3822	7.90495	
2	1.3E+08	1.3E+08	KD2-H4K8la_peak_3823	7.23932	
2	1.3E+08	1.3E+08	KD2-H4K8la_peak_3824	6.31081	
2	1.3E+08	1.3E+08	KD2-H4K8la_peak_3825	8.23729	
2	1.3E+08	1.3E+08	KD2-H4K8la_peak_3826	5.36015	
2	1.3E+08	1.3E+08	KD2-H4K8la_peak_3827	6.64804	
2	1.3E+08	1.3E+08	KD2-H4K8la_peak_3828	6.70626	
2	1.3E+08	1.3E+08	KD2-H4K8la_peak_3829	5.48416	
2	1.3E+08	1.3E+08	KD2-H4K8la_peak_3830	6.01825	
2	1.3E+08	1.3E+08	KD2-H4K8la_peak_3831	6.04899	
2	1.3E+08	1.3E+08	KD2-H4K8la_peak_3832	9.23478	AC079776.1_ENSG00000229536
2	1.3E+08	1.3E+08	KD2-H4K8la_peak_3833	9.0413	AC133785.1_ENSG00000233221;ARHGEF4_ENSG00000136002
2	1.3E+08	1.3E+08	KD2-H4K8la_peak_3834	4.92443	FAM168B_ENSG00000152102
2	1.3E+08	1.3E+08	KD2-H4K8la_peak_3835	8.24908	
2	1.3E+08	1.3E+08	KD2-H4K8la_peak_3836	7.99461	FAM201B_ENSG00000230992
2	1.3E+08	1.3E+08	KD2-H4K8la_peak_3837	7.23116	
2	1.4E+08	1.4E+08	KD2-H4K8la_peak_3838	10.99007	MCM6_ENSG00000076003
2	1.4E+08	1.4E+08	KD2-H4K8la_peak_3839	8.66164	DARS_ENSG00000115866;AC093391.2_ENSG00000231890
2	1.4E+08	1.4E+08	KD2-H4K8la_peak_3840	7.72421	
2	1.5E+08	1.5E+08	KD2-H4K8la_peak_3841	4.86896	ACVR2A_ENSG00000121989
2	1.5E+08	1.5E+08	KD2-H4K8la_peak_3842	8.35736	
2	1.5E+08	1.5E+08	KD2-H4K8la_peak_3843	7.13237	
2	1.5E+08	1.5E+08	KD2-H4K8la_peak_3844	5.05146	PRPF40A_ENSG00000196504;ARL6IP6_ENSG00000177917
2	1.6E+08	1.6E+08	KD2-H4K8la_peak_3845	5.43278	BAZ2B_ENSG00000123636;AC009506.1_ENSG00000224152
2	1.6E+08	1.6E+08	KD2-H4K8la_peak_3846	4.30663	
2	1.6E+08	1.6E+08	KD2-H4K8la_peak_3847	11.06859	RBMS1_ENSG00000153250
2	1.7E+08	1.7E+08	KD2-H4K8la_peak_3848	4.53273	SCN9A_ENSG00000169432
2	1.7E+08	1.7E+08	KD2-H4K8la_peak_3849	6.11881	PPIG_ENSG00000138398
2	1.7E+08	1.7E+08	KD2-H4K8la_peak_3850	4.53273	
2	1.7E+08	1.7E+08	KD2-H4K8la_peak_3851	10.11868	
2	1.7E+08	1.7E+08	KD2-H4K8la_peak_3852	8.39813	HAT1_ENSG00000128708
2	1.7E+08	1.7E+08	KD2-H4K8la_peak_3853	8.59561	MLTK_ENSG00000091436
2	1.7E+08	1.7E+08	KD2-H4K8la_peak_3854	10.87112	SP3_ENSG00000172845
2	1.7E+08	1.7E+08	KD2-H4K8la_peak_3855	8.89364	RP11-394I13.1_ENSG00000260868
2	1.8E+08	1.8E+08	KD2-H4K8la_peak_3856	6.64804	OLA1_ENSG00000138430
2	1.8E+08	1.8E+08	KD2-H4K8la_peak_3857	9.86881	
2	1.8E+08	1.8E+08	KD2-H4K8la_peak_3858	8.07044	
2	1.8E+08	1.8E+08	KD2-H4K8la_peak_3859	6.25288	ATF2_ENSG00000115966;MIR933_ENSG00000215973;AC096649.2_ENSG00000229750
2	1.8E+08	1.8E+08	KD2-H4K8la_peak_3860	6.0136	HOXD9_ENSG00000128709
2	1.8E+08	1.8E+08	KD2-H4K8la_peak_3861	10.47118	LINC01116_ENSG00000163364;LINC01117_ENSG00000224577
2	1.8E+08	1.8E+08	KD2-H4K8la_peak_3862	6.26214	AC079305.10_ENSG00000222043

2	1.8E+08	1.8E+08	KD2-H4K8la_peak_3863	6.3399	AC009948.5_ENSG00000223960
2	1.8E+08	1.8E+08	KD2-H4K8la_peak_3864	8.76116	PRKRA_ENSG00000180228;DFNB59_ENSG00000204311
2	1.8E+08	1.8E+08	KD2-H4K8la_peak_3865	16.13355	AC104076.3_ENSG00000236153
2	1.8E+08	1.8E+08	KD2-H4K8la_peak_3866	4.94337	
2	1.8E+08	1.8E+08	KD2-H4K8la_peak_3867	9.0413	AC013733.3_ENSG00000234595
2	1.9E+08	1.9E+08	KD2-H4K8la_peak_3868	7.52934	WDR75_ENSG00000115368
2	1.9E+08	1.9E+08	KD2-H4K8la_peak_3869	7.8597	
2	1.9E+08	1.9E+08	KD2-H4K8la_peak_3870	8.37064	
2	1.9E+08	1.9E+08	KD2-H4K8la_peak_3871	7.60756	ANKAR_ENSG00000151687
2	1.9E+08	1.9E+08	KD2-H4K8la_peak_3872	4.53273	OSGEPL1_ENSG00000128694;OSGEPL1-AS1_ENSG00000253559
2	1.9E+08	1.9E+08	KD2-H4K8la_peak_3873	8.77936	
2	1.9E+08	1.9E+08	KD2-H4K8la_peak_3874	4.86896	
2	1.9E+08	1.9E+08	KD2-H4K8la_peak_3875	9.77662	TMEM194B_ENSG00000189362;AC093388.3_ENSG00000233654
2	1.9E+08	1.9E+08	KD2-H4K8la_peak_3876	6.96161	
2	2E+08	2E+08	KD2-H4K8la_peak_3877	10.34978	
2	2E+08	2E+08	KD2-H4K8la_peak_3878	8.39919	HECW2_ENSG00000138411
2	2E+08	2E+08	KD2-H4K8la_peak_3879	5.72892	
2	2E+08	2E+08	KD2-H4K8la_peak_3880	9.37576	
2	2E+08	2E+08	KD2-H4K8la_peak_3881	7.61406	
2	2E+08	2E+08	KD2-H4K8la_peak_3882	7.04184	
2	2E+08	2E+08	KD2-H4K8la_peak_3883	8.07102	TYW5_ENSG00000162971;C2orf47_ENSG00000162972
2	2E+08	2E+08	KD2-H4K8la_peak_3884	4.90979	ORC2_ENSG00000115942;AC005037.3_ENSG00000183308
2	2E+08	2E+08	KD2-H4K8la_peak_3885	8.24908	CFLAR_ENSG00000003402
2	2E+08	2E+08	KD2-H4K8la_peak_3886	5.83285	TRAK2_ENSG00000115993
2	2E+08	2E+08	KD2-H4K8la_peak_3887	7.23932	
2	2E+08	2E+08	KD2-H4K8la_peak_3888	8.86558	
2	2E+08	2E+08	KD2-H4K8la_peak_3889	7.13237	SUMO1_ENSG00000116030;AC079354.2_ENSG00000267889
2	2E+08	2E+08	KD2-H4K8la_peak_3890	4.86896	SUMO1_ENSG00000116030;AC079354.2_ENSG00000267889
2	2E+08	2E+08	KD2-H4K8la_peak_3891	7.12014	NOP58_ENSG00000055044
2	2E+08	2E+08	KD2-H4K8la_peak_3892	11.15747	CYP20A1_ENSG00000119004
2	2.1E+08	2.1E+08	KD2-H4K8la_peak_3893	8.50453	PARD3B_ENSG00000116117
2	2.1E+08	2.1E+08	KD2-H4K8la_peak_3894	8.16787	INO80D_ENSG00000114933;AC007383.3_ENSG00000227946
2	2.1E+08	2.1E+08	KD2-H4K8la_peak_3895	5.7631	ADAM23_ENSG00000114948
2	2.1E+08	2.1E+08	KD2-H4K8la_peak_3896	5.16795	
2	2.1E+08	2.1E+08	KD2-H4K8la_peak_3897	7.94013	KLF7_ENSG00000118263
2	2.1E+08	2.1E+08	KD2-H4K8la_peak_3898	11.55297	
2	2.1E+08	2.1E+08	KD2-H4K8la_peak_3899	7.72421	
2	2.1E+08	2.1E+08	KD2-H4K8la_peak_3900	9.11321	AC007879.5_ENSG00000223725;CREB1_ENSG00000118260
2	2.1E+08	2.1E+08	KD2-H4K8la_peak_3901	9.20163	METTL21A_ENSG00000144401
2	2.1E+08	2.1E+08	KD2-H4K8la_peak_3902	6.2278	PLEKHM3_ENSG00000178385
2	2.1E+08	2.1E+08	KD2-H4K8la_peak_3903	10.68716	IDH1_ENSG00000138413;PIKFYVE_ENSG00000115020
2	2.2E+08	2.2E+08	KD2-H4K8la_peak_3904	5.83285	ATIC_ENSG00000138363
2	2.2E+08	2.2E+08	KD2-H4K8la_peak_3905	5.05146	FN1_ENSG00000115414;AC012462.1_ENSG00000230695
2	2.2E+08	2.2E+08	KD2-H4K8la_peak_3906	5.36015	XRCC5_ENSG00000079246
2	2.2E+08	2.2E+08	KD2-H4K8la_peak_3907	7.49467	
2	2.2E+08	2.2E+08	KD2-H4K8la_peak_3908	6.89612	

2	2.2E+08	2.2E+08	KD2-H4K8la_peak_3909	8.62669	
2	2.2E+08	2.2E+08	KD2-H4K8la_peak_3910	6.47589	RUFY4_ENSG00000188282
2	2.2E+08	2.2E+08	KD2-H4K8la_peak_3911	7.83588	ARPC2_ENSG00000163466
2	2.2E+08	2.2E+08	KD2-H4K8la_peak_3912	9.20163	CTDSP1_ENSG00000144579;RP11-378A13.2_ENSG00000273361
2	2.2E+08	2.2E+08	KD2-H4K8la_peak_3913	8.76116	RNF25_ENSG00000163481;STK36_ENSG00000163482
2	2.2E+08	2.2E+08	KD2-H4K8la_peak_3914	6.40658	WNT10A_ENSG00000135925
2	2.2E+08	2.2E+08	KD2-H4K8la_peak_3915	5.43278	AC097468.4_ENSG00000224090
2	2.2E+08	2.2E+08	KD2-H4K8la_peak_3916	7.49467	FAM134A_ENSG00000144567
2	2.2E+08	2.2E+08	KD2-H4K8la_peak_3917	6.64804	CNPPD1_ENSG00000115649
2	2.2E+08	2.2E+08	KD2-H4K8la_peak_3918	9.06396	STK16_ENSG00000115661
2	2.2E+08	2.2E+08	KD2-H4K8la_peak_3919	7.41831	DNAJB2_ENSG00000135924
2	2.2E+08	2.2E+08	KD2-H4K8la_peak_3920	4.30132	
2	2.2E+08	2.2E+08	KD2-H4K8la_peak_3921	10.27876	
2	2.2E+08	2.2E+08	KD2-H4K8la_peak_3922	9.38376	
2	2.2E+08	2.2E+08	KD2-H4K8la_peak_3923	6.11881	
2	2.2E+08	2.2E+08	KD2-H4K8la_peak_3924	5.83285	RP11-316O14.1_ENSG00000268603;GMPPA_ENSG00000144591
2	2.2E+08	2.2E+08	KD2-H4K8la_peak_3925	9.70817	
2	2.2E+08	2.2E+08	KD2-H4K8la_peak_3926	8.76116	
2	2.2E+08	2.2E+08	KD2-H4K8la_peak_3927	5.6499	
2	2.2E+08	2.2E+08	KD2-H4K8la_peak_3928	6.64804	SGPP2_ENSG00000163082
2	2.2E+08	2.2E+08	KD2-H4K8la_peak_3929	5.99671	WDFY1_ENSG00000085449
2	2.3E+08	2.3E+08	KD2-H4K8la_peak_3930	8.86558	CUL3_ENSG00000036257
2	2.3E+08	2.3E+08	KD2-H4K8la_peak_3931	5.72892	
2	2.3E+08	2.3E+08	KD2-H4K8la_peak_3932	7.49467	RHBDD1_ENSG00000144468
2	2.3E+08	2.3E+08	KD2-H4K8la_peak_3933	4.69635	RHBDD1_ENSG00000144468
2	2.3E+08	2.3E+08	KD2-H4K8la_peak_3934	8.487	CAB39_ENSG00000135932
2	2.3E+08	2.3E+08	KD2-H4K8la_peak_3935	6.3399	
2	2.3E+08	2.3E+08	KD2-H4K8la_peak_3936	6.36118	NCL_ENSG00000115053
2	2.3E+08	2.3E+08	KD2-H4K8la_peak_3937	8.13557	
2	2.3E+08	2.3E+08	KD2-H4K8la_peak_3938	10.98662	
2	2.3E+08	2.3E+08	KD2-H4K8la_peak_3939	11.99163	
2	2.3E+08	2.3E+08	KD2-H4K8la_peak_3940	10.16316	
2	2.3E+08	2.3E+08	KD2-H4K8la_peak_3941	10.87112	MGC4771_ENSG00000269363;PTMA_ENSG00000187514
2	2.3E+08	2.3E+08	KD2-H4K8la_peak_3942	11.50321	
2	2.3E+08	2.3E+08	KD2-H4K8la_peak_3943	16.8445	
2	2.3E+08	2.3E+08	KD2-H4K8la_peak_3944	5.52913	
2	2.3E+08	2.3E+08	KD2-H4K8la_peak_3945	8.487	RP11-400N9.1_ENSG00000259793;DGKD_ENSG00000077044;AC019221.4_ENSG00000243637
2	2.3E+08	2.3E+08	KD2-H4K8la_peak_3946	9.0413	
2	2.4E+08	2.4E+08	KD2-H4K8la_peak_3947	6.64804	
2	2.4E+08	2.4E+08	KD2-H4K8la_peak_3948	6.48366	
2	2.4E+08	2.4E+08	KD2-H4K8la_peak_3949	7.51302	
2	2.4E+08	2.4E+08	KD2-H4K8la_peak_3950	12.1221	ARL4C_ENSG00000188042
2	2.4E+08	2.4E+08	KD2-H4K8la_peak_3951	8.93385	SH3BP4_ENSG00000130147
2	2.4E+08	2.4E+08	KD2-H4K8la_peak_3952	6.75892	AGAP1_ENSG00000157985
2	2.4E+08	2.4E+08	KD2-H4K8la_peak_3953	9.73848	AC105760.2_ENSG00000227252;COPS8_ENSG00000198612
2	2.4E+08	2.4E+08	KD2-H4K8la_peak_3954	8.76116	

2	2.4E+08	2.4E+08	KD2-H4K8la_peak_3955	10.58561	
2	2.4E+08	2.4E+08	KD2-H4K8la_peak_3956	13.91009	LRRFIP1_ENSG00000124831
2	2.4E+08	2.4E+08	KD2-H4K8la_peak_3957	6.64804	LRRFIP1_ENSG00000124831
2	2.4E+08	2.4E+08	KD2-H4K8la_peak_3958	6.47589	
2	2.4E+08	2.4E+08	KD2-H4K8la_peak_3959	7.13528	SNORD39_ENSG00000263723
2	2.4E+08	2.4E+08	KD2-H4K8la_peak_3960	5.33721	
2	2.4E+08	2.4E+08	KD2-H4K8la_peak_3961	9.29385	
2	2.4E+08	2.4E+08	KD2-H4K8la_peak_3962	14.25411	
2	2.4E+08	2.4E+08	KD2-H4K8la_peak_3963	5.43278	
2	2.4E+08	2.4E+08	KD2-H4K8la_peak_3964	5.43278	
2	2.4E+08	2.4E+08	KD2-H4K8la_peak_3965	5.33301	SCLY_ENSG00000132330
2	2.4E+08	2.4E+08	KD2-H4K8la_peak_3966	7.3993	ASB1_ENSG00000065802;AC016999.2_ENSG00000229915
2	2.4E+08	2.4E+08	KD2-H4K8la_peak_3967	4.86896	
2	2.4E+08	2.4E+08	KD2-H4K8la_peak_3968	5.40949	
2	2.4E+08	2.4E+08	KD2-H4K8la_peak_3969	5.52913	
2	2.4E+08	2.4E+08	KD2-H4K8la_peak_3970	8.24671	
2	2.4E+08	2.4E+08	KD2-H4K8la_peak_3971	6.3399	
2	2.4E+08	2.4E+08	KD2-H4K8la_peak_3972	7.92775	
2	2.4E+08	2.4E+08	KD2-H4K8la_peak_3973	7.12014	NDUFA10_ENSG00000130414
2	2.4E+08	2.4E+08	KD2-H4K8la_peak_3974	6.14571	AC110619.1_ENSG00000255735
2	2.4E+08	2.4E+08	KD2-H4K8la_peak_3975	6.69599	ANKMY1_ENSG00000144504
2	2.4E+08	2.4E+08	KD2-H4K8la_peak_3976	4.86896	
2	2.4E+08	2.4E+08	KD2-H4K8la_peak_3977	7.64828	AC093585.6_ENSG00000229996
2	2.4E+08	2.4E+08	KD2-H4K8la_peak_3978	7.28641	BOK-AS1_ENSG00000234235;BOK_ENSG00000176720
2	2.4E+08	2.4E+08	KD2-H4K8la_peak_3979	7.47988	BOK-AS1_ENSG00000234235;BOK_ENSG00000176720
2	2.4E+08	2.4E+08	KD2-H4K8la_peak_3980	10.11868	THAP4_ENSG00000176946;ATG4B_ENSG00000168397
2	2.4E+08	2.4E+08	KD2-H4K8la_peak_3981	7.04184	
2	2.4E+08	2.4E+08	KD2-H4K8la_peak_3982	5.24865	
2	2.4E+08	2.4E+08	KD2-H4K8la_peak_3983	9.0516	AC114730.5_ENSG00000215023
2	2.4E+08	2.4E+08	KD2-H4K8la_peak_3984	16.21274	
2	2.4E+08	2.4E+08	KD2-H4K8la_peak_3985	8.44557	
2	2.4E+08	2.4E+08	KD2-H4K8la_peak_3986	7.72421	AC114730.2_ENSG00000235151
2	2.4E+08	2.4E+08	KD2-H4K8la_peak_3987	7.13237	AC114730.2_ENSG00000235151
2	2.4E+08	2.4E+08	KD2-H4K8la_peak_3988	7.2823	
2	2.4E+08	2.4E+08	KD2-H4K8la_peak_3989	7.49467	AC131097.3_ENSG00000233806
20	270738	270990	KD2-H4K8la_peak_3990	13.3197	C20orf96_ENSG00000196476
20	307099	307491	KD2-H4K8la_peak_3991	4.75945	SOX12_ENSG00000177732
20	389008	389385	KD2-H4K8la_peak_3992	8.71024	RBCK1_ENSG00000125826
20	1447129	1447392	KD2-H4K8la_peak_3993	7.81267	
20	2450907	2451278	KD2-H4K8la_peak_3994	8.00569	SNRPB_ENSG00000125835
20	2505365	2505614	KD2-H4K8la_peak_3995	7.83588	ZNF343_ENSG00000088876
20	2632700	2633007	KD2-H4K8la_peak_3996	13.20018	NOP56_ENSG00000101361;MIR1292_ENSG00000221062
20	2672875	2673165	KD2-H4K8la_peak_3997	10.04111	EBF4_ENSG00000088881
20	2820542	2821241	KD2-H4K8la_peak_3998	15.19386	PCED1A_ENSG00000132635;VPS16_ENSG00000215305
20	3027161	3027363	KD2-H4K8la_peak_3999	7.06427	MRPS26_ENSG00000125901
20	3748826	3749062	KD2-H4K8la_peak_4000	9.9701	C20orf27_ENSG00000101220

20	3788646	3789017	KD2-H4K8la_peak_4001	6.11881	
20	3792746	3792956	KD2-H4K8la_peak_4002	6.0136	
20	3800214	3800664	KD2-H4K8la_peak_4003	9.0413	AP5S1_ENSG00000125843
20	4152522	4152942	KD2-H4K8la_peak_4004	5.58883	
20	4741755	4741998	KD2-H4K8la_peak_4005	6.88801	
20	5093370	5093658	KD2-H4K8la_peak_4006	5.58883	TMEM230_ENSG00000089063
20	5484741	5485028	KD2-H4K8la_peak_4007	6.2278	LINC00654_ENSG00000205181
20	5730476	5730697	KD2-H4K8la_peak_4008	5.6499	C20orf196_ENSG00000171984
20	8000072	8000314	KD2-H4K8la_peak_4009	11.87032	TMX4_ENSG00000125827;RP5-971N18.3_ENSG00000229766
20	1.1E+07	1.1E+07	KD2-H4K8la_peak_4010	9.04068	
20	1.4E+07	1.4E+07	KD2-H4K8la_peak_4011	5.40949	ESF1_ENSG00000089048;NDUFAF5_ENSG00000101247
20	1.6E+07	1.6E+07	KD2-H4K8la_peak_4012	7.72421	AL121584.1_ENSG00000212111
20	1.7E+07	1.7E+07	KD2-H4K8la_peak_4013	7.61975	SNRPB2_ENSG00000125870
20	1.8E+07	1.8E+07	KD2-H4K8la_peak_4014	9.86881	
20	1.8E+07	1.8E+07	KD2-H4K8la_peak_4015	6.70626	PET117_ENSG00000232838;CSRP2BP_ENSG00000149474
20	1.8E+07	1.8E+07	KD2-H4K8la_peak_4016	11.45154	PET117_ENSG00000232838;CSRP2BP_ENSG00000149474
20	1.8E+07	1.8E+07	KD2-H4K8la_peak_4017	8.12466	ZNF133_ENSG00000125846
20	2E+07	2E+07	KD2-H4K8la_peak_4018	14.52454	C20orf26_ENSG00000089101
20	2.1E+07	2.1E+07	KD2-H4K8la_peak_4019	9.88732	
20	2.1E+07	2.1E+07	KD2-H4K8la_peak_4020	7.49467	
20	2.1E+07	2.1E+07	KD2-H4K8la_peak_4021	5.58883	LINC00237_ENSG00000225127
20	2.1E+07	2.1E+07	KD2-H4K8la_peak_4022	5.58883	
20	2.3E+07	2.3E+07	KD2-H4K8la_peak_4023	6.2278	NAPB_ENSG00000125814
20	2.4E+07	2.4E+07	KD2-H4K8la_peak_4024	7.45264	CST3_ENSG00000101439
20	2.5E+07	2.5E+07	KD2-H4K8la_peak_4025	5.27164	ENTPD6_ENSG00000197586
20	2.5E+07	2.5E+07	KD2-H4K8la_peak_4026	7.28641	PYGB_ENSG00000100994
20	2.6E+07	2.6E+07	KD2-H4K8la_peak_4027	11.15747	NINL_ENSG00000101004
20	2.6E+07	2.6E+07	KD2-H4K8la_peak_4028	5.36015	RP4-760C5.3_ENSG00000231081
20	3E+07	3E+07	KD2-H4K8la_peak_4029	6.47589	
20	3E+07	3E+07	KD2-H4K8la_peak_4030	11.24937	FRG1B_ENSG00000149531
20	3E+07	3E+07	KD2-H4K8la_peak_4031	9.07681	
20	3E+07	3E+07	KD2-H4K8la_peak_4032	11.84538	COX4I2_ENSG00000131055
20	3E+07	3E+07	KD2-H4K8la_peak_4033	8.944	BCL2L1_ENSG00000171552
20	3E+07	3E+07	KD2-H4K8la_peak_4034	8.07102	
20	3.1E+07	3.1E+07	KD2-H4K8la_peak_4035	7.37741	
20	3.1E+07	3.1E+07	KD2-H4K8la_peak_4036	7.72421	
20	3.1E+07	3.1E+07	KD2-H4K8la_peak_4037	6.20793	TSPY26P_ENSG00000235217
20	3.1E+07	3.1E+07	KD2-H4K8la_peak_4038	10.20357	POFUT1_ENSG00000101346
20	3.1E+07	3.1E+07	KD2-H4K8la_peak_4039	14.30153	
20	3.1E+07	3.1E+07	KD2-H4K8la_peak_4040	10.04111	
20	3.1E+07	3.1E+07	KD2-H4K8la_peak_4041	8.39919	
20	3.1E+07	3.1E+07	KD2-H4K8la_peak_4042	9.27415	
20	3.1E+07	3.1E+07	KD2-H4K8la_peak_4043	5.49688	
20	3.1E+07	3.1E+07	KD2-H4K8la_peak_4044	9.37576	
20	3.1E+07	3.1E+07	KD2-H4K8la_peak_4045	8.12466	MAPRE1_ENSG00000101367
20	3.2E+07	3.2E+07	KD2-H4K8la_peak_4046	5.43278	

20	3.2E+07	3.2E+07	KD2-H4K8la_peak_4047	7.13185	
20	3.2E+07	3.2E+07	KD2-H4K8la_peak_4048	12.14099	PXMP4_ENSG00000101417
20	3.2E+07	3.2E+07	KD2-H4K8la_peak_4049	5.58883	RP4-553F4.2_ENSG00000229188;ZNF341_ENSG00000131061
20	3.2E+07	3.2E+07	KD2-H4K8la_peak_4050	7.39473	
20	3.2E+07	3.2E+07	KD2-H4K8la_peak_4051	9.81238	
20	3.3E+07	3.3E+07	KD2-H4K8la_peak_4052	4.94337	ITCH_ENSG00000078747
20	3.4E+07	3.4E+07	KD2-H4K8la_peak_4053	5.83285	GSS_ENSG00000100983
20	3.4E+07	3.4E+07	KD2-H4K8la_peak_4054	8.07102	
20	3.4E+07	3.4E+07	KD2-H4K8la_peak_4055	5.10388	TRPC4AP_ENSG00000100991
20	3.4E+07	3.4E+07	KD2-H4K8la_peak_4056	6.20793	UQCC1_ENSG00000101019
20	3.4E+07	3.4E+07	KD2-H4K8la_peak_4057	8.10373	NFS1_ENSG00000244005;ROMO1_ENSG00000125995
20	3.5E+07	3.5E+07	KD2-H4K8la_peak_4058	6.75222	
20	3.5E+07	3.5E+07	KD2-H4K8la_peak_4059	6.03964	
20	3.5E+07	3.5E+07	KD2-H4K8la_peak_4060	9.27415	
20	3.5E+07	3.5E+07	KD2-H4K8la_peak_4061	6.28277	
20	3.5E+07	3.5E+07	KD2-H4K8la_peak_4062	6.64804	RP5-977B1.7_ENSG00000232907;TGIF2_ENSG00000118707
20	3.5E+07	3.5E+07	KD2-H4K8la_peak_4063	6.20477	RP5-977B1.7_ENSG00000232907;TGIF2_ENSG00000118707;TGIF2-C20orf24_ENSG00000259399
20	3.5E+07	3.5E+07	KD2-H4K8la_peak_4064	8.37064	
20	3.5E+07	3.5E+07	KD2-H4K8la_peak_4065	5.3107	SOGA1_ENSG00000149639
20	3.6E+07	3.6E+07	KD2-H4K8la_peak_4066	10.90862	SAMHD1_ENSG00000101347
20	3.6E+07	3.6E+07	KD2-H4K8la_peak_4067	6.5403	
20	3.6E+07	3.6E+07	KD2-H4K8la_peak_4068	7.81267	
20	3.6E+07	3.6E+07	KD2-H4K8la_peak_4069	8.50453	
20	3.6E+07	3.6E+07	KD2-H4K8la_peak_4070	11.52197	
20	3.6E+07	3.6E+07	KD2-H4K8la_peak_4071	6.13507	
20	3.6E+07	3.6E+07	KD2-H4K8la_peak_4072	5.83285	
20	3.6E+07	3.6E+07	KD2-H4K8la_peak_4073	9.52279	
20	3.6E+07	3.6E+07	KD2-H4K8la_peak_4074	8.49617	
20	3.6E+07	3.6E+07	KD2-H4K8la_peak_4075	6.77116	
20	3.6E+07	3.6E+07	KD2-H4K8la_peak_4076	9.27415	
20	3.6E+07	3.6E+07	KD2-H4K8la_peak_4077	9.61894	
20	3.6E+07	3.6E+07	KD2-H4K8la_peak_4078	6.3399	
20	3.6E+07	3.6E+07	KD2-H4K8la_peak_4079	8.12466	
20	3.6E+07	3.6E+07	KD2-H4K8la_peak_4080	5.05367	
20	3.6E+07	3.6E+07	KD2-H4K8la_peak_4081	11.15747	
20	3.6E+07	3.6E+07	KD2-H4K8la_peak_4082	13.08108	
20	3.6E+07	3.6E+07	KD2-H4K8la_peak_4083	5.99671	
20	3.6E+07	3.6E+07	KD2-H4K8la_peak_4084	11.50321	BLCAP_ENSG00000166619
20	3.7E+07	3.7E+07	KD2-H4K8la_peak_4085	11.40266	VSTM2L_ENSG00000132821
20	3.7E+07	3.7E+07	KD2-H4K8la_peak_4086	9.67335	SNHG17_ENSG00000196756;SNORA71D_ENSG00000200354
20	3.7E+07	3.7E+07	KD2-H4K8la_peak_4087	9.20163	SNHG11_ENSG00000174365
20	3.8E+07	3.8E+07	KD2-H4K8la_peak_4088	8.79494	RN7SL116P_ENSG00000240474
20	3.8E+07	3.8E+07	KD2-H4K8la_peak_4089	6.77116	
20	3.8E+07	3.8E+07	KD2-H4K8la_peak_4090	6.36118	
20	3.8E+07	3.8E+07	KD2-H4K8la_peak_4091	5.36015	
20	3.9E+07	3.9E+07	KD2-H4K8la_peak_4092	5.67034	MAFB_ENSG00000204103

20	4E+07	4E+07	KD2-H4K8la_peak_4093	8.76116	RP1-1J6.2_ENSG00000226648;PLCG1_ENSG00000124181
20	4E+07	4E+07	KD2-H4K8la_peak_4094	6.77116	RP1-1J6.2_ENSG00000226648;PLCG1_ENSG00000124181
20	4E+07	4E+07	KD2-H4K8la_peak_4095	9.73848	CHD6_ENSG00000124177
20	4.2E+07	4.2E+07	KD2-H4K8la_peak_4096	7.447	
20	4.3E+07	4.3E+07	KD2-H4K8la_peak_4097	9.20163	TOX2_ENSG00000124191
20	4.3E+07	4.3E+07	KD2-H4K8la_peak_4098	6.64804	
20	4.3E+07	4.3E+07	KD2-H4K8la_peak_4099	8.98765	
20	4.3E+07	4.3E+07	KD2-H4K8la_peak_4100	11.15747	
20	4.3E+07	4.3E+07	KD2-H4K8la_peak_4101	11.69149	
20	4.3E+07	4.3E+07	KD2-H4K8la_peak_4102	10.58561	
20	4.3E+07	4.3E+07	KD2-H4K8la_peak_4103	6.20793	
20	4.3E+07	4.3E+07	KD2-H4K8la_peak_4104	5.05146	RP11-445H22.4_ENSG00000244558;KCNK15_ENSG00000124249
20	4.4E+07	4.4E+07	KD2-H4K8la_peak_4105	7.47988	
20	4.4E+07	4.4E+07	KD2-H4K8la_peak_4106	7.9397	SDC4_ENSG00000124145
20	4.4E+07	4.4E+07	KD2-H4K8la_peak_4107	8.12466	PIGT_ENSG00000124155
20	4.4E+07	4.4E+07	KD2-H4K8la_peak_4108	6.2278	
20	4.4E+07	4.4E+07	KD2-H4K8la_peak_4109	8.77936	WFDC3_ENSG00000124116;DNTTIP1_ENSG00000101457
20	4.5E+07	4.5E+07	KD2-H4K8la_peak_4110	5.7631	ZSWIM1_ENSG00000168612
20	4.5E+07	4.5E+07	KD2-H4K8la_peak_4111	6.47589	NEURL2_ENSG00000124257;CTSA_ENSG00000064601
20	4.5E+07	4.5E+07	KD2-H4K8la_peak_4112	14.09647	PCIF1_ENSG00000100982
20	4.5E+07	4.5E+07	KD2-H4K8la_peak_4113	7.61975	ZNF335_ENSG00000198026
20	4.5E+07	4.5E+07	KD2-H4K8la_peak_4114	7.38202	ZNF335_ENSG00000198026
20	4.5E+07	4.5E+07	KD2-H4K8la_peak_4115	5.33512	
20	4.5E+07	4.5E+07	KD2-H4K8la_peak_4116	17.33071	SLC35C2_ENSG00000080189
20	4.5E+07	4.5E+07	KD2-H4K8la_peak_4117	6.97699	ZNF334_ENSG00000198185
20	4.5E+07	4.5E+07	KD2-H4K8la_peak_4118	8.39919	SLC2A10_ENSG00000197496
20	4.6E+07	4.6E+07	KD2-H4K8la_peak_4119	8.17106	
20	4.6E+07	4.6E+07	KD2-H4K8la_peak_4120	5.7631	
20	4.6E+07	4.6E+07	KD2-H4K8la_peak_4121	17.30105	ZMYND8_ENSG00000101040
20	4.6E+07	4.6E+07	KD2-H4K8la_peak_4122	6.13507	ZMYND8_ENSG00000101040
20	4.6E+07	4.6E+07	KD2-H4K8la_peak_4123	6.10429	RNU6-563P_ENSG00000201742
20	4.8E+07	4.8E+07	KD2-H4K8la_peak_4124	6.11881	ARFGEF2_ENSG00000124198
20	4.8E+07	4.8E+07	KD2-H4K8la_peak_4125	9.49266	STAU1_ENSG00000124214
20	4.8E+07	4.8E+07	KD2-H4K8la_peak_4126	5.05146	DDX27_ENSG00000124228
20	4.8E+07	4.8E+07	KD2-H4K8la_peak_4127	6.57319	B4GALT5_ENSG00000158470
20	4.8E+07	4.8E+07	KD2-H4K8la_peak_4128	15.01131	SLC9A8_ENSG00000197818
20	4.9E+07	4.9E+07	KD2-H4K8la_peak_4129	10.28981	
20	4.9E+07	4.9E+07	KD2-H4K8la_peak_4130	10.84057	
20	4.9E+07	4.9E+07	KD2-H4K8la_peak_4131	8.39919	
20	4.9E+07	4.9E+07	KD2-H4K8la_peak_4132	8.93385	
20	4.9E+07	4.9E+07	KD2-H4K8la_peak_4133	10.20357	
20	4.9E+07	4.9E+07	KD2-H4K8la_peak_4134	6.2278	
20	4.9E+07	4.9E+07	KD2-H4K8la_peak_4135	5.0762	BCAS4_ENSG00000124243
20	5E+07	5E+07	KD2-H4K8la_peak_4136	11.5908	ADNP_ENSG00000101126;RP5-914P20.5_ENSG00000259456
20	5E+07	5E+07	KD2-H4K8la_peak_4137	9.90319	
20	5.1E+07	5.1E+07	KD2-H4K8la_peak_4138	8.66164	

20	5.6E+07	5.6E+07	KD2-H4K8la_peak_4139	9.0413	
20	5.6E+07	5.6E+07	KD2-H4K8la_peak_4140	5.36015	
20	5.6E+07	5.6E+07	KD2-H4K8la_peak_4141	6.96161	BMP7_ENSG00000101144;RP4-813D12.3_ENSG00000226308
20	5.6E+07	5.6E+07	KD2-H4K8la_peak_4142	11.97807	RAE1_ENSG00000101146
20	5.6E+07	5.6E+07	KD2-H4K8la_peak_4143	8.12466	
20	5.7E+07	5.7E+07	KD2-H4K8la_peak_4144	7.78147	
20	5.7E+07	5.7E+07	KD2-H4K8la_peak_4145	10.91315	
20	5.7E+07	5.7E+07	KD2-H4K8la_peak_4146	5.28047	ANKRD60_ENSG00000124227
20	5.7E+07	5.7E+07	KD2-H4K8la_peak_4147	7.51302	PPP4R1L_ENSG00000124224;RAB22A_ENSG00000124209
20	5.7E+07	5.7E+07	KD2-H4K8la_peak_4148	9.27415	PPP4R1L_ENSG00000124224;RAB22A_ENSG00000124209
20	5.7E+07	5.7E+07	KD2-H4K8la_peak_4149	8.37064	RP1-309F20.4_ENSG00000270951
20	5.8E+07	5.8E+07	KD2-H4K8la_peak_4150	9.66714	SLMO2_ENSG00000101166
20	5.8E+07	5.8E+07	KD2-H4K8la_peak_4151	8.00569	
20	5.8E+07	5.8E+07	KD2-H4K8la_peak_4152	9.11321	
20	5.8E+07	5.8E+07	KD2-H4K8la_peak_4153	11.06859	
20	5.8E+07	5.8E+07	KD2-H4K8la_peak_4154	4.86896	
20	5.8E+07	5.8E+07	KD2-H4K8la_peak_4155	4.53273	
20	5.9E+07	5.9E+07	KD2-H4K8la_peak_4156	8.13727	SYCP2_ENSG00000196074;FAM217B_ENSG00000196227
20	6.1E+07	6.1E+07	KD2-H4K8la_peak_4157	5.3107	
20	6.1E+07	6.1E+07	KD2-H4K8la_peak_4158	7.3993	
20	6.1E+07	6.1E+07	KD2-H4K8la_peak_4159	7.91061	
20	6.1E+07	6.1E+07	KD2-H4K8la_peak_4160	8.00843	LAMA5_ENSG00000130702
20	6.1E+07	6.1E+07	KD2-H4K8la_peak_4161	6.3399	LAMA5_ENSG00000130702
20	6.1E+07	6.1E+07	KD2-H4K8la_peak_4162	7.12014	RPS21_ENSG00000171858
20	6.1E+07	6.1E+07	KD2-H4K8la_peak_4163	5.98775	
20	6.1E+07	6.1E+07	KD2-H4K8la_peak_4164	5.83285	RP13-30A9.1_ENSG00000232121
20	6.1E+07	6.1E+07	KD2-H4K8la_peak_4165	9.10568	
20	6.1E+07	6.1E+07	KD2-H4K8la_peak_4166	8.16787	
20	6.1E+07	6.1E+07	KD2-H4K8la_peak_4167	7.12014	
20	6.1E+07	6.1E+07	KD2-H4K8la_peak_4168	10.89081	
20	6.1E+07	6.1E+07	KD2-H4K8la_peak_4169	6.2278	
20	6.1E+07	6.1E+07	KD2-H4K8la_peak_4170	8.00701	MRGBP_ENSG00000101189
20	6.1E+07	6.1E+07	KD2-H4K8la_peak_4171	7.12014	
20	6.1E+07	6.1E+07	KD2-H4K8la_peak_4172	5.79108	
20	6.1E+07	6.1E+07	KD2-H4K8la_peak_4173	6.86458	
20	6.1E+07	6.1E+07	KD2-H4K8la_peak_4174	8.76116	
20	6.2E+07	6.2E+07	KD2-H4K8la_peak_4175	5.52913	
20	6.2E+07	6.2E+07	KD2-H4K8la_peak_4176	7.12308	YTHDF1_ENSG00000149658
20	6.2E+07	6.2E+07	KD2-H4K8la_peak_4177	9.0516	
20	6.2E+07	6.2E+07	KD2-H4K8la_peak_4178	7.92775	
20	6.2E+07	6.2E+07	KD2-H4K8la_peak_4179	11.20123	
20	6.2E+07	6.2E+07	KD2-H4K8la_peak_4180	13.47058	
20	6.2E+07	6.2E+07	KD2-H4K8la_peak_4181	14.3505	
20	6.2E+07	6.2E+07	KD2-H4K8la_peak_4182	11.81269	
20	6.2E+07	6.2E+07	KD2-H4K8la_peak_4183	8.84474	
20	6.2E+07	6.2E+07	KD2-H4K8la_peak_4184	4.66473	

20	6.2E+07	6.2E+07	KD2-H4K8la_peak_4185	8.46409	EEF1A2_ENSG00000101210
20	6.2E+07	6.2E+07	KD2-H4K8la_peak_4186	6.98292	EEF1A2_ENSG00000101210
20	6.2E+07	6.2E+07	KD2-H4K8la_peak_4187	7.3993	RP4-697K14.3_ENSG00000230226
20	6.2E+07	6.2E+07	KD2-H4K8la_peak_4188	6.04152	PTK6_ENSG00000101213
20	6.2E+07	6.2E+07	KD2-H4K8la_peak_4189	17.44434	GMEB2_ENSG00000101216;CTD-3184A7.4_ENSG00000232442
20	6.2E+07	6.2E+07	KD2-H4K8la_peak_4190	5.33301	RTEL1_ENSG00000258366
20	6.2E+07	6.2E+07	KD2-H4K8la_peak_4191	7.06427	ARFRP1_ENSG00000101246;ZGPAT_ENSG00000197114
20	6.2E+07	6.2E+07	KD2-H4K8la_peak_4192	9.6708	ARFRP1_ENSG00000101246;ZGPAT_ENSG00000197114;RP4-583P15.15_ENSG00000273154
20	6.2E+07	6.2E+07	KD2-H4K8la_peak_4193	7.49467	
20	6.3E+07	6.3E+07	KD2-H4K8la_peak_4194	8.50453	
20	6.3E+07	6.3E+07	KD2-H4K8la_peak_4195	8.07102	SAMD10_ENSG00000130590;PRPF6_ENSG00000101161
21	1.1E+07	1.1E+07	KD2-H4K8la_peak_4196	5.79108	
21	1.6E+07	1.6E+07	KD2-H4K8la_peak_4197	8.12466	HSPA13_ENSG00000155304
21	1.9E+07	1.9E+07	KD2-H4K8la_peak_4198	8.37064	BTG3_ENSG00000154640
21	1.9E+07	1.9E+07	KD2-H4K8la_peak_4199	10.58561	C21orf91_ENSG00000154642
21	2.7E+07	2.7E+07	KD2-H4K8la_peak_4200	9.86881	ATP5J_ENSG00000154723;GABPA_ENSG00000154727
21	2.8E+07	2.8E+07	KD2-H4K8la_peak_4201	5.83285	ADAMTS1_ENSG00000154734
21	3E+07	3E+07	KD2-H4K8la_peak_4202	7.81267	RP1-100J12.1_ENSG00000273254;USP16_ENSG00000156256
21	3.3E+07	3.3E+07	KD2-H4K8la_peak_4203	5.83285	
21	3.3E+07	3.3E+07	KD2-H4K8la_peak_4204	5.10388	AP000253.1_ENSG00000234509;SOD1_ENSG00000142168
21	3.3E+07	3.3E+07	KD2-H4K8la_peak_4205	6.64804	HUNK_ENSG00000142149
21	3.4E+07	3.4E+07	KD2-H4K8la_peak_4206	10.97039	URB1_ENSG00000142207;C21orf119_ENSG00000256073
21	3.4E+07	3.4E+07	KD2-H4K8la_peak_4207	10.04111	PAXBP1_ENSG00000159086;C21orf49_ENSG00000205930
21	3.5E+07	3.5E+07	KD2-H4K8la_peak_4208	5.84385	IFNAR1_ENSG00000142166
21	3.5E+07	3.5E+07	KD2-H4K8la_peak_4209	10.2573	DNAJC28_ENSG00000177692
21	3.5E+07	3.5E+07	KD2-H4K8la_peak_4210	11.06859	MRPS6_ENSG00000243927;SLC5A3_ENSG00000272962;SLC5A3_ENSG00000198743;AP000320.7_ENSG00000272657
21	3.6E+07	3.6E+07	KD2-H4K8la_peak_4211	9.86881	AP000318.2_ENSG00000214955
21	3.8E+07	3.8E+07	KD2-H4K8la_peak_4212	11.36807	MORC3_ENSG00000159256
21	3.8E+07	3.8E+07	KD2-H4K8la_peak_4213	5.23773	HLCS_ENSG00000159267
21	3.8E+07	3.8E+07	KD2-H4K8la_peak_4214	10.16316	PIGP_ENSG00000185808;TTC3_ENSG00000182670
21	4.1E+07	4.1E+07	KD2-H4K8la_peak_4215	5.67034	BRWD1-IT2_ENSG00000255568
21	4.1E+07	4.1E+07	KD2-H4K8la_peak_4216	12.1221	HMGN1_ENSG00000205581
21	4.1E+07	4.1E+07	KD2-H4K8la_peak_4217	4.6555	HMGN1_ENSG00000205581
21	4.1E+07	4.1E+07	KD2-H4K8la_peak_4218	6.3399	
21	4.3E+07	4.3E+07	KD2-H4K8la_peak_4219	7.13237	PRDM15_ENSG00000141956
21	4.3E+07	4.3E+07	KD2-H4K8la_peak_4220	7.60756	
21	4.4E+07	4.4E+07	KD2-H4K8la_peak_4221	5.67034	
21	4.4E+07	4.4E+07	KD2-H4K8la_peak_4222	4.75945	
21	4.4E+07	4.4E+07	KD2-H4K8la_peak_4223	6.86458	PDE9A_ENSG00000160191
21	4.4E+07	4.4E+07	KD2-H4K8la_peak_4224	7.72421	
21	4.4E+07	4.4E+07	KD2-H4K8la_peak_4225	11.06859	WDR4_ENSG00000160193;NDUFV3_ENSG00000160194
21	4.4E+07	4.4E+07	KD2-H4K8la_peak_4226	8.2051	
21	4.4E+07	4.4E+07	KD2-H4K8la_peak_4227	6.13507	PKNOX1_ENSG00000160199
21	4.5E+07	4.5E+07	KD2-H4K8la_peak_4228	6.36044	
21	4.5E+07	4.5E+07	KD2-H4K8la_peak_4229	14.5688	
21	4.5E+07	4.5E+07	KD2-H4K8la_peak_4230	12.13455	PDXK_ENSG00000160209

21	4.5E+07	4.5E+07	KD2-H4K8la_peak_4231	12.61456	
21	4.5E+07	4.5E+07	KD2-H4K8la_peak_4232	13.63983	AGPAT3_ENSG00000160216
21	4.5E+07	4.5E+07	KD2-H4K8la_peak_4233	8.00569	TRAPPC10_ENSG00000160218
21	4.6E+07	4.6E+07	KD2-H4K8la_peak_4234	4.7338	AP001058.3_ENSG00000232698
21	4.6E+07	4.6E+07	KD2-H4K8la_peak_4235	6.92098	PFKL_ENSG00000141959
21	4.6E+07	4.6E+07	KD2-H4K8la_peak_4236	8.12466	C21orf67_ENSG00000183250;FAM207A_ENSG00000160256
21	4.6E+07	4.6E+07	KD2-H4K8la_peak_4237	5.50657	
21	4.6E+07	4.6E+07	KD2-H4K8la_peak_4238	8.00569	
21	4.6E+07	4.6E+07	KD2-H4K8la_peak_4239	7.19298	
21	4.7E+07	4.7E+07	KD2-H4K8la_peak_4240	7.54643	POFUT2_ENSG00000186866;BX322557.10_ENSG00000215447
21	4.7E+07	4.7E+07	KD2-H4K8la_peak_4241	7.12497	
21	4.7E+07	4.7E+07	KD2-H4K8la_peak_4242	7.31013	
21	4.7E+07	4.7E+07	KD2-H4K8la_peak_4243	7.12014	
21	4.7E+07	4.7E+07	KD2-H4K8la_peak_4244	8.16787	
21	4.7E+07	4.7E+07	KD2-H4K8la_peak_4245	8.80046	
21	4.7E+07	4.7E+07	KD2-H4K8la_peak_4246	17.3629	
21	4.7E+07	4.7E+07	KD2-H4K8la_peak_4247	9.66714	
21	4.7E+07	4.7E+07	KD2-H4K8la_peak_4248	6.96161	
21	4.8E+07	4.8E+07	KD2-H4K8la_peak_4249	8.76082	PRMT2_ENSG00000160310
22	1.8E+07	1.8E+07	KD2-H4K8la_peak_4250	5.83285	CECR5-AS1_ENSG00000185837
22	1.8E+07	1.8E+07	KD2-H4K8la_peak_4251	10.03312	
22	1.8E+07	1.8E+07	KD2-H4K8la_peak_4252	7.45264	
22	1.8E+07	1.8E+07	KD2-H4K8la_peak_4253	7.12014	
22	1.9E+07	1.9E+07	KD2-H4K8la_peak_4254	7.49467	DGCR5_ENSG00000237517
22	1.9E+07	1.9E+07	KD2-H4K8la_peak_4255	17.91109	AC004463.6_ENSG00000260924
22	1.9E+07	1.9E+07	KD2-H4K8la_peak_4256	5.95337	CLTCL1_ENSG00000070371
22	2E+07	2E+07	KD2-H4K8la_peak_4257	5.8369	
22	2E+07	2E+07	KD2-H4K8la_peak_4258	5.97961	GNB1L_ENSG00000185838;C22orf29_ENSG00000215012
22	2E+07	2E+07	KD2-H4K8la_peak_4259	14.15376	DGCR8_ENSG00000128191
22	2E+07	2E+07	KD2-H4K8la_peak_4260	10.74378	
22	2E+07	2E+07	KD2-H4K8la_peak_4261	9.70191	
22	2E+07	2E+07	KD2-H4K8la_peak_4262	5.9366	
22	2.1E+07	2.1E+07	KD2-H4K8la_peak_4263	6.96161	ZNF74_ENSG00000185252
22	2.1E+07	2.1E+07	KD2-H4K8la_peak_4264	5.33301	
22	2.1E+07	2.1E+07	KD2-H4K8la_peak_4265	5.58883	AC002472.13_ENSG00000187905
22	2.2E+07	2.2E+07	KD2-H4K8la_peak_4266	10.36292	
22	2.2E+07	2.2E+07	KD2-H4K8la_peak_4267	10.90862	
22	2.2E+07	2.2E+07	KD2-H4K8la_peak_4268	7.72421	
22	2.3E+07	2.3E+07	KD2-H4K8la_peak_4269	8.37064	
22	2.3E+07	2.3E+07	KD2-H4K8la_peak_4270	8.12047	
22	2.4E+07	2.4E+07	KD2-H4K8la_peak_4271	12.12824	ZDHHC8P1_ENSG00000133519
22	2.4E+07	2.4E+07	KD2-H4K8la_peak_4272	5.3107	
22	2.4E+07	2.4E+07	KD2-H4K8la_peak_4273	6.97699	
22	2.4E+07	2.4E+07	KD2-H4K8la_peak_4274	16.37338	
22	2.4E+07	2.4E+07	KD2-H4K8la_peak_4275	19.16587	
22	2.4E+07	2.4E+07	KD2-H4K8la_peak_4276	5.83285	

22	2.4E+07	2.4E+07	KD2-H4K8la_peak_4277	8.37064	
22	2.4E+07	2.4E+07	KD2-H4K8la_peak_4278	9.20163	GUSBP11_ENSG00000228315;KB-1572G7.2_ENSG00000273000;AP000347.2_ENSG00000272578
22	2.4E+07	2.4E+07	KD2-H4K8la_peak_4279	5.33301	SMARCB1_ENSG00000099956
22	2.4E+07	2.4E+07	KD2-H4K8la_peak_4280	7.41831	
22	2.4E+07	2.4E+07	KD2-H4K8la_peak_4281	8.39919	MIF_ENSG00000240972
22	2.4E+07	2.4E+07	KD2-H4K8la_peak_4282	6.75548	MIF_ENSG00000240972
22	2.4E+07	2.4E+07	KD2-H4K8la_peak_4283	10.99007	
22	2.5E+07	2.5E+07	KD2-H4K8la_peak_4284	10.66289	GGT5_ENSG00000099998
22	2.5E+07	2.5E+07	KD2-H4K8la_peak_4285	4.30663	
22	2.5E+07	2.5E+07	KD2-H4K8la_peak_4286	6.36118	
22	2.5E+07	2.5E+07	KD2-H4K8la_peak_4287	8.98765	
22	2.5E+07	2.5E+07	KD2-H4K8la_peak_4288	6.3399	
22	2.5E+07	2.5E+07	KD2-H4K8la_peak_4289	7.92775	
22	2.5E+07	2.5E+07	KD2-H4K8la_peak_4290	10.02595	
22	2.5E+07	2.5E+07	KD2-H4K8la_peak_4291	5.05146	
22	2.5E+07	2.5E+07	KD2-H4K8la_peak_4292	6.13507	
22	2.6E+07	2.6E+07	KD2-H4K8la_peak_4293	9.20163	
22	2.7E+07	2.7E+07	KD2-H4K8la_peak_4294	8.37064	HPS4_ENSG00000100099;SRRD_ENSG00000100104
22	2.7E+07	2.7E+07	KD2-H4K8la_peak_4295	6.75892	CRYBB1_ENSG00000100122
22	2.7E+07	2.7E+07	KD2-H4K8la_peak_4296	10.16645	
22	2.7E+07	2.7E+07	KD2-H4K8la_peak_4297	4.90979	
22	2.7E+07	2.7E+07	KD2-H4K8la_peak_4298	12.62032	CTA-373H7.7_ENSG00000206028;CTA-211A9.5_ENSG00000244625
22	2.8E+07	2.8E+07	KD2-H4K8la_peak_4299	11.11083	
22	3E+07	3E+07	KD2-H4K8la_peak_4300	5.58883	RHBDD3_ENSG00000100263;EWSR1_ENSG00000182944
22	3E+07	3E+07	KD2-H4K8la_peak_4301	7.33892	NEFH_ENSG00000100285
22	3E+07	3E+07	KD2-H4K8la_peak_4302	4.69635	NIPSNAP1_ENSG00000184117
22	3E+07	3E+07	KD2-H4K8la_peak_4303	9.0413	
22	3E+07	3E+07	KD2-H4K8la_peak_4304	4.30663	ZMAT5_ENSG00000100319;UQCR10_ENSG00000184076
22	3E+07	3E+07	KD2-H4K8la_peak_4305	8.74872	ZMAT5_ENSG00000100319;UQCR10_ENSG00000184076
22	3E+07	3E+07	KD2-H4K8la_peak_4306	8.84474	ASCC2_ENSG00000100325
22	3.1E+07	3.1E+07	KD2-H4K8la_peak_4307	6.13507	
22	3.1E+07	3.1E+07	KD2-H4K8la_peak_4308	15.74238	MORC2_ENSG00000133422
22	3.1E+07	3.1E+07	KD2-H4K8la_peak_4309	9.73848	TUG1_ENSG00000253352
22	3.1E+07	3.1E+07	KD2-H4K8la_peak_4310	9.27415	
22	3.2E+07	3.2E+07	KD2-H4K8la_peak_4311	8.43222	MIR3928_ENSG00000264141;RNF185_ENSG00000138942
22	3.2E+07	3.2E+07	KD2-H4K8la_peak_4312	7.3993	PATZ1_ENSG00000100105;AC005003.1_ENSG00000213888
22	3.2E+07	3.2E+07	KD2-H4K8la_peak_4313	5.89088	
22	3.2E+07	3.2E+07	KD2-H4K8la_peak_4314	4.69635	
22	3.2E+07	3.2E+07	KD2-H4K8la_peak_4315	8.07102	YWHAH_ENSG00000128245
22	3.2E+07	3.2E+07	KD2-H4K8la_peak_4316	7.13237	YWHAH_ENSG00000128245;C22orf24_ENSG00000128254
22	3.3E+07	3.3E+07	KD2-H4K8la_peak_4317	10.04111	
22	3.3E+07	3.3E+07	KD2-H4K8la_peak_4318	9.45339	
22	3.3E+07	3.3E+07	KD2-H4K8la_peak_4319	7.19298	
22	3.6E+07	3.6E+07	KD2-H4K8la_peak_4320	4.72651	HMGXB4_ENSG00000100281
22	3.6E+07	3.6E+07	KD2-H4K8la_peak_4321	9.00481	
22	3.6E+07	3.6E+07	KD2-H4K8la_peak_4322	12.56761	RASD2_ENSG00000100302

22	3.6E+07	3.6E+07	KD2-H4K8la_peak_4323	6.3399	
22	3.6E+07	3.6E+07	KD2-H4K8la_peak_4324	6.57426	
22	3.6E+07	3.6E+07	KD2-H4K8la_peak_4325	14.44261	
22	3.6E+07	3.6E+07	KD2-H4K8la_peak_4326	8.73956	
22	3.6E+07	3.6E+07	KD2-H4K8la_peak_4327	5.80888	
22	3.7E+07	3.7E+07	KD2-H4K8la_peak_4328	4.53273	
22	3.8E+07	3.8E+07	KD2-H4K8la_peak_4329	8.50453	C1QTNF6_ENSG00000133466
22	3.8E+07	3.8E+07	KD2-H4K8la_peak_4330	6.22733	
22	3.8E+07	3.8E+07	KD2-H4K8la_peak_4331	5.05146	CDC42EP1_ENSG00000128283
22	3.8E+07	3.8E+07	KD2-H4K8la_peak_4332	7.43378	
22	3.8E+07	3.8E+07	KD2-H4K8la_peak_4333	10.87112	
22	3.8E+07	3.8E+07	KD2-H4K8la_peak_4334	7.02162	TRIOBP_ENSG00000100106
22	3.8E+07	3.8E+07	KD2-H4K8la_peak_4335	4.69635	
22	3.8E+07	3.8E+07	KD2-H4K8la_peak_4336	6.89612	
22	3.8E+07	3.8E+07	KD2-H4K8la_peak_4337	8.10373	H1F0_ENSG00000189060
22	3.8E+07	3.8E+07	KD2-H4K8la_peak_4338	10.98662	H1F0_ENSG00000189060
22	3.8E+07	3.8E+07	KD2-H4K8la_peak_4339	4.48891	MICALL1_ENSG00000100139
22	3.9E+07	3.9E+07	KD2-H4K8la_peak_4340	5.36015	
22	4E+07	4E+07	KD2-H4K8la_peak_4341	9.86881	ATF4_ENSG00000128272
22	4.1E+07	4.1E+07	KD2-H4K8la_peak_4342	5.72892	MKL1_ENSG00000196588
22	4.1E+07	4.1E+07	KD2-H4K8la_peak_4343	12.14099	EP300_ENSG00000100393;MIR1281_ENSG00000221160
22	4.2E+07	4.2E+07	KD2-H4K8la_peak_4344	7.72421	
22	4.2E+07	4.2E+07	KD2-H4K8la_peak_4345	6.70626	
22	4.2E+07	4.2E+07	KD2-H4K8la_peak_4346	9.72904	FAM109B_ENSG00000177096
22	4.2E+07	4.2E+07	KD2-H4K8la_peak_4347	10.99007	SMDT1_ENSG00000183172
22	4.3E+07	4.3E+07	KD2-H4K8la_peak_4348	5.72892	CYB5R3_ENSG00000100243
22	4.4E+07	4.4E+07	KD2-H4K8la_peak_4349	4.69635	SAMM50_ENSG00000100347
22	4.4E+07	4.4E+07	KD2-H4K8la_peak_4350	6.11881	
22	4.5E+07	4.5E+07	KD2-H4K8la_peak_4351	10.04111	
22	4.5E+07	4.5E+07	KD2-H4K8la_peak_4352	5.27672	
22	4.6E+07	4.6E+07	KD2-H4K8la_peak_4353	9.70191	KIAA0930_ENSG00000100364
22	4.6E+07	4.6E+07	KD2-H4K8la_peak_4354	8.84474	FAM118A_ENSG00000100376
22	4.6E+07	4.6E+07	KD2-H4K8la_peak_4355	6.03112	CITF22-92A6.1_ENSG00000273145
22	4.6E+07	4.6E+07	KD2-H4K8la_peak_4356	8.13727	LINC00899_ENSG00000231711
22	4.6E+07	4.6E+07	KD2-H4K8la_peak_4357	8.43222	LINC00899_ENSG00000231711
22	4.6E+07	4.6E+07	KD2-H4K8la_peak_4358	10.87112	C22orf26_ENSG00000182257;FLJ27365_ENSG00000197182;RP6-109B7.5_ENSG00000273289;RP6-109B7.3_ENSG00000241990
22	4.6E+07	4.6E+07	KD2-H4K8la_peak_4359	6.98743	
22	4.6E+07	4.6E+07	KD2-H4K8la_peak_4360	7.30722	RP6-109B7.4_ENSG00000235159
22	4.7E+07	4.7E+07	KD2-H4K8la_peak_4361	12.13455	GTSE1_ENSG00000075218
22	4.7E+07	4.7E+07	KD2-H4K8la_peak_4362	11.15747	GRAMD4_ENSG00000075240
22	4.7E+07	4.7E+07	KD2-H4K8la_peak_4363	5.0762	
22	4.7E+07	4.7E+07	KD2-H4K8la_peak_4364	5.58883	CERK_ENSG00000100422
22	4.9E+07	4.9E+07	KD2-H4K8la_peak_4365	4.79631	
22	4.9E+07	4.9E+07	KD2-H4K8la_peak_4366	8.12047	
22	4.9E+07	4.9E+07	KD2-H4K8la_peak_4367	9.0516	
22	5E+07	5E+07	KD2-H4K8la_peak_4368	6.22999	

22	5E+07	5E+07	KD2-H4K8la_peak_4369	12.39432	BRD1_ENSG00000100425
22	5E+07	5E+07	KD2-H4K8la_peak_4370	7.49467	
22	5.1E+07	5.1E+07	KD2-H4K8la_peak_4371	7.60756	
22	5.1E+07	5.1E+07	KD2-H4K8la_peak_4372	9.73848	RP3-402G11.26_ENSG00000273253;SELO_ENSG00000073169
22	5.1E+07	5.1E+07	KD2-H4K8la_peak_4373	5.05146	MAPK12_ENSG00000188130
22	5.1E+07	5.1E+07	KD2-H4K8la_peak_4374	9.27415	MAPK12_ENSG00000188130
22	5.1E+07	5.1E+07	KD2-H4K8la_peak_4375	6.89612	MAPK11_ENSG00000185386
22	5.1E+07	5.1E+07	KD2-H4K8la_peak_4376	11.52725	DENND6B_ENSG00000205593
22	5.1E+07	5.1E+07	KD2-H4K8la_peak_4377	8.06229	SBF1_ENSG00000100241
3	4344537	4344786	KD2-H4K8la_peak_4378	5.58883	SETMAR_ENSG00000170364
3	4508669	4508875	KD2-H4K8la_peak_4379	10.04111	SUMF1_ENSG00000144455
3	4509232	4509669	KD2-H4K8la_peak_4380	9.90319	SUMF1_ENSG00000144455
3	4564527	4564777	KD2-H4K8la_peak_4381	6.64643	
3	4575201	4575432	KD2-H4K8la_peak_4382	4.86896	
3	4910483	4910718	KD2-H4K8la_peak_4383	5.28047	
3	5069426	5069653	KD2-H4K8la_peak_4384	7.72421	
3	5164146	5164429	KD2-H4K8la_peak_4385	7.81267	ARL8B_ENSG00000134108
3	8691324	8691584	KD2-H4K8la_peak_4386	4.69635	
3	8699475	8699741	KD2-H4K8la_peak_4387	9.37576	
3	8700745	8701034	KD2-H4K8la_peak_4388	5.83285	
3	9773507	9773731	KD2-H4K8la_peak_4389	10.28981	BRPF1_ENSG00000156983
3	9811990	9812299	KD2-H4K8la_peak_4390	6.3399	CAMK1_ENSG00000134072
3	9851866	9852165	KD2-H4K8la_peak_4391	8.0046	
3	9905797	9906123	KD2-H4K8la_peak_4392	6.36118	
3	9906488	9907002	KD2-H4K8la_peak_4393	8.07102	
3	9931790	9932049	KD2-H4K8la_peak_4394	11.06859	JAGN1_ENSG00000171135
3	9932471	9932771	KD2-H4K8la_peak_4395	8.39919	JAGN1_ENSG00000171135
3	9957455	9957668	KD2-H4K8la_peak_4396	5.43278	
3	9974943	9975240	KD2-H4K8la_peak_4397	9.27415	CRELD1_ENSG00000163703
3	1.1E+07	1.1E+07	KD2-H4K8la_peak_4398	11.15182	
3	1.2E+07	1.2E+07	KD2-H4K8la_peak_4399	5.05146	TAMM41_ENSG00000144559
3	1.3E+07	1.3E+07	KD2-H4K8la_peak_4400	9.78176	RAF1_ENSG00000132155
3	1.4E+07	1.4E+07	KD2-H4K8la_peak_4401	8.76116	
3	1.4E+07	1.4E+07	KD2-H4K8la_peak_4402	11.15747	
3	1.4E+07	1.4E+07	KD2-H4K8la_peak_4403	9.45339	
3	1.4E+07	1.4E+07	KD2-H4K8la_peak_4404	7.3993	CHCHD4_ENSG00000163528;TMEM43_ENSG00000170876
3	1.5E+07	1.5E+07	KD2-H4K8la_peak_4405	6.14571	
3	1.5E+07	1.5E+07	KD2-H4K8la_peak_4406	6.36118	
3	1.7E+07	1.7E+07	KD2-H4K8la_peak_4407	8.37064	
3	1.8E+07	1.8E+07	KD2-H4K8la_peak_4408	6.83387	
3	2.8E+07	2.8E+07	KD2-H4K8la_peak_4409	10.04111	SLC4A7_ENSG00000033867
3	2.8E+07	2.8E+07	KD2-H4K8la_peak_4410	6.70626	AC098614.2_ENSG00000213846
3	2.8E+07	2.8E+07	KD2-H4K8la_peak_4411	13.91009	CMC1_ENSG00000187118
3	2.9E+07	2.9E+07	KD2-H4K8la_peak_4412	5.72892	
3	3.1E+07	3.1E+07	KD2-H4K8la_peak_4413	7.30722	TGFBR2_ENSG00000163513
3	3.2E+07	3.2E+07	KD2-H4K8la_peak_4414	11.15747	

3	3.3E+07	3.3E+07	KD2-H4K8la_peak_4415	7.12014	DYNC1LI1_ENSG00000144635
3	3.7E+07	3.7E+07	KD2-H4K8la_peak_4416	5.42108	
3	3.7E+07	3.7E+07	KD2-H4K8la_peak_4417	7.90495	
3	3.7E+07	3.7E+07	KD2-H4K8la_peak_4418	11.50321	GOLGA4_ENSG00000144674
3	3.7E+07	3.7E+07	KD2-H4K8la_peak_4419	7.90495	ITGA9_ENSG00000144668
3	3.8E+07	3.8E+07	KD2-H4K8la_peak_4420	10.00612	
3	3.8E+07	3.8E+07	KD2-H4K8la_peak_4421	6.64804	
3	3.8E+07	3.8E+07	KD2-H4K8la_peak_4422	8.37064	
3	3.8E+07	3.8E+07	KD2-H4K8la_peak_4423	6.2278	
3	3.8E+07	3.8E+07	KD2-H4K8la_peak_4424	7.90495	MYD88_ENSG00000172936
3	3.8E+07	3.8E+07	KD2-H4K8la_peak_4425	7.94013	ACVR2B-AS1_ENSG00000229589;ACVR2B_ENSG00000114739
3	3.9E+07	3.9E+07	KD2-H4K8la_peak_4426	10.11868	
3	3.9E+07	3.9E+07	KD2-H4K8la_peak_4427	8.76116	SCN5A_ENSG00000183873
3	3.9E+07	3.9E+07	KD2-H4K8la_peak_4428	5.43278	WDR48_ENSG00000114742
3	3.9E+07	3.9E+07	KD2-H4K8la_peak_4429	5.7631	GORASP1_ENSG00000114745;TTC21A_ENSG00000168026
3	3.9E+07	3.9E+07	KD2-H4K8la_peak_4430	7.49467	SLC25A38_ENSG00000144659
3	3.9E+07	3.9E+07	KD2-H4K8la_peak_4431	6.89612	RPSA_ENSG00000168028
3	4.3E+07	4.3E+07	KD2-H4K8la_peak_4432	5.16795	
3	4.3E+07	4.3E+07	KD2-H4K8la_peak_4433	10.04111	POMGNT2_ENSG00000144647
3	4.4E+07	4.4E+07	KD2-H4K8la_peak_4434	8.487	ABHD5_ENSG00000011198
3	4.5E+07	4.5E+07	KD2-H4K8la_peak_4435	9.27415	ZNF502_ENSG00000196653
3	4.6E+07	4.6E+07	KD2-H4K8la_peak_4436	7.60756	
3	4.6E+07	4.6E+07	KD2-H4K8la_peak_4437	11.45154	
3	4.7E+07	4.7E+07	KD2-H4K8la_peak_4438	6.86458	TMIE_ENSG00000181585
3	4.7E+07	4.7E+07	KD2-H4K8la_peak_4439	7.51014	
3	4.7E+07	4.7E+07	KD2-H4K8la_peak_4440	6.28277	NBEAL2_ENSG00000160796
3	4.7E+07	4.7E+07	KD2-H4K8la_peak_4441	14.3505	
3	4.8E+07	4.8E+07	KD2-H4K8la_peak_4442	4.86896	SCAP_ENSG00000114650
3	4.8E+07	4.8E+07	KD2-H4K8la_peak_4443	6.40658	ELP6_ENSG00000163832
3	4.8E+07	4.8E+07	KD2-H4K8la_peak_4444	5.58883	DHX30_ENSG00000132153
3	4.8E+07	4.8E+07	KD2-H4K8la_peak_4445	8.50453	
3	4.8E+07	4.8E+07	KD2-H4K8la_peak_4446	11.15182	CDC25A_ENSG00000164045
3	4.8E+07	4.8E+07	KD2-H4K8la_peak_4447	4.3076	NME6_ENSG00000172113
3	4.9E+07	4.9E+07	KD2-H4K8la_peak_4448	9.67249	TREX1_ENSG00000213689
3	4.9E+07	4.9E+07	KD2-H4K8la_peak_4449	6.97699	
3	4.9E+07	4.9E+07	KD2-H4K8la_peak_4450	12.13455	UQCRC1_ENSG00000010256
3	4.9E+07	4.9E+07	KD2-H4K8la_peak_4451	7.3499	
3	4.9E+07	4.9E+07	KD2-H4K8la_peak_4452	7.51302	
3	4.9E+07	4.9E+07	KD2-H4K8la_peak_4453	19.29514	WDR6_ENSG00000178252
3	4.9E+07	4.9E+07	KD2-H4K8la_peak_4454	18.68203	DALRD3_ENSG00000178149
3	4.9E+07	4.9E+07	KD2-H4K8la_peak_4455	12.7738	USP19_ENSG00000172046
3	4.9E+07	4.9E+07	KD2-H4K8la_peak_4456	9.61894	USP19_ENSG00000172046
3	4.9E+07	4.9E+07	KD2-H4K8la_peak_4457	7.23932	CCDC71_ENSG00000177352
3	5E+07	5E+07	KD2-H4K8la_peak_4458	10.90862	APEH_ENSG00000164062
3	5E+07	5E+07	KD2-H4K8la_peak_4459	5.67596	
3	5E+07	5E+07	KD2-H4K8la_peak_4460	7.51014	MST1_ENSG00000173531;RNF123_ENSG00000164068

3	5E+07	5E+07	KD2-H4K8la_peak_4461	6.64804	AMIGO3_ENSG00000176020;GMPPB_ENSG00000173540
3	5E+07	5E+07	KD2-H4K8la_peak_4462	13.47548	IP6K1_ENSG00000176095
3	5E+07	5E+07	KD2-H4K8la_peak_4463	5.58883	MIR5193_ENSG00000263506
3	5E+07	5E+07	KD2-H4K8la_peak_4464	5.92398	MON1A_ENSG00000164077
3	5E+07	5E+07	KD2-H4K8la_peak_4465	8.37064	RBM6_ENSG00000004534
3	5E+07	5E+07	KD2-H4K8la_peak_4466	9.15937	
3	5E+07	5E+07	KD2-H4K8la_peak_4467	11.81269	
3	5E+07	5E+07	KD2-H4K8la_peak_4468	8.24908	
3	5E+07	5E+07	KD2-H4K8la_peak_4469	8.3574	U73166.2_ENSG00000230454
3	5E+07	5E+07	KD2-H4K8la_peak_4470	12.32641	
3	5E+07	5E+07	KD2-H4K8la_peak_4471	5.66764	
3	5E+07	5E+07	KD2-H4K8la_peak_4472	5.33301	
3	5E+07	5E+07	KD2-H4K8la_peak_4473	8.55715	
3	5E+07	5E+07	KD2-H4K8la_peak_4474	9.0261	
3	5E+07	5E+07	KD2-H4K8la_peak_4475	8.97367	
3	5E+07	5E+07	KD2-H4K8la_peak_4476	5.86422	
3	5E+07	5E+07	KD2-H4K8la_peak_4477	10.31182	
3	5E+07	5E+07	KD2-H4K8la_peak_4478	4.66279	
3	5E+07	5E+07	KD2-H4K8la_peak_4479	10.48003	
3	5E+07	5E+07	KD2-H4K8la_peak_4480	9.20163	
3	5E+07	5E+07	KD2-H4K8la_peak_4481	6.63636	
3	5E+07	5E+07	KD2-H4K8la_peak_4482	6.86458	
3	5E+07	5E+07	KD2-H4K8la_peak_4483	15.18239	
3	5E+07	5E+07	KD2-H4K8la_peak_4484	8.39919	
3	5E+07	5E+07	KD2-H4K8la_peak_4485	10.3389	
3	5E+07	5E+07	KD2-H4K8la_peak_4486	5.27164	
3	5E+07	5E+07	KD2-H4K8la_peak_4487	9.6377	
3	5E+07	5E+07	KD2-H4K8la_peak_4488	7.04068	
3	5.1E+07	5.1E+07	KD2-H4K8la_peak_4489	7.56395	
3	5.1E+07	5.1E+07	KD2-H4K8la_peak_4490	8.8932	
3	5.1E+07	5.1E+07	KD2-H4K8la_peak_4491	6.9399	CISH_ENSG00000114737;MAPKAPK3_ENSG00000114738
3	5.1E+07	5.1E+07	KD2-H4K8la_peak_4492	6.64804	MANF_ENSG00000145050
3	5.2E+07	5.2E+07	KD2-H4K8la_peak_4493	7.13237	
3	5.2E+07	5.2E+07	KD2-H4K8la_peak_4494	6.25288	
3	5.2E+07	5.2E+07	KD2-H4K8la_peak_4495	5.58883	GPR62_ENSG00000180929
3	5.2E+07	5.2E+07	KD2-H4K8la_peak_4496	5.05146	
3	5.2E+07	5.2E+07	KD2-H4K8la_peak_4497	11.90267	PCBP4_ENSG00000090097
3	5.2E+07	5.2E+07	KD2-H4K8la_peak_4498	9.84972	PCBP4_ENSG00000090097
3	5.2E+07	5.2E+07	KD2-H4K8la_peak_4499	9.20163	RP11-155D18.14_ENSG00000272762;RP11-155D18.12_ENSG00000254782
3	5.2E+07	5.2E+07	KD2-H4K8la_peak_4500	9.05604	RP11-155D18.14_ENSG00000272762;RP11-155D18.12_ENSG00000254782;ACY1_ENSG00000243989;ABHD14A-ACY1_ENSG00000114786
3	5.2E+07	5.2E+07	KD2-H4K8la_peak_4501	9.41817	RPL29_ENSG00000162244
3	5.2E+07	5.2E+07	KD2-H4K8la_peak_4502	10.49982	
3	5.2E+07	5.2E+07	KD2-H4K8la_peak_4503	11.15747	
3	5.2E+07	5.2E+07	KD2-H4K8la_peak_4504	6.48828	
3	5.2E+07	5.2E+07	KD2-H4K8la_peak_4505	9.93742	
3	5.2E+07	5.2E+07	KD2-H4K8la_peak_4506	17.22831	

3	5.2E+07	5.2E+07	KD2-H4K8la_peak_4507	10.49982	
3	5.2E+07	5.2E+07	KD2-H4K8la_peak_4508	6.36118	ALAS1_ENSG00000023330
3	5.2E+07	5.2E+07	KD2-H4K8la_peak_4509	11.40266	TLR9_ENSG00000239732;TWF2_ENSG00000247596;RP5-1157M23.2_ENSG00000243224
3	5.2E+07	5.2E+07	KD2-H4K8la_peak_4510	12.13455	WDR82_ENSG00000164091
3	5.2E+07	5.2E+07	KD2-H4K8la_peak_4511	12.25633	
3	5.2E+07	5.2E+07	KD2-H4K8la_peak_4512	6.13507	
3	5.2E+07	5.2E+07	KD2-H4K8la_peak_4513	6.13507	
3	5.2E+07	5.2E+07	KD2-H4K8la_peak_4514	5.33301	
3	5.2E+07	5.2E+07	KD2-H4K8la_peak_4515	6.3399	NISCH_ENSG00000010322
3	5.3E+07	5.3E+07	KD2-H4K8la_peak_4516	7.9076	
3	5.3E+07	5.3E+07	KD2-H4K8la_peak_4517	8.23729	
3	5.4E+07	5.4E+07	KD2-H4K8la_peak_4518	8.76116	CACNA1D_ENSG00000157388
3	5.4E+07	5.4E+07	KD2-H4K8la_peak_4519	9.0413	
3	5.4E+07	5.4E+07	KD2-H4K8la_peak_4520	4.86896	CHDH_ENSG00000016391;IL17RB_ENSG00000056736
3	5.7E+07	5.7E+07	KD2-H4K8la_peak_4521	13.20018	FAM208A_ENSG00000163946
3	5.7E+07	5.7E+07	KD2-H4K8la_peak_4522	8.37064	
3	5.7E+07	5.7E+07	KD2-H4K8la_peak_4523	7.68279	
3	5.7E+07	5.7E+07	KD2-H4K8la_peak_4524	8.13727	IL17RD_ENSG00000144730
3	5.8E+07	5.8E+07	KD2-H4K8la_peak_4525	8.07102	DENND6A_ENSG00000174839;RP11-755B10.3_ENSG00000241933
3	5.8E+07	5.8E+07	KD2-H4K8la_peak_4526	6.64804	RP11-359I18.5_ENSG00000272360;KCTD6_ENSG00000168301
3	5.9E+07	5.9E+07	KD2-H4K8la_peak_4527	10.98662	C3orf67_ENSG00000163689
3	6.1E+07	6.1E+07	KD2-H4K8la_peak_4528	11.15747	FHIT_ENSG00000189283
3	6.5E+07	6.5E+07	KD2-H4K8la_peak_4529	5.36015	
3	6.5E+07	6.5E+07	KD2-H4K8la_peak_4530	9.94613	
3	6.8E+07	6.8E+07	KD2-H4K8la_peak_4531	6.86458	SUCLG2_ENSG00000172340;RP11-81N13.1_ENSG00000241316
3	6.8E+07	6.8E+07	KD2-H4K8la_peak_4532	10.62798	SUCLG2_ENSG00000172340;RP11-81N13.1_ENSG00000241316
3	7.2E+07	7.2E+07	KD2-H4K8la_peak_4533	4.86896	
3	7.2E+07	7.2E+07	KD2-H4K8la_peak_4534	6.89612	LINC00870_ENSG00000243083
3	7.2E+07	7.2E+07	KD2-H4K8la_peak_4535	7.49467	
3	7.2E+07	7.2E+07	KD2-H4K8la_peak_4536	4.86896	
3	7.3E+07	7.3E+07	KD2-H4K8la_peak_4537	5.05146	
3	7.6E+07	7.6E+07	KD2-H4K8la_peak_4538	11.15747	ZNF717_ENSG00000227124
3	8.6E+07	8.6E+07	KD2-H4K8la_peak_4539	8.487	
3	1E+08	1E+08	KD2-H4K8la_peak_4540	7.09021	TBC1D23_ENSG00000036054
3	1.1E+08	1.1E+08	KD2-H4K8la_peak_4541	8.39919	RP11-115H18.1_ENSG00000273125
3	1.1E+08	1.1E+08	KD2-H4K8la_peak_4542	11.7362	RP11-115H18.1_ENSG00000273125
3	1.1E+08	1.1E+08	KD2-H4K8la_peak_4543	10.91646	CD47_ENSG00000196776
3	1.1E+08	1.1E+08	KD2-H4K8la_peak_4544	6.2278	ZBTB20_ENSG00000181722
3	1.2E+08	1.2E+08	KD2-H4K8la_peak_4545	5.47284	RP11-326J18.1_ENSG00000241596
3	1.2E+08	1.2E+08	KD2-H4K8la_peak_4546	8.44557	ARHGAP31_ENSG00000031081
3	1.2E+08	1.2E+08	KD2-H4K8la_peak_4547	7.13237	
3	1.2E+08	1.2E+08	KD2-H4K8la_peak_4548	8.35736	
3	1.2E+08	1.2E+08	KD2-H4K8la_peak_4549	10.52788	
3	1.2E+08	1.2E+08	KD2-H4K8la_peak_4550	8.07102	PARP9_ENSG00000138496;DTX3L_ENSG00000163840
3	1.2E+08	1.2E+08	KD2-H4K8la_peak_4551	6.42754	
3	1.2E+08	1.2E+08	KD2-H4K8la_peak_4552	7.87573	

3	1.2E+08	1.2E+08	KD2-H4K8la_peak_4553	5.88616	
3	1.2E+08	1.2E+08	KD2-H4K8la_peak_4554	9.87983	
3	1.2E+08	1.2E+08	KD2-H4K8la_peak_4555	8.37064	
3	1.2E+08	1.2E+08	KD2-H4K8la_peak_4556	7.06427	
3	1.2E+08	1.2E+08	KD2-H4K8la_peak_4557	7.92775	
3	1.2E+08	1.2E+08	KD2-H4K8la_peak_4558	8.19799	
3	1.2E+08	1.2E+08	KD2-H4K8la_peak_4559	7.51014	
3	1.3E+08	1.3E+08	KD2-H4K8la_peak_4560	9.11321	ZNF148_ENSG00000163848
3	1.3E+08	1.3E+08	KD2-H4K8la_peak_4561	6.3399	
3	1.3E+08	1.3E+08	KD2-H4K8la_peak_4562	9.61894	
3	1.3E+08	1.3E+08	KD2-H4K8la_peak_4563	5.05917	
3	1.3E+08	1.3E+08	KD2-H4K8la_peak_4564	10.18169	
3	1.3E+08	1.3E+08	KD2-H4K8la_peak_4565	6.32736	
3	1.3E+08	1.3E+08	KD2-H4K8la_peak_4566	6.88507	
3	1.3E+08	1.3E+08	KD2-H4K8la_peak_4567	9.9701	TPRA1_ENSG00000163870;MCM2_ENSG00000073111
3	1.3E+08	1.3E+08	KD2-H4K8la_peak_4568	10.04111	PODXL2_ENSG00000114631
3	1.3E+08	1.3E+08	KD2-H4K8la_peak_4569	6.39807	
3	1.3E+08	1.3E+08	KD2-H4K8la_peak_4570	7.78805	
3	1.3E+08	1.3E+08	KD2-H4K8la_peak_4571	10.87112	
3	1.3E+08	1.3E+08	KD2-H4K8la_peak_4572	5.84385	
3	1.3E+08	1.3E+08	KD2-H4K8la_peak_4573	7.4908	
3	1.3E+08	1.3E+08	KD2-H4K8la_peak_4574	8.86558	RUVBL1_ENSG00000175792;EEFSEC_ENSG00000132394
3	1.3E+08	1.3E+08	KD2-H4K8la_peak_4575	9.05604	
3	1.3E+08	1.3E+08	KD2-H4K8la_peak_4576	5.468	
3	1.3E+08	1.3E+08	KD2-H4K8la_peak_4577	7.82875	
3	1.3E+08	1.3E+08	KD2-H4K8la_peak_4578	9.47897	
3	1.3E+08	1.3E+08	KD2-H4K8la_peak_4579	6.56022	
3	1.3E+08	1.3E+08	KD2-H4K8la_peak_4580	5.24027	
3	1.3E+08	1.3E+08	KD2-H4K8la_peak_4581	13.11393	
3	1.3E+08	1.3E+08	KD2-H4K8la_peak_4582	6.69137	
3	1.3E+08	1.3E+08	KD2-H4K8la_peak_4583	6.64804	
3	1.3E+08	1.3E+08	KD2-H4K8la_peak_4584	12.45558	
3	1.3E+08	1.3E+08	KD2-H4K8la_peak_4585	12.35074	
3	1.3E+08	1.3E+08	KD2-H4K8la_peak_4586	6.75222	
3	1.3E+08	1.3E+08	KD2-H4K8la_peak_4587	5.90392	DNAJB8_ENSG00000179407
3	1.3E+08	1.3E+08	KD2-H4K8la_peak_4588	7.23932	
3	1.3E+08	1.3E+08	KD2-H4K8la_peak_4589	9.37943	RP11-475N22.4_ENSG00000244300
3	1.3E+08	1.3E+08	KD2-H4K8la_peak_4590	5.76044	
3	1.3E+08	1.3E+08	KD2-H4K8la_peak_4591	11.91891	
3	1.3E+08	1.3E+08	KD2-H4K8la_peak_4592	14.23294	
3	1.3E+08	1.3E+08	KD2-H4K8la_peak_4593	7.83588	
3	1.3E+08	1.3E+08	KD2-H4K8la_peak_4594	6.48366	
3	1.3E+08	1.3E+08	KD2-H4K8la_peak_4595	4.7338	RPN1_ENSG00000163902
3	1.3E+08	1.3E+08	KD2-H4K8la_peak_4596	9.66714	
3	1.3E+08	1.3E+08	KD2-H4K8la_peak_4597	7.92775	
3	1.3E+08	1.3E+08	KD2-H4K8la_peak_4598	6.86458	CNBP_ENSG00000169714

3	1.3E+08	1.3E+08	KD2-H4K8la_peak_4599	5.22084	H1FX_ENSG00000184897
3	1.3E+08	1.3E+08	KD2-H4K8la_peak_4600	4.30663	RPL32P3_ENSG00000251474
3	1.3E+08	1.3E+08	KD2-H4K8la_peak_4601	4.7338	TMCC1_ENSG00000172765;TMCC1-AS1_ENSG00000271270
3	1.3E+08	1.3E+08	KD2-H4K8la_peak_4602	10.04111	PIK3R4_ENSG00000196455
3	1.3E+08	1.3E+08	KD2-H4K8la_peak_4603	7.49467	RAB6B_ENSG00000154917
3	1.3E+08	1.3E+08	KD2-H4K8la_peak_4604	9.35473	ANAPC13_ENSG00000129055;CEP63_ENSG00000182923
3	1.4E+08	1.4E+08	KD2-H4K8la_peak_4605	11.06859	NME9_ENSG00000181322
3	1.4E+08	1.4E+08	KD2-H4K8la_peak_4606	7.30722	MRAS_ENSG00000158186
3	1.4E+08	1.4E+08	KD2-H4K8la_peak_4607	6.64804	FAIM_ENSG00000158234
3	1.4E+08	1.4E+08	KD2-H4K8la_peak_4608	8.31696	
3	1.4E+08	1.4E+08	KD2-H4K8la_peak_4609	6.01825	
3	1.4E+08	1.4E+08	KD2-H4K8la_peak_4610	7.90495	RBP1_ENSG00000114115
3	1.4E+08	1.4E+08	KD2-H4K8la_peak_4611	5.83285	
3	1.4E+08	1.4E+08	KD2-H4K8la_peak_4612	8.31696	
3	1.4E+08	1.4E+08	KD2-H4K8la_peak_4613	7.51014	GK5_ENSG00000175066
3	1.4E+08	1.4E+08	KD2-H4K8la_peak_4614	9.38376	ATR_ENSG00000175054
3	1.4E+08	1.4E+08	KD2-H4K8la_peak_4615	6.64804	RP11-91G21.1_ENSG00000268129
3	1.5E+08	1.5E+08	KD2-H4K8la_peak_4616	4.98386	GYG1_ENSG00000163754
3	1.6E+08	1.6E+08	KD2-H4K8la_peak_4617	9.70817	LINC00886_ENSG00000240875
3	1.6E+08	1.6E+08	KD2-H4K8la_peak_4618	10.04111	PTX3_ENSG00000163661
3	1.6E+08	1.6E+08	KD2-H4K8la_peak_4619	8.37064	
3	1.6E+08	1.6E+08	KD2-H4K8la_peak_4620	9.70817	RARRES1_ENSG00000118849;MFSD1_ENSG00000118855;RP11-379F4.4_ENSG00000240207
3	1.6E+08	1.6E+08	KD2-H4K8la_peak_4621	5.90392	
3	1.6E+08	1.6E+08	KD2-H4K8la_peak_4622	5.24865	
3	1.6E+08	1.6E+08	KD2-H4K8la_peak_4623	4.30663	
3	1.7E+08	1.7E+08	KD2-H4K8la_peak_4624	8.37064	TERC_ENSG00000270141
3	1.7E+08	1.7E+08	KD2-H4K8la_peak_4625	5.67034	PRKCI_ENSG00000163558
3	1.7E+08	1.7E+08	KD2-H4K8la_peak_4626	4.71418	
3	1.8E+08	1.8E+08	KD2-H4K8la_peak_4627	8.7731	TBL1XR1_ENSG00000177565
3	1.8E+08	1.8E+08	KD2-H4K8la_peak_4628	13.3197	
3	1.8E+08	1.8E+08	KD2-H4K8la_peak_4629	6.56573	MFN1_ENSG00000171109
3	1.8E+08	1.8E+08	KD2-H4K8la_peak_4630	9.05604	RP11-496B10.3_ENSG00000239774;TTC14_ENSG00000163728
3	1.8E+08	1.8E+08	KD2-H4K8la_peak_4631	5.33364	
3	1.8E+08	1.8E+08	KD2-H4K8la_peak_4632	6.3399	
3	1.8E+08	1.8E+08	KD2-H4K8la_peak_4633	10.5885	
3	1.8E+08	1.8E+08	KD2-H4K8la_peak_4634	6.26214	RP11-225N10.1_ENSG00000240063;ATP11B_ENSG00000058063
3	1.8E+08	1.8E+08	KD2-H4K8la_peak_4635	6.61963	LAMP3_ENSG00000078081
3	1.8E+08	1.8E+08	KD2-H4K8la_peak_4636	7.61975	
3	1.8E+08	1.8E+08	KD2-H4K8la_peak_4637	6.0136	
3	1.8E+08	1.8E+08	KD2-H4K8la_peak_4638	7.30722	PARL_ENSG00000175193;RP11-315J22.5_ENSG00000234371
3	1.8E+08	1.8E+08	KD2-H4K8la_peak_4639	8.65116	ABCC5_ENSG00000114770
3	1.8E+08	1.8E+08	KD2-H4K8la_peak_4640	6.07357	ALG3_ENSG00000214160;ECE2_ENSG00000145194
3	1.8E+08	1.8E+08	KD2-H4K8la_peak_4641	8.07102	PSMD2_ENSG00000175166
3	1.8E+08	1.8E+08	KD2-H4K8la_peak_4642	6.3399	EIF4G1_ENSG00000114867
3	1.8E+08	1.8E+08	KD2-H4K8la_peak_4643	8.44557	
3	1.8E+08	1.8E+08	KD2-H4K8la_peak_4644	10.76886	

3	1.8E+08	1.8E+08	KD2-H4K8la_peak_4645	6.25288	
3	1.8E+08	1.8E+08	KD2-H4K8la_peak_4646	8.15454	
3	1.8E+08	1.8E+08	KD2-H4K8la_peak_4647	9.27415	
3	1.8E+08	1.8E+08	KD2-H4K8la_peak_4648	5.84082	
3	1.8E+08	1.8E+08	KD2-H4K8la_peak_4649	9.61894	
3	1.9E+08	1.9E+08	KD2-H4K8la_peak_4650	5.27164	TMEM41A_ENSG00000163900
3	1.9E+08	1.9E+08	KD2-H4K8la_peak_4651	7.96951	
3	1.9E+08	1.9E+08	KD2-H4K8la_peak_4652	5.58883	
3	1.9E+08	1.9E+08	KD2-H4K8la_peak_4653	6.89612	ST6GAL1_ENSG00000073849
3	1.9E+08	1.9E+08	KD2-H4K8la_peak_4654	8.77936	UTS2B_ENSG00000188958;CCDC50_ENSG00000152492
3	1.9E+08	1.9E+08	KD2-H4K8la_peak_4655	5.67034	ATP13A4_ENSG00000127249;RP11-175P19.2_ENSG00000236297;OPA1_ENSG00000198836
3	1.9E+08	1.9E+08	KD2-H4K8la_peak_4656	5.43278	
3	1.9E+08	1.9E+08	KD2-H4K8la_peak_4657	8.76082	
3	1.9E+08	1.9E+08	KD2-H4K8la_peak_4658	6.3399	
3	2E+08	2E+08	KD2-H4K8la_peak_4659	6.3399	
3	2E+08	2E+08	KD2-H4K8la_peak_4660	6.3399	
3	2E+08	2E+08	KD2-H4K8la_peak_4661	6.70626	
3	2E+08	2E+08	KD2-H4K8la_peak_4662	10.27262	SDHAP1_ENSG00000185485
3	2E+08	2E+08	KD2-H4K8la_peak_4663	6.68593	
3	2E+08	2E+08	KD2-H4K8la_peak_4664	11.06859	
3	2E+08	2E+08	KD2-H4K8la_peak_4665	6.86458	RP11-447L10.1_ENSG00000272741;TCTEX1D2_ENSG00000213123;TM4SF19-AS1_ENSG00000235897
3	2E+08	2E+08	KD2-H4K8la_peak_4666	13.3197	RNF168_ENSG00000163961
3	2E+08	2E+08	KD2-H4K8la_peak_4667	5.05146	LINC01063_ENSG00000232065
3	2E+08	2E+08	KD2-H4K8la_peak_4668	8.07102	LINC01063_ENSG00000232065
3	2E+08	2E+08	KD2-H4K8la_peak_4669	6.61963	PIGZ_ENSG00000119227
3	2E+08	2E+08	KD2-H4K8la_peak_4670	8.37064	
3	2E+08	2E+08	KD2-H4K8la_peak_4671	8.76116	
3	2E+08	2E+08	KD2-H4K8la_peak_4672	10.04113	MFI2_ENSG00000163975
3	2E+08	2E+08	KD2-H4K8la_peak_4673	6.51326	DLG1-AS1_ENSG00000227375
3	2E+08	2E+08	KD2-H4K8la_peak_4674	7.19298	
3	2E+08	2E+08	KD2-H4K8la_peak_4675	8.0046	
3	2E+08	2E+08	KD2-H4K8la_peak_4676	8.50453	
3	2E+08	2E+08	KD2-H4K8la_peak_4677	9.38376	AC024560.3_ENSG00000214135
4	330305	330855	KD2-H4K8la_peak_4678	6.57319	RP11-478C6.1_ENSG00000251535;ZNF141_ENSG00000131127
4	467297	467848	KD2-H4K8la_peak_4679	6.2278	ABCA11P_ENSG00000251595
4	493100	493305	KD2-H4K8la_peak_4680	5.10388	ZNF721_ENSG00000182903;PIGG_ENSG00000174227
4	657007	657427	KD2-H4K8la_peak_4681	7.12014	
4	657661	657873	KD2-H4K8la_peak_4682	7.12014	
4	658193	658513	KD2-H4K8la_peak_4683	8.43222	
4	668435	668937	KD2-H4K8la_peak_4684	9.18773	ATP5I_ENSG00000169020
4	674687	674945	KD2-H4K8la_peak_4685	5.58883	
4	699747	700031	KD2-H4K8la_peak_4686	10.87112	PCGF3_ENSG00000185619
4	776842	777137	KD2-H4K8la_peak_4687	8.65988	
4	967589	967809	KD2-H4K8la_peak_4688	10.02595	
4	1187905	1188647	KD2-H4K8la_peak_4689	10.28981	
4	1193593	1193803	KD2-H4K8la_peak_4690	8.07102	

4	1242123	1242427	KD2-H4K8la_peak_4691	5.8369	CTBP1-AS2_ENSG00000196810
4	1243327	1243825	KD2-H4K8la_peak_4692	8.39919	CTBP1_ENSG00000159692;CTBP1-AS2_ENSG00000196810
4	1340097	1340557	KD2-H4K8la_peak_4693	12.76394	UVSSA_ENSG00000163945
4	1686375	1686749	KD2-H4K8la_peak_4694	12.39776	FAM53A_ENSG00000174137
4	1723282	1723807	KD2-H4K8la_peak_4695	4.75945	TMEM129_ENSG00000168936;TACC3_ENSG00000013810
4	1768800	1769014	KD2-H4K8la_peak_4696	5.58883	
4	1872371	1872617	KD2-H4K8la_peak_4697	6.47266	WHSC1_ENSG00000109685
4	2060282	2060608	KD2-H4K8la_peak_4698	6.54311	NAT8L_ENSG00000185818
4	2264431	2264720	KD2-H4K8la_peak_4699	5.22616	MXD4_ENSG00000123933
4	2470078	2470492	KD2-H4K8la_peak_4700	11.87032	
4	2537473	2537732	KD2-H4K8la_peak_4701	10.74378	
4	2757686	2757982	KD2-H4K8la_peak_4702	9.0413	TNIP2_ENSG00000168884
4	2758504	2758797	KD2-H4K8la_peak_4703	6.3399	TNIP2_ENSG00000168884
4	2800458	2800762	KD2-H4K8la_peak_4704	6.47589	
4	2820093	2820610	KD2-H4K8la_peak_4705	14.30428	
4	2936710	2937256	KD2-H4K8la_peak_4706	7.49467	MFSD10_ENSG00000109736;NOP14-AS1_ENSG00000249673
4	3534504	3534724	KD2-H4K8la_peak_4707	5.98775	LRPAP1_ENSG00000163956
4	3659816	3660131	KD2-H4K8la_peak_4708	5.83285	
4	4249683	4249891	KD2-H4K8la_peak_4709	9.0413	TMEM128_ENSG00000132406
4	5958499	5958720	KD2-H4K8la_peak_4710	4.30663	
4	6711929	6712216	KD2-H4K8la_peak_4711	13.14519	MRFAP1L1_ENSG00000178988
4	6784697	6785152	KD2-H4K8la_peak_4712	6.64804	
4	7228543	7229221	KD2-H4K8la_peak_4713	8.56027	
4	7414461	7414677	KD2-H4K8la_peak_4714	9.1923	
4	7882169	7882454	KD2-H4K8la_peak_4715	7.96951	
4	7905041	7905410	KD2-H4K8la_peak_4716	7.12014	
4	7907548	7908000	KD2-H4K8la_peak_4717	7.49467	
4	7908414	7908691	KD2-H4K8la_peak_4718	11.06859	
4	7912357	7912628	KD2-H4K8la_peak_4719	8.07102	
4	8052668	8052899	KD2-H4K8la_peak_4720	8.37064	
4	8609898	8610102	KD2-H4K8la_peak_4721	6.47589	
4	8706019	8706640	KD2-H4K8la_peak_4722	7.81267	
4	9154012	9154269	KD2-H4K8la_peak_4723	16.13355	FAM86KP_ENSG00000163612
4	9382687	9383027	KD2-H4K8la_peak_4724	15.75402	
4	9692910	9693299	KD2-H4K8la_peak_4725	5.40949	FAM86MP_ENSG00000186234
4	1.5E+07	1.5E+07	KD2-H4K8la_peak_4726	12.39776	CPEB2-AS1_ENSG00000247624;CPEB2_ENSG00000137449
4	1.6E+07	1.6E+07	KD2-H4K8la_peak_4727	15.33728	
4	1.6E+07	1.6E+07	KD2-H4K8la_peak_4728	7.8597	FBXL5_ENSG00000118564;FAM200B_ENSG00000237765
4	1.6E+07	1.6E+07	KD2-H4K8la_peak_4729	6.2278	TAPT1_ENSG00000169762;TAPT1-AS1_ENSG00000263327
4	1.7E+07	1.7E+07	KD2-H4K8la_peak_4730	8.76082	
4	1.8E+07	1.8E+07	KD2-H4K8la_peak_4731	8.77936	
4	1.8E+07	1.8E+07	KD2-H4K8la_peak_4732	4.79631	
4	1.8E+07	1.8E+07	KD2-H4K8la_peak_4733	6.2278	LCORL_ENSG00000178177
4	2.5E+07	2.5E+07	KD2-H4K8la_peak_4734	6.71169	DHX15_ENSG00000109606
4	2.5E+07	2.5E+07	KD2-H4K8la_peak_4735	9.72904	DHX15_ENSG00000109606
4	3.9E+07	3.9E+07	KD2-H4K8la_peak_4736	4.69635	KLF3_ENSG00000109787

4	3.9E+07	3.9E+07	KD2-H4K8la_peak_4737	12.31762	RP11-617D20.1_ENSG00000231160;AC021860.1_ENSG00000196355;KLF3_ENSG00000109787
4	4E+07	4E+07	KD2-H4K8la_peak_4738	6.0136	
4	4E+07	4E+07	KD2-H4K8la_peak_4739	9.66714	RP11-333E13.4_ENSG00000205794;N4BP2_ENSG00000078177
4	4E+07	4E+07	KD2-H4K8la_peak_4740	6.86458	RP11-333E13.4_ENSG00000205794;N4BP2_ENSG00000078177
4	4E+07	4E+07	KD2-H4K8la_peak_4741	6.0136	RP11-333E13.4_ENSG00000205794;N4BP2_ENSG00000078177
4	4.1E+07	4.1E+07	KD2-H4K8la_peak_4742	5.83285	
4	4.2E+07	4.2E+07	KD2-H4K8la_peak_4743	6.3399	
4	4.2E+07	4.2E+07	KD2-H4K8la_peak_4744	9.27415	RP11-227F19.2_ENSG00000249122
4	4.2E+07	4.2E+07	KD2-H4K8la_peak_4745	6.01825	
4	4.2E+07	4.2E+07	KD2-H4K8la_peak_4746	7.78021	
4	4.2E+07	4.2E+07	KD2-H4K8la_peak_4747	9.08387	
4	4.2E+07	4.2E+07	KD2-H4K8la_peak_4748	4.86896	
4	4.2E+07	4.2E+07	KD2-H4K8la_peak_4749	13.73095	LINC00682_ENSG00000245870
4	4.2E+07	4.2E+07	KD2-H4K8la_peak_4750	7.83588	
4	4.7E+07	4.7E+07	KD2-H4K8la_peak_4751	5.95337	COMMD8_ENSG00000169019
4	4.8E+07	4.8E+07	KD2-H4K8la_peak_4752	7.22716	NFXL1_ENSG00000170448;NIPAL1_ENSG00000163293
4	4.8E+07	4.8E+07	KD2-H4K8la_peak_4753	4.86896	TEC_ENSG00000135605
4	4.8E+07	4.8E+07	KD2-H4K8la_peak_4754	7.51014	SLC10A4_ENSG00000145248
4	5.4E+07	5.4E+07	KD2-H4K8la_peak_4755	8.37064	USP46_ENSG00000109189;USP46-AS1_ENSG00000248866
4	5.4E+07	5.4E+07	KD2-H4K8la_peak_4756	8.13727	ERVMER34-1_ENSG00000226887;AC104066.1_ENSG00000266656
4	5.4E+07	5.4E+07	KD2-H4K8la_peak_4757	6.70058	
4	5.4E+07	5.4E+07	KD2-H4K8la_peak_4758	7.92775	
4	5.4E+07	5.4E+07	KD2-H4K8la_peak_4759	6.03964	
4	5.4E+07	5.4E+07	KD2-H4K8la_peak_4760	5.40949	FIP1L1_ENSG00000145216
4	5.4E+07	5.4E+07	KD2-H4K8la_peak_4761	7.23116	FIP1L1_ENSG00000145216
4	5.6E+07	5.6E+07	KD2-H4K8la_peak_4762	7.23116	KIT_ENSG00000157404
4	5.6E+07	5.6E+07	KD2-H4K8la_peak_4763	8.76082	SRD5A3-AS1_ENSG00000249700;TMEM165_ENSG00000134851
4	5.7E+07	5.7E+07	KD2-H4K8la_peak_4764	21.33961	
4	5.8E+07	5.8E+07	KD2-H4K8la_peak_4765	6.47589	REST_ENSG00000084093
4	7.2E+07	7.2E+07	KD2-H4K8la_peak_4766	8.12047	GRSF1_ENSG00000132463
4	7.7E+07	7.7E+07	KD2-H4K8la_peak_4767	8.07102	
4	7.7E+07	7.7E+07	KD2-H4K8la_peak_4768	8.07102	FAM47E-STBD1_ENSG00000272414;FAM47E-STBD1_ENSG00000118804
4	7.7E+07	7.7E+07	KD2-H4K8la_peak_4769	6.70626	FAM47E-STBD1_ENSG00000272414;FAM47E-STBD1_ENSG00000118804
4	7.7E+07	7.7E+07	KD2-H4K8la_peak_4770	9.9701	CCDC158_ENSG00000163749
4	7.8E+07	7.8E+07	KD2-H4K8la_peak_4771	9.11321	
4	7.8E+07	7.8E+07	KD2-H4K8la_peak_4772	8.07102	CCNI_ENSG00000118816
4	7.9E+07	7.9E+07	KD2-H4K8la_peak_4773	8.31696	CNOT6L_ENSG00000138767
4	7.9E+07	7.9E+07	KD2-H4K8la_peak_4774	5.43278	FRAS1_ENSG00000138759
4	8.1E+07	8.1E+07	KD2-H4K8la_peak_4775	8.39919	
4	8.1E+07	8.1E+07	KD2-H4K8la_peak_4776	6.64804	FGF5_ENSG00000138675
4	8.3E+07	8.3E+07	KD2-H4K8la_peak_4777	7.12014	HNRNPDL_ENSG00000152795;ENOPH1_ENSG00000145293
4	8.8E+07	8.8E+07	KD2-H4K8la_peak_4778	7.13237	
4	8.8E+07	8.8E+07	KD2-H4K8la_peak_4779	6.26214	
4	8.9E+07	8.9E+07	KD2-H4K8la_peak_4780	4.75945	HERC5_ENSG00000138646
4	9.6E+07	9.6E+07	KD2-H4K8la_peak_4781	4.90581	
4	9.6E+07	9.6E+07	KD2-H4K8la_peak_4782	5.43278	RP11-168E14.1_ENSG00000249599;BMPR1B_ENSG00000138696

4	9.6E+07	9.6E+07	KD2-H4K8la_peak_4783	7.72421	
4	9.6E+07	9.6E+07	KD2-H4K8la_peak_4784	8.77936	
4	9.6E+07	9.6E+07	KD2-H4K8la_peak_4785	5.40949	UNC5C_ENSG00000182168;RP11-710C12.1_ENSG00000271474
4	1E+08	1E+08	KD2-H4K8la_peak_4786	8.07102	
4	1E+08	1E+08	KD2-H4K8la_peak_4787	10.90862	RP11-10L12.4_ENSG00000246560
4	1.1E+08	1.1E+08	KD2-H4K8la_peak_4788	8.86558	TET2_ENSG00000168769
4	1.1E+08	1.1E+08	KD2-H4K8la_peak_4789	8.487	AIMP1_ENSG00000164022
4	1.1E+08	1.1E+08	KD2-H4K8la_peak_4790	5.72892	RP11-602N24.3_ENSG00000272795;GAR1_ENSG00000109534
4	1.1E+08	1.1E+08	KD2-H4K8la_peak_4791	4.80784	RP11-602N24.3_ENSG00000272795;GAR1_ENSG00000109534
4	1.1E+08	1.1E+08	KD2-H4K8la_peak_4792	6.86458	
4	1.1E+08	1.1E+08	KD2-H4K8la_peak_4793	7.51014	
4	1.1E+08	1.1E+08	KD2-H4K8la_peak_4794	6.2278	
4	1.2E+08	1.2E+08	KD2-H4K8la_peak_4795	7.61975	PRDM5_ENSG00000138738
4	1.3E+08	1.3E+08	KD2-H4K8la_peak_4796	7.23116	INTU_ENSG00000164066
4	1.3E+08	1.3E+08	KD2-H4K8la_peak_4797	14.91688	LARP1B_ENSG00000138709
4	1.3E+08	1.3E+08	KD2-H4K8la_peak_4798	8.77936	
4	1.4E+08	1.4E+08	KD2-H4K8la_peak_4799	5.58883	
4	1.4E+08	1.4E+08	KD2-H4K8la_peak_4800	4.86896	NDUFC1_ENSG00000109390;NAA15_ENSG00000164134
4	1.4E+08	1.4E+08	KD2-H4K8la_peak_4801	4.75945	
4	1.4E+08	1.4E+08	KD2-H4K8la_peak_4802	8.23729	
4	1.4E+08	1.4E+08	KD2-H4K8la_peak_4803	9.73848	
4	1.4E+08	1.4E+08	KD2-H4K8la_peak_4804	4.30289	
4	1.4E+08	1.4E+08	KD2-H4K8la_peak_4805	6.64804	
4	1.4E+08	1.4E+08	KD2-H4K8la_peak_4806	13.29476	
4	1.4E+08	1.4E+08	KD2-H4K8la_peak_4807	5.89088	
4	1.4E+08	1.4E+08	KD2-H4K8la_peak_4808	9.19451	
4	1.5E+08	1.5E+08	KD2-H4K8la_peak_4809	7.19298	SMAD1_ENSG00000170365
4	1.5E+08	1.5E+08	KD2-H4K8la_peak_4810	7.51302	MMAA_ENSG00000151611
4	1.5E+08	1.5E+08	KD2-H4K8la_peak_4811	12.13455	
4	1.5E+08	1.5E+08	KD2-H4K8la_peak_4812	5.89483	
4	1.5E+08	1.5E+08	KD2-H4K8la_peak_4813	10.97039	
4	1.5E+08	1.5E+08	KD2-H4K8la_peak_4814	7.61975	RP11-6L6.7_ENSG00000251010
4	1.5E+08	1.5E+08	KD2-H4K8la_peak_4815	5.27672	
4	1.5E+08	1.5E+08	KD2-H4K8la_peak_4816	6.40658	SH3D19_ENSG00000109686
4	1.5E+08	1.5E+08	KD2-H4K8la_peak_4817	6.88801	
4	1.5E+08	1.5E+08	KD2-H4K8la_peak_4818	11.45154	PET112_ENSG00000059691
4	1.5E+08	1.5E+08	KD2-H4K8la_peak_4819	7.56395	FBXW7_ENSG00000109670;MIR4453_ENSG00000268471
4	1.5E+08	1.5E+08	KD2-H4K8la_peak_4820	5.33364	TRIM2_ENSG00000109654
4	1.5E+08	1.5E+08	KD2-H4K8la_peak_4821	9.27415	
4	1.5E+08	1.5E+08	KD2-H4K8la_peak_4822	6.96161	RNF175_ENSG00000145428
4	1.7E+08	1.7E+08	KD2-H4K8la_peak_4823	5.72892	
4	1.7E+08	1.7E+08	KD2-H4K8la_peak_4824	7.13237	RP11-10K16.1_ENSG00000245213;GALNT7_ENSG00000109586
4	1.7E+08	1.7E+08	KD2-H4K8la_peak_4825	6.28277	HMGB2_ENSG00000164104
4	1.7E+08	1.7E+08	KD2-H4K8la_peak_4826	4.53273	
4	1.7E+08	1.7E+08	KD2-H4K8la_peak_4827	7.77341	
4	1.7E+08	1.7E+08	KD2-H4K8la_peak_4828	14.15376	

4	1.7E+08	1.7E+08	KD2-H4K8la_peak_4829	4.53763	
4	1.7E+08	1.7E+08	KD2-H4K8la_peak_4830	6.68593	HAND2_ENSG00000164107
4	1.7E+08	1.7E+08	KD2-H4K8la_peak_4831	6.2624	
4	1.7E+08	1.7E+08	KD2-H4K8la_peak_4832	4.75945	
4	1.8E+08	1.8E+08	KD2-H4K8la_peak_4833	7.49467	AC108142.1_ENSG00000177822;TENM3_ENSG00000218336;RP11-402C9.1_ENSG00000248266
4	1.8E+08	1.8E+08	KD2-H4K8la_peak_4834	5.83285	RP11-188P17.2_ENSG00000272646
4	1.8E+08	1.8E+08	KD2-H4K8la_peak_4835	6.3399	DCTD_ENSG00000129187
4	1.8E+08	1.8E+08	KD2-H4K8la_peak_4836	6.64804	
4	1.8E+08	1.8E+08	KD2-H4K8la_peak_4837	5.89088	CDKN2AIP_ENSG00000168564
4	1.8E+08	1.8E+08	KD2-H4K8la_peak_4838	10.92449	ING2_ENSG00000168556
4	1.8E+08	1.8E+08	KD2-H4K8la_peak_4839	11.40266	
4	1.9E+08	1.9E+08	KD2-H4K8la_peak_4840	8.65116	
4	1.9E+08	1.9E+08	KD2-H4K8la_peak_4841	4.37527	
4	1.9E+08	1.9E+08	KD2-H4K8la_peak_4842	10.49982	
4	1.9E+08	1.9E+08	KD2-H4K8la_peak_4843	4.69635	SNX25_ENSG00000109762
4	1.9E+08	1.9E+08	KD2-H4K8la_peak_4844	6.47266	LRP2BP_ENSG00000109771;ANKRD37_ENSG00000186352
4	1.9E+08	1.9E+08	KD2-H4K8la_peak_4845	9.35473	FAM149A_ENSG00000109794
4	1.9E+08	1.9E+08	KD2-H4K8la_peak_4846	6.3399	AC110771.1_ENSG00000269302;CYP4V2_ENSG00000145476
4	1.9E+08	1.9E+08	KD2-H4K8la_peak_4847	6.3399	AC110771.1_ENSG00000269302;CYP4V2_ENSG00000145476
4	1.9E+08	1.9E+08	KD2-H4K8la_peak_4848	7.13237	
4	1.9E+08	1.9E+08	KD2-H4K8la_peak_4849	5.54909	
4	1.9E+08	1.9E+08	KD2-H4K8la_peak_4850	4.75945	AF146191.4_ENSG00000245685;FRG1_ENSG00000109536
5	91452	91911	KD2-H4K8la_peak_4851	17.87302	CTD-2231H16.1_ENSG00000249430
5	218562	218886	KD2-H4K8la_peak_4852	7.49467	CCDC127_ENSG00000164366;SDHA_ENSG00000073578
5	343793	344014	KD2-H4K8la_peak_4853	7.13237	
5	472838	473088	KD2-H4K8la_peak_4854	6.3399	CTD-2228K2.5_ENSG00000188242;CTD-2228K2.7_ENSG00000225138
5	1345516	1345833	KD2-H4K8la_peak_4855	11.7362	CLPTM1L_ENSG00000049656
5	1524199	1524715	KD2-H4K8la_peak_4856	9.35473	LPCAT1_ENSG00000153395
5	1594810	1595274	KD2-H4K8la_peak_4857	14.09647	SDHAP3_ENSG00000185986;CTD-2012J19.3_ENSG00000271119
5	5026313	5026727	KD2-H4K8la_peak_4858	7.61975	
5	5318233	5318465	KD2-H4K8la_peak_4859	10.04111	
5	5712936	5713146	KD2-H4K8la_peak_4860	8.39919	
5	6197391	6197604	KD2-H4K8la_peak_4861	5.58883	
5	6378291	6378574	KD2-H4K8la_peak_4862	13.11108	MED10_ENSG00000133398
5	1E+07	1E+07	KD2-H4K8la_peak_4863	9.81238	FAM173B_ENSG00000150756;CCT5_ENSG00000150753
5	1E+07	1E+07	KD2-H4K8la_peak_4864	7.05576	CTD-2256P15.2_ENSG00000259802;MARCH6_ENSG00000145495
5	1.1E+07	1.1E+07	KD2-H4K8la_peak_4865	11.36807	
5	1.1E+07	1.1E+07	KD2-H4K8la_peak_4866	6.2278	
5	1.3E+07	1.3E+07	KD2-H4K8la_peak_4867	7.51302	RP11-308B16.2_ENSG00000248783;CT49_ENSG00000248131
5	1.5E+07	1.5E+07	KD2-H4K8la_peak_4868	7.51302	CTD-2165H16.4_ENSG00000261360;FAM105B_ENSG00000154124
5	1.6E+07	1.6E+07	KD2-H4K8la_peak_4869	6.89612	ZNF622_ENSG00000173545
5	3.2E+07	3.2E+07	KD2-H4K8la_peak_4870	5.16795	
5	3.2E+07	3.2E+07	KD2-H4K8la_peak_4871	8.487	
5	3.2E+07	3.2E+07	KD2-H4K8la_peak_4872	6.96161	ZFR_ENSG00000056097
5	3.4E+07	3.4E+07	KD2-H4K8la_peak_4873	26.17414	
5	3.4E+07	3.4E+07	KD2-H4K8la_peak_4874	19.93595	

5	3.4E+07	3.4E+07	KD2-H4K8la_peak_4875	17.41456	
5	3.6E+07	3.6E+07	KD2-H4K8la_peak_4876	8.77936	
5	3.7E+07	3.7E+07	KD2-H4K8la_peak_4877	9.73848	C5orf42_ENSG00000197603
5	4.1E+07	4.1E+07	KD2-H4K8la_peak_4878	6.61495	RPL37_ENSG00000145592
5	4.2E+07	4.2E+07	KD2-H4K8la_peak_4879	8.07102	FBXO4_ENSG00000151876
5	4.2E+07	4.2E+07	KD2-H4K8la_peak_4880	7.13237	FBXO4_ENSG00000151876
5	4.3E+07	4.3E+07	KD2-H4K8la_peak_4881	6.2278	CTD-2201E18.5_ENSG00000271788
5	4.3E+07	4.3E+07	KD2-H4K8la_peak_4882	9.27765	C5orf28_ENSG00000151881;RP11-159F24.3_ENSG00000249492
5	4.4E+07	4.4E+07	KD2-H4K8la_peak_4883	11.06859	
5	4.5E+07	4.5E+07	KD2-H4K8la_peak_4884	6.2278	RP11-53O19.1_ENSG00000251141;MRPS30_ENSG00000112996
5	5E+07	5E+07	KD2-H4K8la_peak_4885	8.89875	CTD-2089N3.2_ENSG00000251573;CTD-2089N3.3_ENSG00000248918
5	5.1E+07	5.1E+07	KD2-H4K8la_peak_4886	7.96951	ISL1_ENSG00000016082
5	5.1E+07	5.1E+07	KD2-H4K8la_peak_4887	6.60114	
5	5.4E+07	5.4E+07	KD2-H4K8la_peak_4888	7.49467	ARL15_ENSG00000185305
5	5.4E+07	5.4E+07	KD2-H4K8la_peak_4889	6.47589	SNX18_ENSG00000178996
5	5.6E+07	5.6E+07	KD2-H4K8la_peak_4890	6.11881	
5	5.6E+07	5.6E+07	KD2-H4K8la_peak_4891	5.05146	
5	5.6E+07	5.6E+07	KD2-H4K8la_peak_4892	5.33364	MAP3K1_ENSG00000095015
5	5.8E+07	5.8E+07	KD2-H4K8la_peak_4893	6.70626	
5	5.9E+07	5.9E+07	KD2-H4K8la_peak_4894	7.23116	
5	6.4E+07	6.4E+07	KD2-H4K8la_peak_4895	6.70626	
5	6.4E+07	6.4E+07	KD2-H4K8la_peak_4896	6.64804	
5	6.4E+07	6.4E+07	KD2-H4K8la_peak_4897	5.05917	
5	6.5E+07	6.5E+07	KD2-H4K8la_peak_4898	5.27164	CTD-2033C11.1_ENSG00000269961;ERBB2IP_ENSG00000112851
5	6.6E+07	6.6E+07	KD2-H4K8la_peak_4899	11.25405	
5	6.7E+07	6.7E+07	KD2-H4K8la_peak_4900	11.15747	
5	6.8E+07	6.8E+07	KD2-H4K8la_peak_4901	10.83384	SLC30A5_ENSG00000145740
5	6.9E+07	6.9E+07	KD2-H4K8la_peak_4902	5.89088	CCDC125_ENSG00000183323;CHCHD2P2_ENSG00000215006
5	6.9E+07	6.9E+07	KD2-H4K8la_peak_4903	6.2278	
5	6.9E+07	6.9E+07	KD2-H4K8la_peak_4904	6.68593	
5	7.1E+07	7.1E+07	KD2-H4K8la_peak_4905	5.05146	
5	7.2E+07	7.2E+07	KD2-H4K8la_peak_4906	10.00612	CTD-2376I4.2_ENSG00000272081;FCHO2_ENSG00000157107
5	7.4E+07	7.4E+07	KD2-H4K8la_peak_4907	4.44972	
5	7.5E+07	7.5E+07	KD2-H4K8la_peak_4908	5.16795	
5	7.5E+07	7.5E+07	KD2-H4K8la_peak_4909	6.26214	POC5_ENSG00000152359
5	7.5E+07	7.5E+07	KD2-H4K8la_peak_4910	5.83285	CTC-235G5.3_ENSG00000248127
5	7.7E+07	7.7E+07	KD2-H4K8la_peak_4911	5.84082	
5	7.8E+07	7.8E+07	KD2-H4K8la_peak_4912	9.96333	
5	7.9E+07	7.9E+07	KD2-H4K8la_peak_4913	4.79631	
5	7.9E+07	7.9E+07	KD2-H4K8la_peak_4914	4.69635	MTX3_ENSG00000177034;THBS4_ENSG00000113296
5	7.9E+07	7.9E+07	KD2-H4K8la_peak_4915	5.69935	
5	8.1E+07	8.1E+07	KD2-H4K8la_peak_4916	6.86458	SSBP2_ENSG00000145687
5	8.6E+07	8.6E+07	KD2-H4K8la_peak_4917	8.07102	COX7C_ENSG00000127184
5	8.7E+07	8.7E+07	KD2-H4K8la_peak_4918	4.79631	
5	9E+07	9E+07	KD2-H4K8la_peak_4919	7.13237	CETN3_ENSG00000153140;AC093510.1_ENSG00000255647
5	9.1E+07	9.1E+07	KD2-H4K8la_peak_4920	5.43278	

5	9.4E+07	9.4E+07	KD2-H4K8la_peak_4921	7.72421	
5	9.5E+07	9.5E+07	KD2-H4K8la_peak_4922	7.81267	GPR150_ENSG00000178015
5	9.5E+07	9.5E+07	KD2-H4K8la_peak_4923	11.15747	
5	9.5E+07	9.5E+07	KD2-H4K8la_peak_4924	7.51302	RFESD_ENSG00000175449
5	9.6E+07	9.6E+07	KD2-H4K8la_peak_4925	8.48488	ERAP1_ENSG00000164307
5	1.1E+08	1.1E+08	KD2-H4K8la_peak_4926	5.36015	
5	1.1E+08	1.1E+08	KD2-H4K8la_peak_4927	10.36046	EFNA5_ENSG00000184349
5	1.1E+08	1.1E+08	KD2-H4K8la_peak_4928	10.04111	FBXL17_ENSG00000145743
5	1.1E+08	1.1E+08	KD2-H4K8la_peak_4929	7.72421	
5	1.1E+08	1.1E+08	KD2-H4K8la_peak_4930	10.28981	STARD4_ENSG00000164211;STARD4-AS1_ENSG00000246859
5	1.1E+08	1.1E+08	KD2-H4K8la_peak_4931	8.39919	
5	1.1E+08	1.1E+08	KD2-H4K8la_peak_4932	6.64804	CTC-487M23.8_ENSG00000272869;SRP19_ENSG00000153037
5	1.1E+08	1.1E+08	KD2-H4K8la_peak_4933	5.83285	
5	1.1E+08	1.1E+08	KD2-H4K8la_peak_4934	12.14099	YTHDC2_ENSG00000047188
5	1.1E+08	1.1E+08	KD2-H4K8la_peak_4935	12.1221	
5	1.1E+08	1.1E+08	KD2-H4K8la_peak_4936	6.64804	FEM1C_ENSG00000145780
5	1.2E+08	1.2E+08	KD2-H4K8la_peak_4937	6.70626	
5	1.2E+08	1.2E+08	KD2-H4K8la_peak_4938	8.487	
5	1.3E+08	1.3E+08	KD2-H4K8la_peak_4939	8.44557	PHAX_ENSG00000164902
5	1.3E+08	1.3E+08	KD2-H4K8la_peak_4940	9.20163	RP11-434D11.4_ENSG00000251072;LMNB1_ENSG00000113368
5	1.3E+08	1.3E+08	KD2-H4K8la_peak_4941	11.36807	
5	1.3E+08	1.3E+08	KD2-H4K8la_peak_4942	6.0136	ADAMTS19-AS1_ENSG00000249421;ADAMTS19_ENSG00000145808
5	1.3E+08	1.3E+08	KD2-H4K8la_peak_4943	5.89088	
5	1.3E+08	1.3E+08	KD2-H4K8la_peak_4944	5.83285	CTC-432M15.3_ENSG00000273217;FNIP1_ENSG00000217128
5	1.3E+08	1.3E+08	KD2-H4K8la_peak_4945	7.49467	
5	1.3E+08	1.3E+08	KD2-H4K8la_peak_4946	7.12014	C5orf56_ENSG00000197536
5	1.3E+08	1.3E+08	KD2-H4K8la_peak_4947	6.53067	
5	1.3E+08	1.3E+08	KD2-H4K8la_peak_4948	6.26214	
5	1.3E+08	1.3E+08	KD2-H4K8la_peak_4949	7.61975	
5	1.3E+08	1.3E+08	KD2-H4K8la_peak_4950	6.13507	SHROOM1_ENSG00000164403
5	1.3E+08	1.3E+08	KD2-H4K8la_peak_4951	5.30989	AFF4_ENSG00000072364;CTC-350I8.1_ENSG00000272023
5	1.3E+08	1.3E+08	KD2-H4K8la_peak_4952	13.47548	AFF4_ENSG00000072364;CTC-350I8.1_ENSG00000272023
5	1.3E+08	1.3E+08	KD2-H4K8la_peak_4953	6.25288	VDAC1_ENSG00000213585;CTB-113I20.2_ENSG00000271737
5	1.3E+08	1.3E+08	KD2-H4K8la_peak_4954	12.48213	
5	1.3E+08	1.3E+08	KD2-H4K8la_peak_4955	8.37064	TCF7_ENSG00000081059
5	1.3E+08	1.3E+08	KD2-H4K8la_peak_4956	10.47452	AC005355.2_ENSG00000251169
5	1.3E+08	1.3E+08	KD2-H4K8la_peak_4957	5.43278	JADE2_ENSG00000043143
5	1.3E+08	1.3E+08	KD2-H4K8la_peak_4958	4.66279	
5	1.3E+08	1.3E+08	KD2-H4K8la_peak_4959	5.30989	
5	1.3E+08	1.3E+08	KD2-H4K8la_peak_4960	7.13528	TXNDC15_ENSG00000113621
5	1.3E+08	1.3E+08	KD2-H4K8la_peak_4961	7.92775	PCBD2_ENSG00000132570
5	1.3E+08	1.3E+08	KD2-H4K8la_peak_4962	15.30845	H2AFY_ENSG00000113648;CTC-203F4.2_ENSG00000270021
5	1.4E+08	1.4E+08	KD2-H4K8la_peak_4963	10.98662	SMAD5_ENSG00000113658
5	1.4E+08	1.4E+08	KD2-H4K8la_peak_4964	7.49467	HNRNPA0_ENSG00000177733
5	1.4E+08	1.4E+08	KD2-H4K8la_peak_4965	8.77936	NME5_ENSG00000112981
5	1.4E+08	1.4E+08	KD2-H4K8la_peak_4966	9.0413	CDC23_ENSG00000094880

5	1.4E+08	1.4E+08	KD2-H4K8la_peak_4967	4.75945	
5	1.4E+08	1.4E+08	KD2-H4K8la_peak_4968	14.15376	
5	1.4E+08	1.4E+08	KD2-H4K8la_peak_4969	10.87112	
5	1.4E+08	1.4E+08	KD2-H4K8la_peak_4970	5.58883	
5	1.4E+08	1.4E+08	KD2-H4K8la_peak_4971	7.61975	HSPA9_ENSG00000113013
5	1.4E+08	1.4E+08	KD2-H4K8la_peak_4972	8.23729	CTNNA1_ENSG00000044115
5	1.4E+08	1.4E+08	KD2-H4K8la_peak_4973	10.74378	AC034243.1_ENSG00000253404
5	1.4E+08	1.4E+08	KD2-H4K8la_peak_4974	7.85401	
5	1.4E+08	1.4E+08	KD2-H4K8la_peak_4975	11.62831	
5	1.4E+08	1.4E+08	KD2-H4K8la_peak_4976	12.19504	
5	1.4E+08	1.4E+08	KD2-H4K8la_peak_4977	8.76116	SIL1_ENSG00000120725
5	1.4E+08	1.4E+08	KD2-H4K8la_peak_4978	10.02595	
5	1.4E+08	1.4E+08	KD2-H4K8la_peak_4979	10.02595	
5	1.4E+08	1.4E+08	KD2-H4K8la_peak_4980	6.18994	
5	1.4E+08	1.4E+08	KD2-H4K8la_peak_4981	10.8046	
5	1.4E+08	1.4E+08	KD2-H4K8la_peak_4982	7.3993	
5	1.4E+08	1.4E+08	KD2-H4K8la_peak_4983	6.14238	
5	1.4E+08	1.4E+08	KD2-H4K8la_peak_4984	10.25348	
5	1.4E+08	1.4E+08	KD2-H4K8la_peak_4985	5.83643	
5	1.4E+08	1.4E+08	KD2-H4K8la_peak_4986	5.36015	CTB-35F21.3_ENSG00000251387;CTB-35F21.5_ENSG00000271792
5	1.4E+08	1.4E+08	KD2-H4K8la_peak_4987	5.83285	NDUFA2_ENSG00000131495;IK_ENSG00000113141;MIR3655_ENSG00000264052
5	1.4E+08	1.4E+08	KD2-H4K8la_peak_4988	13.18631	PCDHA4_ENSG00000204967
5	1.4E+08	1.4E+08	KD2-H4K8la_peak_4989	8.39813	TAF7_ENSG00000178913;AC005618.1_ENSG00000255729
5	1.4E+08	1.4E+08	KD2-H4K8la_peak_4990	5.95337	
5	1.4E+08	1.4E+08	KD2-H4K8la_peak_4991	10.49982	
5	1.4E+08	1.4E+08	KD2-H4K8la_peak_4992	10.04111	
5	1.4E+08	1.4E+08	KD2-H4K8la_peak_4993	6.22733	
5	1.4E+08	1.4E+08	KD2-H4K8la_peak_4994	7.447	
5	1.4E+08	1.4E+08	KD2-H4K8la_peak_4995	8.63473	
5	1.4E+08	1.4E+08	KD2-H4K8la_peak_4996	9.96333	SPRY4_ENSG00000187678;AC005592.2_ENSG00000231185
5	1.4E+08	1.4E+08	KD2-H4K8la_peak_4997	13.13367	
5	1.4E+08	1.4E+08	KD2-H4K8la_peak_4998	8.37064	
5	1.4E+08	1.4E+08	KD2-H4K8la_peak_4999	8.23729	
5	1.4E+08	1.4E+08	KD2-H4K8la_peak_5000	5.5899	
5	1.4E+08	1.4E+08	KD2-H4K8la_peak_5001	7.13237	
5	1.4E+08	1.4E+08	KD2-H4K8la_peak_5002	8.23729	
5	1.5E+08	1.5E+08	KD2-H4K8la_peak_5003	7.92775	TCERG1_ENSG00000113649
5	1.5E+08	1.5E+08	KD2-H4K8la_peak_5004	8.487	SPINK9_ENSG00000204909
5	1.5E+08	1.5E+08	KD2-H4K8la_peak_5005	9.27415	
5	1.5E+08	1.5E+08	KD2-H4K8la_peak_5006	6.89612	
5	1.5E+08	1.5E+08	KD2-H4K8la_peak_5007	7.77341	
5	1.5E+08	1.5E+08	KD2-H4K8la_peak_5008	11.15747	
5	1.5E+08	1.5E+08	KD2-H4K8la_peak_5009	12.49088	TIGD6_ENSG00000164296;HMGXB3_ENSG00000113716
5	1.5E+08	1.5E+08	KD2-H4K8la_peak_5010	4.75945	
5	1.5E+08	1.5E+08	KD2-H4K8la_peak_5011	8.07102	
5	1.5E+08	1.5E+08	KD2-H4K8la_peak_5012	7.61975	TCOF1_ENSG00000070814

5	1.5E+08	1.5E+08	KD2-H4K8la_peak_5013	7.92775	CTC-367J11.1_ENSG00000254333;NDST1_ENSG00000070614
5	1.5E+08	1.5E+08	KD2-H4K8la_peak_5014	10.28981	
5	1.5E+08	1.5E+08	KD2-H4K8la_peak_5015	6.64804	
5	1.5E+08	1.5E+08	KD2-H4K8la_peak_5016	5.10388	
5	1.5E+08	1.5E+08	KD2-H4K8la_peak_5017	8.24908	
5	1.5E+08	1.5E+08	KD2-H4K8la_peak_5018	7.12014	SYNPO_ENSG00000171992
5	1.5E+08	1.5E+08	KD2-H4K8la_peak_5019	12.49088	CTC-345K18.2_ENSG00000250309
5	1.5E+08	1.5E+08	KD2-H4K8la_peak_5020	15.20987	G3BP1_ENSG00000145907
5	1.5E+08	1.5E+08	KD2-H4K8la_peak_5021	6.25288	
5	1.5E+08	1.5E+08	KD2-H4K8la_peak_5022	9.44217	
5	1.5E+08	1.5E+08	KD2-H4K8la_peak_5023	9.88732	
5	1.5E+08	1.5E+08	KD2-H4K8la_peak_5024	10.07942	
5	1.5E+08	1.5E+08	KD2-H4K8la_peak_5025	5.3107	
5	1.5E+08	1.5E+08	KD2-H4K8la_peak_5026	7.23932	
5	1.5E+08	1.5E+08	KD2-H4K8la_peak_5027	10.74378	
5	1.5E+08	1.5E+08	KD2-H4K8la_peak_5028	9.70817	CNOT8_ENSG00000155508
5	1.5E+08	1.5E+08	KD2-H4K8la_peak_5029	6.96161	GEMIN5_ENSG00000082516
5	1.6E+08	1.6E+08	KD2-H4K8la_peak_5030	7.90495	CTB-109A12.1_ENSG00000251405;NIPAL4_ENSG00000172548
5	1.6E+08	1.6E+08	KD2-H4K8la_peak_5031	9.86881	CLINT1_ENSG00000113282
5	1.6E+08	1.6E+08	KD2-H4K8la_peak_5032	8.39919	IL12B_ENSG00000113302
5	1.6E+08	1.6E+08	KD2-H4K8la_peak_5033	6.57319	Y_RNA_ENSG00000199398;TTC1_ENSG00000113312
5	1.7E+08	1.7E+08	KD2-H4K8la_peak_5034	7.49467	
5	1.7E+08	1.7E+08	KD2-H4K8la_peak_5035	7.92775	
5	1.7E+08	1.7E+08	KD2-H4K8la_peak_5036	13.05879	MIR218-2_ENSG00000207739
5	1.7E+08	1.7E+08	KD2-H4K8la_peak_5037	9.34957	
5	1.7E+08	1.7E+08	KD2-H4K8la_peak_5038	7.78805	
5	1.7E+08	1.7E+08	KD2-H4K8la_peak_5039	5.83285	TLX3_ENSG00000164438
5	1.7E+08	1.7E+08	KD2-H4K8la_peak_5040	4.86896	FBXW11_ENSG00000072803
5	1.7E+08	1.7E+08	KD2-H4K8la_peak_5041	5.36015	
5	1.7E+08	1.7E+08	KD2-H4K8la_peak_5042	11.24937	DUSP1_ENSG00000120129
5	1.7E+08	1.7E+08	KD2-H4K8la_peak_5043	7.22716	CTC-308K20.1_ENSG00000204758;RPL26L1_ENSG00000037241
5	1.7E+08	1.7E+08	KD2-H4K8la_peak_5044	8.50453	CTC-308K20.1_ENSG00000204758;RPL26L1_ENSG00000037241
5	1.7E+08	1.7E+08	KD2-H4K8la_peak_5045	7.12014	BNIP1_ENSG00000113734;CTC-209H22.3_ENSG00000253172
5	1.7E+08	1.7E+08	KD2-H4K8la_peak_5046	8.39919	STC2_ENSG00000113739
5	1.7E+08	1.7E+08	KD2-H4K8la_peak_5047	9.73848	STC2_ENSG00000113739
5	1.7E+08	1.7E+08	KD2-H4K8la_peak_5048	8.19799	
5	1.7E+08	1.7E+08	KD2-H4K8la_peak_5049	4.53273	
5	1.7E+08	1.7E+08	KD2-H4K8la_peak_5050	9.38376	CTD-2532K18.1_ENSG00000251670;MIR4634_ENSG00000266890
5	1.7E+08	1.7E+08	KD2-H4K8la_peak_5051	6.64804	
5	1.8E+08	1.8E+08	KD2-H4K8la_peak_5052	8.76116	
5	1.8E+08	1.8E+08	KD2-H4K8la_peak_5053	7.22389	CDHR2_ENSG00000074276
5	1.8E+08	1.8E+08	KD2-H4K8la_peak_5054	9.0413	
5	1.8E+08	1.8E+08	KD2-H4K8la_peak_5055	7.92775	TSPAN17_ENSG00000048140
5	1.8E+08	1.8E+08	KD2-H4K8la_peak_5056	5.83285	TSPAN17_ENSG00000048140
5	1.8E+08	1.8E+08	KD2-H4K8la_peak_5057	10.99007	
5	1.8E+08	1.8E+08	KD2-H4K8la_peak_5058	8.37064	

5	1.8E+08	1.8E+08	KD2-H4K8la_peak_5059	6.64804	
5	1.8E+08	1.8E+08	KD2-H4K8la_peak_5060	5.23773	
5	1.8E+08	1.8E+08	KD2-H4K8la_peak_5061	10.44868	NSD1_ENSG00000165671
5	1.8E+08	1.8E+08	KD2-H4K8la_peak_5062	8.37064	
5	1.8E+08	1.8E+08	KD2-H4K8la_peak_5063	6.21394	
5	1.8E+08	1.8E+08	KD2-H4K8la_peak_5064	9.61894	
5	1.8E+08	1.8E+08	KD2-H4K8la_peak_5065	12.14568	GRK6_ENSG00000198055
5	1.8E+08	1.8E+08	KD2-H4K8la_peak_5066	9.66714	DBN1_ENSG00000113758
5	1.8E+08	1.8E+08	KD2-H4K8la_peak_5067	7.12014	B4GALT7_ENSG00000027847
5	1.8E+08	1.8E+08	KD2-H4K8la_peak_5068	5.99671	
5	1.8E+08	1.8E+08	KD2-H4K8la_peak_5069	5.43278	HNRNPAB_ENSG00000197451
5	1.8E+08	1.8E+08	KD2-H4K8la_peak_5070	11.06859	PHYKPL_ENSG00000175309
5	1.8E+08	1.8E+08	KD2-H4K8la_peak_5071	8.24908	
5	1.8E+08	1.8E+08	KD2-H4K8la_peak_5072	7.72421	ZNF354C_ENSG00000177932
5	1.8E+08	1.8E+08	KD2-H4K8la_peak_5073	4.86896	
5	1.8E+08	1.8E+08	KD2-H4K8la_peak_5074	6.89612	
5	1.8E+08	1.8E+08	KD2-H4K8la_peak_5075	6.20793	
5	1.8E+08	1.8E+08	KD2-H4K8la_peak_5076	15.58875	
5	1.8E+08	1.8E+08	KD2-H4K8la_peak_5077	11.40266	CANX_ENSG00000127022;HMGB3P22_ENSG00000225051
5	1.8E+08	1.8E+08	KD2-H4K8la_peak_5078	10.06702	
5	1.8E+08	1.8E+08	KD2-H4K8la_peak_5079	7.49467	CTC-241N9.1_ENSG00000245317
5	1.8E+08	1.8E+08	KD2-H4K8la_peak_5080	8.07102	MAPK9_ENSG00000050748;CTB-129O4.1_ENSG00000248367
5	1.8E+08	1.8E+08	KD2-H4K8la_peak_5081	9.6708	
5	1.8E+08	1.8E+08	KD2-H4K8la_peak_5082	8.07102	ZFP62_ENSG00000196670
5	1.8E+08	1.8E+08	KD2-H4K8la_peak_5083	6.2278	ZFP62_ENSG00000196670
5	1.8E+08	1.8E+08	KD2-H4K8la_peak_5084	8.16787	
5	1.8E+08	1.8E+08	KD2-H4K8la_peak_5085	6.86458	CTC-338M12.4_ENSG00000233937
5	1.8E+08	1.8E+08	KD2-H4K8la_peak_5086	8.07044	TRIM52_ENSG00000183718;TRIM52-AS1_ENSG00000248275
6	291679	291945	KD2-H4K8la_peak_5087	9.70817	DUSP22_ENSG00000112679
6	1614191	1614400	KD2-H4K8la_peak_5088	7.65547	
6	1838022	1838294	KD2-H4K8la_peak_5089	7.60756	
6	1904891	1905183	KD2-H4K8la_peak_5090	8.73956	
6	2634394	2634659	KD2-H4K8la_peak_5091	9.9701	C6orf195_ENSG00000164385
6	2841802	2842106	KD2-H4K8la_peak_5092	5.98303	SERPINB1_ENSG00000021355
6	2971156	2971589	KD2-H4K8la_peak_5093	7.92775	SERPINB6_ENSG00000124570
6	2988402	2988656	KD2-H4K8la_peak_5094	5.7631	LINC01011_ENSG00000244041;NQO2_ENSG00000124588
6	3000243	3000594	KD2-H4K8la_peak_5095	4.53273	RP1-90J20.8_ENSG00000224846
6	3053680	3053958	KD2-H4K8la_peak_5096	10.20357	
6	3157982	3158309	KD2-H4K8la_peak_5097	15.74238	TUBB2A_ENSG00000137267
6	3228316	3228616	KD2-H4K8la_peak_5098	7.23932	
6	3229402	3229782	KD2-H4K8la_peak_5099	8.24908	
6	3231558	3231839	KD2-H4K8la_peak_5100	12.13455	TUBB2B_ENSG00000137285;PSMG4_ENSG00000180822
6	4020640	4020850	KD2-H4K8la_peak_5101	8.23939	RP3-406P24.3_ENSG00000230648;PRPF4B_ENSG00000112739
6	5004424	5004837	KD2-H4K8la_peak_5102	9.86881	RPP40_ENSG00000124787
6	5747356	5747585	KD2-H4K8la_peak_5103	8.40055	
6	7052710	7052923	KD2-H4K8la_peak_5104	7.72421	

6	8435972	8436180	KD2-H4K8la_peak_5105	7.72421	SLC35B3_ENSG00000124786
6	1E+07	1E+07	KD2-H4K8la_peak_5106	11.50321	
6	1.1E+07	1.1E+07	KD2-H4K8la_peak_5107	7.49467	
6	1.1E+07	1.1E+07	KD2-H4K8la_peak_5108	7.13237	ELOVL2_ENSG00000197977;ELOVL2-AS1_ENSG00000230314
6	1.2E+07	1.2E+07	KD2-H4K8la_peak_5109	8.76082	TMEM170B_ENSG00000205269
6	1.4E+07	1.4E+07	KD2-H4K8la_peak_5110	6.61963	
6	1.4E+07	1.4E+07	KD2-H4K8la_peak_5111	8.39919	
6	1.7E+07	1.7E+07	KD2-H4K8la_peak_5112	5.7631	ATXN1_ENSG00000124788;RP1-151F17.1_ENSG00000229931
6	1.7E+07	1.7E+07	KD2-H4K8la_peak_5113	7.72421	RBM24_ENSG00000112183
6	1.8E+07	1.8E+07	KD2-H4K8la_peak_5114	6.64804	KIF13A_ENSG00000137177
6	2E+07	2E+07	KD2-H4K8la_peak_5115	9.90319	MBOAT1_ENSG00000172197;RP11-239H6.2_ENSG00000227803
6	2E+07	2E+07	KD2-H4K8la_peak_5116	7.3993	E2F3_ENSG00000112242
6	2.2E+07	2.2E+07	KD2-H4K8la_peak_5117	5.58883	
6	2.2E+07	2.2E+07	KD2-H4K8la_peak_5118	5.36015	CASC14_ENSG00000260455
6	2.3E+07	2.3E+07	KD2-H4K8la_peak_5119	7.23116	
6	2.4E+07	2.4E+07	KD2-H4K8la_peak_5120	6.2278	MRS2_ENSG00000124532
6	2.5E+07	2.5E+07	KD2-H4K8la_peak_5121	6.2278	GMNN_ENSG00000112312
6	2.7E+07	2.7E+07	KD2-H4K8la_peak_5122	10.00612	TRNAI2_ENSG00000238621
6	2.8E+07	2.8E+07	KD2-H4K8la_peak_5123	7.4908	
6	2.8E+07	2.8E+07	KD2-H4K8la_peak_5124	11.97807	ZSCAN16-AS1_ENSG00000269293;RP1-265C24.8_ENSG00000261839
6	2.8E+07	2.8E+07	KD2-H4K8la_peak_5125	10.34978	
6	2.8E+07	2.8E+07	KD2-H4K8la_peak_5126	9.72904	
6	2.9E+07	2.9E+07	KD2-H4K8la_peak_5127	8.52281	
6	2.9E+07	2.9E+07	KD2-H4K8la_peak_5128	5.67596	RP5-1186N24.3_ENSG00000246350
6	2.9E+07	2.9E+07	KD2-H4K8la_peak_5129	6.70626	
6	3E+07	3E+07	KD2-H4K8la_peak_5130	11.06859	
6	3E+07	3E+07	KD2-H4K8la_peak_5131	12.01611	HLA-L_ENSG00000243753
6	3E+07	3E+07	KD2-H4K8la_peak_5132	6.98778	HCG17_ENSG00000270604;HCG18_ENSG00000231074;TRIM39_ENSG00000204599
6	3E+07	3E+07	KD2-H4K8la_peak_5133	5.16795	XXbac-BPG249D20.9_ENSG00000235781
6	3.1E+07	3.1E+07	KD2-H4K8la_peak_5134	10.11868	PPP1R18_ENSG00000146112
6	3.1E+07	3.1E+07	KD2-H4K8la_peak_5135	9.48768	TUBB_ENSG00000196230
6	3.1E+07	3.1E+07	KD2-H4K8la_peak_5136	5.38451	
6	3.1E+07	3.1E+07	KD2-H4K8la_peak_5137	6.64804	
6	3.1E+07	3.1E+07	KD2-H4K8la_peak_5138	7.81267	
6	3.2E+07	3.2E+07	KD2-H4K8la_peak_5139	6.61963	
6	3.2E+07	3.2E+07	KD2-H4K8la_peak_5140	8.89364	
6	3.2E+07	3.2E+07	KD2-H4K8la_peak_5141	5.84082	VARS_ENSG00000204394
6	3.2E+07	3.2E+07	KD2-H4K8la_peak_5142	11.77509	EHMT2_ENSG00000204371;C2_ENSG00000166278
6	3.2E+07	3.2E+07	KD2-H4K8la_peak_5143	8.59735	ZBTB12_ENSG00000204366
6	3.2E+07	3.2E+07	KD2-H4K8la_peak_5144	8.13727	FKBPL_ENSG00000204315
6	3.2E+07	3.2E+07	KD2-H4K8la_peak_5145	9.0413	
6	3.2E+07	3.2E+07	KD2-H4K8la_peak_5146	9.06396	GPSM3_ENSG00000213654
6	3.3E+07	3.3E+07	KD2-H4K8la_peak_5147	8.12466	HLA-DMA_ENSG00000204257;BRD2_ENSG00000204256
6	3.3E+07	3.3E+07	KD2-H4K8la_peak_5148	9.92519	BRD2-IT1_ENSG00000223837
6	3.3E+07	3.3E+07	KD2-H4K8la_peak_5149	6.61495	
6	3.4E+07	3.4E+07	KD2-H4K8la_peak_5150	7.94013	LEMD2_ENSG00000161904

6	3.4E+07	3.4E+07	KD2-H4K8la_peak_5151	9.6708	
6	3.4E+07	3.4E+07	KD2-H4K8la_peak_5152	7.92775	HMGA1_ENSG00000137309
6	3.4E+07	3.4E+07	KD2-H4K8la_peak_5153	12.63515	RPS10-NUDT3_ENSG00000270800;RPS10_ENSG00000124614
6	3.5E+07	3.5E+07	KD2-H4K8la_peak_5154	7.13237	C6orf106_ENSG00000196821;RP11-140K17.3_ENSG00000272288
6	3.5E+07	3.5E+07	KD2-H4K8la_peak_5155	8.04432	SNRPC_ENSG00000124562
6	3.5E+07	3.5E+07	KD2-H4K8la_peak_5156	7.12014	SNRPC_ENSG00000124562
6	3.5E+07	3.5E+07	KD2-H4K8la_peak_5157	7.12497	UHRF1BP1_ENSG00000065060
6	3.5E+07	3.5E+07	KD2-H4K8la_peak_5158	8.12466	TAF11_ENSG00000064995;ANKS1A_ENSG00000064999
6	3.5E+07	3.5E+07	KD2-H4K8la_peak_5159	8.37064	RPL10A_ENSG00000198755
6	3.5E+07	3.5E+07	KD2-H4K8la_peak_5160	8.94987	TEAD3_ENSG00000007866
6	3.6E+07	3.6E+07	KD2-H4K8la_peak_5161	9.10568	FKBP5_ENSG00000096060
6	3.6E+07	3.6E+07	KD2-H4K8la_peak_5162	8.49617	RP3-510O8.4_ENSG00000232909;ARMC12_ENSG00000157343
6	3.6E+07	3.6E+07	KD2-H4K8la_peak_5163	8.37064	MAPK14_ENSG00000112062
6	3.6E+07	3.6E+07	KD2-H4K8la_peak_5164	8.24908	
6	3.6E+07	3.6E+07	KD2-H4K8la_peak_5165	11.52725	RP1-179N16.6_ENSG00000246982;BRPF3_ENSG00000096070
6	3.6E+07	3.6E+07	KD2-H4K8la_peak_5166	6.11881	
6	3.7E+07	3.7E+07	KD2-H4K8la_peak_5167	8.4393	
6	3.7E+07	3.7E+07	KD2-H4K8la_peak_5168	6.64804	CMTR1_ENSG00000137200
6	3.8E+07	3.8E+07	KD2-H4K8la_peak_5169	6.96161	
6	3.8E+07	3.8E+07	KD2-H4K8la_peak_5170	7.28641	
6	3.9E+07	3.9E+07	KD2-H4K8la_peak_5171	14.09097	
6	3.9E+07	3.9E+07	KD2-H4K8la_peak_5172	13.5227	
6	3.9E+07	3.9E+07	KD2-H4K8la_peak_5173	6.64804	
6	4E+07	4E+07	KD2-H4K8la_peak_5174	16.08919	KIF6_ENSG00000164627
6	4E+07	4E+07	KD2-H4K8la_peak_5175	12.12133	
6	4E+07	4E+07	KD2-H4K8la_peak_5176	7.49467	
6	4E+07	4E+07	KD2-H4K8la_peak_5177	6.2278	
6	4E+07	4E+07	KD2-H4K8la_peak_5178	7.56395	
6	4E+07	4E+07	KD2-H4K8la_peak_5179	8.07102	
6	4.1E+07	4.1E+07	KD2-H4K8la_peak_5180	6.86458	
6	4.1E+07	4.1E+07	KD2-H4K8la_peak_5181	9.27415	
6	4.1E+07	4.1E+07	KD2-H4K8la_peak_5182	6.68593	NFYA_ENSG00000001167
6	4.1E+07	4.1E+07	KD2-H4K8la_peak_5183	8.07102	
6	4.2E+07	4.2E+07	KD2-H4K8la_peak_5184	5.58883	TFEB_ENSG00000112561
6	4.2E+07	4.2E+07	KD2-H4K8la_peak_5185	4.7338	
6	4.2E+07	4.2E+07	KD2-H4K8la_peak_5186	4.30663	
6	4.2E+07	4.2E+07	KD2-H4K8la_peak_5187	10.28981	
6	4.2E+07	4.2E+07	KD2-H4K8la_peak_5188	11.06787	C6orf132_ENSG00000188112
6	4.2E+07	4.2E+07	KD2-H4K8la_peak_5189	7.49467	MRPS10_ENSG00000048544
6	4.3E+07	4.3E+07	KD2-H4K8la_peak_5190	20.8834	
6	4.3E+07	4.3E+07	KD2-H4K8la_peak_5191	13.01988	
6	4.3E+07	4.3E+07	KD2-H4K8la_peak_5192	11.69149	RPL7L1_ENSG00000146223
6	4.3E+07	4.3E+07	KD2-H4K8la_peak_5193	9.61894	CUL7_ENSG00000044090
6	4.3E+07	4.3E+07	KD2-H4K8la_peak_5194	5.99671	
6	4.3E+07	4.3E+07	KD2-H4K8la_peak_5195	8.07044	DNPH1_ENSG00000112667
6	4.3E+07	4.3E+07	KD2-H4K8la_peak_5196	7.61406	ZNF318_ENSG00000171467

6	4.3E+07	4.3E+07	KD2-H4K8la_peak_5197	5.58883	ABCC10_ENSG00000124574
6	4.3E+07	4.3E+07	KD2-H4K8la_peak_5198	9.0516	ABCC10_ENSG00000124574
6	4.4E+07	4.4E+07	KD2-H4K8la_peak_5199	7.49467	
6	4.4E+07	4.4E+07	KD2-H4K8la_peak_5200	7.3993	
6	4.4E+07	4.4E+07	KD2-H4K8la_peak_5201	5.05146	
6	4.4E+07	4.4E+07	KD2-H4K8la_peak_5202	15.92725	
6	4.4E+07	4.4E+07	KD2-H4K8la_peak_5203	6.04152	RP5-1120P11.1_ENSG00000237686;RP5-1120P11.3_ENSG00000231881
6	4.4E+07	4.4E+07	KD2-H4K8la_peak_5204	6.49887	
6	4.4E+07	4.4E+07	KD2-H4K8la_peak_5205	10.98512	HSP90AB1_ENSG00000096384
6	4.4E+07	4.4E+07	KD2-H4K8la_peak_5206	8.76116	TCTE1_ENSG00000146221
6	4.4E+07	4.4E+07	KD2-H4K8la_peak_5207	8.07102	SPATS1_ENSG00000249481
6	4.4E+07	4.4E+07	KD2-H4K8la_peak_5208	7.92775	CDC5L_ENSG00000096401
6	4.7E+07	4.7E+07	KD2-H4K8la_peak_5209	11.15747	RP11-385F7.1_ENSG00000270761;CD2AP_ENSG00000198087
6	5.1E+07	5.1E+07	KD2-H4K8la_peak_5210	16.12381	TFAP2B_ENSG00000008196
6	5.1E+07	5.1E+07	KD2-H4K8la_peak_5211	7.65547	
6	5.2E+07	5.2E+07	KD2-H4K8la_peak_5212	5.67034	
6	5.2E+07	5.2E+07	KD2-H4K8la_peak_5213	8.37064	
6	5.2E+07	5.2E+07	KD2-H4K8la_peak_5214	8.37064	TRAM2_ENSG00000065308;TRAM2-AS1_ENSG00000225791
6	5.4E+07	5.4E+07	KD2-H4K8la_peak_5215	4.30663	RP13-476E20.1_ENSG00000228614;LRRC1_ENSG00000137269
6	5.4E+07	5.4E+07	KD2-H4K8la_peak_5216	6.0136	RP13-476E20.1_ENSG00000228614;LRRC1_ENSG00000137269
6	7.1E+07	7.1E+07	KD2-H4K8la_peak_5217	6.2278	LMBRD1_ENSG00000168216
6	7.1E+07	7.1E+07	KD2-H4K8la_peak_5218	8.76116	RP11-462G2.2_ENSG00000224349;FAM135A_ENSG00000082269
6	7.4E+07	7.4E+07	KD2-H4K8la_peak_5219	9.0413	AC019205.1_ENSG00000263378;RP11-257K9.8_ENSG00000243501;RP11-398K22.12_ENSG00000229852
6	7.4E+07	7.4E+07	KD2-H4K8la_peak_5220	4.75945	
6	7.4E+07	7.4E+07	KD2-H4K8la_peak_5221	5.51108	
6	7.6E+07	7.6E+07	KD2-H4K8la_peak_5222	4.53273	
6	7.9E+07	7.9E+07	KD2-H4K8la_peak_5223	5.83285	
6	8.4E+07	8.4E+07	KD2-H4K8la_peak_5224	11.40266	ME1_ENSG00000065833
6	8.6E+07	8.6E+07	KD2-H4K8la_peak_5225	8.76116	
6	8.6E+07	8.6E+07	KD2-H4K8la_peak_5226	7.13237	SYNCRIP_ENSG00000135316
6	8.8E+07	8.8E+07	KD2-H4K8la_peak_5227	11.06859	SMIM8_ENSG00000111850;RP1-102H19.8_ENSG00000226524
6	9E+07	9E+07	KD2-H4K8la_peak_5228	6.47589	UBE2J1_ENSG00000198833
6	9.1E+07	9.1E+07	KD2-H4K8la_peak_5229	4.86896	CASP8AP2_ENSG00000118412
6	9.1E+07	9.1E+07	KD2-H4K8la_peak_5230	5.72892	
6	9.6E+07	9.6E+07	KD2-H4K8la_peak_5231	12.1221	MANEA-AS1_ENSG00000261366;MANEA_ENSG00000172469
6	9.7E+07	9.7E+07	KD2-H4K8la_peak_5232	6.88801	GPR63_ENSG00000112218
6	9.9E+07	9.9E+07	KD2-H4K8la_peak_5233	8.07102	FBXL4_ENSG00000112234
6	1E+08	1E+08	KD2-H4K8la_peak_5234	6.0136	FAXC_ENSG00000146267
6	1E+08	1E+08	KD2-H4K8la_peak_5235	8.487	CCNC_ENSG00000112237
6	1E+08	1E+08	KD2-H4K8la_peak_5236	6.60114	
6	1.1E+08	1.1E+08	KD2-H4K8la_peak_5237	8.37064	LINC00577_ENSG00000203809
6	1.1E+08	1.1E+08	KD2-H4K8la_peak_5238	6.26214	RP11-404H14.1_ENSG00000233941
6	1.1E+08	1.1E+08	KD2-H4K8la_peak_5239	7.0396	PDSS2_ENSG00000164494
6	1.1E+08	1.1E+08	KD2-H4K8la_peak_5240	4.86896	
6	1.1E+08	1.1E+08	KD2-H4K8la_peak_5241	8.34387	SNX3_ENSG00000112335
6	1.1E+08	1.1E+08	KD2-H4K8la_peak_5242	7.12014	

6	1.1E+08	1.1E+08	KD2-H4K8la_peak_5243	7.61975	
6	1.1E+08	1.1E+08	KD2-H4K8la_peak_5244	5.58883	METTL24_ENSG00000053328
6	1.1E+08	1.1E+08	KD2-H4K8la_peak_5245	9.38376	METTL24_ENSG00000053328
6	1.1E+08	1.1E+08	KD2-H4K8la_peak_5246	10.65691	GTF3C6_ENSG00000155115
6	1.1E+08	1.1E+08	KD2-H4K8la_peak_5247	6.16656	
6	1.1E+08	1.1E+08	KD2-H4K8la_peak_5248	5.63532	
6	1.1E+08	1.1E+08	KD2-H4K8la_peak_5249	6.70626	
6	1.1E+08	1.1E+08	KD2-H4K8la_peak_5250	11.15747	MARCKS_ENSG00000155130
6	1.2E+08	1.2E+08	KD2-H4K8la_peak_5251	8.07102	KPNA5_ENSG00000196911
6	1.2E+08	1.2E+08	KD2-H4K8la_peak_5252	7.23116	GOPC_ENSG00000047932
6	1.2E+08	1.2E+08	KD2-H4K8la_peak_5253	5.19328	
6	1.3E+08	1.3E+08	KD2-H4K8la_peak_5254	6.86458	
6	1.3E+08	1.3E+08	KD2-H4K8la_peak_5255	9.20163	TRMT11_ENSG00000066651
6	1.3E+08	1.3E+08	KD2-H4K8la_peak_5256	8.07102	SOGA3_ENSG00000214338;SOGA3_ENSG00000255330;C6orf58_ENSG00000184530
6	1.4E+08	1.4E+08	KD2-H4K8la_peak_5257	7.12014	SLC35D3_ENSG00000182747
6	1.4E+08	1.4E+08	KD2-H4K8la_peak_5258	14.33193	
6	1.4E+08	1.4E+08	KD2-H4K8la_peak_5259	9.0413	
6	1.4E+08	1.4E+08	KD2-H4K8la_peak_5260	12.58104	
6	1.4E+08	1.4E+08	KD2-H4K8la_peak_5261	10.04111	
6	1.4E+08	1.4E+08	KD2-H4K8la_peak_5262	5.30989	
6	1.4E+08	1.4E+08	KD2-H4K8la_peak_5263	5.99671	
6	1.4E+08	1.4E+08	KD2-H4K8la_peak_5264	7.72421	
6	1.4E+08	1.4E+08	KD2-H4K8la_peak_5265	15.37939	IL20RA_ENSG00000016402
6	1.4E+08	1.4E+08	KD2-H4K8la_peak_5266	5.43278	REPS1_ENSG00000135597
6	1.4E+08	1.4E+08	KD2-H4K8la_peak_5267	4.53273	ADAT2_ENSG00000189007;RNA5SP221_ENSG00000223203;PEX3_ENSG00000034693
6	1.4E+08	1.4E+08	KD2-H4K8la_peak_5268	12.14099	FUCA2_ENSG00000001036
6	1.4E+08	1.4E+08	KD2-H4K8la_peak_5269	7.51014	PHACTR2_ENSG00000112419
6	1.4E+08	1.4E+08	KD2-H4K8la_peak_5270	8.78735	LTV1_ENSG00000135521
6	1.4E+08	1.4E+08	KD2-H4K8la_peak_5271	10.83873	
6	1.4E+08	1.4E+08	KD2-H4K8la_peak_5272	7.49467	SF3B5_ENSG00000169976
6	1.5E+08	1.5E+08	KD2-H4K8la_peak_5273	7.83588	EPM2A_ENSG00000112425;RP3-466P17.1_ENSG00000270638
6	1.5E+08	1.5E+08	KD2-H4K8la_peak_5274	10.74378	
6	1.5E+08	1.5E+08	KD2-H4K8la_peak_5275	4.75945	PPIL4_ENSG00000131013
6	1.5E+08	1.5E+08	KD2-H4K8la_peak_5276	10.49982	LATS1_ENSG00000131023
6	1.5E+08	1.5E+08	KD2-H4K8la_peak_5277	5.67596	
6	1.5E+08	1.5E+08	KD2-H4K8la_peak_5278	8.37064	
6	1.5E+08	1.5E+08	KD2-H4K8la_peak_5279	8.37064	
6	1.5E+08	1.5E+08	KD2-H4K8la_peak_5280	9.6708	
6	1.5E+08	1.5E+08	KD2-H4K8la_peak_5281	5.58883	
6	1.5E+08	1.5E+08	KD2-H4K8la_peak_5282	6.70626	
6	1.5E+08	1.5E+08	KD2-H4K8la_peak_5283	7.3036	RGS17_ENSG00000091844
6	1.6E+08	1.6E+08	KD2-H4K8la_peak_5284	6.97699	SCAF8_ENSG00000213079
6	1.6E+08	1.6E+08	KD2-H4K8la_peak_5285	7.13237	
6	1.6E+08	1.6E+08	KD2-H4K8la_peak_5286	5.05146	
6	1.6E+08	1.6E+08	KD2-H4K8la_peak_5287	9.9701	SNX9_ENSG00000130340
6	1.6E+08	1.6E+08	KD2-H4K8la_peak_5288	6.88801	

6	1.6E+08	1.6E+08	KD2-H4K8la_peak_5289	5.58883	C6orf99_ENSG00000203711
6	1.6E+08	1.6E+08	KD2-H4K8la_peak_5290	5.58883	
6	1.6E+08	1.6E+08	KD2-H4K8la_peak_5291	10.04111	TCP1_ENSG00000120438;MRPL18_ENSG00000112110
6	1.6E+08	1.6E+08	KD2-H4K8la_peak_5292	7.45264	PARK2_ENSG00000185345;PACRG_ENSG00000112530
6	1.6E+08	1.6E+08	KD2-H4K8la_peak_5293	5.78071	
6	1.7E+08	1.7E+08	KD2-H4K8la_peak_5294	6.3399	
6	1.7E+08	1.7E+08	KD2-H4K8la_peak_5295	11.60966	SFT2D1_ENSG00000198818
6	1.7E+08	1.7E+08	KD2-H4K8la_peak_5296	8.76116	SFT2D1_ENSG00000198818
6	1.7E+08	1.7E+08	KD2-H4K8la_peak_5297	15.42498	
6	1.7E+08	1.7E+08	KD2-H4K8la_peak_5298	8.73956	
6	1.7E+08	1.7E+08	KD2-H4K8la_peak_5299	5.37421	
6	1.7E+08	1.7E+08	KD2-H4K8la_peak_5300	4.66473	
6	1.7E+08	1.7E+08	KD2-H4K8la_peak_5301	12.97881	
6	1.7E+08	1.7E+08	KD2-H4K8la_peak_5302	8.19799	
6	1.7E+08	1.7E+08	KD2-H4K8la_peak_5303	5.83285	
6	1.7E+08	1.7E+08	KD2-H4K8la_peak_5304	8.37064	
6	1.7E+08	1.7E+08	KD2-H4K8la_peak_5305	10.04111	MLLT4-AS1_ENSG00000198221;MLLT4_ENSG00000130396
6	1.7E+08	1.7E+08	KD2-H4K8la_peak_5306	7.64726	
6	1.7E+08	1.7E+08	KD2-H4K8la_peak_5307	10.34978	XXyac-YX65C7_A.2_ENSG00000226445
6	1.7E+08	1.7E+08	KD2-H4K8la_peak_5308	15.75402	PHF10_ENSG00000130024
6	1.7E+08	1.7E+08	KD2-H4K8la_peak_5309	5.83285	
6	1.7E+08	1.7E+08	KD2-H4K8la_peak_5310	9.66714	
7	149749	150591	KD2-H4K8la_peak_5311	13.84838	AC093627.9_ENSG00000242474;AC093627.10_ENSG00000240859
7	495143	495748	KD2-H4K8la_peak_5312	7.55141	
7	533145	534084	KD2-H4K8la_peak_5313	15.50379	
7	751501	752187	KD2-H4K8la_peak_5314	10.87112	
7	752425	752666	KD2-H4K8la_peak_5315	13.14693	
7	765394	765631	KD2-H4K8la_peak_5316	8.44557	HEATR2_ENSG00000164818
7	766541	766836	KD2-H4K8la_peak_5317	6.93796	PRKAR1B_ENSG00000188191;HEATR2_ENSG00000164818
7	808523	808791	KD2-H4K8la_peak_5318	16.1269	
7	810016	810480	KD2-H4K8la_peak_5319	10.16316	
7	941536	941780	KD2-H4K8la_peak_5320	9.61894	
7	949188	949427	KD2-H4K8la_peak_5321	7.12014	
7	1136407	1136616	KD2-H4K8la_peak_5322	6.34062	
7	1178192	1178495	KD2-H4K8la_peak_5323	6.47589	C7orf50_ENSG00000146540
7	1200116	1200703	KD2-H4K8la_peak_5324	15.88392	ZFAND2A_ENSG00000178381;AC091729.9_ENSG00000229043
7	1215450	1215798	KD2-H4K8la_peak_5325	9.61894	
7	1570578	1570832	KD2-H4K8la_peak_5326	8.29917	MAFK_ENSG00000198517
7	1578234	1578474	KD2-H4K8la_peak_5327	4.6555	
7	1608796	1609060	KD2-H4K8la_peak_5328	4.6555	PSMG3-AS1_ENSG00000230487
7	1609386	1609613	KD2-H4K8la_peak_5329	13.84838	PSMG3-AS1_ENSG00000230487
7	1625399	1626123	KD2-H4K8la_peak_5330	10.97465	
7	1626412	1626890	KD2-H4K8la_peak_5331	12.97817	
7	1680229	1681056	KD2-H4K8la_peak_5332	13.18253	
7	1753268	1753524	KD2-H4K8la_peak_5333	5.76341	
7	1878778	1879115	KD2-H4K8la_peak_5334	10.71653	AC110781.3_ENSG00000176349

7	1897104	1897449	KD2-H4K8la_peak_5335	6.77116	
7	2118642	2118886	KD2-H4K8la_peak_5336	8.4237	
7	2143148	2143657	KD2-H4K8la_peak_5337	7.94013	
7	2145298	2145588	KD2-H4K8la_peak_5338	7.63903	
7	2148464	2148871	KD2-H4K8la_peak_5339	10.02511	
7	2177864	2178166	KD2-H4K8la_peak_5340	5.43278	
7	2178472	2178807	KD2-H4K8la_peak_5341	6.68593	
7	2201439	2201766	KD2-H4K8la_peak_5342	10.34952	
7	2281332	2281752	KD2-H4K8la_peak_5343	14.7348	FTSJ2_ENSG00000122687;NUDT1_ENSG00000106268
7	2353763	2353975	KD2-H4K8la_peak_5344	7.23932	
7	2394568	2395105	KD2-H4K8la_peak_5345	8.24908	SNX8_ENSG00000106266;EIF3B_ENSG00000106263
7	2491431	2491780	KD2-H4K8la_peak_5346	6.94579	
7	2535220	2535607	KD2-H4K8la_peak_5347	8.89364	
7	2563878	2564392	KD2-H4K8la_peak_5348	4.6555	
7	2595854	2596136	KD2-H4K8la_peak_5349	9.40293	BRAT1_ENSG00000106009
7	2661557	2661766	KD2-H4K8la_peak_5350	7.01929	
7	2671663	2671986	KD2-H4K8la_peak_5351	14.86256	TTYH3_ENSG00000136295
7	2684642	2684891	KD2-H4K8la_peak_5352	6.36953	
7	2685381	2685643	KD2-H4K8la_peak_5353	7.23932	
7	2969375	2969657	KD2-H4K8la_peak_5354	5.80814	
7	3283202	3283566	KD2-H4K8la_peak_5355	8.83119	
7	4091275	4091596	KD2-H4K8la_peak_5356	13.01988	
7	4183531	4184087	KD2-H4K8la_peak_5357	10.16316	
7	4184867	4185677	KD2-H4K8la_peak_5358	6.37643	
7	4721226	4721739	KD2-H4K8la_peak_5359	12.72886	
7	4814486	4814968	KD2-H4K8la_peak_5360	10.64143	AP5Z1_ENSG00000242802
7	5084813	5085237	KD2-H4K8la_peak_5361	8.48577	RBAK_ENSG00000146587
7	5085635	5085901	KD2-H4K8la_peak_5362	4.9591	RBAK_ENSG00000146587
7	5280263	5280470	KD2-H4K8la_peak_5363	9.29385	
7	5315682	5316343	KD2-H4K8la_peak_5364	7.68525	AC093376.1_ENSG00000263808
7	5464662	5464915	KD2-H4K8la_peak_5365	10.02595	TNRC18_ENSG00000182095;RP11-1275H24.2_ENSG00000272953
7	5466051	5466424	KD2-H4K8la_peak_5366	9.19451	RP11-1275H24.2_ENSG00000272953
7	5466921	5467592	KD2-H4K8la_peak_5367	9.40293	RP11-1275H24.1_ENSG00000234432;RP11-1275H24.3_ENSG00000273084
7	5468939	5469405	KD2-H4K8la_peak_5368	6.47266	RP11-1275H24.1_ENSG00000234432;RP11-1275H24.3_ENSG00000273084
7	5553996	5554212	KD2-H4K8la_peak_5369	9.19451	FBXL18_ENSG00000155034
7	5569805	5570156	KD2-H4K8la_peak_5370	16.3941	
7	5570488	5570957	KD2-H4K8la_peak_5371	12.42246	
7	5595836	5596068	KD2-H4K8la_peak_5372	9.92722	CTB-161C1.1_ENSG00000272719
7	5596323	5596627	KD2-H4K8la_peak_5373	5.43278	CTB-161C1.1_ENSG00000272719
7	5601923	5602256	KD2-H4K8la_peak_5374	7.60503	
7	5610061	5610342	KD2-H4K8la_peak_5375	12.72886	
7	5614698	5615180	KD2-H4K8la_peak_5376	8.39919	
7	5634177	5634420	KD2-H4K8la_peak_5377	7.13246	
7	5674296	5674729	KD2-H4K8la_peak_5378	13.27013	
7	5821511	5821865	KD2-H4K8la_peak_5379	6.40658	RNF216_ENSG00000011275
7	5861789	5861987	KD2-H4K8la_peak_5380	9.52146	ZNF815P_ENSG00000235944

7	5862819	5863285	KD2-H4K8la_peak_5381	8.39677	ZNF815P_ENSG00000235944
7	6048316	6048559	KD2-H4K8la_peak_5382	7.94013	PMS2_ENSG00000122512;AIMP2_ENSG00000106305
7	6098503	6098705	KD2-H4K8la_peak_5383	8.84265	EIF2AK1_ENSG00000086232
7	6099100	6099379	KD2-H4K8la_peak_5384	10.27876	EIF2AK1_ENSG00000086232
7	6143870	6144125	KD2-H4K8la_peak_5385	9.9149	USP42_ENSG00000106346
7	6413294	6413844	KD2-H4K8la_peak_5386	11.15747	RAC1_ENSG00000136238
7	6414294	6414522	KD2-H4K8la_peak_5387	4.86896	RAC1_ENSG00000136238
7	6617176	6617733	KD2-H4K8la_peak_5388	8.39919	ZDHHC4_ENSG00000136247
7	6654695	6655061	KD2-H4K8la_peak_5389	9.7321	ZNF853_ENSG00000236609
7	6655368	6655655	KD2-H4K8la_peak_5390	6.3399	ZNF853_ENSG00000236609
7	6676648	6676854	KD2-H4K8la_peak_5391	8.16916	ZNF316_ENSG00000205903
7	6677568	6677772	KD2-H4K8la_peak_5392	6.68593	ZNF316_ENSG00000205903
7	6703655	6703983	KD2-H4K8la_peak_5393	17.74667	AC073343.13_ENSG00000228010
7	6768640	6769279	KD2-H4K8la_peak_5394	16.13548	
7	6769580	6769797	KD2-H4K8la_peak_5395	8.62939	
7	6894643	6895094	KD2-H4K8la_peak_5396	10.49982	
7	7396818	7397037	KD2-H4K8la_peak_5397	8.12047	
7	7701916	7702219	KD2-H4K8la_peak_5398	6.79079	
7	8300857	8302299	KD2-H4K8la_peak_5399	20.29575	ICA1_ENSG00000003147;AC007009.1_ENSG00000244239;AC007128.1_ENSG00000229970
7	8331799	8332145	KD2-H4K8la_peak_5400	7.41831	
7	1.3E+07	1.3E+07	KD2-H4K8la_peak_5401	7.12972	ARL4A_ENSG00000122644
7	1.6E+07	1.6E+07	KD2-H4K8la_peak_5402	7.06427	ISPD_ENSG00000214960
7	1.8E+07	1.8E+07	KD2-H4K8la_peak_5403	7.16303	SNX13_ENSG00000071189
7	1.9E+07	1.9E+07	KD2-H4K8la_peak_5404	5.19328	
7	1.9E+07	1.9E+07	KD2-H4K8la_peak_5405	14.15376	
7	1.9E+07	1.9E+07	KD2-H4K8la_peak_5406	5.29444	TWIST1_ENSG00000122691
7	1.9E+07	1.9E+07	KD2-H4K8la_peak_5407	7.12014	TWIST1_ENSG00000122691
7	2E+07	2E+07	KD2-H4K8la_peak_5408	8.07102	TWISTNB_ENSG00000105849
7	2E+07	2E+07	KD2-H4K8la_peak_5409	6.56022	CTA-293F17.1_ENSG00000271133;ITGB8_ENSG00000105855
7	2.3E+07	2.3E+07	KD2-H4K8la_peak_5410	11.77509	TOMM7_ENSG00000196683
7	2.3E+07	2.3E+07	KD2-H4K8la_peak_5411	8.12466	TOMM7_ENSG00000196683
7	2.3E+07	2.3E+07	KD2-H4K8la_peak_5412	6.96161	NUPL2_ENSG00000136243
7	2.3E+07	2.3E+07	KD2-H4K8la_peak_5413	7.12308	AC005082.12_ENSG00000226816
7	2.4E+07	2.4E+07	KD2-H4K8la_peak_5414	6.71169	
7	2.4E+07	2.4E+07	KD2-H4K8la_peak_5415	13.11108	IGF2BP3_ENSG00000136231
7	2.4E+07	2.4E+07	KD2-H4K8la_peak_5416	14.22324	
7	2.4E+07	2.4E+07	KD2-H4K8la_peak_5417	9.10568	RPS2P32_ENSG00000232818
7	2.4E+07	2.4E+07	KD2-H4K8la_peak_5418	6.79903	TRA2A_ENSG00000164548
7	2.4E+07	2.4E+07	KD2-H4K8la_peak_5419	14.44261	FAM221A_ENSG00000188732;AC006026.13_ENSG00000234286
7	2.4E+07	2.4E+07	KD2-H4K8la_peak_5420	13.13367	
7	2.4E+07	2.4E+07	KD2-H4K8la_peak_5421	6.64804	
7	2.4E+07	2.4E+07	KD2-H4K8la_peak_5422	6.3399	NPY_ENSG00000122585
7	2.5E+07	2.5E+07	KD2-H4K8la_peak_5423	9.7321	
7	2.5E+07	2.5E+07	KD2-H4K8la_peak_5424	7.65547	OSBPL3_ENSG00000070882
7	2.5E+07	2.5E+07	KD2-H4K8la_peak_5425	6.09229	C7orf31_ENSG00000153790
7	2.6E+07	2.6E+07	KD2-H4K8la_peak_5426	6.57426	

7	2.6E+07	2.6E+07	KD2-H4K8la_peak_5427	12.32641	
7	2.6E+07	2.6E+07	KD2-H4K8la_peak_5428	6.37643	NFE2L3_ENSG00000050344
7	2.6E+07	2.6E+07	KD2-H4K8la_peak_5429	5.43278	HNRNPA2B1_ENSG00000122566;CBX3_ENSG00000122565
7	2.6E+07	2.6E+07	KD2-H4K8la_peak_5430	7.06427	HNRNPA2B1_ENSG00000122566;CBX3_ENSG00000122565
7	2.6E+07	2.6E+07	KD2-H4K8la_peak_5431	12.43915	HNRNPA2B1_ENSG00000122566;CBX3_ENSG00000122565
7	2.6E+07	2.6E+07	KD2-H4K8la_peak_5432	4.66473	AC004540.4_ENSG00000225792
7	2.7E+07	2.7E+07	KD2-H4K8la_peak_5433	4.53237	
7	2.8E+07	2.8E+07	KD2-H4K8la_peak_5434	6.26214	
7	2.9E+07	2.9E+07	KD2-H4K8la_peak_5435	5.78862	AC005013.5_ENSG00000228421
7	2.9E+07	2.9E+07	KD2-H4K8la_peak_5436	6.9399	AC005013.1_ENSG00000255690;TRIL_ENSG00000176734;AC005013.5_ENSG00000228421
7	3E+07	3E+07	KD2-H4K8la_peak_5437	12.62032	ZNRF2P2_ENSG00000225264
7	3E+07	3E+07	KD2-H4K8la_peak_5438	7.77341	
7	3.1E+07	3.1E+07	KD2-H4K8la_peak_5439	7.0396	
7	3.1E+07	3.1E+07	KD2-H4K8la_peak_5440	7.66936	
7	3.1E+07	3.1E+07	KD2-H4K8la_peak_5441	10.37746	FAM188B_ENSG00000106125
7	3.1E+07	3.1E+07	KD2-H4K8la_peak_5442	6.23074	
7	3.1E+07	3.1E+07	KD2-H4K8la_peak_5443	8.84265	
7	3.1E+07	3.1E+07	KD2-H4K8la_peak_5444	7.3993	
7	3.3E+07	3.3E+07	KD2-H4K8la_peak_5445	7.11805	
7	3.3E+07	3.3E+07	KD2-H4K8la_peak_5446	7.7007	FKBP9_ENSG00000122642
7	3.3E+07	3.3E+07	KD2-H4K8la_peak_5447	6.3399	RP9_ENSG00000164610
7	3.5E+07	3.5E+07	KD2-H4K8la_peak_5448	5.10388	DPY19L1_ENSG00000173852
7	3.6E+07	3.6E+07	KD2-H4K8la_peak_5449	5.67596	
7	3.6E+07	3.6E+07	KD2-H4K8la_peak_5450	11.52725	KIAA0895_ENSG00000164542;ANLN_ENSG00000011426
7	3.7E+07	3.7E+07	KD2-H4K8la_peak_5451	4.86896	
7	3.7E+07	3.7E+07	KD2-H4K8la_peak_5452	7.49467	ELMO1_ENSG00000155849
7	3.8E+07	3.8E+07	KD2-H4K8la_peak_5453	4.69635	
7	3.8E+07	3.8E+07	KD2-H4K8la_peak_5454	8.37064	STARD3NL_ENSG00000010270
7	3.9E+07	3.9E+07	KD2-H4K8la_peak_5455	13.2369	
7	4E+07	4E+07	KD2-H4K8la_peak_5456	5.16795	
7	4E+07	4E+07	KD2-H4K8la_peak_5457	9.12246	RALA_ENSG00000006451
7	4E+07	4E+07	KD2-H4K8la_peak_5458	10.92814	LINC00265_ENSG00000188185
7	4E+07	4E+07	KD2-H4K8la_peak_5459	4.86896	RP11-467D6.1_ENSG00000259826;CDK13_ENSG00000065883
7	4.1E+07	4.1E+07	KD2-H4K8la_peak_5460	8.00569	
7	4.3E+07	4.3E+07	KD2-H4K8la_peak_5461	4.30663	
7	4.4E+07	4.4E+07	KD2-H4K8la_peak_5462	4.60174	BLVRA_ENSG00000106605
7	4.4E+07	4.4E+07	KD2-H4K8la_peak_5463	8.39919	BLVRA_ENSG00000106605
7	4.4E+07	4.4E+07	KD2-H4K8la_peak_5464	8.24908	URGCP-MRPS24_ENSG00000270617
7	4.4E+07	4.4E+07	KD2-H4K8la_peak_5465	6.77116	URGCP_ENSG00000106608;UBE2D4_ENSG00000078967
7	4.4E+07	4.4E+07	KD2-H4K8la_peak_5466	6.68593	
7	4.4E+07	4.4E+07	KD2-H4K8la_peak_5467	8.89364	AEBP1_ENSG00000106624
7	4.4E+07	4.4E+07	KD2-H4K8la_peak_5468	8.62002	
7	4.4E+07	4.4E+07	KD2-H4K8la_peak_5469	8.1293	
7	4.4E+07	4.4E+07	KD2-H4K8la_peak_5470	9.70768	
7	4.4E+07	4.4E+07	KD2-H4K8la_peak_5471	7.7007	
7	4.4E+07	4.4E+07	KD2-H4K8la_peak_5472	9.64487	

7	4.5E+07	4.5E+07	KD2-H4K8la_peak_5473	5.22616	
7	4.5E+07	4.5E+07	KD2-H4K8la_peak_5474	10.49982	TMED4_ENSG00000158604
7	4.5E+07	4.5E+07	KD2-H4K8la_peak_5475	7.55141	ZMIZ2_ENSG00000122515
7	4.5E+07	4.5E+07	KD2-H4K8la_peak_5476	8.16787	
7	4.5E+07	4.5E+07	KD2-H4K8la_peak_5477	13.85062	PPIA_ENSG00000196262
7	4.5E+07	4.5E+07	KD2-H4K8la_peak_5478	11.40266	PURB_ENSG00000146676;RP4-673M15.1_ENSG00000272768
7	4.5E+07	4.5E+07	KD2-H4K8la_peak_5479	10.02595	PURB_ENSG00000146676;RP4-673M15.1_ENSG00000272768
7	4.5E+07	4.5E+07	KD2-H4K8la_peak_5480	6.68593	SNHG15_ENSG00000232956
7	4.6E+07	4.6E+07	KD2-H4K8la_peak_5481	8.89364	ADCY1_ENSG00000164742
7	4.6E+07	4.6E+07	KD2-H4K8la_peak_5482	9.78176	
7	4.6E+07	4.6E+07	KD2-H4K8la_peak_5483	7.23932	
7	4.6E+07	4.6E+07	KD2-H4K8la_peak_5484	9.20163	SEPT7P2_ENSG00000214765
7	4.8E+07	4.8E+07	KD2-H4K8la_peak_5485	7.3993	
7	4.8E+07	4.8E+07	KD2-H4K8la_peak_5486	7.60503	
7	4.8E+07	4.8E+07	KD2-H4K8la_peak_5487	8.71024	
7	4.8E+07	4.8E+07	KD2-H4K8la_peak_5488	7.55141	
7	4.8E+07	4.8E+07	KD2-H4K8la_peak_5489	15.63685	
7	4.8E+07	4.8E+07	KD2-H4K8la_peak_5490	8.73956	
7	4.8E+07	4.8E+07	KD2-H4K8la_peak_5491	8.76116	
7	5.1E+07	5.1E+07	KD2-H4K8la_peak_5492	9.17646	FIGNL1_ENSG00000132436
7	5.1E+07	5.1E+07	KD2-H4K8la_peak_5493	8.33263	
7	5.1E+07	5.1E+07	KD2-H4K8la_peak_5494	5.99671	
7	5.6E+07	5.6E+07	KD2-H4K8la_peak_5495	7.13528	VOPP1_ENSG00000154978;RP11-310H4.1_ENSG00000223475
7	5.6E+07	5.6E+07	KD2-H4K8la_peak_5496	8.39919	GBAS_ENSG00000146729;MRPS17_ENSG00000239789
7	5.6E+07	5.6E+07	KD2-H4K8la_peak_5497	6.47266	
7	5.6E+07	5.6E+07	KD2-H4K8la_peak_5498	7.49467	SUMF2_ENSG00000129103
7	6.4E+07	6.4E+07	KD2-H4K8la_peak_5499	8.13557	
7	6.4E+07	6.4E+07	KD2-H4K8la_peak_5500	5.43278	
7	6.4E+07	6.4E+07	KD2-H4K8la_peak_5501	6.96161	ZNF107_ENSG00000196247
7	6.4E+07	6.4E+07	KD2-H4K8la_peak_5502	9.81238	ZNF138_ENSG00000197008
7	6.4E+07	6.4E+07	KD2-H4K8la_peak_5503	4.69635	ZNF117_ENSG00000152926;ERV3-1_ENSG00000213462
7	6.5E+07	6.5E+07	KD2-H4K8la_peak_5504	6.56022	
7	6.5E+07	6.5E+07	KD2-H4K8la_peak_5505	12.83706	GUSB_ENSG00000169919
7	6.6E+07	6.6E+07	KD2-H4K8la_peak_5506	10.87112	CRCP_ENSG00000241258
7	6.6E+07	6.6E+07	KD2-H4K8la_peak_5507	8.02952	
7	6.6E+07	6.6E+07	KD2-H4K8la_peak_5508	7.41831	TPST1_ENSG00000169902
7	6.6E+07	6.6E+07	KD2-H4K8la_peak_5509	5.28646	
7	6.6E+07	6.6E+07	KD2-H4K8la_peak_5510	5.90392	GS1-124K5.3_ENSG00000223473;GS1-124K5.4_ENSG00000237310
7	6.6E+07	6.6E+07	KD2-H4K8la_peak_5511	6.54523	KCTD7_ENSG00000243335
7	6.6E+07	6.6E+07	KD2-H4K8la_peak_5512	9.29385	RP4-756H11.3_ENSG00000226824
7	6.6E+07	6.6E+07	KD2-H4K8la_peak_5513	6.68593	GTF2IRD1P1_ENSG00000230583
7	6.6E+07	6.6E+07	KD2-H4K8la_peak_5514	10.66741	TMEM248_ENSG00000106609
7	6.6E+07	6.6E+07	KD2-H4K8la_peak_5515	9.78176	SBDS_ENSG00000126524;TYW1_ENSG00000198874
7	6.9E+07	6.9E+07	KD2-H4K8la_peak_5516	9.67249	RP5-942I16.1_ENSG00000234215;AUTS2_ENSG00000158321
7	7.2E+07	7.2E+07	KD2-H4K8la_peak_5517	5.78071	
7	7.2E+07	7.2E+07	KD2-H4K8la_peak_5518	5.5899	

7	7.3E+07	7.3E+07	KD2-H4K8la_peak_5519	9.41838	NSUN5_ENSG00000130305
7	7.3E+07	7.3E+07	KD2-H4K8la_peak_5520	11.40266	
7	7.3E+07	7.3E+07	KD2-H4K8la_peak_5521	12.32641	
7	7.3E+07	7.3E+07	KD2-H4K8la_peak_5522	9.67249	
7	7.3E+07	7.3E+07	KD2-H4K8la_peak_5523	12.49088	BAZ1B_ENSG00000009954
7	7.3E+07	7.3E+07	KD2-H4K8la_peak_5524	12.17279	BCL7B_ENSG00000106635
7	7.3E+07	7.3E+07	KD2-H4K8la_peak_5525	7.53542	DNAJC30_ENSG00000176410;WBSCR22_ENSG00000071462
7	7.3E+07	7.3E+07	KD2-H4K8la_peak_5526	7.94013	ABHD11_ENSG00000106077
7	7.3E+07	7.3E+07	KD2-H4K8la_peak_5527	8.78491	WBSCR27_ENSG00000165171
7	7.3E+07	7.3E+07	KD2-H4K8la_peak_5528	9.93607	
7	7.3E+07	7.3E+07	KD2-H4K8la_peak_5529	11.7362	LIMK1_ENSG00000106683
7	7.3E+07	7.3E+07	KD2-H4K8la_peak_5530	5.84385	
7	7.4E+07	7.4E+07	KD2-H4K8la_peak_5531	4.66473	
7	7.4E+07	7.4E+07	KD2-H4K8la_peak_5532	6.3399	
7	7.4E+07	7.4E+07	KD2-H4K8la_peak_5533	5.27164	EIF4H_ENSG00000106682
7	7.4E+07	7.4E+07	KD2-H4K8la_peak_5534	12.12824	EIF4H_ENSG00000106682
7	7.4E+07	7.4E+07	KD2-H4K8la_peak_5535	6.64804	
7	7.4E+07	7.4E+07	KD2-H4K8la_peak_5536	12.14568	
7	7.4E+07	7.4E+07	KD2-H4K8la_peak_5537	4.81796	CLIP2_ENSG00000106665
7	7.4E+07	7.4E+07	KD2-H4K8la_peak_5538	6.50951	CLIP2_ENSG00000106665
7	7.4E+07	7.4E+07	KD2-H4K8la_peak_5539	6.47266	GTF2IRD1_ENSG00000006704
7	7.4E+07	7.4E+07	KD2-H4K8la_peak_5540	5.99671	GTF2IRD1_ENSG00000006704
7	7.4E+07	7.4E+07	KD2-H4K8la_peak_5541	7.94013	
7	7.4E+07	7.4E+07	KD2-H4K8la_peak_5542	5.99671	RP5-1186P10.2_ENSG00000273069
7	7.4E+07	7.4E+07	KD2-H4K8la_peak_5543	10.47118	GTF2I_ENSG00000077809
7	7.4E+07	7.4E+07	KD2-H4K8la_peak_5544	31.11291	GTF2I_ENSG00000077809
7	7.4E+07	7.4E+07	KD2-H4K8la_peak_5545	5.84385	WBSCR16_ENSG00000174374
7	7.4E+07	7.4E+07	KD2-H4K8la_peak_5546	14.15376	WBSCR16_ENSG00000174374
7	7.5E+07	7.5E+07	KD2-H4K8la_peak_5547	10.49982	POM121C_ENSG00000135213;AC006014.7_ENSG00000242073
7	7.5E+07	7.5E+07	KD2-H4K8la_peak_5548	5.80814	PMS2P3_ENSG00000127957
7	7.5E+07	7.5E+07	KD2-H4K8la_peak_5549	7.19244	PMS2P3_ENSG00000127957
7	7.5E+07	7.5E+07	KD2-H4K8la_peak_5550	7.33892	
7	7.5E+07	7.5E+07	KD2-H4K8la_peak_5551	14.44261	HIP1_ENSG00000127946
7	7.6E+07	7.6E+07	KD2-H4K8la_peak_5552	9.27415	
7	7.6E+07	7.6E+07	KD2-H4K8la_peak_5553	6.35027	
7	7.6E+07	7.6E+07	KD2-H4K8la_peak_5554	6.68593	
7	7.6E+07	7.6E+07	KD2-H4K8la_peak_5555	11.04269	
7	7.6E+07	7.6E+07	KD2-H4K8la_peak_5556	11.50321	
7	7.6E+07	7.6E+07	KD2-H4K8la_peak_5557	7.94013	HSPB1_ENSG00000106211
7	7.6E+07	7.6E+07	KD2-H4K8la_peak_5558	8.06101	
7	7.6E+07	7.6E+07	KD2-H4K8la_peak_5559	11.94113	
7	7.6E+07	7.6E+07	KD2-H4K8la_peak_5560	8.0333	
7	7.6E+07	7.6E+07	KD2-H4K8la_peak_5561	9.92226	
7	7.6E+07	7.6E+07	KD2-H4K8la_peak_5562	6.23074	
7	7.6E+07	7.6E+07	KD2-H4K8la_peak_5563	9.44217	
7	7.6E+07	7.6E+07	KD2-H4K8la_peak_5564	8.77936	DTX2_ENSG00000091073

7	7.6E+07	7.6E+07	KD2-H4K8la_peak_5565	6.18829	UPK3B_ENSG00000243566
7	7.6E+07	7.6E+07	KD2-H4K8la_peak_5566	11.40266	AC004980.7_ENSG00000205485
7	7.7E+07	7.7E+07	KD2-H4K8la_peak_5567	5.23773	GSAP_ENSG00000186088;RP5-899E9.1_ENSG00000273341
7	7.7E+07	7.7E+07	KD2-H4K8la_peak_5568	6.57176	RSBN1L-AS1_ENSG00000214293;RSBN1L_ENSG00000187257
7	8.2E+07	8.2E+07	KD2-H4K8la_peak_5569	11.57314	CACNA2D1_ENSG00000153956
7	8.4E+07	8.4E+07	KD2-H4K8la_peak_5570	6.26214	
7	8.5E+07	8.5E+07	KD2-H4K8la_peak_5571	7.13237	
7	8.7E+07	8.7E+07	KD2-H4K8la_peak_5572	6.9399	TMEM243_ENSG00000135185
7	8.7E+07	8.7E+07	KD2-H4K8la_peak_5573	6.64804	
7	9E+07	9E+07	KD2-H4K8la_peak_5574	5.99671	
7	9.2E+07	9.2E+07	KD2-H4K8la_peak_5575	6.13507	MTERF_ENSG00000127989
7	9.2E+07	9.2E+07	KD2-H4K8la_peak_5576	5.8369	CTB-161K23.1_ENSG00000188693
7	9.2E+07	9.2E+07	KD2-H4K8la_peak_5577	14.09647	KRIT1_ENSG00000001631;ANKIB1_ENSG00000001629
7	9.2E+07	9.2E+07	KD2-H4K8la_peak_5578	11.15747	GATAD1_ENSG00000157259
7	9.2E+07	9.2E+07	KD2-H4K8la_peak_5579	9.40293	GATAD1_ENSG00000157259
7	9.2E+07	9.2E+07	KD2-H4K8la_peak_5580	7.90495	
7	9.2E+07	9.2E+07	KD2-H4K8la_peak_5581	10.19807	
7	9.2E+07	9.2E+07	KD2-H4K8la_peak_5582	8.37064	
7	9.4E+07	9.4E+07	KD2-H4K8la_peak_5583	8.13557	GNG11_ENSG00000127920
7	9.4E+07	9.4E+07	KD2-H4K8la_peak_5584	8.39919	CASD1_ENSG00000127995
7	9.4E+07	9.4E+07	KD2-H4K8la_peak_5585	16.42606	
7	9.4E+07	9.4E+07	KD2-H4K8la_peak_5586	7.61975	SGCE_ENSG00000127990;PEG10_ENSG00000242265
7	9.5E+07	9.5E+07	KD2-H4K8la_peak_5587	7.3993	PPP1R9A_ENSG00000158528
7	9.6E+07	9.6E+07	KD2-H4K8la_peak_5588	7.12308	SLC25A13_ENSG00000004864
7	9.7E+07	9.7E+07	KD2-H4K8la_peak_5589	7.12972	
7	9.7E+07	9.7E+07	KD2-H4K8la_peak_5590	6.2278	
7	9.8E+07	9.8E+07	KD2-H4K8la_peak_5591	10.20357	AC004967.7_ENSG00000243554
7	9.8E+07	9.8E+07	KD2-H4K8la_peak_5592	6.3399	AC004967.7_ENSG00000243554
7	9.8E+07	9.8E+07	KD2-H4K8la_peak_5593	11.7362	LMTK2_ENSG00000164715
7	9.8E+07	9.8E+07	KD2-H4K8la_peak_5594	8.03465	
7	9.8E+07	9.8E+07	KD2-H4K8la_peak_5595	9.78176	
7	9.8E+07	9.8E+07	KD2-H4K8la_peak_5596	7.3993	
7	9.8E+07	9.8E+07	KD2-H4K8la_peak_5597	7.61975	
7	9.8E+07	9.8E+07	KD2-H4K8la_peak_5598	10.49982	NPTX2_ENSG00000106236
7	9.8E+07	9.8E+07	KD2-H4K8la_peak_5599	10.64143	TRRAP_ENSG00000196367
7	9.9E+07	9.9E+07	KD2-H4K8la_peak_5600	11.15747	SMURF1_ENSG00000198742
7	9.9E+07	9.9E+07	KD2-H4K8la_peak_5601	7.91061	ARPC1A_ENSG00000241685
7	9.9E+07	9.9E+07	KD2-H4K8la_peak_5602	8.09203	PDAP1_ENSG00000106244;BUD31_ENSG00000106245
7	9.9E+07	9.9E+07	KD2-H4K8la_peak_5603	10.02595	PTCD1_ENSG00000106246;ATP5J2-PTCD1_ENSG00000248919;ATP5J2_ENSG00000241468
7	9.9E+07	9.9E+07	KD2-H4K8la_peak_5604	8.50453	
7	9.9E+07	9.9E+07	KD2-H4K8la_peak_5605	5.78862	ZKSCAN5_ENSG00000196652
7	9.9E+07	9.9E+07	KD2-H4K8la_peak_5606	11.20341	FAM200A_ENSG00000221909;ZNF655_ENSG00000197343;GS1-259H13.10_ENSG00000272647
7	9.9E+07	9.9E+07	KD2-H4K8la_peak_5607	7.30491	
7	9.9E+07	9.9E+07	KD2-H4K8la_peak_5608	9.37943	ZSCAN25_ENSG00000197037
7	1E+08	1E+08	KD2-H4K8la_peak_5609	21.96182	ZSCAN21_ENSG00000166529
7	1E+08	1E+08	KD2-H4K8la_peak_5610	12.20736	ZNF3_ENSG00000166526

7	1E+08	1E+08	KD2-H4K8la_peak_5611	6.47266	MCM7_ENSG00000166508;AP4M1_ENSG00000221838
7	1E+08	1E+08	KD2-H4K8la_peak_5612	9.36765	RP11-506M12.1_ENSG00000242798;MBLAC1_ENSG00000214309
7	1E+08	1E+08	KD2-H4K8la_peak_5613	4.86896	
7	1E+08	1E+08	KD2-H4K8la_peak_5614	7.12308	ZCWPW1_ENSG00000078487;MEPCE_ENSG00000146834
7	1E+08	1E+08	KD2-H4K8la_peak_5615	17.48572	ZCWPW1_ENSG00000078487
7	1E+08	1E+08	KD2-H4K8la_peak_5616	13.95071	RP11-758P17.2_ENSG00000241357;PPP1R35_ENSG00000160813;RP11-758P17.3_ENSG00000240211
7	1E+08	1E+08	KD2-H4K8la_peak_5617	9.41838	PPP1R35_ENSG00000160813;RP11-758P17.3_ENSG00000240211
7	1E+08	1E+08	KD2-H4K8la_peak_5618	9.78176	
7	1E+08	1E+08	KD2-H4K8la_peak_5619	8.48577	
7	1E+08	1E+08	KD2-H4K8la_peak_5620	5.22418	RP11-44M6.3_ENSG00000184414
7	1E+08	1E+08	KD2-H4K8la_peak_5621	5.43278	MOSPD3_ENSG00000106330
7	1E+08	1E+08	KD2-H4K8la_peak_5622	8.48488	GNB2_ENSG00000172354
7	1E+08	1E+08	KD2-H4K8la_peak_5623	11.05839	
7	1E+08	1E+08	KD2-H4K8la_peak_5624	7.88822	
7	1E+08	1E+08	KD2-H4K8la_peak_5625	8.94106	EPHB4_ENSG00000196411;SLC12A9_ENSG00000146828
7	1E+08	1E+08	KD2-H4K8la_peak_5626	5.27164	SRRT_ENSG00000087087
7	1E+08	1E+08	KD2-H4K8la_peak_5627	8.13557	UFSP1_ENSG00000176125
7	1E+08	1E+08	KD2-H4K8la_peak_5628	10.16316	
7	1E+08	1E+08	KD2-H4K8la_peak_5629	10.04113	NAT16_ENSG00000167011
7	1E+08	1E+08	KD2-H4K8la_peak_5630	7.03956	
7	1E+08	1E+08	KD2-H4K8la_peak_5631	6.12632	RP11-132A1.4_ENSG00000232445
7	1E+08	1E+08	KD2-H4K8la_peak_5632	9.98668	
7	1E+08	1E+08	KD2-H4K8la_peak_5633	4.30663	
7	1E+08	1E+08	KD2-H4K8la_peak_5634	28.5812	
7	1E+08	1E+08	KD2-H4K8la_peak_5635	10.00612	PRKRIP1_ENSG00000128563;RP11-163E9.2_ENSG00000239969
7	1E+08	1E+08	KD2-H4K8la_peak_5636	7.77341	
7	1E+08	1E+08	KD2-H4K8la_peak_5637	14.44261	ORAI2_ENSG00000160991
7	1E+08	1E+08	KD2-H4K8la_peak_5638	12.33257	ALKBH4_ENSG00000160993;LRWD1_ENSG00000161036
7	1E+08	1E+08	KD2-H4K8la_peak_5639	7.68525	
7	1E+08	1E+08	KD2-H4K8la_peak_5640	13.13367	
7	1E+08	1E+08	KD2-H4K8la_peak_5641	9.61894	DPY19L2P2_ENSG00000170629
7	1E+08	1E+08	KD2-H4K8la_peak_5642	12.32641	DPY19L2P2_ENSG00000170629
7	1E+08	1E+08	KD2-H4K8la_peak_5643	9.92226	DNAJC2_ENSG00000105821;PSMC2_ENSG00000161057
7	1E+08	1E+08	KD2-H4K8la_peak_5644	8.03615	SLC26A5_ENSG00000170615;CTB-107G13.1_ENSG00000234715
7	1E+08	1E+08	KD2-H4K8la_peak_5645	6.86458	
7	1E+08	1E+08	KD2-H4K8la_peak_5646	6.35646	RELN_ENSG00000189056
7	1E+08	1E+08	KD2-H4K8la_peak_5647	6.86458	RP11-325F22.2_ENSG00000237513
7	1E+08	1E+08	KD2-H4K8la_peak_5648	7.84781	LINC01004_ENSG00000228393;KMT2E-AS1_ENSG00000239569;KMT2E_ENSG00000005483
7	1.1E+08	1.1E+08	KD2-H4K8la_peak_5649	10.59471	SYPL1_ENSG00000008282
7	1.1E+08	1.1E+08	KD2-H4K8la_peak_5650	7.56395	CTB-30L5.1_ENSG00000267052
7	1.1E+08	1.1E+08	KD2-H4K8la_peak_5651	4.6555	CCDC71L_ENSG00000253276;AC004917.1_ENSG00000177820
7	1.1E+08	1.1E+08	KD2-H4K8la_peak_5652	5.33301	COG5_ENSG00000164597;DUS4L_ENSG00000105865
7	1.1E+08	1.1E+08	KD2-H4K8la_peak_5653	7.23932	RP4-593H12.1_ENSG00000272854;BCAP29_ENSG00000075790
7	1.1E+08	1.1E+08	KD2-H4K8la_peak_5654	11.05785	
7	1.1E+08	1.1E+08	KD2-H4K8la_peak_5655	7.59874	
7	1.1E+08	1.1E+08	KD2-H4K8la_peak_5656	6.3399	

7	1.1E+08	1.1E+08	KD2-H4K8la_peak_5657	6.89612	
7	1.1E+08	1.1E+08	KD2-H4K8la_peak_5658	11.15182	
7	1.1E+08	1.1E+08	KD2-H4K8la_peak_5659	10.0408	C7orf60_ENSG00000164603
7	1.1E+08	1.1E+08	KD2-H4K8la_peak_5660	7.13237	
7	1.2E+08	1.2E+08	KD2-H4K8la_peak_5661	10.31182	ST7-AS1_ENSG00000227199;ST7_ENSG00000004866;ST7-OT4_ENSG00000214188
7	1.2E+08	1.2E+08	KD2-H4K8la_peak_5662	9.61235	
7	1.2E+08	1.2E+08	KD2-H4K8la_peak_5663	8.39813	ANKRD7_ENSG00000106013
7	1.2E+08	1.2E+08	KD2-H4K8la_peak_5664	11.76151	ANKRD7_ENSG00000106013
7	1.2E+08	1.2E+08	KD2-H4K8la_peak_5665	5.48079	
7	1.2E+08	1.2E+08	KD2-H4K8la_peak_5666	16.54584	TMEM229A_ENSG00000234224;RP5-921G16.1_ENSG00000242593
7	1.2E+08	1.2E+08	KD2-H4K8la_peak_5667	8.78491	
7	1.3E+08	1.3E+08	KD2-H4K8la_peak_5668	6.75222	
7	1.3E+08	1.3E+08	KD2-H4K8la_peak_5669	13.27013	ARF5_ENSG00000004059
7	1.3E+08	1.3E+08	KD2-H4K8la_peak_5670	6.86458	ARF5_ENSG00000004059
7	1.3E+08	1.3E+08	KD2-H4K8la_peak_5671	9.14592	
7	1.3E+08	1.3E+08	KD2-H4K8la_peak_5672	13.12418	
7	1.3E+08	1.3E+08	KD2-H4K8la_peak_5673	7.30491	
7	1.3E+08	1.3E+08	KD2-H4K8la_peak_5674	8.66164	
7	1.3E+08	1.3E+08	KD2-H4K8la_peak_5675	12.17279	RP11-62J1.4_ENSG00000272915
7	1.3E+08	1.3E+08	KD2-H4K8la_peak_5676	6.94298	
7	1.3E+08	1.3E+08	KD2-H4K8la_peak_5677	18.16096	
7	1.3E+08	1.3E+08	KD2-H4K8la_peak_5678	13.97752	
7	1.3E+08	1.3E+08	KD2-H4K8la_peak_5679	17.35808	PRRT4_ENSG00000224940
7	1.3E+08	1.3E+08	KD2-H4K8la_peak_5680	7.3993	
7	1.3E+08	1.3E+08	KD2-H4K8la_peak_5681	7.92775	
7	1.3E+08	1.3E+08	KD2-H4K8la_peak_5682	6.12473	
7	1.3E+08	1.3E+08	KD2-H4K8la_peak_5683	5.90392	
7	1.3E+08	1.3E+08	KD2-H4K8la_peak_5684	7.52655	RP11-212P7.3_ENSG00000273184;HILPDA_ENSG00000135245;RP11-155G14.6_ENSG00000240758
7	1.3E+08	1.3E+08	KD2-H4K8la_peak_5685	5.43278	METTL2B_ENSG00000165055
7	1.3E+08	1.3E+08	KD2-H4K8la_peak_5686	10.34952	RP11-212P7.2_ENSG00000273270;RP11-274B21.1_ENSG00000242588
7	1.3E+08	1.3E+08	KD2-H4K8la_peak_5687	7.3993	
7	1.3E+08	1.3E+08	KD2-H4K8la_peak_5688	9.38376	RP11-309L24.2_ENSG00000242902;ATP6V1F_ENSG00000128524
7	1.3E+08	1.3E+08	KD2-H4K8la_peak_5689	7.79412	IRF5_ENSG00000128604
7	1.3E+08	1.3E+08	KD2-H4K8la_peak_5690	11.52725	TNPO3_ENSG00000064419;TPI1P2_ENSG00000230359
7	1.3E+08	1.3E+08	KD2-H4K8la_peak_5691	8.62939	SMO_ENSG00000128602
7	1.3E+08	1.3E+08	KD2-H4K8la_peak_5692	6.20793	RP11-448A19.1_ENSG00000273329;NRF1_ENSG00000106459
7	1.3E+08	1.3E+08	KD2-H4K8la_peak_5693	5.99671	
7	1.3E+08	1.3E+08	KD2-H4K8la_peak_5694	9.61894	
7	1.3E+08	1.3E+08	KD2-H4K8la_peak_5695	8.7731	
7	1.3E+08	1.3E+08	KD2-H4K8la_peak_5696	9.35473	ZC3HC1_ENSG00000091732
7	1.3E+08	1.3E+08	KD2-H4K8la_peak_5697	6.64804	
7	1.3E+08	1.3E+08	KD2-H4K8la_peak_5698	5.67596	
7	1.3E+08	1.3E+08	KD2-H4K8la_peak_5699	7.92775	
7	1.3E+08	1.3E+08	KD2-H4K8la_peak_5700	11.32969	CPA1_ENSG00000091704
7	1.3E+08	1.3E+08	KD2-H4K8la_peak_5701	10.31182	
7	1.3E+08	1.3E+08	KD2-H4K8la_peak_5702	12.35598	LINC-PINT_ENSG00000231721;MKLN1_ENSG00000128585

7	1.3E+08	1.3E+08	KD2-H4K8la_peak_5703	11.42932	MKLN1-AS_ENSG00000236753
7	1.3E+08	1.3E+08	KD2-H4K8la_peak_5704	5.50657	
7	1.3E+08	1.3E+08	KD2-H4K8la_peak_5705	13.27013	PODXL_ENSG00000128567
7	1.3E+08	1.3E+08	KD2-H4K8la_peak_5706	7.77341	
7	1.3E+08	1.3E+08	KD2-H4K8la_peak_5707	5.67596	
7	1.3E+08	1.3E+08	KD2-H4K8la_peak_5708	5.33301	
7	1.3E+08	1.3E+08	KD2-H4K8la_peak_5709	9.0413	
7	1.3E+08	1.3E+08	KD2-H4K8la_peak_5710	5.41509	
7	1.3E+08	1.3E+08	KD2-H4K8la_peak_5711	8.84474	
7	1.3E+08	1.3E+08	KD2-H4K8la_peak_5712	4.62617	
7	1.3E+08	1.3E+08	KD2-H4K8la_peak_5713	5.49688	
7	1.3E+08	1.3E+08	KD2-H4K8la_peak_5714	5.33301	
7	1.3E+08	1.3E+08	KD2-H4K8la_peak_5715	5.27164	
7	1.3E+08	1.3E+08	KD2-H4K8la_peak_5716	6.47266	
7	1.3E+08	1.3E+08	KD2-H4K8la_peak_5717	11.15747	
7	1.3E+08	1.3E+08	KD2-H4K8la_peak_5718	8.944	SLC35B4_ENSG00000205060
7	1.3E+08	1.3E+08	KD2-H4K8la_peak_5719	10.92814	AKR1B1_ENSG00000085662
7	1.3E+08	1.3E+08	KD2-H4K8la_peak_5720	9.03222	C7orf49_ENSG00000122783
7	1.4E+08	1.4E+08	KD2-H4K8la_peak_5721	6.76424	
7	1.4E+08	1.4E+08	KD2-H4K8la_peak_5722	7.49467	
7	1.4E+08	1.4E+08	KD2-H4K8la_peak_5723	10.65691	
7	1.4E+08	1.4E+08	KD2-H4K8la_peak_5724	6.86458	
7	1.4E+08	1.4E+08	KD2-H4K8la_peak_5725	8.0333	KIAA1549_ENSG00000122778
7	1.4E+08	1.4E+08	KD2-H4K8la_peak_5726	7.30347	ZC3HAV1_ENSG00000105939
7	1.4E+08	1.4E+08	KD2-H4K8la_peak_5727	10.70284	LUC7L2_ENSG00000269955
7	1.4E+08	1.4E+08	KD2-H4K8la_peak_5728	5.49688	RP11-634H22.1_ENSG00000273391
7	1.4E+08	1.4E+08	KD2-H4K8la_peak_5729	10.70284	RP11-634H22.1_ENSG00000273391
7	1.4E+08	1.4E+08	KD2-H4K8la_peak_5730	6.64804	HIPK2_ENSG00000064393;TBXAS1_ENSG00000059377
7	1.4E+08	1.4E+08	KD2-H4K8la_peak_5731	10.37746	
7	1.4E+08	1.4E+08	KD2-H4K8la_peak_5732	10.02595	
7	1.4E+08	1.4E+08	KD2-H4K8la_peak_5733	8.78491	
7	1.4E+08	1.4E+08	KD2-H4K8la_peak_5734	5.22616	DENND2A_ENSG00000146966;ADCK2_ENSG00000133597
7	1.4E+08	1.4E+08	KD2-H4K8la_peak_5735	6.86458	NDUFB2-AS1_ENSG00000240889
7	1.4E+08	1.4E+08	KD2-H4K8la_peak_5736	12.32641	BRAF_ENSG00000157764
7	1.4E+08	1.4E+08	KD2-H4K8la_peak_5737	8.7731	MRPS33_ENSG00000090263
7	1.4E+08	1.4E+08	KD2-H4K8la_peak_5738	9.32091	
7	1.4E+08	1.4E+08	KD2-H4K8la_peak_5739	12.97817	RP11-744I24.2_ENSG00000261570;AGK_ENSG00000006530
7	1.4E+08	1.4E+08	KD2-H4K8la_peak_5740	16.7435	AC073342.12_ENSG00000231840;CASP2_ENSG00000106144
7	1.4E+08	1.4E+08	KD2-H4K8la_peak_5741	6.85108	AC093673.5_ENSG00000232533;ZYX_ENSG00000159840
7	1.4E+08	1.4E+08	KD2-H4K8la_peak_5742	7.83588	FAM115A_ENSG00000198420
7	1.5E+08	1.5E+08	KD2-H4K8la_peak_5743	7.72421	
7	1.5E+08	1.5E+08	KD2-H4K8la_peak_5744	9.40293	RP5-1136G13.2_ENSG00000273314;CUL1_ENSG00000055130
7	1.5E+08	1.5E+08	KD2-H4K8la_peak_5745	8.89364	RP5-1136G13.2_ENSG00000273314
7	1.5E+08	1.5E+08	KD2-H4K8la_peak_5746	8.73956	EZH2_ENSG00000106462
7	1.5E+08	1.5E+08	KD2-H4K8la_peak_5747	5.58883	RNY1_ENSG00000201098
7	1.5E+08	1.5E+08	KD2-H4K8la_peak_5748	10.94155	ZNF786_ENSG00000197362

7	1.5E+08	1.5E+08	KD2-H4K8la_peak_5749	12.73517	ZNF425_ENSG00000204947;RN7SL521P_ENSG00000240877;ZNF398_ENSG00000197024
7	1.5E+08	1.5E+08	KD2-H4K8la_peak_5750	7.94013	ZNF425_ENSG00000204947;RN7SL521P_ENSG00000240877;ZNF398_ENSG00000197024
7	1.5E+08	1.5E+08	KD2-H4K8la_peak_5751	7.3993	ZNF282_ENSG00000170265
7	1.5E+08	1.5E+08	KD2-H4K8la_peak_5752	11.7362	ZNF783_ENSG00000204946
7	1.5E+08	1.5E+08	KD2-H4K8la_peak_5753	9.03222	ZNF783_ENSG00000204946
7	1.5E+08	1.5E+08	KD2-H4K8la_peak_5754	8.66164	RP11-143I21.1_ENSG00000261842
7	1.5E+08	1.5E+08	KD2-H4K8la_peak_5755	4.75945	RP11-143I21.1_ENSG00000261842
7	1.5E+08	1.5E+08	KD2-H4K8la_peak_5756	9.44217	
7	1.5E+08	1.5E+08	KD2-H4K8la_peak_5757	10.87112	ZNF746_ENSG00000181220
7	1.5E+08	1.5E+08	KD2-H4K8la_peak_5758	9.20163	ZNF767_ENSG00000133624
7	1.5E+08	1.5E+08	KD2-H4K8la_peak_5759	5.83285	
7	1.5E+08	1.5E+08	KD2-H4K8la_peak_5760	13.85062	ZNF862_ENSG00000106479
7	1.5E+08	1.5E+08	KD2-H4K8la_peak_5761	6.27787	REPIN1_ENSG00000214022;ZNF775_ENSG00000196456
7	1.5E+08	1.5E+08	KD2-H4K8la_peak_5762	6.86458	REPIN1_ENSG00000214022;ZNF775_ENSG00000196456
7	1.5E+08	1.5E+08	KD2-H4K8la_peak_5763	7.13528	
7	1.5E+08	1.5E+08	KD2-H4K8la_peak_5764	10.16316	
7	1.5E+08	1.5E+08	KD2-H4K8la_peak_5765	12.32641	
7	1.5E+08	1.5E+08	KD2-H4K8la_peak_5766	11.42772	
7	1.5E+08	1.5E+08	KD2-H4K8la_peak_5767	9.29421	
7	1.5E+08	1.5E+08	KD2-H4K8la_peak_5768	10.12872	
7	1.5E+08	1.5E+08	KD2-H4K8la_peak_5769	10.58805	
7	1.5E+08	1.5E+08	KD2-H4K8la_peak_5770	4.68264	
7	1.5E+08	1.5E+08	KD2-H4K8la_peak_5771	11.40102	
7	1.5E+08	1.5E+08	KD2-H4K8la_peak_5772	6.86458	
7	1.5E+08	1.5E+08	KD2-H4K8la_peak_5773	6.65787	
7	1.5E+08	1.5E+08	KD2-H4K8la_peak_5774	8.33415	CDK5_ENSG00000164885
7	1.5E+08	1.5E+08	KD2-H4K8la_peak_5775	6.23074	FASTK_ENSG00000164896
7	1.5E+08	1.5E+08	KD2-H4K8la_peak_5776	6.4136	FASTK_ENSG00000164896
7	1.5E+08	1.5E+08	KD2-H4K8la_peak_5777	12.72886	ABCF2_ENSG00000033050
7	1.5E+08	1.5E+08	KD2-H4K8la_peak_5778	8.39919	
7	1.5E+08	1.5E+08	KD2-H4K8la_peak_5779	13.27013	
7	1.5E+08	1.5E+08	KD2-H4K8la_peak_5780	14.09647	
7	1.5E+08	1.5E+08	KD2-H4K8la_peak_5781	4.66473	SMARCD3_ENSG00000082014
7	1.5E+08	1.5E+08	KD2-H4K8la_peak_5782	12.73517	NUB1_ENSG00000013374
7	1.5E+08	1.5E+08	KD2-H4K8la_peak_5783	12.32641	WDR86_ENSG00000187260;WDR86-AS1_ENSG00000243836
7	1.5E+08	1.5E+08	KD2-H4K8la_peak_5784	14.7348	RHEB_ENSG00000106615
7	1.5E+08	1.5E+08	KD2-H4K8la_peak_5785	8.15454	
7	1.5E+08	1.5E+08	KD2-H4K8la_peak_5786	7.0396	PRKAG2_ENSG00000106617;PRKAG2-AS1_ENSG00000239911
7	1.5E+08	1.5E+08	KD2-H4K8la_peak_5787	10.02595	GALNT11_ENSG00000178234
7	1.5E+08	1.5E+08	KD2-H4K8la_peak_5788	10.87112	GALNT11_ENSG00000178234
7	1.5E+08	1.5E+08	KD2-H4K8la_peak_5789	7.09232	
7	1.5E+08	1.5E+08	KD2-H4K8la_peak_5790	9.03222	LINC01003_ENSG00000261455
7	1.5E+08	1.5E+08	KD2-H4K8la_peak_5791	7.06427	
7	1.5E+08	1.5E+08	KD2-H4K8la_peak_5792	5.8369	ACTR3B_ENSG00000133627
7	1.5E+08	1.5E+08	KD2-H4K8la_peak_5793	7.62867	
7	1.5E+08	1.5E+08	KD2-H4K8la_peak_5794	17.48572	PAXIP1_ENSG00000157212;PAXIP1-AS1_ENSG00000273344

7	1.6E+08	1.6E+08	KD2-H4K8la_peak_5795	5.33301	
7	1.6E+08	1.6E+08	KD2-H4K8la_peak_5796	11.14458	
7	1.6E+08	1.6E+08	KD2-H4K8la_peak_5797	16.57672	AC144652.1_ENSG00000273117;INSIG1_ENSG00000186480
7	1.6E+08	1.6E+08	KD2-H4K8la_peak_5798	7.11805	
7	1.6E+08	1.6E+08	KD2-H4K8la_peak_5799	9.40293	AC009403.2_ENSG00000216895;RBM33_ENSG00000184863
7	1.6E+08	1.6E+08	KD2-H4K8la_peak_5800	10.00612	
7	1.6E+08	1.6E+08	KD2-H4K8la_peak_5801	39.15503	LINC01006_ENSG00000182648;C7orf13_ENSG00000244291;RNF32_ENSG00000105982
7	1.6E+08	1.6E+08	KD2-H4K8la_peak_5802	8.66164	LINC01006_ENSG00000182648;C7orf13_ENSG00000244291;RNF32_ENSG00000105982
7	1.6E+08	1.6E+08	KD2-H4K8la_peak_5803	8.37064	
7	1.6E+08	1.6E+08	KD2-H4K8la_peak_5804	8.76116	LMBR1_ENSG00000105983
7	1.6E+08	1.6E+08	KD2-H4K8la_peak_5805	5.99671	NOM1_ENSG00000146909
7	1.6E+08	1.6E+08	KD2-H4K8la_peak_5806	8.41487	
7	1.6E+08	1.6E+08	KD2-H4K8la_peak_5807	8.80749	DNAJB6_ENSG00000105993
7	1.6E+08	1.6E+08	KD2-H4K8la_peak_5808	5.16795	
7	1.6E+08	1.6E+08	KD2-H4K8la_peak_5809	5.33721	
7	1.6E+08	1.6E+08	KD2-H4K8la_peak_5810	8.13557	
7	1.6E+08	1.6E+08	KD2-H4K8la_peak_5811	4.85784	
8	182579	182777	KD2-H4K8la_peak_5812	9.05604	RPL23AP53_ENSG00000223508;ZNF596_ENSG00000172748;RP5-855D21.3_ENSG00000272812
8	340874	341120	KD2-H4K8la_peak_5813	8.46409	
8	1104689	1105065	KD2-H4K8la_peak_5814	5.83285	
8	1202940	1203368	KD2-H4K8la_peak_5815	6.70626	
8	1501156	1501363	KD2-H4K8la_peak_5816	6.26214	
8	1712023	1712336	KD2-H4K8la_peak_5817	12.11492	CTD-2336O2.1_ENSG00000253982
8	6263263	6263879	KD2-H4K8la_peak_5818	10.20357	RP11-115C21.2_ENSG00000246089;MCPH1_ENSG00000147316
8	6407952	6408198	KD2-H4K8la_peak_5819	7.51014	
8	7212384	7212801	KD2-H4K8la_peak_5820	10.66741	FAM66B_ENSG00000215374
8	8559629	8559873	KD2-H4K8la_peak_5821	7.3993	CLDN23_ENSG00000253958;AC087269.1_ENSG00000176305
8	8750591	8751063	KD2-H4K8la_peak_5822	7.81267	MFHAS1_ENSG00000147324
8	9041307	9041509	KD2-H4K8la_peak_5823	6.25288	
8	9057728	9057955	KD2-H4K8la_peak_5824	6.70626	
8	9413496	9414001	KD2-H4K8la_peak_5825	8.13557	RP11-375N15.2_ENSG00000272267;TNKS_ENSG00000173273
8	1E+07	1E+07	KD2-H4K8la_peak_5826	10.70061	
8	1.1E+07	1.1E+07	KD2-H4K8la_peak_5827	7.12497	CTD-2135J3.3_ENSG00000248896
8	1.1E+07	1.1E+07	KD2-H4K8la_peak_5828	6.57319	SOX7_ENSG00000171056;SOX7_ENSG00000258724;PINX1_ENSG00000254093;RP11-177H2.2_ENSG00000253695
8	1.1E+07	1.1E+07	KD2-H4K8la_peak_5829	8.15906	
8	1.1E+07	1.1E+07	KD2-H4K8la_peak_5830	6.64804	XKR6_ENSG00000171044
8	1.1E+07	1.1E+07	KD2-H4K8la_peak_5831	4.69635	
8	1.1E+07	1.1E+07	KD2-H4K8la_peak_5832	5.05146	
8	1.1E+07	1.1E+07	KD2-H4K8la_peak_5833	7.13528	
8	1.1E+07	1.1E+07	KD2-H4K8la_peak_5834	12.85404	
8	1.3E+07	1.3E+07	KD2-H4K8la_peak_5835	12.03353	LONRF1_ENSG00000154359
8	1.5E+07	1.5E+07	KD2-H4K8la_peak_5836	10.02595	
8	1.8E+07	1.8E+07	KD2-H4K8la_peak_5837	11.06859	
8	1.9E+07	1.9E+07	KD2-H4K8la_peak_5838	13.63983	
8	2E+07	2E+07	KD2-H4K8la_peak_5839	8.13727	
8	2.2E+07	2.2E+07	KD2-H4K8la_peak_5840	9.1923	

8	2.2E+07	2.2E+07	KD2-H4K8la_peak_5841	7.12014	
8	2.2E+07	2.2E+07	KD2-H4K8la_peak_5842	5.43278	
8	2.2E+07	2.2E+07	KD2-H4K8la_peak_5843	8.73956	
8	2.2E+07	2.2E+07	KD2-H4K8la_peak_5844	4.75945	
8	2.2E+07	2.2E+07	KD2-H4K8la_peak_5845	9.27415	
8	2.2E+07	2.2E+07	KD2-H4K8la_peak_5846	5.83285	NUDT18_ENSG00000173566
8	2.2E+07	2.2E+07	KD2-H4K8la_peak_5847	6.61963	HR_ENSG00000168453
8	2.2E+07	2.2E+07	KD2-H4K8la_peak_5848	5.24865	
8	2.2E+07	2.2E+07	KD2-H4K8la_peak_5849	8.73956	
8	2.2E+07	2.2E+07	KD2-H4K8la_peak_5850	8.07102	PPP3CC_ENSG00000120910
8	2.2E+07	2.2E+07	KD2-H4K8la_peak_5851	9.38376	C8orf58_ENSG00000241852
8	2.2E+07	2.2E+07	KD2-H4K8la_peak_5852	9.68194	CCAR2_ENSG00000158941
8	2.2E+07	2.2E+07	KD2-H4K8la_peak_5853	9.68194	CCAR2_ENSG00000158941
8	2.3E+07	2.3E+07	KD2-H4K8la_peak_5854	6.96161	
8	2.3E+07	2.3E+07	KD2-H4K8la_peak_5855	7.47988	
8	2.3E+07	2.3E+07	KD2-H4K8la_peak_5856	8.07102	
8	2.3E+07	2.3E+07	KD2-H4K8la_peak_5857	6.3399	RP11-177H13.2_ENSG00000253837
8	2.3E+07	2.3E+07	KD2-H4K8la_peak_5858	5.98775	
8	2.5E+07	2.5E+07	KD2-H4K8la_peak_5859	7.19298	DOCK5_ENSG00000147459
8	2.6E+07	2.6E+07	KD2-H4K8la_peak_5860	6.3399	
8	2.6E+07	2.6E+07	KD2-H4K8la_peak_5861	6.04152	
8	2.7E+07	2.7E+07	KD2-H4K8la_peak_5862	9.0413	SCARA3_ENSG00000168077
8	2.8E+07	2.8E+07	KD2-H4K8la_peak_5863	5.83285	
8	2.8E+07	2.8E+07	KD2-H4K8la_peak_5864	5.09794	
8	2.8E+07	2.8E+07	KD2-H4K8la_peak_5865	6.0136	
8	2.8E+07	2.8E+07	KD2-H4K8la_peak_5866	7.96951	
8	2.8E+07	2.8E+07	KD2-H4K8la_peak_5867	10.52788	ZNF395_ENSG00000186918
8	2.9E+07	2.9E+07	KD2-H4K8la_peak_5868	5.6165	
8	2.9E+07	2.9E+07	KD2-H4K8la_peak_5869	9.68194	
8	2.9E+07	2.9E+07	KD2-H4K8la_peak_5870	5.52913	INTS9_ENSG00000104299;HMBOX1_ENSG00000147421
8	2.9E+07	2.9E+07	KD2-H4K8la_peak_5871	4.86896	
8	2.9E+07	2.9E+07	KD2-H4K8la_peak_5872	5.05146	DUSP4_ENSG00000120875
8	2.9E+07	2.9E+07	KD2-H4K8la_peak_5873	7.19298	
8	3E+07	3E+07	KD2-H4K8la_peak_5874	8.4393	
8	3E+07	3E+07	KD2-H4K8la_peak_5875	11.25405	
8	3E+07	3E+07	KD2-H4K8la_peak_5876	5.98303	
8	3E+07	3E+07	KD2-H4K8la_peak_5877	7.51302	
8	3E+07	3E+07	KD2-H4K8la_peak_5878	8.48577	LEPROTL1_ENSG00000104660
8	3.1E+07	3.1E+07	KD2-H4K8la_peak_5879	11.06859	
8	3.8E+07	3.8E+07	KD2-H4K8la_peak_5880	4.75924	
8	3.8E+07	3.8E+07	KD2-H4K8la_peak_5881	9.05604	
8	3.8E+07	3.8E+07	KD2-H4K8la_peak_5882	9.77334	
8	3.8E+07	3.8E+07	KD2-H4K8la_peak_5883	10.74378	LSM1_ENSG00000175324;BAG4_ENSG00000156735
8	3.8E+07	3.8E+07	KD2-H4K8la_peak_5884	6.96161	WHSC1L1_ENSG00000147548;RP11-350N15.5_ENSG00000272092
8	3.8E+07	3.8E+07	KD2-H4K8la_peak_5885	8.13727	C8orf86_ENSG00000196166
8	3.9E+07	3.9E+07	KD2-H4K8la_peak_5886	8.08223	

8	3.9E+07	3.9E+07	KD2-H4K8la_peak_5887	7.92775	
8	3.9E+07	3.9E+07	KD2-H4K8la_peak_5888	9.44217	
8	3.9E+07	3.9E+07	KD2-H4K8la_peak_5889	5.83285	
8	3.9E+07	3.9E+07	KD2-H4K8la_peak_5890	8.76082	
8	4.1E+07	4.1E+07	KD2-H4K8la_peak_5891	7.41438	GINS4_ENSG00000147536
8	4.1E+07	4.1E+07	KD2-H4K8la_peak_5892	10.23822	RP11-360L9.7_ENSG00000253174
8	4.2E+07	4.2E+07	KD2-H4K8la_peak_5893	8.73956	RP11-231D20.2_ENSG00000253408;IKBKB_ENSG00000104365
8	4.3E+07	4.3E+07	KD2-H4K8la_peak_5894	11.40266	POMK_ENSG00000185900
8	4.3E+07	4.3E+07	KD2-H4K8la_peak_5895	5.58883	HGSNAT_ENSG00000165102
8	4.8E+07	4.8E+07	KD2-H4K8la_peak_5896	15.50085	SPIDR_ENSG00000164808
8	4.9E+07	4.9E+07	KD2-H4K8la_peak_5897	9.73848	
8	5.5E+07	5.5E+07	KD2-H4K8la_peak_5898	6.2278	
8	5.5E+07	5.5E+07	KD2-H4K8la_peak_5899	7.22716	
8	5.5E+07	5.5E+07	KD2-H4K8la_peak_5900	5.05146	
8	5.7E+07	5.7E+07	KD2-H4K8la_peak_5901	9.6708	LYN_ENSG00000254087
8	5.7E+07	5.7E+07	KD2-H4K8la_peak_5902	6.2278	
8	5.7E+07	5.7E+07	KD2-H4K8la_peak_5903	5.30989	
8	5.7E+07	5.7E+07	KD2-H4K8la_peak_5904	4.86896	
8	5.9E+07	5.9E+07	KD2-H4K8la_peak_5905	6.83387	
8	5.9E+07	5.9E+07	KD2-H4K8la_peak_5906	5.83285	UBXN2B_ENSG00000215114
8	6E+07	6E+07	KD2-H4K8la_peak_5907	10.04111	NSMAF_ENSG00000035681
8	6E+07	6E+07	KD2-H4K8la_peak_5908	7.51014	TOX_ENSG00000198846;RP11-25K19.1_ENSG00000167912
8	6.2E+07	6.2E+07	KD2-H4K8la_peak_5909	7.55141	AC022182.1_ENSG00000254777
8	6.2E+07	6.2E+07	KD2-H4K8la_peak_5910	4.75945	
8	6.4E+07	6.4E+07	KD2-H4K8la_peak_5911	16.69189	YTHDF3-AS1_ENSG00000270673;YTHDF3_ENSG00000185728
8	6.8E+07	6.8E+07	KD2-H4K8la_peak_5912	5.83285	ARFGEF1_ENSG00000066777;RP11-7F18.2_ENSG00000271966
8	7.6E+07	7.6E+07	KD2-H4K8la_peak_5913	8.23729	CASC9_ENSG00000249395
8	8E+07	8E+07	KD2-H4K8la_peak_5914	6.57319	
8	8E+07	8E+07	KD2-H4K8la_peak_5915	6.70626	
8	8.1E+07	8.1E+07	KD2-H4K8la_peak_5916	5.05917	
8	8.1E+07	8.1E+07	KD2-H4K8la_peak_5917	5.28047	
8	8.1E+07	8.1E+07	KD2-H4K8la_peak_5918	6.13507	
8	8.2E+07	8.2E+07	KD2-H4K8la_peak_5919	6.61963	
8	8.2E+07	8.2E+07	KD2-H4K8la_peak_5920	6.3399	
8	8.2E+07	8.2E+07	KD2-H4K8la_peak_5921	7.49467	
8	8.2E+07	8.2E+07	KD2-H4K8la_peak_5922	5.05146	
8	8.2E+07	8.2E+07	KD2-H4K8la_peak_5923	7.13237	RP11-363E6.3_ENSG00000254027;FABP5_ENSG00000164687
8	8.3E+07	8.3E+07	KD2-H4K8la_peak_5924	15.75402	
8	8.6E+07	8.6E+07	KD2-H4K8la_peak_5925	5.05146	RP11-219B4.7_ENSG00000260493;E2F5_ENSG00000133740;RP11-219B4.3_ENSG00000254208
8	9.1E+07	9.1E+07	KD2-H4K8la_peak_5926	11.15747	
8	9.2E+07	9.2E+07	KD2-H4K8la_peak_5927	10.34978	C8orf88_ENSG00000253250
8	9.5E+07	9.5E+07	KD2-H4K8la_peak_5928	7.13237	LINC00535_ENSG00000246662
8	9.5E+07	9.5E+07	KD2-H4K8la_peak_5929	21.43197	
8	9.8E+07	9.8E+07	KD2-H4K8la_peak_5930	4.53273	
8	9.8E+07	9.8E+07	KD2-H4K8la_peak_5931	6.41605	TSPYL5_ENSG00000180543
8	9.9E+07	9.9E+07	KD2-H4K8la_peak_5932	5.40949	MTDH_ENSG00000147649

8	9.9E+07	9.9E+07	KD2-H4K8la_peak_5933	8.62669	
8	9.9E+07	9.9E+07	KD2-H4K8la_peak_5934	6.13507	
8	9.9E+07	9.9E+07	KD2-H4K8la_peak_5935	5.58883	
8	1E+08	1E+08	KD2-H4K8la_peak_5936	4.30663	
8	1E+08	1E+08	KD2-H4K8la_peak_5937	12.65422	
8	1E+08	1E+08	KD2-H4K8la_peak_5938	6.28277	
8	1E+08	1E+08	KD2-H4K8la_peak_5939	7.22716	ANKRD46_ENSG00000186106
8	1E+08	1E+08	KD2-H4K8la_peak_5940	8.07102	
8	1E+08	1E+08	KD2-H4K8la_peak_5941	6.64804	
8	1E+08	1E+08	KD2-H4K8la_peak_5942	6.40658	
8	1E+08	1E+08	KD2-H4K8la_peak_5943	7.3993	KB-1460A1.1_ENSG00000253395
8	1E+08	1E+08	KD2-H4K8la_peak_5944	7.13237	UBR5_ENSG00000104517
8	1E+08	1E+08	KD2-H4K8la_peak_5945	8.16787	KLF10_ENSG00000155090
8	1E+08	1E+08	KD2-H4K8la_peak_5946	5.58883	
8	1E+08	1E+08	KD2-H4K8la_peak_5947	8.56099	
8	1E+08	1E+08	KD2-H4K8la_peak_5948	7.90495	KB-1732A1.1_ENSG00000253669
8	1E+08	1E+08	KD2-H4K8la_peak_5949	7.12014	
8	1E+08	1E+08	KD2-H4K8la_peak_5950	7.8597	CTHRC1_ENSG00000164932
8	1.2E+08	1.2E+08	KD2-H4K8la_peak_5951	4.30663	
8	1.2E+08	1.2E+08	KD2-H4K8la_peak_5952	8.07102	EIF3H_ENSG00000147677;UTP23_ENSG00000147679
8	1.2E+08	1.2E+08	KD2-H4K8la_peak_5953	6.95423	DERL1_ENSG00000136986;TBC1D31_ENSG00000156787
8	1.3E+08	1.3E+08	KD2-H4K8la_peak_5954	8.67242	
8	1.3E+08	1.3E+08	KD2-H4K8la_peak_5955	11.15747	RP11-473O4.3_ENSG00000253720
8	1.3E+08	1.3E+08	KD2-H4K8la_peak_5956	9.72904	RP11-473O4.3_ENSG00000253720
8	1.3E+08	1.3E+08	KD2-H4K8la_peak_5957	10.07718	
8	1.4E+08	1.4E+08	KD2-H4K8la_peak_5958	7.49467	ZFAT_ENSG00000066827
8	1.4E+08	1.4E+08	KD2-H4K8la_peak_5959	15.56048	
8	1.4E+08	1.4E+08	KD2-H4K8la_peak_5960	5.93538	
8	1.4E+08	1.4E+08	KD2-H4K8la_peak_5961	6.92098	
8	1.4E+08	1.4E+08	KD2-H4K8la_peak_5962	6.86458	CHRAC1_ENSG00000104472
8	1.4E+08	1.4E+08	KD2-H4K8la_peak_5963	6.22733	AGO2_ENSG00000123908
8	1.4E+08	1.4E+08	KD2-H4K8la_peak_5964	9.59371	
8	1.4E+08	1.4E+08	KD2-H4K8la_peak_5965	9.06396	
8	1.4E+08	1.4E+08	KD2-H4K8la_peak_5966	9.37943	
8	1.4E+08	1.4E+08	KD2-H4K8la_peak_5967	6.96161	
8	1.4E+08	1.4E+08	KD2-H4K8la_peak_5968	12.56761	
8	1.4E+08	1.4E+08	KD2-H4K8la_peak_5969	4.6555	CTD-3064M3.4_ENSG00000244998;PTP4A3_ENSG00000184489
8	1.4E+08	1.4E+08	KD2-H4K8la_peak_5970	12.13455	CTD-3064M3.4_ENSG00000244998;PTP4A3_ENSG00000184489
8	1.4E+08	1.4E+08	KD2-H4K8la_peak_5971	7.56395	
8	1.4E+08	1.4E+08	KD2-H4K8la_peak_5972	5.27164	TSNARE1_ENSG00000171045;RP13-467H17.1_ENSG00000261693
8	1.4E+08	1.4E+08	KD2-H4K8la_peak_5973	11.15747	
8	1.4E+08	1.4E+08	KD2-H4K8la_peak_5974	8.56099	
8	1.4E+08	1.4E+08	KD2-H4K8la_peak_5975	9.70817	
8	1.4E+08	1.4E+08	KD2-H4K8la_peak_5976	5.23773	RP11-273G15.2_ENSG00000247317;LY6E_ENSG00000160932
8	1.4E+08	1.4E+08	KD2-H4K8la_peak_5977	16.13355	ZFP41_ENSG00000181638;ZFP41_ENSG00000264668
8	1.4E+08	1.4E+08	KD2-H4K8la_peak_5978	12.61456	

8	1.4E+08	1.4E+08	KD2-H4K8la_peak_5979	15.97418	RP13-582O9.7_ENSG00000272172
8	1.4E+08	1.4E+08	KD2-H4K8la_peak_5980	9.37943	
8	1.4E+08	1.4E+08	KD2-H4K8la_peak_5981	8.23729	
8	1.4E+08	1.4E+08	KD2-H4K8la_peak_5982	5.58883	ZNF623_ENSG00000183309
8	1.4E+08	1.4E+08	KD2-H4K8la_peak_5983	6.18994	PUF60_ENSG00000179950
8	1.5E+08	1.5E+08	KD2-H4K8la_peak_5984	5.98775	PLEC_ENSG00000178209
8	1.5E+08	1.5E+08	KD2-H4K8la_peak_5985	4.92443	CYC1_ENSG00000179091
8	1.5E+08	1.5E+08	KD2-H4K8la_peak_5986	8.76116	DGAT1_ENSG00000185000
8	1.5E+08	1.5E+08	KD2-H4K8la_peak_5987	6.64804	SCRT1_ENSG00000170616
8	1.5E+08	1.5E+08	KD2-H4K8la_peak_5988	7.81267	
8	1.5E+08	1.5E+08	KD2-H4K8la_peak_5989	11.20123	FBXL6_ENSG00000182325
8	1.5E+08	1.5E+08	KD2-H4K8la_peak_5990	16.73797	ADCK5_ENSG00000173137
8	1.5E+08	1.5E+08	KD2-H4K8la_peak_5991	13.10532	
8	1.5E+08	1.5E+08	KD2-H4K8la_peak_5992	6.06941	MFSD3_ENSG00000167700
8	1.5E+08	1.5E+08	KD2-H4K8la_peak_5993	7.61975	MFSD3_ENSG00000167700
8	1.5E+08	1.5E+08	KD2-H4K8la_peak_5994	4.7338	ZNF251_ENSG00000198169
8	1.5E+08	1.5E+08	KD2-H4K8la_peak_5995	10.00612	RPL8_ENSG00000161016
8	1.5E+08	1.5E+08	KD2-H4K8la_peak_5996	11.72919	ZNF517_ENSG00000197363
8	1.5E+08	1.5E+08	KD2-H4K8la_peak_5997	7.12014	ZNF7_ENSG00000147789
8	1.5E+08	1.5E+08	KD2-H4K8la_peak_5998	9.20163	COMMD5_ENSG00000170619;AF235103.1_ENSG00000263640
8	1.5E+08	1.5E+08	KD2-H4K8la_peak_5999	9.38376	ZNF250_ENSG00000196150
8	1.5E+08	1.5E+08	KD2-H4K8la_peak_6000	6.13507	C8orf33_ENSG00000182307
9	470373	470781	KD2-H4K8la_peak_6001	7.13237	KANK1_ENSG00000107104
9	3526135	3526362	KD2-H4K8la_peak_6002	8.37064	RFX3_ENSG00000080298;RP11-509J21.1_ENSG00000232104
9	4297566	4297814	KD2-H4K8la_peak_6003	5.67034	
9	4662457	4662703	KD2-H4K8la_peak_6004	9.49266	PPAPDC2_ENSG00000205808
9	5339646	5339964	KD2-H4K8la_peak_6005	6.2278	RLN1_ENSG00000107018
9	5438272	5438498	KD2-H4K8la_peak_6006	5.36015	PLGRKT_ENSG00000107020
9	6412347	6412966	KD2-H4K8la_peak_6007	16.56946	UHRF2_ENSG00000147854
9	6680930	6681157	KD2-H4K8la_peak_6008	6.64804	
9	1.4E+07	1.4E+07	KD2-H4K8la_peak_6009	10.00612	
9	1.4E+07	1.4E+07	KD2-H4K8la_peak_6010	4.75945	
9	1.4E+07	1.4E+07	KD2-H4K8la_peak_6011	9.0413	
9	1.5E+07	1.5E+07	KD2-H4K8la_peak_6012	12.13455	ZDHHC21_ENSG00000175893
9	1.9E+07	1.9E+07	KD2-H4K8la_peak_6013	9.27415	
9	1.9E+07	1.9E+07	KD2-H4K8la_peak_6014	7.19298	RPS6_ENSG00000137154
9	2.6E+07	2.6E+07	KD2-H4K8la_peak_6015	11.24937	TUSC1_ENSG00000198680
9	2.7E+07	2.7E+07	KD2-H4K8la_peak_6016	13.3197	CAAP1_ENSG00000120159
9	2.8E+07	2.8E+07	KD2-H4K8la_peak_6017	8.77936	C9orf72_ENSG00000147894
9	2.8E+07	2.8E+07	KD2-H4K8la_peak_6018	7.72421	
9	3.4E+07	3.4E+07	KD2-H4K8la_peak_6019	11.20341	PRSS3_ENSG00000010438
9	3.4E+07	3.4E+07	KD2-H4K8la_peak_6020	13.38201	UBE2R2_ENSG00000107341
9	3.4E+07	3.4E+07	KD2-H4K8la_peak_6021	7.60756	
9	3.5E+07	3.5E+07	KD2-H4K8la_peak_6022	6.0136	
9	3.5E+07	3.5E+07	KD2-H4K8la_peak_6023	5.99671	
9	3.5E+07	3.5E+07	KD2-H4K8la_peak_6024	7.56395	CNTFR-AS1_ENSG00000237159

9	3.5E+07	3.5E+07	KD2-H4K8la_peak_6025	13.31601	
9	3.5E+07	3.5E+07	KD2-H4K8la_peak_6026	11.77509	SIGMAR1_ENSG00000147955;GALT_ENSG00000213930
9	3.5E+07	3.5E+07	KD2-H4K8la_peak_6027	4.30663	CCL27_ENSG00000213927;RP11-195F19.30_ENSG00000261215;RP11-195F19.5_ENSG00000187186;RP11-195F19.9_ENSG00000230074
9	3.5E+07	3.5E+07	KD2-H4K8la_peak_6028	8.52349	
9	3.5E+07	3.5E+07	KD2-H4K8la_peak_6029	7.92775	PIGO_ENSG00000165282;RP11-182N22.8_ENSG00000234181
9	3.5E+07	3.5E+07	KD2-H4K8la_peak_6030	11.7362	PIGO_ENSG00000165282;RP11-182N22.8_ENSG00000234181
9	3.5E+07	3.5E+07	KD2-H4K8la_peak_6031	5.40012	RUSC2_ENSG00000198853
9	3.6E+07	3.6E+07	KD2-H4K8la_peak_6032	11.87032	
9	3.6E+07	3.6E+07	KD2-H4K8la_peak_6033	7.79412	ARHGEF39_ENSG00000137135
9	3.6E+07	3.6E+07	KD2-H4K8la_peak_6034	4.75945	MSMP_ENSG00000215183;RP11-112J3.15_ENSG00000228843
9	3.6E+07	3.6E+07	KD2-H4K8la_peak_6035	8.89364	
9	3.6E+07	3.6E+07	KD2-H4K8la_peak_6036	10.49982	
9	3.6E+07	3.6E+07	KD2-H4K8la_peak_6037	4.75945	
9	3.7E+07	3.7E+07	KD2-H4K8la_peak_6038	5.83285	RP11-220I1.5_ENSG00000260100;RP11-220I1.1_ENSG00000233137
9	3.8E+07	3.8E+07	KD2-H4K8la_peak_6039	5.67034	RP11-613M10.8_ENSG00000256966;TOMM5_ENSG00000175768
9	3.8E+07	3.8E+07	KD2-H4K8la_peak_6040	9.63529	
9	3.8E+07	3.8E+07	KD2-H4K8la_peak_6041	13.13367	
9	3.8E+07	3.8E+07	KD2-H4K8la_peak_6042	8.86558	EXOSC3_ENSG00000107371;DCAF10_ENSG00000122741
9	3.8E+07	3.8E+07	KD2-H4K8la_peak_6043	11.15747	EXOSC3_ENSG00000107371;DCAF10_ENSG00000122741
9	3.8E+07	3.8E+07	KD2-H4K8la_peak_6044	10.44353	
9	3.8E+07	3.8E+07	KD2-H4K8la_peak_6045	9.77662	
9	4.5E+07	4.5E+07	KD2-H4K8la_peak_6046	9.86881	AL354718.1_ENSG00000268578
9	6.6E+07	6.6E+07	KD2-H4K8la_peak_6047	5.84303	
9	6.6E+07	6.6E+07	KD2-H4K8la_peak_6048	6.5823	RP11-262H14.1_ENSG00000238113;RNA5SP283_ENSG00000202474
9	6.6E+07	6.6E+07	KD2-H4K8la_peak_6049	5.84385	RP11-262H14.5_ENSG00000229422
9	6.8E+07	6.8E+07	KD2-H4K8la_peak_6050	17.27815	RP11-764K9.4_ENSG00000215548
9	7.1E+07	7.1E+07	KD2-H4K8la_peak_6051	6.61963	FAM122A_ENSG00000187866
9	7.2E+07	7.2E+07	KD2-H4K8la_peak_6052	6.70626	
9	7.5E+07	7.5E+07	KD2-H4K8la_peak_6053	10.11868	ZFAND5_ENSG00000107372
9	7.8E+07	7.8E+07	KD2-H4K8la_peak_6054	13.01988	C9orf40_ENSG00000135045;RP11-197P3.4_ENSG00000203321
9	8E+07	8E+07	KD2-H4K8la_peak_6055	8.48085	
9	8.1E+07	8.1E+07	KD2-H4K8la_peak_6056	4.86896	
9	8.1E+07	8.1E+07	KD2-H4K8la_peak_6057	4.71418	
9	8.2E+07	8.2E+07	KD2-H4K8la_peak_6058	4.86896	TLE4_ENSG00000106829
9	8.2E+07	8.2E+07	KD2-H4K8la_peak_6059	6.97699	
9	8.5E+07	8.5E+07	KD2-H4K8la_peak_6060	8.77936	
9	8.6E+07	8.6E+07	KD2-H4K8la_peak_6061	9.0516	IDNK_ENSG00000148057
9	8.6E+07	8.6E+07	KD2-H4K8la_peak_6062	6.64804	UBQLN1_ENSG00000135018;RP11-522I20.3_ENSG00000254473
9	8.7E+07	8.7E+07	KD2-H4K8la_peak_6063	7.81267	HNRNPK_ENSG00000165119;RMI1_ENSG00000178966
9	8.7E+07	8.7E+07	KD2-H4K8la_peak_6064	4.79631	
9	8.9E+07	8.9E+07	KD2-H4K8la_peak_6065	8.37064	GOLM1_ENSG00000135052
9	8.9E+07	8.9E+07	KD2-H4K8la_peak_6066	4.68652	C9orf153_ENSG00000187753;RN7SKP264_ENSG00000223012
9	8.9E+07	8.9E+07	KD2-H4K8la_peak_6067	7.51302	ZCCHC6_ENSG00000083223
9	8.9E+07	8.9E+07	KD2-H4K8la_peak_6068	10.74378	ZCCHC6_ENSG00000083223
9	9.1E+07	9.1E+07	KD2-H4K8la_peak_6069	7.64726	
9	9.2E+07	9.2E+07	KD2-H4K8la_peak_6070	5.83285	CKS2_ENSG00000123975

9	9.2E+07	9.2E+07	KD2-H4K8la_peak_6071	8.41487	SEMA4D_ENSG00000187764;AL590233.1_ENSG00000264913
9	9.2E+07	9.2E+07	KD2-H4K8la_peak_6072	6.11881	SEMA4D_ENSG00000187764
9	9.2E+07	9.2E+07	KD2-H4K8la_peak_6073	8.73956	
9	9.3E+07	9.3E+07	KD2-H4K8la_peak_6074	7.51302	
9	9.4E+07	9.4E+07	KD2-H4K8la_peak_6075	7.3993	SYK_ENSG00000165025
9	9.4E+07	9.4E+07	KD2-H4K8la_peak_6076	7.81267	
9	9.5E+07	9.5E+07	KD2-H4K8la_peak_6077	7.28641	
9	9.5E+07	9.5E+07	KD2-H4K8la_peak_6078	5.58883	ROR2_ENSG00000169071
9	9.5E+07	9.5E+07	KD2-H4K8la_peak_6079	7.93259	
9	9.5E+07	9.5E+07	KD2-H4K8la_peak_6080	7.72421	OMD_ENSG00000127083
9	9.6E+07	9.6E+07	KD2-H4K8la_peak_6081	6.3399	
9	9.6E+07	9.6E+07	KD2-H4K8la_peak_6082	10.87555	NINJ1_ENSG00000131669
9	9.6E+07	9.6E+07	KD2-H4K8la_peak_6083	10.36292	
9	9.6E+07	9.6E+07	KD2-H4K8la_peak_6084	4.30663	FAM120A_ENSG00000048828
9	9.7E+07	9.7E+07	KD2-H4K8la_peak_6085	5.72892	
9	9.7E+07	9.7E+07	KD2-H4K8la_peak_6086	13.53941	
9	9.7E+07	9.7E+07	KD2-H4K8la_peak_6087	6.22733	
9	9.7E+07	9.7E+07	KD2-H4K8la_peak_6088	7.51302	C9orf3_ENSG00000148120
9	9.8E+07	9.8E+07	KD2-H4K8la_peak_6089	6.64804	
9	9.8E+07	9.8E+07	KD2-H4K8la_peak_6090	6.86458	
9	9.8E+07	9.8E+07	KD2-H4K8la_peak_6091	11.15747	
9	9.9E+07	9.9E+07	KD2-H4K8la_peak_6092	7.19298	AAED1_ENSG00000158122
9	1E+08	1E+08	KD2-H4K8la_peak_6093	7.3993	TDRD7_ENSG00000196116
9	1E+08	1E+08	KD2-H4K8la_peak_6094	8.76082	TMOD1_ENSG00000136842
9	1E+08	1E+08	KD2-H4K8la_peak_6095	7.23116	
9	1E+08	1E+08	KD2-H4K8la_peak_6096	17.33048	ANP32B_ENSG00000136938
9	1E+08	1E+08	KD2-H4K8la_peak_6097	11.20123	ANP32B_ENSG00000136938
9	1E+08	1E+08	KD2-H4K8la_peak_6098	6.88801	TEX10_ENSG00000136891
9	1E+08	1E+08	KD2-H4K8la_peak_6099	6.2278	MSANTD3_ENSG00000066697
9	1E+08	1E+08	KD2-H4K8la_peak_6100	8.77936	MRPL50_ENSG00000136897;ZNF189_ENSG00000136870
9	1.1E+08	1.1E+08	KD2-H4K8la_peak_6101	7.13237	
9	1.1E+08	1.1E+08	KD2-H4K8la_peak_6102	7.13237	
9	1.1E+08	1.1E+08	KD2-H4K8la_peak_6103	8.62669	
9	1.1E+08	1.1E+08	KD2-H4K8la_peak_6104	7.13237	
9	1.1E+08	1.1E+08	KD2-H4K8la_peak_6105	7.75267	TMEM245_ENSG00000106771
9	1.1E+08	1.1E+08	KD2-H4K8la_peak_6106	6.3399	LPAR1_ENSG00000198121
9	1.1E+08	1.1E+08	KD2-H4K8la_peak_6107	8.65116	
9	1.1E+08	1.1E+08	KD2-H4K8la_peak_6108	5.16795	KIAA0368_ENSG00000136813
9	1.1E+08	1.1E+08	KD2-H4K8la_peak_6109	7.72421	ZNF483_ENSG00000173258
9	1.1E+08	1.1E+08	KD2-H4K8la_peak_6110	8.89364	SUSD1_ENSG00000106868
9	1.2E+08	1.2E+08	KD2-H4K8la_peak_6111	6.88801	C9orf147_ENSG00000230185;KIAA1958_ENSG00000165185
9	1.2E+08	1.2E+08	KD2-H4K8la_peak_6112	10.5885	C9orf147_ENSG00000230185;KIAA1958_ENSG00000165185
9	1.2E+08	1.2E+08	KD2-H4K8la_peak_6113	5.83285	
9	1.2E+08	1.2E+08	KD2-H4K8la_peak_6114	10.04111	ATP6V1G1_ENSG00000136888
9	1.2E+08	1.2E+08	KD2-H4K8la_peak_6115	13.23927	C9orf91_ENSG00000157693
9	1.2E+08	1.2E+08	KD2-H4K8la_peak_6116	8.44557	ASTN2_ENSG00000148219

9	1.2E+08	1.2E+08	KD2-H4K8la_peak_6117	6.28277	
9	1.2E+08	1.2E+08	KD2-H4K8la_peak_6118	5.16795	CDK5RAP2_ENSG00000136861
9	1.2E+08	1.2E+08	KD2-H4K8la_peak_6119	16.8445	MEGF9_ENSG00000106780
9	1.2E+08	1.2E+08	KD2-H4K8la_peak_6120	10.74378	PHF19_ENSG00000119403
9	1.2E+08	1.2E+08	KD2-H4K8la_peak_6121	5.47284	STOM_ENSG00000148175
9	1.2E+08	1.2E+08	KD2-H4K8la_peak_6122	6.25288	
9	1.2E+08	1.2E+08	KD2-H4K8la_peak_6123	5.95337	TTLL11_ENSG00000175764
9	1.3E+08	1.3E+08	KD2-H4K8la_peak_6124	6.64804	
9	1.3E+08	1.3E+08	KD2-H4K8la_peak_6125	4.86896	DENND1A_ENSG00000119522
9	1.3E+08	1.3E+08	KD2-H4K8la_peak_6126	7.05576	DENND1A_ENSG00000119522
9	1.3E+08	1.3E+08	KD2-H4K8la_peak_6127	6.2278	NEK6_ENSG00000119408
9	1.3E+08	1.3E+08	KD2-H4K8la_peak_6128	12.80043	
9	1.3E+08	1.3E+08	KD2-H4K8la_peak_6129	11.76151	
9	1.3E+08	1.3E+08	KD2-H4K8la_peak_6130	6.0136	
9	1.3E+08	1.3E+08	KD2-H4K8la_peak_6131	9.35473	RPL35_ENSG00000136942;ARPC5L_ENSG00000136950
9	1.3E+08	1.3E+08	KD2-H4K8la_peak_6132	7.86224	
9	1.3E+08	1.3E+08	KD2-H4K8la_peak_6133	13.38201	MAPKAP1_ENSG00000119487
9	1.3E+08	1.3E+08	KD2-H4K8la_peak_6134	5.11209	RP11-423C15.3_ENSG00000229582;PBX3_ENSG00000167081
9	1.3E+08	1.3E+08	KD2-H4K8la_peak_6135	9.86881	RP11-343J18.1_ENSG00000228392
9	1.3E+08	1.3E+08	KD2-H4K8la_peak_6136	5.05146	MVB12B_ENSG00000196814
9	1.3E+08	1.3E+08	KD2-H4K8la_peak_6137	6.68593	
9	1.3E+08	1.3E+08	KD2-H4K8la_peak_6138	6.47589	AL356309.1_ENSG00000221768
9	1.3E+08	1.3E+08	KD2-H4K8la_peak_6139	9.19451	
9	1.3E+08	1.3E+08	KD2-H4K8la_peak_6140	7.49467	
9	1.3E+08	1.3E+08	KD2-H4K8la_peak_6141	8.76116	
9	1.3E+08	1.3E+08	KD2-H4K8la_peak_6142	12.39776	
9	1.3E+08	1.3E+08	KD2-H4K8la_peak_6143	10.02595	
9	1.3E+08	1.3E+08	KD2-H4K8la_peak_6144	7.61975	
9	1.3E+08	1.3E+08	KD2-H4K8la_peak_6145	17.45665	
9	1.3E+08	1.3E+08	KD2-H4K8la_peak_6146	4.69635	
9	1.3E+08	1.3E+08	KD2-H4K8la_peak_6147	8.16787	
9	1.3E+08	1.3E+08	KD2-H4K8la_peak_6148	6.36753	ST6GALNAC4_ENSG00000136840
9	1.3E+08	1.3E+08	KD2-H4K8la_peak_6149	6.23124	
9	1.3E+08	1.3E+08	KD2-H4K8la_peak_6150	11.29733	
9	1.3E+08	1.3E+08	KD2-H4K8la_peak_6151	10.49982	
9	1.3E+08	1.3E+08	KD2-H4K8la_peak_6152	8.69108	
9	1.3E+08	1.3E+08	KD2-H4K8la_peak_6153	8.76116	PTGES2_ENSG00000148334;AL590708.2_ENSG00000232850
9	1.3E+08	1.3E+08	KD2-H4K8la_peak_6154	6.59301	C9orf16_ENSG00000171159
9	1.3E+08	1.3E+08	KD2-H4K8la_peak_6155	6.0136	
9	1.3E+08	1.3E+08	KD2-H4K8la_peak_6156	14.30153	MIR199B_ENSG00000207581;MIR3154_ENSG00000264823
9	1.3E+08	1.3E+08	KD2-H4K8la_peak_6157	4.86896	
9	1.3E+08	1.3E+08	KD2-H4K8la_peak_6158	7.19244	SWI5_ENSG00000175854;GOLGA2_ENSG00000167110
9	1.3E+08	1.3E+08	KD2-H4K8la_peak_6159	7.3993	SLC27A4_ENSG00000167114
9	1.3E+08	1.3E+08	KD2-H4K8la_peak_6160	9.29385	
9	1.3E+08	1.3E+08	KD2-H4K8la_peak_6161	8.24671	
9	1.3E+08	1.3E+08	KD2-H4K8la_peak_6162	8.07102	

9	1.3E+08	1.3E+08	KD2-H4K8la_peak_6163	7.56395	TBC1D13_ENSG00000107021
9	1.3E+08	1.3E+08	KD2-H4K8la_peak_6164	8.37064	CRAT_ENSG00000095321;PPP2R4_ENSG00000119383
9	1.3E+08	1.3E+08	KD2-H4K8la_peak_6165	14.7348	
9	1.3E+08	1.3E+08	KD2-H4K8la_peak_6166	7.23932	
9	1.3E+08	1.3E+08	KD2-H4K8la_peak_6167	11.77509	IER5L_ENSG00000188483
9	1.3E+08	1.3E+08	KD2-H4K8la_peak_6168	8.43222	
9	1.3E+08	1.3E+08	KD2-H4K8la_peak_6169	7.91061	
9	1.3E+08	1.3E+08	KD2-H4K8la_peak_6170	7.51014	C9orf106_ENSG00000179082
9	1.3E+08	1.3E+08	KD2-H4K8la_peak_6171	11.06859	C9orf106_ENSG00000179082
9	1.3E+08	1.3E+08	KD2-H4K8la_peak_6172	4.86896	
9	1.3E+08	1.3E+08	KD2-H4K8la_peak_6173	5.22418	
9	1.3E+08	1.3E+08	KD2-H4K8la_peak_6174	6.64804	
9	1.3E+08	1.3E+08	KD2-H4K8la_peak_6175	7.19244	
9	1.3E+08	1.3E+08	KD2-H4K8la_peak_6176	15.08271	
9	1.3E+08	1.3E+08	KD2-H4K8la_peak_6177	5.05146	
9	1.3E+08	1.3E+08	KD2-H4K8la_peak_6178	7.03956	
9	1.3E+08	1.3E+08	KD2-H4K8la_peak_6179	6.68593	
9	1.3E+08	1.3E+08	KD2-H4K8la_peak_6180	15.37939	
9	1.3E+08	1.3E+08	KD2-H4K8la_peak_6181	6.64804	
9	1.3E+08	1.3E+08	KD2-H4K8la_peak_6182	17.92782	RP11-65J3.14_ENSG00000261334
9	1.3E+08	1.3E+08	KD2-H4K8la_peak_6183	11.15747	RP11-65J3.14_ENSG00000261334;LINC00963_ENSG00000204054
9	1.3E+08	1.3E+08	KD2-H4K8la_peak_6184	8.39919	RP11-65J3.14_ENSG00000261334;LINC00963_ENSG00000204054
9	1.3E+08	1.3E+08	KD2-H4K8la_peak_6185	8.47841	
9	1.3E+08	1.3E+08	KD2-H4K8la_peak_6186	7.61975	
9	1.3E+08	1.3E+08	KD2-H4K8la_peak_6187	6.06941	C9orf50_ENSG00000179058
9	1.3E+08	1.3E+08	KD2-H4K8la_peak_6188	9.48768	
9	1.3E+08	1.3E+08	KD2-H4K8la_peak_6189	7.59044	ASB6_ENSG00000148331
9	1.3E+08	1.3E+08	KD2-H4K8la_peak_6190	6.4136	
9	1.3E+08	1.3E+08	KD2-H4K8la_peak_6191	7.12014	PRRX2_ENSG00000167157
9	1.3E+08	1.3E+08	KD2-H4K8la_peak_6192	11.15747	
9	1.3E+08	1.3E+08	KD2-H4K8la_peak_6193	5.76341	GPR107_ENSG00000148358
9	1.3E+08	1.3E+08	KD2-H4K8la_peak_6194	7.92775	
9	1.3E+08	1.3E+08	KD2-H4K8la_peak_6195	5.27164	
9	1.3E+08	1.3E+08	KD2-H4K8la_peak_6196	8.76082	
9	1.3E+08	1.3E+08	KD2-H4K8la_peak_6197	8.33263	
9	1.3E+08	1.3E+08	KD2-H4K8la_peak_6198	9.27415	
9	1.3E+08	1.3E+08	KD2-H4K8la_peak_6199	7.25464	
9	1.3E+08	1.3E+08	KD2-H4K8la_peak_6200	9.9701	ASS1_ENSG00000130707
9	1.3E+08	1.3E+08	KD2-H4K8la_peak_6201	5.90392	
9	1.3E+08	1.3E+08	KD2-H4K8la_peak_6202	6.13507	
9	1.3E+08	1.3E+08	KD2-H4K8la_peak_6203	4.75945	
9	1.3E+08	1.3E+08	KD2-H4K8la_peak_6204	4.30663	
9	1.3E+08	1.3E+08	KD2-H4K8la_peak_6205	8.76116	
9	1.3E+08	1.3E+08	KD2-H4K8la_peak_6206	10.16645	EXOSC2_ENSG00000130713
9	1.3E+08	1.3E+08	KD2-H4K8la_peak_6207	4.75945	
9	1.3E+08	1.3E+08	KD2-H4K8la_peak_6208	6.13507	

9	1.3E+08	1.3E+08	KD2-H4K8la_peak_6209	10.97039	PRRC2B_ENSG00000130723
9	1.3E+08	1.3E+08	KD2-H4K8la_peak_6210	19.53802	UCK1_ENSG00000130717
9	1.3E+08	1.3E+08	KD2-H4K8la_peak_6211	9.54333	RAPGEF1_ENSG00000107263
9	1.4E+08	1.4E+08	KD2-H4K8la_peak_6212	11.15747	TSC1_ENSG00000165699;GFI1B_ENSG00000165702;MIR548AW_ENSG00000263816
9	1.4E+08	1.4E+08	KD2-H4K8la_peak_6213	5.468	GTF3C5_ENSG00000148308
9	1.4E+08	1.4E+08	KD2-H4K8la_peak_6214	13.01988	REXO4_ENSG00000148300
9	1.4E+08	1.4E+08	KD2-H4K8la_peak_6215	9.11321	
9	1.4E+08	1.4E+08	KD2-H4K8la_peak_6216	6.28277	
9	1.4E+08	1.4E+08	KD2-H4K8la_peak_6217	7.49467	
9	1.4E+08	1.4E+08	KD2-H4K8la_peak_6218	6.8348	
9	1.4E+08	1.4E+08	KD2-H4K8la_peak_6219	11.42695	
9	1.4E+08	1.4E+08	KD2-H4K8la_peak_6220	11.28887	
9	1.4E+08	1.4E+08	KD2-H4K8la_peak_6221	4.86896	
9	1.4E+08	1.4E+08	KD2-H4K8la_peak_6222	25.55588	
9	1.4E+08	1.4E+08	KD2-H4K8la_peak_6223	4.86896	LL09NC01-254D11.1_ENSG00000261018
9	1.4E+08	1.4E+08	KD2-H4K8la_peak_6224	7.61975	
9	1.4E+08	1.4E+08	KD2-H4K8la_peak_6225	4.3076	
9	1.4E+08	1.4E+08	KD2-H4K8la_peak_6226	9.20163	VAV2_ENSG00000160293
9	1.4E+08	1.4E+08	KD2-H4K8la_peak_6227	8.07044	BRD3_ENSG00000169925
9	1.4E+08	1.4E+08	KD2-H4K8la_peak_6228	9.97166	RNU6ATAC_ENSG00000221676
9	1.4E+08	1.4E+08	KD2-H4K8la_peak_6229	5.09794	
9	1.4E+08	1.4E+08	KD2-H4K8la_peak_6230	5.05146	
9	1.4E+08	1.4E+08	KD2-H4K8la_peak_6231	6.56022	
9	1.4E+08	1.4E+08	KD2-H4K8la_peak_6232	5.99671	
9	1.4E+08	1.4E+08	KD2-H4K8la_peak_6233	10.16645	PPP1R26_ENSG00000196422
9	1.4E+08	1.4E+08	KD2-H4K8la_peak_6234	7.96951	PPP1R26-AS1_ENSG00000225361
9	1.4E+08	1.4E+08	KD2-H4K8la_peak_6235	13.6415	
9	1.4E+08	1.4E+08	KD2-H4K8la_peak_6236	6.01825	
9	1.4E+08	1.4E+08	KD2-H4K8la_peak_6237	7.33815	
9	1.4E+08	1.4E+08	KD2-H4K8la_peak_6238	9.44217	RP11-83N9.5_ENSG00000260193
9	1.4E+08	1.4E+08	KD2-H4K8la_peak_6239	6.13507	C9orf69_ENSG00000238227
9	1.4E+08	1.4E+08	KD2-H4K8la_peak_6240	8.76116	QSOX2_ENSG00000165661
9	1.4E+08	1.4E+08	KD2-H4K8la_peak_6241	7.51014	DKFZP434A062_ENSG00000267845
9	1.4E+08	1.4E+08	KD2-H4K8la_peak_6242	8.76116	GPSM1_ENSG00000160360
9	1.4E+08	1.4E+08	KD2-H4K8la_peak_6243	10.1108	GPSM1_ENSG00000160360
9	1.4E+08	1.4E+08	KD2-H4K8la_peak_6244	14.20958	DNLZ_ENSG00000213221
9	1.4E+08	1.4E+08	KD2-H4K8la_peak_6245	8.13557	DNLZ_ENSG00000213221
9	1.4E+08	1.4E+08	KD2-H4K8la_peak_6246	8.37064	CARD9_ENSG00000187796
9	1.4E+08	1.4E+08	KD2-H4K8la_peak_6247	9.9701	SNAPC4_ENSG00000165684
9	1.4E+08	1.4E+08	KD2-H4K8la_peak_6248	10.02595	SDCCAG3_ENSG00000165689;PMPCA_ENSG00000165688
9	1.4E+08	1.4E+08	KD2-H4K8la_peak_6249	12.93686	INPP5E_ENSG00000148384
9	1.4E+08	1.4E+08	KD2-H4K8la_peak_6250	8.3574	
9	1.4E+08	1.4E+08	KD2-H4K8la_peak_6251	7.92775	
9	1.4E+08	1.4E+08	KD2-H4K8la_peak_6252	5.43278	
9	1.4E+08	1.4E+08	KD2-H4K8la_peak_6253	10.58561	FAM69B_ENSG00000165716
9	1.4E+08	1.4E+08	KD2-H4K8la_peak_6254	9.77334	SNHG7_ENSG00000233016

9	1.4E+08	1.4E+08	KD2-H4K8la_peak_6255	6.78322	
9	1.4E+08	1.4E+08	KD2-H4K8la_peak_6256	4.30663	PHPT1_ENSG00000054148
9	1.4E+08	1.4E+08	KD2-H4K8la_peak_6257	8.39919	EDF1_ENSG00000107223
9	1.4E+08	1.4E+08	KD2-H4K8la_peak_6258	8.39919	MIR4479_ENSG00000266507
9	1.4E+08	1.4E+08	KD2-H4K8la_peak_6259	8.39919	FBXW5_ENSG00000159069;C8G_ENSG00000176919
9	1.4E+08	1.4E+08	KD2-H4K8la_peak_6260	9.61894	LCN12_ENSG00000184925
9	1.4E+08	1.4E+08	KD2-H4K8la_peak_6261	6.18994	NPDC1_ENSG00000107281
9	1.4E+08	1.4E+08	KD2-H4K8la_peak_6262	7.92775	ENTPD2_ENSG00000054179
9	1.4E+08	1.4E+08	KD2-H4K8la_peak_6263	7.94013	SAPCD2_ENSG00000186193
9	1.4E+08	1.4E+08	KD2-H4K8la_peak_6264	7.61975	DPP7_ENSG00000176978
9	1.4E+08	1.4E+08	KD2-H4K8la_peak_6265	6.97699	
9	1.4E+08	1.4E+08	KD2-H4K8la_peak_6266	9.27415	ANAPC2_ENSG00000176248;SSNA1_ENSG00000176101
9	1.4E+08	1.4E+08	KD2-H4K8la_peak_6267	6.68593	
9	1.4E+08	1.4E+08	KD2-H4K8la_peak_6268	11.7362	TUBB4B_ENSG00000188229
9	1.4E+08	1.4E+08	KD2-H4K8la_peak_6269	9.9701	NELFB_ENSG00000188986
9	1.4E+08	1.4E+08	KD2-H4K8la_peak_6270	13.13367	NELFB_ENSG00000188986
9	1.4E+08	1.4E+08	KD2-H4K8la_peak_6271	11.20341	
9	1.4E+08	1.4E+08	KD2-H4K8la_peak_6272	4.6555	
9	1.4E+08	1.4E+08	KD2-H4K8la_peak_6273	6.3399	RP13-122B23.8_ENSG00000260996
9	1.4E+08	1.4E+08	KD2-H4K8la_peak_6274	6.68593	NRARP_ENSG00000198435
9	1.4E+08	1.4E+08	KD2-H4K8la_peak_6275	12.166	
9	1.4E+08	1.4E+08	KD2-H4K8la_peak_6276	9.27415	
9	1.4E+08	1.4E+08	KD2-H4K8la_peak_6277	7.61975	EXD3_ENSG00000187609;NOXA1_ENSG00000188747
9	1.4E+08	1.4E+08	KD2-H4K8la_peak_6278	6.3399	EXD3_ENSG00000187609;NOXA1_ENSG00000188747
9	1.4E+08	1.4E+08	KD2-H4K8la_peak_6279	4.66473	NSMF_ENSG00000165802
9	1.4E+08	1.4E+08	KD2-H4K8la_peak_6280	8.13727	DPH7_ENSG00000148399
9	1.4E+08	1.4E+08	KD2-H4K8la_peak_6281	9.10568	C9orf37_ENSG00000203993;EHMT1_ENSG00000181090
9	1.4E+08	1.4E+08	KD2-H4K8la_peak_6282	11.15747	CACNA1B_ENSG00000148408
00019	114691	114984	KD2-H4K8la_peak_6283	7.79412	
00019	30120	30565	KD2-H4K8la_peak_6284	8.45512	
00019	32160	32359	KD2-H4K8la_peak_6285	9.62441	
00019	33126	33486	KD2-H4K8la_peak_6286	5.64882	
00019	86962	87173	KD2-H4K8la_peak_6287	8.77747	
00019	121310	121535	KD2-H4K8la_peak_6288	5.0364	
00019	138050	138257	KD2-H4K8la_peak_6289	9.7321	
00020	85821	86020	KD2-H4K8la_peak_6290	14.0867	
00020	86960	87232	KD2-H4K8la_peak_6291	23.05476	
00020	87935	88441	KD2-H4K8la_peak_6292	18.13987	
00020	110110	111901	KD2-H4K8la_peak_6293	28.70666	
00021	47457	48007	KD2-H4K8la_peak_6294	9.49266	
00021	29779	30016	KD2-H4K8la_peak_6295	9.58311	
00021	30445	30756	KD2-H4K8la_peak_6296	18.98252	
00021	41305	42170	KD2-H4K8la_peak_6297	7.11805	
00021	44712	44967	KD2-H4K8la_peak_6298	12.69007	
00021	45223	45625	KD2-H4K8la_peak_6299	9.40293	
00021	99837	100403	KD2-H4K8la_peak_6300	34.06538	

00022	6438	7130	KD2-H4K8la_peak_6301	8.45512	
00022	30916	31511	KD2-H4K8la_peak_6302	6.63197	
X	347506	347856	KD2-H4K8la_peak_6303	5.80888	PPP2R3B_ENSG00000167393
X	1511373	1511575	KD2-H4K8la_peak_6304	7.90495	SLC25A6_ENSG00000169100
X	2418326	2418628	KD2-H4K8la_peak_6305	7.12014	ZBED1_ENSG00000214717
X	3733723	3734372	KD2-H4K8la_peak_6306	13.03006	
X	7065695	7066081	KD2-H4K8la_peak_6307	10.97039	HDHD1_ENSG00000130021;MIR4767_ENSG00000264268
X	1.5E+07	1.5E+07	KD2-H4K8la_peak_6308	7.13237	
X	1.7E+07	1.7E+07	KD2-H4K8la_peak_6309	6.2278	CTPS2_ENSG00000047230
X	1.7E+07	1.7E+07	KD2-H4K8la_peak_6310	5.72892	SYAP1_ENSG00000169895
X	1.7E+07	1.7E+07	KD2-H4K8la_peak_6311	8.12466	TXLNG_ENSG00000086712
X	1.7E+07	1.7E+07	KD2-H4K8la_peak_6312	6.26214	RBBP7_ENSG00000102054
X	1.7E+07	1.7E+07	KD2-H4K8la_peak_6313	5.36015	
X	1.9E+07	1.9E+07	KD2-H4K8la_peak_6314	6.64804	PPEF1_ENSG00000086717
X	1.9E+07	1.9E+07	KD2-H4K8la_peak_6315	8.77936	
X	2E+07	2E+07	KD2-H4K8la_peak_6316	6.2278	EIF1AX_ENSG00000173674
X	2.4E+07	2.4E+07	KD2-H4K8la_peak_6317	5.10388	ZFX-AS1_ENSG00000234230;ZFX_ENSG00000005889
X	2.4E+07	2.4E+07	KD2-H4K8la_peak_6318	12.211	ZFX-AS1_ENSG00000234230;ZFX_ENSG00000005889
X	3.9E+07	3.9E+07	KD2-H4K8la_peak_6319	4.94337	
X	4E+07	4E+07	KD2-H4K8la_peak_6320	7.13237	
X	4E+07	4E+07	KD2-H4K8la_peak_6321	6.26214	
X	4E+07	4E+07	KD2-H4K8la_peak_6322	5.67034	BCOR_ENSG00000183337
X	4E+07	4E+07	KD2-H4K8la_peak_6323	7.23116	
X	4.1E+07	4.1E+07	KD2-H4K8la_peak_6324	9.27415	DDX3X_ENSG00000215301
X	4.1E+07	4.1E+07	KD2-H4K8la_peak_6325	6.70626	
X	4.4E+07	4.4E+07	KD2-H4K8la_peak_6326	6.2278	FUNDC1_ENSG00000069509
X	4.5E+07	4.5E+07	KD2-H4K8la_peak_6327	10.99007	KDM6A_ENSG00000147050
X	4.7E+07	4.7E+07	KD2-H4K8la_peak_6328	12.62032	
X	4.9E+07	4.9E+07	KD2-H4K8la_peak_6329	6.42754	WAS_ENSG00000015285
X	4.9E+07	4.9E+07	KD2-H4K8la_peak_6330	7.49467	TFE3_ENSG00000068323
X	4.9E+07	4.9E+07	KD2-H4K8la_peak_6331	8.76082	PPP1R3F_ENSG00000049769
X	5.3E+07	5.3E+07	KD2-H4K8la_peak_6332	7.13237	
X	5.3E+07	5.3E+07	KD2-H4K8la_peak_6333	12.1221	
X	5.3E+07	5.3E+07	KD2-H4K8la_peak_6334	7.61975	KDM5C_ENSG00000126012
X	5.4E+07	5.4E+07	KD2-H4K8la_peak_6335	4.30663	FAM120C_ENSG00000184083
X	5.5E+07	5.5E+07	KD2-H4K8la_peak_6336	5.33364	FAM104B_ENSG00000182518
X	6.8E+07	6.8E+07	KD2-H4K8la_peak_6337	19.63589	YIPF6_ENSG00000181704
X	7E+07	7E+07	KD2-H4K8la_peak_6338	9.05604	
X	7.1E+07	7.1E+07	KD2-H4K8la_peak_6339	6.69599	TAF1_ENSG00000147133
X	7.1E+07	7.1E+07	KD2-H4K8la_peak_6340	8.45079	
X	7.4E+07	7.4E+07	KD2-H4K8la_peak_6341	5.22084	
X	1.1E+08	1.1E+08	KD2-H4K8la_peak_6342	11.45154	RBM41_ENSG00000089682
X	1.1E+08	1.1E+08	KD2-H4K8la_peak_6343	8.59561	
X	1.2E+08	1.2E+08	KD2-H4K8la_peak_6344	53.71253	
X	1.2E+08	1.2E+08	KD2-H4K8la_peak_6345	6.13507	NKAP_ENSG00000101882
X	1.3E+08	1.3E+08	KD2-H4K8la_peak_6346	7.12497	ELF4_ENSG00000102034

X	1.3E+08	1.3E+08	KD2-H4K8la_peak_6347	14.52454	RAP2C-AS1_ENSG00000232160
X	1.3E+08	1.3E+08	KD2-H4K8la_peak_6348	6.83387	
X	1.3E+08	1.3E+08	KD2-H4K8la_peak_6349	9.86881	
X	1.3E+08	1.3E+08	KD2-H4K8la_peak_6350	6.60114	DDX26B-AS1_ENSG00000225235;DDX26B_ENSG00000165359
X	1.5E+08	1.5E+08	KD2-H4K8la_peak_6351	6.57319	
X	1.5E+08	1.5E+08	KD2-H4K8la_peak_6352	5.83285	
X	1.5E+08	1.5E+08	KD2-H4K8la_peak_6353	9.70191	
X	1.5E+08	1.5E+08	KD2-H4K8la_peak_6354	7.06427	
X	1.5E+08	1.5E+08	KD2-H4K8la_peak_6355	5.05146	
